# Mitochondrial Haplogroup Classification of Ancient DNA Samples Using Haplotracker

**DOI:** 10.1155/2022/5344418

**Published:** 2022-03-18

**Authors:** Kijeong Kim, Dong-han Kim, Kyung-yong Kim

**Affiliations:** ^1^Institute of Gene and Genome Research, College of Medicine, Chung-Ang University, Seoul 06974, Republic of Korea; ^2^Faculty of Science, University of Sydney, Sydney NSW 2016, Australia

## Abstract

Mitochondrial DNA haplogroup classification is used to study maternal lineage of ancient human populations. The haplogrouping of ancient DNA is not easy because the DNA is usually found in small pieces in limited quantities. We have developed Haplotracker, a straightforward and efficient high-resolution haplogroup classification tool optimized specifically for ancient DNA samples. Haplotracker offers a user-friendly input interface for multiple mitochondrial DNA sequence fragments in a sample. It provides accurate haplogroup classification with full-length mitochondrial genome sequences and provides high-resolution haplogroup predictions for some fragmented control region sequences using a novel algorithm built on Phylotree mtDNA Build 17 (Phylotree) and our haplotype database (*n* = 118,869). Its performance for accuracy was demonstrated to be high through haplogroup classification using 8,216 Phylotree full-length and control region mitochondrial DNA sequences compared with HaploGrep 2, one of the most accurate current haplogroup classifiers. Haplotracker provides a novel haplogroup tracking solution for fragmented sequences to track subhaplogroups or verify the haplogroups efficiently. Using Haplotracker, we classified mitochondrial haplogroups to the final subhaplogroup level in nine ancient DNA samples extracted from human skeletal remains found in 2,000-year-old elite Xiongnu cemetery in Northeast Mongolia. Haplotracker can be freely accessed at https://haplotracker.cau.ac.kr.

## 1. Introduction

Mitochondrial DNA (mtDNA) haplogroup (HG) classification, known as mtDNA haplogrouping, is important for population genetics. Haplogrouping is usually conducted using a software tool that automatically assigns the HG of a given mtDNA sequence based on Phylotree [1, 2], the tree of global mtDNA variation. Haplogrouping of fresh DNA samples, from which large amounts of sequence information are readily available, is much easier than that of degraded DNA samples such as ancient DNA (aDNA). The aDNA is present in very small amounts and is fragmented into tiny pieces [3], often containing exogenous materials that may inhibit or interfere with PCR [4, 5]. These properties make haplogrouping difficult. Next-generation sequencing (NGS) methods have become a revolutionary tool to study mtDNA sequence variation, particularly from degraded DNA samples at the mtDNA genome (mtGenome) level [6–8]. Sanger dideoxy sequencing method can be applied for full-length mtDNA sequencing, but it is inefficient, costly, laborious, and time-consuming. Some commercial kits for mtDNA NGS are currently available. One of the greatest advantages of using the NGS method is its high-throughput performance enabling large-scale sequencing. However, it has some practical limitations when the number of test samples is small. It preferably requires a minimal number of samples to run together mainly due to the cost issues. A series of expensive reagent packages are required for a set of sample units (e.g., 96 sample units) and should be used upon opening to maintain freshness. This situation makes NGS machines suitable for laboratories that routinely process large numbers of specimens collected for a specific purpose, typically clinically oriented. For haplogrouping a small number of samples, a well-designed HG prediction tool using a few control region (CR) sequence fragments and subsequent verification using coding region information can be a quick and simple alternative that reduces cost, labor, and time. In this case, Sanger sequencing methods can be effective. CR is preferentially targeted due to its high mutation rates, and coding region information is often required for refined haplogrouping when CR sequences are used [1, 9]. Existing software tools include mtDNAmanager [10], MitoTool [11], HaploGrep 2 [12], EMPOP [13, 14], HAPLOFIND [15], and Phy-Mer [16]. HaploGrep 2, MitoTool, and EMPOP have established themselves [17–24] because they represent powerful servers with complete mtGenome sequences and provide useful additional functions for mtDNA variation analysis. However, when using CR sequences, they provide the most probable HGs that often vary from server to server but do not suggest additional solutions to verify the results or to determine sub-HGs. These solutions are important because they require additional confirmation as HG prediction using CR sequences is less accurate than using complete mtGenome sequences and often results in multiple HG estimates.

In the present study, we developed an algorithm that enables a high-resolution prediction of HG and implemented it in a web application, Haplotracker. Haplotracker can process multiple sequence fragments of mtDNA as well as complete mtGenome sequences obtained by NGS methods. Haplotracker provides accurate HG classification with full-length mtDNA sequences, but it can also provide high-resolution predictions of HG with some fragmented CR sequences. We organized the application to efficiently track HGs for verification and subhaplogrouping using coding region sequences. We applied our application to efficiently classify mitochondrial haplogroups of nine Mongolian aDNA samples using fragmented mtDNA sequences.

## 2. Materials and Methods

### 2.1. Algorithm

The HG of a sample was first tracked by comparing the sample variants with variant profiles of all HGs (*n* = 5,434) defined by Phylotree mtDNA Build 17 (Phylotree HGs) (Table S1). The highly probable HGs from the sample were listed in ordered rank groups. A rank group was determined by the variant identity, the number of Phylotree-defined variants (Phylotree variants) of an HG present in the sample variants (Vp) minus the number of Phylotree variants of the HG missing (not present) from the sample variants (Vm) within all the ranges of the fragments, which was divided by the number of sample variants (Vs). The variant identities were calculated using all the Phylotree HGs using the equation below. The HGs with the highest variant identity were grouped as Rank Group 1, the second highest as Rank Group 2, the third highest as Rank Group 3, and the fourth highest as Rank Group 4.

(1) $$\begin{array}{c} \text{Variant identity}=\frac{\text{Vp}-\text{Vm}}{\text{Vs}} \end{array}$$

The HGs were further ranked within each rank group by their scores. The scores were produced by the sum of the frequency rates of the HG carrying the extra and missing variants found in the haplotype DB (*n* = 118,869) of Haplotracker (Tables S2, S3, and S4). The equation is as follows:

(2) $$\begin{array}{c} \text{Score}=\sum \frac{\text{He}}{H}+\sum \frac{\text{Hm}}{H} \end{array}$$

where He is the frequency of the HG carrying the extra variant, Hm is the frequency of the HG carrying the missing variant, and *H* is the frequency of the HG in the DB. The haplotype DB was constructed by repeating HG prediction runs thrice with variant identity and regenerated scores using the mtDNA sequences downloaded from GenBank. Erratic sequences reported in previous papers were not used [9, 25, 26]. The DB contained 49,066 complete or partially complete genome sequences and 69,803 CR sequences. The CR sequences were >400 bp and were collected from sequences published until December 25, 2018, at GenBank. Sequences < 400 bp were filtered out during haplotype DB construction due to lack of information and too many HG predictions. There was no specific categorization of CR sequences in their use. Identical sequence samples with different accession numbers from GenBank were treated as distinct samples. These samples increased the frequencies of their HGs and haplotypes (extra or missing variants if any). Multiple HGs predicted with a given variation profile were all used to calculate He, Hm, and *H*. To process many sequences, we used our local servers with a batch script that serially processes many sequences by using the same haplogrouping algorithm used by the web server. We provided the script sources and test examples on the Haplotracker homepage.

The ranks were determined in each rank group. This process differs from the algorithms employed by other servers. We first valued the variant identities more highly than the scores themselves. In addition to our DB, we integrated HelixMTdb [27] into our server to calculate scores based on the HGs and their private variant frequencies of HelixMTdb. This DB contains 15,035 unique variants from the mtGenomes of 195,983 unrelated individuals. However, it only provides haplotype information for super-HGs. Researchers can optionally use HelixMTdb for HG estimation. Haplotracker uses the scores to rank HGs within the rank group.

Narrowing down and differentiating the estimated HGs for further tracking were based on the tree structures and HG variant profiles of Phylotree. The narrowing down process involved integrating the HGs with their most recently common ancestors (MRCAs). In addition, QC analysis was included in this tool to check for possible artificial recombinations. We implemented the tool as described in HaploGrep 2 (a generic rule-based system).

### 2.2. Implementation, Performance, and Usage

Haplotracker was coded using the active server page script of Internet Information Services Version 8.0 for Windows Server 2012 and JavaScript. It was implemented using Gotoh, an open-source C implementation of the Gotoh algorithm, also known as the Needleman-Wunsch algorithm, with affine gap penalties (https://github.com/oboes/gotoh) to search the variants and nucleotide positions of the sample sequences relative to revised Cambridge reference sequence (rCRS) [28, 29]. The variants were realigned according to the Phylotree notation. Detailed instructions, tutorials, evaluation of quality control, and usage examples are provided on the Haplotracker website. The server works with most of the current versions of web browsers.

### 2.3. HG Classification of aDNA Samples Using Haplotracker

**Figure 1:**
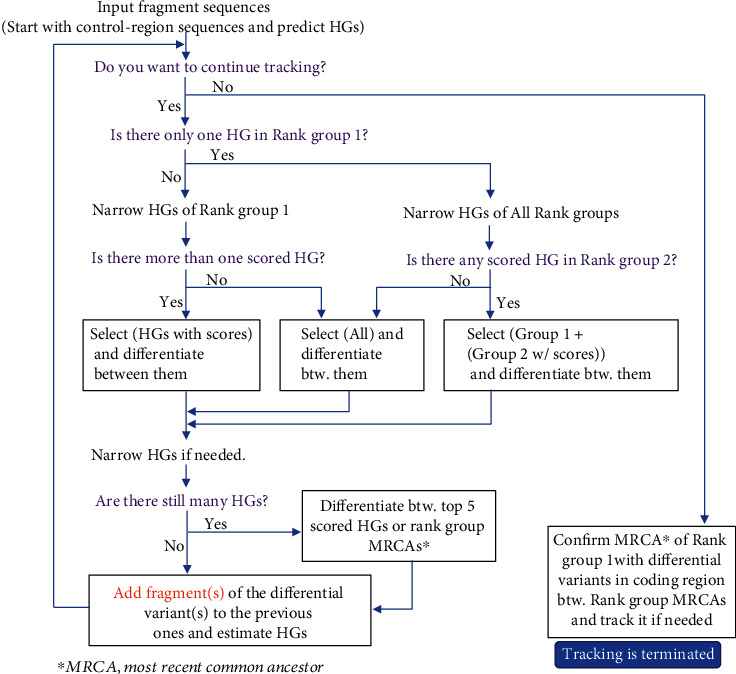
HG tracking flowchart describing a strategy for the use of Haplotracker to track HG confirmation or subhaplogrouping. If there is more than one HG in Rank Group 1 after HG prediction using CR sequences, the narrowing down of the HGs in Rank Group 1 is attempted first. If more than one scored HG (score > 0), it is preferable to track the HGs with scores first. They can be differentiated using specific variants on the server. Then, further narrowing down of the HGs can be conducted if needed. If there are too many HGs to track, differentiation between the top five scored HGs or rank group MRCAs can be conducted. Tracking can be repeated several times to confirm the HGs or track sub-HGs. Tracking ends by verifying the MRCA of Rank Group 1 by differentiating between the rank group MRCAs. HG: haplogroup; CR: control region; MRCA: most recent common ancestor.

We evaluated the utility of Haplotracker for haplogrouping in laboratory experiments with aDNA extracts from human skeletal remains found in 2,000-year-old elite Xiongnu cemetery in Northeast Mongolia. The ancient samples are listed in Table S5. We used the aDNA extracts and mtDNA PCR protocols acquired from the previous studies [30, 31]. Primer information used for mtDNA CR segment amplification is described in the previous report [31]. PCR primers for mtDNA coding region segments are presented in Table S6. We determined the haplogroups of the aDNA samples using Haplotracker with the mtDNA sequence fragments. We designed an HG tracking flowchart to conclude detailed HGs (Figure 1) efficiently.

### 2.4. High-Resolution Melting (HRM) Real-Time PCR for Variant Screening

We designed an HRM real-time PCR method to rapidly screen for HG-specific variants. The protocol consisted of the following three steps: primer design, HRM real-time PCR, and melting temperature analysis for variant identification.

#### 2.4.1. Primer Design

**Figure 2:**
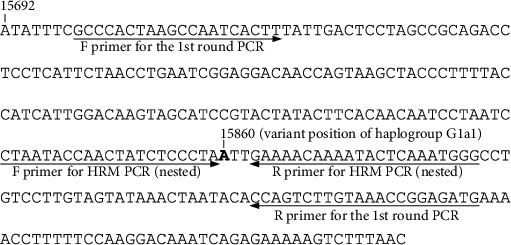
Design of a representative primer pair for variant detection via HRM real-time PCR. The HRM real-time PCR primers to detect the nucleotide changes at the variation site of haplogroup G1a1 (A-to-G transition at nucleotide position 15860 of rCRS) target sites very close to the variation site. The amplicons were as small as 46 bp. The small size conferred large *T*_m_ differences to the amplicons and thereby enhanced the discrimination of single-nucleotide differences. Arrows indicate primer positions. Numbers mark the nucleotide position of rCRS. The base in bold represents a variation site for haplogroup G1a1. HRM: high-resolution melting.

A multiplex strategy was pursued for the first-round PCR to simultaneously amplify fragments each corresponding to a specific variant. Multiple primer pairs were designed toward this end, and another primer pair was designed for the nested HRM real-time PCR to detect variations. All the primer sequences were designed using LightCycler® Probe Design Software 2.0 (LC PDS 2.0) Version 1.0.R.36 (Roche). The software setting for reaction conditions was as follows: 2 mM Mg^2+^, 50 mM monovalent cations, 200 *μ*M dNTPs, and 0% DMSO. This setting was essential to accurately estimate the primer/amplicon melting temperatures (*T*_m_s) and to appropriately adjust the reaction conditions for the *Taq* polymerase used in the experiment. The primers were designed to have no significant 3′-end complementarity with each other by using the “Cross Comp” feature of the software. We used the *T*_m_s provided by this feature to calculate the optimal annealing temperature for PCR and to estimate the amplicon *T*_m_s. For example, to discriminate between the haplogroups of the aDNA sample MNW3 at the third HG-tracking step, we designed a multiplex primer pair for the amplification of the mtDNA segments that contained haplogroup-specific variants for HGs G1a1, G1a1a, and G1a1b. The sequences of the primers used are provided in Table S6. The primers for the HRM real-time PCR were designed to increase the *T*_m_ differences, thereby enabling discrimination of the variants with a single-nucleotide difference. Both forward and reverse primers were located very close to the variation site (Figure 2), and the amplicons were <60 bp. The difference among the *T*_m_s of the amplicons with or without any variation was >0.7°C. The primer design of a representative primer pair for the HRM real-time PCR is presented in Table S7.

#### 2.4.2. Multiplex Real-Time PCR

A LightCycler® 96 (Roche) system was used for the real-time multiplex PCR amplification of coding-region segments. The multiplex PCR reaction mixture (10 *μ*L) consisted of 1× PCR buffer for Ex *Taq* HS (Takara), 0.25 mM dNTP (Takara), 0.4 *μ*M of each primer, 2.5 mM MgCl_2_ (Roche), 0.5 U Ex *Taq* HS, 0.5× SYBR Green I (Sigma), 1.5 mg/mL ultrapure bovine serum albumin (BSA) (ThermoFisher), PCR-grade water (Roche), and 3 *μ*L of aDNA extract. Reaction mixtures for control human-DNA samples were prepared as above. The control human-DNA samples were K562 (Promega) and buccal DNA extracts (from Dr. Kijeong Kim, one of the corresponding authors of this study), and their full-length mtDNA sequences, including the variant profiles, had already been determined in our laboratory. These samples were used as negative control variants in the HRM analysis. The multiplex-PCR cycling conditions were as follows: 60 s at 95°C; 42 cycles of 10 s at 95°C, 40 s at 60°C, 40 s at 68°C, and 40 s at 72°C (with a single fluorescence acquisition at the end of each cycle) and melting-curve analysis with 10 s at 97°C, 60 s at 72°C, and 1 s at 97°C with 0.1°C/s ramp under continuous fluorescence acquisition at a rate of five readings/°C. The primary PCR products were diluted 800 times with PCR-grade water (Roche) to be used as the templates in the HRM real-time PCR.

#### 2.4.3. HRM Real-Time PCR

The HRM real-time PCR samples, each containing a specific primer pair for a target region, were prepared in separate tubes. Each sample consisted of 1× PCR buffer for FastStart *Taq* (Roche), 0.2 mM dNTP (Roche), 0.2 *μ*M of each primer, 2 mM MgCl_2_ (Roche), 0.4 U FastStart *Taq*, 1× Resolight (Roche), 0.5 mg/mL ultrapure BSA (ThermoFisher), 2 *μ*L diluted first-round product, and PCR-grade water (Roche) in 10 *μ*L reaction volume. PCR cycling conditions were as follows: 5 min at 95°C; 25 cycles of 10 s at 95°C, 15 s at 55°C, and 40 s at 60°C (with a single fluorescence acquisition at the end of each cycle) and melting-curve analysis with 60 s at 95°C, 60 s at 40°C, 1 s at 60°C, and 1 s at 85°C with 0.03°C/s ramp under continuous fluorescence acquisition at a rate of 25 readings/°C. This HRM real-time PCR consisted of test samples and negative control samples and was performed in duplicate in a LightCycler® 96 system.

#### 2.4.4. HRM Analysis

HRM analysis to screen for variants was performed using the LightCycler® 96 software (version 1.1.0.1320). The saved run file in the system was opened using this software, and an HRM-analysis protocol was created in “Add analysis.” The melting curves were examined for differences in shape between the test and control samples and between the replicates. A protocol for *T*_m_-calling analysis was created to obtain the *T*_m_ values. *T*_m_ differences > 0.5°C were accepted as significant provided that the melting peaks of each duplicate had the same shape.

## 3. Results and Discussion

### 3.1. HG Determination Using Full-Length and CR mtDNA Sequences

**Figure 3:**
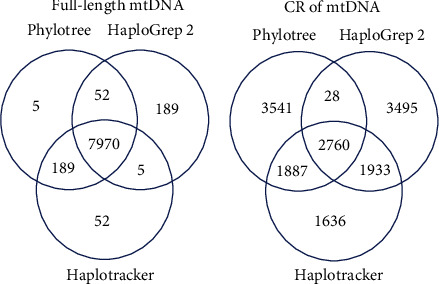
The agreement of HG calls by Haplotracker and HaploGrep 2 with Phylotree HG assignments using full-length (a) and CR (b) mtDNA. HG: haplogroup; mtDNA: mitochondrial DNA; CR: control region.

**Figure 4:**
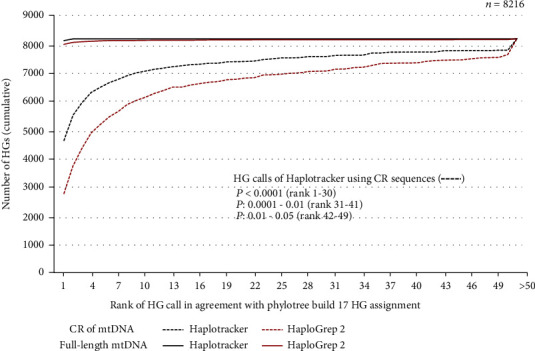
HG assignments with full-length and CR sequences of mtDNA using Haplotracker and HaploGrep 2. These data are based on the cumulative number of estimated HGs consistent with the original Phylotree HG assignments at a rank as tested with 8,216 full-length and CR mtDNA sequences. HG: haplogroup; mtDNA: mitochondrial DNA; CR: control region.

For haplogrouping accuracy evaluation, we ran Haplotracker and HaploGrep 2, on 8,216 full-length mtGenome sequences that were manually assigned to haplogroups when Phylotree was constructed. HaploGrep 2 is the upgraded version of HaploGrep, the most popular and currently one of the most accurate tools for mitochondrial HG assignment [9]. In addition, CR sequences extracted from the mtGenome sequences were tested for evaluation. Results are summarized in Figures 3 and 4. The detailed results can be found in Tables S8 and S9. In total, both Haplotracker and HaploGrep 2 calls had perfect matches with the original Phylotree assignment in 7,970 cases with full-length sequences (97.0%). Specifically, Haplotracker matched with the original Phylotree assignments of 8,159 sequences (99.3%), while HaploGrep 2 matched with the original Phylotree assignments of 8,022 sequences (97.6%). This result shows that Haplotracker and HaploGrep 2 showed comparable performance when the full-length sequences were used. When the CR sequences were used, Haplotracker predicted the HGs with a higher agreement rate (56.6%, *p* < 0.0001) with the Phylotree assignment than HaploGrep 2 (33.9%). The significance of the difference between the two rates was evaluated using MedCalc Version 19.0.5. based on “Test-Based Method” given on page 169 of Sahai and Khurshid [32]. The *p* values were obtained using the chi^2^-statistic, which is based on a normal approximation to a binomial distribution. The 56.6% CR sequences classified by Haplotracker exhibited the following characteristics: the sequences were predominantly scored (39%), indicating that they had private variants in addition to the Phylotree-defined ones (Fig S1). The scores of 22% sequences contributed to HG classification in agreement with the Phylotree HGs. These HG identities would be lost unless the Haplotracker scoring algorithm, which uses a haplotype DB constructed using the frequencies of the private variants, was introduced; the agreement rate of Haplotracker would drop from 56.6% to 34.3%, close to that of HaploGrep 2. In contrast, a small number of scored sequences (7%) were found in the remaining CR sequences that were not haplogrouped in agreement with the Phylotree HGs. A significantly higher Haplotracker agreement than HaploGrep 2 was observed from Ranks 1 to 30. Via Haplotracker, approximately 80% of the CR sequences were assigned to HGs in agreement with the Phylotree HGs in Ranks 1–6 (Figure 4). This result suggests that the further confirmation of up to the top six can determine 80% of the final HGs. This higher HG agreement rate probably stems from our new algorithm. We first isolated the rank groups that met the requirements for Phylotree variants and then used the scores generated from the frequencies of the private variants (extra variants in addition to the HG-defined ones or missing variants) observed in a large haplotype data set to screen the highest-scored HG candidates only within each rank group. Indeed, the value of private mutations for reliable haplogrouping has previously been noted [13]. The performance of Haplotracker was further evaluated using 46,322 CR sequences obtained from GenBank. These sequences included 45,177 and 1,245 CR sequences from the mtGenome sequences published on GenBank before December 25, 2018, and between December 26, 2018, and August 22, 2019, respectively. The latter sequences were downloaded after the Haplotracker algorithm was complete. The CR sequences were derived from mtGenome sequences consistently haplogrouped by both HaploGrep 2 and Haplotracker by using the full-length sequences. The HG results classified from the CR sequences by using Haplotracker and HaploGrep 2 were compared with the haplogroups assigned using full-length sequences from the same samples. The significance of differences was evaluated as described above. Among the 45,177 CR sequences, the HGs identical to full-length-based HGs were predicted by Haplotracker at a rate (54.4%, *p* < 0.0001) higher than that predicted by HaploGrep 2 (24.6%) (Tables S10 and S11). Evaluation with the 1,145 CR sequences also showed that Haplotracker classified the HGs identical to full-length-based HGs at a higher rate (44.5%, *p* < 0.0001) than that predicted by HaploGrep 2 (27.6%) (Table S12).

### 3.2. HG Classification of aDNA Samples Using Haplotracker

To assess the haplogrouping performance of Haplotracker on aDNA samples, we classified the HGs of nine aDNA samples using CR and coding region segment sequences. The sequences are presented in Table S13. Detailed HG tracking results can be found in Table S14. The haplogrouping results and the tracking efficiency are summarized in Table 1. In all samples, we determined the HG to the final sub-HG level of Phylotree Build 17. The results were consistent with our previous studies at higher HG levels [30, 31]. By tracking up to five times and in most cases up to three times, we determined the final HG of all samples. The determined HGs of five samples were found within Rank 2 at the initial tracking using CR sequence segments. The first tracking required four amplicons from CR, and most samples required less than two more amplicons for the remaining track cycles. Sample MNX4 required the highest number of track cycles (five) and amplicons (fourteen).

By using the CR and coding-region sequences, we assessed the minimum number of amplicons required by Haplotracker to discriminate between the HGs in various HG samples. All the distinct HG reference sequences available in Phylotree (*n* = 4680) were tested in silico by using Haplotracker (Fig. S2 and Tables S15 and S16). In 13 samples, Haplotracker classified the HGs (amplicons “0”), which were not identical to the Phylotree HGs even when the full-length sequences were used. Except for these samples, the results were as follows: 95% of the HGs classified by Haplotracker by using up to 12 amplicons were identical to the Phylotree HGs. All the HGs (98.4%), except for some belonging to the two super-HGs (M and H), classified by Haplotracker by using up to 25 amplicons were in agreement with the Phylotree HGs. When the minimum number of amplicons required per super-HG was assessed, HG H was found to require the highest number of amplicons, presumably because HG H had the highest number of sub-HGs and the fewest number of HG-defining variants, particularly in the CR. For example, HG H was defined by Phylotree to have 86 sub-HGs from H1 to H106 at sub-HG level 1 (which can be easily viewed using the Haplotracker accessory tool “HG Database”). Of these 86 sub-HGs, 55 sub-HGs had a single variant 263 in the CR. The lack of HG-defining variants in the CR of these HGs hindered the current CR-priority Haplotracker-tracking approach from discriminating between the HGs. In this case, other tracking strategies can be devised as follows:

**Strategy 1 .**

Tracking HGs that are frequently found in the DB first. H1 is the most frequently found HG (103,895 frequencies) in HG H according to Haplotracker DB. The frequently found HGs are observed in the following order: H1, H3, H13, H7, H5′36, H4, H2, H10, H14, H56, and H6.

**Strategy 2 .**

Establishing an efficient mass-screening method dedicated to this HG discrimination. Multiple probe-based multiplex real-time PCR, multiplex SNaPshot assay, or microarray methods may be considered candidates.

**Strategy 3 .**

Reducing sub-HG tracking to the necessary level appropriate for the study purpose.

**Strategy 4 .**

Complete mtGenome sequencing would be the best option, especially for aDNA samples, if available in the laboratory. The aDNA samples in this study were obtained from Mongolia, where the frequency of HG H is unlikely to be high. HG H samples were not found when the CR sequences of the aDNA samples were used.

Multiplex real-time PCR methods were used to efficiently obtain the multiple amplicons needed for each track cycle. This technique was effective in saving valuable aDNA samples. We directly sequenced the PCR products to track the HGs of all aDNA samples. Alternative methods to sequencing can be used for efficient tracking. Toward this end, we designed an HRM real-time PCR method that immediately identifies variants without sequencing to increase efficiency. This strategy worked effectively, reducing cost, labor, and time (Fig. S3). This strategy may be useful to rapidly screen for variants for HG-tracking purposes in future studies.

## 4. Conclusions

We have developed a novel algorithm for high-resolution and efficient HG classification of aDNA samples and implemented it in our web application, Haplotracker. Haplotracker was designed to process multiple sequence fragments of an mtDNA sample as well as full-length mtGenome sequences. Its haplogrouping accuracy and efficiency were demonstrated to be very high through HG classification using 8,216 mtDNA reference sequences from Phylotree and nine aDNA samples extracted from human skeletal remains found in 2,000-year-old elite Xiongnu cemetery in Northeast Mongolia. HGs of all aDNA samples were efficiently classified to the final sub-HG level in Phylotree by several tracking cycles with a few PCR product sequences.

## Figures and Tables

**Table 1:** HG classification of aDNA samples and its tracking efficiency.

Sample	Haplogroup prediction and no. of amplicons sequenced in each track											Total no. of tested	
Code	1st (CR)		2nd		3rd		4th		5th			Track	Amplicons
MNX2	M D	4	D4j+16311	1	*D4j11*	1						3	6
MNX3	*U2e1a1*	4	*U2e1a1*	1	*U2e1a1*	1						3	6
MNX4	M G D	4	D4	2	D4e	3	D4e4	3	*D4e4a*	2		5	14
MNE1	*G2a1*	4	*G2a1*	1								2	5
MNE2	A+152	4	*A12*	1								2	5
MNE3	D4b1c	4	*D3*	1								2	5
MNW1	W+194	4	W3a	2	*W3a1*	3						3	9
MNW3	*G1a1*	4	*G1a1*	1	*G1a1*	2						3	7
MNW4	*C4a1a+195*	4	*C4a1a+195*	3								2	7

CR: control region; haplogroup in italic indicates the finally determined one.

## Data Availability

The data used in this study are provided in the figures, tables, supplementary materials, and web sites below. Gotoh is an open-source C implementation of the Gotoh algorithm, a.k.a. Needleman-Wunsch with affine gap penalties (https://github.com/oboes/gotoh). Phylotree Build 17 is the most recent version of the phylogenetic tree of global human mitochondrial DNA variation (https://www.phylotree.org). HaploGrep 2 is a fast and free HG classification tool (https://haplogrep.i-med.ac.at).

## Supplementary Materials

Supplementary Materials Fig. S1: characterization of Phylotree-provided control region sequences tested for haplogroup classification by Haplotracker. Fig. S2: minimum number of amplicons required by Haplotracker in discriminating between haplogroups using mtDNA control and coding region sequences. Fig. S3: variant identification of an aDNA sample (MNW3) using an HRM real-time PCR. Table S1: haplogroups and their variant profiles extracted from Phylotree mtDNA Build 17. Table S2: haplogroup frequency carrying an extra variant in 118,869 haplotypes. Table S3: haplogroup frequency carrying a missing variant in 118,869 haplotypes. Table S4: haplogroup frequency in 118,869 haplotypes. Table S5: list of ancient human samples found in 2,000-year-old elite Xiongnu cemetery in Northeast Mongolia. Table S6: primers for the amplification of mtDNA coding region segments for haplogroup determination. Table S7: high-resolution melting real-time PCR primer design for screening variants to differentiate haplogroups G1a1, G1a1a, and G1a1b. Table S8: haplogroup classification of full-length mtGenome sequences from Phylotree (*n* = 8,216). Table S9: haplogroup classification with full-length and control region sequences of mtDNA using Haplotracker and HaploGrep 2. Table S10: comparison of servers using control region sequences from GenBank before December 25, 2018 (*n* = 45,177). Table S11: comparison details for the servers using control region sequences from GenBank before December 25, 2018 (*n* = 45,177). Table S12: comparison of servers using control region sequences downloaded from GenBank from December 26, 2018 to August 22, 2019. Table S13: sequences of mtDNA PCR products from Mongolian ancient DNA samples. Table S14: haplogroup classification of Mongolian ancient DNA samples using Haplotracker. Table S15: minimum number of amplicons required by Haplotracker in discriminating between haplogroups using mtDNA control and coding region sequences. Table S16: minimum number of amplicons per superhaplogroup required by Haplotracker in discriminating between haplogroups using mtDNA control and coding region sequences.
