# Supplementary material for: Mitochondrial Haplogroup Classification of Ancient DNA Samples Using Haplotracker
Source: Biomed Res Int. 2022 Mar 18;2022:5344418. doi: 10.1155/2022/5344418 (PMC8956381; doi:10.1155/2022/5344418)
Supplement: Supplementary Materials — Fig. S1: characterization of Phylotree-provided control region sequences tested for haplogroup classification by Haplotracker. Fig. S2: minimum number of amplicons required by Haplotracker in discriminating between haplogroups using mtDNA control and coding region sequences. Fig. S3: variant identification of an aDNA sample (MNW3) using an HRM real-time PCR. Table S1: haplogroups and their variant profiles extracted from Phylotree mtDNA Build 17. Table S2: haplogroup frequency carrying an extra variant in 118,869 haplotypes. Table S3: haplogroup frequency carrying a missing variant in 118,869 haplotypes. Table S4: haplogroup frequency in 118,869 haplotypes. Table S5: list of ancient human samples found in 2,000-year-old elite Xiongnu cemetery in Northeast Mongolia. Table S6: primers for the amplification of mtDNA coding region segments for haplogroup determination. Table S7: high-resolution melting real-time PCR primer design for screening variants to differentiate haplogroups G1a1, G1a1a, and G1a1b. Table S8: haplogroup classification of full-length mtGenome sequences from Phylotree (n = 8,216). Table S9: haplogroup classification with full-length and control region sequences of mtDNA using Haplotracker and HaploGrep 2. Table S10: comparison of servers using control region sequences from GenBank before December 25, 2018 (n = 45,177). Table S11: comparison details for the servers using control region sequences from GenBank before December 25, 2018 (n = 45,177). Table S12: comparison of servers using control region sequences downloaded from GenBank from December 26, 2018 to August 22, 2019. Table S13: sequences of mtDNA PCR products from Mongolian ancient DNA samples. Table S14: haplogroup classification of Mongolian ancient DNA samples using Haplotracker. Table S15: minimum number of amplicons required by Haplotracker in discriminating between haplogroups using mtDNA control and coding region sequences. Table S16: minimum number of amplicons per superhaplogroup requ [file 5344418.f1.zip › 5344418.f10.pdf]

Table S7. High-resolution melting real-time PCR primer design for screening variants to differentiate haplogroups G1a1, G1a1a, and G1a1b

Haplogroup	Variant[1]	Primer/Amplicon	Sequence (5' → 3')[2]	T_m (ºC)[3]	Size (bp)
G1a1	15860	Forward primer	CTAATACCAACTATCTCCCTA	56.2	21
		Reverse primer	CCCATTTGAGTATTTTGTTTTC	58.2	22
		Amplicon (non-variant)	CTAATACCAACTATCTCCCTAATTGAAAACAAAATACTCAAATGGG	70.0	46
		Amplicon (G1a1)	CTAATACCAACTATCTCCCTA**G**TTGAAAACAAAATACTCAAATGGG	70.8	46
G1a1a	11914	Forward primer	ACTCTCTGTGCTAGTAAC	57.7	18
		Reverse primer	GAGAGTGATATTTGATCAGGA	57.3	21
		Amplicon (non-variant)	ACTCTCTGTGCTAGTAACCACGTTCTCCTGATCAAATATCACTCTC	75.0	46
		Amplicon (G1a1a)	ACTCTCTGTGCTAGTAACCAC**A**TTCTCCTGATCAAATATCACTCTC	74.0	46
G1a1b	12178	Forward primer	CCAAAACATCAGATTGTGAA	57.6	20
		Reverse primer	GTCGTAAGCCTCTGT	56.8	15
		Amplicon (non-variant)	CCAAAACATCAGATTGTGAATCTGACAACAGAGGCTTACGACCC	75.6	44
		Amplicon (G1a1b)	CCAAAACATCAGATTGTGAATCTGA**T**AACAGAGGCTTACGACCC	74.3	44

[1] Shown in Phylotree format.

[2] Base in bold indicates the haplogroup-specific variant

[3] Calculated by using LC PDS software 2.0.
