# Supplementary material for: Mitochondrial Haplogroup Classification of Ancient DNA Samples Using Haplotracker
Source: Biomed Res Int. 2022 Mar 18;2022:5344418. doi: 10.1155/2022/5344418 (PMC8956381; doi:10.1155/2022/5344418)
Supplement: Supplementary Materials — Fig. S1: characterization of Phylotree-provided control region sequences tested for haplogroup classification by Haplotracker. Fig. S2: minimum number of amplicons required by Haplotracker in discriminating between haplogroups using mtDNA control and coding region sequences. Fig. S3: variant identification of an aDNA sample (MNW3) using an HRM real-time PCR. Table S1: haplogroups and their variant profiles extracted from Phylotree mtDNA Build 17. Table S2: haplogroup frequency carrying an extra variant in 118,869 haplotypes. Table S3: haplogroup frequency carrying a missing variant in 118,869 haplotypes. Table S4: haplogroup frequency in 118,869 haplotypes. Table S5: list of ancient human samples found in 2,000-year-old elite Xiongnu cemetery in Northeast Mongolia. Table S6: primers for the amplification of mtDNA coding region segments for haplogroup determination. Table S7: high-resolution melting real-time PCR primer design for screening variants to differentiate haplogroups G1a1, G1a1a, and G1a1b. Table S8: haplogroup classification of full-length mtGenome sequences from Phylotree (n = 8,216). Table S9: haplogroup classification with full-length and control region sequences of mtDNA using Haplotracker and HaploGrep 2. Table S10: comparison of servers using control region sequences from GenBank before December 25, 2018 (n = 45,177). Table S11: comparison details for the servers using control region sequences from GenBank before December 25, 2018 (n = 45,177). Table S12: comparison of servers using control region sequences downloaded from GenBank from December 26, 2018 to August 22, 2019. Table S13: sequences of mtDNA PCR products from Mongolian ancient DNA samples. Table S14: haplogroup classification of Mongolian ancient DNA samples using Haplotracker. Table S15: minimum number of amplicons required by Haplotracker in discriminating between haplogroups using mtDNA control and coding region sequences. Table S16: minimum number of amplicons per superhaplogroup requ [file 5344418.f1.zip › 5344418.f11.pdf]

Table S8. Haplogroup classification of full-length mtGenome sequences from Phylotree (n=8,216)

No.	Accession No.	**Phylotree Build 17** HG	**HaploGrep 2** HG ranked top	Identity 97.6% (n[X]=194)	Rank	**Haplotracker** HG ranked top	Identity 99.3% (n[X]=57)	Rank
1	EU092665	L0a1a	L0a1a	O	1	L0a1a	O	1
2	EU092764	L0a1a	L0a1a	O	1	L0a1a	O	1
3	NA19311	L0a1a+200	L0a1a+200	O	1	L0a1a+200	O	1
4	EU092763	L0a1a+200	L0a1a+200	O	1	L0a1a+200	O	1
5	EU092714	L0a1a1	L0a1a1	O	1	L0a1a1	O	1
6	JQ044893	L0a1a1	L0a1a1	O	1	L0a1a	X	2
7	JX303911	L0a1a2	L0a1a2	O	1	L0a1a2	O	1
8	DQ304901	L0a1a2	L0a1a2	O	1	L0a1a2	O	1
9	JQ044943	L0a1a3	L0a1a3	O	1	L0a1a3	O	1
10	KJ669116	L0a1b1	L0a1b1	O	1	L0a1b1	O	1
11	AF381988	L0a1b1	L0a1b1	O	1	L0a1b1	O	1
12	EU935434	L0a1b1a	L0a1b1a	O	1	L0a1b1a	O	1
13	NA19703	L0a1b1a	L0a1b1a	O	1	L0a1b1a	O	1
14	EU092688	L0a1b1a1	L0a1b1a1	O	1	L0a1b1a1	O	1
15	KC533498	L0a1b1a1	L0a1b1a1	O	1	L0a1b1a1	O	1
16	NA19449	L0a1b1a1a	L0a1b1a1a	O	1	L0a1b1a1a	O	1
17	KJ669120	L0a1b1a1a	L0a1b1a1a	O	1	L0a1b1a1a	O	1
18	DQ304897	L0a1b2	L0a1b2	O	1	L0a1b2	O	1
19	EU092746	L0a1b2a	L0a1b2a	O	1	L0a1b2a	O	1
20	NA19440	L0a1c	L0a1c	O	1	L0a1c	O	1
21	EU092945	L0a1c1	L0a1c1	O	1	L0a1c1	O	1
22	NA19467	L0a1c1	L0a1c1	O	1	L0a1c1	O	1
23	EU092801	L0a1d	L0a1d	O	1	L0a1d	O	1
24	EU092809	L0a1d	L0a1d	O	1	L0a1d	O	1
25	JX303766	L0a1e	L0a1e	O	1	L0a1e	O	1
26	EU092906	L0a4	L0a4	O	1	L0a4	O	1
27	HM771161	L0a2a1	L0a2a1	O	1	L0a2a1	O	1
28	EU092911	L0a2a1a2	L0a2a1a2	O	1	L0a2a1a2	O	1
29	EU092868	L0a2a1a2	L0a2a1a2	O	1	L0a2a1a2	O	1
30	JX303784	L0a2a1b	L0a2a1b	O	1	L0a2a1b	O	1
31	EU092861	L0a2a1b	L0a2a1b	O	1	L0a2a1b	O	1
32	FJ157838	L0a2a2	L0a2a2	O	1	L0a2a2	O	1
33	EU092787	L0a2a2a	L0a2a2a	O	1	L0a2a2a	O	1
34	JX303763	L0a2a2a	L0a2a2a	O	1	L0a2a2a	O	1

35	JX303835	L0a2a2a1	L0a2a2a1	O	1	L0a2a2a1	O	1
36	KJ669122	L0a2a2a1	L0a2a2a1	O	1	L0a2a2a1	O	1
37	JX303786	L0a2a2a2	L0a2a2a2	O	1	L0a2a2a2	O	1
38	KJ669113	L0a2a2a2	L0a2a2a2	O	1	L0a2a2a2	O	1
39	EU597537	L0a2b	L0a2b	O	1	L0a2b	O	1
40	HM771190	L0a2b	L0a2b	O	1	L0a2b	O	1
41	HM771200	L0a2b1	L0a2b1	O	1	L0a2b1	O	1
42	HM771188	L0a2b1	L0a2b1	O	1	L0a2b1	O	1
43	EF556174	L0a2c	L0a2c	O	1	L0a2c	O	1
44	EU092913	L0a2d	L0a2d	O	1	L0a2d	O	1
45	D38112	L0a2d	L0a2d	O	1	L0a2d	O	1
46	EU092900	L0a3	L0a3	O	1	L0a3	O	1
47	NA19039	L0a3	L0a3	O	1	L0a3	O	1
48	KJ669112	L0g	L0g	O	1	L0g	O	1
49	EU092936	L0b	L0b	O	1	L0b	O	1
50	NA19031	L0b	L0b	O	1	L0b	O	1
51	EU092870	L0f1	L0f1	O	1	L0f1	O	1
52	NA19454	L0f1	L0f1	O	1	L0f1	O	1
53	EU092964	L0f2a	L0f2a	O	1	L0f2a	O	1
54	AY963585	L0f2a1	L0f2a1	O	1	L0f2a1	O	1
55	EU092668	L0f2a1	L0f2a1	O	1	L0f2a1	O	1
56	EU092786	L0f2b	L0f2b	O	1	L0f2b	O	1
57	KC345899	L0k1a1	L0k1a1	O	1	L0k1a1	O	1
58	EU092837	L0k1a1	L0k1a1	O	1	L0k1a1	O	1
59	EU092966	L0k1a1a	L0k1a1a	O	1	L0k1a1a	O	1
60	KJ669107	L0k1a1a	L0k1a1a	O	1	L0k1a1a	O	1
61	KC346024	L0k1a1b	L0k1a1b	O	1	L0k1a1b	O	1
62	KJ669111	L0k1a1b	L0k1a1b	O	1	L0k1a1b	O	1
63	KC346108	L0k1a1c	L0k1a1c	O	1	L0k1a1c	O	1
64	AF347009	L0k1a1c	L0k1a1c	O	1	L0k1a1c	O	1
65	KC345966	L0k1a1d	L0k1a1d	O	1	L0k1a1d	O	1
66	KC346048	L0k1a1d	L0k1a1d	O	1	L0k1a1d	O	1
67	EU092855	L0k1a2	L0k1a2	O	1	L0k1a2	O	1
68	KC346083	L0k1a2	L0k1a2	O	1	L0k1a2	O	1
69	KC346199	L0k1a2a	L0k1a2a	O	1	L0k1a2a	O	1
70	KJ669103	L0k1a2a	L0k1a2a	O	1	L0k1a2a	O	1
71	KC346073	L0k1a3	L0k1a3	O	1	L0k1a3	O	1

72	KC345982	L0k1a3	L0k1a3	O	1	L0k1a3	O	1
73	JX303865	L0k1b	L0k1b	O	1	L0k1b	O	1
74	KC346241	L0k1b	L0k1b	O	1	L0k1b	O	1
75	KC346233	L0k2a	L0k2a	O	1	L0k2a	O	1
76	KC345922	L0k2a1	L0k2a1	O	1	L0k2a1	O	1
77	JX303895	L0k2a1a	L0k2a1a	O	1	L0k2a1a	O	1
78	JX303765	L0k2a1a	L0k2a1a	O	1	L0k2a1a	O	1
79	EU092792	L0k2b	L0k2b	O	1	L0k2b	O	1
80	KC346242	L0k2b	L0k2b	O	1	L0k2b	O	1
81	KC345791	L0d1a	L0d1a	O	1	L0d1a	O	1
82	KJ669142	L0d1a1a	L0d1a1a	O	1	L0d1a1a	O	1
83	KC346226	L0d1a1a	L0d1a1a	O	1	L0d1a1a	O	1
84	EU597514	L0d1a1a1	L0d1a1a1	O	1	L0d1a1a1	O	1
85	KC345831	L0d1a1a1	L0d1a1a1	O	1	L0d1a1a1	O	1
86	KC346102	L0d1a1a2	L0d1a1a2	O	1	L0d1a1a2	O	1
87	KJ669143	L0d1a1a2	L0d1a1a2	O	1	L0d1a1a2	O	1
88	EU092863	L0d1a1a3	L0d1a1a3	O	1	L0d1a1a3	O	1
89	KC346227	L0d1a1a3	L0d1a1a3	O	1	L0d1a1a3	O	1
90	EU092833	L0d1a1b	L0d1a1b	O	1	L0d1a1b	O	1
91	KC345969	L0d1a1b1a	L0d1a1b1a	O	1	L0d1a1b1a	O	1
92	KC345775	L0d1a1b1a	L0d1a1b1a	O	1	L0d1a1b1a	O	1
93	KJ669141	L0d1a1b1b	L0d1a1b1b	O	1	L0d1a1b1b	O	1
94	KC533478	L0d1a1b1b	L0d1a1b1b	O	1	L0d1a1b1b	O	1
95	KC346092	L0d1a1c	L0d1a1c	O	1	L0d1a1c	O	1
96	KC533497	L0d1a1c	L0d1a1c	O	1	L0d1a1c	O	1
97	KJ669138	L0d1a1d	L0d1a1d	O	1	L0d1a1d	O	1
98	KC533487	L0d1a1d	L0d1a1d	O	1	L0d1a1d	O	1
99	KJ669137	L0d1d	L0d1d	O	1	L0d1d	O	1
100	KC345764	L0d1c	L0d1c	O	1	L0d1c	O	1
101	KC345920	L0d1c1	L0d1c1	O	1	L0d1c1	O	1
102	KC346144	L0d1c1a	L0d1c1a	O	1	L0d1c1a	O	1
103	KC533490	L0d1c1a	L0d1c1a	O	1	L0d1c1a	O	1
104	KC345933	L0d1c1a1	L0d1c1a1	O	1	L0d1c1a1	O	1
105	KC345934	L0d1c1a1a	L0d1c1a1a	O	1	L0d1c1a1a	O	1
106	EU092856	L0d1c1a1a	L0d1c1a1a	O	1	L0d1c1a1a	O	1
107	KC345835	L0d1c1a1a1	L0d1c1a1a1	O	1	L0d1c1a1a1	O	1
108	KC345860	L0d1c1a1a1	L0d1c1a1a1	O	1	L0d1c1a1a1	O	1

109	KC346231	L0d1c1a1a2	L0d1c1a1a2	O	1	L0d1c1a1a2	O	1
110	KC346026	L0d1c1a1a2	L0d1c1a1a2	O	1	L0d1c1a1a2	O	1
111	EU092832	L0d1c1a1b	L0d1c1a1b	O	1	L0d1c1a1b	O	1
112	KC346101	L0d1c1a1b	L0d1c1a1b	O	1	L0d1c1a1b	O	1
113	KC345824	L0d1c1a2	L0d1c1a2	O	1	L0d1c1a2	O	1
114	KC345854	L0d1c1a2	L0d1c1a2	O	1	L0d1c1a2	O	1
115	KC346238	L0d1c2	L0d1c2	O	1	L0d1c2	O	1
116	KC346183	L0d1c2	L0d1c2	O	1	L0d1c2	O	1
117	KC345852	L0d1c2a	L0d1c2a	O	1	L0d1c2a	O	1
118	KC346081	L0d1c2a1	L0d1c2a1	O	1	L0d1c2a1	O	1
119	KC346015	L0d1c2a1	L0d1c2a1	O	1	L0d1c2a1	O	1
120	KC345944	L0d1c3	L0d1c3	O	1	L0d1c3	O	1
121	EU092831	L0d1c3	L0d1c3	O	1	L0d1c3	O	1
122	KC346074	L0d1b1a	L0d1b1a	O	1	L0d1b1a	O	1
123	KC345788	L0d1b1a1	L0d1b1a1	O	1	L0d1b1a1	O	1
124	KC345918	L0d1b1a1	L0d1b1a1	O	1	L0d1b1a1	O	1
125	KJ669135	L0d1b1b	L0d1b1b	O	1	L0d1b1b	O	1
126	KC346235	L0d1b1b1	L0d1b1b1	O	1	L0d1b1b1	O	1
127	KC345768	L0d1b1b1	L0d1b1b1	O	1	L0d1b1b1	O	1
128	KC345780	L0d1b1+@152	L0d1b1+@152	O	1	L0d1b1+@152	O	1
129	KC346236	L0d1b1c	L0d1b1c	O	1	L0d1b1c	O	1
130	KC346234	L0d1b1c	L0d1b1c	O	1	L0d1b1c	O	1
131	KC346152	L0d1b2a1	L0d1b2a1	O	1	L0d1b2a1	O	1
132	KJ669149	L0d1b2a1	L0d1b2a1	O	1	L0d1b2a1	O	1
133	JX303818	L0d1b2a2	L0d1b2a2	O	1	L0d1b2a2	O	1
134	KC345902	L0d1b2a2	L0d1b2a2	O	1	L0d1b2a2	O	1
135	KC345900	L0d1b2b1a	L0d1b2b1a	O	1	L0d1b2b1a	O	1
136	KC345949	L0d1b2b1a	L0d1b2b1a	O	1	L0d1b2b1a	O	1
137	KC345923	L0d1b2b1b	L0d1b2b1b	O	1	L0d1b2b1b	O	1
138	KC346138	L0d1b2b1b	L0d1b2b1b	O	1	L0d1b2b1b	O	1
139	KJ669153	L0d1b2b1b1	L0d1b2b1b1	O	1	L0d1b2b1b1	O	1
140	KJ669150	L0d1b2b1b1	L0d1b2b1b1	O	1	L0d1b2b1b1	O	1
141	KC345781	L0d1b2b2	L0d1b2b2	O	1	L0d1b2b2	O	1
142	KC345809	L0d1b2b2a	L0d1b2b2a	O	1	L0d1b2b2a	O	1
143	KJ669145	L0d1b2b2a	L0d1b2b2a	O	1	L0d1b2b2a	O	1
144	KC345977	L0d1b2b2b	L0d1b2b2b	O	1	L0d1b2b2b	O	1
145	KC346163	L0d1b2b2b1	L0d1b2b2b1	O	1	L0d1b2b2b1	O	1

146	EU597502	L0d1b2b2b1	L0d1b2b2b1	O	1	L0d1b2b2b1	O	1
147	KC345901	L0d1b2b2c1	L0d1b2b2c1	O	1	L0d1b2b2c1	O	1
148	KC346139	L0d1b2b2c1	L0d1b2b2c1	O	1	L0d1b2b2c1	O	1
149	KC345873	L0d1b2b2c2	L0d1b2b2c2	O	1	L0d1b2b2c2	O	1
150	KC346093	L0d1b2b2c2	L0d1b2b2c2	O	1	L0d1b2b2c2	O	1
151	EU092700	L0d2a1	L0d2a1	O	1	L0d2a1	O	1
152	KC533481	L0d2a1	L0d2a1	O	1	L0d2a1	O	1
153	KC346211	L0d2a1a	L0d2a1a	O	1	L0d2a1a	O	1
154	KC533486	L0d2a1a	L0d2a1a	O	1	L0d2a1a	O	1
155	KC346061	L0d2a1a1	L0d2a1a1	O	1	L0d2a1a1	O	1
156	KC345989	L0d2a1a1	L0d2a1a1	O	1	L0d2a1a1	O	1
157	KC345865	L0d2a1a1a	L0d2a1a1a	O	1	L0d2a1a1a	O	1
158	KC345993	L0d2a1a1a	L0d2a1a1a	O	1	L0d2a1a1a	O	1
159	KJ669171	L0d2a1a2	L0d2a1a2	O	1	L0d2a1a2	O	1
160	KJ669175	L0d2a1a2	L0d2a1a2	O	1	L0d2a1a2	O	1
161	KC346221	L0d2a1a3	L0d2a1a3	O	1	L0d2a1a3	O	1
162	KJ669169	L0d2a1a3	L0d2a1a3	O	1	L0d2a1a3	O	1
163	KC345990	L0d2a1b	L0d2a1b	O	1	L0d2a1b	O	1
164	KC533477	L0d2a1b	L0d2a1b	O	1	L0d2a1b	O	1
165	KC346197	L0d2a1c	L0d2a1c	O	1	L0d2a1c	O	1
166	KC346044	L0d2a1c	L0d2a1c	O	1	L0d2a1c	O	1
167	KC345910	L0d2a2	L0d2a2	O	1	L0d2a2	O	1
168	KC346240	L0d2a2	L0d2a2	O	1	L0d2a2	O	1
169	KC346207	L0d2b1a	L0d2b1a	O	1	L0d2b1a	O	1
170	JX303903	L0d2b1a1	L0d2b1a1	O	1	L0d2b1a1	O	1
171	KC345848	L0d2b1a1a	L0d2b1a1a	O	1	L0d2b1a1a	O	1
172	KC345912	L0d2b1a1a	L0d2b1a1a	O	1	L0d2b1a1a	O	1
173	KC346147	L0d2b1b	L0d2b1b	O	1	L0d2b1b	O	1
174	KC346167	L0d2b1b	L0d2b1b	O	1	L0d2b1b	O	1
175	EU092840	L0d2b2	L0d2b2	O	1	L0d2b2	O	1
176	JX303788	L0d2b2	L0d2b2	O	1	L0d2b2	O	1
177	KJ669167	L0d2d	L0d2d	O	1	L0d2d	O	1
178	KC345891	L0d2d	L0d2d	O	1	L0d2d	O	1
179	EU092708	L0d2c1	L0d2c1	O	1	L0d2c1	O	1
180	KC346104	L0d2c1	L0d2c1	O	1	L0d2c1	O	1
181	KC346086	L0d2c1a	L0d2c1a	O	1	L0d2c1a	O	1
182	KJ669159	L0d2c1a	L0d2c1a	O	1	L0d2c1a	O	1

183	KC346206	L0d2c1a1	L0d2c1a1	O	1	L0d2c1a1	O	1
184	EU092845	L0d2c1a1	L0d2c1a1	O	1	L0d2c1a1	O	1
185	KC346131	L0d2c1b	L0d2c1b	O	1	L0d2c1b	O	1
186	KC345892	L0d2c1b	L0d2c1b	O	1	L0d2c1b	O	1
187	KC346146	L0d2c2	L0d2c2	O	1	L0d2c2	O	1
188	KJ669161	L0d2c2	L0d2c2	O	1	L0d2c2	O	1
189	KC346217	L0d2c2a	L0d2c2a	O	1	L0d2c2a	O	1
190	KJ669164	L0d2c2a1	L0d2c2a1	O	1	L0d2c2a1	O	1
191	KC346053	L0d2c2a1a	L0d2c2a1a	O	1	L0d2c2a1a	O	1
192	KC346052	L0d2c2a1a	L0d2c2a1a	O	1	L0d2c2a1a	O	1
193	KC346218	L0d2c2b	L0d2c2b	O	1	L0d2c2b	O	1
194	KJ669162	L0d2c2b	L0d2c2b	O	1	L0d2c2b	O	1
195	EU092921	L0d3a	L0d3a	O	1	L0d3a	O	1
196	EF184587	L0d3a	L0d3a	O	1	L0d3a	O	1
197	KC533475	L0d3b	L0d3b	O	1	L0d3b	O	1
198	EU092842	L0d3b1	L0d3b1	O	1	L0d3b1	O	1
199	KC346229	L0d3b1	L0d3b1	O	1	L0d3b1	O	1
200	KC345931	L0d3b2	L0d3b2	O	1	L0d3b2	O	1
201	KC346224	L0d3b2	L0d3b2	O	1	L0d3b2	O	1
202	JN214480	L1b1a	L1b1a	O	1	L1b1a	O	1
203	JQ045107	L1b1a4	L1b1a4	O	1	L1b1a4	O	1
204	NA20322	L1b1a4	L1b1a4	O	1	L1b1a4	O	1
205	AY195783	L1b1a4a	L1b1a4a	O	1	L1b1a4a	O	1
206	JQ044792	L1b1a4a	L1b1a4a	O	1	L1b1a4a	O	1
207	EU092672	L1b1a2	L1b1a2	O	1	L1b1a2	O	1
208	EU092775	L1b1a2a	L1b1a2a	O	1	L1b1a2a	O	1
209	EU092952	L1b1a2a	L1b1a2a	O	1	L1b1a2a	O	1
210	JN214471	L1b1a+189	L1b1a+189	O	1	L1b1a+189	O	1
211	DQ304908	L1b1a3	L1b1a3	O	1	L1b1a3	O	1
212	JX303892	L1b1a3	L1b1a3	O	1	L1b1a3	O	1
213	DQ304914	L1b1a3a	L1b1a3a	O	1	L1b1a3a	O	1
214	DQ304905	L1b1a3a	L1b1a3a	O	1	L1b1a3a	O	1
215	DQ304906	L1b1a3a1	L1b1a3a1	O	1	L1b1a3a1	O	1
216	JQ044875	L1b1a3a1	L1b1a3a1	O	1	L1b1a3a1	O	1
217	JQ703036	L1b1a3b	L1b1a3b	O	1	L1b1a3b	O	1
218	DQ304907	L1b1a3b	L1b1a3b	O	1	L1b1a3b	O	1
219	EU092737	L1b1a9	L1b1a9	O	1	L1b1a9	O	1

220	JQ044876	L1b1a9	L1b1a9	O	1	L1b1a9	O	1
221	NA19462	L1b1a15	L1b1a15	O	1	L1b1a15	O	1
222	NA19198	L1b1a15a	L1b1a15a	O	1	L1b1a15a	O	1
223	JQ705587	L1b1a15a	L1b1a15a	O	1	L1b1a15a	O	1
224	JQ704683	L1b1a17	L1b1a17	O	1	L1b1a17	O	1
225	JQ045115	L1b1a17	L1b1a17	O	1	L1b1a17	O	1
226	JQ705606	L1b1a18	L1b1a18	O	1	L1b1a18	O	1
227	NA18501	L1b1a18	L1b1a18	O	1	L1b1a18	O	1
228	NA20753	L1b1a5	L1b1a5	O	1	L1b1a5	O	1
229	EU092928	L1b1a5	L1b1a5	O	1	L1b1a5	O	1
230	EU092716	L1b1a6	L1b1a6	O	1	L1b1a6	O	1
231	JN214461	L1b1a6	L1b1a6	O	1	L1b1a6	O	1
232	DQ304923	L1b1a7	L1b1a7	O	1	L1b1a7	O	1
233	DQ282505	L1b1a7a	L1b1a7a	O	1	L1b1a7a	O	1
234	JQ704825	L1b1a7a	L1b1a7a	O	1	L1b1a7a	O	1
235	JQ704968	L1b1a8	L1b1a8	O	1	L1b1a8	O	1
236	JN214430	L1b1a8	L1b1a8	O	1	L1b1a8	O	1
237	JQ045100	L1b1a10	L1b1a10	O	1	L1b1a10	O	1
238	JQ705832	L1b1a10	L1b1a10	O	1	L1b1a10	O	1
239	EU092893	L1b1a10a	L1b1a10a	O	1	L1b1a10a	O	1
240	EU092884	L1b1a10a	L1b1a10a	O	1	L1b1a10a	O	1
241	EU092852	L1b1a10b	L1b1a10b	O	1	L1b1a10b	O	1
242	JX303846	L1b1a10b	L1b1a10b	O	1	L1b1a10b	O	1
243	FJ460537	L1b1a12a	L1b1a12a	O	1	L1b1a12a	O	1
244	JN214463	L1b1a12a	L1b1a12a	O	1	L1b1a12a	O	1
245	JQ705931	L1b1a12b	L1b1a12b	O	1	L1b1a12b	O	1
246	EU200764	L1b1a12b	L1b1a12b	O	1	L1b1a12b	O	1
247	JN214477	L1b1a13	L1b1a13	O	1	L1b1a13	O	1
248	FJ460522	L1b1a13	L1b1a13	O	1	L1b1a13	O	1
249	JQ045114	L1b1a14	L1b1a14	O	1	L1b1a14	O	1
250	JN214468	L1b1a14	L1b1a14	O	1	L1b1a14	O	1
251	JN214460	L1b1a16	L1b1a16	O	1	L1b1a16	O	1
252	EU092667	L1b1a16	L1b1a16	O	1	L1b1a16	O	1
253	NA19909	L1b2	L1b2	O	1	L1b2	O	1
254	JQ044866	L1b2	L1b2	O	1	L1b2	O	1
255	HM771162	L1b2a	L1b2a	O	1	L1b2a	O	1
256	JN214470	L1b2a	L1b2a	O	1	L1b2a	O	1

257	JQ044936	L1b3	L1b3	O	1	L1b3	O	1
258	JQ045038	L1b3	L1b3	O	1	L1b3	O	1
259	JQ705275	L1c1a	L1c1a	O	1	L1c1a	O	1
260	EU273490	L1c1a1a1a	L1c1a1a1a	O	1	L1c1a1a1a	O	1
261	HM771127	L1c1a1a1a	L1c1a1a1a	O	1	L1c1a1a1a	O	1
262	EU273481	L1c1a1a1b	L1c1a1a1b	O	1	L1c1a1a1b	O	1
263	EU273497	L1c1a1a1b	L1c1a1a1b	O	1	L1c1a1a1b	O	1
264	EU273478	L1c1a1a1b1	L1c1a1a1b1	O	1	L1c1a1a1b1	O	1
265	EU597501	L1c1a1a1b1	L1c1a1a1b1	O	1	L1c1a1a1b1	O	1
266	EU273500	L1c1a1a2	L1c1a1a2	O	1	L1c1a1a2	O	1
267	EU273484	L1c1a1a2	L1c1a1a2	O	1	L1c1a1a2	O	1
268	EU273491	L1c1a1b	L1c1a1b	O	1	L1c1a1b	O	1
269	HM771217	L1c1a2a1	L1c1a2a1	O	1	L1c1a2a1	O	1
270	EU273496	L1c1a2a1	L1c1a2a1	O	1	L1c1a2a1	O	1
271	HM771180	L1c1a2a2	L1c1a2a2	O	1	L1c1a2a2	O	1
272	HM771164	L1c1a2a2	L1c1a2a2	O	1	L1c1a2a2	O	1
273	EU273476	L1c1a2b	L1c1a2b	O	1	L1c1a2b	O	1
274	HM771137	L1c1a2b	L1c1a2b	O	1	L1c1a2b	O	1
275	HM771114	L1c1a2c	L1c1a2c	O	1	L1c1a2c	O	1
276	HM771113	L1c1a2c	L1c1a2c	O	1	L1c1a2c	O	1
277	NA19917	L1c1b	L1c1b	O	1	L1c1b	O	1
278	EU273499	L1c1b1	L1c1b1	O	1	L1c1b1	O	1
279	HM771220	L1c1b1	L1c1b1	O	1	L1c1b1	O	1
280	NA19385	L1c1d	L1c1d	O	1	L1c1d	O	1
281	FJ713601	L1c1d1	L1c1d1	O	1	L1c1d1	O	1
282	AF346987	L1c1d1	L1c1d1	O	1	L1c1d1	O	1
283	EU092717	L1c1c	L1c1c	O	1	L1c1c	O	1
284	JQ701901	L1c1c	L1c1c	O	1	L1c1c	O	1
285	AF346992	L1c2a1a	L1c2a1a	O	1	L1c2a1a	O	1
286	JX303855	L1c2a1a	L1c2a1a	O	1	L1c2a1a	O	1
287	EU273501	L1c2a2	L1c2a2	O	1	L1c2a2	O	1
288	EU092864	L1c2a2	L1c2a2	O	1	L1c2a2	O	1
289	EU092848	L1c2a3	L1c2a3	O	1	L1c2a3	O	1
290	KC533467	L1c2a3a	L1c2a3a	O	1	L1c2a3a	O	1
291	JX303782	L1c2a3a	L1c2a3a	O	1	L1c2a3a	O	1
292	DQ341059	L1c2b1a'b	L1c2b1a'b	O	1	L1c2b1a'b	O	1
293	HM771222	L1c2b1a	L1c2b1a	O	1	L1c2b1a	O	1

294	KC152939	L1c2b1a1	L1c2b1a1	O	1	L1c2b1a1	O	1
295	JQ705864	L1c2b1a1	L1c2b1a1	O	1	L1c2b1a1	O	1
296	EU092849	L1c2b1b	L1c2b1b	O	1	L1c2b1b	O	1
297	JQ705650	L1c2b1b1	L1c2b1b1	O	1	L1c2b1b1	O	1
298	JX303883	L1c2b1b1	L1c2b1b1	O	1	L1c2b1b1	O	1
299	EU092712	L1c2b1c	L1c2b1c	O	1	L1c2b1c	O	1
300	JQ702903	L1c2b1c	L1c2b1c	O	1	L1c2b1c	O	1
301	HM771221	L1c2b2	L1c2b2	O	1	L1c2b2	O	1
302	EU092738	L1c2b2	L1c2b2	O	1	L1c2b2	O	1
303	EU273502	L1c4a	L1c4a	O	1	L1c4a	O	1
304	HM771117	L1c4a	L1c4a	O	1	L1c4a	O	1
305	JF812599	L1c4b	L1c4b	O	1	L1c4b	O	1
306	JQ703773	L1c4b	L1c4b	O	1	L1c4b	O	1
307	EU273489	L1c6	L1c6	O	1	L1c6	O	1
308	JQ044836	L1c6	L1c6	O	1	L1c6	O	1
309	JX303797	L1c5	L1c5	O	1	L1c5	O	1
310	JQ702617	L1c5	L1c5	O	1	L1c5	O	1
311	EU092703	L1c3a	L1c3a	O	1	L1c3a	O	1
312	JX303824	L1c3a	L1c3a	O	1	L1c3a	O	1
313	EU935458	L1c3a1a	L1c3a1a	O	1	L1c3a1a	O	1
314	EU092718	L1c3a1a	L1c3a1a	O	1	L1c3a1a	O	1
315	JX303871	L1c3a1b	L1c3a1b	O	1	L1c3a1b	O	1
316	EU092956	L1c3a1b	L1c3a1b	O	1	L1c3a1b	O	1
317	HG01378	L1c3b1	L1c3b1	O	1	L1c3b1	O	1
318	HG01080	L1c3b1a	L1c3b1a	O	1	L1c3b1a	O	1
319	EU273488	L1c3b1a	L1c3b1a	O	1	L1c3b1a	O	1
320	JQ702600	L1c3b1b	L1c3b1b	O	1	L1c3b1b	O	1
321	EU092689	L1c3b1b	L1c3b1b	O	1	L1c3b1b	O	1
322	NA19256	L1c3b2	L1c3b2	O	1	L1c3b2	O	1
323	NA19163	L1c3b2	L1c3b2	O	1	L1c3b2	O	1
324	EU273493	L1c3c	L1c3c	O	1	L1c3c	O	1
325	EF556173	L5a1a	L5a1a	O	1	L5a1a	O	1
326	DQ341060	L5a1a	L5a1a	O	1	L5a1a	O	1
327	EU092943	L5a1b	L5a1b	O	1	L5a1b	O	1
328	EU092888	L5a1b	L5a1b	O	1	L5a1b	O	1
329	HM771198	L5a1c	L5a1c	O	1	L5a1c	O	1
330	NA19317	L5a1c	L5a1c	O	1	L5a1c	O	1

331	EU092699	L5a2	L5a2	O	1	L5a2	O	1
332	JX303751	L5a2	L5a2	O	1	L5a2	O	1
333	NA19373	L5b1	L5b1	O	1	L5b1	O	1
334	DQ341061	L5b1a	L5b1a	O	1	L5b1a	O	1
335	KC911364	L5b1a	L5b1a	O	1	L5b1a	O	1
336	EU092774	L5b1b	L5b1b	O	1	L5b1b	O	1
337	NA19404	L5b1b	L5b1b	O	1	L5b1b	O	1
338	NA19332	L5b2	L5b2	O	1	L5b2	O	1
339	NA19455	L5b2	L5b2	O	1	L5b2	O	1
340	DQ304928	L2a1a	L2a1a	O	1	L2a1a	O	1
341	EU092916	L2a1a	L2a1a	O	1	L2a1a	O	1
342	NA19266	L2a1a1	L2a1a1	O	1	L2a1a1	O	1
343	DQ304933	L2a1a1	L2a1a1	O	1	L2a1a1	O	1
344	JN214449	L2a1a2	L2a1a2	O	1	L2a1a2	O	1
345	DQ304968	L2a1a2	L2a1a2	O	1	L2a1a2	O	1
346	DQ304975	L2a1a2a	L2a1a2a	O	1	L2a1a2a	O	1
347	NA20287	L2a1a2a1	L2a1a2a1	O	1	L2a1a2a1	O	1
348	JQ044968	L2a1a2a1	L2a1a2a1	O	1	L2a1a2a1	O	1
349	NA19117	L2a1a2a1a	L2a1a2a1a	O	1	L2a1a2a1a	O	1
350	DQ304976	L2a1a2a1a	L2a1a2a1a	O	1	L2a1a2a1a	O	1
351	DQ304972	L2a1a2b	L2a1a2b	O	1	L2a1a2b	O	1
352	JQ045095	L2a1a2b	L2a1a2b	O	1	L2a1a2b	O	1
353	HQ425645	L2a1a2c	L2a1a2c	O	1	L2a1a2c	O	1
354	DQ304927	L2a1a2c	L2a1a2c	O	1	L2a1a2c	O	1
355	EU092711	L2a1a3	L2a1a3	O	1	L2a1a3	O	1
356	EU092890	L2a1a3a	L2a1a3a	O	1	L2a1a3a	O	1
357	EU092905	L2a1a3a	L2a1a3a	O	1	L2a1a3a	O	1
358	DQ304948	L2a1a3b	L2a1a3b	O	1	L2a1a3b	O	1
359	JQ044819	L2a1a3b	L2a1a3b	O	1	L2a1a3b	O	1
360	HM771168	L2a1a3c	L2a1a3c	O	1	L2a1a3c	O	1
361	JQ045101	L2a1a3c	L2a1a3c	O	1	L2a1a3c	O	1
362	EU092806	L2a1+16189_(16192)	L2a1+16189 (16192)	O	1	L2a1+16189_(16192)	O	1
363	EU092761	L2a1b	L2a1b	O	1	L2a1b	O	1
364	NA19129	L2a1b1	L2a1b1	O	1	L2a1b1	O	1
365	EU092690	L2a1b1a	L2a1b1a	O	1	L2a1b1a	O	1
366	JX303858	L2a1b1a	L2a1b1a	O	1	L2a1b1a	O	1
367	JQ044975	L2a1b2	L2a1b2	O	1	L2a1b2	O	1

368	JQ044841	L2a1b2	L2a1b2	O	1	L2a1b2	O	1
369	NA19625	L2a1b+143	L2a1b+143	O	1	L2a1b+143	O	1
370	NA18856	L2a1b3	L2a1b3	O	1	L2a1b3	O	1
371	JQ044837	L2a1b3	L2a1b3	O	1	L2a1b3	O	1
372	DQ304959	L2a1f	L2a1f	O	1	L2a1f	O	1
373	NA19125	L2a1f	L2a1f	O	1	L2a1f	O	1
374	DQ304961	L2a1f1	L2a1f1	O	1	L2a1f1	O	1
375	DQ304955	L2a1f1	L2a1f1	O	1	L2a1f1	O	1
376	DQ304958	L2a1f1a	L2a1f1a	O	1	L2a1f1a	O	1
377	DQ304934	L2a1f1a	L2a1f1a	O	1	L2a1f1a	O	1
378	DQ304957	L2a1f2	L2a1f2	O	1	L2a1f2	O	1
379	KC533500	L2a1f3	L2a1f3	O	1	L2a1f3	O	1
380	JQ044861	L2a1f3	L2a1f3	O	1	L2a1f3	O	1
381	KC533472	L2a1g	L2a1g	O	1	L2a1g	O	1
382	JX303906	L2a1g	L2a1g	O	1	L2a1g	O	1
383	EU092733	L2a1c	L2a1c	O	1	L2a1c	O	1
384	HM771224	L2a1c	L2a1c	O	1	L2a1c	O	1
385	JQ044973	L2a1c+16086	L2a1c+16086	O	1	L2a1c+16086	O	1
386	FJ460560	L2a1c1	L2a1c1	O	1	L2a1c1	O	1
387	JQ705046	L2a1c1a1	L2a1c1a1	O	1	L2a1c1a1	O	1
388	NA18868	L2a1c1a1	L2a1c1a1	O	1	L2a1c1a1	O	1
389	JQ412577	L2a1c1a2	L2a1c1a2	O	1	L2a1c1a2	O	1
390	EU092954	L2a1c1a2	L2a1c1a2	O	1	L2a1c1a2	O	1
391	JN214436	L2a1c6	L2a1c6	O	1	L2a1c6	O	1
392	JQ702659	L2a1c6	L2a1c6	O	1	L2a1c6	O	1
393	JQ045108	L2a1c2	L2a1c2	O	1	L2a1c2	O	1
394	JQ045024	L2a1c2	L2a1c2	O	1	L2a1c2	O	1
395	HM771169	L2a1c2a	L2a1c2a	O	1	L2a1c2a	O	1
396	JQ045025	L2a1c2a	L2a1c2a	O	1	L2a1c2a	O	1
397	EU092663	L2a1c3a	L2a1c3a	O	1	L2a1c3a	O	1
398	EU200762	L2a1c3a	L2a1c3a	O	1	L2a1c3a	O	1
399	JQ705145	L2a1c3a1	L2a1c3a1	O	1	L2a1c3a1	O	1
400	NA19923	L2a1c3a1	L2a1c3a1	O	1	L2a1c3a1	O	1
401	JQ044996	L2a1c3b	L2a1c3b	O	1	L2a1c3b	O	1
402	JQ045110	L2a1c3b1	L2a1c3b1	O	1	L2a1c3b1	O	1
403	EU092720	L2a1c3b1	L2a1c3b1	O	1	L2a1c3b1	O	1
404	NA19096	L2a1c3b2	L2a1c3b2	O	1	L2a1c3b2	O	1

405	JQ045063	L2a1c3b2	L2a1c3b2	O	1	L2a1c3b2	O	1
406	JN214432	L2a1c4	L2a1c4	O	1	L2a1c4	O	1
407	DQ304942	L2a1c4a	L2a1c4a	O	1	L2a1c4a	O	1
408	FJ460549	L2a1c4a	L2a1c4a	O	1	L2a1c4a	O	1
409	DQ304951	L2a1c4a1	L2a1c4a1	O	1	L2a1c4a1	O	1
410	NA18912	L2a1c4a1	L2a1c4a1	O	1	L2a1c4a1	O	1
411	JN858955	L2a1c+16129	L2a1c+16129	O	1	L2a1c+16129	O	1
412	NA18908	L2a1c5	L2a1c5	O	1	L2a1c5	O	1
413	HM771225	L2a1c5	L2a1c5	O	1	L2a1c5	O	1
414	EU092765	L2a1d1	L2a1d1	O	1	L2a1d1	O	1
415	EU092939	L2a1d1	L2a1d1	O	1	L2a1d1	O	1
416	JX303838	L2a1d2	L2a1d2	O	1	L2a1d2	O	1
417	JN858956	L2a1d2	L2a1d2	O	1	L2a1d2	O	1
418	EU092676	L2a1h	L2a1h	O	1	L2a1h	O	1
419	EU092914	L2a1h	L2a1h	O	1	L2a1h	O	1
420	DQ304945	L2a1e	L2a1e	O	1	L2a1e	O	1
421	DQ304930	L2a1e1	L2a1e1	O	1	L2a1e1	O	1
422	NA19116	L2a1e1	L2a1e1	O	1	L2a1e1	O	1
423	FJ460527	L2a1+143+16189_(161	L2a1+143+16189 (16192)	O	1	L2a1+143+16189_(1619	O	1
424	HQ384198	L2a1+143+16189_(161	L2a1+143	X	2	L2a1+143+16189_(1619	O	1
425	EU092793	L2a1+143+16189_(161	L2a1+143+16189 (16192)+	O	1	L2a1+143+16189_(1619	O	1
426	JQ044958	L2a1i	L2a1i	O	1	L2a1i	O	1
427	JQ044905	L2a1i	L2a1i	O	1	L2a1i	O	1
428	JX303853	L2a1i1	L2a1i1	O	1	L2a1i1	O	1
429	JX303870	L2a1q	L2a1q	O	1	L2a1q	O	1
430	NA19381	L2a1q	L2a1q	O	1	L2a1q	O	1
431	EU092756	L2a1j	L2a1j	O	1	L2a1j	O	1
432	EU092816	L2a1j	L2a1j	O	1	L2a1j	O	1
433	EU200763	L2a1k	L2a1k	O	1	L2a1k	O	1
434	EU200760	L2a1k	L2a1k	O	1	L2a1k	O	1
435	JQ044956	L2a1l1	L2a1l1	O	1	L2a1l1	O	1
436	EU092807	L2a1l1a	L2a1l1a	O	1	L2a1l1a	O	1
437	EU092812	L2a1l1a1	L2a1l1a1	O	1	L2a1l1a1	O	1
438	JQ044817	L2a1l1a1	L2a1l1a1	O	1	L2a1l1a1	O	1
439	KF952774	L2a1l1a2	L2a1l1a2	O	1	L2a1l1a2	O	1
440	JQ044897	L2a1l1a2	L2a1l1a2	O	1	L2a1l1a2	O	1
441	NA20332	L2a1l1b	L2a1l1b	O	1	L2a1l1b	O	1

442	JQ044955	L2a1l1b	L2a1l1b	O	1	L2a1l1b	O	1
443	EU092721	L2a1l2	L2a1l2	O	1	L2a1l2	O	1
444	JX266264	L2a1l2a	L2a1l2a	O	1	L2a1l2a	O	1
445	JQ705185	L2a1l2a	L2a1l2a	O	1	L2a1l2a	O	1
446	JX266265	L2a1l2a1	L2a1l2a1	O	1	L2a1l2a1	O	1
447	JQ705589	L2a1l2a1	L2a1l2a1	O	1	L2a1l2a1	O	1
448	JX021728	L2a1l3	L2a1l3	O	1	L2a1l3	O	1
449	NA18487	L2a1l3	L2a1l3	O	1	L2a1l3	O	1
450	JQ045040	L2a1m	L2a1m	O	1	L2a1m	O	1
451	DQ304940	L2a1m1	L2a1m	X	42	L2a1m1	O	1
452	JQ701914	L2a1m1a	L2a1m	X	25	L2a1m1a	O	1
453	DQ304938	L2a1m1a	L2a1m	X	27	L2a1m1a	O	1
454	DQ304941	L2a1n	L2a1+143+16189 (16192)	X	2	L2a1n	O	1
455	NA18519	L2a1n	L2a1+143	X	5	L2a1n	O	1
456	JQ045000	L2a1o	L2a1o	O	1	L2a1o	O	1
457	EU092739	L2a1o	L2a1o	O	1	L2a1o	O	1
458	EU092671	L2a1p	L2a1p	O	1	L2a1p	O	1
459	JQ044822	L2a1p	L2a1p	O	1	L2a1p	O	1
460	EU092896	L2a2a	L2a2a	O	1	L2a2a	O	1
461	EU092902	L2a2a1	L2a2a1	O	1	L2a2a1	O	1
462	HM771205	L2a2a1	L2a2a1	O	1	L2a2a1	O	1
463	NA19443	L2a2b1	L2a2b1	O	1	L2a2b1	O	1
464	HM771208	L2a2b1a	L2a2b1a	O	1	L2a2b1a	O	1
465	HM771192	L2a2b1a	L2a2b1a	O	1	L2a2b1a	O	1
466	HM771191	L2a2b2	L2a2b2	O	1	L2a2b2	O	1
467	HM771207	L2a2b2	L2a2b2	O	1	L2a2b2	O	1
468	HM771206	L2a3	L2a3	O	1	L2a3	O	1
469	HM771212	L2a4a	L2a4a	O	1	L2a4a	O	1
470	HM771196	L2a4a	L2a4a	O	1	L2a4a	O	1
471	NA19393	L2a4b	L2a4b	O	1	L2a4b	O	1
472	NA19376	L2a4b	L2a4b	O	1	L2a4b	O	1
473	NA19045	L2a5	L2a5	O	1	L2a5	O	1
474	HQ384199	L2a5	L2a5	O	1	L2a5	O	1
475	EU092747	L2b1	L2b1	O	1	L2b1	O	1
476	EU092766	L2b1	L2b1	O	1	L2b1	O	1
477	JQ044854	L2b1a	L2b1a	O	1	L2b1a	O	1
478	EU092722	L2b1a2	L2b1a2	O	1	L2b1a2	O	1

479	JQ044890	L2b1a2	L2b1a2	O	1	L2b1a2	O	1
480	DQ304978	L2b1a3	L2b1a3	O	1	L2b1a3	O	1
481	JX303882	L2b1a3	L2b1a3	O	1	L2b1a3	O	1
482	JN214453	L2b1a4	L2b1a4	O	1	L2b1a4	O	1
483	FJ228403	L2b1b	L2b1b	O	1	L2b1b	O	1
484	JQ044800	L2b1b	L2b1b	O	1	L2b1b	O	1
485	EU092692	L2b2	L2b2	O	1	L2b2	O	1
486	KC533513	L2b2a	L2b2a	O	1	L2b2a	O	1
487	JX303841	L2b2a	L2b2a	O	1	L2b2a	O	1
488	JQ702626	L2b3a	L2b3a	O	1	L2b3a	O	1
489	JQ702123	L2b3a	L2b3a	O	1	L2b3a	O	1
490	EU092734	L2b3b	L2b	X	2	L2b3b	O	1
491	JN214443	L2b3b	L2b	X	2	L2b3b	O	1
492	EU092661	L2b3c	L2b	X	2	L2b3c	O	1
493	NA19247	L2b3c	L2b	X	2	L2b3c	O	1
494	JQ044941	L2c	L2c	O	1	L2c	O	1
495	EU092723	L2c	L2c	O	1	L2c	O	1
496	JQ044858	L2c1	L2c1	O	1	L2c1	O	1
497	EU092813	L2c1a	L2c1a	O	1	L2c1a	O	1
498	JQ044887	L2c1a	L2c1a	O	1	L2c1a	O	1
499	NA19229	L2c2	L2c2	O	1	L2c2	O	1
500	EU092697	L2c2	L2c2	O	1	L2c2	O	1
501	DQ304988	L2c2a	L2c2a	O	1	L2c2a	O	1
502	EU092955	L2c2a	L2c2a	O	1	L2c2a	O	1
503	JQ704740	L2c2a1	L2c2a1	O	1	L2c2a1	O	1
504	JX303792	L2c2a1	L2c2a1	O	1	L2c2a1	O	1
505	NA18517	L2c2b1a	L2c2b1a	O	1	L2c2b1a	O	1
506	JQ704094	L2c2b1a	L2c2b1a	O	1	L2c2b1a	O	1
507	KC533455	L2c2b1b	L2c2b1b	O	1	L2c2b1b	O	1
508	EU092710	L2c2b1b	L2c2b1b	O	1	L2c2b1b	O	1
509	JQ044878	L2c2b2	L2c2b2	O	1	L2c2b2	O	1
510	JQ044853	L2c2b2	L2c2b2	O	1	L2c2b2	O	1
511	JQ705626	L2c3	L2c	X	2	L2c3	O	1
512	JQ044810	L2c3	L2c3	O	1	L2c3	O	1
513	AF346995	L2c3a	L2c3a	O	1	L2c3a	O	1
514	JQ045104	L2c3a	L2c3a	O	1	L2c3a	O	1
515	JQ044914	L2c4	L2c4	O	1	L2c4	O	1

516	AY195785	L2c5	L2c5	O	1	L2c5	O	1
517	NA19835	L2c5	L2c5	O	1	L2c5	O	1
518	JQ045050	L2d	L2d	O	1	L2d	O	1
519	JQ045069	L2d+16129	L2d+16129	O	1	L2d+16129	O	1
520	EU092817	L2d+16129	L2d+16129	O	1	L2d+16129	O	1
521	JQ044948	L2d1	L2d1	O	1	L2d1	O	1
522	EU092794	L2d1a	L2d1a	O	1	L2d1a	O	1
523	JQ045011	L2d1a	L2d1a	O	1	L2d1a	O	1
524	EU092724	L2e	L2e	O	1	L2e	O	1
525	JQ044816	L2e1	L2e1	O	1	L2e1	O	1
526	NA19108	L2e1a	L2e1a	O	1	L2e1a	O	1
527	FJ460523	L2e1a	L2e1a	O	1	L2e1a	O	1
528	EU092773	L6a	L6a	O	1	L6a	O	1
529	EU092802	L6a	L6a	O	1	L6a	O	1
530	DQ341063	L6b	L6b	O	1	L6b	O	1
531	EU092686	L6b	L6b	O	1	L6b	O	1
532	FJ460531	L4a1	L4a1	O	1	L4a1	O	1
533	DQ341064	L4a1a	L4a1a	O	1	L4a1a	O	1
534	EU092748	L4a1a	L4a1a	O	1	L4a1a	O	1
535	EU092935	L4a2	L4a2	O	1	L4a2	O	1
536	EU092799	L4a2	L4a2	O	1	L4a2	O	1
537	NA19383	L4b1	L4b1	O	1	L4b1	O	1
538	JQ044811	L4b1a	L4b1a	O	1	L4b1a	O	1
539	EU092808	L4b1a	L4b1a	O	1	L4b1a	O	1
540	EU092942	L4b2a1	L4b2a1	O	1	L4b2a1	O	1
541	DQ341065	L4b2a1	L4b2a1	O	1	L4b2a1	O	1
542	EF184627	L4b2a2	L4b2a2	O	1	L4b2a2	O	1
543	EU092750	L4b2a2	L4b2a2	O	1	L4b2a2	O	1
544	EU092938	L4b2a2a	L4b2a2a	O	1	L4b2a2a	O	1
545	EU092743	L4b2a2a	L4b2a2a	O	1	L4b2a2a	O	1
546	EU092951	L4b2a2b	L4b2a2b	O	1	L4b2a2b	O	1
547	EU092780	L4b2a2b	L4b2a2b	O	1	L4b2a2b	O	1
548	NA19445	L4b2a2c	L4b2a2c	O	1	L4b2a2c	O	1
549	NA19435	L4b2a2c	L4b2a2c	O	1	L4b2a2c	O	1
550	NA19259	L4b2b	L4b2b	O	1	L4b2b	O	1
551	JQ702504	L4b2b1	L4b2b1	O	1	L4b2b1	O	1
552	NA19187	L4b2b1	L4b2b1	O	1	L4b2b1	O	1

553	JN655813	L3a1a	L3a1a	O	1	L3a1a	O	1
554	EF184630	L3a1a	L3a1a	O	1	L3a1a	O	1
555	DQ341081	L3a1b	L3a1b	O	1	L3a1b	O	1
556	JN655774	L3a1b	L3a1b	O	1	L3a1b	O	1
557	JN655803	L3a+709	L3a+709	O	1	L3a+709	O	1
558	NA19438	L3a2	L3a2	O	1	L3a2	O	1
559	JN655805	L3a2a	L3a2a	O	1	L3a2a	O	1
560	EU092941	L3a2a	L3a2a	O	1	L3a2a	O	1
561	EU092726	L3b1a	L3b1a	O	1	L3b1a	O	1
562	JQ705783	L3b1a1	L3b1a1	O	1	L3b1a1	O	1
563	DQ304995	L3b1a1a	L3b1a1a	O	1	L3b1a1a	O	1
564	EU597490	L3b1a1a	L3b1a1a	O	1	L3b1a1a	O	1
565	JN655827	L3b1a2	L3b1a2	O	1	L3b1a2	O	1
566	EU935449	L3b1a2	L3b1a2	O	1	L3b1a2	O	1
567	FJ460529	L3b1a3	L3b1a3	O	1	L3b1a3	O	1
568	DQ304990	L3b1a3	L3b1a3	O	1	L3b1a3	O	1
569	FJ460536	L3b1a4	L3b1a4	O	1	L3b1a4	O	1
570	EU092759	L3b1a4	L3b1a4	O	1	L3b1a4	O	1
571	JQ044986	L3b1a5	L3b1a5	O	1	L3b1a5	O	1
572	EU092962	L3b1a5	L3b1a5	O	1	L3b1a5	O	1
573	NA18865	L3b1a5a	L3b1a5a	O	1	L3b1a5a	O	1
574	NA18907	L3b1a5a	L3b1a5a	O	1	L3b1a5a	O	1
575	JN655790	L3b1a+152	L3b1a+152	O	1	L3b1a+152	O	1
576	EU092958	L3b1a6	L3b1a6	O	1	L3b1a6	O	1
577	JQ045058	L3b1a6	L3b1a6	O	1	L3b1a6	O	1
578	EU092768	L3b1a+@16124	L3b1a+@16124	O	1	L3b1a+@16124	O	1
579	JX666328	L3b1a7	L3b1a7	O	1	L3b1a7	O	1
580	DQ341073	L3b1a7a	L3b1a7a	O	1	L3b1a7a	O	1
581	JQ044820	L3b1a7a	L3b1a7a	O	1	L3b1a7a	O	1
582	NA19222	L3b1a8	L3b1a8	O	1	L3b1a8	O	1
583	NA19175	L3b1a8	L3b1a8	O	1	L3b1a8	O	1
584	JN655791	L3b1a9	L3b1a9	O	1	L3b1a9	O	1
585	JQ045018	L3b1a9	L3b1a9	O	1	L3b1a9	O	1
586	EU092727	L3b1a9a	L3b1a9a	O	1	L3b1a9a	O	1
587	EU092825	L3b1a9a	L3b1a9a	O	1	L3b1a9a	O	1
588	EU092897	L3b1a10	L3b1a10	O	1	L3b1a10	O	1
589	NA19257	L3b1a10	L3b1a10	O	1	L3b1a10	O	1

590	DQ304991	L3b1a11	L3b1a11	O	1	L3b1a11	O	1
591	EU092694	L3b1a11	L3b1a11	O	1	L3b1a11	O	1
592	EU092814	L3b1b	L3b1b	O	1	L3b1b	O	1
593	EU092682	L3b1b1	L3b1b1	O	1	L3b1b1	O	1
594	EU200761	L3b1b1	L3b1b1	O	1	L3b1b1	O	1
595	JQ045029	L3b2	L3b2	O	1	L3b2	O	1
596	EU092725	L3b2a	L3b2a	O	1	L3b2a	O	1
597	EU935440	L3b2a	L3b2a	O	1	L3b2a	O	1
598	NA18916	L3b2b	L3b2b	O	1	L3b2b	O	1
599	JQ703986	L3b3	L3b3	O	1	L3b3	O	1
600	NA18858	L3b3	L3b3	O	1	L3b3	O	1
601	EU092891	L3f1a	L3f1a	O	1	L3f1a	O	1
602	JN655809	L3f1a1	L3f1a1	O	1	L3f1a1	O	1
603	DQ341078	L3f1a1	L3f1a1	O	1	L3f1a1	O	1
604	DQ341077	L3f1b+16292	L3f1b+16292	O	1	L3f1b+16292	O	1
605	JN655783	L3f1b+16292	L3f1b+16292	O	1	L3f1b+16292	O	1
606	JQ044831	L3f1b1	L3f1b1	O	1	L3f1b1	O	1
607	DQ305036	L3f1b1a	L3f1b1a	O	1	L3f1b1a	O	1
608	JQ704670	L3f1b1a	L3f1b1a	O	1	L3f1b1a	O	1
609	JX303774	L3f1b1a1	L3f1b1a1	O	1	L3f1b1a1	O	1
610	EU092857	L3f1b1a1	L3f1b1a1	O	1	L3f1b1a1	O	1
611	EU092805	L3f1b2	L3f1b2	O	1	L3f1b2	O	1
612	EU935451	L3f1b2a	L3f1b2a	O	1	L3f1b2a	O	1
613	EU092953	L3f1b2a	L3f1b2a	O	1	L3f1b2a	O	1
614	NA19172	L3f1b+16292+150	L3f1b+16292+150	O	1	L3f1b+16292+150	O	1
615	EU092883	L3f1b3	L3f1b3	O	1	L3f1b3	O	1
616	NA19213	L3f1b3	L3f1b3	O	1	L3f1b3	O	1
617	EU092865	L3f1b4a	L3f1b4a	O	1	L3f1b4a	O	1
618	EU092912	L3f1b4a1	L3f1b4a1	O	1	L3f1b4a1	O	1
619	EU092791	L3f1b4a1	L3f1b4a1	O	1	L3f1b4a1	O	1
620	JQ045093	L3f1b4b	L3f1b4b	O	1	L3f1b4b	O	1
621	NA18520	L3f1b4b	L3f1b4b	O	1	L3f1b4b	O	1
622	EU092704	L3f1b4c	L3f1b4c	O	1	L3f1b4c	O	1
623	JQ045084	L3f1b4c	L3f1b4c	O	1	L3f1b4c	O	1
624	GU455415	L3f1b5	L3f1b5	O	1	L3f1b5	O	1
625	GU455422	L3f1b5	L3f1b5	O	1	L3f1b5	O	1
626	JN655784	L3f2a	L3f	X	2	L3f2a	O	1

627	JN655841	L3f2a1	L3f2a1	O	1	L3f2a1	O	1
628	DQ341076	L3f2a1a	L3f2a1a	O	1	L3f2a1a	O	1
629	JN655819	L3f2a1a	L3f2a1a	O	1	L3f2a1a	O	1
630	EU092770	L3f2b	L3f2b	O	1	L3f2b	O	1
631	EU092877	L3f2b	L3f2b	O	1	L3f2b	O	1
632	FJ625848	L3f3	L3f3	O	1	L3f3	O	1
633	JN655831	L3f3	L3f3	O	1	L3f3	O	1
634	FJ625855	L3f3a	L3f3a	O	1	L3f3a	O	1
635	FJ625854	L3f3a	L3f3a	O	1	L3f3a	O	1
636	FJ625850	L3f3b	L3f3b	O	1	L3f3b	O	1
637	DQ341075	L3f3b	L3f3b	O	1	L3f3b	O	1
638	EU092660	L3c	L3c	O	1	L3c	O	1
639	DQ341074	L3c	L3c	O	1	L3c	O	1
640	EU092898	L3d1'2'3'4'5'6	L3d1'2'3'4'5'6	O	1	L3d1'2'3'4'5'6	O	1
641	EU092796	L3d1a	L3d1a	O	1	L3d1a	O	1
642	NA19316	L3d1a	L3d1a	O	1	L3d1a	O	1
643	JN655800	L3d1a1a	L3d1a1a	O	1	L3d1a1a	O	1
644	KC911533	L3d1a1a	L3d1a1a	O	1	L3d1a1a	O	1
645	KC533458	L3d1a1a1	L3d1a1a1	O	1	L3d1a1a1	O	1
646	EU092932	L3d1a1a1	L3d1a1a1	O	1	L3d1a1a1	O	1
647	EU092876	L3d1a1b	L3d1a1b	O	1	L3d1a1b	O	1
648	NA18867	L3d1a1b	L3d1a1b	O	1	L3d1a1b	O	1
649	HM771228	L3d1a2	L3d1a2	O	1	L3d1a2	O	1
650	JQ702481	L3d1a2	L3d1a2	O	1	L3d1a2	O	1
651	JQ045112	L3d1b	L3d1b	O	1	L3d1b	O	1
652	EU092899	L3d1b	L3d1b	O	1	L3d1b	O	1
653	DQ341072	L3d1b1	L3d1b1	O	1	L3d1b1	O	1
654	JN214459	L3d1b1a	L3d1b1a	O	1	L3d1b1a	O	1
655	JN214478	L3d1b1a	L3d1b1a	O	1	L3d1b1a	O	1
656	EU200759	L3d1b1b	L3d1b1b	O	1	L3d1b1b	O	1
657	JN655823	L3d1b1b	L3d1b1b	O	1	L3d1b1b	O	1
658	JQ702420	L3d1b2	L3d1b2	O	1	L3d1b2	O	1
659	JQ705077	L3d1b2	L3d1b2	O	1	L3d1b2	O	1
660	JQ045051	L3d1b3	L3d1b3	O	1	L3d1b3	O	1
661	JX303821	L3d1b3a	L3d1b3a	O	1	L3d1b3a	O	1
662	JQ044814	L3d1b3a	L3d1b3a	O	1	L3d1b3a	O	1
663	EU092762	L3d1c	L3d1c	O	1	L3d1c	O	1

664	JQ044862	L3d1c	L3d1c	O	1	L3d1c	O	1
665	EU092830	L3d1c1	L3d1c1	O	1	L3d1c1	O	1
666	JQ044824	L3d1c1	L3d1c1	O	1	L3d1c1	O	1
667	AY195782	L3d1d	L3d1d	O	1	L3d1d	O	1
668	HM771170	L3d2a	L3d2a	O	1	L3d2a	O	1
669	JQ705019	L3d2b	L3d2b	O	1	L3d2b	O	1
670	JN655793	L3d2b	L3d2b	O	1	L3d2b	O	1
671	JX303864	L3d3a1	L3d3a1	O	1	L3d3a1	O	1
672	EU092851	L3d3a1a	L3d3a1a	O	1	L3d3a1a	O	1
673	EU597526	L3d3a1a	L3d3a1a	O	1	L3d3a1a	O	1
674	JX303836	L3d3a1b	L3d3a1b	O	1	L3d3a1b	O	1
675	JX303803	L3d3a1b	L3d3a1b	O	1	L3d3a1b	O	1
676	JN655797	L3d3b	L3d3b	O	1	L3d3b	O	1
677	AF381998	L3d3b	L3d3b	O	1	L3d3b	O	1
678	NA20296	L3d4	L3d4	O	1	L3d4	O	1
679	GU455420	L3d4	L3d4	O	1	L3d4	O	1
680	JN655834	L3d4a	L3d4a	O	1	L3d4a	O	1
681	FJ460540	L3d4a	L3d4a	O	1	L3d4a	O	1
682	JN214479	L3d5	L3d	X	2	L3d5	O	1
683	NA19121	L3d5a	L3d5a	O	1	L3d5a	O	1
684	EU597500	L3d5a	L3d5a	O	1	L3d5a	O	1
685	JQ045091	L3d6	L3d6	O	1	L3d6	O	1
686	NA18864	L3d6	L3d6	O	1	L3d6	O	1
687	EU092827	L3e1	L3e1	O	1	L3e1	O	1
688	EU092887	L3e1	L3e1	O	1	L3e1	O	1
689	EU092749	L3e1a1a	L3e1a1a	O	1	L3e1a1a	O	1
690	KC533483	L3e1a1a	L3e1a1a	O	1	L3e1a1a	O	1
691	JX303878	L3e1a2	L3e1a2	O	1	L3e1a2	O	1
692	EU092867	L3e1a2	L3e1a2	O	1	L3e1a2	O	1
693	KC533488	L3e1a3a	L3e1a3a	O	1	L3e1a3a	O	1
694	HM771173	L3e1a3a	L3e1a3a	O	1	L3e1a3a	O	1
695	NA19114	L3e1a3b	L3e1a3b	O	1	L3e1a3b	O	1
696	EU092960	L3e1a3b	L3e1a3b	O	1	L3e1a3b	O	1
697	JN655794	L3e1b1	L3e1b1	O	1	L3e1b1	O	1
698	EU092681	L3e1b1	L3e1b1	O	1	L3e1b1	O	1
699	KC533453	L3e1b2	L3e1b2	O	1	L3e1b2	O	1
700	JX303759	L3e1b2	L3e1b2	O	1	L3e1b2	O	1

701	EU092879	L3e1c	L3e1c	O	1	L3e1c	O	1
702	EU092740	L3e1c	L3e1c	O	1	L3e1c	O	1
703	HM771171	L3e1d	L3e1d	O	1	L3e1d	O	1
704	EU092862	L3e1d1	L3e1d1	O	1	L3e1d1	O	1
705	JX303847	L3e1d1	L3e1d1	O	1	L3e1d1	O	1
706	EU092693	L3e1d1a	L3e1d1a	O	1	L3e1d1a	O	1
707	JX303886	L3e1d1a	L3e1d1a	O	1	L3e1d1a	O	1
708	JQ045096	L3e1e	L3e1e	O	1	L3e1e	O	1
709	NA19391	L3e1e	L3e1e	O	1	L3e1e	O	1
710	EU092915	L3e1e1	L3e1e1	O	1	L3e1e1	O	1
711	AF346980	L3e1e1	L3e1e1	O	1	L3e1e1	O	1
712	HG01170	L3e1e2	L3e1e2	O	1	L3e1e2	O	1
713	DQ282507	L3e1e2	L3e1e2	O	1	L3e1e2	O	1
714	JQ704728	L3e1f	L3e1	X	16	L3e1f	O	1
715	HM771135	L3e1f1	L3e1	X	16	L3e1f1	O	1
716	HM771172	L3e1f1a	L3e1	X	10	L3e1f1a	O	1
717	KC533470	L3e1f1a	L3e1	X	10	L3e1f1a	O	1
718	JN214448	L3e1f2	L3e1	X	6	L3e1f2	O	1
719	JQ705521	L3e1f2	L3e1	X	6	L3e1f2	O	1
720	JQ044847	L3e1g	L3e1g	O	1	L3e1g	O	1
721	JQ045070	L3e1g	L3e1g	O	1	L3e1g	O	1
722	EU092769	L3e2a	L3e2a	O	1	L3e2a	O	1
723	NA19707	L3e2a	L3e2a	O	1	L3e2a	O	1
724	DQ305026	L3e2a1a	L3e2a1a	O	1	L3e2a1a	O	1
725	DQ305027	L3e2a1a	L3e2a1a	O	1	L3e2a1a	O	1
726	DQ305029	L3e2a1b	L3e2a1b	O	1	L3e2a1b	O	1
727	JQ702533	L3e2a1b	L3e2a1b	O	1	L3e2a1b	O	1
728	DQ305031	L3e2a1b1	L3e2a1b1	O	1	L3e2a1b1	O	1
729	JQ044801	L3e2a1b1	L3e2a1b1	O	1	L3e2a1b1	O	1
730	HM771176	L3e2a1b2	L3e2a1b2	O	1	L3e2a1b2	O	1
731	HM771175	L3e2a1b2	L3e2a1b2	O	1	L3e2a1b2	O	1
732	DQ305025	L3e2a1b3	L3e2a1b3	O	1	L3e2a1b3	O	1
733	HG01456	L3e2a1b3	L3e2a1b3	O	1	L3e2a1b3	O	1
734	JQ045092	L3e2a2	L3e2a2	O	1	L3e2a2	O	1
735	EU092926	L3e2a2	L3e2a2	O	1	L3e2a2	O	1
736	EU092729	L3e2a3	L3e2a3	O	1	L3e2a3	O	1
737	DQ341071	L3e2b	L3e2b	O	1	L3e2b	O	1

738	HM771122	L3e2b	L3e2b	O	1	L3e2b	O	1
739	DQ305022	L3e2b1	L3e2b1	O	1	L3e2b1	O	1
740	JQ703138	L3e2b1a	L3e2b1a	O	1	L3e2b1a	O	1
741	DQ305021	L3e2b1a1	L3e2b1a1	O	1	L3e2b1a1	O	1
742	AF347015	L3e2b1a1	L3e2b1a1	O	1	L3e2b1a1	O	1
743	JX303789	L3e2b1a2	L3e2b1a2	O	1	L3e2b1a2	O	1
744	JQ701829	L3e2b1a2	L3e2b1a2	O	1	L3e2b1a2	O	1
745	EU092784	L3e2b2	L3e2b2	O	1	L3e2b2	O	1
746	EU092777	L3e2b2	L3e2b2	O	1	L3e2b2	O	1
747	DQ304998	L3e2b+152	L3e2b+152	O	1	L3e2b+152	O	1
748	DQ305003	L3e2b+152	L3e2b+152	O	1	L3e2b+152	O	1
749	DQ305008	L3e2b3	L3e2b3	O	1	L3e2b3	O	1
750	DQ305009	L3e2b3	L3e2b3	O	1	L3e2b3	O	1
751	DQ305000	L3e2b4	L3e2b4	O	1	L3e2b4	O	1
752	JN381504	L3e2b4	L3e2b4	O	1	L3e2b4	O	1
753	NA19238	L3e2b5	L3e2b5	O	1	L3e2b5	O	1
754	JQ044972	L3e2b5	L3e2b5	O	1	L3e2b5	O	1
755	JN655795	L3e2b6	L3e2b6	O	1	L3e2b6	O	1
756	NA19147	L3e2b6	L3e2b6	O	1	L3e2b6	O	1
757	AF346994	L3e2b7	L3e2b7	O	1	L3e2b7	O	1
758	EU935465	L3e2b7	L3e2b7	O	1	L3e2b7	O	1
759	DQ305019	L3e2b8	L3e2b8	O	1	L3e2b8	O	1
760	NA19150	L3e2b8	L3e2b8	O	1	L3e2b8	O	1
761	DQ305012	L3e3a	L3e3a	O	1	L3e3a	O	1
762	JN655812	L3e3a	L3e3a	O	1	L3e3a	O	1
763	DQ305011	L3e3b	L3e3b	O	1	L3e3b	O	1
764	DQ305010	L3e3b	L3e3b	O	1	L3e3b	O	1
765	HM771231	L3e3b1	L3e3b1	O	1	L3e3b1	O	1
766	DQ305013	L3e3b1	L3e3b1	O	1	L3e3b1	O	1
767	AF346967	L3e3b2	L3e3b2	O	1	L3e3b2	O	1
768	HM771232	L3e3b2	L3e3b2	O	1	L3e3b2	O	1
769	JQ044850	L3e3b3	L3e3b3	O	1	L3e3b3	O	1
770	JQ044964	L3e3b3	L3e3b3	O	1	L3e3b3	O	1
771	EU092752	L3e4	L3e4	O	1	L3e4	O	1
772	EU092695	L3e4a	L3e4a	O	1	L3e4a	O	1
773	NA18909	L3e4a	L3e4a	O	1	L3e4a	O	1
774	JQ705320	L3e4a1	L3e4a1	O	1	L3e4a1	O	1

775	JQ045125	L3e4a1	L3e4a1	O	1	L3e4a1	O	1
776	KF358485	L3e5	L3e5	O	1	L3e5	O	1
777	JN655822	L3e5	L3e5	O	1	L3e5	O	1
778	JN214452	L3e5a	L3e5a	O	1	L3e5a	O	1
779	EU092821	L3e5a1	L3e5a1	O	1	L3e5a1	O	1
780	KF358475	L3e5a1a	L3e5a1a	O	1	L3e5a1a	O	1
781	JN655798	L3e5a1a	L3e5a1a	O	1	L3e5a1a	O	1
782	EU092959	L3e5b	L3e5b	O	1	L3e5b	O	1
783	KF358486	L3e5b	L3e5b	O	1	L3e5b	O	1
784	JN655828	L3e5+195	L3e5+195	O	1	L3e5+195	O	1
785	KF358482	L3e5c	L3e5c	O	1	L3e5c	O	1
786	KF358474	L3e5c	L3e5c	O	1	L3e5c	O	1
787	FJ460533	L3e5d	L3e5d	O	1	L3e5d	O	1
788	KF358487	L3e5d	L3e5d	O	1	L3e5d	O	1
789	KF358472	L3e5e	L3e5e	O	1	L3e5e	O	1
790	DQ341070	L3e5e	L3e5e	O	1	L3e5e	O	1
791	KF358481	L3e5f	L3e5f	O	1	L3e5f	O	1
792	KF358480	L3e5f	L3e5f	O	1	L3e5f	O	1
793	NA19437	L3i1	L3i1	O	1	L3i1	O	1
794	JN655780	L3i1a	L3i1a	O	1	L3i1a	O	1
795	DQ341069	L3i1b	L3i1b	O	1	L3i1b	O	1
796	EU092923	L3i1b	L3i1b	O	1	L3i1b	O	1
797	DQ341068	L3i2	L3i2	O	1	L3i2	O	1
798	JN655814	L3i2	L3i2	O	1	L3i2	O	1
799	EU092822	L3k	L3k	O	1	L3k	O	1
800	JQ705310	L3k1	L3k1	O	1	L3k1	O	1
801	JN655789	L3k1	L3k1	O	1	L3k1	O	1
802	NA19446	L3x1a1	L3x1a1	O	1	L3x1a1	O	1
803	EF556171	L3x1a1	L3x1a1	O	1	L3x1a1	O	1
804	JN655779	L3x1a2	L3x1a2	O	1	L3x1a2	O	1
805	JN655773	L3x1a2	L3x1a2	O	1	L3x1a2	O	1
806	JN655837	L3x1+16311	L3x1+16311	O	1	L3x1+16311	O	1
807	JN655782	L3x1b	L3x1b	O	1	L3x1b	O	1
808	DQ341067	L3x1b	L3x1b	O	1	L3x1b	O	1
809	EU092944	L3x2a	L3x2a	O	1	L3x2a	O	1
810	JN655776	L3x2a	L3x2a	O	1	L3x2a	O	1
811	DQ341066	L3x2a1	L3x2a1	O	1	L3x2a1	O	1

812	JN655829	L3x2a1a	L3x2a1a	O	1	L3x2a1a	O	1
813	EU092684	L3x2a1a	L3x2a1a	O	1	L3x2a1a	O	1
814	EU092818	L3x2b	L3x2b	O	1	L3x2b	O	1
815	HQ675033	L3x2b	L3x2b	O	1	L3x2b	O	1
816	JN655830	L3h1a1	L3h1a1	O	1	L3h1a1	O	1
817	NA19457	L3h1a1	L3h1a1	O	1	L3h1a1	O	1
818	JN655840	L3h1a2a	L3h1a2a	O	1	L3h1a2a	O	1
819	AF347000	L3h1a2a1	L3h1a2a1	O	1	L3h1a2a1	O	1
820	JN655824	L3h1a2a1	L3h1a2a1	O	1	L3h1a2a1	O	1
821	JN655788	L3h1a2b	L3h1a2b	O	1	L3h1a2b	O	1
822	EU092753	L3h1a2b	L3h1a2b	O	1	L3h1a2b	O	1
823	EU092828	L3h1b1	L3h1b1	O	1	L3h1b1	O	1
824	EU092903	L3h1b1a	L3h1b1a	O	1	L3h1b1a	O	1
825	JN214458	L3h1b1a	L3h1b1a	O	1	L3h1b1a	O	1
826	EU092736	L3h1b2	L3h1b2	O	1	L3h1b2	O	1
827	GU455418	L3h1b2	L3h1b2	O	1	L3h1b2	O	1
828	JN655801	L3h2	L3h2	O	1	L3h2	O	1
829	DQ341080	L3h2	L3h2	O	1	L3h2	O	1
830	JQ702955	M1a	M1a	O	1	M1a	O	1
831	EF060330	M1a1	M1a1	O	1	M1a1	O	1
832	EF060331	M1a1	M1a1	O	1	M1a1	O	1
833	KC152544	M1a1a	M1a1a	O	1	M1a1+16093	X	2
834	JQ704763	M1a1a1	M1a1a1	O	1	M1a1a1	O	1
835	EF060315	M1a1a1	M1a1a1	O	1	M1a1a1	O	1
836	KC152556	M1a1b1	M1a1b1	O	1	M1a1b1	O	1
837	EF060317	M1a1b1	M1a1b1	O	1	M1a1b1	O	1
838	EF060321	M1a1b1a	M1a1b1a	O	1	M1a1b1a	O	1
839	EF060319	M1a1b1a	M1a1b1a	O	1	M1a1b1a	O	1
840	KC152591	M1a1b1b	M1a1b1b	O	1	M1a1b1b	O	1
841	DQ779928	M1a1b1b	M1a1b1b	O	1	M1a1b1b	O	1
842	KC152593	M1a1b1b1	M1a1b1b1	O	1	M1a1b1b1	O	1
843	DQ779930	M1a1b1b1	M1a1b1b1	O	1	M1a1b1b1	O	1
844	JQ705802	M1a1b1c	M1a1b1c	O	1	M1a1b1c	O	1
845	JQ249907	M1a1b1c	M1a1b1c	O	1	M1a1b1c	O	1
846	HQ384200	M1a1b2	M1a1b2	O	1	M1a1b2	O	1
847	KC152571	M1a1b2	M1a1b2	O	1	M1a1b2	O	1
848	EF556180	M1a1c	M1a1c	O	1	M1a1c	O	1

849	EF060322	M1a1c	M1a1c	O	1	M1a1c	O	1
850	EF060325	M1a1d	M1a1+16093	X	30	M1a1d	O	1
851	EF060327	M1a1d	M1a1+16093	X	30	M1a1d	O	1
852	EF060333	M1a1e1	M1a1e1	O	1	M1a1e1	O	1
853	KC152582	M1a1e1	M1a1e1	O	1	M1a1e1	O	1
854	EF060332	M1a1e2	M1a1e2	O	1	M1a1e2	O	1
855	HM852804	M1a1e2	M1a1e2	O	1	M1a1e2	O	1
856	EF060328	M1a1f	M1a1f	O	1	M1a1f	O	1
857	KC152594	M1a1f	M1a1f	O	1	M1a1+16093	X	2
858	KC152575	M1a1g	M1a1g	O	1	M1a1g	O	1
859	DQ341082	M1a1h	M1a1h	O	1	M1a1h	O	1
860	JQ703063	M1a1i	M1a1i	O	1	M1a1i	O	1
861	HG01272	M1a1i	M1a1i	O	1	M1a1i	O	1
862	EF060340	M1a2	M1a2	O	1	M1a2	O	1
863	AF381984	M1a2a	M1a2a	O	1	M1a2a	O	1
864	DQ779927	M1a2a	M1a2a	O	1	M1a2a	O	1
865	EF060337	M1a2b	M1a2b	O	1	M1a2b	O	1
866	KC152570	M1a2b	M1a2b	O	1	M1a2b	O	1
867	EF177443	M1a3a	M1a3a	O	1	M1a3a	O	1
868	EF060342	M1a3a	M1a3a	O	1	M1a3a	O	1
869	GU122999	M1a3b	M1a3b	O	1	M1a3b	O	1
870	KC152560	M1a3b1	M1a3b1	O	1	M1a3b1	O	1
871	EU200765	M1a3b1	M1a3b1	O	1	M1a3b1	O	1
872	EF060343	M1a3b2	M1a3b2	O	1	M1a3b2	O	1
873	JX153066	M1a3b2	M1a3b2	O	1	M1a3b2	O	1
874	EF060348	M1a4	M1a4	O	1	M1a4	O	1
875	EF060349	M1a4	M1a4	O	1	M1a4	O	1
876	EF060346	M1a4a	M1a4a	O	1	M1a4a	O	1
877	EF060347	M1a4a	M1a4a	O	1	M1a4a	O	1
878	EF060351	M1a5	M1a5	O	1	M1a5	O	1
879	EF060350	M1a5	M1a5	O	1	M1a5	O	1
880	KC152573	M1a6	M1a6	O	1	M1a6	O	1
881	EF060344	M1a6	M1a6	O	1	M1a6	O	1
882	KC152588	M1a7	M1a7	O	1	M1a7	O	1
883	EF060345	M1a8	M1a8	O	1	M1a8	O	1
884	JQ702011	M1a8a	M1a8a	O	1	M1a8a	O	1
885	JX154038	M1a8a	M1a8a	O	1	M1a8a	O	1

886	KC152542	M1b1	M1b1	O	1	M1b1	O	1
887	EF060353	M1b1a	M1b1a	O	1	M1b1a	O	1
888	EF060354	M1b1a	M1b1a	O	1	M1b1a	O	1
889	EF060352	M1b1b	M1b1b	O	1	M1b1b	O	1
890	KC152586	M1b1b	M1b1b	O	1	M1b1b	O	1
891	EF060361	M1b2	M1b2	O	1	M1b2	O	1
892	DQ779932	M1b2	M1b2	O	1	M1b2	O	1
893	EF060355	M1b2a	M1b2a	O	1	M1b2a	O	1
894	EF060357	M1b2a	M1b2a	O	1	M1b2a	O	1
895	KC152557	M1b2b	M1b2b	O	1	M1b2b	O	1
896	EF060358	M1b2b	M1b2b	O	1	M1b2b	O	1
897	EF060362	M1b2c	M1b2c	O	1	M1b2c	O	1
898	AP012349	M20	M20	O	1	M20	O	1
899	JX289112	M20	M20	O	1	M20	O	1
900	KC505097	M51a	M51a	O	1	M51a	O	1
901	HM596693	M51a1a	M51a1a	O	1	M51a1a	O	1
902	GQ301874	M51a1a	M51a1a	O	1	M51a1a	O	1
903	EU597554	M51a1b	M51a1b	O	1	M51a1b	O	1
904	KC505102	M51a1b	M51a1b	O	1	M51a1b	O	1
905	GQ301870	M51a2	M51a2	O	1	M51a2	O	1
906	GQ301882	M51a2	M51a2	O	1	M51a2	O	1
907	KC505098	M51b	M51b	O	1	M51b	O	1
908	GQ301879	M51b1a	M51b1a	O	1	M51b1a	O	1
909	KC505099	M51b1a	M51b1a	O	1	M51b1a	O	1
910	KC505100	M51b1b	M51b1b	O	1	M51b1b	O	1
911	KC505101	M51b1b	M51b1b	O	1	M51b1b	O	1
912	EU443499	M2a1	M2a1	O	1	M2a1	O	1
913	EU597558	M2a1	M2a1	O	1	M2a1	O	1
914	EU443460	M2a1a	M2a1a	O	1	M2a1a	O	1
915	EU597516	M2a1a	M2a1a	O	1	M2a1a	O	1
916	EU443472	M2a1a1	M2a1a1	O	1	M2a1a1	O	1
917	EU443508	M2a1a1a	M2a1a1a	O	1	M2a1a1a	O	1
918	FJ383240	M2a1a1a1	M2a1a1a1	O	1	M2a1a1a1	O	1
919	EU443473	M2a1a1a1	M2a1a1a1	O	1	M2a1a1a1	O	1
920	EU443507	M2a1a1b	M2a1a1b	O	1	M2a1a1b	O	1
921	FJ383286	M2a1a1b	M2a1a1b	O	1	M2a1a1b	O	1
922	EU443509	M2a1a1b1	M2a1a1b1	O	1	M2a1a1b1	O	1

923	FJ383285	M2a1a1b1	M2a1a1b1	O	1	M2a1a1b1	O	1
924	EU443493	M2a1a2	M2a1a2	O	1	M2a1a2	O	1
925	EU443494	M2a1a2a	M2a1a2a	O	1	M2a1a2a	O	1
926	FJ383280	M2a1a2a1	M2a1a2a1	O	1	M2a1a2a1	O	1
927	EU443477	M2a1a2a1	M2a1a2a1	O	1	M2a1a2a1	O	1
928	FJ383276	M2a1a2a1a	M2a1a2a1a	O	1	M2a1a2a1a	O	1
929	EU443480	M2a1a2a1a	M2a1a2a1a	O	1	M2a1a2a1a	O	1
930	EU443506	M2a1a+207	M2a1a+207	O	1	M2a1a+207	O	1
931	GU480005	M2a1a+207	M2a1a+207	O	1	M2a1a+207	O	1
932	EU443502	M2a1a3	M2a1a3	O	1	M2a1a3	O	1
933	EU443470	M2a1a3a	M2a1a3a	O	1	M2a1a3a	O	1
934	EU443501	M2a1a3a	M2a1a3a	O	1	M2a1a3a	O	1
935	EU443467	M2a1a3a1	M2a1a3a1	O	1	M2a1a3a1	O	1
936	FJ383273	M2a1a3a1	M2a1a3a1	O	1	M2a1a3a1	O	1
937	FJ383272	M2a1a3b	M2a1a3b	O	1	M2a1a3b	O	1
938	EU443468	M2a1a3b	M2a1a3b	O	1	M2a1a3b	O	1
939	EU443454	M2a1b	M2a1b	O	1	M2a1b	O	1
940	EU443456	M2a1b	M2a1b	O	1	M2a1b	O	1
941	EU443443	M2a1c	M2a1c	O	1	M2a1c	O	1
942	EU443445	M2a1c	M2a1c	O	1	M2a1c	O	1
943	EU443487	M2a2	M2a2	O	1	M2a2	O	1
944	FJ383239	M2a2a	M2a2a	O	1	M2a2a	O	1
945	EU443474	M2a2a	M2a2a	O	1	M2a2a	O	1
946	EU443450	M2a3	M2a3	O	1	M2a3	O	1
947	EU443505	M2a3	M2a3	O	1	M2a3	O	1
948	EU443447	M2a3a	M2a3a	O	1	M2a3a	O	1
949	EU443513	M2a3a	M2a3a	O	1	M2a3a	O	1
950	EU443512	M2b	M2b	O	1	M2b	O	1
951	EU443481	M2b1	M2b1	O	1	M2b1	O	1
952	EU443483	M2b1a	M2b1a	O	1	M2b1a	O	1
953	EU443504	M2b1a	M2b1a	O	1	M2b1a	O	1
954	EU443496	M2b1b	M2b1b	O	1	M2b1b	O	1
955	EU443498	M2b1b	M2b1b	O	1	M2b1b	O	1
956	EU443463	M2b2	M2b2	O	1	M2b2	O	1
957	EU443459	M2b2	M2b2	O	1	M2b2	O	1
958	AY922305	M2b3	M2b3	O	1	M2b3	O	1
959	EU443449	M2b3a	M2b3a	O	1	M2b3a	O	1

960	FJ383267	M2b3a	M2b3a	O	1	M2b3a	O	1
961	EU443484	M2b4	M2b4	O	1	M2b4	O	1
962	EU443486	M2b4	M2b4	O	1	M2b4	O	1
963	KC911426	M2c	M2c	O	1	M2c	O	1
964	DQ408676	M3	M3	O	1	M3	O	1
965	FJ383527	M3a1	M3a1	O	1	M3a1	O	1
966	JX462712	M3a1	M3a1	O	1	M3a1	O	1
967	JQ704107	M3a1+204	M3a1+204	O	1	M3a1+204	O	1
968	FJ383536	M3a1+204	M3a1+204	O	1	M3a1+204	O	1
969	FJ383514	M3a1a	M3a1a	O	1	M3a1a	O	1
970	FJ383518	M3a1a	M3a1a	O	1	M3a1a	O	1
971	KC911530	M3a1b	M3a1b	O	1	M3a1b	O	1
972	JX462700	M3a1b	M3a1b	O	1	M3a1b	O	1
973	AY922266	M3a2	M3a2	O	1	M3a2	O	1
974	JQ702439	M3a2	M3a2	O	1	M3a2	O	1
975	FJ383531	M3a2a	M3a2a	O	1	M3a2a	O	1
976	FJ383530	M3a2a	M3a2a	O	1	M3a2a	O	1
977	FJ383513	M3b	M3b	O	1	M3b	O	1
978	FJ383523	M3b	M3b	O	1	M3b	O	1
979	FJ383542	M3c+152	M3c+152	O	1	M3c+152	O	1
980	JX462722	M3c1a	M3c1a	O	1	M3c1a	O	1
981	FJ770965	M3c1a	M3c1a	O	1	M3c1a	O	1
982	FJ383459	M3c1b	M3c1b	O	1	M3c1b	O	1
983	FJ383468	M3c1b1a	M3c1b1a	O	1	M3c1b1a	O	1
984	FJ383460	M3c1b1a	M3c1b1a	O	1	M3c1b1a	O	1
985	FJ383471	M3c1b1b	M3c1b1b	O	1	M3c1b1b	O	1
986	FJ383462	M3c1b1b	M3c1b1b	O	1	M3c1b1b	O	1
987	GU810074	M3c2	M3c2	O	1	M3c2	O	1
988	FJ383541	M3c2	M3c2	O	1	M3c2	O	1
989	DQ246829	M3d	M3d	O	1	M3d	O	1
990	JF742212	M3d	M3d	O	1	M3d	O	1
991	FJ770946	M3d1	M3d1	O	1	M3d1	O	1
992	JF742206	M3d1a	M3d1a	O	1	M3d1a	O	1
993	KC505095	M3d1a	M3d1a	O	1	M3d1a	O	1
994	KF056282	M3d1a1	M3d1a1	O	1	M3d1a1	O	1
995	KF056281	M3d1a1	M3d1a1	O	1	M3d1a1	O	1
996	KC911394	M4a	M4a	O	1	M4a	O	1

997	FJ383293	M4a	M4a	O	1	M4a	O	1
998	DQ408679	M4b	M4b	O	1	M4b	O	1
999	KC911313	M4b	M4b	O	1	M4b	O	1
1000	AY922291	M65a1	M65a1	O	1	M65a1	O	1
1001	JX462703	M65a1	M65a1	O	1	M65a1	O	1
1002	JX289111	M65a+@16311	M65a+@16311	O	1	M65a+@16311	O	1
1003	HM036536	M65a+@16311	M65a+@16311	O	1	M65a+@16311	O	1
1004	HM036576	M65a2	M65a2	O	1	M65a2	O	1
1005	HM030533	M65a2	M65a2	O	1	M65a2	O	1
1006	FJ383297	M65b	M65b	O	1	M65b	O	1
1007	AY922261	M65b	M65b	O	1	M65b	O	1
1008	FJ383300	M67	M67	O	1	M67	O	1
1009	FJ383294	M67	M67	O	1	M67	O	1
1010	KC911475	M18a	M18a	O	1	M18a	O	1
1011	FJ383336	M18a	M18a	O	1	M18a	O	1
1012	JQ705662	M18b	M18b	O	1	M18b	O	1
1013	FJ383335	M18b	M18b	O	1	M18b	O	1
1014	JX289108	M18c	M18c	O	1	M18c	O	1
1015	FJ383337	M18c	M18c	O	1	M18c	O	1
1016	JX289116	M38	M38	O	1	M38	O	1
1017	AY922286	M38a	M38a	O	1	M38a	O	1
1018	AY922290	M38a	M38a	O	1	M38a	O	1
1019	FJ383403	M38b	M38b	O	1	M38b	O	1
1020	FJ383404	M38b	M38b	O	1	M38b	O	1
1021	FJ383397	M38c	M38c	O	1	M38c	O	1
1022	FJ383395	M38c	M38c	O	1	M38c	O	1
1023	FJ770947	M38d	M38d	O	1	M38d	O	1
1024	FJ770963	M38e	M38e	O	1	M38e	O	1
1025	FJ383657	M30	M30	O	1	M30	O	1
1026	FJ383658	M30	M30	O	1	M30	O	1
1027	FJ383662	M30a	M30	X	3	M30a	O	1
1028	FJ383663	M30a	M30	X	3	M30a	O	1
1029	AY922254	M30a1	M30a1	O	1	M30a1	O	1
1030	AY289072	M30a1	M30a1	O	1	M30a1	O	1
1031	FJ383660	M30a2	M30a2	O	1	M30a2	O	1
1032	FJ383665	M30a2	M30a2	O	1	M30a2	O	1
1033	AY289071	M30b	M30b	O	1	M30b	O	1

1034	JQ702987	M30b	M30b	O	1	M30b	O	1
1035	AF382013	M30c	M30c	O	1	M30c	O	1
1036	AY922268	M30c1	M30c	X	2	M30c1	O	1
1037	HM036539	M30c1a	M30c	X	2	M30c1a	O	1
1038	EF556149	M30c1a1	M30c1a1	O	1	M30c1a1	O	1
1039	AY922257	M30c1a1	M30c	X	3	M30c1	X	2
1040	KC533457	M30d1	M30d1	O	1	M30d1	O	1
1041	FJ383656	M30d1	M30d1	O	1	M30d1	O	1
1042	FJ383668	M30d2	M30d2	O	1	M30d2	O	1
1043	AY922255	M30d2	M30d2	O	1	M30d2	O	1
1044	EU597504	M30+16234	M30+16234	O	1	M30+16234	O	1
1045	AY922258	M30+16234	M30+16234	O	1	M30+16234	O	1
1046	FJ383676	M30e	M30e	O	1	M30e	O	1
1047	FJ383679	M30e	M30e	O	1	M30e	O	1
1048	FJ383674	M30f	M30f	O	1	M30f	O	1
1049	DQ246818	M30f	M30f	O	1	M30f	O	1
1050	JF742213	M30g	M30g	O	1	M30g	O	1
1051	FJ383671	M30g	M30g	O	1	M30g	O	1
1052	FJ383686	M37+152	M37+152	O	1	M37+152	O	1
1053	FJ383685	M37a	M37+152+151	X	5	M37a	O	1
1054	AY922265	M37a	M37+152+151	X	5	M37a	O	1
1055	HQ541751	M37a1	M37+152+151	X	2	M37a1	O	1
1056	FJ383690	M37a1	M37+152+151	X	3	M37a1	O	1
1057	FJ383687	M37d	M37d	O	1	M37d	O	1
1058	FJ383688	M37d	M37d	O	1	M37d	O	1
1059	AY922267	M37e	M37e	O	1	M37e	O	1
1060	FJ383684	M37e2	M37e2	O	1	M37e2	O	1
1061	FJ383694	M37e2	M37e2	O	1	M37e2	O	1
1062	JX289107	M43+16311	M43+16311	O	1	M43+16311	O	1
1063	AY922301	M43a	M43a	O	1	M43a [0.000] M43 (2)	O	1
1064	FJ770954	M43a1	M43a1	O	1	M43a1	O	1
1065	JF742207	M43a1	M43a1	O	1	M43a1	O	1
1066	JF742211	M43b	M43b	O	1	M43b	O	1
1067	FJ383428	M43b	M43b	O	1	M43b	O	1
1068	FJ748709	M45	M45	O	1	M45	O	1
1069	HM030535	M45a	M45a	O	1	M45a	O	1
1070	FJ383437	M45a	M45a	O	1	M45a	O	1

1071	KF056272	M54	M54	O	1	M54	O	1
1072	GU810077	M54	M54	O	1	M54	O	1
1073	FJ383738	M63	M63	O	1	M63	O	1
1074	FJ383813	M64	M64	O	1	M64	O	1
1075	EF556193	M64	M64	O	1	M64	O	1
1076	FJ770971	M66a	M66a	O	1	M66a	O	1
1077	GU480012	M66a	M66a	O	1	M66a	O	1
1078	JX462692	M66b	M66b	O	1	M66b	O	1
1079	AY922283	M66b	M66b	O	1	M66b	O	1
1080	EF556195	M5a	M5a	O	1	M5a	O	1
1081	DQ408675	M5a	M5a	O	1	M5a	O	1
1082	AY922289	M5a1a	M5a1a	O	1	M5a1a	O	1
1083	EF583176	M5a1b	M5a1b	O	1	M5a1b	O	1
1084	FJ383568	M5a1b	M5a1b	O	1	M5a1b	O	1
1085	AY922259	M5a2	M5a2	O	1	M5a2	O	1
1086	FJ383552	M5a2a	M5a2a	O	1	M5a2a	O	1
1087	FJ770949	M5a2a1	M5a2a1	O	1	M5a2a1	O	1
1088	HM030518	M5a2a1a1	M5a2a1a1	O	1	M5a2a1a1	O	1
1089	FJ383564	M5a2a1a1	M5a2a1a1	O	1	M5a2a1a1	O	1
1090	FJ383555	M5a2a1a2	M5a2a1a2	O	1	M5a2a1a2	O	1
1091	AY922285	M5a2a1a2	M5a2a1a2	O	1	M5a2a1a2	O	1
1092	FJ383577	M5a2a2	M5a2a2	O	1	M5a2a2	O	1
1093	AY922273	M5a2a2	M5a2a2	O	1	M5a2a2	O	1
1094	FJ383547	M5a2a3	M5a2a3	O	1	M5a2a3	O	1
1095	FJ383544	M5a2a3	M5a2a3	O	1	M5a2a3	O	1
1096	EU597563	M5a2a4	M5a2a4	O	1	M5a2a4	O	1
1097	KC911557	M5a2a4	M5a2a4	O	1	M5a2a4	O	1
1098	FJ383581	M5a3a	M5a3a	O	1	M5a3a	O	1
1099	FJ383580	M5a3a	M5a3a	O	1	M5a3a	O	1
1100	FJ383559	M5a3b	M5a3b	O	1	M5a3b	O	1
1101	FJ383561	M5a3b	M5a3b	O	1	M5a3b	O	1
1102	FJ383554	M5a4	M5a4	O	1	M5a4	O	1
1103	FJ383590	M5a4	M5a4	O	1	M5a4	O	1
1104	FJ383569	M5a5	M5a5	O	1	M5a5	O	1
1105	FJ383566	M5a5	M5a5	O	1	M5a5	O	1
1106	FJ383557	M5d	M5d	O	1	M5d	O	1
1107	FJ770955	M5d	M5d	O	1	M5d	O	1

1108	KC577360	M5b	M5b	O	1	M5b	O	1
1109	FJ383543	M5b1	M5b1	O	1	M5b1	O	1
1110	FJ383589	M5b1	M5b1	O	1	M5b1	O	1
1111	FJ383575	M5b2a	M5b2a	O	1	M5b2a	O	1
1112	AY922292	M5b2a	M5b2a	O	1	M5b2a	O	1
1113	FJ383596	M5b2b	M5b2b	O	1	M5b2b	O	1
1114	FJ383576	M5b2b	M5b2b	O	1	M5b2b	O	1
1115	FJ383550	M5b2b1	M5b2b	X	2	M5b2b1	O	1
1116	HM036533	M5b2b1a	M5b2b1a	O	1	M5b2b1a	O	1
1117	JF742202	M5b2b1a	M5b2b1a	O	1	M5b2b1a	O	1
1118	FJ383546	M5c1	M5c1	O	1	M5c1	O	1
1119	AY922281	M5c1	M5c1	O	1	M5c1	O	1
1120	FJ770953	M5c2	M5c2	O	1	M5c2	O	1
1121	KF056270	M5c2	M5c2	O	1	M5c2	O	1
1122	JX462705	M6	M6	O	1	M6	O	1
1123	JX462716	M6a1	M6a1	O	1	M6a1	O	1
1124	FJ383308	M6a1a	M6a1a	O	1	M6a1a	O	1
1125	FJ383302	M6a1a	M6a1a	O	1	M6a1a	O	1
1126	JQ703707	M6a1b	M6a1b	O	1	M6a1b	O	1
1127	AY922307	M6a1b	M6a1b	O	1	M6a1b	O	1
1128	FJ383305	M6a2	M6a2	O	1	M6a2	O	1
1129	FJ383301	M6a2	M6a2	O	1	M6a2	O	1
1130	AP008394	M7a+16324	M7a+16324	O	1	M7a+16324	O	1
1131	AP008600	M7a1	M7a+16324	X	2	M7a1	O	1
1132	AP010717	M7a1a	M7a1a	O	1	M7a1a	O	1
1133	AP008402	M7a1a	M7a1a	O	1	M7a1a	O	1
1134	AP009451	M7a1a1	M7a1a1	O	1	M7a1a1	O	1
1135	AP008548	M7a1a1	M7a1a1	O	1	M7a1a1	O	1
1136	AP008249	M7a1a1a	M7a1a1a	O	1	M7a1a1a	O	1
1137	AP010747	M7a1a1a	M7a1a1a	O	1	M7a1a1a	O	1
1138	AP008507	M7a1a2	M7a1a2	O	1	M7a1a2	O	1
1139	AP008731	M7a1a2	M7a1a2	O	1	M7a1a2	O	1
1140	AP008585	M7a1a3	M7a1a3	O	1	M7a1a3	O	1
1141	AP008372	M7a1a3	M7a1a3	O	1	M7a1a3	O	1
1142	AP008367	M7a1a4	M7a1a4	O	1	M7a1a4	O	1
1143	AP008699	M7a1a4a	M7a1a4a	O	1	M7a1a4a	O	1
1144	AP008503	M7a1a4a	M7a1a4a	O	1	M7a1a4a	O	1

1145	AP008280	M7a1a5	M7a1a5	O	1	M7a1a5	O	1
1146	AP013259	M7a1a5a	M7a1a5a	O	1	M7a1a5a	O	1
1147	AP008728	M7a1a5a	M7a1a5a	O	1	M7a1a5a	O	1
1148	AP009423	M7a1a6	M7a1a6	O	1	M7a1a6	O	1
1149	AP008483	M7a1a6a	M7a1a6a	O	1	M7a1a6a	O	1
1150	AP013205	M7a1a6a	M7a1a6a	O	1	M7a1a6a	O	1
1151	AP010750	M7a1a7	M7a1a7	O	1	M7a1a7	O	1
1152	AP008734	M7a1a7	M7a1a7	O	1	M7a1a7	O	1
1153	AP013266	M7a1a8	M7a1a8	O	1	M7a1a8	O	1
1154	NA18965	M7a1a8	M7a1a8	O	1	M7a1a8	O	1
1155	AP008466	M7a1a9	M7a1a9	O	1	M7a1a9	O	1
1156	NA19001	M7a1a9	M7a1a9	O	1	M7a1a9	O	1
1157	AP008266	M7a1b1	M7a1b1	O	1	M7a1b1	O	1
1158	JX987459	M7a1b1	M7a1b1	O	1	M7a1b1	O	1
1159	AP009443	M7a1b2	M7a1b2	O	1	M7a1b2	O	1
1160	AP013148	M7a1b2	M7a1b2	O	1	M7a1b2	O	1
1161	AP010826	M7a2	M7a2	O	1	M7a2	O	1
1162	AP013134	M7a2a	M7a2a	O	1	M7a2a	O	1
1163	AP010824	M7a2a1	M7a2a1	O	1	M7a2a1	O	1
1164	AP008689	M7a2a1	M7a2a1	O	1	M7a2a1	O	1
1165	AP010825	M7a2a2	M7a2a2	O	1	M7a2a2	O	1
1166	AP010763	M7a2a2	M7a2a2	O	1	M7a2a2	O	1
1167	EF153781	M7a2a3	M7a2a3	O	1	M7a2a3	O	1
1168	KF148516	M7a2a3a	M7a2a3a	O	1	M7a2a3a	O	1
1169	KF148524	M7a2a3a	M7a2a3a	O	1	M7a2a3a	O	1
1170	KC569547	M7b1a1	M7b1a1	O	1	M7b1a1 [0.004] M7b1a1-	O	1
1171	KC505115	M7b1a1	M7b1a1	O	1	M7b1a1 [0.016] M7b1a1-	O	1
1172	NA18636	M7b1a1a	M7b1a1a	O	1	M7b1a1a	O	1
1173	AP008365	M7b1a1a1	M7b1a1a1	O	1	M7b1a1a1	O	1
1174	AY255173	M7b1a1a1	M7b1a1a1	O	1	M7b1a1a1	O	1
1175	AP008902	M7b1a1a1a	M7b1a1a1a	O	1	M7b1a1a1a	O	1
1176	AP008354	M7b1a1a1a	M7b1a1a1a	O	1	M7b1a1a1a	O	1
1177	AP008621	M7b1a1a1b	M7b1a1a1b	O	1	M7b1a1a1b	O	1
1178	AP008429	M7b1a1a1b	M7b1a1a1b	O	1	M7b1a1a1b	O	1
1179	NA18952	M7b1a1a1b1	M7b1a1a1b1	O	1	M7b1a1a1b1	O	1
1180	AP013274	M7b1a1a1b1	M7b1a1a1b1	O	1	M7b1a1a1b1	O	1
1181	AP008485	M7b1a1a1c	M7b1a1a1c	O	1	M7b1a1a1c	O	1

1182	AP008625	M7b1a1a1c	M7b1a1a1c	O	1	M7b1a1a1c	O	1
1183	AP013226	M7b1a1a1d	M7b1a1a1d	O	1	M7b1a1a1d	O	1
1184	AP010979	M7b1a1a1d	M7b1a1a1d	O	1	M7b1a1a1d	O	1
1185	JF896798	M7b1a1a2	M7b1a1a2	O	1	M7b1a1a2	O	1
1186	NA18769	M7b1a1a2	M7b1a1a2	O	1	M7b1a1a2	O	1
1187	NA18943	M7b1a1a3	M7b1a1a3	O	1	M7b1a1a3	O	1
1188	HG00512	M7b1a1a3	M7b1a1a3	O	1	M7b1a1a3	O	1
1189	AY255159	M7b1a1b	M7b1a1b	O	1	M7b1a1b	O	1
1190	JX987469	M7b1a1b	M7b1a1b	O	1	M7b1a1b	O	1
1191	EF153820	M7b1a1+(16192)	M7b1a1+(16192)	O	1	M7b1a1+(16192)	O	1
1192	AP012426	M7b1a1+(16192)	M7b1a1+(16192)	O	1	M7b1a1+(16192)	O	1
1193	FJ198219	M7b1a1c	M7b1a1c	O	1	M7b1a1c	O	1
1194	NA18550	M7b1a1c	M7b1a1c	O	1	M7b1a1c	O	1
1195	HM357816	M7b1a1c1	M7b1a1c1	O	1	M7b1a1c1	O	1
1196	FJ198221	M7b1a1c1	M7b1a1c1	O	1	M7b1a1c1	O	1
1197	HG00403	M7b1a1d	M7b1a1d	O	1	M7b1a1d	O	1
1198	GU123012	M7b1a1d1	M7b1a1d1	O	1	M7b1a1d1	O	1
1199	HG00593	M7b1a1d1	M7b1a1d1	O	1	M7b1a1d1	O	1
1200	GU810069	M7b1a1e	M7b1a1e	O	1	M7b1a1e	O	1
1201	HM357821	M7b1a1e1	M7b1a1e1	O	1	M7b1a1e1	O	1
1202	JX987463	M7b1a1e1	M7b1a1e1	O	1	M7b1a1e1	O	1
1203	HG00448	M7b1a1e2	M7b1a1e2	O	1	M7b1a1e2	O	1
1204	JX987447	M7b1a1e2	M7b1a1e2	O	1	M7b1a1e2	O	1
1205	JQ704806	M7b1a1f	M7b1a1f	O	1	M7b1a1f	O	1
1206	JX987450	M7b1a1f	M7b1a1f	O	1	M7b1a1f	O	1
1207	GU392102	M7b1a1g	M7b1a1g	O	1	M7b1a1g	O	1
1208	NA18644	M7b1a1g	M7b1a1g	O	1	M7b1a1g	O	1
1209	AP010993	M7b1a1h	M7b1a1h	O	1	M7b1a1h	O	1
1210	FJ198220	M7b1a1h	M7b1a1h	O	1	M7b1a1h	O	1
1211	KF540506	M7b1a1i	M7b1a1i	O	1	M7b1a1i	O	1
1212	JX987442	M7b1a1i1	M7b1a1i1	O	1	M7b1a1i1	O	1
1213	JX987457	M7b1a1i1	M7b1a1i1	O	1	M7b1a1i1	O	1
1214	NA17969	M7b1a2	M7b1a2	O	1	M7b1a2	O	1
1215	KF849908	M7b1a2a	M7b1a2a	O	1	M7b1a2a	O	1
1216	DQ272126	M7b1a2a	M7b1a2a	O	1	M7b1a2a	O	1
1217	KF540759	M7b1a2a1	M7b1a2a1	O	1	M7b1a2a1	O	1
1218	KF540571	M7b1a2a1	M7b1a2a1	O	1	M7b1a2a1	O	1

1219	KF540556	M7b1a2a1a	M7b1a2a1a	O	1	M7b1a2a1a	O	1
1220	KF540953	M7b1a2a1a	M7b1a2a1a	O	1	M7b1a2a1a	O	1
1221	KF540574	M7b1a2a1b	M7b1a2a1b	O	1	M7b1a2a1b	O	1
1222	KF540567	M7b1a2a1b1	M7b1a2a1b1	O	1	M7b1a2a1b1	O	1
1223	KF540598	M7b1a2a1b1	M7b1a2a1b1	O	1	M7b1a2a1b1	O	1
1224	HM030531	M7b1b	M7b1b	O	1	M7b1b	O	1
1225	EF153817	M7b1b	M7b1b	O	1	M7b1b	O	1
1226	HM030527	M7b2	M7b2	O	1	M7b2	O	1
1227	HM030506	M7b2a	M7b2a	O	1	M7b2a	O	1
1228	JX987443	M7b2a	M7b2a	O	1	M7b2a	O	1
1229	HQ157976	M7c1a	M7c1a	O	1	M7c1a	O	1
1230	KF849978	M7c1a1a	M7c1a1a	O	1	M7c1a1a	O	1
1231	HM036561	M7c1a1a1	M7c1a1a1	O	1	M7c1a1a1	O	1
1232	EU007890	M7c1a1a1	M7c1a1a1	O	1	M7c1a1a1	O	1
1233	HM852807	M7c1a1b	M7c1a1b	O	1	M7c1a1b	O	1
1234	KF148264	M7c1a1b1	M7c1a1b1	O	1	M7c1a1b1	O	1
1235	KF148418	M7c1a1b1	M7c1a1b1	O	1	M7c1a1b1	O	1
1236	HM030547	M7c1a2	M7c1a2	O	1	M7c1a2	O	1
1237	EU597541	M7c1a2a	M7c1a2a	O	1	M7c1a2a	O	1
1238	EF153823	M7c1a2a1	M7c1a2a1	O	1	M7c1a2a1	O	1
1239	AP010681	M7c1a2a1	M7c1a2a1	O	1	M7c1a2a1	O	1
1240	JX987458	M7c1a3	M7c1a3	O	1	M7c1a3	O	1
1241	NA18618	M7c1a3a	M7c1a3	X	3	M7c1a3a	O	1
1242	AP008647	M7c1a3a	M7c1a3	X	3	M7c1a3a	O	1
1243	KF849961	M7c1a4a	M7c1a4a	O	1	M7c1a4a	O	1
1244	NA18756	M7c1a4a	M7c1a4a	O	1	M7c1a4a	O	1
1245	KF541008	M7c1a4b	M7c1a4b	O	1	M7c1a4b	O	1
1246	KC251746	M7c1a4b	M7c1a4b	O	1	M7c1a4b	O	1
1247	AP008755	M7c1a5	M7c1a5	O	1	M7c1a5	O	1
1248	EF397561	M7c1a5	M7c1a5	O	1	M7c1a5	O	1
1249	AY255158	M7c1b	M7c1b	O	1	M7c1b	O	1
1250	EF153782	M7c1b1	M7c1b1	O	1	M7c1b1	O	1
1251	EF153790	M7c1b1	M7c1b1	O	1	M7c1b1	O	1
1252	NA18639	M7c1b2a	M7c1b2a	O	1	M7c1b2a	O	1
1253	EF153818	M7c1b2a	M7c1b2a	O	1	M7c1b2a	O	1
1254	JX987455	M7c1b2b	M7c1b2b	O	1	M7c1b2b	O	1
1255	NA18152	M7c1b2b	M7c1b2b	O	1	M7c1b2b	O	1

1256	JQ702664	M7c1c	M7c1c	O	1	M7c1c	O	1
1257	KF540546	M7c1c1	M7c1c1	O	1	M7c1c1	O	1
1258	KF540664	M7c1c1a	M7c1c1a	O	1	M7c1c1a	O	1
1259	KC994065	M7c1c1a1	M7c1c1a1	O	1	M7c1c1a1	O	1
1260	HM238203	M7c1c1a1	M7c1c1a1	O	1	M7c1c1a1	O	1
1261	JX987460	M7c1c2	M7c1c2	O	1	M7c1c2	O	1
1262	JX987445	M7c1c2	M7c1c2	O	1	M7c1c2	O	1
1263	JX987468	M7c1c2a	M7c1c2a	O	1	M7c1c2a	O	1
1264	DQ272117	M7c1c2a	M7c1c2a	O	1	M7c1c2a	O	1
1265	JX987452	M7c1c3	M7c1c3	O	1	M7c1c3	O	1
1266	HM596650	M7c1c3	M7c1c3	O	1	M7c1c3	O	1
1267	GU733792	M7c1c3a	M7c1c3a	O	1	M7c1c3a	O	1
1268	JX987466	M7c1c3a1	M7c1c3a1	O	1	M7c1c3a1	O	1
1269	HM238218	M7c1c3a1	M7c1c3a1	O	1	M7c1c3a1	O	1
1270	HM596659	M7c1c3b	M7c1c3b	O	1	M7c1c3b	O	1
1271	JX987453	M7c1c3b	M7c1c3b	O	1	M7c1c3b	O	1
1272	NA18674	M7c1c3c	M7c1c3c	O	1	M7c1c3c	O	1
1273	JX987462	M7c1c3c	M7c1c3c	O	1	M7c1c3c	O	1
1274	JQ703844	M7c1c3d	M7c1c3d	O	1	M7c1c3d	O	1
1275	GU733735	M7c1c3d	M7c1c3d	O	1	M7c1c3d	O	1
1276	AP012360	M7c1c3e	M7c1c3e	O	1	M7c1c3e	O	1
1277	AP012363	M7c1c3e	M7c1c3e	O	1	M7c1c3e	O	1
1278	KC994090	M7c1c3f	M7c1c3f	O	1	M7c1c3f	O	1
1279	KC994116	M7c1c3f	M7c1c3f	O	1	M7c1c3f	O	1
1280	KC994102	M7c1c3g	M7c1c3g	O	1	M7c1c3g	O	1
1281	KC994012	M7c1c3g	M7c1c3g	O	1	M7c1c3g	O	1
1282	KC994106	M7c1c3h	M7c1c3h	O	1	M7c1c3h	O	1
1283	KC994099	M7c1c3h	M7c1c3h	O	1	M7c1c3h	O	1
1284	KC994051	M7c1c3i	M7c1c3	X	6	M7c1c3i	O	1
1285	KC994126	M7c1c3i	M7c1c3	X	6	M7c1c3i	O	1
1286	HQ157984	M7c2	M7c2	O	1	M7c2	O	1
1287	HM030509	M7c2a	M7c2a	O	1	M7c2a	O	1
1288	JX987470	M7c2a	M7c2a	O	1	M7c2a	O	1
1289	KF540705	M7c2b	M7c2b	O	1	M7c2b	O	1
1290	EF153810	M7c2b	M7c2b	O	1	M7c2b	O	1
1291	KF540540	M7c3	M7c3	O	1	M7c3	O	1
1292	HM030532	M7c3	M7c3	O	1	M7c3	O	1

1293	KF148510	M8a1	M8a1	O	1	M8a1	O	1
1294	AP008705	M8a1a	M8a1a	O	1	M8a1a	O	1
1295	AP008823	M8a1a	M8a1a	O	1	M8a1a	O	1
1296	NA18538	M8a2	M8a2	O	1	M8a2	O	1
1297	FJ198227	M8a2	M8a2	O	1	M8a2	O	1
1298	AP008531	M8a2+152	M8a2+152	O	1	M8a2+152	O	1
1299	JF824924	M8a2a	M8a2a	O	1	M8a2a	O	1
1300	AP008803	M8a2a1	M8a2a1	O	1	M8a2a1	O	1
1301	AY255150	M8a2a1	M8a2a1	O	1	M8a2a1	O	1
1302	AP008373	M8a2b	M8a2b	O	1	M8a2b	O	1
1303	EU219349	M8a2b	M8a2b	O	1	M8a2b	O	1
1304	AP008727	M8a2c	M8a2c	O	1	M8a2c	O	1
1305	KF849960	M8a2c	M8a2c	O	1	M8a2c	O	1
1306	KC251743	M8a2d	M8a2d	O	1	M8a2d	O	1
1307	AY255161	M8a2d	M8a2d	O	1	M8a2d	O	1
1308	NA18105	M8a2e	M8a2e	O	1	M8a2e	O	1
1309	KF540552	M8a2e	M8a2e	O	1	M8a2e	O	1
1310	NA18646	M8a3	M8a3	O	1	M8a3	O	1
1311	HM460791	M8a3	M8a3	O	1	M8a3	O	1
1312	KF849919	M8a3a	M8a3a	O	1	M8a3a	O	1
1313	KF849962	M8a3a	M8a3a	O	1	M8a3a	O	1
1314	KF849902	M8a3a1	M8a3a1	O	1	M8a3a1	O	1
1315	JF824830	M8a3a1	M8a3a1	O	1	M8a3a1	O	1
1316	AY519496	C1a	C1a	O	1	C1a	O	1
1317	EU007858	C1a	C1a	O	1	C1a	O	1
1318	NA19726	C1b	C	X	2	C1b	O	1
1319	EU095226	C1b	C	X	2	C1b	O	1
1320	AY195759	C1b1	C1b1	O	1	C1b1	O	1
1321	HQ012193	C1b1	C1b1	O	1	C1b1	O	1
1322	DQ282447	C1b2	C1b2	O	1	C1b2	O	1
1323	JN546585	C1b2	C1b2	O	1	C1b2	O	1
1324	DQ282464	C1b3	C1b3	O	1	C1b3	O	1
1325	NA19788	C1b3	C1b3	O	1	C1b3	O	1
1326	EU431085	C1b4	C1b4	O	1	C1b4	O	1
1327	DQ282475	C1b4	C1b4	O	1	C1b4	O	1
1328	HQ012213	C1b5a	C1b5a	O	1	C1b5a	O	1
1329	DQ282469	C1b5a	C1b5a	O	1	C1b5a	O	1

1330	JQ702595	C1b5b	C1b5b	O	1	C1b5b	O	1
1331	JQ702534	C1b5b	C1b5b	O	1	C1b5b	O	1
1332	EU095229	C1b6	C1b6	O	1	C1b6	O	1
1333	EU095230	C1b6	C1b6	O	1	C1b6	O	1
1334	HQ012207	C1b7	C1b7	O	1	C1b7	O	1
1335	HQ012196	C1b7	C1b7	O	1	C1b7	O	1
1336	NA19762	C1b7a	C1b7a	O	1	C1b7a	O	1
1337	HQ012215	C1b7a	C1b7a	O	1	C1b7a	O	1
1338	HQ012198	C1b10	C1b10	O	1	C1b10	O	1
1339	HQ012188	C1b10	C1b10	O	1	C1b10	O	1
1340	HQ012236	C1b8	C	X	2	C1b8	O	1
1341	HQ012210	C1b8a	C1b8a	O	1	C1b8a	O	1
1342	HQ012202	C1b8a	C1b8a	O	1	C1b8a	O	1
1343	HQ012212	C1b9	C1b9	O	1	C1b9	O	1
1344	JQ702984	C1b9	C1b9	O	1	C1b9	O	1
1345	JQ705451	C1b11	C1b11	O	1	C1b11	O	1
1346	JQ701891	C1b11	C	X	2	C1b11	O	1
1347	JQ705153	C1b12	C1b12	O	1	C1b12	O	1
1348	HQ012197	C1b12	C1b12	O	1	C1b12	O	1
1349	JX413055	C1b13	C1b13	O	1	C1b13	O	1
1350	JX413056	C1b13	C1b13	O	1	C1b13	O	1
1351	JX413039	C1b13a	C1b13a	O	1	C1b13a	O	1
1352	JX413038	C1b13a1	C1b13a1	O	1	C1b13a1	O	1
1353	JX413036	C1b13a1	C1b13a1	O	1	C1b13a1	O	1
1354	JX413042	C1b13b	C1b13b	O	1	C1b13b	O	1
1355	JX413041	C1b13b	C1b13b	O	1	C1b13b	O	1
1356	JX413044	C1b13c	C1b13c	O	1	C1b13c	O	1
1357	JX413046	C1b13c1	C1b13c1	O	1	C1b13c1	O	1
1358	JX413048	C1b13c1	C1b13c1	O	1	C1b13c1	O	1
1359	JX413049	C1b13d	C1b13d	O	1	C1b13d	O	1
1360	JX413050	C1b13d	C1b13d	O	1	C1b13d	O	1
1361	JX413051	C1b13e	C1b13e	O	1	C1b13e	O	1
1362	JX413052	C1b13e	C1b13e	O	1	C1b13e	O	1
1363	NA19773	C1b14	C1b14	O	1	C1b14	O	1
1364	HQ012208	C1b14	C1b14	O	1	C1b14	O	1
1365	DQ282459	C1c	C	X	2	C1c	O	1
1366	JQ703840	C1c	C	X	2	C1c	O	1

1367	HQ012229	C1c1	C1c1	O	1	C1c1	O	1
1368	EU597533	C1c1a	C1c1a	O	1	C1c1a	O	1
1369	JQ705676	C1c1b	C1c1b	O	1	C1c1b	O	1
1370	JQ704902	C1c1b	C1c1b	O	1	C1c1b	O	1
1371	DQ282466	C1c2	C1c2	O	1	C1c2	O	1
1372	HQ012232	C1c2	C1c2	O	1	C1c2	O	1
1373	JQ705761	C1c3	C1c3	O	1	C1c3	O	1
1374	EU095544	C1c3	C1c3	O	1	C1c3	O	1
1375	HQ012220	C1c4	C1c4	O	1	C1c4	O	1
1376	EF079875	C1c4	C1c4	O	1	C1c4	O	1
1377	DQ282465	C1c5	C1c5	O	1	C1c5	O	1
1378	HQ012230	C1c5	C1c5	O	1	C1c5	O	1
1379	JQ704040	C1c6	C1c6	O	1	C1c6	O	1
1380	JQ705574	C1c6	C1c6	O	1	C1c6	O	1
1381	JQ703827	C1c7	C1c7	O	1	C1c7	O	1
1382	HQ012223	C1c7	C1c7	O	1	C1c7	O	1
1383	EU327891	C1c8	C1c8	O	1	C1c8	O	1
1384	EU431087	C1c8	C1c8	O	1	C1c8	O	1
1385	HQ012239	C1d	C	X	2	C1d	O	1
1386	HM107306	C1d	C	X	2	C1d	O	1
1387	HM107317	C1d+194	C	X	2	C1d+194	O	1
1388	HM107309	C1d+194	C	X	2	C1d+194	O	1
1389	HM107365	C1d1	C1d1	O	1	C1d1	O	1
1390	HG01462	C1d1	C1d1	O	1	C1d1	O	1
1391	HM107321	C1d1a	C1d1a	O	1	C1d1a	O	1
1392	HM107319	C1d1a1	C1d1a1	O	1	C1d1a1	O	1
1393	HM107320	C1d1a1	C1d1a1	O	1	C1d1a1	O	1
1394	HM107324	C1d1b	C1d1b	O	1	C1d1b	O	1
1395	HM107326	C1d1b1	C1d1b1	O	1	C1d1b1	O	1
1396	HM107332	C1d1b1	C1d1b1	O	1	C1d1b1	O	1
1397	HM107334	C1d1c	C1d1c	O	1	C1d1c	O	1
1398	HQ012235	C1d1c1	C1d1c1	O	1	C1d1c1	O	1
1399	DQ282473	C1d1c1	C1d1c1	O	1	C1d1c1	O	1
1400	HM107355	C1d1d	C1d1d	O	1	C1d1d	O	1
1401	EU095537	C1d1d	C1d1d	O	1	C1d1d	O	1
1402	HM107315	C1d2	C1d2	O	1	C1d2	O	1
1403	HM107314	C1d2a	C1d2a	O	1	C1d2a	O	1

1404	HM107313	C1d2a	C1d2a	O	1	C1d2a	O	1
1405	JQ701741	C1d3	C1d3	O	1	C1d3	O	1
1406	HM804483	C1f	C	X	9	C1f	O	1
1407	FJ951604	C4	C4	O	1	C4	O	1
1408	KC911332	C4a1	C4a1	O	1	C4a1	O	1
1409	FJ951462	C4a1a	C4a1a	O	1	C4a1a	O	1
1410	JX266268	C4a1a1	C4a1a1	O	1	C4a1a1	O	1
1411	FJ383648	C4a1a1a	C4a1a1a	O	1	C4a1a1a	O	1
1412	FJ383597	C4a1a1a	C4a1a1a	O	1	C4a1a1a	O	1
1413	FJ951601	C4a1a+195	C4a1a+195	O	1	C4a1a+195	O	1
1414	EU597517	C4a1a+195	C4a1a+195	O	1	C4a1a+195	O	1
1415	JF824987	C4a1a2	C4a1a2	O	1	C4a1a2	O	1
1416	KF849915	C4a1a2	C4a1a2	O	1	C4a1a2	O	1
1417	AY255174	C4a1a2a	C4a1a2a	O	1	C4a1a2a	O	1
1418	HG00428	C4a1a2a	C4a1a2a	O	1	C4a1a2a	O	1
1419	FJ951442	C4a1a3	C4a1a3	O	1	C4a1a3	O	1
1420	KF148492	C4a1a3	C4a1a3	O	1	C4a1a3	O	1
1421	FJ951463	C4a1a3a	C4a1a3a	O	1	C4a1a3a	O	1
1422	FJ951533	C4a1a3a	C4a1a3a	O	1	C4a1a3a	O	1
1423	EU482371	C4a1a3a1	C4a1a3a1	O	1	C4a1a3a1	O	1
1424	AY615360	C4a1a3a1	C4a1a3a1	O	1	C4a1a3a1	O	1
1425	EU482380	C4a1a3b	C4a1a3b	O	1	C4a1a3b	O	1
1426	FJ951552	C4a1a3b	C4a1a3b	O	1	C4a1a3b	O	1
1427	KF148505	C4a1a3c	C4a1a3c	O	1	C4a1a3c	O	1
1428	KF148229	C4a1a3c	C4a1a3c	O	1	C4a1a3c	O	1
1429	KF148121	C4a1a3d	C4a1a3d	O	1	C4a1a3d	O	1
1430	KF148106	C4a1a3d	C4a1a3d	O	1	C4a1a3d	O	1
1431	FJ951459	C4a1a4	C4a1a4	O	1	C4a1a4	O	1
1432	EU482361	C4a1a4a	C4a1a4a	O	1	C4a1a4a	O	1
1433	FJ951607	C4a1a4a	C4a1a4a	O	1	C4a1a4a	O	1
1434	FJ951612	C4a1a5	C4a1a5	O	1	C4a1a5	O	1
1435	HM036567	C4a1a5	C4a1a5	O	1	C4a1a5	O	1
1436	FJ951475	C4a1a6	C4a1a6	O	1	C4a1a6	O	1
1437	FJ951539	C4a1a6	C4a1a6	O	1	C4a1a+195	X	2
1438	GU392063	C4a1b	C4a1b	O	1	C4a1b	O	1
1439	HQ260971	C4a1b	C4a1b	O	1	C4a1b	O	1
1440	KF148092	C4a2a	C4a2a	O	1	C4a2a	O	1

1441	EU007861	C4a2a1	C4a2a1	O	1	C4a2a1	O	1
1442	KF148221	C4a2a1	C4a2a1	O	1	C4a2a1	O	1
1443	EU482323	C4a2a1a	C4a2a1a	O	1	C4a2a1a	O	1
1444	KF148332	C4a2a1a	C4a2a1a	O	1	C4a2a1a	O	1
1445	KF148469	C4a2a1b	C4a2a1b	O	1	C4a2a1b	O	1
1446	AF346979	C4a2a1b	C4a2a1b	O	1	C4a2a1b	O	1
1447	GQ895170	C4a2b	C4a2b	O	1	C4a2b	O	1
1448	FJ383631	C4a2b1	C4a2b1	O	1	C4a2b1	O	1
1449	FJ383634	C4a2b1	C4a2b1	O	1	C4a2b1	O	1
1450	NA18547	C4a2b2	C4a2b2	O	1	C4a2b2	O	1
1451	KF056249	C4a2b2a	C4a2b2a	O	1	C4a2b2a	O	1
1452	KF056247	C4a2b2a	C4a2b2a	O	1	C4a2b2a	O	1
1453	FJ951548	C4a2c	C4a2c	O	1	C4a2c	O	1
1454	FJ383607	C4a2c1	C4a2c1	O	1	C4a2c1	O	1
1455	FJ383616	C4a2c1	C4a2c1	O	1	C4a2c1	O	1
1456	FJ383647	C4a2c2	C4a2c2	O	1	C4a2c2	O	1
1457	HM036530	C4a2c2a	C4a2c2a	O	1	C4a2c2a	O	1
1458	HM036554	C4a2c2a	C4a2c2a	O	1	C4a2c2a	O	1
1459	EU482314	C4b	C4b	O	1	C4b	O	1
1460	FJ951445	C4b	C4b	O	1	C4b	O	1
1461	FJ951476	C4b1	C4b1	O	1	C4b1	O	1
1462	EU482304	C4b1	C4b1	O	1	C4b1	O	1
1463	AY570526	C4b1a	C4b1a	O	1	C4b1a	O	1
1464	FJ951512	C4b1a	C4b1a	O	1	C4b1a	O	1
1465	KF148250	C4b1b	C4b1b	O	1	C4b1b	O	1
1466	FJ951497	C4b1b	C4b1b	O	1	C4b1b	O	1
1467	KF148135	C4b2	C4b2	O	1	C4b2	O	1
1468	FJ951598	C4b2a	C4b2a	O	1	C4b2a	O	1
1469	EU482333	C4b2a	C4b2a	O	1	C4b2a	O	1
1470	KF148478	C4b3	C4b3	O	1	C4b3	O	1
1471	FJ951447	C4b3	C4b3	O	1	C4b3	O	1
1472	EU482310	C4b3a	C4b3a	O	1	C4b3a	O	1
1473	KF148111	C4b3a	C4b3a	O	1	C4b3a	O	1
1474	EU482324	C4b3a1	C4b3a1	O	1	C4b3a1	O	1
1475	EU482315	C4b3a1	C4b3a1	O	1	C4b3a1	O	1
1476	AF346970	C4b3b	C4b3b	O	1	C4b3b	O	1
1477	KF148228	C4b3b	C4b3b	O	1	C4b3b	O	1

1478	FJ951580	C4b5	C4b5	O	1	C4b5	O	1
1479	FJ951460	C4b5	C4b5	O	1	C4b5	O	1
1480	FJ951451	C4b6	C4b6	O	1	C4b6	O	1
1481	EU482375	C4b6	C4b6	O	1	C4b6	O	1
1482	EU482313	C4b7	C4b7	O	1	C4b7	O	1
1483	EU482326	C4b7	C4b7	O	1	C4b7	O	1
1484	KF148472	C4b8	C4b8	O	1	C4b8	O	1
1485	EU482370	C4b8a	C4b8a	O	1	C4b8a	O	1
1486	AY519490	C4b8a	C4b8a	O	1	C4b8a	O	1
1487	EU095543	C4c	C4c	O	1	C4c	O	1
1488	JF979205	C4c1	C4c1	O	1	C4c1	O	1
1489	GU215075	C4c1	C4c1	O	1	C4c1	O	1
1490	JQ705814	C4c1a	C4c1a	O	1	C4c1a	O	1
1491	JF979198	C4c1a	C4c1a	O	1	C4c1a	O	1
1492	JF979202	C4c1b	C4c1b	O	1	C4c1b	O	1
1493	JF979203	C4c1b	C4c1b	O	1	C4c1b	O	1
1494	JQ705381	C4c2	C4c2	O	1	C4c2	O	1
1495	JF979210	C4c2	C4c2	O	1	C4c2	O	1
1496	GQ895156	C4+152+16093	C4+152+16093	O	1	C4+152+16093	O	1
1497	NA18631	C4d	C4d	O	1	C4d	O	1
1498	GQ895155	C4d	C4d	O	1	C4d	O	1
1499	FJ951611	C4e	C4e	O	1	C4e	O	1
1500	FJ951610	C4e	C4e	O	1	C4e	O	1
1501	FJ951515	C5a1	C5a1	O	1	C5a1	O	1
1502	FJ951439	C5a1	C5a1	O	1	C5a1	O	1
1503	FJ951457	C5a2a	C5a2a	O	1	C5a2a	O	1
1504	KF148083	C5a2a	C5a2a	O	1	C5a2a	O	1
1505	EU482306	C5a2b	C5a2b	O	1	C5a2b	O	1
1506	EU482328	C5a2b	C5a2b	O	1	C5a2b	O	1
1507	KF148150	C5a2b1	C5a2b1	O	1	C5a2b1	O	1
1508	FJ951595	C5a2b1	C5a2b1	O	1	C5a2b1	O	1
1509	FJ951454	C5b	C	X	4	C5b	O	1
1510	FJ951472	C5b1	C	X	4	C5b1	O	1
1511	HM036577	C5b1	C	X	4	C5b1	O	1
1512	FJ951441	C5b1a	C5b1a	O	1	C5b1a	O	1
1513	FJ951566	C5b1a	C5b1a	O	1	C5b1a	O	1
1514	AY615359	C5b1a1	C5b1a	X	2	C5b1a1	O	1

1515	FJ951464	C5b1a1	C5b1a	X	2	C5b1a1	O	1
1516	FJ951496	C5b1b	C5b1b	O	1	C5b1b	O	1
1517	KF148480	C5b1b1	C5b1b1	O	1	C5b1b1	O	1
1518	FJ951438	C5b1b1	C5b1b1	O	1	C5b1b1	O	1
1519	AP008664	C5+16093	C	X	2	C5+16093	O	1
1520	NA18566	C5+16093	C	X	2	C5+16093	O	1
1521	FJ951614	C5c	C5c	O	1	C5c	O	1
1522	KC911293	C5c	C5c	O	1	C5c	O	1
1523	KC911629	C5c+16234	C5c+16234	O	1	C5c+16234	O	1
1524	FJ951452	C5c1	C5c1	O	1	C5c1	O	1
1525	JN315676	C5c1a	C5c1a	O	1	C5c1a	O	1
1526	FJ951605	C5c1a	C5c1a	O	1	C5c1a	O	1
1527	FJ951440	C5d1	C5d1	O	1	C5d1	O	1
1528	EU482303	C5d1	C5d1	O	1	C5d1	O	1
1529	FJ951576	C5d2	C5d2	O	1	C5d2	O	1
1530	JF824965	C5d2	C5d2	O	1	C5d2	O	1
1531	FJ951594	C7	C7	O	1	C7	O	1
1532	KF849951	C7	C7	O	1	C7	O	1
1533	FJ748711	C7a	C7a	O	1	C7a	O	1
1534	GU810045	C7a	C7a	O	1	C7a	O	1
1535	FJ383630	C7a1a1	C7a1a1	O	1	C7a1a1	O	1
1536	FJ383627	C7a1a2	C7a1a2	O	1	C7a1a2	O	1
1537	FJ383622	C7a1a2	C7a1a2	O	1	C7a1a2	O	1
1538	FJ951563	C7a1c	C7a1c	O	1	C7a1c	O	1
1539	AY255176	C7a1c	C7a1c	O	1	C7a1c	O	1
1540	FJ383636	C7a1d	C7a1d	O	1	C7a1d	O	1
1541	JF896797	C7a2	C7a	X	3	C7a2	O	1
1542	EF429136	C7a2a	C7a2a	O	1	C7a2a	O	1
1543	KC251742	C7a2a	C7a2a	O	1	C7a2a	O	1
1544	FJ951540	C7+16051	C7+16051	O	1	C7+16051	O	1
1545	FJ383641	C7b	C7b	O	1	C7b	O	1
1546	JF903929	C7b	C7b	O	1	C7b	O	1
1547	AY519493	Z1	Z1	O	1	Z1	O	1
1548	FJ147318	Z1a	Z1a	O	1	Z1a	O	1
1549	FJ493512	Z1a1a	Z1a1a	O	1	Z1a1a	O	1
1550	DQ902711	Z1a1a	Z1a1a	O	1	Z1a1a	O	1
1551	KC985156	Z1a1b	Z1a1b	O	1	Z1a1b	O	1

1552	FJ493513	Z1a1b	Z1a1b	O	1	Z1a1b	O	1
1553	HM044854	Z1a2	Z1a2	O	1	Z1a2	O	1
1554	AY195761	Z1a2a	Z1a2a	O	1	Z1a2a	O	1
1555	FJ493515	Z1a2a	Z1a2a	O	1	Z1a2a	O	1
1556	KC985155	Z1a3	Z1a3	O	1	Z1a3	O	1
1557	FJ493509	Z1a3	Z1a3	O	1	Z1a3	O	1
1558	AP008426	Z2	Z2	O	1	Z2	O	1
1559	AP011007	Z2	Z2	O	1	Z2	O	1
1560	AP008841	Z3	Z3	O	1	Z3	O	1
1561	EU597518	Z3a1	Z3a1	O	1	Z3a1	O	1
1562	JF824889	Z3a1a	Z3a1a	O	1	Z3a1a	O	1
1563	FJ383642	Z3a1a	Z3a1a	O	1	Z3a1a	O	1
1564	FJ383603	Z3a2	Z3a2	O	1	Z3a2	O	1
1565	FJ383602	Z3a2	Z3a2	O	1	Z3a2	O	1
1566	FJ383644	Z3b	Z3b	O	1	Z3b	O	1
1567	FJ383645	Z3b	Z3b	O	1	Z3b	O	1
1568	KC985166	Z3+709	Z3+709	O	1	Z3+709	O	1
1569	HM852845	Z3c	Z3c	O	1	Z3c	O	1
1570	NA19072	Z3c	Z3c	O	1	Z3c	O	1
1571	JF824858	Z3d	Z3d	O	1	Z3d	O	1
1572	KF540732	Z3d	Z3d	O	1	Z3d	O	1
1573	GU392051	Z4	Z4	O	1	Z4	O	1
1574	KC733271	Z4	Z4	O	1	Z4	O	1
1575	NA19005	Z4a	Z4a	O	1	Z4a	O	1
1576	AY255155	Z4a1	Z4a1	O	1	Z4a1	O	1
1577	HG00436	Z4a1a	Z4a1a	O	1	Z4a1a	O	1
1578	AP013181	Z4a1a1	Z4a1a1	O	1	Z4a1a1	O	1
1579	AP008305	Z4a1a1	Z4a1a1	O	1	Z4a1a1	O	1
1580	FJ383629	Z7	Z7	O	1	Z7	O	1
1581	FJ383625	Z7	Z7	O	1	Z7	O	1
1582	AP008553	Z5	Z5	O	1	Z5	O	1
1583	AP010767	M9a	M9a	O	1	M9a	O	1
1584	HM346891	M9a	M9a	O	1	M9a	O	1
1585	HM346892	M9a1	M9a1	O	1	M9a1	O	1
1586	HM346905	M9a1a	M9a1a	O	1	M9a1a	O	1
1587	HM346903	M9a1a	M9a1a	O	1	M9a1a	O	1
1588	HM346912	M9a1a1	M9a1a1	O	1	M9a1a1	O	1

1589	AP008629	M9a1a1a	M9a1a1a	O	1	M9a1a1a	O	1
1590	AF346972	M9a1a1a	M9a1a1a	O	1	M9a1a1a	O	1
1591	AP008378	M9a1a1b	M9a1a1b	O	1	M9a1a1b	O	1
1592	HM346908	M9a1a1b	M9a1a1b	O	1	M9a1a1b	O	1
1593	HM346913	M9a1a1c	M9a1a1c	O	1	M9a1a1c	O	1
1594	JN857063	M9a1a1c1a	M9a1a1c1a	O	1	M9a1a1c1a	O	1
1595	HM346936	M9a1a1c1a	M9a1a1c1a	O	1	M9a1a1c1a	O	1
1596	HM346915	M9a1a1c1b1	M9a1a1c1b	X	2	M9a1a1c1b1	O	1
1597	HM346924	M9a1a1c1b1a	M9a1a1c1b1a	O	1	M9a1a1c1b1a	O	1
1598	KF056287	M9a1a1c1b1a	M9a1a1c1b1a	O	1	M9a1a1c1b1a	O	1
1599	HM346918	M9a1a1c1b1a1	M9a1a1c1b1a1	O	1	M9a1a1c1b1a1	O	1
1600	HM036540	M9a1a1c1b1a1	M9a1a1c1b1a1	O	1	M9a1a1c1b1a1	O	1
1601	HM036568	M9a1a1c1b1a2	M9a1a1c1b1a2	O	1	M9a1a1c1b1a2	O	1
1602	HM346926	M9a1a1c1b1a2	M9a1a1c1b1a2	O	1	M9a1a1c1b1a2	O	1
1603	FJ383325	M9a1a1c1b2	M9a1a1c1b2	O	1	M9a1a1c1b2	O	1
1604	FJ383324	M9a1a1c1b2	M9a1a1c1b2	O	1	M9a1a1c1b2	O	1
1605	HM346914	M9a1a1c1c	M9a1a1c1c	O	1	M9a1a1c1c	O	1
1606	KF849895	M9a1a1c1c	M9a1a1c1c	O	1	M9a1a1c1c	O	1
1607	HM346910	M9a1a1d	M9a1a1d	O	1	M9a1a1d	O	1
1608	HM346911	M9a1a1d	M9a1a1d	O	1	M9a1a1d	O	1
1609	FJ748758	M9a1a2	M9a1a2	O	1	M9a1a2	O	1
1610	HM346902	M9a1a2	M9a1a2	O	1	M9a1a2	O	1
1611	KF540711	M9a1a3	M9a1a3	O	1	M9a1a3	O	1
1612	JN857051	M9a1a3	M9a1a3	O	1	M9a1a3	O	1
1613	HM346893	M9a1b	M9a1b	O	1	M9a1b	O	1
1614	HM346895	M9a1b+150	M9a1b+150	O	1	M9a1b+150	O	1
1615	GQ895143	M9a1b+150	M9a1b+150	O	1	M9a1b+150	O	1
1616	FJ748735	M9a1b1	M9a1b1	O	1	M9a1b1	O	1
1617	FJ383315	M9a1b1	M9a1b1	O	1	M9a1b1	O	1
1618	FJ383311	M9a1b1a	M9a1b1a	O	1	M9a1b1a	O	1
1619	FJ383310	M9a1b1a1	M9a1b1a1	O	1	M9a1b1a1	O	1
1620	FJ383316	M9a1b1a1	M9a1b1a1	O	1	M9a1b1a1	O	1
1621	FJ383327	M9a1b1b	M9a1b1b	O	1	M9a1b1b	O	1
1622	FJ383326	M9a1b1b	M9a1b1b	O	1	M9a1b1b	O	1
1623	FJ748743	M9a1b1c	M9a1b1c	O	1	M9a1b1c	O	1
1624	GQ895140	M9a1b1c	M9a1b1c	O	1	M9a1b1c	O	1
1625	JN857049	M9a1b2	M9a1b2	O	1	M9a1b2	O	1

1626	HM346898	M9a1b2	M9a1b2	O	1	M9a1b2	O	1
1627	EF093554	M9a4a1	M9a4a1	O	1	M9a4a1	O	1
1628	HM346882	M9a4a1	M9a4a1	O	1	M9a4a1	O	1
1629	HM346885	M9a4a2	M9a4a2	O	1	M9a4a2	O	1
1630	HM346884	M9a4a2	M9a4a2	O	1	M9a4a2	O	1
1631	JN857050	M9a4b	M9a4b	O	1	M9a4b	O	1
1632	HM346886	M9a4b	M9a4b	O	1	M9a4b	O	1
1633	HM346889	M9a5	M9a5	O	1	M9a5	O	1
1634	HM346887	M9a5	M9a5	O	1	M9a5	O	1
1635	KF849927	M9b	M9b	O	1	M9b	O	1
1636	HM346881	M9b	M9b	O	1	M9b	O	1
1637	KF540505	E1	E1	O	1	E1	O	1
1638	GQ119027	E1a1	E1a1	O	1	E1a1	O	1
1639	EF093538	E1a1	E1a1	O	1	E1a1	O	1
1640	EF093539	E1a1a	E1a1a	O	1	E1a1a	O	1
1641	KF540515	E1a1a1	E1a1a1	O	1	E1a1a1	O	1
1642	EF093557	E1a1a1	E1a1a1	O	1	E1a1a1	O	1
1643	EF185804	E1a1a1a	E1a1a1a	O	1	E1a1a1a	O	1
1644	GU810007	E1a1a1a	E1a1a1a	O	1	E1a1a1a	O	1
1645	KF540805	E1a1a1b	E1a1a1b	O	1	E1a1a1b	O	1
1646	KF540812	E1a1a1b	E1a1a1b	O	1	E1a1a1b	O	1
1647	EF093540	E1a1a1b1	E1a1a1b1	O	1	E1a1a1b1	O	1
1648	JQ703727	E1a1a1b1	E1a1a1b1	O	1	E1a1a1b1	O	1
1649	KF540887	E1a1a1b2	E1a1a1b2	O	1	E1a1a1b2	O	1
1650	EF093552	E1a1a1b2	E1a1a1b2	O	1	E1a1a1b2	O	1
1651	KC993911	E1a1a1c	E1a1a1c	O	1	E1a1a1c	O	1
1652	KC993907	E1a1a1c	E1a1a1c	O	1	E1a1a1c	O	1
1653	HQ700849	E1a1b	E1a1b	O	1	E1a1b	O	1
1654	EF185812	E1a1b	E1a1b	O	1	E1a1b	O	1
1655	HQ700847	E1a1b1	E1a1b1	O	1	E1a1b1	O	1
1656	HQ700848	E1a1b1	E1a1b1	O	1	E1a1b1	O	1
1657	EF185805	E1a1b2	E1a1b2	O	1	E1a1b2	O	1
1658	EF185814	E1a1b2	E1a1b2	O	1	E1a1b2	O	1
1659	FJ428235	E1a1b3	E1a1b3	O	1	E1a1b3	O	1
1660	EF093548	E1a1b3	E1a1b3	O	1	E1a1b3	O	1
1661	KJ154942	E1a1b4	E1a1b4	O	1	E1a1b4	O	1
1662	EF061152	E1a1b4	E1a1b4	O	1	E1a1b4	O	1

1663	EF093536	E1a1c	E1a1c	O	1	E1a1c	O	1
1664	KF540532	E1a1c	E1a1c	O	1	E1a1c	O	1
1665	FJ428236	E1a2	E1a2	O	1	E1a2E1a2+(16261) (2)	O	2
1666	EF185795	E1a2+(16261)	E1a2+(16261)	O	1	E1a2+(16261)	O	1
1667	AP012357	E1a2+(16261)	E1a2+(16261)	O	1	E1a2+(16261)	O	1
1668	KJ154697	E1a2a	E1a2a	O	1	E1a2a	O	1
1669	KJ154788	E1a2a	E1a2a	O	1	E1a2a	O	1
1670	EF061150	E1a2a1	E1a2a1	O	1	E1a2a1	O	1
1671	EF061148	E1a2a1	E1a2a1	O	1	E1a2a1	O	1
1672	KJ154892	E1a2a2	E1a2a2	O	1	E1a2a2	O	1
1673	EF061149	E1a2a2	E1a2a2	O	1	E1a2a2	O	1
1674	KJ154845	E1a2a3	E1a2a3	O	1	E1a2a3	O	1
1675	KJ154836	E1a2a3	E1a2a3	O	1	E1a2a3	O	1
1676	KJ154781	E1a2a4	E1a2a4	O	1	E1a2a4	O	1
1677	KJ154653	E1a2a4	E1a2a4	O	1	E1a2a4	O	1
1678	EF093542	E2	E2	O	1	E2	O	1
1679	EF093550	E2a	E2a	O	1	E2a	O	1
1680	HQ700865	E2a	E2a	O	1	E2a	O	1
1681	EF093549	E2a1	E2a1	O	1	E2a1	O	1
1682	GU733741	E2a1a	E2a1a	O	1	E2a1a	O	1
1683	GQ119047	E2a1a	E2a1a	O	1	E2a1a	O	1
1684	EF093541	E2a2	E2a2	O	1	E2a2	O	1
1685	EF185813	E2a2	E2a2	O	1	E2a2	O	1
1686	EF185816	E2b	E2b	O	1	E2b	O	1
1687	GU733775	E2b	E2b	O	1	E2b	O	1
1688	EF185810	E2b1	E2b1	O	1	E2b1	O	1
1689	KF541014	E2b1	E2b1	O	1	E2b1	O	1
1690	EF093537	E2b2	E2b2	O	1	E2b2	O	1
1691	KF540516	E2b2	E2b2	O	1	E2b2	O	1
1692	DQ272116	M10	M10	O	1	M10	O	1
1693	JX289127	M10a	M10a	O	1	M10a	O	1
1694	JX289101	M10a1	M10a1	O	1	M10a1	O	1
1695	JN857060	M10a1+16129	M10a1+16129	O	1	M10a1+16129	O	1
1696	NA18577	M10a1a	M10a1a	O	1	M10a1a	O	1
1697	AP008719	M10a1a1a	M10a1a1a	O	1	M10a1a1a	O	1
1698	AY255178	M10a1a1a	M10a1a1a	O	1	M10a1a1a	O	1
1699	NA18760	M10a1a1b	M10a1a1b	O	1	M10a1a1b	O	1

1700	AP010668	M10a1a1b1	M10a1a1b1	O	1	M10a1a1b1	O	1
1701	JN857013	M10a1a1b1	M10a1a1b1	O	1	M10a1a1b1	O	1
1702	AP013191	M10a1a1b2	M10a1a1b2	O	1	M10a1a1b2	O	1
1703	KF540692	M10a1a1b2	M10a1a1b2	O	1	M10a1a1b2	O	1
1704	AY255154	M10a1b	M10a1b	O	1	M10a1b	O	1
1705	FJ383650	M10a1b	M10a1b	O	1	M10a1b	O	1
1706	JN857058	M10a2	M10a2	O	1	M10a2	O	1
1707	AP008599	M10a2	M10a2	O	1	M10a2	O	1
1708	FJ383334	M11a	M11a	O	1	M11a	O	1
1709	AY255142	M11a	M11a	O	1	M11a	O	1
1710	FJ383332	M11a1	M11a1	O	1	M11a1	O	1
1711	FJ383331	M11a1	M11a1	O	1	M11a1	O	1
1712	FJ748759	M11a2	M11a2	O	1	M11a2	O	1
1713	GQ895157	M11a2	M11a2	O	1	M11a2	O	1
1714	KF540712	M11b1	M11b1	O	1	M11b1	O	1
1715	AP008639	M11b1a	M11b1a	O	1	M11b1a	O	1
1716	AP013153	M11b1a1	M11b1a1	O	1	M11b1a1	O	1
1717	AY255156	M11b1a1	M11b1a1	O	1	M11b1a1	O	1
1718	AP010828	M11b2	M11b2	O	1	M11b2	O	1
1719	JN857010	M11b2	M11b2	O	1	M11b2	O	1
1720	JN857062	M11d	M11d	O	1	M11d	O	1
1721	NA17983	M11d	M11d	O	1	M11d	O	1
1722	AP010716	M11c	M11c	O	1	M11c	O	1
1723	NA18608	M11c	M11c	O	1	M11c	O	1
1724	HQ157977	M12a1a	M12a1a	O	1	M12a1a	O	1
1725	HQ157978	M12a1a1	M12a1a1	O	1	M12a1a1	O	1
1726	HG00626	M12a1a1	M12a1a1	O	1	M12a1a1	O	1
1727	HQ157973	M12a1a2	M12a1a2	O	1	M12a1a2	O	1
1728	EU294322	M12a1a2	M12a1a2	O	1	M12a1a2	O	1
1729	AY255172	M12a1b	M12a1b	O	1	M12a1b	O	1
1730	HQ157983	M12a1b	M12a1b	O	1	M12a1b	O	1
1731	HQ157974	M12a2	M12a2	O	1	M12a2	O	1
1732	JX289125	M12a2	M12a2	O	1	M12a2	O	1
1733	FJ383653	M12b1a1	M12b1a1	O	1	M12b1a1	O	1
1734	FJ383652	M12b1a1	M12b1a1	O	1	M12b1a1	O	1
1735	KC505071	M12b1a2a	M12b1a2a	O	1	M12b1a2a	O	1
1736	KC505077	M12b1a2a	M12b1a2a	O	1	M12b1a2a	O	1

1737	KC505080	M12b1a2b	M12b1a2b	O	1	M12b1a2b	O	1
1738	KC505076	M12b1a2b	M12b1a2b	O	1	M12b1a2b	O	1
1739	KC505084	M12b1b	M12b1b	O	1	M12b1b	O	1
1740	KC505074	M12b1b	M12b1b	O	1	M12b1b	O	1
1741	HQ157972	M12b2	M12b2	O	1	M12b2	O	1
1742	KC505086	M12b2a	M12b2a	O	1	M12b2a	O	1
1743	KC505088	M12b2a	M12b2a	O	1	M12b2a	O	1
1744	NA18634	G1	G1	O	1	G1	O	1
1745	HM460792	G1a1	G1a1	O	1	G1a1	O	1
1746	NA18163	G1a1	G1a1	O	1	G1a1	O	1
1747	AP008511	G1a1a	G1a1a	O	1	G1a1a	O	1
1748	AP010753	G1a1a	G1a1a	O	1	G1a1a	O	1
1749	AP008696	G1a1a1	G1a1a1	O	1	G1a1a1	O	1
1750	AP009447	G1a1a1	G1a1a1	O	1	G1a1a1	O	1
1751	AP013178	G1a1a2	G1a1a2	O	1	G1a1a2	O	1
1752	AP011057	G1a1a2	G1a1a2	O	1	G1a1a2	O	1
1753	AP008909	G1a1a3	G1a1a3	O	1	G1a1a3	O	1
1754	AP008809	G1a1a3	G1a1a3	O	1	G1a1a3	O	1
1755	AP008845	G1a1a4	G1a1a4	O	1	G1a1a4	O	1
1756	AP011049	G1a1a4	G1a1a4	O	1	G1a1a4	O	1
1757	EF153773	G1a1b	G1a1b	O	1	G1a1b	O	1
1758	KF540834	G1a1b	G1a1b	O	1	G1a1b	O	1
1759	DQ272114	G1a2	G1a2	O	1	G1a2	O	1
1760	NA18764	G1a2	G1a2	O	1	G1a2	O	1
1761	AP013126	G1a3	G1a3	O	1	G1a3	O	1
1762	AP008618	G1a3	G1a3	O	1	G1a3	O	1
1763	KF148338	G1b1	G1b1	O	1	G1b1	O	1
1764	EF153826	G1b1	G1b1	O	1	G1b1	O	1
1765	KF148158	G1b+16129	G1b+16129	O	1	G1b+16129	O	1
1766	KF148075	G1b+16129	G1b+16129	O	1	G1b+16129	O	1
1767	EF153829	G1b2	G1b2	O	1	G1b2	O	1
1768	EU007841	G1b2	G1b2	O	1	G1b2	O	1
1769	KF148165	G1b3	G1b3	O	1	G1b3	O	1
1770	EF153805	G1b3	G1b3	O	1	G1b3	O	1
1771	KF148577	G1b4	G1b4	O	1	G1b4	O	1
1772	KF148275	G1b4	G1b4	O	1	G1b4	O	1
1773	AP012400	G1c	G1c	O	1	G1c	O	1

1774	EF153821	G1c	G1c	O	1	G1c	O	1
1775	AY255137	G1c1	G1c1	O	1	G1c1	O	1
1776	FJ748707	G1c1	G1c1	O	1	G1c1	O	1
1777	NA18570	G1c2	G1c2	O	1	G1c2	O	1
1778	FJ198218	G1c2	G1c2	O	1	G1c2	O	1
1779	FJ198217	G2a1	G2a1	O	1	G2a1	O	1
1780	AP008535	G2a1b	G2a1b	O	1	G2a1b	O	1
1781	AP008448	G2a1b	G2a1b	O	1	G2a1b	O	1
1782	AP008515	G2a1+16189	G2a1+16189	O	1	G2a1+16189	O	1
1783	AY255157	G2a1+16189	G2a1+16189	O	1	G2a1+16189	O	1
1784	AP008569	G2a1+16189+16194	G2a1+16189+16194	O	1	G2a1+16189+16194	O	1
1785	AP011017	G2a1c	G2a1c	O	1	G2a1c	O	1
1786	AP008452	G2a1c	G2a1c	O	1	G2a1c	O	1
1787	AP008401	G2a1c1	G2a1c1	O	1	G2a1c1	O	1
1788	AP008294	G2a1c1	G2a1c1	O	1	G2a1c1	O	1
1789	AP008662	G2a1c2	G2a1c2	O	1	G2a1c2	O	1
1790	AP010768	G2a1c2	G2a1c2	O	1	G2a1c2	O	1
1791	AP008427	G2a1d1	G2a1d1	O	1	G2a1d1	O	1
1792	AP008668	G2a1d1	G2a1d1	O	1	G2a1d1	O	1
1793	AP008313	G2a1d1a	G2a1d1a	O	1	G2a1d1a	O	1
1794	NA19003	G2a1d1a	G2a1d1a	O	1	G2a1d1a	O	1
1795	KF849931	G2a1d2	G2a1d2	O	1	G2a1d2	O	1
1796	HM030544	G2a1d2a	G2a1d2a	O	1	G2a1d2a	O	1
1797	NA18526	G2a1d2a	G2a1d2a	O	1	G2a1d2a	O	1
1798	AP008528	G2a1e	G2a1e	O	1	G2a1e	O	1
1799	AP010772	G2a1e	G2a1e	O	1	G2a1e	O	1
1800	NA18635	G2a1f	G2a1f	O	1	G2a1f	O	1
1801	KF849943	G2a1f1	G2a1f1	O	1	G2a1f1	O	1
1802	JF824853	G2a1f1	G2a1f1	O	1	G2a1f1	O	1
1803	KF849910	G2a1g	G2a1g	O	1	G2a1g	O	1
1804	HM460794	G2a1g	G2a1g	O	1	G2a1g	O	1
1805	HM036563	G2a1h	G2a1h	O	1	G2a1h	O	1
1806	FJ383503	G2a1h	G2a1h	O	1	G2a1h	O	1
1807	EU545470	G2a+152	G2a+152	O	1	G2a+152	O	1
1808	JF824917	G2a2	G2a2	O	1	G2a2	O	1
1809	JQ705820	G2a2a	G2a2a	O	1	G2a2a	O	1
1810	JX266266	G2a2a	G2a2a	O	1	G2a2a	O	1

1811	KC911517	G2a3	G2a3	O	1	G2a3	O	1
1812	GU123045	G2a3a	G2a3a	O	1	G2a3a	O	1
1813	EU523127	G2a3a	G2a3a	O	1	G2a3a	O	1
1814	JF824986	G2a4	G2a4	O	1	G2a4	O	1
1815	JX266262	G2a4	G2a4	O	1	G2a4	O	1
1816	AP008897	G2a5	G2a5	O	1	G2a5	O	1
1817	AP008301	G2a5	G2a5	O	1	G2a5	O	1
1818	JF824842	G2c	G2c	O	1	G2c	O	1
1819	KF849950	G2c	G2c	O	1	G2c	O	1
1820	FJ015040	G2b1a	G2b1a	O	1	G2b1a	O	1
1821	KF849921	G2b1a	G2b1a	O	1	G2b1a	O	1
1822	JX289093	G2b1a1	G2b1a1	O	1	G2b1a1	O	1
1823	JX289114	G2b1a1	G2b1a1	O	1	G2b1a1	O	1
1824	KF849956	G2b1a2	G2b1a2	O	1	G2b1a2	O	1
1825	AY255139	G2b1a2	G2b1a2	O	1	G2b1a2	O	1
1826	FJ383505	G2b1b	G2b1b	O	1	G2b1b	O	1
1827	FJ748756	G2b1b	G2b1b	O	1	G2b1b	O	1
1828	EU597571	G2b2	G2b2	O	1	G2b2	O	1
1829	FJ383509	G2b2a	G2b2a	O	1	G2b2a	O	1
1830	FJ383510	G2b2a	G2b2a	O	1	G2b2a	O	1
1831	HQ260973	G2b2b	G2b2b	O	1	G2b2b	O	1
1832	AP010829	G2b2b	G2b2b	O	1	G2b2b	O	1
1833	GU123035	G2b2c	G2b2c	O	1	G2b2c	O	1
1834	JF824915	G2b2c	G2b2c	O	1	G2b2c	O	1
1835	GU014566	G3a1	G3a1	O	1	G3a1	O	1
1836	DQ272108	G3a1a	G3a1a	O	1	G3a1a	O	1
1837	GU014569	G3a1a	G3a1a	O	1	G3a1a	O	1
1838	KF540726	G3a2	G3a2	O	1	G3a2	O	1
1839	JN580300	G3a2+152	G3a2+152	O	1	G3a2+152	O	1
1840	AP008645	G3a2a	G3a2a	O	1	G3a2a	O	1
1841	DQ272111	G3a2a	G3a2a	O	1	G3a2a	O	1
1842	KF849958	G3a3	G3a3	O	1	G3a3	O	1
1843	GU392096	G3a3	G3a3	O	1	G3a3	O	1
1844	FJ748726	G3b1	G3b1	O	1	G3b1	O	1
1845	AF346966	G3b1	G3b1	O	1	G3b1	O	1
1846	DQ272109	G3b2	G3b2	O	1	G3b2	O	1
1847	KF849979	G3b2	G3b2	O	1	G3b2	O	1

1848	AP008911	G4	G4	O	1	G4	O	1
1849	AP013144	G4	G4	O	1	G4	O	1
1850	JX462680	M13a1	M13a1	O	1	M13a1	O	1
1851	AP008495	M13a1a	M13a1a	O	1	M13a1a	O	1
1852	AP008370	M13a1a	M13a1a	O	1	M13a1a	O	1
1853	GU392104	M13a1b	M13a1b	O	1	M13a1b	O	1
1854	GQ895142	M13a1b	M13a1b	O	1	M13a1b	O	1
1855	JN857028	M13a1b1	M13a1b1	O	1	M13a1b1	O	1
1856	KF148509	M13a1b1	M13a1b1	O	1	M13a1b1	O	1
1857	FJ968773	M13a2	M13a2	O	1	M13a2	O	1
1858	FJ748749	M13a2	M13a2	O	1	M13a2	O	1
1859	AY963577	M13b1	M13b1	O	1	M13b1	O	1
1860	FJ770957	M13b1	M13b1	O	1	M13b1	O	1
1861	HM030529	M13b2	M13b2	O	1	M13b2	O	1
1862	FJ383769	M13b2	M13b2	O	1	M13b2	O	1
1863	JX289122	M13c	M13c	O	1	M13c	O	1
1864	GU810056	M13c	M13c	O	1	M13c	O	1
1865	KC505096	M46	M46	O	1	M46	O	1
1866	FJ442939	M46a	M46a	O	1	M46a	O	1
1867	GU810038	M46a	M46a	O	1	M46a	O	1
1868	JX289113	M61	M61	O	1	M61	O	1
1869	HM030538	M61a	M61a	O	1	M61a	O	1
1870	HM030546	M61a	M61a	O	1	M61a	O	1
1871	EF495222	M14	M14	O	1	M14	O	1
1872	DQ904234	M14	M14	O	1	M14	O	1
1873	EF495219	M15	M15	O	1	M15	O	1
1874	GU810076	M17a	M17a	O	1	M17a	O	1
1875	GQ301869	M17a	M17a	O	1	M17a	O	1
1876	GQ301876	M17c	M17c	O	1	M17c	O	1
1877	KC993910	M17c1	M17c1	O	1	M17c1	O	1
1878	GQ352635	M17c1a	M17c1a	O	1	M17c1a	O	1
1879	KC993929	M17c1a1	M17c1a1	O	1	M17c1a1	O	1
1880	KC505090	M17c1a1a	M17c1a1a	O	1	M17c1a1a	O	1
1881	EU597577	M17c1a1a	M17c1a1a	O	1	M17c1a1a	O	1
1882	JF739540	M19	M19	O	1	M19	O	1
1883	AY922302	M53	M53	O	1	M53	O	1
1884	FJ383438	M53b	M53b	O	1	M53b	O	1

1885	FJ383442	M53b	M53b	O	1	M53b	O	1
1886	AP012377	M21a	M21a	O	1	M21a	O	1
1887	AY963576	M21a	M21a	O	1	M21a	O	1
1888	KC505091	M21b1	M21b1	O	1	M21b1	O	1
1889	AY963581	M21b1a	M21b1a	O	1	M21b1a	O	1
1890	JF739541	M21b1a	M21b1a	O	1	M21b1a	O	1
1891	KC505092	M21b+210	M21b+210	O	1	M21b+210	O	1
1892	GQ301866	M21b2	error	X	error	M21b2	O	1
1893	GU810016	M21b2	M21b2	O	1	M21b2	O	1
1894	AY963583	M22a	M22a	O	1	M22a	O	1
1895	GQ301878	M22a	M22a	O	1	M22a	O	1
1896	GQ301880	M22b	M22b	O	1	M22b	O	1
1897	HM030499	M22b	M22b	O	1	M22b	O	1
1898	GQ389777	M23	M23	O	1	M23	O	1
1899	FJ543102	M23	M23	O	1	M23	O	1
1900	HM030540	M75	M75	O	1	M75	O	1
1901	HM030524	M75	M75	O	1	M75	O	1
1902	KC505094	M24a	M24a	O	1	M24a	O	1
1903	JF739543	M24a	M24a	O	1	M24a	O	1
1904	KC505093	M24b	M24b	O	1	M24b	O	1
1905	KF849906	M24b	M24b	O	1	M24b	O	1
1906	DQ513522	M41	M41	O	1	M41	O	1
1907	AY922280	M41a	M41a	O	1	M41a	O	1
1908	FJ383717	M41a1	M41a1	O	1	M41a1	O	1
1909	FJ383716	M41a1	M41a1	O	1	M41a1	O	1
1910	DQ513521	M41b	M41b	O	1	M41b	O	1
1911	FJ383722	M41b	M41b	O	1	M41b	O	1
1912	FJ383720	M41c	M41c	O	1	M41c	O	1
1913	GU480001	M41c	M41c	O	1	M41c	O	1
1914	KJ154681	M25	M25	O	1	M25	O	1
1915	KJ154709	M25	M25	O	1	M25	O	1
1916	HM596710	M26	M26	O	1	M26	O	1
1917	HM596670	M26	M26	O	1	M26	O	1
1918	DQ137411	M27a1a1	M27a1a1	O	1	M27a1a1	O	1
1919	DQ137410	M27a1a1	M27a1a1	O	1	M27a1a1	O	1
1920	KJ154546	M27a1a2	M27a1a2	O	1	M27a1a2	O	1
1921	KJ154498	M27a1a2	M27a1a2	O	1	M27a1a2	O	1

1922	KJ154715	M27a1b	M27a1b	O	1	M27a1b	O	1
1923	KJ154710	M27a1b	M27a1b	O	1	M27a1b	O	1
1924	KJ154533	M27a2a	M27a2a	O	1	M27a2a	O	1
1925	KJ154522	M27a2a	M27a2a	O	1	M27a2a	O	1
1926	KJ154494	M27a2b	M27a2b	O	1	M27a2b	O	1
1927	KJ154601	M27a2b	M27a2b	O	1	M27a2b	O	1
1928	KJ154488	M27a3	M27a3	O	1	M27a3	O	1
1929	KJ154632	M27a3	M27a3	O	1	M27a3	O	1
1930	DQ137403	M27b1	M27b1	O	1	M27b1	O	1
1931	DQ137402	M27b1	M27b1	O	1	M27b1	O	1
1932	KJ154728	M27b2	M27b2	O	1	M27b2	O	1
1933	KJ154720	M27b2	M27b2	O	1	M27b2	O	1
1934	KJ154707	M27b2a	M27b2a	O	1	M27b2a	O	1
1935	KJ154729	M27b2a1	M27b2a1	O	1	M27b2a1	O	1
1936	KJ154665	M27b2a1	M27b2a1	O	1	M27b2a1	O	1
1937	KJ154676	M27b2b	M27b2b	O	1	M27b2b	O	1
1938	KJ154654	M27b2b1	M27b2b1	O	1	M27b2b1	O	1
1939	KJ154792	M27b2b1	M27b2b1	O	1	M27b2b1	O	1
1940	KJ154721	M27b2c	M27b2c	O	1	M27b2c	O	1
1941	KJ154719	M27b2c	M27b2c	O	1	M27b2c	O	1
1942	KJ154770	M27c	M27c	O	1	M27c	O	1
1943	KJ154619	M27c	M27c	O	1	M27c	O	1
1944	DQ372879	M28a1	M28a1	O	1	M28a1	O	1
1945	DQ372883	M28a1	M28a1	O	1	M28a1	O	1
1946	KJ154478	M28a2	M28a2	O	1	M28a2	O	1
1947	EF061145	M28a2a	M28a2a	O	1	M28a2a	O	1
1948	KJ154253	M28a2a	M28a2a	O	1	M28a2a	O	1
1949	KJ154806	M28a3	M28a3	O	1	M28a3	O	1
1950	KJ154798	M28a3	M28a3	O	1	M28a3	O	1
1951	KJ154163	M28a4	M28a4	O	1	M28a4	O	1
1952	KJ154167	M28a4	M28a4	O	1	M28a4	O	1
1953	KJ154451	M28a5	M28a5	O	1	M28a5	O	1
1954	KJ154902	M28a5a	M28a5a	O	1	M28a5a	O	1
1955	KJ154913	M28a5a	M28a5a	O	1	M28a5a	O	1
1956	KJ154832	M28a5b	M28a5b	O	1	M28a5b	O	1
1957	DQ137401	M28a5b	M28a5b	O	1	M28a5b	O	1
1958	KJ154634	M28a6	M28a6	O	1	M28a6	O	1

1959	KJ154481	M28a6a	M28a6a	O	1	M28a6a	O	1
1960	KJ154495	M28a6a	M28a6a	O	1	M28a6a	O	1
1961	DQ137400	M28a7	M28a7	O	1	M28a7	O	1
1962	KJ154668	M28a7a	M28a7a	O	1	M28a7a	O	1
1963	KJ154667	M28a7a	M28a7a	O	1	M28a7a	O	1
1964	KJ154860	M28a7b	M28a7b	O	1	M28a7b	O	1
1965	KJ154859	M28a7b	M28a7b	O	1	M28a7b	O	1
1966	KJ154292	M28b	M28b	O	1	M28b	O	1
1967	KJ154666	M28b1	M28b1	O	1	M28b1	O	1
1968	DQ137399	M28b1	M28b1	O	1	M28b1	O	1
1969	DQ137408	M29a	M29a	O	1	M29a	O	1
1970	DQ137407	M29a	M29a	O	1	M29a	O	1
1971	EF495217	M29b	M29b	O	1	M29b	O	1
1972	KJ154801	M29b	M29b	O	1	M29b	O	1
1973	KJ154923	M29b1	M29b1	O	1	M29b1	O	1
1974	EF061147	M29b1	M29b1	O	1	M29b1	O	1
1975	AY289090	Q1	Q1	O	1	Q1	O	1
1976	AY289085	Q1a	Q1a	O	1	Q1a	O	1
1977	EU597495	Q1a1	Q1a1	O	1	Q1a1	O	1
1978	KJ154590	Q1a1a	Q1a1a	O	1	Q1a1a	O	1
1979	AY289082	Q1a1a	Q1a1a	O	1	Q1a1a	O	1
1980	KJ154866	Q1b	Q1b	O	1	Q1b	O	1
1981	DQ372882	Q1b	Q1b	O	1	Q1b	O	1
1982	KJ154885	Q1c	Q1c	O	1	Q1c	O	1
1983	KJ154631	Q1c1	Q1c1	O	1	Q1c1	O	1
1984	KJ154557	Q1c1a	Q1c1a	O	1	Q1c1a	O	1
1985	EU597543	Q1c1a	Q1c1a	O	1	Q1c1a	O	1
1986	KJ154909	Q1c2	Q1c2	O	1	Q1c2	O	1
1987	KJ154852	Q1c2	Q1c2	O	1	Q1c2	O	1
1988	KJ154875	Q1c2a	Q1c2a	O	1	Q1c2a	O	1
1989	KJ154879	Q1c2a	Q1c2a	O	1	Q1c2a	O	1
1990	KF540947	Q1d	Q1d	O	1	Q1d	O	1
1991	GU733817	Q1d	Q1d	O	1	Q1d	O	1
1992	KJ154939	Q1e	Q1e	O	1	Q1e	O	1
1993	KJ154444	Q1e1	Q1e1	O	1	Q1e1	O	1
1994	KJ154787	Q1e1a	Q1e1a	O	1	Q1e1a	O	1
1995	KJ154833	Q1e1a	Q1e1a	O	1	Q1e1a	O	1

1996	KJ154815	Q1e1a1	Q1e1a1	O	1	Q1e1a1	O	1
1997	KJ154812	Q1e1a1	Q1e1a1	O	1	Q1e1a1	O	1
1998	KJ154774	Q1e1b	Q1e1b	O	1	Q1e1b	O	1
1999	KJ154680	Q1e1b	Q1e1b	O	1	Q1e1b	O	1
2000	KJ154583	Q1e1b1	Q1e1b1	O	1	Q1e1b1	O	1
2001	KJ154603	Q1e1b1	Q1e1b1	O	1	Q1e1b1	O	1
2002	KJ154734	Q1e1c	Q1e1c	O	1	Q1e1c	O	1
2003	KJ154713	Q1e1c	Q1e1c	O	1	Q1e1c	O	1
2004	DQ372885	Q1f1	Q1f1	O	1	Q1f1	O	1
2005	DQ372884	Q1f1	Q1f1	O	1	Q1f1	O	1
2006	KJ154816	Q1f2	Q1f2	O	1	Q1f2	O	1
2007	KJ154797	Q1f2	Q1f2	O	1	Q1f2	O	1
2008	HQ113226	Q2a	Q2a	O	1	Q2a	O	1
2009	KJ154248	Q2a	Q2a	O	1	Q2a	O	1
2010	GQ214525	Q2a1	Q2a1	O	1	Q2a1	O	1
2011	GQ214527	Q2a1	Q2a1	O	1	Q2a1	O	1
2012	GQ214526	Q2a2a	Q2a2a	O	1	Q2a2a	O	1
2013	KJ154936	Q2a2a	Q2a2a	O	1	Q2a2a	O	1
2014	KJ154178	Q2a2b	Q2a2b	O	1	Q2a2b	O	1
2015	GQ214521	Q2a2b	Q2a2b	O	1	Q2a2b	O	1
2016	AY956412	Q2a3a	Q2a3a	O	1	Q2a3a	O	1
2017	KJ154839	Q2a3a	Q2a3a	O	1	Q2a3a	O	1
2018	KJ154799	Q2a3b	Q2a3b	O	1	Q2a3b	O	1
2019	KJ154822	Q2a3b	Q2a3b	O	1	Q2a3b	O	1
2020	KJ154824	Q2a4	Q2a4	O	1	Q2a4	O	1
2021	GQ214522	Q2a4	Q2a4	O	1	Q2a4	O	1
2022	EF495218	Q2b	Q2b	O	1	Q2b	O	1
2023	AY289079	Q3a	Q3a	O	1	Q3a	O	1
2024	AY289089	Q3a1	Q3a1	O	1	Q3a1	O	1
2025	EU597519	Q3a1	Q3a1	O	1	Q3a1	O	1
2026	EF061146	Q3b	Q3b	O	1	Q3b	O	1
2027	AY950298	M31a1a	M31a1a	O	1	M31a1a	O	1
2028	DQ149515	M31a1b	M31a1b	O	1	M31a1b	O	1
2029	DQ408673	M31a1b	M31a1b	O	1	M31a1b	O	1
2030	HQ438684	M31a2	M31a2	O	1	M31a2	O	1
2031	EF060265	M31a2	M31a2	O	1	M31a2	O	1
2032	HQ438686	M31b1	M31b1	O	1	M31b1	O	1

2033	HQ438685	M31b1	M31b1	O	1	M31b1	O	1
2034	FJ770962	M31c	M31c	O	1	M31c	O	1
2035	DQ149512	M32a	M32a	O	1	M32a	O	1
2036	AY950296	M32a	M32a	O	1	M32a	O	1
2037	GQ389779	M32c	M32c	O	1	M32c	O	1
2038	NA19982	M32c	M32c	O	1	M32c	O	1
2039	FJ383487	M56	M56	O	1	M56	O	1
2040	FJ383486	M56	M56	O	1	M56	O	1
2041	FJ770960	M33a	M33a	O	1	M33a	O	1
2042	FJ383339	M33a1a	M33a1a	O	1	M33a1a	O	1
2043	FJ770959	M33a1a	M33a1a	O	1	M33a1a	O	1
2044	FJ383353	M33a1b	M33a1b	O	1	M33a1b	O	1
2045	DQ408680	M33a1b	M33a1b	O	1	M33a1b	O	1
2046	FJ383361	M33a2	M33a2	O	1	M33a2	O	1
2047	FJ770969	M33a2	M33a2	O	1	M33a2	O	1
2048	AY922276	M33a2a	M33a2a	O	1	M33a2a	O	1
2049	JN540042	M33a2a	M33a2a	O	1	M33a2a	O	1
2050	FJ770950	M33a3	M33a3	O	1	M33a3	O	1
2051	JX289104	M33a3a	M33a3a	O	1	M33a3a	O	1
2052	FJ383359	M33a3a	M33a3a	O	1	M33a3a	O	1
2053	FJ770968	M33b	M33b	O	1	M33b	O	1
2054	FJ383362	M33b1	M33b1	O	1	M33b1	O	1
2055	HM030510	M33b1	M33b1	O	1	M33b1	O	1
2056	AY922298	M33b2	M33b2	O	1	M33b2	O	1
2057	JF742198	M33b2	M33b2	O	1	M33b2	O	1
2058	EU148486	M33c	M33c	O	1	M33c	O	1
2059	HM030517	M33c	M33c	O	1	M33c	O	1
2060	FJ383346	M33d	M33d	O	1	M33d	O	1
2061	JX462713	M33d	M33d	O	1	M33d	O	1
2062	JX462718	M34a1	M34a1	O	1	M34a1	O	1
2063	JX289117	M34a1a	M34a1a	O	1	M34a1a	O	1
2064	AY922304	M34a1a	M34a1a	O	1	M34a1a	O	1
2065	FJ383682	M34a2	M34a2	O	1	M34a2	O	1
2066	FJ383680	M34a2	M34a2	O	1	M34a2	O	1
2067	AY922274	M34b	M34b	O	1	M34b	O	1
2068	FJ383681	M34b	M34b	O	1	M34b	O	1
2069	FJ383760	M57+152	M57+152	O	1	M57+152	O	1

2070	FJ383763	M57a	M57a	O	1	M57a	O	1
2071	FJ383764	M57a	M57a	O	1	M57a	O	1
2072	FJ383761	M57b	M57b	O	1	M57b	O	1
2073	FJ383759	M57b1	M57b1	O	1	M57b1	O	1
2074	FJ383758	M57b1	M57b1	O	1	M57b1	O	1
2075	FJ770956	M35	M35	O	1	M29'Q M35	X	2
2076	FJ383365	M35a1	M35a1	O	1	M35a1	O	1
2077	FJ383368	M35a1	M35a1	O	1	M35a1	O	1
2078	FJ383384	M35a1a	M35a1a	O	1	M35a1a	O	1
2079	AY289074	M35a1a	M35a1a	O	1	M35a1a	O	1
2080	FJ383363	M35a2	M35a2	O	1	M35a2	O	1
2081	AY922279	M35a2	M35a2	O	1	M35a2	O	1
2082	AY922272	M35b	M35b	O	1	M35b	O	1
2083	FJ770966	M35b+16304	M35b+16304	O	1	M35b+16304	O	1
2084	FJ383378	M35b1	M35b1	O	1	M35b1	O	1
2085	FJ383374	M35b1	M35b1	O	1	M35b1	O	1
2086	FJ383381	M35b2	M35b2	O	1	M35b2	O	1
2087	FJ383373	M35b2	M35b2	O	1	M35b2	O	1
2088	HM036535	M35b3	M35b3	O	1	M35b3	O	1
2089	HM036559	M35b3	M35b3	O	1	M35b3	O	1
2090	FJ383370	M35b4	M35b4	O	1	M35b4	O	1
2091	JF742208	M35b4	M35b4	O	1	M35b4	O	1
2092	FJ383382	M35c	M35c	O	1	M35c	O	1
2093	FJ383366	M35c	M35c	O	1	M35c	O	1
2094	AY922287	M36a	M36a	O	1	M36a	O	1
2095	AY922284	M36b	M36b	O	1	M36b	O	1
2096	FJ383808	M36c	M36c	O	1	M36c	O	1
2097	FJ383795	M36c	M36c	O	1	M36c	O	1
2098	FJ383807	M36d	M36d	O	1	M36d	O	1
2099	FJ383784	M36d1	M36d1	O	1	M36d1	O	1
2100	FJ383803	M36d1	M36d1	O	1	M36d1	O	1
2101	AY922288	M39	M39'70	X	3	M39	O	1
2102	FJ383714	M39a	M39'70	X	2	M39a	O	1
2103	FJ383710	M39a1	M39a1	O	1	M39a1	O	1
2104	EF556151	M39a1	M39a1	O	1	M39a1	O	1
2105	FJ383705	M39a2	M39a2	O	1	M39a2	O	1
2106	FJ383712	M39a2	M39a2	O	1	M39a2	O	1

2107	FJ383699	M39b1	M39b1	O	1	M39b1	O	1
2108	AY922275	M39b1	M39b1	O	1	M39b1	O	1
2109	FJ383713	M39b2	M39b2	O	1	M39b2	O	1
2110	FJ383702	M39b2	M39b2	O	1	M39b2	O	1
2111	AY922293	M39c	M39c	O	1	M39c	O	1
2112	AY922269	M39c	M39c	O	1	M39c	O	1
2113	HM030534	M70	M70	O	1	M70	O	1
2114	FJ748741	M70	M70	O	1	M70	O	1
2115	AY922294	M40	M40	O	1	M40	O	1
2116	FJ383416	M40a	M40a	O	1	M40a	O	1
2117	AY922271	M40a	M40a	O	1	M40a	O	1
2118	FJ383412	M40a1	M40a1	O	1	M40a1	O	1
2119	FJ383407	M40a1	M40a1	O	1	M40a1	O	1
2120	FJ383413	M40a1a	M40a1a	O	1	M40a1a	O	1
2121	FJ383425	M40a1a	M40a1a	O	1	M40a1a	O	1
2122	FJ383417	M40a1b	M40a1b	O	1	M40a1b	O	1
2123	FJ383419	M40a1b	M40a1b	O	1	M40a1b	O	1
2124	DQ404445	M42a	M42a	O	1	M42a	O	1
2125	HQ260949	M42b1	M42b1	O	1	M42b1	O	1
2126	FJ380213	M42b1	M42b1	O	1	M42b1	O	1
2127	FJ380210	M42b1a	M42b1a	O	1	M42b1a	O	1
2128	FJ383745	M42b1a	M42b1a	O	1	M42b1a	O	1
2129	KC577356	M42b2	M42b2	O	1	M42b2	O	1
2130	FJ380216	M42b2	M42b2	O	1	M42b2	O	1
2131	GU377083	M74a	M74a	O	1	M74a	O	1
2132	HM030539	M74a	M74a	O	1	M74a	O	1
2133	HM030520	M74b	M74b	O	1	M74b	O	1
2134	HM596679	M74b1	M74b1	O	1	M74b1	O	1
2135	JX289120	M74b1	M74b1	O	1	M74b1	O	1
2136	AP012354	M74b2	M74b2	O	1	M74b2	O	1
2137	HM596667	M74b2	M74b2	O	1	M74b2	O	1
2138	FJ383750	M44	M44	O	1	M44	O	1
2139	AY922253	M44a	M44a	O	1	M44a	O	1
2140	FJ383748	M44a	M44a	O	1	M44a	O	1
2141	FJ383751	M44a1	M44a1	O	1	M44a1	O	1
2142	FJ383752	M44a1	M44a1	O	1	M44a1	O	1
2143	HM030526	M49a1	M49a1	O	1	M49a1	O	1

2144	FJ383735	M49a1	M49a1	O	1	M49a1	O	1
2145	FJ383766	M49c	M49c	O	1	M49c	O	1
2146	JX289106	M49c1	M49c1	O	1	M49c1	O	1
2147	FJ383768	M49c1	M49c1	O	1	M49c1	O	1
2148	FJ383765	M49d	M49d	O	1	M49d	O	1
2149	FJ770940	M49d	M49d	O	1	M49d	O	1
2150	JX289133	M49e	M49e	O	1	M49e	O	1
2151	JX289094	M49e1	M49e1	O	1	M49e1	O	1
2152	JX289099	M49e1	M49e1	O	1	M49e1	O	1
2153	GQ301868	M50	M50	O	1	M50	O	1
2154	GU810023	M50a1	M50a1	O	1	M50a1	O	1
2155	GU810018	M50a1	M50a1	O	1	M50a1	O	1
2156	KC994151	M50a2	M50a2	O	1	M50a2	O	1
2157	JX289098	M50a2	M50a2	O	1	M50a2	O	1
2158	JQ703445	M52a	M52a	O	1	M52a	O	1
2159	FJ157841	M52a1a	M52a1a	O	1	M52a1a	O	1
2160	FJ157847	M52a1a	M52a1a	O	1	M52a1a	O	1
2161	JX462717	M52a1b	M52a1b	O	1	M52a1b	O	1
2162	KC993960	M52a1b1	M52a1b1	O	1	M52a1b1	O	1
2163	KC993961	M52a1b1	M52a1b1	O	1	M52a1b1	O	1
2164	JF742203	M52b	M52b	O	1	M52b	O	1
2165	AY922299	M52b	M52b	O	1	M52b	O	1
2166	FJ770964	M52b1	M52b1	O	1	M52b1	O	1
2167	FJ383488	M52b1a	M52b1a	O	1	M52b1a	O	1
2168	FJ383489	M52b1a	M52b1a	O	1	M52b1a	O	1
2169	KC896622	M55	M55	O	1	M55	O	1
2170	AY963582	M55	M55	O	1	M55	O	1
2171	GQ301865	M77	M77	O	1	M77	O	1
2172	FJ383493	M58	M58	O	1	M58	O	1
2173	JX289110	M58	M58	O	1	M58	O	1
2174	JQ702247	M59	M59	O	1	M59	O	1
2175	KC505104	M59	M59	O	1	M59	O	1
2176	FJ383496	M60a1	M60a1	O	1	M60a1	O	1
2177	FJ383495	M60a1	M60a1	O	1	M60a1	O	1
2178	FJ383498	M60a2	M60a2	O	1	M60a2	O	1
2179	FJ383494	M60a2	M60a2	O	1	M60a2	O	1
2180	FJ383500	M60b	M60b	O	1	M60b	O	1

2181	JF742204	M60b	M60b	O	1	M60b	O	1
2182	FJ383501	M62a	M62a	O	1	M62a	O	1
2183	GQ895161	M62a	M62a	O	1	M62a	O	1
2184	FJ544231	M62b	M62b	O	1	M62b	O	1
2185	FJ544239	M62b+204	M62b+204	O	1	M62b+204	O	1
2186	GQ895169	M62b1	M62b1	O	1	M62b1	O	1
2187	FJ544241	M62b1a	M62b1	X	6	M62b1a	O	1
2188	FJ544235	M62b1a1	M62b1	X	5	M62b1a1	O	1
2189	FJ544243	M62b1a1	M62b1	X	5	M62b1a1	O	1
2190	FJ544233	M62b2	M62b2	O	1	M62b2	O	1
2191	FJ748717	M62b2	M62b2	O	1	M62b2	O	1
2192	JX289131	M68	M68	O	1	M68	O	1
2193	KC887488	M68a1	M68a1	O	1	M68a1	O	1
2194	KC887480	M68a1a	M68a1a	O	1	M68a1a	O	1
2195	KC887484	M68a1a	M68a1a	O	1	M68a1a	O	1
2196	KC887491	M68a2	M68a2	O	1	M68a2	O	1
2197	KC505106	M68a2a	M68a2a	O	1	M68a2a	O	1
2198	KC887477	M68a2a	M68a2a	O	1	M68a2a	O	1
2199	HM596653	M69	M69	O	1	M69	O	1
2200	KC887492	M69a	M69a	O	1	M69a	O	1
2201	KC505108	M69a	M69a	O	1	M69a	O	1
2202	KC505110	M71+151	M71+151	O	1	M71+151	O	1
2203	JX289103	M71+151	M71+151	O	1	M71+151	O	1
2204	GQ119008	M71a	M71a	O	1	M71a	O	1
2205	HM030543	M71a1	M71a1	O	1	M71a1	O	1
2206	HM030502	M71a1a	M71a1a	O	1	M71a1a	O	1
2207	KC733256	M71a1a	M71a1a	O	1	M71a1a	O	1
2208	JX289128	M71a2	M71a2	O	1	M71a2	O	1
2209	GQ119038	M71a2	M71a2	O	1	M71a2	O	1
2210	HM030511	M71b	M71b	O	1	M71b	O	1
2211	HM030507	M71b	M71b	O	1	M71b	O	1
2212	GU810039	M71c	M71c	O	1	M71c	O	1
2213	GQ301872	M71c	M71c	O	1	M71c	O	1
2214	HM030501	M72	M72	O	1	M72	O	1
2215	JX289126	M72a	M72a	O	1	M72a	O	1
2216	GQ119044	M72a	M72a	O	1	M72a	O	1
2217	GQ119039	M73a	M73a	O	1	M73a	O	1

2218	GQ119017	M73a1	M73a1	O	1	M73a1	O	1
2219	GQ301883	M73a1	M73a1	O	1	M73a1	O	1
2220	GQ119012	M73b	M73b	O	1	M73b	O	1
2221	KC505111	M73b	M73b	O	1	M73b	O	1
2222	HM030504	M79	M79	O	1	M79	O	1
2223	HM030525	M76	M76	O	1	M76	O	1
2224	HM030541	M76a	M76a	O	1	M76a	O	1
2225	JF824857	M76a	M76a	O	1	M76a	O	1
2226	KC577353	M81	M81	O	1	M81	O	1
2227	JX462697	M81	M81	O	1	M81	O	1
2228	JX289097	M91a	M91a	O	1	M91a	O	1
2229	HM030537	M91a	M91a	O	1	M91a	O	1
2230	KC887489	M91b	M91b	O	1	M91b	O	1
2231	KC505114	M91b	M91b	O	1	M91b	O	1
2232	JF739537	M80	M80	O	1	M80	O	1
2233	JQ704974	D4	D4	O	1	D4	O	1
2234	JN375993	D4	D4	O	1	D4	O	1
2235	JN253391	D1	D1	O	1	D1	O	1
2236	EU095232	D1	D1	O	1	D1	O	1
2237	EU095233	D1a1	D1a1	O	1	D1a1	O	1
2238	EU095234	D1a1	D1a1	O	1	D1a1	O	1
2239	AF346984	D1a2	D1a2	O	1	D1a2	O	1
2240	DQ282479	D1b	D1b	O	1	D1b	O	1
2241	DQ282484	D1b	D1b	O	1	D1b	O	1
2242	HQ012249	D1c	D1c	O	1	D1c	O	1
2243	DQ282477	D1c	D1c	O	1	D1c	O	1
2244	HQ012255	D1d1	D1d1	O	1	D1d1	O	1
2245	DQ282480	D1d1	D1d1	O	1	D1d1	O	1
2246	HQ012245	D1d2	D1d2	O	1	D1d2	O	1
2247	NA19716	D1d2	D1d2	O	1	D1d2	O	1
2248	EU597510	D1e	D1e	O	1	D1e	O	1
2249	EU095236	D1e	D1e	O	1	D1e	O	1
2250	EU095536	D1f	D1f	O	1	D1f	O	1
2251	EU095235	D1f1	D1f1	O	1	D1f1	O	1
2252	EU095240	D1f1	D1f1	O	1	D1f1	O	1
2253	JQ702137	D1f2	D1f2	O	1	D1f2	O	1
2254	HG01441	D1f2	D1f2	O	1	D1f2	O	1

2255	EU431089	D1f+16189	D1f+16189	O	1	D1f+16189	O	1
2256	JQ701868	D1f3	D1f3	O	1	D1f3	O	1
2257	HQ012257	D1f3	D1f3	O	1	D1f3	O	1
2258	JN253393	D1g	D1g	O	1	D1g	O	1
2259	JN253395	D1g1a	D1g1a	O	1	D1g1a	O	1
2260	JN253394	D1g1a	D1g1a	O	1	D1g1a	O	1
2261	JN253399	D1g1b	D1g1b	O	1	D1g1b	O	1
2262	JN253400	D1g1b	D1g1b	O	1	D1g1b	O	1
2263	JN253401	D1g2	D1g2	O	1	D1g2	O	1
2264	JN253404	D1g2a	D1g2a	O	1	D1g2a	O	1
2265	JN253405	D1g2a	D1g2a	O	1	D1g2a	O	1
2266	JN253415	D1g5	D1g5	O	1	D1g5	O	1
2267	JN253414	D1g5	D1g5	O	1	D1g5	O	1
2268	JN253409	D1g3	D1g3	O	1	D1g3	O	1
2269	JN253407	D1g3	D1g3	O	1	D1g3	O	1
2270	JN253411	D1g4	D1g4	O	1	D1g4	O	1
2271	JN253410	D1g4	D1g4	O	1	D1g4	O	1
2272	JN253418	D1g6	D1g6	O	1	D1g6	O	1
2273	JN253417	D1g6	D1g6	O	1	D1g6	O	1
2274	HQ012251	D1h1	D1h1	O	1	D1h1	O	1
2275	NA19661	D1h1	D1h1	O	1	D1h1	O	1
2276	HQ012258	D1h2	D1h2	O	1	D1h2	O	1
2277	NA19728	D1h2	D1h2	O	1	D1h2	O	1
2278	DQ282487	D1i	D1i	O	1	D1i	O	1
2279	NA19747	D1i1	D1i1	O	1	D1i1	O	1
2280	HQ012247	D1i1	D1i1	O	1	D1i1	O	1
2281	NA19741	D1i2	D1i2	O	1	D1i2	O	1
2282	HQ012246	D1i2	D1i2	O	1	D1i2	O	1
2283	JN253419	D1j	D1j	O	1	D1j	O	1
2284	JN253420	D1j	D1j	O	1	D1j	O	1
2285	JN253422	D1j1	D1j1	O	1	D1j1	O	1
2286	JN253421	D1j1	D1j1	O	1	D1j1	O	1
2287	JN253423	D1j1a	D1j1a	O	1	D1j1a	O	1
2288	JN253424	D1j1a	D1j1a	O	1	D1j1a	O	1
2289	JN253431	D1j1a1	D1j1a1	O	1	D1j1a1	O	1
2290	JN253430	D1j1a1	D1j1a1	O	1	D1j1a1	O	1
2291	JN253434	D1j1a2	D1j1a2	O	1	D1j1a2	O	1

2292	JN253435	D1j1a2	D1j1a2	O	1	D1j1a2	O	1
2293	DQ282483	D1k	D1k	O	1	D1k	O	1
2294	NA19755	D1k	D1k	O	1	D1k	O	1
2295	JQ704599	D1m	D1m	O	1	D1m	O	1
2296	HQ012256	D1m	D1m	O	1	D1m	O	1
2297	JQ703865	D1n	D1n	O	1	D1n	O	1
2298	DQ282482	D1n	D1n	O	1	D1n	O	1
2299	AP009430	D4a1	D4a1	O	1	D4a1	O	1
2300	AP008444	D4a1	D4a1	O	1	D4a1	O	1
2301	AP010710	D4a1a	D4a1a	O	1	D4a1a	O	1
2302	AP010723	D4a1a1	D4a1a1	O	1	D4a1a1	O	1
2303	AP008745	D4a1a1	D4a1a1	O	1	D4a1a1	O	1
2304	AP010669	D4a1a1a	D4a1a1a	O	1	D4a1a1a	O	1
2305	AP008398	D4a1a1a	D4a1a1a	O	1	D4a1a1a	O	1
2306	AP008314	D4a1b	D4a1b	O	1	D4a1b	O	1
2307	AP010760	D4a1b	D4a1b	O	1	D4a1b	O	1
2308	AP010762	D4a1b1	D4a1b1	O	1	D4a1b1	O	1
2309	AP008334	D4a1b1	D4a1b1	O	1	D4a1b1	O	1
2310	AP008312	D4a1c	D4a1c	O	1	D4a1c	O	1
2311	AP008449	D4a1c	D4a1c	O	1	D4a1c	O	1
2312	AP008871	D4a1d	D4a1d	O	1	D4a1d	O	1
2313	AP008805	D4a1d	D4a1d	O	1	D4a1d	O	1
2314	KF148380	D4a1e	D4a1e	O	1	D4a1e	O	1
2315	JF824827	D4a1e	D4a1e	O	1	D4a1e	O	1
2316	AP008614	D4a1e1	D4a1e1	O	1	D4a1e1	O	1
2317	AP010713	D4a1e1	D4a1e1	O	1	D4a1e1	O	1
2318	AP010737	D4a1f	D4a1f	O	1	D4a1f	O	1
2319	AP013245	D4a1f1	D4a1f1	O	1	D4a1f1	O	1
2320	JQ702207	D4a1f1	D4a1f1	O	1	D4a1f1	O	1
2321	JF824972	D4a1g	D4a1g	O	1	D4a1g	O	1
2322	FJ951517	D4a1g	D4a1g	O	1	D4a1g	O	1
2323	AP008497	D4a1h	D4a1h	O	1	D4a1h	O	1
2324	AP013113	D4a1h	D4a1h	O	1	D4a1h	O	1
2325	AP013248	D4a2	D4a2	O	1	D4a2	O	1
2326	AP008765	D4a2	D4a2	O	1	D4a2	O	1
2327	AP011008	D4a2a	D4a2a	O	1	D4a2a	O	1
2328	AP008839	D4a2a	D4a2a	O	1	D4a2a	O	1

2329	AP013286	D4a2b	D4a2b	O	1	D4a2b	O	1
2330	AP010663	D4a2b	D4a2b	O	1	D4a2b	O	1
2331	JF824835	D4a3a1	D4a3a1	O	1	D4a3a1	O	1
2332	JF271009	D4a3a1	D4a3a1	O	1	D4a3a1	O	1
2333	AP008862	D4a3a2	D4a3a2	O	1	D4a3a2	O	1
2334	AP008907	D4a3a2	D4a3a2	O	1	D4a3a2	O	1
2335	KF849918	D4a3b	D4a3b	O	1	D4a3b	O	1
2336	AP011047	D4a3b1	D4a3b1	O	1	D4a3b1	O	1
2337	AP009445	D4a3b1	D4a3b1	O	1	D4a3b1	O	1
2338	JF824944	D4a3b2	D4a3b2	O	1	D4a3b2	O	1
2339	AY255160	D4a3b2	D4a3b2	O	1	D4a3b2	O	1
2340	AP008582	D4a+16294	D4a+16294	O	1	D4a+16294	O	1
2341	AP008888	D4a4	D4a4	O	1	D4a4	O	1
2342	FJ748716	D4a5	D4a5	O	1	D4a5	O	1
2343	JX289096	D4a5	D4a5	O	1	D4a5	O	1
2344	KF849929	D4a6	D4a6	O	1	D4a6	O	1
2345	KC577361	D4a6	D4a6	O	1	D4a6	O	1
2346	KF540709	D4a7	D4a7	O	1	D4a7	O	1
2347	KF540686	D4a7	D4a7	O	1	D4a7	O	1
2348	KF540683	D4a8	D4a8	O	1	D4a8	O	1
2349	KF540715	D4a8	D4a8	O	1	D4a8	O	1
2350	AP008876	D4b1a1	D4b1a1	O	1	D4b1a1	O	1
2351	AP010720	D4b1a1	D4b1a1	O	1	D4b1a1	O	1
2352	AP008332	D4b1a1a	D4b1a1a	O	1	D4b1a1a	O	1
2353	AP008767	D4b1a1a	D4b1a1a	O	1	D4b1a1a	O	1
2354	EU482305	D4b1a2	D4b1a2	O	1	D4b1a2	O	1
2355	FJ951581	D4b1a2a	D4b1a2a	O	1	D4b1a2a	O	1
2356	NA18535	D4b1a2a	D4b1a2a	O	1	D4b1a2a	O	1
2357	EU007895	D4b1a2a1	D4b1a2a1	O	1	D4b1a2a1	O	1
2358	FJ951490	D4b1a2a1	D4b1a2a1	O	1	D4b1a2a1	O	1
2359	EU482385	D4b1a2a2	D4b1a2a2	O	1	D4b1a2a2	O	1
2360	FJ951487	D4b1a2a2	D4b1a2a2	O	1	D4b1a2a2	O	1
2361	AP008254	D4b1b1	D4b1b1	O	1	D4b1b1	O	1
2362	AP008440	D4b1b1a	D4b1b1a	O	1	D4b1b1a	O	1
2363	AP008410	D4b1b1a	D4b1b1a	O	1	D4b1b1a	O	1
2364	AP008476	D4b1b1a1	D4b1b1a1	O	1	D4b1b1a1	O	1
2365	AP011029	D4b1b1a1	D4b1b1a1	O	1	D4b1b1a1	O	1

2366	AP008708	D4b1b2	D4b1b	X	6	D4b1b2	O	1
2367	AY255165	D4b1b2	D4b1b	X	6	D4b1b2	O	1
2368	HM030519	D4b1d	D4b1d	O	1	D4b1d	O	1
2369	NA18789	D4b1d	D4b1d	O	1	D4b1d	O	1
2370	JQ703736	D4b1c	D4b1c	O	1	D4b1c	O	1
2371	EU482316	D3	D3	O	1	D3	O	1
2372	EF153800	D3	D3	O	1	D3	O	1
2373	AP011004	D4b2	D4b2	O	1	D4b2	O	1
2374	AP008434	D4b2a	D4b2a	O	1	D4b2a	O	1
2375	AP008286	D4b2a1	D4b2a1	O	1	D4b2a1	O	1
2376	AP008502	D4b2a1	D4b2a1	O	1	D4b2a1	O	1
2377	AP013190	D4b2a2	D4b2a2	O	1	D4b2a2	O	1
2378	AP008703	D4b2a2a	D4b2a2a	O	1	D4b2a2a	O	1
2379	AP008463	D4b2a2a	D4b2a2a	O	1	D4b2a2a	O	1
2380	AP008287	D4b2a2a1	D4b2a2a1	O	1	D4b2a2a1	O	1
2381	AP008868	D4b2a2a1	D4b2a2a1	O	1	D4b2a2a1	O	1
2382	AP013213	D4b2a2a2	D4b2a2a2	O	1	D4b2a2a2	O	1
2383	AP008303	D4b2a2a2	D4b2a2a2	O	1	D4b2a2a2	O	1
2384	AP010697	D4b2a2b	D4b2a2b	O	1	D4b2a2b	O	1
2385	AP008804	D4b2a2b	D4b2a2b	O	1	D4b2a2b	O	1
2386	AP008757	D4b2b	D4b2b	O	1	D4b2b	O	1
2387	NA18702	D4b2b	D4b2b	O	1	D4b2b	O	1
2388	AP008264	D4b2b1	D4b2b1	O	1	D4b2b1	O	1
2389	AF346989	D4b2b1	D4b2b1	O	1	D4b2b1	O	1
2390	AP008467	D4b2b1a	D4b2b1a	O	1	D4b2b1a	O	1
2391	AP008523	D4b2b1a	D4b2b1a	O	1	D4b2b1a	O	1
2392	AP008333	D4b2b1b	D4b2b1b	O	1	D4b2b1b	O	1
2393	AP008302	D4b2b1b	D4b2b1b	O	1	D4b2b1b	O	1
2394	AP013251	D4b2b1c	D4b2b1c	O	1	D4b2b1c	O	1
2395	NA19070	D4b2b1c	D4b2b1c	O	1	D4b2b1c	O	1
2396	AP008292	D4b2b1+146	D4b2b1+146	O	1	D4b2b1+146	O	1
2397	AP008361	D4b2b1+146	D4b2b1+146	O	1	D4b2b1+146	O	1
2398	AP013158	D4b2b1d	D4b2b1d	O	1	D4b2b1d	O	1
2399	EU597488	D4b2b1d	D4b2b1d	O	1	D4b2b1d	O	1
2400	KF849955	D4b2b2	D4b2b2	O	1	D4b2b2	O	1
2401	JF824962	D4b2b2a	D4b2b2a	O	1	D4b2b2a	O	1
2402	AP008457	D4b2b2a1	D4b2b2a1	O	1	D4b2b2a1	O	1

2403	AP008684	D4b2b2a1	D4b2b2a1	O	1	D4b2b2a1	O	1
2404	HM030530	D4b2b2b	D4b2b2b	O	1	D4b2b2b	O	1
2405	NA18647	D4b2b2b	D4b2b2b	O	1	D4b2b2b	O	1
2406	NA18739	D4b2b2c	D4b2b2c	O	1	D4b2b2c	O	1
2407	FJ951498	D4b2b2c	D4b2b2c	O	1	D4b2b2c	O	1
2408	AP013111	D4b2b3	D4b2b3	O	1	D4b2b3	O	1
2409	AP008579	D4b2b3	D4b2b3	O	1	D4b2b3	O	1
2410	FJ383213	D4b2b4	D4b2b4	O	1	D4b2b4	O	1
2411	FJ493502	D4b2b4	D4b2b4	O	1	D4b2b4	O	1
2412	FJ951535	D4b2b5	D4b2b5	O	1	D4b2b5	O	1
2413	FJ951470	D4b2b5	D4b2b5	O	1	D4b2b5	O	1
2414	NA18145	D4b2b6	D4b2b6	O	1	D4b2b6	O	1
2415	NA18625	D4b2b6	D4b2b6	O	1	D4b2b6	O	1
2416	KF540670	D4b2b7	D4b2b7	O	1	D4b2b7	O	1
2417	JQ704956	D4b2b7	D4b2b7	O	1	D4b2b7	O	1
2418	FJ951514	D4b2d	D4b2d	O	1	D4b2d	O	1
2419	FJ951541	D4b2d	D4b2d	O	1	D4b2d	O	1
2420	AP008633	D4c1a	D4c1a	O	1	D4c1a	O	1
2421	AP010983	D4c1a	D4c1a	O	1	D4c1a	O	1
2422	AP009433	D4c1a1	D4c1a1	O	1	D4c1a1	O	1
2423	AP008431	D4c1a1	D4c1a1	O	1	D4c1a1	O	1
2424	AP008251	D4c1b	D4c1b	O	1	D4c1b	O	1
2425	AP008494	D4c1b1	D4c1b1	O	1	D4c1b1	O	1
2426	AP010830	D4c1b1	D4c1b1	O	1	D4c1b1	O	1
2427	AP013273	D4c1b2	D4c1b2	O	1	D4c1b2	O	1
2428	AP008609	D4c1b2	D4c1b2	O	1	D4c1b2	O	1
2429	HM776716	D4c2a	D4c2a	O	1	D4c2a	O	1
2430	FJ951494	D4c2a	D4c2a	O	1	D4c2a	O	1
2431	FJ858889	D4c2b	D4c2b	O	1	D4c2b	O	1
2432	FJ951602	D4c2b	D4c2b	O	1	D4c2b	O	1
2433	AP008348	D4c2c	D4c2c	O	1	D4c2c	O	1
2434	AP010711	D4c2c	D4c2c	O	1	D4c2c	O	1
2435	AP008628	D4d	D4d	O	1	D4d	O	1
2436	NA17981	D4e1	D4e1	O	1	D4e1	O	1
2437	AP008789	D4e1a	D4e1a	O	1	D4e1a	O	1
2438	AP008275	D4e1a1	D4e1a1	O	1	D4e1a1	O	1
2439	AP008624	D4e1a1	D4e1a1	O	1	D4e1a1	O	1

2440	FJ383207	D4e1a2	D4e1a2	O	1	D4e1a2	O	1
2441	AP008772	D4e1a2a	D4e1a2a	O	1	D4e1a2a	O	1
2442	AP008637	D4e1a2a	D4e1a2a	O	1	D4e1a2a	O	1
2443	NA18685	D4e1a3	D4e1a3	O	1	D4e1a3	O	1
2444	HM357813	D4e1a3	D4e1a3	O	1	D4e1a3	O	1
2445	HQ012262	D4e1c	D4e1c	O	1	D4e1c	O	1
2446	HQ012261	D4e1c	D4e1c	O	1	D4e1c	O	1
2447	EU660536	D2a	D2a	O	1	D2a	O	1
2448	EU725621	D2a1	D2a1	O	1	D2a1	O	1
2449	EU660538	D2a1a	D2a1a	O	1	D2a1a	O	1
2450	EU660570	D2a1a	D2a1a	O	1	D2a1a	O	1
2451	EU660553	D2a1b	D2a1b	O	1	D2a1b	O	1
2452	AF347010	D2a1b	D2a1b	O	1	D2a1b	O	1
2453	EU482339	D2a2	D2a2	O	1	D2a2	O	1
2454	EU007835	D2a2	D2a2	O	1	D2a2	O	1
2455	JF824877	D2b1	D2b1	O	1	D2b1	O	1
2456	FJ951588	D2b1	D2b1	O	1	D2b1	O	1
2457	EF153796	D2b1a	D2b1a	O	1	D2b1a	O	1
2458	EU095541	D2b1a	D2b1a	O	1	D2b1a	O	1
2459	EU095542	D2b2	D2b2	O	1	D2b2	O	1
2460	FJ951520	D2b2	D2b2	O	1	D2b2	O	1
2461	FJ951506	D2c	D2c	O	1	D2c	O	1
2462	FJ383174	D4e3	D4e3	O	1	D4e3	O	1
2463	JF824823	D4e3	D4e3	O	1	D4e3	O	1
2464	AP008380	D4e2	D4e2	O	1	D4e2	O	1
2465	AP008896	D4e2	D4e2	O	1	D4e2	O	1
2466	AP008834	D4e2a	D4e2a	O	1	D4e2a	O	1
2467	AP010982	D4e2a	D4e2a	O	1	D4e2a	O	1
2468	AP008352	D4e2b	D4e2b	O	1	D4e2b	O	1
2469	AP008557	D4e2b	D4e2b	O	1	D4e2b	O	1
2470	AP010667	D4e2c	D4e2c	O	1	D4e2c	O	1
2471	AP008825	D4e2c	D4e2c	O	1	D4e2c	O	1
2472	AP008530	D4e2d	D4e2d	O	1	D4e2d	O	1
2473	AP010990	D4e2d	D4e2d	O	1	D4e2d	O	1
2474	KF148448	D4e4	D4e4	O	1	D4e4	O	1
2475	FJ951569	D4e4a	D4e4a	O	1	D4e4a	O	1
2476	FJ858880	D4e4a1	D4e4a1	O	1	D4e4a1	O	1

2477	FJ951564	D4e4a1	D4e4a1	O	1	D4e4a1	O	1
2478	GU122981	D4e4b	D4e4b	O	1	D4e4b	O	1
2479	FJ951616	D4e4b	D4e4b	O	1	D4e4b	O	1
2480	AP008739	D4e5a	D4e5a	O	1	D4e5a	O	1
2481	FJ951450	D4e5a	D4e5a	O	1	D4e5a	O	1
2482	FJ858882	D4e5b	D4e5b	O	1	D4e5b	O	1
2483	NA18108	D4e5b	D4e5b	O	1	D4e5b	O	1
2484	EU007883	D4f	D4f	O	1	D4f	O	1
2485	AP008458	D4f1	D4f1	O	1	D4f1	O	1
2486	AP011012	D4f1	D4f1	O	1	D4f1	O	1
2487	AP008652	D4g1	D4g1	O	1	D4g1	O	1
2488	AP010677	D4g1	D4g1	O	1	D4g1	O	1
2489	AP008544	D4g1a	D4g1a	O	1	D4g1a	O	1
2490	AP008272	D4g1a	D4g1a	O	1	D4g1a	O	1
2491	AP008542	D4g1b	D4g1b	O	1	D4g1b	O	1
2492	AP010985	D4g1b	D4g1b	O	1	D4g1b	O	1
2493	AP008560	D4g1c	D4g1c	O	1	D4g1c	O	1
2494	AP008849	D4g1c	D4g1c	O	1	D4g1c	O	1
2495	JF896799	D4g2	D4g2	O	1	D4g2	O	1
2496	AP008422	D4g2a	D4g2a	O	1	D4g2a	O	1
2497	FJ951522	D4g2a1	D4g2a1	O	1	D4g2a1	O	1
2498	FJ951578	D4g2a1	D4g2a1	O	1	D4g2a1	O	1
2499	AP008447	D4g2a1a	D4g2a1a	O	1	D4g2a1a	O	1
2500	AP008267	D4g2a1a	D4g2a1a	O	1	D4g2a1a	O	1
2501	HM357822	D4g2a1b	D4g2a1b	O	1	D4g2a1b	O	1
2502	GU392078	D4g2a1b	D4g2a1b	O	1	D4g2a1b	O	1
2503	KF849965	D4g2a1c	D4g2a1c	O	1	D4g2a1c	O	1
2504	FJ383179	D4g2a1c	D4g2a1c	O	1	D4g2a1c	O	1
2505	FJ951468	D4g2b	D4g2b	O	1	D4g2b	O	1
2506	HM776717	D4g2b1	D4g2b1	O	1	D4g2b1	O	1
2507	AP008408	D4g2b1a	D4g2b1a	O	1	D4g2b1a	O	1
2508	AP010831	D4g2b1a	D4g2b1a	O	1	D4g2b1a	O	1
2509	AP008524	D4h1a1	D4h1a1	O	1	D4h1a1	O	1
2510	AP008416	D4h1a1	D4h1a1	O	1	D4h1a1	O	1
2511	AP008730	D4h1a2	D4h1a2	O	1	D4h1a2	O	1
2512	AP013233	D4h1a2	D4h1a2	O	1	D4h1a2	O	1
2513	JF271012	D4h1b	D4h1b	O	1	D4h1b	O	1

2514	AP008551	D4h1b	D4h1b	O	1	D4h1b	O	1
2515	KC733262	D4h1c	D4h1c	O	1	D4h1c	O	1
2516	AP009422	D4h1c1	D4h1c1	O	1	D4h1c1	O	1
2517	AP013218	D4h1c1	D4h1c1	O	1	D4h1c1	O	1
2518	FJ951534	D4h1d	D4h1d	O	1	D4h1d	O	1
2519	NA18765	D4h1d	D4h1d	O	1	D4h1d	O	1
2520	EU828638	D4h2	D4h2	O	1	D4h2	O	1
2521	FJ168754	D4h3a	D4h3a	O	1	D4h3a	O	1
2522	EU095531	D4h3a	D4h3a	O	1	D4h3a	O	1
2523	FJ168713	D4h3a1	D4h3a1	O	1	D4h3a1	O	1
2524	FJ168717	D4h3a1a1	D4h3a1a1	O	1	D4h3a1a1	O	1
2525	FJ168715	D4h3a1a1	D4h3a1a1	O	1	D4h3a1a1	O	1
2526	FJ168722	D4h3a1a2	D4h3a1a2	O	1	D4h3a1a2	O	1
2527	FJ168720	D4h3a1a2	D4h3a1a2	O	1	D4h3a1a2	O	1
2528	FJ168727	D4h3a2	D4h3a2	O	1	D4h3a2	O	1
2529	FJ168724	D4h3a2	D4h3a2	O	1	D4h3a2	O	1
2530	FJ168728	D4h3a3	D4h3a	X	5	D4h3a3	O	1
2531	FJ168731	D4h3a3a	D4h3a	X	4	D4h3a3a	O	1
2532	FJ168729	D4h3a3a	D4h3a	X	3	D4h3a3a	O	1
2533	FJ168736	D4h3a4	D4h3a4	O	1	D4h3a4	O	1
2534	FJ168737	D4h3a4	D4h3a4	O	1	D4h3a4	O	1
2535	FJ168739	D4h3a5	D4h3a5	O	1	D4h3a5	O	1
2536	FJ168738	D4h3a5	D4h3a5	O	1	D4h3a5	O	1
2537	FJ168744	D4h3a6	D4h3a6	O	1	D4h3a6	O	1
2538	FJ168743	D4h3a6	D4h3a6	O	1	D4h3a6	O	1
2539	FJ168745	D4h3a+@152	D4h3a+@152	O	1	D4h3a+@152	O	1
2540	KC998701	D4h3a7	D4h3a7	O	1	D4h3a7	O	1
2541	HQ012263	D4h3a8	D4h3a8	O	1	D4h3a8	O	1
2542	FJ168746	D4h3a8	D4h3a8	O	1	D4h3a8	O	1
2543	FJ168747	D4h3a9	D4h3a9	O	1	D4h3a9	O	1
2544	FJ168748	D4h3a9	D4h3a9	O	1	D4h3a9	O	1
2545	FJ168712	D4h3b	D4h3b	O	1	D4h3b	O	1
2546	AP008782	D4h4	D4h4	O	1	D4h4	O	1
2547	FJ951519	D4h4a	D4h4a	O	1	D4h4a	O	1
2548	FJ951484	D4h4a	D4h4a	O	1	D4h4a	O	1
2549	AP013281	D4i	D4i	O	1	D4i	O	1
2550	NA18562	D4i	D4i	O	1	D4i	O	1

2551	AP008428	D4i1	D4i1	O	1	D4i1	O	1
2552	AP008811	D4i1	D4i1	O	1	D4i1	O	1
2553	EU482308	D4i2	D4i2	O	1	D4i2	O	1
2554	EU007867	D4i2	D4i2	O	1	D4i2	O	1
2555	KF540585	D4i3	D4i3	O	1	D4i3	O	1
2556	JF742200	D4i3	D4i3	O	1	D4i3	O	1
2557	AP008780	D4j	D4j	O	1	D4j+(16286)	O	2
2558	AY255134	D4j	D4j	O	1	D4j D4j+(16286)	O	2
2559	FJ383200	D4j1a1	D4j1a1	O	1	D4j1a1	O	1
2560	FJ951584	D4j1a1	D4j1a1	O	1	D4j1a1	O	1
2561	FJ383194	D4j1a1a	D4j1a1a	O	1	D4j1a1a	O	1
2562	FJ383189	D4j1a1a	D4j1a1a	O	1	D4j1a1a	O	1
2563	FJ383225	D4j1a1b	D4j1a1b	O	1	D4j1a1b	O	1
2564	FJ383217	D4j1a1b	D4j1a1b	O	1	D4j1a1b	O	1
2565	GQ895153	D4j1a2	D4j1a2	O	1	D4j1a2	O	1
2566	HM036553	D4j1a2	D4j1a2	O	1	D4j1a2	O	1
2567	FJ383177	D4j1b	D4j1b	O	1	D4j1b	O	1
2568	FJ383199	D4j1b2	D4j1b2	O	1	D4j1b2	O	1
2569	FJ383198	D4j1b2	D4j1b2	O	1	D4j1b2	O	1
2570	FJ415605	D4j2	D4j2	O	1	D4j2	O	1
2571	EU007877	D4j2a	D4j2a	O	1	D4j2a	O	1
2572	EU482362	D4j2a	D4j2a	O	1	D4j2a	O	1
2573	AP011035	D4j3	D4j3	O	1	D4j3	O	1
2574	HM044855	D4j3a	D4j3a	O	1	D4j3a	O	1
2575	EF114278	D4j3a	D4j3a	O	1	D4j3a	O	1
2576	NA18959	D4j3a1	D4j3a1	O	1	D4j3a1	O	1
2577	AP010988	D4j3a1	D4j3a1	O	1	D4j3a1	O	1
2578	FJ951501	D4j11	D4j11	O	1	D4j11	O	1
2579	AP010987	D4j11	D4j11	O	1	D4j11	O	1
2580	EU482369	D4j4	D4j4	O	1	D4j4	O	1
2581	FJ858887	D4j4	D4j4	O	1	D4j4	O	1
2582	HM044856	D4j4a	D4j4a	O	1	D4j4a	O	1
2583	FJ858888	D4j4a	D4j4a	O	1	D4j4a	O	1
2584	KC911603	D4j5	D4j5	O	1	D4j5	O	1
2585	FJ951458	D4j5a	D4j5a	O	1	D4j5a	O	1
2586	EU482325	D4j5a	D4j5a	O	1	D4j5a	O	1
2587	FJ383181	D4j6	D4j6	O	1	D4j6	O	1

2588	FJ951507	D4j6	D4j6	O	1	D4j6	O	1
2589	KF056276	D4j13	D4j13	O	1	D4j13	O	1
2590	GU123036	D4j13	D4j13	O	1	D4j13	O	1
2591	EU482377	D4j7	D4j7	O	1	D4j7	O	1
2592	FJ951492	D4j7a	D4j7a	O	1	D4j7a	O	1
2593	FJ951537	D4j7a	D4j7a	O	1	D4j7a	O	1
2594	EU482337	D4j8	D4j8	O	1	D4j8	O	1
2595	FJ951456	D4j8	D4j8	O	1	D4j8	O	1
2596	KF540701	D4j+(16286)	D4j+(16286)	O	1	D4j+(16286)	O	1
2597	HM153527	D4j+(16286)	D4j+(16286)	O	1	D4j+(16286)	O	1
2598	FJ951555	D4j9	D4j9	O	1	D4j9	O	1
2599	HM153528	D4j9	D4j9	O	1	D4j9	O	1
2600	EU482379	D4j10	D4j10	O	1	D4j10	O	1
2601	FJ951488	D4j10	D4j10	O	1	D4j10	O	1
2602	FJ951553	D4j12	D4j12	O	1	D4j12	O	1
2603	NA20524	D4j12	D4j12	O	1	D4j12	O	1
2604	AP013278	D4j14	D4j14	O	1	D4j14	O	1
2605	AP008611	D4j14	D4j14	O	1	D4j14	O	1
2606	KF849898	D4j15	D4j15	O	1	D4j15	O	1
2607	HG00578	D4j15	D4j15	O	1	D4j+146	X	2
2608	JQ657729	D4j16	D4j16	O	1	D4j16	O	1
2609	KF849900	D4j16	D4j16	O	1	D4j16	O	1
2610	DQ272107	D4k	D4k	O	1	D4k	O	1
2611	AP008390	D4k	D4k	O	1	D4k	O	1
2612	FJ951500	D4o	D4o	O	1	D4o	O	1
2613	EU482383	D4o1	D4o1	O	1	D4o1	O	1
2614	AP008819	D4o1a	D4o1a	O	1	D4o1a	O	1
2615	FJ951483	D4o1a	D4o1a	O	1	D4o1a	O	1
2616	FJ951551	D4o2	D4o2	O	1	D4o2	O	1
2617	DQ272113	D4o2a	D4o2a	O	1	D4o2a	O	1
2618	AY195790	D4o2a	D4o2a	O	1	D4o2a	O	1
2619	JF824814	D4o2a1	D4o2a1	O	1	D4o2a1	O	1
2620	EU482366	D4o2a1	D4o2a1	O	1	D4o2a1	O	1
2621	FJ951448	D4p	D4p	O	1	D4p	O	1
2622	FJ147319	D4p	D4p	O	1	D4p	O	1
2623	AP008424	D4p1	D4p1	O	1	D4p1	O	1
2624	AP010973	D4p1	D4p1	O	1	D4p1	O	1

2625	FJ951528	D4l1	D4l1	O	1	D4l1	O	1
2626	AP008778	D4l1a	D4l1a	O	1	D4l1a	O	1
2627	AP013232	D4l1a1	D4l1a1	O	1	D4l1a1	O	1
2628	AP008916	D4l1a1	D4l1a1	O	1	D4l1a1	O	1
2629	KF148415	D4l2a	D4l2a	O	1	D4l2a	O	1
2630	KF148294	D4l2a1	D4l2a1	O	1	D4l2a1	O	1
2631	KF148488	D4l2a1	D4l2a1	O	1	D4l2a1	O	1
2632	FJ951570	D4l2a2	D4l2a2	O	1	D4l2a2	O	1
2633	EU482318	D4l2a2	D4l2a2	O	1	D4l2a2	O	1
2634	KF849897	D4l2b	D4l2b	O	1	D4l2b	O	1
2635	FJ748734	D4l2b	D4l2b	O	1	D4l2b	O	1
2636	AP008321	D4m1	D4m1	O	1	D4m1	O	1
2637	AP008432	D4m1	D4m1	O	1	D4m1	O	1
2638	EU007866	D4m2	D4m2	O	1	D4m2	O	1
2639	KF148357	D4m2a	D4m2a	O	1	D4m2a	O	1
2640	FJ951461	D4m2a	D4m2a	O	1	D4m2a	O	1
2641	FJ951565	D4m2a1	D4m2a1	O	1	D4m2a1	O	1
2642	KF148254	D4m2a1a	D4m2a1a	O	1	D4m2a1a	O	1
2643	KF148265	D4m2a1a	D4m2a1a	O	1	D4m2a1a	O	1
2644	AP010765	D4n	D4n	O	1	D4n	O	1
2645	AP009469	D4n	D4n	O	1	D4n	O	1
2646	AP008810	D4n1	D4n1	O	1	D4n1	O	1
2647	AP008754	D4n1a	D4n1a	O	1	D4n1a	O	1
2648	AP013290	D4n1a	D4n1a	O	1	D4n1a	O	1
2649	FJ951554	D4n2	D4n2	O	1	D4n2	O	1
2650	JF824981	D4n2	D4n2	O	1	D4n2	O	1
2651	FJ383210	D4q	D4q	O	1	D4q	O	1
2652	FJ383234	D4q1	D4q1	O	1	D4q1	O	1
2653	FJ383220	D4q1a	D4q1a	O	1	D4q1a	O	1
2654	FJ383219	D4q1a	D4q1a	O	1	D4q1a	O	1
2655	FJ748738	D4s	D4s	O	1	D4s	O	1
2656	EU007863	D4s	D4s	O	1	D4s	O	1
2657	JF824902	D4t	D4t	O	1	D4t	O	1
2658	AP009462	D4t	D4t	O	1	D4t	O	1
2659	AP008250	D5a1	D5a1	O	1	D5a1	O	1
2660	AP008315	D5a1	D5a1	O	1	D5a1	O	1
2661	AP008409	D5a1a1	D5a1a1	O	1	D5a1a1	O	1

2662	AP008437	D5a1a1	D5a1a1	O	1	D5a1a1	O	1
2663	AP008619	D5a1a2	D5a1a2	O	1	D5a1a2	O	1
2664	AP011034	D5a1a2	D5a1a2	O	1	D5a1a2	O	1
2665	FJ383201	D5a2	D5a2	O	1	D5a2	O	1
2666	FJ951467	D5a2a	D5a2a	O	1	D5a2a	O	1
2667	AY570525	D5a2a1	D5a2a1	O	1	D5a2a1	O	1
2668	HM460795	D5a2a1	D5a2a1	O	1	D5a2a1	O	1
2669	FJ383180	D5a2a1+@16172	D5a2a1+@16172	O	1	D5a2a1+@16172	O	1
2670	HG00628	D5a2a1+@16172	D5a2a1+@16172	O	1	D5a2a1+@16172	O	1
2671	AP011023	D5a2a1a	D5a2a1a	O	1	D5a2a1a	O	1
2672	AP010743	D5a2a1a	D5a2a1a	O	1	D5a2a1a	O	1
2673	AP009424	D5a2a1a1	D5a2a1a1	O	1	D5a2a1a1	O	1
2674	FJ951453	D5a2a1a1	D5a2a1a1	O	1	D5a2a1a1	O	1
2675	AP008536	D5a2a1a1a	D5a2a1a1a	O	1	D5a2a1a1a	O	1
2676	AP013256	D5a2a1a1a	D5a2a1a1a	O	1	D5a2a1a1a	O	1
2677	AP008854	D5a2a1a2	D5a2a1a2	O	1	D5a2a1a2	O	1
2678	AP013197	D5a2a1a2	D5a2a1a2	O	1	D5a2a1a2	O	1
2679	FJ383195	D5a2a1b	D5a2a1b	O	1	D5a2a1b	O	1
2680	AY255162	D5a2a1b	D5a2a1b	O	1	D5a2a1b	O	1
2681	JF824956	D5a2a1b1	D5a2a1b1	O	1	D5a2a1b1	O	1
2682	KF540717	D5a2a1b1	D5a2a1b1	O	1	D5a2a1b1	O	1
2683	EU482309	D5a2a2	D5a2a2	O	1	D5a2a2	O	1
2684	EU597530	D5a2a2	D5a2a2	O	1	D5a2a2	O	1
2685	KF849928	D5a2b	D5a2b	O	1	D5a2b	O	1
2686	KF056266	D5a2b	D5a2b	O	1	D5a2b	O	1
2687	FJ951589	D5a3	D5a3	O	1	D5a3	O	1
2688	NA17962	D5a3a	D5a3a	O	1	D5a3a	O	1
2689	JF824991	D5a3a1	D5a3a1	O	1	D5a3a1	O	1
2690	FJ951615	D5a3a1a	D5a3a1a	O	1	D5a3a1a	O	1
2691	AY570524	D5a3a1a	D5a3a1a	O	1	D5a3a1a	O	1
2692	JN580302	D5b1	D5b1	O	1	D5b1	O	1
2693	AP008674	D5b1a1	D5b1a1	O	1	D5b1a1	O	1
2694	AP011040	D5b1a1	D5b1a1	O	1	D5b1a1	O	1
2695	AP008572	D5b1a2	D5b1a2	O	1	D5b1a2	O	1
2696	AP010708	D5b1a2	D5b1a2	O	1	D5b1a2	O	1
2697	AP011041	D5b1b	D5b1b	O	1	D5b1b	O	1
2698	AP013198	D5b1b	D5b1b	O	1	D5b1b	O	1

2699	AP008771	D5b1b1	D5b1b1	O	1	D5b1b1	O	1
2700	AP008345	D5b1b1	D5b1b1	O	1	D5b1b1	O	1
2701	AP008260	D5b1b2	D5b1b2	O	1	D5b1b2	O	1
2702	KF540746	D5b1b2	D5b1b2	O	1	D5b1b2	O	1
2703	AY255169	D5b1c	D5b1c	O	1	D5b1c	O	1
2704	JF824949	D5b1c1	D5b1c1	O	1	D5b1c1	O	1
2705	KC994014	D5b1c1a	D5b1c1a	O	1	D5b1c1a	O	1
2706	GQ119025	D5b1c1a	D5b1c1a	O	1	D5b1c1a	O	1
2707	NA18576	D5b1d	D5b1d	O	1	D5b1d	O	1
2708	NA18767	D5b1d	D5b1d	O	1	D5b1d	O	1
2709	AP008289	D5b2	D5b2	O	1	D5b2	O	1
2710	KF540723	D5b3	D5b3	O	1	D5b3	O	1
2711	KF540755	D5b3a	D5b3a	O	1	D5b3a	O	1
2712	KF540902	D5b3a	D5b3a	O	1	D5b3a	O	1
2713	KF540890	D5b3a1	D5b3a1	O	1	D5b3a1	O	1
2714	KF540893	D5b3a1	D5b3a1	O	1	D5b3a1	O	1
2715	KF540841	D5b4	D5b4	O	1	D5b4	O	1
2716	KF540738	D5b4	D5b4	O	1	D5b4	O	1
2717	NA18605	D5c1	D5c1	O	1	D5c1	O	1
2718	AP008519	D5c1a	D5c1a	O	1	D5c1a	O	1
2719	EU482335	D5c1a	D5c1a	O	1	D5c1a	O	1
2720	EU007893	D5c+16311	D5c+16311	O	1	D5c+16311	O	1
2721	HM460797	D5c2	D5c2	O	1	D5c2	O	1
2722	NA18113	D5c2	D5c2	O	1	D5c2	O	1
2723	AP011006	D6a1	D6a1	O	1	D6a1	O	1
2724	JN866825	D6a1	D6a1	O	1	D6a1	O	1
2725	JF824913	D6a1a	D6a1a	O	1	D6a1a	O	1
2726	AP011054	D6a1a	D6a1a	O	1	D6a1a	O	1
2727	GQ119022	D6a2	D6a2	O	1	D6a2	O	1
2728	KF540561	D6a2	D6a2	O	1	D6a2	O	1
2729	AY255151	D6c	D6c	O	1	D6c	O	1
2730	GQ119040	D6c1	D6c1	O	1	D6c1	O	1
2731	GQ119042	D6c1	D6c1	O	1	D6c1	O	1
2732	KC994157	D6c1a	D6c1a	O	1	D6c1a	O	1
2733	KC994145	D6c1a	D6c1a	O	1	D6c1a	O	1
2734	JQ245777	N1a1a	N1a1a	O	1	N1a1a	O	1
2735	JQ245754	N1a1a+152	N1a1a+152	O	1	N1a1a+152	O	1

2736	GU290215	N1a1a1a1	N1a1a1a1	O	1	N1a1a1a1	O	1
2737	EF486519	N1a1a1a1	N1a1a1a1	O	1	N1a1a1a1	O	1
2738	GU290209	N1a1a1a1a	N1a1a1a1a	O	1	N1a1a1a1a	O	1
2739	EF153778	N1a1a1a1a	N1a1a1a1a	O	1	N1a1a1a1a	O	1
2740	FJ348184	N1a1a1a2	N1a1a1a2	O	1	N1a1a1a2	O	1
2741	GU290211	N1a1a1a2	N1a1a1a2	O	1	N1a1a1a2	O	1
2742	GU290216	N1a1a1a3	N1a1a1a3	O	1	N1a1a1a3	O	1
2743	GU290207	N1a1a1b	N1a1a1b	O	1	N1a1a1b	O	1
2744	JQ701924	N1a1a2	N1a1a2	O	1	N1a1a2	O	1
2745	EF486517	N1a1a2	N1a1a2	O	1	N1a1a2	O	1
2746	JQ245794	N1a1a3	N1a1a3	O	1	N1a1a3	O	1
2747	JQ245789	N1a1a3	N1a1a3	O	1	N1a1a3	O	1
2748	JQ245735	N1a1b1	N1a1b1	O	1	N1a1b1	O	1
2749	KF146262	N1a1b1	N1a1b1	O	1	N1a1b1	O	1
2750	JQ245791	I	I	O	1	I	O	1
2751	KF146252	I	I	O	1	I	O	1
2752	JQ705840	I1	I1	O	1	I1	O	1
2753	JQ245776	I1	I1	O	1	I1	O	1
2754	KC911435	I1a	I1a	O	1	I1a	O	1
2755	HM454265	I1a	I1a	O	1	I1a	O	1
2756	FJ460562	I1a1	I1a1	O	1	I1a1	O	1
2757	EF177414	I1a1	I1a1	O	1	I1a1	O	1
2758	KF146236	I1a1a	I1a1a	O	1	I1a1a	O	1
2759	JQ705140	I1a1a	I1a1a	O	1	I1a1a	O	1
2760	JX152986	I1a1a1	I1a1a1	O	1	I1a1a1	O	1
2761	KF899911	I1a1a1	I1a1a1	O	1	I1a1a1	O	1
2762	HG00369	I1a1a2	I1a1a2	O	1	I1a1a2	O	1
2763	AY339509	I1a1a2	I1a1a2	O	1	I1a1a2	O	1
2764	JQ245749	I1a1a3	I1a1a3	O	1	I1a1a3	O	1
2765	JQ705378	I1a1a3	I1a1a3	O	1	I1a1a3	O	1
2766	KJ816752	I1a1a3a	I1a1a3a	O	1	I1a1a3a	O	1
2767	JQ245748	I1a1a3a	I1a1a3a	O	1	I1a1a3a	O	1
2768	JQ704690	I1a1b	I1a1b	O	1	I1a1b	O	1
2769	JQ705595	I1a1b	I1a1b	O	1	I1a1b	O	1
2770	GU123027	I1a1c	I1a1c	O	1	I1a1c	O	1
2771	JQ702023	I1a1c	I1a1c	O	1	I1a1c	O	1
2772	JQ705189	I1a1d	I1a1d	O	1	I1a1d	O	1

2773	JQ702342	I1a1d	I1a1d	O	1	I1a1d	O	1
2774	JQ701900	I1a1e	I1a1e	O	1	I1a1e	O	1
2775	KJ095105	I1a1e	I1a1e	O	1	I1a1e	O	1
2776	FJ968796	I1b	I1b	O	1	I1b	O	1
2777	EF556153	I1b	I1b	O	1	I1b	O	1
2778	JQ705932	I1c	I1c	O	1	I1c	O	1
2779	KF146244	I1c1	I1c1	O	1	I1c1	O	1
2780	JQ705364	I1c1a	I1c1a	O	1	I1c1a	O	1
2781	EU564849	I1c1a	I1c1a	O	1	I1c1a	O	1
2782	KF146245	I1d	I1d	O	1	I1d	O	1
2783	KF146246	I1d	I1d	O	1	I1d	O	1
2784	JX462710	I1e	I1e	O	1	I1e	O	1
2785	HM156684	I1e	I1e	O	1	I1e	O	1
2786	JX153931	I1f	I1f	O	1	I1f	O	1
2787	KF251094	I1f	I1f	O	1	I1f	O	1
2788	EU570217	I2	I2	O	1	I2	O	1
2789	JQ245744	I2	I2	O	1	I2	O	1
2790	HQ695930	I2a	I2a	O	1	I2a	O	1
2791	HQ724528	I2a1	I2a1	O	1	I2a1	O	1
2792	HG00329	I2a1a	I2a1a	O	1	I2a1a	O	1
2793	JQ705921	I2a2	I2a2	O	1	I2a2	O	1
2794	JQ703910	I2a2	I2a2	O	1	I2a2	O	1
2795	JX154048	I2a3	I2a3	O	1	I2a3	O	1
2796	JQ705175	I2a3	I2a3	O	1	I2a3	O	1
2797	AY339498	I2b	I2	X	14	I2b	O	1
2798	AY339499	I2b	I2	X	17	I2b	O	1
2799	JQ705666	I2c	I2c	O	1	I2c	O	1
2800	JQ702253	I2c	I2c	O	1	I2c	O	1
2801	JQ245747	I2d	I2d	O	1	I2d	O	1
2802	JQ705244	I2d	I2d	O	1	I2d	O	1
2803	JQ703106	I2e	I2e	O	1	I2e	O	1
2804	JQ702578	I2e	I2e	O	1	I2e	O	1
2805	GU294854	I2f	I2f	O	1	I2f	O	1
2806	JX152959	I2f	I2f	O	1	I2f	O	1
2807	JQ702041	I3a	I3a	O	1	I3a	O	1
2808	JQ245751	I3a	I3a	O	1	I3a	O	1
2809	JQ704837	I3a1	I3a1	O	1	I3a1	O	1

2810	HQ420832	I3a1	I3a1	O	1	I3a1	O	1
2811	KC257362	I3b	I3b	O	1	I3b	O	1
2812	GU590993	I3b	I3b	O	1	I3b	O	1
2813	JQ703883	I3c	I3c	O	1	I3c	O	1
2814	KJ021060	I3c	I3c	O	1	I3c	O	1
2815	JX440338	I3d	I3d	O	1	I3d	O	1
2816	JQ702647	I3d1	I3d1	O	1	I3d1	O	1
2817	KC754361	I3d1	I3d1	O	1	I3d1	O	1
2818	KJ021059	I4	I4	O	1	I4	O	1
2819	JQ702369	I4a	I4a	O	1	I4a	O	1
2820	JQ245737	I4a	I4a	O	1	I4a	O	1
2821	EF153786	I4a1	I4a1	O	1	I4a1	O	1
2822	KF006401	I4a1	I4a1	O	1	I4a1	O	1
2823	HG00154	I4a2	I4a2	O	1	I4a2	O	1
2824	KF254840	I4a2	I4a2	O	1	I4a2	O	1
2825	JQ704976	I4b	I4b	O	1	I4b	O	1
2826	KF146261	I4b	I4b	O	1	I4b	O	1
2827	JQ245724	I5	I5	O	1	I5	O	1
2828	JQ245807	I5a1	I5a	X	8	I5a1	O	1
2829	JQ705096	I5a1a	I5a	X	4	I5a1a	O	1
2830	KF146248	I5a1a	I5a	X	4	I5a1a	O	1
2831	JQ704713	I5a1b	I5a	X	2	I5a1b	O	1
2832	EF660917	I5a1b	I5a	X	2	I5a1b	O	1
2833	EU597573	I5a1c	I5a	X	5	I5a1c	O	1
2834	KF146247	I5a1c	I5a	X	16	I5a1c	O	1
2835	NA12342	I5a2	I5a2	O	1	I5a2	O	1
2836	JQ701894	I5a2+16086	I5a2+16086	O	1	I5a2+16086	O	1
2837	JQ245781	I5a2a	I5a2a	O	1	I5a2a	O	1
2838	JQ245780	I5a2a	I5a2a	O	1	I5a2a	O	1
2839	JQ245772	I5a3	I5a3	O	1	I5a3	O	1
2840	JN415483	I5a3	I5a3	O	1	I5a3	O	1
2841	KF146249	I5a4	I5a4	O	1	I5a4	O	1
2842	FJ348190	I5a4	I5a4	O	1	I5a4	O	1
2843	KF255549	I5b	I5b	O	1	I5b	O	1
2844	KF146250	I5b1	I5b1	O	1	I5b1	O	1
2845	HM852817	I5b1	I5b1	O	1	I5b1	O	1
2846	KF644562	I5c	I5c	O	1	I5c	O	1

2847	KF146251	I5c1	I5c1	O	1	I5c1	O	1
2848	KC787372	I5c1	I5c1	O	1	I5c1	O	1
2849	JQ705382	I6a	I6a	O	1	I6a	O	1
2850	JQ245773	I6b	I6b	O	1	I6b	O	1
2851	HM852831	I6b	I6b	O	1	I6b	O	1
2852	KF146253	I7	I7	O	1	I7	O	1
2853	JF298212	I7	I7	O	1	I7	O	1
2854	AY714008	N1a2	N1a2	O	1	N1a2	O	1
2855	KC867103	N1a3	N1a3	O	1	N1a3	O	1
2856	KC867116	N1a3a	N1a3a	O	1	N1a3a	O	1
2857	KC867107	N1a3a	N1a3a	O	1	N1a3a	O	1
2858	KC867113	N1a3a1	N1a3a1	O	1	N1a3a1	O	1
2859	JX153442	N1a3a1a	N1a3a1a	O	1	N1a3a1a	O	1
2860	JQ704073	N1a3a1a	N1a3a1a	O	1	N1a3a1a	O	1
2861	KC867115	N1a3a2	N1a3a2	O	1	N1a3a2	O	1
2862	KC867114	N1a3a2	N1a3a2	O	1	N1a3a2	O	1
2863	GU123019	N1a3a3	N1a3a3	O	1	N1a3a3	O	1
2864	EF660937	N1a3a3	N1a3a3	O	1	N1a3a3	O	1
2865	JQ245727	N1b1a	N1b1a	O	1	N1b1a	O	1
2866	JQ705744	N1b1a	N1b1a	O	1	N1b1a	O	1
2867	EU742151	N1b1a1	N1b1a1	O	1	N1b1a1	O	1
2868	JQ705186	N1b1a2	N1b1a2	O	1	N1b1a2	O	1
2869	JQ245725	N1b1a2	N1b1a2	O	1	N1b1a2	O	1
2870	JQ705552	N1b1a2a	N1b1a2a	O	1	N1b1a2a	O	1
2871	HQ315687	N1b1a2a	N1b1a2a	O	1	N1b1a2a	O	1
2872	JQ704068	N1b1a2b	N1b1a2b	O	1	N1b1a2b	O	1
2873	FJ493516	N1b1a2b	N1b1a2b	O	1	N1b1a2b	O	1
2874	JQ245803	N1b1a3	N1b1a3	O	1	N1b1a3	O	1
2875	HM765456	N1b1a3	N1b1a3	O	1	N1b1a3	O	1
2876	EU742150	N1b1a+16129	N1b1a+16129	O	1	N1b1a+16129	O	1
2877	HM236190	N1b1a4	N1b1a4	O	1	N1b1a4	O	1
2878	JX153074	N1b1a4a	N1b1a4a	O	1	N1b1a4a	O	1
2879	EU742149	N1b1a4a	N1b1a4a	O	1	N1b1a4a	O	1
2880	GU122992	N1b1a5	N1b1a5	O	1	N1b1a5	O	1
2881	JQ701805	N1b1a5	N1b1a5	O	1	N1b1a5	O	1
2882	JQ702718	N1b1a6	N1b1a6	O	1	N1b1a6	O	1
2883	JQ245756	N1b1a6	N1b1a6	O	1	N1b1a6	O	1

2884	JF265069	N1b1a7	N1b1a7	O	1	N1b1a7	O	1
2885	HQ384201	N1b1a7	N1b1a7	O	1	N1b1a7	O	1
2886	JQ245742	N1b1a8	N1b1a8	O	1	N1b1a8	O	1
2887	FJ460561	N1b1a8a	N1b1a8a	O	1	N1b1a8a	O	1
2888	JQ702973	N1b1a8a	N1b1a8a	O	1	N1b1a8a	O	1
2889	JX153036	N1b1a8b	N1b1a8b	O	1	N1b1a8b	O	1
2890	JQ245774	N1b1a8b	N1b1a8b	O	1	N1b1a8b	O	1
2891	KF297809	N1b1b	N1b1b	O	1	N1b1b	O	1
2892	KF297808	N1b1b	N1b1b	O	1	N1b1b	O	1
2893	DQ301794	N1b1b1	N1b1b1	O	1	N1b1b1	O	1
2894	EU742154	N1b1b1	N1b1b1	O	1	N1b1b1	O	1
2895	JQ245799	N1b2	N1b2	O	1	N1b2	O	1
2896	JQ245798	N1b2	N1b2	O	1	N1b2	O	1
2897	AY714031	N5	N5	O	1	N5	O	1
2898	GU480015	N5a	N5a	O	1	N5a	O	1
2899	GU480020	N5a	N5a	O	1	N5a	O	1
2900	EU787451	N2a	N2a	O	1	N2a	O	1
2901	JF904935	N2a	N2a	O	1	N2a	O	1
2902	KC911573	N2a1	N2a1	O	1	N2a1	O	1
2903	JF930639	N2a1	N2a1	O	1	N2a1	O	1
2904	JN207845	N2a2	N2a2	O	1	N2a2	O	1
2905	KC911368	N2a2	N2a2	O	1	N2a2	O	1
2906	KF056262	W	W	O	1	W	O	1
2907	JQ702793	W1	W1	O	1	W1	O	1
2908	KF146268	W1	W1	O	1	W1	O	1
2909	AY339475	W1a	W1a	O	1	W1a	O	1
2910	AY339491	W1a	W1a	O	1	W1a	O	1
2911	AY339462	W1b	W1b	O	1	W1b	O	1
2912	AY339465	W1b	W1b	O	1	W1b	O	1
2913	AY339466	W1b1	W1b1	O	1	W1b1	O	1
2914	JQ702545	W1b1	W1b1	O	1	W1b1	O	1
2915	FJ472633	W1+119	W1+119	O	1	W1+119	O	1
2916	JQ705036	W1+119	W1+119	O	1	W1+119	O	1
2917	JQ702450	W1c	W1c	O	1	W1c	O	1
2918	JQ245768	W1c	W1c	O	1	W1c	O	1
2919	JQ702327	W1c1	W1c1	O	1	W1c1	O	1
2920	JQ704790	W1c1	W1c1	O	1	W1c1	O	1

2921	KF146267	W1i	W1i	O	1	W1i	O	1
2922	JQ702332	W1i	W1	X	2	W1i	O	1
2923	EF556154	W1d	W1d	O	1	W1d	O	1
2924	JQ705839	W1e	W1e	O	1	W1e	O	1
2925	FJ543390	W1e1	W1e1	O	1	W1e1	O	1
2926	HM625692	W1e1	W1e1	O	1	W1e1	O	1
2927	JQ702638	W1e1a	W1e1a	O	1	W1e1a	O	1
2928	KF146263	W1e1a	W1e1a	O	1	W1e1a	O	1
2929	FJ384432	W1f	W1f	O	1	W1f	O	1
2930	FJ348225	W1f	W1f	O	1	W1f	O	1
2931	JQ702249	W1g	W1g	O	1	W1g	O	1
2932	JQ704975	W1g	W1g	O	1	W1g	O	1
2933	JQ898579	W1h	W1h	O	1	W1h	O	1
2934	KF146270	W1h	W1h	O	1	W1h	O	1
2935	JQ703970	W1h1	W1h1	O	1	W1h1	O	1
2936	KF146271	W1h1	W1h1	O	1	W1h1	O	1
2937	EU086510	W+194	W+194	O	1	W+194	O	1
2938	JQ705313	W3a	W3a	O	1	W3a	O	1
2939	KF146273	W3a1	W3a1	O	1	W3a1	O	1
2940	JQ245760	W3a1	W3a1	O	1	W3a1	O	1
2941	JQ705286	W3a1a1	W3a1a1	O	1	W3a1a1	O	1
2942	FJ472839	W3a1a1	W3a1a1	O	1	W3a1a1	O	1
2943	GU123000	W3a1a2	W3a1a2	O	1	W3a1a2	O	1
2944	JQ705642	W3a1a2	W3a1a2	O	1	W3a1a2	O	1
2945	FJ821289	W3a1a3	W3a1a3	O	1	W3a1a3	O	1
2946	GU002155	W3a1b	W3a1b	O	1	W3a1b	O	1
2947	AY714043	W3a1b	W3a1b	O	1	W3a1b	O	1
2948	JQ703058	W3a1c	W3a1c	O	1	W3a1c	O	1
2949	JQ702804	W3a1c	W3a1c	O	1	W3a1c	O	1
2950	KF142470	W3a1d	W3a1d	O	1	W3a1d	O	1
2951	JQ704160	W3a1d	W3a1d	O	1	W3a1d	O	1
2952	JQ705487	W3a2	W3a2	O	1	W3a2	O	1
2953	GU147938	W3a2	W3a2	O	1	W3a2	O	1
2954	KF146279	W3b	W3b	O	1	W3b	O	1
2955	JQ245743	W3b	W3b	O	1	W3b	O	1
2956	JQ701824	W3b1	W3b1	O	1	W3b1	O	1
2957	JQ702298	W3b1	W3b1	O	1	W3b1	O	1

2958	KF146281	W4	W4	O	1	W4	O	1
2959	AY714018	W4a	W1+119	X	11	W4a	O	1
2960	HM034304	W4a1	W4a1	O	1	W4a1	O	1
2961	EU400619	W4a1	W	X	5	W4a1	O	1
2962	KF146280	W4b	W4b	O	1	W4b	O	1
2963	FJ348217	W4b	W4b	O	1	W4b	O	1
2964	KF146282	W4c	W4c	O	1	W4c	O	1
2965	NA20503	W4c	W4c	O	1	W4c	O	1
2966	KF146283	W4d	W4d	O	1	W4d	O	1
2967	JQ245758	W4d	W4d	O	1	W4d	O	1
2968	KF146285	W5	W5	O	1	W5	O	1
2969	KC911419	W5	W5	O	1	W5	O	1
2970	JQ701827	W5a	W5a	O	1	W5a	O	1
2971	JN583886	W5a1	W5a1	O	1	W5a1	O	1
2972	EF652811	W5a1a	W5a1a	O	1	W5a1a	O	1
2973	KF146284	W5a1a	W5a1a	O	1	W5a1a	O	1
2974	JF431251	W5a1a1	W5a1a1	O	1	W5a1a1	O	1
2975	EU135972	W5a1a1	W5a1a1	O	1	W5a1a1	O	1
2976	HM057816	W5a1a1a	W5a1a1	X	2	W5a1a1a	O	1
2977	GU828018	W5a1a1a	W5a1a1	X	2	W5a1a1a	O	1
2978	HG00148	W5a2	W5a2	O	1	W5a2	O	1
2979	GU726895	W5a2	W5a2	O	1	W5a2	O	1
2980	JQ705278	W5a2b	W5a2b	O	1	W5a2b	O	1
2981	JF419335	W5a2b	W5a2b	O	1	W5a2b	O	1
2982	JQ703283	W5b	W5	X	2	W5b	O	1
2983	EU744586	W5b1	W5	X	5	W5b1	O	1
2984	JQ701912	W5b1a	W5b1a	O	1	W5b1a	O	1
2985	JX153968	W5b1a	W5b1a	O	1	W5b1a	O	1
2986	JQ245723	W6	W6	O	1	W6	O	1
2987	KF146287	W6	W6	O	1	W6	O	1
2988	NA20804	W6a	W6a	O	1	W6a	O	1
2989	JQ705209	W6a	W6a	O	1	W6a	O	1
2990	JQ245761	W6b	W6b	O	1	W6b	O	1
2991	FJ473381	W6b	W6b	O	1	W6b	O	1
2992	JQ245728	W6b1	W6b1	O	1	W6b1	O	1
2993	KF146290	W6b1	W6b1	O	1	W6b1	O	1
2994	JF275845	W6c	W6c	O	1	W6c	O	1

2995	KF700922	W6c1	W6c1	O	1	W6c1	O	1
2996	KF553923	W6c1a	W6c1a	O	1	W6c1a	O	1
2997	EU515252	W6c1a	W6c1a	O	1	W6c1a	O	1
2998	KF146288	W6d	W6d	O	1	W6d	O	1
2999	JQ245762	W6d	W6d	O	1	W6d	O	1
3000	HM352797	W7	W7	O	1	W7	O	1
3001	JQ702147	W7	W7	O	1	W7	O	1
3002	NA19776	W8	W8	O	1	W8	O	1
3003	JQ245778	W8	W8	O	1	W8	O	1
3004	JQ245759	W9	W9	O	1	W9	O	1
3005	JQ702358	W9	W9	O	1	W9	O	1
3006	KC867130	N3	N3	O	1	N3	O	1
3007	KC867127	N3a	N3a	O	1	N3a	O	1
3008	JQ705752	N3a	N3a	O	1	N3a	O	1
3009	KC867129	N3a1	N3a1	O	1	N3a1	O	1
3010	KC867132	N3a1	N3a1	O	1	N3a1	O	1
3011	KC867123	N3b	N3b	O	1	N3b	O	1
3012	KC867131	N3b	N3b	O	1	N3b	O	1
3013	KC887495	N7a1	N7a1	O	1	N7a1	O	1
3014	KC505118	N7a1	N7a1	O	1	N7a1	O	1
3015	KC505116	N7a2	N7a2	O	1	N7a2	O	1
3016	KC887473	N7a2	N7a2	O	1	N7a2	O	1
3017	KC505117	N7b	N7b	O	1	N7b	O	1
3018	KC505119	N7b	N7b	O	1	N7b	O	1
3019	JX289118	N8	N8	O	1	N8	O	1
3020	HM030548	N8	N8	O	1	N8	O	1
3021	NA18628	N9a	N9a	O	1	N9a	O	1
3022	AP008726	N9a1	N9a1	O	1	N9a1	O	1
3023	HM589048	N9a1	N9a1	O	1	N9a1	O	1
3024	FJ748708	N9a1a	N9a1a	O	1	N9a1a	O	1
3025	AY255141	N9a1a	N9a1a	O	1	N9a1a	O	1
3026	AP008608	N9a3	N9a3	O	1	N9a3	O	1
3027	JN857057	N9a3	N9a3	O	1	N9a3	O	1
3028	JN857033	N9a2	N9a2	O	1	N9a2	O	1
3029	AP008414	N9a2	N9a2	O	1	N9a2	O	1
3030	AP008406	N9a2a	N9a2a	O	1	N9a2a	O	1
3031	AP011046	N9a2a	N9a2a	O	1	N9a2a	O	1

3032	NA18973	N9a2a1	N9a2a1	O	1	N9a2a1	O	1
3033	AP008673	N9a2a1	N9a2a1	O	1	N9a2a1	O	1
3034	JN857061	N9a2a2	N9a2a2	O	1	N9a2a2	O	1
3035	AP010674	N9a2a2	N9a2a2	O	1	N9a2a2	O	1
3036	NA19082	N9a2a3	N9a2a3	O	1	N9a2a3	O	1
3037	AP010995	N9a2a3	N9a2a3	O	1	N9a2a3	O	1
3038	AP008630	N9a2c	N9a2c	O	1	N9a2c	O	1
3039	AP008873	N9a2c	N9a2c	O	1	N9a2c	O	1
3040	AP009434	N9a2d	N9a2d	O	1	N9a2d	O	1
3041	AP008420	N9a2d	N9a2d	O	1	N9a2d	O	1
3042	NA18991	N9a4a	N9a4a	O	1	N9a4a	O	1
3043	AP008558	N9a4a	N9a4a	O	1	N9a4a	O	1
3044	AP008385	N9a4b	N9a4b	O	1	N9a4b	O	1
3045	JF824989	N9a4b	N9a4b	O	1	N9a4b	O	1
3046	HG00531	N9a4b1	N9a4b1	O	1	N9a4b1	O	1
3047	NA18747	N9a4b1	N9a4b1	O	1	N9a4b1	O	1
3048	AP008895	N9a5	N9a5	O	1	N9a5	O	1
3049	AP011045	N9a5	N9a5	O	1	N9a5	O	1
3050	KF540744	N9a11	N9a11	O	1	N9a11	O	1
3051	KF540679	N9a11	N9a11	O	1	N9a11	O	1
3052	HM596703	N9a6	N9a6	O	1	N9a6	O	1
3053	AP012369	N9a6a	N9a6a	O	1	N9a6a	O	1
3054	HM596644	N9a6a	N9a6a	O	1	N9a6a	O	1
3055	AP012413	N9a6b	N9a6b	O	1	N9a6b	O	1
3056	AP012410	N9a6b	N9a6b	O	1	N9a6b	O	1
3057	AP011016	N9a7	N9a7	O	1	N9a7	O	1
3058	AP008400	N9a7	N9a7	O	1	N9a7	O	1
3059	AP008714	N9a8	N9a8	O	1	N9a8	O	1
3060	JN857027	N9a8	N9a8	O	1	N9a8	O	1
3061	NA18563	N9a9	N9a9	O	1	N9a9	O	1
3062	FJ147307	N9a9	N9a9	O	1	N9a9	O	1
3063	GU392084	N9a10	N9a10	O	1	N9a10	O	1
3064	NA18741	N9a10a1	N9a10a1	O	1	N9a10a1	O	1
3065	JN084084	N9a10a1	N9a10a1	O	1	N9a10a1	O	1
3066	HM238208	N9a10a2	N9a10a2	O	1	N9a10a2	O	1
3067	KF540600	N9a10a2a	N9a10a2a	O	1	N9a10a2a	O	1
3068	KF540605	N9a10a2a	N9a10a2a	O	1	N9a10a2a	O	1

3069	FJ748719	N9a10+16311	N9a10+16311	O	1	N9a10+16311	O	1
3070	KF540722	N9a10+16311	N9a10+16311	O	1	N9a10+16311	O	1
3071	KF849926	N9a10b	N9a10b	O	1	N9a10b	O	1
3072	HG00406	N9a10b	N9a10b	O	1	N9a10b	O	1
3073	AP010722	N9b1	N9b1	O	1	N9b1	O	1
3074	AP008610	N9b1a	N9b1a	O	1	N9b1a	O	1
3075	AP008464	N9b1a	N9b1a	O	1	N9b1a	O	1
3076	AP008784	N9b1b	N9b1b	O	1	N9b1b	O	1
3077	AP008635	N9b1b	N9b1b	O	1	N9b1b	O	1
3078	AP008821	N9b1c	N9b1c	O	1	N9b1c	O	1
3079	AP008666	N9b1c	N9b1c	O	1	N9b1c	O	1
3080	AP008529	N9b1c1	N9b1c1	O	1	N9b1c1	O	1
3081	AP010834	N9b1c1	N9b1c1	O	1	N9b1c1	O	1
3082	AP013239	N9b2	N9b2	O	1	N9b2	O	1
3083	AP008790	N9b2a	N9b2a	O	1	N9b2a	O	1
3084	AP008660	N9b2a	N9b2a	O	1	N9b2a	O	1
3085	AP008474	N9b3	N9b3	O	1	N9b3	O	1
3086	AP008901	N9b3	N9b3	O	1	N9b3	O	1
3087	HM776709	N9b4	N9b	X	9	N9b4	O	1
3088	AP008620	N9b4	N9b	X	9	N9b4	O	1
3089	KF540727	Y1	Y1	O	1	Y1	O	1
3090	EF153813	Y1	Y1	O	1	Y1	O	1
3091	EU007892	Y1a	Y1a	O	1	Y1a	O	1
3092	KF148486	Y1a1	Y1a1	O	1	Y1a1	O	1
3093	KF148513	Y1a1	Y1a1	O	1	Y1a1	O	1
3094	KF148339	Y1a+16189	Y1a+16189	O	1	Y1a+16189	O	1
3095	EU007855	Y1a+16189	Y1a+16189	O	1	Y1a+16189	O	1
3096	EF153825	Y1a2	Y1a2	O	1	Y1a2	O	1
3097	KF148143	Y1a2	Y1a2	O	1	Y1a	X	2
3098	GU123044	Y1b	Y1b	O	1	Y1b	O	1
3099	AY255138	Y1b1	Y1b1	O	1	Y1b1	O	1
3100	JF824832	Y1b1	Y1b1	O	1	Y1b1	O	1
3101	JF824992	Y1b1a	Y1b1a	O	1	Y1b1a	O	1
3102	AP008534	Y1b1a	Y1b1a	O	1	Y1b1a	O	1
3103	AP008723	Y2	Y2	O	1	Y2	O	1
3104	EF153812	Y2	Y2	O	1	Y2	O	1
3105	KF540560	Y2a	Y2a	O	1	Y2a	O	1

3106	GQ119016	Y2a	Y2a	O	1	Y2a	O	1
3107	GQ119013	Y2a1	Y2a1	O	1	Y2a1	O	1
3108	HM596675	Y2a1	Y2a1	O	1	Y2a1	O	1
3109	KC994134	Y2a1a	Y2a1a	O	1	Y2a1a	O	1
3110	KC994040	Y2a1a	Y2a1a	O	1	Y2a1a	O	1
3111	AP008764	Y2b	Y2b	O	1	Y2b	O	1
3112	EF153798	Y2b	Y2b	O	1	Y2b	O	1
3113	HM030521	N10a	N10a	O	1	N10a	O	1
3114	HM030542	N10a	N10a	O	1	N10a	O	1
3115	HM030500	N10b	N10b	O	1	N10b	O	1
3116	HM030536	N11a1	N11a1	O	1	N11a1	O	1
3117	HM030513	N11a1	N11a1	O	1	N11a1	O	1
3118	HM030528	N11a2	N11a2	O	1	N11a2	O	1
3119	KF540803	N11a2	N11a2	O	1	N11a2	O	1
3120	GU733740	N11b	N11b	O	1	N11b	O	1
3121	GU733776	N11b	N11b	O	1	N11b	O	1
3122	EF495214	N13	N13	O	1	N13	O	1
3123	JN226143	N13	N13	O	1	N13	O	1
3124	GQ301885	N21	N21	O	1	N21	O	1
3125	GQ301867	N21+195	N21+195	O	1	N21+195	O	1
3126	GQ301887	N21+195	N21+195	O	1	N21+195	O	1
3127	AP012425	N21a	N21a	O	1	N21a	O	1
3128	GQ301877	N21a	N21a	O	1	N21a	O	1
3129	JF739542	N22	N22	O	1	N22	O	1
3130	GQ119034	N22	N22	O	1	N22	O	1
3131	AP012414	N22a	N22a	O	1	N22a	O	1
3132	AY963578	N22a	N22a	O	1	N22a	O	1
3133	AY255144	A+152	A+152	O	1	A+152	O	1
3134	AP013225	A+152+16362	A+152+16362	O	1	A+152+16362	O	1
3135	AP010699	A1	A1	O	1	A1	O	1
3136	EF153833	A1a	A1a	O	1	A1a	O	1
3137	AP008617	A1a	A1a	O	1	A1a	O	1
3138	EF153799	A1a1	A1a1	O	1	A1a1	O	1
3139	EU597529	A1a1	A1a1	O	1	A1a1	O	1
3140	DQ282395	A2	A2	O	1	A2	O	1
3141	HG01187	A2	A2	O	1	A2	O	1
3142	EU007844	A2a	A2a	O	1	A2a	O	1

3143	EU482342	A2a	A2a	O	1	A2a	O	1
3144	EU095547	A2a1	A2a1	O	1	A2a1	O	1
3145	EU007884	A2a1	A2a1	O	1	A2a1	O	1
3146	EU007847	A2a2	A2a2	O	1	A2a2	O	1
3147	EU482349	A2a2	A2a2	O	1	A2a2	O	1
3148	EU007886	A2a3	A2a3	O	1	A2a3	O	1
3149	EU725611	A2a3	A2a3	O	1	A2a3	O	1
3150	KC711001	A2a4	A2a4	O	1	A2a4	O	1
3151	KC711007	A2a4	A2a4	O	1	A2a4	O	1
3152	EU095526	A2a5	A2a5	O	1	A2a5	O	1
3153	KC711017	A2a5	A2a5	O	1	A2a5	O	1
3154	EU095533	A2b	A2b	O	1	A2b	O	1
3155	EU725607	A2b1	A2b1	O	1	A2b1	O	1
3156	EU007894	A2b1	A2b1	O	1	A2b1	O	1
3157	EU095538	A2+(64)	A2+(64)	O	1	A2+(64)	O	1
3158	DQ282394	A2+(64)	A2+(64)	O	1	A2+(64)	O	1
3159	HQ012111	A2c	A2c	O	1	A2c	O	1
3160	KC257400	A2c	A2c	O	1	A2c	O	1
3161	HQ012117	A2d	A2d	O	1	A2+(64)+16129	X	2
3162	HQ012050	A2d	A2d	O	1	A2d	O	1
3163	HQ012083	A2d1	A2d	X	46	A2d1	O	1
3164	HQ012061	A2d1	A2d	X	45	A2d1	O	1
3165	DQ282433	A2d1a	A2d	X	16	A2d1a	O	1
3166	DQ282432	A2d1a	A2d	X	16	A2d1a	O	1
3167	DQ282422	A2d2	A2d2	O	1	A2d2	O	1
3168	DQ282388	A2d2	A2d2	O	1	A2d2	O	1
3169	HQ012105	A2ao	A2ao	O	1	A2ao	O	1
3170	HQ012100	A2ao1	A2ao1	O	1	A2ao1	O	1
3171	JQ702177	A2ao1	A2ao1	O	1	A2ao1	O	1
3172	FJ705809	A2f1a	A2f1a	O	1	A2f1a	O	1
3173	FJ775667	A2f1a	A2f1a	O	1	A2f1a	O	1
3174	HQ012110	A2f2	A2f2	O	1	A2f2	O	1
3175	HQ012126	A2f2	A2f2	O	1	A2f2	O	1
3176	HQ012095	A2f3	A2f3	O	1	A2f3	O	1
3177	HQ012087	A2f3	A2f3	O	1	A2f3	O	1
3178	DQ282392	A2g	A2g	O	1	A2g	O	1
3179	HQ012128	A2g	A2g	O	1	A2g	O	1

3180	DQ282391	A2g1	A2g1	O	1	A2g1	O	1
3181	HQ012076	A2g1	A2g1	O	1	A2g1	O	1
3182	EU095545	A2h	A2h	O	1	A2h	O	1
3183	EU095202	A2h	A2h	O	1	A2h	O	1
3184	HQ012079	A2h1	A2h1	O	1	A2h1	O	1
3185	JQ705534	A2h1	A2h1	O	1	A2h1	O	1
3186	EU431080	A2i	A2i	O	1	A2i	O	1
3187	GQ377757	A2i	A2i	O	1	A2i	O	1
3188	JQ705434	A2j	A2j	O	1	A2j	O	1
3189	HQ012127	A2j	A2j	O	1	A2j	O	1
3190	DQ282398	A2j1	A2j1	O	1	A2j1	O	1
3191	DQ282393	A2j1	A2j1	O	1	A2j1	O	1
3192	DQ282417	A2k	A2k	O	1	A2+(64)+@16111	X	2
3193	EU095552	A2k1	A2k1	O	1	A2k1	O	1
3194	DQ282390	A2k1a	A2k1a	O	1	A2k1a	O	1
3195	HG01097	A2k1a	A2k1a	O	1	A2k1a	O	1
3196	DQ282411	A2+(64)+@16111	A2+(64)+@16111	O	1	A2+(64)+@16111	O	1
3197	HQ012054	A2+(64)+@16111	A2+(64)+@16111	O	1	A2+(64)+@16111	O	1
3198	DQ282409	A2l	A2l	O	1	A2l	O	1
3199	HQ012133	A2l	A2l	O	1	A2l	O	1
3200	HQ012131	A2m	A2m	O	1	A2m	O	1
3201	HQ012062	A2m	A2m	O	1	A2m	O	1
3202	EU431082	A2n	A2n	O	1	A2n	O	1
3203	FJ713602	A2n	A2n	O	1	A2n	O	1
3204	HQ012114	A2o	A2o	O	1	A2o	O	1
3205	DQ282419	A2o	A2o	O	1	A2o	O	1
3206	HQ012107	A2ai	A2ai	O	1	A2ai	O	1
3207	NA19770	A2ai	A2ai	O	1	A2ai	O	1
3208	HQ012056	A2aj	A2aj	O	1	A2aj	O	1
3209	NA19777	A2aj	A2aj	O	1	A2aj	O	1
3210	HQ012065	A2p	A2p	O	1	A2p	O	1
3211	HQ012116	A2p	A2p	O	1	A2p	O	1
3212	HQ012088	A2p1	A2p1	O	1	A2p1	O	1
3213	HQ012067	A2p1	A2p1	O	1	A2p1	O	1
3214	KC257370	A2p2	A2p2	O	1	A2p2	O	1
3215	DQ282403	A2am	A2am	O	1	A2am	O	1
3216	HG01047	A2am	A2am	O	1	A2am	O	1

3217	JQ705973	A2q	A2q	O	1	A2q	O	1
3218	EU431081	A2q	A2	X	2	A2q	O	1
3219	HQ012132	A2q1	A2q1	O	1	A2q1	O	1
3220	JQ705557	A2q1	A2q1	O	1	A2q1	O	1
3221	HQ012052	A2t	A2t	O	1	A2t	O	1
3222	HQ012086	A2t	A2t	O	1	A2t	O	1
3223	HQ012073	A2u1	A2u1	O	1	A2u1	O	1
3224	HQ012057	A2u1	A2u1	O	1	A2u1	O	1
3225	HQ012066	A2u2	A2u2	O	1	A2u2	O	1
3226	HQ012053	A2u2	A2u2	O	1	A2u2	O	1
3227	HG01342	A2v	A2v	O	1	A2v	O	1
3228	AY195786	A2v1	A2v1	O	1	A2v1	O	1
3229	HQ012109	A2v1+152	A2v1+152	O	1	A2v1+152	O	1
3230	JQ702093	A2v1+152	A2v1+152	O	1	A2v1+152	O	1
3231	HQ012085	A2v1a	A2v1a	O	1	A2v1a	O	1
3232	HM589049	A2v1a	A2v1a	O	1	A2v1a	O	1
3233	JQ705501	A2v1b	A2v1b	O	1	A2v1b	O	1
3234	JQ702765	A2v1b	A2v1b	O	1	A2v1b	O	1
3235	HG01375	A2w	A2w	O	1	A2w	O	1
3236	HG01356	A2w	A2w	O	1	A2w	O	1
3237	JQ703875	A2w1	A2w1	O	1	A2w1	O	1
3238	JN100648	A2w1	A2w1	O	1	A2w1	O	1
3239	HQ012071	A2x	A2x	O	1	A2x	O	1
3240	JN419250	A2x	A2x	O	1	A2x	O	1
3241	GQ398480	A2y	A2y	O	1	A2y	O	1
3242	GQ398487	A2y	A2y	O	1	A2y	O	1
3243	EU095196	A2aa	A2aa	O	1	A2aa	O	1
3244	EU095201	A2aa	A2aa	O	1	A2aa	O	1
3245	JX887155	A2ab	A2ab	O	1	A2ab	O	1
3246	JQ702592	A2ab	A2ab	O	1	A2ab	O	1
3247	EU095529	A2ac	A2ac	O	1	A2ac	O	1
3248	JQ702324	A2ac1	A2ac1	O	1	A2ac1	O	1
3249	HG01440	A2ac1	A2ac1	O	1	A2ac1	O	1
3250	EF079873	A2ad	A2ad	O	1	A2ad	O	1
3251	JQ965968	A2ad1	A2ad1	O	1	A2ad1	O	1
3252	JQ965967	A2ad1	A2ad1	O	1	A2ad1	O	1
3253	JQ965970	A2ad2	A2ad2	O	1	A2ad2	O	1

3254	JQ965969	A2ad2	A2ad2	O	1	A2ad2	O	1
3255	JQ704276	A2ae	A2ae	O	1	A2ae	O	1
3256	JQ705314	A2ae	A2ae	O	1	A2ae	O	1
3257	JQ965977	A2af1a	A2af1a	O	1	A2af1a	O	1
3258	JQ965978	A2af1a	A2af1a	O	1	A2af1a	O	1
3259	JQ965972	A2af1a1	A2af1a1	O	1	A2af1a1	O	1
3260	JQ965975	A2af1a1	A2af1a1	O	1	A2af1a1	O	1
3261	JQ965979	A2af1a2	A2af1a2	O	1	A2af1a2	O	1
3262	JQ965980	A2af1a2	A2af1a2	O	1	A2af1a2	O	1
3263	JQ965986	A2af1b	A2af1b	O	1	A2af1b	O	1
3264	JQ965988	A2af1b	A2af1b	O	1	A2af1b	O	1
3265	JQ965983	A2af1b1	A2af1b1	O	1	A2af1b1	O	1
3266	JQ965981	A2af1b1a	A2af1b1a	O	1	A2af1b1a	O	1
3267	JQ965982	A2af1b1a	A2af1b1a	O	1	A2af1b1a	O	1
3268	JQ965984	A2af1b1b	A2af1b1b	O	1	A2af1b1b	O	1
3269	JQ965985	A2af1b1b	A2af1b1b	O	1	A2af1b1b	O	1
3270	HQ012106	A2af1b2	A2af1b2	O	1	A2af1b2	O	1
3271	HQ012104	A2af1b2	A2af1b2	O	1	A2af1b2	O	1
3272	JQ965971	A2af2	A2af2	O	1	A2af2	O	1
3273	KC998702	A2ag	A2ag	O	1	A2ag	O	1
3274	KC998703	A2ag	A2ag	O	1	A2ag	O	1
3275	KC503930	A2ah	A2ah	O	1	A2ah	O	1
3276	KC503929	A2ah	A2ah	O	1	A2ah	O	1
3277	JQ701855	A2ak	A2ak	O	1	A2ak	O	1
3278	NA19663	A2ak	A2ak	O	1	A2ak	O	1
3279	HG01492	A2al	A2al	O	1	A2al	O	1
3280	JQ701976	A2al	A2al	O	1	A2al	O	1
3281	JQ705746	A2an	A2an	O	1	A2+(64)+16189	X	2
3282	EU597486	A2an	A2an	O	1	A2an	O	1
3283	JQ435789	A2+(64)+16189	A2+(64)+16189	O	1	A2+(64)+16189	O	1
3284	HQ012115	A2ap	A2ap	O	1	A2ap	O	1
3285	HM569227	A2ap	A2ap	O	1	A2ap	O	1
3286	KC998705	A2aq	A2aq	O	1	A2aq	O	1
3287	KC998706	A2aq	A2aq	O	1	A2aq	O	1
3288	HQ012092	A2r	A2r	O	1	A2r	O	1
3289	DQ282401	A2r	A2r	O	1	A2r	O	1
3290	HQ012063	A2r1	A2r1	O	1	A2r1	O	1

3291	JQ702393	A2r1	A2r1	O	1	A2r1	O	1
3292	HQ012070	A2s	A2s	O	1	A2s	O	1
3293	DQ282425	A2z	A2z	O	1	A2z	O	1
3294	DQ282424	A2z	A2z	O	1	A2z	O	1
3295	AY963575	A6a	A6a	O	1	A6a	O	1
3296	AY255166	A6a	A6a	O	1	A6a	O	1
3297	FJ748705	A6b	A6b	O	1	A6b	O	1
3298	KF056300	A6b	A6b	O	1	A6b	O	1
3299	EF153771	A12	A12	O	1	A12	O	1
3300	EF397560	A12a	A12a	O	1	A12a	O	1
3301	AY519488	A12a	A12a	O	1	A12a	O	1
3302	EF153794	A23	A23	O	1	A23	O	1
3303	KC911275	A23	A23	O	1	A23	O	1
3304	HM036549	A+152+16362+200	A+152+16362+200	O	1	A+152+16362+200	O	1
3305	EF153819	A+152+16362+200	A+152+16362+200	O	1	A+152+16362+200	O	1
3306	EF114282	A13	A13	O	1	A13	O	1
3307	GU392075	A13	A13	O	1	A13	O	1
3308	EF153775	A14	A14	O	1	A14	O	1
3309	NA18697	A14	A14	O	1	A14	O	1
3310	NA18773	A15a	A15a	O	1	A15a	O	1
3311	JF824996	A15a	A15a	O	1	A15a	O	1
3312	AP010832	A15b	A15b	O	1	A15b	O	1
3313	JN580301	A15b	A15b	O	1	A15b	O	1
3314	JF824895	A15c	A15c	O	1	A15c	O	1
3315	KF056313	A15c1	A15c1	O	1	A15c1	O	1
3316	KF056304	A15c1	A15c1	O	1	A15c1	O	1
3317	EF153780	A16	A16	O	1	A16	O	1
3318	HM852848	A16	A16	O	1	A16	O	1
3319	NA18572	A17	A17	O	1	A17	O	1
3320	HM036566	A17	A17	O	1	A17	O	1
3321	HG00634	A18	A18	O	1	A18	O	1
3322	NA18615	A18	A18	O	1	A18	O	1
3323	GU377085	A19	A19	O	1	A19	O	1
3324	NA18621	A19	A19	O	1	A19	O	1
3325	AP010675	A20	A20	O	1	A20	O	1
3326	NA18164	A20	A20	O	1	A20	O	1
3327	KF056302	A21	A21	O	1	A21	O	1

3328	GQ895144	A21	A21	O	1	A21	O	1
3329	JF824891	A22	A22	O	1	A22	O	1
3330	NA17967	A22	A22	O	1	A22	O	1
3331	EF397559	A24	A24	O	1	A24	O	1
3332	NA18791	A24	A24	O	1	A24	O	1
3333	AP008459	A25	A25	O	1	A25	O	1
3334	KF148411	A25	A25	O	1	A25	O	1
3335	JX153385	A26	A26	O	1	A26	O	1
3336	JX152799	A26	A26	O	1	A26	O	1
3337	AP013217	A3	A3	O	1	A3	O	1
3338	AP010833	A3	A3	O	1	A3	O	1
3339	AP013268	A3a	A3a	O	1	A3a	O	1
3340	AP008741	A3a	A3a	O	1	A3a	O	1
3341	AP008307	A7	A7	O	1	A7	O	1
3342	AP010709	A7	A7	O	1	A7	O	1
3343	GU014565	A11a	A11a	O	1	A11a	O	1
3344	GQ895149	A11a	A11a	O	1	A11a	O	1
3345	GQ895164	A11+16234	A11+16234	O	1	A11+16234	O	1
3346	EU597494	A11b	A11b	O	1	A11b	O	1
3347	FJ748714	A11b	A11b	O	1	A11b	O	1
3348	AP008265	A5a	A5a	O	1	A5a	O	1
3349	AP008743	A5a	A5a	O	1	A5a	O	1
3350	AP009468	A5a1a	A5a1a	O	1	A5a1a	O	1
3351	AP008914	A5a1a	A5a1a	O	1	A5a1a	O	1
3352	DQ272123	A5a1a1	A5a1a1	O	1	A5a1a1	O	1
3353	AP008433	A5a1a1	A5a1a1	O	1	A5a1a1	O	1
3354	AP010701	A5a1a1a	A5a1a1a	O	1	A5a1a1a	O	1
3355	AP008290	A5a1a1a	A5a1a1a	O	1	A5a1a1a	O	1
3356	AP008532	A5a1a1b	A5a1a1b	O	1	A5a1a1b	O	1
3357	AP010976	A5a1a1b	A5a1a1b	O	1	A5a1a1b	O	1
3358	AP011037	A5a1a2	A5a1a2	O	1	A5a1a2	O	1
3359	AP011026	A5a1a2	A5a1a2	O	1	A5a1a2	O	1
3360	AP008538	A5a1a2a	A5a1a2a	O	1	A5a1a2a	O	1
3361	AP008596	A5a1a2a	A5a1a2a	O	1	A5a1a2a	O	1
3362	AP008853	A5a1b	A5a1b	O	1	A5a1b	O	1
3363	AP008562	A5a1b	A5a1b	O	1	A5a1b	O	1
3364	AP011038	A5a2	A5a2	O	1	A5a2	O	1

3365	AP008340	A5a2	A5a2	O	1	A5a2	O	1
3366	NA18997	A5a3	A5a3	O	1	A5a3	O	1
3367	AP013109	A5a3a	A5a3a	O	1	A5a3a	O	1
3368	AP008835	A5a3a	A5a3a	O	1	A5a3a	O	1
3369	AP013122	A5a4	A5a4	O	1	A5a4	O	1
3370	AP009452	A5a4	A5a4	O	1	A5a4	O	1
3371	AP013288	A5a5	A5a5	O	1	A5a5	O	1
3372	EF153824	A5a5	A5a5	O	1	A5a5	O	1
3373	JF824862	A5b	A5	X	2	A5b	O	1
3374	GQ999962	A5b1	A5b1	O	1	A5b1	O	1
3375	NA18611	A5b1	A5b1	O	1	A5b1	O	1
3376	AP008364	A5b1a	A5b1a	O	1	A5b1a	O	1
3377	AP010989	A5b1a	A5b1a	O	1	A5b1a	O	1
3378	JF824875	A5b1b	A5b1b	O	1	A5b1b	O	1
3379	HG00557	A5b1b	A5b1b	O	1	A5b1b	O	1
3380	NA17987	A5b1c	A5b1c	O	1	A5b1c	O	1
3381	KF540620	A5b1c1	A5b1c1	O	1	A5b1c1	O	1
3382	KF540659	A5b1c1	A5b1c1	O	1	A5b1c1	O	1
3383	EF153816	A5c	A5c	O	1	A5c	O	1
3384	AP013209	A5c	A5c	O	1	A5c	O	1
3385	AP008874	A5c1	A5c1	O	1	A5c1	O	1
3386	AP013220	A5c1	A5c1	O	1	A5c1	O	1
3387	EU482363	A8	A8	O	1	A8	O	1
3388	AY519486	A8a	A8a	O	1	A8a	O	1
3389	EF153797	A8a1	A8a1	O	1	A8a1	O	1
3390	JX266267	A8a1	A8a1	O	1	A8a1	O	1
3391	HM569228	A10	A10	O	1	A10	O	1
3392	GU122995	A10	A10	O	1	A10	O	1
3393	AY289059	O	O	O	1	O	O	1
3394	DQ404447	O1	O1	O	1	O1	O	1
3395	AY289056	O1a	O1a	O	1	O1a	O	1
3396	AF346963	S1	S1	O	1	S1	O	1
3397	JN226144	S1a	S1a	O	1	S1a	O	1
3398	DQ404440	S1a	S1a	O	1	S1a	O	1
3399	AY289051	S2	S2	O	1	S2	O	1
3400	AF346964	S2	S2	O	1	S2	O	1
3401	AY289066	S3	S3	O	1	S3	O	1

3402	AY289067	S3	S3	O	1	S3	O	1
3403	AY289062	S4	S4	O	1	S4	O	1
3404	EF495220	S5	S5	O	1	S5	O	1
3405	EU600318	X1a	X1a	O	1	X1a	O	1
3406	EU935456	X1a	X1a	O	1	X1a	O	1
3407	HM765467	X1c	X1c	O	1	X1c	O	1
3408	FJ460521	X1c	X1c	O	1	X1c	O	1
3409	EF177437	X3	X3	O	1	X3	O	1
3410	JQ245804	X3a	X3a	O	1	X3a	O	1
3411	EU600320	X3a	X3a	O	1	X3a	O	1
3412	GQ200588	X2	X2	O	1	X2	O	1
3413	JQ245739	X2	X2	O	1	X2	O	1
3414	EU600328	X2+225	X2+225	O	1	X2+225	O	1
3415	EU439939	X2a1	X2a1	O	1	X2a1	O	1
3416	FJ168765	X2a1	X2a1	O	1	X2a1	O	1
3417	FJ168759	X2a1a	X2a1a	O	1	X2a1a	O	1
3418	EU095249	X2a1a1	X2a1a1	O	1	X2a1a1	O	1
3419	EU095251	X2a1a1	X2a1a1	O	1	X2a1a1	O	1
3420	FJ168761	X2a1b	X2a1b	O	1	X2a1b	O	1
3421	FJ168760	X2a1b	X2a1b	O	1	X2a1b	O	1
3422	FJ168762	X2a1b1	X2a1b1	O	1	X2a1b1	O	1
3423	FJ168763	X2a1b1a	X2a1b1a	O	1	X2a1b1a	O	1
3424	EU095242	X2a1b1a	X2a1b1a	O	1	X2a1b1a	O	1
3425	EU095244	X2a1c	X2a1c	O	1	X2a1c	O	1
3426	FJ168764	X2a1c	X2a1c	O	1	X2a1c	O	1
3427	KC257359	X2a2	X2a2	O	1	X2a2	O	1
3428	AY195787	X2a2	X2a2	O	1	X2a2	O	1
3429	EU935450	X2j	X2j	O	1	X2j	O	1
3430	FJ457949	X2b	X2b	O	1	X2b	O	1
3431	FJ441666	X2b	X2b	O	1	X2b	O	1
3432	JX021501	X2b+226	X2b+226	O	1	X2b+226	O	1
3433	JQ704969	X2b+226	X2b+226	O	1	X2b+226	O	1
3434	EF556159	X2b1	X2b1	O	1	X2b1	O	1
3435	EF556175	X2b1	X2b1	O	1	X2b1	O	1
3436	AF381986	X2b2	X2b2	O	1	X2b2	O	1
3437	DQ523642	X2b3	X2b3	O	1	X2b3	O	1
3438	JQ701946	X2b4	X2b4	O	1	X2b4	O	1

3439	JQ343921	X2b4	X2b4	O	1	X2b4	O	1
3440	EU600321	X2b4a	X2b4a	O	1	X2b4a	O	1
3441	GU945542	X2b4a	X2b4a	O	1	X2b4a	O	1
3442	JQ705458	X2b4a1	X2b4a1	O	1	X2b4a1	O	1
3443	JX153850	X2b4a1	X2b4a1	O	1	X2b4a1	O	1
3444	EU597556	X2b5	X2b5	O	1	X2b5	O	1
3445	JQ705509	X2b5	X2b5	O	1	X2b5	O	1
3446	JQ705179	X2b6	X2b6	O	1	X2b6	O	1
3447	JX153405	X2b6a	X2b6a	O	1	X2b6a	O	1
3448	JQ702893	X2b6a	X2b6a	O	1	X2b6a	O	1
3449	JQ704754	X2b7	X2b7	O	1	X2b7	O	1
3450	JQ705082	X2b7	X2b7	O	1	X2b7	O	1
3451	JQ705154	X2b8	X2b8	O	1	X2b8	O	1
3452	HG00100	X2b8	X2b8	O	1	X2b8	O	1
3453	JQ702096	X2b9	X2b9	O	1	X2b9	O	1
3454	AY339513	X2b9	X2b9	O	1	X2b9	O	1
3455	JQ702482	X2b10	X2b10	O	1	X2b10	O	1
3456	JX153019	X2b10a	X2b10a	O	1	X2b10a	O	1
3457	JX153934	X2b10a	X2b10a	O	1	X2b10a	O	1
3458	JX153084	X2b11	X2b11	O	1	X2b11	O	1
3459	JN415482	X2b11	X2b11	O	1	X2b11	O	1
3460	HG00108	X2b+226+16192	X2b+226+16192	O	1	X2b+226+16192	O	1
3461	JQ702995	X2b+226+16192	X2b+226+16192	O	1	X2b+226+16192	O	1
3462	JQ704859	X2b12	X2b12	O	1	X2b12	O	1
3463	JQ705550	X2b12	X2b12	O	1	X2b12	O	1
3464	JX153312	X2b13	X2b13	O	1	X2b13	O	1
3465	JX153907	X2b13	X2b13	O	1	X2b13	O	1
3466	JQ705795	X2d	X2d	O	1	X2d	O	1
3467	JQ702739	X2d1	X2d1	O	1	X2d1	O	1
3468	GQ231312	X2d1a	X2d1a	O	1	X2d1a	O	1
3469	NA20536	X2d1a	X2d1a	O	1	X2d1a	O	1
3470	HM625691	X2d2	X2d2	O	1	X2d2	O	1
3471	JQ701817	X2d2	X2d2	O	1	X2d2	O	1
3472	JX153623	X2c1	X2c1	O	1	X2c1	O	1
3473	KC257285	X2c1	X2c1	O	1	X2c1	O	1
3474	JQ702817	X2c1a	X2c1a	O	1	X2c1a	O	1
3475	FJ348156	X2c1a	X2c1a	O	1	X2c1a	O	1

3476	HM370114	X2c1b	X2c1b	O	1	X2c1b	O	1
3477	KC257304	X2c1b	X2c1b	O	1	X2c1b	O	1
3478	JQ705612	X2c1c	X2c1c	O	1	X2c1c	O	1
3479	JQ703703	X2c1c1	X2c1c1	O	1	X2c1c1	O	1
3480	JX153379	X2c1c1	X2c1c1	O	1	X2c1c1	O	1
3481	JQ705480	X2c1d	X2c1d	O	1	X2c1d	O	1
3482	JQ702633	X2c1e	X2c1e	O	1	X2c1e	O	1
3483	JX152973	X2c1e	X2c1e	O	1	X2c1e	O	1
3484	DQ523637	X2c2	X2c2	O	1	X2c2	O	1
3485	JQ705155	X2c2	X2c2	O	1	X2c2	O	1
3486	HM852814	X2e1a	X2e1a	O	1	X2e1a	O	1
3487	EF556165	X2e1a1	X2e1a1	O	1	X2e1a1	O	1
3488	JQ705118	X2e1a1	X2e1a1	O	1	X2e1a1	O	1
3489	HM852758	X2e1b	X2e1b	O	1	X2e1b	O	1
3490	JQ245745	X2e2a	X2e2a	O	1	X2e2a	O	1
3491	EU600324	X2e2a	X2e2a	O	1	X2e2a	O	1
3492	FJ147306	X2e2a1	X2e2a1	O	1	X2e2a1	O	1
3493	EF153772	X2e2a1	X2e2a1	O	1	X2e2a1	O	1
3494	JQ245787	X2e2a2	X2e2a2	O	1	X2e2a2	O	1
3495	JQ245779	X2e2a2	X2e2a2	O	1	X2e2a2	O	1
3496	AB626609	X2e2b	X2e2b	O	1	X2e2b	O	1
3497	JQ245775	X2e2b	X2e2b	O	1	X2e2b	O	1
3498	JX153926	X2e2b1	X2e2b1	O	1	X2e2b1	O	1
3499	JQ245752	X2e2b1	X2e2b1	O	1	X2e2b1	O	1
3500	KC911480	X2e2c1	X2e2c1	O	1	X2e2c1	O	1
3501	KC911468	X2e2c1	X2e2c1	O	1	X2e2c1	O	1
3502	FJ168756	X2g	X2g	O	1	X2g	O	1
3503	JQ704985	X2l	X2l	O	1	X2l	O	1
3504	KC911385	X2l	X2l	O	1	X2l	O	1
3505	KC911497	X2+225+@16223	X2+225+@16223	O	1	X2+225+@16223	O	1
3506	EU600325	X2h	X2h	O	1	X2h	O	1
3507	EU600326	X2h	X2h	O	1	X2h	O	1
3508	JQ245730	X2i	X2i	O	1	X2i	O	1
3509	HQ529295	X2i	X2i	O	1	X2i	O	1
3510	JQ245806	X2i+@225	X2i+@225	O	1	X2i+@225	O	1
3511	JQ705067	X2i1	X2i1	O	1	X2i1	O	1
3512	JX153273	X2i1	X2i1	O	1	X2i1	O	1

3513	FJ008043	X2m1	X2m1	O	1	X2m1	O	1
3514	JX153046	X2m1	X2m1	O	1	X2m1	O	1
3515	JQ705389	X2m2	X2m2	O	1	X2m2	O	1
3516	JQ705217	X2m2	X2m2	O	1	X2m2	O	1
3517	EF660942	X2n	X2n	O	1	X2n	O	1
3518	JQ705988	X2n	X2n	O	1	X2n	O	1
3519	JQ245731	X2o	X2o	O	1	X2o	O	1
3520	JQ245770	X2o1	X2o1	O	1	X2o1	O	1
3521	JX153015	X2o1	X2o1	O	1	X2o1	O	1
3522	KC911478	X2f	X2f	O	1	X2f	O	1
3523	EU600323	X2f	X2f	O	1	X2f	O	1
3524	JQ245738	X2f1	X2f1	O	1	X2f1	O	1
3525	HM852756	X2f1	X2f1	O	1	X2f1	O	1
3526	JQ704866	X2k	X2k	O	1	X2k	O	1
3527	JQ701905	X2k	X2k	O	1	X2k	O	1
3528	KC257312	X2p	X2p	O	1	X2p	O	1
3529	JX508853	X2p1	X2p1	O	1	X2p1	O	1
3530	JX508852	X2p1	X2p1	O	1	X2p1	O	1
3531	HQ456226	X4	X4	O	1	X4	O	1
3532	JQ245765	X4	X4	O	1	X4	O	1
3533	JX153281	R0a	R0a	O	1	R0a	O	1
3534	JQ702678	R0a1	R0a1	O	1	R0a1	O	1
3535	HM185216	R0a1a	R0a1a	O	1	R0a1a	O	1
3536	FJ460524	R0a1a	R0a1a	O	1	R0a1a	O	1
3537	HM185217	R0a1a1	R0a1a1	O	1	R0a1a1	O	1
3538	HM185263	R0a1a1	R0a1a1	O	1	R0a1a1	O	1
3539	HM185210	R0a1a1a	R0a1a1a	O	1	R0a1a1a	O	1
3540	HM185208	R0a1a1a	R0a1a1a	O	1	R0a1a1a	O	1
3541	HM185258	R0a1a2	R0a1a2	O	1	R0a1a2	O	1
3542	HM185260	R0a1a2	R0a1a2	O	1	R0a1a2	O	1
3543	HM185213	R0a1a3	R0a1a3	O	1	R0a1a3	O	1
3544	GU592021	R0a1a3	R0a1a3	O	1	R0a1a3	O	1
3545	HM185245	R0a1a4	R0a1a4	O	1	R0a1a4	O	1
3546	HM185233	R0a1a4	R0a1a4	O	1	R0a1a4	O	1
3547	HM185256	R0a1+152	R0a1+152	O	1	R0a1+152	O	1
3548	DQ904236	R0a1b	R0a1b	O	1	R0a1b	O	1
3549	HM185240	R0a1b	R0a1b	O	1	R0a1b	O	1

3550	EU597493	R0a+60.1T	R0a	X	5	R0a+60.1T	O	1
3551	HM185266	R0a2	R0a2	O	1	R0a2	O	1
3552	AY713999	R0a2	R0a2	O	1	R0a2	O	1
3553	EF556170	R0a2a	R0a2a	O	1	R0a2a	O	1
3554	HM185270	R0a2a	R0a2a	O	1	R0a2a	O	1
3555	HM185257	R0a2a1	R0a2a1	O	1	R0a2a1	O	1
3556	JQ702181	R0a2a1	R0a2a1	O	1	R0a2a1	O	1
3557	HM185261	R0a2b	R0a2b	O	1	R0a2b	O	1
3558	EF556172	R0a2b	R0a2b	O	1	R0a2b	O	1
3559	HM185215	R0a2c	R0a2c	O	1	R0a2c	O	1
3560	EF556176	R0a2c	R0a2c	O	1	R0a2c	O	1
3561	JF717359	R0a2d	R0a2d	O	1	R0a2d	O	1
3562	HM185241	R0a2d	R0a2d	O	1	R0a2d	O	1
3563	DQ904242	R0a2e	R0a2e	O	1	R0a2e	O	1
3564	HM185225	R0a2f	R0a2f	O	1	R0a2f	O	1
3565	HM185227	R0a2f1a	R0a2f1a	O	1	R0a2f1a	O	1
3566	HM185221	R0a2f1a	R0a2f1a	O	1	R0a2f1a	O	1
3567	HM185228	R0a2f1b	R0a2f1b	O	1	R0a2f1b	O	1
3568	HM185248	R0a2f1b	R0a2f1b	O	1	R0a2f1b	O	1
3569	HM185271	R0a2g	R0a2g	O	1	R0a2g	O	1
3570	HM185218	R0a2g	R0a2g	O	1	R0a2g	O	1
3571	HM185238	R0a2h	R0a2h	O	1	R0a2h	O	1
3572	DQ904240	R0a2h	R0a2h	O	1	R0a2h	O	1
3573	HM185242	R0a2i	R0a2i	O	1	R0a2i	O	1
3574	DQ904241	R0a2i	R0a2i	O	1	R0a2i	O	1
3575	HM185237	R0a2+195	R0a2+195	O	1	R0a2+195	O	1
3576	HM185246	R0a2j	R0a2j	O	1	R0a2j	O	1
3577	HM185229	R0a2j	R0a2j	O	1	R0a2j	O	1
3578	JF717355	R0a2k	R0a2k	O	1	R0a2k	O	1
3579	HM185254	R0a2k1	R0a2k1	O	1	R0a2k1	O	1
3580	HM185253	R0a2k1	R0a2k1	O	1	R0a2k1	O	1
3581	HM185250	R0a2l	R0a2l	O	1	R0a2l	O	1
3582	HM185244	R0a2l	R0a2l	O	1	R0a2l	O	1
3583	JQ705916	R0a2m	R0a2m	O	1	R0a2m	O	1
3584	JQ705196	R0a2m	R0a2m	O	1	R0a2m	O	1
3585	KC911373	R0a2n	R0a2n	O	1	R0a2n	O	1
3586	JF717356	R0a2n	R0a2n	O	1	R0a2n	O	1

3587	HM185223	R0a3	R0a3	O	1	R0a3	O	1
3588	KC911556	R0a3	R0a3	O	1	R0a3	O	1
3589	HM185265	R0a3a	R0a3a	O	1	R0a3a	O	1
3590	HM185224	R0a3a	R0a3a	O	1	R0a3a	O	1
3591	JQ702940	R0a4	R0a4	O	1	R0a4	O	1
3592	JQ705305	R0a4	R0a4	O	1	R0a4	O	1
3593	JF717361	R0b	R0b	O	1	R0b	O	1
3594	KC911472	HV	HV	O	1	HV	O	1
3595	JQ705599	HV0	HV0	O	1	HV0	O	1
3596	JX153274	HV0	HV0	O	1	HV0	O	1
3597	JQ704188	HV0a	HV0a	O	1	HV0a	O	1
3598	AY738945	HV0a	HV0a	O	1	HV0a	O	1
3599	GU122990	HV0a1	HV0a1	O	1	HV0a1	O	1
3600	JX153134	HV0a1	HV0a1	O	1	HV0a1	O	1
3601	EF649971	HV0a1a	HV0a1a	O	1	HV0a1a	O	1
3602	JQ701930	HV0a1a	HV0a1a	O	1	HV0a1a	O	1
3603	AY495311	V	V	O	1	V	O	1
3604	JX153964	V	V	O	1	V	O	1
3605	JQ702026	V1a	V1a	O	1	V1a	O	1
3606	EF177435	V1a	V1a	O	1	V1a	O	1
3607	AY495325	V1a1	V1a1	O	1	V1a1	O	1
3608	JQ702498	V1a1	V1a1	O	1	V1a1	O	1
3609	AY339438	V1a1a	V1a1a	O	1	V1a1a	O	1
3610	JX153841	V1a1a	V1a1a	O	1	V1a1a	O	1
3611	JX153552	V1a1a1	V1a1a1	O	1	V1a1a1	O	1
3612	GU123003	V1a1a1	V1a1a1	O	1	V1a1a1	O	1
3613	EF661005	V1a1b	V1a1b	O	1	V1a1b	O	1
3614	EF177419	V1a1b	V1a1b	O	1	V1a1b	O	1
3615	JQ702938	V1b	V1b	O	1	V1b	O	1
3616	JQ704342	V1b	V1b	O	1	V1b	O	1
3617	JQ705658	V2	V2	O	1	V2	O	1
3618	JQ703647	V2	V2	O	1	V2	O	1
3619	AY713979	V2a	V2a	O	1	V2a	O	1
3620	AY495322	V2a1	V2a1	O	1	V2a1	O	1
3621	AY495317	V2a1a	V2a1a	O	1	V2a1a	O	1
3622	AY495323	V2a1a	V2a1a	O	1	V2a1a	O	1
3623	AY495313	V2b	V2b	O	1	V2b	O	1

3624	AY495319	V2b1	V2b1	O	1	V2b1	O	1
3625	AY495310	V2b1	V2b1	O	1	V2b1	O	1
3626	JQ704063	V2b2	V2b2	O	1	V2b2	O	1
3627	JX153314	V2b2	V2b2	O	1	V2b2	O	1
3628	JQ705254	V2c	V2c	O	1	V2c	O	1
3629	JX153985	V2c	V2c	O	1	V2c	O	1
3630	JQ703666	V3	V3	O	1	V3	O	1
3631	JQ705789	V3a	V3a	O	1	V3a	O	1
3632	JX297186	V3a	V3a	O	1	V3a	O	1
3633	JQ702667	V3a1	V3a1	O	1	V3a1	O	1
3634	KC257399	V3a1	V3a1	O	1	V3a1	O	1
3635	JQ704036	V3b	V3b	O	1	V3b	O	1
3636	AY495330	V3b	V3b	O	1	V3b	O	1
3637	EU567326	V3c	V3c	O	1	V3c	O	1
3638	JQ705638	V3c	V3c	O	1	V3c	O	1
3639	AY495312	V4	V4	O	1	V4	O	1
3640	FJ384440	V4	V4	O	1	V4	O	1
3641	AY339451	V5	V5	O	1	V5	O	1
3642	AY339452	V5	V5	O	1	V5	O	1
3643	FJ348207	V6	V6	O	1	V+@72	X	2
3644	EF506486	V6	V6	O	1	V6	O	1
3645	JQ705798	V7	V7	O	1	V7	O	1
3646	JQ702803	V7	V7	O	1	V7	O	1
3647	JQ703830	V7a	V7a	O	1	V7a	O	1
3648	EU567454	V7a	V7a	O	1	V7a	O	1
3649	AY339446	V7a1	V7a1	O	1	V7a1	O	1
3650	JQ702569	V7a1	V7a1	O	1	V7a1	O	1
3651	JQ705945	V7b	V7b	O	1	V7b	O	1
3652	JQ704660	V7b	V7b	O	1	V7b	O	1
3653	JQ702025	V8	V8	O	1	V8	O	1
3654	JQ705604	V8	V8	O	1	V8	O	1
3655	EF177445	V9	V9	O	1	V9	O	1
3656	FJ865560	V9a1	V9a1	O	1	V9a1	O	1
3657	DQ658411	V9a1	V9a1	O	1	V9a1	O	1
3658	JQ702145	V9a2	V9a2	O	1	V9a2	O	1
3659	JQ704826	V9a2	V9a2	O	1	V9a2	O	1
3660	JQ704602	V10a	V10a	O	1	V10a	O	1

3661	HQ729918	V10a	V10a	O	1	V10a	O	1
3662	KF142158	V10b	V10b	O	1	V10b	O	1
3663	JQ703770	V10b1	V10b1	O	1	V10b1	O	1
3664	GQ304748	V10b1	V10b1	O	1	V10b1	O	1
3665	HM570048	V10b2	V10b2	O	1	V10b2	O	1
3666	JX153465	V10b2	V10b2	O	1	V10b2	O	1
3667	JQ704405	V11	V11	O	1	V11	O	1
3668	JQ705396	V11	V11	O	1	V11	O	1
3669	AY495321	V12	V12	O	1	V12	O	1
3670	JQ705506	V12	V12	O	1	V12	O	1
3671	GU122979	V13	V13	O	1	V13	O	1
3672	JQ702456	V13	V13	O	1	V13	O	1
3673	JQ702206	V14	V14	O	1	V14	O	1
3674	JQ703215	V14	V14	O	1	V14	O	1
3675	HQ645963	V15	V15	O	1	V15	O	1
3676	JQ703991	V15a	V15a	O	1	V15a	O	1
3677	JQ702391	V15a	V15a	O	1	V15a	O	1
3678	JQ704468	V16	V16	O	1	V16	O	1
3679	KC257385	V16	V16	O	1	V16	O	1
3680	JQ703973	V17	V17	O	1	V17	O	1
3681	GU362082	V17	V17	O	1	V17	O	1
3682	FJ348173	V18	V18	O	1	V18	O	1
3683	JX153154	V18a	V18a	O	1	V18a	O	1
3684	JQ702721	V19	V19	O	1	V19	O	1
3685	JQ703749	V19	V19	O	1	V19	O	1
3686	JX297133	V22	V22	O	1	V22	O	1
3687	JX297137	V22	V22	O	1	V22	O	1
3688	AY495118	V+@16298	V+@16298	O	1	V+@16298	O	1
3689	AY495109	V+@16298	V+@16298	O	1	V+@16298	O	1
3690	JQ705168	V20	V20	O	1	V20	O	1
3691	JQ704703	V20	V20	O	1	V20	O	1
3692	JQ701938	V+@72	V+@72	O	1	V+@72	O	1
3693	JQ705610	V+@72	V+@72	O	1	V+@72	O	1
3694	JQ702689	V21	V21	O	1	V21	O	1
3695	JQ704945	V21	V21	O	1	V21	O	1
3696	JQ704988	V23	V23	O	1	V23	O	1
3697	NA11893	V23	V+@72	X	2	V+@72	X	2

3698	HQ711364	V24	V24	O	1	V24	O	1
3699	JQ704340	V24	V24	O	1	V+@16298	X	2
3700	NA19725	V25	V25	O	1	V25	O	1
3701	AF381990	V25	V25	O	1	V25	O	1
3702	JF502419	V26	V26	O	1	V26	O	1
3703	HQ615879	V26	V26	O	1	V26	O	1
3704	JX153521	V27	V27	O	1	V27	O	1
3705	JQ705885	V27	V27	O	1	V27	O	1
3706	JX153920	V28	V28	O	1	V28	O	1
3707	GU827698	V28	V28	O	1	V28	O	1
3708	JQ703113	HV0+195	HV0+195	O	1	HV0+195	O	1
3709	JQ704255	HV0+195	HV0+195	O	1	HV0+195	O	1
3710	EF556192	HV0b	HV0b	O	1	HV0b	O	1
3711	NA11932	HV0b	HV0b	O	1	HV0b	O	1
3712	JQ704143	HV0c	HV0c	O	1	HV0c	O	1
3713	EF471977	HV0c	HV0c	O	1	HV0c	O	1
3714	JN794568	HV0d	HV0d	O	1	HV0d	O	1
3715	JQ702222	HV0d	HV0d	O	1	HV0d	O	1
3716	JQ705967	HV0e	HV0e	O	1	HV0e	O	1
3717	JX153089	HV0e	HV0e	O	1	HV0e	O	1
3718	JQ705127	HV0f	HV0f	O	1	HV0f	O	1
3719	EF660968	HV0f	HV0f	O	1	HV0f	O	1
3720	JQ704142	HV0g	HV0g	O	1	HV0g	O	1
3721	JX297182	HV0g	HV0g	O	1	HV0g	O	1
3722	JF320654	HV1	HV1	O	1	HV1	O	1
3723	AY738943	HV1a'b'c	HV1a'b'c	O	1	HV1a'b'c	O	1
3724	EF660936	HV1a'b'c	HV1a'b'c	O	1	HV1a'b'c	O	1
3725	HM852761	HV1a1	HV1a1	O	1	HV1a1	O	1
3726	HM575427	HV1a1a	HV1a1a	O	1	HV1a1a	O	1
3727	JF260938	HV1a1a	HV1a1a	O	1	HV1a1a	O	1
3728	FJ210914	HV1a1b	HV1a1b	O	1	HV1a1b	O	1
3729	KC257350	HV1a2	HV1a2	O	1	HV1a2	O	1
3730	JF260943	HV1a2	HV1a2	O	1	HV1a2	O	1
3731	JF260942	HV1a2a	HV1a2a	O	1	HV1a2a	O	1
3732	EU935461	HV1a2a	HV1a2a	O	1	HV1a2a	O	1
3733	JF316743	HV1a2b	HV1a2b	O	1	HV1a2b	O	1
3734	JX153051	HV1a2b	HV1a2b	O	1	HV1a2b	O	1

3735	JF260935	HV1a3	HV1a3	O	1	HV1a3	O	1
3736	JF260937	HV1a3	HV1a3	O	1	HV1a3	O	1
3737	KC911597	HV1a3a	HV1a3a	O	1	HV1a3a	O	1
3738	JF260936	HV1a3a	HV1a3a	O	1	HV1a3a	O	1
3739	AY738942	HV1b	HV1b	O	1	HV1b	O	1
3740	EF556182	HV1b1	HV1b1	O	1	HV1b1	O	1
3741	JF260951	HV1b1a	HV1b1a	O	1	HV1b1a	O	1
3742	JF260949	HV1b1a	HV1b1a	O	1	HV1b1a	O	1
3743	JF260948	HV1b1b	HV1b1b	O	1	HV1b1b	O	1
3744	JF260947	HV1b1b	HV1b1b	O	1	HV1b1b	O	1
3745	JF260944	HV1b+152	HV1b+152	O	1	HV1b+152	O	1
3746	DQ856316	HV1b2	HV1b2	O	1	HV1b2	O	1
3747	EF396958	HV1b2	HV1b2	O	1	HV1b2	O	1
3748	HM998901	HV1b3a	HV1b3a	O	1	HV1b3a	O	1
3749	HQ412622	HV1b3a	HV1b3a	O	1	HV1b3a	O	1
3750	KC911450	HV1b3b	HV1b3b	O	1	HV1b3b	O	1
3751	JQ704284	HV1b3b	HV1b3b	O	1	HV1b3b	O	1
3752	EF556190	HV1c	HV1c	O	1	HV1c	O	1
3753	JQ705526	HV1c	HV1c	O	1	HV1c	O	1
3754	JF260933	HV1d	HV1d	O	1	HV1d	O	1
3755	JF260932	HV1d	HV1d	O	1	HV1d	O	1
3756	KC911408	HV2	HV2	O	1	HV2	O	1
3757	EU567456	HV2a1	HV2a1	O	1	HV2a1	O	1
3758	HM852806	HV2a1	HV2a1	O	1	HV2a1	O	1
3759	AY713986	HV2a2	HV2a2	O	1	HV2a2	O	1
3760	KC911452	HV2a2	HV2a2	O	1	HV2a2	O	1
3761	KC911372	HV2a3	HV2a3	O	1	HV2a3	O	1
3762	HM852860	HV2a3	HV2a3	O	1	HV2a3	O	1
3763	HM036578	HV20	HV20	O	1	HV20	O	1
3764	EU935433	HV20	HV20	O	1	HV20	O	1
3765	EF417833	HV4	HV4	O	1	HV4	O	1
3766	EU545447	HV4	HV4	O	1	HV4	O	1
3767	JN214428	HV4a1	HV4a1	O	1	HV4a1	O	1
3768	EF660939	HV4a1	HV4a1	O	1	HV4a1	O	1
3769	JN214427	HV4a1+16291	HV4a1+16291	O	1	HV4a1+16291	O	1
3770	JN214395	HV4a1a	HV4a1a	O	1	HV4a1a	O	1
3771	JX297154	HV4a1a	HV4a1a	O	1	HV4a1a	O	1

3772	JN214392	HV4a1a1	HV4a1a1	O	1	HV4a1a1	O	1
3773	JN214391	HV4a1a1	HV4a1a1	O	1	HV4a1a1	O	1
3774	JN214397	HV4a1a2	HV4a1a2	O	1	HV4a1a2	O	1
3775	JN214400	HV4a1a2	HV4a1a2	O	1	HV4a1a2	O	1
3776	HQ675034	HV4a1a3	HV4a1a3	O	1	HV4a1a3	O	1
3777	GQ888730	HV4a1a3	HV4a1a3	O	1	HV4a1+16291	X	2
3778	JN214410	HV4a1a4	HV4a1a4	O	1	HV4a1a4	O	1
3779	JN214418	HV4a1a4	HV4a1a4	O	1	HV4a1a4	O	1
3780	GQ888731	HV4a2a	HV4a2a	O	1	HV4a2a	O	1
3781	HM228422	HV4a2a	HV4a2a	O	1	HV4a2a	O	1
3782	EU935457	HV4a2b	HV4a2b	O	1	HV4a2b	O	1
3783	JN214429	HV4a2b	HV4a2b	O	1	HV4a2b	O	1
3784	EF222234	HV4b	HV4b	O	1	HV4b	O	1
3785	HM852851	HV4b	HV4b	O	1	HV4b	O	1
3786	JQ272477	HV4c	HV4c	O	1	HV4c	O	1
3787	AY738941	HV4c	HV4c	O	1	HV4c	O	1
3788	EF419890	HV5a	HV5a	O	1	HV5a	O	1
3789	EU558385	HV5a	HV5a	O	1	HV5a	O	1
3790	KC911476	HV5b	HV5b	O	1	HV5b	O	1
3791	HQ593806	HV5b	HV5b	O	1	HV5b	O	1
3792	KC765916	HV+16311	HV+16311	O	1	HV+16311	O	1
3793	AY713981	HV+16311	HV+16311	O	1	HV+16311	O	1
3794	HQ658738	HV6	HV6	O	1	HV6	O	1
3795	EF222232	HV6	HV6	O	1	HV6	O	1
3796	EU545454	HV6a	HV6a	O	1	HV6a	O	1
3797	EU545439	HV6a	HV6a	O	1	HV6a	O	1
3798	EU545443	HV7	HV7	O	1	HV7	O	1
3799	JQ701908	HV7	HV7	O	1	HV7	O	1
3800	EU545457	HV8	HV8	O	1	HV8	O	1
3801	EF222246	HV8	HV8	O	1	HV8	O	1
3802	JQ703367	HV9	HV9	O	1	HV9	O	1
3803	JX153443	HV9	HV9	O	1	HV9	O	1
3804	EF222253	HV9+152	HV9+152	O	1	HV9+152	O	1
3805	JQ703720	HV9+152	HV9+152	O	1	HV9+152	O	1
3806	EU545422	HV9a	HV9a	O	1	HV9a	O	1
3807	JQ705368	HV9a	HV9a	O	1	HV9a	O	1
3808	KC257361	HV9a1	HV9a1	O	1	HV9a1	O	1

3809	JQ704229	HV9a1	HV9a1	O	1	HV9a1	O	1
3810	EF222247	HV9a1a	HV9a1a	O	1	HV9a1a	O	1
3811	JQ703659	HV9a1a	HV9a1a	O	1	HV9a1a	O	1
3812	FJ147309	HV9b	HV9b	O	1	HV9b	O	1
3813	JQ704799	HV9b	HV9b	O	1	HV9b	O	1
3814	KC911421	HV9c	HV9c	O	1	HV9c	O	1
3815	NA20814	HV9c	HV9c	O	1	HV9c	O	1
3816	EF222245	HV10	HV10	O	1	HV10	O	1
3817	HM008695	HV10	HV10	O	1	HV10	O	1
3818	JQ702048	HV11	HV11	O	1	HV11	O	1
3819	GU592045	HV11a	HV11a	O	1	HV11a	O	1
3820	EF660954	HV11a	HV11a	O	1	HV11a	O	1
3821	KC911391	HV14	HV14	O	1	HV14	O	1
3822	KC911456	HV14a	HV14a	O	1	HV14a	O	1
3823	NA18743	HV14a	HV14a	O	1	HV14a	O	1
3824	JQ704184	HV15	HV15	O	1	HV15	O	1
3825	NA12489	HV15	HV15	O	1	HV15	O	1
3826	HQ658354	HV16	HV16	O	1	HV16	O	1
3827	JQ704124	HV16	HV16	O	1	HV16	O	1
3828	EU545424	HV17	HV17	O	1	HV17	O	1
3829	JQ702596	HV17a	HV17a	O	1	HV17a	O	1
3830	JQ705416	HV17a	HV17a	O	1	HV17a	O	1
3831	EF660945	HV22	HV22	O	1	HV22	O	1
3832	EF660975	HV22	HV22	O	1	HV22	O	1
3833	KC911525	HV23	HV23	O	1	HV23	O	1
3834	FJ460528	HV23	HV23	O	1	HV23	O	1
3835	KC257368	HV24	HV24	O	1	HV24	O	1
3836	NA20506	HV24	HV24	O	1	HV24	O	1
3837	HM852849	HV12a	HV12a	O	1	HV12a	O	1
3838	HQ844516	HV12a1	HV12a1	O	1	HV12a1	O	1
3839	JN053060	HV12a1	HV12a1	O	1	HV12a1	O	1
3840	AY713976	HV12b	HV12b	O	1	HV12b	O	1
3841	HM852785	HV12b1	HV12b1	O	1	HV12b1	O	1
3842	AY713987	HV12b1a	HV12b1a	O	1	HV12b1a	O	1
3843	KC533522	HV12b1a	HV12b1a	O	1	HV12b1a	O	1
3844	HM852828	HV13a	HV13a	O	1	HV13a	O	1
3845	KC911298	HV13a	HV13a	O	1	HV13a	O	1

3846	JX153457	HV13b	HV13b	O	1	HV13b	O	1
3847	JF700125	HV13b	HV13b	O	1	HV13b	O	1
3848	JX153087	HV18	HV18	O	1	HV18	O	1
3849	KC911471	HV18	HV18	O	1	HV18	O	1
3850	HQ436102	HV19	HV19	O	1	HV19	O	1
3851	KC911439	HV19	HV19	O	1	HV19	O	1
3852	KF729951	HV21	HV21	O	1	HV21	O	1
3853	HQ287727	HV21	HV21	O	1	HV21	O	1
3854	JQ705953	H	H	O	1	H	O	1
3855	HQ384174	H1	H1	O	1	H1	O	1
3856	HM103358	H1	H1	O	1	H1	O	1
3857	EU369376	H1a	H1a	O	1	H1a	O	1
3858	JX153520	H1a	H1a	O	1	H1a	O	1
3859	AY495188	H1a1	H1a1	O	1	H1a1	O	1
3860	JQ702187	H1a1	H1a1	O	1	H1a1	O	1
3861	JQ704413	H1a1a	H1a1a	O	1	H1a1a	O	1
3862	JQ703901	H1a1a	H1a1a	O	1	H1a1a	O	1
3863	JQ704265	H1a1a1	H1a1a1	O	1	H1a1a1	O	1
3864	AY495189	H1a1a1	H1a1a1	O	1	H1a1a1	O	1
3865	JQ703424	H1a1b	H1a1b	O	1	H1a1b	O	1
3866	AY495190	H1a1b	H1a1b	O	1	H1a1b	O	1
3867	JX153501	H1a1c	H1a1c	O	1	H1a1c	O	1
3868	JX153539	H1a1c	H1a1c	O	1	H1a1c	O	1
3869	AY339422	H1a2	H1a2	O	1	H1a2	O	1
3870	EU130562	H1a2	H1a2	O	1	H1a2	O	1
3871	EU747355	H1a3	H1a3	O	1	H1a3	O	1
3872	JQ704204	H1a3	H1a3	O	1	H1a3	O	1
3873	JQ704050	H1a3a	H1a3a	O	1	H1a3a	O	1
3874	HQ287892	H1a3a	H1a3a	O	1	H1a3a	O	1
3875	JQ704612	H1a3a1	H1a3a1	O	1	H1a3a1	O	1
3876	JQ705169	H1a3a1	H1a3a1	O	1	H1a3a1	O	1
3877	JQ704838	H1a3a2	H1a3a2	O	1	H1a3a2	O	1
3878	HM589042	H1a3a2	H1a3a2	O	1	H1a3a2	O	1
3879	AY738972	H1a3a3	H1a3a3	O	1	H1a3a3	O	1
3880	NA20768	H1a3a3	H1a3a3	O	1	H1a3a3	O	1
3881	JQ324542	H1a3a4	H1a3a4	O	1	H1a3a4	O	1
3882	EF177447	H1a3a4	H1a3a4	O	1	H1a3a4	O	1

3883	HQ659848	H1a3b	H1a3b	O	1	H1a3b	O	1
3884	JQ703135	H1a3b	H1a3b	O	1	H1a3b	O	1
3885	JQ705533	H1a3b1	H1a3b1	O	1	H1a3b1	O	1
3886	JQ703683	H1a3b1	H1a3b1	O	1	H1a3b1	O	1
3887	JQ702698	H1a3c	H1a3c	O	1	H1a3c	O	1
3888	EU979418	H1a3c	H1a3c	O	1	H1a3c	O	1
3889	JX153342	H1a3c1	H1a3c1	O	1	H1a3c1	O	1
3890	JX153942	H1a3c1	H1a3c1	O	1	H1a3c1	O	1
3891	JX152881	H1a3d	H1a3d	O	1	H1a3d	O	1
3892	JQ704536	H1a3d	H1a3d	O	1	H1a3d	O	1
3893	JQ704223	H1a4	H1a4	O	1	H1a4	O	1
3894	HQ287891	H1a4	H1a4	O	1	H1a4	O	1
3895	JQ702535	H1a5	H1a5	O	1	H1a5	O	1
3896	JQ704525	H1a5	H1a5	O	1	H1a5	O	1
3897	JQ702474	H1a6	H1a6	O	1	H1a6	O	1
3898	JX153364	H1a6	H1a6	O	1	H1a6	O	1
3899	GU797829	H1a7	H1a7	O	1	H1a7	O	1
3900	JQ701944	H1a8	H1a8	O	1	H1a8	O	1
3901	JX153239	H1a8	H1a8	O	1	H1a8	O	1
3902	JQ703758	H1a8a	H1a8a	O	1	H1a8a	O	1
3903	JX153203	H1a8a	H1a8a	O	1	H1a8a	O	1
3904	KF052033	H1a9	H1a9	O	1	H1a9	O	1
3905	KC121273	H1a9	H1a9	O	1	H1a9	O	1
3906	JQ406575	H1+16189	H1+16189	O	1	H1+16189	O	1
3907	JN603189	H1b	H1b	O	1	H1b	O	1
3908	JQ702703	H1b	H1b	O	1	H1b	O	1
3909	KC257358	H1b1	H1b1	O	1	H1b1	O	1
3910	JQ704003	H1b1	H1b1	O	1	H1b1	O	1
3911	EU219920	H1b1+16362	H1b1+16362	O	1	H1b1+16362	O	1
3912	GU724771	H1b1+16362	H1b1+16362	O	1	H1b1+16362	O	1
3913	GU122983	H1b1a	H1b1a	O	1	H1b1a	O	1
3914	AY738975	H1b1a	H1b1a	O	1	H1b1a	O	1
3915	JQ701966	H1b1b	H1b1b	O	1	H1b1b	O	1
3916	JQ703941	H1b1b	H1b1b	O	1	H1b1b	O	1
3917	JQ704159	H1b1c	H1b1c	O	1	H1b1c	O	1
3918	JQ702527	H1b1c	H1b1c	O	1	H1b1c	O	1
3919	JQ705126	H1b1d	H1b1d	O	1	H1b1d	O	1

3920	JQ703500	H1b1h	H1b1h	O	1	H1b1h	O	1
3921	JQ702455	H1b1h	H1b1h	O	1	H1b1h	O	1
3922	JQ703838	H1b1e	H1b1e	O	1	H1b1e	O	1
3923	JQ704913	H1b1e	H1b1e	O	1	H1b1e	O	1
3924	JX152804	H1b1e1	H1b1e1	O	1	H1b1e1	O	1
3925	JQ702763	H1b1e1	H1b1e1	O	1	H1b1e1	O	1
3926	JQ703254	H1b1f	H1b1f	O	1	H1b1f	O	1
3927	GU797136	H1b1f	H1b1f	O	1	H1b1f	O	1
3928	JQ703357	H1b1g	H1b1g	O	1	H1b1g	O	1
3929	JX153222	H1b1i	H1b1i	O	1	H1b1i	O	1
3930	JX153213	H1b1i	H1b1i	O	1	H1b1i	O	1
3931	JQ702529	H1b2	H1b2	O	1	H1b2	O	1
3932	JQ706010	H1b2	H1b2	O	1	H1b2	O	1
3933	JQ702566	H1b2a	H1b2a	O	1	H1b2a	O	1
3934	JQ704894	H1b2a1	H1b2a1	O	1	H1b2a1	O	1
3935	JQ703268	H1b2a1	H1b2a1	O	1	H1b2a1	O	1
3936	JQ704139	H1b3	H1b3	O	1	H1b3	O	1
3937	JX153876	H1b3	H1b3	O	1	H1b3	O	1
3938	JQ324829	H1b4	H1b4	O	1	H1b4	O	1
3939	JQ324933	H1b4	H1b4	O	1	H1b4	O	1
3940	JX152798	H1b5	H1b5	O	1	H1b5	O	1
3941	JQ704486	H1b5	H1b5	O	1	H1b5	O	1
3942	JQ704298	H1f	H1f	O	1	H1f	O	1
3943	JQ703322	H1f+16093	H1f+16093	O	1	H1f+16093	O	1
3944	AY339413	H1f1	H1f1	O	1	H1f1	O	1
3945	KC785095	H1f1	H1f1	O	1	H1f1	O	1
3946	JQ705607	H1f1a	H1f1	X	2	H1f1a	O	1
3947	JQ704916	H1f1a	H1f1	X	2	H1f1a	O	1
3948	JQ701945	H1g1	H1g1	O	1	H1g1	O	1
3949	GU461664	H1g1	H1g1	O	1	H1g1	O	1
3950	JQ703948	H1g2	H1g2	O	1	H1g2	O	1
3951	EU768844	H1k1	H1k1	O	1	H1k1	O	1
3952	EU770202	H1k1a	H1k1a	O	1	H1k1a	O	1
3953	EU779660	H1k1a	H1k1a	O	1	H1k1a	O	1
3954	JQ704157	H1y	H1y	O	1	H1y	O	1
3955	JQ703220	H1y	H1y	O	1	H1y	O	1
3956	JQ703527	H1z	H1z	O	1	H1z	O	1

3957	JQ704562	H1z1	H1z1	O	1	H1z1	O	1
3958	JQ702057	H1aa1	H1aa1	O	1	H1aa1	O	1
3959	JX153082	H1aa1	H1aa1	O	1	H1aa1	O	1
3960	JQ704715	H1ab	H1ab	O	1	H1ab	O	1
3961	AY738982	H1ab1	H1ab1	O	1	H1ab1	O	1
3962	JQ324765	H1ab1	H1ab1	O	1	H1ab1	O	1
3963	JQ704994	H1ac	H1ac	O	1	H1ac	O	1
3964	JQ704571	H1ad	H1ad	O	1	H1ad	O	1
3965	JQ703473	H1ad	H1ad	O	1	H1ad	O	1
3966	JQ705678	H1cc	H1cc	O	1	H1cc	O	1
3967	HQ658466	H1cc	H1cc	O	1	H1cc	O	1
3968	JQ705712	H1c	H1c	O	1	H1c	O	1
3969	JQ702532	H1c	H1c	O	1	H1c	O	1
3970	AY495157	H1c1	H1c1	O	1	H1c1	O	1
3971	JQ703418	H1c1	H1c1	O	1	H1c1	O	1
3972	AY495160	H1c1a	H1c1a	O	1	H1c1a	O	1
3973	AY495163	H1c1a	H1c1a	O	1	H1c1a	O	1
3974	NA12399	H1c1a1	H1c1a1	O	1	H1c1a1	O	1
3975	JQ702436	H1c1a1	H1c1a1	O	1	H1c1a1	O	1
3976	JQ702237	H1c1b	H1c1b	O	1	H1c1b	O	1
3977	JQ702099	H1c1b	H1c1b	O	1	H1c1b	O	1
3978	JX153294	H1c1c	H1c1c	O	1	H1c1c	O	1
3979	JX153899	H1c1c	H1c1c	O	1	H1c1c	O	1
3980	JQ703153	H1c1+16093	H1c1+16093	O	1	H1c1+16093	O	1
3981	JX153157	H1c1+16093	H1c1+16093	O	1	H1c1+16093	O	1
3982	JX153421	H1c1d	H1c1d	O	1	H1c1d	O	1
3983	JX152893	H1c1d	H1c1d	O	1	H1c1d	O	1
3984	HQ681884	H1c2	H1c2	O	1	H1c2	O	1
3985	JQ703166	H1c2	H1c2	O	1	H1c2	O	1
3986	JQ704438	H1c2a	H1c2a	O	1	H1c2a	O	1
3987	AY495096	H1c2a	H1c2a	O	1	H1c2a	O	1
3988	JQ703496	H1c3	H1c3	O	1	H1c3	O	1
3989	JQ702738	H1c3	H1c3	O	1	H1c3	O	1
3990	JQ704119	H1c3a	H1c3a	O	1	H1c3a	O	1
3991	JQ703142	H1c3a	H1c3a	O	1	H1c3a	O	1
3992	GU289555	H1c3b	H1c3b	O	1	H1c3b	O	1
3993	GQ332765	H1c3b	H1c3b	O	1	H1c3b	O	1

3994	JQ705516	H1c4	H1c4	O	1	H1c4	O	1
3995	JX152947	H1c4a	H1c4a	O	1	H1c4a	O	1
3996	JX297157	H1c4a1	H1c4a1	O	1	H1c4a1	O	1
3997	JQ324836	H1c4a1	H1c4a1	O	1	H1c4a1	O	1
3998	JQ704378	H1c4b	H1c4b	O	1	H1c4b	O	1
3999	JQ703499	H1c4b	H1c4b	O	1	H1c4b	O	1
4000	JQ701984	H1c4b1	H1c4b1	O	1	H1c4b1	O	1
4001	GU122996	H1c4b1	H1c4b1	O	1	H1c4b1	O	1
4002	AY495102	H1c5	H1c5	O	1	H1c5	O	1
4003	JQ703745	H1c5a	H1c5	X	2	H1c5a	O	1
4004	JQ702006	H1c5a	H1c5	X	2	H1c5a	O	1
4005	JQ704080	H1c6	H1c6	O	1	H1c6	O	1
4006	GU812902	H1c6	H1c6	O	1	H1c6	O	1
4007	JQ702850	H1c7	H1c7	O	1	H1c7	O	1
4008	JQ702873	H1c7	H1c7	O	1	H1c7	O	1
4009	JQ704294	H1c8	H1c8	O	1	H1c8	O	1
4010	JQ702867	H1c8	H1c8	O	1	H1c8	O	1
4011	JQ701993	H1c+152	H1c+152	O	1	H1c+152	O	1
4012	AY495141	H1c+152	H1c+152	O	1	H1c+152	O	1
4013	JQ703810	H1c9	H1c9	O	1	H1c9	O	1
4014	JQ703637	H1c9a	H1c9a	O	1	H1c9a	O	1
4015	JQ703459	H1c9a	H1c9a	O	1	H1c9a	O	1
4016	JQ705468	H1c10	H1c10	O	1	H1c10	O	1
4017	JQ705495	H1c11	H1c11	O	1	H1c11	O	1
4018	JQ704352	H1c11	H1c11	O	1	H1c11	O	1
4019	HM462218	H1c12	H1c12	O	1	H1c12	O	1
4020	JQ704242	H1c12	H1c12	O	1	H1c12	O	1
4021	JQ702501	H1c13	H1c13	O	1	H1c13	O	1
4022	AY495139	H1c13	H1c13	O	1	H1c+152	X	2
4023	JQ703342	H1c14	H1c14	O	1	H1c14	O	1
4024	JQ703551	H1c15	H1c	X	18	H1c15	O	1
4025	JQ704909	H1c15	H1c+152	X	19	H1c+152	X	2
4026	HQ658607	H1c16	H1c16	O	1	H1c16	O	1
4027	JQ324739	H1c16	H1c16	O	1	H1c+152	X	2
4028	HQ875780	H1c17	H1c17	O	1	H1c17	O	1
4029	JQ705238	H1c17	H1c17	O	1	H1c17	O	1
4030	JQ705757	H1c18	H1c18	O	1	H1c18	O	1

4031	JX153132	H1c18	H1c18	O	1	H1c18	O	1
4032	JQ703126	H1c19	H1c19	O	1	H1c19	O	1
4033	JQ703998	H1c19	H1c19	O	1	H1c19	O	1
4034	JQ703383	H1c20	H1c20	O	1	H1c20	O	1
4035	JQ702607	H1c20	H1c20	O	1	H1c20	O	1
4036	JX153194	H1c22	H1c22	O	1	H1c22	O	1
4037	FJ348214	H1c22	H1c22	O	1	H1c22	O	1
4038	JQ702328	H1e	H1e	O	1	H1e	O	1
4039	DQ523657	H1e	H1e	O	1	H1e	O	1
4040	JQ705024	H1e1	H1e1	O	1	H1e1	O	1
4041	EF177428	H1e1a	H1e1a	O	1	H1e1a	O	1
4042	JQ703438	H1e1a	H1e1a	O	1	H1e1a	O	1
4043	JQ324873	H1e1a1	H1e1a1	O	1	H1e1a1	O	1
4044	JQ324659	H1e1a1	H1e1a1	O	1	H1e1a1	O	1
4045	JQ705496	H1e1a2	H1e1a2	O	1	H1e1a2	O	1
4046	HQ658480	H1e1a3	H1e1a3	O	1	H1e1a3	O	1
4047	EU914954	H1e1a3	H1e1a3	O	1	H1e1a3	O	1
4048	JQ324756	H1e1a4	H1e1a4	O	1	H1e1a4	O	1
4049	JQ324691	H1e1a4	H1e1a4	O	1	H1e1a4	O	1
4050	JQ705884	H1e1a5	H1e1a5	O	1	H1e1a5	O	1
4051	FJ348185	H1e1a5	H1e1a5	O	1	H1e1a5	O	1
4052	JX297199	H1e1a6	H1e1a6	O	1	H1e1a6	O	1
4053	JQ703576	H1e1a6	H1e1a6	O	1	H1e1a6	O	1
4054	JX153958	H1e1a7	H1e1a7	O	1	H1e1a7	O	1
4055	JQ324885	H1e1a7	H1e1a7	O	1	H1e1a7	O	1
4056	JQ704464	H1e1a+16278	H1e1a+16278	O	1	H1e1a+16278	O	1
4057	JX153401	H1e1a8	H1e1a8	O	1	H1e1a8	O	1
4058	JQ705331	H1e1a8	H1e1a8	O	1	H1e1a8	O	1
4059	JQ702833	H1e1b	H1e1b	O	1	H1e1b	O	1
4060	JQ705799	H1e1b1	H1e1b1	O	1	H1e1b1	O	1
4061	JQ705809	H1e1b1	H1e1b1	O	1	H1e1b1	O	1
4062	JQ702649	H1e1b1a	H1e1b1a	O	1	H1e1b1a	O	1
4063	NA20513	H1e1b1a	H1e1b1a	O	1	H1e1b1a	O	1
4064	JQ704656	H1e1b1b	H1e1b1b	O	1	H1e1b1b	O	1
4065	JQ702104	H1e1b1b	H1e1b1b	O	1	H1e1b1b	O	1
4066	JX152857	H1e1c	H1e1c	O	1	H1e1c	O	1
4067	JQ703603	H1e1c	H1e1c	O	1	H1e1c	O	1

4068	HQ880578	H1e2	H1e2	O	1	H1e2	O	1
4069	JQ705717	H1e2	H1e2	O	1	H1e2	O	1
4070	JQ704343	H1e2a	H1e2a	O	1	H1e2a	O	1
4071	JQ704128	H1e2a	H1e2a	O	1	H1e2a	O	1
4072	JQ702992	H1e2b	H1e2b	O	1	H1e2b	O	1
4073	JQ703207	H1e2b	H1e2b	O	1	H1e2b	O	1
4074	JQ703115	H1e2c	H1e2c	O	1	H1e2c	O	1
4075	AY738971	H1e2c	H1e2c	O	1	H1e2c	O	1
4076	JQ702491	H1e2d	H1e2d	O	1	H1e2d	O	1
4077	NA20803	H1e2d	H1e2d	O	1	H1e2d	O	1
4078	EU555475	H1e+16129	H1e+16129	O	1	H1e+16129	O	1
4079	HQ663877	H1e3	H1e3	O	1	H1e3	O	1
4080	JQ324720	H1e3	H1e3	O	1	H1e3	O	1
4081	JQ703196	H1e4	H1e4	O	1	H1e4	O	1
4082	JQ704392	H1e4	H1e4	O	1	H1e4	O	1
4083	EU148452	H1e4a	H1e4a	O	1	H1e4a	O	1
4084	EU262984	H1e4a	H1e4a	O	1	H1e4a	O	1
4085	EF660933	H1e5a	H1e5a	O	1	H1e5a	O	1
4086	JQ704250	H1e5a	H1e5a	O	1	H1e5a	O	1
4087	JX153041	H1e5b	H1e5b	O	1	H1e5b	O	1
4088	JX153088	H1e5b	H1e5b	O	1	H1e5b	O	1
4089	JQ705426	H1e6	H1e6	O	1	H1e6	O	1
4090	JQ702313	H1e7	H1e7	O	1	H1e7	O	1
4091	AY495134	H1e7	H1e7	O	1	H+152	X	2
4092	EF556181	H1e8	H1e8	O	1	H1e8	O	1
4093	JX153138	H1e8a	H1e8a	O	1	H1e8a	O	1
4094	JX153381	H1e8a	H1e8a	O	1	H1e8a	O	1
4095	HQ659703	H1h1	H1h1	O	1	H1h1	O	1
4096	JQ703535	H1h1	H1h1	O	1	H1h1	O	1
4097	JQ704717	H1h2	H1h2	O	1	H1h2	O	1
4098	JQ703305	H1+152	H1+152	O	1	H1+152	O	1
4099	JQ703270	H1i	H1i	O	1	H1i	O	1
4100	FJ940865	H1i1	H1i1	O	1	H1i1	O	1
4101	EU568371	H1i1	H1i1	O	1	H1i1	O	1
4102	JQ703251	H1i2	H1i2	O	1	H1i2	O	1
4103	JQ701978	H1i2	H1i2	O	1	H1i2	O	1
4104	JQ705039	H1i2a	H1i2a	O	1	H1i2a	O	1

4105	JQ702223	H1i2a	H1i2a	O	1	H1i2a	O	1
4106	JQ703403	H1an1	H1an1	O	1	H1an1	O	1
4107	JQ324893	H1an1a	H1an1a	O	1	H1an1a	O	1
4108	JQ324894	H1an1a	H1an1a	O	1	H1an1a	O	1
4109	JQ702335	H1an2	H1an2	O	1	H1an2	O	1
4110	AY495144	H1bb	H1bb	O	1	H1bb	O	1
4111	JQ703170	H1bb	H1bb	O	1	H1bb	O	1
4112	JQ705294	H1j	H1j	O	1	H1j	O	1
4113	JQ701806	H1j	H1j	O	1	H1j	O	1
4114	JQ324705	H1j1	H1j1	O	1	H1j1	O	1
4115	JQ324525	H1j1	H1j1	O	1	H1j1	O	1
4116	JX297163	H1j1a	H1j1a	O	1	H1j1a	O	1
4117	JQ324736	H1j1a	H1j1a	O	1	H1j1a	O	1
4118	JQ324599	H1j1a1	H1j1a1	O	1	H1j1a1	O	1
4119	JQ324650	H1j1a1	H1j1a1	O	1	H1j1a1	O	1
4120	JQ324870	H1j1a2	H1j1a2	O	1	H1j1a2	O	1
4121	JQ324539	H1j1a2	H1j1a2	O	1	H1j1a2	O	1
4122	JQ324604	H1j1b	H1j1b	O	1	H1j1b	O	1
4123	JQ324901	H1j1b	H1j1b	O	1	H1j1b	O	1
4124	JQ324760	H1j1c	H1j1c	O	1	H1j1c	O	1
4125	JQ324864	H1j1c	H1j1c	O	1	H1j1c	O	1
4126	JQ703536	H1j2	H1j2	O	1	H1j2	O	1
4127	JQ324660	H1j2a	H1j2a	O	1	H1j2a	O	1
4128	JQ704358	H1j3	H1j3	O	1	H1j3	O	1
4129	EF491001	H1j3	H1j3	O	1	H1j3	O	1
4130	JQ704398	H1j4	H1j4	O	1	H1j4	O	1
4131	JQ704199	H1j4	H1j4	O	1	H1j4	O	1
4132	AY495119	H1j5	H1j5	O	1	H1j5	O	1
4133	JQ704090	H1j5	H1j5	O	1	H1j5	O	1
4134	JQ703147	H1j6	H1j6	O	1	H1j6	O	1
4135	JQ703631	H1j7	H1j7	O	1	H1j7	O	1
4136	EF177436	H1j8	H1j8	O	1	H1j8	O	1
4137	JQ705914	H1j8	H1j8	O	1	H1j8	O	1
4138	EU372630	H1j9	H1j9	O	1	H1j9	O	1
4139	JQ705493	H1m	H1m	O	1	H1m	O	1
4140	JQ065050	H1m	H1m	O	1	H1m	O	1
4141	JQ703995	H1m1	H1m1	O	1	H1m1	O	1

4142	JQ704459	H1m1	H1m1	O	1	H1m1	O	1
4143	FJ348196	H1n+146	H1n+146	O	1	H1n+146	O	1
4144	JQ701825	H1n+146	H1n+146	O	1	H1n+146	O	1
4145	JQ704655	H1n1	H1n1	O	1	H1n1	O	1
4146	JX152925	H1n1	H1n1	O	1	H1n1	O	1
4147	JQ704566	H1n1a	H1n1a	O	1	H1n1a	O	1
4148	JQ703319	H1n1a	H1n1a	O	1	H1n1a	O	1
4149	JQ705930	H1n1b	H1n1b	O	1	H1n1b	O	1
4150	JQ702009	H1n1b	H1n1b	O	1	H1n1b	O	1
4151	FJ348199	H1n2	H1n2	O	1	H1n2	O	1
4152	JQ702161	H1n2	H1n2	O	1	H1n2	O	1
4153	HQ658482	H1n3	H1n3	O	1	H1n3	O	1
4154	JQ703765	H1n4	H1n4	O	1	H1n4	O	1
4155	JQ702043	H1n4	H1n4	O	1	H1n4	O	1
4156	HQ839859	H1n+146+195	H1n+146+195	O	1	H1n+146+195	O	1
4157	JQ703312	H1n5	H1n5	O	1	H1n5	O	1
4158	JX152889	H1n6	H1n6	O	1	H1n6	O	1
4159	JQ704613	H1n6	H1n6	O	1	H1n6	O	1
4160	JQ702360	H1o	H1o	O	1	H1o	O	1
4161	JQ324594	H1o	H1o	O	1	H1o	O	1
4162	JX153389	H1ck	H1ck	O	1	H1ck	O	1
4163	JX153869	H1ck	H1ck	O	1	H1ck	O	1
4164	EF556184	H1p	H1p	O	1	H1p	O	1
4165	NA07345	H1p	H1p	O	1	H1p	O	1
4166	JQ704630	H1q	H1q	O	1	H1q	O	1
4167	JQ703742	H1q	H1q	O	1	H1q	O	1
4168	JQ704373	H1q1	H1q1	O	1	H1q1	O	1
4169	JX153851	H1q1	H1q1	O	1	H1q1	O	1
4170	JQ324843	H1q1a	H1q1a	O	1	H1q1a	O	1
4171	HQ662225	H1q1a	H1q1a	O	1	H1q1a	O	1
4172	JX152803	H1q2	H1q2	O	1	H1q2	O	1
4173	JQ324580	H1q2	H1q2	O	1	H1q2	O	1
4174	NA20811	H1q3	H1q3	O	1	H1q3	O	1
4175	JX153975	H1q3	H1q3	O	1	H1q3	O	1
4176	AF346981	H1r	H1r	O	1	H1r	O	1
4177	JQ705944	H1r	H1r	O	1	H1r	O	1
4178	JX297164	H1r1	H1r1	O	1	H1r1	O	1

4179	GQ888727	H1r1	H1r1	O	1	H1r1	O	1
4180	GQ478575	H1s	H1s	O	1	H1s	O	1
4181	EU080974	H1s	H1s	O	1	H1s	O	1
4182	JX153137	H1s1	H1s1	O	1	H1s1	O	1
4183	JX154028	H1t	H1t	O	1	H1t	O	1
4184	JQ704194	H1t	H1t	O	1	H1t	O	1
4185	JQ324837	H1t1	H1t1	O	1	H1t1	O	1
4186	JQ324688	H1t1a	H1t1a	O	1	H1t1a	O	1
4187	GQ888724	H1t1a	H1t1a	O	1	H1t1a	O	1
4188	JX297158	H1t1a1	H1t1a1	O	1	H1t1a1	O	1
4189	JQ324838	H1t1a1	H1t1a1	O	1	H1t1a1	O	1
4190	GQ888725	H1t2	H1t2	O	1	H1t2	O	1
4191	EF177427	H1t2	H1t2	O	1	H1t2	O	1
4192	JQ702726	H1u	H1u	O	1	H1u	O	1
4193	JQ705383	H1u	H1u	O	1	H1u	O	1
4194	AY495103	H1u1	H1u1	O	1	H1u1	O	1
4195	JQ702971	H1u1	H1u1	O	1	H1u1	O	1
4196	JN604117	H1u2	H1u2	O	1	H1u2	O	1
4197	JX153139	H1u2	H1u2	O	1	H1u2	O	1
4198	FJ460532	H1v	H1v	O	1	H1v	O	1
4199	JQ703465	H1v	H1v	O	1	H1v	O	1
4200	HM171271	H1v1a	H1v1a	O	1	H1v1a	O	1
4201	HM171273	H1v1b	H1v1b	O	1	H1v1b	O	1
4202	HM171275	H1w	H1w	O	1	H1w	O	1
4203	HM171277	H1w	H1w	O	1	H1w	O	1
4204	HM171279	H1x	H1x	O	1	H1x	O	1
4205	HM171278	H1x	H1x	O	1	H1x	O	1
4206	JQ705436	H1ae	H1ae	O	1	H1ae	O	1
4207	JQ702071	H1ae1	H1ae1	O	1	H1ae1	O	1
4208	JQ704433	H1ae1	H1ae1	O	1	H1ae1	O	1
4209	JQ704032	H1ae2	H1ae2	O	1	H1ae2	O	1
4210	JQ705102	H1ae2	H1ae2	O	1	H1ae2	O	1
4211	JQ702370	H1ae2a	H1ae2a	O	1	H1ae2a	O	1
4212	HQ400746	H1ae2a	H1ae2a	O	1	H1ae2a	O	1
4213	JQ703756	H1ae3a	H1ae3a	O	1	H1ae3a	O	1
4214	JQ704509	H1ae3a	H1ae3a	O	1	H1ae3a	O	1
4215	JQ704303	H1af	H1af	O	1	H1af	O	1

4216	JQ705435	H1af	H1af	O	1	H1af	O	1
4217	GQ888707	H1af1a	H1af1a	O	1	H1af1a	O	1
4218	JQ703089	H1af1a	H1af1a	O	1	H1af1a	O	1
4219	JQ704957	H1af1b	H1af1b	O	1	H1af1b	O	1
4220	JX153763	H1af1b	H1af1b	O	1	H1af1b	O	1
4221	FJ656214	H1af2	H1af2	O	1	H1af2	O	1
4222	KC257371	H1ag	H1ag	O	1	H1ag	O	1
4223	JQ703316	H1ag1	H1ag1	O	1	H1ag1	O	1
4224	JQ704657	H1ag1	H1ag1	O	1	H1ag1	O	1
4225	JQ703938	H1ag1a	H1ag1a	O	1	H1ag1a	O	1
4226	GQ175058	H1ag1a	H1ag1a	O	1	H1ag1a	O	1
4227	JQ704170	H1ag1b	H1ag1b	O	1	H1ag1b	O	1
4228	JQ702557	H1ag1b	H1ag1b	O	1	H1ag1b	O	1
4229	NA19655	H1ah	H1ah	O	1	H1ah	O	1
4230	JQ324708	H1ah1	H1ah1	O	1	H1ah1	O	1
4231	JQ702002	H1ah1	H1ah1	O	1	H1ah1	O	1
4232	JQ703687	H1ah2	H1ah2	O	1	H1ah2	O	1
4233	JQ704293	H1ah2	H1ah2	O	1	H1ah2	O	1
4234	JQ701969	H1ai1	H1ai1	O	1	H1ai1	O	1
4235	EU675941	H1ai1	H1ai1	O	1	H1ai1	O	1
4236	JQ703262	H1aj	H1aj	O	1	H1aj	O	1
4237	NA20525	H1aj	H1aj	O	1	H1aj	O	1
4238	JQ703788	H1aj1a	H1aj1a	O	1	H1aj1a	O	1
4239	JQ702591	H1aj1a	H1aj1a	O	1	H1aj1a	O	1
4240	JQ702623	H1ak	H1ak	O	1	H1ak	O	1
4241	AY738979	H1ak	H1ak	O	1	H1ak	O	1
4242	JQ324892	H1ak1	H1ak1	O	1	H1ak1	O	1
4243	KC533493	H1ak1	H1ak1	O	1	H1ak1	O	1
4244	JQ324884	H1ak2	H1ak2	O	1	H1ak2	O	1
4245	JQ324782	H1ak2	H1ak2	O	1	H1ak2	O	1
4246	JQ703706	H1am	H1am	O	1	H1am	O	1
4247	HM488738	H1am1	H1am1	O	1	H1am1	O	1
4248	JX152833	H1am1	H1am1	O	1	H1am1	O	1
4249	JQ704226	H1ao	H1ao	O	1	H1ao	O	1
4250	JQ704336	H1ao	H1ao	O	1	H1ao	O	1
4251	JQ704235	H1ao1	H1ao1	O	1	H1ao1	O	1
4252	GQ334714	H1ao1	H1ao1	O	1	H1ao1	O	1

4253	JX153703	H1cg	H1cg	O	1	H1cg	O	1
4254	HM625689	H1cg	H1cg	O	1	H1cg	O	1
4255	JQ704669	H1ap1	H1ap1	O	1	H1ap1	O	1
4256	KC257395	H1ap1	H1ap1	O	1	H1ap1	O	1
4257	EU597511	H1aq	H1aq	O	1	H1aq	O	1
4258	JQ324562	H1aq	H1aq	O	1	H1aq	O	1
4259	JQ703371	H1aq1	H1aq1	O	1	H1aq1	O	1
4260	JQ702907	H1aq1	H1aq1	O	1	H1aq1	O	1
4261	JQ324931	H1ar	H1ar	O	1	H1ar	O	1
4262	JQ704291	H1ar1	H1ar1	O	1	H1ar1	O	1
4263	JQ704671	H1ar1	H1ar1	O	1	H1ar1	O	1
4264	JQ702701	H1as	H1as	O	1	H1as	O	1
4265	JQ703561	H1as1	H1as1	O	1	H1as1	O	1
4266	JQ704158	H1as1a	H1as1a	O	1	H1as1a	O	1
4267	EF177421	H1as1a	H1as1a	O	1	H1as1a	O	1
4268	JQ704370	H1as2	H1as2	O	1	H1as2	O	1
4269	JQ702495	H1as2	H1as2	O	1	H1as2	O	1
4270	JQ324928	H1at	H1at	O	1	H1at	O	1
4271	JQ703224	H1at1	H1at1	O	1	H1at1	O	1
4272	JQ704066	H1at1a	H1at1a	O	1	H1at1a	O	1
4273	FJ858266	H1at1a	H1at1a	O	1	H1at1a	O	1
4274	GU981676	H1au	H1au	O	1	H1au	O	1
4275	JQ703256	H1au1a	H1au1a	O	1	H1au1a	O	1
4276	JQ703506	H1au1a	H1au1a	O	1	H1au1a	O	1
4277	GU207871	H1au1b	H1au1b	O	1	H1au1b	O	1
4278	JQ704010	H1au1b	H1au1b	O	1	H1au1b	O	1
4279	JQ705362	H1av	H1av	O	1	H1+16189	X	2
4280	JQ704538	H1av	H1av	O	1	H1av	O	1
4281	JQ324719	H1av1	H1av1	O	1	H1av1	O	1
4282	JQ324841	H1av1	H1av1	O	1	H1av1	O	1
4283	JQ324552	H1av1a	H1av1a	O	1	H1av1a	O	1
4284	JQ324605	H1av1a	H1av1a	O	1	H1av1a	O	1
4285	JQ704411	H1aw	H1aw	O	1	H1aw	O	1
4286	AY495153	H1aw	H1aw	O	1	H1aw	O	1
4287	JQ324713	H1aw1	H1aw1	O	1	H1aw1	O	1
4288	JX153392	H1aw1	H1aw1	O	1	H1aw1	O	1
4289	JQ704285	H1ax	H1ax	O	1	H1ax	O	1

4290	HM625711	H1ax	H1ax	O	1	H1ax	O	1
4291	KF562342	H1ax1	H1ax1	O	1	H1ax1	O	1
4292	JQ704177	H1ax1	H1ax1	O	1	H1ax1	O	1
4293	JQ703923	H1ay	H1ay	O	1	H1+16189	X	2
4294	JQ704587	H1ay	H1ay	O	1	H1+16239	X	2
4295	JQ702269	H1az	H1az	O	1	H1az	O	1
4296	JQ704564	H1az	H1az	O	1	H1az	O	1
4297	JQ702799	H1ba	H1ba	O	1	H1ba	O	1
4298	JQ703888	H1ba	H1ba	O	1	H1ba	O	1
4299	HM765465	H1ba1	H1ba1	O	1	H1ba1	O	1
4300	EF660923	H1ba1	H1ba1	O	1	H1ba1	O	1
4301	JQ704162	H1bc	H1bc	O	1	H1bc	O	1
4302	JQ702634	H1bc	H1bc	O	1	H1bc	O	1
4303	JQ704553	H1bd	H1bd	O	1	H1bd	O	1
4304	JQ703681	H1be	H1be	O	1	H1be	O	1
4305	JQ704424	H1be	H1be	O	1	H1be	O	1
4306	JQ704230	H1+16239	H1+16239	O	1	H1+16239	O	1
4307	JQ705157	H1+16239	H1+16239	O	1	H1+16239	O	1
4308	JQ704186	H1bf	H1bf	O	1	H1bf	O	1
4309	JX297162	H1bf1	H1bf1	O	1	H1bf1	O	1
4310	HM103354	H1bf1	H1bf1	O	1	H1bf1	O	1
4311	JQ703148	H1bg	H1bg	O	1	H1bg	O	1
4312	JQ704588	H1bg	H1bg	O	1	H1bg	O	1
4313	JX153634	H1bh	H1bh	O	1	H1bh	O	1
4314	JQ703668	H1bh	H1bh	O	1	H1bh	O	1
4315	JX153093	H1ch	H1ch	O	1	H1ch	O	1
4316	JQ704465	H1ch	H1ch	O	1	H1ch	O	1
4317	JQ704212	H1bi	H1bi	O	1	H1bi	O	1
4318	JQ704435	H1bi	H1bi	O	1	H1bi	O	1
4319	JQ704756	H1bj	H1bj	O	1	H1bj	O	1
4320	JQ703132	H1bk	H1bk	O	1	H1bk	O	1
4321	JQ705754	H1bk	H1bk	O	1	H1bk	O	1
4322	JQ705579	H1bm	H1bm	O	1	H1bm	O	1
4323	JQ704794	H1bm	H1bm	O	1	H1bm	O	1
4324	JQ703317	H1bn	H1bn	O	1	H1bn	O	1
4325	JQ705138	H1bn	H1bn	O	1	H1bn	O	1
4326	EF556177	H1bo	H1bo	O	1	H1bo	O	1

4327	JQ702570	H1bo	H1bo	O	1	H1bo	O	1
4328	AY738977	H1bp	H1bp	O	1	H1bp	O	1
4329	JX153073	H1bp	H1bp	O	1	H1bp	O	1
4330	JQ703346	H1bq	H1bq	O	1	H1+16189	X	2
4331	JQ324662	H1bq	H1bq	O	1	H1+16311	X	2
4332	JQ704089	H1br	H1br	O	1	H1br	O	1
4333	JQ704606	H1br	H1br	O	1	H1br	O	1
4334	JQ324633	H1bs	H1bs	O	1	H1bs	O	1
4335	JQ703571	H1bs	H1bs	O	1	H1bs	O	1
4336	JQ703244	H1bt1	H1bt1	O	1	H1bt1	O	1
4337	JQ704419	H1bt1	H1bt1	O	1	H1bt1	O	1
4338	JQ703635	H1bu	H1bu	O	1	H1bu	O	1
4339	KC175606	H1bv	H1bv	O	1	H1bv	O	1
4340	JQ324778	H1bv1	H1bv1	O	1	H1bv1	O	1
4341	JQ324723	H1bv1	H1bv1	O	1	H1bv1	O	1
4342	JQ704366	H1bw	H1bw	O	1	H1bw	O	1
4343	JQ702483	H1bw	H1bw	O	1	H1bw	O	1
4344	JQ324627	H1bx	H1bx	O	1	H1bx	O	1
4345	JQ324844	H1bx	H1bx	O	1	H1bx	O	1
4346	JQ898578	H1bz	H1bz	O	1	H1bz	O	1
4347	EU600345	H1ca	H1ca	O	1	H1ca	O	1
4348	KC911277	H1ca	H1ca	O	1	H1ca	O	1
4349	JQ705028	H1+16311	H1+16311	O	1	H1+16311	O	1
4350	JQ324670	H1cd	H1cd	O	1	H1cd	O	1
4351	HG01521	H1cd	H1cd	O	1	H1cd	O	1
4352	KC257391	H1cf	H1cf	O	1	H1cf	O	1
4353	JQ703388	H1cf	H1cf	O	1	H1cf	O	1
4354	JX153783	H1ci	H1ci	O	1	H1ci	O	1
4355	HQ703482	H1cj	H1cj	O	1	H1cj	O	1
4356	JX153943	H1cj	H1cj	O	1	H1cj	O	1
4357	EU597492	H2a	H2a	O	1	H2a	O	1
4358	GU592029	H2a	H2a	O	1	H2a	O	1
4359	FJ238053	H2a1	H2a1	O	1	H2a1	O	1
4360	JQ324929	H2a1	H2a1	O	1	H2a1	O	1
4361	FJ668389	H2a1a	H2a1a	O	1	H2a1a	O	1
4362	HQ667351	H2a1a	H2a1a	O	1	H2a1a	O	1
4363	JQ703397	H2a1a1	H2a1a1	O	1	H2a1a1	O	1

4364	JQ704198	H2a1a1	H2a1a1	O	1	H2a1a1	O	1
4365	JQ701848	H2a1a2	H2a1a2	O	1	H2a1a2	O	1
4366	JQ703509	H2a1a2	H2a1a2	O	1	H2a1a2	O	1
4367	JQ703204	H2a1b1	H2a1b1	O	1	H2a1b1	O	1
4368	JQ703362	H2a1b1	H2a1b1	O	1	H2a1b1	O	1
4369	JQ703223	H2a1b2	H2a1b2	O	1	H2a1b2	O	1
4370	JX152855	H2a1b2	H2a1b2	O	1	H2a1b2	O	1
4371	JQ703582	H2a1c	H2a1c	O	1	H2a1c	O	1
4372	EU597521	H2a1c	H2a1c	O	1	H2a1c	O	1
4373	FJ161702	H2a1d	H2a1d	O	1	H2a1d	O	1
4374	FJ800808	H2a1d	H2a1d	O	1	H2a1d	O	1
4375	JQ703145	H2a1e	H2a1e	O	1	H2a1e	O	1
4376	JQ324896	H2a1e	H2a1e	O	1	H2a1e	O	1
4377	JQ704189	H2a1e1	H2a1e1	O	1	H2a1e1	O	1
4378	FJ842614	H2a1e1a	H2a1e1a	O	1	H2a1e1a	O	1
4379	JQ703366	H2a1e1a	H2a1e1a	O	1	H2a1e1a	O	1
4380	JQ703214	H2a1e1a1	H2a1e1a1	O	1	H2a1e1a1	O	1
4381	HQ698266	H2a1e1a1	H2a1e1a1	O	1	H2a1e1a1	O	1
4382	JQ704847	H2a1e1b	H2a1e1b	O	1	H2a1e1b	O	1
4383	HQ336422	H2a1f	H2a1f	O	1	H2a1f	O	1
4384	JQ705806	H2a1f1	H2a1f1	O	1	H2a1f1	O	1
4385	JX153159	H2a1f1	H2a1f1	O	1	H2a1f1	O	1
4386	KF723706	H2a1f2	H2a1f2	O	1	H2a1f2	O	1
4387	JX153614	H2a1f2	H2a1f2	O	1	H2a1f2	O	1
4388	JQ705411	H2a1g	H2a1g	O	1	H2a1g	O	1
4389	JQ703760	H2a1i	H2a1i	O	1	H2a1i	O	1
4390	KC911386	H2a1i	H2a1i	O	1	H2a1i	O	1
4391	JQ701819	H2a1j	H2a1j	O	1	H2a1j	O	1
4392	JQ705223	H2a1j	H2a1j	O	1	H2a1j	O	1
4393	JQ705370	H2a1k	H2a1k	O	1	H2a1k	O	1
4394	JQ704025	H2a1k	H2a1k	O	1	H2a1k	O	1
4395	JX153259	H2a1m	H2a1m	O	1	H2a1m	O	1
4396	JX153250	H2a1m	H2a1m	O	1	H2a1m	O	1
4397	JQ704434	H2a1+146	H2a1+146	O	1	H2a1+146	O	1
4398	JX153119	H2a1n	H2a1n	O	1	H2a1n	O	1
4399	EU780223	H2a1n	H2a1n	O	1	H2a1n	O	1
4400	JQ704533	H2a2	H2a2	O	1	H2a2 H2a2+(16235)	O	2

4401	JQ703191	H2a2	H2a2	O	1	H2a2 H2a2+(16235)	O	2
4402	GU122997	H2a2a	H2a2a	O	1	H2a2a	O	1
4403	JQ701904	H2a2a	H2a2a	O	1	H2a2a	O	1
4404	NC_012920	H2a2a1	error	X	error	H2a2a1	O	1
4405	EU795361	H2a2a1a	H2a2a1a	O	1	H2a2a1a	O	1
4406	EU157923	H2a2a1a	H2a2a1a	O	1	H2a2a1a	O	1
4407	JQ702287	H2a2a1b	H2a2a1b	O	1	H2a2a1b	O	1
4408	JQ704346	H2a2a1b	H2a2a1b	O	1	H2a2a1b	O	1
4409	JQ704842	H2a2a1d	H2a2a1d	O	1	H2a2a1d	O	1
4410	JQ705139	H2a2a1d	H2a2a1d	O	1	H2a2a1d	O	1
4411	HQ670226	H2a2a1e	H2a2a1e	O	1	H2a2a1e	O	1
4412	NA07346	H2a2a1e	H2a2a1e	O	1	H2a2a1e	O	1
4413	JQ704520	H2a2a1f	J2a1a1a	X	6	H2a2a1f	O	1
4414	HG00127	H2a2a1f	H2a2a1f	O	1	H2a2a1f	O	1
4415	JQ705265	H2a2a1g	J2a1a1a	X	6	H2a2a1g	O	1
4416	JQ704224	H2a2a1g	H2a2a1g	O	1	H2a2a1g	O	1
4417	JQ704532	H2a2a1h	H2a2a1h	O	1	H2a2a1h	O	1
4418	JX153491	H2a2a1h	H2a2a1h	O	1	H2a2a1h	O	1
4419	JQ704269	H2a2a2	H2a2a2	O	1	H2a2a2	O	1
4420	EU716647	H2a2a2	H2a2a2	O	1	H2a2a2	O	1
4421	JQ703290	H2a2+(16235)	H2a2a+(16235)	X	Not found	H2a2 H2a2+(16235)	O	2
4422	JQ704337	H2a2+(16235)	H2a2a+(16235)	X	Not found	H2a2+(16235)	O	1
4423	HQ914650	H2a2b	H2a2b	O	1	H2a2b	O	1
4424	EU719115	H2a2b	H2a2b	O	1	H2a2b	O	1
4425	JQ705563	H2a2b1	H2a2b1	O	1	H2a2b1	O	1
4426	JQ705365	H2a2b1	H2a2b1	O	1	H2a2b1	O	1
4427	KC257390	H2a2b1a	H2a2b1a	O	1	H2a2b1a	O	1
4428	JQ702368	H2a2b1a	H2a2b1a	O	1	H2a2b1a	O	1
4429	EF418606	H2a2b1a1	H2a2b1a1	O	1	H2a2b1a1	O	1
4430	JQ704361	H2a2b1a1	H2a2b1a1	O	1	H2a2b1a1	O	1
4431	JQ704140	H2a2b2	H2a2b2	O	1	H2a2b2	O	1
4432	EU444119	H2a2b2	H2a2b2	O	1	H2a2b2	O	1
4433	JQ702309	H2a2b3	H2a2b3	O	1	H2a2b3	O	1
4434	JX153667	H2a2b3	H2a2b3	O	1	H2a2b3	O	1
4435	JQ705734	H2a2b4	H2a2b4	O	1	H2a2b4	O	1
4436	JQ702900	H2a2b4	H2a2b4	O	1	H2a2b4	O	1
4437	HQ675041	H2a2b5	H2a2b5	O	1	H2a2b5	O	1

4438	JQ704565	H2a2b5a	H2a2b5a	O	1	H2a2b5a	O	1
4439	HG00123	H2a2b5a	H2a2b5a	O	1	H2a2b5a	O	1
4440	AM263180	H2a3	H2a3	O	1	H2a3	O	1
4441	JX153748	H2a3a	H2a3a	O	1	H2a3a	O	1
4442	JQ704220	H2a3a1	H2a3a1	O	1	H2a3a1	O	1
4443	JX153268	H2a3a1	H2a3a1	O	1	H2a3a1	O	1
4444	HQ659870	H2a3b	H2a3b	O	1	H2a3b	O	1
4445	JQ703192	H2a3b	H2a3b	O	1	H2a3b	O	1
4446	AM263179	H2a4	H2a4	O	1	H2a4	O	1
4447	AM263181	H2a4	H2a4	O	1	H2a4	O	1
4448	KC533501	H2a5	H2a5	O	1	H2a5	O	1
4449	JQ704563	H2a5	H2a5	O	1	H2a5	O	1
4450	JQ324555	H2a5a1	H2a5a1	O	1	H2a5a1	O	1
4451	JQ324582	H2a5a1	H2a5a1	O	1	H2a5a1	O	1
4452	FJ527772	H2a5a1a	H2a5a1a	O	1	H2a5a1a	O	1
4453	FJ527777	H2a5a1a	H2a5a1a	O	1	H2a5a1a	O	1
4454	JF284817	H2a5a1b	H2a5a1b	O	1	H2a5a1b	O	1
4455	AY738963	H2a5a1b	H2a5a1b	O	1	H2a5a1b	O	1
4456	JQ701826	H2a5b	H2a5b	O	1	H2a5b	O	1
4457	JQ703081	H2a5b	H2a5b	O	1	H2a5b	O	1
4458	JQ704687	H2a5b1	H2a5b	X	2	H2a5b1	O	1
4459	JQ702205	H2a5b1	H2a5b	X	2	H2a5b1	O	1
4460	HQ153430	H2a5b2	H2a5b2	O	1	H2a5b2	O	1
4461	EU597574	H2b	H2b	O	1	H2b	O	1
4462	HQ659667	H2b	H2b	O	1	H2b	O	1
4463	JQ704503	H2c	H2c	O	1	H2c	O	1
4464	JQ701885	H2c	H2c	O	1	H2c	O	1
4465	JQ704496	H2c1	H2c1	O	1	H2c1	O	1
4466	JQ704499	H2c1	H2c1	O	1	H2c1	O	1
4467	AY738987	H3	H3	O	1	H3	O	1
4468	HQ659688	H3	H3	O	1	H3	O	1
4469	JQ702087	H3+152	H3+152	O	1	H3+152	O	1
4470	JQ703549	H3+152	H3+152	O	1	H3+152	O	1
4471	JQ704834	H3a	H3a	O	1	H3a	O	1
4472	JQ704253	H3a1	H3a1	O	1	H3a1	O	1
4473	AY738986	H3a1	H3a1	O	1	H3a1	O	1
4474	JQ705329	H3a1a	H3a1a	O	1	H3a1a	O	1

4475	JQ704348	H3a1a	H3a1a	O	1	H3a1a	O	1
4476	AY495132	H3g	H3g	O	1	H3g	O	1
4477	AY495142	H3g	H3g	O	1	H3g	O	1
4478	JQ705181	H3g1	H3g1	O	1	H3g1	O	1
4479	JQ704782	H3g1	H3g1	O	1	H3g1	O	1
4480	JQ705694	H3g1a	H3g1a	O	1	H3g1a	O	1
4481	HG00245	H3g1b	H3g1b	O	1	H3g1b	O	1
4482	JQ702329	H3g1b	H3g1b	O	1	H3g1b	O	1
4483	JQ704542	H3g2	H3g2	O	1	H3g2	O	1
4484	JQ704455	H3g2	H3g2	O	1	H3g2	O	1
4485	KC257374	H3g3	H3g3	O	1	H3g3	O	1
4486	JQ705080	H3g3	H3g3	O	1	H3g3	O	1
4487	JQ702762	H3g4	H3g4	O	1	H3g4	O	1
4488	JQ704722	H3g4	H3g4	O	1	H3g4	O	1
4489	JQ703118	H3i	H3i	O	1	H3i	O	1
4490	JN202491	H3i	H3i	O	1	H3i	O	1
4491	GQ165466	H3i1	H3i1	O	1	H3i1	O	1
4492	AY495131	H3i1	H3i1	O	1	H3i1	O	1
4493	HQ696491	H3j	H3j	O	1	H3j	O	1
4494	JQ703265	H3j	H3j	O	1	H3j	O	1
4495	JQ704704	H3k	H3k	O	1	H3k	O	1
4496	JQ324804	H3k	H3k	O	1	H3k	O	1
4497	AY495130	H3k1a	H3k1a	O	1	H3k1a	O	1
4498	JQ705110	H3k1a	H3k1a	O	1	H3k1a	O	1
4499	KC554003	H3b	H3b	O	1	H3b	O	1
4500	JX025434	H3b	H3b	O	1	H3b	O	1
4501	AY495148	H3b+16129	H3b+16129	O	1	H3b+16129	O	1
4502	JQ703467	H3b+16129	H3b+16129	O	1	H3b+16129	O	1
4503	KC533482	H3b1a	H3b1a	O	1	H3b1a	O	1
4504	JQ704748	H3b1a	H3b1a	O	1	H3b1a	O	1
4505	JQ704932	H3b1b	H3b1b	O	1	H3b1b	O	1
4506	JQ704456	H3b1b	H3b1b	O	1	H3b1b	O	1
4507	JQ703382	H3b1b1	H3b1b1	O	1	H3b1b1	O	1
4508	HQ661845	H3b1b1	H3b1b1	O	1	H3b1b1	O	1
4509	JQ704105	H3b1b1a	H3b1b1a	O	1	H3b1b1a	O	1
4510	AY495155	H3b2	H3b2	O	1	H3b2	O	1
4511	JQ702921	H3b2	H3b2	O	1	H3b2	O	1

4512	JQ703291	H3b3	H3b3	O	1	H3b3	O	1
4513	JQ704197	H3b4	H3b4	O	1	H3b4	O	1
4514	JQ705727	H3b4a	H3b4a	O	1	H3b4a	O	1
4515	HG00237	H3b4a	H3b4a	O	1	H3b4a	O	1
4516	HQ384186	H3b5	H3b5	O	1	H3b5	O	1
4517	AY738988	H3b5	H3b5	O	1	H3b	X	2
4518	JQ705735	H3b6	H3b6	O	1	H3b6	O	1
4519	JX153543	H3b6a	H3b6a	O	1	H3b6a	O	1
4520	JX153241	H3b6a	H3b6a	O	1	H3b6a	O	1
4521	JX153702	H3b7	H3b7	O	1	H3b7	O	1
4522	JX153896	H3b7	H3b7	O	1	H3b7	O	1
4523	JQ324618	H3c	H3c	O	1	H3c	O	1
4524	JQ703744	H3c	H3c	O	1	H3c	O	1
4525	AY738990	H3c1	H3c1	O	1	H3c1	O	1
4526	JQ703410	H3c1	H3c1	O	1	H3c1	O	1
4527	JQ705425	H3c2	H3c2	O	1	H3c2	O	1
4528	JQ324828	H3c2a	H3c2a	O	1	H3c2a	O	1
4529	JQ324785	H3c2a	H3c2a	O	1	H3c2a	O	1
4530	JQ324731	H3c2a1	H3c2a1	O	1	H3c2a1	O	1
4531	JQ324776	H3c2a1	H3c2a1	O	1	H3c2a1	O	1
4532	JQ704172	H3c2b	H3c2b	O	1	H3c2b	O	1
4533	JQ705340	H3c2b1	H3c2b1	O	1	H3c2b1	O	1
4534	JX297139	H3c2c	H3c2c	O	1	H3c2c	O	1
4535	JQ324704	H3c2c	H3c2c	O	1	H3c2c	O	1
4536	JQ702603	H3c3	H3c3	O	1	H3c3	O	1
4537	AY495180	H3d	H3d	O	1	H3d	O	1
4538	AY738983	H3d	H3d	O	1	H3d	O	1
4539	JQ324858	H3e	H3e	O	1	H3e	O	1
4540	FJ348206	H3e	H3e	O	1	H3e	O	1
4541	JQ702652	H3+16311	H3+16311	O	1	H3+16311	O	1
4542	KF179063	H3h	H3h	O	1	H3h	O	1
4543	JQ702416	H3h	H3h	O	1	H3h	O	1
4544	JQ086344	H3h1	H3h1	O	1	H3h1	O	1
4545	JQ703677	H3h1	H3h1	O	1	H3h1	O	1
4546	JQ702530	H3h2	H3h2	O	1	H3h2	O	1
4547	KC911606	H3h2a	H3h2a	O	1	H3h2a	O	1
4548	EF661010	H3h2a	H3h2a	O	1	H3h2a	O	1

4549	KF424557	H3h3	H3h3	O	1	H3h3	O	1
4550	JQ704436	H3h3a	H3h3a	O	1	H3h3a	O	1
4551	JQ704979	H3h3a	H3h3a	O	1	H3h3a	O	1
4552	JX153423	H3h3b	H3h3b	O	1	H3h3b	O	1
4553	HM003111	H3h3b	H3h3b	O	1	H3h3b	O	1
4554	JQ704484	H3h4	H3h4	O	1	H3h4	O	1
4555	FJ349555	H3h4	H3h4	O	1	H3h4	O	1
4556	JQ705041	H3h5	H3h5	O	1	H3h5	O	1
4557	JQ324559	H3h6	H3h6	O	1	H3h6	O	1
4558	JQ701864	H3h6	H3h6	O	1	H3h6	O	1
4559	JN129423	H3h7	H3h7	O	1	H3h7	O	1
4560	JQ702635	H3h7	H3h7	O	1	H3h7	O	1
4561	JQ702092	H3m	H3m	O	1	H3m	O	1
4562	JQ704363	H3m	H3m	O	1	H3m	O	1
4563	JQ704633	H3n	H3n	O	1	H3n	O	1
4564	JQ703381	H3n	H3n	O	1	H3n	O	1
4565	JQ704490	H3p	H3p	O	1	H3p	O	1
4566	JQ704133	H3p	H3p	O	1	H3p	O	1
4567	JQ704318	H3q	H3q	O	1	H3q	O	1
4568	JQ703230	H3q	H3q	O	1	H3q	O	1
4569	JX153879	H3q1	H3q1	O	1	H3q1	O	1
4570	GU592027	H3q1	H3q1	O	1	H3q1	O	1
4571	JN112339	H3r	H3r	O	1	H3r	O	1
4572	JQ704887	H3r	H3r	O	1	H3r	O	1
4573	JQ705853	H3r1	H3r1	O	1	H3r1	O	1
4574	EF660938	H3r1	H3r1	O	1	H3r1	O	1
4575	JQ704120	H3s	H3s	O	1	H3s	O	1
4576	JQ324916	H3s	H3s	O	1	H3s	O	1
4577	KC911500	H3t	H3t	O	1	H3t	O	1
4578	JQ702220	H3t	H3t	O	1	H3t	O	1
4579	DQ523647	H3u	H3u	O	1	H3u	O	1
4580	AB626610	H3u1	H3u1	O	1	H3u1	O	1
4581	JQ705759	H3u1	H3u1	O	1	H3u1	O	1
4582	JQ704903	H3v	H3v	O	1	H3v	O	1
4583	JQ703289	H3v1	H3v1	O	1	H3v1	O	1
4584	JQ703185	H3v1	H3v1	O	1	H3v1	O	1
4585	GU997629	H3v+16093	H3v+16093	O	1	H3v+16093	O	1

4586	JQ703387	H3v+16093	H3v+16093	O	1	H3v+16093	O	1
4587	JQ704545	H3v2	H3v2	O	1	H3v2	O	1
4588	JQ703544	H3w	H3w	O	1	H3w	O	1
4589	FJ460550	H3w	H3w	O	1	H3w	O	1
4590	JQ324817	H3x	H3x	O	1	H3x	O	1
4591	JQ324537	H3x	H3x	O	1	H3x	O	1
4592	DQ523662	H3x1	H3x1	O	1	H3x1	O	1
4593	DQ523643	H3x1	H3x1	O	1	H3x1	O	1
4594	JQ703094	H3y	H3y	O	1	H3y	O	1
4595	JQ705116	H3y	H3y	O	1	H3y	O	1
4596	JQ705915	H3z	H3z	O	1	H3z	O	1
4597	JQ702909	H3z	H3z	O	1	H3z	O	1
4598	JQ703690	H3z1	H3z1	O	1	H3z1	O	1
4599	JQ324932	H3z1	H3z1	O	1	H3z1	O	1
4600	JQ704178	H3z2	H3z2	O	1	H3z2	O	1
4601	JX153639	H3z2	H3z2	O	1	H3z2	O	1
4602	JQ704638	H3aa	H3aa	O	1	H3aa	O	1
4603	JQ705422	H3aa	H3aa	O	1	H3aa	O	1
4604	JQ324666	H3ab	H3ab	O	1	H3ab	O	1
4605	JN409387	H3ab	H3ab	O	1	H3ab	O	1
4606	HQ658608	H3ac	H3ac	O	1	H3ac	O	1
4607	JQ702127	H3ac	H3ac	O	1	H3ac	O	1
4608	JQ703272	H3ad	H3ad	O	1	H3ad	O	1
4609	JQ702774	H3ad	H3ad	O	1	H3ad	O	1
4610	JQ705905	H3ae	H3ae	O	1	H3ae	O	1
4611	JQ703598	H3ae	H3ae	O	1	H3ae	O	1
4612	AY495156	H3af	H3af	O	1	H3af	O	1
4613	JQ324794	H3af	H3af	O	1	H3af	O	1
4614	KC866615	H3ag	H3ag	O	1	H3+16311	X	2
4615	HG00252	H3ag	H3ag	O	1	H3ag	O	1
4616	JQ705124	H3ag1	H3ag1	O	1	H3ag1	O	1
4617	NA12283	H3ag1	H3ag1	O	1	H3ag1	O	1
4618	JN107813	H3ah	H3ah	O	1	H3+152	X	2
4619	JQ324638	H3ah	H3ah	O	1	H3ah	O	1
4620	JQ705437	H3ai	H3ai	O	1	H3ai	O	1
4621	JQ324769	H3ai	H3ai	O	1	H3+16189	X	2
4622	JQ701983	H3aj	H3aj	O	1	H3aj	O	1

4623	JQ704135	H3aj	H3aj	O	1	H3aj	O	1
4624	AY195746	H3ak	H3ak	O	1	H3ak	O	1
4625	JQ704482	H3ak	H3ak	O	1	H3ak	O	1
4626	JQ324634	H3am	H3am	O	1	H3am	O	1
4627	JQ324847	H3am	H3am	O	1	H3am	O	1
4628	JQ703304	H3an	H3an	O	1	H3an	O	1
4629	JQ324567	H3ao	H3ao	O	1	H3ao	O	1
4630	JQ704232	H3ao	H3ao	O	1	H3ao	O	1
4631	JQ704183	H3ao1	H3ao1	O	1	H3ao1	O	1
4632	JQ705341	H3ao1	H3ao1	O	1	H3ao1	O	1
4633	JQ703140	H3ap	H3ap	O	1	H3ap	O	1
4634	JQ702411	H3ap	H3ap	O	1	H3ap	O	1
4635	JN674560	H3aq	H3aq	O	1	H3aq	O	1
4636	JQ704383	H3aq	H3aq	O	1	H3aq	O	1
4637	JQ702687	H3ar	H3ar	O	1	H3ar	O	1
4638	JQ704192	H3as	H3as	O	1	H3as	O	1
4639	JQ702769	H3as	H3as	O	1	H3as	O	1
4640	JQ324640	H3at	H3at	O	1	H3at	O	1
4641	JQ324851	H3at1	H3at1	O	1	H3at1	O	1
4642	JQ324906	H3at1	H3at1	O	1	H3at1	O	1
4643	JQ704477	H3au	H3au	O	1	H3au	O	1
4644	JQ324572	H3au	H3au	O	1	H3+152	X	2
4645	JQ324574	H3+16189	H3+16189	O	1	H3+16189	O	1
4646	JQ324687	H3av	H3av	O	1	H3av	O	1
4647	JQ324566	H3av	H3av	O	1	H3av	O	1
4648	JN646688	H4	H4	O	1	H4	O	1
4649	JQ701843	H4	H4	O	1	H4	O	1
4650	JX152997	H4a	H4a	O	1	H4a	O	1
4651	AY495123	H4a1	H4a1	O	1	H4a1	O	1
4652	EF177440	H4a1	H4a1	O	1	H4a1	O	1
4653	HQ860291	H4a1a	H4a1a	O	1	H4a1a	O	1
4654	EU051827	H4a1a	H4a1a	O	1	H4a1a	O	1
4655	AY495095	H4a1a1	H4a1a1	O	1	H4a1a1	O	1
4656	JQ703368	H4a1a1	H4a1a1	O	1	H4a1a1	O	1
4657	AY495178	H4a1a1a	H4a1a1a	O	1	H4a1a1a	O	1
4658	JQ703892	H4a1a1a	H4a1a1a	O	1	H4a1a1a	O	1
4659	EU200347	H4a1a1a1a	H4a1a1a1a	O	1	H4a1a1a1a	O	1

4660	JQ324699	H4a1a1a1a	H4a1a1a1a	O	1	H4a1a1a1a	O	1
4661	AY495186	H4a1a1a1a1	H4a1a1a1a1	O	1	H4a1a1a1a1	O	1
4662	EF609015	H4a1a1a1a1	H4a1a1a1a1	O	1	H4a1a1a1a1	O	1
4663	JQ703532	H4a1a1a2	H4a1a1a2	O	1	H4a1a1a2	O	1
4664	EU719211	H4a1a1a3	H4a1a1a3	O	1	H4a1a1a3	O	1
4665	GU123040	H4a1a1a3	H4a1a1a3	O	1	H4a1a1a3	O	1
4666	JQ324913	H4a1a1a4	H4a1a1a4	O	1	H4a1a1a4	O	1
4667	JQ324855	H4a1a1a4	H4a1a1a4	O	1	H4a1a1a4	O	1
4668	JQ703542	H4a1a2a	H4a1a2a	O	1	H4a1a2a	O	1
4669	EU636711	H4a1a2a	H4a1a2a	O	1	H4a1a2a	O	1
4670	JQ704851	H4a1a2a1	H4a1a2a1	O	1	H4a1a2a1	O	1
4671	JQ702613	H4a1a2a1	H4a1a2a1	O	1	H4a1a2a1	O	1
4672	JQ704883	H4a1a+195	H4a1a+195	O	1	H4a1a+195	O	1
4673	JQ703398	H4a1a3	H4a1a3	O	1	H4a1a3	O	1
4674	JQ702886	H4a1a3a	H4a1a3a	O	1	H4a1a3a	O	1
4675	EF556191	H4a1a3a	H4a1a3a	O	1	H4a1a3a	O	1
4676	JQ703134	H4a1a4a	H4a1a4a	O	1	H4a1a4a	O	1
4677	JX297159	H4a1a4a	H4a1a4a	O	1	H4a1a4a	O	1
4678	HQ659689	H4a1a4b	H4a1a4b	O	1	H4a1a4b	O	1
4679	EU664586	H4a1a4b	H4a1a4b	O	1	H4a1a4b	O	1
4680	JX153972	H4a1a4b1	H4a1a4b1	O	1	H4a1a4b1	O	1
4681	JQ703000	H4a1a4b1	H4a1a4b1	O	1	H4a1a4b1	O	1
4682	JQ702546	H4a1a4b2	H4a1a4b2	O	1	H4a1a4b2	O	1
4683	JQ703210	H4a1a4b2	H4a1a4b2	O	1	H4a1a4b2	O	1
4684	JQ704039	H4a1a5	H4a1a5	O	1	H4a1a5	O	1
4685	JQ703630	H4a1c1	H4a1c1	O	1	H4a1c1	O	1
4686	JQ702631	H4a1c1a	H4a1c1a	O	1	H4a1c1a	O	1
4687	AY738950	H4a1c1a	H4a1c1a	O	1	H4a1c1a	O	1
4688	KF305642	H4a1c2	H4a1c2	O	1	H4a1c2	O	1
4689	DQ523646	H4a1d	H4a1d	O	1	H4a1d	O	1
4690	JQ704648	H4a1d	H4a1d	O	1	H4a1d	O	1
4691	JQ702936	H4a2	H4a2	O	1	H4a2	O	1
4692	JQ703939	H4a2	H4a2	O	1	H4a2	O	1
4693	JQ702580	H4b	H4b	O	1	H4b	O	1
4694	AM263185	H4b	H4b	O	1	H4b	O	1
4695	EU600352	H4b1	H4b1	O	1	H4b1	O	1
4696	EU600350	H4b1	H4b1	O	1	H4b1	O	1

4697	JQ705112	H4c1	H4c1	O	1	H4c1	O	1
4698	JQ702960	H4c1	H4c1	O	1	H4c1	O	1
4699	EU935460	H4d	H4d	O	1	H4d	O	1
4700	JX153118	H4d	H4d	O	1	H4d	O	1
4701	JQ324611	H5'36	H5'36	O	1	H5'36	O	1
4702	GQ983078	H5'36	H5'36	O	1	H5'36	O	1
4703	AY495174	H5	H5	O	1	H5	O	1
4704	JQ703257	H5	H5	O	1	H5	O	1
4705	GQ983068	H5a	H5a	O	1	H5a	O	1
4706	GQ983060	H5a	H5a	O	1	H5a	O	1
4707	AY195747	H5a1	H5a1	O	1	H5a1	O	1
4708	HQ674629	H5a1	H5a1	O	1	H5a1	O	1
4709	HQ659693	H5a1a	H5a1a	O	1	H5a1a	O	1
4710	JQ705767	H5a1a	H5a1a	O	1	H5a1a	O	1
4711	AY495167	H5a1b	H5a1b	O	1	H5a1b	O	1
4712	AY495176	H5a1b	H5a1b	O	1	H5a1b	O	1
4713	HQ663878	H5a1c1a	H5a1c1a	O	1	H5a1c1a	O	1
4714	JQ702653	H5a1c1a	H5a1c1a	O	1	H5a1c1a	O	1
4715	JQ705790	H5a1c2	H5a1c2	O	1	H5a1c2	O	1
4716	JQ705035	H5a1d	H5a1d	O	1	H5a1d	O	1
4717	AY495171	H5a1d	H5a1d	O	1	H5a1d	O	1
4718	AY339431	H5a1e	H5a1e	O	1	H5a1e	O	1
4719	JQ703620	H5a1e	H5a1e	O	1	H5a1e	O	1
4720	JQ703738	H5a1f	H5a1f	O	1	H5a1f	O	1
4721	JQ704730	H5a1f	H5a1f	O	1	H5a1f	O	1
4722	JN008723	H5a1g1	H5a1g1	O	1	H5a1g1	O	1
4723	EU294323	H5a1g1	H5a1g1	O	1	H5a1g1	O	1
4724	JQ704886	H5a1g1a	H5a1g1a	O	1	H5a1g1a	O	1
4725	JQ703513	H5a1g1a	H5a1g1a	O	1	H5a1g1a	O	1
4726	JQ703820	H5a1g2	H5a1g2	O	1	H5a1g2	O	1
4727	JQ705415	H5a1g2	H5a1g2	O	1	H5a1g2	O	1
4728	JQ702502	H5a1h	H5a1h	O	1	H5a1h	O	1
4729	JX128043	H5a1i	H5a1i	O	1	H5a1i	O	1
4730	JQ705787	H5a1i	H5a1i	O	1	H5a1i	O	1
4731	EU372627	H5a1j	H5a1j	O	1	H5a1j	O	1
4732	JQ704060	H5a1j	H5a1j	O	1	H5a1j	O	1
4733	AY495170	H5a1k	H5a1k	O	1	H5a1k	O	1

4734	GQ983064	H5a1k	H5a1k	O	1	H5a1k	O	1
4735	GQ334692	H5a1+152	H5a1+152	O	1	H5a1+152	O	1
4736	JQ705998	H5a1m	H5a1m	O	1	H5a1m	O	1
4737	JQ705412	H5a1m	H5a1m	O	1	H5a1m	O	1
4738	JQ704324	H5a1n	H5a1n	O	1	H5a1n	O	1
4739	HM103356	H5a1n	H13a1b	X	Not found	H13a1b	X	Not found
4740	GQ983075	H5a1+16093	H5a1+16093	O	1	H5a1+16093	O	1
4741	FJ966912	H5a1p	H5a1p	O	1	H5a1p	O	1
4742	HQ677908	H5a1p	H5a1p	O	1	H5a1p	O	1
4743	JQ704766	H5a1q	H5a1q	O	1	H5a1q	O	1
4744	JX128066	H5a1q	H5a1q	O	1	H5a1q	O	1
4745	JQ704415	H5a2	H5a2	O	1	H5a2	O	1
4746	AY738952	H5a2	H5a2	O	1	H5a2	O	1
4747	JQ705582	H5a2a	H5a2a	O	1	H5a2a	O	1
4748	JX128085	H5a2a	H5a2a	O	1	H5a2a	O	1
4749	KC554002	H5a3	H5a3	O	1	H5a3	O	1
4750	GQ983105	H5a3	H5a3	O	1	H5a3	O	1
4751	JQ704246	H5a3a	H5a3a	O	1	H5a3a	O	1
4752	JQ704372	H5a3a	H5a3a	O	1	H5a3a	O	1
4753	JQ324706	H5a3a1	H5a3a1	O	1	H5a3a1	O	1
4754	JX297188	H5a3a1	H5a3a1	O	1	H5a3a1	O	1
4755	GQ983069	H5a3a+152	H5a3a+152	O	1	H5a3a+152	O	1
4756	GQ983073	H5a3a2	H5a3a2	O	1	H5a3a2	O	1
4757	JQ701896	H5a3a2	H5a3a2	O	1	H5a3a2	O	1
4758	JX153276	H5a3a3	H5a3a3	O	1	H5a3a3	O	1
4759	AY495175	H5a3a3	H5a3a3	O	1	H5a3a3	O	1
4760	JQ703824	H5a3b	H5a3b	O	1	H5a3b	O	1
4761	JQ703800	H5a3b	H5a3b	O	1	H5a3b	O	1
4762	JQ703731	H5a4	H5a4	O	1	H5a4	O	1
4763	GQ983092	H5a4a1	H5a4a1	O	1	H5a4a1	O	1
4764	GQ983058	H5a4a1	H5a4a1	O	1	H5a4a1	O	1
4765	JQ702083	H5a4a1a	H5a4a1a	O	1	H5a4a1a	O	1
4766	JX153318	H5a5	H5a5	O	1	H5a5	O	1
4767	JQ702571	H5a5	H5a5	O	1	H5a5	O	1
4768	JQ324545	H5a+152	H5a+152	O	1	H5a+152	O	1
4769	GQ983056	H5a+152	H5a+152	O	1	H5a+152	O	1
4770	JQ705812	H5a6	H5a6	O	1	H5a6	O	1

4771	GQ983096	H5a6	H5a6	O	1	H5a6	O	1
4772	JQ704237	H5a6a	H5a6a	O	1	H5a6a	O	1
4773	JQ704396	H5a7	H5a7	O	1	H5a7	O	1
4774	GQ983106	H5a7	H5a7	O	1	H5a7	O	1
4775	JX153844	H5a8	H5a8	O	1	H5a8	O	1
4776	AY339430	H5a8	H5a8	O	1	H5a8	O	1
4777	JX153930	H5a9	H5a9	O	1	H5a9	O	1
4778	JX128047	H5a9	H5a9	O	1	H5a+152	X	2
4779	NA20533	H5b	H5b	O	1	H5b	O	1
4780	JX128050	H5b	H5b	O	1	H5b	O	1
4781	FJ794473	H5b1	H5b1	O	1	H5b1	O	1
4782	JQ705511	H5b1	H5b1	O	1	H5b1	O	1
4783	HQ700378	H5b2	H5b2	O	1	H5b2	O	1
4784	JQ704264	H5b2	H5b2	O	1	H5b2	O	1
4785	JQ702345	H5b3	H5b3	O	1	H5b3	O	1
4786	EU915472	H5b3	H5b3	O	1	H5b3	O	1
4787	JF795326	H5b4	H5b4	O	1	H5b4	O	1
4788	GQ983088	H5b4	H5b4	O	1	H5b4	O	1
4789	JX128049	H5b5	H5b5	O	1	H5b5	O	1
4790	JX153042	H5b5	H5b5	O	1	H5b5	O	1
4791	JQ702402	H5c	H5c	O	1	H5c	O	1
4792	JQ705748	H5c	H5c	O	1	H5c	O	1
4793	JQ705824	H5c1	H5c1	O	1	H5c1	O	1
4794	JQ705569	H5c1a	H5c1a	O	1	H5c1a	O	1
4795	JQ703627	H5c2	H5c2	O	1	H5c2	O	1
4796	JQ704498	H5d	H5d	O	1	H5d	O	1
4797	GQ983076	H5d	H5d	O	1	H5d	O	1
4798	JQ324661	H5e	H5e	O	1	H5e	O	1
4799	KF830849	H5e1	H5e1	O	1	H5e1	O	1
4800	EU677750	H5e1a	H5e1a	O	1	H5e1a	O	1
4801	JQ704706	H5e1a	H5e1a	O	1	H5e1a	O	1
4802	JX128081	H5e1a1	H5e1a1	O	1	H5e1a1	O	1
4803	JX128091	H5e1a1	H5e1a1	O	1	H5e1a1	O	1
4804	HM636849	H5e1b	H5e1b	O	1	H5e1b	O	1
4805	GQ983082	H5e1b	H5e1b	O	1	H5e1b	O	1
4806	GQ983074	H5f	H5f	O	1	H5f	O	1
4807	GQ983099	H5f	H5f	O	1	H5f	O	1

4808	JQ704716	H5g	H5g	O	1	H5g	O	1
4809	HQ661100	H5g	H5g	O	1	H5g	O	1
4810	JQ703405	H5h	H5h	O	1	H5h	O	1
4811	JQ704341	H5h	H5h	O	1	H5h	O	1
4812	JQ704386	H5j	H5j	O	1	H5j	O	1
4813	JQ324861	H5j	H5j	O	1	H5j	O	1
4814	AY495173	H5k	H5k	O	1	H5k	O	1
4815	HQ864470	H5k	H5k	O	1	H5k	O	1
4816	JQ702895	H5m	H5m	O	1	H5m	O	1
4817	JQ702583	H5m	H5m	O	1	H5m	O	1
4818	GQ983109	H5n	H5n	O	1	H5n	O	1
4819	GQ983079	H5n	H5n	O	1	H5n	O	1
4820	EF420250	H5p	H5p	O	1	H5p	O	1
4821	AY495172	H5p	H5p	O	1	H5p	O	1
4822	JQ703228	H5+16192	H5+16192	O	1	H5+16192	O	1
4823	JQ703278	H5q	H5q	O	1	H5q	O	1
4824	JQ703632	H5q	H5q	O	1	H5q	O	1
4825	JQ324596	H5+16311	H5+16311	O	1	H5+16311	O	1
4826	GQ983083	H5r	H5r	O	1	H5r	O	1
4827	GQ983107	H5r	H5r	O	1	H5r	O	1
4828	FJ384434	H5r1	H5r1	O	1	H5r1	O	1
4829	GQ983103	H5r1	H5r1	O	1	H5r1	O	1
4830	GQ983061	H5r2	H5r2	O	1	H5r2	O	1
4831	GQ983062	H5r2	H5r2	O	1	H5r2	O	1
4832	JQ703547	H5s	H5s	O	1	H5s	O	1
4833	JQ703266	H5s	H5s	O	1	H5s	O	1
4834	HQ661871	H5t	H5t	O	1	H5t	O	1
4835	GQ983098	H5t	H5t	O	1	H5t	O	1
4836	EF661003	H5u	H5u	O	1	H5u	O	1
4837	JX153503	H5u1	H5u1	O	1	H5u1	O	1
4838	JX128090	H5u1	H5u1	O	1	H5u1	O	1
4839	JX153021	H5+709	H5+709	O	1	H5+709	O	1
4840	JX153984	H5v	H5v	O	1	H5v	O	1
4841	EF660986	H5v	H5v	O	1	H5v	O	1
4842	FJ348166	H36	H36	O	1	H36	O	1
4843	FJ348151	H36	H36	O	1	H36	O	1
4844	HM765475	H6a	H6a	O	1	H6a	O	1

4845	JQ324601	H6a1a	H6a1a	O	1	H6a1a	O	1
4846	JQ704299	H6a1a	H6a1a	O	1	H6a1a	O	1
4847	JQ701925	H6a1a1	H6a1a1	O	1	H6a1a1	O	1
4848	AM263177	H6a1a1	H6a1a1	O	1	H6a1a1	O	1
4849	EF556185	H6a1a1a	H6a1a1a	O	1	H6a1a1a	O	1
4850	JQ702509	H6a1a1a	H6a1a1a	O	1	H6a1a1a	O	1
4851	JQ702107	H6a1a2a	H6a1a2a	O	1	H6a1a2a	O	1
4852	JQ704333	H6a1a2a	H6a1a2a	O	1	H6a1a2a	O	1
4853	JQ324568	H6a1a2b	H6a1a2b	O	1	H6a1a2b	O	1
4854	JQ703345	H6a1a2b1	H6a1a2b1	O	1	H6a1a2b1	O	1
4855	JQ701963	H6a1a2b1	H6a1a2b1	O	1	H6a1a2b1	O	1
4856	HQ730608	H6a1a3	H6a1a3	O	1	H6a1a3	O	1
4857	JQ703028	H6a1a3	H6a1a3	O	1	H6a1a3	O	1
4858	JQ703205	H6a1a3a	H6a1a3a	O	1	H6a1a3a	O	1
4859	GU123020	H6a1a4	H6a1a4	O	1	H6a1a4	O	1
4860	JQ704472	H6a1a4	H6a1a4	O	1	H6a1a4	O	1
4861	HQ405756	H6a1a5	H6a1a5	O	1	H6a1a5	O	1
4862	HM019517	H6a1a5	H6a1a5	O	1	H6a1a5	O	1
4863	JQ704195	H6a1a6	H6a1a6	O	1	H6a1a6	O	1
4864	JQ702189	H6a1a6	H6a1a6	O	1	H6a1a6	O	1
4865	HG01489	H6a1a7	H6a1a7	O	1	H6a1a7	O	1
4866	JQ324730	H6a1a7	H6a1a7	O	1	H6a1a7	O	1
4867	JQ705660	H6a1a8a	H6a1a8a	O	1	H6a1a8a	O	1
4868	JQ704469	H6a1a8a	H6a1a8a	O	1	H6a1a8a	O	1
4869	JX153292	H6a1a9	H6a1a9	O	1	H6a1a9	O	1
4870	JQ704380	H6a1a9	H6a1a9	O	1	H6a1a9	O	1
4871	JQ702606	H6a1a10	H6a1a10	O	1	H6a1a10	O	1
4872	JX297423	H6a1a10	H6a1a10	O	1	H6a1a10	O	1
4873	JQ704592	H6a1b	H6a1b	O	1	H6a1b	O	1
4874	JQ701955	H6a1b	H6a1b	O	1	H6a1b	O	1
4875	EF556178	H6a1b1	H6a1b1	O	1	H6a1b1	O	1
4876	JQ704495	H6a1b2	H6a1b2	O	1	H6a1b2	O	1
4877	JQ705873	H6a1b2	H6a1b2	O	1	H6a1b2	O	1
4878	HQ658481	H6a1b2a	H6a1b2a	O	1	H6a1b2a	O	1
4879	JQ704376	H6a1b2a	H6a1b2a	O	1	H6a1b2a	O	1
4880	JQ703391	H6a1b2b	H6a1b2b	O	1	H6a1b2b	O	1
4881	JQ704239	H6a1b2c	H6a1b2c	O	1	H6a1b2c	O	1

4882	JQ324676	H6a1b2d	H6a1b2d	O	1	H6a1b2d	O	1
4883	EU154342	H6a1b2d	H6a1b2d	O	1	H6a1b2d	O	1
4884	JX153784	H6a1b2e	H6a1b2e	O	1	H6a1b2e	O	1
4885	JQ705659	H6a1b2e	H6a1b2e	O	1	H6a1b2e	O	1
4886	JX307108	H6a1b3	H6a1b3	O	1	H6a1b3	O	1
4887	JQ704651	H6a1b3	H6a1b3	O	1	H6a1b3	O	1
4888	JQ702830	H6a1b3a	H6a1b3a	O	1	H6a1b3a	O	1
4889	JQ704543	H6a1b3a	H6a1b3a	O	1	H6a1b3a	O	1
4890	HQ659687	H6a1b3b	H6a1b3b	O	1	H6a1b3b	O	1
4891	JQ703321	H6a1b4	H6a1b4	O	1	H6a1b4	O	1
4892	JQ704270	H6a1b4	H6a1b4	O	1	H6a1b4	O	1
4893	JQ703232	H6a2	H6a2	O	1	H6a2	O	1
4894	JQ704515	H6a2	H6a2	O	1	H6a2	O	1
4895	JQ704208	H6a2a	H6a2a	O	1	H6a2a	O	1
4896	JQ702285	H6b	H6b	O	1	H6b	O	1
4897	AY738956	H6b1	H6b	X	3	H6b1	O	1
4898	JQ703353	H6b1	H6b	X	3	H6b1	O	1
4899	FJ348203	H6b2	H6b2	O	1	H6b2	O	1
4900	JX153090	H6b2	H6b2	O	1	H6b2	O	1
4901	FJ652065	H6c	H6c	O	1	H6c	O	1
4902	JN601518	H6c	H6c	O	1	H6c	O	1
4903	JX307099	H6c1	H6c1	O	1	H6c1	O	1
4904	JX307110	H6c1	H6c1	O	1	H6c1	O	1
4905	AY495120	H7	H7	O	1	H7	O	1
4906	JQ703313	H7	H7	O	1	H+152	X	2
4907	AY495090	H7a	H7a	O	1	H7a	O	1
4908	JQ704362	H7a	H7a	O	1	H7a	O	1
4909	EF177426	H7a1	H7a1	O	1	H7a1	O	1
4910	DQ862537	H7a1	H7a1	O	1	H7a1	O	1
4911	JQ704518	H7a1a	H7a1a	O	1	H7a1a	O	1
4912	EF660947	H7a1a	H7a1a	O	1	H7a1a	O	1
4913	JQ703217	H7a1b	H7a1b	O	1	H7a1b	O	1
4914	JQ705420	H7a1b	H7a1b	O	1	H7a1b	O	1
4915	JQ704524	H7a1c	H7a1c	O	1	H7a1c	O	1
4916	JQ705679	H7a1c	H7a1c	O	1	H7a1c	O	1
4917	JX153387	H7a1d	H7a1d	O	1	H7a1d	O	1
4918	JX153331	H7a1d	H7a1d	O	1	H7a1d	O	1

4919	JQ703233	H7a2	H7a2	O	1	H7a2	O	1
4920	JQ704171	H7a2	H7a2	O	1	H7a2	O	1
4921	JQ703372	H7b	H7b	O	1	H7b	O	1
4922	JQ704360	H7b	H7b	O	1	H7b	O	1
4923	EU600357	H7b1	H7b1	O	1	H7b1	O	1
4924	AY738966	H7b1	H7b1	O	1	H7b1	O	1
4925	JQ702056	H7b2	H7b2	O	1	H7b2	O	1
4926	JX153372	H7b2	H7b2	O	1	H7b2	O	1
4927	JQ703275	H7b2a	H7b2a	O	1	H7b2a	O	1
4928	EF581833	H7b3	H7b3	O	1	H7b3	O	1
4929	JQ704850	H7b4	H7b4	O	1	H7b4	O	1
4930	AY495104	H7b5	H7b5	O	1	H7b5	O	1
4931	HQ658454	H7b5	H7b5	O	1	H7b5	O	1
4932	JQ704428	H7b6	H7b6	O	1	H7b6	O	1
4933	EU600355	H7c1	H7c1	O	1	H7c1	O	1
4934	AY738965	H7c1	H7c1	O	1	H7c1	O	1
4935	JQ704605	H7c2	H7c2	O	1	H7c2	O	1
4936	JQ704714	H7c2	H7c2	O	1	H7c2	O	1
4937	JQ703177	H7c3	H7c3	O	1	H7c3	O	1
4938	HQ658573	H7c3	H7c3	O	1	H7c3	O	1
4939	NA20537	H7c4	H7c4	O	1	H7c4	O	1
4940	JQ702277	H7c4	H7c4	O	1	H7c4	O	1
4941	KC257398	H7c5	H7c5	O	1	H7c5	O	1
4942	GQ422375	H7c5	H7c5	O	1	H7c5	O	1
4943	JX153913	H7c6	H7c6	O	1	H7c6	O	1
4944	JQ703456	H7c6	H7c6	O	1	H7c6	O	1
4945	JQ704712	H7d	H7d	O	1	H7d	O	1
4946	JQ705880	H7d1	H7d1	O	1	H7d1	O	1
4947	JQ704506	H7d1	H7d1	O	1	H7d1	O	1
4948	JQ704711	H7d2	H7d	X	2	H7d2	O	1
4949	JQ702337	H7d2a	H7d2a	O	1	H7d2a	O	1
4950	JQ705230	H7d2a	H7d2a	O	1	H7d2a	O	1
4951	JQ324573	H7d3	H7d3	O	1	H7d3	O	1
4952	JQ703288	H7d3a	H7d3a	O	1	H7d3a	O	1
4953	AY495108	H7d3a	H7d3a	O	1	H7d3a	O	1
4954	JF262142	H7d4	H7d4	O	1	H7d4	O	1
4955	JQ701917	H7d4	H7d4	O	1	H7d4	O	1

4956	JN035288	H7d5	H7d5	O	1	H7d5	O	1
4957	JQ705045	H7d5	H7d5	O	1	H7d5	O	1
4958	HQ267514	H7e	H7e	O	1	H7e	O	1
4959	JQ703437	H7e	H7e	O	1	H7e	O	1
4960	JQ704377	H7f	H7f	O	1	H7f	O	1
4961	JQ704646	H7f	H7f	O	1	H7f	O	1
4962	JQ703479	H7g	H7g	O	1	H7g	O	1
4963	JQ703495	H7h	H7h	O	1	H7h	O	1
4964	JQ702766	H7h	H7h	O	1	H7h	O	1
4965	JX152892	H7h1	H7h1	O	1	H7h1	O	1
4966	AY738964	H7h1	H7h1	O	1	H7h1	O	1
4967	JF795008	H7i	H7i	O	1	H7i	O	1
4968	JX153705	H7i1	H7i1	O	1	H7i1	O	1
4969	JX153277	H7i1	H7i1	O	1	H7i1	O	1
4970	EU600360	H8a	H8a	O	1	H8a	O	1
4971	AY738957	H8a1	H8a1	O	1	H8a1	O	1
4972	JF960238	H8a1	H8a1	O	1	H8a1	O	1
4973	JQ324881	H8b	H8b	O	1	H8b	O	1
4974	FJ147310	H8b1	H8b1	O	1	H8b1	O	1
4975	KF148188	H8b1	H8b1	O	1	H8b1	O	1
4976	EF177429	H8+(114)+152	H8+(114)+152	O	1	H8+(114)+152	O	1
4977	JQ702865	H8c	H8c	O	1	H8c	O	1
4978	JQ703247	H8c	H8c	O	1	H8c	O	1
4979	JQ701943	H8c1	H8c1	O	1	H8c1	O	1
4980	JQ735909	H8c2	H8c2	O	1	H8c2	O	1
4981	AY195758	H8c2	H8c2	O	1	H8c2	O	1
4982	EF660914	H31	H31	O	1	H31	O	1
4983	JN009620	H31	H31	O	1	H31	O	1
4984	JQ028728	H31a	H31a	O	1	H31a	O	1
4985	JQ704510	H31a	H31a	O	1	H31a	O	1
4986	JQ703940	H31b	H31b	O	1	H31b	O	1
4987	JQ702697	H31b	H31b	O	1	H31b	O	1
4988	JQ703684	H11	H11	O	1	H11	O	1
4989	JX152992	H11a	H11a	O	1	H11a	O	1
4990	GU945760	H11a	H11a	O	1	H11a	O	1
4991	JQ703259	H11a1	H11a1	O	1	H11a1	O	1
4992	JQ703453	H11a1	H11a1	O	1	H11a1	O	1

4993	GU949563	H11a2	H11a2	O	1	H11a2	O	1
4994	JQ703175	H11a2	H11a2	O	1	H11a2	O	1
4995	JQ705580	H11a2a	H11a2a	O	1	H11a2a	O	1
4996	JQ704395	H11a2a	H11a2a	O	1	H11a2a	O	1
4997	EF545566	H11a2a1	H11a2a1	O	1	H11a2a1	O	1
4998	JQ703451	H11a2a1	H11a2a1	O	1	H11a2a1	O	1
4999	JQ703071	H11a2a2	H11a2a2	O	1	H11a2a2	O	1
5000	JQ705954	H11a2a2	H11a2a2	O	1	H11a2a2	O	1
5001	JX306646	H11a2a3	H11a2a3	O	1	H11a2a3	O	1
5002	FJ705060	H11a2a3	H11a2a3	O	1	H11a2a3	O	1
5003	JQ703235	H11a3	H11a3	O	1	H11a3	O	1
5004	HQ257449	H11a3	H11a3	O	1	H11a3	O	1
5005	HQ659686	H11a4	H11a	X	3	H11a4	O	1
5006	GU592038	H11a5	H11a5	O	1	H11a5	O	1
5007	JQ703949	H11a5	H11a5	O	1	H11a5	O	1
5008	JQ705740	H11a6	H11a6	O	1	H11a6	O	1
5009	NA12873	H11a6	H11a6	O	1	H11a6	O	1
5010	JQ703253	H11a+152	H11a+152	O	1	H11a+152	O	1
5011	HQ707396	H11a+152	H11a+152	O	1	H11a+152	O	1
5012	JQ703733	H11a7	H11a7	O	1	H11a7	O	1
5013	KF765775	H11a7	H11a7	O	1	H11a7	O	1
5014	JQ705643	H11a8	H11a8	O	1	H11a8	O	1
5015	JX153476	H11a8	H11a8	O	1	H11a8	O	1
5016	JQ704286	H11b1	H11b1	O	1	H11b1	O	1
5017	JQ704081	H11b1	H11b1	O	1	H11b1	O	1
5018	EF660955	H12	H12	O	1	H12	O	1
5019	DQ341083	H12	H12	O	1	H12	O	1
5020	AY738994	H12a	H12a	O	1	H12a	O	1
5021	JX153458	H12a	H12a	O	1	H12a	O	1
5022	JQ703303	H91	H91	O	1	H91	O	1
5023	NA20800	H91	H91	O	1	H91	O	1
5024	JQ324697	H108	H108	O	1	H108	O	1
5025	JX154006	H108	H108	O	1	H108	O	1
5026	JQ703798	H+152	H+152	O	1	H+152	O	1
5027	AY713978	H9	H9	O	1	H9	O	1
5028	AY738969	H9a	H9a	O	1	H9a	O	1
5029	JQ324810	H9a	H9a	O	1	H9a	O	1

5030	DQ523619	H32	H32	O	1	H32	O	1
5031	EF660931	H32	H32	O	1	H32	O	1
5032	AY495140	H46	H46	O	1	H46	O	1
5033	JQ703070	H46a	H46a	O	1	H46a	O	1
5034	JQ705005	H46a	H46a	O	1	H46a	O	1
5035	KC553988	H46b	H46b	O	1	H46b	O	1
5036	JQ703349	H52	H52	O	1	H52	O	1
5037	EU744541	H52	H52	O	1	H52	O	1
5038	KC257357	H69	H69	O	1	H69	O	1
5039	AY495136	H69	H69	O	1	H69	O	1
5040	HM470002	H103	H103	O	1	H103	O	1
5041	JX153034	H103	H103	O	1	H103	O	1
5042	JX153071	H107	H107	O	1	H107	O	1
5043	HM765464	H107	H107	O	1	H107	O	1
5044	JQ701849	H10	H10	O	1	H10 H10+(16093)	O	2
5045	EU156036	H10	H10	O	1	H10	O	1
5046	KC257394	H10a	H10a	O	1	H10a	O	1
5047	AY495101	H10a	H10a	O	1	H10a	O	1
5048	HM021152	H10a1	H10a1	O	1	H10a1	O	1
5049	KF056318	H10a1	H10a1	O	1	H10a1	O	1
5050	JQ704287	H10a1a	H10a1a	O	1	H10a1a	O	1
5051	AY738970	H10a1a	H10a1a	O	1	H10a1a	O	1
5052	EU073971	H10a1a1	H10a1a1	O	1	H10a1a1	O	1
5053	JQ704452	H10a1a1	H10a1a1	O	1	H10a1a1	O	1
5054	JQ703237	H10a1b	H10a1b	O	1	H10a1b	O	1
5055	JQ704531	H10a1b	H10a1b	O	1	H10a1b	O	1
5056	JQ701860	H10b	H10b	O	1	H10b	O	1
5057	AY495145	H10b	H10b	O	1	H10b	O	1
5058	JQ704573	H10b1	H10b1	O	1	H10b1	O	1
5059	AY495093	H10b1	H10b1	O	1	H10b1	O	1
5060	JQ704955	H10c	H10c	O	1	H10c	O	1
5061	GU984044	H10c1	H10c1	O	1	H10c1	O	1
5062	JQ704295	H10c1	H10c1	O	1	H10c1	O	1
5063	JQ703504	H10d	H10d	O	1	H10d	O	1
5064	JQ704561	H10d	H10d	O	1	H10d	O	1
5065	JQ703539	H10+(16093)	H10+(16093)	O	1	H10+(16093)	O	1
5066	JQ705866	H10+(16093)	H10+(16093)	O	1	H10+(16093)	O	1

5067	JQ704209	H10e	H10e	O	1	H10e	O	1
5068	JQ705702	H10e	H10e	O	1	H10e	O	1
5069	JQ703002	H10e1	H10e1	O	1	H10e1	O	1
5070	JQ705937	H10e1	H10e1	O	1	H10e1	O	1
5071	JQ703645	H10e1a	H10e1a	O	1	H10e1a	O	1
5072	JQ704387	H10e1a	H10e1a	O	1	H10e1a	O	1
5073	GU569076	H10e2	H10e2	O	1	H10e2	O	1
5074	HM101252	H10e2	H10e2	O	1	H10e2	O	1
5075	JQ702010	H10e3a	H10e3a	O	1	H10e3a	O	1
5076	JQ702599	H10e3a	H10e3a	O	1	H10e3a	O	1
5077	JQ703010	H10f	H10f	O	1	H10f	O	1
5078	GU122976	H10g	H10g	O	1	H10g	O	1
5079	JQ702818	H10h	H10h	O	1	H10h	O	1
5080	JQ702272	H10h	H10h	O	1	H10h	O	1
5081	AY495107	H13a1a	H13a1a	O	1	H13a1a	O	1
5082	JQ705098	H13a1a	H13a1a	O	1	H13a1a	O	1
5083	JQ704682	H13a1a1	H13a1a1	O	1	H13a1a1	O	1
5084	JQ705399	H13a1a1	H13a1a1	O	1	H13a1a1	O	1
5085	AY495129	H13a1a1a	H13a1a1a	O	1	H13a1a1a	O	1
5086	JQ706004	H13a1a1a	H13a1a1a	O	1	H13a1a1a	O	1
5087	JQ704234	H13a1a1b	H13a1a1b	O	1	H13a1a1b	O	1
5088	JQ703386	H13a1a1b	H13a1a1b	O	1	H13a1a1b	O	1
5089	AM263191	H13a1a1c	H13a1a1c	O	1	H13a1a1c	O	1
5090	JQ703808	H13a1a1d	H13a1a1d	O	1	H13a1a1d	O	1
5091	JQ704841	H13a1a1d1	H13a1a1d1	O	1	H13a1a1d1	O	1
5092	HG00324	H13a1a1d1	H13a1a1d1	O	1	H13a1a1d1	O	1
5093	JQ702022	H13a1a1e	H13a1a1e	O	1	H13a1a1e	O	1
5094	JQ703421	H13a1a1e	H13a1a1e	O	1	H13a1a1e	O	1
5095	AM263186	H13a1a2	H13a1a2	O	1	H13a1a2	O	1
5096	EU370982	H13a1a2a	H13a1a2a	O	1	H13a1a2a	O	1
5097	JQ703208	H13a1a2a	H13a1a2a	O	1	H13a1a2a	O	1
5098	EU600347	H13a1a2b	H13a1a2b	O	1	H13a1a2b	O	1
5099	KC911290	H13a1a3	H13a1a3	O	1	H13a1a3	O	1
5100	JQ703193	H13a1a3	H13a1a3	O	1	H13a1a3	O	1
5101	FJ560455	H13a1a4	H13a1a4	O	1	H13a1a4	O	1
5102	JQ705726	H13a1a5	H13a1a5	O	1	H13a1a5	O	1
5103	JQ704176	H13a1a6	H13a1a6	O	1	H13a1a6	O	1

5104	HM103355	H13a1b	H13a1b	O	1	H13a1b	O	1
5105	HG01173	H13a1b	H13a1b	O	1	H13a1b	O	1
5106	JN712772	H13a1c	H13a1c	O	1	H13a1c	O	1
5107	KF889440	H13a1c	H13a1c	O	1	H13a1c	O	1
5108	EU597515	H13a1+152	H13a1+152	O	1	H13a1+152	O	1
5109	JX153573	H13a1d	H13a1d	O	1	H13a1d	O	1
5110	AM263187	H13a1d	H13a1d	O	1	H13a1d	O	1
5111	KC577357	H13a2a	H13a2a	O	1	H13a2a	O	1
5112	KC911335	H13a2a	H13a2a	O	1	H13a2a	O	1
5113	KC911369	H13a2a1	H13a2a1	O	1	H13a2a1	O	1
5114	KC911458	H13a2a1	H13a2a1	O	1	H13a2a1	O	1
5115	JX153617	H13a2b	H13a2b	O	1	H13a2b	O	1
5116	JQ701816	H13a2b1	H13a2b1	O	1	H13a2b1	O	1
5117	EF660948	H13a2b1	H13a2b1	O	1	H13a2b1	O	1
5118	HQ593813	H13a2b2	H13a2b2	O	1	H13a2b2	O	1
5119	JF828090	H13a2b2a	H13a2b2a	O	1	H13a2b2a	O	1
5120	KC911361	H13a2b2a	H13a2b2a	O	1	H13a2b2a	O	1
5121	JQ705524	H13a2b3	H13a2b3	O	1	H13a2b3	O	1
5122	HQ593810	H13a2b4	H13a2b4	O	1	H13a2b4	O	1
5123	KC911469	H13a2b4	H13a2b4	O	1	H13a2b4	O	1
5124	HQ658464	H13a2b5	H13a2b5	O	1	H13a2b5	O	1
5125	JX153198	H13a2b5	H13a2b5	O	1	H13a2b5	O	1
5126	KC911338	H13a2c	H13a2c	O	1	H13a2c	O	1
5127	JQ703878	H13a2c1	H13a2c1	O	1	H13a2c1	O	1
5128	AM263190	H13a2c1	H13a2c1	O	1	H13a2c1	O	1
5129	AM263188	H13b	H13b	O	1	H13b	O	1
5130	EF660956	H13b1	H13b1	O	1	H13b1	O	1
5131	GU228506	H13b1+200	H13b1+200	O	1	H13b1+200	O	1
5132	JQ704111	H13b1a	H13b1a	O	1	H13b1a	O	1
5133	EU052289	H13b1a	H13b1a	O	1	H13b1a	O	1
5134	NA20771	H13b1b	H13b1b	O	1	H13b1b	O	1
5135	JQ704147	H13b1b	H13b1b	O	1	H13b1b	O	1
5136	JQ704313	H13b2	H13b2	O	1	H13b2	O	1
5137	KC911454	H13b2	H13b2	O	1	H13b2	O	1
5138	JQ703657	H13c1	H13c1	O	1	H13c1	O	1
5139	HG00233	H13c1	H13c1	O	1	H13c1	O	1
5140	KC911462	H13c1a	H13c1a	O	1	H13c1a	O	1

5141	EF660920	H13c1a	H13c1a	O	1	H13c1a	O	1
5142	KC911276	H13c2	H13c2	O	1	H13c2	O	1
5143	HQ234355	H13c2	H13c2	O	1	H13c2	O	1
5144	GQ983090	H14a	H14a	O	1	H14a	O	1
5145	AM263183	H14a	H14a	O	1	H14a	O	1
5146	JQ705084	H14a+146	H14a+146	O	1	H14a+146	O	1
5147	EF556163	H14a1	H14a1	O	1	H14a1	O	1
5148	JQ702157	H14a2	H14a2	O	1	H14a2	O	1
5149	JQ704161	H14a2a	H14a2a	O	1	H14a2a	O	1
5150	JQ705233	H14a2a	H14a2a	O	1	H14a2a	O	1
5151	JQ703871	H14a2b	H14a2b	O	1	H14a2b	O	1
5152	JQ702283	H14a2b	H14a2b	O	1	H14a2b	O	1
5153	JX153559	H14a2c	H14a2c	O	1	H14a2c	O	1
5154	JN609592	H14a2c	H14a2c	O	1	H14a2c	O	1
5155	AM263182	H14b	H14b	O	1	H14b	O	1
5156	JQ704798	H14b	H14b	O	1	H14b	O	1
5157	JQ324608	H14b1	H14b1	O	1	H14b1	O	1
5158	JQ324547	H14b1	H14b1	O	1	H14b1	O	1
5159	KC911300	H14b2	H14b2	O	1	H14b2	O	1
5160	JQ704795	H14b2a	H14b2a	O	1	H14b2a	O	1
5161	KC911545	H14b3	H14b3	O	1	H14b3	O	1
5162	JQ702840	H14b3	H14b3	O	1	H14b3	O	1
5163	JX153284	H14b4	H14b4	O	1	H14b4	O	1
5164	JQ704254	H14b4	H14b4	O	1	H14b4	O	1
5165	KC911292	H15	H15	O	1	H15	O	1
5166	AY495146	H15a	H15a	O	1	H15a	O	1
5167	JQ703079	H15a1	H15a1	O	1	H15a1	O	1
5168	JQ704504	H15a1	H15a1	O	1	H15a1	O	1
5169	JQ704364	H15a1a	H15a1a	O	1	H15a1a	O	1
5170	JQ701892	H15a1a1	H15a1a1	O	1	H15a1a1	O	1
5171	AY713995	H15a1a1	H15a1a1	O	1	H15a1a1	O	1
5172	JQ324533	H15a1b	H15a1b	O	1	H15a1b	O	1
5173	KC911422	H15a1b	H15a1b	O	1	H15a1b	O	1
5174	JQ704870	H15b	H15b	O	1	H15b	O	1
5175	AY738960	H15b	H15b	O	1	H15b	O	1
5176	NA20798	H15b1	H15b1	O	1	H15b1	O	1
5177	FJ384438	H15b1	H15b1	O	1	H15b1	O	1

5178	JN651417	H15b2	H15b2	O	1	H15b2	O	1
5179	JF901940	H15b2	H15b2	O	1	H15b2	O	1
5180	JQ704409	H16	H16	O	1	H16	O	1
5181	JQ703108	H16	H16	O	1	H16	O	1
5182	EF609012	H16+152	H16+152	O	1	H16+152	O	1
5183	AY495135	H16+152	H16+152	O	1	H16+152	O	1
5184	EU170619	H16a	H16a	O	1	H16a	O	1
5185	NA12340	H16a	H16a	O	1	H16a	O	1
5186	AY495133	H16a1	H16a1	O	1	H16a1	O	1
5187	AY495138	H16a1	H16a1	O	1	H16a1	O	1
5188	JQ702103	H16c	H16c	O	1	H16c	O	1
5189	JN037470	H16d	H16d	O	1	H16d	O	1
5190	AY495127	H16d	H16d	O	1	H16d	O	1
5191	EF472971	H16b	H16b	O	1	H16b	O	1
5192	EU715287	H16b	H16b	O	1	H16b	O	1
5193	JX153946	H16e	H16e	O	1	H16e	O	1
5194	JX153305	H16e	H16e	O	1	H16e	O	1
5195	AY495150	H17	H17	O	1	H17	O	1
5196	JQ704083	H17	H17	O	1	H17	O	1
5197	JQ324832	H17a	H17a	O	1	H17a	O	1
5198	JQ702119	H17a	H17a	O	1	H17a	O	1
5199	JQ703188	H17a1	H17a1	O	1	H17a1	O	1
5200	JQ704404	H17a1	H17a1	O	1	H17a1	O	1
5201	AY495154	H17a2	H17a2	O	1	H17a2	O	1
5202	JX153880	H17b	H17b	O	1	H17b	O	1
5203	JQ705146	H17b	H17b	O	1	H17b	O	1
5204	JQ704457	H17c	H17c	O	1	H17c	O	1
5205	JQ705366	H27	H27	O	1	H27	O	1
5206	HQ658132	H27	H27	O	1	H27	O	1
5207	JQ705258	H27+16093	H27+16093	O	1	H27	X	2
5208	JQ704331	H27+16093	H27+16093	O	1	H27+16093	O	1
5209	EU007878	H27a	H27a	O	1	H27a	O	1
5210	GU123038	H27a	H27a	O	1	H27a	O	1
5211	FJ348192	H27b	H27b	O	1	H27b	O	1
5212	FJ348179	H27b	H27b	O	1	H27b	O	1
5213	JQ703092	H27c	H27c	O	1	H27c	O	1
5214	JQ702961	H27d	H27d	O	1	H27d	O	1

5215	JQ703107	H27d	H27d	O	1	H27d	O	1
5216	JQ704541	H27e	H27	X	Not found	H27e	O	1
5217	JQ705074	H27e	H27	X	Not found	H27e	O	1
5218	JQ704644	H27f	H27f	O	1	H27f	O	1
5219	JX153431	H27f	H27f	O	1	H27f	O	1
5220	JQ704443	H18	H18	O	1	H18	O	1
5221	JQ705490	H18	H18	O	1	H18	O	1
5222	JX154019	H18b	H18b	O	1	H18b	O	1
5223	JQ704205	H18b	H18b	O	1	H18b	O	1
5224	KC911570	H19	H19	O	1	H19	O	1
5225	KC911539	H19	H19	O	1	H19	O	1
5226	JX153278	H20	H20	O	1	H20	O	1
5227	KC911430	H20a1	H20a	X	2	H20a1	O	1
5228	HQ436412	H20a1	H20a	X	4	H20a1	O	1
5229	EF556187	H20a1a	H20a1a	O	1	H20a1a	O	1
5230	JQ703578	H20a1a	H20a1a	O	1	H20a1a	O	1
5231	JX153059	H20a2	H20a2	O	1	H20a2	O	1
5232	JX153027	H20a2	H20a2	O	1	H20a2	O	1
5233	EU600331	H20b	H20b	O	1	H20b	O	1
5234	EU600334	H20b	H20b	O	1	H20b	O	1
5235	HG00143	H20c	H20c	O	1	H20c	O	1
5236	JQ324684	H20c	H20c	O	1	H20c	O	1
5237	AM263178	H21	H21	O	1	H21	O	1
5238	GU122977	H23	H23	O	1	H23	O	1
5239	JQ794835	H23	H23	O	1	H23	O	1
5240	JQ324553	H24	H24	O	1	H24	O	1
5241	HM589043	H24	H24	O	1	H24	O	1
5242	JQ705260	H24a	H24a	O	1	H24a	O	1
5243	JQ703982	H24a	H24a	O	1	H24a	O	1
5244	JQ703898	H24a1	H24a1	O	1	H24a1	O	1
5245	HM625710	H24a1	H24a1	O	1	H24a1	O	1
5246	JQ705486	H24a2	H24a2	O	1	H51	X	2
5247	JQ324875	H24b	H24b	O	1	H24b	O	1
5248	JQ704672	H24b	H24b	O	1	H24b	O	1
5249	EF556189	H25	H25	O	1	H25	O	1
5250	JQ703239	H26	H26	O	1	H26	O	1
5251	JQ702217	H26	H26	O	1	H26	O	1

5252	JN415471	H26a	H26a	O	1	H26a	O	1
5253	EU684000	H26a1	H26a1	O	1	H26a1	O	1
5254	EF532800	H26a1	H26a1	O	1	H26a1	O	1
5255	JQ702037	H26a1a	H26a1a	O	1	H26a1a	O	1
5256	JQ703417	H26a1a1	H26a1a1	O	1	H26a1a1	O	1
5257	HG00133	H26a1a1	H26a1a1	O	1	H26a1a1	O	1
5258	JX152941	H26a1b	H26a1b	O	1	H26a1b	O	1
5259	HM625708	H26a1b	H26a1b	O	1	H26a1b	O	1
5260	EU600338	H26b	H26b	O	1	H26b	O	1
5261	HM775971	H26b	H26b	O	1	H26b	O	1
5262	JQ704110	H26c	H26c	O	1	H26c	O	1
5263	JQ703097	H26c	H26c	O	1	H26c	O	1
5264	DQ523654	H28	H28	O	1	H28	O	1
5265	EU670874	H28a	H28a	O	1	H28a	O	1
5266	JQ705312	H28a	H28a	O	1	H28a	O	1
5267	JQ704416	H28a1	H28a1	O	1	H28a1	O	1
5268	JF298814	H28a2	H28a2	O	1	H28a2	O	1
5269	JX153255	H28a2	H28a2	O	1	H28a2	O	1
5270	JQ705134	H29	H29	O	1	H29	O	1
5271	EU600335	H29a	H29a	O	1	H29a	O	1
5272	EU600343	H29a	H29a	O	1	H29a	O	1
5273	JQ704540	H29b	H29b	O	1	H29b	O	1
5274	EF660925	H29b	H29b	O	1	H29b	O	1
5275	EF556162	H30	H30	O	1	H+195	X	2
5276	JQ704227	H30a	H30a	O	1	H30a	O	1
5277	JQ703004	H30a	H30a	O	1	H30a	O	1
5278	JQ704389	H30b	H30b	O	1	H1+16311	X	2
5279	HQ315686	H30b1	H30b1	O	1	H30b1	O	1
5280	JQ324587	H30b1	H30b1	O	1	H30b1	O	1
5281	EU600336	H33	H33	O	1	H33	O	1
5282	JQ702229	H33	H33	O	1	H33	O	1
5283	JQ702297	H33a	H33a	O	1	H33a	O	1
5284	HM765469	H33a	H33a	O	1	H33a	O	1
5285	EU600341	H33b	H33b	O	1	H33b	O	1
5286	JQ703596	H33b	H33b	O	1	H33b	O	1
5287	KC618506	H33c	H33c	O	1	H33c	O	1
5288	JX153043	H33c	H33c	O	1	H33c	O	1

5289	JQ705818	H+16291	H+16291	O	1	H+16291	O	1
5290	EU007846	H34	H34	O	1	H34	O	1
5291	AY738968	H34	H34	O	1	H34	O	1
5292	JQ702918	H64	H64	O	1	H64	O	1
5293	JQ704251	H85	H85	O	1	H85	O	1
5294	HQ699439	H85	H85	O	1	H85	O	1
5295	JQ703157	H35	H35	O	1	H35	O	1
5296	JQ703287	H35	H35	O	1	H35	O	1
5297	AY495316	H35a	H35a	O	1	H35a	O	1
5298	JQ702256	H35a	H35a	O	1	H35a	O	1
5299	AY339402	H39	H39	O	1	H39	O	1
5300	EU677425	H39	H39	O	1	H39	O	1
5301	JQ702834	H39a	H39a	O	1	H39a	O	1
5302	JQ704461	H39a1	H39a1	O	1	H39a1	O	1
5303	JQ703325	H39b	H39b	O	1	H39b	O	1
5304	JQ705710	H39b	H39b	O	1	H39b	O	1
5305	JQ704325	H39c	H39c	O	1	H39c	O	1
5306	JX153830	H39c	H39c	O	1	H39c	O	1
5307	JQ704876	H40	H40	O	1	H40	O	1
5308	FJ985851	H40a	H40	X	4	H40a	O	1
5309	FJ788098	H40a	H40	X	4	H40a	O	1
5310	JQ702933	H40b	H40b	O	1	H40b	O	1
5311	JQ702924	H40b	H40b	O	1	H40b	O	1
5312	JQ702060	H41a	H41a	O	1	H41a	O	1
5313	FJ348209	H41a	H41a	O	1	H41a	O	1
5314	JQ704132	H42	H42	O	1	H42	O	1
5315	JQ324628	H42a	H42a	O	1	H42a	O	1
5316	JQ324734	H42a1	H42a1	O	1	H42a1	O	1
5317	JQ324682	H42a1	H42a1	O	1	H42a1	O	1
5318	JX153894	H42a2	H42a2	O	1	H42a2	O	1
5319	JX153511	H42a2	H42a2	O	1	H42a2	O	1
5320	AY495114	H43	H43	O	1	H43	O	1
5321	AY495098	H43	H43	O	1	H43	O	1
5322	JQ704024	H44a	H44a	O	1	H44a	O	1
5323	JQ703701	H44a	H44a	O	1	H44a	O	1
5324	HM355887	H44a1	H44a1	O	1	H44a1	O	1
5325	JQ705692	H44b	H44b	O	1	H44b	O	1

5326	JQ704290	H44b	H44b	O	1	H44b	O	1
5327	JQ705232	H45a	H45a	O	1	H45a	O	1
5328	JQ705128	H45a	H45a	O	1	H45a	O	1
5329	JQ703821	H45b	H45b	O	1	H45b	O	1
5330	JQ705144	H45b	H45b	O	1	H45b	O	1
5331	GU123005	H47	H47	O	1	H47	O	1
5332	AY738959	H47	H47	O	1	H47	O	1
5333	JQ705143	H47a	H47a	O	1	H47a	O	1
5334	JQ702052	H47a	H47a	O	1	H47a	O	1
5335	HQ676807	H48	H48	O	1	H48	O	1
5336	KC257382	H48	H48	O	1	H48	O	1
5337	HQ660082	H49	H49	O	1	H49	O	1
5338	JQ704535	H49	H49	O	1	H49	O	1
5339	EU131366	H49a	H49a	O	1	H49a	O	1
5340	JQ702841	H49a	H49a	O	1	H49a	O	1
5341	JQ702985	H49a1	H49a1	O	1	H49a1	O	1
5342	JQ702889	H49a2	H49a2	O	1	H49a2	O	1
5343	JQ704556	H49b	H49b	O	1	H49b	O	1
5344	JQ703327	H50	H50	O	1	H50	O	1
5345	JQ704577	H50	H50	O	1	H50	O	1
5346	JQ705176	H51	H51	O	1	H51	O	1
5347	JQ324544	H51	H51	O	1	H51	O	1
5348	JQ703369	H51a	H51a	O	1	H51a	O	1
5349	JQ704144	H51a	H51a	O	1	H51a	O	1
5350	EU597538	H53	H53	O	1	H+152	X	2
5351	JQ324556	H53	H53	O	1	H53	O	1
5352	JQ704987	H54	H54	O	1	H54	O	1
5353	FJ188715	H54	H54	O	1	H54	O	1
5354	JQ703111	H55a	H55a	O	1	H55a	O	1
5355	GQ902958	H55a	H55a	O	1	H55a	O	1
5356	JQ702906	H55+153	H55+153	O	1	H55+153	O	1
5357	JQ705203	H55b	H55b	O	1	H55b	O	1
5358	JQ704460	H55b	H55b	O	1	H55b	O	1
5359	JQ702073	H56	H56	O	1	H56	O	1
5360	JQ703478	H56	H56	O	1	H56	O	1
5361	JQ705859	H56a	H56a	O	1	H56a	O	1
5362	JQ703350	H56b	H56b	O	1	H56b	O	1

5363	HQ287897	H56c	H56c	O	1	H56c	O	1
5364	JQ704371	H56c	H56c	O	1	H56c	O	1
5365	NA20582	H56d	H56d	O	1	H56d	O	1
5366	KC257389	H56d	H56d	O	1	H56d	O	1
5367	JQ704677	H57	H	X	9	H57	O	1
5368	KC911474	H57	H	X	5	H57	O	1
5369	JQ703264	H58	H58	O	1	H58	O	1
5370	JQ324788	H58a	H58a	O	1	H58a	O	1
5371	JQ702375	H58a	H58a	O	1	H58a	O	1
5372	JQ701910	H59	H59	O	1	H59	O	1
5373	JQ704027	H59a	H59a	O	1	H59a	O	1
5374	JQ705583	H60a	H60a	O	1	H60a	O	1
5375	JQ704493	H60a	H60a	O	1	H60a	O	1
5376	AY495185	H61	H61	O	1	H61	O	1
5377	JQ702768	H61	H61	O	1	H61	O	1
5378	JQ703804	H61a	H61a	O	1	H61a	O	1
5379	KC257403	H61a	H61a	O	1	H61a	O	1
5380	EF609014	H62	H62	O	1	H62	O	1
5381	JQ703306	H62	H62	O	1	H+152	X	2
5382	JQ704149	H63	H63	O	1	H63	O	1
5383	JQ704491	H63	H63	O	1	H63	O	1
5384	JQ704853	H63a	H63a	O	1	H63a	O	1
5385	HM625709	H65	H65	O	1	H65	O	1
5386	JQ703909	H65	H65	O	1	H65	O	1
5387	GU592025	H65a	H65a	O	1	H65a	O	1
5388	KC911282	H66	H66	O	1	H+152	X	2
5389	JN415472	H66	H66	O	1	H+16129	X	3
5390	JQ705407	H66a	H66a	O	1	H66a	O	1
5391	NA12842	H66a	H66a	O	1	H66a	O	1
5392	JQ703455	H66a1	H66a1	O	1	H66a1	O	1
5393	JQ703181	H66a1	H66a1	O	1	H66a1	O	1
5394	JQ704151	H67	H67	O	1	H67	O	1
5395	JQ702448	H67a	H67a	O	1	H67a	O	1
5396	FJ348189	H67a	H67a	O	1	H67a	O	1
5397	JQ704792	H70	H70	O	1	H70	O	1
5398	AY738998	H70	H70	O	1	H70	O	1
5399	JQ704813	H71	H71	O	1	H71	O	1

5400	JQ703155	H71	H71	O	1	H71	O	1
5401	JQ704835	H72	H72	O	1	H72	O	1
5402	JQ703178	H72	H72	O	1	H72	O	1
5403	JQ702419	H73	H73	O	1	H73	O	1
5404	JN202547	H73a	H73a	O	1	H73a	O	1
5405	JQ702443	H73a	H73a	O	1	H73a	O	1
5406	EU282416	H73a1	H73a1	O	1	H73a1	O	1
5407	HM625697	H73a1	H73a1	O	1	H73a1	O	1
5408	JQ324578	H74	H74	O	1	H74	O	1
5409	JQ703482	H75	H75	O	1	H75	O	1
5410	JQ705450	H76	H76	O	1	H76	O	1
5411	HQ287896	H76	H76	O	1	H76	O	1
5412	JQ704521	H76a	H76a	O	1	H76a	O	1
5413	JX153108	H76a	H76a	O	1	H76a	O	1
5414	JQ701996	H77	H77	O	1	H77	O	1
5415	EU600333	H78	H78	O	1	H78	O	1
5416	JQ704137	H78	H78	O	1	H78	O	1
5417	JQ702218	H79	H79	O	1	H79	O	1
5418	JQ701998	H79	H79	O	1	H79	O	1
5419	JQ705299	H79a	H79a	O	1	H79a	O	1
5420	JQ702589	H79a	H79a	O	1	H79a	O	1
5421	EF493867	H80	H80	O	1	H80	O	1
5422	JQ705682	H80	H80	O	1	H80	O	1
5423	JQ324727	H81	H81	O	1	H81	O	1
5424	JQ704831	H81a	H81a	O	1	H81a	O	1
5425	JQ702080	H82	H82	O	1	H82	O	1
5426	JQ705403	H83	H83	O	1	H83	O	1
5427	JQ703310	H83	H83	O	1	H83	O	1
5428	JX153022	H86	H86	O	1	H86	O	1
5429	JQ324603	H86	H86	O	1	H86	O	1
5430	JQ705040	H87	H87	O	1	H87	O	1
5431	JQ324534	H87	H87	O	1	H87	O	1
5432	KC553984	H88	H88	O	1	H88	O	1
5433	KC553989	H89	H89	O	1	H89	O	1
5434	JQ703454	H89	H89	O	1	H89	O	1
5435	KC554018	H90	H90	O	1	H90	O	1
5436	EU600340	H92	H92	O	1	H92	O	1

5437	NA20527	H92	H92	O	1	H92	O	1
5438	JQ704394	H93	H93	O	1	H93	O	1
5439	NA20502	H93	H93	O	1	H93	O	1
5440	NA19750	H94	H94	O	1	H94	O	1
5441	JQ705893	H94	H94	O	1	H94	O	1
5442	HQ593807	H95	H95	O	1	H95	O	1
5443	KC964603	H95a	H95a	O	1	H95a	O	1
5444	JQ703248	H95a	H95a	O	1	H95a	O	1
5445	KC810015	H96	H96	O	1	H96	O	1
5446	JQ705172	H96	H96	O	1	H96	O	1
5447	HQ659700	H100	H100	O	1	H100	O	1
5448	HM625681	H100	H100	O	1	H100	O	1
5449	KC911463	H101	H101	O	1	H101	O	1
5450	KC911349	H101	H101	O	1	H101	O	1
5451	JQ703658	H102	H102	O	1	H102	O	1
5452	JQ324570	H104	H104	O	1	H104	O	1
5453	EU600339	H104	H104	O	1	H20	X	2
5454	JX153176	H104a	H104a	O	1	H104a	O	1
5455	JX153822	H104a	H104a	O	1	H104a	O	1
5456	JX153454	H105	H105	O	1	H105	O	1
5457	JN573371	H105	H105	O	1	H105	O	1
5458	JQ324820	H105a	H105a	O	1	H105a	O	1
5459	JX153097	H106	H106	O	1	H106	O	1
5460	JQ701959	H106	H106	O	1	H106	O	1
5461	KC985149	R1a	R1a	O	1	R1a	O	1
5462	KC985147	R1a	R1a	O	1	R1a	O	1
5463	KC985159	R1a1	R1a1	O	1	R1a1	O	1
5464	HQ602771	R1a1	R1a1	O	1	R1a1	O	1
5465	JQ705561	R1a1a	R1a1a	O	1	R1a1a	O	1
5466	AY714045	R1a1a	R1a1a	O	1	R1a1a	O	1
5467	EU545437	R1a1a1	R1a1a1	O	1	R1a1a1	O	1
5468	EF222243	R1a1a1a	R1a1a1a	O	1	R1a1a1a	O	1
5469	EF222244	R1a1a1a	R1a1a1a	O	1	R1a1a1a	O	1
5470	GU451313	R1a1a2	R1a1a2	O	1	R1a1a2	O	1
5471	JQ702484	R1a1a2	R1a1a2	O	1	R1a1a2	O	1
5472	HM030522	R1a1b	R1a1b	O	1	R1a1b	O	1
5473	HM852892	R1a1b	R1a1b	O	1	R1a1b	O	1

5474	KC985148	R1a1c	R1a1c	O	1	R1a1c	O	1
5475	KC985151	R1a1c	R1a1c	O	1	R1a1c	O	1
5476	KC985165	R1b	R1b	O	1	R1b	O	1
5477	JQ703633	R1b1	R1b1	O	1	R1b1	O	1
5478	HM996895	R1b1	R1b1	O	1	R1b1	O	1
5479	KC911379	R2	R2	O	1	R2	O	1
5480	KC911319	R2	R2	O	1	R2	O	1
5481	KC911374	R2+13500	R2+13500	O	1	R2+13500	O	1
5482	AY714007	R2+13500+195	R2+13500+195	O	1	R2+13500+195	O	1
5483	JX155266	R2a	R2a	O	1	R2a	O	1
5484	JX155268	R2a	R2a	O	1	R2a	O	1
5485	JX152991	R2b	R2b	O	1	R2b	O	1
5486	EF556167	R2b1	R2b1	O	1	R2b1	O	1
5487	HM852870	R2b1	R2b1	O	1	R2b1	O	1
5488	JX155271	R2c	R2c	O	1	R2c	O	1
5489	JX155269	R2c	R2c	O	1	R2c	O	1
5490	KC911337	R2d	R2d	O	1	R2d	O	1
5491	KC911627	R2d	R2d	O	1	R2d	O	1
5492	KC911521	J1b	J1b	O	1	J1b	O	1
5493	AY714035	J1b1a1	J1b1a1	O	1	J1b1a1	O	1
5494	JQ705093	J1b1a1	J1b1a1	O	1	J1b1a1	O	1
5495	FJ213450	J1b1a1a	J1b1a1a	O	1	J1b1a1a	O	1
5496	NA12282	J1b1a1a	J1b1a1a	O	1	J1b1a1a	O	1
5497	AY495233	J1b1a1b	J1b1a1b	O	1	J1b1a1b	O	1
5498	AY495236	J1b1a1b	J1b1a1b	O	1	J1b1a1b	O	1
5499	AY495231	J1b1a1c	J1b1a1c	O	1	J1b1a1c	O	1
5500	HQ543056	J1b1a1c	J1b1a1c	O	1	J1b1a1c	O	1
5501	JX153487	J1b1a1d	J1b1a1d	O	1	J1b1a1d	O	1
5502	JQ705588	J1b1a1d	J1b1a1d	O	1	J1b1a1d	O	1
5503	JQ797761	J1b1a1+146	J1b1a1+146	O	1	J1b1a1+146	O	1
5504	HM856621	J1b1a1e	J1b1a1e	O	1	J1b1a1e	O	1
5505	KC911404	J1b1a1e	J1b1a1e	O	1	J1b1a1e	O	1
5506	JQ797762	J1b1a2a	J1b1a2a	O	1	J1b1a2a	O	1
5507	HM852779	J1b1a2a	J1b1a2a	O	1	J1b1a2a	O	1
5508	JQ797763	J1b1a2b	J1b1a2b	O	1	J1b1a2b	O	1
5509	JQ797764	J1b1a2b	J1b1a2b	O	1	J1b1a2b	O	1
5510	EF660916	J1b1a3	J1b1a3	O	1	J1b1a3	O	1

5511	KC911610	J1b1a3	J1b1a3	O	1	J1b1a3	O	1
5512	KC911496	J1b1b1	J1b1b1	O	1	J1b1b1	O	1
5513	JQ797765	J1b1b1	J1b1b1	O	1	J1b1b1	O	1
5514	JQ797766	J1b1b1a	J1b1b1a	O	1	J1b1b1a	O	1
5515	EF397562	J1b1b1a	J1b1b1a	O	1	J1b1b1a	O	1
5516	JQ797769	J1b1b1b	J1b1b1b	O	1	J1b1b1b	O	1
5517	JF939049	J1b1b1b	J1b1b1b	O	1	J1b1b1b	O	1
5518	KC911590	J1b1b1c	J1b1b1c	O	1	J1b1b1c	O	1
5519	JQ797768	J1b1b1c	J1b1b1c	O	1	J1b1b1c	O	1
5520	JQ703656	J1b1b2	J1b1b2	O	1	J1b1b2	O	1
5521	JQ797770	J1b1b2	J1b1b2	O	1	J1b1b2	O	1
5522	AY714033	J1b1b3	J1b1b3	O	1	J1b1b3	O	1
5523	JX153479	J1b1b3	J1b1b3	O	1	J1b1b3	O	1
5524	EF556169	J1b2	J1b2	O	1	J1b2	O	1
5525	DQ282488	J1b2	J1b2	O	1	J1b2	O	1
5526	JQ702001	J1b2a	J1b2a	O	1	J1b2a	O	1
5527	JQ797771	J1b2a	J1b2a	O	1	J1b2a	O	1
5528	KC911407	J1b3	J1b3	O	1	J1b3	O	1
5529	HM594676	J1b3a	J1b3a	O	1	J1b3a	O	1
5530	EF583177	J1b3a	J1b3a	O	1	J1b3a	O	1
5531	JQ797775	J1b3b	J1b3b	O	1	J1b3b	O	1
5532	KC911307	J1b3b1	J1b3b1	O	1	J1b3b1	O	1
5533	KC911461	J1b3b1	J1b3b1	O	1	J1b3b1	O	1
5534	JQ797776	J1b4	J1b4	O	1	J1b4	O	1
5535	JN561091	J1b4a1	J1b4a1	O	1	J1b4a1	O	1
5536	KC911381	J1b4a1	J1b4a1	O	1	J1b4a1	O	1
5537	HQ637485	J1b4a2	J1b4a2	O	1	J1b4a2	O	1
5538	HM992836	J1b4a2	J1b4a2	O	1	J1b4a2	O	1
5539	JX153311	J1b5	J1b5	O	1	J1b5	O	1
5540	KC911544	J1b5a	J1b5a	O	1	J1b5a	O	1
5541	JQ797777	J1b5a1	J1b5a1	O	1	J1b5a1	O	1
5542	JQ797778	J1b5a1	J1b5a1	O	1	J1b5a1	O	1
5543	JQ797782	J1b6	J1b6	O	1	J1b6	O	1
5544	DQ282491	J1b6a	J1b6a	O	1	J1b6a	O	1
5545	JQ797781	J1b6a	J1b6a	O	1	J1b6a	O	1
5546	KC911401	J1b6b	J1b6b	O	1	J1b6b	O	1
5547	JQ797780	J1b6b	J1b6b	O	1	J1b6b	O	1

5548	KC911359	J1b7	J1b7	O	1	J1b7	O	1
5549	JQ797784	J1b7a	J1b7a	O	1	J1b7a	O	1
5550	JQ797783	J1b7a	J1b7a	O	1	J1b7a	O	1
5551	JQ064573	J1b8	J1b8	O	1	J1b8	O	1
5552	HQ914447	J1b8	J1b8	O	1	J1b8	O	1
5553	JQ797779	J1b9	J1b9	O	1	J1b9	O	1
5554	HM852835	J1b9	J1b9	O	1	J1b9	O	1
5555	AY495206	J1c	J1c	O	1	J1c	O	1
5556	JQ797870	J1c	J1c	O	1	J1c	O	1
5557	JX153672	J1c1	J1c1	O	1	J1c1	O	1
5558	JQ797787	J1c1	J1c1	O	1	J1c1	O	1
5559	AY495210	J1c1a	J1c1a	O	1	J1c1a	O	1
5560	FJ447985	J1c1a	J1c1a	O	1	J1c1a	O	1
5561	JQ703783	J1c1b	J1c1b	O	1	J1c1b	O	1
5562	AY495202	J1c1b	J1c1b	O	1	J1c1b	O	1
5563	EF452293	J1c1b1	J1c1b1	O	1	J1c1b1	O	1
5564	JQ703029	J1c1b1	J1c1b1	O	1	J1c1b1	O	1
5565	FJ502349	J1c1b1a	J1c1b1a	O	1	J1c1b1a	O	1
5566	JQ703825	J1c1b1a	J1c1b1a	O	1	J1c1b1a	O	1
5567	JQ703599	J1c1b1a1	J1c1b1a1	O	1	J1c1b1a1	O	1
5568	JQ705164	J1c1b1a1	J1c1b1a1	O	1	J1c1b1a1	O	1
5569	JQ705141	J1c1b2	J1c1b2	O	1	J1c1b2	O	1
5570	JQ702981	J1c1b2a	J1c1b2a	O	1	J1c1b2a	O	1
5571	JQ702020	J1c1b2a	J1c1b2a	O	1	J1c1b2a	O	1
5572	JQ704867	J1c1c	J1c1c	O	1	J1c1c	O	1
5573	JQ703464	J1c1c	J1c1c	O	1	J1c1c	O	1
5574	JX154000	J1c1d	J1c1d	O	1	J1c1d	O	1
5575	JX152930	J1c1d	J1c1d	O	1	J1c1d	O	1
5576	AY495208	J1c1e	J1c1e	O	1	J1c1e	O	1
5577	NA12234	J1c1e	J1c1e	O	1	J1c1e	O	1
5578	JQ705770	J1c1f	J1c1f	O	1	J1c1f	O	1
5579	JN635301	J1c1g	J1c1g	O	1	J1c1g	O	1
5580	JX153741	J1c1g1	J1c1g1	O	1	J1c1g1	O	1
5581	JQ797793	J1c1g1	J1c1g1	O	1	J1c1g1	O	1
5582	NA20787	J1c1h	J1c1h	O	1	J1c1h	O	1
5583	EF660981	J1c1h	J1c1h	O	1	J1c1h	O	1
5584	AY495218	J1c2	J1c2	O	1	J1c2	O	1

5585	EU155191	J1c2	J1c2	O	1	J1c2	O	1
5586	AY495227	J1c2a1	J1c2a1	O	1	J1c2a1	O	1
5587	AY495229	J1c2a1a	J1c2a1a	O	1	J1c2a1a	O	1
5588	JX153394	J1c2a1a	J1c2a1a	O	1	J1c2a1a	O	1
5589	JX153382	J1c2a2	J1c2a2	O	1	J1c2a2	O	1
5590	DQ787109	J1c2a2	J1c2a2	O	1	J1c2a2	O	1
5591	JQ702742	J1c2a3	J1c2a3	O	1	J1c2a3	O	1
5592	AY495220	J1c2a3	J1c2a3	O	1	J1c2a3	O	1
5593	JQ704554	J1c2b	J1c2b	O	1	J1c2b	O	1
5594	JQ703778	J1c2b	J1c2b	O	1	J1c2b	O	1
5595	JQ703613	J1c2b1	J1c2b1	O	1	J1c2b1	O	1
5596	JQ704710	J1c2b1	J1c2b1	O	1	J1c2b1	O	1
5597	JX153336	J1c2b2	J1c2b2	O	1	J1c2b2	O	1
5598	HM776018	J1c2b2	J1c2b2	O	1	J1c2b2	O	1
5599	JQ702706	J1c2b3	J1c2b3	O	1	J1c2b3	O	1
5600	JQ701968	J1c2b3	J1c2b3	O	1	J1c2b3	O	1
5601	JQ703567	J1c2b4	J1c2b4	O	1	J1c2b4	O	1
5602	JQ703672	J1c2b4	J1c2b4	O	1	J1c2b4	O	1
5603	JX153700	J1c2b5	J1c2b5	O	1	J1c2b5	O	1
5604	JX153355	J1c2b5	J1c2b5	O	1	J1c2b5	O	1
5605	GU949564	J1c2c	J1c2c	O	1	J1c2c	O	1
5606	EF177422	J1c2c	J1c2c	O	1	J1c2c	O	1
5607	FJ449571	J1c2c1	J1c2c1	O	1	J1c2c1	O	1
5608	JQ702499	J1c2c1	J1c2c1	O	1	J1c2c1	O	1
5609	JQ705961	J1c2c1a	J1c2c1a	O	1	J1c2c1a	O	1
5610	GQ304746	J1c2c1a	J1c2c1a	O	1	J1c2c1a	O	1
5611	JQ702741	J1c2c2	J1c2c2	O	1	J1c2c2	O	1
5612	FJ348202	J1c2c2	J1c2c2	O	1	J1c2c2	O	1
5613	GU592019	J1c2c2a	J1c2c2a	O	1	J1c2c2a	O	1
5614	HQ336424	J1c2c2a	J1c2c2a	O	1	J1c2c2a	O	1
5615	JQ704965	J1c2d	J1c2d	O	1	J1c2d	O	1
5616	FJ499472	J1c2d	J1c2d	O	1	J1c2d	O	1
5617	JQ797812	J1c2e	J1c2e	O	1	J1c2e	O	1
5618	JQ702311	J1c2e	J1c2e	O	1	J1c2e	O	1
5619	GU123042	J1c2e1	J1c2e1	O	1	J1c2e1	O	1
5620	JX153716	J1c2e1	J1c2e1	O	1	J1c2e1	O	1
5621	JX297168	J1c2e2	J1c2e2	O	1	J1c2e2	O	1

5622	JX401416	J1c2e2	J1c2e2	O	1	J1c2e2	O	1
5623	JQ704432	J1c2f	J1c2f	O	1	J1c2f	O	1
5624	JQ705129	J1c2f	J1c2f	O	1	J1c2f	O	1
5625	JQ704946	J1c2g	J1c2g	O	1	J1c2g	O	1
5626	HQ260985	J1c2g	J1c2g	O	1	J1c2g	O	1
5627	JQ705996	J1c2h	J1c2h	O	1	J1c2h	O	1
5628	JQ701851	J1c2h	J1c2h	O	1	J1c2h	O	1
5629	JQ797821	J1c2i	J1c2i	O	1	J1c2i	O	1
5630	JQ797820	J1c2i	J1c2i	O	1	J1c2i	O	1
5631	JQ797822	J1c2j	J1c2j	O	1	J1c2j	O	1
5632	EU915479	J1c2j	J1c2j	O	1	J1c2j	O	1
5633	JQ797804	J1c2k	J1c2k	O	1	J1c2k	O	1
5634	JQ705224	J1c2k	J1c2k	O	1	J1c2k	O	1
5635	AY495223	J1c2l	J1c2l	O	1	J1c2l	O	1
5636	JQ704829	J1c2l	J1c2l	O	1	J1c2l	O	1
5637	JQ797801	J1c2m	J1c2m	O	1	J1c2m	O	1
5638	KC911595	J1c2m	J1c2m	O	1	J1c2m	O	1
5639	JQ702724	J1c2m1	J1c2m1	O	1	J1c2m1	O	1
5640	JQ702366	J1c2m1	J1c2m1	O	1	J1c2m1	O	1
5641	HG00377	J1c2n	J1c2n	O	1	J1c2n	O	1
5642	AY339585	J1c2n	J1c2n	O	1	J1c2n	O	1
5643	JX153610	J1c2n1	J1c2n1	O	1	J1c2n1	O	1
5644	JX154069	J1c2n1	J1c2n1	O	1	J1c2n1	O	1
5645	JX152877	J1c2o	J1c2o	O	1	J1c2o	O	1
5646	KC257367	J1c2o	J1c2o	O	1	J1c2o	O	1
5647	AY495225	J1c2p	J1c2p	O	1	J1c2p	O	1
5648	JQ797799	J1c2p	J1c2p	O	1	J1c2p	O	1
5649	HG00258	J1c2q	J1c2q	O	1	J1c2q	O	1
5650	AY495226	J1c2q	J1c2q	O	1	J1c2q	O	1
5651	JX153505	J1c2q1	J1c2q1	O	1	J1c2q1	O	1
5652	JX153326	J1c2q1	J1c2q1	O	1	J1c2q1	O	1
5653	FJ190383	J1c2r	J1c2r	O	1	J1c2r	O	1
5654	JQ797819	J1c2r	J1c2r	O	1	J1c2r	O	1
5655	JX153398	J1c2s	J1c2s	O	1	J1c2s	O	1
5656	FJ499471	J1c2s1	J1c2s1	O	1	J1c2s1	O	1
5657	JQ703785	J1c2s1	J1c2s1	O	1	J1c2s1	O	1
5658	JQ705054	J1c2t	J1c2t	O	1	J1c2t	O	1

5659	HG00235	J1c2t	J1c2t	O	1	J1c2t	O	1
5660	JQ702691	J1c3	J1c3	O	1	J1c3	O	1
5661	JQ797823	J1c3	J1c3	O	1	J1c3	O	1
5662	AY495211	J1c3a1	J1c3a1	O	1	J1c3a1	O	1
5663	JQ702646	J1c3a1	J1c3a1	O	1	J1c3a1	O	1
5664	JQ703792	J1c3a2	J1c3a2	O	1	J1c3a2	O	1
5665	AY495213	J1c3a2	J1c3a2	O	1	J1c3a2	O	1
5666	JQ048704	J1c3b	J1c3b	O	1	J1c3b	O	1
5667	JQ705447	J1c3b1	J1c3b1	O	1	J1c3b1	O	1
5668	HQ696458	J1c3b1	J1c3b1	O	1	J1c3b1	O	1
5669	FJ445407	J1c3b1a	J1c3b1a	O	1	J1c3b1a	O	1
5670	JQ705472	J1c3b1a	J1c3b1a	O	1	J1c3b1a	O	1
5671	HM026752	J1c3b2	J1c3b2	O	1	J1c3b2	O	1
5672	HG00103	J1c3b2	J1c3b2	O	1	J1c3b2	O	1
5673	NA12044	J1c3c	J1c3c	O	1	J1c3c	O	1
5674	HQ709168	J1c3c1	J1c3c1	O	1	J1c3c1	O	1
5675	JQ702395	J1c3c1	J1c3c1	O	1	J1c3c1	O	1
5676	JQ703602	J1c3c2	J1c3c2	O	1	J1c3c2	O	1
5677	JQ702727	J1c3c2	J1c3c2	O	1	J1c3c2	O	1
5678	AY495201	J1c3d	J1c3d	O	1	J1c3d	O	1
5679	AY495207	J1c3d	J1c3d	O	1	J1c3d	O	1
5680	JQ705868	J1c3e1	J1c3e1	O	1	J1c3e1	O	1
5681	JQ703895	J1c3e1	J1c3e1	O	1	J1c3e1	O	1
5682	JQ705308	J1c3e2	J1c3e2	O	1	J1c3e2	O	1
5683	NA20785	J1c3e2	J1c3e2	O	1	J1c3e2	O	1
5684	JQ797828	J1c3f	J1c3f	O	1	J1c3f	O	1
5685	JQ701854	J1c3f	J1c3f	O	1	J1c3f	O	1
5686	JQ705212	J1c3g	J1c3g	O	1	J1c3g	O	1
5687	JF703252	J1c3g	J1c3g	O	1	J1c3g	O	1
5688	DQ523659	J1c3h	J1c3h	O	1	J1c3h	O	1
5689	JQ797837	J1c3h	J1c3h	O	1	J1c3h	O	1
5690	JQ703753	J1c3i	J1c3i	O	1	J1c3i	O	1
5691	AY495214	J1c3i	J1c3i	O	1	J1c3i	O	1
5692	FJ603099	J1c3j	J1c3j	O	1	J1c3j	O	1
5693	JQ701961	J1c3j	J1c3j	O	1	J1c3j	O	1
5694	JQ797826	J1c3k	J1c3k	O	1	J1c3k	O	1
5695	JQ702912	J1c3k	J1c3k	O	1	J1c3k	O	1

5696	HG00334	J1c3+189	J1c3+189	O	1	J1c3+189	O	1
5697	JQ702063	J1c3+189	J1c3+189	O	1	J1c3+189	O	1
5698	JQ797836	J1c3m	J1c3m	O	1	J1c3m	O	1
5699	JX153002	J1c3m	J1c3m	O	1	J1c3m	O	1
5700	AY495197	J1c4	J1c4	O	1	J1c4	O	1
5701	FJ538285	J1c4	J1c4	O	1	J1c4	O	1
5702	JQ703916	J1c4b	J1c4b	O	1	J1c4b	O	1
5703	GU808335	J1c4b	J1c4b	O	1	J1c4b	O	1
5704	JQ705439	J1c4c	J1c4c	O	1	J1c4c	O	1
5705	JQ705633	J1c4c	J1c4c	O	1	J1c4c	O	1
5706	HG00120	J1c5	J1c5	O	1	J1c5	O	1
5707	AY495199	J1c5	J1c5	O	1	J1c5	O	1
5708	JQ703803	J1c5a	J1c5a	O	1	J1c5a	O	1
5709	HQ287874	J1c5a1	J1c5a1	O	1	J1c5a1	O	1
5710	JQ704736	J1c5a1	J1c5a1	O	1	J1c5a1	O	1
5711	JQ703604	J1c5b	J1c5b	O	1	J1c5b	O	1
5712	JQ703639	J1c5b	J1c5b	O	1	J1c5b	O	1
5713	JQ704816	J1c5c	J1c5c	O	1	J1c5c	O	1
5714	JX297129	J1c5c1	J1c5c1	O	1	J1c5c1	O	1
5715	JX297126	J1c5c1	J1c5c1	O	1	J1c5c1	O	1
5716	HQ287873	J1c5d	J1c5d	O	1	J1c5d	O	1
5717	AY495204	J1c5d	J1c5d	O	1	J1c5d	O	1
5718	JQ797844	J1c5e	J1c5e	O	1	J1c5e	O	1
5719	AY495209	J1c6	J1c6	O	1	J1c6	O	1
5720	JQ703919	J1c6	J1c6	O	1	J1c6	O	1
5721	EU073970	J1c6a	J1c6a	O	1	J1c6a	O	1
5722	JX297170	J1c6a	J1c6a	O	1	J1c6a	O	1
5723	FJ348153	J1c+16261	J1c+16261	O	1	J1c+16261	O	1
5724	JQ705489	J1c+16261	J1c+16261	O	1	J1c+16261	O	1
5725	JQ797855	J1c7	J1c7	O	1	J1c7	O	1
5726	JQ703682	J1c7	J1c7	O	1	J1c7	O	1
5727	JQ703932	J1c7a	J1c7a	O	1	J1c7a	O	1
5728	JQ797859	J1c7a	J1c7a	O	1	J1c7a	O	1
5729	JN415478	J1c+16261+189	J1c+16261+189	O	1	J1c+16261+189	O	1
5730	EU284668	J1c12	J1c12	O	1	J1c12	O	1
5731	FJ348222	J1c12	J1c12	O	1	J1c12	O	1
5732	JQ703516	J1c12a	J1c12a	O	1	J1c12a	O	1

5733	JQ704581	J1c12a	J1c12a	O	1	J1c12a	O	1
5734	JQ702088	J1c12b	J1c12b	O	1	J1c12b	O	1
5735	JQ702208	J1c12b	J1c12b	O	1	J1c12b	O	1
5736	JQ703584	J1c13	J1c13	O	1	J1c13	O	1
5737	JQ702609	J1c13	J1c13	O	1	J1c13	O	1
5738	JQ705811	J1c14	J1c14	O	1	J1c14	O	1
5739	JQ705891	J1c14	J1c14	O	1	J1c14	O	1
5740	JQ797868	J1c8a	J1c8a	O	1	J1c8a	O	1
5741	JN635299	J1c8a	J1c8a	O	1	J1c8a	O	1
5742	JQ702049	J1c8a1	J1c8a1	O	1	J1c8a1	O	1
5743	JQ797866	J1c8a1	J1c8a1	O	1	J1c8a1	O	1
5744	JQ705760	J1c8a1a	J1c8a1a	O	1	J1c8a1a	O	1
5745	KC421179	J1c8a1a	J1c8a1a	O	1	J1c8a1a	O	1
5746	GU906781	J1c8a2	J1c8a2	O	1	J1c8a2	O	1
5747	JQ797864	J1c8a2	J1c8a2	O	1	J1c8a2	O	1
5748	JQ705449	J1c8b	J1c8b	O	1	J1c8b	O	1
5749	JQ701992	J1c8b	J1c8b	O	1	J1c8b	O	1
5750	AY495205	J1c9	J1c9	O	1	J1c9	O	1
5751	JQ797871	J1c9	J1c9	O	1	J1c9	O	1
5752	JQ704751	J1c10	J1c10	O	1	J1c10	O	1
5753	EF660915	J1c10	J1c10	O	1	J1c10	O	1
5754	JQ797872	J1c10a	J1c10a	O	1	J1c10a	O	1
5755	JQ797873	J1c10a	J1c10a	O	1	J1c10a	O	1
5756	JQ702967	J1c11	J1c11	O	1	J1c11	O	1
5757	HM765470	J1c11a	J1c11a	O	1	J1c11a	O	1
5758	GU592047	J1c11a	J1c11a	O	1	J1c11a	O	1
5759	JQ797881	J1c15	J1c15	O	1	J1c15	O	1
5760	JQ797879	J1c15a	J1c15a	O	1	J1c15a	O	1
5761	JQ797880	J1c15a1	J1c15a1	O	1	J1c15a1	O	1
5762	HM852793	J1c15a1	J1c15a1	O	1	J1c15a1	O	1
5763	JQ797877	J1c15b	J1c15b	O	1	J1c15b	O	1
5764	JQ797878	J1c15b	J1c15b	O	1	J1c15b	O	1
5765	JQ705083	J1c16	J1c16	O	1	J1c16	O	1
5766	JQ797874	J1c16	J1c16	O	1	J1c16	O	1
5767	JQ797882	J1c17	J1c17	O	1	J1c17	O	1
5768	JQ706022	J1c17a	J1c17a	O	1	J1c17a	O	1
5769	JX153275	J1c17a	J1c17a	O	1	J1c17a	O	1

5770	JQ797885	J1+16193	J1+16193	O	1	J1+16193	O	1
5771	JQ797902	J1d	J1d	O	1	J1d	O	1
5772	JQ797886	J1d1a	J1d1a	O	1	J1d1a	O	1
5773	JQ797888	J1d1a1	J1d1a1	O	1	J1d1a1	O	1
5774	JQ705319	J1d1a1	J1d1a1	O	1	J1d1a1	O	1
5775	JQ797890	J1d1a1a	J1d1a1a	O	1	J1d1a1a	O	1
5776	JQ797891	J1d1a1a	J1d1a1a	O	1	J1d1a1a	O	1
5777	AF382001	J1d1b	J1d1b	O	1	J1d1b	O	1
5778	KC911578	J1d1b	J1d1b	O	1	J1d1b	O	1
5779	JQ797892	J1d1b1	J1d1b1	O	1	J1d1b1	O	1
5780	HM852780	J1d1b1	J1d1b1	O	1	J1d1b1	O	1
5781	DQ341088	J1d2	J1d2	O	1	J1d2	O	1
5782	HQ325739	J1d2a	J1d2a	O	1	J1d2a	O	1
5783	JQ797893	J1d2a	J1d2a	O	1	J1d2a	O	1
5784	KC911411	J1d3	J1d3	O	1	J1d3	O	1
5785	JQ797899	J1d3a	J1d3a	O	1	J1d3a	O	1
5786	JQ797897	J1d3a1	J1d3a1	O	1	J1d3a1	O	1
5787	JQ797896	J1d3a1	J1d3a1	O	1	J1d3a1	O	1
5788	EU597552	J1d3a2	J1d3a2	O	1	J1d3a2	O	1
5789	JQ704809	J1d3a2	J1d3a2	O	1	J1d3a2	O	1
5790	HM453206	J1d4	J1d4	O	1	J1d4	O	1
5791	JQ797900	J1d4	J1d4	O	1	J1d4	O	1
5792	JQ703915	J1d5	J1d5	O	1	J1d5	O	1
5793	HM852829	J1d5	J1d5	O	1	J1d5	O	1
5794	KC911498	J1d5a	J1d5a	O	1	J1d5a	O	1
5795	JQ797901	J1d5a	J1d5a	O	1	J1d5a	O	1
5796	GU122987	J1d6	J1d6	O	1	J1d6	O	1
5797	JQ797894	J1d6	J1d6	O	1	J1d6	O	1
5798	JQ797895	J1d6a	J1d6a	O	1	J1d6a	O	1
5799	EU007880	J1d6a	J1d6a	O	1	J1d6a	O	1
5800	JQ704041	J2a1	J2a1	O	1	J2a1	O	1
5801	JQ701807	J2a1a1	J2a1a1	O	1	J2a1a1	O	1
5802	JQ797903	J2a1a1	J2a1a1	O	1	J2a1a1	O	1
5803	GU903270	J2a1a1a	J2a1a1a	O	1	J2a1a1a	O	1
5804	JX153321	J2a1a1a	J2a1a1a	O	1	J2a1a1a	O	1
5805	JX152974	J2a1a1a1	J2a1a1a1	O	1	J2a1a1a1	O	1
5806	JX153821	J2a1a1a1	J2a1a1a1	O	1	J2a1a1a1	O	1

5807	FJ348157	J2a1a1a2	J2a1a1a2	O	1	J2a1a1a2	O	1
5808	JQ705625	J2a1a1a2	J2a1a1a2	O	1	J2a1a1a2	O	1
5809	JX152842	J2a1a1a2a	J2a1a1a2a	O	1	J2a1a1a2a	O	1
5810	JX153676	J2a1a1a2a	J2a1a1a2a	O	1	J2a1a1a2a	O	1
5811	JQ703568	J2a1a1a3	J2a1a1a3	O	1	J2a1a1a3	O	1
5812	JQ705323	J2a1a1a3	J2a1a1a3	O	1	J2a1a1a3	O	1
5813	JQ705390	J2a1a1b	J2a1a1b	O	1	J2a1a1b	O	1
5814	JQ705042	J2a1a1b	J2a1a1b	O	1	J2a1a1b	O	1
5815	JQ703552	J2a1a1c	J2a1a1c	O	1	J2a1a1c	O	1
5816	JQ797904	J2a1a1c	J2a1a1c	O	1	J2a1a1c	O	1
5817	JQ797911	J2a1a1d	J2a1a1d	O	1	J2a1a1d	O	1
5818	JQ797912	J2a1a1d	J2a1a1d	O	1	J2a1a1d	O	1
5819	JX297144	J2a1a1e	J2a1a1e	O	1	J2a1a1e	O	1
5820	KC533492	J2a1a1e	J2a1a1e	O	1	J2a1a1e	O	1
5821	DQ341089	J2a1a2	J2a1a2	O	1	J2a1a2	O	1
5822	JQ797914	J2a1a2a	J2a1a2a	O	1	J2a1a2a	O	1
5823	JQ764985	J2a1a2a1	J2a1a2a1	O	1	J2a1a2a1	O	1
5824	JQ702364	J2a1a2a1a	J2a1a2a1a	O	1	J2a1a2a1a	O	1
5825	JX153453	J2a1a2a1a	J2a1a2a1a	O	1	J2a1a2a1a	O	1
5826	EF660967	J2a2a	J2a2a	O	1	J2a2a	O	1
5827	JQ797915	J2a2a1	J2a2a1	O	1	J2a2a1	O	1
5828	JQ797921	J2a2a1+16311	J2a2a1+16311	O	1	J2a2a1+16311	O	1
5829	JQ797920	J2a2a1a	J2a2a1a	O	1	J2a2a1a	O	1
5830	JQ797919	J2a2a1a1	J2a2a1a1	O	1	J2a2a1a1	O	1
5831	JQ797917	J2a2a1a1	J2a2a1a1	O	1	J2a2a1a1	O	1
5832	JQ797923	J2a2a2	J2a2a2	O	1	J2a2a2	O	1
5833	GU065327	J2a2a2	J2a2a2	O	1	J2a2a2	O	1
5834	FJ460543	J2a2b1	J2a2b1	O	1	J2a2b1	O	1
5835	JQ797926	J2a2b1a	J2a2b1a	O	1	J2a2b1a	O	1
5836	JX153096	J2a2b1a	J2a2b1a	O	1	J2a2b1a	O	1
5837	NA20752	J2a2b2	J2a2b2	O	1	J2a2b2	O	1
5838	JQ703605	J2a2b2	J2a2b2	O	1	J2a2b2	O	1
5839	KF148204	J2a2b3	J2a2b3	O	1	J2a2b3	O	1
5840	KF148206	J2a2b3	J2a2b3	O	1	J2a2b3	O	1
5841	JQ797930	J2a2c	J2a2c	O	1	J2a2c	O	1
5842	JQ797933	J2a2c1	J2a2c1	O	1	J2a2c1	O	1
5843	JQ797931	J2a2c1	J2a2c1	O	1	J2a2c1	O	1

5844	FJ460559	J2a2d	J2a2d	O	1	J2a2d	O	1
5845	JQ797935	J2a2d	J2a2d	O	1	J2a2d	O	1
5846	JX153004	J2a2e	J2a2e	O	1	J2a2e	O	1
5847	JQ797924	J2a2e	J2a2e	O	1	J2a2e	O	1
5848	JQ702563	J2b1	J2b1	O	1	J2b1	O	1
5849	JQ797954	J2b1	J2b1	O	1	J2b1	O	1
5850	JX153193	J2b1a	J2b1a	O	1	J2b1a	O	1
5851	JF915700	J2b1a	J2b1a	O	1	J2b1a	O	1
5852	JQ703580	J2b1a1	J2b1a1	O	1	J2b1a1	O	1
5853	JQ705356	J2b1a1	J2b1a1	O	1	J2b1a1	O	1
5854	FJ445408	J2b1a1a	J2b1a1a	O	1	J2b1a1a	O	1
5855	JQ702863	J2b1a1a	J2b1a1a	O	1	J2b1a1a	O	1
5856	JN635305	J2b1a2	J2b1a2	O	1	J2b1a2	O	1
5857	JQ705677	J2b1a2	J2b1a2	O	1	J2b1a2	O	1
5858	JF938916	J2b1a2a	J2b1a2a	O	1	J2b1a2a	O	1
5859	JQ797936	J2b1a2a	J2b1a2a	O	1	J2b1a2a	O	1
5860	JQ702858	J2b1a3	J2b1a3	O	1	J2b1a3	O	1
5861	AY195778	J2b1a3	J2b1a3	O	1	J2b1a3	O	1
5862	JQ705466	J2b1a4	J2b1a4	O	1	J2b1a4	O	1
5863	JQ703515	J2b1a4	J2b1a4	O	1	J2b1a4	O	1
5864	JX153921	J2b1a+16311	J2b1a+16311	O	1	J2b1a+16311	O	1
5865	JQ797946	J2b1a5	J2b1a5	O	1	J2b1a5	O	1
5866	DQ523653	J2b1a5	J2b1a5	O	1	J2b1a5	O	1
5867	JQ797944	J2b1a6	J2b1a6	O	1	J2b1a6	O	1
5868	JQ704523	J2b1a6	J2b1a6	O	1	J2b1a6	O	1
5869	JQ703784	J2b1b	J2b1b	O	1	J2b1b	O	1
5870	EU862198	J2b1b1	J2b1b1	O	1	J2b1b1	O	1
5871	JX153450	J2b1b1	J2b1b1	O	1	J2b1b1	O	1
5872	JQ797951	J2b1c	J2b1c	O	1	J2b1c	O	1
5873	JQ797952	J2b1c	J2b1c	O	1	J2b1c	O	1
5874	EU807741	J2b1c1	J2b1c1	O	1	J2b1c1	O	1
5875	FJ445409	J2b1d	J2b1d	O	1	J2b1d	O	1
5876	JQ797958	J2b1d	J2b1d	O	1	J2b1d	O	1
5877	JQ701981	J2b1e	J2b1e	O	1	J2b1e	O	1
5878	JQ797964	J2b1e1	J2b1e1	O	1	J2b1e1	O	1
5879	JQ797965	J2b1e1	J2b1e1	O	1	J2b1e1	O	1
5880	JQ797968	J2b1f	J2b1f	O	1	J2b1f	O	1

5881	HQ727682	J2b1f	J2b1f	O	1	J2b1f	O	1
5882	JQ702459	J2b1g	J2b1g	O	1	J2b1g	O	1
5883	JQ702488	J2b1g	J2b1g	O	1	J2b1g	O	1
5884	JQ702424	J2b1h	J2b1h	O	1	J2b1h	O	1
5885	FJ213765	J2b2	J2b2	O	1	J2b2	O	1
5886	JQ797972	J2b2	J2b2	O	1	J2b2	O	1
5887	JQ797976	T1	T1	O	1	T1	O	1
5888	KC405582	T1a	T1a	O	1	T1a	O	1
5889	JQ798024	T1a	T1a	O	1	T1a	O	1
5890	GU122980	T1a1	T1a1	O	1	T1a1	O	1
5891	JQ797984	T1a1	T1a1	O	1	T1a1	O	1
5892	JQ705982	T1a1a	T1a1a	O	1	T1a1a	O	1
5893	JQ703032	T1a1a1	T1a1a1	O	1	T1a1a1	O	1
5894	AY495294	T1a1a1	T1a1a1	O	1	T1a1a1	O	1
5895	HM184912	T1a1b	T1a1b	O	1	T1a1b	O	1
5896	JQ797991	T1a1b	T1a1b	O	1	T1a1b	O	1
5897	JQ797994	T1a1b1	T1a1b1	O	1	T1a1b1	O	1
5898	AY714036	T1a1b1	T1a1b1	O	1	T1a1b1	O	1
5899	JQ704801	T1a1c	T1a1c	O	1	T1a1c	O	1
5900	JQ702923	T1a1c	T1a1c	O	1	T1a1c	O	1
5901	EU007876	T1a1d	T1a1d	O	1	T1a1d	O	1
5902	JQ798005	T1a1d	T1a1d	O	1	T1a1d	O	1
5903	JX152843	T1a1e	T1a1e	O	1	T1a1e	O	1
5904	JQ798006	T1a1e	T1a1e	O	1	T1a1e	O	1
5905	JQ702643	T1a1f	T1a1f	O	1	T1a1f	O	1
5906	JQ797983	T1a1f	T1a1f	O	1	T1a1f	O	1
5907	JF833040	T1a1g	T1a1g	O	1	T1a1g	O	1
5908	JQ704732	T1a1g	T1a1g	O	1	T1a1g	O	1
5909	JN089342	T1a1h	T1a1h	O	1	T1a1h	O	1
5910	JQ702680	T1a1h	T1a1h	O	1	T1a1h	O	1
5911	JQ705441	T1a1i	T1a1i	O	1	T1a1i	O	1
5912	JN021256	T1a1i	T1a1i	O	1	T1a1i	O	1
5913	AY495291	T1a1j	T1a1j	O	1	T1a1j	O	1
5914	JQ703446	T1a1j	T1a1j	O	1	T1a1j	O	1
5915	JQ702728	T1a1k	T1a1k	O	1	T1a1k	O	1
5916	JQ702959	T1a1k1	T1a1k1	O	1	T1a1k1	O	1
5917	JQ705707	T1a1k1	T1a1k1	O	1	T1a1k1	O	1

5918	JX152851	T1a1k2	T1a1k2	O	1	T1a1k2	O	1
5919	JF926125	T1a1k2	T1a1k2	O	1	T1a1k2	O	1
5920	JF929200	T1a1+@152	T1a1+@152	O	1	T1a1+@152	O	1
5921	JQ702988	T1a1+@152	T1a1+@152	O	1	T1a1+@152	O	1
5922	JF979131	T1a1l	T1a1l	O	1	T1a1l	O	1
5923	JQ798004	T1a1l	T1a1l	O	1	T1a1l	O	1
5924	KC911350	T1a1m	T1a1m	O	1	T1a1m	O	1
5925	KC911329	T1a1m1	T1a1m1	O	1	T1a1m1	O	1
5926	JQ797998	T1a1m1	T1a1m1	O	1	T1a1m1	O	1
5927	EF177406	T1a1n	T1a1n	O	1	T1a1n	O	1
5928	JQ702825	T1a1n	T1a1n	O	1	T1a1n	O	1
5929	EU597578	T1a1p	T1a1p	O	1	T1a1p	O	1
5930	JX153687	T1a1p	T1a1p	O	1	T1a1p	O	1
5931	JX153590	T1a1q	T1a1q	O	1	T1a1q	O	1
5932	JQ702173	T1a1q	T1a1q	O	1	T1a1q	O	1
5933	JX153003	T1a1r	T1a1r	O	1	T1a1r	O	1
5934	EF661000	T1a1r	T1a1r	O	1	T1a1r	O	1
5935	JQ798022	T1a3	T1a3	O	1	T1a+152	X	2
5936	AY495296	T1a3a	T1a3a	O	1	T1a3a	O	1
5937	EF645646	T1a3a	T1a3a	O	1	T1a3a	O	1
5938	JQ798012	T1a2	T1a2	O	1	T1a2	O	1
5939	JN083377	T1a2	T1a2	O	1	T1a2	O	1
5940	NA20581	T1a2a	T1a2a	O	1	T1a2a	O	1
5941	FJ348220	T1a2a	T1a2a	O	1	T1a2a	O	1
5942	JQ798013	T1a2b	T1a2b	O	1	T1a2b	O	1
5943	HM852775	T1a2b	T1a2b	O	1	T1a2b	O	1
5944	EU979542	T1a4	T1a4	O	1	T1a4	O	1
5945	JQ702040	T1a4	T1a4	O	1	T1a4	O	1
5946	JQ031816	T1a11	T1a11	O	1	T1a11	O	1
5947	JQ798021	T1a11	T1a11	O	1	T1a11	O	1
5948	JQ798020	T1a12	T1a12	O	1	T1a12	O	1
5949	JQ798019	T1a12	T1a12	O	1	T1a12	O	1
5950	JQ798007	T1a13	T1a13	O	1	T1a13	O	1
5951	JQ798009	T1a13	T1a13	O	1	T1a13	O	1
5952	JQ703724	T1a5	T1a5	O	1	T1a5	O	1
5953	JQ798030	T1a5	T1a5	O	1	T1a5	O	1
5954	JX153408	T1a5a	T1a5a	O	1	T1a5a	O	1

5955	JF937680	T1a5a	T1a5a	O	1	T1a5a	O	1
5956	JN104727	T1a6	T1a6	O	1	T1a6	O	1
5957	EU369395	T1a6	T1a6	O	1	T1a6	O	1
5958	EU935435	T1a7	T1a7	O	1	T1a7	O	1
5959	JQ798027	T1a7	T1a7	O	1	T1a7	O	1
5960	JQ798034	T1a8a	T1a8a	O	1	T1a8a	O	1
5961	JQ798033	T1a8a	T1a8a	O	1	T1a8a	O	1
5962	JQ798035	T1a8b	T1a8b	O	1	T1a8b	O	1
5963	JQ798036	T1a8b	T1a8b	O	1	T1a8b	O	1
5964	JQ798018	T1a9	T1a9	O	1	T1a9	O	1
5965	JQ798017	T1a9	T1a9	O	1	T1a9	O	1
5966	NA20819	T1a10	T1a10	O	1	T1a10	O	1
5967	JX152859	T1a10a	T1a10a	O	1	T1a10a	O	1
5968	JQ798025	T1a10a	T1a10a	O	1	T1a10a	O	1
5969	JQ798046	T1b	T1b	O	1	T1b	O	1
5970	JQ798051	T1b	T1b	O	1	T1b	O	1
5971	JQ798037	T1b1	T1b1	O	1	T1b1	O	1
5972	AY339570	T1b1	T1b1	O	1	T1b1	O	1
5973	JQ798039	T1b2	T1b2	O	1	T1b2	O	1
5974	JQ798041	T1b2	T1b2	O	1	T1b2	O	1
5975	JQ798043	T1b3	T1b3	O	1	T1b3	O	1
5976	JQ705372	T1b3	T1b3	O	1	T1b3	O	1
5977	JQ798050	T1b4	T1b4	O	1	T1b4	O	1
5978	JQ798049	T1b4	T1b4	O	1	T1b4	O	1
5979	JQ798138	T2	T2	O	1	T2	O	1
5980	JF707633	T2	T2	O	1	T2	O	1
5981	JQ798058	T2a1	T2a1	O	1	T2a1	O	1
5982	JF927949	T2a1	T2a1	O	1	T2a1	O	1
5983	AY495298	T2a1a	T2a1a	O	1	T2a1a	O	1
5984	FJ656215	T2a1a	T2a1a	O	1	T2a1a	O	1
5985	FJ348180	T2a1a1	T2a1a1	O	1	T2a1a1	O	1
5986	JN120787	T2a1a2	T2a1a2	O	1	T2a1a2	O	1
5987	GU553285	T2a1a2	T2a1a2	O	1	T2a1a2	O	1
5988	JX297193	T2a1a3	T2a1a3	O	1	T2a1a3	O	1
5989	AY495300	T2a1a3a	T2a1a3a	O	1	T2a1a3a	O	1
5990	JF833041	T2a1a3a	T2a1a3a	O	1	T2a1a3a	O	1
5991	JQ705499	T2a1a5	T2a1a5	O	1	T2a1a5	O	1

5992	JQ702880	T2a1a5	T2a1a5	O	1	T2a1a5	O	1
5993	JQ702036	T2a1a6	T2a1a6	O	1	T2a1a6	O	1
5994	JQ704797	T2a1a6	T2a1a6	O	1	T2a1a6	O	1
5995	JQ701880	T2a1a7	T2a1a7	O	1	T2a1a7	O	1
5996	JQ045864	T2a1a7	T2a1a7	O	1	T2a1a7	O	1
5997	JF958082	T2a1a8	T2a1a8	O	1	T2a1a8	O	1
5998	JQ701972	T2a1a8	T2a1a8	O	1	T2a1a8	O	1
5999	JQ702344	T2a1b	T2a1b	O	1	T2a1b	O	1
6000	JQ798054	T2a1b	T2a1b	O	1	T2a1b	O	1
6001	HM625705	T2a1b1	T2a1b1	O	1	T2a1b1	O	1
6002	JQ046361	T2a1b1a	T2a1b1a	O	1	T2a1b1a	O	1
6003	JQ705641	T2a1b1a	T2a1b1a	O	1	T2a1b1a	O	1
6004	JQ702937	T2a1b1a1	T2a1b1a1	O	1	T2a1b1a1	O	1
6005	JQ703149	T2a1b1a1	T2a1b1a1	O	1	T2a1b1a1	O	1
6006	JQ703868	T2a1b1a1a1	T2a1b1a1a1	O	1	T2a1b1a1a1	O	1
6007	JQ703700	T2a1b1a1a1	T2a1b1a1a1	O	1	T2a1b1a1a1	O	1
6008	JX153849	T2a1b1a1a2	T2a1b1a1a2	O	1	T2a1b1a1a2	O	1
6009	JQ798055	T2a1b1a1a2	T2a1b1a1a2	O	1	T2a1b1a1a2	O	1
6010	JN024624	T2a1b1a1b	T2a1b1a1b	O	1	T2a1b1a1b	O	1
6011	JX153319	T2a1b1a1b	T2a1b1a1b	O	1	T2a1b1a1b	O	1
6012	JX153732	T2a1b1a1b1	T2a1b1a1b1	O	1	T2a1b1a1b1	O	1
6013	JX153948	T2a1b1a1b1	T2a1b1a1b1	O	1	T2a1b1a1b1	O	1
6014	JQ703776	T2a1b1a2	T2a1b1a2	O	1	T2a1b1a2	O	1
6015	JX153440	T2a1b1a2	T2a1b1a2	O	1	T2a1b1a2	O	1
6016	GU123001	T2a1b2a	T2a1b2a	O	1	T2a1b2a	O	1
6017	JQ704821	T2a1b2a	T2a1b2a	O	1	T2a1b2a	O	1
6018	JQ798057	T2a1b2b	T2a1b2b	O	1	T2a1b2b	O	1
6019	JQ798056	T2a1b2b	T2a1b2b	O	1	T2a1b2b	O	1
6020	FJ238094	T2a2	T2a2	O	1	T2a2	O	1
6021	JX912164	T2a2	T2a2	O	1	T2a2	O	1
6022	JX152815	T2a2a	T2a2a	O	1	T2a2a	O	1
6023	JF944823	T2a2a	T2a2a	O	1	T2a2a	O	1
6024	KC911444	T2a3	T2a3	O	1	T2a3	O	1
6025	KC911302	T2a3	T2a3	O	1	T2a3	O	1
6026	AY495273	T2b	T2b	O	1	T2b	O	1
6027	HM055613	T2b	T2b	O	1	T2b	O	1
6028	EF177444	T2b1	T2b1	O	1	T2b1	O	1

6029	AY495267	T2b1	T2b1	O	1	T2b1	O	1
6030	AY714016	T2b2	T2b2	O	1	T2b2	O	1
6031	JN035224	T2b2	T2b2	O	1	T2b2	O	1
6032	AY495299	T2b2b	T2b2b	O	1	T2b2b	O	1
6033	GU183768	T2b2b	T2b2b	O	1	T2b2b	O	1
6034	JQ701832	T2b2b1	T2b2b1	O	1	T2b2b1	O	1
6035	JQ705208	T2b2b1	T2b2b1	O	1	T2b2b1	O	1
6036	HM122274	T2b3	T2b3	O	1	T2b3	O	1
6037	HQ384203	T2b3+151	T2b3+151	O	1	T2b3+151	O	1
6038	JQ703699	T2b3+151	T2b3+151	O	1	T2b3+151	O	1
6039	DQ523649	T2b3a	T2b3a	O	1	T2b3a	O	1
6040	JQ798060	T2b3a1	T2b3a1	O	1	T2b3a1	O	1
6041	JQ798061	T2b3a1	T2b3a1	O	1	T2b3a1	O	1
6042	JQ705786	T2b3c	T2b3c	O	1	T2b3c	O	1
6043	JQ702932	T2b3c	T2b3c	O	1	T2b3c	O	1
6044	JQ705037	T2b3d	T2b3d	O	1	T2b3d	O	1
6045	JQ701927	T2b3d	T2b3d	O	1	T2b3d	O	1
6046	JQ705199	T2b3e	T2b3e	O	1	T2b3e	O	1
6047	JF837334	T2b3e	T2b3e	O	1	T2b3e	O	1
6048	AY495275	T2b3b	T2b3b	O	1	T2b3b	O	1
6049	JX153347	T2b3b	T2b3b	O	1	T2b3b	O	1
6050	JQ703501	T2b4	T2b4	O	1	T2b4	O	1
6051	JN037468	T2b4	T2b4	O	1	T2b4	O	1
6052	JF836084	T2b4a	T2b4a	O	1	T2b4a	O	1
6053	GU123018	T2b4a	T2b4a	O	1	T2b4a	O	1
6054	JQ704738	T2b4a1	T2b4a1	O	1	T2b4a1	O	1
6055	JQ798069	T2b4a1	T2b4a1	O	1	T2b4a1	O	1
6056	JQ702278	T2b4i	T2b4i	O	1	T2b4i	O	1
6057	JX153109	T2b4i	T2b4i	O	1	T2b4i	O	1
6058	JQ705242	T2b4+152	T2b4+152	O	1	T2b4+152	O	1
6059	JQ702464	T2b4+152	T2b4+152	O	1	T2b4+152	O	1
6060	JQ705939	T2b4b	T2b4b	O	1	T2b4b	O	1
6061	JF900491	T2b4b	T2b4b	O	1	T2b4b	O	1
6062	JQ704742	T2b4c	T2b4c	O	1	T2b4c	O	1
6063	JQ702604	T2b4c	T2b4c	O	1	T2b4c	O	1
6064	JQ702158	T2b4d	T2b4d	O	1	T2b4d	O	1
6065	JQ703734	T2b4d	T2b4d	O	1	T2b4d	O	1

6066	JQ798064	T2b4e	T2b4e	O	1	T2b4e	O	1
6067	HM852802	T2b4e	T2b4e	O	1	T2b4e	O	1
6068	JX152937	T2b4f	T2b4f	O	1	T2b4f	O	1
6069	JQ704596	T2b4f	T2b4f	O	1	T2b4f	O	1
6070	EF660982	T2b4g	T2b4g	O	1	T2b4g	O	1
6071	JQ798068	T2b4g	T2b4g	O	1	T2b4g	O	1
6072	JQ798066	T2b4h	T2b4h	O	1	T2b4h	O	1
6073	EU007872	T2b4h	T2b4h	O	1	T2b4h	O	1
6074	JQ702978	T2b5	T2b5	O	1	T2b5	O	1
6075	AY495287	T2b5	T2b5	O	1	T2b5	O	1
6076	JQ703716	T2b5a	T2b5a	O	1	T2b5a	O	1
6077	JQ703712	T2b5a1	T2b5a1	O	1	T2b5a1	O	1
6078	JF833039	T2b5a1	T2b5a1	O	1	T2b5a	X	2
6079	AY495284	T2b6	T2b6	O	1	T2b6	O	1
6080	JQ705494	T2b6a	T2b6a	O	1	T2b6a	O	1
6081	AY495282	T2b6a	T2b6a	O	1	T2b6a	O	1
6082	JQ705357	T2b6+146	T2b6+146	O	1	T2b6+146	O	1
6083	JF891418	T2b6b	T2b6b	O	1	T2b6b	O	1
6084	JQ705972	T2b6b	T2b6b	O	1	T2b6b	O	1
6085	GU170817	T2b7	T2b7	O	1	T2b7	O	1
6086	JQ703294	T2b7a1	T2b7a	X	2	T2b7a1	O	1
6087	JQ702544	T2b7a1	T2b7a	X	2	T2b7a1	O	1
6088	JQ704997	T2b7a2	T2b7a2	O	1	T2b7a2	O	1
6089	JQ704643	T2b7a2	T2b7a2	O	1	T2b7a2	O	1
6090	NA20507	T2b7a3	T2b7a3	O	1	T2b7a3	O	1
6091	JQ798079	T2b7a3	T2b7a3	O	1	T2b7a3	O	1
6092	GU122998	T2b8	T2b35	X	2	T2b8 T2b35	X	2
6093	JQ702965	T2b+150	T2b+150	O	1	T2b+150	O	1
6094	JQ703011	T2b9	T2b9	O	1	T2b9	O	1
6095	JQ702737	T2b9	T2b9	O	1	T2b9	O	1
6096	JF968593	T2b11	T2b11	O	1	T2b11	O	1
6097	JQ798085	T2b11	T2b11	O	1	T2b11	O	1
6098	JQ701982	T2b13	T2b13	O	1	T2b13	O	1
6099	JQ702963	T2b13	T2b13	O	1	T2b13	O	1
6100	JN038393	T2b13a	T2b13a	O	1	T2b13a	O	1
6101	JQ702084	T2b13a	T2b13a	O	1	T2b13a	O	1
6102	JQ703042	T2b13b	T2b13b	O	1	T2b13b	O	1

6103	JQ705492	T2b13b	T2b13b	O	1	T2b13b	O	1
6104	JF975728	T2b15	T2b	X	Not found	T2b15	O	1
6105	EU682394	T2b15	T2b	X	Not found	T2b15	O	1
6106	JN004272	T2b+16362	T2b+16362	O	1	T2b+16362	O	1
6107	JN106183	T2b+16362	T2b+16362	O	1	T2b+16362	O	1
6108	GU123025	T2b16	T2b16	O	1	T2b16	O	1
6109	JQ798077	T2b16	T2b16	O	1	T2b16	O	1
6110	JF957699	T2b17	T2b17	O	1	T2b17	O	1
6111	AY495280	T2b17a	T2b17a	O	1	T2b17a	O	1
6112	JX153685	T2b17a	T2b17a	O	1	T2b17a	O	1
6113	JQ705664	T2b19	T2b19	O	1	T2b19	O	1
6114	EU744542	T2b19	T2b19	O	1	T2b19	O	1
6115	HG00141	T2b19b	T2b19b	O	1	T2b19b	O	1
6116	JQ703009	T2b19b	T2b19b	O	1	T2b19b	O	1
6117	EU747356	T2b+152	T2b+152	O	1	T2b+152	O	1
6118	HQ917079	T2b21	T2b21	O	1	T2b21	O	1
6119	JQ705895	T2b21a	T2b21a	O	1	T2b21a	O	1
6120	JF340114	T2b21a	T2b21a	O	1	T2b21a	O	1
6121	JQ702492	T2b21b	T2b21b	O	1	T2b21b	O	1
6122	JQ705338	T2b21b	T2b21b	O	1	T2b21b	O	1
6123	HQ840646	T2b22	T2b22	O	1	T2b22	O	1
6124	JQ703263	T2b22	T2b22	O	1	T2b22	O	1
6125	JQ798075	T2b23	T2b23	O	1	T2b23	O	1
6126	JQ798074	T2b23	T2b23	O	1	T2b23	O	1
6127	FJ348208	T2b23a	T2b23a	O	1	T2b23a	O	1
6128	JQ704788	T2b23a	T2b23a	O	1	T2b23a	O	1
6129	JQ705038	T2b24	T2b24	O	1	T2b24	O	1
6130	JF946696	T2b24a	T2b24a	O	1	T2b24a	O	1
6131	JQ702547	T2b24a	T2b24a	O	1	T2b24a	O	1
6132	JQ704697	T2b25	T2b25	O	1	T2b25	O	1
6133	JQ705882	T2b25	T2b25	O	1	T2b25	O	1
6134	JQ702312	T2b26	T2b26	O	1	T2b26	O	1
6135	JQ702953	T2b26	T2b26	O	1	T2b26	O	1
6136	JQ705444	T2b27	T2b27	O	1	T2b27	O	1
6137	AY495271	T2b27	T2b27	O	1	T2b27	O	1
6138	JQ701939	T2b28	T2b28	O	1	T2b28	O	1
6139	JQ704973	T2b28	T2b28	O	1	T2b28	O	1

6140	JQ798081	T2b29	T2b29	O	1	T2b29	O	1
6141	JQ798080	T2b29	T2b29	O	1	T2b29	O	1
6142	JN024625	T2b30	T2b30	O	1	T2b30	O	1
6143	HQ399469	T2b30	T2b30	O	1	T2b30	O	1
6144	JQ703715	T2b31	T2b31	O	1	T2b31	O	1
6145	JQ798089	T2b31	T2b31	O	1	T2b31	O	1
6146	KC521456	T2b32	T2b32	O	1	T2b32	O	1
6147	JQ703728	T2b32	T2b32	O	1	T2b32	O	1
6148	KC533521	T2b33	T2b33	O	1	T2b33	O	1
6149	JQ705484	T2b33	T2b33	O	1	T2b33	O	1
6150	EU926622	T2b34	T2b34	O	1	T2b34	O	1
6151	KC911423	T2b34	T2b34	O	1	T2b34	O	1
6152	JX153427	T2b35	T2b35	O	1	T2b35	O	1
6153	JN126047	T2b35	T2b35	O	1	T2b+16362	X	2
6154	JX153715	T2b36	T2b36	O	1	T2b36	O	1
6155	JX153237	T2b36	T2b36	O	1	T2b36	O	1
6156	JQ701867	T2b37	T2b37	O	1	T2b+152	X	2
6157	JX153053	T2b37	T2b37	O	1	T2b37	O	1
6158	JQ798090	T2c	T2c	O	1	T2c	O	1
6159	JQ796696	T2c1a	T2c1a	O	1	T2c1a	O	1
6160	JQ703030	T2c1a1	T2c1a1	O	1	T2c1a1	O	1
6161	EF556160	T2c1a1	T2c1a1	O	1	T2c1a1	O	1
6162	EF660941	T2c1a2	T2c1a2	O	1	T2c1a2	O	1
6163	JQ701934	T2c1a2	T2c1a2	O	1	T2c1a2	O	1
6164	JN202494	T2c1a3	T2c1a3	O	1	T2c1a3	O	1
6165	KC911377	T2c1a3	T2c1a3	O	1	T2c1a3	O	1
6166	JQ798103	T2c1c	T2c1c	O	1	T2c1c	O	1
6167	JQ798101	T2c1c	T2c1c	O	1	T2c1c	O	1
6168	JQ798099	T2c1c1	T2c1c1	O	1	T2c1c1	O	1
6169	JQ798100	T2c1c1	T2c1c1	O	1	T2c1c1	O	1
6170	JQ702795	T2c1c2	T2c1c2	O	1	T2c1c2	O	1
6171	JQ798102	T2c1c2	T2c1c2	O	1	T2c1c2	O	1
6172	JQ798091	T2c1+146	T2c1+146	O	1	T2c1+146	O	1
6173	JQ704020	T2c1d1	T2c1d1	O	1	T2c1d1	O	1
6174	KC911358	T2c1d1	T2c1d1	O	1	T2c1d1	O	1
6175	JN580589	T2c1d1a	T2c1d1a	O	1	T2c1d1a	O	1
6176	JX153536	T2c1d1a	T2c1d1a	O	1	T2c1d1a	O	1

6177	JQ798094	T2c1d+152	T2c1d+152	O	1	T2c1d+152	O	1
6178	JF833037	T2c1d+152	T2c1d+152	O	1	T2c1d+152	O	1
6179	JQ798096	T2c1d2	T2c1d2	O	1	T2c1d2	O	1
6180	DQ523629	T2c1d2a	T2c1d2a	O	1	T2c1d2a	O	1
6181	JQ798095	T2c1d2a	T2c1d2a	O	1	T2c1d2a	O	1
6182	JQ703823	T2c1e	T2c1e	O	1	T2c1e	O	1
6183	JQ705571	T2c1e	T2c1e	O	1	T2c1e	O	1
6184	GU048747	T2c1f	T2c1f	O	1	T2c1f	O	1
6185	JQ798097	T2c1f	T2c1f	O	1	T2c1f	O	1
6186	AY714037	T2d1a	T2d1a	O	1	T2d1a	O	1
6187	JQ798104	T2d1a	T2d1a	O	1	T2d1a	O	1
6188	JQ798105	T2d1b1	T2d1b1	O	1	T2d1b1	O	1
6189	JQ798106	T2d1b1	T2d1b1	O	1	T2d1b1	O	1
6190	KC911414	T2d1b2	T2d1b2	O	1	T2d1b2	O	1
6191	HM765473	T2d1b2	T2d1b2	O	1	T2d1b2	O	1
6192	JQ798108	T2d2	T2d2	O	1	T2d2	O	1
6193	NA20770	T2d2	T2d2	O	1	T2d2	O	1
6194	EF177410	T2e	T2e	O	1	T2e	O	1
6195	JQ703777	T2e	T2e	O	1	T2e	O	1
6196	JQ798113	T2e1	T2e1	O	1	T2e1	O	1
6197	JQ705465	T2e1a	T2e1a	O	1	T2e1a	O	1
6198	KF577587	T2e1a1a	T2e1a1a	O	1	T2e1a1a	O	1
6199	KF657641	T2e1a1a	T2e1a1a	O	1	T2e1a1a	O	1
6200	AF381985	T2e1a1b	T2e1a1b	O	1	T2e1a1b	O	1
6201	JN030346	T2e1a1b1	T2e1a1b1	O	1	T2e1a1b1	O	1
6202	KM007555	T2e1a1b1	T2e1a1b1	O	1	T2e1a1b1	O	1
6203	KF048033	T2e1b	T2e1b	O	1	T2e1b	O	1
6204	KF577586	T2e1b1	T2e1b1	O	1	T2e1b1	O	1
6205	EF556188	T2e1b1	T2e1b1	O	1	T2e1b1	O	1
6206	AY714029	T2e2	T2e2	O	1	T2e2	O	1
6207	JQ798114	T2e2a	T2e2a	O	1	T2e2a	O	1
6208	EF060363	T2e2a	T2e2a	O	1	T2e2a	O	1
6209	JQ702891	T2e5	T2e5	O	1	T2e5	O	1
6210	JQ701985	T2e5	T2e5	O	1	T2e5	O	1
6211	JQ798118	T2e6	T2e6	O	1	T2e6	O	1
6212	JQ798119	T2e6	T2e6	O	1	T2e6	O	1
6213	JQ702210	T2e+152	T2e+152	O	1	T2e+152	O	1

6214	JQ798110	T2e7	T2e7	O	1	T2e7	O	1
6215	JX153029	T2e7	T2e7	O	1	T2e7	O	1
6216	KC911558	T2m	T2m	O	1	T2m	O	1
6217	KC911342	T2m	T2m	O	1	T2m	O	1
6218	JQ798135	T2+16189	T2+16189	O	1	T2+16189	O	1
6219	HM852766	T2+16189	T2+16189	O	1	T2+16189	O	1
6220	JQ703997	T2f	T2+16189	X	Not found	T2f	O	1
6221	JQ704129	T2f	T2+16189	X	Not found	T2f	O	1
6222	JQ798120	T2f1	T2+16189	X	Not found	T2f1	O	1
6223	JQ704807	T2f1a	T2+16189	X	3	T2f1a	O	1
6224	GU123028	T2f1a	T2+16189	X	4	T2f1a	O	1
6225	GU932663	T2f1a1	T2f1a1	O	1	T2f1a1	O	1
6226	JQ798122	T2f1a1	T2f1a1	O	1	T2f1a1	O	1
6227	JQ798125	T2f2	T2+16189	X	Not found	T2f2	O	1
6228	HQ286590	T2f2	T2+16189	X	Not found	T2f2	O	1
6229	JF960209	T2f3	T2+16189	X	Not found	T2f3	O	1
6230	JQ704426	T2f3	T2+16189	X	Not found	T2f3	O	1
6231	JQ705528	T2f4	T2+16189	X	Not found	T2f4	O	1
6232	JN034044	T2f4	T2+16189	X	Not found	T2f4	O	1
6233	JQ702957	T2f5	T2+16189	X	17	T2f5	O	1
6234	JQ702294	T2f5	T2+16189	X	22	T2f5	O	1
6235	JN084792	T2f6	T2+16189	X	Not found	T2f6	O	1
6236	JX153995	T2f7	T2+16189	X	Not found	T2f7	O	1
6237	JQ619780	T2f7a	T2+16189	X	Not found	T2f7a	O	1
6238	KF322082	T2f7a	T2+16189	X	Not found	T2f7a	O	1
6239	JX153048	T2f8	T2+16189	X	Not found	T2f8	O	1
6240	JX153682	T2f8a	T2+16189	X	Not found	T2f8a	O	1
6241	JX152786	T2f8a	T2+16189	X	49	T2f8a	O	1
6242	EU935442	T2g1a	T2g1a	O	1	T2g1a	O	1
6243	HE576983	T2g1a	T2g1a	O	1	T2g1a	O	1
6244	EF556186	T2g1a1	T2g1a1	O	1	T2g1a1	O	1
6245	JQ703123	T2g1a1	T2g1a1	O	1	T2g1a1	O	1
6246	KC911398	T2g1b	T2g1b	O	1	T2g1b	O	1
6247	JQ798126	T2g1b	T2g1b	O	1	T2g1b	O	1
6248	HM625693	T2g2	T2g2	O	1	T2g2	O	1
6249	JQ702710	T2g2a	T2g2a	O	1	T2g2a	O	1
6250	JQ702108	T2g2a	T2g2a	O	1	T2g2a	O	1

6251	JN037469	T2h	T2h	O	1	T2h	O	1
6252	JQ798128	T2h	T2h	O	1	T2h	O	1
6253	JN202724	T2h1	T2h1	O	1	T2h1	O	1
6254	JQ705018	T2h1	T2h1	O	1	T2h1	O	1
6255	JX153755	T2h2	T2h2	O	1	T2h2	O	1
6256	JQ702170	T2h2	T2h2	O	1	T2h2	O	1
6257	JQ798129	T2i	T2i	O	1	T2i	O	1
6258	JQ704055	T2i1	T2i1	O	1	T2i1	O	1
6259	JQ798130	T2i1	T2i1	O	1	T2i1	O	1
6260	JQ798131	T2i2	T2i2	O	1	T2i2	O	1
6261	KC911499	T2i2	T2i2	O	1	T2i2	O	1
6262	JQ704786	T2j	T2j	O	1	T2j	O	1
6263	EF177442	T2j1	T2j1	O	1	T2j1	O	1
6264	HQ877824	T2j1	T2j1	O	1	T2j1	O	1
6265	JQ705690	T2k	T2k	O	1	T2k	O	1
6266	HM852810	T2k	T2k	O	1	T2k	O	1
6267	KC127675	T2l	T2l	O	1	T2l	O	1
6268	JQ798137	T2l	T2l	O	1	T2l	O	1
6269	KC911608	T2n	T2n	O	1	T2n	O	1
6270	KC911397	T2n	T2n	O	1	T2n	O	1
6271	JX462725	T3	T3	O	1	T3	O	1
6272	AY713998	R5	R5	O	1	R5	O	1
6273	FJ004837	R5a1	R5a1	O	1	R5a1	O	1
6274	FJ004822	R5a1a	R5a1a	O	1	R5a1a	O	1
6275	AY713983	R5a1a	R5a1a	O	1	R5a1a	O	1
6276	AY713993	R5a2	R5a2	O	1	R5a2	O	1
6277	AY713985	R5a2a	R5a2a	O	1	R5a2a	O	1
6278	AY713996	R5a2a	R5a2a	O	1	R5a2a	O	1
6279	FJ004815	R5a2b	R5a2b	O	1	R5a2b	O	1
6280	FJ004814	R5a2b	R5a2b	O	1	R5a2b	O	1
6281	AY713991	R5a2b1	R5a2b1	O	1	R5a2b1	O	1
6282	AY714000	R5a2b1	R5a2b1	O	1	R5a2b1	O	1
6283	AY714002	R5a2b2	R5a2b2	O	1	R5a2b2	O	1
6284	FJ004829	R5a2b2	R5a2b2	O	1	R5a2b2	O	1
6285	FJ004833	R5a2b3	R5a2b3	O	1	R5a2b3	O	1
6286	FJ004812	R5a2b3	R5a2b3	O	1	R5a2b3	O	1
6287	FJ004834	R5a2b4	R5a2b4	O	1	R5a2b4	O	1

6288	FJ004828	R5a2b4	R5a2b4	O	1	R5a2b4	O	1
6289	AY714028	R6+16129	R6+16129	O	1	R6+16129	O	1
6290	JF742197	R6+16129	R6+16129	O	1	R6+16129	O	1
6291	FJ004819	R6a1	R6a1	O	1	R6a1	O	1
6292	GU480018	R6a1	R6a1	O	1	R6a1	O	1
6293	JQ704804	R6a2	R6a2	O	1	R6a2	O	1
6294	GU480008	R6a2	R6a2	O	1	R6a2	O	1
6295	AY713994	R6b	R6b	O	1	R6b	O	1
6296	JX289095	R6b	R6b	O	1	R6b	O	1
6297	AY714030	R7a	R7a	O	1	R7a	O	1
6298	JX462727	R7a	R7a	O	1	R7a	O	1
6299	FJ004835	R7a1	R7a1	O	1	R7a1	O	1
6300	FJ004805	R7a1	R7a1	O	1	R7a1	O	1
6301	FJ004823	R7a1a	R7a1a	O	1	R7a1a	O	1
6302	EU597487	R7a1a	R7a1a	O	1	R7a1a	O	1
6303	FJ004831	R7a1b1	R7a1b1	O	1	R7a1b1	O	1
6304	FJ004813	R7a1b1	R7a1b1	O	1	R7a1b1	O	1
6305	FJ004804	R7a1b2	R7a1	X	2	R7a1b2	O	1
6306	FJ004810	R7a1b2	R7a1	X	2	R7a1b2	O	1
6307	FJ004817	R7b1	R7b1	O	1	R7b1	O	1
6308	AY714024	R7b1a	R7b1a	O	1	R7b1a	O	1
6309	FJ004811	R7b1a1	R7b1a1	O	1	R7b1a1	O	1
6310	FJ004821	R7b1a1	R7b1a1	O	1	R7b1a1	O	1
6311	GU170816	R7b2	R7b2	O	1	R7b2	O	1
6312	GU170819	R7b2	R7b2	O	1	R7b2	O	1
6313	FJ467953	R8a1a	R8a1a	O	1	R8a1a	O	1
6314	JX462723	R8a1a	R8a1a	O	1	R8a1a	O	1
6315	FJ467965	R8a1a1	R8a1a1	O	1	R8a1a1	O	1
6316	FJ004808	R8a1a1	R8a1a1	O	1	R8a1a1	O	1
6317	AY714012	R8a1a1a1	R8a1a1a1	O	1	R8a1a1a1	O	1
6318	GU810073	R8a1a1a1	R8a1a1a1	O	1	R8a1a1a1	O	1
6319	FJ467978	R8a1a1a1a	R8a1a1a1a	O	1	R8a1a1a1a	O	1
6320	KC911621	R8a1a1a1a	R8a1a1a1a	O	1	R8a1a1a1a	O	1
6321	FJ467943	R8a1a1a2	R8a1a1a2	O	1	R8a1a1a2	O	1
6322	JF742196	R8a1a1a2	R8a1a1a2	O	1	R8a1a1a2	O	1
6323	FJ467949	R8a1a1b	R8a1a1b	O	1	R8a1a1b	O	1
6324	FJ467951	R8a1a1b	R8a1a1b	O	1	R8a1a1b	O	1

6325	FJ467942	R8a1a1c	R8a1a1c	O	1	R8a1a1c	O	1
6326	FJ467955	R8a1a1c	R8a1a1c	O	1	R8a1a1c	O	1
6327	HM345949	R8a1a1d	R8a1a1d	O	1	R8a1a1d	O	1
6328	FJ467947	R8a1a1d	R8a1a1d	O	1	R8a1a1d	O	1
6329	FJ467952	R8a1a2	R8a1a2	O	1	R8a1a2	O	1
6330	FJ467971	R8a1a2a	R8a1a2a	O	1	R8a1a2a	O	1
6331	FJ467974	R8a1a2a	R8a1a2a	O	1	R8a1a2a	O	1
6332	AY714011	R8a1a3	R8a1a3	O	1	R8a1a3	O	1
6333	FJ467984	R8a1a3	R8a1a3	O	1	R8a1a3	O	1
6334	FJ467972	R8a1+16093	R8a1+16093	O	1	R8a1+16093	O	1
6335	FJ467968	R8a1b	R8a1b	O	1	R8a1b	O	1
6336	FJ467970	R8a1b	R8a1b	O	1	R8a1b	O	1
6337	FJ467946	R8a2	R8a2	O	1	R8a2	O	1
6338	FJ004836	R8a2	R8a2	O	1	R8a2	O	1
6339	FJ467941	R8b1	R8b1	O	1	R8b1	O	1
6340	FJ467948	R8b1	R8b1	O	1	R8b1	O	1
6341	FJ467940	R8b1a	R8b1a	O	1	R8b1a	O	1
6342	AY714009	R8b1a	R8b1a	O	1	R8b1a	O	1
6343	FJ467989	R8b2	R8b2	O	1	R8b2	O	1
6344	FJ467987	R8b2	R8b2	O	1	R8b2	O	1
6345	KC505122	R9b	R9b	O	1	R9b	O	1
6346	KF849983	R9b1	R9b1	O	1	R9b1	O	1
6347	GU733737	R9b1a1	R9b1a1	O	1	R9b1a1	O	1
6348	DQ981466	R9b1a1a	R9b1a1a	O	1	R9b1a1a	O	1
6349	DQ981472	R9b1a1a	R9b1a1a	O	1	R9b1a1a	O	1
6350	KF541026	R9b1a2	R9b1a2	O	1	R9b1a2	O	1
6351	DQ981471	R9b1a2a	R9b1a2a	O	1	R9b1a2a	O	1
6352	FJ147308	R9b1a2a	R9b1a2a	O	1	R9b1a2a	O	1
6353	JN580298	R9b1a2b	R9b1a2b	O	1	R9b1a2b	O	1
6354	KF540702	R9b1a2b	R9b1a2b	O	1	R9b1a2b	O	1
6355	DQ981469	R9b1a3	R9b1a3	O	1	R9b1a3	O	1
6356	JF824932	R9b1a3	R9b1a3	O	1	R9b1a3	O	1
6357	DQ981470	R9b1b	R9b1b	O	1	R9b1b	O	1
6358	EF114273	R9b1b	R9b1b	O	1	R9b1b	O	1
6359	GU810062	R9b2	R9b2	O	1	R9b2	O	1
6360	DQ981475	R9b2	R9b2	O	1	R9b2	O	1
6361	JN857032	R9c	R9c	O	1	R9c	O	1

6362	GQ119010	R9c1a	R9c1a	O	1	R9c1a	O	1
6363	KC994068	R9c1a	R9c1a	O	1	R9c1a	O	1
6364	HQ700872	R9c1a1	R9c1a1	O	1	R9c1a1	O	1
6365	KF540867	R9c1a1	R9c1a1	O	1	R9c1a1	O	1
6366	KF541011	R9c1a2	R9c1a2	O	1	R9c1a2	O	1
6367	KF540625	R9c1a2	R9c1a2	O	1	R9c1a2	O	1
6368	JF739535	R9c1a3	R9c1a3	O	1	R9c1a3	O	1
6369	JF739539	R9c1a3	R9c1a3	O	1	R9c1a3	O	1
6370	JX289135	R9c1b1	R9c1b1	O	1	R9c1b1	O	1
6371	HG00407	R9c1b1	R9c1b1	O	1	R9c1b1	O	1
6372	KF540813	R9c1b2	R9c1b2	O	1	R9c1b2	O	1
6373	KC994001	R9c1b2	R9c1b2	O	1	R9c1b2	O	1
6374	NA17963	F1a	F1a	O	1	F1a	O	1
6375	AP008906	F1a1	F1a1	O	1	F1a1	O	1
6376	AP008667	F1a1	F1a1	O	1	F1a1	O	1
6377	HM357817	F1a1a	F1a1a	O	1	F1a1a	O	1
6378	AY255175	F1a1a	F1a1a	O	1	F1a1a	O	1
6379	AY963572	F1a1a1	F1a1a1	O	1	F1a1a1	O	1
6380	AY195791	F1a1a1	F1a1a1	O	1	F1a1a1	O	1
6381	AP008347	F1a1b	F1a1b	O	1	F1a1b	O	1
6382	AP008279	F1a1b	F1a1b	O	1	F1a1b	O	1
6383	NA18620	F1a1c	F1a1c	O	1	F1a1c	O	1
6384	HM357820	F1a1c	F1a1c	O	1	F1a1c	O	1
6385	GU810005	F1a1c1	F1a1c1	O	1	F1a1c1	O	1
6386	GU810022	F1a1c1	F1a1c1	O	1	F1a1c1	O	1
6387	AP008475	F1a1c2	F1a1c2	O	1	F1a1c2	O	1
6388	NA19078	F1a1c2	F1a1c2	O	1	F1a1c2	O	1
6389	NA18606	F1a1c3	F1a1c3	O	1	F1a1c3	O	1
6390	NA18569	F1a1c3	F1a1c3	O	1	F1a1c3	O	1
6391	GQ119028	F1a1d	F1a1d	O	1	F1a1d	O	1
6392	KF849953	F1a1d	F1a1d	O	1	F1a1d	O	1
6393	HM238209	F1a1d1	F1a1d1	O	1	F1a1d1	O	1
6394	HM238211	F1a1d1	F1a1d1	O	1	F1a1d1	O	1
6395	NA18132	F1a4a	F1a4a	O	1	F1a4a	O	1
6396	KC994022	F1a4a1	F1a4a1	O	1	F1a4a1	O	1
6397	HM238204	F1a4a1	F1a4a1	O	1	F1a4a1	O	1
6398	JF824826	F1a4b	F1a4b	O	1	F1a4b	O	1

6399	KC733251	F1a4b	F1a4b	O	1	F1a4b	O	1
6400	FJ748725	F1a2	F1a2	O	1	F1a2	O	1
6401	KF849920	F1a2a	F1a2a	O	1	F1a2a	O	1
6402	DQ272124	F1a2a	F1a2a	O	1	F1a2a	O	1
6403	KF540917	F1a3+16311	F1a3+16311	O	1	F1a3+16311	O	1
6404	JF739536	F1a3a	F1a3a	O	1	F1a3a	O	1
6405	KC993954	F1a3a	F1a3a	O	1	F1a3a	O	1
6406	KF540654	F1a3a1	F1a3a1	O	1	F1a3a1	O	1
6407	AP008701	F1a3a1a	F1a3a1a	O	1	F1a3a1a	O	1
6408	AP008679	F1a3a1a	F1a3a1a	O	1	F1a3a1a	O	1
6409	KC994062	F1a3a2	F1a3a2	O	1	F1a3a2	O	1
6410	HM238199	F1a3a2	F1a3a2	O	1	F1a3a2	O	1
6411	KC994075	F1a3a3	F1a3a3	O	1	F1a3a3	O	1
6412	HM238205	F1a3a3	F1a3a3	O	1	F1a3a3	O	1
6413	KF540619	F1a3a3a	F1a3a3a	O	1	F1a3a3a	O	1
6414	KF541018	F1a3a3a	F1a3a3a	O	1	F1a3a3a	O	1
6415	NA17977	F1a3b	F1a3b	O	1	F1a3b	O	1
6416	KF540845	F1a3b	F1a3b	O	1	F1a3b	O	1
6417	AP010738	F1c	F1c	O	1	F1c	O	1
6418	AP008616	F1c1	F1c1	O	1	F1c1	O	1
6419	AY255177	F1c1a1	F1c1a1	O	1	F1c1a1	O	1
6420	KF056261	F1c1a1a	F1c1a1a	O	1	F1c1a1a	O	1
6421	FJ748754	F1c1a1a	F1c1a1a	O	1	F1c1a1a	O	1
6422	KF849932	F1c1a1b	F1c1a1b	O	1	F1c1a1b	O	1
6423	HG00472	F1c1a1b	F1c1a1b	O	1	F1c1a1b	O	1
6424	FJ748720	F1c1a2	F1c1a2	O	1	F1c1a2	O	1
6425	JX462682	F1c1a2	F1c1a2	O	1	F1c1a2	O	1
6426	AP012346	F1f	F1f	O	1	F1f	O	1
6427	JF824974	F1f	F1f	O	1	F1f	O	1
6428	AP008498	F1b1a	F1b1a	O	1	F1b1a	O	1
6429	NA19059	F1b1a	F1b1a	O	1	F1b1a	O	1
6430	AP008591	F1b1a1	F1b1a1	O	1	F1b1a1	O	1
6431	AP008421	F1b1a1a	F1b1a1a	O	1	F1b1a1a	O	1
6432	AP008919	F1b1a1a1	F1b1a1a1	O	1	F1b1a1a1	O	1
6433	AP010731	F1b1a1a1	F1b1a1a1	O	1	F1b1a1a1	O	1
6434	AP009474	F1b1a1a1a	F1b1a1a1a	O	1	F1b1a1a1a	O	1
6435	AP008820	F1b1a1a1a	F1b1a1a1a	O	1	F1b1a1a1a	O	1

6436	AP010754	F1b1a1a2	F1b1a1a2	O	1	F1b1a1a2	O	1
6437	AP008490	F1b1a1a2	F1b1a1a2	O	1	F1b1a1a2	O	1
6438	AP013115	F1b1a1a3	F1b1a1a3	O	1	F1b1a1a3	O	1
6439	AP008864	F1b1a1a3	F1b1a1a3	O	1	F1b1a1a3	O	1
6440	AP008577	F1b1a2	F1b1a2	O	1	F1b1a2	O	1
6441	AP010975	F1b1a2	F1b1a2	O	1	F1b1a2	O	1
6442	DQ272125	F1b1b	F1b1b	O	1	F1b1b	O	1
6443	HM852871	F1b1b	F1b1b	O	1	F1b1b	O	1
6444	FJ198216	F1b1c	F1b1c	O	1	F1b1c	O	1
6445	KF849982	F1b1c	F1b1c	O	1	F1b1c	O	1
6446	HM036531	F1b1+@152	F1b1+@152	O	1	F1b1+@152	O	1
6447	HQ108344	F1b1+@152	F1b1+@152	O	1	F1b1+@152	O	1
6448	AP013215	F1b1d	F1b1d	O	1	F1b1d	O	1
6449	AP008446	F1b1d	F1b1d	O	1	F1b1d	O	1
6450	HQ405768	F1b1e	F1b1e	O	1	F1b1e	O	1
6451	KF148437	F1b1e1	F1b1e1	O	1	F1b1e1	O	1
6452	KF148087	F1b1e1	F1b1e1	O	1	F1b1e1	O	1
6453	KF148222	F1b1f	F1b1f	O	1	F1b1f	O	1
6454	JF824901	F1b1f	F1b1f	O	1	F1b1f	O	1
6455	AP008808	F1d	F1d	O	1	F1d	O	1
6456	HG00530	F1d	F1d	O	1	F1d	O	1
6457	AP008328	F1d1	F1d1	O	1	F1d1	O	1
6458	NA18603	F1d1	F1d1	O	1	F1d1	O	1
6459	HG00437	F1e1	F1e1	O	1	F1e1	O	1
6460	AP008508	F1e1a	F1e1a	O	1	F1e1a	O	1
6461	AP010837	F1e1a	F1e1a	O	1	F1e1a	O	1
6462	EU597547	F1e2	F1e2	O	1	F1e2	O	1
6463	GU392056	F1e2	F1e2	O	1	F1e2	O	1
6464	KF849952	F1e3	F1e3	O	1	F1e3	O	1
6465	HG00442	F1e3	F1e3	O	1	F1e3	O	1
6466	JN133516	F1g	F1g	O	1	F1g	O	1
6467	JF824890	F1g	F1g	O	1	F1g	O	1
6468	GU392087	F1g1	F1g1	O	1	F1g1	O	1
6469	FJ748713	F1g1	F1g1	O	1	F1g1	O	1
6470	AP008692	F2a	F2a	O	1	F2a	O	1
6471	KF849969	F2a	F2a	O	1	F2a	O	1
6472	NA18598	F2a+@16291	F2a+@16291	O	1	F2a+@16291	O	1

6473	AY255168	F2a1	F2a1	O	1	F2a1	O	1
6474	KF540771	F2a1	F2a1	O	1	F2a1	O	1
6475	AY255180	F2b	F2b	O	1	F2b	O	1
6476	NA18790	F2b1	F2b1	O	1	F2b1	O	1
6477	JF824880	F2b1	F2b1	O	1	F2b1	O	1
6478	HM036555	F2g	F2g	O	1	F2g	O	1
6479	KF849899	F2g	F2g	O	1	F2g	O	1
6480	KF849911	F2c	F2c	O	1	F2c	O	1
6481	JF824941	F2c1	F2c1	O	1	F2c1	O	1
6482	AP010683	F2c1	F2c1	O	1	F2c1	O	1
6483	NA18583	F2c2	F2c2	O	1	F2c2	O	1
6484	NA18609	F2c2	F2c2	O	1	F2c2	O	1
6485	AP013247	F2d	F2d	O	1	F2d	O	1
6486	GU392094	F2d	F2d	O	1	F2d	O	1
6487	NA18629	F2e	F2e	O	1	F2e	O	1
6488	JN857034	F2e1	F2e1	O	1	F2e1	O	1
6489	KF849967	F2e1	F2e1	O	1	F2e1	O	1
6490	AP013271	F2f	F2f	O	1	F2f	O	1
6491	JF824818	F2f	F2f	O	1	F2+195	X	2
6492	GQ999958	F2h	F2h	O	1	F2h	O	1
6493	FJ748736	F2h	F2h	O	1	F2h	O	1
6494	KF849909	F2i	F2i	O	1	F2i	O	1
6495	KF540840	F2i	F2i	O	1	F2i	O	1
6496	AY255167	F3a	F3a	O	1	F3a	O	1
6497	JF824855	F3a+207	F3a+207	O	1	F3a+207	O	1
6498	KF849966	F3a1	F3a1	O	1	F3a1	O	1
6499	NA18759	F3a1	F3a1	O	1	F3a1	O	1
6500	AP013180	F3b	F3b	O	1	F3b	O	1
6501	AY972053	F3b+152	F3b+152	O	1	F3b+152	O	1
6502	KC994154	F3b1a+16093	F3b1a+16093	O	1	F3b1a+16093	O	1
6503	GQ119009	F3b1a+16093	F3b1a+16093	O	1	F3b1a+16093	O	1
6504	KC993991	F3b1a1	F3b1a1	O	1	F3b1a1	O	1
6505	GQ119011	F3b1a1	F3b1a1	O	1	F3b1a1	O	1
6506	GQ119015	F3b1a2	F3b1a2	O	1	F3b1a2	O	1
6507	KF540752	F3b1a2	F3b1a2	O	1	F3b1a2	O	1
6508	JF739538	F3b1b	F3b1b	O	1	F3b1b	O	1
6509	KC994125	F3b1b1	F3b1b1	O	1	F3b1b1	O	1

6510	KC994009	F3b1b1	F3b1b1	O	1	F3b1b1	O	1
6511	AP008744	F4a1a	F4a1a	O	1	F4a1a	O	1
6512	EU597565	F4a1a	F4a1a	O	1	F4a1a	O	1
6513	AP013200	F4a1b	F4a1b	O	1	F4a1b	O	1
6514	AF346973	F4a1b	F4a1b	O	1	F4a1b	O	1
6515	NA18691	F4a2	F4a2	O	1	F4a2	O	1
6516	JF824892	F4a2	F4a2	O	1	F4a2	O	1
6517	NA18592	F4b	F4b	O	1	F4b	O	1
6518	AY289095	F4b1	F4b1	O	1	F4b1	O	1
6519	KF540575	F4b1	F4b1	O	1	F4b1	O	1
6520	NA18763	R11	R11	O	1	R11	O	1
6521	AP010998	R11a	R11a	O	1	R11a	O	1
6522	JF824897	R11b	R11b	O	1	R11b	O	1
6523	JN857012	R11b1a	R11b1a	O	1	R11b1a	O	1
6524	NA18555	R11b1a	R11b1a	O	1	R11b1a	O	1
6525	AY255163	R11b1b	R11b1b	O	1	R11b1b	O	1
6526	HG00536	R11b1b	R11b1b	O	1	R11b1b	O	1
6527	AY255136	B6a	R11'B6	X	2	B6a	O	1
6528	GQ119045	B6a1	R11'B6	X	2	B6a1	O	1
6529	JX289129	B6a1a	B6a1a	O	1	B6a1a	O	1
6530	AP012415	B6a1a	B6a1a	O	1	B6a1a	O	1
6531	KC417443	B4'5	R+16189	X	Not found	B4'5	O	1
6532	HM030512	B4'5	R+16189	X	Not found	B4'5	O	1
6533	NA17990	B4a	R+16189	X	6	B4a	O	1
6534	DQ372871	B4a1a	B4a1a	O	1	B4a1a	O	1
6535	HM238202	B4a1a	B4a1a	O	1	B4a1a	O	1
6536	HQ873527	B4a1a1	B4a1a1	O	1	B4a1a1	O	1
6537	KJ154569	B4a1a1	B4a1a1	O	1	B4a1a1	O	1
6538	HQ873525	B4a1a1a	B4a1a1a	O	1	B4a1a1a	O	1
6539	KJ154187	B4a1a1a	B4a1a1a	O	1	B4a1a1a	O	1
6540	JX900618	B4a1a1a1	B4a1a1a1	O	1	B4a1a1a1	O	1
6541	JX900565	B4a1a1a1	B4a1a1a1	O	1	B4a1a1a1	O	1
6542	JX900769	B4a1a1a1a	B4a1a1a1a	O	1	B4a1a1a1a	O	1
6543	JX900376	B4a1a1a1a1	B4a1a1a1a1	O	1	B4a1a1a1a1	O	1
6544	JX900472	B4a1a1a1a1	B4a1a1a1a1	O	1	B4a1a1a1a1	O	1
6545	JX900604	B4a1a1a1b	B4a1a1a1b	O	1	B4a1a1a1b	O	1
6546	JX900773	B4a1a1a1b	B4a1a1a1b	O	1	B4a1a1a1b	O	1

6547	KJ154487	B4a1a1a1c	B4a1a1a1c	O	1	B4a1a1a1c	O	1
6548	KJ154627	B4a1a1a1c	B4a1a1a1c	O	1	B4a1a1a1c	O	1
6549	KJ154414	B4a1a1a1d	B4a1a1a1d	O	1	B4a1a1a1d	O	1
6550	KJ154286	B4a1a1a1d	B4a1a1a1d	O	1	B4a1a1a1d	O	1
6551	JX900433	B4a1a1a2	B4a1a1a2	O	1	B4a1a1a2	O	1
6552	HQ873516	B4a1a1a2	B4a1a1a2	O	1	B4a1a1a2	O	1
6553	JX900535	B4a1a1a2a	B4a1a1a2a	O	1	B4a1a1a2a	O	1
6554	JX900548	B4a1a1a2a	B4a1a1a2a	O	1	B4a1a1a2a	O	1
6555	KJ154620	B4a1a1a2b	B4a1a1a2b	O	1	B4a1a1a2b	O	1
6556	KJ154535	B4a1a1a2b	B4a1a1a2b	O	1	B4a1a1a2b	O	1
6557	JX900766	B4a1a1a3	B4a1a1a3	O	1	B4a1a1a3	O	1
6558	JX900480	B4a1a1a3	B4a1a1a3	O	1	B4a1a1a3	O	1
6559	JX900408	B4a1a1a4	B4a1a1a4	O	1	B4a1a1a4	O	1
6560	HQ873490	B4a1a1a4	B4a1a1a4	O	1	B4a1a1a4	O	1
6561	JX900578	B4a1a1a5	B4a1a1a5	O	1	B4a1a1a5	O	1
6562	JX900762	B4a1a1a5	B4a1a1a5	O	1	B4a1a1a5	O	1
6563	JX900564	B4a1a1a6	B4a1a1a6	O	1	B4a1a1a6	O	1
6564	JX900841	B4a1a1a6	B4a1a1a6	O	1	B4a1a1a6	O	1
6565	JX900343	B4a1a1a7	B4a1a1a7	O	1	B4a1a1a7	O	1
6566	JX900331	B4a1a1a7	B4a1a1a7	O	1	B4a1a1a7	O	1
6567	JX900752	B4a1a1a8	B4a1a1a8	O	1	B4a1a1a8	O	1
6568	KJ154435	B4a1a1a8	B4a1a1a8	O	1	B4a1a1a8	O	1
6569	JX900826	B4a1a1a9	B4a1a1a9	O	1	B4a1a1a9	O	1
6570	JX900819	B4a1a1a9	B4a1a1a9	O	1	B4a1a1a9	O	1
6571	JX900412	B4a1a1a10	B4a1a1a10	O	1	B4a1a1a10	O	1
6572	JX900614	B4a1a1a10	B4a1a1a10	O	1	B4a1a1a10	O	1
6573	JX900639	B4a1a1a11	B4a1a1a11	O	1	B4a1a1a11	O	1
6574	HQ873524	B4a1a1a11a	B4a1a1a11a	O	1	B4a1a1a11a	O	1
6575	JX900497	B4a1a1a11a	B4a1a1a11a	O	1	B4a1a1a11a	O	1
6576	JX900344	B4a1a1a11b	B4a1a1a11b	O	1	B4a1a1a11b	O	1
6577	KJ154230	B4a1a1a11b	B4a1a1a11b	O	1	B4a1a1a11b	O	1
6578	JX900373	B4a1a1a12	B4a1a1a12	O	1	B4a1a1a12	O	1
6579	JX900802	B4a1a1a12	B4a1a1a12	O	1	B4a1a1a12	O	1
6580	JX900500	B4a1a1a13	B4a1a1a13	O	1	B4a1a1a13	O	1
6581	AY289093	B4a1a1a13	B4a1a1a13	O	1	B4a1a1a13	O	1
6582	KJ154580	B4a1a1a14	B4a1a1a14	O	1	B4a1a1a14	O	1
6583	KJ154589	B4a1a1a14	B4a1a1a14	O	1	B4a1a1a14	O	1

6584	KJ154242	B4a1a1a15	B4a1a1a15	O	1	B4a1a1a15	O	1
6585	KJ154315	B4a1a1a15	B4a1a1a15	O	1	B4a1a1a15	O	1
6586	JX900734	B4a1a1a16	B4a1a1a16	O	1	B4a1a1a16	O	1
6587	KJ154328	B4a1a1a16	B4a1a1a16	O	1	B4a1a1a16	O	1
6588	KJ154515	B4a1a1a17	B4a1a1a17	O	1	B4a1a1a17	O	1
6589	KJ154591	B4a1a1a17	B4a1a1a17	O	1	B4a1a1a17	O	1
6590	KJ154415	B4a1a1a18	B4a1a1a18	O	1	B4a1a1a18	O	1
6591	KJ154356	B4a1a1a18	B4a1a1a18	O	1	B4a1a1a18	O	1
6592	KJ154865	B4a1a1a19	B4a1a1a19	O	1	B4a1a1a19	O	1
6593	HQ873515	B4a1a1a19	B4a1a1a19	O	1	B4a1a1a19	O	1
6594	KJ154207	B4a1a1a20	B4a1a1a20	O	1	B4a1a1a20	O	1
6595	KJ154294	B4a1a1a20	B4a1a1a20	O	1	B4a1a1a20	O	1
6596	JX900558	B4a1a1a21	B4a1a1a21	O	1	B4a1a1a21	O	1
6597	KJ154218	B4a1a1a21	B4a1a1a21	O	1	B4a1a1a21	O	1
6598	KJ154215	B4a1a1a22	B4a1a1a22	O	1	B4a1a1a22	O	1
6599	KJ154210	B4a1a1a22	B4a1a1a22	O	1	B4a1a1a22	O	1
6600	JX900455	B4a1a1a+195	B4a1a1a+195	O	1	B4a1a1a+195	O	1
6601	JX900848	B4a1a1a+195	B4a1a1a+195	O	1	B4a1a1a+195	O	1
6602	JX900644	B4a1a1a23	B4a1a1a23	O	1	B4a1a1a23	O	1
6603	KJ154480	B4a1a1a23	B4a1a1a23	O	1	B4a1a1a23	O	1
6604	FJ767912	B4a1a1b	B4a1a1b	O	1	B4a1a1b	O	1
6605	FJ767910	B4a1a1b	B4a1a1b	O	1	B4a1a1b	O	1
6606	KJ154275	B4a1a1c	B4a1a1c	O	1	B4a1a1c	O	1
6607	KJ154368	B4a1a1c	B4a1a1c	O	1	B4a1a1c	O	1
6608	JX900453	B4a1a1d	B4a1a1d	O	1	B4a1a1d	O	1
6609	HQ873507	B4a1a1d	B4a1a1d	O	1	B4a1a1d	O	1
6610	JX900634	B4a1a1e	B4a1a1e	O	1	B4a1a1e	O	1
6611	JX900555	B4a1a1e	B4a1a1e	O	1	B4a1a1e	O	1
6612	JX900410	B4a1a1f	B4a1a1f	O	1	B4a1a1f	O	1
6613	JX900411	B4a1a1f	B4a1a1f	O	1	B4a1a1f	O	1
6614	JX900722	B4a1a1g	B4a1a1g	O	1	B4a1a1g	O	1
6615	JX900543	B4a1a1g	B4a1a1g	O	1	B4a1a1g	O	1
6616	JX900348	B4a1a1h	B4a1a1h	O	1	B4a1a1h	O	1
6617	JX900338	B4a1a1h	B4a1a1h	O	1	B4a1a1h	O	1
6618	JX900622	B4a1a1i	B4a1a1i	O	1	B4a1a1i	O	1
6619	JX900779	B4a1a1i	B4a1a1i	O	1	B4a1a1i	O	1
6620	JX900692	B4a1a1j	B4a1a1j	O	1	B4a1a1j	O	1

6621	JX900794	B4a1a1j	B4a1a1j	O	1	B4a1a1j	O	1
6622	KJ154396	B4a1a1k	B4a1a1k	O	1	B4a1a1k	O	1
6623	KJ154220	B4a1a1k	B4a1a1k	O	1	B4a1a1k	O	1
6624	KJ154221	B4a1a1k1	B4a1a1k1	O	1	B4a1a1k1	O	1
6625	KJ154190	B4a1a1k1	B4a1a1k1	O	1	B4a1a1k1	O	1
6626	KJ154269	B4a1a1+151	B4a1a1+151	O	1	B4a1a1+151	O	1
6627	KJ154287	B4a1a1+151	B4a1a1+151	O	1	B4a1a1+151	O	1
6628	KJ154397	B4a1a1m	B4a1a1m	O	1	B4a1a1m	O	1
6629	KJ154364	B4a1a1m	B4a1a1m	O	1	B4a1a1m	O	1
6630	AY289068	B4a1a1m1	B4a1a1m1	O	1	B4a1a1m1	O	1
6631	KJ154402	B4a1a1m1	B4a1a1m1	O	1	B4a1a1m1	O	1
6632	JX900810	B4a1a1n	B4a1a1n	O	1	B4a1a1n	O	1
6633	KJ154312	B4a1a1n	B4a1a1n	O	1	B4a1a1n	O	1
6634	HQ873531	B4a1a1+152	B4a1a1+152	O	1	B4a1a1+152	O	1
6635	AF347007	B4a1a1+152	B4a1a1+152	O	1	B4a1a1+152	O	1
6636	JX900738	B4a1a1o	B4a1a1o	O	1	B4a1a1o	O	1
6637	JX900828	B4a1a1o	B4a1a1o	O	1	B4a1a1o	O	1
6638	JX900384	B4a1a1p	B4a1a1p	O	1	B4a1a1p	O	1
6639	JX900387	B4a1a1p	B4a1a1p	O	1	B4a1a1p	O	1
6640	HQ873532	B4a1a1q	B4a1a1q	O	1	B4a1a1q	O	1
6641	JX900508	B4a1a1q	B4a1a1q	O	1	B4a1a1q	O	1
6642	KJ154410	B4a1a1+16126	B4a1a1+16126	O	1	B4a1a1+16126	O	1
6643	KJ154226	B4a1a1+16126	B4a1a1+16126	O	1	B4a1a1+16126	O	1
6644	KJ154474	B4a1a1s	B4a1a1s	O	1	B4a1a1s	O	1
6645	KJ154645	B4a1a1s	B4a1a1s	O	1	B4a1a1s	O	1
6646	KJ154214	B4a1a1t	B4a1a1t	O	1	B4a1a1t	O	1
6647	KJ154367	B4a1a1t	B4a1a1t	O	1	B4a1a1t	O	1
6648	KJ154439	B4a1a1u	B4a1a1u	O	1	B4a1a1u	O	1
6649	KJ154259	B4a1a1u	B4a1a1u	O	1	B4a1a1u	O	1
6650	KJ154288	B4a1a1v	B4a1a1v	O	1	B4a1a1v	O	1
6651	KJ154257	B4a1a1v	B4a1a1v	O	1	B4a1a1v	O	1
6652	KJ154872	B4a1a1w	B4a1a1w	O	1	B4a1a1w	O	1
6653	KJ154864	B4a1a1w	B4a1a1w	O	1	B4a1a1w	O	1
6654	KJ154158	B4a1a1x	B4a1a1x	O	1	B4a1a1x	O	1
6655	DQ372877	B4a1a1x	B4a1a1x	O	1	B4a1a1x	O	1
6656	JX900630	B4a1a1y	B4a1a1y	O	1	B4a1a1y	O	1
6657	JX900846	B4a1a1y	B4a1a1y	O	1	B4a1a1y	O	1

6658	KJ154929	B4a1a1z	B4a1a1z	O	1	B4a1a1z	O	1
6659	KJ154925	B4a1a1z	B4a1a1z	O	1	B4a1a1z	O	1
6660	AY963574	B4a1a1aa	B4a1a1aa	O	1	B4a1a1aa	O	1
6661	KJ154629	B4a1a1aa	B4a1a1aa	O	1	B4a1a1aa	O	1
6662	JX900575	B4a1a1ab	B4a1a1ab	O	1	B4a1a1ab	O	1
6663	KJ154209	B4a1a1ab	B4a1a1ab	O	1	B4a1a1ab	O	1
6664	JX900514	B4a1a1ac	B4a1a1ac	O	1	B4a1a1ac	O	1
6665	KJ154307	B4a1a1ac	B4a1a1ac	O	1	B4a1a1ac	O	1
6666	KJ154208	B4a1a1ad	B4a1a1ad	O	1	B4a1a1ad	O	1
6667	KJ154233	B4a1a1ad	B4a1a1ad	O	1	B4a1a1ad	O	1
6668	HQ873511	B4a1a1ae	B4a1a1ae	O	1	B4a1a1ae	O	1
6669	HQ873509	B4a1a1ae	B4a1a1ae	O	1	B4a1a1ae	O	1
6670	KJ154891	B4a1a1af	B4a1a1af	O	1	B4a1a1af	O	1
6671	KJ154884	B4a1a1af	B4a1a1af	O	1	B4a1a1af	O	1
6672	AJ842748	B4a1a2	B4a1a2	O	1	B4a1a2	O	1
6673	AJ842745	B4a1a2	B4a1a2	O	1	B4a1a2	O	1
6674	HQ873558	B4a1a3	B4a1a3	O	1	B4a1a3	O	1
6675	HQ873560	B4a1a3a	B4a1a3a	O	1	B4a1a3a	O	1
6676	HQ873543	B4a1a3a1	B4a1a3a1	O	1	B4a1a3a1	O	1
6677	KC994063	B4a1a3a1	B4a1a3a1	O	1	B4a1a3a1	O	1
6678	AJ842744	B4a1a3a1a	B4a1a3a1a	O	1	B4a1a3a1a	O	1
6679	KF540509	B4a1a3a1a	B4a1a3a1a	O	1	B4a1a3a1a	O	1
6680	KC994073	B4a1a4	B4a1a4	O	1	B4a1a4	O	1
6681	AJ842747	B4a1a4	B4a1a4	O	1	B4a1a4	O	1
6682	GQ119021	B4a1a5	B4a1a5	O	1	B4a1a5	O	1
6683	HQ873542	B4a1a5	B4a1a5	O	1	B4a1a5	O	1
6684	KC994105	B4a1a5a	B4a1a5a	O	1	B4a1a5a	O	1
6685	KC994064	B4a1a5a	B4a1a5a	O	1	B4a1a5a	O	1
6686	KC994047	B4a1a6	B4a1a6	O	1	B4a1a6	O	1
6687	KC994101	B4a1a6	B4a1a6	O	1	B4a1a6	O	1
6688	KC994027	B4a1a6a	B4a1a6a	O	1	B4a1a6a	O	1
6689	KC994097	B4a1a6a	B4a1a6a	O	1	B4a1a6a	O	1
6690	HQ873557	B4a1a7	B4a1a7	O	1	B4a1a7	O	1
6691	KF540551	B4a1a7	B4a1a7	O	1	B4a1a7	O	1
6692	EU597506	B4a1+16311	R+16189	X	10	B4a1+16311	O	1
6693	AP008595	B4a1b	B4a1b	O	1	B4a1b	O	1
6694	AP008842	B4a1b1	B4a1b1	O	1	B4a1b1	O	1

6695	AP008640	B4a1b1a	B4a1b1a	O	1	B4a1b1a	O	1
6696	AF346993	B4a1b1a	B4a1b1a	O	1	B4a1b1a	O	1
6697	JN866824	B4a1e	B4a1e	O	1	B4a1e	O	1
6698	KF849945	B4a1e	B4a1e	O	1	B4a1e	O	1
6699	AP008650	B4a1c1	R+16189	X	3	B4a1c1	O	1
6700	AP013242	B4a1c1a	B4a1c1a	O	1	B4a1c1a	O	1
6701	JF824900	B4a1c1a	B4a1c1a	O	1	B4a1c1a	O	1
6702	AP008912	B4a1c1a1	B4a1c1a1	O	1	B4a1c1a1	O	1
6703	NA18975	B4a1c1a1	B4a1c1a1	O	1	B4a1c1a1	O	1
6704	AY519495	B4a1c2	B4a1c2	O	1	B4a1c2	O	1
6705	AY519492	B4a1c2	B4a1c2	O	1	B4a1c2	O	1
6706	EU597505	B4a1c4	B4a1c4	O	1	B4a1c4	O	1
6707	HQ873562	B4a1c4	B4a1c4	O	1	B4a1c4	O	1
6708	HQ873564	B4a1c5	B4a1c5	O	1	B4a1c5	O	1
6709	KF540685	B4a1c5	B4a1c5	O	1	B4a1c5	O	1
6710	AP010705	B4a1c3a	B4a1c3a	O	1	B4a1c3a	O	1
6711	AP008412	B4a1c3a	B4a1c3a	O	1	B4a1c3a	O	1
6712	AP008521	B4a1c3b	B4a1c3b	O	1	B4a1c3b	O	1
6713	KF849971	B4a1c3b	B4a1c3b	O	1	B4a1c3b	O	1
6714	HQ873495	B4a1d	B4a1d	O	1	B4a1d	O	1
6715	HQ873566	B4a1d	B4a1d	O	1	B4a1d	O	1
6716	HQ873538	B4a2a	B4a2a	O	1	B4a2a	O	1
6717	HM596696	B4a2a	B4a2a	O	1	B4a2a	O	1
6718	AJ842751	B4a2a1	B4a2a1	O	1	B4a2a1	O	1
6719	HM238207	B4a2a1	B4a2a1	O	1	B4a2a1	O	1
6720	HQ873563	B4a2a2	B4a2a2	O	1	B4a2a2	O	1
6721	KF540572	B4a2a2	B4a2a2	O	1	B4a2a2	O	1
6722	AJ842750	B4a2a3	B4a2a3	O	1	B4a2a3	O	1
6723	KF540774	B4a2a3	B4a2a3	O	1	B4a2a3	O	1
6724	NA18528	B4a2b	B4a2b	O	1	B4a2b	O	1
6725	JF824854	B4a2b1	B4a2b1	O	1	B4a2b1	O	1
6726	KF849947	B4a2b1	B4a2b1	O	1	B4a2b1	O	1
6727	AP013280	B4a2b1a	B4a2b1a	O	1	B4a2b1a	O	1
6728	AP008257	B4a2b1a	B4a2b1a	O	1	B4a2b1a	O	1
6729	FJ748745	B4a3	B4a3	O	1	B4a3	O	1
6730	AP008567	B4a3	B4a3	O	1	B4a3	O	1
6731	AP008384	B4a4	B4a4	O	1	B4a4	O	1

6732	NA18794	B4a4	B4a4	O	1	B4a4	O	1
6733	KF540699	B4a5	B4a5	O	1	B4a5	O	1
6734	KF540665	B4a5	B4a5	O	1	B4a5	O	1
6735	JQ703874	B4g1	R+16189	X	4	B4g1	O	1
6736	GU810060	B4g1a	B4g1a	O	1	B4g1a	O	1
6737	HQ873489	B4g1a	B4g1a	O	1	B4g1a	O	1
6738	NA18109	B4g1b	B4g1b	O	1	B4g1b	O	1
6739	NA18770	B4g1b	B4g1b	O	1	B4g1b	O	1
6740	KF540983	B4g2	B4g2	O	1	B4g2	O	1
6741	HG00419	B4g2	B4g2	O	1	B4g2	O	1
6742	GU377090	B4h	R+16189	X	12	B4h	O	1
6743	KF849963	B4h1	R+16189	X	5	B4h1	O	1
6744	HQ873569	B4h1	R+16189	X	5	B4h1	O	1
6745	JF824906	B4i	R+16189	X	12	B4i	O	1
6746	KC733274	B4i1	B4i1	O	1	B4i1	O	1
6747	NA18541	B4i1	R+16189	X	2	B4i1	O	1
6748	KF849913	B4k	R+16189	X	3	B4k	O	1
6749	EU597559	B4k	R+16189	X	3	B4k	O	1
6750	KF540707	B4m	R	X	17	B4m	O	1
6751	KF540708	B4m	R	X	18	B4m	O	1
6752	EU095548	B2	B2	O	1	B2	O	1
6753	EF079874	B2	B2	O	1	B2	O	1
6754	KC711022	B2a	B2a	O	1	B2a	O	1
6755	KC711021	B2a	B2a	O	1	B2a	O	1
6756	DQ282442	B2a1	B2a1	O	1	B2a1	O	1
6757	KC711026	B2a1	B2a1	O	1	B2a1	O	1
6758	DQ282444	B2a1a	B2a1a	O	1	B2a1a	O	1
6759	JQ702668	B2a1a1	B2a1a1	O	1	B2a1a1	O	1
6760	KC711024	B2a1a1	B2a1a1	O	1	B2a1a1	O	1
6761	DQ282445	B2a1b	B2a1b	O	1	B2a1b	O	1
6762	KC711025	B2a1b	B2a1b	O	1	B2a1b	O	1
6763	DQ282441	B2a2	B2a2	O	1	B2a2	O	1
6764	KC711028	B2a2	B2a2	O	1	B2a2	O	1
6765	HQ012136	B2a3	B2a3	O	1	B2a3	O	1
6766	KC711031	B2a3	B2a3	O	1	B2a3	O	1
6767	JQ703852	B2a4	B2a4	O	1	B2a4	O	1
6768	KC711033	B2a4a	B2a4a	O	1	B2a4a	O	1

6769	KC711034	B2a4a1	B2a4a1	O	1	B2a4a1	O	1
6770	KC711035	B2a4a1	B2a4a1	O	1	B2a4a1	O	1
6771	AF347001	B2a5	B2a5	O	1	B2a5	O	1
6772	KC711039	B2a5	B2a5	O	1	B2a5	O	1
6773	EU095532	B2b	B2b	O	1	B2b	O	1
6774	HG01437	B2b+152	B2b+152	O	1	B2b+152	O	1
6775	EU095210	B2b+152	B2b+152	O	1	B2b+152	O	1
6776	JF431064	B2b1	B2b1	O	1	B2b1	O	1
6777	KC503926	B2b2	B2b2	O	1	B2b2	O	1
6778	KC503927	B2b2a	B2b2a	O	1	B2b2a	O	1
6779	KC503925	B2b2a	B2b2a	O	1	B2b2a	O	1
6780	EU095221	B2b3	B2b3	O	1	B2b3	O	1
6781	HG00640	B2b3a	B2b3a	O	1	B2b3a	O	1
6782	EU095216	B2b3a	B2b3a	O	1	B2b3a	O	1
6783	HQ012137	B2b4	B2b4	O	1	B2b4	O	1
6784	DQ282436	B2c	B2c	O	1	B2c	O	1
6785	DQ282438	B2c1	B2c1	O	1	B2c1	O	1
6786	DQ282439	B2c1	B2c1	O	1	B2c1	O	1
6787	DQ282434	B2c1a	B2c1a	O	1	B2c1a	O	1
6788	HQ012177	B2c1a	B2c1a	O	1	B2c1a	O	1
6789	HQ012160	B2c1b	B2c1b	O	1	B2c1b	O	1
6790	DQ282437	B2c1b	B2c1b	O	1	B2c1b	O	1
6791	HQ012164	B2c1c	B2c1c	O	1	B2c1c	O	1
6792	NA19731	B2c1c	B2c1c	O	1	B2c1c	O	1
6793	HQ012143	B2c2	B2c2	O	1	B2c2	O	1
6794	HQ012140	B2c2a	B2c2a	O	1	B2c2a	O	1
6795	HQ012158	B2c2a	B2c2a	O	1	B2c2a	O	1
6796	HQ012151	B2c2b	B2c2b	O	1	B2c2b	O	1
6797	JQ705349	B2c2b	B2c2b	O	1	B2c2b	O	1
6798	EU095550	B2d	B2d	O	1	B2d	O	1
6799	HG01494	B2d	B2d	O	1	B2d	O	1
6800	EU597569	B2e	B2e	O	1	B2e	O	1
6801	EU095209	B2e	B2e	O	1	B2e	O	1
6802	EU334872	B2f	B2f	O	1	B2f	O	1
6803	HQ012185	B2g1	B2g1	O	1	B2g1	O	1
6804	HQ012145	B2g1	B2g1	O	1	B2g1	O	1
6805	KC257372	B2g2	B2g2	O	1	B2g2	O	1

6806	JQ702661	B2g2	B2g2	O	1	B2g2	O	1
6807	EU095206	B2h	B2h	O	1	B2h	O	1
6808	EU095215	B2h	B2h	O	1	B2h	O	1
6809	EU095218	B2i1	B2i1	O	1	B2i1	O	1
6810	EU095217	B2i1	B2i1	O	1	B2i1	O	1
6811	JX413035	B2i2	B2i2	O	1	B2i2	O	1
6812	JX413013	B2i2a	B2i2a	O	1	B2i2a	O	1
6813	JX413012	B2i2a	B2i2a	O	1	B2i2a	O	1
6814	JX413023	B2i2a1	B2i2a1	O	1	B2i2a1	O	1
6815	JX413022	B2i2a1	B2i2a1	O	1	B2i2a1	O	1
6816	JX413014	B2i2a1a	B2i2a1a	O	1	B2i2a1a	O	1
6817	JX413019	B2i2a1a	B2i2a1a	O	1	B2i2a1a	O	1
6818	JX413020	B2i2a1b	B2i2a1b	O	1	B2i2a1b	O	1
6819	JX413021	B2i2a1b	B2i2a1b	O	1	B2i2a1b	O	1
6820	JX413027	B2i2b	B2i2b	O	1	B2i2b	O	1
6821	JX174728	B2i2b	B2i2b	O	1	B2i2b	O	1
6822	JX413032	B2i2b1	B2i2b1	O	1	B2i2b1	O	1
6823	JX413033	B2i2b1	B2i2b1	O	1	B2i2b1	O	1
6824	JF431059	B2j	B2j	O	1	B2j	O	1
6825	JF431060	B2j	B2j	O	1	B2j	O	1
6826	HQ012156	B2k	B2k	O	1	B2k	O	1
6827	JF431061	B2k	B2k	O	1	B2k	O	1
6828	JQ702293	B2l	B2l	O	1	B2l	O	1
6829	NA19785	B2l	B2l	O	1	B2l	O	1
6830	HQ012141	B2m	B2m	O	1	B2m	O	1
6831	NA19761	B2m	B2m	O	1	B2m	O	1
6832	HQ012163	B2n	B2n	O	1	B2n	O	1
6833	NA19795	B2n	B2n	O	1	B2n	O	1
6834	JQ703851	B2o	B2o	O	1	B2o	O	1
6835	KC503933	B2o1	B2o1	O	1	B2o1	O	1
6836	KC503931	B2o1a	B2o1a	O	1	B2o1a	O	1
6837	KC503932	B2o1a	B2o1a	O	1	B2o1a	O	1
6838	HQ012138	B2p	B2p	O	1	B2p	O	1
6839	HQ012167	B2p	B2p	O	1	B2p	O	1
6840	HQ012153	B2q	B2q	O	1	B2+16278	X	2
6841	HQ012176	B2q	B2q	O	1	B2q	O	1
6842	HQ012175	B2r	B2	X	10	B2r	O	1

6843	DQ282435	B2r	B2	X	10	B2r	O	1
6844	HQ012165	B2s	B2s	O	1	B2s	O	1
6845	NA19780	B2s	B2s	O	1	B2s	O	1
6846	NA19684	B2t	B2t	O	1	B2t	O	1
6847	JQ705648	B2t	B2t	O	1	B2t	O	1
6848	HQ012183	B2u	B2u	O	1	B2u	O	1
6849	HQ012170	B2u	B2u	O	1	B2u	O	1
6850	HQ012173	B2v	B2v	O	1	B2v	O	1
6851	HQ012159	B2v	B2v	O	1	B2v	O	1
6852	HQ012172	B2+16278	B2+16278	O	1	B2+16278	O	1
6853	JQ705755	B2w	B2w	O	1	B2w	O	1
6854	HQ012168	B2w	B2w	O	1	B2w	O	1
6855	HQ012147	B2x	B2x	O	1	B2x	O	1
6856	NA19681	B2x	B2x	O	1	B2x	O	1
6857	EU597534	B2y	B2y	O	1	B2y	O	1
6858	JQ705259	B2y1	B2y1	O	1	B2y1	O	1
6859	EU431084	B2y1	B2y1	O	1	B2y1	O	1
6860	AY519494	B4b1a	B4b1a	O	1	B4b1a	O	1
6861	AP008325	B4b1a1	B4b1a1	O	1	B4b1a1	O	1
6862	AP010733	B4b1a1	B4b1a1	O	1	B4b1a1	O	1
6863	AP008848	B4b1a1a	B4b1a1a	O	1	B4b1a1a	O	1
6864	AP009471	B4b1a1a	B4b1a1a	O	1	B4b1a1a	O	1
6865	AP009444	B4b1a1b	B4b1a1b	O	1	B4b1a1b	O	1
6866	AP013219	B4b1a1b	B4b1a1b	O	1	B4b1a1b	O	1
6867	AP008900	B4b1a1c	B4b1a1c	O	1	B4b1a1c	O	1
6868	AP013162	B4b1a1c	B4b1a1c	O	1	B4b1a1c	O	1
6869	KC993941	B4b1a2	B4b1a2	O	1	B4b1a2	O	1
6870	AP008856	B4b1a2	B4b1a2	O	1	B4b1a2	O	1
6871	AY255170	B4b1a2a	B4b1a2a	O	1	B4b1a2a	O	1
6872	AP010671	B4b1a2a	B4b1a2a	O	1	B4b1a2a	O	1
6873	KC994146	B4b1a2b1	B4b1a2b1	O	1	B4b1a2b1	O	1
6874	GU733790	B4b1a2b1	B4b1a2b1	O	1	B4b1a2b1	O	1
6875	KF540811	B4b1a2b2	B4b1a2b2	O	1	B4b1a2b2	O	1
6876	KF540644	B4b1a2b2	B4b1a2b2	O	1	B4b1a2b2	O	1
6877	GU733724	B4b1a2c	B4b1a2c	O	1	B4b1a2c	O	1
6878	GU733744	B4b1a2c	B4b1a2c	O	1	B4b1a2c	O	1
6879	GU733814	B4b1a2d	B4b1a2d	O	1	B4b1a2d	O	1

6880	GU733825	B4b1a2d	B4b1a2d	O	1	B4b1a2d	O	1
6881	JF824980	B4b1a2e	B4b1a2e	O	1	B4b1a2e	O	1
6882	EU597553	B4b1a2e	B4b1a2e	O	1	B4b1a2e	O	1
6883	KF540629	B4b1a2f	B4b1a2f	O	1	B4b1a2f	O	1
6884	KF541024	B4b1a2f	B4b1a2f	O	1	B4b1a2f	O	1
6885	KF540610	B4b1a2g	B4b1a2g	O	1	B4b1a2g	O	1
6886	KF540608	B4b1a2g1	B4b1a2g1	O	1	B4b1a2g1	O	1
6887	KF540617	B4b1a2g1	B4b1a2g1	O	1	B4b1a2g1	O	1
6888	KF540519	B4b1a2h	B4b1a2h	O	1	B4b1a2h	O	1
6889	KF540523	B4b1a2h	B4b1a2h	O	1	B4b1a2h	O	1
6890	KJ154674	B4b1a2i	B4b1a2i	O	1	B4b1a2i	O	1
6891	KJ154236	B4b1a2i	B4b1a2i	O	1	B4b1a2i	O	1
6892	NA18689	B4b1a3	B4b1a3	O	1	B4b1a3	O	1
6893	JN857022	B4b1a3	B4b1a3	O	1	B4b1a3	O	1
6894	KF148375	B4b1a3a	B4b1a3a	O	1	B4b1a3a	O	1
6895	JN857043	B4b1a3a	B4b1a3a	O	1	B4b1a3a	O	1
6896	AP008682	B4b1b	B4b1b	O	1	B4b1b	O	1
6897	AP008550	B4b1b	B4b1b	O	1	B4b1b	O	1
6898	HQ873567	B4b1c1	B4b1c1	O	1	B4b1c1	O	1
6899	AP009450	B4b1c1	B4b1c1	O	1	B4b1c1	O	1
6900	DQ272119	B4b1c2	B4b1c2	O	1	B4b1c2	O	1
6901	KF540671	B4b1c2	B4b1c2	O	1	B4b1c2	O	1
6902	AY255135	B4d1	B4d1	O	1	B4d1	O	1
6903	GU392048	B4d1	B4d1	O	1	B4d1	O	1
6904	NA18117	B4d1a	B4d1a	O	1	B4d1a	O	1
6905	JN857029	B4d1a	B4d1a	O	1	B4d1a	O	1
6906	AY255140	B4d2	B4d2	O	1	B4d2	O	1
6907	NA18619	B4d3	B4d3	O	1	B4d3	O	1
6908	JF824975	B4d3a	B4d3a	O	1	B4d3a	O	1
6909	AP010836	B4d3a1	B4d3a1	O	1	B4d3a1	O	1
6910	AP013142	B4d3a1	B4d3a1	O	1	B4d3a1	O	1
6911	AP008492	B4d4	B4d4	O	1	B4d4	O	1
6912	AP013230	B4d4	B4d4	O	1	B4d4	O	1
6913	KC733253	B4e	B4e	O	1	B4e	O	1
6914	AP008436	B4e	B4e	O	1	B4e	O	1
6915	JN857017	B4j	B4j	O	1	B4j	O	1
6916	KC521454	B4c1a	B4c1a	O	1	B4c1a	O	1

6917	NA18982	B4c1a1	B4c1a1	O	1	B4c1a1	O	1
6918	AP008603	B4c1a1	B4c1a1	O	1	B4c1a1	O	1
6919	AP008482	B4c1a1a	B4c1a1a	O	1	B4c1a1a	O	1
6920	AP008540	B4c1a1a	B4c1a1a	O	1	B4c1a1a	O	1
6921	AP008298	B4c1a1a1	B4c1a1a1	O	1	B4c1a1a1	O	1
6922	AP011003	B4c1a1a1a	B4c1a1a1a	O	1	B4c1a1a1a	O	1
6923	AP008604	B4c1a1a1a	B4c1a1a1a	O	1	B4c1a1a1a	O	1
6924	AP008688	B4c1a1a2	B4c1a1a2	O	1	B4c1a1a2	O	1
6925	AP008281	B4c1a1b	B4c1a1b	O	1	B4c1a1b	O	1
6926	AP010696	B4c1a1b	B4c1a1b	O	1	B4c1a1b	O	1
6927	AP010704	B4c1a1c	B4c1a1c	O	1	B4c1a1c	O	1
6928	NA19088	B4c1a1c	B4c1a1c	O	1	B4c1a1c	O	1
6929	JN857019	B4c1a2	B4c1a2	O	1	B4c1a2	O	1
6930	JN857031	B4c1a2a	B4c1a2a	O	1	B4c1a2a	O	1
6931	JN857046	B4c1a2a	B4c1a2a	O	1	B4c1a2a	O	1
6932	AP008570	B4c1b	B4c1b	O	1	B4c1b	O	1
6933	AP008899	B4c1b1	B4c1b1	O	1	B4c1b1	O	1
6934	AP008920	B4c1b1a	B4c1b1a	O	1	B4c1b1a	O	1
6935	AP008393	B4c1b1a	B4c1b1a	O	1	B4c1b1a	O	1
6936	AP008472	B4c1b+16335	B4c1b+16335	O	1	B4c1b+16335	O	1
6937	AY255149	B4c1b2a	B4c1b2a	O	1	B4c1b2a	O	1
6938	GQ161177	B4c1b2a1	B4c1b2a1	O	1	B4c1b2a1	O	1
6939	EF429139	B4c1b2a1	B4c1b2a1	O	1	B4c1b2a1	O	1
6940	HM596646	B4c1b2a2	B4c1b2a2	O	1	B4c1b2a2	O	1
6941	KC994085	B4c1b2a2a	B4c1b2a2a	O	1	B4c1b2a2a	O	1
6942	HM238214	B4c1b2a2a	B4c1b2a2a	O	1	B4c1b2a2a	O	1
6943	KC994083	B4c1b2a2b	B4c1b2a2b	O	1	B4c1b2a2b	O	1
6944	HM238200	B4c1b2a2b	B4c1b2a2b	O	1	B4c1b2a2b	O	1
6945	NA18156	B4c1b2b	B4c1b2b	O	1	B4c1b2b	O	1
6946	KF540737	B4c1b2b	B4c1b2b	O	1	B4c1b2b	O	1
6947	NA18111	B4c1b2c	B4c1b2c	O	1	B4c1b2c	O	1
6948	NA18778	B4c1b2c1	B4c1b2c1	O	1	B4c1b2c1	O	1
6949	AP013284	B4c1b2c1	B4c1b2c1	O	1	B4c1b2c1	O	1
6950	KF540661	B4c1b2c2	B4c1b2c2	O	1	B4c1b2c2	O	1
6951	KF849975	B4c1b2c2	B4c1b2c2	O	1	B4c1b2c2	O	1
6952	AP009461	B4c1c	B4c1c	O	1	B4c1c	O	1
6953	AP009436	B4c1c+16311	B4c1c+16311	O	1	B4c1c+16311	O	1

6954	AP008425	B4c1c1	B4c1c1	O	1	B4c1c1	O	1
6955	AP008450	B4c1c1	B4c1c1	O	1	B4c1c1	O	1
6956	GU810059	B4c2	B4c2	O	1	B4c2	O	1
6957	AY289101	B4c2	B4c2	O	1	B4c2	O	1
6958	AF347011	B4c2a	B4c2a	O	1	B4c2a	O	1
6959	AY289100	B4c2a	B4c2a	O	1	B4c2a	O	1
6960	GU592219	B4c2b	B4c2b	O	1	B4c2b	O	1
6961	AP012407	B4c2b	B4c2b	O	1	B4c2b	O	1
6962	GU592216	B4c2c	B4c2c	O	1	B4c2c	O	1
6963	GU592217	B4c2c	B4c2c	O	1	B4c2c	O	1
6964	JQ703514	B4f	B4f	O	1	B4f	O	1
6965	AP008788	B4f	B4f	O	1	B4f	O	1
6966	AP013140	B4f1	B4f1	O	1	B4f1	O	1
6967	AP008262	B4f1	B4f1	O	1	B4f1	O	1
6968	EU597566	B5a1a	B5a1a	O	1	B5a1a	O	1
6969	GU810057	B5a1a	B5a1a	O	1	B5a1a	O	1
6970	AY950286	B5a1a1	B5a1a1	O	1	B5a1a1	O	1
6971	AY950290	B5a1a1	B5a1a1	O	1	B5a1a1	O	1
6972	AY255145	B5a1b	B5a1b	O	1	B5a1b	O	1
6973	KC505067	B5a1b1	B5a1b1	O	1	B5a1b1	O	1
6974	GQ119031	B5a1b1	B5a1b1	O	1	B5a1b1	O	1
6975	KF540745	B5a1c	B5a1c	O	1	B5a1c	O	1
6976	KF540703	B5a1c1	B5a1c1	O	1	B5a1c1	O	1
6977	JQ731598	B5a1c1a	B5a1c1a	O	1	B5a1c1a	O	1
6978	JQ731601	B5a1c1a	B5a1c1a	O	1	B5a1c1a	O	1
6979	JF896801	B5a1c1a1	B5a1c1a1	O	1	B5a1c1a1	O	1
6980	HG00418	B5a1c1a1	B5a1c1a1	O	1	B5a1c1a1	O	1
6981	KF849949	B5a1c2	B5a1c2	O	1	B5a1c2	O	1
6982	JQ731600	B5a1c2	B5a1c2	O	1	B5a1c2	O	1
6983	GU810075	B5a1d	B5a1d	O	1	B5a1d	O	1
6984	HM596698	B5a1d	B5a1d	O	1	B5a1d	O	1
6985	HG00543	B5a2	R30	X	2	B5a2	O	1
6986	EF114286	B5a2a1a	B5a2a1a	O	1	B5a2a1a	O	1
6987	KF849923	B5a2a1a	B5a2a1a	O	1	B5a2a1a	O	1
6988	JQ731602	B5a2a1+16129	B5a2a1+16129	O	1	B5a2a1+16129	O	1
6989	AP008263	B5a2a1b	B5a2a1b	O	1	B5a2a1b	O	1
6990	AP008451	B5a2a1b	B5a2a1b	O	1	B5a2a1b	O	1

6991	DQ372869	B5a2a2a1	B5a2a2a1	O	1	B5a2a2a1	O	1
6992	KF540936	B5a2a2a1	B5a2a2a1	O	1	B5a2a2a1	O	1
6993	KF540943	B5a2a2a2	B5a2a2a2	O	1	B5a2a2a2	O	1
6994	KF540901	B5a2a2a2	B5a2a2a2	O	1	B5a2a2a2	O	1
6995	KC994087	B5a2a2b1	B5a2a2b1	O	1	B5a2a2b1	O	1
6996	KF540623	B5a2a2b1a	B5a2a2b1a	O	1	B5a2a2b1a	O	1
6997	KF540613	B5a2a2b1a	B5a2a2b1a	O	1	B5a2a2b1a	O	1
6998	KF540807	B5a2a2b2	B5a2a2b2	O	1	B5a2a2b2	O	1
6999	KF540825	B5a2a2b2	B5a2a2b2	O	1	B5a2a2b2	O	1
7000	AP010771	B5b1	B5b1	O	1	B5b1	O	1
7001	AP008284	B5b1	B5b1	O	1	B5b1	O	1
7002	JF824930	B5b1a	B5b1a	O	1	B5b1a	O	1
7003	AP008827	B5b1a1	B5b1a1	O	1	B5b1a1	O	1
7004	AP008631	B5b1a1	B5b1a1	O	1	B5b1a1	O	1
7005	AP008273	B5b1a2	B5b1a2	O	1	B5b1a2	O	1
7006	AP008288	B5b1a2	B5b1a2	O	1	B5b1a2	O	1
7007	AP008518	B5b1a2a	B5b1a2a	O	1	B5b1a2a	O	1
7008	AP008875	B5b1a2a	B5b1a2a	O	1	B5b1a2a	O	1
7009	KC994070	B5b1c	B5b1c	O	1	B5b1c	O	1
7010	GQ119020	B5b1c	B5b1c	O	1	B5b1c	O	1
7011	KC994143	B5b1c1	B5b1c1	O	1	B5b1c1	O	1
7012	KC994058	B5b1c1a	B5b1c1a	O	1	B5b1c1a	O	1
7013	KC994128	B5b1c1a	B5b1c1a	O	1	B5b1c1a	O	1
7014	AY519489	B5b2a	B5b2a	O	1	B5b2a	O	1
7015	AP008546	B5b2a1	B5b2a1	O	1	B5b2a1	O	1
7016	AP008847	B5b2a1	B5b2a1	O	1	B5b2a1	O	1
7017	AP009425	B5b2a2	B5b2a2	O	1	B5b2a2	O	1
7018	AP008371	B5b2a2	B5b2a2	O	1	B5b2a2	O	1
7019	AP008910	B5b2a2a1	B5b2a2a1	O	1	B5b2a2a1	O	1
7020	AP008403	B5b2a2a1	B5b2a2a1	O	1	B5b2a2a1	O	1
7021	KJ154763	B5b2a2a2	B5b2a2a2	O	1	B5b2a2a2	O	1
7022	GQ119041	B5b2a2a2	B5b2a2a2	O	1	B5b2a2a2	O	1
7023	KF148463	B5b2b	B5b2b	O	1	B5b2b	O	1
7024	KF148089	B5b2b	B5b2b	O	1	B5b2b	O	1
7025	GU377081	B5b2+@204	B5b2+@204	O	1	B5b2+@204	O	1
7026	KF540720	B5b2c	B5b2c	O	1	B5b2c	O	1
7027	AP008465	B5b2c1	B5b2c1	O	1	B5b2c1	O	1

7028	HG00404	B5b2c1	B5b2c1	O	1	B5b2c1	O	1
7029	AP013282	B5b3a	B5b3a	O	1	B5b3a	O	1
7030	AP008566	B5b3a	B5b3a	O	1	B5b3a	O	1
7031	AP010761	B5b3b	B5b3b	O	1	B5b3b	O	1
7032	AP013129	B5b3b	B5b3b	O	1	B5b3b	O	1
7033	KF849981	B5b4	B5b4	O	1	B5b4	O	1
7034	JN857015	B5b4	B5b4	O	1	B5b4	O	1
7035	KF540694	B5b5	B5b5	O	1	B5b5	O	1
7036	NA18704	B5b5	B5b5	O	1	B5b5	O	1
7037	GU733726	R24	R24	O	1	R24	O	1
7038	GQ119037	R24a	R24a	O	1	R24a	O	1
7039	GQ119033	R24a	R24a	O	1	R24a	O	1
7040	AP012392	R21	R21	O	1	R21	O	1
7041	AP012370	R21	R21	O	1	R21	O	1
7042	EF495216	R14	R14	O	1	R14	O	1
7043	GQ301886	R22	R22	O	1	R22	O	1
7044	GQ301863	R22	R22	O	1	R22	O	1
7045	GQ301864	R23	R23	O	1	R23	O	1
7046	AY714006	R30	R30	O	1	R30	O	1
7047	JX462702	R30a	R30a	O	1	R30a	O	1
7048	AY714001	R30a1a	R30a1a	O	1	R30a1a	O	1
7049	FJ770961	R30a1a	R30a1a	O	1	R30a1a	O	1
7050	AY714032	R30a1b	R30a1b	O	1	R30a1b	O	1
7051	GU170818	R30a1b1	R30a1b1	O	1	R30a1b1	O	1
7052	JX462715	R30a1b1	R30a1b1	O	1	R30a1b1	O	1
7053	FJ004824	R30a1c	R30a1c	O	1	R30a1c	O	1
7054	KC911324	R30a1c	R30a1c	O	1	R30a1c	O	1
7055	FJ004827	R30b1	R30b1	O	1	R30b1	O	1
7056	AY714047	R30b1	R30b1	O	1	R30b1	O	1
7057	FJ004807	R30b2	R30b2	O	1	R30b2	O	1
7058	EF556148	R30b2a	R30b2a	O	1	R30b2a	O	1
7059	JX462737	R30b2a	R30b2a	O	1	R30b2a	O	1
7060	AY714021	R31a	R31a	O	1	R31a	O	1
7061	JQ705959	R31a	R31a	O	1	R31a	O	1
7062	FJ004825	R31a1	R31a1	O	1	R31a1	O	1
7063	FJ004826	R31a1	R31a1	O	1	R31a1	O	1
7064	AY714046	R31b	R31b	O	1	R31b	O	1

7065	AY714048	R31b	R31b	O	1	R31b	O	1
7066	KC577355	R32	R32	O	1	R32	O	1
7067	AY289087	P1d1	P1d1	O	1	P1d1	O	1
7068	KJ154317	P1d1a	P1d1a	O	1	P1d1a	O	1
7069	EU597507	P1d1a	P1d1a	O	1	P1d1a	O	1
7070	KJ154684	P1d2	P1d2	O	1	P1d2	O	1
7071	KJ154767	P1d2a	P1d2a	O	1	P1d2a	O	1
7072	KJ154172	P1d2a	P1d2a	O	1	P1d2a	O	1
7073	KJ154805	P1+152	P1+152	O	1	P1+152	O	1
7074	KJ154904	P1f	P1f	O	1	P1f	O	1
7075	KJ154181	P1f	P1f	O	1	P1f	O	1
7076	AY289088	P2	P2	O	1	P2	O	1
7077	KC993994	P10	P10	O	1	P10	O	1
7078	GQ119036	P10	P10	O	1	P10	O	1
7079	DQ404446	P8	P8	O	1	P8	O	1
7080	AY289052	P3a	P3a	O	1	P3a	O	1
7081	AY289065	P3a	P3a	O	1	P3a	O	1
7082	AY289091	P3b	P3b	O	1	P3b	O	1
7083	EF061153	P3b1	P3b1	O	1	P3b1	O	1
7084	EF061154	P3b1	P3b1	O	1	P3b1	O	1
7085	EF061158	P4a	P4a	O	1	P4a	O	1
7086	EF061159	P4a	P4a	O	1	P4a	O	1
7087	EF495221	P4a1	P4a1	O	1	P4a1	O	1
7088	EF061156	P4a1	P4a1	O	1	P4a1	O	1
7089	AY289064	P4b	P4b	O	1	P4b	O	1
7090	DQ404444	P4b1	P4b1	O	1	P4b1	O	1
7091	AY289057	P4b1	P4b1	O	1	P4b1	O	1
7092	AY289063	P5	P5	O	1	P5	O	1
7093	AY289053	P6	P6	O	1	P6	O	1
7094	AY289055	P6	P6	O	1	P6	O	1
7095	AY289054	P7	P7	O	1	P7	O	1
7096	KC993944	P9	P9	O	1	P9	O	1
7097	GQ119026	P9a	P9a	O	1	P9a	O	1
7098	KC993934	P9a	P9a	O	1	P9a	O	1
7099	HM852790	U1a1	U1a1	O	1	U1a1	O	1
7100	GU218692	U1a1a	U1a1a	O	1	U1a1a	O	1
7101	HQ615882	U1a1a1	U1a1a1	O	1	U1a1a1	O	1

7102	KC477757	U1a1a1a	U1a1a1a	O	1	U1a1a1a	O	1
7103	KC911328	U1a1a1a	U1a1a1a	O	1	U1a1a1a	O	1
7104	JQ703793	U1a1a2	U1a1a2	O	1	U1a1a2	O	1
7105	JX289842	U1a1a2	U1a1a2	O	1	U1a1a2	O	1
7106	AY882396	U1a1a+16129	U1a1a+16129	O	1	U1a1a+16129	O	1
7107	JQ705601	U1a1a3	U1a1a3	O	1	U1a1a3	O	1
7108	KC911502	U1a1a3	U1a1a3	O	1	U1a1a3	O	1
7109	JQ704153	U1a1b	U1a1b	O	1	U1a1b	O	1
7110	EF556157	U1a1b	U1a1b	O	1	U1a1b	O	1
7111	JX153116	U1a1c	U1a1c	O	1	U1a1c	O	1
7112	FJ748753	U1a1c1a	U1a1c1a	O	1	U1a1c1a	O	1
7113	AY714038	U1a1c1a	U1a1c1a	O	1	U1a1c1a	O	1
7114	JQ704034	U1a1c1b	U1a1c1b	O	1	U1a1c1b	O	1
7115	NA20826	U1a1c1b	U1a1c1b	O	1	U1a1c1b	O	1
7116	KC911365	U1a1c1c	U1a1c1c	O	1	U1a1c1c	O	1
7117	DQ523621	U1a1c1c1	U1a1c1c1	O	1	U1a1c1c1	O	1
7118	DQ523634	U1a1c1c1	U1a1c1c1	O	1	U1a1c1c1	O	1
7119	KC911457	U1a1c1d	U1a1c1d	O	1	U1a1c1d	O	1
7120	KC911527	U1a1c1d1	U1a1c1d1	O	1	U1a1c1d1	O	1
7121	KC911306	U1a1c1d1	U1a1c1d1	O	1	U1a1c1d1	O	1
7122	EF692533	U1a1d	U1a1d	O	1	U1a1d	O	1
7123	HM241417	U1a1d	U1a1d	O	1	U1a1d	O	1
7124	EF556194	U1a2	U1a2	O	1	U1a2	O	1
7125	HM852789	U1a2	U1a2	O	1	U1a2	O	1
7126	KC540656	U1a3	U1a3	O	1	U1a3	O	1
7127	HM852844	U1a3	U1a3	O	1	U1a3	O	1
7128	JQ705704	U1b1	U1b1	O	1	U1b1	O	1
7129	HQ325737	U1b1	U1b1	O	1	U1b1	O	1
7130	KC911628	U1b2	U1b2	O	1	U1b2	O	1
7131	GU123007	U1b2	U1b2	O	1	U1b2	O	1
7132	AY882397	U1b3	U1b3	O	1	U1b3	O	1
7133	JQ705292	U1b3	U1b3	O	1	U1b3	O	1
7134	KC521458	U5	U5	O	1	U5	O	1
7135	GU296570	U5a1	U5a1	O	1	U5a1	O	1
7136	FJ348174	U5a1	U5a1	O	1	U5a1	O	1
7137	EU523128	U5a1a1	U5a1a1	O	1	U5a1a1	O	1
7138	DQ785296	U5a1a1	U5a1a1	O	1	U5a1a1	O	1

7139	GU296601	U5a1a1+152	U5a1a1+152	O	1	U5a1a1+152	O	1
7140	GU296636	U5a1a1+152	U5a1a1+152	O	1	U5a1a1+152	O	1
7141	DQ904330	U5a1a1a	U5a1a1a	O	1	U5a1a1a	O	1
7142	GU296558	U5a1a1a	U5a1a1a	O	1	U5a1a1a	O	1
7143	JQ703937	U5a1a1b	U5a1a1b	O	1	U5a1a1b	O	1
7144	GU296652	U5a1a1b	U5a1a1b	O	1	U5a1a1b	O	1
7145	GQ368895	U5a1a1h	U5a1a1h	O	1	U5a1a1h	O	1
7146	JX153217	U5a1a1h	U5a1a1h	O	1	U5a1a1h	O	1
7147	JQ703590	U5a1a1c	U5a1a1c	O	1	U5a1a1c	O	1
7148	JQ704022	U5a1a1c	U5a1a1c	O	1	U5a1a1c	O	1
7149	JQ704920	U5a1a1+16362	U5a1a1+16362	O	1	U5a1a1+16362	O	1
7150	GU459066	U5a1a1d	U5a1a1d	O	1	U5a1a1d	O	1
7151	GQ160809	U5a1a1d	U5a1a1d	O	1	U5a1a1d	O	1
7152	JQ701866	U5a1a1d1	U5a1a1d1	O	1	U5a1a1d1	O	1
7153	KC257387	U5a1a1d1	U5a1a1d1	O	1	U5a1a1d1	O	1
7154	JQ705621	U5a1a1e	U5a1a1e	O	1	U5a1a1e	O	1
7155	JQ704796	U5a1a1e	U5a1a1e	O	1	U5a1a1e	O	1
7156	JQ705243	U5a1a1g	U5a1a1g	O	1	U5a1a1g	O	1
7157	HG00145	U5a1a1g	U5a1a1g	O	1	U5a1a1+16362	X	2
7158	JQ582984	U5a1a1i	U5a1a1i	O	1	U5a1a1i	O	1
7159	JX153282	U5a1a1i	U5a1a1i	O	1	U5a1a1i	O	1
7160	GU296557	U5a1a2a	U5a1a2a	O	1	U5a1a2a	O	1
7161	HQ588904	U5a1a2a	U5a1a2a	O	1	U5a1a2a	O	1
7162	JQ703926	U5a1a2a1	U5a1a2a1	O	1	U5a1a2a1	O	1
7163	JX153678	U5a1a2a1	U5a1a2a1	O	1	U5a1a2a1	O	1
7164	JQ702568	U5a1a2a1a	U5a1a2a1a	O	1	U5a1a2a1a	O	1
7165	KC602499	U5a1a2a1a	U5a1a2a1a	O	1	U5a1a2a1a	O	1
7166	JQ704701	U5a1a2b	U5a1a2b	O	1	U5a1a2b	O	1
7167	GU296543	U5a1a2b	U5a1a2b	O	1	U5a1a2b	O	1
7168	JQ705279	U5a1a2b1	U5a1a2b1	O	1	U5a1a2b1	O	1
7169	JQ702496	U5a1a2b1	U5a1a2b1	O	1	U5a1a2b1	O	1
7170	GU296640	U5a1g	U5a1g	O	1	U5a1g	O	1
7171	HM765468	U5a1g	U5a1g	O	1	U5a1g	O	1
7172	JQ702913	U5a1g1	U5a1g1	O	1	U5a1g1	O	1
7173	JN412063	U5a1g1	U5a1g1	O	1	U5a1g1	O	1
7174	KC911409	U5a1g2	U5a1g2	O	1	U5a1g2	O	1
7175	KC911325	U5a1g2	U5a1g2	O	1	U5a1g2	O	1

7176	JX141361	U5a1b	U5a1b	O	1	U5a1b	O	1
7177	JX153668	U5a1b	U5a1b	O	1	U5a1b	O	1
7178	JQ702775	U5a1b1	U5a1b1	O	1	U5a1b1	O	1
7179	JN982470	U5a1b1	U5a1b1	O	1	U5a1b1	O	1
7180	JQ703963	U5a1b1a	U5a1b1a	O	1	U5a1b1a	O	1
7181	JQ691414	U5a1b1a	U5a1b1a	O	1	U5a1b1a	O	1
7182	JQ705245	U5a1b1a1	U5a1b1a1	O	1	U5a1b1a1	O	1
7183	JQ706015	U5a1b1a1	U5a1b1a1	O	1	U5a1b1a1	O	1
7184	NA12814	U5a1b1a2	U5a1b1a2	O	1	U5a1b1a2	O	1
7185	JQ703074	U5a1b1a2	U5a1b1a2	O	1	U5a1b1a2	O	1
7186	GU296614	U5a1b1b	U5a1b1b	O	1	U5a1b1b	O	1
7187	GU296619	U5a1b1b1	U5a1b1b1	O	1	U5a1b1b1	O	1
7188	GU296649	U5a1b1b1	U5a1b1b1	O	1	U5a1b1b1	O	1
7189	EU140330	U5a1b1c	U5a1b1c	O	1	U5a1b1c	O	1
7190	GU296610	U5a1b1c	U5a1b1c	O	1	U5a1b1c	O	1
7191	JQ705297	U5a1b1c1	U5a1b1c1	O	1	U5a1b1c1	O	1
7192	FJ493508	U5a1b1c1	U5a1b1c1	O	1	U5a1b1c1	O	1
7193	JQ704028	U5a1b1c2	U5a1b1c2	O	1	U5a1b1c2	O	1
7194	GU296628	U5a1b1c2	U5a1b1c2	O	1	U5a1b1c2	O	1
7195	JQ702400	U5a1b1d	U5a1b1d	O	1	U5a1b1d	O	1
7196	JX152788	U5a1b1d+16093	U5a1b1d+16093	O	1	U5a1b1d+16093	O	1
7197	JQ705693	U5a1b1d1	U5a1b1d1	O	1	U5a1b1d1	O	1
7198	JX153853	U5a1b1d1	U5a1b1d1	O	1	U5a1b1d1	O	1
7199	JX153770	U5a1b1e	U5a1b1e	O	1	U5a1b1e	O	1
7200	JQ704679	U5a1b1e	U5a1b1e	O	1	U5a1b1e	O	1
7201	AY714003	U5a1b1f	U5a1b1f	O	1	U5a1b1f	O	1
7202	JQ702552	U5a1b1f	U5a1b1f	O	1	U5a1b1f	O	1
7203	JN809915	U5a1b1g	U5a1b1g	O	1	U5a1b1g	O	1
7204	HG00104	U5a1b1g	U5a1b1g	O	1	U5a1b1g	O	1
7205	JX153227	U5a1b1h	U5a1b1h	O	1	U5a1b1h	O	1
7206	JX153196	U5a1b1h	U5a1b1h	O	1	U5a1b1h	O	1
7207	GU296562	U5a1b2	U5a1b2	O	1	U5a1b2	O	1
7208	GU296569	U5a1b2	U5a1b2	O	1	U5a1b2	O	1
7209	JQ704045	U5a1b+16362	U5a1b+16362	O	1	U5a1b+16362	O	1
7210	JQ703331	U5a1b3	U5a1b3	O	1	U5a1b3	O	1
7211	GU296592	U5a1b3	U5a1b3	O	1	U5a1b3	O	1
7212	JQ705935	U5a1b3a	U5a1b+16362	X	3	U5a1b+16362	X	2

7213	JQ705101	U5a1b3a1	U5a1b3a1	O	1	U5a1b3a1	O	1
7214	JX153622	U5a1b3a1	U5a1b3a1	O	1	U5a1b3a1	O	1
7215	HM171295	U5a1b4	U5a1b4	O	1	U5a1b4	O	1
7216	KC257373	U5a1b4	U5a1b4	O	1	U5a1b4	O	1
7217	GU296588	U5a1c1	U5a1c1	O	1	U5a1c1	O	1
7218	GU296617	U5a1c1	U5a1c1	O	1	U5a1c1	O	1
7219	JX297176	U5a1c1a	U5a1c1a	O	1	U5a1c1a	O	1
7220	JQ705573	U5a1c1a	U5a1c1a	O	1	U5a1c1a	O	1
7221	GU296589	U5a1c2	U5a1c2	O	1	U5a1c2	O	1
7222	JQ704555	U5a1c2a	U5a1c2a	O	1	U5a1c2a	O	1
7223	JQ705908	U5a1c2a1	U5a1c2a1	O	1	U5a1c2a1	O	1
7224	JX153340	U5a1c2a1	U5a1c2a1	O	1	U5a1c2a1	O	1
7225	GU296542	U5a1d1	U5a1d1	O	1	U5a1d1	O	1
7226	GU296612	U5a1d1	U5a1d1	O	1	U5a1d1	O	1
7227	HM173090	U5a1d2a	U5a1d2a	O	1	U5a1d2a	O	1
7228	JQ704700	U5a1d2a	U5a1d2a	O	1	U5a1d2a	O	1
7229	GU296655	U5a1d2a1	U5a1d2a1	O	1	U5a1d2a1	O	1
7230	GU296599	U5a1d2a1	U5a1d2a1	O	1	U5a1d2a1	O	1
7231	GU123032	U5a1d2b	U5a1d2b	O	1	U5a1d2b	O	1
7232	JQ705737	U5a1d2b	U5a1d2b	O	1	U5a1d2b	O	1
7233	GU296547	U5a1e	U5a1e	O	1	U5a1e	O	1
7234	GU296625	U5a1e	U5a1e	O	1	U5a1e	O	1
7235	JQ702160	U5a1f1	U5a1f1	O	1	U5a1f1	O	1
7236	AY882398	U5a1f1a	U5a1f1a	O	1	U5a1f1a	O	1
7237	JQ704962	U5a1f1a	U5a1f1a	O	1	U5a1f1a	O	1
7238	JQ702735	U5a1f1a1	U5a1f1a1	O	1	U5a1f1a1	O	1
7239	JQ702225	U5a1f1a1	U5a1f1a1	O	1	U5a1f1a1	O	1
7240	GU296603	U5a1f2	U5a1f2	O	1	U5a1f2	O	1
7241	KF921965	U5a1f2	U5a1f2	O	1	U5a1f2	O	1
7242	JQ704026	U5a1h	U5a1h	O	1	U5a1h	O	1
7243	JQ705910	U5a1h	U5a1h	O	1	U5a1h	O	1
7244	KC257306	U5a1i	U5a1i	O	1	U5a1i	O	1
7245	JQ705326	U5a1i1	U5a1i1	O	1	U5a1i1	O	1
7246	KC569552	U5a1i1	U5a1i1	O	1	U5a1i1	O	1
7247	KF647698	U5a1j	U5a1j	O	1	U5a1j	O	1
7248	KF262460	U5a1j	U5a1j	O	1	U5a1j	O	1
7249	JX153018	U5a2+16294	U5a2+16294	O	1	U5a2+16294	O	1

7250	JQ702310	U5a2+16294	U5a2+16294	O	1	U5a2+16294	O	1
7251	JN707685	U5a2a	U5a2a	O	1	U5a2a	O	1
7252	GU296615	U5a2a1	U5a2a1	O	1	U5a2a1	O	1
7253	JQ703936	U5a2a1	U5a2a1	O	1	U5a2a1	O	1
7254	AY339524	U5a2a1a	U5a2a1a	O	1	U5a2a1a	O	1
7255	JX153215	U5a2a1a	U5a2a1a	O	1	U5a2a1a	O	1
7256	GU296626	U5a2a1b	U5a2a1b	O	1	U5a2a1b	O	1
7257	HM246245	U5a2a1b	U5a2a1b	O	1	U5a2a1b	O	1
7258	JQ702355	U5a2a1b1	U5a2a1b1	O	1	U5a2a1b1	O	1
7259	HM765474	U5a2a1b1	U5a2a1b1	O	1	U5a2a1b1	O	1
7260	GU296597	U5a2a1c	U5a2a1c	O	1	U5a2a1c	O	1
7261	GU296650	U5a2a1c	U5a2a1c	O	1	U5a2a1c	O	1
7262	EU124886	U5a2a1d	U5a2a1d	O	1	U5a2a1d	O	1
7263	JQ703988	U5a2a1d	U5a2a1d	O	1	U5a2a1d	O	1
7264	JQ704074	U5a2a1+152	U5a2a1+152	O	1	U5a2a1+152	O	1
7265	JQ703335	U5a2a1+152	U5a2a1+152	O	1	U5a2a1+152	O	1
7266	AY339527	U5a2a1e	U5a2a1e	O	1	U5a2a1e	O	1
7267	JX171113	U5a2a1e	U5a2a1e	O	1	U5a2a1e	O	1
7268	JQ705530	U5a2a2	U5a2a2	O	1	U5a2a2	O	1
7269	JX153225	U5a2a2a	U5a2a2a	O	1	U5a2a2a	O	1
7270	JX153731	U5a2a2a	U5a2a2a	O	1	U5a2a2a	O	1
7271	GU296559	U5a2b	U5a2b	O	1	U5a2b	O	1
7272	GU371930	U5a2b	U5a2b	O	1	U5a2b	O	1
7273	GU296624	U5a2b1	U5a2b	X	4	U5a2b1	O	1
7274	JQ702746	U5a2b1	U5a2b	X	4	U5a2b1	O	1
7275	JQ705696	U5a2b1a	U5a2b1a	O	1	U5a2b1a	O	1
7276	GU296651	U5a2b1a	U5a2b	X	2	U5a2b1a	O	1
7277	JQ704044	U5a2b1b	U5a2b	X	2	U5a2b1b	O	1
7278	GU296607	U5a2b1b	U5a2b	X	2	U5a2b1b	O	1
7279	GU296629	U5a2b1c	U5a2b1c	O	1	U5a2b1c	O	1
7280	GU296600	U5a2b1c	U5a2b1c	O	1	U5a2b1c	O	1
7281	JQ703087	U5a2b1d	U5a2b1d	O	1	U5a2b1d	O	1
7282	GU296656	U5a2b1d	U5a2b1d	O	1	U5a2b1d	O	1
7283	KC246057	U5a2b2	U5a2b2	O	1	U5a2b2	O	1
7284	JQ702184	U5a2b2a	U5a2b2a	O	1	U5a2b2a	O	1
7285	GU296587	U5a2b2a1	U5a2b2a	X	3	U5a2b2a1	O	1
7286	GU296646	U5a2b2a1	U5a2b2a	X	3	U5a2b2a1	O	1

7287	KC661077	U5a2b3	U5a2b3	O	1	U5a2b3	O	1
7288	JQ702847	U5a2b3a	U5a2b3a	O	1	U5a2b3a	O	1
7289	JX153580	U5a2b3a	U5a2b3a	O	1	U5a2b3a	O	1
7290	JQ702230	U5a2b3a1	U5a2b3a1	O	1	U5a2b3a1	O	1
7291	JN899603	U5a2b3a1	U5a2b3a1	O	1	U5a2b3a1	O	1
7292	JQ702320	U5a2b4	U5a2b4	O	1	U5a2b4	O	1
7293	KC146708	U5a2b4a	U5a2b4a	O	1	U5a2b4a	O	1
7294	JX153863	U5a2b4a	U5a2b4a	O	1	U5a2b4a	O	1
7295	NA20760	U5a2b5	U5a2b5	O	1	U5a2b5	O	1
7296	EU597544	U5a2b5	U5a2b5	O	1	U5a2b5	O	1
7297	JQ703187	U5a2c	U5a2c	O	1	U5a2c	O	1
7298	GU012633	U5a2c	U5a2c	O	1	U5a2c	O	1
7299	FJ460558	U5a2c1	U5a2c1	O	1	U5a2c1	O	1
7300	EU151864	U5a2c1	U5a2c1	O	1	U5a2c1	O	1
7301	JQ704067	U5a2c2	U5a2c2	O	1	U5a2c2	O	1
7302	EF660950	U5a2c2	U5a2c2	O	1	U5a2c2	O	1
7303	JX101637	U5a2c3	U5a2c3	O	1	U5a2c3	O	1
7304	JF487827	U5a2c3a	U5a2c3a	O	1	U5a2c3a	O	1
7305	EU049814	U5a2c3a	U5a2c3a	O	1	U5a2c3a	O	1
7306	JQ705779	U5a2c4	U5a2c4	O	1	U5a2c4	O	1
7307	GU296611	U5a2c4	U5a2c4	O	1	U5a2c4	O	1
7308	JQ703280	U5a2d	U5a2d	O	1	U5a2+16294	X	2
7309	JQ702144	U5a2d	U5a2d	O	1	U5a2d	O	1
7310	HM490393	U5a2d1	U5a2d1	O	1	U5a2d1	O	1
7311	JN604831	U5a2d1a	U5a2d1a	O	1	U5a2d1a	O	1
7312	JX153171	U5a2d1a	U5a2d1a	O	1	U5a2d1a	O	1
7313	JQ705111	U5a2+16362	U5a2+16362	O	1	U5a2+16362	O	1
7314	JN819535	U5a2e	U5a2e	O	1	U5a2e	O	1
7315	GU296648	U5a2e	U5a2e	O	1	U5a2e	O	1
7316	JQ704112	U5b1	U5b1	O	1	U5b1	O	1
7317	KC521455	U5b1a	U5b1a	O	1	U5b1a	O	1
7318	JN544933	U5b1a	U5b1a	O	1	U5b1a	O	1
7319	EF420876	U5b1+16189	U5b1+16189	O	1	U5b1+16189	O	1
7320	GU296644	U5b1b	U5b1b	O	1	U5b1+16189+@16192	X	2
7321	GU296566	U5b1b1	U5b1b1	O	1	U5b1b1	O	1
7322	JN897374	U5b1b1+@16192	U5b1b1+@16192	O	1	U5b1b1+@16192	O	1
7323	AY882400	U5b1b1+@16192	U5b1b1+@16192	O	1	U5b1b1+@16192	O	1

7324	AY882403	U5b1b1a	U5b1b1a	O	1	U5b1b1a	O	1
7325	JQ702837	U5b1b1a	U5b1b1a	O	1	U5b1b1a	O	1
7326	DQ902696	U5b1b1a1	U5b1b1a1	O	1	U5b1b1a1	O	1
7327	AY882404	U5b1b1a1	U5b1b1a1	O	1	U5b1b1a1	O	1
7328	HM116534	U5b1b1a1a	U5b1b1a1a	O	1	U5b1b1a1a	O	1
7329	GU296598	U5b1b1a1a	U5b1b1a1a	O	1	U5b1b1a1a	O	1
7330	KF466256	U5b1b1a1a1	U5b1b1a1a1	O	1	U5b1b1a1a1	O	1
7331	KF631316	U5b1b1a1a1	U5b1b1a1a1	O	1	U5b1b1a1a1	O	1
7332	JQ703600	U5b1b1a1b	U5b1b1a1b	O	1	U5b1b1a1b	O	1
7333	JX153265	U5b1b1a1b	U5b1b1a1b	O	1	U5b1b1a1b	O	1
7334	EF420877	U5b1b1a2	U5b1b1a2	O	1	U5b1b1a2	O	1
7335	JX171119	U5b1b1a2	U5b1b1a2	O	1	U5b1b1a2	O	1
7336	JX153170	U5b1b1a3	U5b1b1a3	O	1	U5b1b1a3	O	1
7337	AY882406	U5b1b1a3	U5b1b1a3	O	1	U5b1b1a3	O	1
7338	AY882402	U5b1b1d	U5b1b1d	O	1	U5b1b1d	O	1
7339	AY882401	U5b1b1d	U5b1b1d	O	1	U5b1b1d	O	1
7340	GU296591	U5b1b1f	U5b1b1f	O	1	U5b1b1f	O	1
7341	JQ705418	U5b1b1f	U5b1b1f	O	1	U5b1b1f	O	1
7342	AY882407	U5b1b1b	U5b1b1b	O	1	U5b1b1b	O	1
7343	DQ282508	U5b1b1b	U5b1b1b	O	1	U5b1b1b	O	1
7344	AF381989	U5b1b1+152	U5b1b1+152	O	1	U5b1b1+152	O	1
7345	JQ704517	U5b1b1e	U5b1b1e	O	1	U5b1b1e	O	1
7346	AY882408	U5b1b1e	U5b1b1e	O	1	U5b1b1e	O	1
7347	KC479033	U5b1b1g	U5b1b1g	O	1	U5b1b1g	O	1
7348	KP688570	U5b1b1g1	U5b1b1g1	O	1	U5b1b1g1	O	1
7349	KP835772	U5b1b1g1a	U5b1b1g1a	O	1	U5b1b1g1a	O	1
7350	HM046248	U5b1b1g1a	U5b1b1g1a	O	1	U5b1b1g1a	O	1
7351	AY339536	U5b1b2	U5b1b2	O	1	U5b1b2	O	1
7352	JQ705105	U5b1b2	U5b1b2	O	1	U5b1b2	O	1
7353	HG00357	U5b1b2a	U5b1b2a	O	1	U5b1b2a	O	1
7354	HG00274	U5b1b2a	U5b1b2a	O	1	U5b1b2a	O	1
7355	JX152982	U5b1b2b	U5b1b2b	O	1	U5b1b2b	O	1
7356	JX153220	U5b1b2b	U5b1b2b	O	1	U5b1b2b	O	1
7357	AY882409	U5b1c	U5b1c	O	1	U5b1c	O	1
7358	JQ705870	U5b1c1	U5b1c1	O	1	U5b1c1	O	1
7359	JQ408439	U5b1c1a	U5b1c1a	O	1	U5b1c1a	O	1
7360	JQ705095	U5b1c1a1	U5b1c1a1	O	1	U5b1c1a1	O	1

7361	JX297153	U5b1c1a1	U5b1c1a1	O	1	U5b1c1a1	O	1
7362	JQ703983	U5b1c2	U5b1c2	O	1	U5b1c2	O	1
7363	DQ661681	U5b1c2	U5b1c2	O	1	U5b1c2	O	1
7364	EU597535	U5b1c2a	U5b1c2a	O	1	U5b1c2a	O	1
7365	JQ703944	U5b1c2a	U5b1c2a	O	1	U5b1c2a	O	1
7366	JQ704043	U5b1c2b	U5b1c2b	O	1	U5b1c2b	O	1
7367	JQ705473	U5b1c2b	U5b1c2b	O	1	U5b1c2b	O	1
7368	GU296582	U5b1+16189+@16192	U5b1+16189+@16192	O	1	U5b1+16189+@16192	O	1
7369	JQ702743	U5b1+16189+@16192	U5b1+16189+@16192	O	1	U5b1+16189+@16192	O	1
7370	JX677560	U5b1e	U5b1e	O	1	U5b1e	O	1
7371	KC257380	U5b1e1	U5b1e1	O	1	U5b1e1	O	1
7372	FJ493517	U5b1e1	U5b1e1	O	1	U5b1e1	O	1
7373	FJ499497	U5b1e1a	U5b1e1a	O	1	U5b1e1a	O	1
7374	GU296571	U5b1e1a	U5b1e1a	O	1	U5b1e1a	O	1
7375	JQ705183	U5b1h	U5b1h	O	1	U5b1h	O	1
7376	JQ704053	U5b1h	U5b1h	O	1	U5b1h	O	1
7377	JQ702807	U5b1d1	U5b1d1	O	1	U5b1d1	O	1
7378	JQ702376	U5b1d1a	U5b1d1a	O	1	U5b1d1a	O	1
7379	AY882412	U5b1d1a	U5b1d1a	O	1	U5b1d1a	O	1
7380	AY882411	U5b1d1b	U5b1d1b	O	1	U5b1d1b	O	1
7381	JX156641	U5b1d1b	U5b1d1b	O	1	U5b1d1b	O	1
7382	GU977214	U5b1d1c	U5b1d1c	O	1	U5b1d1c	O	1
7383	HQ287878	U5b1d1c	U5b1d1c	O	1	U5b1d1c	O	1
7384	HM043711	U5b1d2	U5b1d2	O	1	U5b1d2	O	1
7385	JF436855	U5b1d2	U5b1d2	O	1	U5b1d2	O	1
7386	HQ675036	U5b1f1	U5b1f1	O	1	U5b1f1	O	1
7387	JX297131	U5b1f1a	U5b1f1a	O	1	U5b1f1a	O	1
7388	JX286537	U5b1f1a	U5b1f1a	O	1	U5b1f1a	O	1
7389	HQ384206	U5b1g	U5b1g	O	1	U5b1g	O	1
7390	HQ675038	U5b1g	U5b1g	O	1	U5b1g	O	1
7391	JQ681270	U5b1i	U5b1i	O	1	U5b1i	O	1
7392	DQ523645	U5b1i	U5b1i	O	1	U5b1i	O	1
7393	JQ705429	U5b2	U5b2	O	1	U5b2	O	1
7394	JQ703964	U5b2a1a	U5b2a1a	O	1	U5b2a1a	O	1
7395	JQ704726	U5b2a1a+16311	U5b2a1a+16311	O	1	U5b2a1a+16311	O	1
7396	GQ853200	U5b2a1a1	U5b2a1a1	O	1	U5b2a1a1	O	1
7397	GU296568	U5b2a1a1	U5b2a1a1	O	1	U5b2a1a1	O	1

7398	JQ701915	U5b2a1a1a	U5b2a1a1a	O	1	U5b2a1a1a	O	1
7399	JQ702021	U5b2a1a1a	U5b2a1a1a	O	1	U5b2a1a1a	O	1
7400	EU784076	U5b2a1a1b	U5b2a1a1	X	2	U5b2a1a1b	O	1
7401	JQ703942	U5b2a1a1b	U5b2a1a1	X	2	U5b2a1a1b	O	1
7402	EU244000	U5b2a1a1d	U5b2a1a1d	O	1	U5b2a1a1d	O	1
7403	HG00284	U5b2a1a1d	U5b2a1a1d	O	1	U5b2a1a1d	O	1
7404	GU296621	U5b2a1a2	U5b2a1a2	O	1	U5b2a1a2	O	1
7405	AY882413	U5b2a1a2	U5b2a1a2	O	1	U5b2a1a2	O	1
7406	EU182656	U5b2a1b	U5b2a1b	O	1	U5b2a1b	O	1
7407	FJ887848	U5b2a1b	U5b2a1b	O	1	U5b2a1b	O	1
7408	GU296541	U5b2a2	U5b2a2	O	1	U5b2a2	O	1
7409	JQ705158	U5b2a2a	U5b2a2a	O	1	U5b2a2a	O	1
7410	AY882415	U5b2a2a1	U5b2a2a1	O	1	U5b2a2a1	O	1
7411	GU296552	U5b2a2a1	U5b2a2a1	O	1	U5b2a2a1	O	1
7412	JQ702167	U5b2a2a2	U5b2a2a2	O	1	U5b2a2a2	O	1
7413	EF459670	U5b2a2b	U5b2a2b	O	1	U5b2a2b	O	1
7414	JN969984	U5b2a2b	U5b2a2b	O	1	U5b2a2b	O	1
7415	GU296567	U5b2a2b1	U5b2a2b1	O	1	U5b2a2b1	O	1
7416	HM130562	U5b2a2b1	U5b2a2b1	O	1	U5b2a2b1	O	1
7417	JQ702431	U5b2a2c	U5b2a2c	O	1	U5b2a2c	O	1
7418	JQ705688	U5b2a2c	U5b2a2c	O	1	U5b2a2c	O	1
7419	EU682506	U5b2a3	U5b2a3	O	1	U5b2a3	O	1
7420	EF420249	U5b2a3	U5b2a3	O	1	U5b2a3	O	1
7421	JX153147	U5b2a3a	U5b2a3a	O	1	U5b2a3a	O	1
7422	NA20517	U5b2a3a	U5b2a3a	O	1	U5b2a3a	O	1
7423	JQ705337	U5b2a4	U5b2a4	O	1	U5b2a4	O	1
7424	JQ705419	U5b2a4a	U5b2a4a	O	1	U5b2a4a	O	1
7425	JQ705298	U5b2a4a	U5b2a4a	O	1	U5b2a4a	O	1
7426	EU233797	U5b2a5	U5b2a5	O	1	U5b2a5	O	1
7427	JQ290366	U5b2a5	U5b2a5	O	1	U5b2a5	O	1
7428	JX152979	U5b2a5a	U5b2a5a	O	1	U5b2a5a	O	1
7429	JX153565	U5b2a5a	U5b2a5a	O	1	U5b2a5a	O	1
7430	JQ704771	U5b2a6	U5b2a6	O	1	U5b2a	X	3
7431	JX154045	U5b2a6	U5b2a6	O	1	U5b2a6	O	1
7432	JQ706019	U5b2b	U5b2b	O	1	U5b2b	O	1
7433	KC337071	U5b2b1a1	U5b2b1a1	O	1	U5b2b1a1	O	1
7434	JQ703741	U5b2b1a1	U5b2b1a1	O	1	U5b2b1a1	O	1

7435	GU296627	U5b2b1a2	U5b2b1a2	O	1	U5b2b1a2	O	1
7436	JQ703780	U5b2b1a2	U5b2b1a2	O	1	U5b2b1a2	O	1
7437	GU296641	U5b2b1b	U5b2b1b	O	1	U5b2b1b	O	1
7438	JQ702927	U5b2b1b	U5b2b1b	O	1	U5b2b1b	O	1
7439	EF419891	U5b2b2	U5b2b2	O	1	U5b2b2	O	1
7440	GQ132188	U5b2b2	U5b2b2	O	1	U5b2b2	O	1
7441	JF812598	U5b2b3	U5b2b3	O	1	U5b2b3	O	1
7442	JQ702843	U5b2b3a	U5b2b3a	O	1	U5b2b3a	O	1
7443	JQ702046	U5b2b3a1	U5b2b3a1	O	1	U5b2b3a1	O	1
7444	JQ703790	U5b2b3a1a	U5b2b3a1a	O	1	U5b2b3a1a	O	1
7445	FJ916904	U5b2b3a1a	U5b2b3a1a	O	1	U5b2b3a1a	O	1
7446	KC851802	U5b2b3b	U5b2b3b	O	1	U5b2b3b	O	1
7447	JQ702136	U5b2b3b	U5b2b3b	O	1	U5b2b3b	O	1
7448	JQ702585	U5b2b4	U5b2b4	O	1	U5b2b4	O	1
7449	JQ702531	U5b2b4	U5b2b4	O	1	U5b2b4	O	1
7450	JQ705877	U5b2b4a	U5b2b4a	O	1	U5b2b4a	O	1
7451	JQ702209	U5b2b4a	U5b2b4a	O	1	U5b2b4a	O	1
7452	JX153076	U5b2b5	U5b2b5	O	1	U5b2b5	O	1
7453	JQ704572	U5b2b5	U5b2b5	O	1	U5b2b5	O	1
7454	KC847158	U5b2c	U5b2c	O	1	U5b2c	O	1
7455	JX186998	U5b2c1	U5b2c1	O	1	U5b2c1	O	1
7456	JQ702406	U5b2c1	U5b2c1	O	1	U5b2c1	O	1
7457	JF265240	U5b2c2	U5b2c2	O	1	U5b2c2	O	1
7458	JQ705947	U5b2c2a	U5b2c2a	O	1	U5b2c2a	O	1
7459	JQ702625	U5b2c2a	U5b2c2a	O	1	U5b2c2a	O	1
7460	EU490797	U5b2c2b	U5b2c2b	O	1	U5b2c2b	O	1
7461	JQ703980	U5b2c2b	U5b2c2b	O	1	U5b2c2b	O	1
7462	JQ705968	U5b3	U5b3	O	1	U5b3	O	1
7463	GQ129144	U5b3	U5b3	O	1	U5b3	O	1
7464	AF346988	U5b3a1a	U5b3a1a	O	1	U5b3a1a	O	1
7465	GQ129152	U5b3a1a	U5b3a1a	O	1	U5b3a1a	O	1
7466	GQ129157	U5b3a1b	U5b3a1b	O	1	U5b3a1b	O	1
7467	GQ129158	U5b3a1b	U5b3a1b	O	1	U5b3a1b	O	1
7468	GQ129163	U5b3a2	U5b3a2	O	1	U5b3a2	O	1
7469	GQ129164	U5b3a2	U5b3a2	O	1	U5b3a2	O	1
7470	JQ704971	U5b3b	U5b3b	O	1	U5b3b	O	1
7471	GQ129168	U5b3b	U5b3b	O	1	U5b3b	O	1

7472	GQ129167	U5b3b1	U5b3b1	O	1	U5b3b1	O	1
7473	GQ129165	U5b3b1	U5b3b1	O	1	U5b3b1	O	1
7474	JX153697	U5b3b2	U5b3b2	O	1	U5b3b2	O	1
7475	JQ703971	U5b3b2	U5b3b2	O	1	U5b3b2	O	1
7476	GQ129170	U5b3c	U5b3c	O	1	U5b3c	O	1
7477	GQ129172	U5b3c	U5b3c	O	1	U5b3c	O	1
7478	GQ129173	U5b3d	U5b3d	O	1	U5b3d	O	1
7479	GQ129174	U5b3d	U5b3d	O	1	U5b3d	O	1
7480	JQ702295	U5b3e	U5b3e	O	1	U5b3e	O	1
7481	GQ129177	U5b3e	U5b3e	O	1	U5b3e	O	1
7482	GQ129179	U5b3f	U5b3f	O	1	U5b3f	O	1
7483	GQ129180	U5b3f	U5b3f	O	1	U5b3f	O	1
7484	GQ129143	U5b3g	U5b3g	O	1	U5b3g	O	1
7485	JQ689453	U5b3g	U5b3g	O	1	U5b3g	O	1
7486	JX153636	U5b3h	U5b3h	O	1	U5b3h	O	1
7487	JN315868	U5b3h	U5b3h	O	1	U5b3h	O	1
7488	EF064317	U6a	U6a	O	1	U6a	O	1
7489	HQ651694	U6a1	U6a1	O	1	U6a1	O	1
7490	JX120710	U6a1a1	U6a1a1	O	1	U6a1a1	O	1
7491	HQ651688	U6a1a1	U6a1a1	O	1	U6a1a1	O	1
7492	JQ704749	U6a1a2	U6a1a2	O	1	U6a1a2	O	1
7493	JQ704539	U6a1a2	U6a1a2	O	1	U6a1a2	O	1
7494	HQ651685	U6a1b1a	U6a1b1a	O	1	U6a1b1a	O	1
7495	EF064321	U6a1b1a	U6a1b1a	O	1	U6a1b1a	O	1
7496	HQ651693	U6a1b1b	U6a1b1b	O	1	U6a1b1b	O	1
7497	JX120712	U6a1b1b	U6a1b1b	O	1	U6a1b1b	O	1
7498	JQ702816	U6a1b2	U6a1b2	O	1	U6a1b2	O	1
7499	HQ651696	U6a1b2	U6a1b2	O	1	U6a1b2	O	1
7500	HQ651705	U6a1b3	U6a1b3	O	1	U6a1b3	O	1
7501	JX120709	U6a1b3	U6a1b3	O	1	U6a1	X	2
7502	EF064320	U6a1b4	U6a1b4	O	1	U6a1b4	O	1
7503	JX153033	U6a1b4	U6a1b4	O	1	U6a1b4	O	1
7504	HQ651707	U6a+16189	U6a+16189	O	1	U6a+16189 U6a+16189+	O	2
7505	EF064322	U6a2	U6a2	O	1	U6a2	O	1
7506	JX120713	U6a2a1	U6a2a1	O	1	U6a2a1	O	1
7507	EF064323	U6a2a1	U6a2a1	O	1	U6a2a1	O	1
7508	KC152576	U6a2a2	U6a2a2	O	1	U6a2a2	O	1

7509	AY882416	U6a2a2a	U6a2a2a	O	1	U6a2a2a	O	1
7510	JX120776	U6a2a2a	U6a2a2a	O	1	U6a2a2a	O	1
7511	JX120716	U6a2b	U6a2b	O	1	U6a2b	O	1
7512	KC152589	U6a2b1	U6a2b1	O	1	U6a2b1	O	1
7513	JX120714	U6a2b1	U6a2b1	O	1	U6a2b1	O	1
7514	KC152590	U6a2+195	U6a2+195	O	1	U6a2+195	O	1
7515	KC152569	U6a2c	U6a2c	O	1	U6a2c	O	1
7516	HQ651702	U6a2c	U6a2c	O	1	U6a2c	O	1
7517	KC152541	U6a8a	U6a8a	O	1	U6a8a	O	1
7518	KC152552	U6a8a	U6a8a	O	1	U6a8a	O	1
7519	JQ629405	U6a8b	U6a8b	O	1	U6a8b	O	1
7520	KC152578	U6a8b	U6a8b	O	1	U6a8b	O	1
7521	HQ651714	U6a3	U6a3	O	1	U6a3	O	1
7522	HQ651713	U6a3a	U6a3a	O	1	U6a3a	O	1
7523	HQ651686	U6a3a1	U6a3a1	O	1	U6a3a1	O	1
7524	JQ704800	U6a3a1	U6a3a1	O	1	U6a3a1	O	1
7525	HQ585390	U6a3a1a	U6a3a1a	O	1	U6a3a1a	O	1
7526	KC152538	U6a3a1a	U6a3a1a	O	1	U6a3a1a	O	1
7527	KC152549	U6a3a2	U6a3a2	O	1	U6a3a2	O	1
7528	JX120708	U6a3a2a	U6a3a2a	O	1	U6a3a2a	O	1
7529	JQ704099	U6a3a2a	U6a3a2a	O	1	U6a3a2a	O	1
7530	HQ651704	U6a3b	U6a3b	O	1	U6a3b	O	1
7531	HQ651699	U6a3b	U6a3b	O	1	U6a3b	O	1
7532	HQ651697	U6a3b1	U6a3b1	O	1	U6a3b1	O	1
7533	HQ651703	U6a3b1	U6a3b1	O	1	U6a3b1	O	1
7534	JX120720	U6a3e	U6a3e	O	1	U6a3e	O	1
7535	EF064326	U6a3e	U6a3e	O	1	U6a3e	O	1
7536	EF064324	U6a3f1	U6a3f1	O	1	U6a3f1	O	1
7537	KC152584	U6a3f1	U6a3f1	O	1	U6a3f1	O	1
7538	KC152585	U6a3f2	U6a3f2	O	1	U6a3f2	O	1
7539	JQ044807	U6a3f2	U6a3f2	O	1	U6a3f2	O	1
7540	JX120769	U6a3c	U6a3c	O	1	U6a3c	O	1
7541	JX120718	U6a3c	U6a3c	O	1	U6a3c	O	1
7542	HQ651711	U6a3d1a	U6a3d1a	O	1	U6a3d1a	O	1
7543	KC152574	U6a3d1a	U6a3d1a	O	1	U6a3d1a	O	1
7544	EF064327	U6a4	U6a4	O	1	U6a4	O	1
7545	EF064328	U6a4	U6a4	O	1	U6a4	O	1

7546	JQ703902	U6a5	U6a5	O	1	U6a5	O	1
7547	EF064329	U6a5	U6a5	O	1	U6a5	O	1
7548	EF064330	U6a5a	U6a5a	O	1	U6a5a	O	1
7549	HQ592783	U6a5a1	U6a5a1	O	1	U6a5a1	O	1
7550	KC152553	U6a5a1	U6a5a1	O	1	U6a5a1	O	1
7551	JX120722	U6a5b	U6a5b	O	1	U6a5b	O	1
7552	JX120723	U6a5b	U6a5b	O	1	U6a5b	O	1
7553	EF064332	U6a5c	U6a5c	O	1	U6a5c	O	1
7554	JX153008	U6a5c	U6a5c	O	1	U6a5c	O	1
7555	KC152565	U6a6a	U6a6a	O	1	U6a6a	O	1
7556	JX120726	U6a6a1	U6a6a1	O	1	U6a6a1	O	1
7557	KC152562	U6a6a1	U6a6a1	O	1	U6a6a1	O	1
7558	KC152559	U6a6b1	U6a6b1	O	1	U6a6b1	O	1
7559	JX120724	U6a6b1	U6a6b1	O	1	U6a6b1	O	1
7560	EF064334	U6a6b2	U6a6b2	O	1	U6a6b2	O	1
7561	EF064333	U6a6b2	U6a6b2	O	1	U6a6b2	O	1
7562	AY275531	U6a7a1	U6a7a1	O	1	U6a7a1	O	1
7563	FJ979865	U6a7a1	U6a7a1	O	1	U6a7a1	O	1
7564	GU967378	U6a7a1a	U6a7a1a	O	1	U6a7a1a	O	1
7565	JX120766	U6a7a1a	U6a7a1a	O	1	U6a7a1a	O	1
7566	EF064337	U6a7a1b	U6a7a1b	O	1	U6a7a1b	O	1
7567	JX120757	U6a7a1b	U6a7a1b	O	1	U6a7a1b	O	1
7568	EF064335	U6a7a1+@152	U6a7a1+@152	O	1	U6a7a1+@152	O	1
7569	HQ843176	U6a7a1c	U6a7a1c	O	1	U6a7a1c	O	1
7570	EF064338	U6a7a1c	U6a7a1c	O	1	U6a7a1c	O	1
7571	JX120730	U6a7a2	U6a7a2	O	1	U6a7a2	O	1
7572	JX120762	U6a7a2a	U6a7a2a	O	1	U6a7a2a	O	1
7573	JX120728	U6a7a2a	U6a7a2a	O	1	U6a7a2a	O	1
7574	KC152555	U6a7b	U6a7	X	2	U6a7b	O	1
7575	EF064339	U6a7b1	U6a7b1	O	1	U6a7b1	O	1
7576	JX120731	U6a7b1	U6a7b1	O	1	U6a7b1	O	1
7577	JQ702118	U6a7c	U6a7c	O	1	U6a7c	O	1
7578	KC152547	U6a7c1	U6a7c1	O	1	U6a7c1	O	1
7579	JX120765	U6a7c1	U6a7c1	O	1	U6a7c1	O	1
7580	JX120738	U6b	U6b	O	1	U6b	O	1
7581	AY275530	U6b	U6b	O	1	U6b	O	1
7582	HQ651678	U6b1a	U6b1a	O	1	U6b1a	O	1

7583	JQ704896	U6b1a	U6b1a	O	1	U6b1a	O	1
7584	AY275528	U6b1a1	U6b1a1	O	1	U6b1a1	O	1
7585	EF064340	U6b1a1	U6b1a1	O	1	U6b1a1	O	1
7586	KC152548	U6b1b	U6b1b	O	1	U6b1b	O	1
7587	KC152567	U6b1b	U6b1b	O	1	U6b1b	O	1
7588	JQ704008	U6b2	U6b2	O	1	U6b2	O	1
7589	AY275527	U6b2	U6b2	O	1	U6b2	O	1
7590	JX120734	U6b3	U6b3	O	1	U6b3	O	1
7591	JX120735	U6b3a	U6b3a	O	1	U6b3a	O	1
7592	FJ460538	U6b3a	U6b3a	O	1	U6b3a	O	1
7593	JX120741	U6d	U6d	O	1	U6d	O	1
7594	HQ651684	U6d1	U6d1	O	1	U6d1	O	1
7595	GU366066	U6d1a	U6d1a	O	1	U6d1a	O	1
7596	DQ523663	U6d1a	U6d1a	O	1	U6d1a	O	1
7597	EF064342	U6d1b	U6d1b	O	1	U6d1b	O	1
7598	EF064341	U6d1b	U6d1b	O	1	U6d1b	O	1
7599	KC152592	U6d2	U6d2	O	1	U6d2	O	1
7600	EF064343	U6d2	U6d2	O	1	U6d2	O	1
7601	JX120743	U6d3	U6d3	O	1	U6d3	O	1
7602	HQ651681	U6d3a	U6d3a	O	1	U6d3a	O	1
7603	NA19652	U6d3a	U6d3a	O	1	U6d3a	O	1
7604	HQ161773	U6c1	U6c1	O	1	U6c1	O	1
7605	EF064344	U6c1	U6c1	O	1	U6c1	O	1
7606	KC152568	U6c2	U6c2	O	1	U6c2	O	1
7607	HM775494	U6c2	U6c2	O	1	U6c2	O	1
7608	FN600416	U2	U2	O	1	U2	O	1
7609	JX488759	U2a1a	U2a1a	O	1	U2a1a	O	1
7610	AY882379	U2a1a	U2a1a	O	1	U2a1a	O	1
7611	AY713992	U2a1b	U2a1b	O	1	U2a1b	O	1
7612	KF056257	U2a2	U2a2	O	1	U2a2	O	1
7613	KC533515	U2a2	U2a2	O	1	U2a2	O	1
7614	HM156690	U2b1	U2b1	O	1	U2b1	O	1
7615	EU330890	U2b1	U2b1	O	1	U2b1	O	1
7616	AY714020	U2b1a	U2b1a	O	1	U2b1a	O	1
7617	KF056258	U2b1a	U2b1a	O	1	U2b1a	O	1
7618	AY714027	U2b2	U2b2	O	1	U2b2	O	1
7619	AY882380	U2b2	U2b2	O	1	U2b2	O	1

7620	AY714010	U2c	U2c	O	1	U2c	O	1
7621	KC533505	U2c1	U2c1	O	1	U2c1	O	1
7622	JX984460	U2c1	U2c1	O	1	U2c1	O	1
7623	AY714005	U2c1a	U2c1a	O	1	U2c1a	O	1
7624	HM036565	U2c1a	U2c1a	O	1	U2c1a	O	1
7625	AY882381	U2c1b	U2c1b	O	1	U2c1b	O	1
7626	HM036556	U2c1b	U2c1b	O	1	U2c1b	O	1
7627	JQ706062	U2d1	U2d1	O	1	U2d1	O	1
7628	EU440736	U2d1	U2d1	O	1	U2d1	O	1
7629	JQ705999	U2d2	U2d2	O	1	U2d2	O	1
7630	JQ706046	U2d2a	U2d2a	O	1	U2d2a	O	1
7631	KC911405	U2d2a	U2d2a	O	1	U2d2a	O	1
7632	JQ706045	U2d3	U2d3	O	1	U2d3	O	1
7633	JQ706025	U2d3	U2d3	O	1	U2d3	O	1
7634	AY714026	U2e	U2e	O	1	U2e	O	1
7635	JQ703356	U2e1	U2e1	O	1	U2e1	O	1
7636	FJ147311	U2e1	U2e1	O	1	U2e1	O	1
7637	FJ828532	U2e1a	U2e1a	O	1	U2e1a	O	1
7638	AY882382	U2e1a1	U2e1a1	O	1	U2e1a1	O	1
7639	JQ703957	U2e1a1	U2e1a1	O	1	U2e1a1	O	1
7640	JQ705936	U2e1a1a	U2e1a1a	O	1	U2e1a1a	O	1
7641	JQ705117	U2e1a1a	U2e1a1a	O	1	U2e1a1a	O	1
7642	HQ424846	U2e1a1b	U2e1a1b	O	1	U2e1a1b	O	1
7643	JQ703203	U2e1a1b	U2e1a1b	O	1	U2e1a1b	O	1
7644	JQ704817	U2e1a1c	U2e1a1c	O	1	U2e1a1c	O	1
7645	FJ348194	U2e1a1c	U2e1a1c	O	1	U2e1a1c	O	1
7646	AY714049	U2e1b	U2e1b	O	1	U2e1b	O	1
7647	JQ705941	U2e1b	U2e1b	O	1	U2e1b	O	1
7648	JQ702663	U2e1b1	U2e1b1	O	1	U2e1b1	O	1
7649	JQ703917	U2e1b1	U2e1b1	O	1	U2e1b1	O	1
7650	JQ705946	U2e1b2	U2e1b2	O	1	U2e1b2	O	1
7651	EF661006	U2e1b2	U2e1b2	O	1	U2e1b2	O	1
7652	KF142160	U2e1c	U2e1c	O	1	U2e1c	O	1
7653	JQ705933	U2e1c1	U2e1c1	O	1	U2e1c1	O	1
7654	JQ701857	U2e1c1	U2e1c1	O	1	U2e1c1	O	1
7655	JQ702520	U2e1d	U2e1d	O	1	U2e1d	O	1
7656	JQ704268	U2e1d	U2e1d	O	1	U2e1d	O	1

7657	JQ702316	U2e1e	U2e1e	O	1	U2e1e	O	1
7658	EF452294	U2e1e	U2e1e	O	1	U2e1e	O	1
7659	JQ705813	U2e1f	U2e1f	O	1	U2e1f	O	1
7660	AY195764	U2e1f1	U2e1f1	O	1	U2e1f1	O	1
7661	NA12347	U2e1f1	U2e1f1	O	1	U2e1f1	O	1
7662	JQ703491	U2e1g	U2e1g	O	1	U2e1g	O	1
7663	KC464359	U2e1g	U2e1g	O	1	U2e1g	O	1
7664	FJ493504	U2e1h	U2e1h	O	1	U2e1h	O	1
7665	KF849954	U2e1h	U2e1h	O	1	U2e1h	O	1
7666	KC911567	U2e2	U2e2	O	1	U2e2	O	1
7667	JX462708	U2e2a	U2e2a	O	1	U2e2a	O	1
7668	JQ705900	U2e2a	U2e2a	O	1	U2e2a	O	1
7669	JQ703968	U2e2a1a	U2e2a1a	O	1	U2e2a1a	O	1
7670	JX153832	U2e2a1a	U2e2a1a	O	1	U2e2a1a	O	1
7671	JX154009	U2e2a1a1	U2e2a1a1	O	1	U2e2a1a1	O	1
7672	JQ702432	U2e2a1a1	U2e2a1a1	O	1	U2e2a1a1	O	1
7673	JQ705667	U2e2a1a2	U2e2a1a2	O	1	U2e2a1a2	O	1
7674	JX153656	U2e2a1a2	U2e2a1a2	O	1	U2e2a1a2	O	1
7675	FJ984932	U2e2a1b	U2e2a1b	O	1	U2e2a1b	O	1
7676	EF528162	U2e2a1b	U2e2a1b	O	1	U2e2a1b	O	1
7677	JQ705386	U2e2a1c	U2e2a1c	O	1	U2e2a1c	O	1
7678	JQ704627	U2e2a1c	U2e2a1c	O	1	U2e2a1c	O	1
7679	JQ702004	U2e2a1d	U2e2a1d	O	1	U2e2a1d	O	1
7680	JQ701890	U2e2a1d	U2e2a1d	O	1	U2e2a1d	O	1
7681	JX286498	U2e3	U2e3	O	1	U2e3	O	1
7682	JQ704102	U2e3a	U2e3a	O	1	U2e3a	O	1
7683	JQ702389	U2e3a	U2e3a	O	1	U2e3a	O	1
7684	HM852891	U3a	U3a	O	1	U3a	O	1
7685	JX153939	U3a1	U3a1	O	1	U3a1	O	1
7686	JQ705387	U3a1	U3a1	O	1	U3a1	O	1
7687	GU122986	U3a1a	U3a1a	O	1	U3a1a	O	1
7688	JQ703990	U3a1a	U3a1a	O	1	U3a1a	O	1
7689	JN203207	U3a1a1	U3a1a1	O	1	U3a1a1	O	1
7690	JX153017	U3a1a1	U3a1a1	O	1	U3a1a1	O	1
7691	JQ702681	U3a1b	U3a1b	O	1	U3a1b	O	1
7692	JQ702677	U3a1b	U3a1b	O	1	U3a1b	O	1
7693	JQ703769	U3a1c	U3a1c	O	1	U3a1c	O	1

7694	AF381982	U3a1c	U3a1c	O	1	U3a1c	O	1
7695	JQ705739	U3a1c1	U3a1c1	O	1	U3a1c1	O	1
7696	HQ286323	U3a1c1	U3a1c1	O	1	U3a1c1	O	1
7697	KC911305	U3a2	U3a2	O	1	U3a2	O	1
7698	FJ348224	U3a2a	U3a2a	O	1	U3a2a	O	1
7699	AY882383	U3a2a1	U3a2a1	O	1	U3a2a1	O	1
7700	KC911522	U3a2a1a	U3a2a1a	O	1	U3a2a1a	O	1
7701	KC911568	U3a2a1a	U3a2a1a	O	1	U3a2a1a	O	1
7702	JQ704440	U3a3	U3a3	O	1	U3a3	O	1
7703	HM852895	U3a3	U3a3	O	1	U3a3	O	1
7704	HM852803	U3c	U3c	O	1	U3c	O	1
7705	HM852797	U3c	U3c	O	1	U3c	O	1
7706	JN663830	U3b	U3b	O	1	U3b	O	1
7707	AY714023	U3b	U3b	O	1	U3b	O	1
7708	EF177433	U3b1	U3b1	O	1	U3b1	O	1
7709	JQ703911	U3b1	U3b1	O	1	U3b1	O	1
7710	EF556158	U3b1a	U3b1a	O	1	U3b1a	O	1
7711	KC911623	U3b1a1	U3b1a1	O	1	U3b1a1	O	1
7712	AY882385	U3b1a1	U3b1a1	O	1	U3b1a1	O	1
7713	FJ711758	U3b1b	U3b1b	O	1	U3b1b	O	1
7714	JQ705043	U3b1b	U3b1b	O	1	U3b1b	O	1
7715	JQ704130	U3b2	U3b	X	3	U3b2	O	1
7716	JQ702150	U3b2	U3b	X	3	U3b2	O	1
7717	JN969086	U3b2a	U3b2a	O	1	U3b2a	O	1
7718	KC911445	U3b2a1	U3b2a1	O	1	U3b2a1	O	1
7719	FJ348193	U3b2a1	U3b2a1	O	1	U3b2a1	O	1
7720	HQ257369	U3b2a1a	U3b2a1a	O	1	U3b2a1a	O	1
7721	JX153055	U3b2a1a	U3b2a1a	O	1	U3b2a1a	O	1
7722	JQ704981	U3b2b	U3b2b	O	1	U3b2b	O	1
7723	KC250336	U3b2b	U3b2b	O	1	U3b2b	O	1
7724	KC911486	U3b2c	U3b	X	2	U3b2c	O	1
7725	KC911459	U3b2c	U3b	X	2	U3b2c	O	1
7726	HQ384204	U3b3	U3b3	O	1	U3b3	O	1
7727	AY882384	U3b3	U3b3	O	1	U3b3	O	1
7728	JQ703947	U4a1	U4a1	O	1	U4a1	O	1
7729	EU597575	U4a1	U4a1	O	1	U4a1	O	1
7730	HQ659692	U4a1a	U4a1a	O	1	U4a1a	O	1

7731	JQ703929	U4a1a	U4a1a	O	1	U4a1a	O	1
7732	EU545417	U4a1a1	U4a1a	X	3	U4a1a1	O	1
7733	EU545451	U4a1a1	U4a1a	X	3	U4a1a1	O	1
7734	JQ703887	U4a1a2	U4a1a2	O	1	U4a1a2	O	1
7735	JQ705687	U4a1a2	U4a1a2	O	1	U4a1a2	O	1
7736	JQ704345	U4a1a3	U4a1a3	O	1	U4a1a3	O	1
7737	JQ703977	U4a1a3	U4a1a3	O	1	U4a1a3	O	1
7738	JQ702270	U4a1b	U4a1b	O	1	U4a1b	O	1
7739	EU545459	U4a1b1	U4a1b1	O	1	U4a1b1	O	1
7740	GU562438	U4a1b1	U4a1b1	O	1	U4a1b1	O	1
7741	GU252762	U4a1b1a	U4a1b1a	O	1	U4a1b1a	O	1
7742	EU428753	U4a1b1a	U4a1b1a	O	1	U4a1b1a	O	1
7743	EU545432	U4a1b2	U4a1b2	O	1	U4a1b2	O	1
7744	EU545429	U4a1b2	U4a1b2	O	1	U4a1b2	O	1
7745	EU545428	U4a1c	U4a1c	O	1	U4a1c	O	1
7746	EU545427	U4a1c	U4a1c	O	1	U4a1c	O	1
7747	FJ493506	U4a1d	U4a1d	O	1	U4a1d	O	1
7748	GU123031	U4a1d	U4a1d	O	1	U4a1d	O	1
7749	JX021669	U4a1e	U4a1e	O	1	U4a1e	O	1
7750	KF148090	U4a1e	U4a1e	O	1	U4a1e	O	1
7751	EU545430	U4a2	U4a2	O	1	U4a2	O	1
7752	JQ704052	U4a2	U4a2	O	1	U4a2	O	1
7753	EF222249	U4a2a	U4a2a	O	1	U4a2a	O	1
7754	EU545450	U4a2a	U4a2a	O	1	U4a2a	O	1
7755	EF222236	U4a2a1	U4a2a1	O	1	U4a2a1	O	1
7756	EF222250	U4a2a1	U4a2a1	O	1	U4a2a1	O	1
7757	EU545461	U4a2a2	U4a2a2	O	1	U4a2a2	O	1
7758	JX154043	U4a2a2	U4a2a2	O	1	U4a2a2	O	1
7759	EF222239	U4a2a3	U4a2a3	O	1	U4a2a3	O	1
7760	EU545446	U4a2a3	U4a2a3	O	1	U4a2a3	O	1
7761	GU123033	U4a2b	U4a2b	O	1	U4a2b	O	1
7762	EF222241	U4a2b	U4a2b	O	1	U4a2b	O	1
7763	EU545464	U4a2c	U4a2c	O	1	U4a2c	O	1
7764	EF222252	U4a2c1	U4a2c1	O	1	U4a2c1	O	1
7765	EF222242	U4a2c1	U4a2c1	O	1	U4a2c1	O	1
7766	EU545421	U4a2d	U4a2d	O	1	U4a2d	O	1
7767	JQ705152	U4a2d	U4a2d	O	1	U4a2d	O	1

7768	EU545442	U4a2e	U4a2e	O	1	U4a2e	O	1
7769	JQ702862	U4a2e	U4a2e	O	1	U4a2e	O	1
7770	JQ705828	U4a2f	U4a2f	O	1	U4a2f	O	1
7771	JQ704811	U4a2f	U4a2f	O	1	U4a2f	O	1
7772	JQ480650	U4a2g	U4a2g	O	1	U4a2g	O	1
7773	EU545434	U4a2g	U4a2g	O	1	U4a2g	O	1
7774	EF222235	U4a2h	U4a2h	O	1	U4a2h	O	1
7775	JQ702549	U4a2h1	U4a2h1	O	1	U4a2h1	O	1
7776	JX153725	U4a2h1	U4a2h1	O	1	U4a2h1	O	1
7777	EF060364	U4a3	U4a3	O	1	U4a3	O	1
7778	EU545423	U4a3a	U4a3a	O	1	U4a3a	O	1
7779	KF938935	U4a3a	U4a3a	O	1	U4a3a	O	1
7780	EU545444	U4b	U4b	O	1	U4b	O	1
7781	JQ702211	U4b	U4b	O	1	U4b	O	1
7782	HM044301	U4b1a1	U4b1a1	O	1	U4b1a1	O	1
7783	JQ705255	U4b1a1a	U4b1a1a	O	1	U4b1a1a	O	1
7784	JQ703912	U4b1a1a	U4b1a1a	O	1	U4b1a1a	O	1
7785	AY882388	U4b1a1a1	U4b1a1a1	O	1	U4b1a1a1	O	1
7786	KC911524	U4b1a1a1	U4b1a1a1	O	1	U4b1a1a1	O	1
7787	HM041972	U4b1a2	U4b1a2	O	1	U4b1a2	O	1
7788	EU545415	U4b1a2a	U4b1a2a	O	1	U4b1a2a	O	1
7789	JQ704098	U4b1a2a	U4b1a2a	O	1	U4b1a2a	O	1
7790	JX153376	U4b1a2b	U4b1a2b	O	1	U4b1a2b	O	1
7791	JQ701990	U4b1a2b	U4b1a2b	O	1	U4b1a2b	O	1
7792	EU545419	U4b1a3	U4b1a3	O	1	U4b1a3	O	1
7793	EU571946	U4b1a3a	U4b1a3a	O	1	U4b1a3a	O	1
7794	FJ858802	U4b1a3a	U4b1a3a	O	1	U4b1a3a	O	1
7795	FJ147313	U4b1a4	U4b1a4	O	1	U4b1a4	O	1
7796	FJ147316	U4b1a4	U4b1a4	O	1	U4b1a4	O	1
7797	FJ147315	U4b1+146_152	U4b1+146 152	O	1	U4b1+146_152	O	1
7798	JQ705342	U4b1b1	U4b1b1	O	1	U4b1b1	O	1
7799	JN647925	U4b1b1	U4b1b1	O	1	U4b1b1	O	1
7800	HM535647	U4b1b1a	U4b1b1a	O	1	U4b1b1a	O	1
7801	GU797476	U4b1b1a	U4b1b1a	O	1	U4b1b1a	O	1
7802	JQ704155	U4b1b1b	U4b1b1b	O	1	U4b1b1b	O	1
7803	JQ702864	U4b1b1b	U4b1b1b	O	1	U4b1b1b	O	1
7804	FJ858877	U4b1b1+16311	U4b1b1+16311	O	1	U4b1b1+16311	O	1

7805	GU123041	U4b1b1c	U4b1b1c	O	1	U4b1b1c	O	1
7806	KC911460	U4b1b1c	U4b1b1c	O	1	U4b1b1c	O	1
7807	NA12156	U4b1b1d	U4b1b1d	O	1	U4b1b1d	O	1
7808	JX153693	U4b1b1d	U4b1b1d	O	1	U4b1b1d	O	1
7809	JQ704676	U4b1b2	U4b1b2	O	1	U4b1b2	O	1
7810	HG00155	U4b1b2	U4b1b2	O	1	U4b1b2	O	1
7811	EU140898	U4b2	U4b2	O	1	U4b2	O	1
7812	JQ702783	U4b2a	U4b2a	O	1	U4b2a	O	1
7813	JQ701937	U4b2a	U4b2a	O	1	U4b2a	O	1
7814	GQ891957	U4b2a1	U4b2a1	O	1	U4b2a1	O	1
7815	GU365881	U4b2a1a	U4b2a1a	O	1	U4b2a1a	O	1
7816	HQ190906	U4b2a1a	U4b2a1a	O	1	U4b2a1a	O	1
7817	FJ147322	U4b3	U4b3	O	1	U4b3	O	1
7818	JX152874	U4b3	U4b3	O	1	U4b3	O	1
7819	EU545465	U4c1	U4c1	O	1	U4c1	O	1
7820	KC911375	U4c1	U4c1	O	1	U4c1	O	1
7821	JX153870	U4c1a	U4c1a	O	1	U4c1a	O	1
7822	HQ418462	U4c2	U4c2	O	1	U4c2	O	1
7823	EU545460	U4c2a	U4c2a	O	1	U4c2a	O	1
7824	JQ702690	U4c2a	U4c2a	O	1	U4c2a	O	1
7825	HQ167735	U4d1	U4d1	O	1	U4d1	O	1
7826	GU123021	U4d1a	U4d1a	O	1	U4d1a	O	1
7827	JQ702267	U4d1a1	U4d1a1	O	1	U4d1a1	O	1
7828	JQ704741	U4d1a1a	U4d1a1a	O	1	U4d1a1a	O	1
7829	HG00186	U4d1a1a	U4d1a1a	O	1	U4d1a1a	O	1
7830	EU545438	U4d1b	U4d1b	O	1	U4d1b	O	1
7831	GU122982	U4d1b	U4d1b	O	1	U4d1b	O	1
7832	FJ230891	U4d2	U4d2	O	1	U4d2	O	1
7833	EU545426	U4d2	U4d2	O	1	U4d2	O	1
7834	GU727822	U4d3	U4d3	O	1	U4d3	O	1
7835	AY882389	U9a	U9a	O	1	U9a	O	1
7836	GU170820	U9a1	U9a1	O	1	U9a1	O	1
7837	FJ770944	U9a1	U9a1	O	1	U9a1	O	1
7838	GU990521	U9b	U9b	O	1	U9b	O	1
7839	AY882390	U9b1	U9b1	O	1	U9b1	O	1
7840	EU597540	U9b1	U9b1	O	1	U9b1	O	1
7841	AY714004	U7	U7	O	1	U7	O	1

7842	EF556179	U7a	U7a	O	1	U7a	O	1
7843	KC911622	U7a	U7a	O	1	U7a	O	1
7844	KC911620	U7a1	U7a1	O	1	U7a1	O	1
7845	AY714014	U7a1a	U7a1a	O	1	U7a1a	O	1
7846	AY714013	U7a1a	U7a1a	O	1	U7a1a	O	1
7847	FJ858878	U7a2	U7a2	O	1	U7a2	O	1
7848	KC911509	U7a2a	U7a2a	O	1	U7a2a	O	1
7849	EU597503	U7a2a	U7a2a	O	1	U7a2a	O	1
7850	KC911455	U7a3a	U7a3a	O	1	U7a3a	O	1
7851	JQ705966	U7a3a	U7a3a	O	1	U7a3a	O	1
7852	KF418766	U7a3b	U7a3b	O	1	U7a3b	O	1
7853	KC911508	U7a3b	U7a3b	O	1	U7a3b	O	1
7854	HM852777	U7a4	U7a4	O	1	U7a4	O	1
7855	KC911299	U7a4a	U7a4a	O	1	U7a4a	O	1
7856	KC911402	U7a4a1	U7a4a1	O	1	U7a4a1	O	1
7857	HM852823	U7a4a1	U7a4a1	O	1	U7a4a1	O	1
7858	KC911288	U7a4a1a	U7a4a1a	O	1	U7a4a1a	O	1
7859	KC911347	U7a4a1a	U7a4a1a	O	1	U7a4a1a	O	1
7860	JQ703913	U7a5	U7a5	O	1	U7a5	O	1
7861	JQ703978	U7a5	U7a5	O	1	U7a5	O	1
7862	JQ705198	U7b	U7b	O	1	U7b	O	1
7863	AY882391	U7b	U7b	O	1	U7b	O	1
7864	EU445683	U7b1	U7b1	O	1	U7b1	O	1
7865	EU445684	U7b1	U7b1	O	1	U7b1	O	1
7866	JX153191	U7b2	U7b	X	2	U7b2	O	1
7867	JQ704100	U7b2	U7b	X	2	U7b2	O	1
7868	JQ702759	U8a	U8a	O	1	U8a	O	1
7869	JQ705347	U8a1a1	U8a1a1	O	1	U8a1a1	O	1
7870	AY882392	U8a1a1	U8a1a1	O	1	U8a1a1	O	1
7871	GU299344	U8a1a1a	U8a1a1a	O	1	U8a1a1a	O	1
7872	HM765458	U8a1a1a1	U8a1a1a1	O	1	U8a1a1a1	O	1
7873	JQ705281	U8a1a1a1	U8a1a1a1	O	1	U8a1a1a1	O	1
7874	JX273295	U8a1a1b	U8a1a1b	O	1	U8a1a1b	O	1
7875	HQ022823	U8a1a1b1	U8a1a1b1	O	1	U8a1a1b1	O	1
7876	HM008694	U8a1a1b1	U8a1a1b1	O	1	U8a1a1b1	O	1
7877	JQ702761	U8a1a2	U8a1a2	O	1	U8a1a2	O	1
7878	HM113490	U8a1a2	U8a1a2	O	1	U8a1a2	O	1

7879	JX297480	U8a1a3	U8a1a3	O	1	U8a1a3	O	1
7880	HQ336423	U8a1a3	U8a1a3	O	1	U8a1a3	O	1
7881	JX273297	U8a1a4	U8a1a4	O	1	U8a1a4	O	1
7882	DQ200802	U8a1a4	U8a1a4	O	1	U8a1a4	O	1
7883	JQ611709	U8a1b	U8a1b	O	1	U8a1b	O	1
7884	HG00263	U8a1b	U8a1b	O	1	U8a1b	O	1
7885	DQ200801	U8a2	U8a2	O	1	U8a2	O	1
7886	HQ384210	U8a2	U8a2	O	1	U8a2	O	1
7887	KC911451	U8b1a1	U8b1a1	O	1	U8b1a1	O	1
7888	AY882393	U8b1a1	U8b1a1	O	1	U8b1a1	O	1
7889	KC911536	U8b1a2a	U8b1a2a	O	1	U8b1a2a	O	1
7890	DQ200805	U8b1a2a	U8b1a2a	O	1	U8b1a2a	O	1
7891	JX273294	U8b1a2+16311	U8b1a2+16311	O	1	U8b1a2+16311	O	1
7892	JX273296	U8b1a2b	U8b1a2b	O	1	U8b1a2b	O	1
7893	JX153047	U8b1a2b	U8b1a2b	O	1	U8b1a2b	O	1
7894	JQ705831	U8b1b	U8b1b	O	1	U8b1b	O	1
7895	JQ704970	U8b1b1	U8b1b1	O	1	U8b1b1	O	1
7896	NA20786	U8b1b1	U8b1b1	O	1	U8b1b1	O	1
7897	JX273243	U8b1b2	U8b1b2	O	1	U8b1b2	O	1
7898	JX153058	U8b1b2	U8b1b2	O	1	U8b1b2	O	1
7899	JQ706038	K1a	K1a	O	1	K1a	O	1
7900	HM043706	K1a	K1a	O	1	K1a	O	1
7901	JQ705483	K1a1a	K1a1a	O	1	K1a1a	O	1
7902	JQ702802	K1a1a	K1a1a	O	1	K1a1a	O	1
7903	FJ348183	K1a1a1	K1a1a1	O	1	K1a1a1	O	1
7904	GU722598	K1a1a1	K1a1a1	O	1	K1a1a1	O	1
7905	JQ704696	K1a1a2	K1a1a2	O	1	K1a1a2	O	1
7906	GU980957	K1a1a2a	K1a1a2a	O	1	K1a1a2a	O	1
7907	AY495257	K1a1a2a1	K1a1a2a1	O	1	K1a1a2a1	O	1
7908	JQ705393	K1a1a2a1	K1a1a2a1	O	1	K1a1a2a1	O	1
7909	JN048471	K1a1b	K1a1b	O	1	K1a1b	O	1
7910	DQ200804	K1a1b1	K1a1b1	O	1	K1a1b1	O	1
7911	DQ301795	K1a1b1a	K1a1b1a	O	1	K1a1b1a	O	1
7912	HM101136	K1a1b1a	K1a1b1a	O	1	K1a1b1a	O	1
7913	JQ702898	K1a1b1b	K1a1b1b	O	1	K1a1b1b	O	1
7914	JQ704983	K1a1b1b1	K1a1b1b1	O	1	K1a1b1b1	O	1
7915	AY339554	K1a1b1b1	K1a1b1b1	O	1	K1a1b1b1	O	1

7916	GU592026	K1a1b1c	K1a1b1c	O	1	K1a1b1c	O	1
7917	HE576978	K1a1b1c	K1a1b1c	O	1	K1a1b1c	O	1
7918	JQ702782	K1a1b1d	K1a1b1d	O	1	K1a1b1d	O	1
7919	JQ704096	K1a1b1d	K1a1b1d	O	1	K1a1b1d	O	1
7920	JQ704056	K1a1b1e	K1a1b1e	O	1	K1a1b1e	O	1
7921	JQ704207	K1a1b1e	K1a1b1e	O	1	K1a1b1e	O	1
7922	JQ703737	K1a1b1f	K1a1b1f	O	1	K1a1b1f	O	1
7923	JX048065	K1a1b1f	K1a1b1f	O	1	K1a1b1f	O	1
7924	FJ348218	K1a1b1g	K1a1b1g	O	1	K1a1b1g	O	1
7925	JQ668027	K1a1b1g	K1a1b1g	O	1	K1a1b1g	O	1
7926	AY714044	K1a1b2a	K1a1b2a	O	1	K1a1b2a	O	1
7927	HQ586011	K1a1b2a1	K1a1b2a1	O	1	K1a1b2a1	O	1
7928	HQ413154	K1a1b2a1a	K1a1b2a1a	O	1	K1a1b2a1a	O	1
7929	JQ701858	K1a1b2a1a	K1a1b2a1a	O	1	K1a1b2a1a	O	1
7930	FJ865502	K1a1b2b	K1a1b2b	O	1	K1a1b2b	O	1
7931	JQ702624	K1a1b2b	K1a1b2b	O	1	K1a1b2b	O	1
7932	JX153526	K1a1c	K1a1c	O	1	K1a1c	O	1
7933	JQ705697	K1a1c	K1a1c	O	1	K1a1c	O	1
7934	KC911410	K1a2	K1a2	O	1	K1a2	O	1
7935	JQ703309	K1a2	K1a2	O	1	K1a2	O	1
7936	HG01617	K1a2a	K1a2a	O	1	K1a2a	O	1
7937	EU915473	K1a2a	K1a2a	O	1	K1a2a	O	1
7938	JQ702748	K1a2a1	K1a2a1	O	1	K1a2a1	O	1
7939	JQ701856	K1a2a1	K1a2a1	O	1	K1a2a1	O	1
7940	JQ703581	K1a2a2	K1a2a2	O	1	K1a2a2	O	1
7941	HG00346	K1a2a2	K1a2a2	O	1	K1a2a2	O	1
7942	HM041971	K1a2b	K1a2b	O	1	K1a2b	O	1
7943	JQ704017	K1a2b	K1a2b	O	1	K1a2b	O	1
7944	JQ705149	K1a2c	K1a2c	O	1	K1a2c	O	1
7945	KC477768	K1a2c	K1a2c	O	1	K1a2c	O	1
7946	AY495264	K1a3	K1a3	O	1	K1a3	O	1
7947	JQ703347	K1a3	K1a3	O	1	K1a3	O	1
7948	JQ705485	K1a3a	K1a3a	O	1	K1a3a	O	1
7949	EU344156	K1a3a	K1a3a	O	1	K1a3a	O	1
7950	JQ703648	K1a3a1	K1a3a1	O	1	K1a3a1	O	1
7951	JQ703096	K1a3a1	K1a3a1	O	1	K1a3a1	O	1
7952	DQ301814	K1a3a1a	K1a3a1a	O	1	K1a3a1a	O	1

7953	AY495258	K1a3a1b	K1a3a1b	O	1	K1a3a1b	O	1
7954	GU799583	K1a3a1b	K1a3a1b	O	1	K1a3a1b	O	1
7955	JQ705957	K1a3a2	K1a3a2	O	1	K1a3a2	O	1
7956	JQ702693	K1a3a2	K1a3a2	O	1	K1a3a2	O	1
7957	JQ701962	K1a3a3	K1a3a3	O	1	K1a3a3	O	1
7958	HQ839861	K1a3a3	K1a3a3	O	1	K1a3a3	O	1
7959	HQ610202	K1a3a4	K1a3a4	O	1	K1a3a4	O	1
7960	JQ703775	K1a3a4	K1a3a4	O	1	K1a3a4	O	1
7961	AY495261	K1a4	K1a4	O	1	K1a4	O	1
7962	EU603401	K1a4a	K1a4a	O	1	K1a4a	O	1
7963	JQ702521	K1a4a	K1a4a	O	1	K1a4+146	X	2
7964	AY495262	K1a4a1	K1a4a1	O	1	K1a4a1	O	1
7965	JQ703493	K1a4a1	K1a4a1	O	1	K1a4a1	O	1
7966	JQ702175	K1a4a1a	K1a4a1a	O	1	K1a4a1a	O	1
7967	JQ702213	K1a4a1a+195	K1a4a1a+195	O	1	K1a4a1a+195	O	1
7968	JQ703444	K1a4a1a+195	K1a4a1a+195	O	1	K1a4a1a+195	O	1
7969	JQ702811	K1a4a1a1	K1a4a1a1	O	1	K1a4a1a1	O	1
7970	JQ702773	K1a4a1a1	K1a4a1a1	O	1	K1a4a1a1	O	1
7971	GU122978	K1a4a1a3	K1a4a1a3	O	1	K1a4a1a3	O	1
7972	JN122621	K1a4a1a3	K1a4a1a3	O	1	K1a4a1a3	O	1
7973	JQ703168	K1a4a1a2	K1a4a1a2	O	1	K1a4a1a2	O	1
7974	JN559854	K1a4a1a2a	K1a4a1a2a	O	1	K1a4a1a2a	O	1
7975	HM031133	K1a4a1a2a	K1a4a1a2a	O	1	K1a4a1a2a	O	1
7976	JQ702896	K1a4a1a2b	K1a4a1a2b	O	1	K1a4a1a2b	O	1
7977	JQ701810	K1a4a1a2b	K1a4a1a2b	O	1	K1a4a1a2b	O	1
7978	JQ703407	K1a4a1b	K1a4a1b	O	1	K1a4a1b	O	1
7979	GU191795	K1a4a1b1	K1a4a1b1	O	1	K1a4a1b1	O	1
7980	EU677385	K1a4a1b1	K1a4a1b1	O	1	K1a4a1b1	O	1
7981	JQ703171	K1a4a1b2	K1a4a1b2	O	1	K1a4a1b2	O	1
7982	JQ704948	K1a4a1b2	K1a4a1b2	O	1	K1a4a1b2	O	1
7983	JN258704	K1a4a1c	K1a4a1c	O	1	K1a4a1c	O	1
7984	JQ705215	K1a4a1c1	K1a4a1c1	O	1	K1a4a1c1	O	1
7985	EU692798	K1a4a1c1	K1a4a1c1	O	1	K1a4a1c1	O	1
7986	JQ705404	K1a4a1d	K1a4a1d	O	1	K1a4a1d	O	1
7987	JQ705558	K1a4a1d	K1a4a1d	O	1	K1a4a1d	O	1
7988	JQ702341	K1a4a1e	K1a4a1e	O	1	K1a4a1e	O	1
7989	JQ704526	K1a4a1e	K1a4a1e	O	1	K1a4a1e	O	1

7990	JQ705816	K1a4a1f	K1a4a1f	O	1	K1a4a1f	O	1
7991	JQ702302	K1a4a1f1	K1a4a1f1	O	1	K1a4a1f1	O	1
7992	JQ702760	K1a4a1f1	K1a4a1f1	O	1	K1a4a1f1	O	1
7993	JQ704759	K1a4a1g	K1a4a1g	O	1	K1a4a1g	O	1
7994	JX048671	K1a4a1g	K1a4a1g	O	1	K1a4a1g	O	1
7995	JN001930	K1a4a1h	K1a4a1h	O	1	K1a4a1h	O	1
7996	HG01051	K1a4a1h	K1a4a1h	O	1	K1a4a1h	O	1
7997	JQ702315	K1a4a1i	K1a4a1i	O	1	K1a4a1i	O	1
7998	KF417432	K1a4a1i	K1a4a1i	O	1	K1a4a1i	O	1
7999	EU675299	K1a4b	K1a4b	O	1	K1a4b	O	1
8000	EU600366	K1a4b1	K1a4b1	O	1	K1a4b1	O	1
8001	DQ301809	K1a4c	K1a4c	O	1	K1a4c	O	1
8002	JQ703523	K1a4c1	K1a4c1	O	1	K1a4c1	O	1
8003	HQ538515	K1a4c1	K1a4c1	O	1	K1a4c1	O	1
8004	EU926621	K1a4d	K1a4d	O	1	K1a4d	O	1
8005	JF710373	K1a4d	K1a4d	O	1	K1a4d	O	1
8006	JQ704761	K1a4e	K1a4e	O	1	K1a4e	O	1
8007	KC900995	K1a4f	K1a4f	O	1	K1a4f	O	1
8008	JQ704764	K1a4f1	K1a4f1	O	1	K1a4f1	O	1
8009	EF177416	K1a4f1	K1a4f1	O	1	K1a4f1	O	1
8010	JQ705656	K1a4g	K1a4g	O	1	K1a4g	O	1
8011	HG00121	K1a4g	K1a4g	O	1	K1a4g	O	1
8012	JQ703361	K1a4h	K1a4h	O	1	K1a4h	O	1
8013	KC911589	K1a4h1	K1a4h1	O	1	K1a4h1	O	1
8014	JQ702911	K1a4h1	K1a4h1	O	1	K1a4h1	O	1
8015	JX273270	K1a4i	K1a4i	O	1	K1a4i	O	1
8016	JX273269	K1a4i	K1a4i	O	1	K1a4i	O	1
8017	JX273271	K1a4+146	K1a4+146	O	1	K1a4+146	O	1
8018	KC878714	K1a4j	K1a4j	O	1	K1a4j	O	1
8019	KC911477	K1a4j1	K1a4j1	O	1	K1a4j1	O	1
8020	JX273292	K1a4j1	K1a4j1	O	1	K1a4j1	O	1
8021	EU600368	K1a5a	K1a5a	O	1	K1a5a	O	1
8022	JX273274	K1a5a	K1a5a	O	1	K1a5a	O	1
8023	HQ637410	K1a5b	K1a5b	O	1	K1a5b	O	1
8024	JQ706059	K1a5b	K1a5b	O	1	K1a5b	O	1
8025	EU600369	K1a6	K1a6	O	1	K1a6	O	1
8026	JX273288	K1a6	K1a6	O	1	K1a6	O	1

8027	DQ301807	K1a7	K1a7	O	1	K1a7	O	1
8028	JQ706029	K1a7	K1a7	O	1	K1a7	O	1
8029	JQ702902	K1a8	K1a8	O	1	K1a8	O	1
8030	DQ301817	K1a8a	K1a8a	O	1	K1a8a	O	1
8031	DQ301816	K1a8a1	K1a8a1	O	1	K1a8a1	O	1
8032	DQ301818	K1a8a1	K1a8a1	O	1	K1a8a1	O	1
8033	FJ460551	K1a8b	K1a8b	O	1	K1a8b	O	1
8034	JQ706043	K1a8b	K1a8b	O	1	K1a8b	O	1
8035	FJ348175	K1a+195	K1a+195	O	1	K1a+195	O	1
8036	GU471244	K1a+195	K1a+195	O	1	K1a+195	O	1
8037	GU295448	K1a9	K1a9	O	1	K1a9	O	1
8038	JQ704228	K1a9	K1a9	O	1	K1a9	O	1
8039	JQ702740	K1a10	K1a10	O	1	K1a10	O	1
8040	JX535003	K1a10a	K1a10a	O	1	K1a10a	O	1
8041	HM054058	K1a10a	K1a10a	O	1	K1a10a	O	1
8042	JQ702796	K1a13	K1a13	O	1	K1a13	O	1
8043	GU944475	K1a13a	K1a13a	O	1	K1a13a	O	1
8044	JN202723	K1a13a	K1a13a	O	1	K1a13a	O	1
8045	GU471243	K1a14	K1a14	O	1	K1a14	O	1
8046	HQ675035	K1a14	K1a14	O	1	K1a14	O	1
8047	JQ703379	K1a15	K1a15	O	1	K1a15	O	1
8048	JQ702674	K1a15	K1a15	O	1	K1a15	O	1
8049	JQ703365	K1a16	K1a16	O	1	K1a16	O	1
8050	JQ702271	K1a16	K1a16	O	1	K1a16	O	1
8051	AY882395	K1a26	K1a26	O	1	K1a26	O	1
8052	GU811147	K1a26	K1a26	O	1	K1a26	O	1
8053	JQ702273	K1a+150	K1a+150	O	1	K1a+150	O	1
8054	JX193906	K1a11	K1a+150	X	26	K1a11	O	1
8055	JQ705747	K1a11	K1a+150	X	18	K1a11	O	1
8056	JN657206	K1a11a	K1a+150	X	5	K1a11a	O	1
8057	JQ703607	K1a11a	K1a+150	X	4	K1a11a	O	1
8058	JQ705336	K1a11a1	K1a11a1	O	1	K1a11a1	O	1
8059	JX679248	K1a11a1	K1a15	X	2	K1a11a1	O	1
8060	EU523125	K1a11b	K1a+150	X	4	K1a11b	O	1
8061	EU603402	K1a11b	K1a+150	X	4	K1a11b	O	1
8062	JQ703891	K1a24	K1a24	O	1	K1a24	O	1
8063	JQ702717	K1a24a	K1a24a	O	1	K1a24a	O	1

8064	JF489152	K1a24a	K1a24a	O	1	K1a24a	O	1
8065	JQ705751	K1a30	K1a30	O	1	K1a30	O	1
8066	KC878711	K1a30a	K1a30a	O	1	K1a30a	O	1
8067	KC878710	K1a30a	K1a30a	O	1	K1a30a	O	1
8068	JQ706056	K1a31	K1a31	O	1	K1a31	O	1
8069	KC911309	K1a31	K1a31	O	1	K1a31	O	1
8070	JQ703323	K1a12	K1a12	O	1	K1a+195	X	2
8071	EU600362	K1a12a	K1a12a	O	1	K1a12a	O	1
8072	JQ703711	K1a12a	K1a12a	O	1	K1a12a	O	1
8073	JQ706035	K1a12a1	K1a12a1	O	1	K1a12a1	O	1
8074	JQ706066	K1a12a1a	K1a12a1a	O	1	K1a12a1a	O	1
8075	JQ706065	K1a12a1a	K1a12a1a	O	1	K1a12a1a	O	1
8076	JQ706034	K1a17	K1a17	O	1	K1a17	O	1
8077	JQ706064	K1a17a	K1a17a	O	1	K1a17a	O	1
8078	EU600361	K1a17a	K1a17a	O	1	K1a17a	O	1
8079	JQ706048	K1a18	K1a18	O	1	K1a18	O	1
8080	EU600365	K1a18	K1a18	O	1	K1a18	O	1
8081	JQ308836	K1a19	K1a19	O	1	K1a19	O	1
8082	JQ706040	K1a19	K1a19	O	1	K1a19	O	1
8083	JQ706039	K1a19a	K1a19a	O	1	K1a19a	O	1
8084	JQ706063	K1a19a	K1a19a	O	1	K1a19a	O	1
8085	JQ703522	K1a23	K1a23	O	1	K1a23	O	1
8086	JQ706042	K1a23	K1a23	O	1	K1a+195	X	2
8087	HM625696	K1a25	K1a25	O	1	K1a25	O	1
8088	JQ706051	K1a25	K1a25	O	1	K1a25	O	1
8089	JN415475	K1a27	K1a27	O	1	K1a27	O	1
8090	JQ705460	K1a27	K1a27	O	1	K1a27	O	1
8091	JF303729	K1a28	K1a28	O	1	K1a28	O	1
8092	JX273263	K1a28	K1a28	O	1	K1a28	O	1
8093	JX273268	K1a29	K1a29	O	1	K1a29	O	1
8094	JX273252	K1a29a	K1a29a	O	1	K1a29a	O	1
8095	HM852805	K1a29a	K1a29a	O	1	K1a29a	O	1
8096	GU797785	K1b1a1	K1b1a1	O	1	K1b1a1	O	1
8097	JQ703130	K1b1a1	K1b1a1	O	1	K1b1a1	O	1
8098	JQ702168	K1b1a1a	K1b1a1a	O	1	K1b1a1a	O	1
8099	JQ702919	K1b1a1a	K1b1a1a	O	1	K1b1a1a	O	1
8100	JQ702752	K1b1a1b	K1b1a1b	O	1	K1b1a1b	O	1

8101	GU361772	K1b1a1c	K1b1a1c	O	1	K1b1a1c	O	1
8102	EU239477	K1b1a1c1	K1b1a1c1	O	1	K1b1a1c1	O	1
8103	KC158584	K1b1a1c1	K1b1a1c1	O	1	K1b1a1c1	O	1
8104	NA11892	K1b1a1d	K1b1a1d	O	1	K1b1a1d	O	1
8105	JX891380	K1b1a1d1	K1b1a1d1	O	1	K1b1a1d1	O	1
8106	JX154012	K1b1a1d1	K1b1a1d1	O	1	K1b1a1d1	O	1
8107	EU714300	K1b1a2	K1b1a2	O	1	K1b1a2	O	1
8108	EF177415	K1b1a2	K1b1a2	O	1	K1b1a2	O	1
8109	DQ301800	K1b1b	K1b1b	O	1	K1b1b	O	1
8110	AY495260	K1b1b1	K1b1b1	O	1	K1b1b1	O	1
8111	JQ703412	K1b1b1	K1b1b1	O	1	K1b1b1	O	1
8112	EU600370	K1b1c	K1b1c	O	1	K1b1c	O	1
8113	GU455378	K1b1c	K1b1c	O	1	K1b1c	O	1
8114	JQ705467	K1b2a	K1b2a	O	1	K1b2a	O	1
8115	HQ000094	K1b2a1	K1b2a1	O	1	K1b2a1	O	1
8116	FJ348210	K1b2a1	K1b2a1	O	1	K1b2a1	O	1
8117	JQ705289	K1b2a1a	K1b2a1a	O	1	K1b2a1a	O	1
8118	JQ705519	K1b2a1a1	K1b2a1a1	O	1	K1b2a1a1	O	1
8119	JQ702926	K1b2a1a1	K1b2a1a1	O	1	K1b2a1a1	O	1
8120	JQ705044	K1b2a2	K1b2a2	O	1	K1b2a2	O	1
8121	EU372659	K1b2a2	K1b2a2	O	1	K1b2a2	O	1
8122	EU849091	K1b2a2a	K1b2a2a	O	1	K1b2a2a	O	1
8123	JQ702636	K1b2a2a	K1b2a2a	O	1	K1b2a2a	O	1
8124	JQ703021	K1b2a3	K1b2a3	O	1	K1b2a3	O	1
8125	JX273244	K1b2a3	K1b2a3	O	1	K1b2a3	O	1
8126	JQ703876	K1b2b	K1b2b	O	1	K1b2b	O	1
8127	JX273245	K1b2b	K1b2b	O	1	K1b2b	O	1
8128	JX153522	K1b2b1	K1b2b1	O	1	K1b2b1	O	1
8129	KC257396	K1b2b1	K1b2b1	O	1	K1b2b1	O	1
8130	GU936958	K1c1	K1c1	O	1	K1c1	O	1
8131	GU722602	K1c1	K1c1	O	1	K1c1	O	1
8132	HM851442	K1c1a	K1c1a	O	1	K1c1a	O	1
8133	AY882394	K1c1a	K1c1a	O	1	K1c1a	O	1
8134	AY495250	K1c1b	K1c1b	O	1	K1c1b	O	1
8135	AY495243	K1c1b	K1c1b	O	1	K1c1b	O	1
8136	JQ703860	K1c1c	K1c1c	O	1	K1c1c	O	1
8137	EU262720	K1c1c	K1c1c	O	1	K1c1c	O	1

8138	JQ703292	K1c1d	K1c1d	O	1	K1c1d	O	1
8139	JF819714	K1c1d	K1c1d	O	1	K1c1d	O	1
8140	GU123011	K1c1e	K1c1e	O	1	K1c1e	O	1
8141	JQ702901	K1c1e	K1c1e	O	1	K1c1e	O	1
8142	JQ703815	K1c1f	K1c1f	O	1	K1c1f	O	1
8143	HQ398201	K1c1f	K1c1f	O	1	K1c1f	O	1
8144	JQ702722	K1c1g	K1c1g	O	1	K1c1g	O	1
8145	JQ703189	K1c1g	K1c1g	O	1	K1c1g	O	1
8146	KF736454	K1c1h	K1c1h	O	1	K1c1h	O	1
8147	JX153393	K1c1h	K1c1h	O	1	K1c1h	O	1
8148	HM032895	K1c1i	K1c1i	O	1	K1c1i	O	1
8149	JN897373	K1c1i	K1c1i	O	1	K1c1i	O	1
8150	JN620369	K1c2	K1c2	O	1	K1c2	O	1
8151	JQ702129	K1c2	K1c2	O	1	K1c2	O	1
8152	GU323604	K1c2a	K1c2	X	2	K1c2a	O	1
8153	DQ830736	K1c2a	K1c2	X	2	K1c2a	O	1
8154	JQ704724	K1d	K1d	O	1	K1d	O	1
8155	JX152800	K1d1	K1d1	O	1	K1d1	O	1
8156	JQ702254	K1d1	K1d1	O	1	K1d1	O	1
8157	JX293716	K1e	K1e	O	1	K1e	O	1
8158	EU073969	K1e1	K1e1	O	1	K1e1	O	1
8159	JQ705063	K1e1	K1e1	O	1	K1e1	O	1
8160	EU810403	K1f	K1f	O	1	K1f	O	1
8161	AY495241	K2a	K2a	O	1	K2a	O	1
8162	JQ702887	K2a	K2a	O	1	K2a	O	1
8163	JQ665461	K2a1	K2a1	O	1	K2a1	O	1
8164	DQ301815	K2a1a	K2a1a	O	1	K2a1a	O	1
8165	KF408388	K2a2	K2a2	O	1	K2a2	O	1
8166	AY495246	K2a2	K2a2	O	1	K2a2	O	1
8167	HQ154135	K2a2a	K2a2a	O	1	K2a2a	O	1
8168	EU327986	K2a2a1	K2a2a1	O	1	K2a2a1	O	1
8169	JQ703629	K2a2a1	K2a2a1	O	1	K2a2a1	O	1
8170	JQ704707	K2a3	K2a3	O	1	K2a3	O	1
8171	HM103361	K2a3	K2a3	O	1	K2a3	O	1
8172	JQ703540	K2a3a	K2a3a	O	1	K2a3a	O	1
8173	AY495249	K2a3a1	K2a3a1	O	1	K2a3a1	O	1
8174	HM625707	K2a3a1	K2a3a1	O	1	K2a3a1	O	1

8175	AY495242	K2a4	K2a4	O	1	K2a4	O	1
8176	JQ705156	K2a4	K2a4	O	1	K2a4	O	1
8177	EU597528	K2a5	K2a5	O	1	K2a5	O	1
8178	JQ703018	K2a5	K2a5	O	1	K2a5	O	1
8179	JQ704076	K2a5a	K2a5a	O	1	K2a5a	O	1
8180	EU718789	K2a5a1	K2a5a1	O	1	K2a5a1	O	1
8181	JQ704843	K2a5a1	K2a5a1	O	1	K2a5a1	O	1
8182	AY714017	K2a5b	K2a5b	O	1	K2a5b	O	1
8183	KC911393	K2a5b	K2a5b	O	1	K2a5b	O	1
8184	AY495239	K2a6	K2a6	O	1	K2a6	O	1
8185	HQ213857	K2a6	K2a6	O	1	K2a6	O	1
8186	JQ702380	K2a7	K2a7	O	1	K2a7	O	1
8187	EU284177	K2a7	K2a7	O	1	K2a7	O	1
8188	DQ282493	K2a8	K2a8	O	1	K2a8	O	1
8189	DQ282503	K2a8	K2a8	O	1	K2a8	O	1
8190	JQ702282	K2a9	K2a9	O	1	K2a9	O	1
8191	KF030560	K2a10	K2a10	O	1	K2a10	O	1
8192	JQ702134	K2a10	K2a10	O	1	K2a10	O	1
8193	EU884127	K2a11	K2a11	O	1	K2a11	O	1
8194	HG01334	K2a11	K2a11	O	1	K2a11	O	1
8195	JQ704070	K2b1	K2b1	O	1	K2b1	O	1
8196	JQ701883	K2b1a	K2b1a	O	1	K2b1a	O	1
8197	KF644446	K2b1a	K2b1a	O	1	K2b1a	O	1
8198	JQ705092	K2b1a1	K2b1a1	O	1	K2b1a1	O	1
8199	JQ703767	K2b1a1	K2b1a1	O	1	K2b1a1	O	1
8200	JN409346	K2b1a1a	K2b1a1a	O	1	K2b1a1a	O	1
8201	EU770310	K2b1a1a	K2b1a1a	O	1	K2b1a1a	O	1
8202	JF497777	K2b1a2	K2b1a2	O	1	K2b1a2	O	1
8203	JX847130	K2b1a2	K2b1a2	O	1	K2b1a2	O	1
8204	EU294321	K2b1a3	K2b1a3	O	1	K2b1a3	O	1
8205	HG00128	K2b1a3	K2b1a3	O	1	K2b1a3	O	1
8206	JQ702075	K2b1a4	K2b1a4	O	1	K2b1a4	O	1
8207	JX153873	K2b1a4	K2b1a4	O	1	K2b1a4	O	1
8208	JQ704064	K2b1b	K2b1b	O	1	K2b1b	O	1
8209	JQ704900	K2b1b	K2b1b	O	1	K2b1b	O	1
8210	JX273249	K2b2	K2b2	O	1	K2b2	O	1
8211	KC911571	K2b2	K2b2	O	1	K2b2	O	1

8212	DQ301796	K2c	K2c	O	1	K2c	O	1
8213	JX273247	K2c	K2c	O	1	K2c	O	1
8214	NA18539	K3	K3	O	1	K3	O	1
8215	HM852886	K3	K3	O	1	K3	O	1
8216	KC521459	U8c	U8c	O	1	U8c	O	1

HG, haplogroup

O, identical to Phylotree definition; X, non-identical
