# Supplementary material for: Mitochondrial Haplogroup Classification of Ancient DNA Samples Using Haplotracker
Source: Biomed Res Int. 2022 Mar 18;2022:5344418. doi: 10.1155/2022/5344418 (PMC8956381; doi:10.1155/2022/5344418)
Supplement: Supplementary Materials — Fig. S1: characterization of Phylotree-provided control region sequences tested for haplogroup classification by Haplotracker. Fig. S2: minimum number of amplicons required by Haplotracker in discriminating between haplogroups using mtDNA control and coding region sequences. Fig. S3: variant identification of an aDNA sample (MNW3) using an HRM real-time PCR. Table S1: haplogroups and their variant profiles extracted from Phylotree mtDNA Build 17. Table S2: haplogroup frequency carrying an extra variant in 118,869 haplotypes. Table S3: haplogroup frequency carrying a missing variant in 118,869 haplotypes. Table S4: haplogroup frequency in 118,869 haplotypes. Table S5: list of ancient human samples found in 2,000-year-old elite Xiongnu cemetery in Northeast Mongolia. Table S6: primers for the amplification of mtDNA coding region segments for haplogroup determination. Table S7: high-resolution melting real-time PCR primer design for screening variants to differentiate haplogroups G1a1, G1a1a, and G1a1b. Table S8: haplogroup classification of full-length mtGenome sequences from Phylotree (n = 8,216). Table S9: haplogroup classification with full-length and control region sequences of mtDNA using Haplotracker and HaploGrep 2. Table S10: comparison of servers using control region sequences from GenBank before December 25, 2018 (n = 45,177). Table S11: comparison details for the servers using control region sequences from GenBank before December 25, 2018 (n = 45,177). Table S12: comparison of servers using control region sequences downloaded from GenBank from December 26, 2018 to August 22, 2019. Table S13: sequences of mtDNA PCR products from Mongolian ancient DNA samples. Table S14: haplogroup classification of Mongolian ancient DNA samples using Haplotracker. Table S15: minimum number of amplicons required by Haplotracker in discriminating between haplogroups using mtDNA control and coding region sequences. Table S16: minimum number of amplicons per superhaplogroup requ [file 5344418.f1.zip › 5344418.f12.pdf]

Table S9. Haplogroup classification with full-length and control region sequences of mtDNA using Haplotracker and HaploGrep 2

Rank[1]	HaploGrep 2				Haplotracker				
	No. of Samples	%	CUSUM	%	No. of Samples	%	CUSUM	%	P value[2]
1	2788	33.9	2788	33.9	4647	56.6	4647	56.6	<0.0001
2	971	11.8	3759	45.8	886	10.8	5533	67.3	<0.0001
3	620	7.5	4379	53.3	445	5.4	5978	72.8	<0.0001
4	533	6.5	4912	59.8	355	4.3	6333	77.1	<0.0001
5	296	3.6	5208	63.4	179	2.2	6512	79.3	<0.0001
6	272	3.3	5480	66.7	165	2.0	6677	81.3	<0.0001
7	188	2.3	5668	69.0	118	1.4	6795	82.7	<0.0001
8	247	3.0	5915	72.0	129	1.6	6924	84.3	<0.0001
9	139	1.7	6054	73.7	99	1.2	7023	85.5	<0.0001
10	113	1.4	6167	75.1	60	0.7	7083	86.2	<0.0001
11	136	1.7	6303	76.7	63	0.8	7146	87.0	<0.0001
12	99	1.2	6402	77.9	43	0.5	7189	87.5	<0.0001
13	120	1.5	6522	79.4	48	0.6	7237	88.1	<0.0001
14	0	0.0	6522	79.4	33	0.4	7270	88.5	<0.0001
15	73	0.9	6595	80.3	40	0.5	7310	89.0	<0.0001
16	49	0.6	6644	80.9	15	0.2	7325	89.2	<0.0001
17	49	0.6	6693	81.5	31	0.4	7356	89.5	<0.0001
18	21	0.3	6714	81.7	4	0.0	7360	89.6	<0.0001
19	67	0.8	6781	82.5	48	0.6	7408	90.2	<0.0001
20	14	0.2	6795	82.7	4	0.0	7412	90.2	<0.0001
21	45	0.5	6840	83.3	6	0.1	7418	90.3	<0.0001
22	10	0.1	6850	83.4	18	0.2	7436	90.5	<0.0001
23	96	1.2	6946	84.5	50	0.6	7486	91.1	<0.0001
24	8	0.1	6954	84.6	28	0.3	7514	91.5	<0.0001
25	22	0.3	6976	84.9	31	0.4	7545	91.8	<0.0001
26	30	0.4	7006	85.3	2	0.0	7547	91.9	<0.0001
27	15	0.2	7021	85.5	5	0.1	7552	91.9	<0.0001
28	47	0.6	7068	86.0	40	0.5	7592	92.4	<0.0001
29	11	0.1	7079	86.2	0	0.0	7592	92.4	<0.0001
30	7	0.1	7086	86.2	3	0.0	7595	92.4	<0.0001
31	65	0.8	7151	87.0	37	0.5	7632	92.9	0.0001
32	4	0.0	7155	87.1	8	0.1	7640	93.0	0.0001
33	49	0.6	7204	87.7	1	0.0	7641	93.0	0.0004
34	15	0.2	7219	87.9	1	0.0	7642	93.0	0.0006
35	60	0.7	7279	88.6	75	0.9	7717	93.9	0.0004
36	70	0.9	7349	89.4	1	0.0	7718	93.9	0.003
37	9	0.1	7358	89.6	30	0.4	7748	94.3	0.0017
38	6	0.1	7364	89.6	1	0.0	7749	94.3	0.0019
39	4	0.0	7368	89.7	2	0.0	7751	94.3	0.002
40	4	0.0	7372	89.7	2	0.0	7753	94.4	0.0021
41	52	0.6	7424	90.4	1	0.0	7754	94.4	0.0078
42	30	0.4	7454	90.7	0	0.0	7754	94.4	0.0157
43	13	0.2	7467	90.9	44	0.5	7798	94.9	0.0076

44	5	0.1	7472	90.9	1	0.0	7799	94.9	0.0081
45	6	0.1	7478	91.0	3	0.0	7802	95.0	0.0094
46	37	0.5	7515	91.5	1	0.0	7803	95.0	0.0217
47	33	0.4	7548	91.9	0	0.0	7803	95.0	0.0428
48	6	0.1	7554	91.9	0	0.0	7803	95.0	0.048
49	7	0.1	7561	92.0	12	0.1	7815	95.1	0.0446
50	105	1.3	7666	93.3	2	0.0	7817	95.1	0.2406
>50	0	0.0	7666	93.3	379	4.6	8196	99.8	<0.0001
Not foun	550	6.7	8216	100.0	20	0.2	8216	100.0	<0.0001[3]

[1] Rank of haplogroup call in agreement with phylotree Build 17 haplogroup assinment

[2] MedCalc Version 19.0.5 (comparison of two rates)

[3] Comparison between unfound HGs
