# Supplementary material for: Mitochondrial Haplogroup Classification of Ancient DNA Samples Using Haplotracker
Source: Biomed Res Int. 2022 Mar 18;2022:5344418. doi: 10.1155/2022/5344418 (PMC8956381; doi:10.1155/2022/5344418)
Supplement: Supplementary Materials — Fig. S1: characterization of Phylotree-provided control region sequences tested for haplogroup classification by Haplotracker. Fig. S2: minimum number of amplicons required by Haplotracker in discriminating between haplogroups using mtDNA control and coding region sequences. Fig. S3: variant identification of an aDNA sample (MNW3) using an HRM real-time PCR. Table S1: haplogroups and their variant profiles extracted from Phylotree mtDNA Build 17. Table S2: haplogroup frequency carrying an extra variant in 118,869 haplotypes. Table S3: haplogroup frequency carrying a missing variant in 118,869 haplotypes. Table S4: haplogroup frequency in 118,869 haplotypes. Table S5: list of ancient human samples found in 2,000-year-old elite Xiongnu cemetery in Northeast Mongolia. Table S6: primers for the amplification of mtDNA coding region segments for haplogroup determination. Table S7: high-resolution melting real-time PCR primer design for screening variants to differentiate haplogroups G1a1, G1a1a, and G1a1b. Table S8: haplogroup classification of full-length mtGenome sequences from Phylotree (n = 8,216). Table S9: haplogroup classification with full-length and control region sequences of mtDNA using Haplotracker and HaploGrep 2. Table S10: comparison of servers using control region sequences from GenBank before December 25, 2018 (n = 45,177). Table S11: comparison details for the servers using control region sequences from GenBank before December 25, 2018 (n = 45,177). Table S12: comparison of servers using control region sequences downloaded from GenBank from December 26, 2018 to August 22, 2019. Table S13: sequences of mtDNA PCR products from Mongolian ancient DNA samples. Table S14: haplogroup classification of Mongolian ancient DNA samples using Haplotracker. Table S15: minimum number of amplicons required by Haplotracker in discriminating between haplogroups using mtDNA control and coding region sequences. Table S16: minimum number of amplicons per superhaplogroup requ [file 5344418.f1.zip › 5344418.f13.pdf]

Table S10. Comparison of servers using control region sequences from GenBank before December 25, 2018 (n=45,177)

Rank	HaploGrep 2				Haplotracker			
	No of Samples	%	CUSUM	%	No of Samples	%	CUSUM	%
1	11104	24.6	11104	24.6	24588	54.4	24588	54.4*
2	5069	11.2	16173	35.8	4964	11.0	29552	65.4*
3	3752	8.3	19925	44.1	2463	5.5	32015	70.9*
4	3429	7.6	23354	51.7	2529	5.6	34544	76.5*
5	1702	3.8	25056	55.5	1054	2.3	35598	78.8*
6	1945	4.3	27001	59.8	1125	2.5	36723	81.3*
7	1094	2.4	28095	62.2	592	1.3	37315	82.6*
8	1598	3.5	29693	65.7	713	1.6	38028	84.2*
9	940	2.1	30633	67.8	528	1.2	38556	85.3*
10	789	1.7	31422	69.6	439	1.0	38995	86.3*
11-50	10967	24.3	42389	93.8	4267	9.4	43262	95.8**
>51		0.0	42389	93.8	1472	3.3	44734	99*
Not found	2745	6.1	45134	99.9	443	1.0***	45177	100.0
Server error	43	0.1	45177	100.0	0	0.0****	45177	100.0

*P<0.0001, comparison of the concordance rate of Haplotracker and HaploGrep 2 using MedCalc Version 19.0.5 (comparion of two rates)

**P=0.0029, comparison of the concordance rate of Haplotracker and HaploGrep 2 using MedCalc Version 19.0.5 (comparion of two rates)

***P<0.0001, comparison of the unfound HG rate of Haplotracker and HaploGrep 2 using MedCalc Version 19.0.5 (comparion of two rates)

****P<0.0001, comparison of the server error rate of Haplotracker and HaploGrep 2 using MedCalc Version 19.0.5 (comparion of two rates)
