# Supplementary material for: Mitochondrial Haplogroup Classification of Ancient DNA Samples Using Haplotracker
Source: Biomed Res Int. 2022 Mar 18;2022:5344418. doi: 10.1155/2022/5344418 (PMC8956381; doi:10.1155/2022/5344418)
Supplement: Supplementary Materials — Fig. S1: characterization of Phylotree-provided control region sequences tested for haplogroup classification by Haplotracker. Fig. S2: minimum number of amplicons required by Haplotracker in discriminating between haplogroups using mtDNA control and coding region sequences. Fig. S3: variant identification of an aDNA sample (MNW3) using an HRM real-time PCR. Table S1: haplogroups and their variant profiles extracted from Phylotree mtDNA Build 17. Table S2: haplogroup frequency carrying an extra variant in 118,869 haplotypes. Table S3: haplogroup frequency carrying a missing variant in 118,869 haplotypes. Table S4: haplogroup frequency in 118,869 haplotypes. Table S5: list of ancient human samples found in 2,000-year-old elite Xiongnu cemetery in Northeast Mongolia. Table S6: primers for the amplification of mtDNA coding region segments for haplogroup determination. Table S7: high-resolution melting real-time PCR primer design for screening variants to differentiate haplogroups G1a1, G1a1a, and G1a1b. Table S8: haplogroup classification of full-length mtGenome sequences from Phylotree (n = 8,216). Table S9: haplogroup classification with full-length and control region sequences of mtDNA using Haplotracker and HaploGrep 2. Table S10: comparison of servers using control region sequences from GenBank before December 25, 2018 (n = 45,177). Table S11: comparison details for the servers using control region sequences from GenBank before December 25, 2018 (n = 45,177). Table S12: comparison of servers using control region sequences downloaded from GenBank from December 26, 2018 to August 22, 2019. Table S13: sequences of mtDNA PCR products from Mongolian ancient DNA samples. Table S14: haplogroup classification of Mongolian ancient DNA samples using Haplotracker. Table S15: minimum number of amplicons required by Haplotracker in discriminating between haplogroups using mtDNA control and coding region sequences. Table S16: minimum number of amplicons per superhaplogroup requ [file 5344418.f1.zip › 5344418.f15.pdf]

**Table S12. Comparison of servers using control region sequences downloaded from GenBank from December 26, 2018 to August 22, 2019**

| | HaploGrep 2 | | | | Haplotracker | | | |
|---|---|---|---|---|---|---|---|---|
| Rank | No of Samples | % | CUSUM | % | No of Samples | % | CUSUM | % |
| 1 | 316 | 27.6 | 316 | 27.6 | 510 | 44.5 | 510 | 44.5* |
| 2 | 168 | 14.7 | 484 | 42.3 | 186 | 16.2 | 696 | 60.8* |
| 3 | 69 | 6.0 | 553 | 48.3 | 50 | 4.4 | 746 | 65.2* |
| 4 | 69 | 6.0 | 622 | 54.3 | 89 | 7.8 | 835 | 72.9* |
| 5 | 22 | 1.9 | 644 | 56.2 | 21 | 1.8 | 856 | 74.8* |
| 6 | 33 | 2.9 | 677 | 59.1 | 27 | 2.4 | 883 | 77.1* |
| 7 | 20 | 1.7 | 697 | 60.9 | 13 | 1.1 | 896 | 78.3* |
| 8 | 58 | 5.1 | 755 | 65.9 | 19 | 1.7 | 915 | 79.9* |
| 9 | 25 | 2.2 | 780 | 68.1 | 17 | 1.5 | 932 | 81.4* |
| 10 | 84 | 7.3 | 864 | 75.5 | 36 | 3.1 | 968 | 84.5 |
| 11-50 | 224 | 19.6 | 1088 | 95.0 | 110 | 9.6 | 1078 | 94.1 |
| >51 | | 0.0 | 1088 | 95.0 | 34 | 3.0 | 1112 | 97.1 |
| Not found | 57 | 5.0 | 1145 | 100.0 | 33 | 2.9** | 1145 | 100 |

CR sequences used from complete mtGenome sequences downloaded from GenBank (Dec. 26, 2018 - Aug. 22, 2019) (n=1145) .

*P<0.0001, comparison of the concordance rate of Haplotracker and HaploGrep 2 using MedCalc Version 19.0.5 (comparison of two rates)

**P=0.0114, comparison of the unfound HG rate of Haplotracker and HaploGrep 2 using MedCalc Version 19.0.5 (comparison of two rates)
