# Supplementary material for: Mitochondrial Haplogroup Classification of Ancient DNA Samples Using Haplotracker
Source: Biomed Res Int. 2022 Mar 18;2022:5344418. doi: 10.1155/2022/5344418 (PMC8956381; doi:10.1155/2022/5344418)
Supplement: Supplementary Materials — Fig. S1: characterization of Phylotree-provided control region sequences tested for haplogroup classification by Haplotracker. Fig. S2: minimum number of amplicons required by Haplotracker in discriminating between haplogroups using mtDNA control and coding region sequences. Fig. S3: variant identification of an aDNA sample (MNW3) using an HRM real-time PCR. Table S1: haplogroups and their variant profiles extracted from Phylotree mtDNA Build 17. Table S2: haplogroup frequency carrying an extra variant in 118,869 haplotypes. Table S3: haplogroup frequency carrying a missing variant in 118,869 haplotypes. Table S4: haplogroup frequency in 118,869 haplotypes. Table S5: list of ancient human samples found in 2,000-year-old elite Xiongnu cemetery in Northeast Mongolia. Table S6: primers for the amplification of mtDNA coding region segments for haplogroup determination. Table S7: high-resolution melting real-time PCR primer design for screening variants to differentiate haplogroups G1a1, G1a1a, and G1a1b. Table S8: haplogroup classification of full-length mtGenome sequences from Phylotree (n = 8,216). Table S9: haplogroup classification with full-length and control region sequences of mtDNA using Haplotracker and HaploGrep 2. Table S10: comparison of servers using control region sequences from GenBank before December 25, 2018 (n = 45,177). Table S11: comparison details for the servers using control region sequences from GenBank before December 25, 2018 (n = 45,177). Table S12: comparison of servers using control region sequences downloaded from GenBank from December 26, 2018 to August 22, 2019. Table S13: sequences of mtDNA PCR products from Mongolian ancient DNA samples. Table S14: haplogroup classification of Mongolian ancient DNA samples using Haplotracker. Table S15: minimum number of amplicons required by Haplotracker in discriminating between haplogroups using mtDNA control and coding region sequences. Table S16: minimum number of amplicons per superhaplogroup requ [file 5344418.f1.zip › 5344418.f16.pdf]

Table S13. Sequences of mtDNA PCR products from Mongolian ancient DNA samples

Sample code	Determined haplogroup	PCR targets[1]	Region (NP)[2]	Sequnences	GenBank accession No.	Note
MNX2	D4j11	HV1a	16003-16196	TCTAATTTAAACTATTCTCTGTTCTTTCATGGGGAAGCAGATTTGG GTACCACCCAAGTATTGACTCACCCATCAACAACCGCTATGTATTT CGTACATTACTGCCAGCCACCATGAATATTGTACGGTACCATAAAT ACTTGACCACCTGTAGTACATAAAAACCCAATCCACATCAAAACC CCCTCCCCATG	OK042311	Present study
		HV1b	16217-16413	TCAACCTTCAACTATCACACATCAACTGCAACTCCAAAGCCACCC CTCACCCACTAGGATACCAACAAACCTACCCACCCTTAACAGTAC ATAGCACATAAAGCCATTTACCGTACATAGCACATTACAGTCAAA TCCCTTCTCGCCCCCATGGATGACCCCCCTCAGATAGGGGTCCCTT GACCACCATCCTCCGT	OK042312	Present study
		HV2a	35-263	GGAGCTCTCCATGCATTTGGTATTTTCGTCTGGGGGGTGTGCACGC GATAGCATTGCGAGACGCTGGAGCCGGAGCACCCTATGTCGCAGT ATCTGTCTTTGATTCCTGCCTCATCCTATTATTTATCGCACCTACGT TCAATATTACAGGCGAACATACTTACTAAAGTGTGTTAATTAATTA ATGCTTGTAGGACATAATAATAACAATTGAATGTCTGCACAGCCG	OK042313	Present study
		HV2b	184-367	GGCGAACATACTTACTAAAGTGTGTTAATTAATTAATGCTTGTAGG ACATAATAATAACAATTGAATGTCTGCACAGCCGCTTTCCACACA GACATCATAACAAAAAATTTCCACCAAACCCCCCCCTCCCCCCGC TTCTGGCCACAGCACTTAAACACATCTCTGCCAAACCCCAAAAAC AAAGA	OK042314	Present study
		D (5178A) +	5080-5305	ATTTAACTATTTATATTATCCTAACTACTACCGCATTCCTACTACTC AACTTAAACTCCAGCACCACGACCCTACTACTATCTCGCACCTGA AACAAGATAACATGACTAACACCCTTAATTCCATCCACCCTCCTCT CCCTAGGAGGCCTGCCCCCGCTAACCGGCTTTTTGCCCAAATGGG CCATTATCGAAGAATTCACAAAAAACAATAGCCTCATCATCCC	GQ145594	[30]
		D4j11 (11218) +	11157-11276	TCACCCGATGAGGCAACCAGCCAGAACGCCTGAACGCAGGCACAT ACTTCCTATTCTACACTCTAGTAGGCTCCCTTCCCCTACTCATCGC ACTAATTTACACTCACAACACCCTAGGCT	OK042323	Present study

MNX3	U2e1a1	HV1a	16039-16192	GCAGATTTGGGTGCCACCCAAGTATTGACTCACCCATCAACAACC GCTATGTATCTCGTACATTACTGCCAGCCACCATGAATATTGTACC GTACCATAAATACTTGACCACCTGTAGTACATAAAAACCCAATCC ACATCAACCCCCCCCCCC	OK042315	Present study
		HV1b	16217-16413	TCAACCCTCAACTATCACACATCAACTGCAACTCCAAAGCCACCC CTCACCCACTAGGATACCAACAAACCTACCCACCCTTAACAGTAC ATAGTACATAAAGCCATTTACCGTACATAGCACATTACAGTCAAA TCCCTTCTCGCCCCCATGGATGACCCCCCTCAGATAGGGGTCCCTT GACCACCATCCTCCGT	OK042316	Present study
		HV2a	35-263	GGAGCTCTCCATGCATTTGGTATTTTCGTCTGGGGGGTGTGCACGC GATAGCATTGCGAGACGCTGGAGCCGGAGCACCCTATGTCGCAGT ATCTGTCTTTGATTCCTGCCTCATCCCATTATTTATCGCACCTACGT TCAATATTACAGGCGAACATACTTACTAAAGTGTGTTAATTAATCA ATGCTTGTAGGACATAATAATAACAATTGAATGTCTGCACAGCCG	OK042317	Present study
		HV2b	184-392	GGCGAACATACTTACTAAAGTGTGTTAATTAATCAATGCTTGTAGG ACATAATAATAACAATTGAATGTCTGCACAGCCGCTTTCCACACA GACATCATAACAAAAAATTTCCACCAAACCCCCCCTCCCCCCGCT TCTGGCCACAGCACTTAAACATATCTCTGCCAAACCCCAAAAACA AAGAACCCTAACACCAGCCTAACCAGATT	OK042318	Present study
		U2e1a1 (3116) +	3050-3197	TAAAGTCCTACGTGATCTGAGTTCAGACCGGAGTAATCCAGGTCG GTTTCTATCTACTTCAAATTTCTCCCTGTACGAAAGGACAAGAGAA ATAAGGCCTACTTCACAAAGCGCCTTCCCCCGTAAATGATATCATC TCAACTTAGT	OK042324	Present study
		U2e1a1c (10127) -	10034-10162	TTTTGACAACATTCAAAAAAGAGTAATAAACTTCGCCTTAATTTTA ATAATCAACACCCTCCTAGCCTTACTACTAATAATTATTACATTTT GACTACCACAACTCAACGGCTACATAGAAAAATCCAC	OK042325	Present study
MNX4	D4e4a	HV1a	16003-16196	TCTAATTTAAACTATTCTCTGTTCTTTCATGGGGAAGCAGATTTGG GTACCACCCAAGTATTGACTCACCCATCAACAACCGCTATGTATTT CGTACATTACTGCCAGCCACCATGAATATTGTACGGTACCATAAAT ACTTGACCACCTGTAGTACATAAAAACCCAATCCACATCAAAACC CCCTCCCCATG	OK042319	Present study

HV1b	16217-16413	TCAACCTTCAACTATCACACATCAACTGCAACTCCAAAGCCACCC<br>CTCACCCACTAGGATACCAACAAACCTACCCACCCTTAACAGTAC<br>ATAGTACATAAAGCCATTTACCGTACATAGCACATTACAGTCAAA<br>TCCCTTCTCGCCCCCATGGATGACCCCCCTCAGATAGGGGTCCCTT<br>GACCACCATCCTCCGT	OK042320	Present study
HV2a	35-263	GGAGCTCTCCATGCATTTGGTATTTTCGTCTGGGGGGTGTGCACGC<br>GATAGCATTGCGAGACGCTGGAGCCGGAGCACCCTATGTCGCAGT<br>ATCTGTCTTTGATTCCTGCCTCATCCTATTATTTATCGCACCTACGT<br>TCAATATTACAGGCGAACATACTTACTAAAGTGTGTTAATTAATTA<br>ATGCTTGTAGGACATAATAATAACAATTGAATGTCTGCACAGCCG	OK042321	Present study
HV2b	212-369	TTAATTAATGCTTGTAGGACATAATAATAACAATTGAATGTCTGCA<br>CAGCCGCTTTCCACACAGACATCATAACAAAAAATTTCCACCAAA<br>CCCCCCCCTCCCCCCGCTTCTGGCCACAGCACTTAAACACATCTCT<br>GCCAAACCCCAAAAACAAAGAAC	OK042322	Present study
D (5178A) +	5080-5305	ATTTAACTATTTATATTATCCTAACTACTACCGCATTCCTACTACTC<br>AACTTAAACTCCAGCACCACGACCCTACTACTATCTCGCACCTGA<br>AACAAGATAACATGACTAACACCCTTAATTCCATCCACCCTCCTCT<br>CCCTAGGAGGCCTGCCCCCGCTAACCGGCTTTTTGCCCAAATGGG<br>CCATTATCGAAGAATTCACAAAAAACAATAGCCTCATCATCCC	GQ145590	[30]
D4 (3010) +	2956-3217	AGAGTCCATATCAACAATAGGGTTTACGACCTCGATGTTGGATCA<br>GGACATCCCAATGGTGCAGCCGCTATTAAAGGTTCGTTTGTTCAAC<br>GATTAAAGTCCTACGTGATCTGAGTTCAGACCGGAGTAATCCAGG<br>TCGGTTTCTATCTACTTCAAATTCCTCCCTGTACGAAAGGACAAGA<br>GAAATAAGGCCTACTTCACAAAGCGCCTTCCCCCGTAAATGATAT<br>CATCTCAACTTAGTATTATACCCACACCCACCCA	GQ145589	[30]
D4b (8020) -	7951-8070	ACTTCCCCCATTATTCCTAGAACCAGGCGACCTGCGACTCCTTGAC<br>GTTGACAATCGAGTAGTACTCCCGATTGAAGCCCCCATTCGTATAA<br>TAATTACATCACAAGACGTCTTGCACTC	OK042326	Present study
D4e (11215) +	11157-11276	TCACCCGATGAGGCAACCAGCCAGAACGCCTGAACGCAGGCACAT<br>ACTTCCTATTCTATACCCTAGTAGGCTCCCTTCCCCTACTCATCGC<br>ACTAATTTACACTCACAACACCCTAGGCT	OK042327	Present study

		D4j (11696) -	11628-11743	CAATCAGCCACATAGCCCTCGTAGTAACAGCCATTCTCATCCAAA<br>CCCCCTGAAGCTTCACCGGCGCAGTCATTCTCATAATCGCCCACG<br>GACTTACATCCTCATTACTATTCTGC	OK042328	Present study
		D4e1 (3316) -	3262-3370	ACTTAAAACTTTACAGTCAGAGGTTCAATTCCTCTTCTTAACAACA<br>TACCCATGGCCAACCTCCTACTCCTCATTGTACCCATTCTAATCGC<br>AATGGCATTCCTAATGC	OK042329	Present study
		D4e2 (15874) -	15819-15929	ACTTCACAACAATCCTAATCCTAATACCAACTATCTCCCTAATTGA<br>AAACAAAATACTCAAATGGGCCTGTCCTTGTAGTATAAACTAATA<br>CACCAGTCTTGTAAACCGGA	OK042330	Present study
		D4e4 (1935) +	1888-1992	GCCAAAGCTAAGACCCCCGAAACCAGACGAGCTACCTAAGAACA<br>GCTGAAAGAGCACACCCGTCTATGTAGCAAAATAGTGGGAAGATT<br>TATAGGTAGAGGCGAC	OK042331	Present study
		D4e4a (8683) +	8619-8739	CCCCACCTCCAAATATCTCATCAACAACCGACTAATCACCACCCA<br>ACAATGACTAATCAAACTAGCCTCAAAACAAATGATAGCCATACA<br>CAACACTAAAGGACGAACCTGATCTCTTATA	OK042332	Present study
		D4e4b (12882) -	12826-12935	GCCAACACAGCAGCCATTCAAGCAATCCTATACAACCGTATCGGC<br>GATATCGGTTTCATCCTCGCCTTAGCATGATTTATCCTACACTCCA<br>ACTCATGAGACCCACAACA	OK042333	Present study
MNE1	G2a1	HV1a	16003-16196	TCTAATTTAAACTATTCTCTGTTCTTTCATGGGGAAGCAGATTTGG<br>GTACCACCCAAGTATTGACTCACCCATCAACAACCGCTATGTATTT<br>CGTACATTACTGCCAGCCACCATGAATATTGTACGGTACCATAAAT<br>ACTTGACCACCTGTAGTACATAAAAACCCAATCCACATCAAAACC<br>CCCTCCCCATG	MZ702745	[31]
		HV1b	16217-16413	TCAACCTTCAGCTATCATACATCAACTGCAACTCCAAAGCCACCC<br>CTCACCCACTAGGATATCAACAAACCTACCCACCCTTAACAGTAC<br>ATAGTACATAAAGCCATTTACCGTACATAGCACATTACAGTCAAA<br>TCCCTTCTCGCCCCCATGGATGACCCCCCTCAGATAGGGGTCCCTT<br>GACCACCATCCTCCGT	MZ702746	[31]

		HV2a	35-263	GGAGCTCTCCATGCATTTGGTATTTTCGTCTGGGGGGTGTGCACGC<br>GATAGCATTGCGAGACGCTGGAGCCGGAGCACCCTATGTCGCAGT<br>ATCTGTCTTTGATTCCTGCCTCATCCTATTATTTATCGCACCTACGT<br>TCAATATTACAGGCGAACATACTTACTAAAGTGTGTTAATTAATTA<br>ATGCTTGTAGGACATAATAATAACAATTGAATGTCTGCACAGCCG	MZ702747	[31]
		HV2b	212-365	TTAATTAATGCTTGTAGGACATAATAATAACAATTGAATGTCTGCA<br>CAGCCGCTTTCCACACAGACATCATAACAAAAAATTTCCACCAAA<br>CCCCCCCCTCCCCCCGCTTCTGGCCACAGCACTTAAACACATCTCT<br>GCCAAACCCCAAAAACAAA	MZ702748	[31]
		G2a1 (14200) +	14190-14248	TTCCCCCGAGCAATCTCAATTACAATATATACACCAACAAACAAT<br>GTCCAACCAGTAACTACTACTAATCAACGCCCATAATCATACAAA<br>GCCCCC	OK042334	Present study
MNE2	A12	HV1a	16039-16192	GCAGATTTGGGTACCACCCAAGTATTGACTCACCCATCAACAACC<br>GCTATGTATTTCGTACATTACTGCCAGCCACCATGAATATTGTACG<br>GTACCATAAATACTTGACCACCTGTAGTACATAAAAACCCAATCC<br>ACATCAAAACCCCCCCCC	MZ702749	[31]
		HV1b	16217-16413	TCAACCTTCAACTATCACACATCAACTGCAACTCCAAAGCCACCC<br>TTCACCCACTAGGATACCAACAAACCTATCCACCCTTAACAGTAC<br>ATAGTACATAAAACCATTTACCGTACATAGCACATTACAGTCAAA<br>TCCCTTCTCGTCCCCATGGATGACCCCCCTCAGATAGGGGTCCCTT<br>GACCACCATCCTCCGT	MZ702750	[31]
		HV2a	35-263	GGAGCTCTCCATGCATTTGGTATTTTCGTCTGGGGGGTGTGCACGC<br>GATAGCATTGCGAGACGCTGGAGCCGGAGCACCCTATGTCGCAGT<br>ATCTGTCTTTGATTCCTGCCTCATCCCATTATTTATCGCACCTACGT<br>TCAATATTACAGGCGAACATACTTACTAAAGTGTGTTAATTAATTA<br>ATGCTTGTAGGACATAGTAATAACAATTGAATGTCTGCACAGCCG	MZ702751	[31]
		HV2b	212-392	TTAATTAATGCTTGTAGGACATAGTAATAACAATTGAATGTCTGCA<br>CAGCCGCTTTCCACACAGACATCATAACAAAAAATTTCCACCAAA<br>CCCCCCCTCCCCCCGCTTCTGGCCACAGCACTTAAACACATCTCTG<br>CCAAACCCCAAAAACAAAGAACCCTAACACCAGCCTAACCAGATT	MZ702752	[31]

		A12 (12720)	12652-12800	TGATATATAAACTCAGACCCAAACATTAATCAGTTCTTCAAATATC<br>TACTCATTTTCCTAATTACCATGCTAATCTTAGTTACCGCTAACAA<br>CCTATTCCAACTGTTCATCGGCTGAGAGGGCGTAGGAATTATATCC<br>TTCTTGCTCAT	OK042335	Present study
MNE3	D3	HV1a	16003-16196	TCTAATTTAAACTATTCTCTGTTCTTTCATGGGGAAGCAGATTTGG<br>GTACCACCCAAGTATTGACTCACCCATCAACAACCGCTATGTATTT<br>CGTACATTACTGCCAGCCACCATGAATATTGTACGGTACCATAAAT<br>ACTTGACCACCTGTAGTACATAAAAACCCAATCCACATCAAAACC<br>CCCTCCCCATG	MZ702766	[31]
		HV1b	16217-16413	TCAACCTTCAACTATCACACATCAACTGCAACTCCAAAGCCACCC<br>CTCACCCACTAGGATACCAACAAACCTACCCACCCTTAATAGTAC<br>ATAGTACATAAAACCATTTACCGTACATAGCACATTACAGTCAAA<br>TCCCTTCTCGCCCCCATGGATGACCCCCCTCAGATAGGGGTCCCTT<br>GACCACCATCCTCCGT	MZ702767	[31]
		HV2a	35-263	GGGAGCTCTCCA**T**GCATTTGGTATTTTCGTCTGGGGGGTGTGCACG<br>CGATAGCATTGCGAGACGCTGGAGCCGGAGCACCCTATGTCGCAG<br>TATCTGTCTTTGATTCCTGCCTCATCCTATTATTTATCGCACCTACG<br>TTCAATATTACAGGCGAACATACTTACTGAAGTGTGTTAATTAATT<br>AATGCTTGTAGGACATAATAACAACAATTGAATGTCTGCACAGCC<br>G	MZ702768	[31]
		HV2b	184-392	GGCGAACATACTTACTGAAGTGTGTTAATTAATTAATGCTTGTAGG<br>ACATAATAACAACAATTGAATGTCTGCACAGCCGCTTTCCACACA<br>GACATCATAACAAAAAATTTCCGCCAAACCCCCCCTCCCCCCGCT<br>TCTGGCCACAGCACTTAAACACATCTCTGCCAAACCCCAAAAACA<br>AAGAACCCTAACACCAGCCTAACCAGATT	MZ702769	[31]
		D3 (9785)	9692-9828	ACTGCTTATTACAATTTTACTGGGTCTCTATTTTACCCTCCTACAAG<br>CCTCAGAGTACTTCGAGTCTCCCTTCACCATTTCCGACGGCATCTA<br>TGGCTCAACATTTTTTGTAGCCACAGGCTTCCACGGACTTCACG	OK042336	Present study

MNW1	W3a1	HV1a	16003-16196	TCTAATTTAAACTATTCTCTGTTCTTTCATGGGGAAGCAGATTTGG<br>GTACCACCCAAGTATTGACTCACCCATCAACAACCGCTATGTATTT<br>CGTACATTACTGCCAGCCACCATGAATATTGTACGGTACCATAAAT<br>ACTTGACCACCTGTAGTACATAAAAACCCAATCCACATCAAAACC<br>CCCTCCCCATG	MZ702753	[31]
		HV1b	16217-16413	TCAACCTTCAACTATCACACATCAACTGCAACTCCAAAGCCACCC<br>CTCACCCACTAGGATACCAACAAACCTACCTACCCTTAACAGTAC<br>ATAGTACATAAAGCCATTTACCGTACATAGCACATTACAGTCAAA<br>TCCCTTCTCGTCCCCATGGATGACCCCCCTCAGATAGGGGTCCCTT<br>GACCACCATCCTCCGT	MZ702754	[31]
		HV2a	35-263	GGAGCTCTCCATGCATTTGGTATTTTCGTCTGGGGGGTGTGCACGC<br>GATAGCATTGCGAGACGCTGGAGCCGGAGCACCCTATGTCGCAGT<br>ATCTGTCTTTGATTCCTGCCTCATCCTATTATTTATCGCACCTACGT<br>TCAATATTACAGGCGAGCATATCTACTAAAGCGTATTAATTAATTA<br>ATGCTTGTAGGACATAATAATAACAATTGAATGTCTGCACAGCCG	MZ702755	[31]
		HV2b	184-365	GGCGAGCATATTTACTAAAGCGTATTAATTAATTAATGCTTGTAGG<br>ACATAATAATAACAATTGAATGTCTGCACAGCCGCTTTCCACACA<br>GACATCATAACAAAAAATTTCCACCAAACCCCCCCCTCCCCCCGC<br>TTCTGGCCACAGCACTTAAACACATCTCTGCCAAACCCCAAAAAC<br>AAA	MZ702756	[31]
		W3 (1406) +	1339-1467	GCCCATGAGGTGGCAAGAAATGGGCTACATTTTCTACCCCAGAAA<br>ACTACGATAGCCCTTATGAAACCTAAGGGTCGAAGGTGGATTTAG<br>CAGTAAACTGAGAGTAGAGTGCTTAGTTGAACAGGGCCC	OK042337	Present study
		W5 (15775) -<br>W (15884C) +<br>W3a (15784) +	15728-15901	CTAGCCGCAGACCTCCTCATTCTAACCTGAATCGGAGGACAACCA<br>GTAAGCTACCCCTTTACCATCATTGGACAAGTAGCATCCGTACTAT<br>ACTTCACAACAATCCTAATCCTAATACCAACTATCTCCCTAATTGA<br>AAACAAAATACTCAAATGGCCCTGTCCTTGTAGTATA	MZ702773	[31]
		W3a1 (13263) +	13188-13306	CACTCTGTTCGCAGCAGTCTGCGCCCTTACACAAAATGACATCAA<br>AAAAATCGTAGCCTTCTCCACTTCAAGTCAGCTAGGACTCATAATA<br>GTTACAATCGGCATCAACCAACCACACC	OK042338	Present study

		W3a1a (7151) -	7093-7219	TTCCCCTATTCTCAGGCTACACCCTAGACCAAACCTACGCCAAAATCCATTTCACTATCATATTCATCGGCGTAAATCTAACTTTCTTCCCACAACACTTTCTCGGCCTATCCGGAATGCCCCGACG	OK042339	Present study
		W3a1b (10245) -	10193-10281	CCCCGCCCGCGTCCCTTTCTCCATAAAATTCTTCTTAGTAGCTATTACCTTCTTATTATTTGATCTAGAAATTGCCCTCCTTTTACCCC	OK042340	Present study
MNW3	G1a1	HV1a	16003-16196	TCTAATTTAAACTATTCTCTGTTCTTTCATGGGGAAGCAGATTTGGGTACCACCCAAGTATTGACTCACCCATCAACAACCGCTATGTATTTCGTACATTACTGCCAGCCACCATGAATATTGTACGGTACCATAAATACTTGACCACCTGTAGTACATAAAAACCCAATCCACATCAAAACCCCCTCCCCATG	MZ702757	[31]
		HV1b	16217-16413	TCAACCTTCAACTATCACACATCAACTGCAACTCCAAAGCCACCCCTCACCCACTAGGATACCAACAAACCTACCCACCCTTAACAGTACATAGTACATAAAGCCATTCACCGTACATAGCACATTACAGTCAAATCCCTTCTCGTCCCCATGGATGACCCCCCTCAGATAGGGGTCCCTTGACCACCATCCTCCGT	MZ702758	[31]
		HV2a	35-263	GGAGCTCTCCATGCATTTGGTATTTTCGTCTGGGGGGTGTGCACGCGATAGCATTGCGAGACGCTGGAGCCGGAGCACCCTATGTCGCAGTATCTGTCTTTGATTCCTGCCTCATTCTATTATTTATCGCACCTACGTTCAATATTACAGGCGAACATACTTACTAAAGTGTGTTAATTAATTAATGCTTGTAGGACATAATAATAACAATTGAATGTCTGCACAGCCG	MZ702759	[31]
		HV2b	212-365	TTAATTAATGCTTGTAGGACATAATAATAACAATTGAATGTCTGCACAGCCGCTTTCCACACAGACATCATAACAAAAAATTTCCACCAAACCCCCCCTCCCCCCGCTTCTGGCCACAGCACTTAAACACATCTCTGCCAAACCCCAAAAACAAA	MZ702760	[31]
		G1a1 (15860) +	15728-15892	CTAGCCGCAGACCTCCTCATTCTAACCTGAATCGGAGGACAACCAGTAAGCTACCCTTTTACCATCATTGGACAAGTAGCATCCGTACTATACTTCACAACAATCCTAATCCTAATACCAACTATCTCCCTAGTTGAAAACAAAATACTCAAATGGGCCTGTCCT	OK042341	Present study
		G1a1a (11914) -	11867-11960	CCCCCCACTATTAACCTACTGGGAGAACTCTCTGTGCTAGTAACCACGTTCTCCTGATCAAATATCACTCTCCTACTTACAGGACTCAACATAC	OK042342	Present study

		G1a1b (12178) -	12135-12243	CTTGTAAATATAGTTTAACCAAAACATCAGATTGTGAATCTGACAACAGAGGCTTACGACCCCTTATTTACCGAGAAAGCTCACAAGAACTGCTAACTCATGCCCCCAT	OK042343	Present study
MNW4	C4a1a+195	HV1a	16003-16196	TCTAATTTAAACTATTCTCTGTTCTTTCATGGGGAAGCAGATTTGGGTACCACCCAAGTATTGACTCACCCATCAACAACCGCTATGTATTTCGTACATTACTGCCAGCCACCATGAATATTGTACAGTACCATAAATACTTGACCACCTGTAGTACATAAAAACCCAATCCACATCAAAACCCCCTCCCCATG	MZ702761	[31]
		HV1b	16217-16413	TCAACCTTCAACTATCACACATCAACTGCAACTCCAAAGCCACCCCTCACCCACTAGGATACCAACAAACCTACCCACCCTCAACAGTACATAGTACATAAAGCCATTTATCGTACATAGCACATTACAGTCAAATCCCTTCTCGTCCCCATGGATGACCCCCCTCAGATAGGGGTCCCTTGACCACCATCCTCCGT	MZ702762	[31]
		HV2a	35-263	GGAGCTCTCCATGCATTTGGTATTTTCGTCTGGGGGGTGTGCACGCGATAGCATTGCGAGACGCTGGAGCCGGAGCACCCTATGTCGCAGTATCTGTCTTTGATTCCTGCCTCATCCTATTATTTATCGCACCTACGTTCAATATTACAGGCGAACATACCTACTAAAGTGTGTTAATTAATTAATGCTTGTAGGACATAATAATAACAATTGATGTCTGCACAGCCG	MZ702763	[31]
		HV2b	216-365	TTAATGCTTGTAGGACATAATAATAACAATTGATGTCTGCACAGCCGCTTTCCACACAGACATCATAACAAAAAATTTCCACCAAACCCCCCCCTCCCCCCGCTTCTGGCCACAGCACTTAAACACATCTCTGCCAAACCCCAAAAACAAA	MZ702764	[31]
		C4a1a2 (10891) -	10802-10939	ACATGACTTTCCAAAAAACACATAATTTGAATCAACACAACCACCCACAGCCTAATTATTAGCATCATCCCCCTACTATTTTTTAACCAAATCAACAACAACCTATTTAGCTGTTCCCCAACCTTTTCCTCCGACCCC	OK042344	Present study
		C4a1a3 (15607) -	15561-15686	GATATTTCCTATTCGCCTACACAATTCTCCGATCCGTCCCTAACAAACTAGGAGGCGTCCTTGCCCTATTACTATCCATCCTCATCCTAGCAATAATCCCCATCCTCCATATATCCAAACAACAAA	OK042345	Present study

C4a1a4 (12940) -	12868-13002	GGCGATATCGGTTTCATCCTCGCCTTAGCATGATTTATCCTACACT CCAACTCATGAGACCCACAACAAATAGCCCTTCTAAACGCTAATC CAAGCCTCACCCCACTACTAGGCCTCCTCCTAGCAGCAGCAGGC	OK042346	Present study

[1] Variants of the target haplogroup are indicated in parentheses.; +, variant present; -, no variant.

[2] Numbers indicate nucleotide positions based on the revised Cambridge reference sequence [29].
