# Supplementary material for: Mitochondrial Haplogroup Classification of Ancient DNA Samples Using Haplotracker
Source: Biomed Res Int. 2022 Mar 18;2022:5344418. doi: 10.1155/2022/5344418 (PMC8956381; doi:10.1155/2022/5344418)
Supplement: Supplementary Materials — Fig. S1: characterization of Phylotree-provided control region sequences tested for haplogroup classification by Haplotracker. Fig. S2: minimum number of amplicons required by Haplotracker in discriminating between haplogroups using mtDNA control and coding region sequences. Fig. S3: variant identification of an aDNA sample (MNW3) using an HRM real-time PCR. Table S1: haplogroups and their variant profiles extracted from Phylotree mtDNA Build 17. Table S2: haplogroup frequency carrying an extra variant in 118,869 haplotypes. Table S3: haplogroup frequency carrying a missing variant in 118,869 haplotypes. Table S4: haplogroup frequency in 118,869 haplotypes. Table S5: list of ancient human samples found in 2,000-year-old elite Xiongnu cemetery in Northeast Mongolia. Table S6: primers for the amplification of mtDNA coding region segments for haplogroup determination. Table S7: high-resolution melting real-time PCR primer design for screening variants to differentiate haplogroups G1a1, G1a1a, and G1a1b. Table S8: haplogroup classification of full-length mtGenome sequences from Phylotree (n = 8,216). Table S9: haplogroup classification with full-length and control region sequences of mtDNA using Haplotracker and HaploGrep 2. Table S10: comparison of servers using control region sequences from GenBank before December 25, 2018 (n = 45,177). Table S11: comparison details for the servers using control region sequences from GenBank before December 25, 2018 (n = 45,177). Table S12: comparison of servers using control region sequences downloaded from GenBank from December 26, 2018 to August 22, 2019. Table S13: sequences of mtDNA PCR products from Mongolian ancient DNA samples. Table S14: haplogroup classification of Mongolian ancient DNA samples using Haplotracker. Table S15: minimum number of amplicons required by Haplotracker in discriminating between haplogroups using mtDNA control and coding region sequences. Table S16: minimum number of amplicons per superhaplogroup requ [file 5344418.f1.zip › 5344418.f17.pdf]

Table S14. Haplogroup classification of Mongolian ancient DNA samples using Haplotracker

Sample code	Determined Haplogroup	Track No.	PCR targets[1]	Ranges[2]	Haplogroup prediction using Haplotracker						Confirmation
					Variants[3]	Identity	Rank	MRCA	Haplogroups[4]	Super-HG	
MNX2	D4j11	Track 1	HV1<br><br>HV2	16003-16196<br>16217-16413<br>35-263<br>184-367	 16223 16311 16362<br>73 263<br>263 309.1C 315.1C	100% (5/5)	6	M	[0.000] M43a1 M74 M74b M74b2 D4j+16311 **D4j11** {6}	M D	
		Track 2	D (5178A) +	5080-5305	5178A	100% (6/6)	2	D4j+16311	[0.000] D4j+16311 **D4j11** {2}	D	
		Track 3	D4j11 (11218) +	11157-11276	11218	100% (7/7)	1	D4j11	[0.000] **D4j11** {1}		D4j (11696) +<br>D4 (3010) +
MNX3	U2e1a1	Track 1	HV1<br><br><br>HV2	16039-16192<br><br>16217-16413<br>35-263<br>184-392	16051 16093 16129C 16182C<br>16183C 16189 16362<br>73 152 217 263<br>217 263 315.1C 340	90% (9/10)	1	U2e1	[0.101] **U2e1a1** {1}<br>[0.015] U2e1a1c {2}<br>[0.013] U2e1a {3}<br>[0.012] U2e1 {4}<br>[0.000] U2e1a1a U2e1c U2e1c1 U2e1d U2e1g {9}	U	
		Track 2	U2e1a1 (3116) +	3050-3197	3116	90.9% (10/11)	1	U2e1a1	[0.101] **U2e1a1** {1}<br>[0.015] U2e1a1c {2}		
		Track 3	U2e1a1c (10127) -	10034-10162		90.9% (10/11)	1	U2e1a1	[0.101] **U2e1a1** {1}		U2e1a (11197) +<br>U2e (6045) +<br>U (11467) +

MNX4	D4e4a	Track 1	HV1<br><br>HV2	16003-16196<br>16217-16413<br>35-263<br>212-369	 16223 16362<br>73 263<br>263 309.1C 315.1C	100% (4/4)	60	M	[0.000] M6 M9 G G1 G1c G1c2 G2 G2a'c G2b G2b1 G2b1a G2b2 G2b2a M33+16362 D D4 D4b D4b2 D4b2a D4b2a2 D4b2a2b D4b2b D4b2b1 D4b2b1a D4b2b1b D4b2b1c D4b2b2 D4b2b3 D4b2b6 D4e D4e1'3 D4e1 D4e1c D4e3 D4e2 **D4e2a** D4e2b D4e2c D4e2d D4e4 D4e4a D4e4b D4f D4f1 D4g D4h D4h4 D4j D4j1 D4j1b D4j4 D4j5 D4j5a D4j+(16286) D4j9 D4j10 D4j12 D4j15 D4j16 D4m {60}	M G D
		Track 2	D (5178A) +<br>D4 (3010) +	5080-5305<br>2956-3217	5178A<br>3010	100% (6/6)	43	D4	[0.000] D4 D4b D4b2 D4b2a D4b2a2 D4b2a2b D4b2b D4b2b1 D4b2b1a D4b2b1b D4b2b1c D4b2b2 D4b2b3 D4b2b6 D4e D4e1'3 D4e1 D4e1c D4e3 D4e2 D4e2a D4e2b D4e2c D4e2d D4e4 **D4e4a** D4e4b D4f D4f1 D4g D4h D4h4 D4j D4j4 D4j5 D4j5a D4j+(16286) D4j9 D4j10 D4j12 D4j15 D4j16 D4m {43}	D
		Track 3	D4b (8020) -<br>D4e (11215) +<br>D4j (11696) -	7951-8070<br>11157-11276<br>11628-11743	 11215<br>11719	100% (8/8)	13	D4e	[0.000] D4e D4e1'3 D4e1 D4e1c D4e3 D4e2 D4e2a D4e2b D4e2c D4e2d D4e4 **D4e4a** D4e4b {13}	
		Track 4	D4e1 (3316) -<br>D4e2 (15874) -<br>D4e4 (1935) +	3262-3370<br>15819-15929<br>1888-1992	 <br>1935	100% (9/9)	3	D4e4	[0.000] D4e4 **D4e4a** D4e4b {3}	
		Track 5	D4e4a (8683) +<br>D4e4b (12882) -	8619-8739<br>12826-12935	8683 8701	100% (11/11)	1	D4e4a	[0.000] **D4e4a** {1}	

MNE1	G2a1	Track 1	HV1<br>HV2	16003-16196<br>16217-16413<br>35-263<br>212-365	 16223 16227 16234 16278 16362<br>73 263<br>263 309.1C 315.1C	85.7% (6/7)	3	G2a	[0.047] **G2a1** {1}<br>[0.000] G2a G2a5 {3}	G	
		Track 2	G2a1 (14200) +	14190-14248	14200	87.5% (7/8)	1	G2a1	[0.047] **G2a1** {1}		G2a (7600) +<br>G2 (13563) +<br>G (5108) +
MNE2	A12	Track 1	HV1<br>HV2	16039-16192<br>16217-16413<br>35-263<br>212-392	16189<br>16223 16262 16290 16319<br>73 152 235 263<br>235 263 315.1C	77.8% (7/9)	6	A+152	[0.300] A+152 {1}<br>[0.071] **A12** {2}<br>[0.000] A+152+16362+16189 A3 A3a A9 {6}	A	
		Track 2	A12 (12720) +	12652-12800	12705 12720	81.8% (9/11)	1	A12	[0.071] A12 {1}		A12 (14290) +<br>A (1736) +
MNE3	D3	Track 1	HV1<br>HV2	16003-16196<br>16217-16413<br>47-281<br>184-392	 16223 16301 16319 16362<br>73 200 239 263<br>200 239 263 297 315.1C	77.8% (7/9)	2	D4b1c	[0.014] D4b1c {1}<br>[0.000] **D3** {2}	D	
		Track 2	D3 (9785) +	9692-9828	9785	80% (8/10)	1	D3	[0.000] **D3** {1}		D3 (4023) +<br>D4 (8414) +<br>D (5178A) +
MNW1	W3a1	Track 1	HV1<br>HV2	16003-16196<br>16217-16413<br>35-263<br>184-365	 16223 16292<br>73 189 194 195 204 207 263<br>189 194 204 207 263 309.1C 315.1C	100% (9/9)	12	W+194	[0.000] W+194 W3 W3a **W3a1** W3a1a W3a1a1 W3a1a2 W3a1a3 W3a1b W5 W8 W9 {12}	W	
		Track 2	W3 (1406) +<br>W5 (15775)<br>W (15884C) +<br>W3a (15784) +	1339-1467<br>15728-15901	1406 1438<br>15784 15884C	100% (13/13)	7	W3a	[0.000] W3a **W3a1** W3a1a W3a1a1 W3a1a2 W3a1a3 W3a1b {7}		
		Track 3	W3a1 (13263) +<br>W3a1a (7151) -<br>W3a1b (10245) -	13188-13306<br>7093-7219<br>10221-10281	13263	100% (14/14)	1	W3a1	[0.000] **W3a1** {1}		

MNW3	G1a1	Track 1	HV1<br><br>HV2	16003-16196<br>16217-16413<br>35-263<br>212-365	 16223 16325<br>73 150 263<br>263 315.1C	80% (4/5)	12	L3	[0.058] **G1a1** {1}<br>[0.000] L3e'i'k'x L3e L3e3'4'5 L3e3'4 L3i G1a1a G1a1a1 G1a1a2 G1a1a4 G1a1b M62'68 {12}	G {1} L3<br>M {3}	
		Track 2	G1a1 (15860) +	15728-15892	15860	83.3% (5/6)	6	G1a1	[0.058] **G1a1** {1}<br>[0.000] G1a1a G1a1a1 G1a1a2 G1a1a4 G1a1b {6}		
		Track 3	G1a1a (11914) -<br>G1a1b (12178) -	11867-11960<br>12135-12243		83.3% (5/6)	1	G1a1	[0.058] **G1a1** {1}		G1a (7867) +<br>G1 (15323) +<br>G (5108) +
MNW4	C4a1a+195	Track 1	HV1<br><br>HV2	16003-16196<br>16217-16413<br>35-263<br>216-365	16129<br>16223 16298 16327<br>73 195 249d 263<br>249d 263 309.1C 315.1C	100% (8/8)	9	C4a1a+195	[0.000] **C4a1a+195** C4a1a2 C4a1a2a C4a1a3 C4a1a3a C4a1a3a1 C4a1a3b C4a1a3c C4a1a4 {9}	C	
		Track 2	C4a1a2 (10891) -<br>C4a1a3 (15607) -<br>C4a1a4 (12940) -	10802-10939<br>15561-15686<br>12868-13002	10873	100% (9/9)	1	C4a1a+195	[0.000] **C4a1a+195** {1}		C4a1a (1715) +<br>C4a1 (7999) +<br>C4a (12672) +<br>C4 (15204) +<br>C (3552A) +

HV, hypervariable region; MRCA, most recent common ancestor; Super-HG, super-haplogroup.

Haplogroup in boldface indicates the determined one.

Multiple haplogroups are separated by a single space.

[1] Variants of the target haplogroup are indicated in parentheses.; +, variant present; -, no variant.

[2] Numbers indicate nucleotide positions based on the revised Cambridge reference sequence [29].

[3] Shown in PhyloTree format.

[4] Ranks are shown in curly brackets; scores, in square brackets.
