# Supplementary material for: Mitochondrial Haplogroup Classification of Ancient DNA Samples Using Haplotracker
Source: Biomed Res Int. 2022 Mar 18;2022:5344418. doi: 10.1155/2022/5344418 (PMC8956381; doi:10.1155/2022/5344418)
Supplement: Supplementary Materials — Fig. S1: characterization of Phylotree-provided control region sequences tested for haplogroup classification by Haplotracker. Fig. S2: minimum number of amplicons required by Haplotracker in discriminating between haplogroups using mtDNA control and coding region sequences. Fig. S3: variant identification of an aDNA sample (MNW3) using an HRM real-time PCR. Table S1: haplogroups and their variant profiles extracted from Phylotree mtDNA Build 17. Table S2: haplogroup frequency carrying an extra variant in 118,869 haplotypes. Table S3: haplogroup frequency carrying a missing variant in 118,869 haplotypes. Table S4: haplogroup frequency in 118,869 haplotypes. Table S5: list of ancient human samples found in 2,000-year-old elite Xiongnu cemetery in Northeast Mongolia. Table S6: primers for the amplification of mtDNA coding region segments for haplogroup determination. Table S7: high-resolution melting real-time PCR primer design for screening variants to differentiate haplogroups G1a1, G1a1a, and G1a1b. Table S8: haplogroup classification of full-length mtGenome sequences from Phylotree (n = 8,216). Table S9: haplogroup classification with full-length and control region sequences of mtDNA using Haplotracker and HaploGrep 2. Table S10: comparison of servers using control region sequences from GenBank before December 25, 2018 (n = 45,177). Table S11: comparison details for the servers using control region sequences from GenBank before December 25, 2018 (n = 45,177). Table S12: comparison of servers using control region sequences downloaded from GenBank from December 26, 2018 to August 22, 2019. Table S13: sequences of mtDNA PCR products from Mongolian ancient DNA samples. Table S14: haplogroup classification of Mongolian ancient DNA samples using Haplotracker. Table S15: minimum number of amplicons required by Haplotracker in discriminating between haplogroups using mtDNA control and coding region sequences. Table S16: minimum number of amplicons per superhaplogroup requ [file 5344418.f1.zip › 5344418.f18.pdf]

Table S15. Minimum number of amplicons required by Haplotracker in discriminating between haplogroups using mtDNA control and coding region sequences

Phylotree build 17 samples			Haplogroup prediction and number of amplicons used in each track																														Total No. of tested	
No.	Accession	Haplogroup	1st (Control region)		2nd		3rd		4th		5th		6th		7th		8th		9th		10th		11th		12th		13th		14th		15th		Tracks	Aamplicons
1	EU092665	L0a1a	L0a1a	1																													1	1
2	NA19311	L0a1a+200	L0a1a+200	1																													1	1
3	EU092714	L0a1a1	L0a1a1	1																													1	1
4	JX303911	L0a1a2	L0a1a2	1																													1	1
5	JQ044943	L0a1a3	L0a1a3	1																													1	1
6	KJ669116	L0a1b1	L0a1b2	1	L0a1b1	2																											2	3
7	EU935434	L0a1b1a	L0a1b1	1	L0a1b1a	1	L0a1b1a	1																									3	3
8	EU092688	L0a1b1a1	L0a1b1	1	L0a1b1a	1	L0a1b1a1	1	L0a1b1a1	1																							4	4
9	KJ669120	L0a1b1a1a	L0a1b1a1a	1																													1	1
10	DQ304897	L0a1b2	L0a1b2	1																													1	1
11	EU092746	L0a1b2a	L0a1b2	1	L0a1b2a	1																											2	2
12	NA19440	L0a1c	L0a1c	1																													1	1
13	EU092945	L0a1c1	L0a1c1	1																													1	1
14	EU092801	L0a1d	L0a1d	1																													1	1
15	JX303766	L0a1e	L0a1e	1																													1	1
16	EU092906	L0a4	L0a4	1																													1	1
17	HM771161	L0a2a1	L0a2a1b	1	L0a2a1	2																											2	3
18	EU092911	L0a2a1a2	L0a2a1a2	1																													1	1
19	EU092861	L0a2a1b	L0a2a1b	1																													1	1
20	FJ157838	L0a2a2	L0a2a2	1	L0a2a2	1																											2	2
21	JX303763	L0a2a2a	L0a2a2a1	1	L0a2a2a	2																											2	3
22	JX303835	L0a2a2a1	L0a2a2a1	1																													1	1
23	JX303786	L0a2a2a2	L0a2a2	1	L0a2a2a	1	L0a2a2a2	2																									3	4
24	EU597537	L0a2b	L0a2b	1	L0a2b	1																											2	2
25	HM771200	L0a2b1	L0a2b	1	L0a2b1	1																											2	2
26	EF556174	L0a2c	L0a2c	1																													1	1
27	EU092913	L0a2d	L0a2	1	L0a2d	2																											2	3
28	EU092900	L0a3	L0a	1	L0a3	1																											2	2
29	KJ669112	L0g	L0g	1																													1	1
30	EU092936	L0b	L0b	1																													1	1
31	EU092870	L0f1	L0f1	1																													1	1
32	EU092964	L0f2a	L0f2a	1																													1	1
33	AY963585	L0f2a1	L0f2a1	1																													1	1
34	EU092786	L0f2b	L0f2b	1																													1	1
35	KC345899	L0k1a1	L0k1a1	1	L0k1a1	3																											2	4
36	EU092966	L0k1a1a	L0k1a1a	1																													1	1
37	KJ669111	L0k1a1b	L0k1a1b	1																													1	1
38	KC346108	L0k1a1c	L0k1a1	1	L0k1a1c	3																											2	4
39	KC345966	L0k1a1d	L0k1a1d	1																													1	1
40	KC346083	L0k1a2	L0k1a2	1																													1	1
41	KJ669103	L0k1a2a	L0k1a2a	1																													1	1
42	KC346073	L0k1a3	L0k1a3	1																													1	1
43	KC346241	L0k1b	L0k1b	1																													1	1
44	KC346233	L0k2a	L0k2a	1																													1	1
45	KC345922	L0k2a1	L0k2a1	1																													1	1
46	JX303895	L0k2a1a	L0k2a1a	1																													1	1
47	EU092792	L0k2b	L0k2b	1																													1	1
48	KC345791	L0d1a	L0d1a	1	L0d1a	1																											2	2
49	KJ669142	L0d1a1a	L0d1a1a	1																													1	1
50	EU597514	L0d1a1a1	L0d1a1a1	1																													1	1
51	KC346102	L0d1a1a2	L0d1a1a2	1																													1	1
52	EU092863	L0d1a1a3	L0d1a1	1	L0d1a1a	3	L0d1a1a3	1																									3	5
53	EU092833	L0d1a1b	L0d1a1b	1																													1	1

54	KC345969	L0d1a1b1a	L0d1a1b1	1	L0d1a1b1a	2					2	3
55	KJ669141	L0d1a1b1b	L0d1a1b1b	1							1	1
56	KC346092	L0d1a1c	L0d1a1c	1							1	1
57	KJ669138	L0d1a1d	L0d1a1	1	L0d1a1d	3					2	4
58	KJ669137	L0d1d	L0d1d	1							1	1
59	KC345764	L0d1c	L0d1c	1							1	1
60	KC345920	L0d1c1	L0d1c1	1							1	1
61	KC346144	L0d1c1a	L0d1c1a	1							1	1
62	KC345933	L0d1c1a1	L0d1c1a1	1							1	1
63	KC345934	L0d1c1a1a	L0d1c1a1	1	L0d1c1a1a	2					2	3
64	KC345835	L0d1c1a1a1	L0d1c1a1a1	1							1	1
65	KC346231	L0d1c1a1a2	L0d1c1a1	1	L0d1c1a1a	2	L0d1c1a1a2	1			3	4
66	KC346101	L0d1c1a1b	L0d1c1a1	1	L0d1c1a1b	1					2	2
67	KC345824	L0d1c1a2	L0d1c1a2	1							1	1
68	KC346238	L0d1c2	L0d1c2	1							1	1
69	KC345852	L0d1c2a	L0d1c2a	1							1	1
70	KC346081	L0d1c2a1	L0d1c2a1	1							1	1
71	KC345944	L0d1c3	L0d1c3	1							1	1
72	KC346074	L0d1b1a	L0d1b1	1	L0d1b1a	2	L0d1b1a	1			3	4
73	KC345788	L0d1b1a1	L0d1b1	1	L0d1b1a	2	L0d1b1a1	1			3	4
74	KJ669135	L0d1b1b	L0d1b1b	1							1	1
75	KC346235	L0d1b1b1	L0d1b1b1	1							1	1
76	KC345780	L0d1b1+@152	L0d1b1+@152	1							1	1
77	KC346236	L0d1b1c	L0d1b1c	1							1	1
78	KJ669149	L0d1b2a1	L0d1b2a1	1							1	1
79	KC345902	L0d1b2a2	L0d1b2a2	1							1	1
80	KC345949	L0d1b2b1a	L0d1b2b1a	1							1	1
81	KC345923	L0d1b2b1b	L0d1b2b1b	1							1	1
82	KJ669150	L0d1b2b1b1	L0d1b2b1b	1	L0d1b2b1b	1	L0d1b2b1b1	1			3	3
83	KC345781	L0d1b2b2	L0d1b2b2	1							1	1
84	KJ669145	L0d1b2b2a	L0d1b2b2a	1							1	1
85	KC345977	L0d1b2b2b	L0d1b2b2b	1							1	1
86	KC346163	L0d1b2b2b1	L0d1b2b2b1	1							1	1
87	KC345901	L0d1b2b2c1	L0d1b2b2	1	L0d1b2b2c	3	L0d1b2b2c1	1			3	5
88	KC345873	L0d1b2b2c2	L0d1b2b2c2	1							1	1
89	KC533481	L0d2a1	L0d2a1	1							1	1
90	KC533486	L0d2a1a	L0d2a1a	1							1	1
91	KC345989	L0d2a1a1	L0d2a1a1	1							1	1
92	KC345865	L0d2a1a1a	L0d2a1a1a	1							1	1
93	KJ669171	L0d2a1a2	L0d2a1a	1	L0d2a1a	2	L0d2a1a2	1			3	4
94	KC346221	L0d2a1a3	L0d2a1a3	1							1	1
95	KC345990	L0d2a1b	L0d2a1b	1							1	1
96	KC346044	L0d2a1c	L0d2a1c	1							1	1
97	KC345910	L0d2a2	L0d2a2	1							1	1
98	KC346207	L0d2b1a	L0d2b1b	1	L0d2b1a	2					2	3
99	JX303903	L0d2b1a1	L0d2b1a1	1							1	1
100	KC345912	L0d2b1a1a	L0d2b1b	1	L0d2b1a1a	2					2	3
101	KC346147	L0d2b1b	L0d2b1b	1							1	1
102	EU092840	L0d2b2	L0d2b2	1							1	1
103	KJ669167	L0d2d	L0d2d	1							1	1
104	EU092708	L0d2c1	L0d2c1	1							1	1
105	KC346086	L0d2c1a	L0d2c1a	1							1	1
106	KC346206	L0d2c1a1	L0d2c	1	L0d2c1	1	L0d2c1a	1	L0d2c1a1	1	4	4
107	KC346131	L0d2c1b	L0d2c1b	1							1	1
108	KC346146	L0d2c2	L0d2c2	1	L0d2c2	2					2	3
109	KC346217	L0d2c2a	L0d2c2	1	L0d2c2a	2	L0d2c2a	1			3	4
110	KJ669164	L0d2c2a1	L0d2c2	1	L0d2c2a1	2					2	3

111	KC346052	L0d2c2a1a	L0d2c2a1a	1									1	1
112	KJ669162	L0d2c2b	L0d2c2	1	L0d2c2b	2							2	3
113	EU092921	L0d3a	L0d3a	1									1	1
114	KC533475	L0d3b	L0d3b	1									1	1
115	EU092842	L0d3b1	L0d3b1	1									1	1
116	KC346224	L0d3b2	L0d3b2	1									1	1
117	JN214480	L1b1a	L1b	1	L1b1	1	L1b1a	1	L1b1a	7			4	10
118	JQ045107	L1b1a4	L1b1a4	1									1	1
119	AY195783	L1b1a4a	L1b1a4a	1									1	1
120	EU092672	L1b1a2	L1b1a+189	1	L1b1a+189	4	L1b1a2	1					3	6
121	EU092775	L1b1a2a	L1b1a2a	1									1	1
122	JN214471	L1b1a+189	L1b1a+189	1									1	1
123	DQ304908	L1b1a3	L1b1a+189	1	L1b1a3	4	L1b1a3	2					3	7
124	DQ304914	L1b1a3a	L1b1a+189	1	L1b1a3	4	L1b1a3a	2	L1b1a3a	1			4	8
125	JQ044875	L1b1a3a1	L1b1a3a1	1									1	1
126	JQ703036	L1b1a3b	L1b1a+189	1	L1b1a3	4	L1b1a3b	2					3	7
127	JQ044876	L1b1a9	L1b1a9	1									1	1
128	NA19462	L1b1a15	L1b1a15	1									1	1
129	NA19198	L1b1a15a	L1b1a15a	1									1	1
130	JQ045115	L1b1a17	L1b1a17	1									1	1
131	JQ705606	L1b1a18	L1b1a18	1									1	1
132	NA20753	L1b1a5	L1b	1	L1b1	1	L1b1a	1	L1b1a5	7			4	10
133	EU092716	L1b1a6	L1b1a6	1									1	1
134	DQ304923	L1b1a7	L1b	1	L1b1	1	L1b1a	1	L1b1a7	7			4	10
135	DQ282505	L1b1a7a	L1b1a7a	1									1	1
136	JQ704968	L1b1a8	L1b1a8	1									1	1
137	JQ045100	L1b1a10	L1b1a10	1	L1b1a10	2							2	3
138	EU092884	L1b1a10a	L1b1a16	1	L1b1a10a	2							2	3
139	EU092852	L1b1a10b	L1b1a10	1	L1b1a10b	2							2	3
140	JN214463	L1b1a12a	L1b1a12a	1									1	1
141	JQ705931	L1b1a12b	L1b1a12b	1									1	1
142	JN214477	L1b1a13	L1b	1	L1b1	1	L1b1a	1	L1b1a13	7			4	10
143	JQ045114	L1b1a14	L1b1a5	1	L1b	2	L1b1	1	L1b1a	1	L1b1a14	5	5	10
144	JN214460	L1b1a16	L1b1a16	1									1	1
145	NA19909	L1b2	L1b2	1									1	1
146	HM771162	L1b2a	L1b2a	1									1	1
147	JQ044936	L1b3	L1b3	1									1	1
148	JQ705275	L1c1a	L1c1a	1									1	1
149	EU273490	L1c1a1a1a	L1c1a1a1a	1									1	1
150	EU273497	L1c1a1a1b	L1c1a1a1b	1									1	1
151	EU273478	L1c1a1a1b1	L1c1a1a1b	1	L1c1a1a1b1	1							2	2
152	EU273500	L1c1a1a2	L1c1a1a2	1									1	1
153	EU273491	L1c1a1b	L1c1a1b	1									1	1
154	HM771217	L1c1a2a1	L1c1a2	1	L1c1a2a	2	L1c1a2a1	2					3	5
155	HM771180	L1c1a2a2	L1c1a2	1	L1c1a2a	2	L1c1a2a2	2					3	5
156	EU273476	L1c1a2b	L1c1a2b	1									1	1
157	HM771113	L1c1a2c	L1c1a2c	1									1	1
158	NA19917	L1c1b	L1c1b	1									1	1
159	EU273499	L1c1b1	L1c1b1	1									1	1
160	NA19385	L1c1d	L1c1d	1									1	1
161	FJ713601	L1c1d1	L1c1d1	1									1	1
162	EU092717	L1c1c	L1c1c	1									1	1
163	AF346992	L1c2a1a	L1c2a1a	1									1	1
164	EU273501	L1c2a2	L1c2a2	1									1	1
165	EU092848	L1c2a3	L1c2a3	1									1	1
166	KC533467	L1c2a3a	L1c2a3a	1									1	1
167	DQ341059	L1c2b1a'b	L1c2b1a'b	1									1	1

168	HM771222	L1c2b1a	L1c2b1a	1							1	1
169	KC152939	L1c2b1a1	L1c2b1a1	1							1	1
170	EU092849	L1c2b1b	L1c2b1b	1							1	1
171	JQ705650	L1c2b1b1	L1c2b1b1	1							1	1
172	EU092712	L1c2b1c	L1c2b1c	1							1	1
173	HM771221	L1c2b2	L1c2b2	1							1	1
174	EU273502	L1c4a	L1c4a	1							1	1
175	JF812599	L1c4b	L1c4b	1							1	1
176	EU273489	L1c6	L1c6	1							1	1
177	JX303797	L1c5	L1c5	1							1	1
178	EU092703	L1c3a	L1c3a	1							1	1
179	EU092718	L1c3a1a	L1c3a1a	1							1	1
180	JX303871	L1c3a1b	L1c3a1b	1							1	1
181	HG01378	L1c3b1	L1c3b1	1							1	1
182	HG01080	L1c3b1a	L1c3b1a	1							1	1
183	EU092689	L1c3b1b	L1c3b'c	1	L1c3b1b	1					2	2
184	NA19256	L1c3b2	L1c3b2	1							1	1
185	EU273493	L1c3c	L1c3c	1							1	1
186	EF556173	L5a1a	L5a1a	1							1	1
187	EU092943	L5a1b	L5a1b	1							1	1
188	HM771198	L5a1c	L5a1c	1							1	1
189	EU092699	L5a2	L5a2	1							1	1
190	NA19373	L5b1	L5b1	1							1	1
191	DQ341061	L5b1a	L5b1a	1							1	1
192	EU092774	L5b1b	L5b1b	1							1	1
193	NA19332	L5b2	L5b2	1							1	1
194	EU092916	L2a1a	L2a1a	1							1	1
195	NA19266	L2a1a1	L2a1a1	1							1	1
196	JN214449	L2a1a2	L2a1a2	1	L2a1a2	3					2	4
197	DQ304975	L2a1a2a	L2a1a2	1	L2a1a2a	3	L2a1a2a	1	L2a1a2a	1	4	6
198	JQ044968	L2a1a2a1	L2a1a2a1a	1	L2a1a2	1	L2a1a2a1	1			3	3
199	NA19117	L2a1a2a1a	L2a1a2	1	L2a1a2a	3	L2a1a2a1	1	L2a1a2a1a	1	4	6
200	DQ304972	L2a1a2b	L2a1a2	1	L2a1a2b	3					2	4
201	HQ425645	L2a1a2c	L2a1a2c	1							1	1
202	EU092711	L2a1a3	L2a1a3	1							1	1
203	EU092890	L2a1a3a	L2a1a3	1	L2a1a3a	2					2	3
204	JQ044819	L2a1a3b	L2a1a3b	1							1	1
205	HM771168	L2a1a3c	L2a1a3c	1							1	1
206	EU092806	L2a1+16189_(16192)	L2a1+16189_(16192)	1							1	1
207	EU092761	L2a1b	L2a1+16189_(16192)	1	L2a1b	2	L2a1b	2			3	5
208	NA19129	L2a1b1	L2a1+16189_(16192)	1	L2a1b	2	L2a1b1	2			3	5
209	EU092690	L2a1b1a	L2a1b1a	1							1	1
210	JQ044841	L2a1b2	L2a1f	1	L2a1b2	2					2	3
211	NA19625	L2a1b+143	L2a1b+143	1							1	1
212	NA18856	L2a1b3	L2a1b3	1							1	1
213	DQ304959	L2a1f	L2a1b1	1	L2a1f	2					2	3
214	DQ304961	L2a1f1	L2a1+16189_(16192)	1	L2a1f	2	L2a1f1	2	L2a1f1	1	4	6
215	DQ304958	L2a1f1a	L2a1+16189_(16192)	1	L2a1f	2	L2a1f1	2	L2a1f1a	1	4	6
216	DQ304957	L2a1f2	L2a1f2	1							1	1
217	KC533500	L2a1f3	L2a1f3	1							1	1
218	KC533472	L2a1g	L2a1g	1							1	1
219	HM771224	L2a1c	L2a1c	1							1	1

220	JQ044973	L2a1c+16086	L2a1f	1	L2a1+16189_(16192)	2	L2a1c+16086	5							3	8
221	FJ460560	L2a1c1	L2a1c1	1	L2a1c1	1									2	2
222	NA18868	L2a1c1a1	L2a1c1a1	1											1	1
223	JQ412577	L2a1c1a2	L2a1c1	1	L2a1c1a	1	L2a1c1a2	2							3	4
224	JN214436	L2a1c6	L2a1c6	1											1	1
225	JQ045108	L2a1c2	L2a1c2	1											1	1
226	HM771169	L2a1c2a	L2a1c2a	1											1	1
227	EU200762	L2a1c3a	L2a1e	1	L2a1c3a	2									2	3
228	JQ705145	L2a1c3a1	L2a1c3a1	1											1	1
229	JQ044996	L2a1c3b	L2a1c3b	1											1	1
230	JQ045110	L2a1c3b1	L2a1c3b1	1											1	1
231	NA19096	L2a1c3b2	L2a1c3b2	1											1	1
232	JN214432	L2a1c4	L2a1c4	1											1	1
233	DQ304942	L2a1c4a	L2a1	1	L2a1+143	1	L2a1c	2	L2a1c4	2	L2a1c4a	1	L2a1c4a	1	6	8
234	DQ304951	L2a1c4a1	L2a1	1	L2a1+143	1	L2a1c	2	L2a1c4	2	L2a1c4a	1	L2a1c4a1	1	6	8
235	JN858955	L2a1c+16129	L2a1h	1	L2a1c+16129	2									2	3
236	NA18908	L2a1c5	L2a1c+16129	1	L2a1c5	1									2	2
237	EU092765	L2a1d1	L2a1d1	1											1	1
238	JX303838	L2a1d2	L2a1d2	1											1	1
239	EU092914	L2a1h	L2a1h	1											1	1
240	DQ304945	L2a1e	L2a1	1	L2a1+143	1	L2a1e	2							3	4
241	DQ304930	L2a1e1	L2a1	1	L2a1e1	2									2	3
242	FJ460527	L2a1+143+16189_(16192)	L2a1	1	L2a1+143+16189_(16192)	1	L2a1+143+16189_(16192)	5							3	7
243	EU092793	L2a1+143+16189_(16192)+@16309	L2a1+143+16189_(16192)+@16309	1											1	1
244	JQ044958	L2a1i	L2a1i	1	L2a1i	1									2	2
245	JX303853	L2a1i1	L2a1i	1	L2a1i1	1									2	2
246	JX303870	L2a1q	L2a1q	1											1	1
247	EU092756	L2a1j	L2a1	1	L2a1+143+16189_(16192)	1	L2a1j	5							3	7
248	EU200763	L2a1k	L2a1k	1											1	1
249	JQ044956	L2a1l1	L2a1l1	1											1	1
250	EU092807	L2a1l1a	L2a1l	1	L2a1l1	2	L2a1l1a	2	L2a1l1a	2					4	7
251	JQ044817	L2a1l1a1	L2a1l1a1	1											1	1
252	KF952774	L2a1l1a2	L2a1+143+16189_(16192)+@16309	1	L2a1l1a2	2									2	3
253	JQ044955	L2a1l1b	L2a1l1b	1											1	1
254	EU092721	L2a1l2	L2a1l2	1											1	1
255	JX266264	L2a1l2a	L2a1l2a	1	L2a1l2a	1									2	2
256	JQ705589	L2a1l2a1	L2a1l2a1	1											1	1
257	JX021728	L2a1l3	L2a1l3	1											1	1
258	JQ045040	L2a1m	L2a1m	1											1	1
259	DQ304940	L2a1m1	L2a1m1	1											1	1
260	JQ701914	L2a1m1a	L2a1	1	L2a1+143+16189_(16192)	1	L2a1m	5	L2a1m1	1	L2a1m1a	1			5	9
261	DQ304941	L2a1n	L2a1	1	L2a1+143+16189_(16192)	1	L2a1n	5							3	7
262	JQ045000	L2a1o	L2a1m	1	L2a1o	1									2	2
263	JQ044822	L2a1p	L2a1+143+16189_(16192)+@16309	1	L2a1p	6									2	7
264	EU092896	L2a2a	L2a2a	1											1	1
265	HM771205	L2a2a1	L2a2a1	1											1	1

266	NA19443	L2a2b1	L2a2b1	1					1	1
267	HM771208	L2a2b1a	L2a2b1a	1					1	1
268	HM771191	L2a2b2	L2a2b2	1					1	1
269	HM771206	L2a3	L2a3	1					1	1
270	HM771212	L2a4a	L2a4a	1					1	1
271	NA19393	L2a4b	L2a4b	1					1	1
272	NA19045	L2a5	L2a5	1					1	1
273	EU092747	L2b1	L2b1	1					1	1
274	JQ044854	L2b1a	L2b1a	1					1	1
275	EU092722	L2b1a2	L2b1a2	1					1	1
276	DQ304978	L2b1a3	L2b1a	1	L2b1a3	3			2	4
277	JN214453	L2b1a4	L2b1a	1	L2b1a4	3			2	4
278	FJ228403	L2b1b	L2b1b	1					1	1
279	EU092692	L2b2	L2b2	1					1	1
280	KC533513	L2b2a	L2b2a	1					1	1
281	JQ702626	L2b3a	L2b3a	1					1	1
282	JN214443	L2b3b	L2b3b	1					1	1
283	EU092661	L2b3c	L2b3c	1					1	1
284	JQ044941	L2c	L2c	1					1	1
285	JQ044858	L2c1	L2c1	1					1	1
286	EU092813	L2c1a	L2c1a	1					1	1
287	EU092697	L2c2	L2c2	1					1	1
288	EU092955	L2c2a	L2c2a	1					1	1
289	JQ704740	L2c2a1	L2c2	1	L2c2a	1	L2c2a1	1	3	3
290	NA18517	L2c2b1a	L2c2b	1	L2c2b1	2	L2c2b1a	1	3	4
291	KC533455	L2c2b1b	L2c2b1b	1					1	1
292	JQ044853	L2c2b2	L2c2b2	1					1	1
293	JQ705626	L2c3	L2c3	1					1	1
294	AF346995	L2c3a	L2c3a	1					1	1
295	JQ044914	L2c4	L2c4	1					1	1
296	AY195785	L2c5	L2c	1	L2c5	2			2	3
297	JQ045050	L2d	L2d	1					1	1
298	JQ045069	L2d+16129	L2d+16129	1					1	1
299	JQ044948	L2d1	L2d+16129	1	L2d1	1			2	2
300	EU092794	L2d1a	L2d1a	1					1	1
301	EU092724	L2e	L2e	1					1	1
302	JQ044816	L2e1	L2e1	1					1	1
303	NA19108	L2e1a	L2e1a	1					1	1
304	EU092773	L6a	L6a	1					1	1
305	DQ341063	L6b	L6b	1					1	1
306	FJ460531	L4a1	L4a1	1					1	1
307	DQ341064	L4a1a	L4a1a	1					1	1
308	EU092935	L4a2	L4a2	1					1	1
309	NA19383	L4b1	L4b1	1					1	1
310	JQ044811	L4b1a	L4b1a	1					1	1
311	EU092942	L4b2a1	L4b2a1	1					1	1
312	EF184627	L4b2a2	L4b2a2	1					1	1
313	EU092938	L4b2a2a	L4b2a2	1	L4b2a2a	1			2	2
314	EU092951	L4b2a2b	L4b2a2b	1					1	1
315	NA19445	L4b2a2c	L4b2a2c	1					1	1
316	NA19259	L4b2b	L4b2b	1					1	1
317	JQ702504	L4b2b1	L4b2b1	1					1	1
318	JN655813	L3a1a	L3a1a	1					1	1
319	DQ341081	L3a1b	L3a1b	1					1	1
320	JN655803	L3a+709	L3a+709	1					1	1
321	NA19438	L3a2	L3a2	1					1	1
322	JN655805	L3a2a	L3a2a	1					1	1

323	EU092726	L3b1a	L3b	1	L3b1	1	L3b1a	1	L3b1a	5	4	8
324	JQ705783	L3b1a1	L3b1a1a	1	L3b1a1	3					2	4
325	EU597490	L3b1a1a	L3b1a1a	1							1	1
326	EU935449	L3b1a2	L3b1a2	1							1	1
327	FJ460529	L3b1a3	L3b1a3	1							1	1
328	EU092759	L3b1a4	L3b1a+@16124	1	L3b1a+@16124	1	L3b1a4	3			3	5
329	JQ044986	L3b1a5	L3b1a	1	L3b1a	1	L3b1a5	4			3	6
330	NA18865	L3b1a5a	L3b1a1a	1	L3b1a5a	3					2	4
331	JN655790	L3b1a+152	L3b1a+152	1							1	1
332	EU092958	L3b1a6	L3b1	1	L3b1a+152	2	L3b1a6	1			3	4
333	EU092768	L3b1a+@16124	L3b1a+@16124	1	L3b1a+@16124	1					2	2
334	JX666328	L3b1a7	L3b1a7	1							1	1
335	JQ044820	L3b1a7a	L3b1a7a	1							1	1
336	NA19222	L3b1a8	L3b1a8	1							1	1
337	JN655791	L3b1a9	L3b1a9	1							1	1
338	EU092727	L3b1a9a	L3b1a9a	1							1	1
339	EU092897	L3b1a10	L3b	1	L3b1	1	L3b1a	1	L3b1a10	5	4	8
340	EU092694	L3b1a11	L3b1a11	1							1	1
341	EU092814	L3b1b	L3b1	1	L3b1b	2					2	3
342	EU092682	L3b1b1	L3b1b1	1							1	1
343	JQ045029	L3b2	L3b2	1							1	1
344	EU092725	L3b2a	L3b2a	1							1	1
345	NA18916	L3b2b	L3b2b	1							1	1
346	JQ703986	L3b3	L3b3	1							1	1
347	EU092891	L3f1a	L3f	1	L3f	4	L3f1a	1			3	6
348	JN655809	L3f1a1	L3f1a1	1							1	1
349	DQ341077	L3f1b+16292	L3f1b+16292	1	L3f1b+16292	2					2	3
350	JQ044831	L3f1b1	L3f1b1	1							1	1
351	DQ305036	L3f1b1a	L3f1b1a	1							1	1
352	JX303774	L3f1b1a1	L3f1b1a1	1							1	1
353	EU092805	L3f1b2	L3f1b2	1							1	1
354	EU935451	L3f1b2a	L3f1b2a	1							1	1
355	NA19172	L3f1b+16292+150	L3f1b+16292+150	1							1	1
356	EU092883	L3f1b3	L3f1b3	1							1	1
357	EU092865	L3f1b4a	L3f1b4a	1	L3f1b4a	1					2	2
358	EU092791	L3f1b4a1	L3f1b4a1	1							1	1
359	JQ045093	L3f1b4b	L3f1b4b	1							1	1
360	EU092704	L3f1b4c	L3f1b4c	1							1	1
361	GU455415	L3f1b5	L3f1b5	1							1	1
362	JN655784	L3f2a	L3f2a	1							1	1
363	JN655841	L3f2a1	L3f1b	1	L3f1b	1	L3f2a1	5			3	7
364	DQ341076	L3f2a1a	L3f2a1a	1							1	1
365	EU092770	L3f2b	L3f2b	1							1	1
366	FJ625848	L3f3	L3f3	1							1	1
367	FJ625855	L3f3a	L3f3a	1							1	1
368	FJ625850	L3f3b	L3f3b	1							1	1
369	EU092660	L3c	L3c	1							1	1
370	EU092898	L3d1'2'3'4'5'6	L3d1'2'3'4'5'6	1							1	1
371	NA19316	L3d1a	L3d3a	1	L3d3a	1	L3d1a	4			2	6
372	JN655800	L3d1a1a	L3d1a1a	1	L3d1a1a	1					2	2
373	EU092932	L3d1a1a1	L3d1a1a1	1							1	1
374	EU092876	L3d1a1b	L3d1a1b	1							1	1
375	HM771228	L3d1a2	L3d1a2	1							1	1

376	JQ045112	L3d1b	L3d1b	1											1	1
377	DQ341072	L3d1b1	L3d1b2	1	L3d1b1	3									2	4
378	JN214459	L3d1b1a	L3d	1	L3d1'2'3'4'5'6	1	L3d1	3	L3d1b	3	L3d1b1	1	L3d1b1a	1	6	10
379	EU200759	L3d1b1b	L3d1b1b	1											1	1
380	JQ702420	L3d1b2	L3d1b2	1											1	1
381	JQ045051	L3d1b3	L3d1b3	1											1	1
382	JX303821	L3d1b3a	L3d1b3a	1											1	1
383	EU092762	L3d1c	L3d1c	1											1	1
384	JQ044824	L3d1c1	L3d1c1	1											1	1
385	AY195782	L3d1d	L3d1d	1											1	1
386	HM771170	L3d2a	L3d2a	1											1	1
387	JQ705019	L3d2b	L3d2b	1											1	1
388	JX303864	L3d3a1	L3d3a1	1											1	1
389	EU092851	L3d3a1a	L3d3a	1	L3d3a1	1	L3d3a1a	2							3	4
390	JX303836	L3d3a1b	L3d3a	1	L3d3a1	1	L3d3a1b	2							3	4
391	JN655797	L3d3b	L3d3b	1											1	1
392	GU455420	L3d4	L3d4	1											1	1
393	JN655834	L3d4a	L3d4a	1											1	1
394	JN214479	L3d5	L3d5	1											1	1
395	NA19121	L3d5a	L3d5a	1											1	1
396	NA18864	L3d6	L3d1c	1	L3d1c	1	L3d6	3							3	5
397	EU092887	L3e1	L3e1	1											1	1
398	EU092749	L3e1a1a	L3e1a1	1	L3e1a1a	1									2	2
399	JX303878	L3e1a2	L3e1a2	1											1	1
400	KC533488	L3e1a3a	L3e1a	1	L3e1a3	1	L3e1a3a	1							3	3
401	NA19114	L3e1a3b	L3e1a3b	1											1	1
402	JN655794	L3e1b1	L3e1b1	1											1	1
403	KC533453	L3e1b2	L3e1b2	1											1	1
404	EU092879	L3e1c	L3e1c	1											1	1
405	HM771171	L3e1d	L3e1d	1	L3e1d	1									2	2
406	JX303847	L3e1d1	L3e1d	1	L3e1d1	1									2	2
407	EU092693	L3e1d1a	L3e1d1a	1											1	1
408	JQ045096	L3e1e	L3e1e	1											1	1
409	EU092915	L3e1e1	L3e1	1	L3e1e	3	L3e1e1	1							3	5
410	HG01170	L3e1e2	L3e1e2	1											1	1
411	JQ704728	L3e1f	L3e1f	1											1	1
412	HM771135	L3e1f1	L3e1f1	1											1	1
413	HM771172	L3e1f1a	L3e1f1a	1											1	1
414	JN214448	L3e1f2	L3e1f2	1											1	1
415	JQ044847	L3e1g	L3e1g	1											1	1
416	NA19707	L3e2a	L3e2a3	1	L3e2a	1									2	2
417	DQ305026	L3e2a1a	L3e2a1	1	L3e2a1a	2									2	3
418	JQ702533	L3e2a1b	L3e2a1b3	1	L3e2a1b	1									2	2
419	DQ305031	L3e2a1b1	L3e2a1b1	1											1	1
420	HM771176	L3e2a1b2	L3e2a1b2	1											1	1
421	HG01456	L3e2a1b3	L3e2a1b3	1											1	1
422	JQ045092	L3e2a2	L3e2a2	1											1	1
423	EU092729	L3e2a3	L3e2a2	1	L3e2a3	2									2	3
424	DQ341071	L3e2b	L3e2b	1	L3e2b	4									2	5
425	DQ305022	L3e2b1	L3e2b	1	L3e2b1	4	L3e2b1	1							3	6
426	JQ703138	L3e2b1a	L3e2b	1	L3e2b1	4	L3e2b1a	1	L3e2b1a	2					4	8
427	AF347015	L3e2b1a1	L3e2b1a1	1											1	1
428	JQ701829	L3e2b1a2	L3e2b7	1	L3e2b1a2	2									2	3
429	EU092777	L3e2b2	L3e2b2	1											1	1
430	DQ304998	L3e2b+152	L3e2b+152	1	L3e2b+152	3									2	4
431	DQ305008	L3e2b3	L3e2b+152	1	L3e2b3	3									2	4
432	DQ305000	L3e2b4	L3e2b+152	1	L3e2b4	3									2	4

433	NA19238	L3e2b5	L3e2b5	1					1	1
434	JN655795	L3e2b6	L3e2b6	1					1	1
435	EU935465	L3e2b7	L3e2b7	1					1	1
436	NA19150	L3e2b8	L3e2b8	1					1	1
437	DQ305012	L3e3a	L3e3a	1					1	1
438	DQ305011	L3e3b	L3e3	1	L3e3b	1			2	2
439	DQ305013	L3e3b1	L3e3b1	1					1	1
440	AF346967	L3e3b2	L3e3b2	1					1	1
441	JQ044850	L3e3b3	L3e3b3	1					1	1
442	EU092752	L3e4	L3e4	1					1	1
443	EU092695	L3e4a	L3e4a	1					1	1
444	JQ045125	L3e4a1	L3e4a1	1					1	1
445	KF358485	L3e5	L3e5	1	L3e5	3			2	4
446	JN214452	L3e5a	L3e5	1	L3e5a	3	L3e5a	1	3	5
447	EU092821	L3e5a1	L3e5a1	1					1	1
448	KF358475	L3e5a1a	L3e5a1a	1					1	1
449	EU092959	L3e5b	L3e5b	1					1	1
450	JN655828	L3e5+195	L3e5+195	1					1	1
451	KF358474	L3e5c	L3e5c	1					1	1
452	FJ460533	L3e5d	L3e5d	1					1	1
453	KF358472	L3e5e	L3e5e	1					1	1
454	KF358481	L3e5f	L3e5f	1					1	1
455	NA19437	L3i1	L3i1	1					1	1
456	JN655780	L3i1a	L3i1a	1					1	1
457	DQ341069	L3i1b	L3i1b	1					1	1
458	DQ341068	L3i2	L3i2	1					1	1
459	EU092822	L3k	L3k	1					1	1
460	JQ705310	L3k1	L3k1	1					1	1
461	NA19446	L3x1a1	L3x1a1	1					1	1
462	JN655773	L3x1a2	L3x1a2	1					1	1
463	JN655837	L3x1+16311	L3x1+16311	1					1	1
464	JN655782	L3x1b	L3x1b	1					1	1
465	EU092944	L3x2a	L3x2a	1					1	1
466	DQ341066	L3x2a1	L3x2a1	1					1	1
467	JN655829	L3x2a1a	L3x2a1	1	L3x2a1a	1			2	2
468	EU092818	L3x2b	L3x2b	1					1	1
469	JN655830	L3h1a1	L3h1a1	1					1	1
470	JN655840	L3h1a2a	L3h1a2a	1					1	1
471	AF347000	L3h1a2a1	L3h1a2a1	1					1	1
472	JN655788	L3h1a2b	L3h1a2b	1					1	1
473	EU092828	L3h1b1	L3h1b1	1					1	1
474	EU092903	L3h1b1a	L3h1b1a	1					1	1
475	EU092736	L3h1b2	L3h1b2	1					1	1
476	JN655801	L3h2	L3h2	1					1	1
477	JQ702955	M1a	M1a	1					1	1
478	EF060331	M1a1	M1a1	1					1	1
479	KC152544	M1a1a	M1a1+16093	0					0	0
480	JQ704763	M1a1a1	M1a1a1	1					1	1
481	KC152556	M1a1b1	M1a1b1	1					1	1
482	EF060321	M1a1b1a	M1a1b1a	1					1	1
483	KC152591	M1a1b1b	M1a1d	1	M1a1b1b	2			2	3
484	DQ779930	M1a1b1b1	M1a1b1a	1	M1a1	1	M1a1b1b1	2	3	4
485	JQ705802	M1a1b1c	M1a1b1c	1					1	1
486	HQ384200	M1a1b2	M1a1b2	1					1	1
487	EF556180	M1a1c	M1a1+16093	1	M1a1c	1			2	2
488	EF060325	M1a1d	M1a1d	1					1	1
489	EF060333	M1a1e1	M1a1	1	M1a1e	4	M1a1e1	2	3	7

490	HM852804	M1a1e2	M1a1e2	1					1	1
491	EF060328	M1a1f	M1a1f	1					1	1
492	KC152575	M1a1g	M1a1g	1					1	1
493	DQ341082	M1a1h	M1a1h	1					1	1
494	JQ703063	M1a1i	M1a1i	1					1	1
495	EF060340	M1a2	M1a2	1					1	1
496	AF381984	M1a2a	M1a2a	1					1	1
497	EF060337	M1a2b	M1a2b	1					1	1
498	EF177443	M1a3a	M1a3a	1					1	1
499	GU122999	M1a3b	M1a3	1	M1a3b	2	M1a3b	1	3	4
500	KC152560	M1a3b1	M1a3b1	1					1	1
501	EF060343	M1a3b2	M1a3b2	1					1	1
502	EF060349	M1a4	M1a4	1					1	1
503	EF060346	M1a4a	M1a4a	1					1	1
504	EF060351	M1a5	M1a5	1					1	1
505	KC152573	M1a6	M1a6	1					1	1
506	KC152588	M1a7	M1a7	1					1	1
507	EF060345	M1a8	M1a8	1					1	1
508	JX154038	M1a8a	M1a8a	1					1	1
509	KC152542	M1b1	M1b1	1					1	1
510	EF060353	M1b1a	M1b1a	1					1	1
511	EF060352	M1b1b	M1b1b	1					1	1
512	EF060361	M1b2	M1b2	1	M1b2	1			2	2
513	EF060355	M1b2a	M1b2a	1					1	1
514	KC152557	M1b2b	M1b2b	1					1	1
515	EF060362	M1b2c	M1b2c	1					1	1
516	AP012349	M20	M20	1					1	1
517	KC505097	M51a	M51b1	1	M51b1	1	M51a	3	3	5
518	HM596693	M51a1a	M51a1a	1					1	1
519	EU597554	M51a1b	M51a1b	1					1	1
520	GQ301870	M51a2	M51a2	1					1	1
521	KC505098	M51b	M51a2	1	M51b	2			2	3
522	GQ301879	M51b1a	M51b1a	1					1	1
523	KC505100	M51b1b	M51b1b	1					1	1
524	EU443499	M2a1	M2a1	1					1	1
525	EU443460	M2a1a	M2a1a	1					1	1
526	EU443472	M2a1a1	M2a1a	1	M2a1a1	1	M2a1a1	2	3	4
527	EU443508	M2a1a1a	M2a1a	1	M2a1a1	1	M2a1a1a	2	3	4
528	FJ383240	M2a1a1a1	M2a1a1a1	1					1	1
529	EU443507	M2a1a1b	M2a1a	1	M2a1a1	1	M2a1a1b	2	3	4
530	EU443509	M2a1a1b1	M2a1a1b1	1					1	1
531	EU443493	M2a1a2	M2a1a2	1					1	1
532	EU443494	M2a1a2a	M2a1a2a	1					1	1
533	FJ383280	M2a1a2a1	M2a1a2a1	1	M2a1a2a1	1			2	2
534	FJ383276	M2a1a2a1a	M2a1a2a1	1	M2a1a2a1a	1			2	2
535	EU443506	M2a1a+207	M2a1a+207	1					1	1
536	EU443502	M2a1a3	M2a1a3	1					1	1
537	EU443470	M2a1a3a	M2a1a3a	1					1	1
538	EU443467	M2a1a3a1	M2a1a3a1	1					1	1
539	FJ383272	M2a1a3b	M2a1a3+16093	1	M2a1a3b	1			2	2
540	EU443454	M2a1b	M2a1b	1					1	1
541	EU443443	M2a1c	M2a1c	1					1	1
542	EU443487	M2a2	M2a2	1					1	1
543	FJ383239	M2a2a	M2a2	1	M2a2a	1			2	2
544	EU443450	M2a3	M2a3	1					1	1
545	EU443447	M2a3a	M2a3a	1					1	1
546	EU443512	M2b	M2b	1					1	1

547	EU443481	M2b1	M2b1	1					1	1
548	EU443483	M2b1a	M2b1a	1					1	1
549	EU443496	M2b1b	M2b1b	1					1	1
550	EU443463	M2b2	M2b2	1					1	1
551	AY922305	M2b3	M2b3	1					1	1
552	EU443449	M2b3a	M2b3a	1					1	1
553	EU443484	M2b4	M2b4	1					1	1
554	KC911426	M2c	M2c	1					1	1
555	DQ408676	M3	M3	1					1	1
556	FJ383527	M3a1	M3	1	M3a	3	M3a1	2	3	6
557	JQ704107	M3a1+204	M3a1+204	1					1	1
558	FJ383514	M3a1a	M3a1+204	1	M3a1a	2			2	3
559	KC911530	M3a1b	M3a1b	1					1	1
560	AY922266	M3a2	M3a2	1					1	1
561	FJ383531	M3a2a	M3a2a	1					1	1
562	FJ383513	M3b	M3	1	M3b	3			2	4
563	FJ383542	M3c+152	M3c+152	1					1	1
564	FJ770965	M3c1a	M3c1a	1					1	1
565	FJ383459	M3c1b	M45a	1	M3c1b	2			2	3
566	FJ383468	M3c1b1a	M3c1b1a	1					1	1
567	FJ383462	M3c1b1b	M3c1b1b	1					1	1
568	GU810074	M3c2	M3c2	1					1	1
569	DQ246829	M3d	M3d	1					1	1
570	FJ770946	M3d1	M3d1a	1	M3d1	1			2	2
571	JF742206	M3d1a	M3d1a	1					1	1
572	KF056281	M3d1a1	M3d1a1	1					1	1
573	KC911394	M4a	M4a	1					1	1
574	DQ408679	M4b	M4b	1					1	1
575	AY922291	M65a1	M65a	1	M65a1	1			2	2
576	JX289111	M65a+@16311	M65a+@16311	1	M65a+@163 11	1			2	2
577	HM036576	M65a2	M65a2	1					1	1
578	FJ383297	M65b	M65b	1					1	1
579	FJ383300	M67	M67	1					1	1
580	KC911475	M18a	M18a	1					1	1
581	JQ705662	M18b	M18b	1					1	1
582	JX289108	M18c	M18c	1					1	1
583	JX289116	M38	M38	1					1	1
584	AY922286	M38a	M38a	1					1	1
585	FJ383403	M38b	M38b	1					1	1
586	FJ383397	M38c	M38c	1					1	1
587	FJ770947	M38d	M38d	1					1	1
588	FJ770963	M38e	M38e	1					1	1
589	FJ383657	M30	M30	1					1	1
590	FJ383663	M30a	M30a	1					1	1
591	AY922254	M30a1	M30a1	1					1	1
592	FJ383660	M30a2	M30a	1	M30a2	2			2	3
593	AY289071	M30b	M30b	1					1	1
594	AF382013	M30c	M30c	1					1	1
595	AY922268	M30c1	M30c1	1					1	1
596	HM036539	M30c1a	M30c1a	1	M30c1a	1			2	2
597	EF556149	M30c1a1	M30c1a	1	M30c1a1	1			2	2
598	KC533457	M30d1	M30d	1	M30d1	2			2	3
599	AY922255	M30d2	M30d1 M30d2 (2)	1	M30d2	2			2	3
600	EU597504	M30+16234	M30+16234	1					1	1
601	FJ383676	M30e	M30e	1					1	1

602	FJ383674	M30f	M30f	1																			1	1
603	JF742213	M30g	M30g	1																			1	1
604	FJ383686	M37+152	D4a1a	1	D4a	4	D4a	2	M	1	M13'46'61+ 16362	3	M37+152	4									6	15
605	AY922265	M37a	M37a	1																			1	1
606	HQ541751	M37a1	M37+152+151	1	M37a	2	M37a1	1															3	4
607	FJ383688	M37d	M37d	1																			1	1
608	AY922267	M37e	M37e	1																			1	1
609	FJ383684	M37e2	M37e2	1																			1	1
610	JX289107	M43+16311	M43+16311	1																			1	1
611	AY922301	M43a	H3b6	1	M	3	L3	2	L3	3	M	2	M	1	M	5	M	5	M43a	5	M43a	1	10	28
612	JF742207	M43a1	M43a1	1																			1	1
613	FJ383428	M43b	M43b G1b	1	M43b	3																	2	4
614	FJ748709	M45	M45	1																			1	1
615	HM030535	M45a	M45a	1																			1	1
616	KF056272	M54	M54	1																			1	1
617	FJ383738	M63	M63	1																			1	1
618	FJ383813	M64	M64	1																			1	1
619	FJ770971	M66a	M66a	1																			1	1
620	JX462692	M66b	M66b	1																			1	1
621	DQ408675	M5a	M5a	1																			1	1
622	AY922289	M5a1a	M5a1a	1																			1	1
623	EF583176	M5a1b	M5a1b	1																			1	1
624	AY922259	M5a2	M	1	M5	4	M5a'd	2	M5a	1	M5a2	2	M5a2	1									6	11
625	FJ383552	M5a2a	M17	1	M5a2a	2																	2	3
626	FJ770949	M5a2a1	M	1	M5	4	M5a'd	2	M5a	1	M5a2	2	M5a2a	1	M5a2a1	1							7	12
627	HM030518	M5a2a1a1	M5a2a1a	1	M5a2a1a1	1																	2	2
628	FJ383555	M5a2a1a2	M5a2a1a2	1																			1	1
629	FJ383577	M5a2a2	M5a2a2	1																			1	1
630	FJ383547	M5a2a3	M5a2a3	1																			1	1
631	EU597563	M5a2a4	M5a2a4	1																			1	1
632	FJ383581	M5a3a	M5a3	1	M5a3a	1																	2	2
633	FJ383559	M5a3b	M5a3b	1																			1	1
634	FJ383554	M5a4	M5a4	1																			1	1
635	FJ383566	M5a5	M5	1	M5	3	M5a5	1															3	5
636	FJ383557	M5d	M5d	1																			1	1
637	KC577360	M5b	D2	1	M5b	3																	2	4
638	FJ383543	M5b1	M5b1	1																			1	1
639	FJ383575	M5b2a	M5b2a	1																			1	1
640	FJ383576	M5b2b	M5b2b	1																			1	1
641	FJ383550	M5b2b1	M5b2b1	1																			1	1
642	HM036533	M5b2b1a	M5b2b1a	1																			1	1
643	FJ383546	M5c1	M5c1	1																			1	1
644	FJ770953	M5c2	M5c2	1																			1	1
645	JX462705	M6	M6	1																			1	1
646	JX462716	M6a1	M6a1	1																			1	1
647	FJ383308	M6a1a	M6a1a	1																			1	1
648	JQ703707	M6a1b	M6a1b	1																			1	1
649	FJ383305	M6a2	M6a2	1																			1	1
650	AP008394	M7a+16324	M7a+16324	1	M7a+16324	1																	2	2
651	AP008600	M7a1	M7a1	1																			1	1
652	AP010717	M7a1a	M7a1a	1																			1	1
653	AP008548	M7a1a1	M7a1a1	1																			1	1
654	AP008249	M7a1a1a	M7a+16324	1	M7a1	1	M7a1a	2	M7a1a1	6	M7a1a1a	1											5	11
655	AP008507	M7a1a2	M7a1a2	1																			1	1
656	AP008585	M7a1a3	M7a1a3	1																			1	1
657	AP008367	M7a1a4	M7a+16324	1	M7a1	1	M7a1a	2	M7a1a4	6	M7a1a4	1											5	11

658	AP008503	M7a1a4a	M7a1a M7a1a4a (2)	1	M7a1a	1	M7a1a4a	1					3	3
659	AP008280	M7a1a5	M7a+16324	1	M7a1	1	M7a1a	2	M7a1a5	6	M7a1a5	1	5	11
660	AP013259	M7a1a5a	M7a1a5a	1									1	1
661	AP009423	M7a1a6	M7a+16324	1	M7a1	1	M7a1a	2	M7a1a6	6			4	10
662	AP008483	M7a1a6a	M7a1a6a	1									1	1
663	AP010750	M7a1a7	M7a1a7	1									1	1
664	AP013266	M7a1a8	M7a1a8	1									1	1
665	AP008466	M7a1a9	M7a1a9	1									1	1
666	AP008266	M7a1b1	M7a1b1	1									1	1
667	AP009443	M7a1b2	M7a1b2	1									1	1
668	AP010826	M7a2	M49c1	1	M7a2	4							2	5
669	AP013134	M7a2a	M7a2a	1									1	1
670	AP010824	M7a2a1	M7a2a1	1									1	1
671	AP010825	M7a2a2	M7a2a2	1									1	1
672	EF153781	M7a2a3	M7a2a3	1	M7a2a3	1							2	2
673	KF148516	M7a2a3a	M7a2a3	1	M7a2a3a	1							2	2
674	KC569547	M7b1a1	M7b1a1	1									1	1
675	NA18636	M7b1a1a	M7b1a1a	1	M7b1a1a	1							2	2
676	AY255173	M7b1a1a1	M7b1a1a1	1									1	1
677	AP008354	M7b1a1a1a	M7b1a1a1a	1									1	1
678	AP008621	M7b1a1a1b	M7b1a1a1b	1									1	1
679	AP013274	M7b1a1a1b1	M7b1a1a1b1	1									1	1
680	AP008485	M7b1a1a1c	M7b1a1a1c	1									1	1
681	AP010979	M7b1a1a1d	M7b1a1a1d	1									1	1
682	NA18769	M7b1a1a2	M7b1a1a2	1									1	1
683	NA18943	M7b1a1a3	M7b1a1a3	1									1	1
684	AY255159	M7b1a1b	M7b1a1b	1									1	1
685	AP012426	M7b1a1+(16192	M7b1a1+(16192	1									1	1
686	FJ198219	M7b1a1c	M7b1a1c	1									1	1
687	HM357816	M7b1a1c1	M7b1a1c1	1									1	1
688	HG00403	M7b1a1d	M7b1a1	1	M7b1a1d	5	M7b1a1d	1					3	7
689	GU123012	M7b1a1d1	M7b1a1f	1	M7b1a1d1	3							2	4
690	GU810069	M7b1a1e	M7b1a1e	1									1	1
691	JX987463	M7b1a1e1	M7b1a1e1	1									1	1
692	HG00448	M7b1a1e2	M7b1a1e2	1									1	1
693	JX987450	M7b1a1f	M7b1a1f	1									1	1
694	NA18644	M7b1a1g	M7b1a1g	1									1	1
695	AP010993	M7b1a1h	M7b1a1+(16192	1	M7b1a1h	2							2	3
696	KF540506	M7b1a1i	M7b1a1i	1									1	1
697	JX987442	M7b1a1i1	M7b1a1i1	1									1	1
698	NA17969	M7b1a2	M7b1a2	1									1	1
699	KF849908	M7b1a2a	M7b1a2a	1									1	1
700	KF540571	M7b1a2a1	M7b1a2a1	1									1	1
701	KF540953	M7b1a2a1a	M7b1a2a1a	1									1	1
702	KF540574	M7b1a2a1b	M7b1a2a	1	M7b1a2a1	1	M7b1a2a1b	2					3	4
703	KF540567	M7b1a2a1b1	M7b1a2a1b1	1									1	1
704	HM030531	M7b1b	M7b1b	1									1	1
705	HM030527	M7b2	M7b2	1									1	1
706	HM030506	M7b2a	M7b2a	1									1	1
707	HQ157976	M7c1a	M45	1	M7c1a	4							2	5
708	KF849978	M7c1a1a	M7c1a1a	1									1	1
709	HM036561	M7c1a1a1	M7c1a1a1	1									1	1
710	HM852807	M7c1a1b	M7c1a1b	1									1	1
711	KF148264	M7c1a1b1	M7c1a1b1	1									1	1
712	HM030547	M7c1a2	M7c1a2	1									1	1
713	EU597541	M7c1a2a	M7c1a2a	1									1	1

714	AP010681	M7c1a2a1	M7c1a2a1	1						1	1
715	JX987458	M7c1a3	M7c1a3	1						1	1
716	NA18618	M7c1a3a	M7c1a3a	1						1	1
717	KF849961	M7c1a4a	M7c1a4a	1						1	1
718	KF541008	M7c1a4b	M7c1a4b	1						1	1
719	AP008755	M7c1a5	M7c1a5	1						1	1
720	AY255158	M7c1b	M7c1	1	M7c1b	3	M7c1b	2		3	6
721	EF153790	M7c1b1	M7c1b1	1						1	1
722	NA18639	M7c1b2a	M7c1b2a	1						1	1
723	JX987455	M7c1b2b	M7c1a	1	M7c1b2b	3				2	4
724	JQ702664	M7c1c	M7c1a	1	M7c1c	3				2	4
725	KF540546	M7c1c1	M7c1c1	1						1	1
726	KF540664	M7c1c1a	M7c1c1a	1						1	1
727	KC994065	M7c1c1a1	M7c1c1a	1	M7c1c1a1	1				2	2
728	JX987460	M7c1c2	M7c1c2	1						1	1
729	JX987468	M7c1c2a	M7c1c2a	1						1	1
730	JX987452	M7c1c3	M7c1	1	M7c1c3	2	M7c1c3	4		3	7
731	GU733792	M7c1c3a	M7c1c3a	1						1	1
732	HM238218	M7c1c3a1	M7c1c3a1	1						1	1
733	JX987453	M7c1c3b	M7c1c3	1	M7c1c3	2	M7c1c3b	1		3	4
734	JX987462	M7c1c3c	M7c1c3c	1						1	1
735	GU733735	M7c1c3d	M7c1c3	1	M7c1c3	2	M7c1c3d	1		3	4
736	AP012360	M7c1c3e	M7c1c3e	1						1	1
737	KC994090	M7c1c3f	M7c1c3f	1						1	1
738	KC994012	M7c1c3g	M7c1c3g	1						1	1
739	KC994106	M7c1c3h	M7c1c3h	1						1	1
740	KC994051	M7c1c3i	M7c1c3i	1						1	1
741	HQ157984	M7c2	M7c2	1						1	1
742	HM030509	M7c2a	M7c2a	1						1	1
743	EF153810	M7c2b	M7c2b	1						1	1
744	HM030532	M7c3	M73	1	M7c3	2				2	3
745	KF148510	M8a1	M8a1	1						1	1
746	AP008705	M8a1a	M8a1a	1						1	1
747	NA18538	M8a2	M8a2'3	1	M8a2	2	M8a2	2		3	5
748	AP008531	M8a2+152	M8a2+152	1						1	1
749	JF824924	M8a2a	M8a2a	1						1	1
750	AP008803	M8a2a1	M8a2a1	1						1	1
751	EU219349	M8a2b	M8a2b	1						1	1
752	KF849960	M8a2c	M8a2c	1						1	1
753	KC251743	M8a2d	M8a2d	1						1	1
754	KF540552	M8a2e	M8a	1	M8a	1	M8a2e	1		3	3
755	NA18646	M8a3	M8a3	1						1	1
756	KF849919	M8a3a	M8a3a	1	M8a3a	1				2	2
757	JF824830	M8a3a1	M8a3a1	1						1	1
758	AY519496	C1a	C1a	1						1	1
759	EU095226	C1b	C1b	1						1	1
760	HQ012193	C1b1	C1b1	1						1	1
761	DQ282447	C1b2	C1b2	1						1	1
762	NA19788	C1b3	C1b3	1						1	1
763	EU431085	C1b4	C1b4	1						1	1
764	HQ012213	C1b5a	C1b5a	1						1	1
765	JQ702595	C1b5b	C1b5b	1						1	1
766	EU095229	C1b6	C1b6	1						1	1
767	HQ012207	C1b7	C1b7	1						1	1
768	HQ012215	C1b7a	C1b7a	1						1	1
769	HQ012198	C1b10	C1b10	1						1	1
770	HQ012236	C1b8	C1c+195	1	C1b8	1				2	2

771	HQ012210	C1b8a	C1b8a	1							1	1
772	HQ012212	C1b9	C1b9	1							1	1
773	JQ705451	C1b11	C1b11	1							1	1
774	HQ012197	C1b12	C1b	1	C1b12	2					2	3
775	JX413055	C1b13	C1b13	1							1	1
776	JX413039	C1b13a	C1b13a	1							1	1
777	JX413038	C1b13a1	C1b13a1	1							1	1
778	JX413042	C1b13b	C1b13b	1							1	1
779	JX413044	C1b13c	C1b13	1	C1b13c	4	C1b13c	1			3	6
780	JX413048	C1b13c1	C1b13c	1	C1b13c	4	C1b13c1	1			3	6
781	JX413049	C1b13d	C1b13d	1							1	1
782	JX413051	C1b13e	C1b13e	1							1	1
783	NA19773	C1b14	C1b14	1							1	1
784	JQ703840	C1c	C1c	1							1	1
785	HQ012229	C1c1	C1c1	1							1	1
786	EU597533	C1c1a	C1c1	1	C1c1a	2					2	3
787	JQ704902	C1c1b	C1c1b	1							1	1
788	DQ282466	C1c2	C1	1	C1c	1	C1c2	2			3	4
789	JQ705761	C1c3	C1c3	1							1	1
790	HQ012220	C1c4	C1c4	1							1	1
791	DQ282465	C1c5	C1c5	1							1	1
792	JQ704040	C1c6	C1c6	1							1	1
793	JQ703827	C1c7	C1c7	1							1	1
794	EU327891	C1c8	C1c8	1							1	1
795	HQ012239	C1d	C1d	1	C1d	1					2	2
796	HM107309	C1d+194	C1d+194	1							1	1
797	HG01462	C1d1	C1d1	1							1	1
798	HM107321	C1d1a	C1d1a	1							1	1
799	HM107319	C1d1a1	C1d	1	C1d1a	1	C1d1a1	1			3	3
800	HM107324	C1d1b	C1d1b	1	C1d1b	1					2	2
801	HM107332	C1d1b1	C1d1b1	1							1	1
802	HM107334	C1d1c	C1d1c	1							1	1
803	HQ012235	C1d1c1	C1d1c1	1							1	1
804	HM107355	C1d1d	C1d1d	1							1	1
805	HM107315	C1d2	C1d2	1							1	1
806	HM107314	C1d2a	C1d2a	1							1	1
807	JQ701741	C1d3	C1d3	1							1	1
808	HM804483	C1f	C1f	1							1	1
809	FJ951604	C4	C4b	1	C4	1					2	2
810	KC911332	C4a1	C4a1	1	C4a1	1					2	2
811	FJ951462	C4a1a	C4a'b'c	1	C4a1	2	C4a1a	1	C4a1a	3	4	7
812	JX266268	C4a1a1	C4a1a1	1							1	1
813	FJ383648	C4a1a1a	C4a1a1a	1							1	1
814	EU597517	C4a1a+195	C4a1a+195	1							1	1
815	KF849915	C4a1a2	C4a1a2	1							1	1
816	AY255174	C4a1a2a	C4a1a+195	1	C4a1a2	3	C4a1a2a	1			3	5
817	FJ951442	C4a1a3	C4a1a+195	1	C4a1a3	3	C4a1a3	3			3	7
818	FJ951533	C4a1a3a	C4a1a3a	1							1	1
819	AY615360	C4a1a3a1	C4a1a3a1	1							1	1
820	EU482380	C4a1a3b	C4a1a3b	1							1	1
821	KF148505	C4a1a3c	C4a1a+195	1	C4a1a3	3	C4a1a3c	3			3	7
822	KF148121	C4a1a3d	C4a1a3d	1							1	1
823	FJ951459	C4a1a4	C4a1a4	1							1	1
824	EU482361	C4a1a4a	C4a1a4a	1							1	1
825	FJ951612	C4a1a5	C4a1 C4a1a5 (2)	1	C4a1	2	C4a1a5	1			3	4
826	FJ951475	C4a1a6	C4a'b'c	1	C4a1	2	C4a1a	1	C4a1a6	3	4	7
827	GU392063	C4a1b	C4a1b	1							1	1

828	KF148092	C4a2a	C4a2a	1									1	1
829	KF148221	C4a2a1	C4a2a1	1									1	1
830	KF148332	C4a2a1a	C4a2a1a	1									1	1
831	KF148469	C4a2a1b	C4a2a1	1	C4a2a1b	2							2	3
832	GQ895170	C4a2b	C4a2b	1									1	1
833	FJ383631	C4a2b1	C4a2b1	1									1	1
834	NA18547	C4a2b2	C4a2b2	1	C4a2b2	1							2	2
835	KF056247	C4a2b2a	C4a2b2a	1									1	1
836	FJ951548	C4a2c	C4a2c	1									1	1
837	FJ383607	C4a2c1	C4a2c1	1									1	1
838	FJ383647	C4a2c2	C4a2c2	1	C4a2c2	1							2	2
839	HM036530	C4a2c2a	C4a2c2	1	C4a2c2a	1							2	2
840	FJ951445	C4b	C4b	1									1	1
841	FJ951476	C4b1	C	1	C4b1	2	C4b1	1					3	4
842	AY570526	C4b1a	C4b1a	1									1	1
843	FJ951497	C4b1b	C4b1b	1									1	1
844	KF148135	C4b2	C4b2	1									1	1
845	FJ951598	C4b2a	C4b2a	1									1	1
846	KF148478	C4b3	C4b3	1	C4b3	1							2	2
847	KF148111	C4b3a	C4b3a	1									1	1
848	EU482324	C4b3a1	C4b3a1	1									1	1
849	AF346970	C4b3b	C4b3b	1									1	1
850	FJ951460	C4b5	C4b	1	C4b	3	C4b5	2					3	6
851	FJ951451	C4b6	C	1	C4	2	C4a'b'c	1	C4b	3	C4b6	4	5	11
852	EU482313	C4b7	C	1	C4	2	C4a'b'c	1	C4b	3	C4b7	4	5	11
853	KF148472	C4b8	C	1	C4	2	C4a'b'c	1	C4b	3	C4b8	4	5	11
854	EU482370	C4b8a	C4b8a	1									1	1
855	EU095543	C4c	C4c	1									1	1
856	JF979205	C4c1	C4c1 C7	1	C4c1	2							2	3
857	JQ705814	C4c1a	C4c1a	1									1	1
858	JF979203	C4c1b	C4c1b	1									1	1
859	JQ705381	C4c2	C4c2	1									1	1
860	GQ895156	C4+152+16093	C4+152+16093	1									1	1
861	GQ895155	C4d	C4d	1									1	1
862	FJ951611	C4e	C4e	1									1	1
863	FJ951515	C5a1	C5a	1	C5a1	1							2	2
864	KF148083	C5a2a	C5a2a	1									1	1
865	EU482328	C5a2b	C5a2b	1									1	1
866	FJ951595	C5a2b1	C5a2b1	1									1	1
867	FJ951454	C5b	C5b	1									1	1
868	FJ951472	C5b1	C5b1	1									1	1
869	FJ951441	C5b1a	C5b1a	1									1	1
870	FJ951464	C5b1a1	C5b1a1	1									1	1
871	FJ951496	C5b1b	C5b1b	1	C5b1b	1							2	2
872	KF148480	C5b1b1	C5b1b	1	C5b1b1	1							2	2
873	NA18566	C5+16093	C5+16093	1									1	1
874	FJ951614	C5c	C5c	1									1	1
875	KC911629	C5c+16234	C5c+16234	1									1	1
876	FJ951452	C5c1	C5c1	1									1	1
877	FJ951605	C5c1a	C5c1a	1									1	1
878	FJ951440	C5d1	C5d1	1									1	1
879	FJ951576	C5d2	C5d2	1									1	1
880	FJ951594	C7	C4+152	1	C7	4							2	5
881	FJ748711	C7a	C	1	C7	2	C7a	1					3	4
882	FJ383630	C7a1a1	C7a1a1	1									1	1
883	FJ383627	C7a1a2	C7a1a2	1									1	1
884	AY255176	C7a1c	C7a1c	1									1	1

885	FJ383636	C7a1d	C7a1d	1							1	1
886	JF896797	C7a2	C	1	C7a2	2	C7a2	1			3	4
887	KC251742	C7a2a	C7a2a	1							1	1
888	FJ951540	C7+16051	C4+152+16093	1	C4+152+16093	1	C7+16051	4			3	6
889	FJ383641	C7b	C7b	1							1	1
890	AY519493	Z1	Z3a	1	Z3a	1	Z3a	1	Z1	2	4	5
891	FJ147318	Z1a	Z1a	1	Z1a	3					2	4
892	FJ493512	Z1a1a	Z1a	1	Z1a1	3	Z1a1a	2			3	6
893	KC985156	Z1a1b	Z1a	1	Z1a1	3	Z1a1b	2			3	6
894	HM044854	Z1a2	Z1a2a	1	Z1a2	1					2	2
895	FJ493515	Z1a2a	Z1a2a	1							1	1
896	KC985155	Z1a3	Z1a	1	Z1a3	3					2	4
897	AP011007	Z2	Z3+709	1	Z2	2					2	3
898	AP008841	Z3	Z+152	1	Z3	4	Z3	1			3	6
899	EU597518	Z3a1	Z3a	1	Z3a	1	Z3a1	1	Z3a1	1	4	4
900	FJ383642	Z3a1a	Z3a1a	1							1	1
901	FJ383603	Z3a2	Z3a	1	Z3a2	1					2	2
902	FJ383644	Z3b	Z3b	1							1	1
903	KC985166	Z3+709	Z	1	Z3+709	1					2	2
904	NA19072	Z3c	Z3c	1							1	1
905	JF824858	Z3d	Z3d	1							1	1
906	GU392051	Z4	Z4	1							1	1
907	NA19005	Z4a	Z4a	1							1	1
908	AY255155	Z4a1	Z4a1	1							1	1
909	HG00436	Z4a1a	Z4a1	1	Z4a1a	1					2	2
910	AP013181	Z4a1a1	Z4a1a1	1							1	1
911	FJ383629	Z7	Z+152	1	Z7	4					2	5
912	AP008553	Z5	Z5	1							1	1
913	AP010767	M9a	M9a	1							1	1
914	HM346892	M9a1	M9a1	1							1	1
915	HM346905	M9a1a	M9a1a	1							1	1
916	HM346912	M9a1a1	M9a1a1	1							1	1
917	AF346972	M9a1a1a	M9a1a1a	1							1	1
918	AP008378	M9a1a1b	M9a1a1b	1							1	1
919	HM346913	M9a1a1c	M9a1a1c	1	M9a1a1c	1					2	2
920	JN857063	M9a1a1c1a	M9a1a1c	1	M9a1a1c1	1	M9a1a1c1a	2			3	4
921	HM346915	M9a1a1c1b1	M9a1a1c1a	1	M9a1a1c1b1	2	M9a1a1c1b1	1			3	4
922	KF056287	M9a1a1c1b1a	M9a1a1c1b1a	1							1	1
923	HM346918	M9a1a1c1b1a1	M9a1a1c1b1a1	1							1	1
924	HM036568	M9a1a1c1b1a2	M9a1a1c1b1a2	1							1	1
925	FJ383325	M9a1a1c1b2	M9a1a	1	M9a1a1	1	M9a1a1c1b	2	M9a1a1c1b2	1	4	5
926	HM346914	M9a1a1c1c	M9a1a1c1c	1							1	1
927	HM346910	M9a1a1d	M9a1a	1	M9a1a1	1	M9a1a1d	2			3	4
928	FJ748758	M9a1a2	M9a1a2	1							1	1
929	KF540711	M9a1a3	M9a1a3	1							1	1
930	HM346893	M9a1b	M9a1b	1							1	1
931	HM346895	M9a1b+150	M9a1b+150	1	M9a1b+150	1					2	2
932	FJ383315	M9a1b1	M9a1b1	1							1	1
933	FJ383311	M9a1b1a	M9a1b1a	1							1	1
934	FJ383316	M9a1b1a1	M9a1b1a1	1							1	1
935	FJ383327	M9a1b1b	M9a1b1	1	M9a1b1b	3					2	4
936	GQ895140	M9a1b1c	M9a1b1c	1							1	1
937	JN857049	M9a1b2	M9a1b2	1							1	1
938	HM346882	M9a4a1	M9a4a1	1							1	1
939	HM346885	M9a4a2	M9a4a2	1							1	1

940	HM346886	M9a4b	M9a4b	1											1	1
941	HM346889	M9a5	M9a5	1											1	1
942	KF849927	M9b	M9b	1											1	1
943	KF540505	E1	E	1	E1	1	E1	1							3	3
944	EF093538	E1a1	E1a1	1											1	1
945	EF093539	E1a1a	E	1	E1	1	E1a	1	E1a1	1	E1a1	1	E1a1a	1	6	6
946	EF093557	E1a1a1	E1a1a1	1											1	1
947	EF185804	E1a1a1a	E1a1a1	1	E1a1a1a	2									2	3
948	KF540805	E1a1a1b	E1a1a1b	1	E1a1a1b	2									2	3
949	EF093540	E1a1a1b1	E1a1a1b	1	E1a1a1b1	2									2	3
950	KF540887	E1a1a1b2	E1a1a1b	1	E1a1a1b2	2									2	3
951	KC993907	E1a1a1c	E1a1a1c	1											1	1
952	EF185812	E1a1b	E1a1b	1											1	1
953	HQ700847	E1a1b1	E1a1b	1	E1a1b1	1									2	2
954	EF185805	E1a1b2	E1a1b2	1											1	1
955	FJ428235	E1a1b3	E1a1b3	1											1	1
956	KJ154942	E1a1b4	E1a1b4	1											1	1
957	EF093536	E1a1c	E	1	E1	1	E1a	1	E1a1	1	E1a1c	1			5	5
958	FJ428236	E1a2	E1a2	1	E1a2	2	E1a2	1							3	4
959	EF185795	E1a2+(16261)	E1a2+(16261)	1	E1a2+(16261	1									2	2
960	KJ154788	E1a2a	E1a2a	1											1	1
961	EF061148	E1a2a1	E1a2a1	1											1	1
962	KJ154892	E1a2a2	E1a2a2	1											1	1
963	KJ154845	E1a2a3	E1a2+(16261)	1	E1a2a	1	E1a2a3	2							3	4
964	KJ154781	E1a2a4	E1a2a4	1											1	1
965	EF093542	E2	E2	1											1	1
966	HQ700865	E2a	E2a	1											1	1
967	EF093549	E2a1	E2	1	E2a	1	E2a1	2							3	4
968	GQ119047	E2a1a	E2a1a	1											1	1
969	EF185813	E2a2	E2a2	1											1	1
970	EF185816	E2b	E2b	1											1	1
971	EF185810	E2b1	E2b1	1											1	1
972	EF093537	E2b2	E2b2	1											1	1
973	DQ272116	M10	M10	1											1	1
974	JX289127	M10a	M10a	1											1	1
975	JX289101	M10a1	M10a1	1											1	1
976	JN857060	M10a1+16129	M10a1+16129	1											1	1
977	NA18577	M10a1a	M5a2a	1	M10a1a	5									2	6
978	AP008719	M10a1a1a	M10a1a1a	1											1	1
979	NA18760	M10a1a1b	M10a1a1b	1											1	1
980	AP010668	M10a1a1b1	M10a1a1b1	1											1	1
981	AP013191	M10a1a1b2	M10a1a1b	1	M10a1a1b2	1									2	2
982	AY255154	M10a1b	M10a1b	1											1	1
983	JN857058	M10a2	M10a2	1											1	1
984	FJ383334	M11a	M11a	1											1	1
985	FJ383331	M11a1	M11a1	1											1	1
986	FJ748759	M11a2	M11a2	1											1	1
987	KF540712	M11b1	M11	1	M11b1	2									2	3
988	AP008639	M11b1a	M11b1a	1											1	1
989	AP013153	M11b1a1	M11b1a1	1											1	1
990	AP010828	M11b2	M11b2	1											1	1
991	JN857062	M11d	M11d	1											1	1
992	AP010716	M11c	M11c	1											1	1
993	HQ157977	M12a1a	M12a1a	1											1	1
994	HQ157978	M12a1a1	M12a1a1	1											1	1
995	HQ157973	M12a1a2	M12a1a2	1											1	1
996	AY255172	M12a1b	M12a1b	1											1	1

997	HQ157974	M12a2	M12a2	1							1	1
998	FJ383653	M12b1a1	M12b1a1	1							1	1
999	KC505077	M12b1a2a	M12b1a2a	1							1	1
1000	KC505080	M12b1a2b	M12b1a2b	1							1	1
1001	KC505084	M12b1b	M12b1b	1							1	1
1002	HQ157972	M12b2	M12b2	1							1	1
1003	KC505086	M12b2a	M12b2a	1							1	1
1004	NA18634	G1	M	1	G	2	G1	2	G1	1	4	6
1005	HM460792	G1a1	G1a1	1							1	1
1006	AP008511	G1a1a	G1a1	1	G1a1a	2	G1a1a	3			3	6
1007	AP009447	G1a1a1	G1a1a1	1							1	1
1008	AP013178	G1a1a2	G1a1	1	G1a1a	2	G1a1a2	3			3	6
1009	AP008909	G1a1a3	G1a1a3	1							1	1
1010	AP011049	G1a1a4	G1a1a4	1							1	1
1011	KF540834	G1a1b	G1a1b	1							1	1
1012	DQ272114	G1a2	G1a2	1							1	1
1013	AP013126	G1a3	G1a3	1							1	1
1014	KF148338	G1b1	G1b1	1							1	1
1015	KF148075	G1b+16129	G1b+16129	1							1	1
1016	EF153829	G1b2	G1b+16129	1	G1b2	2					2	3
1017	KF148165	G1b3	G1b3	1							1	1
1018	KF148275	G1b4	G1b4	1							1	1
1019	EF153821	G1c	G1c	1							1	1
1020	AY255137	G1c1	G1c1	1							1	1
1021	NA18570	G1c2	G1c2	1							1	1
1022	FJ198217	G2a1	G2a1	1							1	1
1023	AP008535	G2a1b	G2a1b	1							1	1
1024	AP008515	G2a1+16189	G2a1+16189	1							1	1
1025	AP008569	G2a1+16189+16194	G2a1+16189+16194	1							1	1
1026	AP011017	G2a1c	G2a1c	1	G2a1c	1					2	2
1027	AP008401	G2a1c1	G2a1c1	1							1	1
1028	AP008662	G2a1c2	G2a1c2	1							1	1
1029	AP008427	G2a1d1	G2a1d	1	G2a1d1	1	G2a1d1	1			3	3
1030	AP008313	G2a1d1a	G2a1d1a	1							1	1
1031	KF849931	G2a1d2	G2a1d2	1	G2a1d2	1					2	2
1032	HM030544	G2a1d2a	G2a1d2	1	G2a1d2a	1					2	2
1033	AP008528	G2a1e	G2a1e	1							1	1
1034	NA18635	G2a1f	G2a1f	1							1	1
1035	KF849943	G2a1f1	G2a1f1	1							1	1
1036	KF849910	G2a1g	G2a1g	1							1	1
1037	HM036563	G2a1h	G2a1h	1							1	1
1038	EU545470	G2a+152	G2a+152	1	G2a+152	1					2	2
1039	JF824917	G2a2	G2a+152	1	G2a2	1					2	2
1040	JQ705820	G2a2a	G2a2a	1							1	1
1041	KC911517	G2a3	G2a3	1							1	1
1042	GU123045	G2a3a	G2a3a	1							1	1
1043	JF824986	G2a4	G2a4	1							1	1
1044	AP008897	G2a5	G2a5	1							1	1
1045	JF824842	G2c	G2c	1							1	1
1046	FJ015040	G2b1a	G2b1a	1							1	1
1047	JX289093	G2b1a1	G2b1a1	1							1	1
1048	KF849956	G2b1a2	G2b1a2	1							1	1
1049	FJ383505	G2b1b	G2b1b	1							1	1
1050	EU597571	G2b2	G2b2	1							1	1
1051	FJ383510	G2b2a	D4i2	1	D4i2	1	M	1	G2b2a	2	4	5
1052	HQ260973	G2b2b	G2b2b	1							1	1

1053	GU123035	G2b2c	G2b2c	1																									1	1
1054	GU014566	G3a1	G3a1	1																									1	1
1055	GU014569	G3a1a	G3a1a	1																									1	1
1056	KF540726	G3a2	G3a2	1																									1	1
1057	JN580300	G3a2+152	G3a2+152	1																									1	1
1058	AP008645	G3a2a	G3a2a	1																									1	1
1059	KF849958	G3a3	G3a3	1																									1	1
1060	FJ748726	G3b1	G3b1	1																									1	1
1061	DQ272109	G3b2	G3b2	1																									1	1
1062	AP008911	G4	G4	1																									1	1
1063	JX462680	M13a1	M13a1	1																									1	1
1064	AP008495	M13a1a	M13a1a	1																									1	1
1065	GQ895142	M13a1b	M13a1b	1																									1	1
1066	KF148509	M13a1b1	M13a1b1	1																									1	1
1067	FJ968773	M13a2	M13a2	1																									1	1
1068	AY963577	M13b1	M13b1	1																									1	1
1069	HM030529	M13b2	M13b2	1																									1	1
1070	JX289122	M13c	M13c	1																									1	1
1071	KC505096	M46	M7c2	1	M7c2	1	M7c2	1	M74	3	D4d	3	K1f	2	M46	5													7	16
1072	FJ442939	M46a	M46a	1																									1	1
1073	JX289113	M61	M61	1																									1	1
1074	HM030538	M61a	M61a	1																									1	1
1075	EF495222	M14	M14	1																									1	1
1076	EF495219	M15	M15	1																									1	1
1077	GU810076	M17a	M17a	1																									1	1
1078	GQ301876	M17c	D4j+16311	1	M68	2	L3x	3	M	2	M	2	L3x	5	M	5	M	5	M	5	M	5	M	5	M	5	M17c	5	13	50
1079	KC993910	M17c1	M17c1	1																									1	1
1080	GQ352635	M17c1a	M17c1a	1																									1	1
1081	KC993929	M17c1a1	M17c1a1	1	M17c1a1	1																							2	2
1082	EU597577	M17c1a1a	M17c1a1a	1																									1	1
1083	JF739540	M19	M19	1																									1	1
1084	AY922302	M53	M53	1																									1	1
1085	FJ383438	M53b	M53b	1																									1	1
1086	AP012377	M21a	M21a	1																									1	1
1087	KC505091	M21b1	M22a	1	M	2	L3'4	2	M	1	M21b1	9																	5	15
1088	AY963581	M21b1a	M21b1a	1																									1	1
1089	KC505092	M21b+210	M5c1	1	M21b+210	5																							2	6
1090	GQ301866	M21b2	M21b2	1																									1	1
1091	AY963583	M22a	M22a	1																									1	1
1092	HM030499	M22b	M22b	1																									1	1
1093	GQ389777	M23	M23	1																									1	1
1094	HM030540	M75	M75	1																									1	1
1095	KC505094	M24a	M24a	1																									1	1
1096	KC505093	M24b	M24b	1																									1	1
1097	DQ513522	M41	M41	1	M41	2																							2	3
1098	AY922280	M41a	M41a	1																									1	1
1099	FJ383717	M41a1	M41a1	1																									1	1
1100	DQ513521	M41b	M41	1	M41b	2																							2	3
1101	FJ383720	M41c	M41	1	M41c	2																							2	3
1102	KJ154681	M25	M25	1																									1	1
1103	HM596710	M26	M26	1																									1	1
1104	DQ137411	M27a1a1	M27a1a1	1																									1	1
1105	KJ154546	M27a1a2	M27a1a	1	M27a1a2	1																							2	2
1106	KJ154715	M27a1b	M27a1b	1																									1	1
1107	KJ154533	M27a2a	M27a2	1	M27a2a	2																							2	3
1108	KJ154494	M27a2b	M27a2	1	M27a2b	2																							2	3
1109	KJ154488	M27a3	M27a3	1																									1	1

1110	DQ137403	M27b1	M27b1	1					1	1
1111	KJ154728	M27b2	M27b2	1	M27b2	3			2	4
1112	KJ154707	M27b2a	M27b2a	1					1	1
1113	KJ154729	M27b2a1	M27b2a1	1					1	1
1114	KJ154676	M27b2b	M27b2b	1					1	1
1115	KJ154792	M27b2b1	M27b2b1	1					1	1
1116	KJ154721	M27b2c	M27b2	1	M27b2c	3			2	4
1117	KJ154770	M27c	M27c	1					1	1
1118	DQ372883	M28a1	M28a1	1					1	1
1119	KJ154478	M28a2	M28a2	1					1	1
1120	EF061145	M28a2a	M28a2a	1					1	1
1121	KJ154806	M28a3	M28a3	1					1	1
1122	KJ154163	M28a4	M28a4	1					1	1
1123	KJ154451	M28a5	M28a5	1					1	1
1124	KJ154913	M28a5a	M28a5a	1					1	1
1125	KJ154832	M28a5b	M28a5b	1					1	1
1126	KJ154634	M28a6	M28a	1	M28a6	2	M28a6	1	3	4
1127	KJ154481	M28a6a	M28a	1	M28a6	2	M28a6a	1	3	4
1128	DQ137400	M28a7	M28a7	1	M28a7	1			2	2
1129	KJ154667	M28a7a	M28a7a	1					1	1
1130	KJ154860	M28a7b	M28a7b	1					1	1
1131	KJ154292	M28b	M28b	1					1	1
1132	KJ154666	M28b1	M28b1	1					1	1
1133	DQ137408	M29a	M29a	1					1	1
1134	EF495217	M29b	M29b	1					1	1
1135	KJ154923	M29b1	M29b1	1					1	1
1136	AY289090	Q1	Q1	1	Q1	3			2	4
1137	AY289085	Q1a	Q1a	1					1	1
1138	EU597495	Q1a1	Q1a1	1					1	1
1139	KJ154590	Q1a1a	Q1a1a	1					1	1
1140	KJ154866	Q1b	Q1b	1					1	1
1141	KJ154885	Q1c	Q1c	1					1	1
1142	KJ154631	Q1c1	Q1c1	1					1	1
1143	KJ154557	Q1c1a	Q1c1a	1					1	1
1144	KJ154852	Q1c2	Q1c2	1					1	1
1145	KJ154875	Q1c2a	Q1c2a	1					1	1
1146	KF540947	Q1d	Q1d	1					1	1
1147	KJ154939	Q1e	Q1e1c	1	Q1e	1			2	2
1148	KJ154444	Q1e1	Q1d	1	Q1e1	3			2	4
1149	KJ154833	Q1e1a	Q1e1a	1					1	1
1150	KJ154812	Q1e1a1	Q1e1a1	1					1	1
1151	KJ154680	Q1e1b	Q1e1b	1					1	1
1152	KJ154603	Q1e1b1	Q1e1b1	1					1	1
1153	KJ154734	Q1e1c	Q1f2	1	Q1e	2	Q1e1c	1	3	4
1154	DQ372885	Q1f1	Q1f1	1					1	1
1155	KJ154816	Q1f2	Q1f2	1					1	1
1156	HQ113226	Q2a	Q2a	1					1	1
1157	GQ214525	Q2a1	Q2a1	1					1	1
1158	GQ214526	Q2a2a	Q2a2a	1					1	1
1159	KJ154178	Q2a2b	Q2a2b	1					1	1
1160	AY956412	Q2a3a	Q2a3a	1					1	1
1161	KJ154799	Q2a3b	Q2a3b	1					1	1
1162	KJ154824	Q2a4	Q2a4	1					1	1
1163	EF495218	Q2b	Q2b	1					1	1
1164	AY289079	Q3a	Q3a	1					1	1
1165	AY289089	Q3a1	Q3a1	1					1	1
1166	EF061146	Q3b	Q3b	1					1	1

1167	AY950298	M31a1a	M31a1	1	M31a1a	2									2	3
1168	DQ149515	M31a1b	M31a1	1	M31a1b	2									2	3
1169	HQ438684	M31a2	M31a2	1											1	1
1170	HQ438686	M31b1	M31b1	1											1	1
1171	FJ770962	M31c	M31c	1											1	1
1172	DQ149512	M32a	M32a	1											1	1
1173	GQ389779	M32c	M32c	1											1	1
1174	FJ383487	M56	M56	1											1	1
1175	FJ770960	M33a	M24	1	M24	4		1	M	4	M	2	M33a	5	6	17
1176	FJ383339	M33a1a	M33a1a	1											1	1
1177	FJ383353	M33a1b	M33a1b	1											1	1
1178	FJ383361	M33a2	M33a2	1											1	1
1179	AY922276	M33a2a	M33a2a	1											1	1
1180	FJ770950	M33a3	M33a3	1											1	1
1181	JX289104	M33a3a	M33a3a	1											1	1
1182	FJ770968	M33b	M33b	1											1	1
1183	FJ383362	M33b1	M33b1	1											1	1
1184	AY922298	M33b2	M33b2	1											1	1
1185	EU148486	M33c	M33c	1											1	1
1186	FJ383346	M33d	M33d	1											1	1
1187	JX462718	M34a1	M34a1	1											1	1
1188	JX289117	M34a1a	M34a1a	1											1	1
1189	FJ383682	M34a2	M34a2	1											1	1
1190	AY922274	M34b	M34b	1											1	1
1191	FJ383760	M57+152	M31b	1	M57+152	2									2	3
1192	FJ383763	M57a	M57a	1											1	1
1193	FJ383761	M57b	M57b	1											1	1
1194	FJ383759	M57b1	M57b1	1											1	1
1195	FJ770956	M35	M29'Q M35 (2)	1	M	4	M35	1							3	6
1196	FJ383365	M35a1	M35a	1	M35a1	2									2	3
1197	FJ383384	M35a1a	M35a1a	1											1	1
1198	FJ383363	M35a2	M35a2	1											1	1
1199	AY922272	M35b	M33a1b	1	M35b	2									2	3
1200	FJ770966	M35b+16304	M35b+16304	1											1	1
1201	FJ383378	M35b1	M35b1	1											1	1
1202	FJ383381	M35b2	M35b2	1											1	1
1203	HM036559	M35b3	M35b2	1	M35b3	2									2	3
1204	FJ383370	M35b4	M35b4	1											1	1
1205	FJ383382	M35c	M35c	1											1	1
1206	AY922287	M36a	M36a	1											1	1
1207	AY922284	M36b	M36b	1											1	1
1208	FJ383808	M36c	M36c	1											1	1
1209	FJ383807	M36d	M36a	1	M36d	2									2	3
1210	FJ383784	M36d1	M36d1	1											1	1
1211	AY922288	M39	M39	1											1	1
1212	FJ383714	M39a	M39a	1											1	1
1213	EF556151	M39a1	M39a1	1											1	1
1214	FJ383705	M39a2	M39a2	1											1	1
1215	FJ383699	M39b1	M39b1	1											1	1
1216	FJ383713	M39b2	M39b2	1											1	1
1217	AY922293	M39c	M39c	1											1	1
1218	HM030534	M70	M70	1											1	1
1219	AY922294	M40	M40	1											1	1
1220	FJ383416	M40a	M40a	1											1	1
1221	FJ383412	M40a1	M40a1	1	M40a1	1									2	2
1222	FJ383413	M40a1a	M40a1a	1											1	1
1223	FJ383417	M40a1b	M40a1	1	M40a1b	1									2	2

1224	DQ404445	M42a	M42a	1															1	1
1225	HQ260949	M42b1	M42b1	1															1	1
1226	FJ380210	M42b1a	M42b1a	1															1	1
1227	KC577356	M42b2	M42b2	1															1	1
1228	GU377083	M74a	M74a	1															1	1
1229	HM030520	M74b	M74b	1															1	1
1230	HM596679	M74b1	M74b1	1															1	1
1231	AP012354	M74b2	M74b2	1															1	1
1232	FJ383750	M44	M44	1	M44	1													2	2
1233	AY922253	M44a	M44	1	M44a	1	M44a	1											3	3
1234	FJ383751	M44a1	M44	1	M44a	1	M44a1	1											3	3
1235	HM030526	M49a1	M49a1	1															1	1
1236	FJ383766	M49c	M49	1	M49	2	M49c	2	M49c	1									4	6
1237	JX289106	M49c1	M49c1	1															1	1
1238	FJ383765	M49d	M49d	1															1	1
1239	JX289133	M49e	M49e	1															1	1
1240	JX289094	M49e1	M49	1	M49e	2	M49e1	1											3	4
1241	GQ301868	M50	M51a	1	M51	2	M51	1	M50	10									4	14
1242	GU810023	M50a1	M50a1	1															1	1
1243	KC994151	M50a2	M50a2	1															1	1
1244	JQ703445	M52a	M52a	1															1	1
1245	FJ157841	M52a1a	M52a1a	1															1	1
1246	JX462717	M52a1b	M52a1b	1															1	1
1247	KC993960	M52a1b1	M52a1b1	1															1	1
1248	JF742203	M52b	M52b	1															1	1
1249	FJ770964	M52b1	M52b1	1															1	1
1250	FJ383488	M52b1a	M52b1a	1															1	1
1251	KC896622	M55	M55	1															1	1
1252	GQ301865	M77	M77	1															1	1
1253	JX289110	M58	M37e	1	M58	4													2	5
1254	JQ702247	M59	M59	1															1	1
1255	FJ383496	M60a1	M60a	1	M60a1	2													2	3
1256	FJ383498	M60a2	M60a2	1															1	1
1257	FJ383500	M60b	M60b	1															1	1
1258	FJ383501	M62a	M62a	1															1	1
1259	FJ544231	M62b	M62b	1															1	1
1260	FJ544239	M62b+204	M62b+204	1															1	1
1261	GQ895169	M62b1	M62b1	1															1	1
1262	FJ544241	M62b1a	M62b1a	1															1	1
1263	FJ544235	M62b1a1	M62b1a1	1															1	1
1264	FJ544233	M62b2	M62b2	1															1	1
1265	JX289131	M68	U3a2a	1	M	4	M68	2											3	7
1266	KC887488	M68a1	M68a1	1															1	1
1267	KC887480	M68a1a	M68a1a	1															1	1
1268	KC887491	M68a2	M68a2	1															1	1
1269	KC505106	M68a2a	M68a2a	1															1	1
1270	HM596653	M69	D1e	1	M	5	M13'46'61	4	M13'46'61	1	M	2	M	2	M	5	M69	5	8	25
1271	KC887492	M69a	M69a	1															1	1
1272	KC505110	M71+151	M71+151	1															1	1
1273	GQ119008	M71a	M71a	1															1	1
1274	HM030543	M71a1	M71	1	M71	3	M71a1	1											3	5
1275	HM030502	M71a1a	M71a1a	1															1	1
1276	JX289128	M71a2	M71a2	1															1	1
1277	HM030511	M71b	M71b	1															1	1
1278	GU810039	M71c	M71c	1															1	1
1279	HM030501	M72	M72	1															1	1
1280	JX289126	M72a	M72a	1															1	1

1281	GQ119039	M73a	M73a	1									1	1
1282	GQ119017	M73a1	M73a1	1									1	1
1283	GQ119012	M73b	M73b	1									1	1
1284	HM030504	M79	M79	1									1	1
1285	HM030525	M76	M76	1									1	1
1286	HM030541	M76a	M76a	1									1	1
1287	KC577353	M81	M81	1									1	1
1288	JX289097	M91a	M91a	1									1	1
1289	KC887489	M91b	M91b	1									1	1
1290	JF739537	M80	M80	1									1	1
1291	JN375993	D4	D4	1									1	1
1292	JN253391	D1	D1	1									1	1
1293	EU095233	D1a1	D1a1	1									1	1
1294	AF346984	D1a2	D1a2	1									1	1
1295	DQ282479	D1b	D1	1	D1b	6							2	7
1296	HQ012249	D1c	D1	1	D1c	6							2	7
1297	HQ012255	D1d1	D1 D1d1 (2)	1	D1d1	1							2	2
1298	HQ012245	D1d2	D1d2	1									1	1
1299	EU597510	D1e	D1e	1									1	1
1300	EU095536	D1f	D4h2	1	D1f	2							2	3
1301	EU095235	D1f1	D1f1	1									1	1
1302	JQ702137	D1f2	D1f2	1									1	1
1303	EU431089	D1f+16189	D1f+16189	1									1	1
1304	JQ701868	D1f3	D1f3	1									1	1
1305	JN253393	D1g	D1g	1									1	1
1306	JN253394	D1g1a	D1g1a	1									1	1
1307	JN253399	D1g1b	D1g1b	1									1	1
1308	JN253401	D1g2	D1g3	1	D1g2	2							2	3
1309	JN253404	D1g2a	D1g2a	1									1	1
1310	JN253415	D1g5	D1g5	1									1	1
1311	JN253407	D1g3	D1g3	1									1	1
1312	JN253411	D1g4	D1g4	1									1	1
1313	JN253418	D1g6	D1g6	1									1	1
1314	HQ012251	D1h1	D1h1	1									1	1
1315	HQ012258	D1h2	D1h2	1									1	1
1316	DQ282487	D1i	D1i	1	D1i	1							2	2
1317	NA19747	D1i1	D1i1	1									1	1
1318	NA19741	D1i2	D1i2	1									1	1
1319	JN253419	D1j	D1j	1									1	1
1320	JN253422	D1j1	D1j1	1									1	1
1321	JN253424	D1j1a	D1j1a	1									1	1
1322	JN253430	D1j1a1	D1j1a1	1									1	1
1323	JN253434	D1j1a2	D1j1a2	1									1	1
1324	DQ282483	D1k	D1	1	D1k	6							2	7
1325	JQ704599	D1m	D1m	1									1	1
1326	JQ703865	D1n	D1	1	D1n	6							2	7
1327	AP008444	D4a1	D4a1c	1	D4a1	2							2	3
1328	AP010710	D4a1a	D4a1	1	D4a1	3	D4a1a	1					3	5
1329	AP008745	D4a1a1	D4a1a1	1									1	1
1330	AP010669	D4a1a1a	D4a	1	D4a1	3	D4a1a	6	D4a1a1	1	D4a1a1a	1	5	12
1331	AP010760	D4a1b	D4a1b	1									1	1
1332	AP010762	D4a1b1	D4a1b1	1									1	1
1333	AP008449	D4a1c	D4a1c	1									1	1
1334	AP008871	D4a1d	D4a1d	1									1	1
1335	JF824827	D4a1e	D4a1e	1									1	1
1336	AP008614	D4a1e1	D4a1e	1	D4a1e1	1							2	2
1337	AP010737	D4a1f	D4a	1	D4a1	3	D4a1f	6	D4a1f	1			4	11

1338	JQ702207	D4a1f1	D4a1f1	1																	1	1
1339	FJ951517	D4a1g	D4a1g	1																	1	1
1340	AP008497	D4a1h	D4a1h	1																	1	1
1341	AP013248	D4a2	D4a	1	D4a2	3	D4a2	2													3	6
1342	AP008839	D4a2a	D4a2a	1																	1	1
1343	AP013286	D4a2b	D4a2b	1																	1	1
1344	JF824835	D4a3a1	D4a3a1	1																	1	1
1345	AP008862	D4a3a2	D4a3a2	1																	1	1
1346	KF849918	D4a3b	D4a3a2	1	D4a3b	2															2	3
1347	AP011047	D4a3b1	D4a3b1	1																	1	1
1348	JF824944	D4a3b2	D4a3b2	1																	1	1
1349	AP008582	D4a+16294	D4a+16294	1																	1	1
1350	AP008888	D4a4	D4a4	1																	1	1
1351	FJ748716	D4a5	E1a2	1	D4a5	4															2	5
1352	KF849929	D4a6	D4a6	1																	1	1
1353	KF540709	D4a7	D4a7	1																	1	1
1354	KF540683	D4a8	D4a8	1																	1	1
1355	AP010720	D4b1a1	D4b1a1	1																	1	1
1356	AP008767	D4b1a1a	D4b1a1a	1																	1	1
1357	EU482305	D4b1a2	D4b1a2	1																	1	1
1358	FJ951581	D4b1a2a	D4d	1	D4b1a2a	2															2	3
1359	EU007895	D4b1a2a1	D4b1a2a1	1																	1	1
1360	EU482385	D4b1a2a2	D4	1	D4b1	2	D4b1a	1	D4b1a2	1	D4b1a2a	1	D4b1a2a2	1							6	7
1361	AP008254	D4b1b1	D4b1b1	1																	1	1
1362	AP008440	D4b1b1a	D4b1b1a	1	D4b1b1a	1															2	2
1363	AP008476	D4b1b1a1	D4b1b1a	1	D4b1b1a1	1															2	2
1364	AP008708	D4b1b2	D4b1b2	1																	1	1
1365	HM030519	D4b1d	D4b1d	1																	1	1
1366	JQ703736	D4b1c	D4b1c	1																	1	1
1367	EU482316	D3	D4b1c	1	D3	1															2	2
1368	AP011004	D4b2	M	1	D	2	D4	1	D4b	5	D4b2	1	D4b2	2							6	12
1369	AP008434	D4b2a	G2b1a1	1	D4b2a	6															2	7
1370	AP008286	D4b2a1	D4b2a1	1																	1	1
1371	AP013190	D4b2a2	M	1	D	2	D4	1	D4b	5	D4b2	1	D4b2	2	D4b2a	2	D4b2a2	1	D4b2a2	1	9	16
1372	AP008703	D4b2a2a	D	1	D4b2a2a	1	D4b2a2a	1													3	3
1373	AP008287	D4b2a2a1	D4b2a2a1	1																	1	1
1374	AP008303	D4b2a2a2	D4b2a2a2	1																	1	1
1375	AP008804	D4b2a2b	D4b2a2b	1																	1	1
1376	AP008757	D4b2b	D4b2b	1	D4b2b	4															2	5
1377	AF346989	D4b2b1	M33c	1	D4b2b	2	D4b2b1	1													3	4
1378	AP008467	D4b2b1a	D4b2b1a	1																	1	1
1379	AP008333	D4b2b1b	D4b2b	1	D4b2b1	4	D4b2b1b	3													3	8
1380	AP013251	D4b2b1c	D4b2b	1	D4b2b1	4	D4b2b1c	3													3	8
1381	AP008361	D4b2b1+146	D4b2b1+146	1																	1	1
1382	EU597488	D4b2b1d	D4b2b1d	1																	1	1
1383	KF849955	D4b2b2	D4b2b	1	D4b2b2	4															2	5
1384	JF824962	D4b2b2a	D4b2b	1	D4b2b2a	2	D4b2b2a	1													3	4
1385	AP008684	D4b2b2a1	D4b2b2a1	1																	1	1
1386	HM030530	D4b2b2b	D4b2b2b	1																	1	1
1387	NA18739	D4b2b2c	D4b2b2c	1																	1	1
1388	AP008579	D4b2b3	D4b2b3	1																	1	1
1389	FJ383213	D4b2b4	D4b2b4	1																	1	1
1390	FJ951535	D4b2b5	D4b2b5	1																	1	1
1391	NA18145	D4b2b6	D4b2b6	1																	1	1
1392	KF540670	D4b2b7	D4b2b7	1																	1	1
1393	FJ951514	D4b2d	D4b2d	1																	1	1
1394	AP010983	D4c1a	D4c1a	1																	1	1

1395	AP008431	D4c1a1	D4c1a1	1											1	1
1396	AP008251	D4c1b	D4c1b	1	D4c1b	1									2	2
1397	AP008494	D4c1b1	D4c1b1	1											1	1
1398	AP013273	D4c1b2	D4c1b	1	D4c1b2	1									2	2
1399	HM776716	D4c2a	D4c2a	1											1	1
1400	FJ858889	D4c2b	D4c2b	1											1	1
1401	AP010711	D4c2c	D4c2c	1											1	1
1402	AP008628	D4d	D4	1	D4d	2									2	3
1403	NA17981	D4e1	D4e1	1	D4e1	1									2	2
1404	AP008789	D4e1a	D4e1a	1											1	1
1405	AP008275	D4e1a1	D4e1a1	1											1	1
1406	FJ383207	D4e1a2	D4e1a2	1											1	1
1407	AP008772	D4e1a2a	D4e1a2a	1											1	1
1408	NA18685	D4e1a3	D4e1a3	1											1	1
1409	HQ012262	D4e1c	D4e1c	1											1	1
1410	EU660536	D2a	D2a'b	1	D2a	1	D2a	2							3	4
1411	EU725621	D2a1	D2a'b	1	D2a	1	D2a1	2	D2a1	1					4	5
1412	EU660570	D2a1a	D2a1a	1											1	1
1413	EU660553	D2a1b	D2a1b	1											1	1
1414	EU482339	D2a2	D2a'b	1	D2a	1	D2a2	2							3	4
1415	JF824877	D2b1	D2b1	1											1	1
1416	EF153796	D2b1a	D2b1a	1											1	1
1417	EU095542	D2b2	D2b2	1											1	1
1418	FJ951506	D2c	D2c	1											1	1
1419	JF824823	D4e3	D4j+146	1	M	3	D4e3	5							3	9
1420	AP008896	D4e2	D4+195	1	D4e2	5		1							3	1
1421	AP008834	D4e2a	D4e2a	1											1	1
1422	AP008557	D4e2b	D4e2b	1											1	1
1423	AP008825	D4e2c	D4e2c	1											1	1
1424	AP008530	D4e2d	M	1	D	2	D4	1	D4e	5	D4e2	3	D4e2d	3	6	15
1425	KF148448	D4e4	D4+195	1	D4+195	4	D4e4	1							3	6
1426	FJ951569	D4e4a	D4e4a	1											1	1
1427	FJ858880	D4e4a1	D4e4a1	1											1	1
1428	FJ951616	D4e4b	D4e4b	1											1	1
1429	AP008739	D4e5a	D4e5a	1											1	1
1430	NA18108	D4e5b	D4e5b	1											1	1
1431	EU007883	D4f	M	1	M D G	2	D4f	5							3	8
1432	AP011012	D4f1	D4b2b1a	1	D4f1	7									2	8
1433	AP008652	D4g1	D4g1	1	D4g1	3									2	4
1434	AP008544	D4g1a	D4g1	1	D4g1a	3									2	4
1435	AP010985	D4g1b	D4g1b	1											1	1
1436	AP008560	D4g1c	D4g1	1	D4g1c	3									2	4
1437	JF896799	D4g2	D4g2	1											1	1
1438	AP008422	D4g2a	D1h1	1	D4g2a	2									2	3
1439	FJ951522	D4g2a1	D4g2a	1	D4g2a1	1	D4g2a1	1							3	3
1440	AP008447	D4g2a1a	D4g2a1a	1											1	1
1441	HM357822	D4g2a1b	D4g2a1b	1											1	1
1442	KF849965	D4g2a1c	D4g2a1c	1											1	1
1443	FJ951468	D4g2b	D4g2b	1											1	1
1444	HM776717	D4g2b1	D4g2b1	1											1	1
1445	AP008408	D4g2b1a	D4g2b1a	1											1	1
1446	AP008524	D4h1a1	D4h1a1	1											1	1
1447	AP008730	D4h1a2	D4h1a	1	D4h1a2	1									2	2
1448	JF271012	D4h1b	D4h1b	1											1	1
1449	KC733262	D4h1c	D4h1c	1											1	1
1450	AP009422	D4h1c1	D4h1c1	1											1	1
1451	FJ951534	D4h1d	D4h1d	1											1	1

1452	EU828638	D4h2	D4h2	1											1	1
1453	EU095531	D4h3a	D4h3a	1											1	1
1454	FJ168713	D4h3a1	D4h3a1	1											1	1
1455	FJ168717	D4h3a1a1	D4h3a1a1	1											1	1
1456	FJ168720	D4h3a1a2	D4h3a1a2	1											1	1
1457	FJ168727	D4h3a2	D4h3a2	1											1	1
1458	FJ168728	D4h3a3	D4h3a3	1											1	1
1459	FJ168731	D4h3a3a	D4h3a3a	1											1	1
1460	FJ168736	D4h3a4	D4h3a4	1											1	1
1461	FJ168739	D4h3a5	D4h3a5	1											1	1
1462	FJ168744	D4h3a6	D4h3a6	1											1	1
1463	FJ168745	D4h3a+@152	D4h3a+@152	1											1	1
1464	KC998701	D4h3a7	D4h3a+@152	1	D4h3a7	2									2	3
1465	HQ012263	D4h3a8	D4h3a8	1											1	1
1466	FJ168747	D4h3a9	D4h3a9	1											1	1
1467	FJ168712	D4h3b	D4h3b	1											1	1
1468	AP008782	D4h4	D4e3	1	D4h4	2									2	3
1469	FJ951519	D4h4a	D4h4a	1											1	1
1470	AP013281	D4i	D4i	1											1	1
1471	AP008428	D4i1	D4i1	1											1	1
1472	EU007867	D4i2	D4i2	1											1	1
1473	KF540585	D4i3	D4i3	1											1	1
1474	AP008780	D4j	D4j,	1											1	1
1475	FJ383200	D4j1a1	D4j1a	1	D4j1a1	1	D4j1a1	2							3	4
1476	FJ383189	D4j1a1a	D4j1a1a	1											1	1
1477	FJ383225	D4j1a1b	D4j1a1b	1											1	1
1478	GQ895153	D4j1a2	D4j1a2	1											1	1
1479	FJ383177	D4j1b	M	1	D	2	D4	1	D4j	5	D4j1	5	D4j1b	1	6	15
1480	FJ383199	D4j1b2	D4j1b2	1											1	1
1481	FJ415605	D4j2	D4j2	1											1	1
1482	EU482362	D4j2a	D4j2a	1											1	1
1483	AP011035	D4j3	D4j3	1											1	1
1484	EF114278	D4j3a	D4j3a	1											1	1
1485	NA18959	D4j3a1	D4j3a1	1											1	1
1486	AP010987	D4j11	D4j11	1											1	1
1487	EU482369	D4j4	M	1	D	2	D4	1	D4j	5	D4j4	5	D4j4	1	6	15
1488	HM044856	D4j4a	D4j4a	1											1	1
1489	KC911603	D4j5	D1	1	D1k	6	D4j5	2							3	9
1490	EU482325	D4j5a	D4	1	D4j5a	2									2	3
1491	FJ951507	D4j6	D4j6	1											1	1
1492	KF056276	D4j13	D4j13	1											1	1
1493	EU482377	D4j7	D4j7	1											1	1
1494	FJ951537	D4j7a	D4j7a	1											1	1
1495	FJ951456	D4j8	D4j8	1											1	1
1496	KF540701	D4j+(16286)	D4j+(16286)	1	D4j+(16286)	1									2	2
1497	FJ951555	D4j9	D4j+(16286)<br>D4j9 (2)	1	D4j	2	D4j9	1							3	4
1498	EU482379	D4j10	M	1	D	2	D4	1	D4j	5	D4j10	5			5	14
1499	FJ951553	D4j12	M	1	D	2	D4	1	D4j	5	D4j12	5			5	14
1500	AP013278	D4j14	D4j14	1											1	1
1501	KF849898	D4j15	D4j1b2	1	D4j15	3									2	4
1502	JQ657729	D4j16	D1	1	D1	6	D4j16	3							3	10
1503	DQ272107	D4k	D4k	1											1	1
1504	FJ951500	D4o	D4o	1											1	1
1505	EU482383	D4o1	D4o1	1											1	1
1506	AP008819	D4o1a	D4o1	1	D4o1a	1									2	2
1507	FJ951551	D4o2	D4o2	1											1	1

1508	DQ272113	D4o2a	D4o2a	1					1	1
1509	JF824814	D4o2a1	D4o2a1	1					1	1
1510	FJ951448	D4p	D4p	1	D4p	1			2	2
1511	AP008424	D4p1	D4p	1	D4p1	1			2	2
1512	FJ951528	D4l1	D4l1	1					1	1
1513	AP008778	D4l1a	D4l1a	1	D4l1a	1			2	2
1514	AP008916	D4l1a1	D4l1a1	1					1	1
1515	KF148415	D4l2a	D4l2	1	D4l2a	1			2	2
1516	KF148294	D4l2a1	D4l2a1	1					1	1
1517	FJ951570	D4l2a2	D4l2a2	1					1	1
1518	KF849897	D4l2b	D4l2b	1					1	1
1519	AP008321	D4m1	D4m1	1					1	1
1520	EU007866	D4m2	D4m2	1					1	1
1521	KF148357	D4m2a	D4m2a	1	D4m2a	1			2	2
1522	FJ951565	D4m2a1	D4m2a1a	1	D4m2a1	1			2	2
1523	KF148265	D4m2a1a	D4m2a1a	1					1	1
1524	AP010765	D4n	D4n	1					1	1
1525	AP008810	D4n1	D4n	1	D4n1	1	D4n1	1	3	3
1526	AP008754	D4n1a	D4n	1	D4n1	1	D4n1a	1	3	3
1527	FJ951554	D4n2	D4n2	1					1	1
1528	FJ383210	D4q	D4q	1					1	1
1529	FJ383234	D4q1	D4q1	1	D4q1	1			2	2
1530	FJ383220	D4q1a	D4q1	1	D4q1a	1			2	2
1531	EU007863	D4s	D4s	1					1	1
1532	JF824902	D4t	D4t	1					1	1
1533	AP008315	D5a1	D5a1	1					1	1
1534	AP008437	D5a1a1	D5a1a1	1					1	1
1535	AP008619	D5a1a2	D5a1	1	D5a1a	1	D5a1a2	2	3	4
1536	FJ383201	D5a2	D5a2	1	D5a2	1			2	2
1537	FJ951467	D5a2a	D5a2a	1					1	1
1538	HM460795	D5a2a1	D5a2a1	1					1	1
1539	FJ383180	D5a2a1+@1617	D5a2a1+@1617	1					1	1
1540	AP011023	D5a2a1a	D5a2a1a	1	D5a2a1a	1			2	2
1541	AP009424	D5a2a1a1	D5a2a1a1	1	D5a2a1a1	1			2	2
1542	AP013256	D5a2a1a1a	D5a2a1a1a	1					1	1
1543	AP013197	D5a2a1a2	D5a2a1a2	1					1	1
1544	FJ383195	D5a2a1b	D5a2a1	1	D5a2a1b	1			2	2
1545	JF824956	D5a2a1b1	D5a2a1b1	1					1	1
1546	EU482309	D5a2a2	D5a2a+16092	1	D5a2a2	1			2	2
1547	KF056266	D5a2b	D5a2b	1					1	1
1548	FJ951589	D5a3	D5a3	1					1	1
1549	NA17962	D5a3a	D5a3	1	D5a3a	1	D5a3a	1	3	3
1550	JF824991	D5a3a1	D5a3a1	1					1	1
1551	FJ951615	D5a3a1a	D5a3a1a	1					1	1
1552	JN580302	D5b1	D5b	1	D5b1	2	D5b1	3	3	6
1553	AP008674	D5b1a1	D5b1a1	1					1	1
1554	AP008572	D5b1a2	D5b1a2	1					1	1
1555	AP013198	D5b1b	D5b1d	1	D5b1b	2			2	3
1556	AP008771	D5b1b1	D5b1b1	1					1	1
1557	AP008260	D5b1b2	D5b1b2	1					1	1
1558	AY255169	D5b1c	D5b	1	D5b1	2	D5b1c	3	3	6
1559	JF824949	D5b1c1	D5b1c1	1					1	1
1560	KC994014	D5b1c1a	D5b1c1	1	D5b1c1a	2			2	3
1561	NA18576	D5b1d	D5b1d	1					1	1
1562	AP008289	D5b2	D5b2	1					1	1
1563	KF540723	D5b3	D5b3a	1	D5b3	1			2	2
1564	KF540755	D5b3a	D5b3a	1					1	1

1565	KF540890	D5b3a1	D5b3a1	1									1	1
1566	KF540841	D5b4	D5b4	1									1	1
1567	NA18605	D5c1	D5c1	1									1	1
1568	AP008519	D5c1a	D5c1a	1									1	1
1569	EU007893	D5c+16311	D5c+16311	1									1	1
1570	HM460797	D5c2	D5c2	1									1	1
1571	JN866825	D6a1	D6a1	1									1	1
1572	JF824913	D6a1a	D6a1	1	D6a1a	1							2	2
1573	GQ119022	D6a2	D6a2	1									1	1
1574	AY255151	D6c	D6c	1									1	1
1575	GQ119042	D6c1	D6c1	1									1	1
1576	KC994157	D6c1a	D6c1a	1									1	1
1577	JQ245777	N1a1a	N1a1a	1									1	1
1578	JQ245754	N1a1a+152	N1a1a+152	1									1	1
1579	EF486519	N1a1a1a1	N1a1a1a1	1									1	1
1580	GU290209	N1a1a1a1a	N1a1a1a1a	1									1	1
1581	FJ348184	N1a1a1a2	N1a1a1a2	1									1	1
1582	GU290216	N1a1a1a3	N1a1a1a3	1									1	1
1583	GU290207	N1a1a1b	N1a1a1b	1									1	1
1584	EF486517	N1a1a2	N1a1a2	1									1	1
1585	JQ245789	N1a1a3	N1a1a3	1									1	1
1586	JQ245735	N1a1b1	N1a1b1	1									1	1
1587	KF146252	I	I5a	1	I5a	2	I5a	1	I	1	I	1	5	6
1588	JQ705840	I1	I1	1									1	1
1589	KC911435	I1a	I1a	1									1	1
1590	EF177414	I1a1	I1a1a	1	I1a1	4							2	5
1591	KF146236	I1a1a	I1a1	1	I1a1a	4	I1a1a	2					3	7
1592	KF899911	I1a1a1	I1a1d	1	I1a1a1	2							2	3
1593	AY339509	I1a1a2	I1a1a	1	I1a1a	4	I1a1a2	2					3	7
1594	JQ245749	I1a1a3	I1a1a3	1									1	1
1595	KJ816752	I1a1a3a	I1a1a3	1	I1a1a3a	1							2	2
1596	JQ704690	I1a1b	I1a1	1	I1a1b	4							2	5
1597	JQ702023	I1a1c	I1a1c	1									1	1
1598	JQ705189	I1a1d	I1a1d	1									1	1
1599	JQ701900	I1a1e	I1a1e	1									1	1
1600	FJ968796	I1b	I1b	1									1	1
1601	JQ705932	I1c	I1c	1									1	1
1602	KF146244	I1c1	I1c1	1									1	1
1603	JQ705364	I1c1a	I1c1	1	I1c1a	1							2	2
1604	KF146245	I1d	I1d	1									1	1
1605	JX462710	I1e	I1e	1									1	1
1606	JX153931	I1f	I1f	1									1	1
1607	JQ245744	I2	I2	1									1	1
1608	HQ695930	I2a	I2a	1	I2a	1							2	2
1609	HQ724528	I2a1	I2a1	1	I2a1	1							2	2
1610	HG00329	I2a1a	I2a1	1	I2a1a	1							2	2
1611	JQ703910	I2a2	I2a2	1									1	1
1612	JX154048	I2a3	I2a3	1									1	1
1613	AY339498	I2b	I2b	1									1	1
1614	JQ705666	I2c	I2c	1									1	1
1615	JQ705244	I2d	I2d	1									1	1
1616	JQ703106	I2e	I2'3	1	I2e	2							2	3
1617	GU294854	I2f	I2'3	1	I2	1	I2f	3					3	5
1618	JQ245751	I3a	I3a	1									1	1
1619	JQ704837	I3a1	I3a1	1									1	1
1620	KC257362	I3b	I3b	1									1	1
1621	JQ703883	I3c	I3	1	I3c	2							2	3

1622	JX440338	I3d	I3a	1	I3a	1	I3d	1			3	3
1623	JQ702647	I3d1	I3d1	1							1	1
1624	KJ021059	I4	I	1	I4	2					2	3
1625	JQ245737	I4a	I4a	1							1	1
1626	EF153786	I4a1	I4a1	1							1	1
1627	HG00154	I4a2	I	1	I4	3	I4a	2	I4a2	1	4	7
1628	KF146261	I4b	I4b	1							1	1
1629	JQ245724	I5	I	1	I5	3	I5	1			3	5
1630	JQ245807	I5a1	I5a	1	I5a1	2	I5a1	1	I5a1	1	4	5
1631	JQ705096	I5a1a	I5a	1	I5a1	2	I5a1	1	I5a1a	1	4	5
1632	JQ704713	I5a1b	I5a1b	1							1	1
1633	KF146247	I5a1c	I5a	1	I5a1c	1					2	2
1634	NA12342	I5a2	I5a2	1							1	1
1635	JQ701894	I5a2+16086	I5a2+16086	1							1	1
1636	JQ245781	I5a2a	I5a2+16086	1	I5a2a	1					2	2
1637	JQ245772	I5a3	I5a3	1							1	1
1638	KF146249	I5a4	I5a4	1							1	1
1639	KF255549	I5b	I	1	I5b	2					2	3
1640	KF146250	I5b1	I5b1	1							1	1
1641	KF644562	I5c	I5c	1							1	1
1642	KF146251	I5c1	I5c1	1							1	1
1643	JQ705382	I6a	I6a	1							1	1
1644	JQ245773	I6b	I6b	1							1	1
1645	KF146253	I7	I7	1							1	1
1646	AY714008	N1a2	N1a2	1							1	1
1647	KC867103	N1a3	N1a3	1							1	1
1648	KC867116	N1a3a	N1a3a	1							1	1
1649	KC867113	N1a3a1	N1a3a1	1							1	1
1650	JX153442	N1a3a1a	N1a3a1a	1							1	1
1651	KC867115	N1a3a2	N1a3a2	1							1	1
1652	GU123019	N1a3a3	N1a3a3	1							1	1
1653	JQ705744	N1b1a	N1b1a	1							1	1
1654	EU742151	N1b1a1	N1b1a1	1							1	1
1655	JQ245725	N1b1a2	N1b1a2	1							1	1
1656	JQ705552	N1b1a2a	N1b1a+16129	1	N1b1a2a	2					2	3
1657	JQ704068	N1b1a2b	N1b1a2b	1							1	1
1658	JQ245803	N1b1a3	N1b1a3	1							1	1
1659	EU742150	N1b1a+16129	N1b1a+16129	1							1	1
1660	HM236190	N1b1a4	N1b1a4	1							1	1
1661	JX153074	N1b1a4a	N1b1a4a	1							1	1
1662	JQ701805	N1b1a5	N1b1a5	1							1	1
1663	JQ702718	N1b1a6	N1b1	1	N1b1a	1	N1b1a6	4			3	6
1664	JF265069	N1b1a7	N1b1a7	1							1	1
1665	JQ245742	N1b1a8	N1b1a8	1							1	1
1666	FJ460561	N1b1a8a	N1b1a8a	1							1	1
1667	JQ245774	N1b1a8b	N1b1a8b	1							1	1
1668	KF297809	N1b1b	N1b1b	1	N1b1b	1					2	2
1669	DQ301794	N1b1b1	N1b1b	1	N1b1b1	1					2	2
1670	JQ245799	N1b2	N1b2	1							1	1
1671	AY714031	N5	N5	1							1	1
1672	GU480015	N5a	N5a	1							1	1
1673	EU787451	N2a	N2a	1							1	1
1674	KC911573	N2a1	N2a1	1							1	1
1675	KC911368	N2a2	N2a2	1							1	1
1676	KF056262	W	W	1							1	1
1677	KF146268	W1	W1	1							1	1
1678	AY339475	W1a	W	1	W1	1	W1a	3			3	5

1679	AY339465	W1b	W1b	1							1	1
1680	AY339466	W1b1	W1b	1	W1b1	1					2	2
1681	FJ472633	W1+119	W1+119	1							1	1
1682	JQ245768	W1c	W1c	1							1	1
1683	JQ702327	W1c1	W1c1	1							1	1
1684	KF146267	W1i	W1+119	1	W1i	2					2	3
1685	EF556154	W1d	W1d	1							1	1
1686	JQ705839	W1e	W	1	W1	1	W1e	3			3	5
1687	FJ543390	W1e1	W1e1	1							1	1
1688	JQ702638	W1e1a	W1e1a	1							1	1
1689	FJ384432	W1f	W1f	1							1	1
1690	JQ702249	W1g	W1g	1							1	1
1691	JQ898579	W1h	W1h	1							1	1
1692	JQ703970	W1h1	W1h1	1							1	1
1693	EU086510	W+194	W+194	1							1	1
1694	JQ705313	W3a	W+194	1	W3	4	W3a	1	W3a	1	4	7
1695	JQ245760	W3a1	W3a1	1							1	1
1696	FJ472839	W3a1a1	W1h	1	W3a1a1	5					2	6
1697	GU123000	W3a1a2	W3a1a2	1							1	1
1698	FJ821289	W3a1a3	W+194	1	W3	4	W3a	1			3	6
1699	AY714043	W3a1b	W3a1b	1							1	1
1700	JQ703058	W3a1c	W3	1	W3a1+199	2	W3a1c	2			3	5
1701	KF142470	W3a1d	W3	1	W3a1+199	2	W3a1d	2			3	5
1702	JQ705487	W3a2	W3a2	1							1	1
1703	JQ245743	W3b	W3b	1							1	1
1704	JQ701824	W3b1	W3b1	1							1	1
1705	KF146281	W4	W4	1							1	1
1706	AY714018	W4a	W4a	1							1	1
1707	HM034304	W4a1	W4a1	1							1	1
1708	KF146280	W4b	W4b	1							1	1
1709	KF146282	W4c	W4c	1							1	1
1710	KF146283	W4d	W4d	1							1	1
1711	KC911419	W5	W8	1	W5	4					2	5
1712	JQ701827	W5a	W5a	1	W5a	1					2	2
1713	JN583886	W5a1	W5a1	1							1	1
1714	EF652811	W5a1a	W5a1a	1							1	1
1715	EU135972	W5a1a1	W5a1a1	1							1	1
1716	HM057816	W5a1a1a	W5a1a1a	1							1	1
1717	HG00148	W5a2	W5a2	1							1	1
1718	JQ705278	W5a2b	W5a2b	1							1	1
1719	JQ703283	W5b	W5b	1							1	1
1720	EU744586	W5b1	W5b1	1							1	1
1721	JQ701912	W5b1a	W5b1a	1							1	1
1722	JQ245723	W6	W6	1							1	1
1723	NA20804	W6a	W6	1	W6a	4					2	5
1724	FJ473381	W6b	W6b	1							1	1
1725	KF146290	W6b1	W6b1	1							1	1
1726	JF275845	W6c	W6	1	W6c	4	W6c	1			3	6
1727	KF700922	W6c1	W6b	1	W6c1	2					2	3
1728	KF553923	W6c1a	W6c1a	1							1	1
1729	KF146288	W6d	W6d	1							1	1
1730	HM352797	W7	W7	1							1	1
1731	JQ245778	W8	W8	1							1	1
1732	JQ245759	W9	W9	1							1	1
1733	KC867130	N3	N3	1							1	1
1734	JQ705752	N3a	N3a	1							1	1
1735	KC867129	N3a1	N3	1	N3a	2	N3a1	1			3	4

1736	KC867131	N3b	N3b	1						1	1
1737	KC887495	N7a1	N7a1	1						1	1
1738	KC505116	N7a2	N7a2	1						1	1
1739	KC505117	N7b	N7	1	N7b	1				2	2
1740	JX289118	N8	N8	1						1	1
1741	NA18628	N9a	N9a10	1	N9a	2				2	3
1742	AP008726	N9a1	N9a1	1	N9a1	1				2	2
1743	AY255141	N9a1a	N9a1a	1						1	1
1744	AP008608	N9a3	N9a3	1						1	1
1745	JN857033	N9a2	N9a2	1						1	1
1746	AP011046	N9a2a	N9a2a	1						1	1
1747	NA18973	N9a2a1	N9a2a1	1						1	1
1748	JN857061	N9a2a2	N9a2a	1	N9a2a2	2				2	3
1749	NA19082	N9a2a3	N9a2a	1	N9a2a3	2				2	3
1750	AP008630	N9a2c	N9a2c	1						1	1
1751	AP009434	N9a2d	N9a2'4'5'11	1	N9a2'4'5'11	1	N9a2d	1		3	3
1752	NA18991	N9a4a	N9a4a	1						1	1
1753	JF824989	N9a4b	N9a4b	1						1	1
1754	HG00531	N9a4b1	N9a4b1	1						1	1
1755	AP008895	N9a5	N9a5	1						1	1
1756	KF540744	N9a11	N9a11	1						1	1
1757	HM596703	N9a6	N9a6	1						1	1
1758	AP012369	N9a6a	N9a6a	1						1	1
1759	AP012413	N9a6b	N9a6b	1						1	1
1760	AP011016	N9a7	N9a7	1						1	1
1761	AP008714	N9a8	N9a8	1						1	1
1762	FJ147307	N9a9	N9a10+16311	1	N9a9	1				2	2
1763	GU392084	N9a10	N9a10	1						1	1
1764	JN084084	N9a10a1	N9a10a1	1						1	1
1765	HM238208	N9a10a2	N9a10a2	1						1	1
1766	KF540605	N9a10a2a	N9a10a2a	1						1	1
1767	FJ748719	N9a10+16311	N9a10+16311	1						1	1
1768	KF849926	N9a10b	N9a10b	1						1	1
1769	AP010722	N9b1	N9b	1	N9b1	2	N9b1	1		3	4
1770	AP008610	N9b1a	N9b1a	1						1	1
1771	AP008784	N9b1b	N9b1b	1						1	1
1772	AP008821	N9b1c	N9b1c	1						1	1
1773	AP008529	N9b1c1	N9b1c1	1						1	1
1774	AP013239	N9b2	N9b2	1						1	1
1775	AP008790	N9b2a	N9b2a	1						1	1
1776	AP008474	N9b3	N9b3	1						1	1
1777	HM776709	N9b4	N9b4	1						1	1
1778	KF540727	Y1	Y1	1						1	1
1779	EU007892	Y1a	Y1	1	Y1a	2	Y1a	1		3	4
1780	KF148513	Y1a1	Y1a1	1						1	1
1781	EU007855	Y1a+16189	Y1a+16189	1						1	1
1782	EF153825	Y1a2	Y1a+16189	1	Y1a2	1				2	2
1783	GU123044	Y1b	Y1b	1						1	1
1784	JF824832	Y1b1	Y1b1	1						1	1
1785	JF824992	Y1b1a	Y1b1a	1						1	1
1786	AP008723	Y2	Y2	1						1	1
1787	KF540560	Y2a	Y2	1	Y2a	1	Y2a	1		3	3
1788	HM596675	Y2a1	Y2a1	1						1	1
1789	KC994040	Y2a1a	Y2a1	1	Y2a1	1	Y2a1a	1		3	3
1790	AP008764	Y2b	Y2b	1						1	1
1791	HM030521	N10a	N10a	1						1	1
1792	HM030500	N10b	N10b	1						1	1

1793	HM030536	N11a1	N11a1	1					1	1
1794	HM030528	N11a2	N11a2	1					1	1
1795	GU733740	N11b	N11b	1					1	1
1796	EF495214	N13	N13	1					1	1
1797	GQ301885	N21	N21	1					1	1
1798	GQ301867	N21+195	N21+195	1					1	1
1799	AP012425	N21a	N21a	1					1	1
1800	JF739542	N22	N22	1					1	1
1801	AP012414	N22a	N22a	1					1	1
1802	AY255144	A+152	A+152	1	A+152	2			2	3
1803	AP013225	A+152+16362	A17	1	A+152+1636	1			2	2
1804	AP010699	A1	A1	1					1	1
1805	AP008617	A1a	A1a	1					1	1
1806	EU597529	A1a1	A1a1	1					1	1
1807	HG01187	A2	A2	1					1	1
1808	EU482342	A2a	A2a	1					1	1
1809	EU095547	A2a1	A2a1	1					1	1
1810	EU482349	A2a2	A2	1	A2a	2	A2a2		2	3
1811	EU007886	A2a3	A2a3	1					1	1
1812	KC711001	A2a4	A2a4	1					1	1
1813	EU095526	A2a5	A2a5	1					1	1
1814	EU095533	A2b	A2b	1					1	1
1815	EU725607	A2b1	A2b1	1					1	1
1816	DQ282394	A2+(64)	A2+(64)	1					1	1
1817	KC257400	A2c	A2r1	1	A2c	3			2	4
1818	HQ012050	A2d	A2d	1					1	1
1819	HQ012083	A2d1	A2d1	1					1	1
1820	DQ282433	A2d1a	A2d1a	1					1	1
1821	DQ282388	A2d2	A2d	1	A2d	2	A2d2	1	3	4
1822	HQ012105	A2ao	A2ao	1					1	1
1823	HQ012100	A2ao1	A2ao1	1					1	1
1824	FJ705809	A2f1a	A2f1	1	A2f1a	1			2	2
1825	HQ012110	A2f2	A2f2	1					1	1
1826	HQ012095	A2f3	A2f3	1					1	1
1827	DQ282392	A2g	A2g	1	A2g	1			2	2
1828	DQ282391	A2g1	A2g	1	A2g1	1			2	2
1829	EU095545	A2h	A2h	1					1	1
1830	HQ012079	A2h1	A2h1	1					1	1
1831	EU431080	A2i	A2i	1					1	1
1832	JQ705434	A2j	A2j	1					1	1
1833	DQ282398	A2j1	A2+(64)	1	A2j	9	A2j1	1	3	11
1834	DQ282417	A2k	A2+(64)+@16111	0					0	0
1835	EU095552	A2k1	A2k1	1					1	1
1836	HG01097	A2k1a	A2k1a	1					1	1
1837	DQ282411	A2+(64)+@16111	A2+(64)+@16111	1	A2+(64)+@16111	3			2	4
1838	DQ282409	A2l	A2+(64)+@16111	1	A2l	3			2	4
1839	HQ012131	A2m	A2m	1					1	1
1840	FJ713602	A2n	A2+(64)+@16111	1	A2n	1			2	2
1841	HQ012114	A2o	A2+(64)+@16111	1	A2o	3			2	4
1842	HQ012107	A2ai	A2ai	1					1	1
1843	HQ012056	A2aj	A2aj	1					1	1
1844	HQ012116	A2p	A2p	1					1	1

1845	HQ012067	A2p1	A2p1	1							1	1
1846	KC257370	A2p2	A2p2	1							1	1
1847	DQ282403	A2am	A2+(64)+16129	1	A2am	2					2	3
1848	JQ705973	A2q	A2q	1							1	1
1849	JQ705557	A2q1	A2q1	1							1	1
1850	HQ012086	A2t	A2t	1							1	1
1851	HQ012073	A2u1	A2u1	1							1	1
1852	HQ012066	A2u2	A2u2	1							1	1
1853	HG01342	A2v	A2v	1	A2v	1					2	2
1854	AY195786	A2v1	A2v	1	A2v1	1					2	2
1855	JQ702093	A2v1+152	A2v1+152	1							1	1
1856	HM589049	A2v1a	A2v1a	1							1	1
1857	JQ705501	A2v1b	A2v1b	1							1	1
1858	HG01356	A2w	A2+(64)+16189	1	A2+(64)+16189	1	A2w	1			3	3
1859	JQ703875	A2w1	A2w1	1							1	1
1860	JN419250	A2x	A2x	1							1	1
1861	GQ398480	A2y	A2y	1							1	1
1862	EU095196	A2aa	A2aa	1							1	1
1863	JX887155	A2ab	A2ab	1							1	1
1864	EU095529	A2ac	A2ac	1							1	1
1865	JQ702324	A2ac1	A2ac1	1							1	1
1866	EF079873	A2ad	A2ad	1							1	1
1867	JQ965968	A2ad1	A2ad1	1							1	1
1868	JQ965970	A2ad2	A2ad2	1							1	1
1869	JQ704276	A2ae	A2ae	1							1	1
1870	JQ965977	A2af1a	A2af1a	1							1	1
1871	JQ965972	A2af1a1	A2af1a1	1							1	1
1872	JQ965979	A2af1a2	A2af1a2	1							1	1
1873	JQ965986	A2af1b	A2af1b	1							1	1
1874	JQ965983	A2af1b1	A2af	1	A2af1	1	A2af1b	1	A2af1b1	1	4	4
1875	JQ965981	A2af1b1a	A2af1b1a	1							1	1
1876	JQ965984	A2af1b1b	A2af1b1b	1							1	1
1877	HQ012106	A2af1b2	A2af1b2	1							1	1
1878	JQ965971	A2af2	A2af2	1							1	1
1879	KC998702	A2ag	A2ag	1							1	1
1880	KC503930	A2ah	A2ah	1							1	1
1881	JQ701855	A2ak	A2ak	1							1	1
1882	HG01492	A2al	A2al	1							1	1
1883	EU597486	A2an	A2an	1							1	1
1884	JQ435789	A2+(64)+16189	A2+(64)+16189	1							1	1
1885	HQ012115	A2ap	A2ap	1							1	1
1886	KC998705	A2aq	A2aq	1							1	1
1887	HQ012092	A2r	A2	1	A2r	2					2	3
1888	HQ012063	A2r1	A2r1	1							1	1
1889	HQ012070	A2s	A2s	1							1	1
1890	DQ282425	A2z	A2z	1							1	1
1891	AY963575	A6a	A6a	1							1	1
1892	FJ748705	A6b	A6b	1							1	1
1893	EF153771	A12	A+152	1	A12	1					2	2
1894	EF397560	A12a	A12a	1							1	1
1895	EF153794	A23	A23	1							1	1
1896	EF153819	A+152+16362+200	A+152+16362+200	1							1	1
1897	EF114282	A13	A13	1							1	1
1898	EF153775	A14	A14	1							1	1
1899	JF824996	A15a	A15a	1							1	1

1900	AP010832	A15b	A15b	1									1	1
1901	JF824895	A15c	A15a	1	A15c	2							2	3
1902	KF056313	A15c1	A15	1	A15c	2	A15c1	1					3	4
1903	EF153780	A16	A16	1									1	1
1904	NA18572	A17	A17	1									1	1
1905	NA18615	A18	A18	1									1	1
1906	GU377085	A19	A19	1									1	1
1907	NA18164	A20	A+152+16362	1	A20	1							2	2
1908	KF056302	A21	A21	1									1	1
1909	JF824891	A22	A+152+16362	1	A22	7							2	8
1910	EF397559	A24	A24	1									1	1
1911	AP008459	A25	A25	1									1	1
1912	JX153385	A26	A26	1									1	1
1913	AP010833	A3	A3	1									1	1
1914	AP013268	A3a	A+152	1	A3	2	A3a	1					3	4
1915	AP008307	A7	A7	1									1	1
1916	GQ895149	A11a	A11a	1									1	1
1917	GQ895164	A11+16234	A11+16234	1									1	1
1918	EU597494	A11b	A11b	1									1	1
1919	AP008265	A5a	A5a	1	A5a	5							2	6
1920	AP009468	A5a1a	A5a1a	1									1	1
1921	AP008433	A5a1a1	A5a1a1	1									1	1
1922	AP010701	A5a1a1a	A5a	1	A5a1	5	A5a1a	1	A5a1a1	2	A5a1a1a	2	5	11
1923	AP010976	A5a1a1b	A5a1a1b	1									1	1
1924	AP011037	A5a1a2	A5a1a2	1									1	1
1925	AP008596	A5a1a2a	A5a3	1	A5a1a2a	2							2	3
1926	AP008853	A5a1b	A5a1b	1									1	1
1927	AP011038	A5a2	A5a	1	A5a2	5							2	6
1928	NA18997	A5a3	A5a3	1									1	1
1929	AP008835	A5a3a	A5a3a	1									1	1
1930	AP013122	A5a4	A5a	1	A5a4	5							2	6
1931	AP013288	A5a5	A5a5	1									1	1
1932	JF824862	A5b	A5b	1									1	1
1933	NA18611	A5b1	A5b1	1									1	1
1934	AP010989	A5b1a	A5b1a	1									1	1
1935	JF824875	A5b1b	A5b1b	1									1	1
1936	NA17987	A5b1c	A5b1	1	A5b1c	2							2	3
1937	KF540620	A5b1c1	A5b1c1	1									1	1
1938	EF153816	A5c	A5c	1	A5c	1							2	2
1939	AP013220	A5c1	A5c1	1									1	1
1940	EU482363	A8	A8	1									1	1
1941	AY519486	A8a	A8a	1									1	1
1942	EF153797	A8a1	A8a1	1									1	1
1943	HM569228	A10	A10	1									1	1
1944	AY289059	O	N9b2	1	O	3							2	4
1945	DQ404447	O1	O1	1									1	1
1946	AY289056	O1a	O1	1	O1a	1							2	2
1947	AF346963	S1	S1	1									1	1
1948	JN226144	S1a	S1a	1									1	1
1949	AF346964	S2	M7b2	1	S2	2							2	3
1950	AY289066	S3	S3	1									1	1
1951	AY289062	S4	S4	1									1	1
1952	EF495220	S5	S5	1									1	1
1953	EU600318	X1a	X1a	1									1	1
1954	HM765467	X1c	X1c	1									1	1
1955	EF177437	X3	X1'3	1	X3	1							2	2
1956	JQ245804	X3a	X3a	1									1	1

1957	JQ245739	X2	X2	1							1	1
1958	EU600328	X2+225	X2+225	1							1	1
1959	EU439939	X2a1	X2a1	1	X2a1	1					2	2
1960	FJ168759	X2a1a	X2a1a	1							1	1
1961	EU095249	X2a1a1	X2a1a1	1							1	1
1962	FJ168761	X2a1b	X2a1	1	X2a1b	1	X2a1b	1			3	3
1963	FJ168762	X2a1b1	X2a1	1	X2a1b	1	X2a1b1	1			3	3
1964	FJ168763	X2a1b1a	X2a1	1	X2a1b	1	X2a1b1	1	X2a1b1a	1	4	4
1965	EU095244	X2a1c	X2a1c	1							1	1
1966	KC257359	X2a2	X2a2	1							1	1
1967	EU935450	X2j	X2j	1							1	1
1968	FJ457949	X2b	X2+225	1	X2b'd	4	X2b	1			3	6
1969	JQ704969	X2b+226	X2b+226	1							1	1
1970	EF556159	X2b1	X2b1	1							1	1
1971	AF381986	X2b2	X2c	1	X2b	1	X2b2	2			3	4
1972	DQ523642	X2b3	X2+225	1	X2b+226	1	X2b3	8			3	10
1973	JQ343921	X2b4	X2b4	1							1	1
1974	EU600321	X2b4a	X2b4a	1							1	1
1975	JQ705458	X2b4a1	X2b4a1	1							1	1
1976	EU597556	X2b5	X2+225	1	X2b+226	1	X2b5	8			3	10
1977	JQ705179	X2b6	X2b6	1							1	1
1978	JQ702893	X2b6a	X2b6a	1							1	1
1979	JQ705082	X2b7	X2b10	1	X2b7	4					2	5
1980	JQ705154	X2b8	X2b8	1							1	1
1981	AY339513	X2b9	X2b9	1							1	1
1982	JQ702482	X2b10	X2b10	1							1	1
1983	JX153019	X2b10a	X2b10a	1							1	1
1984	JX153084	X2b11	X2b11	1							1	1
1985	JQ702995	X2b+226+16192	X2b+226+1619	1							1	1
1986	JQ705550	X2b12	X2b12	1							1	1
1987	JX153312	X2b13	X2b13	1							1	1
1988	JQ705795	X2d	X2d	1							1	1
1989	JQ702739	X2d1	X2d1	1	X2d1	1					2	2
1990	GQ231312	X2d1a	X2d1a	1							1	1
1991	JQ701817	X2d2	X2d2	1							1	1
1992	JX153623	X2c1	X2c	1	X2c1	2	X2c1	1			3	4
1993	JQ702817	X2c1a	X2c1a	1							1	1
1994	HM370114	X2c1b	X2c1b	1							1	1
1995	JQ705612	X2c1c	X2c1c	1							1	1
1996	JQ703703	X2c1c1	X2c1c1	1							1	1
1997	JQ705480	X2c1d	X2c	1	X2c1	2	X2c1d	1			3	4
1998	JQ702633	X2c1e	X2c1e	1							1	1
1999	JQ705155	X2c2	X2c2	1							1	1
2000	HM852814	X2e1a	X2e1a	1							1	1
2001	EF556165	X2e1a1	X2e1a1	1							1	1
2002	HM852758	X2e1b	X2e1b	1							1	1
2003	JQ245745	X2e2a	X2e2a	1							1	1
2004	FJ147306	X2e2a1	X2	1	X2e2	4	X2e2a	1	X2e2a1	1	4	7
2005	JQ245787	X2e2a2	X2e2a2	1							1	1
2006	AB626609	X2e2b	X2e2b	1							1	1
2007	JX153926	X2e2b1	X2e2b1	1							1	1
2008	KC911468	X2e2c1	X2e2c1	1							1	1
2009	FJ168756	X2g	X2+225+@153	1	X2g	2					2	3
2010	JQ704985	X2l	X2l	1							1	1
2011	KC911497	X2+225+@1622	X2+225+@1622	1							1	1
2012	EU600326	X2h	X2h	1							1	1
2013	JQ245730	X2i	X2i	1							1	1

2014	JQ245806	X2i+@225	X2i+@225	1									1	1
2015	JQ705067	X2i1	X2i1	1									1	1
2016	FJ008043	X2m1	X2m1	1									1	1
2017	JQ705389	X2m2	X2m2	1									1	1
2018	EF660942	X2n	X2n	1									1	1
2019	JQ245731	X2o	X2+225	1	X2o	4							2	5
2020	JQ245770	X2o1	X2o1	1									1	1
2021	KC911478	X2f	X2f	1									1	1
2022	JQ245738	X2f1	X2f1	1									1	1
2023	JQ701905	X2k	X2e2	1	X2k	2							2	3
2024	KC257312	X2p	X2d	1	X2d	1	X2p	2					3	4
2025	JX508853	X2p1	X2p1	1									1	1
2026	HQ456226	X4	X4	1									1	1
2027	JX153281	R0a	R0a1	1	R0a	1							2	2
2028	JQ702678	R0a1	R0a1	1									1	1
2029	FJ460524	R0a1a	R0a1a	1									1	1
2030	HM185263	R0a1a1	R0a1a	1	R0a1a1	1							2	2
2031	HM185210	R0a1a1a	R0a1a1a	1									1	1
2032	HM185258	R0a1a2	R0a1a2	1									1	1
2033	HM185213	R0a1a3	R0a1a3	1									1	1
2034	HM185245	R0a1a4	R0a1a4	1									1	1
2035	HM185256	R0a1+152	R0a1+152	1									1	1
2036	DQ904236	R0a1b	R0a1b	1									1	1
2037	EU597493	R0a+60.1T	R0a'b	1	R0a+60.1T	1							2	2
2038	HM185266	R0a2	R0a2	1									1	1
2039	HM185270	R0a2a	R0a2a	1									1	1
2040	HM185257	R0a2a1	R0a+60.1T	1	R0a2'3	1	R0a2	2	R0a2a	8	R0a2a1	1	5	13
2041	HM185261	R0a2b	R0a2b	1									1	1
2042	HM185215	R0a2c	R0a2c	1									1	1
2043	JF717359	R0a2d	R0a2d	1									1	1
2044	DQ904242	R0a2e	R0a+60.1T	1	R0a2'3	1	R0a2	2	R0a2e	8			4	12
2045	HM185225	R0a2f	R0a1	1	R0a	1	R0a2f	3					3	5
2046	HM185221	R0a2f1a	R0a2f1a	1									1	1
2047	HM185228	R0a2f1b	R0a2f1b	1									1	1
2048	HM185218	R0a2g	R0a2	1	R0a2	1	R0a2g	3					3	5
2049	HM185238	R0a2h	R0a2h	1									1	1
2050	HM185242	R0a2i	R0a2i	1									1	1
2051	HM185237	R0a2+195	R0a2+195	1									1	1
2052	HM185246	R0a2j	R0a2j	1									1	1
2053	JF717355	R0a2k	R0a2k	1									1	1
2054	HM185254	R0a2k1	R0a2k1	1									1	1
2055	HM185250	R0a2l	R0a2l	1									1	1
2056	JQ705196	R0a2m	R0a2f	1	R0a2	1	R0a2	1	R0a2m	1			4	4
2057	KC911373	R0a2n	R0a2n	1									1	1
2058	HM185223	R0a3	R0a3	1									1	1
2059	HM185265	R0a3a	R0a3a	1									1	1
2060	JQ702940	R0a4	R0a4	1									1	1
2061	JF717361	R0b	R0b	1									1	1
2062	KC911472	HV	R0	1	HV	5	HV	4					3	10
2063	JX153274	HV0	HV0	1									1	1
2064	JQ704188	HV0a	HV0a	1									1	1
2065	GU122990	HV0a1	HV0a1	1									1	1
2066	EF649971	HV0a1a	HV0a1a	1									1	1
2067	JX153964	V	V2c	1	V	2							2	3
2068	EF177435	V1a	V2c	1	V	2	V	2	V1a	3			4	8
2069	JQ702498	V1a1	V10b	1	V	1	V	1	V1a1	4			4	7
2070	AY339438	V1a1a	V1a1a	1									1	1

2071	JX153552	V1a1a1	V1a1a1	1													1	1
2072	EF661005	V1a1b	HV0	1	HV0a	1	V	1	V1	5	V1a	1	V1a1	1	V1a1b	1	7	11
2073	JQ702938	V1b	V1b	1													1	1
2074	JQ703647	V2	V19	1	V2	2											2	3
2075	AY713979	V2a	V2a	1													1	1
2076	AY495322	V2a1	HV0	1	HV0a	1	V	1	V2	5	V2a	3	V2a1	1	V2a1	1	7	13
2077	AY495317	V2a1a	HV0	1	HV0a	1	V	1	V2	5	V2a	3	V2a1	1	V2a1a	1	7	13
2078	AY495313	V2b	HV0	1	HV0a	1	V	1	V2	5	V2b	3	V2b	2			6	13
2079	AY495319	V2b1	HV0	1	HV0a	1	V	1	V2	5	V2b	3	V2b1	2			6	13
2080	JQ704063	V2b2	HV0	1	HV0a	1	V	1	V2	5	V2b	3	V2b2	2			6	13
2081	JX153985	V2c	V2c V13	1	V2c	2											2	3
2082	JQ703666	V3	HV0	1	HV0a	1	V	1	V3	5	V3	1					5	9
2083	JQ705789	V3a	V3a	1													1	1
2084	KC257399	V3a1	V3a1	1													1	1
2085	JQ704036	V3b	HV0	1	HV0a	1	V	1	V3	5	V3b	1					5	9
2086	EU567326	V3c	V3c	1													1	1
2087	AY495312	V4	V4	1													1	1
2088	AY339451	V5	V5	1													1	1
2089	EF506486	V6	V6	1													1	1
2090	JQ705798	V7	V7	1													1	1
2091	JQ703830	V7a	V7a	1													1	1
2092	AY339446	V7a1	V7a	1	V7a1	1											2	2
2093	JQ705945	V7b	V7b	1													1	1
2094	JQ705604	V8	V8	1													1	1
2095	EF177445	V9	V9	1													1	1
2096	FJ865560	V9a1	V9a1	1													1	1
2097	JQ702145	V9a2	V9a2	1													1	1
2098	JQ704602	V10a	V10a	1													1	1
2099	KF142158	V10b	V10b	1													1	1
2100	JQ703770	V10b1	V20	1	V10b1	2											2	3
2101	HM570048	V10b2	HV0	1	HV0a	1	V	1	V	5	V10	5	V10b	1	V10b2	2	7	16
2102	JQ704405	V11	HV0	1	HV0a	1	V	1	V	5	V11	5					5	13
2103	AY495321	V12	HV0	1	HV0a	1	V	1	V	5	V12	5					5	13
2104	GU122979	V13	HV0	1	HV0a	1	V	1	V	5	V13	5					5	13
2105	JQ703215	V14	V10b	1	V	1	V	1	V14	4							4	7
2106	HQ645963	V15	V10b	1	V15	2											2	3
2107	JQ703991	V15a	HV0	1	HV0a	1	V	1	V	5	V	5	V15	5	V15a	1	7	19
2108	JQ704468	V16	V16	1													1	1
2109	JQ703973	V17	V17	1													1	1
2110	FJ348173	V18	V18	1	V18	1											2	2
2111	JX153154	V18a	V18	1	V18a	1											2	2
2112	JQ702721	V19	V19	1													1	1
2113	JX297133	V22	V+150	1	V22	1											2	2
2114	AY495118	V+@16298	V+@16298	1													1	1
2115	JQ705168	V20	V20	1													1	1
2116	JQ701938	V+@72	V+@72	1													1	1
2117	JQ702689	V21	V21	1													1	1
2118	JQ704988	V23	HV0	1	HV0a	1	V	1	V	5	V	5	V23	5			6	18
2119	HQ711364	V24	HV0	1	HV0a	1	V	1	V	5	V	5	V24	5			6	18
2120	NA19725	V25	V25	1													1	1
2121	HQ615879	V26	V26	1													1	1
2122	JX153521	V27	V27	1													1	1
2123	GU827698	V28	V28	1													1	1
2124	JQ704255	HV0+195	HV0+195	1													1	1
2125	EF556192	HV0b	HV0b	1													1	1
2126	JQ704143	HV0c	HV0+195	1	HV0c	2											2	3
2127	JQ702222	HV0d	HV0+195	1	HV0d	2											2	3

2128	JQ705967	HV0e	HV0e	1									1	1
2129	JQ705127	HV0f	HV0f	1									1	1
2130	JQ704142	HV0g	HV0g	1									1	1
2131	JF320654	HV1	HV1	1	HV1	1							2	2
2132	EF660936	HV1a'b'c	HV1a'b'c	1									1	1
2133	HM852761	HV1a1	HV1a1	1									1	1
2134	HM575427	HV1a1a	HV1a1a	1									1	1
2135	FJ210914	HV1a1b	HV1a1	1	HV1a1b	2							2	3
2136	KC257350	HV1a2	HV1	1	HV1a'b'c	1	HV1a	3	HV1a2	1	HV1a2	1	5	7
2137	JF260942	HV1a2a	HV1	1	HV1a'b'c	1	HV1a	3	HV1a2	1	HV1a2a	1	5	7
2138	JF316743	HV1a2b	HV1a2b	1									1	1
2139	JF260935	HV1a3	HV1a3	1	HV1a3	1							2	2
2140	JF260936	HV1a3a	HV1a3a	1									1	1
2141	AY738942	HV1b	HV1b	1									1	1
2142	EF556182	HV1b1	HV1b1	1	HV1b1	2							2	3
2143	JF260951	HV1b1a	HV1b1	1	HV1b1a	2							2	3
2144	JF260947	HV1b1b	HV1b1b	1									1	1
2145	JF260944	HV1b+152	HV1b+152	1									1	1
2146	DQ856316	HV1b2	HV1b2	1									1	1
2147	HM998901	HV1b3a	HV1b3a	1									1	1
2148	KC911450	HV1b3b	HV1b3b	1									1	1
2149	EF556190	HV1c	HV1c	1									1	1
2150	JF260933	HV1d	HV1d	1									1	1
2151	KC911408	HV2	HV2	1									1	1
2152	EU567456	HV2a1	HV2a1	1									1	1
2153	AY713986	HV2a2	HV2a2	1									1	1
2154	KC911372	HV2a3	HV2a3	1									1	1
2155	EU935433	HV20	R+16189	1	R+16189	2	HV20	11					3	14
2156	EF417833	HV4	R0	1	HV	5	HV4	4	HV4	1			4	11
2157	JN214428	HV4a1	HV4a1	1									1	1
2158	JN214427	HV4a1+16291	HV4a1+16291	1									1	1
2159	JN214395	HV4a1a	HV4a1+16291	1	HV4a1a	1	HV4a1a	2					3	4
2160	JN214392	HV4a1a1	HV4a1a1	1									1	1
2161	JN214397	HV4a1a2	HV4a1a2	1									1	1
2162	HQ675034	HV4a1a3	HV4a1a3	1									1	1
2163	JN214418	HV4a1a4	HV4a1a4	1									1	1
2164	GQ888731	HV4a2a	HV4a2a	1									1	1
2165	EU935457	HV4a2b	HV4a2b	1									1	1
2166	EF222234	HV4b	HV4b	1									1	1
2167	AY738941	HV4c	H7f	1	HV4c	1							2	2
2168	EF419890	HV5a	R0	1	HV	5	HV5	4	HV5a	1			4	11
2169	HQ593806	HV5b	HV5b	1									1	1
2170	AY713981	HV+16311	H11a2	1	HV+16311	3							2	4
2171	HQ658738	HV6	HV6	1									1	1
2172	EU545454	HV6a	HV6a	1									1	1
2173	EU545443	HV7	HV7	1									1	1
2174	EU545457	HV8	HV8	1									1	1
2175	JX153443	HV9	HV9	1									1	1
2176	JQ703720	HV9+152	H1cd	1	HV9+152	2							2	3
2177	JQ705368	HV9a	HV9a	1									1	1
2178	JQ704229	HV9a1	HV9a1	1									1	1
2179	JQ703659	HV9a1a	HV9a1a	1									1	1
2180	JQ704799	HV9b	HV9b	1									1	1
2181	NA20814	HV9c	H4a1	1	HV9c	3							2	4
2182	EF222245	HV10	H1j1	1	HV+16311	3	HV+16311	1	HV10	1			4	6
2183	JQ702048	HV11	H3h2	1	HV11	4							2	5
2184	EF660954	HV11a	HV+16311	1	HV+16311	2	HV11a	4					3	7

2185	KC911391	HV14	H1e5a	1	H	3	H1e5	1	HV14	2	4	7
2186	KC911456	HV14a	HV14	1	HV14a	1					2	2
2187	JQ704184	HV15	HV15	1							1	1
2188	HQ658354	HV16	HV	1	HV+16311	3	HV+16311	1	HV16	5	4	10
2189	EU545424	HV17	HV17	1							1	1
2190	JQ702596	HV17a	HV17a	1							1	1
2191	EF660945	HV22	HV22	1							1	1
2192	FJ460528	HV23	HV23	1							1	1
2193	KC257368	HV24	HV	1	HV+16311	3	HV+16311	1	HV24	5	4	10
2194	HM852849	HV12a	HV12a	1							1	1
2195	HQ844516	HV12a1	HV12a1	1							1	1
2196	AY713976	HV12b	HV	1	HV12b	5					2	6
2197	HM852785	HV12b1	HV12b1	1							1	1
2198	AY713987	HV12b1a	HV12b1a	1							1	1
2199	HM852828	HV13a	HV13a	1							1	1
2200	JF700125	HV13b	HV13b	1							1	1
2201	KC911471	HV18	H	1	HV	2	HV18	7			3	10
2202	HQ436102	HV19	HV19	1							1	1
2203	KF729951	HV21	HV21	1							1	1
2204	JQ705953	H	R0	1	H	5	H	57	H	1	4	64
2205	HQ384174	H1	R0	1	H	5	H1	48			3	54
2206	EU369376	H1a	H1a	1	H1a	5					2	6
2207	AY495188	H1a1	H1a1	1	H1a1	2					2	3
2208	JQ704413	H1a1a	H1a1a	1							1	1
2209	JQ704265	H1a1a1	H1a1	1	H1a1a	2	H1a1a1	1			3	4
2210	JQ703424	H1a1b	H1a1	1	H1a1b	2					2	3
2211	JX153501	H1a1c	H1a1c	1							1	1
2212	AY339422	H1a2	H1a	1	H1a2	5					2	6
2213	EU747355	H1a3	H1a3	1	H1a3	2					2	3
2214	JQ704050	H1a3a	H1a3	1	H1a3a	2	H1a3a	1			3	4
2215	JQ704612	H1a3a1	H1a3	1	H1a3a	2	H1a3a1	1			3	4
2216	JQ704838	H1a3a2	H1a3a2	1							1	1
2217	AY738972	H1a3a3	H1a3a3	1							1	1
2218	JQ324542	H1a3a4	H1a3a4	1							1	1
2219	JQ703135	H1a3b	H1a3b	1							1	1
2220	JQ705533	H1a3b1	H1a3b1	1							1	1
2221	JQ702698	H1a3c	H1a3c	1							1	1
2222	JX153342	H1a3c1	H1a3c1	1							1	1
2223	JX152881	H1a3d	H1a3	1	H1a3d	2					2	3
2224	JQ704223	H1a4	H1a	1	H1a4	5					2	6
2225	JQ702535	H1a5	H1a	1	H1a5	5					2	6
2226	JQ702474	H1a6	H1a6	1							1	1
2227	GU797829	H1a7	H1a	1	H1a7	5					2	6
2228	JQ701944	H1a8	H1a	1	H1a8	5	H1a8	1			3	7
2229	JQ703758	H1a8a	H1a	1	H1a8	5	H1a8	5	H1a8a	1	4	12
2230	KF052033	H1a9	H1a9	1							1	1
2231	JQ406575	H1+16189	HV	1	H1+16189	3	H1+16189	7			3	11
2232	JQ702703	H1b	H1b	1							1	1
2233	KC257358	H1b1	H1b	1	H1b1	3	H1b1	2			3	6
2234	EU219920	H1b1+16362	H1b1+16362	1	H1b1+16362	3					2	4
2235	GU122983	H1b1a	H1b1+16362	1	H1b1a	3					2	4
2236	JQ701966	H1b1b	H1b1b	1							1	1
2237	JQ704159	H1b1c	H1b1c	1							1	1
2238	JQ705126	H1b1d	H1b1d	1							1	1
2239	JQ703500	H1b1h	H1b1+16362	1	H1b1h	3					2	4
2240	JQ703838	H1b1e	H1b	1	H1b1	3	H1b1e	2	H1b1e	1	4	7
2241	JQ702763	H1b1e1	H1b1e1	1							1	1

2242	JQ703254	H1b1f	H1b1f	1									1	1
2243	JQ703357	H1b1g	H1b1g	1									1	1
2244	JX153222	H1b1i	H1b	1	H1b1	3	H1b1i	2					3	6
2245	JQ702529	H1b2	H1b2	1									1	1
2246	JQ702566	H1b2a	H1b2a	1	H1b2a	1							2	2
2247	JQ704894	H1b2a1	H1b2a	1	H1b2a1	1							2	2
2248	JQ704139	H1b3	H1b3	1									1	1
2249	JQ324829	H1b4	H1b	1	H1b4	3							2	4
2250	JQ704486	H1b5	H1b5	1									1	1
2251	JQ704298	H1f	HV	1	H1+16189	3	H1f	7					3	11
2252	JQ703322	H1f+16093	H1f+16093	1	H1f+16093	1							2	2
2253	AY339413	H1f1	H1f+16093	1	H1f1	1							2	2
2254	JQ705607	H1f1a	H1f1a	1									1	1
2255	JQ701945	H1g1	HV	1	H1+16189	3	H1g	7	H1g1	2			4	13
2256	JQ703948	H1g2	R+16189	1	R+16189	2	H1g2	6					3	9
2257	EU768844	H1k1	H1k	1	H1k1	1	H1k1	1					3	3
2258	EU770202	H1k1a	H1k	1	H1k1	1	H1k1a	1					3	3
2259	JQ704157	H1y	HV	1	H1+16189	3	H1y	7					3	11
2260	JQ703527	H1z	H1z	1									1	1
2261	JQ704562	H1z1	H1z1	1									1	1
2262	JQ702057	H1aa1	HV	1	H1+16189	3	H1aa	7	H1aa1	1			4	12
2263	JQ704715	H1ab	HV	1	H1+16189	3	H1ab	7					3	11
2264	AY738982	H1ab1	H1ab1	1									1	1
2265	JQ704994	H1ac	HV	1	H1+16189	3	H1ac	7					3	11
2266	JQ704571	H1ad	HV	1	H1+16189	3	H1ad	7					3	11
2267	JQ705678	H1cc	H1cc	1									1	1
2268	JQ705712	H1c	H1c	1	H1c	5	H1c	5	H1c	5	H1c	4	5	20
2269	AY495157	H1c1	H1c1	1	H1c1	1							2	2
2270	AY495160	H1c1a	H1c1	1	H1c1a	1	H1c1a	1					3	3
2271	NA12399	H1c1a1	H1c1	1	H1c1a	1	H1c1a1	1					3	3
2272	JQ702237	H1c1b	H1c1b	1									1	1
2273	JX153294	H1c1c	H1c1c	1									1	1
2274	JQ703153	H1c1+16093	H1c1+16093	1	H1c1+16093	1							2	2
2275	JX152893	H1c1d	H1c1d	1									1	1
2276	JQ703166	H1c2	H4a1a4	1	H1c2	2	H1c2	1					3	4
2277	JQ704438	H1c2a	H1c	1	H1c2	2	H1c2a	1					3	4
2278	JQ703496	H1c3	H1c3	1									1	1
2279	JQ704119	H1c3a	H1c3a	1									1	1
2280	GU289555	H1c3b	H1c3b	1									1	1
2281	JQ705516	H1c4	H1c	1	H1c4	2	H1c4	1					3	4
2282	JX152947	H1c4a	H4a1a4	1	H	2	H1c	1	H1c4a	1			4	5
2283	JX297157	H1c4a1	H1c	1	H1c4	2	H1c4a	1	H1c4a1	1			4	5
2284	JQ704378	H1c4b	H1c4b	1	H1c4b	1							2	2
2285	JQ701984	H1c4b1	H1c4b	1	H1c4b1	1							2	2
2286	AY495102	H1c5	H1c	1	H1c	2	H1c5	5					3	8
2287	JQ703745	H1c5a	H1c5a	1									1	1
2288	JQ704080	H1c6	H1c	1	H1c	2	H1c6	5					3	8
2289	JQ702850	H1c7	H1c	1	H1c	2	H1c7	5					3	8
2290	JQ704294	H1c8	H1c	1	H1c	2	H1c	5	H1c8	5			4	13
2291	JQ701993	H1c+152	H1c+152	1	H1c+152	1							2	2
2292	JQ703810	H1c9	H1c+152	1	H1c9	1	H1c9	1					3	3
2293	JQ703459	H1c9a	H1c9a	1									1	1
2294	JQ705468	H1c10	H1c	1	H1c	2	H1c	5	H1c10	5			4	13
2295	JQ705495	H1c11	H1c	1	H1c	2	H1c	5	H1c11	5			4	13
2296	HM462218	H1c12	H1c	1	H1c	2	H1c	5	H1c12	5			4	13
2297	JQ702501	H1c13	H1c	1	H1c	2	H1c	5	H1c13	5			4	13
2298	JQ703342	H1c14	H1c	1	H1c	2	H1c	5	H1c	5	H1c14	5	5	18

2299	JQ703551	H1c15	H1c	1	H1c	2	H1c	5	H1c	5	H1c15	5									5	18
2300	HQ658607	H1c16	H1c	1	H1c	2	H1c	5	H1c	5	H1c16	5									5	18
2301	JQ705238	H1c17	H1c17	1																	1	1
2302	JX153132	H1c18	H1c+152	1	H1c+152	1	H1c18	3													3	5
2303	JQ703126	H1c19	H1c	1	H1c	2	H1c	5	H1c	5	H1c	5	H1c19	5							6	23
2304	JQ703383	H1c20	H1c	1	H1c	2	H1c	5	H1c	5	H1c	5	H1c20	5							6	23
2305	JX153194	H1c22	H1c	1	H1c	2	H1c	5	H1c	5	H1c	5	H1c22	5							6	23
2306	DQ523657	H1e	H1e H1e1	1	H1e	2															2	3
2307	JQ705024	H1e1	H1n2	1	H1+16189	1	H1	4	H1+16189	2	H1+16189	5	H1+16189	2	H1	5	H1e1	4	H1e1	4	9	28
2308	EF177428	H1e1a	R0	1	H	5	H1	4	H1e	5	H1e1	4	H1e1a	2	H1e1a	2					7	23
2309	JQ324659	H1e1a1	H1e1a1	1																	1	1
2310	JQ705496	H1e1a2	H1e1a2	1																	1	1
2311	HQ658480	H1e1a3	H1e1a3	1																	1	1
2312	JQ324756	H1e1a4	HV	1	H1	3	H1e1a4	1													3	5
2313	JQ705884	H1e1a5	H1e1a5	1																	1	1
2314	JX297199	H1e1a6	HV	1	H1e1a6	5															2	6
2315	JX153958	H1e1a7	R0	1	H	5	H1	4	H1e	5	H1e1	4	H1e1a	2	H1e1a7	2					7	23
2316	JQ704464	H1e1a+16278	H	1	H1e1a+16278	2															2	3
2317	JX153401	H1e1a8	H1e1a8	1																	1	1
2318	JQ702833	H1e1b	H1e1b	1	H1e1b	1															2	2
2319	JQ705799	H1e1b1	H1e1b	1	H1e1b1	1	H1e1b1	2													3	4
2320	NA20513	H1e1b1a	H1e1b1a	1																	1	1
2321	JQ702104	H1e1b1b	H1e1b1b	1																	1	1
2322	JQ703603	H1e1c	H1bb	1	H	2	H1+152	3	H1+152	2	H1+152	2	H1+16189	5	H1e1c	3					7	18
2323	JQ705717	H1e2	HV	1	H1	2	H1e1a6	3	H1e2	3											4	9
2324	JQ704343	H1e2a	R0	1	H	5	H1	4	H1e	5	H1e2	4	H1e2a	3							6	22
2325	JQ703207	H1e2b	H1e2b	1																	1	1
2326	AY738971	H1e2c	H1e2c	1																	1	1
2327	NA20803	H1e2d	H13a1c	1	H1e2d	6															2	7
2328	EU555475	H1e+16129	H17	1	H1e+16129	2															2	3
2329	HQ663877	H1e3	H1e3	1																	1	1
2330	JQ704392	H1e4	HV	1	H1	3	H1+16189	1	H1e4	10											4	15
2331	EU148452	H1e4a	H	1	H1e4a	2															2	3
2332	JQ704250	H1e5a	H52	1	H1e5a	4															2	5
2333	JX153088	H1e5b	H1e5b	1																	1	1
2334	JQ705426	H1e6	H1m1	1	H	2	H	1	H	4	H1e6	5									5	13
2335	JQ702313	H1e7	H29b	1	H	5	H1e7	5													3	11
2336	EF556181	H1e8	R0	1	H	5	H1	4	H1e	5	H1e8	4	H1e8	1							6	20
2337	JX153138	H1e8a	R0	1	H	5	H1	4	H1e	5	H1e8	4	H1e8a	1							6	20
2338	JQ703535	H1h1	H1h1	1																	1	1
2339	JQ704717	H1h2	H	1	H	5	H	5	H1h2	4											4	15
2340	JQ703305	H1+152	H	1	H	2	H1+152	2	H1+152	3											4	8
2341	JQ703270	H1i	H1i	1																	1	1
2342	EU568371	H1i1	H3g1a	1	H1i1	4															2	5
2343	JQ703251	H1i2	H1i2	1																	1	1
2344	JQ705039	H1i2a	H	1	H	2	H1+152	2	H1i	3	H1i2	2	H1i2a	1							6	11
2345	JQ703403	H1an1	H1an1	1																	1	1
2346	JQ324894	H1an1a	H1an1a	1																	1	1
2347	JQ702335	H1an2	H1an2	1																	1	1
2348	JQ703170	H1bb	H1bb	1																	1	1
2349	JQ705294	H1j	R0	1	H	5	H1	4	H1j	5	H1j	5									5	20
2350	JQ324705	H1j1	H	1	H1	2	H1j1	3	H1j1	3											4	9
2351	JX297163	H1j1a	H	1	H1	2	H1j1	3	H1j1a	3	H1j1a	1									5	10
2352	JQ324599	H1j1a1	H1j1a1	1																	1	1
2353	JQ324870	H1j1a2	H	1	H1	2	H1j1	3	H1j1a	3	H1j1a2	1									5	10
2354	JQ324604	H1j1b	H	1	H1	2	H1j1	3	H1j1b	3											4	9
2355	JQ324864	H1j1c	H3w	1	H1j1c	2															2	3

2356	JQ703536	H1j2	H	1	H1j2	2													2	3
2357	JQ324660	H1j2a	H1j2a	1															1	1
2358	JQ704358	H1j3	R0	1	H	5	H1	4	H1j	5	H1j3	5							5	20
2359	JQ704398	H1j4	H1j4	1															1	1
2360	AY495119	H1j5	R0	1	H	5	H1	4	H1j	5	H1j5	5							5	20
2361	JQ703147	H1j6	R0	1	H	5	H1	4	H1j	5	H1j6	5							5	20
2362	JQ703631	H1j7	R0	1	H	5	H1	4	H1j	5	H1j7	5							5	20
2363	EF177436	H1j8	H1j8	1															1	1
2364	EU372630	H1j9	HV	1	H1j9	3													2	4
2365	JQ065050	H1m	HV21	1	H1m	2													2	3
2366	JQ703995	H1m1	H	1	H1m1	4													2	5
2367	FJ348196	H1n+146	H	1	H1n+146	4	H1n+146	4											3	9
2368	JQ704655	H1n1	H26c	1	H1n1	11													2	12
2369	JQ704566	H1n1a	H1n1a	1															1	1
2370	JQ705930	H1n1b	H1n1b	1															1	1
2371	JQ702161	H1n2	H26c	1	H1n2	10													2	11
2372	HQ658482	H1n3	H	1	H1n+146	4	H1n3	4											3	9
2373	JQ702043	H1n4	H1n4	1															1	1
2374	HQ839859	H1n+146+195	H1n+146+195	1															1	1
2375	JQ703312	H1n5	H1n5	1															1	1
2376	JX152889	H1n6	H1n6	1															1	1
2377	JQ702360	H1o	H1o	1															1	1
2378	JX153869	H1ck	H1ck	1															1	1
2379	EF556184	H1p	R0	1	H	5	H1	4	H1	5	H1p	5							5	20
2380	JQ703742	H1q	H1q	1															1	1
2381	JX153851	H1q1	H13a1a1e	1	H	2	H1q	1	H1q1	1									4	5
2382	JQ324843	H1q1a	H	1	H1q1a	3													2	4
2383	JQ324580	H1q2	H	1	H1	3	H	3	H1q2	4									4	11
2384	NA20811	H1q3	H1q3	1															1	1
2385	AF346981	H1r	R0	1	H	5	H1	4	H1	5	H1r	5							5	20
2386	JX297164	H1r1	H1r1	1															1	1
2387	EU080974	H1s	H1s	1															1	1
2388	JX153137	H1s1	R0	1	H	5	H1	4	H1	5	H1s	5	H1s1	1					6	21
2389	JX154028	H1t	H3p	1	H1t	2													2	3
2390	JQ324837	H1t1	R0	1	H	5	H1	4	H1	5	H1t	5	H1t1	2	H1t1	1			7	23
2391	JQ324688	H1t1a	R0	1	H	5	H1	4	H1	5	H1t	5	H1t1	2	H1t1a	1	H1t1a	1	8	24
2392	JX297158	H1t1a1	H1t1a1	1															1	1
2393	EF177427	H1t2	H1t2	1															1	1
2394	JQ702726	H1u	H1u H3	1	H1u	2													2	3
2395	JQ702971	H1u1	H1u1	1															1	1
2396	JN604117	H1u2	H1u2	1															1	1
2397	FJ460532	H1v	R0	1	H	5	H1	4	H1	5	H1	5	H1v	5	H1v	1			7	26
2398	HM171271	H1v1a	R0	1	H	5	H1	4	H1	5	H1	5	H1v	5	H1v1	1	H1v1a	2	8	28
2399	HM171273	H1v1b	R0	1	H	5	H1	4	H1	5	H1	5	H1v	5	H1v1	1	H1v1b	2	8	28
2400	HM171275	H1w	R0	1	H	5	H1	4	H1	5	H1	5	H1w	5					6	25
2401	HM171279	H1x	H1x	1															1	1
2402	JQ705436	H1ae	H1j9	1	H1ae	4													2	5
2403	JQ702071	H1ae1	R0	1	H	5	H1	4	H1	5	H1	5	H1ae	5	H1ae1	3			7	28
2404	JQ705102	H1ae2	H1ae2	1															1	1
2405	HQ400746	H1ae2a	H	1	H1aj1	2	H1ae2a	4											3	7
2406	JQ703756	H1ae3a	R0	1	H	5	H1	4	H1	5	H1	5	H1ae	5	H1ae3	3	H1ae3a	1	8	29
2407	JQ705435	H1af	H1af	1															1	1
2408	GQ888707	H1af1a	HV	1	H1af	2	H1af1	2	H1af1a	1									4	6
2409	JQ704957	H1af1b	H1af1b	1															1	1
2410	FJ656214	H1af2	H1af2	1															1	1
2411	KC257371	H1ag	H	1	H	2	H	1	H1ag	6									4	10
2412	JQ703316	H1ag1	R0	1	H	5	H1	4	H1	5	H1	5	H1ag	5	H1ag1	1	H1ag1	2	8	28

2413	JQ703938	H1ag1a	H	1	H1ag1a	2																	2	3
2414	JQ704170	H1ag1b	H	1	H1e4a	2	H1ag1b	2															3	5
2415	NA19655	H1ah	R0	1	H	5	H1	4	H1	5	H1	5	H1	5	H1ah	5	H1ah	2					8	32
2416	JQ324708	H1ah1	H1ah1	1																			1	1
2417	JQ703687	H1ah2	H	1	H1ah2	1																	2	2
2418	JQ701969	H1ai1	R0	1	H	5	H1	4	H1	5	H1	5	H1	5	H1ai	5	H1ai1	1					8	31
2419	JQ703262	H1aj	H1aj	1																			1	1
2420	JQ703788	H1aj1a	H1aj1a	1																			1	1
2421	JQ702623	H1ak	R0	1	H	5	H1	4	H1	5	H1	5	H1	5	H1ak	5	H1ak	2					8	32
2422	KC533493	H1ak1	H26c	1	H1+16311	4	H1+16311	3	H1+16311	5	H1+16311	5	H1	5	H1ak1	1							7	24
2423	JQ324884	H1ak2	R0	1	H	5	H1	4	H1	5	H1	5	H1	5	H1ak	5	H1ak2	2					8	32
2424	JQ703706	H1am	R0	1	H	5	H1	4	H1	5	H1	5	H1	5	H1am	5	H1am	1					8	31
2425	HM488738	H1am1	R0	1	H	5	H1	4	H1	5	H1	5	H1	5	H1am	5	H1am1	1					8	31
2426	JQ704336	H1ao	H1ao	1																			1	1
2427	JQ704235	H1ao1	H1ao1	1																			1	1
2428	HM625689	H1cg	H1cg	1																			1	1
2429	KC257395	H1ap1	H1ap1	1																			1	1
2430	EU597511	H1aq	H	1	R0	2	H	1	H1aq	6													4	10
2431	JQ703371	H1aq1	H	1	H1aq1	2																	2	3
2432	JQ324931	H1ar	H1ar	1																			1	1
2433	JQ704291	H1ar1	H1ar1	1																			1	1
2434	JQ702701	H1as	R0	1	H	5	H1	4	H1	5	H1	5	H1	5	H1	5	H1as	5	H1as	2			9	37
2435	JQ703561	H1as1	H1as1	1																			1	1
2436	JQ704158	H1as1a	H1as1a	1																			1	1
2437	JQ704370	H1as2	R0	1	H	5	H1	4	H1	5	H1	5	H1	5	H1	5	H1as	5	H1as2	2			9	37
2438	JQ324928	H1at	H6a2	1	H1at	3																	2	4
2439	JQ703224	H1at1	R0	1	H	5	H1	4	H1	5	H1	5	H1	5	H1	5	H1at	5	H1at1	1	H1at1	1	10	37
2440	JQ704066	H1at1a	R0	1	H	5	H1	4	H1	5	H1	5	H1	5	H1	5	H1at	5	H1at1	1	H1at1a	1	10	37
2441	GU981676	H1au	H	1	H1au	2																	2	3
2442	JQ703506	H1au1a	H1au1a	1																			1	1
2443	JQ704010	H1au1b	H1au1b	1																			1	1
2444	JQ704538	H1av	R0	1	H	5	H1	4	H1	5	H1	5	H1	5	H1	5	H1av	5	H1av	1			9	36
2445	JQ324719	H1av1	HV	1	H1av1	5	H1av1	1															3	7
2446	JQ324552	H1av1a	HV	1	H1av1	5																	2	6
2447	JQ704411	H1aw	H1aw	1																			1	1
2448	JX153392	H1aw1	H1aw1	1																			1	1
2449	JQ704285	H1ax	H1ax	1																			1	1
2450	KF562342	H1ax1	H13b1a	1	H	4	H1ax1	2															3	7
2451	JQ704587	H1ay	H1+16239	0																			0	0
2452	JQ702269	H1az	R0	1	H	5	H1	4	H1	5	H1	5	H1	5	H1	5	H1	5	H1az	5			9	40
2453	JQ702799	H1ba	H1ba	1																			1	1
2454	HM765465	H1ba1	H1ba1	1																			1	1
2455	JQ704162	H1bc	H	1	H	2	H1bc	2															3	5
2456	JQ704553	H1bd	H13b1a	1	H	2	H1bd	2															3	5
2457	JQ703681	H1be	R0	1	H	5	H1	4	H1	5	H1	5	H1	5	H1	5	H1	5	H1be	5			9	40
2458	JQ704230	H1+16239	H1+16239	1	H1+16239	4																	2	5
2459	JQ704186	H1bf	H	1	H	2	H	2	H1bf	4													4	9
2460	JX297162	H1bf1	H1bf1	1																			1	1
2461	JQ704588	H1bg	H1av1	1	H1bg	2																	2	3
2462	JX153634	H1bh	H1bh	1																			1	1
2463	JQ704465	H1ch	H1ch	1																			1	1
2464	JQ704435	H1bi	H10+(16093)	1	H10+(16093)	1	H	5	H1bi	5													4	12
2465	JQ704756	H1bj	R0	1	H	5	H1	4	H1	5	H1	5	H1	5	H1	5	H1	5	H1	5	H1bj	5	10	45
2466	JQ705754	H1bk	H3	1	H1bk	2																	2	3
2467	JQ705579	H1bm	R0	1	H	5	H1	4	H1	5	H1	5	H1	5	H1	5	H1	5	H1	5	H1bm	5	10	45
2468	JQ703317	H1bn	R0	1	H	5	H1	4	H1	5	H1	5	H1	5	H1	5	H1	5	H1	5	H1bn	5	10	45
2469	EF556177	H1bo	H1bo	1																			1	1

2470	AY738977	H1bp	H	1	H1j2	3	H1bp	4																			3	8
2471	JQ703346	H1bq	HV	0																							0	0
2472	JQ704089	H1br	H10+(16093)	1	H10+(16093)	1	H	5	H1br	5																	4	12
2473	JQ324633	H1bs	H1bs	1																							1	1
2474	JQ703244	H1bt1	H1bt	1	H1bt1	1																					2	2
2475	JQ703635	H1bu	R0	1	H	5	H1	4	H1	5	H1	5	H1	5	H1	5	H1	5	H1	5	H1	5	H1bu	5			11	50
2476	KC175606	H1bv	H7c	1	H	2	H1bv	3																			3	6
2477	JQ324778	H1bv1	H	1	H1bv1	2																					2	3
2478	JQ704366	H1bw	R0	1	H	5	H1	4	H1	5	H1	5	H1	5	H1	5	H1	5	H1	5	H1	5	H1	5	H1bw	4	12	54
2479	JQ324844	H1bx	H	1	H1aj1	2	H1bx	4																			3	7
2480	JQ898578	H1bz	H1	1	H1bz	2																					2	3
2481	EU600345	H1ca	HV	1	H1j2	4	H1+16189	1	H1ca	10																	4	16
2482	JQ705028	H1+16311	HV	1	H1	3	H1+16311	1																			3	5
2483	HG01521	H1cd	H1cd	1																							1	1
2484	KC257391	H1cf	H	1	H1cf	2																					2	3
2485	JX153783	H1ci	R0	1	H	5	H1	4	H1	5	H1	5	H1	5	H1	5	H1	5	H1	5	H1	5	H1	5	H1ci	4	12	54
2486	JX153943	H1cj	H1cj	1																							1	1
2487	EU597492	H2a	H1t1a1	1	H2a	3																					2	4
2488	FJ238053	H2a1	H2a1	1	H2a1	6																					2	7
2489	FJ668389	H2a1a	H2a1a	1																							1	1
2490	JQ704198	H2a1a1	H2a1a1	1																							1	1
2491	JQ703509	H2a1a2	H2a1a2	1																							1	1
2492	JQ703204	H2a1b1	H2a1b1	1																							1	1
2493	JQ703223	H2a1b2	H2a1b2	1																							1	1
2494	JQ703582	H2a1c	H2a1	1	H2a1c	6																					2	7
2495	FJ800808	H2a1d	H2a1d	1																							1	1
2496	JQ703145	H2a1e	H2a1e	1	H2a1e	1																					2	2
2497	JQ704189	H2a1e1	H2a1e1	1																							1	1
2498	FJ842614	H2a1e1a	H2a1e1a	1																							1	1
2499	JQ703214	H2a1e1a1	H2a1e1a1	1																							1	1
2500	JQ704847	H2a1e1b	H2a1e1b	1																							1	1
2501	HQ336422	H2a1f	H2a1f	1																							1	1
2502	JQ705806	H2a1f1	H2a1f1	1																							1	1
2503	KF723706	H2a1f2	H2a1f2	1																							1	1
2504	JQ705411	H2a1g	H2a1	1	H2a1g	6																					2	7
2505	JQ703760	H2a1i	H2a1i	1																							1	1
2506	JQ701819	H2a1j	H2a1j	1																							1	1
2507	JQ705370	H2a1k	H2a1k	1																							1	1
2508	JX153259	H2a1m	H2a1m	1																							1	1
2509	JQ704434	H2a1+146	H2a1+146	1	H2a1+146	1																					2	2
2510	JX153119	H2a1n	H2a1+146	1	H2a1n	1																					2	2
2511	JQ704533	H2a2	H66a	1	H2	6	H2a2	1																			3	8
2512	JQ701904	H2a2a	H108	1	H	2	H+195	2	H+195	1	H2a2a	7															5	13
2513	NC_012920	H2a2a1	H2a2a1	1																							1	1
2514	EU795361	H2a2a1a	H2a2a1a	1																							1	1
2515	JQ704346	H2a2a1b	H2a2a1b	1																							1	1
2516	JQ704842	H2a2a1d	H2a2a1d	1																							1	1
2517	NA07346	H2a2a1e	H2a2a1e	1																							1	1
2518	JQ704520	H2a2a1f	H2a2a1f	1																							1	1
2519	JQ705265	H2a2a1g	H2a2a1g	1																							1	1
2520	JQ704532	H2a2a1h	H2a2a1h	1																							1	1
2521	EU716647	H2a2a2	H17a2	1	H2a2a2	6																					2	7
2522	JQ703290	H2a2+(16235)	H	1	H2a2+(16235	1																					2	2
2523	HQ914650	H2a2b	H2a2b	1																							1	1
2524	JQ705365	H2a2b1	H2a2b1	1																							1	1
2525	KC257390	H2a2b1a	H2a2b1a	1																							1	1
2526	EF418606	H2a2b1a1	H2a2b1a1	1																							1	1

2527	EU444119	H2a2b2	H2a2b2	1													1	1
2528	JX153667	H2a2b3	H2a2b3	1													1	1
2529	JQ705734	H2a2b4	H2a2b	1	H2a2b4	4											2	5
2530	HQ675041	H2a2b5	H2a2b	1	H2a2b5	4	H2a2b5	1									3	6
2531	HG00123	H2a2b5a	H2a2b5a	1													1	1
2532	AM263180	H2a3	H2a3	1													1	1
2533	JX153748	H2a3a	H1n1a	1	H2a3a	2											2	3
2534	JQ704220	H2a3a1	H1n1a	1	H2a3a	2	H2a3a1	1									3	4
2535	HQ659870	H2a3b	H2a3b	1													1	1
2536	AM263181	H2a4	H	1	H	2	H2a4	2									3	5
2537	KC533501	H2a5	R0	1	H	5	H2	5	H2a	1	H2a5	2	H2a5	2			6	16
2538	JQ324582	H2a5a1	H2a5a1	1													1	1
2539	FJ527772	H2a5a1a	H	1	H2a5a1	2	H2a5a1a	2									3	5
2540	JF284817	H2a5a1b	H	1	H2a5a1	2	H2a5a1b	2									3	5
2541	JQ703081	H2a5b	H2a5b	1													1	1
2542	JQ704687	H2a5b1	H2a5b1	1													1	1
2543	HQ153430	H2a5b2	H2a5b2	1													1	1
2544	EU597574	H2b	H2b	1													1	1
2545	JQ701885	H2c	H2c	1													1	1
2546	JQ704496	H2c1	H2c1	1													1	1
2547	AY738987	H3	R0	1	H3	5	H3	5	H3	5	H3	5	H3	5	H3	3	7	29
2548	JQ703549	H3+152	H52	1	H	5	H3+152	1									3	7
2549	JQ704834	H3a	H3a	1	H3a	1											2	2
2550	JQ704253	H3a1	H3a1	1													1	1
2551	JQ705329	H3a1a	H3a	1	H3a1	1	H3a1a	1									3	3
2552	AY495132	H3g	H	1	H3+152	2	H3g	4	H3g	4							4	11
2553	JQ704782	H3g1	H3g1	1													1	1
2554	JQ705694	H3g1a	H3g1a	1													1	1
2555	HG00245	H3g1b	H	1	H3+152	2	H3g	4	H3g1	4	H3g1b	2					5	13
2556	JQ704542	H3g2	H	1	H3+152	2	H3g	4	H3g2	4							4	11
2557	JQ705080	H3g3	H3g3	1													1	1
2558	JQ702762	H3g4	H3g4	1													1	1
2559	JQ703118	H3i	H	1	H3+152	2	H3i	4	H3i	1							4	8
2560	GQ165466	H3i1	H	1	H3+152	2	H3i	4	H3i1	1							4	8
2561	HQ696491	H3j	HV	1	HV9+152	1	H3j	9									3	11
2562	JQ704704	H3k	H3k	1													1	1
2563	AY495130	H3k1a	H	1	H3+152	2	H3k	4	H3k1	2	H3k1a	1					5	10
2564	JX025434	H3b	H	1	R0	2	H3b	2									3	5
2565	AY495148	H3b+16129	H	1	H3	2	H3b+16129	2	H3b+16129	4							4	9
2566	KC533482	H3b1a	H3b1	1	H3b1a	1											2	2
2567	JQ704932	H3b1b	H3b1b	1													1	1
2568	JQ703382	H3b1b1	H3b1b1	1	H3b1b1	1											2	2
2569	JQ704105	H3b1b1a	H3b1b1	1	H3b1b1a	1											2	2
2570	AY495155	H3b2	H	1	H3	2	H3b+16129	2	H3b2	4							4	9
2571	JQ703291	H3b3	H	1	H3	2	H3b+16129	2	H3b3	4							4	9
2572	JQ704197	H3b4	H3b4	1	H3b4	1											2	2
2573	JQ705727	H3b4a	H3b4a	1													1	1
2574	HQ384186	H3b5	H3b5	1													1	1
2575	JQ705735	H3b6	H3b6	1	H3b6	1											2	2
2576	JX153543	H3b6a	H3b6	1	H3b6a	1											2	2
2577	JX153702	H3b7	H	1	H3	2	H3b+16129	2	H3b7	4							4	9
2578	JQ703744	H3c	H29b	1	H3c	3											2	4
2579	AY738990	H3c1	V+@16298	1	HV	2	H3	3	H3c1	2							4	8
2580	JQ705425	H3c2	H3c2	1	H3c2	3											2	4
2581	JQ324828	H3c2a	H3c2	1	H3c2a	3	H3c2a	1									3	5
2582	JQ324776	H3c2a1	H1n+146+195	1	H3c2a1	4											2	5
2583	JQ704172	H3c2b	H3c2b	1													1	1

2584	JQ705340	H3c2b1	H3c2b1	1											1	1
2585	JQ324704	H3c2c	H3c2c	1											1	1
2586	JQ702603	H3c3	H3c3	1											1	1
2587	AY495180	H3d	R	1	H3+73	4	H3d	1							3	6
2588	FJ348206	H3e	H3e	1											1	1
2589	JQ702652	H3+16311	HV	1	H3+16311	3	H3+16311	1							3	5
2590	KF179063	H3h	HV	1	H3+16311	3	H3h	2	H3h	5					4	11
2591	JQ086344	H3h1	HV	1	H3+16311	3	H3h	2	H3h1	5					4	11
2592	JQ702530	H3h2	H3h2 H3h2 (2)	1	H3h2	5									2	6
2593	KC911606	H3h2a	H3h2a	1											1	1
2594	KF424557	H3h3	HV	1	H3+16311	3	H3h	2	H3h3	5	H3h3	2			5	13
2595	JQ704436	H3h3a	HV	1	H3+16311	3	H3h	2	H3h3	5	H3h3a	2			5	13
2596	HM003111	H3h3b	HV6	1	H3h3b	3									2	4
2597	JQ704484	H3h4	H3h4	1											1	1
2598	JQ705041	H3h5	HV	1	H3+16311	3	H3h	2	H3h5	5					4	11
2599	JQ324559	H3h6	HV	1	H3+16311	3	H3h	2	H3h6	5					4	11
2600	JN129423	H3h7	H3h7	1											1	1
2601	JQ704363	H3m	H3m	1											1	1
2602	JQ704633	H3n	H3n	1											1	1
2603	JQ704490	H3p	H3p	1											1	1
2604	JQ704318	H3q	R0	1	H3	5	H3q	5	H3q	1					4	12
2605	GU592027	H3q1	H	1	H3+152	2	H3+152	4	H3q1	2					4	9
2606	JN112339	H3r	R0	1	H3	5	H3r	5	H3r	1					4	12
2607	JQ705853	H3r1	R0	1	H3	5	H3r	5	H3r1	1					4	12
2608	JQ704120	H3s	H3s	1											1	1
2609	KC911500	H3t	R0	1	H3	5	H3	5	H3t	5					4	16
2610	DQ523647	H3u	H1	1	H3u	2									2	3
2611	JQ705759	H3u1	H3u1	1											1	1
2612	JQ704903	H3v	H3v	1	H3v	1									2	2
2613	JQ703289	H3v1	H3v	1	H3v1	1									2	2
2614	GU997629	H3v+16093	H3v+16093	1											1	1
2615	JQ704545	H3v2	H3v2	1											1	1
2616	JQ703544	H3w	H	1	H3w	2									2	3
2617	JQ324817	H3x	H3x	1											1	1
2618	DQ523662	H3x1	H3x1	1											1	1
2619	JQ705116	H3y	H2a2a H3y (2)	1	H3y	1									2	2
2620	JQ705915	H3z	H3z	1											1	1
2621	JQ703690	H3z1	H3z1	1											1	1
2622	JQ704178	H3z2	H3z2	1											1	1
2623	JQ705422	H3aa	H	1	H83	1	H3aa	1							3	3
2624	JN409387	H3ab	H1t1a1	1	H3ab	3									2	4
2625	HQ658608	H3ac	H3ac	1											1	1
2626	JQ703272	H3ad	H55a	1	H3ad	4									2	5
2627	JQ705905	H3ae	R0	1	H3	5	H3	5	H3	5	H3ae	5			5	21
2628	JQ324794	H3af	H3af	1											1	1
2629	HG00252	H3ag	H	1	H	2	H3ag	1							3	4
2630	NA12283	H3ag1	H3ag1	1											1	1
2631	JQ324638	H3ah	H	1	H26c	2	H	3	H3ah	2					4	8
2632	JQ705437	H3ai	R0	1	H3	5	H3	5	H3	5	H3	5	H3ai	5	6	26
2633	JQ701983	H3aj	R0	1	H3	5	H3	5	H3	5	H3	5	H3aj	5	6	26
2634	AY195746	H3ak	H3ak	1											1	1
2635	JQ324634	H3am	H3am	1											1	1
2636	JQ703304	H3an	H3an	1											1	1
2637	JQ704232	H3ao	H3ao	1											1	1
2638	JQ704183	H3ao1	H3ao1	1											1	1
2639	JQ702411	H3ap	H3	1	H3	3	H3ap	2							3	6
2640	JN674560	H3aq	R0	1	H3	5	H3	5	H3	5	H3	5	H3aq	5	6	26

2641	JQ702687	H3ar	R0	1	H3	5	H3	5	H3	5	H3	5	H3ar	5							6	26
2642	JQ702769	H3as	H3as	1																	1	1
2643	JQ324640	H3at	R0	1	H3	5	H3	5	H3	5	H3	5	H3	5	H3at	3	H3at	1			8	30
2644	JQ324851	H3at1	R0	1	H3	5	H3	5	H3	5	H3	5	H3	5	H3at	3	H3at1	1			8	30
2645	JQ704477	H3au	R0	1	H3	5	H3	5	H3	5	H3	5	H3	5	H3au	3					7	29
2646	JQ324574	H3+16189	HV	1	H3+16189	3															2	4
2647	JQ324687	H3av	H	1	H3av	2															2	3
2648	JN646688	H4	H	1	R0	2	R0	2	H4	2											4	7
2649	JX152997	H4a	R0	1	H	5	H4	5	H4a	3	H4a	1									5	15
2650	EF177440	H4a1	H1q3	1	H	2	H4a1	3	H4a1	1											4	7
2651	EU051827	H4a1a	H+152	1	H+152	5	H+152	3	H+152	3	H	4	H4a1	2	H4a1a	3					7	21
2652	AY495095	H4a1a1	R0	1	H	5	H4	5	H4a	3	H4a1	1	H4a1a	3	H4a1a1	3	H4a1a1	1			8	22
2653	JQ703892	H4a1a1a	H4a1a1a	1																	1	1
2654	EU200347	H4a1a1a1a	R	1	H4a1a1a	4	H4a1a1a1	3	H4a1a1a1a	1	H4a1a1a1a	1									5	10
2655	EF609015	H4a1a1a1a1	H4a1a1a1a1	1																	1	1
2656	JQ703532	H4a1a1a2	H4a1a1a2	1																	1	1
2657	EU719211	H4a1a1a3	H4a1a1a3	1																	1	1
2658	JQ324913	H4a1a1a4	R	1	H4a1a1a	4	H4a1a1a4	3													3	8
2659	JQ703542	H4a1a2a	R0	1	H	5	H4	5	H4a	3	H4a1	1	H4a1a	3	H4a1a2	3	H4a1a2a	1	H4a1a2a	1	9	23
2660	JQ704851	H4a1a2a1	H3as	1	R0	2	H	2	H4a1a2a1	3											4	8
2661	JQ704883	H4a1a+195	H4a1a+195	1																	1	1
2662	JQ703398	H4a1a3	H4a1a3	1																	1	1
2663	EF556191	H4a1a3a	H4a1a3a	1																	1	1
2664	JQ703134	H4a1a4a	H4a1a4a	1																	1	1
2665	HQ659689	H4a1a4b	H	1	H4a1a+195	2	H4a1a4	2	H4a1a4b	2	H4a1a4b	1									5	8
2666	JX153972	H4a1a4b1	H4a1a4b1	1																	1	1
2667	JQ702546	H4a1a4b2	H	1	H4a1a+195	2	H4a1a4	2	H4a1a4b	2	H4a1a4b2	1									5	8
2668	JQ704039	H4a1a5	R0	1	H	5	H4	5	H4a	3	H4a1	1	H4a1a	3	H4a1a5	3					7	21
2669	JQ703630	H4a1c1	HV	1	HV	3	H4	5	H4a1	2	H4a1c1	2									5	13
2670	AY738950	H4a1c1a	H4a1c1a	1																	1	1
2671	KF305642	H4a1c2	H4a1c2	1																	1	1
2672	JQ704648	H4a1d	H2a1	1	H2a1d	6	H4a1d	6													3	13
2673	JQ703939	H4a2	H4a2	1																	1	1
2674	JQ702580	H4b	HV	1	HV	3	H4	5	H4b	2											4	11
2675	EU600352	H4b1	H4b1	1																	1	1
2676	JQ705112	H4c1	H4c1	1																	1	1
2677	EU935460	H4d	H4d	1																	1	1
2678	JQ324611	H5'36	H5'36	1																	1	1
2679	AY495174	H5	H5	1	H5	3	H5	5	H5	1											4	10
2680	GQ983068	H5a	H5	1	H5a	3	H5a	2	H5a	3											4	9
2681	AY195747	H5a1	H5	1	H5	3	H5a1	2	H5a1	5	H5a1	3									5	14
2682	HQ659693	H5a1a	H5	1	H5a	3	H5a1	2	H5a1a	5											4	11
2683	AY495167	H5a1b	H5	1	H5a	3	H5a1	2	H5a1b	5											4	11
2684	JQ702653	H5a1c1a	H5a1c1a	1																	1	1
2685	JQ705790	H5a1c2	H5a1c2	1																	1	1
2686	JQ705035	H5a1d	H5	1	H5a	3	H5a1	2	H5a1d	5											4	11
2687	AY339431	H5a1e	H5a1e	1																	1	1
2688	JQ703738	H5a1f	H5	1	H5a	3	H5a1	2	H5a1f	5											4	11
2689	JN008723	H5a1g1	H5a1g1	1	H5a1g1	1															2	2
2690	JQ704886	H5a1g1a	H5a1g1	1	H5a1g1a	1															2	2
2691	JQ703820	H5a1g2	H5a1g2	1																	1	1
2692	JQ702502	H5a1h	H5	1	H5a	3	H5a1	2	H5a1	5	H5a1h	3									5	14
2693	JX128043	H5a1i	H5a1i	1																	1	1
2694	EU372627	H5a1j	H5a1j	1																	1	1
2695	GQ983064	H5a1k	H5a1a H5a1k H5j	1	H5a1k	3															2	4

2696	GQ334692	H5a1+152	H5a	1	H5a1+152	1	H5a1+152	2							3	4
2697	JQ705998	H5a1m	H5a1m	1											1	1
2698	JQ704324	H5a1n	H5a	1	H5a1+152	1	H5a1n	2							3	4
2699	GQ983075	H5a1+16093	H5a1+16093	1	H5a1+16093	1									2	2
2700	FJ966912	H5a1p	H5a1+16093	1	H5a1p	1									2	2
2701	JX128066	H5a1q	H5a1	1	H5a1	1	H5a1q	1							3	3
2702	AY738952	H5a2	H5a1+16093	1	H5a1+16093	1	H5a2	3							3	5
2703	JQ705582	H5a2a	H5	1	H5a	3	H5a2	2	H5a2a	1					4	7
2704	GQ983105	H5a3	H5a3	1											1	1
2705	JQ704246	H5a3a	H5a3	1	H5a3a	2	H5a3a	1							3	4
2706	JQ324706	H5a3a1	H5a3a1	1											1	1
2707	GQ983069	H5a3a+152	H5a3a+152	1	H5a3a+152	1									2	2
2708	GQ983073	H5a3a2	H5a3a+152	1	H5a3a2	1									2	2
2709	AY495175	H5a3a3	H5a3a3	1											1	1
2710	JQ703800	H5a3b	H5a3b	1											1	1
2711	JQ703731	H5a4	H5e1	1	H5a4	2									2	3
2712	GQ983092	H5a4a1	H5a4a1	1											1	1
2713	JQ702083	H5a4a1a	H5a4a1a	1											1	1
2714	JX153318	H5a5	H5	1	H5a5	2									2	3
2715	JQ324545	H5a+152	H5a+152	1											1	1
2716	GQ983096	H5a6	H5a6	1											1	1
2717	JQ704237	H5a6a	H5a	1	H5a+152	1	H5a6	1	H5a6a	1					4	4
2718	GQ983106	H5a7	H5a7	1											1	1
2719	JX153844	H5a8	H5	1	H5a	3	H5a	2	H5a8	3					4	9
2720	JX153930	H5a9	H5	1	H5a	3	H5a	2	H5a9	3					4	9
2721	JX128050	H5b	H5b	1											1	1
2722	FJ794473	H5b1	H5b1	1											1	1
2723	HQ700378	H5b2	H5b2	1											1	1
2724	JQ702345	H5b3	H5b3	1											1	1
2725	JF795326	H5b4	H5b4	1											1	1
2726	JX153042	H5b5	H5e1a	1	H5	3	H5e	1	H5e	1	H5b	3	H5b5	2	6	11
2727	JQ705748	H5c	H5a1a	1	H5c	4									2	5
2728	JQ705824	H5c1	H5+16311	1	H5c1	3									2	4
2729	JQ705569	H5c1a	H5a1	1	H5c1a	4									2	5
2730	JQ703627	H5c2	H5c2	1											1	1
2731	JQ704498	H5d	H5d	1											1	1
2732	JQ324661	H5e	H5	1	H5e	1	H5e	1							3	3
2733	KF830849	H5e1	H5	1	H5e	1	H5e1	1	H5e1	2					4	5
2734	EU677750	H5e1a	H5	1	H5e	1	H5e1	1	H5e1a	2	H5e1a	1			5	6
2735	JX128091	H5e1a1	H5e1a1	1											1	1
2736	HM636849	H5e1b	H5e1b	1											1	1
2737	GQ983074	H5f	H5	1	H5	3	H5f	5							3	9
2738	JQ704716	H5g	H5g	1											1	1
2739	JQ703405	H5h	H5h	1											1	1
2740	JQ324861	H5j	H5j	1											1	1
2741	AY495173	H5k	H5	1	H5	3	H5k	5							3	9
2742	JQ702583	H5m	H5m	1											1	1
2743	GQ983109	H5n	H5n	1											1	1
2744	EF420250	H5p	H5	1	H5	3	H5p	5							3	9
2745	JQ703228	H5+16192	H5+16192	1	H5+16192	1									2	2
2746	JQ703632	H5q	H5q	1											1	1
2747	JQ324596	H5+16311	H5+16311	1											1	1
2748	GQ983083	H5r	H5r	1	H5r	2									2	3
2749	GQ983103	H5r1	H5r	1	H5r1	1									2	2
2750	GQ983061	H5r2	H5r	1	H5r2	2									2	3
2751	JQ703547	H5s	H5s	1											1	1
2752	HQ661871	H5t	H5t	1											1	1

2753	EF661003	H5u	H5u	1													1	1
2754	JX128090	H5u1	H5u1	1													1	1
2755	JX153021	H5+709	H5+709	1													1	1
2756	JX153984	H5v	H5	1	H5	1	H5v	4									3	6
2757	FJ348166	H36	H36	1													1	1
2758	HM765475	H6a	H6a	1													1	1
2759	JQ324601	H6a1a	H6	1	H6a	2	H6a1	2	H6a1a	2	H6a1a	5	H6a1a	2			6	14
2760	AM263177	H6a1a1	H6a1a1	1													1	1
2761	EF556185	H6a1a1a	H6a1a1a	1													1	1
2762	JQ702107	H6a1a2a	H6a1a2a	1													1	1
2763	JQ324568	H6a1a2b	H6a1b2e	1	H6a1a	2	H6a1a2b	2									3	5
2764	JQ703345	H6a1a2b1	H6	1	H6a	2	H6a1	2	H6a1a	2	H6a1a2	5	H6a1a2b	1	H6a1a2b1	1	7	14
2765	HQ730608	H6a1a3	H6	1	H6a	2	H6a1	2	H6a1a	2	H6a1a3	5	H6a1a3	1			6	13
2766	JQ703205	H6a1a3a	H6a1a3a	1													1	1
2767	JQ704472	H6a1a4	H6a2a	1	H6a1a	2	H6a1a	2	H6a1a4	1							4	6
2768	HM019517	H6a1a5	H6a1b2c	1	H6a	1	H6a1a5	2									3	4
2769	JQ704195	H6a1a6	H6a1a6	1													1	1
2770	HG01489	H6a1a7	H6a1a7	1													1	1
2771	JQ705660	H6a1a8a	H6a1a8a	1													1	1
2772	JQ704380	H6a1a9	H6a1a9	1													1	1
2773	JX297423	H6a1a10	H6a1a10	1													1	1
2774	JQ704592	H6a1b	H6a1b	1													1	1
2775	EF556178	H6a1b1	H6a1b1	1													1	1
2776	JQ704495	H6a1b2	H6a1b2	1													1	1
2777	JQ704376	H6a1b2a	H6a1b2a	1													1	1
2778	JQ703391	H6a1b2b	H6	1	H6a	2	H6a1	2	H6a1b	2	H6a1b2	1	H6a1b2b	4			6	12
2779	JQ704239	H6a1b2c	H6a1b2c	1													1	1
2780	JQ324676	H6a1b2d	H6a1b2d	1													1	1
2781	JX153784	H6a1b2e	H6a1b2e	1													1	1
2782	JX307108	H6a1b3	H6a1b3	1	H6a1b3	2											2	3
2783	JQ702830	H6a1b3a	H6a1b3	1	H6a1b3a	2											2	3
2784	HQ659687	H6a1b3b	H6a1b3b	1													1	1
2785	JQ703321	H6a1b4	H6a1b4	1													1	1
2786	JQ704515	H6a2	H6a1a7	1	H6a2	4											2	5
2787	JQ704208	H6a2a	H6a1a	1	H6a2a	4											2	5
2788	JQ702285	H6b	H6b	1													1	1
2789	AY738956	H6b1	H6b1	1													1	1
2790	FJ348203	H6b2	H6b2	1													1	1
2791	JN601518	H6c	H6c	1													1	1
2792	JX307099	H6c1	H6c1	1													1	1
2793	AY495120	H7	R0	1	H	5	H7	5	H7	6							4	17
2794	JQ704362	H7a	H10+(16093)	1	H10+(16093)	1	H7	5	H7a	3							4	10
2795	DQ862537	H7a1	H7a1	1													1	1
2796	JQ704518	H7a1a	H7a1a	1													1	1
2797	JQ705420	H7a1b	H7a1b	1													1	1
2798	JQ705679	H7a1c	H7a1c	1													1	1
2799	JX153387	H7a1d	H	1	H7a1	2	H7a1d	3									3	6
2800	JQ703233	H7a2	H7a2	1													1	1
2801	JQ703372	H7b	R0	1	H	5	H7	5	H7b	6	H7b	6					5	23
2802	EU600357	H7b1	H1	1	H7b1	4											2	5
2803	JX153372	H7b2	HV	1	HV	3	H7	5	H7b2	4							4	13
2804	JQ703275	H7b2a	H3s	1	H7b2a	6											2	7
2805	EF581833	H7b3	R0	1	H	5	H7	5	H7b	6	H7b3	6					5	23
2806	JQ704850	H7b4	R0	1	H	5	H7	5	H7b	6	H7b4	6					5	23
2807	HQ658454	H7b5	HV	1	H	4	HV	2	H	2	H7b5	2					5	11
2808	JQ704428	H7b6	R0	1	H	5	H7	5	H7b	6	H7b6	6					5	23
2809	EU600355	H7c1	H7c1	1													1	1

2810	JQ704714	H7c2	HV	1	H	4	HV	2	H	2	H7c2	2			5	11
2811	JQ703177	H7c3	H10+(16093)	1	H10+(16093)	1	H7c3	4							3	6
2812	NA20537	H7c4	H7c4	1											1	1
2813	KC257398	H7c5	R0	1	H	5	H7	5	H7c	6	H7c5	4			5	21
2814	JX153913	H7c6	H1t1a1	1	H7c6	4									2	5
2815	JQ704712	H7d	H7d	1											1	1
2816	JQ705880	H7d1	H55a	1	H7d1	4									2	5
2817	JQ704711	H7d2	H	1	H7d2	2	H7d2	1							3	4
2818	JQ702337	H7d2a	H	1	H7d2	1	H7d2a	1							3	3
2819	JQ324573	H7d3	H7d3 H73a1	1	H7d3	6									2	7
2820	JQ703288	H7d3a	HV	1	HV	2	H7d3a	3							3	6
2821	JF262142	H7d4	H7d4 H44a1	1	H7d4	3									2	4
2822	JN035288	H7d5	H7d5	1											1	1
2823	HQ267514	H7e	R0	1	H	5	H7	5	H7e	6					4	17
2824	JQ704377	H7f	H7f	1											1	1
2825	JQ703479	H7g	R0	1	H	5	H7	5	H7g	6					4	17
2826	JQ703495	H7h	H7h	1											1	1
2827	AY738964	H7h1	H7h1	1											1	1
2828	JF795008	H7i	H7i	1											1	1
2829	JX153705	H7i1	H7i1	1											1	1
2830	EU600360	H8a	H8a	1											1	1
2831	AY738957	H8a1	H8a1	1											1	1
2832	JQ324881	H8b	H8b	1											1	1
2833	KF148188	H8b1	H8b1	1											1	1
2834	EF177429	H8+(114)+152	H8+(114)+152	1											1	1
2835	JQ702865	H8c	H8c	1											1	1
2836	JQ701943	H8c1	H8+(114)+152	1	H8c	1	H8c1	1							3	3
2837	JQ735909	H8c2	H8c2	1											1	1
2838	JN009620	H31	H31	1											1	1
2839	JQ028728	H31a	H31a	1											1	1
2840	JQ702697	H31b	H12	1	H31b	3									2	4
2841	JQ703684	H11	H11	1	H11	1									2	2
2842	GU945760	H11a	H11a	1											1	1
2843	JQ703259	H11a1	H11a1	1											1	1
2844	GU949563	H11a2	H11a2	1											1	1
2845	JQ705580	H11a2a	H11a2a	1	H11a2a	2									2	3
2846	EF545566	H11a2a1	H11a2a	1	H11a2a1	2									2	3
2847	JQ703071	H11a2a2	H11a2a2	1											1	1
2848	FJ705060	H11a2a3	H11a2a3	1											1	1
2849	JQ703235	H11a3	H11a3	1											1	1
2850	HQ659686	H11a4	H11a4	1											1	1
2851	GU592038	H11a5	H11a	1	H11a5	3									2	4
2852	JQ705740	H11a6	H11a6	1											1	1
2853	JQ703253	H11a+152	H11a+152	1	H11a+152	1									2	2
2854	JQ703733	H11a7	H11a7	1											1	1
2855	JQ705643	H11a8	H11a8	1											1	1
2856	JQ704286	H11b1	H11	1	H11b	1	H11b1	1							3	3
2857	DQ341083	H12	H12	1											1	1
2858	AY738994	H12a	H12a	1											1	1
2859	JQ703303	H91	H91	1											1	1
2860	JQ324697	H108	H108	1											1	1
2861	JQ703798	H+152	H9	1	H+152	2									2	3
2862	AY713978	H9	H26c	1	H	1	H9	5							3	7
2863	AY738969	H9a	H9a	1											1	1
2864	DQ523619	H32	H32	1											1	1
2865	AY495140	H46	H	1	H	2	H	2	H+152	3	H46	3	H46	2	6	13
2866	JQ703070	H46a	H	1	H	2	H	2	H+152	3	H46	3	H46a	2	6	13

2867	KC553988	H46b	R0	1	H	5	H	5	H	53	H46b	5					5	69
2868	EU744541	H52	H52	1													1	1
2869	KC257357	H69	H	1	H	2	H	2	H+152	3	H69	3					5	11
2870	HM470002	H103	H103	1													1	1
2871	JX153071	H107	H107	1													1	1
2872	JQ701849	H10	R0	1	H	5	H10	5	H10	4	H10	1					5	16
2873	KC257394	H10a	R0	1	H	5	H10	5	H10a	4	H10a	1					5	16
2874	HM021152	H10a1	H	1	H10a1	2	H10a1	1									3	4
2875	JQ704287	H10a1a	H	1	H10a1	2	H10a1a	1	H10a1a	1							4	5
2876	EU073971	H10a1a1	H	1	H10a1	2	H10a1a	1	H10a1a1	1							4	5
2877	JQ703237	H10a1b	H10a1b	1													1	1
2878	AY495145	H10b	H	1	H	5	H+152	2	H+152	3	H10	4	H10b	1			6	16
2879	JQ704573	H10b1	R0	1	H	5	H10	5	H10b	4	H10b1	1					5	16
2880	JQ704955	H10c	R0	1	H	5	H10	5	H10c	4	H10c	1					5	16
2881	JQ704295	H10c1	H1t1a1	1	H	4	H10c1	3									3	8
2882	JQ703504	H10d	H10d	1													1	1
2883	JQ703539	H10+(16093)	H10+(16093)	1													1	1
2884	JQ705702	H10e	H10e	1													1	1
2885	JQ703002	H10e1	H10e1	1													1	1
2886	JQ703645	H10e1a	H10e1a	1													1	1
2887	GU569076	H10e2	H10e2	1													1	1
2888	JQ702010	H10e3a	H10e3a	1													1	1
2889	JQ703010	H10f	H10+(16093)	1	H10f	1											2	2
2890	GU122976	H10g	H10g	1													1	1
2891	JQ702818	H10h	R0	1	H	5	H10	5	H10h	4							4	15
2892	JQ705098	H13a1a	H13b1a	1	H13	4	H13a1a	4									3	9
2893	JQ704682	H13a1a1	R0	1	H13	5	H13a	3	H13a1	2	H13a1a	1	H13a1a1	5	H13a1a1	5	7	22
2894	JQ706004	H13a1a1a	H1e1c	1	H	2	H13a1a1a	2									3	5
2895	JQ703386	H13a1a1b	H7d3	1	H13a1a1b	6											2	7
2896	AM263191	H13a1a1c	H10+(16093)	1	H10+(16093)	1	H13a1a1c	4									3	6
2897	JQ703808	H13a1a1d	H13a1a1d	1													1	1
2898	JQ704841	H13a1a1d1	H13a1a1d	1	H13a1a1d1	1											2	2
2899	JQ703421	H13a1a1e	H	1	H	2	H13a1a1e	5									3	8
2900	AM263186	H13a1a2	H1	1	H13a1a2	3											2	4
2901	EU370982	H13a1a2a	H13a1a2a	1													1	1
2902	EU600347	H13a1a2b	H13a1a2b	1													1	1
2903	KC911290	H13a1a3	R0	1	H13	5	H13a	3	H13a1	2	H13a1a	1	H13a1a3	5			6	17
2904	FJ560455	H13a1a4	R0	1	H13	5	H13a	3	H13a1	2	H13a1a	1	H13a1a4	5			6	17
2905	JQ705726	H13a1a5	H3am	1	H13a1a5	6											2	7
2906	JQ704176	H13a1a6	H13a1a6	1													1	1
2907	HG01173	H13a1b	H13a1b	1													1	1
2908	JN712772	H13a1c	H13a1c	1													1	1
2909	EU597515	H13a1+152	H2+152_16311	1	HV	3	H13a1+152	2									3	6
2910	JX153573	H13a1d	H13a1d	1													1	1
2911	KC577357	H13a2a	R0	1	H13	5	H13a	3	H13a2	2	H13a2a	2	H13a2a	1			6	14
2912	KC911458	H13a2a1	H4a1	1	HG	2	H13a2a1	8									3	11
2913	JX153617	H13a2b	R0	1	H13	5	H13a	3	H13a2	2	H13a2b	2	H13a2b	2			6	15
2914	EF660948	H13a2b1	H3h2	1	H13a2b1	5											2	6
2915	HQ593813	H13a2b2	R0	1	H13	5	H13a	3	H13a2	2	H13a2b	2	H13a2b2	2	H13a2b2	1	7	16
2916	JF828090	H13a2b2a	R0	1	H13	5	H13a	3	H13a2	2	H13a2b	2	H13a2b2	2	H13a2b2a	1	7	16
2917	JQ705524	H13a2b3	H13a2b3	1													1	1
2918	KC911469	H13a2b4	H	1	R0	2	H13	4	H13a2b4	2							4	9
2919	JX153198	H13a2b5	H13a2b5	1													1	1
2920	KC911338	H13a2c	R7	1	H13a2c	3											2	4
2921	JQ703878	H13a2c1	H13a2c1	1													1	1
2922	AM263188	H13b	H13b	1													1	1
2923	EF660956	H13b1	H7a1	1	H13b1	2											2	3

2924	GU228506	H13b1+200	H13b1+200	1											1	1
2925	JQ704111	H13b1a	H13b1+200	1	H13b1a	1									2	2
2926	NA20771	H13b1b	H13b1b	1											1	1
2927	JQ704313	H13b2	H	1	H13b	2	H13b2	1							3	4
2928	JQ703657	H13c1	H61a	1	R0	2	H13c1	8							3	11
2929	KC911462	H13c1a	R0	1	H13	5	H13c	3	H13c1	1	H13c1a	1			5	11
2930	KC911276	H13c2	H13c2	1											1	1
2931	GQ983090	H14a	H14a	1											1	1
2932	JQ705084	H14a+146	H14a+146	1	H14a+146	1									2	2
2933	EF556163	H14a1	H14a+146	1	H14a1	1									2	2
2934	JQ702157	H14a2	H14a2	1											1	1
2935	JQ704161	H14a2a	H14a2a	1											1	1
2936	JQ702283	H14a2b	H14a2b	1											1	1
2937	JX153559	H14a2c	H14a2c	1											1	1
2938	AM263182	H14b	H16c	1	H14b	3									2	4
2939	JQ324608	H14b1	H14b1	1											1	1
2940	KC911300	H14b2	H1f+16093	1	H1f+16093	1	H1f+16093	3	H1e1a1	3	H14	5	H14b2	1	6	14
2941	JQ704795	H14b2a	H14b2a	1											1	1
2942	KC911545	H14b3	H14b3	1											1	1
2943	JX153284	H14b4	H3g1a	1	H14b4	4									2	5
2944	KC911292	H15	H15	1											1	1
2945	AY495146	H15a	H15a	1											1	1
2946	JQ703079	H15a1	H15	1	H15a	2	H15a1	1							3	4
2947	JQ704364	H15a1a	H15	1	H15a	2	H15a1	1	H15a1a	1					4	5
2948	JQ701892	H15a1a1	H15a1a1	1											1	1
2949	JQ324533	H15a1b	H15a1b	1											1	1
2950	JQ704870	H15b	H15b	1											1	1
2951	FJ384438	H15b1	H15b1	1											1	1
2952	JN651417	H15b2	H15	1	H15b2	2									2	3
2953	JQ703108	H16	HV	1	HV	4	H16	5							3	10
2954	EF609012	H16+152	H	1	H	2	H	2	H16+152	3	H16+152	3			5	11
2955	EU170619	H16a	H	1	H	2	H	2	H16+152	3	H16a	3	H16a	1	6	12
2956	AY495133	H16a1	H	1	H	2	H	2	H16+152	3	H16a	3	H16a1	1	6	12
2957	JQ702103	H16c	H+195	1	H16c	6									2	7
2958	JN037470	H16d	H	1	H	2	H	2	H16+152	3	H16d	3			5	11
2959	EU715287	H16b	H1	1	H16b	4									2	5
2960	JX153305	H16e	HV	1	H	4	HV	2	HV	4	H	5	H16e	5	6	21
2961	JQ704083	H17	H17	1											1	1
2962	JQ324832	H17a	H	1	H	2	H+16129	1	H17	1	H17a	2	H17a	1	6	8
2963	JQ704404	H17a1	H17a1	1											1	1
2964	AY495154	H17a2	H	1	H	2	H+16129	1	H17	1	H17a	2	H17a2	1	6	8
2965	JQ705146	H17b	H26c	1	H17b	2									2	3
2966	JQ704457	H17c	H17c	1											1	1
2967	HQ658132	H27	H27	1											1	1
2968	JQ704331	H27+16093	H27+16093	1	H27+16093	1									2	2
2969	EU007878	H27a	H27+16093	1	H27a	1									2	2
2970	FJ348192	H27b	H27b	1											1	1
2971	JQ703092	H27c	H27	1	H27c	2									2	3
2972	JQ702961	H27d	H27d	1											1	1
2973	JQ704541	H27e	H27	1	H27e	2									2	3
2974	JQ704644	H27f	H27f	1											1	1
2975	JQ705490	H18	HV	1	HV	4	H18	5							3	10
2976	JX154019	H18b	R0	1	H	5	H	5	H+13708	5	H18	1	H18b	1	6	18
2977	KC911570	H19	H19	1											1	1
2978	JX153278	H20	H20	1											1	1
2979	KC911430	H20a1	H20a1	1											1	1
2980	EF556187	H20a1a	H20a1a	1											1	1

2981	JX153027	H20a2	H20a2	1													1	1
2982	EU600334	H20b	H20b	1													1	1
2983	HG00143	H20c	H20c	1													1	1
2984	AM263178	H21	H21	1													1	1
2985	GU122977	H23	R0	1	H	5	H	5	H23	5							4	16
2986	HM589043	H24	H24	1													1	1
2987	JQ703982	H24a	H24a	1													1	1
2988	JQ703898	H24a1	H24a1	1													1	1
2989	JQ705486	H24a2	H35a	0													0	0
2990	JQ704672	H24b	H24b	1													1	1
2991	EF556189	H25	R0	1	H	5	H	5	H25	5							4	16
2992	JQ703239	H26	R0	1	H	5	H	5	H26	5	H26	1					5	17
2993	JN415471	H26a	H26a	1													1	1
2994	EF532800	H26a1	H3s	1	H	2	H	3	H+152	2	H+152	3	H26a1	5			6	16
2995	JQ702037	H26a1a	R0	1	H	5	H	5	H26	5	H26a	1	H26a1	1	H26a1a	1	7	19
2996	JQ703417	H26a1a1	H26a1a1	1													1	1
2997	JX152941	H26a1b	H26a1b	1													1	1
2998	EU600338	H26b	H	1	H26b	2											2	3
2999	JQ704110	H26c	H26c	1													1	1
3000	DQ523654	H28	H28	1													1	1
3001	JQ705312	H28a	H28a	1													1	1
3002	JQ704416	H28a1	H28	1	H28a	1	H28a1	2									3	4
3003	JX153255	H28a2	H28a2	1													1	1
3004	JQ705134	H29	H29	1													1	1
3005	EU600343	H29a	H29a	1													1	1
3006	JQ704540	H29b	H29b	1													1	1
3007	EF556162	H30	H+195	0													0	0
3008	JQ703004	H30a	H30a	1													1	1
3009	JQ704389	H30b	H1j1	0													0	0
3010	HQ315686	H30b1	H	1	H	2	H30b1	2									3	5
3011	EU600336	H33	R0	1	H	5	H	5	H	5	H33	5	H33	1			6	22
3012	JQ702297	H33a	R0	1	H	5	H	5	H	5	H33	5	H33a	1			6	22
3013	EU600341	H33b	H33b	1													1	1
3014	KC618506	H33c	H33c	1													1	1
3015	JQ705818	H+16291	H34	1	H+16291	1											2	2
3016	AY738968	H34	H34	1													1	1
3017	JQ702918	H64	H64	1													1	1
3018	JQ704251	H85	H85	1													1	1
3019	JQ703287	H35	H	1	H	4	H	2	H35	5							4	12
3020	AY495316	H35a	V+@72	1	H35a	4											2	5
3021	EU677425	H39	H39b	1	H39	1											2	2
3022	JQ702834	H39a	H39a	1	H39a	1											2	2
3023	JQ704461	H39a1	H39a	1	H39a1	1											2	2
3024	JQ703325	H39b	H39	1	H39b	2											2	3
3025	JX153830	H39c	H39c	1													1	1
3026	JQ704876	H40	H2a2a	1	H	2	H40	1									3	4
3027	FJ985851	H40a	R0	1	H	5	H	5	H	5	H40	5	H40a	2			6	23
3028	JQ702924	H40b	H	1	H	5	H+152	2	H+152	3	H+152	4	H+152	5	H40b	3	7	23
3029	JQ702060	H41a	H41a	1													1	1
3030	JQ704132	H42	R0	1	H	5	H	5	H	5	H	5	H42	5	H42	1	7	27
3031	JQ324628	H42a	H42a	1													1	1
3032	JQ324734	H42a1	H42a	1	H42a1	2											2	3
3033	JX153894	H42a2	H42a	1	H42a2	2											2	3
3034	AY495114	H43	R0	1	H	5	H	5	H	5	H	5	H43	5			6	26
3035	JQ704024	H44a	R0	1	H	5	H	5	H	5	H	5	H44	5	H44a	2	7	28
3036	HM355887	H44a1	H7d4 H44a1 (2)	1	H44a1	3											2	4
3037	JQ704290	H44b	H44b	1													1	1

3038	JQ705128	H45a	H3	1	H45a	3																					2	4
3039	JQ703821	H45b	R0	1	H	5	H	5	H	5	H	5	H45	5	H45b	2											7	28
3040	GU123005	H47	H	1	R	2	H	3	H47	6																	4	12
3041	JQ705143	H47a	H	1	H	2	H	2	H47a	3																	4	8
3042	HQ676807	H48	HV12b1	1	H48	4																					2	5
3043	JQ704535	H49	HV12b1	1	H	2	H49	5																			3	8
3044	JQ702841	H49a	H49a	1																							1	1
3045	JQ702985	H49a1	R0	1	H	5	H	5	H	5	H	5	H	5	H49	5	H49a	2	H49a1	2							9	35
3046	JQ702889	H49a2	H	1	H49a2	2																					2	3
3047	JQ704556	H49b	H57	1	H49b	4																					2	5
3048	JQ704577	H50	H66a	1	H50	2																					2	3
3049	JQ324544	H51	H1g	1	HV	3	HV	5	HV	5	H51	5															5	19
3050	JQ703369	H51a	R0	1	H	5	H	5	H	5	H	5	H	5	H51	5	H51a	1									8	32
3051	JQ324556	H53	R0	1	H	5	H	5	H	5	H	5	H	5	H53	5											7	31
3052	JQ704987	H54	H3am	1	H54	6																					2	7
3053	JQ703111	H55a	H55a	1																							1	1
3054	JQ702906	H55+153	H	1	H55+153	4																					2	5
3055	JQ705203	H55b	H55b	1																							1	1
3056	JQ703478	H56	H10+(16093)	1	H10+(16093)	1	H10+(16093)	4	R0	3	R0	2															5	11
3057	JQ705859	H56a	R0	1	H	5	H	5	H	5	H	5	H	5	H	5	H56	5	H56a	3	H56a	1					10	40
3058	JQ703350	H56b	H7a H56b (2)	1	H56b	2																					2	3
3059	HQ287897	H56c	H	1	H	2	H+16129	1	H+16129	1	H56c	5															5	10
3060	KC257389	H56d	H56d	1																							1	1
3061	JQ704677	H57	H57	1																							1	1
3062	JQ703264	H58	H	1	H26c	2	H26c	3	H	5	H	5	H58	3													6	19
3063	JQ702375	H58a	H61a	1	H	4	H	3	H+152	1	H+152	3	H+152	5	H	5	H58a	1									8	23
3064	JQ701910	H59	H29b	1	H	5	H59	4																			3	10
3065	JQ704027	H59a	R0	1	H	5	H	5	H	5	H	5	H	5	H	5	H59	5	H59a	1							9	37
3066	JQ705583	H60a	HV	1	H96	3	H96	4	H60a	5																	4	13
3067	JQ702768	H61	H10f	1	H61	2																					2	3
3068	JQ703804	H61a	H	1	H61a	2																					2	3
3069	EF609014	H62	H3s	1	H62	6																					2	7
3070	JQ704491	H63	HV	1	H63	2																					2	3
3071	JQ704853	H63a	H63a	1																							1	1
3072	JQ703909	H65	H	1	R	2	H	5	H65	7																	4	15
3073	GU592025	H65a	R0	1	H	5	H	5	H	5	H	5	H	5	H	5	H	5	H65	5	H65a	1					10	42
3074	KC911282	H66	H3k	0																							0	0
3075	JQ705407	H66a	H66a	1																							1	1
3076	JQ703455	H66a1	H66a1	1																							1	1
3077	JQ704151	H67	H10+(16093)	1	H10+(16093)	1	R0	5	R0	2	R0	5	H67	3													6	17
3078	JQ702448	H67a	R0	1	H	5	H	5	H	5	H	5	H	5	H	5	H	5	H	5	H67	5	H67a	1			11	47
3079	AY738998	H70	H13a1a1e	1	H70	7																					2	8
3080	JQ704813	H71	H16	1	H	2	H71	1																			3	4
3081	JQ704835	H72	R0	1	H	5	H	5	H	5	H	5	H	5	H	5	H	5	H	5	H72	5					10	46
3082	JQ702419	H73	H7h	1	H7h	1	H73	1																			3	3
3083	JN202547	H73a	R0	1	H	5	H	5	H	5	H	5	H	5	H	5	H	5	H	5	H	5	H73	5	H73a	1	12	52
3084	HM625697	H73a1	H7d3 H73a1 (2)	1	H73a1	6																					2	7
3085	JQ324578	H74	H74	1																							1	1
3086	JQ703482	H75	H	1	H	5	H+152	2	H+152	3	H75	5															5	16
3087	JQ705450	H76	HV+16311	1	H76	4																					2	5
3088	JQ704521	H76a	H76a	1																							1	1
3089	JQ701996	H77	H	1	H77	2																					2	3
3090	EU600333	H78	H1bz	1	H26c	2	H	3	H78	5																	4	11
3091	JQ702218	H79	R0	1	H	5	H	5	H	5	H	5	H	5	H	5	H	5	H	5	H	5	H79	5	H79	1	12	52
3092	JQ705299	H79a	R0	1	H	5	H	5	H	5	H	5	H	5	H	5	H	5	H	5	H	5	H79	5	H79a	1	12	52
3093	EF493867	H80	H80	1																							1	1
3094	JQ324727	H81	R0	1	H	5	H	5	H	5	H	5	H	5	H	5	H	5	H	5	H	5	H81	5	H81	1	12	52

3095	JQ704831	H81a	R0	1	H	5	H	5	H	5	H	5	H	5	H	5	H	5	H	5	H	5	H81	5	H81a	1							12	52
3096	JQ702080	H82	H82	1																													1	1
3097	JQ705403	H83	H83	1																													1	1
3098	JX153022	H86	R0	1	H	5	H	5	H	5	H	5	H	5	H	5	H	5	H	5	H	5	H	5	H86	5							12	56
3099	JQ705040	H87	HV	1	HV	2	HV	3	H87	5																							4	11
3100	KC553984	H88	R0	1	H	5	H	5	H	5	H	5	H	5	H	5	H	5	H	5	H	5	H	5	H88	5							12	56
3101	KC553989	H89	R0	1	H	5	H	5	H	5	H	5	H	5	H	5	H	5	H	5	H	5	H	5	H89	5							12	56
3102	KC554018	H90	R0	1	H	5	H	5	H	5	H	5	H	5	H	5	H	5	H	5	H	5	H	5	H	5	H90	5					13	61
3103	NA20527	H92	H3x	1	H	3	H92	1																									3	5
3104	NA20502	H93	H	1	H93	1																											2	2
3105	NA19750	H94	H	1	H94	2																											2	3
3106	HQ593807	H95	H	1	H95	6																											2	7
3107	JQ703248	H95a	H7c	1	R0	5	H95a	3																									3	9
3108	KC810015	H96	H96	1																													1	1
3109	HQ659700	H100	H	1	R0	2	R0	1	H	3	H100	9																					5	16
3110	KC911463	H101	H101	1																													1	1
3111	JQ703658	H102	H13a2b5	1	H	2	H102	4																									3	7
3112	JQ324570	H104	R0	1	H	5	H	5	H	5	H	5	H	5	H	5	H	5	H	5	H	5	H	5	H	5	H	5	H104	3	H104	1	15	65
3113	JX153176	H104a	H104a	1																													1	1
3114	JX153454	H105	H105	1																													1	1
3115	JQ324820	H105a	R0	1	H	5	H	5	H	5	H	5	H	5	H	5	H	5	H	5	H	5	H	5	H	5	H	5	H105	3	H105a	1	15	65
3116	JX153097	H106	H106	1																													1	1
3117	KC985147	R1a	R1a	1																													1	1
3118	KC985159	R1a1	R1a	1	R1a1	1	R1a1	3																									3	5
3119	AY714045	R1a1a	R1a1a	1																													1	1
3120	EU545437	R1a1a1	R1a1a1	1																													1	1
3121	EF222244	R1a1a1a	R1a1a1a	1																													1	1
3122	GU451313	R1a1a2	R1a	1	R1a1	1	R1a1a	3	R1a1a2	2																							4	7
3123	HM030522	R1a1b	R1a1b	1																													1	1
3124	KC985148	R1a1c	R1a1c	1																													1	1
3125	KC985165	R1b	R1b	1																													1	1
3126	JQ703633	R1b1	R1b1	1																													1	1
3127	KC911379	R2	R2	1																													1	1
3128	KC911374	R2+13500	R2	1	R2+13500	1	R2+13500	2																									3	4
3129	AY714007	R2+13500+195	R2+13500+195	1																													1	1
3130	JX155268	R2a	R2a	1																													1	1
3131	JX152991	R2b	R2b	1																													1	1
3132	EF556167	R2b1	R2b1	1																													1	1
3133	JX155271	R2c	R2c	1																													1	1
3134	KC911337	R2d	R2	1	R2+13500	1	R2d	2																									3	4
3135	KC911521	J1b	J1b	1																													1	1
3136	AY714035	J1b1a1	J1b1a1	1	J1b1a1	1																											2	2
3137	FJ213450	J1b1a1a	J1b1a1a	1																													1	1
3138	AY495233	J1b1a1b	J1b1a1	1	J1b1a1b	1																											2	2
3139	AY495231	J1b1a1c	J1b1a1c	1																													1	1
3140	JX153487	J1b1a1d	J1b1a1d	1																													1	1
3141	JQ797761	J1b1a1+146	J1b1a1+146	1	J1b1a1+146	1																											2	2
3142	HM856621	J1b1a1e	J1b1a1+146	1	J1b1a1e	1																											2	2
3143	HM852779	J1b1a2a	J1b1a2a	1																													1	1
3144	JQ797763	J1b1a2b	J1b1a2b	1																													1	1
3145	EF660916	J1b1a3	J1b1a3	1																													1	1
3146	KC911496	J1b1b1	J1b1b1	1																													1	1
3147	JQ797766	J1b1b1a	J1b1b1a	1																													1	1
3148	JQ797769	J1b1b1b	J1b1b1b	1																													1	1
3149	KC911590	J1b1b1c	J1b1b1c	1																													1	1
3150	JQ703656	J1b1b2	J1b1b2	1																													1	1
3151	AY714033	J1b1b3	J1b1b3	1																													1	1

3152	EF556169	J1b2	J1b	1	J1b2	5					2	6
3153	JQ702001	J1b2a	J1b2a	1							1	1
3154	KC911407	J1b3	J1b3b	1	J1b3	4					2	5
3155	HM594676	J1b3a	J1b3a	1							1	1
3156	JQ797775	J1b3b	J1b3b	1							1	1
3157	KC911307	J1b3b1	J1b3b1	1							1	1
3158	JQ797776	J1b4	J1b4	1							1	1
3159	JN561091	J1b4a1	J1b4a	1	J1b4a1	2					2	3
3160	HM992836	J1b4a2	J1b4a2	1							1	1
3161	JX153311	J1b5	J1b5	1							1	1
3162	KC911544	J1b5a	J1b5a	1	J1b5a	1					2	2
3163	JQ797778	J1b5a1	J1b5a1	1							1	1
3164	JQ797782	J1b6	J1b	1	J1b6	5	J1b6	1			3	7
3165	JQ797781	J1b6a	J1c3c1	1	J1b6a	3					2	4
3166	KC911401	J1b6b	J1b6b	1							1	1
3167	KC911359	J1b7	J1b7	1							1	1
3168	JQ797784	J1b7a	J1b7a	1							1	1
3169	JQ064573	J1b8	J1b8	1							1	1
3170	JQ797779	J1b9	J1b9	1							1	1
3171	JQ797870	J1c	J1c8a	1	J1c8a	1	J1c	1			3	3
3172	JX153672	J1c1	J1c1	1	J1c1	4					2	5
3173	AY495210	J1c1a	J1c1	1	J1c1a	4					2	5
3174	JQ703783	J1c1b	J1c1	1	J1c1b	4	J1c1b	2			3	7
3175	EF452293	J1c1b1	J1c1b1	1							1	1
3176	FJ502349	J1c1b1a	J1c1b1a	1							1	1
3177	JQ705164	J1c1b1a1	J1c1b1a1	1							1	1
3178	JQ705141	J1c1b2	J1c1	1	J1c1b	4	J1c1b2	2	J1c1b2	1	4	8
3179	JQ702981	J1c1b2a	J1c1	1	J1c1b	4	J1c1b2	2	J1c1b2a	1	4	8
3180	JQ704867	J1c1c	J1c1c	1							1	1
3181	JX154000	J1c1d	J1c1d	1							1	1
3182	AY495208	J1c1e	J1c1e	1							1	1
3183	JQ705770	J1c1f	J1c1f	1							1	1
3184	JN635301	J1c1g	J1c1c	1	J1c1g	3					2	4
3185	JQ797793	J1c1g1	J1c1g1	1							1	1
3186	NA20787	J1c1h	J1c1h	1							1	1
3187	AY495218	J1c2	J1c2	1	J1c2	3	J1c2	5	J1c2	5	4	14
3188	AY495227	J1c2a1	J1c2	1	J1c2a	3	J1c2a1	2	J1c2a1	1	4	7
3189	JX153394	J1c2a1a	J1c2a1a	1							1	1
3190	JX153382	J1c2a2	J1c2a2	1							1	1
3191	JQ702742	J1c2a3	J1c2	1	J1c2a	3	J1c2a3	2			3	6
3192	JQ703778	J1c2b	J1c2b	1							1	1
3193	JQ704710	J1c2b1	J1c2b1	1							1	1
3194	JX153336	J1c2b2	J1c2	1	J1c2b	3	J1c2b2	3			3	7
3195	JQ702706	J1c2b3	J1c2b3	1							1	1
3196	JQ703567	J1c2b4	J1c2b4	1							1	1
3197	JX153355	J1c2b5	J1c2b5	1							1	1
3198	GU949564	J1c2c	J1c2c	1							1	1
3199	FJ449571	J1c2c1	J1c2c1	1							1	1
3200	JQ705961	J1c2c1a	J1c2c1a	1							1	1
3201	JQ702741	J1c2c2	J1c2c	1	J1c2c2	2	J1c2c2	1			3	4
3202	GU592019	J1c2c2a	J1c2c	1	J1c2c2	2	J1c2c2a	1			3	4
3203	FJ499472	J1c2d	J1c2d	1							1	1
3204	JQ797812	J1c2e	J1c2e	1							1	1
3205	GU123042	J1c2e1	J1c2e1	1							1	1
3206	JX297168	J1c2e2	J1c2e2	1							1	1
3207	JQ704432	J1c2f	J1c2	1	J1c2	3	J1c2f	5			3	9
3208	HQ260985	J1c2g	J1c2a2	1	J1c2g	2					2	3

3209	JQ701851	J1c2h	J1c2h	1							1	1
3210	JQ797821	J1c2i	J1c2i	1							1	1
3211	JQ797822	J1c2j	J1c2j	1							1	1
3212	JQ797804	J1c2k	J1c2	1	J1c2	3	J1c2k	5			3	9
3213	AY495223	J1c2l	J1c2	1	J1c2	3	J1c2l	5			3	9
3214	JQ797801	J1c2m	J1c2m	1							1	1
3215	JQ702724	J1c2m1	J1c2m1	1							1	1
3216	HG00377	J1c2n	J1c2	1	J1c2	3	J1c2	5	J1c2n	5	4	14
3217	JX154069	J1c2n1	J1c2a1a	1	J1c2n1	4					2	5
3218	JX152877	J1c2o	J1c2o	1							1	1
3219	JQ797799	J1c2p	J1c2c	1	J1c2c	2	J1c2p	3			3	6
3220	HG00258	J1c2q	J1c2c2a	1	J1c2q	2					2	3
3221	JX153326	J1c2q1	J1c2b4	1	J1c2q1	2					2	3
3222	FJ190383	J1c2r	J1c2r	1							1	1
3223	JX153398	J1c2s	J1c2	1	J1c2	3	J1c2	5	J1c2s	5	4	14
3224	FJ499471	J1c2s1	J1c2s1	1							1	1
3225	HG00235	J1c2t	J1c2t	1							1	1
3226	JQ702691	J1c3	J1c	1	J1c3	5	J1c3	5	J1c3	2	4	13
3227	AY495211	J1c3a1	J1c	1	J1c3	5	J1c3a	5	J1c3a1	2	4	13
3228	JQ703792	J1c3a2	J1c	1	J1c3	5	J1c3a	5	J1c3a2	2	4	13
3229	JQ048704	J1c3b	J1c	1	J1c3	5	J1c3b	5	J1c3b	2	4	13
3230	JQ705447	J1c3b1	J1c	1	J1c3	5	J1c3b	5	J1c3b1	2	4	13
3231	JQ705472	J1c3b1a	J1c3b1a	1							1	1
3232	HG00103	J1c3b2	J1c3e	1	J1c3	1	J1c3	3	J1c3b2	1	4	6
3233	NA12044	J1c3c	J1c3c	1							1	1
3234	HQ709168	J1c3c1	J1c3c	1	J1c3c1	2					2	3
3235	JQ703602	J1c3c2	J1c3c	1	J1c3c2	2					2	3
3236	AY495201	J1c3d	J1c	1	J1c3	5	J1c3d	5			3	11
3237	JQ703895	J1c3e1	J1c3e1	1							1	1
3238	NA20785	J1c3e2	J1c3e2	1							1	1
3239	JQ797828	J1c3f	J1c3f	1							1	1
3240	JF703252	J1c3g	J1c4	1	J1c3g	5					2	6
3241	DQ523659	J1c3h	J1c	1	J1c3	5	J1c3h	5			3	11
3242	JQ703753	J1c3i	J1c	1	J1c3	5	J1c3	5	J1c3i	2	4	13
3243	JQ701961	J1c3j	J1c3j	1							1	1
3244	JQ797826	J1c3k	J1c	1	J1c3	5	J1c3	5	J1c3k	2	4	13
3245	HG00334	J1c3+189	J1c3+189	1							1	1
3246	JQ797836	J1c3m	J1c3m	1							1	1
3247	FJ538285	J1c4	J1c4	1							1	1
3248	JQ703916	J1c4b	J1c	1	J1c4	5	J1c4b	1			3	7
3249	JQ705439	J1c4c	J1c4c	1							1	1
3250	HG00120	J1c5	J1c	1	J1c5	5	J1c5	5	J1c5	1	4	12
3251	JQ703803	J1c5a	J1c5a	1							1	1
3252	HQ287874	J1c5a1	J1c	1	J1c5	5	J1c5a	5	J1c5a1	1	4	12
3253	JQ703639	J1c5b	J1c	1	J1c5b	5					2	6
3254	JQ704816	J1c5c	J1c	1	J1c5	5	J1c5c	5			3	11
3255	JX297129	J1c5c1	J1c5c1	1							1	1
3256	HQ287873	J1c5d	J1c3	1	J1c5d	2					2	3
3257	JQ797844	J1c5e	J1c5e	1							1	1
3258	JQ703919	J1c6	J1c5a	1	J1c6	2					2	3
3259	EU073970	J1c6a	J1c	1	J1c6	5	J1c6a	1			3	7
3260	FJ348153	J1c+16261	J1c+16261	1							1	1
3261	JQ797855	J1c7	J1c7	1	J1c7	1					2	2
3262	JQ797859	J1c7a	J1c7a	1							1	1
3263	JN415478	J1c+16261+189	J1c+16261+189	1	J1c+16261+189	1					2	2
3264	EU284668	J1c12	J1c+16261+189	1	J1c12	1	J1c12	2			3	4

3265	JQ703516	J1c12a	J1c+16261+189	1	J1c12	1	J1c12a	2					3	4
3266	JQ702088	J1c12b	J1c+16261+189	1	J1c12	1	J1c12b	2					3	4
3267	JQ703584	J1c13	J1c13	1									1	1
3268	JQ705811	J1c14	J1c14	1									1	1
3269	JQ797868	J1c8a	J1c8a	1									1	1
3270	JQ702049	J1c8a1	J1c8a1	1									1	1
3271	JQ705760	J1c8a1a	J1c8a1a	1									1	1
3272	GU906781	J1c8a2	J1c8a2	1									1	1
3273	JQ705449	J1c8b	J1c	1	J1c	5	J1c	5	J1c8	1	J1c8b	1	5	13
3274	JQ797871	J1c9	J1c9	1									1	1
3275	JQ704751	J1c10	J1c10	1									1	1
3276	JQ797873	J1c10a	J1c8a	1	J1c10a	3							2	4
3277	JQ702967	J1c11	J1c11	1	J1c11	1							2	2
3278	HM765470	J1c11a	J1c11	1	J1c11a	1							2	2
3279	JQ797881	J1c15	J1c15	1									1	1
3280	JQ797879	J1c15a	J1c7	1	J1c15a	5							2	6
3281	JQ797880	J1c15a1	J1c5b	1	J1c15a1	3							2	4
3282	JQ797877	J1c15b	J1c15b	1									1	1
3283	JQ705083	J1c16	J1c16	1									1	1
3284	JQ797882	J1c17	J1c17	1									1	1
3285	JQ706022	J1c17a	J1c17a	1									1	1
3286	JQ797885	J1+16193	J1+16193	1									1	1
3287	JQ797902	J1d	J1d	1									1	1
3288	JQ797886	J1d1a	J1d1a1	1	J1d1a	1							2	2
3289	JQ705319	J1d1a1	J1d1a1	1									1	1
3290	JQ797890	J1d1a1a	J1d1a1a	1									1	1
3291	AF382001	J1d1b	J1d1b	1									1	1
3292	HM852780	J1d1b1	J1d1b1	1									1	1
3293	DQ341088	J1d2	J1d2	1									1	1
3294	JQ797893	J1d2a	J1d2a	1									1	1
3295	KC911411	J1d3	J1+16193	1	J1d3	2							2	3
3296	JQ797899	J1d3a	J1d3a	1									1	1
3297	JQ797897	J1d3a1	J1d3a1	1									1	1
3298	JQ704809	J1d3a2	J1d3a2	1									1	1
3299	HM453206	J1d4	J1d	1	J1d4	2							2	3
3300	JQ703915	J1d5	J1d5	1									1	1
3301	KC911498	J1d5a	J1d5a	1									1	1
3302	JQ797894	J1d6	J1d	1	J1d6	1							2	2
3303	JQ797895	J1d6a	J1d6a	1									1	1
3304	JQ704041	J2a1	J2a	1	J2a1	1							2	2
3305	JQ701807	J2a1a1	J2a1a1	1									1	1
3306	GU903270	J2a1a1a	J2a1a1a	1	J2a1a1a	1							2	2
3307	JX152974	J2a1a1a1	J2a1a1a1	1									1	1
3308	JQ705625	J2a1a1a2	J2a1a1a	1	J2a1a1a2	1							2	2
3309	JX152842	J2a1a1a2a	J2a1a1a	1	J2a1a1a2	1	J2a1a1a2a	1					3	3
3310	JQ703568	J2a1a1a3	J2a1a1a3	1									1	1
3311	JQ705042	J2a1a1b	J2a1a1b	1									1	1
3312	JQ797904	J2a1a1c	J2a1a1c	1									1	1
3313	JQ797912	J2a1a1d	J2a1a1d	1									1	1
3314	JX297144	J2a1a1e	J2a1a1e	1									1	1
3315	DQ341089	J2a1a2	J2a1a2	1									1	1
3316	JQ797914	J2a1a2a	J2a1a2a	1									1	1
3317	JQ764985	J2a1a2a1	J2a1a2	1	J2a1a2a	1	J2a1a2a1	1					3	3
3318	JQ702364	J2a1a2a1a	J2a1a2	1	J2a1a2a	1	J2a1a2a1	1	J2a1a2a1a	1			4	4
3319	EF660967	J2a2a	J2a2a	1									1	1
3320	JQ797915	J2a2a1	J2a2a1	1									1	1
3321	JQ797921	J2a2a1+16311	J2a2a1+16311	1	J2a2a1+1631	1							2	2

3322	JQ797920	J2a2a1a	J2a2a1a1	1	J2a2a1a	1					2	2
3323	JQ797919	J2a2a1a1	J2a2a1a1	1							1	1
3324	JQ797923	J2a2a2	J2a2a2	1							1	1
3325	FJ460543	J2a2b1	J2a2b1	1							1	1
3326	JQ797926	J2a2b1a	J2a2b1a	1							1	1
3327	NA20752	J2a2b2	J2a2b2	1							1	1
3328	KF148204	J2a2b3	J2a2b	1	J2a2b3	1					2	2
3329	JQ797930	J2a2c	J2a2c	1							1	1
3330	JQ797933	J2a2c1	J2a2c1	1							1	1
3331	FJ460559	J2a2d	J2a2d	1							1	1
3332	JX153004	J2a2e	J2a2e	1							1	1
3333	JQ702563	J2b1	J2b	1	J2b1	2	J2b1	6			3	9
3334	JF915700	J2b1a	J2b1a	1							1	1
3335	JQ703580	J2b1a1	J2b1a	1	J2b1a1	4					2	5
3336	FJ445408	J2b1a1a	J2b1a1a	1							1	1
3337	JQ705677	J2b1a2	J2b1a2	1							1	1
3338	JQ797936	J2b1a2a	J2b1a2a	1							1	1
3339	AY195778	J2b1a3	J2b1a3	1							1	1
3340	JQ705466	J2b1a4	J2b1a	1	J2b1a4	4					2	5
3341	JX153921	J2b1a+16311	J2b1a+16311	1	J2b1a+16311	1					2	2
3342	JQ797946	J2b1a5	J2b1a+16311	1	J2b1a5	1					2	2
3343	JQ797944	J2b1a6	J2b1a6	1							1	1
3344	JQ703784	J2b1b	J2b	1	J2b1	2	J2b1b	6	J2b1b	1	4	10
3345	JX153450	J2b1b1	J2b1b1	1							1	1
3346	JQ797951	J2b1c	J2b1c	1							1	1
3347	EU807741	J2b1c1	J2b1c1	1							1	1
3348	FJ445409	J2b1d	J2b1d	1							1	1
3349	JQ701981	J2b1e	J2b1	1	J2b1	2	J2b1e	1			3	4
3350	JQ797964	J2b1e1	J2b1e1	1							1	1
3351	HQ727682	J2b1f	J2b1f	1							1	1
3352	JQ702488	J2b1g	J2b1g	1							1	1
3353	JQ702424	J2b1h	J2b1h	1							1	1
3354	FJ213765	J2b2	J2b2	1							1	1
3355	JQ797976	T1	T1	1							1	1
3356	JQ798024	T1a	T1a	1							1	1
3357	JQ797984	T1a1	T1a1	1							1	1
3358	JQ705982	T1a1a	T1a1'3	1	T1a1	2	T1a1a	11	T1a1a	1	4	15
3359	JQ703032	T1a1a1	T1a1'3	1	T1a1	2	T1a1a	11	T1a1a1	1	4	15
3360	JQ797991	T1a1b	T1a4	1	T1a1b	2					2	3
3361	AY714036	T1a1b1	T1a1b1	1							1	1
3362	JQ702923	T1a1c	T1a1c	1							1	1
3363	EU007876	T1a1d	T1a1d	1							1	1
3364	JX152843	T1a1e	T1a1e	1							1	1
3365	JQ702643	T1a1f	T1a1'3	1	T1a1	2	T1a1f	11			3	14
3366	JQ704732	T1a1g	T1a1b	1	T1a1g	2					2	3
3367	JN089342	T1a1h	T1a1h	1							1	1
3368	JN021256	T1a1i	T1a1+@152	1	T1a1+@152	1	T1a1i	2			3	4
3369	JQ703446	T1a1j	T1a1j	1							1	1
3370	JQ702728	T1a1k	T1a1k	1	T1a1k	1					2	2
3371	JQ702959	T1a1k1	T1a1k1	1							1	1
3372	JX152851	T1a1k2	T1a1k	1	T1a1k2	1					2	2
3373	JF929200	T1a1+@152	T1a1+@152	1	T1a1+@152	1					2	2
3374	JF979131	T1a1l	T1a1+@152	1	T1a1l	1					2	2
3375	KC911350	T1a1m	T1a1m	1	T1a1m	2					2	3
3376	JQ797998	T1a1m1	T1a1m1	1							1	1
3377	EF177406	T1a1n	T1a1n	1							1	1
3378	EU597578	T1a1p	T1a1'3	1	T1a1	2	T1a1p	11			3	14

3379	JX153590	T1a1q	T1a1'3	1	T1a1	2	T1a1q	11					3	14
3380	JX153003	T1a1r	T1a1'3	1	T1a1	2	T1a1r	11					3	14
3381	JQ798022	T1a3	T1a+152	0									0	0
3382	EF645646	T1a3a	T1a1b1	1	T1a3a	2							2	3
3383	JQ798012	T1a2	T1a2	1									1	1
3384	NA20581	T1a2a	T1a2a	1									1	1
3385	JQ798013	T1a2b	T1a2b	1									1	1
3386	EU979542	T1a4	T1a4	1									1	1
3387	JQ031816	T1a11	T1a11	1									1	1
3388	JQ798020	T1a12	T1a12	1									1	1
3389	JQ798007	T1a13	T1a13	1									1	1
3390	JQ703724	T1a5	T1a6	1	T1a5	2							2	3
3391	JX153408	T1a5a	T1a5a	1									1	1
3392	JN104727	T1a6	T1a6	1									1	1
3393	EU935435	T1a7	T1a7	1									1	1
3394	JQ798034	T1a8a	T1a8a	1									1	1
3395	JQ798036	T1a8b	T1a8b	1									1	1
3396	JQ798018	T1a9	T1a9	1									1	1
3397	NA20819	T1a10	T1a10	1									1	1
3398	JX152859	T1a10a	T1a10a	1									1	1
3399	JQ798051	T1b	T1b	1									1	1
3400	AY339570	T1b1	T1b1	1									1	1
3401	JQ798041	T1b2	T1b2	1									1	1
3402	JQ705372	T1b3	T1b3	1									1	1
3403	JQ798050	T1b4	T1b4	1									1	1
3404	JF707633	T2	T2	1									1	1
3405	JF927949	T2a1	T2a1	1									1	1
3406	FJ656215	T2a1a	T2a1a	1									1	1
3407	FJ348180	T2a1a1	T2a1a1	1									1	1
3408	JN120787	T2a1a2	T2	1	T2a	2	T2a1	1	T2a1a	1	T2a1a2	5	5	10
3409	JX297193	T2a1a3	T2a1a3	1									1	1
3410	JF833041	T2a1a3a	T2	1	T2	2	T2a1a3a	5					3	8
3411	JQ705499	T2a1a5	T2	1	T2a	2	T2a1	1	T2a1a	1	T2a1a5	5	5	10
3412	JQ704797	T2a1a6	T2a1a6	1									1	1
3413	JQ045864	T2a1a7	T2a1a7	1									1	1
3414	JQ701972	T2a1a8	T2a1a8	1									1	1
3415	JQ798054	T2a1b	T2a1b	1									1	1
3416	HM625705	T2a1b1	T2a1b1	1									1	1
3417	JQ705641	T2a1b1a	T2a1b1a	1									1	1
3418	JQ702937	T2a1b1a1	T2a1b	1	T2a1b1	2	T2a1b1a	1	T2a1b1a1	1			4	5
3419	JQ703868	T2a1b1a1a1	T2a1b1a1a1	1									1	1
3420	JQ798055	T2a1b1a1a2	T2a1b1a1a2	1									1	1
3421	JX153319	T2a1b1a1b	T2a1b1a1b	1									1	1
3422	JX153948	T2a1b1a1b1	T2a1b1	1	T2a1b1	2	T2a1b1a1b1	1					3	4
			T2a1b1a1b1 (2)											
3423	JQ703776	T2a1b1a2	T2a1b1a2	1									1	1
3424	JQ704821	T2a1b2a	T2a1b2a	1									1	1
3425	JQ798057	T2a1b2b	T2a1b2b	1									1	1
3426	FJ238094	T2a2	T2a2	1									1	1
3427	JX152815	T2a2a	T2a2a	1									1	1
3428	KC911302	T2a3	T2a3	1									1	1
3429	HM055613	T2b	T2b	1									1	1
3430	EF177444	T2b1	T2b	1	T2b1	5							2	6
3431	AY714016	T2b2	T2b	1	T2b2	5							2	6
3432	AY495299	T2b2b	T2	1	T2	2	T2b2b	5					3	8
3433	JQ701832	T2b2b1	T2b2b1	1									1	1
3434	HM122274	T2b3	T2a+195	1	T2b	2	T2b3	7					3	10

3435	HQ384203	T2b3+151	T2b3+151	1	T2b3+151	1					2	2
3436	DQ523649	T2b3a	T2b3a	1							1	1
3437	JQ798060	T2b3a1	T2b3a1	1							1	1
3438	JQ702932	T2b3c	T2b3c	1							1	1
3439	JQ705037	T2b3d	T2b3d	1							1	1
3440	JQ705199	T2b3e	T2b3e	1							1	1
3441	JX153347	T2b3b	T2	1	T2	2	T2	5	T2b3b	3	4	11
3442	JN037468	T2b4	T2b2b1	1	T2b4	3					2	4
3443	JF836084	T2b4a	T2b4a	1							1	1
3444	JQ704738	T2b4a1	T2b4a	1	T2b4a1	1					2	2
3445	JQ702278	T2b4i	T2b4i	1							1	1
3446	JQ705242	T2b4+152	T2b	1	T2b4+152	1	T2b4+152	5			3	7
3447	JQ705939	T2b4b	T2b4b	1							1	1
3448	JQ704742	T2b4c	T2b	1	T2b4+152	1	T2b4c	5			3	7
3449	JQ702158	T2b4d	T2b	1	T2b4+152	1	T2b4d	5			3	7
3450	HM852802	T2b4e	T2b4+152	1	T2b4+152	1	T2b4e	2			3	4
3451	JQ704596	T2b4f	T2b4f	1							1	1
3452	JQ798068	T2b4g	T2b4g	1							1	1
3453	JQ798066	T2b4h	T2b4h	1							1	1
3454	AY495287	T2b5	T2b5	1							1	1
3455	JQ703716	T2b5a	T2b	1	T2b5	5	T2b5a	1			3	7
3456	JQ703712	T2b5a1	T2b5a1	1							1	1
3457	AY495284	T2b6	T2b6	1	T2b6	1					2	2
3458	JQ705494	T2b6a	T2b6	1	T2b6a	1					2	2
3459	JQ705357	T2b6+146	T2b6+146	1							1	1
3460	JF891418	T2b6b	T2b6b	1							1	1
3461	GU170817	T2b7	T2b	1	T2b	5	T2b7	5			3	11
3462	JQ703294	T2b7a1	T2b7a1	1							1	1
3463	JQ704997	T2b7a2	T2b7a2	1							1	1
3464	NA20507	T2b7a3	T2b7a3	1							1	1
3465	GU122998	T2b8	T2b	0							0	0
3466	JQ702965	T2b+150	T2b+150	1	T2b+150	1					2	2
3467	JQ703011	T2b9	T2b+150	1	T2b9	1					2	2
3468	JF968593	T2b11	T2b11	1							1	1
3469	JQ702963	T2b13	T2a+195	1	T2a+195	2	T2b13	9			3	12
3470	JN038393	T2b13a	T2b13a	1							1	1
3471	JQ705492	T2b13b	T2b+16362	1	T2b+16362	1	T2b+16362	1	T2b13b	2	4	5
3472	JF975728	T2b15	T2b	1	T2b	5	T2b15	5			3	11
3473	JN004272	T2b+16362	T2b+16362	1	T2b+16362	1					2	2
3474	JQ798077	T2b16	T2b+16362	1	T2b16	1					2	2
3475	JF957699	T2b17	T2b	1	T2b	5	T2b17	5			3	11
3476	JX153685	T2b17a	T2b	1	T2b+152	1	T2b17a	4			3	6
3477	JQ705664	T2b19	T2b	1	T2b	5	T2b	5	T2b19	5	4	16
3478	HG00141	T2b19b	T2b19b	1							1	1
3479	EU747356	T2b+152	T2b	1	T2b4+152	1	T2b+152	2			3	4
3480	HQ917079	T2b21	T2b4h	1	T2b21	2					2	3
3481	JQ705895	T2b21a	T2b21a	1							1	1
3482	JQ705338	T2b21b	T2b21b	1							1	1
3483	HQ840646	T2b22	T2b	1	T2b+152	1	T2b22	2			3	4
3484	JQ798075	T2b23	T2b23	1							1	1
3485	FJ348208	T2b23a	T2b23a	1							1	1
3486	JQ705038	T2b24	T2b24	1							1	1
3487	JF946696	T2b24a	T2b24	1	T2b24a	1					2	2
3488	JQ704697	T2b25	T2b25	1							1	1
3489	JQ702312	T2b26	T2b	1	T2b26	1					2	2
3490	JQ705444	T2b27	T2b25	1	T2b27	2					2	3
3491	JQ704973	T2b28	T2b28	1							1	1

3492	JQ798081	T2b29	T2b	1	T2b	5	T2b	5	T2b	5	T2b29	5	5	21
3493	JN024625	T2b30	T2b	1	T2b	5	T2b	5	T2b	5	T2b30	5	5	21
3494	JQ798089	T2b31	T2b31	1									1	1
3495	KC521456	T2b32	T2b32	1									1	1
3496	KC533521	T2b33	T2b33	1									1	1
3497	EU926622	T2b34	T2b34	1									1	1
3498	JX153427	T2b35	T2b	1	T2b	5	T2b	5	T2b	5	T2b35	5	5	21
3499	JX153237	T2b36	T2b36	1									1	1
3500	JX153053	T2b37	T2a+195	1	T2b	2	T2b37	7					3	10
3501	JQ798090	T2c	T2g1a1	1	T2c	2							2	3
3502	JQ796696	T2c1a	T2i	1	T2c1a	5							2	6
3503	JQ703030	T2c1a1	T2c1a1	1									1	1
3504	EF660941	T2c1a2	T2c1	1	T2c1a2	2							2	3
3505	JN202494	T2c1a3	T2c1a	1	T2c1a3	2							2	3
3506	JQ798103	T2c1c	T2c1c	1									1	1
3507	JQ798099	T2c1c1	T2c1c1	1									1	1
3508	JQ702795	T2c1c2	T2c1c2	1									1	1
3509	JQ798091	T2c1+146	T2c1+146	1									1	1
3510	JQ704020	T2c1d1	T2c1d1	1									1	1
3511	JX153536	T2c1d1a	T2c1d1a	1									1	1
3512	JQ798094	T2c1d+152	T2c1d+152	1	T2c1d+152	1							2	2
3513	JQ798096	T2c1d2	T2c1d+152	1	T2c1d2	1	T2c1d2	1					3	3
3514	JQ798095	T2c1d2a	T2c1d2a	1									1	1
3515	JQ705571	T2c1e	T2c1e	1									1	1
3516	GU048747	T2c1f	T2c1f	1									1	1
3517	AY714037	T2d1a	T2d1a	1									1	1
3518	JQ798106	T2d1b1	T2d1b1	1									1	1
3519	KC911414	T2d1b2	T2d1	1	T2d1	2	T2d1b2	1					3	4
3520	JQ798108	T2d2	T2d2 T2h	1	T2d2	2							2	3
3521	JQ703777	T2e	T2e	1									1	1
3522	JQ798113	T2e1	T2e1	1									1	1
3523	JQ705465	T2e1a	T2e1a	1									1	1
3524	KF577587	T2e1a1a	T2e1a1a	1									1	1
3525	AF381985	T2e1a1b	T2e1a1b	1									1	1
3526	JN030346	T2e1a1b1	T2e1a1b1	1									1	1
3527	KF048033	T2e1b	T2e1	1	T2e1b	2	T2e1b	1					3	4
3528	KF577586	T2e1b1	T2e1b1	1									1	1
3529	AY714029	T2e2	T2e2	1									1	1
3530	EF060363	T2e2a	T2e2a	1									1	1
3531	JQ702891	T2e5	T2e5	1									1	1
3532	JQ798118	T2e6	T2e6	1									1	1
3533	JQ702210	T2e+152	T2e+152	1									1	1
3534	JX153029	T2e7	T2e7	1									1	1
3535	KC911558	T2m	T2m	1									1	1
3536	HM852766	T2+16189	T2f7	1	T2+16189	2							2	3
3537	JQ704129	T2f	T2f	1									1	1
3538	JQ798120	T2f1	T2f1	1									1	1
3539	GU123028	T2f1a	T2f1a	1									1	1
3540	GU932663	T2f1a1	T2f1a	1	T2f1a1	1							2	2
3541	JQ798125	T2f2	T2f2	1									1	1
3542	JF960209	T2f3	T2f3	1									1	1
3543	JQ705528	T2f4	T2+16189	1	T2f	1	T2f4	4					3	6
3544	JQ702957	T2f5	T2f5	1									1	1
3545	JN084792	T2f6	T2+16189	1	T2f	1	T2f6	4					3	6
3546	JX153995	T2f7	T2f7	1									1	1
3547	JQ619780	T2f7a	T2f7a	1									1	1
3548	JX153048	T2f8	T2f8	1									1	1

3549	JX153682	T2f8a	T2f8a	1							1	1
3550	HE576983	T2g1a	T2g1a	1							1	1
3551	EF556186	T2g1a1	T2g1a1	1							1	1
3552	KC911398	T2g1b	T2g1b	1							1	1
3553	HM625693	T2g2	T2g2	1							1	1
3554	JQ702710	T2g2a	T2g2a	1							1	1
3555	JQ798128	T2h	T2h	1							1	1
3556	JQ705018	T2h1	T2k	1	T2h1	3					2	4
3557	JX153755	T2h2	T2h2	1							1	1
3558	JQ798129	T2i	T2i	1							1	1
3559	JQ704055	T2i1	T2i1	1							1	1
3560	JQ798131	T2i2	T2i2	1							1	1
3561	JQ704786	T2j	T2j	1							1	1
3562	EF177442	T2j1	T2j1	1							1	1
3563	JQ705690	T2k	T2k	1							1	1
3564	KC127675	T2l	T	1	T2	1	T2	2	T2l	5	4	9
3565	KC911608	T2n	T2	1	T2n	2					2	3
3566	JX462725	T3	T3	1							1	1
3567	AY713998	R5	R5	1							1	1
3568	FJ004837	R5a1	R5a1	1							1	1
3569	FJ004822	R5a1a	R5a1	1	R5a1a	1					2	2
3570	AY713993	R5a2	R5a2	1							1	1
3571	AY713985	R5a2a	R5a2a	1							1	1
3572	FJ004814	R5a2b	R5a2b	1							1	1
3573	AY714000	R5a2b1	R5a2b1	1							1	1
3574	AY714002	R5a2b2	R5a2b2	1							1	1
3575	FJ004812	R5a2b3	R5a2b3	1							1	1
3576	FJ004834	R5a2b4	R5a2b	1	R5a2b4	3					2	4
3577	AY714028	R6+16129	R6+16129	1							1	1
3578	FJ004819	R6a1	R6a1	1							1	1
3579	JQ704804	R6a2	R6a2	1							1	1
3580	AY713994	R6b	R6b	1							1	1
3581	AY714030	R7a	R7a	1							1	1
3582	FJ004805	R7a1	R7a1	1							1	1
3583	FJ004823	R7a1a	R7a1a	1							1	1
3584	FJ004831	R7a1b1	R7a1b1	1							1	1
3585	FJ004804	R7a1b2	R7a1b2	1							1	1
3586	FJ004817	R7b1	R7b	1	R7b1	2					2	3
3587	AY714024	R7b1a	R7b1a	1							1	1
3588	FJ004811	R7b1a1	R7b1a1	1							1	1
3589	GU170816	R7b2	R7b1a	1	R7b1a	1	R7b2	2			3	4
3590	FJ467953	R8a1a	R8a1a	1							1	1
3591	FJ467965	R8a1a1	R8a1a1	1							1	1
3592	GU810073	R8a1a1a1	R8a1a1a1	1							1	1
3593	KC911621	R8a1a1a1a	R8a1a1a1a	1							1	1
3594	FJ467943	R8a1a1a2	R8a1a1a2	1							1	1
3595	FJ467949	R8a1a1b	R8a1a1b	1							1	1
3596	FJ467942	R8a1a1c	R8a1a1c	1							1	1
3597	HM345949	R8a1a1d	R8a1a1d	1							1	1
3598	FJ467952	R8a1a2	R8a1a2	1	R8a1a2	1					2	2
3599	FJ467971	R8a1a2a	R8a1a2	1	R8a1a2a	1					2	2
3600	AY714011	R8a1a3	R8a1a3	1							1	1
3601	FJ467972	R8a1+16093	R8a1+16093	1							1	1
3602	FJ467968	R8a1b	R8a1+16093	1	R8a1b	1					2	2
3603	FJ467946	R8a2	R8a2	1							1	1
3604	FJ467941	R8b1	R8b1	1							1	1
3605	AY714009	R8b1a	R8b1a	1							1	1

3606	FJ467989	R8b2	R8b2	1							1	1
3607	KC505122	R9b	H1af1b	1	R9b	7					2	8
3608	KF849983	R9b1	R9b1	1							1	1
3609	GU733737	R9b1a1	R9b1a1	1							1	1
3610	DQ981466	R9b1a1a	R9b1a1a	1							1	1
3611	KF541026	R9b1a2	R9b1a2	1							1	1
3612	DQ981471	R9b1a2a	R9b1a2a	1							1	1
3613	JN580298	R9b1a2b	R9b1a2b	1							1	1
3614	JF824932	R9b1a3	R9b1a3	1							1	1
3615	DQ981470	R9b1b	R9b1b	1							1	1
3616	GU810062	R9b2	R9b2	1							1	1
3617	JN857032	R9c	U2b1a	1	U2b	2	U2b	1	R9c	6	4	10
3618	KC994068	R9c1a	R9c1a	1							1	1
3619	KF540867	R9c1a1	R9c1a2	1	R9c1a1	2					2	3
3620	KF541011	R9c1a2	R9c1a2	1							1	1
3621	JF739539	R9c1a3	R9c1a3	1							1	1
3622	JX289135	R9c1b1	R9c1b1	1							1	1
3623	KC994001	R9c1b2	R9c1b2	1							1	1
3624	NA17963	F1a	F1a	1							1	1
3625	AP008906	F1a1	F1a1	1							1	1
3626	HM357817	F1a1a	F1a1a	1							1	1
3627	AY963572	F1a1a1	F1a1a	1	F1a1a1	1					2	2
3628	AP008347	F1a1b	F1a1b	1							1	1
3629	HM357820	F1a1c	F1a1c	1							1	1
3630	GU810005	F1a1c1	F1a1c1	1							1	1
3631	NA19078	F1a1c2	F1a1c2	1							1	1
3632	NA18606	F1a1c3	F1a1c	1	F1a1c3	2					2	3
3633	KF849953	F1a1d	F1a1d	1							1	1
3634	HM238211	F1a1d1	F1a1d1	1							1	1
3635	NA18132	F1a4a	F1a4a	1							1	1
3636	KC994022	F1a4a1	F1a4a1	1							1	1
3637	JF824826	F1a4b	F1a4b	1							1	1
3638	FJ748725	F1a2	F1a2	1							1	1
3639	KF849920	F1a2a	F1a2a	1							1	1
3640	KF540917	F1a3+16311	F1a3a	1	F1a3+16311	1					2	2
3641	JF739536	F1a3a	F1a3+16311	1	F1a3a	1	F1a3a	1			3	3
3642	KF540654	F1a3a1	F1a3a1	1							1	1
3643	AP008701	F1a3a1a	F1a3a1a	1							1	1
3644	KC994062	F1a3a2	F1a3a2	1							1	1
3645	HM238205	F1a3a3	F1a3a3	1							1	1
3646	KF540619	F1a3a3a	F1a3a	1	F1a3a	1	F1a3a3a	1			3	3
3647	NA17977	F1a3b	F1a3b	1							1	1
3648	AP010738	F1c	F1c	1							1	1
3649	AP008616	F1c1	F1c1	1							1	1
3650	AY255177	F1c1a1	F1c1a1	1	F1c1a1	2					2	3
3651	KF056261	F1c1a1a	F1c1a1a	1							1	1
3652	KF849932	F1c1a1b	F1c1a1	1	F1c1a1b	2					2	3
3653	FJ748720	F1c1a2	F1c1a	1	F1c1a2	1					2	2
3654	AP012346	F1f	F1a	1	F1a	2	F1f	3			3	6
3655	AP008498	F1b1a	F1b1a	1	F1b1a	2					2	3
3656	AP008591	F1b1a1	F1b1a1	1							1	1
3657	AP008421	F1b1a1a	F1b1a	1	F1b1a1	2	F1b1a1a	1	F1b1a1a	3	4	7
3658	AP010731	F1b1a1a1	F1b1a1a1	1							1	1
3659	AP009474	F1b1a1a1a	F1b1a1a1a	1							1	1
3660	AP010754	F1b1a1a2	F1b1a	1	F1b1a1	2	F1b1a1a	1	F1b1a1a2	3	4	7
3661	AP013115	F1b1a1a3	F1b1a1a3	1							1	1
3662	AP010975	F1b1a2	F1b1a2	1							1	1

3663	DQ272125	F1b1b	F1b1b	1												1	1
3664	FJ198216	F1b1c	F1b1c	1												1	1
3665	HM036531	F1b1+@152	F1b1+@152	1	F1b1+@152	2										2	3
3666	AP013215	F1b1d	F1b1d	1												1	1
3667	HQ405768	F1b1e	F1b1a1a1a	1	F1b	1	F1b1+@152	1	F1b	2	F1b	2	F1b1e	1		6	8
3668	KF148437	F1b1e1	F1b1+@152	1	F1b1e	2	F1b1e1	1								3	4
3669	KF148222	F1b1f	F1b1+@152	1	F1b1f	1										2	2
3670	AP008808	F1d	F1d	1												1	1
3671	NA18603	F1d1	F1d1	1												1	1
3672	HG00437	F1e1	F2g	1	F1e1	4										2	5
3673	AP008508	F1e1a	F1e1a	1												1	1
3674	EU597547	F1e2	F1e2	1												1	1
3675	KF849952	F1e3	F1e3	1												1	1
3676	JF824890	F1g	F1g	1												1	1
3677	GU392087	F1g1	F1g1	1												1	1
3678	AP008692	F2a	F2a	1												1	1
3679	NA18598	F2a+@16291	F1a'c'f	1	F2a+@16291	3										2	4
3680	AY255168	F2a1	F2a1	1												1	1
3681	AY255180	F2b	F2b	1												1	1
3682	NA18790	F2b1	F2b1	1												1	1
3683	HM036555	F2g	F2+16291	1	F2g	1										2	2
3684	KF849911	F2c	F	1	F2	3	F2c	3								3	7
3685	AP010683	F2c1	F1e1a	1	F2c1	2										2	3
3686	NA18583	F2c2	F2c2	1												1	1
3687	AP013247	F2d	F2d	1												1	1
3688	NA18629	F2e	H2a5b1	1	F2e	1										2	2
3689	JN857034	F2e1	F2e1	1												1	1
3690	AP013271	F2f	F	1	F2	3	F2f	3								3	7
3691	FJ748736	F2h	F2h	1												1	1
3692	KF849909	F2i	F2i	1												1	1
3693	AY255167	F3a	F3a	1												1	1
3694	JF824855	F3a+207	F3a+207	1												1	1
3695	KF849966	F3a1	F3a1	1												1	1
3696	AP013180	F3b	F3b	1												1	1
3697	AY972053	F3b+152	F3b+152	1												1	1
3698	KC994154	F3b1a+16093	F3b1a+16093	1												1	1
3699	KC993991	F3b1a1	F3b1a1	1												1	1
3700	GQ119015	F3b1a2	F3b1a2	1												1	1
3701	JF739538	F3b1b	F3b1	1	F3b1b	1	F3b1b	1								3	3
3702	KC994125	F3b1b1	F3b1	1	F3b1b	1	F3b1b1	1								3	3
3703	AP008744	F4a1a	F4a1a	1												1	1
3704	AP013200	F4a1b	F4a1b	1												1	1
3705	NA18691	F4a2	F4a2	1												1	1
3706	NA18592	F4b	F4b	1												1	1
3707	AY289095	F4b1	F4b1	1												1	1
3708	NA18763	R11	R11	1												1	1
3709	AP010998	R11a	R11a	1												1	1
3710	JF824897	R11b	R11b	1												1	1
3711	JN857012	R11b1a	R11b1a	1												1	1
3712	AY255163	R11b1b	R11b1b	1												1	1
3713	AY255136	B6a	B6a	1												1	1
3714	GQ119045	B6a1	B6a1	1												1	1
3715	JX289129	B6a1a	B6a1a	1												1	1
3716	KC417443	B4'5	B4'5	1												1	1
3717	NA17990	B4a	B4h	1	B4a	3										2	4
3718	DQ372871	B4a1a	B4a1	1	B4a1a	2	B4a1a	5	B4a1a	2						4	10
3719	KJ154569	B4a1a1	B4a1a1	1												1	1

3720	KJ154187	B4a1a1a	B4a1a1a	1									1	1
3721	JX900618	B4a1a1a1	B4a1a1	1	B4a1a1a	2	B4a1a1a1	1	B4a1a1a1	1			4	5
3722	JX900769	B4a1a1a1a	B4a1a1a1a	1	B4a1a1a1a	1							2	2
3723	JX900376	B4a1a1a1a1	B4a1a1a1a1	1									1	1
3724	JX900604	B4a1a1a1b	B4a1a1a1b	1									1	1
3725	KJ154487	B4a1a1a1c	B4a1a1a1c	1									1	1
3726	KJ154414	B4a1a1a1d	B4a1a1	1	B4a1a1a1d	1							2	2
3727	JX900433	B4a1a1a2	B4a1a1a2	1									1	1
3728	JX900535	B4a1a1a2a	B4a1a1a2	1	B4a1a1a2a	1							2	2
3729	KJ154620	B4a1a1a2b	B4a1a1a2b	1									1	1
3730	JX900766	B4a1a1a3	B4a1a1	1	B4a1a1a	2	B4a1a1a	1	B4a1a1a3	5			4	9
3731	JX900408	B4a1a1a4	B4a1a1a4	1									1	1
3732	JX900762	B4a1a1a5	B4a1a1a5	1									1	1
3733	JX900841	B4a1a1a6	B4a1a1	1	B4a1a1a	13	B4a1a1a6	4					3	18
3734	JX900343	B4a1a1a7	B4a1a1a7	1									1	1
3735	JX900752	B4a1a1a8	B4a1a1a8	1									1	1
3736	JX900826	B4a1a1a9	B4a1a1a9	1									1	1
3737	JX900614	B4a1a1a10	B4a1a1a10	1									1	1
3738	JX900639	B4a1a1a11	B4a1a1	1	B4a1a1a	2	B4a1a1a11	1	B4a1a1a11	1			4	5
3739	HQ873524	B4a1a1a11a	B4a1a1a11a	1									1	1
3740	JX900344	B4a1a1a11b	B4a1a1a11b	1									1	1
3741	JX900373	B4a1a1a12	B4a1a1a12	1									1	1
3742	JX900500	B4a1a1a13	B4a1a1	1	B4a1a1a	2	B4a1a1a	1	B4a1a1a13	7			4	11
3743	KJ154580	B4a1a1a14	B4a1a1a14	1									1	1
3744	KJ154315	B4a1a1a15	B4a1a1a7	1	B4a1a1a15	5							2	6
3745	JX900734	B4a1a1a16	B4a1a1a16	1									1	1
3746	KJ154591	B4a1a1a17	B4a1a1a7	1	B4a1a1	2	B4a1a1a	1	B4a1a1a17	1			4	5
3747	KJ154415	B4a1a1a18	B4a1a1a18	1									1	1
3748	KJ154865	B4a1a1a19	B4a1a1a19	1									1	1
3749	KJ154207	B4a1a1a20	B4a1a1a20	1									1	1
3750	KJ154218	B4a1a1a21	B4a1a1a21	1									1	1
3751	KJ154210	B4a1a1a22	B4a1a1	1	B4a1a1a1d	1	B4a1a1	1	B4a1a1a	1	B4a1a1a22	1	5	5
3752	JX900848	B4a1a1a+195	B4a1a1a+195	1									1	1
3753	JX900644	B4a1a1a23	B4a1a1a+195	1	B4a1a1a23	1							2	2
3754	FJ767910	B4a1a1b	B4a1a1b	1									1	1
3755	KJ154368	B4a1a1c	B4a1a1c	1									1	1
3756	HQ873507	B4a1a1d	B4a1a1d<br>B4a1a1w	1	B4a1a1d	2							2	3
3757	JX900634	B4a1a1e	B4a1a1	1	B4a1a1	2	B4a1a1e	5					3	8
3758	JX900410	B4a1a1f	B4a1a1a	1	B4a1a1f	13							2	14
3759	JX900543	B4a1a1g	B4a1a1g	1									1	1
3760	JX900348	B4a1a1h	B4a1a1h	1									1	1
3761	JX900779	B4a1a1i	B4a1a1i	1									1	1
3762	JX900692	B4a1a1j	B4a1a1j	1									1	1
3763	KJ154396	B4a1a1k	B4a1a1k	1									1	1
3764	KJ154221	B4a1a1k1	B4a1a1k1	1									1	1
3765	KJ154269	B4a1a1+151	B4a1a1+151	1	B4a1a1+151	1							2	2
3766	KJ154364	B4a1a1m	B4a1a1m	1									1	1
3767	KJ154402	B4a1a1m1	B4a1a1m1	1									1	1
3768	JX900810	B4a1a1n	B4a1a1	1	B4a1a1n	8							2	9
3769	AF347007	B4a1a1+152	B4a1a1+152	1									1	1
3770	JX900828	B4a1a1o	B4a1a1o	1									1	1
3771	JX900384	B4a1a1p	B4a1a1a1b	1	B4a1a1p	2							2	3
3772	HQ873532	B4a1a1q	B4a1a1q	1									1	1
3773	KJ154410	B4a1a1+16126	B4a1a1+16126	1	B4a1a1+1612	1							2	2
3774	KJ154645	B4a1a1s	B4	1	B4a1a1a11b	2	B4a1a1s	6					3	9
3775	KJ154367	B4a1a1t	B4a1a1a18	1	B4a1a1t	3							2	4

3776	KJ154259	B4a1a1u	B4a1a1u	1											1	1
3777	KJ154288	B4a1a1v	B4a1a1v	1											1	1
3778	KJ154872	B4a1a1w	B4a1a1	1	B4a1a1	2	B4a1a1	5	B4a1a1	5	B4a1a1w	5			5	18
3779	DQ372877	B4a1a1x	B4a1a1a+195	1	B4a1a1a+195	1	B4a1a1	2	B4a1a1x	3					4	7
3780	JX900846	B4a1a1y	B4a1a1	1	B4a1a1	3	B4a1a1y	15							3	19
3781	KJ154929	B4a1a1z	B4a1a1	1	B4a1a1	2	B4a1a1	5	B4a1a1	5	B4a1a1	5	B4a1a1z	5	6	23
3782	AY963574	B4a1a1aa	B4a1a1aa	1											1	1
3783	JX900575	B4a1a1ab	B4a1a1ab	1											1	1
3784	KJ154307	B4a1a1ac	B4a1a1ac	1											1	1
3785	KJ154208	B4a1a1ad	B4a1a1	1	B4a1a1	2	B4a1a1	5	B4a1a1	5	B4a1a1	5	B4a1a1ad	5	6	23
3786	HQ873511	B4a1a1ae	B4a1a1	1	B4a1a1	2	B4a1a1	5	B4a1a1	5	B4a1a1	5	B4a1a1ae	5	6	23
3787	KJ154891	B4a1a1af	B4a1a1af	1											1	1
3788	AJ842748	B4a1a2	B4a1a1a20	1	B4a1	1	B4a1a	2	B4a1a2	4					4	8
3789	HQ873558	B4a1a3	B4a1	1	B4a1a	2	B4a1a3	5							3	8
3790	HQ873560	B4a1a3a	B4a1a3a	1	B4a1a3a	1									2	2
3791	KC994063	B4a1a3a1	B4a1a3a1	1											1	1
3792	AJ842744	B4a1a3a1a	B4a1a3a1a	1											1	1
3793	AJ842747	B4a1a4	B4a1a4	1											1	1
3794	GQ119021	B4a1a5	B4a1a5	1											1	1
3795	KC994064	B4a1a5a	B4a1a5a	1											1	1
3796	KC994101	B4a1a6	B4a1a1a11b	1	B4a1a6	5									2	6
3797	KC994097	B4a1a6a	B4a1a1	1	B4a1a	2	B4a1a6	2	B4a1a6a	1					4	6
3798	HQ873557	B4a1a7	B4a1a1aa	1	B4a1a7	4									2	5
3799	EU597506	B4a1+16311	B4c1a1b	1	B4c1a1	1	B4a1+16311	4							3	6
3800	AP008595	B4a1b	B4a1b	1											1	1
3801	AP008842	B4a1b1	B4a1b1	1	B4a1b1	1									2	2
3802	AP008640	B4a1b1a	B4a1b1	1	B4a1b1a	1									2	2
3803	JN866824	B4a1e	B4a1e	1											1	1
3804	AP008650	B4a1c1	B4a1c1	1											1	1
3805	JF824900	B4a1c1a	B4i1	1	B4a1c1a	3									2	4
3806	AP008912	B4a1c1a1	B4a1c1a1	1											1	1
3807	AY519495	B4a1c2	B4a1c2	1											1	1
3808	EU597505	B4a1c4	B4a1c4	1											1	1
3809	KF540685	B4a1c5	B4a1a	1	B4a1c5	2									2	3
3810	AP010705	B4a1c3a	B4a1c3a	1											1	1
3811	AP008521	B4a1c3b	B4a1c3b	1											1	1
3812	HQ873495	B4a1d	B4a1d	1											1	1
3813	HQ873538	B4a2a	B4a2a	1											1	1
3814	AJ842751	B4a2a1	B4a2a1	1											1	1
3815	HQ873563	B4a2a2	B4a2a2	1											1	1
3816	AJ842750	B4a2a3	B4a2a3	1											1	1
3817	NA18528	B4a2b	B4a2b	1											1	1
3818	KF849947	B4a2b1	B4a2b1	1											1	1
3819	AP013280	B4a2b1a	B4a2b1a	1											1	1
3820	FJ748745	B4a3	B4a3	1											1	1
3821	AP008384	B4a4	B4a4	1											1	1
3822	KF540699	B4a5	B4a5	1											1	1
3823	JQ703874	B4g1	B4g1	1											1	1
3824	GU810060	B4g1a	B4g1	1	B4g1	1	B4g1a	1							3	3
3825	NA18109	B4g1b	B4g1b	1											1	1
3826	KF540983	B4g2	B4g2	1											1	1
3827	GU377090	B4h	B4a1+16311	1	B4h	2									2	3
3828	KF849963	B4h1	B4h1	1											1	1
3829	JF824906	B4i	B4a2b	1	B4a2b	1	B4i	3							3	5
3830	KC733274	B4i1	B4i1	1											1	1
3831	KF849913	B4k	B4k	1											1	1
3832	KF540707	B4m	B4m	1											1	1

3833	EU095548	B2	B2f	1	B2	2							2	3
3834	KC711022	B2a	B2a	1									1	1
3835	DQ282442	B2a1	B2a1	1									1	1
3836	DQ282444	B2a1a	B2a	1	B2a1	2	B2a1	1	B2a1a	1	B2a1a	1	5	6
3837	KC711024	B2a1a1	B2a1a1	1									1	1
3838	KC711025	B2a1b	B2a1b	1									1	1
3839	DQ282441	B2a2	B2a2	1									1	1
3840	HQ012136	B2a3	B2a3	1									1	1
3841	JQ703852	B2a4	B2a4	1									1	1
3842	KC711033	B2a4a	B2a4a	1									1	1
3843	KC711034	B2a4a1	B2a4a1	1									1	1
3844	AF347001	B2a5	B2a5	1									1	1
3845	EU095532	B2b	B4b	1	B2b	2							2	3
3846	HG01437	B2b+152	B2b+152	1									1	1
3847	JF431064	B2b1	B2b1	1									1	1
3848	KC503926	B2b2	B2b2	1									1	1
3849	KC503927	B2b2a	B2b2a	1									1	1
3850	EU095221	B2b3	B2b4	1	B2b	2	B2b	1	B2b3	1			4	5
3851	HG00640	B2b3a	B2b3a	1									1	1
3852	HQ012137	B2b4	B2b4	1									1	1
3853	DQ282436	B2c	B2c1	1	B2c	2							2	3
3854	DQ282439	B2c1	B2c1	1									1	1
3855	HQ012177	B2c1a	B2c1a	1									1	1
3856	HQ012160	B2c1b	B4b	1	B2	1	B2c	9	B2c1	1	B2c1b	3	5	15
3857	HQ012164	B2c1c	B2c1c	1									1	1
3858	HQ012143	B2c2	B2	1	B2c2	2							2	3
3859	HQ012140	B2c2a	B2c2a	1									1	1
3860	HQ012151	B2c2b	B2c2b	1									1	1
3861	EU095550	B2d	B2d	1									1	1
3862	EU597569	B2e	B2e	1									1	1
3863	EU334872	B2f	B4b	1	B2	1	B2f	9					3	11
3864	HQ012185	B2g1	B2g1	1									1	1
3865	JQ702661	B2g2	B2g2	1									1	1
3866	EU095206	B2h	B2h	1									1	1
3867	EU095218	B2i1	B2i1	1									1	1
3868	JX413035	B2i2	B2i2	1									1	1
3869	JX413013	B2i2a	B2i2a	1									1	1
3870	JX413022	B2i2a1	B2i2a1	1									1	1
3871	JX413014	B2i2a1a	B2i2a1	1	B2i2a1a	1							2	2
3872	JX413021	B2i2a1b	B2i2a1b	1									1	1
3873	JX413027	B2i2b	B2i2b	1									1	1
3874	JX413032	B2i2b1	B2i2b1	1									1	1
3875	JF431059	B2j	B2j	1									1	1
3876	JF431061	B2k	B2k	1									1	1
3877	JQ702293	B2l	B2l	1									1	1
3878	HQ012141	B2m	B2m	1									1	1
3879	NA19795	B2n	B2b+152	1	B4b	2	B2n	1					3	4
3880	JQ703851	B2o	B2o	1									1	1
3881	KC503933	B2o1	B2o1	1									1	1
3882	KC503931	B2o1a	B2o1a	1									1	1
3883	HQ012167	B2p	B2p	1									1	1
3884	HQ012176	B2q	B2q	1									1	1
3885	HQ012175	B2r	B2b+152	1	B4b	1	B2	1	B2r	4			4	7
3886	HQ012165	B2s	B2s	1									1	1
3887	NA19684	B2t	B2t	1									1	1
3888	HQ012183	B2u	B2u	1									1	1
3889	HQ012173	B2v	B2v	1									1	1

3890	HQ012172	B2+16278	B2+16278	1									1	1
3891	JQ705755	B2w	B2w	1									1	1
3892	HQ012147	B2x	B2x	1									1	1
3893	EU597534	B2y	B2y	1									1	1
3894	EU431084	B2y1	B2y1	1									1	1
3895	AY519494	B4b1a	B4b1a3a	1	B4b	2	B4b1a	1					3	4
3896	AP010733	B4b1a1	B4b1a1	1									1	1
3897	AP009471	B4b1a1a	B4b1a1a	1									1	1
3898	AP009444	B4b1a1b	B4b1a1b	1									1	1
3899	AP008900	B4b1a1c	B4b1a1c	1									1	1
3900	AP008856	B4b1a2	B4b1a2	1									1	1
3901	AY255170	B4b1a2a	B4b1a2a	1									1	1
3902	KC994146	B4b1a2b1	B4b1a2b1	1									1	1
3903	KF540811	B4b1a2b2	B4b1a2b2	1									1	1
3904	GU733724	B4b1a2c	B4b1a2c	1									1	1
3905	GU733814	B4b1a2d	B4b1a2d	1									1	1
3906	EU597553	B4b1a2e	B4b1a2e	1									1	1
3907	KF540629	B4b1a2f	B4b1a2f	1									1	1
3908	KF540610	B4b1a2g	B4b1a2g	1	B4b1a2g	1							2	2
			B4b1a2g1											
3909	KF540608	B4b1a2g1	B4b1a2g	1	B4b1a2g1	1							2	2
3910	KF540519	B4b1a2h	B4b1a2f	1	B4b1a2h	1							2	2
3911	KJ154674	B4b1a2i	B4b1a2i	1									1	1
3912	NA18689	B4b1a3	B4b1a3	1									1	1
3913	KF148375	B4b1a3a	B4b1a3a	1									1	1
3914	AP008682	B4b1b	B4b1b	1									1	1
3915	HQ873567	B4b1c1	B4b1b'c	1	B4b1c	1	B4b1c1	2					3	4
3916	DQ272119	B4b1c2	B4b1c2	1									1	1
3917	GU392048	B4d1	B4d1	1									1	1
3918	NA18117	B4d1a	B4d1a	1									1	1
3919	AY255140	B4d2	B4	1	B4b'd'e'j	2	B4d	1	B4d1'2'3	1	B4d2	2	5	7
3920	NA18619	B4d3	B4d3	1	B4d3	1							2	2
3921	JF824975	B4d3a	B4d3	1	B4d3a	1	B4d3a	1					3	3
3922	AP010836	B4d3a1	B4d3a1	1									1	1
3923	AP008492	B4d4	B4d4	1									1	1
3924	KC733253	B4e	B4e	1									1	1
3925	JN857017	B4j	B4j	1									1	1
3926	KC521454	B4c1a	B4	1	B4c	2	B4c1	1	B4c1a'b	1	B4c1a	1	5	6
3927	NA18982	B4c1a1	B4c1a1	1									1	1
3928	AP008482	B4c1a1a	B4c1a1a	1	B4c1a1a	1							2	2
3929	AP008298	B4c1a1a1	B4c1a1a1	1									1	1
3930	AP011003	B4c1a1a1a	B4c1a1a1a	1									1	1
3931	AP008688	B4c1a1a2	B4c1a1a	1	B4c1a1a2	2							2	3
3932	AP008281	B4c1a1b	B4c1a1b	1									1	1
3933	AP010704	B4c1a1c	B4c1a1c	1									1	1
3934	JN857019	B4c1a2	B4c1a2	1									1	1
3935	JN857031	B4c1a2a	B4c1a2a	1									1	1
3936	AP008570	B4c1b	B4c1b	1									1	1
3937	AP008899	B4c1b1	B4c1b1	1									1	1
3938	AP008920	B4c1b1a	B4c1b1	1	B4c1b1a	1							2	2
3939	AP008472	B4c1b+16335	B4c1b+16335	1									1	1
3940	AY255149	B4c1b2a	B4c1b	1	B4c1b	1	B4c1b2a	1					3	3
3941	GQ161177	B4c1b2a1	B4c1b2a1	1									1	1
3942	HM596646	B4c1b2a2	B4c1b2a2	1	B4c1b2a2	2							2	3
3943	KC994085	B4c1b2a2a	B4c1b2a2	1	B4c1b2a2a	2							2	3
3944	KC994083	B4c1b2a2b	B4c1b2a2	1	B4c1b2a2b	2							2	3
3945	NA18156	B4c1b2b	B4c1b2b	1									1	1

3946	NA18111	B4c1b2c	B4c1b+16335	1	B4c1b2	1	B4c1b2c	1			3	3
3947	NA18778	B4c1b2c1	B4c1b2c1	1							1	1
3948	KF540661	B4c1b2c2	B4c1b2c2	1							1	1
3949	AP009461	B4c1c	B4c1c	1							1	1
3950	AP009436	B4c1c+16311	B4c1c+16311	1							1	1
3951	AP008425	B4c1c1	B4c1c1	1							1	1
3952	GU810059	B4c2	B4c2	1	B4c2	3					2	4
3953	AY289100	B4c2a	B4c2a	1							1	1
3954	AP012407	B4c2b	B4c2b	1							1	1
3955	GU592217	B4c2c	B4c2c	1							1	1
3956	JQ703514	B4f	B4f	1							1	1
3957	AP013140	B4f1	B4f1	1							1	1
3958	EU597566	B5a1a	B5a1	1	B5a1	1	B5a1a	2			3	4
3959	AY950290	B5a1a1	B5a1a1	1							1	1
3960	AY255145	B5a1b	B5a	1	B5a1	1	B5a1b	3	B5a1b	1	4	6
3961	GQ119031	B5a1b1	B5a1b1	1							1	1
3962	KF540745	B5a1c	B5a1c1a	1	B5a	2	B5a1c	1			3	4
3963	KF540703	B5a1c1	B5a1b1	1	B5a	3	B5a1c	1	B5a1c1	1	4	6
3964	JQ731601	B5a1c1a	B5a1c1a	1							1	1
3965	JF896801	B5a1c1a1	B5a1c1a1	1							1	1
3966	JQ731600	B5a1c2	B5a1c2	1							1	1
3967	GU810075	B5a1d	B5a1d	1							1	1
3968	HG00543	B5a2	B5a2	1							1	1
3969	EF114286	B5a2a1a	B5a2a1a	1							1	1
3970	JQ731602	B5a2a1+16129	B5a2a1+16129	1							1	1
3971	AP008263	B5a2a1b	B5a2a1b	1							1	1
3972	KF540936	B5a2a2a1	B5a2a2a1	1							1	1
3973	KF540943	B5a2a2a2	B5a2a2a2	1							1	1
3974	KC994087	B5a2a2b1	B5a2a2b1	1							1	1
3975	KF540623	B5a2a2b1a	B5a2a2b1a	1							1	1
3976	KF540807	B5a2a2b2	B5a2a2b2	1							1	1
3977	AP010771	B5b1	B5b1	1							1	1
3978	JF824930	B5b1a	B5b1a	1							1	1
3979	AP008827	B5b1a1	B5b1a1	1							1	1
3980	AP008273	B5b1a2	B5b1a2	1	B5b1a2	1					2	2
3981	AP008875	B5b1a2a	B5b1a2a	1							1	1
3982	GQ119020	B5b1c	B5b1c	1							1	1
3983	KC994143	B5b1c1	B5b1c1	1							1	1
3984	KC994058	B5b1c1a	B5b1c	1	B5b1c1	1	B5b1c1a	1			3	3
3985	AY519489	B5b2a	B5b2a	1							1	1
3986	AP008546	B5b2a1	B5b2a1	1							1	1
3987	AP009425	B5b2a2	B5b2a2	1							1	1
3988	AP008910	B5b2a2a1	B5b2a2	1	B5b2a2a	1	B5b2a2a1	1			3	3
3989	KJ154763	B5b2a2a2	B5b2a2a2	1							1	1
3990	KF148463	B5b2b	B5b2b	1							1	1
3991	GU377081	B5b2+@204	B5b2+@204	1							1	1
3992	KF540720	B5b2c	B5b2c	1							1	1
3993	AP008465	B5b2c1	B5b2c1	1							1	1
3994	AP013282	B5b3a	B5b3a	1							1	1
3995	AP013129	B5b3b	B5b3b	1							1	1
3996	KF849981	B5b4	B5b4	1							1	1
3997	KF540694	B5b5	B5b5	1							1	1
3998	GU733726	R24	R24	1							1	1
3999	GQ119037	R24a	R24a	1							1	1
4000	AP012392	R21	R21	1							1	1
4001	EF495216	R14	R14	1							1	1
4002	GQ301886	R22	R22	1							1	1

4003	GQ301864	R23	R23	1									1	1
4004	AY714006	R30	R8a1a1d	1	R8a	5	R	3	P9a	2	R30	4	5	15
4005	JX462702	R30a	R	1	R	4	R30	4	R30a	1	R30a	1	5	11
4006	FJ770961	R30a1a	R30a1a	1									1	1
4007	AY714032	R30a1b	R30a1b	1									1	1
4008	GU170818	R30a1b1	R30a1b1	1									1	1
4009	FJ004824	R30a1c	R30a1c	1									1	1
4010	FJ004827	R30b1	R30b1	1									1	1
4011	FJ004807	R30b2	U5b2a1a2	1	R30b2	5							2	6
4012	JX462737	R30b2a	R30b2a	1									1	1
4013	AY714021	R31a	R31a	1									1	1
4014	FJ004825	R31a1	R31a1	1									1	1
4015	AY714046	R31b	R31b	1									1	1
4016	KC577355	R32	R32	1									1	1
4017	AY289087	P1d1	P1d1	1									1	1
4018	KJ154317	P1d1a	P1d1a	1									1	1
4019	KJ154684	P1d2	P1	1	P1d2	1							2	2
4020	KJ154767	P1d2a	P1d2a	1									1	1
4021	KJ154805	P1+152	P1+152	1									1	1
4022	KJ154904	P1f	P1f	1									1	1
4023	AY289088	P2	H13a2b3	1	P2	4							2	5
4024	KC993994	P10	P10	1									1	1
4025	DQ404446	P8	P8	1									1	1
4026	AY289052	P3a	P3a	1									1	1
4027	AY289091	P3b	P3b	1									1	1
4028	EF061153	P3b1	P3b1	1									1	1
4029	EF061158	P4a	P4a	1									1	1
4030	EF495221	P4a1	P4a1	1									1	1
4031	AY289064	P4b	P4b	1									1	1
4032	DQ404444	P4b1	P4b1	1									1	1
4033	AY289063	P5	P5	1									1	1
4034	AY289053	P6	P6	1									1	1
4035	AY289054	P7	P7	1									1	1
4036	KC993944	P9	P9	1									1	1
4037	GQ119026	P9a	P2	1	P9a	5							2	6
4038	HM852790	U1a1	U1a1	1									1	1
4039	GU218692	U1a1a	U1a1a	1									1	1
4040	HQ615882	U1a1a1	U1a1a1	1									1	1
4041	KC911328	U1a1a1a	U1a1a1a	1									1	1
4042	JQ703793	U1a1a2	U1a1a2	1									1	1
4043	AY882396	U1a1a+16129	U1a1a+16129	1									1	1
4044	JQ705601	U1a1a3	U1a1a3	1									1	1
4045	JQ704153	U1a1b	U1a1b	1									1	1
4046	JX153116	U1a1c	U1a1c1d1	1	U1a	1	U1a1c	2					3	4
4047	FJ748753	U1a1c1a	U1a1c1a	1									1	1
4048	JQ704034	U1a1c1b	U1a1c1b	1									1	1
4049	KC911365	U1a1c1c	U1a3	1	U1a1c1c	2							2	3
4050	DQ523621	U1a1c1c1	U1a1c1c1	1									1	1
4051	KC911457	U1a1c1d	U1a	1	U1a1	1	U1a1c	1	U1a1c1	1	U1a1c1d	3	5	7
4052	KC911527	U1a1c1d1	U1a1c1d1	1									1	1
4053	EF692533	U1a1d	U1a1d	1									1	1
4054	EF556194	U1a2	U1a2	1									1	1
4055	KC540656	U1a3	U1a3	1									1	1
4056	JQ705704	U1b1	U1b1	1									1	1
4057	KC911628	U1b2	U1b2	1									1	1
4058	AY882397	U1b3	U1b3	1									1	1
4059	KC521458	U5	U5	1	U5	1							2	2

4060	FJ348174	U5a1	U5a1	1							1	1
4061	DQ785296	U5a1a1	U5a1a1e	1	U5a1a1	1					2	2
4062	GU296636	U5a1a1+152	U5a1a1+152	1							1	1
4063	DQ904330	U5a1a1a	U5a1a1+152	1	U5a1a1a	2					2	3
4064	JQ703937	U5a1a1b	U5a1a1+152	1	U5a1a1b	2					2	3
4065	GQ368895	U5a1a1h	U5a1a1h	1							1	1
4066	JQ703590	U5a1a1c	U5a1+@16192	1	U5a1a	2	U5a1a1	1	U5a1a1c	4	4	8
4067	JQ704920	U5a1a1+16362	U5a1a1+16362	1							1	1
4068	GU459066	U5a1a1d	U5a1a1d	1							1	1
4069	JQ701866	U5a1a1d1	U5a1a1d1	1							1	1
4070	JQ704796	U5a1a1e	U5a1a1e	1							1	1
4071	JQ705243	U5a1a1g	U5a1+@16192	1	U5a1a	2	U5a1a1	1	U5a1a1g	4	4	8
4072	JX153282	U5a1a1i	U5a1a1i	1							1	1
4073	HQ588904	U5a1a2a	U5a1a2a	1							1	1
4074	JQ703926	U5a1a2a1	U5a1a2a1	1							1	1
4075	JQ702568	U5a1a2a1a	U5a1a2	1	U5a1a2a	2	U5a1a2a1	1	U5a1a2a1a	1	4	5
4076	GU296543	U5a1a2b	U5a1a2b	1							1	1
4077	JQ705279	U5a1a2b1	U5a1a2	1	U5a1a2b	2	U5a1a2b1	1			3	4
4078	HM765468	U5a1g	U5a1g	1							1	1
4079	JQ702913	U5a1g1	U5a1g1	1							1	1
4080	KC911409	U5a1g2	U5a1g2	1							1	1
4081	JX141361	U5a1b	U5a1	1	U5a1b	6	U5a1b	1			3	8
4082	JQ702775	U5a1b1	U5a1b1	1							1	1
4083	JQ691414	U5a1b1a	U5a1b1a	1							1	1
4084	JQ705245	U5a1b1a1	U5a1b1	1	U5a1b1a	7	U5a1b1a1	2			3	10
4085	JQ703074	U5a1b1a2	U5a1b1d+16093	1	U5a1b1a2	4					2	5
4086	GU296614	U5a1b1b	U5a1b1d+16093	1	U5a1b1b	4					2	5
4087	GU296619	U5a1b1b1	U5a1b1b1	1							1	1
4088	EU140330	U5a1b1c	U5a1b1	1	U5a1b1c	7	U5a1b1c	1			3	9
4089	JQ705297	U5a1b1c1	U5a1b1	1	U5a1b1c	7	U5a1b1c1	1			3	9
4090	GU296628	U5a1b1c2	U5a1b1c2	1							1	1
4091	JQ702400	U5a1b1d	U5a1b1	1	U5a1b1d	7					2	8
4092	JX152788	U5a1b1d+16093	U5a1b1d+16093	1							1	1
4093	JQ705693	U5a1b1d1	U5a1b1d1	1							1	1
4094	JX153770	U5a1b1e	U5a1b1e	1							1	1
4095	JQ702552	U5a1b1f	U5a1b1f	1							1	1
4096	HG00104	U5a1b1g	U5a1b1	1	U5a1b1	2	U5a1b1g	4			3	7
4097	JX153196	U5a1b1h	U5a1b1d+16093	1	U5a1b1h	4					2	5
4098	GU296569	U5a1b2	U5a1b2	1							1	1
4099	JQ704045	U5a1b+16362	U5a1b+16362	1	U5a1b+16362	1					2	2
4100	JQ703331	U5a1b3	U5a1b3	1	U5a1b3	1					2	2
4101	JQ705935	U5a1b3a	U5a1b+16362	0							0	0
4102	JQ705101	U5a1b3a1	U5a1b3a1	1							1	1
4103	HM171295	U5a1b4	U5a1b4	1							1	1
4104	GU296588	U5a1c1	U5a1c1	1							1	1
4105	JX297176	U5a1c1a	U5a1c1a	1							1	1
4106	GU296589	U5a1c2	U5a1c2	1							1	1
4107	JQ704555	U5a1c2a	U5a1c2a	1							1	1
4108	JQ705908	U5a1c2a1	U5a1c2a1	1							1	1
4109	GU296542	U5a1d1	U5a1d1	1							1	1
4110	HM173090	U5a1d2a	U5a1d2a	1	U5a1d2a	1					2	2
4111	GU296599	U5a1d2a1	U5a1d2a1	1							1	1
4112	GU123032	U5a1d2b	U5a1d2b	1							1	1
4113	GU296625	U5a1e	U5a1e	1							1	1
4114	JQ702160	U5a1f1	U5a1f1	1							1	1
4115	AY882398	U5a1f1a	U5a1f1a	1							1	1
4116	JQ702735	U5a1f1a1	U5a1f1a1	1							1	1

4117	GU296603	U5a1f2	U5a1f2	1							1	1
4118	JQ704026	U5a1h	U5a1h	1							1	1
4119	KC257306	U5a1i	U5a1a1	1	U5a1+@16192	1	U5a1+@16192	2	U5a1i	2	4	6
4120	JQ705326	U5a1i1	U5a1i1	1							1	1
4121	KF262460	U5a1j	U5a1j	1							1	1
4122	JX153018	U5a2+16294	U5a2+16294	1							1	1
4123	JN707685	U5a2a	U5a2a	1	U5a2a	1					2	2
4124	GU296615	U5a2a1	U5a2a	1	U5a2a1	1	U5a2a1	3			3	5
4125	AY339524	U5a2a1a	U5a2a1a	1							1	1
4126	GU296626	U5a2a1b	U5a2a	1	U5a2a1	1	U5a2a1b	3			3	5
4127	JQ702355	U5a2a1b1	U5a2a1b1	1							1	1
4128	GU296597	U5a2a1c	U5a2a1c	1							1	1
4129	EU124886	U5a2a1d	U5a2a1+152	1	U5a2a1+152	1	U5a2a1d	1			3	3
4130	JQ704074	U5a2a1+152	U5a2a1+152	1	U5a2a1+152	1					2	2
4131	JX171113	U5a2a1e	U5a2a1e	1							1	1
4132	JQ705530	U5a2a2	U5a2a2	1							1	1
4133	JX153731	U5a2a2a	U5a2a2a	1							1	1
4134	GU371930	U5a2b	U5a2b	1							1	1
4135	JQ702746	U5a2b1	U5a2	1	U5a2	2	U5a2b1	3			3	6
4136	GU296651	U5a2b1a	U5a2b1a	1							1	1
4137	JQ704044	U5a2b1b	U5a2b1b	1							1	1
4138	GU296600	U5a2b1c	U5a2b1c	1							1	1
4139	JQ703087	U5a2b1d	U5a2b	1	U5a2b	2	U5a2b1	1	U5a2b1d	2	4	6
4140	KC246057	U5a2b2	U5a2b2	1							1	1
4141	JQ702184	U5a2b2a	U5a2b2a	1							1	1
4142	GU296587	U5a2b2a1	U5a2b2a1	1							1	1
4143	KC661077	U5a2b3	U5a2b3	1							1	1
4144	JQ702847	U5a2b3a	U5a2b3a	1							1	1
4145	JQ702230	U5a2b3a1	U5a2b3a1	1							1	1
4146	JQ702320	U5a2b4	U5a2b4	1							1	1
4147	JX153863	U5a2b4a	U5a2b4a	1							1	1
4148	NA20760	U5a2b5	U5a2b5	1							1	1
4149	JQ703187	U5a2c	U5a2c	1							1	1
4150	FJ460558	U5a2c1	U5a2	1	U5a2c	3	U5a2c1	3			3	7
4151	EF660950	U5a2c2	U5a	1	U5a2c2	5					2	6
4152	JX101637	U5a2c3	U5a2c3	1							1	1
4153	JF487827	U5a2c3a	U5a2	1	U5a2c3	2	U5a2c3a	1			3	4
4154	JQ705779	U5a2c4	U5a2	1	U5a2c	3	U5a2c4	3			3	7
4155	JQ702144	U5a2d	U5a2d	1							1	1
4156	HM490393	U5a2d1	U5a2d1	1							1	1
4157	JX153171	U5a2d1a	U5a2d1a	1							1	1
4158	JQ705111	U5a2+16362	U5a2+16362	1							1	1
4159	JN819535	U5a2e	U5a2e	1							1	1
4160	JQ704112	U5b1	U5b1	1							1	1
4161	KC521455	U5b1a	U5b	1	U5b1	2	U5b1a	3			3	6
4162	EF420876	U5b1+16189	U5b	1	U5b1+16189	2	U5b1+16189	1			3	4
4163	GU296644	U5b1b	U5b1+16189+@16192	0							0	0
4164	GU296566	U5b1b1	U5b	1	U5b1+16189	2	U5b1b	1			3	4
4165	AY882400	U5b1b1+@16192	U5b1b1+@16192	1							1	1
4166	AY882403	U5b1b1a	U5b1b1a	1	U5b1b1a	2					2	3
4167	DQ902696	U5b1b1a1	U5b1b1a	1	U5b1b1a1	2	U5b1b1a1	2			3	5
4168	HM116534	U5b1b1a1a	U5b1b1a1a	1							1	1
4169	KF466256	U5b1b1a1a1	U5b1b1a1a1	1							1	1
4170	JQ703600	U5b1b1a1b	U5b1b1a	1	U5b1b1a1	2	U5b1b1a1b	2			3	5

4171	EF420877	U5b1b1a2	U5b1b1a2	1					1	1
4172	JX153170	U5b1b1a3	U5b1b1a3	1					1	1
4173	AY882402	U5b1b1d	U5b	1	U5b1b1+@16192	3	U5b1b1d	2	3	6
4174	GU296591	U5b1b1f	U5b1b1+@16192	1	U5b1b1+@16192	2	U5b1b1f	1	3	4
4175	AY882407	U5b1b1b	U5b1b1b	1					1	1
4176	AF381989	U5b1b1+152	U5b1b1+152	1	U5b1b1+152	1			2	2
4177	JQ704517	U5b1b1e	U5b1b1+152	1	U5b1b1e	1			2	2
4178	KC479033	U5b1b1g	U5b1b1g	1					1	1
4179	KP688570	U5b1b1g1	U5b1b1g1	1					1	1
4180	KP835772	U5b1b1g1a	U5b1b1g1a	1					1	1
4181	AY339536	U5b1b2	U5b1b2	1	U5b1b2	2			2	3
4182	HG00357	U5b1b2a	U5b1b2	1	U5b1b2a	2			2	3
4183	JX152982	U5b1b2b	U5b1b2b	1					1	1
4184	AY882409	U5b1c	U5b1c	1					1	1
4185	JQ705870	U5b1c1	U5b1c1	1					1	1
4186	JQ408439	U5b1c1a	U5b1c1a	1					1	1
4187	JX297153	U5b1c1a1	U5b1c1a1	1					1	1
4188	DQ661681	U5b1c2	U5b1c2	1					1	1
4189	EU597535	U5b1c2a	U5b1c2a	1					1	1
4190	JQ704043	U5b1c2b	U5b1c2	1	U5b1c2b	1			2	2
4191	JQ702743	U5b1+16189+@16192	U5b1+16189+@16192	1					1	1
4192	JX677560	U5b1e	U5b1e	1					1	1
4193	FJ493517	U5b1e1	U5b1e1	1					1	1
4194	GU296571	U5b1e1a	U5b1e1a	1					1	1
4195	JQ705183	U5b1h	U5b1h	1					1	1
4196	JQ702807	U5b1d1	U5b1d1	1					1	1
4197	JQ702376	U5b1d1a	U5b	1	U5b1d1a	2			2	3
4198	AY882411	U5b1d1b	U5b1d1b	1					1	1
4199	GU977214	U5b1d1c	U5b1d1c	1					1	1
4200	HM043711	U5b1d2	U5b1d2	1					1	1
4201	HQ675036	U5b1f1	U5b1f1	1					1	1
4202	JX297131	U5b1f1a	U5b1f1a	1					1	1
4203	HQ384206	U5b1g	U5b1g	1					1	1
4204	JQ681270	U5b1i	U5b1i	1					1	1
4205	JQ705429	U5b2	U5b2	1					1	1
4206	JQ703964	U5b2a1a	U5b2a1a2	1	L3	6	U5b2a1a	2	3	9
4207	JQ704726	U5b2a1a+16311	U5b2a1a+16311	1					1	1
4208	GQ853200	U5b2a1a1	U5b2a1a1	1					1	1
4209	JQ701915	U5b2a1a1a	U5b2a1a+16311	1	U5b2a1a1	1	U5b2a1a1a	2	3	4
4210	EU784076	U5b2a1a1b	U5b2a1a1b	1					1	1
4211	HG00284	U5b2a1a1d	U5b2a1a1d	1					1	1
4212	GU296621	U5b2a1a2	U5b2a1a2	1					1	1
4213	EU182656	U5b2a1b	U5b2a1b	1					1	1
4214	GU296541	U5b2a2	U5b2a2	1					1	1
4215	JQ705158	U5b2a2a	U5b2a2a	1					1	1
4216	AY882415	U5b2a2a1	U5b2a2	1	U5b2a2a	2	U5b2a2a1	2	3	5
4217	JQ702167	U5b2a2a2	U5b2a2	1	U5b2a2a	2	U5b2a2a2	2	3	5
4218	JN969984	U5b2a2b	U5b2a2b	1					1	1
4219	GU296567	U5b2a2b1	U5b2a2	1	U5b2a2b	2	U5b2a2b1	1	3	4
4220	JQ702431	U5b2a2c	U5b2a2c	1					1	1
4221	EU682506	U5b2a3	U5b2a3	1					1	1
4222	NA20517	U5b2a3a	U5b2a3a	1					1	1
4223	JQ705337	U5b2a4	U5b2a4	1					1	1
4224	JQ705298	U5b2a4a	U5b2a4a	1					1	1

4225	JQ290366	U5b2a5	U5b2a5	1							1	1
4226	JX152979	U5b2a5a	U5b2a5	1	U5b2a5a	1					2	2
4227	JX154045	U5b2a6	U5b2a5	1	U5b2a5	1	U5b2a6	3			3	5
4228	JQ706019	U5b2b	R	1	L3	2	U5b2b	6			3	9
4229	KC337071	U5b2b1a1	U5b2b1a1	1							1	1
4230	GU296627	U5b2b1a2	U5b2b1a2	1							1	1
4231	GU296641	U5b2b1b	U5b2b1b	1							1	1
4232	EF419891	U5b2b2	U5b2b2	1							1	1
4233	JF812598	U5b2b3	U5b2b3	1							1	1
4234	JQ702843	U5b2b3a	U5b2b3a	1							1	1
4235	JQ702046	U5b2b3a1	U5b2b3a1	1							1	1
4236	JQ703790	U5b2b3a1a	U5b2b3a1a	1							1	1
4237	KC851802	U5b2b3b	U5b2b3b	1							1	1
4238	JQ702531	U5b2b4	U5b2b4	1							1	1
4239	JQ705877	U5b2b4a	U5b2b4a	1							1	1
4240	JX153076	U5b2b5	U5b2b5	1							1	1
4241	KC847158	U5b2c	U5b	1	U5b2	2	U5b2c	1	U5b2c	2	4	6
4242	JX186998	U5b2c1	U5b2c1	1							1	1
4243	JF265240	U5b2c2	U5b	1	U5b2	2	U5b2c	1			3	4
4244	JQ702625	U5b2c2a	U5b2	1	U5b2	2	U5b2c	1	U5b2c2a	1	4	5
4245	EU490797	U5b2c2b	U5b2c2b	1							1	1
4246	JQ705968	U5b3	U5b3	1	U5b3	4					2	5
4247	AF346988	U5b3a1a	U5b3a1a	1							1	1
4248	GQ129157	U5b3a1b	U5b3a1b	1							1	1
4249	GQ129163	U5b3a2	U5b3a2	1							1	1
4250	GQ129168	U5b3b	U5b3h	1	U5b3b	2					2	3
4251	GQ129167	U5b3b1	U5b3b1	1							1	1
4252	JX153697	U5b3b2	U5b3b2	1							1	1
4253	GQ129170	U5b3c	U5b3	1	U5b3c	4					2	5
4254	GQ129173	U5b3d	U5b3d	1							1	1
4255	JQ702295	U5b3e	U5b3	1	U5b3e	4					2	5
4256	GQ129179	U5b3f	U5b3f	1							1	1
4257	GQ129143	U5b3g	U5b3g	1							1	1
4258	JX153636	U5b3h	U5b3h	1							1	1
4259	EF064317	U6a	R30a1c	1	U6a	2					2	3
4260	HQ651694	U6a1	U6a7a	1	U6a7a	1	U6a1	3			3	5
4261	JX120710	U6a1a1	U6a1a1	1							1	1
4262	JQ704749	U6a1a2	U6a1a2	1							1	1
4263	HQ651685	U6a1b1a	U6a1b1a	1							1	1
4264	HQ651693	U6a1b1b	U6a1b1b	1							1	1
4265	JQ702816	U6a1b2	U6a1b2	1							1	1
4266	HQ651705	U6a1b3	U6a1b3	1							1	1
4267	EF064320	U6a1b4	U6a1b4	1							1	1
4268	HQ651707	U6a+16189	U6a+16189<br>U6a+16189+(103)	1	U6a'b'd	4	U6a+16189	2			3	7
4269	EF064322	U6a2	U6a2	1							1	1
4270	JX120713	U6a2a1	U6a2a1	1							1	1
4271	KC152576	U6a2a2	U6a2a2	1							1	1
4272	AY882416	U6a2a2a	U6a2a2a	1							1	1
4273	JX120716	U6a2b	U6a2b	1							1	1
4274	KC152589	U6a2b1	U6a2b1	1							1	1
4275	KC152590	U6a2+195	U6a2+195	1	U6a2+195	1					2	2
4276	KC152569	U6a2c	U6a2c	1							1	1
4277	KC152541	U6a8a	U6a8a	1							1	1
4278	JQ629405	U6a8b	U6a8b	1							1	1
4279	HQ651714	U6a3	U6a3	1							1	1

4280	HQ651713	U6a3a	U6a3a	1											1	1
4281	HQ651686	U6a3a1	U6a	1	U6a+16189	3	U6a3	1	U6a3a	1	U6a3a1	1	U6a3a1	1	6	8
4282	KC152538	U6a3a1a	U6a3a1a	1											1	1
4283	KC152549	U6a3a2	U6a3a2	1											1	1
4284	JX120708	U6a3a2a	U6a3a2a	1											1	1
4285	HQ651704	U6a3b	U6a3b	1											1	1
4286	HQ651697	U6a3b1	U6a3b1	1											1	1
4287	JX120720	U6a3e	U6a3e	1											1	1
4288	KC152584	U6a3f1	U6a3f1	1											1	1
4289	KC152585	U6a3f2	U6a3f2	1											1	1
4290	JX120769	U6a3c	U6a3c	1											1	1
4291	HQ651711	U6a3d1a	U6a3d1a	1											1	1
4292	EF064327	U6a4	U6a4	1											1	1
4293	EF064329	U6a5	U6a5	1											1	1
4294	EF064330	U6a5a	U6a	1	U6a5c	3	U6a5a	2							3	6
4295	HQ592783	U6a5a1	U6a	1	U6a5	4	U6a5a	1	U6a5a1	1					4	7
4296	JX120722	U6a5b	U6a5b	1											1	1
4297	EF064332	U6a5c	U6a2a2	1	U6a5c	4									2	5
4298	KC152565	U6a6a	U6a6a	1	U6a6a	1									2	2
4299	JX120726	U6a6a1	U6a6a1	1											1	1
4300	JX120724	U6a6b1	U6a6b1	1											1	1
4301	EF064334	U6a6b2	U6a6b2	1											1	1
4302	FJ979865	U6a7a1	U6a7a1	1											1	1
4303	GU967378	U6a7a1a	U6a7a	1	U6a7a1	1	U6a7a1a	1							3	3
4304	EF064337	U6a7a1b	U6a7a1b	1											1	1
4305	EF064335	U6a7a1+@152	U6a'b'd	1	U6a7a1+@152	1									2	2
4306	EF064338	U6a7a1c	U6a7a1c	1											1	1
4307	JX120730	U6a7a2	U6a7a2	1	U6a7a2	1									2	2
4308	JX120762	U6a7a2a	U6a7a2	1	U6a7a2a	1									2	2
4309	KC152555	U6a7b	U6a7b	1											1	1
4310	EF064339	U6a7b1	U6a7b1	1											1	1
4311	JQ702118	U6a7c	U6a7c	1											1	1
4312	JX120765	U6a7c1	U6a7c1	1											1	1
4313	AY275530	U6b	U6b	1											1	1
4314	HQ651678	U6b1a	U6b1a	1	U6b1a	1									2	2
4315	EF064340	U6b1a1	U6b1a1	1											1	1
4316	KC152548	U6b1b	U6b1b	1											1	1
4317	JQ704008	U6b2	U6b2	1											1	1
4318	JX120734	U6b3	U6b3	1											1	1
4319	JX120735	U6b3a	U6b3a	1											1	1
4320	JX120741	U6d	U6d	1											1	1
4321	HQ651684	U6d1	U6d1	1	U6d1	2									2	3
4322	GU366066	U6d1a	U6d1	1	U6d1a	2									2	3
4323	EF064341	U6d1b	U6d1b	1											1	1
4324	KC152592	U6d2	U6+16311	1	U6d	2	U6d2	1							3	4
4325	JX120743	U6d3	U6d3	1	U6d3	1									2	2
4326	HQ651681	U6d3a	U6d3a	1											1	1
4327	HQ161773	U6c1	U6c1	1											1	1
4328	KC152568	U6c2	U6c2	1											1	1
4329	FN600416	U2	U2	1											1	1
4330	JX488759	U2a1a	U2a1a	1											1	1
4331	AY713992	U2a1b	U2a1	1	U2a1b	1									2	2
4332	KF056257	U2a2	U2a2	1											1	1
4333	EU330890	U2b1	U2b	1	U2b1	1									2	2
4334	KF056258	U2b1a	U2b1a	1											1	1
4335	AY714027	U2b2	U2b2	1											1	1

4336	AY714010	U2c	U2c	1									1	1
4337	KC533505	U2c1	U2c1	1									1	1
4338	AY714005	U2c1a	U2c1a	1									1	1
4339	HM036556	U2c1b	U2c1b	1									1	1
4340	JQ706062	U2d1	U2d1	1									1	1
4341	JQ705999	U2d2	U2d2	1									1	1
4342	JQ706046	U2d2a	U2d	1	U2d2	1	U2d2a	1					3	3
4343	JQ706045	U2d3	U2d3	1									1	1
4344	AY714026	U2e	U2e	1									1	1
4345	FJ147311	U2e1	U2e1	1									1	1
4346	FJ828532	U2e1a	U2e1a1	1	U2e1	1	U2e1a	1					3	3
4347	JQ703957	U2e1a1	U2e1a1	1									1	1
4348	JQ705117	U2e1a1a	U2e1a1a U2e1g	1	U2e1a1a	2							2	3
4349	HQ424846	U2e1a1b	U2e1a1b	1									1	1
4350	FJ348194	U2e1a1c	U2e1a1c	1									1	1
4351	AY714049	U2e1b	U2e1b	1									1	1
4352	JQ702663	U2e1b1	U2e1b1	1									1	1
4353	JQ705946	U2e1b2	U2e1b2	1									1	1
4354	KF142160	U2e1c	U2e1c	1									1	1
4355	JQ701857	U2e1c1	U2e1	1	U2e1c1	1							2	2
4356	JQ702520	U2e1d	U2e1d	1									1	1
4357	JQ702316	U2e1e	U2e1e	1									1	1
4358	JQ705813	U2e1f	U2e1f	1									1	1
4359	AY195764	U2e1f1	U2e1f1	1									1	1
4360	KC464359	U2e1g	U2e1g	1									1	1
4361	FJ493504	U2e1h	U2e1h	1									1	1
4362	KC911567	U2e2	U2e2	1	U2e2	1							2	2
4363	JQ705900	U2e2a	U2e2a	1									1	1
4364	JQ703968	U2e2a1a	U2e2	1	U2e2a	1	U2e2a1	1	U2e2a1a	3	U2e2a1a	2	5	8
4365	JQ702432	U2e2a1a1	U2e2a1a1	1									1	1
4366	JQ705667	U2e2a1a2	U2e2a1a2	1									1	1
4367	FJ984932	U2e2a1b	U2e2a1b	1									1	1
4368	JQ704627	U2e2a1c	U2e2a1a1	1	U2e2a1c	2							2	3
4369	JQ702004	U2e2a1d	U2e2	1	U2e2a	1	U2e2a1	1	U2e2a1d	3			4	6
4370	JX286498	U2e3	U2e3	1									1	1
4371	JQ704102	U2e3a	U2e3a	1									1	1
4372	HM852891	U3a	U3a	1									1	1
4373	JX153939	U3a1	U3a	1	U3a1	2	U3a1	2					3	5
4374	GU122986	U3a1a	U3a	1	U3a1	2	U3a1a	2	U3a1a	1			4	6
4375	JX153017	U3a1a1	U3a1a1	1									1	1
4376	JQ702681	U3a1b	U3a	1	U3a1	2	U3a1b	2					3	5
4377	JQ703769	U3a1c	U3a1c	1									1	1
4378	JQ705739	U3a1c1	U3a1c1	1									1	1
4379	KC911305	U3a2	U3a2	1									1	1
4380	FJ348224	U3a2a	U3a2a	1									1	1
4381	AY882383	U3a2a1	U3a2a1	1									1	1
4382	KC911522	U3a2a1a	U3a2a1a	1									1	1
4383	JQ704440	U3a3	U3a3	1									1	1
4384	HM852803	U3c	U3c	1									1	1
4385	AY714023	U3b	U3b	1									1	1
4386	EF177433	U3b1	U3b2a1	1	U3b1	2							2	3
4387	EF556158	U3b1a	U3b1a	1									1	1
4388	KC911623	U3b1a1	U3b1a1	1									1	1
4389	FJ711758	U3b1b	U3	1	U3b	2	U3b1	2	U3b1b	1			4	6
4390	JQ702150	U3b2	U3b2	1									1	1
4391	JN969086	U3b2a	U3b2a1	1	U3	1	U3b2a	1					3	3
4392	FJ348193	U3b2a1	U3b2a1	1									1	1

4393	HQ257369	U3b2a1a	U3b2a1a	1											1	1
4394	JQ704981	U3b2b	U3b2b	1											1	1
4395	KC911486	U3b2c	U3	1	U3b	2	U3b2	2	U3b2c	2					4	7
4396	HQ384204	U3b3	U3b3	1											1	1
4397	EU597575	U4a1	U4a1	1											1	1
4398	JQ703929	U4a1a	U4a1a	1											1	1
4399	EU545417	U4a1a1	U4a1	1	U4a1a	2	U4a1a1	3							3	6
4400	JQ703887	U4a1a2	U4a1	1	U4a1a	2	U4a1a2	3							3	6
4401	JQ704345	U4a1a3	U4a1	1	U4a1a	2	U4a1a3	3							3	6
4402	JQ702270	U4a1b	U4a1	1	U4a1b	2									2	3
4403	EU545459	U4a1b1	U4a1b1	1											1	1
4404	GU252762	U4a1b1a	U4a1b1a	1											1	1
4405	EU545432	U4a1b2	U4a1b2	1											1	1
4406	EU545428	U4a1c	U4a1c	1											1	1
4407	FJ493506	U4a1d	U4a1d	1											1	1
4408	JX021669	U4a1e	U4a1e	1											1	1
4409	JQ704052	U4a2	U4a2	1											1	1
4410	EU545450	U4a2a	U4a2a	1											1	1
4411	EF222250	U4a2a1	U4a2a1	1											1	1
4412	EU545461	U4a2a2	U4a2a	1	U4a2a2	2									2	3
4413	EF222239	U4a2a3	U4a2a3	1											1	1
4414	GU123033	U4a2b	U4a2b	1											1	1
4415	EU545464	U4a2c	U4a2	1	U4a2c	5									2	6
4416	EF222252	U4a2c1	U4a2c1	1											1	1
4417	EU545421	U4a2d	U4a2d	1											1	1
4418	EU545442	U4a2e	U4a2	1	U4a2e	5									2	6
4419	JQ705828	U4a2f	U4a2f	1											1	1
4420	JQ480650	U4a2g	U4a2g	1											1	1
4421	EF222235	U4a2h	U4a2	1	U4a2h	5									2	6
4422	JQ702549	U4a2h1	U4a2h1	1											1	1
4423	EF060364	U4a3	U4a3	1											1	1
4424	EU545423	U4a3a	U4a3a	1											1	1
4425	EU545444	U4b	U4	1	U4b	4	U4b	1							3	6
4426	HM044301	U4b1a1	U4	1	U4b	4	U4b1	1	U4b1a	1	U4b1a1	3	U4b1a1	1	6	11
4427	JQ703912	U4b1a1a	U4b1a1a	1											1	1
4428	AY882388	U4b1a1a1	U4b1a1a1	1											1	1
4429	HM041972	U4b1a2	U4	1	U4b	4	U4b1	1	U4b1a	1	U4b1a2	3	U4b1a2	2	6	12
4430	JQ704098	U4b1a2a	U4b1a2a	1											1	1
4431	JX153376	U4b1a2b	U4	1	U4b	4	U4b1	1	U4b1a	1	U4b1a2	3	U4b1a2b	2	6	12
4432	EU545419	U4b1a3	U4b1a3	1											1	1
4433	FJ858802	U4b1a3a	U4b1a3a	1											1	1
4434	FJ147316	U4b1a4	U4b1a4	1											1	1
4435	FJ147315	U4b1+146_152	U4b1b1+16311	1	U4b1b1+16311	1	U4b1+146_152	1							3	3
4436	JN647925	U4b1b1	U4b1b1	1											1	1
4437	HM535647	U4b1b1a	U4b1b1a	1											1	1
4438	JQ704155	U4b1b1b	U4b1b	1	U4b1b1b	2									2	3
4439	FJ858877	U4b1b1+16311	U4b1b1+16311	1	U4b1b1+16311	1									2	2
4440	KC911460	U4b1b1c	U4b1b1c	1											1	1
4441	NA12156	U4b1b1d	U4b1+146_152	1	U4b1b	1	U4b1b1	1	U4b1b1d	1					4	4
4442	HG00155	U4b1b2	U4b1b2	1											1	1
4443	EU140898	U4b2	U4b2	1											1	1
4444	JQ701937	U4b2a	U4b2a	1											1	1
4445	GQ891957	U4b2a1	U4b2a1	1											1	1
4446	GU365881	U4b2a1a	U4b2a1	1	U4b2a1a	1									2	2

4447	FJ147322	U4b3	U4b3	1											1	1
4448	KC911375	U4c1	U4c1	1											1	1
4449	JX153870	U4c1a	U4c1	1	U4c1a	1									2	2
4450	HQ418462	U4c2	U4b1a3	1	U4b1a3	1	U4c2	3							3	5
4451	EU545460	U4c2a	U4c2a	1											1	1
4452	HQ167735	U4d1	U4d1	1											1	1
4453	GU123021	U4d1a	U4	1	U4d	4	U4d1	1	U4d1a	2	U4d1a	1			5	9
4454	JQ702267	U4d1a1	U4	1	U4d	4	U4d1	1	U4d1a	2	U4d1a1	1	U4d1a1	1	6	10
4455	JQ704741	U4d1a1a	U4	1	U4d	4	U4d1	1	U4d1a	2	U4d1a1	1	U4d1a1a	1	6	10
4456	EU545438	U4d1b	U4	1	U4d	4	U4d1	1	U4d1b	2					4	8
4457	FJ230891	U4d2	U4d2	1											1	1
4458	GU727822	U4d3	U4d3	1											1	1
4459	AY882389	U9a	U9a	1											1	1
4460	GU170820	U9a1	U9a1	1											1	1
4461	GU990521	U9b	U9b	1											1	1
4462	AY882390	U9b1	U9b1	1											1	1
4463	AY714004	U7	U7	1											1	1
4464	KC911622	U7a	U7a	1											1	1
4465	KC911620	U7a1	U7a1	1											1	1
4466	AY714014	U7a1a	U7a1a	1											1	1
4467	FJ858878	U7a2	U7a	1	U7a2	2									2	3
4468	KC911509	U7a2a	U7a2a	1											1	1
4469	JQ705966	U7a3a	U7a3a	1											1	1
4470	KF418766	U7a3b	U7a3b	1											1	1
4471	HM852777	U7a4	U7a4	1											1	1
4472	KC911299	U7a4a	U7a4a	1											1	1
4473	HM852823	U7a4a1	U7a4a1	1											1	1
4474	KC911288	U7a4a1a	U7a4a1a	1											1	1
4475	JQ703913	U7a5	U7a5	1											1	1
4476	JQ705198	U7b	U7b	1											1	1
4477	EU445683	U7b1	U7b1	1											1	1
4478	JX153191	U7b2	U7b2	1											1	1
4479	JQ702759	U8a	U8a	1											1	1
4480	AY882392	U8a1a1	U8a1a1	1											1	1
4481	GU299344	U8a1a1a	U8a1a1a	1											1	1
4482	HM765458	U8a1a1a1	U8a1a	1	U8a1a1	3	U8a1a1a	2	U8a1a1a1	1					4	7
4483	JX273295	U8a1a1b	U8a1a	1	U8a1a1	3	U8a1a1b	2	U8a1a1b	1					4	7
4484	HQ022823	U8a1a1b1	U8a1a	1	U8a1a1	3	U8a1a1b	2	U8a1a1b1	1					4	7
4485	HM113490	U8a1a2	U8a1a2	1											1	1
4486	HQ336423	U8a1a3	U8a1a3	1											1	1
4487	JX273297	U8a1a4	U8a1a4	1											1	1
4488	JQ611709	U8a1b	U8a1b	1											1	1
4489	DQ200801	U8a2	U8a2	1											1	1
4490	KC911451	U8b1a1	U8b1a1	1											1	1
4491	KC911536	U8b1a2a	U8b1a2a	1											1	1
4492	JX273294	U8b1a2+16311	U8b1a2+16311	1											1	1
4493	JX273296	U8b1a2b	U8b1a2b	1											1	1
4494	JQ705831	U8b1b	U8b1b	1											1	1
4495	NA20786	U8b1b1	U8b1b1	1											1	1
4496	JX273243	U8b1b2	U8b1b2	1											1	1
4497	HM043706	K1a	K1a	1											1	1
4498	JQ705483	K1a1a	K1a1a	1											1	1
4499	FJ348183	K1a1a1	K1a1b2a	1	K1a1b2a	1	K1a1a1	3							3	5
4500	JQ704696	K1a1a2	K1a3a1	1	K1a1a2	3									2	4
4501	GU980957	K1a1a2a	K1a1a2a	1											1	1
4502	AY495257	K1a1a2a1	K1a	1	K1a1	5	K1a1a	2	K1a1a2	2	K1a1a2a	1	K1a1a2a1	1	6	12
4503	JN048471	K1a1b	K1a1c	1	K1a1b	2									2	3

4504	DQ200804	K1a1b1	H13a1a2a	1	K1a1b1	7	K1a1b1	4							3	12
4505	DQ301795	K1a1b1a	K1a1b1a	1											1	1
4506	JQ702898	K1a1b1b	K1a1b1b	1											1	1
4507	JQ704983	K1a1b1b1	K1a	1	K1a1	5	K1a1b	2	K1a1b1	2	K1a1b1b	4	K1a1b1b1	1	6	15
4508	GU592026	K1a1b1c	K1a1b1c	1											1	1
4509	JQ702782	K1a1b1d	K1a1b1d	1											1	1
4510	JQ704207	K1a1b1e	K1a1b1	1	K1a1b1	2	K1a1b1e	1							3	4
4511	JQ703737	K1a1b1f	K1a1	1	K1a1b	2	K1a1b1	2	K1a1b1f	4					4	9
4512	FJ348218	K1a1b1g	K1a1b1g	1											1	1
4513	AY714044	K1a1b2a	K1a1b2a	1	K1a1b2a	1									2	2
4514	HQ586011	K1a1b2a1	K1a1b2a1	1											1	1
4515	HQ413154	K1a1b2a1a	K1a1b2a	1	K1a1b2a1	1	K1a1b2a1a	1							3	3
4516	FJ865502	K1a1b2b	K1a1b2b	1											1	1
4517	JX153526	K1a1c	K1a1c	1											1	1
4518	KC911410	K1a2	K1a	1	K1a	3	K1a	7	K1a2	2	K1a2	2			5	15
4519	EU915473	K1a2a	K1a8b	1	K1a2a	4									2	5
4520	JQ702748	K1a2a1	K1a2a1	1											1	1
4521	HG00346	K1a2a2	K1a2a2	1											1	1
4522	JQ704017	K1a2b	K1a	1	K1a	3	K1a2b	9							3	13
4523	JQ705149	K1a2c	K1a4+146	1	K1a4+146	2	K1a4+146	1	K1a2c	1					4	5
4524	JQ703347	K1a3	K1a	1	K1a	3	K1a3	9							3	13
4525	EU344156	K1a3a	K1a3a	1											1	1
4526	JQ703096	K1a3a1	K1a3a1	1											1	1
4527	DQ301814	K1a3a1a	K1a	1	K1a3	5	K1a3a	1	K1a3a1	4	K1a3a1a	2			5	13
4528	GU799583	K1a3a1b	K1a2c	1	K1a3a1b	4									2	5
4529	JQ705957	K1a3a2	K1a3a2	1											1	1
4530	JQ701962	K1a3a3	K1a1b1a	1	K1a3a3	3									2	4
4531	HQ610202	K1a3a4	K1a	1	K1a3	5	K1a3a	1	K1a3a4	4					4	11
4532	AY495261	K1a4	K1a	1	K1a4	5	K1a4	5	K1a4	2					4	13
4533	EU603401	K1a4a	K1a	1	K1a+195	1	K1a+195	4	K1a4a	7					4	13
4534	JQ703493	K1a4a1	K1a4+146	1	K1a4+146	1	K1a4a1	7							3	9
4535	JQ702175	K1a4a1a	K1a4a1a	1											1	1
4536	JQ702213	K1a4a1a+195	K1a	1	K1a4a1a+195	1	K1a4a1a+195	2							3	4
4537	JQ702811	K1a4a1a1	K1a	1	K1a4a1a+195	1	K1a4a1a1	2							3	4
4538	JN122621	K1a4a1a3	K1a4a1a3	1											1	1
4539	JQ703168	K1a4a1a2	K1a4a1a2	1	K1a4a1a2	1									2	2
4540	HM031133	K1a4a1a2a	K1a4a1a2a	1											1	1
4541	JQ702896	K1a4a1a2b	K1a4a1a2b	1											1	1
4542	JQ703407	K1a4a1b	K1a4+146	1	K1a4+146	1	K1a4a1b	7							3	9
4543	EU677385	K1a4a1b1	K1a	1	K1a+195	1	K1a4a1	7	K1a4a1	1	K1a4a1b1	2			5	12
4544	JQ703171	K1a4a1b2	K1a4a1b2	1											1	1
4545	JN258704	K1a4a1c	K1a	1	K1a4	5	K1a4a	5	K1a4a1	1	K1a4a1c	5			5	17
4546	JQ705215	K1a4a1c1	K1a4a1c1	1											1	1
4547	JQ705404	K1a4a1d	K1a	1	K1a4	5	K1a4a	5	K1a4a1	1	K1a4a1d	5			5	17
4548	JQ702341	K1a4a1e	K1a4a1e	1											1	1
4549	JQ705816	K1a4a1f	K1a4a1f	1											1	1
4550	JQ702302	K1a4a1f1	K1a4a1f1	1											1	1
4551	JQ704759	K1a4a1g	K1a4a1g	1											1	1
4552	JN001930	K1a4a1h	K1a	1	K1a+195	1	K1a4a1	7	K1a4a1	1	K1a4a1h	2			5	12
4553	KF417432	K1a4a1i	K1a	1	K1a29a	2	K1a4a1i	5							3	8
4554	EU675299	K1a4b	K1a4b	1	K1a4b	1									2	2
4555	EU600366	K1a4b1	K1a4b1	1											1	1
4556	DQ301809	K1a4c	K1a1b1a	1	K1a4c	2									2	3
4557	JQ703523	K1a4c1	K1a4c1	1											1	1
4558	EU926621	K1a4d	K1a	1	K1a4	5	K1a4d	5							3	11
4559	JQ704761	K1a4e	K1a4e	1											1	1
4560	KC900995	K1a4f	K1a4f	1											1	1

4561	JQ704764	K1a4f1	K1a	1	K1a4	5	K1a4f	5	K1a4f1	1					4	12
4562	JQ705656	K1a4g	K1a4g	1											1	1
4563	JQ703361	K1a4h	K1a	1	K1a4	5	K1a4h	5	K1a4h	2					4	13
4564	KC911589	K1a4h1	K1a4h1	1											1	1
4565	JX273269	K1a4i	K1a2a1	1	K1a	1	K1a+195	2	K1a+195	3	K1a	10	K1a4i	1	6	18
4566	JX273271	K1a4+146	K1a13	1	K1a13	1	K1	5	K1a4+146	2					4	9
4567	KC878714	K1a4j	K1a1b1c	1	K1a4j	3									2	4
4568	KC911477	K1a4j1	K1a4j1	1											1	1
4569	EU600368	K1a5a	K1a5a	1											1	1
4570	HQ637410	K1a5b	K1a5b	1											1	1
4571	EU600369	K1a6	K1a6	1											1	1
4572	DQ301807	K1a7	K1a7	1											1	1
4573	JQ702902	K1a8	K1a8	1											1	1
4574	DQ301817	K1a8a	K1a8a	1	K1a8a	1									2	2
4575	DQ301816	K1a8a1	K1a8a	1	K1a8a1	1									2	2
4576	FJ460551	K1a8b	K1a8b	1											1	1
4577	GU471244	K1a+195	K1a+195	1											1	1
4578	GU295448	K1a9	K1a9	1											1	1
4579	JQ702740	K1a10	K1a10	1											1	1
4580	JX535003	K1a10a	K1a10a	1											1	1
4581	JQ702796	K1a13	K1a13	1	K1a13	1									2	2
4582	GU944475	K1a13a	K1a13	1	K1a13a	1									2	2
4583	HQ675035	K1a14	K1a2a1	1	K1a+195	1	K1a+195	1	K1a14	1					4	4
4584	JQ702674	K1a15	K1a15	1											1	1
4585	JQ703365	K1a16	K1a	1	K1a+195	1	K1a16	4							3	6
4586	GU811147	K1a26	K1a26	1											1	1
4587	JQ702273	K1a+150	K1a+150	1											1	1
4588	JQ705747	K1a11	K1a11	1											1	1
4589	JQ703607	K1a11a	K1a11a	1											1	1
4590	JQ705336	K1a11a1	K1a11a	1	K1a11a1	1									2	2
4591	EU523125	K1a11b	K1a11	1	K1a11b	1									2	2
4592	JQ703891	K1a24	K1a+150	1	K1a24	3									2	4
4593	JQ702717	K1a24a	K1a24a	1											1	1
4594	JQ705751	K1a30	K1a5a	1	K1a30	2									2	3
4595	KC878711	K1a30a	K1a30a	1											1	1
4596	KC911309	K1a31	K1a+150	1	K1a+150	3	K1a31	1							3	5
4597	JQ703323	K1a12	K1a+195	0											0	0
4598	EU600362	K1a12a	K1a	1	K1a12	5	K1a12a	1	K1a12a	1					4	8
4599	JQ706035	K1a12a1	K1a	1	K1a12	5	K1a12a	1	K1a12a1	1					4	8
4600	JQ706066	K1a12a1a	K1a1a2a	1											1	1
4601	JQ706034	K1a17	K1a17	1											1	1
4602	JQ706064	K1a17a	K1a17a	1											1	1
4603	EU600365	K1a18	K1a4d	1	K1a18	3									2	4
4604	JQ308836	K1a19	K1a	1	K1a	5	K1a19	5							3	11
4605	JQ706039	K1a19a	K1a19a	1											1	1
4606	JQ703522	K1a23	K1a23	1											1	1
4607	HM625696	K1a25	K1a25	1											1	1
4608	JN415475	K1a27	K1a27	1											1	1
4609	JF303729	K1a28	K1a28	1											1	1
4610	JX273268	K1a29	K1a27	1	K1a29	3									2	4
4611	JX273252	K1a29a	K1a29a	1											1	1
4612	GU797785	K1b1a1	K1b1a	1	K1b1a1	1	K1b1a1	2							3	4
4613	JQ702168	K1b1a1a	K1b1a1+199	1	K1b1a1a	2									2	3
4614	JQ702752	K1b1a1b	K1b1a1b	1											1	1
4615	GU361772	K1b1a1c	K1b1a1c	1											1	1
4616	KC158584	K1b1a1c1	K1b1a1c1	1											1	1
4617	NA11892	K1b1a1d	K1b1a	1	K1b1a1	1	K1b1a1d	2	K1b1a1d	1					4	5

4618	JX891380	K1b1a1d1	K1b1a1d1	1									1	1
4619	EU714300	K1b1a2	K1b1a2	1									1	1
4620	DQ301800	K1b1b	K1b1b	1									1	1
4621	JQ703412	K1b1b1	K1b1b1	1									1	1
4622	EU600370	K1b1c	K1b1c	1									1	1
4623	JQ705467	K1b2a	K1b2	1	K1b2a	2	K1b2a	3					3	6
4624	HQ000094	K1b2a1	K1b2	1	K1b2a	2	K1b2a1	3	K1b2a1	1			4	7
4625	JQ705289	K1b2a1a	K1b2	1	K1b2a	2	K1b2a1	3	K1b2a1a	1			4	7
4626	JQ705519	K1b2a1a1	K1b2a1a1	1									1	1
4627	JQ705044	K1b2a2	K1b2	1	K1b2a	2	K1b2a2	3	K1b2a2	1			4	7
4628	EU849091	K1b2a2a	K1b2a2a	1									1	1
4629	JX273244	K1b2a3	K1b2a3	1									1	1
4630	JQ703876	K1b2b	K1b2	1	K1b2b	2							2	3
4631	JX153522	K1b2b1	K1b2b1	1									1	1
4632	GU936958	K1c1	K1c	1	K1c1	1	K1c1	8					3	10
4633	AY882394	K1c1a	K1c1a	1									1	1
4634	AY495250	K1c1b	K1c	1	K1c1	1	K1c1b	8					3	10
4635	JQ703860	K1c1c	K1c	1	K1c1	1	K1c1c	8					3	10
4636	JQ703292	K1c1d	K1c	1	K1c1	1	K1c1d	8					3	10
4637	GU123011	K1c1e	K1c1e	1									1	1
4638	JQ703815	K1c1f	K1c	1	K1c1	1	K1c1f	8					3	10
4639	JQ702722	K1c1g	K1c	1	K1c1	1	K1c1g	8					3	10
4640	JX153393	K1c1h	K1c1h	1									1	1
4641	JN897373	K1c1i	K1c1i	1									1	1
4642	JN620369	K1c2	K1c2	1	K1c2	1							2	2
4643	DQ830736	K1c2a	K1c2a	1									1	1
4644	JQ704724	K1d	K1d	1	K1d	1							2	2
4645	JX152800	K1d1	K1d	1	K1d1	1							2	2
4646	JX293716	K1e	K1e	1									1	1
4647	EU073969	K1e1	K1e1	1									1	1
4648	EU810403	K1f	K1+16362	1	K1f	1							2	2
4649	JQ702887	K2a	K1b1b1	1	K2a	7							2	8
4650	JQ665461	K2a1	K2a	1	K2a1	8							2	9
4651	DQ301815	K2a1a	K2a1a	1									1	1
4652	AY495246	K2a2	K2a2	1									1	1
4653	HQ154135	K2a2a	K2a2a	1									1	1
4654	EU327986	K2a2a1	K2a2a1	1									1	1
4655	JQ704707	K2a3	K2a	1	K2a3	8	K2a3	1					3	10
4656	JQ703540	K2a3a	K2a	1	K2a3	8	K2a3a	1	K2a3a	1			4	11
4657	AY495249	K2a3a1	K2a	1	K2a3	8	K2a3a	1	K2a3a1	1			4	11
4658	JQ705156	K2a4	K2b1b	1	K2a4	1							2	2
4659	EU597528	K2a5	K2a5	1									1	1
4660	JQ704076	K2a5a	K2a5	1	K2a5a	2							2	3
4661	EU718789	K2a5a1	K2a5a1	1									1	1
4662	AY714017	K2a5b	K2a5b	1									1	1
4663	AY495239	K2a6	K2a	1	K2a6	8							2	9
4664	EU284177	K2a7	K2a7	1									1	1
4665	DQ282493	K2a8	K2a8	1									1	1
4666	JQ702282	K2a9	K2	1	K2	2	K2a9	2					3	5
4667	KF030560	K2a10	K2a	1	K2a10	8							2	9
4668	EU884127	K2a11	K2a11	1									1	1
4669	JQ704070	K2b1	K2a	1	K2a	5	K2a	3	K2b1	3			4	12
4670	KF644446	K2b1a	K2b1a	1									1	1
4671	JQ705092	K2b1a1	K2b1a1	1									1	1
4672	JN409346	K2b1a1a	K2b1a1a	1									1	1
4673	JF497777	K2b1a2	K2	1	K2b	2	K2b1	2	K2b1a	1	K2b1a2	2	5	8

4674	HG00128	K2b1a3	K2b1a3	1	1	1
4675	JQ702075	K2b1a4	K2b1a4	1	1	1
4676	JQ704064	K2b1b	K2b1b	1	1	1
4677	KC911571	K2b2	K2b2	1	1	1
4678	DQ301796	K2c	K2c	1	1	1
4679	NA18539	K3	K3	1	1	1
4680	KC521459	U8c	U8c	1	1	1
