# Supplementary material for: Mitochondrial Haplogroup Classification of Ancient DNA Samples Using Haplotracker
Source: Biomed Res Int. 2022 Mar 18;2022:5344418. doi: 10.1155/2022/5344418 (PMC8956381; doi:10.1155/2022/5344418)
Supplement: Supplementary Materials — Fig. S1: characterization of Phylotree-provided control region sequences tested for haplogroup classification by Haplotracker. Fig. S2: minimum number of amplicons required by Haplotracker in discriminating between haplogroups using mtDNA control and coding region sequences. Fig. S3: variant identification of an aDNA sample (MNW3) using an HRM real-time PCR. Table S1: haplogroups and their variant profiles extracted from Phylotree mtDNA Build 17. Table S2: haplogroup frequency carrying an extra variant in 118,869 haplotypes. Table S3: haplogroup frequency carrying a missing variant in 118,869 haplotypes. Table S4: haplogroup frequency in 118,869 haplotypes. Table S5: list of ancient human samples found in 2,000-year-old elite Xiongnu cemetery in Northeast Mongolia. Table S6: primers for the amplification of mtDNA coding region segments for haplogroup determination. Table S7: high-resolution melting real-time PCR primer design for screening variants to differentiate haplogroups G1a1, G1a1a, and G1a1b. Table S8: haplogroup classification of full-length mtGenome sequences from Phylotree (n = 8,216). Table S9: haplogroup classification with full-length and control region sequences of mtDNA using Haplotracker and HaploGrep 2. Table S10: comparison of servers using control region sequences from GenBank before December 25, 2018 (n = 45,177). Table S11: comparison details for the servers using control region sequences from GenBank before December 25, 2018 (n = 45,177). Table S12: comparison of servers using control region sequences downloaded from GenBank from December 26, 2018 to August 22, 2019. Table S13: sequences of mtDNA PCR products from Mongolian ancient DNA samples. Table S14: haplogroup classification of Mongolian ancient DNA samples using Haplotracker. Table S15: minimum number of amplicons required by Haplotracker in discriminating between haplogroups using mtDNA control and coding region sequences. Table S16: minimum number of amplicons per superhaplogroup requ [file 5344418.f1.zip › 5344418.f19.pdf]

Table S16. Minimum number of amplicons per super-haplogroup required by Haplotracker in discriminating between haplogroups using mtDNA control and coding region sequences

HG	No. of HGs including sub-HGs		Minimum number of amplicons for HG discrimination			
	Phylotree-provided	Phylotree-defined	100%	98%	95%	90%
L*	476	625	10	8	6	4
M*	539	689	50	11	5	4
CZ*	0	2				
C	132	147	11	7	6	4
Z	23	26	6		6	5
E	30	31	6	5	5	4
G	59	72	6	6	5	5
Q	31	39	4	4	4	2
D	286	333	16	12	8	5
N*	96	128	6	4	3	2
I	59	62	7	7	6	5
W	57	59	7	6	6	5
Y	13	13	4	4	3	3
A	142	157	11	6	4	3
O	3	2	4	4	4	3
S	6	7	3	3	3	1
X	74	88	10	10	5	4
R*	134	171	15	11	5	4
HV*	85	95	14	11	10	7
V	57	60	19	18	16	13
H	913	976	69	46	28	20
JT*	0	2				
J	220	239	14	13	12	8
T	212	229	21	15	11	8
F	84	103	8	7	7	4
B	285	320	23	15	8	5
P	21	27	5	5	2	1
U*	460	533	12	9	7	5
K	183	197	18	15	13	11
Total	4680	5432	69	23	13	8

HG, haplogroup

* Number of sub-HGs listed in the table was excluded from sub-HG counting.
