# Supplementary material for: Mitochondrial Haplogroup Classification of Ancient DNA Samples Using Haplotracker
Source: Biomed Res Int. 2022 Mar 18;2022:5344418. doi: 10.1155/2022/5344418 (PMC8956381; doi:10.1155/2022/5344418)
Supplement: Supplementary Materials — Fig. S1: characterization of Phylotree-provided control region sequences tested for haplogroup classification by Haplotracker. Fig. S2: minimum number of amplicons required by Haplotracker in discriminating between haplogroups using mtDNA control and coding region sequences. Fig. S3: variant identification of an aDNA sample (MNW3) using an HRM real-time PCR. Table S1: haplogroups and their variant profiles extracted from Phylotree mtDNA Build 17. Table S2: haplogroup frequency carrying an extra variant in 118,869 haplotypes. Table S3: haplogroup frequency carrying a missing variant in 118,869 haplotypes. Table S4: haplogroup frequency in 118,869 haplotypes. Table S5: list of ancient human samples found in 2,000-year-old elite Xiongnu cemetery in Northeast Mongolia. Table S6: primers for the amplification of mtDNA coding region segments for haplogroup determination. Table S7: high-resolution melting real-time PCR primer design for screening variants to differentiate haplogroups G1a1, G1a1a, and G1a1b. Table S8: haplogroup classification of full-length mtGenome sequences from Phylotree (n = 8,216). Table S9: haplogroup classification with full-length and control region sequences of mtDNA using Haplotracker and HaploGrep 2. Table S10: comparison of servers using control region sequences from GenBank before December 25, 2018 (n = 45,177). Table S11: comparison details for the servers using control region sequences from GenBank before December 25, 2018 (n = 45,177). Table S12: comparison of servers using control region sequences downloaded from GenBank from December 26, 2018 to August 22, 2019. Table S13: sequences of mtDNA PCR products from Mongolian ancient DNA samples. Table S14: haplogroup classification of Mongolian ancient DNA samples using Haplotracker. Table S15: minimum number of amplicons required by Haplotracker in discriminating between haplogroups using mtDNA control and coding region sequences. Table S16: minimum number of amplicons per superhaplogroup requ [file 5344418.f1.zip › 5344418.f3.pdf]

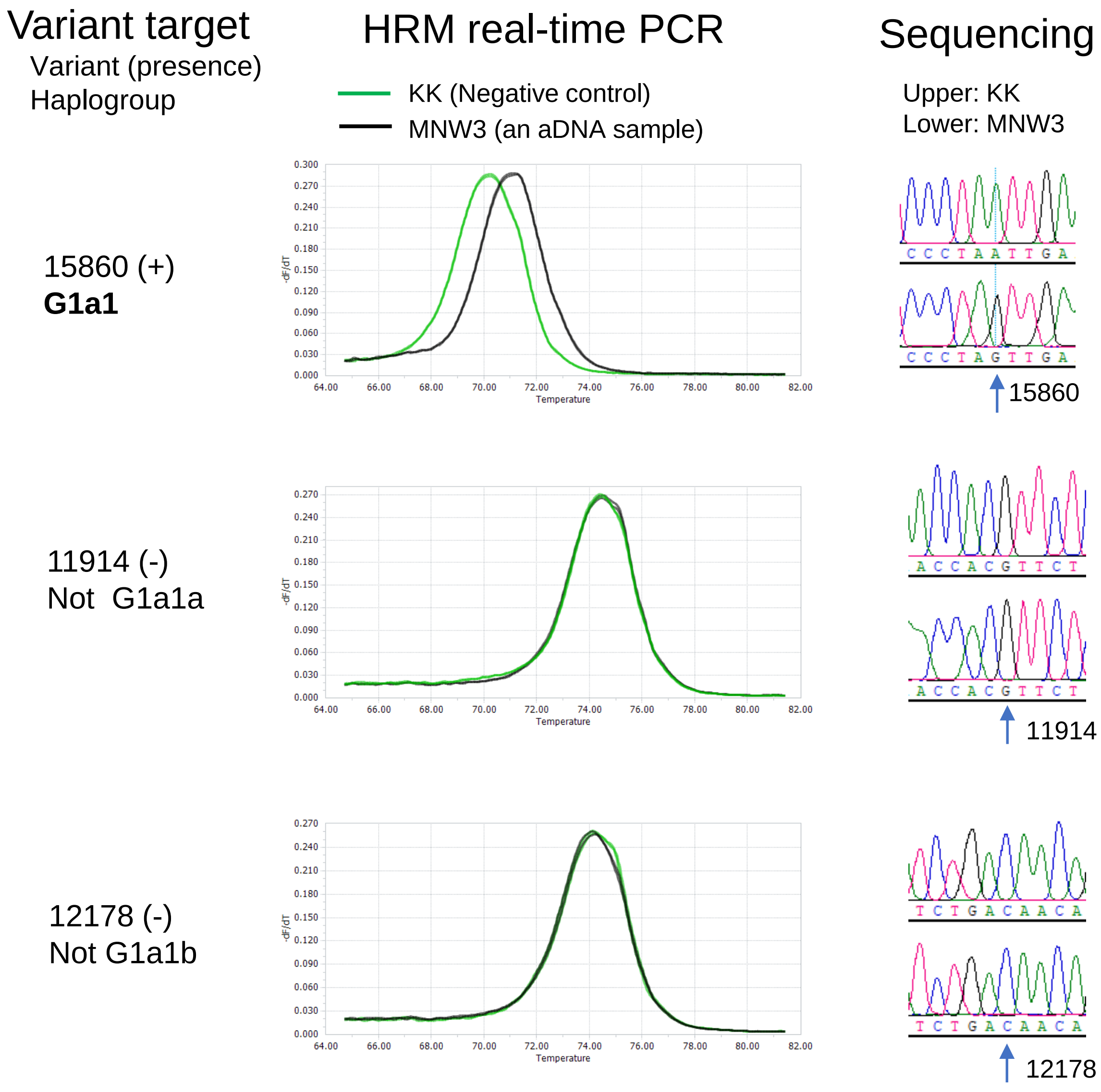

Fig. S3. Variant identification of an aDNA sample (MNW3) using an HRM real-time PCR. Small mtDNA segments carrying haplogroup-specific variant sites were amplified from an aDNA sample (MNW3) and a negative control DNA sample (KK), followed by comparison of melting temperatures to identify variants in the aDNA sample. Three haplogroups and their specific variant site segments were tested to differentiate between them. HRM real-time PCR identified variant 15860 (haplogroup G1a1-specific) in MNW3 by showing a melting peak distinctively different from that of the negative control sample. There were no significant differences in melting peaks for the other haplogroup-specific variants (11914 for G1a1a and 12178 for G1a1b) between MNW3 and negative control. These results demonstrate that the haplogroup of MNW3 was successfully differentiated into G1a1 using the HRM real-time PCR method. The sequencing results were consistent with those of HRM real-time PCR. It is suggested that HRM real-time PCR method can be used for the screening of the presence of variants in mtDNA haplogroup tracking to reduce the number of DNA sequencing. The finally determined haplogroup should be confirmed by DNA sequencing.
