# Supplementary material for: Mitochondrial Haplogroup Classification of Ancient DNA Samples Using Haplotracker
Source: Biomed Res Int. 2022 Mar 18;2022:5344418. doi: 10.1155/2022/5344418 (PMC8956381; doi:10.1155/2022/5344418)
Supplement: Supplementary Materials — Fig. S1: characterization of Phylotree-provided control region sequences tested for haplogroup classification by Haplotracker. Fig. S2: minimum number of amplicons required by Haplotracker in discriminating between haplogroups using mtDNA control and coding region sequences. Fig. S3: variant identification of an aDNA sample (MNW3) using an HRM real-time PCR. Table S1: haplogroups and their variant profiles extracted from Phylotree mtDNA Build 17. Table S2: haplogroup frequency carrying an extra variant in 118,869 haplotypes. Table S3: haplogroup frequency carrying a missing variant in 118,869 haplotypes. Table S4: haplogroup frequency in 118,869 haplotypes. Table S5: list of ancient human samples found in 2,000-year-old elite Xiongnu cemetery in Northeast Mongolia. Table S6: primers for the amplification of mtDNA coding region segments for haplogroup determination. Table S7: high-resolution melting real-time PCR primer design for screening variants to differentiate haplogroups G1a1, G1a1a, and G1a1b. Table S8: haplogroup classification of full-length mtGenome sequences from Phylotree (n = 8,216). Table S9: haplogroup classification with full-length and control region sequences of mtDNA using Haplotracker and HaploGrep 2. Table S10: comparison of servers using control region sequences from GenBank before December 25, 2018 (n = 45,177). Table S11: comparison details for the servers using control region sequences from GenBank before December 25, 2018 (n = 45,177). Table S12: comparison of servers using control region sequences downloaded from GenBank from December 26, 2018 to August 22, 2019. Table S13: sequences of mtDNA PCR products from Mongolian ancient DNA samples. Table S14: haplogroup classification of Mongolian ancient DNA samples using Haplotracker. Table S15: minimum number of amplicons required by Haplotracker in discriminating between haplogroups using mtDNA control and coding region sequences. Table S16: minimum number of amplicons per superhaplogroup requ [file 5344418.f1.zip › 5344418.f4.pdf]

**Table S1. Haplogroups and their variant profiles extracted from Phylotree mtDNA Build 17**

| Haplogroup | Variant profile |
|---|---|
| L0 | 73 146 152 195 247 750 769 825A 1018 1048 1438 2706 2758 2885 3516A 3594 4104 4312 4769 5442 6185 7028 7146 7256 7521 8468 8655 8701 8860 9042 9347 9540 10398 10589 10664 10688 10810 10873 10915 11719 11914 12007 12705 12720 13105 13276 13506 13650 14766 15326 16129 16187 16189 16223 16230 16278 16311 |
| L0a'b'f'g'k | 73 146 152 189 195 247 750 769 825A 1018 1048 1438 2706 2758 2885 3516A 3594 4104 4312 4586 4769 5442 6185 7028 7146 7256 7521 8468 8655 8701 8860 9042 9347 9540 9818 10398 10589 10664 10688 10810 10873 10915 11719 11914 12007 12705 12720 13105 13276 13506 13650 14766 15326 16129 16172 16187 16189 16223 16230 16278 16311 |
| L0a'b'f'g | 146 152 185 189 247 263 750 769 825A 1018 1048 1438 2245 2706 2758 2885 3516A 3594 4104 4312 4586 4769 5442 5603 6185 7028 7146 7256 7521 8468 8655 8701 8860 9042 9347 9540 9818 10398 10589 10664 10688 10810 10873 10915 11641 11719 11914 12007 12705 12720 13105 13276 13506 13650 14766 15136 15326 15431 16129 16172 16187 16189 16223 16230 16278 16311 |
| L0a'b'g | 93 (95C) 146 152 185 189 236 247 263 750 769 825A 1018 1048 1438 2245 2706 2758 2885 3516A 3594 4104 4312 4586 4769 5442 5603 6185 7028 7146 7256 7521 8428 8468 8566 8655 8701 8860 9042 9347 9540 9755 9818 10398 10589 10664 10688 10810 10873 10915 11641 11719 11914 12007 12705 12720 13105 13276 13506 13650 14766 15136 15326 15431 16129 16148 16172 16187 16189 16223 16230 16278 16311 |
| L0a'g | 93 (95C) 146 152 185 189 236 247 263 750 769 825A 1018 1048 1438 2245 2706 2758 2885 3516A 3594 4104 4312 4586 4769 5442 5603 6185 7028 7146 7256 7521 8428 8468 8566 8655 8701 8860 9042 9347 9540 9755 9818 10398 10589 10664 10688 10810 10873 10915 11176 11641 11719 11914 12007 12705 12720 13105 13276 13506 13650 14766 15136 15326 15431 16129 16148 16172 16187 16188G 16189 16223 16230 16278 16311 |
| L0a | 93 (95C) 152 185 189 236 247 263 750 769 825A 1018 1048 1438 2245 2706 2758 2885 3516A 3594 4104 4312 4586 4769 5231 5442 5460 5603 6185 7028 7146 7256 7521 8428 8468 8566 8655 8701 8860 9042 9347 9540 9755 9818 10398 10589 10664 10688 10810 10873 10915 11176 11641 11719 11914 12007 12705 12720 13105 13276 13506 13650 14308 14766 15136 15326 15431 16129 16148 16172 16187 16188G 16189 16223 16230 16311 16320 |
| L0a1'4 | 93 (95C) 152 185 189 236 247 263 750 769 825A 1018 1048 1438 2245 2706 2758 2885 3516A 3594 4104 4312 4586 4769 5231 5442 5460 5603 6185 7028 7146 7256 7521 8428 8468 8566 8655 8701 8860 9042 9347 9540 9755 9818 10398 10589 10664 10688 10810 10873 10915 11176 11641 11719 11914 12007 12705 12720 13105 13276 13506 13650 14308 14766 15136 15326 15431 16129 16148 16168 16172 16187 16188G 16189 16223 16230 16311 16320 |

| | |
|---|---|
| L0a1 | 93 (95C) 152 185 189 236 247 263 750 769 825A 1018 1048 1438 2245 2706 2758 2885 3516A 3594 4104 4312 4586 4769 5096 5231 5442 5460 5603 6185 7028 7146 7256 7521 8428 8468 8566 8655 8701 8860 9042 9347 9540 9755 9818 10398 10589 10664 10688 10810 10873 10915 11176 11641 11719 11914 12007 12705 12720 13105 13276 13506 13650 14308 14766 15136 15326 15431 16129 16148 16168 16172 16187 16188G 16189 16223 16230 16311 16320 |
| L0a1a | (64) 93 (95C) 152 185 189 236 247 263 750 769 825A 1018 1048 1438 2245 2706 2758 2885 3516A 3594 3866 4104 4312 4586 4769 5096 5231 5442 5460 5603 6185 7028 7146 7256 7521 8428 8468 8566 8655 8701 8860 9042 9347 9540 9755 9818 10398 10589 10664 10688 10810 10873 10915 11176 11641 11719 11914 12007 12705 12720 13105 13276 13506 13650 14308 14766 15136 15326 15431 16129 16148 16168 16172 16187 16188G 16189 16223 16230 16311 16320 |
| L0a1a+200 | (64) 93 (95C) 152 185 189 200 236 247 263 750 769 825A 1018 1048 1438 2245 2706 2758 2885 3516A 3594 3866 4104 4312 4586 4769 5096 5231 5442 5460 5603 6185 7028 7146 7256 7521 8428 8468 8566 8655 8701 8860 9042 9347 9540 9755 9818 10398 10589 10664 10688 10810 10873 10915 11176 11641 11719 11914 12007 12705 12720 13105 13276 13506 13650 14308 14766 15136 15326 15431 16129 16148 16168 16172 16187 16188G 16189 16223 16230 16311 16320 |
| L0a1a1 | (64) 93 (95C) 152 185 189 200 236 247 263 750 769 825A 1018 1048 1438 2245 2706 2758 2759 2885 3516A 3594 3866 4104 4312 4586 4769 5096 5231 5442 5460 5603 6185 7028 7146 7256 7521 8428 8468 8566 8655 8701 8860 9042 9347 9540 9755 9818 10398 10589 10664 10688 10810 10873 10915 11176 11641 11719 11914 12007 12705 12720 13105 13276 13506 13650 14308 14766 15136 15326 15431 16129 16148 16168 16172 16187 16188G 16189 16223 16230 16311 16320 |
| L0a1a2 | (64) 93 (95C) 185 189 200 236 247 263 750 769 825A 1018 1048 1438 2245C 2706 2758 2885 3516A 3594 3866 4104 4312 4586 4769 5096 5231 5442 5460 5603 6185 7028 7146 7256 7521 8428 8468 8566 8655 8701 8860 9042 9347 9540 9755 9818 10398 10589 10664 10688 10810 10873 10915 11176 11641 11719 11914 12007 12705 12720 13105 13276 13506 13650 14308 14766 15136 15326 15431 16129 16148 16168 16172 16187 16188G 16189 16223 16230 16311 16320 |
| L0a1a3 | (64) 93 (95C) 152 185 189 200 236 247 263 750 769 825A 1018 1048 1438 2245 2706 2758 2885 3516A 3594 3866 4104 4312 4586 4769 5096 5231 5442 5460 5603 6185 7028 7146 7256 7521 8428 8468 8566 8655 8701 8860 9042 9181 9347 9540 9755 9818 10398 10589 10664 10688 10810 10873 10915 11176 11641 11719 11914 12007 12705 12720 13105 13276 13506 13650 14308 14766 15136 15326 15431 16129 16148 16168 16172 16187 16188G 16189 16223 16230 16311 16320 |

| | |
|---|---|
| L0a1+16293 | 93 (95C) 152 185 189 236 247 263 750 769 825A 1018 1048 1438 2245 2706 2758 2885 3516A 3594 4104 4312 4586 4769 5096 5231 5442 5460 5603 6185 7028 7146 7256 7521 8428 8468 8566 8655 8701 8860 9042 9347 9540 9755 9818 10398 10589 10664 10688 10810 10873 10915 11176 11641 11719 11914 12007 12705 12720 13105 13276 13506 13650 14308 14766 15136 15326 15431 16129 16148 16168 16172 16187 16188G 16189 16223 16230 16293 16311 16320 |
| L0a1b | 93 (95C) 152 185 189 236 247 263 750 769 825A 1018 1048 1438 2245 2706 2758 2885 3516A 3594 4104 4312 4586 4769 5096 5231 5442 5460 5603 5911 6185 7028 7146 7256 7521 8428 8468 8566 8655 8701 8860 9042 9347 9540 9755 9818 10398 10589 10664 10688 10810 10873 10915 11176 11641 11719 11914 12007 12705 12720 13105 13276 13506 13650 14007 14308 14766 15136 15326 15431 16129 16148 16168 16172 16187 16188G 16189 16223 16230 16278 16293 16311 16320 |
| L0a1b1 | 93 (95C) 185 189 236 247 263 750 769 825A 1018 1048 1438 2245 2706 2758 2885 3516A 3594 4104 4312 4586 4769 5096 5231 5442 5460 5603 5911 6185 7028 7146 7256 7521 8191 8428 8468 8566 8655 8701 8860 9042 9347 9540 9755 9818 10398 10589 10664 10688 10810 10873 10915 11176 11641 11719 11914 12007 12705 12720 13105 13276 13506 13650 14007 14308 14766 15136 15326 15431 16129 16148 16168 16172 16187 16188G 16189 16223 16230 16278 16293 16311 16320 |
| L0a1b1a | 93 (95C) 185 189 236 247 263 750 769 825A 1018 1048 1438 2245 2706 2758 2885 3516A 3594 4104 4312 4586 4769 5096 5231 5442 5460 5603 5911 6185 7028 7146 7256 7521 8191 8428 8468 8566 8655 8701 8860 9042 9347 9540 9755 9818 10398 10589 10664 10688 10810 10873 10915 11176 11641 11719 11914 12007 12127 12705 12720 13105 13276 13506 13650 14007 14308 14766 15136 15326 15431 16129 16148 16168 16172 16187 16188G 16189 16223 16230 16278 16293 16311 16320 |
| L0a1b1a1 | 93 (95C) 185 189 236 247 263 750 769 825A 961 1018 1048 1438 2245 2706 2758 2885 3516A 3594 4104 4312 4586 4769 5096 5231 5442 5460 5603 5911 6185 7028 7146 7256 7521 8191 8428 8468 8566 8655 8701 8860 9042 9347 9540 9755 9818 10398 10589 10664 10688 10810 10873 10915 11176 11641 11719 11914 12007 12127 12705 12720 13105 13276 13506 13650 14007 14308 14766 15136 15326 15431 16129 16148 16168 16172 16187 16188G 16189 16223 16230 16278 16293 16311 16320 |
| L0a1b1a1a | 93 (95C) 185 189 236 247 263 750 769 825A 961 1018 1048 1438 2245 2706 2758 2885 3516A 3594 4104 4312 4586 4769 5096 5231 5442 5460 5603 5911 6185 7028 7146 7256 7521 8191 8428 8468 8566 8655 8701 8860 9042 9347 9540 9755 9818 10398 10589 10664 10688 10810 10873 10915 11017 11176 11641 11719 11914 12007 12127 12705 12720 13105 13276 13506 13650 14007 14308 14766 15136 15326 15381 15431 16129 16148 16168 16172 16187 16188G 16189 16223 16230 16278 16293 16311 16320 |

| | |
|---|---|
| L0a1b2 | 93 (95C) 152 185 189 236 247 263 750 769 825A 1018 1048 1438 2245 2706 2758 2885 3516A 3594 4104 4312 4586 4769 5096 5231 5442 5460 5563 5603 5911 6185 7028 7146 7256 7521 8428 8468 8566 8655 8701 8860 9042 9347 9540 9755 9818 10398 10589 10664 10688 10810 10873 10915 11176 11641 11719 11914 12007 12705 12720 13105 13276 13506 13650 14007 14106 14308 14766 15136 15326 15431 16093 16129 16148 16168 16172 16187 16188G 16189 16223 16230 16278 16293 16311 16320 |
| L0a1b2a | 93 (95C) 152 185 189 236 247 263 750 769 825A 1018 1048 1438 2245 2706 2758 2885 3516A 3594 4104 4312 4586 4769 5096 5231 5442 5460 5563 5603 5911 6185 7028 7146 7256 7521 8428 8468 8566 8655 8701 8860 9042 9347 9540 9755 9818 10398 10589 10664 10688 10810 10873 10915 11176 11641 11719 11914 12007 12705 12720 13105 13276 13506 13650 14007 14106 14308 14766 15099 15136 15326 15431 16093 16129 16148 16168 16172 16187 16188G 16189 16223 16230 16278 16293 16311 16320 |
| L0a1c | 93 (95C) 152 185 189 236 247 263 750 769 825A 1018 1048 1438 2245 2706 2758 2885 3516A 3594 4104 4312 4586 4769 5096 5231 5442 5460 5603 6185 7028 7146 7256 7521 8428 8468 8566 8655 8701 8860 9042 9347 9540 9755 9818 10398 10589 10664 10688 10810 10873 10915 11176 11641 11719 11914 12007 12705 12720 13105 13276 13506 13650 14308 14569 14766 15136 15326 15431 16129 16148 16168 16172 16187 16188G 16189 16223 16230 16293 16311 16320 |
| L0a1c1 | 93 (95C) 152 185 189 236 247 263 750 769 825A 1018 1048 1438 2245 2706 2758 2885 3516A 3594 4104 4312 4586 4769 5096 5231 5442 5460 5603 6185 7028 7146 7256 7521 8188 8428 8468 8566 8655 8701 8860 9042 9347 9540 9755 9818 10398 10589 10664 10688 10810 10873 10915 11176 11641 11719 11914 12007 12705 12720 13105 13276 13506 13650 14308 14569 14766 15136 15326 15431 16148 16168 16172 16187 16188G 16189 16223 16230 16287 16293 16311 16320 |
| L0a1d | 93 (95C) 152 185 189 236 247 263 553 750 769 825A 1018 1048 1438 2245 2706 2758 2885 3516A 3594 4104 4312 4586 4769 5096 5231 5442 5460 5603 6185 7028 7146 7256 7521 8428 8468 8566 8655 8701 8860 9042 9347 9540 9755 9818 10398 10589 10664 10688 10810 10873 10915 11176 11641 11719 11914 12007 12557 12705 12720 13105 13276 13506 13650 14308 14766 15136 15326 15431 16129 16148 16168 16172 16187 16188G 16189 16223 16230 16293 16311 |
| L0a1e | 93 (95C) 185 189 236 247 263 750 769 825A 1018 1048 1438 2245 2706 2758 2885 3516A 3594 4104 4312 4586 4769 5096 5147 5231 5442 5460 5603 5608 6185 7028 7146 7256 7521 7568 8428 8468 8566 8655 8701 8860 9042 9347 9540 9755 9818 10398 10589 10664 10688 10810 10873 10915 11176 11641 11719 11914 12007 12705 12720 13105 13276 13506 13650 14308 14766 15136 15326 15431 16129 16148 16172 16187 16188G 16189 16223 16230 16311 16320 |

| | |
|---|---|
| L0a4 | 93 (95C) 185 189 236 247 263 750 769 825A 1018 1048 1438 2245 2706 2758 2885 2887 3010 3516A 3594 4104 4312 4586 4769 5060 5231 5442 5460 5603 6185 7028 7146 7256 7521 7830 8428 8468 8545 8566 8655 8701 8860 8870 9042 9347 9540 9755 9818 10398 10589 10664 10688 10810 10873 10915 11176 11380 11641 11719 11809 11914 12007 12705 12720 12780 13105 13276 13506 13650 14001 14142 14308 14766 15136 15326 15431 16129 16148 16168 16172 16187 16188G 16189 16192 16223 16230 16234 16259A 16311 16319 16320 |
| L0a2 | 64 93 (95C) 152 189 236 247 263 750 769 825A 1018 1048 1438 2245 2706 2758 2885 3516A 3594 4104 4312 4586 4769 5147 5231 5442 5460 5603 5711 6185 6257 7028 7146 7256 7521 8281-8289d 8428 8460 8468 8566 8655 8701 8860 9042 9347 9540 9755 9818 10398 10589 10664 10688 10810 10873 10915 11172 11176 11641 11719 11914 12007 12705 12720 13105 13276 13506 13650 14308 14766 15136 15326 15431 16148 16172 16187 16188G 16189 16223 16230 16311 16320 |
| L0a2a | 64 93 (95C) 152 189 236 247 263 750 769 825A 1018 1048 1438 2245 2706 2758 2885 3516A 3594 4104 4312 4586 4769 5147 5231 5442 5460 5603 5711 6185 6257 7028 7146 7256 7521 8281-8289d 8428 8460 8468 8566 8655 8701 8860 9042 9347 9540 9755 9818 10398 10589 10664 10688 10810 10873 10915 11143 11172 11176 11641 11719 11914 12007 12705 12720 13105 13276 13506 13650 14308 14755 14766 15136 15326 15431 16148 16172 16187 16188G 16189 16223 16230 16311 16320 |
| L0a2a1 | 64 93 (95C) 152 189 236 247 263 750 769 825A 1018 1048 1438 2245 2706 2758 2885 3516A 3594 4104 4312 4586 4769 5147 5231 5442 5460 5603 5711 6185 6257 7028 7146 7256 7521 8281-8289d 8428 8460 8468 8566 8655 8701 8860 9042 9347 9540 9755 9818 10398 10589 10664 10688 10810 10873 10915 11143 11172 11176 11641 11719 11914 12007 12705 12720 13105 13276 13506 13650 14182 14308 14755 14766 15136 15326 15431 16148 16172 16187 16188G 16189 16223 16230 16311 16320 |
| L0a2a1a | 64 93 (95C) 152 189 236 247 263 750 769 825A 1018 1048 1438 2245 2706 2758 2885 3516A 3594 4104 4312 4586 4598 4769 5147 5231 5442 5460 5603 5711 6185 6257 7028 7146 7256 7521 8281-8289d 8428 8460 8468 8566 8655 8701 8860 9042 9347 9540 9755 9818 10398 10589 10664 10688 10810 10873 10915 11143 11172 11176 11641 11719 11914 12007 12705 12720 13105 13276 13506 13650 14182 14308 14755 14766 15136 15326 15431 16148 16172 16187 16188G 16189 16223 16230 16311 16320 |
| L0a2a1a1 | 64 93 (95C) 152 189 236 247 263 750 769 825A 1018 1048 1438 2245 2706 2758 2885 3516A 3594 4104 4312 4586 4598 4769 5147 5231 5442 5460 5603 5711 6185 6257 7028 7146 7256 7521 8264 8281-8289d 8428 8460 8468 8566 8655 8701 8860 9042 9178 9347 9540 9755 9818 10398 10589 10664 10688 10810 10873 10915 11143 11172 11176 11641 11719 11914 12007 12705 12720 13105 13276 13506 13650 14182 14308 14755 14766 15136 15326 15431 16148 16172 16187 16188G 16189 16223 16230 16311 16320 |

| | |
|---|---|
| L0a2a1a2 | 64 93 (95C) 152 189 236 247 263 750 769 825A 1018 1048 1438 2245 2706 2758 2885 3516A 3594 4104 4312 4586 4598 4769 5147 5231 5442 5460 5603 5711 6185 6257 7028 7146 7256 7424 7521 8281-8289d 8428 8460 8468 8566 8655 8701 8860 9042 9347 9540 9755 9818 10398 10589 10664 10688 10810 10873 10915 11143 11172 11176 11641 11719 11914 12007 12705 12720 13105 13276 13506 13650 14182 14308 14755 14766 15136 15326 15431 15617 16148 16172 16187 16188G 16189 16223 16230 16311 16320 |
| L0a2a1b | 64 93 (95C) 152 189 236 247 263 750 769 825A 1018 1048 1438 2245 2706 2758 2885 3516A 3594 3834 4104 4312 4586 4769 5147 5231 5442 5460 5558 5603 5711 6185 6257 7028 7146 7256 7521 8281-8289d 8428 8460 8468 8566 8655 8701 8860 9042 9347 9540 9755 9818 10398 10589 10664 10688 10810 10873 10915 11143 11172 11176 11641 11719 11914 12007 12705 12720 13105 13276 13506 13650 14182 14308 14755 14766 15136 15326 15431 16148 16172 16187 16188G 16189 16223 16230 16311 16320 |
| L0a2a2 | 64 93 (95C) 152 189 204 207 236 247 263 750 769 825A 1018 1048 1438 2245 2706 2758 2885 3516A 3594 4104 4312 4586 4769 5147 5231 5442 5460 5603 5711 6185 6257 7028 7146 7256 7521 8281-8289d 8428 8460 8468 8566 8655 8701 8860 9042 9347 9540 9545 9554 9755 9818 10398 10589 10664 10688 10810 10873 10915 11143 11172 11176 11641 11719 11914 12007 12705 12720 13105 13276 13506 13650 14308 14755 14766 15136 15326 15431 16148 16172 16187 16188G 16189 16223 16230 16311 16320 |
| L0a2a2a | 64 93 (95C) 152 189 204 207 236 247 263 750 769 825A 1018 1048 1438 2245 2706 2758 2885 3516A 3594 4104 4312 4586 4769 5147 5231 5442 5460 5603 5711 6185 6257 7028 7146 7256 7521 8281-8289d 8428 8460 8468 8566 8655 8701 8860 9042 9347 9540 9545 9554 9755 9818 10398 10589 10664 10688 10810 10873 10915 11143 11172 11176 11641 11719 11914 12007 12705 12720 13105 13116 13276 13506 13650 14308 14755 14766 15136 15326 15431 16148 16172 16187 16188G 16189 16223 16230 16311 16320 |
| L0a2a2a1 | 64 93 (95C) 152 189 204 207 236 247 263 750 769 825A 1018 1048 1438 2245 2706 2758 2885 3516A 3594 4104 4312 4586 4769 5147 5231 5442 5460 5603 5711 6185 6257 7028 7146 7256 7521 8281-8289d 8428 8460 8468 8566 8655 8860 9042 9347 9540 9545 9554 9755 9818 10398 10589 10664 10688 10810 10873 10915 11143 11172 11176 11641 11719 11914 12007 12705 12720 13105 13116 13276 13506 13650 14308 14755 14766 14905 15136 15326 15431 16148 16172 16187 16188G 16189 16223 16230 16311 16320 |
| L0a2a2a2 | 64 93 (95C) 152 189 204 207 236 247 263 750 769 825A 1018 1048 1438 2245 2706 2758 2885 3516A 3594 4104 4312 4586 4769 5147 5231 5442 5460 5603 5711 6185 6257 7028 7146 7256 7521 8281-8289d 8428 8460 8468 8566 8655 8701 8860 9042 9347 9540 9545 9554 9734T 9755 9818 10398 10589 10664 10688 10810 10873 10915 11143 11172 11176 11641 11719 11914 12007 12705 12720 13105 13116 13276 13506 13650 14308 14755 14766 15136 15326 15431 16148 16172 16187 16188G 16189 16223 16230 16311 16320 |

| | |
|---|---|
| L0a2b | 64 93 (95C) 236 247 263 750 769 825A 1018 1048 1438 2245 2706 2758 2885 3372 3516A 3594 4104 4312 4586 4769 5147 5231 5237 5442 5460 5603 5711 6185 6257 7028 7146 7256 7521 8281-8289d 8428 8460 8468 8566 8655 8701 8860 9042 9347 9540 9755 9818 10398 10589 10664 10688 10810 10873 10915 11172 11176 11269 11641 11719 11914 12007 12172 12705 12720 13105 13276 13281 13506 13650 14308 14766 15136 15326 15431 16148 16172 16187 16188A 16189 16223 16230 16242 16311 16320 16390 |
| L0a2b1 | 64 93 (95C) 236 247 263 750 769 825A 1018 1048 1438 1598 2245 2706 2758 2885 3372 3516A 3594 4104 4312 4586 4769 5147 5231 5237 5442 5460 5603 5711 6185 6257 7028 7146 7256 7521 8281-8289d 8428 8460 8468 8566 8655 8701 8860 9042 9347 9540 9755 9818 10398 10589 10664 10688 10810 10873 10915 11172 11176 11269 11641 11719 11914 12007 12172 12705 12720 13105 13276 13281 13506 13650 14308 14766 15136 15326 15431 16148 16172 16187 16188A 16189 16223 16230 16242 16311 16320 16390 |
| L0a2c | 64 93 (95C) 152 189 194 236 247 263 750 769 825A 1018 1048 1438 2245 2706 2758 2885 3516A 3594 3693 3738 4012 4104 4312 4586 4769 5147 5231 5442 5460 5603 5711 6185 6257 7028 7146 7256 7521 8281-8289d 8389 8428 8460 8468 8566 8655 8701 8860 9042 9347 9540 9755 9818 10398 10589 10664 10688 10810 10873 10915 11009 11172 11176 11641 11719 11887 11914 12007 12705 12720 13105 13276 13506 13650 13984 14308 14560 14766 15136 15326 15431 16148 16172 16187 16188A 16189 16214 16223 16230 16234 16311 |
| L0a2d | 64 93 (95C) 152 189 236 247 263 750 769 825A 1018 1048 1438 2245 2706 2758 2885 3516A 3594 4104 4312 4586 4769 5147 5231 5442 5460 5581 5603 5711 6185 6257 7028 7146 7256 7521 8281-8289d 8428 8460 8468 8566 8655 8701 8860 9042 9347 9540 9755 9818 10398 10589 10664 10688 10810 10873 10915 11172 11176 11641 11719 11914 12007 12705 12720 13105 13276 13506 13650 14308 14766 15136 15317 15326 15431 16148 16172 16187 16188G 16189 16223 16230 16311 16320 |
| L0a3 | 93 (95C) 152 185 189 236 247 263 750 769 825A 1018 1048 1438 2245 2706 2758 2885 3516A 3594 4104 4312 4586 4769 5231 5442 5460 5603 6185 7028 7146 7256 7521 8428 8468 8566 8655 8701 8860 9042 9347 9540 9755 9818 10398 10589 10664 10688 10810 10873 10915 11176 11641 11719 11914 12007 12705 12720 13105 13276 13506 13650 14308 14581 14766 15136 15326 15431 16129 16148 16172 16187 16188G 16189 16223 16230 16311 16320 |
| L0g | 93 (95C) 146 152 185 189 195 198 204 207 236 247 263 499 750 769 825A 1018 1048 1415 1438 2245 2706 2758 2885 3516A 3594 4104 4312 4466 4586 4769 4814 4943 5141 5442 5656 5782 6185 7028 7146 7256 7521 8047 8224 8428 8468 8566 8655 8701 8860 9042 9347 9540 9755 9818 10039 10398 10427 10589 10664 10688 10810 10873 10915 11176 11641 11719 11914 12007 12528 12720 13105 13276 13431 13506 13650 13767A 14002 14110 14766 15136 15326 15431 15803 16129 16148 16169 16172 16187 16188G 16189 16223 16230 16278 16311 |

| | |
|---|---|
| L0b | 93 (95C) 146 152 185 189 236 247 263 750 769 825A 1018 1048 1438 2245 2706 2758 2885 3516A 3594 4104 4312 4586 4769 5442 5603 6185 6719 7028 7146 7256 7521 8428 8468 8566 8655 8701 8860 9042 9347 9540 9755 9818 10398 10589 10664 10688 10810 10873 10915 11641 11719 11914 12007 12705 12720 13105 13276 13506 13650 14766 15106 15136 15326 15431 15622 16051 16129 16148 16164 16172 16189 16223 16230 16278 16311 |
| L0f | 146 152 185 189 (207) 247 263 750 769 825A 1018 1048 1438 2245 2706 2758 2885 3516A 3594 4104 4312 4586 4769 4964 5442 5603 6185 7028 7146 7148 7256 7521 8468 8655 8701 8860 9042 9347 9540 9581 9620 9818 10398 10589 10664 10688 10810 10873 10915 11641 11719 11914 12007 12705 12720 13105 13276 13470 13506 13650 14109 14620 14766 15136 15326 15431 15852 16129 16169 16172 16187 16189 16223 16230 16278 16311 16327 16368 |
| L0f1 | 151 152 185 189 (207) 247 263 750 769 825A 1018 1048 1438 2245 2706 2758 2885 3516A 3594 4104 4312 4586 4769 4964 5442 5603 6185 7028 7146 7148 7256 7364 7521 8468 8655 8701 8860 9042 9347 9540 9581 9620 9818 10398 10589 10664 10688 10810 10873 10915 11641 11719 11914 12007 12705 12720 13105 13276 13470 13506 13650 14109 14620 14766 15136 15326 15431 15852 16129 16169 16172 16189 16223 16230 16278 16311 16327 16354 16368 |
| L0f2 | 146 152 185 189 (207) 247 263 750 769 825A 1018 1048 1438 2245 2706 2758 2885 3516A 3594 4104 4194 4312 4586 4769 4964 5442 5603 6185 7028 7146 7148 7256 7521 8468 8655 8701 8860 9042 9347 9540 9581 9620 9818 10398 10589 10664 10688 10810 10873 10915 11287 11641 11719 11914 12007 12705 12720 13105 13276 13470 13506 13650 13680 13928C 14109 14620 14766 15136 15326 15431 15852 16129 16169 16172 16187 16189 16223 16230 16278 16311 16327 16368 |
| L0f2a | 146 152 185 189 (207) 247 263 750 769 825A 1018 1048 1438 1719 2245 2706 2758 2885 3516A 3594 4104 4194 4312 4562 4586 4769 4964 5442 5603 6185 7028 7146 7148 7256 7521 8468 8655 8701 8860 9042 9347 9540 9581 9620 9818 10398 10589 10664 10688 10790 10810 10873 10915 11287 11641 11719 11914 12007 12705 12720 13105 13276 13470 13506 13650 13680 13928C 14109 14620 14766 15136 15326 15431 15852 16129 16169 16172 16187 16189 16223 16230 16278 16311 16327 16368 |
| L0f2a1 | 143 146 152 185 189 (207) 247 263 750 769 825A 978 1018 1048 1438 1719 2245 2706 2758 2772 2789 2885 3516A 3594 3852 4104 4194 4312 4562 4586 4769 4964 5321 5442 5603 6185 7028 7146 7148 7256 7521 8468 8655 8701 8860 9042 9347 9540 9581 9620 9818 10143 10398 10589 10664 10688 10790 10810 10873 10915 11287 11299 11641 11719 11914 12007 12705 13105 13276 13470 13506 13650 13680 13708 13928C 14109 14620 14766 15136 15326 15431 15852 16129 16169 16172 16173 16187 16189 16223 16230 16239 16278 16311 16327 16368 |

| | |
|---|---|
| L0f2b | 146 183 184 185 189 (207) 247 263 750 769 783 825A 1018 1048 1438 2245 2706 2758 2885 3027 3516A 3531 3592 3594 4104 4194 4312 4586 4769 4964 5442 5603 6152 6176 6185 7028 7146 7148 7256 7419 7521 8020 8468 8655 8701 8748 8860 9042 9347 9540 9581 9620 9725 9818 10398 10589 10664 10688 10810 10873 10915 11287 11641 11719 11914 11974 12007 12705 12720 12855 13105 13145 13276 13317 13470 13506 13650 13680 13928C 14109 14620 14683 14766 15136 15326 15431 15852 15884 15927 16169 16172 16187 16189 16218 16223 16230 16278 16291 16311 16327 16354 16368 |
| L0k | 73 146 152 189 195 199 247 750 769 825A 850 1018 1048 1243 1438 2706 2758 2885 3516A 3594 4104 4312 4541 4586 4769 4907 5442 5811 6185 7028 7146 7256 7257 7521 8468 8655 8701 8860 8911 8922 8994 9042 9136 9347 9540 9818 10398 10499 10589 10664 10688 10810 10873 10876 10915 10920 11296 11299 11653 11719 11914 12007 12705 12720 13105 13276 13506 13590 13650 13819 13928C 14020 14182 14371 14374 14766 15326 16166C 16172 16187 16189 16214 16223 16230 16278 16291G 16311 |
| L0k1 | 73 146 152 189 195 199 247 750 769 825A 850 1018 1048 1243 1438 2706 2758 2836A 2885 3516A 3594 4104 4312 4541 4586 4769 4907 5442 5811 6185 6938 7028 7146 7256 7257 7521 8468 8655 8701 8860 8911 8922 8994 9042 9136 9347 9540 9818 10398 10499 10589 10664 10688 10810 10873 10876 10915 10920 11296 11299 11653 11719 11914 12007 12705 12720 13105 13276 13506 13590 13650 13819 13928C 14020 14182 14371 14374 14766 15326 16166C 16172 16187 16189 16209 16214 16223 16230 16278 16291G 16311 |
| L0k1a | 73 146 152 189 195 198 247 750 769 825A 850 1018 1048 1243 1438 2706 2758 2836A 2885 3516A 3594 4104 4312 4541 4586 4769 4907 5442 5811 6185 6938 7028 7146 7256 7257 7521 8468 8655 8701 8860 8911 8922 8994 9042 9136 9347 9540 9818 10398 10499 10589 10664 10688 10810 10873 10876 10915 10920 10939 11296 11299 11653 11719 11914 12007 12070 12705 13020 13105 13276 13506 13590 13650 13819 13928C 14020 14182 14371 14374 14766 15326 16166C 16172 16187 16189 16209 16214 16223 16230 16278 16291G 16311 |
| L0k1a1 | 73 146 152 189 195 198 207 247 750 769 825A 850 1018 1048 1243 1438 2706 2758 2836A 2885 3516A 3594 4104 4312 4541 4586 4769 4907 5442 5811 6185 6938 7028 7146 7256 7257 7521 8468 8655 8701 8860 8911 8994 9042 9136 9347 9540 9818 10398 10499 10589 10664 10688 10810 10873 10876 10915 10920 10939 11296 11299 11653 11719 11914 12007 12070 12705 13020 13105 13276 13506 13590 13650 13819 13928C 14020 14182 14371 14374 14766 15326 16166C 16172 16187 16189 16209 16214 16223 16230 16278 16291G 16311 |
| L0k1a1a | 73 146 152 189 195 198 204 207 247 750 769 825A 850 1018 1048 1243 1438 2706 2758 2836A 2885 3516A 3594 4104 4312 4541 4586 4769 4907 5442 5811 6185 6938 7028 7146 7256 7257 7521 8251 8468 8655 8701 8860 8911 8994 9042 9136 9347 9540 9818 10398 10499 10589 10664 10688 10810 10873 10876 10915 10920 10939 11296 11299 11653 11719 11914 12007 12070 12705 13020 13105 13276 13506 13590 13650 13819 13928C 14020 14182 14371 14374 14766 15326 16166C 16172 16187 16189 16209 16214 16223 16230 16278 16291G 16311 |

| | |
|---|---|
| L0k1a1b | 73 146 152 189 195 198 207 247 750 769 825A 850 1018 1048 1243 1438 2706 2758 2836A 2885 3516A 3594 4104 4312 4541 4586 4769 4907 5442 5811 6185 6938 7028 7146 7256 7257 7521 8468 8655 8701 8860 8911 8994 9042 9136 9347 9540 9818 10398 10499 10589 10664 10688 10810 10873 10876 10915 10920 10939 11296 11299 11653 11719 11914 12007 12070 12705 13020 13105 13276 13506 13590 13650 13819 13928C 14020 14182 14371 14374 14569 14766 15326 16166C 16172 16187 16189 16209 16214 16223 16230 16278 16291G 16311 |
| L0k1a1c | 73 146 152 189 195 198 207 247 750 769 825A 850 1018 1048 1243 1438 2060 2706 2758 2836A 2885 3516A 3594 4104 4312 4541 4586 4769 4907 5442 5811 6185 6938 7028 7146 7256 7257 7521 8468 8655 8701 8860 8911 8994 9042 9136 9347 9540 9818 10398 10499 10589 10664 10688 10810 10873 10876 10915 10920 10939 11296 11299 11653 11719 11914 12007 12070 12705 13020 13105 13276 13506 13590 13650 13819 13928C 14020 14182 14371 14374 14766 15326 16166C 16172 16187 16189 16209 16214 16223 16230 16278 16291G 16311 |
| L0k1a1d | 73 146 152 189 195 198 207 247 750 769 825A 850 1018 1048 1243 1438 2706 2758 2836A 2885 3516A 3594 4104 4312 4541 4586 4769 4907 5442 5582 5811 6185 6938 7028 7146 7256 7257 7521 8468 8655 8701 8860 8911 8994 9042 9136 9347 9540 9818 10398 10499 10589 10664 10688 10810 10873 10876 10915 10920 10939 11296 11299 11653 11719 11914 12007 12070 12705 13020 13105 13276 13506 13590 13650 13819 13928C 14020 14182 14371 14374 14766 15326 16166C 16172 16187 16189 16209 16214 16223 16230 16278 16291G 16311 |
| L0k1a2 | 73 146 152 189 195 198 247 750 769 825A 850 1018 1048 1243 1438 2706 2758 2836A 2885 3516A 3594 4104 4312 4541 4586 4769 4907 5442 5811 6185 6938 7028 7146 7256 7257 7521 8222 8468 8655 8701 8860 8911 8922 8994 9042 9136 9347 9540 9818 10398 10499 10589 10664 10688 10810 10873 10876 10915 10920 10939 11296 11299 11653 11719 11914 12007 12070 12705 13020 13105 13276 13506 13590 13650 13819 13928C 14020 14182 14371 14374 14766 15326 16166C 16172 16187 16189 16209 16214 16223 16230 16278 16291G 16311 |
| L0k1a2a | 73 146 152 189 195 198 247 750 769 825A 850 1018 1048 1243 1438 2706 2758 2836A 2885 3516A 3594 4104 4312 4541 4586 4769 4907 5442 5811 6185 6938 7028 7146 7256 7257 7521 8222 8468 8655 8701 8860 8911 8922 8994 9042 9136 9347 9540 9818 10398 10499 10589 10664 10688 10810 10873 10876 10915 10920 10939 10978 11296 11299 11653 11719 11914 12007 12070 12705 13020 13105 13276 13506 13590 13650 13819 13928C 14020 14182 14371 14374 14766 15326 16166C 16172 16187 16189 16209 16214 16223 16230 16278 16291G 16311 |
| L0k1a3 | 73 146 152 189 195 198 247 750 769 825A 850 1018 1048 1243 1438 2706 2758 2836A 2885 3516A 3594 4104 4312 4541 4586 4769 4907 5442 5811 6185 6938 7028 7146 7256 7257 7521 8468 8655 8701 8860 8911 8922 8994 9042 9136 9347 9540 9818 10398 10499 10589 10664 10688 10810 10873 10876 10915 10920 10939 11296 11299 11653 11719 11914 12007 12070 12705 13020 13105 13276 13506 13590 13650 13819 13928C 14020 14182 14371 14374 14766 15326 16129 16166C 16172 16187 16189 16209 16214 16223 16230 16242 16269 16274 16278 16291G 16311 |

| | |
|---|---|
| L0k1b | 73 146 152 189 195 199 247 593 750 769 825A 850 1018 1048 1243 1438 2706 2758 2836A 2885 3516A 3594 3777 4104 4312 4541 4586 4769 4907 5442 5811 6185 6938 7028 7146 7256 7257 7521 8468 8655 8701 8860 8911 8922 8994 9042 9136 9347 9540 9818 10321 10398 10499 10589 10664 10688 10810 10873 10876 10915 10920 11296 11299 11653 11719 11914 12007 12705 12720 13105 13276 13506 13590 13650 13819 13928C 14020 14182 14364 14371 14374 14766 15326 15721 15884 16166C 16172 16187 16189 16209 16214 16223 16230 16278 16291G 16311 |
| L0k2 | 73 146 152 189 195 199 204 247 750 769 825A 850 1018 1048 1243 1438 2706 2758 2885 3516A 3594 4104 4312 4541 4586 4769 4907 5442 5811 6185 7028 7146 7256 7257 7521 8468 8655 8701 8860 8911 8922 8994 9042 9136 9540 9818 9884 10398 10499 10589 10664 10688 10810 10873 10876 10915 10920 11296 11299 11653 11719 11914 12007 12705 12720 13105 13276 13506 13590 13650 13819 13928C 14020 14182 14371 14374 14766 15221 15326 16166C 16172 16187 16189 16214 16230 16278 16291A 16311 |
| L0k2a | 73 146 152 189 195 199 204 247 750 769 825A 850 1018 1048 1243 1438 2706 2758 2885 3516A 3594 4012 4104 4312 4541 4586 4769 4907 5442 5811 6185 7028 7146 7256 7257 7521 8468 8655 8701 8860 8911 8922 8994 9042 9540 9818 9884 10398 10499 10589 10664 10688 10810 10873 10876 10915 10920 11296 11299 11653 11719 11914 12007 12705 12720 13105 13276 13506 13590 13650 13819 13928C 14020 14182 14371 14374 14766 15221 15299 15326 16166C 16172 16187 16189 16214 16230 16278 16291A 16311 16527 |
| L0k2a1 | 73 146 152 189 195 199 204 247 750 769 825A 850 1018 1048 1243 1438 2706 2758 2885 3516A 3594 4012 4104 4312 4541 4586 4769 4907 5442 5811 6185 7028 7146 7256 7257 7521 8468 8655 8701 8860 8911 8922 8994 9042 9540 9818 9884 10398 10499 10589 10664 10688 10810 10873 10876 10915 10920 11296 11299 11653 11719 11914 12007 12705 12720 13105 13276 13506 13590 13650 13819 13928C 14020 14182 14371 14374 14766 15221 15299 15326 16166C 16171 16172 16187 16189 16214 16230 16278 16291A 16311 16527 |
| L0k2a1a | 73 146 152 189 195 199 204 247 750 769 825A 850 1018 1048 1243 1438 2706 2758 2885 3516A 3594 4012 4104 4312 4541 4586 4769 4907 5442 5811 6185 7028 7146 7256 7257 7521 8468 8655 8701 8838 8860 8911 8922 8994 9042 9540 9818 9884 10398 10499 10589 10664 10688 10810 10873 10876 10915 10920 11296 11299 11653 11719 11893 11914 12007 12705 12720 13105 13276 13506 13590 13650 13819 13928C 14020 14182 14371 14374 14766 15221 15299 15326 16166C 16171 16172 16187 16189 16214 16230 16278 16291A 16311 16360 16527 |
| L0k2b | 73 146 152 189 195 199 204 247 750 769 825A 850 1018 1048 1243 1438 2706 2758 2885 3516A 3594 4104 4312 4541 4586 4769 4907 5442 5811 6185 7028 7146 7256 7257 7521 8468 8655 8701 8860 8911 8922 8994 9042 9136 9540 9818 9884 10398 10499 10589 10664 10688 10810 10873 10876 10915 10920 11296 11299 11653 11719 11914 12007 12705 12720 13105 13276 13506 13590 13650 13819 13928C 14020 14182 14371 14374 14766 15221 15326 16166C 16172 16187 16189 16214 16230 16278 16287 16291A 16311 |

| | |
|---|---|
| L0d | 73 146 152 195 247 750 769 825A 1018 1048 2706 2758 2885 3516A 3594 4104 4232 4312 4769 5442 6185 6815 7028 7146 7256 7521 8113A 8152 8251 8468 8655 8701 8860 9042 9347 9540 10398 10589 10664 10688 10810 10873 10915 11719 11914 12007 12121 12705 12720 13105 13276 13506 13650 14766 15326 15466 15930 15941 16129 16187 16189 16223 16230 16243 16278 16311 |
| L0d1'2 | 73 146 152 195 247 498d 750 769 825A 1018 1048 2706 2758 2885 3516A 3594 3756 4104 4232 4312 4769 5442 6185 6815 7028 7146 7256 7521 8113A 8152 8251 8468 8655 8701 8860 9042 9347 9540 9755 10398 10589 10664 10688 10810 10873 10915 11719 11914 12007 12121 12705 12720 13105 13276 13506 13650 14766 15326 15466 15930 15941 16129 16187 16189 16223 16230 16243 16311 |
| L0d1 | 73 146 152 195 247 498d 719 750 769 825A 1018 1048 2758 2885 3438 3516A 3594 3756 4104 4232 4312 4769 5442 6185 6266 6815 7028 7146 7256 7521 8113A 8152 8251 8468 8655 8701 8860 9042 9347 9540 9755 10398 10589 10664 10688 10810 10873 10915 11719 11914 12007 12121 12705 12720 13105 13276 13506 13650 13759 14766 15326 15466 15930 15941 16129 16187 16189 16223 16230 16243 16311 |
| L0d1a'c'd | 73 146 152 195 247 498d 719 750 769 825A 1018 1048 2758 2885 3438 3516A 3594 3756 4104 4232 4312 4769 5442 6185 6266 6815 7028 7146 7256 7521 8113A 8152 8251 8468 8655 8701 8860 9042 9347 9540 9755 10398 10589 10664 10688 10810 10873 10915 11719 11914 12007 12121 12705 12720 13105 13276 13506 13650 13759 14766 15326 15466 15930 15941 16129 16187 16189 16223 16230 16234 16243 16311 |
| L0d1a'd | 73 146 195 247 498d 719 750 769 825A 1018 1048 2758 2885 3438 3516A 3594 3756 4104 4232 4312 4769 5442 6185 6266 6815 7028 7146 7256 7521 8113A 8152 8251 8468 8655 8701 8860 9042 9347 9540 9755 10398 10589 10664 10688 10810 10873 10915 11719 11914 12007 12121 12705 12720 13105 13276 13506 13650 13759 14766 15326 15466 15930 15941 16129 16187 16189 16230 16234 16243 16311 |
| L0d1a | 73 146 195 247 498d 719 750 769 825A 1018 1048 1243 2758 2885 3438 3516A 3594 3756 4104 4232 4312 4769 5442 6185 6266 6815 7028 7146 7256 7521 8113A 8152 8251 8468 8655 8701 8860 9042 9347 9540 9755 9950 10398 10589 10664 10688 10810 10873 10915 11719 11914 12007 12121 12142 12705 12720 13105 13276 13506 13650 13759 14766 15326 15466 15930 15941 16129 16187 16189 16230 16234 16243 16266G 16311 |
| L0d1a1 | 73 146 195 199 247 498d 719 750 769 825A 1018 1048 1243 2758 2885 3438 3516A 3594 3756 4104 4232 4312 4769 5442 6185 6266 6815 7028 7146 7256 7521 8113A 8152 8251 8468 8655 8701 8860 9042 9347 9540 9755 9950 10398 10589 10664 10688 10810 10873 10915 11719 11914 12007 12121 12142 12705 12720 12798 13105 13276 13506 13650 13759 14766 15326 15466 15930 15941 16129 16187 16189 16230 16234 16243 16266G (16266A) 16311 |

| | |
|---|---|
| L0d1a1a | 73 146 195 199 247 498d 719 750 769 825A 1018 1048 1243 2758 2885 3438 3516A 3594 3756 4104 4232 4312 4769 5442 6185 6266 6815 7028 7146 7256 7389 7521 8113A 8152 8251 8468 8655 8701 8860 9042 9347 9540 9755 9950 10398 10589 10664 10688 10810 10873 10915 11719 11914 12007 12121 12142 12696 12705 12720 12798 13105 13276 13506 13650 13759 14766 15326 15466 15930 15941 16129 16187 16189 16230 16234 16243 16266G (16266A) 16311 |
| L0d1a1a1 | 73 146 195 247 498d 719 750 769 825A 1018 1048 1243 2758 2885 3438 3516A 3594 3756 4104 4232 4312 4769 5442 6185 6266 6815 7028 7146 7256 7389 7521 8113A 8152 8251 8468 8655 8701 8860 9042 9347 9540 9755 9950 10398 10589 10664 10688 10810 10873 10915 11719 11914 12007 12121 12142 12696 12705 12720 12798 13105 13276 13506 13650 13759 14766 15326 15466 15930 15941 16129 16187 16189 16230 16234 16243 16266G (16266A) 16311 |
| L0d1a1a2 | 73 146 195 199 247 318 498d 719 750 769 825A 1018 1048 1243 2758 2885 3438 3516A 3594 3756 4104 4232 4312 4769 5442 6185 6266 6815 7028 7146 7256 7389 7521 8113A 8152 8251 8468 8655 8701 8860 9042 9347 9540 9755 9950 10398 10589 10664 10688 10810 10873 10915 11719 11914 12007 12121 12142 12696 12705 12720 12798 13105 13276 13506 13650 13759 14766 15326 15466 15930 15941 16129 16187 16189 16230 16234 16243 16266G (16266A) 16311 |
| L0d1a1a3 | 73 146 195 199 247 498d 719 750 769 825A 1018 1048 1243 2758 2885 3438 3516A 3594 3756 4104 4232 4312 4769 5442 6185 6266 6815 7028 7146 7256 7389 7521 8113A 8152 8251 8468 8655 8701 8860 9042 9347 9540 9755 9950 10398 10589 10664 10688 10810 10873 10915 11719 11914 12007 12121 12142 12696 12705 12720 12798 13105 13276 13506 13650 13759 14750 14766 15326 15466 15930 15941 16129 16187 16189 16230 16234 16243 16266G (16266A) 16311 |
| L0d1a1b | 73 146 195 199 247 498d 719 750 769 825A 1018 1048 1243 2758 2885 3438 3516A 3594 3756 4104 4232 4312 4769 5442 6185 6266 6815 7028 7146 7256 7521 8113A 8152 8251 8468 8655 8701 8860 9042 9347 9540 9755 9950 10398 10589 10664 10688 10810 10873 10915 11719 11914 12007 12121 12142 12705 12720 12798 13105 13276 13506 13650 13759 13855 13967 14766 15326 15466 15930 15941 16129 16187 16189 16230 16234 16243 16266G (16266A) 16311 |
| L0d1a1b1 | 73 146 153 195 199 247 498d 719 750 769 825A 1018 1048 1243 2758 2885 3438 3516A 3594 3756 4104 4232 4312 4769 5442 6185 6266 6815 7028 7146 7256 7521 8113A 8152 8251 8468 8655 8701 8860 9042 9347 9540 9755 9950 10398 10589 10664 10688 10810 10873 10915 11719 11914 12007 12121 12142 12705 12720 12798 13105 13276 13506 13650 13759 13855 13967 14766 15326 15466 15930 15941 16129 16187 16189 16209 16230 16234 16243 16266G (16266A) 16311 |

| | |
|---|---|
| L0d1a1b1a | 73 146 153 195 199 247 498d 719 750 769 825A 1018 1048 1243 2758 2885 3438 3516A 3594 3756 4104 4232 4312 4769 5442 6185 6266 6815 7028 7146 7256 7521 8113A 8152 8251 8468 8655 8701 8860 9042 9347 9540 9755 9950 10319 10398 10589 10664 10688 10810 10873 10915 11719 11914 12007 12121 12142 12705 12720 12798 13105 13276 13506 13650 13759 13855 13967 14766 15326 15466 15930 15941 16129 16187 16189 16209 16230 16234 16243 16266G (16266A) 16311 |
| L0d1a1b1b | 73 146 153 195 199 247 498d 719 750 769 825A 1018 1048 1243 2758 2885 3438 3516A 3594 3756 4104 4232 4312 4769 5442 6185 6266 6815 7028 7146 7256 7521 8113A 8152 8251 8468 8655 8701 8860 9042 9347 9540 9755 9950 10398 10589 10664 10688 10810 10873 10915 11719 11914 12007 12121 12142 12705 12720 12798 13105 13276 13506 13650 13759 13855 13967 14766 14953 15326 15466 15930 15941 16129 16187 16189 16209 16230 16234 16243 16266G (16266A) 16311 |
| L0d1a1c | 73 146 152 199 247 498d 719 750 769 825A 1018 1048 1243 1719 2758 2885 3438 3516A 3594 3756 4104 4232 4312 4769 5442 6185 6266 6815 7028 7146 7256 7283 7521 8113A 8152 8251 8468 8655 8701 8860 9042 9347 9540 9755 9950 10237 10398 10589 10664 10688 10810 10873 10915 11719 11914 12007 12121 12142 12705 12720 12798 13105 13276 13506 13650 13759 14766 15326 15466 15924 15930 15941 16129 16187 16189 16230 16234 16243 16266G (16266A) 16311 |
| L0d1a1d | 73 146 195 199 247 498d 719 750 769 825A 1018 1048 1243 2758 2885 3438 3516A 3594 3756 3849 4104 4232 4312 4769 5442 6185 6266 6815 7028 7146 7256 7521 8113A 8152 8251 8468 8655 8701 8793 8860 9042 9347 9540 9755 9950 10398 10589 10664 10688 10810 10873 10915 11719 11914 12007 12121 12142 12705 12720 12798 13105 13276 13506 13650 13759 14766 15326 15466 15930 15941 16129 16187 16189 16230 16234 16243 16266G (16266A) 16311 |
| L0d1d | 73 146 195 247 498d 719 750 769 825A 1018 1048 2491.1C 2758 2885 3438 3516A 3594 3756 4104 4232 4312 4688 4769 5104 5442 6185 6266 6815 7028 7146 7256 7521 8113A 8152 8251 8270 8468 8655 8701 8860 9042 9347 9540 9755 10398 10589 10664 10688 10810 10873 10915 11447 11611 11719 11914 12007 12121 12705 12720 13105 13276 13650 13759 14560 14766 15326 15459 15466 15930 15941 16172 16187 16189 16218 16230 16234 16243 16311 |
| L0d1c | 73 146 152 195 247 456 498d 719 750 769 825A 1018 1048 2758 2885 3438 3516A 3594 3756 4104 4197 4232 4312 4769 5442 6185 6266 6815 7028 7146 7256 7521 8113A 8152 8251 8468 8655 8701 8860 9042 9150 9347 9540 9755 10398 10589 10664 10688 10810 10873 10915 11437 11719 11914 12007 12121 12235 12705 12720 13105 13129 13276 13506 13650 13759 14766 15326 15466 15930 15941 15951 16187 16189 16223 16230 16234 16243 16311 |

| | |
|---|---|
| L0d1c1 | 73 146 152 195 247 456 498d 719 750 769 825A 1018 1048 2758 2885 3438 3516A 3594 3756 4104 4197 4232 4312 4769 5442 6185 6266 6815 7028 7146 7256 7521 8113A 8152 8251 8468 8655 8701 8860 9042 9150 9347 9540 9755 10398 10589 10664 10688 10810 10873 10915 11437 11719 11914 12007 12121 12235 12705 12720 13105 13129 13276 13506 13650 13759 14766 15326 15466 15550 15930 15941 15951 16187 16189 16223 16230 16234 16242 16243 16311 |
| L0d1c1a | 73 146 152 195 247 456 498d 719 750 769 825A 1018 1048 2758 2885 3438 3516A 3594 3756 4104 4197 4232 4312 4769 5442 6185 6266 6815 7028 7146 7256 7521 7828 8113A 8152 8251 8468 8655 8701 8860 9042 9150 9347 9438 9540 9755 10398 10589 10664 10688 10810 10873 10915 11437 11719 11914 12007 12121 12235 12705 12720 13105 13129 13276 13506 13650 13759 14766 15326 15466 15550 15930 15941 15951 16187 16189 16223 16230 16234 16242 16243 16311 |
| L0d1c1a1 | 73 146 152 195 198 247 456 498d 719 750 769 825A 1018 1048 2758 2885 3438 3516A 3594 3756 4104 4197 4232 4312 4769 5442 6185 6266 6815 7028 7146 7256 7521 7828 8113A 8152 8251 8468 8655 8701 8860 9042 9150 9347 9438 9540 9755 10398 10589 10664 10688 10810 10873 10915 11437 11719 11914 12007 12121 12235 12705 12720 13105 13129 13276 13506 13650 13759 14766 15326 15466 15550 15930 15941 15951 16167 16187 16189 16223 16230 16234 16242 16243 16311 |
| L0d1c1a1a | 73 146 152 195 198 247 456 498d 719 750 769 825A 1018 1048 2758 2885 3438 3516A 3594 3666 3756 4104 4197 4232 4312 4769 5442 6185 6266 6815 7028 7146 7256 7521 7828 8113A 8152 8251 8468 8655 8701 8860 9042 9150 9347 9438 9540 9755 10398 10589 10664 10688 10810 10873 10915 11437 11719 11914 12007 12121 12235 12705 12720 13105 13129 13276 13506 13650 13759 14766 15326 15466 15550 15930 15941 15951 16167 16187 16189 16223 16230 16234 16242 16243 16311 |
| L0d1c1a1a1 | 73 146 152 195 198 247 294 456 498d 719 750 769 825A 1018 1048 2758 2885 3438 3516A 3594 3666 3756 4104 4197 4232 4312 4769 5442 5460 6185 6266 6815 7028 7146 7256 7521 7828 8113A 8152 8251 8468 8655 8701 8860 9042 9150 9347 9438 9540 9755 10398 10589 10664 10688 10810 10873 10915 11437 11719 11914 12007 12121 12235 12705 12720 13105 13129 13276 13506 13650 13759 14766 15326 15466 15550 15731 15930 15941 15951 16167 16187 16189 16223 16230 16234 16242 16243 16311 |
| L0d1c1a1a2 | 73 146 152 195 198 247 456 498d 719 750 769 825A 1018 1048 2758 2885 3438 3516A 3594 3666 3756 4104 4197 4232 4312 4769 5442 6185 6266 6815 7028 7146 7256 7521 7828 8113A 8152 8251 8468 8655 8701 8860 9042 9105 9150 9347 9438 9540 9755 10398 10589 10664 10688 10810 10873 10915 11437 11719 11914 12007 12121 12235 12705 12720 13105 13129 13276 13506 13650 13759 14766 15326 15466 15550 15930 15941 15951 16167 16187 16189 16223 16230 16234 16242 16243 16311 |

| | |
|---|---|
| L0d1c1a1b | 73 146 152 195 198 247 456 498d 719 750 769 825A 1018 1048 2758 2885 3438 3516A 3594 3756 4104 4197 4232 4312 4769 5442 6185 6266 6815 7028 7146 7256 7521 7828 7967 8113A 8152 8251 8468 8655 8701 8860 9042 9150 9347 9438 9540 9755 10398 10589 10664 10688 10810 10873 10915 11437 11719 11914 12007 12121 12235 12705 12720 13105 13129 13276 13506 13650 13759 14766 15326 15466 15550 15930 15941 15951 16167 16187 16189 16223 16230 16234 16242 16243 16311 |
| L0d1c1a2 | 73 114A 146 152 195 247 456 498d 719 750 769 825A 1018 1048 2758 2885 3438 3516A 3594 3756 4104 4197 4232 4312 4769 5442 5460 6185 6266 6815 7028 7146 7256 7521 7828 8113A 8152 8251 8468 8655 8701 8860 9042 9150 9347 9438 9540 9755 10398 10589 10664 10688 10810 10873 10915 11437 11719 11914 12007 12121 12235 12705 12720 13105 13129 13203 13276 13506 13650 13759 14766 15326 15466 15550 15930 15941 15951 16187 16189 16223 16230 16234 16242 16243 16311 |
| L0d1c2 | 73 146 152 195 247 456 498d 719 750 769 825A 1018 1048 2758 2885 3438 3516A 3594 3756 4104 4197 4232 4312 4769 5442 6185 6266 6815 7028 7146 7256 7521 8113A 8152 8251 8468 8655 8701 8860 9042 9150 9347 9540 9755 10398 10589 10664 10688 10810 10873 10915 11437 11719 11914 12007 12121 12235 12705 12720 13105 13129 13276 13506 13650 13759 14766 15326 15466 15930 15941 15951 16187 16189 16223 16230 16234 16243 16294G 16311 |
| L0d1c2a | 73 146 152 195 200 247 456 498d 719 750 769 825A 1018 1048 2758 2885 3438 3516A 3594 3756 4104 4197 4232 4312 4769 5442 6185 6266 6815 6929 7028 7146 7256 7521 8113A 8152 8251 8468 8655 8701 8860 9042 9150 9347 9540 9755 10398 10589 10664 10688 10810 10873 10915 11437 11719 11914 12007 12121 12235 12705 12720 13105 13129 13276 13506 13650 13759 14766 15326 15466 15930 15941 15951 16187 16189 16223 16230 16234 16243 16256 16294A 16311 |
| L0d1c2a1 | 73 146 152 195 200 247 456 498d 719 750 769 825A 1018 1048 2758 2885 3438 3516A 3594 3756 4104 4197 4232 4312 4769 5442 6185 6266 6815 6929 7028 7146 7256 7521 8113A 8152 8251 8468 8655 8701 8860 9042 9150 9180 9347 9540 9755 10398 10589 10664 10688 10810 10873 10915 11437 11719 11914 12007 12121 12235 12609A 12705 12720 13105 13129 13276 13506 13650 13759 14766 15326 15466 15470 15930 15941 15951 16187 16189 16223 16230 16234 16243 16256 16274 16294A 16311 |
| L0d1c3 | 73 146 152 195 247 265 456 498d 719 750 769 825A 1018 1048 2758 2885 3438 3516A 3591 3594 3756 4104 4197 4232 4312 4769 5442 6185 6266 6815 7028 7146 7256 7521 8113A 8152 8251 8468 8655 8701 8860 9042 9150 9347 9540 9755 10398 10589 10664 10688 10810 10873 10915 11437 11719 11914 12007 12121 12235 12705 12720 13105 13129 13276 13506 13650 13759 14766 15326 15466 15519 15930 15941 15951 16093 16187 16189 16223 16230 16234 16243 16311 |
| L0d1b | 73 146 152 195 247 498d 719 750 769 825A 1018 1048 2758 2885 3438 3516A 3594 3618 3756 4104 4232 4312 4769 5442 6185 6266 6815 7028 7146 7256 7521 8113A 8152 8251 8468 8655 8701 8860 9042 9347 9540 9755 10398 10589 10664 10688 10810 10873 10915 11719 11914 12007 12121 12705 12720 13105 13276 13506 13650 13759 14766 15326 15466 15930 15941 16129 16187 16189 16223 16230 16243 16311 |

| | |
|---|---|
| L0d1b1 | 73 146 152 188 195 247 498d 719 750 769 825A 1018 1048 2484.1C 2758 2885 3438 3516A 3594 3618 3756 4104 4232 4312 4562C 4769 5442 6185 6266 6815 7028 7146 7256 7521 8113A 8152 8251 8468 8655 8701 8860 9042 9347 9540 9755 10589 10664 10688 10810 10873 10915 11719 11914 12007 12121 12705 12720 13105 13276 13506 13650 13759 14766 15326 15466 15930 15941 16129 16179 16187 16189 16223 16230 16243 16311 |
| L0d1b1a | 73 146 152 188 195 247 498d 719 750 769 825A 1018 1048 2484.1C 2758 2885 3438 3516A 3531 3594 3618 3756 4104 4232 4312 4562C 4769 5442 6185 6266 6815 7028 7146 7256 7521 8027 8113A 8152 8251 8468 8655 8701 8860 9042 9347 9540 9755 10589 10664 10688 10707G 10810 10873 10915 11719 11914 12007 12121 12705 12720 13105 13276 13506 13650 13759 14766 15217 15326 15466 15479 15930 15941 16129 16179 16187 16189 16223 16230 16243 16311 |
| L0d1b1a1 | 73 146 152 188 195 247 498d 719 750 769 825A 1018 1048 2484.1C 2758 2885 3438 3516A 3531 3594 3618 3756 4104 4232 4312 4562C 4769 5442 6185 6266 6815 7028 7146 7256 7521 8027 8113A 8152 8251 8468 8655 8701 8860 9042 9347 9540 9755 10589 10664 10688 10707G 10810 10873 10915 11719 11914 12007 12121 12705 12720 13105 13276 13506 13650 13759 14766 15217 15314 15326 15466 15479 15930 15941 16129 16179 16187 16189 16223 16230 16243 16311 |
| L0d1b1b | 73 146 152 188 195 247 498d 719 750 769 825A 1018 1048 2484.1C 2758 2885 3438 3516A 3594 3618 3756 4104 4232 4312 4562C 4769 5442 6185 6266 6815 7028 7146 7256 7521 8113A 8152 8251 8468 8655 8701 8860 9042 9347 9540 9755 10589 10664 10688 10810 10873 10915 11341 11719 11914 12007 12121 12705 12720 13105 13276 13506 13650 13759 14766 15326 15466 15930 15941 16129 16179 16187 16189 16223 16230 16243 16311 |
| L0d1b1b1 | 73 146 152 188 195 204 207 247 498d 719 750 769 825A 1018 1048 2484.1C 2758 2885 3438 3516A 3594 3618 3756 4104 4232 4312 4562C 4769 5442 6185 6266 6815 7028 7146 7256 7521 8113A 8152 8251 8468 8655 8701 8860 9042 9347 9540 9755 10589 10664 10688 10810 10873 10915 11341 11719 11914 12007 12121 12705 12720 13105 13276 13506 13650 13759 14766 15326 15466 15930 15941 16129 16140 16179 16187 16189 16223 16230 16243 16293 16311 |
| L0d1b1+@152 | 73 146 188 195 247 498d 719 750 769 825A 1018 1048 2484.1C 2758 2885 3438 3516A 3594 3618 3756 4104 4232 4312 4562C 4769 5442 6185 6266 6815 7028 7146 7256 7521 8113A 8152 8251 8468 8655 8701 8860 9042 9347 9540 9755 10589 10664 10688 10810 10873 10915 11719 11914 12007 12121 12705 12720 13105 13276 13506 13650 13759 14766 15326 15466 15930 15941 16129 16179 16187 16189 16223 16230 16243 16311 |
| L0d1b1c | 73 146 188 195 247 498d 719 731 750 769 825A 1018 1048 2285 2484.1C 2882 2885 3438 3516A 3594 3618 3756 4104 4232 4312 4562C 4769 5442 6185 6266 6815 7028 7146 7256 7521 8113A 8152 8251 8468 8655 8701 8860 9042 9347 9540 9755 10589 10664 10688 10810 10873 10915 11016 11719 11914 12007 12121 12705 12720 13105 13276 13506 13650 13759 14766 15326 15466 15930 15941 16129 16179 16187 16189 16223 16230 16243 16311 16390T |

| | |
|---|---|
| L0d1b2 | 73 146 152 195 247 498d 719 750 769 825A 1018 1048 2758 2885 3438 3516A 3594 3618 3756 4104 4232 4312 4769 5442 6185 6266 6815 7028 7146 7256 7283 7521 8113A 8152 8251 8468 8655 8701 8860 9042 9347 9540 9755 10398 10589 10664 10688 10810 10873 10915 11719 11914 12007 12121 12705 12720 13105 13276 13506 13650 13759 14315 14659 14766 15326 15466 15930 15941 16129 16187 16189 16223 16230 16243 16294 16311 |
| L0d1b2a | 73 146 152 195 247 498d 719 750 769 825A 1018 1048 2758 2885 3438 3516A 3594 3618 3756 4104 4232 4312 4769 5442 6185 6266 6692 6815 7028 7146 7256 7283 7521 8113A 8152 8251 8468 8655 8701 8790 8860 9042 9347 9540 9755 10398 10589 10664 10688 10810 10873 10915 11719 11914 12007 12121 12705 12720 13105 13276 13506 13650 13759 14280 14315 14560 14659 14766 15326 15449 15466 15930 15941 16129 16153 16187 16189 16223 16230 16243 16294 16311 (16474T) |
| L0d1b2a1 | 73 146 152 195 247 498d 719 750 769 825A 1018 1048 2758 2885 3438 3516A 3594 3618 3756 4104 4232 4312 4769 5442 6185 6266 6692 6815 7028 7146 7256 7283 7521 8113A 8152 8251 8468 8655 8701 8790 8860 9042 9347 9540 9755 9887 10398 10589 10664 10688 10810 10873 10915 11719 11914 12007 12121 12705 12720 13105 13276 13506 13650 13759 14280 14315 14560 14659 14766 15326 15449 15466 15930 15941 16129 16153 16187 16189 16223 16230 16243 16294 16311 (16474T) |
| L0d1b2a2 | 73 146 152 195 247 498d 719 750 769 825A 1018 1048 2758 2885 2951 3438 3516A 3594 3618 3756 4104 4232 4312 4769 5442 6185 6266 6692 6815 7028 7146 7256 7283 7521 8113A 8152 8251 8388 8468 8655 8701 8790 8860 9042 9347 9540 9755 10398 10589 10664 10688 10810 10873 10915 11719 11914 12007 12121 12705 12720 13062 13105 13276 13506 13650 13759 14280 14315 14560 14659 14766 15326 15449 15466 15930 15941 16129 16153 16187 16189 16223 16230 16243 16294 16311 (16474T) |
| L0d1b2b | 73 146 152 195 247 498d 719 750 769 825A 1018 1048 2758 2885 3438 3516A 3594 3618 3756 4104 4232 4312 4769 5442 6185 6266 6815 7028 7146 7256 7283 7521 8113A 8152 8251 8468 8655 8701 8860 9042 9347 9540 9755 10398 10589 10664 10688 10810 10873 10915 11719 11914 12007 12121 12705 12720 13105 13276 13506 13650 13759 14315 14659 14766 15326 15466 15930 15941 16129 16187 16189 16223 16230 16239 16243 16294 16311 |
| L0d1b2b1 | 73 146 152 195 247 498d 719 750 769 825A 1018 1048 2758 2885 3438 3516A 3594 3618 3756 4104 4232 4312 4769 5442 6185 6266 6815 7028 7146 7256 7283 7521 8113A 8152 8251 8468 8655 8701 8860 9042 9111 9347 9540 9755 10398 10589 10664 10688 10810 10873 10915 10920 11719 11914 12007 12121 12705 12720 13105 13276 13506 13650 13759 14315 14659 14766 15326 15466 15930 15941 16129 16187 16189 16223 16230 16239 16243 16294 16311 |

| | |
|---|---|
| L0d1b2b1a | 73 146 152 195 247 498d 719 750 769 825A 957 1018 1048 2758 2885 3438 3516A 3594 3618 3756 4104 4232 4312 4769 5442 5460 6185 6266 6815 7028 7146 7256 7283 7521 8113A 8152 8251 8468 8655 8701 8715 8860 8970 9042 9111 9347 9540 9755 10398 10589 10664 10688 10810 10873 10915 10920 11087 11719 11914 12007 12121 12303 12705 12720 13105 13276 13506 13650 13759 14211 14315 14659 14766 15106 15326 15466 15930 15941 16129 16187 16189 16223 16230 16239 16243 16294 16311 |
| L0d1b2b1b | 73 146 152 195 247 498d 719 750 769 825A 1018 1048 2758 2885 3438 3516A 3594 3618 3756 4104 4232 4312 4769 5442 6185 6266 6383 6815 7028 7146 7256 7283 7521 8113A 8152 8251 8468 8655 8701 8860 9042 9111 9347 9540 9755 10398 10589 10664 10688 10810 10873 10915 10920 11719 11914 12007 12121 12436 12705 12720 13105 13276 13506 13650 13759 14315 14659 14766 15326 15337 15466 15930 15941 16129 16187 16189 16223 16230 16239 16243 16294 16311 |
| L0d1b2b1b1 | 73 146 152 195 247 498d 719 750 769 825A 1018 1048 2758 2885 3438 3516A 3594 3618 3756 4104 4232 4312 4769 5442 6185 6266 6383 6815 7028 7146 7256 7283 7521 8113A 8152 8251 8468 8655 8701 8860 9042 9111 9347 9540 9755 10084 10398 10589 10664 10688 10810 10873 10915 10920 11719 11914 12007 12121 12436 12705 12720 13105 13276 13506 13650 13759 14315 14659 14766 15326 15337 15466 15930 15941 16129 16187 16189 16223 16230 16239 16243 16294 16311 |
| L0d1b2b2 | 73 146 152 195 247 498d 573.XC 719 750 769 825A 1018 1048 2758 2885 3438 3516A 3594 3618 3756 4104 4232 4312 4769 5442 6185 6266 6815 7028 7146 7256 7283 7521 8113A 8152 8251 8383 8468 8655 8701 8860 9042 9347 9540 9755 10398 10589 10664 10688 10810 10873 10915 11719 11914 12007 12121 12705 12720 13105 13276 13506 13650 13759 14315 14659 14766 15326 15466 15692 15930 15941 16129 16187 16189 16223 16239 16243 16294 16311 |
| L0d1b2b2a | 73 146 152 195 247 498d 573.XC 719 750 769 825A 1018 1048 2758 2885 2887 3438 3516A 3594 3618 3756 4104 4232 4312 4769 5442 6185 6266 6815 7028 7146 7256 7283 7521 8113A 8152 8251 8383 8468 8655 8701 8860 9042 9347 9540 9755 10398 10589 10664 10688 10810 10873 10915 11719 11914 12007 12121 12705 12720 13105 13276 13506 13650 13759 14315 14659 14766 15326 15466 15692 15930 15941 16129 16187 16189 16223 16239 16243 16294 16311 |
| L0d1b2b2b | 73 146 152 195 247 498d 573.XC 719 750 769 825A 1018 1048 2758 2885 3438 3516A 3594 3618 3756 4104 4232 4312 4769 5442 6185 6266 6815 7028 7146 7256 7283 7521 8113A 8152 8251 8383 8468 8655 8701 8860 9042 9347 9540 9755 10398 10589 10664 10688 10810 10873 10915 11719 11914 12007 12121 12705 12720 13105 13276 13506 13650 13759 14315 14659 14766 15326 15466 15692 15697 15930 15941 16129 16187 16189 16223 16239 16243 16294 16311 |

| | |
|---|---|
| L0d1b2b2b1 | 73 146 152 195 247 498d 573.XC 719 750 769 825A 1018 1048 2758 2885 3438 3516A 3594 3618 3756 4104 4232 4312 4769 5442 6185 6266 6815 7028 7146 7256 7283 7521 8113A 8152 8251 8383 8468 8655 8701 8860 9042 9347 9540 9755 10398 10589 10664 10688 10810 10873 10915 11719 11914 12007 12121 12501 12705 12720 13105 13276 13506 13650 13759 14315 14659 14766 15326 15466 15692 15697 15930 15941 16129 16187 16189 16218 16223 16239 16243 16294 16311 |
| L0d1b2b2c | 73 146 152 195 247 498d 573.XC 719 750 769 825A 1018 1048 2758 2885 3438 3516A 3594 3618 3756 4104 4232 4312 4769 5029 5442 6185 6266 6815 7028 7146 7256 7283 7521 8113A 8152 8251 8383 8468 8655 8701 8860 9042 9347 9540 9755 10398 10589 10664 10688 10810 10873 10915 11719 11914 12007 12121 12705 12720 13105 13276 13506 13650 13759 14315 14659 14766 15326 15466 15692 15930 15941 16129 16187 16189 16223 16239 16243 16294 16311 |
| L0d1b2b2c1 | 73 146 152 195 247 498d 573.XC 719 750 769 825A 1018 1048 2758 2885 3438 3516A 3594 3618 3756 4104 4232 4312 4769 5029 5442 6185 6266 6815 7028 7146 7256 7283 7521 8113A 8152 8251 8383 8468 8655 8701 8860 8937 9042 9347 9540 9755 10398 10589 10664 10688 10810 10873 10915 11719 11914 12007 12121 12705 12720 13105 13276 13506 13650 13759 14315 14659 14766 15326 15466 15692 15930 15941 16129 16187 16189 16223 16239 16243 16294 16311 |
| L0d1b2b2c2 | 73 146 152 195 247 498d 573.XC 719 750 769 825A 1018 1048 2758 2885 3438 3516A 3594 3618 3666 3756 4104 4232 4312 4769 5029 5442 6185 6266 6815 7028 7146 7256 7283 7521 8113A 8152 8251 8383 8468 8655 8701 8860 9042 9347 9540 9755 10398 10589 10664 10688 10810 10873 10915 11719 11914 12007 12121 12705 12720 12816 13105 13276 13506 13650 13759 14315 14659 14766 15326 15466 15692 15930 15941 16129 16157 16187 16189 16223 16239 16243 16294 16311 |
| L0d2 | 73 146 152 195 247 498d 750 769 825A 1018 1048 2706 2758 2885 3516A 3594 3756 3981 4025 4044 4104 4232 4312 4769 5442 6185 6815 7028 7146 7154 7256 7521 8113A 8152 8251 8468 8655 8701 8860 9042 9347 9540 9755 10398 10589 10664 10688 10810 10873 10915 11719 11854 11914 12007 12121 12705 12720 13105 13276 13506 13650 14766 15326 15466 15766 15930 15941 16129 16187 16189 16223 16230 16243 16311 |
| L0d2a'b'd | 73 146 152 195 247 498d 750 769 825A 1018 1048 2706 2758 2885 3516A 3594 3756 3981 4025 4044 4104 4232 4312 4769 5442 6185 6815 7028 7146 7154 7256 7521 8113A 8152 8251 8468 8655 8701 8860 9042 9347 9540 9755 10398 10589 10664 10688 10810 10873 10915 11719 11854 11914 12007 12121 12705 12720 13105 13276 13506 13650 14766 15326 15466 15766 15930 15941 16129 16187 16189 16212 16223 16230 16243 16311 |
| L0d2a | 73 146 152 195 247 498d 750 769 825A 1018 1048 2706 2758 2885 3516A 3594 3756 3981 4025 4044 4104 4232 4312 4769 5153 5442 6185 6815 7028 7146 7154 7256 7521 8113A 8152 8251 8468 8655 8701 8860 9042 9347 9540 9755 10398 10589 10664 10688 10810 10873 10915 11719 11854 11914 12007 12121 12172 12705 12720 13105 13276 13506 13650 14221 14766 15326 15466 15766 15930 15941 16129 16187 16189 16212 16223 16230 16243 16311 16390 |

| | |
|---|---|
| L0d2a1 | 73 146 152 195 198 247 498d 597 750 769 825A 1018 1048 2706 2758 2885 3516A 3594 3756 3981 4025 4044 4104 4225 4232 4312 4769 5153 5442 6185 6815 7028 7146 7154 7256 7521 8113A 8152 8251 8392 8468 8655 8701 8860 9042 9347 9540 9755 10398 10589 10664 10688 10810 10873 10915 11719 11854 11914 12007 12121 12172 12234 12705 12720 12810 13105 13276 13506 13650 14221 14766 15326 15466 15766 15930 15941 16129 16187 16189 16212 16223 16230 16243 16311 16390 |
| L0d2a1a | 73 146 152 195 198 247 498d 597 750 769 825A 1018 1048 2706 2758 2885 3516A 3594 3756 3981 4025 4044 4104 4225 4232 4312 4769 5153 5442 6185 6815 7028 7146 7154 7256 7521 8113A 8152 8251 8392 8468 8545 8655 8701 8860 9042 9347 9540 9755 10398 10589 10664 10688 10810 10873 10915 11719 11854 11914 12007 12121 12172 12234 12705 12720 12810 13105 13276 13506 13650 14221 14766 15326 15466 15766 15930 15941 16129 16187 16189 16212 16223 16230 16243 16311 16390 |
| L0d2a1a1 | 73 146 152 195 198 247 498d 597 750 769 825A 1018 1048 2072 2706 2758 2885 3516A 3594 3756 3981 4025 4044 4104 4225 4232 4312 4769 5153 5442 6185 6815 7028 7146 7154 7256 7521 8113A 8152 8251 8392 8468 8545 8655 8701 8860 9042 9347 9540 9755 10398 10589 10664 10688 10810 10873 10915 11719 11854 11914 12007 12121 12172 12234 12705 12720 12810 13105 13276 13506 13650 14221 14766 15326 15466 15766 15930 15941 16129 16187 16189 16212 16223 16230 16243 16311 16390 |
| L0d2a1a1a | 73 146 152 195 198 247 498d 597 750 769 825A 1018 1048 2072 2706 2758 2885 3516A 3594 3756 3981 4025 4044 4104 4225 4232 4312 4769 5153 5442 6185 6815 7028 7146 7154 7256 7521 8113A 8152 8251 8392 8468 8545 8655 8701 8860 9042 9347 9540 9755 10398 10589 10664 10688 10810 10873 10915 11719 11854 11914 12007 12121 12172 12234 12705 12720 12810 13105 13276 13506 13650 14221 14766 15326 15466 15766 15930 15941 16129 16187 16189 16212 16223 16230 16243 16292 16311 16390 |
| L0d2a1a2 | 73 146 152 195 198 247 498d 597 750 769 825A 1018 1048 2706 2758 2885 3516A 3594 3756 3981 4025 4044 4104 4225 4232 4312 4769 4896 5153 5442 6185 6815 7028 7146 7154 7256 7521 8113A 8152 8251 8392 8468 8545 8655 8701 8860 9042 9347 9540 9755 10398 10589 10664 10688 10810 10873 10915 11719 11854 11914 12007 12121 12172 12234 12705 12720 12810 13105 13276 13506 13650 14221 14766 15326 15466 15766 15930 15941 16129 16187 16189 16212 16223 16230 16243 16311 16390 |
| L0d2a1a3 | 73 146 152 195 198 247 498d 597 750 769 825A 1018 1048 2706 2758 2885 3516A 3594 3756 3981 4025 4044 4104 4225 4232 4312 4769 5153 5442 6185 6815 7028 7146 7154 7256 7521 8113A 8152 8251 8392 8468 8545 8655 8701 8860 9042 9347 9540 9755 10398 10589 10664 10688 10810 10873 10915 11719 11854 11914 12007 12121 12172 12234 12705 12720 12810 13105 13276 13506 13650 14221 14766 15326 15466 15766 15930 15941 16129 16172 16187 16189 16212 16223 16230 16243 16311 16390 |

| | |
|---|---|
| L0d2a1b | 73 146 152 195 198 247 463 498d 597 750 769 825A 1018 1048 2706 2758 2885 3516A 3594 3756 3981 4025 4044 4104 4225 4232 4312 4769 5153 5442 6185 6815 7028 7146 7154 7256 7521 7861 8113A 8152 8251 8392 8468 8655 8701 8860 9042 9347 9540 9755 10398 10589 10664 10688 10810 10873 10915 11719 11854 11914 12007 12121 12172 12234 12705 12720 12810 13105 13276 13506 13650 14221 14766 15326 15466 15766 15930 15941 16129 16189 16212 16223 16230 16243 16311 16390 |
| L0d2a1c | 73 146 152 195 198 247 498d 597 750 769 825A 1018 1048 2609 2623 2706 2758 2885 3516A 3594 3756 3981 4025 4044 4104 4225 4232 4312 4769 5153 5442 6185 6815 7028 7146 7154 7256 7521 8113A 8152 8251 8392 8468 8655 8701 8860 9042 9347 9540 9656 9755 10398 10589 10664 10688 10810 10873 10915 11150 11719 11854 11914 12007 12121 12172 12234 12705 12720 12810 13105 13276 13506 13650 14221 14766 15326 15466 15766 15930 15941 16129 16187 16189 16212 16223 16230 16243 16311 16390 |
| L0d2a2 | 73 93 95C 146 152 195 247 498d 750 769 825A 1018 1048 2706 2758 2885 3105 3290 3516A 3594 3756 3981 4025 4044 4104 4232 4312 4574 4769 5153 5442 6185 6815 7028 7146 7154 7256 7412 7521 8113A 8152 8251 8468 8655 8701 8860 9042 9347 9540 9755 10398 10589 10664 10688 10810 10873 10915 11719 11854 11914 12007 12121 12172 12705 12720 13105 13276 13506 13650 14221 14766 15326 15466 15766 15930 15941 16129 16187 16189 16212 16223 16230 16243 16256 16289 16311 16390 |
| L0d2b | 73 146 195 247 498d 750 769 825A 1018 1048 1386 2706 2758 2885 3516A 3594 3756 3981 4025 4044 4104 4232 4312 4769 5442 6185 6815 7028 7146 7154 7256 7521 8113A 8152 8251 8468 8655 8701 8860 9042 9347 9540 9755 9932 10084 10398 10589 10664 10688 10810 10873 10915 11719 11854 11914 12007 12121 12705 12720 13105 13276 13506 13650 14766 15326 15466 15766 15930 15941 16069 16129 16169 16187 16189 16212 16223 16230 16243 16311 |
| L0d2b1 | 73 146 195 247 265 498d 750 769 825A 1018 1048 1386 2706 2758 2885 3516A 3594 3756 3981 4025 4044 4104 4232 4312 5442 5515 6185 6815 7028 7146 7154 7256 7521 8113A 8152 8251 8468 8655 8701 8860 8865 8994 9042 9347 9540 9755 9932 10084 10398 10589 10664 10688 10810 10873 10915 11719 11854 11914 12007 12121 12705 12720 13105 13276 13386 13506 13650 14766 14770 15326 15466 15766 15930 15941 16069 16126 16129 16169 16189 16212 16223 16230 16243 16258C 16291 16311 |
| L0d2b1a | 73 146 195 247 265 498d 709 750 769 825A 1018 1048 1386 2706 2758 2885 3516A 3594 3981 4025 4044 4104 4232 4312 5442 5515 6185 6815 7028 7146 7154 7256 7521 8113A 8152 8251 8468 8655 8701 8860 8865 8994 9042 9347 9540 9755 9932 10084 10398 10589 10664 10688 10810 10873 10915 11719 11854 11914 12007 12121 12705 12720 13105 13276 13386 13506 13650 14766 14770 15326 15466 15766 15930 15941 16069 16126 16129 16169 16189 16212 16223 16230 16243 16258C 16291 16311 |

| | |
|---|---|
| L0d2b1a1 | 73 146 195 247 265 498d 709 750 769 825A 1018 1048 1386 2706 2758 2885 3516A 3594 3981 4025 4044 4104 4232 4312 5442 5515 6185 6815 7028 7146 7154 7256 7521 8113A 8152 8251 8468 8563 8655 8701 8860 8865 8994 9042 9347 9540 9755 9776 9932 10084 10398 10589 10664 10688 10810 10873 10915 11719 11854 11914 12007 12121 12705 12720 13105 13276 13386 13506 13650 14766 14770 15326 15466 15766 15930 15941 16069 16126 16129 16169 16189 16212 16223 16230 16243 16258C 16291 16311 |
| L0d2b1a1a | 73 146 195 247 265 498d 709 750 769 825A 1018 1048 1386 2706 2758 2885 3516A 3594 3981 4025 4044 4104 4232 4312 5442 5515 6185 6815 7028 7146 7154 7256 7521 8113A 8152 8251 8468 8563 8655 8701 8860 8865 8994 9042 9347 9540 9755 9776 9932 10084 10398 10589 10664 10688 10810 10873 10915 11719 11854 11914 12007 12121 12705 12720 13105 13276 13386 13506 13650 14766 14770 15326 15466 15596 15766 15930 15941 16069 16126 16129 16169 16189 16212 16223 16230 16243 16258C 16291 16311 |
| L0d2b1b | 73 146 195 247 265 498d 750 769 825A 1018 1048 1386 2706 2758 2885 3516A 3594 3756 3981 4025 4044 4104 4232 4312 5442 5515 6185 6815 7028 7146 7154 7256 7521 8113A 8152 8251 8468 8655 8701 8860 8865 8994 9042 9347 9540 9755 9932 10084 10398 10589 10664 10688 10810 10873 10915 11719 11854 11914 12007 12121 12705 12720 13105 13276 13386 13466C 13506 13650 14302 14693 14766 14770 15326 15466 15766 15930 15941 15944d 16069 16126 16129 16169 16189 16212 16223 16230 16243 16258C 16291 16311 |
| L0d2b2 | 73 146 195 247 498d 750 769 794 825A 1018 1048 1386 2706 2758 2885 3516A 3594 3756 3981 4025 4044 4104 4232 4312 4769 5054 5442 6185 6815 7028 7146 7154 7256 7521 8113A 8152 8251 8468 8655 8701 8860 9042 9347 9540 9755 9932 10084 10398 10589 10664 10688 10810 10873 10915 11719 11854 11914 12007 12121 12705 12720 13105 13276 13506 13650 13768 14326 14766 15326 15466 15766 15930 15941 16069 16129 16169 16187 16189 16212 16223 16230 16243 16311 |
| L0d2d | 73 125 127 146 150 152 188 195 247 498d 750 769 825A 1018 1048 2706 2758 2885 3516A 3594 3756 3981 4025 4044 4104 4232 4312 4769 5147 5231 5442 6185 6776 6815 7028 7146 7154 7256 7521 8113A 8152 8251 8434 8468 8655 8701 8860 9042 9254 9347 9476 9540 9755 10398 10589 10664 10688 10745 10810 10873 10915 11719 11854 11914 12007 12121 12705 12720 13105 13276 13506 13650 14094 14766 15326 15466 15766 15884 15930 15941 16129 16187 16189 16212 16223 16230 16243 16311 16390T |
| L0d2c | 73 146 152 195 247 294A 498d 750 769 825A 1018 1048 2706 2758 2885 3516A 3594 3756 3981 4025 4038 4044 4104 4204 4232 4312 4769 4937 5442 6185 6644 6815 7028 7146 7154 7256 7521 8113A 8152 8251 8284 8420 8468 8655 8701 8860 9042 9230 9305 9347 9540 9755 10398 10589 10664 10688 10810 10873 10915 11719 11854 11914 11974 12007 12121 12705 12720 13105 13276 13506 13650 13827 14007 14766 15326 15346 15466 15766 15930 15941 16129 16187 16189 16223 16230 16243 16311 |

| | |
|---|---|
| L0d2c1 | 73 146 152 195 247 294A 498d 750 769 825A 1018 1048 2706 2758 2885 3516A 3594 3756 3981 4025 4038 4044 4104 4204 4232 4312 4769 4937 5442 6185 6249 6644 6815 7028 7146 7154 7256 7521 8113A 8152 8251 8284 8420 8468 8655 8701 8860 9042 9230 9305 9347 9540 9755 10398 10589 10664 10688 10810 10873 10915 11719 11854 11914 11974 12007 12121 12705 12720 13105 13276 13506 13650 13827 14007 14766 15326 15346 15466 15766 15930 15941 16129 16187 16189 16223 16230 16243 16311 |
| L0d2c1a | 73 146 152 195 247 294A 498d 750 769 825A 1018 1048 2706 2758 2885 3516A 3594 3756 3981 4025 4038 4044 4052 4104 4204 4232 4312 4395 4769 4937 5442 5899.XC 6185 6249 6644 6815 7028 7146 7154 7256 7521 8113A 8152 8251 8284 8420 8468 8655 8701 8860 9042 9230 9305 9347 9540 9755 10398 10589 10664 10688 10810 10873 10915 11719 11854 11914 11974 12007 12121 12705 12720 13105 13276 13506 13650 13827 14007 14766 15326 15346 15466 15766 15930 15941 16129 16187 16189 16223 16230 16243 16311 |
| L0d2c1a1 | 73 146 152 195 247 294A 498d 750 769 825A 930 1018 1048 2483 2706 2758 2885 3516A 3594 3756 3981 4025 4038 4044 4052 4104 4204 4232 4312 4395 4769 4937 5442 5899.XC 6134 6185 6249 6644 6815 7028 7146 7154 7256 7521 8113A 8152 8251 8284 8420 8468 8655 8701 8860 9042 9230 9305 9347 9540 9554 9755 10398 10589 10664 10688 10810 10873 10915 11719 11854 11914 11974 12007 12121 12705 12720 13105 13276 13506 13650 13827 14007 14766 15326 15346 15466 15766 15930 15941 16129 16187 16189 16223 16230 16243 16311 |
| L0d2c1b | 73 146 152 195 247 294A 498d 750 769 825A 1018 1048 2706 2758 2885 3516A 3594 3756 3981 4025 4038 4044 4104 4204 4232 4312 4769 4937 5442 6185 6249 6644 6815 7028 7146 7154 7256 7521 8113A 8152 8251 8284 8420 8468 8655 8701 8860 9042 9230 9305 9347 9540 9755 10398 10589 10664 10688 10810 10873 10915 11719 11854 11914 11974 12007 12121 12562 12705 12720 13105 13276 13506 13650 13827 14007 14766 15326 15346 15466 15766 15930 15941 16129 16187 16189 16223 16230 16243 16311 16482 |
| L0d2c2 | 73 94 146 195 247 294A 498d 750 769 825A 1018 1048 2706 2758 2885 3516A 3594 3756 3981 4025 4038 4044 4104 4204 4232 4312 4769 4937 5442 6185 6644 6815 7028 7146 7154 7256 7521 8113A 8152 8251 8284 8420 8468 8655 8701 8725 9042 9230 9305 9347 9531 9540 9755 10373 10398 10589 10664 10688 10810 10873 10915 11719 11854 11914 11974 12007 12121 12705 12720 13105 13276 13506 13650 13827 14007 14766 15326 15346 15466 15766 15930 15941 16129 16187 16189 16223 16230 16243 16311 |
| L0d2c2a | 73 94 146 195 247 294A 498d 750 769 825A 1018 1048 2706 2758 2885 3516A 3594 3756 3981 4025 4038 4044 4104 4204 4232 4312 4646 4769 4937 5442 6185 6644 6815 7028 7146 7154 7256 7521 8113A 8152 8251 8284 8420 8468 8655 8701 8725 9042 9230 9305 9347 9531 9540 9755 10373 10398 10589 10664 10688 10810 10873 10915 11719 11854 11914 11974 12007 12121 12705 12720 13105 13276 13506 13650 13827 14007 14766 15326 15346 15466 15766 15930 15941 16129 16187 16189 16223 16230 16243 16311 |

| | |
|---|---|
| L0d2c2a1 | 73 94 146 195 247 294A 498d 750 769 825A 1018 1048 2706 2758 2885 3516A 3594 3756 3981 4025 4038 4044 4104 4204 4232 4312 4646 4769 4937 5442 6185 6644 6815 7028 7146 7154 7256 7521 8113A 8152 8251 8284 8420 8468 8655 8701 8725 9042 9230 9305 9347 9531 9540 9755 10373 10398 10589 10664 10688 10810 10873 10915 11719 11854 11914 11974 12007 12121 12234 12705 12720 13105 13276 13506 13650 13827 14007 14766 15326 15346 15466 15766 15930 15941 16129 16187 16189 16223 16230 16243 16311 |
| L0d2c2a1a | 73 94 146 195 247 294A 498d 750 769 825A 1018 1048 2706 2758 2885 3516A 3594 3756 3981 4025 4038 4044 4104 4204 4232 4312 4646 4769 4937 5442 6185 6644 6815 7028 7146 7154 7256 7521 8113A 8152 8251 8284 8420 8468 8655 8701 8725 9042 9230 9305 9347 9531 9540 9755 10373 10398 10589 10664 10688 10810 10873 10915 11719 11854 11914 11974 12007 12121 12234 12705 12720 13105 13276 13506 13650 13827 14007 14693 14766 15326 15346 15466 15541 15766 15930 15941 16129 16187 16189 16223 16230 16243 16311 |
| L0d2c2b | 73 94 146 195 247 294A 498d 750 769 825A 1018 1048 2706 2758 2885 3516A 3594 3756 3981 4025 4038 4044 4104 4204 4232 4312 4769 4937 5442 6185 6644 6815 7028 7146 7154 7256 7521 8113A 8152 8251 8284 8420 8468 8655 8701 8725 9042 9072 9090A 9230 9305 9347 9531 9540 9755 10373 10398 10589 10664 10688 10810 10873 10915 11719 11854 11914 11974 12007 12121 12705 12720 13105 13276 13506 13650 13827 14007 14766 15326 15346 15466 15766 15930 15941 16129 16187 16189 16223 16230 16243 16311 |
| L0d3 | 73 146 150 152 195 247 316 721 750 769 825A 1018 1048 1243 2706 2755 2758 2885 3516A 3594 4104 4232 4312 4769 5442 5460 5773 6185 6377 6815 7028 7146 7256 7521 8113A 8152 8251 8459 8468 8598 8655 8701 8860 9027 9042 9347 9488 9540 10398 10589 10664 10688 10810 10873 10915 11061 11719 11914 12007 12121 12705 12720 13105 13276 13359 13506 13650 14766 15236 15312 15326 15466 15930 15941 16129 16187 16189 16223 16230 16243 16278 16290 16300 16311 |
| L0d3a | 73 146 150 152 195 247 316 721 750 769 825A 1018 1048 1243 2706 2755 2758 2885 3516A 3594 4104 4232 4312 4769 5442 5460 5773 6185 6377 6815 7028 7146 7256 7521 8113A 8152 8251 8459 8468 8598 8655 8701 8860 9027 9042 9347 9488 9540 10398 10589 10664 10688 10810 10873 10915 11061 11653 11719 11914 12007 12121 12705 12720 13105 13276 13359 13506 13650 14766 15236 15312 15326 15461 15466 15586 15930 15941 16129 16187 16189 16223 16230 16243 16278 16290 16300 16311 16399 |
| L0d3b | 73 146 150 195 247 316 721 750 769 825A 1018 1048 1243 2706 2755 2758 2885 3516A 3594 4104 4232 4312 4769 5442 5460 5773 6170 6185 6377 6815 7028 7119 7146 7256 7521 8113A 8152 8251 8290 8459 8468 8598 8655 8701 8860 9027 9042 9347 9488 9540 10114 10128A 10398 10589 10664 10688 10810 10873 10915 11061 11719 11914 12007 12121 12705 12720 13105 13276 13359 13506 13650 14766 15236 15312 15326 15466 15930 15941 16187 16189 16223 16230 16243 16274 16278 16290 16300 16311 |

| | |
|---|---|
| L0d3b1 | 73 146 150 195 247 316 721 750 769 825A 1018 1048 1243 2706 2755 2758 2885 3516A 3594 4104 4232 4312 4769 5442 5460 5773 6170 6185 6377 6815 7028 7119 7146 7256 7521 8113A 8152 8251 8290 8459 8468 8598 8655 8701 8860 9027 9042 9347 9488 9540 10114 10128A 10398 10589 10664 10688 10810 10873 10915 11061 11719 11914 12007 12121 12705 12720 12978 13105 13276 13359 13506 13650 14766 15236 15312 15326 15466 15930 15941 16187 16189 16223 16230 16243 16274 16278 16290 16300 16311 |
| L0d3b2 | 73 146 150 195 247 316 721 750 769 825A 1018 1048 1243 2706 2755 2758 2885 3516A 3594 4104 4232 4312 4769 5442 5460 5773 6152 6170 6185 6377 6815 7028 7119 7146 7256 7521 8113A 8152 8251 8290 8459 8468 8598 8655 8701 8860 9027 9042 9347 9488 9540 10114 10128A 10398 10589 10664 10688 10810 10873 10915 11061 11719 11914 12007 12121 12705 12720 13105 13276 13359 13506 13650 14766 15236 15312 15326 15466 15930 15941 16187 16189 16223 16230 16243 16274 16278 16290 16300 16311 |
| L1'2'3'4'5'6 | 73 152 182 195 247 263 750 769 825A 1018 1438 2706 2758 2885 3594 4104 4769 7028 7146 7256 7521 8468 8655 8701 8860 9540 10398 10688 10810 10873 11719 12705 13105 13506 13650 14766 15326 16129 16187 16189 16223 16278 16311 |
| L1 | 73 152 182 195 247 263 750 769 825A 1018 1438 2706 2758 2885 3594 3666 4104 4769 7028 7055 7146 7256 7389 7521 8468 8655 8701 8860 9540 10398 10688 10810 10873 11719 12705 13105 13506 13650 13789 14178 14560 14766 15326 16129 16187 16189 16223 16278 16311 |
| L1b | 73 152 182 185T 195 247 263 357 709 710 750 769 825A 1018 1738 2352 2706 2758 2768 2885 3308 3594 3666 3693 4104 4769 6548 6827 6989 7028 7055 7146 7256 7389 7521 7867 8248 8468 8655 8701 8860 9540 10398 10688 10810 10873 11719 12519 12705 13105 13506 13650 13789 14178 14560 14766 14769 15115 15326 16126 16187 16189 16223 16264 16270 16278 (16293) 16311 |
| L1b1 | 73 152 182 185T 195 247 263 357 709 710 750 769 825A 1018 1738 2352 2706 2758 2768 2885 3308 3594 3666 3693 4104 4769 5036 5046 5655 6548 6827 6989 7028 7055 7146 7256 7389 7521 7867 8248 8468 8655 8701 8860 9540 10398 10688 10810 10873 11719 12519 12705 13105 13506 13650 13789 13880A 14178 14203 14560 14766 14769 15115 15326 16126 16187 16189 16223 16264 16270 16278 (16293) 16311 |
| L1b1a | 73 152 182 185T 195 247 263 357 709 710 750 769 825A 1018 1738 2352 2706 2758 2768 2885 3308 3594 3666 3693 4104 4769 5036 5046 5393 5655 6548 6827 6989 7028 7055 7146 7256 7389 7521 7867 8248 8468 8655 8701 8860 9540 10398 10688 10810 10873 11719 12519 12705 13105 13506 13650 13789 13880A 14178 14203 14560 14766 14769 15115 15326 16126 16187 16189 16223 16264 16270 16278 (16293) 16311 |
| L1b1a1'4 | 73 152 182 185T 195 247 263 357 709 710 750 769 825A 1018 1738 2352 2706 2758 2768 2885 3308 3594 3666 3693 4104 4769 5036 5046 5393 5655 6548 6827 6989 7028 7055 7146 7256 7389 7521 7867 8248 8468 8655 8701 8860 9540 10398 10688 10810 10873 11719 12519 12705 13105 13506 13650 13789 13880A 14178 14203 14560 14766 14769 15115 15326 16114A 16126 16187 16189 16223 16264 16270 16278 (16293) 16311 |

| | |
|---|---|
| L1b1a1 | 73 152 182 185T 195 247 263 264 357 709 710 750 769 825A 1018 1738 2352 2706 2758 2768 2885 3308 3396 3594 3666 3693 4104 4769 5036 5046 5393 5655 6548 6827 6989 7028 7055 7146 7256 7389 7521 7867 8248 8468 8655 8701 8860 9540 10398 10688 10810 10873 11719 12519 12705 13105 13506 13650 13789 13880A 14178 14203 14560 14766 14769 15115 15326 16114A 16126 16187 16189 16215 16223 16264 16270 16278 (16293) 16311 |
| L1b1a4 | 73 152 182 185T 195 247 263 357 709 710 750 769 825A 1018 1738 2352 2706 2758 2768 2885 3308 3594 3666 3693 4104 4769 5036 5046 5393 5655 6548 6827 6989 7028 7055 7146 7256 7389 7521 7867 8248 8468 8655 8701 8790 8860 9540 10398 10688 10810 10873 11719 12519 12705 13105 13506 13650 13789 13880A 14178 14203 14560 14766 14769 15115 15326 16114A 16126 16187 16189 16223 16264 16270 16278 (16293) 16311 |
| L1b1a4a | 73 152 182 185T 195 247 263 357 709 710 750 769 825A 1018 1738 2352 2706 2758 2768 2885 3308 3594 3666 3693 4104 4769 5036 5046 5393 5655 6548 6827 6989 7028 7055 7146 7256 7389 7521 7867 8248 8468 8655 8701 8790 8860 9540 10398 10688 10810 10873 11719 12519 12705 13105 13194 13506 13650 13789 13880A 14178 14203 14560 14766 14769 15115 15326 16114A 16126 16187 16189 16223 16264 16270 16278 (16293) 16311 |
| L1b1a2 | 73 152 182 185T 195 247 263 357 709 710 750 769 825A 1018 1738 2352 2706 2758 2768 2885 3308 3594 3666 3693 4104 4769 5036 5046 5393 5655 6548 6827 6989 7028 7055 7146 7256 7389 7521 7867 7954 8248 8468 8655 8701 8860 9540 10398 10688 10810 10873 11719 12519 12705 13105 13506 13650 13789 13880A 14178 14203 14560 14766 14769 15115 15326 16126 16187 16189 16223 16264 16270 16278 (16293) 16311 |
| L1b1a2a | 73 152 182 185T 195 247 263 357 709 710 750 769 825A 1018 1738 2352 2706 2758 2768 2885 3308 3594 3666 3693 4104 4769 5036 5046 5393 5655 6548 6827 6989 7028 7055 7146 7256 7389 7521 7867 7954 8248 8468 8655 8701 8860 9540 10398 10688 10810 10873 11719 12519 12705 13105 13506 13650 13789 13880A 14178 14203 14560 14766 14769 15115 15326 16126 16187 16189 16223 16264 16270 16278 16289 (16293) 16311 |
| L1b1a+189 | 73 152 182 185T 189 195 247 263 357 709 710 750 769 825A 1018 1738 2352 2706 2758 2768 2885 3308 3594 3666 3693 4104 4769 5036 5046 5393 5655 6548 6827 6989 7028 7055 7146 7256 7389 7521 7867 8248 8468 8655 8701 8860 9540 10398 10688 10810 10873 11719 12519 12705 13105 13506 13650 13789 13880A 14178 14203 14560 14766 14769 15115 15326 16126 16187 16189 16223 16264 16270 16278 (16293) 16311 |
| L1b1a3 | 73 152 182 185T 189 195 247 263 357 709 710 750 769 825A 1018 1738 2352 2706 2758 2768 2885 3308 3594 3666 3693 4104 4769 5036 5046 5393 5655 6548 6827 6989 7028 7055 7146 7256 7389 7521 7867 8248 8468 8655 8701 8860 9540 10398 10688 10810 10873 11719 12519 12705 13105 13506 13650 13789 13880A 13980 14178 14203 14560 14766 14769 15115 15326 16126 16187 16189 16223 16264 16270 16278 (16293) 16311 |

| | |
|---|---|
| L1b1a3a | 73 152 182 185T 189 195 247 263 357 709 710 750 769 825A 1018 1738 2352 2706 2758 2768 2885 3308 3594 3666 3693 4104 4769 5036 5046 5393 5655 6548 6827 6989 7028 7055 7146 7256 7389 7521 7867 7915 8248 8468 8655 8701 8860 9540 10398 10688 10810 10873 11719 12519 12705 13105 13506 13650 13789 13880A 13980 14178 14203 14560 14766 14769 15115 15326 16126 16187 16189 16223 16264 16270 16278 (16293) 16311 |
| L1b1a3a1 | 73 152 182 185T 189 195 247 263 357 709 710 750 769 825A 1018 1738 2352 2706 2758 2768 2885 3308 3594 3666 3693 4104 4769 5036 5046 5393 5655 6548 6827 6989 7028 7055 7146 7256 7389 7521 7867 7915 8248 8468 8655 8701 8860 9540 10398 10688 10810 10873 11254 11719 12519 12705 13105 13506 13650 13789 13880A 13980 14178 14203 14560 14766 14769 15115 15326 16126 16187 16189 16223 16264 16270 16278 (16293) 16311 |
| L1b1a3b | 73 152 182 185T 189 195 247 263 357 709 710 750 769 825A 1018 1719 1738 2352 2706 2758 2768 2885 3308 3594 3666 3693 4104 4216 4769 5036 5046 5393 5655 6548 6827 6989 7028 7055 7146 7256 7389 7521 7867 8248 8468 8655 8701 8860 9540 10398 10688 10810 10873 11719 12519 12705 13105 13506 13650 13789 13880A 13980 14178 14203 14560 14766 14769 15115 15326 16126 16187 16189 16223 16264 16270 16278 (16293) 16311 |
| L1b1a9 | 73 152 182 185T 189 195 247 263 357 709 710 750 769 825A 1018 1738 2352 2706 2758 2768 2885 3308 3594 3666 3693 4104 4769 5036 5046 5393 5655 6446 6548 6827 6989 7028 7055 7146 7256 7389 7521 7867 8248 8468 8655 8701 8860 9540 10398 10688 10810 10873 11719 12519 12705 13105 13506 13650 13789 13880A 14178 14203 14560 14766 14769 15115 15326 16126 16187 16189 16223 16264 16270 16278 (16293) 16311 |
| L1b1a15 | 73 152 182 185T 189 195 247 263 357 709 710 750 769 825A 1018 1738 2352 2706 2758 2768 2885 3308 3594 3666 3693 4104 4769 5036 5046 5393 5655 6548 6827 6989 7028 7055 7146 7256 7389 7521 7867 8248 8468 8655 8701 8860 9540 10398 10688 10810 10873 11719 12519 12705 13105 13506 13650 13789 13880A 14178 14203 14560 14766 14769 15077 15103 15115 15326 16126 16187 16189 16223 16264 16270 16278 (16293) 16311 |
| L1b1a15a | 73 152 182 185T 189 195 247 263 357 709 710 750 769 825A 1018 1738 2352 2706 2758 2768 2885 3308 3594 3666 3693 4104 4769 5036 5046 5393 5655 6548 6827 6989 7028 7055 7146 7256 7389 7521 7867 8248 8468 8655 8701 8860 9540 10398 10688 10810 10873 11719 12519 12705 13105 13506 13650 13789 13880A 14178 14203 14560 14766 14769 15077 15103 15115 15326 16126 16187 16189 16223 16264 16270 16278 (16293) 16311 16355 |
| L1b1a17 | 73 152 182 185T 189 195 247 263 357 709 710 750 769 825A 1018 1738 2352 2706 2758 2768 2885 3308 3594 3666 3693 4104 4769 5036 5046 5393 5655 6548 6827 6989 7028 7055 7146 7256 7389 7521 7867 8248 8468 8655 8701 8860 9540 10398 10688 10810 10873 11719 12519 12705 13105 13506 13650 13789 13880A 14178 14203 14560 14766 14769 15115 15326 15629 16126 16187 16189 16223 16264 16270 16278 (16293) 16311 |
| L1b1a18 | 73 152 185T 189 195 247 263 357 709 710 750 769 825A 1018 1738 2352 2706 2758 2768 2885 3308 3594 3666 3693 4104 4769 5036 5046 5393 5655 6548 6827 6989 7028 7055 7146 7256 7389 7521 7867 8248 8468 8655 8701 8860 9540 10398 10688 10810 10873 11719 12519 12609 12696 12705 13105 13506 13650 13789 13880A 14178 14203 14560 14766 14769 15115 15326 16126 16187 16189 16223 16264 16270 16278 (16293) 16311 |

| | |
|---|---|
| L1b1a5 | 73 152 182 185T 195 247 263 357 709 710 750 769 825A 1018 1738 2352 2706 2758 2768 2885 3308 3594 3666 3693 4104 4769 5036 5046 5393 5655 6548 6827 6989 7028 7055 7146 7256 7389 7521 7867 8248 8468 8655 8701 8860 9540 10398 10688 10810 10873 11719 12519 12705 13105 13506 13650 13789 13880A 14178 14203 14560 14766 14769 14812 15115 15326 16126 16187 16189 16223 16264 16270 16278 (16293) 16311 |
| L1b1a6 | 73 152 182 185T 195 247 263 357 709 710 750 769 825A 1018 1738 2352 2706 2758 2768 2885 3308 3594 3666 3693 4104 4769 5036 5046 5393 5655 6548 6827 6989 7028 7055 7146 7256 7389 7521 7867 8248 8468 8655 8701 8860 9540 9755 10398 10688 10810 10873 11719 12519 12705 13105 13506 13650 13789 13880A 14110 14178 14203 14560 14766 14769 15115 15326 16126 16187 16189 16223 16264 16270 16278 (16293) 16311 |
| L1b1a7 | 73 152 182 185T 195 247 263 357 709 710 750 769 825A 1018 1738 2352 2706 2758 2768 2885 3308 3594 3666 3693 4104 4769 5036 5046 5393 5655 6378 6548 6827 6989 7028 7055 7146 7256 7389 7521 7867 8248 8468 8655 8701 8860 9540 10398 10688 10810 10873 11719 12519 12705 13105 13506 13650 13789 13880A 14178 14203 14560 14766 14769 15115 15326 16126 16187 16189 16223 16264 16270 16278 (16293) 16311 |
| L1b1a7a | 73 152 182 185T 195 228 247 263 357 709 710 750 769 825A 1018 1738 2352 2706 2758 2768 2885 3308 3594 3666 3693 4104 4769 5036 5046 5393 5655 6378 6548 6827 6989 7028 7055 7146 7256 7389 7521 7867 8248 8468 8655 8701 8860 9540 10398 10688 10810 10873 11719 12519 12705 13105 13506 13650 13789 13880A 14053 14178 14203 14560 14766 14769 15115 15326 16126 16187 16189 16223 16264 16270 16278 (16293) 16311 |
| L1b1a8 | 73 152 182 185T 195 247 263 357 709 710 750 769 825A 1018 1738 2352 2706 2758 2768 2885 3308 3594 3666 3693 4104 4769 5036 5046 5393 5655 6548 6827 6989 7028 7055 7146 7256 7298 7389 7521 7867 8248 8468 8655 8701 8860 9540 10398 10688 10810 10873 11719 12519 12705 13105 13506 13650 13789 13880A 14178 14203 14560 14766 14769 15115 15326 16126 16187 16189 16223 16264 16270 16278 (16293) 16311 |
| L1b1a10 | 73 151 152 182 185T 195 247 263 357 709 710 750 769 825A 1018 1738 2352 2706 2758 2768 2885 3308 3594 3666 3693 4104 4769 5036 5046 5393 5655 6548 6827 6989 7028 7055 7146 7256 7389 7521 7867 8248 8468 8655 8701 8860 9540 10398 10688 10810 10873 11719 12519 12705 13105 13506 13650 13789 13880A 14178 14203 14560 14766 14769 15115 15326 16126 16187 16189 16223 16264 16270 16278 (16293) 16311 |
| L1b1a10a | 73 151 152 182 185T 195 247 263 357 709 710 750 769 825A 1018 1738 2352 2706 2758 2768 2885 3308 3594 3666 3693 4104 4769 5036 5046 5393 5655 6548 6827 6989 7028 7055 7146 7256 7389 7521 7867 8248 8468 8655 8701 8860 9540 10398 10688 10810 10873 11719 12519 12705 13105 13506 13650 13789 13880A 14178 14203 14560 14766 14769 15115 15326 15763 16126 16187 16189 16223 16264 16270 16278 (16293) 16311 |
| L1b1a10b | 73 151 152 182 185T 195 247 263 357 709 710 750 769 825A 1018 1738 2352 2706 2758 2768 2885 3308 3594 3666 3693 4104 4769 5036 5046 5393 5655 6548 6827 6989 7028 7055 7146 7256 7389 7521 7867 8248 8468 8655 8701 8860 9540 10398 10688 10810 10873 11506 11719 12519 12705 13105 13506 13650 13789 13880A 14178 14203 14560 14766 14769 15115 15326 16126 16187 16189 16223 16264 16270 16278 (16293) 16311 |

| | |
|---|---|
| L1b1a12 | 73 152 182 185T 195 247 263 357 709 710 750 769 825A 1018 1462 1738 2352 2706 2758 2768 2885 3308 3594 3666 3693 4104 4769 5036 5046 5393 5655 6548 6827 6989 7028 7055 7146 7256 7389 7521 7867 8248 8468 8655 8701 8860 9540 10398 10688 10810 10873 11719 12519 12705 13105 13506 13650 13789 13880A 14178 14203 14560 14766 14769 15115 15326 16126 16187 16189 16223 16264 16270 16278 (16293) 16311 16400 |
| L1b1a12a | 73 152 182 185T 195 247 263 357 709 710 750 769 825A 1018 1462 1738 2352 2706 2758 2768 2885 3308 3594 3666 3693 4104 4769 5036 5046 5393 5655 6548 6827 6989 7028 7055 7146 7256 7389 7521 7867 8248 8468 8655 8701 8860 9540 10398 10688 10810 10873 11002 11719 12519 12705 13105 13506 13650 13789 13880A 14178 14203 14560 14766 14769 15115 15326 16126 16187 16189 16223 16264 16270 16278 (16293) 16311 16400 |
| L1b1a12b | 73 152 182 185T 195 247 263 357 709 710 750 769 825A 1018 1462 1738 2352 2706 2758 2768 2885 3308 3594 3666 3693 4104 4769 5036 5046 5393 5655 6548 6827 6989 7028 7055 7146 7256 7298 7389 7521 7867 8248 8468 8655 8701 8860 9540 10398 10688 10810 10873 11719 12519 12705 13105 13506 13650 13789 13880A 14178 14203 14560 14766 14769 15115 15326 16126 16189 16223 16264 16270 16278 (16293) 16311 16400 |
| L1b1a13 | 73 152 182 185T 195 247 263 357 709 710 750 769 825A 1018 1738 2352 2706 2758 2768 2885 3308 3594 3666 3693 4104 4769 5036 5046 5393 5509 5655 6548 6827 6989 7028 7055 7146 7256 7389 7521 7867 8248 8468 8655 8701 8860 9540 10398 10688 10810 10873 11719 12519 12705 13105 13506 13650 13789 13880A 14178 14203 14560 14766 14769 15115 15326 16126 16187 16189 16223 16264 16270 16278 (16293) 16311 |
| L1b1a14 | 73 152 182 185T 195 247 263 357 709 710 750 769 825A 1018 1738 2352 2706 2758 2768 2885 3308 3594 3666 3693 4104 4769 5036 5046 5393 5655 6548 6827 6989 7028 7055 7146 7256 7389 7521 7867 8248 8468 8655 8701 8860 9540 10398 10688 10810 10873 11719 12512T 12519 12705 13105 13506 13650 13789 13880A 14178 14203 14560 14766 14769 15115 15326 16126 16187 16189 16223 16264 16270 16278 (16293) 16311 |
| L1b1a16 | 73 152 182 185C 195 247 263 357 709 710 750 769 825A 1018 1738 2352 2706 2758 2768 2885 3308 3594 3666 3693 4104 4769 5036 5046 5393 5655 6548 6827 6989 7028 7055 7146 7256 7389 7521 7867 8248 8468 8655 8701 8860 9540 10398 10688 10810 10873 11719 12519 12705 13105 13506 13650 13789 13880A 14016 14040 14178 14203 14560 14766 14769 15115 15326 16126 16187 16189 16223 16264 16270 16278 (16293) 16311 |
| L1b2'3 | 73 152 182 185T 247 263 357 709 710 750 769 825A 1018 1738 2352 2706 2758 2768 2885 3308 3594 3666 3693 4104 4769 6548 6827 6989 7028 7055 7146 7256 7389 7521 7867 8248 8468 8655 8701 8860 9540 10398 10688 10810 10873 11719 12519 12705 13105 13506 13650 13789 14178 14560 14766 14769 15115 15326 16126 16187 16189 16223 16264 16270 16278 (16293) 16311 |
| L1b2 | 73 152 182 185T 189 247 263 357 709 710 750 769 825A 1018 1738 2352 2706 2758 2768 2885 3308 3594 3666 3693 4104 4769 6548 6827 6989 7028 7055 7146 7256 7389 7521 7867 8248 8468 8655 8701 8860 9540 10398 10688 10810 10873 11719 12519 12705 12891 13105 13506 13650 13789 13893 14178 14323 14560 14766 14769 15115 15326 16126 16187 16189 16223 16239 16264 16270 16278 (16293) 16311 |

| | |
|---|---|
| L1b2a | 73 146 152 182 185T 189 247 263 357 709 710 750 769 825A 1018 1738 2352 2706 2758 2768 2885 3308 3594 3666 3693 4104 4769 6548 6827 6989 7028 7055 7146 7256 7389 7521 7867 8248 8468 8655 8701 8860 9540 10398 10688 10810 10873 11719 12171 12519 12705 12891 13105 13506 13650 13789 13893 14178 14323 14560 14766 14769 15115 15326 16111 16126 16187 16189 16223 16239 16270 16278 (16293) 16311 |
| L1b3 | 73 152 182 185T 247 263 357 709 710 723 750 769 825A 1018 1738 2352 2706 2758 2768 2885 3308 3594 3666 3693 4104 4562 4769 6548 6827 6989 7028 7055 7146 7256 7389 7521 7867 7972 8248 8468 8655 8701 8860 9540 10398 10688 10810 10873 11719 12519 12705 13105 13506 13650 13789 14178 14560 14766 14769 14800 15115 15326 16126 16187 16189 16223 16264 16270 16278 (16293) 16311 |
| L1c | 73 151 152 182 186A 189C 195 247 263 316 750 769 825A 1018 1438 2395d 2706 2758 2885 3594 3666 4104 4769 5951 6071 7028 7055 7146 7256 7389 7521 8027 8468 8655 8701 8860 9072 9540 10398 10586 10688 10810 10873 11719 12705 12810 13105 13485 13506 13650 13789 14000A 14178 14560 14766 14911 15326 16129 16187 16189 16223 16278 16294 16311 16360 |
| L1c1'2'4'5'6 | 73 151 152 182 186A 189C 195 247 263 297 316 750 769 825A 1018 1438 2395d 2706 2758 2885 3594 3666 4104 4769 5951 6071 7028 7055 7146 7256 7389 7521 8027 8468 8655 8701 8860 9072 9540 10398 10586 10688 10810 10873 11719 12705 12810 13105 13485 13506 13650 13789 14000A 14178 14560 14766 14911 15326 16129 16187 16189 16223 16278 16294 16311 16360 |
| L1c1'2'4'6 | 73 151 152 182 186A 189C 195 198 247 263 297 316 750 769 825A 1018 1438 2395d 2706 2758 2885 3594 3666 4104 4769 5951 6071 7028 7055 7146 7256 7389 7521 8027 8468 8655 8701 8860 9072 9540 10321 10398 10586 10688 10810 10873 11719 12705 12810 13105 13485 13506 13650 13789 14000A 14178 14560 14766 14911 15326 16129 16187 16189 16223 16278 16294 16311 16360 |
| L1c1 | 73 151 152 182 186A 189C 195 198 247 263 297 316 750 769 825A 1018 1438 2395d 2706 2758 2885 3594 3666 3796T 3843 4104 4769 5951 6071 7028 7055 7146 7256 7389 7521 8027 8468 8655 8701 8860 9072 9540 10321 10398 10586 10688 10810 10873 11719 12705 12810 13105 13485 13506 13650 13789 14000A 14148 14178 14560 14766 14911 15326 16129 16187 16189 16223 16278 16293 16294 16311 16360 |
| L1c1a'b'd | 73 151 152 182 186A 189C 195 198 247 263 297 316 750 769 825A 1018 1438 2395d 2706 2758 2885 3594 3666 3796T 3843 4104 4769 5951 6071 7028 7055 7146 7256 7389 7521 8027 8468 8655 8701 8860 9072 9540 10321 10398 10586 10688 10810 10873 11719 11899 12705 12810 13105 13485 13506 13650 13789 14000A 14148 14178 14560 14766 14911 15326 16129 16187 16189 16223 16278 16293 16294 16311 16360 |
| L1c1a | 73 151 152 182 186A 189C 195 198 247 263 297 316 750 769 825A 1018 1438 2395d 2706 2758 2885 3594 3666 3796T 3843 4104 4454A 4769 5951 6071 7028 7055 7146 7256 7389 7521 8027 8087 8468 8655 8701 8860 9072 9540 10321 10398 10586 10688 10810 10873 11719 11899 12705 12810 13105 13485 13506 13650 13789 14000A 14088 14148 14178 14560 14766 14911 15326 16129 16187 16189 16223 16278 16293 16294 16311 16360 |

| | |
|---|---|
| L1c1a+@198 | 73 151 152 182 186A 189C 195 247 263 297 316 750 769 825A 1018 1438 2395d 2706 2758 2885 3594 3666 3796T 3843 4104 4454A 4769 5951 6071 7028 7055 7146 7256 7389 7521 8027 8087 8468 8655 8701 8860 9072 9540 10321 10398 10586 10688 10810 10873 11719 11899 12705 12810 13105 13485 13506 13650 13789 14000A 14088 14148 14178 14560 14766 14911 15326 16129 16187 16189 16223 16278 16293 16294 16311 16360 |
| L1c1a1 | 73 151 152 182 186A 189C 195 247 263 297 316 750 769 825A 1018 1438 2395d 2706 2758 2885 3594 3666 3796T 3843 4104 4454A 4769 5951 6071 7028 7055 7146 7256 7389 7521 8027 8087 8468 8655 8701 8860 9072 9540 10321 10586 10688 10810 10873 11719 11899 12705 12810 13105 13485 13506 13650 13789 14000A 14034 14088 14148 14178 14560 14766 14911 15326 16129 16187 16189 16278 16293 16294 16311 16360 |
| L1c1a1a | 44.1C 73 151 152 182 186A 189C 195 204 247 263 297 316 750 769 825A 1018 1438 2395d 2706 2758 2885 3594 3666 3796T 3843 4104 4454A 4769 5951 6071 7028 7055 7146 7256 7389 7521 8027 8087 8468 8655 8701 8860 9072 9540 10321 10586 10688 10810 10873 11257 11719 11899 12705 12810 13105 13485 13506 13650 13789 14000A 14034 14088 14148 14178 14560 14766 14911 15326 16129 16187 16189 16214 16234 16249 16278 16293 16294 16311 16360 |
| L1c1a1a1 | 44.1C 73 151 152 186A 189C 195 204 247 263 297 316 467 750 769 825A 1018 1438 2308 2395d 2706 2758 2885 3594 3666 3796T 3843 4104 4454A 4769 5951 5984 6071 7028 7055 7146 7256 7389 7521 8027 8087 8468 8655 8701 8860 9072 9540 10321 10586 10688 10810 10873 11167 11257 11719 11899 12705 12810 12930T 13105 13485 13506 13650 13789 14000A 14034 14088 14148 14178 14560 14766 14911 15326 16129 16187 16189 16214 16234 16249 16274 16278 16293 16294 16311 16360 |
| L1c1a1a1a | 44.1C 73 151 152 186A 189C 195 204 247 263 297 316 467 750 769 825A 1018 1438 2308 2395d 2706 2758 2885 3594 3666 3796T 3843 4104 4454A 4769 5951 5984 6071 6182 7028 7055 7146 7256 7389 7521 8027 8087 8468 8655 8701 8860 8928 9072 9311 9540 10321 10586 10688 10810 10873 11167 11257 11719 11899 12705 12810 12930T 13105 13485 13506 13650 13789 14000A 14034 14088 14148 14178 14560 14766 14911 15326 15663 16129 16187 16189 16214 16234 16249 16274 16278 16294 16311 16360 |
| L1c1a1a1b | 44.1C 73 151 152 186A 189C 195 247 263 297 316 467 750 769 825A 1018 1438 2308 2395d 2706 2758 2885 3594 3666 3796T 3843 4104 4454A 4769 5951 5984 6071 7028 7055 7146 7256 7389 7521 8027 8087 8468 8655 8701 8860 9072 9540 10321 10586 10688 10810 10873 11167 11257 11719 11899 12705 12810 12930T 13105 13485 13506 13650 13789 14000A 14034 14088 14148 14178 14560 14766 14911 15326 16051 16129 16187 16189 16214 16234 16249 16258 16274 16278 16293 16294 16311 16360 |
| L1c1a1a1b1 | 44.1C 73 151 152 186A 189C 195 247 263 297 316 467 750 769 825A 1018 1438 2308 2395d 2706 2758 2885 3594 3666 3796T 3843 4104 4454A 4769 5300 5951 5984 6071 7028 7055 7146 7256 7389 7521 8027 8087 8468 8655 8701 8860 9072 9540 10321 10586 10688 10810 10873 11167 11257 11719 11899 12705 12810 12930T 13105 13485 13506 13650 13789 14000A 14034 14088 14148 14178 14560 14766 14911 15326 16051 16129 16187 16189 16214 16234 16249 16258 16274 16278 16293 16294 16311 16360 |

| | |
|---|---|
| L1c1a1a2 | 44.1C 73 151 152 182 186A 189C 195 204 247 263 297 316 750 769 825A 1018 1438 2395d 2706 2758 2885 3594 3666 3796T 3843 4104 4454A 4491 4769 5951 6071 7028 7055 7146 7256 7389 7521 8027 8087 8468 8655 8701 8860 9072 9540 10321 10586 10667 10688 10810 10873 11257 11719 11899 12705 12810 13105 13194 13485 13506 13650 13789 14000A 14034 14088 14148 14178 14560 14766 14911 15326 16187 16189 16214 16234 16249 16278 16293 16294 16311 16360 |
| L1c1a1b | 73 151 152 182 186A 189C 247 263 297 316 750 769 825A 1018 1438 2141 2395d 2706 2758 2885 3594 3666 3796T 3843 4104 4454A 4769 5277 5951 6071 7028 7055 7146 7256 7389 7521 8027 8087 8468 8655 8701 8769 8860 9072 9540 9580 10321 10586 10688 10810 10873 11719 11899 12164 12705 12810 13105 13174 13485 13506 13650 13789 14000A 14034 14040 14088 14148 14178 14560 14766 14911 15326 15777 16184 16187 16189 16278 16293 16294 16311 |
| L1c1a2 | 73 93 95C 152 182 186A 189C 195 236 247 263 297 316 750 769 825A 1018 1438 2395d 2706 2755 2758 2863 2885 3513 3594 3666 3796T 3843 3927 4104 4454A 4506 4769 5951 6071 7028 7055 7146 7202 7256 7389 7521 8027 8087 8468 8655 8701 8860 9072 9540 9647 10321 10398 10586 10688 10810 10873 11719 11899 12705 12768 12810 13105 13485 13506 13650 13789 14000A 14088 14148 14178 14560 14766 14911 15326 16129 16187 16189 16223 16274 16278 16293 16294 16311 16360 |
| L1c1a2a | 73 93 95C 152 182 186A 189C 195 236 247 263 297 316 750 769 825A 1018 1438 2395d 2706 2755 2758 2863 2885 3513 3594 3666 3796T 3843 3927 4104 4454A 4506 4634 4769 5951 6071 7028 7055 7146 7202 7256 7389 7521 8027 8087 8468 8655 8701 8860 9072 9336 9540 9647 10321 10398 10586 10688 10810 10873 11719 11899 12477 12705 12768 12810 13105 13485 13506 13650 13789 14000A 14088 14148 14178 14560 14766 14911 15326 16129 16187 16189 16223 16274 16278 16293 16294 16311 16360 |
| L1c1a2a1 | 73 93 95C 152 182 186A 189C 195 236 247 263 297 316 750 769 825A 1018 1438 2395d 2706 2755 2758 2863 2885 3513 3594 3666 3796T 3843 3927 4104 4454A 4506 4634 4769 5951 6071 7028 7055 7146 7202 7256 7389 7521 7692 8027 8087 8468 8655 8701 8860 9072 9336 9540 9647 10321 10398 10586 10688 10810 10873 11719 11899 12477 12705 12768 12810 13105 13485 13506 13650 13789 14000A 14088 14148 14178 14560 14766 14911 15326 16129 16187 16189 16223 16274 16278 16293 16294 16311 16360 |
| L1c1a2a2 | 73 93 95C 152 182 186A 189C 195 236 247 263 297 316 750 769 825A 1018 1438 2395d 2706 2755 2758 2863 2885 3513 3594 3666 3796T 3843 3927 4104 4454A 4506 4634 4769 5951 6071 7028 7055 7146 7202 7256 7389 7521 8027 8087 8468 8655 8701 8860 9072 9336 9540 9647 10321 10398 10586 10688 10810 10873 11719 11899 12477 12705 12768 12810 13105 13485 13506 13650 13789 14000A 14088 14148 14178 14560 14766 14911 15289 15326 16129 16187 16189 16223 16274 16278 16293 16294 16311 16360 |

| | |
|---|---|
| L1c1a2b | 73 93 95C 182 186A 189C 195 236 247 263 297 316 750 769 825A 1018 1438 2395d 2706 2755 2758 2863 2885 3513 3594 3666 3796T 3843 3927 4104 4454A 4506 4769 5951 6071 6752 7028 7055 7146 7202 7256 7389 7521 7660 7693 8027 8087 8468 8655 8701 8860 9072 9272 9540 9647 10321 10398 10586 10688 10810 10873 11719 11899 12705 12768 12810 13105 13485 13506 13650 13789 14000A 14088 14148 14178 14560 14766 14911 15326 16129 16187 16189 16223 16274 16278 16293 16294 16311 16360 |
| L1c1a2c | 73 93 95C 152 182 186A 189C 195 236 247 263 297 316 750 769 825A 1018 1438 2395d 2706 2755 2758 2863 2885 3513 3594 3666 3796T 3843 3927 4104 4454A 4506 4769 5951 6071 7028 7055 7146 7202 7256 7389 7521 8027 8087 8266 8468 8655 8701 8860 9072 9540 9647 10321 10398 10586 10688 10810 10873 11719 11899 12007 12705 12768 12810 13105 13485 13506 13650 13789 13879 14000A 14088 14148 14178 14560 14766 14911 15326 16129 16187 16189 16223 16274 16278 16293 16294 16311 16360 |
| L1c1b'd | 73 151 152 182 186A 189C 195 198 247 263 297 316 750 769 825A 1018 1438 2395d 2706 2758 2885 3594 3666 3796T 3843 4104 4769 5951 6071 7028 7055 7146 7256 7389 7521 8027 8468 8655 8701 8860 9072 9540 10321 10398 10586 10688 10810 10873 11719 11899 12705 12810 13105 13485 13506 13650 13789 14000A 14148 14178 14560 14766 14911 15326 16086 16129 16187 16189 16223 16278 16293 16294 16311 16360 |
| L1c1b | 73 151 152 182 186A 189C 195 198 247 263 297 316 750 769 825A 1018 1291 1438 2395d 2706 2758 2885 3594 3666 3796T 3843 4104 4688 4769 5553 5951 6071 7028 7055 7146 7256 7389 7521 8027 8468 8619 8655 8701 8860 9072 9540 9861 10084 10321 10398 10586 10688 10810 10873 11719 11899 12681 12705 12810 13105 13485 13506 13650 13789 14000A 14148 14178 14393 14560 14766 14911 15326 16086 16129 16187 16189 16223 16241 16278 16293 16294 16311 16360 |
| L1c1b1 | 73 151 152 182 186A 189C 195 198 247 263 297 316 750 769 825A 1018 1291 1438 2395d 2706 2758 2885 3594 3666 3796T 3843 4104 4688 4769 4824 5553 5951 6071 7028 7055 7146 7256 7389 7521 8027 8277 8468 8619 8655 8701 8860 9072 9540 9861 10084 10321 10398 10586 10688 10810 10873 11719 11899 12681 12705 12810 13105 13485 13506 13650 13789 14000A 14148 14178 14393 14560 14766 14911 15025 15326 16086 16129 16187 16189 16223 16241 16278 16291 16294 16311 16360 |
| L1c1d | 73 151 152 182 186A 189C 195 198 247 263 297 316 750 769 825A 1018 1438 2395d 2706 2758 2885 3594 3666 3796T 3843 4104 4769 5460 5951 6071 7028 7055 7146 7256 7389 7521 8027 8468 8655 8701 8860 9072 9540 10321 10398 10586 10688 10810 10873 11719 11899 11914 12705 12810 13105 13485 13506 13650 13789 14000A 14148 14178 14560 14766 14911 15301 15326 15626 16038 16086 16129 16187 16189 16223 16278 16293 16294 16311 16360 |

| | |
|---|---|
| L1c1d1 | 73 151 152 182 186A 189C 195 198 247 263 297 316 750 769 825A 1018 1438 2395d 2706 2758 2885 3594 3666 3796T 3843 4104 4769 5108 5460 5951 6071 7028 7055 7146 7256 7389 7521 7948 8027 8468 8655 8701 8860 9072 9540 10321 10398 10586 10688 10810 10873 11719 11899 11914 12705 12810 13105 13485 13506 13650 13789 14000A 14148 14178 14560 14766 14911 15301 15326 15626 16038 16086 16129 16187 16189 16223 16278 16293 16294 16311 16360 |
| L1c1c | 73 151 152 182 186A 189C 195 247 249d 263 297 316 750 769 825A 1018 1438 2395d 2706 2758 2885 3594 3666 3796T 3843 4104 4769 5951 6071 6267 7028 7055 7146 7256 7389 7521 8027 8387 8389 8468 8655 8701 8860 9072 9233 9540 10321 10398 10586 10688 10810 10873 11335 11719 12705 12810 12879 13105 13485 13506 13650 13789 14000A 14148 14178 14560 14766 14911 15326 16129 16172 16189 16223 16278 16293 16311 16360 |
| L1c2'4 | 73 151 152 182 186A 189C 195 198 247 263 297 316 750 769 825A 1018 1438 2395d 2706 2758 2885 3594 3666 4104 4769 5899.1C 5951 6071 7028 7055 7146 7256 7389 7521 8027 8468 8655 8701 8860 9072 9540 10321 10398 10586 10688 10810 10873 11719 12049 12705 12810 13105 13149 13485 13506 13650 13789 14000A 14178 14560 14766 14911 15326 16129 16187 16189 16223 16278 16294 16311 16360 |
| L1c2 | 73 151 152 182 186A 189C 195 198 247 263 297 316 750 769 825A 1018 1438 2395d 2706 2758 2885 3594 3666 4104 4769 5899.1C 5951 6071 6150 6253 7028 7055 7076 7146 7256 7337 7389 7521 8027 8468 8655 8701 8784 8860 8877 9072 9540 10321 10398 10586 10688 10792 10793 10810 10873 11654 11719 12049 12705 12810 13105 13149 13485 13506 13650 13789 14000A 14178 14560 14766 14911 15326 16129 16187 16189 16223 16265C 16278 16286G 16294 16311 16360 16527 |
| L1c2a | 73 151 152 182 186A 189C 195 198 247 263 297 316 750 769 825A 1018 1420 1438 2156.1A 2395d 2706 2758 2885 3594 3666 4104 4769 5899.1C 5951 6071 6150 6253 7028 7055 7076 7146 7256 7337 7389 7521 8027 8468 8655 8701 8784 8860 8877 9072 9540 10321 10398 10586 10688 10792 10793 10810 10873 11654 11719 12049 12705 12810 13105 13149 13485 13506 13650 13789 14000A 14178 14560 14766 14911 15016 15326 15784 16129 16187 16189 16223 16265C 16278 16286G 16294 16311 16360 16527 |
| L1c2a1 | 73 151 152 182 186A 189C 195 198 247 263 297 316 750 769 825A 1018 1420 1438 2156.1A 2395d 2706 2758 2885 3594 3666 4104 4769 5899.1C 5951 6071 6150 6253 7028 7055 7076 7146 7256 7337 7389 7521 8027 8468 8655 8701 8784 8860 8877 9072 9540 10321 10398 10586 10688 10792 10793 10810 10873 11654 11719 12049 12705 12810 13105 13149 13485 13506 13650 13789 14000A 14178 14560 14766 14911 15016 15326 15784 16129 16145 16187 16189 16213 16223 16265C 16278 16286G 16294 16311 16360 16527 |

| | |
|---|---|
| L1c2a1a | 73 151 152 182 186A 189C 195 198 247 263 297 316 750 769 825A 1018 1420 1438 2156.1A 2395d 2706 2758 2885 3594 3666 4104 4769 5899.1C 5951 6071 6150 6253 7028 7055 7076 7146 7256 7337 7389 7521 7744 8027 8251 8468 8655 8701 8784 8860 8877 9072 9540 10321 10398 10586 10688 10792 10793 10810 10873 11654 11719 12049 12705 12810 13105 13149 13212 13281 13485 13506 13650 13789 14000A 14178 14560 14766 14812 14911 15016 15326 15784 16071 16129 16145 16187 16189 16213 16223 16234 16265C 16278 16286G 16294 16311 16360 16527 |
| L1c2a1b | 73 151 152 182 186A 189C 195 198 247 263 297 316 750 769 825A 1018 1420 1438 2156.1A 2395d 2706 2758 2885 3594 3666 3777 4104 4769 5237 5899.1C 5951 6071 6150 6253 7028 7055 7076 7146 7256 7337 7389 7521 8027 8468 8655 8701 8784 8860 8877 9072 9540 10321 10398 10586 10688 10792 10793 10810 10873 11654 11719 12049 12705 12810 13105 13149 13485 13506 13650 13789 14000A 14178 14560 14766 14911 15016 15326 15784 16129 16145 16187 16189 16213 16223 16265C 16278 16286G 16294 16311 16360 16527 |
| L1c2a2 | 73 151 152 182 186A 189C 195 198 247 263 297 316 709 750 769 825A 1018 1420 1438 2156.1A 2395d 2706 2758 2885 3438 3594 3666 4104 4769 5951 6071 6150 6253 7028 7055 7070 7076 7146 7160 7256 7301 7337 7389 7521 8027 8468 8655 8784 8860 8877 9018 9072 9540 10321 10398 10586 10688 10792 10793 10810 10873 11654 11719 12049 12705 12810 13105 13149 13437 13485 13506 13650 13789 14000A 14178 14560 14766 14911 15016 15326 15784 16129 16187 16189 16223 16265C 16278 16286G 16288 16294 16311 16357 16527 |
| L1c2a3 | 73 151 152 182 186A 189C 195 198 247 263 297 316 471 750 769 825A 1018 1420 1438 2156.1A 2395d 2706 2758 2885 3594 3666 4104 4769 5899.1C 5951 6071 6150 6253 6723 7028 7055 7076 7146 7256 7337 7389 7521 8027 8468 8655 8701 8784 8860 8877 9072 9540 10321 10398 10586 10688 10792 10793 10810 10873 11329 11654 11719 12049 12705 12810 13105 13149 13485 13506 13650 13789 14000A 14178 14560 14766 14861 14911 15016 15326 15784 16129 16187 16189 16223 16265C 16278 16286G 16294 16311 16360 16527 |
| L1c2a3a | 73 151 152 182 186A 189C 195 198 247 263 297 316 385 471 750 769 825A 1018 1048 1420 1438 2156.1A 2395d 2706 2758 2885 3594 3666 4104 4769 5899.1C 5951 6071 6150 6253 6723 7028 7055 7076 7146 7256 7337 7389 7521 7741 8027 8468 8655 8701 8784 8860 8877 9072 9540 10321 10398 10586 10688 10792 10793 10810 10873 11329 11654 11719 12049 12705 12810 13105 13149 13485 13506 13650 13789 14000A 14127 14178 14560 14766 14861 14911 15016 15326 15784 16172 16187 16189 16223 16265C 16278 16286G 16294 16311 16360 16527 |
| L1c2b | 73 151 152 182 186A 189C 195 198 247 263 297 316 750 769 825A 1018 1438 2395d 2706 2758 2885 3594 3666 4104 4769 5899.1C 5951 6071 6150 6253 7028 7055 7076 7146 7256 7337 7389 7521 8027 8468 8655 8701 8784 8860 8877 9072 9540 10321 10398 10586 10688 10792 10793 10810 10873 11164 11654 11719 12049 12705 12810 13105 13149 13485 13506 13650 13789 14000A 14178 14560 14766 14911 15326 (16093) 16129 16187 16189 16223 16265C 16278 16286G (16286A) 16294 16311 16360 16527 |

| | |
|---|---|
| L1c2b1 | 73 151 152 182 186A 189C 195 198 247 263 297 316 750 769 825A 1018 1438 2395d 2706 2758 2885 3594 3666 4104 4769 5899.1C 5951 6071 6150 6253 7028 7055 7076 7146 7256 7337 7389 7521 8027 8468 8655 8701 8784 8860 8877 9072 9540 10031 10321 10398 10586 10688 10792 10793 10810 10873 11164 11654 11719 12049 12705 12810 13105 13149 13485 13506 13650 13789 14000A 14178 14560 14766 14911 15326 (16093) 16129 16187 16189 16223 16265C 16278 16286G (16286A) 16294 16311 16360 16527 |
| L1c2b1a'b | 73 151 152 182 186A 189C 195 198 247 263 297 316 750 769 825A 1018 1438 2220 2395d 2706 2758 2885 3594 3666 4104 4769 5899.1C 5951 6071 6150 6253 7028 7055 7076 7146 7256 7337 7389 7521 8027 8468 8655 8701 8784 8860 8877 9072 9540 10031 10321 10398 10586 10688 10792 10793 10810 10873 11164 11654 11719 12049 12705 12810 13105 13149 13485 13506 13650 13789 14000A 14178 14560 14766 14911 15326 (16093) 16129 16187 16189 16223 16265C 16278 16286G (16286A) 16294 16311 16360 16527 |
| L1c2b1a | 73 151 152 182 186A 189C 195 198 247 263 297 316 750 769 825A 1018 1438 2220 2395d 2706 2758 2885 3594 3666 4104 4769 5580 5899.1C 5951 6071 6150 6209 6253 7028 7055 7076 7146 7256 7337 7389 7521 8027 8468 8655 8701 8784 8860 8877 9072 9540 10031 10321 10398 10586 10688 10792 10793 10810 10873 11164 11654 11719 12049 12705 12810 13105 13149 13485 13506 13650 13789 14000A 14071 14178 14560 14766 14911 15326 (16093) 16129 16187 16189 16223 16265C 16278 16286G (16286A) 16294 16311 16360 16527 |
| L1c2b1a1 | 73 151 152 182 186A 189C 195 198 247 263 297 316 750 769 825A 1018 1438 2220 2395d 2706 2758 2885 3349 3594 3666 4104 4769 5580 5899.1C 5951 6071 6150 6209 6253 7028 7055 7076 7146 7256 7337 7389 7521 7675 8027 8468 8655 8701 8784 8860 8877 9072 9540 10031 10321 10398 10586 10688 10792 10793 10810 10873 11164 11654 11719 12049 12705 12771 12810 13105 13149 13485 13506 13650 13789 14000A 14071 14178 14560 14766 14911 15326 (16093) 16129 16187 16189 16223 16265C 16278 16286G (16286A) 16294 16311 16360 16527 |
| L1c2b1b | 73 151 152 182 186A 189C 195 198 247 263 297 316 750 769 825A 1018 1438 2220 2395d 2706 2758 2885 3594 3666 4104 4769 5087 5899.1C 5951 6071 6150 6253 6480 7028 7055 7076 7146 7256 7337 7389 7521 8027 8468 8655 8701 8784 8860 8877 9072 9108 9540 10031 10321 10398 10586 10688 10792 10793 10810 10873 11164 11654 11719 12049 12669 12705 12810 13105 13149 13485 13506 13650 13789 14000A 14178 14560 14766 14911 15326 (16093) 16129 16187 16189 16223 16265C 16278 16286G (16286A) 16294 16311 16360 16527 |
| L1c2b1b1 | 73 151 152 182 186A 189C 195 198 247 263 264 297 316 750 769 825A 1018 1438 2220 2395d 2706 2758 2885 3275G 3594 3666 4104 4769 5087 5899.1C 5951 6071 6150 6173 6253 6480 7028 7055 7076 7146 7256 7337 7389 7521 8027 8468 8655 8701 8784 8860 8877 9072 9108 9540 10031 10321 10398 10586 10688 10792 10793 10810 10873 11164 11654 11719 12049 12669 12705 12810 13105 13149 13485 13506 13650 13789 14000A 14178 14560 14766 14911 15326 (16093) 16129 16187 16189 16223 16265C 16278 16286G (16286A) 16294 16311 16360 16527 |

| | |
|---|---|
| L1c2b1c | 73 151 152 182 186A 189C 195 198 247 263 297 316 633 723 750 769 825A 1018 1438 2395d 2706 2758 2885 3421 3594 3666 3777 4104 4769 5580 5894d 5899.1C 5951 6071 6150 6253 7028 7055 7076 7146 7256 7337 7389 7521 8027 8468 8655 8701 8784 8860 8877 9072 9540 10031 10071 10321 10398 10586 10688 10792 10793 10810 10873 11164 11654 11719 12049 12705 12810 13105 13149 13485 13506 13650 13789 14000A 14178 14560 14766 14911 15326 15672 (16093) 16129 16187 16189 16223 16265C 16278 16286G (16286A) 16292 16294 16311 16360 16527 |
| L1c2b2 | 73 151 152 182 186A 189C 195 198 247 263 297 316 750 769 825A 1018 1438 2395d 2706 2758 2885 3594 3666 4104 4769 5899.1C 5951 6071 6150 6253 7028 7055 7076 7146 7256 7337 7389 7521 8027 8468 8567 8655 8701 8784 8860 8877 9072 9540 10321 10398 10586 10688 10792 10793 10810 10873 11164 11654 11719 12049 12705 12810 13105 13149 13269 13485 13506 13650 13789 14000A 14178 14560 14766 14911 15326 (16093) 16129 16187 16189 16223 16265C 16278 16286G (16286A) 16294 16311 16320 16360 16527 |
| L1c4 | 73 152 182 186A 189C 195 247 263 297 316 750 769 825A 1018 1438 2395d 2706 2758 2885 3594 3666 4104 4769 5196 5899.1C 5951 6071 6629 7028 7055 7146 7256 7389 7521 7673 7960 8027 8468 8655 8701 8860 9072 9266 9540 9545 10321 10398 10586 10688 10810 10873 11719 12049 12705 12810 13105 13149 13485 13506 13650 13741 13789 13879 14000A 14178 14560 14766 14911 15326 16129 16184 16187 16189 16223 16278 16294 16301 16311 16360 |
| L1c4a | 73 152 182 186A 189C 195 247 263 280 297 316 750 769 825A 1018 1438 1503 2395d 2706 2758 2885 3594 3666 4104 4596 4769 4937 4959 5054 5196 5899.1C 5951 6071 6629 7028 7055 7146 7256 7389 7521 7673 7960 8027 8468 8655 8701 8860 9072 9266 9540 9545 10321 10398 10586 10688 10810 10873 11337 11719 11854 12049 12705 12810 12940 13105 13149 13485 13506 13650 13741 13789 13879 14000A 14178 14182 14560 14766 14911 15191 15326 16037 16093 16129 16140 16184 16187 16189 16223 16278 16294 16301 16311 16360 |
| L1c4b | 73 152 182 186A 189C 195 247 263 297 316 750 769 825A 1018 1438 2395d 2706 2758 2885 3594 3666 4104 4706 4769 5196 5899.1C 5951 6071 6629 7028 7055 7146 7256 7389 7521 7673 7960 8027 8468 8655 8701 8860 9072 9266 9540 9545 10321 10398 10586 10688 10810 10873 11002 11719 12049 12705 12810 13105 13149 13485 13506 13650 13741 13789 13879 14000A 14178 14560 14766 14911 15326 16129 16184 16187 16189 16223 16278 16294 16301 16311 16360 |
| L1c6 | 73 151 152 182 186A 189C 195 198 247 263 297 316 750 769 825A 930 1018 1438 2251 2395d 2706 2758 2885 3594 3666 4104 4769 5231 5951 6071 7028 7055 7146 7256 7389 7521 8027 8468 8655 8701 8860 9072 9540 10321 10398 10586 10688 10810 10873 11719 12705 12810 13105 13485 13506 13650 13789 14000A 14178 14470 14560 14766 14911 15326 16129 16187 16189 16223 16278 16294 16311 16360 |

| | |
|---|---|
| L1c5 | 73 151 152 182 186A 189C 195 247 263 291T 297 316 709 750 769 825A 1018 1438 2395d 2706 2758 2885 3594 3666 4104 4769 5390 5951 6071 7028 7055 7146 7256 7389 7521 7762 8027 8143 8281-8289d 8468 8655 8701 8860 9072 9540 9899 10398 10586 10688 10810 10873 11150 11719 12425 12630 12705 12810 13105 13359 13368 13485 13506 13650 13789 14000A 14178 14560 14766 14911 15326 15449 15553 15941 (16114A) 16129 16187 16189 16223 16261 16278 16311 16360 |
| L1c3 | 73 151 152 182 186A 189C 247 263 316 750 769 825A 1018 1438 2395d 2706 2758 2885 3594 3666 4104 4769 5951 6071 6221A 6917 7028 7146 7256 7389 7521 8027 8468 8655 8701 8860 9072 9540 10398 10586 10688 10810 10873 11302 11719 12705 12810 13105 13485 13506 13650 13789 14000A 14178 14560 14766 14911 15226 15326 15905 15978 16129 16187 16189 16223 16278 16294 16311 16360 |
| L1c3a | 73 151 152 182 186A 189C 247 263 316 750 769 825A 1018 1438 2395d 2706 2758 2885 3594 3666 4104 4769 5951 6071 6221A 6260 6917 7028 7146 7256 7389 7498 7521 7789 8027 8468 8655 8701 8860 9072 9540 9966 10398 10586 10688 10810 10873 11302 11719 12019 12501 12705 12810 13105 13485 13506 13650 13789 14000A 14178 14560 14766 14911 15226 15326 15905 15978 16129 16189 16215 16223 16278 16294 16311 16360 |
| L1c3a1 | 73 151 152 182 186A 189C 247 263 316 750 769 825A 1018 1438 2395d 2706 2758 2885 3105 3594 3666 4104 4769 5951 6071 6221A 6260 6917 7028 7146 7256 7389 7498 7521 7789 8027 8468 8655 8701 8860 9072 9540 9966 10398 10586 10688 10810 10873 11302 11719 12019 12501 12705 12810 13105 13485 13506 13650 13789 14000A 14178 14560 14766 14911 15226 15326 15905 15978 16129 16189 16215 16223 16278 16294 16311 16360 |
| L1c3a1a | 73 151 152 182 186A 189C 247 263 316 750 769 825A 1018 1438 2387 2395d 2706 2758 2885 3105 3594 3666 4104 4769 5951 6071 6221A 6260 6917 7028 7146 7256 7389 7498 7521 7789 8027 8468 8655 8701 8860 9072 9540 9966 10398 10586 10688 10810 10873 11302 11719 12019 12501 12616 12705 12810 13105 13485 13506 13650 13789 14000A 14178 14560 14766 14911 15226 15326 15905 15978 16129 16189 16215 16223 16278 16294 16311 16360 |
| L1c3a1b | 73 151 152 182 186A 189C 247 263 316 750 769 825A 1018 1438 2395d 2706 2758 2885 3105 3594 3666 4104 4769 5951 6071 6221A 6260 6917 7028 7146 7256 7389 7498 7521 7789 8027 8468 8655 8668 8701 8860 9072 9540 9966 10398 10586 10688 10810 10873 11302 11719 12019 12501 12705 12810 13105 13485 13506 13650 13789 14000A 14178 14560 14766 14911 15226 15326 15905 15978 16129 16189 16215 16223 16278 16294 16311 16355 16360 |
| L1c3b'c | 73 151 152 182 186A 189C 247 263 316 750 769 825A 1018 1438 2283 2395d 2706 2758 2885 3594 3666 4104 4769 5951 6071 6221A 6917 7028 7146 7256 7389 7521 8027 8468 8655 8701 8860 9072 9540 10398 10586 10688 10810 10873 11302 11719 12705 12810 13105 13485 13506 13650 13789 14000A 14178 14560 14766 14911 15226 15326 15905 15978 16129 16187 16189 16223 16278 16293 16294 16311 16360 |

| | |
|---|---|
| L1c3b | 73 151 152 182 186A 189C 247 263 316 750 769 825A 1018 1438 2283 2395d 2706 2758 2885 3594 3666 4104 4769 5951 6071 6221A 6917 7028 7146 7256 7389 7521 8027 8251 8468 8655 8701 8860 9072 9540 10398 10586 10688 10810 10873 11302 11719 12705 12810 13105 13485 13506 13650 13789 13981 14000A 14178 14560 14766 14794 14911 15226 15326 15905 15978 16129 16163 16187 16189 16223 16278 16293 16294 16311 16360 |
| L1c3b1 | 73 151 152 182 186A 189C 247 263 316 629 750 769 825A 1018 1438 2283 2395d 2706 2758 2885 3210 3434 3594 3666 4104 4755 4769 5951 6071 6221A 6917 7028 7146 7256 7389 7521 8027 8251 8417 8468 8655 8701 8860 9072 9540 10398 10586 10688 10810 10873 11302 11719 12400 12542 12705 12810 13105 13485 13506 13650 13789 13981 14000A 14178 14560 14766 14794 14911 15226 15326 15905 15978 16017 16129 16163 16187 16189 16223 16278 16293 16294 16311 16360 |
| L1c3b1a | 73 151 152 182 186A 189C 247 263 316 629 750 769 825A 1018 1438 2283 2395d 2706 2758 2885 3210 3434 3594 3666 4104 4755 4769 5951 6071 6221A 6917 7028 7146 7256 7389 7521 8027 8251 8417 8468 8655 8701 8860 9072 9540 10398 10586 10688 10810 10873 11302 11317 11719 12400 12542 12705 12810 13105 13485 13506 13650 13708 13789 13981 14000A 14178 14560 14766 14794 14911 15226 15326 15905 15978 16017 16129 16163 16187 16189 16209 16223 16278 16293 16294 16311 16360 |
| L1c3b1b | 73 151 152 182 186A 189C 247 263 316 629 750 769 825A 1018 1438 1686 2083 2283 2395d 2706 2758 2885 3210 3434 3594 3666 4104 4755 4769 5951 6071 6221A 6917 7028 7146 7256 7389 7521 8027 8251 8417 8468 8655 8701 8860 9072 9540 10398 10586 10688 10810 10873 11302 11719 12400 12542 12705 12810 13105 13485 13506 13650 13789 13981 14000A 14178 14560 14766 14794 14911 15226 15326 15905 15978 16017 16129 16163 16187 16189 16223 16278 16293 16294 16311 16360 |
| L1c3b2 | 73 151 152 182 186A 189C 247 263 316 750 769 825A 1018 1438 2395d 2706 2758 2885 3096 3594 3666 4104 4769 5951 6071 6221A 6297 6353 6917 7028 7146 7256 7389 7521 7805 7844 8027 8251 8468 8655 8701 8860 9072 9540 10398 10586 10688 10810 10873 11302 11719 12705 12810 13105 13485 13506 13650 13789 13981 14000A 14128 14178 14560 14766 14794 14831 14911 15226 15244 15326 15905 15924 15978 16086 16129 16163 16187 16189 16223 16278 16293 16294 16311 16360 |
| L1c3c | 73 93 151 152 182 186A 189C 195 247 248 263 316 458 745.1T 750 769 825A 1018 1438 2283 2395d 2706 2758 2885 3027 3525 3594 3600 3666 4104 4769 4853 5951 6071 6221A 6917 7028 7146 7256 7389 7521 8027 8468 8655 8701 8860 9072 9540 10398 10586 10688 10810 10873 11302 11719 11852 11984 12507 12705 12810 13105 13485 13506 13650 13789 14000A 14178 14560 14669 14766 14911 15226 15326 15905 15978 16129 16187 16189 16223 16278 16293 16294 16311 16360 |
| L2'3'4'5'6 | 73 182 195 247 263 750 769 825A 1018 1438 2706 3594 4104 4769 7028 7256 7521 8655 8701 8860 9540 10398 10688 10810 10873 11719 12705 13105 13506 13650 14766 15326 16129 16187 16189 16223 16278 16311 |

| | |
|---|---|
| L5 | 73 182 195 247 263 459.1C 750 769 825A 1018 1438 2706 3423 3594 4104 4769 7028 7256 7521 7972 8655 8701 8860 9540 10398 10688 10810 10873 11719 12432 12705 12950 13105 13506 13650 14766 15326 16129 16148 16166 16187 16189 16223 16278 16311 |
| L5a | 73 152 182 195 247 263 455.1T 459.1C 709 750 769 825A 851 1018 1438 1822 2706 3423 3594 4104 4769 5111 5147 5656 6182 6297 7028 7256 7424 7521 7972 8155 8188 8582 8655 8701 8860 9305 9329 9540 10398 10688 10810 10873 11025 11719 11881 12236 12432 12705 12950 13105 13506 13650 13722 14212 14239 14581 14766 14905 14971 15217 15326 15884 16129 16148 16166 16187 16189 16223 16278 16311 16355 16362 |
| L5a1 | 73 152 182 195 247 263 455.2T 459.1C 709 750 769 825A 851 930 1018 1438 1822 2706 3423 3594 4104 4496 4769 5111 5147 5656 6182 6297 7028 7256 7424 7521 7972 8155 8188 8582 8655 8701 8754 8860 9305 9329 9540 10398 10688 10810 10873 11025 11719 11881 12236 12432 12705 12950 13105 13506 13650 13722 14212 14239 14581 14766 14905 14971 15217 15326 15884 16129 16148 16166 16187 16189 16223 16278 16311 16355 16362 |
| L5a1a | 73 152 182 195 247 263 455.2T 459.1C 709 750 769 825A 851 930 1018 1438 1822 2706 3423 3594 4104 4496 4769 5004 5111 5147 5656 6182 6297 7028 7256 7424 7521 7972 8155 8188 8582 8655 8701 8754 8860 9305 9329 9540 9899 10398 10688 10810 10873 11025 11719 11881 12236 12432 12705 12950 13105 13506 13650 13722 14212 14239 14581 14766 14905 14971 15217 15326 15884 16129 16148 16166 16187 16189 16223 16278 16311 16355 16362 |
| L5a1b | 73 152 182 195 247 263 455.2T 459.1C 709 750 769 825A 851 930 1018 1438 1822 2706 3423 3594 4104 4496 4769 5111 5147 5656 6182 6297 7028 7256 7424 7521 7972 8155 8188 8582 8655 8701 8754 8860 9305 9329 9540 10398 10688 10810 10873 11025 11719 11881 12236 12432 12705 12950 13105 13506 13650 13722 14212 14239 14581 14668 14766 14819 14905 14971 15217 15326 15884 16129 16148 16166 16187 16189 16223 16278 16311 16355 16362 |
| L5a1c | 73 152 182 195 247 263 455.2T 459.1C 513 709 750 769 825A 851 930 1018 1438 1822 2706 3423 3594 4104 4137 4496 4769 5111 5147 5656 6182 6297 7028 7256 7424 7521 7972 8155 8188 8582 8655 8701 8754 8860 9305 9329 9540 10398 10688 10810 10873 11025 11719 11881 12236 12432 12705 12950 13105 13506 13650 13722 14212 14239 14581 14766 14905 14971 15217 15326 15884 16129 16148 16166 16187 16189 16223 16278 16311 16355 16362 |
| L5a2 | 73 152 182 195 247 263 455.1T 459.1C 527 709 750 769 825A 851 1018 1438 1822 2706 3423 3594 4104 4769 5111 5147 5656 6182 6297 7028 7256 7424 7521 7972 8155 8188 8582 8655 8701 8856 8860 9305 9329 9540 10398 10688 10810 10873 11025 11719 11881 12236 12432 12705 12950 13105 13506 13650 13722 14212 14239 14581 14766 14905 14971 15217 15326 15884 16129 16148 16166 16187 16189 16223 16278 16311 16355 16362 |

| | |
|---|---|
| L5b | 73 195 247 263 459.1C 750 769 825A 1018 1438 2706 3423 3594 4104 4769 7028 7256 7521 7972 8655 8701 8860 9540 10398 10688 10810 10873 11719 12432 12705 12950 13506 13650 14766 15326 16129 16148 16166 16187 16189 16223 16254 16278 |
| L5b1 | 73 195 247 249d 263 459.1C 535 750 769 825A 1018 1438 2417G 2706 3027 3423 3594 3720 4104 4769 4976 5213 7028 7256 7521 7972 8655 8701 8860 9540 9809C 10398 10493 10688 10810 10873 11701 11719 12188 12432 12546T 12705 12714 12810 12950 13506 13569 13650 13830 14766 15326 16111 16129 16148 16166 16187 16189 16223 16254 16278 [illegible] |
| L5b1a | 73 182 195 247 249d 263 459.1C 535 750 769 825A 1018 1438 2417G 2706 3027 3423 3594 3720 4104 4769 4976 5213 7028 7256 7521 7972 8152 8655 8701 8860 9540 9809C 10398 10493 10688 10810 10873 11065 11701 11719 12188 12215 12432 12546T 12705 12714 12810 12950 13506 13569 13650 13830 14766 15326 16111 16129 16148 16166 16187 16189 16223 16254 16278 16360 |
| L5b1b | 73 195 247 249d 263 459.1C 535 750 769 825A 1018 1438 2417G 2706 3027 3423 3594 3720 4104 4769 4976 5213 7028 7256 7521 7972 8655 8701 8860 9540 9809C 10398 10493 10688 10810 10873 11701 11719 12188 12432 12546T 12705 12714 12810 12950 13506 13569 13650 13830 14581 14766 15326 16111 16129 16148 16166 16187 16189 16223 16254 16278 16360 |
| L5b2 | 73 247 263 750 769 825A 1018 1438 1717 1719 2080 2484 2706 3423 3564 3594 3918 4092 4104 4655 4676 4769 4967 6527 7028 7256 7521 7765 7789 8655 8701 8860 9018 9540 9653 10101 10398 10688 10810 10873 11266 11287 11719 11809 12432 12507 12705 12940 13050 13383 13506 13602 13650 14766 15236 15326 15418 16129 16148 16166 16187 16223 16254 16278 16368 16527 |
| L2'3'4'6 | 73 182 263 750 769 1018 1438 2706 3594 4104 4769 7028 7256 7521 8701 8860 9540 10398 10873 11719 12705 13650 14766 15301 15326 16223 16278 16311 |
| L2 | 73 146 150 152 182 263 750 769 1018 1438 2416 2706 3594 4104 4769 7028 7256 7521 8206 8701 8860 9221 9540 10115 10398 10873 11719 12705 13590 13650 14766 15301 15326 16223 16278 16390 |
| L2a'b'c'd | 73 146 150 152 182 195 263 750 769 1018 1438 2416 2706 3594 4104 4769 7028 7256 7521 8206 8701 8860 9221 9540 10115 10398 10873 11719 11944 12705 13590 13650 14766 15301 15326 16223 16278 16390 |
| L2a | 73 146 152 182 195 263 750 769 1018 1438 2416 2706 3594 4104 4769 7028 7175 7256 7521 8206 8701 8860 9221 9540 10115 10398 10873 11719 11944 12705 13590 13650 14766 15301 15326 16223 16278 16390 |
| L2a1'2'3'4 | 73 146 152 182 195 263 750 769 1018 1438 2416 2706 2789 3594 4104 4769 7028 7175 7256 7274 7521 7771 8206 8701 8860 9221 9540 10115 10398 10873 11719 11914 11944 12705 13590 13650 13803 14566 14766 15301 15326 16223 16278 16294 16390 |
| L2a1 | 73 146 152 195 263 750 769 1018 1438 2416 2706 2789 3594 4104 4769 7028 7175 7256 7274 7521 7771 8206 8701 8860 9221 9540 10115 10398 10873 11719 11914 11944 12693 12705 13590 13650 13803 14566 14766 15301 15326 15784 16223 16278 16294 16309 16390 |

| | |
|---|---|
| L2a1a | 73 146 152 195 263 750 769 1018 1438 2416 2706 2789 3594 3918 4104 4769 5285 7028 7175 7256 7274 7521 7771 8206 8701 8860 9221 9540 10115 10398 10873 11719 11914 11944 12693 12705 13590 13650 13803 14566 14766 15244 15301 15326 15629 15784 16223 16278 16294 16309 16390 |
| L2a1a1 | 73 146 152 195 263 750 769 1018 1438 2416 2706 2789 3594 3918 4104 4769 5285 6152 7028 7175 7256 7274 7521 7771 8206 8701 8860 9221 9540 10115 10398 10873 11719 11914 11944 12693 12705 13590 13650 13803 14566 14766 15244 15301 15326 15391 15629 15784 16223 16278 16294 16309 16368 16390 |
| L2a1a2 | 73 146 152 195 263 750 769 1018 1438 2416 2706 2789 3594 3918 4104 4769 5285 7028 7175 7256 7274 7521 7771 8206 8701 8860 9221 9540 10115 10398 10873 11719 11914 11944 12693 12705 13590 13650 13803 14566 14766 15244 15301 15326 15629 15784 16223 16278 16286 16294 16309 16390 |
| L2a1a2a | 73 146 152 195 263 750 769 1018 1438 2416 2706 2789 3594 3918 4104 4769 5285 7028 7175 7256 7274 7521 7771 8206 8701 8860 9221 9540 10115 10398 10454 10873 11719 11914 11944 12693 12705 13590 13650 13803 14566 14766 15244 15301 15326 15629 15784 16223 16278 16286 16294 16309 16390 |
| L2a1a2a1 | 73 146 152 195 263 750 769 1018 1438 2416 2706 2789 3594 3918 4104 4769 5285 7028 7175 7256 7274 7521 7771 8206 8701 8860 9221 9540 10115 10398 10454 10873 11719 11914 11944 12693 12705 13590 13650 13803 14566 14766 15211 15244 15301 15326 15629 15784 16223 16278 16286 16294 16309 16390 |
| L2a1a2a1a | 73 146 152 195 263 750 769 1018 1438 2416 2706 2789 3594 3918 4104 4769 5285 7028 7175 7256 7274 7521 7771 8206 8701 8860 9221 9540 10115 10398 10454 10873 11719 11914 11944 12693 12705 13590 13650 13803 14566 14766 15211 15244 15301 15326 15421 15629 15784 16223 16278 16286 16294 16309 16390 |
| L2a1a2b | 73 146 152 195 263 750 769 1018 1438 2416 2706 2789 3336 3594 3918 4104 4769 5285 7028 7175 7256 7274 7521 7771 8206 8701 8860 9221 9540 10115 10398 10873 11719 11914 11944 12693 12705 13590 13650 13803 14566 14766 15244 15301 15326 15629 15784 16223 16278 16286 16294 16309 16390 |
| L2a1a2c | 73 146 152 195 263 750 769 1018 1438 2416 2706 2789 3594 3918 4104 4769 5285 5492 7028 7175 7256 7274 7521 7771 8206 8701 8841 8860 9221 9540 10115 10398 10873 11719 11914 11944 12693 12705 13590 13615 13650 13803 14566 14766 15244 15301 15326 15629 15784 16223 16278 16286 16294 16309 16390 |
| L2a1a3 | 73 143 146 152 195 263 750 769 1018 1438 2416 2706 2789 3594 3918 4104 4769 5285 7028 7175 7256 7274 7521 7771 8206 8701 8860 9221 9540 10115 10398 10873 11719 11914 11944 12693 12705 13590 13650 13803 14566 14766 15244 15301 15326 15629 15784 (16093) 16223 16278 16294 16309 16390 |

| | |
|---|---|
| L2a1a3a | 73 143 146 152 195 263 750 769 1018 1438 2416 2706 2789 3594 3918 4104 4769 5285 7028 7175 7256 7274 7521 7771 8206 8701 8860 9165 9221 9540 10115 10398 10873 11719 11914 11944 12693 12705 13590 13650 13803 14566 14766 15244 15301 15326 15629 15784 (16093) 16223 16278 16294 16309 16390 |
| L2a1a3b | 73 143 146 152 195 263 750 769 1018 1438 2416 2706 2789 3316 3594 3918 4104 4769 5285 7028 7175 7256 7274 7521 7771 8206 8701 8860 9221 9540 10115 10398 10873 11719 11914 11944 12693 12705 13590 13650 13803 14566 14766 15244 15301 15326 15629 15784 (16093) 16223 16278 16294 16309 16390 |
| L2a1a3c | 73 143 146 152 195 263 750 769 1018 1438 2416 2706 2789 3594 3918 4104 4769 5285 7028 7175 7256 7274 7521 7771 8206 8701 8860 9221 9449 9540 10115 10398 10873 11719 11914 11944 12693 12705 13590 13650 13803 14566 14766 15244 15301 15326 15629 15784 (16093) 16223 16256 16278 16294 16309 16390 |
| L2a1+16189_(16192) | 73 146 152 195 263 750 769 1018 1438 2416 2706 2789 3594 4104 4769 7028 7175 7256 7274 7521 7771 8206 8701 8860 9221 9540 10115 10398 10873 11719 11914 11944 12693 12705 13590 13650 13803 14566 14766 15301 15326 15784 16189 (16192) 16223 16278 16294 16309 16390 |
| L2a1b | 73 146 152 195 263 750 769 1018 1438 2416 2706 2789 3594 4104 4769 7028 7175 7256 7274 7521 7771 8206 8701 8860 9221 9540 10115 10143 10398 10873 11719 11914 11944 12693 12705 13590 13650 13803 14566 14766 15301 15326 15784 16189 (16192) 16223 16278 16294 16309 16390 |
| L2a1b1 | 73 146 152 195 263 750 769 1018 1438 2416 2706 2789 3594 4104 4769 7028 7175 7256 7274 7521 7771 8206 8701 8860 9221 9540 10115 10143 10398 10873 11719 11914 11944 12693 12705 13590 13650 13803 14566 14766 15301 15326 15735 15784 16189 (16192) 16223 16278 16294 16309 16390 |
| L2a1b1a | 73 146 152 195 263 750 769 1018 1438 2416 2706 2789 3594 4104 4769 5090 7028 7175 7256 7274 7521 7771 8206 8701 8860 9221 9540 10115 10143 10398 10873 11719 11914 11944 12693 12705 13590 13650 13803 14566 14766 15301 15326 15735 15784 16189 (16192) 16223 16278 16290 16294 16309 16390 |
| L2a1b2 | 73 146 152 195 263 750 769 1018 1438 2416 2706 2789 3594 4104 4769 7028 7175 7256 7274 7521 7771 8116 8206 8701 8860 9221 9540 10115 10143 10398 10873 11719 11914 11944 12693 12705 13590 13650 13803 14566 14766 15301 15326 15784 16189 (16192) 16223 16278 16294 16309 16390 |
| L2a1b+143 | 73 143 146 152 195 263 750 769 1018 1438 2416 2706 2789 3594 4104 4769 7028 7175 7256 7274 7521 7771 8206 8701 8860 9221 9540 10115 10143 10398 10873 11719 11914 11944 12693 12705 13590 13650 13803 14566 14766 15301 15326 15784 16189 (16192) 16223 16278 16294 16309 [illegible] |
| L2a1b3 | 73 143 146 152 195 207 263 750 769 1018 1189 1438 2416 2706 2789 3594 4104 4769 7028 7175 7256 7274 7521 7771 8206 8701 8860 9221 9540 10115 10143 10398 10873 11719 11914 11944 12693 12705 13590 13650 13803 14566 14766 15301 15326 15784 16189 (16192) 16223 16278 16294 16309 16390 |

| | |
|---|---|
| L2a1f | 73 146 152 195 263 750 769 1018 1438 2416 2706 2789 3594 4104 4769 5581 7028 7175 7256 7274 7521 7771 8206 8701 8860 9221 9540 10115 10398 10873 11719 11914 11944 12693 12705 13590 13650 13803 14566 14766 15301 15326 15784 16189 (16192) 16223 16278 16294 16309 16390 |
| L2a1f1 | 73 146 152 195 263 731 750 769 1018 1438 2416 2706 2789 3594 4104 4769 5581 7028 7175 7256 7274 7521 7771 8206 8701 8860 9221 9540 10115 10398 10873 11719 11914 11944 12693 12705 13590 13650 13803 14566 14766 15301 15326 15784 16189 (16192) 16223 16278 16294 16309 [illegible] |
| L2a1f1a | 73 146 152 195 263 731 750 769 1018 1438 2416 2706 2789 3594 4104 4769 5581 7028 7175 7256 7274 7394 7521 7771 8206 8701 8860 9221 9540 10115 10398 10873 11719 11914 11944 12693 12705 13590 13650 13803 14566 14766 15301 15326 15784 16189 (16192) 16223 16278 16294 16309 16390 |
| L2a1f2 | 73 146 152 195 263 750 769 1018 1438 2416 2706 2789 3594 4104 4769 5581 7028 7175 7256 7274 7521 7771 8206 8701 8860 9221 9540 10115 10398 10873 11692 11719 11914 11944 12693 12705 13590 13650 13803 14566 14766 15301 15326 15784 16189 (16192) 16223 16278 16294 16309 16390 |
| L2a1f3 | 73 146 152 195 198 263 750 769 1018 1438 2416 2706 2789 3594 4104 4769 5581 7028 7175 7256 7274 7521 7771 8206 8701 8860 9221 9540 10115 10398 10873 11719 11914 11944 12693 12705 13590 13650 13803 14566 14766 15301 15326 15784 16189 (16192) 16223 16278 16294 16309 [illegible] |
| L2a1g | 73 146 152 195 263 750 769 1018 1438 2416 2706 2789 3594 4104 4769 7028 7175 7256 7274 7521 7771 8014 8206 8701 8860 9221 9540 10115 10398 10873 11719 11914 11944 12693 12705 13590 13650 13803 14281 14566 14766 15301 15326 15784 16131 16189 (16192) 16223 16225 16234 16278 16294 16309 16390 |
| L2a1+143 | 73 143 146 152 195 263 750 769 1018 1438 2416 2706 2789 3594 4104 4769 7028 7175 7256 7274 7521 7771 8206 8701 8860 9221 9540 10115 10398 10873 11719 11914 11944 12693 12705 13590 13650 13803 14566 14766 15301 15326 15784 16223 16278 16294 16309 16390 |
| L2a1c | 73 143 146 152 195 263 750 769 1018 1438 2416 2706 2789 3010 3594 4104 4769 6663 7028 7175 7256 7274 7521 7771 8206 8701 8860 9221 9540 10115 10398 10873 11719 11914 11944 12693 12705 13590 13650 13803 14566 14766 15301 15326 15784 16223 16278 16294 16309 16390 |
| L2a1c+16086 | 73 143 146 152 195 263 750 769 1018 1438 2416 2706 2789 3010 3594 4104 4769 6663 7028 7175 7256 7274 7521 7771 8206 8701 8860 9221 9540 10115 10398 10873 11719 11914 11944 12693 12705 13590 13650 13803 14566 14766 15301 15326 15784 16086 16223 16278 16294 16309 [illegible] |
| L2a1c1 | 73 143 146 152 195 198 263 750 769 930 1018 1438 2416 2706 2789 3010 3308 3594 4104 4769 6663 7028 7175 7256 7274 7521 7771 8206 8604 8701 8860 9221 9540 10115 10398 10873 11719 11914 11944 12693 12705 13590 13650 13803 14566 14766 15301 15326 15784 16086 16223 16278 16294 16309 16390 |

| | |
|---|---|
| L2a1c1a | 73 143 146 152 195 198 263 750 769 930 1018 1438 2416 2706 2789 3010 3308 3594 4104 4769 6311 6663 7028 7175 7256 7274 7521 7771 8206 8604 8701 8860 9221 9540 10115 10398 10873 11719 11914 11944 12693 12705 13590 13650 13803 14566 14766 15301 15326 15784 16086 16223 16278 16294 16309 16390 |
| L2a1c1a1 | 73 143 146 152 195 198 263 750 769 930 1018 1438 2416 2706 2789 3010 3308 3338 3594 4104 4769 6311 6663 7028 7175 7256 7274 7521 7771 8206 8604 8701 8860 9221 9540 10115 10398 10873 11719 11914 11944 12693 12705 13590 13650 13803 14566 14766 15301 15326 15784 16086 16223 16278 16294 16309 16390 |
| L2a1c1a2 | 73 143 146 152 195 198 263 750 769 930 1018 1438 2416 2706 2789 3010 3308 3594 4104 4769 6164 6311 6663 7028 7175 7256 7274 7521 7771 8206 8604 8701 8860 9221 9540 10115 10398 10873 11719 11914 11944 12693 12705 13590 13650 13803 14566 14766 15301 15326 15784 16086 16223 16278 16294 16309 16390 |
| L2a1c6 | 73 143 146 152 189 195 263 750 769 1018 1438 2416 2706 2789 3010 3594 4104 4164 4769 6663 7028 7175 7256 7274 7521 7771 8206 8701 8860 9221 9540 10115 10398 10873 10954 11719 11914 11944 12693 12705 13590 13650 13803 14566 14766 15301 15326 15784 16086 16169 16223 16278 16294 16309 16390 |
| L2a1c2 | 73 143 146 152 195 263 750 769 1018 1438 2416 2706 2789 3010 3594 4104 4769 6663 7028 7175 7256 7274 7521 7771 8206 8701 8860 9221 9540 10115 10398 10873 10903 11719 11914 11944 12693 12705 13590 13650 13803 14566 14766 15301 15326 15784 15924 16213 16223 16278 16294 16309 16390 |
| L2a1c2a | 73 143 146 152 195 263 513 750 769 1018 1438 2416 2706 2789 3010 3594 4104 4769 6663 7028 7175 7256 7274 7521 7771 8206 8701 8860 9221 9540 10115 10398 10873 10903 11719 11914 11944 12693 12705 13590 13650 13803 14566 14766 15301 15326 15784 15924 16193 16213 16223 16239 16278 16294 16309 16390 |
| L2a1c3 | 73 143 146 152 195 263 750 769 1018 1438 2416 2706 2789 3010 3594 4104 4769 6663 7028 7175 7256 7274 7521 7771 8206 8701 8860 9221 9540 9932 10115 10398 10873 11719 11914 11944 12693 12705 13590 13650 13803 14566 14766 15301 15326 15784 16223 16278 16294 16309 [illegible] |
| L2a1c3a | 73 143 146 152 195 263 750 769 1018 1438 2416 2706 2789 3010 3594 4104 4769 6663 7028 7175 7256 7274 7521 7771 7858 8206 8701 8860 9221 9540 9932 10115 10398 10873 11719 11914 11944 12693 12705 13590 13650 13803 14566 14766 15301 15326 15784 16223 16278 16294 [illegible] |
| L2a1c3a1 | 73 143 146 152 195 198 263 750 769 1018 1438 2416 2706 2789 3010 3594 4104 4769 5843 6663 7028 7175 7256 7274 7521 7771 7858 8206 8701 8860 9221 9540 9932 10115 10398 10873 11719 11914 11944 12693 12705 13590 13650 13803 14566 14766 15115 15301 15326 15784 16223 16278 16290 16294 16309 16390 |
| L2a1c3b | 73 143 146 152 195 263 750 769 1018 1438 2416 2706 2789 3010 3594 4104 4769 6663 7028 7175 7256 7274 7521 7771 8206 8701 8860 9221 9540 9932 10115 10398 10873 11719 11914 11944 12693 12705 13590 13650 13803 14566 14766 15301 15326 15784 16223 16278 16294 16355 [illegible] |

| | |
|---|---|
| L2a1c3b1 | 73 143 146 152 195 263 750 769 1018 1438 2416 2706 2789 3010 3594 4104 4769 6663 7028 7175 7256 7274 7521 7771 8206 8701 8860 9221 9540 9932 10115 10398 10410 10873 11719 11914 11944 12693 12705 13590 13650 13803 14566 14766 15301 15326 15784 16223 16278 16294 [illegible] |
| L2a1c3b2 | 73 143 146 152 195 263 750 769 1018 1438 2416 2706 2789 3010 3594 4104 4769 6663 7028 7175 7256 7274 7521 7771 8206 8701 8860 9221 9540 9854 9932 10115 10398 10873 11719 11914 11944 12693 12705 13590 13650 13803 14384 14566 14766 14965 15301 15326 15784 16223 16278 16294 16311 16355 16390 |
| L2a1c4 | 73 143 146 152 195 263 750 769 1018 1438 2416 2706 2789 3010 3594 4104 4769 6663 7028 7175 7256 7274 7521 7771 8206 8701 8860 9221 9540 10115 10398 10873 11719 11914 11944 12172 12693 12705 13590 13650 13803 14566 14766 15301 15326 15784 16223 16278 16294 16309 [illegible] |
| L2a1c4a | 73 143 146 152 195 263 750 769 1018 1438 2416 2706 2789 3010 3594 4104 4769 6663 7028 7175 7256 7274 7521 7771 8206 8701 8860 9221 9540 10115 10398 10873 11719 11914 11944 12172 12354 12693 12705 13590 13650 13803 14566 14766 15301 15326 15784 16223 16278 16294 16309 16390 |
| L2a1c4a1 | 73 143 146 152 195 263 750 769 1018 1438 2416 2706 2789 3010 3594 4104 4769 5252 6663 7028 7175 7256 7274 7521 7771 8206 8701 8860 9221 9540 10115 10398 10873 11719 11914 11944 12172 12354 12693 12705 13590 13650 13803 14566 14766 15301 15326 15784 16223 16278 16294 16309 16390 |
| L2a1c+16129 | 73 143 146 152 195 263 750 769 1018 1438 2416 2706 2789 3010 3594 4104 4769 6663 7028 7175 7256 7274 7521 7771 8206 8701 8860 9221 9540 10115 10398 10873 11719 11914 11944 12693 12705 13590 13650 13803 14566 14766 15301 15326 15784 16129 16223 16278 16294 16309 [illegible] |
| L2a1c5 | 73 143 146 152 195 263 750 769 1018 1438 2416 2706 2789 3010 3594 4104 4769 6663 7028 7175 7256 7274 7521 7771 8206 8701 8860 9221 9540 10115 10398 10873 11719 11914 11944 12693 12705 13260 13590 13650 13803 14566 14766 15301 15326 15784 16129 16223 16278 16294 16309 16390 |
| L2a1+143+@16309 | 73 143 146 152 195 263 750 769 1018 1438 2416 2706 2789 3594 4104 4769 7028 7175 7256 7274 7521 7771 8206 8701 8860 9221 9540 10115 10398 10873 11719 11914 11944 12693 12705 13590 13650 13803 14566 14766 15301 15326 15784 16223 16278 16294 16390 |
| L2a1d | 73 143 146 152 182 195 263 750 769 1018 1438 2416 2706 2789 3594 4104 4769 5196 7028 7175 7256 7274 7521 7771 8206 8701 8860 9221 9530 9540 10115 10398 10873 11386 11719 11914 11944 12612 12693 12705 13590 13650 13803 13934 14566 14766 15301 15326 15784 16223 16278 16294 16390 |
| L2a1d1 | 73 143 146 152 182 195 263 750 769 1018 1438 2416 2706 2789 3594 4104 4769 5196 7028 7175 7256 7274 7521 7771 8206 8701 8860 9221 9530 9540 10115 10398 10873 11386 11719 11914 11944 12007 12612 12693 12705 13395 13590 13650 13803 13934 14566 14766 15301 15326 15784 16209 16223 16278 16294 16301 16354 16390 |

| | |
|---|---|
| L2a1d2 | 73 143 146 152 182 195 263 750 769 1018 1438 2416 2706 2789 3594 4104 4769 5196 7028 7175 7256 7274 7521 7771 8206 8701 8860 9221 9356 9530 9540 10115 10398 10873 11386 11719 11914 11944 12612 12693 12705 13590 13650 13803 13934 14566 14766 15301 15326 15784 16223 16278 16294 16390 |
| L2a1h | 73 143 146 152 195 263 750 769 1018 1438 2416 2706 2789 3505 3594 4104 4769 4772 7028 7175 7256 7274 7521 7771 8206 8701 8860 9221 9540 10115 10398 10873 11719 11914 11944 12693 12705 12976 13590 13650 13803 14566 14766 15301 15326 15784 16223 16278 16294 16390 |
| L2a1e | 73 143 146 152 195 263 750 769 1018 1438 2416 2706 2789 3495A 3594 4104 4769 7028 7175 7256 7274 7521 7771 8206 8701 8790 8860 9221 9540 10115 10398 10873 11719 11914 11944 12630 12693 12705 13590 13650 13803 14566 14766 15301 15326 15784 16223 16278 16294 16309 [illegible] |
| L2a1e1 | 73 146 152 195 263 750 769 1018 1438 2416 2706 2789 3495A 3594 4104 4769 7028 7175 7256 7274 7521 7771 8206 8541 8701 8790 8860 9221 9540 10115 10398 10873 11719 11914 11944 12630 12693 12705 13590 13650 13803 14566 14599 14766 15301 15326 15784 16223 16278 16294 16309 16390 |
| L2a1+143+16189_(16192) | 73 143 146 152 195 263 750 769 1018 1438 2416 2706 2789 3594 4104 4769 7028 7175 7256 7274 7521 7771 8206 8701 8860 9221 9540 10115 10398 10873 11719 11914 11944 12693 12705 13590 13650 13803 14566 14766 15301 15326 15784 16189 (16192) 16223 16278 16294 16309 16390 |
| L2a1+143+16189_(16192)+@ | 73 143 146 152 195 263 750 769 1018 1438 2416 2706 2789 3594 4104 4769 7028 7175 7256 7274 7521 7771 8206 8701 8860 9221 9540 10115 10398 10873 11719 11914 11944 12693 12705 13590 13650 13803 14566 14766 15301 15326 15784 16189 (16192) 16223 16278 16294 16390 |
| L2a1i | 73 143 146 152 195 263 750 769 1018 1438 2416 2706 2789 3594 4104 4769 7028 7175 7256 7274 7521 7771 8206 8701 8860 9221 9540 10115 10398 10873 11719 11914 11944 12693 12705 13590 13650 13803 14566 14766 15229 15301 15326 15784 16189 (16192) 16223 16278 16294 16362 [illegible] |
| L2a1i1 | 73 143 146 152 195 263 750 769 1018 1438 2416 2706 2789 3594 4104 4769 6164 7028 7175 7256 7274 7521 7771 8206 8701 8860 9221 9540 10115 10398 10873 10920 11719 11914 11944 12693 12705 13590 13650 13803 14566 14766 15229 15301 15326 15784 16189 (16192) 16223 16278 16294 16362 16390 |
| L2a1q | 73 143 146 152 195 263 750 769 1018 1438 2416 2706 2746 2789 3594 4104 4769 7028 7175 7256 7274 7521 7771 8206 8701 8860 9221 9540 10115 10398 10873 11719 11914 11944 12693 12705 13590 13650 13803 13917 14566 14766 15301 15326 15784 16189 (16192) 16223 16245 16278 16294 16390 |
| L2a1j | 73 143 146 152 195 263 750 769 1018 1438 2416 2706 2789 3594 4104 4769 7028 7175 7256 7274 7521 7771 8206 8701 8764 8860 9221 9540 10115 10398 10873 11719 11914 11944 12693 12705 13590 13650 13803 14464 14566 14766 15301 15326 15784 16189 (16192) 16223 16278 16294 16309 16390 |

| | |
|---|---|
| L2a1k | 73 143 146 152 195 263 750 769 1018 1438 2416 2706 2789 3594 4104 4769 6722 7028 7175 7256 7274 7521 7771 8206 8701 8860 9221 9540 10115 10398 10873 11719 11914 11944 12693 12705 12903 13590 13650 13803 14566 14766 15301 15326 15784 16189 (16192) 16218 16223 16278 16294 16309 16390 |
| L2a1l | 73 143 146 152 195 263 534 750 769 1018 1438 2416 2706 2789 3594 4104 4769 7028 7175 7256 7274 7521 7771 8206 8701 8860 9221 9540 10115 10398 10873 11719 11914 11944 12693 12705 13590 13650 13803 14566 14766 15301 15326 15784 16189 (16192) 16223 16278 16294 16309 16390 |
| L2a1l1 | 73 143 146 152 195 263 534 750 769 1018 1438 2416 2706 2789 3594 4104 4769 7028 7175 7256 7274 7521 7771 8206 8701 8860 9221 9540 10115 10398 10873 11719 11914 11944 12408 12693 12705 13590 13650 13803 14566 14766 15301 15326 15784 16189 (16192) 16223 16278 16294 16309 16390 |
| L2a1l1a | 73 143 146 152 195 263 534 750 769 1018 1438 2416 2706 2789 3594 4104 4769 5580 7028 7175 7256 7274 7521 7771 8206 8701 8860 9221 9540 10115 10398 10873 11719 11914 11944 12408 12693 12705 13590 13650 13803 14566 14766 15301 15326 15784 16189 (16192) 16223 16278 16294 16309 16390 |
| L2a1l1a1 | 73 143 146 152 195 263 534 750 769 1018 1438 2416 2706 2789 3594 4104 4769 5580 7028 7175 7256 7274 7521 7771 8206 8701 8860 9221 9540 10115 10398 10873 11059 11719 11914 11944 12408 12693 12705 13590 13650 13803 14566 14766 15301 15326 15784 16189 (16192) 16223 16278 16294 16309 16390 |
| L2a1l1a2 | 73 143 146 152 195 263 534 750 769 1018 1438 2416 2706 2789 3345 3594 4104 4769 5580 7028 7175 7256 7274 7521 7771 8206 8701 8860 9221 9540 10115 10398 10873 11719 11914 11944 12408 12693 12705 13590 13650 13803 14566 14766 15301 15326 15784 16189 (16192) 16223 16278 16294 16309 16390 |
| L2a1l1b | 73 143 146 152 195 263 534 750 769 1018 1438 2416 2706 2789 3594 4104 4769 7028 7175 7256 7274 7521 7771 8206 8701 8860 9221 9540 10115 10398 10873 11719 11914 11944 12408 12693 12705 13590 13650 13803 14566 14766 15301 15326 15784 15880 16189 (16192) 16223 16278 16294 16309 16390 |
| L2a1l2 | 73 143 146 152 195 263 534 750 769 1018 1438 2416 2706 2789 3594 4104 4769 5366A 7028 7175 7256 7274 7521 7771 8206 8701 8860 9221 9540 10115 10398 10873 11719 11914 11944 12693 12705 13590 13650 13803 14566 14766 15301 15326 15784 16189 (16192) 16223 16278 16294 16309 16390 |
| L2a1l2a | 73 146 152 195 263 534 750 769 1018 1438 2416 2706 2789 3594 4104 4769 5366A 7028 7175 7256 7274 7521 7771 8206 8701 8860 9221 9540 10115 10398 10873 11719 11914 11944 12693 12705 13590 13650 13803 14180 14566 14766 15301 15326 15784 16189 (16192) 16223 16278 16294 16309 16390 |

| | |
|---|---|
| L2a1l2a1 | 73 146 152 195 263 534 750 769 1018 1438 2416 2706 2789 3573A 3594 4104 4769 5366A 7028 7175 7256 7274 7521 7771 8206 8701 8860 9221 9540 10115 10398 10873 11719 11914 11944 12693 12705 13590 13650 13803 14180 14566 14766 15301 15326 15784 16189 (16192) 16223 16278 16294 16309 16390 |
| L2a1l3 | 73 143 146 152 195 263 534 750 769 1018 1438 2416 2706 2789 3594 4104 4769 7028 7175 7256 7274 7521 7771 8206 8701 8860 9221 9540 10115 10398 10873 11719 11914 11944 12693 12705 13590 13650 13803 14566 14766 14905 15301 15326 15784 16189 (16192) 16223 16278 16294 16309 16357 16390 |
| L2a1m | 73 143 146 152 195 263 750 769 1018 1438 2416 2706 2789 3594 4104 4769 7028 7175 7256 7274 7521 7771 8206 8701 8860 9221 9540 10115 10398 10873 11719 11914 11944 12693 12705 13590 13650 13803 13884 14566 14766 15301 15326 15784 16189 (16192) 16223 16278 16294 16309 [illegible] |
| L2a1m1 | 73 143 146 152 195 263 750 769 1018 1438 2416 2706 2789 3594 4104 4769 7028 7175 7256 7274 7521 7771 8206 8281-8289d 8701 8860 9221 9540 10115 10398 10873 11719 11914 11944 12693 12705 13590 13650 13803 13884 14566 14766 15301 15326 15784 16189 (16192) 16223 16278 16294 16309 16390 |
| L2a1m1a | 73 143 146 152 195 263 750 769 1018 1438 2416 2706 2789 3594 4104 4769 7028 7175 7256 7274 7521 7771 8206 8281-8289d 8553 8701 8860 9221 9540 10115 10398 10873 11719 11914 11944 12693 12705 13590 13650 13803 13884 14566 14766 15301 15326 15784 16189 (16192) 16223 16278 16294 16309 16390 |
| L2a1n | 73 143 146 152 195 263 750 769 1018 1438 2416 2706 2789 3594 4104 4317d 4769 5147 7028 7175 7256 7274 7521 7771 8206 8701 8860 9221 9540 10115 10398 10873 11719 11914 11944 12693 12705 13590 13650 13803 14566 14766 15301 15326 15784 16189 (16192) 16223 16278 16294 16309 16390 |
| L2a1o | 73 143 146 152 195 263 750 769 1018 1438 2416 2706 2789 3594 4104 4769 7028 7175 7256 7274 7521 7771 8206 8701 8860 9221 9540 10115 10398 10873 11719 11914 11944 12438 12693 12705 13590 13650 13803 14566 14766 15301 15326 15784 16189 (16192) 16223 16278 16294 16309 [illegible] |
| L2a1p | 73 143 146 152 195 263 750 769 1018 1438 2416 2706 2789 3594 4104 4769 7028 7175 7256 7274 7521 7771 8206 8701 8860 9221 9410 9540 10115 10398 10873 11719 11914 11944 12693 12705 13590 13650 13803 13818 14566 14766 15301 15326 15626 15784 16189 (16192) 16223 16278 16294 16309 16390 |
| L2a2'3'4 | 73 152 182 195 263 750 769 1018 1438 2416 2706 2789 3594 4104 4769 6752 7028 7175 7256 7274 7521 7771 8206 8701 8860 9221 9540 10115 10398 10873 11719 11914 11944 12705 13590 13650 13803 14566 14766 15301 15326 16189 16223 16229 16278 16294 16311 16390 |
| L2a2'3 | 73 152 182 195 263 709 750 769 1018 1438 2416 2706 2789 3594 4104 4769 6752 7028 7175 7256 7274 7521 7771 8206 8701 8860 9221 9540 10115 10398 10873 11719 11914 11944 12705 13590 13650 13803 14566 14766 15301 15326 15939 16189 16223 16229 16278 16291 16294 16311 [illegible] |

| | |
|---|---|
| L2a2 | 73 152 182 195 263 709 750 769 1018 1438 2416 2706 2789 3594 4104 4769 6752 7028 7175 7256 7274 7521 7771 8206 8701 8860 9221 9540 9932 10115 10398 10873 11719 11914 11944 12705 13590 13650 13803 14566 14766 15301 15326 15939 16189 16223 16229 16278 16291 16294 16311 16390 |
| L2a2a | 73 152 182 195 263 709 750 769 1018 1438 2416 2706 2789 3594 4104 4769 6752 7028 7175 7256 7274 7521 7771 8206 8701 8860 9083 9221 9438 9540 9932 10115 10398 10873 11719 11914 11944 12705 13590 13650 13803 14566 14766 15301 15326 15803 15939 16189 16223 16229 16278 16291 16294 16311 16390 |
| L2a2a1 | 73 152 182 195 263 709 750 769 1018 1438 2416 2706 2789 3594 4104 4769 6752 7028 7175 7256 7274 7521 7771 8206 8701 8727 8860 9083 9221 9438 9540 9932 10115 10398 10873 11719 11914 11944 12705 13590 13650 13803 14566 14766 15301 15326 15803 15939 16189 16223 16229 16278 16291 16294 16311 16390 |
| L2a2b | 73 152 182 263 709 750 769 1018 1438 2416 2706 2789 3594 4104 4769 6752 7028 7175 7256 7274 7521 7771 8206 8701 8860 9221 9540 9932 10115 10398 10873 11719 11767 11914 11944 12705 13590 13650 13803 14118 14566 14766 15301 15326 15939 16189 16223 16229 16278 16291 16294 16311 16390 |
| L2a2b1 | 73 152 182 263 709 750 769 1018 1438 2416 2706 2789 3594 4104 4769 6752 7028 7175 7256 7274 7521 7771 8206 8701 8860 9221 9540 9932 10115 10398 10873 11719 11767 11914 11944 12705 13590 13650 13803 13827 14118 14566 14766 15301 15326 15939 16189 16223 16229 16278 16291 16294 16311 16390 |
| L2a2b1a | 73 152 263 709 750 769 1018 1438 2416 2706 2789 3594 4104 4769 6752 7028 7175 7256 7274 7521 7664 7771 8206 8701 8721 8860 9221 9540 9932 10115 10398 10873 11719 11767 11914 11944 12705 13068 13590 13650 13803 13827 14118 14566 14766 15301 15326 15939 16172 16189 16223 16229 16278 16291 16294 16311 16390 |
| L2a2b2 | 73 152 182 235 263 709 750 769 1018 1438 2416 2706 2789 3594 4104 4769 6383 6752 7028 7175 7256 7274 7521 7771 8206 8701 8860 9077 9221 9540 9932 10115 10398 10873 11719 11767 11914 11944 12705 13590 13650 13803 14118 14566 14766 15301 15326 15939 16189 16223 16229 16278 16291 16294 16311 16390 |
| L2a3 | 73 152 182 257 263 709 750 769 1018 1438 2416 2706 2789 3594 4104 4769 6752 7028 7175 7256 7274 7521 7771 8206 8701 8860 9221 9540 9797 10115 10398 10873 11719 11914 11944 12705 13590 13650 13803 14566 14766 14894 15301 15326 15939 16086 16189 16215 16223 16229 16278 16291 16294 16311 16390 |
| L2a4 | 73 152 182 195 263 513 593A 750 769 1018 1438 2416 2706 2789 3594 4104 4769 6752 7028 7175 7256 7274 7521 7771 8206 8701 8860 9221 9438 9540 10115 10398 10873 11719 11914 11944 12705 13125 13590 13650 13803 14566 14766 15301 15326 16170 16189 16223 16229 16278 16294 16311 16390 |

| | |
|---|---|
| L2a4a | 73 152 182 195 263 513 573.XC 593A 750 769 1018 1438 2416 2706 2789 3594 4104 4769 5147 6752 6959 7028 7175 7256 7274 7521 7771 7897 8206 8614 8701 8860 9221 9438 9540 10115 10398 10873 11719 11914 11944 12480 12705 13125 13590 13650 13803 14566 14766 15301 15326 15812 16170 16189 16223 16229 16278 16294 16311 16390 |
| L2a4b | 73 152 182 263 513 593A 750 769 1018 1438 2416 2706 2780 2789 3594 3834 4104 4703 4769 6752 7028 7175 7256 7274 7521 7771 8206 8701 8860 9221 9438 9540 10115 10398 10873 11719 11914 11944 12612 12705 13125 13590 13650 13803 14566 14766 15301 15326 16170 16189 16223 16229 16264 16278 16294 16311 16390 |
| L2a5 | 73 146 152 182 263 750 769 1018 1438 2416 2626 2706 3594 3654 4104 4769 5263 6040C 6497 7028 7175 7256 7521 8206 8701 8860 9221 9540 10115 10398 10873 11719 11944 12705 12879 13590 13650 14766 14890 15301 15326 16223 16224 16278 16390 |
| L2b'c'd | 73 146 150 152 182 195 263 750 769 1018 1438 2332 2416 2706 3594 4104 4769 7028 7256 7521 8206 8701 8860 9221 9540 10115 10398 10873 11719 11944 12705 13590 13650 14766 15301 15326 16223 16278 16390 |
| L2b'c | 73 146 150 152 182 195 198 263 750 769 1018 1438 1442 2332 2416 2706 3594 4104 4769 7028 7256 7521 7624A 8206 8701 8860 9221 9540 10115 10398 10873 11719 11944 12236 12705 13590 13650 14766 15110 15217 15301 15326 16223 16278 16390 |
| L2b | 73 146 150 152 182 195 198 204 263 750 769 1018 1438 1442 1706 2332 2358 2416 2706 3594 4104 4158 4370 4767 4769 5027 5331A 5814 6713 7028 7256 7521 7624A 8080 8206 8387 8701 8860 9221 9540 10115 10398 10873 11719 11944 12236 12705 12948 13590 13650 14059 14766 15110 15217 15301 15326 16114A 16129 16213 16223 16278 16390 |
| L2b1 | 73 146 150 152 182 195 198 204 263 418 750 769 1018 1438 1442 1706 2332 2358 2416 2706 3594 4104 4158 4370 4767 4769 5027 5331A 5814 6026 6713 7028 7256 7521 7624A 8080 8206 8387 8701 8860 9221 9540 10115 10398 10828 10873 11719 11944 12236 12705 12948 13590 13650 13924 14059 14766 15110 15217 15301 15326 16114A 16129 16213 16223 16278 16362 16390 |
| L2b1a | 73 150 152 182 195 198 204 263 418 750 769 1018 1438 1442 1706 2332 2358 2416 2706 3594 4104 4158 4370 4767 4769 5027 5331A 5814 6026 6713 7028 7256 7521 7624A 8080 8206 8387 8701 8860 9221 9540 10115 10398 10828 10873 11719 11944 12236 12705 12948 13590 13650 13924 14059 14766 15110 15217 15301 15326 16114A 16129 16213 16223 16278 16355 16362 16390 |
| L2b1a2 | 73 150 152 182 195 198 204 263 418 750 769 1018 1438 1442 1706 2332 2358 2416 2706 3594 4104 4158 4370 4767 4769 5027 5331A 5814 6026 6713 7028 7256 7521 7569 7624A 8080 8206 8387 8701 8860 9221 9540 10115 10398 10828 10873 11719 11944 12236 12705 12948 13590 13650 13924 14059 14766 15110 15217 15301 15326 16114A 16129 16213 16223 16278 16355 16362 16390 |

| | |
|---|---|
| L2b1a3 | 73 150 152 182 195 198 204 263 418 750 769 1018 1438 1442 1706 2332 2358 2416 2706 3594 4104 4158 4370 4767 4769 5027 5331A 5814 6026 6713 7028 7256 7521 7624A 8080 8206 8387 8701 8856 8860 9221 9540 10115 10398 10828 10873 11719 11944 12236 12705 12948 13590 13650 13924 14059 14766 15110 15217 15301 15326 16114A 16129 16213 16223 16278 16355 16362 16390 |
| L2b1a4 | 73 150 152 182 195 198 204 263 418 750 769 1018 1438 1442 1706 2332 2358 2416 2706 3594 4104 4158 4370 4767 4769 5027 5331A 5814 6026 6713 7028 7256 7521 7624A 8080 8206 8387 8701 8860 9221 9540 10115 10398 10828 10873 11719 11944 12236 12406 12705 12948 13590 13650 13924 14059 14766 15110 15217 15301 15326 16114A 16129 16213 16223 16278 16355 16362 16390 |
| L2b1b | 73 146 150 152 182 195 198 204 263 385 418 750 769 1018 1438 1442 1706 2332 2358 2416 2706 3594 4104 4158 4370 4767 4769 5027 5331A 5814 6026 6629 6713 7028 7256 7521 7624A 8080 8206 8387 8701 8860 9221 9540 10115 10398 10828 10873 11719 11944 12236 12705 12948 13590 13650 13924 14059 14766 15110 15217 15301 15326 16114A 16129 16213 16223 16278 16362 16390 |
| L2b2 | 73 146 150 152 182 195 198 204 263 750 769 1018 1438 1442 1706 2332 2358 2416 2706 3594 4104 4158 4370 4767 4769 5027 5331A 5814 6614 6713 6806 7028 7256 7521 7624A 8080 8206 8387 8503 8701 8860 9221 9540 10115 10398 10873 11719 11944 12236 12705 12948 13590 13650 14059 14766 15110 15217 15301 15326 16114A 16129 16213 16223 16278 16390 |
| L2b2a | 73 146 150 152 182 195 198 204 263 709 750 769 1018 1438 1442 1706 2332 2358 2416 2706 3594 4104 4158 4370 4767 4769 5027 5331A 5814 6614 6713 6806 7028 7256 7521 7624A 8080 8206 8387 8503 8701 8790 8860 9221 9350 9540 10115 10398 10873 11719 11944 12236 12705 12948 13590 13650 13966 14059 14407 14766 15110 15217 15301 15326 16114A 16129 16213 16223 16278 16354 16390 |
| L2b3 | 73 146 150 152 182 195 198 204 263 750 769 1018 1438 1442 1706 2332 2358 2416 2706 3594 4104 4158 4370 4767 4769 5027 5331A 5814 6713 7028 7256 7521 7624A 8080 8206 8387 8701 8860 9221 9540 10115 10398 10873 11719 11944 12236 12705 12948 13590 13650 14059 14766 15110 15217 15301 15326 15944.1T 16114A 16129 16213 16223 16278 16390 |
| L2b3a | 73 146 150 152 182 195 198 204 263 750 769 1018 1438 1442 1706 2332 2358 2416 2706 3594 4104 4158 4185 4370 4767 4769 5027 5331A 5744 5814 6713 7028 7256 7521 7624A 8080 8206 8387 8701 8860 8925 9221 9540 10115 10398 10873 11719 11944 12236 12705 12948 13590 13650 14059 14544 14766 15110 15217 15236 15301 15944.1T 16114A 16129 16213 16223 16278 16390 |
| L2b3b | 73 146 150 152 182 195 198 204 263 750 769 1018 1438 1442 1706 2332 2358 2416 2626 2706 3594 4104 4158 4370 4767 4769 5027 5331A 5814 6713 7028 7256 7521 7624A 8080 8206 8387 8701 8860 9221 9540 10115 10398 10873 11719 11944 12236 12705 12948 13590 13650 14059 14766 15110 15217 15301 15326 15944.1T 16114A 16129 16213 16223 16278 16390 |

| | |
|---|---|
| L2b3c | 73 146 150 152 182 195 198 204 263 750 769 1018 1438 1442 1706 2332 2358 2416 2706 3594 4104 4158 4370 4767 4769 5027 5331A 5814 6713 7028 7256 7521 7624A 8080 8206 8387 8701 8860 9221 9540 10115 10398 10873 11719 11944 12011 12236 12705 12948 13590 13650 14059 14766 15110 15217 15301 15326 15944.1T 16114A 16129 16213 16223 16278 16390 |
| L2c | 73 93 146 150 152 182 195 198 263 325 680 709 750 769 1018 1438 1442 2332 2416 2706 3200A 3594 4104 4769 7028 7256 7521 7624A 8206 8701 8860 9221 9540 10115 10398 10873 11719 11944 12236 12705 13590 13650 13928C 13958C 14766 15110 15217 15301 15326 15849 16223 16278 16390 |
| L2c1 | 73 93 146 150 152 182 195 198 263 325 680 709 750 769 1018 1438 1442 2332 2416 2706 3200A 3594 4104 4769 7028 7256 7521 7624A 8206 8701 8860 9221 9540 10115 10398 10873 11719 11944 12236 12705 13590 13650 13928C 13958C 14766 15110 15217 15301 15326 15849 16223 16278 16318 16390 |
| L2c1a | 73 146 150 152 182 195 198 263 325 680 709 750 769 1018 1438 1442 2332 2416 2706 3200A 3594 4104 4769 5201 7028 7256 7521 7624A 8206 8701 8860 9221 9540 10115 10398 10873 11719 11944 12236 12705 13590 13650 13928C 13958C 14766 15110 15217 15301 15326 15849 16223 16278 16318 16390 |
| L2c2 | 73 93 146 150 152 182 195 198 263 325 680 709 750 769 1018 1040 1438 1442 2332 2416 2706 3200A 3594 4104 4769 7028 7256 7521 7624A 8206 8701 8860 9221 9540 10115 10398 10873 11719 11944 12236 12705 13590 13650 13928C 13958C 14766 15110 15217 15301 15326 15849 16223 16264 16278 16390 |
| L2c2a | 73 93 146 150 152 182 195 198 263 325 680 709 750 769 1018 1040 1438 1442 2332 2416 2706 3200A 3594 4104 4769 7028 7256 7521 7624A 8206 8701 8860 9221 9540 10115 10398 10873 11719 11944 12236 12705 13590 13650 13928C 13958C 14766 15043 15110 15217 15301 15326 15849 16223 16264 16278 16390 |
| L2c2a1 | 73 93 146 150 152 182 195 198 263 325 680 709 750 769 869 1018 1040 1438 1442 2332 2416 2706 3200A 3594 4104 4769 5823 7028 7256 7521 7624A 8206 8701 8860 9221 9540 10115 10398 10873 11719 11944 12236 12705 13184 13590 13650 13928C 13958C 14766 15043 15110 15217 15301 15326 15849 16223 16264 16278 16390 |
| L2c2b | 73 93 146 150 152 182 183 195 198 263 325 680 709 750 769 1018 1040 1438 1442 2332 2416 2706 3200A 3594 4104 4769 7028 7256 7521 7624A 8206 8701 8860 9221 9540 10115 10398 10873 11719 11944 12236 12705 13590 13650 13928C 13958C 14766 15110 15217 15301 15326 15849 16223 16264 16278 16390 |
| L2c2b1 | 73 93 146 150 152 182 183 195 198 263 325 680 709 750 769 1018 1040 1438 1442 2332 2416 2706 3200A 3594 4104 4769 7028 7256 7521 7624A 8206 8701 8772 8860 9221 9540 10115 10398 10873 11719 11944 12236 12705 13590 13650 13928C 13958C 14766 15110 15217 15301 15313 15326 15849 16223 16264 16278 16390 |

| | |
|---|---|
| L2c2b1a | 73 93 146 150 152 182 183 195 198 263 325 680 709 750 769 1018 1040 1438 1442 1888 2332 2416 2706 3200A 3594 4104 4769 7028 7256 7521 7624A 8206 8701 8772 8860 9221 9540 10115 10398 10873 11719 11944 12236 12705 13590 13650 13928C 13958C 14766 15110 15217 15301 15313 15326 15574 15849 16223 16264 16278 16390 |
| L2c2b1b | 73 93 146 150 152 182 183 195 198 263 325 680 709 750 769 1018 1040 1438 1442 2332 2416 2706 3200A 3594 4104 4769 7028 7256 7521 7624A 8206 8567 8701 8772 8860 9063 9221 9540 10115 10398 10790 10873 11719 11944 12236 12705 13590 13650 13928C 13958C 14766 15110 15217 15301 15313 15326 15849 16223 16264 16278 16311 16390 |
| L2c2b2 | 73 93 146 150 152 182 183 195 198 263 325 680 709 750 769 1018 1040 1438 1442 2332 2416 2706 3200A 3594 4104 4769 7028 7256 7521 7624A 8206 8701 8860 9221 9540 9758 10115 10398 10873 11719 11944 12236 12705 13590 13650 13928C 13958C 14766 15110 15217 15301 15326 15849 16223 16264 16278 16390 |
| L2c3 | 73 93 146 150 152 182 195 198 263 325 513 680 709 750 769 1018 1438 1442 2332 2416 2706 3200A 3594 4104 4769 7028 7256 7521 7624A 8206 8701 8860 9221 9540 10115 10398 10873 11719 11944 12236 12705 13590 13650 13928C 13958C 14766 15110 15217 15301 15326 15849 16223 16278 16390 |
| L2c3a | 73 93 146 150 152 182 195 198 263 325 513 680 709 750 769 1018 1438 1442 2332 2416 2706 3200A 3594 4104 4769 5255 7028 7256 7521 7624A 8206 8701 8733 8860 9221 9540 10115 10398 10873 11719 11944 12236 12705 13590 13650 13928C 13958C 14766 15110 15217 15301 15326 15849 16223 16278 16390 |
| L2c4 | 73 93 146 150 152 182 195 198 263 325 680 709 750 769 1018 1438 1442 2332 2416 2706 3200A 3594 4104 4769 7028 7256 7521 7624A 8206 8701 8860 9221 9540 10115 10398 10873 11719 11944 12236 12705 13440 13590 13650 13928C 13958C 14766 15110 15217 15301 15326 15849 16223 16278 16390 |
| L2c5 | 73 93 146 150 152 182 195 198 263 325 680 709 750 769 1018 1438 1442 2332 2416 2706 3200A 3594 4104 4596 4769 7028 7256 7521 7624A 8206 8701 8860 9221 9540 10115 10398 10873 11719 11944 12236 12705 13590 13650 13928C 13958C 14766 15110 15217 15301 15326 15849 16223 16278 16390 |
| L2d | 73 146 150 195 263 456 750 769 870 1018 1438 2159 2332 2416 2706 3254A 3434 3594 3693 4104 4769 6231 7028 7256 7521 8206 8701 8860 9221 9540 9554 9941 10115 10398 10873 10955 11353 11719 11944 12705 13590 13650 14766 14845 15301 15326 15777C 16189 16278 16300 16354 16390 16399 |
| L2d+16129 | 73 146 150 195 263 456 750 769 870 1018 1438 2159 2332 2416 2706 3254A 3434 3594 3693 4104 4769 6231 7028 7256 7521 8206 8701 8860 9221 9540 9554 9941 10115 10398 10873 10955 11353 11719 11944 12705 13590 13650 14766 14845 15301 15326 15777C 16129 16189 16278 16300 16354 16390 16399 |

| | |
|---|---|
| L2d1 | 73 146 150 195 263 456 750 769 870 1018 1438 2159 2332 2416 2706 3254A 3434 3594 3693 4104 4769 6231 7028 7256 7521 8206 8701 8860 9221 9540 9554 9941 10115 10398 10700 10873 10955 11353 11719 11944 12705 13590 13650 14766 14845 15301 15326 15777C 16129 16189 16278 16300 16354 16390 16399 |
| L2d1a | 73 146 150 195 263 456 750 769 870 1018 1438 2159 2332 2416 2706 3254A 3434 3594 3693 4104 4769 6231 7028 7256 7521 8206 8701 8856 8860 9221 9540 9554 9941 10115 10398 10700 10873 10955 11353 11719 11944 12705 13590 13650 14766 14845 15263 15301 15326 15458 15703 15777C 16129 16189 16278 16300 16354 16390 16399 |
| L2e | 73 146 150 152 182 263 479 719 750 769 1018 1211 1438 2416 2706 3537 3594 4104 4562 4769 5069T 6014 7028 7256 7521 8206 8383 8701 8860 9221 9377 9540 9971 10115 10398 10873 11719 11935 12189 12705 13590 13650 13708 14299 14766 15301 15326 15697 15734 15889 16111A 16145 16184 16223 16239 16278 16292 16355 16390 16399 16400 |
| L2e1 | 73 146 150 152 182 263 479 719 750 769 954 1018 1211 1438 2416 2706 3537 3594 4104 4562 4769 5069T 6014 7028 7256 7521 8206 8383 8701 8860 9221 9377 9540 9971 10115 10398 10873 11719 11935 12189 12705 13590 13650 13708 14299 14766 15301 15326 15697 15734 15889 16111A 16145 16184 16223 16239 16278 16292 16355 16390 16399 16400 |
| L2e1a | 73 146 150 152 182 263 479 719 750 769 954 1018 1211 1438 2416 2706 3537 3594 4104 4562 4769 5069T 6014 7028 7256 7521 8206 8383 8701 8860 8994 9221 9377 9540 9971 10115 10398 10873 11149 11719 11935 12189 12705 13194 13590 13650 13708 14299 14766 15301 15326 15697 15734 15889 16111A 16145 16184 16223 16239 16278 16292 16355 16390 16399 16400 |
| L3'4'6 | 73 182 263 750 769 1018 1438 2706 3594 4769 7028 7256 8701 8860 9540 10398 10873 11719 12705 13650 14766 15301 15326 16223 16278 16311 |
| L6 | 73 146 152 182 185C 263 709 750 769 770 961 1018 1438 1461 2706 3594 4769 4964 5267 6002 6284 7028 7256 8701 8860 9332 9540 10398 10873 10978 11116 11719 11743 12705 12771 13650 13710 14766 14791 14959 15244 15289 15301 15326 15499 16048 16223 16224 16278 16311 |
| L6a | 73 146 152 182 185C 207 263 265 709 750 769 770 961 1018 1438 1461 2706 3594 4769 4964 5267 6002 6284 7028 7256 8701 8860 9332 9540 10398 10873 10978 11116 11719 11743 12705 12771 13650 13710 14766 14791 14959 15244 15289 15301 15326 15499 16048 16223 16224 16278 16311 |
| L6b | 73 146 152 182 185C 263 709 750 769 770 961 1018 1438 1461 2706 3594 4769 4964 5267 6002 6284 7028 7256 8701 8860 9332 9540 10398 10873 10978 11116 11719 11743 12705 12771 13650 13710 14533A 14766 14791 14959 15244 15289 15301 15326 15499 16048 16223 16224 16278 16311 |
| L3'4 | 73 263 750 769 1018 1438 2706 4769 7028 8701 8860 9540 10398 10873 11719 12705 14766 15301 15326 16223 16311 |
| L4 | 73 195 263 750 769 1018 1438 2706 4769 5460 7028 8701 8860 9540 10398 10873 11719 12705 14766 15301 15326 16223 16311 16362 |
| L4a | 73 195 198 263 750 769 1018 1438 2706 3357 4769 5460 7028 8701 8860 9540 10373 10398 10873 11253 11344 11485 11719 12414 12705 13174 14302 14766 15301 15326 16223 16260 16311 16362 |

| | |
|---|---|
| L4a1 | 73 195 198 263 325 750 769 1018 1438 2706 3357 4769 5460 6167 7028 7376 7775 8701 8860 9540 10373 10398 10873 11253 11344 11485 11653 11719 12414 12705 13174 14000A 14302 14766 15301 15326 16207T 16223 16260 16311 16362 |
| L4a1a | 73 (150) 195 198 263 325 750 769 1018 1438 2706 3357 4769 5460 6167 7028 7376 7762 7775 8473 8631 8701 8860 9540 10373 10398 10873 11253 11344 11485 11653 11719 12414 12705 13174 14000A 14302 14766 15301 15326 16207T 16223 16260 16311 16362 |
| L4a2 | 73 195 198 263 750 769 1018 1438 2706 3357 4769 5460 7028 8701 8860 9540 10373 10398 10873 11253 11344 11485 11719 12361 12414 12705 13174 14302 14766 15301 15326 16223 16260 16264 16311 16362 |
| L4b | 73 195 263 709 750 769 1018 1438 2706 3918 4769 5460 7028 8701 8860 9540 10398 10873 11719 12705 14766 15301 15326 16223 16311 16362 |
| L4b1 | 73 150 204 263 709 750 769 1018 1438 2706 3918 4769 4977 5460 7028 8701 8860 9540 10398 10813 10873 11719 12705 14766 15301 15326 16223 16311 16362 |
| L4b1a | 73 150 199 204 263 513 709 750 769 1018 1438 1804 2706 3010 3505 3918 4017 4029A 4216 4232 4769 4977 5460 7028 7624 8614 8701 8860 8974G 9248 9540 9986 10398 10813 10873 11719 12661T 12705 13497 14016 14766 14905 15301 15326 16179 16189 16223 16239 16311 16320 16362 |
| L4b2 | 73 146 195 244 263 709 750 769 1018 1438 2706 3918 4769 6260 7028 8104 8701 8860 9540 9855 10398 10873 11719 12609 12705 13470 14766 15301 15326 16223 16293T 16311 16355 16362 16399 |
| L4b2a | 73 146 195 244 263 750 769 1018 1413 1438 2706 3918 4769 6260 7028 8104 8701 8860 9540 9855 10398 10873 11719 12609 12705 13470 14766 15301 15326 16223 16293T 16311 16355 16362 16399 |
| L4b2a1 | 73 146 152 195 244 263 471 547 750 769 1018 1413 1438 2706 3918 4769 5471 5580 5746 7028 8104 8701 8860 9540 9855 10398 10873 11719 12609 12705 13470 14766 15301 15326 16223 16274 16293T 16311 16355 16362 16399 |
| L4b2a2 | 73 146 244 263 750 769 1018 1413 1438 1694 2706 3918 4769 4949 6260 7028 8104 8701 8860 9540 9855 10398 10783C 10873 11719 12609 12705 13470 14766 15301 15326 16172 16223 16293T 16311 16355 16362 16399 |
| L4b2a2a | 73 146 244 263 750 769 1018 1413 1438 1694 2483 2706 3918 4769 4949 6260 6620 7028 8104 8701 8860 9540 9855 10398 10783C 10873 11137 11719 12609 12705 13470 14766 15301 15326 16172 16223 16293T 16311 16355 16362 16399 |
| L4b2a2b | 73 146 244 263 750 769 1018 1413 1438 1694 2706 3918 4769 4949 6260 6956 7028 8104 8485 8701 8860 9540 9855 10398 10783C 10873 11719 12609 12705 13470 14766 15301 15326 15970 16172 16223 16287 16293T 16311 16355 16362 16399 |
| L4b2a2c | 73 146 244 263 391 750 769 1018 1413 1438 1694 2706 3918 4769 4949 6260 7028 8104 8478 8701 8860 9540 9855 10398 10783C 10873 11719 12609 12705 13470 14766 15301 15326 15454 16172 16223 16293T 16311 16355 16362 16399 |
| L4b2b | 73 146 195 244 263 709 750 769 1018 1438 2706 3918 4769 5128 6260 7028 7805 8104 8701 8860 9540 9855 10265 10398 10873 11719 12438 12609 12705 13470 14766 15301 15326 16223 16293T 16311 16355 16362 |

| | |
|---|---|
| L4b2b1 | 73 146 195 244 263 340 709 750 769 1018 1438 2220T 2706 3918 4206 4769 5128 6260 7028 7805 8104 8701 8860 9540 9855 10265 10398 10873 11719 12438 12609 12705 13470 14766 15301 15326 16223 16293T 16311 16316 16355 16362 16399 |
| L3 | 73 263 750 1438 2706 4769 7028 8701 8860 9540 10398 10873 11719 12705 14766 15301 15326 16223 |
| L3a | 73 151 152 263 750 1438 2706 4769 7028 8701 8860 9540 10398 10873 11719 12705 12816 14766 15301 15326 16223 (16254) 16316 |
| L3a1 | 73 151 152 263 721 750 1438 2706 4769 7028 8701 8860 9540 10314 10398 10873 11719 12705 12816 14461 14766 14851 15301 15326 15553 16223 (16254) 16311 16316 |
| L3a1a | 73 152 263 721 750 1438 2706 4088 4769 7028 8701 8860 9540 10314 10398 10873 11719 12705 12816 14461 14766 14851 15301 15326 15553 16223 (16254) 16311 16316 |
| L3a1b | 73 151 152 195 198 263 721 750 1438 2357 2706 4769 7028 8701 8860 9540 10184 10314 10398 10873 11719 12618 12705 12816 13708 14461 14566 14766 14851 15301 15326 15553 16223 (16254) 16311 16316 |
| L3a+709 | 73 151 152 263 709 750 1438 2706 4769 7028 8701 8860 9540 10398 10873 11719 12705 12816 14766 15301 15326 16223 (16254) 16316 |
| L3a2 | 73 151 152 263 573 709 750 1438 2706 4769 7028 7364 8701 8860 9540 10398 10873 11719 12705 12816 14766 15301 15326 16223 (16254) 16316 |
| L3a2a | 73 152 263 573 709 750 1438 2706 3996 4769 7028 7364 8701 8860 9540 10398 10873 11719 12705 12816 13887 14766 15301 15326 (16254) 16316 |
| L3b'f | 73 263 750 1438 2706 4769 7028 8701 8860 9540 10398 10873 11719 12705 14766 15301 15326 15944d 16223 |
| L3b | 73 263 750 1438 2706 3450 4769 5773 6221 7028 8701 8860 9449 9540 10086 10398 10873 11719 12705 13105 13914A 14766 15301 15311 15326 15824 15944d 16124 16223 16278 16362 |
| L3b1 | 73 263 750 1438 2706 3450 4769 5773 6221 7028 8701 8860 9449 9540 10086 10373 10398 10873 11719 12705 13105 13914A 14766 15301 15311 15326 15824 15944d 16124 16223 16278 16362 |
| L3b1a | 73 263 750 1438 2706 3450 4769 5773 6221 7028 8701 8860 9449 9540 10086 10373 10398 10873 11002 11719 12705 13105 13914A 14766 15301 15311 15326 15824 15944d 16124 16223 16278 16362 |
| L3b1a1 | 73 263 750 1438 2706 3450 4769 5773 6221 7028 8701 8860 9449 9540 10086 10373 10398 10873 11002 11719 11800 12705 13105 13914A 14766 15301 15311 15326 15824 15944d 16124 16223 16278 16362 |
| L3b1a1a | 73 263 750 1438 2706 3450 4769 5773 6221 7028 8701 8860 9449 9540 10086 10373 10398 10873 11002 11719 11800 12705 13105 13914A 14766 15301 15311 15326 15824 15883 15944d 16124 16223 16278 16362 |
| L3b1a2 | 73 263 750 1438 2706 3450 4769 5773 6221 7028 8701 8860 9300 9449 9540 10086 10373 10398 10873 11002 11719 12705 13105 13914A 14766 15301 15311 15326 15824 15944d 16124 16223 16278 16362 |
| L3b1a3 | 73 263 750 1438 2706 3450 4769 5773 6221 7028 8701 8860 9449 9540 10086 10373 10398 10873 11002 11719 12705 13105 13914A 13933 14766 15301 15311 15326 15824 15944d 16124 16223 16278 16311 16362 |

| | |
|---|---|
| L3b1a4 | 73 263 750 1438 1710C 2706 3450 4769 5773 6221 7028 8701 8860 9449 9540 9605 10086 10373 10398 10873 11002 11719 12705 13105 13914A 14766 15301 15311 15326 15824 15944d 16124 16223 16278 16362 |
| L3b1a5 | 73 263 750 1438 2706 3450 4769 5063 5773 6221 7028 8701 8860 9449 9540 10086 10373 10398 10873 11002 11719 12705 13105 13914A 14766 15301 15311 15326 15824 15944d 16124 16223 16278 16362 |
| L3b1a5a | 73 263 750 1438 2706 3450 4769 5063 5417 5773 6221 7028 8701 8860 9449 9540 10086 10373 10398 10873 11002 11719 12705 13105 13914A 14766 15301 15311 15326 15824 15944d 16124 16223 16278 16362 |
| L3b1a+152 | 73 152 263 750 1438 2706 3450 4769 5773 6221 7028 8701 8860 9449 9540 10086 10373 10398 10873 11002 11719 12705 13105 13914A 14766 15301 15311 15326 15824 15944d 16124 16223 16278 16362 |
| L3b1a6 | 73 152 263 750 1438 2706 3385 3450 4769 5255 5773 6221 7028 8155 8701 8860 9449 9540 10086 10373 10398 10873 11002 11719 12705 13105 13914A 14766 15301 15311 15326 15824 15944d 16124 16223 16278 |
| L3b1a+@16124 | 73 263 750 1438 2706 3450 4769 5773 6221 7028 8701 8860 9449 9540 10086 10373 10398 10873 11002 11719 12705 13105 13914A 14766 15301 15311 15326 15824 15944d 16223 16278 16362 |
| L3b1a7 | 73 263 750 1438 2706 3441 3450 4769 5211 5581 5773 6221 7028 8701 8860 9449 9540 10086 10373 10398 10873 11002 11719 12705 13105 13914A 14766 15299 15301 15311 15326 15824 15944d 16223 16278 |
| L3b1a7a | 73 263 750 1438 2706 3441 3450 4769 5211 5581 5773 6221 7028 8701 8860 9449 9477 9540 10086 10373 10398 10873 11002 11719 12705 13105 13914A 14766 15299 15301 15311 15326 15824 15944d 16223 16278 |
| L3b1a8 | 73 263 750 1438 2332 2706 3450 4769 5773 6221 7028 8701 8860 9449 9540 10086 10373 10398 10873 11002 11719 12705 13105 13914A 14766 15301 15311 15326 15824 15944d 16145 16223 16278 16362 |
| L3b1a9 | 73 263 750 1438 2706 3450 4769 5773 6221 7028 8701 8860 9449 9540 10086 10373 10398 10873 11002 11719 12705 13105 13914A 14766 15301 15311 15326 15824 15944d 16051 16223 16278 16362 |
| L3b1a9a | 73 263 750 1438 2706 3450 4769 5773 6221 7028 8701 8860 9449 9540 10086 10373 10398 10873 11002 11719 12705 13105 13914A 14766 15301 15311 15326 15824 15944d 16051 16223 16278 16318 16362 |
| L3b1a10 | 73 263 750 1438 2706 3450 4659 4769 5773 6221 7028 7859 8701 8860 9449 9540 10086 10373 10398 10873 11002 11719 12705 13105 13914A 14766 15301 15311 15326 15824 15944d 16124 16223 16278 16362 |
| L3b1a11 | 73 263 750 1438 2706 3450 4769 5773 6221 7028 8701 8860 9449 9540 10086 10373 10398 10873 11002 11719 12705 13105 13790 13914A 14766 15301 15311 15326 15824 15944d 16124 16223 16278 16311 16362 |
| L3b1b | 73 152 263 750 1438 2706 3450 4769 5773 6221 7028 8701 8860 9079 9449 9540 10086 10373 10398 10873 11719 12705 13105 13914A 14766 15301 15311 15326 15664 15824 15944d 16124 16223 16278 16362 |
| L3b1b1 | 73 152 217 263 750 1438 2706 3450 4769 5773 6221 7028 8701 8860 9079 9449 9540 10086 10373 10398 10873 11719 12705 13105 13914A 14766 15301 15311 15326 15664 15824 15944d 16124 16223 16278 16362 |
| L3b2 | 73 263 750 1438 2706 3420 3450 4769 5773 6221 7028 8701 8860 9449 9540 10086 10398 10640 10873 11719 12705 13105 13914A 14766 15301 15311 15326 15824 15944d 16124 16223 16278 16362 16527 |

| | |
|---|---|
| L3b2a | 73 263 750 1438 2706 3420 3450 4769 5250 5773 6221 7028 8701 8860 9449 9540 10086 10398 10640 10873 11719 12705 13105 13914A 14766 15301 15311 15326 15824 15944d 16124 16189 16223 16278 16362 16527 |
| L3b2b | 73 263 750 1438 2706 3420 3450 4769 5773 6221 7028 8701 8860 9053 9067 9449 9540 10086 10398 10640 10873 11719 12705 13105 13914A 14766 15301 15311 15326 15550 15824 15944d 16124 16223 16278 16362 16527 |
| L3b3 | 73 185 189 263 750 1438 2706 3450 4769 5773 6221 6527 7028 8701 8860 9007 9449 9540 10086 10398 10873 11719 12705 13105 13914A 13934 14182 14766 15301 15311 15326 15824 15944d 16048 16124 16223 16278 16362 |
| L3f | 73 263 750 1438 2706 3396 4218 4769 7028 8701 8860 9540 10398 10873 11719 12705 14766 15301 15326 15514 15944d 16209 16223 |
| L3f1 | 73 263 750 1438 2706 3396 4218 4769 5601 7028 8701 8860 9540 9950 10398 10873 11719 12705 14766 15301 15326 15514 15944d 16209 16223 |
| L3f1a | 73 263 750 1438 2706 3396 3693 4218 4350 4769 5194 5601 7028 8701 8860 9540 9950 10398 10873 11719 12705 14148 14766 15106 15301 15326 15514 15944d 16209 16223 |
| L3f1a1 | 73 263 750 1438 2706 3197 3396 3693 4218 4350 4769 5194 5601 7028 8701 8860 9540 9950 10398 10873 11719 12507 12705 14148 14766 15106 15301 15326 15514 15944d 16209 16223 |
| L3f1b | 73 189 (200) 263 750 1438 1822 2706 3396 4218 4769 5601 7028 7819A 8527 8701 8860 8932 9540 9950 10398 10873 11440 11719 12705 14766 14769 15301 15326 15514 15944d 16209 16223 16311 |
| L3f1b+16292 | 73 189 (200) 263 750 1438 1822 2706 3396 4218 4769 5601 7028 7819A 8527 8701 8860 8932 9540 9950 10398 10873 11440 11719 12705 14766 14769 15301 15326 15514 15944d 16209 16223 16292 16311 |
| L3f1b1 | 73 189 (200) 263 750 1438 1822 2706 3396 4218 4769 5601 7028 7819A 8527 8701 8860 8932 9540 9950 10398 10873 11440 11719 12705 14766 14769 15301 15326 15514 15944d 16209 16223 16292 16295 16311 |
| L3f1b1a | 73 189 (200) 263 750 1438 1822 2706 3396 4218 4769 5601 7028 7819A 8410 8527 8701 8860 8932 9540 9950 10070 10398 10873 11440 11719 12705 14766 14769 15301 15326 15514 15944d 16129 16209 16223 16292 16295 16311 |
| L3f1b1a1 | 73 189 (200) 263 272 750 1438 1822 2706 3396 4218 4769 5601 7028 7819A 8410 8527 8701 8860 8932 9540 9950 10070 10398 10873 11440 11719 12705 14766 14769 15301 15326 15514 15944d 16129 16209 16223 16292 16295 16311 |
| L3f1b2 | 73 189 (200) 263 750 1438 1822 2706 3396 4218 4769 5601 7028 7235A 7819A 8527 8701 8860 8932 9540 9950 10398 10873 11440 11719 12705 14766 14769 15301 15326 15514 15944d 16209 16223 16292 16311 |
| L3f1b2a | 73 189 (200) 263 750 1438 1822 2706 3396 4218 4769 5601 7028 7235A 7819A 8527 8701 8860 8932 9540 9950 10398 10873 11440 11719 12705 14766 14769 15301 15326 15514 15944d 16172 16209 16223 16266A 16292 16311 |
| L3f1b+16292+150 | 73 150 189 (200) 263 750 1438 1822 2706 3396 4218 4769 5601 7028 7819A 8527 8701 8860 8932 9540 9950 10398 10873 11440 11719 12705 14766 14769 15301 15326 15514 15944d 16209 16223 16292 16311 |

| | |
|---|---|
| L3f1b3 | 73 150 189 (200) 263 711 750 1438 1822 2706 3396 4218 4769 5601 6806 7028 7158 7819A 8527 8701 8860 8932 9540 9950 10398 10873 11440 11719 12705 14766 14769 15301 15326 15514 15944d 16209 16223 16292 16311 |
| L3f1b4 | 73 150 189 (200) 263 750 1438 1822 2706 3396 3505 4218 4769 5601 7028 7819A 8527 8701 8860 8932 9540 9950 10398 10873 11440 11719 12705 14766 14769 15301 15326 15514 15944d 16209 16223 16292 16311 |
| L3f1b4a | 73 150 189 (200) 263 750 1438 1822 2706 3396 3505 4218 4769 5601 7028 7819A 8527 8701 8860 8932 9540 9950 10398 10873 11440 11719 12705 13167 14766 14769 15301 15326 15514 15944d 16209 16223 16311 |
| L3f1b4a1 | 73 150 189 (200) 263 750 1438 1822 2706 3396 3505 4218 4769 5601 7028 7819A 8527 8701 8799 8860 8932 9540 9950 10398 10873 11440 11719 12705 13167 14766 14769 15301 15326 15514 15944d 16209 16223 16311 |
| L3f1b4b | 73 150 189 (200) 263 750 1438 1822 2706 3396 3505 4218 4769 5601 7028 7819A 8527 8701 8860 8932 9540 9950 10398 10873 11440 11719 12705 14766 14769 15301 15326 15514 15930 15944d 16209 16223 16292 16311 |
| L3f1b4c | 73 150 189 (200) 263 750 1438 1822 2706 3396 3505 4218 4769 5601 7028 7819A 8527 8701 8860 8932 9540 9950 10398 10873 11440 11719 12705 14766 14769 15301 15326 15514 15944d 16209 16218 16223 16292 16311 |
| L3f1b5 | 73 189 (200) 263 750 1438 1822 2706 3396 4218 4769 5601 7028 7819A 8527 8701 8860 8932 9540 9950 10398 10873 11440 11582 11719 12705 14766 14769 15301 15326 15514 15944d 16209 16223 16292 16311 |
| L3f2 | 73 263 (745.1T) 750 1438 2706 3396 4218 4769 7028 8701 8860 9540 10398 10873 11719 12705 14766 15301 15326 15514 15944d 16209 16223 16311 |
| L3f2a | 73 152 263 (745.1T) 750 1438 2706 3396 4218 4769 7028 8701 8860 9540 10398 10873 11719 12705 14766 15301 15326 15479 15514 15944d 16209 16223 16311 |
| L3f2a1 | 73 152 263 (745.1T) 750 1438 2706 3396 4218 4769 5821 7028 8701 8860 9365 9540 10398 10873 11719 12705 14766 15301 15314 15326 15479 15514 15944d 16209 16223 16311 |
| L3f2a1a | 73 152 263 (745.1T) 750 1438 1842 2706 3396 4218 4769 5821 7028 8701 8860 9365 9540 10398 10873 11719 12705 14766 15301 15314 15326 15479 15514 15944d 16209 16223 16266 16311 |
| L3f2b | 73 263 374.1A (745.1T) 750 1438 2442 2706 3396 4218 4769 5342 7028 8701 8860 9540 10398 10873 11016 11719 12338 12705 14766 15301 15326 15466 15514 15658 15944d 16209 16223 16311 |
| L3f3 | 73 189 263 318 750 959 1438 2706 3396 4218 4643 4769 5181 6602 7028 8158 8251 8701 8860 9540 9932 10398 10604 10873 11719 11770 12705 14766 15301 15326 15514 15940 15944d 16176 16209 16223 16234 |
| L3f3a | 73 189 263 318 750 959 1438 2706 3396 4218 4643 4769 5181 6602 7028 8158 8251 8701 8860 9540 9932 10398 10604 10873 11719 11770 12705 14766 15301 15326 15514 15940 15944d 16176 16209 16223 16234 16284 |
| L3f3b | 73 189 263 318 750 959 1438 2706 3396 4218 4643 4769 5181 6602 7028 8158 8251 8701 8860 9540 9932 10398 10604 10873 11719 11770 12705 14766 15301 15326 15514 15940 15944d 16176 16188 16209 16223 16234 |
| L3c'd | 73 152 263 750 1438 2706 4769 7028 8701 8860 9540 10398 10873 11719 12705 13105 14766 15301 15326 16223 |

| | |
|---|---|
| L3c | 73 152 195 263 498.1C 678 750 1438 2706 3582 4491 4769 5393 7028 7394 8701 8860 9337 9540 9682 10398 10873 11719 12373 12705 13105 14221 14371 14560 14587 14766 15301 15326 16223 16311 16362 |
| L3d | 73 152 263 750 1438 2706 4769 5147 7028 7424 8618 8701 8860 9540 10398 10873 11719 12705 13105 13886 14284 14766 15301 15326 16124 16223 |
| L3d1'2'3'4'5'6 | 73 152 263 750 921 1438 2706 4769 5147 7028 7424 8618 8701 8860 9540 10398 10873 11719 12705 13105 13886 14284 14766 15301 15326 16124 16223 |
| L3d1 | 73 152 263 750 921 1438 2706 4769 5147 6680 7028 7424 8618 8701 8860 9540 10398 10873 11719 12705 13105 13886 14284 14766 15301 15326 16124 16223 |
| L3d1a | 73 152 263 750 921 1438 2706 4048 4769 5147 6680 7028 7424 7648 8618 8701 8860 9540 10398 10873 11719 11887 12705 13105 13886 14284 14766 15301 15326 16124 16223 |
| L3d1a1'2 | 73 152 263 750 921 1438 2706 4048 4769 5147 6680 7028 7424 7648 8618 8701 8860 9540 10398 10873 11719 11887 12705 13105 13886 14284 14766 15301 15326 16124 16223 16319 |
| L3d1a1 | 73 152 263 750 921 1438 1503 2706 4048 4769 5147 6680 7028 7424 7648 8618 8701 8860 9540 10398 10873 11719 11887 12705 13105 13886 14284 14766 15301 15326 16124 16223 16319 |
| L3d1a1a | 73 150 152 263 750 921 1438 1503 2706 4048 4203 4769 5147 5471 6680 7028 7424 7648 8618 8701 8860 9540 10398 10640 10873 10915 11719 11887 12705 13105 13886 14284 14766 15301 15326 16124 16223 16319 |
| L3d1a1a1 | 73 150 152 263 750 921 1438 1503 2706 3579 4048 4203 4769 5147 5471 6680 7028 7424 7648 8618 8701 8860 9540 10398 10640 10873 10915 11719 11887 12705 13105 13886 14284 14766 15301 15326 16124 16223 16319 |
| L3d1a1b | 73 146 152 195 263 750 921 1438 1503 2706 4048 4769 5147 5162 6680 7028 7424 7648 8618 8701 8860 9452 9540 10398 10837 10873 11719 11887 12406 12705 13105 13884 13886 14284 14766 15106 15301 15326 16124 16223 16319 |
| L3d1a2 | 73 152 263 750 921 1438 2706 4048 4769 5147 6680 7028 7424 7648 8618 8701 8860 9540 10398 10873 11719 11887 12705 13105 13886 14284 14766 15061 15301 15326 16124 16223 16319 |
| L3d1b | 73 152 263 750 921 1438 2706 4769 5046 5147 6680 7028 7424 8618 8701 8860 9540 10398 10873 11719 12705 13105 13886 14284 14766 15301 15326 16124 16223 |
| L3d1b1 | 73 152 263 750 921 1438 2706 4769 5046 5147 6272 6680 7028 7424 8618 8701 8860 9540 10398 10873 11719 12705 13105 13886 14284 14766 15301 15326 16124 16223 |
| L3d1b1a | 73 152 263 750 921 1438 2706 4769 5046 5147 6272 6680 7028 7424 8014C 8618 8701 8860 9540 10398 10873 11719 12705 13105 13886 14284 14766 15301 15326 16124 16223 |
| L3d1b1b | 73 151 152 195 263 750 921 1438 2706 3957 4769 5046 5147 6272 6680 7028 7424 8618 8701 8860 9540 10398 10873 11719 12705 13105 13886 14284 14766 15301 15326 16124 16223 |

| | |
|---|---|
| L3d1b2 | 73 150 152 263 750 921 1438 2706 4553 4769 5046 5147 6680 7028 7424 8618 8701 8860 9540 10398 10873 11719 12705 13105 13886 14284 14766 15301 15326 15514 16124 16223 |
| L3d1b3 | 73 146 152 263 750 921 1438 2706 4769 5046 5147 6680 7028 7424 8618 8701 8860 9540 10398 10873 11719 12705 13105 13886 14284 14634 14766 15110 15301 15326 16124 16223 |
| L3d1b3a | 73 146 152 263 750 921 1438 2706 4769 5046 5147 6680 7028 7424 8618 8701 8860 9540 10398 10873 11719 12705 13105 13886 14284 14634 14766 15110 15301 15326 16124 16223 16256 |
| L3d1c | 73 152 263 750 921 1438 2706 3203 4769 5147 6680 7028 7424 8618 8701 8860 9111 9540 10398 10873 11239 11719 12705 12870 13105 13542 13886 14284 14766 15301 15326 16124 16166 16223 |
| L3d1c1 | 73 152 263 750 921 1438 2706 3203 4769 5147 5372 6680 7028 7424 8618 8701 8860 9111 9254 9540 10398 10873 11239 11719 12705 12870 13105 13542 13886 14284 14766 15301 15326 16124 16166 16223 |
| L3d1d | 73 152 263 750 921 1438 2706 4769 5147 6680 7028 7424 7765 8618 8701 8860 9151 9540 10398 10873 11719 12705 13105 13886 14284 14766 15301 15326 16124 16223 (16256) (16368) |
| L3d2 | 73 152 263 750 921 1438 2706 4769 5147 7028 7424 8618 8701 8860 9540 10398 10873 11719 12705 13105 13886 14272 14284 14584 14766 15301 15326 16111 16124 16223 |
| L3d2a | 73 152 263 750 921 1438 2706 4769 5147 7028 7424 8618 8701 8860 9540 10398 10873 11719 12705 13105 13886 14272 14284 14584 14766 15115 15301 15326 16111 16124 (16189) 16223 |
| L3d2b | 73 152 199 263 750 921 1438 2706 4769 5147 7028 7424 8618 8701 8860 9540 10398 10873 11719 12705 13105 13886 14272 14284 14584 14766 15301 15326 16111 16124 16223 |
| L3d3 | 73 152 263 750 921 1438 1719 2706 4688 4769 5147 7028 7424 8618 8701 8860 9540 10398 10873 11719 12705 13105 13886 14284 14766 15061 15301 15326 16124 16223 |
| L3d3a | 73 152 263 750 921 1438 1719 2706 3498G 4688 4769 5147 7028 7424 8251 8618 8701 8860 9540 10398 10873 10899 11404 11719 12705 13105 13886 14284 14766 15061 15301 15326 16124 (16189) 16223 (16278) (16304) (16311) |
| L3d3a1 | 73 152 263 750 921 1438 1719 2706 3498G 4688 4769 5147 7028 7424 8251 8618 8701 8860 9540 10398 10873 10899 11404 11719 12705 13105 13886 14284 14766 15061 15208 15301 15326 16124 (16189) 16223 (16278) (16304) (16311) |
| L3d3a1a | 73 152 263 750 921 1438 1719 2416 2706 3498G 4688 4769 5147 7028 7424 8251 8618 8701 8860 9540 10398 10873 10899 11404 11719 12705 13105 13886 14284 14766 15061 15208 15301 15326 16124 (16189) 16223 (16278) (16304) (16311) |
| L3d3a1b | 73 152 263 750 921 1438 1719 2706 3498G 4688 4769 5147 7028 7424 8251 8618 8701 8860 9540 10398 10873 10899 11404 11719 12705 13105 13886 14284 14766 15061 15208 15301 15326 16000 16124 (16189) 16223 (16278) (16304) (16311) |

| | |
|---|---|
| L3d3b | 73 152 263 750 921 1438 1719 2706 4688 4769 5147 7028 7389 7424 8618 8701 8860 9540 10398 10873 11719 12705 13105 13886 14284 14766 15061 15301 15326 16124 16223 |
| L3d4 | 73 152 189 195 263 750 921 1438 2706 4769 5147 7028 7424 8618 8701 8860 9540 10398 10873 11719 12705 13105 13886 14284 14766 15301 15326 16124 16223 |
| L3d4a | 73 152 189 195 263 750 921 1393 1438 2706 4769 5147 7028 7424 8618 8701 8860 9540 10398 10873 11719 12280 12705 13105 13886 14284 14766 15301 15326 16124 16223 |
| L3d5 | 73 152 263 750 921 1438 2706 4769 5147 7028 7424 8618 8701 8860 9540 10398 10873 11719 12705 13105 13886 14284 14766 15301 15326 15799 16124 16223 16362 |
| L3d5a | 73 152 263 750 921 1438 2706 4769 5147 7028 7158 7424 8618 8701 8860 9540 10398 10873 11719 12311 12705 13105 13886 14284 14766 15301 15326 15799 15930 16124 16223 16362 |
| L3d6 | 73 152 263 750 921 1438 2706 4769 5147 5492 7028 7424 8473 8618 8658 8701 8860 9300 9540 10398 10873 11719 12705 13105 13886 14284 14766 15223 15301 15326 16124 16223 |
| L3e'i'k'x | 73 150 263 750 1438 2706 4769 7028 8701 8860 9540 10398 10819 10873 11719 12705 14766 15301 15326 16223 |
| L3e | 73 150 263 750 1438 2352 2706 4769 7028 8701 8860 9540 10398 10819 10873 11719 12705 14212 14766 15301 15326 16223 |
| L3e1 | 73 150 189 200 263 750 1438 2352 2706 4769 6221 6587 7028 8701 8860 9540 10398 10819 10873 11719 12705 14152 14212 14766 15301 15326 15670 15942 16223 16327 |
| L3e1a | 73 150 189 200 263 750 1438 2352 2706 4769 6221 6587 7028 8701 8860 9540 10398 10819 10873 11719 12705 14152 14212 14766 15301 15326 15670 15942 16185 16223 16327 |
| L3e1a1 | 73 150 185 189 200 263 750 1438 2352 2706 4769 6221 6587 7028 8650 8701 8860 9540 10398 10819 10873 11719 12705 14152 14212 14766 15301 15326 15670 15942 16185 16223 16311 16327 |
| L3e1a1a | 73 150 185 189 200 263 750 1438 2352 2706 3438 4769 6221 6587 7028 8650 8701 8860 9540 10398 10819 10873 11719 12705 14152 14212 14766 15301 15326 15670 15942 16185 16223 16311 16327 |
| L3e1a2 | 73 150 152 189 195 200 207 263 750 1438 2352 2706 4769 5774A 6221 6587 7028 8701 8860 9254 9540 10398 10819 10873 11024 11719 12705 14152 14212 14569 14766 15301 15326 15670 15942 16185 16209 16223 16327 |
| L3e1a3 | 73 150 189 200 263 750 1438 2352 2706 4769 6221 6587 7028 8701 8860 9540 10819 10873 11719 12705 14152 14212 14766 15301 15326 15670 15942 16185 16223 16327 |
| L3e1a3a | 73 150 189 200 263 750 1438 2352 2706 4769 5255 6221 6587 7028 7337 8701 8860 9540 10819 10873 11719 12705 14152 14212 14766 15301 15326 15670 15942 16185 16223 16327 |
| L3e1a3b | 73 150 189 200 263 750 1438 2352 2706 4769 6221 6587 7028 8701 8860 9540 10172 10819 10873 11719 12438 12705 14152 14212 14766 15301 15326 15670 15942 16093 16185 16223 16327 |

| | |
|---|---|
| L3e1b | 73 150 189 200 263 750 1438 2352 2706 4769 6221 6587 7028 8701 8860 9540 10398 10819 10873 11719 12705 14152 14212 14766 14926 15301 15326 15670 15942 16223 16325d 16327 |
| L3e1b1 | 73 150 189 200 263 750 1438 2352 2706 4769 6221 6587 7028 8701 8860 9540 10398 10819 10873 11719 12705 14152 14212 14766 14926 15301 15326 15670 15942 16223 16256 16325d 16327 |
| L3e1b2 | 73 150 185 189 263 750 1438 2352 2706 4769 6221 6587 7028 8577 8701 8860 9540 10398 10819 10873 11719 12192 12705 14152 14212 14766 14926 15301 15326 15670 15942 16223 16325d 16327 |
| L3e1c | 73 150 189 200 263 750 1438 2352 2706 3675 4769 5460 6221 6587 7028 8289.1CCCCCTCTACCCCCTCTA 8860 9540 10398 10819 10873 11719 12705 14152 14212 14323 14766 15301 15326 15670 15942 16223 16327 |
| L3e1d | 73 150 152 189 200 263 750 1438 2352 2706 4769 6221 6587 7028 8701 8860 9540 10398 10819 10873 11719 12705 14152 14212 14766 15301 15326 15670 15942 16176 16223 16327 |
| L3e1d1 | 73 150 152 189 200 263 750 1438 2352 2706 4769 6221 6587 7028 8701 8703 8860 9300 9540 10398 10819 10873 11719 12705 12738 14152 14212 14766 15301 15326 15670 15942 16176 16223 16327 |
| L3e1d1a | 73 150 152 189 200 263 750 1438 2352 2706 4769 6221 6587 7028 8701 8703 8860 9300 9327 9540 10398 10819 10873 11719 12705 12738 14152 14212 14766 15301 15326 15670 15942 16176 16223 16327 |
| L3e1e | 73 150 189 200 263 750 1438 2352 2706 4769 6221 6587 7028 8701 8860 9540 10370 10398 10819 10873 11719 12705 14152 14212 14766 15301 15326 15670 15942 16223 16327 |
| L3e1e1 | 73 150 189 200 263 750 1438 2352 2706 4769 6221 6587 7028 8701 8860 9540 10370 10398 10819 10873 11719 12705 14152 14212 14571A 14766 15301 15326 15670 15942 16223 16327 |
| L3e1e2 | 90 97 106-111d 150 189 200 263 750 1438 2352 2706 4562 4769 6221 6587 7028 8701 8860 9098 9540 10370 10398 10819 10873 11719 12705 14152 14212 14766 15301 15326 15670 15942 16223 16327 |
| L3e1f | 73 150 189 200 263 750 1438 2352 2706 4769 6221 6587 7028 8281-8289d 8701 8860 9540 10398 10819 10873 11719 12705 14152 14212 14766 15301 15326 15670 15942 16223 16327 |
| L3e1f1 | 73 150 189 200 263 750 1438 2352 2706 4769 6221 6587 7028 8281-8289d 8701 8860 9540 10398 10819 10873 11719 12705 14152 14212 14766 15301 15326 15670 15942 16189 16223 16327 |
| L3e1f1a | 73 150 189 200 263 750 1438 2352 2706 4395 4769 6221 6587 7028 8281-8289d 8701 8860 9540 10398 10819 10873 11719 12705 14152 14212 14766 15301 15326 15670 15942 16189 16223 16260 16327 |
| L3e1f2 | 73 150 189 195 200 207 263 750 1438 2352 2706 4219 4769 6221 6587 7028 7805 8281-8289d 8701 8860 9540 10398 10819 10873 11719 12705 14152 14212 14766 15301 15326 15670 15942 16223 16327 |
| L3e1g | 73 150 189 200 263 750 1438 1442 2352 2706 4769 4844 5498 5618 6221 6587 7028 8093 8701 8860 9540 10398 10490 10819 10873 11318 11719 12215 12705 14152 14212 14766 15301 15326 15670 15942 16172 16223 16327 16399 |
| L3e2 | 73 150 195 263 750 1438 2352 2706 4769 7028 8701 8860 9540 10398 10819 10873 11719 12705 14212 14766 14905 15301 15326 16223 16320 |

| | |
|---|---|
| L3e2a | 73 150 195 263 750 1438 2352 2706 4769 4823 7028 8701 8860 9540 10398 10819 10873 11719 12705 13105 14212 14766 14869 14905 15301 15326 16223 16320 |
| L3e2a1 | 73 150 195 198 263 750 1438 2352 2706 4769 4823 7028 8701 8860 9540 10398 10819 10873 11719 12705 13105 14212 14766 14869 14905 15301 15326 16223 16320 |
| L3e2a1a | 73 150 195 198 263 750 1438 1737 2352 2706 3852 4769 4823 7028 7196 8701 8860 9540 10398 10819 10873 11719 12705 13105 14212 14766 14869 14905 15301 15326 15924 16223 16320 |
| L3e2a1b | 73 150 195 198 263 750 1438 2352 2706 4769 4823 6413 7028 8701 8860 9540 10398 10819 10873 11719 12705 13105 14212 14766 14869 14905 15301 15326 16223 16320 |
| L3e2a1b1 | 73 150 195 198 263 750 1438 2352 2706 4769 4823 6413 7028 8701 8860 9540 10398 10819 10873 11719 12705 13105 14212 14766 14869 14905 15301 15326 16223 16320 16399 |
| L3e2a1b2 | 73 150 195 198 263 750 1438 2352 2706 4769 4823 6413 7028 8701 8860 9540 10398 10819 10873 11719 12705 13105 14212 14766 14869 14905 15076 15301 15326 16223 16311 16320 |
| L3e2a1b3 | 73 150 195 198 263 750 1438 2352 2706 4769 4823 6413 7028 8701 8860 9540 10398 10819 10873 11719 12705 13105 14212 14750 14766 14869 14905 15301 15326 16223 16320 |
| L3e2a2 | 73 150 195 263 750 1438 2352 2706 4769 4823 7028 8701 8860 9540 10335 10398 10819 10873 11719 12705 13105 14212 14766 14869 14905 15301 15326 16223 16320 |
| L3e2a3 | 73 150 195 263 750 1438 2352 2706 4769 4823 5102 7028 8701 8860 9540 10398 10819 10873 11719 12414 12705 13105 14212 14766 14869 14905 15301 15326 16223 16320 |
| L3e2b | 73 150 195 263 750 1438 2352 2706 4769 7028 8701 8860 9540 10398 10819 10873 11719 12705 14212 14766 14905 15301 15326 16172 16189 16223 16320 |
| L3e2b1 | 73 150 195 263 750 1438 2352 2706 4769 7028 8701 8860 9377 9540 10398 10819 10873 11719 12705 14212 14766 14905 15301 15326 16172 16189 16223 16320 |
| L3e2b1a | 73 150 195 263 750 1438 2352 2483 2706 4769 7028 8701 8860 9377 9540 10398 10819 10873 11719 12705 14212 14766 14905 15301 15326 16172 16189 16223 16320 |
| L3e2b1a1 | 73 150 195 263 750 1438 2352 2483 2706 4769 5580 7028 8701 8860 9377 9540 10398 10819 10873 11719 12705 14212 14766 14905 15301 15326 16172 16189 16223 16320 |
| L3e2b1a2 | 73 150 195 263 750 1438 2352 2483 2706 3277 4769 7028 8701 8860 9377 9540 10398 10819 10873 11719 12406 12705 14212 14766 14905 15301 15326 16172 16189 16223 16320 |
| L3e2b2 | 73 150 195 263 750 1438 2352 2706 4769 7028 8701 8860 9540 10398 10819 10873 11377 11719 12705 14212 14766 14905 15301 15326 16172 16189 16223 16320 |
| L3e2b+152 | 73 150 152 195 263 750 1438 2352 2706 4769 7028 8701 8860 9540 10398 10819 10873 11719 12705 14212 14766 14905 15301 15326 16172 16189 16223 16320 |

| | |
|---|---|
| L3e2b3 | 73 150 152 195 263 750 1438 2352 2706 4769 6932 7028 8701 8860 9540 10398 10819 10873 11719 12705 14212 14766 14905 15301 15326 16172 16189 16223 16320 |
| L3e2b4 | 73 150 152 195 263 750 1438 2352 2706 4769 7028 8701 8860 9540 10398 10819 10861 10873 11719 12705 14212 14766 14905 15301 15326 16172 16189 16223 16320 |
| L3e2b5 | 73 150 152 195 263 750 1438 2352 2706 4769 6261 7028 8701 8860 9540 10398 10819 10873 11719 12705 14212 14766 14905 15301 15326 16172 16189 16223 16320 |
| L3e2b6 | 73 146 150 152 195 263 750 1438 2352 2706 4769 7028 7370 8701 8860 9540 10398 10819 10873 11719 12705 14212 14766 14905 15301 15326 16172 16189 16223 16320 |
| L3e2b7 | 73 150 195 263 750 1438 1927 2352 2706 4769 7028 8701 8860 9540 10398 10819 10873 11719 12705 14212 14766 14905 15301 15326 16172 16189 16223 16320 |
| L3e2b8 | 73 150 195 263 750 1438 2352 2706 4769 7028 8701 8860 9196 9540 10398 10819 10873 11719 12705 14212 14766 14905 15301 15326 16172 16189 16223 16320 |
| L3e3'4'5 | 73 150 263 1438 2352 2706 4769 7028 8701 8860 9540 10398 10819 10873 11719 12705 14212 14766 15301 15326 16223 |
| L3e3'4 | 73 150 263 1438 2352 2706 4769 5262 7028 8701 8860 9540 10398 10819 10873 11719 12705 14212 14766 15301 15326 16223 |
| L3e3 | 73 150 195 263 1438 2000 2352 2706 4769 5262 6524 7028 8701 8860 9540 9554 10398 10667 10816 10819 10873 11719 12705 13101C 14212 14766 15301 15326 16223 16265T |
| L3e3a | 73 150 195 263 573.XC 1438 2000 2352 2706 4769 5262 6524 7028 8701 8860 9540 9554 10286 10398 10667 10816 10819 10873 11719 12397 12705 13101C 14212 14766 15301 15326 16223 16265T |
| L3e3b | 73 150 195 263 1438 2000 2352 2706 4655 4769 5262 6524 7028 8701 8860 9540 9554 10398 10667 10816 10819 10873 11719 12248 12705 13101C 13197 13651 14212 14766 15301 15326 15812 16223 16265T |
| L3e3b1 | 73 150 195 263 1438 2000 2352 2706 4655 4769 5262 6261 6524 7028 8701 8860 9540 9554 10398 10667 10816 10819 10873 11719 12248 12705 13101C 13197 13651 14212 14766 15301 15326 15812 16223 16265T |
| L3e3b2 | 73 150 195 263 1438 2000 2352 2706 4655 4769 5262 6524 7028 8701 8860 9540 9554 10398 10667 10816 10819 10873 11719 12248 12705 13101C 13197 13651 14212 14766 15301 15326 15812 16223 16265T |
| L3e3b3 | 73 150 195 263 1438 2000 2352 2706 4655 4769 5262 6524 7028 8701 8860 9540 9554 10398 10667 10816 10819 10873 11719 12248 12705 13101C 13197 13651 14212 14766 15301 15326 15812 16223 16265T |
| L3e4 | 73 150 263 1438 2352 2706 3915 4769 5262 5584 7028 8701 8860 9540 10398 10819 10873 11257 11719 12705 13749 14212 14766 15301 15326 16051 16223 |
| L3e4a | 73 150 263 1438 2352 2706 3915 4769 5262 5584 7028 8701 8860 9540 10398 10819 10873 11257 11719 12705 13749 14212 14766 15301 15326 16051 16223 16264 |

| | |
|---|---|
| L3e4a1 | 73 150 263 1438 2352 2706 3915 4769 5262 5584 7028 8701 8860 9490 9540 10398 10819 10873 11257 11719 12705 13749 14212 14766 15301 15326 16051 16223 16264 |
| L3e5 | 73 150 263 398 1438 2352 2706 4769 7028 8392 8701 8860 9540 10398 10819 10873 11719 12705 14212 14766 15301 15326 16041 16223 |
| L3e5a | 73 150 263 398 1438 2352 2706 4769 7028 8392 8701 8860 9540 10398 10819 10873 11719 12705 13317 14212 14766 15301 15326 16041 16223 |
| L3e5a1 | 73 150 263 398 1438 2352 2706 2833 4769 7028 8392 8701 8860 9540 10398 10819 10873 11719 12705 13317 14212 14766 15301 15326 16041 16223 |
| L3e5a1a | 73 150 263 398 431A 1438 2352 2706 2833 4769 7028 8392 8701 8860 9540 10398 10819 10873 11719 12705 13317 14212 14766 15301 15326 16041 16223 |
| L3e5b | 73 150 263 398 1438 2352 2706 4769 5483 7028 8392 8701 8860 9540 10398 10819 10873 11719 12705 14212 14766 15301 15326 16041 16223 |
| L3e5+195 | 73 150 195 263 398 1438 2352 2706 4769 7028 8392 8701 8860 9540 10398 10819 10873 11719 12705 14212 14766 15301 15326 16041 16223 |
| L3e5c | 73 150 195 263 398 1438 2352 2706 4769 7028 8392 8701 8860 9540 10398 10819 10873 11548 11719 12705 14212 14766 15301 15326 16041 |
| L3e5d | 73 150 263 398 1438 2352 2706 4769 7028 8392 8701 8860 8869 9540 10398 10819 10873 11719 12591 12705 14212 14766 15301 15326 16041 16223 |
| L3e5e | 73 150 263 398 1438 2352 2706 4769 7028 8392 8701 8860 9540 10398 10819 10873 11719 12705 14212 14766 15301 15326 15367 16037 16041 16223 |
| L3e5f | 73 150 263 398 1438 2352 2706 4769 7028 8392 8701 8860 9540 10398 10819 10873 11719 12173 12705 14212 14766 15301 15326 15734 16041 16223 16343 |
| L3i | 73 150 263 750 1438 2706 4769 7028 7645 8701 8860 9540 10398 10819 10873 11719 12705 14766 15301 15326 16223 |
| L3i1 | 73 150 263 750 1438 2706 4769 7028 7645 8701 8860 9540 10398 10679 10819 10873 11260 11719 12705 13800A 14766 15301 15326 16153 16223 |
| L3i1a | 73 150 263 750 1438 2706 4769 7028 7645 8701 8860 9540 10398 10679 10819 10873 11260 11719 12705 13800A 14766 15301 15326 15758 (16129) 16153 16223 |
| L3i1b | 73 150 263 750 1438 2158 2706 4769 7028 7645 8701 8860 9540 10398 10679 10819 10873 11260 11719 12705 13687 13800A 14766 15301 15326 16153 16223 |
| L3i2 | 73 150 152 189 263 750 1438 2706 4769 5441 7028 7645 8222 8701 8860 9540 10398 10819 10873 11719 12705 14766 14818 15301 15326 15388 16223 16260 16311 |
| L3k | 73 150 152 235 263 494 750 1438 2706 3918 4769 6620G 7028 8649 8701 8860 9467 9540 10398 10819 10873 11719 12705 13135 13992 14766 15301 15314 15326 16223 |
| L3k1 | 73 150 152 235 263 494 735 750 1438 2706 3918 4313 4769 6620G 7028 8649 8701 8860 9007T 9329 9467 9540 10398 10819 10873 11719 12705 13135 13542 13862 13992 14766 15301 15314 15326 16223 16355 |

| | |
|---|---|
| L3x | 73 150 263 750 1438 2706 3483 4769 5899.XC 6401 7028 8311 8701 8817 8860 9540 10398 10819 10873 11719 12705 13708 14766 15301 15326 16169 16223 |
| L3x1 | 73 150 204 263 750 1438 2706 3483 4769 5899.XC 6401 7028 8311 8701 8817 8860 9540 10398 10819 10873 11719 12705 13708 14766 15172 15301 15326 16169 16223 16278 |
| L3x1a | 73 150 204 263 750 1438 2706 3483 4769 5460 5899.XC 6401 7028 8311 8701 8817 8860 9540 10398 10819 10873 11719 12705 13708 14766 15172 15301 15326 16169 16223 16278 |
| L3x1a1 | 73 150 189 199 204 263 750 1438 2706 3483 4769 5460 6401 7028 8311 8701 8817 8860 9540 10398 10819 10873 11719 12705 13708 14766 15172 15301 15326 16169 16223 16278 |
| L3x1a2 | 73 150 204 263 750 1438 2706 3483 4769 5460 5899.XC 6401 7028 8311 8701 8817 8860 9540 10398 10819 10873 11719 12705 13392 13708 14766 15172 15301 15326 16169 16223 16278 |
| L3x1+16311 | 73 150 204 263 750 1438 2706 3483 4769 5899.XC 6401 7028 8311 8701 8817 8860 9540 10398 10819 10873 11719 12705 13708 14766 15172 15301 15326 16169 16223 16278 16311 |
| L3x1b | 73 150 204 263 750 1438 2706 3483 4769 5899.XC 6401 7028 8311 8701 8817 8860 9540 10398 10819 10873 11719 12705 13708 14766 15172 15301 15326 16169 16223 16256 16278 16311 |
| L3x2 | 73 150 (200) 249d 263 494 750 1438 2706 3483 4769 5899.XC 6401 7028 8311 8701 8817 8860 9540 9941 10398 10819 10873 11719 12705 13708 14766 15301 15326 16169 16193 16195 16223 |
| L3x2a | 73 150 (200) 249d 263 494 750 1438 2706 3435 3483 4769 5899.XC 6401 7028 8311 8701 8817 8860 9540 9941 10398 10819 10873 11719 12705 13708 14766 15301 15326 16169 16193 16195 |
| L3x2a1 | 73 150 (200) 249d 263 368.1AGAA 494 750 1438 2706 3435 3483 4769 5899.XC 6392 6401 7028 7129 8311 8701 8817 8860 9540 9941 10398 10819 10873 11719 12705 13708 14766 15301 15326 16086 16169 16193 16195 |
| L3x2a1a | 73 150 (200) 249d 263 368.1AGAA 494 750 1438 2706 3435 3483 4769 5899.XC 6392 6401 7028 7129 8311 8701 8817 8860 9180 9540 9941 10398 10819 10873 11719 12705 13681 13708 13722 14766 15301 15326 16086 16169 16193 16195 |
| L3x2b | 73 150 (200) 249d 263 494 650 750 1438 2706 3483 4769 5899.XC 6401 7028 8311 8701 8817 8860 9254 9540 9941 10398 10819 10873 11719 12705 13708 14766 15301 15326 15758 15928 16169 16193 16195 16223 16243 |
| L3h | 73 263 750 1438 2706 4769 7028 7861 8701 8860 9540 9575 10398 10873 11719 12705 14766 15301 15326 16223 |
| L3h1 | 73 263 750 1438 1719 2706 4388 4769 5300 7028 7861 8701 8860 9509 9540 9575 10398 10873 11590 11719 12705 14766 15301 15326 16223 16311 |
| L3h1a | 73 263 750 1438 1719 2706 4388 4769 5300 5492 7028 7861 8701 8860 9509 9540 9575 10398 10873 11590 11719 12705 14766 15301 15326 16223 16311 |

| | |
|---|---|
| L3h1a1 | 73 263 750 1438 1719 2706 4388 4769 5300 5492 7028 7861 8047 8485 8701 8860 9509 9540 9575 10398 10873 11590 11719 12432 12705 13708 13926 14110 14766 15301 15326 16192 16223 16298 16311 |
| L3h1a2 | 73 263 750 1438 1719 2706 4388 4769 5300 5492 7028 7861 8701 8781A 8860 9509 9540 9575 10398 10873 11590 11719 12705 14766 15301 15326 16223 16311 |
| L3h1a2a | 73 146 263 750 1438 1719 2706 4388 4688 4742 4769 5300 5492 7028 7861 8701 8781A 8860 8943 9509 9540 9575 10398 10873 11590 11719 12175 12519 12705 14587 14766 15301 15326 15646 16223 16311 16399 |
| L3h1a2a1 | 73 146 263 750 1438 1719 2706 4388 4688 4742 4769 5300 5492 7028 7861 8701 8781A 8860 8943 9509 9540 9575 10398 10873 11590 11719 12175 12236 12519 12705 14587 14766 14862 15301 15326 15646 16223 16311 16399 |
| L3h1a2b | 73 263 750 1438 1719 2706 4388 4769 5300 5492 7028 7861 8701 8781A 8860 9509 9540 9575 10398 10873 11590 11719 12705 14766 15301 15326 16223 16270 16311 |
| L3h1b | 73 189C 195 263 750 1438 1719 2706 4388 4769 5300 7028 7861 8701 8860 9509 9540 9575 10044 10398 10873 11590 11719 12705 14410 14766 15301 15326 16223 16256A 16311 |
| L3h1b1 | 73 189C 195 263 750 1438 1719 2706 4388 4769 5300 7028 7861 8701 8860 9509 9540 9575 10044 10398 10873 11590 11719 12705 14410 14766 15301 15326 16223 16256A 16284 16311 |
| L3h1b1a | 73 189C 195 263 750 1438 1719 2706 3777 4388 4769 5300 7028 7861 8701 8860 9509 9540 9575 10044 10398 10873 11590 11719 12705 14410 14766 15301 15326 16179 16223 16256A 16284 16311 |
| L3h1b2 | 73 151 152 189C 195 263 294 606 750 990 1438 1719 2706 4388 4769 5300 7028 7861 8701 8842 8860 9509 9540 9575 9758 10044 10398 10873 11590 11719 12705 12882 13437 14410 14766 15301 15326 16129 16223 16256A 16311 16362 |
| L3h2 | 73 150 195 263 318 750 1438 2706 3879 4769 5460 5813 5930 7028 7861 8020 8701 8860 9098 9380 9540 9575 9965 10398 10873 11440 11719 12469 12705 13080 13755 14766 15301 15326 16111 16184 16223 16304 |
| M | 73 263 489 750 1438 2706 4769 7028 8701 8860 9540 10398 10400 10873 11719 12705 14766 14783 15043 15301 15326 16223 |
| M1'20'51 | 73 263 489 750 1438 2706 4769 7028 8701 8860 9540 10398 10400 10873 11719 12705 14110 14766 14783 15043 15301 15326 16223 |
| M1 | 73 195 263 489 750 1438 2706 4769 6446 6680 7028 8701 8860 9540 10398 10400 10873 11719 12403 12705 12950C 14110 14766 14783 15043 15301 15326 16129 16189 16223 16249 16311 |
| M1a | 73 195 263 489 750 813 1438 2706 4769 6446 6671 6680 7028 8701 8860 9540 10398 10400 10873 11719 12403 12705 12950C 14110 14766 14783 15043 15301 15326 16129 16189 16223 16249 16311 |
| M1a1 | 73 195 263 489 750 813 1438 2706 3705 4769 6446 6671 6680 7028 8701 8860 9540 10398 10400 10873 11719 12346 12403 12705 12950C 14110 14766 14783 15043 15301 15326 16129 16189 16223 16249 16311 16359 |

| | |
|---|---|
| M1a1a | 73 195 263 489 750 813 1438 2706 3705 4769 6446 6671 6680 7028 8701 8860 9540 10398 10400 10873 11719 12346 12403 12705 12950C 14110 14182 14766 14783 15043 15301 15326 16129 16189 16223 16249 16311 16359 |
| M1a1a1 | 73 195 263 489 750 813 1438 2706 3705 4011 4769 6446 6671 6680 7028 8521 8701 8860 9540 10398 10400 10873 11044 11719 12346 12403 12705 12950C 14110 14182 14766 14783 15043 15301 15326 16129 16189 16223 16311 16359 |
| M1a1b | 73 195 263 489 750 813 930 1438 2706 3705 4769 6446 6671 6680 7028 7853 8701 8860 9540 10398 10400 10873 11719 12346 12403 12705 12950C 14110 14766 14783 15043 15301 15326 16129 16189 16223 16249 16311 16359 |
| M1a1b1 | 73 195 263 489 750 813 930 1438 2706 3705 4769 6446 6671 6680 7028 7853 8701 8860 9540 10398 10400 10873 11719 12346 12403 12705 12950C 14110 14766 14769 14783 15043 15301 15326 16129 16189 16223 16249 16311 16359 |
| M1a1b1a | 73 195 263 489 750 813 930 1438 2706 3705 4769 5319 6446 6671 6680 7028 7853 8701 8860 9540 10398 10400 10873 11719 12346 12403 12705 12950C 14110 14766 14769 14783 15043 15301 15326 16129 16189 16223 16249 16311 16359 |
| M1a1b1b | 73 195 263 489 750 813 930 1438 2706 3705 4769 6446 6671 6680 7028 7853 8701 8860 9540 10398 10400 10873 11719 12346 12403 12705 12950C 13152 14110 14766 14769 14783 15043 15301 15326 16129 16189 16223 16249 16311 16359 |
| M1a1b1b1 | 73 195 263 489 750 813 930 1438 2706 3705 4769 6446 6671 6680 7028 7853 8701 8860 9053 9540 10398 10400 10873 11719 12346 12403 12705 12950C 13152 14110 14766 14769 14783 15043 15301 15326 16129 16189 16223 16249 16311 16359 |
| M1a1b1c | 73 195 263 375G 489 750 813 930 1438 2706 3705 4769 6446 6671 6680 7028 7853 8701 8860 9540 10398 10400 10873 11719 12346 12403 12705 12950C 14110 14766 14769 14783 15043 15301 15326 16129 16189 16223 16249 16289 16311 16359 |
| M1a1b2 | 73 195 263 489 750 813 930 1438 1820 2706 3705 4769 6446 6671 6680 7028 7853 8701 8860 9540 10398 10400 10873 11719 12346 12403 12705 12950C 14110 14766 14783 15043 15301 15326 16129 16189 16223 16249 16311 16359 |
| M1a1+16093 | 73 195 263 489 750 813 1438 2706 3705 4769 6446 6671 6680 7028 8701 8860 9540 10398 10400 10873 11719 12346 12403 12705 12950C 14110 14766 14783 15043 15301 15326 16093 16129 16189 16223 16249 16311 16359 |
| M1a1c | 73 195 263 489 750 813 1438 1719 2706 3705 4769 6446 6671 6680 6962 7028 8701 8860 9540 9966 10398 10400 10873 11719 12346 12373 12403 12705 12950C 13542C 14110 14766 14783 15043 15301 15326 16093 16129 16189 16223 16249 16311 16359 |
| M1a1d | 73 150 195 263 489 750 813 1438 2706 3705 4769 6446 6671 6680 7028 8270 8281-8289d 8701 8860 9540 10398 10400 10873 11719 12346 12403 12705 12950C 14110 14766 14783 15043 15301 15326 16093 16129 16189 16223 16249 16311 16359 |

| | |
|---|---|
| M1a1e | 73 195 263 489 750 813 1438 2706 3705 3906A 4769 6446 6671 6680 7028 8701 8860 9540 10398 10400 10873 11719 12346 12403 12705 12950C 14110 14766 14783 15043 15301 15326 16129 16189 16223 16249 16311 16359 |
| M1a1e1 | 73 195 263 489 750 813 1438 2706 3705 3906A 4769 6446 6671 6680 7028 8701 8860 9540 9629 10398 10400 10873 11719 12346 12403 12705 12950C 14110 14766 14783 15043 15301 15326 16129 16189 16223 16249 16311 16359 |
| M1a1e2 | 73 195 263 489 750 813 1438 2706 3705 3906A 4769 6446 6671 6680 7028 8701 8860 9540 10398 10400 10873 11719 11914 12346 12403 12705 12950C 14110 14766 14783 15043 15301 15326 16129 16189 16223 16249 16311 16359 |
| M1a1f | 73 195 263 489 750 813 1438 2706 3705 4769 6378 6446 6671 6680 7028 8701 8860 9540 10398 10400 10873 11719 12346 12403 12705 12950C 14110 14766 14783 15043 15301 15326 16129 16189 16223 16249 16311 16359 |
| M1a1g | 73 195 263 489 750 813 1438 2706 3705 4769 6446 6671 6680 7028 8701 8860 9540 10398 10400 10873 11719 12346 12403 12705 12873 12950C 14110 14766 14783 14793 15043 15287 15301 15326 16129 16189 16223 16249 16261 16311 16359 |
| M1a1h | 73 195 263 489 750 813 1438 2706 3705 4769 6446 6671 6680 7028 8701 8860 9540 10398 10400 10873 11719 12346 12403 12705 12950C 14110 14766 14783 15043 15301 15326 15514 16129 16189 16223 16249 16278 16311 16359 |
| M1a1i | 73 195 204 489 750 813 1438 2706 3705 4769 6446 6671 6680 7028 8701 8860 9540 9845 10398 10400 10873 11719 12346 12403 12705 12950C 14110 14766 14783 15043 15301 15326 16129 16189 16223 16249 16311 16359 16527 |
| M1a2 | 73 195 263 489 750 813 1438 2706 4769 6446 6671 6680 7028 8701 8860 9540 10398 10400 10873 11719 12403 12705 12950C 14110 14766 14783 15043 15301 15326 15884 16129 16189 16223 16249 16311 |
| M1a2a | 73 152 195 263 489 (513) 750 813 1438 2706 4769 6446 6671 6680 7028 8701 8860 9540 10398 10400 10873 11719 12403 12705 12950C 14110 14766 14783 15043 15172 15301 15326 15884 16129 16189 16223 16249 16311 |
| M1a2b | 73 195 263 489 593 750 813 1438 2706 4769 6446 6671 6680 7028 8701 8860 9540 10398 10400 10873 11719 12403 12705 12950C 14110 14766 14783 15043 15301 15326 15884 16129 16189 16223 16249 16260 16311 16320 |
| M1a3 | 73 195 263 489 750 813 1438 2706 4769 6446 6671 6680 7028 8701 8860 9540 10398 10400 10873 11719 12403 12705 12950C 13637 14110 14766 14783 15043 15301 15326 16129 16189 16249 16311 |
| M1a3a | 73 195 263 489 750 813 1438 2706 4769 6446 6473 6671 6680 7028 8701 8860 9540 10398 10400 10873 11719 12403 12705 12950C 13637 14110 14766 14783 15043 15301 15326 16129 16189 16249 16311 |
| M1a3b | 73 195 263 489 750 813 1438 2706 4769 6446 6671 6680 7028 8701 8860 9540 10398 10400 10873 11719 12403 12414 12705 12950C 13637 14110 14766 14783 15043 15301 15326 16129 16189 16249 16311 |

| | |
|---|---|
| M1a3b1 | 73 195 263 489 750 813 1438 1503 2706 4769 6253 6446 6671 6680 7028 8701 8860 9540 10398 10400 10873 11719 12403 12414 12705 12950C 13637 14110 14766 14783 15043 15301 15326 16189 16249 16311 |
| M1a3b2 | 73 195 263 489 750 813 1438 2706 4769 6446 6671 6680 7028 8701 8860 9540 9740 10398 10400 10873 11719 12403 12414 12705 12950C 13637 14110 14766 14783 15043 15301 15326 16129 16189 16249 16311 |
| M1a4 | 73 195 263 489 750 813 1438 2706 3513 4769 6446 6671 6680 7028 8607 8701 8860 9540 10398 10400 10873 11719 12403 12705 12950C 14110 14766 14783 15043 15301 15326 16129 16189 16223 16249 16311 |
| M1a4a | 73 195 263 489 750 813 1438 2706 3513 4769 6446 6671 6680 7028 8607 8701 8860 9540 10398 10400 10667 10873 11719 12403 12705 12950C 14110 14766 14783 15043 15301 15326 16129 16189 16223 16249 16311 16465 |
| M1a5 | 73 195 263 489 750 813 1438 2706 4769 6446 6671 6680 7028 8701 8860 9540 10398 10400 10694 10873 11719 12403 12705 12950C 13215 13722 14110 14323 14515 14766 14783 15043 15301 15326 15770 15799 16189 16223 16249 16311 |
| M1a6 | 73 195 263 489 750 813 954 1438 2706 4769 6446 6671 6680 7028 8701 8860 9540 10398 10400 10873 11719 12403 12705 12950C 14110 14766 14783 15043 15301 15326 16129 16189 16223 16249 16311 |
| M1a7 | 73 195 263 489 750 813 1438 2706 4769 6446 6671 6680 7028 8701 8860 9540 10398 10400 10873 11719 12403 12705 12950C 14110 14766 14783 15043 15301 15326 16129 16189 16223 16249 16311 16357 |
| M1a8 | 73 195 263 489 750 813 1438 2706 4769 5773 6446 6671 6680 7028 8701 8860 9540 10398 10400 10873 11719 12403 12705 12950C 14110 14766 14783 15043 15301 15326 16129 16189 16223 16249 16311 |
| M1a8a | 73 195 263 489 750 813 1438 2706 4769 5054 5773 6446 6671 6680 7028 8701 8860 9540 10398 10400 10873 11719 12403 12705 12950C 14110 14766 14783 15043 15217 15301 15326 16129 16189 16223 16249 16311 |
| M1b | 73 195 263 489 750 1438 2706 4769 6446 6680 7028 8701 8860 9540 10398 10400 10873 11719 12403 12705 12950C 13111 14110 14766 14783 15043 15301 15326 16129 16189 16223 16249 16311 |
| M1b1 | 73 195 263 466 489 750 1438 2706 4769 4936 6446 6680 7028 8701 8860 8868 9540 10398 10400 10873 11719 12403 12705 12950C 13111 14110 14766 14783 15043 15247G 15301 15326 16129 16185 16189 16223 16249 16311 |
| M1b1a | 73 195 200 263 466 489 750 1438 2706 4769 4936 6446 6680 7028 8701 8860 8868 9540 10398 10400 10873 11671 11719 12403 12705 12950C 13111 14110 14766 14783 15043 15247G 15301 15326 16129 16185 16189 16223 16249 16311 |
| M1b1b | 73 195 263 466 489 750 1438 2706 4769 4936 6179 6446 6680 6815 7028 8701 8860 8868 9540 10398 10400 10873 11719 12403 12705 12950C 13111 14110 14766 14783 15043 15247G 15301 15326 16129 16185 16189 16223 16249 16311 |
| M1b2 | 73 195 263 489 750 1438 2706 4769 6446 6680 7028 8701 8860 9540 10398 10400 10873 10895 11719 12403 12705 12950C 13111 14110 14766 14783 15043 15301 15326 16129 16189 16223 16249 16311 16399 |

| | |
|---|---|
| M1b2a | 73 195 263 489 750 1438 2706 4769 6446 6680 7028 8701 8860 9540 10398 10400 10873 10895 11377 11719 12403 12705 12950C 13111 14110 14766 14783 15043 15301 15326 16129 16189 16223 16249 16291 16311 |
| M1b2b | 73 195 263 489 750 1438 2706 3705 4769 6446 6680 7028 8701 8860 9540 10398 10400 10873 10895 11719 12007 12403 12705 12950C 13111 14110 14766 14783 15043 15301 15326 16129 16189 16223 16249 16311 16362 16399 |
| M1b2c | 73 195 263 489 750 1007 1438 2706 4769 6446 6680 7028 8701 8860 9540 10398 10400 10873 10895 11719 12403 12705 12950C 13111 14110 14766 14783 15043 15301 15326 16129 16189 16223 16249 16311 16399 |
| M20 | 73 152 249d 263 316 489 750 1438 2706 3200 3714 4385T 4769 4772 7028 7433 8701 8853 8860 9127 9512 9540 10274 10398 10400 10679 10873 11719 11914 12354 12705 14110 14766 14783 14974 15043 15301 15326 15691 16129 16209 16223 16272 |
| M51 | 73 150 263 489 750 1438 2706 4697 4769 4973 7028 8701 8860 9509 9540 10398 10400 10873 11719 12705 14110 14356 14687 14766 14783 15043 15301 15317 15326 16223 16278 |
| M51a | 73 150 263 489 750 1438 2706 4697 4769 4973 7028 8701 8860 9509 9540 10398 10400 10873 11719 12236 12705 14110 14356 14687 14766 14783 15043 15301 15317 15326 16223 16278 |
| M51a1 | 73 150 263 489 732 750 1438 2706 4697 4769 4973 7028 8701 8860 9509 9540 10398 10400 10873 11719 12236 12705 12715 14110 14356 14687 14766 14783 15043 15301 15317 15326 16223 16278 16294 |
| M51a1a | 73 150 185 263 489 732 750 1438 2045 2706 3105 4697 4769 4973 7028 8701 8860 9509 9540 9716 10398 10400 10873 11719 12236 12468 12705 12715 14110 14356 14687 14766 14783 15043 15301 15317 15326 16129 16223 16278 16294 |
| M51a1b | 73 150 263 489 732 750 1438 2706 4697 4769 4973 7028 7738 8701 8860 9233 9509 9540 10398 10400 10873 11719 12025 12236 12705 12715 14110 14315 14323 14356 14687 14766 14783 15043 15301 15317 15326 15862 16223 16234 16278 16294 |
| M51a2 | 73 150 263 489 750 1438 1822 2706 4136 4697 4769 4973 5461 7028 8701 8860 9100 9509 9540 10398 10400 10873 11719 12236 12705 14110 14356 14527 14687 14766 14783 15043 15301 15317 15326 15383 16189 16223 16278 |
| M51b | 73 150 263 489 750 1438 2706 2833 4697 4769 4973 7028 8701 8860 9509 9540 10398 10400 10873 11719 12705 14110 14356 14687 14766 14783 15043 15301 15317 15326 16223 16278 |
| M51b1 | 73 150 263 489 750 1438 2706 2833 4697 4769 4973 7028 8701 8860 9509 9540 10398 10400 10873 11719 12012 12609 12705 14110 14356 14687 14766 14783 15043 15301 15317 15326 16223 16278 16311 |
| M51b1a | 73 150 263 489 750 1438 2706 2833 4697 4769 4973 7028 8119 8701 8860 9509 9540 10398 10400 10873 11470 11719 12012 12609 12705 14110 14356 14687 14766 14783 15043 15301 15317 15326 16223 16278 16311 |
| M51b1b | 73 150 263 489 750 1438 2706 2833 3565 3999 4336 4697 4769 4973 7028 7642 8701 8860 9509 9540 10398 10400 10873 11719 12012 12609 12705 13105 13768 13928C 14110 14356 14417 14687 14766 14783 15043 15301 15317 15326 16145 16223 16278 16311 |

| | |
|---|---|
| M2 | 73 263 447G 489 750 1438 1780 2706 4769 7028 8701 8860 9540 10398 10400 10873 11083 11719 12705 14766 14783 15043 15301 15326 15670 16223 16274 |
| M2a'b | 73 263 447G 489 750 1438 1780 2706 4769 7028 8502 8701 8860 9540 10398 10400 10873 11083 11719 12705 14766 14783 15043 15301 15326 15670 16223 16274 16319 |
| M2a | 73 263 447G 489 750 1438 1780 2706 4769 7028 7961 8502 8701 8860 9540 10398 10400 10873 11083 11719 12705 12810 14766 14783 15043 15301 15326 15670 16223 16274 16319 |
| M2a1 | 73 204 263 447G 489 750 1438 1780 2706 4769 5252 7028 7961 8396 8502 8701 8860 9540 9758 10398 10400 10873 11083 11719 12705 12810 14766 14783 15043 15301 15326 15670 16223 16270 16274 16319 16352 |
| M2a1a | 73 195 204 263 447G 489 750 1438 1780 2706 4769 5252 7028 7961 8396 8502 8701 8860 9540 9758 10398 10400 10873 11083 11719 12705 12810 14766 14783 15043 15301 15326 15670 16223 16270 16319 16352 |
| M2a1a1 | 73 195 204 263 447G 489 750 1438 1780 2706 4769 5252 7028 7472 7961 8396 8502 8701 8860 9540 9758 10398 10400 10873 11083 11719 12705 12810 14693 14766 14783 15043 15301 15326 15670 16223 16270 16319 16352 |
| M2a1a1a | 73 195 204 263 447G 489 750 1438 1780 2706 4769 5252 7028 7472 7961 8396 8502 8701 8860 9540 9758 10398 10400 10873 11083 11084 11719 12705 12810 14693 14766 14783 15043 15301 15326 15670 16223 16270 16319 16352 |
| M2a1a1a1 | 73 195 204 263 447G 489 750 1438 1780 2706 4769 5252 7028 7472 7961 8396 8502 8701 8860 9540 9758 10398 10400 10873 11083 11084 11719 12705 12810 14693 14766 14783 15043 15301 15326 15670 16172 16223 16234 16270 16319 16352 |
| M2a1a1b | 73 195 204 263 447G 489 750 1438 1780 2706 4769 5252 7028 7472 7961 8396 8502 8701 8860 9540 9758 10398 10400 10873 11083 11339 11719 12705 12810 14693 14766 14783 15043 15301 15326 15670 16223 16270 16319 16352 |
| M2a1a1b1 | 73 195 204 263 447G 489 750 1438 1780 2706 4769 5252 7028 7472 7961 8396 8502 8701 8860 9540 9758 10398 10400 10873 11083 11339 11719 12705 12810 14693 14766 14783 15043 15301 15326 15670 16223 16256 16270 16319 16352 |
| M2a1a2 | 73 195 204 263 447G 489 750 1438 1780 2706 4769 5252 7028 7961 8396 8502 8701 8860 9540 9758 10398 10400 10873 11083 11719 12705 12810 14766 14783 15043 15301 15326 15670 16223 16243 16270 16319 16352 |
| M2a1a2a | 73 195 204 263 447G 489 750 1438 1780 2706 4769 5252 7028 7961 8396 8502 8701 8860 9540 9758 10398 10400 10873 11083 11719 12501 12705 12810 14766 14783 15043 15301 15326 15670 16223 16243 16270 16319 16352 |
| M2a1a2a1 | 73 195 199 204 263 447G 489 750 1438 1780 2706 4769 5252 7028 7961 8396 8502 8701 8860 9540 9758 10398 10400 10819 10873 11083 11719 12501 12705 12810 14766 14783 15043 15301 15326 15670 16223 16230 16243 16270 16319 16352 |

| | |
|---|---|
| M2a1a2a1a | 73 195 199 204 263 447G 489 750 961 1438 1780 2706 4769 5252 7028 7961 8396 8502 8701 8860 9540 9758 10398 10400 10819 10873 11083 11719 12501 12705 12810 14766 14783 15043 15301 15326 15670 16223 16230 16243 16270 16319 16352 |
| M2a1a+207 | 73 195 204 207 263 447G 489 750 1438 1780 2706 4769 5252 7028 7961 8396 8502 8701 8860 9540 9758 10398 10400 10873 11083 11719 12705 12810 14766 14783 15043 15301 15326 15670 16223 16270 16319 16352 |
| M2a1a3 | 73 195 198 204 207 263 447G 482 489 750 1438 1780 2706 4769 5252 7028 7961 8396 8502 8701 8860 9540 9758 10398 10400 10873 11083 11719 12705 12810 14766 14783 15043 15301 15326 15670 16223 16270 16319 16352 |
| M2a1a3+16093 | 73 195 198 204 207 263 447G 482 489 750 1438 1780 2706 4769 5252 7028 7961 8396 8502 8701 8860 9540 9758 10398 10400 10873 11083 11719 12705 12810 14766 14783 15043 15301 15326 15670 16093 16223 16270 16319 16352 |
| M2a1a3a | 73 195 198 204 207 263 447G 482 489 750 1438 1780 2706 4769 5252 7028 7961 8396 8502 8701 8860 9540 9758 10398 10400 10873 11083 11719 12705 12810 14766 14783 15043 15301 15326 15670 16093 16223 16270 16295 16319 16352 |
| M2a1a3a1 | 73 195 198 204 207 263 447G 482 489 750 1438 1780 2706 4769 5252 7028 7961 8396 8502 8701 8860 9540 9758 10398 10400 10873 11083 11719 12705 12810 14766 14783 15043 15301 15326 15670 16093 16147 16223 16270 16295 16319 16352 |
| M2a1a3b | 73 195 198 204 207 263 447G 482 489 750 1438 1780 2706 4769 5252 7028 7961 8396 8502 8701 8860 9540 9758 10398 10400 10873 11083 11719 11864 12705 12810 14766 14783 15043 15301 15326 15670 16093 16223 16270 16319 16352 |
| M2a1b | 73 204 263 447G 489 750 1438 1462 1780 2706 4216 4769 5252 7028 7961 8396 8502 8701 8860 9540 9758 10398 10400 10873 11083 11719 12705 12810 14766 14783 15043 15301 15326 15670 15700 15924 16017 16075 16223 16270 16274 16278 16319 16352 |
| M2a1c | 73 204 263 335 447G 489 526 750 1438 1780 2706 4769 5252 7028 7961 8396 8502 8701 8860 9540 9758 10398 10400 10873 11083 11719 12705 12810 14766 14783 15043 15301 15326 15670 16223 16270 16274 16319 16352 |
| M2a2 | 73 263 447G 489 750 1438 1780 2706 4769 7028 7702 7961 8502 8701 8860 9540 10398 10400 10873 11041A 11083 11719 12657 12705 12810 13708 14766 14783 15043 15301 15326 15670 16223 16240C 16274 16311 |
| M2a2a | 73 263 447G 489 750 1438 1780 2706 4769 6035 7028 7702 7961 8395 8502 8701 8860 9540 10398 10400 10873 11041A 11083 11719 12657 12705 12810 13708 14766 14783 15043 15301 15326 15670 16223 16240C 16274 16311 16319 |
| M2a3 | 73 146 263 447G 489 750 1438 1780 2706 4769 5426 5774 7028 7762 7961 8502 8701 8860 9540 10398 10400 10873 11083 11719 12705 12810 14766 14783 15043 15301 15326 15670 16223 16274 16319 |
| M2a3a | 73 146 263 447G 489 750 1438 1780 2706 4769 5426 5774 7028 7762 7961 8502 8701 8860 9540 10398 10400 10873 11083 11719 12705 12810 14766 14783 15043 15301 15326 15670 16223 16265C 16274 16319 |

| | |
|---|---|
| M2b | 73 152 182 195 263 447G 489 750 1438 1453 1780 2706 2831T 3630 4769 5744 6647 7028 8502 8701 8860 9540 9899 10398 10400 10873 11083 11719 12705 13254 14783 15043 15301 15326 15670 16169.1C 16189 16223 16274 16319 16320 |
| M2b1 | 73 152 182 195 263 447G 489 750 1438 1453 1780 2706 2831T 3630 4769 5420 5744 6260 6647 7028 8502 8701 8860 9540 9899 10398 10400 10873 11083 11719 12705 13254 14783 15043 15301 15326 15670 16169.1C 16189 16223 16274 16319 16320 |
| M2b1a | 73 152 182 195 214 263 447G 489 750 1438 1453 1677 1780 2392 2706 2707C 2831T 3630 4769 5420 5744 6260 6647 7028 8502 8701 8860 9540 9899 10398 10400 10873 11016 11083 11719 12705 13254 14783 15043 15301 15326 15670 16169.1C 16189 16223 16256 16274 16319 16320 |
| M2b1b | 73 152 182 195 263 447G 471 489 549 750 1438 1453 1780 2706 2831T 3630 4769 5263 5420 5480 5744 5747 6260 6647 7028 7337 8502 8632 8701 8860 9233 9540 9899 10398 10400 10873 11083 11719 12705 13254 14783 15043 15301 15326 15670 15777C 16169.1C 16189 16223 16274 16319 16320 |
| M2b2 | 73 152 182 195 263 447G 489 750 1438 1453 1780 2706 2831T 3630 4769 5744 6647 7028 8502 8701 8860 9540 9899 10398 10400 10873 11083 11719 12705 13254 14783 15043 15301 15326 15670 16169.1C 16189 16223 16274 16295 16319 16320 |
| M2b3 | 73 152 182 195 263 447G 489 750 1438 1453 1780 2706 2831T 3630 4769 5744 6647 7028 8502 8701 8860 9540 9899 10398 10400 10873 11083 11377 11719 12705 13254 14783 15043 15301 15326 15670 16169.1C 16189 16223 16274 16319 16320 |
| M2b3a | 73 146 152 182 195 263 447G 489 750 1438 1453 1780 2706 2831T 3630 4769 5744 6647 7028 8502 8701 8860 9540 9899 10398 10400 10873 11083 11377 11719 12705 13254 14783 15043 15301 15326 15670 16136 16169.1C 16189 16223 16274 16319 16320 |
| M2b4 | 73 152 182 195 263 447G 459d 489 750 1438 1453 1780 2706 2831T 3630 4769 4823 5744 6128 6647 7028 8502 8556 8701 8860 9540 9899 10398 10400 10873 11083 11719 12705 13254 14783 15043 15301 15326 15670 16169.1C 16189 16223 16240C 16274 16319 16320 |
| M2c | 73 263 447G 489 750 1438 1598 1780 2706 4216 4769 7028 8701 8860 9540 10398 10400 10873 11083 11719 12705 13212 14766 14783 14800 15043 15301 15326 15670 16223 16274 |
| M3 | 73 263 482 489 750 1438 2706 4769 7028 8701 8860 9540 10398 10400 10873 11719 12705 14766 14783 15043 15301 15326 16126 16223 |
| M3a | 73 263 482 489 750 1438 2706 4580 4769 7028 8701 8860 9540 10398 10400 10873 11719 12705 14766 14783 15043 15301 15326 16126 16223 |
| M3a1 | 73 263 482 489 750 1438 2706 4580 4703 4769 7028 8701 8860 9540 10398 10400 10873 11719 12705 14766 14783 15043 15301 15326 16126 |
| M3a1+204 | 73 204 263 482 489 750 1438 2706 4580 4703 4769 7028 8701 8860 9540 10398 10400 10873 11719 12705 14766 14783 15043 15301 15326 16126 16223 |
| M3a1a | 73 204 263 482 489 750 1438 2706 4580 4703 4769 7028 8701 8860 9540 10398 10400 10873 11719 12705 14476 14766 14783 15043 15301 15326 16126 16223 |

| | |
|---|---|
| M3a1b | 73 204 263 482 489 750 1438 2706 4580 4703 4769 7028 8701 8860 9540 10398 10400 10845 10873 11719 12705 14766 14783 15043 15301 15326 16126 16223 |
| M3a2 | 73 263 482 489 750 1438 2706 4580 4769 5783 7028 8701 8860 9540 10398 10400 10727 10873 11719 12705 14766 14783 15043 15301 15326 16126 16223 |
| M3a2a | 73 150 263 482 489 750 1438 2706 4562 4580 4769 5783 7028 8701 8860 8988 9540 10398 10400 10727 10873 11719 12705 14766 14783 15043 15301 15326 16126 16223 |
| M3b | 73 263 482 489 750 1438 2706 4769 6353 7028 8701 8860 9010 9540 10398 10400 10873 11719 12031A 12705 14766 14783 15043 15301 15326 16126 16223 |
| M3c | 73 263 482 489 750 1438 2706 4769 7028 8701 8860 9064 9540 10398 10400 10873 11719 12705 14766 14783 15043 15301 15326 16126 16223 |
| M3c+152 | 73 152 263 482 489 750 1438 2706 4769 7028 8701 8860 9064 9540 10398 10400 10873 11719 12705 14766 14783 15043 15301 15326 16126 16223 |
| M3c1 | 73 152 263 482 489 750 1438 2706 4769 7028 8701 8860 9064 9540 10365 10398 10400 10632 10873 11719 12705 14440 14766 14783 15043 15301 15326 16189 16223 16294 |
| M3c1a | 73 152 263 482 489 750 1438 2706 4769 7028 8701 8860 9064 9540 10365 10398 10400 10632 10873 11719 12705 14440 14766 14783 15043 15097 15301 15326 16189 16223 16294 |
| M3c1b | 73 152 263 482 489 750 1438 2706 4769 6366 7028 7598 8701 8860 9064 9540 10365 10398 10400 10632 10873 11719 12705 14440 14587 14766 14783 15043 15301 15326 16179 16189 16223 16294 |
| M3c1b1 | 73 152 263 482 489 750 1438 2706 4769 6366 7028 7598 8701 8860 9064 9540 10365 10398 10400 10632 10873 11016 11719 12705 14440 14587 14766 14783 15043 15301 15326 16124 16179 16189 16223 16294 |
| M3c1b1a | 73 152 263 482 489 750 1438 2706 4769 6366 7028 7598 8701 8856 8860 9064 9540 10365 10398 10400 10632 10873 11016 11719 12705 14440 14587 14766 14783 15043 15301 15326 16124 16179 16189 16223 16294 16319 |
| M3c1b1b | 73 152 263 482 489 750 1438 2706 4769 6366 7028 7598 8701 8860 9064 9540 10365 10398 10400 10632 10873 11016 11719 12705 14440 14587 14766 14783 15043 15301 15323 15326 16124 16179 16189 16223 16294 |
| M3c2 | 73 263 482 489 750 1438 2706 4769 7028 8701 8860 9064 9540 10398 10400 10873 11719 12705 14766 14783 15043 15301 15326 16126 16154 16223 |
| M3d | 73 263 482 489 750 1438 2706 4769 7028 8701 8860 9540 10398 10400 10873 11719 11827 12705 14766 14783 15043 15301 15326 16126 16223 16344 |
| M3d1 | 73 263 482 489 750 1438 2706 4769 7028 8701 8860 9540 10238 10398 10400 10873 11719 11827 12705 13820 14766 14783 15043 15301 15326 16126 16223 16344 |
| M3d1a | 73 263 482 489 750 1438 2706 4769 7028 8701 8860 9540 10238 10398 10400 10873 11719 11827 12705 12813 13820 14766 14783 15043 15301 15326 16126 16223 16344 |

| | |
|---|---|
| M3d1a1 | 73 263 482 489 750 1438 2706 4769 7028 8701 8860 9540 10238 10398 10400 10873 11719 11827 12705 12813 13820 13836 14766 14783 15043 15301 15326 16126 16223 16344 |
| M4"67 | 73 263 489 750 1438 2706 4769 7028 8701 8860 9540 10398 10400 10873 11719 12007 12705 14766 14783 15043 15301 15326 16223 |
| M4"67+16311 | 73 263 489 750 1438 2706 4769 7028 8701 8860 9540 10398 10400 10873 11719 12007 12705 14766 14783 15043 15301 15326 16223 16311 |
| M4 | 73 263 489 750 1438 2706 4769 6620 7028 7859 8701 8860 9540 10398 10400 10873 11719 12007 12705 14766 14783 15043 15301 15326 16145 16223 16261 16311 |
| M4a | 73 263 489 750 1438 2706 4769 6620 7028 7859 8701 8860 9540 10398 10400 10873 11719 12007 12705 14766 14783 15043 15301 15326 16145 16176 16223 16261 16311 |
| M4b | 73 152 263 489 750 1438 2706 4769 6620 7028 7859 8701 8860 9540 10398 10400 10873 11719 12007 12705 14766 14783 15043 15301 15326 15466 16086 16145 16223 16261 16311 |
| M65 | 73 263 489 511 750 1438 2706 4769 7028 8701 8860 9540 10398 10400 10873 11719 12007 12705 14766 14783 15043 15301 15326 16223 16311 |
| M65a | 73 263 489 511 750 1438 2706 4769 4916 7028 8701 8860 9540 10398 10400 10873 11719 12007 12705 13651 14766 14783 15043 15301 15326 16223 16289 16311 |
| M65a1 | 73 263 489 511 750 1438 1664 2706 4769 4916 6584 7028 8251 8701 8860 9540 10398 10400 10873 11413 11719 12007 12705 13651 14766 14783 15043 15301 15326 15924 16223 16289 16311 |
| M65a+@16311 | 73 263 489 511 750 1438 2706 4769 4916 7028 8701 8860 9540 10398 10400 10873 11719 12007 12705 13651 14766 14783 15043 15301 15326 16223 16289 |
| M65a2 | 73 263 489 511 750 1438 2706 4655 4769 4916 5302 7028 8701 8860 9540 10398 10400 10873 11719 12007 12705 13651 14766 14783 15043 15301 15326 16223 16289 |
| M65b | 73 241 263 489 511 750 1438 2706 3398 4769 7028 8701 8860 8865 9540 10398 10400 10873 11719 12007 12705 14766 14783 15043 15301 15326 16223 16311 |
| M67 | 73 143 263 489 750 1438 2706 2833 4227 4769 5979 6267 6366 7028 8701 8860 9540 9863 10398 10400 10873 11719 12007 12406 12705 13708 14766 14783 15043 15301 15326 15769 15833 16075 16169 16223 16311 |
| M18'38 | 73 246 263 489 750 1438 2706 4769 7028 8701 8860 9540 10398 10400 10873 11719 12007 12705 14766 14783 15043 15301 15326 16223 |
| M18 | 73 246 263 489 750 1438 2706 4769 7028 8701 8860 9540 10398 10400 10873 11719 12007 12498 12705 13135 14766 14783 15043 15301 15326 16223 16318T |
| M18a | 73 93 194 246 263 489 750 1438 2706 4769 7028 8701 8860 9540 10398 10400 10873 11719 12007 12498 12705 13135 14766 14783 15043 15301 15326 16223 16318T |
| M18b | 73 246 263 489 750 1438 2706 4769 7028 8277 8701 8860 9540 10398 10400 10873 11719 12007 12498 12705 13135 14587 14766 14783 15043 15301 15326 16223 16318T 16325 |

| | |
|---|---|
| M18c | 73 246 263 489 750 1438 2706 4769 7028 7853 8206 8701 8860 9540 10398 10400 10873 11719 12007 12498 12705 13135 14766 14783 15043 15301 15326 16223 16311 16318T |
| M38 | 73 246 263 489 750 1438 2706 4099 4769 7028 8701 8860 9540 10398 10400 10873 11719 12007 12705 14766 14783 15043 15301 15314 15326 15487 16223 |
| M38a | 73 189 246 263 489 750 1438 1808 2706 4099 4769 6267 6899 7028 8701 8860 9540 9966 10398 10400 10873 11719 12007 12705 14766 14783 15043 15301 15314 15326 15487 16223 |
| M38+195 | 73 195 246 263 489 750 1438 2706 4099 4769 7028 8701 8860 9540 10398 10400 10873 11719 12007 12705 14766 14783 15043 15301 15314 15326 15487 16223 |
| M38b | 73 146 153 195 246 263 489 644 750 1438 2706 3591 3987 4099 4769 6881 7028 7844 8047 8701 8860 9540 10398 10400 10873 11050 11719 12007 12705 14766 14783 15043 15301 15314 15326 15487 16189 16223 |
| M38c | 73 195 246 263 489 629 750 1438 1503 2706 4099 4769 7028 8701 8860 9186 9540 10398 10400 10748 10873 11719 12007 12103 12705 14569 14766 14783 14914 15043 15301 15314 15326 15487 16223 |
| M38+199 | 73 199 246 263 489 750 1438 2706 4099 4769 7028 8701 8860 9540 10398 10400 10873 11719 12007 12705 14766 14783 15043 15301 15314 15326 15487 16223 |
| M38d | 73 199 246 263 489 750 1438 2706 4099 4769 6297 7028 7356 8701 8860 9540 10398 10400 10873 11719 12007 12705 14766 14783 14944 15043 15301 15314 15326 15487 16093 16184 16223 16260A 16266 |
| M38e | 73 199 246 263 488 489 750 1438 2706 2833 4099 4654 4769 6815 7028 8289.1CCCCCTCTA 8701 8860 9438 9540 10398 10400 10873 11719 12007 12705 14706 14766 14783 15043 15301 15314 15326 15487 16223 16311 |
| M30 | 73 195A 263 489 750 1438 2706 4769 7028 8701 8860 9540 10398 10400 10873 11719 12007 12705 14766 14783 15043 15301 15326 15431 16223 |
| M30a | 73 195A 263 489 513 750 1438 2706 4769 7028 8701 8860 9540 10398 10400 10873 11719 12007 12705 14766 14783 15043 15301 15326 15431 16223 |
| M30a1 | 73 195A 263 489 513 750 1438 2706 4769 6366 7028 8701 8860 9540 10398 10400 10873 11719 12007 12705 14766 14783 15043 15301 15326 15431 16223 |
| M30a2 | 73 195A 263 489 513 750 1438 2706 4769 7028 8701 8860 9540 10398 10400 10873 11719 11935 12007 12705 14766 14783 15043 15301 15326 15431 16223 |
| M30b | 73 152 195A 263 489 750 1438 2706 4769 5147 7028 8701 8860 9540 10398 10400 10873 11719 12007 12705 14766 14783 15043 15301 15326 15431 16223 16278 16311 |
| M30c | 73 146 195A 263 489 750 1438 2706 4769 7028 8701 8860 9540 10398 10400 10873 11719 12007 12234 12705 14766 14783 15043 15301 15326 15431 16223 |
| M30c1 | 73 146 195A 263 489 750 1438 2706 4769 7028 8701 8860 9540 10398 10400 10873 11719 12007 12234 12705 14766 14783 15043 15301 15326 15431 16166d 16223 |

| | |
|---|---|
| M30c1a | 73 146 195A 263 489 750 1438 2706 4769 7028 8701 8860 9540 10398 10400 10873 11719 12007 12234 12705 14766 14783 15043 15301 15326 15431 16069 16166d 16223 |
| M30c1a1 | 73 146 195A 263 489 750 1438 2706 4769 7028 8701 8860 9540 9966 10398 10400 10873 11719 12007 12234 12705 14766 14783 15043 15301 15326 15431 16069 16166d 16223 |
| M30d | 73 195A 263 489 750 1438 2706 4769 7028 8701 8860 9540 10398 10400 10873 11719 12007 12705 14766 14783 15043 15259 15301 15326 15431 16223 (16302) |
| M30d1 | 73 195A 263 489 750 1438 1598 2706 4769 7028 8701 8860 9540 10398 10400 10873 11719 12007 12705 14766 14783 15043 15259 15301 15326 15431 16223 (16302) |
| M30d2 | 73 195A 263 489 750 1438 2706 4769 7028 8701 8860 9540 10160 10398 10400 10873 11719 12007 12705 14766 14783 15043 15259 15301 15326 15431 16223 (16302) |
| M30+16234 | 73 195A 263 489 750 1438 2706 4769 7028 8701 8860 9540 10398 10400 10873 11719 12007 12705 14766 14783 15043 15301 15326 15431 16223 16234 |
| M30e | 73 152 195A 263 489 750 1438 2706 4769 6620 7028 8701 8860 9540 10398 10400 10873 11719 12007 12705 13303 14766 14783 15043 15301 15326 15431 16223 16234 |
| M30f | 73 195A 263 489 750 1438 2706 4769 5894 7028 8701 8860 9540 10398 10400 10873 11719 12007 12705 14766 14783 15043 15301 15326 15431 16223 16368 |
| M30g | 73 195A 204 263 489 750 1438 2706 4769 6119 7028 8701 8860 9540 10398 10400 10873 11719 12007 12705 14766 14783 15043 15301 15326 15431 16223 |
| M37 | 73 263 489 750 1438 2706 4769 7028 8701 8860 9540 10398 10400 10556 10873 11719 12007 12705 14766 14783 15043 15301 15326 16223 |
| M37+152 | 73 152 263 489 750 1438 2706 4769 7028 8701 8860 9540 10398 10400 10556 10873 11719 12007 12705 14766 14783 15043 15301 15326 16223 |
| M37+152+151 | 73 151 152 263 489 750 1438 2706 4769 7028 8701 8860 9540 10398 10400 10556 10873 11719 12007 12705 14766 14783 15043 15301 15326 |
| M37a | 73 151 152 263 489 750 1438 2074d 2706 4769 7028 8701 8860 9540 10398 10400 10556 10873 11719 12007 12705 14766 14783 15043 15301 15326 16223 |
| M37a1 | 73 151 152 263 489 750 1438 2074d 2706 4769 7028 8701 8860 9055 9540 10398 10400 10556 10873 11719 12007 12705 14766 14783 15043 15301 15326 16223 |
| M37d | 73 151 152 263 489 750 1438 2706 4769 7028 8701 8860 9287 9540 10398 10400 10556 10873 11719 12007 12705 14766 14783 15043 15301 15326 16223 |
| M37e | 73 263 489 750 1438 2706 4769 7028 8701 8860 9540 10398 10400 10556 10873 11050 11719 12007 12705 14766 14783 15043 15301 15326 16111 16189 16223 16295 |
| M37e2 | 73 263 489 750 1438 2706 4769 7028 8701 8860 9540 10398 10400 10556 10873 11050 11719 12007 12705 14766 14783 15043 15301 15326 16111 16189 16223 16224 16295 |

| | |
|---|---|
| M43 | 73 263 489 709 750 1438 2706 4769 7028 8701 8860 9540 10316 10398 10400 10873 11696 11719 12007 12636 12705 14766 14783 15043 15301 15326 16223 |
| M43+16311 | 73 263 489 709 750 1438 2706 4769 7028 8701 8860 9540 10316 10398 10400 10873 11696 11719 12007 12636 12705 14766 14783 15043 15301 15326 16223 16311 |
| M43a | 73 263 489 709 750 1438 2706 4769 7028 8701 8860 9540 10316 10398 10400 10873 11696 11719 12007 12636 12705 14305 14766 14783 15043 15301 15326 16223 16311 |
| M43a1 | 73 263 489 709 750 1438 2706 4769 7028 8701 8860 9540 10316 10398 10400 10873 11696 11719 11963 12007 12636 12705 13656 13966 14305 14766 14783 15043 15301 15326 16223 16311 16362 |
| M43b | 73 263 489 709 750 1438 2706 4769 7028 8701 8860 9540 10316 10398 10400 10873 11696 11719 12007 12636 12705 14766 14783 15043 15148 15301 15326 16000T 16223 |
| M45 | 73 146 263 489 750 1438 2706 4734 4769 7028 8701 8860 9540 10398 10400 10873 11719 12007 12705 14766 14783 15043 15301 15326 16189 16223 |
| M45a | 73 143 146 152 263 489 750 1438 4734 4769 7028 7049 8701 8860 9180 9540 10398 10400 10873 11719 12007 12705 14766 14783 15043 15301 15326 16189 16223 16300 |
| M54 | 73 263 489 750 1438 2706 4769 7028 8701 8860 9374 9540 10398 10400 10873 11719 12007 12414 12705 14766 14783 15043 15301 15326 15848 16223 16304 |
| M63 | 73 214 263 489 (709) 750 1438 2706 4769 4991 5046 7028 8701 8860 9540 10398 10400 10873 11437 11719 12007 12705 12897 13089 13194 14766 14783 15043 15301 15326 16172 16192 16223 (16248) |
| M64 | 73 152 263 489 750 1438 2706 4769 5201 7028 8701 8843 8860 9540 9947 10398 10400 10685 10873 11719 12007 12705 13105 14766 14783 15043 15301 15326 15355 15968 16223 16263 16527 |
| M66 | 73 195 263 489 750 1438 1888 2706 4541 4769 6827 7028 8701 8860 9061 9540 10398 10400 10873 11719 12007 12705 14766 14783 15043 15301 15326 16184 16223 |
| M66a | 73 195 263 489 750 1438 1888 2706 4541 4769 6221 6827 7028 8701 8860 9061 9540 10398 10400 10873 11719 12007 12501 12705 14766 14783 15043 15301 15326 16184 16223 |
| M66b | 73 195 198 263 489 750 1438 1888 2706 4541 4769 6827 7028 8701 8860 9061 9540 10398 10400 10873 11719 12007 12705 14766 14783 15043 15301 15326 16184 16223 |
| M5 | 73 263 489 750 1438 1888 2706 4769 7028 8701 8860 9540 10398 10400 10873 11719 12705 14766 14783 15043 15301 15326 16129 16223 |
| M5a'd | 73 263 489 709 750 1438 1888 2706 3921 4769 7028 8701 8860 9540 10398 10400 10873 11719 12705 14323 14766 14783 15043 15301 15326 16129 16223 |
| M5a | 73 263 489 709 750 1438 1888 2706 3921 4769 7028 8701 8860 9540 10398 10400 10873 11719 12477 12705 14323 14766 14783 15043 15301 15326 16129 16223 |

| | |
|---|---|
| M5a1 | 73 263 489 709 750 1438 1888 2706 3921 4769 4916 7028 8701 8860 9540 10398 10400 10873 11719 12477 12705 14323 14766 14783 15043 15287 15301 15326 16129 16223 16291 |
| M5a1a | 73 (185) 263 (334) 489 709 750 1438 1888 2706 3921 4769 4916 7028 8701 8860 9064 9540 10398 10400 10873 11016 11719 12477 12705 14323 14766 14783 15043 15287 15301 15326 16129 16223 16291 |
| M5a1b | 73 263 489 709 750 1303 1438 1888 2706 3921 4769 4916 6461 7028 8701 8860 9540 10398 10400 10873 11719 12477 12705 14323 14766 14783 15043 15287 15301 15326 16129 16223 16291 |
| M5a2 | 73 263 489 709 750 1438 1888 2706 3921 4454 4769 7028 8701 8860 9540 10398 10400 10873 11719 12477 12705 14323 14766 14783 15043 15301 15326 16129 16223 |
| M5a2a | 73 263 489 709 750 1438 1888 2706 3921 4454 4769 7028 8701 8860 9540 10398 10400 10873 11719 12477 12705 14323 14766 14783 15043 15262 15301 15326 16129 16223 |
| M5a2a1 | 73 263 489 709 750 1438 1888 2706 3921 4454 4769 7028 8701 8860 8886 9540 10398 10400 10873 11719 12477 12705 14323 14766 14783 15043 15262 15301 15326 16129 16223 |
| M5a2a1a | 73 263 489 709 750 1438 1888 2706 3921 4454 4769 7028 8701 8860 8886 9540 10398 10400 10873 11719 12477 12705 14323 14766 14783 15043 15262 15301 15326 16129 16223 16265C |
| M5a2a1a1 | 73 263 489 709 750 1438 1888 2706 3921 4454 4769 7028 8701 8860 8886 9540 10398 10400 10873 11719 12372 12477 12705 14323 14766 14783 15043 15262 15301 15326 16129 16223 16265C |
| M5a2a1a2 | 73 263 489 709 750 1438 1888 2706 3921 4454 4769 7028 8701 8860 8886 9540 10398 10400 10873 11719 12477 12705 14323 14766 14783 15043 15262 15301 15326 16129 16223 16264 16265C |
| M5a2a2 | 73 263 489 709 750 1438 1888 2706 3399 3921 4454 4769 7028 8701 8860 9540 10398 10400 10873 11719 12477 12705 14323 14766 14783 15043 15262 15301 15326 16129 16144A 16223 |
| M5a2a3 | 73 263 489 709 750 1438 1888 2706 3921 4454 4769 7028 8701 8860 9540 10398 10400 10873 11719 12477 12705 13590 14323 14766 14783 15043 15262 15301 15326 16114A 16129 16223 |
| M5a2a4 | 73 199 263 489 709 750 1438 1888 2706 3921 4454 4769 4991 7028 8158 8701 8860 9540 10398 10400 10685 10873 11719 12477 12705 14323 14766 14783 15043 15262 15301 15326 16129 16223 |
| M5a3 | 73 194 263 489 709 750 1438 1888 2706 3921 4769 7028 8701 8860 9540 10398 10400 10873 11719 12477 12705 14323 14766 14783 15043 15301 15326 16129 16223 16295 |
| M5a3a | 73 194 263 489 709 750 1438 1888 2706 3921 4769 6378 7028 8547 8701 8860 9540 10398 10400 10873 11719 12477 12705 14323 14766 14783 15043 15301 15326 16129 16223 16295 |
| M5a3b | 73 194 263 489 709 750 1438 1888 2706 3921 4769 7028 7421 8701 8860 9540 10398 10400 10873 11719 12477 12705 14323 14766 14783 15043 15301 15326 16129 16189 16223 16295 |
| M5a4 | 73 146 263 489 709 750 1438 1888 2706 3921 4769 7028 8701 8860 9540 10398 10400 10547 10873 11719 12477 12705 14323 14766 14783 15043 15301 15326 16129 16223 16224 16311 |

| | |
|---|---|
| M5a5 | 73 263 489 709 750 1438 1888 2706 3921 4769 7028 8701 8860 9540 10398 10400 10873 11719 11914 12477 12705 14323 14766 14783 15043 15301 15326 15672 16129 16223 |
| M5d | 73 146 263 489 709 750 1438 1888 2706 3826 3921 4769 7028 8701 8860 9540 10398 10400 10873 11719 12705 13215 14323 14766 14783 15043 15301 15326 16129 16223 16311 |
| M5b'c | 73 263 489 750 1438 1888 2706 4769 7028 8701 8860 9540 10398 10400 10873 11719 12705 13368 14766 14783 15043 15301 15326 16129 16223 |
| M5b | 73 263 489 750 1438 1888 2706 4769 7028 8701 8784T 8860 9540 10398 10400 10873 11719 12705 13368 14766 14783 15043 15301 15326 16129 16223 |
| M5b1 | 73 263 489 750 1438 1888 2706 4769 5147 7028 8701 8784T 8860 9540 10398 10400 10873 11719 12705 13368 14066 14766 14783 15043 15301 15326 16086 16108 16129 16223 16278 |
| M5b2 | 73 263 489 750 1438 1888 2706 3744 4769 7028 8701 8784T 8860 9540 10398 10400 10873 11719 12705 13368 14766 14783 15043 15301 15326 16048 16129 16223 |
| M5b2a | 73 263 489 750 1438 1888 2706 3744 4769 7028 8005 8701 8784T 8860 9540 10398 10400 10873 11719 12705 13368 14766 14783 15043 15301 15326 16048 16129 16223 16390 |
| M5b2b | 73 263 489 750 1438 1888 2706 3744 4769 6293 7028 8701 8784T 8860 9540 10398 10400 10873 11719 12705 13368 14766 14783 15043 15301 15326 16048 16129 16223 |
| M5b2b1 | 73 263 455.1T 460 489 750 1438 1888 2706 3744 4769 6293 7028 8701 8784T 8860 9540 10398 10400 10873 11719 12705 13368 14766 14783 15043 15301 15326 16048 16129 16223 |
| M5b2b1a | 73 263 455.1T 460 489 750 1438 1888 2706 3744 4769 6293 7028 8701 8784T 8860 9540 10166 10398 10400 10873 11719 12279 12705 13368 14410C 14766 14783 15043 15301 15326 16048 16129 16223 |
| M5c | 73 263 489 575 750 1438 1888 2706 4769 5319 7028 8701 8860 9540 10398 10400 10873 11719 12705 13368 14766 14783 15043 15301 15326 16129 16223 |
| M5c1 | 73 150 263 489 575 750 1438 1888 2706 4769 4851 5319 6413 7028 8701 8860 9540 10398 10400 10873 11719 12705 13708 14766 14783 15043 15301 15326 16129 16223 |
| M5c2 | 73 263 472 489 575 750 1438 2706 4769 5319 7028 8701 8860 9540 10398 10400 10873 11719 12705 13368 14766 14783 15043 15301 15326 15896 16129 16223 16240 16291 |
| M6 | 73 263 461 489 750 1438 2706 4769 5301 5558 7028 8701 8860 9540 10398 10400 10640 10873 11719 12705 14128 14766 14783 15043 15301 15326 16223 16362 |
| M6a | 73 263 461 489 750 1438 2706 3537 4769 5082 5301 5558 7028 8701 8860 9540 10398 10400 10640 10873 11719 12705 14128 14766 14783 15043 15301 15326 16223 16231 16362 |
| M6a1 | 73 263 461 489 750 1438 2706 3486 3537 4769 5082 5301 5558 7028 8701 8860 9329 9540 10398 10400 10640 10873 11719 12705 13966 14128 14766 14783 15043 15301 15326 16223 16231 16362 |

| | |
|---|---|
| M6a1a | 73 263 461 489 750 1438 2706 3486 3537 4769 5082 5301 5558 7028 8701 8860 9329 9540 10398 10400 10640 10873 11719 12507 12705 13966 14128 14766 14783 15043 15301 15326 16223 16231 16356 16362 |
| M6a1b | 73 146 152 263 461 489 750 1438 2706 3486 3537 4769 5082 5301 5319 5558 5585 7028 8110 8701 8860 9329 9540 10398 10400 10640 10873 11314 11719 12705 13966 14128 14766 14783 15043 15094 15301 15326 16188 16223 16231 16362 |
| M6a2 | 73 228 263 461 489 750 1438 2706 3537 4769 5082 5301 5558 7028 7118 8701 8860 9540 10398 10400 10640 10873 11719 12705 14128 14766 14783 15043 15301 15326 16223 16231 16291 16319 16362 |
| M6b | 73 263 461 489 750 1438 2706 4769 5301 5558 7028 8281-8289d 8701 8860 9540 10398 10400 10640 10873 11719 12705 14128 14766 14783 15043 15301 15326 16184 16223 16256G 16311 16362 |
| M7 | 73 263 489 750 1438 2706 4769 6455 7028 8701 8860 9540 9824 10398 10400 10873 11719 12705 14766 14783 15043 15301 15326 16223 |
| M7a | 73 263 489 750 1438 2626 2706 2772 4386 4769 4958 6455 7028 8701 8860 9540 9824 10398 10400 10873 11719 12705 12771 14766 14783 15043 15301 15326 16209 16223 |
| M7a+16324 | 73 263 489 750 1438 2626 2706 2772 4386 4769 4958 6455 7028 8701 8860 9540 9824 10398 10400 10873 11719 12705 12771 14766 14783 15043 15301 15326 16209 16223 16324 |
| M7a1 | 73 263 489 750 1438 2626 2706 2772 4386 4769 4958 5899.XC 6455 7028 8701 8860 9540 9824 10398 10400 10873 11719 12705 12771 14364 14766 14783 15043 15301 15326 16209 16223 16324 |
| M7a1a | 73 263 489 750 1438 2626 2706 2772 4386 4769 4958 5899.XC 6455 7028 8701 8860 9540 9824 10398 10400 10873 11017 11084 11719 12705 12771 14364 14766 14783 15043 15301 15326 16209 16223 16324 |
| M7a1a1 | 73 263 489 750 1438 2626 2706 2772 4386 4769 4958 5899.XC 6455 7028 8701 8860 9540 9824 10398 10400 10873 11017 11084 11719 11722 12705 12771 14364 14766 14783 15043 15301 15326 16209 16223 16324 |
| M7a1a1a | 73 263 489 750 1438 2626 2706 2772 4386 4769 4958 5899.XC 6455 7028 8701 8860 9299 9540 9824 10398 10400 10873 11017 11084 11719 11722 12705 12771 14364 14766 14783 15043 15301 15326 16209 16223 16324 |
| M7a1a2 | 73 207 263 489 750 1438 1442 2626 2706 2772 4386 4769 4958 5899.XC 6455 7028 8701 8860 9540 9824 10398 10400 10873 11017 11084 11719 12705 12771 14364 14766 14783 15043 15218 15301 15326 16189 16209 16223 16324 |
| M7a1a3 | 73 263 489 750 1438 2626 2706 2772 4386 4769 4958 5899.XC 6455 7028 8701 8860 9540 9824 10398 10400 10873 11017 11084 11167 11701 11719 12705 12771 14364 14766 14783 15043 15301 15326 16209 16223 16324 |
| M7a1a4 | 73 263 489 750 1438 2626 2706 2772 4047 4386 4769 4958 5899.XC 6455 7028 8701 8860 9540 9824 10398 10400 10873 11017 11084 11719 12705 12771 14185T 14364 14766 14783 15043 15301 15326 16209 16223 16324 |
| M7a1a4a | 73 263 489 750 1438 2626 2706 2772 4047 4386 4769 4958 5899.XC 6455 7028 8701 8860 9540 9824 10398 10400 10873 11017 11084 11368 11719 12705 12771 14148 14185T 14364 14766 14783 15043 15301 15326 15886 16209 16223 16324 |

| | |
|---|---|
| M7a1a5 | 73 263 489 750 1438 2626 2706 2772 4386 4769 4958 5899.XC 6455 7028 8701 8860 9540 9824 10398 10400 10873 11017 11084 11719 12705 12771 13768 14364 14766 14783 15043 15301 15326 16209 16223 16324 |
| M7a1a5a | 73 263 489 750 1438 2626 2706 2772 4386 4769 4958 5899.XC 6455 7028 7852 8701 8860 9540 9824 10398 10400 10873 11017 11084 11719 12705 12771 13768 14364 14766 14783 15043 15301 15326 16209 16223 16324 |
| M7a1a6 | 73 263 489 750 1438 2626 2706 2772 3756 4386 4769 4958 5899.XC 6455 7028 8701 8860 9540 9824 10398 10400 10873 11017 11084 11719 12705 12771 14364 14766 14783 15043 15301 15326 16209 16223 16324 |
| M7a1a6a | 73 152 263 489 750 1438 2626 2706 2772 3756 4386 4769 4958 5899.XC 6455 7028 8701 8860 9540 9824 10398 10400 10873 11017 11084 11719 12705 12771 14364 14766 14783 15043 15301 15326 16209 16324 |
| M7a1a7 | 73 263 489 750 1438 2626 2706 2772 4386 4769 4958 5899.XC 6455 7028 8701 8860 9540 9824 10398 10400 10873 11017 11084 11719 12705 12771 14364 14766 14783 15043 15301 15326 16209 16223 16291 16324 |
| M7a1a8 | 73 263 489 750 1438 2626 2706 2772 4386 4769 4958 5899.XC 6455 7028 8701 8860 9540 9824 10398 10400 10873 11017 11084 11719 12705 12771 13500 14364 14766 14783 15043 15301 15326 16209 16223 16233 16324 |
| M7a1a9 | 73 263 489 750 1438 2626 2706 2772 4048 4386 4769 4958 5899.XC 6455 7028 8701 8860 9540 9824 10398 10400 10873 11017 11084 11719 12705 12771 14364 14766 14783 15043 15301 15326 16209 16223 16324 |
| M7a1b | 73 263 489 750 1438 2626 2706 2772 4386 4769 4958 5899.XC 6018 6455 7028 7403 8701 8860 9540 9824 10398 10400 10873 11719 12705 12771 13110 14364 14766 14783 15043 15301 15326 16209 16223 16324 |
| M7a1b1 | 73 204 207 263 408A 489 750 1438 1977 2626 2706 2772 4386 4769 4958 5899.XC 6018 6455 7028 7403 8701 8860 9540 9824 10398 10400 10873 11719 12705 12771 13110 14364 14766 14783 15043 15301 15326 16209 16223 16324 |
| M7a1b2 | 73 152 263 489 750 1438 2626 2706 2772 3316 4386 4769 4958 5899.XC 6018 6455 7028 7403 7784 8701 8860 9540 9824 10398 10400 10873 11719 12705 12771 13032 13110 13788 14364 14766 14783 15043 15301 15326 15844 16209 16223 16324 |
| M7a2 | 73 263 489 750 1438 2626 2706 2772 4386 4769 4958 6455 7028 8701 8860 9540 9824 10398 10400 10873 11719 12705 12771 14766 14783 15043 15301 15326 15422 16209 16223 |
| M7a2a | 73 146 263 489 750 1438 2626 2706 2772 4386 4769 4958 6455 7028 8701 8860 9540 9824 10398 10400 10873 11719 12705 12771 14766 14783 15043 15301 15326 15422 16140 16209 16223 |
| M7a2a1 | 73 146 263 489 750 1438 2626 2706 2772 4386 4769 4958 6455 7028 8176 8701 8860 9540 9824 10398 10400 10873 11719 12234 12705 12771 14766 14783 15043 15301 15326 15422 16093 16140 16209 16223 |
| M7a2a2 | 73 146 263 489 750 961 965.XC 1438 2626 2706 2772 4386 4769 4958 6455 7028 8005 8701 8860 9540 9824 10398 10400 10873 11719 12705 12771 14766 14783 15043 15301 15326 15422 16140 16209 16223 |
| M7a2a3 | 73 146 263 489 750 1438 1888 2626 2706 2772 4093 4386 4769 4958 6455 7028 8701 8860 9540 9824 10398 10400 10873 10966 11719 12705 12771 14766 14783 15043 15301 15326 15422 16140 16187 16209 16223 |

| | |
|---|---|
| M7a2a3a | 73 146 263 489 750 1438 1888 2626 2706 2772 4093 4386 4769 4958 6455 7028 8701 8860 9540 9824 10398 10400 10873 10966 11719 12705 12771 14766 14783 15043 15301 15326 15422 15449 16140 16187 16209 16223 |
| M7b'c | 73 263 489 750 1438 2706 4071 4769 6455 7028 8701 8860 9540 9824 10398 10400 10873 11719 12705 14766 14783 15043 15301 15326 16223 |
| M7b | 73 263 489 750 1438 2706 4071 4769 6455 7028 8701 8860 9540 9824 10398 10400 10873 11719 12405 12705 14766 14783 15043 15301 15326 16223 |
| M7b1 | 73 263 489 750 1438 2706 4071 4769 5351 5460 6455 7028 7684 7853 8701 8860 9540 9824 10398 10400 10873 11719 12405 12705 14766 14783 15043 15301 15326 16129 16223 |
| M7b1a | 73 199 263 489 750 1438 2706 4048 4071 4164 4769 5351 5460 6455 6680 7028 7684 7853 8701 8860 9540 9824 10398 10400 10873 11719 12405 12705 14766 14783 15043 15301 15326 16129 16223 16297 |
| M7b1a1 | 73 150 199 263 489 750 1438 2706 4048 4071 4164 4769 5351 5460 6455 6680 7028 7684 7853 8701 8860 9540 9824 10398 10400 10873 11719 12405 12705 12811 14766 14783 15043 15301 15326 16129 16223 16297 |
| M7b1a1a | 73 150 199 263 489 750 1438 2706 4048 4071 4164 4769 5351 5460 6455 6680 7028 7684 7853 8701 8860 9540 9824 10398 10400 10873 11719 12405 12705 12811 14766 14783 15043 15301 15326 16129 16189 16223 16297 |
| M7b1a1a1 | 73 150 199 263 489 750 1438 2706 4048 4071 4164 4769 5351 5460 6455 6680 7028 7684 7853 8701 8860 9540 9824 10345 10398 10400 10873 11719 12405 12705 12811 14766 14783 15043 15301 15326 16129 16189 16223 16297 16298 |
| M7b1a1a1a | 73 150 199 263 489 750 1438 2706 4048 4071 4164 4769 5351 5460 6455 6680 7028 7684 7853 8020 8701 8860 9540 9824 10345 10398 10400 10873 11719 12405 12705 12811 14766 14783 15043 15301 15326 16129 16189 16223 16297 16298 |
| M7b1a1a1b | 73 150 199 263 489 750 1438 2706 4048 4071 4113 4164 4769 5351 5460 6455 6680 7028 7684 7853 8701 8860 9540 9824 10345 10398 10400 10873 11719 12405 12705 12811 14766 14783 15043 15301 15326 16129 16189 16223 16297 16298 |
| M7b1a1a1b1 | 73 150 199 263 489 750 1438 2706 4048 4071 4113 4164 4769 5351 5460 6455 6680 7028 7269 7684 7853 8701 8860 9540 9824 10345 10398 10400 10873 11719 12405 12705 12811 14766 14783 15043 15301 15326 16129 16189 16223 16297 16298 |
| M7b1a1a1c | 73 150 195A 199 263 489 750 1438 2706 4048 4071 4164 4769 5351 5460 6455 6680 7028 7684 7853 8701 8860 9540 9824 10345 10398 10400 10873 11719 12405 12705 12811 14766 14783 15043 15301 15326 16129 16189 16223 16297 16298 |
| M7b1a1a1d | 73 150 199 263 489 750 1438 2706 4048 4071 4164 4769 5351 5460 6455 6680 7028 7684 7853 7961 8701 8860 9540 9824 10345 10398 10400 10873 11719 12405 12705 12811 14766 14783 15043 15301 15326 16129 16189 16223 16297 16298 |

| | |
|---|---|
| M7b1a1a2 | 73 150 199 263 489 750 1438 1694 2706 4048 4071 4164 4769 5351 5460 6455 6680 7028 7684 7853 8701 8860 9540 9824 10398 10400 10873 11659 11719 12405 12705 12811 14766 14783 15043 15301 15326 16129 16189 16223 16297 |
| M7b1a1a3 | 73 150 199 204 263 (456) 489 750 1438 2706 4048 4071 4164 4769 5351 5460 6455 6680 7028 7684 7853 8701 8860 9540 9824 10398 10400 10873 11719 12405 12705 12811 14766 14783 14978 15043 15301 15326 16129 16189 16223 16297 |
| M7b1a1b | 73 150 199 204 263 489 750 1438 2706 3483 4048 4071 4164 4769 5351 5460 6455 6680 7028 7684 7853 8701 8860 9540 9824 10398 10400 10873 11719 12405 12705 12811 14766 14783 15043 15301 15326 16223 16297 |
| M7b1a1+(16192) | 73 150 199 263 489 750 1438 2706 4048 4071 4164 4769 5351 5460 6455 6680 7028 7684 7853 8701 8860 9540 9824 10398 10400 10873 11719 12405 12705 12811 14766 14783 15043 15301 15326 16129 (16192) 16223 16297 |
| M7b1a1c | 73 150 159 199 263 489 750 1438 2706 4048 4071 4164 4769 5351 5460 6455 6680 7028 7684 7853 8701 8860 9540 9824 10159 10398 10400 10873 11719 11944 12405 12705 12811 14766 14783 15043 15301 15326 16092 16129 (16192) 16223 16297 |
| M7b1a1c1 | 73 150 159 182 199 263 489 750 1438 2706 4048 4071 4164 4769 5351 5460 6455 6680 7028 7684 7853 8701 8860 9540 9824 10159 10398 10400 10873 11719 11944 12405 12705 12811 14766 14783 15043 15301 15326 16092 16129 (16192) 16223 16254 16297 |
| M7b1a1d | 73 150 199 263 489 750 1438 2706 4048 4071 4164 4769 5351 5460 6125 6455 6680 7028 7684 7853 8701 8860 9540 9824 10398 10400 10873 11719 12405 12705 12811 14766 14783 15043 15301 15326 16129 (16192) 16223 16297 |
| M7b1a1d1 | 73 150 199 263 489 750 980 1438 2706 4048 4071 4164 4769 5351 5460 6125 6455 6680 7028 7684 7853 8701 8860 9540 9824 10398 10400 10873 11719 12405 12705 12811 14766 14783 15043 15301 15326 16129 (16192) 16223 16297 |
| M7b1a1e | 73 150 199 263 489 750 1438 2706 4048 4071 4164 4769 5351 5460 6228 6455 6680 7028 7684 7853 8701 8860 9540 9824 10398 10400 10873 11719 12405 12705 12811 14766 14783 15043 15301 15326 16129 (16192) 16223 16297 |
| M7b1a1e1 | 73 150 199 263 489 750 1438 2706 4048 4071 4164 4769 5351 5460 6228 6455 6680 7028 7684 7853 8701 8860 9540 9824 10232 10398 10400 10873 11719 12405 12705 12811 14766 14783 15043 15301 15326 16129 (16192) 16223 16297 |
| M7b1a1e2 | 73 150 199 263 489 750 1438 2706 4048 4071 4164 4769 5351 5460 6228 6455 6680 7028 7684 7853 8701 8860 9540 9824 10398 10400 10873 11407 11719 12405 12705 12811 14766 14783 15043 15301 15326 16129 (16192) 16223 16297 16527 |
| M7b1a1f | 73 150 199 263 489 750 1438 2706 4048 4071 4164 4769 5351 5460 6455 6680 7028 7684 7853 8110 8701 8860 9540 9824 10398 10400 10873 11719 12405 12705 12811 14766 14783 15043 15301 15326 16129 (16192) 16223 16297 |

| | |
|---|---|
| M7b1a1g | 73 150 199 263 489 750 1438 2706 4048 4071 4164 4769 5252 5351 5460 6455 6680 7028 7598 7684 7853 8701 8860 9540 9824 10398 10400 10873 11719 12405 12705 12811 14766 14783 15043 15301 15326 16129 (16192) 16223 16297 |
| M7b1a1h | 73 150 199 263 489 750 1438 2706 4048 4071 4164 4769 5351 5460 6455 6680 7028 7684 7853 8701 8860 9540 9824 10397 10398 10400 10873 11719 12405 12705 12811 14766 14783 15043 15301 15326 16129 (16192) 16223 16297 |
| M7b1a1i | 73 150 199 263 489 750 1438 2706 4048 4071 4164 4769 5351 5460 6455 6680 7028 7684 7853 8701 8860 9540 9824 10398 10400 10873 11719 12405 12705 12811 14766 14783 15043 15301 15326 16126 16129 (16192) 16223 16297 |
| M7b1a1i1 | 73 146 150 199 263 489 750 1438 2706 3995 4048 4071 4164 4769 5351 5460 6455 6680 7028 7684 7853 8701 8860 9540 9824 10398 10400 10873 11719 12405 12705 12811 14766 14783 15043 15301 15326 16126 16129 (16192) 16223 16297 |
| M7b1a2 | 73 199 263 489 750 1438 1664 2706 4048 4071 4164 4769 5351 5460 6351 6455 6680 7028 7684 7853 8701 8860 9468 9540 9824 10398 10400 10497 10873 11719 12121 12405 12705 14115 14766 14783 15043 15301 15326 16086 16129 16297 |
| M7b1a2a | 73 199 263 489 750 1438 1664 2706 4048 4071 4164 4769 5351 5460 6351 6455 6680 7028 7684 7853 8701 8860 9468 9540 9824 10398 10400 10497 10873 11719 12121 12405 12705 14115 14766 14783 15043 15301 15326 16086 16129 16297 16324 |
| M7b1a2a1 | 73 199 263 489 750 1438 1664 2706 4048 4071 4164 4454 4769 5351 5460 6351 6455 6680 7028 7684 7853 8701 8860 9468 9540 9824 10398 10400 10497 10873 11719 12121 12405 12705 14115 14766 14783 15043 15301 15326 16086 16129 16297 16324 |
| M7b1a2a1a | 73 199 263 489 709 750 1438 1664 2706 4048 4071 4164 4454 4769 5351 5460 6351 6455 6680 7028 7175 7684 7853 8701 8860 9468 9540 9824 10398 10400 10497 10873 11719 12121 12405 12705 14115 14766 14783 15043 15301 15326 15912 16086 16129 16297 16324 |
| M7b1a2a1b | 73 199 263 489 750 1438 1664 2706 4048 4071 4164 4454 4769 5351 5460 6351 6455 6680 7028 7684 7853 8701 8860 9468 9540 9824 10398 10400 10497 10873 11719 12121 12405 12705 13965 14115 14766 14783 15043 15301 15326 16086 16129 16297 16324 |
| M7b1a2a1b1 | 73 199 263 471 489 750 1438 1664 2706 4048 4071 4164 4454 4769 5351 5460 6351 6455 6680 7028 7684 7853 8701 8860 9468 9540 9824 10398 10400 10497 10873 11719 12121 12405 12705 13965 14115 14766 14783 15043 15301 15326 16086 16129 16297 16324 |
| M7b1b | 73 263 489 750 958 1438 2706 4071 4769 5351 5460 6455 7028 7684 7853 8701 8860 9540 9824 10398 10400 10873 11719 12358 12405 12705 14053 14314 14766 14783 15043 15301 15326 16129 16152 16179 16192 16223 16362 |
| M7b2 | 73 263 489 723 750 1438 2706 3140 4071 4769 5324 6455 7028 8027 8701 8860 9540 9824 10398 10400 10873 11719 12405 12705 14284 14766 14783 15043 15301 15326 16184 16223 |

| | |
|---|---|
| M7b2a | 73 263 489 723 750 1438 2706 3140 4071 4769 5324 6455 7028 8027 8701 8860 9540 9824 10398 10400 10873 11719 12405 12705 14284 14766 14783 15043 15301 15326 16086 16184 16223 16235 |
| M7c | 73 146 263 489 750 1438 2706 4071 4769 4850 6455 7028 8701 8860 9540 9824 10398 10400 10873 11665 11719 12091 12705 14766 14783 15043 15301 15326 16223 |
| M7c1 | 73 146 199 263 489 750 1438 2706 4071 4769 4850 5442 6455 7028 8701 8860 9540 9824 10398 10400 10873 11665 11719 12091 12705 14766 14783 15043 15301 15326 16223 (16295) |
| M7c1a | 73 146 199 263 489 750 1438 2706 3882 4071 4769 4850 5442 6455 7028 8701 8860 9540 9824 10398 10400 10873 11665 11719 12091 12705 14766 14783 15043 15301 15326 16223 (16295) |
| M7c1a1 | 73 146 199 263 489 750 1438 2706 3882 4071 4769 4850 5442 6455 7028 8701 8860 9540 9824 10398 10400 10873 11665 11719 12091 12372 12705 13759 14766 14783 15043 15301 15326 16223 (16295) |
| M7c1a1a | 73 146 199 263 489 750 1438 2706 3882 4071 4769 4850 5442 6455 7028 8701 8860 9540 9824 10398 10400 10873 11665 11719 12091 12372 12705 12810 13759 14766 14783 15043 15301 15326 16223 (16295) |
| M7c1a1a1 | 73 146 199 263 489 750 1438 2706 3882 4071 4769 4850 5442 6455 7028 8701 8860 9266 9540 9824 10398 10400 10873 11665 11719 12091 12372 12705 12810 13759 14766 14783 15043 15301 15326 16223 (16295) |
| M7c1a1b | 73 199 263 489 750 1438 2706 3736 3882 4071 4769 4850 5442 6455 7028 8701 8860 9540 9824 10398 10400 10586 10873 11665 11719 12091 12372 12705 13759 14766 14783 15043 15301 15326 16223 (16295) 16304 |
| M7c1a1b1 | 73 199 263 489 750 1438 2706 3736 3882 4071 4769 4850 5442 6455 7028 8701 8860 9540 9824 10398 10400 10586 10873 11665 11719 12091 12372 12705 13759 14766 14783 15043 15301 15326 15769 16145 16223 (16295) 16304 |
| M7c1a2 | 73 146 199 263 489 750 1438 2706 3882 4071 4769 4850 5442 6455 7028 8701 8860 9540 9824 10398 10400 10873 11665 11719 12091 12705 14766 14783 15043 15301 15326 16223 16294 (16295) |
| M7c1a2a | 73 146 199 263 489 750 1438 2706 3882 4071 4769 4850 5442 6053 6455 7028 8701 8860 9540 9824 10398 10400 10873 11665 11719 12091 12705 12804 14755T 14766 14783 15043 15301 15326 16223 16294 (16295) |
| M7c1a2a1 | 73 146 199 263 489 750 1438 2706 3882 4071 4769 4850 5442 6053 6455 7028 7961 8701 8860 9540 9824 10398 10400 10873 11665 11719 12091 12705 12804 14755T 14766 14783 15043 15301 15326 16223 16294 |
| M7c1a3 | 73 146 199 263 489 750 1438 2706 3882 4071 4769 4850 5442 6455 7028 8701 8860 9540 9824 10398 10400 10873 11665 11719 12091 12705 14766 14783 15043 15301 15326 16223 (16295) 16319 |
| M7c1a3a | 73 146 199 263 489 513 750 1438 2706 3882 4071 4769 4850 5442 6455 7028 8701 8860 9540 9824 10398 10400 10873 11665 11719 12091 12705 14766 14783 15043 15301 15326 16223 (16295) 16319 |
| M7c1a4 | 73 146 199 263 489 750 1438 2706 3882 4071 4769 4850 5442 6455 7028 8701 8860 9540 9824 10398 10400 10873 11665 11719 12091 12705 12906A 14766 14783 15043 15301 15326 16223 (16295) |

| | |
|---|---|
| M7c1a4a | 73 146 199 263 489 750 1438 2706 3882 4071 4769 4850 5442 6455 7028 8701 8860 9540 9797 9824 10398 10400 10873 11665 11719 11815A 12091 12705 12906A 14766 14783 15043 15301 15326 16223 (16295) |
| M7c1a4b | 73 146 199 263 334 489 750 1438 2706 3882 4071 4769 4850 5442 5894 6455 7028 8701 8860 9540 9824 10398 10400 10873 11665 11719 12091 12705 12906A 14766 14783 15043 15301 15326 16223 (16295) |
| M7c1a5 | 73 146 199 263 373 489 750 1438 2706 3882 4071 4452 4769 4850 5442 6455 7028 7960 8701 8860 9540 9824 10398 10400 10873 11389 11665 11719 12091 12705 14766 14783 15043 15301 15326 15787 15924 16223 (16295) |
| M7c1b | 73 146 199 263 489 750 1438 2706 4071 4769 4850 5442 6455 7028 8701 8860 9540 9824 10398 10400 10873 11665 11719 12091 12561 12705 14766 14783 15043 15301 15326 16223 (16295) |
| M7c1b1 | 73 146 199 263 489 750 1438 2706 4071 4769 4850 5442 6455 7028 8701 8860 9540 9824 10398 10400 10873 11665 11719 12091 12561 12705 14766 14783 15043 15301 15326 15884 16223 (16295) |
| M7c1b2 | 73 146 199 263 489 750 1438 2706 4071 4769 4850 5442 6455 7028 8701 8860 9540 9824 9957 10398 10400 10873 11665 11719 12091 12561 12705 13590 14766 14783 15043 15301 15326 16223 (16295) |
| M7c1b2a | 73 146 199 263 489 750 1438 2706 4071 4769 4850 5442 6455 7028 8701 8860 9540 9824 9957 10398 10400 10873 11665 11719 12091 12561 12705 13590 14766 14783 15043 15301 15326 16172 16223 (16295) |
| M7c1b2b | 73 146 199 263 489 750 1438 2706 4071 4769 4850 5442 6455 7028 7337 8701 8860 9540 9824 9957 10398 10400 10861 10873 11665 11719 12091 12561 12705 13590 14766 14783 15043 15301 15326 16223 (16295) |
| M7c1c | 73 146 199 263 489 750 1438 2706 3606 4071 4769 4850 5442 6455 7028 8701 8860 9540 9824 10398 10400 10873 11665 11719 12091 12705 14766 14783 15043 15236 15301 15326 16223 (16295) |
| M7c1c1 | 73 146 199 263 489 750 1438 2706 3606 4071 4769 4850 5442 6455 7028 8701 8860 9540 9824 10398 10400 10873 11665 11719 12091 12705 13204 14766 14783 15043 15236 15301 15326 16223 16249 (16295) 16319 |
| M7c1c1a | 73 146 199 263 489 750 1438 2706 3606 4071 4769 4850 5442 6455 7028 8701 8860 9540 9824 10398 10400 10873 11665 11719 12091 12705 13204 14766 14783 15043 15236 15301 15326 16223 16242 16249 (16295) 16319 |
| M7c1c1a1 | 73 146 199 263 489 750 1438 2706 3606 4071 4769 4850 5442 6455 7028 8701 8860 9540 9824 10398 10400 10873 11665 11719 12049 12091 12705 13204 14766 14783 15043 15236 15301 15326 16223 16242 16249 (16295) 16319 |
| M7c1c2 | 73 146A 199 263 489 750 1438 2706 3606 4071 4769 4850 5442 6455 7028 8701 8860 9540 9824 10398 10400 10873 11665 11719 12091 12705 14766 14783 15043 15236 15301 15326 (16295) |
| M7c1c2a | 73 146A 199 263 489 750 1438 2706 3606 4071 4769 4850 5442 6455 7028 8701 8860 9540 9824 10398 10400 10873 11665 11719 12091 12705 14766 14783 15043 15236 15301 15326 16147 (16295) |
| M7c1c3 | 73 146 199 263 489 750 1438 2706 3606 4071 4769 4850 5442 6455 7028 8701 8860 9540 9824 10398 10400 10873 11665 11719 12091 12705 14766 14783 15043 15236 15301 15326 16223 (16295) 16362 |

| | |
|---|---|
| M7c1c3a | 73 146 199 263 489 750 1438 2706 3606 4071 4769 4850 5442 5775 6455 7028 8701 8860 9540 9824 10398 10400 10873 11665 11719 12091 12705 14766 14783 15043 15236 15301 15326 16168 16223 (16295) 16362 |
| M7c1c3a1 | 73 146 199 263 489 750 1438 2706 2887 3606 4071 4769 4850 5442 5775 6455 7028 8701 8860 9540 9824 10398 10400 10873 11665 11719 11893 12091 12705 14766 14783 15043 15236 15301 15326 16168 16223 (16295) 16362 |
| M7c1c3b | 73 146 199 263 489 630 750 1438 2706 3606 4071 4769 4850 5442 6455 7028 8701 8860 9540 9824 10398 10400 10873 11665 11719 12091 12705 14766 14783 15043 15236 15301 15326 16223 (16295) 16362 |
| M7c1c3c | 73 146 199 263 489 750 1438 2706 3606 4071 4769 4850 5442 6260 6455 6681 7028 8701 8860 9540 9824 10398 10400 10873 11665 11719 11908 12091 12705 13563 14766 14783 15043 15236 15301 15326 16223 (16295) 16362 |
| M7c1c3d | 73 146 199 263 489 750 1438 2706 3606 3915 4071 4769 4850 5442 6455 7028 7853 8701 8860 9540 9824 10398 10400 10873 11665 11719 12091 12705 13768 14766 14783 15043 15236 15301 15326 16223 (16295) 16362 |
| M7c1c3e | 73 146 199 263 489 750 1438 2706 3606 4071 4769 4850 5442 6455 7028 8701 8860 9540 9824 10398 10400 10873 11665 11719 12091 12705 14766 14783 15043 15236 15301 15326 16086 16223 (16295) 16362 |
| M7c1c3f | 73 146 199 263 489 750 1438 2706 3606 4071 4769 4850 5442 6455 7028 8023 8701 8860 9540 9824 10398 10400 10873 11665 11719 12091 12705 14766 14783 15043 15236 15301 15326 16223 (16295) 16362 16392 |
| M7c1c3g | 73 146 199 263 489 750 1438 2706 3606 4071 4769 4850 5442 6455 7028 8701 8856 8860 9540 9824 10398 10400 10873 11665 11719 12091 12705 14766 14783 15043 15236 15301 15326 16223 (16295) 16362 |
| M7c1c3h | 73 146 199 263 489 750 1438 2706 3606 4071 4769 4850 5442 6455 7028 8701 8860 9540 9824 10398 10400 10873 11665 11719 12091 12705 14766 14783 15043 15236 15301 15326 16223 (16295) 16324 16362 |
| M7c1c3i | 73 146 199 263 455.1T 489 750 1438 2706 3606 4071 4769 4850 5442 6455 7028 8701 8860 9540 9824 10398 10400 10873 11665 11719 12091 12705 14766 14783 15043 15236 15301 15326 16223 (16295) 16362 |
| M7c2 | 73 146 263 489 750 1438 2706 3912 4071 4769 4850 5378 6455 7028 8701 8860 9449 9540 9824 10398 10400 10873 10897 11665 11719 11932 12091 12705 14053 14766 14783 15043 15301 15326 15338 16172 16223 16311 |
| M7c2a | 73 146 263 489 750 1438 2706 3912 4071 4769 4850 5378 6455 7028 8701 8860 9449 9540 9824 10398 10400 10873 10897 11665 11719 11932 12091 12705 14053 14766 14783 15043 15301 15326 15338 16172 16223 16291 16311 |
| M7c2b | 73 146 263 489 750 1438 2706 3912 4071 4769 4850 5378 6455 7028 8701 8860 9449 9540 9824 10398 10400 10873 10897 11084 11665 11719 11932 12091 12705 14053 14766 14783 15043 15301 15326 15338 16172 16223 16311 |
| M7c3 | 73 146 263 489 750 1438 2706 4071 4769 4850 4856 5460 6455 7028 8701 8860 9540 9824 10398 10400 10873 11527 11665 11719 11875 12091 12705 12950 14118 14766 14783 15043 15301 15326 16223 |

| | |
|---|---|
| M8 | 73 263 489 750 1438 2706 4715 4769 7028 7196A 8584 8701 8860 9540 10398 10400 10873 11719 12705 14766 14783 15043 15301 15326 15487T 16223 16298 |
| M8a | 73 263 489 750 1438 2706 4715 4769 6179 7028 7196A 8584 8684 8701 8860 9540 10398 10400 10873 11719 12705 14470 14766 14783 15043 15301 15326 15487T 16223 16298 16319 |
| M8a1 | 73 263 489 750 1438 2706 4715 4769 6179 7028 7196A 8453 8584 8684 8701 8860 9540 10398 10400 10873 11719 12705 14470 14766 14783 15043 15047 15148 15301 15326 15355 15487T 15697 (16086) 16223 |
| M8a1a | 73 263 489 750 1438 2706 4715 4769 6179 6719 7028 7196A 8453 8584 8684 8701 8860 9540 10398 10400 10873 11719 12705 14470 14766 14783 15043 15047 15148 15301 15326 15355 15487T 15697 (16086) 16223 16298 16311 16319 |
| M8a2'3 | 73 263 489 750 1438 2706 4715 4769 6179 7028 7196A 8584 8684 8701 8860 9540 10398 10400 10873 11719 12705 14470 14766 14783 15043 15301 15326 15487T 16184 16223 16298 16319 |
| M8a2 | 73 263 489 750 1438 2706 2835 4715 4769 6179 7028 7196A 8584 8684 8701 8860 9540 10398 10400 10873 11719 12705 14470 14766 14783 15043 15301 15326 15487T 16184 16223 16298 16319 |
| M8a2+152 | 73 152 263 489 750 1438 2706 2835 4715 4769 6179 7028 7196A 8584 8684 8701 8860 9540 10398 10400 10873 11719 12705 14470 14766 14783 15043 15301 15326 15487T 16184 16223 16298 16319 |
| M8a2a | 73 152 263 489 750 1438 2706 2835 4715 4769 6179 7028 7196A 8584 8684 8701 8860 9540 10398 10400 10873 11176 11719 12705 14470 14766 14783 15043 15301 15326 15487T 16184 16189 16223 16298 16319 |
| M8a2a1 | 73 152 263 489 750 1438 2706 2835 4715 4769 6179 7028 7196A 8584 8684 8701 8860 9540 10398 10400 10873 11176 11719 12705 14470 14766 14783 15043 15301 15326 15487T 16184 16189 16223 16298 16319 16470 |
| M8a2b | 73 152 263 489 750 1438 2706 2835 4670 4715 4769 6179 6671 7028 7196A 8584 8684 8701 8860 9540 10398 10400 10873 11719 12705 13050 14470 14766 14783 15043 15301 15326 15487T 16184 16223 16298 16319 |
| M8a2c | 73 263 489 750 1438 2706 2835 4715 4769 6179 7028 7196A 8584 8684 8701 8860 9540 10398 10400 10873 11719 12705 14470 14766 14783 15043 15244 15301 15326 15487T 16184 16223 16298 16319 |
| M8a2d | 73 234 263 489 750 1438 2706 2835 4715 4769 6179 7028 7196A 8584 8684 8701 8860 9540 9861 10398 10400 10646 10873 11719 12375 12705 13993 14470 14766 14783 15043 15301 15326 15487T 16184 16223 16298 |
| M8a2e | 73 263 489 750 1438 2706 2835 4715 4769 6179 7028 7196A 8584 8684 8701 8860 9540 10398 10400 10873 11719 12705 14470 14766 14783 15043 15217 15301 15326 15487T 16184 16223 16298 16319 |
| M8a3 | 73 263 489 750 1438 2706 4715 4769 5100 6179 7028 7196A 8584 8684 8701 8860 9540 10398 10400 10873 11719 12705 14470 14766 14783 15043 15301 15326 15487T 16184 16223 16298 16319 |
| M8a3a | 73 263 489 750 1438 2706 4715 4769 5100 6179 7028 7196A 8584 8684 8701 8860 9540 10398 10400 10873 11719 12705 14470 14766 14783 15043 15301 15326 15487T 16134 16184 16223 16298 16319 |

| | |
|---|---|
| M8a3a1 | 73 263 489 750 1438 2706 4715 4769 5100 6179 7028 7196A 8584 8684 8701 8860 9540 9758 10398 10400 10873 11719 12705 14470 14766 14783 15043 15301 15326 15487T 16134 16184 16223 16298 16319 |
| CZ | 73 249d 263 489 750 1438 2706 4715 4769 7028 7196A 8584 8701 8860 9540 10398 10400 10873 11719 12705 14766 14783 15043 15301 15326 15487T 16223 16298 |
| C | 73 249d 263 489 750 1438 2706 3552A 4715 4769 7028 7196A 8584 8701 8860 9540 9545 10398 10400 10873 11719 11914 12705 13263 14318 14766 14783 15043 15301 15326 15487T 16223 16298 16327 |
| C1 | 73 249d 263 290-291d 489 750 1438 2706 3552A 4715 4769 7028 7196A 8584 8701 8860 9540 9545 10398 10400 10873 11719 11914 12705 13263 14318 14766 14783 15043 15301 15326 15487T 16223 16298 16325 16327 |
| C1a | 73 249d 263 290-291d 489 750 1438 2706 3552A 3826 4715 4769 7028 7196A 7598 8584 8701 8860 9540 9545 10398 10400 10873 11719 11914 12705 13263 14318 14766 14783 15043 15301 15326 15487T 16223 16298 16325 16327 16356 |
| C1b | 73 249d 263 290-291d 489 493 750 1438 2706 3552A 4715 4769 7028 7196A 8584 8701 8860 9540 9545 10398 10400 10873 11719 11914 12705 13263 14318 14766 14783 15043 15301 15326 15487T 16223 16298 16325 16327 |
| C1b1 | 73 249d 263 290-291d 489 493 750 1438 2706 3552A 4715 4769 7028 7196A 8584 8701 8860 9540 9545 10398 10400 10873 11147 11719 11914 12705 13263 14318 14766 14783 15043 15301 15326 15487T 16223 16298 16325 16327 |
| C1b2 | 73 249d 290-291d 489 493 750 1438 2706 3552A 4242 4715 4769 7013 7028 7196A 8584 8701 8860 9540 9545 9557 10398 10400 10873 11719 11914 12454 12705 13263 14318 14766 14783 15043 15301 15326 15487T 16223 16298 16325 16327 |
| C1b3 | 73 249d 263 290-291d 489 493 750 1438 2706 3552A 4715 4769 7028 7196A 8584 8701 8860 9540 9545 10398 10400 10873 11719 11914 12630 12705 13263 14318 14766 14783 15043 15301 15326 15487T 16223 16298 16325 16327 |
| C1b4 | 73 143 249d 263 290-291d 489 493 750 2706 3552A 4167 4715 4769 7028 7196A 8584 8701 8860 9540 9545 10398 10400 10873 11719 11914 12705 13263 14318 14524 14766 14783 15043 15301 15326 15487T 16086 16189 16223 16278 16298 16325 16327 |
| C1b5 | 73 249d 263 290-291d 489 493 750 1438 2706 3552A 4715 4769 7028 7196A 8584 8701 8860 9540 9545 10398 10400 10873 11719 11914 11989 12705 13263 14318 14766 14783 15043 15119 15301 15326 15487T 16223 16298 16325 16327 |
| C1b5a | 73 249d 263 290-291d 489 493 750 1005 1438 2706 3552A 4715 4769 7028 7196A 8584 8701 8860 9540 9545 10398 10400 10873 11719 11914 11989 12705 13263 14318 14766 14783 15043 15119 15301 15326 15487T 16223 16298 16325 16327 |
| C1b5b | 73 249d 263 290-291d 489 493 750 1438 2706 3552A 4715 4769 5157 7028 7196A 8584 8701 8860 9540 9545 10398 10400 10873 11719 11914 11989 12705 13263 14318 14766 14783 15043 15119 15301 15326 15487T 16223 16249 16298 16325 16327 |

| | |
|---|---|
| C1b6 | 73 249d 263 290-291d 489 493 750 1438 2706 3552A 4715 4769 7028 7196A 8584 8701 8848 8860 9540 9545 10398 10400 10873 11719 11914 12705 13263 13326 14318 14766 14783 15043 15301 15326 15487T 16223 16271 16298 16325 16327 16357 |
| C1b+16311 | 73 249d 263 290-291d 489 493 750 1438 2706 3552A 4715 4769 7028 7196A 8584 8701 8860 9540 9545 10398 10400 10873 11719 11914 12705 13263 14318 14766 14783 15043 15301 15326 15487T 16223 16298 16311 16325 16327 |
| C1b7 | 73 249d 263 290-291d 489 493 750 1438 2706 3552A 4715 4769 7028 7196A 8584 8701 8860 9540 9545 10398 10400 10873 11719 11914 12705 13263 13635 14318 14766 14783 15043 15301 15326 15487T 16223 16298 16311 16325 16327 |
| C1b7a | 73 249d 263 290-291d 489 493 750 1438 2706 3552A 4715 4769 7028 7196A 8584 8701 8860 9540 9545 10398 10400 10873 11719 11914 12705 13263 13635 14318 14766 14783 15043 15301 15326 15470 15487T 16223 16298 16311 16325 16327 |
| C1b10 | 73 146 249d 263 290-291d 385 489 493 750 1438 2706 3552A 4715 4769 6284 7028 7196A 8584 8701 8860 9540 9545 10398 10400 10873 11719 11914 12705 13263 14318 14766 14783 15043 15301 15326 15487T 16129 16172 16223 16298 16311 16325 16327 |
| C1b8 | 73 249d 263 290-291d 489 493 750 1438 2706 3552A 4715 4769 7028 7196A 8584 8701 8860 9540 9545 10398 10400 10873 11719 11914 12705 13263 14318 14766 14783 15043 15301 15326 15487T 15613 16223 16298 16325 16327 16362 |
| C1b8a | 73 249d 263 290-291d 489 493 750 1438 2706 3552A 3736 4381 4715 4769 4911G 7028 7196A 8584 8701 8860 9130 9540 9545 10398 10400 10873 11719 11812 11914 12705 13263 14318 14766 14783 15043 15301 15326 15487T 15613 16223 16298 16325 16327 16362 |
| C1b9 | 73 249d 263 290-291d 489 493 750 1438 2706 3552A 4715 4769 6297 7028 7196A 8047 8584 8701 8860 9540 9545 10398 10400 10873 11719 11914 12705 13263 14318 14766 14783 15043 15301 15326 15487T 16223 16298 16325 16327 |
| C1b11 | 73 249d 263 290-291d 489 493 750 1438 2706 3552A 4715 4769 7028 7196A 8584 8701 8860 9540 9545 10398 10400 10873 11719 11914 12705 13263 14318 14766 14783 15043 15301 15326 15487T 16223 16295 16298 16325 16327 |
| C1b12 | 73 249d 263 290-291d 489 493 750 1438 2706 3552A 4715 4769 7028 7196A 8584 8701 8860 9540 9545 10398 10400 10873 11025 11719 11914 12705 13263 14318 14766 14783 15043 15301 15326 15487T 16223 16298 16325 16327 |
| C1b13 | 73 249d 258 263 290-291d 489 493 750 1438 2706 3552A 4715 4769 7028 7091 7196A 8584 8701 8860 9540 9545 10398 10400 10873 11719 11914 12705 13263 14318 14766 14783 15043 15301 15326 15487T 16223 16298 16325 16327 |
| C1b13a | 73 249d 258 263 290-291d 489 493 750 1193 1438 2706 3552A 4715 4769 7028 7091 7196A 8584 8701 8860 9540 9545 10398 10400 10873 11719 11914 12705 13263 14318 14766 14783 15043 15301 15326 15487T 16223 16298 16325 16327 |

| | |
|---|---|
| C1b13a1 | 73 249d 258 263 290-291d 489 493 750 1193 1438 2706 3552A 4715 4769 7028 7091 7196A 8584 8701 8860 9540 9545 10398 10400 10873 11084 11719 11914 12411 12705 13263 14318 14766 14783 15043 15301 15326 15487T 16223 16298 16325 16327 |
| C1b13b | 73 249d 258 263 290-291d 489 493 750 1438 1842 2706 3552A 4715 4769 7028 7091 7196A 8584 8701 8860 9540 9545 10101 10398 10400 10873 11719 11914 12705 13263 14318 14766 14783 15043 15301 15326 15487T 16223 16298 16325 16327 |
| C1b13c | 73 249d 258 263 290-291d 489 493 750 1438 2706 3552A 4715 4769 7028 7091 7196A 8584 8701 8860 9540 9545 10398 10400 10873 11719 11914 12705 13263 14318 14766 14783 15043 15244 15301 15326 15487T 16223 16298 16325 16327 |
| C1b13c1 | 73 249d 258 263 290-291d 489 493 750 1438 2706 3552A 4715 4769 7028 7091 7196A 8584 8701 8860 9540 9545 10398 10400 10873 11719 11914 12705 13263 13392 14318 14766 14783 15043 15244 15301 15326 15487T 16223 16298 16325 16327 |
| C1b13d | 73 249d 258 263 290-291d 489 493 750 1438 2706 3552A 4715 4769 7028 7091 7196A 8584 8701 8860 9540 9545 10398 10400 10873 11719 11914 12705 13263 13965 14318 14766 14783 15043 15301 15326 15487T 16051 16223 16298 16325 16327 |
| C1b13e | 73 249d 258 263 290-291d 489 493 750 1438 2706 3552A 4715 4769 7028 7091 7196A 8584 8701 8860 9540 9545 10398 10400 10873 11719 11914 12705 13263 14318 14766 14783 14944 15043 15301 15326 15487T 16223 16298 16325 16327 |
| C1b14 | 73 249d 263 290-291d 489 493 750 1438 2706 3552A 4715 4769 5894 7028 7196A 8584 8701 8860 9540 9545 10397 10398 10400 10873 11719 11914 12705 13263 14318 14766 14783 15043 15301 15326 15487T 16181 16223 16298 16325 16327 |
| C1c | 73 249d 263 290-291d 489 750 1438 1888 2706 3552A 4715 4769 7028 7196A 8584 8701 8860 9540 9545 10398 10400 10873 11719 11914 12705 13263 14318 14766 14783 15043 15301 15326 15487T 15930 16223 16298 16325 16327 |
| C1c1 | 73 215 249d 263 290-291d 489 750 1438 1888 2706 3552A 4715 4769 7028 7196A 8584 8701 8860 9540 9545 10398 10400 10873 11719 11914 12705 13263 14318 14766 14783 15043 15301 15326 15487T 15930 16223 16298 16325 16327 |
| C1c1a | 73 215 249d 263 290-291d 489 750 1438 1888 2706 3552A 4715 4769 7028 7196A 8584 8701 8860 9540 9545 10398 10400 10873 11719 11914 12705 12978 13263 14318 14766 14783 15043 15301 15326 15487T 15930 16223 16298 16325 16327 |
| C1c1b | 73 215 249d 263 290-291d 489 750 1438 1888 2706 3552A 4715 4769 5773 7028 7196A 8584 8701 8860 9540 9545 10398 10400 10873 11719 11914 12705 13263 14318 14766 14783 15043 15301 15326 15487T 15930 16223 16298 16325 16327 |
| C1c2 | 73 249d 263 290-291d 489 750 1438 1888 2706 3010 3552A 4715 4769 7028 7196A 8584 8701 8860 9540 9545 10398 10400 10873 11440 11719 11794 11914 12705 13263 14318 14356 14766 14783 15043 15301 15326 15487T 15930 16223 16298 16325 16327 |

| | |
|---|---|
| C1c3 | 73 249d 263 290-291d 489 750 1438 1888 2706 3140 3552A 3705 4715 4769 6815 7022A 7028 7196A 8584 8701 8860 9540 9545 10398 10400 10873 11719 11914 12705 13263 14318 14766 14783 15043 15301 15326 15487T 15670 15930 16223 16265 16298 16319 16325 16327 |
| C1c4 | 73 214 249d 263 290-291d 489 750 1438 1888 2706 3552A 4715 4769 7028 7196A 8584 8701 8860 9540 9545 10398 10400 10873 11719 11914 12705 13263 14318 14766 14783 15043 15301 15326 15487T 15930 16223 16274 16298 16325 16327 |
| C1c5 | 73 249d 263 290-291d 489 750 1438 1888 2706 3552A 4715 4769 7028 7196A 8584 8701 8860 9540 9545 10398 10400 10873 11617 11719 11914 12705 13263 14318 14766 14783 15043 15301 15326 15487T 15930 16223 16298 16325 16327 16526 |
| C1c+195 | 73 195 249d 263 290-291d 489 750 1438 1888 2706 3552A 4715 4769 7028 7196A 8584 8701 8860 9540 9545 10398 10400 10873 11719 11914 12705 13263 14318 14766 14783 15043 15301 15326 15487T 15930 16223 16298 16325 16327 |
| C1c6 | 73 195 249d 263 290-291d 489 750 1438 1888 2706 3552A 4715 4769 7028 7196A 8584 8701 8860 9540 9545 10398 10400 10873 11719 11914 12414 12705 13263 14318 14766 14783 15043 15301 15326 15487T 15930 16153 16223 16298 16325 16327 |
| C1c7 | 73 195 249d 263 290-291d 489 750 1303 1438 1888 2706 3552A 4715 4769 7028 7196A 8584 8701 8860 9540 9545 9932 10398 10400 10873 11719 11914 12705 13263 14318 14766 14783 15043 15301 15326 15487T 15930 16092 16176 16218 16223 16298 16325 16327 |
| C1c8 | 73 249d 263 290-291d 489 750 1438 1888 2706 3552A 4715 4769 7028 7196A 8584 8701 8860 9254 9540 9545 10398 10400 10873 11719 11914 12705 13263 14318 14766 14783 15043 15301 15326 15487T 15930 16114 16223 16298 16325 16327 |
| C1d | 73 249d 263 290-291d 489 750 1438 2706 3552A 4715 4769 7028 7196A 8584 8701 8860 9540 9545 10398 10400 10873 11719 11914 12705 13263 14318 14766 14783 15043 15301 15326 15487T 16051 16223 16298 16325 16327 |
| C1d+194 | 73 194 249d 263 290-291d 489 750 1438 2706 3552A 4715 4769 7028 7196A 8584 8701 8860 9540 9545 10398 10400 10873 11719 11914 12705 13263 14318 14766 14783 15043 15301 15326 15487T 16051 16223 16298 16325 16327 |
| C1d1 | 73 194 249d 263 290-291d 489 750 1438 2706 3552A 4715 4769 7028 7196A 7697 8584 8701 8860 9540 9545 10398 10400 10873 11719 11914 12705 13263 14318 14766 14783 15043 15301 15326 15487T 16051 16223 16298 16325 16327 |
| C1d1a | 73 249d 263 290-291d 489 750 1415 1438 2706 3552A 4715 4769 7028 7196A 7343 7697 8584 8701 8860 9540 9545 10398 10400 10873 11719 11914 12705 13263 14318 14766 14783 15043 15301 15326 15487T 16051 16223 16298 16325 16327 |
| C1d1a1 | 73 249d 263 290-291d 489 750 1415 1438 2706 3552A 4715 4769 6297 7028 7196A 7343 7697 8584 8701 8860 9540 9545 10398 10400 10873 11719 11914 12705 13263 14305 14318 14766 14783 15043 15301 15326 15487T 16051 16223 16298 16325 16327 |

| | |
|---|---|
| C1d1b | 73 194 195 249d 263 290-291d 489 750 928 1438 2706 3552A 4715 4769 7028 7196A 7697 8584 8701 8860 9540 9545 10398 10400 10873 11719 11914 12705 13263 14318 14766 14783 15043 15301 15326 15487T 16051 16223 16298 16325 16327 |
| C1d1b1 | 73 194 195 249d 263 290-291d 489 750 928 1438 2706 3552A 4715 4769 7028 7196A 7337 7697 8584 8701 8860 9540 9545 10398 10400 10873 11719 11914 12705 13263 14318 14766 14783 15043 15301 15326 15487T 16051 16223 16298 16325 16327 |
| C1d1c | 73 194 249d 263 290-291d 489 750 1438 2706 3552A 4715 4769 7028 7196A 7697 8584 8701 8860 9540 9545 10398 10400 10873 11719 11914 12705 13263 14318 14766 14783 15043 15301 15326 15487T 16051 16188 16223 16298 16325 16327 16362 |
| C1d1c1 | 73 249d 263 290-291d 489 750 1438 2706 3552A 4715 4769 7028 7196A 7697 8584 8701 8860 9540 9545 10398 10400 10873 11719 11914 12705 13263 14318 14766 14783 15043 15301 15326 15487T 16051 16188 16223 16325 16327 16362 |
| C1d1d | 73 194 249d 263 290-291d 489 750 1438 2706 3552A 4715 4769 7028 7196A 7697 8584 8701 8860 9540 9545 10398 10400 10873 11719 11914 12705 13263 14318 14766 14783 15043 15301 15326 15487T 16051 16172 16223 16298 16325 16327 |
| C1d2 | 73 194 249d 263 290-291d 489 750 1438 2706 3552A 4715 4769 7028 7196A 8584 8701 8860 9540 9545 10398 10400 10834 10873 11719 11914 12705 13263 14318 14766 14783 15043 15301 15326 15487T 16051 16223 16298 16325 16327 |
| C1d2a | 73 194 249d 263 290-291d 489 593 750 1438 2706 3552A 4715 4769 7028 7196A 8584 8701 8860 9540 9545 10398 10400 10834 10873 11719 11914 12705 13263 14318 14766 14783 15043 15301 15326 15487T 16051 16209 16223 16298 16325 16327 |
| C1d3 | 73 194 249d 263 290-291d 489 750 1438 2706 3552A 4715 4769 7028 7196A 8584 8701 8860 9540 9545 10398 10400 10873 11719 11914 12378 12705 13263 14318 14766 14783 15043 15301 15326 15487T 16051 16140 16223 16288 16298 16325 16327 |
| C1e | 73 152 249d 263 290-291d 489 534 750 1438 2706 3395 3507 3552A 4715 4769 5899.XC 7028 7196A 7331 8584 8701 8860 9540 9545 10398 10400 10873 11719 11914 12705 13263 13651 13966 14318 14324 14766 14783 15043 15301 15326 15487T 15613 16223 16298 16311 16325 16327 |
| C1f | 73 247 249d 263 290-291d 489 750 1438 2706 3552A 4715 4769 7028 7196A 8584 8701 8860 9540 9545 10398 10400 10873 11719 11914 12705 13263 14318 14766 14783 15043 15301 15326 15487T 16223 16298 16325 16327 |
| C4 | 73 249d 263 489 750 1438 2706 3552A 4715 4769 6026 7028 7196A 8584 8701 8860 9540 9545 10398 10400 10873 11719 11914 11969 12705 13263 14318 14766 14783 15043 15204 15301 15326 15487T 16223 16298 16327 |
| C4a'b'c | 73 249d 263 489 750 1438 2232.1A 2706 3552A 4715 4769 6026 7028 7196A 8584 8701 8860 9540 9545 10398 10400 10873 11719 11914 11969 12705 13263 14318 14766 14783 15043 15204 15301 15326 15487T 16223 16298 16327 |

| | |
|---|---|
| C4a | 73 249d 263 489 750 1438 2232.1A 2706 3552A 4715 4769 6026 7028 7196A 8584 8701 8860 9540 9545 10398 10400 10873 11719 11914 11969 12672 12705 13263 14318 14766 14783 15043 15204 15301 15326 15487T 16223 16298 16327 |
| C4a1 | 73 249d 263 489 750 1438 2232.1A 2706 3552A 4715 4769 6026 7028 7196A 7999 8584 8701 8860 9540 9545 10398 10400 10873 11719 11914 11969 12672 12705 13263 14318 14766 14783 15043 15204 15301 15326 15487T (16093) (16129) 16223 16298 16327 |
| C4a1a | 73 249d 263 489 750 1438 1715 2232.1A 2706 3552A 4715 4769 6026 7028 7196A 7999 8584 8701 8860 9540 9545 10398 10400 10873 11719 11914 11969 12672 12705 13263 14318 14766 14783 15043 15204 15301 15326 15487T 15968 (16093) (16129) 16223 16298 16327 |
| C4a1a1 | 73 249d 263 489 750 1438 1715 2232.2A 2706 3552A 4715 4769 6026 7028 7196A 7999 8508 8584 8701 8860 9540 9545 10398 10400 10873 11719 11914 11969 12672 12705 13263 14318 14766 14783 15043 15204 15301 15326 15487T 15968 (16093) (16129) 16223 16298 16327 |
| C4a1a1a | 73 249d 263 489 750 1438 1715 2232.2A 2706 3552A 3576 4715 4769 4884 4958 6026 7028 7196A 7999 8508 8584 8701 8860 9540 9545 10398 10400 10873 11719 11914 11969 12672 12705 13263 14318 14766 14783 15043 15204 15301 15326 15487T 15968 (16093) (16129) 16223 16298 [illegible] |
| C4a1a+195 | 73 195 249d 263 489 750 1438 1715 2232.1A 2706 3552A 4715 4769 6026 7028 7196A 7999 8584 8701 8860 9540 9545 10398 10400 10873 11719 11914 11969 12672 12705 13263 14318 14766 14783 15043 15204 15301 15326 15487T 15968 (16093) (16129) 16223 16298 16327 |
| C4a1a2 | 73 195 249d 263 489 750 1438 1715 2232.1A 2706 3552A 4715 4769 6026 7028 7196A 7999 8584 8701 8860 9540 9545 10398 10400 10873 10891 11719 11914 11969 12672 12705 13263 14318 14766 14783 15043 15204 15301 15326 15487T 15968 (16093) (16129) 16223 16298 16327 |
| C4a1a2a | 73 195 249d 263 489 750 1438 1715 2232.1A 2706 3552A 4715 4769 6026 7028 7196A 7999 8584 8701 8860 9540 9545 10398 10400 10685 10873 10891 11719 11914 11969 12672 12705 13263 14318 14766 14783 15043 15204 15301 15326 15487T 15968 (16093) (16129) 16223 16298 16327 |
| C4a1a3 | 73 195 249d 263 489 750 1438 1715 2232.1A 2706 3552A 4715 4769 6026 7028 7196A 7999 8584 8701 8860 9540 9545 10398 10400 10873 11719 11914 11969 12672 12705 13263 14318 14766 14783 15043 15204 15301 15326 15487T 15607 15968 (16093) (16129) 16223 16298 16327 |
| C4a1a3a | 73 195 249d 263 489 750 1438 1715 2232.1A 2706 3552A 4715 4769 6026 7028 7196A 7999 8389 8584 8701 8860 9540 9545 10398 10400 10873 11719 11914 11969 12672 12705 13263 14318 14766 14783 15043 15204 15301 15326 15487T 15607 15968 (16093) (16129) 16223 16298 16327 |
| C4a1a3a1 | 73 195 249d 263 489 750 1438 1715 2232.1A 2706 3552A 4715 4769 6026 7028 7196A 7999 8389 8584 8701 8860 9540 9545 10398 10400 10873 11476 11719 11914 11969 12672 12705 13263 14318 14766 14783 15043 15204 15301 15326 15487T 15607 15968 (16093) (16129) 16223 16298 16327 |

| | |
|---|---|
| C4a1a3b | 73 195 249d 263 489 750 1438 1715 2232.1A 2706 3552A 4715 4769 6026 7028 7196A 7999 8584 8701 8860 9540 9545 10398 10400 10873 11719 11914 11969 12397 12672 12705 13263 14318 14766 14783 15043 15204 15301 15326 15487T 15607 15968 (16093) (16129) 16223 16298 16327 |
| C4a1a3c | 73 195 249d 263 489 750 1438 1715 2232.1A 2706 3552A 3796 4715 4769 6026 7028 7196A 7999 8584 8701 8860 9540 9545 10398 10400 10873 11719 11914 11969 12672 12705 13263 14318 14766 14783 15043 15204 15301 15326 15487T 15607 15968 (16093) (16129) 16223 16298 16327 |
| C4a1a3d | 73 195 249d 263 489 750 1438 1715 2232.1A 2706 3552A 4715 4769 6026 7028 7196A 7999 8584 8701 8860 9540 9545 10398 10400 10873 11719 11914 11969 12672 12705 13263 14318 14766 14783 15043 15204 15301 15326 15487T 15607 15968 (16093) (16129) 16223 16327 |
| C4a1a4 | 73 195 249d 263 489 750 1438 1715 2232.1A 2706 3552A 4715 4769 6026 7028 7196A 7999 8584 8701 8860 9540 9545 10398 10400 10873 11719 11914 11969 12672 12705 12940 13263 14318 14766 14783 15043 15204 15301 15326 15487T 15968 (16093) (16129) 16223 16298 16327 |
| C4a1a4a | 73 195 249d 263 489 750 1438 1715 2232.1A 2706 3552A 4715 4769 6026 7028 7196A 7999 8584 8701 8860 9540 9545 10398 10400 10873 11719 11914 11969 12672 12705 12940 13263 14318 14766 14783 15043 15204 15301 15326 15487T 15968 (16093) (16129) 16150 16223 16298 16327 |
| C4a1a5 | 73 249d 263 489 750 1438 1715 2232.1A 2706 3552A 4080 4715 4769 5460 6026 6261 7028 7196A 7999 8584 8701 8860 9540 9545 10398 10400 10873 11719 11914 11969 12672 12705 13263 14318 14766 14783 15043 15204 15301 15326 15487T 15968 (16093) (16129) 16223 16298 16327 |
| C4a1a6 | 73 249d 263 489 750 1438 1715 2232.1A 2706 3552A 4715 4769 6026 6221 7028 7196A 7999 8584 8701 8860 9540 9545 10398 10400 10873 11719 11914 11969 12672 12705 13263 14318 14766 14783 15043 15204 15301 15326 15487T 15968 (16093) (16129) 16223 16298 16327 |
| C4a1b | 73 249d 263 489 750 1438 2232.1A 2706 3552A 4715 4769 5846 6026 7028 7196A 7999 8584 8701 8860 9540 9545 10398 10400 10873 11719 11914 11969 12672 12705 13263 14318 14766 14783 15043 15204 15301 15326 15487T (16093) (16129) 16192 16223 16298 16327 |
| C4a2 | 73 249d 263 489 750 1438 2232.1A 2706 3552A 4715 4769 6026 7028 7196A 8584 8701 8860 9540 9545 10398 10400 10873 11719 11914 11969 12672 12705 13263 14318 14766 14783 15043 15204 15301 15326 15487T 16223 16298 16327 16357 |
| C4a2a | 73 249d 263 489 750 1438 2232.1A 2706 3552A 4715 4769 6026 7028 7196A 8485 8584 8701 8793 8860 9540 9545 10398 10400 10873 11719 11914 11969 12672 12705 13263 14318 14766 14783 15043 15204 15301 15326 15487T 16223 16298 16327 16344 16357 |
| C4a2a1 | 73 249d 263 489 750 1438 2232.1A 2706 3552A 4715 4769 6026 7028 7196A 8485 8584 8701 8793 8860 9540 9545 10398 10400 10873 11719 11914 11969 12672 12705 13263 14318 14766 14783 15043 15204 15301 15326 15487T 16171 16223 16298 16327 16344 16357 |
| C4a2a1a | 73 249d 263 489 750 1438 2232.1A 2706 3552A 4715 4769 6026 7028 7196A 8485 8584 8701 8793 8860 8869 9540 9545 10398 10400 10873 11719 11914 11969 12672 12705 13263 14318 14766 14783 15043 15204 15301 15326 15487T 16171 16223 16298 16327 16344 16357 |

| | |
|---|---|
| C4a2a1b | 73 249d 263 489 750 1438 2232.1A 2706 3552A 4715 4769 6026 7028 7196A 8014 8485 8584 8701 8793 8860 9263 9540 9545 10398 10400 10873 11719 11914 11969 12672 12705 13263 14318 14766 14783 15043 15204 15301 15326 15487T 16171 16223 16298 16327 16344 16357 |
| C4a2b | 73 249d 263 489 750 1438 2232.1A 2706 2887 3552A 4696 4715 4769 6026 7028 7196A 8584 8701 8860 9540 9545 10398 10400 10873 11719 11914 11969 12624 12672 12705 13263 14318 14766 14783 15043 15204 15301 15326 15487T 16223 16298 16327 16357 |
| C4a2b1 | 73 249d 263 489 750 1438 2232.1A 2706 2887 3552A 4696 4715 4769 5036 6026 7028 7196A 8584 8701 8860 9540 9545 10398 10400 10873 11719 11914 11969 12624 12672 12705 13263 14318 14766 14783 15043 15204 15301 15326 15487T 16086 16223 16278 16298 16327 16357 |
| C4a2b2 | 73 249d 263 310 489 750 1438 1677 2232.1A 2706 2887 3552A 4696 4715 4769 6026 7028 7196A 8584 8701 8860 9540 9545 10304 10398 10400 10873 11719 11914 11969 12624 12672 12705 13263 14318 14766 14783 15043 15204 15301 15326 15487T 16223 16298 16311 16327 16357 |
| C4a2b2a | 73 249d 263 310 489 750 1438 1677 2232.1A 2706 2887 3552A 3745 4696 4715 4769 5899.1C 6026 7028 7196A 8584 8701 8860 9540 9545 10304 10398 10400 10873 11155 11719 11914 11969 12624 12672 12705 13263 13563 14318 14766 14783 15043 15204 15301 15326 15487T 16223 16298 16311 16327 16357 |
| C4a2c | 47 73 249d 263 489 750 1438 2232.1A 2706 3394 3552A 4715 4769 6026 7028 7196A 8584 8701 8860 9540 9545 10398 10400 10873 11719 11914 11969 12672 12705 13263 13967 14318 14766 14783 15043 15204 15301 15326 15487T 16223 16298 16327 16357 |
| C4a2c1 | 47 73 207 249d 263 489 750 1438 2232.1A 2706 3394 3552A 4715 4769 6026 6929 7028 7196A 8584 8701 8860 9540 9545 10398 10400 10724 10873 11719 11914 11969 12672 12705 13145 13263 13967 14318 14766 14783 15043 15204 15301 15326 15487T 16223 16239 16298 16327 16357 |
| C4a2c2 | 47 73 249d 263 489 750 1438 2232.1A 2706 3394 3552A 4715 4769 6026 7028 7196A 8584 8701 8860 9540 9545 10398 10400 10873 11719 11914 11969 12672 12705 13263 13967 14318 14766 14783 15043 15204 15301 15487T 16086 16223 16298 16327 16357 |
| C4a2c2a | 47 73 249d 263 489 750 1438 2232.1A 2706 3394 3552A 4715 4769 6026 7028 7196A 8584 8701 8860 9540 9545 10398 10400 10873 11719 11914 11969 12672 12705 13263 13967 14318 14668 14766 14783 15043 15204 15301 15487T 16086 16223 16298 16327 16357 |
| C4b | 73 249d 263 489 750 1438 2232.1A 2706 3552A 3816 4715 4769 6026 7028 7196A 8584 8701 8860 9540 9545 10398 10400 10873 11719 11914 11969 12705 13263 14318 14766 14783 15043 15204 15301 15326 15487T 16223 16298 16327 |
| C4b1 | 73 146 249d 263 489 750 1438 2232.1A 2706 3552A 3816 4715 4769 6026 7028 7196A 8251 8584 8701 8860 9540 9545 10398 10400 10873 11719 11914 11969 12705 13263 14318 14766 14783 15043 15204 15301 15326 15487T 16223 16298 16327 |

| | |
|---|---|
| C4b1a | 73 146 249d 263 489 710 750 1438 2232.1A 2706 3552A 3816 4715 4769 6026 7028 7196A 8251 8584 8701 8860 9540 9545 10398 10400 10873 11719 11914 11969 12705 13263 14318 14766 14783 15043 15204 15301 15326 15487T 16223 16259.1A 16298 16327 |
| C4b1b | 73 146 249d 263 489 750 1438 2232.1A 2706 3552A 3816 4715 4769 6026 7028 7196A 8251 8292 8584 8701 8860 9540 9545 10398 10400 10873 11719 11914 11969 12705 13263 14318 14766 14783 15043 15204 15301 15326 15487T 16223 16298 16327 |
| C4b2 | 73 249d 263 489 750 1438 2232.1A 2706 3552A 3816 4715 4769 6026 7028 7196A 8584 8701 8860 9540 9545 10398 10400 10873 11719 11914 11969 12705 13263 14318 14766 14783 15043 15204 15301 15326 15487T 16124 16223 16298 16327 |
| C4b2a | 73 249d 263 489 750 1438 2232.1A 2706 3552A 3816 4715 4769 6026 7028 7196A 8584 8701 8860 9540 9545 10398 10400 10873 11719 11914 11969 12705 13263 14318 14766 14783 15043 15204 15301 15326 15487T 16124 16223 16298 16318T 16327 |
| C4b3 | 73 249d 263 489 750 1438 2232.1A 2706 3552A 3816 4715 4769 6026 7028 7196A 8584 8701 8860 9540 9545 10398 10400 10873 11719 11914 11969 12705 13263 14318 14766 14783 15043 15204 15301 15326 15487T 16223 16291 16298 16327 |
| C4b3a | 73 249d 263 489 750 1438 2232.1A 2706 3552A 3816 4715 4769 6026 7028 7196A 8584 8701 8860 9540 9545 10398 10400 10873 11719 11914 11969 12705 13263 14318 14766 14783 15043 15145 15204 15301 15326 15487T 16223 16291 16298 16327 |
| C4b3a1 | 73 249d 263 489 750 1438 2232.1A 2706 3552A 3816 4715 4769 6026 7028 7196A 8584 8701 8860 9540 9545 10398 10400 10873 11719 11914 11969 12705 13263 14318 14766 14783 15043 15145 15204 15301 15326 15487T 16223 16291 16298 16327 16399 |
| C4b3b | 73 249d 263 489 750 1438 2232.1A 2706 3552A 3816 4715 4769 6026 7028 7196A 8584 8701 8860 9248 9540 9545 10398 10400 10873 11719 11914 11969 12705 13263 14318 14766 14783 15043 15204 15301 15326 15487T 16223 16249 16291 16298 16327 |
| C4b5 | 73 249d 263 489 750 1438 2232.1A 2706 3552A 3816 4715 4769 6026 7028 7196A 8584 8701 8860 9540 9545 10398 10400 10873 11377 11719 11914 11969 12705 13263 14318 14766 14783 15043 15204 15301 15326 15487T 16223 16298 16327 |
| C4b6 | 73 249d 263 489 750 1438 1788 2232.1A 2706 3552A 3816 4715 4769 6026 7028 7196A 8584 8701 8860 9540 9545 10398 10400 10873 11719 11914 11969 12705 13263 14318 14766 14783 15043 15204 15301 15326 15487T 16223 16298 16327 |
| C4b7 | 73 249d 263 489 750 1438 2232.1A 2706 3552A 3816 4136 4715 4769 6026 7028 7196A 8584 8701 8860 9540 9545 10398 10400 10873 11719 11914 11969 12705 13263 14318 14766 14783 15043 15204 15301 15326 15487T 15924 16223 16298 16327 |
| C4b8 | 73 249d 263 489 750 1438 2232.1A 2706 3392C 3552A 3816 4715 4769 6026 7028 7196A 8584 8701 8860 9540 9545 10398 10400 10873 11719 11914 11969 12705 13263 14318 14766 14783 15043 15204 15301 15326 15487T 16223 16298 16327 |

| | |
|---|---|
| C4b8a | 73 249d 263 489 750 1438 2232.1A 2706 3392C 3552A 3816 4715 4769 6026 7028 7196A 8584 8701 8860 9540 9545 10398 10400 10873 11254 11719 11914 11969 12705 13263 14318 14766 14783 15043 15204 15301 15326 15487T 16093 16223 16298 16327 |
| C4c | 73 249d 263 489 750 1438 2232.1A 2706 3552A 4715 4769 6026 7028 7196A 8584 8701 8860 9540 9545 10398 10400 10873 11719 11914 11969 12705 13263 14318 14433 14766 14783 15043 15148 15204 15301 15326 15487T 16223 16298 16327 |
| C4c1 | 73 249d 263 489 750 1243 1438 2232.1A 2706 3552A 4715 4769 6026 7028 7196A 8584 8701 8860 9540 9545 10398 10400 10873 11719 11914 11969 12705 13263 14318 14433 14766 14783 15043 15148 15204 15301 15326 15487T 16223 16298 16327 |
| C4c1a | 73 249d 263 489 750 1243 1438 2232.1A 2706 3552A 4715 4769 6026 7028 7196A 8584 8701 8860 9540 9545 10398 10400 10873 11719 11914 11969 12705 13263 14318 14433 14766 14783 15043 15148 15204 15301 15326 15487T 15629 16223 16241 16298 16327 |
| C4c1b | 73 249d 263 489 750 1243 1438 2232.1A 2706 3552A 4715 4769 6026 7028 7196A 8584 8701 8860 9540 9545 10398 10400 10873 11719 11914 11969 12705 13263 14318 14433 14766 14783 15043 15148 15204 15301 15326 15487T 16189 16223 16298 16327 |
| C4c2 | 96 105-110d 114 189 249d 263 473 489 750 2232.1A 2706 3552A 4715 4769 6026 7028 7196A 8584 8701 8860 9540 9545 10398 10400 10873 11719 11914 11969 12705 13263 14318 14433 14766 14783 15043 15148 15204 15301 15326 15487T 15523 16223 16298 16327 |
| C4+152 | 73 152 249d 263 489 750 1438 2706 3552A 4715 4769 6026 7028 7196A 8584 8701 8860 9540 9545 10398 10400 10873 11719 11914 11969 12705 13263 14318 14766 14783 15043 15204 15301 15326 15487T 16223 16298 16327 |
| C4+152+16093 | 73 152 249d 263 489 750 1438 2706 3552A 4715 4769 6026 7028 7196A 8584 8701 8860 9540 9545 10398 10400 10873 11719 11914 11969 12705 13263 14318 14766 14783 15043 15204 15301 15326 15487T 16093 16223 16298 16327 |
| C4d | 73 152 249d 263 489 750 1438 2706 3552A 4715 4769 6026 7028 7100 7196A 8584 8701 8860 9540 9545 10398 10400 10873 11719 11914 11969 12705 12780 13263 14318 14766 14783 15043 15204 15236 15301 15326 15487T 16093 16223 16298 16327 |
| C4e | 73 151 152 249d 263 489 750 1438 2706 3552A 4715 4769 6026 7028 7196A 7307 8584 8701 8860 9540 9545 10398 10400 10873 11719 11914 11969 12705 13263 14318 14766 14783 15043 15204 15301 15326 15479 15487T 16223 16298 16327 |
| C5 | 73 249d 263 489 595.1C 750 1438 2706 3552A 4715 4769 7028 7196A 8584 8701 8860 9540 9545 10398 10400 10873 11719 11914 12705 13263 14318 14766 14783 15043 15301 15326 15487T 16223 16288 16298 16327 |
| C5a | 73 249d 263 489 595.1C 750 1438 2706 3552A 3591 4715 4769 7028 7196A 8584 8701 8860 9540 9545 10398 10400 10873 11719 11914 12705 13263 14318 14766 14783 15043 15301 15326 15487T 16223 16261 16288 |

| | |
|---|---|
| C5a1 | 73 249d 263 489 595.1C 750 1438 2706 3552A 3591 4715 4769 4904 7028 7196A 8140 8584 8701 8860 9540 9545 10398 10400 10873 11719 11914 12705 13263 14318 14766 14783 15043 15301 15326 15487T 16223 16261 16288 16298 |
| C5a2 | 73 249d 263 489 595.1C 750 1438 2706 3552A 3591 4715 4769 7028 7196A 8584 8701 8860 9540 9545 10084 10398 10400 10873 11719 11914 11928 12705 13263 14318 14766 14783 15043 15301 15326 15487T 16093 16223 16261 16288 16298 |
| C5a2a | 73 249d 263 489 595.1C 750 1438 1462 2706 3552A 3591 4715 4769 7028 7196A 8584 8701 8860 9540 9545 10084 10398 10400 10873 11719 11914 11928 12705 13263 14318 14766 14783 15043 15301 15326 15487T 16093 16223 16261 16288 16298 |
| C5a2b | 73 249d 263 489 595.1C 750 1438 2706 3552A 3591 4715 4769 7028 7196A 8584 8701 8860 9540 9545 10084 10398 10400 10873 11719 11914 11928 12705 13263 14318 14766 14783 15043 15301 15326 15487T 16093 16189 16223 16261 16288 16298 |
| C5a2b1 | 73 249d 263 489 595.1C 750 1438 2706 3552A 3591 4715 4769 7028 7196A 8440 8584 8701 8860 9540 9545 10084 10398 10400 10873 11719 11914 11928 12705 13263 14318 14766 14783 15043 15301 15326 15487T 16093 16189 16223 16261 16288 16298 |
| C5b | 73 249d 263 489 595.1C 750 1438 1719.1G 2706 3552A 4715 4769 7028 7196A 8584 8701 8860 9540 9545 10398 10400 10873 11719 11914 12705 13263 14318 14766 14783 15043 15301 15326 15487T 16223 16288 16298 16327 |
| C5b1 | 73 249d 263 489 595.1C 750 1438 1719.1G 2706 3552A 4715 4769 7028 7196A 8584 8701 8860 9540 9545 10398 10400 10873 11719 11914 12705 13263 14318 14766 14783 15043 15301 15326 15487T 16148 16223 16288 16298 16327 |
| C5b1a | 73 249d 263 489 595.1C 750 1438 1719.1G 2706 3397 3552A 4715 4769 7028 7196A 8584 8701 8860 9233 9540 9545 10398 10400 10873 11719 11914 12705 13263 14318 14766 14783 15043 15301 15326 15487T 16148 16223 16288 16298 16327 |
| C5b1a1 | 73 249d 263 489 595.1C 750 1438 1719 1719.1G 2706 3397 3552A 4715 4769 7028 7196A 8584 8701 8860 9233 9540 9545 10398 10400 10873 11719 11914 12705 13263 14318 14766 14783 15043 15301 15326 15487T 16148 16223 16288 16298 16327 |
| C5b1b | 73 249d 263 489 595.1C 750 1438 1719.1G 2706 3552A 4715 4769 4937 7028 7196A 8584 8701 8860 9540 9545 10398 10400 10873 11719 11914 12705 13263 14318 14344 14766 14783 15043 15301 15326 15487T 15734 16148 16164 16223 16288 16298 16327 |
| C5b1b1 | 73 249d 263 489 595.1C 750 1438 1719.1G 2706 3552A 4715 4769 4937 7028 7196A 8584 8701 8860 9540 9545 10398 10400 10873 11719 11914 12705 13263 14318 14344 14766 14783 15043 15055 15301 15326 15487T 15734 16148 16164 16223 16288 16298 16327 |
| C5+16093 | 73 249d 263 489 595.1C 750 1438 2706 3552A 4715 4769 7028 7196A 8584 8701 8860 9540 9545 10398 10400 10873 11719 11914 12705 13263 14318 14766 14783 15043 15301 15326 15487T 16093 16223 16288 16298 |

| | |
|---|---|
| C5c | 73 249d 263 489 595.1C 750 1438 2706 3552A 4715 4769 7028 7196A 8584 8701 8860 9540 9545 10398 10400 10454 10873 11719 11914 12705 13263 14318 14766 14783 15043 15301 15326 15487T 16093 16223 16288 16298 16327 16518T 16527 |
| C5c+16234 | 73 249d 263 489 595.1C 750 1438 2706 3552A 4715 4769 7028 7196A 8584 8701 8860 9540 9545 10398 10400 10454 10873 11719 11914 12705 13263 14318 14766 14783 15043 15301 15326 15487T 16093 16223 16234 16288 16298 16327 16518T 16527 |
| C5c1 | 73 249d 263 489 595.1C 750 1438 1670T 2706 3552A 4715 4769 7028 7196A 8584 8701 8860 9540 9545 10398 10400 10454 10873 11719 11914 12705 13263 14318 14766 14783 15043 15301 15326 15487T 16093 16223 16234 16288 16298 16327 16518T 16527 |
| C5c1a | 73 249d 263 489 595.1C 750 1438 1670T 2706 3552A 4715 4769 7028 7196A 7694 8584 8701 8860 9540 9545 10398 10400 10454 10873 11719 11914 12705 13263 14318 14766 14783 15043 15301 15326 15487T 16093 16223 16234 16288 16298 16327 16518T 16527 |
| C5d | 73 249d 263 489 595.1C 750 1438 2706 3552A 4715 4769 7028 7196A 8584 8701 8860 9540 9545 10398 10400 10873 11719 11914 12705 13263 14318 14766 14783 15043 15080 15301 15326 15487T 16093 16223 16288 16298 16327 |
| C5d1 | 73 249d 263 489 595.1C 750 1415 1438 2706 3552A 4715 4769 7028 7196A 8188 8584 8701 8860 9540 9545 10398 10400 10873 11719 11914 12705 13263 14318 14766 14783 15043 15080 15301 15326 15487T 16093 16223 16288 16298 16327 16390 |
| C5d2 | 73 249d 263 489 595.1C 750 1438 2706 3552A 4715 4769 7028 7196A 8584 8701 8860 9540 9545 10398 10400 10682 10873 11719 11914 12705 13263 13968 14318 14766 14783 15043 15080 15301 15326 15487T 16093 16223 16288 16298 16327 |
| C7 | 73 249d 263 489 750 1438 2706 3552A 4715 4769 5821 6338 7028 7196A 8584 8701 8860 9540 9545 10398 10400 10873 11719 11914 12705 13263 14318 14766 14783 15043 15301 15326 15487T 16223 16298 16327 |
| C7a | 73 249d 263 489 750 1438 2706 3552A 4715 4769 5821 6338 7028 7196A 7853 8584 8701 8860 9540 9545 10398 10400 10873 11719 11914 12705 13263 14318 14766 14783 15043 15301 15326 15487T 16223 16298 16327 |
| C7a1 | 73 146 249d 263 489 750 1438 2706 3552A 4715 4769 5821 6338 7028 7196A 7853 8584 8701 8860 9540 9545 10398 10400 10873 11719 11914 12705 12957 13263 14318 14766 14783 14978 15043 15301 15326 15487T 16223 16298 16327 |
| C7a1a | 73 146 153 249d 263 489 750 1438 2706 3552A 4715 4769 5821 6338 7028 7196A 7853 8584 8701 8860 9540 9545 10295 10398 10400 10873 11719 11914 12705 12957 13263 14318 14766 14783 14978 15043 15301 15326 15487T 16223 16298 16327 |
| C7a1a1 | 73 146 151 153 249d 263 333 489 750 1438 2706 3552A 4715 4769 5821 6338 6791 7028 7196A 7853 8584 8701 8860 9540 9545 10295 10398 10400 10873 11719 11914 12705 12957 13263 14318 14766 14783 14978 15043 15301 15326 15487T 16223 16327 |

| | |
|---|---|
| C7a1a2 | 73 146 153 249d 263 489 750 1438 2706 3345 3552A 4715 4769 5821 6338 7028 7196A 7598 7853 8584 8701 8860 9540 9545 10295 10398 10400 10873 11719 11914 12477 12705 12957 13263 13708 14318 14766 14783 14978 15043 15301 15326 15487T 16223 16298 16311 16327 |
| C7a1c | 73 146 249d 263 489 750 1438 2706 3552A 4047 4715 4769 5821 5987 6338 7028 7196A 7853 8584 8701 8860 9540 9545 10398 10400 10873 11719 11914 12705 12957 13263 14318 14766 14783 14978 15043 15301 15326 15487T 16223 16298 16327 |
| C7a1d | 73 146 249d 263 489 750 1438 2706 2905 3552A 4715 4769 5821 6338 7028 7196A 7853 8584 8701 8860 9540 9545 10398 10400 10873 11204 11719 11914 12705 12957 13263 14318 14766 14783 14978 15043 15301 15326 15487T 16223 16242 16256 16298 16327 |
| C7a2 | 73 249d 263 489 750 1438 2232.1A 2706 3552A 4715 4769 5821 6338 7028 7196A 7853 8584 8701 8860 9540 9545 10398 10400 10873 11719 11914 12705 13263 14318 14766 14783 15043 15301 15326 15487T 16189 16223 16298 16327 |
| C7a2a | 73 249d 263 489 750 1438 2232.1A 2706 3552A 4715 4769 5821 6338 7028 7196A 7853 8584 8701 8860 9540 9545 10398 10400 10873 11719 11914 12705 12810 12909 13263 13635G 14318 14470 14766 14783 15043 15301 15326 15487T 16189 16223 16298 16327 |
| C7+16051 | 73 249d 263 489 750 1438 2706 3552A 4715 4769 5821 6338 7028 7196A 8584 8701 8860 9540 9545 10398 10400 10873 11719 11914 12705 13263 14318 14766 14783 15043 15301 15326 15487T 16051 16223 16298 16327 |
| C7b | 73 249d 263 489 750 1438 2706 3552A 4715 4769 5821 5918 6338 7028 7196A 8584 8701 8860 9540 9545 10398 10400 10873 11101 11719 11914 12705 13263 14318 14766 14783 15043 15301 15326 15487T 15928 16051 16223 16298 16327 |
| Z | 73 249d 263 489 750 1438 2706 4715 4769 6752 7028 7196A 8584 8701 8860 9090 9540 10398 10400 10873 11719 12705 14766 14783 15043 15301 15326 15487T 15784 16185 16223 16260 16298 |
| Z+152 | 73 152 249d 263 489 750 1438 2706 4715 4769 6752 7028 7196A 8584 8701 8860 9090 9540 10398 10400 10873 11719 12705 14766 14783 15043 15301 15326 15487T 15784 16185 16223 16260 16298 |
| Z1 | 73 152 249d 263 489 750 1438 2706 4715 4769 6752 7028 7196A 8584 8701 8860 9090 9540 10398 10400 10873 11719 12705 14766 14783 15043 15261 15301 15326 15487T 15784 16185 16223 16260 16298 |
| Z1a | 73 151 152 249d 263 489 750 1438 2706 4715 4769 6752 7028 7196A 8584 8701 8860 9090 9540 10325 10398 10400 10873 11719 12705 14766 14783 15043 15261 15301 15326 15487T 15784 16129 16185 16223 16224 16260 16298 |
| Z1a1 | 73 151 152 249d 263 489 750 1438 2706 4715 4769 6752 7028 7196A 8584 8701 8860 9090 9494 9540 10325 10398 10400 10873 11719 12705 14766 14783 15043 15261 15301 15326 15487T 15784 16129 16185 16223 16224 16260 16298 |
| Z1a1a | 73 151 152 249d 263 489 740 750 1438 2706 4715 4769 6752 7028 7196A 8584 8701 8860 9090 9494 9540 10325 10398 10400 10873 11719 12705 12930 14766 14783 15043 15261 15301 15326 15487T 15784 16129 16185 16223 16224 16260 16298 |

| | |
|---|---|
| Z1a1b | 73 151 152 249d 263 489 750 1438 2706 4715 4769 6752 7028 7196A 7521 8251 8584 8701 8860 9090 9494 9540 10325 10398 10400 10873 11719 12705 14766 14783 15043 15261 15301 15326 15487T 15784 16129 16185 16223 16224 16260 16298 |
| Z1a2 | 73 151 152 249d 263 489 750 1438 2706 4715 4769 6752 7028 7196A 8584 8701 8860 9090 9540 10325 10398 10400 10873 11590 11719 12705 14766 14783 15043 15261 15301 15326 15487T 15784 16129 16185 16223 16224 16260 16298 |
| Z1a2a | 73 151 152 249d 263 489 750 1438 2706 4715 4769 6752 7028 7196A 8584 8701 8860 9090 9540 10325 10398 10400 10873 11078 11590 11719 12705 14766 14783 15043 15261 15301 15326 15487T 15784 16129 16185 16223 16224 16260 16298 |
| Z1a3 | 73 151 152 249d 263 489 750 1438 2706 4715 4769 6752 7028 7196A 8584 8701 8860 9090 9540 10325 10398 10400 10873 11252 11719 12705 14766 14783 15043 15261 15301 15326 15487T 15784 16129 16185 16223 16224 16260 16298 |
| Z2 | 73 152 249d 263 489 750 1438 2706 3145 4715 4769 6752 7028 7196A 8188 8584 8701 8860 9090 9540 10398 10400 10873 11719 12705 14668 14766 14783 15043 15184 15301 15326 15487T 15784 16185 16223 16260 |
| Z3 | 73 152 249d 263 489 750 1438 2706 4715 4769 6752 7028 7196A 8584 8701 8860 9090 9540 10208 10398 10400 10873 11719 12705 14766 14783 15043 15301 15326 15487T 15784 16185 16223 16260 16298 |
| Z3a | 73 152 207 249d 263 489 750 1438 2706 4715 4769 6752 7028 7196A 8584 8701 8860 9090 9540 9713 10208 10398 10400 10873 11719 12705 13620 14766 14783 15043 15301 15326 15487T 15784 15928 16185 16223 16260 16298 |
| Z3a1 | 73 152 207 249d 263 489 750 1438 2706 4715 4769 6752 7028 7196A 8584 8701 8860 9090 9540 9713 10208 10398 10400 10873 11719 12705 13620 14766 14783 15043 15301 15487T 15784 15928 16185 16223 16260 16298 |
| Z3a1a | 73 152 207 249d 263 489 750 1438 2706 4715 4769 6752 7028 7196A 8584 8701 8860 8931 9090 9540 9713 10208 10398 10400 10873 11075 11719 12705 13620 14766 14783 15043 15301 15487T 15784 15928 16185 16223 16260 16298 |
| Z3a2 | 73 152 207 249d 263 489 750 1438 2706 4715 4769 6378 6752 7028 7196A 8584 8701 8860 9090 9540 9713 10208 10398 10400 10873 11719 11902 12477 12705 13620 14476 14766 14783 15043 15301 15326 15487T 15784 15884 15928 16185 16223 16260 16298 |
| Z3b | 73 146 152 249d 263 489 750 1438 2706 4715 4769 6752 7028 7196A 8584 8701 8860 9055 9090 9540 10208 10398 10400 10873 11719 12501 12705 14766 14783 15043 15301 15326 15487T 15784 16185 16223 16260 16298 |
| Z3+709 | 73 152 249d 263 489 709 750 1438 2706 4715 4769 6752 7028 7196A 8584 8701 8860 9090 9540 10208 10398 10400 10873 11719 12705 14766 14783 15043 15301 15326 15487T 15784 16185 16223 16260 16298 |
| Z3c | 73 152 249d 263 489 709 750 1438 2706 4715 4769 4853 6752 7028 7196A 8584 8701 8860 9090 9540 10208 10398 10400 10873 11719 12705 14766 14783 15043 15301 15326 15487T 15784 16185 16223 16260 16298 |

| | |
|---|---|
| Z3d | 73 152 249d 263 489 709 750 1438 2706 4715 4769 6752 7028 7196A 8584 8701 8860 9090 9540 10208 10398 10400 10873 11719 11782 12705 14766 14783 15043 15301 15326 15487T 15784 16185 16223 16260 16298 |
| Z4 | 73 151 152 249d 263 489 750 1438 2706 4715 4769 6752 7028 7196A 8584 8701 8860 9090 9540 10398 10400 10873 11719 12705 14766 14783 15043 15301 15326 15475 15487T 15784 15944d 16185 16223 16260 16298 |
| Z4a | 73 151 152 249d 263 489 750 1438 2706 4715 4769 6752 7028 7196A 8584 8701 8860 9090 9540 10398 10400 10873 11719 12705 14766 14783 15043 15301 15326 15475 15487T 15784 15944d 16185 16223 16260 16298 |
| Z4a1 | 73 152 249d 263 489 750 1438 2706 4715 4769 5492 6752 7028 7196A 8584 8701 8860 9090 9540 10398 10400 10873 11719 12705 14766 14783 15043 15301 15326 15475 15487T 15784 15944d 16185 16189 16223 16260 16298 16302 |
| Z4a1a | 73 152 249d 263 489 750 1438 2706 4715 4769 5492 5894 6752 7028 7196A 8584 8701 8860 9090 9540 10398 10400 10873 11719 12705 14766 14783 15043 15301 15326 15475 15487T 15784 15944d 16185 16189 16223 16260 16298 16302 |
| Z4a1a1 | 73 152 249d 263 489 750 1438 2706 3520 4715 4769 5492 5894 6752 7028 7196A 7270 8584 8654 8701 8860 9090 9540 10398 10400 10873 12705 14766 14783 15043 15301 15326 15475 15487T 15784 15944d 16185 16189 16193d 16223 16260 16294 16298 16302 |
| Z7 | 73 152 249d 263 489 750 1438 2352 2706 2780 4363 4715 4769 4841 6752 7028 7196A 7471d 8348 8584 8598 8701 8860 9090 9540 10398 10400 10653 10873 11719 12705 14766 14783 15043 15301 15326 15487T 15784 16185 16223 16260 16298 |
| Z5 | 73 249d 255 263 489 750 814 1438 2056 2706 4712 4715 4769 6752 7028 7196A 8251 8584 8701 8860 9053 9090 9540 10398 10400 10873 11719 12705 14052 14766 14783 15043 15301 15326 15487T 15553 15784 16185 16223 16260 16298 |
| M9 | 73 263 489 750 1438 2706 4491 4769 7028 8701 8860 9540 10398 10400 10873 11719 12705 14766 14783 15043 15301 15326 16223 16362 |
| M9a'b | 73 153 263 489 750 1438 2706 3394 4491 4769 7028 8701 8860 9540 10398 10400 10873 11719 12705 14766 14783 15043 15301 15326 16223 |
| M9a | 73 153 263 489 750 1438 2706 3394 4491 4769 7028 8701 8860 9540 10398 10400 10873 11719 12705 14308 14766 14783 15043 15301 15326 16223 16234 16362 |
| M9a1 | 73 153 263 489 750 1041 1438 2706 3394 4491 4769 7028 8701 8860 9540 10398 10400 10873 11719 12705 14308 14766 14783 15043 15301 15326 16223 16234 16362 |
| M9a1a | 73 153 263 489 750 1041 1438 2706 3394 4491 4769 7028 8701 8860 9540 10398 10400 10873 11719 12705 14308 14766 14783 15043 15301 15326 16223 16234 16316 16362 |
| M9a1a1 | 73 153 263 489 750 1041 1438 2706 3394 4491 4769 7028 8701 8860 9242 9540 10398 10400 10873 11719 12705 14308 14766 14783 15043 15301 15326 16223 16234 16316 16362 |
| M9a1a1a | 73 263 489 750 1041 1438 2706 3394 4491 4769 7028 8701 8860 9242 9540 10398 10400 10873 11719 11963 12705 14308 14766 14783 15043 15301 15326 16223 16234 16316 16362 |

| | |
|---|---|
| M9a1a1b | 73 153 263 489 750 1041 1438 2706 3394 4491 4769 5951 7028 8701 8860 9115 9242 9540 10398 10400 10873 11719 12705 14308 14766 14783 15043 15301 15326 16223 16234 16300 16316 16362 |
| M9a1a1c | 73 153 263 489 750 1041 1438 2706 3394 4491 4769 7028 7142 8701 8860 9242 9540 10398 10400 10873 11719 12705 14308 14766 14783 15043 15301 15326 16223 16234 16291 16316 16362 |
| M9a1a1c1 | 73 153 263 489 750 1041 1438 2706 3394 4491 4769 7028 7142 8701 8860 9242 9540 10398 10400 10873 11719 12705 14308 14417 14766 14783 15043 15301 15326 16223 16234 16291 16316 16362 |
| M9a1a1c1a | 73 153 263 489 750 1041 1438 2706 3394 4491 4769 7028 7142 7861 8701 8860 9242 9540 10398 10400 10873 11719 12705 14308 14417 14766 14783 15043 15301 15326 16223 16234 16291 16316 16362 |
| M9a1a1c1b | 73 153 263 489 750 1041 1438 2706 3394 4491 4769 7028 7142 7697 8701 8860 9242 9540 10398 10400 10873 11719 12705 14308 14417 14766 14783 15043 15301 15326 16223 16234 16316 16362 |
| M9a1a1c1b1 | 73 263 489 750 1041 1438 2706 3394 4491 4769 5899.XC 7028 7142 7697 8701 8860 9242 9540 10398 10400 10873 11719 12705 14308 14417 14766 14783 15043 15301 15326 16223 16234 16316 16362 |
| M9a1a1c1b1a | 73 263 489 711 750 1041 1438 2706 3394 4491 4769 5899.XC 7028 7142 7697 8701 8860 9242 9540 10398 10400 10873 11719 12705 14308 14417 14766 14783 15043 15301 15326 16223 16234 16316 16362 |
| M9a1a1c1b1a1 | 73 263 489 711 750 1041 1438 2706 3394 4491 4769 5899.XC 7028 7142 7697 8567 8701 8860 9242 9540 10398 10400 10873 11719 12705 14308 14417 14766 14783 15043 15301 15326 16223 16234 16316 16362 |
| M9a1a1c1b1a2 | 73 146 263 489 711 750 1041 1438 2706 3394 4491 4769 5899.XC 6446 7028 7142 7697 8701 8860 9242 9540 10398 10400 10873 11719 12705 14308 14417 14766 14783 15043 15301 15326 16223 16234 16316 16362 |
| M9a1a1c1b2 | 73 153 263 489 750 1041 1438 2706 3394 4491 4769 7028 7142 7697 8701 8860 9242 9540 10398 10400 10873 11719 12705 14308 14417 14766 14783 15043 15301 15326 15839 16223 16234 16316 16362 |
| M9a1a1c1c | 73 153 263 489 750 1041 1438 2706 3394 4491 4769 7028 7142 8631 8701 8860 9242 9540 10398 10400 10873 11719 12705 14308 14417 14766 14783 15043 15301 15326 16223 16234 16291 16316 16362 |
| M9a1a1d | 73 153 263 489 750 1041 1438 2706 3394 4491 4769 7028 8701 8860 9242 9540 10398 10400 10873 11719 12705 14308 14766 14783 15043 15175 15301 15326 16223 16234 16316 16362 |
| M9a1a2 | 73 153 263 489 750 1041 1438 2706 3394 4491 4769 7028 7256 8701 8860 9540 10398 10400 10873 11719 12705 14308 14766 14783 15043 15301 15326 16145 16223 16234 16316 16362 |
| M9a1a3 | 73 153 263 489 750 1041 1438 2706 3394 4491 4769 7028 8701 8860 9540 10398 10400 10873 11719 12705 14308 14766 14783 15043 15301 15326 15884 16223 16234 16248 16265C 16316 16362 |
| M9a1b | 73 152 153 263 489 750 1041 1438 2706 3394 4491 4769 7028 8701 8860 9540 10398 10400 10873 11719 12362 12705 14308 14766 14783 15043 15301 15326 15671 16223 16234 16362 |
| M9a1b+150 | 73 150 152 153 263 489 750 1041 1438 2706 3394 4491 4769 7028 8701 8860 9540 10398 10400 10873 11719 12362 12705 14308 14766 14783 15043 15301 15326 15671 16223 16234 16362 |

| | |
|---|---|
| M9a1b1 | 73 150 152 153 263 489 750 1041 1438 2706 3394 4491 4769 7028 8701 8860 9540 10398 10400 10873 11719 12362 12705 14308 14766 14783 15043 15301 15326 15671 16158 16223 16234 16362 |
| M9a1b1a | 73 150 152 153 263 489 750 1041 1438 2706 3394 4491 4769 7028 8701 8860 9540 10398 10400 10873 11719 11872 12362 12705 14308 14766 14783 15043 15301 15326 15671 16158 16223 16234 16362 |
| M9a1b1a1 | 73 150 152 153 263 489 750 1041 1438 2706 3394 4491 4769 7028 8557C 8701 8860 9540 10398 10400 10873 11623 11719 11872 12362 12705 14308 14766 14783 15043 15301 15326 15671 16158 16223 16234 16362 |
| M9a1b1b | 73 150 152 153 263 489 750 1041 1438 2706 3394 3522 4491 4769 7028 8701 8860 9540 10398 10400 10873 11719 12362 12705 14308 14683 14766 14783 15043 15301 15326 15671 16158 16223 16234 16362 |
| M9a1b1c | 73 150 152 153 263 489 750 1041 1438 2706 3394 4491 4769 7028 8701 8860 9540 10398 10400 10454 10873 11719 12362 12705 14308 14766 14783 15043 15301 15326 15671 16158 16223 16234 16362 |
| M9a1b2 | 73 150 152 153 263 489 750 1041 1438 2706 3394 4491 4769 7028 8701 8860 9540 10398 10400 10873 10951A 11719 12362 12705 14308 14766 14783 15043 15301 15326 15671 16223 16234 16362 |
| M9a4 | 73 153 263 489 750 1438 2706 3394 4491 4769 6366 7028 8701 8860 9540 10398 10400 10873 11719 12705 14308 14766 14783 15043 15301 15326 16223 16234 16362 |
| M9a4a | 73 153 263 489 750 1438 2706 3394 4491 4769 6366 7028 8701 8860 9540 10398 10400 10873 11719 12705 14308 14766 14783 15043 15301 15326 16223 16234 16271 16362 |
| M9a4a1 | 73 153 263 489 750 1120 1438 2706 3394 4491 4769 5899.XC 6366 7028 8701 8860 9540 10398 10400 10873 11719 12705 14308 14766 14783 15043 15301 15326 16223 16234 16271 16362 |
| M9a4a2 | 73 151 153 263 489 750 1438 1719 2706 3394 4491 4769 6366 7028 8701 8860 9540 10398 10400 10873 11719 12705 14308 14766 14783 15043 15301 15326 16223 16234 16271 16362 |
| M9a4b | 73 153 263 489 750 1438 2706 3394 4491 4769 6366 6815 7028 8701 8860 9540 10398 10400 10873 11719 12705 14308 14766 14783 15043 15301 15326 16223 16234 16362 |
| M9a5 | 73 153 263 385 489 750 1438 2706 3394 4491 4769 7028 8155 8701 8860 9540 10398 10400 10873 11719 12237 12705 14308 14766 14783 15043 15301 15326 16223 16234 16362 |
| M9b | 73 153 263 489 573.XC 750 1438 2706 3394 4491 4769 5964 6842 7028 7609 7789 8701 8860 9540 10398 10400 10873 11719 12705 12864 14766 14783 15043 15301 15326 16051 16209 16223 16362 |
| E | 73 263 489 750 1438 2706 3027 3705 4491 4769 7028 7598 8701 8860 9540 10398 10400 10873 11719 12705 13626 14766 14783 15043 15301 15326 16223 16362 16390 |
| E1 | 73 263 489 750 1438 2706 3027 3705 4491 4769 7028 7598 8701 8860 9540 10398 10400 10873 11719 12705 13254 13626 14577 14766 14783 15043 15301 15326 16223 16362 16390 |
| E1a | 73 263 489 750 1438 2706 3027 3705 4248 4491 4769 7028 7598 8701 8860 9540 10398 10400 10834 10873 11719 12705 13254 13626 14577 14766 14783 15043 15301 15326 16223 16362 16390 |

| | |
|---|---|
| E1a1 | 73 263 489 750 1438 2706 3027 3705 4248 4491 4769 6620 7028 7598 8701 8860 9540 10398 10400 10834 10873 11719 12705 13254 13626 14577 14766 14783 15043 15301 15326 16223 16362 16390 |
| E1a1a | 73 263 489 750 1438 2706 3027 3705 4248 4491 4769 6620 7028 7598 8701 8860 9540 10398 10400 10834 10873 11719 12705 13254 13626 14577 14783 15043 15301 15326 16223 16362 16390 |
| E1a1a1 | 73 263 489 750 1438 2706 3027 3705 4248 4491 4769 6620 7028 7598 8701 8860 9540 10398 10400 10834 10873 11719 12705 13254 13626 14577 14783 15043 15301 15326 16223 16291 16362 16390 |
| E1a1a1a | 73 263 489 750 1438 2706 3027 3705 4248 4491 4769 6620 7028 7598 8701 8843 8860 9540 10398 10400 10834 10873 11719 12705 13254 13626 14577 14783 15043 15301 15326 16223 16291 16362 16390 |
| E1a1a1b | 73 131 263 489 750 1438 2706 3027 3705 4248 4491 4769 6620 7028 7598 8577 8701 8860 9540 10398 10400 10834 10873 11719 12705 13254 13626 14577 14783 15043 15301 15326 16223 16291 16362 16390 |
| E1a1a1b1 | 73 131 263 489 750 1438 2706 3027 3705 4248 4491 4769 6620 7028 7598 8577 8701 8860 9020 9540 10398 10400 10834 10873 11719 12705 13254 13626 14577 14783 15043 15301 15326 16223 16291 16362 16390 |
| E1a1a1b2 | 73 131 263 489 750 1438 2706 3027 3705 4102A 4248 4491 4769 6620 7028 7598 8577 8701 8860 9540 10398 10400 10834 10873 11719 12705 13254 13626 14577 14783 15043 15301 15326 16223 16291 16362 16390 |
| E1a1a1c | 73 263 489 723T 750 1438 2706 3027 3705 4248 4491 4769 6620 7028 7598 8701 8860 9540 9861 10398 10400 10834 10873 11719 12705 13254 13626 14577 14783 15043 15301 15326 16223 16291 16362 16390 |
| E1a1b | 73 263 373 489 750 1438 2706 3027 3705 4248 4491 4769 6620 7028 7598 8701 8860 9540 10398 10400 10834 10873 11719 12705 13254 13626 14577 14766 14783 15043 15301 15326 16223 16362 16390 |
| E1a1b1 | 73 263 373 489 750 1438 2706 3027 3705 4248 4491 4769 6620 6680 7028 7598 8701 8860 9540 10398 10400 10834 10873 11719 12705 13254 13626 14577 14766 14783 15043 15301 15326 16223 16362 16390 |
| E1a1b2 | 73 263 373 489 750 1438 2706 3027 3705 4248 4491 4769 6620 7028 7598 8701 8860 9540 10398 10400 10834 10873 11719 12705 13254 13626 14577 14766 14783 15043 15301 15326 16223 16324 16362 16390 |
| E1a1b3 | 73 263 373 489 750 1438 2706 3027 3705 4248 4491 4769 6620 7028 7598 8701 8860 9540 10398 10400 10834 10873 11719 12705 13254 13626 14577 14766 14783 15043 15301 15326 16362 16390 |
| E1a1b4 | 73 263 373 489 750 1438 2706 3027 3705 4248 4491 4769 6620 7028 7598 8701 8860 9540 10398 10400 10834 10873 11719 12705 13254 13626 14577 14766 14783 15043 15301 15326 16223 16255 16362 16390 |
| E1a1c | 73 263 489 750 1438 2706 3027 3705 4248 4491 4769 6340 6620 7028 7598 8701 8860 9540 10398 10400 10834 10873 11719 12705 13254 13626 14577 14766 14783 15043 15301 15326 16223 16362 16390 |
| E1a2 | 73 152 263 489 750 869 1438 2706 3027 3705 4248 4491 4769 7028 7598 8701 8860 9192 9540 10398 10400 10834 10873 11719 12705 13254 13626 14577 14766 14783 15043 15301 15326 16223 16362 16390 |
| E1a2+(16261) | 73 152 263 489 750 869 1438 2706 3027 3705 4248 4491 4769 7028 7598 8701 8860 9192 9540 10398 10400 10834 10873 11719 12705 13254 13626 14577 14766 14783 15043 15301 15326 16223 (16261) 16362 16390 |

| | |
|---|---|
| E1a2a | 73 152 263 489 750 869 1047 1438 2706 3027 3705 4248 4491 4769 7028 7598 8701 8860 9192 9540 10398 10400 10834 10873 11719 12705 13254 13626 14577 14766 14783 15043 15301 15326 16223 (16261) 16362 16390 |
| E1a2a1 | 73 152 263 489 750 869 1047 1438 2706 3027 3705 4248 4491 4586 4769 7028 7598 8701 8860 9192 9540 10398 10400 10834 10873 11719 12705 13254 13626 14577 14766 14783 15043 15301 15326 16223 (16261) 16362 16390 |
| E1a2a2 | 73 152 263 489 750 869 1047 1438 2706 3027 3705 4248 4491 4769 7028 7598 8701 8860 9192 9540 10398 10400 10834 10873 11719 12705 13254 13626 14577 14766 14783 15043 15301 15326 16176 16223 (16261) 16362 16390 |
| E1a2a3 | 73 152 263 489 750 869 1047 1438 1452 2706 3027 3705 4248 4491 4769 7028 7598 7678 8158 8251 8701 8860 9192 9540 10398 10400 10834 10873 11016 11719 12705 13254 13626 14577 14766 14783 15043 15301 15326 16223 (16261) 16362 16390 |
| E1a2a4 | 73 152 263 310 489 750 869 1047 1438 2706 3027 3705 4248 4491 4541 4769 7028 7598 8701 8860 9192 9540 10398 10400 10834 10873 11719 12705 13254 13626 14577 14766 14783 15043 15301 15326 16223 (16261) 16362 16390 |
| E2 | 73 195 263 489 750 1438 2706 3027 3705 4491 4769 7028 7598 8440 8701 8860 9080 9540 10398 10400 10873 11719 12705 13626 14766 14783 15043 15178 15301 15326 16051 (16185) 16223 16362 16390 |
| E2a | 73 195 263 489 750 1438 2706 3027 3705 4491 4769 7028 7598 8440 8701 8730 8860 9080 9540 10398 10400 10873 11719 12705 13626 14766 14783 15043 15178 15301 15326 16051 (16185) 16223 16362 16390 |
| E2a1 | 73 195 263 489 750 1438 2706 3027 3705 4491 4769 7028 7598 8440 8701 8730 8860 9080 9254 9540 10398 10400 10873 11719 12705 13626 14766 14783 15043 15178 15301 15326 16051 (16185) 16223 16362 16390 |
| E2a1a | 73 195 263 489 750 1438 2706 3027 3421 3705 4454 4491 4769 7028 7598 8440 8701 8730 8860 9080 9254 9540 10398 10400 10742 10873 11719 12705 13626 14766 14783 15043 15178 15301 15326 16051 (16185) 16223 16362 16390 |
| E2a2 | 73 195 263 489 750 1438 2706 3027 3705 4491 4769 7028 7598 8440 8701 8730 8860 9063 9080 9540 10398 10400 10873 11719 12705 13626 14766 14783 15043 15178 15301 15326 16051 (16185) 16223 16362 16390 |
| E2b | 73 195 263 489 750 1438 2706 3027 3705 4491 4769 7028 7598 8440 8701 8860 9080 9540 10398 10400 10873 11719 12705 13626 14766 14783 15043 15178 15301 15326 16051 16086 (16185) 16223 16362 16390 |
| E2b1 | 73 195 263 489 750 1438 2706 3027 3705 4491 4769 7028 7598 8440 8701 8860 9080 9540 10398 10400 10873 11719 12705 13626 14766 14783 15043 15178 15301 15326 16037 16051 16086 (16185) 16223 16362 16390 |
| E2b2 | 73 195 246 263 489 750 1438 2706 3027 3705 4491 4769 7028 7598 8440 8701 8860 9080 9540 10398 10400 10873 11719 12705 13626 14766 14783 15043 15178 15301 15326 16051 16086 (16185) 16223 16362 16390 |
| M10 | 73 263 489 573.XC 709 750 1438 2706 4140 4769 7028 8701 8793 8860 9540 10398 10400 10873 11719 12549 12705 13152 14502 14766 14783 15040 15043 15071 15301 15326 16223 16311 |

| | |
|---|---|
| M10a | 73 263 489 573.XC 709 750 1438 2706 3172.1C 4140 4769 7028 7250 8701 8793 8856 8860 9540 10398 10400 10646 10873 11719 12549 12705 13152 14502 14766 14783 15040 15043 15071 15301 15326 16223 16311 |
| M10a1 | 73 263 489 573.XC 709 750 1438 2706 3172.1C 4140 4769 7028 7250 8701 8793 8856 8860 9540 10398 10400 10646 10873 11719 12549 12705 13152 14502 14766 14783 15040 15043 15071 15218 15301 15326 16223 16311 |
| M10a1+16129 | 73 263 489 573.XC 709 750 1438 2706 3172.1C 4140 4769 7028 7250 8701 8793 8856 8860 9540 10398 10400 10646 10873 11719 12549 12705 13152 14502 14766 14783 15040 15043 15071 15218 15301 15326 16129 16223 16311 |
| M10a1a | 73 263 489 573.XC 709 750 1438 2706 3172.1C 4140 4769 7028 7250 8701 8793 8856 8860 9540 10398 10400 10646 10873 11719 12549 12705 13135 13152 14502 14766 14783 15040 15043 15071 15218 15301 15326 16129 16223 16311 |
| M10a1a1 | 73 263 489 573.XC 709 750 1438 2706 3172.1C 4140 4769 7028 7250 8701 8793 8856 8860 9540 10398 10400 10646 10873 11719 12549 12705 13135 13152 14502 14766 14783 15040 15043 15071 15218 15301 15326 16093 16129 16223 16311 16357 16497 |
| M10a1a1a | 73 263 489 573.XC 709 750 1438 2706 3083 3172.1C 4140 4769 7028 7250 7948 8701 8793 8856 8860 9540 10398 10400 10646 10873 11719 12549 12705 13135 13152 14337 14502 14766 14783 15040 15043 15071 15218 15301 15326 16093 16129 16223 16311 16357 16497 |
| M10a1a1b | 73 146 263 489 573.XC 709 750 1438 2706 3172.1C 4140 4769 7028 7250 8701 8793 8856 8860 9540 10398 10400 10646 10873 11719 12549 12705 13135 13152 14502 14766 14783 15040 15043 15071 15218 15301 15326 16093 16129 16193 16223 16311 16357 16497 |
| M10a1a1b1 | 73 146 263 489 573.XC 709 750 1438 2706 3172.1C 4140 4769 7028 7250 8701 8793 8856 8860 9540 10398 10400 10529 10646 10873 11719 12549 12705 13135 13152 14502 14766 14783 15040 15043 15071 15218 15301 15326 16093 16193 16223 16311 16357 16497 |
| M10a1a1b2 | 73 146 263 489 573.XC 709 750 1438 2706 3172.1C 4140 4769 7028 7250 8701 8793 8856 8860 9540 10289 10398 10400 10646 10873 11719 12549 12705 13135 13152 14502 14766 14783 15040 15043 15071 15218 15301 15326 16093 16129 16193 16223 16311 16357 16497 |
| M10a1b | 73 263 489 573.XC 709 750 1438 2706 3172.1C 4140 4769 7028 7250 8701 8793 8856 8860 9540 10398 10400 10646 10873 11719 12549 12705 13152 14502 14766 14783 15040 15043 15071 15218 15301 15326 16066 16223 16311 |
| M10a2 | 73 200 263 489 573.XC 709 750 1438 2706 3172.1C 4140 4769 7028 7250 8701 8793 8856 8860 9540 10398 10400 10646 10873 11719 12549 12705 13152 14502 14766 14783 15040 15043 15071 15301 15326 16066 16223 16311 |
| M11 | 73 215 263 318 326 489 750 1095 1438 2706 4769 6531 7028 7642 8108 8701 8860 9540 9950 10398 10400 10873 11719 11969 12705 14766 14783 15043 15301 15326 16223 |
| M11+200 | 73 200 215 263 318 326 489 750 1095 1438 2706 4769 6531 7028 7642 8108 8701 8860 9540 9950 10398 10400 10873 11719 11969 12705 14766 14783 15043 15301 15326 16223 |

| | |
|---|---|
| M11a'b | 73 200 215 263 318 326 489 750 1095 1438 2706 4769 6531 7028 7642 8108 8701 8860 9540 9950 10398 10400 10873 11719 11969 12705 13074 14766 14783 15043 15301 15326 16223 |
| M11a | 73 (198) 200 215 263 318 326 489 750 1095 1438 2706 4769 6531 7028 7642 8108 8701 8860 9540 9950 10398 10400 10873 11719 11969 12705 13074 14340 14766 14783 15043 15301 15326 16223 |
| M11a1 | 73 (198) 200 215 263 318 326 489 750 1095 1438 2706 4769 6531 6632 7028 7642 8108 8701 8860 9540 9950 10398 10400 10873 11719 11969 12705 13074 14340 14766 14783 15043 15301 15326 16223 |
| M11a2 | 73 146 (198) 200 215 263 318 326 489 750 1095 1438 2706 4769 6531 7028 7642 8108 8701 8860 9540 9950 10398 10400 10873 11719 11969 12705 13074 14340 14766 14783 15043 15301 15326 16173 16223 |
| M11b | 73 200 215 263 318 326 489 750 1095 1438 2706 4769 6531 7028 7642 8108 8701 8860 9540 9950 10398 10400 10873 11719 11969 12705 13074 13890 14766 14783 15043 15301 15326 16223 |
| M11b1 | 73 200 215 263 318 326 489 750 1095 1438 2706 4769 6531 7028 7642 8108 8701 8860 9540 9950 10398 10400 10873 11719 11969 12705 13074 13890 14766 14783 14790 15043 15301 15326 16223 |
| M11b1a | 73 146 200 215 263 318 326 489 750 1095 1438 2706 4769 6531 7028 7642 8108 8701 8860 9540 9950 10398 10400 10685 10873 11719 11969 12705 13074 13890 14766 14783 14790 15043 15301 15326 16223 |
| M11b1a1 | 73 146 215 263 318 326 489 750 1095 1438 2706 4769 6531 7028 7642 8108 8701 8860 9540 9950 10398 10400 10685 10873 11719 11969 12705 13074 13890 13928T 14766 14783 14790 15043 15301 15326 16223 16497 |
| M11b2 | 73 200 215 263 318 326 459.1C 489 750 1095 1438 2706 4769 5192 6531 7028 7642 8108 8701 8860 9540 9950 10398 10400 10873 11719 11969 12705 13074 13890 14766 14783 15043 15301 15326 16223 |
| M11d | 73 200 215 263 318 326 471 489 750 1095 1438 2706 3523 4769 6383 6531 7028 7642 8108 8701 8860 9540 9950 10398 10400 10873 11350 11719 11969 12705 14766 14783 15043 15301 15326 16223 16295 |
| M11c | 73 215 263 318 326 489 750 1095 1289 1438 2706 4769 6531 7028 7642 8108 8701 8860 9540 9950 10398 10400 10873 11719 11969 12705 14502 14766 14783 15043 15301 15326 16169 16223 |
| M12'G | 73 263 489 750 1438 2706 4769 7028 8701 8860 9540 10398 10400 10873 11719 12705 14569 14766 14783 15043 15301 15326 16223 |
| M12 | 73 263 489 750 1438 2706 4170 4769 5580 7028 8701 8860 9540 10398 10400 10873 11719 12030 12372 12705 14569 14727 14766 14783 15010 15043 15301 15326 16223 16234 16290 |
| M12a | 73 263 318 489 750 1438 2706 4170 4769 5580 7028 8701 8860 9540 10398 10400 10873 11719 12030 12358 12372 12705 14569 14727 14766 14783 15010 15043 15301 15326 16223 16234 16290 |
| M12a1 | 73 125 127 128 263 318 489 750 1438 2706 4170 4769 5580 7028 8701 8860 9540 10398 10400 10873 11719 12030 12358 12372 12705 14569 14727 14766 14783 15010 15043 15301 15326 16223 16234 16290 |
| M12a1a | 73 125 127 128 263 318 489 750 1438 2706 4170 4769 5580 7028 8701 8860 9540 10398 10400 10873 11719 12030 12358 12372 12705 14569 14727 14766 14783 15010 15043 15301 15326 15463 15651 16223 16234 |

| | |
|---|---|
| M12a1a1 | 73 125 127 128 263 318 489 513 750 1438 2706 4170 4769 5580 7028 8701 8860 9490 9540 10398 10400 10873 11719 12030 12358 12372 12705 14569 14727 14766 14783 15010 15043 15301 15326 15463 15651 16223 16234 16290 16362 |
| M12a1a2 | 73 125 127 128 150 263 318 489 750 1438 2706 4170 4769 5580 7028 8005 8701 8860 9540 10398 10400 10873 11719 12030 12358 12372 12705 14569 14693 14727 14766 14783 15010 15043 15301 15326 15463 15651 16093 16223 16290 |
| M12a1b | 73 125 127 128 263 489 750 1438 2706 4170 4769 5492 5580 7028 8701 8860 9540 10398 10400 10873 11353 11719 12030 12358 12372 12705 14569 14727 14766 14783 15010 15043 15301 15326 16172 16223 16234 16290 |
| M12a2 | 73 263 318 463 489 573.XC 750 1438 2706 4170 4769 5580 7028 8701 8860 9540 10398 10400 10873 11719 12030 12358 12372 12705 14569 14727 14766 14783 15010 15043 15301 15326 16223 16234 16261 16290 |
| M12b | 73 263 489 750 1438 2706 4170 4769 5580 7028 8701 8860 9540 10398 10400 10873 11359 11719 12030 12372 12705 14569 14727 14766 14783 15010 15043 15301 15326 16129 16172 16223 16234 16290 |
| M12b1 | 73 263 489 750 1438 2706 4170 4769 5580 7028 8701 8860 9540 10398 10400 10873 11359 11719 12030 12372 12705 14569 14727 14766 14783 14788 15010 15043 15301 15326 16129 16172 16223 16234 16290 |
| M12b1a | 73 263 489 750 1331 1438 2706 4170 4769 5580 7028 8701 8860 9540 10398 10400 10873 11359 11719 12030 12372 12705 14569 14727 14766 14783 14788 15010 15043 15236 15301 15326 15951 16129 16172 16223 16234 16290 |
| M12b1a1 | 73 263 489 750 1331 1438 2706 4170 4769 5580 7028 7080 8701 8860 9540 10398 10400 10873 11359 11719 12030 12372 12705 13708 14569 14727 14766 14783 14788 15010 15043 15236 15301 15326 15951 16129 16172 16223 16234 16290 |
| M12b1a2 | 73 263 489 750 1331 1438 2706 4170 4769 5580 7028 8701 8860 9540 10398 10400 10873 11260 11359 11719 12030 12372 12705 14569 14727 14766 14783 14788 15010 15043 15236 15301 15326 15951 16129 16172 16223 16234 16290 16319 |
| M12b1a2a | 73 263 489 750 1438 2706 4170 4769 5580 7028 8701 8749 8860 9540 10398 10400 10873 11260 11359 11719 12030 12372 12705 14569 14727 14766 14783 14788 15010 15043 15236 15301 15326 15951 16129 16172 16223 16234 16290 16319 |
| M12b1a2b | 73 263 489 750 1331 1438 2706 4170 4769 5580 7028 8701 8860 9540 10398 10400 10873 11260 11359 11719 12030 12372 12705 14569 14727 14766 14783 14788 15010 15043 15236 15301 15326 15951 16129 16172 16223 16234 16260 16290 16319 |
| M12b1b | 73 185 189 263 489 750 1438 2706 3897 4170 4769 5336 5580 7028 8701 8860 9540 10398 10400 10873 11359 11719 12030 12372 12705 14569 14727 14766 14783 14788 14869 15010 15043 15301 15326 16108 16129 16172 16223 16234 16290 |
| M12b2 | 73 152 263 489 750 1438 2706 4170 4769 5580 7028 7337 8701 8860 9540 10398 10400 10873 11359 11719 12030 12372 12705 14569 14727 14766 14783 15010 15043 15301 15326 15884T 16129 16172 16223 16290 16305 |

| | |
|---|---|
| M12b2a | 73 151 152 263 489 750 1438 2706 4170 4769 5580 7028 7337 8701 8860 9540 9604 10398 10400 10873 11359 11719 12030 12372 12705 14569 14727 14766 14783 15010 15043 15301 15326 15884T 16129 16172 16223 16290 16305 16311 |
| G | 73 263 489 709 750 1438 2706 4769 4833 5108 7028 8701 8860 9540 10398 10400 10873 11719 12705 14569 14766 14783 15043 15301 15326 16223 16362 |
| G1 | 73 263 489 709 750 1438 2706 4769 4833 5108 7028 8200 8701 8860 9540 10398 10400 10873 11719 12705 14569 14766 14783 15043 15301 15323 15326 15497 16223 16362 |
| G1a | 73 150 263 489 709 750 1438 2706 4769 4833 5108 7028 7867 8200 8701 8860 9540 10398 10400 10873 11719 12705 14569 14766 14783 15043 15301 15323 15326 15497 16223 16362 |
| G1a1 | 73 150 263 489 709 750 1438 2706 4769 4833 5108 7028 7867 8200 8701 8860 9540 10398 10400 10873 11719 12705 14569 14766 14783 15043 15301 15323 15326 15497 15860 16223 16325 16362 |
| G1a1a | 73 150 263 489 709 750 1438 2706 4769 4793 4833 5108 7028 7867 8200 8701 8860 9540 10398 10400 10873 11719 11914 12705 14569 14766 14783 15043 15301 15323 15326 15497 15860 16223 16325 16362 |
| G1a1a1 | 73 150 263 489 709 750 827 1438 2706 4769 4793 4833 5108 7028 7867 8200 8701 8860 9540 10398 10400 10873 11719 11914 12705 14569 14766 14783 15043 15301 15323 15326 15497 15860 16223 16325 16362 |
| G1a1a2 | 73 150 263 489 709 750 1299 1438 2706 4769 4793 4833 5108 7028 7867 8200 8701 8860 9540 10398 10400 10873 11719 11914 12705 14569 14766 14783 15043 15301 15323 15326 15497 15860 16223 16325 16362 |
| G1a1a3 | 73 150 263 489 709 750 1438 2706 4769 4793 4833 5108 5263 7028 7867 8200 8566 8701 8860 9540 10398 10400 10873 11719 11914 12705 14569 14766 14783 15043 15301 15323 15326 15497 15860 16187 16223 16325 16362 |
| G1a1a4 | 73 150 263 489 709 750 1438 2706 4769 4793 4833 5108 6167G 7028 7867 8200 8701 8860 9540 10398 10400 10873 11719 11914 12705 14569 14766 14783 15043 15301 15323 15326 15497 15860 16223 16325 16362 |
| G1a1b | 73 150 263 489 709 750 1438 2706 4769 4833 5108 7028 7867 8200 8701 8860 9540 10398 10400 10873 11719 12178 12705 14569 14766 14783 15043 15301 15323 15326 15497 15860 16223 16325 16362 |
| G1a2'3 | 73 150 263 489 709 750 1438 2706 4769 4833 5108 7028 7867 8200 8701 8860 9540 10398 10400 10873 11719 12705 14569 14766 14783 15043 15301 15323 15326 15497 16184 16223 16362 |
| G1a2 | 73 150 263 489 709 750 1438 2706 4769 4833 5108 7028 7867 8200 8701 8860 9540 10398 10400 10873 11719 12040 12705 14569 14766 14783 15043 15301 15323 15326 15497 16184 16223 16290 16362 |
| G1a3 | 73 263 489 709 750 1438 2706 4769 5108 7028 7867 8200 8701 8860 9540 10398 10400 10873 11719 12705 14569 14766 14783 15043 15301 15323 15326 15497 16184 16214 16223 16362 |
| G1b | 73 (207) 263 489 709 750 1438 2706 4769 4833 5108 7028 8200 8701 8860 9540 10398 10400 10873 11719 12361 12705 12972 14569 14766 14783 15043 15301 15323 15326 15497 16017 (16093) 16223 |

| | |
|---|---|
| G1b1 | 73 (207) 263 489 709 750 1438 2706 4769 4833 5108 7028 8200 8701 8860 9540 10398 10400 10873 11719 12361 12705 12972 14569 14766 14783 15043 15301 15323 15326 15497 16017 (16093) 16207 16223 |
| G1b+16129 | 73 (207) 263 489 709 750 1438 2706 4769 4833 5108 7028 8200 8701 8860 9540 10398 10400 10873 11719 12361 12705 12972 14569 14766 14783 15043 15301 15323 15326 15497 16017 (16093) 16129 16223 |
| G1b2 | 73 (207) 263 489 709 750 1438 2706 4646 4769 4833 5108 7028 8200 8701 8860 9540 10143 10398 10400 10873 11719 12361 12705 12972 14569 14766 14783 15043 15301 15323 15326 15497 16017 (16093) 16129 16223 |
| G1b3 | 73 (207) 263 489 709 750 1438 2706 4769 4833 5108 7028 8200 8701 8860 9540 10398 10400 10873 11719 12361 12705 12972 14569 14766 14783 15043 15301 15323 15326 15497 16017 (16093) 16129 16172 16223 16265 |
| G1b4 | 73 (207) 263 489 709 750 1438 2706 3456 4769 4833 5108 7028 8200 8701 8860 9540 10398 10400 10873 11719 12361 12705 12972 14569 14766 14783 15043 15301 15323 15326 15497 16017 (16093) 16129 16223 |
| G1c | 73 263 489 593 709 750 1438 2706 4769 4833 5108 7028 8200 8701 8860 9540 9966 10398 10400 10873 11719 12705 14569 14766 14783 15043 15301 15323 15326 15497 16223 16362 |
| G1c1 | 73 263 489 593 709 750 1438 2226 2706 4769 4833 5108 7028 8200 8701 8860 9540 9966 10398 10400 10873 11719 12705 14569 14766 14783 15043 15301 15323 15326 15497 15565 16220 16223 16362 |
| G1c2 | 73 263 489 593 709 750 1438 2706 4769 4833 4973 5108 7028 8161 8200 8701 8860 9540 9966 10398 10400 10873 11719 12705 14569 14766 14783 15043 15301 15323 15326 15497 16223 16362 |
| G2 | 73 263 489 709 750 1438 2706 4769 4833 5108 5601 7028 8701 8860 9540 10398 10400 10873 11719 12705 13563 14569 14766 14783 15043 15301 15326 16223 16362 |
| G2a'c | 73 263 489 709 750 1438 2706 4769 4833 5108 5601 7028 8701 8860 9540 9575 10398 10400 10873 11719 12705 13563 14569 14766 14783 15043 15301 15326 16223 16362 |
| G2a | 73 263 489 709 750 1438 2706 4769 4833 5108 5601 7028 7600 8701 8860 9377 9540 9575 10398 10400 10873 11719 12705 13563 14569 14766 14783 15043 15301 15326 16223 (16227) 16278 16362 |
| G2a1 | 73 263 489 709 750 1438 2706 4769 4833 5108 5601 7028 7600 8701 8860 9377 9540 9575 10398 10400 10873 11719 12705 13563 14200 14569 14766 14783 15043 15301 15326 16223 (16227) 16278 16362 |
| G2a1b | 73 146 207 263 489 709 750 1438 2706 4769 4833 5108 5601 7028 7600 8063 8701 8860 9266 9377 9540 9575 10398 10400 10873 11719 12705 12753 13563 14200 14569 14766 14783 15043 15301 15326 15758 16223 (16227) 16278 16362 |
| G2a1+16189 | 73 263 489 709 750 1438 2706 4769 4833 5108 5601 7028 7600 8701 8860 9377 9540 9575 10398 10400 10873 11719 12705 13563 14200 14569 14766 14783 15043 15301 15326 16189 16223 (16227) 16278 16362 |
| G2a1+16189+16194 | 73 263 489 709 750 1438 2706 4769 4833 5108 5601 7028 7600 8701 8860 9377 9540 9575 10398 10400 10873 11719 12705 13563 14200 14569 14766 14783 15043 15301 15326 16189 16194 16223 (16227) 16278 16362 |

| | |
|---|---|
| G2a1c | 73 263 489 709 750 1438 2706 4769 4833 5108 5601 7028 7600 8701 8860 9377 9540 9575 10398 10400 10873 11719 12705 13563 14200 14569 14766 14783 15043 15301 15326 16189 16194 16195 16223 (16227) 16278 |
| G2a1c1 | 73 263 489 709 750 1438 2706 4769 4833 5108 5601 7028 7600 8701 8860 9377 9540 9575 10398 10400 10873 11719 12705 13563 14200 14569 14766 14783 15043 15301 15326 16189 16194C 16195 16223 (16227) |
| G2a1c2 | 73 263 489 709 750 1438 2706 4769 4833 5108 5601 7028 7337 7600 8701 8860 9377 9540 9575 10398 10400 10873 11719 12705 13563 14200 14569 14766 14783 15043 15301 15326 16189 16194 16195 16223 (16227) 16278 16362 |
| G2a1d | 73 260 263 489 709 750 1438 2706 4769 4833 5108 5601 6737 7028 7600 8701 8860 9377 9540 9575 10398 10400 10873 11719 12311 12705 13563 14200 14569 14766 14783 15043 15301 15326 16189 16223 (16227) 16278 16362 |
| G2a1d1 | 73 260 263 489 709 750 1438 2706 4505 4769 4833 5108 5601 6737 7028 7600 8701 8860 9377 9540 9575 10398 10400 10873 11719 12311 12705 13563 14200 14281 14569 14766 14783 15043 15301 15326 16189 16223 (16227) 16278 16362 |
| G2a1d1a | 73 260 263 489 709 750 1438 2706 4505 4769 4833 5108 5601 6737 7028 7600 8701 8860 9165 9377 9540 9575 10398 10400 10873 11719 12311 12705 13563 14200 14281 14569 14766 14783 15043 15301 15326 16189 16223 (16227) 16278 16362 |
| G2a1d2 | 73 260 263 489 709 750 1438 2706 4769 5108 5601 6737 7028 7600 8701 8748 8860 9377 9540 9575 10398 10400 10873 11719 12311 12705 13563 14200 14569 14766 14783 15043 15301 15326 16223 (16227) 16278 16362 |
| G2a1d2a | 73 260 263 489 709 750 1438 2706 4769 5108 5601 6737 7028 7600 8108 8440 8701 8748 8860 9377 9540 9575 10398 10400 10873 11719 12311 12705 13563 14200 14569 14766 14783 15043 15301 15326 16223 (16227) 16278 16362 |
| G2a1e | 73 263 489 709 750 1438 2706 4769 4833 5108 5601 7028 7600 8701 8860 9377 9540 9575 10398 10400 10873 11719 12705 13563 14200 14569 14766 14783 15043 15301 15326 16051 16150 16223 (16227) 16278 16362 |
| G2a1f | 73 263 489 709 750 1438 2706 4769 4833 5108 5601 7028 7600 8701 8860 9377 9540 9575 10398 10400 10873 11719 12705 13563 14200 14569 14766 14783 15043 15301 15326 16114 16223 (16227) 16278 16362 |
| G2a1f1 | 73 199 263 489 709 750 1438 2706 4769 4833 4843 5108 5601 7028 7600 8701 8860 9377 9540 9575 10248 10398 10400 10873 11719 12705 13563 14200 14569 14766 14783 15043 15301 15326 16114 16223 (16227) 16278 16362 |
| G2a1g | 73 263 318 489 709 750 1438 2706 4769 4833 5108 5601 7028 7600 8701 8860 9377 9540 9575 10398 10400 10873 11719 12705 13563 14200 14569 14766 14783 15043 15301 15326 16223 (16227) 16278 16293C 16362 |
| G2a1h | 73 263 489 709 750 1438 2706 3777 4769 4833 5108 5601 7028 7600 8701 8860 9377 9540 9575 10398 10400 10873 11719 12705 13194 13563 14200 14569 14766 14783 15043 15301 15326 16223 (16227) 16278 16311 16362 |
| G2a+152 | 73 152 263 489 709 750 1438 2706 4769 4833 5108 5601 7028 7600 8701 8860 9377 9540 9575 10398 10400 10873 11719 12705 13563 14569 14766 14783 15043 15301 15326 16223 (16227) 16278 16362 |

| | |
|---|---|
| G2a2 | 73 152 263 489 709 711 750 1438 2706 4769 4833 5108 5601 7028 7600 8701 8860 8943 9377 9540 9575 10398 10400 10873 11719 12705 13563 14569 14766 14783 15043 15110 15301 15326 16223 (16227) 16278 16362 |
| G2a2a | 73 152 207 263 489 709 711 750 1438 2706 4769 4833 5108 5601 7028 7600 8701 8860 8943 9377 9540 9575 10398 10400 10873 11719 12705 13395 13563 14569 14766 14783 15043 15110 15301 15326 16223 (16227) 16278 16362 |
| G2a3 | 73 152 185 263 489 709 750 1438 2706 3882 4769 4833 5108 5601 7028 7600 8701 8860 9377 9540 9575 10398 10400 10873 11719 12705 13563 14569 14766 14783 15043 15301 15326 16223 (16227) 16262 16278 16294 16362 |
| G2a3a | 73 152 185 263 489 709 750 1438 2706 3882 4769 4833 5108 5601 7028 7600 8701 8860 9377 9540 9575 10398 10400 10873 11719 12705 13563 14569 14766 14783 15043 15301 15326 16169 16223 (16227) 16262 16278 16294 16318 16362 16526 |
| G2a4 | 73 152 263 489 709 750 1438 2706 4769 4833 5108 5601 7028 7600 8701 8860 9377 9540 9575 10398 10400 10873 11719 12280 12705 13563 14569 14766 14783 15043 15301 15326 16223 (16227) 16272 16278 16319 16362 |
| G2a5 | 73 152 263 489 709 750 1438 2706 4688 4769 4833 5108 5601 7028 7600 8701 8860 9377 9540 9575 10398 10400 10873 11719 12192 12705 13383 13563 14569 14766 14783 14861 15043 15301 15326 15562 16223 (16227) 16234 16278 16362 |
| G2c | 73 152 195 263 489 709 750 1438 2706 4769 4833 5108 5601 5782 7028 7503d 8701 8860 9540 9575 10398 10400 10873 11719 12441 12705 13145 13563 14569 14766 14783 14839 15043 15301 15326 16223 16362 |
| G2b | 73 263 489 709 750 1438 2706 4769 4833 5108 5601 7028 8701 8860 8877 9540 10398 10400 10873 11719 12705 13563 14569 14766 14783 15043 15301 15326 16223 16362 |
| G2b1 | 73 263 489 709 750 1438 2706 4769 4833 4853 5108 5601 7028 8701 8860 8877 9540 10398 10400 10873 11719 12705 13563 14569 14766 14783 15043 15301 15326 16223 16362 |
| G2b1a | 73 263 489 709 750 1438 2706 4769 4833 4853 5108 5601 7028 8701 8860 8877 9540 10398 10400 10873 11151 11719 12705 13563 14569 14766 14783 15043 15301 15326 16223 16362 |
| G2b1a1 | 73 207 263 489 709 750 1438 2706 3398 4048 4769 4833 4853 5108 5601 7028 8701 8860 8877 9540 10398 10400 10873 11151 11719 12705 13191 13563 14182 14569 14766 14783 15043 15301 15326 16223 16362 |
| G2b1a2 | 73 489 709 750 1438 2706 3593 4769 4833 4853 5108 5601 7028 8701 8860 8877 9540 10398 10400 10873 11151 11719 12705 13563 14569 14766 14783 15043 15301 15326 16172 16223 16362 |
| G2b1b | 73 263 489 709 750 1438 2706 4769 4833 4853 5108 5601 7028 8701 8860 8877 9540 10398 10400 10873 11719 12375 12705 13563 14569 14766 14783 15043 15301 15326 16223 16269 16362 |
| G2b2 | 73 263 489 709 750 1438 2706 4769 4833 5108 5601 6932 7028 8701 8860 8877 9540 10398 10400 10873 11719 12705 13563 14569 14766 14783 15043 15301 15326 16223 16362 |

| | |
|---|---|
| G2b2a | 73 263 489 709 750 1438 1692 2706 4680A 4769 4833 5108 5601 6932 7028 8701 8860 8877 9540 10398 10400 10873 11719 12705 13563 14569 14766 14783 15043 15301 15326 16223 16362 |
| G2b2b | 73 263 489 709 750 1438 2706 4769 4833 5108 5601 6932 7028 8701 8860 8877 9540 10398 10400 10873 11719 12705 13563 14569 14766 14783 15043 15301 15326 15930 16223 16260 16362 |
| G2b2c | 73 263 489 709 750 1438 2706 3591 4769 4833 5108 5601 6932 7028 8701 8860 8877 9540 10398 10400 10873 11719 12705 13563 14569 14766 14783 15043 15301 15326 16183 16223 16362 |
| G3 | 73 263 489 709 750 1438 2706 4769 4833 5108 7028 8701 8860 9540 10398 10400 10873 11719 12705 14569 14766 14783 15043 15301 15326 16223 16274 16362 |
| G3a | 73 263 489 709 750 1438 2706 4769 4833 5108 7028 8701 8860 9540 10398 10400 10873 11719 12705 14569 14766 14783 15043 15301 15326 15746 16223 16274 16362 |
| G3a1'2 | 73 143 263 489 709 750 1438 2706 4769 4833 5108 7028 8701 8860 9540 10398 10400 10873 11719 12705 14569 14766 14783 15043 15301 15326 15746 16223 16274 16362 |
| G3a1 | 16T 73 143 150 263 489 709 750 1438 2706 4769 4833 5108 7028 8701 8860 9540 10398 10400 10873 11719 11914 12705 14569 14766 14783 15043 15301 15326 15746 16215 16223 16274 |
| G3a1a | 16T 73 143 150 263 489 709 750 1438 2706 4769 4833 5108 7028 8701 8860 8861 9540 10398 10400 10873 11719 11914 12705 14569 14766 14783 15043 15301 15326 15746 16215 16223 16274 |
| G3a2 | 73 143 263 489 709 750 1438 2706 4769 4833 5108 7028 7621 8701 8860 9540 10398 10400 10873 11719 12705 14569 14766 14783 15043 15301 15326 15746 16223 16274 16362 |
| G3a2+152 | 73 143 152 263 489 709 750 1438 2706 4769 4833 5108 7028 7621 8701 8860 9540 10398 10400 10873 11719 12705 14569 14766 14783 15043 15301 15326 15746 16223 16274 16362 |
| G3a2a | 73 143 152 263 489 573.XC 709 750 1438 2706 4769 4833 5108 6086 6221 7028 7621 8701 8860 9540 10398 10400 10873 11719 12705 14569 14766 14783 15043 15301 15326 15746 16189 16223 16265C 16274 16362 |
| G3a3 | 73 263 489 709 750 1438 2706 4769 4833 5108 7028 8701 8860 9540 10398 10400 10873 11719 12408 12705 14569 14766 14783 15043 15301 15326 15746 16223 16274 16362 16390 |
| G3b | 73 263 489 709 750 1438 2706 4769 4833 5108 7028 8701 8860 9540 10398 10400 10873 11719 12705 13477 14569 14605 14766 14783 15043 15301 15326 15927 16223 16274 16362 |
| G3b1 | 73 195 263 489 709 750 1438 2706 4769 4833 5108 7028 8701 8860 9540 9599 10398 10400 10873 11719 12705 13477 14569 14605 14766 14783 15043 15301 15326 15927 16223 16274 16362 |
| G3b2 | 73 263 489 709 750 1438 2706 4769 4833 5108 6896 7028 8701 8860 9540 10398 10400 10873 11719 12705 13477 14569 14605 14766 14783 15043 15301 15326 15927 16223 16274 16362 |

| | |
|---|---|
| G4 | 73 191.1A 194 263 489 709 750 1438 2706 4769 4833 5051 5108 5460 6216 7028 7521 7660 8701 8860 9540 9670 10398 10400 10873 11383 11719 12705 14569 14766 14783 15043 15301 15326 15940 (16093) 16114A 16223 16362 |
| M13'46'61 | 73 152 263 489 750 1438 2706 4769 6253 7028 8701 8860 9540 10398 10400 10873 11719 12705 14766 14783 15043 15301 15326 16223 |
| M13 | 73 152 263 489 750 1438 2706 4769 6023 6253 7028 8701 8860 9540 10398 10400 10873 11719 12705 14766 14783 15043 15301 15326 16223 |
| M13a'b | 73 152 263 489 750 1438 2706 4769 6023 6253 7028 8701 8860 9540 10398 10400 10873 11719 12705 14766 14783 15043 15301 15326 15924 |
| M13a | 73 152 263 489 750 1438 2706 3644 4769 5773 6023 6253 6620 7028 8701 8860 9540 10398 10400 10411 10790 10873 11719 12705 14766 14783 15043 15301 15326 15924 16145 16188 16189 16223 |
| M13a1 | 73 152 263 489 750 1438 2706 3644 4769 5773 6023 6253 6620 7028 8701 8860 9540 10398 10400 10411 10790 10873 11719 12705 13135 14766 14783 15043 15301 15326 15924 16145 16188 16189 16223 16381 |
| M13a1a | 73 152 263 489 750 1438 2706 3644 4769 5773 6023 6253 6620 7028 7673 8701 8860 9540 10398 10400 10411 10790 10873 11719 11959 12705 13135 14766 14783 15043 15301 15326 15924 16145 16188 16189 16223 16311 16381 |
| M13a1b | 73 152 263 489 750 1438 2706 3644 4769 5773 6023 6253 6620 7028 8701 8860 9540 10398 10400 10411 10790 10873 11719 12705 13135 14766 14783 15043 15301 15326 15924 16145 16148 16188 16189 16223 16381 |
| M13a1b1 | 73 152 263 489 750 1438 2706 3644 4769 5045 5773 6023 6253 6620 7028 8701 8860 9540 10398 10400 10411 10790 10873 11719 12705 13135 14766 14783 15043 15301 15326 15924 16145 16148 16188 16189 16223 |
| M13a2 | 73 152 263 489 513 750 1438 2706 3644 4769 5773 6023 6253 6620 7028 8405 8701 8860 9540 10373 10398 10400 10411 10790 10873 11719 12705 14766 14783 15043 15301 15326 15924 16145 16168 16188 16189 16223 16257 16311 |
| M13b | 73 263 489 750 1438 2706 4769 6023 6253 7028 8701 8860 9182 9540 10398 10400 10873 11665A 11719 12705 14766 14783 15043 15301 15326 15468 15924 15930 16129 16223 |
| M13b1 | 73 263 489 709 750 1438 2706 4769 6023 6253 7028 8701 8860 9182 9540 10172 10398 10400 10873 11665A 11719 12705 14766 14783 15043 15301 15326 15468 15924 15930 16129 16223 16263 |
| M13b2 | 73 263 489 750 1211 1438 2706 4742 4769 6023 6253 7028 8701 8860 9012G 9182 9540 10398 10400 10873 11506 11665A 11719 12705 14766 14783 15043 15301 15326 15468 15930 16129 16172 16195 16223 16270 |
| M13c | 73 152 263 489 750 1438 2706 3666 4769 5910 6023 6253 7028 8701 8860 9540 10398 10400 10873 11719 11944 12705 14766 14783 15043 15301 15326 16223 16381 16390 |
| M13'46'61+16362 | 73 152 263 489 750 1438 2706 4769 6253 7028 8701 8860 9540 10398 10400 10873 11719 12705 14766 14783 15043 15301 15326 16223 16362 |
| M46 | 73 146 152 263 489 750 1438 2706 3588 4769 6253 7028 8701 8860 9540 10398 10400 10873 11719 12705 14766 14783 15043 15301 15326 16223 16362 |

| | |
|---|---|
| M46a | 73 146 152 263 489 750 1393 1438 2706 3588 4394 4769 6032 6253 7028 7828 8701 8860 8886 9115 9540 10130 10398 10400 10873 11008 11151 11719 12705 13434 14766 14783 15043 15301 15326 16223 16278 16300 16343T 16362 |
| M61 | 73 152 263 489 750 1193 1342 1438 2706 4769 6253 7028 8269 8701 8860 9540 10398 10400 10873 11719 11810 12705 12732 14766 14783 15043 15301 15326 16223 16362 16381 |
| M61a | 73 152 263 489 750 980 1193 1342 1438 2706 3438 4769 5582C 6253 7028 8269 8701 8790 8860 9540 10398 10400 10873 11719 11810 12705 12732 14766 14783 15043 15301 15326 16223 16270 16362 16381 |
| M14 | 73 234 263 489 750 1438 2706 4216 4769 6962 7028 8701 8860 9540 10398 10400 10873 11719 12705 14766 14783 15043 15301 15326 16223 |
| M15 | 64 73 183 263 489 750 1438 2706 4769 7028 8167 8701 8860 9540 10398 10400 10873 11002 11629T 11719 12662 12705 13659 14284 14766 14783 15043 15301 15326 15412 16147A 16193 16223 16249 16362 |
| M17 | 73 263 489 750 930C 1438 2706 4769 7028 8701 8860 9540 10398 10400 10873 11719 12705 12973 14766 14783 15043 15301 15326 16209 16223 |
| M17a | 73 263 489 750 862 930C 1438 2706 4769 7028 8701 8860 9540 10324 10398 10400 10873 11016 11719 11908 12705 12711 12804 12973 14766 14783 15043 15301 15326 15530 15802 16129 16209 16223 16325 |
| M17c | 73 263 489 750 930C 1438 1598 2706 4769 7028 8701 8860 9540 10398 10400 10873 11719 12705 12973 14766 14783 15043 15301 15326 16209 16223 |
| M17c1 | 73 143 263 489 750 930C 1438 1598 2706 4769 7028 8701 8860 9540 10398 10400 10873 11719 12705 12973 14766 14783 15043 15301 15326 16209 16223 16304 |
| M17c1a | 73 143 263 489 750 930C 1438 1598 2706 4769 7028 8701 8860 9540 10398 10400 10873 11719 12705 12973 13651 14766 14783 15043 15301 15326 16209 16223 16304 |
| M17c1a1 | 73 143 263 489 750 930C 1438 1598 2706 4769 7028 8701 8860 9540 10398 10400 10873 11719 12705 12973 13651 14766 14783 15043 15301 15326 15853 16209 16223 16233 16274 16304 |
| M17c1a1a | 73 143 263 489 750 930C 1438 1598 2706 2833 4769 7028 8701 8860 9540 10398 10400 10873 11719 12705 12973 13651 14766 14783 15043 15301 15326 15853 16209 16223 16233 16274 16304 |
| M19'53 | 73 263 489 750 1438 2706 4769 7028 8701 8860 9540 10398 10400 10873 11167 11719 12705 14766 14783 15043 15301 15326 16223 |
| M19 | 73 152 263 489 527 750 1438 2392 2706 3828 4769 5046 5250 6620 6896 7028 8701 8860 9540 10398 10400 10873 11167 11719 12397 12465 12705 14766 14783 15043 15301 15326 15935 16223 16249 16362 |
| M53 | 73 240 263 390T 489 572 750 1438 2706 4769 5493 5821 7028 8701 8860 9302 9540 10398 10400 10873 11167 11560 11719 12705 14766 14783 15043 15301 15326 16051 16189 16223 16316 |
| M53b | 73 240 263 390T 489 572 750 1438 1462 2706 4769 5493 5821 6815 7028 8701 8860 9302 9540 10398 10400 10873 11023 11167 11560 11719 12705 14766 14783 15043 15301 15326 16051 16189 16223 16316 16399 |
| M21 | 73 263 489 750 1438 2706 4769 7028 8701 8860 9540 10398 10400 10873 11482 11719 12705 14766 14783 15043 15301 15326 16223 |

| | |
|---|---|
| M21a | 73 263 489 709 750 1438 2706 3796 4769 7028 8701 8860 9540 10202 10398 10400 10873 11287 11482 11611 11719 12705 14766 14783 15043 15301 15326 15924 16129 16223 16256 16271 16362 |
| M21b | 73 263 489 750 1438 2706 3915 4769 5108 7028 7861 8701 8860 9540 10398 10400 10873 11482 11719 12705 14766 14783 15043 15301 15326 16223 |
| M21b1 | 73 263 489 750 1438 2706 3915 4769 5108 7028 7861 8701 8860 9116 9540 10398 10400 10873 11482 11719 12705 12940 14766 14783 15043 15301 15326 16223 |
| M21b1a | 73 263 489 750 1438 2706 3915 4769 5108 7028 7765 7861 8701 8860 9116 9540 10398 10400 10873 11482 11719 12705 12940 14766 14783 15043 15301 15326 16223 (16319) |
| M21b+210 | 73 210 263 489 750 1438 2706 3915 4769 5108 7028 7861 8701 8860 9540 10398 10400 10873 11482 11719 12705 14766 14783 15043 15301 15326 16223 |
| M21b2 | 73 210 263 489 709 750 (965d) 1438 1598 1763 2080 2706 3663 3819 3915 4769 5108 6231 7028 7861 8509 8701 8860 9540 10398 10400 10873 11482 11719 12705 14766 14783 15043 15236 15301 15326 16145 16181 16223 16304 |
| M22 | 73 263 489 710 750 1438 2706 4639 4769 6776 7028 8701 8860 9540 10398 10400 10873 11719 12705 14766 14783 15043 15301 15326 16223 |
| M22a | 73 263 489 710 750 1438 2706 4639 4769 6071 6776 7028 8701 8860 9316 9540 10398 10400 10873 11719 12705 14766 14783 15043 15301 15326 16223 16290 |
| M22b | 73 263 489 710 750 1438 2706 4639 4769 6776 7028 8701 8860 9540 10398 10400 10873 11719 12705 13681 14766 14783 15043 15301 15326 |
| M23'75 | 73 263 489 750 1438 2706 4769 7028 8701 8860 9540 10398 10400 10873 11719 12279 12705 14766 14783 15043 15301 15326 16223 |
| M23 | 73 152 195 204 263 417 489 533 750 1438 4769 7028 8360 8701 8860 9438 9540 9545 10142 10295 10398 10400 10873 11569 11719 11899 12279 12618 12705 14766 14783 15025 15043 15301 15326 16223 16263 16311 |
| M75 | 73 146 150 263 489 750 1438 2706 3316 3453 4769 5147 7028 7313 7765 8701 8860 9540 10398 10400 10873 11719 12279 12705 14110 14766 14783 15043 15109 15301 15326 16068 16189 16223 |
| M24'41 | 73 263 489 750 1438 2706 4769 7028 8701 8860 9540 10398 10400 10873 11719 12705 14766 14783 15043 15301 15326 15601 16223 |
| M24 | 73 146 195 263 489 750 (951) 1438 2706 4769 5773 7028 8701 8860 9540 10398 10400 10873 11719 12705 13359 14766 14783 15043 15301 15326 15601 16223 |
| M24a | 73 146 152 195 263 489 750 (951) 1438 2706 4769 5773 7028 7040 8701 8860 9540 10398 10400 10873 11719 12705 13359 14766 14783 15043 15301 15326 15601 16223 16311 |
| M24b | 73 146 195 263 489 750 (951) 1438 2140 2706 4769 5773 7028 8701 8860 9540 10101 10398 10400 10873 11719 12651 12705 13359 13812 14766 14783 15043 15301 15326 15601 16086 16223 |
| M41 | 73 263 375 489 750 870 1438 2706 4769 6297 7028 8701 8860 9540 10398 10400 10873 11719 12398 12469 12705 13656 14766 14783 15043 15301 15326 15601 16223 16327 16330 |

| | |
|---|---|
| M41a | 73 263 375 489 750 870 1438 2706 4769 6297 7028 8701 8860 9540 10398 10400 10873 11719 12398 12469 12705 13656 14766 14783 15043 15301 15326 15601 16189 16223 16327 16330 |
| M41a1 | 73 113 195 263 375 489 750 870 1438 2706 4769 6297 7028 8701 8860 9540 10398 10400 10873 11719 12398 12469 12705 13656 14766 14783 15043 15301 15326 15601 16189 16223 16327 16330 16545 |
| M41b | 73 263 375 489 750 870 1438 2706 4769 6297 7028 8701 8860 9540 10398 10400 10873 11719 12398 12469 12705 13656 14766 14783 15043 15301 15326 15442 15601 16223 16327 16330 |
| M41c | 73 263 375 489 750 870 1438 2706 4769 6297 7028 8701 8860 9540 10325 10398 10400 10873 11719 12398 12469 12705 13113 13656 14766 14783 15043 15301 15326 15601 16223 16327 16330 |
| M25 | 73 150 263 316 489 750 869 1406 1438 2706 3511 4769 7028 7954 8701 8776 8860 9540 10398 10400 10873 11719 11734 12705 13144T 13350 13708 14766 14783 15043 15301 15326 16129 16189 16290A 16368 |
| M26 | 73 152 263 489 750 1438 2706 4769 5656 7028 8701 8860 9540 9899 10398 10400 10873 11428 11521 11719 12190 12705 13708 13932 14218 14311 14766 14783 15043 15109 15263 15301 15326 16214A 16223 16256 |
| M27 | 73 263 489 750 1438 2706 4769 5375 7028 8701 8860 9201 9540 10398 10400 10873 11719 12358 12705 14766 14783 15043 15301 15326 16223 |
| M27a | 73 195 234 263 489 537 739 750 1438 2404 2706 4769 5375 7028 8406 8701 8860 9201 9540 10042 10398 10400 10873 11020 11554G 11719 12358 12705 14766 14783 15043 15301 15326 15451 16048 16077T 16172 16223 16311 16320 |
| M27a1 | 73 195 228 234 263 489 537 739 750 1438 2404 2706 4769 5375 5585 7028 7337 8406 8701 8860 9201 9540 10042 10398 10400 10873 11020 11554G 11719 12358 12705 14766 14783 15043 15301 15326 15451 16048 16077T 16172 16223 16311 16320 |
| M27a1a | 73 150 195 204 228 234 263 489 537 739 750 1438 2404 2706 4769 5375 5585 7028 7337 8406 8701 8860 9201 9389 9540 10042 10398 10400 10873 11020 11554G 11719 12358 12705 14766 14783 15043 15301 15326 15451 16048 16077T 16136 16172 16223 16311 16320 |
| M27a1a1 | 73 150 195 204 228 234 263 489 537 739 750 980 1438 2404 2706 4769 5177 5375 5585 7028 7337 8406 8701 8860 9201 9389 9540 10042 10398 10400 10873 11020 11554G 11719 12358 12705 14766 14783 15043 15301 15326 15451 16048 16077T 16136 16172 16189 16223 16311 16320 |
| M27a1a2 | 73 150 195 204 228 234 263 489 537 739 750 1438 2404 2706 4769 5375 5585 7028 7337 8406 8701 8839 8860 9201 9389 9540 10042 10398 10400 10873 11020 11554G 11719 12358 12705 14766 14783 15043 15301 15326 15451 16048 16077T 16136 16172 16223 16311 16320 |
| M27a1b | 73 195 228 234 263 489 537 739 750 1438 2404 2706 4769 5375 5585 7028 7337 8406 8701 8860 9201 9540 10042 10398 10400 10873 11020 11554G 11719 12358 12705 14766 14783 15043 15301 15326 15451 16048 16077T 16172 16223 16311 |
| M27a2 | 73 189 195 234 263 489 537 739 750 1438 2404 2706 4769 4924 5375 7028 8406 8701 8860 9181 9201 9540 10042 10398 10400 10807 10873 11020 11554G 11719 12358 12705 13434 13934 14766 14783 15043 15193 15301 15326 15451 16048 16077T 16172 16223 16311 16320 |

| | |
|---|---|
| M27a2a | 73 189 195 234 263 489 537 739 750 1438 2404 2706 4769 4924 5375 7028 8406 8701 8860 9181 9201 9540 10042 10398 10400 10807 10873 11020 11554G 11719 12358 12414 12705 13434 13934 14766 14783 15043 15193 15301 15326 15451 16048 16077T 16172 16223 16311 16320 |
| M27a2b | 73 189 195 234 263 489 537 739 750 1438 2404 2706 4769 4924 5375 7028 8406 8701 8860 9181 9201 9540 10042 10398 10400 10807 10873 11020 11554G 11719 12358 12705 13145 13434 13934 14766 14783 15043 15193 15301 15326 15451 16048 16077T 16172 16223 16311 16320 |
| M27a3 | 73 195 234 263 489 537 739 750 1438 2404 2706 4212 4769 5375 7028 8406 8701 8857 8860 9201 9540 10042 10398 10400 10873 11020 11554G 11719 12358 12705 13830 14766 14783 15043 15301 15326 15451 16048 16077T 16172 16223 16264 16311 16320 |
| M27b | 73 152 263 489 656 750 1438 1719 2315 2706 3204 3591 3693 4342 4769 5375 5892 7028 8701 8860 9201 9540 10166 10398 10400 10493 10873 11719 12358 12705 14137 14370 14766 14783 15043 15301 15326 16209 16223 16299 16390 |
| M27b1 | 64 73 152 199 236 263 489 656 750 1438 1598 1719 2315 2706 3204 3591 3693 4342 4769 4775 5375 5788 5892 7028 8701 8860 9201 9540 10166 10398 10400 10493 10873 11719 12358 12705 14137 14370 14674 14766 14783 15043 15301 15326 16145 16209 16299 16390 |
| M27b2 | 73 152 263 489 656 750 1438 1719 2315 2416 2706 3204 3591 3693 3768 4342 4769 5375 5892 7028 8701 8860 9201 9540 10166 10398 10400 10493 10873 11719 12358 12705 14137 14370 14766 14783 15043 15301 15326 16086 16209 16223 16299 16390 |
| M27b2a | 73 152 263 489 656 750 1053 1438 1719 2315 2416 2706 3204 3591 3693 3768 4342 4769 5375 5892 7028 8701 8860 9201 9540 10166 10398 10400 10493 10873 11719 12358 12705 14137 14370 14766 14783 15043 15301 15326 16086 16209 16223 16299 16390 |
| M27b2a1 | 73 152 263 489 656 750 1053 1438 1719 2315 2416 2706 3204 3591 3693 3768 4342 4769 5375 5892 7028 8701 8860 9201 9540 10166 10398 10400 10493 10873 11719 12358 12705 14137 14370 14766 14783 15043 15172 15301 15326 16086 16104 16209 16223 16299 16390 |
| M27b2b | 73 152 263 489 656 750 1438 1719 2315 2416 2706 3204 3591 3693 3768 4342 4769 5375 5892 7028 8603 8701 8860 9201 9540 10166 10398 10400 10493 10873 11719 12358 12705 14137 14370 14766 14783 15043 15301 15326 16086 16209 16223 16299 16390 |
| M27b2b1 | 73 152 263 489 656 750 1438 1719 2315 2416 2706 3204 3591 3693 3768 4198 4342 4769 5375 5892 7028 8603 8701 8860 9201 9540 10166 10398 10400 10493 10873 11719 12358 12705 14137 14370 14766 14783 15043 15301 15326 16086 16209 16223 16299 16390 |
| M27b2c | 73 152 263 489 656 750 1438 1719 1780 2315 2416 2706 3204 3591 3693 3768 4342 4769 5375 5892 7028 8701 8860 9201 9540 10166 10398 10400 10493 10873 11719 12358 12705 14137 14370 14766 14783 15043 15301 15326 16086 16209 16223 16299 16390 |
| M27c | 73 146 (151) (186) (204) 263 489 750 1438 1692 2706 3397 4769 5375 7028 8503 8701 8860 9033 9127 9201 9540 10398 10400 10873 11719 11959 12358 12370 12705 14766 14783 14870 15043 15301 15326 16223 16301 16304 |

| | |
|---|---|
| M28 | 73 152 195 263 489 750 1438 1719 2706 4769 6281 6374 7028 8701 8860 9540 10245 10398 10400 10873 11719 12705 14766 14783 15043 15067 15301 15326 16148 16223 16362 16468 |
| M28a | 73 152 195 263 489 750 1438 1598 1719 2706 4769 6281 6374 7028 8701 8860 9540 10245 10398 10400 10658 10873 11719 12705 14766 14783 15043 15067 15301 15326 16086 16129 16148 16223 16362 16468 |
| M28a1 | 73 152 195 263 489 750 1438 1598 1719 2706 4769 6281 6374 6962 7028 8701 8860 9540 10245 10398 10400 10658 10873 11719 12705 14766 14783 15043 15067 15301 15326 16086 16129 16148 16223 16362 16468 |
| M28a+204 | 73 152 195 204 263 489 750 1438 1598 1719 2706 4769 6281 6374 7028 8701 8860 9540 10245 10398 10400 10658 10873 11719 12705 14766 14783 15043 15067 15301 15326 16086 16129 16148 16223 16362 16468 |
| M28a2 | 73 152 195 204 207 263 431A 489 750 1438 1598 1719 2706 4769 6281 6374 7028 8701 8860 9540 10245 10398 10400 10658 10873 11084 11719 12705 14766 14783 15043 15067 15301 15326 16051 16086 16129 16148 16223 16362 16468 |
| M28a2a | 73 152 195 198 204 207 263 431A 489 750 1438 1598 1719 2706 4769 6281 6374 7028 8701 8860 9540 9590 10245 10398 10400 10658 10873 11084 11719 12705 13803 14766 14783 15043 15067 15301 15326 16051 16086 16129 16148 16223 16362 16468 |
| M28a3 | 73 152 195 204 263 489 750 1438 1598 1719 2706 4769 5063 6281 6374 7028 7145 8701 8860 9540 10245 10398 10400 10658 10691 10873 11719 12705 14766 14783 15043 15067 15301 15326 16086 16129 16148 16223 16261 16362 16468 |
| M28a4 | 73 151 152 195 199 204 263 489 750 1438 1598 1719 2706 4769 6281 6374 7028 8701 8860 9540 10245 10398 10400 10658 10873 11719 12705 14766 14783 15043 15067 15301 15326 15363 16129 16148 16223 16362 16468 |
| M28a5 | 73 152 195 263 489 750 1185 1438 1598 1719 2706 4769 6281 6374 7028 8701 8860 9540 10245 10398 10400 10658 10873 11719 12705 14766 14783 15043 15067 15301 15326 16086 16129 16148 16223 16362 16429 |
| M28a5a | 73 152 195 263 489 750 1185 1438 1598 1719 2706 4769 5249 6281 6374 7028 8701 8860 9540 9663 10245 10398 10400 10658 10873 11719 12705 14766 14783 15043 15067 15301 15326 16086 16129 16148 16223 16362 16429 16468 |
| M28a5b | 73 152 195 234 263 489 750 1185 1438 1598 1719 2706 3651 4769 6281 6374 7028 8701 8860 9540 10245 10398 10400 10658 10873 11719 12705 14766 14783 15043 15067 15301 15326 16086 16129 16148 16223 16362 16429 16468 |
| M28a6 | 73 152 195 263 489 750 789 1438 1598 1719 2706 4310 4769 6275 6281 6374 7028 8701 8860 9540 10245 10398 10400 10658 10873 11719 12705 14766 14783 15043 15067 15301 15326 15481A 16086 16129 16148 16223 16362 16468 |
| M28a6a | 73 152 195 263 489 750 789 1438 1598 1719 2706 4167 4310 4769 6257 6275 6281 6374 7028 7424 8701 8860 9540 10245 10398 10400 10658 10873 11719 12705 13386 14766 14783 15043 15067 15301 15326 15481A 16086 16129 16148 16223 16362 16468 |

| | |
|---|---|
| M28a7 | 73 152 195 263 489 750 1438 1598 1719 2706 4769 6281 6374 7028 8701 8860 9540 10245 10398 10400 10658 10873 11719 12705 14766 14783 15043 15067 15301 15326 16086 16129 16148 16223 16320 16362 16468 |
| M28a7a | 73 152 195 263 489 750 1438 1598 1719 2706 4769 6281 6374 7028 8701 8756 8860 9540 10245 10398 10400 10658 10873 11719 12705 13246 14122 14766 14783 15043 15067 15301 15326 16086 16129 16148 16223 16320 16362 16468 |
| M28a7b | 73 152 195 263 489 750 1438 1598 1719 2706 3565 4769 6281 6374 7028 8093 8701 8860 9540 10245 10398 10400 10658 10873 11719 12705 14766 14783 15043 15067 15301 15326 16086 16129 16148 16223 16320 16362 16468 16471 |
| M28b | 73 94 152 195 204 263 489 750 1438 1719 2706 4769 6281 6374 7028 8701 8860 9540 10245 10398 10400 10873 11719 12172 12705 14766 14783 15043 15067 15301 15326 16148 16223 16318T 16362 16468 |
| M28b1 | 73 94 152 195 204 263 489 750 1438 1719 2135 2706 4769 5951 6281 6374 7028 8701 8860 9540 10245 10398 10400 10873 11719 12172 12373 12507 12705 13754 14766 14783 15043 15067 15301 15326 16148 16223 16291 16318T 16362 16468 |
| M29'Q | 73 263 489 750 1438 2706 4769 7028 8701 8860 9540 10398 10400 10873 11719 12705 13500 14766 14783 15043 15301 15326 16223 |
| M29 | 73 151 152 211 263 464 489 513 750 1048 1438 1598 2706 4230 4769 7028 7711 8701 8860 9540 10398 10400 10873 11719 12705 13065 13500 14766 14783 15043 15301 15326 16189 16223 |
| M29a | 73 151 152 211 263 310 464 489 513 750 1048 1438 1598 2706 4050 4230 4769 5263 7028 7711 8701 8860 9540 9852 10398 10400 10873 11719 12127 12366 12705 13065 13452 13500 14766 14783 15043 15301 15326 16189 16223 16311 |
| M29b | 73 151 152 211 263 464 489 513 750 1048 1438 1598 2706 4230 4769 7028 7711 8701 8860 9540 10398 10400 10873 11719 12705 13065 13500 14766 14783 15043 15301 15326 16189 16223 16294 |
| M29b1 | 73 151 152 211 263 464 489 513 750 1048 1438 1598 2706 4230 4769 7028 7711 8701 8860 9540 10398 10400 10873 11719 12705 13065 13500 14766 14783 15043 15301 15326 16104 16189 16223 16294 |
| Q | 73 263 489 750 1438 2706 4117 4769 5843 7028 8701 8790 8860 9540 10398 10400 10873 11719 12705 12940 13500 14766 14783 15043 15301 15326 16129 16223 16241 16311 |
| Q1'2 | 73 263 489 750 1438 2706 4117 4769 5460 5843 7028 8701 8790 8860 9540 10398 10400 10873 11719 12705 12940 13500 14766 14783 15043 15301 15326 16129 16223 16241 16311 |
| Q1 | 73 89 92 146 263 489 750 1438 2706 4117 4769 5460 5843 7028 8701 8790 8860 8964 9540 10398 10400 10873 11719 12705 12940 13500 14025 14766 14783 15043 15301 15326 16129 16144 16148 16223 16241 16265C 16311 16343 |
| Q1+@16223 | 73 89 92 146 263 489 750 1438 2706 4117 4769 5460 5843 7028 8701 8790 8860 8964 9540 10398 10400 10873 11719 12705 12940 13500 14025 14766 14783 15043 15301 15326 16129 16144 16148 16241 16265C 16311 |

| | |
|---|---|
| Q1a | 73 89 92 146 263 489 750 1438 2706 4117 4769 5460 5843 7028 7681 8701 8790 8860 8964 9540 10398 10400 10873 11719 12705 12940 13500 14025 14766 14783 15043 15301 15326 16129 16144 16148 16241 16265C 16311 16343 |
| Q1a1 | 73 89 92 146 208 263 489 750 1438 2706 4117 4769 5460 5843 7028 7681 8701 8790 8860 8964 9540 10398 10400 10873 11719 12705 12940 13500 14025 14766 14783 15043 15301 15326 16129 16144 16148 16241 16265C 16311 16343 |
| Q1a1a | 73 89 92 146 208 263 489 750 1438 2706 4117 4769 5460 5843 7028 7681 8701 8790 8860 8964 9266 9540 10398 10400 10873 11719 12705 12940 13500 14025 14766 14783 15043 15301 15326 16129 16144 16148 16222 16241 16265C 16311 16343 |
| Q1b | 73 89 146 207 263 489 750 1375 1438 2706 4117 4769 5460 5843 7028 7993 8701 8790 8860 8964 9540 10256 10398 10400 10873 11314 11719 12705 12940 13500 14025 14766 14783 15043 15301 15326 16129 16144 16148 16265C 16311 16343 |
| Q1c | 73 89 92 146 263 489 750 1391 1438 2706 4117 4769 5460 5843 6221 7028 8701 8790 8860 8964 9540 10398 10400 10873 11719 12705 12940 13368 13500 14025 14766 14783 15043 15301 15326 16129 16144 16148 16241 16265C 16311 16343 |
| Q1c1 | 73 89 92 146 263 489 750 1391 1438 2706 4117 4707 4769 5460 5843 6221 7028 8701 8790 8860 8964 9540 10398 10400 10873 11719 12705 12940 13368 13500 14025 14404 14766 14783 15043 15301 15326 16129 16144 16148 16241 16265C 16311 16343 |
| Q1c1a | 73 89 92 146 263 489 750 1391 1438 2706 4117 4707 4769 5460 5843 6221 7028 8701 8790 8860 8964 9540 10398 10400 10801 10873 11719 12705 12940 13368 13500 14025 14404 14766 14783 15043 15301 15326 16129 16144 16148 16241 16265C 16311 16343 16488 |
| Q1c2 | 73 89 92 146 263 489 750 1391 1438 2706 4117 4769 5460 5843 6221 7028 8701 8790 8860 8964 9540 10398 10400 10873 10914 11719 12705 12940 13368 13500 14025 14766 14783 15043 15115 15301 15326 16129 16144 16148 16241 16265C 16311 16343 |
| Q1c2a | 73 89 92 146 235 263 489 750 1391 1438 1692 2706 4117 4769 5460 5843 6221 7028 8701 8790 8860 8964 9540 10398 10400 10873 10914 11719 12705 12940 13368 13500 14025 14766 14783 15043 15115 15301 15326 16129 16144 16148 16241 16265C 16311 16343 |
| Q1d | 73 89 92 146 263 489 750 1438 2706 4117 4769 5460 5843 7028 8701 8790 8860 8964 9111 9540 10398 10400 10873 11719 12705 12940 13500 14025 14766 14783 15043 15301 15326 16129 16144 16148 16223 16241 16265C 16311 16343 |
| Q1e | 73 89 92 146 263 489 750 1438 2359 2706 4117 4769 5460 5843 7028 8701 8790 8860 8964 9540 10398 10400 10873 11719 11902 12358 12705 12940 13500 14025 14766 14783 15043 15301 15326 16129 16144 16148 16223 16241 16265C 16311 16343 |
| Q1e1 | 73 89 92 146 263 489 750 1438 2359 2706 4117 4769 5414 5460 5843 7028 8701 8790 8860 8964 9540 10398 10400 10873 11719 11902 12358 12705 12940 13500 14025 14766 14783 15043 15301 15326 16129 16144 16148 16223 16241 16265C 16311 16343 |

| | |
|---|---|
| Q1e1a | 73 89 92 146 263 489 750 1438 2359 2706 4117 4769 5414 5460 5843 7028 8701 8790 8860 8964 9540 10398 10400 10873 10924 11719 11902 12358 12705 12940 13500 14025 14766 14783 15043 15110 15301 15326 16129 16144 16148 16176 16223 16241 16265C 16311 16343 |
| Q1e1a1 | 73 89 92 146 263 489 750 1438 2359 2706 4117 4769 5414 5460 5843 6164 7028 8701 8790 8860 8964 9540 10398 10400 10873 10924 11719 11902 12358 12705 12940 13500 14025 14766 14783 15043 15110 15301 15326 16129 16144 16148 16176 16223 16241 16265C 16311 16343 |
| Q1e1b | 73 89 92 146 263 489 750 1438 2359 2706 4117 4769 5414 5460 5843 7028 8701 8790 8860 8964 9540 10398 10400 10873 11719 11902 12358 12705 12940 13500 14025 14766 14783 15043 15301 15326 16129 16144 16148 16223 16241 16264 16265C 16311 16343 |
| Q1e1b1 | 73 89 92 146 263 489 750 1438 2359 2706 4117 4769 5414 5460 5843 7028 7816 8701 8790 8860 8964 9540 10398 10400 10873 11719 11902 12358 12705 12940 13500 14025 14766 14783 15043 15301 15326 16129 16144 16148 16223 16241 16264 16265C 16311 16343 |
| Q1e1c | 73 89 92 146 263 489 750 827 1438 2359 2706 4117 4769 5414 5460 5843 7028 8440 8701 8790 8860 8964 9540 10398 10400 10873 11719 11902 11914 12358 12705 12940 13500 14025 14766 14783 15043 15301 15326 16129 16144 16148 16223 16241 16265C 16311 16343 |
| Q1f | 73 89 92 146 263 489 750 1438 2706 4117 4769 5460 5843 7028 8701 8790 8860 8964 9540 10398 10400 10873 11719 11884 12705 12940 13500 14025 14766 14783 15043 15301 15326 16129 16144 16148 16223 16241 16265C 16311 16343 |
| Q1f1 | 73 89 92 146 263 489 750 1407A 1438 2706 3834 4117 4769 4913C 5460 5843 7028 8701 8790 8860 8964 9101 9540 10398 10400 10873 11719 11884 12705 12940 13047 13500 14025 14766 14783 14798 15043 15301 15326 16129 16144 16148 16223 16241 16265C 16311 16343 16526 |
| Q1f2 | 73 89 92 146 263 489 750 1438 2706 4025 4117 4769 5460 5843 7028 8701 8790 8860 8964 9037 9540 10172 10398 10400 10873 11719 11884 12705 12940 13500 14025 14766 14783 15043 15301 15326 16129 16144 16148 16223 16241 16265C 16311 16343 |
| Q2 | 73 228T 263 489 750 1438 2706 4117 4769 5460 5843 7028 8701 8790 8860 9540 10398 10400 10873 11061 11719 12705 12940 13500 14766 14783 15043 15301 15326 16066 16129 16223 16241 |
| Q2a | 73 195 228T 263 489 750 1438 2706 4117 4769 5460 5557 5843 7028 8701 8790 8860 9540 10214 10283 10398 10400 10873 11061 11719 12705 12940 13500 14766 14783 15043 15301 15326 15519 16066 16129 16223 |
| Q2a1 | 73 195 228T 263 489 750 1438 2706 4117 4769 5460 5557 5843 7028 8701 8790 8860 9377 9540 10214 10283 10398 10400 10873 11061 11719 12705 12940 12957 13500 14766 14783 15043 15301 15326 15519 15712 16066 16129 16223 16241 16355 |
| Q2a2 | 73 195 228T 263 489 750 1438 2706 4117 4310 4769 5460 5557 5843 7028 8701 8790 8860 9540 10214 10283 10398 10400 10873 11061 11719 12705 12940 13500 14766 14783 15043 15301 15326 15519 15930 16066 16129 16223 16241 |

| | |
|---|---|
| Q2a2a | 73 195 228T 263 489 750 1438 2706 4117 4310 4769 5460 5557 5843 7028 8701 8790 8860 9540 10214 10283 10398 10400 10873 10978C 11061 11719 12705 12940 13500 14305 14766 14783 15043 15301 15326 15519 15930 16066 16129 16223 16241 |
| Q2a2b | 73 143 195 228T 263 489 750 789 1438 2706 4117 4310 4769 5075 5460 5557 5843 7028 8701 8790 8860 9192 9540 10214 10283 10398 10400 10873 11061 11719 12705 12940 13500 14766 14783 15043 15301 15326 15519 15930 16066 16129 16223 16241 16362 |
| Q2a3 | 73 195 228T 263 489 750 1438 2706 4117 4769 5460 5557 5843 6260 7028 8701 8790 8860 9540 10214 10283 10398 10400 10873 11061 11719 12705 12940 13500 14766 14783 15043 15301 15326 15519 16066 16129 16223 16241 |
| Q2a3a | 73 152 195 228T 263 489 750 1438 2706 4117 4769 4787 4917 5460 5557 5843 6260 7028 8701 8790 8860 9540 10214 10283 10398 10400 10873 11061 11719 12675 12705 12940 13500 14766 14783 15043 15301 15326 15519 16066 16129 16223 16241 16390 |
| Q2a3b | 73 195 198 228T 263 489 750 1438 2706 4117 4769 5460 5557 5843 6260 7028 8701 8790 8860 9540 10214 10283 10398 10400 10873 11061 11719 12705 12940 13500 14766 14783 15043 15301 15326 15516 15519 16066 16129 16223 16241 16261 16292 |
| Q2a4 | 73 195 228T 263 489 750 1438 2706 4117 4769 5460 5557 5843 7028 8701 8790 8860 9540 10214 10283 10398 10400 10873 11061 11554 11719 12705 12940 13500 14766 14783 15043 15301 15326 15519 16066 16129 16223 16241 |
| Q2b | 73 152 228T 263 489 744 750 1438 1462 2706 4117 4769 5456 5843 7028 8701 8790 8860 9540 10398 10400 10598 10873 11061 11719 12267G 12268 12705 12940 13500 14766 14783 15043 15070 15301 15326 16066 16129 16145 16223 16286 |
| Q3 | 73 263 489 750 1438 2706 4117 4335 4769 5843 7028 8701 8790 8860 9540 10398 10400 10873 11719 12705 12940 13500 14766 14783 15043 15172 15301 15326 16129 16223 16241 16311 |
| Q3a | 73 143 263 489 750 1438 2706 2768 4117 4335 4769 5843 7028 8701 8790 8860 9540 10398 10400 10873 11719 12705 12940 13500 14766 14783 15043 15172 15301 15326 16129 16223 16241 16311 |
| Q3a+61_62 | 61 62 73 143 263 489 750 1438 2706 2768 4117 4335 4769 5843 7028 8701 8790 8860 9540 10398 10400 10873 11719 12705 12940 13500 14766 14783 15043 15172 15301 15326 16129 16223 16241 16311 |
| Q3a1 | 61 62 73 143 263 489 750 1438 2706 2768 4117 4335 4769 5843 7028 8701 8790 8860 9254 9540 10398 10400 10873 11719 12705 12940 13500 14766 14783 15043 15172 15301 15326 16129 16209 16223 16241 16311 |
| Q3b | 73 263 489 750 1438 2706 4117 4335 4769 5460 5843 7028 8454 8701 8790 8860 9254 9540 10398 10400 10750 10873 11719 12684 12705 12940 13500 14484 14766 14783 15043 15172 15301 15326 16129 16223 16241 16249 16311 16362 |
| M31 | 73 263 489 750 1438 2706 4769 4907 7028 8701 8860 9540 10398 10400 10873 11176 11719 12705 14766 14783 15043 15301 15326 15440 15530 16223 |

| | |
|---|---|
| M31a | 73 263 489 750 1438 2156.1A 2706 3999 4769 4907 7028 8701 8860 9540 10398 10400 10873 11176 11719 12705 12876 14766 14783 15043 15301 15326 15440 15530 16223 |
| M31a1 | 73 249d 263 489 750 1438 1524 2045 2156.1A 2706 3975 3999 4769 4907 7028 8701 8860 8973 9540 9581 10398 10400 10873 11014 11176 11719 12705 12876 14407 14766 14783 15043 15301 15326 15440 15530 16223 16311 |
| M31a1a | 73 249d 263 489 750 1438 1524 2045 2156.1A 2706 3975 3999 4769 4907 7028 8701 8860 8973 9540 9581 9617 10398 10400 10873 11014 11176 11719 12705 12876 14407 14766 14783 15043 15301 15326 15440 15530 16223 16311 |
| M31a1b | 73 249d 263 489 750 1438 1524 2045 2156.1A 2706 3975 3999 4769 4907 7028 8701 8860 8973 9540 9581 10398 10400 10873 11014 11176 11719 12705 12876 13710 14407 14766 14783 15043 15301 15326 15440 15530 16223 16311 |
| M31a2 | 73 195 263 489 750 868 1438 2156.1A 2706 3999 4769 4775 4907 7028 8701 8860 9540 9966 10398 10400 10873 11023T 11176 11719 12705 12876 14766 14783 15043 15258 15301 15326 15440 15530 16017 16093 16126 16145 16223 |
| M31b'c | 73 263 489 750 1438 2706 4769 4907 7028 8701 8860 9540 10398 10400 10873 11176 11719 12705 14766 14783 15043 15301 15326 15440 15530 (16093) 16136 16223 |
| M31b | 73 152 263 489 750 808 1438 2706 4769 4907 7028 8093 8701 8860 9540 10398 10400 10873 11176 11719 12705 14766 14783 15043 15301 15326 15440 15530 (16093) 16136 16223 |
| M31b1 | 73 152 263 489 750 808 1438 2706 4769 4907 7028 8093 8701 8860 9540 10398 10400 10873 11176 11719 12705 14766 14783 15043 15301 15326 15440 15530 15676 (16093) 16136 16223 |
| M31b2 | 73 152 263 489 750 808 1438 2706 3777 3826 4769 4907 6299 7028 8093 8701 8860 9540 10398 10400 10873 11176 11719 12705 14766 14783 15043 15301 15326 15440 15530 (16093) 16136 16223 |
| M31c | 73 188 234 263 282 489 750 1438 2706 4769 4907 7028 8701 8860 9269 9540 10398 10400 10873 11176 11719 12705 14152 14766 14783 15043 15301 15326 15440 15530 15935 (16093) 16136 16223 16311 |
| M32'56 | 73 263 489 750 2706 4769 7028 8701 8860 9540 10398 10400 10873 11719 12705 14766 14783 15043 15301 15326 16223 |
| M32 | 73 263 489 750 2706 4769 7028 8701 8860 9540 10398 10400 10873 11719 12705 14766 14783 15043 15301 15326 16223 16319 16526 |
| M32a | 73 195 207 263 489 750 2156.1A 2706 3817 4769 7028 8701 8860 9064 9540 10398 10400 10873 11719 12189 12705 14766 14783 15043 15301 15326 15754 16223 16319 16344 16357 16526 |
| M32c | 73 150 200 263 489 750 2706 3588 4769 7028 7960 8701 8768 8860 9540 10398 10400 10873 11719 12705 13308 13590 13708 14130 14260 14527 14766 14783 15043 15172 15301 15326 15625 16086 16148 16223 16259 16278 16319 16399 16526 |
| M56 | 61 73 263 489 750 2706 4769 7028 8701 8860 9540 10398 10400 10873 11719 12705 13753 14766 14783 15043 15301 15326 16223 16311 |

| | |
|---|---|
| M33 | 73 263 489 750 1438 2361 2706 4769 7028 8701 8860 9540 10398 10400 10873 11719 12705 14766 14783 15043 15301 15326 16223 |
| M33a | 73 263 489 750 1438 2361 2706 4769 7028 8562 8701 8860 9540 10398 10400 10873 11719 12705 14766 14783 15043 15301 15326 15908 16223 |
| M33a1 | 73 263 489 750 1438 2361 2706 4769 7028 8562 8701 8860 9540 10398 10400 10873 11719 12153 12705 14766 14783 15043 15301 15326 15908 16223 |
| M33a1a | 73 195 263 489 573.XC 750 1438 2361 2706 4769 7028 8562 8572 8701 8860 9540 10398 10400 10873 11719 12153 12705 14053 14766 14783 15043 15052 15301 15326 15466 15908 16223 16294 |
| M33a1b | 73 199 263 489 750 1438 2361 2706 4769 7028 8562 8701 8705 8860 9540 10398 10400 10598 10873 11569 11719 12153 12696 12705 13105 14766 14783 15043 15301 15326 15908 16223 |
| M33a2'3 | 73 263 489 750 1438 2361 2706 4769 7028 8562 8701 8860 9540 10398 10400 10873 11719 12705 14766 14783 15043 15301 15326 15908 16172 16223 |
| M33a2 | 73 263 489 750 1438 2361 2706 4769 5423 7028 8562 8701 8860 9540 10398 10400 10873 11719 12705 13731 14766 14783 15043 15301 15326 15908 16169 16172 16223 |
| M33a2a | 73 150 263 462 489 750 1438 2361 2706 4769 5124A 5423 7028 8562 8701 8860 9540 10398 10400 10873 11719 12705 13731 14766 14783 15043 15301 15326 15908 16169 16172 16223 |
| M33a3 | 73 146 263 489 750 1438 2361 2706 4769 7028 8562 8701 8860 9540 10398 10400 10873 11719 12705 14766 14783 15043 15301 15326 15796 15908 16172 16223 |
| M33a3a | 73 146 263 489 750 1438 2361 2706 4769 7028 8562 8701 8860 9540 10398 10400 10873 11719 11914 12236 12705 14766 14783 15043 15301 15326 15796 15908 16136 16172 16223 |
| M33+16362 | 73 263 489 750 1438 2361 2706 4769 7028 8701 8860 9540 10398 10400 10873 11719 12705 14766 14783 15043 15301 15326 16223 16362 |
| M33b | 73 263 489 676 750 1438 1719 2361 2706 3221 4769 6293 7028 8701 8860 9540 10398 10400 10873 11719 12705 14766 14783 15043 15301 15326 16223 16324 16362 |
| M33b1 | 73 263 489 676 750 1438 1719 2361 2706 3221 4769 4814 (6092) 6293 7028 8701 8860 9540 10398 10400 10724 10873 11719 12705 14766 14783 15043 15301 15326 16223 16259 16324 16362 |
| M33b2 | 73 263 489 676 750 1438 1719 2361 2706 3221 4769 6293 7028 8701 8860 9344 9540 10398 10400 10873 11719 12705 14766 14783 15043 15301 15326 16223 16324 16362 |
| M33c | 73 263 489 750 1438 2361 2706 3316 4079 4769 5894 7028 8227 8701 8848 8860 9540 10398 10400 10873 11719 12705 14766 14783 15043 15301 15326 16111 16223 16362 |
| M33d | 73 152 204 207 263 489 513 750 1438 2361 2706 3116 4024 4769 6563 7028 8668 8701 8860 9540 9966 10398 10400 10873 10969 11719 12705 14766 14783 14950 15043 15301 15326 (15924) 16178 16223 16288 |
| M34'57 | 73 263 489 750 1438 2706 4769 7028 8701 8860 9540 10398 10400 10873 11101 11719 12705 14766 14783 15043 15301 15326 16223 |

| | |
|---|---|
| M34 | 73 263 489 569 750 1438 2706 3010 4769 6794 7028 8701 8860 9540 10398 10400 10873 11101 11719 12705 14766 14783 15043 15301 15326 15865 16223 16249 |
| M34a | 73 263 489 569 750 1438 2706 3010 4769 5108 6794 7028 8404 8701 8860 9540 10398 10400 10873 11101 11719 12705 14094 14766 14783 15043 15301 15326 15865 16223 16249 16359 |
| M34a1 | 73 146 263 489 569 750 1438 2706 3010 4769 5108 6794 7028 8404 8701 8860 9540 10361 10398 10400 10873 11101 11719 11992 12705 14094 14766 14783 15043 15301 15326 15865 16095 16223 16249 16359 |
| M34a1a | 73 146 263 489 569 750 1438 2706 3010 3447 4769 5108 6794 7028 8404 8701 8860 9540 10361 10398 10400 10873 11101 11719 11992 12311 12705 14094 14766 14783 15043 15301 15326 15865 16095 16223 16249 |
| M34a2 | 73 114 263 489 569 750 1438 2706 3010 4769 5108 5585 6794 7028 8404 8548 8701 8860 9540 10398 10400 10873 11101 11719 12705 14094 14766 14783 15043 15301 15326 15865 16223 16249 16359 |
| M34b | 73 263 489 569 750 1438 2706 3010 4769 6367 6794 7028 7406 8701 8860 9540 10398 10400 10873 11101 11719 11969 12705 14766 14783 15043 15301 15326 15865 16051 16223 16249 16250 |
| M57 | 73 263 489 750 1438 2706 3483 4020 4769 7028 8701 8860 9540 10398 10400 10873 11101 11719 12705 13651 14766 14783 15043 15301 15326 16223 16311 |
| M57+152 | 73 152 263 489 750 1438 2706 3483 4020 4769 7028 8701 8860 9540 10398 10400 10873 11101 11719 12705 13651 14766 14783 15043 15301 15326 16223 16311 |
| M57a | 73 152 263 489 750 1438 2706 3483 4020 4769 7028 8701 8860 9540 10398 10400 10873 11101 11719 12705 13651 14766 14783 15043 15301 15326 16111 16223 16311 |
| M57b | 73 146 189 263 489 750 1438 2706 3483 4020 4132 4769 7028 8701 8860 9098 9540 9656 10398 10400 10873 11101 11719 12705 13651 14743 14766 14783 15043 15301 15326 16223 16311 |
| M57b1 | 73 146 189 263 489 502 750 1438 2706 3483 4020 4132 4769 7028 8701 8860 9098 9540 9656 10398 10400 10873 10915 11101 11719 12705 13651 13708 14743 14766 14783 15043 15301 15326 16223 16311 |
| M35 | 73 263 489 750 1438 2706 4769 7028 8701 8860 9540 10398 10400 10873 11719 12561 12705 14766 14783 15043 15301 15326 16223 |
| M35+199 | 73 199 263 489 750 1438 2706 4769 7028 8701 8860 9540 10398 10400 10873 11719 12561 12705 14766 14783 15043 15301 15326 16223 |
| M35a | 73 199 263 482 489 750 1438 2706 4769 5432 7028 8701 8860 9540 10398 10400 10670 10873 11719 12561 12705 14766 14783 15043 15301 15326 15924 16093 16223 |
| M35a1 | 73 199 263 482 489 750 1438 2706 4769 5426 5432 7028 8701 8860 9540 10398 10400 10670 10873 11719 12561 12705 14766 14783 15043 15301 15326 15924 16093 16223 |
| M35a1a | 73 146 199 263 482 489 750 1438 2706 4769 5426 5432 7028 8701 8860 9540 10398 10400 10670 10873 11719 12561 12705 13928C 14766 14783 15043 15301 15326 15924 16093 16223 16319 |

| | |
|---|---|
| M35a2 | 73 199 263 482 489 750 1438 2706 4769 5118 5432 7028 8056 8701 8860 9540 10398 10400 10670 10873 11719 12561 12705 14766 14783 15043 15301 15326 15924 16093 16223 |
| M35b | 73 199 263 489 750 1438 2706 4769 7028 8701 8860 9540 10398 10400 10873 11719 12561 12705 14766 14783 15043 15301 15326 15928 16223 |
| M35b+16304 | 73 199 263 489 750 1438 2706 4769 7028 8701 8860 9540 10398 10400 10873 11719 12561 12705 14766 14783 15043 15301 15326 15928 16223 16304 |
| M35b1 | 73 199 204 263 489 750 1438 2706 4234T 4769 7028 8701 8860 9540 10398 10400 10873 11719 12561 12705 13500 14766 14783 15043 15301 15326 15928 16223 16304 |
| M35b2 | 73 199 263 489 750 1438 2706 4769 7028 8701 8860 9540 10398 10400 10873 11719 12561 12705 14766 14783 15043 15289 15301 15326 15928 16223 16304 |
| M35b3 | 73 199 263 489 750 1438 2706 4769 7028 8701 8860 9300 9540 10398 10400 10873 11224 11719 12561 12705 14766 14783 15043 15301 15326 15328 15928 16223 16304 |
| M35b4 | 73 199 263 489 750 1438 2706 4769 7028 7961 8701 8860 9540 10398 10400 10565 10873 11719 11887 12561 12705 14766 14783 15043 15301 15326 15928 16223 |
| M35c | 73 263 489 750 1438 2706 4769 7028 8701 8860 9540 10398 10400 10873 11719 11984 12561 12705 14766 14783 15043 15301 15326 16145 16189 16223 16290 16381 |
| M36 | 73 239 263 489 750 1438 2706 4769 7028 7271 8701 8860 9540 10398 10400 10873 11719 12705 14766 14783 15043 15110 15301 15326 16223 |
| M36a | 73 152 239 263 489 750 1438 2706 4769 6320 6917 7028 7271 8701 8860 9540 10398 10400 10873 11719 12346 12705 14203 14302 14766 14783 15043 15110 15301 15326 16193 16223 |
| M36b | 73 146 151 153 239 263 489 750 1438 2706 4769 7028 7271 8701 8860 9036 9540 10398 10400 10873 11719 12362 12410 12705 12954 13371 14766 14783 15043 15110 15301 15326 15670 16223 16325 16357 |
| M36c | 73 239 263 489 750 1438 2706 4101 4102 4135 4769 7028 7271 8701 8860 9094 9540 10335 10398 10400 10873 11143 11719 12705 13708 14766 14783 15043 15110 15301 15326 15352 16223 16234 16262 |
| M36d | 73 151 152 239 263 489 750 1438 2706 4769 5483 7028 7271 8701 8860 9540 10398 10400 10873 11719 12705 14766 14783 15043 15110 15301 15326 16223 |
| M36d1 | 73 151 152 239 249d 263 489 750 1438 2706 3523T 4769 5483 7028 7271 8701 8860 9540 10398 10400 10873 11719 11827 12705 13926 14766 14783 15043 15110 15301 15326 15937 16223 |
| M39'70 | 73 263 489 750 1438 2706 4769 7028 8679 8701 8860 9540 10398 10400 10873 11719 12705 14766 14783 15043 15301 15326 16223 |
| M39 | 55.1T 59-60d 65.1T (66T) 73 263 489 750 1438 1811 2706 4769 7028 8679 8701 8860 9540 10398 10400 10873 11719 12705 14766 14783 15043 15301 15326 15938 16223 |
| M39a | 55.1T 59-60d 65.1T (66T) 73 207 263 489 750 1120 1438 1811 2706 4769 4928 5773 7028 8679 8701 8704 8860 9540 10398 10400 10873 11719 12705 14766 14783 15043 15301 15326 15938 16223 |

| | |
|---|---|
| M39a1 | 55.1T 59-60d 65.1T (66T) 73 207 263 489 750 1120 1438 1811 2706 4769 4928 5773 7028 8679 8701 8704 8860 9383 9540 10398 10400 10873 11719 12705 14766 14783 15043 15301 15326 15938 16223 |
| M39a2 | 55.1T 59-60d 65.1T (66T) 73 207 263 489 750 1120 1438 1811 2706 4769 4928 5773 7028 8679 8701 8704 8860 9540 10398 10400 10654 10873 11719 12705 14766 14783 15043 15301 15326 15938 16111 16353 |
| M39b | 55.1T 59-60d 65.1T (66T) 73 153 263 485 489 750 1438 1811 2706 4769 7028 8679 8701 8860 9540 10398 10400 10873 11719 12705 14766 14783 15043 15301 15326 15938 16223 |
| M39b1 | 55.1T 59-60d 65.1T (66T) 73 153 263 463 485 489 750 1438 1811 2706 4769 7028 8679 8701 8860 9374 9540 10398 10400 10873 11719 12705 14766 14783 15043 15301 15326 15938 16223 |
| M39b2 | 55.1T 59-60d 65.1T (66T) 73 153 263 485 489 750 1438 1811 2706 4769 7028 8679 8701 8860 9540 10398 10400 10873 11719 12705 14766 14783 15043 15301 15326 15508 15938 16223 |
| M39c | 55.1T 59-60d 65.1T (66T) 73 182 263 489 750 1438 1811 2706 4769 7028 8679 8701 8860 9540 10188 10398 10400 10619 10873 11719 12705 14766 14783 15043 15301 15326 15589A 15938 16223 16270 16325 |
| M70 | 73 236 263 489 750 1438 2706 4769 7028 8679 8701 8860 8867 9297 9540 10398 10400 10873 11719 12705 14766 14783 15043 15061 15148 15301 15326 16214 16223 16297 16342 16381 |
| M40 | 73 263 489 750 1438 2706 4769 7028 8701 8860 8925 9540 10398 10400 10873 11719 12705 14766 14783 15043 15301 15326 15721 15954 16223 16463 |
| M40a | 73 200 263 489 750 1438 2706 4769 7028 8701 8860 8925 9540 10398 10400 10873 11719 12705 13542 14766 14783 15043 15301 15326 15721 15954 16179 16223 16294 16463 |
| M40a1 | 73 200 263 489 750 1438 2706 4769 7028 8701 8860 8925 9540 10398 10400 10873 11719 12705 13542 14766 14783 15043 15301 15326 15721 15954 16179 16223 16294 16319 16356 16463 |
| M40a1a | 73 200 249 263 489 750 1438 2083 2706 4769 7028 8701 8860 8925 9540 10398 10400 10873 11719 12705 13542 14766 14783 15043 15301 15326 15721 15954 16179 16223 16289 16294 16319 16356 16463 |
| M40a1b | 73 200 263 489 750 1438 2706 4769 7028 8701 8860 8925 9540 10398 10400 10873 11719 12705 13542 14323 14766 14783 15043 15301 15326 15721 15954 16179 16223 16294 16319 16356 16463 |
| M42'74 | 73 263 489 750 1438 2706 4769 7028 8251 8701 8860 9540 10398 10400 10873 11719 12705 14766 14783 15043 15301 15326 16223 |
| M42 | 73 263 489 750 1438 2706 4769 7028 8251 8701 8860 9156 9540 10398 10400 10873 11719 12705 14766 14783 15043 15301 15326 16223 |
| M42a | 73 263 489 750 1438 1598 2706 4508 4769 7028 8251 8701 8793 8860 9156 9540 10398 10400 10873 11719 12705 12771 14766 14783 15043 15301 15326 16223 16287 16356 |
| M42b | 73 234 263 489 750 1438 2706 2880 4769 7028 8251 8701 8860 9156 9540 10398 10400 10873 11719 12705 14766 14783 15043 15301 15326 16189 16223 |

| | |
|---|---|
| M42b1 | 73 93 234 263 489 750 1438 2706 2880 4769 7028 7229 7533 8251 8597 8701 8860 9156 9165 9540 10310C 10398 10400 10873 11719 12705 14766 14783 15043 15301 15326 16189 16223 |
| M42b1a | 73 93 143 234 263 489 750 1438 2706 2880 4769 6083 6131 7028 7229 7533 8251 8597 8701 8860 9156 9165 9540 10310C 10398 10400 10873 11719 12705 14766 14783 15043 15301 15326 16189 16223 |
| M42b2 | 73 234 263 489 750 1053 1438 2706 2880 4197 4769 7028 8251 8701 8860 9156 9540 10398 10400 10873 11719 12705 14766 14783 15043 15241 15301 15326 15562 16189 16223 16278 |
| M74 | 73 263 489 750 1438 2706 4769 5054 7028 8251 8701 8860 9540 10268 10398 10400 10873 11719 12705 14766 14783 15043 15301 15326 16223 16311 16362 |
| M74a | 63 64 66 73 263 489 750 1438 2706 4769 5054 6185 6575 7028 8251 8701 8860 9540 10268 10398 10400 10873 11719 12705 12850 14766 14783 15043 15301 15326 16223 16311 16362 16381 |
| M74b | 73 263 489 750 1438 2706 4769 5054 7028 8080A 8167 8251 8701 8860 9540 10268 10398 10400 10873 11719 12651 12705 14766 14783 15043 15301 15326 15908 16223 16311 16362 |
| M74b1 | 73 195 263 489 750 1438 2706 4769 5054 7028 8080A 8155 8167 8251 8616 8701 8860 9540 10268 10398 10400 10873 11719 12651 12705 14766 14783 15043 15226 15301 15326 15908 16223 16246T 16311 16362 |
| M74b2 | 73 263 489 750 1438 2706 4769 5054 6755 6884 7028 7775 8080A 8167 8701 8860 9540 10268 10398 10400 10873 10993 11719 12007 12651 12705 14766 14783 15043 15301 15326 15908 16223 16311 16362 |
| M44 | 73 146 263 489 750 961 1438 2706 4769 7028 8701 8860 9540 10398 10400 10873 11719 12705 14766 14783 15043 15301 15326 16223 16301 |
| M44a | 73 146 263 489 750 930 961 1438 2706 4769 7028 8179 8701 8860 9540 10398 10400 10873 11719 12705 14766 14783 15043 15301 15326 16223 16301 |
| M44a1 | 73 146 263 489 750 930 961 1438 2706 4769 7028 8179 8554 8701 8860 9540 10398 10400 10873 11719 12705 14766 14783 15043 15301 15326 16223 16301 |
| M47 | 73 263 489 750 1438 2706 4769 7028 8701 8860 9540 10398 10400 10873 11719 12705 14766 14783 15043 15301 15326 16129 16166C 16189 16223 16287 16319 |
| M48 | 73 199 263 489 750 1438 2706 4769 6366 7028 8701 8860 9540 10398 10400 10873 11719 12705 14766 14783 15043 15301 15326 15900 16223 16225 16234 16390 |
| M49 | 73 263 489 750 1438 2706 3780 4769 7028 8701 8860 9540 10398 10400 10873 11719 12705 14766 14783 15043 15301 15326 16223 16234 |
| M49a | 73 263 489 750 1438 2706 3780 4769 7028 7598 8701 8860 9540 10398 10400 10873 11263 11719 12346 12705 14384 14766 14783 15043 15301 15326 16153 16223 16234 |
| M49a1 | 73 263 489 750 1438 2706 3780 4769 7028 7598 8701 8860 9540 10398 10400 10873 11263 11719 12346 12705 12880 13934 14384 14766 14783 15043 15301 15326 16153 16223 16234 |

| | |
|---|---|
| M49a2 | 73 263 489 750 1438 2706 4769 7028 7598 8701 8860 9540 10398 10400 10873 11263 11719 12346 12705 14384 14766 14783 15043 15301 15326 15511 15647 16153 16223 16234 |
| M49c | 73 263 489 750 1438 2706 3780 4769 7028 8701 8860 9540 10398 10400 10514 10873 11719 12705 13032 14766 14783 15043 15301 15326 16223 16234 |
| M49c1 | 73 263 489 750 1438 2706 3780 4769 6605 7028 8701 8860 9540 10398 10400 10514 10873 11719 12705 13032 14766 14783 15043 15301 15326 16223 16234 |
| M49d | 73 263 489 750 1438 2706 3780 4769 7028 8701 8860 9540 10373 10398 10400 10873 11542 11719 12705 14766 14783 15043 15184 15301 15326 16223 16234 16243 |
| M49e | 73 263 489 750 1438 2706 3780 4735A 4769 7028 8701 8860 9540 10398 10400 10700 10873 11061 11719 12705 14766 14783 15043 15301 15326 16223 16234 |
| M49e1 | 73 263 489 750 1438 2706 3780 4735A 4769 6515 7028 8701 8860 9540 10398 10400 10700 10873 11061 11719 12705 14766 14783 15043 15301 15326 16223 16234 |
| M50 | 73 263 489 750 1438 2706 4769 7028 7226 8701 8860 9540 10398 10400 10873 11719 12705 14766 14783 15043 15301 15326 15663 16223 16263 |
| M50a | 73 204 207 263 489 750 1438 2706 4769 5054 6620 7028 7226 8701 8860 9540 10398 10400 10873 11719 12705 14766 14783 15043 15301 15326 15663 16129 16223 16263 16526 |
| M50a1 | 73 204 207 263 466 489 750 1438 2706 4688G 4769 5054 6293 6620 6962 7028 7226 7642 8297 8701 8860 9540 10398 10400 10873 11719 12705 13802 14766 14783 15043 15301 15326 15663 16129 16153 16223 16263 16309 16526 |
| M50a2 | 73 204 207 263 489 750 1438 2010 2706 3834 4769 5054 6620 7028 7226 8701 8860 9540 10398 10400 10873 11719 12705 12879 14766 14783 15043 15301 15326 15663 16129 16223 16263 16526 |
| M52 | 73 263 489 750 1438 1462T 1598 2706 4769 5460 (6020) 7028 8701 8860 9540 10398 10400 10873 11719 12705 14766 14783 15043 15301 15326 16223 (16275) |
| M52a | 73 263 489 573.XC 750 1438 1462T 1598 2706 4769 5460 (6020) 7028 8701 8860 9540 10398 10400 10873 11719 12705 14766 14783 15043 15301 15326 15349 16223 (16275) 16390 |
| M52a1 | 73 263 489 573.XC 750 1438 1462T 1598 2706 4769 5460 (6020) 6755 7028 8701 8860 9540 10398 10400 10873 11719 12705 14766 14783 15043 15301 15326 15349 16223 (16275) 16327A 16390 |
| M52a1a | 73 263 489 573.XC 750 1438 1462T 1598 2706 4525 4769 5460 (6020) 6755 7028 8701 8860 9540 10398 10400 10873 11719 12705 14766 14783 15043 15301 15326 15349 16223 (16275) 16327A 16390 |
| M52a1b | 73 263 489 573.XC 750 1438 1462T 1598 2706 3591 4769 5460 (6020) 6755 7028 8701 8860 9540 10398 10400 10873 11719 11914 12705 14766 14783 15043 15301 15326 15349 16223 (16275) 16327A 16390 |

| | |
|---|---|
| M52a1b1 | 73 263 489 573.XC 614 750 1438 1462T 1598 2706 3591 4769 5460 (6020) 6755 7028 8158 8701 8860 9540 10398 10400 10685 10873 11084 11719 11914 12705 13135 14766 14783 15043 15301 15326 15349 16093 16270 (16275) 16327A 16390 |
| M52b | 73 263 489 750 1438 1462T 1598 2706 4769 5460 (6020) 7028 8701 8860 9540 10398 10400 10750 10873 11719 12705 14766 14783 15043 15301 15326 16223 (16275) |
| M52b1 | 73 263 489 750 1438 1462T 1598 2706 4769 5460 (6020) 7028 8701 8860 9540 10398 10400 10750 10873 11719 12705 14766 14783 15043 15301 15326 16223 (16275) 16438 |
| M52b1a | 63 64 73 146 263 489 750 1438 1462T 1598 2706 3434 3552 3866 4218 4769 5460 (6020) 7028 7954 8701 8860 9540 10398 10400 10750 10873 11719 12705 14766 14783 15043 15301 15326 16209 16223 (16275) 16438 |
| M55'77 | 73 263 489 750 1393 1438 2706 4769 7028 8701 8860 9540 10398 10400 10873 11719 12705 14766 14783 15043 15301 15326 16223 |
| M55 | 73 94 173 204 263 482 489 709 750 1393 1438 2706 3540 4491 4655 4769 5899.1C 6752 7028 8279.1T 8701 8860 9477 9540 9938 10398 10400 10873 11314 11719 12705 14766 14783 15043 15301 15326 16136 16217 16223 16319 16381 |
| M77 | 73 150 263 489 750 1393 1409d 1438 2706 4065 4769 7028 8701 8860 9540 10398 10400 10873 11719 11914 12618 12705 13407 13542 14178 14544 14766 14783 15043 15301 15326 16129 16189 16213 16218 16223 |
| M58 | 73 263 489 750 1438 2706 3209 4769 5460 7028 8701 8860 9540 10398 10400 10873 11719 12705 14766 14783 15043 15301 15326 16223 |
| M59 | 73 249 263 489 750 1438 2706 4769 7028 8701 8860 9380 9540 10256 10398 10400 10873 11140 11719 12705 14040 14766 14783 15043 15301 15326 16140 16223 16278 |
| M60 | 73 263 489 750 1438 2706 4769 7028 7912 8345 8701 8860 9540 10398 10400 10873 11719 12705 14766 14783 15043 15301 15326 16223 |
| M60a | 73 263 489 750 1438 2706 4769 7028 7912 8345 8701 8860 9540 10398 10400 10873 11719 12705 14766 14783 15043 15301 15326 16223 16284 16319 |
| M60a1 | 73 263 489 750 1438 2706 4769 7028 7912 8345 8701 8860 9540 10398 10400 10873 11719 12705 14766 14783 15043 15301 15326 15760 16223 16284 16319 |
| M60a2 | 73 263 489 750 1438 2442 2706 4769 6728 7028 7912 8345 8701 8860 9540 10398 10400 10873 11719 12705 14766 14783 15043 15301 15326 15910 16223 16284 16319 |
| M60b | 73 189 263 489 750 1438 2706 3592 3894 4047 4769 5471 7028 7853 7912 8345 8701 8723 8860 9540 10398 10400 10873 11719 12705 14766 14783 15043 15301 15326 16223 16266 16357 |
| M62'68 | 73 150 263 489 750 1438 2706 4561 4769 7028 7664 8701 8860 9540 10398 10400 10873 11719 12705 14766 14783 15043 15301 15326 16223 |
| M62 | 73 150 263 489 750 1438 2706 2735 3511 4561 4769 7028 7664 8149 8701 8860 9540 10398 10400 10873 11719 12705 13708 14766 14783 15043 15301 15326 15510 15520 15629 15721 16223 16295 |

| | |
|---|---|
| M62a | 73 146 150 263 310 489 750 1438 2706 2735 3511 4561 4763 4769 7028 7664 8149 8701 8860 9540 9935 10398 10400 10873 11719 12705 13708 14766 14783 15043 15301 15326 15510 15520 15629 15721 16147 16223 16295 |
| M62b | 73 150 263 489 750 1438 2706 2735 3511 4561 4769 7028 7664 8149 8701 8860 9540 10398 10400 10873 11719 12705 13708 14766 14783 15043 15301 15326 15510 15520 15629 15721 16223 16260 16295 |
| M62b+204 | 73 150 204 263 489 750 1438 2706 2735 3511 4561 4769 7028 7664 8149 8701 8860 9540 10398 10400 10873 11719 12705 13708 14766 14783 15043 15301 15326 15510 15520 15629 15721 16223 16260 16295 |
| M62b1 | 73 150 203 204 263 489 750 1438 2706 2735 3511 4561 4769 7028 7664 8149 8701 8860 9540 10398 10400 10873 11719 12705 13708 14766 14783 15043 15301 15326 15510 15520 15629 15721 16223 16260 16295 |
| M62b1a | 73 150 203 204 263 489 750 1438 2706 2735 3511 4561 4769 7028 7664 8149 8270 8281-8289d 8701 8860 9540 10398 10400 10873 10978 11431 11719 12705 13708 14766 14783 15043 15301 15326 15510 15520 15629 15721 16223 16260 16295 |
| M62b1a1 | 73 150 203 204 263 489 750 1438 2706 2735 3511 4561 4769 7028 7664 7844 8149 8270 8281-8289d 8701 8860 9540 10398 10400 10873 10978 11431 11719 12705 13708 14766 14783 15043 15301 15326 15510 15520 15629 15721 16169 16223 16260 16295 |
| M62b2 | 73 150 204 263 489 750 1438 2706 2735 3511 3693 4561 4769 6305 7028 7664 8149 8701 8860 9540 10398 10400 10873 11719 12705 13708 14766 14783 15043 15301 15326 15510 15520 15629 15721 16223 16260 16295 |
| M68 | 73 150 263 489 750 1438 2706 4561 4769 7028 7664 8701 8860 9540 10398 10400 10873 11719 12705 14766 14783 15043 15301 15326 16223 |
| M68a | 73 263 291-294d 489 750 1438 2626 2706 3828 4561 4688 4769 4902C 5489A 7028 7664 8093 8701 8860 9540 9644 10398 10400 10873 11719 12705 12950 13401 13806 14233 14766 14783 15043 15301 15326 16223 16255 16259 16278 16362 |
| M68a1 | 73 152 178 263 291-294d 489 750 1438 2626 2706 3828 4561 4688 4769 4902C 5489A 7028 8093 8701 8860 9540 9644 10203 10398 10400 10496 10873 11719 12705 12950 13401 13806 14233 14766 14783 15043 15301 15326 16223 16255 16259 16278 16362 |
| M68a1a | 73 152 178 263 291-294d 489 750 1438 2626 2706 3828 4561 4688 4769 4902C 5489A 7028 8093 8251 8701 8860 9540 9644 10203 10398 10400 10496 10873 11719 12705 12950 13401 13806 14233 14766 14783 15043 15301 15326 16169 16223 16255 16257 16259 16278 16362 |
| M68a2 | 73 263 291-294d 489 736 750 1438 2626 2706 3828 4561 4688 4769 4902C 5489A 7028 7664 7765 8093 8701 8853 8860 9540 9644 10398 10400 10873 11719 12705 12950 13401 13806 14233 14766 14783 15043 15301 15326 16223 16255 16259 16278 16362 16488 |
| M68a2a | 73 263 291-294d 489 736 750 1438 2626 2706 3828 4561 4688 4769 4902C 5489A 7028 7664 7765 8093 8701 8853 8860 9041 9540 9644 10398 10400 10873 11719 12705 12950 13401 13806 14233 14766 14783 15043 15301 15326 16223 16255 16259 16278 16311 16362 16488 |
| M69 | 73 263 489 750 1438 2706 4392 4769 7028 8701 8860 9540 10398 10400 10873 11365 11719 12705 14766 14783 15043 15301 15326 16223 |

| | |
|---|---|
| M69a | 73 195 263 489 553 750 1438 2706 3546 4392 4769 5499 6050 6899 7028 8701 8860 8964 9078 9210 9540 10398 10400 10873 11365 11719 12705 14110 14544 14766 14783 15043 15301 15326 15924 16124 16148 16223 |
| M71 | 73 263 489 750 1438 2706 4769 7028 8701 8718 8860 9540 10398 10400 10873 11719 12705 14766 14783 15043 15301 15326 15458 16223 16271 |
| M71+151 | 73 151 263 489 750 1438 2706 4769 7028 8701 8718 8860 9540 10398 10400 10873 11719 12705 14766 14783 15043 15301 15326 15458 16223 16271 |
| M71a | 73 151 263 489 750 1438 2706 4769 7028 8440 8701 8718 8860 9540 10398 10400 10873 11719 12705 14766 14783 15043 15301 15326 15458 16223 16271 |
| M71a1 | 73 150 151 263 489 750 1438 2706 4769 7028 7202 8440 8701 8718 8860 9540 9554 10398 10400 10873 11719 12705 14766 14783 15043 15257 15301 15326 15458 16223 16271 |
| M71a1a | 73 150 151 263 489 750 1438 2706 4769 7028 7202 8440 8701 8718 8860 9540 9554 10398 10400 10873 11719 12705 14766 14783 15043 15257 15301 15326 15458 16223 16269 16271 |
| M71a2 | 73 143 146 151 263 489 750 1438 2706 4769 7028 8440 8701 8718 8838 8860 9540 9615 10398 10400 10873 11719 12705 13759 14605 14766 14783 15043 15301 15326 15458 16129 16140 16223 16271 |
| M71b | 73 151 263 489 750 1438 2706 3777 4769 7028 7055 8701 8718 8860 9540 9548 10398 10400 10873 11719 12705 13149 14766 14783 15043 15301 15326 15458 16223 16260 16264 16271 |
| M71c | 73 143 195 204 263 489 750 1438 2706 3894 4769 7028 8701 8718 8860 9055 9380 9540 10398 10400 10873 11017 11719 12705 13401 14766 14783 15043 15301 15326 15458 15734 16223 16271 16362 |
| M72 | 73 263 489 750 1438 1872 2706 4769 7028 8701 8860 9540 10398 10400 10873 11719 12705 14233 14766 14783 15043 15301 15326 15644 15820 16166d 16214 16223 |
| M72a | 73 263 489 750 1438 1872 2706 4769 7028 8701 8860 9540 10398 10400 10873 11719 12705 12753 14233 14766 14783 15043 15301 15326 15644 15820 16166d 16214 16223 |
| M73'79 | 73 263 489 750 1438 2706 4769 7028 8701 8860 9540 10398 10400 10873 11719 12705 14034 14766 14783 15043 15301 15326 16223 16278 |
| M73 | 73 263 489 750 1438 2263A 2352 2706 3396 3399T 4769 5655 7028 8701 8860 9540 10398 10400 10873 11719 12705 14034 14766 14783 15043 15301 15326 16223 16278 |
| M73a | 73 263 489 750 1438 2263A 2352 2706 3396 3399T 4769 4856 5655 7028 8701 8860 9540 10398 10400 10685 10873 11476 11719 12705 14034 14766 14783 15043 15301 15326 16184A 16223 16278 |
| M73a1 | 73 263 489 750 1438 2263A 2352 2706 3396 3399T 3533 4769 4856 5655 7028 8701 8860 9540 10398 10400 10685 10873 11476 11719 12705 12950 14034 14766 14783 15043 15301 15326 16093 16184A 16223 16278 |
| M73b | 73 199 263 489 709 750 1438 2263A 2352 2706 3396 3399T 3453 4491 4769 5655 7028 8701 8860 9064 9540 10398 10400 10873 11708 11719 12705 14034 14766 14783 15043 15236 15301 15326 15663 16223 16278 16354 |

| | |
|---|---|
| M79 | 73 151 152 263 489 499 750 1438 2706 3442 3588 3669 3972 4769 7028 7661 8437 8701 8860 9540 10398 10400 10873 11719 12705 14034 14766 14783 15043 15301 15326 16223 16243 16260 16262 16278 16311 16319 |
| M76 | 73 263 489 513 750 1438 2706 3753 4769 7028 7521 8701 8860 9540 10325 10398 10400 10873 11719 12406 12705 14341 14766 14783 15043 15301 15326 16189 16293C 16362 |
| M76a | 73 146 234 263 489 513 750 1438 1709 2706 3438 3753 4769 7028 7353 7521 7793T 8470 8701 8860 9055 9540 10325 10398 10400 10873 11009 11719 12406 12603 12705 14341 14766 14783 15043 15301 15326 15547 16189 16293C 16325 16362 |
| M81 | 73 215 263 489 750 1438 2706 4254 4769 6620 7028 8701 8860 9540 10398 10400 10873 11719 12705 13590 14766 14783 15043 15301 15326 16129 16223 16311 |
| M91 | 73 93 263 489 750 1438 2706 3447 4164 4769 7028 8701 8860 9540 10398 10400 10873 11719 12705 14766 14783 15043 15301 15326 15652 16223 16287 16327A |
| M91a | 73 93 200 263 489 750 1438 2706 3447 4164 4769 5999 7028 7064 8701 8860 9540 10398 10400 10873 11719 11887 12705 13194 14766 14783 14902 15043 15301 15326 15622 15652 16223 16287 16327A |
| M91b | 73 93 263 489 647 750 1438 2706 3438 3447 4164 4769 5147 5592 7028 8701 8860 9540 10398 10400 10873 11389 11719 11914 12705 14766 14783 15043 15236 15301 15326 15652 15940 16069 16223 16287 16299 |
| M80'D | 73 263 489 750 1438 2706 4769 4883 7028 8701 8860 9540 10398 10400 10873 11719 12705 14766 14783 15043 15301 15326 16223 |
| M80 | 73 249 263 489 709 750 1352A 1438 2695 2706 4197 4769 4823 4883 5492 5894 7028 7939 8429 8701 8775 8860 9254 9540 10398 10400 10873 11719 11732 12705 14118 14766 14783 15043 15301 15326 16134 16189 16223 16325 |
| D | 73 263 489 750 1438 2706 4769 4883 5178A 7028 8701 8860 9540 10398 10400 10873 11719 12705 14766 14783 15043 15301 15326 16223 16362 |
| D4 | 73 263 489 750 1438 2706 3010 4769 4883 5178A 7028 8414 8701 8860 9540 10398 10400 10873 11719 12705 14668 14766 14783 15043 15301 15326 16223 16362 |
| D1 | 73 263 489 750 1438 2092 2706 3010 4769 4883 5178A 7028 8414 8701 8860 9540 10398 10400 10873 11719 12705 14668 14766 14783 15043 15301 15326 16223 16325 16362 |
| D1a | 73 263 489 750 1438 2092 2706 3010 4769 4883 5178A 5821 7028 8414 8701 8860 9540 10398 10400 10873 11719 12705 14668 14766 14783 15043 15301 15326 16223 16325 16362 |
| D1a1 | 57G 73 489 750 1438 2092 2706 3010 4769 4883 5178A 5821 7028 8414 8701 8860 9540 10398 10400 10873 11719 12705 14668 14766 14783 15043 15301 15313 15326 15719 16189 16223 16325 16362 |
| D1a2 | 73 (114) (143) 263 489 750 1438 2092 2706 3010 4769 4883 5178A 5821 7028 8414 8701 8860 9540 10398 10400 10816T 10873 11719 11914 12705 13059 14668 14766 14783 15043 15301 15326 15514 16223 16325 |
| D1b | 73 263 489 750 1438 2092 2706 3010 3335 4769 4883 5178A 6314 7028 8414 8701 8860 9540 10398 10400 10873 11719 12705 14047 14364 14668 14766 14783 15043 15301 15326 16223 16325 16362 |

| | |
|---|---|
| D1c | 73 263 489 750 1438 2092 2706 3010 4769 4883 5178A 7028 8414 8674 8701 8860 9540 10398 10400 10873 11719 12705 14668 14766 14783 15043 15301 15326 15805 16223 16325 16362 |
| D1d | 73 263 489 750 1438 2092 2706 3010 4769 4790 4883 5178A 7028 8414 8701 8860 9540 10398 10400 10873 11719 12414 12705 14668 14766 14783 15043 15301 15326 16223 16325 16362 |
| D1d1 | 73 263 489 750 1438 2092 2706 3010 4769 4790 4883 5178A 7028 8414 8701 8860 9540 10398 10400 10873 11719 12414 12705 14569 14668 14766 14783 15043 15301 15326 16223 16325 16362 |
| D1d2 | 73 263 489 750 1438 2092 2706 3010 4769 4790 4883 5147 5178A 7028 8414 8701 8860 9540 10398 10400 10873 11719 12414 12705 14668 14766 14783 15043 15301 15326 16093 16223 16325 16362 |
| D1e | 73 146 263 489 750 1438 2092 2706 3010 3316 4769 4883 5178A 7028 8414 8701 8860 9540 10398 10400 10873 11248 11719 12705 14668 14766 14783 15043 15301 15326 16223 16325 16362 |
| D1f | 73 263 489 750 1438 2092 2706 3010 4769 4883 5178A 7028 8414 8701 8860 9540 10398 10400 10873 11719 12705 14668 14766 14783 15043 15301 15326 16142 16223 16325 16362 |
| D1f1 | 73 263 489 750 1438 2092 2706 3010 4769 4883 5178A 7028 8414 8701 8860 9540 10398 10400 10873 10874 11719 12705 14668 14766 14783 15043 15301 15326 16142 16179 16223 16295 16325 16362 16497 |
| D1f2 | 73 152 263 489 750 1438 2092 2706 3010 3834 4769 4883 5178A 7028 8414 8701 8860 9540 10398 10400 10873 11719 12705 14668 14766 14783 15043 15301 15326 16142 16223 16325 16362 |
| D1f+16189 | 73 263 489 750 1438 2092 2706 3010 4769 4883 5178A 7028 8414 8701 8860 9540 10398 10400 10873 11719 12705 14668 14766 14783 15043 15301 15326 16142 16189 16223 16325 16362 |
| D1f3 | 73 263 489 750 1438 2092 2706 3010 4769 4883 5178A (5460) 7028 8414 8701 8860 9540 10398 10400 10644 10873 11719 12705 14668 14766 14783 15043 15301 15326 16142 16189 16223 16325 16362 |
| D1g | 73 263 489 750 1438 2092 2706 3010 4769 4883 5178A 7028 8116 8414 8701 8860 9540 10398 10400 10873 11719 12705 14668 14766 14783 15043 15301 15326 16187 16223 16325 16362 |
| D1g1 | 73 146 152 263 489 750 1438 2092 2706 3010 4769 4883 5178A 7028 8116 8414 8433 8701 8860 9540 10398 10400 10873 11719 12705 14027T 14668 14766 14783 15043 15301 15326 16187 16223 16325 16362 16390 |
| D1g1a | 73 146 152 263 489 750 1438 2092 2706 3010 4769 4883 5178A 7028 8116 8414 8433 8701 8860 9540 10398 10400 10873 11719 12705 14027T 14470 14668 14766 14783 15043 15301 15326 16187 16223 16325 16362 |
| D1g1b | 73 146 152 263 489 750 1438 2092 2706 3010 4769 4883 5178A 7028 8116 8414 8433 8701 8860 9540 10398 10400 10873 11719 12705 14027T 14668 14766 14783 15043 15301 15326 16187 16223 16245 16325 16362 |
| D1g+16189 | 73 263 489 750 1438 2092 2706 3010 4769 4883 5178A 7028 8116 8414 8701 8860 9540 10398 10400 10873 11719 12705 14668 14766 14783 15043 15301 15326 16187 16189 16223 16325 16362 |
| D1g2 | 73 143 263 489 750 1438 2092 2706 3010 4769 4883 5178A 7028 8116 8414 8701 8860 9540 10398 10400 10873 11719 12705 14668 14766 14783 15043 15301 15326 15769 16187 16189 16223 16325 16362 |

| | |
|---|---|
| D1g2a | 73 143 263 489 709 750 1438 2092 2706 3010 4769 4883 5178A 7028 8116 8414 8557 8701 8860 9540 10398 10400 10873 11719 12705 14668 14766 14783 15043 15301 15326 15769 15930 16187 16189 16223 16362 |
| D1g5 | 55 56 73 263 489 499 750 1438 2092 2706 3010 3505 4769 4883 5178A 7028 8116 8414 8701 8860 9540 10398 10400 10595 10873 11719 12705 14668 14693 14766 14783 15043 15301 15326 16187 16189 16209 16223 16325 16362 |
| D1g3 | 73 263 489 750 1438 2092 2706 2885 3010 4769 4883 5178A 7028 8116 8414 8701 8860 9540 10398 10400 10873 11719 12705 14668 14766 14783 15043 15301 15326 16187 16223 16325 16362 |
| D1g4 | 73 151 204 245 263 489 750 1438 2092 2706 3010 4769 4883 5178A 6179 7028 8116 8414 8701 8860 9419 9540 9545 10398 10400 10873 11719 11914 12705 14668 14766 14783 15043 15301 15326 15924 16187 16223 16290 16325 16362 |
| D1g6 | 73 150 199 263 374 489 750 960.1C 1438 2092 2706 3010 4769 4883 5178A 7028 7340 8116 8414 8701 8860 9540 10398 10400 10873 11719 12705 14668 14766 14783 15043 15301 15326 16178 16187 16223 16325 |
| D1h | 73 263 489 750 1438 2092 2706 3010 4769 4883 5178A 7028 8414 8701 8860 9540 10398 10400 10873 11719 12705 14668 14766 14783 15043 15301 15326 16093 16223 16274 16325 16362 |
| D1h1 | 73 204 263 489 750 1438 2092 2706 3010 4769 4883 5178A 7028 7861 8414 8701 8860 9540 10398 10400 10873 11719 12705 13635 14668 14766 14783 15043 15301 15326 16093 16223 16274 16325 16362 |
| D1h2 | 73 263 489 750 1438 2092 2706 3010 3349 3402 4769 4883 5178A 7028 8414 8701 8860 9540 10398 10400 10873 11719 12705 14668 14766 14783 15043 15301 15326 16093 16223 16239 16260 16274 16325 16362 |
| D1i | 73 263 417 489 750 1438 2092 2706 3010 4769 4883 5178A 7028 8414 8701 8860 9540 10398 10400 10873 11719 12280 12705 14668 14766 14783 15043 15301 15326 16223 16325 16362 |
| D1i1 | 73 263 417 489 750 1438 1943 2092 2706 3010 3828 4769 4883 5178A 7028 8277 8414 8701 8860 9540 10398 10400 10873 11719 12280 12705 14668 14766 14783 15043 15301 15326 16223 16325 16362 |
| D1i2 | 73 263 417 489 551 709 750 1438 2092 2706 3010 4769 4883 5178A 7028 8414 8701 8860 9540 10398 10400 10873 11314 11719 12280 12705 14668 14766 14783 15043 15301 15326 15877A 16223 16325 16362 16368 |
| D1j | 73 152 263 489 750 1438 2092 2706 3010 4769 4883 5178A 7028 8414 8701 8860 9540 10398 10400 10873 11719 12705 14668 14766 14783 15043 15301 15326 16223 16242 16311 16325 16362 |
| D1j1 | 73 152 263 489 750 1438 2092 2706 3010 4769 4883 5178A 7028 8414 8701 8860 9540 10398 10400 10873 11719 12705 14668 14766 14783 15043 15301 15326 15868 16223 16242 16311 16325 16362 |
| D1j1a | 73 152 263 489 750 1438 2092 2706 3010 4212 4769 4883 5004 5178A 7028 8414 8701 8860 9540 10398 10400 10873 11719 12705 14668 14766 14783 15043 15301 15326 15644 15868 16223 16242 16311 16325 16362 |
| D1j1a1 | 73 152 263 489 750 1438 2092 2706 3010 4212 4769 4883 5004 5178A 5325T 7028 8414 8701 8860 9540 10398 10400 10873 11719 12705 14668 14766 14783 15043 15301 15326 15644 15868 16223 16242 16311 16325 16362 |

| | |
|---|---|
| D1j1a2 | 73 106-111d 152 212 263 489 750 1438 2092 2706 3010 4212 4769 4883 5004 5178A 6392 7028 8414 8701 8860 9540 10398 10400 10873 11719 12705 14668 14766 14783 15043 15301 15644 15868 16223 16242 16311 16325 16362 |
| D1k | 73 263 489 750 1438 2092 2706 3010 4769 4883 5178A 7028 8414 8701 8860 9540 10398 10400 10873 11719 12705 13368 14668 14766 14783 15043 15301 15326 16223 16325 16362 |
| D1m | 73 263 489 750 1438 2092 2706 3010 4769 4823 4883 5178A 7028 8414 8548 8701 8860 9540 10398 10400 10754 10873 11719 12705 14668 14766 14783 15043 15301 15326 16223 16316 16325 16362 |
| D1n | 73 263 489 750 1438 2092 2706 3010 4769 4883 5178A 7028 8414 8701 8860 9180 9540 10398 10400 10873 11719 12705 14668 14766 14783 15043 15301 15326 16223 16325 16362 |
| D4a | 73 152 263 489 750 1438 2706 3010 3206 4769 4883 5178A 7028 8414 8473 8701 8860 9540 10398 10400 10873 11719 12705 14668 14766 14783 14979 15043 15301 15326 16129 16223 16362 |
| D4a1 | 73 152 263 489 750 1438 2706 3010 3206 4769 4883 5178A 7028 8414 8473 8701 8860 9540 10398 10400 10410 10873 11719 12705 14668 14766 14783 14979 15043 15301 15326 16129 16223 16362 |
| D4a1a | 73 152 263 489 750 1438 2706 3010 3206 4769 4883 5178A 5261 7028 8414 8473 8701 8860 9540 10398 10400 10410 10873 11719 12705 14668 14766 14783 14979 15043 15301 15326 16129 16223 16362 |
| D4a1a1 | 73 152 263 489 750 1438 2706 3010 3206 4769 4883 5178A 5261 7028 8414 8473 8701 8860 9540 10398 10400 10410 10873 11719 12705 14668 14766 14783 14979 15043 15301 15314 15326 16129 16223 16362 |
| D4a1a1a | 73 152 263 489 750 1438 2706 3010 3206 4769 4883 5178A 5261 7028 8414 8473 8701 8860 9540 10398 10400 10410 10873 11719 12705 14668 14766 14783 14979 15043 15301 15314 15326 15757 16129 16223 16362 |
| D4a1b | 73 152 263 489 750 1438 2706 3010 3206 4769 4883 5178A 7028 8414 8473 8701 8860 9540 10398 10400 10410 10873 11719 12705 13651 14668 14766 14783 14979 15043 15301 15326 16129 16223 16362 |
| D4a1b1 | 73 152 263 489 750 1438 2706 3010 3206 4769 4883 5178A 7028 8414 8473 8701 8860 9540 10398 10400 10410 10873 11719 12705 13651 14668 14766 14783 14979 15043 15301 15326 16129 16223 16309 16362 |
| D4a1c | 73 152 263 489 750 1438 2706 3010 3206 4769 4883 5178A 7028 7822 8414 8473 8701 8860 9540 10398 10400 10410 10873 11719 12705 14668 14766 14783 14979 15043 15301 15326 16129 16223 16362 |
| D4a1d | 73 152 263 489 750 1438 2706 3010 3206 4769 4883 5178A 7028 8414 8473 8701 8860 9540 10398 10400 10410 10873 11719 12705 14668 14766 14783 14979 15043 15301 15326 16129 16223 16286 16362 |
| D4a1e | 73 152 263 489 750 1438 2706 3010 3206 4769 4883 5178A 7028 7364 8414 8473 8701 8860 9540 10398 10400 10410 10873 11719 12705 14668 14766 14783 14979 15043 15301 15326 16129 16223 16256 16362 |
| D4a1e1 | 73 152 263 489 750 1438 2706 3010 3206 4769 4883 5178A 7028 7364 8414 8473 8614 8701 8860 9254 9540 10398 10400 10410 10873 11719 12705 14668 14766 14783 14979 15043 15301 15326 16129 16223 16256 |

| | |
|---|---|
| D4a1f | 73 152 263 489 750 1438 2706 3010 3206 4313 4769 4883 5178A 7028 8414 8473 8701 8860 9540 10398 10400 10410 10873 11719 12705 14668 14766 14783 14979 15043 15301 15326 16129 16223 16362 |
| D4a1f1 | 73 152 263 489 750 1438 2706 3010 3206 4313 4769 4883 5178A 7028 8414 8473 8701 8860 9540 10398 10400 10410 10873 11719 12705 14467 14668 14766 14783 14979 15043 15301 15326 16129 16223 16362 |
| D4a1g | 73 152 263 489 750 1438 2706 3010 3206 4769 4883 5178A 5459 7028 8414 8473 8701 8860 9540 10398 10400 10410 10873 11719 12705 14668 14766 14783 14979 15043 15301 15326 16129 16223 16362 |
| D4a1h | 73 152 263 489 750 1438 2706 3010 3206 4769 4883 5178A 7028 8414 8473 8701 8860 9540 10398 10400 10410 10873 11719 12705 14053 14668 14766 14783 14979 15043 15301 15326 16129 16223 16362 |
| D4a2 | 73 152 263 489 750 1438 2706 3010 3206 4769 4883 5178A 7028 8414 8473 8701 8860 9540 10398 10400 10873 11719 12705 12957 14668 14766 14783 14979 15043 15301 15326 16129 16223 16362 |
| D4a2a | 73 152 263 489 750 1438 2706 3010 3206 3531 4769 4883 5178A 7028 8296 8414 8473 8701 8860 9540 10005 10398 10400 10873 11719 12705 12957 14668 14766 14783 14979 15043 15301 15326 16129 16223 16362 |
| D4a2b | 73 152 263 489 750 1438 2706 3010 3206 4769 4883 5178A 6285 7028 8414 8473 8701 8860 9540 10398 10400 10873 11719 12705 12957 14668 14766 14783 14979 15043 15301 15326 16129 16223 16362 |
| D4a3 | 73 152 263 489 750 1438 2706 3010 3206 4769 4883 5178A 5466 7028 8414 8473 8701 8860 9540 10398 10400 10873 11719 12705 14668 14766 14783 14979 15043 15301 15326 16129 16223 16249 16362 |
| D4a3a | 73 152 263 489 750 1438 2706 3010 3206 4769 4883 5178A 5466 7028 8414 8473 8701 8860 9540 10398 10400 10873 11719 12705 14668 14766 14783 14979 15043 15301 15326 15412 16129 16223 16249 16362 |
| D4a3a1 | 73 152 228T 263 489 750 1438 2706 3010 3206 3866 4769 4883 5178A 5466 7028 8414 8473 8701 8860 9540 10398 10400 10873 11719 12705 14668 14766 14783 14979 15043 15301 15326 15412 16129 16223 16249 16362 |
| D4a3a2 | 73 152 263 489 750 1438 2706 3010 3206 3396 4769 4883 5178A 5466 7028 8414 8473 8701 8860 9540 10398 10400 10873 11719 12705 14013 14577 14668 14766 14783 14979 15043 15301 15326 15412 15448 16093 16129 16223 16249 16362 |
| D4a3b | 73 152 263 489 750 1438 2706 3010 3206 4769 4883 5178A 5466 7028 7912 8414 8473 8701 8860 9540 10398 10400 10873 11719 12705 14668 14766 14783 14979 15043 15301 15326 16129 16223 16249 16362 |
| D4a3b1 | 73 152 263 489 750 1438 2706 3010 3206 4769 4883 5178A 5466 7028 7912 8414 8473 8701 8860 9540 10398 10400 10601 10873 11719 12705 14668 14766 14783 14979 15043 15301 15326 15440 16129 16223 16249 16266 16362 |
| D4a3b2 | 73 152 263 489 750 1438 1520 2706 3010 3206 4769 4883 5178A 5466 7028 7912 8414 8473 8701 8860 9540 10398 10400 10873 11719 12705 13834 14668 14766 14783 14979 15043 15301 15326 16129 16223 16249 16311 16362 |

| | |
|---|---|
| D4a+16294 | 73 152 263 489 750 1438 2706 3010 3206 4769 4883 5178A 7028 8414 8473 8701 8860 9540 10398 10400 10873 11719 12705 14668 14766 14783 14979 15043 15301 15326 16129 16223 16294 16362 |
| D4a4 | 73 152 263 469G 489 750 1438 2706 3010 3206 4248 4769 4883 5178A 7028 8414 8473 8659 8701 8860 9109 9540 10398 10400 10873 11719 12705 14668 14766 14783 14979 15043 15301 15326 16129 16223 16294 16362 |
| D4a5 | 73 152 263 489 750 1438 2706 3010 3206 4769 4883 5178A 7028 8414 8473 8701 8860 9540 10398 10400 10873 11719 12468 12705 14668 14766 14783 14979 15043 15301 15326 15758 16129 16223 16362 |
| D4a6 | 73 152 217 263 489 750 1438 1811 2706 3010 3206 4769 4883 5178A 7028 7444 8414 8473 8701 8860 9540 10398 10400 10873 11719 12705 14668 14766 14783 14979 15043 15301 15326 16129 16223 16362 |
| D4a7 | 73 152 263 489 750 1438 2706 3010 3206 4769 4883 5178A 7028 8414 8473 8701 8860 9540 9845 10398 10400 10873 11719 12705 13650 13966 14668 14766 14783 14979 15043 15301 15326 15889 16129 16223 16263 |
| D4a8 | 73 146 152 263 489 750 1438 2706 3010 3206 4769 4883 5178A 7028 8414 8473 8701 8860 9540 10398 10400 10873 11719 12705 13350 14668 14766 14783 14979 15043 15301 15326 16129 16223 16270 16362 |
| D4b | 73 263 489 750 1438 2706 3010 4769 4883 5178A 7028 8020 8414 8701 8860 9540 10398 10400 10873 11719 12705 14668 14766 14783 15043 15301 15326 16223 16362 |
| D4b1 | 73 263 489 750 1438 2706 3010 4769 4883 5178A 7028 8020 8414 8701 8860 9540 10181 10398 10400 10873 11719 12705 14668 14766 14783 15043 15301 15326 15440 15951 16223 16319 16362 |
| D4b1a | 73 263 489 750 1438 2706 3010 4769 4883 5178A 7028 8020 8414 8701 8860 9540 10181 10398 10400 10873 11719 12705 14668 14766 14783 14927 15043 15301 15326 15440 15951 16223 16319 16362 |
| D4b1a1 | 73 152 263 489 750 1438 2246 2706 3010 4769 4883 5178A 7028 8020 8414 8450 8701 8860 9540 10181 10398 10400 10873 11719 12705 13827 14091 14668 14766 14783 14927 15043 15217 15301 15326 15440 15805 15951 16223 16319 16362 |
| D4b1a1a | 73 152 263 489 750 1438 2246 2706 3010 4769 4883 5178A 7028 8020 8414 8450 8701 8860 9540 10181 10398 10400 10873 11719 12705 13827 14091 14180 14668 14766 14783 14927 15043 15217 15301 15326 15440 15805 15951 16223 16319 16362 |
| D4b1a2 | 73 263 489 750 1438 2706 3010 4769 4883 5178A 6881 7028 8020 8414 8701 8860 9540 10181 10398 10400 10873 11719 12705 14668 14766 14783 14815 14927 15043 15301 15326 15440 15951 16223 16319 16362 |
| D4b1a2a | 73 263 489 750 1438 2706 3010 4769 4883 5178A 6881 7028 8020 8414 8701 8860 9540 10181 10398 10400 10873 11719 12705 13720 14668 14766 14783 14815 14927 15043 15301 15326 15440 15951 16223 16319 |
| D4b1a2a1 | 73 263 489 750 1438 2706 3010 4769 4883 5178A 6881 7028 8020 8414 8701 8860 9540 10181 10398 10400 10873 11719 12705 13720 14668 14766 14783 14815 14927 15043 15301 15326 15440 15951 16173 16223 16319 16362 |

| | |
|---|---|
| D4b1a2a2 | 73 263 489 750 1438 2706 3010 4592 4769 4883 5178A 6881 7028 8020 8414 8701 8860 9540 10181 10398 10400 10873 11719 12705 13720 14668 14766 14783 14815 14927 15043 15244 15301 15326 15440 15951 16223 16319 16362 |
| D4b1b'd | 73 263 489 750 1438 2706 3010 4769 4883 5178A 7028 8020 8414 8701 8860 9540 10181 10398 10400 10873 11719 12705 14668 14766 14783 15043 15301 15326 15440 15951 16223 16287 16319 16362 |
| D4b1b | 73 263 431 489 750 1438 2706 3010 4769 4883 5178A 6689 7028 8020 8414 8701 8860 9540 10181 10398 10400 10873 11719 12705 14668 14766 14783 15043 15301 15326 15440 15951 16223 16287 16319 16362 |
| D4b1b1 | 73 263 431 489 750 1438 2706 3010 4769 4859 4883 5178A 6410 6689 7028 8020 8414 8701 8860 9540 10181 10398 10400 10873 11719 12705 14668 14766 14783 15043 15301 15326 15440 15951 16223 16287 16319 |
| D4b1b1a | 73 263 431 489 750 1438 2706 3010 4769 4859 4883 5178A 6410 6689 7028 8020 8414 8701 8860 9540 10084 10181 10398 10400 10873 11719 12705 14668 14766 14783 15043 15301 15326 15440 15951 16223 16287 16319 16362 16399 |
| D4b1b1a1 | 73 263 431 489 750 1438 2706 3010 4769 4859 4883 5178A 6410 6689 7028 8020 8414 8701 8860 9540 10084 10181 10398 10400 10873 11719 12172 12705 14668 14766 14783 15043 15301 15326 15440 15951 16223 16287 16319 16362 16399 |
| D4b1b2 | 73 263 431 489 750 1438 2706 3010 4769 4883 5178A 6689 7028 8020 8281-8289d 8414 8701 8860 9540 10181 10398 10400 10873 11719 12705 14560C 14668 14766 14783 15043 15301 15326 15440 15951 16223 16287 16319 16362 |
| D4b1d | 73 152 263 489 750 1438 2706 3010 4769 4883 5178A 6578 7028 8020 8414 8701 8860 9540 10181 10398 10400 10873 11116 11719 11992 12705 14668 14766 14783 15043 15301 15326 15370 15440 15951 16158 16223 16284 16287 16319 |
| D4b1c | 73 239 263 297 489 750 951 1438 2706 3010 4769 4883 5178A 7028 8020 8414 8701 8860 9540 10181 10398 10400 10873 11719 12705 14668 14766 14783 15043 15301 15326 15440 15951 16223 16319 16362 |
| D3 | 73 239 263 297 489 722 750 951 1438 2706 3010 4023 4769 4883 5178A 6374 7028 8020 8414 8701 8860 9540 9785 10181 10398 10400 10873 11719 12705 14668 14766 14783 15043 15301 15326 15440 15951 16223 16319 16362 |
| D4b2 | 73 263 489 750 1382C 1438 2706 3010 4769 4883 5178A 7028 8020 8414 8701 8860 8964 9540 9824A 10398 10400 10873 11719 12705 14668 14766 14783 15043 15301 15326 16223 16362 |
| D4b2a | 73 263 489 750 1382C 1438 2706 3010 4769 4883 5178A 7028 8020 8414 8701 8860 8964 9540 9824A 10398 10400 10873 11719 12705 14668 14766 14783 15043 15301 15326 15524 16223 16362 |
| D4b2a1 | 73 263 280 489 750 1382C 1438 2706 3010 4769 4883 5178A 7028 8020 8414 8701 8860 8964 9540 9824A 10398 10400 10873 11719 12705 13708 14668 14766 14783 15043 15301 15326 15524 16223 16355 16362 |
| D4b2a2 | 73 263 489 750 1382C 1438 2706 3010 4769 4883 5178A 7028 8020 8251 8414 8701 8860 8964 9540 9824A 10104 10398 10400 10873 11719 12705 14668 14766 14783 15043 15301 15326 15524 16223 16362 |

| | |
|---|---|
| D4b2a2a | 73 263 489 750 1382C 1438 2706 3010 4769 4883 5178A 7028 8020 8251 8414 8701 8860 8964 9540 9824A 10104 10398 10400 10873 11719 12705 14287 14668 14766 14783 15043 15301 15326 15524 16189 16223 16362 |
| D4b2a2a1 | 73 199 263 489 750 1382C 1438 2706 3010 4769 4883 5178A 7028 8020 8251 8414 8701 8860 8964 9540 9824A 10104 10398 10400 10873 11719 12705 14287 14668 14766 14783 15043 15301 15326 15524 16189 16223 16362 |
| D4b2a2a2 | 73 263 489 750 1382C 1438 2706 3010 3591 4769 4883 5178A 7028 8020 8251 8414 8701 8860 8964 9540 9824A 10104 10398 10400 10873 11719 12705 14287 14668 14766 14783 15043 15301 15326 15524 16189 16223 16362 |
| D4b2a2b | 73 263 489 750 1382C 1438 2706 3010 4769 4883 5178A 6005 7028 8020 8251 8414 8701 8860 8964 9540 9824A 10104 10398 10400 10873 11719 12705 14668 14766 14783 15043 15301 15326 15524 16223 16362 |
| D4b2b | 73 (194) 263 489 750 1382C 1438 2706 3010 4769 4883 5178A 7028 8020 8414 8701 8860 8964 9296 9540 9824A 10398 10400 10873 11719 12705 14668 14766 14783 15043 15301 15326 16223 16362 |
| D4b2b1 | 73 (194) 263 489 750 1382C 1438 2706 3010 4769 4883 5178A 7028 8020 8414 8701 8860 8964 9296 9540 9824A 10398 10400 10873 11719 12705 14605 14668 14766 14783 15043 15301 15326 16223 16362 |
| D4b2b1a | 73 (194) 263 489 750 1382C 1438 2706 3010 4769 4883 5178A 7028 8020 8414 8701 8860 8964 9296 9540 9824A 10398 10400 10873 11719 12705 14605 14668 14766 14783 15043 15244 15301 15326 16223 16362 |
| D4b2b1b | 73 (194) 263 489 750 1382C 1438 2706 3010 3696 4769 4883 5178A 7028 8020 8414 8701 8812 8860 8964 9296 9540 9824A 10398 10400 10873 11719 12705 14605 14668 14766 14783 15043 15301 15326 16223 16362 |
| D4b2b1c | 73 (194) 263 489 750 1382C 1438 2706 3010 4769 4883 5178A 6176 7028 8020 8414 8701 8860 8964 9296 9540 9824A 10398 10400 10873 11719 12705 14605 14668 14766 14783 15043 15301 15326 16223 16362 |
| D4b2b1+146 | 73 146 (194) 263 489 750 1382C 1438 2706 3010 4769 4883 5178A 7028 8020 8414 8701 8860 8964 9296 9540 9824A 10398 10400 10873 11719 12705 14605 14668 14766 14783 15043 15301 15326 16223 16362 |
| D4b2b1d | 73 146 (194) 263 489 750 1382C 1438 2706 3010 4117 4769 4883 5178A 7028 8020 8414 8701 8860 8964 9296 9540 9824A 10398 10400 10873 11719 12705 14605 14668 14766 14783 15043 15301 15326 16223 16362 |
| D4b2b2 | 73 (194) 263 489 750 1382C 1438 2706 3010 4769 4883 5178A 7028 8020 8414 8701 8860 8964 9296 9540 9824A 10398 10400 10873 11719 12358 12705 14668 14766 14783 15043 15301 15326 16223 16362 |
| D4b2b2a | 73 150 (194) 263 489 750 1382C 1438 2706 3010 4769 4883 5178A 7028 8020 8414 8701 8860 8964 9296 9540 9824A 10398 10400 10873 11719 12358 12705 14668 14766 14783 15043 15301 15326 16223 16362 |
| D4b2b2a1 | 73 150 (194) 263 489 750 1382C 1438 2706 3010 4769 4883 5178A 7028 8020 8414 8701 8860 8964 9296 9540 9824A 10398 10400 10873 11719 12358 12705 14668 14766 14783 14867 15043 15301 15326 16223 16362 |
| D4b2b2b | 73 (194) 263 489 750 1382C 1438 2706 3010 4769 4883 5178A 7028 8020 8414 8577 8701 8860 8964 9296 9540 9824A 10398 10400 10873 11719 12358 12705 14668 14766 14783 15043 15301 15326 16172 16362 |

| | |
|---|---|
| D4b2b2c | 73 (194) 263 489 750 1382C 1438 2706 3010 4769 4883 5178A 7028 8020 8414 8701 8860 8964 9296 9540 9824A 10398 10400 10873 11719 12358 12705 13707 14668 14766 14783 15043 15301 15326 16189 16223 16356 16362 |
| D4b2b3 | 73 (194) 263 489 750 1382C 1438 2706 3010 4209 4769 4883 5178A 7028 8020 8414 8701 8860 8964 9296 9540 9824A 10373 10398 10400 10873 11719 12705 14325 14668 14766 14783 15043 15301 15326 15331A 16223 16362 |
| D4b2b4 | 73 150 (194) 263 489 750 1382C 1438 2706 3010 4769 4883 5178A 7028 8020 8414 8701 8860 8964 9296 9540 9824A 10398 10400 10873 11719 12705 14668 14766 14783 15001 15043 15301 15326 16223 16362 |
| D4b2b5 | 73 (194) 263 279 489 750 1382C 1438 2706 3010 4769 4883 5178A 7028 8020 8414 8701 8860 8964 9296 9540 9824A 10398 10400 10873 11719 12705 14668 14766 14783 15043 15301 15326 16223 16362 |
| D4b2b6 | 73 (194) 263 489 750 1382C 1438 1978 2706 3010 4769 4883 5178A 7028 8020 8414 8701 8860 8964 9296 9540 9824A 10398 10400 10873 11719 12705 13182 14668 14766 14783 15043 15301 15326 15832 16223 16362 |
| D4b2b7 | 73 (194) 263 489 750 1382C 1438 2706 3010 4769 4883 5178A 7028 8020 8414 8701 8860 8964 9296 9540 9824A 10398 10400 10873 11719 12705 14668 14766 14783 15043 15301 15326 15896 16209 16223 16266 16362 |
| D4b2d | 73 263 489 750 1382C 1438 2706 3010 4769 4883 5178A 6407 7028 7816 8020 8414 8701 8860 8964 9540 9824A 10398 10400 10873 11719 12705 13174 13239 14668 14766 14783 15043 15301 15326 16223 16287 16362 |
| D4c | 73 263 489 750 1438 2706 3010 4769 4883 5178A 7028 8414 8701 8860 9540 10398 10400 10873 11719 12705 14668 14766 14783 15043 15301 15326 16223 16245 16362 |
| D4c1 | 73 263 489 750 1438 2706 2766 3010 4769 4883 5178A 7028 8414 8701 8860 9540 10398 10400 10873 11719 12705 14668 14766 14783 15043 15301 15326 16245 16362 |
| D4c1a | 73 191.1A 194 199 207 263 489 750 1438 2706 2766 3010 3391 4769 4883 5178A 7028 8414 8701 8860 9540 9755 10398 10400 10873 11719 12705 14668 14766 14783 15043 15301 15326 16245 16362 |
| D4c1a1 | 73 191.1A 194 199 207 263 489 750 1438 2706 2766 3010 3391 4769 4883 5178A 7028 8414 8701 8860 9477 9540 9755 10398 10400 10873 11719 12705 14668 14766 14783 15043 15301 15326 16245 16362 |
| D4c1b | 73 263 489 750 856 1438 2706 2766 3010 4769 4883 5178A 7028 8414 8701 8860 9540 10398 10400 10873 11719 12705 14668 14766 14783 15043 15301 15326 16224 16245 16292 16362 |
| D4c1b1 | 73 146 263 489 750 856 1438 2706 2766 3010 4769 4883 5178A 7028 8414 8701 8860 9540 10398 10400 10873 11719 12705 14668 14692 14766 14783 15043 15301 15326 16223 16224 16245 16292 16362 |
| D4c1b2 | 73 263 489 750 856 1438 2706 2766 3010 4769 4883 5178A 7028 8414 8701 8860 9540 10398 10400 10873 11719 12705 14063 14668 14766 14783 15043 15301 15326 16224 16245 16292 16362 |
| D4c2 | 73 263 489 750 1438 2706 3010 4769 4883 5178A 7028 8383 8414 8701 8860 9431 9540 10398 10400 10873 11719 12705 14668 14766 14783 15043 15301 15326 16223 16245 16362 |

| | |
|---|---|
| D4c2a | 73 263 489 750 1438 2706 3010 4233 4769 4883 5178A 7028 8383 8414 8701 8860 9431 9540 10398 10400 10873 11719 12705 14668 14766 14783 15043 15301 15326 15391A 16223 16245 16311 16362 16368 |
| D4c2b | 73 263 489 534 750 1438 1462 2706 3010 4769 4883 5178A 7028 8383 8414 8701 8860 9431 9540 10398 10400 10873 11719 12705 14668 14766 14783 15043 15301 15326 16223 16245 16362 |
| D4c2c | 73 263 489 750 1438 2706 3010 4769 4883 5178A 7028 8383 8414 8419 8701 8860 9431 9540 10398 10400 10873 11719 12705 14668 14766 14783 15043 15301 15326 16223 16245 16362 |
| D4d | 73 263 489 750 1438 2706 3010 3593 4769 4883 5178A 5539 6503C 7028 7757 8414 8701 8860 9540 9938 10398 10400 10873 11719 12085 12705 13879 14668 14766 14783 15043 15301 15326 16223 16319 16362 |
| D4e | 73 263 489 750 1438 2706 3010 4769 4883 5178A 7028 8414 8701 8860 9540 10398 10400 10873 11215 11719 12705 14668 14766 14783 15043 15301 15326 16223 16362 |
| D4e1'3 | 73 263 489 750 1438 2706 3010 4769 4883 5178A 7028 8414 8701 8860 9536 9540 10398 10400 10873 11215 11719 12705 14668 14766 14783 15043 15301 15326 16223 16362 |
| D4e1 | 73 263 489 750 1438 2706 3010 3316 4769 4883 5178A 7028 8414 8701 8860 9536 9540 10398 10400 10873 11215 11719 12705 14668 14766 14783 15043 15301 15326 (16092) 16223 16362 |
| D4e1a | 73 94 263 489 750 1438 2706 3010 3316 4769 4883 5178A 5964 7028 8414 8701 8860 9536 9540 10398 10400 10873 11215 11719 12705 14470 14668 14766 14783 15043 15301 15326 15924 (16092) 16223 16362 |
| D4e1a1 | 73 94 194 263 489 750 1438 2706 3010 3316 4769 4883 5178A 5964 7028 8414 8701 8860 9536 9540 10398 10400 10873 11215 11719 12705 14470 14668 14766 14783 15043 15301 15326 15924 (16092) 16093 16176 16223 16362 |
| D4e1a2 | 73 94 214 263 489 750 1438 2706 3010 3316 4769 4883 5178A 5964 7028 8414 8701 8860 9536 9540 10398 10400 10873 11215 11719 12705 14470 14668 14766 14783 15043 15301 15326 15924 (16092) 16223 16362 |
| D4e1a2a | 73 94 214 263 334 489 750 1438 2706 3010 3316 4769 4883 4959 5178A 5964 7028 8414 8701 8860 9495 9536 9540 10398 10400 10873 11215 11719 12705 14470 14668 14766 14783 15043 15301 15326 15924 (16092) 16223 16362 |
| D4e1a3 | 73 94 263 489 750 1438 2706 3010 3316 4769 4883 5178A 5964 7028 8119 8414 8701 8860 9536 9540 10398 10400 10873 11215 11719 12705 14470 14668 14766 14783 15043 15301 15326 15924 (16092) 16223 16362 |
| D4e1c | 73 263 489 750 1438 2706 3010 3316 4769 4883 5178A 7028 8414 8701 8860 9536 9540 10398 10400 10873 11215 11719 12705 14207 14668 14766 14783 15043 15301 15326 (16092) 16223 16362 |
| D2 | 73 263 489 750 1438 2706 3010 3316 4769 4883 5178A 7028 8414 8701 8703 8860 9536 9540 10398 10400 10873 11215 11719 12705 14668 14766 14783 15043 15301 15326 (16092) 16129 16223 16362 |
| D2a'b | 73 263 489 750 1438 2706 3010 3316 4769 4883 5178A 7028 7493 8414 8701 8703 8860 9536 9540 10398 10400 10873 11215 11719 12705 14668 14766 14783 15043 15301 15326 (16092) 16129 16223 16271 16362 |

| | |
|---|---|
| D2a | 73 263 489 750 1438 2706 3010 3316 4769 4883 5178A 7028 7493 8414 8701 8703 8860 9536 9540 10398 10400 10873 11215 11719 11959 12705 14668 14766 14783 15043 15301 15326 (16092) 16129 16223 16271 16362 |
| D2a1 | 73 263 489 750 1438 2706 3010 3316 4769 4883 5178A 7028 7493 8414 8701 8703 8860 9536 9540 9667 10398 10400 10873 11215 11719 11959 12705 14668 14766 14783 15043 15301 15326 (16092) 16129 16223 16271 16362 |
| D2a1a | 73 263 489 750 1438 2706 3010 3316 4769 4883 5178A 7028 7493 8414 8701 8703 8860 8910A 9536 9540 9667 10398 10400 10873 11215 11719 11959 12705 14668 14766 14783 15043 15301 15326 (16092) 16129 16223 16271 16362 |
| D2a1b | 73 263 489 750 1438 2706 3010 3316 4769 4883 5178A 7028 7493 8414 8701 8703 8860 9536 9540 9667 10398 10400 10873 11215 11719 11959 12705 14668 14766 14783 15043 15301 15326 (16092) 16111 16129 16223 16271 16362 16366 |
| D2a2 | 73 263 489 750 1438 2706 3010 3316 4769 4883 4991 5178A 7028 7493 8414 8701 8703 8860 9536 9540 10398 10400 10873 11215 11719 11959 12705 14668 14766 14783 15043 15301 15326 (16092) 16129 16223 16271 16362 |
| D2b | 73 195 263 489 750 1438 2706 3010 3316 4769 4883 5178A 7028 7493 8414 8701 8703 8860 9181 9536 9540 10398 10400 10873 11215 11719 12705 14668 14766 14783 15043 15301 15326 (16092) 16129 16223 16271 |
| D2b1 | 73 195 263 489 750 1106 1438 2706 3010 3316 4769 4883 5178A 7028 7493 8414 8701 8703 8860 9181 9536 9540 10398 10400 10873 11215 11719 12705 14668 14766 14783 15043 15301 15326 (16092) 16129 16223 16271 16362 |
| D2b1a | 73 195 263 489 750 1106 1438 2706 3010 3316 4769 4883 5004 5178A 7028 7493 8414 8701 8703 8860 9181 9536 9540 10398 10400 10873 11215 11719 12705 14668 14766 14783 15043 15301 15326 (16092) 16129 16223 16271 16362 |
| D2b2 | 73 195 263 489 750 1438 2706 3010 3316 4769 4883 5178A 7028 7493 8414 8701 8703 8860 9181 9536 9540 10084 10398 10400 10873 11215 11719 12705 14668 14766 14783 15043 15301 15326 15836 (16092) 16129 16223 16271 16362 |
| D2c | 73 263 310 489 750 1438 2706 3010 3316 4769 4883 5178A 7028 8414 8701 8703 8860 9042 9536 9540 10398 10400 10586 10873 11215 11719 12468 12705 14668 14766 14783 15043 15301 15326 (16092) 16129 16223 |
| D4e3 | 73 263 489 750 1438 2706 3010 4769 4883 5178A 7028 8414 8701 8860 9536 9540 10398 10400 10873 11215 11719 12705 13135 14668 14766 14783 14857 15043 15301 15326 16223 16362 |
| D4e2 | 73 263 489 750 1438 2706 3010 4769 4883 5178A 7028 8414 8701 8860 9540 10398 10400 10873 11215 11719 12705 14668 14766 14783 15043 15301 15326 15874 16223 16362 |
| D4e2a | 73 263 489 573.XC 750 1438 2706 3010 4769 4883 5178A 7028 8414 8701 8860 9540 10398 10400 10873 11215 11719 12705 13716 14668 14766 14783 15043 15301 15326 15874 16223 16362 |

| | |
|---|---|
| D4e2b | 73 263 489 750 1438 2706 3010 4769 4883 5178A 7028 8149 8414 8701 8860 9540 10398 10400 10873 11215 11719 12705 14668 14766 14783 15043 15301 15326 15874 16223 16362 |
| D4e2c | 73 263 489 750 1438 2706 3010 4769 4883 5178A 7028 8414 8701 8860 9540 10398 10400 10873 11215 11719 12705 14053 14668 14766 14783 15043 15301 15326 15874 16223 16362 |
| D4e2d | 73 263 489 750 1438 2706 3010 4769 4883 5178A 7028 8414 8701 8860 9540 10398 10400 10873 11215 11719 11764 12705 14668 14766 14783 15043 15301 15326 15874 16223 16362 |
| D4e4 | 73 263 489 750 1438 1935 2706 3010 4769 4883 5178A 7028 8414 8701 8860 9540 10398 10400 10873 11215 11719 12705 14668 14766 14783 14905 15043 15301 15326 16223 16362 |
| D4e4a | 73 263 489 750 1438 1935 2706 3010 4769 4883 5178A 7028 8414 8683 8701 8860 9540 10398 10400 10873 11215 11719 12705 14668 14766 14783 14905 15043 15301 15326 16223 16362 |
| D4e4a1 | 73 263 310 453A 489 750 1438 1935 2706 3010 4769 4883 5178A 7028 8414 8683 8701 8860 9540 10398 10400 10873 11215 11719 12705 14668 14766 14783 14905 15043 15301 15326 16189 16223 16362 |
| D4e4b | 73 263 489 750 1438 1935 2706 3010 4769 4883 5178A 7028 8414 8701 8860 9540 10310 10398 10400 10873 11215 11719 12705 12882 14668 14766 14783 14905 15043 15301 15326 16223 16362 |
| D4e5 | 73 263 489 750 1438 2706 3010 4769 4883 5021 5178A 7028 8414 8701 8860 9540 10398 10400 10873 11215 11719 12705 14668 14766 14783 15043 15106 15184 15301 15326 16223 16274 16362 |
| D4e5a | 73 152 263 489 573.XC 750 1438 2706 3010 4769 4883 5021 5178A 7028 8414 8701 8860 9540 10398 10400 10873 11215 11719 12705 14668 14755 14766 14783 15043 15106 15184 15301 15326 16223 16274 16362 |
| D4e5b | 73 263 489 750 1438 2706 3010 4769 4883 5021 5178A 7028 8414 8701 8860 9540 10398 10400 10873 11215 11719 12705 14384 14668 14766 14783 15043 15106 15184 15301 15326 16223 16274 16291 16362 |
| D4f | 73 263 489 750 1438 2706 3010 4769 4883 5178A 7028 7270 8414 8701 8860 9540 10398 10400 10873 11719 12705 14668 14766 14783 15043 15301 15326 16223 16362 |
| D4f1 | 73 263 489 750 1438 3010 4538 4769 4883 5178A 7028 7270 8414 8701 8764 8860 9540 10398 10400 10873 11255 11719 12705 14668 14766 14783 15043 15301 15326 16223 16362 |
| D4g | 73 263 489 750 1438 2706 3010 4769 4883 5178A 7028 8414 8701 8860 9540 10398 10400 10873 11719 12705 13104 14668 14766 14783 15043 15301 15326 16223 16362 |
| D4g1 | 73 263 489 573.XC 750 1438 2706 3010 4343 4769 4883 5178A 7028 8414 8860 9540 10398 10400 10873 11719 12705 13104 14668 14766 14783 15043 15301 15326 15518 16223 16278 16362 |
| D4g1a | 73 263 489 573.XC 750 1438 2706 3010 3394 4343 4769 4883 5178A 7028 8414 8860 9540 10398 10400 10873 11719 12705 13104 13887 14668 14766 14783 15043 15301 15326 15518 16223 16278 16362 |
| D4g1b | 73 263 489 573.XC 750 1438 2706 3010 4343 4769 4883 5178A 7028 8414 8860 9004 9540 10398 10400 10873 11719 12705 13104 13512 14668 14766 14783 15043 15301 15326 15518 16223 16278 16362 |

| | |
|---|---|
| D4g1c | 73 263 489 573.XC 709 750 1438 2706 3010 4343 4769 4883 5178A 7028 8414 8860 9540 10398 10400 10873 11719 12705 13104 14668 14766 14783 15043 15301 15326 15518 16223 16278 16362 |
| D4g2 | 73 263 298 489 750 1438 2706 3010 4769 4883 5178A 5231 7028 8414 8701 8860 9540 10398 10400 10873 11719 12705 13104 14668 14766 14783 15043 15301 15326 16223 16362 |
| D4g2a | 73 263 298 489 750 1438 2706 3010 4394 4769 4883 5178A 5231 7028 8414 8701 8860 9540 10398 10400 10873 11719 12705 13104 14668 14766 14783 15043 15301 15326 16223 16274 16362 |
| D4g2a1 | 73 263 298 489 750 1438 2706 3010 4394 4769 4883 5178A 5231 7028 8414 8701 8860 9540 10398 10400 10873 11059 11719 12705 13104 14668 14766 14783 15043 15301 15326 16223 16274 16362 |
| D4g2a1a | 73 195 263 298 489 546 750 1438 2706 3010 4394 4769 4883 5178A 5231 7028 8414 8701 8860 8994 9540 10398 10400 10873 11059 11719 12705 13104 14668 14766 14783 14793 15043 15301 15326 16274 16362 |
| D4g2a1b | 73 263 298 459d 489 750 1438 2706 3010 4394 4769 4883 5081 5178A 5231 6164 7028 8414 8701 8860 9540 10398 10400 10873 11059 11719 12705 13104 14668 14766 14783 15043 15301 15326 16223 16274 16362 |
| D4g2a1c | 73 263 298 489 750 1438 2706 3010 4394 4769 4883 5178A 5231 7028 8414 8701 8860 9540 10398 10400 10873 11059 11719 12705 12966A 13104 14668 14766 14783 15043 15301 15326 16223 16274 16362 |
| D4g2b | 73 263 298 489 750 1438 2706 3010 4131 4769 4883 5178A 5231 7028 8414 8701 8860 9540 10398 10400 10873 11719 12705 13104 14668 14766 14783 15043 15301 15326 16223 16362 |
| D4g2b1 | 73 263 298 489 750 1438 2706 3010 4131 4769 4883 5178A 5231 7028 8414 8701 8860 9540 10398 10400 10873 11719 12705 13104 13359 14668 14766 14783 15043 15301 15326 16223 16362 |
| D4g2b1a | 73 195 298 489 504 750 1438 2706 3010 4131 4769 4883 5178A 5231 5460 6719 7028 8414 8701 8860 9386 9540 10398 10400 10873 11719 12705 13104 13359 14668 14766 14783 15043 15301 15326 16223 16362 |
| D4h | 73 263 489 750 1438 2706 3010 3336 3644 4769 4883 5048 5178A 7028 8414 8701 8860 9540 10398 10400 10873 11719 12705 14668 14766 14783 15043 15301 15326 16223 16362 |
| D4h1 | 73 263 489 750 1438 2706 3010 3336 3644 4769 4883 5048 5178A 7028 8414 8701 8860 9540 10398 10400 10873 11719 12705 14668 14766 14783 15043 15301 15326 16174 16223 16362 |
| D4h1a | 73 146 183 263 489 750 1438 2706 3010 3336 3644 4769 4883 5048 5178A 7028 8414 8701 8860 9540 10398 10400 10873 11719 12396 12705 14668 14766 14783 15043 15301 15326 16174 16223 16362 |
| D4h1a1 | 73 146 183 263 489 750 1438 2706 3010 3336 3644 4769 4883 5048 5178A 7028 8414 8701 8860 9540 10398 10400 10873 11719 12372 12396 12705 14668 14766 14783 15043 15301 15326 16174 16223 16362 16497 |
| D4h1a2 | 73 146 183 263 489 750 827 1009 1438 2706 3010 3336 3644 4769 4883 5048 5178A 6962 7028 8414 8701 8860 9540 10398 10400 10873 11719 12396 12705 13819 14668 14766 14783 15043 15301 15326 16174 16223 16362 |

| | |
|---|---|
| D4h1b | 73 263 489 750 1438 2706 3010 3336 3644 4769 4883 5048 5178A 7028 8414 8701 8860 9540 10398 10400 10873 11092 11719 12705 13708 14668 14766 14783 15043 15301 15326 16174 16223 16362 |
| D4h1c | 73 152 263 489 750 1438 2706 3010 3336 3644 4769 4883 5048 5178A 7028 7181 7673 8414 8701 8860 9540 10398 10400 10873 11719 12705 13914A 14668 14766 14783 15043 15301 15326 16174 16223 16311 16362 |
| D4h1c1 | 73 152 263 489 750 1438 2706 3010 3336 3644 4769 4883 5048 5178A 7028 7181 7673 8414 8701 8860 9540 10398 10400 10873 11719 12705 13914A 14470 14668 14766 14783 15043 15301 15326 16174 16223 16311 16317 16362 |
| D4h1d | 73 263 489 750 1438 2706 3010 3336 3644 4769 4883 5048 5178A 5262 7028 8387 8414 8701 8860 9540 10042 10398 10400 10873 11719 12705 14668 14766 14783 15043 15301 15326 16167 16172 16174 16223 16287 16362 |
| D4h2 | 73 263 489 750 961 1438 2706 3010 3336 3644 4769 4883 5048 5178A 7028 8269 8414 8701 8860 9540 10398 10400 10873 11719 12705 13879 14668 14766 14783 15043 15236 15301 15326 16223 16278 16325 16362 |
| D4h3 | 73 152 263 489 750 1438 2706 3010 3336 3644 4769 4883 5048 5178A 7028 8414 8701 8860 9540 10398 10400 10873 11719 12705 13135 14668 14766 14783 15043 15301 15326 16223 16301 16342 16362 |
| D4h3a | 73 152 263 489 750 1438 2706 3010 3336 3396 3644 4025 4769 4883 5048 5178A 6285 7028 8414 8701 8860 8946 9458 9540 10398 10400 10873 11719 12705 13135 14668 14766 14783 15043 15301 15326 16223 16241 16301 16342 16362 |
| D4h3a1 | 73 152 263 489 750 1438 2706 3010 3336 3396 3644 4025 4769 4883 5048 5178A 5442 6285 7028 8414 8701 8860 8946 9458 9540 10398 10400 10873 11719 12705 12990 13135 14668 14766 14783 15043 15301 15326 16223 16241 16301 16342 16362 |
| D4h3a1a | 73 152 263 489 750 930 1438 1719 2706 3010 3336 3396 3644 4025 4769 4883 5048 5178A 5442 6285 7028 8414 8701 8860 8946 9458 9540 10398 10400 10873 11719 12705 12990 13135 14668 14766 14783 15043 15301 15326 16223 16241 16301 16342 16362 |
| D4h3a1a1 | 73 146 152 263 489 750 930 1438 1719 2706 3010 3336 3396 3644 4025 4769 4883 5048 5178A 5442 6285 7028 8414 8701 8860 8946 9458 9540 10398 10400 10873 11701 11719 12705 12990 13135 14668 14766 14783 15043 15301 15326 16223 16241 16301 16342 16362 |
| D4h3a1a2 | 73 152 263 489 750 930 1438 1719 2706 3010 3336 3396 3644 4025 4769 4883 5048 5178A 5442 6285 7028 8414 8701 8860 8946 9458 9540 10398 10400 10873 11719 11914 12705 12990 13135 14668 14766 14783 15043 15301 15326 16223 16241 16301 16342 16362 |
| D4h3a2 | 73 152 263 489 573.XC 750 1438 2706 3010 3336 3396 3644 4025 4769 4883 5048 5178A 6285 7028 8414 8701 8860 8946 9458 9540 10398 10400 10733 10873 11719 12705 13135 14668 14766 14783 15043 15301 15326 16223 16241 16301 16342 16362 |
| D4h3a3 | 71d 73 152 263 489 750 1438 2706 3010 3336 3396 3644 4025 4769 4883 5048 5178A 6285 7028 8414 8701 8860 8946 9458 9540 10398 10400 10873 11719 12705 13135 14668 14766 14783 15043 15301 15326 16223 16241 16301 16342 16362 |

| | |
|---|---|
| D4h3a3a | 71d 73 152 263 489 533 750 1438 2706 3010 3336 3396 3644 4025 4769 4883 5048 5178A 6285 7028 8414 8701 8860 8946 9458 9540 10398 10400 10873 11719 12705 13135 14668 14766 14783 15043 15301 15326 16223 16241 16301 16342 16362 |
| D4h3a4 | 73 152 263 489 750 1438 2706 3010 3336 3396 3644 4025 4769 4883 4959 5048 5178A 6285 7028 8414 8701 8860 8946 9458 9540 9851 10398 10400 10873 11719 12705 13135 14668 14766 14783 15043 15301 15326 16223 16241 16294 16301 16342 16362 |
| D4h3a5 | 73 152 263 489 750 1438 2706 3010 3336 3396 3644 4025 4769 4883 5048 5178A 6285 7028 8414 8701 8860 8946 9458 9540 10398 10400 10873 11719 12705 13135 14668 14766 14783 15043 15301 15326 16223 16241 16342 16362 |
| D4h3a6 | 73 143 152 263 489 750 1438 2706 3010 3336 3396 3644 4025 4363 4769 4883 5048 5178A 6285 7028 8414 8701 8860 8946 9458 9540 10398 10400 10873 11719 12705 13135 14668 14766 14783 15043 15301 15326 16185 16223 16241 16301 16342 16362 16463 |
| D4h3a+@152 | 73 263 489 750 1438 2706 3010 3336 3396 3644 4025 4769 4883 5048 5178A 6285 7028 8414 8701 8860 8946 9458 9540 10398 10400 10873 11719 12705 13135 14668 14766 14783 15043 15301 15326 16223 16241 16301 16342 16362 |
| D4h3a7 | 73 263 489 750 1438 2706 3010 3336 3396 3644 4025 4769 4883 5048 5178A 6285 7028 8414 8701 8860 8946 9458 9540 9962 10398 10400 10873 11719 12705 13135 14668 14766 14783 15043 15301 15326 16223 16241 16301 16342 16362 |
| D4h3a8 | 73 263 489 750 1438 2706 3010 3336 3396 3644 4025 4769 4883 5048 5178A 6285 7028 8414 8701 8860 8946 9458 9540 10398 10400 10873 11719 11809 12705 13065 13135 14668 14766 14783 15043 15301 15326 16223 16234G 16241 16301 16342 16362 |
| D4h3a9 | 73 263 489 750 1438 2706 3010 3336 3396 3644 4025 4769 4883 5048 5178A 6023 6285 7028 8414 8701 8860 8946 9458 9540 10398 10400 10873 11719 12705 13135 14668 14766 14783 14861 15043 15301 15326 16223 16241 16301 16342 16362 |
| D4h3b | 73 152 263 489 750 990 1438 1810 2416 2706 3010 3336 3644 4769 4883 5048 5178A 7028 7785 8414 8701 8860 9046 9540 10398 10400 10873 11150 11719 12705 13135 14668 14766 14783 15043 15301 15326 15346 16148 16223 16249 16301 16342 16362 |
| D4h4 | 73 263 489 750 1438 2706 3010 3336 3644 4769 4883 5048 5178A 7028 8414 8701 8860 9540 10398 10400 10873 11719 12705 14668 14766 14783 15022 15043 15301 15326 16223 16362 |
| D4h4a | 73 152 263 489 644 750 1438 2706 3010 3336 3558 3644 4769 4883 5048 5178A 7028 8414 8701 8860 9540 10373 10398 10400 10873 11719 12705 14668 14766 14783 15022 15043 15301 15326 16223 16311 16362 |
| D4i | 73 263 489 750 1438 2706 3010 4769 4883 5178A 7028 8414 8701 8860 9540 10398 10400 10873 11719 12705 14668 14766 14783 15043 15301 15326 16223 16294 16362 |
| D4i1 | 73 263 489 750 1438 2706 3010 4769 4883 5178A 5773 7028 8414 8701 8860 9540 10398 10400 10873 11719 12705 14668 14766 14783 15043 15301 15326 16223 16263 16294 16362 |

| | |
|---|---|
| D4i2 | 73 263 489 750 1438 2706 3010 4769 4833 4883 5178A 7028 8414 8701 8860 9540 10398 10400 10873 11719 12705 14668 14766 14783 15043 15301 15326 16223 16294 16362 |
| D4i3 | 73 195 263 489 750 1438 2706 3010 4769 4883 4936 5178A 7028 8414 8701 8860 9540 10398 10400 10873 11719 12705 14668 14766 14783 15043 15301 15326 16114 16223 16294 16318 16362 |
| D4j | 73 263 489 750 1438 2706 3010 4769 4883 5178A 7028 8414 8701 8860 9540 10398 10400 10873 11696 11719 12705 14668 14766 14783 15043 15301 15326 16223 16362 |
| D4j1 | 73 263 489 750 1438 2706 3010 4769 4883 5178A 5262 7028 8414 8701 8860 9540 10398 10400 10873 11696 11719 12705 14668 14766 14783 15043 15301 15326 16223 16362 |
| D4j1a | 73 263 489 750 1438 2706 3010 4769 4883 5178A 5262 7028 7783 8414 8701 8860 9540 10398 10400 10873 11696 11719 12130 12358 12705 14668 14766 14783 15043 15301 15326 16086 16223 16362 |
| D4j1a1 | 73 263 489 750 1438 2706 3010 4769 4883 5178A 5262 7028 7581 7783 8414 8701 8860 9540 10398 10400 10873 11696 11719 12130 12358 12705 14668 14766 14783 15043 15295 15301 15326 16086 16223 16362 |
| D4j1a1a | 73 263 489 750 1438 2706 3010 4769 4883 5178A 5262 7028 7581 7783 8414 8701 8860 9540 10398 10400 10873 11696 11719 12130 12358 12681 12705 14668 14766 14783 15043 15295 15301 15326 16086 16223 16362 |
| D4j1a1b | 73 263 489 750 1438 2706 3010 4769 4883 5178A 5262 7028 7581 7783 7980C 8414 8701 8860 9540 10398 10400 10873 11696 11719 12130 12358 12705 14668 14766 14783 15043 15295 15301 15326 16086 16223 |
| D4j1a2 | 73 263 489 750 1438 2706 3010 4769 4883 5178A 5262 7028 7783 8414 8701 8860 9540 10398 10400 10873 11696 11719 12130 12358 12705 13635 14668 14766 14783 15043 15301 15326 16086 16223 16271 16362 |
| D4j1b | 73 263 489 750 1438 1734 2706 3010 4769 4883 5178A 5262 7028 7055 8414 8701 8860 9540 10398 10400 10873 11696 11719 12705 14668 14766 14783 15043 15301 15326 16223 16362 |
| D4j1b2 | 73 185 263 489 750 1438 1734 2706 3010 4769 4883 5178A 5262 6852 7028 7055 8414 8701 8860 9540 10398 10400 10873 11696 11719 12705 14668 14766 14783 15043 15301 15326 16223 16362 |
| D4j2 | 73 152 263 489 750 1438 2706 3010 4769 4883 5178A 5563 5824 7028 8414 8701 8860 9540 10398 10400 10873 11696 11719 12501 12705 14668 14766 14783 15043 15301 15326 16223 16291 16362 |
| D4j2a | 73 152 263 489 750 1438 2706 3010 3693 4769 4883 5178A 5563 5824 7028 8414 8701 8860 9540 10398 10400 10873 11696 11719 12501 12705 14668 14766 14783 15043 15301 15326 16223 16291 16362 |
| D4j+16311 | 73 263 489 750 1438 2706 3010 4769 4883 5178A 7028 8414 8701 8860 9540 10398 10400 10873 11696 11719 12705 14668 14766 14783 15043 15301 15326 16223 16311 16362 |
| D4j3 | 73 263 489 750 1438 2706 3010 4769 4883 5178A 7028 8414 8701 8860 9540 10398 10400 10873 11696 11719 12705 14668 14766 14783 15043 15301 15326 16184 16223 16311 16362 |
| D4j3a | 73 263 489 750 1438 1709 2706 3010 4769 4883 5178A 7028 8414 8701 8860 9540 10398 10400 10873 11696 11719 12705 14668 14766 14783 15043 15301 15326 16184 16223 16311 16362 |

| | |
|---|---|
| D4j3a1 | 73 263 489 750 1438 1709 2706 3010 4769 4883 5178A 7028 8414 8701 8860 9540 10398 10400 10873 11696 11719 12705 13656 14668 14766 14783 15043 15139 15301 15326 16184 16223 16311 16362 16525 |
| D4j11 | 73 263 489 750 1438 2706 3010 4769 4883 5178A 7028 8414 8701 8860 9540 10398 10400 10873 11218 11696 11719 12630 12705 14668 14766 14783 15043 15301 15326 16223 16311 16362 |
| D4j4 | 73 263 489 750 1438 2706 3010 4769 4883 5178A 7028 8414 8701 8860 9540 10245 10398 10400 10873 11696 11719 12705 14668 14766 14783 15043 15301 15326 16223 16362 |
| D4j4a | 73 263 489 750 1438 2706 3010 4769 4883 5178A 7028 8414 8701 8860 9540 10245 10398 10400 10873 11696 11719 12705 14668 14766 14783 15043 15301 15326 16223 16263 16362 |
| D4j5 | 73 263 489 750 1438 2706 3010 4769 4883 5178A 7028 8414 8701 8860 9540 10398 10400 10873 11696 11719 12705 13368 14128 14668 14766 14783 15043 15301 15326 16223 16362 |
| D4j5a | 73 263 489 750 1438 2706 3010 4769 4883 5178A 7028 7445C 8414 8701 8860 9540 10398 10400 10873 11696 11719 12705 13086 13368 14128 14668 14766 14783 15043 15301 15326 16223 16362 |
| D4j+146 | 73 146 263 489 750 1438 2706 3010 4769 4883 5178A 7028 8414 8701 8860 9540 10398 10400 10873 11696 11719 12705 14668 14766 14783 15043 15301 15326 16223 16362 |
| D4j6 | 73 146 263 489 750 1438 2706 3010 4769 4883 5178A 7028 7805 8414 8701 8860 9540 10398 10400 10873 11696 11719 12705 14668 14766 14783 15043 15301 15326 16223 16362 |
| D4j13 | 73 146 263 485 489 750 1438 2706 3010 4655 4769 4883 5075 5178A 7028 8414 8701 8860 9540 10398 10400 10873 11696 11719 12705 14668 14766 14783 15043 15301 15326 16223 16362 |
| D4j7 | 73 263 489 750 1438 2706 3010 4769 4883 5178A 7028 8414 8701 8860 9540 9578 10398 10400 10873 11696 11719 12705 13753 14668 14766 14783 15043 15301 15326 16082 16223 16362 |
| D4j7a | 73 263 489 750 1438 2706 3010 4769 4883 5178A 7028 8414 8434 8701 8860 9540 9578 10398 10400 10873 11696 11719 12705 13753 14000A 14668 14766 14783 15043 15301 15326 16082 16223 16362 |
| D4j8 | 73 263 489 750 1438 2706 3010 4769 4883 5178A 7028 8414 8701 8860 9540 10398 10400 10873 11696 11719 12705 14668 14766 14783 15043 15301 15326 16174 16223 16362 |
| D4j+(16286) | 73 263 489 750 1438 2706 3010 4769 4883 5178A 7028 8414 8701 8860 9540 10398 10400 10873 11696 11719 12705 14668 14766 14783 15043 15301 15326 16223 (16286) 16362 |
| D4j9 | 73 263 489 750 1438 2706 3010 4769 4883 5178A 7028 8414 8701 8860 9540 10398 10400 10873 11696 11719 12705 13573A 14668 14766 14783 15043 15301 15326 16223 (16286) 16362 |
| D4j10 | 73 263 489 750 1438 2706 3010 4769 4883 5178A 7028 8414 8701 8860 9540 10398 10400 10873 11696 11719 12705 14488 14668 14766 14783 15043 15301 15326 16223 16362 |
| D4j12 | 73 263 489 750 1438 2706 3010 4769 4883 5178A 7028 7353 8273 8414 8701 8860 9540 10398 10400 10873 11696 11719 12705 14668 14766 14783 15043 15301 15326 15470 16223 16362 |

| | |
|---|---|
| D4j14 | 73 263 489 750 1438 2706 3010 4769 4883 5178A 7028 8414 8701 8860 9540 10398 10400 10873 11696 11719 12705 14668 14766 14783 15043 15301 15326 16223 16231 16362 |
| D4j15 | 73 263 489 750 1438 2706 3010 4769 4883 5178A 7028 8414 8701 8860 9540 10398 10400 10873 11696 11719 12705 14668 14766 14783 15043 15301 15326 15529A 16223 16362 |
| D4j16 | 73 263 489 750 1438 2706 3010 4769 4883 5178A 6752 7028 8414 8701 8860 9540 10398 10400 10873 11696 11719 12705 12771 14668 14766 14783 15043 15301 15326 16223 16362 |
| D4+195 | 73 195 263 489 750 1438 2706 3010 4769 4883 5178A 7028 8414 8701 8860 9540 10398 10400 10873 11719 12705 14668 14766 14783 15043 15301 15326 16223 16362 |
| D4k | 73 195 263 489 750 1438 2706 3010 4769 4883 5178A 7028 8414 8701 8860 9540 10398 10400 10873 11719 12092A 12705 14668 14766 14783 15043 15301 15326 16223 |
| D4o | 73 195 263 489 750 1438 2706 3010 4769 4883 5178A 7028 8414 8701 8860 9540 10398 10400 10646 10873 11719 12705 14668 14766 14783 15043 15301 15326 16223 16290 16362 |
| D4o1 | 73 195 263 489 750 1438 2706 3010 4769 4883 5178A 7028 8414 8701 8860 9540 9833 10398 10400 10646 10873 11719 12705 13500 14668 14766 14783 15043 15301 15326 16183 16223 16274 16290 16319 16362 |
| D4o1a | 73 195 263 489 750 1438 2330 2706 3010 4769 4883 5178A 7028 8414 8701 8860 9540 9833 10398 10400 10646 10873 11719 12705 13500 14668 14766 14783 15043 15301 15326 16183 16223 16274 16290 16319 16362 |
| D4o2 | 73 195 263 489 750 1438 2706 3010 4769 4883 5178A 7028 8414 8701 8860 9077 9540 10398 10400 10646 10873 11719 12705 13812 14668 14766 14783 15043 15301 15326 16093 16223 16290 16362 |
| D4o2a | 73 195 263 489 750 1438 2706 3010 4769 4883 5178A 7028 8414 8701 8860 9077 9540 10398 10400 10646 10873 11719 12705 13812 14668 14766 14783 15043 15301 15326 16093 16223 16232 16290 16362 |
| D4o2a1 | 73 185 195 263 489 533 750 1438 2706 3010 4769 4883 5178A 7028 8414 8701 8860 9077 9540 10398 10400 10646 10873 11719 12705 13812 14668 14766 14783 15043 15301 15326 16093 16223 16232 16290 16362 |
| D4p | 73 195 198 263 489 750 1438 2706 3010 4769 4883 5178A 7028 8414 8701 8860 9540 10398 10400 10873 11719 12705 14668 14766 14783 15043 15106 15301 15326 16223 16362 |
| D4p1 | 73 195 198 263 489 750 1438 2178 2706 3010 4769 4883 5178A 7028 8414 8701 8860 9540 10398 10400 10873 11719 12705 14668 14766 14783 15043 15106 15301 15326 16223 16362 |
| D4l | 73 263 489 750 1438 2706 3010 4769 4883 5178A 7028 8414 8701 8860 9540 10398 10400 10427 10873 11719 12705 14668 14766 14783 15043 15301 15326 16223 16362 16368 |
| D4l1 | 73 263 489 750 1310 1438 2706 3010 4769 4883 5178A 7028 8414 8701 8860 9540 10398 10400 10427 10873 11719 12705 14668 14766 14783 15043 15301 15326 16223 16362 16368 |
| D4l1a | 73 263 489 750 1310 1438 2706 3010 3535 4769 4883 5178A 7028 8414 8701 8860 9540 10398 10400 10427 10873 11719 12705 14668 14766 14783 15043 15301 15326 16145 16223 16362 16368 |

| | |
|---|---|
| D4l1a1 | 73 263 489 750 1310 1438 2706 3010 3535 4769 4883 5178A 7028 8414 8701 8860 9355 9540 10398 10400 10427 10873 11719 12705 14668 14766 14783 15043 15301 15326 16145 16223 16362 16368 |
| D4l2 | 125 127 263 489 750 1438 2706 3010 4769 4883 5178A 7028 8414 8701 8860 9540 10398 10400 10427 10873 11719 12705 14668 14766 14783 15043 15301 15326 16223 16362 16368 |
| D4l2a | 125 127 263 489 750 1438 2706 3010 4769 4883 5178A 7028 8414 8701 8860 9540 10398 10400 10427 10873 11719 12705 13651 14668 14766 14783 15043 15301 15326 16223 16362 16368 |
| D4l2a1 | 125 127 263 489 750 1438 2706 3010 4769 4883 5147 5178A 7028 8414 8701 8860 9540 10398 10400 10427 10873 11719 12705 13651 14668 14766 14783 15043 15301 15326 16145 16223 16311 16362 16368 |
| D4l2a2 | 125 127 263 489 750 1438 2706 3010 4769 4883 5178A 7028 8414 8701 8860 9540 10398 10400 10427 10873 11719 12705 13651 14668 14766 14783 15043 15301 15326 16223 16274 16362 16368 |
| D4l2b | 125 127 263 318 489 750 1438 2706 3010 4769 4883 5178A 7028 8414 8503 8701 8860 9540 10398 10400 10427 10873 11719 12705 14668 14766 14783 15043 15301 15326 16223 16362 16368 |
| D4m | 73 263 489 750 1438 2706 3010 4769 4883 5178A 7028 8414 8701 8762 8860 9540 10398 10400 10873 11719 12651C 12705 14668 14766 14783 15043 15301 15326 16223 16362 |
| D4m1 | 73 263 489 750 1148 1438 2706 3010 4769 4883 5178A 6620 7028 8414 8701 8762 8860 9540 9667 10398 10400 10873 11719 12088 12651C 12705 14668 14766 14783 15043 15301 15326 16244 16362 |
| D4m2 | 73 263 489 750 1222 1438 1719 2706 3010 3492 4769 4883 5178A 7028 8414 8701 8762 8860 9540 10398 10400 10873 11719 12651C 12705 14668 14766 14783 15043 15301 15326 16042 16223 16362 |
| D4m2a | 73 263 489 750 1222 1438 1719 2706 3010 3492 4769 4883 5178A 7028 8414 8701 8762 8860 9540 10398 10400 10873 11719 12651C 12705 14668 14766 14783 15043 15301 15326 16042 16214 16223 16362 |
| D4m2a1 | 73 263 489 750 1222 1438 1719 2706 3010 3492 4769 4883 5178A 7028 8414 8701 8762 8860 9540 10398 10400 10873 11719 12651C 12705 14668 14766 14783 15043 15301 15313 15326 16042 16214 16223 16362 |
| D4m2a1a | 73 263 489 750 1222 1438 1719 2706 3010 3492 4769 4883 5178A 7028 8414 8701 8762 8860 9540 10398 10400 10873 11719 12651C 12705 12750 14668 14766 14783 15043 15301 15313 15326 16042 16214 16223 |
| D4n | 73 263 489 750 1438 2706 3010 3421 4769 4883 5178A 7028 8414 8701 8860 9540 10398 10400 10873 11719 12705 14668 14766 14783 15043 15301 15326 16223 16355A 16362 |
| D4n1 | 73 263 489 750 1438 2706 3010 3421 4769 4883 5178A 7028 8414 8701 8860 9540 10398 10400 10873 11719 12705 14668 14766 14783 15043 15301 15326 15924 16223 16355A 16362 |
| D4n1a | 73 263 489 750 951 1438 2706 3010 3421 4769 4883 5178A 7028 8414 8701 8860 9540 10398 10400 10873 11719 12705 14668 14766 14783 15043 15301 15326 15924 16223 16355A 16362 |
| D4n2 | 73 195 263 489 750 1438 2706 3010 3421 4769 4883 5178A 6704 7028 8414 8701 8860 9540 10398 10400 10873 11719 12705 14668 14766 14783 15043 15301 15326 16223 16355A 16362 |

| | |
|---|---|
| D4q | 73 200 263 489 750 1438 2706 3010 4769 4883 5178A 7028 8414 8701 8860 9540 10398 10400 10873 11719 12705 14668 14766 14783 15043 15301 15326 16223 16256 16311 16362 |
| D4q1 | 73 200 263 489 750 1438 2706 3010 4769 4883 5178A 7028 8414 8701 8860 9540 10398 10400 10873 11719 12705 14668 14766 14783 15043 15301 15326 16140 16223 16256 16311 16362 |
| D4q1a | 73 200 263 489 750 1438 2706 3010 4769 4883 4973 5178A 7028 8414 8701 8860 9540 10398 10400 10873 11719 12705 14668 14766 14783 15043 15301 15326 15791 16140 16223 16256 16311 16362 |
| D4s | 73 199 263 489 750 813 1438 2706 3010 4769 4883 5178A 7028 8414 8701 8860 9540 10398 10400 10873 11719 12662 12705 14668 14766 14783 15043 15301 15326 16223 16362 |
| D4t | 73 489 750 1438 2706 3010 4769 4883 5178A 7028 8414 8701 8860 9540 10398 10400 10873 11719 12705 14668 14766 14783 15043 15301 15326 15901 16223 16362 |
| D+16189 | 73 263 489 750 1438 2706 4769 4883 5178A 7028 8701 8860 9540 10398 10400 10873 11719 12705 14766 14783 15043 15301 15326 16189 16223 16362 |
| D5 | 73 150 263 489 750 1107 1438 2706 4769 4883 5178A 5301 7028 8701 8860 9540 10397 10398 10400 10873 11719 12705 14766 14783 15043 15301 15326 16189 16223 16362 |
| D5a'b | 73 150 263 489 750 1107 1438 2706 4769 4883 5178A 5301 7028 8701 8860 9180 9540 10397 10398 10400 10873 11719 12705 14766 14783 15043 15301 15326 16189 16223 16362 |
| D5a | 73 150 263 489 750 752 1107 1438 2706 4769 4883 5178A 5301 7028 8701 8860 9180 9540 10397 10398 10400 10873 11719 11944 12026 12705 14766 14783 15043 15301 15326 16189 16223 16362 |
| D5a1 | 68 73 150 263 309d 489 750 752 1107 1438 2706 3496T 4769 4883 5178A 5301 7028 8701 8860 9180 9540 10397 10398 10400 10873 11719 11944 12026 12705 13708 14766 14783 15043 15301 15326 16189 16223 16362 16390 |
| D5a1a | 68 73 150 263 309d 489 750 752 1107 1438 2706 3496T 4769 4883 5178A 5301 6185 7028 8701 8860 9180 9540 10397 10398 10400 10873 11719 11944 12026 12705 13708 14766 14783 15043 15301 15326 16189 16223 16362 16390 |
| D5a1a1 | 68 73 150 263 309d 489 750 752 1107 1438 2706 3496T 4769 4883 5178A 5301 6185 7028 8701 8860 9180 9540 10397 10398 10400 10873 11719 11944 12026 12705 13708 14371 14766 14783 15043 15301 15326 16189 16223 16362 16390 |
| D5a1a2 | 68 73 150 263 309d 489 750 752 1107 1438 2706 3337 3496T 4769 4883 5178A 5301 6185 7028 8701 8860 9180 9540 10397 10398 10400 10873 11719 11944 12026 12705 13708 14766 14783 15043 15301 15326 16189 16223 16362 16390 |
| D5a2 | 73 150 263 489 750 752 1107 2706 4769 4883 5178A 5301 7028 8701 8860 9180 9540 10397 10398 10400 10873 11719 11944 12026 12705 14766 14783 15043 15301 15326 16172 16189 16223 16362 |

| | |
|---|---|
| D5a2a | 73 150 263 489 750 752 1107 2706 4769 4883 5178A 5301 7028 8701 8860 9180 9540 10397 10398 10400 10873 11719 11944 12026 12705 14766 14783 15043 15301 15326 16172 16189 16223 16266 16362 |
| D5a2a+16092 | 73 150 263 489 750 752 1107 2706 4769 4883 5178A 5301 7028 8701 8860 9180 9540 10397 10398 10400 10873 11719 11944 12026 12705 14766 14783 15043 15301 15326 16092 16172 16189 16223 16266 16362 |
| D5a2a1 | 73 150 263 489 750 752 1107 2706 4769 4883 5178A 5301 7028 8701 8860 9180 9540 10397 10398 10400 10873 11719 11944 12026 12705 14766 14783 15043 15301 15326 16092 16164 16172 16189 16223 16266 16362 |
| D5a2a1+@16172 | 73 150 263 489 750 752 1107 2706 4769 4883 5178A 5301 7028 8701 8860 9180 9540 10397 10398 10400 10873 11719 11944 12026 12705 14766 14783 15043 15301 15326 16092 16164 16189 16223 16266 16362 |
| D5a2a1a | 44.1C 73 150 263 489 750 752 1107 1310 2706 4769 4883 5178A 5301 7028 8701 8860 9180 9540 10397 10398 10400 10873 11719 11944 12026 12705 13278 14766 14783 15043 15301 15326 16092 16164 16189 16223 16266 16362 |
| D5a2a1a1 | 44.1C 73 150 263 489 750 752 1107 1310 2706 4769 4883 5178A 5301 7028 8701 8860 9180 9540 10397 10398 10400 10873 11719 11944 12026 12705 13278 14766 14783 15043 15301 15326 16092 16102 16164 16189 16223 16266 16362 |
| D5a2a1a1a | 44.1C 73 150 263 489 750 752 1107 1310 2706 4769 4883 5178A 5301 7028 8701 8860 9180 9540 10397 10398 10400 10589 10873 11719 11944 12026 12705 13278 14766 14783 15043 15301 15326 16092 16102 16164 16189 16223 16266 16362 |
| D5a2a1a2 | 44.1C 73 150 263 489 750 752 1107 1310 2706 4769 4883 5178A 5301 7028 8071 8701 8860 9180 9540 10397 10398 10400 10873 11719 11944 12026 12705 13278 14766 14783 15043 15301 15326 16092 16164 16189 16223 16266 16362 |
| D5a2a1b | 73 150 263 489 750 752 1107 2706 3528 4769 4883 5178A 5301 7028 8701 8860 9180 9540 10397 10398 10400 10873 11719 11944 12026 12705 14766 14783 15043 15301 15326 16092 16164 16172 16189 16223 16266 |
| D5a2a1b1 | 73 150 263 489 750 752 1107 2706 3528 4769 4883 5178A 5301 7028 8701 8860 9180 9540 10397 10398 10400 10873 11719 11944 12026 12705 14766 14783 15043 15301 15326 16092 16164 16167 16172 16189 16223 16266 16362 |
| D5a2a2 | 73 150 263 489 750 752 1107 2706 4769 4883 5178A 5301 7028 8479 8701 8860 9180 9540 10397 10398 10400 10873 11719 11944 12026 12705 14766 14783 15043 15301 15326 16092 16172 16189 16223 16266 16362 |
| D5a2b | 73 150 263 489 750 752 1107 2706 4769 4883 5178A 5301 7028 8701 8860 9180 9540 10397 10398 10400 10873 11719 11944 12026 12705 13356 14766 14783 15043 15301 15326 16172 16189 16223 16362 |
| D5a3 | 73 150 263 489 750 752 1107 1438 2706 4769 4883 5178A 5301 7028 8701 8860 9180 9540 10397 10398 10400 10873 11719 11944 12026 12705 14766 14783 15043 15301 15326 16189 16223 16360 16362 |
| D5a3a | 73 150 263 489 750 752 1107 1438 2706 4769 4883 5178A 5301 7028 8701 8860 9180 9540 10397 10398 10400 10873 11719 11944 12026 12705 13759 14766 14783 15043 15301 15326 16189 16223 16360 16362 |

| | |
|---|---|
| D5a3a1 | 73 150 263 489 750 752 1107 1438 2706 3702 4769 4883 5178A 5301 7028 8701 8860 9180 9540 10397 10398 10400 10873 11719 11944 12026 12705 13759 14766 14783 15043 15301 15326 16189 16223 16360 16362 |
| D5a3a1a | 73 150 263 489 750 752 1107 1438 2706 3702 4769 4883 5178A 5301 7028 8701 8838 8860 9180 9540 10397 10398 10400 10873 11719 11944 12026 12705 13759 14766 14783 15043 15301 15326 16126 16136 16189 16223 16360 16362 |
| D5b | 73 150 263 456 489 681 750 1048 1107 1438 2706 4769 4883 5153 5178A 5301 7028 8701 8860 9180 9540 10397 10398 10400 10873 11719 12705 14766 14783 15043 15301 15326 15724 16189 16223 16362 |
| D5b1 | 73 150 263 456 489 681 750 1048 1107 1438 2706 4769 4883 5153 5178A 5301 6253 7028 8701 8860 9180 9540 10397 10398 10400 10873 11719 12705 14766 14783 15043 15301 15326 15724 16189 16223 16362 |
| D5b1a | 73 150 263 456 489 681 750 1048 1107 1438 2706 4769 4883 5153 5178A 5301 6253 7028 8701 8860 9180 9540 10397 10398 10400 10873 11719 12705 13437 14766 14783 15043 15301 15326 15724 16189 16223 16362 |
| D5b1a1 | 73 150 263 456 489 681 750 1048 1107 1438 2706 4769 4883 5153 5178A 5301 6253 7028 8701 8860 9180 9540 10397 10398 10400 10873 11719 12705 13437 14766 14783 15043 15301 15326 15724 16167 16189 16223 16362 |
| D5b1a2 | 73 150 263 456 489 681 750 1048 1107 1438 2706 4769 4883 5153 5178A 5301 6253 7028 8701 8860 9180 9540 10397 10398 10400 10873 11719 12705 13437 14766 14783 15043 15301 15326 15724 16172 16189 16223 16355 16362 |
| D5b1b | 73 150 263 456 489 681 750 1048 1107 1438 2706 3759 4769 4883 5153 5178A 5301 6253 7028 8701 8860 9180 9540 10397 10398 10400 10873 11719 12705 14766 14783 15043 15301 15326 15724 16189 16223 16362 |
| D5b1b1 | 73 150 263 456 489 681 750 1048 1107 1438 2416 2706 3759 4769 4883 5153 5178A 5301 6253 7028 7220 8701 8860 9180 9540 10397 10398 10400 10873 11719 12705 14766 14783 15043 15301 15326 15724 16189 16216 16223 16362 |
| D5b1b2 | 73 150 263 456 489 681 750 1048 1107 1438 2706 3759 4769 4883 5147 5153 5178A 5301 6253 7028 7403 8701 8860 9180 9540 10397 10398 10400 10873 11719 12705 14766 14783 15043 15301 15326 15724 16189 16223 16357 16362 |
| D5b1c | 73 150 263 456 489 681 750 1048 1107 1438 2706 4769 4883 5153 5178A 5301 6253 7028 8701 8860 9180 9540 10397 10398 10400 10873 11719 12666C 12705 14766 14783 15043 15301 15326 15724 16189 16223 16362 |
| D5b1c1 | 73 150 152 185 263 456 489 681 750 1048 1107 1438 2706 4769 4883 5153 5178A 5301 5899.1C 6253 7028 8701 8860 9180 9540 10397 10398 10400 10873 11719 12666C 12705 14766 14783 15043 15301 15326 15470 15724 16148 16189 16223 16362 |
| D5b1c1a | 73 150 152 185 263 456 489 681 750 1048 1107 1438 2706 4203 4769 4883 5153 5178A 5301 5899.1C 6253 7028 8701 8860 9180 9540 10397 10398 10400 10873 11719 12666C 12705 14766 14783 15043 15301 15326 15470 16148 16189 16223 16362 |

| | |
|---|---|
| D5b1d | 73 146 150 263 456 489 681 750 1048 1107 1438 2706 4048 4769 4883 5153 5178A 5301 6253 7028 8701 8860 9180 9540 10397 10398 10400 10810 10873 11719 12705 14766 14783 15043 15301 15326 15724 16189 16223 16362 |
| D5b2 | 73 150 263 281 456 489 681 750 983 1048 1107 1438 2706 4769 4883 5153 5178A 5301 5539 6146 7028 8701 8860 9180 9377 9540 10397 10398 10400 10873 11719 12705 13614 13617 14766 14783 15043 15301 15326 15724 15769C 16176 16189 16223 16292G 16300 16362 |
| D5b3 | 73 150 263 456 489 681 750 1048 1107 1438 2706 4092 4769 4883 5153 5178A 5301 7028 8701 8860 9180 9540 10397 10398 10400 10873 11719 12705 14766 14783 15043 15301 15326 15724 16189 16223 16362 |
| D5b3a | 73 150 263 456 489 681 750 1048 1107 1438 2706 4092 4769 4883 5153 5178A 5301 7028 7241 8701 8860 9180 9540 10397 10398 10400 10652 10873 11719 12705 14766 14783 15043 15301 15326 15724 16189 16223 16362 |
| D5b3a1 | 73 150 263 456 489 681 750 1048 1107 1438 2706 4092 4769 4883 5153 5178A 5301 7028 7241 8701 8860 9180 9540 10397 10398 10400 10652 10873 11719 12705 14766 14783 15043 15301 15326 15724 16069 16189 16223 16362 |
| D5b4 | 73 150 456 489 681 750 1048 1107 1438 2706 4048 4769 4883 5153 5178A 5301 7028 8860 9180 9540 9667 9992 10397 10398 10400 10873 11176 11719 12705 13954 14766 14783 15043 15301 15326 15724 16189 16223 16362 |
| D5c | 73 150 151 152 263 489 750 1107 1438 2706 4200T 4216 4769 4883 5178A 5301 7028 8701 8860 9540 10397 10398 10400 10873 11719 12705 14766 14783 14927 15043 15301 15326 15622 16189 16190 16223 16362 |
| D5c1 | 73 146 150 151 152 263 489 750 1107 1438 2706 3546 4200T 4216 4769 4883 5178A 5301 7028 8701 8860 9540 10397 10398 10400 10873 11719 12705 13105 14766 14783 14927 15043 15301 15326 15622 16189 16190 16223 16362 16390 |
| D5c1a | 73 146 150 151 152 182 217 263 489 750 1107 1438 2706 3546 4200T 4216 4769 4883 5178A 5301 7028 8701 8860 9540 10397 10398 10400 10873 11719 12705 13105 14766 14783 14927 15043 15301 15326 15622 16189 16190 16362 16390 |
| D5c+16311 | 73 150 151 152 263 489 750 1107 1438 2706 4200T 4216 4769 4883 5178A 5301 7028 8701 8860 9540 10397 10398 10400 10873 11719 12705 14766 14783 14927 15043 15301 15326 15622 16189 16190 16223 16311 16362 |
| D5c2 | 73 150 151 152 263 489 750 1107 1438 2706 4200T 4216 4769 4883 5178A 5301 7028 7129 7669 8580 8701 8860 9540 10397 10398 10400 10873 11719 12705 12810 13984 14766 14783 14927 15043 15301 15326 15622 15737 16189 16190 16223 16311 16316 16362 |
| D6 | 73 263 489 709 750 1438 1719 2706 3714 4769 4883 5178A 7028 8701 8860 9540 10398 10400 10873 11719 12654 12705 14766 14783 15043 15301 15326 16189 16223 16311 16362 |
| D6a | 73 263 489 709 750 1438 1719 2706 3714 4769 4883 5178A 7028 8701 8860 9540 10398 10400 10873 11719 12654 12705 14766 14783 15043 15301 15326 16189 16223 16274 16311 16362 |

| | |
|---|---|
| D6a1 | 73 263 489 709 750 1438 1719 2706 3714 4769 4883 5178A 6701 7028 7424 7879 8701 8860 9540 10398 10400 10873 11719 12654 12705 13194 14766 14783 15043 15301 15326 16189 16223 16274 16362 |
| D6a1a | 73 263 489 709 750 1438 1719 2706 3714 4769 4883 5178A 6701 7028 7424 7879 8020 8701 8860 9540 10398 10400 10873 11719 12654 12705 13194 14766 14783 15043 15301 15326 16189 16223 16274 16362 |
| D6a2 | 73 263 489 709 750 1438 1719 2706 3714 4679 4769 4883 4937 5178A 7028 8701 8860 9540 10398 10400 10873 11719 12654 12705 14530 14766 14783 15043 15301 15326 16129 16223 16274 16311 16317 16362 |
| D6c | 73 152 263 489 709 750 1438 1719 2706 3714 4769 4883 5178A 5441T 7028 8389 8521 8701 8860 9540 10398 10400 10873 11719 12654 12705 14766 14783 15043 15301 15326 16189 16223 16311 16362 |
| D6c1 | 73 152 263 489 709 750 1438 1719 2706 3714 4769 4883 5178A 5441T 7028 8389 8521 8701 8860 9540 10398 10400 10873 11719 12654 12705 14371 14766 14783 15043 15301 15326 16189 16223 16311 16362 |
| D6c1a | 73 152 263 489 709 750 1438 1719 2706 3714 4769 4883 5178A 5441T 7028 8389 8521 8701 8860 9540 10007 10398 10400 10873 11719 12654 12705 14371 14766 14783 15043 15301 15326 15662 16189 16223 16311 16362 |
| N | 73 263 750 1438 2706 4769 7028 8860 11719 12705 14766 15326 16223 |
| N1'5 | 73 263 750 1438 1719 2706 4769 7028 8860 11719 12705 14766 15326 16223 |
| N1 | 73 263 750 1438 1719 2706 4769 7028 8860 10238 11719 12501 12705 14766 15326 16223 |
| N1a | 73 204 263 750 1438 1719 2706 4769 7028 8860 10238 11719 12501 12705 13780 14766 15326 16223 |
| N1a1'2 | 73 199 204 263 750 1438 1719 2706 4769 7028 8860 10238 11719 12501 12705 13780 14766 15326 16223 |
| N1a1 | 73 199 204 263 573.XC 750 1438 1719 2706 4769 7028 8860 10238 10398 11719 12501 12705 13780 14766 15043 15326 16223 |
| N1a1a | 73 199 204 263 573.XC 669 750 1438 1719 2702 2706 4769 5315 7028 8860 8901 10238 10398 11719 12501 12705 13780 14766 15043 15326 16147G 16172 16223 16248 16355 |
| N1a1a+152 | 73 152 199 204 263 573.XC 669 750 1438 1719 2702 2706 4769 5315 7028 8860 8901 10238 10398 11719 12501 12705 13780 14766 15043 15326 16147G 16172 16223 16248 16355 |
| N1a1a1 | 73 152 199 204 263 573.XC 669 750 1438 1719 2702 2706 3336 4769 5315 7028 8860 8901 10238 10398 11719 12501 12705 13780 14766 15043 15326 16147A 16172 16223 16248 16355 |
| N1a1a1a | 73 152 199 204 263 573.XC 669 750 1438 1719 2702 2706 3336 4769 5315 7028 8860 8901 10238 10398 11719 12501 12705 13780 14766 15043 15326 16147A 16172 16223 16248 16320 16355 |
| N1a1a1a1 | 73 152 199 204 263 573.XC 669 750 1438 1719 2706 3336 4769 5315 7028 8164 8860 8901 9300 10238 10398 11719 12501 12705 13780 14766 15043 15326 16147A 16172 16223 16248 16320 16355 |
| N1a1a1a1a | 73 152 199 204 263 573.XC 669 750 1438 1719 2706 3336 4769 5315 6641 7028 8164 8860 8901 9300 10238 10398 11719 12501 12705 13780 14766 15043 15326 16147A 16172 16189 16223 16248 16320 16355 |

| | |
|---|---|
| N1a1a1a2 | 73 152 199 204 207 263 573.XC 669 750 1438 1719 2702 2706 3336 4769 5315 7028 8485 8860 8901 10238 10398 11719 12501 12705 13780 14766 15043 15299 15326 16086 16147A 16223 16248 16320 16355 |
| N1a1a1a3 | 73 152 199 204 263 573.XC 669 750 1438 1719 2702 2706 3336 4769 5315 7028 8860 8901 10238 10398 11719 12501 12705 13780 14766 15043 15326 16147A 16154 16172 16223 16248 16320 16355 |
| N1a1a1b | 73 199 204 263 573.XC 669 750 1438 1711 1719 2702 2706 3336 4769 5315 7028 7750 8227 8573 8860 8901 10143 10238 10398 10454 11719 11884 12358 12501 12705 13780 14766 15043 15326 16093 16147A 16172 16223 16248 16355 |
| N1a1a2 | 73 152 199 204 263 573.XC 669 750 1438 1719 2702 2706 4721 4769 5315 7028 8860 8901 10238 10398 11719 12501 12705 13780 14766 15043 15326 16147G 16172 16223 16248 16355 |
| N1a1a3 | 73 199 204 263 573.XC 669 750 1438 1719 2702 2706 2758 4769 5315 7028 8860 8901 10238 10398 11719 12501 12705 13780 14766 15043 15326 16147G 16172 16223 16248 16355 |
| N1a1b | 73 199 204 250 263 573.XC 750 1438 1719 2706 4529T 4769 7028 8251 8860 10238 10398 11719 12501 12705 13780 14766 15043 15326 15924 16223 16391 |
| N1a1b1 | 73 143 199 204 250 263 573.XC 710 750 1438 1719 2706 4529T 4769 7028 8251 8860 10238 10398 10790 11719 12501 12705 13780 14766 15043 15326 15924 16223 16309 16311 16391 |
| I | 73 199 204 250 263 573.XC 750 1438 1719 2706 4529T 4769 7028 8251 8860 10034 10238 10398 11719 12501 12705 13780 14766 15043 15326 15924 16129 16223 16391 |
| I1 | 73 199 204 250 263 455.1T 573.XC 750 1438 1719 2706 4529T 4769 6734 7028 8251 8860 9966 10034 10238 10398 11719 12501 12705 13780 14766 15043 15326 15924 16129 16223 16311 16391 |
| I1a | 73 199 204 250 263 455.1T 573.XC 750 1438 1719 2706 3447 4529T 4769 6734 7028 8251 8616T 8860 9966 10034 10238 10398 11719 12501 12705 13780 14766 15043 15326 15924 16129 16172 16223 16311 16391 |
| I1a1 | 73 199 203 204 250 263 455.1T 573.XC 750 1438 1719 2706 3447 3990 4529T 4769 6734 7028 8251 8616T 8860 9947 10034 10238 10398 10915 11719 12501 12705 13780 14766 15043 15326 15924 16129 16172 16223 16311 16391 |
| I1a1a | 73 199 203 204 250 263 455.1T 573.XC 750 1438 1719 2706 3447 3990 4529T 4769 6734 7028 8251 8616T 8860 9053 9947 10034 10238 10398 10915 11719 12501 12705 13780 14766 15043 15326 15924 16129 16172 16223 16311 16391 |
| I1a1a1 | 73 199 203 204 250 263 455.1T 573.XC 750 1438 1719 2706 3447 3990 4529T 4769 6734 7028 8251 8616T 8860 9053 9947 10034 10238 10398 10915 11719 12501 12705 13780 14766 15043 15326 15547 15924 16129 16172 16223 16311 16391 |
| I1a1a2 | 73 199 203 204 250 263 455.1T 573.XC 750 1342 1438 1719 2706 3447 3990 4529T 4769 6734 7028 8251 8616T 8860 9053 9947 10034 10238 10398 10915 11719 12501 12705 13780 14766 15043 15326 15924 16129 16172 16223 16311 16391 |

| | |
|---|---|
| I1a1a3 | 73 199 203 204 250 263 455.1T 573.XC 750 1438 1719 2706 3447 3990 4529T 4769 6734 7028 8251 8616T 8860 9053 9947 10034 10238 10398 10915 11719 12501 12705 13780 14766 15043 15326 15924 16129 16172 16223 16311 16319 16391 |
| I1a1a3a | 73 199 203 204 250 263 455.1T 573.XC 750 1438 1719 2706 3447 3990 4454 4529T 4769 6734 7028 8251 8616T 8860 9053 9947 10034 10238 10398 10915 11719 12501 12705 13780 14766 15043 15326 15924 16129 16172 16223 16311 16319 16391 |
| I1a1b | 73 199 203 204 250 263 455.1T 573.XC 750 1438 1719 2706 3447 3990 4529T 4769 6734 7028 8251 8616T 8860 9947 10034 10238 10398 10915 11719 12501 12705 13780 14182 14766 15043 15326 15924 16129 16172 16223 16311 16391 |
| I1a1c | 73 199 203 204 250 263 455.1T 573.XC 750 1438 1719 2706 3447 3990 4529T 4769 6620 6734 7028 8251 8616T 8860 9947 10034 10238 10398 10915 11719 12501 12705 13780 14766 15043 15326 15924 16129 16172 16223 16311 16391 |
| I1a1d | 73 199 203 204 250 263 455.1T 573.XC 750 1438 1719 1836 2706 3447 3990 4023 4529T 4769 6734 7028 8251 8616T 8860 9947 10034 10238 10398 10915 11719 12501 12705 13488 13780 14766 15043 15326 15924 16129 16172 16189 16223 16311 16391 |
| I1a1e | 73 199 203 204 250 263 455.1T 573.XC 750 1438 1719 2706 3447 3990 4529T 4769 6734 7028 8251 8616T 8860 9947 10034 10238 10398 10915 11719 12501 12705 13404 13780 14766 15043 15326 15924 16129 16172 16223 16311 16391 |
| I1b | 73 199 204 250 263 455.1T 573.XC 750 1438 1719 2706 4529T 4769 6227 6734 7028 8251 8860 9966 10034 10238 10398 11719 12501 12705 13780 14766 15043 15326 15924 16129 16223 16311 16391 |
| I1c | 73 199 204 250 263 455.1T 573.XC 750 1438 1719 2706 4529T 4769 6734 7028 8251 8573 8860 9966 10034 10238 10398 11719 12501 12705 13780 14766 15043 15326 15924 16129 16223 16264 16311 16319 16362 16391 |
| I1c1 | 73 199 204 250 263 455.1T 573.XC 750 1438 1719 2706 4529T 4769 6734 7028 8251 8573 8860 9386 9966 10034 10238 10398 11719 12501 12705 13780 14766 15043 15326 15924 16129 16223 16264 16270 16311 16319 16362 16391 |
| I1c1a | 73 199 204 250 263 455.1T 573.XC 750 1438 1719 2706 4529T 4769 6267 6359 6734 7028 8251 8573 8860 9386 9966 10034 10238 10398 11719 12501 12705 13780 14766 15043 15326 15924 16129 16223 16264 16270 16311 16319 16362 16391 |
| I1d | 73 199 204 250 263 455.1T 573.XC 750 1438 1719 2706 4529T 4769 6734 7028 8161 8251 8860 9966 10034 10238 10398 11719 12501 12705 13614 13780 14766 15043 15326 15924 16129 16223 16311 |
| I1e | 73 199 204 250 263 455.1T 573.XC 750 1438 1719 2706 4529T 4769 6524 6734 6755 7028 7796 8251 8860 9966 10034 10238 10398 11719 12501 12705 13780 14766 15043 15326 15924 16129 16223 16311 16391 |
| I1f | 73 199 204 250 263 455.1T 573.XC 750 1438 1719 2706 4529T 4769 6734 7028 8251 8860 9966 10034 10238 10398 11719 12501 12705 13780 14766 14971 15043 15326 15924 16129 16171 16223 16293C 16311 16391 |

| | |
|---|---|
| I2'3 | 73 152 199 204 207 250 263 573.XC 750 1438 1719 2706 4529T 4769 7028 8251 8860 10034 10238 10398 11719 12501 12705 13780 14766 15043 15326 15924 16129 16223 16391 |
| I2 | 73 152 199 204 207 250 263 573.XC 750 1438 1719 2706 4529T 4769 7028 8251 8860 10034 10238 10398 11719 12501 12705 13780 14766 15043 15326 15758 15924 16129 16223 16391 |
| I2a | 73 152 199 204 207 250 263 573.XC 750 1438 1719 2706 4529T 4769 7028 8251 8860 10034 10238 10398 11065 11719 12501 12705 13780 14766 15043 15326 15758 15924 16129 16145 16223 16391 |
| I2a1 | 73 152 199 204 207 250 263 533 573.XC 750 1438 1719 2706 3398 4529T 4769 7028 8251 8860 10034 10238 10398 11065 11719 12501 12705 13780 14766 15043 15326 15758 15924 16129 16145 16223 16391 |
| I2a1a | 73 152 199 204 207 250 263 533 573.XC 750 1438 1719 2706 3398 4529T 4769 5922 7028 8251 8860 10034 10238 10398 11065 11719 12501 12705 13780 14766 15043 15326 15758 15924 16129 16145 16223 16391 |
| I2a2 | 73 152 199 204 207 250 263 573.XC 750 1438 1719 2706 4529T 4769 7028 8251 8860 9266 10034 10238 10398 11065 11719 12501 12705 13780 14766 15043 15326 15758 15924 16129 16145 16223 16391 |
| I2a3 | 73 152 199 204 207 250 263 573.XC 750 1438 1719 2706 2835A 4529T 4769 7028 8251 8860 10034 10238 10398 11065 11719 12501 12705 13780 14766 15043 15326 15758 15924 16074 16129 16145 16223 16391 |
| I2b | 73 152 199 204 207 250 263 573.XC 750 1438 1719 2706 4529T 4769 6515 7028 8251 8281-8289d 8860 10034 10238 10398 11719 12501 12705 13780 14766 15043 15326 15758 15924 16129 16166C 16223 16391 |
| I2c | 73 152 199 204 207 250 263 460 573.XC 750 1438 1719 2706 4529T 4769 7028 8251 8860 9438 10034 10238 10398 11719 12501 12705 13780 14766 15043 15326 15758 15924 16129 16223 16391 |
| I2d | 73 152 199 204 207 250 263 573.XC 750 1438 1719 2706 4529T 4769 6480 7028 8251 8860 10034 10238 10398 11719 12501 12705 13780 14766 15043 15326 15758 15924 16129 16223 16391 |
| I2e | 73 152 199 204 207 250 263 573.XC 750 1438 1719 2706 3591 4529T 4769 7028 8251 8860 10034 10238 10398 11719 12501 12705 13780 14766 15043 15326 15758 15924 16129 16223 16391 |
| I2f | 73 152 199 204 207 250 263 573.XC 750 1438 1719 2706 4418 4529T 4769 7028 8251 8860 10034 10238 10398 11719 12501 12705 13780 14766 15043 15326 15758 15924 16129 16223 16391 |
| I3 | 73 152 199 204 207 239 250 263 573.XC 750 1438 1719 2706 4529T 4769 7028 8251 8860 10034 10238 10398 11719 12501 12705 13780 14766 15043 15326 15924 16129 16223 16391 |
| I3a | 73 152 199 204 207 239 250 263 573.XC 750 1438 1719 2706 4529T 4769 7028 8251 8860 10034 10238 10398 11719 12501 12705 13780 14766 15043 15326 15924 16086 16129 16223 16391 |
| I3a1 | 73 152 199 204 207 239 250 263 573.XC 750 1438 1719 2706 2849 4529T 4769 7028 8251 8860 10034 10238 10398 11719 12501 12705 13780 14766 15043 15326 15924 16086 16129 16223 16391 |
| I3b | 73 152 199 204 207 239 250 263 573.XC 750 1438 1719 2706 4529T 4769 7028 8251 8860 10034 10238 10398 11719 12501 12705 13780 14766 15043 15326 15924 16129 16223 16391 16491 16494 |

| | |
|---|---|
| I3c | 73 152 199 204 207 239 250 263 573.XC 750 1438 1719 2628 2706 4529T 4769 7028 8251 8860 10034 10238 10398 11719 12501 12705 13780 14766 15043 15326 15924 16129 16223 16391 |
| I3d | 73 152 199 204 207 239 250 263 573.XC 750 1438 1719 2706 4529T 4769 7028 8251 8260 8860 10034 10238 10398 11719 12501 12705 13780 14766 15043 15326 15924 16129 16223 16391 |
| I3d1 | 73 152 183 199 204 207 239 250 263 382 573.XC 750 1438 1719 1900 2706 4529T 4769 7028 8251 8260 8860 10034 10238 10398 11719 12501 12705 13780 14299 14766 15043 15326 15924 16129 16223 16391 |
| I4 | 73 199 204 250 263 573.XC 750 1438 1719 2706 4529T 4769 7028 8251 8519 8860 10034 10238 10398 11719 12501 12705 13780 14766 15043 15326 15924 16129 16223 16391 |
| I4a | 73 199 204 250 263 573.XC 750 1438 1719 2706 4529T 4769 7028 8251 8519 8860 10034 10238 10398 10819 11719 12501 12705 13780 14766 15043 15326 15924 16129 16223 16391 |
| I4a1 | 73 199 204 250 263 573.XC 750 1438 1719 2706 4529T 4769 7028 8251 8519 8860 10034 10238 10398 10819 11719 12501 12705 13780 14766 15043 15326 15924 16129 16223 16304 16391 |
| I4a2 | 73 199 204 250 263 573.XC 750 1438 1719 2706 4529T 4769 7028 8251 8519 8860 9851 10034 10238 10398 10819 11719 12501 12705 13780 14766 15043 15326 15924 16129 16223 16391 |
| I4b | 73 199 204 250 263 573.XC 750 1438 1719 2308 2706 4113 4529T 4769 7028 8251 8519 8860 10034 10238 10398 11719 12501 12705 13662 13780 14766 15043 15326 15924 16129 16223 16391 |
| I5 | 73 199 204 250 263 573.XC 750 1438 1719 2706 4529T 4769 7028 8251 8860 10034 10238 10398 11719 12501 12705 13780 14233 14766 15043 15326 15924 16129 16223 16391 |
| I5a | 73 199 204 250 263 573.XC 750 1438 1719 2706 4529T 4769 5074 7028 8251 8860 10034 10238 10398 11719 12501 12705 13780 14233 14766 15043 15326 15924 16129 16148 16223 16391 |
| I5a1 | 73 199 204 250 263 573.XC 750 1438 1719 2706 4529T 4769 5074 7028 8251 8281-8289d 8860 10034 10238 10398 11719 12501 12705 12961 13780 14233 14766 15043 15326 15924 16129 16148 16223 16391 |
| I5a1a | 73 199 204 250 263 573.XC 750 1438 1719 2706 4529T 4769 5074 7028 8251 8281-8289d 10034 10238 10398 11719 12501 12705 12961 13780 14233 14766 15043 15326 15924 16129 16148 16223 16391 |
| I5a1b | 73 199 250 263 573.XC 750 1438 1719 2706 4529T 4769 5074 7028 8251 8281-8289d 8860 10034 10238 10398 11719 12501 12705 12961 13602 13780 14233 14766 15043 15326 15924 15968 16129 16148 16223 16391 |
| I5a1c | 73 199 204 250 263 573.XC 750 1438 1719 2706 4529T 4769 5074 6182 7028 8251 8281-8289d 8860 10034 10238 10398 11719 12501 12705 12961 13780 14233 14766 15043 15326 15924 16129 16148 16223 16391 |
| I5a2 | 73 199 204 250 263 573.XC 750 1438 1719 2706 3615 4529T 4769 5074 7028 8251 8742 8860 10034 10238 10398 11719 12501 12705 13780 14233 14766 15043 15326 15924 16129 16148 16223 16391 |
| I5a2+16086 | 73 199 204 250 263 573.XC 750 1438 1719 2706 3615 4529T 4769 5074 7028 8251 8742 8860 10034 10238 10398 11719 12501 12705 13780 14233 14766 15043 15326 15924 16086 16129 16148 16223 16391 |

| | |
|---|---|
| I5a2a | 73 199 204 250 263 573.XC 750 1438 1719 2706 3615 3705 4529T 4769 5074 5096 5773 7028 8251 8562 8742 8860 10034 10238 10398 11719 12501 12705 13780 14233 14766 15043 15326 15924 15941 16086 16129 16148 16223 16391 |
| I5a3 | 73 150 199 204 250 263 573.XC 750 1438 1719 2706 4529T 4769 5074 5231 6278 7028 8251 8860 9190 10034 10238 10398 11719 12073 12501 12705 13780 14233 14766 15043 15052 15326 15924 16129 16148 16223 16301 16391 |
| I5a4 | 73 199 204 250 263 573.XC 574C 750 1438 1719 2706 4529T 4769 5074 7028 8251 8860 10034 10238 10398 11719 12408A 12501 12705 13780 14233 14766 15043 15244 15326 15924 16129 16148 16192 16223 16294 16391 |
| I5b | 73 199 204 250 263 573.XC 709 750 1438 1719 2706 4529T 4769 6506 7028 8251 8860 10034 10238 10398 11719 12501 12705 13780 14233 14766 15043 15326 15924 16129 16223 16391 |
| I5b1 | 73 204 250 263 459d 573.XC 709 750 1438 1719 2706 4529T 4769 6506 7028 8251 8860 10034 10238 10398 11719 12501 12705 13780 14233 14766 15043 15326 15924 16017 16129 16223 16391 |
| I5c | 73 199 204 250 263 573.XC 750 1438 1719 2706 4529T 4769 7028 8251 8860 10034 10238 10398 11719 12501 12705 13780 14233 14766 15043 15326 15924 16129 16169 16223 16391 |
| I5c1 | 73 199 204 250 263 573.XC 750 1719 2706 4529T 4769 7028 8251 8860 10034 10238 10398 11719 12501 12705 13780 14233 14766 15043 15326 15924 16075 16129 16169 16223 16260 16391 |
| I6 | 73 199 204 250 263 573.XC 750 1438 1719 2706 3645 4529T 4769 7028 8251 8860 10034 10238 10398 11719 12501 12705 13780 14766 15043 15326 15924 16129 16223 16391 |
| I6a | 73 199 (203) 204 250 263 573.XC 750 1438 1719 2706 3645 3915 4529T 4769 6116 7028 7804 8251 8860 10034 10238 10398 11719 12501 12705 13780 14766 15043 15287 15326 15924 16129 16223 (16293C) 16391 |
| I6b | 73 199 204 250 263 573.XC 750 1438 1719 2706 3645 4529T 4769 7028 8251 8860 10034 10238 10398 10955 11719 12501 12705 13780 14766 15043 15326 15924 16129 16185 16223 16391 |
| I7 | 73 199 204 250 263 573.XC 750 1438 1719 2706 3534 4529T 4769 4829 7028 8251 8860 10034 10238 10398 11719 12501 12705 13780 14766 15043 15326 15924 16129 16223 16324 16391 |
| N1a2 | 73 199 204 263 750 953 1438 1719 2706 3783A 4769 6713 7028 7830 8860 9947 10238 11719 12501 12705 13437 13780 14766 15326 16223 16274 16301 16356 |
| N1a3 | 73 189 195 204 207 210 263 750 1438 1719 2706 4769 7028 8222 8860 10238 11025 11437 11719 11914 12501 12705 13637 13780 14766 15326 16223 16265 |
| N1a3a | 73 189 195 204 207 210 263 750 1438 1719 2706 4769 7028 8222 8860 10238 11025 11437 11719 11914 12501 12705 13637 13780 14766 15326 16201 16223 16265 |
| N1a3a1 | 73 189 195 204 207 263 750 1438 1719 2706 4769 7028 8222 8860 10238 11025 11437 11719 11914 12501 12705 13637 13780 14766 15326 16201 16223 16259 16265 16270G 16319 |

| | |
|---|---|
| N1a3a1a | 73 152 189 195 204 207 263 750 1438 1719 2706 3672T 4769 5460 7028 7861 8222 8860 10238 11025 11437 11719 11914 12501 12705 13637 13780 14766 15326 16201 16223 16259 16265 16270G 16319 |
| N1a3a2 | 73 189 195 204 207 210 263 750 1438 1719 2706 4769 5981 7028 8222 8860 10238 11025 11437 11719 11914 12501 12705 13637 13780 14766 15326 16111 16201 16223 16265 |
| N1a3a3 | 73 152 189 195 207 210 263 750 1438 1719 2706 4769 7028 8222 8308 8860 8943 10238 11025 11437 11719 11914 12501 12612 12705 13637 13780 14560 14766 15326 16086 16201 16223 16265 |
| N1b | 73 152 263 750 1438 1598 1719 2639 2706 4769 5471 7028 8251 8836 8860 10238 11719 12501 12705 14766 15326 16176G 16223 16390 |
| N1b1 | 73 152 263 750 1438 1598 1703 1719 2639 2706 3921A 4769 4960 5471 7028 8251 8472 8836 8860 10238 11719 12501 12705 12822 14766 15326 16145 16176G 16223 16390 |
| N1b1a | 73 152 263 750 1438 1598 1703 1719 2639 2706 3921A 4769 4960 5471 7028 8251 8472 8836 8860 9335 10238 11362 11719 12501 12705 12822 14766 15326 16145 16176G 16223 16390 |
| N1b1a1 | 73 152 263 455.1T 750 1438 1598 1719 2639 2706 3921A 4769 4960 5471 7028 8084 8251 8472 8836 8860 9335 10238 11362 11719 12501 12705 12822 14766 15326 16145 16176G 16223 16390 |
| N1b1a2 | 73 152 263 750 1438 1598 1703 1719 2639 2706 3921A 4769 4904 4960 5471 7028 8251 8472 8836 8860 9335 10238 11362 11719 12501 12705 12822 14766 15326 16145 16176G 16223 16390 |
| N1b1a2a | 73 152 263 750 1438 1598 1703 1719 2639 2706 3921A 4769 4904 4960 5471 7028 8251 8264 8472 8836 8860 9335 10238 11362 11719 12501 12705 12822 14766 15326 16145 16176G 16223 16390 |
| N1b1a2b | 73 152 263 750 1438 1598 1703 1719 2639 2706 3221 3921A 4769 4820 4904 4960 5291 5471 7028 8251 8309 8472 8836 8860 9335 10238 11362 11719 12501 12705 12822 14766 15326 16145 16176G 16223 16390 |
| N1b1a3 | 73 152 263 750 1438 1598 1703 1719 2639 2706 3921A 4769 4960 5471 7028 8251 8472 8836 8860 9335 9957 10238 11362 11719 12501 12705 12822 14766 15326 16145 16176G 16223 16390 |
| N1b1a+16129 | 73 152 263 750 1438 1598 1703 1719 2639 2706 3921A 4769 4960 5471 7028 8251 8472 8836 8860 9335 10238 11362 11719 12501 12705 12822 14766 15326 16129 16145 16176G 16223 16390 |
| N1b1a4 | 73 152 185 188 263 750 1438 1598 1703 1719 2639 2706 3921A 4769 4960 5471 7028 8251 8472 8763 8836 8860 9335 10238 11362 11719 12501 12705 12822 14766 15326 16129 16145 16176G 16223 16390 |
| N1b1a4a | 73 152 185 188 263 750 1438 1598 1703 1719 2639 2706 3921A 4769 4960 5471 7028 8251 8261 8410 8472 8763 8836 8860 9335 10238 11362 11719 12501 12705 12822 14766 15326 16129 16145 16176G 16223 16291 16390 |
| N1b1a5 | 73 152 263 750 1438 1598 1703 1719 2639 2706 3921A 4769 4960 4967 5471 7028 8251 8472 8836 8860 9335 10238 11362 11719 12501 12705 12822 14766 15326 16145 16176G 16223 16390 |
| N1b1a6 | 73 152 263 750 1438 1598 1703 1719 2639 2706 3921A 4769 4960 5471 7010 7028 8251 8469 8472 8836 8860 9335 10238 11362 11719 12501 12705 12822 14766 15326 16145 16176G 16223 16390 |

| | |
|---|---|
| N1b1a+195 | 73 152 195 263 750 1438 1598 1703 1719 2639 2706 3921A 4769 4960 5471 7028 8251 8472 8836 8860 9335 10238 11362 11719 12501 12705 12822 14766 15326 16145 16176G 16223 16390 |
| N1b1a7 | 73 152 195 263 750 1406 1438 1598 1703 1719 2639 2706 3921A 4769 4960 5471 7028 8251 8472 8836 8860 9335 10238 11362 11719 12501 12705 12822 14766 15326 16145 16176G 16223 16390 |
| N1b1a8 | 73 152 195 263 750 1438 1598 1703 1719 2639 2706 3921A 4769 4960 5471 7028 8251 8472 8836 8860 9335 10238 11362 11719 12501 12705 12822 14766 15326 16126 16145 16176G 16223 16390 |
| N1b1a8a | 73 152 195 263 750 1438 1598 1703 1719 2639 2706 3921A 4769 4960 5471 7028 8251 8472 8836 8860 9335 10238 11362 11719 12297 12501 12705 12822 14766 15326 16126 16145 16176G 16390 |
| N1b1a8b | 73 152 195 263 750 1438 1598 1703 1719 2639 2706 3921A 4769 4960 5471 7028 8251 8472 8836 8860 9335 10238 11362 11719 12501 12705 12822 13419 14766 15326 16126 16145 16176G 16223 16390 |
| N1b1b | 73 152 263 750 1438 1598 1703 1719 2639 2706 3921A 4735A 4769 4917 4960 5471 7028 8251 8472 8836 8860 10238 11719 11928 12092 12501 12705 12822 13129 13710 14766 15326 16145 16176A 16223 16390 |
| N1b1b1 | 73 152 263 750 1438 1598 1703 1719 2639 2706 3921A 4735A 4769 4917 4960 5471 7028 8251 8472 8836 8860 10238 11719 11928 12092 12501 12705 12822 13129 13710 14581 14766 15326 16145 16176A 16223 16390 |
| N1b2 | 73 152 263 750 1438 1598 2639 2706 4769 5471 5507A 7028 8251 8836 8860 10238 11719 12026 12501 12705 14766 15326 15784 16176G 16223 16258C 16390 |
| N5 | 73 263 750 1438 1719 2706 4769 5063 7028 7076 8860 9545 11626 11719 12705 (13434) 14766 15326 16111 16223 16311 |
| N5a | 73 131 199 228 234 263 374 750 1438 1719 2706 4769 5063 6974 7028 7076 8860 9545 11626 11719 12705 (13434) (13830) 14766 15119 15326 16111 16144 16185 16223 16261 16311 16319 |
| N2 | 73 189 263 709 750 1438 2706 4769 5046 7028 8860 11674 11719 12414 12705 14766 15326 16223 |
| N2a | 73 189 199 263 709 739 750 1438 2706 4769 5046 7028 7581 8860 11674 11719 12414 12705 14766 15106 15326 16153 16223 16319 |
| N2a1 | 73 189 199 263 709 739 750 1438 2706 4769 5046 7028 7581 8860 11674 11719 12414 12705 14152 14766 15106 15326 16111 16153 16223 16319 |
| N2a2 | 73 189 199 263 709 739 750 1438 2706 4065 4769 5046 7028 7581 8860 11674 11719 12414 12705 14766 15106 15326 16153 16223 16319 |
| W | 73 189 195 204 207 263 709 750 1243 1438 2706 3505 4769 5046 5460 7028 8251 8860 8994 11674 11719 11947 12414 12705 14766 15326 15884C 16223 16292 |
| W1 | 73 189 195 204 207 263 709 750 1243 1438 2706 3505 4769 5046 5460 7028 7864 8251 8860 8994 11674 11719 11947 12414 12705 14766 15326 15884C 16223 16292 |
| W1a | 73 189 195 204 207 263 709 750 1243 1438 2706 3505 4769 5046 5460 5495 7028 7864 8251 8860 8994 11674 11719 11947 12414 12669 12705 14766 15326 15884C 16223 16292 |

| | |
|---|---|
| W1b | 73 189 195 204 207 227 263 709 750 1243 1438 2706 3505 4769 4928 5046 5460 7028 7864 8251 8860 8994 9612 11674 11719 11947 12414 12705 14766 15326 15884C 16223 16292 |
| W1b1 | 73 189 195 204 207 227 263 709 750 1243 1438 2706 3505 4769 4928 5046 5460 7028 7864 8251 8860 8994 9612 10086 11674 11719 11947 12414 12705 14766 15326 15884C 16223 16292 |
| W1+119 | 73 119 189 195 204 207 263 709 750 1243 1438 2706 3505 4769 5046 5460 7028 7864 8251 8860 8994 11674 11719 11947 12414 12705 14766 15326 15884C 16223 16292 |
| W1c | 73 119 189 195 204 207 263 709 750 1243 1438 2706 3505 4769 5046 5460 7028 7864 8251 8860 8994 11674 11719 11947 12414 12705 14148 14766 15326 15884C 16223 16292 |
| W1c1 | 73 119 189 195 204 207 263 709 750 1243 1438 2706 3505 4769 5046 5460 7028 7864 8251 8860 8994 11204 11674 11719 11947 12414 12648 12705 14148 14766 15326 15884C 16184 16223 16292 |
| W1i | 73 119 189 195 204 207 263 709 750 1243 1438 2706 3505 4769 5046 5460 5580 7028 7864 8251 8860 8994 11674 11719 11947 12414 12705 14766 15326 15884C 16223 16292 |
| W1d | 73 194 195 200 204 207 263 709 750 1243 1438 2706 3505 4769 5046 5460 7028 7864 8251 8383 8860 8994 9278 11674 11719 11947 12414 12705 14766 14981C 15326 15884C 16223 16260 16298 |
| W1e | 73 189 195 204 207 263 709 750 1243 1438 2706 3505 4769 5046 5460 7028 7864 8251 8659 8860 8887 8994 11674 11719 11947 12414 12705 14766 15326 15884C 16223 16292 |
| W1e1 | 73 189 195 204 207 263 709 750 1243 1438 2706 3505 4769 5046 5460 7028 7864 8251 8659 8860 8887 8994 11674 11719 11947 12414 12705 14766 15326 15884C 16223 16292 16295 |
| W1e1a | 73 189 195 204 207 263 709 750 1243 1438 2706 3505 4769 5046 5460 7028 7864 8251 8659 8860 8887 8994 10398 11674 11719 11947 12414 12705 14766 15326 15884C 16223 16292 16295 16324 |
| W1f | 73 189 195 204 207 263 709 750 1243 1438 2706 3505 4769 5046 5460 7028 7864 8251 8860 8994 9950 11674 11719 11947 12414 12705 14766 15326 15884C 16223 16292 |
| W1g | 73 189 195 204 207 263 709 750 1243 1438 2706 3505 4769 5046 5460 7028 7864 8251 8860 8994 11674 11719 11947 12414 12705 14766 15326 15884C 16223 16292 16320 |
| W1h | 73 189 195 204 207 263 709 750 1243 1438 2706 3505 4769 5046 5460 7028 7864 8251 8860 8994 11674 11719 11947 12414 12705 14766 15326 15884C 16145 16223 16292 |
| W1h1 | 73 189 195 204 207 263 513C 709 750 1243 1438 2706 3505 4769 5046 5460 7028 7864 8251 8860 8994 11674 11719 11947 12414 12705 14766 15326 15884C 16145 16223 16292 |
| W+194 | 73 189 194 195 204 207 263 709 750 1243 1438 2706 3505 4769 5046 5460 7028 8251 8860 8994 11674 11719 11947 12414 12705 14766 15326 15884C 16223 16292 |
| W3 | 73 189 194 195 204 207 263 709 750 1243 1406 1438 2706 3505 4769 5046 5460 7028 8251 8860 8994 11674 11719 11947 12414 12705 14766 15326 15884C 16223 16292 |

| | |
|---|---|
| W3a | 73 189 194 195 204 207 263 709 750 1243 1406 1438 2706 3505 4769 5046 5460 7028 8251 8860 8994 11674 11719 11947 12414 12705 14766 15326 15784 15884C 16223 16292 |
| W3a1 | 73 189 194 195 204 207 263 709 750 1243 1406 1438 2706 3505 4769 5046 5460 7028 8251 8860 8994 11674 11719 11947 12414 12705 13263 14766 15326 15784 15884C 16223 16292 |
| W3a1a | 73 189 194 195 204 207 263 709 750 1243 1406 1438 2706 3505 4769 5046 5460 7028 7151 8251 8860 8994 11674 11719 11947 12414 12705 13263 14766 15326 15784 15884C 16223 16292 |
| W3a1a1 | 73 189 194 195 204 207 263 709 750 1243 1406 1438 2706 3421 3505 4769 5046 5460 7028 7151 8251 8860 8994 11674 11719 11947 12414 12705 13263 14766 15326 15784 15884C 16223 16292 |
| W3a1a2 | 73 189 194 195 204 207 263 709 750 1243 1406 1438 2706 3505 4769 5046 5460 5585 7028 7151 8251 8860 8994 9127 11674 11719 11947 12414 12705 13263 14766 15326 15784 15884C 16223 16292 |
| W3a1a3 | 73 189 194 195 204 207 263 709 750 1243 1406 1438 2706 3505 4769 5046 5460 7028 7151 8251 8860 8994 11674 11719 11947 12414 12705 13263 14766 15109 15326 15784 15884C 16223 16292 |
| W3a1b | 73 189 194 195 204 207 263 709 750 1243 1406 1438 2706 3505 4769 5046 5460 7028 8251 8860 8994 10245 11674 11719 11947 12414 12705 13263 14766 15326 15784 15884C 16223 16292 |
| W3a1+199 | 73 189 194 195 199 204 207 263 709 750 1243 1406 1438 2706 3505 4769 5046 5460 7028 8251 8860 8994 11674 11719 11947 12414 12705 13263 14766 15326 15784 15884C 16223 16292 |
| W3a1c | 73 189 194 195 199 204 207 263 709 750 1243 1406 1438 2706 3505 4769 5046 5460 7028 7269 8251 8860 8994 11674 11719 11947 12414 12705 13263 14766 15326 15784 15884C 16223 16292 |
| W3a1d | 73 189 194 195 199 204 207 263 709 750 1243 1406 1438 2706 3505 4769 5046 5460 7028 8251 8860 8994 10398 11674 11719 11947 12414 12705 13263 14766 15326 15784 15884C 16223 16292 |
| W3a2 | 73 189 194 195 204 207 263 709 750 1243 1406 1438 2706 3505 4769 5046 5291 5460 7028 8251 8860 8994 11674 11719 11947 12414 12705 14766 15326 15784 15884C 16209 16223 16255 16292 |
| W3b | 73 189 194 195 199 204 207 263 709 750 1243 1406 1438 2706 3505 4769 5046 5460 7028 8251 8860 8994 11674 11719 11947 12414 12705 12923T 14766 15326 15884C 16223 16292 |
| W3b1 | 73 194 195 199 204 207 263 709 750 1243 1406 1438 2706 3505 4769 5046 5460 7028 7058 8251 8860 8994 11674 11719 11947 12414 12705 12923T 14766 15326 15884C 16223 16292 |
| W4 | 73 143 189 194 195 196 204 207 263 709 750 1243 1438 2706 3505 4769 5046 5460 7028 8251 8860 8994 11674 11719 11947 12414 12705 14766 15326 15884C 16223 (16286) 16292 |
| W4a | 73 143 189 192 194 195 196 204 207 263 709 750 1243 1438 2706 3505 4769 5046 5460 7028 8251 8860 8994 11674 11719 11947 12414 12705 14766 15326 15884C 16223 (16286) 16292 |
| W4a1 | 73 143 189 192 194 195 196 204 207 263 709 750 1243 1438 2706 3505 3531 4769 5046 5460 7028 8251 8860 8994 11674 11719 11947 12414 12705 14766 15326 15884C 16223 (16286) |

| | |
|---|---|
| W4b | 73 143 189 194 195 196 204 207 263 709 750 1243 1438 2706 3505 4769 5046 5460 7028 7444 8251 8860 8994 11674 11719 11947 12414 12705 14766 15326 15884C 16223 (16286) 16292 |
| W4c | 73 143 189 194 195 196 204 207 263 709 750 1243 1438 2706 3505 4769 5046 5460 7028 8251 8860 8994 11674 11719 12414 12705 14766 15326 15884C 16223 (16286) 16292 |
| W4d | 73 143 189 194 195 196 204 207 263 709 750 1243 1438 2706 3505 4769 5046 5460 7028 8251 8860 8994 11674 11719 11947 12414 12705 14766 15326 15884C 16082 16223 (16286) 16292 |
| W5 | 73 189 194 195 204 207 263 709 750 1243 1438 2706 3505 4769 5046 5460 6528 7028 8251 8860 8994 11674 11719 11947 12414 12705 14766 15326 15775 15884C 16223 16292 |
| W5a | 73 189 194 195 204 207 263 709 750 1243 1438 2706 3505 4769 5046 5460 6528 7028 8251 8860 8994 10097 11674 11719 11947 12414 12705 14766 15326 15775 15884C 16223 16292 16362 |
| W5a1 | 73 189 194 195 204 207 263 709 750 1243 1438 2706 3505 4769 5046 5460 6528 7028 8251 8860 8994 10097 10410 11674 11719 11947 12414 12705 14766 15326 15775 15884C 16223 16292 16362 |
| W5a1a | 73 189 194 195 204 207 263 709 750 1243 1438 2706 3505 4363 4769 5046 5460 6528 7028 8251 8860 8994 10097 10410 11674 11719 11947 12414 12705 14766 15326 15775 15884C 16223 16292 16362 |
| W5a1a1 | 73 189 194 195 204 207 263 709 750 1243 1438 2706 3505 4363 4769 5046 5460 6528 7028 8251 8860 8994 9275 10097 10410 11674 11719 11947 12414 12705 14766 15326 15775 15884C 16223 16292 16362 |
| W5a1a1a | 57 60.1T 73 139 189 194 195 204 207 263 709 750 1243 1438 2706 3505 4363 4769 5046 5460 6528 7028 8251 8860 8994 9275 10097 10410 11674 11719 11947 12414 12705 14766 15326 15775 15884C 16223 16292 16362 |
| W5a2 | 73 150 189 194 195 204 207 263 709 750 1243 1438 2706 3505 4769 5046 5460 6528 7028 8251 8860 8994 10097 11674 11719 11947 12414 12705 14766 15326 15775 15884C 16223 16292 16362 |
| W5a2b | 73 150 189 194 195 204 207 263 709 750 1243 1438 2706 3505 3644 4769 5046 5460 6528 7028 8251 8860 8994 10097 11674 11719 11947 12414 12705 14766 15326 15775 15884C 16177 16223 16292 16362 |
| W5b | 60.2T 64-65d 73 189 194 195 204 207 263 709 750 1243 1438 2706 3505 4769 5046 5460 6528 7028 8251 8860 8994 11674 11696 11719 11947 12414 12705 14766 15326 15775 15884C 16223 16292 |
| W5b1 | 58 60.2T 64-65d 66C 73 189 194 195 204 207 263 709 750 1243 1438 2706 3505 4769 5046 5460 6528 7028 8251 8860 8994 11674 11696 11719 11947 12414 12705 14766 15326 15775 15884C 16223 16292 |
| W5b1a | 58 60.2T 64-65d 66C 73 189 194 204 207 263 709 750 1243 1438 2706 3505 4769 5046 5460 6528 7028 8251 8618 8860 8994 9809 11674 11696 11719 11947 12414 12705 14766 15326 15775 15884C 16223 16292 |
| W6 | 73 189 194 195 204 207 263 709 750 1243 1438 2706 3505 4093 4769 5046 5460 7028 8251 8614 8860 8994 11674 11719 11947 12414 12705 14766 15326 15884C (16192) 16223 16292 16325 |
| W6a | 73 189 194 195 204 207 263 709 750 1243 1438 2706 3505 4093 4769 5046 5460 7028 8251 8610 8614 8860 8994 11674 11719 11947 12414 12705 14766 15326 15884C (16192) 16223 16292 16325 |

| | |
|---|---|
| W6b | 73 189 194 195 204 207 263 709 750 1243 1438 2706 3505 4093 4646 4769 5046 5460 6297 7028 8251 8614 8705 8860 8994 11674 11719 11947 12414 12705 14766 15326 15884C (16192) 16223 16292 16325 |
| W6b1 | 73 189 194 195 204 207 263 709 750 1243 1438 2706 3505 4093 4646 4769 5046 5460 6297 7028 7269 8251 8614 8705 8860 8994 11674 11719 11947 12414 12705 14766 15326 15884C (16192) 16223 16292 16325 |
| W6c | 73 189 194 195 204 207 263 709 750 1243 1438 2706 3505 4093 4769 5046 5460 7028 8002 8251 8614 8658 8860 8994 11674 11719 11947 12414 12705 14766 15326 15884C (16192) 16223 16292 16325 |
| W6c1 | 73 189 194 195 204 207 263 709 750 1243 1438 2706 3505 4093 4769 5046 5460 7028 8002 8149 8251 8614 8658 8860 8994 11674 11719 11947 12414 12705 14766 15326 15884C (16192) 16223 16292 16325 |
| W6c1a | 73 189 194 195 204 207 263 709 750 1243 1438 2706 3505 4093 4769 5046 5460 7028 8002 8149 8251 8614 8658 8860 8994 11674 11719 11947 12414 12705 14766 15326 15884C (16192) 16211 16223 16292 16325 |
| W6d | 73 189 194 195 204 207 263 709 750 1243 1438 2706 3505 4093 4769 5046 5460 7028 8251 8614 8860 8994 9211 11674 11719 11947 12414 12705 14766 15326 15884C (16192) 16223 16292 16325 |
| W7 | 73 185 189 194 195 204 207 263 709 750 1243 1438 2706 3505 4769 5046 5460 7028 8251 8860 8994 11674 11719 11947 12414 12705 14766 15326 15884C 16223 16292 |
| W8 | 73 189 194 195 204 207 263 709 750 1243 1438 2706 3505 4769 5046 5147 5460 7028 8251 8697 8860 8994 11674 11719 11947 12414 12705 14766 15326 15884C 16223 16292 |
| W9 | 73 189 194 195 204 207 263 709 750 1243 1438 2706 3505 4769 5046 5460 7028 8251 8860 8994 11674 11719 11947 12414 12705 14097 14766 15326 15884C 16223 16292 |
| N3 | 73 146 210 263 571 750 1005 1438 2706 3357 4769 6366 6806 7028 7897 8653 8860 8937 10586 11719 12705 14634 14766 15326 16086 16172 16187 16189 16217 16223 |
| N3a | 73 146 210 263 571 750 1005 1438 2706 3357 4769 5048 6366 6806 7028 7897 8653 8860 8937 9815 10586 11128 11719 12705 14634 14766 15326 16086 16172 16187 16189 16217 16223 |
| N3a1 | 73 146 210 263 571 750 1005 1438 2706 3357 4769 5048 6366 6806 7028 7897 8653 8860 8937 9815 10586 11128 11719 12705 14569 14634 14766 15326 16086 16172 16187 16189 16217 16223 |
| N3b | 73 146 210 263 571 750 1005 1438 2706 3357 4769 5553 6366 6806 7028 7897 8653 8860 8937 9211 10586 11719 12705 14634 14766 15326 15670 16086 16172 16187 16189 16217 16223 |
| N7 | 73 263 723 750 1438 2706 4769 6570T 7028 8860 11617 11719 12705 13542 14668 14766 15326 15945 16129 16223 (16318) |
| N7a | 73 75T 152 263 279 508 723 750 1438 2706 3396 4769 5261 5390 6570T 6710 7028 8860 9254 9438 11617 11719 12705 13542 14668 14766 15326 15945 16051 16063 16129 16223 (16318) |
| N7a1 | 73 75T 152 263 279 508 709 723 750 1438 2706 3396 4769 5261 5390 6570T 6710 7028 8860 9254 9438 11617 11719 12705 13542 14668 14766 15326 15945 16051 16063 16129 16166d 16223 (16318) 16463 16466 |

| | |
|---|---|
| N7a2 | 73 75T 152 263 279 508 723 750 1438 2706 3396 4769 5261 5390 6570T 6710 7028 8860 9254 9438 10370 11617 11719 12705 13542 14668 14766 15326 15945 16051 16063 16129 16173 16223 (16318) 16355 |
| N7b | 73 263 723 750 1438 1503 1888 2706 3391 4769 5135 6570T 7028 8483 8860 11617 11719 12705 12906 13542 14485 14668 14766 15326 15945 16129 16223 (16318) |
| N8 | 73 263 750 1438 2706 2760 3027 4769 7028 7885 7961 8188 8860 10398 11719 12705 13710 14766 15211 15326 16223 16263 16274 16311 16343 |
| N9 | 73 263 750 1438 2706 4769 5417 7028 8860 11719 12705 14766 15326 16223 |
| N9a | 73 150 263 750 1438 2706 4769 5231 5417 7028 8860 11719 12358 12372 12705 14766 15326 16223 16257A 16261 |
| N9a1'3 | 73 150 263 750 1438 2706 4769 5231 5417 7028 8860 11719 12358 12372 12705 14766 15326 16129 16223 16257A 16261 |
| N9a1 | 73 150 263 750 1438 2706 4386 4769 5231 5417 7028 8860 11719 12007 12358 12372 12705 14766 15326 16111 16129 16223 16257A 16261 |
| N9a1a | 73 150 263 750 1438 2706 4386 4769 5231 5417 7028 8860 11719 12007 12358 12372 12705 14766 15095 15326 16111 16129 16223 16257A 16261 |
| N9a3 | 73 150 263 750 1438 2706 4129 4769 5231 5417 7028 8860 11719 12354 12358 12372 12612 12705 14766 15326 16129 16223 16257A 16261 |
| N9a2'4'5'11 | 73 150 263 750 1438 2706 4769 5231 5417 7028 8860 11719 12358 12372 12705 14766 15326 16172 16223 16257A 16261 |
| N9a2 | 73 150 263 750 961 1438 2706 4769 5231 5417 7028 8860 11719 12358 12372 12705 14766 15067 15326 16172 16223 16257A 16261 |
| N9a2a | 73 150 263 750 961 1438 2706 4769 5231 5417 7028 8860 11719 12358 12372 12705 14766 15067 15326 16172 16223 16257A 16261 16497 |
| N9a2a1 | 73 150 263 338 750 961 1438 2706 4769 5231 5417 7028 8860 11719 12358 12372 12705 14766 14968 15067 15326 16172 16223 16257A 16261 16304 16497 |
| N9a2a2 | 73 150 263 750 961 1438 2706 4769 5231 5417 7028 8860 11719 12358 12372 12705 14148 14766 15067 15326 16172 16223 16257A 16261 16497 |
| N9a2a3 | 73 150 263 750 961 1438 2706 4688A 4769 5231 5417 5553G 7028 8860 11719 12358 12372 12705 14766 15067 15326 16172 16223 16257A 16261 16497 |
| N9a2c | 73 150 263 750 961 1438 2706 4769 5231 5417 7028 8860 10685 11719 12358 12372 12705 13857 14766 15067 15326 16172 16223 16250 16257A 16261 |
| N9a2d | 73 150 263 750 961 1438 2706 3537 4769 5231 5417 7028 8860 11016 11719 12358 12372 12705 14766 15067 15326 16172 16223 16257A 16261 |
| N9a4 | 73 150 263 750 1438 1664 2706 4769 5231 5417 7028 8860 11719 12358 12372 12705 14766 15326 16145 16172 16223 16245 16257A 16261 |
| N9a4a | 73 150 263 750 1438 1664 2706 4769 4820 5231 5417 7028 8860 11719 12358 12372 12705 14766 15326 16145 16172 16223 16245 16257A 16261 |
| N9a4b | 73 150 263 750 1438 1664 2706 4769 5231 5417 7028 8860 11719 12358 12372 12705 14766 15326 16092 16145 16172 16223 16245 16257A 16261 |
| N9a4b1 | 73 150 263 750 1438 1664 2706 4769 5231 5417 7028 8860 9156 11719 12358 12372 12705 14766 15326 16092 16145 16172 16223 16245 16257A 16261 |

| | |
|---|---|
| N9a5 | 73 150 263 750 1438 2706 3729 4769 5231 5417 7028 8860 11719 12358 12372 12705 14766 15326 15883 16172 16189 16209 16223 16257A 16261 |
| N9a11 | 73 150 195 263 453A 750 1438 2706 4769 5231 5417 6092 7028 8860 9180 10801 11719 12358 12372 12705 13934 14581 14766 15326 16172 16223 16257A 16261 16311 |
| N9a6 | 73 150 263 750 1438 2706 4769 4856 5231 5417 7028 8860 11719 12358 12372 12705 14766 15326 16223 16257A 16261 16292 |
| N9a6a | 73 150 263 750 1438 2706 4769 4856 5231 5417 7028 8860 11719 12358 12372 12705 13742 14766 15080 15326 16223 16257A 16261 16292 16294 |
| N9a6b | 73 150 263 750 1438 2706 4769 4856 5231 5417 7028 8860 10530C 11719 12358 12372 12705 14364 14766 15326 16223 16257A 16261 16292 16342 |
| N9a7 | 73 150 263 750 1438 2706 4769 5231 5417 7028 8860 11719 12358 12372 12705 14016 14766 15326 16223 16257A 16261 16291 |
| N9a8 | 73 150 263 750 1438 2706 4769 5231 5417 7028 8860 11368 11719 12358 12372 12705 14766 15090 15326 16223 16257A 16261 |
| N9a9 | 73 150 263 750 1438 2706 2887 4769 5231 5417 7028 8860 11719 12358 12372 12705 14766 15326 16223 16257A 16261 |
| N9a10 | 73 150 263 750 1438 2706 4769 5231 5417 7028 8860 11719 12358 12372 12705 12771 14766 15326 16223 16257A 16261 |
| N9a10a | 73 150 263 750 1438 2706 4769 5231 5417 7028 8860 9755 11719 11968T 12358 12372 12705 12771 14766 15326 16223 16257A |
| N9a10a1 | 73 150 263 750 1438 2706 4769 5231 5417 7028 8860 9755 9821 11719 11968T 12358 12372 12705 12771 14766 15326 16223 16257A |
| N9a10a2 | 73 150 263 750 1438 2706 4769 5231 5417 7028 8860 9428 9755 11719 11968T 12358 12372 12705 12771 14766 15326 16223 16257A |
| N9a10a2a | 73 150 263 750 1438 2706 4769 5231 5417 5773 7028 8860 9428 9755 11719 11968T 12358 12372 12705 12771 14766 15326 16223 16257A |
| N9a10+16311 | 73 150 263 750 1438 2706 4769 5231 5417 7028 8860 11719 12358 12372 12705 12771 14766 15326 16223 16257A 16261 16311 |
| N9a10b | 42.1G 73 150 263 750 1438 2706 4769 5231 5417 6899 7028 8860 11719 12358 12372 12705 12771 14766 15326 16223 16257A 16261 16311 |
| N9b | 73 263 750 1438 2706 4769 5147 5417 7028 8860 10607 11016 11719 12705 13183 14766 14893 15326 16189 16223 |
| N9b1 | 73 263 750 1438 2706 4769 5147 5417 7028 8860 10607 11016 11719 12501 12705 13183 14766 14893 15326 16189 16223 |
| N9b1a | 73 263 750 1438 2706 4769 5147 5417 7028 8860 9410 10607 11016 11719 12501 12705 13183 14766 14893 15326 16189 16223 |
| N9b1b | 73 263 750 1438 2706 4769 5147 5417 7028 7830 8860 10607 11016 11719 12501 12705 13183 14766 14893 15326 16129 16189 16223 16390 |
| N9b1c | 73 94 263 750 1438 2706 4769 5147 5417 7028 8860 10607 11016 11719 12501 12705 13183 14766 14893 15326 16189 16223 |
| N9b1c1 | 73 94 207 263 750 1438 2706 4769 5147 5417 7028 8860 10607 11016 11719 12501 12705 13183 14766 14893 15326 16189 16223 |
| N9b2 | 73 263 750 1438 2706 4769 5147 5417 7028 8860 10607 11016 11719 12705 13183 14766 14893 15326 16189 16223 16294 |
| N9b2a | 73 263 750 1438 2706 4769 5147 5417 7028 8860 10607 11016 11719 12705 13183 14766 14893 15326 16189 16223 16294 16309 |

| | |
|---|---|
| N9b3 | 73 263 750 1438 2706 4769 5147 5417 7028 8860 10607 11016 11719 12705 13183 14766 14893 14996 15326 16189 16223 |
| N9b4 | 73 263 750 1438 2706 4769 5147 5417 7028 8860 10607 11016 11719 12705 13183 14766 14893 15326 16187d 16188 16189 16223 |
| Y | 73 263 750 1438 2706 4769 5417 7028 8392 8860 10398 11719 12705 14178 14693 14766 15326 16126 16231 |
| Y1 | 73 146 263 750 1438 2706 3834 4769 5417 7028 8392 8860 10398 11719 12705 14178 14693 14766 15326 16126 16231 (16266) |
| Y1a | 73 146 263 750 1438 2706 3834 4769 5417 7028 7933 8392 8860 10398 11719 12705 14178 14693 14766 15326 16126 16231 (16266) |
| Y1a1 | 73 146 263 750 1438 2706 3834 4769 5417 7028 7933 8392 8860 10398 11719 12705 12732 14178 14693 14766 15326 16126 16231 (16266) |
| Y1a+16189 | 73 146 263 750 1438 2706 3834 4769 5417 7028 7933 8392 8860 10398 11719 12705 14178 14693 14766 15326 16126 16189 16231 (16266) |
| Y1a2 | 73 146 263 750 1438 2706 3834 4769 5417 7028 7933 8392 8860 10398 11719 12397 12705 14178 14693 14766 15326 16126 16189 16231 (16266) |
| Y1b | 73 146 263 750 1438 2706 3834 4769 5417 7028 8392 8860 10097 10398 11719 12705 14178 14693 14766 15326 15460 16126 16231 (16266) |
| Y1b1 | 73 146 263 750 1438 2706 3834 4769 5417 7028 8392 8860 10097 10398 11719 12705 14178 14693 14766 15221 15326 15460 16126 16231 (16266) |
| Y1b1a | 73 146 263 750 1438 2706 3834 4769 5417 7028 8392 8860 9278 10097 10398 11719 12705 14178 14693 14766 15221 15326 15460 16126 16231 (16266) |
| Y2 | 73 263 482 750 1438 2706 4769 5147 5417 6941 7028 7859 8392 8860 10398 11719 12705 14178 14693 14766 14914 15244 15326 16126 16231 16311 |
| Y2a | 73 263 482 750 1438 2706 4769 5147 5417 6941 7028 7859 8392 8860 10398 11719 12161 12705 14178 14693 14766 14914 15244 15326 16126 16231 16311 |
| Y2a1 | 73 263 482 750 1438 2706 4769 5147 5417 6941 7028 7859 8392 8860 10398 11299 11719 12161 12705 14178 14693 14766 14914 15244 15326 16126 16231 16311 |
| Y2a1a | 73 263 482 750 1438 2706 2856 4769 5147 5417 6941 7028 7859 8392 8860 10398 11299 11719 12161 12705 13135 14178 14693 14766 14914 15244 15326 16126 16231 16311 |
| Y2b | 73 263 338 482 750 1438 2706 4769 5147 5417 6941 7028 7859 8392 8860 10398 11719 12705 14178 14693 14766 14914 15244 15326 16126 16231 16311 |
| N10 | 73 263 1438 2706 4769 5581 7028 8860 11719 12705 14766 15326 16172 16223 16362 |
| N10a | 73 185 189 263 1438 2706 3543 3873 4562 4769 5581 7028 7853 8860 10373 11719 12136 12705 13434 13928C 14766 14953 15211 15326 16172 16189 16223 16362 |
| N10b | 73 150 152 199 263 709 1438 2010 2706 2707C 4769 4977 5581 6245 7028 8130 8167 8860 9459 10345 11719 12092A 12705 14053 14766 15226 15326 16069 16172 16223 16278 16291A 16298 16362 |
| N11 | 73 263 750 1438 2706 4769 7028 8860 11581A 11719 12705 14766 15326 16223 |

| | |
|---|---|
| N11a | 73 195 263 750 813 1438 2706 4769 6674 7028 7975 8618 8860 10289 10908 11581A 11719 12705 14311 14431 14502 14766 15326 15514 15900 16223 16355 |
| N11a1 | 73 195 263 750 813 1438 2706 4769 6674 7028 7975 8618 8860 10289 10908 11581A 11719 12634 12705 14311 14431 14502 14766 15326 15514 15900 16189 16223 16355 |
| N11a2 | 73 195 263 750 813 1438 2706 4769 6674 7028 7975 8618 8860 8928 9180 10289 10908 11581A 11719 12705 14220 14311 14431 14502 14766 15326 15514 15900 16176 16223 16232 16355 |
| N11b | 73 150 261 263 750 1438 2706 3736 4080 4769 5234 6092 6620 7028 7356 8512 8860 8867 9503 9596 10321 11204 11581A 11719 11728 12401 12705 14357 14766 15326 16129 16193 16209 16223 16270 16291 |
| N13 | 73 146 152 178 195 263 750 1438 2706 2831 4769 5704 6896 7028 8860 10101 11020 11719 12705 13263 14766 15326 16066 16223 16261 16290 |
| N14 | 73 263 750 1438 1598 1888 2706 4769 4917 5291 7028 8251 8269 8860 11491 11719 12705 14384C 14766 15040 15326 15884C 16223 |
| N21 | 73 150 263 337d 750 1438 2706 4769 6752 7028 8701 8860 10583 11719 12705 13437 14560 14766 15326 16193 16223 |
| N21+195 | 73 150 195 263 337d 750 1438 2706 4769 6752 7028 8701 8860 10583 11719 12705 13437 14560 14766 15326 16193 16223 |
| N21a | 73 150 195 263 337d 750 1438 2706 4769 6752 7028 8701 8860 9512 10583 11719 12705 13135 13437 14560 14766 15326 16193 16223 16291 |
| N22 | 73 150 263 750 942 1438 2706 4769 7028 7158 8860 9254 11365 11719 12705 14766 15326 16168 16223 16249 |
| N22a | 73 150 263 292.1AT 750 942 1438 2706 4769 7028 7158 8860 9159 9254 11365 11719 12025 12705 14587 14766 15326 16017 16168 16223 16249 |
| A | 73 235 263 663 750 1438 1736 2706 4248 4769 4824 7028 8794 8860 11719 12705 14766 15326 16223 16290 16319 |
| A+152 | 73 152 235 263 663 750 1438 1736 2706 4248 4769 4824 7028 8794 8860 11719 12705 14766 15326 16223 16290 16319 |
| A+152+16362 | 73 152 235 263 663 750 1438 1736 2706 4248 4769 4824 7028 8794 8860 11719 12705 14766 15326 16223 16290 16319 16362 |
| A1 | 73 152 235 263 663 750 1438 1442 1736 2706 4248 4769 4824 7028 8794 8860 11719 12705 14766 15326 16223 16290 16319 16362 |
| A1a | 73 152 235 263 663 750 1438 1442 1736 2706 4248 4769 4824 7028 8794 8860 9713 11719 12705 14766 15326 16223 16249 16290 16319 16362 |
| A1a1 | 73 152 235 263 663 750 1438 1442 1736 2706 4248 4769 4824 4928 7028 8794 8860 9713 11719 12705 14766 15326 16223 16249 16290 16319 |
| A2 | 73 146 153 235 263 663 750 1438 1736 2706 4248 4769 4824 7028 8027 8794 8860 11719 12007 12705 14766 15326 16111 16223 16290 16319 16362 |
| A2a | 73 146 153 235 263 663 750 1438 1736 2706 3330 4248 4769 4824 7028 8027 8794 8860 11719 12007 12705 14766 15326 16111 16192 16223 16290 16319 16362 |
| A2a1 | 73 146 153 235 263 663 750 1438 1736 2706 3330 4248 4769 4824 7028 8027 8794 8860 11719 12007 12705 14766 15326 16111 16192 16223 16261 16290 16319 16362 |

| | |
|---|---|
| A2a2 | 73 146 153 235 263 663 750 1438 1736 2706 3330 4248 4769 4824 7028 8027 8794 8860 9301 11719 12007 12705 14766 15326 16111 16192 16223 16290 16319 16362 |
| A2a3 | 73 146 153 235 263 663 750 1438 1736 2706 3330 4248 4769 4824 7028 8027 8794 8860 11719 12007 12705 14766 15326 16111 16192 16223 16290 16311 16319 16362 |
| A2a4 | 73 146 153 235 263 663 750 1438 1736 2706 3330 4248 4769 4824 5460 7028 8027 8794 8860 11719 12007 12705 14766 15326 16093 16111 16192 16223 16290 16319 16362 |
| A2a5 | 73 146 153 235 263 663 750 1438 1736 2706 3330 3552 4248 4769 4824 7028 8027 8794 8860 11719 12007 12166 12705 14766 15326 16111 16192 16223 16233 16290 16319 16331 |
| A2b | 73 146 153 235 263 663 750 1438 1736 2706 4248 4769 4824 7028 8027 8794 8860 11365 11719 12007 12705 14766 15326 16111 16223 16290 16319 16362 |
| A2b1 | 73 146 153 235 263 663 750 1438 1736 2706 4248 4769 4824 7028 8027 8794 8860 11365 11719 12007 12705 14766 15326 16111 16223 16265 16290 16319 16362 |
| A2+(64) | (64) 73 146 153 235 263 663 750 1438 1736 2706 4248 4769 4824 7028 8027 8794 8860 11719 12007 12705 14766 15326 16111 16223 16290 16319 16362 |
| A2c | (64) 73 146 153 235 263 663 750 1438 1736 2706 4248 4769 4824 7028 8027 8794 8860 11719 12007 12468 12705 14364 14766 15326 16111 16223 16290 16319 16362 |
| A2d | (64) 73 146 153 235 263 663 750 1438 1736 2706 4248 4769 4824 6308 7028 8027 8794 8860 11719 12007 12705 14766 15326 16111 16223 16290 16319 16362 |
| A2d1 | (64) 73 146 153 235 263 663 750 1438 1736 2706 4248 4769 4824 6308 7028 8027 8281-8289d 8794 8860 11314 11719 12007 12705 14766 15326 16111 16223 16290 16319 16362 |
| A2d1a | (64) 73 146 153 235 263 663 750 1438 1736 2706 4248 4769 4824 6308 7028 8027 8281-8289d 8548 8794 8860 11314 11719 12007 12705 14766 15326 16111 16223 16274 16290 16319 16362 |
| A2d2 | (64) 73 146 153 235 263 663 750 1438 1736 2706 4248 4769 4824 5081 6308 7028 8027 8794 8860 11719 12007 12705 14766 15326 16111 16223 16290 16319 16362 |
| A2+(64)+@153 | (64) 73 146 235 263 663 750 1438 1736 2706 4248 4769 4824 7028 8027 8794 8860 11719 12007 12705 14766 15326 16111 16223 16290 16319 16362 |
| A2e | (64) 73 146 235 263 663 750 1438 1736 2706 4248 4769 4824 7028 7112 8027 8794 8860 11719 12007 12705 13708 14766 15326 16111 16189 16223 16290 16319 16336 16362 |
| A2ao | (64) 73 146 235 263 663 750 1438 1736 2706 4248 4769 4824 7028 8027 8794 8860 9650 11719 12007 12705 14766 15326 16111 16223 16290 16319 16362 |
| A2ao1 | (64) 73 146 235 263 663 750 1438 1736 2706 3394 4248 4769 4824 7028 8027 8794 8860 9650 11719 12007 12705 14766 15326 16111 16223 16264 16290 16319 16362 |

| | |
|---|---|
| A2f | (64) 73 146 153 235 263 663 750 1438 1736 2706 4248 4769 4824 7028 8027 8794 8860 11719 12007 12705 12940 14766 15326 16111 16223 16290 16319 16362 |
| A2f1 | (64) 73 146 153 235 263 663 750 1438 1736 2638 2706 3316 4248 4769 4824 7028 7897 8027 8794 8860 11719 12007 12705 12940 14766 15326 15670 16111 (16192) 16223 16290 16319 16362 |
| A2f1a | (64) 73 146 153 235 263 663 750 1438 1736 1809 2638 2706 3316 4248 4769 4824 7028 7897 8027 8794 8860 11719 12007 12092A 12705 12940 14766 14911 15326 15670 16111 (16192) 16223 16290 16319 16362 |
| A2f2 | (64) 73 153 235 263 663 676 750 1438 1736 2706 4248 4769 4824 7028 8027 8794 8860 11719 12007 12705 12940 14766 15326 16111 16223 16290 16319 16357 16362 |
| A2f3 | (64) 73 146 151 153 235 263 663 750 1438 1736 2706 4248 4769 4824 5585 7028 8027 8794 8860 9156 11719 11914 12007 12705 12940 14275 14766 15323 15326 16111 16223 16290 16319 16362 |
| A2g | (64) 73 146 153 235 263 663 750 1438 1736 2706 4248 4769 4824 7028 7724T 8027 8794 8860 11719 12007 12705 14766 15326 16111 16223 16290 16319 16362 16391 |
| A2g1 | (64) 73 146 153 235 263 663 750 1438 1736 2706 4248 4769 4824 4970 7028 7724T 8027 8794 8860 11719 12007 12705 13855 14766 15326 16111 16223 16290 16319 16362 16391 |
| A2h | (64) 73 146 153 235 263 663 750 1438 1736 2706 4248 4769 4824 7028 8027 8794 8860 11719 12007 12705 14766 15326 16111 16223 16290 16319 16362 16526 |
| A2h1 | (64) 73 146 153 235 263 663 750 1438 1598 1736 1888 2706 4248 4769 4824 7028 8027 8794 8860 11719 12007 12705 12811 14766 15326 16111 16223 16290 16319 16335 16362 16526 |
| A2i | (64) 73 94 146 153 235 263 663 750 960.XC 1438 1736 2706 3307.1A 3308 4248 4769 4824 5165 6620 7028 8027 8794 8860 11719 12007 12705 14280 14470 14766 15326 15386 16111 16223 16290 16319 16325 16362 |
| A2j | (64) 73 146 153 235 263 663 750 1438 1736 2706 4248 4769 4824 7028 8027 8794 8860 10595 11548 11719 12007 12705 14766 15326 16111 16223 16290 16319 16362 |
| A2j1 | (64) 73 146 153 235 263 663 750 1438 1736 2706 4248 4769 4824 7028 8027 8794 8860 10595 11314 11548 11719 12007 12705 14766 15326 16111 16223 16290 16319 16362 |
| A2k | (64) 73 146 153 235 263 663 750 1438 1736 2706 3202 4248 4769 4824 7028 8027 8794 8860 11719 12007 12705 14766 15326 16111 16223 16290 16319 16362 |
| A2k1 | (64) 73 146 153 235 263 663 750 1438 1736 2706 3202 4248 4769 4824 7028 8027 8794 8860 11719 12007 12705 14766 15326 15924 16111 16223 16290 16319 16362 |
| A2k1a | (64) 73 146 153 235 263 663 750 1438 1736 2706 3202 4248 4769 4824 7028 8027 8460 8794 8860 11719 12007 12705 14766 15326 15924 16111 16223 16290 16319 16362 |
| A2+(64)+@16111 | (64) 73 146 153 235 263 663 750 1438 1736 2706 4248 4769 4824 7028 8027 8794 8860 11719 12007 12705 14766 15326 16223 16290 16319 |

| | |
|---|---|
| A2l | (64) 73 146 153 235 263 663 750 1438 1736 2706 4248 4769 4824 7028 8027 8794 8860 11719 12007 12705 14766 15326 15629 16223 16290 16319 16362 |
| A2m | (64) 73 146 153 235 263 663 750 1438 1736 2706 4248 4769 4824 7028 8027 8794 8860 8947 9039 11719 12007 12705 14766 15172 15326 16223 16240 16290 16319 16362 |
| A2n | (64) 73 146 153 235 263 663 750 1438 1736 2706 3849 4248 4769 4824 7028 8027 8794 8860 9344 10700 11719 12007 12705 14766 15326 16223 16290 16319 16362 |
| A2o | (64) 73 146 153 235 263 663 750 1438 1736 2706 3972 4248 4769 4824 7028 8027 8794 8860 11719 12007 12705 14766 15326 16223 16290 16319 16362 |
| A2ai | (64) 73 146 153 235 263 663 750 1438 1736 2706 4248 4769 4824 5494G 7028 8027 8794 8860 11248 11719 12007 12705 14766 15326 16213 16223 16290 16319 16362 |
| A2aj | (64) 73 146 153 235 263 663 750 1438 1442 1736 1738 2706 4248 4769 4824 5378 7028 8027 8794 8860 10373 11719 12007 12705 14766 15326 16223 16270 16290 16319 16362 |
| A2+(64)+16129 | (64) 73 146 153 235 263 663 750 1438 1736 2706 4248 4769 4824 7028 8027 8794 8860 11719 12007 12705 14766 15326 16111 16129 16223 16290 16319 16362 |
| A2p | (64) 73 146 153 235 263 663 750 1438 1736 2706 4248 4769 4824 7028 8027 8794 8860 10199 11719 12007 12705 14766 15326 16111 16129 16223 16290 16319 16362 |
| A2p1 | (64) 73 146 153 235 263 663 750 1438 1736 2706 4248 4769 4824 5585 6488 7028 8027 8537 8794 8860 10199 11719 12007 12705 14766 15326 16111 16129 16223 16290 16319 16362 |
| A2p2 | (64) 73 146 153 235 263 663 750 1438 1736 2706 3591 4248 4769 4824 7028 8027 8794 8860 10199 11719 12007 12705 14766 14791 15326 16111 16129 (16178G) (16209) 16223 (16274) 16290 16319 16362 |
| A2am | (64) 73 146 153 235 263 663 750 1438 1736 2706 4248 4769 4824 6253 7028 8027 8794 8860 11719 12007 12705 14766 15229 15314 15326 16111 16129 16223 16290 16319 16362 |
| A2q | (64) 73 146 153 235 263 663 750 1438 1736 2706 4248 4769 4824 7028 8027 8794 8860 11719 12007 12705 14766 15326 16111 16209 16223 16290 16319 16362 |
| A2q1 | (64) 73 146 153 235 263 663 750 1438 1736 2706 4248 4769 4824 7028 7604 7861 8027 8794 8860 11719 12007 12705 14766 15326 16111 16209 16223 16290 16319 16362 |
| A2t | (64) 73 146 153 235 263 663 750 1438 1736 2071 2706 4248 4769 4824 7028 8027 8794 8860 9053 11404 11719 12007 12705 14766 15236 15326 16111 16223 16290 16319 16362 |
| A2u | (64) 73 146 153 235 263 663 750 1438 1736 2706 4248 4769 4824 7028 8027 8794 8860 11719 12007 12705 14766 15326 16111 16136 16223 16290 16319 16362 |
| A2u1 | (64) 73 146 153 235 263 663 750 1438 1736 2706 4248 4769 4824 7028 8027 8794 8860 11719 12007 12705 12906 14766 15326 16111 16136 16223 16257 16290 16319 16344 16362 |

| | |
|---|---|
| A2u2 | (64) 73 146 153 235 263 663 750 1438 1736 2706 4248 4769 4824 7028 8027 8794 8860 11719 12007 12705 14766 15326 16111 16136 16223 16260 16290 16319 16362 |
| A2v | (64) 73 146 153 235 263 663 750 1438 1736 2706 4248 4769 4824 7028 8027 8794 8860 11719 12007 12705 14766 15326 16111 16223 16239 16290 16319 16362 |
| A2v1 | (64) 73 146 153 235 263 663 750 1438 1736 2706 4248 4769 4824 6491A 7028 8027 8794 8860 11719 12007 12705 14766 15326 16111 16223 16239 16290 16319 16362 |
| A2v1+152 | (64) 73 146 152 153 235 263 663 750 1438 1736 2706 4248 4769 4824 6491A 7028 8027 8794 8860 11719 12007 12705 14766 15326 16111 16223 16239 16290 16319 16362 |
| A2v1a | (64) 73 146 152 153 235 263 663 750 1438 1736 2706 4248 4769 4824 6491A 7028 7403 8027 8794 8860 11719 12007 12705 14766 15326 16111 16223 16239 16290 16319 16362 |
| A2v1b | (64) 73 146 152 153 235 263 663 750 1438 1736 2706 4248 4769 4824 6491A 7028 8027 8794 8860 11719 12007 12705 14766 15326 16111 16223 16234 16239 16290 16319 16362 |
| A2w | (64) 73 146 153 235 263 663 750 1438 1736 2706 4248 4769 4824 7028 7124 8027 8794 8860 11016 11719 12007 12705 14766 15326 16111 16223 16290 16319 16362 |
| A2w1 | (64) 73 146 153 235 263 573.XC 663 750 1438 1736 2706 4248 4769 4824 7028 7124 8027 8794 8860 11016 11719 12007 12705 14766 15326 16111 16187 16223 16290 16319 16362 |
| A2x | (64) 73 146 153 235 263 663 750 1438 1736 2706 4248 4769 4824 7028 8027 8794 8860 9055 11719 12007 12705 14766 15326 15924 16111 16223 16290 16319 16362 |
| A2y | (64) 73 146 153 189 207 235 263 663 750 1438 1736 2706 4248 4769 4824 5093 5910 6641 7028 8027 8794 8860 11719 12007 12705 14766 15326 16111 16223 16290 16293 16304 16319 16362 |
| A2aa | (64) 73 146 153 235 263 663 750 1438 1736 2706 4248 4769 4824 7028 8027 8794 8860 11719 12007 12705 14766 15326 16111 16223 16239A 16266 16290 16319 16362 |
| A2ab | (64) 73 146 153 235 263 663 750 1438 1736 2706 4248 4769 4824 6216 7028 8027 8794 8860 11719 12007 12705 14476 14766 15326 16111 16223 16290 16291 16319 16362 |
| A2ac | (64) 73 146 153 235 263 663 750 1438 1736 2706 4248 4769 4824 7028 8027 8794 8860 9377 11719 12007 12705 14766 15074 15326 16111 16213 16223 16290 16319 16362 |
| A2ac1 | (64) 73 146 153 235 263 663 750 1438 1736 2706 4248 4769 4824 7028 8027 8794 8860 9055 9377 9473C 11719 12007 12705 14687 14766 15074 15326 16111 16213 16223 16290 16319 16362 |
| A2ad | (64) 73 146 153 235 263 663 750 1438 1736 2706 4248 4769 4824 7028 8027 8471 8794 8860 11719 12007 12705 14766 15326 16111 16175 16223 16290 16300 16319 16362 |
| A2ad1 | (64) 73 146 153 235 263 663 750 1438 1736 2706 4248 4769 4824 5298 7028 8027 8471 8794 8860 11719 12007 12372 12705 14766 14798 15326 15734 16175 16223 16290 16300 16319 16362 |

| | |
|---|---|
| A2ad2 | (64) 73 146 153 235 263 663 750 1438 1736 2706 4248 4769 4824 6182 7028 8027 8471 8794 8860 10993 11719 12007 12705 14766 15326 16111 16175 16223 16290 16300 16319 16362 |
| A2ae | (64) 73 146 152 153 235 263 487 663 750 1438 1736 2706 3565 4248 4769 4824 7028 8027 8794 8860 11719 12007 12705 14766 15326 16111 16223 (16284) 16290 16319 16362 |
| A2af | (64) 106-111d 146 153 235 263 663 750 1438 1736 2706 4248 4769 4824 5460 7028 8027 8794 8860 11719 12007 12705 14766 15326 16111 16223 16290 16319 16360 16362 |
| A2af1 | (64) 106-111d 146 153 235 263 663 750 1438 1736 2706 4248 4769 4824 5460 6794 7028 7960 8027 8794 8860 11719 12007 12705 14766 15326 16111 16223 16290 16319 16360 16362 |
| A2af1a | (64) 89 106-111d 146 153 235 263 663 750 1438 1736 2706 4248 4769 4824 5460 6794 7028 7960 8027 8794 8860 11719 12007 12705 14766 15326 16111 16223 16290 16319 16360 16362 |
| A2af1a1 | (64) 89 106-111d 146 153 198 235 263 663 750 1438 1736 2706 4248 4769 4824 5460 6794 7028 7960 8027 8794 8860 11719 12007 12705 14766 15326 16111 16223 16290 16319 16360 16362 |
| A2af1a2 | (64) 89 106-111d 146 153 235 263 499 663 750 1438 1736 2706 2762A 4248 4769 4824 5460 6794 7028 7960 8027 8794 8860 11719 12007 12705 14766 15326 16111 16175T 16223 16290 16319 16360 16362 |
| A2af1b | (64) 106-111d 146 153 235 263 663 750 1438 1736 2706 4248 4769 4824 5460 6794 7028 7960 8027 8794 8860 11482 11719 12007 12705 14766 15326 16111 16223 16290 16319 16360 16362 |
| A2af1b1 | (64) 106-111d 146 153 235 263 663 750 1438 1736 2706 3840 4248 4769 4824 5460 6794 7028 7960 8027 8794 8860 10343 11482 11719 12007 12705 12771 14766 15326 16111 16223 16290 16319 16360 16362 |
| A2af1b1a | (64) 106-111d 146 153 200 235 263 663 750 1438 1736 2706 3840 4248 4769 4824 5460 6794 7028 7960 8027 8794 8860 9053 10343 11482 11719 12007 12705 12771 13443 14766 15326 15847T 16223 16290 16319 16360 16362 |
| A2af1b1b | (64) 106-111d 146 153 235 263 663 750 1438 1736 2706 3840 4248 4769 4824 5460 6794 7028 7960 8027 8794 8860 10343 11482 11719 12007 12705 12771 14766 15326 16111 16168 16209 16223 16290 16319 16360 16362 |
| A2af1b2 | (64) 92 93.1T 106-111d 146 153 235 263 663 750 1438 1736 2706 4248 4769 4824 6794 7028 7269 7960 8027 8152 8794 8860 9944 11482 11719 12007 12705 13359 14766 15326 16111 16223 16290 16319 16360 16362 |
| A2af2 | (64) 97C 106-111d 146 153 204 211 235 263 663 750 1438 1736 2706 4248 4769 4824 5460 7028 8027 8563 8794 8860 10344 11719 12007 12705 14211 14766 15326 16111 16189 16223 16290 16319 16360 16362 |
| A2ag | (64) 73 146 153 195 235 263 663 750 1438 1736 2706 4248 4769 4824 7028 8027 8794 8860 9653 11719 12007 12705 14766 15326 16111 16223 16290 16319 16362 |
| A2ah | (64) 73 146 153 235 263 663 750 1438 1736 2706 4248 4769 4824 7028 8027 8794 8860 11719 12007 12705 14766 15326 16097 16098 16111 16223 16290 16319 16362 |

| | |
|---|---|
| A2ak | (64) 73 146 153 235 263 663 750 1438 1736 2706 4248 4769 4824 7028 8027 8654 8794 8860 9668 11719 12007 12705 14766 15326 15458 16111 16223 16290 16319 16362 16466C |
| A2al | (64) 73 146 153 235 263 663 750 1438 1736 2706 3516 4248 4769 4824 7028 8027 8794 8860 11152 11719 12007 12705 14766 15326 16111 16126 16223 16290 16319 16362 |
| A2an | (64) 73 146 153 235 263 663 750 1438 1736 2706 4248 4769 4824 7028 8027 8772 8794 8860 11719 12007 12705 14766 15326 16111 16223 16290 16319 16362 |
| A2+(64)+16189 | (64) 73 146 153 235 263 663 750 1438 1736 2706 4248 4769 4824 7028 8027 8794 8860 11719 12007 12705 14766 15326 16111 16189 16223 16290 16319 16362 |
| A2ap | (64) 73 146 153 235 263 663 750 1438 1736 2706 4248 4769 4824 7028 8027 8794 8860 9809 11719 12007 12705 14766 15326 16111 16189 16223 16290 16319 16362 |
| A2aq | (64) 73 143 146 153 235 263 663 750 808 1438 1736 2706 3421 4248 4769 4824 6267 6374 7028 8027 8794 8860 11719 12007 12705 14766 15326 16111 16223 16290 16319 16355 16362 |
| A2r | 73 146 153 235 263 663 750 1438 1736 2706 4248 4769 4824 6935 7028 8027 8794 8860 11719 12007 12705 14766 15326 16111 16223 16290 16319 16362 |
| A2r1 | 73 103 146 153 235 263 663 750 1438 1736 2706 4248 4769 4824 6935 7028 8027 8794 8860 9518 11719 12007 12705 14766 15326 16111 16223 16290 16319 16362 |
| A2s | 73 146 153 235 263 466 663 750 1438 1736 2706 3145 3766 4248 4769 4824 7028 7269 8027 8552 8794 8860 11719 11809 12007 12346 12705 14766 15326 16111 16207 16223 16290 16311 16319 16362 16400 |
| A2z | 73 146 152 153 214 235 263 663 750 1438 1736 2706 2836 3744 4248 4769 4824 6632 7028 8027 8794 8860 11719 12007 12705 14766 15326 16083 16111 16223 16256 16290 16319 16362 |
| A6 | 73 152 235 263 654 663 750 1438 1736 2706 3687 4248 4769 4824 7028 8794 8860 11719 12705 13287 14766 15326 16223 16290 16319 16362 |
| A6a | 73 152 235 263 654 663 750 1438 1736 2706 3687 4248 4257 4769 4824 7028 8794 8860 11719 12705 13287 14766 15326 16223 16290 16319 |
| A6b | 73 146 152 235 263 654 663 750 1438 1736 2706 3687 4248 4769 4824 6707 7028 8531 8794 8860 10993 11719 12705 13287 14766 15326 15670 16223 16290 16319 16362 |
| A+152+16362+16189 | 73 152 235 263 663 750 1438 1736 2706 4248 4769 4824 7028 8794 8860 11719 12705 14766 15326 16189 16223 16290 16319 16362 |
| A12 | 73 152 235 263 663 750 1438 1736 2706 4248 4769 4824 7028 8794 8860 11719 12705 12720 14290 14766 15326 16189 16223 16290 16319 16362 |
| A12a | 73 152 235 263 663 750 1438 1709 1736 2706 4248 4769 4824 7028 8794 8860 9754 11719 12705 12720 14290 14766 15326 16039 16189 16223 16290 16319 16356 16362 |
| A23 | 73 97T 105-110d 150 152 235 263 663 750 1438 1736 2706 3213 4248 4769 4824 5153 6101 6602 7028 8794 8860 11719 12705 14766 15326 16189 16223 16290 16319 16362 |

| | |
|---|---|
| A+152+16362+200 | 73 152 200 235 263 663 750 1438 1736 2706 4248 4769 4824 7028 8794 8860 11719 12705 14766 15326 16223 16290 16319 16362 |
| A13 | 73 152 200 235 263 663 750 1438 1736 2706 3504 4248 4769 4824 7028 8794 8860 11719 12705 14766 15326 16223 16290 16319 16362 |
| A14 | 73 151 152 200 235 263 663 735 750 1438 1736 2706 4248 4769 4824 7028 8794 8860 11719 12705 14766 15326 16223 16290 16319 16362 |
| A15 | 73 152 (207) 235 663 750 1438 1736 2706 4248 4769 4824 7028 8459 8794 8860 11719 12705 14067 14766 15326 16223 16290 16319 16362 |
| A15a | 73 152 (207) 235 663 750 1438 1736 2706 4248 4769 4824 7028 8459 8794 8860 11084 11719 12705 14067 14766 15326 16223 16290 16319 16362 |
| A15b | 73 152 (207) 235 663 750 1438 1736 2706 3408 4248 4769 4824 7028 8409 8459 8794 8860 11719 12705 14067 14766 15326 16223 16234 16290 16319 16362 |
| A15c | 73 152 (207) 235 663 750 1438 1736 2706 4248 4769 4824 7028 8459 8794 8860 11719 12705 14067 14766 15262 15326 16223 16290 16319 16362 |
| A15c1 | 73 152 (207) 235 663 750 1438 1736 2706 4216 4248 4769 4824 7028 8459 8794 8860 9052 11719 12705 13111 14067 14766 15262 15326 15924 16223 16290 16319 16362 |
| A16 | 73 152 235 263 298A 663 750 1438 1736 4248 4769 4824 6023 7028 8794 8860 11557 11719 12705 14766 14929 15326 16223 16290 16292A 16319 16362 |
| A17 | 73 152 235 263 663 750 1438 1736 2706 4113 4248 4769 4824 5514 7028 8794 8860 9126 11719 12705 14766 15217 15326 16223 16290 16319 |
| A18 | 73 152 235 263 663 750 1438 1736 2706 4248 4769 4824 7028 8794 8860 11465 11719 12705 14766 15326 16223 16290 16319 16362 |
| A19 | 73 152 199 235 263 663 750 1438 1736 2706 4248 4769 4824 7028 8794 8860 11719 12705 14766 15326 15436 16223 16290 16311 16319 16362 |
| A20 | 73 152 235 263 663 750 1438 1736 2706 4248 4769 4824 7028 8794 8860 11719 12705 14696 14766 15326 16223 16290 16319 16362 |
| A21 | 73 152 235 263 663 750 1438 1736 2706 4248 4769 4824 7028 8794 8860 11719 12705 14364 14766 15326 16223 16290 16319 16362 |
| A22 | 73 152 235 263 663 750 1438 1736 2706 4248 4769 4824 6378 7028 8393 8794 8860 9123 9128 11719 12705 14766 15326 16223 16290 16319 16362 |
| A24 | 73 152 235 263 449 663 750 1438 1736 2706 4248 4769 4824 7028 8794 8860 9524 11719 12705 14766 15172 15326 16223 16290 16319 16362 |
| A25 | 73 152 235 263 663 750 1438 1736 2706 4248 4769 4824 7028 8794 8860 11719 12705 14766 15326 16179 16223 16290 16319 16362 |
| A26 | 73 152 235 263 663 750 1438 1736 2706 4248 4769 4824 4924 7028 8794 8860 9716A 11719 11809 12705 13650 13752 14766 14968 15326 16144 16223 16290 16319 16362 |
| A3 | 73 152 235 263 663 750 1438 1736 2706 2857 4248 4769 4824 7028 8794 8860 8962 9711 11719 12705 14766 15326 16223 16290 16319 |
| A3a | 73 152 235 263 663 750 1438 1736 2706 2857 4248 4769 4824 7028 8794 8860 8962 9711 11719 12705 13140 14766 15326 16223 16290 16319 |
| A7 | 73 146 152 235 263 663 750 1438 1736 2706 4248 4769 4824 7028 8413 8794 8860 10172 11719 12705 14766 15326 15379 16051 16129 16189 16223 16290 16319 |

| | |
|---|---|
| A9 | 73 152 235 263 663 750 1438 1736 2706 4248 4769 4824 6950 7028 7228 8340 8794 8860 10700 11719 12705 14766 15326 15874 16223 16290 |
| A11 | 73 152 235 263 663 750 1438 1736 2706 4248 4769 4824 7028 8794 8860 9650 11719 12705 14766 15326 16223 16290 16293C 16319 |
| A11a | 73 152 235 263 663 750 1005 1438 1736 2706 4248 4769 4824 5899.XC 6755 7028 8794 8843 8860 9650 11719 12705 14766 15326 16223 16290 16293C 16319 |
| A11+16234 | 73 152 235 263 663 750 1438 1736 2706 4248 4769 4824 7028 8794 8860 9650 11719 12705 14766 15326 16223 16234 16290 16293C 16319 |
| A11b | 73 152 235 263 663 750 1438 1736 2706 3290 4248 4769 4824 7028 8794 8860 9650 11719 12705 14766 15326 16223 16234 16290 16293C 16319 16527 |
| A5 | 73 235 263 663 750 1438 1736 2706 4248 4769 4824 7028 8563 8794 8860 11536 11719 12705 14766 15326 16223 16290 16319 |
| A5a | 73 235 263 663 750 1438 1736 2156.1A 2706 4248 4655 4769 4824 7028 8563 8794 8860 11536 11647 11719 12705 14766 15326 16187 16223 16290 16319 |
| A5a1 | 73 235 263 663 750 1438 1736 2156.1A 2706 4248 4655 4769 4824 7028 8563 8794 8860 11536 11647 11719 12705 14766 14944 15326 16187 16223 16290 16319 |
| A5a1a | 73 235 263 663 750 1438 1736 2156.1A 2706 4248 4655 4769 4824 7028 8563 8794 8860 10801 11536 11647 11719 12705 14766 14944 15326 16187 16223 16290 16319 |
| A5a1a1 | 73 235 263 663 750 1438 1736 2156.1A 2706 4248 4655 4769 4824 5773 7028 8563 8794 8860 10801 11536 11647 11719 12705 12880 14766 14944 15326 16187 16223 16290 16319 |
| A5a1a1a | 73 235 263 663 750 1438 1736 2156.1A 2706 4248 4655 4769 4824 5773 7028 8563 8794 8860 10801 11536 11647 11719 12705 12880 13221 14766 14944 15326 16187 16223 16290 16319 |
| A5a1a1b | 73 235 263 663 750 1438 1736 2156.1A 2706 4248 4655 4736 4769 4824 5773 7028 8563 8794 8860 10801 11536 11647 11719 12705 12880 14766 14944 15326 16187 16223 16290 16319 |
| A5a1a2 | 73 235 263 663 750 1438 1736 2156.1A 2706 4248 4655 4769 4824 7028 8563 8628 8794 8860 10801 11536 11647 11719 12705 14766 14944 15326 16187 16223 16290 16319 |
| A5a1a2a | 73 235 263 663 750 1438 1736 2156.1A 2706 4248 4655 4769 4824 6956 7028 8563 8628 8794 8860 10801 11536 11647 11719 12705 14766 14944 15326 16187 16223 16290 16319 |
| A5a1b | 73 235 263 663 750 1438 1736 2156.1A 2706 4248 4655 4769 4824 7028 8563 8794 8860 11536 11647 11719 12705 14766 14944 15326 16160 16187 16223 16290 16319 |
| A5a2 | 73 235 263 663 750 1438 1736 2156.1A 2706 4248 4655 4769 4824 7028 8563 8794 8860 11536 11647 11719 12705 13461 14766 15326 16187 16223 16290 16319 |
| A5a3 | 73 235 263 663 750 1438 1736 2156.1A 2706 4248 4655 4769 4824 7028 8563 8794 8860 11536 11647 11719 12705 12909 14766 15326 16187 16223 16290 16319 |

| | |
|---|---|
| A5a3a | 73 235 263 663 750 1438 1736 2156.1A 2706 4248 4655 4769 4824 7028 8563 8794 8860 11536 11647 11719 12705 12909 14766 15326 15341 16187 16223 16290 16319 |
| A5a4 | 73 235 263 663 750 1438 1736 2156.1A 2706 4248 4655 4769 4824 7028 8563 8794 8860 10325 11536 11647 11719 12705 14766 15326 16187 16223 16290 16319 |
| A5a5 | 73 235 263 663 750 1438 1736 2156.1A 2706 4248 4655 4769 4824 7028 7694 8563 8794 8860 11536 11647 11719 12705 14766 15326 16187 16223 16290 16319 |
| A5b | 73 235 263 663 750 961 965.XC 1438 1736 2706 4248 4769 4824 7028 8563 8794 8860 11536 11719 12705 14766 15326 16223 16290 16319 |
| A5b1 | 73 235 263 663 750 961 965.XC 1438 1709 1736 2706 4248 4769 4824 7028 8563 8794 8860 11536 11719 12705 14766 15326 16126 16223 16235 16290 16319 |
| A5b1a | 73 235 263 663 750 961 965.XC 1438 1709 1736 2706 4248 4769 4824 7028 8563 8794 8860 10637 11536 11719 12705 14766 15326 16126 16223 16235 16290 16319 |
| A5b1b | 73 235 263 663 750 961 965.XC 1438 1709 1736 2706 4248 4769 4824 7028 8563 8794 8860 11536 11719 12705 14766 15326 16126 16223 16234 16235 16290 16319 |
| A5b1c | 73 235 263 663 750 961 965.XC 1438 1709 1736 2706 4248 4769 4824 7028 8260 8563 8794 8860 11536 11719 12705 14766 15326 16126 16223 16235 16290 16319 |
| A5b1c1 | 73 152 235 263 663 750 961 965.XC 1438 1709 1736 2706 4248 4769 4824 5054C 7028 8260 8563 8794 8860 11536 11719 12705 14766 15326 15442 16126 16223 16235 16290 16319 |
| A5c | 73 152 235 263 663 750 1438 1736 2706 4248 4769 4824 7028 8563 8794 8860 11536 11719 12705 14766 15326 16129 16213 16223 16290 16319 |
| A5c1 | 73 152 235 263 663 750 1438 1736 2706 4248 4769 4824 7028 8563 8794 8860 11536 11719 12705 12816 14766 15326 16129 16213 16223 16290 16319 |
| A8 | 73 235 263 663 750 1438 1736 2706 4248 4769 4824 7028 8794 8860 11719 12705 14766 15326 16223 16242 16290 16319 |
| A8a | 64 73 146 235 263 663 750 1438 1736 2706 4248 4769 4824 7028 8794 8860 11719 12705 14766 15326 16223 16242 16290 16319 |
| A8a1 | 64 73 146 152 235 263 663 750 1438 1736 2706 4248 4769 4824 6962 7028 8794 8860 8865 11719 12705 12777 14766 15326 16223 16242 16290 |
| A10 | 73 235 263 663 750 1438 1736 2706 4248 4769 4824 5393 7028 7468 8794 8860 9948 10094 11719 12705 14766 15326 16223 16227C 16290 16311 16319 |
| O | 73 263 750 1438 2706 4769 6755 7028 8860 9140 11719 12705 14766 15326 16213 16223 |
| O1 | 73 152 263 509 750 1438 2706 4769 5563 6221 6755 7028 8860 9140 11719 12705 14766 15300 15326 15852 15885 16213 16223 16303 16362 |
| O1a | 73 152 263 509 750 1438 2706 4769 5460 5563 6221 6755 7028 8860 9140 11719 12705 14766 15300 15326 15852 15885 15924 16213 16223 16303 16362 |

| | |
|---|---|
| S | 73 263 750 1438 2706 4769 7028 8404 8860 11719 12705 14766 15326 16223 |
| S1 | 73 263 750 1438 2706 4769 7028 8404 8860 11719 12705 14384C 14766 15326 16075 16223 |
| S1a | 73 150G 189 215 263 271G 750 1438 2706 4769 7028 8251 8404 8506 8860 11719 12705 14384C 14766 15040 15326 16075 16172 16223 16311 |
| S2 | 73 263 750 1438 2380 2706 3438 4769 6167 7028 8404 8860 11719 12705 14766 15326 16223 |
| S+152 | 73 152 263 750 1438 2706 4769 7028 8404 8860 11719 12705 14766 15326 16223 |
| S3 | 63 64 66 73 151 152 195 263 710 750 1438 2706 4769 5302 5492 7028 8277 8278.XC 8404 8860 9938 10398 10786 11151 11719 12705 14766 15204 15317 15326 15663 15664 16140 16187 16189 16223 16287 16311 |
| S4 | 73 152 263 750 1007 1438 2706 4181 4769 5090 6528 7028 7498 8404 8860 9866 11719 12705 14766 15326 16172 16192 16223 16319 |
| S5 | 73 152 234 249d 263 508 750 1375 1438 2706 3169 3593 3996 4769 7028 8027 8404 8860 10188 11470 11719 12705 13770A 14121 14766 15326 16223 16243 16256 |
| X | 73 263 750 1438 2706 4769 6221 6371 7028 8860 11719 12705 13966 14470 14766 15326 16189 16223 16278 |
| X1'2'3 | 73 153 263 750 1438 2706 4769 6221 6371 7028 8860 11719 12705 13966 14470 14766 15326 16189 16223 16278 |
| X1'3 | 73 146 153 263 750 1438 2706 4769 6221 6371 7028 8860 11719 12705 13966 14470 14766 15326 16189 16223 16278 |
| X1 | 73 146 153 263 750 1438 2706 4769 5302 6221 6371 7028 8860 11719 12705 13966 14470 14587 14766 15326 15654 (16104) 16189 16223 |
| X1a | 73 146 153 263 750 1438 2706 4769 5302 6221 6359 6371 7028 7533 8140 8860 11719 12705 13966 14470 14587 14766 15326 15654 (16104) 16189 16223 |
| X1c | 73 146 153 263 750 1438 2706 4769 5302 6221 6371 7028 7337 8860 9615 11719 12705 13966 14470 14587 14766 15326 15654 (16104) 16189 16223 |
| X3 | 73 146 153 263 750 1438 2706 3531 4769 6221 6371 7028 8860 11719 12705 13785 13879 13966 14470 14560 14766 15326 15672 16189 16223 16278 |
| X3a | 73 146 153 256 263 750 1438 2706 3531 4769 6221 6371 7028 8860 11719 12705 13785 13879 13966 14470 14560 14766 15326 15672 16189 16223 16278 |
| X2 | 73 153 195 263 750 1438 1719 2706 4769 6221 6371 7028 8860 11719 12705 13966 14470 14766 15326 16189 16223 16278 |
| X2+225 | 73 153 195 225 263 750 1438 1719 2706 4769 6221 6371 7028 8860 11719 12705 13966 14470 14766 15326 16189 16223 16278 |
| X2a'j | 73 153 195 225 263 750 1438 1719 2706 4769 6221 6371 7028 8860 11719 12397 12705 13966 14470 14766 15326 16189 16223 16278 |
| X2a | 73 153 195 200 225 263 750 1438 1719 2706 4769 6221 6371 7028 8860 8913 11719 12397 12705 13966 14470 14502 14766 15326 16189 16213 16223 16278 |

| | |
|---|---|
| X2a1 | 73 143 153 195 200 263 750 1438 1719 2706 3552 4769 6221 6371 7028 8860 8913 11719 12397 12705 13966 14470 14502 14766 15326 16093 16189 16213 16223 16278 |
| X2a1a | 73 143 153 195 200 263 750 1438 1719 2706 3552 4769 6113 6221 6371 7028 8860 8913 11719 12397 12705 13966 14470 14502 14766 15326 16093 16189 16213 16223 16278 16357 |
| X2a1a1 | 73 143 195 200 204 263 750 1438 1719 2706 3552 4769 6113 6221 6371 7028 8860 8913 11719 12397 12705 13966 14470 14502 14766 15326 16189 16223 16278 16319 16357 |
| X2a1b | 73 143 153 195 200 263 750 1438 1719 2706 3552 4769 6221 6371 7028 8422 8860 8913 11719 12397 12705 13966 14470 14502 14766 15326 16093 16189 16213 16223 16278 |
| X2a1b1 | 73 143 153 195 200 263 750 1438 1719 2706 3552 4769 6221 6371 7028 7299 8422 8860 8913 11719 12397 12705 13966 14470 14502 14766 15326 16093 16189 16213 16223 16278 |
| X2a1b1a | 73 143 153 195 200 263 750 1438 1719 2706 3552 4769 6221 6371 6680 7028 7299 8422 8860 8913 11719 12397 12705 13966 14470 14502 14766 15326 16093 16189 16213 16223 16278 |
| X2a1c | 73 143 153 195 200 263 750 1438 1719 2706 3552 4769 6221 6371 7028 8842 8860 8913 11719 12397 12705 13966 14470 14502 14766 15326 16093 16104 16147 16189 16213 16223 16278 |
| X2a2 | 73 153 195 200 225 263 750 1415 1438 1719 2706 4769 6221 6371 7028 8860 8913 10256 11719 12397 12705 13966 14470 14502 14560 14766 15326 16189 16213 16223 16254C 16278 |
| X2j | 44.1C 73 153 195 225 263 750 794 1438 1719 2120 2706 4769 6221 6371 7028 8860 9548 11719 12397 12705 13966 14470 14766 15326 15469 16179 16189 16223 16278 16357 |
| X2b'd | 73 153 195 225 263 750 1438 1719 2706 4769 6221 6371 7028 8860 11719 12705 13708 13966 14470 14766 15326 16189 16223 16278 |
| X2b | 73 153 195 225 263 750 1438 1719 2706 4769 6221 6371 7028 8393 8860 11719 12705 13708 13966 14470 14766 15326 15927 16189 16223 16278 |
| X2b+226 | 73 153 195 225 226 263 750 1438 1719 2706 4769 6221 6371 7028 8393 8860 11719 12705 13708 13966 14470 14766 15326 15927 16189 16223 16278 |
| X2b1 | 73 153 195 225 226 263 750 1438 1719 2706 4769 6221 6371 7028 8393 8814 8860 11719 12705 13708 13966 14470 14766 15326 15927 16189 16223 16248 16278 |
| X2b2 | 73 153 195 225 226 263 750 1438 1719 2706 4722 4769 6221 6371 7028 7400A 8393 8860 11719 11908 12705 13708 13966 14470 14766 15326 15927 16189 16223 16278 |
| X2b3 | 73 153 195 225 226 263 750 1438 1719 2706 4769 6221 6371 7028 8269 8393 8860 11719 12705 13708 13966 14470 14766 14818C 15326 15927 16189 16223 16278 |
| X2b4 | 73 153 195 225 226 263 750 1438 1719 2706 3705 4769 6221 6371 7028 8393 8860 11719 12705 13708 13966 14470 14766 15326 15927 16189 16223 16278 |

| | |
|---|---|
| X2b4a | 73 153 195 225 226 263 750 1438 1719 2706 3705 4769 6221 6371 7028 8393 8592 8860 11719 12705 13708 13966 14470 14766 15326 15927 16189 16223 16278 |
| X2b4a1 | 73 153 195 225 226 263 750 1438 1719 2706 3705 4769 5375 6221 6371 7028 8393 8592 8860 11719 12705 13708 13966 14470 14766 15326 15927 16189 16223 16278 |
| X2b5 | 73 153 195 225 226 263 750 1438 1719 2706 4769 6221 6371 7028 8393 8860 10532 11719 12705 13251 13708 13966 14470 14766 15326 15927 16189 16223 16278 |
| X2b6 | 73 153 195 225 226 263 750 1438 1719 2706 4577 4769 6221 6371 7028 7570 8393 8860 11719 11914 12705 13708 13966 14016 14470 14766 15326 15927 16189 16223 16278 |
| X2b6a | 73 153 195 225 226 263 750 1438 1719 2706 4577 4769 5427 6221 6371 7028 7570 8393 8860 11719 11914 12705 13708 13966 14016 14470 14766 15326 15927 16189 16223 16278 |
| X2b7 | 73 153 195 225 226 263 750 1438 1719 2706 4216 4769 6221 6371 7028 8393 8860 11719 12705 13708 13966 14470 14766 15326 15927 16189 16223 16278 |
| X2b8 | 73 153 195 225 226 263 750 1438 1719 2706 4769 4820 6221 6371 7028 8393 8860 11719 12705 13708 13966 14470 14766 15326 15927 16189 16223 16278 |
| X2b9 | 73 153 195 225 226 263 750 1438 1719 2706 4769 6221 6371 7028 8393 8860 9830 11719 12705 13708 13966 14470 14766 15326 15927 16189 16223 16278 |
| X2b10 | 73 146 153 195 225 226 263 750 1438 1719 2706 4769 6221 6371 7028 8393 8425 8860 11719 12705 13708 13966 14470 14766 15326 15466 15927 16189 16223 16278 |
| X2b10a | 73 146 153 195 225 226 263 750 1438 1719 2706 4769 6221 6371 7028 8393 8425 8860 11719 12705 13708 13966 14470 14766 15326 15466 15927 16189 16223 16278 16365 |
| X2b11 | 73 153 189 195 225 226 263 750 1438 1719 2706 4769 6221 6371 7028 8393 8860 8910 11719 12705 13708 13966 14470 14766 15326 15927 16189 16223 16278 |
| X2b+226+16192 | 73 153 195 225 226 263 750 1438 1719 2706 4769 6221 6371 7028 8393 8860 11719 12705 13708 13966 14470 14766 15326 15927 16189 16192 16223 16278 |
| X2b12 | 73 153 195 225 226 263 750 1438 1719 2706 4769 6221 6371 7028 8393 8860 9151 11719 12705 13708 13966 14470 14766 15326 15927 16189 16192 16223 16278 |
| X2b13 | 73 153 195 225 226 263 750 1438 1719 2706 4769 6221 6371 7028 8393 8860 11719 12705 13708 13966 14470 14766 15326 15927 16189 16192 16223 16278 16320 |
| X2d | 73 195 263 750 1438 1719 2706 4769 6221 6371 6791 7028 8503 8860 11719 12705 13708 13966 14470 14766 15326 16189 16223 16278 |
| X2d1 | 73 195 204 207 263 750 1438 1719 2706 4769 5186 6221 6371 6791 7028 8503 8860 11719 11878 12705 13708 13966 14470 14766 15326 16189 16223 16278 |

| | |
|---|---|
| X2d1a | 73 195 204 207 263 750 1438 1719 2706 4769 5186 6221 6371 6626 6791 7028 8503 8860 10084 11465 11719 11878 12705 13708 13966 14470 14766 15326 16189 16223 16278 |
| X2d2 | 73 195 263 750 1438 1719 2706 4769 6221 6371 6791 7028 8503 8860 11719 12705 13708 13966 14470 14766 15300 15326 16189 16223 16278 |
| X2c | 73 153 195 225 227 263 750 1438 1719 2706 4769 6221 6371 7028 8860 11719 12705 13966 14470 14766 15326 16189 16223 16255 16278 |
| X2c1 | 73 153 195 225 227 263 750 1438 1719 2706 4769 6221 6371 7028 8705 8860 11719 12705 13966 14470 14766 15326 16189 16223 16255 16278 |
| X2c1a | 73 153 195 225 227 263 750 1438 1719 2706 4769 6221 6371 7028 8705 8860 11719 12705 13966 14470 14766 15326 16108 16189 16223 16255 16278 |
| X2c1b | 73 153 195 225 227 263 750 1438 1719 2706 4769 6221 6371 7028 8463 8705 8860 11719 12705 13966 14470 14766 15326 16189 16223 16255 16278 16300 |
| X2c1c | 73 153 195 200 225 227 263 750 1438 1719 2706 4769 6221 6371 7028 8705 8860 11719 12705 13966 14470 14766 15326 16189 16223 16255 |
| X2c1c1 | 73 153 195 200 225 227 263 750 1438 1719 2706 4769 6221 6371 7028 8705 8860 11719 12705 13966 14470 14766 15326 16189 16209 16223 16255 16278 |
| X2c1d | 73 153 195 225 227 263 750 1438 1719 2706 4769 6221 6371 7028 8705 8860 11719 12705 13966 14470 14766 15313 15326 16189 16223 16255 16278 |
| X2c1e | 73 153 195 225 227 263 750 1438 1719 2706 4541 4769 6221 6371 7028 8705 8860 11719 12705 13966 14470 14766 15326 16189 16223 16248 16255 16278 |
| X2c2 | 73 153 195 225 227 263 750 1438 1719 2706 4769 6221 6371 7028 8860 11719 12705 13966 14470 14766 15314 15326 16189 16223 16255 16278 |
| X2e | 73 153 195 225 263 750 1438 1719 2706 4769 6221 6371 7028 8860 11719 12705 13966 14470 14766 15310 15326 16189 16223 16278 |
| X2e1 | 73 153 195 225 263 750 1438 1719 2706 4769 6221 6371 7028 8860 11719 12705 13966 14470 14766 15310 15326 16189A 16223 16278 |
| X2e1a | 73 153 195 225 263 750 1438 1719 2706 4769 6221 6371 7028 8860 9380 11719 12705 13966 14470 14766 15310 15326 16189A 16223 16278 |
| X2e1a1 | 73 153 195 225 263 750 1438 1719 2706 4769 6221 6371 7028 8860 9380 11719 12705 13966 14470 14766 15310 15326 16134 16189A 16223 16278 16311 |
| X2e1b | 73 153 195 263 750 1438 1719 2706 4769 6221 6371 7028 8860 11719 12705 13966 14470 14766 15310 15326 16126 16189A 16223 16278 |
| X2e2 | 73 153 195 263 750 1438 1719 2706 4769 6221 6371 7028 8860 11719 12084 12705 13966 14470 14766 15310 15326 16189 16223 16278 |
| X2e2a | 73 153 195 263 750 1438 1719 2706 3948 4769 6221 6371 7028 8860 11719 12084 12705 13966 14470 14766 15310 15326 16189 16223 16278 |
| X2e2a1 | 73 153 195 263 750 1438 1719 2706 3948 4769 6221 6371 7028 8860 11719 12084 12705 13327 13966 14470 14766 15310 15326 16189 16223 |
| X2e2a2 | 73 152 153 195 263 750 1438 1719 2706 3948 4769 6221 6371 7028 8856 8860 11719 12084 12705 13966 14470 14766 15310 15326 16189 16223 16278 |

| | |
|---|---|
| X2e2b | 73 153 195 263 750 1438 1719 2706 4769 6221 6371 7028 8860 11719 12084 12705 13966 14470 14766 15310 15326 16189 16223 16265 16278 |
| X2e2b1 | 73 153 195 263 750 1438 1719 2706 4769 6221 6371 7028 7853 8860 11719 11875 12084 12705 13966 14470 14766 15310 15326 16189 16223 16265 16278 |
| X2e2c | 73 195 263 750 1438 1719 2706 4769 6221 6371 7028 8860 11719 12084 12705 13966 14470 14766 14861 15310 15326 16189 16223 16278 16316 |
| X2e2c1 | 73 195 263 750 1438 1719 2232.1A 2706 4769 6221 6371 7028 8406 8860 11719 12084 12705 13966 14025 14470 14766 14861 15310 15326 16189 16223 16278 16316 |
| X2+225+@153 | 73 195 225 263 750 1438 1719 2706 4769 6221 6371 7028 8860 11719 12705 13966 14470 14766 15326 16189 16223 16278 |
| X2g | 73 195 225 263 750 769 1438 1719 2706 4769 5111 6221 6371 7028 8860 9722 11719 12705 13368 13966 14470 14766 15326 15650 15924 16189 16223 16278 |
| X2l | 73 195 225 263 750 1438 1719 2706 4769 6221 6371 7028 8860 9899 11197 11719 12705 13966 14470 14766 15326 15358 16189 16223 16278 |
| X2+225+@16223 | 73 153 195 225 263 750 1438 1719 2706 4769 6221 6371 7028 8860 11719 12705 13966 14470 14766 15326 16189 16278 |
| X2h | 73 153 195 225 263 750 1438 1719 2706 4769 6221 6371 7028 8467 8860 9033 11719 12705 13966 14470 14766 15326 16189 16278 |
| X2i | 73 153 195 225 263 750 1438 1719 2706 4769 6221 6371 7028 8860 11719 12705 13966 14470 14766 15326 16189 16223 16248 16278 |
| X2i+@225 | 73 153 195 263 750 1438 1719 2706 4769 6221 6371 7028 8860 11719 12705 13966 14470 14766 15326 16189 16223 16248 16278 |
| X2i1 | 73 153 195 263 750 1438 1719 2706 4769 6221 6371 7028 8860 11719 12705 13966 14470 14766 15326 16189 16223 16248 16278 16527 |
| X2m'n | 73 153 195 225 226 263 750 1438 1719 2706 4769 6221 6371 7028 8860 11719 12705 13966 14470 14766 15326 16189 16223 16278 |
| X2m | 73 143 195 225 226 263 750 1438 1719 2706 4769 6221 6371 7028 8860 11719 12705 13966 14470 14766 15326 16189 16223 16278 |
| X2m1 | 73 143 195 225 226 235 263 750 1438 1719 2706 4769 6221 6371 7028 8860 11719 12705 13966 14470 14766 15326 16189 16223 16278 16292 |
| X2m2 | 73 143 195 225 226 263 750 1438 1719 2706 4059 4769 6221 6371 6713 7028 8473 8860 11719 12705 13966 14470 14766 15326 16189 16217 16223 16278 |
| X2n | 64 73 153 195 225 226 750 1438 1719 2706 4659 4769 6221 6371 7028 8860 9903 11719 12705 13966 14470 14766 15326 15793 16189 16223 16266 16278 |
| X2o | 73 153 195 225 263 750 1438 1656d 1719 2706 4769 6221 6371 7028 8860 9840A 11719 12705 13656 13966 14470 14766 15326 16189 16223 16278 |
| X2o1 | 73 153 195 263 750 1438 1656d 1719 2706 4769 6221 6371 7028 8860 9840A 11719 12705 13035A 13656 13966 14470 14766 15326 16189 16223 16278 |
| X2f | 73 153 195 257 263 750 1438 1719 2706 4769 6221 6371 7028 8860 11719 12705 13966 14470 14766 15326 16189 16223 16278 |
| X2f1 | 73 153 195 257 263 750 1438 1719 2392 2706 4769 6221 6371 7028 8860 10398 11719 12705 13966 14470 14766 15326 16189 16223 16278 16311 |

| | |
|---|---|
| X2k | 73 153 195 263 750 1438 1719 2706 4769 6221 6371 7028 8860 11719 12705 13212 13966 14470 14766 15326 16189 16223 16278 |
| X2p | 73 153 195 263 750 1438 1719 2706 4769 6221 6371 7028 7109 8860 11719 12705 13966 14470 14766 15326 16189 16223 16278 |
| X2p1 | 73 153 195 263 513 750 1438 1719 2706 4093 4769 6221 6371 7028 7109 8645 8860 9708 11719 11778 12705 13966 14470 14766 15326 16189 16223 16278 16291 |
| X4 | 73 152 195 263 750 1438 2706 4769 6221 6227 6371 7028 8860 11719 12406 12705 13966 14470 14766 15326 16189 16223 16266 16274 16278 16390 |
| R | 73 263 750 1438 2706 4769 7028 8860 11719 14766 15326 |
| R0 | 263 750 1438 2706 4769 7028 8860 14766 15326 |
| R0a'b | (58) 64 263 750 1438 2442 2706 4769 7028 8860 14766 15326 16362 |
| R0a | (58) 64 263 750 1438 2442 2706 3847 4769 7028 8860 13188 14766 15326 16126 16362 |
| R0a1 | (58) 64 263 750 827 1438 2442 2706 3847 4769 7028 8860 13188 14766 15326 16126 16362 |
| R0a1a | (58) 64 146 263 750 827 1438 2442 2706 3847 4769 7028 8292 8860 11761 13188 14766 15326 16126 16355 16362 |
| R0a1a1 | (58) 64 146 263 750 827 1438 2442 2706 3847 4769 7028 8292 8860 11761 13188 13708 14766 15326 16126 16355 16362 |
| R0a1a1a | (58) 64 146 263 750 827 1438 2442 2706 3847 4769 7028 8292 8860 11761 13188 13708 14766 15326 16126 16172 16355 16362 |
| R0a1a2 | (58) 64 93 95C 146 263 750 827 1438 2442 2706 3847 3972 4769 7028 8020 8292 8860 10652 11761 12741 13188 14766 15326 16126 16355 |
| R0a1a3 | (58) 64 146 263 750 827 1438 2069 2442 2706 3847 4703 4769 7028 8292 8860 9647 11761 13188 13479T 14766 15326 16126 16355 16362 |
| R0a1a4 | (58) 64 146 263 750 827 1438 2442 2706 3438 3847 4769 5120 5333 7028 8292 8860 11761 13188 14766 15326 16126 16355 16362 |
| R0a1+152 | (58) 64 152 263 750 827 1438 2442 2706 3847 4769 7028 8860 13188 14766 15326 16126 16362 |
| R0a1b | 26 (58) 64 152 263 750 827 1438 2442 2706 3847 4769 7028 8860 13188 14766 15326 16093 16126 16189 16362 |
| R0a+60.1T | (58) 60.1T 64 263 750 1438 2442 2706 3847 4769 7028 8860 13188 14766 15326 16126 16362 |
| R0a2'3 | (58) 60.1T 64 263 750 1438 2442 2706 3847 4769 7028 8860 13188 14766 15326 15674 16126 16362 |
| R0a2 | (58) 60.1T 64 263 750 1438 2355 2442 2706 3847 4769 7028 8860 13188 14766 15326 15674 16126 16362 |
| R0a2a | (58) 60.1T 64 263 750 1438 2355 2442 2706 3847 4769 7028 8860 13188 14544 14766 15326 15674 16126 16362 |
| R0a2a1 | (58) 60.1T 64 263 750 1438 2355 2442 2706 3847 4769 7028 7249 8860 13188 14544 14766 15326 15674 16126 16362 |
| R0a2b | (58) 60.1T 64 263 750 1438 2355 2442 2706 3847 4769 5237 7028 8860 13188 14766 15326 15674 15924 16126 16305T 16362 |
| R0a2c | (58) 60.1T 64 263 750 1438 2355 2442 2706 3847 4769 7028 8860 13188 14766 15326 15674 16126 16304 16362 |

| | |
|---|---|
| R0a2d | (58) 60.1T 64 263 750 1438 2355 2442 2706 3438 3847 4769 7028 8860 10728 13188 14766 15326 15674 16126 16362 |
| R0a2e | (58) 60.1T 64 263 750 1438 2355 2442 2706 3847 4106 4769 7028 7256 8701 8860 10493G 13188 14766 15326 15674 16126 16362 |
| R0a2f | (58) 60.1T 64 263 750 1438 2355 2442 2706 3847 4769 7028 8251 8860 13188 14766 15326 15674 16126 16362 |
| R0a2f1 | (58) 60.1T 64 131 263 750 1438 2355 2442 2706 3847 4769 7028 7837 8251 8860 12542 13188 13708 13827 14766 15326 15674 16126 16362 |
| R0a2f1a | (58) 60.1T 64 131 263 750 1438 2355 2442 2706 3847 4769 7028 7837 8251 8860 11365 12542 13188 13708 13827 14766 15326 15674 16126 |
| R0a2f1b | (58) 60.1T 64 131 263 750 1438 2355 2442 2706 3847 4769 7028 7837 8251 8860 12542 13188 13708 13827 14766 15326 15674 16126 16207 |
| R0a2g | (58) 60.1T 64 263 750 1438 2355 2442 2706 3847 4769 7028 8860 9128 13188 14766 15326 15674 16126 16362 |
| R0a2h | (58) 60.1T 64 263 750 1438 2355 2442 2706 3847 4769 7028 8860 13188 14766 15326 15674 16126 16172 16184A 16362 |
| R0a2i | (58) 60.1T 64 263 750 1438 2355 2442 2706 3847 4769 7028 8860 9337 11944 13188 14766 15326 15674 16092 16126 (16278) 16362 |
| R0a2+195 | (58) 60.1T 64 195 263 750 1438 2355 2442 2706 3847 4769 7028 8860 13188 14766 15326 15674 16126 16362 |
| R0a2j | (58) 60.1T 64 152 195 263 750 1438 2355 2442 2706 3397 3847 4769 7028 8860 13188 14766 15326 15674 16126 16362 |
| R0a2k | (58) 60.1T 64 263 750 1438 2355 2442 2706 3847 4769 7028 8860 12295 13188 14766 15326 15674 16126 16362 |
| R0a2k1 | (58) 60.1T 64 263 750 1438 2355 2442 2706 3531 3644 3847 3918 4769 5557 7028 8860 10398 12295 13188 14766 15326 15674 16114 16126 16325 16362 |
| R0a2l | (58) 60.1T 64 263 750 1438 2179 2355 2442 2706 3847 4769 7028 7471d 8860 13188 14392 14766 15326 15674 16126 16362 |
| R0a2m | (58) 60.1T 64 263 750 1438 2355 2442 2706 3847 4767 4769 7028 8860 13188 14766 15326 15674 16126 16362 |
| R0a2n | (58) 60.1T 64 263 750 1438 2355 2442 2706 3847 4769 7028 8860 11914 13188 14766 15326 15674 16126 16266 16362 |
| R0a3 | (58) 60.1T 64 263 750 1438 2442 2706 3847 4769 7028 8860 13188 14766 15326 15466 15674 16126 16362 |
| R0a3a | (58) 60.1T 64 263 750 1438 2442 2706 3847 4769 7028 8860 12474 13188 14766 15326 15466 15674 16126 16362 |
| R0a4 | (58) 60.1T 64 150 263 750 1438 2351 2442 2706 3847 4769 7028 8860 9531 13188 14766 15326 16126 16362 |
| R0b | (58) 64 93 152 263 750 1438 1719 2442 2706 4769 4924 5205 7028 8772 8860 9055 10101 10775 11764 14766 15326 15613 15628 16301 16362 |
| HV | 263 750 1438 2706 4769 7028 8860 15326 |
| HV0 | 72 263 750 1438 2706 4769 7028 8860 15326 16298 |
| HV0a | 72 263 750 1438 2706 4769 7028 8860 15326 15904 16298 |
| HV0a1 | 72 263 750 1438 2706 4769 7028 8860 10196 15326 15904 16126 16298 |
| HV0a1a | 72 200 263 750 1438 2706 3200 4769 7028 8860 10196 15326 15904 16126 16298 16346C |
| V | 72 263 750 1438 2706 4580 4769 7028 8860 15326 15904 16298 |

| | |
|---|---|
| V1 | 72 263 750 1438 2706 4580 4769 7028 8860 8869 15326 15904 16298 |
| V1a | 72 263 750 1438 2706 4580 4639 4769 7028 8860 8869 15326 15904 16298 |
| V1a1 | 72 263 750 1438 2706 4580 4639 4769 5263 7028 8860 8869 15326 15904 16298 |
| V1a1a | 72 263 485 750 1438 2706 4580 4639 4769 5263 7028 8860 8869 15326 15904 16183 16298 |
| V1a1a1 | 72 227 263 485 750 1438 2706 4580 4639 4769 5263 7028 8860 8869 15326 15904 16183 16298 |
| V1a1b | 72 263 750 1438 2706 4580 4639 4769 5263 7028 8860 8869 12490 15326 15904 16298 |
| V1b | 72 263 750 1438 2706 4580 4769 7028 8860 8869 15326 15904 16290 |
| V2 | 72 263 750 1438 2706 4580 4769 7028 8860 13105 15326 15904 16298 |
| V2a | 72 263 750 1438 2706 4580 4769 7028 8860 12438 13105 15326 15904 16298 |
| V2a1 | 72 263 750 1438 2706 4580 4769 7028 8860 9088 12438 13105 14793 15326 15904 16298 |
| V2a1a | 72 263 750 1438 2706 4580 4769 5250 7028 8860 9088 12438 13105 14793 15326 15904 16298 |
| V2b | 72 263 750 1438 2706 4580 4769 7028 8860 13105 14770 15326 15904 16298 |
| V2b1 | 72 263 750 1438 2706 4580 4769 7028 8860 13105 14770 15326 15773 15904 16298 |
| V2b2 | 72 263 750 1438 2706 4580 4769 5420 7028 8860 13105 14770 15326 15904 16298 |
| V2c | 72 263 750 1438 2706 4580 4769 7028 8860 9210 13105 15326 15904 |
| V3 | 72 263 750 1438 2706 4580 4769 7028 8860 12810 15326 15904 16298 |
| V3a | 72 263 750 1438 2706 4580 4769 7028 8860 12810 15326 15904 16240 16298 |
| V3a1 | 72 263 750 1438 2706 4580 4769 7028 8860 11337 12810 15326 15904 16240 16298 |
| V3b | 72 263 750 1438 2706 4550 4580 4769 7028 8860 12810 15326 15346 15904 16298 |
| V3c | 72 263 750 1438 2706 4580 4769 7028 8860 12810 15326 15904 16216 16298 |
| V4 | 72 263 750 1438 2706 4580 4769 7028 8860 15250 15326 15904 16298 |
| V5 | 72 263 750 1438 2706 4580 4769 7028 8860 14550 15326 15904 16203 16298 |
| V6 | 72 263 750 1438 2706 4580 4769 7028 8860 15326 15904 16162 16298 |
| V7 | 72 93 263 750 1438 2706 4580 4769 7028 7444 8860 15326 15904 16298 |
| V7a | 72 93 263 750 1438 2706 4580 4769 7028 7444 8860 11899 15326 15904 16153 16298 |
| V7a1 | 72 93 263 750 1438 2706 3549 4580 4769 7028 7444 8860 11899 15326 15904 16153 16298 |
| V7b | 72 93 263 750 1438 2706 4580 4769 7028 7444 8860 8910 14121 15326 15904 16114 |
| V8 | 72 263 750 1438 2706 4580 4769 7028 8860 13350 14016 15326 15904 16298 |
| V9 | 72 204 207 263 750 1438 2706 4580 4769 7028 8860 15326 15904 16298 |

| | |
|---|---|
| V9a | 72 204 207 263 750 1438 2706 4580 4769 7028 8179 8860 11581 13759 15326 15904 |
| V9a1 | 72 204 207 263 750 1438 2706 4580 4769 7028 8179 8860 11581 13759 15326 15904 16192 16219 |
| V9a2 | 72 204 207 263 750 1438 2706 4580 4769 7028 8179 8860 9887 11581 13759 15326 15904 |
| V10 | 72 263 750 1438 2706 4580 4769 7028 8860 9368 15326 15904 16298 |
| V10a | 72 263 750 1438 2706 4580 4769 7028 8860 9368 15326 15904 16261 16298 16311 |
| V10b | 72 263 750 1438 2706 4580 4769 7028 8860 9254 9368 15326 15904 16298 |
| V10b1 | 72 263 750 1438 2706 4580 4769 7028 8860 9254 9368 9932 15326 15904 16298 |
| V10b2 | 72 263 750 1438 2706 4580 4769 7028 8860 9098G 9254 9368 15326 15904 16298 |
| V11 | 72 263 750 1438 2706 4580 4769 7028 8251 8860 11620 15326 15904 |
| V12 | 72 263 750 1438 2706 4580 4769 7028 7270 8860 15326 15904 16298 |
| V13 | 72 263 750 1438 2706 4580 4769 7028 8860 12795 15326 15784 15904 16298 |
| V14 | 72 263 750 1438 2706 4580 4769 7028 8843 8860 15326 15904 16298 |
| V15 | 72 263 750 1438 2706 4221 4580 4769 7028 8860 15326 15904 16298 |
| V15a | 72 263 750 1438 2706 4221 4580 4769 7028 8860 10308 15326 15904 |
| V16 | 72 263 750 1438 2706 4580 4769 7028 8860 15326 15904 16298 16301 |
| V17 | 72 263 750 1438 1537 2706 4580 4769 7028 8860 8982 15326 15904 16298 |
| V18 | 72 263 508 750 1438 2706 4580 4769 7028 8860 15326 15904 16298 |
| V18a | 72 263 508 750 1438 2706 4580 4769 7028 8860 14914C 15326 15904 16298 |
| V+150 | 72 150 263 750 1438 2706 4580 4769 7028 8860 15326 15904 16298 |
| V19 | 72 150 263 750 1438 2706 4580 4769 7028 8860 15326 15904 16298 16362 |
| V22 | 72 150 263 709 750 1438 2706 4580 4769 7028 7765 8860 15326 15904 16298 |
| V+@16298 | 72 263 750 1438 2706 4580 4769 7028 8860 15326 15904 |
| V20 | 72 263 750 1438 2706 4580 4769 7028 8584 8860 15326 15904 16256 |
| V+@72 | 263 750 1438 2706 4580 4769 7028 8860 15326 15904 16298 |
| V21 | 263 750 1438 2706 4580 4769 7028 8860 15326 15431 15904 16298 16390 |
| V23 | 72 263 750 1438 2706 4580 4769 6734 7028 8860 15326 15904 16298 |
| V24 | 72 263 750 1438 2706 3573 4580 4769 7028 8860 15326 15904 16298 |
| V25 | 72 263 750 1438 2706 4580 4769 7028 8860 14629 15326 15904 16298 |
| V26 | 72 263 750 1438 2706 4580 4769 7028 8860 12681 15326 15904 16298 |
| V27 | 72 263 569 750 1438 2706 4580 4769 7028 8860 15326 15904 16298 |
| V28 | 72 263 750 1438 2706 4580 4769 7028 8860 10256 15326 15904 16298 |
| HV0+195 | 72 195 263 750 1438 2706 4769 7028 8860 15326 16298 |
| HV0b | 72 195 198 263 750 1438 2706 4769 7028 8860 15326 16298 |
| HV0c | 72 195 263 750 1438 2706 4769 6413 7028 8860 15326 16298 |
| HV0d | 72 195 263 750 1438 2706 4769 7028 8860 15110 15326 16298 |
| HV0e | 72 195 263 750 1438 2706 4769 7028 8860 10609 15326 15454 16298 |
| HV0f | 195 263 750 1438 2706 4769 7028 8706 8860 15326 16298 |
| HV0g | 72 195 263 750 1438 2706 4047 4769 7028 8860 15172 15326 16129 16298 |
| HV1 | 263 750 1438 2706 4769 7028 8014T 8860 15326 16067 |

| | |
|---|---|
| HV1a'b'c | 263 750 1438 2706 4769 7028 8014T 8860 15218 15326 16067 |
| HV1a | 263 750 1438 2706 4769 7028 8014T 8277 8860 15218 15326 16067 |
| HV1a1 | 150 263 750 1438 2706 4769 7028 8014T 8277 8860 15218 15326 15927 16067 16355 |
| HV1a1a | 150 263 750 1438 2706 4227 4769 7028 8014T 8277 8860 9554 15218 15326 15927 16067 16355 |
| HV1a1b | 150 263 750 1438 2706 4769 6150 7028 7379 8014T 8277 8860 15218 15326 15927 16067 16355 |
| HV1a2 | 263 750 1438 2706 4596 4769 7028 8014T 8277 8860 15218 15326 16067 |
| HV1a2a | 263 750 1438 2706 4596 4769 7028 8014T 8277 8860 14587 15218 15326 16067 |
| HV1a2b | 93 263 750 1438 2706 4596 4769 7028 8014T 8277 8860 15218 15326 16067 |
| HV1a3 | 263 750 1438 2706 3421 4769 7028 8014T 8277 8860 15218 15326 16067 16327A |
| HV1a3a | 263 750 1438 2706 3421 4769 7028 8014T 8277 8860 14443 15218 15326 16067 16327A |
| HV1b | 263 750 1438 2706 4769 7028 8014T 8860 12696 15218 15326 16067 |
| HV1b1 | 263 750 1438 2626 2706 4739 4769 7028 7598 8014T 8860 12696 15218 15326 16067 16274 |
| HV1b1a | 263 750 1438 2626 2706 4739 4769 7028 7598 8014T 8860 11314 12696 14861 15218 15326 16067 16274 |
| HV1b1b | 263 750 1438 2626 2706 4739 4769 5656 7028 7598 8014T 8860 12696 15218 15326 16067 16274 |
| HV1b+152 | 152 263 750 1438 2706 4769 7028 8014T 8860 12696 15218 15326 16067 |
| HV1b2 | 152 263 750 1438 2706 3547 4769 6023 7028 8014T 8860 12696 15218 15326 16067 16189 |
| HV1b3 | 152 195 263 750 1438 2706 4769 5250 7028 8014T 8860 12696 15218 15326 16067 |
| HV1b3a | 151 152 183 195 263 750 1438 2706 4769 5250 7028 7664 8014T 8860 12696 15172 15218 15236 15326 15519 16067 |
| HV1b3b | 152 195 263 750 1438 2706 4769 5250 7028 8014T 8860 10295 10750 12696 14161 15218 15326 16067 16311 |
| HV1c | 263 750 1438 2706 4769 7028 8014T 8860 13933 15218 15326 16067 |
| HV1d | 146 249d 263 750 1438 2308 2706 4769 7028 7372 8014T 8860 11476 12279 13143 13650 13708 15326 16067 16278 16362 |
| HV+73 | 73 263 750 1438 2706 4769 7028 8860 15326 |
| HV2 | 73 152 263 750 1438 2706 4769 7028 8860 15326 16217 |
| HV2a | (72) 73 152 195 263 750 1438 2706 4769 7028 7193 8860 9336 11935 12061 15326 16217 |
| HV2a1 | (72) 73 152 195 263 750 1438 2706 4769 5147 7028 7193 8860 9336 11935 12061 15326 16214 16217 16335 |
| HV2a2 | (72) 73 152 195 263 750 1438 2706 4769 5153 7028 7193 7861 8860 9336 11935 12061 15326 16217 |
| HV2a3 | (72) 73 152 195 263 739 750 1005 1438 2706 4769 7028 7193 8860 9336 11935 12061 15326 16217 |
| HV20 | 73 263 750 1438 2706 4769 7028 8860 13866 15326 |
| HV4 | 263 750 1438 2706 4769 7028 7094 8860 15326 |

| | |
|---|---|
| HV4a | 263 750 1438 2706 4769 7028 7094 8860 15326 16221 |
| HV4a1 | 263 750 1438 2706 4769 7028 7094 8860 13680 15326 16221 |
| HV4a1+16291 | 263 750 1438 2706 4769 7028 7094 8860 13680 15326 16221 16291 |
| HV4a1a | 263 750 1438 2706 4769 7028 7094 8860 9950 13680 15326 16221 16291 |
| HV4a1a1 | 152 263 750 1438 2706 4769 7028 7094 7645 8860 9950 11365 13680 15326 16221 16291 |
| HV4a1a2 | 73 263 750 1438 2706 4769 7028 7094 7843 8860 9950 13680 15326 16221 16291 |
| HV4a1a3 | 263 750 1438 2706 4769 7028 7094 8860 9950 12972 13680 15326 16221 16291 |
| HV4a1a4 | 263 750 1438 2706 4452 4769 7028 7094 8860 9950 13680 15326 16221 16291 |
| HV4a2 | 263 750 1438 2706 4769 7028 7094 7805 8860 15326 16129 16221 |
| HV4a2a | 263 750 1438 2706 4769 7028 7094 7805 8860 15326 16129 16287 16311 |
| HV4a2b | 263 750 1438 2706 4769 7028 7094 7805 8860 11671 15326 16129 16221 |
| HV4b | 263 750 1438 1715 2706 4769 7028 7094 8860 15326 16069 |
| HV4c | 263 750 1438 2706 2758 3834 4769 7028 7094 8860 10356 15326 |
| HV5 | 263 750 1438 2706 4769 7028 8860 13105 15326 |
| HV5a | 263 750 1438 2706 4769 7028 8860 12133 13105 15326 |
| HV5b | 150 263 750 1438 2706 4769 7028 8860 13105 15326 15766 |
| HV+16311 | 263 750 1438 2706 4769 7028 8860 15326 16311 |
| HV6 | 263 750 1438 2706 4769 6755 7028 8860 15326 16172 16311 |
| HV6a | 263 750 1438 2706 3507 4769 6755 7028 8545 8860 9266 15326 16113C 16172 16311 |
| HV7 | 263 750 1438 2706 4769 5471 7028 8860 14560 15326 16278 16311 |
| HV8 | 263 750 1438 2706 4769 7028 8860 15326 16311 16354 |
| HV9 | 263 750 1438 2706 4769 7028 8860 8994 15326 16311 |
| HV9+152 | 152 263 750 1438 2706 4769 7028 8860 8994 15326 16311 |
| HV9a | 131 152 263 750 1438 2706 4769 7028 8860 8994 15326 16311 |
| HV9a1 | 131 152 263 750 1438 2706 4769 5480 7028 8860 8994 11257 15326 16311 |
| HV9a1a | 131 152 263 750 930 1438 2706 4769 5480 7028 8860 8994 11257 15326 16311 |
| HV9b | 263 750 1438 2706 4769 7028 8860 8994 15326 16249 16311 |
| HV9c | 263 750 1438 2706 4769 6248 7028 8860 8994 15326 |
| HV10 | 263 750 1438 2706 4769 7028 8860 13449 15326 16311 |
| HV11 | 263 750 1438 2706 4769 6040 7028 8860 15326 16311 |
| HV11a | 263 750 1438 2706 4769 6040 7028 8860 9000 10283 15326 16311 |
| HV14 | 263 480 750 1438 2706 4769 7028 8860 15115 15326 16311 |
| HV14a | 263 480 750 1438 2706 4655 4769 7028 8860 15115 15326 16311 |
| HV15 | 263 750 1438 2706 4769 5746 7028 8860 15326 16234 16311 |
| HV16 | 263 750 1438 2706 4769 7028 8860 12492T 15326 16311 |
| HV17 | 263 750 1438 2706 3397 4769 7028 8860 15326 16111 16311 |
| HV17a | 263 549 750 1438 2706 3397 4769 7028 8860 15326 16111 16311 |
| HV22 | 146 263 750 1438 2706 4769 4997 7028 8860 15326 16038 16311 |
| HV23 | 263 750 1438 2706 4769 7028 8860 9548 15326 16311 |
| HV24 | 263 750 1438 1601 2706 4769 7028 8860 15326 16311 |
| HV12 | 263 750 1438 2706 4769 7028 8860 13889 15326 |
| HV12a | 263 750 1438 2706 4769 7028 8860 13889 15326 16220C 16292 |

| | |
|---|---|
| HV12a1 | 152 263 750 1438 2706 3892 4254 4769 7028 8860 13889 14344 15326 16220C 16243 16292 16342 |
| HV12b | 150 263 750 1438 2706 4769 7028 8860 13889 15326 15682 |
| HV12b1 | 150 263 750 1438 2706 4769 7028 8860 13889 15326 15682 16356 |
| HV12b1a | 150 263 337 594 750 1438 2706 4769 7028 8860 13572 13889 14569 15326 15682 16356 |
| HV13 | 263 750 1438 2706 4769 7028 8860 15326 16357 |
| HV13a | (72) 263 750 1438 2706 4769 7028 8774 8860 9027 10143 11101 12879 15326 16311 16357 |
| HV13b | 263 750 1438 2706 3290 4769 7028 8860 15326 16357 |
| HV18 | 263 750 1438 2706 4769 7028 8860 9039 15326 16189 |
| HV19 | 263 750 1438 2706 4769 4823 7028 8860 15326 16260 16399 |
| HV21 | 263 750 1438 2706 4769 7028 8860 15326 16169 |
| H | 263 750 1438 4769 8860 15326 |
| H1 | 263 750 1438 3010 4769 8860 15326 |
| H1a | 73 263 750 1438 3010 4769 8860 15326 16162 |
| H1a1 | 73 263 750 1438 3010 4769 6365 8860 15326 16162 16209 |
| H1a1a | 73 263 750 1438 3010 4639 4769 6365 8860 15326 16162 16209 |
| H1a1a1 | 73 263 750 1438 3010 4639 4769 6365 8860 10993 15326 16162 16209 |
| H1a1b | 73 263 750 1438 3010 4769 6365 7961 8860 15326 16162 16209 |
| H1a1c | 73 263 750 1438 3010 4769 6365 8860 15326 16162 16172 16209 |
| H1a2 | 73 263 750 1438 3010 4769 8271T 8860 15326 16162 |
| H1a3 | 73 263 750 1438 3010 4769 8860 15326 16051 16162 |
| H1a3a | 73 263 750 1438 3010 4769 8860 14978 15326 16051 16162 |
| H1a3a1 | 73 263 750 1438 3010 3396 4769 8860 14978 15326 16051 16162 |
| H1a3a2 | 42.1G 73 263 750 1438 3010 4769 8860 14978 15326 16051 16162 |
| H1a3a3 | 73 263 750 1438 3010 4769 8468 8860 14978 15326 16051 16162 16259 |
| H1a3a4 | 73 263 534 750 1438 3010 4769 8860 14978 15326 16051 16162 |
| H1a3b | 73 263 750 1438 3010 4769 8860 15326 16051 16162 16465 |
| H1a3b1 | 73 263 750 1438 3010 4769 8860 9053 15326 16051 16162 16465 |
| H1a3c | 73 263 750 1438 3010 4769 8860 15326 16051 16162 16266 |
| H1a3c1 | 73 263 750 1438 3010 4769 8860 15326 16051 16162 16213 16266 |
| H1a3d | 73 263 750 1438 3010 4769 8860 11950 15326 16051 16162 |
| H1a4 | 73 263 750 1438 3010 4769 8860 9341T 15326 16162 |
| H1a5 | 73 263 750 1438 3010 4769 8839 8860 15326 15789 16162 |
| H1a6 | 73 151 263 750 1438 3010 4769 8860 11893 15326 16162 16189 |
| H1a7 | 73 263 750 1438 3010 4769 5747 8860 15326 16162 |
| H1a8 | 73 263 750 1438 3010 4769 8537 8860 15326 16162 |
| H1a8a | 73 263 750 1438 3010 4769 7080 8537 8860 15326 16162 |
| H1a9 | 73 263 750 1438 3010 4769 8334 8860 15326 16162 16167 |
| H1+16189 | 263 750 1438 3010 4769 8860 15326 16189 |
| H1b | 263 750 1438 3010 4769 8860 15326 16189 16356 |
| H1b1 | 263 750 1438 3010 3796 4769 8860 15326 16189 16356 |
| H1b1+16362 | 263 750 1438 3010 3796 4769 8860 15326 16189 16356 16362 |
| H1b1a | 263 750 1438 3010 3796 4769 5899.XC 8348 8860 15326 16189 16356 16362 |
| H1b1b | 263 750 1438 3010 3796 4769 8860 15326 16129 16189 16355 16356 |
| H1b1c | 263 750 1438 3010 3796 4769 8860 10455 15326 16189 16356 16362 |

| | |
|---|---|
| H1b1d | 93 263 750 1438 3010 3796 4769 8860 15326 16189 16356 16362 |
| H1b1h | 263 750 1438 3010 3796 4769 7960 8860 15326 16189 16356 16362 |
| H1b1e | 263 750 1438 3010 3796 4769 8860 9698 15326 16189 16356 |
| H1b1e1 | 263 750 1438 3010 3796 4769 8860 9698 11962 15326 16189 16356 |
| H1b1f | 263 750 1438 2145 3010 3796 4769 8860 15326 16114 16189 16356 |
| H1b1g | 64 195 263 750 3010 3796 4769 8860 15326 16189 16356 |
| H1b1i | 263 750 1438 3010 3796 4769 4823 8860 15326 16189 16356 |
| H1b2 | 263 750 1438 3010 4769 8251 8860 15326 16080 16189 16356 |
| H1b2a | 183 263 750 1438 3010 4769 8251 8286 8860 15326 16080 16189 16356 16360 |
| H1b2a1 | 183 263 750 1438 3010 4769 7691 8251 8286 8860 15326 16080 16189 16356 16360 |
| H1b3 | 263 750 1438 3010 4769 8860 15326 16075 16189 16356 |
| H1b4 | 263 750 1438 3010 4769 8860 11029 15326 16189 16356 |
| H1b5 | 263 750 1438 3010 4769 8860 12130 15326 16189 16356 |
| H1f | 263 750 1438 3010 4452 4769 8860 9066 15326 16189 |
| H1f+16093 | 263 750 1438 3010 4452 4769 8860 9066 15326 16093 16189 |
| H1f1 | 263 750 1438 3010 4452 4769 7309 8860 9066 15326 16093 16189 |
| H1f1a | 263 459d 750 1438 3010 4452 4769 7309 8860 9066 15326 16093 16189 |
| H1g | 263 750 1438 3010 4769 8860 14212 15326 16189 |
| H1g1 | 263 750 1438 3010 4769 8602 8860 14212 15326 16189 |
| H1g2 | 263 750 1438 3010 4769 8860 9230 14212 15326 16189 |
| H1k | 263 750 1438 3010 4769 8860 15326 16189 16290 |
| H1k1 | 263 750 1438 3010 4769 5206 8860 15326 15766 16189 16290 |
| H1k1a | 263 750 1438 2887 3010 4769 5206 8860 15326 15766 16189 16290 |
| H1y | 263 750 1438 3010 4769 8860 15299 15326 16189 |
| H1z | 263 327 750 1438 3010 4769 8860 11428 15326 16189 |
| H1z1 | 263 327 750 1438 3010 4769 8860 10632 11428 15326 16189 16311 |
| H1aa | 263 750 1438 3010 4131 4769 8860 15326 16189 |
| H1aa1 | 263 750 1438 3010 4131 4769 8860 11914 15326 16189 |
| H1ab | 263 750 1438 3010 4769 8860 15047 15326 16189 |
| H1ab1 | 263 750 1438 3010 4769 8860 15047 15326 16189 16234 |
| H1ac | 263 750 1438 3010 4769 8860 11893 15326 16189 |
| H1ad | 263 750 1438 3010 3504 4769 8860 15326 16189 |
| H1cc | 72 263 750 1438 3010 4769 6261 8860 15326 16189 16291 |
| H1c | 263 477 750 1438 3010 4769 8860 15326 |
| H1c1 | 263 477 750 1438 3010 4769 8860 9150 15326 16263 |
| H1c1a | 263 477 750 1438 3010 4769 8860 9150 9380 15326 16263 |
| H1c1a1 | 263 477 750 1438 3010 4769 8860 9007 9150 9380 15326 16263 |
| H1c1b | 263 477 750 1438 3010 4769 8860 9150 15326 16263 16316 |
| H1c1c | 263 477 750 1438 1926 3010 4769 8860 9150 15326 16263 16295 |
| H1c1+16093 | 263 477 750 1438 3010 4769 8860 9150 15326 16093 16263 |
| H1c1d | 263 477 750 1438 3010 4769 8860 9150 10192 15326 16093 16263 |
| H1c2 | 263 477 750 1438 3010 4769 8860 12858 15326 |
| H1c2a | 263 477 750 1438 3010 4769 8764 8860 12858 15326 |
| H1c3 | 257 263 477 750 1438 3010 4769 8473 8860 15326 |
| H1c3a | 146 257 263 477 750 1438 3010 4769 8473 8860 15326 16176 16219 |
| H1c3b | 257 263 477 750 1438 3010 4769 8473 8860 15326 16189 16362 |

| | |
|---|---|
| H1c4 | 263 477 750 1438 3010 4769 8860 13759 15326 |
| H1c4a | 263 477 750 1438 3010 4769 7299 8860 13759 15326 |
| H1c4a1 | 263 477 750 1438 3010 4769 7299 8860 13759 15315 15326 |
| H1c4b | 263 477 750 1438 3010 4769 8860 13759 15326 16355 |
| H1c4b1 | 263 477 750 1438 3010 4769 6074 8860 13759 15326 16355 |
| H1c5 | 263 477 750 1438 3010 4769 8803 8860 15326 |
| H1c5a | 152 249d 263 477 750 1438 3010 4769 8803 8860 15326 |
| H1c6 | 263 477 750 1438 3010 4769 5979 8860 15326 |
| H1c7 | 263 477 750 1438 3010 4769 8860 14350 15326 |
| H1c8 | 263 477 750 1438 1462 3010 4769 8860 14182A 15326 |
| H1c+152 | 152 263 477 750 1438 3010 4769 8860 15326 |
| H1c9 | 152 263 477 750 1438 3010 4769 8860 13203 15326 |
| H1c9a | 152 263 477 750 1438 3010 4769 8860 9377 13203 15326 |
| H1c10 | 263 477 750 1438 3010 3531 4769 8417A 8860 15326 |
| H1c11 | 263 477 750 1438 3010 4769 6629 8860 15326 |
| H1c12 | 263 477 750 1438 3010 4484 4769 8860 15326 |
| H1c13 | 263 477 750 1438 3010 4769 8645 8860 15326 |
| H1c14 | 263 477 750 1438 3010 4769 8860 10365 12627 15326 15758 |
| H1c15 | 263 477 750 1438 3010 4769 8860 12310.1A 15326 |
| H1c16 | 263 477 750 1438 3010 4769 6267 8860 15326 |
| H1c17 | 263 477 750 1438 3010 4769 8860 15325C 15326 |
| H1c18 | 263 477 750 1438 3010 4562 4769 8860 12636 15326 |
| H1c19 | 263 477 750 1438 3010 4769 7379 8860 15326 |
| H1c20 | 263 477 750 1438 3010 4769 8730 8860 15326 |
| H1c21 | 263 477 750 1438 3010 4767 4769 8860 15326 |
| H1c22 | 263 477 750 1438 3010 4769 8860 10679 15326 |
| H1e | 263 750 1438 3010 4769 5460 8860 15326 |
| H1e1 | 263 750 1438 3010 4769 5460 8512 8860 15326 |
| H1e1a | 263 750 1438 3010 4769 5460 8512 8860 14902 15326 |
| H1e1a1 | 93 263 750 1438 3010 4769 5301 5387 5460 8512 8860 14491 14902 15326 |
| H1e1a2 | 263 750 1438 3010 4769 5460 8512 8860 13326 14902 15326 |
| H1e1a3 | 263 750 1438 3010 4769 5460 8512 8860 14902 15326 16111A |
| H1e1a4 | 263 709 750 1438 3010 4769 5460 7196 8512 8860 11113 11371 14902 15326 16311 |
| H1e1a5 | 263 750 1438 1900C 2320 3010 4769 4823 5460 6216 8512 8860 12915A 14902 15326 15884 16066 |
| H1e1a6 | 150 263 750 1438 3010 4769 5460 8512 8860 14902 15326 |
| H1e1a7 | 263 750 1438 3010 4769 5460 8512 8860 14581 14902 15326 |
| H1e1a+16278 | 263 750 1438 3010 4769 5460 8512 8860 14902 15326 16278 |
| H1e1a8 | 195 263 750 1438 3010 4769 4781 5460 8512 8860 14902 15326 16278 |
| H1e1b | 263 453 750 1438 3010 4769 5460 8512 8860 15326 |
| H1e1b1 | 263 453 750 1438 3010 4769 5460 8512 8860 13590 15326 |
| H1e1b1a | 263 453 750 1438 3010 4769 5460 8023 8512 8860 13590 15326 |
| H1e1b1b | 263 453 750 1438 1812 3010 4673 4769 5460 8512 8860 13590 15326 |
| H1e1c | 263 750 1438 3010 3745 4769 5460 8512 8860 15326 |
| H1e2 | 263 750 1438 3010 4769 5460 8860 15326 15817 |
| H1e2a | 263 750 1438 3010 4769 5460 8860 12070 15326 15817 |
| H1e2b | 263 750 983 1438 3010 4769 5460 8860 15326 15817 |

| | |
|---|---|
| H1e2c | 73 263 750 1438 3010 4769 5460 8860 15326 15817 |
| H1e2d | 263 750 1438 3010 3548 4769 5460 8860 15326 15817 |
| H1e+16129 | 263 750 1438 3010 4769 5460 8860 15326 16129 |
| H1e3 | 93 263 750 960.XC 1438 1462 3010 4769 4883 5460 8860 15326 16129 |
| H1e4 | 263 750 1438 3010 3316 4769 5460 8860 15326 |
| H1e4a | 263 750 1438 3010 3316 4769 5460 8860 15326 16114 |
| H1e5 | 263 750 1438 3010 4769 5460 8860 15326 16256 |
| H1e5a | 263 750 1438 3010 4769 5460 8860 9110 15326 16256 |
| H1e5b | 263 750 1438 3010 4769 5460 8860 10922 15326 16256 |
| H1e6 | 263 750 1438 3010 4769 5460 8860 9554 15326 |
| H1e7 | 263 750 1438 3010 3511 4769 5460 8860 15326 |
| H1e8 | 263 750 1438 3010 4769 5460 5746 8860 15326 |
| H1e8a | 263 750 1438 3010 4769 5460 5746 7119 8860 15326 |
| H1h | 263 750 1438 3010 4769 7013 8860 15326 |
| H1h1 | 263 750 1438 3010 4769 7013 8860 11914 15326 |
| H1h2 | 263 750 1438 2851 3010 4769 7013 8860 14148 14420 15326 |
| H1+152 | 152 263 750 1438 3010 4769 8860 15326 |
| H1i | 152 263 750 1438 3010 4769 6237A 8860 15326 |
| H1i1 | 152 263 750 1438 3010 4769 6237A 8860 9300 15326 |
| H1i2 | 152 263 750 1438 3010 4769 6237A 8860 12346 15326 |
| H1i2a | 152 263 750 1438 3010 4769 6237A 8860 12026 12346 15326 |
| H1an | 152 263 750 1438 3010 4769 8860 15326 15394 |
| H1an1 | 152 263 750 1438 3010 4769 8860 13050 15326 15394 |
| H1an1a | 152 263 750 1438 3010 4454 4769 8860 13050 15326 15394 |
| H1an2 | 151 152 263 750 1438 3010 4769 8860 15326 15394 16325 |
| H1bb | 152 263 750 1438 3010 4769 8860 11864 15326 |
| H1j | 263 750 1438 3010 4733 4769 8860 15326 |
| H1j1 | 263 750 1438 3010 4733 4769 8860 15326 16129 |
| H1j1a | 263 750 1438 3010 3849 4733 4769 8860 15326 16129 |
| H1j1a1 | 263 447G 750 1438 3010 3849 4733 4769 8860 15326 16129 |
| H1j1a2 | 263 750 1438 3010 3849 4733 4769 8860 11144T 15326 16129 |
| H1j1b | 263 750 1438 3010 4733 4769 8860 11087 15326 16129 |
| H1j1c | 263 750 1438 3010 4733 4769 8860 9181 11963C 15326 16129 |
| H1j2 | 263 750 1438 1821 3010 4733 4769 8860 13708 15326 16291 |
| H1j2a | 263 316 750 1438 1821 3010 4733 4769 8860 13708 15326 16291 |
| H1j3 | 263 750 1438 3010 4733 4769 5249 8860 15326 |
| H1j4 | 263 750 1438 3010 4733 4769 8860 15326 16312 |
| H1j5 | 263 750 1438 3010 4733 4769 8860 9290A 10256 15326 |
| H1j6 | 263 709 750 1438 3010 3084 4733 4769 8860 9653 15326 |
| H1j7 | 263 750 1438 3010 4733 4769 6353 8860 14178 15326 15497 |
| H1j8 | 263 750 1438 2222G 3010 4733 4769 8860 15326 16240 |
| H1j9 | 263 750 1438 2626 3010 4733 4769 8860 15326 (16189) |
| H1m | 263 750 1438 3010 4769 8860 15323 15326 |
| H1m1 | 146 263 750 1438 3010 4171 4769 8860 9468 15323 15326 |
| H1n | 263 750 1438 2098 3010 4769 8860 15326 |
| H1n+146 | 146 263 750 1438 2098 3010 4769 8860 15326 |
| H1n1 | 146 263 750 1438 2098 3010 4769 8860 15326 15742A |
| H1n1a | 146 263 750 1438 2098 3010 4769 8860 11339 15326 15742A |

| | |
|---|---|
| H1n1b | 146 263 750 1438 2098 3010 4769 8860 15326 15742A 15924 16468 |
| H1n2 | 146 263 750 1438 2098 3010 4769 5483 8860 13431 15326 |
| H1n3 | 146 263 750 1438 2098 3010 4769 7295 8860 15326 |
| H1n4 | 146 263 750 1438 2098 3010 4769 8860 11914 15326 |
| H1n+146+195 | 146 195 263 750 1438 2098 3010 4769 8860 15326 |
| H1n5 | 146 195 263 471 750 1438 2098 3010 4769 8860 15326 |
| H1n6 | 263 552A 750 1438 2098 3010 4769 8860 11551 14221 15326 |
| H1+16355 | 263 750 1438 3010 4769 8860 15326 16355 |
| H1o | 150 263 750 1438 3010 4769 8860 14053 15326 16355 |
| H1ck | 263 750 1438 3010 4769 7604 8860 15326 16355 |
| H1p | 263 750 1438 3010 4769 8860 13470 15326 |
| H1q | 263 750 1438 3010 4769 4859 8860 15326 |
| H1q1 | 263 750 1438 3010 4769 4859 8860 12366 15326 |
| H1q1a | 263 750 1438 3010 4769 4859 8860 12366 15326 16256 |
| H1q2 | 263 750 1438 3010 4769 4859 8860 15326 15941 |
| H1q3 | 263 750 1438 3010 4769 4859 8856 8860 14258 15326 16037 |
| H1r | 263 750 1438 3010 4769 8860 9356 15326 |
| H1r1 | 146 263 750 1438 3010 4769 8860 9356 15326 16278 |
| H1s | 263 750 1438 3010 4769 8572 8860 15326 |
| H1s1 | 263 750 1438 3010 4769 8572 8860 12172 15326 |
| H1t | 263 750 1438 3010 4769 8860 9986 15326 |
| H1t1 | 263 750 1438 3010 4769 8860 9986 14129 15326 |
| H1t1a | 263 750 1438 3010 4769 8860 9986 14129 14224 15326 |
| H1t1a1 | 263 750 1438 3010 4769 8860 9986 14129 14224 15326 16266 |
| H1t2 | 263 750 1438 3010 4769 5978 8860 9986 15326 |
| H1u | 263 750 1438 3010 4769 8860 9923 15326 |
| H1u1 | 263 750 1438 3010 4769 7975 8860 9923 15326 |
| H1u2 | 263 750 1438 3010 3483 4769 8860 9923 15326 16320 |
| H1v | 263 750 1438 3010 4769 8860 10314 15326 |
| H1v1 | 263 750 1438 3010 4313 4769 8860 10314 15326 |
| H1v1a | 263 750 1438 3010 4313 4769 8860 9148 10314 15326 |
| H1v1b | 263 750 1438 3010 4313 4769 8860 10314 14560 15326 |
| H1w | 263 750 1438 3010 4769 8860 8966 15326 |
| H1x | 263 750 1438 3010 4769 7765 8860 10410A 15326 16037 16256 |
| H1ae | 263 750 1438 3010 4769 8860 15326 15553 |
| H1ae1 | 263 750 1438 3010 4769 8470 8860 15326 15553 |
| H1ae2 | 263 750 1438 3010 4769 8860 11696 15326 15553 |
| H1ae2a | 263 750 1438 3010 4769 8843 8860 11696 15326 15553 |
| H1ae3 | 263 750 1438 3010 4769 8723 8860 12771 15326 15553 |
| H1ae3a | 263 750 1438 3010 4769 5231 8723 8860 12771 15326 15553 |
| H1af | 263 750 1438 3010 4769 8860 15326 16357 |
| H1af1 | 263 750 1438 3010 4769 8860 15326 15646 16357 |
| H1af1a | 263 750 1438 3010 4769 8860 9078 15326 15646 16357 |
| H1af1b | 263 750 1438 3010 4769 8860 15326 15646 16129 16357 |
| H1af2 | 263 750 1438 3010 4137 4769 8860 15326 16357 |
| H1ag | 263 750 1438 3010 4769 8860 14869 15326 |
| H1ag1 | 263 750 1438 3010 4769 6272 8860 14869 15326 |
| H1ag1a | 263 750 1438 3010 4769 6272 8860 12940 14869 15326 16218 |

| | |
|---|---|
| H1ag1b | 263 750 1438 3010 4769 6272 6734 8860 14869 15326 |
| H1ah | 263 750 1438 3010 4769 8860 8950 12507 15326 |
| H1ah1 | (200) 263 750 1438 3010 3411 4769 7412 8860 8950 12507 15326 16248 |
| H1ah2 | 263 750 1438 3010 4769 6261 8860 8950 12507 15326 16212 |
| H1ai | 263 750 1438 3010 4769 8860 15088 15326 |
| H1ai1 | 263 750 1438 3010 4769 6722 8860 15088 15326 |
| H1aj | 263 750 1438 3010 4769 8860 14133 15326 |
| H1aj1 | 263 750 1438 3010 4769 8618 8860 14133 15326 16192 |
| H1aj1a | 207 263 750 1438 3010 4769 8618 8860 9621 14133 15326 16172 16192 16456 |
| H1ak | 263 750 1438 3010 3666 4769 8860 15326 |
| H1ak1 | 263 750 1438 3010 3666 4769 5393 8860 15326 |
| H1ak2 | 263 750 1438 3010 3666 4435 4769 8860 15326 |
| H1am | 263 750 1438 3010 4763A 4769 8860 15326 |
| H1am1 | 263 750 1438 3010 4763A 4769 5309G 8860 15326 |
| H1+16278 | 263 750 1438 3010 4769 8860 15326 16278 |
| H1ao | 93 146 263 750 1438 3010 4769 8860 11809 15326 16278 |
| H1ao1 | 93 146 263 750 1438 3010 4769 8860 11809 11914 15326 16278 |
| H1cg | 263 750 1438 3010 4767 4769 8860 15326 16278 |
| H1ap | 263 750 1438 3010 4769 8410 8860 15326 |
| H1ap1 | 152 263 750 1438 3010 4769 5780 8410 8860 15326 16189 |
| H1aq | 263 750 1438 3010 4769 8860 13386 15326 |
| H1aq1 | 263 750 1438 3010 4769 8860 13386 15304A 15326 16218 |
| H1ar | 183 263 750 1438 3010 4769 8860 15326 16468 |
| H1ar1 | 183 263 750 1438 3010 4769 8860 11890 15326 16302 16468 |
| H1as | 263 750 1438 3010 4688 4769 8860 15326 |
| H1as1 | 150 263 750 1438 3010 4688 4769 6216 8860 15326 |
| H1as1a | 150 263 750 1438 3010 4688 4769 6216 8860 15326 16261 |
| H1as2 | 263 750 980 1438 3010 4688 4769 8860 15326 |
| H1at | 263 750 1438 3010 4769 8860 15326 15758 |
| H1at1 | 263 750 1438 3010 4769 7337 8860 15326 15758 |
| H1at1a | 263 750 1438 3010 4769 7337 8860 12136 15326 15758 |
| H1au | 263 750 1438 3010 4769 8860 15326 16148 |
| H1au1 | 263 750 1438 3010 4769 8860 11515A 15326 16148 |
| H1au1a | 263 750 1438 1664 3010 4769 8860 11515A 15326 16148 |
| H1au1b | 263 750 1438 1719 3010 4769 8860 11515A 15326 16148 |
| H1av | 263 750 1438 3010 4769 7762 8860 15326 |
| H1av1 | 150 263 750 1438 3010 4769 7762 8860 15326 |
| H1av1a | 150 263 750 1438 3010 4674 4769 7762 8860 15326 |
| H1aw | 263 460 750 1438 3010 4769 8701 8860 15326 16129 |
| H1aw1 | 263 460 750 1438 3010 4769 7403 8701 8860 15326 16129 |
| H1ax | 263 750 1438 3010 4769 5054C 7471 8429 8860 15326 |
| H1ax1 | 263 750 1438 3010 4769 5054C 7471 8429 8860 14861 15326 |
| H1ay | 263 750 1438 3010 4769 8860 9708 15326 |
| H1az | 263 750 1438 3010 4769 8308 8860 15326 |
| H1ba | 263 750 1438 3010 4769 8860 15326 16270 |
| H1ba1 | 263 750 1438 3010 4769 8860 15326 16270 16276 |
| H1bc | 152 263 750 1438 3010 4769 8860 10325 15326 |

| | |
|---|---|
| H1bd | 263 750 1438 3010 4769 8860 9966 15326 |
| H1be | 263 750 1438 3010 4769 8860 10750 13035 15326 |
| H1+16239 | 263 750 1438 3010 4769 8860 15326 16239 |
| H1bf | 263 750 1438 3010 4769 8860 13768 15326 16239 |
| H1bf1 | 263 750 1438 3010 4769 8860 13768 15326 16239 16242 |
| H1bg | 263 750 1438 3010 4769 5054 7849 8860 15326 16239 |
| H1bh | 263 750 1438 3010 4769 8860 11377 15326 16239 |
| H1ch | 263 750 1438 1462 3010 4769 8860 15326 16239 |
| H1bi | 263 750 1438 3010 3745 4769 8860 15326 |
| H1bj | 263 750 1438 3010 4769 8860 10295 15326 |
| H1bk | 263 750 1438 3010 4769 8860 11084 15326 |
| H1bm | 263 750 1438 3010 4216 4769 8860 15326 |
| H1bn | 263 750 1438 3010 4769 8245 8860 15326 |
| H1bo | 263 267 485 750 1438 3010 4769 6446T 8860 11002 15326 15844 |
| H1bp | 263 750 1438 3010 4769 8860 10003 15326 |
| H1bq | 263 750 1438 3010 4769 8860 14467 15326 |
| H1br | 263 750 1438 3010 4769 8860 10454 15326 |
| H1bs | 263 750 1438 3010 4769 8860 15326 16220C |
| H1bt | 263 750 1438 3010 4769 8860 15326 16527 |
| H1bt1 | 263 750 1438 3010 4769 8860 10348 12007 15326 16527 |
| H1bu | 263 750 1438 1811 3010 4769 5558 8860 15326 |
| H1bv | 263 750 1438 3010 4769 8860 12681 15326 |
| H1bv1 | 263 750 1438 3010 4769 8164 8860 12681 15326 16362 |
| H1bw | 263 750 1438 3010 4769 8478 8860 15326 |
| H1bx | 263 750 789 1438 3010 4769 8740 8860 15326 |
| H1bz | 263 750 1438 1531 3010 4769 4924 8860 15326 16240 |
| H1ca | 263 750 1438 3010 4316 4769 6515G 8860 15326 |
| H1+16311 | 263 750 1438 3010 4769 8860 15326 16311 |
| H1cd | 263 750 1438 3010 4769 8860 9722 10316 12373 15326 16217 16311 |
| H1cf | 263 750 1438 3010 4769 8860 15326 16260 |
| H1ci | 263 750 1438 3010 4769 8860 12454 14914 15016 15326 |
| H1cj | 263 750 1438 3010 3221 4769 8705 8860 9581 11204 15326 16129 |
| H2 | 263 750 4769 8860 15326 |
| H2a | 263 750 8860 15326 |
| H2a1 | 263 750 951 8860 15326 16354 |
| H2a1a | 263 750 951 6173 8860 13095 15326 16354 |
| H2a1a1 | 263 750 951 979 3567 6173 8860 12127 13095 13272 15326 16354 |
| H2a1a2 | 263 750 951 6173 6620 8860 9862A 11077 13095 14494 15326 16354 |
| H2a1b | 263 750 951 5978 8860 13395 15326 16354 |
| H2a1b1 | 210 263 750 951 5978 8860 13395 15326 16354 |
| H2a1b2 | 72G 263 750 951 2195 5978 8860 13395 15326 16256 16354 |
| H2a1c | 263 750 951 3834 8860 15326 16354 |
| H2a1d | 263 750 951 4769 6260 8679 8860 15326 16354 |
| H2a1e | 263 575 750 951 8860 15326 16354 |
| H2a1e1 | 263 575 750 751 951 8860 15326 16354 |
| H2a1e1a | 263 575 750 751 951 8860 15326 16124 16354 |
| H2a1e1a1 | 263 575 750 751 951 8860 15326 16124 16148 16166 16354 |
| H2a1e1b | 263 575 750 751 951 4715 5899.XC 8860 15326 16354 |

| | | |
|---|---|---|
| H2a1f | 263 750 951 8860 15326 16193 16354 | |
| H2a1f1 | 127 263 750 951 8860 15326 16193 16354 | |
| H2a1f2 | 150 263 750 951 8860 15326 16193 16354 | |
| H2a1g | 263 750 951 8065 8860 15326 16354 | |
| H2a1i | 113 263 750 951 8860 15326 16145 16354 | |
| H2a1j | 263 444 750 951 8860 14189 15326 16354 | |
| H2a1k | 263 750 951 3396 8860 15326 16354 | |
| H2a1m | 195 263 750 951 2442 8860 15326 16354 | |
| H2a1+146 | 146 263 750 951 8860 15326 16354 | |
| H2a1n | 146 263 750 951 4659 8860 15326 16354 | |
| H2a2 | 263 8860 15326 | |
| H2a2a | | 263 |
| H2a2a1 | | |
| H2a2a1a | | 15314 |
| H2a2a1b | | 9299 |
| H2a2a1c | 6632 16051 | |
| H2a2a1d | | 16172 |
| H2a2a1e | | 8182 |
| H2a2a1f | | 93 |
| H2a2a1g | | 16189 |
| H2a2a1h | | 16320 |
| H2a2a2 | 152 263 6716 | |
| H2a2+(16235) | 263 8860 15326 (16235) | |
| H2a2b | 263 8860 15326 (16235) 16291 | |
| H2a2b1 | 263 8860 15326 (16235) 16291 16293 | |
| H2a2b1a | 263 3388A 8860 15326 (16235) 16291 16293 | |
| H2a2b1a1 | 263 3388A 8860 15326 (16235) 16291 16293 16400 | |
| H2a2b2 | 263 8634 8860 15326 (16235) 16291 | |
| H2a2b3 | 263 4080 8860 15326 (16235) 16291 | |
| H2a2b4 | 263 5460 8860 15326 (16235) 16291 | |
| H2a2b5 | 263 8860 14305 15326 (16235) 16291 | |
| H2a2b5a | 263 8860 11914 12954 14305 15326 (16235) 16291 | |
| H2a3 | 263 750 8860 10810 15326 16274 | |
| H2a3a | 263 750 1462 8860 10810 15326 16274 | |
| H2a3a1 | 263 750 1462 2749 8860 10810 15326 16274 | |
| H2a3b | 263 750 8860 10810 14693 15326 16274 | |
| H2a4 | 263 750 8860 11140 15326 | |
| H2a5 | 263 750 8860 13708 15326 | |
| H2a5a | 263 750 1842 8860 13708 15326 | |
| H2a5a1 | 263 750 1842 8860 13708 15326 16291 | |
| H2a5a1a | 263 750 1842 4592 8860 13708 15326 16291 | |
| H2a5a1b | 263 750 1842 8860 11233 13708 15326 16291 | |
| H2a5b | 263 750 8843 8860 13708 15326 | |
| H2a5b1 | 249d 263 750 8843 8860 13708 15326 | |
| H2a5b2 | 263 750 8843 8860 11086 13708 15326 16183 | |
| H2+152_16311 | 152 263 750 4769 8860 15326 16311 | |
| H2b | 152 263 750 4769 8598 8860 15326 16311 | |
| H2c | 152 263 750 4769 8860 13708 13934 15326 16311 | |

| | |
|---|---|
| H2c1 | 194 205 263 750 3334 4769 8860 13708 13934 15326 16311 |
| H3 | 263 750 1438 4769 6776 8860 15326 |
| H3+152 | 152 263 750 1438 4769 6776 8860 15326 |
| H3a | 152 263 750 1438 4769 6776 8860 15326 16239G |
| H3a1 | 152 263 750 1438 4769 6776 8860 13404 15326 16239G |
| H3a1a | 152 263 750 1438 4769 6776 8860 13404 14125 15326 16239G |
| H3g | 152 263 750 1438 4769 6776 8860 10754C 15326 |
| H3g1 | 152 263 750 1438 3992 4418 4769 6776 8860 10754C 15326 |
| H3g1a | 152 263 750 1438 3992 4418 4769 6776 8860 10754C 15326 15377 |
| H3g1b | 152 263 750 1438 3992 4418 4769 6776 8860 8950 10754C 15326 |
| H3g2 | 152 263 735 750 1438 4769 6776 8860 10754C 15326 |
| H3g3 | 152 263 750 1438 4769 6776 8860 9196 10754C 15326 |
| H3g4 | 152 263 750 1438 4769 6776 8860 10754C 13611 15326 |
| H3i | 152 263 750 1438 4769 5893 6776 8860 15326 |
| H3i1 | 152 263 750 1438 4769 5213 5893 6776 8860 15326 |
| H3j | 152 263 750 1438 4769 6351 6776 8860 15326 |
| H3k | 152 263 750 1438 4769 6776 8860 14687 15326 |
| H3k1 | 152 263 750 1438 4769 6776 8860 11590 14687 15326 |
| H3k1a | 152 263 750 1438 4769 6776 8860 11590 12217 14687 15326 |
| H3b | 263 750 1438 2581 4769 6776 8860 15326 |
| H3b+16129 | 263 750 1438 2581 4769 6776 8860 15326 16129 |
| H3b1 | 263 750 1438 2581 4769 5147 6776 8860 15326 16129 16256 |
| H3b1a | 263 750 1290 1438 2581 4769 5147 6776 8860 15326 16129 16256 |
| H3b1b | 153 263 750 1438 2581 4769 5147 6776 8860 15326 16129 16256 |
| H3b1b1 | 153 263 750 1438 2581 4769 5147 6776 8860 13813 15326 16111 16129 16256 |
| H3b1b1a | 153 263 750 1438 2581 3832A 4769 5147 6776 8860 13813 15326 16111 16129 16256 |
| H3b2 | 263 750 1438 2581 4769 6638 6776 8860 15326 16129 |
| H3b3 | 263 750 1438 2581 4769 4924 6776 8860 15326 16129 |
| H3b4 | 263 417 750 1438 2581 4769 6776 8860 15326 16129 |
| H3b4a | 263 417 750 1438 2581 4769 6776 8860 12447 15326 16129 |
| H3b5 | 263 750 1438 2581 4769 5911 6776 8860 15326 16129 |
| H3b6 | 73 263 750 1438 2581 4769 6776 8860 15326 16129 |
| H3b6a | 73 263 750 1438 2581 3396 4769 6776 8860 15326 16129 |
| H3b7 | 263 750 1438 2581 4769 5471 6776 8860 15326 16129 |
| H3c | 263 750 1438 4769 6776 8860 12957 15326 |
| H3c1 | 263 750 1438 1536 4769 6776 8860 12957 15326 |
| H3c2 | 195 263 750 1438 4769 6776 8860 12957 15326 16176 |
| H3c2a | 195 263 750 1438 4769 6776 8860 12957 14200 15326 16176 |
| H3c2a1 | 195 263 750 1438 4769 6425 6776 8860 12957 14200 15326 16176 |
| H3c2b | 195 263 750 1438 4769 6776 8860 12957 14305 15326 16176 |
| H3c2b1 | 195 263 750 1211 1438 4769 6776 8860 12957 14305 15326 16176 |
| H3c2c | 195 263 750 1438 4769 5896 6776 8860 12957 15326 16176 |
| H3c3 | 260 263 750 1438 4769 6776 8860 12957 15326 15769 16278 |
| H3+73 | 73 263 750 1438 4769 6776 8860 15326 |
| H3d | 73 263 750 761 1438 4769 6776 8860 15326 |
| H3e | 263 750 1438 1618 4769 6776 8860 15326 15592 |

| | |
|---|---|
| H3+16311 | 263 750 1438 4769 6776 8860 15326 16311 |
| H3h | 263 750 1438 4769 6776 8860 12811 15326 16311 |
| H3h1 | 263 750 1438 4769 6776 8705 8860 12811 15326 16311 |
| H3h2 | 263 750 1438 4769 5960 6776 8860 12811 15326 (16093) 16311 |
| H3h2a | 263 750 1438 4769 5960 6776 8860 12811 15326 (16093) 16266 16311 |
| H3h3 | 263 750 1438 4769 6776 8860 12811 13967 15326 16311 |
| H3h3a | 263 750 1438 4769 6776 8860 12811 13967 15326 15470 16311 |
| H3h3b | 263 750 1438 3915 4769 6776 8860 12811 13967 15326 16311 |
| H3h4 | 146 217 263 750 1438 4769 6776 8860 11944 12811 13713 15326 16311 |
| H3h5 | 263 750 1438 4769 6776 8860 10589 12811 15326 16311 |
| H3h6 | 263 750 1438 4025 4769 6776 8860 12811 15326 16311 |
| H3h7 | 93 263 750 1438 4769 6776 8860 12811 15326 16311 |
| H3m | 263 750 1438 4769 6776 8860 14501 15326 16311 |
| H3n | 263 750 1438 4769 6776 8860 15326 15781 16104 16176 16311 |
| H3p | 263 750 1438 4769 6776 8860 15326 16222 |
| H3q | 263 750 1438 3970 4769 6776 8860 15326 |
| H3q1 | 263 750 1438 3970 4769 6776 8860 12236 15326 |
| H3r | 263 750 961 1438 4769 6776 8860 15326 |
| H3r1 | 263 750 961 1438 1888 4769 6776 8860 15326 |
| H3s | 194 263 750 1438 4769 6776 6953 8860 13105 15326 |
| H3t | 263 750 1438 4769 6776 7148 8860 15326 |
| H3u | 263 750 1438 4769 6776 8860 9966 15326 |
| H3u1 | 263 750 1438 4769 5899.XC 6776 8860 9966 14319 15326 15380 16148 |
| H3v | 263 408A 750 1438 4769 6776 8860 15326 |
| H3v1 | 263 408A 750 1438 3345 4769 6776 8841 8860 9117 10685 15326 |
| H3v+16093 | 263 408A 750 1438 4769 6776 8860 15326 16093 |
| H3v2 | 73 263 408A 750 1438 4769 6776 8860 8939 12401 14148 15326 16093 |
| H3w | 263 750 1438 3613 4769 5999 6776 8860 15326 16248 |
| H3x | 263 750 1438 4769 6776 8860 15326 16368 |
| H3x1 | 263 750 1438 3615 4769 6776 8860 8937 11914 15326 16293 16368 |
| H3y | 263 750 1438 2352 4512 4769 5480 6776 8860 15326 |
| H3z | 263 293 750 1438 4769 6776 8860 15326 |
| H3z1 | 263 293 750 1438 4769 6776 8860 12346 15326 15530 |
| H3z2 | 263 293 493 750 1438 4315 4769 6776 8860 15326 15917 |
| H3aa | 263 750 1438 4769 6776 8860 10535 15326 |
| H3ab | 263 750 1438 4386 4769 6776 8860 10724 15326 |
| H3ac | 263 750 1438 4769 6266 6776 8860 15326 |
| H3ad | 263 750 1438 4769 5054C 6776 8860 15326 |
| H3ae | 263 750 1438 4769 6776 8860 12441 15326 |
| H3af | 263 750 1438 4769 6776 8470 8860 15326 16129 |
| H3ag | 263 750 1438 4769 6293 6776 8860 15326 |
| H3ag1 | 263 750 1438 4769 6293 6776 8860 10410 15204 15326 |
| H3ah | 263 750 1438 4769 6776 8860 10247 15326 |
| H3ai | 263 750 1438 4769 6776 8860 9693 15326 |
| H3aj | 263 750 1438 4769 6776 8718 8860 15326 |
| H3ak | 143 263 750 1438 4769 6776 8860 9325 15326 16362 |
| H3am | 182 263 750 1438 4769 6776 8860 15326 |
| H3an | 251 263 750 1438 4769 6776 8860 15326 |

| | |
|---|---|
| H3ao | 263 750 1438 4769 6776 8860 15326 16256 |
| H3ao1 | 263 750 1438 4769 6776 8860 15326 16170 16256 |
| H3ap | 263 750 1438 4769 6776 8860 10915 15326 |
| H3aq | 263 750 1018 1438 4769 6776 8860 15326 |
| H3ar | 263 750 1438 4769 6776 8860 10310 15326 |
| H3as | 263 750 1438 4769 6776 8860 9316 15326 |
| H3at | 263 750 1438 4769 6776 8860 15224 15326 |
| H3at1 | 263 750 1438 2156.1A 4769 6776 8860 9525 15224 15326 |
| H3au | 263 750 1438 4769 6776 8860 12372 15326 |
| H3+16189 | 263 750 1438 4769 6776 8860 15326 16189 |
| H3av | 152 263 750 1438 4769 6776 7849 8383 8860 15326 16189 |
| H4 | 263 750 1438 3992 4769 5004 8860 9123 15326 |
| H4a | 263 750 1438 3992 4024 4769 5004 8860 9123 14582 15326 |
| H4a1 | 263 750 1438 3992 4024 4769 5004 8860 9123 14365 14582 15326 |
| H4a1a | 263 750 1438 3992 4024 4769 5004 8269 8860 9123 14365 14582 15326 |
| H4a1a1 | 263 750 1438 3992 4024 4769 5004 8269 8860 9123 10044 14365 14582 15326 |
| H4a1a1a | 73 263 750 1438 3992 4024 4769 5004 8269 8860 9123 10044 14365 14582 15326 |
| H4a1a1a1 | 73 263 750 1438 3992 4024 4769 5004 8269 8860 9123 10034 10044 14365 14582 15326 |
| H4a1a1a1a | 73 263 750 1438 3992 4024 4769 5004 8269 8860 9123 10007 10034 10044 14365 14582 15326 |
| H4a1a1a1a1 | 73 263 750 1438 1656 3992 4024 4769 5004 8269 8860 9123 10007 10034 10044 11440 14365 14582 15326 |
| H4a1a1a2 | 73 263 750 1438 3992 4024 4769 5004 8269 8860 9123 10044 14325 14365 14582 15326 |
| H4a1a1a3 | 73 263 750 1438 3992 4024 4769 5004 8269 8860 9123 10044 14365 14582 15244 15326 16287 |
| H4a1a1a4 | 73 263 750 960.XC 1438 3992 4024 4769 5004 7870 8269 8860 9123 10044 14365 14582 15326 |
| H4a1a2 | 263 750 1438 3992 4024 4769 5004 8269 8860 9123 13708 14365 14582 15326 |
| H4a1a2a | 263 750 1438 3992 4024 4769 5004 8269 8860 9123 10124 13708 14365 14582 14956 15326 |
| H4a1a2a1 | 263 750 1438 3992 4024 4769 5004 6040 8269 8860 9123 10124 13708 14365 14582 14956 15326 |
| H4a1a+195 | 195 263 750 1438 3992 4024 4769 5004 8269 8860 9123 14365 14582 |
| H4a1a3 | 195 263 750 1438 3992 4024 4769 5004 8269 8860 9123 13889 14365 14582 15326 |
| H4a1a3a | 195 263 750 1438 3992 4024 4769 5004 5773 8269 8860 9123 13889 14365 14582 15326 |
| H4a1a4 | 195 263 750 1438 3992 4024 4769 5004 8269 8860 9123 14365 14569 14582 15326 |
| H4a1a4a | 195 263 750 1438 3992 4024 4769 5004 8269 8860 9123 9615 14365 14569 14582 15326 |
| H4a1a4b | 195 263 750 1438 3992 4024 4769 5004 8269 8860 9123 12642 14365 14569 14582 15326 15884 |

| | |
|---|---|
| H4a1a4b1 | 195 263 750 1438 3992 4024 4769 5004 8269 8860 9123 12642 14365 14569 14582 15326 15884 16286G |
| H4a1a4b2 | 195 263 750 1438 3992 4024 4769 5004 6951 8269 8860 9123 12642 14365 14569 14582 15326 15884 |
| H4a1a5 | 263 750 1438 3992 4024 4769 5004 8269 8380 8860 9123 14365 14582 15326 |
| H4a1c | 263 750 1438 3992 4024 4769 5004 8860 9123 14365 14582 15326 15930 |
| H4a1c1 | 263 750 1438 3992 4024 4769 5004 8860 9123 14365 14582 15326 15497 15930 |
| H4a1c1a | 263 750 1438 3992 4024 4769 5004 7581 8860 9123 14365 14582 15326 15497 15930 |
| H4a1c2 | 263 750 1438 3992 4024 4769 5004 7859 8860 9123 14365 14582 15326 15930 16179A |
| H4a1d | 263 750 1438 3992 4024 4769 5004 7356 7521 8860 9123 14365 14582 15326 |
| H4a2 | 263 750 1438 3624 3992 4024 4769 5004 8860 9123 14582 15326 16235 |
| H4b | 263 750 1438 3992 4769 5004 8860 9123 10166 15326 |
| H4b1 | 153 (195) 263 750 1438 3992 4769 5004 8860 9123 10166 15326 (16093) |
| H4c | 263 750 1438 3992 4769 5004 8860 9123 9276 15326 |
| H4c1 | 73 263 750 1438 3992 4769 5004 8860 9123 9276 15326 16248 |
| H4d | 263 482 750 1438 3992 4769 5004 8860 9123 15326 |
| H5'36 | 263 456 750 1438 4769 8860 15326 |
| H5 | 263 456 750 1438 4769 8860 15326 16304 |
| H5a | 263 456 750 1438 4336 4769 8860 15326 16304 |
| H5a1 | 263 456 750 1438 4336 4769 8860 15326 15833 16304 |
| H5a1a | 263 456 721 750 1438 4336 4769 8860 15326 15833 16304 |
| H5a1b | 263 456 750 1438 4336 4769 8860 11719 15326 15833 16304 |
| H5a1c | 263 456 750 1438 4095 4336 4769 8860 13194 15326 15833 16304 |
| H5a1c1 | 263 456 750 1438 4095 4336 4769 8860 9055 13194 15326 15833 16304 |
| H5a1c1a | 263 456 750 1438 2851 4095 4336 4769 8860 9055 13194 15326 15833 16304 |
| H5a1c2 | 263 456 750 1438 4095 4336 4769 8860 9386 13194 15326 15833 16243 16304 |
| H5a1d | 263 456 750 1438 4336 4769 8803T 8860 15326 15833 16304 |
| H5a1e | 263 456 750 1438 4336 4769 8860 15326 15833 16166 16304 |
| H5a1f | 263 456 750 961 1438 4336 4769 8860 15326 15833 16304 |
| H5a1g | 263 456 750 1438 4336 4769 8860 15326 15833 16172 16304 |
| H5a1g1 | 263 444 456 750 1438 4336 4769 8860 9804 15326 15833 16172 16304 16311 |
| H5a1g1a | 263 444 456 750 1284 1438 4336 4769 7517 8860 9804 15326 15833 16172 16304 16311 |
| H5a1g2 | 263 456 750 1438 4336 4769 8860 15326 15833 16172 16291 16304 |
| H5a1h | 263 456 750 1438 4336 4769 8860 9392T 15326 15833 16304 |
| H5a1i | 263 456 750 1438 4336 4769 5051 8860 15326 15833 16153 16304 |
| H5a1j | 263 456 750 1438 4336 4769 8860 15326 15833 16209 16304 |
| H5a1k | 263 456 750 1438 4336 4769 8860 12864 15326 15833 16304 |
| H5a1+152 | 152 263 456 750 1438 4336 4769 8860 15326 15833 16304 |
| H5a1m | 152 263 456 750 1438 4336 4769 8860 11221 15326 15833 16304 |

| | |
|---|---|
| H5a1n | 152 263 456 710 750 1438 4336 4769 8860 15326 15833 16304 |
| H5a1+16093 | 263 456 750 1438 4336 4769 8860 15326 15833 16093 16304 |
| H5a1p | 263 456 750 1438 4336 4769 8860 15326 15638T 15833 16093 16304 |
| H5a1q | 263 456 750 1438 4336 4769 4916 8860 15326 15833 16304 |
| H5a2 | 263 456 750 1438 4336 4769 5839 8860 15326 16304 |
| H5a2a | 263 456 750 1438 3310 4336 4769 5839 8860 15326 16304 |
| H5a3 | 263 456 513 750 1438 4336 4769 8860 15326 15884 16304 |
| H5a3a | 263 456 513 750 1438 4336 4769 8860 10915 15326 15884 16304 |
| H5a3a1 | 93 263 456 513 750 1438 4336 4769 8860 9123 10915 15326 15884 16304 |
| H5a3a+152 | 152 263 456 513 750 1438 4336 4769 8860 10915 15326 15884 16304 |
| H5a3a2 | 152 263 456 513 750 1438 4336 4769 8860 9515 10915 15326 15884 16304 |
| H5a3a3 | 263 456 513 750 1438 4336 4769 8860 10915 12384 15326 15884 16304 |
| H5a3b | 263 456 513 750 1438 4336 4769 8860 12648 15326 15884 16304 |
| H5a4 | 263 456 750 1438 3753 4336 4769 8860 15326 16294 16304 |
| H5a4a | 263 456 750 1438 3753 4336 4769 8860 15326 16294 16304 16320 |
| H5a4a1 | 263 456 593 750 1438 3753 4336 4769 8860 15139 15326 16294 16304 16320 |
| H5a4a1a | 263 456 593 750 1438 1520 3753 4336 4769 8860 15139 15326 16294 16304 16320 |
| H5a5 | 146 263 456 750 1438 4336 4769 7025 8860 15326 16304 |
| H5a+152 | 152 263 456 750 1438 4336 4769 8860 15326 16304 |
| H5a6 | 152 263 456 750 1438 4336 4769 5319 8563 8860 15326 16304 |
| H5a6a | 152 263 456 750 769 1438 4336 4769 5319 5450 8563 8860 15326 16304 |
| H5a7 | 263 456 750 1438 4336 4769 8557 8860 15175 15326 16304 |
| H5a8 | 263 456 750 1438 4336 4769 8860 11969 15326 16304 |
| H5a9 | 263 456 750 1438 4336 4769 5821 8860 15326 16304 |
| H5b | 263 456 750 1438 4769 5471 8860 15326 16304 |
| H5b1 | 146 195 263 456 750 1438 4769 5471 8860 14497 15326 16304 |
| H5b2 | 263 327 456 750 1438 4769 5471 8860 12864 15326 16304 |
| H5b3 | 263 456 750 1438 4769 5471 6923 8860 15326 16304 |
| H5b4 | 189 263 456 750 1438 4769 5471 8011 8188 8860 14311G 15326 16304 |
| H5b5 | 263 456 750 1438 4769 5471 8860 14488 15326 16304 |
| H5c | 263 456 750 1438 4769 8860 12127 15326 16304 |
| H5c1 | 263 456 750 1438 4769 8860 11944 12127 15326 16304 |
| H5c1a | 263 456 750 1438 4769 8860 11944 12127 15157 15326 16304 |
| H5c2 | 263 456 750 1438 3819 4769 8860 12127 15326 16213 16304 |
| H5d | 263 456 750 1438 4769 5082 8860 15326 16304 |
| H5e | 263 456 750 1438 4769 8860 15326 16294 16304 |
| H5e1 | 263 456 750 1438 4769 8343 8860 12771 15326 16294 16304 |
| H5e1a | 263 456 750 1438 4769 8343 8860 12771 13434 15326 16294 16304 |
| H5e1a1 | 263 456 722 750 1438 4769 8343 8860 12771 13434 15326 16294 16304 |
| H5e1b | 263 456 750 1438 4769 7474 8343 8860 12771 15326 16294 16304 |
| H5f | 263 456 750 1438 4769 6425 8860 15326 16304 |
| H5g | 263 456 750 1438 4769 8860 15326 16304 16325 |
| H5h | 263 456 750 1438 4769 4796 8860 15326 16235 16304 |
| H5j | 263 456 750 1005 1438 4769 8860 9468 10754 13614 15082 15244 15326 16304 |
| H5k | 263 456 750 1438 2626 4769 8020 8860 15326 16304 |

| | |
|---|---|
| H5m | 146 263 456 750 1438 4769 8860 15326 15883 16304 |
| H5n | 263 373 456 750 1438 4769 8860 15326 16304 |
| H5p | 263 456 750 1438 4769 8860 15326 15930 16304 |
| H5+16192 | 263 456 750 1438 4769 8860 15326 16192 16304 |
| H5q | 263 456 750 1438 4769 8860 8975 15326 16192 16304 |
| H5+16311 | 263 456 750 1438 4769 8860 15326 16304 16311 |
| H5r | 207 263 456 750 1438 4769 8860 10410 13725 15326 16304 16311 |
| H5r1 | 207 263 456 750 1438 4769 7961 8860 10410 13725 15326 16304 16311 |
| H5r2 | 207 263 456 750 1438 4769 8860 10410 13725 15326 15930 16304 16311 |
| H5s | 263 456 750 1438 4769 5302 8860 15326 16304 16311 16391 |
| H5t | 263 456 750 1438 4769 8473 8593 8860 15326 16124 16304 16311 |
| H5u | 263 456 750 1438 4769 8860 15326 16304 16400 |
| H5u1 | 263 456 750 1438 4769 8860 10595 11914 12855 15326 16304 16400 |
| H5+709 | 263 456 709 750 1438 4769 8860 15326 16304 |
| H5v | 263 456 709 750 1438 4769 8860 9325 15326 16304 |
| H36 | 152 263 456 750 1438 3525A 4769 8578 8860 13056 15326 16070 |
| H6 | 239 263 750 1438 4769 8860 15326 16362 (16482) |
| H6a | 239 263 750 1438 3915 4769 8860 9380 15326 16362 (16482) |
| H6a1 | 239 263 750 1438 3915 4727 4769 8860 9380 15326 16362 (16482) |
| H6a1a | 239 263 750 1438 3915 4727 4769 8860 9380 11253 15326 16362 (16482) |
| H6a1a1 | 239 263 750 1438 3915 4727 4769 5460 8860 9380 11253 15326 16362 (16482) |
| H6a1a1a | 239 263 750 1438 3915 4727 4769 5460 7325 8860 9362 9380 11253 11611 15326 16311 16362 (16482) |
| H6a1a2 | 239 263 750 1438 3915 4727 4769 7202 8860 9380 11253 15326 16362 (16482) |
| H6a1a2a | 41 239 263 750 1438 3915 4727 4769 7202 8860 9380 11253 15326 16362 (16482) |
| H6a1a2b | 239 263 750 1438 3915 4727 4769 7202 8860 9380 9773 11253 11662 15326 16362 (16482) |
| H6a1a2b1 | 239 263 750 1438 3705 3915 4727 4769 7202 8860 9380 9773 11253 11662 15326 16362 (16482) |
| H6a1a3 | 239 263 750 1438 3915 4727 4769 5785 8860 9380 11253 15326 16362 (16482) |
| H6a1a3a | 239 263 750 827 1438 3915 4727 4769 5785 8860 9380 11253 15326 16362 (16482) |
| H6a1a4 | 239 263 750 1438 3915 4727 4769 8860 9380 10237 11253 15326 16362 (16482) |
| H6a1a5 | 239 263 750 1438 3915 4727 4769 8860 9380 10936 11253 15326 16362 (16482) |
| H6a1a6 | 239 263 288 750 1438 3915 4727 4769 8860 9380 11253 15326 16362 (16482) |
| H6a1a7 | 150 239 263 750 1438 3915 4727 4769 7805 8860 9380 11253 15326 16362 (16482) |
| H6a1a8 | 239 263 750 1438 3915 4727 4769 8860 9380 11253 15326 16298 16362 (16482) |
| H6a1a8a | 239 263 750 1438 3915 4727 4769 6185 8860 9380 11253 15326 16145 16298 16362 (16482) |

| | |
|---|---|
| H6a1a9 | 239 263 750 1438 3915 4727 4769 8860 9380 11253 13953 15326 16362 (16482) |
| H6a1a10 | 239 263 750 1438 3548 3915 4727 4769 8860 9380 11253 15326 16362 (16482) |
| H6a1b | 239 263 750 1438 3915 4727 4769 8860 9380 10589 15326 16362 (16482) |
| H6a1b1 | 239 263 750 1438 3915 4727 4769 6218 7859 8563 8860 9380 10589 15326 15930 16284 16362 (16482) |
| H6a1b2 | 239 263 750 1438 3915 4727 4769 8860 9254 9380 10589 15326 16362 (16482) |
| H6a1b2a | 239 263 750 1438 3915 4727 4769 8860 9254 9380 10589 12975 15326 16362 (16482) |
| H6a1b2b | 239 263 750 1438 3915 4727 4769 6261 8860 9254 9380 10589 15326 16362 (16482) |
| H6a1b2c | 239 263 750 1438 3915 4727 4769 8860 9254 9380 10589 13869 15326 16362 (16482) |
| H6a1b2d | 239 263 750 1438 3915 4727 4769 8860 9254 9380 10589 15326 16066 16362 (16482) |
| H6a1b2e | 239 263 750 1438 3915 4727 4769 8860 9254 9380 10589 15326 15928 16362 (16482) |
| H6a1b3 | 204 239 263 750 1438 3915 4727 4769 8860 9380 10589 15326 16193 16219 16362 (16482) |
| H6a1b3a | 204 239 263 750 1438 3915 4727 4769 8860 9380 10463 10589 13768 15326 16193 16219 16362 (16482) |
| H6a1b3b | 204 239 263 750 1438 3915 4727 4769 8860 9380 10589 13359 15326 16193 16219 16362 (16482) |
| H6a1b4 | 239 263 750 1438 3915 4727 4769 8860 9380 10589 15326 16249 16362 (16482) |
| H6a2 | 239 263 750 1438 3915 4769 8860 9380 11155 15326 16362 (16482) |
| H6a2a | 239 263 750 1438 3915 4769 8860 9380 11155 15326 15940 16362 (16482) |
| H6b | 239 263 750 1438 4769 8860 14040 15326 16300 16362 (16482) |
| H6b1 | 44.1C 152 239 263 750 1438 4769 8860 14040 15326 16300 16362 (16482) |
| H6b2 | 239 263 750 1438 4769 8860 14040 15326 16300 16325 16362 (16482) |
| H6c | 239 263 750 1438 4769 6869 8860 9804 15326 16362 (16482) |
| H6c1 | 239 263 750 1438 4769 6869 8860 9804 14185T 15326 16362 16400 |
| H7 | 263 750 1438 4769 4793 8860 15326 |
| H7a | 263 750 1438 1719 4769 4793 8860 15326 |
| H7a1 | 263 750 1438 1719 4769 4793 8860 15326 16261 |
| H7a1a | 93 263 750 1438 1719 4769 4793 8860 11167 15326 16261 |
| H7a1b | 263 750 1393 1438 1719 4769 4793 8860 15326 16261 |
| H7a1c | 263 750 1438 1719 4769 4793 8860 9833 15326 16261 |
| H7a1d | 263 750 1438 1719 4769 4793 8286 8860 15326 16261 |
| H7a2 | 263 750 1438 1719 4769 4793 8860 10253 15326 16176 |
| H7b | 263 750 1438 4769 4793 5348 8860 15326 |
| H7b1 | 263 750 1438 4769 4793 5348 8860 12351 15326 |
| H7b2 | 263 750 1438 4769 4793 5348 8860 13323 15326 |
| H7b2a | 263 750 1438 4769 4793 5348 8860 13323 15326 15862 |
| H7b3 | 263 750 1438 3335 4769 4793 5348 8860 15326 |
| H7b4 | 263 750 1438 4688 4769 4793 5348 8860 15326 |

| | |
|---|---|
| H7b5 | 263 750 1438 4769 4793 5348 8860 11314 15326 |
| H7b6 | 263 750 1438 4769 4793 5348 8860 15326 15942 |
| H7c | 263 750 1438 4769 4793 6296A 8860 15326 |
| H7c1 | 263 750 1438 4769 4793 6296A 8860 15326 16265 |
| H7c2 | 263 750 1438 4769 4793 6296A 8860 13959 15326 |
| H7c3 | 263 750 1438 4769 4793 6296A 8860 9329 15326 15459 |
| H7c4 | 263 750 1438 4769 4793 6296A 8860 15326 16265C |
| H7c5 | 263 750 1438 4769 4793 6296A 8860 11020 11992 15326 |
| H7c6 | 263 750 1438 4769 4793 6296A 8557 8860 15326 15497 |
| H7d | 263 750 1438 4769 4793 8860 15326 15409 |
| H7d1 | 263 750 1438 2283 4769 4793 8860 13032 15326 15409 |
| H7d2 | 263 291.1A 750 1438 4769 4793 8860 11887 15326 15409 |
| H7d2a | 263 291.1A 750 1438 4769 4793 8860 11887 14871 15326 15409 |
| H7d3 | 263 750 1438 4769 4793 8860 12630 15326 15409 |
| H7d3a | 263 750 1438 4769 4793 8860 12630 14405 15326 15409 |
| H7d4 | 263 750 1438 4769 4793 6152 7299 8860 11827 14162 15326 15409 |
| H7d5 | 263 750 1438 4769 4793 6962 8639G 8860 9380 15326 15409 16342 |
| H7e | 263 750 1438 4769 4793 8026T 8860 9527 15326 |
| H7f | 263 750 1438 4769 4793 8860 15326 16168 |
| H7g | 263 750 1438 4769 4793 7472T 8860 15326 |
| H7h | 263 750 1438 4769 4793 8860 15326 16213 |
| H7h1 | 263 750 1438 4769 4793 8573 8860 15326 16213 |
| H7i | 228 263 750 1438 3984 4769 4793 8860 11016 11971 15326 |
| H7i1 | 143 228 263 750 1438 3984 4769 4793 8860 11016 11971 15326 |
| H+195 | 195 263 750 1438 4769 8860 15326 |
| H+195+146 | 146 195 263 750 1438 4769 8860 15326 |
| H8 | 146 195 263 709 750 1438 4769 8860 13101C 15326 16288 16362 |
| H8a | 146 195 263 709 750 1438 4769 8860 13101C 15326 16111 16288 16362 |
| H8a1 | 146 195 263 709 750 1438 4769 8860 13101C 15326 16111 16167 16288 16362 |
| H8+(114) | (114) 146 195 263 709 750 1438 4769 8860 13101C 15326 16288 16362 |
| H8b | (114) 146 195 263 709 750 1438 4769 8860 12490 13101C 15326 16288 16362 |
| H8b1 | (114) 146 195 263 709 750 1438 4769 7765 8860 12490 13101C 15326 16288 16362 |
| H8+(114)+152 | (114) 146 152 195 263 709 750 1438 4769 8860 13101C 15326 16288 |
| H8c | (114) 146 152 195 263 709 750 1438 4769 8860 13101C 13711 15326 16288 16362 |
| H8c1 | (114) 146 152 195 263 709 750 1438 4769 5082 8860 13101C 13711 14071 15326 16288 16362 |
| H8c2 | (114) 146 152 195 210 263 709 750 1438 4769 8860 9114 13101C 13711 15326 16153 16288 16362 |
| H31 | 146 195 263 750 1438 4769 7930T 8860 10771 15326 |
| H31a | 72G 146 195 263 750 1438 4769 7930T 8860 10771 15326 |
| H31b | 146 195 263 750 1438 4769 7930T 8860 10771 15247 15326 |
| H11 | 195 263 750 1438 4769 8448 8860 13759 15326 16311 |
| H11a | 195 263 750 961G 1438 4769 8448 8860 13759 15326 16293 16311 |

| | |
|---|---|
| H11a1 | 195 263 750 961G 1438 4769 8448 8860 8898 13759 15326 16278 16293 16311 |
| H11a2 | 195 263 750 961G 1438 4769 8448 8860 13759 14587 15326 (16092) 16293 16311 |
| H11a2a | 195 263 750 961G 1438 4769 8448 8860 13759 14587 15326 (16092) 16140 16293 16311 |
| H11a2a1 | 195 263 750 961G 1438 3145 4769 8448 8860 13759 14587 15326 (16092) 16140 16293 16311 |
| H11a2a2 | 195 263 750 961G 1438 4769 5585 8448 8860 13759 14587 15326 15670 (16092) 16140 16265 16293 16311 |
| H11a2a3 | 195 263 750 961G 1438 4769 8448 8860 9521 13759 14587 15326 (16092) 16140 16293 16311 |
| H11a3 | 195 263 750 961G 1438 4769 8448 8860 13759 15326 16243 16293 16311 |
| H11a4 | 195 263 750 961G 1438 4769 5899.XC 8448 8860 13759 15326 16111 16293 16311 |
| H11a5 | 195 263 750 961G 1438 4769 8448 8860 13759 15040 15326 16293 16311 |
| H11a6 | 195 263 750 961G 1438 1719 4769 5979 8448 8860 13759 15326 16293 16311 16525 |
| H11a+152 | 152 195 263 750 961G 1438 4769 8448 8860 13759 15326 16293 16311 |
| H11a7 | 152 195 263 750 961G 1438 4769 8448 8860 9911 13759 15326 16293 16311 |
| H11a8 | 195 263 750 961G 1438 4769 8448 8860 13759 14325 15326 16293 16311 |
| H11b | 195 263 750 1438 4769 8448 8860 13572 13759 15326 16311 |
| H11b1 | 195 263 750 1438 4769 7645 8448 8860 13572 13759 15326 16311 |
| H12 | 195 263 750 1438 3936 4769 8860 14552 15326 |
| H12a | 195 263 750 1438 3936 4769 8860 14552 15326 16287 |
| H91 | 195 263 335 750 1438 4769 8860 15326 |
| H108 | 195 263 750 1438 3808 4769 8860 15326 |
| H+152 | 152 263 750 1438 4769 8860 15326 |
| H9 | 152 263 750 1438 4769 8860 13020 15326 |
| H9a | 152 263 750 1438 3591 4310 4769 8860 13020 15326 16168 |
| H32 | 73 152 263 750 1438 4769 8557 8860 15326 |
| H46 | 152 263 750 1438 2772 4769 8860 15326 |
| H46a | 152 263 750 1438 2772 4769 6743 8860 10754 15326 |
| H46b | 152 263 750 1438 2772 4769 8860 11893 15326 |
| H52 | 152 263 750 1438 4769 8860 14220 15326 |
| H69 | 152 263 750 1438 4646 4769 8860 15326 |
| H103 | 152 263 750 1438 2352 3834 4243 4769 7299 8860 15326 16295 |
| H107 | 152 263 750 1438 4769 8860 15326 16209 16261 |
| H10 | 263 750 1438 4769 8860 14470A 15326 |
| H10a | 263 750 1438 4216 4769 8860 14470A 15326 |
| H10a1 | 263 750 1438 4216 4769 8860 14470A 15326 16114 |
| H10a1a | 263 750 1438 4216 4769 8860 14470A 14548 15326 16114 |
| H10a1a1 | 263 750 1438 4216 4769 8860 13711 14470A 14548 15326 16114 |
| H10a1b | 263 513 750 1438 4216 4769 7220 8860 10325 14470A 15326 16114 16344 |
| H10b | 263 750 1438 4769 8860 9091 14470A 15326 |
| H10b1 | 263 750 1438 4769 8860 9091 12153 14470A 15326 |
| H10c | 263 709 750 1438 4769 6734 8860 14470A 15326 |

| | |
|---|---|
| H10c1 | 263 709 750 1438 1888 4769 6734 8860 14470A 15287 15326 |
| H10d | 263 533 750 1438 4769 8860 14470A 15326 |
| H10+(16093) | 263 750 1438 4769 8860 14470A 15326 (16093) |
| H10e | 263 750 1438 4769 8860 14470A 15326 (16093) 16221 |
| H10e1 | 263 750 1438 4769 8860 13830 14470A 15326 (16093) 16221 |
| H10e1a | 263 750 1438 4769 8860 13830 14470A 15326 (16093) 16221 16266 |
| H10e2 | 263 750 1438 4769 8860 14470A 14602 15326 (16093) 16221 |
| H10e3 | 263 750 1438 4769 5918 8860 14470A 15326 (16093) 16221 |
| H10e3a | 263 750 961 1438 4769 5918 8860 14470A 15326 (16093) 16221 |
| H10f | 263 750 1438 1842 4769 8860 14470A 15326 (16093) |
| H10g | 263 750 1438 4769 8860 14470A 15326 (16093) 16272 |
| H10h | 263 750 1438 3381 4769 8860 8888 9966 14470A 15326 |
| H13 | 263 750 1438 4769 8860 14872 15326 |
| H13a | 263 750 1438 2259 4769 8860 14872 15326 |
| H13a1 | 263 750 1438 2259 4745 4769 8860 14872 15326 |
| H13a1a | 263 750 1438 2259 4745 4769 8860 13680 14872 15326 |
| H13a1a1 | 263 750 1438 2259 4745 4769 7337 8860 13326 13680 14872 15326 |
| H13a1a1a | 263 750 1438 2259 4745 4769 7337 8860 13326 13680 14831 14872 15326 |
| H13a1a1b | 263 750 1438 2259 4745 4769 7337 8860 13326 13680 14727 14872 15326 |
| H13a1a1c | 263 750 1438 2259 4745 4769 7337 8860 13326 13680 14872 15326 15412 |
| H13a1a1d | 263 709 750 1438 2259 4745 4769 7337 8842 8860 13326 13680 14872 15326 16261 16311 |
| H13a1a1d1 | 263 709 750 1438 2259 4745 4769 7337 8842 8860 10101 13326 13680 14872 15326 16261 16311 |
| H13a1a1e | 263 750 1438 2259 4745 4769 7337 7830 8860 13326 13680 14872 15326 |
| H13a1a2 | 263 750 1438 2259 4745 4769 8860 13542 13680 14872 15326 |
| H13a1a2+16311 | 263 750 1438 2259 4745 4769 8860 13542 13680 14872 15326 16311 |
| H13a1a2a | 263 750 1438 2259 4745 4769 8860 9449 13542 13680 14872 15326 15712 16278 16311 |
| H13a1a2b | 146 263 750 1438 2259 2701 4745 4769 8860 12651 13542 13680 14872 15326 15758 |
| H13a1a3 | 263 750 1438 2259 4745 4769 8860 13680 14239 14872 15326 |
| H13a1a4 | 263 750 1438 2259 4745 4769 8381 8860 13680 14872 15326 |
| H13a1a5 | 263 750 1438 2259 4745 4769 8860 9801 13680 14872 15326 |
| H13a1a6 | 228 263 750 1438 2259 4745 4769 8860 13680 14872 15326 16207 |
| H13a1b | 153 263 750 1438 2259 4231 4745 4769 7903 8860 14872 15326 |
| H13a1c | 263 750 1438 2259 3744 4745 4769 8860 14872 15326 16244 |
| H13a1+152 | 152 263 750 1438 2259 4745 4769 8860 14872 15326 |
| H13a1d | 152 263 750 1438 2259 4745 4769 8860 14872 15326 16234 |
| H13a2 | 263 709 750 1438 2259 4769 8860 14872 15326 |
| H13a2a | 263 709 750 1008 1438 2259 4769 8860 14872 15326 |
| H13a2a1 | 263 709 750 1008 1438 2259 4011 4769 8860 12662 14872 15326 |
| H13a2b | 263 709 750 1438 2259 4769 8860 13762G 14872 15326 |
| H13a2b1 | 263 709 750 1438 2259 4639 4769 8860 13762G 14872 15001 15326 16311 |
| H13a2b2 | 263 709 750 1438 2259 4769 8860 10463 13575 13762G 14872 15326 |
| H13a2b2a | 263 709 750 1438 2259 4769 8860 10463 13575 13762G 14872 15326 15713G |
| H13a2b3 | 73 143 263 709 750 1438 2259 4769 8860 13762G 14872 15326 |

| | |
|---|---|
| H13a2b4 | 263 709 750 2259 4769 8266 8860 9548 13762G 14872 15217 15326 |
| H13a2b5 | 263 709 750 1438 2259 4769 8860 13762G 14863 14872 15326 16278 |
| H13a2c | 263 709 750 1438 2259 4769 6827 8860 14872 15218 15326 16256 16319 |
| H13a2c1 | 189 263 709 750 1438 2259 4769 6827 8860 14872 15218 15326 16148 16256 16319 |
| H13b | 263 750 1438 4107 4769 8860 14872 15326 (16362) |
| H13b1 | 263 750 1438 4107 4769 8860 11485 14872 15326 16261 (16362) |
| H13b1+200 | 200 263 750 1438 4107 4769 8860 11485 14872 15326 16261 (16362) |
| H13b1a | 200 263 750 1438 4107 4769 8860 11485 13620 14872 15326 16261 |
| H13b1b | 200 263 750 1438 4107 4769 7403 8860 11440 11485 14872 15326 16261 16291 (16362) |
| H13b2 | 263 750 1438 2713 4107 4769 8860 14872 15109 15326 (16362) |
| H13c | 263 750 1438 4769 8206 8860 14872 15326 |
| H13c1 | 263 750 1438 4769 5568 8206 8860 14872 15326 |
| H13c1a | 263 750 1438 4769 5568 8206 8860 10202 11314 14872 15326 |
| H13c2 | 143 263 750 1438 4769 8206 8860 14872 15326 16129 16300 |
| H14 | 263 750 1438 4769 7645 8860 10217 15326 |
| H14a | 263 750 1438 4769 7645 8860 10217 15326 16256 16352 |
| H14a+146 | 146 263 750 1438 4769 7645 8860 10217 15326 16256 16352 |
| H14a1 | 146 263 750 1438 4769 7645 8860 10217 12870 15326 16256 16352 |
| H14a2 | 263 750 1438 4769 6182 7645 8860 10217 15326 16256 16352 |
| H14a2a | 263 750 1438 4769 6182 7645 8296 8860 10217 15326 16256 16294 16352 |
| H14a2b | 263 750 1438 1462 4769 6182 7645 8227 8860 10217 15326 16256 16352 |
| H14a2c | 263 750 4769 6182 7645 8860 10217 14569 15326 16256 16352 |
| H14b | (152) 263 750 1438 3197 4769 7645 8860 10217 15326 |
| H14b1 | (152) 263 750 1438 3197 3906 4769 7645 8860 10217 11864 15326 16126 |
| H14b2 | (152) 263 750 1438 3197 4769 7610 7645 8860 9689 10217 15326 |
| H14b2a | (152) 197 263 750 1438 3197 4769 7610 7645 8860 9148 9689 10217 |
| H14b3 | (152) 263 750 1438 3197 4769 5319 7645 8860 10217 15326 16260 |
| H14b4 | (152) 263 750 1438 3197 4769 7645 8860 10217 12834 15326 |
| H15 | 55 57 263 750 1438 4769 6253 8860 15326 |
| H15a | 55 57 263 750 1438 4769 6253 8860 11410 15326 |
| H15a1 | 55 57 263 750 1438 4769 6253 8860 11410 14953 15326 |
| H15a1a | 55 57 263 750 1438 4769 6253 8860 11143 11410 14953 15326 |
| H15a1a1 | 55 57 263 750 1438 4769 6253 8860 11143 11410 14953 15326 16366 |
| H15a1b | 44.1C 55 57 146 263 750 1438 4769 6253 8860 11410 11914 14953 15326 16184 |
| H15b | 55 57 263 750 1438 3847 4769 6253 8860 15326 |
| H15b1 | 55 57 263 750 1438 3847 4769 6253 8860 15326 15715 |
| H15b2 | 55 57 263 750 1438 3847 4769 6253 8699 8860 13818 15326 |
| H16 | 263 750 1438 4769 8860 10394 15326 |
| H16+152 | 152 263 750 1438 4769 8860 10394 15326 |
| H16a | 152 263 750 1438 4769 8592 8860 10394 15326 |
| H16a1 | 152 263 750 1438 4769 8592 8860 10394 15326 15340 |
| H16c | 152 263 750 1438 4769 8860 9071 10394 15326 |
| H16d | 152 263 750 1438 4769 8860 10394 14155 14869C 15326 |
| H16b | 263 750 1438 4769 8860 9129 10394 15326 |
| H16e | 263 750 1438 4769 8860 10394 14249 14364 15326 |

| | |
|---|---|
| H+16129 | 263 750 1438 4769 8860 15326 16129 |
| H17 | 263 750 1438 3915 4769 8860 15326 16129 |
| H17a | 263 750 1438 3915 4769 6296 8860 15326 16129 |
| H17a1 | 263 750 1438 3915 4769 6296 8860 12346 15326 16129 16291 |
| H17a2 | 263 593 750 1438 3915 4769 6296 8860 15326 16129 |
| H17b | 263 750 1438 3915 4769 8860 8895A 15326 16129 |
| H17c | 73 263 750 1438 3915 4769 8860 12397 15326 16129 16239 |
| H27 | 263 750 1438 4769 8860 11719 15326 16129 16316 |
| H27+16093 | 263 750 1438 4769 8860 11719 15326 16093 16129 16316 |
| H27a | 263 750 1438 4769 8860 9391 11719 15326 16093 16129 16316 |
| H27b | 263 750 1438 4769 8860 11719 15326 16093 16129 16258C 16316 |
| H27c | 263 750 1438 4769 4838 8860 11719 15326 16129 16316 |
| H27d | 263 380 750 1438 4769 8860 11719 15326 16129 16316 |
| H27e | 263 750 1438 4769 8281-8289d 8860 11719 15326 16129 16316 |
| H27f | 263 750 1438 4769 8860 11719 13827 15326 16129 16291 16316 |
| H+13708 | 263 750 1438 4769 8860 13708 15326 |
| H18 | 263 750 1438 4769 8860 13708 14364 15326 |
| H18b | 263 750 1438 4769 8860 13105 13708 14364 15326 |
| H19 | 85 263 750 1438 4769 7598 8860 13708 15326 |
| H20 | 263 750 1438 4769 8860 15326 16218 |
| H20a | 263 750 1438 4769 8860 15326 16218 16328A |
| H20a1 | 249d 263 750 1438 4769 8860 15326 16218 16328A |
| H20a1a | 249d 263 292 750 1438 4769 8860 15326 16218 16328A 16362 |
| H20a2 | 263 750 1438 4769 8860 9756G 15326 16218 16328A |
| H20b | 263 750 1438 2835 4769 8860 10115 14968 15326 15562 15758 16218 |
| H20c | 263 750 1438 4769 7334 8860 15326 16218 |
| H21 | 186 263 750 1438 3822 4769 8860 8994 15326 16192 |
| H22 | 263 750 1438 4769 8860 15326 16145 16227 |
| H23 | 263 750 1438 4769 8860 10211 15326 |
| H24 | 263 750 1438 4769 8860 15326 16293 |
| H24a | 263 750 1438 3333 4769 8860 15326 16293 |
| H24a1 | 263 750 1438 3333 4769 5261 8860 15326 16234 16293 |
| H24a2 | 263 750 1438 3333 4769 8860 11440 15326 16293 |
| H24b | 263 750 1438 1719 4769 8860 15326 16293 |
| H25 | 263 750 1438 4769 8860 9620 15326 |
| H26 | 263 750 1438 4769 8860 11152 15326 |
| H26a | 263 750 1438 4769 8860 9545 11152 15326 |
| H26a1 | 263 750 1438 4769 8860 9545 11152 13500 15326 |
| H26a1a | 263 750 1438 4769 8860 9545 11152 13500 14203 15326 |
| H26a1a1 | 196 263 750 1438 4769 8860 9545 11152 12651 13500 14203 15326 |
| H26a1b | 263 750 1438 4769 8860 9545 11152 13500 15326 16170 16390 |
| H26b | 263 750 1438 4769 8860 11152 11399 12810 15326 16320 |
| H26c | 146 263 750 1438 4769 8860 10562 11152 15326 |
| H28 | 186A 263 750 1438 4769 8860 15326 |
| H28a | 186A 263 750 1438 4769 8715 8860 11191 15326 |
| H28a1 | 186A 263 750 1438 2239 4769 8715 8860 11191 15326 15758 |
| H28a2 | 186A 263 750 1438 4769 5495 8715 8860 11191 15326 |
| H29 | 93 263 573.XC 750 1438 4769 5582 8860 15326 |

| | |
|---|---|
| H29a | 93 263 573.XC 750 1438 4769 5582 8860 9156 15326 |
| H29b | 93 263 573.XC 750 1438 4769 5582 8860 13635A 15326 |
| H30 | 263 750 1438 4769 8200 8860 15326 |
| H30a | 263 750 1438 4769 8200 8860 12133 14979 15326 |
| H30b | 263 750 1438 3010 4769 8200 8860 15326 |
| H30b1 | 152 263 709 750 1438 3010 4769 8200 8860 9101 15326 |
| H33 | 263 750 1438 4769 8860 11447 15326 |
| H33a | 263 750 1438 3507 4769 8860 11447 15326 |
| H33b | 263 750 1438 4769 8860 11447 15326 16172 16400 |
| H33c | 263 750 1438 4769 8860 10373 11447 15326 16188 |
| H+16291 | 263 750 1438 4769 8860 15326 16291 |
| H34 | 263 750 1438 4769 8860 15326 15519 16291 |
| H64 | 263 750 1438 4769 8860 11150 15326 16291 |
| H85 | 263 750 1438 4769 6323 8860 15326 16291 16390 |
| H35 | 263 750 1438 3342 4769 8860 15326 |
| H35a | 263 750 1438 3342 4769 8860 11253 15326 |
| H39 | 263 750 1438 4769 8860 15326 16299 |
| H39a | 263 709 750 1438 4769 8860 15326 16169 16299 |
| H39a1 | 263 709 750 1438 4769 8860 14553 15326 16169 16299 |
| H39b | 263 750 1438 4769 5892 8860 15326 16299 |
| H39c | 263 750 1438 4769 8860 13362 15326 16299 |
| H40 | 263 750 1438 4769 7621 8860 15326 |
| H40a | 263 750 1438 4769 5752.1A 7621 8860 15326 |
| H40b | 263 750 1438 4769 7444 7621 7678 8860 15326 |
| H41 | 263 750 1438 4769 8860 15326 15617 |
| H41a | 262 263 750 1438 4769 5460 8860 10124 14118 15326 15617 |
| H42 | 263 750 1438 4769 8860 9758 15326 |
| H42a | 263 750 1438 4769 5498 8860 9758 15326 16294 |
| H42a1 | 263 750 1438 4769 5498 8860 9758 11458 15326 16294 |
| H42a2 | 263 750 1438 4769 5498 8860 9758 11989 14502 15326 16294 |
| H43 | 263 750 1438 4769 7202 8860 15326 |
| H44 | 263 750 1438 4769 5442 8860 15326 |
| H44a | 263 750 1438 4769 5442 8860 9932 14149 15326 |
| H44a1 | 263 750 1438 4769 5442 8843 8860 9932 11044 14149 15326 |
| H44b | 195 263 750 1438 4769 5442 8860 13662 15326 |
| H45 | 263 750 1438 4769 8843 8860 15326 |
| H45a | 263 750 1438 4769 8843 8860 9039 12192 15326 |
| H45b | 263 750 1438 4164 4769 8843 8860 12130 15326 |
| H47 | 263 750 1438 4769 8860 9530 12633 15326 |
| H47a | 152 263 750 1438 4769 8756 8860 9530 12633 13020A 15326 |
| H48 | 263 750 1438 4769 8860 11016 15326 |
| H49 | 263 750 1438 4769 8860 11560 15326 |
| H49a | 263 750 1438 4769 8860 11560 15058 15326 |
| H49a1 | 263 750 1438 4769 5082 8860 11560 15058 15326 |
| H49a2 | 93 263 750 1438 4769 8860 9612 11560 15058 15326 |
| H49b | 263 750 1438 4769 8860 11560 15223 15326 |
| H50 | 263 750 1438 4769 8860 14831 15326 |
| H51 | 263 750 1438 4769 8860 11440 15326 |

| | |
|---|---|
| H51a | 263 750 1438 4769 8860 10704 11440 14239 15326 |
| H53 | 263 750 1438 4769 8860 9380 15326 |
| H54 | 263 750 1438 4769 5585 8860 15326 |
| H55 | 263 750 1438 4769 8860 10646 15326 |
| H55a | 263 750 1438 3387 4709 4769 8860 10646 15326 |
| H55+153 | 153 263 750 1438 4769 8860 10646 15326 |
| H55b | 153 263 750 1438 4769 8860 10646 15326 16189 |
| H56 | 263 750 1438 4769 8860 11788 15326 |
| H56a | 263 750 1438 4769 8860 11788 13145 15326 |
| H56a1 | 263 750 1438 4654 4769 8860 11788 13145 15326 |
| H56b | 263 750 1438 4769 8860 11788 12696 15326 |
| H56c | 263 750 1438 4769 8860 11788 14129 15326 |
| H56d | 153 263 750 1438 4769 8860 11788 15326 |
| H57 | 64 263 750 1438 4317d 4769 8860 15326 |
| H58 | 263 750 1438 4769 8860 15326 15466 |
| H58a | 263 750 1438 4769 8860 15326 15466 15721 |
| H59 | 263 750 1438 4769 8860 12674 15326 |
| H59a | 263 750 1438 4769 8860 10283 12674 15326 |
| H60 | 263 750 1438 4769 5108 8860 15326 |
| H60a | 263 750 1438 4769 5108 6971 8860 15326 |
| H61 | 263 750 1438 1842 4769 8860 11641 15326 |
| H61a | 263 750 1438 1842 3509 4769 8860 11641 15326 16192 |
| H62 | 263 750 1438 4769 8860 14344 15326 |
| H63 | 263 750 1438 2159 4769 8860 14193 15326 |
| H63a | 263 750 1438 2159 4769 8860 12904 14193 15326 16129 |
| H65 | 263 750 1438 4769 8860 15326 15784 |
| H65a | 263 750 1438 3010 4769 7805 8860 15326 15784 |
| H66 | 263 750 1438 4769 7337 8860 15326 |
| H66a | 263 750 1438 4769 7337 8860 15326 16172 |
| H66a1 | 263 750 1438 4769 7337 8860 15326 16172 16527 |
| H67 | 263 750 1438 4769 8860 13147 15326 |
| H67a | 263 750 1438 4769 5174 8347 8860 13147 15326 |
| H70 | 263 750 1438 4769 8860 9033 15326 |
| H71 | 263 750 1438 4769 6164 8860 15326 |
| H72 | 263 750 1438 4769 6647 8860 13785 15326 15927 |
| H73 | 263 750 1438 4221A 4769 8860 15326 |
| H73a | 263 750 1438 4221A 4769 8860 12477 13500 15326 15658 |
| H73a1 | 263 750 1438 4221A 4769 8860 11590 12477 13500 15326 15658 |
| H74 | 72G 263 750 1438 4769 8860 12753 14488 15326 |
| H75 | 263 750 1438 4769 5821 8592 8860 15326 |
| H76 | 263 750 1438 4769 8572 8860 15326 16311 |
| H76a | 152 263 750 1438 4769 8572 8860 15326 16093 16311 |
| H77 | 263 291.1A 750 1438 4769 8387 8860 15326 |
| H78 | 263 750 1438 4769 7002 8860 15326 |
| H79 | 263 750 1438 4769 8860 12397 15326 |
| H79a | 263 750 1438 3010 4769 8860 12397 15326 |
| H80 | 263 750 1438 2361 4769 8860 15326 16311 |
| H81 | 263 750 1438 4769 8860 15172 15326 |

| | |
|---|---|
| H81a | 263 750 1438 2056 3421 4769 7382 8860 9677 12063 15172 15326 |
| H82 | 263 750 1438 4769 8860 15326 16220 |
| H83 | 263 750 1438 4769 8860 15326 16212 |
| H84 | 263 750 1438 1888 4769 8860 15326 |
| H86 | 263 750 1438 1809 4769 8860 15326 |
| H87 | 263 750 1438 4769 8188 8860 15326 |
| H88 | 263 750 1438 4769 8596 8860 15326 |
| H89 | 263 750 1438 4769 6932 8068 8860 12696 15326 |
| H90 | 263 750 1438 4769 5435 8860 8911 10237 15109 15326 |
| H92 | 263 750 1438 4769 8860 9497 15326 |
| H93 | 263 750 1438 3535 4769 8860 15326 |
| H94 | 263 750 1438 4769 8860 14323 15326 16355 |
| H95 | 263 750 1438 4769 8860 11069 15326 |
| H95a | 263 750 1438 4769 8860 10403 11069 14025 15314 15326 |
| H96 | 185 189 263 709 750 1438 4769 8860 13269 14040 15326 16189 |
| H100 | 263 750 1438 4769 8860 12358 15326 |
| H101 | 263 750 1438 4769 8860 9230 15326 16184 |
| H102 | 263 750 1438 3798 4769 8860 14180 15326 |
| H104 | 263 750 1438 4769 8723 8860 15326 |
| H104a | 263 750 1438 4769 5618 8723 8812 8860 15326 16258 16311 16325 |
| H105 | 263 750 1438 4769 8860 9921 15326 |
| H105a | 263 750 1438 3010 4769 8860 9921 15326 |
| H106 | 263 750 1438 4769 6410 8860 15326 16245 |
| R1 | 73 263 750 1391 1438 2706 4769 7028 8860 11719 14766 15326 16311 |
| R1a | 73 263 295A 750 1391 1438 2706 3360 4769 4917 5586 5823 6557 6671 7028 7547 8388 8860 8887 10658 10825 11719 13948 14632 14766 15326 15721 16311 |
| R1a1 | 73 263 295A 750 1391 1438 2706 3360 4026 4769 4917 5586 5823 6557 6671 7028 7424 7547 8388 8860 8887 10658 10825 11719 13948 14632 14766 15326 15721 16311 |
| R1a1a | 73 263 295A 750 1391 1438 2706 3360 4026 4769 4917 5378 5586 5823 6557 6671 7028 7424 7547 8388 8860 8887 10658 10825 11719 13948 14632 14766 15326 15721 16311 |
| R1a1a1 | 73 263 295A 750 1391 1438 2706 3360 4026 4769 4917 5378 5586 5823 6557 6671 7028 7424 7547 8388 8860 8887 10658 10825 11719 13105 13368 13948 14632 14766 15326 15721 16311 |
| R1a1a1a | 73 263 295A 750 1391 1438 2706 3360 4026 4769 4917 5378 5586 5823 6261 6557 6671 7028 7424 7547 8388 8860 8887 10658 10825 11719 13105 13368 13948 14632 14766 15326 15721 16311 |
| R1a1a2 | 73 263 295A 750 1391 1438 2706 3360 4026 4769 4917 5378 5492 5586 5823 6557 6671 7028 7424 7547 8388 8765 8860 8887 10658 10825 11719 11977 13948 14632 14766 15326 15721 16311 |
| R1a1b | 73 263 295A 750 1391 1438 2706 3360 3796 4026 4769 4917 5586 5823 6557 6671 7028 7424 7547 8388 8860 8887 9101 10658 10825 11719 13948 14632 14766 15326 15721 16311 |
| R1a1c | 73 263 295A 750 1391 1438 2706 3360 4026 4769 4917 5586 5823 6260 6557 6671 7028 7424 7547 8388 8860 8887 10658 10825 11719 13948 14632 14766 15326 15721 16311 |

| | |
|---|---|
| R1b | 73 150 241 263 750 1095 1391 1438 2706 4769 7028 8860 11151 11719 12245 13167 13359 14239 14766 15326 15940 16311 |
| R1b1 | 73 150 241 263 513 750 1095 1391 1438 2706 4769 6683 7028 8860 11122A 11151 11719 12245 13167 13359 14239 14766 15326 15940 16311 |
| R2'JT | 73 263 750 1438 2706 4216 4769 7028 8860 11719 14766 15326 |
| R2 | 73 152 263 750 1438 2706 4216 7028 7657 8473 8860 9932 10685 11719 12654 14305 14766 15326 16071 |
| R2+13500 | 73 152 263 750 1438 2706 4216 7028 7657 8473 8860 9932 10685 11719 12654 13500 14305 14766 15326 16071 |
| R2+13500+195 | 73 152 195 263 750 1438 2706 4216 7028 7657 8473 8860 9932 10685 11719 12654 13500 14305 14766 15326 16071 |
| R2a | 73 152 195 263 750 769 1438 2706 4216 7028 7657 8473 8860 9932 10685 11719 12519 12654 13500 14305 14766 15326 16071 |
| R2b | 73 152 263 750 1438 2706 4216 7028 7657 8473 8790 8860 9738 9932 10685 11719 12654 13500 14062 14305 14323 14766 15326 16071 |
| R2b1 | 73 146 152 263 750 1438 2706 4216 7028 7657 8473 8790 8860 9738 9932 10685 11719 12654 13500 14062 14305 14323 14766 15204 15326 16071 |
| R2c | 73 146 152 263 573.XC 750 1438 2706 4216 7028 7657 8473 8860 8994 9861 9932 10685 11719 12654 12793 13500 14185 14305 14766 15326 16071 16320 |
| R2d | 73 152 263 750 1438 2706 4216 7028 7657 8143 8473 8860 9932 10685 11719 12654 13434 13500 13914A 14305 14766 15326 16071 |
| JT | 73 263 750 1438 2706 4216 4769 7028 8860 11251 11719 14766 15326 15452A 16126 |
| J | 73 263 295 489 750 1438 2706 4216 4769 7028 8860 10398 11251 11719 12612 13708 14766 15326 15452A 16069 16126 |
| J1 | 73 263 295 462 489 750 1438 2706 3010 4216 4769 7028 8860 10398 11251 11719 12612 13708 14766 15326 15452A 16069 16126 |
| J1b | 73 263 295 462 489 750 1438 2706 3010 4216 4769 7028 8269 8860 10398 11251 11719 12612 13708 14766 15326 15452A 16069 16126 16145 (16222) 16261 |
| J1b1 | 73 263 295 462 489 750 1438 2706 3010 4216 4769 5460 7028 8269 8860 10398 11251 11719 12612 13708 13879 14766 15326 15452A 16069 16126 16145 (16222) 16261 |
| J1b1a | 73 242 263 295 462 489 750 1438 2158 2706 3010 4216 4769 5460 7028 8269 8557 8860 10398 11251 11719 12007 12612 13708 13879 14766 15326 15452A 16069 16126 16145 (16222) 16261 |
| J1b1a1 | 73 242 263 295 462 489 750 1438 2158 2706 3010 4216 4769 5460 7028 8269 8557 8860 10398 11251 11719 12007 12612 13708 13879 14766 15326 15452A 16069 16126 16145 16172 (16222) 16261 |
| J1b1a1a | 73 242 263 295 462 489 750 1438 2158 2706 3010 4216 4769 5460 5463 6911 7028 8269 8557 8860 10398 11251 11719 12007 12612 13708 13879 14766 15326 15452A 16069 16126 16145 16172 16192 (16222) 16261 |
| J1b1a1b | 73 242 263 295 462 489 750 1438 2158 2706 3010 4216 4769 5460 7028 8269 8557 8860 10398 11251 11719 12007 12612 13708 13879 14766 15067 15326 15452A 16069 16126 16145 16172 (16222) 16261 |

| | |
|---|---|
| J1b1a1c | 10 73 242 263 295 462 489 750 1438 2158 2706 3010 4216 4769 5460 7028 8269 8557 8860 10398 11251 11719 12007 12612 13708 13879 14766 15326 15452A 16069 16126 16145 16172 (16222) 16261 |
| J1b1a1d | 73 185 242 263 295 462 489 750 1438 2158 2706 3010 4216 4769 5460 6345 7028 7299 8269 8557 8860 10398 11251 11719 12007 12612 13708 13879 14766 15326 15452A 16069 16126 16145 16172 (16222) 16261 |
| J1b1a1+146 | 73 146 242 263 295 462 489 750 1438 2158 2706 3010 4216 4769 5460 7028 8269 8557 8860 10398 11251 11719 12007 12612 13708 13879 14766 15326 15452A 16069 16126 16145 16172 (16222) 16261 |
| J1b1a1e | 73 146 242 263 295 462 489 750 1438 2158 2706 3010 4216 4769 5460 7028 8269 8557 8860 10398 11251 11719 12007 12612 13708 13879 14470 14766 15326 15452A 16069 16126 16145 16172 (16222) 16261 |
| J1b1a2 | 73 199 242 263 295 462 489 750 1438 2158 2706 3010 4216 4769 5460 5964 7028 8269 8557 8860 10398 11251 11719 12007 12396 12612 13308 13708 13879 14766 15326 15452A 16069 16126 16145 16187 (16222) |
| J1b1a2a | 73 199 242 263 295 462 489 750 1438 2158 2706 3010 4216 4769 5460 5585 5964 7028 8269 8557 8860 9494 10398 11251 11719 12007 12396 12612 13308 13708 13879 14766 15326 15452A 16069 16126 16145 16187 (16222) 16261 |
| J1b1a2b | 73 199 242 263 295 462 489 750 1438 2158 2706 3010 4216 4769 4772 5460 5964 7028 8269 8557 8860 10398 11251 11719 12007 12396 12612 13308 13708 13879 14766 15326 15452A 16069 16126 16145 16187 (16222) 16261 |
| J1b1a3 | 73 242 263 295 462 489 750 1438 2158 2706 3010 4216 4769 5460 7028 8269 8557 8860 10398 11251 11719 12007 12612 13708 13879 14766 15326 15442 15452A 16069 16126 16145 (16222) 16261 |
| J1b1b | 73 263 271 295 462 489 750 1438 2706 3010 4216 4769 5460 7028 8269 8860 10398 11251 11719 12612 13708 13879 14766 15326 15452A 16069 16126 16145 (16222) 16261 |
| J1b1b1 | 73 263 271 295 462 489 750 1438 2706 3010 4216 4769 5460 7028 8269 8860 10398 10410A 11251 11719 12612 13708 13879 14766 15326 15452A 16069 16126 16145 (16222) 16261 |
| J1b1b1a | 73 263 271 295 462 489 750 1438 2706 2707C 3010 4216 4769 5460 7028 8269 8860 10398 10410A 11251 11719 12612 13708 13879 14766 15326 15452A 16069 16126 16145 (16222) 16261 16290 |
| J1b1b1b | 73 263 271 295 462 489 750 1438 2706 3010 4216 4769 5460 5582 7028 8269 8860 10398 10410A 11251 11719 12612 13708 13879 14766 15326 15452A 16069 16126 16145 (16222) 16261 |
| J1b1b1c | 73 204 207 263 271 295 462 489 750 2706 3010 4216 4769 5460 7028 7747 8269 8860 10398 10410A 10942 11251 11719 12612 13708 13879 14766 15326 15452A 16069 16126 16145 (16222) 16256 16261 |
| J1b1b2 | 73 152 263 271 295 462 489 750 1438 2706 3010 4216 4769 5460 7028 8269 8860 9083 10398 11251 11719 12612 13708 13879 14766 15326 15452A 16069 16126 16145 (16222) 16235 16261 |
| J1b1b3 | 73 263 271 295 462 489 750 1438 2706 3010 4216 4769 5460 7028 8269 8860 10398 11251 11719 12612 13708 13879 14766 15326 15452A 15941 16069 16126 16145 (16222) 16261 |

| | |
|---|---|
| J1b2 | 73 263 295 462 489 750 1438 1733 2706 3010 4216 4769 7028 8269 8860 10398 11251 11719 12612 13708 14766 15326 15452A 16069 16126 16145 (16222) 16261 |
| J1b2a | 73 263 295 462 489 750 1438 1733 2706 3010 4216 4769 7028 8269 8860 9667 10398 11251 11719 12612 13708 14766 15326 15452A 15466 16069 16126 16136 16145 (16222) 16261 |
| J1b3 | 73 263 295 462 489 750 1438 2706 3010 4216 4769 7028 8269 8460 8860 10398 11251 11719 12612 13708 14766 15326 15452A 16069 16126 16145 (16222) (16235) 16261 |
| J1b3a | 73 263 295 462 489 750 1438 2706 3010 4216 4769 7028 8269 8460 8860 10398 11251 11719 12612 13708 14766 15326 15452A 16069 16126 16145 (16222) (16235) 16261 16271 |
| J1b3b | 73 263 295 462 489 750 1438 2706 3010 4216 4769 7028 8269 8460 8860 10398 11251 11719 12612 13708 14766 15326 15452A 15530 16069 16126 16145 (16222) (16235) 16261 |
| J1b3b1 | 73 263 295 462 489 750 1438 2706 3010 4216 4769 7028 8269 8460 8860 10398 11251 11719 12612 13708 14766 15172T 15326 15452A 15490 15530 16069 16126 16145 (16222) (16235) 16261 |
| J1b4 | 73 263 295 462 489 750 1438 2706 3010 3290 4216 4769 7028 8269 8860 10398 11251 11719 12612 13708 14766 15326 15452A 16069 16126 16145 (16222) 16261 |
| J1b4a | 73 263 295 462 489 750 769 1438 2706 3010 3290 4216 4769 7028 8269 8860 10398 11084 11251 11719 12612 13708 14766 15326 15452A 16069 16126 16145 (16222) 16261 16287 |
| J1b4a1 | 73 263 295 462 489 750 769 1438 2706 3010 3290 4013 4216 4769 7028 8269 8860 10398 11084 11251 11719 12612 13708 14766 15326 15452A 16069 16126 16145 (16222) 16261 16287 |
| J1b4a2 | 73 263 295 462 489 750 769 1438 2706 3010 3290 4216 4769 7028 8222 8269 8860 10398 11084 11251 11719 12612 13708 14766 15326 15452A 16069 16126 16145 (16222) 16261 16287 |
| J1b5 | 73 263 295 462 489 750 1438 2706 3010 4216 4769 7028 8269 8860 10274 10398 11251 11719 12612 13145 13708 14766 15326 15452A 16069 16126 16145 (16222) 16261 16274 |
| J1b5a | 73 199 263 295 462 489 709 750 1438 2706 3010 4216 4769 7028 8269 8860 9324 10274 10398 11251 11719 12612 13145 13708 14455 14766 15326 15452A 16069 16126 16145 (16222) 16261 16274 |
| J1b5a1 | 73 199 263 295 462 489 709 750 1438 2706 3010 4216 4769 6045A 7028 8269 8860 9324 10274 10398 11251 11719 12612 13145 13708 14455 14766 15326 15452A 16069 16126 16145 (16222) 16261 16274 |
| J1b6 | 73 263 295 462 489 750 1438 2706 3010 4216 4769 5501 7028 8269 8860 10398 11251 11719 12612 13708 14766 15326 15452A 16069 16126 16145 (16222) 16261 |
| J1b6a | 73 263 295 462 489 750 1438 2706 3010 4216 4769 4991 5501 7028 8269 8860 10398 11251 11719 12612 13708 14766 15326 15452A 16069 16126 16145 (16222) 16261 |
| J1b6b | 73 203C 263 295 462 489 750 1438 2706 3010 4216 4769 5501 7028 8269 8860 10398 11251 11719 12612 13708 14766 15326 15452A 16069 16126 16145 16189 (16222) 16261 16288 |

| | |
|---|---|
| J1b7 | 73 150 263 266 295 462 489 750 1438 2706 3010 4216 4769 7028 8269 8860 10398 11251 11719 12612 13708 14766 15326 15452A 16069 16126 16145 (16222) 16261 |
| J1b7a | 73 150 263 266 295 462 489 750 1438 2706 3010 4216 4769 7028 8269 8860 10398 11251 11719 12612 13708 14766 15326 15452A 16069 16086 16126 16145 (16222) 16261 |
| J1b8 | 73 152 263 295 462 489 750 1438 2706 3010 4216 4696 4769 7028 8269 8860 9932 10398 11251 11719 12612 13708 14766 15326 15452A 16069 16126 16145 (16222) 16261 |
| J1b9 | 73 263 295 462 489 709 750 1438 2706 3010 4216 4769 7028 8269 8860 10398 10601A 11251 11719 12612 13708 14766 15326 15452A 16069 16126 16145 (16222) 16261 16290 |
| J1c | 73 (185) (228) 263 295 462 489 750 1438 2706 3010 4216 4769 7028 8860 10398 11251 11719 12612 13708 14766 14798 15326 15452A 16069 16126 |
| J1c1 | 73 (185) (228) 263 295 462 482 489 750 1438 2706 3010 3394 4216 4769 7028 8860 10398 11251 11719 12612 13708 14766 14798 15326 15452A 16069 16126 |
| J1c1a | 73 (185) (228) 263 295 462 482 489 750 1438 2706 3010 3394 4216 4769 7028 8860 9635C 10398 11251 11623 11719 12612 13708 13899 14766 14798 15326 15452A 16069 16126 |
| J1c1b | 73 (185) (228) 263 295 462 482 489 750 1438 2706 3010 3394 4216 4769 7028 7184 8860 10398 11251 11719 12612 13708 14766 14798 15326 15452A 16069 16126 |
| J1c1b1 | 73 (185) (228) 263 295 462 482 489 750 1438 2706 3010 3394 4216 4769 5773 7028 7184 8860 10398 11251 11719 12612 13708 14766 14798 15326 15452A 16069 16126 |
| J1c1b1a | 73 (185) (228) 263 295 462 482 489 750 1438 2706 3010 3394 4216 4769 5773 7028 7184 8860 10398 10463 11251 11719 12612 13708 14766 14798 15326 15452A 16069 16126 |
| J1c1b1a1 | 73 (185) (228) 263 295 462 482 489 750 1438 2706 3010 3394 4216 4769 5773 7028 7184 8860 10398 10463 11251 11719 12612 13708 14766 14798 15326 15452A 15617 16069 16126 |
| J1c1b2 | 73 (185) (228) 263 295 462 482 489 750 1438 2706 3010 3394 4216 4769 7028 7184 8860 10398 11251 11719 12612 13708 13953 14766 14798 15326 15452A 15725 16069 16126 |
| J1c1b2a | 73 (185) (228) 263 295 462 482 489 750 1005 1438 2706 3010 3394 4216 4769 7028 7184 8860 10398 11251 11719 12612 13398 13708 13953 14766 14798 15326 15452A 15725 16069 16126 |
| J1c1c | 73 150 (185) (228) 263 295 462 482 489 750 1438 2706 3010 3394 4216 4769 7028 8860 10398 11251 11719 12612 13708 14766 14798 15326 15452A 16069 16126 16145 |
| J1c1d | 73 (185) (228) 263 295 462 482 489 750 1438 2706 3010 3394 4216 4769 7028 8860 10398 11251 11719 12612 13708 14766 14798 15326 15452A 16069 16126 16213 |
| J1c1e | 73 (185) (228) 263 295 462 482 489 750 1438 2706 3010 3394 4216 4769 7028 8860 10398 10454 11251 11719 12612 13708 14766 14798 15326 15452A 16069 16126 16368 |

| | |
|---|---|
| J1c1f | 73 (185) (228) 263 295 462 482 489 750 1438 2706 3010 3394 4216 4769 5276 7028 8860 10398 11227 11251 11719 12612 13708 14766 14798 15326 15452A 16069 16126 |
| J1c1g | 73 (185) (228) 263 295 462 482 489 750 1438 2706 3010 3394 4216 4769 7028 8860 10398 11251 11287 11719 12612 13708 14766 14798 15326 15452A 16069 16126 |
| J1c1g1 | 73 (185) (228) 263 295 462 482 489 750 1438 2706 3010 3394 4216 4769 7028 8020 8860 10398 11251 11287 11719 12612 13708 14766 14798 15326 15452A 16069 16126 |
| J1c1h | 73 (185) (228) 263 295 462 482 489 750 1438 2706 3010 3394 4216 4769 7028 8860 9192 10398 11251 11719 12612 13708 14766 14798 15326 15452A 16069 16126 16169 |
| J1c2 | 73 (185) 188 (228) 263 295 462 489 750 1438 2706 3010 4216 4769 7028 8860 10398 11251 11719 12612 13708 14766 14798 15326 15452A 16069 16126 |
| J1c2a | 73 (185) 188 (228) 263 295 462 489 750 1438 2706 3010 4216 4769 6293 7028 8860 10398 11251 11719 12612 13708 14766 14798 15326 15452A 16069 16126 |
| J1c2a1 | 73 (185) 188 (228) 263 295 462 489 750 1438 2706 3010 4216 4769 6293 7028 7245 8839 8860 10398 11251 11719 12612 13708 14766 14798 15326 15452A 16069 16126 |
| J1c2a1a | 73 (185) 188 (228) 263 295 462 489 750 1438 2706 3010 4216 4769 6293 7028 7245 8839 8860 9181 10398 11251 11719 12612 13708 14766 14798 15326 15452A 16069 16126 |
| J1c2a2 | 73 (185) 188 (228) 263 295 462 489 750 1438 2706 3010 4216 4769 6293 6915 7028 8860 10398 11251 11719 12612 13708 14766 14798 15326 15452A 16069 16126 16256 |
| J1c2a3 | 73 (185) 188 (228) 263 295 462 489 750 1438 2706 3010 4216 4769 4790 6293 7028 8860 10398 11251 11719 12612 13708 14766 14798 15043 15326 15452A 16069 16126 |
| J1c2b | 73 (185) 188 (228) 263 295 462 489 750 1438 2706 3010 4216 4454 4769 7028 8860 10398 11251 11719 12612 13708 14766 14798 15326 15452A 16069 16126 |
| J1c2b1 | 73 (185) 188 (228) 263 295 462 489 750 1438 2706 3010 4216 4454 4769 7028 8853 8860 10398 11251 11719 12612 13506 13708 14470 14766 14798 15326 15452A 16069 16126 |
| J1c2b2 | 73 (185) 188 (228) 263 295 462 489 750 1438 2706 3010 4216 4454 4769 7028 8860 10398 11251 11719 12612 13708 14766 14798 15326 15452A 15511 16069 16126 |
| J1c2b3 | 73 (185) 188 (228) 263 295 462 489 750 1438 2706 3010 4216 4454 4769 5108 7028 8860 10398 11251 11719 12612 13708 14766 14798 15326 15452A 16066 16069 16126 16370 |
| J1c2b4 | 73 (185) 188 (228) 263 295 462 489 750 1438 2706 3010 4216 4454 4769 7028 8860 10398 11251 11719 12612 13708 14766 14798 15326 15452A 16069 16086 16126 |
| J1c2b5 | 73 (185) 188 (228) 263 295 462 489 750 1438 2706 3010 4216 4454 4769 6383 7028 8860 10398 11251 11719 12612 13708 14766 14798 15326 15452A 16069 16126 |

| | |
|---|---|
| J1c2c | 73 (146) (185) 188 (228) 263 295 462 489 750 1438 2706 3010 4216 4769 7028 8860 10398 10685 11251 11719 12612 13281 13708 13933 14766 14798 15326 15452A 16069 16126 |
| J1c2c1 | 73 (146) (185) 188 222 (228) 263 295 462 489 750 1438 2706 3010 4216 4769 7028 8860 10398 10685 11251 11719 12612 13281 13708 13933 14766 14798 15326 15452A 16069 16126 |
| J1c2c1a | 73 (146) (185) 188 222 (228) 263 295 462 489 750 1438 2706 3010 3456 4216 4769 7028 8860 9145 10398 10685 11251 11719 12612 13281 13708 13933 14766 14798 15326 15452A 16069 16126 16261A |
| J1c2c2 | 73 (146) (185) 188 (228) 263 295 462 489 750 1438 2706 3010 4216 4769 7028 8860 8865 10398 10685 11251 11719 12612 13281 13708 13933 14766 14798 15326 15452A 16069 16126 |
| J1c2c2a | 73 (146) (185) 188 (228) 263 295 462 489 750 1438 2706 3010 4216 4769 7028 8860 8865 10398 10685 11251 11719 12020 12612 13281 13708 13933 14766 14798 15326 15452A 16069 16126 |
| J1c2c3 | 73 (146) (185) 188 (228) 263 295 462 489 750 1438 2706 3010 4216 4769 7028 8860 10398 10685 11251 11719 12397 12612 13281 13708 13933 14766 14798 15326 15452A 16069 16126 |
| J1c2d | 73 (185) 188 (228) 263 295 462 489 750 1438 2706 3010 4216 4769 6620 7028 8860 8868 10398 11251 11719 12612 13708 14766 14798 15326 15452A 16069 16126 |
| J1c2e | 73 (185) 188 (228) 263 295 462 489 750 1438 2706 3010 4216 4769 7028 8860 10398 11251 11719 12612 13708 14766 14798 15326 15452A 16069 16126 16366 |
| J1c2e1 | 73 (185) 188 (228) 263 295 462 489 750 1438 2706 3010 4216 4769 7028 8860 10398 11251 11719 12612 13708 14766 14798 15326 15452A 16069 16126 16366 16390 |
| J1c2e2 | 73 (185) 188 (228) 263 295 462 489 709 750 1438 2706 3010 4216 4232 4769 7028 8404 8860 10398 11251 11719 12612 13708 14766 14798 15326 15452A 16069 16126 16278 16366 |
| J1c2f | 73 (185) 188 (228) 263 295 462 489 750 1438 2706 3010 4216 4769 7028 8860 9055 10398 11251 11719 12612 13708 14766 14798 15326 15452A 16069 16126 |
| J1c2g | 73 (185) 188 (228) 263 295 462 489 750 1438 2706 3010 4216 4769 7028 8860 10398 11251 11719 12311 12379 12612 13708 14766 14798 15119 15326 15452A 16069 16126 |
| J1c2h | 73 (185) 188 222 (228) 263 295 462 489 750 1438 2706 3010 4216 4769 7028 8860 10398 11251 11719 12612 13708 14766 14798 15326 15452A 16069 16126 |
| J1c2i | 73 (185) 188 (228) 263 295 462 489 750 1438 2706 3010 4216 4769 5162 7028 7870 8149 8860 10398 11251 11719 12612 13708 14766 14798 15326 15452A 16069 16126 16147 16242 |
| J1c2j | 73 (185) 188 (228) 263 295 462 489 750 1438 2706 3010 4216 4769 7028 8727 8860 10398 11251 11719 12612 13708 14766 14798 15326 15452A 16069 16126 16362 16390 |
| J1c2k | 73 (185) 188 (228) 263 295 462 489 750 1438 2706 3010 3866 4216 4769 7028 8860 10398 11251 11719 12612 13708 14766 14798 15326 15452A 16069 16126 |

| | |
|---|---|
| J1c2l | 73 (185) 188 (228) 263 295 462 489 750 1438 2706 3010 4216 4769 7028 7891 8860 10398 11251 11719 12612 13708 14766 14798 15199 15326 15452A 15936T 16069 16126 |
| J1c2m | 73 (185) 188 (228) 263 295 462 489 750 1438 2706 3010 4216 4769 7028 8860 10398 11251 11719 12612 13708 14766 14798 15326 15452A 16069 16126 16148 |
| J1c2m1 | 73 (185) 188 (228) 263 295 462 489 750 1438 2706 3010 4216 4769 7028 8860 10398 11251 11719 12612 13708 14766 14798 15326 15452A 16069 16126 16148 16235 16368 |
| J1c2n | 73 (185) 188 (228) 263 295 462 489 750 1438 2706 3010 4216 4769 7028 8860 8923 10398 11251 11719 12612 13708 14766 14798 15326 15452A 16069 16126 |
| J1c2n1 | 73 (185) 188 (228) 263 295 462 489 750 1438 2706 3010 4216 4769 7028 8860 8923 10101 10398 11251 11719 12612 12891 13708 14766 14798 15326 15452A 16069 16126 |
| J1c2o | 73 (185) 188 (228) 263 295 462 489 750 1438 2706 3010 4216 4769 7028 8860 10398 11251 11719 12612 13708 14766 14798 15326 15452A 16069 16126 16163 |
| J1c2p | 73 (185) 188 (228) 263 295 462 489 750 1438 2706 3010 4216 4769 7028 8860 10398 11177 11251 11719 12612 13708 14766 14798 15326 15452A 16069 16126 |
| J1c2q | 73 (185) 188 (228) 263 295 462 489 750 1438 2706 3010 4216 4769 7028 8558 8860 10398 11251 11719 12612 13557 13708 14766 14798 15326 15452A 16069 16126 |
| J1c2q1 | 73 (185) 188 (228) 263 295 462 489 750 1438 2706 3010 4216 4769 7028 8558 8860 10398 11251 11719 12612 13145 13557 13708 14766 14798 15326 15452A 16069 16126 |
| J1c2r | 73 (185) 188 (228) 263 295 462 489 750 1438 2706 3010 4216 4769 7028 8860 10398 11002 11251 11719 12612 13032 13708 14325 14766 14798 15326 15452A 16069 16126 16186 |
| J1c2s | 73 (185) 188 (228) 263 295 462 489 750 1438 2706 3010 4216 4769 7028 8860 10398 11016 11251 11719 12612 13708 14766 14798 15326 15452A 16069 16126 |
| J1c2s1 | 73 114 (185) 188 (228) 263 295 462 489 750 1438 2706 3010 4216 4769 7028 7364 8860 10398 11016 11251 11719 12612 13708 14766 14798 15326 15452A 16069 16126 |
| J1c2t | 73 (185) 188 (228) 263 295 462 489 750 1438 2706 3010 4216 4769 7028 8860 10398 11251 11719 12612 13708 14766 14798 15326 15452A 15947 16069 16126 |
| J1c3 | 73 (185) (228) 263 295 462 489 750 1438 2706 3010 4216 4769 7028 8860 10398 11251 11719 12612 13708 13934 14766 14798 15326 15452A 16069 16126 |
| J1c3a | 73 (185) (228) 263 295 462 489 750 1438 2706 3010 4216 4769 7028 8860 9548 10398 11251 11719 12612 13708 13934 14766 14798 15326 15452A 16069 16126 |
| J1c3a1 | 73 (185) (228) 263 295 462 489 750 1438 2706 3010 4216 4769 7028 7711 8860 9548 10398 11251 11719 12612 13708 13934 14766 14798 15326 15452A 16069 16126 |

| | |
|---|---|
| J1c3a2 | 73 (185) (228) 263 295 462 489 750 1438 2706 3010 4216 4769 7028 8860 9548 9836 10398 11251 11719 12612 13708 13934 14766 14798 15326 15452A 16069 16126 |
| J1c3b | 73 (185) (228) 263 295 462 489 750 1438 2706 3010 4216 4769 7028 8860 10398 11251 11719 12612 13708 13934 14766 14798 15326 15367 15452A 16069 16126 |
| J1c3b1 | 73 (185) (228) 263 295 462 489 750 1438 2706 3010 4216 4769 5237 7028 8860 10398 11251 11719 12612 13708 13934 14766 14798 15326 15367 15452A 16069 16126 |
| J1c3b1a | 73 (185) (228) 263 295 462 489 750 1438 2706 3010 4216 4769 5237 6261 7028 8860 10398 11251 11719 12612 13708 13934 14766 14798 15326 15367 15452A 16069 16126 |
| J1c3b2 | 73 (185) (228) 263 295 462 489 750 1438 2706 3010 4216 4769 4829 7028 8860 10398 11251 11719 12612 13708 13934 14766 14798 15326 15367 15452A 16069 16126 |
| J1c3c | 73 (185) (228) 263 295 462 489 750 1438 3010 4216 4769 7028 8860 10398 11251 11719 12612 13708 13934 14766 14798 15326 15452A 16069 16126 16222 |
| J1c3c1 | 73 (185) (228) 263 295 462 489 750 1438 3010 4216 4769 7028 8847 8860 10398 11251 11719 12612 13708 13934 14766 14798 15326 15452A 16069 16126 16222 |
| J1c3c2 | 73 (185) (228) 263 295 462 489 750 1438 3010 4216 4769 7028 8860 10227 10398 11251 11719 12612 13708 13934 14766 14798 15326 15452A 16069 16126 16222 |
| J1c3d | 73 (185) (228) 263 295 462 489 750 1438 1811 2706 3010 4216 4769 7028 8860 10398 11251 11719 12612 13708 13934 14766 14798 15326 15452A 16069 16126 |
| J1c3e | 73 (185) (228) 263 295 462 489 750 1438 2706 3010 4216 4769 7028 8860 10398 11251 11719 12612 13708 13934 14766 14798 15326 15452A 16069 16126 16390 |
| J1c3e1 | 73 (185) (228) 263 295 462 489 750 1438 2706 3010 4216 4769 7028 8860 10398 11251 11719 12612 13145 13708 13934 14296 14766 14798 15326 15452A 16069 16126 16390 |
| J1c3e2 | 73 (185) (228) 263 295 462 489 750 1438 2706 3010 4216 4769 7028 8860 8865 10398 11251 11719 12612 13708 13934 14766 14798 15326 15452A 16069 16126 16390 |
| J1c3f | 73 (185) (228) 263 295 462 489 750 1438 2706 3010 4216 4769 7028 8860 10398 11251 11719 12477 12612 13708 13934 14766 14798 15326 15452A 16063 16069 16126 |
| J1c3g | 73 (185) (228) 263 295 462 489 750 1438 2706 3010 4216 4769 7028 8860 9755 10398 11251 11719 12612 13708 13934 14766 14798 15326 15452A 16069 16126 |
| J1c3h | 73 (185) (228) 263 295 462 489 750 1438 2706 3010 4216 4769 7028 8659 8860 10398 11251 11719 12612 13708 13934 14766 14798 15326 15452A 16069 16126 |
| J1c3i | 73 (185) (228) 263 295 462 489 750 1438 2706 3010 4216 4769 5442 7028 8860 10398 11251 11719 12612 13708 13934 14766 14798 15326 15452A 15758 16069 16126 |

| | |
|---|---|
| J1c3j | 73 (185) (228) 263 295 462 489 750 1438 2706 3010 4216 4769 7028 8860 10398 11251 11719 12358 12612 13708 13934 14766 14798 15326 15452A 16069 16126 16311 |
| J1c3k | 73 (185) (228) 263 295 462 489 750 1438 2706 3010 4216 4769 7028 8860 10398 10497 11251 11719 12612 13708 13934 14766 14798 15326 15452A 16069 16126 |
| J1c3+189 | 73 (185) 189 (228) 263 295 462 489 750 1438 2706 3010 4216 4769 7028 8860 10398 11251 11719 12612 13708 13934 14766 14798 15326 15452A 16069 16126 |
| J1c3m | 73 (185) 189 (228) 263 295 462 489 750 1438 2706 3010 4216 4769 7028 8860 10398 11251 11719 12612 13708 13934 14766 14798 15326 15452A 16069 16114 16126 16145 |
| J1c4 | 73 (185) (228) 263 295 462 489 750 1438 2706 3010 4216 4769 7028 8860 9632 10398 11251 11719 12083G 12612 13708 14766 14798 15326 15452A 16069 16126 |
| J1c4b | 73 (185) (228) 263 295 462 489 750 1438 2706 3010 4216 4769 7028 8860 9120 9632 10398 11251 11719 12083G 12612 13708 14766 14798 15326 15452A 16069 16126 |
| J1c4c | 73 (185) (228) 263 295 462 489 750 1438 2706 3010 4216 4769 7028 8860 9632 10398 11251 11719 12083G 12612 13708 14766 14798 15326 15452A 16069 16126 16278 |
| J1c5 | 73 (185) (228) 263 295 462 489 750 1438 2706 3010 4216 4769 5198 7028 8860 10398 11251 11719 12612 13708 14766 14798 15326 15452A 16069 16126 |
| J1c5a | 73 (185) (228) 263 295 462 489 750 1438 2387 2706 3010 4216 4769 5198 7028 8860 10192 10398 11251 11719 12612 13708 14766 14798 15326 15452A 16069 16126 |
| J1c5a1 | 73 (185) (228) 263 295 462 489 750 1438 2387 2706 3010 4216 4769 5198 7028 8860 10192 10398 10598 11251 11719 12612 13708 14766 14798 15326 15452A 16069 16126 |
| J1c5b | 73 (185) (228) 263 295 462 489 750 1438 2706 3010 4216 4769 5198 7028 8596 8860 10334 10398 11251 11719 12612 13708 14766 14798 15326 15452A 16069 16126 |
| J1c5c | 73 (185) (228) 263 295 462 489 750 1438 2706 3010 3531 4216 4769 5198 7028 8734A 8860 10398 11251 11719 12612 13708 14766 14798 15326 15452A 16069 16126 |
| J1c5c1 | 73 (185) (228) 263 295 462 489 750 1438 2706 3010 3531 3721 4216 4769 5198 7028 8734A 8860 8994 10398 11251 11719 12612 13708 14766 14798 15326 15452A 16069 16126 16300 |
| J1c5d | 73 (185) (228) 263 295 462 489 750 1438 2706 3010 4216 4688 4769 5198 7028 8860 10398 11251 11719 12612 13434 13708 14766 14798 15326 15452A 16069 16126 |
| J1c5e | 73 (185) (228) 263 295 462 489 750 1438 2706 3010 4216 4769 5198 7028 8860 10398 11251 11719 12612 13708 14766 14798 15326 15452A 15916 16069 16126 (16309) |
| J1c5f | 73 (185) (228) 263 295 462 489 750 1438 2706 3010 4216 4769 5198 7028 8860 10398 11087 11251 11719 12612 13708 14766 14798 15326 15452A 15924 16069 16126 (16311) |

| | |
|---|---|
| J1c6 | 73 (185) (228) 263 295 462 489 750 1438 2706 3010 4025 4216 4769 7028 8860 10398 11251 11719 12612 13708 14766 14798 15326 15452A 16069 16126 |
| J1c6a | 73 (185) (228) 263 295 462 489 750 1438 2706 2969 3010 3398 4025 4216 4769 7028 8860 10398 10410 11251 11719 12561 12612 13708 14766 14798 15326 15452A 15913 16069 16126 |
| J1c+16261 | 73 (185) (228) 263 295 462 489 750 1438 2706 3010 4216 4769 7028 8860 10398 11251 11719 12612 13708 14766 14798 15326 15452A 16069 16126 16261 |
| J1c7 | 73 (185) (228) 263 295 462 489 750 1438 2706 3010 4216 4769 6554 7028 8860 10398 11251 11719 12127 12612 13708 14766 14798 15326 15452A 16069 (16092) 16126 16261 |
| J1c7a | 73 (185) (228) 263 295 462 489 750 1438 2706 3010 4216 4769 6464A 6554 7028 8860 10398 11251 11719 12127 12612 13681 13708 14766 14798 15326 15452A 16069 (16092) 16126 16261 |
| J1c+16261+189 | 73 (185) 189 (228) 263 295 462 489 750 1438 2706 3010 4216 4769 7028 8860 10398 11251 11719 12612 13708 14766 14798 15326 15452A 16069 16126 16261 |
| J1c12 | 73 (185) 189 (228) 263 295 462 489 750 789 1438 2706 3010 4216 4769 7028 8860 10398 11251 11719 12612 13708 14766 14798 15326 15452A 16069 16126 16261 |
| J1c12a | 73 (185) 189 (228) 263 295 462 489 750 789 1438 2706 3010 4084 4216 4769 7028 8860 10398 11251 11719 12612 13708 14766 14798 15326 15452A 16069 16126 16261 |
| J1c12b | 73 (185) 189 (228) 263 295 462 489 750 789 1438 2706 3010 4216 4769 7028 8860 10398 11251 11719 12612 13581 13708 14766 14798 15326 15452A 16069 16126 16261 |
| J1c13 | 73 (185) 189 204 (228) 263 295 462 489 750 1438 2706 3010 4216 4769 6249 7028 8860 9219G 10398 11251 11503 11719 12612 13708 14766 14798 15326 15452A 16069 16126 16261 |
| J1c14 | 73 (185) 195 (228) 263 295 462 489 750 1438 2706 3010 4216 4491 4769 7028 8860 8940 9120 10398 11251 11719 12612 13708 14766 14798 15326 15452A 16069 16126 16261 16274 16355 |
| J1c8 | 73 (185) (228) 263 295 462 489 750 1438 2706 3010 4216 4769 7028 8860 11251 11719 12612 13708 14766 14798 15326 15452A 16069 16126 |
| J1c8a | 73 (185) (228) 263 295 462 489 750 1438 2706 3010 4216 4769 7028 8860 10084 11251 11719 12612 13708 14766 14798 15326 15452A 16069 16126 16319 |
| J1c8a1 | 73 (185) (228) 263 295 462 489 750 1438 2706 3010 4216 4769 7028 8860 9052 10084 11251 11719 12612 13708 14766 14798 15326 15452A 16069 16126 16265 16319 |
| J1c8a1a | 73 (185) (228) 263 295 462 489 750 1438 2706 3010 4216 4769 7028 8860 9052 10084 11251 11719 12612 13708 14766 14798 15326 15452A 16069 16126 16189 16265 16319 |
| J1c8a2 | 73 (185) (228) 263 295 462 489 750 1438 2706 3010 4216 4769 7028 8860 10084 11251 11719 12612 13708 14766 14798 15326 15452A 16069 16126 16265C 16319 |

| | |
|---|---|
| J1c8b | 73 (185) (228) 263 295 462 489 750 1438 2706 3010 4216 4769 7028 8860 10345 11251 11719 12612 13708 14766 14798 15326 15452A 16069 16126 |
| J1c9 | 73 (185) (228) 263 295 462 489 750 1438 2706 3010 4216 4769 6887 7028 8860 10398 11251 11719 12612 13708 14766 14798 15326 15452A 16069 16126 |
| J1c10 | 73 (185) (228) 263 295 462 489 750 1438 2706 3010 4216 4769 5024 7028 8860 10398 11251 11719 12612 13708 14766 14798 15326 15452A 16069 16126 |
| J1c10a | 73 (185) (228) 263 295 462 489 750 1438 2706 3010 4216 4769 5024 6716 7028 8860 10398 11251 11719 12612 13708 14766 14798 15326 15452A 16069 16126 |
| J1c11 | 73 (185) (228) 263 295 462 489 750 1438 2706 3010 4216 4769 7028 8860 10398 11251 11719 12612 13708 14766 14798 15326 15452A 16069 16126 16224 |
| J1c11a | 73 (185) (228) 263 295 462 489 750 1438 2706 3010 4216 4769 7028 8860 10398 11251 11719 12612 13708 14766 14798 15262 15326 15452A 16069 16126 16224 |
| J1c15 | 73 (185) (228) 263 295 462 489 750 1438 2706 3010 4216 4769 7028 8860 10398 11251 11719 12453 12612 13708 14766 14798 15326 15452A 16069 16126 |
| J1c15a | 73 (185) (228) 263 295 462 489 750 1438 2706 3010 4216 4769 7028 8860 10398 11251 11719 12453 12612 13708 14766 14798 15113 15326 15452A 16069 16126 |
| J1c15a1 | 73 (185) (228) 263 295 462 489 750 1438 2706 3010 4216 4769 5231 7028 8860 10398 11251 11252 11719 12453 12612 13708 14766 14798 15113 15326 15452A 16069 16126 |
| J1c15b | 73 146 (185) (228) 263 295 462 489 750 1438 2706 3010 4216 4769 7028 8152 8860 9117 10398 11251 11719 12453 12612 13708 13917 14182 14766 14798 15326 15452A 15514 16069 16126 16527 |
| J1c16 | 73 152 (185) (228) 263 295 462 489 750 1438 2706 3010 4216 4769 7028 8860 10398 11251 11719 12123 12612 13708 14766 14798 15326 15452A 15670 16069 16126 16218 16256 |
| J1c17 | 73 (185) (228) 263 295 462 489 750 1438 2706 3010 3847 4216 4769 7028 8860 10398 11251 11719 12612 13708 14766 14798 15326 15452A 16069 16126 |
| J1c17a | 73 (185) (228) 263 295 462 489 750 1438 2140 2706 3010 3847 4216 4769 7028 8860 10398 11251 11719 12612 12634 13708 14766 14798 15326 15452A 16069 16311 |
| J1+16193 | 73 263 295 462 489 750 1438 2706 3010 4216 4769 7028 8860 10398 11251 11719 12612 13708 14766 15326 15452A 16069 16126 16193 |
| J1d | 73 152 263 295 462 489 750 1438 2706 3010 4216 4769 7028 7789 7963 8860 10398 11251 11719 12612 13708 14766 15326 15452A 16069 16126 16193 |
| J1d1 | 73 152 263 295 462 489 750 1438 2706 3010 4216 4769 7028 7789 7963 8860 10398 11251 11719 12612 13708 14766 15326 15452A 16069 16126 16193 16300 |

| | |
|---|---|
| J1d1a | 73 152 263 295 462 489 750 1007 1438 2706 3010 4216 4769 7028 7789 7963 8860 10398 11251 11719 12612 13708 14766 15326 15452A 16069 16126 16193 16300 16309 |
| J1d1a1 | 73 152 263 295 462 489 750 1007 1438 2706 3010 4216 4769 7028 7789 7963 8860 10398 11251 11719 12612 13392 13708 14766 15326 15452A 16069 16126 16193 16300 16309 |
| J1d1a1a | 73 151 152 263 295 462 489 750 1007 1438 2706 3010 4216 4769 7028 7789 7963 8860 10398 11251 11719 12612 13392 13500 13708 14766 15326 15452A 16069 16126 16193 16300 16309 |
| J1d1b | 73 152 263 295 462 489 750 1438 2706 3010 4216 4769 7028 7789 7963 8633 8860 10398 11251 11719 12612 13708 14766 15326 15452A 16069 16126 16193 16300 |
| J1d1b1 | 73 152 263 295 462 489 750 1438 2706 3010 3213 4216 4769 7028 7789 7963 8633 8860 10398 11251 11719 12612 13708 14766 15326 15452A 16069 16126 16193 16300 |
| J1d2 | 73 152 263 295 462 489 750 1438 2706 3010 4216 4769 7028 7789 7963 8860 10166 10398 11251 11719 12612 13708 14766 15326 15452A 16069 16126 16186 16193 |
| J1d2a | 73 152 263 295 462 489 750 1438 2706 3010 4216 4769 7028 7789 7963 8860 10166 10398 11251 11719 12612 13188 13708 14766 15326 15452A 16069 16126 16186 16193 |
| J1d3 | 73 152 263 295 462 489 750 1438 2706 3010 4216 4769 5262 7028 7789 7963 8860 10398 11251 11719 12612 13708 14766 15326 15452A 16069 16126 16193 |
| J1d3a | 73 152 263 295 462 489 750 1438 2706 3010 4216 4769 5262 7028 7521 7789 7963 8860 10398 11251 11719 12612 13708 14766 15326 15452A 16069 16126 16193 |
| J1d3a1 | 73 152 263 295 462 489 750 1438 2706 3010 4216 4769 5262 5297 5774 7028 7521 7789 7963 8860 10398 11251 11719 12612 13708 14766 15326 15452A 15836 15894 16069 16126 16193 16335 |
| J1d3a2 | 73 152 263 295 462 489 750 1438 2706 3010 4011 4216 4769 5262 7028 7521 7789 7963 8860 10398 11251 11719 12612 13708 14766 15326 15452A 16069 16126 16193 |
| J1d4 | 73 152 263 295 462 489 750 1438 2706 3010 4216 4769 7028 7789 7963 8860 9725 10398 11251 11719 12561 12612 13135 13708 14766 15326 15452A 16069 16126 16193 |
| J1d5 | 73 152 263 295 462 489 750 1438 2706 3010 4216 4769 4853 7028 7789 7963 8860 10398 11251 11719 12612 13708 14766 15326 15452A 16069 16126 16193 16274 |
| J1d5a | 73 152 204 263 295 462 489 750 1438 2706 3010 4216 4769 4853 7028 7789 7963 8860 9090 10398 11251 11719 12612 13708 14766 15326 15452A 16069 16126 16193 16274 |
| J1d6 | 73 152 263 295 462 489 750 1438 2706 3010 4216 4769 7028 7789 7963 8860 9041 10398 11251 11719 12612 13708 14766 15326 15452A 16069 16126 16193 |
| J1d6a | 73 152 263 295 462 489 750 1438 2706 3010 4216 4769 7028 7789 7963 8860 9041 10398 11251 11719 12612 13708 14766 15326 15452A 16069 16126 16193 16301 |

| | |
|---|---|
| J2 | 73 (150) 152 263 295 489 750 1438 2706 4216 4769 7028 7476 8860 10398 11251 11719 12612 13708 14766 15257 15326 15452A 16069 16126 |
| J2a | 73 (150) 152 195 263 295 489 750 1438 2706 4216 4769 7028 7476 8860 10398 10499 11251 11377 11719 12612 13708 14766 15257 15326 15452A 16069 16126 |
| J2a1 | 73 (150) 152 195 263 295 489 750 1438 2706 4216 4769 7028 7476 8860 10398 10499 11251 11377 11719 12612 13708 14133 14766 15257 15326 15452A 16069 16126 |
| J2a1a | 73 (150) 152 195 (215) 263 295 (319) 489 513 750 1438 2706 4216 4769 7028 7476 7789 8860 10398 10499 11251 11377 11719 12612 13708 14133 14766 15257 15326 15452A 16069 16126 16145 16231 |
| J2a1a1 | 73 (150) 152 195 (215) 263 295 (319) 489 513 750 1438 2706 4216 4769 7028 7476 7789 8860 10398 10499 11251 11377 11719 12612 13708 13722 14133 14766 15257 15326 15452A 16069 16126 16145 16231 16261 |
| J2a1a1a | 73 (150) 152 195 (215) 263 295 310.1T (319) 489 513 750 1438 1850 2706 4216 4769 7028 7476 7789 8860 10398 10499 11251 11377 11719 12612 13708 13722 14133 14766 15257 15326 15452A 16069 16126 16145 16231 16261 |
| J2a1a1a1 | 73 (150) 152 189 195 (215) 263 295 310.1T (319) 489 513 750 1438 1850 2706 4216 4769 7028 7476 7789 8860 10237 10398 10499 11251 11377 11719 12612 13708 13722 14133 14766 15191 15257 15326 15452A 16069 16126 16145 16172 16231 16261 |
| J2a1a1a2 | 73 (150) 152 195 (215) 263 295 310.1T (319) 489 513 750 1438 1850 2706 3447 4216 4769 7028 7476 7789 8860 10398 10499 11251 11377 11719 12612 13708 13722 14133 14766 15257 15326 15452A 16069 16126 16145 16231 16261 |
| J2a1a1a2a | 73 (150) 152 195 (215) 263 295 310.1T (319) 489 513 750 1438 1850 2706 3447 4216 4769 7028 7476 7501 7789 8860 10398 10499 11251 11377 11719 12612 13708 13722 14133 14766 15257 15326 15452A 16069 16126 16145 16231 16261 |
| J2a1a1a3 | 73 (150) 152 195 (215) 263 295 310.1T (319) 430 489 513 750 1438 1850 2706 4216 4769 7028 7476 7789 8860 10398 10499 11251 11377 11719 12612 13708 13722 14133 14766 15257 15326 15452A 16069 16126 16145 16231 16261 |
| J2a1a1b | 73 (150) 152 195 (215) 263 295 (319) 489 513 750 1438 2706 4216 4769 7028 7476 7789 8860 10398 10499 11251 11377 11518 11719 12612 13708 13722 14133 14766 14793 15257 15326 15452A 16069 16126 16145 16231 16261 |
| J2a1a1c | 73 (150) 152 195 (215) 263 295 (319) 489 513 750 1438 2706 4216 4769 7028 7476 7789 8860 10398 10499 11251 11377 11719 12612 13708 13722 14133 14766 15257 15326 15452A 15596 16069 16126 16145 16231 16261 |
| J2a1a1d | 73 (150) 152 195 (215) 263 295 (319) 489 513 750 1438 2706 4216 4769 7028 7476 7789 8860 10398 10499 11251 11377 11719 12612 13708 13722 13753 14133 14766 15257 15326 15452A 16069 16126 16145 16231 16261 |
| J2a1a1e | 73 (150) 152 195 (215) 263 295 (319) 489 513 750 1438 2706 4216 4769 7028 7476 7789 8860 10398 10499 11251 11377 11719 12612 13708 13722 14133 14766 15257 15326 15452A 16069 16126 16145 16189 16231 16261 |

| | |
|---|---|
| J2a1a2 | 73 (150) 152 195 (215) 263 295 (319) 489 513 750 1438 2706 4216 4769 6635 7028 7476 7789 8860 10398 10499 11251 11377 11719 12612 13708 14133 14766 15257 15326 15452A 16069 16126 16145 16231 16284 |
| J2a1a2a | 73 (150) 152 195 (215) 263 295 (319) 489 513 750 1438 2706 4216 4769 6635 7028 7476 7789 8860 10398 10499 11251 11377 11719 11857 12612 13708 14133 14766 15257 15326 15452A 16069 16126 16145 16231 16284 |
| J2a1a2a1 | 73 (150) 152 195 (215) 263 295 (319) 489 513 750 1438 2706 4216 4769 6635 7028 7476 7789 8860 10398 10499 11251 11377 11386 11719 11857 12612 13708 14133 14766 15257 15326 15452A 16069 16126 16145 16231 16284 |
| J2a1a2a1a | 73 (150) 152 195 (215) 263 295 (319) 489 513 750 1438 2706 4216 4769 6635 7028 7476 7789 8860 10398 10499 11204 11251 11377 11386 11719 11857 12612 13708 14133 14766 15257 15326 15452A 16069 16126 16145 16231 16284 |
| J2a2 | 73 (150) 195 263 295 489 750 1438 2706 4216 4769 6671 7028 7476 8860 10398 10499 11002 11251 11377 11719 12570 12612 13708 14766 15257 15326 15452A 15679 16069 16126 |
| J2a2a | 73 (150) 195 235 263 295 489 750 1438 2706 4216 4769 6671 7028 7476 8386 8860 10398 10499 11002 11251 11377 11719 12570 12612 13708 14766 15257 15326 15452A 15679 16069 16126 |
| J2a2a1 | 73 (150) 195 235 263 295 489 750 1438 2706 4216 4769 6671 7028 7476 8386 8860 10398 10499 11002 11251 11377 11440 11719 12171 12570 12612 13708 14766 15257 15326 15452A 15679 16069 16126 |
| J2a2a1+16311 | 73 (150) 195 235 263 295 489 750 1438 2706 4216 4769 6671 7028 7476 8386 8860 10398 10499 11002 11251 11377 11440 11719 12171 12570 12612 13708 14766 15257 15326 15452A 15679 16069 16126 16311 |
| J2a2a1a | 73 (150) 195 235 263 295 489 750 1438 2706 4216 4769 6671 7028 7476 8386 8860 9103 10398 10499 11002 11251 11377 11440 11719 12171 12570 12612 13708 14766 15257 15326 15452A 15679 16069 16126 16311 |
| J2a2a1a1 | 73 (150) 195 235 263 295 489 750 1438 2706 4216 4769 6671 7028 7476 8386 8860 9103 10398 10499 11002 11251 11377 11440 11719 12171 12570 12612 13708 13813 14766 15257 15326 15452A 15679 16069 16126 |
| J2a2a2 | 73 (150) 195 235 263 295 489 750 1438 2706 4216 4769 6671 7028 7476 8386 8860 10398 10499 11002 11251 11377 11719 11797 12570 12612 13419 13708 14766 15257 15326 15452A 15679 16069 16126 16169 |
| J2a2b | 73 (150) 195 263 295 489 750 1438 2706 4216 4769 6671 7028 7476 8860 10398 10499 11002 11251 11377 11719 12570 12612 13708 14766 15257 15326 15452A 15679 16069 16126 16241 |
| J2a2b1 | 73 (150) 189 195 263 295 489 750 1438 2706 4216 4769 6671 7028 7476 8860 10398 10499 11002 11251 11377 11719 12570 12612 13708 14766 15257 15326 15452A 15679 16069 16126 16241 |
| J2a2b1a | 73 (150) 189 195 263 295 489 750 1438 2706 4216 4769 5915 6671 7028 7476 8860 10398 10499 11002 11251 11377 11719 12570 12612 13708 14061 14766 15257 15326 15452A 15679 16069 16126 16241 |
| J2a2b2 | 73 (150) 195 263 295 456 489 750 1438 2706 4216 4769 6671 7028 7476 8860 10398 10499 11002 11251 11377 11719 12570 12612 13015 13708 13830 14766 15257 15326 15452A 15679 16069 16126 16241 |

| | |
|---|---|
| J2a2b3 | 73 (150) 195 263 295 489 750 1438 2706 4216 4688 4769 4802 6671 7028 7476 8078 8860 10398 10499 11002 11251 11377 11416 11719 12570 12612 13708 14766 15257 15326 15452A 15672 15679 16069 16126 16241 |
| J2a2c | 73 (150) 195 263 295 489 750 1438 2706 4216 4769 6671 7028 7476 8860 10398 10499 11002 11251 11377 11719 12570 12612 13708 14766 15257 15326 15452A 15679 16069 16126 16231 |
| J2a2c1 | 73 (150) 195 263 295 489 750 1438 2706 4216 4769 6671 7028 7476 8860 10398 10499 11002 11251 11377 11719 12570 12612 12672 13708 14766 15257 15326 15452A 15679 16069 16126 16214 16231 |
| J2a2d | 73 (150) 195 263 295 489 750 1438 2706 4216 4769 5493 6671 7028 7076 7476 8860 8958 10398 10499 11002 11016 11251 11377 11719 12570 12612 13708 13759 14766 15148 15257 15326 15452A 15679 16069 16126 |
| J2a2e | 73 (150) 195 263 295 489 750 1438 2706 4216 4769 6671 7028 7476 8860 10398 10499 10685 11002 11251 11377 11719 12570 12612 13708 14364 14766 15257 15326 15452A 15679 16069 16126 |
| J2b | 73 (150) 152 263 295 489 750 1438 2706 4216 4769 5633 7028 7476 8860 10398 11251 11719 12612 13708 14766 15257 15326 15452A 15812 16069 16126 16193 |
| J2b1 | 73 (150) 152 263 295 489 750 1438 2706 4216 4769 5633 7028 7476 8860 10172 10398 11251 11719 12612 13708 14766 15257 15326 15452A 15812 16069 16126 16193 |
| J2b1a | 73 (150) 152 263 295 489 750 1438 2706 4216 4769 5633 7028 7476 8860 10172 10398 11251 11719 12612 13708 14766 15257 15326 15452A 15812 16069 16126 16193 16278 |
| J2b1a1 | 73 (150) 152 263 295 489 750 1438 2706 4216 4769 5633 7028 7476 8860 10172 10398 11251 11719 12612 13708 14569 14766 15257 15326 15452A 15812 16069 16126 16193 16278 |
| J2b1a1a | 73 (150) 152 263 295 489 750 1438 2706 4216 4769 5633 7028 7476 8860 10172 10398 11251 11719 12612 13708 14569 14766 15257 15326 15452A 15812 16069 16193 16278 16362 |
| J2b1a2 | 73 (150) 152 263 295 489 750 1438 2706 4216 4769 5633 6216 7028 7476 8860 10172 10398 11251 11719 12612 13708 14766 15257 15326 15452A 15812 16069 16126 16193 16278 |
| J2b1a2a | 73 (150) 152 263 295 489 750 1438 2706 4216 4769 5633 6216 6893 7028 7476 8860 10172 10398 11251 11719 12612 13708 14766 15257 15326 15452A 15812 16069 16126 16193 16278 |
| J2b1a3 | 73 (150) 152 263 295 489 750 1438 2706 4216 4769 5633 7028 7476 8860 10172 10398 10966 11251 11719 12612 13708 14766 15257 15326 15452A 15812 16069 16126 16193 16278 |
| J2b1a4 | 73 (150) 152 263 295 489 750 1438 2706 4216 4769 5633 7028 7302 7476 8860 10172 10398 11251 11719 12612 13708 14766 15014 15257 15326 15452A 15812 16069 16126 16193 16278 |
| J2b1a+16311 | 73 (150) 152 263 295 489 750 1438 2706 4216 4769 5633 7028 7476 8860 10172 10398 11251 11719 12612 13708 14766 15257 15326 15452A 15812 16069 16126 16193 16278 16311 |
| J2b1a5 | 73 (150) 152 263 295 489 750 1438 2706 4216 4769 5633 6491 7028 7476 8860 10172 10398 11251 11719 12612 13708 14766 15257 15326 15452A 15812 16069 16126 16193 16278 16311 |

| | |
|---|---|
| J2b1a6 | 73 (150) 263 295 489 750 1438 2706 4216 4769 5633 7028 7476 8860 9016 9494 10172 10398 11251 11719 12612 13708 14766 15257 15326 15452A 15662 15812 16069 16126 16193 16278 |
| J2b1b | 73 (150) 152 263 295 489 750 1438 2404 2706 4216 4769 5633 6962T 7028 7476 8860 10172 10389 10398 11251 11719 12612 13708 14766 15257 15326 15452A 15812 16069 16126 16193 |
| J2b1b1 | 73 (150) 152 263 295 489 750 1438 2404 2706 4216 4769 5633 6962T 7028 7211 7476 8860 10172 10389 10398 11251 11719 12612 13708 14766 15257 15326 15452A 15812 16069 16126 16193 |
| J2b1c | 73 (150) 152 263 295 489 750 1438 2706 4216 4769 5633 7028 7476 8860 10172 10398 11251 11719 12612 13708 13899 14766 15257 15326 15452A 15812 16069 16126 16193 |
| J2b1c1 | 73 (150) 152 263 295 489 750 1438 2706 4216 4769 5633 7028 7476 8860 10172 10398 11251 11719 12612 13708 13899 14766 15257 15326 15452A 15812 16069 16126 16193 16319 |
| J2b1d | 73 (150) 152 263 295 489 750 1438 2706 4216 4769 5633 7028 7476 8860 10172 10398 11251 11719 12612 13708 14766 14769 15257 15326 15452A 15812 16069 16126 16193 |
| J2b1e | 73 (150) 152 263 295 489 750 1438 2706 4216 4769 5633 7028 7476 8843 8860 10172 10398 11251 11719 12612 13708 14766 15257 15326 15452A 15812 16069 16126 16193 |
| J2b1e1 | 73 (150) 152 263 295 489 750 1438 2706 4216 4769 5633 7028 7476 8290 8843 8860 10172 10398 11251 11719 12612 13708 14766 14950 15257 15326 15452A 15812 16069 16126 16193 16438 |
| J2b1f | 73 (150) 152 263 295 489 750 1438 2706 4216 4769 5633 7028 7476 8860 10172 10398 11251 11719 12612 13708 14766 15257 15326 15452A 15453 15812 16069 16126 16193 |
| J2b1g | 73 (150) 152 263 295 489 750 1438 2706 4216 4769 5633 7028 7476 8860 9872 10172 10398 11251 11719 12612 13708 14766 15257 15326 15452A 15812 16069 16126 16193 |
| J2b1h | 73 (150) 152 159 263 295 489 750 1438 2706 4216 4769 5633 6830A 7028 7476 8860 10172 10398 11251 11719 12612 13612T 13708 14766 15257 15326 15452A 15812 16069 16126 16193 16195 16242A 16526 |
| J2b2 | 73 (150) 152 263 295 489 750 1438 2706 4216 4769 5460 5633 7028 7391 7476 8860 10398 11251 11719 12612 13708 14029 14766 15257 15326 15452A 15812 16069 16126 16193 |
| T | 73 263 709 750 1438 1888 2706 4216 4769 4917 7028 8697 8860 10463 11251 11719 13368 14766 14905 15326 15452A 15607 15928 16126 16294 |
| T1 | 73 263 709 750 1438 1888 2706 4216 4769 4917 7028 8697 8860 10463 11251 11719 12633A 13368 14766 14905 15326 15452A 15607 15928 16126 16163 16189 16294 |
| T1a | 73 263 709 750 1438 1888 2706 4216 4769 4917 7028 8697 8860 10463 11251 11719 12633A 13368 14766 14905 15326 15452A 15607 15928 16126 16163 16186 16189 16294 |
| T1a+152 | 73 152 263 709 750 1438 1888 2706 4216 4769 4917 7028 8697 8860 10463 11251 11719 12633A 13368 14766 14905 15326 15452A 15607 15928 16126 16163 16186 16189 16294 |

| | |
|---|---|
| T1a1'3 | 73 152 195 263 709 750 1438 1888 2706 4216 4769 4917 7028 8697 8860 10463 11251 11719 12633A 13368 14766 14905 15326 15452A 15607 15928 16126 16163 16186 16189 16294 |
| T1a1 | 73 152 195 263 709 750 1438 1888 2706 4216 4769 4917 7028 8697 8860 9899 10463 11251 11719 12633A 13368 14766 14905 15326 15452A 15607 15928 16126 16163 16186 16189 16294 |
| T1a1a | 73 152 195 263 709 750 1438 1888 2706 4216 4769 4917 5558 7028 8697 8860 9899 10463 11251 11719 12633A 13368 14766 14905 15326 15452A 15607 15928 16126 16163 16186 16189 16294 |
| T1a1a1 | 73 152 195 263 709 750 1438 1888 2706 4216 4769 4917 5414 5558 7028 8697 8860 9899 10463 11251 11719 12633A 13368 14766 14905 15326 15452A 15607 15928 16126 16163 16186 16189 16294 |
| T1a1b | 73 152 195 263 709 750 1438 1888 2706 4216 4769 4917 7028 8697 8860 9899 10143 10463 11251 11719 12633A 13368 14281 14766 14905 15326 15452A 15607 15928 16126 16163 16186 16189 16294 |
| T1a1b1 | 73 152 195 263 709 750 1438 1888 2706 4216 4769 4917 7028 8697 8860 9548 9899 10143 10463 11251 11719 12028 12633A 13368 14281 14766 14905 15326 15452A 15607 15928 16126 16163 16186 16189 16294 |
| T1a1c | 73 152 195 263 709 750 1438 1888 2706 4216 4769 4917 7028 8697 8860 9120 9899 10463 11251 11719 12633A 13368 14766 14905 15326 15452A 15607 15928 15965 16126 16163 16186 16189 16294 |
| T1a1d | 73 152 195 263 593 709 750 1438 1888 2706 4216 4769 4917 7028 8697 8860 9899 10463 11251 11719 12633A 13132 13368 13785 14766 14905 15326 15452A 15607 15928 16126 16163 16186 16189 16294 |
| T1a1e | 73 152 195 263 709 750 1438 1888 2380 2706 4155 4216 4769 4917 5054 7028 8697 8860 9899 10463 11251 11719 12308 12633A 13368 14766 14905 15326 15452A 15607 15928 16126 16163 16186 16189 16256 16294 |
| T1a1f | 73 152 195 263 709 750 1438 1503 1888 2706 4216 4769 4917 7028 8697 8860 9899 10463 11251 11719 12633A 13368 14766 14905 15326 15452A 15607 15928 16126 16163 16186 16189 16294 |
| T1a1g | 73 152 195 263 709 750 1438 1888 2706 4216 4769 4917 7028 8697 8860 9899 10463 11251 11719 12633A 13368 14766 14905 15213 15326 15452A 15607 15928 16126 16163 16186 16189 16294 |
| T1a1h | 73 152 195 263 709 750 1438 1888 2706 4216 4769 4917 7028 8697 8860 9899 10463 11251 11719 12633A 13368 14766 14905 15326 15452A 15607 15928 16126 16163 16186 16189 16269C 16294 |
| T1a1i | 73 152 195 263 709 750 1438 1888 2706 3308G 4216 4769 4917 7028 8697 8860 9899 10463 11251 11719 11944 12633A 13368 14766 14905 15326 15452A 15607 15928 16126 16163 16186 16189 16294 |
| T1a1j | 73 152 195 263 709 750 1438 1888 2706 4216 4769 4917 7028 8530 8697 8860 9899 10463 11251 11719 12633A 13368 14766 14905 15326 15452A 15607 15928 16126 16163 16186 16189 16294 |
| T1a1k | 73 152 195 263 469 709 750 1438 1888 2706 4216 4769 4917 7028 8697 8860 9899 10463 11251 11719 12633A 13368 14766 14905 15326 15452A 15607 15928 16126 16163 16186 16189 16294 |
| T1a1k1 | 73 146 152 195 263 469 709 750 1438 1888 2706 4216 4769 4917 7028 8697 8860 9899 10463 11251 11719 11914 12633A 12753 13368 14766 14905 15326 15452A 15607 15928 16126 16163 16186 16189 16294 |

| | |
|---|---|
| T1a1k2 | 73 152 195 263 469 709 750 1438 1888 2706 4216 4769 4917 7028 8697 8860 8974 9899 10463 11251 11719 12633A 13368 14766 14905 15326 15452A 15607 15928 16126 16163 16186 16189 16294 |
| T1a1+@152 | 73 195 263 709 750 1438 1888 2706 4216 4769 4917 7028 8697 8860 9899 10463 11251 11719 12633A 13368 14766 14905 15326 15452A 15607 15928 16126 16163 16186 16189 16294 |
| T1a1l | 73 195 263 709 750 1438 1888 2706 3705 4216 4769 4917 7028 8697 8860 9899 10463 11251 11719 12633A 13368 14766 14905 15326 15452A 15607 15928 16126 16163 16186 16189 16294 |
| T1a1m | 73 152 195 263 709 750 1438 1888 2706 4216 4769 4917 7028 7783 8697 8860 9899 10463 11251 11719 12633A 13368 14766 14905 15326 15452A 15607 15928 16126 16163 16186 16189 16294 16325 |
| T1a1m1 | 73 152 195 263 709 750 1438 1888 2706 4216 4769 7028 7783 8697 8860 9899 10463 11251 11719 12633A 13368 13590 14766 14905 15326 15452A 15607 15928 16126 16163 16186 16189 16294 16325 |
| T1a1n | 73 152 195 263 709 750 1438 1888 2706 4216 4769 4917 7028 8697 8860 9899 10463 11251 11719 12633A 13368 14766 14905 15326 15452A 15607 15928 16126 16163 16186 16189 16294 16304 |
| T1a1p | 73 152 195 263 709 750 1438 1888 2706 4216 4769 4917 7028 8697 8860 9545 9899 10463 11251 11719 12633A 13368 14766 14905 15326 15452A 15607 15928 16126 16163 16186 16189 16294 |
| T1a1q | 73 152 195 263 709 750 1438 1888 2706 4216 4769 4917 7028 8697 8860 9899 10463 11251 11266 11719 12633A 13368 14766 14905 15326 15452A 15607 15928 16126 16163 16186 16189 16294 |
| T1a1r | 73 152 195 263 709 750 1438 1888 2706 4216 4769 4917 7028 8697 8860 9899 10463 11251 11719 12633A 13368 14274 14766 14905 15326 15452A 15928 16126 16163 16186 16189 16294 |
| T1a3 | 73 152 195 263 709 750 1438 1888 2706 4216 4769 4917 7028 8697 8860 10321 10463 11251 11719 12633A 13368 14766 14905 15326 15452A 15607 15928 16126 16163 16186 16189 16294 |
| T1a3a | 73 152 195 263 709 750 1438 1888 2706 4216 4769 4917 7028 7258 8697 8860 10321 10463 11251 11719 12633A 13368 14766 14905 15326 15452A 15607 15928 16126 16163 16186 16189 16294 |
| T1a2 | 73 152 263 709 750 1438 1888 2706 4216 4769 4917 7028 7853 8697 8860 10463 11251 11719 12633A 13368 14766 14905 15326 15452A 15607 15928 16126 16163 16186 16189 16294 |
| T1a2a | 73 152 263 384 709 750 1438 1888 2706 4216 4769 4917 4959 5558 7028 7853 8697 8860 9300 10463 11251 11719 12633A 13368 14766 14905 15326 15452A 15607 15928 16126 16163 16186 16189 16261 16294 |
| T1a2b | 73 152 263 709 750 1438 1888 2706 4216 4769 4917 7028 7853 8697 8860 9111 10463 11251 11719 12633A 12957 13368 14766 14905 15326 15452A 15607 15928 16126 16163 16186 16189 16248 16266 16294 |
| T1a4 | 73 152 263 709 750 1438 1888 2706 4216 4769 4917 7001 7028 8697 8860 10463 11251 11719 12633A 13368 14766 14905 15326 15452A 15607 15928 16126 16163 16186 16189 16263 16294 |
| T1a11 | 91 97 152 263 709 750 1438 1888 2706 4216 4769 4917 7028 8412 8697 8860 10463 11251 11719 12633A 13368 13759 13791 14284 14766 14905 15326 15452A 15607 15928 16126 16163 16186 16189 16294 |

| | |
|---|---|
| T1a12 | 73 152 263 709 750 1438 1888 2706 4216 4769 4917 7028 8697 8860 10463 11251 11719 12633A 13368 14766 14905 15326 15452A 15607 15884 15928 16126 16163 16186 16189 16294 16355 |
| T1a13 | 73 152 263 709 750 1438 1888 2706 4216 4769 4917 7028 8697 8860 10463 11251 11719 12633A 13368 14766 14905 15326 15452A 15607 15928 16126 16129 16163 16186 16189 16294 16362 |
| T1a5 | 73 263 709 750 1438 1888 2706 4216 4769 4917 6152 7028 8697 8860 10463 11251 11719 12633A 13368 14766 14905 15326 15452A 15607 15928 16126 16163 16186 16189 16294 |
| T1a5a | 73 263 709 750 1438 1888 2706 4216 4769 4917 5378 6152 7028 8697 8860 10463 11251 11719 12406 12633A 13368 14766 14905 15326 15452A 15607 15928 16126 16163 16186 16189 16218 16294 |
| T1a6 | 73 263 709 750 1438 1888 2706 3867 4216 4769 4917 7028 8697 8860 10376 10463 11251 11719 12633A 13368 14766 14905 15326 15452A 15607 15928 16126 16163 16186 16189 16294 |
| T1a7 | 73 263 512 709 750 1438 1888 2706 4216 4769 4917 7028 8697 8860 10463 11251 11719 12633A 13368 14500 14766 14905 15326 15452A 15607 15928 16126 16163 16186 16189 16274 16294 |
| T1a8 | 73 263 709 750 1438 1888 2706 4216 4769 4917 4991 7028 8697 8860 10463 11251 11719 12633A 13368 14766 14905 15326 15452A 15607 15928 16126 16163 16186 16189 16294 |
| T1a8a | 73 263 709 750 1438 1888 2706 4107 4216 4769 4917 4991 7028 8697 8860 10463 11251 11719 11914 12633A 13368 14766 14905 15326 15452A 15607 15928 16126 16163 16186 16189 16294 16325 |
| T1a8b | 73 263 709 750 1438 1888 2706 4216 4769 4917 4991 7028 8697 8860 9438 10463 11251 11719 12633A 13368 14323 14766 14905 15326 15452A 15607 15928 16126 16163 16186 16189 16294 |
| T1a9 | 73 263 709 750 1438 1888 2706 4216 4769 4917 7028 8697 8860 10463 11251 11719 12633A 12879 13368 14766 14905 15326 15452A 15607 15928 16126 16163 16186 16189 16249 16294 |
| T1a10 | 73 263 709 750 1438 1888 2706 4216 4769 4917 7028 8697 8860 10463 11251 11719 12633A 13368 13470 14766 14905 15326 15452A 15607 15928 16126 16163 16186 16189 16294 |
| T1a10a | 73 150 214 263 709 750 1438 1888 2706 4216 4769 4917 7028 8697 8860 10463 11251 11719 12633A 13368 13470 13708 14766 14905 15326 15452A 15607 15928 16126 16163 16186 16189 16294 |
| T1b | 73 263 709 750 1438 1888 2706 4216 4769 4917 7028 8697 8860 10463 11251 11719 12633A 13368 14766 14905 15326 15452A 15607 15928 16126 16163 16189 16243 16294 |
| T1b1 | 73 263 709 750 1438 1888 2706 4216 4769 4917 7028 8697 8860 10463 11251 11647 11719 12633A 13368 14766 14905 15326 15452A 15607 15928 16126 16163 16189 16243 16294 |
| T1b2 | 73 263 709 750 1438 1888 2706 4216 4739 4769 4917 7028 8697 8860 9254 10463 11251 11719 12633A 13368 14766 14905 15326 15452A 15607 15928 16126 16163 16189 16243 16294 |
| T1b3 | 73 263 709 750 1438 1888 2706 4216 4769 4917 7028 8697 8860 10463 11251 11719 12633A 13368 14766 14905 15326 15452A 15607 15853 15928 16126 16163 16189 16243 16294 |

| | |
|---|---|
| T1b4 | 73 263 709 750 1438 1888 2706 4216 4769 4917 7028 7310 8697 8860 10463 11251 11719 12633A 13368 14766 14905 15326 15452A 15607 15928 16126 16163 16189 16243 16294 |
| T2 | 73 263 709 750 1438 1888 2706 4216 4769 4917 7028 8697 8860 10463 11251 11719 11812 13368 14233 14766 14905 15326 15452A 15607 15928 16126 16294 (16296) |
| T2a | 73 263 709 750 1438 1888 2706 4216 4769 4917 7028 8697 8860 10463 11251 11719 11812 13368 13965 14233 14766 14905 15326 15452A 15607 15928 16126 16294 (16296) |
| T2a1 | 73 263 709 750 1438 1888 2706 4216 4769 4917 7028 8697 8860 10463 11251 11719 11812 13368 13965 14233 14687 14766 14905 15326 15452A 15607 15928 16126 16294 (16296) |
| T2a1a | 73 263 709 750 1438 1888 2706 2850 4216 4769 4917 7022 7028 8697 8860 10463 11251 11719 11812 13368 13965 14233 14687 14766 14905 15326 15452A 15607 15928 16126 16294 (16296) |
| T2a1a1 | 73 (143) 263 709 750 1438 1888 2706 2850 4216 4769 4917 7022 7028 8697 8715 8860 10463 11251 11719 11812 13368 13965 14233 14687 14766 14905 15326 15452A 15607 15928 16126 16294 (16296) |
| T2a1a2 | 73 263 709 750 1438 1888 2706 2850 4216 4688 4769 4917 7022 7028 8697 8860 10463 11251 11719 11812 13368 13965 14233 14687 14766 14905 15326 15452A 15607 15928 16126 16294 (16296) |
| T2a1a3 | 73 263 709 750 1438 1888 2706 2850 4216 4769 4917 5498 7022 7028 8697 8860 10463 11251 11719 11812 13368 13965 14233 14687 14766 14905 15326 15452A 15607 15928 16126 16294 (16296) |
| T2a1a3a | 73 263 709 750 1438 1888 2706 2850 4216 4769 4808 4917 5498 7022 7028 8697 8860 10463 11251 11719 11812 13368 13965 14233 14687 14766 14905 15326 15452A 15607 15928 16126 16294 (16296) |
| T2a1a5 | 73 263 709 750 1438 1888 2706 2850 4216 4769 4917 7022 7028 8697 8860 10463 11251 11719 11812 13368 13965 14233 14687 14766 14905 15326 15452A 15607 15928 15935 16126 16294 (16296) |
| T2a1a6 | 73 263 709 750 1438 1888 2706 2850 4216 4769 4917 6425 7022 7028 8697 8860 10463 11251 11719 11812 13368 13965 14233 14687 14766 14905 15326 15452A 15607 15928 16126 16294 (16296) |
| T2a1a7 | 73 263 709 750 1438 1888 2706 2850 4216 4769 4917 7022 7028 7268 8697 8860 10463 11251 11719 11812 13368 13965 14233 14687 14766 14905 15326 15452A 15607 15928 16126 16294 (16296) |
| T2a1a8 | 73 263 709 750 1438 1888 2706 2850 4216 4769 4917 4931 7022 7028 8697 8860 10463 11251 11719 11812 13368 13965 14233 14687 14766 14905 15326 15452A 15607 15928 16126 16294 (16296) |
| T2a1b | 73 263 709 750 1438 1888 2706 4216 4769 4917 7028 8697 8860 10463 11251 11719 11812 13368 13965 14233 14687 14766 14905 15326 15452A 15607 15928 16126 16294 (16296) 16324 |
| T2a1b1 | 73 263 709 750 1438 1888 2141 2706 4216 4769 4917 7028 8697 8860 10463 11251 11719 11812 13368 13965 13966 14233 14687 14766 14905 15326 15452A 15607 15928 16126 16294 (16296) 16324 |
| T2a1b1a | 73 263 709 750 1438 1888 2141 2706 4216 4769 4917 7028 8697 8860 9117 10463 11251 11719 11812 13368 13965 13966 14233 14687 14766 14905 15326 15452A 15607 15928 16126 16294 (16296) 16324 |

| | |
|---|---|
| T2a1b1a1 | 73 263 709 750 1438 1888 2141 2706 4216 4769 4917 7028 8697 8860 9117 10463 11251 11719 11812 12741 13368 13965 13966 14233 14687 14766 14905 15326 15452A 15607 15928 16126 16294 (16296) 16324 |
| T2a1b1a1a | 73 263 709 750 1438 1888 2141 2706 4216 4769 4917 6524 7028 8697 8860 9117 10463 11251 11719 11812 12741 13368 13965 13966 14233 14687 14766 14905 15326 15452A 15607 15928 16126 16294 (16296) |
| T2a1b1a1a1 | 73 263 709 750 1438 1888 2141 2706 4216 4769 4917 6524 7028 8697 8860 9117 9948 10463 11251 11719 11812 12741 13368 13965 13966 14233 14687 14766 14905 15326 15452A 15607 15928 16126 16294 (16296) 16324 |
| T2a1b1a1a2 | 73 263 709 750 1420 1438 1888 2141 2706 4216 4769 4917 6249 6524 7028 8697 8860 9117 10463 11251 11719 11812 12741 13368 13965 13966 14233 14687 14766 14905 15326 15452A 15607 15928 16126 16294 (16296) 16324 |
| T2a1b1a1b | 73 263 709 750 1438 1888 2141 2706 3350 4216 4769 4917 7028 8697 8860 9117 10463 11251 11719 11812 12741 13368 13965 13966 14233 14687 14766 14905 15326 15452A 15607 15928 16126 16294 (16296) |
| T2a1b1a1b1 | 73 263 709 750 1438 1888 2141 2706 3350 4216 4769 4917 7028 8256 8697 8860 9117 10463 11251 11719 11812 12741 13368 13965 13966 14233 14687 14766 14905 15326 15452A 15607 15928 16126 16294 (16296) 16324 |
| T2a1b1a2 | 73 263 709 750 1438 1888 2141 2706 4216 4769 4917 7028 8697 8860 9117 10463 11251 11719 11812 13368 13965 13966 14233 14687 14766 14905 15110 15326 15452A 15607 15928 16126 16292 16294 (16296) |
| T2a1b2 | 73 263 709 750 1438 1888 2706 3882 4216 4769 4917 7028 8697 8860 10463 11251 11719 11812 13368 13965 14233 14687 14766 14905 15326 15452A 15607 15928 16126 16294 (16296) 16324 |
| T2a1b2a | 73 263 709 750 1438 1888 2706 3882 4216 4769 4917 7028 8697 8860 10463 11251 11719 11812 13368 13965 14233 14687 14766 14905 15326 15452A 15454 15607 15928 16126 16294 (16296) 16324 |
| T2a1b2b | 73 263 709 750 1438 1888 2706 3882 4216 4769 4917 7028 8697 8860 10463 11251 11719 11812 13368 13965 14233 14687 14766 14905 15326 15452A 15607 15928 16126 16256 16294 (16296) 16324 |
| T2a+195 | 73 195 263 709 750 1438 1888 2706 4216 4769 4917 7028 8697 8860 10463 11251 11719 11812 13368 13965 14233 14766 14905 15326 15452A 15607 15928 16126 16294 (16296) |
| T2a2 | 73 195 198 263 709 750 1438 1888 2706 4216 4769 4917 7028 8697 8860 10463 11251 11719 11812 13020 13368 13965 14233 14766 14905 15326 15452A 15607 15928 16126 16294 (16296) |
| T2a2a | 73 195 198 215 263 709 750 1438 1888 2706 4216 4769 4917 7028 8697 8860 10463 11251 11719 11812 13020 13368 13965 14233 14766 14905 15326 15452A 15607 15928 16126 16294 (16296) |
| T2a3 | 73 195 263 709 750 1438 1888 2706 4216 4464 4769 4917 7028 8697 8860 10463 11251 11719 11812 13368 13965 14233 14766 14905 15326 15452A 15607 15928 16126 16294 (16296) |
| T2b | 73 263 709 750 930 1438 1888 2706 4216 4769 4917 5147 7028 8697 8860 10463 11251 11719 11812 13368 14233 14766 14905 15326 15452A 15607 15928 16126 16294 (16296) 16304 |

| | |
|---|---|
| T2b1 | 73 263 709 750 930 1438 1888 2706 4216 4769 4917 5147 7028 8697 8860 10463 11251 11719 11812 13368 14016 14233 14766 14905 15326 15452A 15607 15928 16126 16294 (16296) 16304 |
| T2b2 | 73 263 709 750 930 1438 1888 2706 4216 4769 4917 5147 7028 8697 8860 10463 11242G 11251 11719 11812 13368 14233 14766 14905 15326 15452A 15607 15928 16126 16294 (16296) 16304 |
| T2b2b | 73 263 709 750 930 1438 1888 2706 4216 4769 4917 5147 7028 8697 8860 10463 11242G 11251 11719 11812 13368 14233 14766 14905 15326 15452A 15607 15928 16126 16294 (16296) |
| T2b2b1 | 73 263 709 750 930 1438 1888 2706 4216 4769 4917 5147 7028 8697 8860 10463 11242G 11251 11719 11812 12171 13368 14233 14766 14905 15326 15452A 15607 15928 16126 16192 16294 (16296) |
| T2b3 | 73 263 709 750 930 1438 1888 2706 4216 4769 4917 5147 7028 8697 8860 10463 10750 11251 11719 11812 13368 14233 14766 14905 15326 15452A 15607 15928 16126 16294 (16296) 16304 |
| T2b3+151 | 73 151 263 709 750 930 1438 1888 2706 4216 4769 4917 5147 7028 8697 8860 10463 10750 11251 11719 11812 13368 14233 14766 14905 15326 15452A 15607 15928 16126 16294 (16296) 16304 |
| T2b3a | 73 151 152 263 709 750 930 1438 1888 2706 4216 4769 4917 5147 5656 7028 8697 8860 10463 10750 11251 11719 11812 13368 14233 14766 14905 15326 15452A 15607 15928 16126 16292 16294 (16296) 16304 |
| T2b3a1 | 55.1T 57 59 73 151 152 263 709 750 930 1438 1888 2706 4216 4769 4917 5147 5656 7028 8697 8860 10463 10750 11251 11719 11812 13368 14233 14766 14905 15326 15452A 15607 15928 16126 16292 16294 (16296) 16304 |
| T2b3c | 73 151 263 709 750 930 1438 1888 2706 4216 4769 4917 5147 7028 7150 8697 8860 10463 10750 11251 11719 11812 13368 14233 14766 14905 15326 15452A 15607 15928 16126 16294 (16296) 16304 |
| T2b3d | 73 151 199 263 709 750 930 1438 1888 2706 4216 4769 4917 5147 7028 8697 8860 10463 10750 11251 11719 11812 13368 14233 14544 14766 14905 15326 15452A 15607 15928 16126 16294 (16296) 16304 |
| T2b3e | 73 151 263 709 750 930 1438 1888 2706 4216 4769 4917 5147 7028 8697 8860 10463 10750 11251 11719 11812 13368 14233 14766 14905 15326 15452A 15607 15928 16126 16187 16294 (16296) 16304 |
| T2b3b | 73 263 709 750 930 1438 1888 2706 4216 4769 4917 5147 7028 8697 8860 10463 10750 11251 11719 11812 13368 13722 14233 14766 14905 15326 15452A 15607 15928 16126 16294 (16296) 16304 |
| T2b4 | 73 263 709 750 930 1438 1888 2706 4216 4769 4917 5147 7028 8697 8860 9254 10463 11251 11719 11812 13368 14233 14766 14905 15326 15452A 15607 15928 16126 16294 (16296) 16304 |
| T2b4a | 73 263 709 750 930 1438 1888 2706 4216 4769 4917 5147 7028 8697 8860 9254 10463 11251 11719 11812 13368 14233 14766 14905 15326 15452A 15607 15928 16126 16172 16294 (16296) 16304 |
| T2b4a1 | 73 263 709 750 930 1438 1888 2706 4216 4769 4917 5147 5527 7028 8697 8860 9254 10463 11251 11719 11812 13368 14233 14766 14905 15326 15452A 15607 15928 16126 16172 16294 (16296) 16304 |

| | |
|---|---|
| T2b4i | 73 263 709 750 930 1438 1888 2706 4216 4769 4917 5147 7028 8152 8697 8860 9254 10463 11251 11719 11812 13368 14233 14766 14905 15326 15452A 15607 15928 16126 16218 16294 (16296) 16304 |
| T2b4+152 | 73 152 263 709 750 930 1438 1888 2706 4216 4769 4917 5147 7028 8697 8860 9254 10463 11251 11719 11812 13368 14233 14766 14905 15326 15452A 15607 15928 16126 16294 (16296) 16304 |
| T2b4b | 73 152 263 709 750 930 1438 1888 2706 4216 4769 4917 5147 7028 8697 8860 9254 10463 11251 11719 11812 13368 14233 14766 14905 15326 15452A 15607 15928 16104 16126 16294 (16296) 16304 |
| T2b4c | 73 152 263 709 750 930 1438 1888 2706 3992 4216 4769 4917 5147 7028 8697 8860 9254 10463 11251 11719 11812 13368 14233 14766 14905 15326 15452A 15607 15928 16126 16294 (16296) 16304 |
| T2b4d | 73 152 263 709 750 930 1438 1888 2706 4216 4769 4917 5147 7028 8697 8711 8860 9254 10463 11251 11719 11812 13368 14233 14766 14905 15326 15452A 15607 15928 16126 16294 (16296) 16304 |
| T2b4e | 73 152 263 709 750 930 1438 1888 2706 4216 4769 4917 5147 7028 8697 8860 9254 10463 11251 11719 11812 13368 13743 14233 14766 14905 15326 15452A 15607 15928 16126 16294 (16296) 16304 |
| T2b4f | 73 152 263 709 750 930 1438 1888 2706 4216 4769 4917 5147 7028 8697 8860 9254 10463 11251 11719 11812 12172 13368 14233 14766 14905 15326 15452A 15607 15928 16126 16294 (16296) 16304 |
| T2b4g | 73 152 263 709 750 930 1438 1888 2706 4216 4769 4917 5147 7028 8697 8860 9254 10463 11251 11719 11812 13368 14233 14766 14905 15326 15445 15452A 15607 15928 16126 16294 (16296) 16304 |
| T2b4h | 73 152 214 263 709 750 930 1438 1888 2706 4216 4769 4917 5147 7028 8697 8860 9254 10463 11251 11719 11812 13368 14233 14766 14905 15326 15452A 15607 15928 16126 16294 (16296) 16304 |
| T2b5 | 73 263 709 750 930 1438 1888 2706 3826 4216 4769 4917 5147 7028 8697 8860 10463 11251 11719 11812 13368 14233 14766 14905 15326 15452A 15607 15928 16126 16294 (16296) 16304 |
| T2b5a | 73 263 709 750 930 1438 1888 2706 3826 4216 4769 4917 5147 5201 7028 8697 8860 10463 11251 11719 11812 13368 14233 14766 14905 15326 15452A 15607 15928 16126 16294 (16296) 16304 |
| T2b5a1 | 73 263 573.XC 709 750 930 1438 1888 2706 3826 4216 4769 4917 5147 5201 7028 8504 8697 8860 10463 11251 11719 11812 13368 14233 14766 14905 15326 15452A 15607 15928 16126 16294 (16296) 16304 |
| T2b6 | 73 263 458 709 750 930 1438 1709 1888 2706 4216 4769 4917 5147 7028 8697 8860 9300 10463 11251 11533 11719 11812 13368 14233 14766 14905 15326 15452A 15607 15928 16126 16294 (16296) 16304 |
| T2b6a | 73 263 458 709 750 930 1438 1709 1888 2706 4216 4769 4917 5147 7028 8697 8860 9300 10463 11251 11533 11719 11812 12007 13368 14233 14766 14905 15326 15452A 15607 15928 16126 16294 (16296) 16304 |
| T2b6+146 | 73 146 263 458 709 750 930 1438 1709 1888 2706 4216 4769 4917 5147 7028 8697 8860 9300 10463 11251 11533 11719 11812 13368 14233 14766 14905 15326 15452A 15607 15928 16126 16294 (16296) 16304 |

| | |
|---|---|
| T2b6b | 73 146 263 458 709 750 930 1438 1709 1888 2706 4216 4769 4917 5147 7028 8697 8730 8860 9300 10463 11251 11533 11719 11812 13368 14016 14233 14766 14905 15326 15452A 15607 15928 16126 16218 16287 16294 (16296) 16304 |
| T2b7 | 73 263 709 750 930 1438 1888 2706 4216 4769 4917 5147 7028 8697 8860 9966 10463 11251 11719 11812 13368 14233 14766 14905 15326 15452A 15607 15928 16126 16294 (16296) 16304 |
| T2b7a | 73 263 709 750 930 1438 1888 2706 4216 4769 4917 5147 7028 8697 8860 9180 9966 10463 11251 11719 11812 13368 14233 14766 14905 15326 15452A 15607 15928 16126 16294 (16296) 16304 |
| T2b7a1 | 73 152 263 709 750 930 1438 1888 2706 4216 4769 4917 5147 7028 8697 8860 9180 9966 10463 11251 11719 11812 13368 13768 14233 14766 14905 15326 15452A 15607 15928 16126 16257d 16294 (16296) 16304 |
| T2b7a2 | 73 263 709 750 930 1438 1836 1888 2706 4216 4769 4917 5147 7028 8697 8860 9180 9966 10463 11251 11719 11812 13368 14233 14766 14905 15326 15452A 15607 15928 16126 16239 16294 (16296) 16304 |
| T2b7a3 | 73 263 709 750 930 1438 1888 2706 4216 4769 4917 5147 7028 8697 8860 9180 9966 10463 11251 11440 11719 11812 13368 14233 14766 14905 15326 15452A 15607 15928 16126 16292 16294 (16296) 16304 |
| T2b8 | 73 263 709 750 930 1438 1888 2706 3338 4216 4769 4917 5147 7028 8697 8860 10463 11251 11719 11812 13368 14233 14766 14905 15326 15452A 15607 15928 16126 16294 (16296) 16304 |
| T2b+150 | 73 150 263 709 750 930 1438 1888 2706 4216 4769 4917 5147 7028 8697 8860 10463 11251 11719 11812 13368 14233 14766 14905 15326 15452A 15607 15928 16126 16294 (16296) 16304 |
| T2b9 | 73 150 263 709 750 930 1438 1888 2706 4216 4769 4917 5147 5580 7028 8697 8860 10463 10559 11251 11719 11812 13368 14233 14766 14905 15326 15452A 15607 15928 16126 16294 (16296) 16304 |
| T2b11 | 73 207 263 709 750 930 1438 1888 2706 3398 4216 4769 4917 5147 7028 8697 8860 10463 11251 11719 11812 13368 14233 14766 14905 15326 15452A 15607 15928 16126 16294 (16296) 16304 |
| T2b13 | 73 263 709 750 930 1438 1888 2706 4216 4769 4917 5147 7028 8697 8860 10463 11251 11719 11812 13368 14233 14766 14861 14905 15326 15452A 15607 15928 16126 16294 (16296) 16304 |
| T2b13a | 73 263 709 750 930 1438 1888 2706 4216 4769 4917 5147 7028 8697 8860 10463 11251 11719 11812 13368 14233 14766 14861 14905 15326 15452A 15607 15928 16051 16126 16294 (16296) 16304 |
| T2b13b | 73 263 709 750 930 1438 1888 2706 4216 4769 4917 5147 7028 7269 8697 8860 10463 11251 11719 11812 13368 14233 14766 14861 14905 15326 15452A 15607 15928 16126 16294 (16296) 16304 |
| T2b15 | 73 263 709 750 930 1438 1888 2706 4216 4769 4917 5147 5836 7028 8281-8289d 8697 8860 10463 11251 11719 11812 13368 14233 14766 14905 15326 15452A 15607 15928 16126 16294 (16296) 16304 |
| T2b+16362 | 73 263 709 750 930 1438 1888 2706 4216 4769 4917 5147 7028 8697 8860 10463 11251 11719 11812 13368 14233 14766 14905 15326 15452A 15607 15928 16126 16294 (16296) 16304 16362 |

| | |
|---|---|
| T2b16 | 73 263 634 709 750 930 1438 1888 2706 4216 4769 4917 5147 7028 8697 8860 10463 11251 11719 11812 13368 14233 14766 14905 15326 15452A 15607 15928 16126 16294 (16296) 16304 16362 |
| T2b17 | 73 263 709 750 930 1438 1888 2706 4216 4769 4917 5147 7028 8697 8860 10463 11251 11719 11812 13368 13692 14233 14766 14905 15326 15452A 15607 15928 16126 16294 (16296) 16304 |
| T2b17a | 73 263 709 750 930 1438 1888 2706 4216 4688 4769 4917 5147 7028 7891 8697 8860 10463 11251 11719 11812 13368 13692 14233 14766 14905 15326 15452A 15607 15928 16126 16294 (16296) 16304 |
| T2b19 | 73 263 709 750 930 1438 1888 2706 4216 4769 4917 5147 7028 8697 8860 10463 11251 11719 11812 13368 13928C 14233 14766 14905 15326 15452A 15607 15928 16126 16294 (16296) 16304 |
| T2b19b | 73 263 709 750 930 1438 1888 2706 4216 4769 4917 5147 7028 8697 8860 10463 11251 11719 11812 13368 13681 13928C 14233 14766 14905 15326 15452A 15607 15928 16126 16248 16294 (16296) 16304 |
| T2b+152 | 73 152 263 709 750 930 1438 1888 2706 4216 4769 4917 5147 7028 8697 8860 10463 11251 11719 11812 13368 14233 14766 14905 15326 15452A 15607 15928 16126 16294 (16296) 16304 |
| T2b21 | 73 152 263 709 750 930 1438 1888 2706 4216 4769 4917 5147 7028 8697 8860 10463 11251 11719 11812 13368 14233 14766 14836 14905 15326 15452A 15607 15928 16126 16294 (16296) 16304 |
| T2b21a | 73 152 263 709 750 930 1438 1888 2706 4216 4769 4917 5147 7028 8697 8860 10463 10490 11251 11719 11812 12557 13368 14233 14766 14836 14905 15326 15452A 15607 15928 16126 16169 16294 (16296) 16304 |
| T2b21b | 73 152 263 709 750 930 1438 1888 2706 4216 4769 4917 5147 7028 8697 8860 10463 11251 11719 11812 13368 14233 14766 14836 14905 15090 15326 15452A 15607 15928 16126 16294 (16296) 16304 |
| T2b22 | 73 152 263 709 750 930 1438 1888 2706 3820 4216 4769 4917 5147 7028 8697 8860 10463 11251 11719 11812 12223 13368 13500 14233 14766 14905 15326 15452A 15607 15928 16126 16294 (16296) 16304 |
| T2b23 | 73 263 709 750 930 1438 1888 2706 4216 4769 4917 5147 7028 8697 8860 10463 11251 11719 11812 12441 13368 14233 14766 14905 15326 15452A 15607 15928 16126 16147 16294 (16296) 16297 16304 |
| T2b23a | 73 263 709 750 930 1438 1888 2706 4216 4769 4917 5147 7028 8697 8860 9224 10463 11251 11719 11812 12441 13368 14233 14766 14905 15326 15452A 15607 15928 16126 16147 16294 (16296) 16297 16304 |
| T2b24 | 73 263 321 709 750 930 1438 1888 2706 4216 4769 4917 5147 7028 8697 8860 10463 11251 11719 11812 13368 14233 14766 14905 15326 15452A 15607 15928 16126 16294 (16296) 16304 |
| T2b24a | 73 263 321 709 750 930 1438 1888 2706 4216 4769 4917 5147 5426 7028 8572 8697 8860 10463 11251 11719 11812 13368 14233 14766 14905 15326 15452A 15607 15928 16126 16294 (16296) 16304 |
| T2b25 | 73 263 709 750 930 1438 1888 2706 4216 4769 4917 5147 7028 7521 8697 8860 8934 10463 11251 11719 11812 13368 14233 14766 14905 15326 15452A 15607 15928 16126 16294 (16296) 16304 |
| T2b26 | 73 263 709 750 930 1438 1888 2706 4216 4769 4917 5147 7028 8697 8860 10463 11251 11719 11812 13368 14180 14233 14766 14905 15326 15452A 15607 15928 16126 16294 (16296) 16304 |

| | |
|---|---|
| T2b27 | 73 263 709 750 930 1438 1888 2706 4216 4769 4917 5147 7028 7897 8697 8860 10463 11251 11719 11812 13368 14233 14766 14905 15326 15452A 15607 15928 16126 16294 (16296) 16304 |
| T2b28 | 73 263 709 750 930 1438 1888 2706 4216 4769 4917 5147 7028 8697 8860 10463 11176 11251 11719 11812 11914 13368 14233 14766 14905 15326 15452A 15607 15928 16126 16294 (16296) 16304 |
| T2b29 | 73 263 709 750 930 1438 1888 2706 4216 4769 4917 5147 5483 7028 8419 8697 8860 10463 11251 11719 11812 13368 14233 14766 14905 15326 15452A 15607 15928 16126 16294 (16296) 16304 |
| T2b30 | 73 263 709 750 930 1438 1888 2706 4216 4769 4917 5147 6026 7028 8697 8860 10463 11251 11719 11812 13368 14233 14766 14905 15326 15452A 15607 15928 16126 16294 (16296) 16304 |
| T2b31 | 73 263 709 750 930 1438 1888 2706 4216 4769 4917 5147 5811 7028 8697 8860 10463 11251 11719 11812 13368 14233 14766 14905 15326 15452A 15607 15928 16126 16294 (16296) 16304 |
| T2b32 | 73 263 709 750 930 1438 1871 1888 2706 4216 4769 4917 5147 6620 7028 8697 8860 10463 11251 11719 11812 13368 14233 14766 14905 15326 15452A 15607 15884 15928 16126 16294 (16296) 16304 |
| T2b33 | 73 263 385 709 750 930 1438 1530 1888 2706 4216 4769 4917 5147 7028 8697 8860 10463 11251 11719 11812 13368 14233 14766 14905 15326 15452A 15607 15928 16126 16266 16294 (16296) 16304 |
| T2b34 | 41 73 263 319 709 750 930 1438 1888 2706 4216 4769 4917 5147 7028 8697 8860 9861 10463 11251 11719 11812 13368 14233 14766 14905 15326 15452A 15607 15928 16126 16294 (16296) 16304 |
| T2b35 | 73 263 709 750 930 1438 1888 2706 4216 4769 4917 5147 7028 8697 8860 9843 10463 11251 11719 11812 13368 14233 14766 14905 15326 15452A 15607 15928 16126 16294 (16296) 16304 |
| T2b36 | 73 263 709 750 930 1438 1888 2706 4216 4769 4917 5147 7028 8697 8860 10463 11251 11719 11812 13368 14233 14766 14905 15326 15452A 15607 15758 15928 16126 16294 (16296) 16304 |
| T2b37 | 73 263 709 750 930 1438 1888 2706 4216 4769 4917 5147 7028 8697 8860 10463 11251 11719 11812 13368 14233 14569 14766 14905 15326 15452A 15607 15928 16126 16294 (16296) 16304 |
| T2c | 73 263 709 750 1438 1888 2706 4216 4769 4917 7028 8697 8860 10463 10822 11251 11719 11812 13368 14233 14766 14905 15326 15452A 15607 15928 16126 16294 (16296) |
| T2c1 | 73 263 709 750 1438 1888 2706 4216 4769 4917 6261 7028 8697 8860 10463 10822 11251 11719 11812 13368 14233 14766 14905 15326 15452A 15607 15928 16126 16292 16294 (16296) |
| T2c1a | 73 263 573.XC 709 750 1438 1888 2706 4216 4769 4917 6261 7028 8455 8697 8860 10463 10822 11251 11719 11812 13368 13973T 14233 14766 14905 15326 15452A 15607 15928 16126 16292 16294 (16296) |
| T2c1a1 | 73 152 263 499 573.XC 709 750 1438 1888 2706 4216 4769 4917 6261 6998 7028 8455 8697 8838 8860 10463 10822 11251 11719 11812 11914 13368 13973T 14233 14766 14905 15326 15452A 15607 15928 16126 16292 16294 (16296) |

| | |
|---|---|
| T2c1a2 | 73 263 573.XC 709 750 1438 1888 2706 4216 4769 4917 5817 6261 7028 8455 8697 8860 10463 10822 11251 11719 11812 13368 13973T 14233 14766 14905 15326 15452A 15607 15928 16126 16292 16294 (16296) |
| T2c1a3 | 73 263 573.XC 709 750 1438 1888 2706 4216 4769 4917 6261 7028 8455 8697 8860 9612 10463 10822 11251 11719 11800 11812 13368 13973T 14233 14766 14905 15326 15452A 15607 15928 16126 16292 16294 (16296) |
| T2c1c | 73 263 709 750 1438 1888 2706 4216 4769 4823 4917 6261 7028 8697 8860 10463 10822 11251 11719 11812 13368 14233 14766 14905 15326 15452A 15607 15928 16126 16146 16292 16294 (16296) |
| T2c1c1 | 73 150 263 709 750 1438 1888 2706 3834 3915 4216 4769 4823 4917 6261 7028 8697 8860 10463 10822 11251 11719 11812 13368 14233 14766 14905 15326 15452A 15454 15601 15607 15928 16126 16146 16292 16294 (16296) |
| T2c1c2 | 73 263 709 750 1438 1888 2706 4216 4769 4823 4917 6261 7028 7283 8697 8775 8860 10463 10822 11251 11719 11812 13368 14233 14766 14905 15326 15452A 15607 15928 16126 16146 16284 16292 16294 |
| T2c1+146 | 73 146 263 709 750 1438 1888 2706 4216 4769 4917 6261 7028 8697 8860 10463 10822 11251 11719 11812 13368 14233 14766 14905 15326 15452A 15607 15928 16126 16292 16294 (16296) |
| T2c1d | 73 146 263 279 709 750 1438 1888 2706 4216 4769 4917 5187 6261 7028 7873 8697 8860 10463 10822 11251 11719 11812 13368 14233 14766 14905 15326 15452A 15607 15928 16126 16292 16294 (16296) |
| T2c1d1 | 73 146 263 279 709 750 1438 1888 2706 4216 4769 4917 5187 6261 7028 7873 8697 8860 10463 10822 11251 11719 11812 11914 13368 14233 14766 14905 15326 15452A 15607 15928 16126 16292 16294 (16296) |
| T2c1d1a | 73 146 263 279 709 750 1438 1888 2706 4216 4769 4917 5187 6261 7028 7873 8697 8860 10463 10822 11251 11719 11812 11914 12363 13368 14233 14766 14905 15326 15452A 15607 15928 16126 16292 16294 |
| T2c1d+152 | 73 146 152 263 279 709 750 1438 1888 2706 4216 4769 4917 5187 6261 7028 7873 8697 8860 10463 10822 11251 11719 11812 13368 14233 14766 14905 15326 15452A 15607 15928 16126 16292 16294 (16296) |
| T2c1d2 | 73 146 152 263 279 709 750 1438 1888 2706 4216 4769 4917 5187 6261 7028 7873 8697 8860 10463 10822 11251 11719 11812 13368 14233 14766 14905 15326 15452A 15607 15784 15928 16126 16292 16294 (16296) |
| T2c1d2a | 73 146 152 263 279 709 750 1438 1888 2706 4216 4769 4917 5187 6261 7028 7679 7873 8697 8860 10463 10822 11251 11719 11812 13368 14233 14766 14905 15326 15452A 15607 15784 15928 16126 16292 16294 (16296) |
| T2c1e | 73 146 263 709 750 1438 1888 2706 4216 4769 4917 6261 7028 8697 8860 10289 10463 10822 11251 11719 11812 13368 14233 14766 14905 15326 15452A 15607 15928 16126 16292 16294 (16296) |
| T2c1f | 73 146 263 709 750 1438 1888 2706 4216 4769 4917 6261 7028 8697 8860 10463 10822 11251 11719 11812 13368 14233 14766 14905 15326 15452A 15607 15928 16126 16292 16294 (16296) 16438 |
| T2d | 73 263 709 750 1438 1888 2706 4216 4769 4917 7028 8697 8860 10463 11251 11719 11812 13260 13368 14233 14766 14905 15326 15452A 15607 15928 16126 16294 (16296) |

| | |
|---|---|
| T2d1 | 73 152 263 709 750 1438 1888 2706 4216 4769 4917 5747 7028 8697 8860 10463 11251 11719 11812 13260 13368 13708 14233 14766 14905 15326 15452A 15607 15928 16126 16294 (16296) |
| T2d1a | 73 152 263 709 750 1438 1888 2706 4216 4769 4917 5747 7028 8697 8860 10463 11251 11719 11812 13260 13368 13708 14233 14766 14905 15326 15452A 15607 15928 16086 16126 16294 (16296) |
| T2d1b | 73 152 194 200 263 709 750 1438 1888 2706 4216 4769 4917 5747 7028 7295 8697 8860 10463 11251 11719 11812 12373 13260 13368 13708 14233 14766 14905 15326 15452A 15607 15928 16126 16294 (16296) |
| T2d1b1 | 73 152 194 200 263 709 750 1438 1888 2706 4216 4769 4917 5747 7028 7295 8697 8860 9617 10463 11251 11719 11812 11827 12373 13260 13368 13708 14233 14766 14905 15326 15452A 15607 15928 16126 16294 (16296) |
| T2d1b2 | 73 152 194 200 263 709 750 1438 1888 2706 4216 4769 4917 5747 7028 7295 8697 8860 9233 10463 11251 11719 11812 12373 13260 13368 13708 14233 14766 14905 15326 15452A 15607 15928 16126 16294 (16296) |
| T2d2 | 73 263 709 750 1438 1888 2706 4216 4769 4917 5471 6445 7028 8697 8860 10463 11251 11719 11812 12408 13260 13368 14233 14323 14605 14766 14905 15326 15452A 15607 15928 16126 16294 (16296) |
| T2+150 | 73 150 263 709 750 1438 1888 2706 4216 4769 4917 7028 8697 8860 10463 11251 11719 11812 13368 14233 14766 14905 15326 15452A 15607 15928 16126 16294 (16296) |
| T2e | 73 150 263 709 750 1438 1888 2706 4216 4769 4917 7028 8697 8860 10463 11251 11719 11812 13368 14233 14766 14905 15326 15452A 15607 15928 16126 16153 16294 (16296) |
| T2e1 | 41 73 150 263 709 750 1438 1888 2706 4216 4769 4917 7028 8697 8860 10463 11251 11719 11812 13368 14233 14766 14905 15326 15452A 15607 15928 16126 16153 16294 (16296) |
| T2e1a | 41 73 150 263 709 750 1438 1888 2308 2706 4216 4769 4917 7028 8697 8860 10463 11251 11719 11812 13368 14233 14766 14905 15326 15452A 15607 15928 16126 16153 16294 (16296) |
| T2e1a1 | 41 73 150 263 709 750 1438 1888 2308 2706 4216 4769 4917 7028 8697 8860 10463 11251 11719 11812 13368 14233 14766 14905 15326 15452A 15499 15607 15928 16126 16153 16294 (16296) |
| T2e1a1a | 73 150 263 709 750 1438 1888 2308 2706 4216 4769 4917 7028 8697 8860 10463 11251 11719 11812 13368 14233 14766 14905 15326 15452A 15499 15607 15928 (16114) 16126 16153 16192 16294 (16296) |
| T2e1a1b | 41 73 150 263 709 750 1438 1888 2308 2706 4216 4769 4917 7028 8697 8860 10463 11251 11719 11812 13368 14233 14766 14905 15326 15452A 15499 15607 15928 16126 16153 16189 16294 (16296) |
| T2e1a1b1 | 41 73 150 247 263 709 750 1438 1888 2308 2706 4216 4769 4917 4924 7028 8697 8860 8864 10463 10682T 11251 11719 11812 13368 14233 14766 14905 15326 15452A 15499 15607 15928 16126 16153 16189 16294 (16296) |
| T2e1b | 41 73 150 263 709 750 1438 1888 2706 4216 4769 4917 7028 8697 8860 9181 10463 11251 11719 11812 13368 14233 14766 14905 15326 15452A 15607 15928 16126 16153 16294 (16296) |

| | |
|---|---|
| T2e1b1 | 41 73 150 263 709 750 1438 1888 2706 4216 4769 4917 7028 8697 8860 9181 10463 11251 11719 11812 13368 14233 14766 14905 15326 15452A 15607 15787 15928 16126 16153 16294 (16296) |
| T2e2 | 73 150 263 709 750 1438 1888 2706 4216 4769 4917 7028 8697 8860 9139 10463 11251 11719 11812 13368 14233 14766 14905 15326 15452A 15607 15928 16126 16153 16294 (16296) |
| T2e2a | 73 150 263 709 750 1438 1888 2706 4216 4769 4917 6026 7028 8697 8860 9139 10463 11251 11719 11812 13368 14233 14766 14905 15326 15452A 15607 15928 16126 16153 16294 (16296) |
| T2e5 | 73 150 200 263 709 750 1438 1888 2706 4216 4769 4917 7028 8230 8697 8860 10463 11251 11719 11812 13368 14233 14766 14905 15326 15452A 15607 15928 16126 16153 16294 (16296) |
| T2e6 | 73 150 263 709 750 1438 1888 2706 4216 4769 4917 7028 8697 8860 10463 11251 11719 11812 13368 14233 14766 14905 15326 15452A 15607 15928 16126 16153 16240C 16294 (16296) |
| T2e+152 | 73 150 152 263 709 750 1438 1888 2706 4216 4769 4917 7028 8697 8860 10463 11251 11719 11812 13368 14233 14766 14905 15326 15452A 15607 15928 16126 16153 16294 (16296) |
| T2e7 | 73 150 152 263 709 750 1438 1888 2706 3203 4216 4769 4917 7028 8697 8860 10463 11251 11719 11812 13368 14233 14766 14905 15326 15452A 15607 15928 16126 16153 16294 (16296) |
| T2m | 73 150 153 263 709 750 1438 1888 2706 4216 4769 4917 7028 8404 8697 8860 10463 11251 11719 11812 13368 14233 14766 14905 15326 15452A 15607 15928 16126 16270 16294 (16296) 16357 |
| T2+16189 | 73 263 709 750 1438 1888 2706 4216 4769 4917 7028 8697 8860 10463 11251 11719 11812 13368 14233 14766 14905 15326 15452A 15607 15928 16126 16189 16294 (16296) |
| T2f | 73 263 709 750 1438 1888 2706 4216 4769 4917 7028 8270 8281-8289d 8697 8860 10463 11251 11719 11812 13368 14233 14766 14905 15326 15452A 15607 15928 16126 16189 16294 (16296) |
| T2f1 | 73 195 263 709 750 1438 1888 2706 4216 4769 4917 6489A 7028 8270 8281-8289d 8697 8860 10463 11251 11719 11812 13368 14233 14766 14905 15326 15452A 15607 15928 16126 16189 16294 (16296) |
| T2f1a | 73 195 263 709 750 1438 1888 2706 4216 4769 4917 5277 5426 6489A 7028 8270 8281-8289d 8697 8860 10463 11251 11719 11812 13368 14233 14766 14905 15043 15326 15452A 15607 15928 16126 16189 16294 (16296) 16298 |
| T2f1a1 | 73 195 263 709 750 1438 1888 2706 4216 4769 4917 5277 5426 6489A 7028 8270 8281-8289d 8697 8860 10463 11251 11719 11812 13368 14233 14766 14905 15028A 15043 15326 15452A 15607 15928 16126 16189 16294 (16296) 16298 |
| T2f2 | 73 263 709 750 1438 1888 2706 4216 4769 4917 7028 7984 8270 8281-8289d 8697 8860 10463 11251 11719 11812 13368 14233 14766 14905 15326 15452A 15607 15928 16126 16140 16189 16294 (16296) 16311 |
| T2f3 | 73 263 709 750 1438 1888 2706 4216 4769 4917 7028 8270 8281-8289d 8697 8854 8860 10463 11251 11719 11812 13368 14233 14766 14905 15326 15452A 15607 15928 16126 16189 16278 16294 (16296) |

| | |
|---|---|
| T2f4 | 73 263 709 750 1438 1888 2706 4216 4769 4917 6392 7028 8270 8281-8289d 8697 8860 10463 11251 11719 11812 13368 14233 14766 14905 15326 15452A 15607 15928 16126 16189 16294 (16296) |
| T2f5 | 73 263 499 709 750 751T 1438 1888 2706 4216 4769 4917 7028 8270 8281-8289d 8419 8697 8860 10463 11251 11719 11812 13368 14233 14766 14905 15326 15452A 15607 15928 16126 16189 16294 (16296) |
| T2f6 | 73 263 709 750 1438 1888 2706 4216 4769 4917 7028 8270 8281-8289d 8697 8860 10463 11251 11719 11812 11818 13368 14233 14766 14905 15326 15452A 15607 15928 16126 16189 16294 (16296) |
| T2f7 | 73 263 709 750 1438 1888 2706 4216 4769 4917 7028 8270 8281-8289d 8697 8860 10463 10496 11251 11719 11812 13368 14233 14766 14905 15326 15452A 15607 15928 16126 16189 16294 (16296) |
| T2f7a | 73 263 709 750 1438 1888 2706 4216 4769 4917 7028 8270 8281-8289d 8697 8860 10463 10496 11251 11719 11812 13368 14233 14766 14905 15326 15452A 15607 15928 16126 16189 16292 16294 (16296) |
| T2f8 | 73 263 709 750 1438 1888 2706 4216 4769 4917 7028 8270 8281-8289d 8697 8860 10463 11251 11719 11812 13368 14118 14233 14766 14905 15326 15452A 15607 15928 16126 16189 16294 (16296) |
| T2f8a | 73 263 709 750 1438 1888 2706 4216 4769 4917 5322C 7028 8270 8281-8289d 8697 8860 10463 11251 11719 11812 13368 14118 14233 14766 14905 15326 15452A 15607 15928 16126 16189 16294 (16296) |
| T2g | 73 263 709 750 1438 1888 2706 4216 4769 4917 7028 8697 8860 10463 11251 11719 11812 13368 14233 14766 14798 14905 15326 15452A 15607 15928 16126 16294 (16296) |
| T2g1 | 73 (200) 263 709 750 1438 1888 2706 3834 4216 4769 4917 7028 8697 8860 10463 11251 11719 11812 13368 14233 14766 14798 14839 14905 15326 15452A 15607 15928 16126 16294 (16296) |
| T2g1a | 73 (200) 263 709 750 1438 1888 1977 2706 3834 4216 4769 4917 7028 8697 8860 10463 11251 11719 11812 13368 14233 14766 14798 14839 14905 15326 15452A 15607 15928 16126 16294 (16296) |
| T2g1a1 | 73 (200) 263 709 750 1438 1888 1977 2706 3834 4216 4769 4917 7028 8697 8860 10463 11251 11719 11812 13368 14233 14766 14798 14839 14905 15326 15452A 15607 15928 16126 16148 16294 (16296) |
| T2g1b | 73 (200) 263 709 750 1438 1888 2706 3834 4216 4769 4917 7028 8697 8860 10463 11251 11719 11812 13368 14233 14766 14798 14839 14905 15326 15452A 15607 15928 16051 16126 16294 (16296) |
| T2g2 | 73 263 507 709 750 1438 1766 1888 2706 4216 4769 4917 7028 7337 8697 8860 10463 11251 11719 11812 13368 13834 14233 14766 14798 14905 15326 15452A 15607 15928 16126 16294 (16296) |
| T2g2a | 73 263 507 709 750 1438 1766 1888 2706 4216 4769 4917 7028 7337 8697 8860 10463 11251 11719 11812 13368 13834 14233 14766 14798 14905 15326 15452A 15607 15928 16126 16188 16257 16294 (16296) |
| T2h | 73 263 709 750 1438 1888 2706 4216 4769 4917 7028 8697 8860 10463 11251 11719 11812 12397 13368 14233 14766 14905 15326 15452A 15607 15928 16126 16294 (16296) |
| T2h1 | 73 263 709 750 1438 1888 2706 4216 4769 4917 6260 7028 8697 8860 9234 10463 11251 11719 11812 12397 13368 14233 14766 14905 15326 15452A 15607 15928 16126 16294 (16296) |

| | |
|---|---|
| T2h2 | 73 263 709 750 1438 1888 2706 4216 4769 4917 7028 8041 8697 8860 10463 11251 11719 11812 12397 12771 13368 14233 14766 14905 15326 15452A 15607 15928 16126 16172 16294 (16296) |
| T2i | 73 263 709 750 1438 1888 2706 4216 4769 4917 7028 8155 8697 8860 9422C 10463 11251 11719 11812 13368 14233 14766 14905 15326 15452A 15607 15928 16126 16294 (16296) 16362 |
| T2i1 | 73 263 709 750 1438 1888 2706 4216 4769 4917 7028 8155 8697 8860 9422C 10463 11251 11719 11812 13368 14233 14766 14905 15326 15452A 15607 15928 16126 16286 16294 (16296) 16362 |
| T2i2 | 73 263 709 750 1438 1888 2706 4216 4769 4917 7028 8155 8697 8860 9422C 10463 11251 11719 13368 14233 14766 14905 15326 15452A 15607 15928 16126 (16296) 16362 |
| T2j | 73 263 709 750 1438 1888 2706 4216 4769 4917 7028 8697 8860 10463 11251 11719 11812 13368 14233 14766 14905 15326 15452A 15607 15928 16126 16260 16294 (16296) |
| T2j1 | 73 263 709 750 1438 1888 2706 4216 4695 4769 4917 6620 7028 8697 8860 10463 11251 11719 11812 11914 13368 14040 14233 14766 14905 15326 15452A 15607 15928 16126 16260 16294 (16296) |
| T2k | 73 263 709 750 1438 1888 2706 4216 4769 4917 7028 8271 8697 8860 10446 10463 11062 11251 11719 11812 13368 14233 14766 14905 15326 15452A 15607 15928 16126 16291 16294 (16296) |
| T2l | 73 263 709 750 1438 1888 2706 4216 4769 4917 7028 8697 8860 10463 11251 11383 11719 11812 13368 14233 14766 14905 15326 15452A 15607 15928 16126 16294 (16296) |
| T2n | 73 152 263 709 750 1438 1888 2706 4216 4769 4917 7028 8697 8860 10463 10750 11251 11719 11812 13368 14233 14766 14905 15326 15452A 15607 15928 16126 16294 (16296) |
| T3 | 73 195 263 709 750 1406 1438 1829 1888 2706 4216 4225 4769 4917 7028 8697 8860 10463 11251 11467 11719 11778 13368 13956 13980 14766 14905 15326 15452A 15509 15607 15928 16126 16294 16325 |
| R5 | 73 263 750 1438 2706 4769 7028 8594 8860 10754 11719 14544 14766 15326 16304 16524 |
| R5a | 73 263 750 1438 2706 4769 7028 8594 8860 10754 11719 14544 14766 15326 16266 16304 16524 |
| R5a1 | 73 93 200 263 750 1438 2706 2833 4769 7028 8594 8860 10754 11719 14544 14766 15326 16266 16304 16524 |
| R5a1a | 73 93 200 263 750 1438 2706 2833 4769 7028 8594 8860 8987 9708 10084 10754 11719 14544 14766 15326 16266 16304 16524 |
| R5a2 | 73 152 263 750 1438 2706 4769 7028 8594 8860 10754 11293 11719 13635 14040 14544 14766 15326 15385 16266 16304 16356 16524 |
| R5a2a | 73 152 263 750 1438 2706 4769 7028 8594 8860 10754 11293 11719 13635 14040 14544 14766 15326 15385 16304 16311 16356 16524 |
| R5a2b | 73 152 263 597.1T 750 1438 2706 4769 7028 8594 8860 10754 11293 11719 13635 14040 14544 14766 14990 15326 15385 16266 16304 16309 16325 16356 16524 |
| R5a2b1 | 73 152 263 597.1T 750 1438 2706 4769 6629 7028 8594 8860 10754 11293 11719 13635 14040 14544 14766 14990 15326 15385 16266 16304 16309 16325 16356 16524 |

| | |
|---|---|
| R5a2b2 | 73 152 263 597.1T 750 1438 2706 4769 7028 8594 8860 10754 11293 11719 13635 14040 14544 14766 14990 15326 15385 16304 16309 16325 16356 16524 |
| R5a2b3 | 73 152 263 597.1T 750 1438 2706 4769 7028 8594 8860 10754 11293 11409 11719 13635 14040 14544 14766 14990 15326 15385 16266 16304 16309 16325 16356 16524 |
| R5a2b4 | 73 152 263 597.1T 750 1438 2706 4769 7028 8594 8860 10754 11293 11719 13635 13827 14040 14544 14766 14990 15326 15385 16266 16304 16309 16325 16356 16524 |
| R6 | 73 (195) 263 750 1438 2706 4769 7028 8860 11719 12285 14766 15326 (16266) 16362 |
| R6+16129 | 73 (195) 263 750 1438 2706 4769 7028 8860 11719 12285 14766 15326 16129 (16266) 16362 |
| R6a | 73 (195) 228 263 750 1438 2706 4769 7028 8860 11075 11719 12285 14058 14766 15326 16129 (16266) 16362 |
| R6a1 | 73 (195) 228 263 750 1438 2706 4769 6305 7028 8584 8650 8860 11075 11719 12285 14058 14766 15326 16129 (16266) 16318 16320 16362 |
| R6a2 | 73 (195) 228 263 750 1438 2706 4769 7028 8860 11075 11719 12133 12285 14058 14766 15067 15326 16129 (16266) 16362 |
| R6b | 73 (195) 246 263 750 1438 2706 4769 4991 7028 7364 8860 9254 11719 12285 14766 15326 16179 16227 16245 (16266) 16278 16362 |
| R7 | 73 263 750 1438 2706 4769 7028 8860 11719 13105 14766 15326 16319 16362 |
| R7a'b | 73 263 750 1438 1442 2706 4769 6248 7028 7870 8860 9051 9110 10289 11719 13105 13830 14766 15326 16260 16261 16319 16362 |
| R7a | 73 263 750 1438 1442 2706 4769 6248 7028 7870 8860 9051 9110 10143 10289 10915 11719 13105 13404 13830 14766 15326 15346 16260 16261 16319 16362 |
| R7a1 | 73 263 750 1438 1442 2706 4769 6248 7028 7870 8860 9051 9110 10143 10289 10915 11719 12406 13105 13404 13674 13830 14766 15326 15346 16260 16261 16319 16362 |
| R7a1a | 73 263 750 1438 1442 2706 4769 5585 6248 7028 7870 8860 9051 9110 10143 10289 10915 11719 12406 13105 13404 13674 13830 14766 15326 15346 15497 16146 16260 16261 16319 16362 |
| R7a1b | 73 263 750 1438 1442 2706 3105 4769 6248 7028 7870 8860 9051 9110 10143 10289 10915 11719 12406 13105 13404 13674 13830 14766 15326 15346 16260 16261 16319 16362 |
| R7a1b1 | 73 263 750 1438 1442 2706 3105 4596 4769 6248 7028 7870 8860 9051 9110 10143 10289 10915 11719 12406 13105 13404 13674 13830 14766 15326 15346 16075 16260 16261 16319 16362 |
| R7a1b2 | 73 263 750 1438 1442 2706 3105 4769 6248 7028 7870 8860 9051 9110 10143 10289 10915 11719 12406 13105 13404 13674 13830 14766 15326 15346 16260 16261 16319 16362 16390 |
| R7b | 73 146 263 750 1438 1442 2282 2706 4769 6248 7028 7870 8557 8860 9051 9110 10289 11719 13105 13830 14064 14766 15326 16260 16261 16311 16319 16362 |

| | |
|---|---|
| R7b1 | 73 146 263 750 1438 1442 1804 2282 2706 4769 6248 7028 7870 8557 8860 9051 9110 10289 11719 12432 13105 13830 14064 14766 15326 15942 16260 16261 16311 16319 16362 |
| R7b1a | 73 146 152 263 750 1438 1442 1804 2282 2706 4769 6248 6254 7028 7870 8557 8860 9051 9110 10289 11719 12432 13105 13830 14064 14766 15942 16260 16261 16311 16319 16362 |
| R7b1a1 | 73 146 152 263 750 1438 1442 1804 2282 2706 4769 6248 6254 7028 7870 8557 8860 9051 9110 9325 10289 11719 11776 12432 13105 13830 14064 14766 15942 16260 16261 16311 16319 16362 |
| R7b2 | 73 146 263 750 1438 1442 2282 2706 3817 4769 6248 7028 7310 7711 7870 8557 8860 9051 9110 10289 11380 11719 12246 12384 12940 13105 13830 14064 14766 15326 16260 16261 16311 16319 16362 |
| R8 | 73 195 263 750 1438 2706 2755 3384 4769 7028 7759 8860 9449 11719 13215 14766 15326 |
| R8a | 73 195 263 750 1438 2706 2755 3384 4769 5510 7028 7759 8860 9449 11719 13215 14766 |
| R8a1 | 73 195 263 709 750 1438 2706 2755 3384 4769 5510 5911 7028 7759 8860 9449 11719 13215 13782 14766 |
| R8a1a | 73 195 263 709 750 1438 2706 2755 3384 4769 5510 5911 7028 7759 8860 9449 9716 11719 13215 13782 14766 |
| R8a1a1 | 73 195 263 709 750 1438 2706 2755 3384 4769 5510 5911 7028 7759 8646 8860 9449 9716 11719 13215 13782 14766 |
| R8a1a1a | 73 195 263 709 750 1438 2706 2755 3384 4769 5510 5911 6131 7028 7759 8646 8860 9449 9716 11719 13215 13782 14766 |
| R8a1a1a1 | 73 195 243 263 709 750 1438 2706 2755 3384 4769 5510 5911 6131 7028 7759 8646 8860 9449 9716 11719 13215 13782 14766 |
| R8a1a1a1a | 73 195 243 263 709 750 1438 2706 2755 3384 3483 4769 5298 5510 5911 6131 7028 7759 8646 8860 9449 9716 11050 11719 13215 13782 14766 |
| R8a1a1a2 | 73 195 263 709 750 1438 2706 2755 3384 4695 4769 5510 5911 6131 7028 7444 7759 8646 8860 9449 9716 11719 13215 13782 14766 16185 |
| R8a1a1b | 73 195 263 709 750 1438 2706 2755 3384 4769 5510 5911 7028 7759 8646 8860 9449 9716 11719 13215 13782 14766 16153 |
| R8a1a1c | 73 195 263 709 750 1438 2706 2755 3384 4769 5510 5911 7028 7759 8646 8860 9449 9716 11719 11914 13215 13782 14766 |
| R8a1a1d | 73 195 263 709 750 1438 2706 2755 3384 4769 5510 5911 7028 7759 8646 8860 9210 9449 9716 11719 12462A 13215 13782 14766 16390 |
| R8a1a2 | 73 195 263 709 750 1438 2706 2755 3384 4205 4769 5510 5911 7028 7759 8860 9449 9716 11719 13215 13782 14766 16324 |
| R8a1a2a | 73 195 263 709 750 1438 2706 2755 3384 4205 4769 5510 5911 7028 7364 7759 8860 9449 9716 11172 11497 11719 12127 13215 13782 14766 16324 |
| R8a1a3 | 73 195 263 709 750 1438 2706 2755 3384 4769 5510 5911 7028 7759 8860 9449 9716 11719 13215 13782 14766 16292 |
| R8a1+16093 | 73 195 263 709 750 1438 2706 2755 3384 4769 5510 5911 7028 7759 8860 9449 11719 13215 13782 14766 16093 |
| R8a1b | 73 195 263 709 750 1438 2706 2755 3384 4769 5510 5911 7028 7759 8860 9449 11719 13212 13215 13782 14041 14766 16093 |
| R8a2 | 73 185 189 263 750 1438 2706 2755 3384 4769 5510 7028 7759 8860 9449 11719 11864 13215 14766 |

| | |
|---|---|
| R8b | 73 195 263 456 750 1438 2706 2755 3384 4769 6485 7028 7759 8860 9449 11719 12007 13194 13215 14766 15326 16390 |
| R8b1 | 73 154 195 263 456 750 1438 2706 2755 3384 4769 6485 7028 7759 8860 9449 11719 12007 13194 13215 14766 15326 16390 |
| R8b1a | 73 154 195 263 456 750 1438 2392 2706 2755 3384 4769 6485 7028 7759 8860 9449 11719 12007 13194 13215 14766 15326 16390 |
| R8b2 | 73 150 195 263 456.1T 750 1438 2706 2755 3384 4136 4769 6485 7028 7759 8860 9449 11719 12007 13194 13215 14502 14766 15250 15326 16172 16390 |
| R9 | 73 263 750 1438 2706 3970 4769 7028 8860 11719 13928C 14766 15326 16304 |
| R9b | 73 263 750 1438 1541 2706 3970 4769 7028 8860 11719 13928C 14766 15326 16304 |
| R9b1 | 73 263 750 1438 1541 2706 3970 4769 7028 8860 11719 12714 13928C 14766 15326 16304 16309 16390 |
| R9b1a | 73 183 263 750 1438 1541 2706 3970 4769 7028 8860 11719 12714 13928C 14766 15326 16192 16304 16309 16390 |
| R9b1a1 | 73 143 183 263 750 1438 1541 2706 3970 4017 4769 7028 7849 8860 11719 12714 13928C 14766 15326 16192 16304 16309 16390 |
| R9b1a1a | 73 143 183 263 750 1438 1541 2706 3970 4017 4769 7028 7849 8860 11719 12714 13928C 14766 15326 16192 16288 16304 16309 16390 |
| R9b1a2 | 73 183 263 750 1438 1541 2706 3970 4769 7028 8860 10736 11719 12714 13928C 14766 15326 16192 16304 16309 16390 |
| R9b1a2a | 73 183 263 750 1438 1541 2706 3970 4769 7028 8860 10736 11719 12172 12714 13928C 14766 15326 16145 16192 16243 16304 16309 16390 |
| R9b1a2b | 73 183 263 750 1438 1541 2706 3970 4769 6620 7028 8860 10736 11719 12714 13928C 14766 15326 16192 16304 16309 16390 |
| R9b1a3 | 73 183 263 750 1438 1541 2706 3204 3316 3970 4769 6815 7028 8860 11719 12714 13928C 14766 15326 16192 16304 16309 16390 |
| R9b1b | 73 263 750 1438 1541 2322A 2706 3970 4769 7028 8860 11719 12714 13928C 14766 15326 16124 16148 16304 16309 16390 |
| R9b2 | 73 263 750 1438 1541 2706 3666 3970 4769 6647 7028 8724 8860 11299 11422 11719 13191 13722 13928C 14766 15314 15326 15883 16304 16362 |
| R9c | 73 263 750 1438 2706 3970 4769 7028 7861 8860 11002 11719 13928C 14766 15326 15479 16304 |
| R9c1 | 73 263 750 1438 2706 3970 4769 7028 7861 8860 11002 11719 13928C 14766 15326 15479 16157 16304 |
| R9c1a | 73 249d 263 750 951 1438 2706 3970 4769 6446 7028 7861 8440 8860 11002 11308 11719 13928C 14227 14766 15326 15479 15734 16157 16256 16304 16335 |
| R9c1a1 | 73 249d 263 750 951 1438 1462 2706 3970 4769 6446 7028 7861 8440 8860 11002 11308 11719 13928C 14227 14766 15326 15479 15734 16157 16256 16304 16335 |
| R9c1a2 | 73 249d 263 750 951 1438 2706 3970 4769 6446 7028 7861 8440 8860 11002 11197 11308 11719 13928C 14227 14766 15326 15479 15734 16157 16256 16304 16335 |

| | |
|---|---|
| R9c1a3 | 73 249d 263 750 951 1438 2706 3970 4769 6446 7028 7861 8440 8860 11002 11308 11719 12123 13928C 14227 14766 15326 15479 15734 16157 16256 16304 16335 |
| R9c1b | 73 263 750 1438 2706 3970 4769 7028 7861 8860 11002 11719 12618 13928C 14766 15326 15479 16157 16304 |
| R9c1b1 | 73 151 263 479 750 1438 2706 3970 4769 7028 7684 7861 8860 11002 11719 12618 13928C 14766 15326 15479 16157 16304 |
| R9c1b2 | 73 263 750 1438 2706 3970 4769 7028 7299 7861 8860 11002 11719 12406 12618 13928C 14766 15326 15479 16157 16304 |
| F | 73 249d 263 750 1438 2706 3970 4769 6392 7028 8860 10310 11719 13928C 14766 15326 16304 |
| F1 | 73 249d 263 750 1438 2706 3970 4769 6392 6962 7028 8860 10310 10609 11719 12406 12882 13928C 14766 15326 16304 |
| F1a'c'f | 73 249d 263 750 1438 2706 3970 4769 6392 6962 7028 8860 9053 10310 10609 11719 12406 12882 13759 13928C 14766 15326 16129 16304 |
| F1a | 73 249d 263 750 1438 2706 3970 4086 4769 6392 6962 7028 8860 9053 10310 10609 11719 12406 12882 13759 13928C 14766 15326 16129 16172 16304 |
| F1a1'4 | 73 249d 263 750 1438 2706 3970 4086 4769 6392 6962 7028 8860 9053 9548 10310 10609 11719 12406 12882 13759 13928C 14766 15326 16129 16172 16304 |
| F1a1 | 73 249d 263 750 1438 2706 3970 4086 4769 6392 6962 7028 8860 9053 9548 10310 10609 11719 12406 12882 13759 13928C 14766 15326 16129 16162 16172 16304 |
| F1a1a | 73 249d 263 750 1438 2706 3970 4086 4769 6392 6962 7028 8149 8860 9053 9548 10310 10609 11719 12406 12882 13759 13928C 14766 15326 16108 16129 16162 16172 16304 |
| F1a1a1 | 73 249d 263 750 1438 2706 3970 4086 4769 6392 6962 7028 8149 8860 9053 9548 10310 10609 11215 11719 12406 12882 13759 13928C 14766 15326 16108 16129 16162 16172 16304 |
| F1a1b | 64 73 249d 263 750 1438 2706 3970 4086 4769 6392 6962 7028 8860 9053 9548 10310 10609 11719 12406 12882 13759 13928C 14002 14766 15326 16129 16162 16172 16304 |
| F1a1c | 73 249d 263 548 750 1438 2706 3970 4086 4769 6392 6962 7028 8860 9053 9548 10211 10310 10609 11719 12406 12882 13759 13928C 14766 15326 16129 16162 16172 16304 |
| F1a1c1 | 73 195 249d 263 548 750 1438 2706 3666 3970 4086 4769 6392 6962 7028 8860 9053 9548 10211 10283 10310 10609 11719 12406 12882 13759 13928C 14766 15326 16129 16162 16172 16224 16304 |
| F1a1c2 | 73 249d 263 548 750 1438 2706 3970 4086 4769 6392 6962 7028 8860 9053 9548 10211 10310 10609 11719 12406 12882 13759 13928C 14766 15314 15326 15629 16129 16162 16172 16304 |
| F1a1c3 | 73 249d 263 548 750 1438 3970 4086 4769 6098 6392 6962 7028 8860 9053 9548 10211 10310 10609 11719 12406 12882 13759 13928C 14766 15326 16129 16162 16172 16304 |
| F1a1d | 73 249d 263 750 1438 2706 3970 4086 4769 6392 6962 7028 8860 9053 9548 10310 10609 11380 11719 12406 12882 13759 13928C 14766 15326 16129 16162 16172 16304 16399 |

| | |
|---|---|
| F1a1d1 | 73 249d 263 750 1438 2706 3970 4086 4769 6392 6962 7028 8860 9053 9548 10042 10310 10609 11380 11719 12406 12882 13759 13928C 14305 14766 15326 16129 16162 16172 16304 16399 |
| F1a4 | 73 249d 263 750 1438 2706 3970 4086 4769 6392 6962 7028 8860 9053 9548 10310 10609 11719 12406 12882 13759 13928C 14766 15326 15445 16129 16172 16304 |
| F1a4a | 73 152 249d 263 750 1438 2706 3970 4086 4769 5985 6392 6962 7028 8860 9053 9548 10310 10609 11719 12406 12882 13759 13928C 14766 15326 15445 16129 16172 16304 16362 |
| F1a4a1 | 73 152 249d 263 750 1438 2706 3970 4086 4769 5985 6392 6962 7028 8277 8860 9053 9548 10310 10609 11719 12406 12882 13422 13759 13928C 14766 15326 15445 16129 16172 16294 16304 16362 |
| F1a4b | 73 249d 263 466 750 1438 2706 3970 4086 4769 6392 6962 7028 8251 8860 9053 9548 10310 10609 11719 12406 12882 13759 13928C 14766 15326 15445 16129 16172 16304 |
| F1a2 | 73 249d 263 750 1438 2706 3970 4086 4769 6392 6962 7028 8860 9053 10034 10310 10609 11719 12406 12882 13759 13928C 14766 15326 16172 16304 |
| F1a2a | 73 249d 263 750 1438 2706 3970 4086 4769 6392 6962 7028 8860 9053 10034 10310 10609 11719 12406 12882 13759 13928C 14766 15326 16172 16304 16465 |
| F1a3 | 73 249d 263 750 1438 2706 3970 4086 4769 6392 6962 7028 8860 9053 10310 10609 11719 12406 12882 13759 13928C 14766 15326 15565 16129 16172 16304 |
| F1a3+16311 | 73 249d 263 750 1438 2706 3970 4086 4769 6392 6962 7028 8860 9053 10310 10609 11719 12406 12882 13759 13928C 14766 15326 15565 16129 16172 16304 16311 |
| F1a3a | 73 249d 263 750 1438 2706 3970 4086 4769 6392 6962 7028 8860 9053 9554 9944 10310 10609 11719 12406 12882 13748 13759 13928C 14766 15326 15565 16129 16172 16304 16311 |
| F1a3a1 | 73 249d 263 750 1438 2706 3970 4086 4769 6392 6962 7028 8860 9053 9554 9944 10310 10609 11719 12406 12882 13748 13759 13928C 14766 15326 15565 16172 16304 16311 16390 |
| F1a3a1a | 73 249d 263 750 1438 2706 3970 4086 4769 6392 6962 7028 8705 8860 9053 9554 9944 10310 10609 11719 12406 12882 13748 13759 13928C 14766 15326 15565 16172 16284 16304 16311 16390 |
| F1a3a2 | 73 150 249d 263 750 1438 2706 3970 4011 4086 4769 6392 6962 7028 8860 9053 9554 9944 10310 10609 11719 12406 12882 13748 13759 13928C 14766 15326 15565 16129 16172 16189 16304 16311 |
| F1a3a3 | 73 249d 263 750 1438 2706 3970 4086 4769 6392 6962 7028 8860 9053 9554 9944 10310 10609 11719 12406 12882 13748 13759 13928C 14766 15326 15452 15565 16129 16172 16304 16311 |
| F1a3a3a | 73 249d 263 750 1438 2706 3970 4086 4769 6392 6962 7028 8860 9053 9554 9944 10310 10609 11719 12406 12882 13748 13759 13928C 14323 14766 15326 15452 15565 16129 16172 16304 16311 |
| F1a3b | 53 54C 73 249d 263 318 750 1438 2706 3970 4086 4769 6392 6962 7028 8718 8860 9053 10310 10609 11719 12406 12882 13044 13759 13928C 14766 15326 15565 16129 16172 16304 |

| | |
|---|---|
| F1c | 73 249d 263 709 750 1438 2706 3970 4769 6392 6962 7028 8860 9053 10310 10454 10609 11719 12406 12882 13759 13928C 14766 15326 16129 16304 |
| F1c1 | 73 152 249d 263 709 750 1438 2706 3970 4769 6392 6599 6962 7028 8860 9053 10310 10454 10609 11719 12406 12882 13759 13928C 14766 15326 16129 16304 |
| F1c1a | 73 152 249d 263 709 750 1438 2706 3970 4769 6392 6599 6962 7028 8860 9053 10310 10454 10609 11719 12406 12882 13759 13928C 14766 15326 16111 16129 16304 |
| F1c1a1 | 73 152 249d 263 709 750 1438 2706 3970 4769 6392 6599 6962 7028 8860 9053 10310 10454 10609 11719 12406 12882 13759 13928C 14766 15326 16111 16129 16266 16304 |
| F1c1a1a | 73 152 249d 263 709 750 1438 2706 3970 4769 6392 6599 6962 7028 8860 9053 9647 10310 10454 10609 11719 12406 12882 13759 13928C 14766 15326 16111 16129 16266 16304 |
| F1c1a1b | 73 152 249d 263 709 750 1438 2706 3970 4769 6392 6599 6962 7028 8772 8860 9053 10310 10454 10609 11719 12406 12882 13759 13928C 14766 15326 16111 16129 16266 16304 |
| F1c1a2 | 73 152 249d 263 709 750 1438 1927 2706 3970 4769 6392 6599 6962 7028 8860 9053 10310 10454 10609 11719 12406 12882 13759 13928C 14766 15326 16111 16129 16304 |
| F1f | 73 249d 263 750 1438 2706 3970 4715 4769 6392 6515 6962 7028 8860 9053 10310 10609 11719 12406 12771 12882 13759 13928C 14766 15326 16129 16304 |
| F1+16189 | 73 249d 263 750 1438 2706 3970 4769 6392 6962 7028 8860 10310 10609 11719 12406 12882 13928C 14766 15326 16189 16304 |
| F1b | 73 152 249d 263 750 1438 2706 3970 4769 6392 6962 7028 8860 10310 10609 10976 11719 12406 12633 12882 13928C 14476 14766 15326 16189 16232A 16249 16304 16311 |
| F1b1 | 73 152 249d 263 750 1438 2706 3970 4732 4769 5147 6392 6962 7028 8860 10310 10609 10976 11719 12406 12633 12882 13928C 14476 14766 15326 16189 16232A 16249 16304 16311 |
| F1b1a | 73 152 249d 263 750 1438 2706 3970 4732 4769 5147 6392 6962 7028 8860 10310 10609 10976 11719 12406 12633 12882 13928C 14476 14766 15326 16129 16189 16232A 16249 16304 16311 16344 |
| F1b1a1 | 73 152 249d 263 750 1438 2706 3970 4732 4769 5147 6392 6962 7028 8860 10310 10609 10976 11719 12406 12633 12882 13928C 14476 14766 15326 15954 16129 16189 16232A 16249 16304 16311 16344 |
| F1b1a1a | 73 152 249d 263 750 1438 2706 3970 4732 4769 5049 5147 6392 6962 7028 8860 10310 10609 10976 11719 12406 12633 12882 13928C 14476 14766 15326 15954 16129 16189 16232A 16249 16304 16311 16344 |
| F1b1a1a1 | 73 152 249d 263 750 1438 2706 3970 4705 4732 4769 5049 5147 6392 6962 7028 8860 10310 10609 10976 11719 12406 12633 12882 13928C 14476 14766 15326 15954 16129 16189 16232A 16249 16304 16311 16344 |
| F1b1a1a1a | 73 152 249d 263 750 1438 2706 3970 4705 4732 4769 5049 5147 6392 6962 7028 8860 10310 10609 10976 11719 12406 12633 12882 13928C 14103A 14476 14766 15326 15954 16129 16189 16232A 16249 16304 |

| | |
|---|---|
| F1b1a1a2 | 73 152 249d 263 750 1438 2706 3970 4732 4769 5049 5147 6392 6962 7028 8860 9389 10310 10609 10976 11719 12406 12633 12882 13928C 14476 14766 15326 15954 16129 16189 16232A 16249 16304 16311 16344 |
| F1b1a1a3 | 73 152 249d 263 750 1438 2706 3970 4732 4769 5049 5147 6392 6962 7028 8860 10310 10609 10976 11719 12406 12633 12882 13928C 14476 14766 15193 15326 15954 16129 16189 16232A 16249 16304 16311 16344 |
| F1b1a2 | 73 152 249d 263 750 1438 2706 3970 4732 4769 5147 6392 6962 7028 8860 10310 10609 10976 11719 12406 12633 12882 13928C 14476 14629 14766 15326 16129 16189 16232A 16249 16304 16311 16344 |
| F1b1b | 73 152 204 249d 263 750 1438 2706 3970 4732 4769 5147 6392 6962 7028 8772 8860 10310 10609 10976 11719 12406 12633 12882 13928C 14476 14766 15326 16172 16189 16232A 16249 16304 16311 |
| F1b1c | 73 152 249d 263 750 1438 2706 3970 4732 4769 5147 5508 6392 6962 7028 8860 10235 10310 10609 10976 11719 12406 12882 13928C 14476 14766 15326 16189 16232A 16249 16304 |
| F1b1+@152 | 73 249d 263 750 1438 2706 3970 4732 4769 5147 6392 6962 7028 8860 10310 10609 10976 11719 12406 12633 12882 13928C 14476 14766 15326 16189 16232A 16249 16304 16311 |
| F1b1d | 73 153 249d 263 750 1438 2706 3970 4732 4769 5147 6392 6962 7028 8860 10310 10609 10976 11719 12406 12633 12882 13928C 14476 14766 15326 15459 16189 16232A 16249 16304 16311 |
| F1b1e | 73 249d 263 750 1438 1709T 2706 3970 4732 4769 5147 6392 6962 7028 8104 8860 10310 10609 10976 11719 12406 12633 12882 13928C 14476 14766 15326 16189 16232A 16249 16304 16311 |
| F1b1e1 | 73 249d 263 750 1438 1709T 2706 3202 3970 4732 4769 5147 6392 6962 7028 8104 8860 9007 10310 10609 10976 11719 12406 12633 12882 13928C 14476 14766 15326 16189 16232A 16249 16304 16311 |
| F1b1f | 73 249d 263 750 1438 2706 3970 4732 4769 5147 6392 6962 7028 8860 10045 10310 10609 10976 11719 12406 12633 12882 13928C 14476 14766 15326 16189 16232A 16249 16304 16311 |
| F1d | 73 146 249d 263 750 1438 1734 2706 3970 4769 5628 6392 6962 7028 7738 8860 10310 10609 11719 12406 12882 13928C 14766 15326 15402 16189 16304 |
| F1d1 | 73 146 249d 263 750 1438 1734 2706 3970 4769 5628 6392 6962 7028 7738 8860 10310 10609 11719 12406 12882 13135 13928C 14766 15326 15402 16189 16304 |
| F1e | 73 249d 263 750 1438 2706 3970 4769 5587 6392 6962 7028 8860 10310 10609 11719 12406 12882 13928C 14766 15024 15326 16189 16304 |
| F1e1 | 73 249d 263 750 1438 2706 3970 4769 5587 6392 6962 7028 8860 10310 10609 11719 12406 12618 12882 13928C 14766 15024 15326 16189 16304 |
| F1e1a | 73 249d 263 750 1438 2706 3027 3970 4769 5587 6392 6962 7028 8860 10007 10310 10609 11719 12406 12618 12882 13260 13749 13928C 14766 15022 15024 15326 15496 16189 16304 16355 |
| F1e2 | 73 189 195 249d 263 750 1438 2706 3970 4769 5587 6392 6962 7028 8860 9078 10310 10609 11326 11719 12406 12882 13928C 14218 14766 15024 15326 16189 16234 16242 16304 |

| | |
|---|---|
| F1e3 | 73 150 249d 263 750 1438 2706 3970 4769 5587 6392 6962 7028 8860 10310 10609 11719 12406 12882 13928C 14766 15024 15326 16189 16300 16304 |
| F1g | 73 249d 263 750 1438 2389 2706 3398 3970 4769 6392 6962 7028 8860 10310 10609 11719 12406 12882 13928C 14766 15326 16189 16304 |
| F1g1 | 73 249d 263 750 1438 2389 2706 3398 3621 3970 4769 6392 6962 7028 8860 10310 10609 11719 12406 12882 13928C 14766 15326 16189 16304 |
| F2 | 73 249d 263 750 1005 1438 1824 2706 3970 4769 6392 7028 7828 8860 10310 10535 10586 11719 12338 13708 13928C 14766 15326 16304 |
| F2+16291 | 73 249d 263 750 1005 1438 1824 2706 3970 4769 6392 7028 7828 8860 10310 10535 10586 11719 12338 13708 13928C 14766 15326 16291 16304 |
| F2a | 73 249d 263 750 1005 1438 1824 2706 3970 4769 6392 7028 7828 8860 10310 10535 10586 11719 12338 13708 13928C 14766 15326 16203 16291 16304 |
| F2a+@16291 | 73 249d 263 750 1005 1438 1824 2706 3970 4769 6392 7028 7828 8860 10310 10535 10586 11719 12338 13708 13928C 14766 15326 16203 16304 |
| F2a1 | 73 249d 263 750 1005 1438 1824 2706 3970 4769 6392 7028 7828 8860 10310 10535 10586 10810 11719 12338 13708 13928C 14766 15326 16203 16304 |
| F2b | 73 249d 263 750 1005 1438 1824 2706 3970 4769 4811 6392 7028 7828 8860 10310 10535 10586 11719 12338 13708 13928C 14766 15326 16291 16304 |
| F2b1 | 73 249d 263 750 1005 1438 1824 2706 3970 4769 4811 6392 7028 7828 8860 10310 10535 10586 11719 12338 13708 13928C 14766 15326 16092A 16291 16304 |
| F2g | 73 249d 263 747 750 1005 1438 1824 2706 3970 4769 6392 7028 7828 8860 10310 10535 10586 11719 12338 13708 13928C 14766 15326 16291 |
| F2c | 73 249d 263 750 1005 1438 1824 2706 3970 4769 6392 7028 7828 8860 10310 10535 10586 11719 12338 13708 13722 13928C 14766 15326 16304 |
| F2c1 | 73 249d 263 750 1005 1438 1824 2706 3970 4769 6392 7028 7828 8860 10310 10535 10586 11150 11719 12338 13708 13722 13928C 14766 15326 16189 16304 |
| F2c2 | 73 249d 263 750 761 1005 1438 1824 2706 3970 4769 5978 6156 6392 6570T 7028 7828 8152 8860 10310 10535 10586 11719 12338 13708 13722 13928C 14133 14766 15326 15418 16067 16271 16304 |
| F2d | 73 235 249d 263 750 1005 1009 1438 1824 2706 3970 4769 6392 7028 7828 8860 10310 10535 10586 11719 12338 13708 13928C 14766 15326 |
| F2e | 73 249d 263 750 1005 1438 1824 2281 2706 3010 3970 4769 6392 7028 7828 8860 10310 10535 10586 11719 12338 13708 13928C 14766 14769 15326 16304 |
| F2e1 | 64 73 153 249d 263 750 1005 1438 1824 2281 2706 3010 3970 4769 4820 6392 7028 7828 8020 8473 8860 10310 10535 10586 11719 12338 13708 13782A 13928C 14766 14769 15326 16260 16304 |
| F2f | 73 249d 263 750 1005 1438 1824 2706 3970 4769 6392 7028 7828 8860 10310 10535 10586 11339 11719 12338 13708 13928C 14766 15326 16304 |
| F2+195 | 73 195 249d 263 750 1005 1438 1824 2706 3970 4769 6392 7028 7828 8860 10310 10535 10586 11719 12338 13708 13928C 14766 15326 16304 |

| | |
|---|---|
| F2h | 73 195 249d 263 750 1005 1438 1824 2706 3970 4769 6392 7028 7828 8860 9612C 10005 10310 10535 10586 11696 11719 12338 13708 13928C 14002 14560 14766 15326 15862 16304 |
| F2i | 73 152 195 249d 263 275 750 1005 1438 1824 2706 3753 3970 4769 6392 7028 7828 8860 10084 10310 10535 10586 11323 11410 11719 12338 13708 13928C 14766 15326 16221 16304 |
| F3 | 73 249d 263 750 1438 2706 3434 3970 4769 5585 5913 5978 6392 7028 8860 10310 10320 11065 11719 13928C 14766 15326 16298 16362 |
| F3a | 73 249d 263 750 1438 2706 3434 3970 4769 5585 5913 5978 6392 7028 8860 10310 10320 11065 11719 12621 13928C 14766 15326 16298 16355 16362 |
| F3a+207 | 73 207 249d 263 750 1438 2706 3434 3970 4769 5585 5913 5978 6392 7028 8860 10310 10320 11065 11719 12621 13928C 14766 15326 16298 16355 16362 |
| F3a1 | 73 207 249d 263 709 750 1438 2706 3434 3970 4769 5585 5894 5913 5978 6392 7028 8860 10310 10320 11065 11719 12621 13928C 14766 14971 15326 16260 16298 16355 16362 |
| F3b | 73 249d 263 750 1438 2706 3434 3970 4769 5585 5913 5978 6392 7028 8860 9947 10310 10320 11065 11719 13928C 14766 15326 16220C 16298 16362 |
| F3b+152 | 73 152 249d 263 750 1438 2706 3434 3970 4769 5585 5913 5978 6392 7028 8860 9947 10310 10320 11065 11719 13928C 14766 15326 16220C 16298 16362 |
| F3b1 | 73 150 152 249d 263 750 1438 2706 3434 3970 4769 5076 5585 5913 5978 6392 7028 8838 8860 9947 10310 10320 11719 13928C 14766 15326 15784 16220C 16265 16298 16362 |
| F3b1a | 73 150 152 249d 263 750 1438 2706 3434 3970 4769 5076 5585 5913 5978 6392 6791 7028 8838 8860 9947 10310 10320 11719 13928C 14766 15326 15784 16220C 16265 16298 16311 16362 |
| F3b1a+16093 | 73 150 152 249d 263 750 1438 2706 3434 3970 4769 5076 5585 5913 5978 6392 6791 7028 8838 8860 9947 10310 10320 11719 13928C 14766 15326 15784 16093 16220C 16265 16298 16311 16362 |
| F3b1a1 | 73 150 152 249d 263 750 1438 2706 3434 3970 4769 5076 5585 5913 5978 6392 6791 7028 8838 8860 9947 10310 10320 11719 13928C 14766 15326 15784 16093 16220C 16265 16274 16298 16311 16362 |
| F3b1a2 | 73 150 249d 263 750 1438 2706 3434 3970 4769 5076 5585 5913 5978 6392 6791 7028 8838 8860 9947 10310 10320 11719 13928C 14766 15326 15784 16220C 16298 16311 16362 |
| F3b1b | 73 150 152 249d 263 750 1438 2706 3434 3970 4769 5076 5585 5899.XC 5913 5978 6392 7028 8337 8838 8860 9947 10310 10320 11719 13928C 14133 14766 15326 15784 16220C 16265 16298 16362 |
| F3b1b1 | 73 150 152 249d 263 750 1438 2706 3434 3970 4769 5076 5585 5899.XC 5913 5978 6392 7028 8337 8838 8860 9947 10310 10320 11719 13928C 14013 14133 14766 15326 15784 16220C 16265 16298 16362 |
| F4 | 73 249d 263 750 1438 2706 3970 4769 5263 6392 7028 8860 10310 11719 12630 13928C 14766 15326 15670 16304 |
| F4a | 73 146 152 249d 263 750 1438 2706 3290 3970 4769 5263 6392 7028 8860 10310 10915 11719 12630 13928C 14766 15326 15670 16207 16304 16399 |

| | |
|---|---|
| F4a1 | 73 146 152 249d 263 750 1438 2706 3290 3970 4769 5263 6392 7028 8860 10310 10915 11719 12153 12630 13602 13928C 14766 15326 15670 16207 16304 16362 16399 |
| F4a1a | 73 146 152 207 249d 263 750 1438 2706 3290 3970 4769 5263 6392 7028 7861 8860 10310 10915 11038 11719 12153 12396 12408 12630 13602 13928C 14766 15326 15670 16207 16304 16362 16399 16497 |
| F4a1b | 73 146 249d 263 317A 750 1438 2706 3290 3316 3970 4769 5263 6392 7028 7561 8860 10310 10915 11719 12153 12630 13602 13928C 14766 15326 15670 16126 16207 16304 16362 16399 |
| F4a2 | 73 146 152 249d 263 281 750 1438 2706 3290 3970 4769 5263 6392 7028 8860 10310 10915 11719 11776 12612 12630 13928C 14016 14766 15326 15670 15908 16207 16304 16399 |
| F4b | 73 249d 263 573.XC 750 1438 2706 3970 4769 5263 6392 6653 7028 8020 8575 8603 8860 10310 11719 12630 13928C 14766 15326 15670 16218 16304 16311 |
| F4b1 | 73 249d 263 573.XC 750 1438 2706 3970 4769 5263 6392 6653 7028 8020 8575 8603 8860 10097C 10310 11719 12630 13928C 14766 15326 15670 16218 16304 16311 |
| R+16189 | 73 263 750 1438 2706 4769 7028 8860 11719 14766 15326 16189 |
| R11'B6 | 73 263 750 1438 2706 4769 7028 8860 11719 12950 14766 15326 16189 |
| R11 | 73 185 189 263 709 750 1438 2706 4769 7028 8277 8278.XC 8860 10031 10398 11061 11719 12950 13681 14766 15326 16189 16311 |
| R11a | 73 185 189 263 709 750 1438 2706 4769 7028 8277 8278.XC 8860 10031 10398 10978 11061 11719 12950 13681 14766 15326 16189 16311 (16365) |
| R11b | 73 185 189 263 709 750 1438 2706 4769 7028 8277 8278.XC 8860 10031 10398 11061 11719 12950 13269 13681 14766 15326 16189 16311 |
| R11b1 | 73 185 189 263 709 750 1438 2706 4769 7028 8277 8278.XC 8860 10031 10398 11061 11719 12950 13269 13681 14766 15326 16189 16311 16390 |
| R11b1a | 73 185 189 263 709 750 1438 2706 4769 7028 8277 8278.XC 8860 10031 10398 11061 11719 12950 13215 13269 13681 14766 15326 16092 16189 16311 16390 |
| R11b1b | 73 185 189 234 263 709 750 1438 2706 4769 7028 8277 8278.XC 8860 10031 10398 11061 11719 12950 13269 13681 14766 15326 16189 16311 16390 |
| B6 | 73 150 263 750 1438 2706 4769 7028 8281-8289d 8860 9452 11719 12950 13928C 14766 15326 16189 |
| B6a | 73 150 263 750 1438 2706 4769 5894C 7028 8281-8289d 8860 9452 11719 11914 12950 13928C 14305 14766 15326 16093 16189 |
| B6a1 | 73 263 356.1C 750 1438 2706 4093 4769 5894C 7028 8281-8289d 8860 9452 11719 11914 12950 13928C 14305 14766 15326 16051 16093 16189 |
| B6a1a | 73 263 356.1C 750 1438 1719 2706 4093 4769 5894C 7028 8281-8289d 8860 9452 11719 11914 12950 13928C 14305 14766 15326 16051 16189 16527 |
| B4'5 | 73 263 750 1438 2706 4769 7028 8281-8289d 8860 11719 14766 15326 16189 |
| B4 | 73 263 750 1438 2706 4769 7028 8281-8289d 8860 11719 14766 15326 16189 16217 |

| | |
|---|---|
| B4+16261 | 73 263 750 1438 2706 4769 7028 8281-8289d 8860 11719 14766 15326 16189 16217 16261 |
| B4a | 73 263 750 1438 2706 4769 5465 7028 8281-8289d 8860 9123 11719 14766 15326 16189 16217 16261 |
| B4a1 | 73 263 750 1438 2706 4769 5465 7028 8281-8289d 8860 9123 10238 11719 14766 15326 16189 16217 16261 |
| B4a1a | 73 146 263 750 1438 2706 4769 5465 6719 7028 8281-8289d 8860 9123 10238 11719 12239 14766 15326 15746 16189 16217 16261 |
| B4a1a1 | 73 146 263 750 1438 2706 4769 5465 6719 7028 8281-8289d 8860 9123 10238 11719 12239 14022 14766 15326 15746 16189 16217 (16247) 16261 |
| B4a1a1a | 73 146 263 750 1438 2706 4769 5465 6719 6905 7028 8281-8289d 8860 9123 10238 11719 12239 14022 14766 15326 15746 16189 16217 (16247) 16261 |
| B4a1a1a1 | 73 146 263 750 1438 2706 4769 5465 6719 6905 7028 8281-8289d 8860 9123 10238 11719 12239 13479 14022 14766 15326 15746 16189 16217 (16247) 16261 |
| B4a1a1a1a | 73 146 263 373 750 1438 2706 4769 5465 6719 6905 7028 8281-8289d 8860 9123 10238 11719 12239 13479 14022 14766 15326 15746 16189 16217 (16247) 16261 |
| B4a1a1a1a1 | 73 146 263 373 750 1438 2706 4769 5465 6719 6905 7028 8281-8289d 8842 8860 9123 10238 11719 12239 13479 14022 14766 15326 15746 16189 16217 (16247) 16261 |
| B4a1a1a1b | 73 146 263 750 1438 2706 4769 5465 6719 6905 7028 7598 8281-8289d 8860 9123 10238 11719 12239 13479 14022 14766 15326 15746 16189 16217 (16247) 16261 |
| B4a1a1a1c | 73 94 146 263 750 1438 2706 4769 5465 6719 6905 7028 8281-8289d 8860 9123 10084 10238 11719 12239 13479 14022 14766 15326 15746 16189 16217 (16247) 16261 |
| B4a1a1a1d | 73 146 152 263 750 1438 2706 4769 5465 6719 6905 7028 8281-8289d 8860 9123 10238 11719 12239 12940 13479 14022 14766 15326 15746 16189 16217 (16247) 16261 |
| B4a1a1a2 | 73 146 263 750 1438 2706 4769 5465 6719 6905 7028 8281-8289d 8860 9123 10238 11719 12239 14022 14766 15326 15746 16189 16217 (16247) 16261 16292 |
| B4a1a1a2a | 73 146 263 750 1438 2706 4769 5465 6719 6905 7028 8281-8289d 8860 9123 10238 11719 12239 14022 14766 15326 15746 15905 16189 16217 (16247) 16261 16292 |
| B4a1a1a2b | 73 146 263 750 1438 2706 4769 5465 6719 6905 7028 8281-8289d 8860 9123 10238 11719 12239 14022 14766 15326 15746 16136 16189 16217 (16247) 16261 16292 |
| B4a1a1a3 | 73 146 263 750 1438 2706 4769 5465 6719 6905 7028 8281-8289d 8860 9123 10238 11719 12239 14022 14470 14766 15326 15746 16189 16217 (16247) 16261 |
| B4a1a1a4 | 73 146 263 750 1438 2706 4769 5465 6719 6905 7028 8281-8289d 8860 9123 10238 11719 12239 14022 14766 15326 15746 16189 16217 (16247) 16249 16261 |

| | |
|---|---|
| B4a1a1a5 | 73 146 263 750 1438 2706 4769 5465 6719 6905 7028 8281-8289d 8860 9123 10238 11719 12239 14022 14560 14766 15326 15355 15746 16189 16217 (16247) 16261 |
| B4a1a1a6 | 73 146 263 750 1438 2706 4769 5465 6719 6905 7028 8281-8289d 8860 9123 10238 10931 11719 12239 14022 14766 15326 15746 16189 16217 (16247) 16261 |
| B4a1a1a7 | 73 146 263 750 1438 2706 4769 5465 6719 6905 7028 8281-8289d 8860 9123 10238 11719 12239 14022 14540 14766 15326 15746 (16093) 16189 16217 (16247) 16261 |
| B4a1a1a8 | 73 146 235 263 750 1438 2706 4769 5465 6261 6719 6905 7028 8281-8289d 8860 9123 10238 11719 12239 12468 14022 14766 15326 15746 16189 16217 (16247) 16261 |
| B4a1a1a9 | 73 146 263 750 1438 4769 5465 6719 6905 7028 8281-8289d 8860 9123 10238 11719 12239 14022 14766 15326 15746 16163 16189 16217 (16247) 16261 |
| B4a1a1a10 | 73 146 263 750 1438 2706 4769 5465 6719 6905 7028 8281-8289d 8860 9123 10238 10364 11719 12239 14022 14766 15326 15746 16189 16217 (16247) 16261 |
| B4a1a1a11 | 73 146 263 750 1438 2706 4769 5465 6719 6905 7028 8281-8289d 8860 9123 10238 11719 12239 13943 14022 14766 15326 15746 16189 16217 (16247) 16261 |
| B4a1a1a11a | 73 146 263 750 1438 2706 4769 5465 6719 6905 7028 8281-8289d 8860 9123 10238 10966 11719 12239 13943 14022 14766 15326 15746 16189 16217 (16247) 16261 |
| B4a1a1a11b | 73 146 263 750 1438 2706 4769 5465 6719 6905 7028 8281-8289d 8860 9123 10238 11719 12239 13943 14022 14766 15326 15746 16189 16217 (16247) |
| B4a1a1a12 | 73 146 263 750 1438 2706 4769 5465 6719 6905 7028 8281-8289d 8860 9123 10238 11719 12239 14022 14766 15326 15746 16189 16217 (16247) 16261 16305 |
| B4a1a1a13 | 73 146 263 750 1438 2706 4093 4769 5465 6719 6905 7028 8281-8289d 8860 9123 10238 11719 12239 14022 14766 15326 15746 16189 16217 (16247) 16261 |
| B4a1a1a14 | 73 146 263 750 1438 2706 4769 5465 6719 6905 7028 8281-8289d 8860 9123 10238 11719 12239 14022 14766 15326 15746 16189 16217 (16247) 16261 16390 |
| B4a1a1a15 | 73 146 263 750 1438 2706 4769 5465 6719 6905 7028 8281-8289d 8860 9123 10238 11719 12239 13759 14022 14766 15326 15746 16189 16217 (16247) 16261 |
| B4a1a1a16 | 73 146 263 750 1438 2706 4769 5465 6719 6905 7028 8281-8289d 8860 9123 10238 11719 12239 14022 14766 15326 15746 16182T 16189 16217 (16247) 16261 |
| B4a1a1a17 | 73 146 263 750 1438 2706 4769 5465 6719 6905 7028 7534 8281-8289d 8860 9123 10238 11719 12239 14022 14766 15326 15746 16189 16217 (16247) 16261 |
| B4a1a1a18 | 73 146 263 750 1438 2706 4769 5465 6719 6905 7028 8281-8289d 8860 9123 9145 10238 11719 12239 14022 14766 15326 15746 16051 16189 16217 (16247) 16261 |

| | |
|---|---|
| B4a1a1a19 | 73 146 263 750 1438 2706 4769 5465 6719 6905 7028 8281-8289d 8860 9123 10238 11719 12239 14022 14766 15326 15746 16189 16217 (16247) 16261 16302 |
| B4a1a1a20 | 73 146 263 750 1438 2706 4769 5465 6719 6905 7028 8281-8289d 8860 9123 10238 11016 11719 12239 14022 14766 15326 15746 16189 16217 (16247) 16261 16274 |
| B4a1a1a21 | 73 146 263 750 1438 2706 4216 4769 5465 6719 6905 7028 8281-8289d 8860 9123 10238 11719 12239 14022 14766 15326 15746 16189 16217 (16247) 16261 |
| B4a1a1a22 | 73 146 263 750 1438 2706 3398 4769 5465 6719 6905 7028 8281-8289d 8860 9123 10238 11719 12239 14022 14766 15326 15746 16189 16217 (16247) 16261 |
| B4a1a1a+195 | 73 146 195 263 750 1438 2706 4769 5465 6719 6905 7028 8281-8289d 8860 9123 10238 11719 12239 14022 14766 15326 15746 16189 16217 (16247) 16261 |
| B4a1a1a23 | 73 146 195 263 750 1438 2706 4769 5465 6719 6905 7028 8281-8289d 8860 9123 9734 10238 11719 12239 14022 14766 15326 15746 16189 16217 (16247) 16261 |
| B4a1a1b | 73 146 263 750 1438 1473 2706 3423A 4769 5465 6719 7028 8281-8289d 8860 9123 10238 11719 12239 14022 14766 15326 15746 16189 16217 (16247) 16261 |
| B4a1a1c | 73 146 263 750 1185 1438 2706 5465 6719 7028 8281-8289d 8860 9123 10238 11719 12239 14022 14766 15326 15746 16189 16217 (16247) 16261 |
| B4a1a1d | 73 146 263 750 1438 2706 4769 5465 6581 6719 7028 8281-8289d 8860 9123 10238 11719 12239 14022 14766 15326 15746 16189 16217 (16247) 16261 |
| B4a1a1e | 73 146 263 750 1438 2706 3391 4769 5465 6719 7028 8281-8289d 8860 9123 10238 11719 12239 14022 14766 15326 15746 16189 16217 (16247) 16261 |
| B4a1a1f | 73 146 263 750 1438 2706 4769 5465 6719 7028 7740 8281-8289d 8860 9123 10238 11719 12239 14022 14766 15326 15746 16189 16217 (16247) 16261 |
| B4a1a1g | 73 146 263 750 1438 2706 4769 5465 6719 7028 8281-8289d 8338 8860 9123 10238 11719 12239 14022 14766 15326 15746 16189 16217 (16247) 16261 |
| B4a1a1h | 73 146 263 318 750 1438 2706 4769 5465 6719 7028 8281-8289d 8860 9123 10031 10238 11719 12239 14022 14766 15326 15746 16189 16217 (16247) 16261 |
| B4a1a1i | 73 146 263 750 1438 2706 4769 5465 6719 7028 8281-8289d 8860 9123 10238 11399 11719 11887 12239 14022 14766 15326 15746 16189 16217 (16247) 16261 |
| B4a1a1j | 73 146 263 750 1438 2706 4769 5465 6719 7028 8281-8289d 8860 9123 10238 11719 12239 14022 14766 15326 15746 16189 16217 (16247) 16261 16289T |
| B4a1a1k | 73 146 263 750 1438 2706 4769 5465 6719 7028 8281-8289d 8860 9123 10238 11719 12239 14022 14766 15326 15746 16189 16217 (16247) 16261 16399 |

| | |
|---|---|
| B4a1a1k1 | 70 73 146 263 750 1438 2706 4769 5465 6719 7028 8281-8289d 8860 9123 10238 11719 12239 14022 14766 15326 15746 16189 16217 (16247) 16261 16352 16399 |
| B4a1a1+151 | 73 146 151 263 750 1438 2706 4769 5465 6719 7028 8281-8289d 8860 9123 10238 11719 12239 14022 14766 15326 15746 16189 16217 (16247) |
| B4a1a1m | 73 146 151 263 750 1438 2416 2706 4769 5465 6719 7028 8281-8289d 8860 9123 10238 11719 12239 14022 14766 15326 15746 16189 16217 (16247) 16261 |
| B4a1a1m1 | 73 146 151 263 750 1438 1692 2416 2706 4769 5465 6719 7028 8281-8289d 8860 9123 10238 11719 12239 14022 14766 15326 15746 16189 16217 (16247) 16261 |
| B4a1a1n | 73 146 263 750 1438 2706 4769 5465 6719 7028 8281-8289d 8572 8860 9123 10238 11719 12239 14022 14766 15326 15746 16189 16217 (16247) 16261 |
| B4a1a1+152 | 73 146 152 263 750 1438 2706 4769 5465 6719 7028 8281-8289d 8860 9123 10238 11719 12239 14022 14766 15326 15746 16189 16217 (16247) |
| B4a1a1o | 73 146 152 263 750 1438 2706 4769 5465 6719 7028 8281-8289d 8860 9123 10238 11203 11719 12239 14022 14766 15326 15746 16189 16217 (16247) 16261 |
| B4a1a1p | 73 146 263 750 1438 2706 4769 5465 6719 7028 8281-8289d 8860 9123 10238 11719 12239 13416 14022 14766 15326 15746 16189 16217 (16247) 16261 |
| B4a1a1q | 73 146 263 750 1438 2706 4769 5465 6719 7028 8281-8289d 8860 9123 10238 11719 12239 14022 14766 15326 15746 16189 16217 (16247) 16255 16261 |
| B4a1a1+16126 | 73 146 263 750 1438 2706 4769 5465 6719 7028 8281-8289d 8860 9123 10238 11719 12239 14022 14766 15326 15746 16126 16189 16217 (16247) 16261 |
| B4a1a1r | 73 146 263 750 1438 2706 3909 4769 5465 6719 7028 8281-8289d 8860 9123 10238 11719 12239 14022 14766 15326 15746 16126 16189 16217 (16247) 16261 |
| B4a1a1s | 73 146 263 750 1438 2706 4769 5465 6719 7028 8281-8289d 8860 9123 10238 11407 11719 12239 14022 14766 15326 15746 16189 16217 (16247) 16261 |
| B4a1a1t | 73 146 263 750 1438 2706 4769 5465 6719 7028 8281-8289d 8860 9123 10238 11719 12239 14022 14766 15221 15326 15746 16189 16217 (16247) 16261 |
| B4a1a1u | 73 146 263 750 1438 2706 4769 5465 6719 7028 8281-8289d 8860 9123 10238 10325 11719 12239 14022 14766 15326 15746 16189 16217 (16247) 16261 |
| B4a1a1v | 73 146 263 750 1438 2706 4769 5465 6719 7028 7346 8281-8289d 8860 9123 10238 11719 12239 13908 14022 14766 15326 15746 16189 16217 (16247) 16261 |
| B4a1a1w | 73 146 263 750 1438 2706 4769 5465 6719 7028 7389 8281-8289d 8860 9123 10238 11719 12239 14022 14766 15326 15746 16189 16217 (16247) 16261 |

| | |
|---|---|
| B4a1a1x | 73 146 263 750 1438 2706 4769 5465 6719 7028 8281-8289d 8860 9123 10238 11719 12239 14022 14766 15326 15746 15924 16189 16217 (16247) 16261 |
| B4a1a1y | 73 146 263 750 1438 2706 4769 5465 6719 7028 8281-8289d 8860 9123 10238 11719 12239 14022 14766 15326 15601 15746 16189 16217 (16247) 16261 |
| B4a1a1z | 73 146 263 750 1438 2706 4769 5465 6719 7028 8281-8289d 8860 9123 10238 11719 12239 14022 14683 14766 15326 15746 16189 16217 (16247) 16261 |
| B4a1a1aa | 73 146 263 750 1438 2706 4742 4769 5465 6719 7028 8281-8289d 8860 9123 10238 11719 12239 14022 14766 15326 15746 16189 16217 (16247) 16261 16311 |
| B4a1a1ab | 73 146 263 750 1438 2706 4769 5465 6719 7028 8281-8289d 8860 9123 9809 10238 11719 12239 14022 14766 15326 15746 16189 16217 (16247) 16261 |
| B4a1a1ac | 73 146 263 750 1438 2706 4769 5465 6719 7028 8281-8289d 8790 8860 9123 10238 11719 12239 14022 14766 15326 15746 16189 16217 (16247) 16261 |
| B4a1a1ad | 73 146 263 750 1438 2706 4769 5460 5465 6719 7028 8281-8289d 8860 9123 10238 11719 12239 14022 14766 15061 15326 15746 16189 16217 (16247) 16261 |
| B4a1a1ae | 73 146 263 750 1438 2706 4769 5465 6719 7028 7058 8281-8289d 8860 9123 10238 11719 12239 14022 14766 15326 15746 16189 16217 (16247) 16261 |
| B4a1a1af | 73 146 263 750 1438 2706 4769 5465 6719 7028 8281-8289d 8860 9123 10238 11719 12239 14022 14766 15326 15746 16189 16212 16217 (16247) 16261 |
| B4a1a2 | 73 146 263 750 1438 2706 4733 4769 5465 6366 6719 7028 8281-8289d 8860 9123 10238 11719 12239 12519 14766 15326 15746 16189 16217 16261 |
| B4a1a3 | 73 146 263 750 1438 2706 4769 5465 6719 7028 8281-8289d 8860 9123 10238 11719 12239 14449 14766 15326 15746 16189 16217 16261 |
| B4a1a3a | 73 146 263 750 1438 2706 4769 5465 6719 7028 8281-8289d 8860 9123 10238 11719 12239 14449 14766 15326 15746 16189 16217 16223 16261 |
| B4a1a3a1 | 73 146 263 750 1438 2706 4769 5465 6719 7028 8281-8289d 8525 8860 9123 10238 11719 12239 14449 14766 15326 15746 16189 16217 16223 16261 |
| B4a1a3a1a | 73 146 195 263 750 1438 2706 4769 5465 6719 7028 8281-8289d 8525 8860 9123 10238 11719 12239 14290 14449 14766 15326 15746 16189 16217 16223 16261 |
| B4a1a4 | 73 146 263 750 1438 2706 4025 5465 6719 7028 8281-8289d 8860 9123 10238 11719 12239 14766 15326 15746 16189 16217 16261 |
| B4a1a5 | 73 146 263 750 1438 2706 4048 4769 5465 6719 7028 8281-8289d 8860 9123 10238 11719 12239 14766 15326 15746 16189 16217 16261 |
| B4a1a5a | 73 146 263 750 1438 2706 4048 4769 5465 5539 6719 7028 8281-8289d 8860 9123 10238 11719 12239 14766 15326 15746 16189 16217 16261 |
| B4a1a6 | 73 146 263 750 1438 2706 4769 5465 6719 7028 8281-8289d 8860 9123 10238 11719 11928 12239 14766 15326 15746 16189 16217 16261 |

| | |
|---|---|
| B4a1a6a | 73 146 263 750 1438 2706 3483 4769 5465 6719 7028 8281-8289d 8860 9123 10238 11719 11928 12239 14766 15326 15746 16189 16217 16261 |
| B4a1a7 | 73 146 263 750 1438 2706 4769 5465 6492 6719 7028 8281-8289d 8860 9123 10238 11719 12239 13350 14766 15326 15746 16189 16217 16261 |
| B4a1+16311 | 73 263 750 1438 2706 4769 5465 7028 8281-8289d 8860 9123 10238 11719 14766 15326 16189 16217 16261 16311 |
| B4a1b | 73 263 750 1438 2706 4769 5465 7028 8281-8289d 8860 9123 9932 10238 10915 11719 13143 14766 15326 16189 16217 16261 16288 16311 |
| B4a1b1 | 73 263 750 1438 2706 4769 5465 7028 8281-8289d 8860 9123 9932 10238 10915 11719 13143 14766 15326 16189 16217 16261 16268 16288 16311 |
| B4a1b1a | 73 263 750 1438 2706 4769 5465 7028 8281-8289d 8860 9123 9932 9977 10238 10915 11719 13143 14766 15326 16189 16217 16261 16268 16288 16311 |
| B4a1e | 73 263 750 1438 2706 3548 4769 5465 7028 8281-8289d 8860 9123 10238 11719 14766 15326 16168 16189 16217 16261 16311 |
| B4a1c | 73 263 709 750 1438 2706 4769 5465 7028 8281-8289d 8860 9123 10238 11719 14766 15326 16189 16217 16261 |
| B4a1c1 | 73 263 709 750 1438 2706 4769 5465 7028 8281-8289d 8860 9123 9254 10238 11719 14766 15326 16189 16217 16261 |
| B4a1c1a | 73 263 709 750 1438 2706 4769 5465 7028 8281-8289d 8860 9123 9254 10238 11719 14766 15292 15326 16189 16217 16261 |
| B4a1c1a1 | 73C 89 263 709 750 1438 2706 4769 5465 7028 8281-8289d 8860 9123 9254 10238 11719 14766 15292 15326 16189 16217 16261 |
| B4a1c+146 | 73 146 263 709 750 1438 2706 4769 5465 7028 8281-8289d 8860 9123 10238 11719 14766 15326 16189 16217 16261 |
| B4a1c2 | 73 146 263 709 750 1438 2706 4769 5465 7028 7052 7271 8281-8289d 8860 9123 9822A 10238 11719 14766 15326 15661 16167 16189 16217 16261 16317 |
| B4a1c4 | 73 146 263 709 750 1438 2706 4769 5465 7028 8281-8289d 8860 9123 10238 11719 12904 14766 15326 16189 16217 16261 |
| B4a1c5 | 73 146 263 709 750 1438 2706 4769 5465 7028 8281-8289d 8860 9123 10238 11632 11719 12237 14766 15038 15326 16189 16217 16261 |
| B4a1c3 | 73 263 709 750 1438 2706 4769 5465 7028 8281-8289d 8860 9123 10238 11719 14766 15326 16189 16194C 16195 16217 16261 |
| B4a1c3a | 73 263 310 709 750 1438 2706 3960 4203 4769 5263 5465 7028 7927 8281-8289d 8860 9123 10238 11719 14766 15326 15479 16000 16189 16194C 16195 16217 16261 16266G |
| B4a1c3b | 73 263 709 750 1438 2706 4218 4769 4907 5465 7028 8281-8289d 8860 9123 10238 11719 14766 15326 16189 16194C 16195 16217 16261 |
| B4a1d | 73 263 750 1438 2706 4769 5465 5894 7028 8281-8289d 8860 9123 10238 11719 14766 15326 16189 16217 16261 |
| B4a2 | 73 263 750 1438 2706 4769 5465 6620 7028 8281-8289d 8860 9123 9812 11719 14766 15326 16189 16217 16261 |
| B4a2a | 73 263 750 1438 2706 4769 4823 5465 6620 7028 8281-8289d 8860 9123 9812 11719 14766 15326 16189 16217 16261 16324 |
| B4a2a1 | 73 263 750 1438 2706 4769 4823 5465 5824 6131 6620 7028 8281-8289d 8860 9123 9812 11719 14766 15326 15777 16129 16189 16217 16261 |

| | |
|---|---|
| B4a2a2 | 73 263 750 1438 2706 3693 4769 4823 5465 6620 7028 8281-8289d 8860 9123 9812 11719 14766 15326 16154 16189 16217 16261 16324 |
| B4a2a3 | 73 263 750 1438 2706 4769 4823 5465 6620 7028 8281-8289d 8860 9123 9812 11719 11992 12366 14766 15326 16189 16217 16261 16272 16324 |
| B4a2b | 73 263 750 1438 2706 4769 5465 6620 7028 8281-8289d 8860 9123 9812 11719 14766 15326 16189 16217 16261 16360 |
| B4a2b1 | 73 263 750 1438 2706 4769 5465 6620 7028 8281-8289d 8860 9123 9812 10876 11719 14766 15326 16189 16217 16261 16360 |
| B4a2b1a | 73 263 750 1438 2706 4769 5465 6620 7028 8281-8289d 8860 9069 9123 9812 10876 11719 13413 14766 15326 16189 16217 16261 16360 |
| B4a3 | 73 263 750 1438 2706 4769 5465 7028 8281-8289d 8860 9123 11719 14766 15326 15355 15632A 15940 15941 15983 16092 16189 16217 16261 16399 |
| B4a4 | 73 193 263 750 1438 2706 4769 5465 7028 8281-8289d 8860 9123 11719 14751 14766 15326 16189 16217 16261 16299 |
| B4a5 | 73 263 750 1438 2706 4769 5465 5814 7028 8281-8289d 8860 9123 11719 12732 14518 14766 15326 16189 16217 16261 |
| B4g | 73 263 750 1438 2706 4769 7028 8281-8289d 8860 9968 11719 14766 15326 16189 16213 16217 16261 16292 |
| B4g1 | 73 263 750 1438 2706 4769 7028 7789 8281-8289d 8860 9968 11719 14766 15326 16189 16213 16217 16261 16292 |
| B4g1a | 73 263 750 1438 2706 4769 5108 7028 7789 8281-8289d 8860 9968 11719 14766 14905 15326 16189 16213 16217 16261 16292 |
| B4g1b | 73 263 750 1438 2706 4769 7028 7789 8281-8289d 8860 9968 11353 11719 13547 14766 15326 16153 16189 16213 16217 16261 16292 |
| B4g2 | 62 73 263 750 1438 2378 2706 4769 6482 7028 8281-8289d 8860 9968 11719 14766 15326 16189 16213 16217 16261 16292 16301 |
| B4h | 73 263 750 1438 2706 4769 5093 7028 8281-8289d 8860 11719 13269 14766 15326 16129 16189 16217 16261 |
| B4h1 | 73 263 750 1438 2706 4703 4769 5093 7028 8281-8289d 8860 11719 13269 14766 15326 16129 16189 16261 |
| B4i | 73 263 750 1438 2706 4769 6302 7028 8281-8289d 8860 11719 14766 15326 16189 16217 16261 |
| B4i1 | 73 263 750 1438 2706 4769 6302 6497 7028 8281-8289d 8860 9329 11719 14766 15326 16189 16217 16261 |
| B4k | 73 263 750 1438 2706 4769 5483 7028 8281-8289d 8860 11719 12741 14766 15326 16093 16189 16217 16261 |
| B4m | 73 263 750 1438 2706 4769 7028 8281-8289d 8860 10398 11719 14766 15326 16189 16217 16240 16261 |
| B4b'd'e'j | 73 263 750 827 1438 2706 4769 7028 8281-8289d 8860 11719 14766 15326 15535 16189 16217 |
| B4b | 73 263 499 750 827 1438 2706 4769 4820 7028 8281-8289d 8860 11719 13590 14766 15326 15535 16189 16217 |
| B2 | 73 263 499 750 827 1438 2706 3547 4769 4820 4977 6473 7028 8281-8289d 8860 9950 11177 11719 13590 14766 15326 15535 16189 16217 |
| B2a | 73 263 499 750 827 1438 2706 3547 4769 4820 4977 6473 7028 8281-8289d 8860 9950 11177 11719 13590 14766 15326 15535 16111 16189 16217 16483 |

| | |
|---|---|
| B2a1 | 73 263 499 750 827 1438 2706 3547 4769 4820 4977 6473 7028 8281-8289d 8860 9950 10895 11177 11719 13590 14766 15326 15535 16111 16189 16217 16483 |
| B2a1a | 73 263 499 750 827 1438 2706 3547 4769 4820 4977 6473 7028 8281-8289d 8860 9950 10895 11177 11719 13590 15326 15535 16111 16189 16217 16483 |
| B2a1a1 | 73 263 499 750 827 1438 2706 3547 4769 4820 4977 6473 7028 8281-8289d 8860 9950 10895 11177 11719 12729 13590 15326 15535 16111 16189 16217 16483 |
| B2a1b | 73 263 499 750 827 1438 2706 3027 3547 4769 4820 4977 6473 7028 8281-8289d 8860 9950 10895 11177 11719 12890 13590 14766 15326 15535 16111 16189 16217 16483 |
| B2a2 | 73 263 499 750 827 1438 2706 3547 4769 4820 4977 6473 7028 8281-8289d 8860 9097 9950 11177 11719 13590 14766 15326 15535 16111 16189 16217 16483 |
| B2a3 | 73 263 499 551 750 827 1438 2706 3547 4769 4820 4977 5054 6473 7028 8281-8289d 8860 9950 11177 11719 13590 14766 15326 15535 16111 16189 16217 16483 |
| B2a4 | 73 263 499 750 827 1438 2706 3547 4769 4820 4977 6473 7028 8281-8289d 8860 9950 11177 11719 13590 14766 15326 15535 16092 16111 16189 16217 16483 |
| B2a4a | 73 228 263 499 750 827 1438 2706 3547 4769 4820 4977 6473 7028 8281-8289d 8860 9950 11177 11719 13590 14766 15326 15535 16092 16111 16189 16217 16483 |
| B2a4a1 | 73 228 263 499 750 827 1438 2706 3547 3663 4769 4820 4977 6473 7028 8281-8289d 8860 9950 10685 11177 11719 13590 14766 15326 15535 16092 16111 16189 16217 16325 16483 |
| B2a5 | 73 189 263 499 750 827 1438 2706 3547 4769 4820 4977 5987 6473 7028 8281-8289d 8860 9950 11177 11719 11884 13221 13590 14766 15326 15535 16111 16189 16217 16278 16483 |
| B2b | 73 263 499 750 827 1438 2706 3547 4769 4820 4977 6473 6755 7028 8281-8289d 8860 9950 11177 11719 13590 14766 15326 15535 16189 16217 |
| B2b+152 | 73 152 263 499 750 827 1438 2706 3547 4769 4820 4977 6473 6755 7028 8281-8289d 8860 9950 11177 11719 13590 14766 15326 15535 16189 |
| B2b1 | 73 152 207 263 499 750 827 1041 1438 1842 2706 3547 4226 4769 4814 4820 4977 6473 6755 7028 8281-8289d 8860 9950 11177 11719 13590 14766 15326 15535 16093 16175 16189 16217 |
| B2b2 | 73 263 499 750 827 1438 2706 3547 4769 4820 4977 6473 6755 7028 8281-8289d 8860 9950 11177 11719 13590 14766 15326 15535 16145 16189 16217 |
| B2b2a | 73 209 263 499 750 827 1438 2706 3394 3547 4769 4820 4977 6260 6473 6755 7028 8281-8289d 8860 9233 9950 10915 11177 11719 11968 13590 14766 15326 15535 16145 16189 16217 16320 |
| B2b3 | 73 263 499 750 827 1438 2706 3547 4769 4820 4977 6473 6755 7028 8281-8289d 8860 9950 11177 11719 13590 13708 14766 15326 15535 16189 16217 |

| | |
|---|---|
| B2b3a | 73 152 263 271 499 750 827 1438 2706 3547 3918 4232 4769 4820 4977 6473 6755 7028 8281-8289d 8860 9950 11177 11719 13590 13708 14766 15326 15535 15784 16189 16217 16249 16312 |
| B2b4 | 73 (159) (195) 263 499 750 827 1438 2706 3547 4769 4820 4977 6473 6755 7028 8281-8289d 8641 8860 9605 9950 11177 11569 11719 13590 14766 15326 15521 15535 16189 16217 (16239) (16353) |
| B2c | 73 263 499 750 827 1438 2706 3547 4769 4820 4977 6473 7028 7241 8281-8289d 8860 9950 11177 11719 13590 14766 15326 15535 16189 16217 |
| B2c1 | 73 263 499 750 827 1438 2706 3547 4769 4820 4977 6473 7028 7241 8281-8289d 8860 9098 9950 11177 11719 13590 14766 15326 15535 16189 |
| B2c1a | 73 263 499 750 827 1438 2706 3547 4769 4820 4977 6473 6722 7028 7241 8281-8289d 8860 9098 9950 11177 11719 13590 14766 15326 15535 16189 16217 |
| B2c1b | 73 263 499 750 827 1438 2706 3547 4435 4769 4820 4977 6473 7028 7241 7262 7822 8281-8289d 8860 9098 9950 11177 11719 13590 14766 15326 15535 16189 16217 |
| B2c1c | 73 263 499 750 827 1438 2706 3547 4769 4820 4977 6473 7028 7241 8281-8289d 8860 9098 9950 11177 11719 13590 14063 14766 15326 15535 16189 16217 |
| B2c2 | 73 146 263 499 750 827 1438 2706 3547 4755 4769 4820 4977 6473 7028 7241 8281-8289d 8860 9950 11177 11719 13590 14757 14766 15326 15535 16189 16217 |
| B2c2a | 73 146 263 499 750 827 1438 2706 3547 4755 4769 4820 4977 6473 7028 7241 8281-8289d 8702 8860 9950 11177 11719 13590 14757 14766 15326 15535 16189 16217 16319 |
| B2c2b | 73 146 152 263 499 750 827 1438 2706 3547 4755 4769 4820 4977 6473 7028 7241 8281-8289d 8860 9682 9950 11177 11719 13590 13661 14757 14766 15326 15535 16189 16217 16295 |
| B2d | 73 263 498d 499 750 827 1438 2706 3547 4122 4123 4769 4820 4977 6473 7028 8281-8289d 8860 8875 9682 9950 11177 11719 13590 14766 15326 15535 16189 16217 |
| B2e | 73 263 499 750 827 1438 2706 3547 4769 4820 4977 6119 6473 7028 8281-8289d 8860 9950 11177 11719 13590 14049 14766 15326 15535 16189 16217 |
| B2f | 73 263 499 750 827 1438 2706 3547 3796 3996 4769 4820 4977 6473 7028 8281-8289d 8860 9950 10535 11177 11719 13590 13833 14766 15326 15535 16189 16217 |
| B2g | 73 114G 263 499 750 827 1438 2706 3547 3766 4769 4820 4977 6164 6473 7028 8281-8289d 8860 9950 11177 11719 13590 14766 15326 15535 16189 16217 |
| B2g1 | 73 114G 263 499 750 827 1002 1438 2706 3547 3766 4769 4820 4977 6164 6473 7028 8281-8289d 8860 9950 11177 11719 13590 14766 15326 15535 16189 16217 16298 |
| B2g2 | 73 114G 263 499 750 827 1438 2706 3547 3766 4769 4820 4977 6164 6473 7028 7340 8281-8289d 8860 9950 11177 11647 11719 11875 13590 14766 15326 15535 16189 16217 |

| | |
|---|---|
| B2h | 73 263 499 750 827 1438 2706 3547 4769 4820 4977 6473 7028 8281-8289d 8860 9950 11177 11719 11821 13590 14766 15326 15535 16189 16217 16468 |
| B2i | 73 263 499 750 827 1438 2706 3547 4769 4820 4977 6272 6473 7028 8281-8289d 8860 9950 11177 11719 13590 14766 15326 15535 16189 16217 |
| B2i1 | 73 263 430 485 499 750 827 961 1438 2706 3547 4769 4820 4977 6272 6473 7028 8281-8289d 8860 9950 11177 11719 13590 14766 15326 15535 16189 16217 16311 |
| B2i2 | 73 263 470 499 750 827 1438 2706 3547 4769 4820 4977 6272 6473 7028 8281-8289d 8860 9950 11177 11611 11719 13590 14766 15077 15326 15535 16189 16217 |
| B2i2a | 73 263 470 499 750 827 1438 2706 3547 4769 4820 4977 6272 6473 7028 8281-8289d 8860 9950 11177 11611 11719 13590 14766 15077 15326 15535 16189 16207 16217 |
| B2i2a1 | 73 263 470 499 750 827 1438 2706 3547 4769 4820 4977 6272 6473 7028 8281-8289d 8860 9950 10248 11177 11611 11719 13590 14766 15077 15326 15535 16189 16207 16217 16291 |
| B2i2a1a | 73 263 470 499 750 827 1438 2706 3547 4259 4769 4820 4977 6272 6473 7028 8281-8289d 8860 9950 10248 11177 11611 11719 12400 13590 14766 15077 15326 15535 16189 16207 16217 16291 |
| B2i2a1b | 73 263 470 499 750 827 1438 2706 3547 3843 4769 4820 4977 6272 6473 7028 8281-8289d 8860 9950 10248 11177 11611 11719 13590 14766 15077 15326 15535 16189 16207 16217 16291 |
| B2i2b | 73 207 263 470 499 750 827 1438 2706 3547 4769 4820 4977 6272 6473 7028 8281-8289d 8860 9950 11177 11611 11719 13590 14766 15077 15326 15535 16189 16217 |
| B2i2b1 | 73 153 207 263 470 499 750 827 1438 2706 3547 4769 4820 4977 6272 6473 7028 8281-8289d 8860 9950 11177 11611 11719 13590 14766 15077 15326 15535 16189 16217 16249 |
| B2j | 73 131 183 263 499 750 827 1438 2706 3547 4769 4820 4977 5270 6473 7028 8281-8289d 8860 9950 11177 11719 13590 14766 15326 15535 15924 16166 16189 16217 16361 |
| B2k | 73 146 263 499 750 827 1438 2706 3547 4371 4769 4820 4977 6473 7028 8281-8289d 8860 9950 11177 11719 13590 14766 15326 15535 16189 |
| B2l | 73 263 499 750 827 1438 2706 3547 4769 4820 4977 6473 7028 8281-8289d 8860 9950 11177 11719 13590 14766 15326 15535 16189 16217 |
| B2m | 73 263 499 750 827 1438 2706 3547 4769 4820 4977 6473 7028 8281-8289d 8860 9950 11177 11719 13590 14766 15326 15535 15766 16164 16189 16217 16519A |
| B2n | 73 263 499 750 827 1438 2706 3547 4191 4769 4820 4977 6383 6473 7028 8281-8289d 8860 9950 11177 11719 13590 14766 15326 15535 16189 |
| B2o | 73 263 499 750 827 1438 2706 3547 4769 4820 4977 6473 7028 8281-8289d 8860 9950 11177 11719 13590 14766 15326 15535 16092 16189 |
| B2o1 | 73 263 499 750 827 1438 2706 3547 4769 4820 4977 6473 7028 7270 8281-8289d 8860 9950 11177 11719 13590 14766 15326 15535 16092 16145 16189 16217 |

| | |
|---|---|
| B2o1a | 73 152 263 499 750 827 1438 2706 3547 4769 4820 4977 6473 7028 7270 8281-8289d 8860 9950 11177 11719 13590 14500 14766 15326 15535 16092 16145 16189 16217 |
| B2p | 73 263 499 750 827 1438 2380 2706 3547 4769 4820 4977 6473 7028 8222 8281-8289d 8860 9950 11177 11696 11719 13590 14766 15326 15535 16189 16217 |
| B2q | 73 263 499 750 827 1438 2706 3547 4047 4769 4820 4977 6473 7028 8281-8289d 8860 9950 11177 11719 13590 14766 15326 15535 16189 16217 |
| B2r | 73 263 499 750 827 1438 2706 3547 4769 4820 4977 5899d 6473 7028 8281-8289d 8860 9950 11177 11719 13590 14766 15326 15535 16189 |
| B2s | 73 263 310 499 750 827 1438 2706 3547 4769 4820 4977 6473 7028 8281-8289d 8567 8860 9950 11177 11719 12616 13590 13740 14766 15326 15535 16152 16189 16217 16325 |
| B2t | 73 263 499 750 827 1438 2706 3547 4769 4820 4977 6473 7028 8281-8289d 8860 9950 10792 11177 11719 13590 14766 15244 15326 15535 15884 16189 16217 16259 16357 16467 |
| B2u | 73 152 182 263 499 750 827 1438 2706 3547 3927 4769 4820 4977 5492 6473 7028 8146 8281-8289d 8860 9950 11177 11719 13590 14766 15326 15535 16189 16217 16256 |
| B2v | 73 263 499 750 827 1438 2706 3547 4769 4820 4977 6473 7028 7376 8281-8289d 8860 9950 11177 11719 13590 14766 15326 15535 15661 16140 16189 16217 |
| B2+16278 | 73 263 499 750 827 1438 2706 3547 4769 4820 4977 6473 7028 8281-8289d 8860 9950 11177 11719 13590 14766 15326 15535 16189 16217 |
| B2w | 73 146 263 499 750 827 1438 2706 3547 4769 4820 4977 6473 7028 8281-8289d 8860 9950 11177 11719 11950 13590 14569 14766 15326 15535 16189 16217 16270G 16278 |
| B2x | 73 263 499 750 827 1438 2706 3547 4129 4769 4820 4977 6473 7028 8281-8289d 8860 9950 10646 11177 11389 11719 12346 13590 14766 15326 15535 16189 16217 16323 |
| B2y | 73 263 499 750 827 1438 2706 3547 4769 4820 4977 6473 7028 8281-8289d 8860 9950 11177 11719 13590 14766 15326 15535 16189 16217 |
| B2y1 | 73 263 499 750 827 1438 2706 3480 3547 4769 4820 4977 6473 7028 8281-8289d 8860 9950 11177 11719 13590 14766 15326 15535 16189 16217 16261 |
| B4b1 | 73 263 499 750 827 1438 2706 4769 4820 7028 8281-8289d 8860 11719 13590 14766 15326 15535 16136 16189 16217 |
| B4b1a | 73 263 499 750 827 1438 2706 4769 4820 6023 6413 7028 8281-8289d 8860 11719 13590 14766 15326 15535 16136 16189 16217 |
| B4b1a+207 | 73 207 263 499 750 827 1438 2706 4769 4820 6023 6413 7028 8281-8289d 8860 11719 13590 14766 15326 15535 16136 16189 16217 |
| B4b1a1 | 73 199 202 207 263 499 750 827 1438 2706 2831 4117 4769 4820 6023 6413 7028 8206 8281-8289d 8860 11719 13590 14766 15236 15326 15535 16136 16189 16217 16284 |
| B4b1a1a | 73 199 202 207 263 499 750 827 1438 2706 2831 4117 4769 4820 6023 6413 7028 7664 8206 8281-8289d 8860 11719 13590 14766 15236 15326 15535 16136 16189 16217 16284 |

| | |
|---|---|
| B4b1a1b | 73 199 202 207 263 499 750 827 1438 2706 2831 4117 4769 4820 6023 6413 7028 8206 8281-8289d 8860 10685 11719 13590 14766 15236 15326 15535 16136 16189 16217 16284 |
| B4b1a1c | 73 199 202 207 263 499 750 827 1438 2706 2831 4117 4769 4820 6023 6413 7028 8206 8281-8289d 8860 11719 13590 14766 15236 15326 15535 16136 16189 16217 16284 16357 |
| B4b1a2 | 73 207 263 499 750 827 1438 2706 4769 4820 6023 6216 6413 7028 8281-8289d 8860 11719 13590 14766 15326 15535 16136 16189 16217 |
| B4b1a2a | 73 207 263 499 750 827 1438 1719 2220 2706 4769 4820 5899.XC 6023 6216 6413 7028 8281-8289d 8860 11719 13590 14766 15326 15535 16136 16189 16217 |
| B4b1a2b | 73 207 263 499 750 827 1438 2706 4769 4820 6023 6216 6413 7028 8281-8289d 8860 11719 13590 14766 15326 15535 16136 16189 16217 16300 |
| B4b1a2b1 | 73 207 263 499 750 827 1438 2706 4769 4820 6023 6216 6413 7028 8281-8289d 8860 9305 11719 13590 14766 15326 15535 16136 16189 16217 16300 |
| B4b1a2b2 | 73 207 263 499 750 827 1438 2706 4769 4820 6023 6216 6413 7028 8281-8289d 8659 8860 11719 13105 13590 14766 15326 15535 16136 16189 16217 16300 |
| B4b1a2c | 73 207 263 499 750 827 1438 2706 4769 4820 6023 6216 6413 7028 8281-8289d 8860 11719 13590 13599 14766 15326 15535 16136 16189 16217 16311 |
| B4b1a2d | 73 207 263 499 750 827 1438 2706 2836 4769 4820 6023 6216 6413 7028 8281-8289d 8860 11719 13590 14004 14053 14766 15326 15459 15535 16131 16136 16189 16217 |
| B4b1a2e | 73 207 263 499 750 827 1438 1452 2706 3648 4769 4820 6023 6216 6413 7028 8281-8289d 8860 11719 13590 14766 15326 15535 16136 16189 |
| B4b1a2f | 73 207 263 499 709 750 827 1438 2706 4769 4820 6023 6216 6413 7028 8281-8289d 8860 11719 13590 14110 14766 15326 15535 16136 16189 16217 |
| B4b1a2g | 73 207 263 499 750 827 1438 2706 4769 4820 6023 6216 6413 7028 8281-8289d 8860 11719 13590 14766 15326 15535 16136 16189 16217 16365 |
| B4b1a2g1 | 73 207 263 499 750 827 1438 2706 4769 4820 6023 6216 6413 7028 7843 8281-8289d 8860 11719 13590 14766 15326 15535 16136 16189 16217 16365 |
| B4b1a2h | 73 207 263 499 750 827 1438 2706 4769 4820 6023 6216 6413 7028 8281-8289d 8860 11719 13590 14766 14783 15326 15535 16136 16189 16217 |
| B4b1a2i | 73 207 263 499 750 827 1438 2706 4769 4820 6023 6216 6413 7028 8281-8289d 8860 11719 13590 14766 15326 15535 16067 16136 16189 16217 |
| B4b1a3 | 73 207 263 408A 499 750 827 1438 2706 4769 4820 6023 6413 7028 8281-8289d 8860 9055 9338T 9615 11719 13590 14766 15326 15535 16136 16189 16217 |
| B4b1a3a | 73 146 207 263 408A 499 750 827 1438 2706 4769 4820 6023 6413 7028 8281-8289d 8860 9055 9338T 9615 11719 13590 14133 14766 15326 15535 16086 16136 16189 16217 |
| B4b1b'c | 73 263 499 750 827 1438 2706 4769 4820 7028 8281-8289d 8860 11719 13590 14766 15326 15535 16136 16189 16217 16218 |

| | |
|---|---|
| B4b1b | 73 152 263 499 750 827 1391 1438 2706 3981 4769 4820 5585 7028 8281-8289d 8860 11719 13590 13934 14569 14766 15326 15535 16136 16189 16217 16218 16298 16362 |
| B4b1c | 73 263 499 750 827 1438 2706 4769 4820 7028 8281-8289d 8860 11719 13590 14587 14766 15326 15535 16136 16189 16217 16218 |
| B4b1c1 | 73 263 499 750 827 1438 2706 4769 4820 7028 7521 8281-8289d 8860 9101G 11239 11719 11914 13590 14587 14766 15326 15535 16136 16189 16217 16218 |
| B4b1c2 | 73 263 499 750 827 1438 2706 4769 4820 7028 8281-8289d 8343 8860 11719 13590 14587 14766 15326 15535 16136 16189 16217 16218 |
| B4d | 73 263 750 827 1438 2706 4769 7028 8281-8289d 8860 11719 11914 13942 14766 15326 15535 16189 16217 |
| B4d1'2'3 | 73 263 750 827 1438 2706 4769 7028 8281-8289d 8860 11719 11914 13942 14766 15326 15535 15930 16189 16217 |
| B4d1 | 73 263 750 827 1438 2706 4769 7028 8281-8289d 8860 11719 11914 13942 14766 15038 15326 15535 15930 16189 16217 |
| B4d1a | 73 263 750 827 1438 2706 4769 7028 8281-8289d 8860 11719 11914 12732 13942 14766 15038 15326 15535 15930 16189 16217 |
| B4d2 | 73 263 750 827 1438 2706 4769 6722 7028 8281-8289d 8379 8855 8860 9701 11719 11914 13942 14766 15326 15512 15535 15930 16189 16217 |
| B4d3 | 73 151 197 263 546 750 827 1438 2706 4769 7028 8281-8289d 8860 9120 11719 11914 12135 13942 14766 15326 15535 15930 16185 16189 (16193d) 16217 16234 |
| B4d3a | 73 151 197 263 546 750 827 1438 2706 4769 5418 7028 8281-8289d 8860 9120 11719 11914 12135 13942 14766 15326 15535 15930 16185 16189 (16193d) 16217 16234 |
| B4d3a1 | 73 151 197 263 546 750 827 1438 2706 4769 5084 5418 7028 8281-8289d 8860 9120 11719 11914 12135 13942 14766 15326 15535 15930 16185 16189 (16193d) 16217 16234 |
| B4d4 | 73 263 750 827 1438 2706 4769 6040 6836 7028 8281-8289d 8860 9212 11314 11719 11914 13942 14053 14687 14766 15326 15470 15535 16155 16189 16217 16278 |
| B4e | 73 146 185 189 195 263 513 750 827 1438 2706 4769 6026 6614 7028 8281-8289d 8860 9921 10750 11719 14766 15326 15535 16189 16217 |
| B4j | 73 263 750 827 1438 2706 3548 4080 4769 5300 6122 7028 8281-8289d 8860 11719 11893 11941 13911 14248 14766 15172 15326 15535 16189 16217 16223 16362 |
| B4c | 73 263 750 1119 1438 2706 4769 7028 8281-8289d 8860 11719 14766 15326 15346 16189 16217 |
| B4c1 | 73 263 750 1119 1438 2706 3497 4769 7028 8281-8289d 8860 11719 14766 15326 15346 16189 16217 |
| B4c1a'b | 73 263 709 750 1119 1438 2706 3497 4769 7028 8281-8289d 8860 11719 14766 15326 15346 16189 16217 |
| B4c1a | 73 263 709 750 1119 1438 2706 3497 4769 7028 8281-8289d 8860 10310 11719 14766 15326 15346 16189 16217 |
| B4c1a1 | 73 263 709 750 1119 1438 2706 3497 4769 7028 8281-8289d 8860 10310 11719 14133 14766 15326 15346 16189 16217 16311 |

| | |
|---|---|
| B4c1a1a | 73 263 709 750 1119 1438 2706 3497 4769 7028 8281-8289d 8860 10310 11719 14133 14766 15326 15346 16086 16189 16217 16311 |
| B4c1a1a1 | 73 263 709 750 1119 1438 2706 3497 4769 7028 8281-8289d 8844 8860 10310 11719 14133 14766 15326 15346 16086 16189 16217 16311 |
| B4c1a1a1a | 73 263 709 750 1119 1438 2706 3497 4769 5592 7028 8281-8289d 8844 8860 10310 11719 14133 14766 15326 15346 16086 16129 16189 16217 16311 |
| B4c1a1a2 | 73 263 709 750 1119 1438 2706 3497 4769 6164 7028 8281-8289d 8860 10310 11719 14133 14766 15326 15346 16086 16189 16217 16311 |
| B4c1a1b | 73 263 709 750 1119 1438 2706 3497 4769 6416 7028 8281-8289d 8860 10310 11719 14133 14766 15326 15346 16189 16217 16311 |
| B4c1a1c | 73 152 263 709 750 1119 1438 2706 3497 4769 7028 7319 8281-8289d 8860 10310 11719 14133 14452 14766 15326 15346 16189 16217 16311 |
| B4c1a2 | 73 263 709 750 1119 1438 2706 3497 4769 7028 8281-8289d 8860 10310 11719 14766 15326 15346 16189 16217 16527 |
| B4c1a2a | 73 263 709 750 1119 1438 2706 3497 4769 7028 8281-8289d 8860 10310 11719 12373 14766 15326 15346 16092 16189 16217 16527 |
| B4c1b | 73 150 263 709 750 1119 1438 2706 3497 4769 7028 8281-8289d 8860 11719 14766 15326 15346 16140 16189 16217 16274 |
| B4c1b1 | 73 150 211 263 455.1T 709 750 1119 1438 2706 3221 3497 4742 4769 5826 7028 7909 8281-8289d 8860 11719 13879 14162 14766 15326 15346 16111 16140 16154 16189 16217 16274 16497 |
| B4c1b1a | 73 150 211 263 455.1T 709 750 1119 1438 2706 3221 3497 4742 4769 5826 7028 7909 8167A 8281-8289d 8860 11719 13879 14162 14766 15148 15326 15346 15391 16111 16140 16154 16189 16217 16274 16497 |
| B4c1b+16335 | 73 150 263 709 750 1119 1438 2706 3497 4769 7028 8281-8289d 8860 11719 14766 15326 15346 16140 16189 16217 16274 16335 |
| B4c1b2 | 73 150 263 709 750 1119 1438 2706 3497 3571 4769 7028 8281-8289d 8860 11719 14766 15326 15346 16140 16189 16217 16274 16335 |
| B4c1b2a | 73 146 150 263 709 750 1119 1438 2706 3497 3571 4769 7028 8281-8289d 8772 8860 11719 14766 15326 15346 16140 16189 16217 16274 16335 |
| B4c1b2a1 | 73 146 150 263 709 750 1119 1438 1709 1716 2706 3497 3571 4769 7028 8281-8289d 8772 8860 11719 14766 15326 15346 16140 16189 16217 16274 16335 |
| B4c1b2a2 | 73 146 150 195 263 709 750 1119 1438 2706 3497 3571 4769 7028 8281-8289d 8772 8860 11719 14766 15301 15326 15346 16140 16189 16217 16274 16335 |
| B4c1b2a2a | 73 146 150 195 263 709 750 1119 1438 2706 3497 3571 4769 4937 7028 8281-8289d 8772 8860 11719 14766 15301 15326 15346 16140 16189 16217 16274 16335 |
| B4c1b2a2b | 73 146 150 195 263 709 750 1119 1438 2706 3497 3571 4769 7028 7598 8281-8289d 8772 8860 11719 14766 15301 15326 15346 16140 16189 16217 16274 16335 |
| B4c1b2b | 73 150 195 263 709 750 1119 1438 2706 3497 3571 4769 7028 8200 8281-8289d 8860 11719 14766 15326 15346 16140 16189 16217 16274 16335 |
| B4c1b2c | 73 150 263 709 750 1119 1438 2706 3435 3497 3571 4769 7028 8281-8289d 8860 9128 11440 11719 14766 15326 15346 16140 16189 16217 16274 16335 |

| | |
|---|---|
| B4c1b2c1 | 73 150 263 709 750 1119 1438 2706 3435 3497 3571 4769 7028 8281-8289d 8860 9128 9575 10493 11440 11719 14766 15326 15346 16136 16140 16189 16217 16249 16274 16291 16335 |
| B4c1b2c2 | 73 150 263 709 750 1119 1438 2706 3435 3497 3571 4769 7028 8281-8289d 8860 9128 11440 11719 14766 15326 15346 16129 16140 16166 16189 16217 16274 16335 |
| B4c1c | 73 150 195 263 750 1119 1438 2706 3497 4769 5441 7028 8281-8289d 8860 10398 11719 13629 14766 15326 15346 15941 16189 16217 |
| B4c1c+16311 | 73 150 195 263 750 1119 1438 2706 3497 4769 5441 7028 8281-8289d 8860 10398 11719 13629 14766 15326 15346 15941 16189 16217 16311 |
| B4c1c1 | 73 150 195 214 263 750 1119 1438 2706 3497 4769 5441 7028 8281-8289d 8860 10398 11719 13629 14766 15326 15346 15941 16189 16217 16311 |
| B4c2 | 73 263 750 1119 1438 2706 4769 5108 7028 8281-8289d 8860 11719 14088 14209 14766 15326 15346 16147 16184A 16189 16217 16235 |
| B4c2a | 73 263 750 1119 1438 2706 4769 5108 7028 8281-8289d 8860 9103 11719 14088 14209 14766 15326 15346 16147 16184A 16189 16217 16235 |
| B4c2b | 73 263 750 1119 1438 2706 4769 5108 6221 7028 8281-8289d 8860 11719 14088 14209 14766 15326 15346 16147 16184A 16189 16217 16235 |
| B4c2c | 73 263 750 1119 1438 2706 4769 5108 7028 8281-8289d 8823 8860 9053 11719 14088 14209 14766 15326 15346 16147 16184A 16189 16217 16235 |
| B4f | 73 200 263 573.XC 750 1438 1442 2706 4612 4769 5127 6332 7028 7389 8281-8289d 8860 11719 11969 13105 14766 15326 16168 16172 16189 16217 16249 16325 16390 |
| B4f1 | 73 200 257 263 573.XC 750 1438 1442 2706 4612 4769 5127 6332 7028 7389 8281-8289d 8291 8860 11719 11969 13105 14766 15326 16168 16172 16189 16217 16249 16325 16390 16523 |
| B5 | 73 263 709 750 1438 2706 4769 7028 8281-8289d 8584 8860 9950 10398 11719 14766 15326 16140 16189 |
| B5a | 73 210 263 709 750 1438 2706 3537 4769 7028 8281-8289d 8584 8860 9950 10398 11719 14766 15235 15326 16140 16189 16266A |
| B5a1 | 73 210 263 709 750 1438 2706 3537 4769 6960 7028 8281-8289d 8584 8860 9950 10398 11719 14766 15235 15326 16140 16189 16266A |
| B5a1a | 73 210 263 709 750 1438 2706 3537 4769 6960 7028 8281-8289d 8584 8860 9950 10398 11719 13145 13395 14766 15235 15326 16140 16189 |
| B5a1a1 | 73 210 263 709 750 1438 2706 3537 4769 6960 7028 8281-8289d 8584 8860 9950 10398 11719 11881 13145 13395 14766 15235 15326 16140 16189 16266A |
| B5a1b | 73 210 263 709 750 1438 2706 3537 4769 6960 7028 8281-8289d 8584 8860 9950 10398 10754 11719 14766 14989 15235 15326 16140 16189 |
| B5a1b1 | 73 210 263 709 750 1438 2706 3537 4769 6960 7028 7852 8281-8289d 8584 8860 9950 10398 10754 11719 14766 14989 15235 15326 16140 16189 16266A |
| B5a1c | 73 210 263 709 750 1438 2706 3537 4769 6960 7028 8281-8289d 8584 8860 9950 10325 10398 11719 14766 15235 15326 16140 16189 16266A |
| B5a1c1 | 73 210 263 709 750 1438 2706 3537 4769 6960 7028 8281-8289d 8584 8860 9950 10325 10398 10523 11719 14766 15235 15326 16140 16189 |

| | |
|---|---|
| B5a1c1a | 73 210 263 593 709 750 1438 2706 3537 4769 5237 6960 7028 8281-8289d 8584 8860 9950 10325 10398 10523 11719 14766 15235 15326 16140 16189 16266A |
| B5a1c1a1 | 73 210 263 593 709 750 1438 2706 3537 4769 5237 6960 7028 8281-8289d 8584 8860 9950 10325 10398 10523 11719 14766 15235 15326 16140 16189 16262 16266A |
| B5a1c2 | 73 210 263 709 750 1438 2706 3537 4562 4769 6960 7028 8281-8289d 8584 8860 9950 10325 10398 11719 14766 15235 15326 15514 16140 16189 16266A |
| B5a1d | 73 152 210 263 709 750 1438 2706 3537 4086 4769 6960 7028 8281-8289d 8584 8860 9950 10398 11465 11719 14766 15235 15326 16140 16189 16261 16266A |
| B5a2 | 73 210 263 709 750 1438 2706 3537 4769 7028 8281-8289d 8584 8860 9950 9962 10398 11151 11719 14766 15235 15326 16140 16189 16266G |
| B5a2a | 73 93 210 263 709 750 1438 2706 3537 4769 7028 8281-8289d 8584 8860 9950 9962 10398 11149 11151 11719 14149 14766 15235 15326 16140 16189 16266G |
| B5a2a1 | 73 93 210 263 709 750 1438 2706 3537 4769 7028 8281-8289d 8584 8860 9950 9962 10398 11149 11151 11719 12234 14149 14766 15235 15326 16140 16187 16189 16266G |
| B5a2a1a | 73 93 210 263 709 750 1438 2706 3537 4769 7028 8281-8289d 8584 8860 9189 9950 9962 10398 11149 11151 11719 12234 13422 14149 14766 14861 15235 15326 16140 16187 16189 16256 16266G |
| B5a2a1+16129 | 73 93 210 263 709 750 1438 2706 3537 4769 7028 8281-8289d 8584 8860 9950 9962 10398 11149 11151 11719 12234 14149 14766 15235 15326 16129 16140 16187 16189 16266G |
| B5a2a1b | 73 93 210 263 709 750 1438 2706 3537 4769 7028 8281-8289d 8584 8860 9950 9962 10398 11149 11151 11719 12234 14149 14158 14766 15235 15326 16129 16140 16187 16189 16266G |
| B5a2a2 | 73 93 210 263 709 750 1438 2706 3537 4769 7028 8281-8289d 8584 8614 8860 9950 9962 10398 11149 11151 11719 14149 14766 15235 15326 16140 16189 16266G |
| B5a2a2a | 73 93 (204) 210 263 709 750 1438 2706 3537 4769 7028 8281-8289d 8584 8614 8860 9950 9962 10398 11149 11151 11719 14149 14766 15046 15235 15326 16140 16189 16266G 16362 |
| B5a2a2a1 | 73 93 (204) 210 263 709 750 1438 2706 3537 4769 7028 8281-8289d 8557 8584 8614 8860 9950 9962 10398 11149 11151 11719 14149 14766 15046 15235 15301 15326 16140 16189 16266G 16362 |
| B5a2a2a2 | 73 93 (204) 210 263 709 750 1438 2706 3537 4769 7028 8281-8289d 8584 8614 8860 9950 9962 10398 11149 11151 11719 14149 14766 15046 15235 15326 16140 16189 16245 16266G 16362 |
| B5a2a2b | 73 93 210 263 709 750 1438 2706 3537 4769 5027 7028 8059 8281-8289d 8584 8614 8860 9950 9962 10398 11149 11151 11719 14149 14766 15235 15326 16140 16189 16266G |
| B5a2a2b1 | 73 93 210 263 709 750 1438 2706 3537 4769 4824 5027 7028 8059 8281-8289d 8584 8614 8860 9950 9962 10398 11149 11151 11719 14149 14766 15235 15326 16140 16189 16266G |

| | |
|---|---|
| B5a2a2b1a | 73 93 210 263 709 750 1438 2706 3537 4769 4824 5027 7028 8059 8281-8289d 8584 8614 8860 9554 9950 9962 10398 11149 11151 11719 14149 14766 15235 15326 16140 16189 16266G |
| B5a2a2b2 | 73 93 210 263 709 750 1438 2706 3537 4769 5027 7028 8059 8281-8289d 8584 8614 8860 9950 9962 10398 11149 11151 11719 14149 14766 15235 15326 16140 16189 16243A 16266G |
| B5b | 73 103 204 263 709 750 1438 1598 2706 4769 7028 8281-8289d 8584 8829 8860 9950 10398 11719 12361 14766 15223 15326 15508 15662 15851 15927 16140 16189 16243 |
| B5b1 | 73 103 204 263 709 750 (960.1C) 1438 1598 2706 4769 7028 8281-8289d 8584 8784 8829 8860 9950 10398 11719 12361 14766 15223 15326 15508 15662 15851 15927 16140 16189 16243 |
| B5b1a | 73 103 204 263 709 750 (960.1C) 1438 1598 2706 4769 7028 8281-8289d 8584 8784 8829 8860 9950 10398 11146 11719 12361 14470 14766 15223 15326 15508 15662 15851 15927 16140 16189 16243 |
| B5b1a1 | 73 195 204 263 709 750 (960.1C) 1438 1598 2706 4769 7028 8281-8289d 8584 8784 8829 8860 9950 10398 11146 11719 12361 14470 14766 15223 15326 15508 15662 15851 15927 16140 16189 16243 16278 16324 |
| B5b1a2 | 73 103 199 204 263 709 750 (960.1C) 1438 1598 2706 4769 7028 8281-8289d 8584 8784 8829 8860 9950 10398 11146 11719 11914 12192 12361 14470 14766 15223 15326 15508 15662 15851 15927 16140 16189 16223 16243 16318T 16319 |
| B5b1a2a | 73 103 199 204 263 709 750 (960.1C) 1438 1598 2706 4769 7028 8281-8289d 8584 8784 8829 8860 9950 10398 11146 11719 11914 12192 12361 14470 14766 14959 15223 15326 15508 15662 15851 15927 16140 16189 16223 16243 16318T 16319 |
| B5b1c | 73 103 152 204 263 709 750 (960.1C) 1438 1598 2706 3480 3819 4769 5836 7028 7771 8281-8289d 8467 8584 8784 8829 8860 9950 10274 10398 11719 12361 14766 15223 15326 15508 15662 15851 15927 16140 16189 |
| B5b1c1 | 73 103 152 204 263 709 750 (960.1C) 1438 1598 2706 3480 3819 4769 5836 6116 7028 7771 8281-8289d 8467 8584 8784 8829 8860 9950 10274 10398 11719 12361 14233 14766 15223 15326 15508 15662 15851 15927 16140 16189 16243 |
| B5b1c1a | 73 103 152 204 263 709 750 (960.1C) 1438 1598 2706 3480 3819 4769 5836 6116 7028 7771 8281-8289d 8467 8584 8784 8829 8860 9950 10274 10398 11207 11719 12361 14233 14766 15223 15326 15508 15662 15851 15927 16140 16189 16243 |
| B5b2 | 73 103 131 204 263 709 750 1438 1598 2706 4769 4895 7028 8281-8289d 8584 8829 8860 9950 10398 11719 12361 14766 15223 15326 15508 15662 15850 15851 15927 16111 16140 16189 16234 16243 16463 |
| B5b2a | 73 131 204 263 709 750 1438 1598 2706 4769 4895 7028 8281-8289d 8584 8829 8860 9950 10398 11719 12361 14766 15223 15326 15508 15662 15850 15851 15927 16111 16140 16189 16234 16243 16463 |
| B5b2a1 | 73 131 204 263 709 750 1438 1598 2706 4769 4895 5744 7028 8281-8289d 8584 8829 8860 9950 10398 11437 11719 12361 14766 15223 15326 15508 15662 15850 15851 15927 16111 16140 16189 16234 16243 16291 16463 |

| | |
|---|---|
| B5b2a2 | 73 131 204 207 263 709 750 1438 1598 2706 4769 4895 7028 8281-8289d 8584 8829 8860 9950 10398 11719 12361 14766 15223 15326 15508 15662 15758 15850 15851 15927 16111 16140 16189 16234 16243 16463 |
| B5b2a2a | 73 131 204 207 263 709 750 1438 1598 2706 4769 4895 7028 8281-8289d 8584 8829 8860 9950 10398 11719 12361 14750 14766 15223 15326 15508 15662 15758 15850 15851 15927 16111 16140 16189 16234 16243 16463 |
| B5b2a2a1 | 73 131 204 207 263 709 750 1438 1598 2706 4769 4895 7028 8281-8289d 8584 8829 8860 8894T 9950 10398 11719 12361 14750 14766 15223 15326 15508 15662 15758 15850 15851 15927 16111 16140 16189 16234 16243 16463 |
| B5b2a2a2 | 73 103 131 204 207 263 709 750 1438 1598 2706 4769 4895 7028 7542 7853 8281-8289d 8584 8829 8860 9950 10398 10807 11719 12361 14750 14766 15223 15326 15508 15662 15850 15851 15927 16111 16140 16189 16234 16243 16399 16463 |
| B5b2b | 73 103 131 204 263 709 750 1438 1598 2706 4769 4895 5964 7028 8281-8289d 8584 8829 8860 9950 10398 11719 12361 14766 15223 15326 15508 15662 15850 15851 15927 16111 16140 16189 16234 16243 16463 |
| B5b2+@204 | 73 103 131 263 709 750 1438 1598 2706 4769 4895 7028 8281-8289d 8584 8829 8860 9950 10398 11719 12361 14766 15223 15326 15508 15662 15850 15851 15927 16111 16140 16189 16234 16243 16463 |
| B5b2c | 73 103 131 263 481 709 750 1438 1598 2706 4161 4769 4895 7028 8281-8289d 8584 8829 8860 9950 10398 11101 11719 12361 14766 15223 15326 15508 15662 15850 15851 15927 16111 16140 16189 16234 16243 16463 |
| B5b2c1 | 73 103 263 481 709 750 1438 1598 2361 2706 4161 4769 4895 7028 8281-8289d 8584 8829 8860 9950 10398 11101 11719 12361 12451 14766 15223 15326 15508 15662 15850 15851 15927 16111 16140 16189 16234 16243 16463 |
| B5b3 | 73 103 189 203 204 263 709 750 1438 1598 2626 2706 4769 6962 7028 8281-8289d 8584 8829 8856 8860 9950 10103 10398 11719 12361 14766 15223 15326 15508 15662 15851 15927 16140 16189 16243 |
| B5b3a | 73 103 189 199 203 204 263 709 750 1438 1598 2626 2706 4769 6962 7028 8281-8289d 8584 8829 8856 8860 9950 10103 10398 11719 12361 13477 14766 15223 15326 15508 15662 15851 15927 16140 16189 16243 16256 |
| B5b3b | 73 103 189 203 204 263 709 750 1438 1598 2626 2706 4769 6962 7028 8281-8289d 8584 8829 8856 8860 9950 10103 10398 11719 12361 14766 15223 15326 15508 15662 15758 15851 15927 16140 16189 16243 |
| B5b4 | 73 103 146 204 263 709 750 1438 1598 2706 4703 4769 7028 7170 8281-8289d 8584 8829 8860 9950 10398 11719 12361 14766 15223 15326 15508 15662 15851 15927 16140 16189 16243 16274 |
| B5b5 | 73 103 152 195 204 263 709 750 1438 1598 2706 3834 4769 4924 7028 8281-8289d 8584 8829 8860 9950 10398 11719 12361 14028 14410 14766 14831 15223 15326 15508 15662 15851 15927 16140 16166 16189 16243 |
| R24 | 73 146 263 750 1438 2706 3565 4260 4769 7028 8860 9759 11710 11719 12483 14766 15326 16051 16185 16189 16193d |
| R24a | 73 146 263 750 1438 2706 3565 4260 4769 7028 8772 8860 9759 11710 11719 12483 13434 14766 14902 15326 16051 16185 16189 16193d 16324 |
| R12'21 | 73 263 750 1438 2706 4769 7028 8860 10398 11404 11719 14766 15326 16295 |

| | |
|---|---|
| R12 | 73 263 750 1438 2706 4011 4200 4695 4703 4769 5417 7028 8027 8860 8906 9386 9644 9881 10398 11404 11719 12172 13708 13759 14305 14766 14989 15326 15562 15884 16295 |
| R21 | 73 146 152 199 249 263 750 1438 1709 1719 2397 2706 4711 4769 7028 8279 8860 9067 9077 9109 10398 10610T 11404 11719 12234 12510 13145 14766 15326 15613 16168 16295 16296 16304 |
| R14 | 56T 73 182 204 234 263 573.XC 750 1438 2706 4002 4769 7022G 7028 9128 10172 11321 11560 11719 12612 13281 14002 14766 14800 15326 15394 15458 16187 16288 16304G 16362 |
| R22 | 73 152 263 329 750 1040 1438 2706 4769 6770 7028 8860 10316 10609 11719 13359 14766 15326 16249 16288 16304 |
| R23 | 73 195 263 634A 750 1438 2706 4769 6216 7007 7028 7158 7388 7822 8614 8860 10987 11719 11914 12151 12459 13885A 14687 14766 15326 15412 16256 16290 16465 |
| R30 | 73 263 750 1438 2706 4769 7028 8584 8860 11719 14766 15326 |
| R30a | 73 263 750 1438 2706 3316 4769 6764 7028 8584 8860 11047A 11719 12714 14766 15055 15326 |
| R30a1 | 73 (152) 263 750 1438 2056 2706 3316 4232 4769 5442 6764 7028 8584 8860 9156 9242 11047A 11719 12714 14766 15055 15326 |
| R30a1a | 73 (152) 263 750 1438 2056 2706 3316 4232 4769 5442 6764 7028 8584 8860 9156 9242 10688 11047A 11719 12714 14766 15055 15326 |
| R30a1b | 73 (152) 263 750 1438 2056 2706 3316 4225 4232 4769 5442 6764 7028 8584 8860 9156 9242 11047A 11719 12714 14766 15055 15326 16209 |
| R30a1b1 | 73 (152) 263 750 1438 2056 2706 3316 4225 4232 4769 5442 6764 7028 8584 8860 9156 9242 11047A 11719 11735 12714 14766 15055 15326 16209 16256 |
| R30a1c | 73 (152) 263 750 1438 2056 2706 3316 4232 4769 5442 6764 7028 8584 8860 9142 9156 9242 9869G 11047A 11719 12714 13161 13773 14766 15055 15326 16172 16278 |
| R30b | 73 263 373 750 1438 2706 4769 7028 8584 8860 11719 14766 15326 |
| R30b1 | 73 152 263 299d 373 480 750 1438 1598 2706 4062 4225 4769 5836 7028 7268G 7490 8584 8805 8860 9174 10631A 11009 11719 12406 13236 14194 14305 14766 15307 15326 16189 16298 16299 |
| R30b2 | 73 263 373 750 1438 2706 4769 6290 7028 7280 7843 8584 8860 11719 13539 14766 15148 15326 16292 |
| R30b2a | 73 263 373 750 1438 2706 4769 6290 7028 7280 7843 8584 8860 11719 13539 14000A 14766 15148 15326 16292 |
| R31 | 73 263 750 1438 2706 4769 7028 8860 11719 14766 15326 15884 (16362) |
| R31a | 73 146 263 338 750 1438 1531 2706 4769 5348 6293 6480 7028 7981 8860 10373 10631 11719 13557 13768A 14553 14766 15326 15334 15884 16172 16304 (16362) |
| R31a1 | 73 146 152 237 263 338 750 1438 1531 2248 2706 4769 5348 6293 6480 7028 7981 8842 8860 10373 10631 11719 13557 13768A 14290 14553 14766 15326 15334 15884 16172 16304 (16362) 16463 |
| R31b | 73 207 228 234 263 750 1309 1438 1452 2706 2763 3849 3882 4769 5503 6827 7028 7046 8400 8860 8988 11719 12007 12651 14488 14766 15148 15326 15884 16051 16093 16189 16218 16292 (16362) |

| | |
|---|---|
| R32 | 73 152 263 750 1438 2706 3816 4769 4820 7028 8281-8289d 8464 8743 8784 8860 8887 9116 11719 12771 14766 15326 15712 16145 16185 16239 16311 16325 |
| P | 73 263 750 1438 2706 4769 7028 8860 11719 14766 15326 15607 |
| P+16176 | 73 263 750 1438 2706 4769 7028 8860 11719 14766 15326 15607 16176 |
| P1 | 73 212 263 750 1438 2706 4769 6077 7028 8860 10118 11719 14766 15326 15607 16176 16266 16357 |
| P1d | 73 212 263 750 1438 2706 4769 6077 7028 8860 10118 11719 12346 14766 15326 15607 16176 16266 16357 |
| P1d1 | 73 212 263 750 1438 2706 4769 6077 7028 8860 10118 11719 12346 14766 15326 15607 16176 16266 16270 16357 |
| P1d1a | 73 212 263 750 1438 2706 4769 5969 6077 7028 8860 10118 11719 12346 14766 15326 15607 16176 16266 16270 16357 |
| P1d2 | 73 212 263 750 1438 2706 4769 6077 7028 8860 10118 11719 12346 12711 14766 15326 15479 15607 16176 16266 16357 |
| P1d2a | 73 212 263 750 1438 2706 4769 6077 7028 7624 8860 10118 11719 12346 12711 14766 15326 15479 15607 16176 16209 16266 16357 |
| P1+152 | 73 152 212 263 750 1438 2706 4769 6077 7028 8860 10118 11719 14766 15326 15607 16176 16266 16357 |
| P1f | 73 146 152 212 263 750 1438 2706 4769 6077 6671 7028 7569 8860 10118 11719 12361 14766 15326 15607 16176 16266 16357 16438 |
| P2'10 | 73 263 750 1438 2706 3882 4769 7028 8860 11719 14766 15326 15607 16176 |
| P2 | 73 263 750 2706 3203 3882 4122 4769 7028 8859 8860 11719 14766 14890 15326 15607 |
| P10 | 73 263 710 750 1438 1673 2706 3738 3882 4314 4769 5915 6131 6446 6740 7028 8158T 8664 8860 9077 11719 11914 12642 13810 13958C 14766 15326 15607 15811 15839 15924 16145 16176 16224 16233 16311 16456 16527 |
| P8 | 73 152 263 750 1438 2706 3391 4769 5237 7028 7805 8167 8614 8860 11065 11353 11404 11719 12414 12756 14766 15110 15326 15511 15521C 15607 16176 16241T 16261 16362 |
| P3 | 73 127 128 263 573.XC 750 1438 2706 3645 4769 7028 8860 11719 14338 14766 15326 15607 15748 15937T |
| P3a | 73 125 127 128 263 573.XC 750 1438 2706 3645 4769 5899d 6734 7028 8860 11719 13651 14338 14766 15326 15607 15748 15937T 16145 16278 |
| P3b | 73 127 128 236 263 573.XC 750 1438 2706 3645 4769 7028 8860 11719 12121 13934 14338 14766 15326 15607 15748 15937T |
| P3b1 | 73 127 128 152 236 259 573.XC 750 1438 2706 3645 4769 7028 8860 11719 12121 13934 14053 14338 14766 15326 15607 15748 15937C 16327 |
| P4 | 73 263 750 1438 1719 2706 4769 5460 7028 8860 10398 11719 14766 15326 15607 |
| P4a | 73 152 263 507 517 750 1438 1719 2706 3915 4216 4769 5460 7028 7852 8860 9587 9755 10398 10463 11167 11641 11719 12358 13135 14512 14766 15314 15326 15607 16319 |
| P4a1 | 73 152 263 507 517 750 1438 1719 2706 3915 4216 4769 4814 5460 7028 7852 8860 9587 9755 10398 10463 11167 11641 11719 12358 13135 13468 14512 14696 14766 15314 15326 15607 16319 |

| | |
|---|---|
| P4b | 73 263 750 1438 1719 2706 4769 5460 7028 8860 10398 10914 11016 11288 11719 14766 15326 15607 16274 16291 |
| P4b1 | 73 263 591A 750 1438 1719 2706 3010 4769 5276 6881 7028 8860 10088 10398 10914 11016 11288 11719 14766 15326 15607 16274 16291 16337 16362 |
| P5 | 73 152 263 750 2706 4769 7028 7419 7657 8860 9509 11719 12406 12831 13368 14766 15326 15412 15607 15970 16192 16311 |
| P6 | 73 263 750 1438 2706 4769 6719 7028 8860 11719 14766 15326 15607 16311 16362 |
| P7 | 73 152 263 374 750 1438 1598 2283 2706 4769 6249 7028 7325 8387 8860 10373 10810 11719 12026 12235 12361 14502 14766 15326 15607 15613 16148 16234 |
| P9 | 73 263 750 1438 2706 3172A 4769 7028 8860 11719 14766 14788 15326 15607 |
| P9a | 73 263 750 1438 2706 3172A 4769 7028 8860 11719 13452A 14766 14788 15326 15607 |
| U | 73 263 750 1438 2706 4769 7028 8860 11467 11719 12308 12372 14766 15326 |
| U1 | 73 263 285 750 1438 2706 4769 7028 8860 11467 11719 12308 12372 12879 13104 14070 14766 15148 15326 15954C 16249 |
| U1a | 73 263 285 750 1438 2218 2706 4769 7028 8860 11467 11719 12308 12372 12879 13104 14070 14364 14766 15148 15326 15954C 16189 16249 |
| U1a1 | 73 263 285 750 1438 2218 2706 4769 4991 6026 7028 7581 8860 11467 11719 12308 12372 12879 13104 14070 14364 14766 15148 15326 15954C 16189 16249 |
| U1a1a | 73 263 285 385 750 1438 2218 2706 3158.1T 3591 4769 4991 6026 7028 7581 8860 11467 11719 12308 12372 12879 13104 13422 14070 14364 14766 15148 15326 15954C 16189 16249 |
| U1a1a1 | 73 263 285 385 573.XC 750 1438 2218 2706 3158.1T 3591 4769 4991 6026 7028 7581 8860 9575 11467 11719 12308 12372 12879 13104 13422 14070 14364 14766 15148 15326 15954C 16189 16249 |
| U1a1a1a | 73 263 285 385 573.XC 750 1438 2218 2706 2836 3158.1T 3591 4769 4991 6026 7028 7581 8860 9575 11467 11719 12308 12372 12879 13104 13422 14070 14364 14766 15148 15326 15954C 16189 16249 |
| U1a1a2 | 73 262 263 285 385 750 1438 2218 2706 3158.1T 3591 4561 4769 4991 6026 7028 7581 8860 11467 11719 12308 12372 12879 13104 13422 14070 14364 14766 15148 15326 15954C 16189 16249 |
| U1a1a+16129 | 73 263 285 385 750 1438 2218 2706 3158.1T 3591 4769 4991 6026 7028 7581 8860 11467 11719 12308 12372 12879 13104 13422 14070 14364 14766 15148 15326 15954C 16129 16189 16249 |
| U1a1a3 | 73 195 263 285 385 750 1438 2218 2706 3158.1T 3591 4769 4991 5899.1C 6026 7028 7581 8860 11467 11719 12308 12372 12879 13104 13422 14070 14364 14766 15148 15954C 16129 16189 16249 16288 |
| U1a1b | 73 152 263 285 750 1438 2218 2706 4769 4991 6026 7028 7581 8730 8860 11467 11719 12308 12372 12879 13104 14070 14364 14766 15148 15326 15954C 16166d 16189 16249 16311 |

| | |
|---|---|
| U1a1c | 73 263 285 750 1438 2218 2706 4769 4991 6026 7028 7581 8860 11467 11719 12308 12372 12879 13104 14070 14364 14766 15148 15217 15326 15954C 16189 16249 |
| U1a1c1 | 73 263 285 750 1438 2218 2706 4769 4991 6026 7028 7581 8860 11467 11719 12308 12372 12879 13104 14070 14364 14766 15115 15148 15217 15326 15954C 16189 16249 |
| U1a1c1a | 73 263 285 750 1438 2218 2706 4769 4991 6026 7028 7581 8766 8860 11467 11719 12308 12372 12879 13104 14070 14364 14766 15115 15148 15217 15326 15954C 16189 16249 16465 |
| U1a1c1b | 73 263 285 750 1438 2218 2706 4769 4991 6026 6041 6293 7028 7253 7581 8860 11467 11719 12308 12372 12879 13104 14070 14364 14766 15106 15115 15148 15217 15326 15954C 16189 16249 |
| U1a1c1c | 73 263 285 750 1438 2218 2706 4769 4991 6026 7028 7403 7581 8860 11467 11719 12308 12372 12879 13104 14070 14364 14766 15115 15148 15217 15326 15954C 16189 16249 |
| U1a1c1c1 | 73 195 263 285 663 750 1438 2218 2706 4769 4991 6026 7028 7403 7581 8860 11116 11467 11719 12308 12372 12879 13104 13656 14070 14364 14766 15115 15148 15217 15326 15954C 16145 16189 16249 |
| U1a1c1d | 73 263 285 750 1438 2218 2706 4769 4991 6026 7028 7581 8860 10253 11467 11719 12308 12372 12879 13104 14070 14364 14766 15115 15148 15217 15326 15954C 16189 16249 |
| U1a1c1d1 | 73 263 285 750 1438 2218 2706 4769 4991 6026 7028 7581 8860 9716 10253 11467 11719 12308 12372 12879 13104 14070 14364 14766 15115 15148 15217 15326 15954C 16129 16189 16249 |
| U1a1d | 73 263 285 750 1438 2218 2706 4769 4991 6026 7028 7581 8860 11467 11719 12308 12372 12879 13104 14070 14364 14766 15148 15326 15954C 16189 16242 16249 16362 |
| U1a2 | 73 263 285 455.1T 750 1438 2218 2706 4769 7028 8860 11467 11719 12136 12308 12372 12879 13104 14070 14364 14766 15148 15326 15954C 16129 16189 16249 |
| U1a3 | 73 146 263 285 750 1438 2218 2706 4769 7028 8860 10203 11467 11719 12308 12372 12879 13104 13681 14063 14070 14364 14766 15148 15326 15954C 16189 16249 |
| U1b | 73 146 263 285 750 1438 2387 2706 4769 7028 8395 8860 10885 11467 11566 11719 12308 12372 12879 13104 14070 14766 15148 15172 15326 15954C (16111) 16249 16327 |
| U1b1 | 73 146 263 285 750 1438 2387 2706 4769 7028 8395 8860 10885 11467 11566 11719 12308 12372 12879 13104 14070 14766 15110 15148 15172 15326 15954C (16111) 16249 16327 |
| U1b2 | 73 146 263 285 750 1438 2387 2706 4769 7028 8395 8860 10885 11467 11566 11719 12308 12372 12879 13104 14070 14766 15148 15172 15326 15954C (16111) 16214A 16249 16327 |
| U1b3 | 73 146 195 263 285 750 1438 2387 2706 4769 7028 8395 8860 10885 11467 11566 11719 12308 12372 12879 13104 14070 14766 15148 15172 15326 15954C (16111) 16249 16311 16327 |
| U5 | 73 263 750 1438 2706 4769 7028 8860 11467 11719 12308 12372 14766 15326 16192 16270 |

| | |
|---|---|
| U5a'b | 73 263 750 1438 2706 3197 4769 7028 8860 9477 11467 11719 12308 12372 13617 14766 15326 16192 16270 |
| U5a | 73 263 750 1438 2706 3197 4769 7028 8860 9477 11467 11719 12308 12372 13617 14766 14793 15326 16192 16256 16270 |
| U5a1 | 73 263 750 1438 2706 3197 4769 7028 8860 9477 11467 11719 12308 12372 13617 14766 14793 15218 15326 16192 16256 16270 16399 |
| U5a1+@16192 | 73 263 750 1438 2706 3197 4769 7028 8860 9477 11467 11719 12308 12372 13617 14766 14793 15218 15326 16256 16270 16399 |
| U5a1a | 73 263 750 1438 1700 2706 3197 4769 7028 8860 9477 11467 11719 12308 12372 13617 14766 14793 15218 15326 16256 16270 16399 |
| U5a1a1 | 73 263 750 1438 1700 2706 3197 4769 5495 7028 8860 9477 11467 11719 12308 12372 13617 14766 14793 15218 15326 15924 16256 16270 16399 |
| U5a1a1+152 | 73 152 263 750 1438 1700 2706 3197 4769 5495 7028 8860 9477 11467 11719 12308 12372 13617 14766 14793 15218 15326 15924 16256 16270 16399 |
| U5a1a1a | 73 152 263 750 1438 1700 2706 3197 3816 4769 5495 7028 8860 9477 11467 11719 12308 12372 13617 14766 14793 15218 15326 15924 16256 16270 16399 |
| U5a1a1b | 73 152 263 750 1438 1700 2706 3197 4769 5495 7028 8860 9477 11467 11719 12308 12372 13617 14766 14793 15110 15218 15326 15924 16256 16270 16399 |
| U5a1a1h | 73 152 263 750 1438 1700 2706 3197 4769 5495 7028 8860 9477 11467 11719 12308 12372 13617 14766 14793 15218 15326 15924 16256 16270 16294 16399 |
| U5a1a1c | 73 263 750 1438 1700 2706 3197 4769 5495 6905 7028 8860 9477 11467 11719 12308 12372 13015 13617 14766 14793 15218 15326 15924 16256 16270 16399 |
| U5a1a1+16362 | 73 263 750 1438 1700 2706 3197 4769 5495 7028 8860 9477 11467 11719 12308 12372 13617 14766 14793 15218 15326 15924 16256 16270 16362 16399 |
| U5a1a1d | 73 185 204 263 750 1438 1700 2706 3197 4769 5495 7028 8860 9477 11467 11719 12308 12372 13617 14766 14793 15218 15326 15924 16256 16270 16362 16399 |
| U5a1a1d1 | 73 185 189 204 263 750 1438 1700 2706 3197 4769 5495 7028 8860 9477 11467 11719 12308 12372 13617 14766 14793 15218 15326 15924 16256 16270 16362 16399 |
| U5a1a1e | 73 263 750 1438 1700 2706 3197 4769 5495 7028 8860 9477 9682 11467 11719 12308 12372 13617 14766 14793 15218 15326 15924 16256 16270 16399 |
| U5a1a1g | 73 263 750 1438 1700 2706 3197 4224 4769 5495 7028 8860 9477 11467 11719 12308 12372 13617 14766 14793 15218 15326 15924 16256 16270 16399 |
| U5a1a1i | 73 263 750 1438 1700 2706 3197 3388A 4769 5495 7028 8860 9477 11467 11719 12308 12372 13617 14766 14793 15218 15326 15924 16256 16270 16399 |
| U5a1a2 | 73 263 573.XC 750 1438 1700 2706 3197 4769 7028 8860 9477 11467 11719 12308 12346 12372 13617 14766 14793 15218 15326 16256 16270 16399 |

| | |
|---|---|
| U5a1a2a | 73 263 573.XC 750 1438 1700 2706 3197 4769 5319 6629 6719 7028 8860 9477 11467 11719 12308 12346 12372 13617 14766 14793 15218 15326 16256 16270 16399 |
| U5a1a2a1 | 73 263 573.XC 750 1438 1700 2706 3197 4769 5319 6293 6629 6719 7028 8860 9477 11467 11719 12308 12346 12372 13617 14766 14793 15218 15326 16256 16270 16399 |
| U5a1a2a1a | 73 263 573.XC 750 1438 1700 2706 3197 4769 5319 6293 6629 6719 7028 8860 9477 10972 11467 11719 12308 12346 12372 13617 14766 14793 15218 15326 16256 16270 16399 |
| U5a1a2b | 73 263 573.XC 750 1438 1700 2706 3197 4769 7028 8860 9477 11467 11719 12308 12346 12372 13105 13617 14766 14793 15218 15326 16256 16270 16399 |
| U5a1a2b1 | 73 263 573.XC 750 1438 1700 2706 3197 4769 7028 8269 8860 9477 10326A 11467 11719 12308 12346 12372 13105 13617 14278 14766 14793 15218 15326 16256 16270 16399 |
| U5a1g | 73 263 750 1438 2706 3197 4769 7028 7792 8860 9477 11467 11719 12308 12372 13617 14766 14793 15218 15326 16256 16270 16399 |
| U5a1g1 | 73 263 750 1438 2706 3197 4769 7028 7792 8860 9477 11467 11719 11914 12308 12372 13617 14766 14793 15218 15326 16256 16270 16293 16399 |
| U5a1g2 | 64 73 263 750 1438 2706 3197 4769 7028 7792 8860 9477 11467 11719 12308 12372 13617 14766 14793 15218 15326 16256 16270 16399 |
| U5a1b | 73 263 750 1438 2706 3197 4769 7028 8860 9477 9667 11467 11719 12308 12372 13617 14766 14793 15218 15326 16192 16256 16270 16399 |
| U5a1b1 | 73 263 750 1438 2706 3197 4769 7028 8860 9477 9667 11467 11719 12308 12372 13617 14766 14793 15218 15326 16192 16256 16270 16291 16399 |
| U5a1b1a | 73 263 750 1438 2706 3197 4553 4769 7028 8860 9477 9667 11467 11719 12308 12372 13617 14766 14793 15218 15326 16192 16256 16270 16291 16399 |
| U5a1b1a1 | 73 263 750 1438 2706 3197 4553 4769 7028 8860 9477 9667 11467 11719 12308 12372 13617 14574 14766 14793 15218 15326 16192 16256 16270 16291 16399 |
| U5a1b1a2 | 73 263 750 1438 2706 3197 4553 4769 7028 8860 9477 9667 11467 11719 12308 12372 13617 14766 14793 15218 15326 15883 16192 16256 16270 16291 16399 |
| U5a1b1b | 73 263 750 1438 2706 3197 4769 7028 8119 8860 9477 9667 11467 11719 12308 12372 13617 14766 14793 15218 15326 16192 16256 16270 16291 16399 |
| U5a1b1b1 | 73 263 750 1438 2706 3197 4769 7028 8119 8860 9477 9667 11467 11719 12308 12372 13617 14766 14793 15218 15326 16092 16192 16256 16270 16291 16399 |
| U5a1b1c | 73 263 750 1438 2706 3197 4769 7028 8860 9055 9477 9667 11467 11719 12308 12372 13617 14766 14793 15218 15326 16192 16256 16270 16291 16399 |
| U5a1b1c1 | 73 263 750 1187 1438 2706 3197 4769 7028 8860 9055 9477 9667 11467 11719 12308 12372 13617 14766 14793 15218 15326 16192 16256 16270 16291 16399 |

| | |
|---|---|
| U5a1b1c2 | 73 263 750 1438 2706 3197 3705 4769 7028 8860 9055 9477 9667 11467 11719 12308 12372 13617 14766 14793 15218 15326 16129 16192 16256 16270 16291 16399 |
| U5a1b1d | 73 263 750 1438 2706 3197 4769 7028 8860 9477 9667 11467 11719 12308 12358 12372 13617 14766 14793 15218 15326 16192 16256 16270 16291 16399 |
| U5a1b1d+16093 | 73 263 750 1438 2706 3197 4769 7028 8860 9477 9667 11467 11719 12308 12358 12372 13617 14766 14793 15218 15326 16093 16192 16256 16270 16291 16399 |
| U5a1b1d1 | 73 198 263 750 1438 2706 3197 4769 7028 8860 9477 9667 11467 11719 12308 12358 12372 13617 14766 14793 15218 15326 16093 16192 16256 16270 16291 16399 |
| U5a1b1e | 73 263 750 1438 2706 3197 4769 7028 8860 9477 9667 11467 11719 12308 12372 12582 13617 14766 14793 15218 15326 16192 16256 16270 16291 16399 |
| U5a1b1f | 73 263 750 1438 2706 3197 4769 7028 8860 9477 9667 11467 11719 12308 12372 13617 14766 14793 15218 15326 15596 16129 16192 16256 16270 16291 16399 |
| U5a1b1g | 73 263 750 1438 2706 3197 4769 7028 8860 9477 9667 11353 11467 11719 12308 12372 13617 14766 14793 15218 15326 16192 16256 16270 16291 16399 |
| U5a1b1h | 73 263 750 1438 2706 3197 4769 7028 8860 9477 9667 10754 11467 11719 12308 12372 13617 14766 14793 15218 15326 16192 16256 16270 16291 16399 |
| U5a1b2 | 73 263 750 1438 2706 3197 4769 7028 8860 9477 9632 9667 11467 11719 12308 12372 13617 14766 14793 15218 15326 16192 16256 16270 16399 |
| U5a1b+16362 | 73 263 750 1438 2706 3197 4769 7028 8860 9477 9667 11467 11719 12308 12372 13617 14766 14793 15218 15326 16192 16256 16270 16362 16399 |
| U5a1b3 | 73 263 750 1438 2706 3197 4769 7028 8860 9477 9667 11467 11719 12308 12372 13617 14766 14793 15218 15326 16192 16256 16270 16362 16399 16428 |
| U5a1b3a | 73 263 750 1438 2706 3197 4769 5153 7028 8860 9477 9667 11467 11719 12308 12372 13617 14766 14793 15218 15326 16192 16256 16270 16362 16399 16428 |
| U5a1b3a1 | 73 249 263 750 1438 2706 3197 4769 5153 7028 8860 9477 9667 11467 11719 12308 12372 13617 14766 14793 15218 15326 16192 16256 16270 16362 16399 16428 |
| U5a1b4 | 73 263 750 1438 2706 3197 4769 7028 8860 9477 9667 11467 11719 12308 12331 12372 13617 14766 14793 15218 15326 16192 16256 16270 16362 16399 |
| U5a1c | 73 263 750 1438 2706 3197 4769 7028 8860 9477 11467 11719 12308 12372 13617 14766 14793 15218 15326 16192 16256 16270 16320 16399 |
| U5a1c1 | 73 195 263 750 1438 2706 3197 4769 7028 8860 9477 11467 11719 12308 12372 13617 13802 14766 14793 15218 15326 16192 16256 16270 16320 16399 |
| U5a1c1a | 73 153 195 263 750 1438 2706 3197 4769 7028 8860 9477 11467 11719 12308 12372 13617 13802 14766 14793 15218 15326 16192 16256 16270 16320 16399 |

| | |
|---|---|
| U5a1c2 | 73 263 750 961 1438 2706 3197 4769 7028 8860 9477 11467 11719 12308 12372 13617 14766 14793 15218 15326 16192 16256 16270 16320 16399 |
| U5a1c2a | 73 183 263 750 961 1438 2706 3197 4769 7028 8860 9477 10544 11467 11719 12308 12372 13617 14766 14793 15218 15326 16192 16256 16270 16320 16399 |
| U5a1c2a1 | 73 183 263 750 961 1438 2706 3197 4769 7028 8860 9477 10544 11467 11719 12308 12372 13617 14766 14793 15218 15326 16192 16256 16270 16286 16320 16399 |
| U5a1d | 73 263 750 1438 2706 3027 3197 4769 7028 8860 9477 11467 11719 12308 12372 13617 14766 14793 15218 15326 16192 16256 16270 16399 |
| U5a1d1 | 73 263 750 1438 2706 3027 3197 4769 5263 7028 8860 9477 11467 11719 12308 12372 13002A 13617 14766 14793 15218 15326 16192 16256 16270 16399 |
| U5a1d2 | 73 263 573.XC 750 1438 2706 3027 3197 3552 4769 7028 8860 9477 11467 11719 12308 12372 13617 14766 14793 15218 15326 16192 16256 16270 16399 |
| U5a1d2a | 73 195 263 573.XC 750 1438 2706 3027 3197 3552 4769 4823 5583 7028 8860 9477 11467 11719 12308 12372 13617 14766 14793 15218 15326 16145 16189 16192 16256 16270 16399 |
| U5a1d2a1 | 73 195 263 573.XC 750 1438 2706 3027 3197 3552 4769 4823 5583 6584 6836 7028 8860 9477 11467 11719 12308 12372 13617 14766 14793 15218 15326 16145 16189 16192 16256 16270 16399 |
| U5a1d2b | 73 263 573.XC 750 1438 2706 3027 3197 3552 4769 4924C 7028 8860 9477 10858 11467 11719 12308 12372 13617 14110 14766 14793 15217 15218 15326 16192 16256 16270 16304 16399 |
| U5a1e | 73 263 750 1438 2706 3197 3564 4769 7028 8610 8860 9477 11467 11719 12308 12372 13617 14766 14793 15218 15326 16192 16256 16270 16399 |
| U5a1f | 73 263 750 1438 2706 3197 4769 6023 7028 8860 9477 11467 11719 12308 12372 13617 14766 14793 15218 15326 16192 16256 16270 16399 |
| U5a1f1 | 73 263 750 1438 2706 3197 4769 5585 6023 7028 7569 8860 9477 11467 11719 12308 12372 13617 14766 14793 15218 15326 16192 16256 16270 16311 16399 |
| U5a1f1a | 73 199 263 750 1438 2706 3197 4769 5585 6023 7028 7569 8251 8860 9477 11467 11719 12308 12372 13617 14766 14793 15218 15326 16192 16256 16270 16311 16399 |
| U5a1f1a1 | 73 199 263 750 1438 2706 3197 4769 5585 6023 7028 7569 8251 8860 9477 11467 11719 12308 12372 13617 14766 14793 15218 15326 16192 16256 16270 16311 16399 16526 |
| U5a1f2 | 73 263 750 1438 2706 3197 4769 5557 6023 7028 8860 8994 9477 11467 11719 11935 12308 12372 12491 13617 14766 14793 15218 15326 16192 16222 16256 16270 16399 |
| U5a1h | 73 150 263 750 1303 1438 2706 3192 3197 3591 4592 4769 7028 8860 9477 11296 11467 11719 11938 12308 12372 12618 13617 14766 14793 15218 15326 16192 16239 16256 16270 16399 |
| U5a1i | 73 263 750 1438 2706 3197 4769 4796 7028 8860 9477 11467 11719 12308 12372 13617 14766 14793 14893 14971 15218 15326 16192 16256 16270 16399 |

| | |
|---|---|
| U5a1i1 | 73 263 750 1438 2706 3197 4769 4796 7028 8860 9477 11467 11719 12103A 12308 12372 13617 14003 14766 14793 14893 14971 15218 15326 16192 16256 16270 16399 |
| U5a1j | 73 263 750 1438 2706 3197 3198 4745T 4769 7028 8860 8994 9477 11467 11719 12308 12372 13617 14766 14793 15218 15326 16192 16256 16270 16399 |
| U5a2 | 73 263 750 1438 2706 3197 4769 7028 8860 9477 11467 11719 12308 12372 13617 14766 14793 15326 16192 16256 16270 16526 |
| U5a2+16294 | 73 263 750 1438 2706 3197 4769 7028 8860 9477 11467 11719 12308 12372 13617 14766 14793 15326 16192 16256 16270 16294 16526 |
| U5a2a | 73 263 750 1438 2706 3197 4769 7028 8860 9477 11467 11719 12308 12372 13617 14766 14793 15326 16114A 16192 16256 16270 16294 16526 |
| U5a2a1 | 73 263 750 1438 2706 3197 4769 7028 8860 9477 11467 11719 12308 12372 13617 13827 13928C 14766 14793 15326 16114A 16192 16256 16270 16294 16526 |
| U5a2a1a | 73 263 750 1438 2706 3197 4769 7028 8860 9477 11467 11719 12308 12372 12630 13617 13827 13928C 14766 14793 15326 16114A 16192 16256 16270 16271 16294 16526 |
| U5a2a1b | 73 263 750 1438 2706 3197 4769 7028 8860 9477 11467 11719 12308 12372 13015 13617 13827 13928C 14766 14793 15326 16114A 16192 16256 16270 16294 16526 |
| U5a2a1b1 | 73 252 263 750 1438 2706 3197 4769 7028 8860 9477 11467 11719 12308 12372 13015 13617 13827 13928C 14766 14793 15326 16114A 16192 16256 16270 16294 16526 |
| U5a2a1c | 73 263 750 1438 2706 3197 4769 7028 8860 9293 9477 11467 11719 12308 12372 13617 13827 13928C 14766 14793 15326 16114A 16192 16256 16270 16294 16526 |
| U5a2a1d | 73 263 750 1438 2706 3197 4232 4655 4769 7028 8860 9477 11467 11719 11893 12308 12372 13617 13827 13928C 14766 14793 15326 16114A 16192 16256 16270 16294 16526 |
| U5a2a1+152 | 73 152 263 750 1438 2706 3197 4769 7028 8860 9477 11467 11719 12308 12372 13617 13827 13928C 14766 14793 15326 16114A 16192 16256 16270 16294 16526 |
| U5a2a1e | 73 152 263 750 1438 2706 3197 4769 7028 8860 9477 11467 11719 12308 12372 13617 13827 13928C 14198 14766 14793 15326 16114A 16192 16256 16270 16294 16526 |
| U5a2a2 | 73 263 750 1438 2706 3197 4769 7028 7853 8860 9477 11467 11719 12308 12372 12406 13617 14766 14793 15326 16114A 16256 16270 16294 16526 |
| U5a2a2a | 73 263 750 1438 2706 3197 4769 7028 7853 8860 9477 11467 11719 12308 12372 12406 13145 13617 14518 14766 14793 15326 16114A 16256 16270 16294 16526 |
| U5a2b | 73 263 750 1438 2706 3197 4769 7028 8860 9477 9548 11467 11719 12308 12372 13617 14766 14793 15326 16192 16256 16270 16526 |
| U5a2b1 | 73 263 750 960.XC 1438 2706 3197 4769 7028 8860 9477 9548 11467 11719 12308 12372 13617 14766 14793 15326 16192 16256 16270 16526 |
| U5a2b1a | 73 263 750 960.XC 1438 2706 3197 4769 7028 8860 9477 9548 11467 11719 12308 12372 12441 13617 14766 14793 15326 16192 16256 16270 16526 |

| | |
|---|---|
| U5a2b1b | 73 263 750 960.XC 1438 2706 3197 4769 7028 8860 9477 9548 11467 11719 12308 12372 13617 14766 14793 15326 16192 16256 16270 16465 16526 |
| U5a2b1c | 73 263 750 960.XC 1438 2706 3197 4769 7028 8860 9477 9548 11467 11719 12308 12372 13617 14766 14793 15326 15903 16192 16256 16270 16526 |
| U5a2b1d | 73 263 750 960.XC 1438 2706 3197 4655 4769 7028 8860 8933 9477 9548 11467 11719 12308 12372 13617 14766 14793 15326 16192 16256 16270 16526 |
| U5a2b2 | 73 263 750 1438 2706 3197 4769 7028 8860 9477 9548 10685 11467 11719 11984 12372 12654 13285 13617 14766 14793 15326 16192 16256 16270 16526 |
| U5a2b2a | 73 263 750 1438 2706 3197 4769 7028 8860 9477 9548 10685 11467 11719 11984 12372 12654 13269 13285 13617 14766 14793 15326 16192 16256 16270 16526 |
| U5a2b2a1 | 73 263 750 1438 2706 3197 4769 7028 8281-8289d 8860 9477 9548 10685 11467 11719 11984 12372 12654 13269 13285 13617 14766 14793 15326 16192 16234 16256 16270 16526 |
| U5a2b3 | 73 263 455d 750 1438 2706 3197 4769 7028 8860 9477 9548 11467 11719 12308 12372 13617 14684 14766 14793 15326 16168 16192 16256 16270 16526 |
| U5a2b3a | 73 263 455d 750 1438 2706 3197 4769 7028 8860 9477 9548 11467 11719 12308 12372 13351 13617 14684 14766 14793 15326 16168 16192 16256 16270 16526 |
| U5a2b3a1 | 73 152 263 455d 750 1438 2706 3197 3666 4769 7028 8860 9477 9548 11467 11719 12308 12372 13351 13617 14684 14766 14793 15326 16168 16192 16256 16270 16526 |
| U5a2b4 | 73 263 750 1438 2706 3197 4769 7028 8860 9477 9548 11467 11719 12308 12372 13617 14766 14793 15301 15326 16192 16256 16270 16526 |
| U5a2b4a | 73 263 750 1438 2706 3197 4769 7028 8860 9477 9548 11467 11719 12308 12372 13617 13759 14766 14793 15301 15326 16192 16256 16270 16526 |
| U5a2b5 | 73 207 263 750 1438 2706 3197 3504 4769 7028 7471 8860 9477 9548 11467 11719 12308 12372 13617 14766 14793 15326 16192 16256 16270 16526 |
| U5a2c | 73 263 750 1438 2706 3197 4769 7028 8860 9477 10619 11467 11719 12308 12372 13617 14766 14793 15326 16192 16256 16270 16526 |
| U5a2c1 | 73 263 750 1438 2706 3197 4769 7028 8860 9469 9477 10619 11467 11719 12308 12372 13617 14766 14793 15326 16192 16256 16270 16526 |
| U5a2c2 | 73 263 750 1438 2706 3197 4769 7028 8860 9477 10619 10768 11467 11719 12308 12372 13617 14766 14793 15326 16192 16256 16270 16526 |
| U5a2c3 | 73 263 750 1438 2706 3197 4769 7028 8860 9477 10619 11465 11467 11719 12308 12372 13617 14766 14793 15326 16256 16270 16526 |
| U5a2c3a | 73 263 750 1438 2706 3197 4769 7028 7960C 8860 9477 10619 10709C 11465 11467 11719 12308 12372 13617 14766 14793 15326 16256 16270 16526 |
| U5a2c4 | 73 263 750 1438 2706 3197 3531 4769 7028 8860 9477 10619 11467 11719 12308 12372 13617 14766 14793 15326 16192 16256 16270 16526 |

| | |
|---|---|
| U5a2d | 73 263 750 1438 2706 3197 4769 7028 7843 8860 9477 11467 11719 12308 12372 13617 14766 14793 15326 16192 16256 16270 16526 |
| U5a2d1 | 73 263 750 1438 2706 3197 3750 4769 7028 7843 7978 8104 8860 9477 11107 11467 11719 12308 12372 13617 14766 14793 15326 16256 16270 16526 |
| U5a2d1a | 73 263 750 1438 2706 3197 3750 4769 7028 7843 7978 8104 8860 9148 9477 11107 11467 11719 12308 12372 13617 14577G 14766 14793 15326 16256 16270 16526 |
| U5a2+16362 | 73 263 750 1438 2706 3197 4769 7028 8860 9477 11467 11719 12308 12372 13617 14766 14793 15326 16192 16256 16270 16362 16526 |
| U5a2e | 73 151 152 263 750 1438 2706 3197 3768 4769 7028 8860 9477 11467 11719 12308 12372 13617 14766 14793 15289 15326 16192 16256 16270 16311 16362 16526 |
| U5b | 73 150 263 750 1438 2706 3197 4769 7028 7768 8860 9477 11467 11719 12308 12372 13617 14182 14766 15326 16192 16270 |
| U5b1 | 73 150 263 750 1438 2706 3197 4769 5656 7028 7768 8860 9477 11467 11719 12308 12372 13617 14182 14766 15326 16192 16270 |
| U5b1a | 73 150 263 750 1438 2706 3197 4769 5656 7768 8860 9477 11467 11719 12308 12372 13617 14182 14766 15097 15326 16192 16270 |
| U5b1+16189 | 73 150 263 750 1438 2706 3197 4769 5656 7028 7768 8860 9477 11467 11719 12308 12372 13617 14182 14766 15326 16189 16192 16270 |
| U5b1b | 73 150 263 750 1438 2706 3197 4769 5656 7028 7768 8860 9477 11467 11719 12308 12372 12618 13617 14182 14766 15326 16189 16192 16270 |
| U5b1b1 | 73 150 263 750 1438 2706 3197 4769 5656 7028 7385 7768 8860 9477 10927 11467 11719 12308 12372 12618 13617 14182 14766 15326 16189 16192 16270 |
| U5b1b1+@16192 | 73 150 263 750 1438 2706 3197 4769 5656 7028 7385 7768 8860 9477 10927 11467 11719 12308 12372 12618 13617 14182 14766 15326 16189 16270 |
| U5b1b1a | 73 150 263 750 1438 2706 3197 4769 5656 7028 7385 7768 8860 9477 10927 11467 11719 12308 12372 12618 13617 14182 14766 15326 16144 16189 16270 |
| U5b1b1a1 | 73 150 263 750 1438 2706 3197 4059 4769 5656 7028 7385 7768 8860 9477 10927 11467 11719 12308 12372 12618 13617 14182 14766 15326 16144 16189 16270 |
| U5b1b1a1a | 73 150 263 750 1341 1438 2706 3197 4059 4769 5656 7028 7385 7768 8860 9477 10927 11467 11719 12308 12372 12618 13617 14182 14766 15326 16144 16189 16270 |
| U5b1b1a1a1 | 73 150 263 750 1341 1438 2706 3197 4059 4769 5656 7028 7385 7768 8860 9477 10927 11467 11719 12308 12372 12618 13617 14182 14766 15326 16144 16189 16270 16301 |
| U5b1b1a1b | 73 150 263 750 1438 2706 3197 4059 4769 5656 7028 7385 7768 8428A 8860 9477 10927 11467 11719 12308 12372 12618 13617 14182 14766 15326 16144 16189 16270 |
| U5b1b1a2 | 73 150 263 750 1438 2706 3197 4769 5656 7028 7385 7768 8860 9477 10907 10927 11467 11719 12308 12372 12618 13617 14182 14766 15326 16144 16189 16270 |

| | |
|---|---|
| U5b1b1a3 | 73 150 263 750 1438 1850 2706 3197 4769 5656 7028 7385 7768 8860 9477 10927 11467 11719 12308 12372 12618 13617 14182 14766 15326 16144 16148 16189 16270 |
| U5b1b1d | 73 150 263 750 1438 2706 3197 4769 5656 7028 7385 7768 8860 9477 10927 11467 11719 12308 12372 12618 13617 14182 14766 15326 15884 16189 16270 |
| U5b1b1f | 73 150 263 750 1047 1438 2706 3197 4769 5656 7028 7385 7768 8860 9477 10927 11467 11719 12308 12372 12618 13617 14182 14766 15326 16189 16270 |
| U5b1b1b | 73 150 263 750 1438 2706 3197 4769 5656 7028 7385 7768 8413 8860 9477 10927 11467 11719 12308 12372 12618 13617 14182 14766 15326 16189 16192 16270 16320 |
| U5b1b1+152 | 73 150 152 263 750 1438 2706 3197 4769 5656 7028 7385 7768 8860 9477 10927 11467 11719 12308 12372 12618 13617 14182 14766 15326 16189 16192 16270 |
| U5b1b1e | 73 150 152 263 750 1438 2387 2706 3197 4769 5656 7028 7385 7768 8860 9477 10927 11467 11719 12308 12372 12618 13617 14182 14766 15326 16189 16192 16270 |
| U5b1b1g | 73 150 263 750 1438 1822 2706 3197 4769 5656 5788 7028 7385 7768 8860 9477 10301 10927 11467 11719 12308 12372 12618 13617 14182 14470 14766 15326 16189 16192 16270 |
| U5b1b1g1 | 73 150 263 750 1438 1822 2706 3197 4769 5656 5788 7028 7385 7768 8860 9477 10301 10927 11467 11719 12308 12372 12618 13617 14182 14470 14766 15326 15533 16074 16189 16192 16270 |
| U5b1b1g1a | 73 150 242 263 750 1438 1822 2706 3197 4769 5656 5788 7028 7385 7768 8860 9477 10301 10927 11467 11719 12308 12372 12618 13617 14182 14470 14766 15326 15533 16074 16189 16192 16270 |
| U5b1b2 | 73 150 217 263 (573.XC) 750 1438 2706 3197 4769 5656 7028 7768 8860 9477 11467 11719 12308 12372 12618 13617 14182 14766 15326 16189 16192 16270 |
| U5b1b2a | 73 150 217 263 (573.XC) 750 1438 2706 3197 3434 4769 5656 7028 7768 8860 9477 11467 11719 12308 12372 12618 13617 14182 14766 15326 16189 16192 16270 |
| U5b1b2b | 73 150 217 263 (573.XC) 750 1438 2706 3197 4769 5656 7028 7768 8230 8860 9477 11467 11719 12308 12372 12618 13617 14182 14766 15326 16189 16192 16270 |
| U5b1c | 73 150 263 750 1438 2706 3197 4769 5656 7028 7768 8860 9477 11467 11719 12308 12372 13617 14182 14766 15191 15326 16189 16192 16270 16311 |
| U5b1c1 | 73 150 263 750 1438 2706 3197 4769 5656 7028 7768 8860 9477 11467 11719 12308 12372 13617 14182 14766 15191 15326 16189 16192 16270 16311 16336 |
| U5b1c1a | 55A 73 150 263 750 1438 2706 3197 4769 5656 7028 7768 8860 9477 11467 11719 12308 12372 13617 14182 14766 15191 15326 16189 16192 16270 16311 16336 |
| U5b1c1a1 | 55A 73 150 263 750 1438 2706 3197 4769 5656 7028 7768 8860 9477 11467 11719 12308 12372 13617 14182 14420 14470 14766 15191 15326 16189 16192 16270 16311 16336 |

| | |
|---|---|
| U5b1c2 | 73 150 263 516 750 1438 2706 3197 4769 5656 7028 7768 8860 9477 11467 11719 12308 12372 13617 14182 14766 15191 15326 16174 16189 16192 16270 16311 |
| U5b1c2a | 73 150 263 516 750 1438 2706 3197 4769 5656 7028 7768 8860 9477 11467 11719 12308 12372 13617 14182 14766 15191 15326 16140 16174 16189 16270 16288 16311 |
| U5b1c2b | 73 150 263 516 750 1438 2706 3197 4769 5656 6341 7028 7768 8860 9477 11467 11719 12308 12372 13194 13617 14182 14766 15191 15326 16174 16189 16192 16270 16311 |
| U5b1+16189+@16192 | 73 150 263 750 1438 2706 3197 4769 5656 7028 7768 8860 9477 11467 11719 12308 12372 13617 14182 14766 15326 16189 16270 |
| U5b1e | 73 150 152 263 750 1438 2706 3197 4769 5656 7028 7768 8860 9477 11467 11719 12308 12372 13617 14182 14766 15326 16189 16270 16465 |
| U5b1e1 | 73 150 152 263 750 1438 2706 2757 3197 4769 5656 7028 7768 8860 9477 10283 11467 11719 12308 12372 12616 13617 14182 14766 15326 16189 16270 16465 |
| U5b1e1a | 73 150 152 263 750 1438 2706 2757 3197 4209 4769 5656 7028 7768 8860 9477 10283 11467 11719 12308 12372 12616 13617 14182 14766 15326 16189 16270 16465 |
| U5b1h | 73 150 263 384 750 1438 2706 3197 4769 5656 7028 7768 8860 9477 11467 11719 12308 12372 13617 14182 14766 15326 16189 16270 |
| U5b1d | 73 150 263 750 1438 2706 3197 4769 5437 5656 7028 7768 8860 9477 11467 11719 12308 12372 13617 14182 14766 15326 16192 16270 |
| U5b1d1 | 73 150 263 750 1438 2706 3197 4769 5437 5656 7028 7768 8860 9477 11467 11719 12308 12372 13617 14182 14766 15326 15721 16192 16270 |
| U5b1d1a | 73 150 263 742 750 1438 2706 3197 4769 5437 5656 7028 7768 8860 9477 11467 11719 12308 12372 13617 14182 14766 15326 15355 15721 16270 |
| U5b1d1b | 73 150 199 263 750 1438 2225A 2706 3197 3507 4769 5437 5656 7028 7768 8277 8860 9477 10084 11467 11719 12136 12308 12372 13617 14182 14766 15326 15721 16192 16270 |
| U5b1d1c | 73 150 263 750 1438 2706 3197 4769 5437 5656 7028 7768 7912 8860 9477 11467 11719 12308 12372 13617 14182 14766 15326 15631 15721 16192 16218 16270 16320 |
| U5b1d2 | 73 150 263 750 1438 2706 3197 4769 5437 5656 7028 7085 7768 8860 9477 11467 11719 12308 12372 13617 14182 14766 15326 16192 16239 |
| U5b1f | 73 150 263 750 1438 2706 3197 3507A 4769 5656 7028 7768 8860 9477 11467 11719 12308 12372 13617 14182 14766 15326 16192 16270 |
| U5b1f1 | 73 150 263 533 750 1438 2706 3197 3507A 4769 5656 7028 7768 8860 9477 11467 11719 12308 12372 13617 14182 14766 15326 16192 16270 |
| U5b1f1a | 73 150 263 533 750 1438 2706 3197 3507A 4769 5656 7028 7768 8860 9477 11467 11719 12308 12372 13617 14182 14766 15326 16192 16270 |
| U5b1g | 73 150 151 228 263 573.XC 750 1438 2706 3197 4769 5656 7028 7768 8860 9477 10654 11467 11719 12308 12372 13617 13759 14182 14577 14766 15326 16192 16270 |
| U5b1i | 73 150 263 750 1438 2706 3105 3197 3498 4769 5656 6674 7028 7768 8860 9477 11467 11719 12308 12372 13617 14182 14766 15326 15777 16167 16192 16270 16311 16356 |

| | |
|---|---|
| U5b2 | 73 150 263 750 1438 1721 2706 3197 4769 7028 7768 8860 9477 11467 11719 12308 12372 13617 13637 14182 14766 15326 16192 16270 |
| U5b2a | 73 150 263 750 1438 1721 2706 3197 4732 4769 7028 7768 8860 9477 11467 11719 12308 12372 13617 13637 14182 14766 15326 16189 16192 16270 |
| U5b2a1 | 73 150 263 750 1438 1721 2706 3197 4732 4769 7028 7768 8860 9477 11467 11719 12308 12372 13617 13637 14182 14766 15326 16189 16192 |
| U5b2a1a | 73 150 263 750 1438 1721 2706 3197 4732 4769 7028 7768 8860 9477 11467 11719 12308 12372 13617 13637 14182 14766 15326 15511 16192 |
| U5b2a1a+16311 | 73 150 263 750 1438 1721 2706 3197 4732 4769 7028 7768 8860 9477 11467 11719 12308 12372 13617 13637 14182 14766 15326 15511 16192 16311 |
| U5b2a1a1 | 73 150 263 750 896 1438 1721 2706 3197 4732 4769 7028 7768 8860 9477 11467 11719 12308 12372 13617 13637 14182 14766 15326 15511 16192 16311 |
| U5b2a1a1a | 73 150 263 750 896 1438 1721 2706 3197 4732 4769 7028 7768 8860 9477 11467 11719 12308 12372 12406 13617 13637 14182 14766 15326 15511 16192 16311 |
| U5b2a1a1b | 73 150 249d 263 750 896 1438 1721 2706 3197 4732 4769 7028 7768 8860 9477 11467 11719 12308 12372 13617 13637 14182 14766 15326 15511 16192 16311 |
| U5b2a1a1d | 73 150 263 750 896 1438 1721 2706 3197 4732 4769 7028 7768 8860 9477 11329 11467 11719 12308 12372 13617 13637 14182 14766 15326 15511 16192 16311 |
| U5b2a1a2 | 73 150 263 750 1438 1721 1834 2706 3197 4732 4769 5452 7028 7768 8705 8860 9477 11467 11719 12308 12372 13617 13637 14182 14766 15326 15511 15924 |
| U5b2a1b | 73 150 152 263 750 1438 1721 2706 3197 4732 4769 5918 7028 7768 8860 9477 11467 11719 12308 12372 13617 13637 14182 14323 14766 15326 16189 16325 |
| U5b2a2 | 73 150 263 750 1438 1721 2706 3197 3212 4732 4769 7028 7768 8860 9477 11467 11719 12308 12372 13617 13637 14182 14766 15326 16189 16192 16270 16398 |
| U5b2a2a | 73 150 263 750 1438 1721 2706 3197 3212 4732 4769 7028 7768 8860 9477 11467 11719 12136 12308 12372 13617 13637 14182 14766 15326 16189 16192 16270 16398 |
| U5b2a2a1 | 73 150 263 750 1438 1721 2706 3197 3212 4732 4769 7028 7768 8860 9477 9682 11467 11719 12136 12308 12372 13617 13637 14182 14766 15326 16189 16192 16270 16398 |
| U5b2a2a2 | 73 150 263 750 1438 1721 2706 3197 3212 4732 4769 5508 7028 7768 8860 9477 10589 11467 11719 12136 12308 12372 13617 13637 14182 14766 15326 16189 16192 16270 16398 |
| U5b2a2b | 73 150 263 750 1438 1721 2706 2757 3197 3212 4732 4769 7028 7768 8860 9477 11467 11719 12308 12372 13617 13637 14182 14766 14956 15326 16189 16192 16270 16398 |
| U5b2a2b1 | 73 150 263 750 1438 1721 2706 2757 3197 3212 4732 4769 4843 7028 7768 8860 9477 11467 11719 12308 12372 13617 13637 14182 14766 14956 15326 16189 16192 16270 16398 |

| | |
|---|---|
| U5b2a2c | 73 150 263 750 1438 1721 2706 3197 3212 4732 4769 7028 7768 8860 9477 11467 11719 12308 12372 12678 13617 13637 14182 14766 15326 16189 16261 16270 16304 16398 |
| U5b2a3 | 73 150 263 750 1438 1721 2706 3197 4732 4769 7028 7768 8860 9477 10031 11467 11719 12308 12372 13617 13637 14182 14766 15326 16189 16192 16270 |
| U5b2a3a | 73 150 263 750 1438 1721 2706 3197 4732 4769 6899 7028 7768 8860 9477 10031 11467 11719 12308 12372 13617 13637 14182 14766 15326 16189 16192 16270 |
| U5b2a+@16192 | 73 150 263 750 1438 1721 2706 3197 4732 4769 7028 7768 8860 9477 11467 11719 12308 12372 13617 13637 14182 14766 15326 16189 16270 |
| U5b2a4 | 73 150 263 750 1438 1721 2706 3197 3397 4732 4769 7028 7768 8860 9477 11467 11719 12308 12372 13368 13617 13637 14182 14766 14767 15326 16189 16270 |
| U5b2a4a | 73 150 263 750 1438 1721 2706 3197 3397 4732 4769 6293 7028 7768 8860 9477 11467 11719 12308 12372 13368 13617 13637 14182 14766 14767 15326 16189 16270 |
| U5b2a5 | 73 150 263 750 1438 1721 2706 3197 4732 4769 7028 7768 8706 8860 9477 10654 11467 11719 11725 12308 12372 13617 13637 14182 14766 15326 16189 16270 16311 |
| U5b2a5a | 73 150 263 750 1438 1721 2706 3197 3394 4732 4769 7028 7768 8706 8860 9477 10654 11467 11719 11725 12308 12372 13617 13637 14182 14766 15326 16189 16270 16311 |
| U5b2a6 | 73 150 263 750 1438 1721 2706 3197 4129 4732 4769 7028 7768 8860 9477 11467 11719 12308 12372 13617 13637 14182 14766 15326 16189 |
| U5b2b | 73 150 263 750 1438 1721 2706 3197 4769 7028 7768 8860 9477 11467 11653 11719 12308 12372 12634 13617 13630 13637 14182 14766 15326 16270 |
| U5b2b1 | 73 150 263 750 1438 1721 2706 3197 4769 7028 7768 8860 9477 11467 11653 11719 12308 12372 12634 13617 13630 13637 14182 14766 15326 15497 16270 |
| U5b2b1a | 73 150 263 750 1438 1721 2706 3197 3861 4769 7028 7768 8860 9477 11467 11653 11719 12308 12372 12634 13617 13630 13637 14182 14766 15326 15497 16270 16362 |
| U5b2b1a1 | 73 150 263 750 1438 1721 2706 3197 3861 4769 7028 7768 8860 9477 11467 11653 11719 12308 12372 12634 13617 13630 13637 14182 14766 15326 15497 16270 16292 16362 |
| U5b2b1a2 | 73 150 263 750 1438 1721 2706 3197 3861 4769 7028 7768 8860 9477 11467 11653 11719 12308 12372 12634 12696 13617 13630 13637 14182 14766 15326 15497 16240C 16270 16362 |
| U5b2b1b | 73 150 263 750 1438 1721 2706 3197 4769 7028 7768 8860 9477 11467 11653 11719 12308 12372 12634 13420 13617 13630 13637 14182 14766 15326 15497 16270 16526 |
| U5b2b2 | 73 150 263 750 1438 1721 2706 3197 4616 4769 7028 7768 8027 8860 9477 11467 11653 11719 12308 12372 12634 13617 13630 13637 14182 14766 15326 16270 |

| | |
|---|---|
| U5b2b3 | 73 150 263 517T 750 1438 1721 2706 2755 3197 4769 7028 7768 8860 9477 11467 11653 11719 12308 12372 12634 13617 13630 13637 14182 14766 15326 15905 16224 16270 |
| U5b2b3a | 73 150 263 279 517T 750 1438 1721 2706 2755 3197 3338 4769 5261 7028 7768 8860 9477 11467 11653 11719 12308 12372 12634 13617 13630 13637 14182 14766 15326 15905 16224 16270 |
| U5b2b3a1 | 73 150 263 279 517T 750 1438 1721 2706 2755 3197 3338 4769 5261 7028 7768 8860 9477 9494 11467 11653 11719 12308 12372 12634 13617 13630 13637 14182 14766 15326 15905 16224 16270 |
| U5b2b3a1a | 73 150 263 279 517T 750 1438 1721 2706 2755 3197 3338 4769 5261 7028 7768 8860 9477 9494 11467 11653 11719 12308 12372 12634 13617 13630 13637 14182 14766 15326 15905 16114 16224 16270 |
| U5b2b3b | 73 150 263 517T 750 1438 1721 2706 2755 3197 4769 7028 7768 8860 9477 11467 11653 11719 12308 12372 12634 13617 13630 13637 14182 14766 15326 15905 16224 16242 16270 |
| U5b2b4 | 73 150 263 750 1438 1721 2706 3197 4769 7028 7768 8860 9477 9670 11467 11653 11719 12308 12372 12634 13617 13630 13637 14182 14766 15326 16189 16270 |
| U5b2b4a | 73 150 185 263 750 1438 1721 2706 3197 4769 7028 7768 8860 9477 9670 11467 11653 11719 12308 12372 12634 13617 13630 13637 14182 14766 15326 16189 16270 |
| U5b2b5 | 73 150 263 750 1438 1721 2706 3197 4769 7028 7768 8860 9477 11467 11653 11719 12308 12372 12634 13161 13617 13630 13637 14182 14766 15326 16270 16296 |
| U5b2c | 73 150 263 723 750 960.XC 1438 1721 2706 3197 4769 7028 7768 8860 9477 11467 11719 12308 12372 13017 13617 13637 14182 14766 15326 16192 16270 |
| U5b2c1 | 73 150 263 723 750 960.XC 1438 1721 2706 3197 4769 6920A 7028 7768 8860 9477 11467 11719 12308 12372 13017 13434 13617 13637 14182 14766 15326 16192 16270 |
| U5b2c2 | 73 150 263 723 750 960.XC 1438 1721 2706 3197 3861 4769 5836 7028 7768 8860 9477 10262 11467 11719 12308 12372 13017 13617 13637 14182 14766 15326 16192 16270 |
| U5b2c2a | 73 150 263 723 750 960.XC 1438 1721 2706 3197 3861 4769 5836 7028 7768 8860 9477 10262 11467 11719 12308 12372 13017 13617 13637 14182 14259 14766 15326 16192 16270 |
| U5b2c2b | 73 150 263 723 750 960.XC 1438 1721 2706 3197 3861 4769 5836 7028 7768 8860 9477 10262 11467 11719 12308 12372 13017 13617 13637 14182 14766 15326 16192 16249 16270 |
| U5b3 | 73 150 (228) 263 750 1438 2706 3197 4769 7028 7226 7768 8860 9477 11467 11719 12308 12372 13617 14182 14766 15326 16192 16270 16304 |
| U5b3a | 73 150 (228) 263 750 1438 2706 3197 4769 7028 7226 7768 8860 9477 11467 11719 12308 12372 13617 14182 14766 15326 16192 16235 16270 16304 |
| U5b3a1 | 73 150 (228) 263 750 1438 2706 3197 4769 7028 7226 7768 8860 9477 11467 11719 12308 12372 13617 14182 14766 15326 16169A 16192 16235 16270 16304 |

| | |
|---|---|
| U5b3a1a | 73 150 (228) 263 373 750 1438 2706 3197 4769 7028 7226 7768 8860 9477 11177 11467 11719 12308 12372 13617 14182 14766 15326 16169A 16192 16235 16270 |
| U5b3a1b | 73 150 (228) 263 750 1438 2706 3197 4769 7028 7226 7768 8860 9477 11467 11719 12308 12372 13617 14182 14766 15326 15781 16169A 16192 16235 16270 16304 |
| U5b3a2 | 73 150 (228) 263 750 1438 2706 3197 4769 7028 7226 7768 8860 9477 10978 11467 11719 12308 12372 13617 14182 14766 15326 16192 16235 16270 16304 |
| U5b3b | 73 150 (228) 263 750 1438 2706 3197 4769 7028 7226 7768 8860 9196 9477 11467 11719 12308 12372 13617 14182 14766 15326 16192 16270 |
| U5b3b1 | 73 150 189 (228) 263 750 1438 2706 3197 4769 7028 7226 7768 8860 9196 9477 11467 11719 12308 12372 13617 14182 14766 15326 16192 16270 16304 |
| U5b3b2 | 73 150 (228) 263 750 1438 2706 3197 4769 7028 7226 7768 8860 9196 9477 11467 11719 12308 12372 13617 14182 14766 15326 16192 16270 16304 16526 |
| U5b3c | 73 150 (228) 263 750 1438 2706 3197 4769 4775 5557 6461 7028 7226 7768 8860 9477 11467 11719 12308 12372 13617 14182 14766 15326 16192 16270 16304 |
| U5b3d | 73 150 (228) 263 750 1438 2706 3197 4769 7028 7226 7768 8860 9477 11467 11719 12308 12372 13617 13830 14182 14766 15326 16067 16192 16270 16304 16311 |
| U5b3e | 73 150 (228) 263 750 1438 2706 3197 3535A 4769 7028 7226 7768 8701 8860 9477 11467 11719 12308 12372 13617 14182 14766 15326 16192 16270 16304 |
| U5b3f | 73 150 (228) 263 750 1438 2706 3197 4769 7028 7226 7768 8860 9477 11467 11719 12308 12372 13617 14182 14766 15326 16129 16192 16270 16304 |
| U5b3g | 73 150 (228) 263 750 1438 2706 3197 4769 5147 7028 7226 7768 8860 9477 11467 11719 12308 12372 13617 14182 14766 15326 16192 16270 |
| U5b3h | 73 150 (228) 263 750 1438 2706 3197 4769 6527 7028 7226 7768 8860 9477 11467 11719 12308 12372 13617 14182 14766 15326 16270 16304 |
| U6 | 73 263 750 1438 2706 3348 4769 7028 8860 11467 11719 12308 12372 14766 15326 16172 |
| U6a'b'd | 73 263 750 1438 2706 3348 4769 7028 8860 11467 11719 12308 12372 14766 15326 16172 16219 |
| U6a | 73 263 750 1438 2706 3348 4769 7028 7805 8860 11467 11719 12308 12372 14179 14766 15326 16172 16219 16278 |
| U6a1 | 73 263 750 1438 2706 3348 4769 7028 7805 8860 11467 11719 12308 12372 14179 14766 14927 15326 16172 16219 16278 |
| U6a1a | 73 263 750 1438 2706 3348 3969 4769 7028 7805 8860 11467 11719 12308 12372 14179 14766 14927 15326 16172 16189 16219 16278 |
| U6a1a1 | 73 263 750 1438 2706 3348 3969 4172A 4769 7028 7805 8860 11467 11719 11938 12308 12372 14179 14766 14927 15326 16172 16189 16219 16239 16278 |
| U6a1a2 | 73 263 750 1438 1472 2706 3348 3969 4769 7028 7805 8860 11467 11719 12308 12372 13759 14179 14766 14927 15326 16172 16189 16219 16278 |

| | |
|---|---|
| U6a1b | 73 263 750 1438 2706 3348 4769 7028 7805 8860 11467 11719 12308 12372 14179 14766 14927 15326 16172 16219 16235 16278 |
| U6a1b1 | 73 263 750 1438 2706 3348 4769 7028 7805 8860 11176 11467 11719 12308 12372 12376G 12727 14179 14766 14927 15244 15326 16172 16219 16235 16278 |
| U6a1b1a | 73 146 263 750 1438 2706 3348 4769 7028 7805 8860 11176 11467 11719 12308 12372 12376G 12727 14179 14766 14927 15244 15326 16172 16219 16235 16278 16355 |
| U6a1b1b | 73 263 750 1438 2158 2706 3348 4769 7028 7805 8860 10336 11176 11467 11719 12308 12372 12376G 12727 14034 14179 14766 14927 15244 15326 16145 16172 16219 16235 16278 |
| U6a1b2 | 73 195 263 750 1438 2706 3348 4769 7028 7805 8860 10364 11467 11719 12308 12372 14179 14562 14766 14927 15326 16172 16219 16235 16278 |
| U6a1b3 | 73 263 750 1438 2706 3348 4769 7028 7805 8860 11467 11719 11971 12308 12372 14179 14766 14927 15326 16172 16219 16235 16278 |
| U6a1b4 | 73 263 750 1438 2706 3348 4769 7028 7805 8860 8865 9377 9896 10784 11467 11719 12022 12308 12372 13834 14179 14766 14927 15067 15326 16086 16172 16219 16235 16278 |
| U6a+16189 | 73 263 750 1438 2706 3348 4769 7028 7805 8860 11467 11719 12308 12372 14179 14766 15326 16172 16189 16219 16278 |
| U6a+16189+(103) | 73 (103) 263 750 1438 2706 3348 4769 7028 7805 8860 11467 11719 12308 12372 14179 14766 15326 16172 16189 16219 16278 |
| U6a2 | 73 (103) 263 750 1438 2706 3348 4769 7028 7805 8860 11467 11719 12308 12372 14179 14766 15326 15626 16172 16189 16219 16278 |
| U6a2a | 73 (103) 263 750 960d 1438 2706 3348 4769 6359 7028 7805 8860 11204 11467 11719 12308 12372 14179 14766 15326 15626 16172 16189 16219 16278 |
| U6a2a1 | 73 (103) 263 750 960d 1438 2706 3348 4769 6359 7028 7805 8860 9128 9891 11204 11467 11719 12308 12372 14179 14766 15326 15626 16172 16189 16219 16278 |
| U6a2a2 | 73 (103) 146 263 750 960d 1438 2706 3348 4769 6359 7028 7805 8860 11204 11467 11719 12308 12372 13359 14179 14766 15326 15626 16172 16189 16219 16278 |
| U6a2a2a | 73 (103) 146 263 337 750 960d 1438 2706 3348 4769 6359 7028 7805 8860 11204 11467 11719 12308 12372 13359 14179 14766 15326 15626 16172 16189 16219 16235 16278 |
| U6a2b | 73 (103) 263 750 1438 2706 3348 4769 7028 7805 8860 11467 11719 12308 12372 14179 14766 15326 15383 15626 16172 16189 16219 16278 |
| U6a2b1 | 73 (103) 263 750 1438 2706 3348 4769 7028 7805 8860 11467 11719 12308 12372 14179 14766 15314 15326 15383 15626 16172 16184 16189 16219 16278 16354 |
| U6a2+195 | 73 (103) 195 263 750 1438 2706 3348 4769 7028 7805 8860 11467 11719 12308 12372 14179 14766 15326 15626 16172 16189 16219 16278 |
| U6a2c | 73 (103) 195 263 750 1438 1709 2706 3348 4769 7028 7805 8860 11467 11719 12308 12372 14179 14766 15326 15626 16172 16189 16219 16278 |
| U6a8 | 73 (103) 263 750 1438 2706 3348 4769 7028 7805 8860 10172 11467 11719 12308 12372 14179 14766 15326 16172 16189 16219 16278 |

| | |
|---|---|
| U6a8a | 73 (103) 143 263 1438 2706 3348 4769 7028 7805 8282 8860 10172 11467 11539 11719 12308 12372 14179 14766 15326 16172 16189 16219 16278 |
| U6a8b | 73 (103) 263 750 1438 2706 3348 4769 4936 5894C 7028 7805 8860 9100 9128 9335A 10172 11467 11719 12308 12372 14179 14766 15326 16172 16189 16219 16278 16295 |
| U6a3 | 73 263 750 1438 2706 3348 4769 7028 7805 8860 11467 11719 12308 12372 14179 14766 15326 15790 16172 16189 16219 16278 |
| U6a3a | 73 263 750 1438 2706 3348 4769 4820 7028 7805 8860 11467 11719 12308 12372 14179 14766 15326 15790 16172 16189 16219 16278 |
| U6a3a1 | 73 263 750 1438 2706 3348 4769 4820 7028 7805 8860 11467 11719 12308 12372 14179 14364 14766 15326 15790 16172 16189 16219 16278 |
| U6a3a1a | 73 263 750 1438 2706 3348 4769 4820 7028 7805 8598 8860 11467 11719 12308 12372 14179 14364 14766 15326 15790 16172 16189 16219 16278 |
| U6a3a2 | 73 185 263 750 1438 2706 3348 4769 4820 7028 7805 8860 11467 11719 12308 12372 14179 14766 15326 15790 16093 16172 16189 16219 16278 |
| U6a3a2a | 73 185 263 709 750 1438 2706 3348 3847 4769 4820 7028 7805 8860 11467 11719 12308 12372 14179 14766 15326 15790 16093 16172 16189 16219 16278 |
| U6a3+185 | 73 185 263 750 1438 2706 3348 4769 7028 7805 8860 11467 11719 12308 12372 14179 14766 15326 15790 16172 16189 16219 16278 |
| U6a3b | 73 146 152 185 188 263 750 1211 1438 2706 3348 4769 7028 7805 8860 11268 11467 11719 12308 12372 13431 14179 14766 15326 15634 15790 16172 16189 16219 16278 |
| U6a3b1 | 73 146 152 185 188 263 750 1211 1438 2706 3348 4769 7028 7805 8860 11268 11467 11719 12308 12372 13431 14179 14766 15326 15634 15790 16172 16189 16219 16278 16311 |
| U6a3e | 73 185 263 750 1438 2706 3337 3348 4021 4769 7028 7805 8705 8860 11467 11719 12097 12308 12372 13569 13928 14179 14766 15326 15790 16172 16189 16219 16278 16362 16399 |
| U6a3f | 73 150 185 263 750 1438 2706 3348 4769 7028 7805 8860 11467 11719 12308 12372 14179 14766 15326 15790 16172 16189 16219 16278 |
| U6a3f1 | 73 150 185 263 750 1438 2706 3348 3826 4769 7028 7805 8860 11467 11719 12308 12372 14179 14766 15326 15790 16172 16189 16219 16278 |
| U6a3f2 | 73 150 185 263 750 1438 2706 3348 4769 7028 7805 8860 11467 11719 12308 12372 14179 14766 15326 15790 15941 16172 16189 16219 16278 |
| U6a3c | 73 146 263 291.1A 750 960d 1438 1809 2706 3348 4769 5554A 6182 7028 7805 8860 11272 11467 11719 12308 12372 14179 14766 15326 15380 15790 16172 16189 16219 16278 |
| U6a3d | 73 263 750 1438 2706 3348 4769 7028 7805 8860 11467 11719 12308 12372 14179 14766 15326 15790 16147 16172 16189 16219 16278 |
| U6a3d1 | 73 263 750 1438 2706 3348 4769 7028 7805 8860 11467 11719 12308 12372 13635 14179 14766 15326 15790 16147 16172 16189 16219 16278 |
| U6a3d1a | 73 263 385 750 1438 2706 3348 4674 4769 7028 7805 8860 10203 10927 11467 11719 12308 12372 13635 14179 14766 15067 15326 15790 16147 16172 16189 16219 16278 |
| U6a4 | 73 150 263 750 1438 2706 3348 4769 7028 7805 8860 11467 11719 12246 12308 12372 13674 14179 14215 14766 15326 15862 16134 16172 16219 16278 |

| | |
|---|---|
| U6a5 | 73 263 750 1438 2706 3348 3591 4769 7028 7642 7805 8860 11467 11719 12308 12372 13590 14179 14766 15326 15927 16172 16219 16278 |
| U6a5a | 73 263 750 1438 2706 3348 3591 4769 7028 7642 7805 8860 11467 11590 11719 12308 12372 13590 14179 14766 15326 15927 16172 16219 16278 |
| U6a5a1 | 73 263 750 1438 2706 3348 3591 4769 7028 7642 7805 8860 11191 11467 11590 11719 12308 12372 13590 14179 14766 15326 15927 16172 16219 16278 |
| U6a5b | 73 263 750 1438 2706 3348 3591 3714 4769 7028 7642 7805 8860 11467 11719 12308 12372 13590 14179 14766 15326 15927 16172 16184 16219 16234 16278 |
| U6a5c | 73 263 750 1438 2706 3348 3591 4769 6407 7028 7642 7805 8860 10398 11467 11719 12308 12372 13590 14179 14766 15326 15927 16172 16189 16219 16278 |
| U6a6 | 73 263 750 1438 2706 3348 4769 7028 7805 8557C 8860 11467 11719 12308 12372 14179 14766 15326 16172 16219 16278 |
| U6a6a | 73 263 750 1438 2706 3348 3714 4769 7028 7805 8557C 8860 11467 11719 12172 12308 12372 14179 14766 15326 16079 16172 16189 16219 16274 16278 |
| U6a6a1 | 73 263 750 1438 2706 3348 3714 4769 7028 7805 8557C 8860 9031 11467 11719 12172 12308 12372 14179 14766 15326 16079 16172 16189 16219 16274 16278 |
| U6a6b | 73 263 750 1438 2706 3348 4769 7028 7805 8407A 8557C 8860 (10685) 11467 11719 12308 12372 13440 14179 14766 15326 16172 16219 16278 |
| U6a6b1 | 73 263 750 1438 2706 3348 4769 7028 7391 7805 8407A 8557C 8860 (10685) 11467 11719 12308 12372 13440 14179 14766 15326 16172 16219 16278 |
| U6a6b2 | 73 263 750 1438 2706 3348 4769 7028 7805 8407A 8557C 8860 9779 (10685) 10700 11467 11719 11734 12308 12372 13440 14179 14766 15326 16172 16219 16278 |
| U6a7 | 73 263 750 1438 2706 3348 4769 7028 7805 8860 11467 11719 12308 12372 14179 14766 15043 15326 16172 16219 16278 |
| U6a7a | 73 152 263 750 794A 1193 1438 1692T 2706 3348 4769 5471 7028 7805 8473 8860 11467 11719 12308 12372 14179 14766 15043 15326 15530 15632 16172 16219 16278 |
| U6a7a1 | 73 152 263 750 794A 1193 1438 1692T 2706 3348 4769 5120 5471 7028 7805 8473 8860 11467 11719 12308 12372 14179 14766 15043 15326 15530 15632 16172 16219 16278 |
| U6a7a1a | 73 152 263 750 794A 1193 1438 1692T 2672 2706 3348 4769 5120 5471 7028 7805 8473 8860 11467 11719 11929 12308 12372 14179 14766 15043 15326 15530 15632 16172 16219 16278 |
| U6a7a1b | 73 150 152 263 750 794A 1193 1438 1692T 2706 3348 4769 5120 5471 7028 7805 8473 8860 11467 11719 12308 12372 14179 14766 15043 15326 15530 15632 16172 16219 16278 |
| U6a7a1+@152 | 73 263 750 794A 1193 1438 1692T 2706 3348 4769 5120 5471 7028 7805 8473 8860 11467 11719 12308 12372 14179 14766 15043 15326 15530 15632 16172 16219 16278 |

| | |
|---|---|
| U6a7a1c | 73 263 750 794A 1193 1438 1692T 2706 3348 4769 5120 5471 7028 7805 8473 8860 11038 11467 11719 12308 12372 14179 14766 15043 15326 15530 15632 16172 16219 16278 |
| U6a7a2 | 73 152 263 750 794A 1193 1438 1692T 2706 3348 4769 5471 7028 7805 8473 8860 11467 11719 12308 12372 14034 14179 14766 15043 15326 15530 15632 16172 16219 |
| U6a7a2a | 73 152 263 750 794A 1193 1438 1692T 2706 3348 4769 5471 7028 7805 8473 8860 11467 11719 11941 12308 12372 14034 14179 14766 15043 15326 15530 15632 16172 16219 |
| U6a7b | 73 195 198 263 750 960.1C 1438 2706 3348 4769 7028 7805 8860 11467 11719 12308 12372 14179 14766 15043 15326 16172 16219 16278 |
| U6a7b1 | 73 195 198 263 709 750 960.1C 1438 1842 2706 3348 4769 7028 7735 7805 8860 11467 11719 12308 12372 12950C 14179 14766 15043 15326 16172 16278 |
| U6a7c | 73 263 750 1438 2706 3348 4769 7028 7337 7805 8860 11467 11719 12308 12372 14179 14766 15043 15326 16172 16189 16219 16278 |
| U6a7c1 | 73 263 750 1438 2706 3348 4769 7028 7337 7805 8860 11467 11719 12308 12372 14766 15043 15326 16172 16189 16219 16278 |
| U6+16311 | 73 263 750 1438 2706 3348 4769 7028 8860 11467 11719 12308 12372 14766 15326 16172 16219 16311 |
| U6b | 73 263 750 1438 2706 3348 4769 7028 8860 9438 11467 11719 12308 12372 14766 15326 16172 16219 16311 |
| U6b1 | 73 263 750 1438 2706 3348 4769 7028 8860 9438 9738 11467 11719 12308 12372 14766 15326 15431 16172 16219 16311 |
| U6b1a | 73 263 750 1438 2352 2706 3348 4769 7028 8860 9438 9738 11467 11719 12308 12372 14766 15326 15431 16163 16172 16219 16311 |
| U6b1a1 | 73 263 750 1438 2352 2706 3348 4769 7028 7700 8860 9438 9738 11467 11719 12308 12372 14766 15326 15431 16163 16172 16219 16311 |
| U6b1b | 73 263 750 1438 1520 2706 3348 4113 4769 5821 7028 8860 9438 9571 9738 11467 11719 12308 12372 14766 15326 15431 16172 16189 16219 16311 |
| U6b2 | 73 263 750 1438 2706 3348 4062 4769 7028 8860 9438 11467 11719 12308 12372 12535 13637 14766 15326 15355 16172 16219 16311 |
| U6b3 | 73 263 750 1438 2706 3348 4769 7028 8860 9438 11467 11719 12308 12372 14766 15326 16172 16219 16278 16311 |
| U6b3a | 73 235 263 750 1438 2706 3348 4769 7028 8860 9438 11467 11719 12308 12372 14766 15326 16172 16219 16278 16311 |
| U6d | 73 263 750 1438 2706 3348 4336 4769 7028 8860 11467 11719 12308 12372 12501 14518 14766 15326 16172 16219 16311 |
| U6d1 | 73 263 750 1438 2706 3348 4336 4454 4769 7028 8860 11467 11719 12308 12372 12501 14470 14518 14766 15326 16172 16219 16261 16311 |
| U6d1a | 73 263 750 1438 2706 3348 4336 4454 4769 5147 7028 8860 11467 11719 12308 12372 12501 14470 14518 14766 15326 16172 16219 16261 16311 |
| U6d1b | 73 263 750 1438 2706 3348 4336 4454 4769 7028 8860 11467 11719 12308 12372 12501 12603 14470 14518 14766 15326 16172 16219 16261 16311 |
| U6d2 | 73 263 750 1438 2706 3348 4336 4769 5460 7028 8860 11467 11719 12308 12372 12501 12842 14518 14766 15326 15892 16172 16219 16311 |

| | |
|---|---|
| U6d3 | 73 263 750 1438 2706 3348 4336 4769 7028 8860 11467 11719 12308 12372 12501 14518 14766 15326 16172 16174 16219 16311 |
| U6d3a | 73 263 750 1438 2706 3348 4336 4769 7028 8860 10397 11467 11719 11947 12308 12372 12501 12530 14518 14766 15326 16172 16174 16219 |
| U6c | 73 150 263 437 750 793 1438 2706 3348 3688C 4769 4965 5081 7028 8860 11013 11467 11719 12308 12372 13879 14766 15244 15326 16169 16172 16189 |
| U6c1 | 73 150 263 437 750 793 1438 2706 3348 3688C 4769 4965 5081 7028 8860 11013 11467 11719 12308 12372 13879 14766 15244 15326 15553 16129 16169 16172 16189 |
| U6c2 | 73 150 194 263 437 750 793 1438 2706 3348 3688C 4769 4965 5081 7028 8860 11013 11467 11719 12308 12372 13879 14766 15244 15326 16169 16172 16189 |
| U2'3'4'7'8'9 | 73 263 750 1438 1811 2706 4769 7028 8860 11467 11719 12308 12372 14766 15326 |
| U2 | 73 263 750 1438 1811 2706 4769 7028 8860 11467 11719 12308 12372 14766 15326 16051 |
| U2a | 73 263 750 1438 1811 2706 4769 7028 8860 11467 11719 12308 12372 14766 15326 16051 16206C |
| U2a1 | 73 (195) 263 750 1438 1811 2706 4769 7028 8860 11467 11719 12308 12372 14766 15326 16051 16206C 16230 16311 |
| U2a1a | 73 (195) 263 750 1438 1811 2706 4769 7028 8572 8860 11467 11719 12308 12372 14766 15326 16051 16154 16206C 16230 16311 |
| U2a1b | 73 (195) 263 750 1438 1811 2706 4769 7028 8860 11383 11467 11719 12308 12372 14766 15326 16051 16206C 16230 16311 |
| U2a2 | 73 194 263 750 1438 1811 2706 3316 4769 4970 5201 7028 7382 7853 7859 8860 11151 11299 11467 11719 12308 12372 12477 12561 14766 14883 15326 15734 16051 16206C 16271 |
| U2b | 73 146 263 750 1438 1811 4769 5186T 7028 8860 11467 11719 12106 12308 12372 13194 14766 15049 15326 16051 |
| U2b1 | 73 146 263 750 1438 1811 3915 4093 4769 5186T 7028 8860 11467 11719 12106 12308 12372 13194 14766 15049 15326 16051 (16168) |
| U2b1a | 73 146 263 750 1438 1811 3915 4093 4769 5186T 7028 8860 11467 11719 12106 12308 12372 13194 13708 14766 15049 15326 16051 (16168) |
| U2b2 | 73 146 152 234 263 750 1438 1811 1888 4769 5186T 7028 8860 9094 9614 11467 11719 12106 12308 12372 12793 13194 13656 14766 15049 15326 15930 16051 16209 16239 16352 16353 |
| U2+152 | 73 152 263 750 1438 1811 2706 4769 7028 8860 11467 11719 12308 12372 14766 15326 16051 |
| U2c'd | 73 152 263 750 1438 1811 2706 4769 7028 8860 11467 11719 12308 12372 14766 15326 16051 16234 |
| U2c | 73 152 263 750 1438 1811 2706 4769 5790A 7028 8860 11467 11719 12308 12372 14766 14935 15061 15326 16051 16234 |
| U2c1 | 73 152 263 750 1438 1811 2706 4769 5790A 7028 8023 8676 8860 9767 11467 11719 12308 12372 14766 14935 15061 15326 16051 16234 |
| U2c1a | 73 152 263 750 1438 1811 2706 4769 5790A 7028 8023 8676 8860 9767 11467 11719 12308 12372 14766 14935 15043 15061 15326 16051 16179 16234 16240C |

| | |
|---|---|
| U2c1b | 73 152 263 750 1438 1811 2706 4769 5790A 7028 8023 8676 8860 9767 11467 11719 12172 12308 12372 14766 14935 15061 15326 16051 16234 |
| U2d | 73 152 199 471 750 1438 1700 1811 2706 4025 4769 7028 8860 8938 11467 11719 11893 12308 12372 14766 14926 15326 16051 16189 16234 |
| U2d1 | 73 152 199 471 750 1438 1700 1811 2706 4025 4769 7028 8860 8938 8982 11467 11719 11893 12308 12372 14766 14926 15326 16051 16184 16189 16234 16294 16342 |
| U2d2 | 73 152 199 471 750 1438 1700 1811 2706 4025 4769 5147 5460 6956 7028 8296 8860 8938 11467 11719 11893 12308 12372 13789 14766 14926 15326 16051 16189 16234 16294 |
| U2d2a | 73 152 199 471 750 1438 1700 1811 2706 4025 4769 5147 5460 6956 7028 8296 8860 8938 11467 11719 11893 12308 12372 13789 14766 14926 15326 15883 16051 16189 16234 16294 |
| U2d3 | 73 152 199 471 750 1040 1438 1700 1811 2706 4025 4769 6023 7028 7277 7894 8860 8938 11467 11719 11893 12308 12372 14766 14926 15326 16051 16189 16234 16266 16294 |
| U2e | 73 152 263 508 750 1438 1811 2706 3720 4769 5390 5426 6045 6152 7028 8860 10876 11467 11719 12308 12372 13020 13734 14766 15326 15907 16051 16129C 16189 16362 |
| U2e1'2'3 | 73 152 217 263 508 750 1438 1811 2706 3720 4769 5390 5426 6045 6152 7028 8860 10876 11467 11719 12308 12372 13020 13734 14766 15326 15907 16051 16129C 16189 16362 |
| U2e1 | 73 152 217 263 340 508 750 1438 1811 2706 3720 4769 5390 5426 6045 6152 7028 8860 10876 11467 11719 12308 12372 13020 13734 14766 15326 15907 16051 16129C 16189 16362 |
| U2e1a | 73 152 217 263 340 508 750 1438 1811 2706 3720 4769 5390 5426 6045 6152 7028 8860 10876 11197 11467 11719 11732 12308 12372 13020 13734 14766 15326 15907 16051 16129C 16189 16362 |
| U2e1a1 | 73 152 217 263 340 508 750 1438 1811 2706 3116 3720 4769 5390 5426 6045 6152 7028 8860 10876 11197 11467 11719 11732 12308 12372 13020 13734 14766 15326 15907 16051 16129C 16189 16362 |
| U2e1a1a | 73 152 217 263 340 508 750 1438 1811 2706 3116 3197 3720 4769 5390 5426 6045 6152 7028 8860 10876 11197 11467 11719 11732 12308 12372 13020 13734 14766 15326 15907 16051 16129C 16189 16362 |
| U2e1a1b | 73 108 152 217 263 340 508 750 1438 1811 2706 3116 3720 4769 5390 5426 6045 6152 7028 8860 10876 11197 11467 11719 11732 12308 12372 13020 13734 14766 15326 15907 16051 16129C 16189 16362 |
| U2e1a1c | 73 152 217 263 340 508 750 1438 1811 2706 3116 3720 4769 5390 5426 6045 6152 7028 8860 10127 10876 11197 11467 11719 11732 12308 12372 13020 13734 14766 15326 15907 16051 16129C 16189 16362 |
| U2e1b | 73 152 217 263 340 508 750 988 1438 1811 2706 3720 4769 5390 5426 6045 6152 7028 8860 10876 11467 11719 12308 12372 13020 13734 14766 15326 15907 16051 16129C 16189 16256 16362 |
| U2e1b1 | 73 152 217 263 340 508 750 988 1438 1811 2706 3720 4769 5390 5426 6045 6152 7028 7109 8860 10876 11467 11719 12308 12372 13020 13734 14766 15326 15784 15907 16051 16129C 16189 16256 |

| | |
|---|---|
| U2e1b2 | 73 152 217 263 340 508 750 988 1438 1811 2706 3229.1A 3720 4769 5390 5426 6045 6152 7028 8860 10876 11467 11719 12308 12372 13020 13734 14766 15326 15661 15907 16051 16129C 16189 16256 16362 |
| U2e1c | 73 152 217 263 340 508 750 1438 1811 2706 3720 4769 5390 5426 6045 6152 7028 7151 8860 10876 11467 11719 12308 12346 12372 13020 13734 14766 15326 15907 16051 16129C 16189 16362 |
| U2e1c1 | 73 152 217 263 340 508 750 1438 1811 2706 3720 4769 5390 5426 6045 6152 7028 7151 8860 10876 11176 11467 11719 12308 12346 12372 13020 13734 14766 15326 15907 16051 16129C 16189 16362 |
| U2e1d | 73 152 217 263 340 508 750 1438 1811 2706 3720 4132 4769 5390 5426 6045 6152 7028 8860 10876 11467 11719 12308 12372 13020 13734 14766 15326 15907 16051 16129C 16189 16362 |
| U2e1e | 73 152 217 263 340 508 750 1438 1811 2526 2706 3720 4769 5390 5426 6045 6152 7028 8860 10876 11467 11719 12308 12372 12618 13020 13734 14766 15326 15907 16051 16129C 16145 16189 16362 |
| U2e1f | 73 152 217 263 340 508 750 1438 1811 2706 3720 4769 5390 5426 6045 6152 7028 8860 10876 11467 11719 12308 12372 13020 13734 14766 15326 15519 15907 16051 16129C 16189 16311 16362 |
| U2e1f1 | 73 146 152 217 263 340 489 508 750 1438 1811 2706 3720 4769 5390 5426 6045 6152 7028 8155 8860 9101 10876 11467 11719 12308 12358 12372 13020 13734 14766 15326 15519 15907 16051 16129C 16189 16311 16362 |
| U2e1g | 73 152 217 263 340 508 750 1438 1811 2706 3720 4769 5390 5426 6045 6152 7028 8860 10876 11233 11467 11719 12308 12372 13020 13734 14766 15326 15907 16051 16129C 16189 16362 |
| U2e1h | 73 217 263 340 508 750 1438 1811 2706 3720 4769 5390 5426 6045 6152 7028 8860 10876 11467 11719 12308 12372 13020 13734 14766 15326 15907 16051 16129C 16362 |
| U2e2 | 73 152 217 263 508 750 1438 1811 2706 3720 4769 5390 5426 6045 6152 7028 8473 8860 10876 11467 11719 12308 12372 13020 13734 14766 15326 15907 16051 (16092) 16129C 16189 16362 |
| U2e2a | 73 152 217 263 508 750 1438 1811 2706 3720 4769 5390 5426 6045 6152 7028 8473 8860 10876 11467 11719 12308 12372 12557 13020 13734 14766 15326 15907 16051 (16092) 16129C 16189 16362 |
| U2e2a1 | 73 152 217 263 508 750 1438 1811 2706 3720 3849 4553 4769 5390 5426 6045 6152 7028 8473 8860 10876 11467 11719 12308 12372 12557 13020 13734 14766 15326 15907 16051 (16092) 16129C 16189 16362 |
| U2e2a1a | 73 152 217 263 508 750 1438 1811 2706 3720 3849 4553 4736 4769 5390 5426 6045 6152 7028 8473 8860 10876 11467 11719 12308 12372 12557 13020 13734 14766 15326 15907 16051 (16092) 16129C 16189 16362 |
| U2e2a1a1 | 73 152 217 263 508 750 1438 1811 2706 3720 3849 4553 4736 4769 5390 5426 6045 6152 7028 8473 8860 9664 10876 11467 11719 12308 12372 12557 13020 13734 14766 15326 15907 16051 (16092) 16129C 16189 |
| U2e2a1a2 | 73 152 217 263 508 750 1438 1811 2706 3720 3849 4553 4736 4769 5390 5426 6045 6152 7028 8473 8860 10876 11467 11719 12308 12372 12557 13020 13734 14766 15326 15891 15907 16051 (16092) 16129C 16189 |
| U2e2a1b | 73 152 217 263 508 750 1438 1811 2706 3720 3849 4553 4769 5390 5426 6045 6152 7028 7624 8473 8860 10876 11467 11719 12308 12372 12557 13020 13734 14766 15326 15907 16051 (16092) 16129C 16189 16362 |

| | |
|---|---|
| U2e2a1c | 73 152 217 263 508 750 1438 1811 2706 3720 3849 4553 4769 5390 5426 5686T 6045 6152 7028 8473 8860 10876 11467 11719 12308 12372 12557 13020 13734 14766 15326 15907 16051 (16092) 16129C 16189 16362 |
| U2e2a1d | 73 152 217 263 508 750 1438 1811 2706 3720 3849 4553 4769 5390 5426 5746 6045 6152 7028 8473 8860 10876 11467 11719 12308 12372 12557 13020 13734 14766 15326 15907 16051 (16092) 16129C 16189 16362 |
| U2e3 | 73 152 217 263 394 508 750 1438 1811 2706 3720 4769 5390 5426 6045 6152 7028 8860 10876 11467 11719 12308 12372 13020 13734 14766 15326 15907 16051 16129C 16189 16260 16356 16362 |
| U2e3a | 73 152 217 263 309d 394 508 575 750 1438 1811 2706 3170A 3720 4769 5390 5426 6045 6152 7028 8860 10876 11467 11719 12308 12372 13020 13734 14766 15326 15721 15907 16051 16129C 16181 16189 16260 16356 16362 |
| U3 | 73 150 263 750 1438 1811 2706 4769 7028 8860 11467 11719 12308 12372 14139 14766 15326 15454 16343 |
| U3a'c | 73 150 263 750 1438 1811 2294 2706 4703 4769 7028 8860 9266 11467 11719 12308 12372 14139 14766 15326 15454 16343 |
| U3a | 73 150 263 750 1438 1811 2294 2706 4703 4769 6518 7028 8860 9266 10506 11467 11719 12308 12372 13934 14139 14766 15326 15454 16343 16390 |
| U3a1 | 73 150 263 750 1438 1811 2294 2706 3010 4703 4769 6518 7028 8860 9266 10506 11467 11719 12308 12372 13934 14139 14766 15326 15454 16343 16390 |
| U3a1a | 73 150 263 750 1438 1811 2294 2706 3010 4703 4769 6518 7028 7521 8860 9266 10506 11467 11719 12308 12372 13934 14139 14766 15326 15454 16343 16390 |
| U3a1a1 | 73 150 263 750 1438 1811 2294 2706 3010 4703 4769 6518 7028 7521 8860 9266 10506 11467 11719 12308 12372 13934 14139 14258 14766 15326 15454 16343 16390 |
| U3a1b | 73 150 263 723C 750 1438 1811 2294 2706 3010 4703 4769 6518 7028 8860 9266 10506 11467 11719 12308 12372 13934 14139 14766 15326 15454 16343 16390 |
| U3a1c | 73 150 263 750 1438 1811 2294 2706 3010 4703 4769 6518 7028 8860 9266 10506 11467 11719 12308 12372 13934 14139 14766 15326 15454 16343 16356 16390 |
| U3a1c1 | 73 150 263 750 1438 1811 2294 2706 3010 4703 4769 6518 7028 8860 9266 10506 11467 11719 12308 12372 13934 14139 14766 15326 15454 16301 16343 16356 16390 |
| U3a2 | 73 150 263 750 1438 1811 2294 2706 4703 4769 6518 7028 8860 9266 10506 11050 11467 11719 12308 12372 13934 14139 14766 15326 15454 16343 16390 |
| U3a2a | 73 150 200 263 750 1438 1811 2294 2706 4703 4769 6518 7028 8860 9266 10506 11050 11467 11719 12308 12372 13934 14139 14766 15326 15454 16343 16390 |
| U3a2a1 | 73 150 189 200 263 750 1438 1811 2294 2706 4703 4769 6518 7028 8860 9266 10506 11050 11467 11719 12308 12372 13934 14139 14766 15326 15454 16343 16390 |

| | |
|---|---|
| U3a2a1a | 73 150 189 200 263 750 1438 1811 2294 2706 4703 4769 6452 6518 7028 8860 9266 10506 11050 11467 11719 12308 12372 13934 14139 14766 14921 15326 15454 16245 16343 16390 |
| U3a3 | 73 150 263 750 1438 1811 2294 2706 2766A 4703 4769 6518 7028 8860 9266 10143 10506 11467 11719 12308 12372 13934 14139 14766 15326 15454 16189 16343 16390 |
| U3c | 73 150 263 750 1438 1811 2294 2706 4703 4769 7028 8860 9266 11467 11719 12308 12372 12843 14139 14766 15326 15454 15613 16193 16249 16343 16526 |
| U3b | 73 150 263 750 1438 1811 2706 4188 4640A 4769 7028 8860 9656 11467 11719 12308 12372 13743 14139 14766 15326 15454 16343 |
| U3b1 | 73 150 263 750 1438 1811 2706 3546A 4188 4640A 4769 7028 8860 9656 11467 11719 12308 12372 13743 14139 14766 15326 15454 16343 |
| U3b1a | 73 150 263 750 1438 1811 2706 3546A 4188 4640A 4769 7028 8812 8860 9656 11467 11719 12308 12372 13743 14139 14766 15326 15454 16086 16343 |
| U3b1a1 | 73 150 152 263 750 1438 1811 2706 3546A 4188 4562 4640A 4654 4769 5465 7028 8778 8812 8860 9656 11467 11719 12308 12372 13743 14139 14766 15326 15454 16086 16343 |
| U3b1b | 73 150 263 750 1438 1811 2706 3546A 4188 4640A 4769 6359 7028 8860 9656 11467 11719 12308 12372 12720 13743 14139 14766 15326 15454 16343 |
| U3b2 | 73 150 263 750 1438 1811 2706 4188 4640A 4769 7028 8860 9656 11467 11719 12308 12372 13743 14139 14766 15326 15454 15944d 16343 |
| U3b2a | 73 150 263 750 1438 1811 2706 2707 4188 4640A 4769 7028 8860 9656 9770 11467 11719 12308 12372 13743 14139 14766 15326 15454 15944d 16343 |
| U3b2a1 | 73 150 263 750 1438 1811 2706 2707 4188 4640A 4769 7028 8860 8950 9656 9770 11467 11719 12308 12372 13743 14139 14766 15326 15454 15944d 16343 |
| U3b2a1a | 73 150 263 334 750 1438 1811 2706 2707 4188 4640A 4769 7028 8860 8950 9656 9770 11467 11719 12308 12372 13743 14139 14766 15326 15454 15944d 16343 |
| U3b2b | 73 150 263 750 1438 1811 2706 4188 4640A 4769 7028 8490 8860 9656 11467 11719 12308 12372 13743 14139 14766 15326 15327 15454 15944d 16343 16362 |
| U3b2c | 73 150 263 750 1438 1811 2706 4188 4640A 4769 6383 7028 8860 9656 11467 11719 12308 12372 13743 14139 14766 15326 15454 15944d 16343 |
| U3b3 | 73 150 263 750 1438 1811 2706 4188 4640A 4769 7028 8860 9656 11467 11719 12308 12372 13743 14139 14766 15326 15454 16168 16343 |
| U4'9 | 73 195 263 499 750 1438 1811 2706 4769 5999 7028 8860 11467 11719 12308 12372 14766 15326 |
| U4 | 73 195 263 499 750 1438 1811 2706 4646 4769 5999 6047 7028 8860 11332 11467 11719 12308 12372 14620 14766 15326 15693 16356 |
| U4a | 73 195 263 499 750 1438 1811 2706 4646 4769 5999 6047 7028 8818 8860 11332 11467 11719 12308 12372 14620 14766 15326 15693 16356 |

| | |
|---|---|
| U4a1 | 73 152 195 263 499 750 1438 1811 2706 4646 4769 5999 6047 7028 8818 8860 11332 11467 11719 12308 12372 12937 14620 14766 15326 15693 16134 16356 |
| U4a1a | 73 152 195 263 499 750 961 1438 1811 2706 4646 4769 5999 6047 7028 8818 8860 11332 11467 11719 12308 12372 12937 14620 14766 15326 15693 16134 16356 |
| U4a1a1 | 73 152 195 263 499 750 961 965.XC 1438 1811 2706 4646 4769 5999 6047 7028 8818 8860 11332 11467 11719 12308 12372 12937 14620 14766 15326 15693 16134 16356 |
| U4a1a2 | 73 152 195 263 499 750 961 1438 1811 2706 4646 4769 5250 5999 6047 7028 8818 8860 11332 11467 11719 12308 12372 12630 12937 14620 14766 15326 15693 16134 16356 |
| U4a1a3 | 73 152 195 263 499 750 961 1438 1811 2706 4646 4769 5999 6047 6216 7028 8818 8860 11332 11467 11719 12308 12372 12937 14620 14766 15326 15544A 15693 16134 16356 |
| U4a1b | 73 152 195 263 499 745 750 1438 1811 2706 3204 4646 4769 5999 6047 7028 8818 8860 11332 11467 11719 12308 12372 12937 14620 14766 15326 15693 16134 16356 |
| U4a1b1 | 73 152 195 263 296 499 745 750 1438 1811 2706 3204 4646 4769 5999 6047 7028 8818 8860 11332 11467 11719 12308 12372 12937 14620 14766 15326 15693 16134 16356 |
| U4a1b1a | 73 152 195 263 296 499 745 750 1438 1811 2706 3204 4646 4769 5999 6047 7028 8705 8818 8860 11332 11467 11719 12308 12372 12937 14620 14766 15326 15693 16129 16134 16356 |
| U4a1b2 | 73 152 195 263 499 745 750 1438 1811 2706 3204 4646 4769 5999 6047 7028 8818 8860 11332 11467 11719 12308 12372 12937 14620 14766 15326 15693 16134 16301 16356 |
| U4a1c | 73 152 195 263 499 750 1438 1811 2706 4646 4769 5999 6047 7028 8155 8818 8860 11332 11467 11719 12308 12372 12937 13158 14110 14620 14766 15326 15693 16134 16234 16356 |
| U4a1d | 73 152 195 198 263 499 750 1438 1811 2706 4646 4769 5999 6047 7028 8818 8860 11332 11467 11719 12308 12372 12937 14620 14766 15326 15693 16134 16356 |
| U4a1e | 73 152 195 263 499 750 1438 1811 2706 4646 4769 5999 6047 7028 8818 8860 11332 11467 11719 12308 12372 12937 14620 14766 15326 15693 16093 16134 16311 16356 |
| U4a2 | 73 195 263 310 499 750 1438 1811 2706 4646 4769 5999 6047 7028 8818 8860 11332 11467 11719 12308 12372 14620 14766 15326 15693 16356 |
| U4a2a | 73 195 263 310 499 750 1438 1811 2706 4646 4769 5999 6047 7028 8818 8860 11332 11467 11719 12308 12372 14620 14766 15326 15693 |
| U4a2a1 | 73 195 263 310 499 750 1438 1811 2706 4646 4769 5999 6047 7028 8496 8818 8860 11332 11467 11719 12308 12372 14620 14766 15326 15693 |
| U4a2a2 | 73 195 263 310 499 750 1438 1811 2706 4646 4769 5999 6047 7028 8818 8860 11332 11467 11719 12308 12372 13759 14620 14766 15326 15693 |
| U4a2a3 | 73 195 263 310 499 750 1438 1811 2706 4646 4769 5999 6047 7028 8818 8860 11332 11467 11719 12308 12372 14319 14620 14766 15326 15693 16294 |

| | |
|---|---|
| U4a2b | 73 195 263 310 499 750 1438 1811 2706 4646 4769 5999 6047 7028 8818 8860 11332 11467 11719 12308 12372 14620 14766 15326 15693 16223 16356 |
| U4a2c | 73 195 263 310 499 750 1438 1811 2706 4646 4769 5999 6047 7028 8567 8818 8860 11332 11467 11719 12308 12372 14620 14766 15326 15693 16356 |
| U4a2c1 | 73 195 263 310 499 750 1438 1811 2706 4646 4769 5999 7028 8567 8818 8860 10654 11332 11467 11719 12308 12372 14620 14766 15326 15693 16242A 16288 16356 16362 |
| U4a2d | 73 195 263 310 499 750 1438 1811 2706 4646 4769 5999 6047 7028 8818 8860 11332 11467 11719 12308 12372 14620 14766 15326 15693 16295 16356 |
| U4a2e | 73 195 263 310 499 750 1438 1811 2246 2706 4646 4769 5999 6047 6383 7028 8818 8860 11332 11467 11719 12308 12372 14620 14766 15326 15693 16356 |
| U4a2f | 73 195 263 310 499 750 1189 1438 1811 1978 2706 4646 4769 5999 6047 7028 8818 8860 11332 11467 11719 12308 12372 12397 14620 14766 15172 15326 15693 16356 |
| U4a2g | 73 195 263 310 499 750 1438 1811 2706 4646 4769 5999 6047 6164 7028 8818 8860 11332 11467 11719 12308 12372 14620 14766 15326 15693 16356 |
| U4a2h | 73 195 263 310 499 750 1438 1811 2706 4646 4769 5261 5999 6047 7028 8818 8860 11332 11467 11719 12308 12372 14620 14766 15326 15693 16356 |
| U4a2h1 | 73 195 263 310 499 750 1438 1811 2706 4646 4769 5261 5999 6047 7028 8818 8860 11332 11467 11719 12308 12372 14620 14766 15326 15693 16173 16356 |
| U4a3 | 73 195 247 263 499 750 1438 1811 2706 2792 4646 4769 5999 6047 6665 6929 7028 8065 8818 8860 11332 11467 11719 12308 12372 14620 14766 15326 15693 16356 16362 |
| U4a3a | 73 195 247 263 499 750 1438 1811 2706 2792 4646 4769 5999 6047 6665 6929 7028 8065 8818 8860 11332 11467 11719 12308 12372 14620 14766 15326 15693 16265 16356 16362 |
| U4b | 73 195 263 499 750 1438 1811 2706 4646 4769 5999 6047 7028 7705 8860 11332 11467 11719 12308 12372 14620 14766 15326 15693 16356 |
| U4b1 | 73 195 263 499 750 1438 1811 2706 4646 4769 5999 6047 7028 7705 8860 11332 11339 11467 11719 12308 12372 14620 14766 15326 15693 16356 |
| U4b1a | 73 195 263 499 750 1438 1811 2706 3672 4646 4769 5999 6047 7028 7705 8860 11332 11339 11467 11719 12308 12372 14620 14766 15326 15693 16356 |
| U4b1a1 | 73 195 263 499 750 1438 1811 2083 2706 3672 4646 4769 5999 6047 7028 7705 8860 11332 11339 11467 11719 12297 12308 12372 14620 14766 15326 15693 16356 |
| U4b1a1a | 73 195 263 499 750 1438 1811 2083 2706 3672 4646 4769 5999 6047 7028 7705 8860 11332 11339 11467 11719 12297 12308 12372 14620 14766 15326 15693 15789 16356 |

| | |
|---|---|
| U4b1a1a1 | 73 195 263 499 750 1438 1811 2083 2706 3672 4646 4769 5999 6047 7028 7705 8642 8860 11332 11339 11467 11719 12297 12308 12372 14620 14766 15326 15693 15789 16356 16362 |
| U4b1a2 | 73 195 263 499 750 1438 1811 2706 3672 4646 4769 5999 6047 7028 7705 8860 11143 11176 11332 11339 11467 11719 12308 12372 12609 13708 14620 14766 15326 15693 16356 |
| U4b1a2a | 73 195 263 499 750 1438 1811 2706 3672 4646 4769 5999 6047 7028 7705 8860 11143 11176 11332 11339 11467 11719 12308 12372 12609 13708 14620 14766 15326 15632 15693 16356 |
| U4b1a2b | 73 195 263 499 750 1438 1811 2706 3672 4646 4769 5999 6047 7028 7705 8860 10876 11143 11176 11332 11339 11467 11719 12308 12372 12609 13708 14620 14766 15067 15326 15693 16356 |
| U4b1a3 | 73 146 195 263 499 750 1438 1811 2706 3672 4646 4769 5752d 5999 6047 7028 7705 8860 9989 11332 11339 11467 11674 11719 12308 12372 14620 14766 15326 15693 16356 |
| U4b1a3a | 73 146 195 263 499 750 1438 1811 2706 3672 4646 4769 5752d 5999 6047 7028 7265 7705 8860 9989 11332 11339 11467 11674 11719 12308 12372 14620 14766 15326 15693 16356 |
| U4b1a4 | 73 195 263 499 750 1438 1811 2706 3672 4646 4769 5999 6047 7028 7705 7853 8860 11332 11339 11467 11719 11965 12308 12372 12957 14620 14766 15326 15693 16356 |
| U4b1+146_152 | 73 146 152 195 263 499 750 1438 1811 2706 4646 4769 5999 6047 7028 7705 8860 11332 11339 11467 11719 12308 12372 14620 14766 15326 15693 16356 |
| U4b1b | 73 146 152 195 263 499 750 1438 1811 2706 4646 4769 5999 6047 7028 7705 8860 11332 11339 11467 11719 12308 12372 13528 14620 14766 15326 15693 16356 |
| U4b1b1 | 73 146 152 195 263 499 750 1438 1811 2706 4646 4769 5999 6047 7028 7705 8860 11332 11339 11467 11719 12308 12372 13528 13565 14620 14766 15326 15693 16356 |
| U4b1b1a | 73 146 152 195 263 499 750 1438 1811 2706 4646 4769 5999 6047 7028 7705 8860 11332 11339 11467 11719 12308 12372 13528 13565 14620 14766 15326 15693 16111 16140 16356 |
| U4b1b1b | 73 152 195 263 499 750 1438 1811 2706 4646 4769 5999 6047 7028 7705 8308 8860 9389 10819 11332 11339 11467 11719 12308 12372 13528 13565 14620 14766 15326 15373 15693 15758 16356 |
| U4b1b1+16311 | 73 146 152 195 263 499 750 1438 1811 2706 4646 4769 5999 6047 7028 7705 8860 11332 11339 11467 11719 12308 12372 13528 13565 14620 14766 15326 15693 16311 16356 |
| U4b1b1c | 73 146 152 195 263 499 750 1438 1811 2483 2706 4646 4769 5999 6047 7028 7705 8860 11332 11339 11467 11719 12308 12372 13528 13565 14620 14766 15326 15693 16311 16356 |
| U4b1b1d | 73 146 152 195 263 499 750 1438 1811 2706 4646 4769 5262 5999 6047 7028 7705 8860 11332 11339 11467 11719 12308 12372 13528 13565 14620 14766 15326 15693 16356 |
| U4b1b2 | 73 152 195 263 499 750 1438 1811 2706 4646 4769 5999 6047 7028 7705 8860 11332 11339 11467 11719 11788 12308 12372 13528 14620 14766 15514 15693 16356 |

| | |
|---|---|
| U4b2 | 73 195 263 499 750 1438 1811 2706 4646 4769 5999 6047 7028 7705 8860 11332 11467 11719 12308 12372 14620 14766 15326 15693 16136 16356 |
| U4b2a | 73 195 263 499 750 1438 1811 2706 4646 4769 5999 6047 7028 7705 8860 11332 11467 11719 12308 12372 14620 14766 15326 15693 15883 16136 16356 |
| U4b2a1 | 73 195 263 499 750 1438 1811 2706 4646 4769 5999 6047 7028 7705 8860 11332 11467 11719 12308 12372 14620 14766 15326 15693 15883 16136 16278 16356 |
| U4b2a1a | 73 195 263 499 750 1438 1811 2706 4646 4769 5999 6047 7028 7705 8433 8860 11332 11467 11719 12308 12372 14178 14620 14766 15326 15693 15883 16136 16278 16356 |
| U4b3 | 73 195 215 263 499 750 1438 1811 2706 4646 4769 5999 6047 7028 7705 8860 11332 11467 11719 12308 12372 14620 14766 15326 15693 16356 |
| U4c | 73 195 263 499 750 1438 1811 2706 4646 4769 5999 6047 7028 8860 10907 11332 11467 11719 12308 12372 14620 14766 15326 15693 16356 |
| U4c1 | 73 195 263 499 750 1438 1811 2706 4646 4769 4811 5999 6047 6146 7028 8860 9070G 10907 11009 11332 11467 11719 12308 12372 14620 14766 14866 15326 15693 16179 16356 |
| U4c1a | 73 195 263 499 750 1438 1811 2706 3654 4646 4769 4811 5999 6047 6146 7028 8860 9070G 10907 11009 11332 11467 11719 12308 12372 14620 14766 14866 15326 15693 16179 16356 |
| U4c2 | 73 195 263 499 750 1438 1811 2706 4646 4769 5999 6047 7028 8705 8860 10676 10907 11332 11467 11719 12308 12372 14620 14766 15326 15693 16356 |
| U4c2a | 73 195 263 499 750 1438 1811 2706 4646 4769 5999 6047 7028 8705 8860 10676 10907 11332 11467 11719 12308 12372 14620 14766 15326 15693 16261 16356 |
| U4d | 73 195 263 499 629 750 1438 1811 2405.1C 2706 4646 4769 5999 6047 7028 8860 11332 11467 11719 12308 12372 14620 14766 15326 15693 16356 |
| U4d1 | 73 195 263 499 629 750 1438 1811 2405.1C 2706 2772 4646 4769 5999 6047 7028 8860 11332 11467 11719 12308 12372 14620 14766 15326 15693 16356 |
| U4d1a | 73 195 263 499 629 750 1438 1811 2405.1C 2706 2772 4646 4769 5984 5999 6047 6938 7028 8860 11332 11467 11719 12308 12372 14620 14766 15326 15693 16356 |
| U4d1a1 | 73 195 263 499 629 750 1438 1811 2405.1C 2706 2772 4646 4769 5984 5999 6047 6938 7028 8260 8860 11332 11467 11719 12308 12372 14620 14766 15326 15693 16356 |
| U4d1a1a | 73 195 263 499 629 750 1438 1811 2405.1C 2706 2772 4646 4769 5319T 5984 5999 6047 6938 7028 8260 8860 11332 11467 11719 12308 12372 14620 14766 15326 15693 16356 |
| U4d1b | 73 195 263 499 629 750 1438 1811 2405.1C 2706 2772 4646 4769 5999 6047 7028 8860 11332 11467 11719 12308 12372 13398 14620 14766 15326 15693 16356 |
| U4d2 | 73 195 263 499 629 750 1438 1811 2405.1C 2706 4646 4769 5567 5999 6047 7028 8860 10692 11326 11332 11467 11518 11719 12308 12372 13105 14620 14766 15326 15693 16189 16356 |

| | |
|---|---|
| U4d3 | 73 195 263 499 573.XC 629 750 1438 1811 2405.1C 2706 4646 4769 5999 6047 7028 8860 11332 11467 11719 12308 12372 14577 14620 14766 15326 15693 16356 |
| U9 | 73 195 263 499 750 1438 1811 2706 3531 3834 4769 5999 6386 7028 8860 11467 11719 12308 12372 14094 14766 15326 |
| U9a | 73 263 499 750 1438 1811 2706 3531 3834 4769 5999 6386 7028 8860 11467 11719 12308 12372 14094 14766 15326 16051 16278 |
| U9a1 | 73 263 499 750 1438 1811 2706 3290 3531 3834 4769 5999 6386 7028 8860 11467 11719 12308 12372 14094 14766 15077 15326 16051 16193 16278 16357 |
| U9b | 73 195 263 499 750 1438 1811 2706 3531 3834 4769 5999 6386 7028 8860 11467 11719 12308 12372 12615 14094 14766 15326 |
| U9b1 | 73 195 263 499 573.XC 750 1438 1811 2706 3531 3834 4769 5999 6386 7028 8860 9299 11350 11467 11719 12308 12372 12615 13111 14094 14766 15326 |
| U7 | 73 152 263 750 980 1438 1811 2706 3741 4769 5360 7028 8137 8684 8860 10142 11467 11719 12308 12372 13500 14569 14766 15326 (16309) |
| U7a | 73 151 152 263 750 980 1438 1811 2706 3741 4769 5360 7028 8137 8684 8860 10142 11467 11719 12308 12372 13500 14569 14766 15326 (16309) 16318T |
| U7a1 | 73 151 152 263 750 980 1438 1811 2706 3741 4769 5360 7028 8137 8684 8860 10142 11467 11719 12308 12372 13500 14569 14766 15326 16167 (16309) 16318T |
| U7a1a | 73 151 152 263 270 750 980 1438 1811 2706 3741 4769 5360 5486 7028 8137 8684 8860 10142 11467 11719 12308 12372 13281 13500 14569 14766 15326 16167 (16309) 16318T |
| U7a2 | 73 151 152 263 750 980 1438 1811 2706 3741 4502 4769 5360 7028 8137 8684 8860 10142 11467 11719 12308 12372 13500 14569 14766 15326 (16309) 16318T |
| U7a2a | 73 151 152 263 750 980 1438 1811 2706 3741 4502 4769 5265 5360 7028 8137 8684 8860 10142 11467 11719 12094 12308 12372 13500 14533 14569 14766 15326 16129 (16309) 16318T |
| U7a3 | 73 151 152 263 750 980 1438 1811 2706 3741 4769 5360 7028 8137 8684 8860 10142 11467 11719 12308 12372 12618 13500 14569 14766 15326 (16309) 16318T |
| U7a3a | 73 151 152 263 750 980 1438 1811 2706 3741 4769 5360 7028 8137 8684 8860 9852 10142 11467 11719 12308 12372 12618 13500 14569 14766 15326 (16309) 16318T |
| U7a3b | 73 151 152 263 750 980 1438 1811 2706 3741 3834 4769 5360 7028 8137 8684 8860 10142 11467 11719 12308 12372 12618 13500 14569 14766 15326 16207 (16309) 16318T |
| U7a4 | 73 146 151 152 263 750 980 1438 1811 2706 3741 4769 5360 7028 8137 8684 8860 10142 11467 11719 12308 12372 13500 14569 14766 15326 16126 (16309) 16318T |
| U7a4a | 73 146 151 152 263 750 980 1438 1811 2706 3741 4769 5360 7028 8137 8684 8860 10142 11467 11719 12308 12372 13500 14569 14766 15326 16126 16148 (16309) 16318T |

| | |
|---|---|
| U7a4a1 | 73 146 151 152 195 263 750 980 1438 1811 2706 3741 4769 5360 6221 7028 8137 8684 8860 10142 11467 11719 12308 12372 13500 14569 14766 15326 16126 16148 (16309) 16318T |
| U7a4a1a | 73 146 151 152 195 263 750 980 1438 1811 2706 3741 4769 5360 6221 7028 8137 8574 8684 8860 10142 11467 11719 12308 12372 13500 14569 14766 15326 16126 16148 (16309) 16318T |
| U7a5 | 73 151 152 263 573.XC 750 980 1438 1811 2706 3741 4769 5360 7028 8137 8684 8860 9300 10142 11467 11719 12308 12372 13500 13966 14245 14569 14766 14869C 15326 16291 16304 (16309) 16318T |
| U7b | 73 152 263 750 980 1438 1811 2706 3741 4769 5360 7028 8137 8684 8860 10084 10142 11467 11719 12308 12372 13500 14569 14766 15326 (16309) 16318T |
| U7b1 | 73 152 263 750 980 1438 1811 2706 3741 4769 5360 7028 8137 8684 8860 10084 10142 11467 11719 12308 12372 13395 13500 14569 14766 15326 16271 (16309) 16318T |
| U7b2 | 73 152 263 291.1A 750 980 1438 1811 2706 3741 4769 5360 7028 8137 8684 8860 9480 10084 10142 11467 11719 12308 12372 13500 14569 14766 15326 16166d (16309) 16318T |
| U8 | 73 263 750 1438 1811 2706 4769 7028 8860 9698 11467 11719 12308 12372 14766 15326 |
| U8a | 73 263 282 750 1438 1811 2706 4769 6392 6455 7028 7055 8860 9365 9698 11467 11719 12308 12372 13145 14766 15326 |
| U8a1 | 73 263 282 750 1438 1811 2706 3738 4769 6392 6455 7028 7055 8860 9365 9698 11467 11719 12308 12372 13145 14766 15326 |
| U8a1a | 73 263 282 750 1438 1811 2706 3738 4769 5240 6392 6455 7028 7055 8860 9365 9698 10733 11467 11719 12308 12372 13145 14766 15326 (16146) 16342 |
| U8a1a1 | 73 263 282 750 1438 1811 2706 3738 4769 5240 6392 6455 7028 7055 8860 9365 9698 10733 11467 11719 12135A 12308 12372 13145 14766 15326 (16146) 16342 |
| U8a1a1a | 73 263 282 750 1438 1811 2706 3738 4129 4769 5240 6392 6455 7028 7055 8860 9365 9698 10733 11467 11719 12135A 12308 12372 13145 14766 15326 (16146) 16342 |
| U8a1a1a1 | 73 263 282 750 1438 1811 2706 3738 4053 4129 4769 5240 6392 6455 7028 7055 8860 9365 9698 10733 11467 11719 12135A 12308 12372 13145 14766 15326 (16146) 16342 |
| U8a1a1b | 73 263 282 750 1438 1811 2706 3738 4769 5240 6392 6455 7028 7055 8860 9365 9698 9777 10733 11467 11719 12135A 12308 12372 13145 14766 15326 (16146) 16342 |
| U8a1a1b1 | 73 263 282 750 1438 1811 2706 3738 4769 5240 6392 6455 7028 7055 8860 9338 9365 9698 9777 10733 11467 11719 12135A 12308 12372 13145 14766 15326 (16146) 16342 |
| U8a1a2 | 73 263 282 750 1438 1811 2706 3738 4769 5240 6392 6455 7028 7055 8860 9365 9698 10733 11465 11467 11719 12308 12372 13145 14766 15326 (16146) 16342 |
| U8a1a3 | 73 263 282 750 1438 1503 1811 2706 3738 4769 5240 6392 6455 7028 7055 7340 8860 9365 9698 10733 11467 11719 12308 12372 13145 14766 15326 (16146) 16342 |

| | |
|---|---|
| U8a1a4 | 73 263 282 750 1438 1811 2706 3738 4769 5240 6392 6455 7028 7055 8860 9365 9698 10733 11467 11719 12308 12372 12768 13145 14766 15326 (16146) 16189 16342 |
| U8a1b | 73 263 282 709 750 1438 1811 2706 3738 4491 4769 6392 6455 7028 7055 8860 9365 9698 11467 11719 12308 12372 13145 14766 15326 16167 |
| U8a2 | 73 195 263 282 750 827 1438 1700 1811 2706 4769 6392 6455 7028 7055 8860 9365 9698 11467 11719 12308 12372 13145 14766 15326 |
| U8b'c | 73 263 750 1438 1811 2706 3480 4769 7028 8860 9698 11467 11719 12308 12372 14766 15326 |
| U8b | 73 263 750 1438 1811 2706 3480 4769 7028 8860 9055 9698 11467 11719 12308 12372 14167 14766 15326 |
| U8b1 | 73 195 263 750 1438 1811 2706 3480 4769 7028 8860 9055 9698 11467 11719 12308 12372 14167 14766 15326 16189 16234 |
| U8b1a | 73 195 263 750 1438 1811 2706 3480 4769 6546 6599 7028 8860 9055 9698 11467 11719 12308 12372 12771 14167 14766 15326 16189 16234 |
| U8b1a1 | 73 195 263 750 1438 1811 2706 3480 4769 6546 6599 7028 8860 9055 9698 9948 11467 11719 11914 12308 12372 12771 14167 14766 15326 16129 16189 16234 |
| U8b1a2 | 73 195 263 750 1438 1811 2706 3480 4769 6546 6599 7028 8860 9055 9698 11467 11719 12308 12372 12771 14167 14766 14831 15326 16189 |
| U8b1a2a | 73 195 263 750 1438 1811 2706 3480 4769 6546 6599 7028 8860 9055 9698 11467 11719 12308 12372 12771 14167 14364 14766 14831 15326 16189 16234 16259 |
| U8b1a2+16311 | 73 195 263 750 1438 1811 2706 3480 4769 6546 6599 7028 8860 9055 9698 11467 11719 12308 12372 12771 14167 14766 14831 15326 16189 16234 16311 |
| U8b1a2b | 73 195 263 750 1438 1811 2706 3480 4769 6546 6599 7028 8860 9055 9698 11467 11719 12308 12372 12771 14167 14766 14831 15326 16172 16189 16234 16311 |
| U8b1b | 73 195 263 750 1438 2706 3480 4769 5165 7028 8860 9055 9698 11467 11719 12308 12372 14167 14766 15326 16189 16234 16324 |
| U8b1b1 | 73 195 263 750 1438 2706 3480 4769 5165 7028 8860 9055 9698 11467 11719 12308 12372 14053 14167 14766 15326 16189 16234 16324 |
| U8b1b2 | 73 195 263 750 1438 2706 3480 3696 4769 5165 7028 8860 9055 9698 11467 11582 11719 12308 12372 12557 14167 14766 15326 16189 16234 16324 |
| K | 73 263 750 1438 1811 2706 3480 4769 7028 8860 9055 9698 10550 11299 11467 11719 12308 12372 14167 14766 14798 15326 16224 16311 |
| K1 | 73 263 750 1189 1438 1811 2706 3480 4769 7028 8860 9055 9698 10398 10550 11299 11467 11719 12308 12372 14167 14766 14798 15326 16224 16311 |
| K1a | 73 263 497 750 1189 1438 1811 2706 3480 4769 7028 8860 9055 9698 10398 10550 11299 11467 11719 12308 12372 14167 14766 14798 15326 (16093) 16224 16311 |
| K1a1 | 73 (114) 263 497 750 1189 1438 1811 2706 3480 4769 7028 8860 9055 9698 10398 10550 11299 11467 11719 11914 12308 12372 14167 14766 14798 15326 (16093) 16224 16311 |

| | |
|---|---|
| K1a1a | 73 (114) 263 497 750 1189 1438 1811 2706 3480 3552 4769 7028 8860 9055 9698 10398 10550 11299 11467 11719 11914 12308 12372 14167 14766 14798 15326 (16093) 16224 16311 |
| K1a1a1 | 73 (114) 263 497 750 1189 1438 1811 2706 3480 3552 4769 7028 8860 9055 9698 9801 10398 10550 11299 11467 11719 11914 12308 12372 14167 14766 14798 15326 (16093) 16224 16311 |
| K1a1a2 | 73 (114) 263 497 750 1189 1438 1811 2706 3480 3552 4769 7028 8860 9055 9698 10398 10550 11299 11467 11719 11914 12308 12372 14167 14766 14798 15326 15799 (16093) 16224 16311 |
| K1a1a2a | 73 (114) 263 497 750 1189 1438 1811 2706 3480 3552 4769 6060 7028 8276.1C 8860 9055 9698 10398 10550 11299 11467 11719 11914 12308 12372 14167 14766 14798 15326 15799 (16093) 16224 16311 |
| K1a1a2a1 | 73 (114) 263 497 750 1189 1438 1811 2706 3480 3552 4769 4959 6060 7028 8276.1C 8860 9055 9698 10398 10550 11299 11467 11719 11914 12308 12372 14167 14766 14798 15326 15799 (16093) 16224 16311 |
| K1a1b | 73 (114) 263 497 750 1189 1438 1811 2706 3480 4769 7028 8860 9055 9698 10398 10550 11299 11467 11719 11914 12308 12372 14167 14766 14798 15326 15924 (16093) 16224 16311 |
| K1a1b1 | 73 (114) 263 497 750 1189 1438 1811 2706 3480 4769 7028 8860 9055 9698 10398 10550 11299 11467 11470 11719 11914 12308 12372 14167 14766 14798 15326 15924 (16093) 16224 16311 |
| K1a1b1a | 73 (114) 263 497 750 1189 1438 1811 2706 3480 4769 7028 8860 9055 9698 10398 10550 10978 11299 11467 11470 11719 11914 12308 12372 12954 14167 14766 14798 15326 15924 (16093) 16224 16234 16311 |
| K1a1b1b | 73 (114) 263 497 593 750 1189 1438 1811 2483 2706 3480 4769 7028 8860 9055 9698 10398 10550 11299 11467 11470 11719 11914 12308 12372 14167 14766 14798 15326 15924 (16093) 16224 16311 |
| K1a1b1b1 | 73 (114) 263 497 593 750 789 1189 1438 1811 2483 2706 3480 4769 7028 8860 9055 9698 10398 10550 11299 11467 11470 11620 11719 11914 12308 12372 14167 14766 14798 15326 15924 (16093) 16224 16311 |
| K1a1b1c | 73 (114) 263 497 750 1189 1438 1811 2706 3480 4769 5585 7028 8860 9055 9698 10398 10550 11299 11467 11470 11719 11914 12308 12372 14167 14766 14798 15326 15924 (16093) 16222 16224 16311 |
| K1a1b1d | 73 (114) 263 497 750 1189 1438 1811 2706 3480 4769 7028 8860 9055 9698 10398 10550 11299 11467 11470 11719 11914 12308 12372 14167 14388 14766 14798 15326 15924 16092 (16093) 16223 16224 16311 |
| K1a1b1e | 73 (114) 263 497 750 1189 1438 1811 2706 3480 4769 7028 8860 9055 9698 9932 10398 10550 11299 11467 11470 11719 11914 12308 12372 14167 14766 14798 15326 15924 (16093) 16224 16311 |
| K1a1b1f | 73 (114) 263 497 750 1189 1438 1811 2706 3480 4769 4823 6528 7028 8842C 8860 9055 9698 10398 10550 11299 11467 11470 11719 11914 12308 12372 14167 14766 14798 15326 15924 (16093) 16224 16311 |
| K1a1b1g | 73 (114) 263 497 750 1189 1438 1811 2706 3480 4769 5583 7028 8860 9055 9698 10398 10550 11299 11467 11470 11719 11914 12007 12308 12372 14167 14766 14798 15326 15924 (16093) 16224 16311 |
| K1a1b2 | 73 (114) 263 497 750 1189 1438 1811 2706 3480 4769 7028 8860 9055 9698 10398 10550 11299 11467 11719 11914 12308 12372 13326 14167 14766 14798 15326 15924 (16093) 16224 16311 |

| | |
|---|---|
| K1a1b2a | 73 (114) 152 263 497 750 1189 1438 1811 2706 3480 4769 7028 7729 8860 9055 9698 9800 10398 10550 11299 11467 11719 11914 12308 12372 13326 14167 14766 14798 15326 15924 (16093) 16224 16311 |
| K1a1b2a1 | 73 (114) 152 263 497 750 1189 1438 1811 2706 3480 3777 4769 7028 7729 8860 9055 9698 9800 10398 10550 11299 11467 11719 11914 12308 12372 13326 14167 14766 14798 15326 15758 15924 (16093) 16224 16311 |
| K1a1b2a1a | 73 (114) 152 263 497 750 1189 1438 1811 2706 3480 3777 4769 7028 7278 7729 8860 9055 9698 9800 10398 10550 11299 11467 11719 11914 12308 12372 13326 14167 14766 14798 15326 15758 15924 (16093) 16224 16311 |
| K1a1b2b | 73 (114) 189 263 497 750 1189 1438 1811 2706 3480 4769 7028 8860 9055 9698 10202 10398 10550 11299 11467 11719 11914 12308 12372 13326 14167 14766 14798 15326 15924 (16093) 16224 16311 |
| K1a1c | 73 (114) 263 497 709 750 1189 1438 1694 1811 2706 3480 4769 7028 8860 9055 9698 10398 10550 11299 11467 11719 11914 12308 12372 14167 14766 14798 15326 (16093) 16224 16311 16362 16400 |
| K1a2 | 73 263 497 750 1189 1438 1811 2706 3480 4769 7028 8860 9055 9698 10398 10550 11025 11299 11467 11719 12308 12372 14167 14766 14798 15326 (16093) 16224 16311 |
| K1a2a | 73 263 497 750 1189 1438 1811 2706 3480 4769 5773 7028 8860 9055 9698 10398 10550 11025 11299 11467 11719 12308 12372 14167 14766 14798 15326 (16093) 16224 16311 |
| K1a2a1 | 73 263 497 750 1189 1438 1811 2706 3480 4748 4769 5773 7028 8860 9055 9698 10398 10550 11025 11299 11467 11719 12308 12372 13368 14167 14766 14798 15326 (16093) 16189 16224 16311 |
| K1a2a2 | 73 263 497 750 1189 1438 1811 2706 3480 4769 5773 7028 7918 8860 9055 9698 10398 10550 11025 11299 11467 11719 12308 12372 14167 14766 14798 15326 (16093) 16224 16311 |
| K1a2b | 73 263 497 750 1189 1438 1811 2706 3480 4769 7028 8005 8860 9055 9698 10398 10550 11025 11299 11467 11719 12308 12372 14167 14766 14798 15326 (16093) 16224 16311 |
| K1a2c | 73 263 497 750 1189 1438 1811 2706 3480 4216 4769 7028 7775 8860 9055 9698 10398 10550 11025 11299 11467 11719 12308 12372 14167 14766 14798 15326 (16093) 16224 16311 |
| K1a3 | 73 263 497 750 1189 1438 1811 2706 3480 4769 7028 8860 9055 9698 10398 10550 11299 11467 11719 12308 12372 13117 14167 14766 14798 15326 (16093) 16224 16311 |
| K1a3a | 73 263 497 750 1189 1438 1811 2706 3480 4769 7028 7559 8860 9055 9698 10398 10550 11299 11467 11719 12308 12372 13117 14167 14766 14798 15326 (16093) 16224 16311 |
| K1a3a1 | 73 263 497 750 1189 1438 1811 2706 3480 4769 7028 7559 8440 8860 9055 9698 10398 10550 11299 11467 11719 12308 12372 13117 14167 14766 14798 15326 (16093) 16224 16311 |
| K1a3a1a | 73 263 497 750 1189 1438 1811 2706 3480 4769 7028 7559 8440 8860 9055 9548 9698 10398 10550 10697 11299 11467 11719 12308 12372 13117 14167 14530 14766 14798 15326 (16093) 16224 16311 |
| K1a3a1b | 73 263 497 750 1189 1438 1811 2706 3480 4769 7028 7559 8440 8522 8860 9055 9698 10398 10550 11299 11467 11719 12308 12372 13117 14167 14766 14798 15326 (16093) 16224 16311 |

| | |
|---|---|
| K1a3a2 | 73 263 497 750 1189 1438 1811 2706 3480 4769 5580 7028 7559 8860 9055 9698 10398 10550 11299 11467 11719 12308 12372 13117 14167 14766 14798 15326 (16093) 16224 16311 |
| K1a3a3 | 73 263 497 750 1189 1438 1811 2706 3480 4769 7028 7559 8860 9055 9698 10398 10550 11299 11467 11719 12308 12372 13117 13590 14167 14766 14798 15326 (16093) 16224 16311 |
| K1a3a4 | 73 263 497 750 1189 1438 1811 2706 3480 4769 6257 7028 7559 8860 9055 9698 10398 10550 11299 11467 11719 12308 12372 13117 14094 14167 14766 14798 15326 (16093) 16224 16311 |
| K1a4 | 73 263 497 750 1189 1438 1811 2706 3480 4769 7028 8860 9055 9698 10398 10550 11299 11467 11485 11719 12308 12372 14167 14766 14798 15326 (16093) 16224 16311 |
| K1a4a | 73 263 497 750 1189 1438 1811 2706 3480 4769 6260 7028 8860 9055 9698 10398 10550 11299 11467 11485 11719 12308 12372 14167 14766 14798 15326 (16093) 16224 16311 |
| K1a4a1 | 73 263 497 750 1189 1438 1811 2706 3480 4769 6260 7028 8860 9055 9698 10398 10550 11299 11467 11485 11719 11840 12308 12372 13740 14167 14766 14798 15326 (16093) 16224 16311 |
| K1a4a1a | 73 263 497 750 1189 1438 1811 2706 3480 4295 4769 6260 7028 8860 9055 9698 10398 10550 11299 11467 11485 11719 11840 12308 12372 13740 14167 14766 14798 15326 15884 (16093) 16224 16311 |
| K1a4a1a+195 | 73 195 263 497 750 1189 1438 1811 2706 3480 4295 4769 6260 7028 8860 9055 9698 10398 10550 11299 11467 11485 11719 11840 12308 12372 13740 14167 14766 14798 15326 15884 (16093) 16224 16311 |
| K1a4a1a1 | 73 195 263 497 750 1189 1438 1811 2706 3480 3867 4295 4769 6260 7028 8860 9055 9698 10398 10550 11299 11467 11485 11719 11840 12308 12372 13740 14167 14766 14798 15326 15884 (16093) 16224 16311 |
| K1a4a1a3 | 73 195 263 497 750 1189 1438 1811 2706 3480 4295 4769 5177 6260 7028 8860 9055 9698 10398 10550 11299 11467 11485 11719 11840 12308 12372 13740 14167 14766 14798 15326 15884 (16093) 16224 16311 |
| K1a4a1a2 | 73 263 497 750 1189 1438 1811 2706 3480 4295 4769 6260 7028 8860 9055 9698 10398 10550 11299 11467 11485 11719 11840 12308 12372 13740 14167 14766 14798 15326 15884 (16093) 16224 16245 16311 |
| K1a4a1a2a | 73 263 497 750 1189 1438 1811 2706 3480 4295 4769 6260 7028 8860 9055 9377 9698 10398 10550 11299 11467 11485 11719 11840 12308 12372 13740 14167 14766 14798 15326 15884 (16093) 16224 16245 16311 |
| K1a4a1a2b | 73 146 263 497 750 1189 1438 1811 2706 3480 4295 4769 6260 7028 8860 9055 9698 10398 10550 11299 11467 11485 11719 11840 12308 12372 13401 13740 14167 14766 14798 15326 15884 (16093) 16224 16245 16311 |
| K1a4a1b | 73 263 497 750 1189 1438 1811 2706 3480 4769 6260 7028 8098 8860 9055 9698 10398 10550 11299 11467 11485 11719 11840 12308 12372 13740 14167 14766 14798 15326 (16093) 16224 16311 |
| K1a4a1b1 | 73 263 497 750 1189 1438 1811 2706 3480 4769 6260 7028 8098 8856C 8860 9055 9698 10398 10550 11299 11467 11485 11719 11840 12308 12372 13740 14167 14766 14798 15326 (16093) 16224 16311 |
| K1a4a1b2 | 73 263 497 750 1189 1438 2706 3480 4769 6260 7028 8098 8860 9055 9698 10398 10550 11299 11467 11485 11719 11840 12308 12372 13740 14167 14766 14798 15326 (16093) 16224 16261 16311 |

| | |
|---|---|
| K1a4a1c | 73 263 497 750 1189 1438 1811 2706 3480 4769 6260 7028 8860 9055 9698 10398 10550 11299 11467 11485 11719 11840 12308 12372 13413 13740 14167 14766 14798 15326 (16093) 16224 16311 |
| K1a4a1c1 | 73 199 263 497 750 1189 1438 1811 2706 3480 4769 6260 7028 8860 9055 9698 10398 10550 11299 11467 11485 11719 11840 12308 12372 13413 13740 14167 14766 14798 15326 (16093) 16224 16311 |
| K1a4a1d | 73 263 497 750 1189 1438 1811 2706 3394 3480 4769 6260 7028 8860 9055 9698 10398 10550 11299 11467 11485 11719 11840 12308 12372 13740 14167 14766 14798 15326 (16093) 16224 16311 |
| K1a4a1e | 73 152 263 497 750 1189 1438 1811 2706 3480 4769 6260 7028 8860 9055 9698 10398 10550 11299 11467 11485 11719 11840 12308 12372 13740 14167 14766 14798 15326 (16093) 16224 |
| K1a4a1f | 73 152 263 325 497 750 1189 1438 1811 2706 3480 4769 6260 7028 8860 9055 9698 10029 10398 10550 11299 11467 11485 11719 11840 12308 12372 13740 14167 14766 14798 15326 (16093) 16224 16311 |
| K1a4a1f1 | 73 152 217 263 325 497 750 1189 1438 1811 2706 3480 4769 5774 6260 7028 8860 9055 9698 10029 10398 10550 11299 11467 11485 11719 11840 12308 12372 13740 14167 14766 14798 15326 (16093) 16224 16311 |
| K1a4a1g | 73 263 497 750 1189 1438 1811 2706 3480 4769 6260 7028 8149 8860 9055 9698 10398 10550 11299 11467 11485 11719 11840 12308 12372 13740 14167 14766 14798 15326 (16093) 16224 16287 16311 |
| K1a4a1h | 73 263 497 750 1189 1438 1811 2706 3480 4769 6260 7028 8860 9055 9698 10398 10550 11299 11467 11485 11719 11840 12308 12372 13404 13740 14167 14766 14798 15326 (16093) 16224 16311 |
| K1a4a1i | 73 263 497 750 1189 1438 1811 2706 3480 4769 7028 8860 9055 9698 10398 10550 11299 11467 11485 11719 11840 12308 12372 13740 14167 14766 14798 15326 15880 (16093) 16224 16311 |
| K1a4b | 73 263 280G 497 750 1189 1438 1811 2706 3480 4769 7028 8860 9055 9448 9698 10398 10550 11299 11467 11485 11719 12308 12372 14167 14766 14798 15326 (16093) 16224 16311 |
| K1a4b1 | 73 263 280G 497 750 1189 1438 1811 2706 3480 4769 6975 7028 7388 8410 8860 9055 9448 9698 10398 10550 11299 11467 11485 11719 12308 12372 13105 14167 14766 14798 15326 (16093) 16224 16311 |
| K1a4c | 73 152 263 497 750 1189 1438 1811 2706 3480 4769 7028 8860 9055 9698 10398 10550 11299 11467 11485 11719 12308 12372 12612 13827 14167 14766 14798 15326 (16093) 16224 16311 |
| K1a4c1 | 73 152 263 497 750 1189 1438 1811 2706 3480 4769 5264 7028 8860 9055 9698 10398 10550 11299 11467 11485 11719 12308 12372 12612 13710 13827 14167 14766 14798 15326 (16093) 16224 16246T 16311 |
| K1a4d | 73 263 497 723C 750 1189 1438 1811 2706 3480 4769 7028 8860 9055 9698 10398 10550 11071 11299 11467 11485 11719 12308 12372 14167 14766 14798 15326 15355 (16093) 16224 16311 |
| K1a4e | 73 263 497 750 942 1189 1438 1811 2706 3480 4769 7028 8860 9055 9698 10398 10550 11299 11467 11485 11719 12308 12372 14167 14766 14798 15326 (16093) 16224 16311 |
| K1a4f | 73 263 497 750 1189 1438 1811 2706 3480 4769 7028 7118 8860 9055 9698 10398 10550 11299 11467 11485 11719 12308 12372 14167 14766 14798 15326 (16093) 16224 16311 |

| | |
|---|---|
| K1a4f1 | 73 263 497 750 1189 1438 1811 2706 3480 4769 7028 7118 8860 9055 9698 10398 10550 11299 11467 11485 11719 12017 12308 12372 14167 14766 14798 15326 (16093) 16224 16311 |
| K1a4g | 73 263 497 750 1189 1438 1811 2706 3480 4769 6518 7028 8860 9055 9698 10398 10550 11299 11467 11485 11719 11914 12308 12372 14167 14766 14798 15326 (16093) 16224 16311 |
| K1a4h | 73 263 497 750 1189 1438 1811 2706 3480 4769 5237 7028 8531 8860 9055 9698 10398 10550 11299 11467 11485 11719 12308 12372 14167 14766 14798 15326 (16093) 16224 16311 |
| K1a4h1 | 73 263 497 750 1189 1438 1811 2706 3480 4137 4769 5237 7028 8531 8860 9055 9698 10398 10550 11299 11467 11485 11719 12308 12372 14167 14766 14798 15326 (16093) 16224 16311 |
| K1a4i | 73 263 497 750 1189 1438 1811 1926T 2706 3480 4769 7028 8860 9055 9698 10398 10550 11299 11467 11485 11719 12308 12372 14167 14766 14798 15326 (16093) 16224 16311 |
| K1a4+146 | 73 146 263 497 750 1189 1438 1811 2706 3480 4769 7028 8860 9055 9698 10398 10550 11299 11467 11485 11719 12308 12372 14167 14766 14798 15326 (16093) 16224 16311 |
| K1a4j | 73 146 263 497 750 1189 1438 1811 2706 3480 4769 7028 8860 9055 9698 10398 10550 11299 11467 11485 11719 12308 12372 12681 14167 14766 14798 15326 (16093) 16224 16311 |
| K1a4j1 | 73 146 152 263 497 750 1189 1438 1811 2706 3480 4769 7028 8860 9055 9698 10398 10550 11167 11299 11467 11485 11719 12308 12372 12681 14167 14766 14798 15326 (16093) 16224 16311 |
| K1a5 | 73 263 497 750 1189 1438 1811 2706 3480 4769 7028 8860 9055 9698 10398 10550 11017 11299 11467 11719 12308 12372 14167 14766 14798 15326 (16093) 16224 16311 |
| K1a5a | 73 263 497 750 1189 1438 1811 2706 3480 4640 4769 7028 8860 9055 9647 9698 10398 10550 11017 11299 11467 11719 12308 12372 14167 14766 14798 15326 (16093) 16224 16311 16362 |
| K1a5b | 73 153 263 408A 497 750 1189 1438 1811 2706 3480 4769 6407 7028 8860 9055 9428 9698 10398 10550 11017 11299 11467 11719 12308 12372 14167 14766 14798 15326 (16093) 16224 16311 |
| K1a6 | 73 263 497 750 1189 1438 1811 2706 3480 4769 7028 8790 8860 9055 9698 10398 10454 10550 11299 11467 11719 12308 12372 14167 14766 14798 15326 (16093) 16224 16311 16527 |
| K1a7 | 73 263 431A 497 750 1189 1438 1811 2706 3480 4769 7028 8860 9055 9698 10398 10550 11299 11467 11719 12308 12372 14167 14766 14798 15326 (16093) 16224 16311 |
| K1a8 | 73 263 295A 497 750 1189 1438 1811 2706 3480 4769 7028 8860 9055 9698 10398 10550 11299 11467 11719 12308 12372 14167 14766 14798 15326 (16093) 16224 16311 |
| K1a8a | 73 263 295A 497 750 1189 1438 1811 2706 3480 4769 7028 7927G 8860 9055 9698 10398 10550 11299 11467 11719 12308 12372 14167 14766 14798 15326 (16093) 16224 16234 16311 |
| K1a8a1 | 73 263 295A 497 750 1189 1438 1811 2706 3480 4769 7028 7927G 8860 9055 9698 10398 10550 11299 11467 11719 12308 12372 14167 14766 14798 15326 15850G (16093) 16224 16234 16311 |

| | |
|---|---|
| K1a8b | 73 263 295A 497 750 1189 1438 1811 2706 3480 4769 7028 8860 9055 9698 10398 10550 11299 11467 11719 11818 12308 12372 14167 14766 14798 15326 (16093) 16224 16311 16497 |
| K1a+195 | 73 195 263 497 750 1189 1438 1811 2706 3480 4769 7028 8860 9055 9698 10398 10550 11299 11467 11719 12308 12372 14167 14766 14798 15326 (16093) 16224 16311 |
| K1a9 | 73 195 263 497 750 1189 1438 1811 2706 3480 4769 7028 8860 9055 9698 10398 10550 11299 11467 11719 12308 12372 14167 14766 14798 15326 (16093) 16224 16311 16524 |
| K1a10 | 73 195 263 497 750 1189 1438 1811 2706 3480 4769 7028 8860 9055 9698 10398 10550 11299 11467 11719 12308 12372 14167 14766 14798 15326 16048 (16093) 16224 16311 |
| K1a10a | 73 195 263 497 750 1189 1438 1811 2706 3480 4769 7028 8860 9055 9698 10398 10550 11299 11467 11719 12308 12372 14167 14766 14798 15326 16048 (16093) 16224 16291 16311 |
| K1a13 | 73 146 195 263 497 750 1189 1438 1709 1811 2706 3480 4769 7028 8860 9055 9545 9698 10398 10550 11299 11467 11719 12308 12372 14167 14766 14798 15326 (16093) 16224 16311 |
| K1a13a | 73 146 195 263 497 750 1189 1438 1709 1811 2706 3480 4769 7028 8860 8870 9055 9545 9698 10398 10550 11299 11467 11719 12308 12372 14167 14766 14798 15326 (16093) 16224 16311 |
| K1a14 | 73 195 263 497 750 1189 1438 1811 2706 3480 4769 7028 8020 8860 9055 9698 9977 10398 10550 11299 11467 11719 12308 12372 14167 14766 14798 15326 15626 (16093) 16224 16311 |
| K1a15 | 73 195 263 497 750 1189 1438 1811 2706 3480 4769 7028 8155 8860 9055 9698 10398 10550 11299 11467 11719 12308 12372 14167 14766 14798 15326 (16093) 16224 16311 |
| K1a16 | 73 195 263 497 750 1189 1438 1811 2706 3480 4769 6367 7028 8860 9055 9698 10398 10550 11299 11467 11719 12308 12372 14167 14766 14798 15326 (16093) 16224 16311 |
| K1a26 | 73 195 263 497 750 1189 1438 1811 2706 3480 4113 4769 7028 8860 9055 9698 10398 10550 11299 11467 11719 12308 12372 14167 14766 14798 15326 (16093) 16224 16311 |
| K1a+150 | 73 150 263 497 750 1189 1438 1811 2706 3480 4769 7028 8860 9055 9698 10398 10550 11299 11467 11719 12308 12372 14167 14766 14798 15326 (16093) 16224 16311 |
| K1a11 | 16T 73 150 199 263 497 750 1189 1438 1811 2706 3480 4769 7028 8281-8289d 8860 9055 9698 9938 10398 10550 11299 11467 11719 12308 12372 14167 14766 14798 15326 (16093) 16129 16224 16311 |
| K1a11a | 16T 73 150 152 199 263 497 750 1189 1438 1811 2706 3480 4769 7028 8281-8289d 8860 9055 9698 9938 10398 10550 10966 11299 11467 11719 12308 12372 14167 14766 14798 15326 (16093) 16129 16224 16311 |
| K1a11a1 | 16T 73 150 152 199 263 497 750 1189 1438 1811 2706 3480 4769 7028 8281-8289d 8702 8860 9055 9698 9938 10398 10550 10966 11299 11467 11719 12308 12372 14167 14766 14798 15326 (16093) 16129 16224 16311 |
| K1a11b | 16T 73 150 199 263 497 750 1189 1438 1811 2706 3480 4769 7028 8281-8289d 8860 9055 9698 9938 10398 10550 11299 11467 11719 12308 12372 14167 14605T 14766 14798 15326 (16093) 16129 16224 16311 |

| | |
|---|---|
| K1a24 | 73 150 263 497 750 1189 1438 1811 2706 3480 4769 7028 8860 9055 9698 10398 10550 11299 11467 11719 12308 12372 14167 14766 14798 15326 15625A (16093) 16224 16311 |
| K1a24a | 73 150 195 263 497 750 1189 1438 1811 2706 3480 4769 5964 7028 8860 9055 9698 10398 10550 11299 11467 11719 12308 12372 14167 14766 14798 15326 15625A 15791 (16093) 16145 16224 16311 |
| K1a30 | 73 150 263 497 750 1189 1438 1811 2706 3480 4769 7028 8860 9055 9698 10398 10550 11299 11467 11719 12308 12372 14167 14766 14798 15326 15650 (16093) 16224 16311 |
| K1a30a | 73 150 263 497 750 1189 1438 1811 2706 3480 4769 6392 7028 8860 9055 9698 10398 10550 11299 11467 11719 12308 12372 14167 14766 14798 15326 15650 (16093) 16224 16311 |
| K1a31 | 73 150 263 497 750 1189 1438 1811 2706 3480 4769 7028 8860 8952 9055 9698 10398 10550 11299 11467 11719 12308 12372 14167 14766 14798 15326 (16093) 16224 16311 |
| K1a12 | 73 263 497 750 1189 1438 1811 2706 3480 4769 5460 7028 8860 9055 9698 10398 10550 11299 11467 11719 12308 12372 14167 14766 14798 15326 (16093) 16224 16311 |
| K1a12a | 73 263 497 750 1189 1438 1811 2706 3480 4769 5460 7028 8860 9055 9698 10398 10550 11299 11467 11719 11923 12308 12372 14167 14766 14798 15326 (16093) 16224 16311 |
| K1a12a1 | 73 263 497 750 1189 1438 1811 2706 3480 4769 5460 6284 7028 8860 9055 9698 10398 10550 11299 11467 11719 11923 12308 12372 14167 14766 14798 15326 (16093) 16224 16311 |
| K1a12a1a | 73 263 497 750 1189 1438 1811 2706 3480 4769 5460 6284 7028 8860 9055 9698 10398 10550 11299 11467 11497 11719 11923 12308 12372 14167 14766 14798 15326 (16093) 16224 16311 |
| K1a17 | 73 247 263 497 750 1189 1438 1811 2706 3480 4561 4769 7028 8860 9055 9698 10398 10550 11299 11467 11719 12308 12372 14167 14766 14798 15326 15670 (16093) 16224 16311 |
| K1a17a | 73 247 263 497 750 1189 1438 1719 1811 2706 3480 4561 4769 7028 8860 9055 9698 10398 10550 11299 11467 11719 12308 12372 14167 14766 14798 15326 15670 (16093) 16224 16260 16311 |
| K1a18 | 73 263 497 750 1189 1438 1811 2706 3480 4769 6680 6881 7028 8860 9055 9698 10398 10550 11299 11467 11719 12308 12372 14167 14766 14798 15326 (16093) 16224 16311 |
| K1a19 | 73 263 497 750 1189 1438 1811 2706 3480 4769 7028 8860 9055 9698 10398 10550 11299 11467 11719 12308 12338 12372 14167 14766 14798 15326 (16093) 16224 16311 |
| K1a19a | 63 64 73 263 497 750 1189 1438 1811 2706 3480 4769 7028 8277 8860 9055 9698 10398 10550 11299 11467 11719 11878 12134 12308 12338 12372 12397 14167 14766 14798 15031 15326 (16093) 16224 16311 |
| K1a23 | 73 263 497 750 1189 1438 1811 2706 3480 4769 5093 7028 8860 9055 9698 10398 10550 11299 11467 11719 12308 12372 14167 14766 14798 15326 (16093) 16224 16311 |
| K1a25 | 73 152 263 497 750 1189 1438 1811 2706 3480 4158 4769 7028 8860 9055 9698 10398 10550 11299 11467 11719 12308 12372 14167 14308 14766 14798 15326 (16093) 16224 16311 |

| | |
|---|---|
| K1a27 | 73 263 497 750 1189 1438 1811 2706 3480 4769 7028 7202 8860 9055 9698 10398 10550 11299 11467 11719 12308 12372 14167 14766 14798 15326 (16093) 16176 16224 16311 |
| K1a28 | 73 263 466 497 750 1189 1438 1811 2706 3480 4769 6071 7028 8860 9055 9287 9698 10398 10550 11299 11467 11719 12308 12372 14167 14766 14798 15326 15544 (16093) 16224 16311 |
| K1a29 | 73 263 497 750 1189 1438 1811 2706 3480 4769 7028 8167 8860 9055 9698 10398 10550 11299 11467 11719 12308 12372 14167 14766 14798 15326 (16093) 16224 16311 |
| K1a29a | 73 263 497 750 1189 1438 1811 2706 3480 3702 4769 7028 8167 8860 9055 9698 10398 10550 11299 11467 11719 12308 12372 14167 14766 14798 15326 (16093) 16224 16265 16311 |
| K1b | 73 263 750 1189 1438 1811 2706 3480 4769 5913 7028 8860 9055 9698 10398 10550 11299 11467 11719 12308 12372 14167 14766 14798 15326 16224 16311 |
| K1b1 | 73 263 750 1189 1438 1811 2706 3480 4769 5913 7028 8860 9055 9698 9962 10289 10398 10550 11299 11467 11719 12308 12372 14167 14766 14798 15326 15946 16224 16311 |
| K1b1+(16093) | 73 263 750 1189 1438 1811 2706 3480 4769 5913 7028 8860 9055 9698 9962 10289 10398 10550 11299 11467 11719 12308 12372 14167 14766 14798 15326 15946 (16093) 16224 16311 |
| K1b1a | 73 152 263 750 1189 1438 1811 2706 3480 4769 5913 7028 8860 9055 9698 9962 10289 10398 10550 11299 11467 11719 11923 12308 12372 14167 14766 14798 15257 15326 15946 (16093) 16224 16311 16319 16463 |
| K1b1a1 | 73 152 263 750 1189 1438 1811 2706 3480 4769 5913 7028 8860 9055 9698 9962 10289 10398 10550 11299 11467 11719 11923 12308 12372 13967 14167 14766 14798 15257 15326 15946 (16093) 16224 16311 16319 |
| K1b1a1+199 | 73 152 199 263 750 1189 1438 1811 2706 3480 4769 5913 7028 8860 9055 9698 9962 10289 10398 10550 11299 11467 11719 11923 12308 12372 13967 14167 14766 14798 15257 15326 15946 (16093) 16224 16311 16319 16463 |
| K1b1a1a | 73 152 199 263 750 1189 1438 1811 2706 3480 4646 4769 5913 7028 8860 9055 9698 9962 10289 10398 10550 11299 11467 11719 11923 12308 12372 13967 14167 14766 14798 15257 15326 15946 (16093) 16224 16311 16319 16463 |
| K1b1a1b | 73 152 199 263 750 1189 1438 1811 2706 3480 4769 5913 7028 8860 9055 9698 9962 10289 10398 10550 11299 11467 11719 11923 12308 12372 13967 14167 14308 14766 14798 15257 15326 15946 (16093) (16189) 16224 16311 16319 16463 |
| K1b1a1c | 73 152 263 750 1189 1438 1811 2706 3480 3834 4769 4775 5913 7028 8860 9055 9698 9962 10289 10398 10550 11299 11467 11719 11923 12308 12372 13967 14167 14766 14798 15257 15326 15946 (16093) 16224 16311 16319 16463 |
| K1b1a1c1 | 73 152 263 750 1189 1438 1811 2706 3480 3834 4769 4775 5298 5913 7028 8860 9055 9698 9962 10289 10398 10550 11299 11467 11719 11923 12308 12372 13967 14167 14766 14798 15257 15326 15946 (16093) 16224 16311 16319 16463 |

| | |
|---|---|
| K1b1a1d | 73 152 263 750 1189 1438 1811 2706 3480 4769 5913 7028 8860 9055 9698 9962 10289 10398 10550 10861 11299 11467 11719 11923 12308 12372 13967 14167 14766 14798 15257 15326 15946 (16093) 16224 16311 16319 16463 |
| K1b1a1d1 | 73 152 263 750 1189 1438 1811 2706 3480 4769 5913 7028 8860 9055 9698 9962 10289 10398 10550 10861 11299 11467 11719 11923 12308 12372 13967 14167 14766 14798 15148 15257 15326 15946 (16093) 16224 16311 16319 16463 |
| K1b1a2 | 73 152 195 263 750 1189 1438 1811 2706 3480 4769 5913 7028 8260 8860 9055 9371 9698 9962 10289 10398 10550 11299 11467 11719 11923 12308 12372 14167 14766 14798 15257 15326 15381 15946 (16093) 16224 16311 16318T 16319 16463 |
| K1b1b | 73 263 750 1189 1438 1811 2706 3480 4769 5913 6053 7028 8164 8860 9055 9698 9962 10289 10398 10550 11299 11467 11719 12308 12372 14167 14766 14798 15326 15946 (16093) 16224 16311 |
| K1b1b1 | 73 263 750 1189 1438 1811 2706 3480 4769 5913 6053 7028 8164 8860 9055 9698 9962 10289 10398 10550 11299 11467 11719 12308 12372 14063 14167 14384 14766 14798 15326 15946 (16093) 16224 16311 |
| K1b1c | 73 94 263 750 1189 1438 1811 2706 3337 3480 4769 5913 7028 8860 9055 9698 9962 10289 10398 10550 11299 11467 11719 11914 12308 12372 14167 14766 14798 15326 15946 16224 16311 |
| K1b2 | 73 146 195 263 750 1189 1438 1811 2706 3480 4769 5913 7028 8860 9055 9698 10398 10550 11299 11467 11719 12308 12372 12738G 14167 14766 14798 15326 16224 16311 |
| K1b2a | 73 146 195 263 750 1189 1438 1811 2706 3480 4769 5913 7028 8860 9055 9698 10398 10550 11299 11467 11719 12308 12372 12738G 12771 14167 14766 14798 15326 16224 16311 |
| K1b2a1 | 73 146 195 263 750 1189 1438 1811 2706 3480 4769 5913 7028 8860 9055 9698 10398 10550 11299 11467 11719 12308 12372 12738G 12771 13759 14167 14766 14798 15326 16224 16311 |
| K1b2a1a | 73 146 195 263 750 1189 1438 1811 2706 3480 4769 5913 7028 8860 9055 9698 10398 10550 11299 11467 11719 12019 12308 12372 12738G 12771 13759 14167 14766 14798 15326 16224 16311 |
| K1b2a1a1 | 73 146 195 263 750 1189 1438 1811 2706 3480 4769 5913 7028 8860 9055 9698 10398 10550 11299 11467 11719 12019 12308 12372 12738G 12771 13759 14167 14766 14798 15326 16224 16311 16524 |
| K1b2a2 | 73 146 195 263 750 1189 1438 1811 2706 3480 4769 5913 7028 7521 8860 9055 9698 10398 10550 11299 11467 11719 12308 12372 12738G 12771 14167 14766 14798 15326 16224 16311 |
| K1b2a2a | 73 146 195 263 750 1189 1438 1811 2706 3480 4769 5913 7028 7521 8860 9055 9698 10398 10550 11299 11467 11719 12308 12372 12738G 12771 14167 14687 14766 14798 15326 16224 16311 |
| K1b2a3 | 73 146 195 263 750 1189 1438 1811 2706 3480 4769 5913 7028 8860 9055 9698 10398 10550 11299 11467 11719 12188 12308 12372 12738G 12771 14167 14766 14798 15326 16224 16311 |
| K1b2b | 73 146 195 263 750 1189 1438 1811 2706 3480 4769 5237 5913 6845 7028 8860 9055 9698 10154 10398 10550 11299 11467 11719 12308 12372 12738G 14167 14766 14798 15301 15326 16224 16311 |

| | |
|---|---|
| K1b2b1 | 73 146 195 263 368 750 1189 1438 1811 2706 3480 4769 5237 5913 6845 7028 8860 9055 9698 10154 10398 10550 11299 11467 11719 12308 12372 12738G 14167 14766 14798 15301 15326 16224 16311 |
| K1c | 73 146 152 263 498d 750 1189 1438 1811 2706 3480 4769 7028 8860 9055 9698 10398 10550 11299 11467 11719 12308 12372 14167 14766 14798 15326 16224 16311 |
| K1c1 | 73 146 152 263 498d 750 1189 1438 1811 2706 3480 4769 7028 8860 9055 9093 9698 10398 10550 11299 11377 11467 11719 12308 12372 14167 14766 14798 15326 16224 16311 |
| K1c1a | 73 146 152 263 498d 750 1189 1438 1811 2706 3480 4769 7028 7082 8860 9055 9093 9698 10398 10550 11299 11377 11467 11719 12308 12372 14167 14766 14798 15326 16224 16311 |
| K1c1b | 73 146 152 263 498d 750 1189 1438 1811 2706 3480 4769 7028 8860 9055 9093 9698 10398 10550 11299 11377 11467 11719 12308 12372 14167 14766 14798 15326 15900 16224 16311 |
| K1c1c | 73 146 152 263 498d 750 1189 1438 1811 2706 3480 4769 7028 8860 9055 9093 9698 9903 10398 10550 11299 11377 11467 11719 12308 12372 14167 14766 14798 15326 16224 16311 |
| K1c1d | 73 146 152 263 498d 750 1189 1438 1442 1811 2706 3480 3720 4769 7028 8860 9055 9093 9698 10398 10550 11299 11377 11467 11719 12308 12372 13602 14167 14766 14798 15326 16224 16311 |
| K1c1e | 73 146 152 263 498d 750 1189 1438 1811 2706 3480 4769 7028 8860 9055 9093 9698 10398 10550 11299 11377 11467 11719 12308 12372 14167 14766 14798 15326 16224 16311 16368 |
| K1c1f | 73 146 152 263 498d 750 1189 1438 1811 2706 3480 4769 7028 8860 9055 9093 9698 10398 10550 10698 11299 11377 11467 11719 12308 12372 14167 14766 14798 15326 16224 16311 |
| K1c1g | 73 146 152 263 498d 750 1189 1438 1811 2706 3480 4769 7028 8860 9055 9093 9698 10398 10550 11299 11377 11467 11719 12161 12308 12372 14167 14766 14798 15326 16224 16311 |
| K1c1h | 73 146 152 263 498d 750 1189 1438 1811 2706 3480 4769 7028 8473 8860 9055 9093 9698 10398 10550 11299 11377 11467 11719 12308 12372 14167 14766 14798 15326 16224 16311 |
| K1c1i | 73 146 152 263 498d 750 1189 1438 1811 2706 3480 4769 7028 7202 8860 9055 9093 9698 10398 10550 11299 11377 11467 11719 12308 12372 14167 14766 14798 15326 16224 16311 |
| K1c2 | 73 146 152 263 498d 750 1189 1438 1811 2706 3480 4769 7028 8860 9006 9055 9698 10398 10550 11299 11467 11719 12308 12372 14002 14040 14167 14766 14798 15326 16224 16311 16320 |
| K1c2a | 73 146 152 263 498d 750 1189 1438 1811 2706 3480 4769 7028 8860 9006 9055 9698 10398 10550 11299 11467 11719 12308 12372 14002 14040 14167 14766 14798 15326 15944d 16224 16311 16320 |
| K1+16362 | 73 263 750 1189 1438 1811 2706 3480 4769 7028 8860 9055 9698 10398 10550 11299 11467 11719 12308 12372 14167 14766 14798 15326 16224 16311 16362 |
| K1d | 73 195 263 573.XC 750 1189 1438 1811 2706 3480 4769 4856 5592 7028 8860 8901 9055 9698 10398 10550 11299 11467 11719 12308 12372 14167 14766 14798 15326 16224 16311 16362 |

| | |
|---|---|
| K1d1 | 73 195 263 573.XC 750 1189 1438 1811 2706 3480 4769 4856 5592 6890 7028 8860 8901 9055 9698 10398 10550 11299 11467 11719 12308 12372 14167 14766 14798 15326 16224 16311 16362 |
| K1e | 73 152 263 750 1189 1438 1811 1819 2706 3480 4769 7028 8251 8860 9055 9698 10398 10478 10550 11299 11467 11719 12308 12372 14142G 14167 14766 14798 15326 16224 16311 16362 |
| K1e1 | 73 151 152 263 750 1189 1438 1811 1819 2706 3480 4769 6413 7028 8251 8860 9055 9698 10398 10478 10550 11299 11467 11719 12308 12372 14142G 14167 14766 14798 15326 16189A 16224 16274 16311 16362 |
| K1f | 73 263 750 1189 1438 1811 2706 3480 3513 4769 7028 8137 8860 9055 9698 10398 10550 11299 11467 11719 12308 12372 14167 14766 14798 15326 16224 16311 16362 |
| K2 | 73 146 263 750 1438 1811 2706 3480 4769 7028 8860 9055 9698 9716 10550 11299 11467 11719 12308 12372 14167 14766 14798 15326 16224 16311 |
| K2a | 73 146 152 263 709 750 1438 1811 2706 3480 4561 4769 7028 8860 9055 9698 9716 10550 11299 11467 11719 12308 12372 14167 14766 14798 15326 16224 16311 |
| K2a1 | 73 146 152 263 709 750 1438 1811 2706 3480 4561 4769 7028 7930 8860 9055 9698 9716 10550 11299 11467 11719 12308 12372 14167 14766 14798 15325 15326 15652 16224 16311 |
| K2a1a | 73 146 152 263 709 750 1438 1811 2706 3480 4561 4769 7028 7930 8860 9055 9698 9716 10550 11299 11467 11719 11881 12308 12372 14167 14766 14798 15325 15326 15652 16224 |
| K2a2 | 73 146 152 263 709 750 1438 1811 2706 3480 4561 4769 7028 8697 8860 9055 9698 9716 10550 11299 11467 11719 12308 12372 14167 14766 14798 15326 16224 16311 |
| K2a2a | 73 146 152 263 709 750 1438 1811 2706 3480 4561 4769 7028 8697 8860 9055 9698 9716 10550 11299 11348 11467 11719 12308 12372 14167 14766 14798 15326 16224 16311 |
| K2a2a1 | 73 146 152 263 512C 709 750 1438 1811 2706 3480 4561 4769 7028 8697 8860 9055 9254 9698 9716 10550 11299 11348 11467 11719 11914 12308 12372 14167 14766 14798 15326 16224 16311 |
| K2a3 | 73 146 152 263 709 750 1438 1811 2706 3480 4561 4769 7028 8860 9055 9698 9716 10550 11299 11467 11719 12308 12372 13293 14167 14766 14798 15326 16224 16311 |
| K2a3a | 73 146 152 263 709 750 1438 1811 2706 3480 4561 4769 7028 8860 9055 9698 9716 10550 11299 11467 11719 12308 12372 12795 13293 14167 14766 14798 15326 16224 16311 |
| K2a3a1 | 73 146 152 263 709 750 1438 1811 2706 3480 4561 4769 7028 8860 9055 9698 9716 10550 11299 11467 11719 12308 12372 12795 13293 13708 14167 14766 14798 15326 16224 16311 |
| K2a4 | 73 146 152 263 709 750 1438 1811 2706 3480 4561 4769 7028 8860 9055 9698 9716 10550 11299 11467 11549 11719 12308 12372 14167 14766 14798 15326 16224 16311 |
| K2a5 | 73 146 152 263 324 709 750 1438 1811 2706 3480 4561 4769 7028 8860 9055 9698 9716 10550 11299 11467 11719 12308 12372 14167 14766 14798 15326 16224 16311 |

| | |
|---|---|
| K2a5a | 73 146 152 263 324 709 750 1438 1811 2706 3480 4561 4769 7028 8860 9055 9698 9716 10550 11299 11467 11719 12308 12372 14167 14766 14798 15326 15803 16224 16311 |
| K2a5a1 | 73 146 152 263 324 709 750 1438 1811 2706 3480 4561 4769 7028 8860 9055 9698 9716 10550 11299 11467 11491 11719 12308 12372 14167 14766 14798 15326 15803 16224 16235 16311 |
| K2a5b | 73 146 152 263 324 709 750 1438 1811 2706 2831 3480 4561 4769 7028 8860 9055 9698 9716 10550 11299 11467 11719 12308 12372 14167 14766 14798 15326 16224 16311 |
| K2a6 | 73 146 152 263 709 750 1438 1811 2706 3480 4561 4769 7028 8860 9055 9698 9716 10550 11299 11467 11719 12308 12372 14167 14305 14766 14798 15326 16224 16311 |
| K2a7 | 73 146 152 263 709 750 1438 1811 2706 3480 4561 4769 7028 8860 9055 9698 9716 10550 11299 11467 11719 12308 12346 12372 14167 14766 14798 15326 16224 16311 |
| K2a8 | 73 146 152 207 263 709 750 1438 1811 2706 3480 4561 4769 7028 8860 9055 9698 9716 10550 11299 11467 11719 12308 12372 14167 14766 14798 15326 16189 16224 16311 |
| K2a9 | 73 146 152 263 709 750 1438 1811 2706 3480 4561 4769 5081 5130 7028 8860 9055 9698 9716 10550 11299 11467 11719 12308 12372 14167 14766 14798 15326 16224 16311 |
| K2a10 | 73 146 152 263 709 750 1438 1811 2706 3480 4561 4769 7028 8860 9055 9698 9716 10550 11299 11467 11719 11923 12308 12372 14167 14766 14798 15326 16224 16311 |
| K2a11 | 73 146 152 263 709 750 1438 1811 2706 3480 4561 4769 7028 8860 9055 9698 9716 10398 10550 11204 11299 11467 11719 12308 12372 12870 14167 14766 14798 15326 16224 16293 16311 |
| K2b | 73 146 263 750 1438 1811 2217 2706 3480 4769 5231 7028 8860 9055 9698 9716 10550 11299 11467 11719 12308 12372 14037 14167 14766 14798 15326 16224 16311 |
| K2b1 | 73 146 263 750 1438 1811 2217 2706 3480 4769 5231 7028 8860 9055 9698 9716 10550 11299 11467 11719 11869A 12308 12372 14037 14167 14766 14798 15326 16224 16311 |
| K2b1a | 73 146 263 750 1438 1811 2217 2706 3480 4769 5231 7028 8860 9055 9698 9716 10550 11299 11467 11719 11869A 12308 12372 13135 14037 14167 14766 14798 15326 16224 16311 |
| K2b1a1 | 73 146 263 750 1438 1811 2217 2706 3480 4769 5231 7028 8860 9055 9698 9716 10550 11299 11467 11719 11869A 12308 12372 13135 14037 14167 14766 14798 15326 16224 16270 16311 |
| K2b1a1a | 73 146 263 750 1438 1811 2217 2706 3480 4769 5231 7028 8860 9055 9698 9716 10550 11299 11467 11719 11869A 12308 12372 13135 14037 14167 14766 14798 15326 16222 16224 16270 16311 |
| K2b1a2 | 73 146 263 750 1438 1811 2217 2706 3480 4769 5231 7028 8860 9055 9698 9716 10550 11299 11374 11467 11719 11869A 12308 12372 13135 14037 14167 14766 14798 15326 16224 16311 |
| K2b1a3 | 73 146 263 723 750 1438 1811 2217 2706 3480 4769 5231 7028 8860 9055 9698 9716 10550 11299 11467 11719 11869A 12308 12372 13135 14037 14167 14766 14798 15326 16224 16311 |

| | |
|---|---|
| K2b1a4 | 73 93 146 207 263 750 1438 1811 2217 2706 3480 4769 5231 7028 8860 9055 9698 9716 10550 11167 11299 11467 11719 11869A 12308 12372 13135 14037 14167 14766 14798 15326 16224 16311 |
| K2b1b | 73 146 263 750 1438 1811 2217 2706 3480 4769 5054 5231 7028 8860 9055 9698 9716 10550 11016 11299 11467 11719 11869A 12308 12372 12501 14037 14167 14766 14798 15326 16129 16224 16311 |
| K2b2 | 73 146 263 750 1438 1811 2217 2706 3480 4769 5231 7028 7310 8860 9055 9698 9716 10550 11299 11467 11719 12308 12372 14037 14167 14766 14798 15326 16224 16311 |
| K2c | 73 146 263 750 1438 1811 2706 3480 4769 7028 8860 9055 9698 9716 10550 11172 11299 11467 11719 12308 12372 14167 14766 14798 15326 16224 16311 |
| K3 | 73 150 195 235 263 560 750 1438 1811 2706 3480 4769 7028 7657 8188 8860 9055 9698 9852T 10550 11299 11467 11719 12308 12372 14167 14212 14766 14798 15326 16093 16148 16153 16224 16311 |
| U8c | 73 151 263 750 1438 1811 2706 3480 4769 7028 7031 8860 9698 10398 11467 11719 12308 12372 14766 15326 16189 |
