# Supplementary material for: Mitochondrial Haplogroup Classification of Ancient DNA Samples Using Haplotracker
Source: Biomed Res Int. 2022 Mar 18;2022:5344418. doi: 10.1155/2022/5344418 (PMC8956381; doi:10.1155/2022/5344418)
Supplement: Supplementary Materials — Fig. S1: characterization of Phylotree-provided control region sequences tested for haplogroup classification by Haplotracker. Fig. S2: minimum number of amplicons required by Haplotracker in discriminating between haplogroups using mtDNA control and coding region sequences. Fig. S3: variant identification of an aDNA sample (MNW3) using an HRM real-time PCR. Table S1: haplogroups and their variant profiles extracted from Phylotree mtDNA Build 17. Table S2: haplogroup frequency carrying an extra variant in 118,869 haplotypes. Table S3: haplogroup frequency carrying a missing variant in 118,869 haplotypes. Table S4: haplogroup frequency in 118,869 haplotypes. Table S5: list of ancient human samples found in 2,000-year-old elite Xiongnu cemetery in Northeast Mongolia. Table S6: primers for the amplification of mtDNA coding region segments for haplogroup determination. Table S7: high-resolution melting real-time PCR primer design for screening variants to differentiate haplogroups G1a1, G1a1a, and G1a1b. Table S8: haplogroup classification of full-length mtGenome sequences from Phylotree (n = 8,216). Table S9: haplogroup classification with full-length and control region sequences of mtDNA using Haplotracker and HaploGrep 2. Table S10: comparison of servers using control region sequences from GenBank before December 25, 2018 (n = 45,177). Table S11: comparison details for the servers using control region sequences from GenBank before December 25, 2018 (n = 45,177). Table S12: comparison of servers using control region sequences downloaded from GenBank from December 26, 2018 to August 22, 2019. Table S13: sequences of mtDNA PCR products from Mongolian ancient DNA samples. Table S14: haplogroup classification of Mongolian ancient DNA samples using Haplotracker. Table S15: minimum number of amplicons required by Haplotracker in discriminating between haplogroups using mtDNA control and coding region sequences. Table S16: minimum number of amplicons per superhaplogroup requ [file 5344418.f1.zip › 5344418.f5.pdf]

Table S2. Haplogroup frequency carrying an extra variant in 118,869 haplotypes

HG	Extra variant	Frequency	Rate
A	11081T	1	0.028
A	1215	1	0.028
A	146	3	0.083
A	150	1	0.028
A	16051	3	0.083
A	16079G	1	0.028
A	16124	2	0.056
A	16125	3	0.083
A	16126	1	0.028
A	16196	1	0.028
A	16214	1	0.028
A	16220C	2	0.056
A	16287	2	0.056
A	16304	1	0.028
A	16311	14	0.389
A	16311G	1	0.028
A	16317	2	0.056
A	16362	5	0.139
A	16555G	1	0.028
A	1709	1	0.028
A	195	2	0.056
A	215	1	0.028
A	248	1	0.028
A	318	1	0.028
A	376	1	0.028
A	3915	1	0.028
A	4224	1	0.028
A	4232	1	0.028
A	489	1	0.028
A	5093	1	0.028
A	576	3	0.083
A	8563	1	0.028
A	93	1	0.028
A+152	10646	3	0.05
A+152	10936	1	0.017
A+152	11243	1	0.017
A+152	11260	1	0.017
A+152	12561	2	0.033
A+152	12883	1	0.017
A+152	13590	3	0.05
A+152	13622A	1	0.017
A+152	13879	1	0.017
A+152	151	13	0.217
A+152	16093	9	0.15
A+152	16111A	1	0.017
A+152	16179A	3	0.05
A+152	16189	18	0.3
A+152	16192	2	0.033
A+152	16241	2	0.033
A+152	16259	1	0.017
A+152	16293T	5	0.083
A+152	16311	1	0.017
A+152	16465	2	0.033
A+152	200	1	0.017
A+152	2563	2	0.033
A+152	279	1	0.017
A+152	292	2	0.033
A+152	315.2C	1	0.017
A+152	318	1	0.017
A+152	3200	1	0.017
A+152	4227	1	0.017
A+152	4314	1	0.017
A+152	5512	1	0.017
A+152	5585	2	0.033
A+152	5774	2	0.033
A+152	64	9	0.15
A+152	7298	1	0.017
A+152	7493	1	0.017
A+152	8038	2	0.033
A+152	81	1	0.017
A+152	8270	2	0.033
A+152	8281-8289d	2	0.033
A+152	8383	1	0.017
A+152	8531	1	0.017
A+152	8572	1	0.017
A+152	8713	1	0.017
A+152	9058	1	0.017
A+152	9148	1	0.017
A+152	9300	2	0.033
A+152	9380	1	0.017
A+152	9548	1	0.017
A+152+16362	10310	1	0.002
A+152+16362	10320	1	0.002
A+152+16362	10335	1	0.002
A+152+16362	10724	1	0.002
A+152+16362	10967	2	0.004
A+152+16362	11914	1	0.002
A+152+16362	12172	2	0.004
A+152+16362	12768	1	0.002
A+152+16362	13086	1	0.002
A+152+16362	13488	1	0.002
A+152+16362	13602	1	0.002
A+152+16362	13707	1	0.002
A+152+16362	13711	1	0.002
A+152+16362	13759	1	0.002
A+152+16362	14256	1	0.002
A+152+16362	143	1	0.002
A+152+16362	146	2	0.004
A+152+16362	14693.1T	1	0.002
A+152+16362	14788	1	0.002
A+152+16362	14833	1	0.002
A+152+16362	151	12	0.023
A+152+16362	15142	1	0.002
A+152+16362	15258	1	0.002
A+152+16362	15412	1	0.002

A+152+16362 | 15497 | 1 | 0.002
A+152+16362 | 15727 | 2 | 0.004
A+152+16362 | 15814 | 1 | 0.002
A+152+16362 | 15930 | 1 | 0.002
A+152+16362 | 16067 | 2 | 0.004
A+152+16362 | 16086 | 1 | 0.002
A+152+16362 | 16092 | 1 | 0.002
A+152+16362 | 16093 | 1 | 0.002
A+152+16362 | 16124 | 1 | 0.002
A+152+16362 | 16125 | 1 | 0.002
A+152+16362 | 16148 | 6 | 0.012
A+152+16362 | 16166 | 1 | 0.002
A+152+16362 | 16175 | 1 | 0.002
A+152+16362 | 16186 | 1 | 0.002
A+152+16362 | 16192 | 2 | 0.004
A+152+16362 | 16201 | 1 | 0.002
A+152+16362 | 16209 | 1 | 0.002
A+152+16362 | 16214 | 2 | 0.004
A+152+16362 | 16216 | 1 | 0.002
A+152+16362 | 16248 | 53 | 0.103
A+152+16362 | 16256 | 1 | 0.002
A+152+16362 | 16261 | 1 | 0.002
A+152+16362 | 16270 | 1 | 0.002
A+152+16362 | 16274 | 28 | 0.054
A+152+16362 | 16289 | 2 | 0.004
A+152+16362 | 16293C | 3 | 0.006
A+152+16362 | 16293T | 2 | 0.004
A+152+16362 | 16294 | 1 | 0.002
A+152+16362 | 16295 | 2 | 0.004
A+152+16362 | 16304 | 1 | 0.002
A+152+16362 | 16309 | 4 | 0.008
A+152+16362 | 16311 | 2 | 0.004
A+152+16362 | 16325 | 1 | 0.002
A+152+16362 | 16344 | 1 | 0.002
A+152+16362 | 16352 | 1 | 0.002
A+152+16362 | 16356 | 4 | 0.008
A+152+16362 | 16357 | 2 | 0.004
A+152+16362 | 16400 | 1 | 0.002
A+152+16362 | 16525C | 1 | 0.002
A+152+16362 | 16527 | 25 | 0.048
A+152+16362 | 182 | 10 | 0.019
A+152+16362 | 185 | 1 | 0.002
A+152+16362 | 188 | 3 | 0.006
A+152+16362 | 195 | 1 | 0.002
A+152+16362 | 204 | 1 | 0.002
A+152+16362 | 232C | 1 | 0.002
A+152+16362 | 249d | 1 | 0.002
A+152+16362 | 2831 | 1 | 0.002
A+152+16362 | 315.2C | 2 | 0.004
A+152+16362 | 316C | 5 | 0.01
A+152+16362 | 3483 | 2 | 0.004
A+152+16362 | 366 | 1 | 0.002
A+152+16362 | 3666 | 1 | 0.002
A+152+16362 | 3732 | 2 | 0.004
A+152+16362 | 3828 | 1 | 0.002
A+152+16362 | 3834 | 1 | 0.002
A+152+16362 | 389 | 1 | 0.002
A+152+16362 | 3892 | 1 | 0.002
A+152+16362 | 4083 | 3 | 0.006
A+152+16362 | 4232 | 1 | 0.002
A+152+16362 | 450 | 1 | 0.002
A+152+16362 | 455d | 1 | 0.002
A+152+16362 | 464C | 1 | 0.002
A+152+16362 | 4658 | 1 | 0.002
A+152+16362 | 4917 | 1 | 0.002
A+152+16362 | 523d | 1 | 0.002
A+152+16362 | 549 | 1 | 0.002
A+152+16362 | 573.1C | 1 | 0.002
A+152+16362 | 576 | 1 | 0.002
A+152+16362 | 592 | 1 | 0.002
A+152+16362 | 5951 | 3 | 0.006
A+152+16362 | 6482 | 1 | 0.002
A+152+16362 | 6827 | 1 | 0.002
A+152+16362 | 6890 | 1 | 0.002
A+152+16362 | 711 | 2 | 0.004
A+152+16362 | 7269 | 1 | 0.002
A+152+16362 | 7705 | 1 | 0.002
A+152+16362 | 7861 | 2 | 0.004
A+152+16362 | 824 | 1 | 0.002
A+152+16362 | 8623 | 1 | 0.002
A+152+16362 | 8686 | 3 | 0.006
A+152+16362 | 8908 | 1 | 0.002
A+152+16362 | 9033 | 1 | 0.002
A+152+16362 | 9037 | 1 | 0.002
A+152+16362 | 9477 | 2 | 0.004
A+152+16362 | 9482 | 1 | 0.002
A+152+16362 | 9545 | 1 | 0.002
A+152+16362+16189 | 11914 | 4 | 0.103
A+152+16362+16189 | 1243 | 4 | 0.103
A+152+16362+16189 | 12662 | 4 | 0.103
A+152+16362+16189 | 14410 | 1 | 0.026
A+152+16362+16189 | 150 | 15 | 0.385
A+152+16362+16189 | 15064 | 4 | 0.103
A+152+16362+16189 | 15497 | 2 | 0.051
A+152+16362+16189 | 15718 | 1 | 0.026
A+152+16362+16189 | 1585 | 4 | 0.103
A+152+16362+16189 | 15924 | 1 | 0.026
A+152+16362+16189 | 16086 | 2 | 0.051
A+152+16362+16189 | 16092 | 4 | 0.103
A+152+16362+16189 | 16093 | 1 | 0.026
A+152+16362+16189 | 16125 | 1 | 0.026
A+152+16362+16189 | 16164 | 1 | 0.026
A+152+16362+16189 | 16190 | 5 | 0.128
A+152+16362+16189 | 16196 | 1 | 0.026
A+152+16362+16189 | 16266 | 1 | 0.026
A+152+16362+16189 | 16311 | 6 | 0.154
A+152+16362+16189 | 204 | 1 | 0.026
A+152+16362+16189 | 2831 | 2 | 0.051
A+152+16362+16189 | 318 | 1 | 0.026
A+152+16362+16189 | 3213 | 3 | 0.077
A+152+16362+16189 | 338 | 1 | 0.026

A+152+16362+16189 | 3796 | 2 | 0.051
A+152+16362+16189 | 576 | 1 | 0.026
A+152+16362+16189 | 6392 | 2 | 0.051
A+152+16362+16189 | 683 | 1 | 0.026
A+152+16362+16189 | 6890 | 2 | 0.051
A+152+16362+200 | 1005 | 1 | 0.002
A+152+16362+200 | 10084 | 1 | 0.002
A+152+16362+200 | 10410 | 2 | 0.005
A+152+16362+200 | 10565 | 2 | 0.005
A+152+16362+200 | 10750 | 6 | 0.014
A+152+16362+200 | 10882 | 2 | 0.005
A+152+16362+200 | 1094 | 2 | 0.005
A+152+16362+200 | 11150 | 2 | 0.005
A+152+16362+200 | 11447 | 1 | 0.002
A+152+16362+200 | 11914 | 1 | 0.002
A+152+16362+200 | 1243 | 1 | 0.002
A+152+16362+200 | 12477 | 2 | 0.005
A+152+16362+200 | 12603 | 1 | 0.002
A+152+16362+200 | 13437 | 2 | 0.005
A+152+16362+200 | 13590 | 3 | 0.007
A+152+16362+200 | 13602 | 1 | 0.002
A+152+16362+200 | 14053 | 2 | 0.005
A+152+16362+200 | 14110 | 1 | 0.002
A+152+16362+200 | 14151 | 1 | 0.002
A+152+16362+200 | 146 | 6 | 0.014
A+152+16362+200 | 146G | 1 | 0.002
A+152+16362+200 | 151 | 4 | 0.009
A+152+16362+200 | 15262 | 1 | 0.002
A+152+16362+200 | 15930 | 1 | 0.002
A+152+16362+200 | 16063 | 1 | 0.002
A+152+16362+200 | 16076d | 1 | 0.002
A+152+16362+200 | 16126 | 1 | 0.002
A+152+16362+200 | 16129 | 13 | 0.03
A+152+16362+200 | 16147A | 1 | 0.002
A+152+16362+200 | 16168 | 5 | 0.012
A+152+16362+200 | 16172 | 2 | 0.005
A+152+16362+200 | 16176 | 1 | 0.002
A+152+16362+200 | 16184 | 1 | 0.002
A+152+16362+200 | 16188 | 3 | 0.007
A+152+16362+200 | 16192 | 3 | 0.007
A+152+16362+200 | 16214 | 1 | 0.002
A+152+16362+200 | 16216 | 6 | 0.014
A+152+16362+200 | 16221 | 1 | 0.002
A+152+16362+200 | 16234 | 1 | 0.002
A+152+16362+200 | 16239 | 2 | 0.005
A+152+16362+200 | 16241C | 1 | 0.002
A+152+16362+200 | 16243 | 1 | 0.002
A+152+16362+200 | 16266 | 1 | 0.002
A+152+16362+200 | 16287 | 2 | 0.005
A+152+16362+200 | 16291 | 1 | 0.002
A+152+16362+200 | 16294 | 12 | 0.028
A+152+16362+200 | 16296 | 1 | 0.002
A+152+16362+200 | 16297 | 1 | 0.002
A+152+16362+200 | 16304 | 1 | 0.002
A+152+16362+200 | 16311 | 3 | 0.007
A+152+16362+200 | 16325 | 1 | 0.002
A+152+16362+200 | 16326T | 1 | 0.002
A+152+16362+200 | 16335 | 2 | 0.005
A+152+16362+200 | 16368 | 1 | 0.002
A+152+16362+200 | 16478G | 1 | 0.002
A+152+16362+200 | 16497 | 3 | 0.007
A+152+16362+200 | 1811 | 1 | 0.002
A+152+16362+200 | 182 | 2 | 0.005
A+152+16362+200 | 185 | 1 | 0.002
A+152+16362+200 | 194 | 1 | 0.002
A+152+16362+200 | 195 | 6 | 0.014
A+152+16362+200 | 204 | 10 | 0.023
A+152+16362+200 | 2156d | 1 | 0.002
A+152+16362+200 | 310 | 1 | 0.002
A+152+16362+200 | 3316 | 2 | 0.005
A+152+16362+200 | 3337 | 1 | 0.002
A+152+16362+200 | 3594 | 1 | 0.002
A+152+16362+200 | 374 | 1 | 0.002
A+152+16362+200 | 3918 | 1 | 0.002
A+152+16362+200 | 4231 | 1 | 0.002
A+152+16362+200 | 4314 | 2 | 0.005
A+152+16362+200 | 5046 | 1 | 0.002
A+152+16362+200 | 5360 | 1 | 0.002
A+152+16362+200 | 5426 | 2 | 0.005
A+152+16362+200 | 573.1C | 1 | 0.002
A+152+16362+200 | 574C | 1 | 0.002
A+152+16362+200 | 576C | 1 | 0.002
A+152+16362+200 | 709 | 2 | 0.005
A+152+16362+200 | 7298 | 2 | 0.005
A+152+16362+200 | 735 | 1 | 0.002
A+152+16362+200 | 789 | 1 | 0.002
A+152+16362+200 | 7954 | 2 | 0.005
A+152+16362+200 | 8251 | 2 | 0.005
A+152+16362+200 | 8382 | 1 | 0.002
A+152+16362+200 | 8531 | 2 | 0.005
A+152+16362+200 | 8682 | 2 | 0.005
A+152+16362+200 | 8812 | 2 | 0.005
A+152+16362+200 | 9036 | 2 | 0.005
A1 | 156 | 11 | 0.027
A1 | 159 | 1 | 0.002
A1 | 16124 | 1 | 0.002
A1 | 16157 | 3 | 0.007
A1 | 16209 | 1 | 0.002
A1 | 16264 | 1 | 0.002
A1 | 16270 | 1 | 0.002
A1 | 16462 | 1 | 0.002
A1 | 16525C | 1 | 0.002
A1 | 16538 | 1 | 0.002
A1 | 210 | 1 | 0.002
A1 | 232C | 1 | 0.002
A1 | 341C | 1 | 0.002
A1 | 407A | 1 | 0.002
A1 | 450 | 1 | 0.002
A1 | 464C | 1 | 0.002
A1 | 471 | 2 | 0.005
A1 | 573.1C | 1 | 0.002

A1 | 6266 | 1 | 0.002
A1 | 824 | 1 | 0.002
A10 | 10289 | 1 | 0.111
A10 | 16129 | 1 | 0.111
A10 | 16230 | 1 | 0.111
A10 | 16256 | 1 | 0.111
A10 | 16295 | 1 | 0.111
A10 | 2831 | 1 | 0.111
A10 | 310 | 1 | 0.111
A10 | 544 | 2 | 0.222
A10 | 64 | 4 | 0.444
A10 | 8995 | 1 | 0.111
A10 | 9524 | 1 | 0.111
A10 | 9911 | 1 | 0.111
A11 | 151 | 1 | 0.007
A11 | 16129 | 5 | 0.034
A11 | 16169 | 1 | 0.007
A11 | 16189 | 2 | 0.013
A11 | 16192 | 1 | 0.007
A11 | 16212 | 1 | 0.007
A11 | 16221 | 6 | 0.04
A11 | 16241 | 1 | 0.007
A11 | 16261 | 1 | 0.007
A11 | 16291 | 1 | 0.007
A11 | 16291A | 1 | 0.007
A11 | 16294 | 6 | 0.04
A11 | 16300 | 1 | 0.007
A11 | 16304 | 1 | 0.007
A11 | 16309 | 1 | 0.007
A11 | 16355 | 1 | 0.007
A11 | 16359 | 1 | 0.007
A11 | 16362 | 4 | 0.027
A11 | 252 | 1 | 0.007
A11 | 316C | 8 | 0.054
A11 | 64 | 1 | 0.007
A11 | 650 | 1 | 0.007
A11+16234 | 13638 | 1 | 0.009
A11+16234 | 14110 | 1 | 0.009
A11+16234 | 14527 | 1 | 0.009
A11+16234 | 151 | 1 | 0.009
A11+16234 | 16051 | 1 | 0.009
A11+16234 | 16093 | 13 | 0.123
A11+16234 | 16184 | 2 | 0.019
A11+16234 | 16192 | 3 | 0.028
A11+16234 | 16193 | 1 | 0.009
A11+16234 | 16278 | 2 | 0.019
A11+16234 | 16309 | 1 | 0.009
A11+16234 | 16318T | 5 | 0.047
A11+16234 | 16370C | 1 | 0.009
A11+16234 | 186 | 1 | 0.009
A11+16234 | 316C | 2 | 0.019
A11+16234 | 385 | 1 | 0.009
A11+16234 | 4901 | 1 | 0.009
A11+16234 | 571 | 1 | 0.009
A11+16234 | 573.1C | 1 | 0.009
A11+16234 | 625 | 1 | 0.009
A11a | 10511 | 1 | 0.002
A11a | 10908 | 1 | 0.002
A11a | 10978 | 1 | 0.002
A11a | 12397 | 1 | 0.002
A11a | 12523 | 3 | 0.007
A11a | 12634 | 2 | 0.005
A11a | 13246 | 1 | 0.002
A11a | 13790 | 1 | 0.002
A11a | 14182 | 1 | 0.002
A11a | 146 | 1 | 0.002
A11a | 14782 | 1 | 0.002
A11a | 14809 | 1 | 0.002
A11a | 151 | 8 | 0.018
A11a | 15924 | 1 | 0.002
A11a | 16037 | 1 | 0.002
A11a | 16051 | 2 | 0.005
A11a | 16069 | 1 | 0.002
A11a | 16093 | 312 | 0.707
A11a | 16111 | 5 | 0.011
A11a | 16126 | 5 | 0.011
A11a | 16129 | 13 | 0.029
A11a | 16168 | 1 | 0.002
A11a | 16171 | 1 | 0.002
A11a | 16172 | 5 | 0.011
A11a | 16173 | 1 | 0.002
A11a | 16179 | 1 | 0.002
A11a | 16180 | 3 | 0.007
A11a | 16184 | 2 | 0.005
A11a | 16186 | 1 | 0.002
A11a | 16189 | 1 | 0.002
A11a | 16207 | 1 | 0.002
A11a | 16212 | 4 | 0.009
A11a | 16215 | 1 | 0.002
A11a | 16221 | 5 | 0.011
A11a | 16243 | 6 | 0.014
A11a | 16256 | 1 | 0.002
A11a | 16263 | 1 | 0.002
A11a | 16274 | 7 | 0.016
A11a | 16278 | 1 | 0.002
A11a | 16286 | 1 | 0.002
A11a | 16289.1T | 1 | 0.002
A11a | 16291 | 1 | 0.002
A11a | 16291A | 1 | 0.002
A11a | 16292 | 4 | 0.009
A11a | 16294 | 4 | 0.009
A11a | 16300 | 2 | 0.005
A11a | 16302 | 6 | 0.014
A11a | 16311 | 7 | 0.016
A11a | 16353 | 1 | 0.002
A11a | 16355 | 1 | 0.002
A11a | 16357 | 1 | 0.002
A11a | 16360 | 1 | 0.002
A11a | 16361C | 1 | 0.002
A11a | 16362 | 5 | 0.011
A11a | 16374C | 3 | 0.007

A11a | 16400 | 1 | 0.002
A11a | 16422 | 2 | 0.005
A11a | 16456 | 1 | 0.002
A11a | 195 | 1 | 0.002
A11a | 207 | 4 | 0.009
A11a | 2483 | 2 | 0.005
A11a | 279 | 4 | 0.009
A11a | 316C | 8 | 0.018
A11a | 4048 | 1 | 0.002
A11a | 4113 | 1 | 0.002
A11a | 489 | 1 | 0.002
A11a | 4952 | 2 | 0.005
A11a | 5105 | 3 | 0.007
A11a | 5162 | 3 | 0.007
A11a | 5414 | 1 | 0.002
A11a | 5581 | 1 | 0.002
A11a | 6164 | 3 | 0.007
A11a | 6497 | 1 | 0.002
A11a | 749 | 2 | 0.005
A11a | 7674 | 1 | 0.002
A11a | 8600 | 1 | 0.002
A11a | 9055 | 1 | 0.002
A11a | 9098 | 1 | 0.002
A11a | 9103 | 1 | 0.002
A11b | 11914 | 1 | 0.011
A11b | 16066 | 1 | 0.011
A11b | 16094 | 1 | 0.011
A11b | 16157 | 1 | 0.011
A11b | 16189 | 3 | 0.033
A11b | 16209 | 1 | 0.011
A11b | 16240 | 1 | 0.011
A11b | 16311 | 12 | 0.13
A11b | 16318 | 1 | 0.011
A11b | 189 | 5 | 0.054
A11b | 194 | 9 | 0.098
A11b | 207 | 6 | 0.065
A11b | 316C | 1 | 0.011
A11b | 3204 | 7 | 0.076
A11b | 4463 | 1 | 0.011
A11b | 5460 | 1 | 0.011
A11b | 6164 | 3 | 0.033
A11b | 6962 | 3 | 0.033
A11b | 709 | 7 | 0.076
A11b | 8279 | 2 | 0.022
A11b | 8843 | 2 | 0.022
A12 | 16075 | 1 | 0.036
A12 | 16086 | 1 | 0.036
A12 | 16168 | 1 | 0.036
A12 | 16172 | 1 | 0.036
A12 | 16184 | 1 | 0.036
A12 | 16184A | 4 | 0.143
A12 | 16188 | 1 | 0.036
A12 | 16193d | 1 | 0.036
A12 | 16209 | 1 | 0.036
A12 | 16230 | 1 | 0.036
A12 | 16274 | 3 | 0.107
A12 | 16399 | 3 | 0.107
A12 | 16527 | 1 | 0.036
A12 | 200 | 2 | 0.071
A12 | 204 | 1 | 0.036
A12 | 3010 | 1 | 0.036
A12 | 636 | 1 | 0.036
A12 | 654 | 2 | 0.071
A12 | 8496 | 2 | 0.071
A12 | 8874A | 2 | 0.071
A12a | 10718 | 1 | 0.04
A12a | 1113 | 1 | 0.04
A12a | 13758 | 1 | 0.04
A12a | 15355 | 1 | 0.04
A12a | 15712 | 3 | 0.12
A12a | 16000 | 1 | 0.04
A12a | 16176 | 1 | 0.04
A12a | 16188 | 7 | 0.28
A12a | 16218 | 1 | 0.04
A12a | 16256 | 1 | 0.04
A12a | 3310 | 1 | 0.04
A12a | 4770 | 1 | 0.04
A12a | 4794 | 1 | 0.04
A12a | 4812C | 1 | 0.04
A12a | 7308 | 1 | 0.04
A12a | 7337 | 1 | 0.04
A12a | 8839 | 2 | 0.08
A12a | 9758 | 1 | 0.04
A12a | 9804 | 2 | 0.08
A13 | 11809 | 3 | 0.007
A13 | 12131 | 2 | 0.005
A13 | 13215 | 3 | 0.007
A13 | 13500 | 1 | 0.002
A13 | 13780 | 5 | 0.011
A13 | 146G | 1 | 0.002
A13 | 15043 | 1 | 0.002
A13 | 151 | 2 | 0.005
A13 | 15672 | 11 | 0.025
A13 | 16086 | 1 | 0.002
A13 | 16093 | 9 | 0.02
A13 | 16124 | 57 | 0.128
A13 | 16126 | 1 | 0.002
A13 | 16166d | 11 | 0.025
A13 | 16176 | 1 | 0.002
A13 | 16192 | 3 | 0.007
A13 | 16214 | 1 | 0.002
A13 | 16221 | 1 | 0.002
A13 | 16245 | 2 | 0.005
A13 | 16256 | 1 | 0.002
A13 | 16266 | 1 | 0.002
A13 | 16293T | 2 | 0.005
A13 | 16300 | 1 | 0.002
A13 | 16304 | 1 | 0.002
A13 | 16391 | 1 | 0.002
A13 | 16418 | 1 | 0.002
A13 | 16478G | 1 | 0.002

A13	182	1	0.002
A13	185	1	0.002
A13	204	2	0.005
A13	207	2	0.005
A13	2593	1	0.002
A13	310	1	0.002
A13	3150	11	0.025
A13	316.1G	1	0.002
A13	316C	1	0.002
A13	3342	1	0.002
A13	3645	1	0.002
A13	4086	1	0.002
A13	460	1	0.002
A13	6950	2	0.005
A13	789	1	0.002
A13	8756	11	0.025
A13	9091	5	0.011
A13	922A	1	0.002
A13	9804	5	0.011
A14	10864	1	0.002
A14	114	2	0.004
A14	11821C	1	0.002
A14	11929	2	0.004
A14	12004	2	0.004
A14	12855	2	0.004
A14	12990	2	0.004
A14	13803T	1	0.002
A14	13866	1	0.002
A14	15388	1	0.002
A14	1598	1	0.002
A14	16086	3	0.006
A14	16093	10	0.02
A14	16111	1	0.002
A14	16124	1	0.002
A14	16126	1	0.002
A14	16129	1	0.002
A14	16136	1	0.002
A14	16172	2	0.004
A14	16176	7	0.014
A14	16185	3	0.006
A14	16187	4	0.008
A14	16189	1	0.002
A14	16201	1	0.002
A14	16231	2	0.004
A14	16239	2	0.004
A14	16243	1	0.002
A14	16246	2	0.004
A14	16256	11	0.022
A14	16263	2	0.004
A14	16264	4	0.008
A14	16269	1	0.002
A14	16287	2	0.004
A14	16291	1	0.002
A14	16292	2	0.004
A14	16293	1	0.002
A14	16309	1	0.002
A14	16311	3	0.006
A14	16365	3	0.006
A14	16381	2	0.004
A14	16446	2	0.004
A14	16463	16	0.032
A14	182	4	0.008
A14	207	3	0.006
A14	2080	1	0.002
A14	2158	2	0.004
A14	260	1	0.002
A14	279	2	0.004
A14	291.2A	7	0.014
A14	294	2	0.004
A14	3027	1	0.002
A14	310	1	0.002
A14	315.2C	1	0.002
A14	316C	1	0.002
A14	3363	9	0.018
A14	4048	1	0.002
A14	408G	1	0.002
A14	4336	9	0.018
A14	438.1C	1	0.002
A14	456	2	0.004
A14	458	1	0.002
A14	4580	1	0.002
A14	471	1	0.002
A14	5493	1	0.002
A14	5663	1	0.002
A14	573.1C	1	0.002
A14	600d	1	0.002
A14	604d	1	0.002
A14	6227	1	0.002
A14	6671	1	0.002
A14	6968	2	0.004
A14	709	3	0.006
A14	714T	2	0.004
A14	723	1	0.002
A14	7805	3	0.006
A14	7854	1	0.002
A14	8005	9	0.018
A14	8053C	1	0.002
A14	8167	3	0.006
A14	8251	1	0.002
A14	8334	2	0.004
A14	8765	1	0.002
A14	8975	3	0.006
A14	9182	1	0.002
A14	93	2	0.004
A14	9750	1	0.002
A14	9830	9	0.018
A15	140	1	0.002
A15	151	24	0.059
A15	15314	1	0.002
A15	16021A	1	0.002
A15	16092	1	0.002

A15 | 16106 | 1 | 0.002
A15 | 16126 | 1 | 0.002
A15 | 16127 | 2 | 0.005
A15 | 16148 | 1 | 0.002
A15 | 16153 | 3 | 0.007
A15 | 16154 | 1 | 0.002
A15 | 16192 | 1 | 0.002
A15 | 16193d | 1 | 0.002
A15 | 16194T | 1 | 0.002
A15 | 16207 | 2 | 0.005
A15 | 16249 | 1 | 0.002
A15 | 16255 | 1 | 0.002
A15 | 16258 | 1 | 0.002
A15 | 16267 | 1 | 0.002
A15 | 16286 | 1 | 0.002
A15 | 16287 | 1 | 0.002
A15 | 16292 | 1 | 0.002
A15 | 16295 | 1 | 0.002
A15 | 16304 | 1 | 0.002
A15 | 16356 | 1 | 0.002
A15 | 16526 | 22 | 0.054
A15 | 16T | 1 | 0.002
A15 | 183 | 1 | 0.002
A15 | 188 | 2 | 0.005
A15 | 195 | 1 | 0.002
A15 | 247 | 1 | 0.002
A15 | 254 | 1 | 0.002
A15 | 263T | 1 | 0.002
A15 | 316 | 1 | 0.002
A15 | 341 | 1 | 0.002
A15 | 3531 | 1 | 0.002
A15 | 364 | 1 | 0.002
A15 | 372 | 1 | 0.002
A15 | 3849 | 1 | 0.002
A15 | 430 | 3 | 0.007
A15 | 444 | 1 | 0.002
A15 | 448 | 1 | 0.002
A15 | 513 | 1 | 0.002
A15 | 529 | 1 | 0.002
A15 | 554 | 3 | 0.007
A15 | 556 | 1 | 0.002
A15 | 573.1C | 1 | 0.002
A15 | 7316 | 1 | 0.002
A15 | 8659 | 1 | 0.002
A15a | 1 | 1 | 0.002
A15a | 11974 | 1 | 0.002
A15a | 13535 | 1 | 0.002
A15a | 13788 | 1 | 0.002
A15a | 139 | 1 | 0.002
A15a | 13943 | 1 | 0.002
A15a | 140 | 1 | 0.002
A15a | 146 | 8 | 0.018
A15a | 152d | 1 | 0.002
A15a | 159 | 1 | 0.002
A15a | 15984 | 1 | 0.002
A15a | 16025 | 1 | 0.002
A15a | 16028 | 1 | 0.002
A15a | 16032 | 1 | 0.002
A15a | 16068 | 2 | 0.004
A15a | 16086 | 2 | 0.004
A15a | 16092 | 1 | 0.002
A15a | 16093 | 6 | 0.013
A15a | 16094 | 1 | 0.002
A15a | 16111 | 1 | 0.002
A15a | 16117 | 1 | 0.002
A15a | 16126 | 1 | 0.002
A15a | 16129 | 4 | 0.009
A15a | 16148 | 1 | 0.002
A15a | 16169 | 4 | 0.009
A15a | 16172 | 3 | 0.007
A15a | 16177 | 1 | 0.002
A15a | 16179 | 1 | 0.002
A15a | 16183 | 1 | 0.002
A15a | 16192 | 1 | 0.002
A15a | 16209 | 2 | 0.004
A15a | 16241 | 1 | 0.002
A15a | 16249 | 1 | 0.002
A15a | 16257 | 1 | 0.002
A15a | 16261 | 3 | 0.007
A15a | 16263 | 1 | 0.002
A15a | 16265 | 1 | 0.002
A15a | 16274 | 9 | 0.02
A15a | 16277 | 1 | 0.002
A15a | 16286 | 1 | 0.002
A15a | 16287 | 1 | 0.002
A15a | 16289T | 1 | 0.002
A15a | 16292 | 1 | 0.002
A15a | 16320 | 3 | 0.007
A15a | 16333 | 1 | 0.002
A15a | 16356 | 1 | 0.002
A15a | 16399T | 1 | 0.002
A15a | 16413 | 1 | 0.002
A15a | 16439G | 1 | 0.002
A15a | 16476T | 1 | 0.002
A15a | 16487 | 1 | 0.002
A15a | 16497 | 1 | 0.002
A15a | 16500 | 1 | 0.002
A15a | 16522 | 1 | 0.002
A15a | 16545 | 1 | 0.002
A15a | 1719 | 1 | 0.002
A15a | 178 | 2 | 0.004
A15a | 183 | 1 | 0.002
A15a | 189.1A | 1 | 0.002
A15a | 195 | 1 | 0.002
A15a | 199 | 1 | 0.002
A15a | 200 | 4 | 0.009
A15a | 202d | 1 | 0.002
A15a | 204 | 54 | 0.119
A15a | 2121 | 1 | 0.002
A15a | 307-309d | 3 | 0.007
A15a | 309.3C | 5 | 0.011

A15a | 309.4C | 1 | 0.002
A15a | 310 | 2 | 0.004
A15a | 313-315d | 1 | 0.002
A15a | 316C | 2 | 0.004
A15a | 329 | 3 | 0.007
A15a | 341 | 1 | 0.002
A15a | 346 | 1 | 0.002
A15a | 372 | 1 | 0.002
A15a | 375 | 1 | 0.002
A15a | 397d | 1 | 0.002
A15a | 424d | 1 | 0.002
A15a | 444 | 1 | 0.002
A15a | 482 | 2 | 0.004
A15a | 484 | 1 | 0.002
A15a | 502d | 1 | 0.002
A15a | 513 | 1 | 0.002
A15a | 554 | 4 | 0.009
A15a | 5899.2C | 2 | 0.004
A15a | 59 | 1 | 0.002
A15a | 6239 | 1 | 0.002
A15a | 679 | 1 | 0.002
A15a | 68 | 1 | 0.002
A15a | 684A | 1 | 0.002
A15a | 68C | 1 | 0.002
A15a | 71.1G | 2 | 0.004
A15a | 71d | 1 | 0.002
A15a | 7220 | 1 | 0.002
A15a | 7609 | 1 | 0.002
A15a | 7849 | 1 | 0.002
A15a | 959 | 2 | 0.004
A15b | 11016 | 2 | 0.08
A15b | 14062 | 1 | 0.04
A15b | 146 | 2 | 0.08
A15b | 15097 | 2 | 0.08
A15b | 151 | 1 | 0.04
A15b | 16093 | 5 | 0.2
A15b | 16117 | 1 | 0.04
A15b | 16172 | 3 | 0.12
A15b | 16221 | 1 | 0.04
A15b | 16230 | 1 | 0.04
A15b | 16249 | 2 | 0.08
A15b | 16292 | 1 | 0.04
A15b | 16293C | 1 | 0.04
A15b | 16316 | 2 | 0.08
A15b | 16325G | 1 | 0.04
A15b | 200 | 2 | 0.08
A15b | 263 | 1 | 0.04
A15b | 315.2C | 1 | 0.04
A15b | 366 | 1 | 0.04
A15b | 564 | 1 | 0.04
A15b | 64 | 1 | 0.04
A15b | 8348 | 1 | 0.04
A15b | 8656 | 2 | 0.08
A15b | 9912 | 1 | 0.04
A15c | 13111 | 4 | 0.01
A15c | 140 | 1 | 0.002
A15c | 146 | 2 | 0.005
A15c | 153 | 14 | 0.034
A15c | 156 | 3 | 0.007
A15c | 159 | 3 | 0.007
A15c | 16092 | 1 | 0.002
A15c | 16126 | 1 | 0.002
A15c | 16127 | 2 | 0.005
A15c | 16153 | 3 | 0.007
A15c | 16192 | 10 | 0.024
A15c | 16207 | 4 | 0.01
A15c | 16224 | 3 | 0.007
A15c | 16267 | 1 | 0.002
A15c | 16274 | 2 | 0.005
A15c | 16287 | 1 | 0.002
A15c | 16292 | 1 | 0.002
A15c | 16295 | 4 | 0.01
A15c | 16304 | 1 | 0.002
A15c | 16355 | 1 | 0.002
A15c | 16356 | 1 | 0.002
A15c | 183 | 1 | 0.002
A15c | 188 | 2 | 0.005
A15c | 195 | 1 | 0.002
A15c | 2582 | 1 | 0.002
A15c | 316 | 1 | 0.002
A15c | 316C | 6 | 0.014
A15c | 341 | 1 | 0.002
A15c | 372 | 1 | 0.002
A15c | 3849 | 2 | 0.005
A15c | 430 | 3 | 0.007
A15c | 489 | 1 | 0.002
A15c | 513 | 1 | 0.002
A15c | 554 | 3 | 0.007
A15c | 573.1C | 1 | 0.002
A15c | 6324 | 1 | 0.002
A15c | 709 | 3 | 0.007
A15c | 9052 | 4 | 0.01
A15c1 | 13191 | 1 | 0.002
A15c1 | 13928C | 1 | 0.002
A15c1 | 140 | 1 | 0.002
A15c1 | 151 | 1 | 0.002
A15c1 | 16092 | 1 | 0.002
A15c1 | 16126 | 1 | 0.002
A15c1 | 16153 | 3 | 0.007
A15c1 | 16287 | 1 | 0.002
A15c1 | 16292 | 1 | 0.002
A15c1 | 16311 | 6 | 0.014
A15c1 | 16356 | 1 | 0.002
A15c1 | 183 | 1 | 0.002
A15c1 | 195 | 1 | 0.002
A15c1 | 341 | 1 | 0.002
A15c1 | 368 | 1 | 0.002
A15c1 | 372 | 1 | 0.002
A15c1 | 430 | 3 | 0.007
A15c1 | 44.1C | 1 | 0.002
A15c1 | 5355 | 3 | 0.007

A15c1 | 554 | 3 | 0.007
A15c1 | 573.1C | 1 | 0.002
A15c1 | 6956 | 1 | 0.002
A15c1 | 7692 | 1 | 0.002
A15c1 | 8959 | 1 | 0.002
A15c1 | 8998 | 4 | 0.009
A16 | 11914 | 1 | 0.059
A16 | 15110 | 1 | 0.059
A16 | 16325 | 2 | 0.118
A16 | 199 | 5 | 0.294
A16 | 310 | 1 | 0.059
A16 | 5267 | 1 | 0.059
A17 | 10101 | 4 | 0.007
A17 | 10604 | 1 | 0.002
A17 | 10790 | 1 | 0.002
A17 | 10873 | 2 | 0.003
A17 | 11172 | 1 | 0.002
A17 | 11935 | 1 | 0.002
A17 | 12136 | 4 | 0.007
A17 | 134 | 1 | 0.002
A17 | 13788 | 1 | 0.002
A17 | 13965 | 3 | 0.005
A17 | 140 | 1 | 0.002
A17 | 14180 | 2 | 0.003
A17 | 14251 | 1 | 0.002
A17 | 1452 | 1 | 0.002
A17 | 14577 | 9 | 0.015
A17 | 14581 | 9 | 0.015
A17 | 14861 | 11 | 0.019
A17 | 14924 | 1 | 0.002
A17 | 151 | 2 | 0.003
A17 | 15106 | 2 | 0.003
A17 | 153 | 1 | 0.002
A17 | 15346 | 1 | 0.002
A17 | 15442 | 1 | 0.002
A17 | 15454 | 2 | 0.003
A17 | 15497 | 1 | 0.002
A17 | 15519 | 1 | 0.002
A17 | 15574 | 1 | 0.002
A17 | 16037 | 1 | 0.002
A17 | 16051 | 4 | 0.007
A17 | 16086 | 62 | 0.107
A17 | 16093 | 83 | 0.143
A17 | 16124 | 2 | 0.003
A17 | 16129 | 41 | 0.07
A17 | 16136 | 7 | 0.012
A17 | 16145 | 2 | 0.003
A17 | 16150 | 3 | 0.005
A17 | 16151A | 1 | 0.002
A17 | 16157 | 1 | 0.002
A17 | 16172 | 17 | 0.029
A17 | 16195 | 1 | 0.002
A17 | 16230 | 1 | 0.002
A17 | 16235 | 9 | 0.015
A17 | 16248 | 1 | 0.002
A17 | 16260 | 4 | 0.007
A17 | 16266 | 12 | 0.021
A17 | 16271 | 1 | 0.002
A17 | 16274 | 2 | 0.003
A17 | 16278 | 2 | 0.003
A17 | 16284 | 44 | 0.076
A17 | 16287 | 2 | 0.003
A17 | 16292 | 24 | 0.041
A17 | 16293T | 1 | 0.002
A17 | 16294 | 1 | 0.002
A17 | 16295 | 10 | 0.017
A17 | 16304 | 1 | 0.002
A17 | 16311 | 12 | 0.021
A17 | 16325 | 7 | 0.012
A17 | 16338 | 4 | 0.007
A17 | 16356 | 3 | 0.005
A17 | 16365 | 1 | 0.002
A17 | 16390 | 1 | 0.002
A17 | 16391 | 1 | 0.002
A17 | 16399 | 2 | 0.003
A17 | 16465 | 4 | 0.007
A17 | 16480T | 1 | 0.002
A17 | 16526 | 1 | 0.002
A17 | 16527 | 3 | 0.005
A17 | 179 | 2 | 0.003
A17 | 1833 | 3 | 0.005
A17 | 189 | 1 | 0.002
A17 | 195 | 1 | 0.002
A17 | 199 | 3 | 0.005
A17 | 2056 | 1 | 0.002
A17 | 215 | 1 | 0.002
A17 | 234 | 6 | 0.01
A17 | 238T | 1 | 0.002
A17 | 294 | 2 | 0.003
A17 | 309 | 2 | 0.003
A17 | 313 | 1 | 0.002
A17 | 315.2C | 1 | 0.002
A17 | 316C | 1 | 0.002
A17 | 3693 | 1 | 0.002
A17 | 41 | 3 | 0.005
A17 | 4136 | 2 | 0.003
A17 | 4512 | 2 | 0.003
A17 | 4561 | 1 | 0.002
A17 | 460 | 1 | 0.002
A17 | 471 | 1 | 0.002
A17 | 476 | 1 | 0.002
A17 | 482 | 1 | 0.002
A17 | 5049 | 2 | 0.003
A17 | 5186C | 1 | 0.002
A17 | 5301C | 2 | 0.003
A17 | 5351 | 2 | 0.003
A17 | 573.1C | 5 | 0.009
A17 | 5951 | 3 | 0.005
A17 | 6221A | 1 | 0.002
A17 | 6239 | 2 | 0.003
A17 | 751T | 1 | 0.002

A17 | 8675 | 2 | 0.003
A17 | 8701 | 2 | 0.003
A17 | 9095 | 1 | 0.002
A17 | 930 | 1 | 0.002
A17 | 9344 | 1 | 0.002
A17 | 9410 | 2 | 0.003
A17 | 9540 | 2 | 0.003
A17 | 9554 | 2 | 0.003
A18 | 12975T | 1 | 0.002
A18 | 14965 | 1 | 0.002
A18 | 16124 | 1 | 0.002
A18 | 16157 | 3 | 0.007
A18 | 16230 | 9 | 0.022
A18 | 16262 | 1 | 0.002
A18 | 16264 | 1 | 0.002
A18 | 16270 | 1 | 0.002
A18 | 16462 | 1 | 0.002
A18 | 16525C | 1 | 0.002
A18 | 16538 | 1 | 0.002
A18 | 232C | 1 | 0.002
A18 | 3010 | 1 | 0.002
A18 | 341C | 1 | 0.002
A18 | 3891 | 1 | 0.002
A18 | 407A | 1 | 0.002
A18 | 450 | 1 | 0.002
A18 | 464C | 1 | 0.002
A18 | 573.1C | 1 | 0.002
A18 | 824 | 1 | 0.002
A18 | 8440 | 1 | 0.002
A18 | 8939 | 1 | 0.002
A19 | 140 | 1 | 0.009
A19 | 150 | 2 | 0.019
A19 | 151 | 1 | 0.009
A19 | 156 | 1 | 0.009
A19 | 16042 | 1 | 0.009
A19 | 16086 | 1 | 0.009
A19 | 16092 | 3 | 0.028
A19 | 16093 | 1 | 0.009
A19 | 16125 | 16 | 0.15
A19 | 16148 | 1 | 0.009
A19 | 16150 | 1 | 0.009
A19 | 16158 | 1 | 0.009
A19 | 16169 | 1 | 0.009
A19 | 16192 | 1 | 0.009
A19 | 16196 | 3 | 0.028
A19 | 16209 | 2 | 0.019
A19 | 16216 | 1 | 0.009
A19 | 16234 | 4 | 0.037
A19 | 16235 | 37 | 0.346
A19 | 16241T | 1 | 0.009
A19 | 16271 | 1 | 0.009
A19 | 16274 | 3 | 0.028
A19 | 16278 | 2 | 0.019
A19 | 16286 | 1 | 0.009
A19 | 16287 | 3 | 0.028
A19 | 16291 | 2 | 0.019
A19 | 16292 | 2 | 0.019
A19 | 16294 | 2 | 0.019
A19 | 16297 | 1 | 0.009
A19 | 16299 | 2 | 0.019
A19 | 16304 | 2 | 0.019
A19 | 16335 | 1 | 0.009
A19 | 16365 | 1 | 0.009
A19 | 16527 | 8 | 0.075
A19 | 195 | 1 | 0.009
A19 | 195A | 1 | 0.009
A19 | 234 | 31 | 0.29
A19 | 256 | 1 | 0.009
A19 | 259 | 2 | 0.019
A19 | 310 | 2 | 0.019
A19 | 315d | 2 | 0.019
A19 | 316C | 5 | 0.047
A19 | 455d | 1 | 0.009
A19 | 456 | 1 | 0.009
A19 | 4977 | 1 | 0.009
A19 | 5460 | 1 | 0.009
A19 | 573.2C | 2 | 0.019
A19 | 573.3C | 1 | 0.009
A19 | 573.4C | 2 | 0.019
A19 | 573.5C | 1 | 0.009
A19 | 573.7C | 1 | 0.009
A19 | 574C | 1 | 0.009
A19 | 576 | 6 | 0.056
A19 | 629 | 2 | 0.019
A19 | 72 | 1 | 0.009
A19 | 729 | 1 | 0.009
A19 | 7325 | 1 | 0.009
A1a | 11147 | 1 | 0.025
A1a | 13768 | 1 | 0.025
A1a | 1462 | 2 | 0.05
A1a | 15049 | 1 | 0.025
A1a | 16084 | 2 | 0.05
A1a | 16111 | 2 | 0.05
A1a | 16176 | 8 | 0.2
A1a | 16181 | 2 | 0.05
A1a | 16215 | 2 | 0.05
A1a | 16234 | 1 | 0.025
A1a | 16352 | 1 | 0.025
A1a | 1664 | 1 | 0.025
A1a | 384 | 1 | 0.025
A1a | 9477 | 2 | 0.05
A1a1 | 10915 | 1 | 0.025
A1a1 | 15043 | 8 | 0.2
A1a1 | 310 | 1 | 0.025
A1a1 | 315.3C | 1 | 0.025
A1a1 | 771 | 3 | 0.075
A1a1 | 9756G | 4 | 0.1
A1a1 | 9776 | 1 | 0.025
A2 | 10292 | 1 | 0.009
A2 | 10586 | 1 | 0.009
A2 | 10873 | 4 | 0.037

A2 | 10885 | 3 | 0.028
A2 | 11009 | 1 | 0.009
A2 | 11288 | 1 | 0.009
A2 | 11416 | 1 | 0.009
A2 | 11653 | 9 | 0.083
A2 | 12170 | 1 | 0.009
A2 | 12406 | 1 | 0.009
A2 | 12616 | 1 | 0.009
A2 | 13126 | 3 | 0.028
A2 | 13191 | 2 | 0.019
A2 | 13326 | 1 | 0.009
A2 | 13419 | 3 | 0.028
A2 | 13731 | 9 | 0.083
A2 | 139 | 5 | 0.046
A2 | 14025 | 1 | 0.009
A2 | 14178 | 7 | 0.065
A2 | 14587 | 1 | 0.009
A2 | 1462 | 3 | 0.028
A2 | 14668 | 1 | 0.009
A2 | 14755 | 7 | 0.065
A2 | 14861 | 7 | 0.065
A2 | 14999T | 1 | 0.009
A2 | 15043 | 1 | 0.009
A2 | 15586 | 1 | 0.009
A2 | 15670 | 1 | 0.009
A2 | 15747 | 1 | 0.009
A2 | 15930 | 1 | 0.009
A2 | 16051 | 18 | 0.167
A2 | 16086 | 1 | 0.009
A2 | 16098 | 1 | 0.009
A2 | 16106 | 1 | 0.009
A2 | 16179 | 1 | 0.009
A2 | 16218 | 3 | 0.028
A2 | 16249 | 1 | 0.009
A2 | 16295 | 2 | 0.019
A2 | 16303 | 3 | 0.028
A2 | 16326 | 2 | 0.019
A2 | 16327 | 1 | 0.009
A2 | 16356 | 7 | 0.065
A2 | 16368 | 10 | 0.093
A2 | 16443 | 5 | 0.046
A2 | 16448 | 1 | 0.009
A2 | 179 | 5 | 0.046
A2 | 188 | 1 | 0.009
A2 | 195 | 1 | 0.009
A2 | 207 | 2 | 0.019
A2 | 214 | 5 | 0.046
A2 | 2246 | 1 | 0.009
A2 | 310 | 1 | 0.009
A2 | 3330 | 1 | 0.009
A2 | 3398 | 1 | 0.009
A2 | 3435 | 8 | 0.074
A2 | 3547 | 1 | 0.009
A2 | 3548 | 1 | 0.009
A2 | 3666 | 1 | 0.009
A2 | 384 | 3 | 0.028
A2 | 385 | 5 | 0.046
A2 | 3865 | 3 | 0.028
A2 | 3999 | 13 | 0.12
A2 | 4011 | 1 | 0.009
A2 | 4087 | 1 | 0.009
A2 | 4122 | 1 | 0.009
A2 | 4226 | 1 | 0.009
A2 | 4616 | 13 | 0.12
A2 | 466 | 1 | 0.009
A2 | 492 | 1 | 0.009
A2 | 5147 | 1 | 0.009
A2 | 5267 | 2 | 0.019
A2 | 551 | 1 | 0.009
A2 | 57 | 1 | 0.009
A2 | 573.5C | 1 | 0.009
A2 | 57G | 1 | 0.009
A2 | 5824 | 1 | 0.009
A2 | 60.1T | 1 | 0.009
A2 | 6023 | 1 | 0.009
A2 | 63d | 1 | 0.009
A2 | 65.1C | 1 | 0.009
A2 | 6752 | 1 | 0.009
A2 | 7196A | 1 | 0.009
A2 | 7581 | 5 | 0.046
A2 | 7678 | 5 | 0.046
A2 | 7775 | 1 | 0.009
A2 | 794A | 1 | 0.009
A2 | 8289.1CCCCCTCTA | 1 | 0.009
A2 | 8540 | 1 | 0.009
A2 | 8602 | 12 | 0.111
A2 | 9053 | 1 | 0.009
A2 | 9055 | 1 | 0.009
A2 | 93 | 10 | 0.093
A2 | 9327 | 1 | 0.009
A2 | 9468 | 1 | 0.009
A2 | 9947 | 2 | 0.019
A2 | 9966 | 12 | 0.111
A2+(64) | 10018 | 1 | 0.003
A2+(64) | 1008 | 1 | 0.003
A2+(64) | 10143 | 1 | 0.003
A2+(64) | 1018 | 1 | 0.003
A2+(64) | 10237 | 4 | 0.013
A2+(64) | 10238 | 3 | 0.009
A2+(64) | 10268 | 4 | 0.013
A2+(64) | 103 | 1 | 0.003
A2+(64) | 10398 | 1 | 0.003
A2+(64) | 10406 | 1 | 0.003
A2+(64) | 10410 | 7 | 0.022
A2+(64) | 10511 | 1 | 0.003
A2+(64) | 10535 | 2 | 0.006
A2+(64) | 10577 | 1 | 0.003
A2+(64) | 10586 | 10 | 0.031
A2+(64) | 10649 | 1 | 0.003
A2+(64) | 10673 | 1 | 0.003
A2+(64) | 10780 | 1 | 0.003

A2+(64) | 10793 | 1 | 0.003
A2+(64) | 10810 | 1 | 0.003
A2+(64) | 10887 | 2 | 0.006
A2+(64) | 10915 | 1 | 0.003
A2+(64) | 1100 | 1 | 0.003
A2+(64) | 11009 | 2 | 0.006
A2+(64) | 11016 | 5 | 0.016
A2+(64) | 11092 | 1 | 0.003
A2+(64) | 11107 | 1 | 0.003
A2+(64) | 11140 | 1 | 0.003
A2+(64) | 11150 | 1 | 0.003
A2+(64) | 11176 | 15 | 0.047
A2+(64) | 11177 | 1 | 0.003
A2+(64) | 1119 | 1 | 0.003
A2+(64) | 11221 | 4 | 0.013
A2+(64) | 11383 | 10 | 0.031
A2+(64) | 114 | 1 | 0.003
A2+(64) | 11401 | 4 | 0.013
A2+(64) | 11453 | 1 | 0.003
A2+(64) | 11485 | 1 | 0.003
A2+(64) | 114G | 7 | 0.022
A2+(64) | 11533 | 1 | 0.003
A2+(64) | 11665 | 15 | 0.047
A2+(64) | 1189 | 5 | 0.016
A2+(64) | 11914 | 4 | 0.013
A2+(64) | 12109 | 1 | 0.003
A2+(64) | 12127 | 1 | 0.003
A2+(64) | 12180T | 1 | 0.003
A2+(64) | 12223 | 1 | 0.003
A2+(64) | 12280 | 2 | 0.006
A2+(64) | 12311 | 11 | 0.034
A2+(64) | 12351 | 1 | 0.003
A2+(64) | 12372 | 1 | 0.003
A2+(64) | 12373 | 1 | 0.003
A2+(64) | 12390 | 1 | 0.003
A2+(64) | 12406 | 1 | 0.003
A2+(64) | 12435 | 1 | 0.003
A2+(64) | 125 | 3 | 0.009
A2+(64) | 12519 | 7 | 0.022
A2+(64) | 12568 | 1 | 0.003
A2+(64) | 12609 | 1 | 0.003
A2+(64) | 12634 | 1 | 0.003
A2+(64) | 127 | 3 | 0.009
A2+(64) | 128 | 2 | 0.006
A2+(64) | 12825 | 2 | 0.006
A2+(64) | 12858 | 4 | 0.013
A2+(64) | 12894 | 1 | 0.003
A2+(64) | 12939 | 4 | 0.013
A2+(64) | 12975 | 10 | 0.031
A2+(64) | 13042 | 1 | 0.003
A2+(64) | 13092 | 1 | 0.003
A2+(64) | 131 | 1 | 0.003
A2+(64) | 13105 | 1 | 0.003
A2+(64) | 13135 | 3 | 0.009
A2+(64) | 13145 | 4 | 0.013
A2+(64) | 13191 | 1 | 0.003
A2+(64) | 13419 | 5 | 0.016
A2+(64) | 13434 | 1 | 0.003
A2+(64) | 13474 | 1 | 0.003
A2+(64) | 13563 | 1 | 0.003
A2+(64) | 13590 | 1 | 0.003
A2+(64) | 13606 | 3 | 0.009
A2+(64) | 13614 | 1 | 0.003
A2+(64) | 13651 | 3 | 0.009
A2+(64) | 13681 | 2 | 0.006
A2+(64) | 13692 | 6 | 0.019
A2+(64) | 13708 | 5 | 0.016
A2+(64) | 13740 | 1 | 0.003
A2+(64) | 13753 | 2 | 0.006
A2+(64) | 13762A | 1 | 0.003
A2+(64) | 1377G | 1 | 0.003
A2+(64) | 13879 | 2 | 0.006
A2+(64) | 139 | 1 | 0.003
A2+(64) | 13905 | 1 | 0.003
A2+(64) | 13934 | 1 | 0.003
A2+(64) | 13942 | 2 | 0.006
A2+(64) | 13966 | 2 | 0.006
A2+(64) | 13985 | 1 | 0.003
A2+(64) | 14020 | 2 | 0.006
A2+(64) | 14025 | 1 | 0.003
A2+(64) | 14106 | 1 | 0.003
A2+(64) | 14115 | 2 | 0.006
A2+(64) | 14127C | 1 | 0.003
A2+(64) | 14148 | 3 | 0.009
A2+(64) | 14154G | 1 | 0.003
A2+(64) | 14248 | 1 | 0.003
A2+(64) | 14272 | 1 | 0.003
A2+(64) | 14290 | 3 | 0.009
A2+(64) | 143 | 8 | 0.025
A2+(64) | 14302 | 1 | 0.003
A2+(64) | 14305 | 1 | 0.003
A2+(64) | 14398 | 3 | 0.009
A2+(64) | 14443 | 2 | 0.006
A2+(64) | 14514 | 1 | 0.003
A2+(64) | 14551 | 1 | 0.003
A2+(64) | 14566 | 1 | 0.003
A2+(64) | 14587 | 1 | 0.003
A2+(64) | 14662 | 2 | 0.006
A2+(64) | 14769 | 1 | 0.003
A2+(64) | 14790 | 1 | 0.003
A2+(64) | 14800A | 3 | 0.009
A2+(64) | 14812 | 1 | 0.003
A2+(64) | 14839 | 5 | 0.016
A2+(64) | 14861 | 1 | 0.003
A2+(64) | 14905 | 1 | 0.003
A2+(64) | 14929 | 1 | 0.003
A2+(64) | 14956 | 2 | 0.006
A2+(64) | 14978 | 1 | 0.003
A2+(64) | 150 | 7 | 0.022
A2+(64) | 15031 | 5 | 0.016
A2+(64) | 15032 | 1 | 0.003

A2+(64) | 15043 | 8 | 0.025
A2+(64) | 15077 | 2 | 0.006
A2+(64) | 150A | 1 | 0.003
A2+(64) | 151 | 2 | 0.006
A2+(64) | 15106 | 5 | 0.016
A2+(64) | 15110 | 1 | 0.003
A2+(64) | 152 | 28 | 0.088
A2+(64) | 15221 | 11 | 0.034
A2+(64) | 15282 | 1 | 0.003
A2+(64) | 15289 | 6 | 0.019
A2+(64) | 15344 | 2 | 0.006
A2+(64) | 15355 | 1 | 0.003
A2+(64) | 15544A | 1 | 0.003
A2+(64) | 15553 | 3 | 0.009
A2+(64) | 15565 | 1 | 0.003
A2+(64) | 15596 | 1 | 0.003
A2+(64) | 15670 | 1 | 0.003
A2+(64) | 15703 | 3 | 0.009
A2+(64) | 15734 | 1 | 0.003
A2+(64) | 15747 | 1 | 0.003
A2+(64) | 15758 | 1 | 0.003
A2+(64) | 15762 | 1 | 0.003
A2+(64) | 15769 | 1 | 0.003
A2+(64) | 15784 | 1 | 0.003
A2+(64) | 15826 | 3 | 0.009
A2+(64) | 15891 | 1 | 0.003
A2+(64) | 15924 | 4 | 0.013
A2+(64) | 15951 | 1 | 0.003
A2+(64) | 15968 | 1 | 0.003
A2+(64) | 16051 | 12 | 0.038
A2+(64) | 16086 | 21 | 0.066
A2+(64) | 16090 | 1 | 0.003
A2+(64) | 16092 | 6 | 0.019
A2+(64) | 16093 | 1 | 0.003
A2+(64) | 16095 | 11 | 0.034
A2+(64) | 16103 | 1 | 0.003
A2+(64) | 16104 | 1 | 0.003
A2+(64) | 16124 | 1 | 0.003
A2+(64) | 16127C | 1 | 0.003
A2+(64) | 16131 | 2 | 0.006
A2+(64) | 16134 | 2 | 0.006
A2+(64) | 16138 | 1 | 0.003
A2+(64) | 16145 | 15 | 0.047
A2+(64) | 16157 | 1 | 0.003
A2+(64) | 16162 | 1 | 0.003
A2+(64) | 16171 | 11 | 0.034
A2+(64) | 16172 | 3 | 0.009
A2+(64) | 16176 | 2 | 0.006
A2+(64) | 16185 | 1 | 0.003
A2+(64) | 16187 | 1 | 0.003
A2+(64) | 16192 | 7 | 0.022
A2+(64) | 16193 | 1 | 0.003
A2+(64) | 16207 | 13 | 0.041
A2+(64) | 16214 | 1 | 0.003
A2+(64) | 16216 | 1 | 0.003
A2+(64) | 16217 | 4 | 0.013
A2+(64) | 16218 | 1 | 0.003
A2+(64) | 16221 | 4 | 0.013
A2+(64) | 16222 | 1 | 0.003
A2+(64) | 16231 | 4 | 0.013
A2+(64) | 16242 | 7 | 0.022
A2+(64) | 16242A | 1 | 0.003
A2+(64) | 16243 | 3 | 0.009
A2+(64) | 16259 | 1 | 0.003
A2+(64) | 16266 | 2 | 0.006
A2+(64) | 16266A | 1 | 0.003
A2+(64) | 16267 | 1 | 0.003
A2+(64) | 16270 | 1 | 0.003
A2+(64) | 16274 | 1 | 0.003
A2+(64) | 16278 | 1 | 0.003
A2+(64) | 1628 | 1 | 0.003
A2+(64) | 16286 | 2 | 0.006
A2+(64) | 16291 | 1 | 0.003
A2+(64) | 16293C | 1 | 0.003
A2+(64) | 16294 | 1 | 0.003
A2+(64) | 16295 | 1 | 0.003
A2+(64) | 16300 | 1 | 0.003
A2+(64) | 16304 | 1 | 0.003
A2+(64) | 16305 | 1 | 0.003
A2+(64) | 16311 | 18 | 0.056
A2+(64) | 16325 | 1 | 0.003
A2+(64) | 16326 | 1 | 0.003
A2+(64) | 16343 | 2 | 0.006
A2+(64) | 16356 | 1 | 0.003
A2+(64) | 16357 | 2 | 0.006
A2+(64) | 16360 | 1 | 0.003
A2+(64) | 16390 | 3 | 0.009
A2+(64) | 16391 | 1 | 0.003
A2+(64) | 16399 | 1 | 0.003
A2+(64) | 16422 | 1 | 0.003
A2+(64) | 16445 | 1 | 0.003
A2+(64) | 16448 | 1 | 0.003
A2+(64) | 16452 | 6 | 0.019
A2+(64) | 16463 | 1 | 0.003
A2+(64) | 16468 | 1 | 0.003
A2+(64) | 16474T | 2 | 0.006
A2+(64) | 16477 | 2 | 0.006
A2+(64) | 16497 | 1 | 0.003
A2+(64) | 16518 | 1 | 0.003
A2+(64) | 16524 | 1 | 0.003
A2+(64) | 16525 | 2 | 0.006
A2+(64) | 16527 | 1 | 0.003
A2+(64) | 16533 | 1 | 0.003
A2+(64) | 16566 | 1 | 0.003
A2+(64) | 1664 | 1 | 0.003
A2+(64) | 1677 | 2 | 0.006
A2+(64) | 1719 | 11 | 0.034
A2+(64) | 1766 | 1 | 0.003
A2+(64) | 1819 | 1 | 0.003
A2+(64) | 183 | 6 | 0.019
A2+(64) | 185 | 1 | 0.003

A2+(64) | 1888 | 1 | 0.003
A2+(64) | 189 | 1 | 0.003
A2+(64) | 1913 | 1 | 0.003
A2+(64) | 194 | 4 | 0.013
A2+(64) | 1943 | 1 | 0.003
A2+(64) | 195 | 8 | 0.025
A2+(64) | 198 | 1 | 0.003
A2+(64) | 199 | 2 | 0.006
A2+(64) | 200 | 5 | 0.016
A2+(64) | 2010 | 1 | 0.003
A2+(64) | 204 | 1 | 0.003
A2+(64) | 207 | 2 | 0.006
A2+(64) | 212 | 4 | 0.013
A2+(64) | 2120 | 2 | 0.006
A2+(64) | 214 | 1 | 0.003
A2+(64) | 2140 | 1 | 0.003
A2+(64) | 215 | 6 | 0.019
A2+(64) | 217 | 1 | 0.003
A2+(64) | 2222 | 6 | 0.019
A2+(64) | 226 | 8 | 0.025
A2+(64) | 234 | 1 | 0.003
A2+(64) | 2416 | 4 | 0.013
A2+(64) | 2442 | 1 | 0.003
A2+(64) | 247 | 1 | 0.003
A2+(64) | 280 | 2 | 0.006
A2+(64) | 282 | 1 | 0.003
A2+(64) | 2850 | 1 | 0.003
A2+(64) | 2872 | 1 | 0.003
A2+(64) | 2887 | 4 | 0.013
A2+(64) | 2905 | 1 | 0.003
A2+(64) | 3 | 9 | 0.028
A2+(64) | 3083 | 1 | 0.003
A2+(64) | 309d | 1 | 0.003
A2+(64) | 310 | 10 | 0.031
A2+(64) | 3140 | 1 | 0.003
A2+(64) | 315.2C | 1 | 0.003
A2+(64) | 315.3C | 1 | 0.003
A2+(64) | 315d | 2 | 0.006
A2+(64) | 317 | 2 | 0.006
A2+(64) | 318 | 2 | 0.006
A2+(64) | 3248 | 1 | 0.003
A2+(64) | 3354 | 2 | 0.006
A2+(64) | 338 | 1 | 0.003
A2+(64) | 3394 | 2 | 0.006
A2+(64) | 3396 | 1 | 0.003
A2+(64) | 3421 | 7 | 0.022
A2+(64) | 3423G | 1 | 0.003
A2+(64) | 3438 | 1 | 0.003
A2+(64) | 3495G | 1 | 0.003
A2+(64) | 3531 | 11 | 0.034
A2+(64) | 3547 | 1 | 0.003
A2+(64) | 3579 | 1 | 0.003
A2+(64) | 36 | 2 | 0.006
A2+(64) | 368 | 1 | 0.003
A2+(64) | 3693 | 12 | 0.038
A2+(64) | 373 | 2 | 0.006
A2+(64) | 374 | 1 | 0.003
A2+(64) | 3744 | 3 | 0.009
A2+(64) | 3786 | 3 | 0.009
A2+(64) | 3826 | 2 | 0.006
A2+(64) | 3834 | 1 | 0.003
A2+(64) | 3843 | 2 | 0.006
A2+(64) | 3865 | 5 | 0.016
A2+(64) | 3892 | 1 | 0.003
A2+(64) | 3910 | 1 | 0.003
A2+(64) | 3915 | 3 | 0.009
A2+(64) | 3960 | 1 | 0.003
A2+(64) | 398 | 1 | 0.003
A2+(64) | 3984 | 1 | 0.003
A2+(64) | 3995C | 2 | 0.006
A2+(64) | 4052 | 1 | 0.003
A2+(64) | 4087 | 1 | 0.003
A2+(64) | 4092 | 1 | 0.003
A2+(64) | 4113 | 1 | 0.003
A2+(64) | 4122 | 9 | 0.028
A2+(64) | 4129 | 2 | 0.006
A2+(64) | 4216 | 3 | 0.009
A2+(64) | 4284 | 1 | 0.003
A2+(64) | 4343 | 3 | 0.009
A2+(64) | 4373 | 2 | 0.006
A2+(64) | 4375 | 1 | 0.003
A2+(64) | 4452 | 1 | 0.003
A2+(64) | 4456 | 1 | 0.003
A2+(64) | 447 | 2 | 0.006
A2+(64) | 4481 | 2 | 0.006
A2+(64) | 4491 | 1 | 0.003
A2+(64) | 4505 | 7 | 0.022
A2+(64) | 4511 | 2 | 0.006
A2+(64) | 4512 | 1 | 0.003
A2+(64) | 455.1T | 1 | 0.003
A2+(64) | 4550 | 1 | 0.003
A2+(64) | 4561 | 14 | 0.044
A2+(64) | 4580 | 1 | 0.003
A2+(64) | 459A | 1 | 0.003
A2+(64) | 4612 | 1 | 0.003
A2+(64) | 4655 | 4 | 0.013
A2+(64) | 4659 | 1 | 0.003
A2+(64) | 4811 | 1 | 0.003
A2+(64) | 4820 | 1 | 0.003
A2+(64) | 4848 | 1 | 0.003
A2+(64) | 485 | 2 | 0.006
A2+(64) | 498d | 2 | 0.006
A2+(64) | 4991 | 1 | 0.003
A2+(64) | 5030 | 1 | 0.003
A2+(64) | 5033 | 1 | 0.003
A2+(64) | 5104 | 1 | 0.003
A2+(64) | 5114 | 3 | 0.009
A2+(64) | 5147 | 1 | 0.003
A2+(64) | 5177 | 1 | 0.003
A2+(64) | 52 | 2 | 0.006
A2+(64) | 5222 | 4 | 0.013

A2+(64) | 5258 | 3 | 0.009
A2+(64) | 53 | 1 | 0.003
A2+(64) | 533 | 4 | 0.013
A2+(64) | 5417 | 1 | 0.003
A2+(64) | 5460 | 1 | 0.003
A2+(64) | 551 | 1 | 0.003
A2+(64) | 5567 | 1 | 0.003
A2+(64) | 5585 | 1 | 0.003
A2+(64) | 5628 | 1 | 0.003
A2+(64) | 5634 | 1 | 0.003
A2+(64) | 573.1C | 1 | 0.003
A2+(64) | 573.4C | 1 | 0.003
A2+(64) | 5752.1A | 1 | 0.003
A2+(64) | 5765 | 1 | 0.003
A2+(64) | 5894C | 1 | 0.003
A2+(64) | 5899.1C | 2 | 0.006
A2+(64) | 5899.2C | 1 | 0.003
A2+(64) | 5915 | 1 | 0.003
A2+(64) | 596 | 2 | 0.006
A2+(64) | 5978 | 1 | 0.003
A2+(64) | 5990 | 1 | 0.003
A2+(64) | 6 | 1 | 0.003
A2+(64) | 60 | 3 | 0.009
A2+(64) | 6032 | 1 | 0.003
A2+(64) | 6094G | 1 | 0.003
A2+(64) | 6137 | 1 | 0.003
A2+(64) | 6179 | 1 | 0.003
A2+(64) | 62 | 1 | 0.003
A2+(64) | 6216 | 3 | 0.009
A2+(64) | 6221 | 1 | 0.003
A2+(64) | 6260 | 1 | 0.003
A2+(64) | 6284 | 2 | 0.006
A2+(64) | 6293 | 1 | 0.003
A2+(64) | 62T | 1 | 0.003
A2+(64) | 630 | 1 | 0.003
A2+(64) | 6340 | 1 | 0.003
A2+(64) | 6371 | 1 | 0.003
A2+(64) | 6378 | 1 | 0.003
A2+(64) | 6437 | 1 | 0.003
A2+(64) | 643T | 1 | 0.003
A2+(64) | 6527 | 1 | 0.003
A2+(64) | 6575 | 1 | 0.003
A2+(64) | 6638 | 2 | 0.006
A2+(64) | 6719 | 1 | 0.003
A2+(64) | 6723 | 1 | 0.003
A2+(64) | 6725 | 1 | 0.003
A2+(64) | 675 | 3 | 0.009
A2+(64) | 6911 | 1 | 0.003
A2+(64) | 6956 | 1 | 0.003
A2+(64) | 6962 | 1 | 0.003
A2+(64) | 7 | 1 | 0.003
A2+(64) | 7049 | 1 | 0.003
A2+(64) | 7059 | 1 | 0.003
A2+(64) | 709 | 3 | 0.009
A2+(64) | 7226 | 2 | 0.006
A2+(64) | 7271 | 1 | 0.003
A2+(64) | 7278 | 1 | 0.003
A2+(64) | 7372 | 1 | 0.003
A2+(64) | 7403 | 1 | 0.003
A2+(64) | 7498 | 2 | 0.006
A2+(64) | 756 | 2 | 0.006
A2+(64) | 7673 | 1 | 0.003
A2+(64) | 7697 | 1 | 0.003
A2+(64) | 7702 | 3 | 0.009
A2+(64) | 7846 | 1 | 0.003
A2+(64) | 7853 | 1 | 0.003
A2+(64) | 7861 | 1 | 0.003
A2+(64) | 794A | 1 | 0.003
A2+(64) | 7960 | 1 | 0.003
A2+(64) | 8021 | 1 | 0.003
A2+(64) | 8047 | 4 | 0.013
A2+(64) | 8062 | 1 | 0.003
A2+(64) | 8251 | 2 | 0.006
A2+(64) | 8260 | 2 | 0.006
A2+(64) | 8276d | 1 | 0.003
A2+(64) | 8289.1CCCCCTCTA | 1 | 0.003
A2+(64) | 8292 | 1 | 0.003
A2+(64) | 8345 | 1 | 0.003
A2+(64) | 8347 | 3 | 0.009
A2+(64) | 8381 | 2 | 0.006
A2+(64) | 8389 | 1 | 0.003
A2+(64) | 8400 | 1 | 0.003
A2+(64) | 8440 | 1 | 0.003
A2+(64) | 8477 | 2 | 0.006
A2+(64) | 8485 | 1 | 0.003
A2+(64) | 8491 | 2 | 0.006
A2+(64) | 8504 | 1 | 0.003
A2+(64) | 8566 | 1 | 0.003
A2+(64) | 8572 | 2 | 0.006
A2+(64) | 8602 | 1 | 0.003
A2+(64) | 8603 | 1 | 0.003
A2+(64) | 8618 | 3 | 0.009
A2+(64) | 8623 | 3 | 0.009
A2+(64) | 8648 | 1 | 0.003
A2+(64) | 869 | 1 | 0.003
A2+(64) | 8738 | 1 | 0.003
A2+(64) | 8745 | 1 | 0.003
A2+(64) | 8764 | 3 | 0.009
A2+(64) | 8812 | 1 | 0.003
A2+(64) | 8838 | 1 | 0.003
A2+(64) | 8870 | 6 | 0.019
A2+(64) | 8910A | 1 | 0.003
A2+(64) | 8932 | 1 | 0.003
A2+(64) | 8979 | 1 | 0.003
A2+(64) | 9012 | 3 | 0.009
A2+(64) | 9039 | 2 | 0.006
A2+(64) | 9055 | 1 | 0.003
A2+(64) | 9058 | 1 | 0.003
A2+(64) | 9095 | 1 | 0.003
A2+(64) | 9096 | 1 | 0.003
A2+(64) | 9163 | 1 | 0.003

A2+(64) | 9266 | 1 | 0.003
A2+(64) | 9311 | 1 | 0.003
A2+(64) | 9377 | 2 | 0.006
A2+(64) | 9389 | 1 | 0.003
A2+(64) | 9392 | 1 | 0.003
A2+(64) | 9452 | 1 | 0.003
A2+(64) | 9476 | 1 | 0.003
A2+(64) | 951 | 2 | 0.006
A2+(64) | 9530 | 1 | 0.003
A2+(64) | 9571 | 1 | 0.003
A2+(64) | 960.1C | 1 | 0.003
A2+(64) | 9667 | 1 | 0.003
A2+(64) | 9698 | 1 | 0.003
A2+(64) | 9699 | 1 | 0.003
A2+(64) | 9854 | 1 | 0.003
A2+(64) | 9893 | 1 | 0.003
A2+(64) | 9899 | 3 | 0.009
A2+(64) | 9966 | 1 | 0.003
A2+(64) | 9967 | 1 | 0.003
A2+(64) | 9973 | 1 | 0.003
A2+(64)+@153 | 10370 | 1 | 0.01
A2+(64)+@153 | 10586 | 1 | 0.01
A2+(64)+@153 | 10685 | 2 | 0.02
A2+(64)+@153 | 10963 | 13 | 0.127
A2+(64)+@153 | 11023 | 1 | 0.01
A2+(64)+@153 | 11167 | 2 | 0.02
A2+(64)+@153 | 111C | 1 | 0.01
A2+(64)+@153 | 11206A | 2 | 0.02
A2+(64)+@153 | 11914 | 1 | 0.01
A2+(64)+@153 | 12408 | 2 | 0.02
A2+(64)+@153 | 12618 | 1 | 0.01
A2+(64)+@153 | 12927 | 2 | 0.02
A2+(64)+@153 | 13191 | 1 | 0.01
A2+(64)+@153 | 13416 | 1 | 0.01
A2+(64)+@153 | 13419 | 3 | 0.029
A2+(64)+@153 | 13474 | 8 | 0.078
A2+(64)+@153 | 13651 | 1 | 0.01
A2+(64)+@153 | 13674 | 1 | 0.01
A2+(64)+@153 | 13677 | 1 | 0.01
A2+(64)+@153 | 13753 | 3 | 0.029
A2+(64)+@153 | 13806 | 2 | 0.02
A2+(64)+@153 | 14004A | 2 | 0.02
A2+(64)+@153 | 14280T | 1 | 0.01
A2+(64)+@153 | 152 | 2 | 0.02
A2+(64)+@153 | 15258 | 1 | 0.01
A2+(64)+@153 | 15314 | 2 | 0.02
A2+(64)+@153 | 15317 | 1 | 0.01
A2+(64)+@153 | 15451 | 1 | 0.01
A2+(64)+@153 | 15550 | 1 | 0.01
A2+(64)+@153 | 15746 | 1 | 0.01
A2+(64)+@153 | 15793 | 1 | 0.01
A2+(64)+@153 | 15884 | 3 | 0.029
A2+(64)+@153 | 15924 | 2 | 0.02
A2+(64)+@153 | 15969A | 15 | 0.147
A2+(64)+@153 | 16069 | 1 | 0.01
A2+(64)+@153 | 16092 | 13 | 0.127
A2+(64)+@153 | 16093 | 1 | 0.01
A2+(64)+@153 | 16094 | 1 | 0.01
A2+(64)+@153 | 16124 | 3 | 0.029
A2+(64)+@153 | 16129 | 1 | 0.01
A2+(64)+@153 | 16145 | 15 | 0.147
A2+(64)+@153 | 16172 | 7 | 0.069
A2+(64)+@153 | 16189 | 1 | 0.01
A2+(64)+@153 | 16192 | 3 | 0.029
A2+(64)+@153 | 16213 | 1 | 0.01
A2+(64)+@153 | 16218 | 1 | 0.01
A2+(64)+@153 | 16220 | 1 | 0.01
A2+(64)+@153 | 16265T | 1 | 0.01
A2+(64)+@153 | 16266 | 1 | 0.01
A2+(64)+@153 | 16274 | 1 | 0.01
A2+(64)+@153 | 16287 | 1 | 0.01
A2+(64)+@153 | 16294 | 1 | 0.01
A2+(64)+@153 | 16310 | 1 | 0.01
A2+(64)+@153 | 16320 | 1 | 0.01
A2+(64)+@153 | 16487 | 1 | 0.01
A2+(64)+@153 | 16497 | 1 | 0.01
A2+(64)+@153 | 16527 | 3 | 0.029
A2+(64)+@153 | 188 | 1 | 0.01
A2+(64)+@153 | 189 | 3 | 0.029
A2+(64)+@153 | 195 | 2 | 0.02
A2+(64)+@153 | 211 | 9 | 0.088
A2+(64)+@153 | 2158 | 1 | 0.01
A2+(64)+@153 | 226 | 14 | 0.137
A2+(64)+@153 | 2387 | 2 | 0.02
A2+(64)+@153 | 2971T | 1 | 0.01
A2+(64)+@153 | 297-298d | 1 | 0.01
A2+(64)+@153 | 3084 | 1 | 0.01
A2+(64)+@153 | 309d | 3 | 0.029
A2+(64)+@153 | 310 | 2 | 0.02
A2+(64)+@153 | 3316 | 1 | 0.01
A2+(64)+@153 | 3339 | 2 | 0.02
A2+(64)+@153 | 3531 | 15 | 0.147
A2+(64)+@153 | 3843 | 3 | 0.029
A2+(64)+@153 | 3865 | 3 | 0.029
A2+(64)+@153 | 41 | 3 | 0.029
A2+(64)+@153 | 4122 | 1 | 0.01
A2+(64)+@153 | 4135 | 1 | 0.01
A2+(64)+@153 | 4370 | 1 | 0.01
A2+(64)+@153 | 4562 | 1 | 0.01
A2+(64)+@153 | 480 | 2 | 0.02
A2+(64)+@153 | 4820 | 2 | 0.02
A2+(64)+@153 | 5319 | 2 | 0.02
A2+(64)+@153 | 5573 | 1 | 0.01
A2+(64)+@153 | 5580 | 1 | 0.01
A2+(64)+@153 | 68 | 1 | 0.01
A2+(64)+@153 | 720 | 1 | 0.01
A2+(64)+@153 | 7389 | 2 | 0.02
A2+(64)+@153 | 7444 | 1 | 0.01
A2+(64)+@153 | 7852 | 1 | 0.01
A2+(64)+@153 | 8440 | 15 | 0.147
A2+(64)+@153 | 8461A | 2 | 0.02

A2+(64)+@153 | 8598 | 1 | 0.01
A2+(64)+@153 | 8618 | 2 | 0.02
A2+(64)+@153 | 8681 | 1 | 0.01
A2+(64)+@153 | 8710 | 1 | 0.01
A2+(64)+@153 | 8865 | 1 | 0.01
A2+(64)+@153 | 8946 | 6 | 0.059
A2+(64)+@153 | 8975 | 1 | 0.01
A2+(64)+@153 | 8987 | 8 | 0.078
A2+(64)+@153 | 90C | 1 | 0.01
A2+(64)+@153 | 93 | 1 | 0.01
A2+(64)+@153 | 9316 | 1 | 0.01
A2+(64)+@153 | 94 | 1 | 0.01
A2+(64)+@153 | 9525 | 2 | 0.02
A2+(64)+@153 | 9755 | 1 | 0.01
A2+(64)+@153 | 98 | 1 | 0.01
A2+(64)+@16111 | 10128 | 3 | 0.007
A2+(64)+@16111 | 10586 | 1 | 0.002
A2+(64)+@16111 | 11290 | 8 | 0.018
A2+(64)+@16111 | 113 | 1 | 0.002
A2+(64)+@16111 | 12172 | 8 | 0.018
A2+(64)+@16111 | 12509C | 1 | 0.002
A2+(64)+@16111 | 12618 | 3 | 0.007
A2+(64)+@16111 | 13785 | 1 | 0.002
A2+(64)+@16111 | 14148 | 2 | 0.005
A2+(64)+@16111 | 14245 | 1 | 0.002
A2+(64)+@16111 | 143 | 12 | 0.027
A2+(64)+@16111 | 14364 | 1 | 0.002
A2+(64)+@16111 | 14551 | 3 | 0.007
A2+(64)+@16111 | 14587 | 1 | 0.002
A2+(64)+@16111 | 14777 | 2 | 0.005
A2+(64)+@16111 | 15043 | 1 | 0.002
A2+(64)+@16111 | 150A | 3 | 0.007
A2+(64)+@16111 | 152 | 14 | 0.032
A2+(64)+@16111 | 15218 | 1 | 0.002
A2+(64)+@16111 | 15244 | 1 | 0.002
A2+(64)+@16111 | 15519 | 3 | 0.007
A2+(64)+@16111 | 159 | 1 | 0.002
A2+(64)+@16111 | 15929 | 3 | 0.007
A2+(64)+@16111 | 15930 | 3 | 0.007
A2+(64)+@16111 | 15968 | 6 | 0.014
A2+(64)+@16111 | 16075 | 1 | 0.002
A2+(64)+@16111 | 16076d | 1 | 0.002
A2+(64)+@16111 | 16086 | 2 | 0.005
A2+(64)+@16111 | 16092 | 4 | 0.009
A2+(64)+@16111 | 16126 | 20 | 0.045
A2+(64)+@16111 | 16148 | 1 | 0.002
A2+(64)+@16111 | 16184 | 4 | 0.009
A2+(64)+@16111 | 16234A | 1 | 0.002
A2+(64)+@16111 | 16256 | 3 | 0.007
A2+(64)+@16111 | 16261 | 1 | 0.002
A2+(64)+@16111 | 16274 | 1 | 0.002
A2+(64)+@16111 | 16278 | 21 | 0.048
A2+(64)+@16111 | 16293 | 9 | 0.02
A2+(64)+@16111 | 16310 | 2 | 0.005
A2+(64)+@16111 | 16311 | 2 | 0.005
A2+(64)+@16111 | 16325 | 1 | 0.002
A2+(64)+@16111 | 16390 | 3 | 0.007
A2+(64)+@16111 | 16391 | 5 | 0.011
A2+(64)+@16111 | 16526 | 1 | 0.002
A2+(64)+@16111 | 1719 | 1 | 0.002
A2+(64)+@16111 | 182 | 3 | 0.007
A2+(64)+@16111 | 186 | 2 | 0.005
A2+(64)+@16111 | 189 | 7 | 0.016
A2+(64)+@16111 | 195 | 4 | 0.009
A2+(64)+@16111 | 207 | 8 | 0.018
A2+(64)+@16111 | 2128 | 1 | 0.002
A2+(64)+@16111 | 215 | 1 | 0.002
A2+(64)+@16111 | 2231 | 2 | 0.005
A2+(64)+@16111 | 2267G | 1 | 0.002
A2+(64)+@16111 | 279 | 1 | 0.002
A2+(64)+@16111 | 295G | 1 | 0.002
A2+(64)+@16111 | 297 | 12 | 0.027
A2+(64)+@16111 | 3202 | 3 | 0.007
A2+(64)+@16111 | 3316 | 8 | 0.018
A2+(64)+@16111 | 3535 | 2 | 0.005
A2+(64)+@16111 | 3669 | 8 | 0.018
A2+(64)+@16111 | 3834 | 1 | 0.002
A2+(64)+@16111 | 4122 | 1 | 0.002
A2+(64)+@16111 | 5094 | 1 | 0.002
A2+(64)+@16111 | 51 | 1 | 0.002
A2+(64)+@16111 | 5147 | 3 | 0.007
A2+(64)+@16111 | 5460 | 8 | 0.018
A2+(64)+@16111 | 572.1ACCCCCCCCC | 1 | 0.002
A2+(64)+@16111 | 573.1C | 2 | 0.005
A2+(64)+@16111 | 573.3C | 1 | 0.002
A2+(64)+@16111 | 573.4C | 1 | 0.002
A2+(64)+@16111 | 573.5C | 1 | 0.002
A2+(64)+@16111 | 573.6C | 1 | 0.002
A2+(64)+@16111 | 5824 | 1 | 0.002
A2+(64)+@16111 | 59 | 13 | 0.029
A2+(64)+@16111 | 61A | 1 | 0.002
A2+(64)+@16111 | 62 | 1 | 0.002
A2+(64)+@16111 | 62T | 2 | 0.005
A2+(64)+@16111 | 6308 | 1 | 0.002
A2+(64)+@16111 | 6473 | 1 | 0.002
A2+(64)+@16111 | 6842 | 1 | 0.002
A2+(64)+@16111 | 7001T | 8 | 0.018
A2+(64)+@16111 | 7229 | 8 | 0.018
A2+(64)+@16111 | 7389 | 2 | 0.005
A2+(64)+@16111 | 7669 | 2 | 0.005
A2+(64)+@16111 | 7961 | 3 | 0.007
A2+(64)+@16111 | 7964 | 2 | 0.005
A2+(64)+@16111 | 8281-8289d | 1 | 0.002
A2+(64)+@16111 | 8790 | 1 | 0.002
A2+(64)+@16111 | 9157 | 2 | 0.005
A2+(64)+@16111 | 9559G | 1 | 0.002
A2+(64)+@16111 | 959 | 1 | 0.002
A2+(64)+16129 | 10045 | 1 | 0.023
A2+(64)+16129 | 10289 | 1 | 0.023
A2+(64)+16129 | 10373 | 1 | 0.023
A2+(64)+16129 | 10775 | 1 | 0.023

A2+(64)+16129 | 11224 | 1 | 0.023
A2+(64)+16129 | 1189 | 1 | 0.023
A2+(64)+16129 | 12242 | 1 | 0.023
A2+(64)+16129 | 125 | 2 | 0.045
A2+(64)+16129 | 127 | 2 | 0.045
A2+(64)+16129 | 13474 | 1 | 0.023
A2+(64)+16129 | 14053 | 1 | 0.023
A2+(64)+16129 | 143 | 4 | 0.091
A2+(64)+16129 | 14971 | 2 | 0.045
A2+(64)+16129 | 152 | 6 | 0.136
A2+(64)+16129 | 15289 | 1 | 0.023
A2+(64)+16129 | 16086 | 1 | 0.023
A2+(64)+16129 | 16092 | 5 | 0.114
A2+(64)+16129 | 16093 | 3 | 0.068
A2+(64)+16129 | 16189 | 3 | 0.068
A2+(64)+16129 | 16233 | 2 | 0.045
A2+(64)+16129 | 16274 | 2 | 0.045
A2+(64)+16129 | 16311 | 8 | 0.182
A2+(64)+16129 | 16354 | 1 | 0.023
A2+(64)+16129 | 2056 | 1 | 0.023
A2+(64)+16129 | 215 | 5 | 0.114
A2+(64)+16129 | 264 | 2 | 0.045
A2+(64)+16129 | 297 | 1 | 0.023
A2+(64)+16129 | 309d | 1 | 0.023
A2+(64)+16129 | 310 | 1 | 0.023
A2+(64)+16129 | 3397 | 1 | 0.023
A2+(64)+16129 | 356.1C | 1 | 0.023
A2+(64)+16129 | 4123 | 1 | 0.023
A2+(64)+16129 | 4310 | 1 | 0.023
A2+(64)+16129 | 459.1C | 1 | 0.023
A2+(64)+16129 | 4625G | 1 | 0.023
A2+(64)+16129 | 4655 | 2 | 0.045
A2+(64)+16129 | 499 | 1 | 0.023
A2+(64)+16129 | 513 | 1 | 0.023
A2+(64)+16129 | 5222 | 1 | 0.023
A2+(64)+16129 | 57 | 2 | 0.045
A2+(64)+16129 | 57.1G | 1 | 0.023
A2+(64)+16129 | 60.1T | 1 | 0.023
A2+(64)+16129 | 6249 | 1 | 0.023
A2+(64)+16129 | 6308 | 1 | 0.023
A2+(64)+16129 | 6503 | 1 | 0.023
A2+(64)+16129 | 6716 | 1 | 0.023
A2+(64)+16129 | 6914 | 1 | 0.023
A2+(64)+16129 | 709 | 1 | 0.023
A2+(64)+16129 | 7732 | 1 | 0.023
A2+(64)+16129 | 8281-8289d | 1 | 0.023
A2+(64)+16129 | 8292 | 2 | 0.045
A2+(64)+16129 | 8387 | 1 | 0.023
A2+(64)+16129 | 8757 | 1 | 0.023
A2+(64)+16189 | 10083T | 1 | 0.028
A2+(64)+16189 | 10203 | 1 | 0.028
A2+(64)+16189 | 10271 | 1 | 0.028
A2+(64)+16189 | 10410 | 1 | 0.028
A2+(64)+16189 | 10631 | 1 | 0.028
A2+(64)+16189 | 11025 | 1 | 0.028
A2+(64)+16189 | 11177 | 1 | 0.028
A2+(64)+16189 | 11305 | 1 | 0.028
A2+(64)+16189 | 12285 | 1 | 0.028
A2+(64)+16189 | 12351 | 1 | 0.028
A2+(64)+16189 | 12406 | 1 | 0.028
A2+(64)+16189 | 12441 | 1 | 0.028
A2+(64)+16189 | 125 | 2 | 0.056
A2+(64)+16189 | 12634 | 1 | 0.028
A2+(64)+16189 | 127 | 2 | 0.056
A2+(64)+16189 | 13020 | 1 | 0.028
A2+(64)+16189 | 13287 | 1 | 0.028
A2+(64)+16189 | 13437 | 1 | 0.028
A2+(64)+16189 | 13584 | 1 | 0.028
A2+(64)+16189 | 13614 | 1 | 0.028
A2+(64)+16189 | 13651 | 1 | 0.028
A2+(64)+16189 | 13708 | 2 | 0.056
A2+(64)+16189 | 139 | 1 | 0.028
A2+(64)+16189 | 14053 | 4 | 0.111
A2+(64)+16189 | 14059 | 1 | 0.028
A2+(64)+16189 | 14064 | 4 | 0.111
A2+(64)+16189 | 14112A | 1 | 0.028
A2+(64)+16189 | 143 | 4 | 0.111
A2+(64)+16189 | 14687 | 4 | 0.111
A2+(64)+16189 | 14857 | 1 | 0.028
A2+(64)+16189 | 14861 | 1 | 0.028
A2+(64)+16189 | 14881 | 1 | 0.028
A2+(64)+16189 | 15098 | 1 | 0.028
A2+(64)+16189 | 15106 | 1 | 0.028
A2+(64)+16189 | 15553 | 1 | 0.028
A2+(64)+16189 | 15703 | 1 | 0.028
A2+(64)+16189 | 15924 | 1 | 0.028
A2+(64)+16189 | 15954 | 1 | 0.028
A2+(64)+16189 | 15968 | 1 | 0.028
A2+(64)+16189 | 16066 | 1 | 0.028
A2+(64)+16189 | 16092 | 2 | 0.056
A2+(64)+16189 | 16140 | 5 | 0.139
A2+(64)+16189 | 16148 | 1 | 0.028
A2+(64)+16189 | 16172 | 1 | 0.028
A2+(64)+16189 | 16188 | 1 | 0.028
A2+(64)+16189 | 16190 | 1 | 0.028
A2+(64)+16189 | 16231 | 1 | 0.028
A2+(64)+16189 | 16240 | 1 | 0.028
A2+(64)+16189 | 16264 | 5 | 0.139
A2+(64)+16189 | 16287 | 1 | 0.028
A2+(64)+16189 | 16291 | 1 | 0.028
A2+(64)+16189 | 16293 | 1 | 0.028
A2+(64)+16189 | 16294 | 1 | 0.028
A2+(64)+16189 | 16304 | 1 | 0.028
A2+(64)+16189 | 16311 | 4 | 0.111
A2+(64)+16189 | 16320 | 3 | 0.083
A2+(64)+16189 | 16325 | 1 | 0.028
A2+(64)+16189 | 16326 | 1 | 0.028
A2+(64)+16189 | 16381 | 2 | 0.056
A2+(64)+16189 | 16468 | 2 | 0.056
A2+(64)+16189 | 1806 | 1 | 0.028
A2+(64)+16189 | 185 | 2 | 0.056

A2+(64)+16189 | 189 | 3 | 0.083
A2+(64)+16189 | 1891 | 1 | 0.028
A2+(64)+16189 | 195 | 1 | 0.028
A2+(64)+16189 | 215 | 2 | 0.056
A2+(64)+16189 | 2850 | 1 | 0.028
A2+(64)+16189 | 309d | 3 | 0.083
A2+(64)+16189 | 338 | 3 | 0.083
A2+(64)+16189 | 3438 | 1 | 0.028
A2+(64)+16189 | 345 | 3 | 0.083
A2+(64)+16189 | 4048 | 1 | 0.028
A2+(64)+16189 | 4129 | 1 | 0.028
A2+(64)+16189 | 4216 | 1 | 0.028
A2+(64)+16189 | 4310 | 1 | 0.028
A2+(64)+16189 | 4541 | 1 | 0.028
A2+(64)+16189 | 4547 | 1 | 0.028
A2+(64)+16189 | 4596 | 1 | 0.028
A2+(64)+16189 | 499 | 1 | 0.028
A2+(64)+16189 | 4994 | 4 | 0.111
A2+(64)+16189 | 5093 | 1 | 0.028
A2+(64)+16189 | 51 | 1 | 0.028
A2+(64)+16189 | 5249 | 1 | 0.028
A2+(64)+16189 | 5456 | 1 | 0.028
A2+(64)+16189 | 5460 | 1 | 0.028
A2+(64)+16189 | 573.2C | 1 | 0.028
A2+(64)+16189 | 573.5C | 1 | 0.028
A2+(64)+16189 | 6110 | 1 | 0.028
A2+(64)+16189 | 6249 | 1 | 0.028
A2+(64)+16189 | 6734 | 1 | 0.028
A2+(64)+16189 | 6899 | 1 | 0.028
A2+(64)+16189 | 7202 | 1 | 0.028
A2+(64)+16189 | 7657 | 1 | 0.028
A2+(64)+16189 | 7805 | 1 | 0.028
A2+(64)+16189 | 7822C | 3 | 0.083
A2+(64)+16189 | 8010 | 1 | 0.028
A2+(64)+16189 | 8188 | 1 | 0.028
A2+(64)+16189 | 8265 | 1 | 0.028
A2+(64)+16189 | 8347 | 1 | 0.028
A2+(64)+16189 | 8393 | 1 | 0.028
A2+(64)+16189 | 8577 | 4 | 0.111
A2+(64)+16189 | 8772 | 1 | 0.028
A2+(64)+16189 | 8879 | 1 | 0.028
A2+(64)+16189 | 9053 | 1 | 0.028
A2+(64)+16189 | 9095 | 1 | 0.028
A2+(64)+16189 | 9424 | 1 | 0.028
A2+(64)+16189 | 9509 | 1 | 0.028
A2+(64)+16189 | 961 | 1 | 0.028
A2+(64)+16189 | 965.2C | 1 | 0.028
A2+(64)+16189 | 9682 | 3 | 0.083
A2+(64)+16189 | 9719A | 1 | 0.028
A2+(64)+16189 | 9761 | 1 | 0.028
A20 | 11212 | 1 | 0.002
A20 | 12903 | 1 | 0.002
A20 | 14178 | 1 | 0.002
A20 | 156 | 11 | 0.027
A20 | 159 | 1 | 0.002
A20 | 16124 | 1 | 0.002
A20 | 16148 | 1 | 0.002
A20 | 16157 | 3 | 0.007
A20 | 16209 | 1 | 0.002
A20 | 16264 | 1 | 0.002
A20 | 16270 | 1 | 0.002
A20 | 16462 | 1 | 0.002
A20 | 16525C | 1 | 0.002
A20 | 16538 | 1 | 0.002
A20 | 210 | 1 | 0.002
A20 | 232C | 1 | 0.002
A20 | 341C | 1 | 0.002
A20 | 3492 | 1 | 0.002
A20 | 407A | 1 | 0.002
A20 | 450 | 1 | 0.002
A20 | 464C | 1 | 0.002
A20 | 573.1C | 1 | 0.002
A20 | 824 | 1 | 0.002
A20 | 8289.1CCCCCTCTA | 1 | 0.002
A20 | 8886 | 1 | 0.002
A21 | 12603 | 2 | 0.004
A21 | 12634 | 1 | 0.002
A21 | 13020 | 1 | 0.002
A21 | 13145 | 1 | 0.002
A21 | 13478 | 1 | 0.002
A21 | 13934 | 1 | 0.002
A21 | 143 | 3 | 0.006
A21 | 14798 | 1 | 0.002
A21 | 14962 | 1 | 0.002
A21 | 151 | 1 | 0.002
A21 | 16086 | 2 | 0.004
A21 | 16092 | 108 | 0.21
A21 | 16094 | 1 | 0.002
A21 | 16104 | 3 | 0.006
A21 | 16124 | 2 | 0.004
A21 | 16145 | 1 | 0.002
A21 | 16174 | 2 | 0.004
A21 | 16217 | 1 | 0.002
A21 | 16224 | 1 | 0.002
A21 | 16256 | 1 | 0.002
A21 | 16257 | 10 | 0.019
A21 | 16270 | 1 | 0.002
A21 | 16288 | 1 | 0.002
A21 | 16289 | 1 | 0.002
A21 | 16294 | 1 | 0.002
A21 | 16295 | 1 | 0.002
A21 | 16298 | 2 | 0.004
A21 | 16327 | 2 | 0.004
A21 | 16355 | 1 | 0.002
A21 | 16400 | 2 | 0.004
A21 | 16462 | 1 | 0.002
A21 | 16525C | 1 | 0.002
A21 | 16527 | 1 | 0.002
A21 | 16538 | 1 | 0.002
A21 | 208 | 1 | 0.002
A21 | 232C | 1 | 0.002

A21 | 310 | 2 | 0.004
A21 | 3106A | 2 | 0.004
A21 | 315.2C | 1 | 0.002
A21 | 316C | 4 | 0.008
A21 | 341C | 1 | 0.002
A21 | 407A | 1 | 0.002
A21 | 450 | 1 | 0.002
A21 | 464C | 1 | 0.002
A21 | 573.1C | 1 | 0.002
A21 | 573d | 1 | 0.002
A21 | 632 | 1 | 0.002
A21 | 66T | 2 | 0.004
A21 | 824 | 1 | 0.002
A21 | 8506 | 1 | 0.002
A22 | 12200 | 1 | 0.002
A22 | 16124 | 1 | 0.002
A22 | 16157 | 3 | 0.007
A22 | 16264 | 1 | 0.002
A22 | 16270 | 1 | 0.002
A22 | 16462 | 1 | 0.002
A22 | 16525C | 1 | 0.002
A22 | 16538 | 1 | 0.002
A22 | 232C | 1 | 0.002
A22 | 341C | 1 | 0.002
A22 | 407A | 1 | 0.002
A22 | 450 | 1 | 0.002
A22 | 464C | 1 | 0.002
A22 | 573.1C | 1 | 0.002
A22 | 824 | 1 | 0.002
A22 | 9150 | 1 | 0.002
A23 | 16169 | 1 | 0.043
A23 | 16183 | 2 | 0.087
A23 | 16215 | 1 | 0.043
A23 | 16248 | 6 | 0.261
A23 | 16286G | 1 | 0.043
A23 | 200 | 1 | 0.043
A23 | 207 | 1 | 0.043
A23 | 523d | 1 | 0.043
A23 | 8494 | 1 | 0.043
A24 | 11087 | 1 | 0.003
A24 | 16093 | 1 | 0.003
A24 | 16120T | 1 | 0.003
A24 | 16127 | 2 | 0.005
A24 | 16131 | 1 | 0.003
A24 | 16217 | 1 | 0.003
A24 | 16222A | 1 | 0.003
A24 | 16256 | 1 | 0.003
A24 | 16258C | 1 | 0.003
A24 | 16280T | 1 | 0.003
A24 | 16298 | 1 | 0.003
A24 | 16316 | 1 | 0.003
A24 | 16326 | 4 | 0.01
A24 | 16354 | 1 | 0.003
A24 | 310 | 1 | 0.003
A24 | 316C | 3 | 0.008
A24 | 366 | 1 | 0.003
A24 | 389 | 1 | 0.003
A24 | 5162 | 1 | 0.003
A24 | 8921 | 1 | 0.003
A24 | 9440 | 1 | 0.003
A25 | 10724 | 1 | 0.04
A25 | 11086 | 1 | 0.04
A25 | 13359 | 1 | 0.04
A25 | 13708 | 2 | 0.08
A25 | 146 | 1 | 0.04
A25 | 14692.1T | 1 | 0.04
A25 | 15142 | 1 | 0.04
A25 | 15346 | 2 | 0.08
A25 | 15562 | 2 | 0.08
A25 | 15856 | 2 | 0.08
A25 | 16129 | 1 | 0.04
A25 | 16192 | 1 | 0.04
A25 | 16311 | 14 | 0.56
A25 | 3828 | 1 | 0.04
A25 | 3834 | 1 | 0.04
A25 | 3892 | 1 | 0.04
A25 | 4136 | 1 | 0.04
A25 | 4917 | 1 | 0.04
A25 | 522 | 3 | 0.12
A25 | 5794 | 2 | 0.08
A25 | 7711 | 2 | 0.08
A26 | 15911T | 4 | 0.286
A26 | 16365 | 2 | 0.143
A26 | 5063 | 1 | 0.071
A2a | 11800 | 1 | 0.007
A2a | 11914 | 3 | 0.022
A2a | 12397 | 1 | 0.007
A2a | 14162 | 2 | 0.015
A2a | 14560 | 9 | 0.067
A2a | 16176 | 2 | 0.015
A2a | 310 | 1 | 0.007
A2a | 3338 | 3 | 0.022
A2a | 6018 | 1 | 0.007
A2a | 7633 | 5 | 0.037
A2a | 7775 | 1 | 0.007
A2a | 8613 | 1 | 0.007
A2a1 | 16129 | 2 | 0.069
A2a1 | 8520 | 1 | 0.034
A2a2 | 16124 | 1 | 0.008
A2a2 | 16134 | 1 | 0.008
A2a2 | 16212 | 3 | 0.024
A2a2 | 16256 | 1 | 0.008
A2a2 | 16271 | 1 | 0.008
A2a2 | 16359 | 2 | 0.016
A2a2 | 226 | 1 | 0.008
A2a2 | 6071 | 2 | 0.016
A2a2 | 9068 | 2 | 0.016
A2a3 | 16207 | 1 | 0.02
A2a3 | 16212 | 1 | 0.02
A2a3 | 16274 | 1 | 0.02
A2a3 | 16352 | 1 | 0.02

A2a3 | 2857 | 1 | 0.02
A2a3 | 3395 | 1 | 0.02
A2a4 | 9673 | 1 | 0.111
A2a5 | 152 | 1 | 0.029
A2a5 | 15664 | 5 | 0.147
A2a5 | 159 | 2 | 0.059
A2a5 | 16147 | 2 | 0.059
A2a5 | 16189 | 6 | 0.176
A2a5 | 16193 | 2 | 0.059
A2a5 | 207 | 1 | 0.029
A2a5 | 272 | 5 | 0.147
A2a5 | 7797 | 2 | 0.059
A2aa | 1117 | 1 | 0.031
A2aa | 11548 | 3 | 0.094
A2aa | 11944 | 2 | 0.062
A2aa | 14530 | 1 | 0.031
A2aa | 15110 | 1 | 0.031
A2aa | 152 | 2 | 0.062
A2aa | 15470 | 1 | 0.031
A2aa | 15946 | 1 | 0.031
A2aa | 16048 | 1 | 0.031
A2aa | 16153 | 2 | 0.062
A2aa | 16189 | 1 | 0.031
A2aa | 16218 | 2 | 0.062
A2aa | 16222 | 2 | 0.062
A2aa | 16274 | 2 | 0.062
A2aa | 16284 | 2 | 0.062
A2aa | 16300 | 6 | 0.188
A2aa | 16318T | 2 | 0.062
A2aa | 16325 | 3 | 0.094
A2aa | 1888 | 1 | 0.031
A2aa | 215 | 2 | 0.062
A2aa | 5063 | 1 | 0.031
A2aa | 508 | 3 | 0.094
A2aa | 6351 | 4 | 0.125
A2aa | 709 | 1 | 0.031
A2aa | 7673 | 3 | 0.094
A2aa | 7762 | 1 | 0.031
A2aa | 8286 | 3 | 0.094
A2aa | 8387 | 1 | 0.031
A2aa | 8654 | 1 | 0.031
A2aa | 9022 | 1 | 0.031
A2aa | 9098 | 1 | 0.031
A2aa | 9531 | 1 | 0.031
A2ab | 10 | 2 | 0.1
A2ab | 10589 | 1 | 0.05
A2ab | 125 | 1 | 0.05
A2ab | 127 | 1 | 0.05
A2ab | 1287 | 1 | 0.05
A2ab | 139 | 2 | 0.1
A2ab | 14065 | 1 | 0.05
A2ab | 16066 | 1 | 0.05
A2ab | 16129 | 1 | 0.05
A2ab | 16189 | 1 | 0.05
A2ab | 16278 | 3 | 0.15
A2ab | 16311 | 1 | 0.05
A2ab | 16465 | 2 | 0.1
A2ab | 194 | 1 | 0.05
A2ab | 207 | 2 | 0.1
A2ab | 215 | 1 | 0.05
A2ab | 2246 | 2 | 0.1
A2ab | 310.1T | 1 | 0.05
A2ab | 317G | 1 | 0.05
A2ab | 489 | 1 | 0.05
A2ab | 5228 | 1 | 0.05
A2ab | 7732 | 1 | 0.05
A2ac | 10005 | 1 | 0.015
A2ac | 10034 | 6 | 0.09
A2ac | 11275A | 1 | 0.015
A2ac | 11644 | 2 | 0.03
A2ac | 12070 | 5 | 0.075
A2ac | 12079 | 1 | 0.015
A2ac | 125 | 18 | 0.269
A2ac | 127 | 18 | 0.269
A2ac | 12714 | 2 | 0.03
A2ac | 128 | 3 | 0.045
A2ac | 12950 | 4 | 0.06
A2ac | 13656 | 6 | 0.09
A2ac | 13902 | 1 | 0.015
A2ac | 13934 | 2 | 0.03
A2ac | 14007 | 2 | 0.03
A2ac | 14053 | 6 | 0.09
A2ac | 14097 | 2 | 0.03
A2ac | 14110 | 3 | 0.045
A2ac | 1413 | 1 | 0.015
A2ac | 14258 | 1 | 0.015
A2ac | 14614 | 1 | 0.015
A2ac | 1503 | 1 | 0.015
A2ac | 15077 | 1 | 0.015
A2ac | 15113 | 1 | 0.015
A2ac | 152 | 3 | 0.045
A2ac | 15217 | 1 | 0.015
A2ac | 15457 | 1 | 0.015
A2ac | 15884 | 2 | 0.03
A2ac | 15927 | 1 | 0.015
A2ac | 16037 | 1 | 0.015
A2ac | 16108 | 1 | 0.015
A2ac | 16126 | 2 | 0.03
A2ac | 16129 | 1 | 0.015
A2ac | 16172 | 3 | 0.045
A2ac | 16189 | 2 | 0.03
A2ac | 16192 | 1 | 0.015
A2ac | 16209 | 5 | 0.075
A2ac | 16214 | 1 | 0.015
A2ac | 16244 | 1 | 0.015
A2ac | 16261 | 1 | 0.015
A2ac | 16291 | 1 | 0.015
A2ac | 16292 | 3 | 0.045
A2ac | 16311 | 1 | 0.015
A2ac | 16320 | 2 | 0.03
A2ac | 16356 | 3 | 0.045

A2ac | 16360 | 1 | 0.015
A2ac | 1760 | 17 | 0.254
A2ac | 195 | 3 | 0.045
A2ac | 215 | 1 | 0.015
A2ac | 2416 | 1 | 0.015
A2ac | 249 | 4 | 0.06
A2ac | 249d | 1 | 0.015
A2ac | 2755 | 2 | 0.03
A2ac | 279 | 8 | 0.119
A2ac | 291 | 5 | 0.075
A2ac | 310 | 1 | 0.015
A2ac | 3340 | 2 | 0.03
A2ac | 3364 | 6 | 0.09
A2ac | 3375 | 2 | 0.03
A2ac | 3438 | 1 | 0.015
A2ac | 3525 | 1 | 0.015
A2ac | 3565 | 1 | 0.015
A2ac | 3624 | 2 | 0.03
A2ac | 3993T | 1 | 0.015
A2ac | 3995 | 2 | 0.03
A2ac | 4025 | 1 | 0.015
A2ac | 4158 | 1 | 0.015
A2ac | 449 | 1 | 0.015
A2ac | 455d | 1 | 0.015
A2ac | 4767 | 9 | 0.134
A2ac | 4790 | 6 | 0.09
A2ac | 4826 | 6 | 0.09
A2ac | 573.1C | 1 | 0.015
A2ac | 6050 | 1 | 0.015
A2ac | 6272 | 3 | 0.045
A2ac | 6734 | 1 | 0.015
A2ac | 6755 | 6 | 0.09
A2ac | 6875 | 2 | 0.03
A2ac | 7094 | 1 | 0.015
A2ac | 711 | 1 | 0.015
A2ac | 7199A | 1 | 0.015
A2ac | 7270 | 1 | 0.015
A2ac | 7649 | 1 | 0.015
A2ac | 7897 | 6 | 0.09
A2ac | 8281-8289d | 1 | 0.015
A2ac | 8426 | 4 | 0.06
A2ac | 8440 | 1 | 0.015
A2ac | 8473 | 2 | 0.03
A2ac | 8480 | 1 | 0.015
A2ac | 8485 | 1 | 0.015
A2ac | 8567 | 1 | 0.015
A2ac | 8626 | 6 | 0.09
A2ac | 8635 | 6 | 0.09
A2ac | 8676 | 1 | 0.015
A2ac | 8812 | 1 | 0.015
A2ac | 8836 | 9 | 0.134
A2ac | 8848 | 1 | 0.015
A2ac | 8865 | 2 | 0.03
A2ac | 8937 | 6 | 0.09
A2ac | 8950 | 2 | 0.03
A2ac | 8994 | 1 | 0.015
A2ac | 9064 | 1 | 0.015
A2ac | 93 | 3 | 0.045
A2ac | 9344 | 9 | 0.134
A2ac | 961 | 14 | 0.209
A2ac | 965.2C | 1 | 0.015
A2ac | 9755 | 1 | 0.015
A2ac1 | 14110 | 1 | 0.062
A2ac1 | 15706 | 1 | 0.062
A2ac1 | 16294 | 1 | 0.062
A2ac1 | 16304 | 1 | 0.062
A2ac1 | 16474 | 1 | 0.062
A2ac1 | 182 | 1 | 0.062
A2ac1 | 204 | 3 | 0.188
A2ac1 | 215 | 1 | 0.062
A2ac1 | 249 | 2 | 0.125
A2ac1 | 3083 | 3 | 0.188
A2ac1 | 573.3C | 1 | 0.062
A2ac1 | 9591 | 2 | 0.125
A2ad | 14470 | 1 | 0.111
A2ad | 16075 | 1 | 0.111
A2ad | 16166d | 1 | 0.111
A2ad | 16187 | 1 | 0.111
A2ad | 16259 | 2 | 0.222
A2ad | 189.1A | 1 | 0.111
A2ad | 194 | 3 | 0.333
A2ad | 337 | 1 | 0.111
A2ad | 4991 | 1 | 0.111
A2ad | 5483 | 1 | 0.111
A2ad | 6305 | 1 | 0.111
A2ad | 8093 | 1 | 0.111
A2ad | 8478 | 1 | 0.111
A2ad | 9233 | 1 | 0.111
A2ad | 9837 | 1 | 0.111
A2ad1 | 13135 | 1 | 0.143
A2ad1 | 152 | 1 | 0.143
A2ad1 | 16260 | 1 | 0.143
A2ad1 | 194 | 2 | 0.286
A2ad1 | 5460 | 1 | 0.143
A2ad2 | 1420 | 1 | 0.125
A2ad2 | 15328 | 1 | 0.125
A2ad2 | 16086 | 2 | 0.25
A2ad2 | 16209 | 1 | 0.125
A2ad2 | 16259 | 2 | 0.25
A2ad2 | 16311 | 2 | 0.25
A2ad2 | 16398 | 1 | 0.125
A2ad2 | 1790 | 1 | 0.125
A2ad2 | 194 | 4 | 0.5
A2ad2 | 7960 | 1 | 0.125
A2ad2 | 8553 | 1 | 0.125
A2ae | 13105 | 1 | 0.029
A2ae | 16160 | 1 | 0.029
A2ae | 16325 | 3 | 0.088
A2ae | 16352 | 2 | 0.059
A2ae | 3531 | 1 | 0.029
A2ae | 437 | 5 | 0.147

A2ae | 5021 | 3 | 0.088
A2ae | 7307 | 4 | 0.118
A2af1a | 101 | 4 | 0.571
A2af1a | 102 | 4 | 0.571
A2af1a | 106.2C | 4 | 0.571
A2af1a | 106T | 4 | 0.571
A2af1a | 111C | 4 | 0.571
A2af1a | 11224 | 1 | 0.143
A2af1a | 13135 | 1 | 0.143
A2af1a | 152 | 4 | 0.571
A2af1a | 4539 | 1 | 0.143
A2af1a | 90 | 1 | 0.143
A2af1a1 | 10694 | 1 | 0.125
A2af1a1 | 11940 | 1 | 0.125
A2af1a1 | 13148 | 1 | 0.125
A2af1a1 | 1462 | 1 | 0.125
A2af1a1 | 15760 | 1 | 0.125
A2af1a1 | 16104 | 1 | 0.125
A2af1a1 | 16172 | 1 | 0.125
A2af1a1 | 16213 | 1 | 0.125
A2af1a1 | 16297 | 1 | 0.125
A2af1a1 | 16526 | 1 | 0.125
A2af1a1 | 195 | 2 | 0.25
A2af1a1 | 340 | 1 | 0.125
A2af1a1 | 811 | 1 | 0.125
A2af1a1 | 867 | 1 | 0.125
A2af1a1 | 895 | 1 | 0.125
A2af1a1 | 90 | 1 | 0.125
A2af1a1 | 93.1T | 1 | 0.125
A2af1a1 | 96 | 1 | 0.125
A2af1a1 | 97 | 4 | 0.5
A2af1a1 | 97C | 1 | 0.125
A2af1a1 | 9909 | 1 | 0.125
A2af1a2 | 11245 | 1 | 0.5
A2af1b | 10084 | 1 | 0.25
A2af1b | 15071 | 1 | 0.25
A2af1b | 151 | 1 | 0.25
A2af1b | 16189 | 2 | 0.5
A2af1b | 44.1C | 1 | 0.25
A2af1b | 456 | 2 | 0.5
A2af1b | 709 | 2 | 0.5
A2af1b | 7515 | 1 | 0.25
A2af1b | 97 | 1 | 0.25
A2af1b | 9947 | 1 | 0.25
A2af1b | 9972 | 2 | 0.5
A2af1b1 | 12280 | 1 | 0.5
A2af1b1 | 13191 | 1 | 0.5
A2af1b1a | 1958 | 1 | 0.5
A2af1b1b | 114 | 1 | 0.5
A2af1b1b | 1371.1T | 1 | 0.5
A2af1b2 | 15047 | 1 | 0.333
A2af1b2 | 8392 | 1 | 0.333
A2ag | 10304 | 2 | 0.032
A2ag | 10358 | 2 | 0.032
A2ag | 111 | 6 | 0.097
A2ag | 12501 | 1 | 0.016
A2ag | 12720 | 1 | 0.016
A2ag | 13665 | 1 | 0.016
A2ag | 13722T | 1 | 0.016
A2ag | 13768 | 1 | 0.016
A2ag | 13928C | 2 | 0.032
A2ag | 14449 | 1 | 0.016
A2ag | 1556 | 1 | 0.016
A2ag | 16086 | 1 | 0.016
A2ag | 16093 | 1 | 0.016
A2ag | 16126 | 1 | 0.016
A2ag | 16132 | 1 | 0.016
A2ag | 16169 | 1 | 0.016
A2ag | 16235 | 2 | 0.032
A2ag | 16256 | 1 | 0.016
A2ag | 16260 | 6 | 0.097
A2ag | 16265 | 1 | 0.016
A2ag | 16266 | 3 | 0.048
A2ag | 16301 | 3 | 0.048
A2ag | 16311 | 3 | 0.048
A2ag | 16325 | 1 | 0.016
A2ag | 16390 | 6 | 0.097
A2ag | 16463 | 2 | 0.032
A2ag | 171 | 2 | 0.032
A2ag | 174 | 1 | 0.016
A2ag | 182 | 3 | 0.048
A2ag | 185 | 1 | 0.016
A2ag | 194 | 3 | 0.048
A2ag | 297 | 1 | 0.016
A2ag | 3306 | 4 | 0.065
A2ag | 3339 | 1 | 0.016
A2ag | 3372 | 2 | 0.032
A2ag | 493d | 2 | 0.032
A2ag | 515d | 3 | 0.048
A2ag | 573.1C | 2 | 0.032
A2ag | 573d | 1 | 0.016
A2ag | 7521 | 1 | 0.016
A2ag | 7669 | 5 | 0.081
A2ag | 7897 | 1 | 0.016
A2ag | 8567 | 1 | 0.016
A2ag | 8602 | 1 | 0.016
A2ah | 12373 | 1 | 0.091
A2ah | 13368 | 1 | 0.091
A2ah | 14757 | 1 | 0.091
A2ah | 16189 | 3 | 0.273
A2ah | 16260 | 1 | 0.091
A2ah | 16286 | 1 | 0.091
A2ah | 16320 | 4 | 0.364
A2ah | 16356 | 1 | 0.091
A2ah | 16483 | 1 | 0.091
A2ah | 4634 | 1 | 0.091
A2ah | 512T | 1 | 0.091
A2ai | 10307 | 1 | 0.091
A2ai | 11665 | 1 | 0.091
A2ai | 11827 | 1 | 0.091
A2ai | 15586 | 1 | 0.091

A2ai | 16129 | 2 | 0.182
A2ai | 16325 | 3 | 0.273
A2ai | 215 | 1 | 0.091
A2ai | 4991 | 1 | 0.091
A2aj | 16111 | 1 | 0.125
A2aj | 16189 | 1 | 0.125
A2aj | 6065 | 1 | 0.125
A2ak | 10604 | 2 | 0.2
A2ak | 12810 | 1 | 0.1
A2ak | 16212 | 1 | 0.1
A2ak | 16214 | 2 | 0.2
A2ak | 16256 | 2 | 0.2
A2ak | 16311 | 2 | 0.2
A2ak | 16390 | 1 | 0.1
A2ak | 4664 | 1 | 0.1
A2ak | 5095 | 1 | 0.1
A2ak | 8365 | 1 | 0.1
A2ak | 980 | 1 | 0.1
A2al | 1005 | 1 | 0.059
A2al | 10398 | 1 | 0.059
A2al | 111 | 1 | 0.059
A2al | 11770 | 1 | 0.059
A2al | 14142A | 1 | 0.059
A2al | 15736 | 1 | 0.059
A2al | 15951 | 6 | 0.353
A2al | 16172 | 1 | 0.059
A2al | 16189 | 2 | 0.118
A2al | 16190 | 1 | 0.059
A2al | 16209 | 1 | 0.059
A2al | 16259 | 7 | 0.412
A2al | 16267 | 1 | 0.059
A2al | 16300 | 1 | 0.059
A2al | 16311 | 3 | 0.176
A2al | 16327 | 2 | 0.118
A2al | 16390 | 6 | 0.353
A2al | 195 | 1 | 0.059
A2al | 93 | 1 | 0.059
A2al | 9456 | 1 | 0.059
A2am | 15106 | 1 | 0.034
A2am | 16224 | 2 | 0.069
A2am | 16294 | 1 | 0.034
A2am | 311 | 1 | 0.034
A2am | 315.2C | 1 | 0.034
A2am | 7842 | 1 | 0.034
A2an | 10398 | 5 | 0.041
A2an | 14941 | 5 | 0.041
A2an | 152 | 5 | 0.041
A2an | 16104 | 1 | 0.008
A2an | 16198 | 9 | 0.074
A2an | 16352 | 16 | 0.131
A2an | 194 | 1 | 0.008
A2an | 215 | 2 | 0.016
A2an | 361.1A | 10 | 0.082
A2an | 573.5C | 1 | 0.008
A2ao | 103 | 9 | 0.117
A2ao | 143 | 1 | 0.013
A2ao | 151 | 1 | 0.013
A2ao | 152 | 6 | 0.078
A2ao | 16067 | 1 | 0.013
A2ao | 16075 | 1 | 0.013
A2ao | 16092 | 10 | 0.13
A2ao | 16104 | 1 | 0.013
A2ao | 16148 | 1 | 0.013
A2ao | 16180T | 1 | 0.013
A2ao | 16196 | 1 | 0.013
A2ao | 16257 | 4 | 0.052
A2ao | 16263A | 4 | 0.052
A2ao | 16265 | 6 | 0.078
A2ao | 16271 | 1 | 0.013
A2ao | 16325 | 1 | 0.013
A2ao | 16356 | 1 | 0.013
A2ao | 16360 | 3 | 0.039
A2ao | 198 | 1 | 0.013
A2ao | 206 | 1 | 0.013
A2ao | 215 | 3 | 0.039
A2ao | 250 | 4 | 0.052
A2ao | 3867 | 1 | 0.013
A2ao | 393 | 1 | 0.013
A2ao | 4988A | 1 | 0.013
A2ao | 59 | 1 | 0.013
A2ao | 90C | 1 | 0.013
A2ao | 9524 | 1 | 0.013
A2ao | 98 | 1 | 0.013
A2ao1 | 152 | 1 | 0.333
A2ao1 | 153 | 1 | 0.333
A2ao1 | 15454 | 1 | 0.333
A2ao1 | 16136 | 1 | 0.333
A2ao1 | 3349 | 1 | 0.333
A2ap | 11002 | 1 | 0.029
A2ap | 11422 | 1 | 0.029
A2ap | 143 | 1 | 0.029
A2ap | 151 | 1 | 0.029
A2ap | 16157 | 3 | 0.088
A2ap | 16185 | 1 | 0.029
A2ap | 16192 | 1 | 0.029
A2ap | 16193d | 1 | 0.029
A2ap | 16212 | 1 | 0.029
A2ap | 16234G | 3 | 0.088
A2ap | 16265 | 3 | 0.088
A2ap | 16310 | 1 | 0.029
A2ap | 189 | 1 | 0.029
A2ap | 210 | 1 | 0.029
A2ap | 217 | 1 | 0.029
A2ap | 310 | 1 | 0.029
A2ap | 315.2C | 1 | 0.029
A2ap | 385 | 6 | 0.176
A2ap | 4736 | 1 | 0.029
A2ap | 489 | 1 | 0.029
A2ap | 4925 | 7 | 0.206
A2ap | 513-516d | 6 | 0.176
A2ap | 6842 | 1 | 0.029

A2ap | 7705 | 1 | 0.029
A2ap | 7918 | 1 | 0.029
A2ap | 9746 | 1 | 0.029
A2aq | 14757 | 1 | 0.333
A2aq | 16189 | 2 | 0.667
A2aq | 315.3C | 3 | 1
A2aq | 515d | 2 | 0.667
A2b | 114G | 1 | 0.013
A2b | 139 | 3 | 0.039
A2b | 143 | 2 | 0.026
A2b | 16086 | 2 | 0.026
A2b | 16092 | 3 | 0.039
A2b | 16104 | 1 | 0.013
A2b | 16150 | 1 | 0.013
A2b | 16157 | 1 | 0.013
A2b | 16172 | 1 | 0.013
A2b | 16184 | 1 | 0.013
A2b | 16188 | 2 | 0.026
A2b | 16196 | 1 | 0.013
A2b | 16214 | 1 | 0.013
A2b | 16217 | 1 | 0.013
A2b | 16241 | 2 | 0.026
A2b | 16261 | 1 | 0.013
A2b | 16263 | 1 | 0.013
A2b | 16278 | 1 | 0.013
A2b | 16296 | 1 | 0.013
A2b | 16300 | 1 | 0.013
A2b | 16311 | 7 | 0.092
A2b | 16325 | 1 | 0.013
A2b | 16343T | 1 | 0.013
A2b | 16512 | 3 | 0.039
A2b | 16527 | 1 | 0.013
A2b | 16547 | 3 | 0.039
A2b | 16551 | 2 | 0.026
A2b | 16551.1G | 3 | 0.039
A2b | 194 | 1 | 0.013
A2b | 200 | 1 | 0.013
A2b | 210 | 1 | 0.013
A2b | 217 | 1 | 0.013
A2b | 227T | 1 | 0.013
A2b | 260 | 1 | 0.013
A2b | 294.1T | 1 | 0.013
A2b | 310 | 1 | 0.013
A2b | 456 | 1 | 0.013
A2b | 459d | 1 | 0.013
A2b | 570 | 1 | 0.013
A2b1 | 10007 | 1 | 0.004
A2b1 | 10609 | 3 | 0.012
A2b1 | 10892T | 1 | 0.004
A2b1 | 11164C | 1 | 0.004
A2b1 | 12651 | 1 | 0.004
A2b1 | 150 | 1 | 0.004
A2b1 | 15731 | 1 | 0.004
A2b1 | 16051 | 1 | 0.004
A2b1 | 16092 | 2 | 0.008
A2b1 | 16129 | 2 | 0.008
A2b1 | 16176 | 4 | 0.016
A2b1 | 16185 | 1 | 0.004
A2b1 | 16189 | 1 | 0.004
A2b1 | 16212 | 21 | 0.083
A2b1 | 16278 | 1 | 0.004
A2b1 | 16294 | 3 | 0.012
A2b1 | 16304 | 5 | 0.02
A2b1 | 16320 | 1 | 0.004
A2b1 | 16381G | 1 | 0.004
A2b1 | 16399 | 5 | 0.02
A2b1 | 207 | 1 | 0.004
A2b1 | 225 | 1 | 0.004
A2b1 | 228 | 2 | 0.008
A2b1 | 310 | 1 | 0.004
A2b1 | 3308 | 1 | 0.004
A2b1 | 4991 | 1 | 0.004
A2b1 | 533 | 1 | 0.004
A2b1 | 547 | 1 | 0.004
A2b1 | 709 | 3 | 0.012
A2b1 | 7130 | 1 | 0.004
A2b1 | 7762 | 1 | 0.004
A2b1 | 8280 | 1 | 0.004
A2b1 | 8281-8289d | 1 | 0.004
A2b1 | 8857 | 3 | 0.012
A2b1 | 9099 | 1 | 0.004
A2c | 10192 | 1 | 0.007
A2c | 103 | 1 | 0.007
A2c | 13707 | 1 | 0.007
A2c | 14460G | 1 | 0.007
A2c | 15107 | 1 | 0.007
A2c | 152 | 1 | 0.007
A2c | 15940 | 3 | 0.021
A2c | 16086 | 2 | 0.014
A2c | 16092 | 32 | 0.221
A2c | 16104 | 2 | 0.014
A2c | 16142 | 2 | 0.014
A2c | 16155 | 3 | 0.021
A2c | 16174 | 1 | 0.007
A2c | 16180 | 1 | 0.007
A2c | 16235 | 2 | 0.014
A2c | 16292 | 1 | 0.007
A2c | 16293T | 1 | 0.007
A2c | 16311 | 2 | 0.014
A2c | 16381 | 2 | 0.014
A2c | 16448 | 3 | 0.021
A2c | 16468 | 1 | 0.007
A2c | 182 | 3 | 0.021
A2c | 183 | 1 | 0.007
A2c | 185 | 1 | 0.007
A2c | 214 | 1 | 0.007
A2c | 215 | 1 | 0.007
A2c | 266 | 1 | 0.007
A2c | 297C | 1 | 0.007
A2c | 316 | 3 | 0.021
A2c | 3311 | 1 | 0.007

A2c | 374 | 2 | 0.014
A2c | 482 | 4 | 0.028
A2c | 551 | 3 | 0.021
A2c | 573.1C | 1 | 0.007
A2c | 573.5C | 1 | 0.007
A2c | 6632 | 1 | 0.007
A2c | 7941 | 1 | 0.007
A2c | 9017 | 1 | 0.007
A2c | 93 | 1 | 0.007
A2c | 961 | 1 | 0.007
A2c | 966.2C | 1 | 0.007
A2c | 9947 | 1 | 0.007
A2d | 10 | 1 | 0.008
A2d | 12879 | 1 | 0.008
A2d | 15043 | 1 | 0.008
A2d | 15951 | 1 | 0.008
A2d | 16093 | 2 | 0.016
A2d | 16151A | 1 | 0.008
A2d | 16153 | 2 | 0.016
A2d | 16184 | 1 | 0.008
A2d | 16287 | 1 | 0.008
A2d | 16468 | 9 | 0.072
A2d | 183 | 1 | 0.008
A2d | 210 | 1 | 0.008
A2d | 26 | 1 | 0.008
A2d | 3316 | 1 | 0.008
A2d | 450 | 1 | 0.008
A2d | 456 | 1 | 0.008
A2d | 457 | 1 | 0.008
A2d | 4655 | 1 | 0.008
A2d | 5183 | 1 | 0.008
A2d | 522 | 1 | 0.008
A2d | 5972 | 1 | 0.008
A2d | 629 | 1 | 0.008
A2d | 6366 | 1 | 0.008
A2d | 8108 | 1 | 0.008
A2d | 8281-8289d | 1 | 0.008
A2d | 8557 | 1 | 0.008
A2d | 8746G | 1 | 0.008
A2d | 9160 | 1 | 0.008
A2d | 961 | 1 | 0.008
A2d | 9612C | 1 | 0.008
A2d | 966.1C | 1 | 0.008
A2d1 | 10 | 1 | 0.008
A2d1 | 11696 | 1 | 0.008
A2d1 | 13401 | 1 | 0.008
A2d1 | 14152 | 2 | 0.017
A2d1 | 15436A | 1 | 0.008
A2d1 | 15951 | 1 | 0.008
A2d1 | 16093 | 1 | 0.008
A2d1 | 16151A | 1 | 0.008
A2d1 | 16177 | 1 | 0.008
A2d1 | 16184 | 1 | 0.008
A2d1 | 16234 | 2 | 0.017
A2d1 | 16265C | 1 | 0.008
A2d1 | 16390 | 5 | 0.042
A2d1 | 210 | 1 | 0.008
A2d1 | 26 | 1 | 0.008
A2d1 | 450 | 1 | 0.008
A2d1 | 456 | 1 | 0.008
A2d1 | 457 | 1 | 0.008
A2d1 | 522 | 1 | 0.008
A2d1 | 5423 | 1 | 0.008
A2d1 | 8233 | 1 | 0.008
A2d1 | 930 | 1 | 0.008
A2d1 | 9449 | 3 | 0.025
A2d1a | 10712 | 2 | 0.125
A2d1a | 12358 | 1 | 0.062
A2d1a | 12696 | 1 | 0.062
A2d1a | 16192 | 1 | 0.062
A2d1a | 23 | 1 | 0.062
A2d1a | 5558 | 1 | 0.062
A2d1a | 8122 | 1 | 0.062
A2d2 | 10 | 1 | 0.008
A2d2 | 13827 | 3 | 0.025
A2d2 | 15951 | 1 | 0.008
A2d2 | 16093 | 1 | 0.008
A2d2 | 16151A | 1 | 0.008
A2d2 | 16184 | 1 | 0.008
A2d2 | 16234 | 3 | 0.025
A2d2 | 16256 | 1 | 0.008
A2d2 | 16468 | 1 | 0.008
A2d2 | 210 | 1 | 0.008
A2d2 | 26 | 1 | 0.008
A2d2 | 450 | 1 | 0.008
A2d2 | 456 | 1 | 0.008
A2d2 | 457 | 1 | 0.008
A2d2 | 522 | 1 | 0.008
A2d2 | 8369 | 3 | 0.025
A2d2 | 8705 | 1 | 0.008
A2e | 16094 | 1 | 0.167
A2e | 16266 | 1 | 0.167
A2e | 195 | 3 | 0.5
A2e | 573.1C | 1 | 0.167
A2e | 8032 | 1 | 0.167
A2e | 90C | 1 | 0.167
A2e | 93 | 1 | 0.167
A2e | 98 | 1 | 0.167
A2e | 9836 | 1 | 0.167
A2f | 143 | 21 | 0.168
A2f | 152 | 1 | 0.008
A2f | 15968 | 10 | 0.08
A2f | 16129 | 1 | 0.008
A2f | 16150 | 2 | 0.016
A2f | 676 | 1 | 0.008
A2f1 | 16051 | 1 | 0.005
A2f1 | 16092 | 1 | 0.005
A2f1 | 16212 | 1 | 0.005
A2f1 | 16234 | 2 | 0.009
A2f1 | 16304 | 1 | 0.005
A2f1 | 16399 | 2 | 0.009

A2f1a | 12714 | 1 | 0.005
A2f1a | 15850 | 2 | 0.009
A2f1a | 16051 | 1 | 0.005
A2f1a | 16212 | 1 | 0.005
A2f1a | 16234 | 2 | 0.009
A2f1a | 16304 | 1 | 0.005
A2f1a | 16324G | 2 | 0.009
A2f1a | 4013G | 2 | 0.009
A2f1a | 5471 | 4 | 0.018
A2f1a | 709 | 1 | 0.005
A2f1a | 8108 | 1 | 0.005
A2f2 | 143 | 1 | 0.167
A2f2 | 14569 | 1 | 0.167
A2f2 | 146 | 1 | 0.167
A2f2 | 14687 | 1 | 0.167
A2f2 | 1508 | 1 | 0.167
A2f2 | 15236 | 1 | 0.167
A2f2 | 15992 | 1 | 0.167
A2f2 | 15993C | 1 | 0.167
A2f2 | 15994 | 1 | 0.167
A2f2 | 15999 | 1 | 0.167
A2f2 | 16000T | 1 | 0.167
A2f2 | 16002A | 1 | 0.167
A2f2 | 16007d | 1 | 0.167
A2f2 | 16013d | 1 | 0.167
A2f2 | 16129 | 1 | 0.167
A2f2 | 16248 | 1 | 0.167
A2f2 | 16431A | 1 | 0.167
A2f2 | 199 | 1 | 0.167
A2f2 | 2887 | 3 | 0.5
A2f2 | 3394 | 1 | 0.167
A2f2 | 6962 | 1 | 0.167
A2f3 | 10463A | 1 | 0.029
A2f3 | 12217 | 1 | 0.029
A2f3 | 15028 | 3 | 0.086
A2f3 | 16124 | 1 | 0.029
A2f3 | 16234 | 4 | 0.114
A2f3 | 16304 | 2 | 0.057
A2f3 | 16348 | 1 | 0.029
A2f3 | 183 | 1 | 0.029
A2f3 | 2140 | 1 | 0.029
A2f3 | 249d | 1 | 0.029
A2g | 12033 | 1 | 0.067
A2g | 14215 | 2 | 0.133
A2g | 152 | 2 | 0.133
A2g | 16129 | 1 | 0.067
A2g | 16172 | 2 | 0.133
A2g | 16184A | 2 | 0.133
A2g | 16189 | 2 | 0.133
A2g | 1709 | 1 | 0.067
A2g | 3399 | 2 | 0.133
A2g | 3609 | 1 | 0.067
A2g | 6011 | 1 | 0.067
A2g | 6305 | 1 | 0.067
A2g | 7502 | 1 | 0.067
A2g | 7759 | 1 | 0.067
A2g | 834 | 1 | 0.067
A2g | 8567 | 1 | 0.067
A2g | 9128 | 1 | 0.067
A2g | 9374 | 2 | 0.133
A2g | 961 | 1 | 0.067
A2g1 | 15040G | 1 | 0.056
A2g1 | 16094 | 4 | 0.222
A2g1 | 16192 | 1 | 0.056
A2g1 | 16526 | 1 | 0.056
A2g1 | 1790d | 1 | 0.056
A2g1 | 5913 | 1 | 0.056
A2h | 10694 | 1 | 0.042
A2h | 10775 | 2 | 0.083
A2h | 1189 | 3 | 0.125
A2h | 13830 | 1 | 0.042
A2h | 13928C | 2 | 0.083
A2h | 13967 | 1 | 0.042
A2h | 14053 | 2 | 0.083
A2h | 143 | 3 | 0.125
A2h | 15289 | 3 | 0.125
A2h | 15315 | 2 | 0.083
A2h | 15317 | 2 | 0.083
A2h | 16069 | 1 | 0.042
A2h | 16129 | 2 | 0.083
A2h | 16140 | 1 | 0.042
A2h | 16212.1A | 1 | 0.042
A2h | 16247 | 1 | 0.042
A2h | 16286 | 1 | 0.042
A2h | 16297 | 1 | 0.042
A2h | 16324 | 2 | 0.083
A2h | 16327 | 2 | 0.083
A2h | 199 | 2 | 0.083
A2h | 2056 | 2 | 0.083
A2h | 211 | 2 | 0.083
A2h | 2370 | 3 | 0.125
A2h | 3535 | 1 | 0.042
A2h | 3693 | 2 | 0.083
A2h | 4454 | 2 | 0.083
A2h | 459.1C | 2 | 0.083
A2h | 5222 | 3 | 0.125
A2h | 59 | 3 | 0.125
A2h | 6308 | 1 | 0.042
A2h | 6503 | 2 | 0.083
A2h | 6707 | 1 | 0.042
A2h | 6716 | 2 | 0.083
A2h | 6935 | 1 | 0.042
A2h | 709 | 2 | 0.083
A2h | 7673 | 1 | 0.042
A2h | 7732 | 2 | 0.083
A2h | 7854 | 1 | 0.042
A2h | 9156 | 1 | 0.042
A2h | 9218 | 1 | 0.042
A2h1 | 10274 | 1 | 0.062
A2h1 | 13317 | 1 | 0.062
A2h1 | 14227G | 1 | 0.062

A2h1 | 14385G | 1 | 0.062
A2h1 | 14463G | 1 | 0.062
A2h1 | 16187 | 1 | 0.062
A2h1 | 310 | 3 | 0.188
A2h1 | 315d | 2 | 0.125
A2h1 | 4454 | 1 | 0.062
A2h1 | 5839 | 2 | 0.125
A2h1 | 7702 | 5 | 0.312
A2h1 | 7927G | 1 | 0.062
A2h1 | 7985G | 1 | 0.062
A2h1 | 8047 | 1 | 0.062
A2h1 | 9545 | 1 | 0.062
A2i | 12444 | 1 | 0.048
A2i | 12996 | 1 | 0.048
A2i | 16096 | 1 | 0.048
A2i | 16110 | 1 | 0.048
A2i | 16189 | 1 | 0.048
A2i | 16327 | 2 | 0.095
A2i | 16410 | 1 | 0.048
A2i | 3308.1C | 15 | 0.714
A2i | 3308A | 16 | 0.762
A2i | 3312.1C | 1 | 0.048
A2i | 513 | 8 | 0.381
A2i | 6315 | 1 | 0.048
A2i | 6527 | 1 | 0.048
A2i | 8098 | 8 | 0.381
A2i | 8566 | 1 | 0.048
A2i | 8702 | 8 | 0.381
A2i | 8854 | 1 | 0.048
A2i | 8959 | 1 | 0.048
A2j | 10 | 1 | 0.008
A2j | 10084 | 1 | 0.008
A2j | 10685 | 2 | 0.017
A2j | 11641 | 1 | 0.008
A2j | 1193 | 1 | 0.008
A2j | 12561 | 1 | 0.008
A2j | 15431 | 1 | 0.008
A2j | 15924 | 2 | 0.017
A2j | 15951 | 1 | 0.008
A2j | 16086 | 2 | 0.017
A2j | 16093 | 1 | 0.008
A2j | 16151A | 1 | 0.008
A2j | 16156 | 1 | 0.008
A2j | 16184 | 1 | 0.008
A2j | 16234 | 3 | 0.025
A2j | 16311 | 1 | 0.008
A2j | 210 | 1 | 0.008
A2j | 234 | 1 | 0.008
A2j | 26 | 1 | 0.008
A2j | 385 | 2 | 0.017
A2j | 450 | 1 | 0.008
A2j | 456 | 1 | 0.008
A2j | 4562 | 2 | 0.017
A2j | 457 | 1 | 0.008
A2j | 4966 | 1 | 0.008
A2j | 522 | 1 | 0.008
A2j | 5465 | 2 | 0.017
A2j | 6307 | 1 | 0.008
A2j1 | 10 | 1 | 0.008
A2j1 | 15951 | 1 | 0.008
A2j1 | 16086 | 1 | 0.008
A2j1 | 16093 | 1 | 0.008
A2j1 | 16129 | 1 | 0.008
A2j1 | 16151A | 1 | 0.008
A2j1 | 16184 | 1 | 0.008
A2j1 | 16209 | 1 | 0.008
A2j1 | 16234 | 3 | 0.025
A2j1 | 16316 | 1 | 0.008
A2j1 | 210 | 1 | 0.008
A2j1 | 26 | 1 | 0.008
A2j1 | 3338 | 1 | 0.008
A2j1 | 4080 | 1 | 0.008
A2j1 | 450 | 1 | 0.008
A2j1 | 456 | 1 | 0.008
A2j1 | 457 | 1 | 0.008
A2j1 | 522 | 1 | 0.008
A2j1 | 6575 | 1 | 0.008
A2j1 | 7594 | 1 | 0.008
A2j1 | 8852T | 1 | 0.008
A2k | 143 | 20 | 0.16
A2k | 152 | 1 | 0.008
A2k | 15968 | 10 | 0.08
A2k | 16129 | 1 | 0.008
A2k | 16150 | 1 | 0.008
A2k | 16294 | 2 | 0.016
A2k1 | 16156 | 9 | 0.078
A2k1 | 16176 | 1 | 0.009
A2k1 | 16215 | 4 | 0.035
A2k1 | 16249 | 2 | 0.017
A2k1 | 16263 | 9 | 0.078
A2k1 | 16286 | 1 | 0.009
A2k1 | 16438 | 9 | 0.078
A2k1 | 183 | 4 | 0.035
A2k1 | 198 | 2 | 0.017
A2k1 | 215 | 1 | 0.009
A2k1 | 3397 | 1 | 0.009
A2k1 | 374 | 2 | 0.017
A2k1 | 472 | 2 | 0.017
A2k1 | 482 | 1 | 0.009
A2k1 | 61A | 1 | 0.009
A2k1 | 62 | 1 | 0.009
A2k1 | 8884 | 1 | 0.009
A2k1a | 155 | 4 | 0.031
A2k1a | 16172 | 2 | 0.016
A2k1a | 16249 | 1 | 0.008
A2k1a | 16259 | 5 | 0.039
A2k1a | 16285.1A | 1 | 0.008
A2k1a | 16299 | 3 | 0.023
A2k1a | 16355 | 2 | 0.016
A2k1a | 197 | 1 | 0.008
A2k1a | 239 | 3 | 0.023

A2k1a | 3109d | 1 | 0.008
A2k1a | 466 | 5 | 0.039
A2k1a | 472 | 1 | 0.008
A2k1a | 6853C | 1 | 0.008
A2k1a | 7269 | 1 | 0.008
A2k1a | 7961 | 1 | 0.008
A2l | 10151 | 1 | 0.003
A2l | 10807 | 1 | 0.003
A2l | 152 | 1 | 0.003
A2l | 16114A | 1 | 0.003
A2l | 16127 | 2 | 0.005
A2l | 16399 | 1 | 0.003
A2l | 3483 | 1 | 0.003
A2l | 8152 | 1 | 0.003
A2m | 10097 | 6 | 0.154
A2m | 10274 | 1 | 0.026
A2m | 10993 | 7 | 0.179
A2m | 11914 | 1 | 0.026
A2m | 12528 | 1 | 0.026
A2m | 12561 | 1 | 0.026
A2m | 13135 | 3 | 0.077
A2m | 13656 | 1 | 0.026
A2m | 14016 | 6 | 0.154
A2m | 14530 | 1 | 0.026
A2m | 15427 | 1 | 0.026
A2m | 15784 | 1 | 0.026
A2m | 16086 | 4 | 0.103
A2m | 16104 | 16 | 0.41
A2m | 16129 | 7 | 0.179
A2m | 16153 | 8 | 0.205
A2m | 16172 | 9 | 0.231
A2m | 16215 | 9 | 0.231
A2m | 16249 | 1 | 0.026
A2m | 16256 | 1 | 0.026
A2m | 16266 | 1 | 0.026
A2m | 16278 | 1 | 0.026
A2m | 16324 | 9 | 0.231
A2m | 16449 | 1 | 0.026
A2m | 16451 | 1 | 0.026
A2m | 16455 | 1 | 0.026
A2m | 185 | 1 | 0.026
A2m | 195 | 2 | 0.051
A2m | 204 | 10 | 0.256
A2m | 236 | 3 | 0.077
A2m | 248 | 1 | 0.026
A2m | 273 | 1 | 0.026
A2m | 3745 | 2 | 0.051
A2m | 485 | 1 | 0.026
A2m | 4856 | 1 | 0.026
A2m | 5628 | 8 | 0.205
A2m | 59 | 1 | 0.026
A2m | 629 | 1 | 0.026
A2m | 6689 | 3 | 0.077
A2m | 7148 | 1 | 0.026
A2m | 7245 | 1 | 0.026
A2m | 7337 | 4 | 0.103
A2m | 8639 | 4 | 0.103
A2m | 8785G | 4 | 0.103
A2m | 8995 | 1 | 0.026
A2m | 9804 | 2 | 0.051
A2n | 12172 | 1 | 0.003
A2n | 12280 | 2 | 0.005
A2n | 12972 | 2 | 0.005
A2n | 13356 | 2 | 0.005
A2n | 152 | 2 | 0.005
A2n | 15346 | 4 | 0.01
A2n | 15777 | 2 | 0.005
A2n | 15924 | 2 | 0.005
A2n | 16111 | 4 | 0.01
A2n | 16126 | 2 | 0.005
A2n | 16127 | 2 | 0.005
A2n | 16129 | 2 | 0.005
A2n | 16145 | 1 | 0.003
A2n | 16177 | 3 | 0.008
A2n | 16324 | 2 | 0.005
A2n | 16482 | 4 | 0.01
A2n | 1824 | 1 | 0.003
A2n | 189 | 1 | 0.003
A2n | 207 | 1 | 0.003
A2n | 4137 | 1 | 0.003
A2n | 4232 | 4 | 0.01
A2n | 4742 | 2 | 0.005
A2n | 5105 | 2 | 0.005
A2n | 63A | 2 | 0.005
A2n | 709 | 3 | 0.008
A2o | 1007 | 3 | 0.008
A2o | 13032 | 3 | 0.008
A2o | 16120T | 1 | 0.003
A2o | 16127 | 2 | 0.005
A2o | 16131 | 1 | 0.003
A2o | 16184 | 1 | 0.003
A2o | 16185 | 1 | 0.003
A2o | 16195 | 1 | 0.003
A2o | 16217 | 1 | 0.003
A2o | 16222A | 1 | 0.003
A2o | 16258C | 1 | 0.003
A2o | 16261 | 1 | 0.003
A2o | 16280T | 1 | 0.003
A2o | 16298 | 1 | 0.003
A2o | 16316 | 1 | 0.003
A2o | 16354 | 1 | 0.003
A2o | 16360 | 1 | 0.003
A2o | 16527 | 1 | 0.003
A2o | 238 | 1 | 0.003
A2o | 61 | 1 | 0.003
A2o | 62 | 1 | 0.003
A2o | 723C | 3 | 0.008
A2o | 8744 | 1 | 0.003
A2o | 8970 | 1 | 0.003
A2o | 9167 | 1 | 0.003
A2p | 151 | 2 | 0.069

A2p | 16145 | 3 | 0.103
A2p | 16172 | 2 | 0.069
A2p | 16209 | 5 | 0.172
A2p | 16224 | 2 | 0.069
A2p | 16234 | 6 | 0.207
A2p | 16260 | 1 | 0.034
A2p | 310 | 1 | 0.034
A2p | 315.2C | 1 | 0.034
A2p | 8907 | 1 | 0.034
A2p1 | 11914 | 1 | 0.04
A2p1 | 16069 | 1 | 0.04
A2p1 | 16092 | 2 | 0.08
A2p1 | 16400 | 2 | 0.08
A2p1 | 195 | 3 | 0.12
A2p1 | 4766 | 1 | 0.04
A2p1 | 8588G | 1 | 0.04
A2p2 | 152 | 1 | 0.031
A2p2 | 15657 | 1 | 0.031
A2p2 | 16004 | 2 | 0.062
A2p2 | 16093 | 2 | 0.062
A2p2 | 16126 | 1 | 0.031
A2p2 | 16145 | 1 | 0.031
A2p2 | 16162 | 1 | 0.031
A2p2 | 16243 | 1 | 0.031
A2p2 | 16259 | 1 | 0.031
A2p2 | 16260 | 3 | 0.094
A2p2 | 16267 | 1 | 0.031
A2p2 | 16284 | 3 | 0.094
A2p2 | 16324 | 1 | 0.031
A2p2 | 16361 | 1 | 0.031
A2p2 | 16446 | 1 | 0.031
A2p2 | 2708 | 1 | 0.031
A2p2 | 5277 | 1 | 0.031
A2p2 | 5655 | 1 | 0.031
A2q | 10084 | 4 | 0.143
A2q | 10310 | 5 | 0.179
A2q | 10373 | 1 | 0.036
A2q | 10398 | 3 | 0.107
A2q | 10993 | 1 | 0.036
A2q | 11172 | 1 | 0.036
A2q | 11253 | 3 | 0.107
A2q | 1151 | 4 | 0.143
A2q | 13287 | 4 | 0.143
A2q | 13448 | 1 | 0.036
A2q | 1375 | 1 | 0.036
A2q | 14154G | 1 | 0.036
A2q | 14372A | 1 | 0.036
A2q | 14374 | 8 | 0.286
A2q | 15047 | 1 | 0.036
A2q | 15205 | 1 | 0.036
A2q | 15298 | 5 | 0.179
A2q | 15314 | 3 | 0.107
A2q | 15629 | 1 | 0.036
A2q | 15784 | 1 | 0.036
A2q | 15851 | 4 | 0.143
A2q | 1598 | 3 | 0.107
A2q | 16095 | 4 | 0.143
A2q | 16124 | 1 | 0.036
A2q | 16129 | 2 | 0.071
A2q | 16131 | 5 | 0.179
A2q | 16249 | 1 | 0.036
A2q | 16265T | 1 | 0.036
A2q | 16287 | 1 | 0.036
A2q | 16311 | 10 | 0.357
A2q | 16483 | 3 | 0.107
A2q | 16566 | 4 | 0.143
A2q | 1700 | 4 | 0.143
A2q | 1746 | 1 | 0.036
A2q | 215 | 4 | 0.143
A2q | 3211 | 8 | 0.286
A2q | 3633 | 1 | 0.036
A2q | 3867 | 8 | 0.286
A2q | 4232 | 8 | 0.286
A2q | 437 | 1 | 0.036
A2q | 4688 | 8 | 0.286
A2q | 4961 | 1 | 0.036
A2q | 4976 | 1 | 0.036
A2q | 548 | 1 | 0.036
A2q | 5480 | 1 | 0.036
A2q | 54C | 1 | 0.036
A2q | 573.5C | 1 | 0.036
A2q | 5899.1C | 1 | 0.036
A2q | 6221 | 1 | 0.036
A2q | 6719 | 1 | 0.036
A2q | 7142 | 5 | 0.179
A2q | 8393 | 4 | 0.143
A2q | 8946T | 1 | 0.036
A2q | 8961 | 1 | 0.036
A2q | 9122 | 1 | 0.036
A2q | 9205 | 1 | 0.036
A2q | 9591 | 4 | 0.143
A2q | 9893 | 1 | 0.036
A2q1 | 14551 | 1 | 0.042
A2q1 | 15978 | 1 | 0.042
A2q1 | 16086 | 3 | 0.125
A2q1 | 16095 | 9 | 0.375
A2q1 | 16311 | 12 | 0.5
A2q1 | 16318 | 1 | 0.042
A2q1 | 190 | 1 | 0.042
A2q1 | 215 | 12 | 0.5
A2q1 | 318 | 1 | 0.042
A2q1 | 385 | 1 | 0.042
A2q1 | 4853 | 2 | 0.083
A2q1 | 5054T | 2 | 0.083
A2q1 | 5291 | 1 | 0.042
A2q1 | 9067 | 1 | 0.042
A2q1 | 9254 | 1 | 0.042
A2q1 | 9300 | 1 | 0.042
A2q1 | 94 | 2 | 0.083
A2r | 12535 | 1 | 0.015
A2r | 16104 | 2 | 0.03

A2r | 16189 | 1 | 0.015
A2r | 16217 | 1 | 0.015
A2r | 16278 | 1 | 0.015
A2r | 16288 | 5 | 0.075
A2r | 16294 | 7 | 0.104
A2r | 16296 | 1 | 0.015
A2r | 16343T | 1 | 0.015
A2r | 16348 | 5 | 0.075
A2r | 16399 | 7 | 0.104
A2r | 210 | 1 | 0.015
A2r | 260 | 1 | 0.015
A2r | 294.1T | 1 | 0.015
A2r | 3654 | 2 | 0.03
A2r | 456 | 1 | 0.015
A2r | 459d | 1 | 0.015
A2r | 570 | 1 | 0.015
A2r | 573.5C | 1 | 0.015
A2r | 8334 | 1 | 0.015
A2r | 8581 | 1 | 0.015
A2r1 | 16084 | 1 | 0.027
A2r1 | 16215 | 1 | 0.027
A2r1 | 16288 | 7 | 0.189
A2r1 | 3591 | 1 | 0.027
A2r1 | 5773 | 1 | 0.027
A2r1 | 7521 | 1 | 0.027
A2s | 16124 | 1 | 0.5
A2s | 16465 | 1 | 0.5
A2t | 10 | 1 | 0.008
A2t | 1211 | 1 | 0.008
A2t | 12372 | 2 | 0.017
A2t | 13503 | 1 | 0.008
A2t | 15951 | 1 | 0.008
A2t | 16093 | 1 | 0.008
A2t | 16151A | 1 | 0.008
A2t | 16184 | 1 | 0.008
A2t | 16185 | 1 | 0.008
A2t | 16234 | 2 | 0.017
A2t | 16263 | 1 | 0.008
A2t | 16356 | 5 | 0.041
A2t | 1842 | 2 | 0.017
A2t | 1842T | 1 | 0.008
A2t | 210 | 1 | 0.008
A2t | 245 | 2 | 0.017
A2t | 26 | 1 | 0.008
A2t | 309d | 1 | 0.008
A2t | 3918 | 1 | 0.008
A2t | 450 | 1 | 0.008
A2t | 456 | 1 | 0.008
A2t | 457 | 1 | 0.008
A2t | 5068 | 1 | 0.008
A2t | 522 | 1 | 0.008
A2u | 10310 | 3 | 0.136
A2u | 16093 | 7 | 0.318
A2u | 16129 | 6 | 0.273
A2u | 16189 | 1 | 0.045
A2u | 16284 | 6 | 0.273
A2u | 16295 | 3 | 0.136
A2u | 16311 | 11 | 0.5
A2u | 200 | 3 | 0.136
A2u | 309.3C | 1 | 0.045
A2u | 55 | 6 | 0.273
A2u | 57 | 6 | 0.273
A2u | 593 | 6 | 0.273
A2u1 | 11935 | 1 | 0.067
A2u1 | 12477 | 1 | 0.067
A2u1 | 15817 | 1 | 0.067
A2u1 | 16092 | 2 | 0.133
A2u1 | 16147 | 1 | 0.067
A2u1 | 16247 | 1 | 0.067
A2u1 | 16274 | 1 | 0.067
A2u1 | 16278 | 1 | 0.067
A2u1 | 16299 | 1 | 0.067
A2u1 | 16311 | 2 | 0.133
A2u1 | 16318 | 1 | 0.067
A2u1 | 16468 | 4 | 0.267
A2u1 | 16474.1G | 2 | 0.133
A2u1 | 1694 | 1 | 0.067
A2u1 | 1806 | 1 | 0.067
A2u1 | 210 | 6 | 0.4
A2u1 | 214 | 1 | 0.067
A2u1 | 269 | 1 | 0.067
A2u1 | 2743 | 1 | 0.067
A2u1 | 504 | 2 | 0.133
A2u1 | 7702 | 1 | 0.067
A2u1 | 870 | 1 | 0.067
A2u1 | 9801 | 2 | 0.133
A2u2 | 10007 | 1 | 0.111
A2u2 | 10031 | 1 | 0.111
A2u2 | 10310 | 1 | 0.111
A2u2 | 10370 | 1 | 0.111
A2u2 | 10398 | 1 | 0.111
A2u2 | 11084 | 1 | 0.111
A2u2 | 11347 | 1 | 0.111
A2u2 | 12879 | 1 | 0.111
A2u2 | 13359 | 1 | 0.111
A2u2 | 14798 | 1 | 0.111
A2u2 | 15872 | 1 | 0.111
A2u2 | 16093 | 1 | 0.111
A2u2 | 16269 | 1 | 0.111
A2u2 | 16311 | 1 | 0.111
A2u2 | 16318 | 1 | 0.111
A2u2 | 316 | 1 | 0.111
A2u2 | 3316 | 1 | 0.111
A2u2 | 59 | 1 | 0.111
A2u2 | 5964 | 1 | 0.111
A2u2 | 6260 | 1 | 0.111
A2u2 | 709 | 1 | 0.111
A2u2 | 8681 | 1 | 0.111
A2u2 | 961 | 1 | 0.111
A2u2 | 980 | 1 | 0.111
A2v | 8056 | 1 | 0.25

A2v1 | 11377 | 1 | 0.111
A2v1 | 15172 | 1 | 0.111
A2v1 | 16086 | 1 | 0.111
A2v1 | 16175 | 1 | 0.111
A2v1 | 16209 | 1 | 0.111
A2v1 | 16213 | 1 | 0.111
A2v1 | 16234 | 1 | 0.111
A2v1 | 16284 | 1 | 0.111
A2v1 | 16311 | 1 | 0.111
A2v1 | 249 | 1 | 0.111
A2v1 | 7403 | 1 | 0.111
A2v1 | 8285d | 1 | 0.111
A2v1+152 | 12235 | 1 | 0.1
A2v1+152 | 14405 | 1 | 0.1
A2v1+152 | 15290 | 1 | 0.1
A2v1+152 | 15679 | 3 | 0.3
A2v1+152 | 15776 | 1 | 0.1
A2v1+152 | 16259 | 2 | 0.2
A2v1+152 | 16325 | 1 | 0.1
A2v1+152 | 214 | 2 | 0.2
A2v1+152 | 310 | 1 | 0.1
A2v1+152 | 5127T | 1 | 0.1
A2v1+152 | 6045 | 1 | 0.1
A2v1+152 | 7051 | 3 | 0.3
A2v1+152 | 8975 | 1 | 0.1
A2v1a | 11377 | 2 | 0.143
A2v1a | 13104 | 1 | 0.071
A2v1a | 16075 | 1 | 0.071
A2v1a | 16172 | 1 | 0.071
A2v1a | 16286 | 1 | 0.071
A2v1a | 16291 | 1 | 0.071
A2v1a | 16352 | 1 | 0.071
A2v1a | 16497 | 3 | 0.214
A2v1a | 16512 | 3 | 0.214
A2v1a | 16516 | 1 | 0.071
A2v1a | 16541 | 3 | 0.214
A2v1a | 16550 | 1 | 0.071
A2v1a | 44.1C | 3 | 0.214
A2v1a | 93 | 2 | 0.143
A2v1b | 12507 | 1 | 0.333
A2v1b | 15815 | 1 | 0.333
A2v1b | 16189 | 1 | 0.333
A2v1b | 16286 | 1 | 0.333
A2v1b | 16294 | 1 | 0.333
A2v1b | 8661 | 1 | 0.333
A2w | 10211 | 1 | 0.008
A2w | 11690 | 1 | 0.008
A2w | 12285 | 1 | 0.008
A2w | 12903 | 2 | 0.016
A2w | 14290 | 1 | 0.008
A2w | 15497 | 1 | 0.008
A2w | 15945 | 1 | 0.008
A2w | 16069.1G | 1 | 0.008
A2w | 16104 | 2 | 0.016
A2w | 16129 | 1 | 0.008
A2w | 16189 | 4 | 0.031
A2w | 16292A | 1 | 0.008
A2w | 16360 | 2 | 0.016
A2w | 16465 | 2 | 0.016
A2w | 1966 | 1 | 0.008
A2w | 226 | 1 | 0.008
A2w | 4082 | 1 | 0.008
A2w | 485 | 4 | 0.031
A2w | 5093 | 1 | 0.008
A2w | 573.5C | 1 | 0.008
A2w | 6286 | 2 | 0.016
A2w | 6896 | 1 | 0.008
A2w | 7258 | 1 | 0.008
A2w | 7444 | 1 | 0.008
A2w | 7521 | 2 | 0.016
A2w | 7684 | 1 | 0.008
A2w | 7786 | 2 | 0.016
A2w | 829 | 1 | 0.008
A2w | 8605 | 1 | 0.008
A2w | 895 | 2 | 0.016
A2w | 9098 | 2 | 0.016
A2w | 9377 | 2 | 0.016
A2w | 9626C | 1 | 0.008
A2w | 9893 | 1 | 0.008
A2w1 | 10031 | 1 | 0.037
A2w1 | 1005 | 1 | 0.037
A2w1 | 10364 | 4 | 0.148
A2w1 | 10907 | 1 | 0.037
A2w1 | 12366 | 4 | 0.148
A2w1 | 12398 | 2 | 0.074
A2w1 | 125 | 2 | 0.074
A2w1 | 127 | 2 | 0.074
A2w1 | 13681 | 4 | 0.148
A2w1 | 14353 | 1 | 0.037
A2w1 | 14693 | 4 | 0.148
A2w1 | 15115 | 2 | 0.074
A2w1 | 152 | 8 | 0.296
A2w1 | 16006T | 1 | 0.037
A2w1 | 16030d | 1 | 0.037
A2w1 | 16086 | 1 | 0.037
A2w1 | 16189 | 3 | 0.111
A2w1 | 16311 | 1 | 0.037
A2w1 | 16342 | 6 | 0.222
A2w1 | 16352 | 2 | 0.074
A2w1 | 16390 | 1 | 0.037
A2w1 | 16422 | 1 | 0.037
A2w1 | 16427d | 1 | 0.037
A2w1 | 214 | 1 | 0.037
A2w1 | 234 | 1 | 0.037
A2w1 | 4029 | 2 | 0.074
A2w1 | 4245 | 4 | 0.148
A2w1 | 485 | 1 | 0.037
A2w1 | 538C | 2 | 0.074
A2w1 | 5821 | 1 | 0.037
A2w1 | 6221 | 8 | 0.296
A2w1 | 7888 | 1 | 0.037

A2w1 | 8347 | 1 | 0.037
A2w1 | 8538 | 4 | 0.148
A2w1 | 8572 | 4 | 0.148
A2w1 | 8706 | 1 | 0.037
A2w1 | 8896 | 13 | 0.481
A2w1 | 9128 | 1 | 0.037
A2x | 12196 | 1 | 0.009
A2x | 143 | 2 | 0.018
A2x | 16172 | 2 | 0.018
A2x | 16217 | 2 | 0.018
A2x | 16249 | 1 | 0.009
A2x | 16262 | 2 | 0.018
A2x | 16272 | 1 | 0.009
A2x | 16285.1A | 1 | 0.009
A2x | 16287 | 2 | 0.018
A2x | 16287G | 1 | 0.009
A2x | 16299 | 3 | 0.026
A2x | 16305 | 1 | 0.009
A2x | 16477 | 2 | 0.018
A2x | 197 | 1 | 0.009
A2x | 212 | 2 | 0.018
A2x | 215 | 4 | 0.035
A2x | 222G | 1 | 0.009
A2x | 309d | 1 | 0.009
A2x | 356.1C | 2 | 0.018
A2x | 472 | 1 | 0.009
A2x | 499 | 5 | 0.044
A2y | 14668 | 1 | 0.056
A2y | 15253 | 1 | 0.056
A2y | 15262 | 1 | 0.056
A2y | 16267 | 1 | 0.056
A2y | 8447 | 1 | 0.056
A2z | 12194 | 1 | 0.067
A2z | 16126 | 2 | 0.133
A2z | 4924C | 1 | 0.067
A2z | 5978 | 1 | 0.067
A2z | 8901 | 1 | 0.067
A3 | 150 | 1 | 0.028
A3 | 16086 | 1 | 0.028
A3 | 16209 | 1 | 0.028
A3 | 16294 | 1 | 0.028
A3 | 16356 | 2 | 0.056
A3 | 8781 | 1 | 0.028
A3a | 12771 | 1 | 0.027
A3a | 13737 | 1 | 0.027
A3a | 15286 | 1 | 0.027
A3a | 16086 | 1 | 0.027
A3a | 16177 | 2 | 0.054
A3a | 16294 | 1 | 0.027
A3a | 16311 | 2 | 0.054
A3a | 2755 | 1 | 0.027
A3a | 499 | 1 | 0.027
A3a | 9148 | 1 | 0.027
A3a | 9477 | 1 | 0.027
A5 | 13260 | 1 | 0.036
A5 | 14599 | 1 | 0.036
A5 | 16032d | 1 | 0.036
A5 | 16042d | 1 | 0.036
A5 | 16076d | 1 | 0.036
A5 | 16110d | 1 | 0.036
A5 | 16124 | 1 | 0.036
A5 | 16129T | 3 | 0.107
A5 | 16162 | 1 | 0.036
A5 | 16172 | 3 | 0.107
A5 | 16188 | 3 | 0.107
A5 | 16214 | 1 | 0.036
A5 | 16248 | 1 | 0.036
A5 | 16256 | 1 | 0.036
A5 | 16264 | 1 | 0.036
A5 | 16286 | 1 | 0.036
A5 | 16287 | 1 | 0.036
A5 | 16527 | 1 | 0.036
A5 | 318 | 1 | 0.036
A5 | 3826 | 1 | 0.036
A5 | 7352 | 1 | 0.036
A5 | 8659 | 1 | 0.036
A5a | 13759 | 1 | 0.028
A5a | 14094 | 1 | 0.028
A5a | 14757 | 1 | 0.028
A5a | 153 | 1 | 0.028
A5a | 15519 | 1 | 0.028
A5a | 16129 | 1 | 0.028
A5a | 16176 | 1 | 0.028
A5a | 16209 | 1 | 0.028
A5a | 16295 | 1 | 0.028
A5a | 195 | 3 | 0.083
A5a | 5582 | 1 | 0.028
A5a | 629 | 1 | 0.028
A5a | 9386 | 1 | 0.028
A5a | 9488 | 1 | 0.028
A5a1 | 150 | 4 | 0.133
A5a1a | 10685 | 1 | 0.026
A5a1a | 13928C | 1 | 0.026
A5a1a | 152 | 2 | 0.053
A5a1a | 16233T | 1 | 0.026
A5a1a | 16287 | 3 | 0.079
A5a1a | 16295 | 1 | 0.026
A5a1a | 16296 | 8 | 0.211
A5a1a | 16357 | 1 | 0.026
A5a1a | 8281-8289d | 1 | 0.026
A5a1a1 | 12280 | 1 | 0.018
A5a1a1 | 12816 | 1 | 0.018
A5a1a1 | 146 | 1 | 0.018
A5a1a1 | 15133 | 1 | 0.018
A5a1a1 | 15427 | 1 | 0.018
A5a1a1 | 16076d | 1 | 0.018
A5a1a1 | 16092 | 2 | 0.036
A5a1a1 | 16093 | 1 | 0.018
A5a1a1 | 16174 | 3 | 0.055
A5a1a1 | 16311 | 3 | 0.055
A5a1a1 | 195 | 1 | 0.018

A5a1a1 | 291.1A | 1 | 0.018
A5a1a1 | 318 | 1 | 0.018
A5a1a1 | 3368 | 1 | 0.018
A5a1a1 | 5147 | 1 | 0.018
A5a1a1 | 5396 | 1 | 0.018
A5a1a1 | 5821 | 1 | 0.018
A5a1a1 | 7235 | 1 | 0.018
A5a1a1 | 7964 | 1 | 0.018
A5a1a1 | 8020 | 1 | 0.018
A5a1a1 | 8757 | 1 | 0.018
A5a1a1 | 9174 | 1 | 0.018
A5a1a1a | 13225 | 1 | 0.033
A5a1a1a | 16147 | 1 | 0.033
A5a1a1a | 16390 | 1 | 0.033
A5a1a1a | 5460 | 1 | 0.033
A5a1a1a | 7492 | 1 | 0.033
A5a1a1b | 13889 | 1 | 0.033
A5a1a1b | 16354 | 1 | 0.033
A5a1a1b | 16390 | 1 | 0.033
A5a1a1b | 5237 | 1 | 0.033
A5a1a1b | 5894 | 1 | 0.033
A5a1a2 | 10496 | 1 | 0.03
A5a1a2 | 11914 | 1 | 0.03
A5a1a2 | 16189 | 3 | 0.091
A5a1a2 | 16209 | 1 | 0.03
A5a1a2 | 16399 | 2 | 0.061
A5a1a2 | 189 | 1 | 0.03
A5a1a2 | 3397 | 1 | 0.03
A5a1a2 | 8723 | 1 | 0.03
A5a1a2a | 115 | 1 | 0.03
A5a1a2a | 143 | 1 | 0.03
A5a1a2a | 16075.1A | 1 | 0.03
A5a1a2a | 16172 | 1 | 0.03
A5a1a2a | 186 | 1 | 0.03
A5a1a2a | 309.3C | 1 | 0.03
A5a1a2a | 8167 | 1 | 0.03
A5a1a2a | 8701 | 1 | 0.03
A5a1b | 16189 | 1 | 0.167
A5a1b | 16291 | 1 | 0.167
A5a1b | 1892 | 1 | 0.167
A5a1b | 200 | 2 | 0.333
A5a1b | 369 | 1 | 0.167
A5a1b | 702 | 1 | 0.167
A5a2 | 1420 | 1 | 0.03
A5a2 | 16086 | 3 | 0.091
A5a2 | 16356 | 3 | 0.091
A5a3 | 16093 | 3 | 0.094
A5a3 | 16172 | 1 | 0.031
A5a3 | 16184 | 1 | 0.031
A5a3 | 207 | 4 | 0.125
A5a3 | 9909 | 1 | 0.031
A5a3a | 14118 | 3 | 0.1
A5a3a | 16291 | 1 | 0.033
A5a3a | 7637 | 1 | 0.033
A5a3a | 9422T | 1 | 0.033
A5a4 | 10235 | 2 | 0.065
A5a4 | 7297 | 1 | 0.032
A5a5 | 15109 | 1 | 0.031
A5a5 | 15894 | 1 | 0.031
A5a5 | 16360 | 2 | 0.062
A5b | 13260 | 4 | 0.133
A5b | 14635 | 2 | 0.067
A5b | 16124 | 10 | 0.333
A5b | 16189 | 7 | 0.233
A5b | 16190 | 7 | 0.233
A5b | 16317 | 1 | 0.033
A5b | 16344 | 1 | 0.033
A5b | 16390 | 3 | 0.1
A5b | 207 | 2 | 0.067
A5b | 310 | 1 | 0.033
A5b | 318 | 12 | 0.4
A5b | 8659 | 4 | 0.133
A5b1 | 10398 | 1 | 0.034
A5b1 | 10400 | 1 | 0.034
A5b1 | 10873 | 1 | 0.034
A5b1 | 11172 | 5 | 0.172
A5b1 | 12372 | 1 | 0.034
A5b1 | 14016 | 1 | 0.034
A5b1 | 153 | 4 | 0.138
A5b1 | 16153 | 1 | 0.034
A5b1 | 3338 | 2 | 0.069
A5b1 | 3460 | 1 | 0.034
A5b1 | 3915 | 1 | 0.034
A5b1 | 5021 | 1 | 0.034
A5b1 | 525.1C | 1 | 0.034
A5b1 | 64 | 1 | 0.034
A5b1 | 6788 | 1 | 0.034
A5b1 | 8701 | 1 | 0.034
A5b1 | 9016 | 4 | 0.138
A5b1 | 9938 | 1 | 0.034
A5b1a | 14983 | 1 | 0.036
A5b1a | 15043 | 4 | 0.143
A5b1a | 15793 | 4 | 0.143
A5b1a | 15806 | 4 | 0.143
A5b1a | 2863 | 1 | 0.036
A5b1a | 6852 | 1 | 0.036
A5b1a | 9545 | 3 | 0.107
A5b1a | 9959 | 1 | 0.036
A5b1b | 11204 | 6 | 0.128
A5b1b | 12948 | 2 | 0.043
A5b1b | 13908 | 2 | 0.043
A5b1b | 13999 | 2 | 0.043
A5b1b | 14194 | 2 | 0.043
A5b1b | 14207C | 3 | 0.064
A5b1b | 146 | 3 | 0.064
A5b1b | 152 | 6 | 0.128
A5b1b | 15262 | 2 | 0.043
A5b1b | 15355 | 1 | 0.021
A5b1b | 16076d | 1 | 0.021
A5b1b | 16093 | 9 | 0.191
A5b1b | 16155 | 1 | 0.021

A5b1b | 16189 | 1 | 0.021
A5b1b | 16239 | 1 | 0.021
A5b1b | 16248 | 3 | 0.064
A5b1b | 16259 | 1 | 0.021
A5b1b | 16289 | 3 | 0.064
A5b1b | 16311 | 4 | 0.085
A5b1b | 16445 | 5 | 0.106
A5b1b | 16509 | 1 | 0.021
A5b1b | 1961 | 2 | 0.043
A5b1b | 2404 | 2 | 0.043
A5b1b | 262 | 2 | 0.043
A5b1b | 310 | 1 | 0.021
A5b1b | 320 | 1 | 0.021
A5b1b | 333 | 1 | 0.021
A5b1b | 4316 | 2 | 0.043
A5b1b | 6366 | 1 | 0.021
A5b1b | 6513 | 1 | 0.021
A5b1b | 6671 | 2 | 0.043
A5b1b | 7498 | 2 | 0.043
A5b1b | 7990A | 2 | 0.043
A5b1b | 8251 | 1 | 0.021
A5b1b | 965.0C | 2 | 0.043
A5b1b | 9797 | 3 | 0.064
A5b1c | 16297 | 1 | 0.038
A5b1c | 16311 | 1 | 0.038
A5b1c | 16362 | 1 | 0.038
A5b1c | 16474 | 3 | 0.115
A5b1c | 515 | 1 | 0.038
A5b1c1 | 143 | 1 | 0.026
A5b1c1 | 146 | 2 | 0.053
A5b1c1 | 1495 | 1 | 0.026
A5b1c1 | 1498 | 1 | 0.026
A5b1c1 | 1508 | 1 | 0.026
A5b1c1 | 151 | 1 | 0.026
A5b1c1 | 16093 | 7 | 0.184
A5b1c1 | 16129 | 1 | 0.026
A5b1c1 | 16234 | 7 | 0.184
A5b1c1 | 16278 | 1 | 0.026
A5b1c1 | 16286 | 4 | 0.105
A5b1c1 | 3380 | 1 | 0.026
A5b1c1 | 3393A | 1 | 0.026
A5b1c1 | 3684A | 1 | 0.026
A5b1c1 | 5893 | 1 | 0.026
A5b1c1 | 9010 | 1 | 0.026
A5b1c1 | 9145 | 1 | 0.026
A5c | 2643 | 1 | 0.043
A5c | 9621 | 1 | 0.043
A5c1 | 146 | 1 | 0.04
A5c1 | 14757 | 1 | 0.04
A5c1 | 16051 | 3 | 0.12
A5c1 | 16185 | 1 | 0.04
A5c1 | 16256 | 1 | 0.04
A5c1 | 16318T | 1 | 0.04
A5c1 | 2109 | 1 | 0.04
A5c1 | 262A | 1 | 0.04
A5c1 | 8251 | 1 | 0.04
A6 | 120 | 1 | 0.002
A6 | 126 | 1 | 0.002
A6 | 142 | 1 | 0.002
A6 | 151 | 2 | 0.005
A6 | 16025 | 1 | 0.002
A6 | 16076d | 2 | 0.005
A6 | 16090 | 1 | 0.002
A6 | 16094 | 1 | 0.002
A6 | 16095 | 1 | 0.002
A6 | 16109 | 1 | 0.002
A6 | 16114 | 3 | 0.007
A6 | 16152 | 1 | 0.002
A6 | 16201 | 2 | 0.005
A6 | 16203T | 1 | 0.002
A6 | 16224 | 22 | 0.051
A6 | 16234 | 5 | 0.012
A6 | 16268d | 1 | 0.002
A6 | 16270 | 1 | 0.002
A6 | 16274 | 2 | 0.005
A6 | 16299 | 1 | 0.002
A6 | 16304 | 18 | 0.042
A6 | 16355 | 26 | 0.06
A6 | 16357 | 1 | 0.002
A6 | 16383 | 1 | 0.002
A6 | 16399 | 1 | 0.002
A6 | 16400 | 2 | 0.005
A6 | 16486T | 1 | 0.002
A6 | 16525 | 1 | 0.002
A6 | 16528d | 1 | 0.002
A6 | 16539 | 1 | 0.002
A6 | 16550 | 1 | 0.002
A6 | 175 | 1 | 0.002
A6 | 185 | 1 | 0.002
A6 | 188 | 2 | 0.005
A6 | 214 | 4 | 0.009
A6 | 227 | 1 | 0.002
A6 | 232 | 1 | 0.002
A6 | 232C | 1 | 0.002
A6 | 237 | 1 | 0.002
A6 | 249d | 1 | 0.002
A6 | 309d | 1 | 0.002
A6 | 318 | 1 | 0.002
A6 | 325 | 1 | 0.002
A6 | 334 | 1 | 0.002
A6 | 35 | 1 | 0.002
A6 | 365 | 1 | 0.002
A6 | 377 | 1 | 0.002
A6 | 398 | 1 | 0.002
A6 | 406 | 1 | 0.002
A6 | 432 | 1 | 0.002
A6 | 458 | 1 | 0.002
A6 | 489 | 2 | 0.005
A6 | 499 | 5 | 0.012
A6 | 515T | 2 | 0.005
A6 | 551C | 1 | 0.002

A6 | 560d | 1 | 0.002
A6 | 573.1C | 1 | 0.002
A6 | 6040 | 1 | 0.002
A6 | 6392 | 1 | 0.002
A6 | 6962 | 1 | 0.002
A6 | 69T | 1 | 0.002
A6 | 72 | 1 | 0.002
A6 | 95 | 1 | 0.002
A6 | 9705 | 5 | 0.012
A6 | 99 | 1 | 0.002
A6a | 1168d | 1 | 0.003
A6a | 14776 | 1 | 0.003
A6a | 1494 | 1 | 0.003
A6a | 151 | 2 | 0.005
A6a | 16076d | 1 | 0.003
A6a | 16090 | 1 | 0.003
A6a | 16095 | 1 | 0.003
A6a | 16201 | 2 | 0.005
A6a | 16209 | 4 | 0.01
A6a | 16214 | 5 | 0.013
A6a | 16270 | 1 | 0.003
A6a | 16274 | 2 | 0.005
A6a | 16294 | 5 | 0.013
A6a | 16400 | 2 | 0.005
A6a | 188 | 2 | 0.005
A6a | 214 | 4 | 0.01
A6a | 232C | 1 | 0.003
A6a | 499 | 4 | 0.01
A6a | 511 | 1 | 0.003
A6a | 5628 | 3 | 0.008
A6a | 573.1C | 1 | 0.003
A6a | 64 | 7 | 0.018
A6a | 8551 | 1 | 0.003
A6a | 8563 | 2 | 0.005
A6a | 8567 | 1 | 0.003
A6b | 114 | 2 | 0.005
A6b | 14687 | 1 | 0.003
A6b | 15930 | 1 | 0.003
A6b | 16104 | 2 | 0.005
A6b | 16120T | 1 | 0.003
A6b | 16126 | 2 | 0.005
A6b | 16127 | 2 | 0.005
A6b | 16131 | 1 | 0.003
A6b | 16217 | 1 | 0.003
A6b | 16222A | 1 | 0.003
A6b | 16234 | 1 | 0.003
A6b | 16258C | 1 | 0.003
A6b | 16261 | 1 | 0.003
A6b | 16278 | 2 | 0.005
A6b | 16280T | 1 | 0.003
A6b | 16293 | 1 | 0.003
A6b | 16295 | 2 | 0.005
A6b | 16298 | 1 | 0.003
A6b | 16316 | 1 | 0.003
A6b | 16344 | 4 | 0.011
A6b | 16354 | 1 | 0.003
A6b | 16355 | 1 | 0.003
A6b | 188 | 1 | 0.003
A6b | 217.1T | 1 | 0.003
A6b | 246.1T | 1 | 0.003
A6b | 297 | 2 | 0.005
A6b | 310 | 1 | 0.003
A6b | 314-315d | 1 | 0.003
A6b | 315.2C | 1 | 0.003
A6b | 316C | 1 | 0.003
A6b | 459d | 2 | 0.005
A6b | 573.1C | 2 | 0.005
A6b | 59 | 1 | 0.003
A6b | 61 | 1 | 0.003
A6b | 62 | 1 | 0.003
A6b | 62T | 1 | 0.003
A6b | 64 | 1 | 0.003
A6b | 7034 | 1 | 0.003
A6b | 7202 | 1 | 0.003
A6b | 8793 | 1 | 0.003
A7 | 10745 | 1 | 0.034
A7 | 12397 | 1 | 0.034
A7 | 12411 | 5 | 0.172
A7 | 14178 | 1 | 0.034
A7 | 143 | 2 | 0.069
A7 | 16086 | 1 | 0.034
A7 | 16093 | 3 | 0.103
A7 | 16169 | 2 | 0.069
A7 | 16178 | 1 | 0.034
A7 | 16194C | 5 | 0.172
A7 | 16195 | 5 | 0.172
A7 | 16243 | 1 | 0.034
A7 | 16259A | 4 | 0.138
A7 | 200 | 1 | 0.034
A7 | 309.3C | 4 | 0.138
A7 | 316C | 8 | 0.276
A7 | 4314A | 2 | 0.069
A7 | 7136 | 1 | 0.034
A7 | 8621 | 1 | 0.034
A7 | 9128 | 2 | 0.069
A8 | 12175 | 6 | 0.375
A8 | 16093 | 1 | 0.062
A8 | 310 | 2 | 0.125
A8 | 5824 | 6 | 0.375
A8a | 151 | 6 | 0.162
A8a | 15229 | 1 | 0.027
A8a | 16092 | 1 | 0.027
A8a | 16189 | 1 | 0.027
A8a | 16260 | 2 | 0.054
A8a | 16291A | 1 | 0.027
A8a | 16293C | 14 | 0.378
A8a | 16296 | 9 | 0.243
A8a | 16311 | 5 | 0.135
A8a | 195 | 3 | 0.081
A8a | 253 | 1 | 0.027
A8a | 3144 | 1 | 0.027

A8a | 316C | 1 | 0.027
A8a | 317 | 1 | 0.027
A8a | 3395 | 1 | 0.027
A8a | 4508 | 1 | 0.027
A8a | 466 | 1 | 0.027
A8a | 5046 | 1 | 0.027
A8a | 5067 | 1 | 0.027
A8a | 593 | 2 | 0.054
A8a | 6779 | 2 | 0.054
A8a | 722 | 2 | 0.054
A8a | 7711 | 1 | 0.027
A8a | 7762 | 1 | 0.027
A8a | 7859 | 1 | 0.027
A8a | 9007 | 1 | 0.027
A8a | 9531 | 1 | 0.027
A8a1 | 13708 | 1 | 0.032
A8a1 | 15301 | 2 | 0.065
A8a1 | 15814 | 1 | 0.032
A8a1 | 16066 | 7 | 0.226
A8a1 | 16189 | 1 | 0.032
A8a1 | 16278 | 5 | 0.161
A8a1 | 16289.1T | 1 | 0.032
A8a1 | 16290G | 1 | 0.032
A8a1 | 16293C | 2 | 0.065
A8a1 | 199 | 7 | 0.226
A8a1 | 204 | 1 | 0.032
A8a1 | 5811 | 1 | 0.032
A8a1 | 9318 | 1 | 0.032
A9 | 146 | 3 | 0.071
A9 | 16169 | 1 | 0.024
A9 | 16179d | 1 | 0.024
A9 | 16213 | 2 | 0.048
A9 | 16260 | 3 | 0.071
A9 | 16311 | 1 | 0.024
A9 | 16434 | 1 | 0.024
A9 | 195 | 1 | 0.024
A9 | 200 | 1 | 0.024
A9 | 318 | 10 | 0.238
B2 | 10084 | 2 | 0.004
B2 | 10101 | 2 | 0.004
B2 | 10124 | 1 | 0.002
B2 | 10193 | 27 | 0.057
B2 | 10214 | 1 | 0.002
B2 | 103 | 2 | 0.004
B2 | 10313C | 1 | 0.002
B2 | 10373 | 1 | 0.002
B2 | 10397 | 1 | 0.002
B2 | 105-110d | 5 | 0.011
B2 | 106-111d | 2 | 0.004
B2 | 10658 | 1 | 0.002
B2 | 10688 | 1 | 0.002
B2 | 10751 | 1 | 0.002
B2 | 10763 | 1 | 0.002
B2 | 10790 | 1 | 0.002
B2 | 10909 | 2 | 0.004
B2 | 10915 | 1 | 0.002
B2 | 10975 | 1 | 0.002
B2 | 10978 | 1 | 0.002
B2 | 11026 | 1 | 0.002
B2 | 11084 | 1 | 0.002
B2 | 11087 | 2 | 0.004
B2 | 11119 | 3 | 0.006
B2 | 11176 | 20 | 0.042
B2 | 11242 | 1 | 0.002
B2 | 11253 | 1 | 0.002
B2 | 11335 | 1 | 0.002
B2 | 11339 | 1 | 0.002
B2 | 11353 | 3 | 0.006
B2 | 11383 | 1 | 0.002
B2 | 114 | 1 | 0.002
B2 | 11425 | 2 | 0.004
B2 | 11440 | 2 | 0.004
B2 | 11447 | 2 | 0.004
B2 | 11453 | 1 | 0.002
B2 | 11455 | 1 | 0.002
B2 | 11545 | 1 | 0.002
B2 | 11569 | 1 | 0.002
B2 | 11575 | 3 | 0.006
B2 | 11770 | 2 | 0.004
B2 | 11854 | 1 | 0.002
B2 | 118C | 2 | 0.004
B2 | 119 | 4 | 0.008
B2 | 11908 | 1 | 0.002
B2 | 11914 | 1 | 0.002
B2 | 1193 | 2 | 0.004
B2 | 12033 | 2 | 0.004
B2 | 12130 | 2 | 0.004
B2 | 12172 | 2 | 0.004
B2 | 12191 | 1 | 0.002
B2 | 12192 | 3 | 0.006
B2 | 12235 | 3 | 0.006
B2 | 12358 | 4 | 0.008
B2 | 12381 | 1 | 0.002
B2 | 12406 | 1 | 0.002
B2 | 12561 | 3 | 0.006
B2 | 12616 | 1 | 0.002
B2 | 12618 | 1 | 0.002
B2 | 12630 | 1 | 0.002
B2 | 12684 | 2 | 0.004
B2 | 12696 | 1 | 0.002
B2 | 12879 | 2 | 0.004
B2 | 12904 | 2 | 0.004
B2 | 1291 | 1 | 0.002
B2 | 13014 | 3 | 0.006
B2 | 13015 | 1 | 0.002
B2 | 13035 | 2 | 0.004
B2 | 1307 | 2 | 0.004
B2 | 131 | 1 | 0.002
B2 | 13135 | 16 | 0.034
B2 | 13145 | 1 | 0.002
B2 | 13194 | 1 | 0.002

B2 | 13350 | 1 | 0.002
B2 | 1341 | 2 | 0.004
B2 | 13434 | 2 | 0.004
B2 | 13437 | 1 | 0.002
B2 | 13443 | 1 | 0.002
B2 | 13500 | 4 | 0.008
B2 | 13594 | 1 | 0.002
B2 | 13635 | 3 | 0.006
B2 | 13686 | 3 | 0.006
B2 | 13701 | 6 | 0.013
B2 | 13708 | 3 | 0.006
B2 | 13713A | 2 | 0.004
B2 | 13810 | 1 | 0.002
B2 | 13813 | 1 | 0.002
B2 | 13828 | 1 | 0.002
B2 | 13884 | 1 | 0.002
B2 | 139 | 1 | 0.002
B2 | 13907 | 1 | 0.002
B2 | 13909T | 1 | 0.002
B2 | 1393 | 2 | 0.004
B2 | 13934 | 1 | 0.002
B2 | 13945 | 1 | 0.002
B2 | 13967 | 2 | 0.004
B2 | 13981 | 1 | 0.002
B2 | 14020 | 1 | 0.002
B2 | 14041 | 13 | 0.027
B2 | 14043 | 1 | 0.002
B2 | 14053 | 4 | 0.008
B2 | 14110 | 3 | 0.006
B2 | 14129 | 1 | 0.002
B2 | 14178 | 1 | 0.002
B2 | 14185T | 5 | 0.011
B2 | 14197G | 1 | 0.002
B2 | 14198 | 1 | 0.002
B2 | 14233 | 1 | 0.002
B2 | 143 | 16 | 0.034
B2 | 14323 | 1 | 0.002
B2 | 14364 | 1 | 0.002
B2 | 14392 | 2 | 0.004
B2 | 14446 | 1 | 0.002
B2 | 14485 | 6 | 0.013
B2 | 14560 | 2 | 0.004
B2 | 14569 | 2 | 0.004
B2 | 14587 | 13 | 0.027
B2 | 146 | 17 | 0.036
B2 | 14602 | 1 | 0.002
B2 | 14791 | 1 | 0.002
B2 | 14992 | 1 | 0.002
B2 | 14997A | 1 | 0.002
B2 | 150 | 4 | 0.008
B2 | 15061 | 1 | 0.002
B2 | 15098 | 1 | 0.002
B2 | 151 | 2 | 0.004
B2 | 15148 | 1 | 0.002
B2 | 152 | 44 | 0.093
B2 | 15265 | 3 | 0.006
B2 | 15289 | 1 | 0.002
B2 | 153 | 1 | 0.002
B2 | 15314 | 2 | 0.004
B2 | 15323 | 1 | 0.002
B2 | 15355 | 2 | 0.004
B2 | 15401 | 1 | 0.002
B2 | 1547.1T | 1 | 0.002
B2 | 15479 | 2 | 0.004
B2 | 15571 | 2 | 0.004
B2 | 15596 | 1 | 0.002
B2 | 15610 | 1 | 0.002
B2 | 15632 | 2 | 0.004
B2 | 15661 | 3 | 0.006
B2 | 15670 | 1 | 0.002
B2 | 15671 | 1 | 0.002
B2 | 15731 | 4 | 0.008
B2 | 15777 | 1 | 0.002
B2 | 15883 | 54 | 0.114
B2 | 15884 | 4 | 0.008
B2 | 15892 | 1 | 0.002
B2 | 15914C | 1 | 0.002
B2 | 15924 | 14 | 0.029
B2 | 15928 | 1 | 0.002
B2 | 15932 | 1 | 0.002
B2 | 15937 | 1 | 0.002
B2 | 15942 | 1 | 0.002
B2 | 15951 | 1 | 0.002
B2 | 1598 | 1 | 0.002
B2 | 16043T | 1 | 0.002
B2 | 16051 | 30 | 0.063
B2 | 16066 | 2 | 0.004
B2 | 16075 | 2 | 0.004
B2 | 16083 | 3 | 0.006
B2 | 16084 | 1 | 0.002
B2 | 16086 | 16 | 0.034
B2 | 16093 | 6 | 0.013
B2 | 16111 | 1 | 0.002
B2 | 16126 | 4 | 0.008
B2 | 16129 | 5 | 0.011
B2 | 16129C | 2 | 0.004
B2 | 16140 | 3 | 0.006
B2 | 16144 | 1 | 0.002
B2 | 16148 | 3 | 0.006
B2 | 16150 | 3 | 0.006
B2 | 16167 | 1 | 0.002
B2 | 16168 | 36 | 0.076
B2 | 16169 | 9 | 0.019
B2 | 16172 | 4 | 0.008
B2 | 16173 | 1 | 0.002
B2 | 16178 | 1 | 0.002
B2 | 16179 | 4 | 0.008
B2 | 16185 | 6 | 0.013
B2 | 16187 | 1 | 0.002
B2 | 16188 | 98 | 0.206
B2 | 1619 | 2 | 0.004

B2 | 16190 | 1 | 0.002
B2 | 16192 | 3 | 0.006
B2 | 16192-16193d | 2 | 0.004
B2 | 16193.3C | 1 | 0.002
B2 | 16193d | 16 | 0.034
B2 | 16194C | 1 | 0.002
B2 | 16195 | 1 | 0.002
B2 | 16196 | 1 | 0.002
B2 | 16198 | 5 | 0.011
B2 | 16214 | 2 | 0.004
B2 | 16216 | 1 | 0.002
B2 | 16218 | 1 | 0.002
B2 | 16221 | 1 | 0.002
B2 | 16230 | 1 | 0.002
B2 | 16234 | 2 | 0.004
B2 | 16235 | 2 | 0.004
B2 | 16239 | 1 | 0.002
B2 | 16242 | 2 | 0.004
B2 | 16243 | 1 | 0.002
B2 | 16245 | 1 | 0.002
B2 | 16249 | 1 | 0.002
B2 | 16256 | 2 | 0.004
B2 | 16259 | 1 | 0.002
B2 | 16265 | 1 | 0.002
B2 | 16266 | 3 | 0.006
B2 | 16268 | 1 | 0.002
B2 | 16268A | 8 | 0.017
B2 | 16270A | 1 | 0.002
B2 | 16274 | 3 | 0.006
B2 | 16287 | 1 | 0.002
B2 | 16288 | 2 | 0.004
B2 | 16289 | 2 | 0.004
B2 | 16290 | 3 | 0.006
B2 | 16292 | 6 | 0.013
B2 | 16293 | 2 | 0.004
B2 | 16295 | 3 | 0.006
B2 | 16297 | 1 | 0.002
B2 | 16301 | 1 | 0.002
B2 | 16301A | 5 | 0.011
B2 | 16303 | 1 | 0.002
B2 | 16304 | 21 | 0.044
B2 | 16309 | 3 | 0.006
B2 | 16310 | 1 | 0.002
B2 | 16311 | 19 | 0.04
B2 | 16316 | 2 | 0.004
B2 | 16319 | 4 | 0.008
B2 | 16320 | 1 | 0.002
B2 | 16325 | 1 | 0.002
B2 | 16330 | 3 | 0.006
B2 | 16330A | 3 | 0.006
B2 | 16352 | 1 | 0.002
B2 | 16353 | 2 | 0.004
B2 | 16354 | 17 | 0.036
B2 | 16355 | 1 | 0.002
B2 | 16356 | 1 | 0.002
B2 | 16357 | 8 | 0.017
B2 | 16360 | 13 | 0.027
B2 | 16362 | 22 | 0.046
B2 | 16364.1A | 1 | 0.002
B2 | 16381 | 1 | 0.002
B2 | 16390 | 9 | 0.019
B2 | 16391 | 2 | 0.004
B2 | 16400 | 1 | 0.002
B2 | 16428 | 3 | 0.006
B2 | 16437 | 3 | 0.006
B2 | 16443 | 1 | 0.002
B2 | 16454 | 3 | 0.006
B2 | 16455 | 3 | 0.006
B2 | 16456 | 1 | 0.002
B2 | 16464 | 1 | 0.002
B2 | 16465 | 2 | 0.004
B2 | 16497 | 1 | 0.002
B2 | 16512 | 1 | 0.002
B2 | 16524 | 2 | 0.004
B2 | 16526 | 1 | 0.002
B2 | 1654 | 1 | 0.002
B2 | 16566 | 2 | 0.004
B2 | 1676 | 2 | 0.004
B2 | 1719 | 1 | 0.002
B2 | 174 | 1 | 0.002
B2 | 1772T | 1 | 0.002
B2 | 181 | 1 | 0.002
B2 | 1811 | 1 | 0.002
B2 | 182 | 2 | 0.004
B2 | 1822 | 1 | 0.002
B2 | 183 | 6 | 0.013
B2 | 185 | 30 | 0.063
B2 | 186 | 20 | 0.042
B2 | 188 | 1 | 0.002
B2 | 189 | 5 | 0.011
B2 | 19 | 1 | 0.002
B2 | 193 | 1 | 0.002
B2 | 194 | 1 | 0.002
B2 | 195 | 3 | 0.006
B2 | 197 | 14 | 0.029
B2 | 198 | 4 | 0.008
B2 | 199 | 14 | 0.029
B2 | 203 | 3 | 0.006
B2 | 204 | 25 | 0.053
B2 | 207 | 32 | 0.067
B2 | 209 | 4 | 0.008
B2 | 210 | 7 | 0.015
B2 | 2109 | 1 | 0.002
B2 | 212 | 3 | 0.006
B2 | 214 | 2 | 0.004
B2 | 215 | 9 | 0.019
B2 | 226 | 1 | 0.002
B2 | 227 | 5 | 0.011
B2 | 2281C | 1 | 0.002
B2 | 228T | 1 | 0.002
B2 | 234 | 1 | 0.002

B2 | 2349 | 1 | 0.002
B2 | 235 | 1 | 0.002
B2 | 236 | 1 | 0.002
B2 | 2361 | 1 | 0.002
B2 | 241 | 2 | 0.004
B2 | 2413 | 2 | 0.004
B2 | 247 | 2 | 0.004
B2 | 249d | 1 | 0.002
B2 | 2528 | 1 | 0.002
B2 | 257 | 1 | 0.002
B2 | 2581 | 1 | 0.002
B2 | 260 | 2 | 0.004
B2 | 269 | 1 | 0.002
B2 | 2731G | 1 | 0.002
B2 | 2755 | 1 | 0.002
B2 | 279 | 1 | 0.002
B2 | 2831 | 2 | 0.004
B2 | 284 | 1 | 0.002
B2 | 2844 | 2 | 0.004
B2 | 2857 | 14 | 0.029
B2 | 2880 | 8 | 0.017
B2 | 2885 | 1 | 0.002
B2 | 292 | 14 | 0.029
B2 | 292.1AT | 2 | 0.004
B2 | 2951 | 1 | 0.002
B2 | 3006G | 1 | 0.002
B2 | 3027 | 1 | 0.002
B2 | 302d | 1 | 0.002
B2 | 308-309d | 1 | 0.002
B2 | 309.3C | 3 | 0.006
B2 | 309.4C | 1 | 0.002
B2 | 309d | 10 | 0.021
B2 | 310 | 9 | 0.019
B2 | 311 | 1 | 0.002
B2 | 3140 | 14 | 0.029
B2 | 315.1T | 3 | 0.006
B2 | 315.2C | 1 | 0.002
B2 | 315.3C | 1 | 0.002
B2 | 315d | 1 | 0.002
B2 | 316 | 1 | 0.002
B2 | 316C | 1 | 0.002
B2 | 316d | 1 | 0.002
B2 | 3290 | 14 | 0.029
B2 | 3316 | 4 | 0.008
B2 | 3336 | 1 | 0.002
B2 | 3357 | 8 | 0.017
B2 | 3372 | 2 | 0.004
B2 | 3375 | 2 | 0.004
B2 | 3390 | 1 | 0.002
B2 | 3391 | 1 | 0.002
B2 | 3395 | 1 | 0.002
B2 | 3434 | 6 | 0.013
B2 | 3483 | 1 | 0.002
B2 | 3504 | 2 | 0.004
B2 | 3523 | 2 | 0.004
B2 | 3531 | 3 | 0.006
B2 | 356.1C | 2 | 0.004
B2 | 3591 | 2 | 0.004
B2 | 3592 | 6 | 0.013
B2 | 3612 | 1 | 0.002
B2 | 3615 | 2 | 0.004
B2 | 3696 | 1 | 0.002
B2 | 3705 | 1 | 0.002
B2 | 372 | 2 | 0.004
B2 | 373 | 1 | 0.002
B2 | 3732 | 3 | 0.006
B2 | 3760G | 3 | 0.006
B2 | 3777 | 1 | 0.002
B2 | 3795 | 1 | 0.002
B2 | 3826 | 1 | 0.002
B2 | 383 | 1 | 0.002
B2 | 385 | 1 | 0.002
B2 | 3866 | 1 | 0.002
B2 | 393 | 1 | 0.002
B2 | 3978 | 1 | 0.002
B2 | 4038 | 3 | 0.006
B2 | 4136 | 1 | 0.002
B2 | 4181 | 5 | 0.011
B2 | 42 | 2 | 0.004
B2 | 4232 | 2 | 0.004
B2 | 4245 | 1 | 0.002
B2 | 4314A | 1 | 0.002
B2 | 4317 | 3 | 0.006
B2 | 4417 | 1 | 0.002
B2 | 446C | 1 | 0.002
B2 | 4502 | 9 | 0.019
B2 | 4512 | 1 | 0.002
B2 | 458 | 7 | 0.015
B2 | 4586 | 1 | 0.002
B2 | 46 | 2 | 0.004
B2 | 460d | 1 | 0.002
B2 | 4655 | 7 | 0.015
B2 | 4691 | 1 | 0.002
B2 | 4695 | 1 | 0.002
B2 | 4715 | 6 | 0.013
B2 | 4767 | 1 | 0.002
B2 | 4775 | 1 | 0.002
B2 | 482 | 1 | 0.002
B2 | 4829 | 1 | 0.002
B2 | 4886G | 6 | 0.013
B2 | 4890 | 2 | 0.004
B2 | 4924 | 2 | 0.004
B2 | 4928 | 1 | 0.002
B2 | 4938A | 1 | 0.002
B2 | 4960 | 2 | 0.004
B2 | 4973 | 3 | 0.006
B2 | 501 | 3 | 0.006
B2 | 5021 | 2 | 0.004
B2 | 5027 | 1 | 0.002
B2 | 5033 | 1 | 0.002
B2 | 504 | 1 | 0.002

B2 | 5054 | 1 | 0.002
B2 | 5082 | 1 | 0.002
B2 | 5095 | 3 | 0.006
B2 | 51 | 1 | 0.002
B2 | 513 | 1 | 0.002
B2 | 5134 | 2 | 0.004
B2 | 5186 | 2 | 0.004
B2 | 5196 | 1 | 0.002
B2 | 5237 | 3 | 0.006
B2 | 525.1CA | 1 | 0.002
B2 | 5277 | 8 | 0.017
B2 | 5295A | 1 | 0.002
B2 | 5301 | 1 | 0.002
B2 | 5322C | 1 | 0.002
B2 | 5375 | 2 | 0.004
B2 | 538 | 25 | 0.053
B2 | 5423 | 1 | 0.002
B2 | 5465 | 20 | 0.042
B2 | 5471 | 2 | 0.004
B2 | 5483 | 1 | 0.002
B2 | 5493 | 3 | 0.006
B2 | 55 | 1 | 0.002
B2 | 55.1T | 1 | 0.002
B2 | 5510 | 1 | 0.002
B2 | 5530 | 1 | 0.002
B2 | 5628 | 1 | 0.002
B2 | 5634 | 20 | 0.042
B2 | 569 | 1 | 0.002
B2 | 57 | 1 | 0.002
B2 | 573.1C | 5 | 0.011
B2 | 573.2C | 1 | 0.002
B2 | 5746 | 1 | 0.002
B2 | 5752d | 1 | 0.002
B2 | 5777 | 1 | 0.002
B2 | 57A | 1 | 0.002
B2 | 57G | 2 | 0.004
B2 | 5807 | 1 | 0.002
B2 | 5815 | 1 | 0.002
B2 | 5894 | 1 | 0.002
B2 | 5895 | 2 | 0.004
B2 | 5899.2C | 5 | 0.011
B2 | 5899.5C | 1 | 0.002
B2 | 593 | 1 | 0.002
B2 | 59-60d | 1 | 0.002
B2 | 5964 | 1 | 0.002
B2 | 5988A | 1 | 0.002
B2 | 5C | 1 | 0.002
B2 | 60.1T | 2 | 0.004
B2 | 6002 | 1 | 0.002
B2 | 6026C | 2 | 0.004
B2 | 6056 | 16 | 0.034
B2 | 606 | 2 | 0.004
B2 | 61 | 1 | 0.002
B2 | 6182 | 27 | 0.057
B2 | 62 | 1 | 0.002
B2 | 6221 | 2 | 0.004
B2 | 6245 | 1 | 0.002
B2 | 6252 | 1 | 0.002
B2 | 6260 | 5 | 0.011
B2 | 6261 | 1 | 0.002
B2 | 6267 | 1 | 0.002
B2 | 6293 | 2 | 0.004
B2 | 63 | 29 | 0.061
B2 | 6305 | 2 | 0.004
B2 | 632 | 1 | 0.002
B2 | 6345 | 1 | 0.002
B2 | 6351 | 1 | 0.002
B2 | 6353 | 1 | 0.002
B2 | 6395 | 1 | 0.002
B2 | 64 | 37 | 0.078
B2 | 6446 | 1 | 0.002
B2 | 6480 | 2 | 0.004
B2 | 65 | 2 | 0.004
B2 | 6510 | 1 | 0.002
B2 | 6570T | 2 | 0.004
B2 | 6587 | 1 | 0.002
B2 | 66 | 8 | 0.017
B2 | 6663 | 1 | 0.002
B2 | 6680 | 1 | 0.002
B2 | 66T | 5 | 0.011
B2 | 6722 | 1 | 0.002
B2 | 6815 | 1 | 0.002
B2 | 6833 | 1 | 0.002
B2 | 6869 | 1 | 0.002
B2 | 6896 | 2 | 0.004
B2 | 6917 | 1 | 0.002
B2 | 6929C | 1 | 0.002
B2 | 6956 | 1 | 0.002
B2 | 709 | 3 | 0.006
B2 | 7094 | 6 | 0.013
B2 | 71 | 1 | 0.002
B2 | 7109 | 1 | 0.002
B2 | 72 | 1 | 0.002
B2 | 7202 | 1 | 0.002
B2 | 7226 | 1 | 0.002
B2 | 7227T | 1 | 0.002
B2 | 7251 | 1 | 0.002
B2 | 7260 | 1 | 0.002
B2 | 7269 | 1 | 0.002
B2 | 7340 | 1 | 0.002
B2 | 7349 | 1 | 0.002
B2 | 7419 | 1 | 0.002
B2 | 7471d | 1 | 0.002
B2 | 7521 | 1 | 0.002
B2 | 7533 | 2 | 0.004
B2 | 7598 | 1 | 0.002
B2 | 7664 | 1 | 0.002
B2 | 7754 | 2 | 0.004
B2 | 7756 | 9 | 0.019
B2 | 7786 | 13 | 0.027
B2 | 7792A | 9 | 0.019

B2 | 7813 | 3 | 0.006
B2 | 7830 | 2 | 0.004
B2 | 7859 | 2 | 0.004
B2 | 8020 | 2 | 0.004
B2 | 8022 | 1 | 0.002
B2 | 8194 | 1 | 0.002
B2 | 8251 | 1 | 0.002
B2 | 8269-8270d | 2 | 0.004
B2 | 8271d | 1 | 0.002
B2 | 8277 | 2 | 0.004
B2 | 8279 | 2 | 0.004
B2 | 8280C | 2 | 0.004
B2 | 8285d | 1 | 0.002
B2 | 8286 | 1 | 0.002
B2 | 8288 | 1 | 0.002
B2 | 8290 | 2 | 0.004
B2 | 8308d | 1 | 0.002
B2 | 8308T | 28 | 0.059
B2 | 8343 | 1 | 0.002
B2 | 8389 | 2 | 0.004
B2 | 8407 | 2 | 0.004
B2 | 8455 | 1 | 0.002
B2 | 8460 | 1 | 0.002
B2 | 8465 | 1 | 0.002
B2 | 8531 | 2 | 0.004
B2 | 8540 | 1 | 0.002
B2 | 8545 | 1 | 0.002
B2 | 8602 | 1 | 0.002
B2 | 8643 | 1 | 0.002
B2 | 8659 | 2 | 0.004
B2 | 8673 | 3 | 0.006
B2 | 8701 | 1 | 0.002
B2 | 8718 | 1 | 0.002
B2 | 8720C | 1 | 0.002
B2 | 8736 | 2 | 0.004
B2 | 8749 | 1 | 0.002
B2 | 8853 | 54 | 0.114
B2 | 8857 | 1 | 0.002
B2 | 8871 | 1 | 0.002
B2 | 8875 | 1 | 0.002
B2 | 8897 | 2 | 0.004
B2 | 8901 | 1 | 0.002
B2 | 8950 | 6 | 0.013
B2 | 8992 | 1 | 0.002
B2 | 8994 | 2 | 0.004
B2 | 9027 | 1 | 0.002
B2 | 9054 | 2 | 0.004
B2 | 9055 | 1 | 0.002
B2 | 9060 | 1 | 0.002
B2 | 9064 | 1 | 0.002
B2 | 9082 | 1 | 0.002
B2 | 9095 | 6 | 0.013
B2 | 9123 | 1 | 0.002
B2 | 9128 | 1 | 0.002
B2 | 9139 | 3 | 0.006
B2 | 9142 | 1 | 0.002
B2 | 9186 | 2 | 0.004
B2 | 9188C | 2 | 0.004
B2 | 9210 | 2 | 0.004
B2 | 9221T | 1 | 0.002
B2 | 9242 | 1 | 0.002
B2 | 9254 | 2 | 0.004
B2 | 9266 | 1 | 0.002
B2 | 9284 | 1 | 0.002
B2 | 93 | 7 | 0.015
B2 | 9300 | 1 | 0.002
B2 | 9308 | 1 | 0.002
B2 | 9316 | 9 | 0.019
B2 | 9325 | 1 | 0.002
B2 | 94 | 5 | 0.011
B2 | 9438 | 1 | 0.002
B2 | 9452 | 16 | 0.034
B2 | 9478 | 1 | 0.002
B2 | 9497 | 3 | 0.006
B2 | 9531 | 3 | 0.006
B2 | 9545 | 3 | 0.006
B2 | 9554 | 9 | 0.019
B2 | 9632 | 1 | 0.002
B2 | 9682 | 2 | 0.004
B2 | 97 | 5 | 0.011
B2 | 9716 | 1 | 0.002
B2 | 9780 | 2 | 0.004
B2 | 9813 | 3 | 0.006
B2 | 9833 | 1 | 0.002
B2 | 9894C | 1 | 0.002
B2 | 9932 | 1 | 0.002
B2 | 994 | 1 | 0.002
B2 | 9965 | 1 | 0.002
B2 | 9986 | 1 | 0.002
B2 | 9995 | 1 | 0.002
B2+16278 | 10166 | 2 | 0.049
B2+16278 | 103 | 3 | 0.073
B2+16278 | 10420 | 1 | 0.024
B2+16278 | 10914 | 1 | 0.024
B2+16278 | 11009 | 1 | 0.024
B2+16278 | 11071 | 1 | 0.024
B2+16278 | 11218 | 1 | 0.024
B2+16278 | 11611T | 1 | 0.024
B2+16278 | 11807 | 1 | 0.024
B2+16278 | 11893 | 1 | 0.024
B2+16278 | 12061 | 3 | 0.073
B2+16278 | 12633 | 1 | 0.024
B2+16278 | 12678 | 1 | 0.024
B2+16278 | 127 | 1 | 0.024
B2+16278 | 13014 | 1 | 0.024
B2+16278 | 13105 | 1 | 0.024
B2+16278 | 13651 | 1 | 0.024
B2+16278 | 13708 | 2 | 0.049
B2+16278 | 146 | 13 | 0.317
B2+16278 | 152 | 9 | 0.22
B2+16278 | 15218 | 1 | 0.024

B2+16278 | 15450 | 1 | 0.024
B2+16278 | 15671 | 2 | 0.049
B2+16278 | 15734 | 1 | 0.024
B2+16278 | 15924 | 3 | 0.073
B2+16278 | 16092 | 2 | 0.049
B2+16278 | 16124 | 1 | 0.024
B2+16278 | 16126 | 1 | 0.024
B2+16278 | 16129 | 3 | 0.073
B2+16278 | 16136 | 1 | 0.024
B2+16278 | 16168 | 2 | 0.049
B2+16278 | 16176 | 1 | 0.024
B2+16278 | 16186 | 1 | 0.024
B2+16278 | 16193d | 1 | 0.024
B2+16278 | 16218 | 1 | 0.024
B2+16278 | 16234 | 1 | 0.024
B2+16278 | 16241 | 2 | 0.049
B2+16278 | 16256 | 2 | 0.049
B2+16278 | 16260 | 1 | 0.024
B2+16278 | 16261 | 3 | 0.073
B2+16278 | 16266 | 3 | 0.073
B2+16278 | 16270 | 1 | 0.024
B2+16278 | 16270G | 1 | 0.024
B2+16278 | 16274 | 1 | 0.024
B2+16278 | 16291 | 1 | 0.024
B2+16278 | 16295 | 1 | 0.024
B2+16278 | 16319 | 1 | 0.024
B2+16278 | 16324 | 2 | 0.049
B2+16278 | 16354A | 3 | 0.073
B2+16278 | 16360 | 1 | 0.024
B2+16278 | 16362 | 1 | 0.024
B2+16278 | 16390 | 3 | 0.073
B2+16278 | 16391 | 2 | 0.049
B2+16278 | 16456 | 1 | 0.024
B2+16278 | 16503 | 2 | 0.049
B2+16278 | 178 | 1 | 0.024
B2+16278 | 183 | 1 | 0.024
B2+16278 | 200 | 1 | 0.024
B2+16278 | 204 | 2 | 0.049
B2+16278 | 215 | 2 | 0.049
B2+16278 | 2352 | 1 | 0.024
B2+16278 | 2401 | 1 | 0.024
B2+16278 | 2702 | 1 | 0.024
B2+16278 | 289 | 1 | 0.024
B2+16278 | 306-309d | 1 | 0.024
B2+16278 | 307-309d | 1 | 0.024
B2+16278 | 313 | 1 | 0.024
B2+16278 | 3306 | 1 | 0.024
B2+16278 | 3337 | 2 | 0.049
B2+16278 | 3434 | 2 | 0.049
B2+16278 | 3483 | 2 | 0.049
B2+16278 | 3511 | 1 | 0.024
B2+16278 | 3666 | 1 | 0.024
B2+16278 | 4038 | 1 | 0.024
B2+16278 | 4047 | 2 | 0.049
B2+16278 | 4231 | 1 | 0.024
B2+16278 | 4363 | 1 | 0.024
B2+16278 | 455.1T | 2 | 0.049
B2+16278 | 4853 | 1 | 0.024
B2+16278 | 4914 | 1 | 0.024
B2+16278 | 4973G | 1 | 0.024
B2+16278 | 544 | 3 | 0.073
B2+16278 | 55 | 1 | 0.024
B2+16278 | 56 | 1 | 0.024
B2+16278 | 5655 | 1 | 0.024
B2+16278 | 6755 | 5 | 0.122
B2+16278 | 6779 | 1 | 0.024
B2+16278 | 6803 | 2 | 0.049
B2+16278 | 7888 | 1 | 0.024
B2+16278 | 7912 | 1 | 0.024
B2+16278 | 7961 | 3 | 0.073
B2+16278 | 8206 | 2 | 0.049
B2+16278 | 8292 | 2 | 0.049
B2+16278 | 8994 | 1 | 0.024
B2+16278 | 9186 | 1 | 0.024
B2+16278 | 9287 | 1 | 0.024
B2+16278 | 9962 | 1 | 0.024
B2a | 10795 | 1 | 0.014
B2a | 12810 | 1 | 0.014
B2a | 146 | 2 | 0.028
B2a | 16224 | 1 | 0.014
B2a | 16399 | 1 | 0.014
B2a | 16512 | 1 | 0.014
B2a | 5054 | 1 | 0.014
B2a | 8843 | 1 | 0.014
B2a | 9804 | 1 | 0.014
B2a1 | 10256 | 1 | 0.011
B2a1 | 10694 | 1 | 0.011
B2a1 | 10899 | 1 | 0.011
B2a1 | 12850 | 1 | 0.011
B2a1 | 14225 | 1 | 0.011
B2a1 | 15514 | 1 | 0.011
B2a1 | 15884 | 1 | 0.011
B2a1 | 15924 | 1 | 0.011
B2a1 | 16195 | 1 | 0.011
B2a1 | 16274 | 13 | 0.143
B2a1 | 16278 | 1 | 0.011
B2a1 | 16319 | 2 | 0.022
B2a1 | 16357 | 2 | 0.022
B2a1 | 1719 | 1 | 0.011
B2a1 | 186.1C | 2 | 0.022
B2a1 | 302C | 1 | 0.011
B2a1 | 309.3C | 5 | 0.055
B2a1 | 3339 | 1 | 0.011
B2a1 | 3882 | 1 | 0.011
B2a1 | 4732 | 1 | 0.011
B2a1 | 4757 | 1 | 0.011
B2a1 | 5790A | 1 | 0.011
B2a1 | 6179 | 1 | 0.011
B2a1 | 6260 | 1 | 0.011
B2a1 | 709 | 1 | 0.011
B2a1 | 8119 | 1 | 0.011

B2a1 | 8614 | 1 | 0.011
B2a1 | 9077 | 1 | 0.011
B2a1 | 951 | 1 | 0.011
B2a1a | 13350 | 2 | 0.027
B2a1a | 14527 | 1 | 0.013
B2a1a | 16324 | 1 | 0.013
B2a1a | 16400 | 1 | 0.013
B2a1a | 16471 | 1 | 0.013
B2a1a | 183 | 1 | 0.013
B2a1a | 185 | 1 | 0.013
B2a1a | 189 | 1 | 0.013
B2a1a | 204 | 1 | 0.013
B2a1a | 295 | 1 | 0.013
B2a1a | 310 | 1 | 0.013
B2a1a | 8987 | 2 | 0.027
B2a1a1 | 103 | 5 | 0.065
B2a1a1 | 10813 | 1 | 0.013
B2a1a1 | 13135 | 1 | 0.013
B2a1a1 | 146 | 1 | 0.013
B2a1a1 | 152 | 1 | 0.013
B2a1a1 | 16291 | 2 | 0.026
B2a1a1 | 16566 | 1 | 0.013
B2a1a1 | 195 | 2 | 0.026
B2a1a1 | 57A | 1 | 0.013
B2a1a1 | 8275.1A | 1 | 0.013
B2a1b | 11938A | 1 | 0.01
B2a1b | 150 | 9 | 0.094
B2a1b | 15052 | 1 | 0.01
B2a1b | 16124 | 14 | 0.146
B2a1b | 16131 | 1 | 0.01
B2a1b | 16153 | 1 | 0.01
B2a1b | 16300 | 2 | 0.021
B2a1b | 16342 | 9 | 0.094
B2a1b | 186.1C | 3 | 0.031
B2a1b | 207 | 2 | 0.021
B2a1b | 309.3C | 4 | 0.042
B2a1b | 3316 | 1 | 0.01
B2a1b | 396 | 4 | 0.042
B2a1b | 41 | 1 | 0.01
B2a1b | 4320 | 1 | 0.01
B2a1b | 8407 | 2 | 0.021
B2a1b | 9948 | 2 | 0.021
B2a2 | 11884 | 1 | 0.013
B2a2 | 13035 | 5 | 0.067
B2a2 | 16193d | 1 | 0.013
B2a2 | 16311 | 1 | 0.013
B2a2 | 346 | 2 | 0.027
B2a2 | 4180 | 5 | 0.067
B2a2 | 6383 | 2 | 0.027
B2a2 | 64 | 2 | 0.027
B2a2 | 8348 | 5 | 0.067
B2a2 | 9007 | 2 | 0.027
B2a3 | 10586 | 3 | 0.6
B2a3 | 16086 | 2 | 0.4
B2a3 | 16086A | 1 | 0.2
B2a3 | 16180T | 1 | 0.2
B2a3 | 309d | 1 | 0.2
B2a4 | 13434 | 1 | 0.125
B2a4 | 13879A | 1 | 0.125
B2a4 | 14485 | 1 | 0.125
B2a4 | 15394 | 1 | 0.125
B2a4 | 15481 | 1 | 0.125
B2a4 | 15852 | 1 | 0.125
B2a4 | 15884 | 1 | 0.125
B2a4 | 2804 | 1 | 0.125
B2a4 | 3434 | 1 | 0.125
B2a4 | 8292 | 1 | 0.125
B2a4 | 9095 | 1 | 0.125
B2a4a | 1393 | 1 | 0.143
B2a4a | 16051 | 1 | 0.143
B2a4a | 16129 | 1 | 0.143
B2a4a | 16320 | 1 | 0.143
B2a4a | 16527 | 3 | 0.429
B2a4a | 204 | 1 | 0.143
B2a4a | 215 | 1 | 0.143
B2a4a | 8817 | 1 | 0.143
B2a4a | 8957 | 1 | 0.143
B2a4a | 93 | 4 | 0.571
B2a4a | 9770 | 1 | 0.143
B2a4a1 | 11150 | 1 | 0.083
B2a4a1 | 15439G | 1 | 0.083
B2a4a1 | 15499G | 1 | 0.083
B2a4a1 | 16148 | 2 | 0.167
B2a4a1 | 16352 | 3 | 0.25
B2a4a1 | 204 | 1 | 0.083
B2a4a1 | 234 | 1 | 0.083
B2a4a1 | 3915 | 1 | 0.083
B2a4a1 | 6746 | 1 | 0.083
B2a4a1 | 7954 | 3 | 0.25
B2a5 | 11302 | 1 | 0.053
B2a5 | 16086 | 1 | 0.053
B2a5 | 16140 | 1 | 0.053
B2a5 | 16220 | 1 | 0.053
B2a5 | 16260 | 1 | 0.053
B2a5 | 16318 | 1 | 0.053
B2a5 | 16476 | 4 | 0.211
B2a5 | 204 | 1 | 0.053
B2a5 | 234 | 1 | 0.053
B2a5 | 9827 | 1 | 0.053
B2b | 10032 | 1 | 0.004
B2b | 10245 | 1 | 0.004
B2b | 10293 | 1 | 0.004
B2b | 103 | 18 | 0.073
B2b | 10499 | 4 | 0.016
B2b | 10530 | 4 | 0.016
B2b | 10581 | 1 | 0.004
B2b | 10653 | 1 | 0.004
B2b | 10793 | 1 | 0.004
B2b | 10849 | 6 | 0.024
B2b | 10864 | 1 | 0.004
B2b | 10978 | 1 | 0.004

B2b | 11016 | 1 | 0.004
B2b | 11039 | 2 | 0.008
B2b | 11252 | 1 | 0.004
B2b | 11314 | 2 | 0.008
B2b | 11383 | 1 | 0.004
B2b | 114 | 1 | 0.004
B2b | 11536 | 1 | 0.004
B2b | 11653 | 1 | 0.004
B2b | 11677A | 1 | 0.004
B2b | 11807 | 1 | 0.004
B2b | 11839 | 2 | 0.008
B2b | 11866 | 1 | 0.004
B2b | 11962 | 4 | 0.016
B2b | 11963 | 2 | 0.008
B2b | 11977 | 1 | 0.004
B2b | 12061 | 18 | 0.073
B2b | 12127 | 1 | 0.004
B2b | 12141 | 1 | 0.004
B2b | 12193 | 1 | 0.004
B2b | 12217 | 2 | 0.008
B2b | 12309 | 1 | 0.004
B2b | 12414 | 1 | 0.004
B2b | 12474 | 2 | 0.008
B2b | 12618 | 1 | 0.004
B2b | 12715 | 1 | 0.004
B2b | 12720 | 1 | 0.004
B2b | 12810 | 2 | 0.008
B2b | 12972 | 1 | 0.004
B2b | 13017 | 1 | 0.004
B2b | 131 | 2 | 0.008
B2b | 13145 | 1 | 0.004
B2b | 13182 | 2 | 0.008
B2b | 13215 | 1 | 0.004
B2b | 1324 | 1 | 0.004
B2b | 13434 | 1 | 0.004
B2b | 13488 | 1 | 0.004
B2b | 13565 | 11 | 0.045
B2b | 13681 | 1 | 0.004
B2b | 13692 | 2 | 0.008
B2b | 13722 | 1 | 0.004
B2b | 13753A | 1 | 0.004
B2b | 13835 | 1 | 0.004
B2b | 139 | 6 | 0.024
B2b | 14053 | 14 | 0.057
B2b | 14059 | 1 | 0.004
B2b | 14110 | 1 | 0.004
B2b | 14133 | 1 | 0.004
B2b | 14180 | 1 | 0.004
B2b | 14182 | 1 | 0.004
B2b | 14215 | 26 | 0.106
B2b | 14221 | 2 | 0.008
B2b | 143 | 16 | 0.065
B2b | 14417 | 2 | 0.008
B2b | 14428 | 3 | 0.012
B2b | 14560C | 1 | 0.004
B2b | 14590 | 1 | 0.004
B2b | 146 | 13 | 0.053
B2b | 14693 | 1 | 0.004
B2b | 14757 | 7 | 0.028
B2b | 14803 | 1 | 0.004
B2b | 14861 | 1 | 0.004
B2b | 14902 | 1 | 0.004
B2b | 14905 | 1 | 0.004
B2b | 14933 | 1 | 0.004
B2b | 14936d | 1 | 0.004
B2b | 150 | 13 | 0.053
B2b | 15080 | 4 | 0.016
B2b | 15119 | 1 | 0.004
B2b | 15191A | 1 | 0.004
B2b | 15236 | 2 | 0.008
B2b | 15254 | 1 | 0.004
B2b | 15313 | 1 | 0.004
B2b | 15344 | 2 | 0.008
B2b | 15470 | 3 | 0.012
B2b | 15526 | 1 | 0.004
B2b | 15553 | 3 | 0.012
B2b | 15556A | 1 | 0.004
B2b | 15629 | 1 | 0.004
B2b | 15647 | 1 | 0.004
B2b | 15758 | 1 | 0.004
B2b | 15784 | 4 | 0.016
B2b | 15811 | 1 | 0.004
B2b | 15849 | 1 | 0.004
B2b | 15884 | 1 | 0.004
B2b | 15901 | 1 | 0.004
B2b | 15924 | 1 | 0.004
B2b | 15927 | 2 | 0.008
B2b | 15934.1T | 1 | 0.004
B2b | 15948 | 1 | 0.004
B2b | 15954 | 1 | 0.004
B2b | 15968 | 1 | 0.004
B2b | 15994 | 1 | 0.004
B2b | 16000 | 1 | 0.004
B2b | 16078 | 1 | 0.004
B2b | 16086 | 1 | 0.004
B2b | 16093 | 6 | 0.024
B2b | 16096T | 1 | 0.004
B2b | 16114 | 1 | 0.004
B2b | 16124 | 4 | 0.016
B2b | 16126 | 1 | 0.004
B2b | 16129 | 9 | 0.037
B2b | 16134 | 5 | 0.02
B2b | 16148 | 1 | 0.004
B2b | 16153 | 1 | 0.004
B2b | 16157 | 1 | 0.004
B2b | 16168 | 1 | 0.004
B2b | 16169.1C | 1 | 0.004
B2b | 16172 | 2 | 0.008
B2b | 16176 | 4 | 0.016
B2b | 16183T | 1 | 0.004
B2b | 16184 | 1 | 0.004

B2b | 16188 | 1 | 0.004
B2b | 16190 | 1 | 0.004
B2b | 16193d | 2 | 0.008
B2b | 16194C | 6 | 0.024
B2b | 16195 | 5 | 0.02
B2b | 16195d | 1 | 0.004
B2b | 16209 | 2 | 0.008
B2b | 16223 | 1 | 0.004
B2b | 16224 | 1 | 0.004
B2b | 16233 | 1 | 0.004
B2b | 16234 | 10 | 0.041
B2b | 16234G | 3 | 0.012
B2b | 16235 | 4 | 0.016
B2b | 16240 | 1 | 0.004
B2b | 16249 | 2 | 0.008
B2b | 16250 | 2 | 0.008
B2b | 16254 | 1 | 0.004
B2b | 16258 | 2 | 0.008
B2b | 16258C | 1 | 0.004
B2b | 16258T | 4 | 0.016
B2b | 16263A | 1 | 0.004
B2b | 16265 | 2 | 0.008
B2b | 16266 | 26 | 0.106
B2b | 16270 | 4 | 0.016
B2b | 16271 | 1 | 0.004
B2b | 16274 | 3 | 0.012
B2b | 16287 | 1 | 0.004
B2b | 16289 | 3 | 0.012
B2b | 16290 | 1 | 0.004
B2b | 16291 | 1 | 0.004
B2b | 16295 | 1 | 0.004
B2b | 16298 | 1 | 0.004
B2b | 16300 | 3 | 0.012
B2b | 16309 | 3 | 0.012
B2b | 16311 | 3 | 0.012
B2b | 16316 | 2 | 0.008
B2b | 16319 | 9 | 0.037
B2b | 16324 | 1 | 0.004
B2b | 16325 | 5 | 0.02
B2b | 16327 | 1 | 0.004
B2b | 16343 | 4 | 0.016
B2b | 16344 | 2 | 0.008
B2b | 16352 | 2 | 0.008
B2b | 16353 | 1 | 0.004
B2b | 16355 | 1 | 0.004
B2b | 16356 | 3 | 0.012
B2b | 16357 | 5 | 0.02
B2b | 16362 | 16 | 0.065
B2b | 16390 | 7 | 0.028
B2b | 16438 | 1 | 0.004
B2b | 16448 | 1 | 0.004
B2b | 16456 | 1 | 0.004
B2b | 16468 | 10 | 0.041
B2b | 16485 | 7 | 0.028
B2b | 165 | 2 | 0.008
B2b | 16516 | 4 | 0.016
B2b | 16524 | 3 | 0.012
B2b | 16526 | 1 | 0.004
B2b | 16527 | 3 | 0.012
B2b | 173 | 1 | 0.004
B2b | 1734 | 1 | 0.004
B2b | 1735 | 2 | 0.008
B2b | 1766 | 3 | 0.012
B2b | 183 | 11 | 0.045
B2b | 189 | 5 | 0.02
B2b | 195 | 10 | 0.041
B2b | 199 | 5 | 0.02
B2b | 204 | 1 | 0.004
B2b | 2056 | 8 | 0.033
B2b | 2066 | 2 | 0.008
B2b | 210 | 2 | 0.008
B2b | 214 | 5 | 0.02
B2b | 215 | 2 | 0.008
B2b | 217 | 1 | 0.004
B2b | 2224 | 1 | 0.004
B2b | 234 | 1 | 0.004
B2b | 239 | 3 | 0.012
B2b | 240 | 3 | 0.012
B2b | 243 | 1 | 0.004
B2b | 2896 | 1 | 0.004
B2b | 293 | 1 | 0.004
B2b | 307-309d | 1 | 0.004
B2b | 309.3C | 1 | 0.004
B2b | 309d | 1 | 0.004
B2b | 310 | 10 | 0.041
B2b | 310.1T | 1 | 0.004
B2b | 313 | 3 | 0.012
B2b | 314-315d | 5 | 0.02
B2b | 315d | 3 | 0.012
B2b | 317G | 1 | 0.004
B2b | 318 | 5 | 0.02
B2b | 319 | 1 | 0.004
B2b | 3337 | 3 | 0.012
B2b | 3397 | 2 | 0.008
B2b | 3438 | 2 | 0.008
B2b | 3528 | 1 | 0.004
B2b | 3540 | 2 | 0.008
B2b | 3666 | 2 | 0.008
B2b | 3705 | 1 | 0.004
B2b | 3714 | 8 | 0.033
B2b | 3777 | 1 | 0.004
B2b | 3866 | 2 | 0.008
B2b | 3915 | 2 | 0.008
B2b | 4013 | 11 | 0.045
B2b | 4136 | 3 | 0.012
B2b | 4232 | 2 | 0.008
B2b | 4385 | 1 | 0.004
B2b | 480 | 1 | 0.004
B2b | 4853 | 1 | 0.004
B2b | 4886 | 1 | 0.004
B2b | 4917 | 8 | 0.033

B2b | 4943 | 1 | 0.004
B2b | 495 | 1 | 0.004
B2b | 5046 | 1 | 0.004
B2b | 5082 | 1 | 0.004
B2b | 513 | 1 | 0.004
B2b | 522 | 2 | 0.008
B2b | 524 | 1 | 0.004
B2b | 5261 | 1 | 0.004
B2b | 53 | 1 | 0.004
B2b | 536A | 1 | 0.004
B2b | 5394 | 1 | 0.004
B2b | 540 | 1 | 0.004
B2b | 5417 | 10 | 0.041
B2b | 545 | 2 | 0.008
B2b | 5460 | 1 | 0.004
B2b | 5492 | 1 | 0.004
B2b | 55 | 2 | 0.008
B2b | 55.1T | 1 | 0.004
B2b | 5581 | 4 | 0.016
B2b | 56 | 1 | 0.004
B2b | 57 | 2 | 0.008
B2b | 573.1C | 13 | 0.053
B2b | 573.2C | 5 | 0.02
B2b | 573.3C | 6 | 0.024
B2b | 573.4C | 3 | 0.012
B2b | 573.5C | 1 | 0.004
B2b | 573.6C | 1 | 0.004
B2b | 5746 | 10 | 0.041
B2b | 5899.1C | 1 | 0.004
B2b | 59 | 3 | 0.012
B2b | 5964 | 2 | 0.008
B2b | 60 | 4 | 0.016
B2b | 60.1T | 3 | 0.012
B2b | 6205 | 1 | 0.004
B2b | 6261 | 3 | 0.012
B2b | 629 | 1 | 0.004
B2b | 62T | 2 | 0.008
B2b | 63 | 1 | 0.004
B2b | 6305 | 1 | 0.004
B2b | 64 | 2 | 0.008
B2b | 6464 | 1 | 0.004
B2b | 6488 | 2 | 0.008
B2b | 650 | 1 | 0.004
B2b | 6536 | 1 | 0.004
B2b | 6620 | 2 | 0.008
B2b | 6644 | 2 | 0.008
B2b | 6680 | 2 | 0.008
B2b | 6701 | 1 | 0.004
B2b | 6713 | 1 | 0.004
B2b | 6779 | 1 | 0.004
B2b | 6791 | 2 | 0.008
B2b | 709 | 8 | 0.033
B2b | 7142 | 8 | 0.033
B2b | 71d | 1 | 0.004
B2b | 7202 | 1 | 0.004
B2b | 7245 | 1 | 0.004
B2b | 7278 | 1 | 0.004
B2b | 7369G | 10 | 0.041
B2b | 7598 | 1 | 0.004
B2b | 7630 | 1 | 0.004
B2b | 7740 | 2 | 0.008
B2b | 7762 | 3 | 0.012
B2b | 7810 | 1 | 0.004
B2b | 7830 | 1 | 0.004
B2b | 7831 | 1 | 0.004
B2b | 7961 | 19 | 0.077
B2b | 8092 | 2 | 0.008
B2b | 8095 | 2 | 0.008
B2b | 8152 | 1 | 0.004
B2b | 8185 | 1 | 0.004
B2b | 8276.3C | 1 | 0.004
B2b | 8277 | 1 | 0.004
B2b | 8290d | 1 | 0.004
B2b | 8291 | 2 | 0.008
B2b | 8296 | 1 | 0.004
B2b | 8412 | 1 | 0.004
B2b | 8613 | 1 | 0.004
B2b | 8664 | 1 | 0.004
B2b | 8673 | 3 | 0.012
B2b | 8701 | 1 | 0.004
B2b | 8784 | 1 | 0.004
B2b | 8861 | 1 | 0.004
B2b | 8877 | 2 | 0.008
B2b | 8994 | 1 | 0.004
B2b | 9055 | 4 | 0.016
B2b | 9067 | 1 | 0.004
B2b | 9078 | 1 | 0.004
B2b | 9083 | 1 | 0.004
B2b | 9084 | 3 | 0.012
B2b | 9110 | 3 | 0.012
B2b | 9287 | 1 | 0.004
B2b | 93 | 2 | 0.008
B2b | 9319 | 1 | 0.004
B2b | 9377 | 1 | 0.004
B2b | 9425 | 1 | 0.004
B2b | 9541 | 1 | 0.004
B2b | 9695 | 1 | 0.004
B2b | 9938 | 1 | 0.004
B2b+152 | 10095 | 1 | 0.008
B2b+152 | 10169 | 1 | 0.008
B2b+152 | 103 | 23 | 0.174
B2b+152 | 105-110d | 1 | 0.008
B2b+152 | 10685 | 1 | 0.008
B2b+152 | 10873 | 1 | 0.008
B2b+152 | 10961 | 1 | 0.008
B2b+152 | 11084 | 6 | 0.045
B2b+152 | 11204 | 1 | 0.008
B2b+152 | 11314 | 1 | 0.008
B2b+152 | 114 | 1 | 0.008
B2b+152 | 11977 | 1 | 0.008
B2b+152 | 12061 | 2 | 0.015

B2b+152 | 12127 | 1 | 0.008
B2b+152 | 12366 | 1 | 0.008
B2b+152 | 125 | 1 | 0.008
B2b+152 | 12630 | 1 | 0.008
B2b+152 | 127 | 1 | 0.008
B2b+152 | 13543 | 1 | 0.008
B2b+152 | 13788 | 7 | 0.053
B2b+152 | 13928C | 1 | 0.008
B2b+152 | 13932 | 1 | 0.008
B2b+152 | 14041 | 1 | 0.008
B2b+152 | 14053 | 1 | 0.008
B2b+152 | 14163 | 1 | 0.008
B2b+152 | 14182 | 1 | 0.008
B2b+152 | 14215 | 6 | 0.045
B2b+152 | 143 | 4 | 0.03
B2b+152 | 14428 | 1 | 0.008
B2b+152 | 14470 | 8 | 0.061
B2b+152 | 14482 | 1 | 0.008
B2b+152 | 14587 | 1 | 0.008
B2b+152 | 146 | 17 | 0.129
B2b+152 | 151 | 3 | 0.023
B2b+152 | 15115 | 1 | 0.008
B2b+152 | 15236 | 1 | 0.008
B2b+152 | 15299 | 3 | 0.023
B2b+152 | 15758 | 1 | 0.008
B2b+152 | 16051 | 8 | 0.061
B2b+152 | 16086 | 1 | 0.008
B2b+152 | 16092 | 6 | 0.045
B2b+152 | 16093 | 4 | 0.03
B2b+152 | 16094 | 1 | 0.008
B2b+152 | 16104 | 1 | 0.008
B2b+152 | 16129 | 5 | 0.038
B2b+152 | 16131 | 1 | 0.008
B2b+152 | 16137 | 1 | 0.008
B2b+152 | 16140 | 1 | 0.008
B2b+152 | 16142 | 2 | 0.015
B2b+152 | 16150 | 2 | 0.015
B2b+152 | 16153 | 1 | 0.008
B2b+152 | 16167 | 2 | 0.015
B2b+152 | 16168 | 1 | 0.008
B2b+152 | 16169 | 2 | 0.015
B2b+152 | 16176 | 1 | 0.008
B2b+152 | 16188 | 1 | 0.008
B2b+152 | 16192-16193d | 2 | 0.015
B2b+152 | 16193d | 1 | 0.008
B2b+152 | 16234 | 4 | 0.03
B2b+152 | 16241 | 27 | 0.205
B2b+152 | 16241C | 2 | 0.015
B2b+152 | 16255 | 1 | 0.008
B2b+152 | 16258C | 1 | 0.008
B2b+152 | 16263 | 1 | 0.008
B2b+152 | 16266 | 3 | 0.023
B2b+152 | 16269 | 1 | 0.008
B2b+152 | 16274 | 1 | 0.008
B2b+152 | 1627A | 1 | 0.008
B2b+152 | 16282G | 1 | 0.008
B2b+152 | 16283 | 1 | 0.008
B2b+152 | 16287 | 1 | 0.008
B2b+152 | 16289 | 3 | 0.023
B2b+152 | 16291 | 3 | 0.023
B2b+152 | 16292 | 1 | 0.008
B2b+152 | 16295 | 1 | 0.008
B2b+152 | 16316 | 4 | 0.03
B2b+152 | 16319 | 2 | 0.015
B2b+152 | 16325 | 3 | 0.023
B2b+152 | 16342 | 1 | 0.008
B2b+152 | 16360 | 3 | 0.023
B2b+152 | 16362 | 1 | 0.008
B2b+152 | 16390 | 2 | 0.015
B2b+152 | 16456 | 1 | 0.008
B2b+152 | 16483 | 1 | 0.008
B2b+152 | 16506 | 1 | 0.008
B2b+152 | 1750 | 1 | 0.008
B2b+152 | 1797 | 1 | 0.008
B2b+152 | 183 | 1 | 0.008
B2b+152 | 1842 | 1 | 0.008
B2b+152 | 189 | 6 | 0.045
B2b+152 | 195 | 7 | 0.053
B2b+152 | 199 | 1 | 0.008
B2b+152 | 200 | 3 | 0.023
B2b+152 | 204 | 2 | 0.015
B2b+152 | 2056 | 1 | 0.008
B2b+152 | 207 | 2 | 0.015
B2b+152 | 209 | 1 | 0.008
B2b+152 | 211 | 1 | 0.008
B2b+152 | 215 | 3 | 0.023
B2b+152 | 2222 | 1 | 0.008
B2b+152 | 234 | 2 | 0.015
B2b+152 | 2352 | 1 | 0.008
B2b+152 | 252 | 1 | 0.008
B2b+152 | 260 | 1 | 0.008
B2b+152 | 262 | 1 | 0.008
B2b+152 | 308-309d | 1 | 0.008
B2b+152 | 309.3C | 1 | 0.008
B2b+152 | 309d | 1 | 0.008
B2b+152 | 3339 | 6 | 0.045
B2b+152 | 3351 | 1 | 0.008
B2b+152 | 373 | 1 | 0.008
B2b+152 | 4025 | 1 | 0.008
B2b+152 | 4226 | 1 | 0.008
B2b+152 | 4232 | 1 | 0.008
B2b+152 | 455.1T | 2 | 0.015
B2b+152 | 4690 | 1 | 0.008
B2b+152 | 46G | 3 | 0.023
B2b+152 | 4917 | 2 | 0.015
B2b+152 | 500 | 1 | 0.008
B2b+152 | 525.1AC | 1 | 0.008
B2b+152 | 5291 | 1 | 0.008
B2b+152 | 55 | 1 | 0.008
B2b+152 | 559A | 1 | 0.008
B2b+152 | 56 | 1 | 0.008

B2b+152 | 56.1A | 1 | 0.008
B2b+152 | 5656 | 1 | 0.008
B2b+152 | 567C | 1 | 0.008
B2b+152 | 573.1C | 1 | 0.008
B2b+152 | 573.2C | 1 | 0.008
B2b+152 | 5746 | 2 | 0.015
B2b+152 | 57G | 2 | 0.015
B2b+152 | 593 | 1 | 0.008
B2b+152 | 5978 | 1 | 0.008
B2b+152 | 60 | 1 | 0.008
B2b+152 | 6286 | 1 | 0.008
B2b+152 | 63 | 2 | 0.015
B2b+152 | 64 | 3 | 0.023
B2b+152 | 66 | 1 | 0.008
B2b+152 | 66T | 2 | 0.015
B2b+152 | 7043 | 3 | 0.023
B2b+152 | 7082 | 1 | 0.008
B2b+152 | 71.2G | 1 | 0.008
B2b+152 | 7142 | 2 | 0.015
B2b+152 | 71d | 1 | 0.008
B2b+152 | 7257 | 1 | 0.008
B2b+152 | 7369G | 2 | 0.015
B2b+152 | 7498 | 1 | 0.008
B2b+152 | 75 | 1 | 0.008
B2b+152 | 7808 | 1 | 0.008
B2b+152 | 7961 | 2 | 0.015
B2b+152 | 7964 | 1 | 0.008
B2b+152 | 8008 | 3 | 0.023
B2b+152 | 8047 | 1 | 0.008
B2b+152 | 81 | 1 | 0.008
B2b+152 | 8270 | 1 | 0.008
B2b+152 | 8380 | 1 | 0.008
B2b+152 | 8545 | 1 | 0.008
B2b+152 | 8573 | 1 | 0.008
B2b+152 | 8715A | 1 | 0.008
B2b+152 | 8861 | 2 | 0.015
B2b+152 | 8883 | 1 | 0.008
B2b+152 | 9067 | 2 | 0.015
B2b+152 | 9377 | 1 | 0.008
B2b2 | 1007 | 1 | 0.034
B2b2 | 10978 | 2 | 0.069
B2b2 | 125 | 1 | 0.034
B2b2 | 127 | 1 | 0.034
B2b2 | 13254 | 1 | 0.034
B2b2 | 14034 | 1 | 0.034
B2b2 | 143 | 1 | 0.034
B2b2 | 152 | 1 | 0.034
B2b2 | 15784 | 1 | 0.034
B2b2 | 16001T | 1 | 0.034
B2b2 | 16006 | 1 | 0.034
B2b2 | 16007 | 1 | 0.034
B2b2 | 16010 | 1 | 0.034
B2b2 | 16011C | 1 | 0.034
B2b2 | 16129 | 4 | 0.138
B2b2 | 16148 | 1 | 0.034
B2b2 | 16156 | 7 | 0.241
B2b2 | 16157 | 7 | 0.241
B2b2 | 16187 | 1 | 0.034
B2b2 | 16222 | 1 | 0.034
B2b2 | 16278 | 1 | 0.034
B2b2 | 16299 | 1 | 0.034
B2b2 | 16319 | 1 | 0.034
B2b2 | 16323 | 1 | 0.034
B2b2 | 16357 | 1 | 0.034
B2b2 | 16362 | 1 | 0.034
B2b2 | 16381 | 1 | 0.034
B2b2 | 16516 | 1 | 0.034
B2b2 | 195 | 1 | 0.034
B2b2 | 198 | 1 | 0.034
B2b2 | 209 | 1 | 0.034
B2b2 | 309 | 1 | 0.034
B2b2 | 3960 | 1 | 0.034
B2b2 | 4013 | 1 | 0.034
B2b2 | 55 | 1 | 0.034
B2b2 | 60 | 1 | 0.034
B2b2 | 6026 | 1 | 0.034
B2b2 | 63 | 1 | 0.034
B2b2 | 6340 | 1 | 0.034
B2b2 | 64 | 1 | 0.034
B2b2 | 70 | 1 | 0.034
B2b2 | 8152 | 1 | 0.034
B2b2 | 9083 | 1 | 0.034
B2b2a | 12945 | 1 | 0.5
B2b2a | 2222 | 1 | 0.5
B2b2a | 5794 | 1 | 0.5
B2b3 | 131 | 3 | 0.028
B2b3 | 14634 | 3 | 0.028
B2b3 | 14873A | 2 | 0.019
B2b3 | 15106 | 1 | 0.009
B2b3 | 155 | 2 | 0.019
B2b3 | 15784 | 3 | 0.028
B2b3 | 16221A | 2 | 0.019
B2b3 | 16231 | 1 | 0.009
B2b3 | 16241 | 1 | 0.009
B2b3 | 16259 | 1 | 0.009
B2b3 | 16264 | 2 | 0.019
B2b3 | 16497 | 1 | 0.009
B2b3 | 1709 | 1 | 0.009
B2b3 | 195 | 1 | 0.009
B2b3 | 249d | 1 | 0.009
B2b3 | 283 | 1 | 0.009
B2b3 | 4013 | 3 | 0.028
B2b3 | 417 | 1 | 0.009
B2b3 | 54 | 1 | 0.009
B2b3 | 574 | 2 | 0.019
B2b3 | 8251 | 1 | 0.009
B2b3a | 10 | 1 | 0.026
B2b3a | 10604 | 2 | 0.051
B2b3a | 13780 | 1 | 0.026
B2b3a | 143 | 1 | 0.026
B2b3a | 150 | 1 | 0.026

B2b3a | 16092 | 11 | 0.282
B2b3a | 16093 | 1 | 0.026
B2b3a | 16095 | 3 | 0.077
B2b3a | 16166 | 1 | 0.026
B2b3a | 16168 | 5 | 0.128
B2b3a | 16178 | 1 | 0.026
B2b3a | 16179 | 1 | 0.026
B2b3a | 16192-16193d | 2 | 0.051
B2b3a | 16261 | 1 | 0.026
B2b3a | 16344 | 35 | 0.897
B2b3a | 16381 | 1 | 0.026
B2b3a | 1719 | 2 | 0.051
B2b3a | 204 | 11 | 0.282
B2b3a | 309.3C | 2 | 0.051
B2b3a | 310 | 1 | 0.026
B2b3a | 316C | 1 | 0.026
B2b3a | 317.1C | 1 | 0.026
B2b3a | 4012 | 9 | 0.231
B2b3a | 454 | 4 | 0.103
B2b3a | 455 | 7 | 0.179
B2b3a | 455.1T | 2 | 0.051
B2b3a | 455.2T | 3 | 0.077
B2b3a | 459.1C | 1 | 0.026
B2b3a | 460 | 14 | 0.359
B2b3a | 460d | 3 | 0.077
B2b3a | 463.2C | 1 | 0.026
B2b3a | 4695 | 1 | 0.026
B2b3a | 5899.1C | 2 | 0.051
B2b3a | 63 | 2 | 0.051
B2b3a | 64 | 2 | 0.051
B2b3a | 8276.1T | 1 | 0.026
B2b3a | 8552 | 3 | 0.077
B2b3a | 9531 | 1 | 0.026
B2b4 | 139 | 1 | 0.021
B2b4 | 143 | 2 | 0.042
B2b4 | 153 | 1 | 0.021
B2b4 | 16067 | 2 | 0.042
B2b4 | 16093 | 3 | 0.062
B2b4 | 16142 | 1 | 0.021
B2b4 | 16147A | 1 | 0.021
B2b4 | 16150 | 1 | 0.021
B2b4 | 16156 | 3 | 0.062
B2b4 | 16168 | 1 | 0.021
B2b4 | 16188 | 2 | 0.042
B2b4 | 16189 | 24 | 0.5
B2b4 | 16193d | 1 | 0.021
B2b4 | 16256 | 1 | 0.021
B2b4 | 16257 | 2 | 0.042
B2b4 | 16260 | 1 | 0.021
B2b4 | 16261 | 3 | 0.062
B2b4 | 16266 | 1 | 0.021
B2b4 | 16274 | 2 | 0.042
B2b4 | 16301A | 1 | 0.021
B2b4 | 16319 | 1 | 0.021
B2b4 | 16325 | 1 | 0.021
B2b4 | 16352 | 4 | 0.083
B2b4 | 16357 | 4 | 0.083
B2b4 | 16360 | 2 | 0.042
B2b4 | 16437 | 1 | 0.021
B2b4 | 183 | 1 | 0.021
B2b4 | 185 | 1 | 0.021
B2b4 | 214 | 1 | 0.021
B2b4 | 309d | 1 | 0.021
B2b4 | 513 | 1 | 0.021
B2b4 | 523 | 1 | 0.021
B2b4 | 546 | 4 | 0.083
B2b4 | 5899.2C | 1 | 0.021
B2b4 | 59 | 1 | 0.021
B2b4 | 5901.3C | 1 | 0.021
B2b4 | 60.1T | 1 | 0.021
B2b4 | 64 | 1 | 0.021
B2b4 | 93 | 1 | 0.021
B2c | 10646 | 2 | 0.019
B2c | 11253 | 1 | 0.01
B2c | 11930 | 2 | 0.019
B2c | 14178 | 2 | 0.019
B2c | 14693 | 1 | 0.01
B2c | 14842 | 1 | 0.01
B2c | 152 | 1 | 0.01
B2c | 153 | 1 | 0.01
B2c | 16104 | 2 | 0.019
B2c | 16126 | 8 | 0.077
B2c | 16193d | 1 | 0.01
B2c | 16241T | 1 | 0.01
B2c | 16254 | 1 | 0.01
B2c | 16260 | 1 | 0.01
B2c | 16372 | 6 | 0.058
B2c | 183 | 1 | 0.01
B2c | 2414A | 1 | 0.01
B2c | 309.3C | 1 | 0.01
B2c | 4310 | 2 | 0.019
B2c | 7849 | 2 | 0.019
B2c | 8394 | 1 | 0.01
B2c1 | 10978 | 1 | 0.01
B2c1 | 12223 | 1 | 0.01
B2c1 | 12561 | 1 | 0.01
B2c1 | 12732 | 1 | 0.01
B2c1 | 152 | 1 | 0.01
B2c1 | 16109C | 5 | 0.052
B2c1 | 16311 | 1 | 0.01
B2c1 | 16362 | 1 | 0.01
B2c1 | 16559 | 1 | 0.01
B2c1 | 309.3C | 6 | 0.062
B2c1 | 3316 | 1 | 0.01
B2c1 | 4182 | 1 | 0.01
B2c1 | 4227 | 1 | 0.01
B2c1 | 8247 | 1 | 0.01
B2c1 | 94 | 1 | 0.01
B2c1a | 10335 | 1 | 0.009
B2c1a | 115 | 1 | 0.009
B2c1a | 155 | 1 | 0.009

B2c1a | 16173 | 4 | 0.034
B2c1a | 16221A | 2 | 0.017
B2c1a | 16223 | 4 | 0.034
B2c1a | 16231 | 1 | 0.009
B2c1a | 16241 | 1 | 0.009
B2c1a | 16259 | 1 | 0.009
B2c1a | 16264 | 1 | 0.009
B2c1a | 16301 | 4 | 0.034
B2c1a | 16357 | 3 | 0.026
B2c1a | 16463 | 2 | 0.017
B2c1a | 16525 | 4 | 0.034
B2c1a | 195 | 1 | 0.009
B2c1a | 200 | 4 | 0.034
B2c1a | 234 | 1 | 0.009
B2c1a | 235 | 1 | 0.009
B2c1a | 249d | 1 | 0.009
B2c1a | 283 | 1 | 0.009
B2c1a | 306-309d | 2 | 0.017
B2c1a | 309d | 1 | 0.009
B2c1a | 417 | 1 | 0.009
B2c1a | 54 | 1 | 0.009
B2c1a | 573.1C | 2 | 0.017
B2c1a | 574 | 2 | 0.017
B2c1b | 10922 | 1 | 0.011
B2c1b | 15261 | 2 | 0.022
B2c1b | 16218 | 1 | 0.011
B2c1b | 16316 | 2 | 0.022
B2c1b | 4136 | 1 | 0.011
B2c1b | 513 | 1 | 0.011
B2c1b | 57G | 2 | 0.022
B2c1b | 60.1T | 1 | 0.011
B2c1c | 12717 | 1 | 0.011
B2c1c | 14007 | 2 | 0.022
B2c1c | 153 | 4 | 0.043
B2c1c | 16192-16193d | 1 | 0.011
B2c1c | 16214 | 4 | 0.043
B2c1c | 16221 | 2 | 0.022
B2c1c | 16271 | 1 | 0.011
B2c1c | 16293 | 1 | 0.011
B2c1c | 7278 | 2 | 0.022
B2c2 | 103 | 2 | 0.022
B2c2 | 106-111d | 3 | 0.032
B2c2 | 1117 | 2 | 0.022
B2c2 | 11170G | 1 | 0.011
B2c2 | 11914 | 2 | 0.022
B2c2 | 143 | 12 | 0.129
B2c2 | 151 | 2 | 0.022
B2c2 | 152 | 10 | 0.108
B2c2 | 153 | 10 | 0.108
B2c2 | 16086 | 3 | 0.032
B2c2 | 16093 | 3 | 0.032
B2c2 | 16188 | 3 | 0.032
B2c2 | 16223 | 1 | 0.011
B2c2 | 16249 | 1 | 0.011
B2c2 | 16266 | 1 | 0.011
B2c2 | 16294 | 3 | 0.032
B2c2 | 16295 | 5 | 0.054
B2c2 | 16311 | 4 | 0.043
B2c2 | 16336 | 1 | 0.011
B2c2 | 16359 | 2 | 0.022
B2c2 | 16365 | 1 | 0.011
B2c2 | 16465 | 1 | 0.011
B2c2 | 16485 | 1 | 0.011
B2c2 | 16497 | 2 | 0.022
B2c2 | 168 | 9 | 0.097
B2c2 | 195 | 2 | 0.022
B2c2 | 203 | 3 | 0.032
B2c2 | 204 | 1 | 0.011
B2c2 | 215 | 20 | 0.215
B2c2 | 309.3C | 3 | 0.032
B2c2 | 310 | 1 | 0.011
B2c2 | 315.2C | 1 | 0.011
B2c2 | 455.1T | 20 | 0.215
B2c2 | 63 | 13 | 0.14
B2c2 | 6380 | 2 | 0.022
B2c2 | 64 | 17 | 0.183
B2c2 | 66 | 3 | 0.032
B2c2a | 10133 | 1 | 0.071
B2c2a | 125G | 1 | 0.071
B2c2a | 16136 | 2 | 0.143
B2c2a | 16566 | 1 | 0.071
B2c2a | 2672 | 1 | 0.071
B2c2a | 287 | 1 | 0.071
B2c2a | 309.3C | 1 | 0.071
B2c2a | 4440.1GA | 1 | 0.071
B2c2a | 64 | 1 | 0.071
B2c2a | 8542G | 1 | 0.071
B2c2b | 10411 | 1 | 0.077
B2c2b | 16051 | 1 | 0.077
B2c2b | 16126 | 1 | 0.077
B2c2b | 16324 | 1 | 0.077
B2c2b | 16360 | 1 | 0.077
B2c2b | 3243 | 1 | 0.077
B2c2b | 7746 | 1 | 0.077
B2c2b | 8843 | 1 | 0.077
B2c2b | 9761 | 1 | 0.077
B2d | 10398 | 3 | 0.034
B2d | 11075 | 1 | 0.011
B2d | 11225 | 1 | 0.011
B2d | 11884 | 1 | 0.011
B2d | 11914 | 3 | 0.034
B2d | 11986 | 2 | 0.022
B2d | 12738 | 1 | 0.011
B2d | 13038 | 1 | 0.011
B2d | 13928C | 1 | 0.011
B2d | 14287G | 1 | 0.011
B2d | 143 | 2 | 0.022
B2d | 150 | 2 | 0.022
B2d | 152 | 2 | 0.022
B2d | 15208 | 1 | 0.011
B2d | 16129 | 1 | 0.011

B2d | 16179 | 2 | 0.022
B2d | 16186 | 1 | 0.011
B2d | 16192-16193d | 2 | 0.022
B2d | 16193d | 4 | 0.045
B2d | 16216 | 1 | 0.011
B2d | 16218 | 1 | 0.011
B2d | 16242 | 1 | 0.011
B2d | 16256 | 1 | 0.011
B2d | 16324 | 5 | 0.056
B2d | 16325 | 1 | 0.011
B2d | 16335 | 1 | 0.011
B2d | 16360 | 1 | 0.011
B2d | 1719 | 2 | 0.022
B2d | 180 | 1 | 0.011
B2d | 195 | 1 | 0.011
B2d | 2056 | 6 | 0.067
B2d | 310 | 2 | 0.022
B2d | 315d | 1 | 0.011
B2d | 3786 | 2 | 0.022
B2d | 3796 | 1 | 0.011
B2d | 4113 | 1 | 0.011
B2d | 7269 | 6 | 0.067
B2d | 8270.1T | 1 | 0.011
B2d | 8485 | 2 | 0.022
B2d | 8659 | 1 | 0.011
B2d | 9548 | 2 | 0.022
B2d | 9725 | 3 | 0.034
B2e | 10101 | 1 | 0.008
B2e | 10644 | 1 | 0.008
B2e | 10646 | 2 | 0.016
B2e | 11419 | 1 | 0.008
B2e | 14308 | 1 | 0.008
B2e | 152 | 2 | 0.016
B2e | 16093 | 1 | 0.008
B2e | 16114A | 1 | 0.008
B2e | 16172 | 2 | 0.016
B2e | 16173 | 2 | 0.016
B2e | 16188 | 3 | 0.023
B2e | 16274 | 2 | 0.016
B2e | 16304 | 1 | 0.008
B2e | 16362 | 1 | 0.008
B2e | 16378 | 1 | 0.008
B2e | 16380 | 1 | 0.008
B2e | 16437 | 1 | 0.008
B2e | 1901A | 1 | 0.008
B2e | 194 | 26 | 0.202
B2e | 199 | 32 | 0.248
B2e | 207 | 2 | 0.016
B2e | 225 | 1 | 0.008
B2e | 226 | 1 | 0.008
B2e | 310 | 1 | 0.008
B2e | 438.1C | 1 | 0.008
B2e | 497A | 2 | 0.016
B2e | 55 | 1 | 0.008
B2e | 56T | 2 | 0.016
B2e | 573.5C | 1 | 0.008
B2e | 7403 | 1 | 0.008
B2e | 7444 | 2 | 0.016
B2e | 8272G | 1 | 0.008
B2e | 9182 | 1 | 0.008
B2e | 9380 | 1 | 0.008
B2e | 982 | 2 | 0.016
B2e | 986 | 2 | 0.016
B2e | 9881 | 1 | 0.008
B2e | 9899 | 2 | 0.016
B2f | 16093 | 6 | 0.061
B2f | 16179 | 6 | 0.061
B2f | 16295 | 6 | 0.061
B2f | 16398 | 1 | 0.01
B2f | 178 | 6 | 0.061
B2f | 196 | 6 | 0.061
B2f | 309.3C | 1 | 0.01
B2f | 709 | 1 | 0.01
B2f | 7129 | 1 | 0.01
B2f | 7202 | 1 | 0.01
B2f | 9212 | 3 | 0.031
B2g | 16147 | 1 | 0.023
B2g | 16172 | 2 | 0.045
B2g | 16205 | 1 | 0.023
B2g | 16391 | 2 | 0.045
B2g | 16478 | 1 | 0.023
B2g1 | 10598 | 5 | 0.333
B2g1 | 10792 | 1 | 0.067
B2g1 | 10882 | 2 | 0.133
B2g1 | 11308 | 1 | 0.067
B2g1 | 12280 | 1 | 0.067
B2g1 | 12535 | 2 | 0.133
B2g1 | 13135 | 1 | 0.067
B2g1 | 13710C | 1 | 0.067
B2g1 | 13905 | 1 | 0.067
B2g1 | 14251C | 2 | 0.133
B2g1 | 14296 | 1 | 0.067
B2g1 | 146 | 2 | 0.133
B2g1 | 15055 | 1 | 0.067
B2g1 | 152 | 4 | 0.267
B2g1 | 15221 | 1 | 0.067
B2g1 | 15616 | 2 | 0.133
B2g1 | 16051 | 2 | 0.133
B2g1 | 16194C | 1 | 0.067
B2g1 | 16195 | 1 | 0.067
B2g1 | 16278 | 2 | 0.133
B2g1 | 16353 | 1 | 0.067
B2g1 | 16362 | 1 | 0.067
B2g1 | 16391 | 1 | 0.067
B2g1 | 1709 | 2 | 0.133
B2g1 | 1811 | 1 | 0.067
B2g1 | 207 | 2 | 0.133
B2g1 | 309d | 2 | 0.133
B2g1 | 3892 | 1 | 0.067
B2g1 | 4096 | 2 | 0.133
B2g1 | 4167 | 2 | 0.133

B2g1 | 567C | 2 | 0.133
B2g1 | 573.2C | 1 | 0.067
B2g1 | 61 | 1 | 0.067
B2g1 | 62C | 6 | 0.4
B2g1 | 64 | 6 | 0.4
B2g1 | 66T | 5 | 0.333
B2g1 | 6935 | 1 | 0.067
B2g1 | 709 | 1 | 0.067
B2g1 | 7158 | 2 | 0.133
B2g1 | 7962 | 2 | 0.133
B2g1 | 8460 | 2 | 0.133
B2g1 | 8994 | 2 | 0.133
B2g1 | 9139 | 1 | 0.067
B2g1 | 9377 | 1 | 0.067
B2g1 | 960.1C | 2 | 0.133
B2g2 | 11623 | 1 | 0.022
B2g2 | 14470 | 1 | 0.022
B2g2 | 150 | 1 | 0.022
B2g2 | 15994 | 1 | 0.022
B2g2 | 16148 | 2 | 0.044
B2g2 | 279 | 1 | 0.022
B2g2 | 4937 | 1 | 0.022
B2g2 | 6040 | 2 | 0.044
B2g2 | 9 | 1 | 0.022
B2g2 | 94 | 1 | 0.022
B2h | 14094 | 3 | 0.107
B2h | 14410 | 3 | 0.107
B2h | 153 | 1 | 0.036
B2h | 16168 | 1 | 0.036
B2h | 16192 | 1 | 0.036
B2h | 16193d | 1 | 0.036
B2h | 310.1T | 2 | 0.071
B2h | 311 | 1 | 0.036
B2h | 573.1C | 2 | 0.071
B2h | 7626 | 1 | 0.036
B2i | 127 | 1 | 0.008
B2i | 151 | 2 | 0.016
B2i | 16142 | 2 | 0.016
B2i | 16146 | 2 | 0.016
B2i | 16178 | 16 | 0.126
B2i | 16218 | 3 | 0.024
B2i | 16290 | 1 | 0.008
B2i | 16316 | 3 | 0.024
B2i | 16381 | 16 | 0.126
B2i | 293 | 2 | 0.016
B2i | 42.1G | 2 | 0.016
B2i | 513 | 3 | 0.024
B2i | 57G | 3 | 0.024
B2i | 60.1T | 3 | 0.024
B2i | 71d | 2 | 0.016
B2i1 | 10890T | 1 | 0.033
B2i1 | 10954 | 1 | 0.033
B2i1 | 10976 | 1 | 0.033
B2i1 | 15914C | 1 | 0.033
B2i1 | 16150 | 1 | 0.033
B2i1 | 16223 | 1 | 0.033
B2i1 | 16234 | 8 | 0.267
B2i1 | 16256 | 3 | 0.1
B2i1 | 16320 | 2 | 0.067
B2i1 | 16361 | 3 | 0.1
B2i1 | 16362 | 5 | 0.167
B2i1 | 16390 | 3 | 0.1
B2i1 | 195 | 1 | 0.033
B2i1 | 316C | 1 | 0.033
B2i1 | 575-576d | 1 | 0.033
B2i1 | 8736 | 1 | 0.033
B2i2 | 16051 | 2 | 0.038
B2i2 | 16181 | 2 | 0.038
B2i2 | 16213 | 5 | 0.096
B2i2 | 16234 | 1 | 0.019
B2i2 | 16249 | 5 | 0.096
B2i2a | 146 | 2 | 0.4
B2i2a | 14927 | 2 | 0.4
B2i2a | 16278 | 2 | 0.4
B2i2a | 6284 | 1 | 0.2
B2i2a | 7650 | 1 | 0.2
B2i2a1 | 11632 | 1 | 0.111
B2i2a1 | 1368 | 1 | 0.111
B2i2a1 | 16075 | 6 | 0.667
B2i2a1 | 16319 | 1 | 0.111
B2i2a1 | 195 | 6 | 0.667
B2i2a1 | 504 | 1 | 0.111
B2i2a1 | 6599 | 1 | 0.111
B2i2a1 | 7364 | 1 | 0.111
B2i2a1a | 14356 | 1 | 0.091
B2i2a1a | 16075 | 2 | 0.182
B2i2a1a | 16086 | 1 | 0.091
B2i2a1a | 16296 | 5 | 0.455
B2i2a1a | 16320 | 1 | 0.091
B2i2a1a | 195 | 1 | 0.091
B2i2a1a | 4659 | 1 | 0.091
B2i2a1a | 9162 | 1 | 0.091
B2i2a1b | 10292 | 1 | 0.111
B2i2a1b | 13962 | 1 | 0.111
B2i2a1b | 16075 | 6 | 0.667
B2i2a1b | 16325 | 1 | 0.111
B2i2a1b | 195 | 6 | 0.667
B2i2a1b | 3866 | 1 | 0.111
B2i2b | 10101 | 9 | 0.127
B2i2b | 10751 | 2 | 0.028
B2i2b | 11002 | 9 | 0.127
B2i2b | 12406 | 2 | 0.028
B2i2b | 14569 | 2 | 0.028
B2i2b | 152 | 3 | 0.042
B2i2b | 15778 | 2 | 0.028
B2i2b | 16075A | 2 | 0.028
B2i2b | 16077 | 2 | 0.028
B2i2b | 16154 | 3 | 0.042
B2i2b | 16167 | 4 | 0.056
B2i2b | 16218 | 2 | 0.028
B2i2b | 16233 | 1 | 0.014

B2i2b | 16274 | 1 | 0.014
B2i2b | 16291 | 2 | 0.028
B2i2b | 16294 | 2 | 0.028
B2i2b | 16311 | 2 | 0.028
B2i2b | 16316 | 9 | 0.127
B2i2b | 16390 | 2 | 0.028
B2i2b | 16456 | 1 | 0.014
B2i2b | 16465 | 1 | 0.014
B2i2b | 165 | 9 | 0.127
B2i2b | 195 | 1 | 0.014
B2i2b | 204 | 3 | 0.042
B2i2b | 211 | 1 | 0.014
B2i2b | 316 | 1 | 0.014
B2i2b | 335 | 1 | 0.014
B2i2b | 55 | 1 | 0.014
B2i2b | 56 | 1 | 0.014
B2i2b | 8668 | 2 | 0.028
B2i2b | 9300 | 2 | 0.028
B2i2b | 9629 | 1 | 0.014
B2i2b1 | 11465 | 1 | 0.091
B2i2b1 | 12756 | 1 | 0.091
B2i2b1 | 146 | 1 | 0.091
B2i2b1 | 16136 | 6 | 0.545
B2i2b1 | 16289 | 1 | 0.091
B2i2b1 | 16391 | 1 | 0.091
B2i2b1 | 195 | 1 | 0.091
B2i2b1 | 204 | 1 | 0.091
B2i2b1 | 574 | 1 | 0.091
B2j | 10754 | 1 | 0.167
B2j | 12295 | 1 | 0.167
B2j | 12950 | 1 | 0.167
B2j | 16156 | 2 | 0.333
B2j | 16167 | 2 | 0.333
B2j | 16283 | 1 | 0.167
B2j | 291.1A | 1 | 0.167
B2j | 46 | 2 | 0.333
B2k | 10076 | 1 | 0.014
B2k | 10166 | 8 | 0.11
B2k | 10637 | 3 | 0.041
B2k | 10876 | 1 | 0.014
B2k | 11150 | 1 | 0.014
B2k | 12657 | 1 | 0.014
B2k | 12861 | 1 | 0.014
B2k | 13708 | 1 | 0.014
B2k | 13812 | 1 | 0.014
B2k | 13965 | 3 | 0.041
B2k | 140 | 1 | 0.014
B2k | 14059 | 1 | 0.014
B2k | 14148 | 1 | 0.014
B2k | 14182 | 3 | 0.041
B2k | 143 | 4 | 0.055
B2k | 14788 | 1 | 0.014
B2k | 150 | 3 | 0.041
B2k | 15671 | 8 | 0.11
B2k | 15777 | 1 | 0.014
B2k | 16178 | 2 | 0.027
B2k | 16195G | 1 | 0.014
B2k | 16240 | 4 | 0.055
B2k | 16242 | 3 | 0.041
B2k | 16278 | 2 | 0.027
B2k | 16286 | 2 | 0.027
B2k | 16289 | 2 | 0.027
B2k | 16301 | 1 | 0.014
B2k | 16324 | 3 | 0.041
B2k | 16344 | 1 | 0.014
B2k | 16357 | 1 | 0.014
B2k | 16362 | 1 | 0.014
B2k | 1673 | 1 | 0.014
B2k | 1763 | 3 | 0.041
B2k | 199 | 2 | 0.027
B2k | 200 | 1 | 0.014
B2k | 203 | 1 | 0.014
B2k | 207 | 1 | 0.014
B2k | 2124 | 1 | 0.014
B2k | 215 | 12 | 0.164
B2k | 228 | 1 | 0.014
B2k | 2280A | 1 | 0.014
B2k | 2356 | 2 | 0.027
B2k | 309.3C | 1 | 0.014
B2k | 310 | 1 | 0.014
B2k | 3321 | 3 | 0.041
B2k | 3392C | 1 | 0.014
B2k | 3434 | 1 | 0.014
B2k | 3463 | 1 | 0.014
B2k | 3483 | 8 | 0.11
B2k | 3591 | 3 | 0.041
B2k | 3786 | 1 | 0.014
B2k | 4136 | 1 | 0.014
B2k | 4245 | 1 | 0.014
B2k | 454 | 1 | 0.014
B2k | 455.1T | 7 | 0.096
B2k | 5237 | 3 | 0.041
B2k | 5450 | 3 | 0.041
B2k | 546 | 1 | 0.014
B2k | 5460 | 3 | 0.041
B2k | 5655 | 1 | 0.014
B2k | 5888.1T | 1 | 0.014
B2k | 5981 | 1 | 0.014
B2k | 6284 | 1 | 0.014
B2k | 63 | 3 | 0.041
B2k | 64 | 3 | 0.041
B2k | 64d | 1 | 0.014
B2k | 8170 | 1 | 0.014
B2k | 8435 | 1 | 0.014
B2k | 8697 | 1 | 0.014
B2k | 8718 | 1 | 0.014
B2k | 8841 | 1 | 0.014
B2k | 8933 | 1 | 0.014
B2k | 954 | 5 | 0.068
B2k | 9995 | 1 | 0.014
B2l | 10700 | 1 | 0.042

B2l | 11914 | 1 | 0.042
B2l | 12490 | 2 | 0.083
B2l | 13200T | 2 | 0.083
B2l | 14221 | 2 | 0.083
B2l | 146 | 1 | 0.042
B2l | 1462 | 1 | 0.042
B2l | 15301 | 1 | 0.042
B2l | 15344 | 2 | 0.083
B2l | 159 | 1 | 0.042
B2l | 16051 | 11 | 0.458
B2l | 16085G | 1 | 0.042
B2l | 16102 | 7 | 0.292
B2l | 16111 | 1 | 0.042
B2l | 16126 | 1 | 0.042
B2l | 16129 | 1 | 0.042
B2l | 16176 | 3 | 0.125
B2l | 16195d | 1 | 0.042
B2l | 16257 | 1 | 0.042
B2l | 16258C | 1 | 0.042
B2l | 16263 | 1 | 0.042
B2l | 16280 | 1 | 0.042
B2l | 16288 | 2 | 0.083
B2l | 16302 | 1 | 0.042
B2l | 16311 | 2 | 0.083
B2l | 16319 | 3 | 0.125
B2l | 16438 | 9 | 0.375
B2l | 16465 | 1 | 0.042
B2l | 195 | 2 | 0.083
B2l | 207 | 2 | 0.083
B2l | 210 | 1 | 0.042
B2l | 2220 | 1 | 0.042
B2l | 226 | 1 | 0.042
B2l | 293 | 3 | 0.125
B2l | 309d | 2 | 0.083
B2l | 3426 | 1 | 0.042
B2l | 3721 | 2 | 0.083
B2l | 480 | 1 | 0.042
B2l | 513 | 2 | 0.083
B2l | 5147 | 1 | 0.042
B2l | 5580 | 2 | 0.083
B2l | 592 | 1 | 0.042
B2l | 6755 | 2 | 0.083
B2l | 6791 | 2 | 0.083
B2l | 7238 | 2 | 0.083
B2l | 7424 | 1 | 0.042
B2l | 9938 | 2 | 0.083
B2m | 12135A | 1 | 0.2
B2m | 12544 | 1 | 0.2
B2m | 15805 | 1 | 0.2
B2m | 16212 | 1 | 0.2
B2m | 16304 | 1 | 0.2
B2m | 16325 | 2 | 0.4
B2m | 16520 | 1 | 0.2
B2m | 199 | 1 | 0.2
B2m | 207 | 1 | 0.2
B2m | 2707 | 1 | 0.2
B2m | 310 | 1 | 0.2
B2m | 430 | 1 | 0.2
B2m | 489 | 1 | 0.2
B2m | 5585 | 1 | 0.2
B2m | 8865 | 1 | 0.2
B2n | 11465 | 1 | 0.009
B2n | 131 | 2 | 0.017
B2n | 143 | 3 | 0.026
B2n | 150 | 5 | 0.043
B2n | 152 | 1 | 0.009
B2n | 16093 | 2 | 0.017
B2n | 16148 | 7 | 0.06
B2n | 16157 | 6 | 0.052
B2n | 16167 | 1 | 0.009
B2n | 16185 | 26 | 0.224
B2n | 16193d | 28 | 0.241
B2n | 16240 | 3 | 0.026
B2n | 16304 | 1 | 0.009
B2n | 16317T | 5 | 0.043
B2n | 16320 | 2 | 0.017
B2n | 16343 | 2 | 0.017
B2n | 16355 | 1 | 0.009
B2n | 16400 | 2 | 0.017
B2n | 16437 | 2 | 0.017
B2n | 182 | 3 | 0.026
B2n | 210 | 3 | 0.026
B2n | 214 | 2 | 0.017
B2n | 227 | 2 | 0.017
B2n | 3504 | 1 | 0.009
B2n | 42 | 1 | 0.009
B2n | 57 | 1 | 0.009
B2n | 573.2C | 1 | 0.009
B2n | 573.3C | 3 | 0.026
B2n | 573.4C | 1 | 0.009
B2n | 58 | 1 | 0.009
B2n | 60.1T | 1 | 0.009
B2n | 8020 | 1 | 0.009
B2n | 9254 | 1 | 0.009
B2o | 10601 | 1 | 0.014
B2o | 10849 | 4 | 0.055
B2o | 10914 | 4 | 0.055
B2o | 1187 | 1 | 0.014
B2o | 11963 | 1 | 0.014
B2o | 11992 | 1 | 0.014
B2o | 12007 | 1 | 0.014
B2o | 12136 | 4 | 0.055
B2o | 12630 | 1 | 0.014
B2o | 12880 | 1 | 0.014
B2o | 12996C | 1 | 0.014
B2o | 13083 | 1 | 0.014
B2o | 13477 | 1 | 0.014
B2o | 13651 | 6 | 0.082
B2o | 13708 | 1 | 0.014
B2o | 1375 | 1 | 0.014
B2o | 13782A | 2 | 0.027

B2o | 13879A | 3 | 0.041
B2o | 139 | 10 | 0.137
B2o | 14053 | 2 | 0.027
B2o | 14094 | 1 | 0.014
B2o | 14272G | 1 | 0.014
B2o | 146 | 12 | 0.164
B2o | 150 | 8 | 0.11
B2o | 152 | 10 | 0.137
B2o | 15314 | 1 | 0.014
B2o | 15631 | 1 | 0.014
B2o | 159 | 6 | 0.082
B2o | 16037T | 1 | 0.014
B2o | 16038d | 1 | 0.014
B2o | 16042.1T | 1 | 0.014
B2o | 16051 | 3 | 0.041
B2o | 16072A | 1 | 0.014
B2o | 16073 | 1 | 0.014
B2o | 16073A | 1 | 0.014
B2o | 16095 | 1 | 0.014
B2o | 16104 | 6 | 0.082
B2o | 16111G | 1 | 0.014
B2o | 16126 | 1 | 0.014
B2o | 16129 | 2 | 0.027
B2o | 16142 | 1 | 0.014
B2o | 16148 | 1 | 0.014
B2o | 16157 | 1 | 0.014
B2o | 16167 | 1 | 0.014
B2o | 16185 | 4 | 0.055
B2o | 16193d | 5 | 0.068
B2o | 16214 | 1 | 0.014
B2o | 16223 | 1 | 0.014
B2o | 16230 | 1 | 0.014
B2o | 16249 | 1 | 0.014
B2o | 16257 | 2 | 0.027
B2o | 16261 | 6 | 0.082
B2o | 16274 | 14 | 0.192
B2o | 16289 | 14 | 0.192
B2o | 16295 | 4 | 0.055
B2o | 16296 | 2 | 0.027
B2o | 16301 | 14 | 0.192
B2o | 16311 | 3 | 0.041
B2o | 16335 | 2 | 0.027
B2o | 16348 | 2 | 0.027
B2o | 16355A | 1 | 0.014
B2o | 16362 | 5 | 0.068
B2o | 16381 | 3 | 0.041
B2o | 16411A | 1 | 0.014
B2o | 16414 | 1 | 0.014
B2o | 16414d | 1 | 0.014
B2o | 16416 | 1 | 0.014
B2o | 16416T | 1 | 0.014
B2o | 16417 | 1 | 0.014
B2o | 16468 | 2 | 0.027
B2o | 16483 | 1 | 0.014
B2o | 16512 | 1 | 0.014
B2o | 183 | 3 | 0.041
B2o | 1949 | 1 | 0.014
B2o | 207 | 1 | 0.014
B2o | 228 | 1 | 0.014
B2o | 234 | 2 | 0.027
B2o | 244 | 3 | 0.041
B2o | 2755 | 1 | 0.014
B2o | 2804 | 4 | 0.055
B2o | 296 | 1 | 0.014
B2o | 309d | 3 | 0.041
B2o | 310 | 3 | 0.041
B2o | 313 | 1 | 0.014
B2o | 315.4C | 1 | 0.014
B2o | 315d | 1 | 0.014
B2o | 3553 | 2 | 0.027
B2o | 374 | 1 | 0.014
B2o | 3826 | 1 | 0.014
B2o | 3834 | 1 | 0.014
B2o | 4375 | 1 | 0.014
B2o | 4512 | 2 | 0.027
B2o | 455.2T | 1 | 0.014
B2o | 460d | 1 | 0.014
B2o | 4824 | 1 | 0.014
B2o | 4952 | 2 | 0.027
B2o | 5153 | 2 | 0.027
B2o | 5746 | 1 | 0.014
B2o | 5954C | 2 | 0.027
B2o | 6056 | 1 | 0.014
B2o | 64 | 2 | 0.027
B2o | 6485 | 1 | 0.014
B2o | 6647C | 4 | 0.055
B2o | 6755 | 8 | 0.11
B2o | 6899 | 1 | 0.014
B2o | 71 | 2 | 0.027
B2o | 71d | 1 | 0.014
B2o | 7269 | 2 | 0.027
B2o | 7369G | 1 | 0.014
B2o | 7493 | 1 | 0.014
B2o | 7674 | 1 | 0.014
B2o | 7705 | 1 | 0.014
B2o | 8292 | 3 | 0.041
B2o | 8381 | 1 | 0.014
B2o | 8387 | 2 | 0.027
B2o | 8531 | 1 | 0.014
B2o | 8736 | 1 | 0.014
B2o | 8812 | 2 | 0.027
B2o | 9055 | 2 | 0.027
B2o | 9214C | 1 | 0.014
B2o | 9548C | 1 | 0.014
B2o | 9559G | 1 | 0.014
B2o1 | 146 | 1 | 0.167
B2o1 | 14999 | 1 | 0.167
B2o1 | 15080 | 2 | 0.333
B2o1 | 15978 | 2 | 0.333
B2o1 | 16017 | 2 | 0.333
B2o1 | 16157 | 3 | 0.5

B2o1 | 16274 | 3 | 0.5
B2o1 | 16294 | 3 | 0.5
B2o1 | 199 | 1 | 0.167
B2o1 | 2223 | 1 | 0.167
B2o1 | 3394 | 2 | 0.333
B2o1 | 514 | 3 | 0.5
B2o1 | 62.1C | 1 | 0.167
B2o1 | 6278 | 2 | 0.333
B2o1 | 737 | 2 | 0.333
B2o1 | 765G | 1 | 0.167
B2o1a | 14066 | 1 | 0.048
B2o1a | 150 | 2 | 0.095
B2o1a | 16218 | 1 | 0.048
B2o1a | 16271 | 3 | 0.143
B2o1a | 204 | 1 | 0.048
B2o1a | 4025 | 1 | 0.048
B2o1a | 4232 | 1 | 0.048
B2o1a | 8573 | 1 | 0.048
B2o1a | 8856 | 1 | 0.048
B2o1a | 8939 | 1 | 0.048
B2p | 12599 | 1 | 0.011
B2p | 15684 | 1 | 0.011
B2p | 15937 | 1 | 0.011
B2p | 16186 | 3 | 0.033
B2p | 16218 | 1 | 0.011
B2p | 16316 | 2 | 0.022
B2p | 16360 | 1 | 0.011
B2p | 16457.1G | 1 | 0.011
B2p | 513 | 1 | 0.011
B2p | 57G | 2 | 0.022
B2p | 60.1T | 1 | 0.011
B2p | 6261 | 1 | 0.011
B2p | 9695 | 1 | 0.011
B2q | 14605 | 1 | 0.008
B2q | 152 | 15 | 0.126
B2q | 15400 | 1 | 0.008
B2q | 15670 | 3 | 0.025
B2q | 15900 | 5 | 0.042
B2q | 15989 | 1 | 0.008
B2q | 16035 | 1 | 0.008
B2q | 16051 | 23 | 0.193
B2q | 16086 | 2 | 0.017
B2q | 16126 | 1 | 0.008
B2q | 16129 | 1 | 0.008
B2q | 16190 | 6 | 0.05
B2q | 16249 | 5 | 0.042
B2q | 16270A | 1 | 0.008
B2q | 16293C | 1 | 0.008
B2q | 16360 | 19 | 0.16
B2q | 16429 | 1 | 0.008
B2q | 214 | 1 | 0.008
B2q | 226 | 1 | 0.008
B2q | 228 | 2 | 0.017
B2q | 279 | 5 | 0.042
B2q | 315.2C | 1 | 0.008
B2q | 44 | 1 | 0.008
B2q | 4994 | 1 | 0.008
B2q | 5051 | 17 | 0.143
B2q | 547 | 1 | 0.008
B2q | 573.1C | 1 | 0.008
B2q | 6205 | 1 | 0.008
B2q | 6305C | 1 | 0.008
B2q | 7786 | 17 | 0.143
B2q | 8046 | 1 | 0.008
B2q | 8323 | 1 | 0.008
B2q | 8950 | 1 | 0.008
B2r | 10915 | 1 | 0.011
B2r | 14178 | 1 | 0.011
B2r | 14974G | 1 | 0.011
B2r | 152 | 3 | 0.032
B2r | 15650 | 1 | 0.011
B2r | 16218 | 1 | 0.011
B2r | 16316 | 2 | 0.022
B2r | 1664 | 3 | 0.032
B2r | 1888 | 3 | 0.032
B2r | 2010 | 2 | 0.022
B2r | 3338 | 1 | 0.011
B2r | 513 | 1 | 0.011
B2r | 57G | 2 | 0.022
B2r | 60.1T | 1 | 0.011
B2r | 6734 | 1 | 0.011
B2r | 7055 | 1 | 0.011
B2r | 7403 | 1 | 0.011
B2r | 8276.1TCC | 1 | 0.011
B2r | 9545 | 1 | 0.011
B2r | 9615 | 2 | 0.022
B2r | 9932 | 1 | 0.011
B2s | 11731 | 1 | 0.125
B2s | 16111 | 1 | 0.125
B2s | 16126 | 2 | 0.25
B2s | 313-315d | 1 | 0.125
B2s | 314-315d | 2 | 0.25
B2s | 419 | 1 | 0.125
B2s | 93 | 1 | 0.125
B2s | 930 | 1 | 0.125
B2t | 11380 | 2 | 0.154
B2t | 16095 | 2 | 0.154
B2t | 16129 | 1 | 0.077
B2t | 16194C | 1 | 0.077
B2t | 16195 | 1 | 0.077
B2t | 16468 | 2 | 0.154
B2t | 308-309d | 1 | 0.077
B2t | 310 | 4 | 0.308
B2t | 315d | 1 | 0.077
B2t | 316C | 1 | 0.077
B2t | 319 | 1 | 0.077
B2t | 4973 | 1 | 0.077
B2t | 5786 | 1 | 0.077
B2t | 596 | 3 | 0.231
B2t | 8772 | 1 | 0.077
B2u | 12397 | 1 | 0.2

B2u | 14461 | 1 | 0.2
B2u | 16048 | 1 | 0.2
B2u | 16093 | 1 | 0.2
B2u | 16311 | 1 | 0.2
B2u | 8113 | 1 | 0.2
B2u | 94 | 1 | 0.2
B2v | 131 | 1 | 0.091
B2v | 13194 | 1 | 0.091
B2v | 16172 | 1 | 0.091
B2v | 16261 | 1 | 0.091
B2v | 16311 | 1 | 0.091
B2v | 16335 | 5 | 0.455
B2v | 16437 | 1 | 0.091
B2v | 5752d | 1 | 0.091
B2w | 14097 | 2 | 0.25
B2w | 15790G | 1 | 0.125
B2w | 16465 | 1 | 0.125
B2w | 195 | 1 | 0.125
B2w | 5298 | 1 | 0.125
B2x | 14527 | 1 | 0.167
B2x | 15511 | 1 | 0.167
B2x | 16000 | 1 | 0.167
B2x | 16129 | 1 | 0.167
B2x | 16145 | 1 | 0.167
B2x | 16187 | 1 | 0.167
B2x | 195 | 1 | 0.167
B2x | 267 | 1 | 0.167
B2x | 9260 | 1 | 0.167
B2x | 9317A | 1 | 0.167
B2y | 10101 | 1 | 0.005
B2y | 10595 | 2 | 0.01
B2y | 11125 | 1 | 0.005
B2y | 12390 | 1 | 0.005
B2y | 12771 | 1 | 0.005
B2y | 14581 | 1 | 0.005
B2y | 146 | 2 | 0.01
B2y | 14659 | 1 | 0.005
B2y | 152 | 1 | 0.005
B2y | 15244 | 1 | 0.005
B2y | 15483 | 1 | 0.005
B2y | 1555 | 1 | 0.005
B2y | 16073 | 1 | 0.005
B2y | 16076d | 1 | 0.005
B2y | 16145 | 1 | 0.005
B2y | 16168 | 2 | 0.01
B2y | 16172 | 1 | 0.005
B2y | 16173 | 1 | 0.005
B2y | 16181C | 1 | 0.005
B2y | 16187 | 1 | 0.005
B2y | 16212 | 6 | 0.031
B2y | 16254 | 1 | 0.005
B2y | 16270 | 1 | 0.005
B2y | 1627A | 1 | 0.005
B2y | 16284 | 1 | 0.005
B2y | 16319 | 14 | 0.073
B2y | 16335 | 1 | 0.005
B2y | 16362 | 6 | 0.031
B2y | 16405T | 1 | 0.005
B2y | 16428C | 1 | 0.005
B2y | 16429G | 1 | 0.005
B2y | 16465 | 4 | 0.021
B2y | 1719 | 2 | 0.01
B2y | 207 | 1 | 0.005
B2y | 234 | 1 | 0.005
B2y | 309 | 1 | 0.005
B2y | 311 | 1 | 0.005
B2y | 315.2C | 1 | 0.005
B2y | 3593 | 1 | 0.005
B2y | 3645 | 4 | 0.021
B2y | 3907 | 1 | 0.005
B2y | 507 | 1 | 0.005
B2y | 5442 | 1 | 0.005
B2y | 5460 | 1 | 0.005
B2y | 5899.1C | 1 | 0.005
B2y | 59 | 1 | 0.005
B2y | 6471 | 1 | 0.005
B2y | 65G | 5 | 0.026
B2y | 6839 | 4 | 0.021
B2y | 709 | 8 | 0.042
B2y | 71.1G | 3 | 0.016
B2y | 73C | 1 | 0.005
B2y | 75 | 4 | 0.021
B2y | 8691 | 3 | 0.016
B2y | 8959 | 1 | 0.005
B2y | 8962 | 1 | 0.005
B2y | 8989 | 1 | 0.005
B2y | 94 | 1 | 0.005
B2y1 | 114G | 1 | 0.005
B2y1 | 146 | 1 | 0.005
B2y1 | 16073 | 1 | 0.005
B2y1 | 16076d | 1 | 0.005
B2y1 | 16172 | 2 | 0.009
B2y1 | 16187 | 1 | 0.005
B2y1 | 16188 | 7 | 0.033
B2y1 | 16311 | 2 | 0.009
B2y1 | 16325 | 2 | 0.009
B2y1 | 16352 | 3 | 0.014
B2y1 | 16405T | 1 | 0.005
B2y1 | 16413G | 1 | 0.005
B2y1 | 16428C | 4 | 0.019
B2y1 | 16429G | 4 | 0.019
B2y1 | 195 | 34 | 0.158
B2y1 | 215 | 2 | 0.009
B2y1 | 228 | 1 | 0.005
B2y1 | 6221 | 1 | 0.005
B2y1 | 9563 | 1 | 0.005
B4 | 11527 | 8 | 0.065
B4 | 11827 | 8 | 0.065
B4 | 12975 | 8 | 0.065
B4 | 13896 | 8 | 0.065
B4 | 14319 | 8 | 0.065

B4 | 14440 | 8 | 0.065
B4 | 152 | 10 | 0.081
B4 | 15748 | 8 | 0.065
B4 | 15928 | 8 | 0.065
B4 | 16075 | 1 | 0.008
B4 | 16076d | 1 | 0.008
B4 | 16092 | 3 | 0.024
B4 | 16129 | 1 | 0.008
B4 | 16172 | 1 | 0.008
B4 | 16179 | 2 | 0.016
B4 | 16274 | 25 | 0.203
B4 | 16289 | 19 | 0.154
B4 | 16301 | 19 | 0.154
B4 | 16302 | 1 | 0.008
B4 | 183 | 17 | 0.138
B4 | 2361 | 8 | 0.065
B4 | 244 | 8 | 0.065
B4 | 310 | 16 | 0.13
B4 | 313-315d | 2 | 0.016
B4 | 314-315d | 1 | 0.008
B4 | 315d | 10 | 0.081
B4 | 374 | 16 | 0.13
B4 | 3948 | 8 | 0.065
B4 | 41 | 1 | 0.008
B4 | 4961 | 8 | 0.065
B4 | 7389 | 8 | 0.065
B4 | 8027 | 8 | 0.065
B4 | 9623 | 8 | 0.065
B4+16261 | 16068 | 1 | 0.004
B4+16261 | 16094 | 1 | 0.004
B4+16261 | 16126 | 1 | 0.004
B4+16261 | 16150 | 2 | 0.008
B4+16261 | 16192-16193d | 1 | 0.004
B4+16261 | 16193d | 1 | 0.004
B4+16261 | 16266 | 1 | 0.004
B4+16261 | 16274 | 1 | 0.004
B4+16261 | 16294 | 4 | 0.015
B4+16261 | 16305T | 6 | 0.023
B4+16261 | 207 | 1 | 0.004
B4+16261 | 3083 | 2 | 0.008
B4+16261 | 310 | 1 | 0.004
B4+16261 | 316.1G | 1 | 0.004
B4+16261 | 466 | 3 | 0.011
B4+16261 | 481 | 1 | 0.004
B4+16261 | 521 | 1 | 0.004
B4+16261 | 5483 | 4 | 0.015
B4+16261 | 573.5C | 1 | 0.004
B4+16261 | 6962 | 2 | 0.008
B4+16261 | 732 | 1 | 0.004
B4+16261 | 8764 | 2 | 0.008
B4'5 | 10589 | 1 | 0.008
B4'5 | 10595 | 1 | 0.008
B4'5 | 11020 | 1 | 0.008
B4'5 | 11257 | 1 | 0.008
B4'5 | 12438 | 1 | 0.008
B4'5 | 13098 | 1 | 0.008
B4'5 | 14364 | 1 | 0.008
B4'5 | 14560 | 1 | 0.008
B4'5 | 150 | 1 | 0.008
B4'5 | 152 | 1 | 0.008
B4'5 | 16086 | 1 | 0.008
B4'5 | 16129 | 1 | 0.008
B4'5 | 16150 | 1 | 0.008
B4'5 | 16181C | 3 | 0.025
B4'5 | 16184 | 1 | 0.008
B4'5 | 16193d | 1 | 0.008
B4'5 | 16260 | 1 | 0.008
B4'5 | 16261 | 2 | 0.016
B4'5 | 16266G | 1 | 0.008
B4'5 | 16291 | 1 | 0.008
B4'5 | 16292 | 3 | 0.025
B4'5 | 16293C | 1 | 0.008
B4'5 | 16298 | 1 | 0.008
B4'5 | 16304 | 1 | 0.008
B4'5 | 16352 | 1 | 0.008
B4'5 | 16355 | 1 | 0.008
B4'5 | 16368 | 1 | 0.008
B4'5 | 183 | 1 | 0.008
B4'5 | 185 | 1 | 0.008
B4'5 | 189 | 1 | 0.008
B4'5 | 242 | 1 | 0.008
B4'5 | 249d | 1 | 0.008
B4'5 | 295 | 1 | 0.008
B4'5 | 309d | 1 | 0.008
B4'5 | 310 | 2 | 0.016
B4'5 | 324G | 1 | 0.008
B4'5 | 356-357d | 1 | 0.008
B4'5 | 443C | 1 | 0.008
B4'5 | 489 | 2 | 0.016
B4'5 | 5348 | 1 | 0.008
B4'5 | 573.6C | 1 | 0.008
B4'5 | 5836 | 1 | 0.008
B4'5 | 7825 | 1 | 0.008
B4'5 | 8595 | 1 | 0.008
B4a | 10410 | 1 | 0.004
B4a | 10495 | 2 | 0.007
B4a | 11227 | 2 | 0.007
B4a | 11914 | 1 | 0.004
B4a | 12239 | 1 | 0.004
B4a | 13098T | 1 | 0.004
B4a | 13500 | 2 | 0.007
B4a | 13781 | 2 | 0.007
B4a | 13995 | 1 | 0.004
B4a | 14053 | 2 | 0.007
B4a | 14110 | 1 | 0.004
B4a | 14569 | 1 | 0.004
B4a | 14675 | 1 | 0.004
B4a | 15232 | 1 | 0.004
B4a | 15718 | 1 | 0.004
B4a | 15769 | 2 | 0.007
B4a | 15877 | 1 | 0.004

B4a | 15926 | 2 | 0.007
B4a | 16054C | 1 | 0.004
B4a | 16129 | 2 | 0.007
B4a | 16154 | 6 | 0.021
B4a | 16192-16193d | 1 | 0.004
B4a | 16234 | 9 | 0.032
B4a | 16248 | 1 | 0.004
B4a | 16256 | 9 | 0.032
B4a | 16299 | 9 | 0.032
B4a | 16355 | 13 | 0.046
B4a | 16390 | 9 | 0.032
B4a | 2308 | 1 | 0.004
B4a | 236 | 2 | 0.007
B4a | 310 | 3 | 0.011
B4a | 315.3C | 1 | 0.004
B4a | 315d | 1 | 0.004
B4a | 573.3C | 1 | 0.004
B4a | 573.5C | 1 | 0.004
B4a | 5960 | 1 | 0.004
B4a | 6386 | 2 | 0.007
B4a | 723 | 1 | 0.004
B4a | 7325 | 1 | 0.004
B4a | 813 | 1 | 0.004
B4a | 868 | 1 | 0.004
B4a | 93 | 1 | 0.004
B4a | 94 | 2 | 0.007
B4a | 9953 | 1 | 0.004
B4a1 | 150 | 1 | 0.004
B4a1 | 16068 | 1 | 0.004
B4a1 | 16084 | 2 | 0.008
B4a1 | 16094 | 2 | 0.008
B4a1 | 16126 | 1 | 0.004
B4a1 | 16150 | 4 | 0.015
B4a1 | 16266 | 1 | 0.004
B4a1 | 16274 | 1 | 0.004
B4a1 | 16362 | 4 | 0.015
B4a1 | 16399 | 6 | 0.023
B4a1 | 189 | 2 | 0.008
B4a1 | 204 | 1 | 0.004
B4a1 | 207 | 1 | 0.004
B4a1 | 316.1G | 1 | 0.004
B4a1 | 466 | 4 | 0.015
B4a1 | 481 | 1 | 0.004
B4a1 | 573.5C | 1 | 0.004
B4a1 | 732 | 1 | 0.004
B4a1+16311 | 1000G | 2 | 0.02
B4a1+16311 | 10084 | 6 | 0.061
B4a1+16311 | 114 | 2 | 0.02
B4a1+16311 | 125G | 1 | 0.01
B4a1+16311 | 13386 | 6 | 0.061
B4a1+16311 | 13581 | 6 | 0.061
B4a1+16311 | 143 | 1 | 0.01
B4a1+16311 | 150 | 1 | 0.01
B4a1+16311 | 151 | 2 | 0.02
B4a1+16311 | 15119 | 6 | 0.061
B4a1+16311 | 152 | 2 | 0.02
B4a1+16311 | 15595 | 2 | 0.02
B4a1+16311 | 16051 | 2 | 0.02
B4a1+16311 | 16071G | 1 | 0.01
B4a1+16311 | 16075 | 1 | 0.01
B4a1+16311 | 16075A | 1 | 0.01
B4a1+16311 | 16076d | 1 | 0.01
B4a1+16311 | 16129 | 23 | 0.235
B4a1+16311 | 16148 | 2 | 0.02
B4a1+16311 | 16192-16193d | 1 | 0.01
B4a1+16311 | 16193d | 1 | 0.01
B4a1+16311 | 16219 | 1 | 0.01
B4a1+16311 | 16224 | 1 | 0.01
B4a1+16311 | 16256 | 1 | 0.01
B4a1+16311 | 16293 | 1 | 0.01
B4a1+16311 | 16299 | 2 | 0.02
B4a1+16311 | 16355 | 2 | 0.02
B4a1+16311 | 16362 | 7 | 0.071
B4a1+16311 | 16390 | 2 | 0.02
B4a1+16311 | 16405C | 1 | 0.01
B4a1+16311 | 185 | 1 | 0.01
B4a1+16311 | 195 | 1 | 0.01
B4a1+16311 | 199 | 2 | 0.02
B4a1+16311 | 214 | 1 | 0.01
B4a1+16311 | 258 | 2 | 0.02
B4a1+16311 | 309.4C | 1 | 0.01
B4a1+16311 | 309d | 2 | 0.02
B4a1+16311 | 310 | 2 | 0.02
B4a1+16311 | 366 | 1 | 0.01
B4a1+16311 | 368 | 1 | 0.01
B4a1+16311 | 459A | 5 | 0.051
B4a1+16311 | 4703 | 6 | 0.061
B4a1+16311 | 489 | 1 | 0.01
B4a1+16311 | 499 | 2 | 0.02
B4a1+16311 | 513 | 1 | 0.01
B4a1+16311 | 71d | 1 | 0.01
B4a1+16311 | 9083 | 2 | 0.02
B4a1+16311 | 93 | 1 | 0.01
B4a1+16311 | 9575 | 2 | 0.02
B4a1a | 11542 | 1 | 0.004
B4a1a | 11830 | 1 | 0.004
B4a1a | 12215 | 1 | 0.004
B4a1a | 12236 | 2 | 0.008
B4a1a | 12290 | 1 | 0.004
B4a1a | 12715 | 1 | 0.004
B4a1a | 12738 | 5 | 0.02
B4a1a | 13008 | 1 | 0.004
B4a1a | 13500 | 2 | 0.008
B4a1a | 13527 | 1 | 0.004
B4a1a | 13926 | 2 | 0.008
B4a1a | 14470 | 1 | 0.004
B4a1a | 14587 | 1 | 0.004
B4a1a | 14750 | 3 | 0.012
B4a1a | 15181 | 1 | 0.004
B4a1a | 15199A | 1 | 0.004
B4a1a | 152 | 3 | 0.012

B4a1a | 15283 | 1 | 0.004
B4a1a | 15712 | 1 | 0.004
B4a1a | 15748 | 3 | 0.012
B4a1a | 15773 | 1 | 0.004
B4a1a | 15916 | 1 | 0.004
B4a1a | 1601 | 1 | 0.004
B4a1a | 16086 | 1 | 0.004
B4a1a | 16092 | 1 | 0.004
B4a1a | 16093 | 2 | 0.008
B4a1a | 16094 | 7 | 0.028
B4a1a | 16129 | 1 | 0.004
B4a1a | 16166d | 1 | 0.004
B4a1a | 16178 | 1 | 0.004
B4a1a | 16192-16193d | 5 | 0.02
B4a1a | 16193d | 3 | 0.012
B4a1a | 16194C | 1 | 0.004
B4a1a | 16242 | 2 | 0.008
B4a1a | 16288 | 1 | 0.004
B4a1a | 16293 | 1 | 0.004
B4a1a | 16311 | 1 | 0.004
B4a1a | 16325 | 4 | 0.016
B4a1a | 16478 | 2 | 0.008
B4a1a | 1842 | 2 | 0.008
B4a1a | 185 | 3 | 0.012
B4a1a | 194 | 1 | 0.004
B4a1a | 200 | 1 | 0.004
B4a1a | 203 | 1 | 0.004
B4a1a | 234 | 1 | 0.004
B4a1a | 2442 | 2 | 0.008
B4a1a | 248 | 1 | 0.004
B4a1a | 309d | 5 | 0.02
B4a1a | 310 | 8 | 0.032
B4a1a | 313-315d | 1 | 0.004
B4a1a | 314-315d | 1 | 0.004
B4a1a | 315d | 3 | 0.012
B4a1a | 3357 | 1 | 0.004
B4a1a | 385 | 3 | 0.012
B4a1a | 4117 | 1 | 0.004
B4a1a | 4316 | 3 | 0.012
B4a1a | 4910 | 1 | 0.004
B4a1a | 5004 | 1 | 0.004
B4a1a | 503 | 1 | 0.004
B4a1a | 5460 | 6 | 0.024
B4a1a | 573.1C | 2 | 0.008
B4a1a | 573.2C | 1 | 0.004
B4a1a | 5978 | 1 | 0.004
B4a1a | 6113 | 1 | 0.004
B4a1a | 6267 | 1 | 0.004
B4a1a | 6713 | 2 | 0.008
B4a1a | 6878 | 1 | 0.004
B4a1a | 6990 | 1 | 0.004
B4a1a | 7051 | 1 | 0.004
B4a1a | 7214A | 1 | 0.004
B4a1a | 7853 | 2 | 0.008
B4a1a | 793 | 1 | 0.004
B4a1a | 8470 | 1 | 0.004
B4a1a | 8667 | 1 | 0.004
B4a1a | 8870 | 1 | 0.004
B4a1a | 9025 | 1 | 0.004
B4a1a | 9107 | 1 | 0.004
B4a1a | 9540 | 1 | 0.004
B4a1a | 9865 | 1 | 0.004
B4a1a1 | 1005 | 2 | 0.003
B4a1a1 | 10398 | 1 | 0.001
B4a1a1 | 10484 | 2 | 0.003
B4a1a1 | 10529 | 1 | 0.001
B4a1a1 | 11025 | 1 | 0.001
B4a1a1 | 11149 | 1 | 0.001
B4a1a1 | 11176 | 1 | 0.001
B4a1a1 | 11363 | 1 | 0.001
B4a1a1 | 11377 | 1 | 0.001
B4a1a1 | 114 | 2 | 0.003
B4a1a1 | 11453 | 1 | 0.001
B4a1a1 | 11464A | 1 | 0.001
B4a1a1 | 11485 | 1 | 0.001
B4a1a1 | 11654 | 1 | 0.001
B4a1a1 | 11731 | 1 | 0.001
B4a1a1 | 11812 | 1 | 0.001
B4a1a1 | 11827 | 1 | 0.001
B4a1a1 | 11914 | 9 | 0.012
B4a1a1 | 11929 | 1 | 0.001
B4a1a1 | 11931 | 1 | 0.001
B4a1a1 | 12100 | 3 | 0.004
B4a1a1 | 12164 | 1 | 0.001
B4a1a1 | 121C | 1 | 0.001
B4a1a1 | 12351 | 1 | 0.001
B4a1a1 | 12397 | 1 | 0.001
B4a1a1 | 12501 | 4 | 0.005
B4a1a1 | 12528C | 1 | 0.001
B4a1a1 | 12540 | 1 | 0.001
B4a1a1 | 12543A | 1 | 0.001
B4a1a1 | 12561 | 1 | 0.001
B4a1a1 | 12681 | 2 | 0.003
B4a1a1 | 12729 | 2 | 0.003
B4a1a1 | 13036 | 1 | 0.001
B4a1a1 | 13161 | 1 | 0.001
B4a1a1 | 13224 | 2 | 0.003
B4a1a1 | 13327 | 1 | 0.001
B4a1a1 | 13590 | 1 | 0.001
B4a1a1 | 13681 | 1 | 0.001
B4a1a1 | 13692 | 2 | 0.003
B4a1a1 | 13708 | 1 | 0.001
B4a1a1 | 13710 | 2 | 0.003
B4a1a1 | 13746 | 1 | 0.001
B4a1a1 | 13834 | 4 | 0.005
B4a1a1 | 13933 | 1 | 0.001
B4a1a1 | 14 | 3 | 0.004
B4a1a1 | 14025 | 1 | 0.001
B4a1a1 | 14110 | 2 | 0.003
B4a1a1 | 14249 | 1 | 0.001
B4a1a1 | 14384C | 1 | 0.001

B4a1a1 | 14434 | 1 | 0.001
B4a1a1 | 14662 | 2 | 0.003
B4a1a1 | 14690 | 1 | 0.001
B4a1a1 | 14954 | 1 | 0.001
B4a1a1 | 14956 | 2 | 0.003
B4a1a1 | 150 | 5 | 0.006
B4a1a1 | 15043 | 1 | 0.001
B4a1a1 | 15077 | 2 | 0.003
B4a1a1 | 15172 | 1 | 0.001
B4a1a1 | 15190 | 2 | 0.003
B4a1a1 | 15244 | 1 | 0.001
B4a1a1 | 15257 | 2 | 0.003
B4a1a1 | 153 | 1 | 0.001
B4a1a1 | 15301 | 1 | 0.001
B4a1a1 | 15314 | 1 | 0.001
B4a1a1 | 15622 | 1 | 0.001
B4a1a1 | 15727 | 1 | 0.001
B4a1a1 | 15730 | 1 | 0.001
B4a1a1 | 15813 | 1 | 0.001
B4a1a1 | 15884 | 1 | 0.001
B4a1a1 | 15886 | 1 | 0.001
B4a1a1 | 15917 | 3 | 0.004
B4a1a1 | 15937 | 1 | 0.001
B4a1a1 | 15942 | 1 | 0.001
B4a1a1 | 16084 | 1 | 0.001
B4a1a1 | 16092 | 4 | 0.005
B4a1a1 | 16093 | 8 | 0.01
B4a1a1 | 16114 | 2 | 0.003
B4a1a1 | 16129 | 2 | 0.003
B4a1a1 | 16145 | 2 | 0.003
B4a1a1 | 16148 | 2 | 0.003
B4a1a1 | 16173 | 1 | 0.001
B4a1a1 | 16181C | 1 | 0.001
B4a1a1 | 16192-16193d | 61 | 0.079
B4a1a1 | 16193d | 45 | 0.058
B4a1a1 | 16222 | 1 | 0.001
B4a1a1 | 16223 | 1 | 0.001
B4a1a1 | 16233 | 1 | 0.001
B4a1a1 | 16234 | 1 | 0.001
B4a1a1 | 16248 | 2 | 0.003
B4a1a1 | 16249 | 2 | 0.003
B4a1a1 | 16256 | 1 | 0.001
B4a1a1 | 16271 | 4 | 0.005
B4a1a1 | 16287 | 3 | 0.004
B4a1a1 | 16290 | 1 | 0.001
B4a1a1 | 16292 | 1 | 0.001
B4a1a1 | 16294 | 1 | 0.001
B4a1a1 | 16298 | 2 | 0.003
B4a1a1 | 16311 | 7 | 0.009
B4a1a1 | 16319 | 2 | 0.003
B4a1a1 | 16335 | 1 | 0.001
B4a1a1 | 16360 | 2 | 0.003
B4a1a1 | 16362 | 12 | 0.015
B4a1a1 | 16391 | 6 | 0.008
B4a1a1 | 16468 | 1 | 0.001
B4a1a1 | 16555 | 1 | 0.001
B4a1a1 | 1664 | 1 | 0.001
B4a1a1 | 178 | 1 | 0.001
B4a1a1 | 185 | 3 | 0.004
B4a1a1 | 1909 | 1 | 0.001
B4a1a1 | 195 | 16 | 0.021
B4a1a1 | 198 | 2 | 0.003
B4a1a1 | 204 | 1 | 0.001
B4a1a1 | 207 | 1 | 0.001
B4a1a1 | 2071 | 2 | 0.003
B4a1a1 | 210 | 1 | 0.001
B4a1a1 | 214 | 1 | 0.001
B4a1a1 | 215 | 1 | 0.001
B4a1a1 | 2156.1A | 1 | 0.001
B4a1a1 | 227 | 1 | 0.001
B4a1a1 | 228 | 1 | 0.001
B4a1a1 | 2280 | 1 | 0.001
B4a1a1 | 2700T | 1 | 0.001
B4a1a1 | 2780 | 2 | 0.003
B4a1a1 | 279 | 1 | 0.001
B4a1a1 | 2836 | 1 | 0.001
B4a1a1 | 3027 | 4 | 0.005
B4a1a1 | 309d | 1 | 0.001
B4a1a1 | 310 | 61 | 0.079
B4a1a1 | 313-315d | 1 | 0.001
B4a1a1 | 3136d | 1 | 0.001
B4a1a1 | 318 | 5 | 0.006
B4a1a1 | 319d | 1 | 0.001
B4a1a1 | 3213 | 1 | 0.001
B4a1a1 | 3423A | 2 | 0.003
B4a1a1 | 345A | 2 | 0.003
B4a1a1 | 3660 | 6 | 0.008
B4a1a1 | 3744 | 1 | 0.001
B4a1a1 | 3849 | 2 | 0.003
B4a1a1 | 385 | 3 | 0.004
B4a1a1 | 3887T | 1 | 0.001
B4a1a1 | 3918 | 1 | 0.001
B4a1a1 | 3948 | 1 | 0.001
B4a1a1 | 4025 | 3 | 0.004
B4a1a1 | 4137 | 1 | 0.001
B4a1a1 | 414G | 9 | 0.012
B4a1a1 | 4232 | 1 | 0.001
B4a1a1 | 4314 | 1 | 0.001
B4a1a1 | 437 | 1 | 0.001
B4a1a1 | 449 | 1 | 0.001
B4a1a1 | 452 | 4 | 0.005
B4a1a1 | 460 | 1 | 0.001
B4a1a1 | 4688 | 1 | 0.001
B4a1a1 | 4728 | 1 | 0.001
B4a1a1 | 4742 | 1 | 0.001
B4a1a1 | 4991 | 2 | 0.003
B4a1a1 | 5086 | 1 | 0.001
B4a1a1 | 5093 | 1 | 0.001
B4a1a1 | 5147 | 1 | 0.001
B4a1a1 | 5252 | 1 | 0.001
B4a1a1 | 5390 | 3 | 0.004

B4a1a1 | 5393 | 1 | 0.001
B4a1a1 | 5483 | 1 | 0.001
B4a1a1 | 55 | 1 | 0.001
B4a1a1 | 5558 | 1 | 0.001
B4a1a1 | 5563 | 1 | 0.001
B4a1a1 | 5728 | 1 | 0.001
B4a1a1 | 573.1C | 1 | 0.001
B4a1a1 | 574C | 1 | 0.001
B4a1a1 | 576C | 1 | 0.001
B4a1a1 | 5822 | 1 | 0.001
B4a1a1 | 5899.1C | 2 | 0.003
B4a1a1 | 6018 | 1 | 0.001
B4a1a1 | 6026 | 1 | 0.001
B4a1a1 | 603 | 1 | 0.001
B4a1a1 | 6083 | 1 | 0.001
B4a1a1 | 6136G | 1 | 0.001
B4a1a1 | 614 | 2 | 0.003
B4a1a1 | 6185 | 1 | 0.001
B4a1a1 | 6253 | 1 | 0.001
B4a1a1 | 6257 | 1 | 0.001
B4a1a1 | 6267 | 1 | 0.001
B4a1a1 | 64 | 1 | 0.001
B4a1a1 | 6437 | 1 | 0.001
B4a1a1 | 6480 | 1 | 0.001
B4a1a1 | 6563 | 1 | 0.001
B4a1a1 | 6647 | 1 | 0.001
B4a1a1 | 6734 | 1 | 0.001
B4a1a1 | 6891 | 1 | 0.001
B4a1a1 | 7041 | 1 | 0.001
B4a1a1 | 7080 | 1 | 0.001
B4a1a1 | 709 | 3 | 0.004
B4a1a1 | 710 | 1 | 0.001
B4a1a1 | 7138 | 1 | 0.001
B4a1a1 | 7158 | 1 | 0.001
B4a1a1 | 7302 | 1 | 0.001
B4a1a1 | 7419 | 1 | 0.001
B4a1a1 | 7433 | 1 | 0.001
B4a1a1 | 7598 | 1 | 0.001
B4a1a1 | 7609 | 2 | 0.003
B4a1a1 | 7702 | 1 | 0.001
B4a1a1 | 7738 | 1 | 0.001
B4a1a1 | 7830 | 1 | 0.001
B4a1a1 | 7873 | 2 | 0.003
B4a1a1 | 7876 | 1 | 0.001
B4a1a1 | 7930 | 2 | 0.003
B4a1a1 | 8149 | 3 | 0.004
B4a1a1 | 8167 | 1 | 0.001
B4a1a1 | 8251 | 1 | 0.001
B4a1a1 | 8254A | 3 | 0.004
B4a1a1 | 8266 | 1 | 0.001
B4a1a1 | 8279 | 3 | 0.004
B4a1a1 | 8380 | 1 | 0.001
B4a1a1 | 8410 | 1 | 0.001
B4a1a1 | 8514 | 2 | 0.003
B4a1a1 | 8764 | 2 | 0.003
B4a1a1 | 9022 | 1 | 0.001
B4a1a1 | 9053 | 1 | 0.001
B4a1a1 | 9103 | 1 | 0.001
B4a1a1 | 9211 | 4 | 0.005
B4a1a1 | 9214 | 1 | 0.001
B4a1a1 | 93 | 1 | 0.001
B4a1a1 | 9325 | 1 | 0.001
B4a1a1 | 94 | 1 | 0.001
B4a1a1 | 9449 | 4 | 0.005
B4a1a1 | 9535A | 1 | 0.001
B4a1a1 | 9540 | 1 | 0.001
B4a1a1 | 9721 | 1 | 0.001
B4a1a1 | 9782 | 2 | 0.003
B4a1a1 | 9966 | 2 | 0.003
B4a1a1 | 9983 | 1 | 0.001
B4a1a1+151 | 14233 | 1 | 0.003
B4a1a1+151 | 152 | 1 | 0.003
B4a1a1+151 | 16022G | 1 | 0.003
B4a1a1+151 | 16075 | 3 | 0.008
B4a1a1+151 | 16104 | 2 | 0.006
B4a1a1+151 | 16138T | 1 | 0.003
B4a1a1+151 | 16142 | 1 | 0.003
B4a1a1+151 | 16209 | 1 | 0.003
B4a1a1+151 | 16241 | 1 | 0.003
B4a1a1+151 | 16242A | 2 | 0.006
B4a1a1+151 | 16245 | 1 | 0.003
B4a1a1+151 | 16247 | 1 | 0.003
B4a1a1+151 | 16263 | 1 | 0.003
B4a1a1+151 | 16293 | 5 | 0.014
B4a1a1+151 | 16396A | 2 | 0.006
B4a1a1+151 | 16465 | 1 | 0.003
B4a1a1+151 | 16478G | 1 | 0.003
B4a1a1+151 | 16512 | 1 | 0.003
B4a1a1+151 | 16554 | 1 | 0.003
B4a1a1+151 | 310 | 1 | 0.003
B4a1a1+151 | 7754 | 1 | 0.003
B4a1a1+152 | 10084 | 1 | 0.003
B4a1a1+152 | 11065 | 3 | 0.008
B4a1a1+152 | 12280 | 1 | 0.003
B4a1a1+152 | 151 | 1 | 0.003
B4a1a1+152 | 15758 | 1 | 0.003
B4a1a1+152 | 16075 | 3 | 0.008
B4a1a1+152 | 16092 | 1 | 0.003
B4a1a1+152 | 16093 | 1 | 0.003
B4a1a1+152 | 16111 | 1 | 0.003
B4a1a1+152 | 16124 | 1 | 0.003
B4a1a1+152 | 16138T | 1 | 0.003
B4a1a1+152 | 16142 | 1 | 0.003
B4a1a1+152 | 16167 | 2 | 0.005
B4a1a1+152 | 16173 | 1 | 0.003
B4a1a1+152 | 16181C | 1 | 0.003
B4a1a1+152 | 16192-16193d | 2 | 0.005
B4a1a1+152 | 16193d | 3 | 0.008
B4a1a1+152 | 16209 | 1 | 0.003
B4a1a1+152 | 16218 | 6 | 0.016
B4a1a1+152 | 16224 | 3 | 0.008

B4a1a1+152 | 16234 | 1 | 0.003
B4a1a1+152 | 16241 | 1 | 0.003
B4a1a1+152 | 16243 | 1 | 0.003
B4a1a1+152 | 16245 | 1 | 0.003
B4a1a1+152 | 16263 | 1 | 0.003
B4a1a1+152 | 16266 | 1 | 0.003
B4a1a1+152 | 16317T | 1 | 0.003
B4a1a1+152 | 16324 | 1 | 0.003
B4a1a1+152 | 16478G | 1 | 0.003
B4a1a1+152 | 16506 | 1 | 0.003
B4a1a1+152 | 16512 | 1 | 0.003
B4a1a1+152 | 16527 | 1 | 0.003
B4a1a1+152 | 16554 | 1 | 0.003
B4a1a1+152 | 186 | 4 | 0.011
B4a1a1+152 | 195 | 1 | 0.003
B4a1a1+152 | 198 | 1 | 0.003
B4a1a1+152 | 214 | 1 | 0.003
B4a1a1+152 | 2140 | 3 | 0.008
B4a1a1+152 | 2755 | 1 | 0.003
B4a1a1+152 | 310 | 2 | 0.005
B4a1a1+152 | 3316 | 3 | 0.008
B4a1a1+152 | 350C | 1 | 0.003
B4a1a1+152 | 4973 | 1 | 0.003
B4a1a1+152 | 499 | 2 | 0.005
B4a1a1+152 | 593 | 1 | 0.003
B4a1a1+152 | 6905 | 6 | 0.016
B4a1a1+152 | 7840 | 2 | 0.005
B4a1a1+152 | 789 | 1 | 0.003
B4a1a1+152 | 8322 | 2 | 0.005
B4a1a1+152 | 8843 | 1 | 0.003
B4a1a1+152 | 9698 | 1 | 0.003
B4a1a1+16126 | 10451 | 1 | 0.042
B4a1a1+16126 | 10736 | 1 | 0.042
B4a1a1+16126 | 12290 | 1 | 0.042
B4a1a1+16126 | 152 | 1 | 0.042
B4a1a1+16126 | 15244 | 1 | 0.042
B4a1a1+16126 | 217 | 1 | 0.042
B4a1a1+16126 | 309d | 3 | 0.125
B4a1a1+16126 | 310 | 3 | 0.125
B4a1a1+16126 | 3737 | 1 | 0.042
B4a1a1+16126 | 5664 | 1 | 0.042
B4a1a1+16126 | 6905 | 1 | 0.042
B4a1a1+16126 | 8486 | 1 | 0.042
B4a1a1+16126 | 9007 | 1 | 0.042
B4a1a1a | 1095 | 1 | 0.002
B4a1a1a | 10969 | 1 | 0.002
B4a1a1a | 11485 | 4 | 0.007
B4a1a1a | 11767 | 1 | 0.002
B4a1a1a | 11778 | 1 | 0.002
B4a1a1a | 11818 | 1 | 0.002
B4a1a1a | 11887 | 2 | 0.003
B4a1a1a | 12245 | 1 | 0.002
B4a1a1a | 12397 | 2 | 0.003
B4a1a1a | 12406 | 1 | 0.002
B4a1a1a | 12432 | 1 | 0.002
B4a1a1a | 125 | 1 | 0.002
B4a1a1a | 127 | 1 | 0.002
B4a1a1a | 12750 | 2 | 0.003
B4a1a1a | 12904 | 2 | 0.003
B4a1a1a | 12987 | 1 | 0.002
B4a1a1a | 13062 | 1 | 0.002
B4a1a1a | 13224 | 1 | 0.002
B4a1a1a | 13227 | 1 | 0.002
B4a1a1a | 13590 | 3 | 0.005
B4a1a1a | 13711 | 1 | 0.002
B4a1a1a | 13966 | 1 | 0.002
B4a1a1a | 1398.1T | 1 | 0.002
B4a1a1a | 14060 | 1 | 0.002
B4a1a1a | 14162 | 1 | 0.002
B4a1a1a | 14221 | 1 | 0.002
B4a1a1a | 14236 | 1 | 0.002
B4a1a1a | 14272 | 5 | 0.009
B4a1a1a | 14587 | 1 | 0.002
B4a1a1a | 14757 | 1 | 0.002
B4a1a1a | 150 | 1 | 0.002
B4a1a1a | 15043 | 1 | 0.002
B4a1a1a | 15139 | 1 | 0.002
B4a1a1a | 15199 | 1 | 0.002
B4a1a1a | 152 | 5 | 0.009
B4a1a1a | 153 | 4 | 0.007
B4a1a1a | 15314 | 1 | 0.002
B4a1a1a | 15812 | 1 | 0.002
B4a1a1a | 15927 | 3 | 0.005
B4a1a1a | 16000 | 1 | 0.002
B4a1a1a | 16093 | 1 | 0.002
B4a1a1a | 16124 | 1 | 0.002
B4a1a1a | 16129 | 4 | 0.007
B4a1a1a | 16145 | 1 | 0.002
B4a1a1a | 16147 | 4 | 0.007
B4a1a1a | 16148 | 1 | 0.002
B4a1a1a | 16168 | 1 | 0.002
B4a1a1a | 16192-16193d | 49 | 0.084
B4a1a1a | 16193d | 9 | 0.015
B4a1a1a | 16218 | 1 | 0.002
B4a1a1a | 16223 | 3 | 0.005
B4a1a1a | 16259 | 6 | 0.01
B4a1a1a | 16260 | 5 | 0.009
B4a1a1a | 16287 | 1 | 0.002
B4a1a1a | 16311 | 13 | 0.022
B4a1a1a | 16317 | 3 | 0.005
B4a1a1a | 16318 | 5 | 0.009
B4a1a1a | 16319 | 1 | 0.002
B4a1a1a | 16335 | 1 | 0.002
B4a1a1a | 16342 | 1 | 0.002
B4a1a1a | 16356 | 1 | 0.002
B4a1a1a | 16361 | 1 | 0.002
B4a1a1a | 16362 | 2 | 0.003
B4a1a1a | 16391 | 7 | 0.012
B4a1a1a | 16400 | 5 | 0.009
B4a1a1a | 16524 | 1 | 0.002
B4a1a1a | 1766 | 2 | 0.003

B4a1a1a | 185 | 2 | 0.003
B4a1a1a | 189 | 1 | 0.002
B4a1a1a | 1958 | 1 | 0.002
B4a1a1a | 199 | 1 | 0.002
B4a1a1a | 2140 | 2 | 0.003
B4a1a1a | 226 | 1 | 0.002
B4a1a1a | 234 | 1 | 0.002
B4a1a1a | 239 | 1 | 0.002
B4a1a1a | 310 | 29 | 0.05
B4a1a1a | 3158.1T | 1 | 0.002
B4a1a1a | 316C | 1 | 0.002
B4a1a1a | 319 | 1 | 0.002
B4a1a1a | 3203 | 1 | 0.002
B4a1a1a | 3705 | 1 | 0.002
B4a1a1a | 3892 | 3 | 0.005
B4a1a1a | 4080 | 1 | 0.002
B4a1a1a | 4092 | 2 | 0.003
B4a1a1a | 4186 | 1 | 0.002
B4a1a1a | 4856 | 4 | 0.007
B4a1a1a | 5081 | 1 | 0.002
B4a1a1a | 5095 | 1 | 0.002
B4a1a1a | 5096 | 1 | 0.002
B4a1a1a | 5105 | 2 | 0.003
B4a1a1a | 5258 | 1 | 0.002
B4a1a1a | 533 | 4 | 0.007
B4a1a1a | 5363 | 1 | 0.002
B4a1a1a | 538 | 1 | 0.002
B4a1a1a | 539 | 1 | 0.002
B4a1a1a | 5628 | 1 | 0.002
B4a1a1a | 573.1C | 1 | 0.002
B4a1a1a | 573.3C | 1 | 0.002
B4a1a1a | 5894 | 3 | 0.005
B4a1a1a | 5987 | 5 | 0.009
B4a1a1a | 6297 | 1 | 0.002
B4a1a1a | 6848 | 1 | 0.002
B4a1a1a | 6891 | 1 | 0.002
B4a1a1a | 709 | 1 | 0.002
B4a1a1a | 7278 | 2 | 0.003
B4a1a1a | 7366 | 1 | 0.002
B4a1a1a | 7805 | 1 | 0.002
B4a1a1a | 789 | 1 | 0.002
B4a1a1a | 8075 | 1 | 0.002
B4a1a1a | 8146 | 1 | 0.002
B4a1a1a | 822 | 1 | 0.002
B4a1a1a | 8269-8270d | 2 | 0.003
B4a1a1a | 8563 | 1 | 0.002
B4a1a1a | 8681 | 1 | 0.002
B4a1a1a | 8865 | 1 | 0.002
B4a1a1a | 8879 | 1 | 0.002
B4a1a1a | 8950 | 3 | 0.005
B4a1a1a | 9368 | 3 | 0.005
B4a1a1a | 9452 | 1 | 0.002
B4a1a1a | 9494 | 2 | 0.003
B4a1a1a | 9548 | 2 | 0.003
B4a1a1a | 960d | 1 | 0.002
B4a1a1a | 9716 | 1 | 0.002
B4a1a1a | 9802 | 1 | 0.002
B4a1a1a | 9944 | 1 | 0.002
B4a1a1a | 9967 | 2 | 0.003
B4a1a1a+195 | 16075 | 3 | 0.008
B4a1a1a+195 | 16138T | 1 | 0.003
B4a1a1a+195 | 16142 | 1 | 0.003
B4a1a1a+195 | 16192-16193d | 1 | 0.003
B4a1a1a+195 | 16193d | 3 | 0.008
B4a1a1a+195 | 16209 | 1 | 0.003
B4a1a1a+195 | 16215 | 1 | 0.003
B4a1a1a+195 | 16241 | 1 | 0.003
B4a1a1a+195 | 16245 | 1 | 0.003
B4a1a1a+195 | 16263 | 1 | 0.003
B4a1a1a+195 | 16266 | 3 | 0.008
B4a1a1a+195 | 16293 | 5 | 0.014
B4a1a1a+195 | 16357 | 1 | 0.003
B4a1a1a+195 | 16396A | 2 | 0.006
B4a1a1a+195 | 16478G | 1 | 0.003
B4a1a1a+195 | 16512 | 1 | 0.003
B4a1a1a+195 | 16554 | 1 | 0.003
B4a1a1a+195 | 309.3C | 2 | 0.006
B4a1a1a+195 | 310 | 3 | 0.008
B4a1a1a+195 | 492 | 1 | 0.003
B4a1a1a+195 | 499 | 2 | 0.006
B4a1a1a+195 | 9007 | 3 | 0.008
B4a1a1a1 | 10398 | 2 | 0.005
B4a1a1a1 | 12996 | 2 | 0.005
B4a1a1a1 | 13191 | 1 | 0.002
B4a1a1a1 | 13692 | 2 | 0.005
B4a1a1a1 | 152d | 1 | 0.002
B4a1a1a1 | 1598 | 1 | 0.002
B4a1a1a1 | 16093 | 1 | 0.002
B4a1a1a1 | 16192-16193d | 5 | 0.011
B4a1a1a1 | 16239 | 2 | 0.005
B4a1a1a1 | 16274 | 1 | 0.002
B4a1a1a1 | 16286 | 1 | 0.002
B4a1a1a1 | 16355 | 9 | 0.021
B4a1a1a1 | 1673 | 2 | 0.005
B4a1a1a1 | 310 | 1 | 0.002
B4a1a1a1 | 315.3C | 1 | 0.002
B4a1a1a1 | 456 | 1 | 0.002
B4a1a1a1 | 573.1C | 1 | 0.002
B4a1a1a1 | 6023 | 1 | 0.002
B4a1a1a1 | 7894 | 1 | 0.002
B4a1a1a1 | 9389 | 1 | 0.002
B4a1a1a1 | 9696 | 2 | 0.005
B4a1a1a10 | 15244 | 2 | 0.005
B4a1a1a10 | 16092 | 5 | 0.012
B4a1a1a10 | 16193d | 1 | 0.002
B4a1a1a10 | 16286 | 1 | 0.002
B4a1a1a10 | 16355 | 9 | 0.021
B4a1a1a10 | 315.3C | 1 | 0.002
B4a1a1a10 | 3483 | 2 | 0.005
B4a1a1a10 | 456 | 1 | 0.002
B4a1a1a10 | 573.1C | 1 | 0.002

B4a1a1a11 | 16129 | 2 | 0.005
B4a1a1a11 | 16286 | 1 | 0.002
B4a1a1a11 | 16290 | 1 | 0.002
B4a1a1a11 | 16355 | 9 | 0.021
B4a1a1a11 | 16362 | 1 | 0.002
B4a1a1a11 | 214 | 1 | 0.002
B4a1a1a11 | 309.3C | 4 | 0.009
B4a1a1a11 | 315.3C | 1 | 0.002
B4a1a1a11 | 456 | 1 | 0.002
B4a1a1a11 | 573.1C | 1 | 0.002
B4a1a1a11 | 6120 | 1 | 0.002
B4a1a1a11 | 71 | 1 | 0.002
B4a1a1a11a | 12375 | 4 | 0.009
B4a1a1a11a | 13135 | 1 | 0.002
B4a1a1a11a | 14845 | 4 | 0.009
B4a1a1a11a | 15784 | 1 | 0.002
B4a1a1a11a | 16193d | 1 | 0.002
B4a1a1a11a | 16219T | 1 | 0.002
B4a1a1a11a | 16286 | 1 | 0.002
B4a1a1a11a | 16355 | 9 | 0.021
B4a1a1a11a | 199 | 1 | 0.002
B4a1a1a11a | 315.3C | 1 | 0.002
B4a1a1a11a | 456 | 1 | 0.002
B4a1a1a11a | 573.1C | 1 | 0.002
B4a1a1a11a | 8998 | 1 | 0.002
B4a1a1a11a | 9377 | 1 | 0.002
B4a1a1a11b | 150 | 14 | 0.165
B4a1a1a11b | 15462 | 3 | 0.035
B4a1a1a11b | 16051 | 2 | 0.024
B4a1a1a11b | 16086 | 2 | 0.024
B4a1a1a11b | 16108 | 3 | 0.035
B4a1a1a11b | 16129 | 6 | 0.071
B4a1a1a11b | 16153 | 5 | 0.059
B4a1a1a11b | 16188 | 1 | 0.012
B4a1a1a11b | 16192-16193d | 3 | 0.035
B4a1a1a11b | 16223 | 5 | 0.059
B4a1a1a11b | 16246 | 1 | 0.012
B4a1a1a11b | 16274C | 1 | 0.012
B4a1a1a11b | 16320 | 5 | 0.059
B4a1a1a11b | 16343 | 9 | 0.106
B4a1a1a11b | 16362G | 3 | 0.035
B4a1a1a11b | 188 | 1 | 0.012
B4a1a1a11b | 195 | 1 | 0.012
B4a1a1a11b | 198 | 1 | 0.012
B4a1a1a11b | 214 | 1 | 0.012
B4a1a1a11b | 309d | 1 | 0.012
B4a1a1a11b | 310 | 3 | 0.035
B4a1a1a11b | 8301 | 3 | 0.035
B4a1a1a12 | 144A | 1 | 0.083
B4a1a1a12 | 152 | 1 | 0.083
B4a1a1a12 | 16092 | 2 | 0.167
B4a1a1a12 | 16192-16193d | 4 | 0.333
B4a1a1a12 | 310 | 4 | 0.333
B4a1a1a12 | 316 | 1 | 0.083
B4a1a1a12 | 5029 | 1 | 0.083
B4a1a1a12 | 5156 | 1 | 0.083
B4a1a1a13 | 16129 | 2 | 0.005
B4a1a1a13 | 16192-16193d | 1 | 0.002
B4a1a1a13 | 16286 | 1 | 0.002
B4a1a1a13 | 16290 | 1 | 0.002
B4a1a1a13 | 16355 | 9 | 0.021
B4a1a1a13 | 214 | 1 | 0.002
B4a1a1a13 | 309.3C | 4 | 0.009
B4a1a1a13 | 310 | 1 | 0.002
B4a1a1a13 | 315.3C | 1 | 0.002
B4a1a1a13 | 456 | 1 | 0.002
B4a1a1a13 | 4688 | 1 | 0.002
B4a1a1a13 | 4700A | 1 | 0.002
B4a1a1a13 | 573.1C | 1 | 0.002
B4a1a1a13 | 71 | 1 | 0.002
B4a1a1a14 | 16192-16193d | 1 | 0.053
B4a1a1a14 | 16311 | 7 | 0.368
B4a1a1a14 | 217 | 1 | 0.053
B4a1a1a14 | 222 | 1 | 0.053
B4a1a1a14 | 310 | 1 | 0.053
B4a1a1a14 | 310d | 1 | 0.053
B4a1a1a14 | 316 | 1 | 0.053
B4a1a1a14 | 64 | 3 | 0.158
B4a1a1a14 | 8715 | 1 | 0.053
B4a1a1a15 | 103 | 2 | 0.005
B4a1a1a15 | 151 | 1 | 0.002
B4a1a1a15 | 16093 | 5 | 0.012
B4a1a1a15 | 16286 | 1 | 0.002
B4a1a1a15 | 16355 | 9 | 0.021
B4a1a1a15 | 310 | 1 | 0.002
B4a1a1a15 | 315.3C | 1 | 0.002
B4a1a1a15 | 456 | 1 | 0.002
B4a1a1a15 | 573.1C | 1 | 0.002
B4a1a1a15 | 7202 | 2 | 0.005
B4a1a1a15 | 9288 | 2 | 0.005
B4a1a1a16 | 103 | 2 | 0.051
B4a1a1a16 | 12245 | 3 | 0.077
B4a1a1a16 | 12406 | 3 | 0.077
B4a1a1a16 | 14871 | 1 | 0.026
B4a1a1a16 | 150 | 1 | 0.026
B4a1a1a16 | 151 | 2 | 0.051
B4a1a1a16 | 16092 | 1 | 0.026
B4a1a1a16 | 16129 | 1 | 0.026
B4a1a1a16 | 16203 | 1 | 0.026
B4a1a1a16 | 16213 | 1 | 0.026
B4a1a1a16 | 16214 | 1 | 0.026
B4a1a1a16 | 16295 | 1 | 0.026
B4a1a1a16 | 16298 | 1 | 0.026
B4a1a1a16 | 16319 | 1 | 0.026
B4a1a1a16 | 16361 | 1 | 0.026
B4a1a1a16 | 16532d | 1 | 0.026
B4a1a1a16 | 16536G | 1 | 0.026
B4a1a1a16 | 196 | 5 | 0.128
B4a1a1a16 | 200 | 3 | 0.077
B4a1a1a16 | 307 | 1 | 0.026
B4a1a1a16 | 308 | 3 | 0.077

B4a1a1a16 | 309.3C | 1 | 0.026
B4a1a1a16 | 309.4C | 1 | 0.026
B4a1a1a16 | 309d | 2 | 0.051
B4a1a1a16 | 310 | 5 | 0.128
B4a1a1a16 | 310d | 2 | 0.051
B4a1a1a16 | 314-315d | 1 | 0.026
B4a1a1a16 | 357C | 1 | 0.026
B4a1a1a16 | 37d | 1 | 0.026
B4a1a1a16 | 385 | 2 | 0.051
B4a1a1a16 | 40-44d | 1 | 0.026
B4a1a1a16 | 432C | 1 | 0.026
B4a1a1a16 | 47.1G | 1 | 0.026
B4a1a1a16 | 475C | 1 | 0.026
B4a1a1a16 | 479.1A | 1 | 0.026
B4a1a1a16 | 485A | 1 | 0.026
B4a1a1a16 | 486A | 1 | 0.026
B4a1a1a16 | 489A | 1 | 0.026
B4a1a1a16 | 491A | 1 | 0.026
B4a1a1a16 | 5492 | 1 | 0.026
B4a1a1a16 | 5806 | 1 | 0.026
B4a1a1a16 | 7501 | 1 | 0.026
B4a1a1a16 | 841 | 1 | 0.026
B4a1a1a16 | 9157 | 1 | 0.026
B4a1a1a17 | 153 | 1 | 0.002
B4a1a1a17 | 16129 | 2 | 0.005
B4a1a1a17 | 16286 | 1 | 0.002
B4a1a1a17 | 16290 | 1 | 0.002
B4a1a1a17 | 16355 | 9 | 0.021
B4a1a1a17 | 214 | 1 | 0.002
B4a1a1a17 | 309.3C | 4 | 0.009
B4a1a1a17 | 315.3C | 1 | 0.002
B4a1a1a17 | 456 | 1 | 0.002
B4a1a1a17 | 573.1C | 1 | 0.002
B4a1a1a17 | 686 | 1 | 0.002
B4a1a1a17 | 71 | 1 | 0.002
B4a1a1a17 | 8659 | 1 | 0.002
B4a1a1a18 | 14793 | 1 | 0.018
B4a1a1a18 | 16174 | 1 | 0.018
B4a1a1a18 | 16213 | 1 | 0.018
B4a1a1a18 | 16241 | 2 | 0.035
B4a1a1a18 | 16299 | 1 | 0.018
B4a1a1a18 | 310 | 3 | 0.053
B4a1a1a18 | 5177 | 1 | 0.018
B4a1a1a18 | 9512 | 3 | 0.053
B4a1a1a19 | 12627 | 1 | 0.111
B4a1a1a19 | 12958A | 1 | 0.111
B4a1a1a19 | 16129 | 3 | 0.333
B4a1a1a19 | 16304G | 3 | 0.333
B4a1a1a19 | 16367C | 3 | 0.333
B4a1a1a19 | 16413A | 3 | 0.333
B4a1a1a19 | 16414T | 3 | 0.333
B4a1a1a19 | 16439G | 3 | 0.333
B4a1a1a19 | 5752d | 1 | 0.111
B4a1a1a1a | 15159 | 1 | 0.003
B4a1a1a1a | 16181C | 1 | 0.003
B4a1a1a1a1 | 159 | 2 | 0.005
B4a1a1a1a1 | 16181C | 1 | 0.003
B4a1a1a1a1 | 16192-16193d | 1 | 0.003
B4a1a1a1a1 | 16193d | 1 | 0.003
B4a1a1a1a1 | 16362 | 2 | 0.005
B4a1a1a1a1 | 214 | 3 | 0.008
B4a1a1a1a1 | 2223 | 1 | 0.003
B4a1a1a1a1 | 310 | 1 | 0.003
B4a1a1a1a1 | 3644 | 1 | 0.003
B4a1a1a1a1 | 4227 | 2 | 0.005
B4a1a1a1a1 | 430 | 1 | 0.003
B4a1a1a1a1 | 6216 | 2 | 0.005
B4a1a1a1a1 | 8387 | 1 | 0.003
B4a1a1a1b | 11878 | 1 | 0.002
B4a1a1a1b | 16129 | 2 | 0.005
B4a1a1a1b | 16192-16193d | 2 | 0.005
B4a1a1a1b | 16286 | 1 | 0.002
B4a1a1a1b | 16290 | 1 | 0.002
B4a1a1a1b | 16300 | 3 | 0.007
B4a1a1a1b | 16355 | 9 | 0.021
B4a1a1a1b | 16360 | 3 | 0.007
B4a1a1a1b | 214 | 1 | 0.002
B4a1a1a1b | 309.3C | 4 | 0.009
B4a1a1a1b | 310 | 1 | 0.002
B4a1a1a1b | 315.3C | 1 | 0.002
B4a1a1a1b | 3808 | 1 | 0.002
B4a1a1a1b | 456 | 1 | 0.002
B4a1a1a1b | 573.1C | 1 | 0.002
B4a1a1a1b | 71 | 1 | 0.002
B4a1a1a1c | 12624 | 1 | 0.003
B4a1a1a1c | 13746 | 1 | 0.003
B4a1a1a1c | 16022G | 1 | 0.003
B4a1a1a1c | 16075 | 3 | 0.008
B4a1a1a1c | 16104 | 2 | 0.006
B4a1a1a1c | 16138T | 1 | 0.003
B4a1a1a1c | 16142 | 1 | 0.003
B4a1a1a1c | 16193d | 1 | 0.003
B4a1a1a1c | 16209 | 1 | 0.003
B4a1a1a1c | 16241 | 1 | 0.003
B4a1a1a1c | 16242A | 2 | 0.006
B4a1a1a1c | 16245 | 1 | 0.003
B4a1a1a1c | 16263 | 1 | 0.003
B4a1a1a1c | 16293 | 4 | 0.011
B4a1a1a1c | 16311 | 1 | 0.003
B4a1a1a1c | 16396A | 1 | 0.003
B4a1a1a1c | 16465 | 1 | 0.003
B4a1a1a1c | 16478G | 1 | 0.003
B4a1a1a1c | 16512 | 1 | 0.003
B4a1a1a1c | 16554 | 1 | 0.003
B4a1a1a1c | 310 | 1 | 0.003
B4a1a1a1c | 7419 | 2 | 0.006
B4a1a1a1d | 15043 | 2 | 0.006
B4a1a1a1d | 151 | 1 | 0.003
B4a1a1a1d | 16075 | 3 | 0.009
B4a1a1a1d | 16092 | 1 | 0.003
B4a1a1a1d | 16111 | 1 | 0.003

B4a1a1a1d | 16124 | 1 | 0.003
B4a1a1a1d | 16138T | 1 | 0.003
B4a1a1a1d | 16142 | 1 | 0.003
B4a1a1a1d | 16167 | 2 | 0.006
B4a1a1a1d | 16181C | 1 | 0.003
B4a1a1a1d | 16209 | 1 | 0.003
B4a1a1a1d | 16234 | 1 | 0.003
B4a1a1a1d | 16241 | 1 | 0.003
B4a1a1a1d | 16245 | 1 | 0.003
B4a1a1a1d | 16263 | 1 | 0.003
B4a1a1a1d | 16293 | 4 | 0.011
B4a1a1a1d | 16317T | 1 | 0.003
B4a1a1a1d | 16396A | 1 | 0.003
B4a1a1a1d | 16478G | 1 | 0.003
B4a1a1a1d | 16512 | 1 | 0.003
B4a1a1a1d | 16527 | 1 | 0.003
B4a1a1a1d | 16554 | 1 | 0.003
B4a1a1a1d | 214 | 1 | 0.003
B4a1a1a1d | 350C | 1 | 0.003
B4a1a1a1d | 4767 | 1 | 0.003
B4a1a1a2 | 11914 | 3 | 0.088
B4a1a1a2 | 16092 | 2 | 0.059
B4a1a1a2 | 16192-16193d | 7 | 0.206
B4a1a1a2 | 16193d | 2 | 0.059
B4a1a1a2 | 16260 | 1 | 0.029
B4a1a1a2 | 16311 | 5 | 0.147
B4a1a1a2 | 16505 | 1 | 0.029
B4a1a1a2 | 195 | 2 | 0.059
B4a1a1a2 | 228 | 1 | 0.029
B4a1a1a2 | 234 | 1 | 0.029
B4a1a1a2 | 2626A | 2 | 0.059
B4a1a1a2 | 310 | 4 | 0.118
B4a1a1a2 | 4227 | 1 | 0.029
B4a1a1a2 | 5147C | 2 | 0.059
B4a1a1a2 | 5964 | 1 | 0.029
B4a1a1a2 | 64 | 2 | 0.059
B4a1a1a2 | 9139 | 2 | 0.059
B4a1a1a20 | 16051 | 1 | 0.038
B4a1a1a20 | 16092 | 1 | 0.038
B4a1a1a20 | 16172 | 1 | 0.038
B4a1a1a20 | 16173 | 1 | 0.038
B4a1a1a20 | 16193d | 1 | 0.038
B4a1a1a20 | 16223 | 13 | 0.5
B4a1a1a20 | 16289 | 3 | 0.115
B4a1a1a20 | 16301 | 1 | 0.038
B4a1a1a20 | 16311 | 1 | 0.038
B4a1a1a20 | 16325 | 13 | 0.5
B4a1a1a20 | 16405T | 1 | 0.038
B4a1a1a20 | 183 | 2 | 0.077
B4a1a1a20 | 195 | 1 | 0.038
B4a1a1a20 | 309.3C | 1 | 0.038
B4a1a1a20 | 310 | 2 | 0.077
B4a1a1a20 | 314-315d | 1 | 0.038
B4a1a1a20 | 315.2C | 1 | 0.038
B4a1a1a20 | 315d | 1 | 0.038
B4a1a1a20 | 3394 | 1 | 0.038
B4a1a1a20 | 374 | 2 | 0.077
B4a1a1a21 | 10907 | 1 | 0.002
B4a1a1a21 | 16129 | 2 | 0.005
B4a1a1a21 | 16158 | 1 | 0.002
B4a1a1a21 | 16286 | 1 | 0.002
B4a1a1a21 | 16290 | 1 | 0.002
B4a1a1a21 | 16355 | 9 | 0.021
B4a1a1a21 | 16422 | 1 | 0.002
B4a1a1a21 | 16463 | 1 | 0.002
B4a1a1a21 | 214 | 1 | 0.002
B4a1a1a21 | 309.3C | 4 | 0.009
B4a1a1a21 | 315.3C | 1 | 0.002
B4a1a1a21 | 3645 | 1 | 0.002
B4a1a1a21 | 456 | 1 | 0.002
B4a1a1a21 | 573.1C | 1 | 0.002
B4a1a1a21 | 71 | 1 | 0.002
B4a1a1a21 | 7789 | 1 | 0.002
B4a1a1a22 | 10451 | 1 | 0.002
B4a1a1a22 | 12618 | 1 | 0.002
B4a1a1a22 | 152 | 1 | 0.002
B4a1a1a22 | 16218 | 1 | 0.002
B4a1a1a22 | 16286 | 1 | 0.002
B4a1a1a22 | 16384 | 14 | 0.032
B4a1a1a22 | 310 | 3 | 0.007
B4a1a1a22 | 315.3C | 1 | 0.002
B4a1a1a22 | 456 | 1 | 0.002
B4a1a1a22 | 5302 | 1 | 0.002
B4a1a1a22 | 573.1C | 1 | 0.002
B4a1a1a22 | 6038A | 1 | 0.002
B4a1a1a23 | 1598 | 1 | 0.003
B4a1a1a23 | 16075 | 3 | 0.008
B4a1a1a23 | 16138T | 1 | 0.003
B4a1a1a23 | 16142 | 1 | 0.003
B4a1a1a23 | 16209 | 1 | 0.003
B4a1a1a23 | 16215 | 1 | 0.003
B4a1a1a23 | 16241 | 1 | 0.003
B4a1a1a23 | 16245 | 1 | 0.003
B4a1a1a23 | 16263 | 1 | 0.003
B4a1a1a23 | 16293 | 5 | 0.014
B4a1a1a23 | 16396A | 2 | 0.006
B4a1a1a23 | 16478G | 1 | 0.003
B4a1a1a23 | 16512 | 1 | 0.003
B4a1a1a23 | 16554 | 1 | 0.003
B4a1a1a23 | 309.3C | 2 | 0.006
B4a1a1a23 | 499 | 2 | 0.006
B4a1a1a23 | 5460 | 1 | 0.003
B4a1a1a2a | 103 | 1 | 0.029
B4a1a1a2a | 14693 | 1 | 0.029
B4a1a1a2a | 14927 | 1 | 0.029
B4a1a1a2a | 15076 | 1 | 0.029
B4a1a1a2a | 16093 | 2 | 0.057
B4a1a1a2a | 16108 | 2 | 0.057
B4a1a1a2a | 16181C | 4 | 0.114
B4a1a1a2a | 16192-16193d | 5 | 0.143
B4a1a1a2a | 16193d | 2 | 0.057

B4a1a1a2a | 16203 | 2 | 0.057
B4a1a1a2a | 16219 | 1 | 0.029
B4a1a1a2a | 16325 | 1 | 0.029
B4a1a1a2a | 16355A | 1 | 0.029
B4a1a1a2a | 310 | 1 | 0.029
B4a1a1a2a | 375 | 1 | 0.029
B4a1a1a2a | 5021 | 1 | 0.029
B4a1a1a2a | 7316 | 1 | 0.029
B4a1a1a2a | 7572 | 1 | 0.029
B4a1a1a2a | 7759 | 1 | 0.029
B4a1a1a2a | 8639 | 1 | 0.029
B4a1a1a2a | 8893 | 2 | 0.057
B4a1a1a2a | 9078 | 2 | 0.057
B4a1a1a2b | 12750 | 1 | 0.125
B4a1a1a2b | 151d | 1 | 0.125
B4a1a1a2b | 16456 | 1 | 0.125
B4a1a1a2b | 310 | 3 | 0.375
B4a1a1a2b | 5563 | 1 | 0.125
B4a1a1a2b | 8547 | 1 | 0.125
B4a1a1a2b | 8715 | 1 | 0.125
B4a1a1a3 | 10586 | 2 | 0.005
B4a1a1a3 | 16000 | 1 | 0.002
B4a1a1a3 | 16192-16193d | 1 | 0.002
B4a1a1a3 | 16286 | 1 | 0.002
B4a1a1a3 | 16355 | 9 | 0.021
B4a1a1a3 | 315.3C | 1 | 0.002
B4a1a1a3 | 456 | 1 | 0.002
B4a1a1a3 | 573.1C | 1 | 0.002
B4a1a1a3 | 5979 | 1 | 0.002
B4a1a1a3 | 709 | 1 | 0.002
B4a1a1a4 | 11986 | 1 | 0.062
B4a1a1a4 | 16141 | 3 | 0.188
B4a1a1a4 | 16192-16193d | 4 | 0.25
B4a1a1a4 | 9756G | 1 | 0.062
B4a1a1a5 | 16129 | 2 | 0.005
B4a1a1a5 | 16192-16193d | 1 | 0.002
B4a1a1a5 | 16193d | 1 | 0.002
B4a1a1a5 | 16286 | 1 | 0.002
B4a1a1a5 | 16290 | 1 | 0.002
B4a1a1a5 | 16295 | 1 | 0.002
B4a1a1a5 | 16355 | 9 | 0.021
B4a1a1a5 | 214 | 1 | 0.002
B4a1a1a5 | 309.3C | 4 | 0.009
B4a1a1a5 | 310 | 2 | 0.005
B4a1a1a5 | 315.3C | 1 | 0.002
B4a1a1a5 | 456 | 1 | 0.002
B4a1a1a5 | 573.1C | 1 | 0.002
B4a1a1a5 | 71 | 1 | 0.002
B4a1a1a5 | 8260 | 3 | 0.007
B4a1a1a6 | 10915 | 1 | 0.002
B4a1a1a6 | 14259 | 1 | 0.002
B4a1a1a6 | 16192-16193d | 2 | 0.005
B4a1a1a6 | 16193d | 1 | 0.002
B4a1a1a6 | 16286 | 1 | 0.002
B4a1a1a6 | 16355 | 9 | 0.021
B4a1a1a6 | 315.3C | 1 | 0.002
B4a1a1a6 | 456 | 1 | 0.002
B4a1a1a6 | 573.1C | 1 | 0.002
B4a1a1a7 | 14325 | 2 | 0.004
B4a1a1a7 | 153 | 6 | 0.013
B4a1a1a7 | 16129 | 1 | 0.002
B4a1a1a7 | 16145 | 5 | 0.011
B4a1a1a7 | 16174 | 4 | 0.009
B4a1a1a7 | 16187 | 2 | 0.004
B4a1a1a7 | 16188 | 9 | 0.019
B4a1a1a7 | 16192-16193d | 6 | 0.013
B4a1a1a7 | 16197G | 1 | 0.002
B4a1a1a7 | 16214 | 12 | 0.026
B4a1a1a7 | 16216 | 1 | 0.002
B4a1a1a7 | 16240 | 3 | 0.006
B4a1a1a7 | 16287 | 1 | 0.002
B4a1a1a7 | 16296G | 1 | 0.002
B4a1a1a7 | 199 | 3 | 0.006
B4a1a1a7 | 309.4C | 9 | 0.019
B4a1a1a7 | 309d | 1 | 0.002
B4a1a1a7 | 310 | 5 | 0.011
B4a1a1a7 | 573.1C | 1 | 0.002
B4a1a1a7 | 94 | 9 | 0.019
B4a1a1a8 | 15024 | 1 | 0.003
B4a1a1a8 | 15941 | 2 | 0.006
B4a1a1a8 | 16022G | 1 | 0.003
B4a1a1a8 | 16075 | 3 | 0.008
B4a1a1a8 | 16104 | 2 | 0.006
B4a1a1a8 | 16138T | 1 | 0.003
B4a1a1a8 | 16142 | 1 | 0.003
B4a1a1a8 | 16192-16193d | 3 | 0.008
B4a1a1a8 | 16193d | 1 | 0.003
B4a1a1a8 | 16209 | 1 | 0.003
B4a1a1a8 | 16241 | 1 | 0.003
B4a1a1a8 | 16242A | 2 | 0.006
B4a1a1a8 | 16245 | 1 | 0.003
B4a1a1a8 | 16263 | 1 | 0.003
B4a1a1a8 | 16293 | 4 | 0.011
B4a1a1a8 | 16342 | 2 | 0.006
B4a1a1a8 | 16362 | 1 | 0.003
B4a1a1a8 | 16396A | 1 | 0.003
B4a1a1a8 | 16465 | 1 | 0.003
B4a1a1a8 | 16478G | 1 | 0.003
B4a1a1a8 | 16512 | 1 | 0.003
B4a1a1a8 | 16554 | 1 | 0.003
B4a1a1a8 | 310 | 2 | 0.006
B4a1a1a8 | 8974 | 1 | 0.003
B4a1a1a9 | 16192-16193d | 1 | 0.071
B4a1a1a9 | 16319 | 2 | 0.143
B4a1a1a9 | 310 | 2 | 0.143
B4a1a1a9 | 6011 | 1 | 0.071
B4a1a1aa | 152 | 1 | 0.019
B4a1a1aa | 16092 | 6 | 0.113
B4a1a1aa | 16093 | 1 | 0.019
B4a1a1aa | 16129 | 2 | 0.038
B4a1a1aa | 16179 | 1 | 0.019

B4a1a1aa | 16192-16193d | 2 | 0.038
B4a1a1aa | 16271 | 6 | 0.113
B4a1a1aa | 189 | 1 | 0.019
B4a1a1aa | 200 | 1 | 0.019
B4a1a1aa | 309.3C | 1 | 0.019
B4a1a1aa | 309.4C | 1 | 0.019
B4a1a1aa | 310 | 1 | 0.019
B4a1a1aa | 374 | 1 | 0.019
B4a1a1aa | 5655 | 2 | 0.038
B4a1a1aa | 8934 | 1 | 0.019
B4a1a1aa | 94 | 1 | 0.019
B4a1a1ab | 10688 | 1 | 0.002
B4a1a1ab | 16169 | 2 | 0.005
B4a1a1ab | 16192-16193d | 1 | 0.002
B4a1a1ab | 16193d | 2 | 0.005
B4a1a1ab | 16239 | 2 | 0.005
B4a1a1ab | 16286 | 1 | 0.002
B4a1a1ab | 16291 | 13 | 0.03
B4a1a1ab | 16355 | 9 | 0.021
B4a1a1ab | 16478 | 1 | 0.002
B4a1a1ab | 310 | 2 | 0.005
B4a1a1ab | 315.3C | 1 | 0.002
B4a1a1ab | 4225 | 1 | 0.002
B4a1a1ab | 456 | 1 | 0.002
B4a1a1ab | 573.1C | 1 | 0.002
B4a1a1ab | 5899.1C | 1 | 0.002
B4a1a1ac | 16111 | 7 | 0.016
B4a1a1ac | 16178 | 1 | 0.002
B4a1a1ac | 16192-16193d | 1 | 0.002
B4a1a1ac | 16286 | 1 | 0.002
B4a1a1ac | 16355 | 9 | 0.021
B4a1a1ac | 2071 | 1 | 0.002
B4a1a1ac | 315.3C | 1 | 0.002
B4a1a1ac | 456 | 1 | 0.002
B4a1a1ac | 4917 | 1 | 0.002
B4a1a1ac | 573.1C | 1 | 0.002
B4a1a1ac | 7513d | 1 | 0.002
B4a1a1ac | 8572 | 1 | 0.002
B4a1a1ad | 15448 | 2 | 0.005
B4a1a1ad | 16286 | 1 | 0.002
B4a1a1ad | 16355 | 9 | 0.021
B4a1a1ad | 310 | 1 | 0.002
B4a1a1ad | 315.3C | 1 | 0.002
B4a1a1ad | 456 | 1 | 0.002
B4a1a1ad | 573.1C | 1 | 0.002
B4a1a1ad | 723 | 2 | 0.005
B4a1a1ad | 8563 | 1 | 0.002
B4a1a1ae | 16129 | 2 | 0.005
B4a1a1ae | 16286 | 1 | 0.002
B4a1a1ae | 16290 | 1 | 0.002
B4a1a1ae | 16355 | 9 | 0.021
B4a1a1ae | 214 | 1 | 0.002
B4a1a1ae | 309.3C | 4 | 0.009
B4a1a1ae | 315.3C | 1 | 0.002
B4a1a1ae | 456 | 1 | 0.002
B4a1a1ae | 5147 | 1 | 0.002
B4a1a1ae | 573.1C | 1 | 0.002
B4a1a1ae | 71 | 1 | 0.002
B4a1a1af | 16192-16193d | 1 | 0.143
B4a1a1af | 16193d | 1 | 0.143
B4a1a1af | 16362 | 1 | 0.143
B4a1a1af | 5581 | 1 | 0.143
B4a1a1af | 9554 | 1 | 0.143
B4a1a1b | 10325 | 2 | 0.002
B4a1a1b | 10373 | 1 | 0.001
B4a1a1b | 10398 | 3 | 0.003
B4a1a1b | 10601 | 1 | 0.001
B4a1a1b | 10700 | 1 | 0.001
B4a1a1b | 10828 | 3 | 0.003
B4a1a1b | 10873 | 1 | 0.001
B4a1a1b | 1116 | 7 | 0.007
B4a1a1b | 11194 | 1 | 0.001
B4a1a1b | 11204 | 1 | 0.001
B4a1a1b | 11361 | 1 | 0.001
B4a1a1b | 11605 | 1 | 0.001
B4a1a1b | 11647 | 1 | 0.001
B4a1a1b | 12043 | 1 | 0.001
B4a1a1b | 12070 | 3 | 0.003
B4a1a1b | 12127 | 1 | 0.001
B4a1a1b | 12696 | 1 | 0.001
B4a1a1b | 12864 | 1 | 0.001
B4a1a1b | 13105 | 1 | 0.001
B4a1a1b | 13145 | 5 | 0.005
B4a1a1b | 13194 | 1 | 0.001
B4a1a1b | 13269 | 1 | 0.001
B4a1a1b | 13359 | 2 | 0.002
B4a1a1b | 13371A | 1 | 0.001
B4a1a1b | 13594 | 1 | 0.001
B4a1a1b | 13602 | 1 | 0.001
B4a1a1b | 13650 | 1 | 0.001
B4a1a1b | 13651 | 1 | 0.001
B4a1a1b | 13802 | 1 | 0.001
B4a1a1b | 13827 | 3 | 0.003
B4a1a1b | 13969T | 1 | 0.001
B4a1a1b | 14016 | 1 | 0.001
B4a1a1b | 14019 | 2 | 0.002
B4a1a1b | 14161-14162d | 1 | 0.001
B4a1a1b | 14180 | 4 | 0.004
B4a1a1b | 14189 | 1 | 0.001
B4a1a1b | 14200 | 2 | 0.002
B4a1a1b | 14207 | 1 | 0.001
B4a1a1b | 14209 | 1 | 0.001
B4a1a1b | 14279 | 2 | 0.002
B4a1a1b | 14351 | 1 | 0.001
B4a1a1b | 14388 | 1 | 0.001
B4a1a1b | 14393 | 1 | 0.001
B4a1a1b | 14470 | 4 | 0.004
B4a1a1b | 14501 | 1 | 0.001
B4a1a1b | 1472 | 1 | 0.001
B4a1a1b | 14769 | 1 | 0.001
B4a1a1b | 15109 | 1 | 0.001

B4a1a1b | 15152 | 1 | 0.001
B4a1a1b | 15191 | 1 | 0.001
B4a1a1b | 152 | 3 | 0.003
B4a1a1b | 15236 | 2 | 0.002
B4a1a1b | 15268 | 1 | 0.001
B4a1a1b | 153 | 1 | 0.001
B4a1a1b | 15498 | 1 | 0.001
B4a1a1b | 15534 | 1 | 0.001
B4a1a1b | 15541 | 1 | 0.001
B4a1a1b | 15575 | 2 | 0.002
B4a1a1b | 15657 | 1 | 0.001
B4a1a1b | 15706 | 1 | 0.001
B4a1a1b | 15708C | 6 | 0.006
B4a1a1b | 16038 | 1 | 0.001
B4a1a1b | 16051 | 1 | 0.001
B4a1a1b | 16092 | 5 | 0.005
B4a1a1b | 16093 | 10 | 0.01
B4a1a1b | 16129 | 6 | 0.006
B4a1a1b | 16136 | 5 | 0.005
B4a1a1b | 16145 | 1 | 0.001
B4a1a1b | 16174A | 1 | 0.001
B4a1a1b | 16176 | 1 | 0.001
B4a1a1b | 16185 | 1 | 0.001
B4a1a1b | 16188 | 2 | 0.002
B4a1a1b | 16191-16193d | 1 | 0.001
B4a1a1b | 16192-16193d | 2 | 0.002
B4a1a1b | 16193d | 14 | 0.014
B4a1a1b | 16212 | 1 | 0.001
B4a1a1b | 16223 | 2 | 0.002
B4a1a1b | 16231 | 3 | 0.003
B4a1a1b | 16239 | 1 | 0.001
B4a1a1b | 16240 | 1 | 0.001
B4a1a1b | 16241T | 1 | 0.001
B4a1a1b | 16246 | 2 | 0.002
B4a1a1b | 16260 | 2 | 0.002
B4a1a1b | 16264 | 1 | 0.001
B4a1a1b | 16278 | 4 | 0.004
B4a1a1b | 16285d | 1 | 0.001
B4a1a1b | 16291 | 1 | 0.001
B4a1a1b | 16294 | 1 | 0.001
B4a1a1b | 16295 | 1 | 0.001
B4a1a1b | 16309 | 2 | 0.002
B4a1a1b | 16320 | 2 | 0.002
B4a1a1b | 16325 | 1 | 0.001
B4a1a1b | 16327 | 1 | 0.001
B4a1a1b | 16355 | 1 | 0.001
B4a1a1b | 16357 | 5 | 0.005
B4a1a1b | 16362 | 2 | 0.002
B4a1a1b | 16368 | 8 | 0.008
B4a1a1b | 16390 | 1 | 0.001
B4a1a1b | 16525C | 2 | 0.002
B4a1a1b | 16527 | 1 | 0.001
B4a1a1b | 1717 | 1 | 0.001
B4a1a1b | 1738 | 1 | 0.001
B4a1a1b | 1760 | 1 | 0.001
B4a1a1b | 178 | 6 | 0.006
B4a1a1b | 1811 | 1 | 0.001
B4a1a1b | 183 | 2 | 0.002
B4a1a1b | 1888 | 1 | 0.001
B4a1a1b | 195 | 11 | 0.011
B4a1a1b | 199 | 3 | 0.003
B4a1a1b | 200 | 1 | 0.001
B4a1a1b | 207 | 2 | 0.002
B4a1a1b | 215 | 1 | 0.001
B4a1a1b | 2412 | 1 | 0.001
B4a1a1b | 2442 | 1 | 0.001
B4a1a1b | 2755 | 1 | 0.001
B4a1a1b | 279 | 1 | 0.001
B4a1a1b | 285 | 1 | 0.001
B4a1a1b | 2875 | 1 | 0.001
B4a1a1b | 297 | 1 | 0.001
B4a1a1b | 3010 | 2 | 0.002
B4a1a1b | 306-309d | 1 | 0.001
B4a1a1b | 307-309d | 7 | 0.007
B4a1a1b | 309.3C | 3 | 0.003
B4a1a1b | 309d | 5 | 0.005
B4a1a1b | 310 | 20 | 0.02
B4a1a1b | 310G | 1 | 0.001
B4a1a1b | 312 | 1 | 0.001
B4a1a1b | 313 | 1 | 0.001
B4a1a1b | 315.2C | 1 | 0.001
B4a1a1b | 3184 | 2 | 0.002
B4a1a1b | 3196 | 3 | 0.003
B4a1a1b | 3204 | 1 | 0.001
B4a1a1b | 3308 | 1 | 0.001
B4a1a1b | 3310 | 1 | 0.001
B4a1a1b | 3316 | 4 | 0.004
B4a1a1b | 3438 | 2 | 0.002
B4a1a1b | 3480 | 1 | 0.001
B4a1a1b | 374 | 1 | 0.001
B4a1a1b | 408A | 1 | 0.001
B4a1a1b | 425 | 1 | 0.001
B4a1a1b | 453 | 3 | 0.003
B4a1a1b | 4639 | 2 | 0.002
B4a1a1b | 4694A | 1 | 0.001
B4a1a1b | 471 | 2 | 0.002
B4a1a1b | 4728 | 1 | 0.001
B4a1a1b | 4761 | 1 | 0.001
B4a1a1b | 4824 | 5 | 0.005
B4a1a1b | 489 | 1 | 0.001
B4a1a1b | 498d | 1 | 0.001
B4a1a1b | 513 | 1 | 0.001
B4a1a1b | 5177 | 2 | 0.002
B4a1a1b | 5288 | 1 | 0.001
B4a1a1b | 5442 | 1 | 0.001
B4a1a1b | 5460 | 3 | 0.003
B4a1a1b | 573.1C | 2 | 0.002
B4a1a1b | 5836 | 1 | 0.001
B4a1a1b | 596 | 1 | 0.001
B4a1a1b | 6071 | 1 | 0.001
B4a1a1b | 6242 | 1 | 0.001

B4a1a1b | 6256 | 1 | 0.001
B4a1a1b | 629 | 1 | 0.001
B4a1a1b | 6425 | 1 | 0.001
B4a1a1b | 644 | 2 | 0.002
B4a1a1b | 6605 | 2 | 0.002
B4a1a1b | 6663 | 2 | 0.002
B4a1a1b | 6755 | 1 | 0.001
B4a1a1b | 6758 | 1 | 0.001
B4a1a1b | 6818 | 1 | 0.001
B4a1a1b | 6852 | 1 | 0.001
B4a1a1b | 6917 | 1 | 0.001
B4a1a1b | 6956 | 2 | 0.002
B4a1a1b | 7179 | 1 | 0.001
B4a1a1b | 721 | 1 | 0.001
B4a1a1b | 725 | 1 | 0.001
B4a1a1b | 7259 | 1 | 0.001
B4a1a1b | 7377 | 1 | 0.001
B4a1a1b | 7444C | 3 | 0.003
B4a1a1b | 7598 | 1 | 0.001
B4a1a1b | 7754 | 5 | 0.005
B4a1a1b | 8014 | 1 | 0.001
B4a1a1b | 8027 | 2 | 0.002
B4a1a1b | 8152 | 1 | 0.001
B4a1a1b | 8222 | 1 | 0.001
B4a1a1b | 8290 | 1 | 0.001
B4a1a1b | 8387 | 3 | 0.003
B4a1a1b | 8472 | 2 | 0.002
B4a1a1b | 8506 | 2 | 0.002
B4a1a1b | 8512d | 1 | 0.001
B4a1a1b | 8555 | 2 | 0.002
B4a1a1b | 8639 | 1 | 0.001
B4a1a1b | 8654 | 1 | 0.001
B4a1a1b | 8680A | 2 | 0.002
B4a1a1b | 8701 | 1 | 0.001
B4a1a1b | 8817T | 1 | 0.001
B4a1a1b | 8839 | 1 | 0.001
B4a1a1b | 8854T | 1 | 0.001
B4a1a1b | 8939 | 1 | 0.001
B4a1a1b | 8946 | 1 | 0.001
B4a1a1b | 8994 | 1 | 0.001
B4a1a1b | 9034 | 1 | 0.001
B4a1a1b | 9046 | 2 | 0.002
B4a1a1b | 9052 | 1 | 0.001
B4a1a1b | 9056 | 1 | 0.001
B4a1a1b | 9084 | 1 | 0.001
B4a1a1b | 9188 | 1 | 0.001
B4a1a1b | 9210 | 1 | 0.001
B4a1a1b | 9299 | 1 | 0.001
B4a1a1b | 9371 | 1 | 0.001
B4a1a1b | 9638 | 3 | 0.003
B4a1a1b | 9804 | 1 | 0.001
B4a1a1b | 9863G | 1 | 0.001
B4a1a1b | 9948 | 1 | 0.001
B4a1a1b | 9996 | 1 | 0.001
B4a1a1c | 10451 | 2 | 0.004
B4a1a1c | 11084 | 1 | 0.002
B4a1a1c | 11930 | 1 | 0.002
B4a1a1c | 11969 | 1 | 0.002
B4a1a1c | 12164 | 1 | 0.002
B4a1a1c | 12358 | 1 | 0.002
B4a1a1c | 13708 | 1 | 0.002
B4a1a1c | 13934 | 1 | 0.002
B4a1a1c | 14461 | 1 | 0.002
B4a1a1c | 152 | 1 | 0.002
B4a1a1c | 15496 | 1 | 0.002
B4a1a1c | 16129 | 1 | 0.002
B4a1a1c | 16188 | 1 | 0.002
B4a1a1c | 16193d | 1 | 0.002
B4a1a1c | 16222 | 1 | 0.002
B4a1a1c | 16291 | 8 | 0.016
B4a1a1c | 195 | 2 | 0.004
B4a1a1c | 1992 | 1 | 0.002
B4a1a1c | 234 | 1 | 0.002
B4a1a1c | 310 | 43 | 0.086
B4a1a1c | 3396 | 1 | 0.002
B4a1a1c | 344 | 1 | 0.002
B4a1a1c | 3639 | 2 | 0.004
B4a1a1c | 3766 | 1 | 0.002
B4a1a1c | 4659 | 1 | 0.002
B4a1a1c | 5592 | 1 | 0.002
B4a1a1c | 5628 | 1 | 0.002
B4a1a1c | 573.1C | 1 | 0.002
B4a1a1c | 709 | 1 | 0.002
B4a1a1c | 7513d | 1 | 0.002
B4a1a1c | 7774 | 1 | 0.002
B4a1a1c | 8137G | 1 | 0.002
B4a1a1c | 841 | 8 | 0.016
B4a1a1c | 8812 | 1 | 0.002
B4a1a1c | 8962 | 1 | 0.002
B4a1a1c | 9804 | 1 | 0.002
B4a1a1d | 12557 | 1 | 0.002
B4a1a1d | 14302 | 1 | 0.002
B4a1a1d | 150 | 1 | 0.002
B4a1a1d | 15223 | 1 | 0.002
B4a1a1d | 16086 | 8 | 0.018
B4a1a1d | 16093 | 1 | 0.002
B4a1a1d | 16181C | 1 | 0.002
B4a1a1d | 16192-16193d | 3 | 0.007
B4a1a1d | 16193d | 1 | 0.002
B4a1a1d | 16286 | 1 | 0.002
B4a1a1d | 16355 | 9 | 0.021
B4a1a1d | 204 | 2 | 0.005
B4a1a1d | 207 | 1 | 0.002
B4a1a1d | 315.3C | 1 | 0.002
B4a1a1d | 456 | 1 | 0.002
B4a1a1d | 573.1C | 1 | 0.002
B4a1a1d | 6887 | 1 | 0.002
B4a1a1d | 8222 | 1 | 0.002
B4a1a1e | 16192-16193d | 2 | 0.005
B4a1a1e | 16286 | 1 | 0.002
B4a1a1e | 16311 | 1 | 0.002

B4a1a1e | 16355 | 9 | 0.021
B4a1a1e | 310 | 3 | 0.007
B4a1a1e | 315.3C | 1 | 0.002
B4a1a1e | 4216 | 6 | 0.014
B4a1a1e | 456 | 1 | 0.002
B4a1a1e | 573.1C | 1 | 0.002
B4a1a1e | 6465 | 2 | 0.005
B4a1a1f | 15924 | 1 | 0.002
B4a1a1f | 16172 | 3 | 0.007
B4a1a1f | 16192-16193d | 3 | 0.007
B4a1a1f | 16234 | 1 | 0.002
B4a1a1f | 16286 | 1 | 0.002
B4a1a1f | 16355 | 9 | 0.021
B4a1a1f | 1719 | 1 | 0.002
B4a1a1f | 200 | 1 | 0.002
B4a1a1f | 315.3C | 1 | 0.002
B4a1a1f | 456 | 1 | 0.002
B4a1a1f | 573.1C | 1 | 0.002
B4a1a1f | 8071 | 1 | 0.002
B4a1a1f | 9804 | 2 | 0.005
B4a1a1g | 11339 | 1 | 0.002
B4a1a1g | 11659 | 1 | 0.002
B4a1a1g | 12172 | 1 | 0.002
B4a1a1g | 14560C | 2 | 0.005
B4a1a1g | 14831 | 2 | 0.005
B4a1a1g | 16048 | 2 | 0.005
B4a1a1g | 16178 | 1 | 0.002
B4a1a1g | 16192-16193d | 3 | 0.007
B4a1a1g | 16193d | 3 | 0.007
B4a1a1g | 16253 | 2 | 0.005
B4a1a1g | 16286 | 1 | 0.002
B4a1a1g | 16355 | 9 | 0.021
B4a1a1g | 197 | 1 | 0.002
B4a1a1g | 214 | 3 | 0.007
B4a1a1g | 310 | 4 | 0.009
B4a1a1g | 315.3C | 1 | 0.002
B4a1a1g | 3209 | 1 | 0.002
B4a1a1g | 456 | 1 | 0.002
B4a1a1g | 569 | 1 | 0.002
B4a1a1g | 573.1C | 1 | 0.002
B4a1a1g | 6620 | 1 | 0.002
B4a1a1g | 7861 | 1 | 0.002
B4a1a1g | 8614 | 2 | 0.005
B4a1a1h | 16192-16193d | 17 | 0.043
B4a1a1h | 16193d | 1 | 0.003
B4a1a1h | 16213 | 1 | 0.003
B4a1a1h | 16256 | 1 | 0.003
B4a1a1h | 16258 | 1 | 0.003
B4a1a1h | 195 | 2 | 0.005
B4a1a1h | 310 | 17 | 0.043
B4a1a1h | 6216 | 1 | 0.003
B4a1a1i | 16093 | 2 | 0.005
B4a1a1i | 16150 | 5 | 0.012
B4a1a1i | 16278 | 1 | 0.002
B4a1a1i | 16286 | 1 | 0.002
B4a1a1i | 16355 | 9 | 0.021
B4a1a1i | 310 | 1 | 0.002
B4a1a1i | 315.3C | 1 | 0.002
B4a1a1i | 456 | 1 | 0.002
B4a1a1i | 573.1C | 1 | 0.002
B4a1a1j | 11437 | 1 | 0.042
B4a1a1j | 11626 | 1 | 0.042
B4a1a1j | 12504 | 2 | 0.083
B4a1a1j | 13011A | 1 | 0.042
B4a1a1j | 16093 | 1 | 0.042
B4a1a1j | 16192-16193d | 5 | 0.208
B4a1a1j | 16299 | 3 | 0.125
B4a1a1j | 1692 | 1 | 0.042
B4a1a1j | 310 | 10 | 0.417
B4a1a1j | 318 | 5 | 0.208
B4a1a1j | 3398 | 1 | 0.042
B4a1a1j | 4314 | 2 | 0.083
B4a1a1j | 5774 | 1 | 0.042
B4a1a1j | 7258 | 1 | 0.042
B4a1a1j | 8538 | 2 | 0.083
B4a1a1j | 9535A | 1 | 0.042
B4a1a1k | 12279 | 2 | 0.077
B4a1a1k | 152 | 1 | 0.038
B4a1a1k | 16167 | 1 | 0.038
B4a1a1k | 16192-16193d | 4 | 0.154
B4a1a1k | 16193d | 2 | 0.077
B4a1a1k | 16213 | 1 | 0.038
B4a1a1k | 16274 | 1 | 0.038
B4a1a1k | 16298 | 1 | 0.038
B4a1a1k | 195 | 2 | 0.077
B4a1a1k | 2361 | 9 | 0.346
B4a1a1k | 310 | 2 | 0.077
B4a1a1k | 8889 | 1 | 0.038
B4a1a1k1 | 215 | 1 | 0.111
B4a1a1k1 | 310 | 1 | 0.111
B4a1a1k1 | 6623 | 2 | 0.222
B4a1a1k1 | 6747 | 1 | 0.111
B4a1a1k1 | 709 | 2 | 0.222
B4a1a1m | 143 | 1 | 0.003
B4a1a1m | 152 | 1 | 0.003
B4a1a1m | 16075 | 3 | 0.008
B4a1a1m | 16126 | 1 | 0.003
B4a1a1m | 16138T | 1 | 0.003
B4a1a1m | 16142 | 1 | 0.003
B4a1a1m | 16209 | 1 | 0.003
B4a1a1m | 16241 | 1 | 0.003
B4a1a1m | 16245 | 1 | 0.003
B4a1a1m | 16247 | 1 | 0.003
B4a1a1m | 16263 | 1 | 0.003
B4a1a1m | 16293 | 5 | 0.014
B4a1a1m | 16362 | 1 | 0.003
B4a1a1m | 16396A | 2 | 0.006
B4a1a1m | 16478G | 1 | 0.003
B4a1a1m | 16512 | 1 | 0.003
B4a1a1m | 16554 | 1 | 0.003
B4a1a1m | 7711 | 1 | 0.003

B4a1a1m | 94 | 1 | 0.003
B4a1a1m1 | 10451 | 1 | 0.002
B4a1a1m1 | 10586 | 1 | 0.002
B4a1a1m1 | 13111 | 1 | 0.002
B4a1a1m1 | 13349 | 1 | 0.002
B4a1a1m1 | 14007 | 1 | 0.002
B4a1a1m1 | 14053 | 1 | 0.002
B4a1a1m1 | 14226 | 1 | 0.002
B4a1a1m1 | 14431 | 1 | 0.002
B4a1a1m1 | 14744 | 1 | 0.002
B4a1a1m1 | 14839 | 6 | 0.013
B4a1a1m1 | 15661 | 2 | 0.004
B4a1a1m1 | 15671 | 1 | 0.002
B4a1a1m1 | 15776 | 2 | 0.004
B4a1a1m1 | 1598 | 1 | 0.002
B4a1a1m1 | 16086 | 1 | 0.002
B4a1a1m1 | 16129 | 1 | 0.002
B4a1a1m1 | 16168 | 2 | 0.004
B4a1a1m1 | 16192-16193d | 24 | 0.052
B4a1a1m1 | 16193d | 10 | 0.022
B4a1a1m1 | 16276 | 1 | 0.002
B4a1a1m1 | 16278 | 5 | 0.011
B4a1a1m1 | 228 | 1 | 0.002
B4a1a1m1 | 310 | 64 | 0.138
B4a1a1m1 | 3109d | 1 | 0.002
B4a1a1m1 | 316 | 1 | 0.002
B4a1a1m1 | 3531 | 1 | 0.002
B4a1a1m1 | 4012 | 1 | 0.002
B4a1a1m1 | 4164 | 1 | 0.002
B4a1a1m1 | 4725C | 1 | 0.002
B4a1a1m1 | 5535 | 1 | 0.002
B4a1a1m1 | 5618 | 1 | 0.002
B4a1a1m1 | 5764 | 1 | 0.002
B4a1a1m1 | 5900C | 1 | 0.002
B4a1a1m1 | 6116 | 1 | 0.002
B4a1a1m1 | 6365 | 1 | 0.002
B4a1a1m1 | 7754 | 1 | 0.002
B4a1a1m1 | 8176A | 1 | 0.002
B4a1a1m1 | 8338 | 1 | 0.002
B4a1a1m1 | 8376 | 1 | 0.002
B4a1a1m1 | 841 | 4 | 0.009
B4a1a1m1 | 8433 | 1 | 0.002
B4a1a1m1 | 9060G | 1 | 0.002
B4a1a1m1 | 9477 | 1 | 0.002
B4a1a1m1 | 9554 | 1 | 0.002
B4a1a1m1 | 9682 | 1 | 0.002
B4a1a1n | 12372 | 4 | 0.009
B4a1a1n | 12471 | 3 | 0.007
B4a1a1n | 16192-16193d | 2 | 0.005
B4a1a1n | 16193d | 2 | 0.005
B4a1a1n | 16286 | 1 | 0.002
B4a1a1n | 16355 | 9 | 0.021
B4a1a1n | 2071 | 1 | 0.002
B4a1a1n | 310 | 3 | 0.007
B4a1a1n | 315.3C | 1 | 0.002
B4a1a1n | 3330 | 1 | 0.002
B4a1a1n | 456 | 1 | 0.002
B4a1a1n | 4917 | 1 | 0.002
B4a1a1n | 4960 | 4 | 0.009
B4a1a1n | 573.1C | 1 | 0.002
B4a1a1n | 709 | 4 | 0.009
B4a1a1n | 7513d | 1 | 0.002
B4a1a1n | 8790 | 1 | 0.002
B4a1a1o | 151 | 1 | 0.003
B4a1a1o | 16075 | 3 | 0.008
B4a1a1o | 16092 | 1 | 0.003
B4a1a1o | 16111 | 1 | 0.003
B4a1a1o | 16124 | 1 | 0.003
B4a1a1o | 16138T | 1 | 0.003
B4a1a1o | 16142 | 1 | 0.003
B4a1a1o | 16167 | 2 | 0.005
B4a1a1o | 16181C | 1 | 0.003
B4a1a1o | 16192-16193d | 9 | 0.025
B4a1a1o | 16193d | 2 | 0.005
B4a1a1o | 16209 | 1 | 0.003
B4a1a1o | 16234 | 1 | 0.003
B4a1a1o | 16241 | 1 | 0.003
B4a1a1o | 16245 | 1 | 0.003
B4a1a1o | 16263 | 1 | 0.003
B4a1a1o | 16293 | 4 | 0.011
B4a1a1o | 16317T | 1 | 0.003
B4a1a1o | 16396A | 1 | 0.003
B4a1a1o | 16409 | 1 | 0.003
B4a1a1o | 16478G | 1 | 0.003
B4a1a1o | 16512 | 1 | 0.003
B4a1a1o | 16554 | 1 | 0.003
B4a1a1o | 214 | 1 | 0.003
B4a1a1o | 310 | 4 | 0.011
B4a1a1o | 350C | 1 | 0.003
B4a1a1p | 125G | 1 | 0.002
B4a1a1p | 15751 | 1 | 0.002
B4a1a1p | 16129 | 2 | 0.005
B4a1a1p | 16192-16193d | 1 | 0.002
B4a1a1p | 16286 | 1 | 0.002
B4a1a1p | 16290 | 1 | 0.002
B4a1a1p | 16300 | 2 | 0.005
B4a1a1p | 16355 | 9 | 0.021
B4a1a1p | 214 | 1 | 0.002
B4a1a1p | 309.3C | 4 | 0.009
B4a1a1p | 315.3C | 1 | 0.002
B4a1a1p | 456 | 1 | 0.002
B4a1a1p | 573.1C | 1 | 0.002
B4a1a1p | 71 | 1 | 0.002
B4a1a1q | 12775 | 1 | 0.143
B4a1a1q | 14198 | 1 | 0.143
B4a1a1q | 1824 | 1 | 0.143
B4a1a1r | 16278 | 1 | 0.143
B4a1a1r | 16478 | 1 | 0.143
B4a1a1s | 10256 | 2 | 0.004
B4a1a1s | 10310 | 1 | 0.002
B4a1a1s | 10321 | 1 | 0.002

B4a1a1s | 10921 | 1 | 0.002
B4a1a1s | 11314 | 2 | 0.004
B4a1a1s | 12976 | 2 | 0.004
B4a1a1s | 16192-16193d | 1 | 0.002
B4a1a1s | 16232A | 1 | 0.002
B4a1a1s | 16286 | 1 | 0.002
B4a1a1s | 16319 | 2 | 0.004
B4a1a1s | 16355 | 9 | 0.02
B4a1a1s | 16472 | 2 | 0.004
B4a1a1s | 310 | 3 | 0.007
B4a1a1s | 315.3C | 1 | 0.002
B4a1a1s | 456 | 1 | 0.002
B4a1a1s | 573.1C | 1 | 0.002
B4a1a1t | 152 | 1 | 0.002
B4a1a1t | 15663 | 2 | 0.005
B4a1a1t | 16051 | 1 | 0.002
B4a1a1t | 16286 | 1 | 0.002
B4a1a1t | 16355 | 9 | 0.021
B4a1a1t | 310 | 1 | 0.002
B4a1a1t | 315.3C | 1 | 0.002
B4a1a1t | 3398 | 2 | 0.005
B4a1a1t | 456 | 1 | 0.002
B4a1a1t | 573.1C | 1 | 0.002
B4a1a1u | 14883 | 1 | 0.002
B4a1a1u | 16207 | 1 | 0.002
B4a1a1u | 16286 | 1 | 0.002
B4a1a1u | 16355 | 9 | 0.021
B4a1a1u | 183 | 1 | 0.002
B4a1a1u | 310 | 1 | 0.002
B4a1a1u | 315.3C | 1 | 0.002
B4a1a1u | 456 | 1 | 0.002
B4a1a1u | 573.1C | 1 | 0.002
B4a1a1v | 12684 | 1 | 0.002
B4a1a1v | 15218 | 2 | 0.005
B4a1a1v | 16129 | 2 | 0.005
B4a1a1v | 16248 | 3 | 0.007
B4a1a1v | 16286 | 1 | 0.002
B4a1a1v | 16287 | 2 | 0.005
B4a1a1v | 16290 | 1 | 0.002
B4a1a1v | 16355 | 9 | 0.021
B4a1a1v | 214 | 1 | 0.002
B4a1a1v | 309.3C | 4 | 0.009
B4a1a1v | 315.3C | 1 | 0.002
B4a1a1v | 456 | 1 | 0.002
B4a1a1v | 573.1C | 1 | 0.002
B4a1a1v | 71 | 1 | 0.002
B4a1a1v | 7935 | 2 | 0.005
B4a1a1w | 16086 | 8 | 0.018
B4a1a1w | 16181C | 1 | 0.002
B4a1a1w | 16192-16193d | 1 | 0.002
B4a1a1w | 16286 | 1 | 0.002
B4a1a1w | 16325 | 1 | 0.002
B4a1a1w | 16355 | 9 | 0.021
B4a1a1w | 204 | 2 | 0.005
B4a1a1w | 207 | 1 | 0.002
B4a1a1w | 310 | 2 | 0.005
B4a1a1w | 315.3C | 1 | 0.002
B4a1a1w | 456 | 1 | 0.002
B4a1a1w | 573.1C | 1 | 0.002
B4a1a1w | 6644 | 2 | 0.005
B4a1a1w | 8854 | 6 | 0.014
B4a1a1x | 14076 | 2 | 0.005
B4a1a1x | 16203 | 9 | 0.022
B4a1a1x | 16286 | 1 | 0.002
B4a1a1x | 16326T | 8 | 0.02
B4a1a1x | 195 | 1 | 0.002
B4a1a1x | 302C | 2 | 0.005
B4a1a1x | 315.3C | 1 | 0.002
B4a1a1x | 456 | 1 | 0.002
B4a1a1x | 573.1C | 1 | 0.002
B4a1a1x | 6216 | 1 | 0.002
B4a1a1y | 14587 | 1 | 0.002
B4a1a1y | 16192-16193d | 2 | 0.005
B4a1a1y | 16193d | 3 | 0.007
B4a1a1y | 16286 | 1 | 0.002
B4a1a1y | 16355 | 9 | 0.021
B4a1a1y | 310 | 2 | 0.005
B4a1a1y | 315.3C | 1 | 0.002
B4a1a1y | 456 | 1 | 0.002
B4a1a1y | 573.1C | 1 | 0.002
B4a1a1z | 13020 | 1 | 0.002
B4a1a1z | 16286 | 1 | 0.002
B4a1a1z | 16355 | 9 | 0.021
B4a1a1z | 315.3C | 1 | 0.002
B4a1a1z | 456 | 1 | 0.002
B4a1a1z | 573.1C | 1 | 0.002
B4a1a2 | 14272G | 1 | 0.005
B4a1a2 | 16073 | 1 | 0.005
B4a1a2 | 16076d | 1 | 0.005
B4a1a2 | 16192-16193d | 1 | 0.005
B4a1a2 | 16239 | 5 | 0.025
B4a1a2 | 16265 | 4 | 0.02
B4a1a2 | 16274 | 1 | 0.005
B4a1a2 | 16405T | 1 | 0.005
B4a1a2 | 16428C | 1 | 0.005
B4a1a2 | 16429G | 1 | 0.005
B4a1a2 | 184 | 1 | 0.005
B4a1a2 | 201 | 1 | 0.005
B4a1a2 | 309.3C | 3 | 0.015
B4a1a2 | 310 | 1 | 0.005
B4a1a2 | 3394 | 1 | 0.005
B4a1a2 | 573.1C | 1 | 0.005
B4a1a3 | 16073 | 1 | 0.005
B4a1a3 | 16076d | 1 | 0.005
B4a1a3 | 16239 | 5 | 0.025
B4a1a3 | 16265 | 4 | 0.02
B4a1a3 | 16405T | 1 | 0.005
B4a1a3 | 16428C | 1 | 0.005
B4a1a3 | 16429G | 1 | 0.005
B4a1a3 | 184 | 1 | 0.005
B4a1a3 | 201 | 1 | 0.005

B4a1a3 | 309.3C | 3 | 0.015
B4a1a3 | 573.1C | 1 | 0.005
B4a1a3a | 16086 | 1 | 0.029
B4a1a3a | 16093 | 1 | 0.029
B4a1a3a | 16185 | 1 | 0.029
B4a1a3a | 16257A | 1 | 0.029
B4a1a3a | 16259 | 1 | 0.029
B4a1a3a | 16266 | 1 | 0.029
B4a1a3a | 16271 | 1 | 0.029
B4a1a3a | 16290 | 1 | 0.029
B4a1a3a | 16304 | 2 | 0.059
B4a1a3a | 16353 | 1 | 0.029
B4a1a3a | 189 | 1 | 0.029
B4a1a3a | 196 | 1 | 0.029
B4a1a3a | 469 | 1 | 0.029
B4a1a3a | 470T | 1 | 0.029
B4a1a3a | 472T | 1 | 0.029
B4a1a3a | 513 | 1 | 0.029
B4a1a3a | 7510 | 1 | 0.029
B4a1a3a | 827 | 1 | 0.029
B4a1a3a1 | 10742 | 1 | 0.028
B4a1a3a1 | 11465 | 1 | 0.028
B4a1a3a1 | 12127 | 2 | 0.056
B4a1a3a1 | 12414 | 2 | 0.056
B4a1a3a1 | 131 | 1 | 0.028
B4a1a3a1 | 13149 | 2 | 0.056
B4a1a3a1 | 152 | 1 | 0.028
B4a1a3a1 | 16188 | 5 | 0.139
B4a1a3a1 | 16355 | 5 | 0.139
B4a1a3a1 | 176 | 1 | 0.028
B4a1a3a1 | 310 | 2 | 0.056
B4a1a3a1 | 314-315d | 1 | 0.028
B4a1a3a1 | 316T | 1 | 0.028
B4a1a3a1 | 318-319d | 1 | 0.028
B4a1a3a1 | 3316 | 2 | 0.056
B4a1a3a1 | 3705 | 1 | 0.028
B4a1a3a1 | 456 | 1 | 0.028
B4a1a3a1 | 6524 | 2 | 0.056
B4a1a3a1 | 8628 | 1 | 0.028
B4a1a3a1a | 11810 | 1 | 0.022
B4a1a3a1a | 13437 | 1 | 0.022
B4a1a3a1a | 14954 | 1 | 0.022
B4a1a3a1a | 16092 | 7 | 0.152
B4a1a3a1a | 16093 | 11 | 0.239
B4a1a3a1a | 16186 | 4 | 0.087
B4a1a3a1a | 16216C | 1 | 0.022
B4a1a3a1a | 16259 | 4 | 0.087
B4a1a3a1a | 16271 | 4 | 0.087
B4a1a3a1a | 16291A | 1 | 0.022
B4a1a3a1a | 16547 | 1 | 0.022
B4a1a3a1a | 16549 | 1 | 0.022
B4a1a3a1a | 16552T | 1 | 0.022
B4a1a3a1a | 455.1T | 1 | 0.022
B4a1a3a1a | 589A | 1 | 0.022
B4a1a3a1a | 594.1G | 1 | 0.022
B4a1a3a1a | 596A | 1 | 0.022
B4a1a3a1a | 600C | 1 | 0.022
B4a1a4 | 16073 | 1 | 0.005
B4a1a4 | 16076d | 1 | 0.005
B4a1a4 | 16192-16193d | 2 | 0.01
B4a1a4 | 16360A | 6 | 0.03
B4a1a4 | 16405T | 1 | 0.005
B4a1a4 | 16428C | 1 | 0.005
B4a1a4 | 16429G | 1 | 0.005
B4a1a4 | 184 | 1 | 0.005
B4a1a4 | 195 | 1 | 0.005
B4a1a4 | 201 | 1 | 0.005
B4a1a4 | 573.1C | 1 | 0.005
B4a1a5 | 11077 | 1 | 0.005
B4a1a5 | 11305 | 1 | 0.005
B4a1a5 | 12441 | 1 | 0.005
B4a1a5 | 13191 | 1 | 0.005
B4a1a5 | 13577 | 1 | 0.005
B4a1a5 | 15479 | 1 | 0.005
B4a1a5 | 15930 | 1 | 0.005
B4a1a5 | 16073 | 1 | 0.005
B4a1a5 | 16076d | 1 | 0.005
B4a1a5 | 16192-16193d | 1 | 0.005
B4a1a5 | 16265 | 4 | 0.02
B4a1a5 | 16405T | 1 | 0.005
B4a1a5 | 16428C | 1 | 0.005
B4a1a5 | 16429G | 1 | 0.005
B4a1a5 | 184 | 1 | 0.005
B4a1a5 | 201 | 1 | 0.005
B4a1a5 | 309.3C | 2 | 0.01
B4a1a5 | 324G | 1 | 0.005
B4a1a5 | 4373 | 1 | 0.005
B4a1a5 | 573.1C | 1 | 0.005
B4a1a5a | 16048 | 1 | 0.003
B4a1a5a | 16117 | 1 | 0.003
B4a1a5a | 16181C | 2 | 0.007
B4a1a5a | 16188 | 1 | 0.003
B4a1a5a | 16192-16193d | 2 | 0.007
B4a1a5a | 16193d | 1 | 0.003
B4a1a5a | 16219 | 11 | 0.036
B4a1a5a | 16222 | 1 | 0.003
B4a1a5a | 16284 | 1 | 0.003
B4a1a5a | 16286 | 9 | 0.03
B4a1a5a | 16286A | 1 | 0.003
B4a1a5a | 16289 | 1 | 0.003
B4a1a5a | 16325 | 1 | 0.003
B4a1a5a | 16348 | 11 | 0.036
B4a1a5a | 185 | 1 | 0.003
B4a1a5a | 200 | 1 | 0.003
B4a1a5a | 204 | 1 | 0.003
B4a1a5a | 227 | 1 | 0.003
B4a1a5a | 307-309d | 1 | 0.003
B4a1a5a | 309.3C | 10 | 0.033
B4a1a5a | 309d | 2 | 0.007
B4a1a5a | 310 | 1 | 0.003
B4a1a5a | 315d | 1 | 0.003

B4a1a5a | 3471A | 1 | 0.003
B4a1a5a | 3684A | 1 | 0.003
B4a1a5a | 513 | 1 | 0.003
B4a1a5a | 573.1C | 1 | 0.003
B4a1a5a | 573.5C | 1 | 0.003
B4a1a5a | 573.6C | 1 | 0.003
B4a1a5a | 574C | 2 | 0.007
B4a1a5a | 576C | 2 | 0.007
B4a1a5a | 577C | 2 | 0.007
B4a1a5a | 593 | 1 | 0.003
B4a1a5a | 9647 | 3 | 0.01
B4a1a6 | 10993 | 1 | 0.005
B4a1a6 | 16073 | 1 | 0.005
B4a1a6 | 16076d | 1 | 0.005
B4a1a6 | 16129 | 1 | 0.005
B4a1a6 | 16193d | 2 | 0.01
B4a1a6 | 16213 | 4 | 0.02
B4a1a6 | 16311 | 4 | 0.02
B4a1a6 | 16405T | 1 | 0.005
B4a1a6 | 16428C | 1 | 0.005
B4a1a6 | 16429G | 1 | 0.005
B4a1a6 | 184 | 1 | 0.005
B4a1a6 | 201 | 1 | 0.005
B4a1a6 | 308-309d | 1 | 0.005
B4a1a6 | 573.1C | 1 | 0.005
B4a1a6 | 7642 | 1 | 0.005
B4a1a6a | 10586 | 1 | 0.005
B4a1a6a | 16073 | 1 | 0.005
B4a1a6a | 16076d | 4 | 0.02
B4a1a6a | 16193d | 2 | 0.01
B4a1a6a | 16405T | 1 | 0.005
B4a1a6a | 16428C | 1 | 0.005
B4a1a6a | 16429G | 1 | 0.005
B4a1a6a | 184 | 1 | 0.005
B4a1a6a | 201 | 1 | 0.005
B4a1a6a | 309.3C | 2 | 0.01
B4a1a6a | 310 | 1 | 0.005
B4a1a6a | 573.1C | 1 | 0.005
B4a1a6a | 8701 | 3 | 0.015
B4a1a6a | 930 | 1 | 0.005
B4a1a7 | 14659 | 7 | 0.034
B4a1a7 | 152 | 1 | 0.005
B4a1a7 | 16073 | 1 | 0.005
B4a1a7 | 16076d | 4 | 0.02
B4a1a7 | 16311 | 7 | 0.034
B4a1a7 | 16405T | 1 | 0.005
B4a1a7 | 16428C | 1 | 0.005
B4a1a7 | 16429G | 1 | 0.005
B4a1a7 | 184 | 1 | 0.005
B4a1a7 | 201 | 1 | 0.005
B4a1a7 | 4838 | 1 | 0.005
B4a1a7 | 573.1C | 1 | 0.005
B4a1b | 15936 | 1 | 0.019
B4a1b | 8251 | 1 | 0.019
B4a1b1 | 150 | 1 | 0.016
B4a1b1 | 16193.4C | 1 | 0.016
B4a1b1 | 16266 | 1 | 0.016
B4a1b1 | 16336 | 1 | 0.016
B4a1b1 | 37d | 1 | 0.016
B4a1b1 | 40-42d | 1 | 0.016
B4a1b1 | 43G | 1 | 0.016
B4a1b1 | 44G | 1 | 0.016
B4a1b1 | 45 | 1 | 0.016
B4a1b1 | 46d | 1 | 0.016
B4a1b1 | 474A | 1 | 0.016
B4a1b1 | 475C | 2 | 0.032
B4a1b1 | 477.1T | 1 | 0.016
B4a1b1 | 47C | 1 | 0.016
B4a1b1 | 485A | 2 | 0.032
B4a1b1 | 486A | 3 | 0.048
B4a1b1 | 48G | 1 | 0.016
B4a1b1 | 5004 | 2 | 0.032
B4a1b1 | 56d | 1 | 0.016
B4a1b1a | 16193.4C | 1 | 0.016
B4a1b1a | 16266 | 1 | 0.016
B4a1b1a | 16336 | 1 | 0.016
B4a1b1a | 4695 | 1 | 0.016
B4a1b1a | 7083 | 1 | 0.016
B4a1c | 10915 | 3 | 0.012
B4a1c | 12792 | 3 | 0.012
B4a1c | 13431 | 1 | 0.004
B4a1c | 13488 | 1 | 0.004
B4a1c | 13608 | 1 | 0.004
B4a1c | 14867 | 1 | 0.004
B4a1c | 15038 | 1 | 0.004
B4a1c | 15766C | 3 | 0.012
B4a1c | 15950 | 1 | 0.004
B4a1c | 16000 | 1 | 0.004
B4a1c | 16092 | 1 | 0.004
B4a1c | 16129 | 3 | 0.012
B4a1c | 16153 | 2 | 0.008
B4a1c | 16207 | 1 | 0.004
B4a1c | 16214 | 2 | 0.008
B4a1c | 16274 | 4 | 0.016
B4a1c | 2380 | 3 | 0.012
B4a1c | 279 | 1 | 0.004
B4a1c | 310 | 6 | 0.024
B4a1c | 313-315d | 2 | 0.008
B4a1c | 314-315d | 2 | 0.008
B4a1c | 315d | 2 | 0.008
B4a1c | 316 | 1 | 0.004
B4a1c | 455d | 1 | 0.004
B4a1c | 489 | 1 | 0.004
B4a1c | 513 | 1 | 0.004
B4a1c | 8277G | 1 | 0.004
B4a1c | 8485 | 1 | 0.004
B4a1c | 8603 | 3 | 0.012
B4a1c+146 | 16073 | 1 | 0.005
B4a1c+146 | 16076d | 1 | 0.005
B4a1c+146 | 16150 | 1 | 0.005
B4a1c+146 | 16179 | 1 | 0.005

B4a1c+146 | 16327 | 8 | 0.04
B4a1c+146 | 16405T | 1 | 0.005
B4a1c+146 | 16428C | 1 | 0.005
B4a1c+146 | 16429G | 1 | 0.005
B4a1c+146 | 184 | 1 | 0.005
B4a1c+146 | 201 | 1 | 0.005
B4a1c+146 | 309d | 5 | 0.025
B4a1c+146 | 89 | 1 | 0.005
B4a1c1 | 14148 | 1 | 0.004
B4a1c1 | 14569 | 1 | 0.004
B4a1c1 | 150 | 1 | 0.004
B4a1c1 | 15663 | 1 | 0.004
B4a1c1 | 16092 | 1 | 0.004
B4a1c1 | 16169 | 2 | 0.008
B4a1c1 | 16207 | 1 | 0.004
B4a1c1 | 16239 | 2 | 0.008
B4a1c1 | 16274 | 4 | 0.017
B4a1c1 | 4679 | 1 | 0.004
B4a1c1 | 7533 | 1 | 0.004
B4a1c1 | 8679 | 1 | 0.004
B4a1c1a | 16092 | 1 | 0.004
B4a1c1a | 16207 | 1 | 0.004
B4a1c1a | 16218 | 2 | 0.008
B4a1c1a | 16274 | 4 | 0.017
B4a1c1a | 195 | 2 | 0.008
B4a1c1a | 207 | 2 | 0.008
B4a1c1a | 228 | 3 | 0.012
B4a1c1a | 309.3C | 1 | 0.004
B4a1c1a | 5811 | 2 | 0.008
B4a1c1a | 65G | 3 | 0.012
B4a1c1a | 6891 | 1 | 0.004
B4a1c1a | 8595 | 2 | 0.008
B4a1c1a1 | 150A | 1 | 0.005
B4a1c1a1 | 152 | 2 | 0.011
B4a1c1a1 | 16073 | 1 | 0.005
B4a1c1a1 | 16076d | 1 | 0.005
B4a1c1a1 | 16085A | 1 | 0.005
B4a1c1a1 | 16086 | 4 | 0.022
B4a1c1a1 | 16093 | 1 | 0.005
B4a1c1a1 | 16147G | 1 | 0.005
B4a1c1a1 | 16148 | 1 | 0.005
B4a1c1a1 | 16178 | 1 | 0.005
B4a1c1a1 | 16181 | 1 | 0.005
B4a1c1a1 | 16192-16193d | 1 | 0.005
B4a1c1a1 | 16318 | 1 | 0.005
B4a1c1a1 | 16400 | 1 | 0.005
B4a1c1a1 | 16405T | 1 | 0.005
B4a1c1a1 | 16428C | 1 | 0.005
B4a1c1a1 | 16429G | 1 | 0.005
B4a1c1a1 | 16439A | 1 | 0.005
B4a1c1a1 | 1888 | 1 | 0.005
B4a1c1a1 | 279 | 1 | 0.005
B4a1c1a1 | 308-309d | 1 | 0.005
B4a1c1a1 | 5252 | 1 | 0.005
B4a1c1a1 | 71.1G | 3 | 0.016
B4a1c1a1 | 75 | 5 | 0.027
B4a1c2 | 10 | 1 | 0.032
B4a1c2 | 13188 | 2 | 0.065
B4a1c2 | 1440 | 1 | 0.032
B4a1c2 | 152 | 5 | 0.161
B4a1c2 | 15799 | 1 | 0.032
B4a1c2 | 16092 | 1 | 0.032
B4a1c2 | 16124 | 1 | 0.032
B4a1c2 | 16129 | 3 | 0.097
B4a1c2 | 16179 | 1 | 0.032
B4a1c2 | 16188 | 1 | 0.032
B4a1c2 | 16218 | 4 | 0.129
B4a1c2 | 16311 | 1 | 0.032
B4a1c2 | 16317T | 6 | 0.194
B4a1c2 | 16390 | 1 | 0.032
B4a1c2 | 185 | 1 | 0.032
B4a1c2 | 227T | 1 | 0.032
B4a1c2 | 308-309d | 1 | 0.032
B4a1c2 | 310 | 1 | 0.032
B4a1c2 | 315.2C | 1 | 0.032
B4a1c2 | 316.1G | 1 | 0.032
B4a1c2 | 316C | 1 | 0.032
B4a1c2 | 3202 | 1 | 0.032
B4a1c2 | 350C | 5 | 0.161
B4a1c2 | 3621 | 1 | 0.032
B4a1c2 | 366 | 1 | 0.032
B4a1c2 | 44.1C | 4 | 0.129
B4a1c2 | 4625 | 1 | 0.032
B4a1c2 | 63A | 1 | 0.032
B4a1c2 | 683 | 1 | 0.032
B4a1c2 | 8584 | 1 | 0.032
B4a1c3 | 16092 | 1 | 0.016
B4a1c3 | 16114A | 1 | 0.016
B4a1c3 | 200 | 1 | 0.016
B4a1c3a | 12210 | 1 | 0.077
B4a1c3a | 16212 | 1 | 0.077
B4a1c3a | 16264 | 1 | 0.077
B4a1c3a | 16319 | 1 | 0.077
B4a1c3a | 195 | 1 | 0.077
B4a1c3a | 314-315d | 5 | 0.385
B4a1c3a | 315d | 3 | 0.231
B4a1c3a | 3221 | 1 | 0.077
B4a1c3a | 374 | 1 | 0.077
B4a1c3a | 41 | 1 | 0.077
B4a1c3a | 6278 | 1 | 0.077
B4a1c3b | 10907 | 1 | 0.015
B4a1c3b | 14053 | 1 | 0.015
B4a1c3b | 14560 | 1 | 0.015
B4a1c3b | 146 | 1 | 0.015
B4a1c3b | 150 | 4 | 0.059
B4a1c3b | 152 | 1 | 0.015
B4a1c3b | 15218 | 1 | 0.015
B4a1c3b | 15530 | 1 | 0.015
B4a1c3b | 16092 | 1 | 0.015
B4a1c3b | 16190 | 3 | 0.044
B4a1c3b | 211 | 1 | 0.015

B4a1c3b | 302C | 1 | 0.015
B4a1c3b | 309.3C | 2 | 0.029
B4a1c3b | 393 | 3 | 0.044
B4a1c3b | 813 | 2 | 0.029
B4a1c3b | 8345 | 1 | 0.015
B4a1c4 | 11017 | 1 | 0.004
B4a1c4 | 11050 | 1 | 0.004
B4a1c4 | 11465 | 1 | 0.004
B4a1c4 | 11527 | 3 | 0.012
B4a1c4 | 11818 | 1 | 0.004
B4a1c4 | 11914 | 1 | 0.004
B4a1c4 | 12091 | 1 | 0.004
B4a1c4 | 12396 | 1 | 0.004
B4a1c4 | 13063 | 1 | 0.004
B4a1c4 | 13134 | 2 | 0.008
B4a1c4 | 13224 | 1 | 0.004
B4a1c4 | 143 | 1 | 0.004
B4a1c4 | 14587 | 1 | 0.004
B4a1c4 | 14783 | 1 | 0.004
B4a1c4 | 150 | 7 | 0.028
B4a1c4 | 15096 | 1 | 0.004
B4a1c4 | 15115 | 1 | 0.004
B4a1c4 | 152 | 3 | 0.012
B4a1c4 | 153 | 3 | 0.012
B4a1c4 | 15580 | 5 | 0.02
B4a1c4 | 16093 | 42 | 0.166
B4a1c4 | 16104 | 1 | 0.004
B4a1c4 | 16141 | 1 | 0.004
B4a1c4 | 16145 | 8 | 0.032
B4a1c4 | 16150 | 5 | 0.02
B4a1c4 | 16164C | 1 | 0.004
B4a1c4 | 16164T | 1 | 0.004
B4a1c4 | 16174 | 3 | 0.012
B4a1c4 | 16188 | 25 | 0.099
B4a1c4 | 16193d | 4 | 0.016
B4a1c4 | 16214 | 25 | 0.099
B4a1c4 | 16216 | 2 | 0.008
B4a1c4 | 16243 | 11 | 0.043
B4a1c4 | 16278 | 6 | 0.024
B4a1c4 | 16296d | 6 | 0.024
B4a1c4 | 16325 | 1 | 0.004
B4a1c4 | 16344 | 9 | 0.036
B4a1c4 | 16391 | 1 | 0.004
B4a1c4 | 185 | 1 | 0.004
B4a1c4 | 281 | 1 | 0.004
B4a1c4 | 309.3C | 1 | 0.004
B4a1c4 | 310 | 1 | 0.004
B4a1c4 | 3396 | 1 | 0.004
B4a1c4 | 3531 | 1 | 0.004
B4a1c4 | 3535 | 1 | 0.004
B4a1c4 | 3795 | 2 | 0.008
B4a1c4 | 3866 | 1 | 0.004
B4a1c4 | 4084 | 1 | 0.004
B4a1c4 | 4093 | 3 | 0.012
B4a1c4 | 533 | 2 | 0.008
B4a1c4 | 571 | 1 | 0.004
B4a1c4 | 6182 | 1 | 0.004
B4a1c4 | 633T | 1 | 0.004
B4a1c4 | 6891 | 1 | 0.004
B4a1c4 | 7299 | 1 | 0.004
B4a1c4 | 8837 | 4 | 0.016
B4a1c4 | 909 | 1 | 0.004
B4a1c4 | 9116 | 3 | 0.012
B4a1c4 | 9880 | 1 | 0.004
B4a1c4 | 9956 | 1 | 0.004
B4a1c5 | 16073 | 1 | 0.005
B4a1c5 | 16076d | 1 | 0.005
B4a1c5 | 16129 | 1 | 0.005
B4a1c5 | 16131 | 2 | 0.01
B4a1c5 | 16179 | 4 | 0.02
B4a1c5 | 16405T | 1 | 0.005
B4a1c5 | 16428C | 1 | 0.005
B4a1c5 | 16429G | 1 | 0.005
B4a1c5 | 184 | 1 | 0.005
B4a1c5 | 201 | 1 | 0.005
B4a1c5 | 204 | 1 | 0.005
B4a1c5 | 281 | 1 | 0.005
B4a1c5 | 310 | 4 | 0.02
B4a1c5 | 314-315d | 1 | 0.005
B4a1c5 | 315d | 1 | 0.005
B4a1c5 | 316 | 3 | 0.015
B4a1c5 | 5301 | 1 | 0.005
B4a1c5 | 55 | 1 | 0.005
B4a1c5 | 6078 | 1 | 0.005
B4a1c5 | 89 | 1 | 0.005
B4a1d | 10810 | 1 | 0.004
B4a1d | 16068 | 1 | 0.004
B4a1d | 16094 | 1 | 0.004
B4a1d | 16126 | 1 | 0.004
B4a1d | 16150 | 2 | 0.008
B4a1d | 16266 | 1 | 0.004
B4a1d | 16274 | 4 | 0.015
B4a1d | 16408 | 1 | 0.004
B4a1d | 16551A | 1 | 0.004
B4a1d | 207 | 1 | 0.004
B4a1d | 316.1G | 1 | 0.004
B4a1d | 3438 | 1 | 0.004
B4a1d | 4080 | 1 | 0.004
B4a1d | 408A | 1 | 0.004
B4a1d | 4164 | 1 | 0.004
B4a1d | 466 | 3 | 0.012
B4a1d | 481 | 1 | 0.004
B4a1d | 573.5C | 1 | 0.004
B4a1d | 732 | 1 | 0.004
B4a1e | 10680 | 1 | 0.01
B4a1e | 13145 | 2 | 0.021
B4a1e | 13863 | 1 | 0.01
B4a1e | 146 | 11 | 0.115
B4a1e | 15479 | 3 | 0.031
B4a1e | 15544A | 1 | 0.01
B4a1e | 15745 | 3 | 0.031

B4a1e | 16129 | 1 | 0.01
B4a1e | 16136 | 1 | 0.01
B4a1e | 16193d | 1 | 0.01
B4a1e | 16244C | 1 | 0.01
B4a1e | 16256 | 3 | 0.031
B4a1e | 16258C | 10 | 0.104
B4a1e | 16318T | 1 | 0.01
B4a1e | 16327 | 1 | 0.01
B4a1e | 16362 | 1 | 0.01
B4a1e | 16390 | 1 | 0.01
B4a1e | 182 | 5 | 0.052
B4a1e | 185 | 5 | 0.052
B4a1e | 199 | 1 | 0.01
B4a1e | 226 | 1 | 0.01
B4a1e | 2352 | 4 | 0.042
B4a1e | 308-309d | 1 | 0.01
B4a1e | 309.3C | 2 | 0.021
B4a1e | 310 | 1 | 0.01
B4a1e | 3540 | 3 | 0.031
B4a1e | 356.1C | 6 | 0.062
B4a1e | 3766 | 3 | 0.031
B4a1e | 379 | 1 | 0.01
B4a1e | 4343 | 3 | 0.031
B4a1e | 456 | 1 | 0.01
B4a1e | 4772 | 1 | 0.01
B4a1e | 5147 | 3 | 0.031
B4a1e | 574 | 1 | 0.01
B4a1e | 5892 | 2 | 0.021
B4a1e | 5981 | 3 | 0.031
B4a1e | 676 | 1 | 0.01
B4a1e | 794 | 2 | 0.021
B4a1e | 8521 | 3 | 0.031
B4a1e | 8572 | 3 | 0.031
B4a1e | 94 | 1 | 0.01
B4a1e | 9477 | 3 | 0.031
B4a1e | 9740 | 2 | 0.021
B4a1e | 9861 | 1 | 0.01
B4a2 | 150 | 1 | 0.004
B4a2 | 16068 | 1 | 0.004
B4a2 | 16084 | 2 | 0.008
B4a2 | 16094 | 2 | 0.008
B4a2 | 16126 | 1 | 0.004
B4a2 | 16150 | 4 | 0.015
B4a2 | 16266 | 1 | 0.004
B4a2 | 16274 | 1 | 0.004
B4a2 | 16362 | 4 | 0.015
B4a2 | 16399 | 6 | 0.023
B4a2 | 189 | 2 | 0.008
B4a2 | 204 | 1 | 0.004
B4a2 | 207 | 1 | 0.004
B4a2 | 316.1G | 1 | 0.004
B4a2 | 466 | 4 | 0.015
B4a2 | 481 | 1 | 0.004
B4a2 | 573.5C | 1 | 0.004
B4a2 | 732 | 1 | 0.004
B4a2a | 11404 | 1 | 0.014
B4a2a | 14025 | 1 | 0.014
B4a2a | 14364 | 2 | 0.028
B4a2a | 1598 | 2 | 0.028
B4a2a | 16111 | 1 | 0.014
B4a2a | 16180C | 1 | 0.014
B4a2a | 16278 | 2 | 0.028
B4a2a | 1760 | 1 | 0.014
B4a2a | 291d | 1 | 0.014
B4a2a | 309.3C | 1 | 0.014
B4a2a | 310 | 1 | 0.014
B4a2a | 3209 | 2 | 0.028
B4a2a | 5147 | 2 | 0.028
B4a2a | 65G | 2 | 0.028
B4a2a | 709 | 2 | 0.028
B4a2a | 9129A | 2 | 0.028
B4a2a1 | 16111 | 1 | 0.016
B4a2a1 | 16192-16193d | 2 | 0.031
B4a2a1 | 3736 | 1 | 0.016
B4a2a1 | 5054 | 1 | 0.016
B4a2a1 | 6167 | 1 | 0.016
B4a2a2 | 12634 | 1 | 0.017
B4a2a2 | 5773 | 2 | 0.034
B4a2a2 | 8251 | 2 | 0.034
B4a2a3 | 146 | 2 | 0.021
B4a2a3 | 14821 | 1 | 0.01
B4a2a3 | 151 | 1 | 0.01
B4a2a3 | 16197 | 1 | 0.01
B4a2a3 | 16288 | 3 | 0.031
B4a2a3 | 16390 | 1 | 0.01
B4a2a3 | 320A | 1 | 0.01
B4a2a3 | 321G | 1 | 0.01
B4a2a3 | 324G | 1 | 0.01
B4a2a3 | 332A | 1 | 0.01
B4a2a3 | 343 | 1 | 0.01
B4a2a3 | 345 | 1 | 0.01
B4a2a3 | 545 | 5 | 0.052
B4a2a3 | 8218 | 1 | 0.01
B4a2b | 150 | 2 | 0.027
B4a2b | 152 | 7 | 0.096
B4a2b | 16093 | 1 | 0.014
B4a2b | 16166C | 1 | 0.014
B4a2b | 16254 | 1 | 0.014
B4a2b | 16256 | 4 | 0.055
B4a2b | 16257 | 1 | 0.014
B4a2b | 16274 | 1 | 0.014
B4a2b | 16298 | 3 | 0.041
B4a2b | 16326 | 3 | 0.041
B4a2b | 226 | 1 | 0.014
B4a2b | 308-309d | 1 | 0.014
B4a2b | 508 | 3 | 0.041
B4a2b | 6890 | 1 | 0.014
B4a2b | 7278 | 1 | 0.014
B4a2b | 8950 | 1 | 0.014
B4a2b1 | 10223 | 1 | 0.014
B4a2b1 | 12190 | 3 | 0.042

B4a2b1 | 152 | 2 | 0.028
B4a2b1 | 16153 | 1 | 0.014
B4a2b1 | 16247 | 1 | 0.014
B4a2b1 | 16257 | 3 | 0.042
B4a2b1 | 16284 | 1 | 0.014
B4a2b1 | 16300 | 1 | 0.014
B4a2b1 | 16311 | 5 | 0.07
B4a2b1 | 16355 | 1 | 0.014
B4a2b1 | 3335 | 1 | 0.014
B4a2b1 | 5772 | 2 | 0.028
B4a2b1 | 7379 | 3 | 0.042
B4a2b1a | 13359 | 1 | 0.015
B4a2b1a | 16093 | 2 | 0.03
B4a2b1a | 16186 | 1 | 0.015
B4a2b1a | 16193d | 1 | 0.015
B4a2b1a | 16380 | 2 | 0.03
B4a2b1a | 303 | 1 | 0.015
B4a2b1a | 316C | 1 | 0.015
B4a2b1a | 317.1C | 1 | 0.015
B4a2b1a | 326C | 1 | 0.015
B4a2b1a | 366 | 1 | 0.015
B4a2b1a | 385 | 1 | 0.015
B4a2b1a | 612 | 1 | 0.015
B4a3 | 12033 | 1 | 0.011
B4a3 | 131 | 1 | 0.011
B4a3 | 152 | 4 | 0.046
B4a3 | 16086 | 2 | 0.023
B4a3 | 16094 | 4 | 0.046
B4a3 | 16126 | 1 | 0.011
B4a3 | 16129 | 1 | 0.011
B4a3 | 16145 | 3 | 0.034
B4a3 | 16224 | 1 | 0.011
B4a3 | 16266 | 1 | 0.011
B4a3 | 16357 | 1 | 0.011
B4a3 | 200 | 10 | 0.115
B4a3 | 309.3C | 1 | 0.011
B4a3 | 309d | 1 | 0.011
B4a3 | 316C | 2 | 0.023
B4a3 | 3399 | 1 | 0.011
B4a3 | 3744 | 1 | 0.011
B4a3 | 485 | 1 | 0.011
B4a3 | 64 | 1 | 0.011
B4a4 | 12681 | 2 | 0.013
B4a4 | 13281 | 1 | 0.007
B4a4 | 13834 | 3 | 0.02
B4a4 | 14049 | 3 | 0.02
B4a4 | 14323 | 1 | 0.007
B4a4 | 150 | 1 | 0.007
B4a4 | 150A | 1 | 0.007
B4a4 | 152 | 19 | 0.126
B4a4 | 15262 | 4 | 0.026
B4a4 | 153 | 3 | 0.02
B4a4 | 15944 | 4 | 0.026
B4a4 | 16048 | 1 | 0.007
B4a4 | 16092 | 21 | 0.139
B4a4 | 16093 | 1 | 0.007
B4a4 | 16107 | 1 | 0.007
B4a4 | 16126 | 1 | 0.007
B4a4 | 16145 | 1 | 0.007
B4a4 | 16158 | 1 | 0.007
B4a4 | 16169 | 1 | 0.007
B4a4 | 16179 | 1 | 0.007
B4a4 | 16184 | 1 | 0.007
B4a4 | 16186 | 2 | 0.013
B4a4 | 16192-16193d | 2 | 0.013
B4a4 | 16214 | 2 | 0.013
B4a4 | 16223 | 2 | 0.013
B4a4 | 16247 | 1 | 0.007
B4a4 | 16264 | 3 | 0.02
B4a4 | 16266 | 2 | 0.013
B4a4 | 16274 | 1 | 0.007
B4a4 | 16291 | 1 | 0.007
B4a4 | 16294 | 5 | 0.033
B4a4 | 16305 | 1 | 0.007
B4a4 | 16311 | 2 | 0.013
B4a4 | 16327 | 1 | 0.007
B4a4 | 16355 | 1 | 0.007
B4a4 | 16360 | 2 | 0.013
B4a4 | 16390 | 5 | 0.033
B4a4 | 16446G | 1 | 0.007
B4a4 | 16469d | 7 | 0.046
B4a4 | 16524 | 1 | 0.007
B4a4 | 182 | 1 | 0.007
B4a4 | 189 | 6 | 0.04
B4a4 | 194 | 4 | 0.026
B4a4 | 195 | 1 | 0.007
B4a4 | 198 | 2 | 0.013
B4a4 | 204 | 4 | 0.026
B4a4 | 207 | 1 | 0.007
B4a4 | 2222G | 4 | 0.026
B4a4 | 228T | 1 | 0.007
B4a4 | 3010 | 2 | 0.013
B4a4 | 309d | 4 | 0.026
B4a4 | 310 | 7 | 0.046
B4a4 | 314-315d | 1 | 0.007
B4a4 | 316C | 3 | 0.02
B4a4 | 320 | 1 | 0.007
B4a4 | 324G | 1 | 0.007
B4a4 | 3915 | 1 | 0.007
B4a4 | 4086 | 1 | 0.007
B4a4 | 480 | 1 | 0.007
B4a4 | 4841 | 4 | 0.026
B4a4 | 489 | 1 | 0.007
B4a4 | 5201 | 3 | 0.02
B4a4 | 5498 | 4 | 0.026
B4a4 | 573.1C | 1 | 0.007
B4a4 | 573.3C | 1 | 0.007
B4a4 | 5999 | 1 | 0.007
B4a4 | 6293 | 3 | 0.02
B4a4 | 709 | 38 | 0.252
B4a4 | 719 | 1 | 0.007

B4a4 | 7858 | 1 | 0.007
B4a4 | 8143 | 1 | 0.007
B4a4 | 8194 | 1 | 0.007
B4a4 | 8715 | 1 | 0.007
B4a4 | 93 | 1 | 0.007
B4a4 | 9932 | 1 | 0.007
B4a5 | 14040 | 2 | 0.008
B4a5 | 15236 | 10 | 0.038
B4a5 | 15244 | 10 | 0.038
B4a5 | 16093 | 11 | 0.041
B4a5 | 16136 | 3 | 0.011
B4a5 | 16169 | 2 | 0.008
B4a5 | 16212 | 1 | 0.004
B4a5 | 16248 | 1 | 0.004
B4a5 | 16295 | 4 | 0.015
B4a5 | 16319 | 3 | 0.011
B4a5 | 16357 | 12 | 0.045
B4a5 | 16457d | 4 | 0.015
B4a5 | 309.3C | 2 | 0.008
B4a5 | 310 | 1 | 0.004
B4a5 | 5147 | 3 | 0.011
B4a5 | 573.5C | 1 | 0.004
B4a5 | 5821 | 2 | 0.008
B4a5 | 8155 | 4 | 0.015
B4a5 | 8490 | 4 | 0.015
B4a5 | 9027 | 2 | 0.008
B4a5 | 9428 | 1 | 0.004
B4a5 | 9657 | 2 | 0.008
B4b | 155 | 2 | 0.018
B4b | 16221A | 2 | 0.018
B4b | 16231 | 1 | 0.009
B4b | 16241 | 1 | 0.009
B4b | 16259 | 1 | 0.009
B4b | 16264 | 2 | 0.018
B4b | 249d | 1 | 0.009
B4b | 283 | 1 | 0.009
B4b | 417 | 1 | 0.009
B4b | 54 | 1 | 0.009
B4b | 574 | 2 | 0.018
B4b1 | 146 | 1 | 0.006
B4b1 | 150 | 21 | 0.121
B4b1 | 152 | 31 | 0.179
B4b1 | 16092 | 1 | 0.006
B4b1 | 16093 | 2 | 0.012
B4b1 | 16235 | 1 | 0.006
B4b1 | 16239 | 4 | 0.023
B4b1 | 16261 | 5 | 0.029
B4b1 | 16270 | 14 | 0.081
B4b1 | 16298 | 37 | 0.214
B4b1 | 16300 | 2 | 0.012
B4b1 | 16309 | 2 | 0.012
B4b1 | 16311 | 1 | 0.006
B4b1 | 16354 | 3 | 0.017
B4b1 | 16355 | 1 | 0.006
B4b1 | 16360 | 2 | 0.012
B4b1 | 1819 | 3 | 0.017
B4b1 | 195 | 1 | 0.006
B4b1 | 200 | 4 | 0.023
B4b1 | 228 | 2 | 0.012
B4b1 | 309.4C | 1 | 0.006
B4b1 | 310 | 7 | 0.04
B4b1 | 312-315d | 1 | 0.006
B4b1 | 314-315d | 2 | 0.012
B4b1 | 315d | 3 | 0.017
B4b1 | 333 | 3 | 0.017
B4b1 | 357 | 14 | 0.081
B4b1 | 451d | 1 | 0.006
B4b1 | 471 | 4 | 0.023
B4b1 | 498 | 1 | 0.006
B4b1 | 5301C | 1 | 0.006
B4b1 | 6482 | 1 | 0.006
B4b1 | 737 | 1 | 0.006
B4b1 | 9377 | 1 | 0.006
B4b1 | 9947 | 1 | 0.006
B4b1a | 11 | 1 | 0.006
B4b1a | 11254 | 3 | 0.019
B4b1a | 12397 | 1 | 0.006
B4b1a | 13183 | 2 | 0.013
B4b1a | 13779 | 1 | 0.006
B4b1a | 146 | 6 | 0.038
B4b1a | 15007 | 1 | 0.006
B4b1a | 152 | 1 | 0.006
B4b1a | 15688 | 3 | 0.019
B4b1a | 15758 | 3 | 0.019
B4b1a | 15908 | 1 | 0.006
B4b1a | 16051 | 1 | 0.006
B4b1a | 16075A | 1 | 0.006
B4b1a | 16086 | 1 | 0.006
B4b1a | 16126 | 2 | 0.013
B4b1a | 16129 | 1 | 0.006
B4b1a | 16187A | 1 | 0.006
B4b1a | 16241 | 1 | 0.006
B4b1a | 16260 | 16 | 0.102
B4b1a | 16287 | 4 | 0.025
B4b1a | 16290A | 2 | 0.013
B4b1a | 16291A | 1 | 0.006
B4b1a | 16292 | 2 | 0.013
B4b1a | 16293C | 2 | 0.013
B4b1a | 16325 | 9 | 0.057
B4b1a | 16360 | 1 | 0.006
B4b1a | 16399 | 1 | 0.006
B4b1a | 16400 | 1 | 0.006
B4b1a | 16412T | 1 | 0.006
B4b1a | 16474d | 1 | 0.006
B4b1a | 16527 | 1 | 0.006
B4b1a | 199 | 3 | 0.019
B4b1a | 200 | 1 | 0.006
B4b1a | 2069 | 2 | 0.013
B4b1a | 235 | 1 | 0.006
B4b1a | 310 | 1 | 0.006
B4b1a | 315d | 1 | 0.006

B4b1a | 4655 | 1 | 0.006
B4b1a | 4907 | 1 | 0.006
B4b1a | 5063 | 3 | 0.019
B4b1a | 5237 | 2 | 0.013
B4b1a | 6216 | 7 | 0.045
B4b1a | 6908 | 1 | 0.006
B4b1a | 8014 | 1 | 0.006
B4b1a | 8149 | 1 | 0.006
B4b1a | 8155 | 3 | 0.019
B4b1a+207 | 16066.1A | 1 | 0.006
B4b1a+207 | 16086 | 2 | 0.012
B4b1a+207 | 16111 | 1 | 0.006
B4b1a+207 | 16223 | 1 | 0.006
B4b1a+207 | 16257 | 3 | 0.019
B4b1a+207 | 16258T | 1 | 0.006
B4b1a+207 | 16271 | 2 | 0.012
B4b1a+207 | 309.3C | 3 | 0.019
B4b1a+207 | 388 | 1 | 0.006
B4b1a+207 | 440 | 1 | 0.006
B4b1a+207 | 93 | 1 | 0.006
B4b1a1 | 13194 | 1 | 0.032
B4b1a1 | 13359 | 1 | 0.032
B4b1a1 | 13768 | 1 | 0.032
B4b1a1 | 146 | 7 | 0.226
B4b1a1 | 150 | 1 | 0.032
B4b1a1 | 151 | 1 | 0.032
B4b1a1 | 15773 | 1 | 0.032
B4b1a1 | 16298 | 1 | 0.032
B4b1a1 | 204 | 3 | 0.097
B4b1a1 | 245 | 1 | 0.032
B4b1a1 | 309.3C | 2 | 0.065
B4b1a1 | 309d | 1 | 0.032
B4b1a1 | 4248 | 1 | 0.032
B4b1a1 | 6116 | 1 | 0.032
B4b1a1 | 7238 | 1 | 0.032
B4b1a1 | 8510 | 1 | 0.032
B4b1a1 | 8515 | 1 | 0.032
B4b1a1 | 8730 | 1 | 0.032
B4b1a1 | 8793 | 1 | 0.032
B4b1a1 | 9325 | 1 | 0.032
B4b1a1 | 9332 | 1 | 0.032
B4b1a1 | 9477 | 1 | 0.032
B4b1a1a | 10320 | 1 | 0.043
B4b1a1a | 1189 | 1 | 0.043
B4b1a1a | 12696 | 1 | 0.043
B4b1a1a | 16140 | 1 | 0.043
B4b1a1a | 16194C | 1 | 0.043
B4b1a1a | 16195 | 1 | 0.043
B4b1a1a | 16324 | 1 | 0.043
B4b1a1a | 16355 | 1 | 0.043
B4b1a1a | 2080 | 1 | 0.043
B4b1a1a | 310 | 1 | 0.043
B4b1a1a | 3592 | 1 | 0.043
B4b1a1a | 4025 | 1 | 0.043
B4b1a1a | 508 | 1 | 0.043
B4b1a1a | 633T | 1 | 0.043
B4b1a1b | 15001 | 1 | 0.05
B4b1a1b | 15884 | 1 | 0.05
B4b1a1b | 16093 | 1 | 0.05
B4b1a1b | 16390 | 1 | 0.05
B4b1a1b | 2685G | 1 | 0.05
B4b1a1b | 4967 | 1 | 0.05
B4b1a1c | 146 | 1 | 0.083
B4b1a1c | 309.3C | 1 | 0.083
B4b1a1c | 441 | 1 | 0.083
B4b1a1c | 530 | 1 | 0.083
B4b1a1c | 7119 | 1 | 0.083
B4b1a1c | 7664 | 1 | 0.083
B4b1a1c | 8581 | 1 | 0.083
B4b1a2 | 11453 | 1 | 0.005
B4b1a2 | 12121 | 1 | 0.005
B4b1a2 | 12561 | 1 | 0.005
B4b1a2 | 12771 | 1 | 0.005
B4b1a2 | 1313C | 4 | 0.021
B4b1a2 | 146 | 2 | 0.011
B4b1a2 | 1462 | 1 | 0.005
B4b1a2 | 152 | 4 | 0.021
B4b1a2 | 15313 | 1 | 0.005
B4b1a2 | 15734 | 1 | 0.005
B4b1a2 | 15884 | 3 | 0.016
B4b1a2 | 16092 | 6 | 0.032
B4b1a2 | 16093 | 1 | 0.005
B4b1a2 | 16172 | 2 | 0.011
B4b1a2 | 16192-16193d | 1 | 0.005
B4b1a2 | 16193d | 1 | 0.005
B4b1a2 | 16194C | 1 | 0.005
B4b1a2 | 16195 | 1 | 0.005
B4b1a2 | 16197 | 2 | 0.011
B4b1a2 | 16214 | 3 | 0.016
B4b1a2 | 16261 | 1 | 0.005
B4b1a2 | 16465 | 5 | 0.026
B4b1a2 | 16497 | 2 | 0.011
B4b1a2 | 178 | 2 | 0.011
B4b1a2 | 189 | 1 | 0.005
B4b1a2 | 204 | 3 | 0.016
B4b1a2 | 2056 | 1 | 0.005
B4b1a2 | 244 | 2 | 0.011
B4b1a2 | 291d | 1 | 0.005
B4b1a2 | 309d | 1 | 0.005
B4b1a2 | 310 | 5 | 0.026
B4b1a2 | 315d | 1 | 0.005
B4b1a2 | 3308 | 1 | 0.005
B4b1a2 | 4084 | 2 | 0.011
B4b1a2 | 4639 | 1 | 0.005
B4b1a2 | 4655 | 1 | 0.005
B4b1a2 | 5147 | 1 | 0.005
B4b1a2 | 525.2C | 1 | 0.005
B4b1a2 | 5460 | 1 | 0.005
B4b1a2 | 6560 | 1 | 0.005
B4b1a2 | 7245 | 1 | 0.005
B4b1a2 | 7337 | 2 | 0.011

B4b1a2 | 7533 | 1 | 0.005
B4b1a2 | 7775 | 2 | 0.011
B4b1a2 | 8225 | 1 | 0.005
B4b1a2 | 8270 | 1 | 0.005
B4b1a2 | 8485 | 3 | 0.016
B4b1a2 | 8557 | 1 | 0.005
B4b1a2 | 8558 | 1 | 0.005
B4b1a2 | 9098 | 1 | 0.005
B4b1a2 | 9554 | 3 | 0.016
B4b1a2 | 9668 | 1 | 0.005
B4b1a2 | 9744 | 1 | 0.005
B4b1a2 | 9947 | 1 | 0.005
B4b1a2a | 10166 | 1 | 0.004
B4b1a2a | 10586 | 1 | 0.004
B4b1a2a | 11152 | 1 | 0.004
B4b1a2a | 1119 | 1 | 0.004
B4b1a2a | 12338 | 1 | 0.004
B4b1a2a | 12397 | 1 | 0.004
B4b1a2a | 14097 | 1 | 0.004
B4b1a2a | 14470 | 3 | 0.011
B4b1a2a | 14502 | 1 | 0.004
B4b1a2a | 146 | 2 | 0.007
B4b1a2a | 14696 | 2 | 0.007
B4b1a2a | 150 | 9 | 0.032
B4b1a2a | 15077 | 3 | 0.011
B4b1a2a | 152 | 2 | 0.007
B4b1a2a | 15301 | 37 | 0.132
B4b1a2a | 15314 | 1 | 0.004
B4b1a2a | 16075A | 1 | 0.004
B4b1a2a | 16092 | 3 | 0.011
B4b1a2a | 16093 | 16 | 0.057
B4b1a2a | 16114 | 1 | 0.004
B4b1a2a | 16168 | 1 | 0.004
B4b1a2a | 16169 | 1 | 0.004
B4b1a2a | 16172 | 2 | 0.007
B4b1a2a | 16179 | 13 | 0.046
B4b1a2a | 16192 | 1 | 0.004
B4b1a2a | 16234 | 9 | 0.032
B4b1a2a | 16278 | 2 | 0.007
B4b1a2a | 16294 | 9 | 0.032
B4b1a2a | 16309 | 85 | 0.302
B4b1a2a | 16318C | 1 | 0.004
B4b1a2a | 16318T | 1 | 0.004
B4b1a2a | 16354 | 82 | 0.292
B4b1a2a | 16360 | 1 | 0.004
B4b1a2a | 16363A | 1 | 0.004
B4b1a2a | 16402T | 1 | 0.004
B4b1a2a | 16422 | 1 | 0.004
B4b1a2a | 195 | 1 | 0.004
B4b1a2a | 199 | 1 | 0.004
B4b1a2a | 204 | 18 | 0.064
B4b1a2a | 241 | 1 | 0.004
B4b1a2a | 2831 | 7 | 0.025
B4b1a2a | 294 | 1 | 0.004
B4b1a2a | 309.3C | 1 | 0.004
B4b1a2a | 310 | 1 | 0.004
B4b1a2a | 314-315d | 1 | 0.004
B4b1a2a | 316T | 1 | 0.004
B4b1a2a | 318-319d | 1 | 0.004
B4b1a2a | 3434 | 1 | 0.004
B4b1a2a | 389 | 1 | 0.004
B4b1a2a | 4161 | 1 | 0.004
B4b1a2a | 498 | 22 | 0.078
B4b1a2a | 4991 | 2 | 0.007
B4b1a2a | 522A | 1 | 0.004
B4b1a2a | 524A | 1 | 0.004
B4b1a2a | 5899 | 1 | 0.004
B4b1a2a | 7184 | 1 | 0.004
B4b1a2a | 7419 | 1 | 0.004
B4b1a2a | 7855 | 1 | 0.004
B4b1a2a | 7993 | 28 | 0.1
B4b1a2a | 8020T | 3 | 0.011
B4b1a2a | 8279 | 1 | 0.004
B4b1a2a | 8567 | 1 | 0.004
B4b1a2a | 8632 | 1 | 0.004
B4b1a2a | 8683 | 2 | 0.007
B4b1a2a | 8839 | 2 | 0.007
B4b1a2a | 9016 | 1 | 0.004
B4b1a2a | 9128 | 1 | 0.004
B4b1a2a | 9575 | 2 | 0.007
B4b1a2b | 16051 | 1 | 0.077
B4b1a2b | 16260 | 5 | 0.385
B4b1a2b | 195 | 1 | 0.077
B4b1a2b | 9449 | 2 | 0.154
B4b1a2b1 | 14133 | 1 | 0.083
B4b1a2b1 | 16092 | 1 | 0.083
B4b1a2b1 | 309d | 1 | 0.083
B4b1a2b1 | 310 | 2 | 0.167
B4b1a2b1 | 315d | 2 | 0.167
B4b1a2b1 | 8276.1T | 1 | 0.083
B4b1a2b2 | 146 | 1 | 0.083
B4b1a2b2 | 6297 | 2 | 0.167
B4b1a2b2 | 7754 | 1 | 0.083
B4b1a2c | 11809 | 4 | 0.444
B4b1a2c | 310 | 5 | 0.556
B4b1a2c | 315d | 5 | 0.556
B4b1a2d | 15055 | 1 | 0.125
B4b1a2d | 310 | 5 | 0.625
B4b1a2d | 315d | 4 | 0.5
B4b1a2e | 10410 | 1 | 0.006
B4b1a2e | 13146 | 3 | 0.018
B4b1a2e | 14467 | 2 | 0.012
B4b1a2e | 146 | 2 | 0.012
B4b1a2e | 152 | 10 | 0.06
B4b1a2e | 16111 | 1 | 0.006
B4b1a2e | 16258T | 2 | 0.012
B4b1a2e | 310 | 1 | 0.006
B4b1a2e | 3169 | 1 | 0.006
B4b1a2e | 388 | 1 | 0.006
B4b1a2e | 440 | 1 | 0.006
B4b1a2e | 6255 | 1 | 0.006

B4b1a2e | 9141 | 3 | 0.018
B4b1a2e | 93 | 1 | 0.006
B4b1a2e | 9554 | 1 | 0.006
B4b1a2f | 10313C | 4 | 0.025
B4b1a2f | 14 | 1 | 0.006
B4b1a2f | 14470 | 2 | 0.013
B4b1a2f | 16086 | 2 | 0.013
B4b1a2f | 16145 | 1 | 0.006
B4b1a2f | 16153 | 1 | 0.006
B4b1a2f | 16456 | 3 | 0.019
B4b1a2f | 16555 | 2 | 0.013
B4b1a2f | 204 | 1 | 0.006
B4b1a2f | 3197 | 2 | 0.013
B4b1a2f | 6527 | 6 | 0.038
B4b1a2f | 8296 | 2 | 0.013
B4b1a2g | 12828 | 2 | 0.083
B4b1a2g | 14530 | 1 | 0.042
B4b1a2g | 16037 | 1 | 0.042
B4b1a2g | 16086 | 1 | 0.042
B4b1a2g | 16228 | 1 | 0.042
B4b1a2g | 16483 | 2 | 0.083
B4b1a2g | 3144 | 1 | 0.042
B4b1a2g | 525.2C | 1 | 0.042
B4b1a2g | 6260 | 7 | 0.292
B4b1a2g1 | 16037 | 1 | 0.042
B4b1a2g1 | 4435 | 5 | 0.208
B4b1a2g1 | 9632 | 3 | 0.125
B4b1a2h | 16111 | 1 | 0.006
B4b1a2h | 16258T | 2 | 0.012
B4b1a2h | 204 | 1 | 0.006
B4b1a2h | 388 | 1 | 0.006
B4b1a2h | 440 | 1 | 0.006
B4b1a2h | 8839 | 1 | 0.006
B4b1a2h | 93 | 1 | 0.006
B4b1a2i | 11929 | 5 | 0.2
B4b1a2i | 146 | 1 | 0.04
B4b1a2i | 15883 | 4 | 0.16
B4b1a2i | 16092 | 1 | 0.04
B4b1a2i | 16171T | 1 | 0.04
B4b1a2i | 16172 | 4 | 0.16
B4b1a2i | 16175C | 1 | 0.04
B4b1a2i | 16193d | 1 | 0.04
B4b1a2i | 16274 | 1 | 0.04
B4b1a2i | 16278 | 6 | 0.24
B4b1a2i | 16325d | 1 | 0.04
B4b1a2i | 16343 | 1 | 0.04
B4b1a2i | 16362A | 1 | 0.04
B4b1a2i | 16364 | 1 | 0.04
B4b1a2i | 204 | 6 | 0.24
B4b1a2i | 8852C | 2 | 0.08
B4b1a2i | 9145 | 1 | 0.04
B4b1a2i | 9526 | 4 | 0.16
B4b1a3 | 12945 | 5 | 0.035
B4b1a3 | 150 | 2 | 0.014
B4b1a3 | 15479 | 1 | 0.007
B4b1a3 | 16225 | 1 | 0.007
B4b1a3 | 16242G | 1 | 0.007
B4b1a3 | 16265C | 1 | 0.007
B4b1a3 | 16278 | 1 | 0.007
B4b1a3 | 16287 | 1 | 0.007
B4b1a3 | 16318 | 2 | 0.014
B4b1a3 | 16360 | 4 | 0.028
B4b1a3 | 1766 | 1 | 0.007
B4b1a3 | 3010 | 1 | 0.007
B4b1a3 | 4561 | 1 | 0.007
B4b1a3 | 5843 | 4 | 0.028
B4b1a3 | 9966 | 2 | 0.014
B4b1a3a | 1005 | 1 | 0.025
B4b1a3a | 11084 | 1 | 0.025
B4b1a3a | 14905 | 2 | 0.05
B4b1a3a | 152 | 1 | 0.025
B4b1a3a | 15813 | 1 | 0.025
B4b1a3a | 16197G | 1 | 0.025
B4b1a3a | 16198G | 1 | 0.025
B4b1a3a | 16327 | 2 | 0.05
B4b1a3a | 1819 | 1 | 0.025
B4b1a3a | 188 | 1 | 0.025
B4b1a3a | 309.3C | 1 | 0.025
B4b1a3a | 310 | 3 | 0.075
B4b1a3a | 311 | 1 | 0.025
B4b1a3a | 313 | 1 | 0.025
B4b1a3a | 315.2C | 1 | 0.025
B4b1a3a | 4026T | 2 | 0.05
B4b1a3a | 4435 | 1 | 0.025
B4b1a3a | 4562 | 2 | 0.05
B4b1a3a | 6089 | 1 | 0.025
B4b1a3a | 612 | 1 | 0.025
B4b1a3a | 7364 | 1 | 0.025
B4b1a3a | 7389 | 1 | 0.025
B4b1a3a | 8473 | 1 | 0.025
B4b1b | 146 | 1 | 0.053
B4b1b | 14971 | 1 | 0.053
B4b1b | 15221 | 1 | 0.053
B4b1b | 16295 | 1 | 0.053
B4b1b | 16320 | 1 | 0.053
B4b1b | 16399 | 1 | 0.053
B4b1b | 333 | 1 | 0.053
B4b1b'c | 116 | 1 | 0.015
B4b1b'c | 126 | 1 | 0.015
B4b1b'c | 133 | 1 | 0.015
B4b1b'c | 135.1T | 1 | 0.015
B4b1b'c | 150 | 3 | 0.044
B4b1b'c | 16038 | 1 | 0.015
B4b1b'c | 16086 | 1 | 0.015
B4b1b'c | 16105 | 1 | 0.015
B4b1b'c | 16131 | 1 | 0.015
B4b1b'c | 16139 | 1 | 0.015
B4b1b'c | 16154 | 9 | 0.132
B4b1b'c | 16169 | 1 | 0.015
B4b1b'c | 16179 | 2 | 0.029
B4b1b'c | 16193d | 2 | 0.029

B4b1b'c | 16194C | 1 | 0.015
B4b1b'c | 16195 | 1 | 0.015
B4b1b'c | 16223 | 20 | 0.294
B4b1b'c | 16224G | 1 | 0.015
B4b1b'c | 16240 | 1 | 0.015
B4b1b'c | 16249 | 1 | 0.015
B4b1b'c | 16260 | 1 | 0.015
B4b1b'c | 16292 | 3 | 0.044
B4b1b'c | 16297 | 1 | 0.015
B4b1b'c | 16338 | 1 | 0.015
B4b1b'c | 16344 | 1 | 0.015
B4b1b'c | 16352 | 19 | 0.279
B4b1b'c | 16355 | 19 | 0.279
B4b1b'c | 16399 | 2 | 0.029
B4b1b'c | 16497 | 1 | 0.015
B4b1b'c | 16512 | 1 | 0.015
B4b1b'c | 16521 | 1 | 0.015
B4b1b'c | 16550A | 1 | 0.015
B4b1b'c | 16559 | 1 | 0.015
B4b1b'c | 195 | 1 | 0.015
B4b1b'c | 200 | 2 | 0.029
B4b1b'c | 217-220d | 1 | 0.015
B4b1b'c | 220 | 1 | 0.015
B4b1b'c | 227 | 4 | 0.059
B4b1b'c | 235C | 1 | 0.015
B4b1b'c | 245A | 1 | 0.015
B4b1b'c | 246d | 1 | 0.015
B4b1b'c | 249d | 11 | 0.162
B4b1b'c | 291.1A | 1 | 0.015
B4b1b'c | 309.3C | 2 | 0.029
B4b1b'c | 309.4C | 1 | 0.015
B4b1b'c | 309d | 6 | 0.088
B4b1b'c | 321 | 1 | 0.015
B4b1b'c | 408 | 1 | 0.015
B4b1b'c | 430 | 1 | 0.015
B4b1b'c | 456 | 3 | 0.044
B4b1b'c | 473 | 1 | 0.015
B4b1b'c | 492 | 1 | 0.015
B4b1b'c | 52A | 1 | 0.015
B4b1b'c | 553A | 1 | 0.015
B4b1b'c | 561 | 1 | 0.015
B4b1b'c | 574C | 1 | 0.015
B4b1b'c | 636 | 7 | 0.103
B4b1b'c | 709 | 1 | 0.015
B4b1b'c | 75 | 1 | 0.015
B4b1c | 116 | 1 | 0.015
B4b1c | 126 | 1 | 0.015
B4b1c | 133 | 1 | 0.015
B4b1c | 135.1T | 1 | 0.015
B4b1c | 150 | 3 | 0.044
B4b1c | 16038 | 1 | 0.015
B4b1c | 16086 | 1 | 0.015
B4b1c | 16105 | 1 | 0.015
B4b1c | 16131 | 1 | 0.015
B4b1c | 16139 | 1 | 0.015
B4b1c | 16154 | 9 | 0.132
B4b1c | 16169 | 1 | 0.015
B4b1c | 16179 | 2 | 0.029
B4b1c | 16193d | 2 | 0.029
B4b1c | 16194C | 1 | 0.015
B4b1c | 16195 | 1 | 0.015
B4b1c | 16223 | 20 | 0.294
B4b1c | 16224G | 1 | 0.015
B4b1c | 16240 | 1 | 0.015
B4b1c | 16249 | 1 | 0.015
B4b1c | 16260 | 1 | 0.015
B4b1c | 16292 | 3 | 0.044
B4b1c | 16297 | 1 | 0.015
B4b1c | 16338 | 1 | 0.015
B4b1c | 16344 | 1 | 0.015
B4b1c | 16352 | 19 | 0.279
B4b1c | 16355 | 19 | 0.279
B4b1c | 16399 | 2 | 0.029
B4b1c | 16497 | 1 | 0.015
B4b1c | 16512 | 1 | 0.015
B4b1c | 16521 | 1 | 0.015
B4b1c | 16550A | 1 | 0.015
B4b1c | 16559 | 1 | 0.015
B4b1c | 195 | 1 | 0.015
B4b1c | 200 | 2 | 0.029
B4b1c | 217-220d | 1 | 0.015
B4b1c | 220 | 1 | 0.015
B4b1c | 227 | 4 | 0.059
B4b1c | 235C | 1 | 0.015
B4b1c | 245A | 1 | 0.015
B4b1c | 246d | 1 | 0.015
B4b1c | 249d | 11 | 0.162
B4b1c | 291.1A | 1 | 0.015
B4b1c | 309.3C | 2 | 0.029
B4b1c | 309.4C | 1 | 0.015
B4b1c | 309d | 6 | 0.088
B4b1c | 321 | 1 | 0.015
B4b1c | 408 | 1 | 0.015
B4b1c | 430 | 1 | 0.015
B4b1c | 456 | 3 | 0.044
B4b1c | 473 | 1 | 0.015
B4b1c | 492 | 1 | 0.015
B4b1c | 52A | 1 | 0.015
B4b1c | 553A | 1 | 0.015
B4b1c | 561 | 1 | 0.015
B4b1c | 574C | 1 | 0.015
B4b1c | 636 | 7 | 0.103
B4b1c | 709 | 1 | 0.015
B4b1c | 75 | 1 | 0.015
B4b1c1 | 11809G | 2 | 0.042
B4b1c1 | 13748 | 1 | 0.021
B4b1c1 | 146 | 1 | 0.021
B4b1c1 | 152 | 2 | 0.042
B4b1c1 | 15412 | 1 | 0.021
B4b1c1 | 15670 | 1 | 0.021
B4b1c1 | 16093 | 3 | 0.062

B4b1c1 | 16147 | 2 | 0.042
B4b1c1 | 16172 | 1 | 0.021
B4b1c1 | 16175 | 20 | 0.417
B4b1c1 | 16222 | 1 | 0.021
B4b1c1 | 16223.1C | 1 | 0.021
B4b1c1 | 16311 | 10 | 0.208
B4b1c1 | 16324 | 6 | 0.125
B4b1c1 | 16357 | 1 | 0.021
B4b1c1 | 16360 | 1 | 0.021
B4b1c1 | 16362 | 1 | 0.021
B4b1c1 | 16428 | 1 | 0.021
B4b1c1 | 193 | 1 | 0.021
B4b1c1 | 195 | 1 | 0.021
B4b1c1 | 199 | 4 | 0.083
B4b1c1 | 204 | 1 | 0.021
B4b1c1 | 308-309d | 1 | 0.021
B4b1c1 | 309.3C | 3 | 0.062
B4b1c1 | 309d | 2 | 0.042
B4b1c1 | 3338 | 1 | 0.021
B4b1c1 | 3450 | 1 | 0.021
B4b1c1 | 4435 | 2 | 0.042
B4b1c1 | 455d | 1 | 0.021
B4b1c1 | 5129 | 1 | 0.021
B4b1c1 | 5460 | 1 | 0.021
B4b1c1 | 55.1T | 2 | 0.042
B4b1c1 | 59-60d | 2 | 0.042
B4b1c1 | 6962 | 1 | 0.021
B4b1c1 | 71.1G | 2 | 0.042
B4b1c1 | 7202 | 1 | 0.021
B4b1c1 | 7927 | 1 | 0.021
B4b1c1 | 8994 | 1 | 0.021
B4b1c1 | 9311 | 1 | 0.021
B4b1c1 | 9727 | 1 | 0.021
B4b1c2 | 11053 | 1 | 0.02
B4b1c2 | 12398 | 1 | 0.02
B4b1c2 | 13674 | 2 | 0.04
B4b1c2 | 13899 | 2 | 0.04
B4b1c2 | 152 | 7 | 0.14
B4b1c2 | 16051 | 8 | 0.16
B4b1c2 | 16076d | 2 | 0.04
B4b1c2 | 16145 | 3 | 0.06
B4b1c2 | 16172 | 1 | 0.02
B4b1c2 | 16183T | 1 | 0.02
B4b1c2 | 16239 | 6 | 0.12
B4b1c2 | 16240 | 6 | 0.12
B4b1c2 | 16248 | 5 | 0.1
B4b1c2 | 16249 | 1 | 0.02
B4b1c2 | 16311 | 3 | 0.06
B4b1c2 | 16320 | 1 | 0.02
B4b1c2 | 16362 | 2 | 0.04
B4b1c2 | 16390T | 1 | 0.02
B4b1c2 | 16428 | 1 | 0.02
B4b1c2 | 16484-16489d | 1 | 0.02
B4b1c2 | 185 | 1 | 0.02
B4b1c2 | 195 | 1 | 0.02
B4b1c2 | 227 | 1 | 0.02
B4b1c2 | 2772 | 1 | 0.02
B4b1c2 | 310 | 6 | 0.12
B4b1c2 | 312-315d | 1 | 0.02
B4b1c2 | 511 | 2 | 0.04
B4b1c2 | 636 | 1 | 0.02
B4b1c2 | 8338 | 2 | 0.04
B4b1c2 | 9065 | 1 | 0.02
B4b1c2 | 9899 | 2 | 0.04
B4c | 10192A | 1 | 0.008
B4c | 10310 | 1 | 0.008
B4c | 12630 | 4 | 0.032
B4c | 150 | 1 | 0.008
B4c | 15010 | 21 | 0.167
B4c | 15055 | 1 | 0.008
B4c | 152 | 1 | 0.008
B4c | 16079 | 1 | 0.008
B4c | 16093 | 1 | 0.008
B4c | 16108 | 48 | 0.381
B4c | 16129 | 2 | 0.016
B4c | 16168 | 2 | 0.016
B4c | 16222 | 1 | 0.008
B4c | 16228 | 1 | 0.008
B4c | 16234 | 7 | 0.056
B4c | 16243 | 1 | 0.008
B4c | 16293C | 1 | 0.008
B4c | 16311 | 3 | 0.024
B4c | 16319 | 1 | 0.008
B4c | 16324 | 4 | 0.032
B4c | 16362 | 41 | 0.325
B4c | 16362A | 1 | 0.008
B4c | 16362G | 21 | 0.167
B4c | 16381 | 1 | 0.008
B4c | 1888 | 9 | 0.071
B4c | 199 | 5 | 0.04
B4c | 200 | 2 | 0.016
B4c | 207 | 1 | 0.008
B4c | 214 | 1 | 0.008
B4c | 247 | 4 | 0.032
B4c | 2483 | 2 | 0.016
B4c | 2884 | 21 | 0.167
B4c | 308-309d | 1 | 0.008
B4c | 309.3C | 1 | 0.008
B4c | 372 | 1 | 0.008
B4c | 5585 | 1 | 0.008
B4c | 6293 | 6 | 0.048
B4c | 6520 | 6 | 0.048
B4c | 6960 | 9 | 0.071
B4c | 7783 | 1 | 0.008
B4c | 9024 | 21 | 0.167
B4c | 9030 | 21 | 0.167
B4c | 9151 | 21 | 0.167
B4c | 9254 | 9 | 0.071
B4c1 | 150 | 3 | 0.034
B4c1 | 16086 | 2 | 0.023
B4c1 | 16093 | 1 | 0.011

B4c1 | 16094 | 2 | 0.023
B4c1 | 16129 | 23 | 0.261
B4c1 | 16188 | 1 | 0.011
B4c1 | 16278 | 1 | 0.011
B4c1 | 16296 | 1 | 0.011
B4c1 | 16354 | 1 | 0.011
B4c1 | 16354A | 1 | 0.011
B4c1 | 189 | 2 | 0.023
B4c1 | 309.3C | 1 | 0.011
B4c1 | 338 | 3 | 0.034
B4c1 | 466 | 1 | 0.011
B4c1 | 523d | 1 | 0.011
B4c1 | 722 | 1 | 0.011
B4c1a | 10790 | 8 | 0.079
B4c1a | 11377 | 8 | 0.079
B4c1a | 12406 | 8 | 0.079
B4c1a | 139 | 4 | 0.04
B4c1a | 143 | 1 | 0.01
B4c1a | 146 | 2 | 0.02
B4c1a | 152 | 1 | 0.01
B4c1a | 15948d | 1 | 0.01
B4c1a | 16031T | 1 | 0.01
B4c1a | 16093 | 4 | 0.04
B4c1a | 16173 | 1 | 0.01
B4c1a | 16178 | 12 | 0.119
B4c1a | 16190 | 1 | 0.01
B4c1a | 16192-16193d | 1 | 0.01
B4c1a | 16193d | 2 | 0.02
B4c1a | 16274 | 1 | 0.01
B4c1a | 16278 | 1 | 0.01
B4c1a | 16293C | 2 | 0.02
B4c1a | 16357 | 2 | 0.02
B4c1a | 189 | 1 | 0.01
B4c1a | 214 | 30 | 0.297
B4c1a | 309.3C | 3 | 0.03
B4c1a | 309d | 9 | 0.089
B4c1a | 315.2C | 2 | 0.02
B4c1a | 368 | 25 | 0.248
B4c1a | 3745 | 1 | 0.01
B4c1a | 3918 | 8 | 0.079
B4c1a | 455.1T | 1 | 0.01
B4c1a | 6815 | 1 | 0.01
B4c1a | 8548 | 6 | 0.059
B4c1a | 8764 | 6 | 0.059
B4c1a | 9359 | 6 | 0.059
B4c1a1 | 10373 | 2 | 0.047
B4c1a1 | 11353 | 1 | 0.023
B4c1a1 | 12366 | 1 | 0.023
B4c1a1 | 146 | 3 | 0.07
B4c1a1 | 16233 | 1 | 0.023
B4c1a1 | 16368 | 1 | 0.023
B4c1a1 | 310 | 1 | 0.023
B4c1a1 | 6164 | 1 | 0.023
B4c1a1 | 9163 | 1 | 0.023
B4c1a1a | 12505 | 1 | 0.033
B4c1a1a | 146 | 1 | 0.033
B4c1a1a | 15703 | 1 | 0.033
B4c1a1a | 5899.2C | 1 | 0.033
B4c1a1a | 8950 | 1 | 0.033
B4c1a1a | 9479 | 1 | 0.033
B4c1a1a1 | 10972 | 1 | 0.032
B4c1a1a1 | 348 | 1 | 0.032
B4c1a1a1 | 351C | 1 | 0.032
B4c1a1a1 | 368 | 1 | 0.032
B4c1a1a1 | 380C | 1 | 0.032
B4c1a1a1 | 382G | 1 | 0.032
B4c1a1a1 | 385T | 1 | 0.032
B4c1a1a1 | 3963A | 1 | 0.032
B4c1a1a1 | 506 | 1 | 0.032
B4c1a1a1 | 54.1G | 1 | 0.032
B4c1a1a1 | 62.1G | 1 | 0.032
B4c1a1a1 | 765G | 1 | 0.032
B4c1a1a1a | 11296 | 1 | 0.03
B4c1a1a1a | 152 | 1 | 0.03
B4c1a1a1a | 16156 | 1 | 0.03
B4c1a1a1a | 16162 | 1 | 0.03
B4c1a1a1a | 16256 | 1 | 0.03
B4c1a1a1a | 1822 | 1 | 0.03
B4c1a1a1a | 309.3C | 3 | 0.091
B4c1a1a1a | 5004 | 1 | 0.03
B4c1a1a1a | 5099A | 1 | 0.03
B4c1a1a1a | 8843 | 1 | 0.03
B4c1a1a1a | 9498 | 1 | 0.03
B4c1a1a1a | 9604 | 1 | 0.03
B4c1a1a2 | 54.1G | 1 | 0.032
B4c1a1a2 | 62.1G | 1 | 0.032
B4c1a1a2 | 765G | 1 | 0.032
B4c1a1b | 11770 | 1 | 0.013
B4c1a1b | 127 | 1 | 0.013
B4c1a1b | 127G | 1 | 0.013
B4c1a1b | 14001 | 1 | 0.013
B4c1a1b | 14203 | 1 | 0.013
B4c1a1b | 143 | 1 | 0.013
B4c1a1b | 153 | 1 | 0.013
B4c1a1b | 159 | 1 | 0.013
B4c1a1b | 16048 | 1 | 0.013
B4c1a1b | 16093 | 12 | 0.152
B4c1a1b | 16172 | 1 | 0.013
B4c1a1b | 16186 | 1 | 0.013
B4c1a1b | 16193.3C | 1 | 0.013
B4c1a1b | 16193d | 1 | 0.013
B4c1a1b | 16195 | 1 | 0.013
B4c1a1b | 16223 | 1 | 0.013
B4c1a1b | 16260 | 1 | 0.013
B4c1a1b | 16274 | 8 | 0.101
B4c1a1b | 16287 | 1 | 0.013
B4c1a1b | 16298 | 2 | 0.025
B4c1a1b | 16319 | 1 | 0.013
B4c1a1b | 16320 | 1 | 0.013
B4c1a1b | 16355 | 1 | 0.013
B4c1a1b | 16361 | 1 | 0.013

B4c1a1b | 16362 | 2 | 0.025
B4c1a1b | 16390 | 1 | 0.013
B4c1a1b | 16494 | 1 | 0.013
B4c1a1b | 16510 | 1 | 0.013
B4c1a1b | 204 | 11 | 0.139
B4c1a1b | 207 | 1 | 0.013
B4c1a1b | 210 | 1 | 0.013
B4c1a1b | 214 | 13 | 0.165
B4c1a1b | 230 | 1 | 0.013
B4c1a1b | 256 | 1 | 0.013
B4c1a1b | 309.3C | 2 | 0.025
B4c1a1b | 316 | 1 | 0.013
B4c1a1b | 368 | 13 | 0.165
B4c1a1b | 455d | 1 | 0.013
B4c1a1b | 469 | 1 | 0.013
B4c1a1b | 485 | 1 | 0.013
B4c1a1b | 489 | 1 | 0.013
B4c1a1b | 499 | 11 | 0.139
B4c1a1b | 513 | 12 | 0.152
B4c1a1b | 519 | 1 | 0.013
B4c1a1b | 5379 | 1 | 0.013
B4c1a1c | 10978 | 1 | 0.02
B4c1a1c | 16295 | 1 | 0.02
B4c1a1c | 16365 | 1 | 0.02
B4c1a1c | 16445 | 1 | 0.02
B4c1a1c | 183 | 1 | 0.02
B4c1a1c | 249d | 1 | 0.02
B4c1a1c | 489 | 1 | 0.02
B4c1a1c | 499 | 7 | 0.137
B4c1a1c | 961 | 1 | 0.02
B4c1a1c | 965.5C | 1 | 0.02
B4c1a2 | 11914 | 1 | 0.015
B4c1a2 | 13488 | 1 | 0.015
B4c1a2 | 14180 | 1 | 0.015
B4c1a2 | 16094 | 1 | 0.015
B4c1a2 | 16172 | 1 | 0.015
B4c1a2 | 16174 | 1 | 0.015
B4c1a2 | 16176 | 1 | 0.015
B4c1a2 | 16193d | 1 | 0.015
B4c1a2 | 16234 | 1 | 0.015
B4c1a2 | 16362 | 1 | 0.015
B4c1a2 | 214 | 1 | 0.015
B4c1a2 | 368 | 1 | 0.015
B4c1a2 | 7915 | 1 | 0.015
B4c1a2 | 7984 | 1 | 0.015
B4c1a2 | 8388 | 1 | 0.015
B4c1a2 | 8634 | 1 | 0.015
B4c1a2a | 15424 | 1 | 0.031
B4c1a2a | 16319 | 1 | 0.031
B4c1a2a | 16354 | 1 | 0.031
B4c1a2a | 16510 | 1 | 0.031
B4c1a2a | 16511G | 1 | 0.031
B4c1a2a | 16515T | 1 | 0.031
B4c1a2a | 310 | 1 | 0.031
B4c1a2a | 312 | 1 | 0.031
B4c1a2a | 3146 | 1 | 0.031
B4c1a'b | 16184A | 6 | 0.079
B4c1b | 12853 | 1 | 0.016
B4c1b | 13437 | 1 | 0.016
B4c1b | 14502 | 1 | 0.016
B4c1b | 146 | 14 | 0.222
B4c1b | 14692 | 3 | 0.048
B4c1b | 147.1C | 1 | 0.016
B4c1b | 152 | 1 | 0.016
B4c1b | 16076d | 1 | 0.016
B4c1b | 16093 | 1 | 0.016
B4c1b | 16113C | 1 | 0.016
B4c1b | 16126 | 1 | 0.016
B4c1b | 16129 | 3 | 0.048
B4c1b | 16169 | 1 | 0.016
B4c1b | 16193d | 1 | 0.016
B4c1b | 16214 | 2 | 0.032
B4c1b | 16234d | 1 | 0.016
B4c1b | 16235 | 2 | 0.032
B4c1b | 16242A | 2 | 0.032
B4c1b | 16243 | 5 | 0.079
B4c1b | 16249 | 1 | 0.016
B4c1b | 16261 | 1 | 0.016
B4c1b | 16292 | 1 | 0.016
B4c1b | 16293 | 1 | 0.016
B4c1b | 16304 | 1 | 0.016
B4c1b | 16310 | 1 | 0.016
B4c1b | 16311 | 7 | 0.111
B4c1b | 16336.1G | 1 | 0.016
B4c1b | 16362 | 5 | 0.079
B4c1b | 16452A | 1 | 0.016
B4c1b | 16486C | 1 | 0.016
B4c1b | 16527 | 2 | 0.032
B4c1b | 185 | 4 | 0.063
B4c1b | 188 | 1 | 0.016
B4c1b | 275 | 1 | 0.016
B4c1b | 291.1A | 2 | 0.032
B4c1b | 309.3C | 1 | 0.016
B4c1b | 3394 | 2 | 0.032
B4c1b | 420.1C | 1 | 0.016
B4c1b | 455d | 1 | 0.016
B4c1b | 456 | 1 | 0.016
B4c1b | 4625 | 1 | 0.016
B4c1b | 466 | 3 | 0.048
B4c1b | 4704C | 1 | 0.016
B4c1b | 513 | 1 | 0.016
B4c1b | 515C | 1 | 0.016
B4c1b | 5246A | 1 | 0.016
B4c1b | 7091 | 1 | 0.016
B4c1b+16335 | 14180 | 1 | 0.007
B4c1b+16335 | 14502 | 5 | 0.035
B4c1b+16335 | 152 | 2 | 0.014
B4c1b+16335 | 16086 | 1 | 0.007
B4c1b+16335 | 16093 | 1 | 0.007
B4c1b+16335 | 16129 | 1 | 0.007
B4c1b+16335 | 16145 | 5 | 0.035

B4c1b+16335 | 16256 | 1 | 0.007
B4c1b+16335 | 16266 | 1 | 0.007
B4c1b+16335 | 16304 | 2 | 0.014
B4c1b+16335 | 16310 | 9 | 0.062
B4c1b+16335 | 16311 | 2 | 0.014
B4c1b+16335 | 16342 | 1 | 0.007
B4c1b+16335 | 185 | 1 | 0.007
B4c1b+16335 | 194 | 1 | 0.007
B4c1b+16335 | 200 | 4 | 0.028
B4c1b+16335 | 210 | 1 | 0.007
B4c1b+16335 | 2380 | 4 | 0.028
B4c1b+16335 | 307-309d | 1 | 0.007
B4c1b+16335 | 310 | 1 | 0.007
B4c1b+16335 | 315.2C | 1 | 0.007
B4c1b+16335 | 319 | 1 | 0.007
B4c1b+16335 | 408A | 1 | 0.007
B4c1b+16335 | 513 | 1 | 0.007
B4c1b+16335 | 5246A | 5 | 0.035
B4c1b+16335 | 9848 | 1 | 0.007
B4c1b1 | 16452 | 1 | 1
B4c1b1a | 15550 | 1 | 0.333
B4c1b1a | 8664 | 1 | 0.333
B4c1b1a | 9336C | 1 | 0.333
B4c1b2 | 16092 | 2 | 0.014
B4c1b2 | 16180 | 2 | 0.014
B4c1b2 | 16228 | 1 | 0.007
B4c1b2 | 16291 | 2 | 0.014
B4c1b2 | 16319 | 15 | 0.101
B4c1b2 | 16375 | 1 | 0.007
B4c1b2 | 16390 | 1 | 0.007
B4c1b2 | 228 | 3 | 0.02
B4c1b2a | 10203 | 8 | 0.048
B4c1b2a | 11293 | 1 | 0.006
B4c1b2a | 12882 | 2 | 0.012
B4c1b2a | 13105 | 1 | 0.006
B4c1b2a | 15301 | 1 | 0.006
B4c1b2a | 15511 | 1 | 0.006
B4c1b2a | 15852 | 1 | 0.006
B4c1b2a | 16075A | 1 | 0.006
B4c1b2a | 16093 | 1 | 0.006
B4c1b2a | 16129 | 1 | 0.006
B4c1b2a | 16134 | 1 | 0.006
B4c1b2a | 16136 | 1 | 0.006
B4c1b2a | 16145 | 1 | 0.006
B4c1b2a | 16172 | 1 | 0.006
B4c1b2a | 16187 | 1 | 0.006
B4c1b2a | 16193d | 1 | 0.006
B4c1b2a | 16194C | 2 | 0.012
B4c1b2a | 16195 | 2 | 0.012
B4c1b2a | 16211 | 1 | 0.006
B4c1b2a | 16234G | 1 | 0.006
B4c1b2a | 16239G | 1 | 0.006
B4c1b2a | 16242A | 28 | 0.167
B4c1b2a | 16289 | 2 | 0.012
B4c1b2a | 16309 | 1 | 0.006
B4c1b2a | 16311 | 1 | 0.006
B4c1b2a | 16316 | 8 | 0.048
B4c1b2a | 16330A | 1 | 0.006
B4c1b2a | 16356 | 1 | 0.006
B4c1b2a | 16362 | 2 | 0.012
B4c1b2a | 16372G | 1 | 0.006
B4c1b2a | 16400 | 4 | 0.024
B4c1b2a | 16483 | 5 | 0.03
B4c1b2a | 199 | 1 | 0.006
B4c1b2a | 234 | 1 | 0.006
B4c1b2a | 291.1A | 1 | 0.006
B4c1b2a | 3084 | 1 | 0.006
B4c1b2a | 309.3C | 2 | 0.012
B4c1b2a | 3116 | 9 | 0.054
B4c1b2a | 320 | 1 | 0.006
B4c1b2a | 324 | 1 | 0.006
B4c1b2a | 340 | 1 | 0.006
B4c1b2a | 3438 | 1 | 0.006
B4c1b2a | 3537 | 1 | 0.006
B4c1b2a | 383 | 1 | 0.006
B4c1b2a | 42.1G | 2 | 0.012
B4c1b2a | 4232 | 1 | 0.006
B4c1b2a | 456 | 3 | 0.018
B4c1b2a | 518G | 1 | 0.006
B4c1b2a | 6620 | 8 | 0.048
B4c1b2a | 7424 | 1 | 0.006
B4c1b2a | 9389 | 1 | 0.006
B4c1b2a | 9621 | 1 | 0.006
B4c1b2a1 | 11611 | 3 | 0.017
B4c1b2a1 | 14207 | 1 | 0.006
B4c1b2a1 | 152 | 2 | 0.011
B4c1b2a1 | 16092 | 10 | 0.057
B4c1b2a1 | 16093 | 5 | 0.029
B4c1b2a1 | 16111 | 1 | 0.006
B4c1b2a1 | 16145 | 1 | 0.006
B4c1b2a1 | 16187 | 1 | 0.006
B4c1b2a1 | 16188 | 1 | 0.006
B4c1b2a1 | 16194C | 2 | 0.011
B4c1b2a1 | 16195 | 2 | 0.011
B4c1b2a1 | 16214 | 5 | 0.029
B4c1b2a1 | 16257 | 1 | 0.006
B4c1b2a1 | 16265 | 1 | 0.006
B4c1b2a1 | 16266 | 2 | 0.011
B4c1b2a1 | 16284 | 1 | 0.006
B4c1b2a1 | 16290 | 1 | 0.006
B4c1b2a1 | 16309 | 1 | 0.006
B4c1b2a1 | 16311 | 33 | 0.19
B4c1b2a1 | 16362 | 5 | 0.029
B4c1b2a1 | 16390 | 4 | 0.023
B4c1b2a1 | 16414T | 2 | 0.011
B4c1b2a1 | 16497 | 1 | 0.006
B4c1b2a1 | 16527 | 1 | 0.006
B4c1b2a1 | 16T | 1 | 0.006
B4c1b2a1 | 182 | 1 | 0.006
B4c1b2a1 | 199 | 1 | 0.006
B4c1b2a1 | 234 | 1 | 0.006

B4c1b2a1 | 291.1A | 1 | 0.006
B4c1b2a1 | 309.3C | 2 | 0.011
B4c1b2a1 | 320 | 1 | 0.006
B4c1b2a1 | 324 | 1 | 0.006
B4c1b2a1 | 340 | 1 | 0.006
B4c1b2a1 | 383 | 1 | 0.006
B4c1b2a1 | 42.1G | 2 | 0.011
B4c1b2a1 | 4890 | 1 | 0.006
B4c1b2a1 | 513 | 1 | 0.006
B4c1b2a1 | 518G | 1 | 0.006
B4c1b2a1 | 5493 | 2 | 0.011
B4c1b2a1 | 6167 | 3 | 0.017
B4c1b2a1 | 7819 | 3 | 0.017
B4c1b2a1 | 8951 | 3 | 0.017
B4c1b2a2 | 10160 | 1 | 0.006
B4c1b2a2 | 10858 | 1 | 0.006
B4c1b2a2 | 11150 | 1 | 0.006
B4c1b2a2 | 11172 | 1 | 0.006
B4c1b2a2 | 11299 | 2 | 0.013
B4c1b2a2 | 11368 | 1 | 0.006
B4c1b2a2 | 11788 | 1 | 0.006
B4c1b2a2 | 11887 | 1 | 0.006
B4c1b2a2 | 12142 | 1 | 0.006
B4c1b2a2 | 12192 | 3 | 0.019
B4c1b2a2 | 12810 | 1 | 0.006
B4c1b2a2 | 13105 | 2 | 0.013
B4c1b2a2 | 13708 | 2 | 0.013
B4c1b2a2 | 13720 | 1 | 0.006
B4c1b2a2 | 13782 | 2 | 0.013
B4c1b2a2 | 13827 | 1 | 0.006
B4c1b2a2 | 13934 | 4 | 0.026
B4c1b2a2 | 14180 | 1 | 0.006
B4c1b2a2 | 14182 | 2 | 0.013
B4c1b2a2 | 14332 | 1 | 0.006
B4c1b2a2 | 14452 | 1 | 0.006
B4c1b2a2 | 15172 | 2 | 0.013
B4c1b2a2 | 152 | 5 | 0.032
B4c1b2a2 | 15743 | 4 | 0.026
B4c1b2a2 | 15774 | 1 | 0.006
B4c1b2a2 | 15884 | 2 | 0.013
B4c1b2a2 | 15943G | 1 | 0.006
B4c1b2a2 | 16051 | 1 | 0.006
B4c1b2a2 | 16093 | 2 | 0.013
B4c1b2a2 | 16111G | 1 | 0.006
B4c1b2a2 | 16129 | 3 | 0.019
B4c1b2a2 | 16154 | 1 | 0.006
B4c1b2a2 | 16178 | 1 | 0.006
B4c1b2a2 | 16179 | 1 | 0.006
B4c1b2a2 | 16193d | 2 | 0.013
B4c1b2a2 | 16213 | 2 | 0.013
B4c1b2a2 | 16220 | 1 | 0.006
B4c1b2a2 | 16240 | 1 | 0.006
B4c1b2a2 | 16278 | 2 | 0.013
B4c1b2a2 | 16291 | 1 | 0.006
B4c1b2a2 | 16311 | 6 | 0.038
B4c1b2a2 | 16319 | 1 | 0.006
B4c1b2a2 | 16346 | 1 | 0.006
B4c1b2a2 | 16362 | 1 | 0.006
B4c1b2a2 | 16497 | 1 | 0.006
B4c1b2a2 | 16T | 1 | 0.006
B4c1b2a2 | 1719 | 1 | 0.006
B4c1b2a2 | 1738 | 1 | 0.006
B4c1b2a2 | 189 | 2 | 0.013
B4c1b2a2 | 199 | 4 | 0.026
B4c1b2a2 | 2626 | 2 | 0.013
B4c1b2a2 | 308-309d | 2 | 0.013
B4c1b2a2 | 309d | 1 | 0.006
B4c1b2a2 | 310 | 14 | 0.09
B4c1b2a2 | 312 | 1 | 0.006
B4c1b2a2 | 314-315d | 1 | 0.006
B4c1b2a2 | 315d | 6 | 0.038
B4c1b2a2 | 320 | 1 | 0.006
B4c1b2a2 | 3202 | 1 | 0.006
B4c1b2a2 | 321 | 1 | 0.006
B4c1b2a2 | 3221 | 4 | 0.026
B4c1b2a2 | 332A | 1 | 0.006
B4c1b2a2 | 3496 | 1 | 0.006
B4c1b2a2 | 3666 | 2 | 0.013
B4c1b2a2 | 4226 | 3 | 0.019
B4c1b2a2 | 4820 | 1 | 0.006
B4c1b2a2 | 5147 | 1 | 0.006
B4c1b2a2 | 6221 | 1 | 0.006
B4c1b2a2 | 6257 | 1 | 0.006
B4c1b2a2 | 6383 | 2 | 0.013
B4c1b2a2 | 6737 | 2 | 0.013
B4c1b2a2 | 7079 | 1 | 0.006
B4c1b2a2 | 7270 | 1 | 0.006
B4c1b2a2 | 739 | 1 | 0.006
B4c1b2a2 | 7741 | 1 | 0.006
B4c1b2a2 | 8149 | 1 | 0.006
B4c1b2a2 | 8293 | 1 | 0.006
B4c1b2a2 | 8507 | 1 | 0.006
B4c1b2a2 | 8974 | 1 | 0.006
B4c1b2a2 | 9777 | 1 | 0.006
B4c1b2a2 | 9959 | 1 | 0.006
B4c1b2a2 | 9962 | 3 | 0.019
B4c1b2a2a | 14305 | 1 | 0.007
B4c1b2a2a | 16166C | 2 | 0.014
B4c1b2a2a | 16257 | 3 | 0.021
B4c1b2a2a | 16300 | 7 | 0.049
B4c1b2a2a | 16357 | 2 | 0.014
B4c1b2a2a | 204 | 2 | 0.014
B4c1b2a2a | 547 | 2 | 0.014
B4c1b2a2a | 7328 | 1 | 0.007
B4c1b2a2b | 16051 | 2 | 0.014
B4c1b2a2b | 16126 | 1 | 0.007
B4c1b2a2b | 16136 | 1 | 0.007
B4c1b2a2b | 16164 | 1 | 0.007
B4c1b2a2b | 16166C | 2 | 0.014
B4c1b2a2b | 16193d | 1 | 0.007
B4c1b2a2b | 16264 | 1 | 0.007

B4c1b2a2b | 16300 | 7 | 0.05
B4c1b2a2b | 16324 | 3 | 0.021
B4c1b2a2b | 16375 | 2 | 0.014
B4c1b2a2b | 189 | 4 | 0.029
B4c1b2a2b | 234 | 1 | 0.007
B4c1b2a2b | 309.3C | 1 | 0.007
B4c1b2a2b | 310 | 1 | 0.007
B4c1b2a2b | 4707A | 1 | 0.007
B4c1b2a2b | 537 | 1 | 0.007
B4c1b2a2b | 547 | 2 | 0.014
B4c1b2b | 10157 | 1 | 0.007
B4c1b2b | 14793 | 1 | 0.007
B4c1b2b | 16092 | 1 | 0.007
B4c1b2b | 16129 | 17 | 0.123
B4c1b2b | 16278 | 1 | 0.007
B4c1b2b | 16291 | 1 | 0.007
B4c1b2b | 16305C | 3 | 0.022
B4c1b2b | 16305T | 9 | 0.065
B4c1b2b | 16319 | 1 | 0.007
B4c1b2b | 16369 | 1 | 0.007
B4c1b2b | 16399 | 1 | 0.007
B4c1b2b | 16527 | 2 | 0.014
B4c1b2b | 217 | 1 | 0.007
B4c1b2b | 228 | 1 | 0.007
B4c1b2b | 310 | 1 | 0.007
B4c1b2b | 3738 | 2 | 0.014
B4c1b2b | 513 | 4 | 0.029
B4c1b2b | 525.1CACC | 1 | 0.007
B4c1b2b | 5899.1C | 1 | 0.007
B4c1b2b | 8257 | 2 | 0.014
B4c1b2b | 8634 | 1 | 0.007
B4c1b2c | 10790 | 2 | 0.014
B4c1b2c | 13934 | 3 | 0.021
B4c1b2c | 14509 | 1 | 0.007
B4c1b2c | 14544 | 1 | 0.007
B4c1b2c | 14818 | 1 | 0.007
B4c1b2c | 152 | 2 | 0.014
B4c1b2c | 16042 | 1 | 0.007
B4c1b2c | 16093 | 7 | 0.049
B4c1b2c | 16249 | 2 | 0.014
B4c1b2c | 16266 | 1 | 0.007
B4c1b2c | 16305T | 1 | 0.007
B4c1b2c | 16342 | 1 | 0.007
B4c1b2c | 16527 | 1 | 0.007
B4c1b2c | 1842 | 1 | 0.007
B4c1b2c | 194 | 1 | 0.007
B4c1b2c | 195 | 1 | 0.007
B4c1b2c | 310 | 1 | 0.007
B4c1b2c | 3254 | 2 | 0.014
B4c1b2c | 3666 | 2 | 0.014
B4c1b2c | 408A | 1 | 0.007
B4c1b2c | 4592 | 2 | 0.014
B4c1b2c | 5821 | 1 | 0.007
B4c1b2c | 593 | 2 | 0.014
B4c1b2c | 7990 | 1 | 0.007
B4c1b2c | 8188 | 1 | 0.007
B4c1b2c | 8994 | 1 | 0.007
B4c1b2c | 9123 | 1 | 0.007
B4c1b2c | 9299 | 1 | 0.007
B4c1b2c | 9656 | 2 | 0.014
B4c1b2c1 | 152 | 1 | 0.022
B4c1b2c1 | 16126 | 1 | 0.022
B4c1b2c1 | 16239 | 1 | 0.022
B4c1b2c1 | 16265 | 1 | 0.022
B4c1b2c1 | 16280 | 6 | 0.13
B4c1b2c1 | 16294 | 1 | 0.022
B4c1b2c1 | 16356 | 2 | 0.043
B4c1b2c1 | 195 | 1 | 0.022
B4c1b2c1 | 315.2C | 1 | 0.022
B4c1b2c1 | 4300 | 1 | 0.022
B4c1b2c1 | 5054C | 2 | 0.043
B4c1b2c1 | 8269 | 1 | 0.022
B4c1b2c2 | 10250 | 2 | 0.021
B4c1b2c2 | 10728A | 1 | 0.011
B4c1b2c2 | 11204 | 1 | 0.011
B4c1b2c2 | 11908 | 1 | 0.011
B4c1b2c2 | 12011 | 1 | 0.011
B4c1b2c2 | 13434 | 2 | 0.021
B4c1b2c2 | 14025 | 1 | 0.011
B4c1b2c2 | 14182 | 1 | 0.011
B4c1b2c2 | 146 | 18 | 0.191
B4c1b2c2 | 152 | 1 | 0.011
B4c1b2c2 | 15430 | 1 | 0.011
B4c1b2c2 | 15829 | 1 | 0.011
B4c1b2c2 | 15954 | 2 | 0.021
B4c1b2c2 | 16037 | 1 | 0.011
B4c1b2c2 | 16041 | 1 | 0.011
B4c1b2c2 | 16051 | 3 | 0.032
B4c1b2c2 | 16076d | 1 | 0.011
B4c1b2c2 | 16092 | 2 | 0.021
B4c1b2c2 | 16093 | 1 | 0.011
B4c1b2c2 | 16126 | 1 | 0.011
B4c1b2c2 | 16138 | 1 | 0.011
B4c1b2c2 | 16145 | 14 | 0.149
B4c1b2c2 | 16148 | 1 | 0.011
B4c1b2c2 | 16172 | 2 | 0.021
B4c1b2c2 | 16179 | 3 | 0.032
B4c1b2c2 | 16187 | 4 | 0.043
B4c1b2c2 | 16193d | 1 | 0.011
B4c1b2c2 | 16256 | 1 | 0.011
B4c1b2c2 | 16257 | 1 | 0.011
B4c1b2c2 | 16292 | 1 | 0.011
B4c1b2c2 | 16293 | 1 | 0.011
B4c1b2c2 | 16311 | 6 | 0.064
B4c1b2c2 | 16357 | 3 | 0.032
B4c1b2c2 | 16390 | 3 | 0.032
B4c1b2c2 | 16400 | 1 | 0.011
B4c1b2c2 | 16465 | 1 | 0.011
B4c1b2c2 | 16566 | 2 | 0.021
B4c1b2c2 | 189 | 4 | 0.043
B4c1b2c2 | 194 | 1 | 0.011

B4c1b2c2 | 195 | 6 | 0.064
B4c1b2c2 | 199 | 2 | 0.021
B4c1b2c2 | 200 | 1 | 0.011
B4c1b2c2 | 204 | 1 | 0.011
B4c1b2c2 | 207 | 1 | 0.011
B4c1b2c2 | 210 | 1 | 0.011
B4c1b2c2 | 211 | 3 | 0.032
B4c1b2c2 | 235 | 3 | 0.032
B4c1b2c2 | 282 | 2 | 0.021
B4c1b2c2 | 2833 | 1 | 0.011
B4c1b2c2 | 2903 | 1 | 0.011
B4c1b2c2 | 292 | 1 | 0.011
B4c1b2c2 | 294 | 1 | 0.011
B4c1b2c2 | 306-309d | 1 | 0.011
B4c1b2c2 | 309.3C | 2 | 0.021
B4c1b2c2 | 309d | 2 | 0.021
B4c1b2c2 | 316C | 1 | 0.011
B4c1b2c2 | 329T | 1 | 0.011
B4c1b2c2 | 3394 | 5 | 0.053
B4c1b2c2 | 356.1C | 2 | 0.021
B4c1b2c2 | 3777 | 1 | 0.011
B4c1b2c2 | 384 | 3 | 0.032
B4c1b2c2 | 4092 | 2 | 0.021
B4c1b2c2 | 445 | 1 | 0.011
B4c1b2c2 | 4454 | 2 | 0.021
B4c1b2c2 | 5057 | 2 | 0.021
B4c1b2c2 | 5563 | 2 | 0.021
B4c1b2c2 | 573.1C | 1 | 0.011
B4c1b2c2 | 573.2C | 1 | 0.011
B4c1b2c2 | 5744 | 1 | 0.011
B4c1b2c2 | 574C | 1 | 0.011
B4c1b2c2 | 576C | 1 | 0.011
B4c1b2c2 | 577C | 1 | 0.011
B4c1b2c2 | 5899d | 2 | 0.021
B4c1b2c2 | 6935 | 1 | 0.011
B4c1b2c2 | 6941 | 1 | 0.011
B4c1b2c2 | 7119 | 5 | 0.053
B4c1b2c2 | 7325 | 2 | 0.021
B4c1b2c2 | 794 | 1 | 0.011
B4c1b2c2 | 8592 | 1 | 0.011
B4c1b2c2 | 8609 | 2 | 0.021
B4c1b2c2 | 939T | 1 | 0.011
B4c1b2c2 | 9469 | 1 | 0.011
B4c1b2c2 | 9621 | 1 | 0.011
B4c1c | 10310 | 1 | 0.012
B4c1c | 11716 | 1 | 0.012
B4c1c | 131 | 1 | 0.012
B4c1c | 13934 | 1 | 0.012
B4c1c | 146 | 1 | 0.012
B4c1c | 152 | 2 | 0.025
B4c1c | 16025 | 1 | 0.012
B4c1c | 16036 | 1 | 0.012
B4c1c | 16051 | 1 | 0.012
B4c1c | 16088G | 1 | 0.012
B4c1c | 16093 | 1 | 0.012
B4c1c | 16112d | 1 | 0.012
B4c1c | 16120 | 1 | 0.012
B4c1c | 16126 | 2 | 0.025
B4c1c | 16194C | 2 | 0.025
B4c1c | 16261 | 1 | 0.012
B4c1c | 16295 | 6 | 0.074
B4c1c | 16299 | 1 | 0.012
B4c1c | 16304 | 1 | 0.012
B4c1c | 16357 | 1 | 0.012
B4c1c | 16362 | 4 | 0.049
B4c1c | 16424 | 1 | 0.012
B4c1c | 16522A | 1 | 0.012
B4c1c | 16563 | 2 | 0.025
B4c1c | 172 | 1 | 0.012
B4c1c | 187 | 1 | 0.012
B4c1c | 1C | 1 | 0.012
B4c1c | 204 | 17 | 0.21
B4c1c | 207 | 17 | 0.21
B4c1c | 211 | 1 | 0.012
B4c1c | 234T | 1 | 0.012
B4c1c | 279 | 22 | 0.272
B4c1c | 280 | 1 | 0.012
B4c1c | 309d | 1 | 0.012
B4c1c | 3169 | 1 | 0.012
B4c1c | 32C | 1 | 0.012
B4c1c | 350 | 1 | 0.012
B4c1c | 374 | 1 | 0.012
B4c1c | 392 | 3 | 0.037
B4c1c | 408 | 1 | 0.012
B4c1c | 49 | 1 | 0.012
B4c1c | 52G | 1 | 0.012
B4c1c | 56 | 1 | 0.012
B4c1c | 569 | 1 | 0.012
B4c1c | 573d | 1 | 0.012
B4c1c+16311 | 1117 | 1 | 0.032
B4c1c+16311 | 11329 | 1 | 0.032
B4c1c+16311 | 127 | 1 | 0.032
B4c1c+16311 | 146 | 1 | 0.032
B4c1c+16311 | 152 | 1 | 0.032
B4c1c+16311 | 155d | 1 | 0.032
B4c1c+16311 | 16038 | 1 | 0.032
B4c1c+16311 | 16046 | 1 | 0.032
B4c1c+16311 | 16094 | 1 | 0.032
B4c1c+16311 | 16160C | 1 | 0.032
B4c1c+16311 | 16190 | 1 | 0.032
B4c1c+16311 | 16192-16193d | 2 | 0.065
B4c1c+16311 | 16193d | 1 | 0.032
B4c1c+16311 | 16206 | 1 | 0.032
B4c1c+16311 | 16245 | 1 | 0.032
B4c1c+16311 | 16297 | 1 | 0.032
B4c1c+16311 | 16547G | 1 | 0.032
B4c1c+16311 | 16561 | 1 | 0.032
B4c1c+16311 | 173d | 1 | 0.032
B4c1c+16311 | 204 | 1 | 0.032
B4c1c+16311 | 207 | 1 | 0.032
B4c1c+16311 | 238T | 1 | 0.032

B4c1c+16311 | 242d | 1 | 0.032
B4c1c+16311 | 257.1A | 1 | 0.032
B4c1c+16311 | 352 | 1 | 0.032
B4c1c+16311 | 379 | 1 | 0.032
B4c1c+16311 | 385T | 1 | 0.032
B4c1c+16311 | 386A | 1 | 0.032
B4c1c+16311 | 460 | 1 | 0.032
B4c1c+16311 | 498d | 1 | 0.032
B4c1c+16311 | 503 | 1 | 0.032
B4c1c+16311 | 52.1T | 1 | 0.032
B4c1c+16311 | 5268 | 1 | 0.032
B4c1c+16311 | 60.1T | 1 | 0.032
B4c1c+16311 | 9425C | 1 | 0.032
B4c1c1 | 1007 | 2 | 0.053
B4c1c1 | 14178 | 1 | 0.026
B4c1c1 | 146 | 4 | 0.105
B4c1c1 | 15236 | 1 | 0.026
B4c1c1 | 16093 | 1 | 0.026
B4c1c1 | 16129 | 2 | 0.053
B4c1c1 | 16140 | 1 | 0.026
B4c1c1 | 16214 | 3 | 0.079
B4c1c1 | 16218 | 1 | 0.026
B4c1c1 | 16234 | 1 | 0.026
B4c1c1 | 16261 | 1 | 0.026
B4c1c1 | 16265 | 4 | 0.105
B4c1c1 | 16280 | 1 | 0.026
B4c1c1 | 16304 | 3 | 0.079
B4c1c1 | 16399 | 1 | 0.026
B4c1c1 | 16422 | 1 | 0.026
B4c1c1 | 16526 | 1 | 0.026
B4c1c1 | 2056 | 1 | 0.026
B4c1c1 | 279 | 8 | 0.211
B4c1c1 | 309.3C | 1 | 0.026
B4c1c1 | 3531 | 1 | 0.026
B4c1c1 | 488 | 1 | 0.026
B4c1c1 | 489 | 1 | 0.026
B4c1c1 | 64 | 1 | 0.026
B4c1c1 | 7752 | 1 | 0.026
B4c1c1 | 7870 | 1 | 0.026
B4c2 | 10954A | 1 | 0.007
B4c2 | 10966 | 1 | 0.007
B4c2 | 11087 | 1 | 0.007
B4c2 | 12477 | 1 | 0.007
B4c2 | 13986 | 1 | 0.007
B4c2 | 14002 | 5 | 0.037
B4c2 | 14178 | 1 | 0.007
B4c2 | 14329 | 1 | 0.007
B4c2 | 14560 | 1 | 0.007
B4c2 | 15077 | 2 | 0.015
B4c2 | 15323 | 1 | 0.007
B4c2 | 15924 | 1 | 0.007
B4c2 | 16129 | 10 | 0.075
B4c2 | 16162 | 4 | 0.03
B4c2 | 16168 | 1 | 0.007
B4c2 | 16234 | 10 | 0.075
B4c2 | 16290 | 6 | 0.045
B4c2 | 16294 | 10 | 0.075
B4c2 | 16295 | 2 | 0.015
B4c2 | 16309 | 1 | 0.007
B4c2 | 16362 | 1 | 0.007
B4c2 | 16390 | 1 | 0.007
B4c2 | 16527 | 2 | 0.015
B4c2 | 1719 | 5 | 0.037
B4c2 | 185 | 1 | 0.007
B4c2 | 3010 | 1 | 0.007
B4c2 | 3150 | 1 | 0.007
B4c2 | 3316 | 1 | 0.007
B4c2 | 3335 | 5 | 0.037
B4c2 | 44.1C | 1 | 0.007
B4c2 | 5090 | 1 | 0.007
B4c2 | 5379 | 1 | 0.007
B4c2 | 5508 | 1 | 0.007
B4c2 | 5539 | 1 | 0.007
B4c2 | 5978 | 1 | 0.007
B4c2 | 6216 | 1 | 0.007
B4c2 | 6465 | 1 | 0.007
B4c2 | 6755 | 1 | 0.007
B4c2 | 6932 | 1 | 0.007
B4c2 | 7004 | 1 | 0.007
B4c2 | 7340 | 1 | 0.007
B4c2 | 7598 | 1 | 0.007
B4c2 | 8038 | 1 | 0.007
B4c2 | 8581 | 2 | 0.015
B4c2 | 8622 | 1 | 0.007
B4c2 | 8838 | 1 | 0.007
B4c2 | 8854 | 6 | 0.045
B4c2 | 8958 | 1 | 0.007
B4c2 | 9548C | 1 | 0.007
B4c2 | 9647 | 1 | 0.007
B4c2a | 11017 | 1 | 0.007
B4c2a | 11653 | 1 | 0.007
B4c2a | 14319 | 3 | 0.02
B4c2a | 146 | 1 | 0.007
B4c2a | 150 | 1 | 0.007
B4c2a | 152 | 3 | 0.02
B4c2a | 16048 | 1 | 0.007
B4c2a | 16093 | 3 | 0.02
B4c2a | 16168 | 2 | 0.013
B4c2a | 16169 | 1 | 0.007
B4c2a | 16219 | 1 | 0.007
B4c2a | 16231 | 2 | 0.013
B4c2a | 16234 | 1 | 0.007
B4c2a | 16249 | 1 | 0.007
B4c2a | 16260 | 2 | 0.013
B4c2a | 16274 | 1 | 0.007
B4c2a | 16293 | 1 | 0.007
B4c2a | 16294G | 16 | 0.107
B4c2a | 16311 | 11 | 0.073
B4c2a | 16399 | 1 | 0.007
B4c2a | 16400 | 1 | 0.007
B4c2a | 16496 | 1 | 0.007

B4c2a | 16497 | 1 | 0.007
B4c2a | 194 | 1 | 0.007
B4c2a | 195 | 1 | 0.007
B4c2a | 198 | 1 | 0.007
B4c2a | 199 | 3 | 0.02
B4c2a | 200 | 1 | 0.007
B4c2a | 204 | 1 | 0.007
B4c2a | 207 | 1 | 0.007
B4c2a | 269 | 1 | 0.007
B4c2a | 310 | 1 | 0.007
B4c2a | 401d | 2 | 0.013
B4c2a | 489 | 1 | 0.007
B4c2a | 507 | 1 | 0.007
B4c2a | 8935 | 1 | 0.007
B4c2a | 94 | 1 | 0.007
B4c2b | 103T | 2 | 0.013
B4c2b | 10876 | 1 | 0.007
B4c2b | 13488 | 1 | 0.007
B4c2b | 16092 | 9 | 0.06
B4c2b | 16104 | 9 | 0.06
B4c2b | 16150 | 1 | 0.007
B4c2b | 16154 | 9 | 0.06
B4c2b | 16162 | 1 | 0.007
B4c2b | 16167 | 7 | 0.047
B4c2b | 16172 | 1 | 0.007
B4c2b | 16193d | 1 | 0.007
B4c2b | 16201 | 1 | 0.007
B4c2b | 16213 | 1 | 0.007
B4c2b | 16232 | 1 | 0.007
B4c2b | 16265C | 6 | 0.04
B4c2b | 16266A | 1 | 0.007
B4c2b | 16270 | 1 | 0.007
B4c2b | 16292 | 1 | 0.007
B4c2b | 16294 | 1 | 0.007
B4c2b | 16294G | 1 | 0.007
B4c2b | 16336 | 2 | 0.013
B4c2b | 16362 | 8 | 0.053
B4c2b | 16394 | 1 | 0.007
B4c2b | 16465 | 7 | 0.047
B4c2b | 709 | 2 | 0.013
B4c2c | 10978 | 1 | 0.007
B4c2c | 13327 | 1 | 0.007
B4c2c | 13942 | 1 | 0.007
B4c2c | 15160 | 1 | 0.007
B4c2c | 1598 | 1 | 0.007
B4c2c | 16081 | 1 | 0.007
B4c2c | 16093 | 1 | 0.007
B4c2c | 16154 | 27 | 0.182
B4c2c | 16172 | 2 | 0.014
B4c2c | 16186 | 1 | 0.007
B4c2c | 16239 | 1 | 0.007
B4c2c | 16252C | 1 | 0.007
B4c2c | 16258C | 1 | 0.007
B4c2c | 16269C | 1 | 0.007
B4c2c | 16287 | 1 | 0.007
B4c2c | 16293C | 2 | 0.014
B4c2c | 16319 | 1 | 0.007
B4c2c | 16360 | 5 | 0.034
B4c2c | 16362 | 4 | 0.027
B4c2c | 16384 | 1 | 0.007
B4c2c | 16386A | 1 | 0.007
B4c2c | 16434 | 1 | 0.007
B4c2c | 195A | 1 | 0.007
B4c2c | 197 | 1 | 0.007
B4d | 16188 | 3 | 0.046
B4d1 | 10680 | 1 | 0.007
B4d1 | 12372 | 1 | 0.007
B4d1 | 13419 | 1 | 0.007
B4d1 | 13449 | 2 | 0.015
B4d1 | 13708 | 3 | 0.022
B4d1 | 140 | 1 | 0.007
B4d1 | 14040 | 6 | 0.045
B4d1 | 14239 | 2 | 0.015
B4d1 | 14274 | 1 | 0.007
B4d1 | 14484 | 1 | 0.007
B4d1 | 146 | 2 | 0.015
B4d1 | 150 | 1 | 0.007
B4d1 | 15136 | 1 | 0.007
B4d1 | 152 | 8 | 0.06
B4d1 | 15229 | 1 | 0.007
B4d1 | 15262 | 1 | 0.007
B4d1 | 15307 | 1 | 0.007
B4d1 | 15779 | 1 | 0.007
B4d1 | 15948d | 1 | 0.007
B4d1 | 16051 | 1 | 0.007
B4d1 | 16086 | 2 | 0.015
B4d1 | 16092 | 2 | 0.015
B4d1 | 16093 | 2 | 0.015
B4d1 | 161 | 1 | 0.007
B4d1 | 16168 | 1 | 0.007
B4d1 | 16169.1C | 1 | 0.007
B4d1 | 16172 | 15 | 0.112
B4d1 | 16176 | 1 | 0.007
B4d1 | 16180 | 1 | 0.007
B4d1 | 16185 | 2 | 0.015
B4d1 | 16186 | 1 | 0.007
B4d1 | 16192 | 2 | 0.015
B4d1 | 16193d | 2 | 0.015
B4d1 | 16195 | 2 | 0.015
B4d1 | 16215T | 4 | 0.03
B4d1 | 16242 | 1 | 0.007
B4d1 | 16256 | 1 | 0.007
B4d1 | 16261 | 1 | 0.007
B4d1 | 16274 | 4 | 0.03
B4d1 | 16287 | 1 | 0.007
B4d1 | 16287G | 2 | 0.015
B4d1 | 16291 | 1 | 0.007
B4d1 | 16320 | 1 | 0.007
B4d1 | 16352 | 1 | 0.007
B4d1 | 16354 | 1 | 0.007
B4d1 | 16357 | 1 | 0.007

B4d1 | 16362 | 4 | 0.03
B4d1 | 16527 | 1 | 0.007
B4d1 | 182 | 1 | 0.007
B4d1 | 185 | 2 | 0.015
B4d1 | 189 | 1 | 0.007
B4d1 | 195 | 1 | 0.007
B4d1 | 200 | 4 | 0.03
B4d1 | 207 | 13 | 0.097
B4d1 | 214 | 3 | 0.022
B4d1 | 2244 | 1 | 0.007
B4d1 | 228 | 1 | 0.007
B4d1 | 2360 | 1 | 0.007
B4d1 | 272 | 2 | 0.015
B4d1 | 309d | 2 | 0.015
B4d1 | 310 | 2 | 0.015
B4d1 | 315-316d | 1 | 0.007
B4d1 | 316 | 52 | 0.388
B4d1 | 319 | 1 | 0.007
B4d1 | 328 | 1 | 0.007
B4d1 | 345G | 2 | 0.015
B4d1 | 3460 | 1 | 0.007
B4d1 | 366 | 2 | 0.015
B4d1 | 371G | 2 | 0.015
B4d1 | 385 | 1 | 0.007
B4d1 | 404 | 1 | 0.007
B4d1 | 4092 | 4 | 0.03
B4d1 | 4363 | 2 | 0.015
B4d1 | 464C | 1 | 0.007
B4d1 | 489 | 1 | 0.007
B4d1 | 55 | 13 | 0.097
B4d1 | 56 | 13 | 0.097
B4d1 | 569 | 1 | 0.007
B4d1 | 571 | 3 | 0.022
B4d1 | 6287 | 1 | 0.007
B4d1 | 6413 | 3 | 0.022
B4d1 | 7598 | 1 | 0.007
B4d1 | 8450 | 3 | 0.022
B4d1 | 8745 | 3 | 0.022
B4d1 | 8992 | 1 | 0.007
B4d1 | 9926 | 1 | 0.007
B4d1 | 9947 | 1 | 0.007
B4d1'2'3 | 10757 | 1 | 0.006
B4d1'2'3 | 11428 | 1 | 0.006
B4d1'2'3 | 11701 | 2 | 0.013
B4d1'2'3 | 12732 | 1 | 0.006
B4d1'2'3 | 14003 | 2 | 0.013
B4d1'2'3 | 146 | 1 | 0.006
B4d1'2'3 | 150 | 2 | 0.013
B4d1'2'3 | 15083 | 1 | 0.006
B4d1'2'3 | 152 | 7 | 0.045
B4d1'2'3 | 16051 | 2 | 0.013
B4d1'2'3 | 16093 | 12 | 0.076
B4d1'2'3 | 16129 | 4 | 0.025
B4d1'2'3 | 16140 | 3 | 0.019
B4d1'2'3 | 16150 | 12 | 0.076
B4d1'2'3 | 16158 | 2 | 0.013
B4d1'2'3 | 16167 | 3 | 0.019
B4d1'2'3 | 16215 | 1 | 0.006
B4d1'2'3 | 16234 | 91 | 0.58
B4d1'2'3 | 16240 | 1 | 0.006
B4d1'2'3 | 16249 | 6 | 0.038
B4d1'2'3 | 16266 | 1 | 0.006
B4d1'2'3 | 16316 | 1 | 0.006
B4d1'2'3 | 16360 | 1 | 0.006
B4d1'2'3 | 194 | 1 | 0.006
B4d1'2'3 | 195 | 1 | 0.006
B4d1'2'3 | 252 | 1 | 0.006
B4d1'2'3 | 262 | 1 | 0.006
B4d1'2'3 | 2755 | 1 | 0.006
B4d1'2'3 | 309.3C | 1 | 0.006
B4d1'2'3 | 316C | 2 | 0.013
B4d1'2'3 | 4841 | 2 | 0.013
B4d1'2'3 | 5372 | 1 | 0.006
B4d1'2'3 | 556T | 15 | 0.096
B4d1'2'3 | 5894C | 1 | 0.006
B4d1'2'3 | 600d | 1 | 0.006
B4d1'2'3 | 6324C | 1 | 0.006
B4d1'2'3 | 6524 | 2 | 0.013
B4d1'2'3 | 709 | 1 | 0.006
B4d1'2'3 | 7154 | 2 | 0.013
B4d1'2'3 | 8392 | 2 | 0.013
B4d1a | 10757G | 3 | 0.046
B4d1a | 11428A | 3 | 0.046
B4d1a | 152 | 3 | 0.046
B4d1a | 16176 | 1 | 0.015
B4d1a | 16240 | 3 | 0.046
B4d1a | 310 | 3 | 0.046
B4d1a | 312 | 3 | 0.046
B4d1a | 7864 | 1 | 0.015
B4d2 | 16188 | 3 | 0.046
B4d3 | 152 | 2 | 0.286
B4d3 | 16186 | 1 | 0.143
B4d3 | 16245 | 2 | 0.286
B4d3 | 189 | 1 | 0.143
B4d3 | 3010 | 1 | 0.143
B4d3 | 508 | 2 | 0.286
B4d3 | 52A | 1 | 0.143
B4d3 | 68 | 1 | 0.143
B4d3 | 6891 | 1 | 0.143
B4d3a | 13759 | 2 | 0.667
B4d3a | 15763 | 2 | 0.667
B4d3a | 9804 | 2 | 0.667
B4d3a1 | 12236 | 2 | 0.143
B4d3a1 | 1299 | 1 | 0.071
B4d3a1 | 146 | 1 | 0.071
B4d3a1 | 152 | 1 | 0.071
B4d3a1 | 153 | 1 | 0.071
B4d3a1 | 16086 | 2 | 0.143
B4d3a1 | 16150 | 9 | 0.643
B4d3a1 | 16325 | 1 | 0.071
B4d3a1 | 16355 | 1 | 0.071

B4d3a1 | 204 | 1 | 0.071
B4d3a1 | 573.1C | 1 | 0.071
B4d3a1 | 6989 | 1 | 0.071
B4d4 | 15972 | 1 | 0.045
B4d4 | 16269 | 1 | 0.045
B4d4 | 709 | 1 | 0.045
B4d4 | 8074 | 1 | 0.045
B4e | 10 | 1 | 0.013
B4e | 10410 | 6 | 0.076
B4e | 10912 | 5 | 0.063
B4e | 11353 | 1 | 0.013
B4e | 12358 | 1 | 0.013
B4e | 13135 | 6 | 0.076
B4e | 13194 | 5 | 0.063
B4e | 13464 | 1 | 0.013
B4e | 14133 | 1 | 0.013
B4e | 150 | 12 | 0.152
B4e | 15190 | 3 | 0.038
B4e | 15218 | 5 | 0.063
B4e | 15514 | 1 | 0.013
B4e | 15824 | 1 | 0.013
B4e | 15944d | 6 | 0.076
B4e | 16086 | 1 | 0.013
B4e | 16093 | 1 | 0.013
B4e | 16126 | 2 | 0.025
B4e | 16129 | 14 | 0.177
B4e | 16153 | 1 | 0.013
B4e | 16169 | 2 | 0.025
B4e | 16186 | 2 | 0.025
B4e | 16193d | 2 | 0.025
B4e | 16223 | 36 | 0.456
B4e | 16235 | 3 | 0.038
B4e | 16247 | 2 | 0.025
B4e | 16291 | 5 | 0.063
B4e | 16296 | 1 | 0.013
B4e | 16299 | 1 | 0.013
B4e | 16304 | 2 | 0.025
B4e | 16311 | 8 | 0.101
B4e | 16316 | 2 | 0.025
B4e | 16319 | 1 | 0.013
B4e | 16320 | 2 | 0.025
B4e | 16527 | 2 | 0.025
B4e | 1719 | 1 | 0.013
B4e | 194 | 1 | 0.013
B4e | 196 | 15 | 0.19
B4e | 198 | 1 | 0.013
B4e | 199 | 8 | 0.101
B4e | 200 | 1 | 0.013
B4e | 204 | 1 | 0.013
B4e | 215 | 2 | 0.025
B4e | 234 | 2 | 0.025
B4e | 307-309d | 4 | 0.051
B4e | 308-309d | 2 | 0.025
B4e | 309.3C | 1 | 0.013
B4e | 315.2C | 1 | 0.013
B4e | 348G | 1 | 0.013
B4e | 4048 | 1 | 0.013
B4e | 4232 | 1 | 0.013
B4e | 489 | 1 | 0.013
B4e | 4926 | 1 | 0.013
B4e | 5295 | 3 | 0.038
B4e | 5460 | 1 | 0.013
B4e | 7503d | 1 | 0.013
B4e | 7657 | 7 | 0.089
B4e | 8764 | 1 | 0.013
B4e | 9288 | 1 | 0.013
B4e | 9758 | 6 | 0.076
B4f | 12134 | 1 | 0.167
B4f | 13581 | 1 | 0.167
B4f | 13720 | 1 | 0.167
B4f | 14305 | 1 | 0.167
B4f | 153 | 1 | 0.167
B4f | 16189A | 1 | 0.167
B4f | 513 | 1 | 0.167
B4f | 5824 | 1 | 0.167
B4f | 6969 | 1 | 0.167
B4f | 8158 | 1 | 0.167
B4f | 9856 | 1 | 0.167
B4f1 | 10403 | 1 | 0.167
B4f1 | 11151 | 1 | 0.167
B4f1 | 13928 | 1 | 0.167
B4f1 | 15325 | 1 | 0.167
B4f1 | 4155 | 1 | 0.167
B4f1 | 5747 | 2 | 0.333
B4g1 | 11963 | 1 | 0.007
B4g1 | 12406 | 5 | 0.034
B4g1 | 12630 | 1 | 0.007
B4g1 | 13167 | 1 | 0.007
B4g1 | 13191 | 1 | 0.007
B4g1 | 13488 | 1 | 0.007
B4g1 | 14287 | 1 | 0.007
B4g1 | 146 | 4 | 0.028
B4g1 | 150 | 2 | 0.014
B4g1 | 152 | 8 | 0.055
B4g1 | 15346 | 1 | 0.007
B4g1 | 16059C | 1 | 0.007
B4g1 | 16075A | 1 | 0.007
B4g1 | 16086 | 1 | 0.007
B4g1 | 16093 | 4 | 0.028
B4g1 | 16111 | 1 | 0.007
B4g1 | 16129 | 6 | 0.041
B4g1 | 16129C | 1 | 0.007
B4g1 | 16169 | 2 | 0.014
B4g1 | 16172 | 1 | 0.007
B4g1 | 16180C | 6 | 0.041
B4g1 | 16181C | 85 | 0.586
B4g1 | 16188 | 1 | 0.007
B4g1 | 16193d | 7 | 0.048
B4g1 | 16201 | 3 | 0.021
B4g1 | 16209 | 1 | 0.007
B4g1 | 16230 | 1 | 0.007

B4g1 | 16232 | 1 | 0.007
B4g1 | 16248 | 2 | 0.014
B4g1 | 16249 | 1 | 0.007
B4g1 | 16265 | 2 | 0.014
B4g1 | 16266 | 1 | 0.007
B4g1 | 16270 | 3 | 0.021
B4g1 | 16270G | 1 | 0.007
B4g1 | 16278 | 2 | 0.014
B4g1 | 16294 | 1 | 0.007
B4g1 | 16295 | 1 | 0.007
B4g1 | 16311 | 2 | 0.014
B4g1 | 16319 | 1 | 0.007
B4g1 | 16356 | 3 | 0.021
B4g1 | 16361.1C | 1 | 0.007
B4g1 | 16362 | 7 | 0.048
B4g1 | 16367T | 1 | 0.007
B4g1 | 16370d | 1 | 0.007
B4g1 | 16398 | 2 | 0.014
B4g1 | 16402T | 1 | 0.007
B4g1 | 16405T | 1 | 0.007
B4g1 | 16464 | 1 | 0.007
B4g1 | 183 | 6 | 0.041
B4g1 | 189 | 1 | 0.007
B4g1 | 195 | 3 | 0.021
B4g1 | 215 | 1 | 0.007
B4g1 | 279 | 2 | 0.014
B4g1 | 302C | 2 | 0.014
B4g1 | 309.3C | 4 | 0.028
B4g1 | 309d | 7 | 0.048
B4g1 | 3613 | 1 | 0.007
B4g1 | 372 | 2 | 0.014
B4g1 | 455.1T | 2 | 0.014
B4g1 | 455d | 1 | 0.007
B4g1 | 479d | 1 | 0.007
B4g1 | 488d | 1 | 0.007
B4g1 | 491G | 1 | 0.007
B4g1 | 492T | 1 | 0.007
B4g1 | 493C | 1 | 0.007
B4g1 | 495G | 1 | 0.007
B4g1 | 496 | 1 | 0.007
B4g1 | 497 | 1 | 0.007
B4g1 | 5231 | 1 | 0.007
B4g1 | 61A | 1 | 0.007
B4g1 | 6485 | 1 | 0.007
B4g1 | 71d | 1 | 0.007
B4g1 | 8188 | 1 | 0.007
B4g1 | 9488 | 1 | 0.007
B4g1 | 9742 | 1 | 0.007
B4g1a | 10042C | 7 | 0.095
B4g1a | 10433 | 2 | 0.027
B4g1a | 10589 | 1 | 0.014
B4g1a | 11386 | 2 | 0.027
B4g1a | 11770 | 1 | 0.014
B4g1a | 1187 | 2 | 0.027
B4g1a | 11914 | 1 | 0.014
B4g1a | 12372 | 1 | 0.014
B4g1a | 12705 | 1 | 0.014
B4g1a | 12747.1GTA | 1 | 0.014
B4g1a | 12804 | 1 | 0.014
B4g1a | 13053 | 1 | 0.014
B4g1a | 13780 | 1 | 0.014
B4g1a | 14323 | 1 | 0.014
B4g1a | 152 | 1 | 0.014
B4g1a | 15973 | 1 | 0.014
B4g1a | 16093 | 2 | 0.027
B4g1a | 16169 | 1 | 0.014
B4g1a | 16180C | 2 | 0.027
B4g1a | 16181C | 10 | 0.135
B4g1a | 16193d | 8 | 0.108
B4g1a | 16223 | 3 | 0.041
B4g1a | 16290 | 1 | 0.014
B4g1a | 16319 | 1 | 0.014
B4g1a | 16362 | 1 | 0.014
B4g1a | 1664 | 1 | 0.014
B4g1a | 195 | 1 | 0.014
B4g1a | 309d | 1 | 0.014
B4g1a | 310 | 1 | 0.014
B4g1a | 315d | 1 | 0.014
B4g1a | 321G | 1 | 0.014
B4g1a | 3504 | 1 | 0.014
B4g1a | 3666 | 1 | 0.014
B4g1a | 3918 | 3 | 0.041
B4g1a | 411G | 1 | 0.014
B4g1a | 4248 | 1 | 0.014
B4g1a | 460.1T | 1 | 0.014
B4g1a | 6542 | 3 | 0.041
B4g1a | 702 | 1 | 0.014
B4g1a | 8251 | 3 | 0.041
B4g1a | 8389 | 1 | 0.014
B4g1a | 8723 | 11 | 0.149
B4g1a | 8794 | 1 | 0.014
B4g1a | 9091 | 1 | 0.014
B4g1a | 9111 | 11 | 0.149
B4g1a | 9177 | 1 | 0.014
B4g1a | 9395 | 1 | 0.014
B4g1a | 9804 | 1 | 0.014
B4g1b | 11207 | 1 | 0.014
B4g1b | 152 | 1 | 0.014
B4g1b | 16076d | 1 | 0.014
B4g1b | 16093 | 2 | 0.027
B4g1b | 16103 | 2 | 0.027
B4g1b | 16181C | 16 | 0.219
B4g1b | 16193d | 2 | 0.027
B4g1b | 16295 | 2 | 0.027
B4g1b | 16298 | 1 | 0.014
B4g1b | 16311 | 1 | 0.014
B4g1b | 16362 | 17 | 0.233
B4g1b | 16446G | 1 | 0.014
B4g1b | 1658 | 1 | 0.014
B4g1b | 196 | 1 | 0.014
B4g1b | 267 | 1 | 0.014

B4g1b | 297 | 1 | 0.014
B4g1b | 3221 | 2 | 0.027
B4g1b | 3483 | 2 | 0.027
B4g1b | 5237 | 1 | 0.014
B4g1b | 5703 | 1 | 0.014
B4g1b | 8485 | 1 | 0.014
B4g2 | 11242 | 2 | 0.025
B4g2 | 11453 | 1 | 0.013
B4g2 | 13752 | 2 | 0.025
B4g2 | 13926 | 2 | 0.025
B4g2 | 1413 | 1 | 0.013
B4g2 | 1462 | 1 | 0.013
B4g2 | 150 | 3 | 0.038
B4g2 | 152 | 1 | 0.013
B4g2 | 15244 | 1 | 0.013
B4g2 | 15951 | 1 | 0.013
B4g2 | 16086 | 2 | 0.025
B4g2 | 16090 | 1 | 0.013
B4g2 | 16093 | 4 | 0.051
B4g2 | 16095A | 3 | 0.038
B4g2 | 16102 | 1 | 0.013
B4g2 | 16129 | 3 | 0.038
B4g2 | 16154G | 1 | 0.013
B4g2 | 16173 | 1 | 0.013
B4g2 | 16180C | 5 | 0.063
B4g2 | 16181C | 75 | 0.949
B4g2 | 16192-16193d | 1 | 0.013
B4g2 | 16193d | 9 | 0.114
B4g2 | 16220C | 1 | 0.013
B4g2 | 16235 | 1 | 0.013
B4g2 | 16242 | 20 | 0.253
B4g2 | 16245 | 1 | 0.013
B4g2 | 16249 | 1 | 0.013
B4g2 | 16250 | 1 | 0.013
B4g2 | 16260 | 1 | 0.013
B4g2 | 16274 | 1 | 0.013
B4g2 | 16278 | 1 | 0.013
B4g2 | 16287 | 4 | 0.051
B4g2 | 16289 | 1 | 0.013
B4g2 | 16299 | 1 | 0.013
B4g2 | 16304 | 2 | 0.025
B4g2 | 16309 | 1 | 0.013
B4g2 | 16312 | 1 | 0.013
B4g2 | 16354 | 1 | 0.013
B4g2 | 16355 | 4 | 0.051
B4g2 | 16359 | 1 | 0.013
B4g2 | 16362 | 11 | 0.139
B4g2 | 16C | 1 | 0.013
B4g2 | 182 | 8 | 0.101
B4g2 | 183 | 8 | 0.101
B4g2 | 195 | 3 | 0.038
B4g2 | 207 | 1 | 0.013
B4g2 | 307-309d | 1 | 0.013
B4g2 | 308-309d | 3 | 0.038
B4g2 | 309.3C | 6 | 0.076
B4g2 | 309d | 3 | 0.038
B4g2 | 310 | 3 | 0.038
B4g2 | 315d | 1 | 0.013
B4g2 | 316 | 1 | 0.013
B4g2 | 316C | 2 | 0.025
B4g2 | 318 | 1 | 0.013
B4g2 | 319 | 1 | 0.013
B4g2 | 329 | 1 | 0.013
B4g2 | 455d | 1 | 0.013
B4g2 | 471 | 2 | 0.025
B4g2 | 487T | 1 | 0.013
B4g2 | 488.1GTCG | 1 | 0.013
B4g2 | 491G | 1 | 0.013
B4g2 | 61 | 1 | 0.013
B4g2 | 61A | 71 | 0.899
B4g2 | 709 | 2 | 0.025
B4g2 | 7424 | 1 | 0.013
B4g2 | 8215 | 7 | 0.089
B4g2 | 8931 | 1 | 0.013
B4g2 | 9592G | 1 | 0.013
B4h | 10007 | 2 | 0.012
B4h | 10410 | 3 | 0.018
B4h | 11016 | 1 | 0.006
B4h | 11950 | 1 | 0.006
B4h | 12007 | 1 | 0.006
B4h | 12130 | 1 | 0.006
B4h | 12390 | 1 | 0.006
B4h | 12634 | 1 | 0.006
B4h | 13967 | 3 | 0.018
B4h | 13988A | 1 | 0.006
B4h | 14122 | 2 | 0.012
B4h | 14388 | 1 | 0.006
B4h | 146 | 17 | 0.1
B4h | 14643A | 1 | 0.006
B4h | 14769 | 1 | 0.006
B4h | 14968 | 4 | 0.024
B4h | 150 | 1 | 0.006
B4h | 15043 | 1 | 0.006
B4h | 152 | 1 | 0.006
B4h | 15616 | 1 | 0.006
B4h | 15784 | 1 | 0.006
B4h | 15924 | 2 | 0.012
B4h | 16051 | 1 | 0.006
B4h | 16076d | 1 | 0.006
B4h | 16082A | 1 | 0.006
B4h | 16083G | 1 | 0.006
B4h | 16086 | 1 | 0.006
B4h | 16093 | 3 | 0.018
B4h | 16140 | 1 | 0.006
B4h | 16145 | 2 | 0.012
B4h | 16150 | 1 | 0.006
B4h | 16153 | 1 | 0.006
B4h | 16172 | 2 | 0.012
B4h | 16181C | 2 | 0.012
B4h | 16197 | 1 | 0.006
B4h | 16221A | 1 | 0.006

B4h | 16242 | 1 | 0.006
B4h | 16245 | 1 | 0.006
B4h | 16287 | 1 | 0.006
B4h | 16289 | 1 | 0.006
B4h | 16291 | 1 | 0.006
B4h | 16292 | 1 | 0.006
B4h | 16293 | 1 | 0.006
B4h | 16299 | 3 | 0.018
B4h | 16311 | 1 | 0.006
B4h | 16319 | 1 | 0.006
B4h | 16354 | 11 | 0.065
B4h | 16356 | 13 | 0.076
B4h | 16362 | 4 | 0.024
B4h | 16413A | 1 | 0.006
B4h | 16422 | 1 | 0.006
B4h | 16488 | 3 | 0.018
B4h | 16497 | 1 | 0.006
B4h | 185 | 1 | 0.006
B4h | 194 | 1 | 0.006
B4h | 195 | 3 | 0.018
B4h | 199 | 4 | 0.024
B4h | 257 | 1 | 0.006
B4h | 272 | 1 | 0.006
B4h | 279 | 1 | 0.006
B4h | 302d | 1 | 0.006
B4h | 308-309d | 2 | 0.012
B4h | 309.3C | 3 | 0.018
B4h | 310 | 9 | 0.053
B4h | 313-315d | 5 | 0.029
B4h | 314-315d | 2 | 0.012
B4h | 316C | 1 | 0.006
B4h | 338 | 1 | 0.006
B4h | 3395 | 1 | 0.006
B4h | 3548 | 1 | 0.006
B4h | 3736 | 1 | 0.006
B4h | 3745 | 1 | 0.006
B4h | 3954 | 3 | 0.018
B4h | 455d | 1 | 0.006
B4h | 458 | 2 | 0.012
B4h | 4612 | 1 | 0.006
B4h | 469A | 1 | 0.006
B4h | 477 | 2 | 0.012
B4h | 487.1T | 1 | 0.006
B4h | 488.1GTG | 1 | 0.006
B4h | 493 | 1 | 0.006
B4h | 4937 | 1 | 0.006
B4h | 513 | 5 | 0.029
B4h | 5201 | 1 | 0.006
B4h | 5237 | 1 | 0.006
B4h | 5302 | 1 | 0.006
B4h | 5471 | 1 | 0.006
B4h | 5480 | 1 | 0.006
B4h | 6641 | 1 | 0.006
B4h | 6881 | 1 | 0.006
B4h | 709 | 8 | 0.047
B4h | 71.1G | 2 | 0.012
B4h | 8045 | 1 | 0.006
B4h | 8248 | 1 | 0.006
B4h | 8825 | 1 | 0.006
B4h | 93 | 13 | 0.076
B4h | 9477 | 1 | 0.006
B4h | 9530 | 1 | 0.006
B4h | 9554 | 3 | 0.018
B4h | 960d | 1 | 0.006
B4h | 9659 | 1 | 0.006
B4h1 | 11254 | 2 | 0.02
B4h1 | 13158 | 1 | 0.01
B4h1 | 13368 | 8 | 0.079
B4h1 | 13912 | 2 | 0.02
B4h1 | 143 | 1 | 0.01
B4h1 | 146 | 1 | 0.01
B4h1 | 14788 | 1 | 0.01
B4h1 | 15388 | 1 | 0.01
B4h1 | 16051 | 1 | 0.01
B4h1 | 16076d | 1 | 0.01
B4h1 | 16086 | 1 | 0.01
B4h1 | 16111G | 1 | 0.01
B4h1 | 16181 | 1 | 0.01
B4h1 | 16181C | 1 | 0.01
B4h1 | 16187A | 1 | 0.01
B4h1 | 16192-16193d | 1 | 0.01
B4h1 | 16207 | 1 | 0.01
B4h1 | 16221 | 1 | 0.01
B4h1 | 16223 | 2 | 0.02
B4h1 | 16224 | 1 | 0.01
B4h1 | 16227 | 1 | 0.01
B4h1 | 16274 | 1 | 0.01
B4h1 | 16293C | 1 | 0.01
B4h1 | 16297 | 1 | 0.01
B4h1 | 16304 | 1 | 0.01
B4h1 | 16311 | 8 | 0.079
B4h1 | 16362 | 2 | 0.02
B4h1 | 16497 | 1 | 0.01
B4h1 | 189 | 1 | 0.01
B4h1 | 195 | 2 | 0.02
B4h1 | 200 | 1 | 0.01
B4h1 | 214 | 1 | 0.01
B4h1 | 227 | 1 | 0.01
B4h1 | 2320 | 2 | 0.02
B4h1 | 2363 | 2 | 0.02
B4h1 | 306-309d | 1 | 0.01
B4h1 | 309 | 1 | 0.01
B4h1 | 309.3C | 5 | 0.05
B4h1 | 310 | 2 | 0.02
B4h1 | 314 | 1 | 0.01
B4h1 | 316C | 1 | 0.01
B4h1 | 328 | 1 | 0.01
B4h1 | 513 | 1 | 0.01
B4h1 | 5201 | 1 | 0.01
B4h1 | 573.1C | 1 | 0.01
B4h1 | 5783 | 2 | 0.02

B4h1 | 6485 | 2 | 0.02
B4h1 | 8832 | 1 | 0.01
B4h1 | 93 | 2 | 0.02
B4h1 | 9739 | 2 | 0.02
B4i | 107 | 1 | 0.004
B4i | 11176 | 1 | 0.004
B4i | 12654 | 2 | 0.007
B4i | 135d | 1 | 0.004
B4i | 152 | 29 | 0.107
B4i | 16050 | 1 | 0.004
B4i | 16136 | 1 | 0.004
B4i | 16180 | 1 | 0.004
B4i | 16181C | 1 | 0.004
B4i | 16192-16193d | 1 | 0.004
B4i | 16193d | 5 | 0.018
B4i | 16212 | 1 | 0.004
B4i | 16219 | 1 | 0.004
B4i | 16246 | 2 | 0.007
B4i | 16286 | 1 | 0.004
B4i | 16293 | 1 | 0.004
B4i | 16319 | 1 | 0.004
B4i | 16327 | 2 | 0.007
B4i | 16335 | 1 | 0.004
B4i | 16355 | 4 | 0.015
B4i | 16360 | 2 | 0.007
B4i | 16567 | 1 | 0.004
B4i | 252 | 1 | 0.004
B4i | 290 | 1 | 0.004
B4i | 290C | 1 | 0.004
B4i | 291d | 1 | 0.004
B4i | 3 | 1 | 0.004
B4i | 309.3C | 2 | 0.007
B4i | 336C | 1 | 0.004
B4i | 40 | 1 | 0.004
B4i | 438.1C | 1 | 0.004
B4i | 475 | 1 | 0.004
B4i | 492 | 1 | 0.004
B4i | 573.5C | 1 | 0.004
B4i | 71d | 5 | 0.018
B4i | 801 | 1 | 0.004
B4i1 | 11150 | 2 | 0.008
B4i1 | 14581 | 1 | 0.004
B4i1 | 14953G | 2 | 0.008
B4i1 | 152 | 1 | 0.004
B4i1 | 15262 | 1 | 0.004
B4i1 | 16129 | 1 | 0.004
B4i1 | 16222 | 1 | 0.004
B4i1 | 16287 | 1 | 0.004
B4i1 | 16290 | 1 | 0.004
B4i1 | 16304 | 3 | 0.012
B4i1 | 16311 | 1 | 0.004
B4i1 | 16355 | 1 | 0.004
B4i1 | 16362 | 4 | 0.016
B4i1 | 1836 | 1 | 0.004
B4i1 | 195 | 11 | 0.043
B4i1 | 200 | 3 | 0.012
B4i1 | 204 | 1 | 0.004
B4i1 | 214 | 1 | 0.004
B4i1 | 228 | 1 | 0.004
B4i1 | 252 | 1 | 0.004
B4i1 | 308-309d | 1 | 0.004
B4i1 | 309d | 1 | 0.004
B4i1 | 3337T | 1 | 0.004
B4i1 | 3866 | 2 | 0.008
B4i1 | 4093 | 1 | 0.004
B4i1 | 466 | 2 | 0.008
B4i1 | 5656 | 1 | 0.004
B4i1 | 573.5C | 1 | 0.004
B4i1 | 6524 | 3 | 0.012
B4i1 | 65G | 1 | 0.004
B4i1 | 6791 | 1 | 0.004
B4i1 | 8380 | 1 | 0.004
B4i1 | 8848 | 1 | 0.004
B4i1 | 980 | 3 | 0.012
B4j | 12358 | 1 | 0.032
B4j | 13896 | 1 | 0.032
B4j | 14274 | 1 | 0.032
B4j | 150 | 1 | 0.032
B4j | 16093 | 2 | 0.065
B4j | 16272 | 1 | 0.032
B4j | 16304 | 1 | 0.032
B4j | 16319 | 3 | 0.097
B4j | 200 | 1 | 0.032
B4j | 573.4C | 1 | 0.032
B4j | 573.5C | 1 | 0.032
B4j | 573.6C | 1 | 0.032
B4j | 6179 | 1 | 0.032
B4j | 709 | 1 | 0.032
B4j | 9007 | 1 | 0.032
B4k | 14410 | 1 | 0.009
B4k | 150 | 1 | 0.009
B4k | 152 | 2 | 0.017
B4k | 16104A | 1 | 0.009
B4k | 16111A | 3 | 0.026
B4k | 16129 | 3 | 0.026
B4k | 16136 | 1 | 0.009
B4k | 16169 | 2 | 0.017
B4k | 16179 | 2 | 0.017
B4k | 16181C | 1 | 0.009
B4k | 16192-16193d | 1 | 0.009
B4k | 16193d | 3 | 0.026
B4k | 16213 | 1 | 0.009
B4k | 16219 | 1 | 0.009
B4k | 16248 | 1 | 0.009
B4k | 16257A | 1 | 0.009
B4k | 16271 | 2 | 0.017
B4k | 16286 | 1 | 0.009
B4k | 16287 | 2 | 0.017
B4k | 16290 | 1 | 0.009
B4k | 16299 | 2 | 0.017
B4k | 16303 | 2 | 0.017

B4k | 16319 | 1 | 0.009
B4k | 16327 | 1 | 0.009
B4k | 16355 | 1 | 0.009
B4k | 16357 | 12 | 0.103
B4k | 16390 | 2 | 0.017
B4k | 16465 | 1 | 0.009
B4k | 16474d | 1 | 0.009
B4k | 16526 | 3 | 0.026
B4k | 183 | 1 | 0.009
B4k | 195 | 5 | 0.043
B4k | 204 | 1 | 0.009
B4k | 207 | 1 | 0.009
B4k | 308-309d | 1 | 0.009
B4k | 309.3C | 3 | 0.026
B4k | 316C | 1 | 0.009
B4k | 486 | 1 | 0.009
B4k | 499 | 1 | 0.009
B4k | 709 | 2 | 0.017
B4k | 71.1G | 1 | 0.009
B4k | 711 | 1 | 0.009
B4k | 75 | 1 | 0.009
B4k | 89 | 1 | 0.009
B4k | 9181 | 3 | 0.026
B4k | 9531 | 2 | 0.017
B4m | 12358 | 1 | 0.01
B4m | 13563 | 1 | 0.01
B4m | 14097 | 5 | 0.048
B4m | 143 | 1 | 0.01
B4m | 14410 | 1 | 0.01
B4m | 146 | 2 | 0.019
B4m | 14883 | 1 | 0.01
B4m | 152 | 1 | 0.01
B4m | 15381 | 1 | 0.01
B4m | 16075A | 1 | 0.01
B4m | 16086 | 1 | 0.01
B4m | 16092 | 1 | 0.01
B4m | 16093 | 1 | 0.01
B4m | 16129 | 4 | 0.038
B4m | 16136 | 2 | 0.019
B4m | 16150 | 12 | 0.115
B4m | 16154 | 4 | 0.038
B4m | 16166 | 1 | 0.01
B4m | 16167 | 1 | 0.01
B4m | 16169 | 1 | 0.01
B4m | 16181C | 2 | 0.019
B4m | 16221 | 7 | 0.067
B4m | 16231 | 1 | 0.01
B4m | 16239 | 1 | 0.01
B4m | 16260 | 1 | 0.01
B4m | 16262 | 1 | 0.01
B4m | 16287 | 3 | 0.029
B4m | 16294 | 1 | 0.01
B4m | 16311 | 1 | 0.01
B4m | 16342 | 1 | 0.01
B4m | 16350C | 1 | 0.01
B4m | 16357 | 1 | 0.01
B4m | 16374C | 1 | 0.01
B4m | 16378A | 1 | 0.01
B4m | 16399 | 1 | 0.01
B4m | 195 | 3 | 0.029
B4m | 204 | 1 | 0.01
B4m | 2352 | 1 | 0.01
B4m | 307-309d | 1 | 0.01
B4m | 309.3C | 3 | 0.029
B4m | 309d | 1 | 0.01
B4m | 310 | 1 | 0.01
B4m | 315d | 1 | 0.01
B4m | 3423 | 6 | 0.058
B4m | 382 | 1 | 0.01
B4m | 4679 | 1 | 0.01
B4m | 521 | 1 | 0.01
B4m | 6863 | 1 | 0.01
B4m | 736 | 1 | 0.01
B4m | 8400 | 1 | 0.01
B5 | 105d | 1 | 0.019
B5 | 146 | 2 | 0.037
B5 | 150 | 1 | 0.019
B5 | 151 | 2 | 0.037
B5 | 152 | 3 | 0.056
B5 | 16038 | 1 | 0.019
B5 | 16074T | 1 | 0.019
B5 | 16075 | 1 | 0.019
B5 | 16104 | 1 | 0.019
B5 | 16129 | 6 | 0.111
B5 | 16145 | 1 | 0.019
B5 | 16148 | 1 | 0.019
B5 | 16177T | 1 | 0.019
B5 | 16187 | 1 | 0.019
B5 | 16218 | 1 | 0.019
B5 | 16223 | 1 | 0.019
B5 | 16243 | 5 | 0.093
B5 | 16243G | 1 | 0.019
B5 | 16258C | 1 | 0.019
B5 | 16260 | 4 | 0.074
B5 | 16266A | 8 | 0.148
B5 | 16267 | 1 | 0.019
B5 | 16275 | 3 | 0.056
B5 | 16311 | 4 | 0.074
B5 | 16316 | 1 | 0.019
B5 | 16330 | 1 | 0.019
B5 | 16343 | 1 | 0.019
B5 | 16355 | 4 | 0.074
B5 | 16387T | 1 | 0.019
B5 | 16399 | 3 | 0.056
B5 | 16478G | 1 | 0.019
B5 | 16538 | 1 | 0.019
B5 | 189 | 4 | 0.074
B5 | 200 | 4 | 0.074
B5 | 205 | 2 | 0.037
B5 | 210 | 3 | 0.056
B5 | 215 | 1 | 0.019

B5 | 235 | 1 | 0.019
B5 | 294 | 4 | 0.074
B5 | 295 | 1 | 0.019
B5 | 315.2C | 1 | 0.019
B5 | 363 | 1 | 0.019
B5 | 480G | 1 | 0.019
B5 | 515d | 1 | 0.019
B5a | 103 | 3 | 0.006
B5a | 150 | 1 | 0.002
B5a | 152 | 5 | 0.01
B5a | 15229 | 4 | 0.008
B5a | 15263 | 1 | 0.002
B5a | 15287 | 3 | 0.006
B5a | 153 | 5 | 0.01
B5a | 15663 | 1 | 0.002
B5a | 15777 | 1 | 0.002
B5a | 16032d | 1 | 0.002
B5a | 16038d | 2 | 0.004
B5a | 16042C | 1 | 0.002
B5a | 16042d | 1 | 0.002
B5a | 16061G | 1 | 0.002
B5a | 16079 | 1 | 0.002
B5a | 16093 | 47 | 0.092
B5a | 16102A | 1 | 0.002
B5a | 16110d | 1 | 0.002
B5a | 16117 | 1 | 0.002
B5a | 16145 | 3 | 0.006
B5a | 16153 | 5 | 0.01
B5a | 16167 | 1 | 0.002
B5a | 16178 | 3 | 0.006
B5a | 16194C | 2 | 0.004
B5a | 16207 | 3 | 0.006
B5a | 16209 | 1 | 0.002
B5a | 16220 | 1 | 0.002
B5a | 16223 | 3 | 0.006
B5a | 16224 | 2 | 0.004
B5a | 16235T | 1 | 0.002
B5a | 16248 | 2 | 0.004
B5a | 16257 | 6 | 0.012
B5a | 16258C | 2 | 0.004
B5a | 16260 | 7 | 0.014
B5a | 16262.1C | 1 | 0.002
B5a | 16265 | 1 | 0.002
B5a | 16274 | 8 | 0.016
B5a | 16288 | 1 | 0.002
B5a | 16289 | 1 | 0.002
B5a | 16291 | 1 | 0.002
B5a | 16304 | 1 | 0.002
B5a | 16315 | 2 | 0.004
B5a | 16361 | 10 | 0.02
B5a | 16399 | 14 | 0.027
B5a | 16456 | 1 | 0.002
B5a | 183 | 1 | 0.002
B5a | 189 | 7 | 0.014
B5a | 200 | 7 | 0.014
B5a | 204 | 5 | 0.01
B5a | 23 | 1 | 0.002
B5a | 236 | 1 | 0.002
B5a | 249d | 1 | 0.002
B5a | 26 | 2 | 0.004
B5a | 279 | 1 | 0.002
B5a | 291.1A | 1 | 0.002
B5a | 294 | 5 | 0.01
B5a | 42.1G | 3 | 0.006
B5a | 499 | 1 | 0.002
B5a | 511 | 2 | 0.004
B5a | 547 | 2 | 0.004
B5a | 573.1C | 2 | 0.004
B5a | 648 | 1 | 0.002
B5a | 6663 | 1 | 0.002
B5a | 7001 | 4 | 0.008
B5a | 9100 | 1 | 0.002
B5a | 93 | 2 | 0.004
B5a1 | 10304 | 8 | 0.015
B5a1 | 10523 | 2 | 0.004
B5a1 | 10807 | 3 | 0.006
B5a1 | 11204 | 1 | 0.002
B5a1 | 11732 | 2 | 0.004
B5a1 | 12216 | 8 | 0.015
B5a1 | 12372 | 2 | 0.004
B5a1 | 14053 | 2 | 0.004
B5a1 | 143 | 1 | 0.002
B5a1 | 14587 | 2 | 0.004
B5a1 | 15043 | 2 | 0.004
B5a1 | 15106 | 7 | 0.013
B5a1 | 152 | 17 | 0.032
B5a1 | 15236 | 1 | 0.002
B5a1 | 15299 | 1 | 0.002
B5a1 | 153 | 1 | 0.002
B5a1 | 15862 | 8 | 0.015
B5a1 | 15885 | 3 | 0.006
B5a1 | 16032d | 1 | 0.002
B5a1 | 16042d | 1 | 0.002
B5a1 | 16061G | 1 | 0.002
B5a1 | 16075 | 1 | 0.002
B5a1 | 16079 | 1 | 0.002
B5a1 | 16092 | 30 | 0.056
B5a1 | 16110d | 1 | 0.002
B5a1 | 16117 | 1 | 0.002
B5a1 | 16129 | 5 | 0.009
B5a1 | 16145 | 1 | 0.002
B5a1 | 16148 | 1 | 0.002
B5a1 | 16167 | 1 | 0.002
B5a1 | 16172 | 12 | 0.022
B5a1 | 16195G | 1 | 0.002
B5a1 | 16197G | 1 | 0.002
B5a1 | 16209 | 1 | 0.002
B5a1 | 16212 | 1 | 0.002
B5a1 | 16219 | 1 | 0.002
B5a1 | 16220 | 1 | 0.002
B5a1 | 16223 | 3 | 0.006

B5a1 | 16235T | 1 | 0.002
B5a1 | 16256A | 2 | 0.004
B5a1 | 16264 | 1 | 0.002
B5a1 | 16265 | 1 | 0.002
B5a1 | 16267 | 1 | 0.002
B5a1 | 16274 | 2 | 0.004
B5a1 | 16284 | 2 | 0.004
B5a1 | 16286 | 3 | 0.006
B5a1 | 16288 | 1 | 0.002
B5a1 | 16289 | 1 | 0.002
B5a1 | 16291 | 7 | 0.013
B5a1 | 16293C | 1 | 0.002
B5a1 | 16294 | 7 | 0.013
B5a1 | 16295 | 1 | 0.002
B5a1 | 16296 | 1 | 0.002
B5a1 | 16326 | 5 | 0.009
B5a1 | 16342 | 5 | 0.009
B5a1 | 16354 | 1 | 0.002
B5a1 | 16361 | 7 | 0.013
B5a1 | 16362 | 43 | 0.08
B5a1 | 16371 | 4 | 0.007
B5a1 | 16399 | 12 | 0.022
B5a1 | 16456 | 1 | 0.002
B5a1 | 16527 | 6 | 0.011
B5a1 | 183 | 1 | 0.002
B5a1 | 189 | 2 | 0.004
B5a1 | 191.1A | 1 | 0.002
B5a1 | 195 | 15 | 0.028
B5a1 | 204 | 10 | 0.019
B5a1 | 228 | 3 | 0.006
B5a1 | 256 | 1 | 0.002
B5a1 | 279 | 1 | 0.002
B5a1 | 291.1A | 1 | 0.002
B5a1 | 307-309d | 1 | 0.002
B5a1 | 309.3C | 1 | 0.002
B5a1 | 310 | 2 | 0.004
B5a1 | 3316 | 1 | 0.002
B5a1 | 356.1C | 1 | 0.002
B5a1 | 374 | 1 | 0.002
B5a1 | 3892 | 1 | 0.002
B5a1 | 4164 | 2 | 0.004
B5a1 | 4454 | 2 | 0.004
B5a1 | 455.1T | 2 | 0.004
B5a1 | 455d | 1 | 0.002
B5a1 | 4742 | 7 | 0.013
B5a1 | 502 | 1 | 0.002
B5a1 | 573.1C | 2 | 0.004
B5a1 | 6098 | 1 | 0.002
B5a1 | 6261 | 2 | 0.004
B5a1 | 648 | 1 | 0.002
B5a1 | 6755 | 1 | 0.002
B5a1 | 6975 | 1 | 0.002
B5a1 | 712 | 2 | 0.004
B5a1 | 7325 | 3 | 0.006
B5a1 | 8272 | 1 | 0.002
B5a1 | 8281 | 1 | 0.002
B5a1 | 8727 | 1 | 0.002
B5a1 | 8806 | 1 | 0.002
B5a1 | 8856 | 1 | 0.002
B5a1 | 9455 | 2 | 0.004
B5a1a | 1007 | 1 | 0.001
B5a1a | 10166 | 1 | 0.001
B5a1a | 10463 | 4 | 0.006
B5a1a | 10646 | 3 | 0.004
B5a1a | 10703 | 2 | 0.003
B5a1a | 1106 | 1 | 0.001
B5a1a | 11065 | 9 | 0.013
B5a1a | 11075 | 1 | 0.001
B5a1a | 11167 | 5 | 0.007
B5a1a | 11197 | 1 | 0.001
B5a1a | 11339 | 2 | 0.003
B5a1a | 11377 | 1 | 0.001
B5a1a | 11465 | 3 | 0.004
B5a1a | 11779 | 1 | 0.001
B5a1a | 1189 | 1 | 0.001
B5a1a | 12134 | 2 | 0.003
B5a1a | 12193 | 2 | 0.003
B5a1a | 12483 | 1 | 0.001
B5a1a | 12588 | 7 | 0.01
B5a1a | 12618 | 1 | 0.001
B5a1a | 12793 | 1 | 0.001
B5a1a | 13020 | 2 | 0.003
B5a1a | 13105 | 1 | 0.001
B5a1a | 13174 | 2 | 0.003
B5a1a | 13320 | 1 | 0.001
B5a1a | 13708 | 1 | 0.001
B5a1a | 13768 | 1 | 0.001
B5a1a | 13812 | 1 | 0.001
B5a1a | 13879 | 6 | 0.009
B5a1a | 14041 | 1 | 0.001
B5a1a | 14180 | 2 | 0.003
B5a1a | 14198 | 2 | 0.003
B5a1a | 14209 | 1 | 0.001
B5a1a | 1423 | 1 | 0.001
B5a1a | 14290 | 1 | 0.001
B5a1a | 14314 | 1 | 0.001
B5a1a | 14384 | 1 | 0.001
B5a1a | 14443A | 1 | 0.001
B5a1a | 14569 | 1 | 0.001
B5a1a | 14580 | 1 | 0.001
B5a1a | 146 | 38 | 0.056
B5a1a | 1462 | 1 | 0.001
B5a1a | 14755 | 1 | 0.001
B5a1a | 14927 | 2 | 0.003
B5a1a | 150 | 6 | 0.009
B5a1a | 1503 | 1 | 0.001
B5a1a | 15043 | 3 | 0.004
B5a1a | 151 | 1 | 0.001
B5a1a | 152 | 4 | 0.006
B5a1a | 15229 | 1 | 0.001
B5a1a | 15401 | 1 | 0.001

B5a1a | 15728 | 1 | 0.001
B5a1a | 15886 | 1 | 0.001
B5a1a | 15930 | 7 | 0.01
B5a1a | 15964 | 1 | 0.001
B5a1a | 16092 | 5 | 0.007
B5a1a | 16093 | 2 | 0.003
B5a1a | 16111 | 5 | 0.007
B5a1a | 16114A | 1 | 0.001
B5a1a | 16126 | 10 | 0.015
B5a1a | 16129 | 21 | 0.031
B5a1a | 16131 | 4 | 0.006
B5a1a | 16136 | 1 | 0.001
B5a1a | 16148 | 13 | 0.019
B5a1a | 16166 | 3 | 0.004
B5a1a | 16169A | 2 | 0.003
B5a1a | 16170 | 3 | 0.004
B5a1a | 16178 | 1 | 0.001
B5a1a | 16186 | 1 | 0.001
B5a1a | 16187 | 2 | 0.003
B5a1a | 16188 | 2 | 0.003
B5a1a | 16195 | 1 | 0.001
B5a1a | 16197G | 2 | 0.003
B5a1a | 16203 | 1 | 0.001
B5a1a | 16209 | 1 | 0.001
B5a1a | 16212 | 1 | 0.001
B5a1a | 16213 | 1 | 0.001
B5a1a | 16219 | 3 | 0.004
B5a1a | 16227 | 7 | 0.01
B5a1a | 16234 | 45 | 0.067
B5a1a | 16235 | 1 | 0.001
B5a1a | 16238 | 1 | 0.001
B5a1a | 16242 | 3 | 0.004
B5a1a | 16243 | 9 | 0.013
B5a1a | 16261 | 7 | 0.01
B5a1a | 16267 | 2 | 0.003
B5a1a | 16274 | 1 | 0.001
B5a1a | 16278 | 3 | 0.004
B5a1a | 16286 | 1 | 0.001
B5a1a | 16292 | 5 | 0.007
B5a1a | 16293 | 4 | 0.006
B5a1a | 16295 | 5 | 0.007
B5a1a | 16296 | 1 | 0.001
B5a1a | 16300 | 1 | 0.001
B5a1a | 16304 | 1 | 0.001
B5a1a | 16311 | 6 | 0.009
B5a1a | 16316 | 1 | 0.001
B5a1a | 16317 | 1 | 0.001
B5a1a | 16318 | 1 | 0.001
B5a1a | 16320 | 3 | 0.004
B5a1a | 16325 | 7 | 0.01
B5a1a | 16327 | 3 | 0.004
B5a1a | 16356 | 21 | 0.031
B5a1a | 16357 | 2 | 0.003
B5a1a | 16359 | 3 | 0.004
B5a1a | 16360 | 1 | 0.001
B5a1a | 16362 | 3 | 0.004
B5a1a | 16390 | 1 | 0.001
B5a1a | 16391 | 4 | 0.006
B5a1a | 16399 | 1 | 0.001
B5a1a | 16465 | 1 | 0.001
B5a1a | 16482 | 24 | 0.036
B5a1a | 16525 | 1 | 0.001
B5a1a | 16527 | 2 | 0.003
B5a1a | 1900C | 1 | 0.001
B5a1a | 195 | 2 | 0.003
B5a1a | 204 | 1 | 0.001
B5a1a | 2213 | 1 | 0.001
B5a1a | 226 | 1 | 0.001
B5a1a | 227T | 1 | 0.001
B5a1a | 228 | 7 | 0.01
B5a1a | 234 | 1 | 0.001
B5a1a | 267 | 1 | 0.001
B5a1a | 2772A | 1 | 0.001
B5a1a | 285 | 3 | 0.004
B5a1a | 2863 | 1 | 0.001
B5a1a | 291d | 1 | 0.001
B5a1a | 309.3C | 1 | 0.001
B5a1a | 310 | 4 | 0.006
B5a1a | 315.3C | 1 | 0.001
B5a1a | 315d | 1 | 0.001
B5a1a | 316C | 1 | 0.001
B5a1a | 3202 | 1 | 0.001
B5a1a | 3335 | 2 | 0.003
B5a1a | 3391 | 1 | 0.001
B5a1a | 3397 | 1 | 0.001
B5a1a | 372 | 1 | 0.001
B5a1a | 385 | 3 | 0.004
B5a1a | 3930A | 1 | 0.001
B5a1a | 4068 | 1 | 0.001
B5a1a | 4092 | 4 | 0.006
B5a1a | 418 | 1 | 0.001
B5a1a | 4216 | 2 | 0.003
B5a1a | 455d | 1 | 0.001
B5a1a | 458 | 1 | 0.001
B5a1a | 4703 | 2 | 0.003
B5a1a | 471 | 1 | 0.001
B5a1a | 482 | 2 | 0.003
B5a1a | 4856 | 1 | 0.001
B5a1a | 488.1GTG | 1 | 0.001
B5a1a | 488T | 1 | 0.001
B5a1a | 4988 | 3 | 0.004
B5a1a | 503 | 2 | 0.003
B5a1a | 5033 | 1 | 0.001
B5a1a | 5093 | 1 | 0.001
B5a1a | 5168 | 3 | 0.004
B5a1a | 5263 | 1 | 0.001
B5a1a | 5460 | 1 | 0.001
B5a1a | 5498 | 1 | 0.001
B5a1a | 5553 | 3 | 0.004
B5a1a | 5561 | 1 | 0.001
B5a1a | 5673 | 1 | 0.001

B5a1a | 573.1C | 2 | 0.003
B5a1a | 573.4C | 1 | 0.001
B5a1a | 574C | 1 | 0.001
B5a1a | 576C | 1 | 0.001
B5a1a | 5814 | 1 | 0.001
B5a1a | 593 | 1 | 0.001
B5a1a | 595 | 1 | 0.001
B5a1a | 6261 | 1 | 0.001
B5a1a | 6389 | 3 | 0.004
B5a1a | 6408 | 1 | 0.001
B5a1a | 6465 | 1 | 0.001
B5a1a | 6563 | 1 | 0.001
B5a1a | 6599 | 2 | 0.003
B5a1a | 6681 | 3 | 0.004
B5a1a | 6689 | 1 | 0.001
B5a1a | 7082 | 3 | 0.004
B5a1a | 7609 | 2 | 0.003
B5a1a | 7645A | 1 | 0.001
B5a1a | 7859 | 1 | 0.001
B5a1a | 8251 | 3 | 0.004
B5a1a | 8519 | 2 | 0.003
B5a1a | 8793 | 1 | 0.001
B5a1a | 8835 | 2 | 0.003
B5a1a | 8857 | 2 | 0.003
B5a1a | 8896 | 1 | 0.001
B5a1a | 8976 | 2 | 0.003
B5a1a | 9007 | 1 | 0.001
B5a1a | 9055 | 1 | 0.001
B5a1a | 9192 | 1 | 0.001
B5a1a | 93 | 3 | 0.004
B5a1a | 930 | 1 | 0.001
B5a1a | 9300 | 1 | 0.001
B5a1a | 963 | 3 | 0.004
B5a1a | 9647 | 1 | 0.001
B5a1a | 9682 | 1 | 0.001
B5a1a | 9803 | 7 | 0.01
B5a1a | 9899 | 1 | 0.001
B5a1a1 | 16032d | 1 | 0.002
B5a1a1 | 16038d | 2 | 0.004
B5a1a1 | 16042C | 1 | 0.002
B5a1a1 | 16042d | 1 | 0.002
B5a1a1 | 16061G | 1 | 0.002
B5a1a1 | 16110d | 1 | 0.002
B5a1a1 | 16117 | 1 | 0.002
B5a1a1 | 16167 | 1 | 0.002
B5a1a1 | 16207 | 6 | 0.013
B5a1a1 | 16220 | 1 | 0.002
B5a1a1 | 16235T | 1 | 0.002
B5a1a1 | 16248 | 2 | 0.004
B5a1a1 | 16258C | 2 | 0.004
B5a1a1 | 16262.1C | 1 | 0.002
B5a1a1 | 16265 | 1 | 0.002
B5a1a1 | 16288 | 1 | 0.002
B5a1a1 | 16289 | 1 | 0.002
B5a1a1 | 16304 | 4 | 0.009
B5a1a1 | 16315 | 5 | 0.011
B5a1a1 | 16497 | 1 | 0.002
B5a1a1 | 183 | 1 | 0.002
B5a1a1 | 23 | 1 | 0.002
B5a1a1 | 235 | 3 | 0.007
B5a1a1 | 26 | 2 | 0.004
B5a1a1 | 279 | 1 | 0.002
B5a1a1 | 291.1A | 1 | 0.002
B5a1a1 | 309d | 3 | 0.007
B5a1a1 | 511 | 2 | 0.004
B5a1a1 | 515 | 1 | 0.002
B5a1a1 | 547 | 2 | 0.004
B5a1a1 | 573.1C | 2 | 0.004
B5a1a1 | 6392 | 1 | 0.002
B5a1a1 | 648 | 1 | 0.002
B5a1a1 | 93 | 2 | 0.004
B5a1b | 15926 | 1 | 0.002
B5a1b | 16032d | 1 | 0.002
B5a1b | 16038d | 2 | 0.004
B5a1b | 16042C | 1 | 0.002
B5a1b | 16042d | 1 | 0.002
B5a1b | 16051 | 1 | 0.002
B5a1b | 16061G | 1 | 0.002
B5a1b | 16076d | 2 | 0.004
B5a1b | 16110d | 1 | 0.002
B5a1b | 16117 | 1 | 0.002
B5a1b | 16167 | 1 | 0.002
B5a1b | 16192 | 1 | 0.002
B5a1b | 16207 | 6 | 0.013
B5a1b | 16209 | 21 | 0.044
B5a1b | 16220 | 1 | 0.002
B5a1b | 16220T | 1 | 0.002
B5a1b | 16230 | 1 | 0.002
B5a1b | 16233 | 1 | 0.002
B5a1b | 16235T | 1 | 0.002
B5a1b | 16248 | 2 | 0.004
B5a1b | 16258C | 2 | 0.004
B5a1b | 16260 | 1 | 0.002
B5a1b | 16262.1C | 1 | 0.002
B5a1b | 16265 | 1 | 0.002
B5a1b | 16288 | 1 | 0.002
B5a1b | 16289 | 1 | 0.002
B5a1b | 16304 | 4 | 0.008
B5a1b | 16309 | 1 | 0.002
B5a1b | 16315 | 5 | 0.011
B5a1b | 16384 | 1 | 0.002
B5a1b | 16390 | 3 | 0.006
B5a1b | 16434 | 1 | 0.002
B5a1b | 183 | 1 | 0.002
B5a1b | 23 | 1 | 0.002
B5a1b | 235 | 3 | 0.006
B5a1b | 26 | 2 | 0.004
B5a1b | 279 | 1 | 0.002
B5a1b | 291.1A | 1 | 0.002
B5a1b | 309d | 3 | 0.006
B5a1b | 3540 | 1 | 0.002

B5a1b | 511 | 2 | 0.004
B5a1b | 533 | 1 | 0.002
B5a1b | 547 | 2 | 0.004
B5a1b | 573.1C | 2 | 0.004
B5a1b | 5773 | 1 | 0.002
B5a1b | 648 | 1 | 0.002
B5a1b | 93 | 2 | 0.004
B5a1b1 | 10310 | 1 | 0.002
B5a1b1 | 10364 | 1 | 0.002
B5a1b1 | 10410 | 2 | 0.004
B5a1b1 | 10634 | 2 | 0.004
B5a1b1 | 10724 | 2 | 0.004
B5a1b1 | 11016 | 1 | 0.002
B5a1b1 | 11686 | 18 | 0.034
B5a1b1 | 12007 | 3 | 0.006
B5a1b1 | 12034 | 1 | 0.002
B5a1b1 | 12358 | 1 | 0.002
B5a1b1 | 13362 | 2 | 0.004
B5a1b1 | 13488 | 7 | 0.013
B5a1b1 | 13602 | 1 | 0.002
B5a1b1 | 13928C | 1 | 0.002
B5a1b1 | 14088 | 1 | 0.002
B5a1b1 | 14182 | 1 | 0.002
B5a1b1 | 14924 | 2 | 0.004
B5a1b1 | 150 | 2 | 0.004
B5a1b1 | 1503 | 1 | 0.002
B5a1b1 | 15172 | 1 | 0.002
B5a1b1 | 152 | 1 | 0.002
B5a1b1 | 15524 | 2 | 0.004
B5a1b1 | 15662 | 1 | 0.002
B5a1b1 | 15978 | 1 | 0.002
B5a1b1 | 16032d | 1 | 0.002
B5a1b1 | 16038d | 2 | 0.004
B5a1b1 | 16042C | 1 | 0.002
B5a1b1 | 16042d | 1 | 0.002
B5a1b1 | 16061G | 1 | 0.002
B5a1b1 | 16086 | 2 | 0.004
B5a1b1 | 16110d | 1 | 0.002
B5a1b1 | 16117 | 1 | 0.002
B5a1b1 | 16120 | 1 | 0.002
B5a1b1 | 16138 | 1 | 0.002
B5a1b1 | 16149C | 1 | 0.002
B5a1b1 | 16160T | 1 | 0.002
B5a1b1 | 16161 | 1 | 0.002
B5a1b1 | 16162 | 1 | 0.002
B5a1b1 | 16165C | 1 | 0.002
B5a1b1 | 16166T | 1 | 0.002
B5a1b1 | 16167 | 1 | 0.002
B5a1b1 | 16172 | 1 | 0.002
B5a1b1 | 16188 | 1 | 0.002
B5a1b1 | 16212 | 1 | 0.002
B5a1b1 | 16213 | 1 | 0.002
B5a1b1 | 16218 | 14 | 0.027
B5a1b1 | 16220 | 1 | 0.002
B5a1b1 | 16222 | 1 | 0.002
B5a1b1 | 16234 | 1 | 0.002
B5a1b1 | 16235T | 1 | 0.002
B5a1b1 | 16243 | 1 | 0.002
B5a1b1 | 16248 | 2 | 0.004
B5a1b1 | 16249 | 1 | 0.002
B5a1b1 | 16258C | 2 | 0.004
B5a1b1 | 16260 | 1 | 0.002
B5a1b1 | 16261 | 1 | 0.002
B5a1b1 | 16262.1C | 1 | 0.002
B5a1b1 | 16265 | 1 | 0.002
B5a1b1 | 16274 | 4 | 0.008
B5a1b1 | 16287 | 2 | 0.004
B5a1b1 | 16288 | 1 | 0.002
B5a1b1 | 16289 | 1 | 0.002
B5a1b1 | 16292 | 1 | 0.002
B5a1b1 | 16311 | 23 | 0.044
B5a1b1 | 16315 | 2 | 0.004
B5a1b1 | 16318 | 1 | 0.002
B5a1b1 | 16319 | 3 | 0.006
B5a1b1 | 16344 | 5 | 0.009
B5a1b1 | 16355 | 1 | 0.002
B5a1b1 | 16390 | 2 | 0.004
B5a1b1 | 16400 | 4 | 0.008
B5a1b1 | 16469G | 1 | 0.002
B5a1b1 | 183 | 1 | 0.002
B5a1b1 | 1888 | 3 | 0.006
B5a1b1 | 23 | 1 | 0.002
B5a1b1 | 2404 | 1 | 0.002
B5a1b1 | 26 | 2 | 0.004
B5a1b1 | 279 | 1 | 0.002
B5a1b1 | 291.1A | 1 | 0.002
B5a1b1 | 294 | 1 | 0.002
B5a1b1 | 310 | 1 | 0.002
B5a1b1 | 338 | 1 | 0.002
B5a1b1 | 3396 | 1 | 0.002
B5a1b1 | 3540 | 10 | 0.019
B5a1b1 | 3691 | 1 | 0.002
B5a1b1 | 3714 | 1 | 0.002
B5a1b1 | 3834 | 1 | 0.002
B5a1b1 | 4104 | 3 | 0.006
B5a1b1 | 4219 | 1 | 0.002
B5a1b1 | 46 | 3 | 0.006
B5a1b1 | 4787 | 1 | 0.002
B5a1b1 | 511 | 2 | 0.004
B5a1b1 | 547 | 2 | 0.004
B5a1b1 | 5601 | 1 | 0.002
B5a1b1 | 573.1C | 2 | 0.004
B5a1b1 | 5773 | 10 | 0.019
B5a1b1 | 5864 | 1 | 0.002
B5a1b1 | 6266 | 1 | 0.002
B5a1b1 | 648 | 1 | 0.002
B5a1b1 | 6485 | 1 | 0.002
B5a1b1 | 7278 | 1 | 0.002
B5a1b1 | 7363 | 1 | 0.002
B5a1b1 | 813 | 1 | 0.002
B5a1b1 | 8531 | 2 | 0.004

B5a1b1 | 8634 | 1 | 0.002
B5a1b1 | 8705 | 1 | 0.002
B5a1b1 | 8965T | 1 | 0.002
B5a1b1 | 9 | 1 | 0.002
B5a1b1 | 9056 | 1 | 0.002
B5a1b1 | 9299 | 1 | 0.002
B5a1b1 | 93 | 2 | 0.004
B5a1b1 | 9494 | 4 | 0.008
B5a1b1 | 9770 | 1 | 0.002
B5a1b1 | 9813 | 1 | 0.002
B5a1c | 11914 | 2 | 0.004
B5a1c | 11959 | 1 | 0.002
B5a1c | 12427G | 5 | 0.01
B5a1c | 13145 | 1 | 0.002
B5a1c | 13521 | 1 | 0.002
B5a1c | 13896 | 1 | 0.002
B5a1c | 14314 | 1 | 0.002
B5a1c | 14857 | 1 | 0.002
B5a1c | 150 | 1 | 0.002
B5a1c | 16032d | 1 | 0.002
B5a1c | 16038d | 2 | 0.004
B5a1c | 16042C | 1 | 0.002
B5a1c | 16042d | 1 | 0.002
B5a1c | 16061G | 1 | 0.002
B5a1c | 16110d | 1 | 0.002
B5a1c | 16117 | 1 | 0.002
B5a1c | 16150 | 1 | 0.002
B5a1c | 16167 | 1 | 0.002
B5a1c | 16188 | 5 | 0.01
B5a1c | 16194C | 2 | 0.004
B5a1c | 16195 | 1 | 0.002
B5a1c | 16197G | 2 | 0.004
B5a1c | 16198 | 1 | 0.002
B5a1c | 16218A | 1 | 0.002
B5a1c | 16220 | 1 | 0.002
B5a1c | 16235 | 1 | 0.002
B5a1c | 16235T | 1 | 0.002
B5a1c | 16248 | 2 | 0.004
B5a1c | 16252C | 1 | 0.002
B5a1c | 16258 | 1 | 0.002
B5a1c | 16258C | 2 | 0.004
B5a1c | 16262.1C | 1 | 0.002
B5a1c | 16265 | 1 | 0.002
B5a1c | 16267 | 1 | 0.002
B5a1c | 16269C | 1 | 0.002
B5a1c | 16287 | 1 | 0.002
B5a1c | 16288 | 1 | 0.002
B5a1c | 16289 | 1 | 0.002
B5a1c | 16298 | 1 | 0.002
B5a1c | 16315 | 3 | 0.006
B5a1c | 16318 | 1 | 0.002
B5a1c | 16327 | 1 | 0.002
B5a1c | 16362 | 1 | 0.002
B5a1c | 183 | 1 | 0.002
B5a1c | 189 | 1 | 0.002
B5a1c | 191.1A | 1 | 0.002
B5a1c | 194 | 1 | 0.002
B5a1c | 200 | 1 | 0.002
B5a1c | 204 | 2 | 0.004
B5a1c | 228 | 1 | 0.002
B5a1c | 23 | 1 | 0.002
B5a1c | 235 | 3 | 0.006
B5a1c | 26 | 2 | 0.004
B5a1c | 279 | 1 | 0.002
B5a1c | 291.1A | 1 | 0.002
B5a1c | 309.3C | 15 | 0.031
B5a1c | 309d | 3 | 0.006
B5a1c | 455.1T | 1 | 0.002
B5a1c | 511 | 2 | 0.004
B5a1c | 547 | 2 | 0.004
B5a1c | 573.1C | 2 | 0.004
B5a1c | 6216 | 1 | 0.002
B5a1c | 648 | 1 | 0.002
B5a1c | 81 | 1 | 0.002
B5a1c | 8711 | 1 | 0.002
B5a1c | 8881 | 1 | 0.002
B5a1c | 9021 | 1 | 0.002
B5a1c | 93 | 2 | 0.004
B5a1c1 | 12191 | 1 | 0.002
B5a1c1 | 14470 | 1 | 0.002
B5a1c1 | 16032d | 1 | 0.002
B5a1c1 | 16038d | 2 | 0.004
B5a1c1 | 16042C | 1 | 0.002
B5a1c1 | 16042d | 1 | 0.002
B5a1c1 | 16061G | 1 | 0.002
B5a1c1 | 16110d | 1 | 0.002
B5a1c1 | 16117 | 1 | 0.002
B5a1c1 | 16167 | 1 | 0.002
B5a1c1 | 16207 | 6 | 0.013
B5a1c1 | 16218 | 1 | 0.002
B5a1c1 | 16220 | 1 | 0.002
B5a1c1 | 16234 | 1 | 0.002
B5a1c1 | 16235T | 1 | 0.002
B5a1c1 | 16248 | 2 | 0.004
B5a1c1 | 16258C | 2 | 0.004
B5a1c1 | 16262.1C | 1 | 0.002
B5a1c1 | 16265 | 1 | 0.002
B5a1c1 | 16288 | 1 | 0.002
B5a1c1 | 16289 | 1 | 0.002
B5a1c1 | 16304 | 4 | 0.009
B5a1c1 | 16315 | 5 | 0.011
B5a1c1 | 183 | 1 | 0.002
B5a1c1 | 23 | 1 | 0.002
B5a1c1 | 235 | 3 | 0.007
B5a1c1 | 26 | 2 | 0.004
B5a1c1 | 279 | 1 | 0.002
B5a1c1 | 291.1A | 1 | 0.002
B5a1c1 | 309d | 3 | 0.007
B5a1c1 | 3666 | 1 | 0.002
B5a1c1 | 511 | 2 | 0.004
B5a1c1 | 5177 | 1 | 0.002

B5a1c1 | 547 | 2 | 0.004
B5a1c1 | 5567 | 1 | 0.002
B5a1c1 | 573.1C | 2 | 0.004
B5a1c1 | 648 | 1 | 0.002
B5a1c1 | 6524 | 1 | 0.002
B5a1c1 | 9290 | 1 | 0.002
B5a1c1 | 93 | 2 | 0.004
B5a1c1a | 103 | 1 | 0.002
B5a1c1a | 11194 | 1 | 0.002
B5a1c1a | 114 | 2 | 0.005
B5a1c1a | 12880 | 1 | 0.002
B5a1c1a | 14598 | 1 | 0.002
B5a1c1a | 14745 | 1 | 0.002
B5a1c1a | 150 | 1 | 0.002
B5a1c1a | 152 | 1 | 0.002
B5a1c1a | 15924 | 1 | 0.002
B5a1c1a | 16032d | 1 | 0.002
B5a1c1a | 16038d | 2 | 0.005
B5a1c1a | 16042d | 1 | 0.002
B5a1c1a | 16059 | 1 | 0.002
B5a1c1a | 16061G | 1 | 0.002
B5a1c1a | 16110d | 1 | 0.002
B5a1c1a | 16117 | 1 | 0.002
B5a1c1a | 16129 | 1 | 0.002
B5a1c1a | 16145 | 2 | 0.005
B5a1c1a | 16153 | 1 | 0.002
B5a1c1a | 16154 | 4 | 0.009
B5a1c1a | 16167 | 1 | 0.002
B5a1c1a | 16178 | 1 | 0.002
B5a1c1a | 16179 | 1 | 0.002
B5a1c1a | 16194C | 3 | 0.007
B5a1c1a | 16195 | 1 | 0.002
B5a1c1a | 16207 | 1 | 0.002
B5a1c1a | 16217 | 1 | 0.002
B5a1c1a | 16235T | 1 | 0.002
B5a1c1a | 16240 | 1 | 0.002
B5a1c1a | 16242A | 1 | 0.002
B5a1c1a | 16245 | 1 | 0.002
B5a1c1a | 16248 | 2 | 0.005
B5a1c1a | 16249 | 3 | 0.007
B5a1c1a | 16265 | 1 | 0.002
B5a1c1a | 16270 | 2 | 0.005
B5a1c1a | 16294 | 3 | 0.007
B5a1c1a | 16295 | 1 | 0.002
B5a1c1a | 16297 | 16 | 0.036
B5a1c1a | 16298 | 4 | 0.009
B5a1c1a | 16311 | 1 | 0.002
B5a1c1a | 16315 | 2 | 0.005
B5a1c1a | 16318C | 6 | 0.014
B5a1c1a | 16355 | 1 | 0.002
B5a1c1a | 183 | 1 | 0.002
B5a1c1a | 198 | 2 | 0.005
B5a1c1a | 252 | 3 | 0.007
B5a1c1a | 316C | 3 | 0.007
B5a1c1a | 391 | 2 | 0.005
B5a1c1a | 42.1G | 1 | 0.002
B5a1c1a | 471 | 1 | 0.002
B5a1c1a | 501 | 2 | 0.005
B5a1c1a | 5087 | 1 | 0.002
B5a1c1a | 5453T | 1 | 0.002
B5a1c1a | 650 | 1 | 0.002
B5a1c1a | 8269 | 1 | 0.002
B5a1c1a | 827 | 1 | 0.002
B5a1c1a | 9758 | 2 | 0.005
B5a1c1a1 | 11778 | 2 | 0.039
B5a1c1a1 | 11908 | 2 | 0.039
B5a1c1a1 | 146 | 1 | 0.02
B5a1c1a1 | 14797 | 5 | 0.098
B5a1c1a1 | 14894 | 2 | 0.039
B5a1c1a1 | 16093 | 1 | 0.02
B5a1c1a1 | 16256 | 1 | 0.02
B5a1c1a1 | 16274 | 1 | 0.02
B5a1c1a1 | 16278 | 1 | 0.02
B5a1c1a1 | 16297 | 1 | 0.02
B5a1c1a1 | 16311 | 2 | 0.039
B5a1c1a1 | 16408 | 1 | 0.02
B5a1c1a1 | 46 | 2 | 0.039
B5a1c1a1 | 5471 | 1 | 0.02
B5a1c1a1 | 5964 | 1 | 0.02
B5a1c1a1 | 6167 | 1 | 0.02
B5a1c1a1 | 64 | 31 | 0.608
B5a1c1a1 | 81 | 1 | 0.02
B5a1c1a1 | 9288 | 1 | 0.02
B5a1c1a1 | 9371 | 2 | 0.039
B5a1c2 | 12612 | 1 | 0.002
B5a1c2 | 146 | 1 | 0.002
B5a1c2 | 16032d | 1 | 0.002
B5a1c2 | 16038d | 2 | 0.004
B5a1c2 | 16042C | 1 | 0.002
B5a1c2 | 16042d | 1 | 0.002
B5a1c2 | 16061G | 1 | 0.002
B5a1c2 | 16066 | 4 | 0.009
B5a1c2 | 16110d | 1 | 0.002
B5a1c2 | 16111 | 1 | 0.002
B5a1c2 | 16117 | 1 | 0.002
B5a1c2 | 16167 | 1 | 0.002
B5a1c2 | 16207 | 6 | 0.013
B5a1c2 | 16220 | 1 | 0.002
B5a1c2 | 16235T | 1 | 0.002
B5a1c2 | 16248 | 2 | 0.004
B5a1c2 | 16258C | 2 | 0.004
B5a1c2 | 16262.1C | 1 | 0.002
B5a1c2 | 16265 | 1 | 0.002
B5a1c2 | 16288 | 1 | 0.002
B5a1c2 | 16289 | 1 | 0.002
B5a1c2 | 16304 | 4 | 0.009
B5a1c2 | 16315 | 5 | 0.011
B5a1c2 | 16465 | 1 | 0.002
B5a1c2 | 183 | 1 | 0.002
B5a1c2 | 2219.1C | 1 | 0.002
B5a1c2 | 23 | 1 | 0.002

B5a1c2 | 235 | 3 | 0.007
B5a1c2 | 26 | 2 | 0.004
B5a1c2 | 279 | 1 | 0.002
B5a1c2 | 291.1A | 1 | 0.002
B5a1c2 | 309d | 3 | 0.007
B5a1c2 | 3866 | 1 | 0.002
B5a1c2 | 4092 | 1 | 0.002
B5a1c2 | 511 | 2 | 0.004
B5a1c2 | 5418 | 1 | 0.002
B5a1c2 | 547 | 2 | 0.004
B5a1c2 | 573.1C | 2 | 0.004
B5a1c2 | 5964 | 1 | 0.002
B5a1c2 | 648 | 1 | 0.002
B5a1c2 | 8701 | 1 | 0.002
B5a1c2 | 93 | 2 | 0.004
B5a1d | 10550 | 1 | 0.003
B5a1d | 10589 | 9 | 0.031
B5a1d | 12127 | 2 | 0.007
B5a1d | 12172 | 1 | 0.003
B5a1d | 12696 | 1 | 0.003
B5a1d | 12756C | 1 | 0.003
B5a1d | 13005 | 1 | 0.003
B5a1d | 13395 | 1 | 0.003
B5a1d | 13928C | 4 | 0.014
B5a1d | 14198 | 1 | 0.003
B5a1d | 14209 | 1 | 0.003
B5a1d | 14221 | 1 | 0.003
B5a1d | 14530 | 1 | 0.003
B5a1d | 146 | 2 | 0.007
B5a1d | 14696 | 8 | 0.028
B5a1d | 15460 | 1 | 0.003
B5a1d | 15465 | 1 | 0.003
B5a1d | 15769 | 1 | 0.003
B5a1d | 15784 | 1 | 0.003
B5a1d | 15828 | 15 | 0.052
B5a1d | 15857 | 3 | 0.01
B5a1d | 15911 | 1 | 0.003
B5a1d | 16014 | 1 | 0.003
B5a1d | 16016T | 1 | 0.003
B5a1d | 16019 | 1 | 0.003
B5a1d | 16021 | 1 | 0.003
B5a1d | 16023T | 1 | 0.003
B5a1d | 16026 | 1 | 0.003
B5a1d | 16032G | 1 | 0.003
B5a1d | 16092 | 3 | 0.01
B5a1d | 16093 | 8 | 0.028
B5a1d | 16126 | 1 | 0.003
B5a1d | 16129 | 116 | 0.4
B5a1d | 16150 | 1 | 0.003
B5a1d | 16162T | 3 | 0.01
B5a1d | 16173 | 4 | 0.014
B5a1d | 16174 | 1 | 0.003
B5a1d | 16178 | 3 | 0.01
B5a1d | 16188 | 1 | 0.003
B5a1d | 16192-16193d | 1 | 0.003
B5a1d | 16193d | 1 | 0.003
B5a1d | 16194C | 1 | 0.003
B5a1d | 16197G | 1 | 0.003
B5a1d | 16212 | 1 | 0.003
B5a1d | 16223 | 3 | 0.01
B5a1d | 16224 | 3 | 0.01
B5a1d | 16234 | 1 | 0.003
B5a1d | 16240 | 22 | 0.076
B5a1d | 16242A | 3 | 0.01
B5a1d | 16250 | 3 | 0.01
B5a1d | 16259 | 1 | 0.003
B5a1d | 16260 | 1 | 0.003
B5a1d | 16262 | 1 | 0.003
B5a1d | 16272T | 3 | 0.01
B5a1d | 16274 | 2 | 0.007
B5a1d | 16284 | 1 | 0.003
B5a1d | 16293 | 3 | 0.01
B5a1d | 16293C | 1 | 0.003
B5a1d | 16304 | 6 | 0.021
B5a1d | 16305C | 1 | 0.003
B5a1d | 16306 | 1 | 0.003
B5a1d | 16309 | 3 | 0.01
B5a1d | 16311 | 7 | 0.024
B5a1d | 16325 | 1 | 0.003
B5a1d | 16344 | 1 | 0.003
B5a1d | 16362 | 2 | 0.007
B5a1d | 16527 | 1 | 0.003
B5a1d | 16555A | 1 | 0.003
B5a1d | 189 | 2 | 0.007
B5a1d | 195 | 15 | 0.052
B5a1d | 1978T | 2 | 0.007
B5a1d | 2069 | 1 | 0.003
B5a1d | 228 | 3 | 0.01
B5a1d | 257 | 8 | 0.028
B5a1d | 2756 | 1 | 0.003
B5a1d | 2833 | 2 | 0.007
B5a1d | 308-309d | 1 | 0.003
B5a1d | 309.3C | 6 | 0.021
B5a1d | 309d | 7 | 0.024
B5a1d | 310 | 2 | 0.007
B5a1d | 312 | 1 | 0.003
B5a1d | 313 | 1 | 0.003
B5a1d | 315.2C | 1 | 0.003
B5a1d | 316C | 1 | 0.003
B5a1d | 3197 | 1 | 0.003
B5a1d | 3316 | 3 | 0.01
B5a1d | 3392C | 1 | 0.003
B5a1d | 345 | 1 | 0.003
B5a1d | 3591 | 1 | 0.003
B5a1d | 383 | 1 | 0.003
B5a1d | 4032 | 3 | 0.01
B5a1d | 4092 | 1 | 0.003
B5a1d | 456 | 1 | 0.003
B5a1d | 498 | 1 | 0.003
B5a1d | 5054 | 1 | 0.003
B5a1d | 5366A | 1 | 0.003

B5a1d | 5417 | 1 | 0.003
B5a1d | 5460 | 15 | 0.052
B5a1d | 57 | 2 | 0.007
B5a1d | 5894 | 1 | 0.003
B5a1d | 61 | 2 | 0.007
B5a1d | 6227 | 1 | 0.003
B5a1d | 6249 | 1 | 0.003
B5a1d | 6392 | 2 | 0.007
B5a1d | 71 | 2 | 0.007
B5a1d | 7304 | 1 | 0.003
B5a1d | 7424 | 1 | 0.003
B5a1d | 7762 | 1 | 0.003
B5a1d | 7861 | 2 | 0.007
B5a1d | 7934 | 2 | 0.007
B5a1d | 8222 | 1 | 0.003
B5a1d | 870 | 1 | 0.003
B5a1d | 8897 | 15 | 0.052
B5a1d | 9053 | 1 | 0.003
B5a1d | 909T | 1 | 0.003
B5a1d | 930 | 1 | 0.003
B5a1d | 9305 | 1 | 0.003
B5a1d | 9425 | 1 | 0.003
B5a1d | 9591 | 1 | 0.003
B5a2 | 10388 | 1 | 0.016
B5a2 | 11087 | 2 | 0.032
B5a2 | 12438 | 1 | 0.016
B5a2 | 12501 | 2 | 0.032
B5a2 | 13281 | 2 | 0.032
B5a2 | 1393 | 6 | 0.095
B5a2 | 14178 | 2 | 0.032
B5a2 | 146 | 1 | 0.016
B5a2 | 15110 | 1 | 0.016
B5a2 | 152 | 2 | 0.032
B5a2 | 15229 | 6 | 0.095
B5a2 | 15236 | 6 | 0.095
B5a2 | 15263 | 2 | 0.032
B5a2 | 15671 | 2 | 0.032
B5a2 | 15777 | 2 | 0.032
B5a2 | 16066 | 3 | 0.048
B5a2 | 16093 | 21 | 0.333
B5a2 | 16129 | 3 | 0.048
B5a2 | 16178 | 1 | 0.016
B5a2 | 16188 | 2 | 0.032
B5a2 | 16193 | 1 | 0.016
B5a2 | 16223 | 1 | 0.016
B5a2 | 16224 | 1 | 0.016
B5a2 | 16234 | 1 | 0.016
B5a2 | 16241 | 1 | 0.016
B5a2 | 16260 | 32 | 0.508
B5a2 | 16266A | 1 | 0.016
B5a2 | 16274 | 4 | 0.063
B5a2 | 16278 | 1 | 0.016
B5a2 | 16287 | 1 | 0.016
B5a2 | 16291 | 15 | 0.238
B5a2 | 16311 | 3 | 0.048
B5a2 | 16325 | 1 | 0.016
B5a2 | 16520.1C | 1 | 0.016
B5a2 | 185 | 1 | 0.016
B5a2 | 189 | 1 | 0.016
B5a2 | 200 | 2 | 0.032
B5a2 | 249d | 2 | 0.032
B5a2 | 262 | 1 | 0.016
B5a2 | 294 | 29 | 0.46
B5a2 | 3565 | 1 | 0.016
B5a2 | 4435 | 1 | 0.016
B5a2 | 564T | 1 | 0.016
B5a2 | 6663 | 2 | 0.032
B5a2 | 7001 | 6 | 0.095
B5a2 | 9100 | 2 | 0.032
B5a2 | 9686 | 1 | 0.016
B5a2a | 16082 | 1 | 0.067
B5a2a | 16158 | 1 | 0.067
B5a2a | 16287 | 1 | 0.067
B5a2a | 16527 | 1 | 0.067
B5a2a1 | 375 | 1 | 1
B5a2a1+16129 | 14581 | 1 | 0.091
B5a2a1+16129 | 15058 | 1 | 0.091
B5a2a1+16129 | 151 | 1 | 0.091
B5a2a1+16129 | 15172 | 1 | 0.091
B5a2a1+16129 | 15758 | 1 | 0.091
B5a2a1+16129 | 16093 | 2 | 0.182
B5a2a1+16129 | 16180 | 1 | 0.091
B5a2a1+16129 | 16261 | 1 | 0.091
B5a2a1+16129 | 16278 | 1 | 0.091
B5a2a1+16129 | 16311 | 1 | 0.091
B5a2a1+16129 | 16319 | 2 | 0.182
B5a2a1+16129 | 228 | 1 | 0.091
B5a2a1+16129 | 508 | 2 | 0.182
B5a2a1+16129 | 9293 | 1 | 0.091
B5a2a1+16129 | 9531 | 1 | 0.091
B5a2a1+16129 | 95C | 2 | 0.182
B5a2a1a | 11890 | 1 | 0.026
B5a2a1a | 13857 | 1 | 0.026
B5a2a1a | 13928C | 1 | 0.026
B5a2a1a | 150 | 1 | 0.026
B5a2a1a | 16111A | 5 | 0.132
B5a2a1a | 16184 | 2 | 0.053
B5a2a1a | 16189A | 1 | 0.026
B5a2a1a | 16207 | 1 | 0.026
B5a2a1a | 16231 | 1 | 0.026
B5a2a1a | 16240 | 1 | 0.026
B5a2a1a | 16246T | 1 | 0.026
B5a2a1a | 16266A | 1 | 0.026
B5a2a1a | 16267 | 2 | 0.053
B5a2a1a | 16325 | 1 | 0.026
B5a2a1a | 16327 | 1 | 0.026
B5a2a1a | 16355 | 1 | 0.026
B5a2a1a | 16362 | 1 | 0.026
B5a2a1a | 16391 | 1 | 0.026
B5a2a1a | 16441C | 1 | 0.026
B5a2a1a | 2275 | 1 | 0.026

B5a2a1a | 257 | 1 | 0.026
B5a2a1a | 4917 | 1 | 0.026
B5a2a1a | 523d | 1 | 0.026
B5a2a1a | 6029 | 1 | 0.026
B5a2a1a | 6366 | 1 | 0.026
B5a2a1a | 784d | 1 | 0.026
B5a2a1b | 127 | 1 | 0.071
B5a2a1b | 146 | 1 | 0.071
B5a2a1b | 152 | 1 | 0.071
B5a2a1b | 16111G | 1 | 0.071
B5a2a1b | 16223 | 1 | 0.071
B5a2a1b | 16266A | 10 | 0.714
B5a2a1b | 16304 | 1 | 0.071
B5a2a1b | 2550T | 1 | 0.071
B5a2a1b | 5021 | 1 | 0.071
B5a2a1b | 89 | 1 | 0.071
B5a2a1b | 95C | 1 | 0.071
B5a2a2 | 127 | 1 | 0.056
B5a2a2 | 152 | 1 | 0.056
B5a2a2 | 16037 | 1 | 0.056
B5a2a2 | 16111G | 1 | 0.056
B5a2a2 | 16129 | 2 | 0.111
B5a2a2 | 16234 | 1 | 0.056
B5a2a2 | 16245 | 1 | 0.056
B5a2a2 | 16355 | 1 | 0.056
B5a2a2 | 16357 | 1 | 0.056
B5a2a2 | 849 | 1 | 0.056
B5a2a2 | 8854 | 1 | 0.056
B5a2a2 | 89 | 2 | 0.111
B5a2a2 | 95C | 1 | 0.056
B5a2a2a | 16037 | 1 | 0.2
B5a2a2a | 16287 | 1 | 0.2
B5a2a2a1 | 14226 | 1 | 0.056
B5a2a2a1 | 15948d | 1 | 0.056
B5a2a2a1 | 16158 | 1 | 0.056
B5a2a2a1 | 16266A | 2 | 0.111
B5a2a2a1 | 16287 | 2 | 0.111
B5a2a2a1 | 16391 | 1 | 0.056
B5a2a2a1 | 3948 | 2 | 0.111
B5a2a2a1 | 4047 | 1 | 0.056
B5a2a2a1 | 723 | 2 | 0.111
B5a2a2a1 | 7594 | 1 | 0.056
B5a2a2a2 | 11167 | 1 | 0.083
B5a2a2a2 | 16266A | 3 | 0.25
B5a2a2a2 | 438 | 1 | 0.083
B5a2a2a2 | 9296 | 1 | 0.083
B5a2a2a2 | 942 | 1 | 0.083
B5a2a2b | 16082 | 1 | 0.067
B5a2a2b | 16158 | 1 | 0.067
B5a2a2b | 16287 | 1 | 0.067
B5a2a2b | 16527 | 1 | 0.067
B5a2a2b1 | 12398 | 1 | 0.059
B5a2a2b1 | 152 | 3 | 0.176
B5a2a2b1 | 16193d | 1 | 0.059
B5a2a2b1 | 310 | 1 | 0.059
B5a2a2b1 | 315.3C | 1 | 0.059
B5a2a2b1 | 7142 | 1 | 0.059
B5a2a2b1 | 9368 | 1 | 0.059
B5a2a2b1a | 16129 | 1 | 0.053
B5a2a2b1a | 7805 | 5 | 0.263
B5a2a2b2 | 8706 | 1 | 0.333
B5b | 131 | 1 | 0.006
B5b | 143 | 1 | 0.006
B5b | 151 | 1 | 0.006
B5b | 152 | 1 | 0.006
B5b | 16017 | 2 | 0.012
B5b | 16154 | 1 | 0.006
B5b | 16209 | 3 | 0.018
B5b | 16216 | 2 | 0.012
B5b | 16227 | 1 | 0.006
B5b | 16234 | 2 | 0.012
B5b | 16257 | 1 | 0.006
B5b | 16261 | 1 | 0.006
B5b | 16263 | 1 | 0.006
B5b | 16269 | 1 | 0.006
B5b | 16291 | 1 | 0.006
B5b | 16293C | 2 | 0.012
B5b | 16309 | 2 | 0.012
B5b | 16318T | 1 | 0.006
B5b | 16327 | 1 | 0.006
B5b | 16362 | 2 | 0.012
B5b | 16439A | 1 | 0.006
B5b | 16465 | 1 | 0.006
B5b | 189 | 1 | 0.006
B5b | 203 | 2 | 0.012
B5b | 222 | 1 | 0.006
B5b | 2483 | 1 | 0.006
B5b | 357 | 2 | 0.012
B5b | 3635 | 1 | 0.006
B5b | 3846 | 1 | 0.006
B5b | 408A | 1 | 0.006
B5b | 537 | 1 | 0.006
B5b | 5773 | 1 | 0.006
B5b | 6704 | 1 | 0.006
B5b | 7256 | 2 | 0.012
B5b | 8790 | 2 | 0.012
B5b | 9275 | 1 | 0.006
B5b | 93 | 1 | 0.006
B5b | 9536 | 1 | 0.006
B5b1 | 10101 | 1 | 0.004
B5b1 | 10274 | 1 | 0.004
B5b1 | 10400 | 1 | 0.004
B5b1 | 10746 | 2 | 0.007
B5b1 | 114 | 1 | 0.004
B5b1 | 11518 | 1 | 0.004
B5b1 | 11854 | 2 | 0.007
B5b1 | 11928 | 1 | 0.004
B5b1 | 12331 | 2 | 0.007
B5b1 | 12338 | 1 | 0.004
B5b1 | 12354 | 3 | 0.011
B5b1 | 124 | 1 | 0.004

B5b1 | 12585 | 1 | 0.004
B5b1 | 128 | 1 | 0.004
B5b1 | 131 | 1 | 0.004
B5b1 | 13920 | 1 | 0.004
B5b1 | 13933 | 1 | 0.004
B5b1 | 139d | 1 | 0.004
B5b1 | 14148 | 5 | 0.018
B5b1 | 14302 | 4 | 0.015
B5b1 | 14384 | 6 | 0.022
B5b1 | 14527 | 1 | 0.004
B5b1 | 14544 | 1 | 0.004
B5b1 | 14560 | 4 | 0.015
B5b1 | 14566 | 1 | 0.004
B5b1 | 14632 | 1 | 0.004
B5b1 | 14693 | 1 | 0.004
B5b1 | 150 | 13 | 0.047
B5b1 | 15077 | 2 | 0.007
B5b1 | 15172 | 1 | 0.004
B5b1 | 152 | 1 | 0.004
B5b1 | 153T | 1 | 0.004
B5b1 | 15470 | 1 | 0.004
B5b1 | 15603 | 1 | 0.004
B5b1 | 15734 | 4 | 0.015
B5b1 | 15758 | 1 | 0.004
B5b1 | 15973 | 1 | 0.004
B5b1 | 16030 | 1 | 0.004
B5b1 | 16044 | 1 | 0.004
B5b1 | 16053d | 1 | 0.004
B5b1 | 16067 | 1 | 0.004
B5b1 | 16092 | 1 | 0.004
B5b1 | 16093 | 2 | 0.007
B5b1 | 16105 | 1 | 0.004
B5b1 | 16111 | 1 | 0.004
B5b1 | 16136 | 1 | 0.004
B5b1 | 16142 | 1 | 0.004
B5b1 | 16158 | 2 | 0.007
B5b1 | 16166 | 1 | 0.004
B5b1 | 16172 | 1 | 0.004
B5b1 | 16192-16193d | 1 | 0.004
B5b1 | 16193.3C | 1 | 0.004
B5b1 | 16193d | 3 | 0.011
B5b1 | 16194C | 1 | 0.004
B5b1 | 16209 | 1 | 0.004
B5b1 | 16213 | 1 | 0.004
B5b1 | 16224 | 4 | 0.015
B5b1 | 16244 | 1 | 0.004
B5b1 | 16245 | 1 | 0.004
B5b1 | 16248 | 2 | 0.007
B5b1 | 16249 | 1 | 0.004
B5b1 | 16261 | 2 | 0.007
B5b1 | 16266 | 1 | 0.004
B5b1 | 16269 | 1 | 0.004
B5b1 | 16278 | 8 | 0.029
B5b1 | 16288 | 2 | 0.007
B5b1 | 16292 | 1 | 0.004
B5b1 | 16300 | 1 | 0.004
B5b1 | 16311 | 48 | 0.175
B5b1 | 16316 | 1 | 0.004
B5b1 | 16318T | 2 | 0.007
B5b1 | 16319 | 7 | 0.025
B5b1 | 16342 | 3 | 0.011
B5b1 | 16353 | 1 | 0.004
B5b1 | 16354 | 1 | 0.004
B5b1 | 16355 | 30 | 0.109
B5b1 | 16356 | 1 | 0.004
B5b1 | 16360 | 1 | 0.004
B5b1 | 16390 | 1 | 0.004
B5b1 | 16394 | 1 | 0.004
B5b1 | 16413 | 1 | 0.004
B5b1 | 16441 | 1 | 0.004
B5b1 | 16461 | 1 | 0.004
B5b1 | 16462 | 1 | 0.004
B5b1 | 16476 | 1 | 0.004
B5b1 | 16500 | 1 | 0.004
B5b1 | 16568 | 1 | 0.004
B5b1 | 172 | 1 | 0.004
B5b1 | 175 | 1 | 0.004
B5b1 | 176 | 1 | 0.004
B5b1 | 1832 | 2 | 0.007
B5b1 | 185 | 2 | 0.007
B5b1 | 189 | 4 | 0.015
B5b1 | 195 | 8 | 0.029
B5b1 | 198 | 1 | 0.004
B5b1 | 199 | 1 | 0.004
B5b1 | 200 | 3 | 0.011
B5b1 | 207 | 12 | 0.044
B5b1 | 235 | 1 | 0.004
B5b1 | 2833 | 2 | 0.007
B5b1 | 3 | 1 | 0.004
B5b1 | 3083 | 1 | 0.004
B5b1 | 309.3C | 4 | 0.015
B5b1 | 316C | 4 | 0.015
B5b1 | 3744 | 1 | 0.004
B5b1 | 376C | 1 | 0.004
B5b1 | 393d | 1 | 0.004
B5b1 | 3987 | 1 | 0.004
B5b1 | 4086 | 2 | 0.007
B5b1 | 4216 | 1 | 0.004
B5b1 | 43 | 1 | 0.004
B5b1 | 4418 | 6 | 0.022
B5b1 | 446C | 1 | 0.004
B5b1 | 4673 | 1 | 0.004
B5b1 | 4973 | 1 | 0.004
B5b1 | 5063 | 1 | 0.004
B5b1 | 559A | 1 | 0.004
B5b1 | 570 | 1 | 0.004
B5b1 | 573.2C | 1 | 0.004
B5b1 | 5979 | 1 | 0.004
B5b1 | 6101 | 2 | 0.007
B5b1 | 6216 | 1 | 0.004
B5b1 | 6386 | 2 | 0.007

B5b1 | 6446 | 5 | 0.018
B5b1 | 6480 | 1 | 0.004
B5b1 | 6530 | 2 | 0.007
B5b1 | 6719 | 1 | 0.004
B5b1 | 6843 | 1 | 0.004
B5b1 | 7181 | 2 | 0.007
B5b1 | 7286 | 1 | 0.004
B5b1 | 7775 | 1 | 0.004
B5b1 | 7822 | 1 | 0.004
B5b1 | 8467 | 1 | 0.004
B5b1 | 8557 | 1 | 0.004
B5b1 | 8943 | 1 | 0.004
B5b1 | 8966 | 2 | 0.007
B5b1 | 9053 | 1 | 0.004
B5b1 | 9299 | 1 | 0.004
B5b1 | 93 | 1 | 0.004
B5b1 | 960.0C | 1 | 0.004
B5b1 | 960.2C | 3 | 0.011
B5b1 | 960.3C | 1 | 0.004
B5b1 | 961 | 1 | 0.004
B5b1 | 9785 | 1 | 0.004
B5b1 | 9804 | 3 | 0.011
B5b1 | 9932 | 1 | 0.004
B5b1a | 12840 | 1 | 0.006
B5b1a | 143 | 1 | 0.006
B5b1a | 16209 | 3 | 0.018
B5b1a | 16216 | 2 | 0.012
B5b1a | 16257 | 1 | 0.006
B5b1a | 16261 | 1 | 0.006
B5b1a | 16263 | 1 | 0.006
B5b1a | 16269 | 1 | 0.006
B5b1a | 16291 | 1 | 0.006
B5b1a | 16293C | 2 | 0.012
B5b1a | 16309 | 2 | 0.012
B5b1a | 16318T | 1 | 0.006
B5b1a | 16327 | 1 | 0.006
B5b1a | 16362 | 2 | 0.012
B5b1a | 16439A | 1 | 0.006
B5b1a | 217 | 2 | 0.012
B5b1a | 357 | 2 | 0.012
B5b1a | 369 | 1 | 0.006
B5b1a | 537 | 1 | 0.006
B5b1a | 5581 | 2 | 0.012
B5b1a | 8108 | 1 | 0.006
B5b1a | 9128 | 2 | 0.012
B5b1a1 | 14693 | 1 | 0.143
B5b1a1 | 14696 | 1 | 0.143
B5b1a1 | 15758 | 1 | 0.143
B5b1a1 | 16294 | 1 | 0.143
B5b1a1 | 1766 | 1 | 0.143
B5b1a1 | 545 | 1 | 0.143
B5b1a1 | 6398 | 1 | 0.143
B5b1a1 | 7298 | 1 | 0.143
B5b1a2 | 10308 | 1 | 0.056
B5b1a2 | 10463 | 1 | 0.056
B5b1a2 | 11158 | 1 | 0.056
B5b1a2 | 12408 | 1 | 0.056
B5b1a2 | 14180 | 1 | 0.056
B5b1a2 | 14326 | 1 | 0.056
B5b1a2 | 146 | 1 | 0.056
B5b1a2 | 1547.1T | 1 | 0.056
B5b1a2 | 15650 | 1 | 0.056
B5b1a2 | 16162 | 1 | 0.056
B5b1a2 | 16265 | 2 | 0.111
B5b1a2 | 16294 | 1 | 0.056
B5b1a2 | 16362 | 1 | 0.056
B5b1a2 | 189 | 2 | 0.111
B5b1a2 | 2248 | 1 | 0.056
B5b1a2 | 315d | 1 | 0.056
B5b1a2 | 4500 | 1 | 0.056
B5b1a2 | 4767 | 1 | 0.056
B5b1a2 | 6216 | 1 | 0.056
B5b1a2 | 6577d | 1 | 0.056
B5b1a2 | 6827 | 1 | 0.056
B5b1a2 | 8074 | 1 | 0.056
B5b1a2 | 9540 | 1 | 0.056
B5b1a2 | 955d | 1 | 0.056
B5b1a2 | 9968A | 1 | 0.056
B5b1a2a | 152 | 2 | 0.167
B5b1a2a | 16193.3C | 1 | 0.083
B5b1a2a | 16311 | 2 | 0.167
B5b1a2a | 309.3C | 1 | 0.083
B5b1a2a | 6929 | 2 | 0.167
B5b1c | 10 | 2 | 0.011
B5b1c | 11506 | 1 | 0.005
B5b1c | 11930 | 1 | 0.005
B5b1c | 1275 | 2 | 0.011
B5b1c | 12858 | 1 | 0.005
B5b1c | 15172 | 1 | 0.005
B5b1c | 15211 | 1 | 0.005
B5b1c | 15514 | 1 | 0.005
B5b1c | 1555 | 1 | 0.005
B5b1c | 15712 | 1 | 0.005
B5b1c | 15966d | 1 | 0.005
B5b1c | 16037 | 1 | 0.005
B5b1c | 16067 | 2 | 0.011
B5b1c | 16113C | 1 | 0.005
B5b1c | 16147 | 1 | 0.005
B5b1c | 16149C | 1 | 0.005
B5b1c | 16213 | 1 | 0.005
B5b1c | 16220C | 1 | 0.005
B5b1c | 16249 | 4 | 0.022
B5b1c | 16254 | 1 | 0.005
B5b1c | 16262 | 1 | 0.005
B5b1c | 16263 | 1 | 0.005
B5b1c | 16294 | 1 | 0.005
B5b1c | 16304 | 2 | 0.011
B5b1c | 16354 | 1 | 0.005
B5b1c | 16362 | 1 | 0.005
B5b1c | 189 | 1 | 0.005
B5b1c | 2056 | 1 | 0.005

B5b1c | 234 | 2 | 0.011
B5b1c | 310 | 1 | 0.005
B5b1c | 315d | 1 | 0.005
B5b1c | 3335 | 2 | 0.011
B5b1c | 3395 | 1 | 0.005
B5b1c | 3396 | 1 | 0.005
B5b1c | 3565 | 1 | 0.005
B5b1c | 4204 | 1 | 0.005
B5b1c | 5822 | 2 | 0.011
B5b1c | 8943 | 1 | 0.005
B5b1c | 960.3C | 3 | 0.016
B5b1c1 | 13708 | 2 | 0.012
B5b1c1 | 146 | 1 | 0.006
B5b1c1 | 15502 | 2 | 0.012
B5b1c1 | 16147 | 1 | 0.006
B5b1c1 | 16216 | 2 | 0.012
B5b1c1 | 16262 | 1 | 0.006
B5b1c1 | 16263 | 1 | 0.006
B5b1c1 | 16439A | 1 | 0.006
B5b1c1 | 182 | 2 | 0.012
B5b1c1 | 189 | 1 | 0.006
B5b1c1 | 207 | 2 | 0.012
B5b1c1 | 310 | 4 | 0.023
B5b1c1 | 315.2C | 2 | 0.012
B5b1c1 | 513 | 1 | 0.006
B5b1c1 | 573.6C | 1 | 0.006
B5b1c1 | 5985 | 1 | 0.006
B5b1c1 | 6260 | 1 | 0.006
B5b1c1 | 8257 | 1 | 0.006
B5b1c1a | 13063 | 1 | 0.006
B5b1c1a | 16129 | 3 | 0.017
B5b1c1a | 16147 | 1 | 0.006
B5b1c1a | 16173 | 1 | 0.006
B5b1c1a | 16262 | 1 | 0.006
B5b1c1a | 16263 | 1 | 0.006
B5b1c1a | 16311 | 5 | 0.028
B5b1c1a | 16497 | 2 | 0.011
B5b1c1a | 16500 | 2 | 0.011
B5b1c1a | 189 | 1 | 0.006
B5b1c1a | 203 | 3 | 0.017
B5b1c1a | 309d | 1 | 0.006
B5b1c1a | 310 | 1 | 0.006
B5b1c1a | 9010 | 1 | 0.006
B5b1c1a | 9139 | 1 | 0.006
B5b1c1a | 9455 | 2 | 0.011
B5b2 | 10181 | 1 | 0.045
B5b2 | 11437 | 1 | 0.045
B5b2 | 1187 | 1 | 0.045
B5b2 | 12950 | 2 | 0.091
B5b2 | 13500 | 2 | 0.091
B5b2 | 143 | 3 | 0.136
B5b2 | 16129 | 3 | 0.136
B5b2 | 16239 | 1 | 0.045
B5b2 | 16248 | 1 | 0.045
B5b2 | 16261 | 2 | 0.091
B5b2 | 16304 | 3 | 0.136
B5b2 | 189 | 1 | 0.045
B5b2 | 195 | 1 | 0.045
B5b2 | 207 | 1 | 0.045
B5b2 | 228 | 1 | 0.045
B5b2 | 316 | 1 | 0.045
B5b2 | 3434 | 1 | 0.045
B5b2 | 5744 | 1 | 0.045
B5b2 | 7598 | 1 | 0.045
B5b2 | 8477A | 1 | 0.045
B5b2 | 9058 | 3 | 0.136
B5b2+@204 | 10589 | 1 | 0.032
B5b2+@204 | 12223 | 1 | 0.032
B5b2+@204 | 12280 | 2 | 0.065
B5b2+@204 | 12332 | 1 | 0.032
B5b2+@204 | 12781 | 2 | 0.065
B5b2+@204 | 13650 | 1 | 0.032
B5b2+@204 | 13928 | 2 | 0.065
B5b2+@204 | 146 | 8 | 0.258
B5b2+@204 | 150 | 1 | 0.032
B5b2+@204 | 152 | 2 | 0.065
B5b2+@204 | 16076d | 1 | 0.032
B5b2+@204 | 16092 | 3 | 0.097
B5b2+@204 | 16093 | 1 | 0.032
B5b2+@204 | 16108 | 1 | 0.032
B5b2+@204 | 16129 | 4 | 0.129
B5b2+@204 | 16145 | 1 | 0.032
B5b2+@204 | 16166 | 1 | 0.032
B5b2+@204 | 16188 | 1 | 0.032
B5b2+@204 | 16193d | 1 | 0.032
B5b2+@204 | 16209 | 1 | 0.032
B5b2+@204 | 16242 | 4 | 0.129
B5b2+@204 | 16256 | 2 | 0.065
B5b2+@204 | 16266A | 3 | 0.097
B5b2+@204 | 16297 | 3 | 0.097
B5b2+@204 | 16304 | 1 | 0.032
B5b2+@204 | 16346C | 2 | 0.065
B5b2+@204 | 16399 | 1 | 0.032
B5b2+@204 | 16400 | 1 | 0.032
B5b2+@204 | 195 | 1 | 0.032
B5b2+@204 | 2078 | 2 | 0.065
B5b2+@204 | 2246 | 1 | 0.032
B5b2+@204 | 246 | 2 | 0.065
B5b2+@204 | 310 | 1 | 0.032
B5b2+@204 | 3399 | 1 | 0.032
B5b2+@204 | 347d | 1 | 0.032
B5b2+@204 | 3579 | 1 | 0.032
B5b2+@204 | 3640 | 1 | 0.032
B5b2+@204 | 3766 | 1 | 0.032
B5b2+@204 | 4161 | 1 | 0.032
B5b2+@204 | 6179 | 1 | 0.032
B5b2+@204 | 6395 | 1 | 0.032
B5b2+@204 | 6716 | 1 | 0.032
B5b2+@204 | 8065 | 1 | 0.032
B5b2+@204 | 9103 | 2 | 0.065
B5b2+@204 | 9755 | 1 | 0.032

B5b2a | 10602T | 1 | 0.059
B5b2a | 10915 | 1 | 0.059
B5b2a | 10972 | 1 | 0.059
B5b2a | 1193 | 1 | 0.059
B5b2a | 1440 | 1 | 0.059
B5b2a | 16039 | 1 | 0.059
B5b2a | 16108 | 1 | 0.059
B5b2a | 16129 | 6 | 0.353
B5b2a | 16154 | 1 | 0.059
B5b2a | 16184 | 1 | 0.059
B5b2a | 16213 | 1 | 0.059
B5b2a | 16223 | 1 | 0.059
B5b2a | 16244 | 1 | 0.059
B5b2a | 16249 | 2 | 0.118
B5b2a | 16250 | 2 | 0.118
B5b2a | 16266 | 1 | 0.059
B5b2a | 16287 | 1 | 0.059
B5b2a | 16309 | 1 | 0.059
B5b2a | 16399 | 1 | 0.059
B5b2a | 16465 | 1 | 0.059
B5b2a | 195 | 1 | 0.059
B5b2a | 199 | 1 | 0.059
B5b2a | 203 | 2 | 0.118
B5b2a | 292 | 1 | 0.059
B5b2a | 366 | 1 | 0.059
B5b2a | 408A | 1 | 0.059
B5b2a | 4561 | 1 | 0.059
B5b2a | 493 | 1 | 0.059
B5b2a | 5246 | 1 | 0.059
B5b2a | 55 | 1 | 0.059
B5b2a | 6032 | 1 | 0.059
B5b2a | 6681 | 1 | 0.059
B5b2a | 7590G | 1 | 0.059
B5b2a | 93 | 1 | 0.059
B5b2a1 | 11647G | 1 | 0.034
B5b2a1 | 11908 | 1 | 0.034
B5b2a1 | 13708 | 1 | 0.034
B5b2a1 | 14693 | 1 | 0.034
B5b2a1 | 15209 | 1 | 0.034
B5b2a1 | 16104 | 2 | 0.069
B5b2a1 | 16129 | 1 | 0.034
B5b2a1 | 16182T | 1 | 0.034
B5b2a1 | 16193d | 1 | 0.034
B5b2a1 | 16301 | 1 | 0.034
B5b2a1 | 16319 | 1 | 0.034
B5b2a1 | 16362 | 1 | 0.034
B5b2a1 | 16456C | 1 | 0.034
B5b2a1 | 183 | 3 | 0.103
B5b2a1 | 195 | 2 | 0.069
B5b2a1 | 207 | 1 | 0.034
B5b2a1 | 3972 | 1 | 0.034
B5b2a1 | 6249 | 1 | 0.034
B5b2a1 | 6767 | 3 | 0.103
B5b2a1 | 8551 | 1 | 0.034
B5b2a2 | 12609 | 2 | 0.118
B5b2a2 | 12696 | 2 | 0.118
B5b2a2 | 13788 | 1 | 0.059
B5b2a2 | 16051 | 1 | 0.059
B5b2a2 | 16145 | 1 | 0.059
B5b2a2 | 16265 | 1 | 0.059
B5b2a2 | 3398 | 2 | 0.118
B5b2a2 | 4251 | 1 | 0.059
B5b2a2 | 573.3C | 1 | 0.059
B5b2a2 | 5918 | 2 | 0.118
B5b2a2 | 6340 | 1 | 0.059
B5b2a2 | 7058 | 1 | 0.059
B5b2a2 | 7678 | 3 | 0.176
B5b2a2 | 7852 | 2 | 0.118
B5b2a2 | 93 | 3 | 0.176
B5b2a2 | 9398 | 1 | 0.059
B5b2a2a | 103 | 4 | 0.2
B5b2a2a | 146 | 2 | 0.1
B5b2a2a | 16153 | 6 | 0.3
B5b2a2a | 16169 | 1 | 0.05
B5b2a2a | 16223 | 1 | 0.05
B5b2a2a | 16311 | 3 | 0.15
B5b2a2a | 16319 | 5 | 0.25
B5b2a2a | 199 | 1 | 0.05
B5b2a2a | 234 | 1 | 0.05
B5b2a2a | 309.3C | 3 | 0.15
B5b2a2a | 316C | 2 | 0.1
B5b2a2a | 455d | 1 | 0.05
B5b2a2a1 | 16193.3C | 1 | 0.083
B5b2a2a1 | 1819 | 3 | 0.25
B5b2a2a1 | 310 | 1 | 0.083
B5b2a2a1 | 314-315d | 1 | 0.083
B5b2a2a1 | 322 | 1 | 0.083
B5b2a2a2 | 14016 | 2 | 0.154
B5b2a2a2 | 16129 | 3 | 0.231
B5b2a2a2 | 16244 | 1 | 0.077
B5b2a2a2 | 16356 | 1 | 0.077
B5b2a2a2 | 16362 | 1 | 0.077
B5b2a2a2 | 16497 | 1 | 0.077
B5b2a2a2 | 310 | 1 | 0.077
B5b2a2a2 | 3335 | 1 | 0.077
B5b2a2a2 | 5774G | 2 | 0.154
B5b2a2a2 | 8021 | 1 | 0.077
B5b2a2a2 | 8959 | 1 | 0.077
B5b2b | 143 | 2 | 0.105
B5b2b | 146 | 1 | 0.053
B5b2b | 16017 | 1 | 0.053
B5b2b | 16051 | 1 | 0.053
B5b2b | 16311 | 1 | 0.053
B5b2b | 16346C | 1 | 0.053
B5b2b | 16390 | 1 | 0.053
B5b2b | 183C | 1 | 0.053
B5b2b | 195 | 1 | 0.053
B5b2b | 199 | 4 | 0.211
B5b2b | 246 | 1 | 0.053
B5b2b | 310 | 1 | 0.053
B5b2b | 3305 | 3 | 0.158

B5b2b | 3398 | 3 | 0.158
B5b2b | 573.5C | 1 | 0.053
B5b2b | 6507 | 2 | 0.105
B5b2b | 6737 | 3 | 0.158
B5b2b | 7211 | 3 | 0.158
B5b2b | 7990 | 3 | 0.158
B5b2b | 8994 | 3 | 0.158
B5b2b | 9152 | 3 | 0.158
B5b2c | 10846 | 2 | 0.083
B5b2c | 11016 | 1 | 0.042
B5b2c | 12880 | 1 | 0.042
B5b2c | 1310 | 2 | 0.083
B5b2c | 13263 | 1 | 0.042
B5b2c | 13590 | 1 | 0.042
B5b2c | 13722 | 2 | 0.083
B5b2c | 14221 | 1 | 0.042
B5b2c | 14557 | 1 | 0.042
B5b2c | 1555 | 1 | 0.042
B5b2c | 15727 | 1 | 0.042
B5b2c | 15884 | 1 | 0.042
B5b2c | 16076d | 1 | 0.042
B5b2c | 16093 | 1 | 0.042
B5b2c | 16145 | 1 | 0.042
B5b2c | 16162 | 2 | 0.083
B5b2c | 16169.1C | 2 | 0.083
B5b2c | 16172A | 2 | 0.083
B5b2c | 16178 | 3 | 0.125
B5b2c | 16223 | 1 | 0.042
B5b2c | 16245 | 1 | 0.042
B5b2c | 16249 | 1 | 0.042
B5b2c | 16288 | 1 | 0.042
B5b2c | 16319 | 2 | 0.083
B5b2c | 16344 | 5 | 0.208
B5b2c | 3010 | 2 | 0.083
B5b2c | 309 | 3 | 0.125
B5b2c | 4227C | 1 | 0.042
B5b2c | 4233 | 1 | 0.042
B5b2c | 8392 | 2 | 0.083
B5b2c | 94 | 4 | 0.167
B5b2c | 9804 | 1 | 0.042
B5b2c1 | 10020 | 1 | 0.043
B5b2c1 | 10427 | 2 | 0.087
B5b2c1 | 11539 | 1 | 0.043
B5b2c1 | 146 | 1 | 0.043
B5b2c1 | 150 | 1 | 0.043
B5b2c1 | 15019 | 1 | 0.043
B5b2c1 | 15766 | 1 | 0.043
B5b2c1 | 16037 | 1 | 0.043
B5b2c1 | 16051 | 3 | 0.13
B5b2c1 | 16129 | 2 | 0.087
B5b2c1 | 16145 | 1 | 0.043
B5b2c1 | 16311 | 1 | 0.043
B5b2c1 | 16319 | 1 | 0.043
B5b2c1 | 16325 | 3 | 0.13
B5b2c1 | 3786 | 1 | 0.043
B5b2c1 | 4200 | 1 | 0.043
B5b2c1 | 4907 | 1 | 0.043
B5b2c1 | 866 | 1 | 0.043
B5b2c1 | 9299 | 2 | 0.087
B5b2c1 | 93 | 1 | 0.043
B5b2c1 | 9438 | 1 | 0.043
B5b3 | 151 | 2 | 0.011
B5b3 | 152 | 6 | 0.034
B5b3 | 16017 | 4 | 0.023
B5b3 | 16096T | 1 | 0.006
B5b3 | 16166d | 1 | 0.006
B5b3 | 16172 | 1 | 0.006
B5b3 | 16173 | 1 | 0.006
B5b3 | 16188 | 1 | 0.006
B5b3 | 16201 | 1 | 0.006
B5b3 | 16216 | 2 | 0.011
B5b3 | 16218 | 1 | 0.006
B5b3 | 16223A | 1 | 0.006
B5b3 | 16227 | 6 | 0.034
B5b3 | 16234 | 9 | 0.051
B5b3 | 16343 | 1 | 0.006
B5b3 | 16380 | 1 | 0.006
B5b3 | 16439A | 2 | 0.011
B5b3 | 16449 | 1 | 0.006
B5b3 | 222 | 6 | 0.034
B5b3 | 310 | 2 | 0.011
B5b3 | 315d | 1 | 0.006
B5b3 | 324G | 1 | 0.006
B5b3 | 411G | 1 | 0.006
B5b3a | 10003 | 1 | 0.143
B5b3a | 10907 | 1 | 0.143
B5b3a | 11881 | 1 | 0.143
B5b3a | 152 | 3 | 0.429
B5b3a | 16136 | 3 | 0.429
B5b3a | 16147 | 1 | 0.143
B5b3a | 16170 | 3 | 0.429
B5b3a | 16193d | 1 | 0.143
B5b3a | 16194C | 1 | 0.143
B5b3a | 16195 | 1 | 0.143
B5b3a | 16209 | 1 | 0.143
B5b3a | 16254 | 1 | 0.143
B5b3a | 16311 | 1 | 0.143
B5b3a | 3915 | 1 | 0.143
B5b3b | 12070 | 1 | 0.006
B5b3b | 14189 | 1 | 0.006
B5b3b | 146 | 1 | 0.006
B5b3b | 16096T | 1 | 0.006
B5b3b | 16166d | 1 | 0.006
B5b3b | 16173 | 1 | 0.006
B5b3b | 16188 | 1 | 0.006
B5b3b | 16216 | 2 | 0.011
B5b3b | 16218 | 1 | 0.006
B5b3b | 16223A | 1 | 0.006
B5b3b | 16352 | 4 | 0.023
B5b3b | 16356 | 3 | 0.017
B5b3b | 16380 | 1 | 0.006

B5b3b | 16439A | 2 | 0.011
B5b3b | 16449 | 1 | 0.006
B5b3b | 185 | 1 | 0.006
B5b3b | 199 | 1 | 0.006
B5b3b | 324G | 1 | 0.006
B5b3b | 3745 | 1 | 0.006
B5b3b | 411G | 1 | 0.006
B5b4 | 11017 | 1 | 0.062
B5b4 | 13455 | 1 | 0.062
B5b4 | 15001 | 1 | 0.062
B5b4 | 152 | 3 | 0.188
B5b4 | 15746 | 1 | 0.062
B5b4 | 16093 | 3 | 0.188
B5b4 | 16145 | 1 | 0.062
B5b4 | 16148 | 1 | 0.062
B5b4 | 16209 | 1 | 0.062
B5b4 | 16257 | 3 | 0.188
B5b4 | 16278 | 1 | 0.062
B5b4 | 16298 | 2 | 0.125
B5b4 | 16311 | 1 | 0.062
B5b4 | 16362 | 4 | 0.25
B5b4 | 1709 | 1 | 0.062
B5b4 | 189 | 2 | 0.125
B5b4 | 203 | 1 | 0.062
B5b4 | 228 | 3 | 0.188
B5b4 | 3396 | 1 | 0.062
B5b4 | 3951 | 1 | 0.062
B5b4 | 5459 | 1 | 0.062
B5b4 | 6340 | 2 | 0.125
B5b4 | 7310 | 1 | 0.062
B5b4 | 7903 | 1 | 0.062
B5b4 | 8349 | 2 | 0.125
B5b4 | 8930 | 1 | 0.062
B5b4 | 9266 | 1 | 0.062
B5b4 | 9477 | 1 | 0.062
B5b5 | 16193d | 1 | 0.077
B5b5 | 16194C | 2 | 0.154
B5b5 | 16195 | 2 | 0.154
B5b5 | 16262 | 1 | 0.077
B5b5 | 16266A | 1 | 0.077
B5b5 | 16311 | 1 | 0.077
B5b5 | 16359 | 3 | 0.231
B5b5 | 198 | 1 | 0.077
B5b5 | 309.3C | 1 | 0.077
B5b5 | 3308 | 1 | 0.077
B5b5 | 55G | 1 | 0.077
B5b5 | 573.2C | 1 | 0.077
B5b5 | 573.3C | 4 | 0.308
B5b5 | 573.4C | 1 | 0.077
B5b5 | 574C | 1 | 0.077
B5b5 | 576C | 1 | 0.077
B5b5 | 868 | 1 | 0.077
B5b5 | 9980 | 1 | 0.077
B6 | 13824 | 1 | 0.012
B6 | 146 | 1 | 0.012
B6 | 14783 | 1 | 0.012
B6 | 152 | 2 | 0.025
B6 | 16051 | 1 | 0.012
B6 | 16129 | 1 | 0.012
B6 | 16145 | 1 | 0.012
B6 | 16150 | 1 | 0.012
B6 | 16157 | 2 | 0.025
B6 | 16172 | 1 | 0.012
B6 | 16179 | 7 | 0.086
B6 | 16185 | 1 | 0.012
B6 | 16193d | 1 | 0.012
B6 | 16209 | 6 | 0.074
B6 | 16249 | 1 | 0.012
B6 | 16278 | 1 | 0.012
B6 | 16312C | 1 | 0.012
B6 | 16319 | 5 | 0.062
B6 | 16466T | 1 | 0.012
B6 | 16468 | 2 | 0.025
B6 | 238 | 1 | 0.012
B6 | 249d | 3 | 0.037
B6 | 275 | 1 | 0.012
B6 | 309.3C | 2 | 0.025
B6 | 3763 | 1 | 0.012
B6a | 10849 | 21 | 0.208
B6a | 1193 | 21 | 0.208
B6a | 12094 | 1 | 0.01
B6a | 12172 | 1 | 0.01
B6a | 12280 | 1 | 0.01
B6a | 12346 | 4 | 0.04
B6a | 12454 | 4 | 0.04
B6a | 13227 | 17 | 0.168
B6a | 13611 | 17 | 0.168
B6a | 14025 | 3 | 0.03
B6a | 14256 | 2 | 0.02
B6a | 14392 | 1 | 0.01
B6a | 14569 | 12 | 0.119
B6a | 151 | 1 | 0.01
B6a | 152 | 2 | 0.02
B6a | 15508 | 1 | 0.01
B6a | 15883 | 1 | 0.01
B6a | 16086 | 1 | 0.01
B6a | 16092 | 1 | 0.01
B6a | 16124 | 2 | 0.02
B6a | 16129 | 1 | 0.01
B6a | 16179 | 74 | 0.733
B6a | 16243 | 2 | 0.02
B6a | 16266 | 1 | 0.01
B6a | 16291 | 1 | 0.01
B6a | 16297 | 1 | 0.01
B6a | 16311 | 1 | 0.01
B6a | 16335 | 1 | 0.01
B6a | 16342 | 6 | 0.059
B6a | 16357 | 2 | 0.02
B6a | 16390 | 17 | 0.168
B6a | 1694 | 1 | 0.01
B6a | 207 | 1 | 0.01

B6a | 234 | 1 | 0.01
B6a | 279 | 1 | 0.01
B6a | 3010 | 2 | 0.02
B6a | 309d | 1 | 0.01
B6a | 310 | 1 | 0.01
B6a | 3434 | 17 | 0.168
B6a | 4117 | 2 | 0.02
B6a | 4245 | 1 | 0.01
B6a | 4561 | 1 | 0.01
B6a | 489 | 1 | 0.01
B6a | 547 | 5 | 0.05
B6a | 5892 | 1 | 0.01
B6a | 5892.1TTTTTCC | 1 | 0.01
B6a | 5892.3T | 1 | 0.01
B6a | 5893 | 7 | 0.069
B6a | 5894-5895d | 7 | 0.069
B6a | 5894T | 7 | 0.069
B6a | 61.1A | 1 | 0.01
B6a | 6278 | 1 | 0.01
B6a | 64 | 1 | 0.01
B6a | 7046 | 17 | 0.168
B6a | 7472C | 2 | 0.02
B6a | 7562 | 3 | 0.03
B6a | 7775 | 1 | 0.01
B6a | 8149 | 3 | 0.03
B6a | 8485 | 2 | 0.02
B6a | 8701 | 21 | 0.208
B6a | 930 | 1 | 0.01
B6a | 9545 | 4 | 0.04
B6a1 | 13708 | 4 | 0.154
B6a1 | 14016 | 4 | 0.154
B6a1 | 14311 | 1 | 0.038
B6a1 | 146 | 3 | 0.115
B6a1 | 14989 | 1 | 0.038
B6a1 | 151 | 2 | 0.077
B6a1 | 152 | 7 | 0.269
B6a1 | 153 | 1 | 0.038
B6a1 | 16075A | 1 | 0.038
B6a1 | 16083G | 1 | 0.038
B6a1 | 16086 | 1 | 0.038
B6a1 | 16092 | 2 | 0.077
B6a1 | 16126 | 2 | 0.077
B6a1 | 16145 | 1 | 0.038
B6a1 | 16220 | 8 | 0.308
B6a1 | 16224 | 1 | 0.038
B6a1 | 16237C | 1 | 0.038
B6a1 | 16260 | 1 | 0.038
B6a1 | 16274 | 1 | 0.038
B6a1 | 16278 | 2 | 0.077
B6a1 | 16288 | 1 | 0.038
B6a1 | 16298 | 1 | 0.038
B6a1 | 16342 | 1 | 0.038
B6a1 | 16345C | 1 | 0.038
B6a1 | 16350C | 1 | 0.038
B6a1 | 16355 | 1 | 0.038
B6a1 | 16356 | 5 | 0.192
B6a1 | 16362 | 1 | 0.038
B6a1 | 204 | 1 | 0.038
B6a1 | 214 | 3 | 0.115
B6a1 | 2416 | 2 | 0.077
B6a1 | 29.1G | 1 | 0.038
B6a1 | 306-309d | 2 | 0.077
B6a1 | 309.3C | 1 | 0.038
B6a1 | 309d | 1 | 0.038
B6a1 | 32.1G | 1 | 0.038
B6a1 | 489 | 1 | 0.038
B6a1 | 498d | 1 | 0.038
B6a1 | 5892.1TTTCCCCC | 1 | 0.038
B6a1 | 5893 | 1 | 0.038
B6a1 | 7642 | 1 | 0.038
B6a1 | 8258 | 1 | 0.038
B6a1 | 8865 | 1 | 0.038
B6a1 | 9758 | 1 | 0.038
B6a1a | 11267 | 2 | 0.028
B6a1a | 12459 | 20 | 0.282
B6a1a | 13707 | 7 | 0.099
B6a1a | 14110 | 6 | 0.085
B6a1a | 14188 | 1 | 0.014
B6a1a | 14484 | 1 | 0.014
B6a1a | 146 | 3 | 0.042
B6a1a | 150 | 1 | 0.014
B6a1a | 1547.1T | 1 | 0.014
B6a1a | 16093 | 1 | 0.014
B6a1a | 16145 | 1 | 0.014
B6a1a | 16176 | 10 | 0.141
B6a1a | 16218 | 1 | 0.014
B6a1a | 16258C | 1 | 0.014
B6a1a | 16260 | 1 | 0.014
B6a1a | 16271 | 1 | 0.014
B6a1a | 16272 | 1 | 0.014
B6a1a | 16293C | 1 | 0.014
B6a1a | 16293T | 1 | 0.014
B6a1a | 16304 | 2 | 0.028
B6a1a | 16309 | 1 | 0.014
B6a1a | 16311 | 4 | 0.056
B6a1a | 16322T | 1 | 0.014
B6a1a | 16354 | 29 | 0.408
B6a1a | 16356 | 1 | 0.014
B6a1a | 16362 | 2 | 0.028
B6a1a | 16384 | 1 | 0.014
B6a1a | 16405C | 1 | 0.014
B6a1a | 16413G | 1 | 0.014
B6a1a | 16428C | 1 | 0.014
B6a1a | 16434 | 1 | 0.014
B6a1a | 16496 | 1 | 0.014
B6a1a | 16558 | 1 | 0.014
B6a1a | 204 | 1 | 0.014
B6a1a | 205.1G | 1 | 0.014
B6a1a | 258 | 1 | 0.014
B6a1a | 272C | 1 | 0.014
B6a1a | 310 | 1 | 0.014

B6a1a | 3591 | 1 | 0.014
B6a1a | 4904A | 2 | 0.028
B6a1a | 5899.3C | 1 | 0.014
B6a1a | 6170 | 27 | 0.38
B6a1a | 6758 | 1 | 0.014
B6a1a | 8027 | 1 | 0.014
B6a1a | 8838 | 1 | 0.014
B6a1a | 987T | 1 | 0.014
C | 11533 | 1 | 0.004
C | 11778 | 1 | 0.004
C | 14697 | 1 | 0.004
C | 16086 | 6 | 0.024
C | 16104 | 1 | 0.004
C | 16126 | 2 | 0.008
C | 16147 | 1 | 0.004
C | 16207 | 1 | 0.004
C | 16243 | 1 | 0.004
C | 16309 | 2 | 0.008
C | 16318T | 1 | 0.004
C | 16319 | 1 | 0.004
C | 16325 | 3 | 0.012
C | 16359 | 1 | 0.004
C | 16399 | 5 | 0.02
C | 4026 | 1 | 0.004
C | 4216 | 1 | 0.004
C | 513 | 1 | 0.004
C | 574 | 1 | 0.004
C | 7196 | 1 | 0.004
C | 8152 | 1 | 0.004
C1 | 143 | 45 | 0.218
C1 | 14397 | 1 | 0.005
C1 | 143T | 2 | 0.01
C1 | 14502 | 1 | 0.005
C1 | 150 | 4 | 0.019
C1 | 151 | 2 | 0.01
C1 | 152 | 2 | 0.01
C1 | 16071 | 11 | 0.053
C1 | 16086 | 2 | 0.01
C1 | 16092 | 3 | 0.015
C1 | 16095 | 3 | 0.015
C1 | 16103 | 1 | 0.005
C1 | 16124 | 11 | 0.053
C1 | 16145 | 2 | 0.01
C1 | 16178 | 1 | 0.005
C1 | 16187 | 1 | 0.005
C1 | 16189 | 1 | 0.005
C1 | 16209 | 1 | 0.005
C1 | 16218 | 5 | 0.024
C1 | 16243 | 6 | 0.029
C1 | 16261 | 3 | 0.015
C1 | 16274 | 1 | 0.005
C1 | 16293 | 1 | 0.005
C1 | 16311 | 10 | 0.049
C1 | 16319 | 1 | 0.005
C1 | 16344G | 9 | 0.044
C1 | 16355 | 1 | 0.005
C1 | 16362 | 2 | 0.01
C1 | 18 | 1 | 0.005
C1 | 185 | 2 | 0.01
C1 | 194 | 1 | 0.005
C1 | 207 | 2 | 0.01
C1 | 20A | 1 | 0.005
C1 | 275 | 1 | 0.005
C1 | 289 | 4 | 0.019
C1 | 29G | 1 | 0.005
C1 | 310 | 2 | 0.01
C1 | 3116 | 1 | 0.005
C1 | 316 | 1 | 0.005
C1 | 3203T | 1 | 0.005
C1 | 408A | 2 | 0.01
C1 | 606 | 1 | 0.005
C1 | 93 | 1 | 0.005
C1a | 13590 | 1 | 0.007
C1a | 15773 | 1 | 0.007
C1a | 16093 | 16 | 0.119
C1a | 16126 | 1 | 0.007
C1a | 16166 | 3 | 0.022
C1a | 16176 | 1 | 0.007
C1a | 16181 | 1 | 0.007
C1a | 16185 | 2 | 0.015
C1a | 16192 | 1 | 0.007
C1a | 16224 | 4 | 0.03
C1a | 16260 | 3 | 0.022
C1a | 16311 | 1 | 0.007
C1a | 16527 | 1 | 0.007
C1a | 193 | 3 | 0.022
C1a | 204 | 1 | 0.007
C1a | 2887 | 1 | 0.007
C1a | 295 | 1 | 0.007
C1a | 375 | 1 | 0.007
C1a | 5774 | 2 | 0.015
C1a | 9099A | 2 | 0.015
C1a | 93 | 13 | 0.097
C1a | 9750 | 2 | 0.015
C1b | 10031 | 7 | 0.026
C1b | 1005 | 1 | 0.004
C1b | 10094 | 1 | 0.004
C1b | 1018 | 1 | 0.004
C1b | 10293 | 1 | 0.004
C1b | 103 | 1 | 0.004
C1b | 10310 | 16 | 0.06
C1b | 10364 | 4 | 0.015
C1b | 10370 | 2 | 0.008
C1b | 1040 | 3 | 0.011
C1b | 10420 | 2 | 0.008
C1b | 10456 | 1 | 0.004
C1b | 10586 | 2 | 0.008
C1b | 10601 | 1 | 0.004
C1b | 10616 | 3 | 0.011
C1b | 10748 | 16 | 0.06
C1b | 10754 | 5 | 0.019

C1b | 10798 | 1 | 0.004
C1b | 10915 | 1 | 0.004
C1b | 10987 | 1 | 0.004
C1b | 10993 | 5 | 0.019
C1b | 11026 | 1 | 0.004
C1b | 11084 | 2 | 0.008
C1b | 11150 | 3 | 0.011
C1b | 11152 | 3 | 0.011
C1b | 11243 | 1 | 0.004
C1b | 11253 | 2 | 0.008
C1b | 11296 | 1 | 0.004
C1b | 11335 | 1 | 0.004
C1b | 11383 | 1 | 0.004
C1b | 11389 | 3 | 0.011
C1b | 114 | 2 | 0.008
C1b | 11410 | 3 | 0.011
C1b | 11431A | 1 | 0.004
C1b | 11447 | 1 | 0.004
C1b | 11518 | 1 | 0.004
C1b | 11779 | 1 | 0.004
C1b | 11864 | 2 | 0.008
C1b | 1193 | 1 | 0.004
C1b | 12071 | 1 | 0.004
C1b | 1211 | 1 | 0.004
C1b | 12112 | 2 | 0.008
C1b | 12127 | 1 | 0.004
C1b | 12193 | 2 | 0.008
C1b | 12302 | 3 | 0.011
C1b | 12429 | 1 | 0.004
C1b | 12477 | 1 | 0.004
C1b | 125 | 5 | 0.019
C1b | 12505 | 1 | 0.004
C1b | 12535 | 10 | 0.038
C1b | 125G | 1 | 0.004
C1b | 12612 | 1 | 0.004
C1b | 12631G | 1 | 0.004
C1b | 127 | 5 | 0.019
C1b | 12717 | 3 | 0.011
C1b | 12771 | 2 | 0.008
C1b | 12807 | 1 | 0.004
C1b | 12813 | 10 | 0.038
C1b | 1285d | 1 | 0.004
C1b | 12870 | 2 | 0.008
C1b | 12879 | 2 | 0.008
C1b | 12975 | 1 | 0.004
C1b | 12976 | 2 | 0.008
C1b | 13135 | 24 | 0.09
C1b | 13145 | 1 | 0.004
C1b | 13191 | 1 | 0.004
C1b | 13197 | 1 | 0.004
C1b | 13281 | 1 | 0.004
C1b | 13326 | 1 | 0.004
C1b | 13368 | 1 | 0.004
C1b | 13422 | 4 | 0.015
C1b | 13482T | 1 | 0.004
C1b | 13521 | 2 | 0.008
C1b | 13545 | 1 | 0.004
C1b | 13608 | 1 | 0.004
C1b | 13637 | 2 | 0.008
C1b | 13656 | 1 | 0.004
C1b | 13681 | 1 | 0.004
C1b | 13695A | 1 | 0.004
C1b | 13701 | 1 | 0.004
C1b | 13708 | 1 | 0.004
C1b | 13710C | 1 | 0.004
C1b | 13711 | 4 | 0.015
C1b | 13734 | 2 | 0.008
C1b | 13752 | 2 | 0.008
C1b | 13766A | 1 | 0.004
C1b | 13781 | 1 | 0.004
C1b | 13782 | 1 | 0.004
C1b | 13802 | 2 | 0.008
C1b | 13827C | 1 | 0.004
C1b | 13830 | 1 | 0.004
C1b | 13836 | 4 | 0.015
C1b | 13860 | 1 | 0.004
C1b | 1391 | 1 | 0.004
C1b | 13966 | 1 | 0.004
C1b | 14003 | 1 | 0.004
C1b | 14053 | 1 | 0.004
C1b | 14118 | 1 | 0.004
C1b | 14224 | 1 | 0.004
C1b | 143 | 6 | 0.023
C1b | 14302 | 2 | 0.008
C1b | 14308 | 4 | 0.015
C1b | 14348 | 1 | 0.004
C1b | 14359 | 1 | 0.004
C1b | 14374 | 2 | 0.008
C1b | 14384 | 1 | 0.004
C1b | 14386 | 5 | 0.019
C1b | 14397 | 1 | 0.004
C1b | 143C | 1 | 0.004
C1b | 143T | 1 | 0.004
C1b | 14428 | 1 | 0.004
C1b | 14502 | 1 | 0.004
C1b | 14560 | 1 | 0.004
C1b | 14569 | 1 | 0.004
C1b | 14587 | 8 | 0.03
C1b | 146 | 16 | 0.06
C1b | 14602 | 1 | 0.004
C1b | 1462 | 1 | 0.004
C1b | 14659 | 3 | 0.011
C1b | 14687 | 1 | 0.004
C1b | 14693 | 2 | 0.008
C1b | 14706 | 2 | 0.008
C1b | 14751 | 3 | 0.011
C1b | 14788 | 1 | 0.004
C1b | 14832 | 1 | 0.004
C1b | 14833 | 3 | 0.011
C1b | 14863 | 1 | 0.004
C1b | 14871 | 2 | 0.008

C1b | 14998 | 4 | 0.015
C1b | 150 | 9 | 0.034
C1b | 15056 | 1 | 0.004
C1b | 15061 | 2 | 0.008
C1b | 151 | 2 | 0.008
C1b | 152 | 35 | 0.132
C1b | 15236 | 2 | 0.008
C1b | 15244 | 1 | 0.004
C1b | 15283 | 1 | 0.004
C1b | 153 | 2 | 0.008
C1b | 1530 | 1 | 0.004
C1b | 15313 | 10 | 0.038
C1b | 15314 | 1 | 0.004
C1b | 15315 | 1 | 0.004
C1b | 15323 | 1 | 0.004
C1b | 15431 | 1 | 0.004
C1b | 15454 | 1 | 0.004
C1b | 15462 | 4 | 0.015
C1b | 15465 | 2 | 0.008
C1b | 15478 | 1 | 0.004
C1b | 15519 | 2 | 0.008
C1b | 15530 | 5 | 0.019
C1b | 15574 | 1 | 0.004
C1b | 15643 | 1 | 0.004
C1b | 15670 | 4 | 0.015
C1b | 15706 | 1 | 0.004
C1b | 15728 | 2 | 0.008
C1b | 15734 | 1 | 0.004
C1b | 15740 | 1 | 0.004
C1b | 15758 | 4 | 0.015
C1b | 15774 | 1 | 0.004
C1b | 15905 | 5 | 0.019
C1b | 15924 | 4 | 0.015
C1b | 15930 | 1 | 0.004
C1b | 15941 | 1 | 0.004
C1b | 15968 | 1 | 0.004
C1b | 15970 | 2 | 0.008
C1b | 16037 | 3 | 0.011
C1b | 16048 | 1 | 0.004
C1b | 16071 | 1 | 0.004
C1b | 16086 | 1 | 0.004
C1b | 16092 | 13 | 0.049
C1b | 16093 | 8 | 0.03
C1b | 16103 | 1 | 0.004
C1b | 16111 | 2 | 0.008
C1b | 16114 | 1 | 0.004
C1b | 16124 | 1 | 0.004
C1b | 16126 | 7 | 0.026
C1b | 16127 | 1 | 0.004
C1b | 16129 | 11 | 0.041
C1b | 16136 | 4 | 0.015
C1b | 16139T | 1 | 0.004
C1b | 16140 | 1 | 0.004
C1b | 16145 | 4 | 0.015
C1b | 16146 | 2 | 0.008
C1b | 16147A | 2 | 0.008
C1b | 16156 | 4 | 0.015
C1b | 16166C | 5 | 0.019
C1b | 16166d | 1 | 0.004
C1b | 16170 | 1 | 0.004
C1b | 16176 | 1 | 0.004
C1b | 16178 | 3 | 0.011
C1b | 16179 | 1 | 0.004
C1b | 16181 | 1 | 0.004
C1b | 16182 | 1 | 0.004
C1b | 16183 | 1 | 0.004
C1b | 16185 | 6 | 0.023
C1b | 16187 | 3 | 0.011
C1b | 16189 | 16 | 0.06
C1b | 16190 | 3 | 0.011
C1b | 16192 | 2 | 0.008
C1b | 16193 | 2 | 0.008
C1b | 16198 | 1 | 0.004
C1b | 16199 | 1 | 0.004
C1b | 16207 | 1 | 0.004
C1b | 16209 | 3 | 0.011
C1b | 16227 | 1 | 0.004
C1b | 16234 | 1 | 0.004
C1b | 16240 | 2 | 0.008
C1b | 16243 | 1 | 0.004
C1b | 16244T | 2 | 0.008
C1b | 16248 | 1 | 0.004
C1b | 16248A | 3 | 0.011
C1b | 16249 | 3 | 0.011
C1b | 16254 | 1 | 0.004
C1b | 16256 | 7 | 0.026
C1b | 16258 | 1 | 0.004
C1b | 16266 | 4 | 0.015
C1b | 16270 | 2 | 0.008
C1b | 16274 | 4 | 0.015
C1b | 16287 | 1 | 0.004
C1b | 16291 | 1 | 0.004
C1b | 16292 | 3 | 0.011
C1b | 16293 | 1 | 0.004
C1b | 16293C | 1 | 0.004
C1b | 16294 | 5 | 0.019
C1b | 16297 | 2 | 0.008
C1b | 16309 | 3 | 0.011
C1b | 16317 | 2 | 0.008
C1b | 16318 | 3 | 0.011
C1b | 16318C | 1 | 0.004
C1b | 16319 | 1 | 0.004
C1b | 16335 | 2 | 0.008
C1b | 16342 | 1 | 0.004
C1b | 16344 | 10 | 0.038
C1b | 16348 | 1 | 0.004
C1b | 16355 | 2 | 0.008
C1b | 16356 | 1 | 0.004
C1b | 16357 | 1 | 0.004
C1b | 16359 | 1 | 0.004
C1b | 16360 | 3 | 0.011

C1b | 16362 | 1 | 0.004
C1b | 16365 | 1 | 0.004
C1b | 16368 | 1 | 0.004
C1b | 16390 | 5 | 0.019
C1b | 16399 | 1 | 0.004
C1b | 16400 | 1 | 0.004
C1b | 16428 | 1 | 0.004
C1b | 16430 | 1 | 0.004
C1b | 16440 | 1 | 0.004
C1b | 16445 | 1 | 0.004
C1b | 16463 | 1 | 0.004
C1b | 16465 | 1 | 0.004
C1b | 16468 | 2 | 0.008
C1b | 16471 | 1 | 0.004
C1b | 16505 | 1 | 0.004
C1b | 16526 | 13 | 0.049
C1b | 16540A | 4 | 0.015
C1b | 16540G | 6 | 0.023
C1b | 16555 | 1 | 0.004
C1b | 1692C | 2 | 0.008
C1b | 1709T | 3 | 0.011
C1b | 1717 | 1 | 0.004
C1b | 18 | 1 | 0.004
C1b | 1808 | 3 | 0.011
C1b | 1842 | 4 | 0.015
C1b | 185 | 5 | 0.019
C1b | 188 | 3 | 0.011
C1b | 194 | 3 | 0.011
C1b | 195 | 6 | 0.023
C1b | 199 | 3 | 0.011
C1b | 200 | 2 | 0.008
C1b | 203 | 1 | 0.004
C1b | 204 | 12 | 0.045
C1b | 207 | 11 | 0.041
C1b | 214 | 12 | 0.045
C1b | 215 | 1 | 0.004
C1b | 2220 | 1 | 0.004
C1b | 2270 | 4 | 0.015
C1b | 235 | 3 | 0.011
C1b | 236 | 2 | 0.008
C1b | 2360 | 2 | 0.008
C1b | 2370 | 1 | 0.004
C1b | 2393 | 4 | 0.015
C1b | 248 | 1 | 0.004
C1b | 2526 | 1 | 0.004
C1b | 259 | 1 | 0.004
C1b | 2626G | 1 | 0.004
C1b | 2757 | 1 | 0.004
C1b | 2835 | 1 | 0.004
C1b | 287C | 1 | 0.004
C1b | 291d | 1 | 0.004
C1b | 310 | 3 | 0.011
C1b | 3116 | 1 | 0.004
C1b | 315.2C | 1 | 0.004
C1b | 315d | 1 | 0.004
C1b | 316C | 1 | 0.004
C1b | 317.1C | 1 | 0.004
C1b | 319 | 1 | 0.004
C1b | 3197 | 3 | 0.011
C1b | 3203T | 1 | 0.004
C1b | 3316 | 3 | 0.011
C1b | 3335 | 1 | 0.004
C1b | 3357 | 1 | 0.004
C1b | 3369 | 1 | 0.004
C1b | 3394 | 3 | 0.011
C1b | 340 | 1 | 0.004
C1b | 3531 | 1 | 0.004
C1b | 3768 | 2 | 0.008
C1b | 382A | 5 | 0.019
C1b | 3866 | 1 | 0.004
C1b | 3882 | 4 | 0.015
C1b | 3891 | 1 | 0.004
C1b | 4014 | 1 | 0.004
C1b | 4117 | 1 | 0.004
C1b | 4219 | 1 | 0.004
C1b | 4225 | 1 | 0.004
C1b | 4233 | 1 | 0.004
C1b | 4353 | 1 | 0.004
C1b | 4454 | 3 | 0.011
C1b | 4491 | 1 | 0.004
C1b | 45 | 2 | 0.008
C1b | 4512 | 1 | 0.004
C1b | 4529 | 13 | 0.049
C1b | 4547 | 1 | 0.004
C1b | 462 | 1 | 0.004
C1b | 4676A | 4 | 0.015
C1b | 46G | 1 | 0.004
C1b | 4736 | 1 | 0.004
C1b | 485 | 2 | 0.008
C1b | 4967 | 2 | 0.008
C1b | 4991 | 1 | 0.004
C1b | 4994 | 1 | 0.004
C1b | 5 | 1 | 0.004
C1b | 5015 | 1 | 0.004
C1b | 504 | 1 | 0.004
C1b | 5054C | 1 | 0.004
C1b | 5057 | 2 | 0.008
C1b | 508 | 1 | 0.004
C1b | 51 | 1 | 0.004
C1b | 513 | 1 | 0.004
C1b | 5147 | 1 | 0.004
C1b | 514d | 1 | 0.004
C1b | 5201A | 2 | 0.008
C1b | 5238 | 1 | 0.004
C1b | 5351 | 1 | 0.004
C1b | 5360 | 1 | 0.004
C1b | 5417 | 1 | 0.004
C1b | 5438 | 1 | 0.004
C1b | 5441 | 1 | 0.004
C1b | 5442 | 10 | 0.038
C1b | 5444 | 1 | 0.004

C1b | 5459 | 1 | 0.004
C1b | 5460 | 2 | 0.008
C1b | 5470 | 1 | 0.004
C1b | 549 | 2 | 0.008
C1b | 5492 | 1 | 0.004
C1b | 5493 | 1 | 0.004
C1b | 550T | 1 | 0.004
C1b | 5530 | 10 | 0.038
C1b | 5553 | 1 | 0.004
C1b | 5580 | 2 | 0.008
C1b | 5581 | 10 | 0.038
C1b | 5585 | 1 | 0.004
C1b | 5600 | 2 | 0.008
C1b | 5628 | 1 | 0.004
C1b | 5655 | 1 | 0.004
C1b | 56T | 1 | 0.004
C1b | 57 | 3 | 0.011
C1b | 573.1C | 4 | 0.015
C1b | 5745 | 1 | 0.004
C1b | 5821 | 1 | 0.004
C1b | 5824 | 1 | 0.004
C1b | 5899.3C | 4 | 0.015
C1b | 59 | 1 | 0.004
C1b | 593 | 1 | 0.004
C1b | 5981 | 1 | 0.004
C1b | 60 | 1 | 0.004
C1b | 60.1T | 1 | 0.004
C1b | 6002 | 1 | 0.004
C1b | 6125 | 1 | 0.004
C1b | 62 | 1 | 0.004
C1b | 6228 | 1 | 0.004
C1b | 6249 | 1 | 0.004
C1b | 6253 | 4 | 0.015
C1b | 6267 | 6 | 0.023
C1b | 6278 | 2 | 0.008
C1b | 63 | 1 | 0.004
C1b | 636 | 1 | 0.004
C1b | 64 | 5 | 0.019
C1b | 6456 | 1 | 0.004
C1b | 6476 | 1 | 0.004
C1b | 6480 | 4 | 0.015
C1b | 6494 | 1 | 0.004
C1b | 6497 | 1 | 0.004
C1b | 654 | 1 | 0.004
C1b | 66 | 1 | 0.004
C1b | 6605 | 2 | 0.008
C1b | 6629 | 2 | 0.008
C1b | 6635 | 1 | 0.004
C1b | 6719 | 1 | 0.004
C1b | 6758 | 2 | 0.008
C1b | 68 | 1 | 0.004
C1b | 6872 | 2 | 0.008
C1b | 6959 | 1 | 0.004
C1b | 7076 | 1 | 0.004
C1b | 7080 | 1 | 0.004
C1b | 709 | 2 | 0.008
C1b | 71 | 1 | 0.004
C1b | 7184 | 13 | 0.049
C1b | 719 | 1 | 0.004
C1b | 72 | 1 | 0.004
C1b | 7202 | 2 | 0.008
C1b | 7211 | 3 | 0.011
C1b | 723C | 2 | 0.008
C1b | 7292 | 1 | 0.004
C1b | 7334 | 2 | 0.008
C1b | 7337 | 1 | 0.004
C1b | 7354 | 1 | 0.004
C1b | 7424 | 1 | 0.004
C1b | 7498 | 1 | 0.004
C1b | 7598 | 1 | 0.004
C1b | 76 | 1 | 0.004
C1b | 7664 | 3 | 0.011
C1b | 7759 | 1 | 0.004
C1b | 7762 | 1 | 0.004
C1b | 7766T | 1 | 0.004
C1b | 7789 | 1 | 0.004
C1b | 7853 | 3 | 0.011
C1b | 789 | 10 | 0.038
C1b | 796 | 1 | 0.004
C1b | 79T | 1 | 0.004
C1b | 8020 | 2 | 0.008
C1b | 8131 | 1 | 0.004
C1b | 8134 | 1 | 0.004
C1b | 8155 | 1 | 0.004
C1b | 8194 | 1 | 0.004
C1b | 8210 | 1 | 0.004
C1b | 8270 | 2 | 0.008
C1b | 8281-8289d | 2 | 0.008
C1b | 8289 | 1 | 0.004
C1b | 8380 | 1 | 0.004
C1b | 8383 | 2 | 0.008
C1b | 8400 | 1 | 0.004
C1b | 8474A | 1 | 0.004
C1b | 8508 | 1 | 0.004
C1b | 8516 | 1 | 0.004
C1b | 8566 | 1 | 0.004
C1b | 8580 | 1 | 0.004
C1b | 8634 | 3 | 0.011
C1b | 8637 | 4 | 0.015
C1b | 8649T | 1 | 0.004
C1b | 8655 | 1 | 0.004
C1b | 8685A | 1 | 0.004
C1b | 8739 | 1 | 0.004
C1b | 8856 | 1 | 0.004
C1b | 8865 | 1 | 0.004
C1b | 8896 | 2 | 0.008
C1b | 8901 | 3 | 0.011
C1b | 8931 | 1 | 0.004
C1b | 8952 | 1 | 0.004
C1b | 9 | 2 | 0.008
C1b | 9055 | 1 | 0.004

C1b | 9058 | 2 | 0.008
C1b | 9084 | 1 | 0.004
C1b | 9142 | 1 | 0.004
C1b | 9210 | 1 | 0.004
C1b | 93 | 1 | 0.004
C1b | 9316 | 2 | 0.008
C1b | 9329 | 2 | 0.008
C1b | 9359 | 2 | 0.008
C1b | 9368 | 1 | 0.004
C1b | 9380 | 1 | 0.004
C1b | 9398 | 1 | 0.004
C1b | 9468 | 3 | 0.011
C1b | 9500 | 1 | 0.004
C1b | 9536 | 1 | 0.004
C1b | 960d | 1 | 0.004
C1b | 9686 | 2 | 0.008
C1b | 9692 | 2 | 0.008
C1b | 9755 | 3 | 0.011
C1b | 980 | 1 | 0.004
C1b | 9804 | 1 | 0.004
C1b | 9845 | 3 | 0.011
C1b | 9869A | 4 | 0.015
C1b | 9912 | 1 | 0.004
C1b | 9944 | 1 | 0.004
C1b+16311 | 10084 | 1 | 0.034
C1b+16311 | 10094 | 1 | 0.034
C1b+16311 | 1018 | 1 | 0.034
C1b+16311 | 11026 | 1 | 0.034
C1b+16311 | 11253 | 10 | 0.345
C1b+16311 | 11447 | 1 | 0.034
C1b+16311 | 11578 | 6 | 0.207
C1b+16311 | 12173 | 1 | 0.034
C1b+16311 | 12358 | 1 | 0.034
C1b+16311 | 12441 | 1 | 0.034
C1b+16311 | 12678 | 10 | 0.345
C1b+16311 | 12813 | 1 | 0.034
C1b+16311 | 13135 | 3 | 0.103
C1b+16311 | 13145 | 1 | 0.034
C1b+16311 | 13401 | 1 | 0.034
C1b+16311 | 13608 | 1 | 0.034
C1b+16311 | 13681 | 1 | 0.034
C1b+16311 | 13711 | 1 | 0.034
C1b+16311 | 13836C | 1 | 0.034
C1b+16311 | 13943 | 1 | 0.034
C1b+16311 | 143 | 1 | 0.034
C1b+16311 | 14325 | 1 | 0.034
C1b+16311 | 14359 | 1 | 0.034
C1b+16311 | 14587 | 1 | 0.034
C1b+16311 | 146 | 2 | 0.069
C1b+16311 | 150 | 5 | 0.172
C1b+16311 | 152 | 7 | 0.241
C1b+16311 | 15355 | 1 | 0.034
C1b+16311 | 15670 | 1 | 0.034
C1b+16311 | 15924 | 1 | 0.034
C1b+16311 | 16126 | 1 | 0.034
C1b+16311 | 16163T | 6 | 0.207
C1b+16311 | 16176 | 3 | 0.103
C1b+16311 | 16179A | 10 | 0.345
C1b+16311 | 16185 | 1 | 0.034
C1b+16311 | 16189 | 1 | 0.034
C1b+16311 | 16239 | 1 | 0.034
C1b+16311 | 16243 | 2 | 0.069
C1b+16311 | 16274 | 1 | 0.034
C1b+16311 | 16284 | 1 | 0.034
C1b+16311 | 16294 | 1 | 0.034
C1b+16311 | 16356 | 6 | 0.207
C1b+16311 | 16365 | 1 | 0.034
C1b+16311 | 16540G | 2 | 0.069
C1b+16311 | 1719 | 1 | 0.034
C1b+16311 | 194 | 1 | 0.034
C1b+16311 | 195 | 1 | 0.034
C1b+16311 | 226 | 1 | 0.034
C1b+16311 | 2280 | 1 | 0.034
C1b+16311 | 235 | 1 | 0.034
C1b+16311 | 240 | 1 | 0.034
C1b+16311 | 258 | 1 | 0.034
C1b+16311 | 259 | 1 | 0.034
C1b+16311 | 3010 | 1 | 0.034
C1b+16311 | 3398 | 8 | 0.276
C1b+16311 | 3531 | 1 | 0.034
C1b+16311 | 3816 | 1 | 0.034
C1b+16311 | 382A | 1 | 0.034
C1b+16311 | 419 | 1 | 0.034
C1b+16311 | 4928 | 1 | 0.034
C1b+16311 | 512 | 1 | 0.034
C1b+16311 | 513 | 1 | 0.034
C1b+16311 | 5442 | 2 | 0.069
C1b+16311 | 5530 | 2 | 0.069
C1b+16311 | 5580 | 1 | 0.034
C1b+16311 | 5581 | 2 | 0.069
C1b+16311 | 5981 | 1 | 0.034
C1b+16311 | 709 | 10 | 0.345
C1b+16311 | 7337 | 1 | 0.034
C1b+16311 | 7521 | 1 | 0.034
C1b+16311 | 7678 | 1 | 0.034
C1b+16311 | 7746 | 1 | 0.034
C1b+16311 | 789 | 2 | 0.069
C1b+16311 | 8251 | 1 | 0.034
C1b+16311 | 9037 | 1 | 0.034
C1b+16311 | 9048 | 8 | 0.276
C1b+16311 | 9254 | 1 | 0.034
C1b+16311 | 9389 | 1 | 0.034
C1b+16311 | 961 | 1 | 0.034
C1b+16311 | 965.1C | 1 | 0.034
C1b+16311 | 9755 | 1 | 0.034
C1b+16311 | 9770 | 1 | 0.034
C1b+16311 | 9782 | 1 | 0.034
C1b+16311 | 9801 | 1 | 0.034
C1b1 | 10151 | 1 | 0.014
C1b1 | 11314 | 3 | 0.041
C1b1 | 12285 | 1 | 0.014

C1b1 | 14040 | 3 | 0.041
C1b1 | 143 | 7 | 0.096
C1b1 | 146 | 4 | 0.055
C1b1 | 150 | 11 | 0.151
C1b1 | 15924 | 4 | 0.055
C1b1 | 16092 | 3 | 0.041
C1b1 | 16129 | 12 | 0.164
C1b1 | 16224 | 1 | 0.014
C1b1 | 16241 | 4 | 0.055
C1b1 | 16274 | 18 | 0.247
C1b1 | 16400 | 2 | 0.027
C1b1 | 16463 | 1 | 0.014
C1b1 | 210 | 4 | 0.055
C1b1 | 228 | 3 | 0.041
C1b1 | 46G | 1 | 0.014
C1b1 | 5493 | 2 | 0.027
C1b1 | 6962 | 1 | 0.014
C1b1 | 8389 | 1 | 0.014
C1b1 | 8392 | 2 | 0.027
C1b1 | 8555 | 2 | 0.027
C1b1 | 93 | 1 | 0.014
C1b10 | 10184 | 2 | 0.2
C1b10 | 14482 | 5 | 0.5
C1b10 | 152 | 6 | 0.6
C1b10 | 15622 | 5 | 0.5
C1b10 | 15772 | 1 | 0.1
C1b10 | 16181 | 2 | 0.2
C1b10 | 16189 | 4 | 0.4
C1b10 | 16362 | 1 | 0.1
C1b10 | 310 | 1 | 0.1
C1b10 | 316C | 1 | 0.1
C1b10 | 5414 | 1 | 0.1
C1b10 | 857 | 1 | 0.1
C1b10 | 9099 | 1 | 0.1
C1b11 | 10514 | 44 | 0.44
C1b11 | 12172 | 3 | 0.03
C1b11 | 146 | 15 | 0.15
C1b11 | 150 | 1 | 0.01
C1b11 | 1517 | 1 | 0.01
C1b11 | 152 | 1 | 0.01
C1b11 | 15670 | 1 | 0.01
C1b11 | 15924 | 43 | 0.43
C1b11 | 16092 | 1 | 0.01
C1b11 | 16093 | 3 | 0.03
C1b11 | 16129 | 7 | 0.07
C1b11 | 16148G | 1 | 0.01
C1b11 | 16201 | 1 | 0.01
C1b11 | 16232 | 1 | 0.01
C1b11 | 16234 | 22 | 0.22
C1b11 | 16249 | 3 | 0.03
C1b11 | 16274 | 1 | 0.01
C1b11 | 16311 | 6 | 0.06
C1b11 | 16319 | 1 | 0.01
C1b11 | 16433 | 1 | 0.01
C1b11 | 194 | 83 | 0.83
C1b11 | 3010 | 1 | 0.01
C1b11 | 3397 | 1 | 0.01
C1b11 | 33G | 15 | 0.15
C1b11 | 345 | 1 | 0.01
C1b11 | 4370 | 1 | 0.01
C1b11 | 485 | 5 | 0.05
C1b11 | 4925 | 1 | 0.01
C1b11 | 525d | 1 | 0.01
C1b11 | 5899.1C | 1 | 0.01
C1b11 | 60.1T | 62 | 0.62
C1b11 | 6086 | 3 | 0.03
C1b11 | 63d | 63 | 0.63
C1b11 | 72 | 28 | 0.28
C1b11 | 7757 | 42 | 0.42
C1b11 | 8149 | 3 | 0.03
C1b11 | 8334 | 1 | 0.01
C1b11 | 8512 | 1 | 0.01
C1b11 | 8848 | 3 | 0.03
C1b11 | 9533 | 2 | 0.02
C1b11 | 9554 | 1 | 0.01
C1b12 | 11419 | 2 | 0.028
C1b12 | 12406 | 1 | 0.014
C1b12 | 16186 | 3 | 0.042
C1b12 | 16291 | 1 | 0.014
C1b12 | 16293 | 3 | 0.042
C1b12 | 16384 | 3 | 0.042
C1b12 | 16385 | 7 | 0.097
C1b12 | 16399 | 1 | 0.014
C1b12 | 16509 | 12 | 0.167
C1b12 | 182 | 1 | 0.014
C1b12 | 185 | 1 | 0.014
C1b12 | 199 | 3 | 0.042
C1b12 | 222 | 1 | 0.014
C1b12 | 266 | 1 | 0.014
C1b12 | 288 | 1 | 0.014
C1b12 | 316d | 1 | 0.014
C1b12 | 3405 | 1 | 0.014
C1b12 | 9456 | 1 | 0.014
C1b13 | 105-110d | 1 | 0.026
C1b13 | 11270 | 1 | 0.026
C1b13 | 16067 | 1 | 0.026
C1b13 | 16311 | 17 | 0.447
C1b13 | 16343 | 2 | 0.053
C1b13 | 188 | 1 | 0.026
C1b13 | 198 | 1 | 0.026
C1b13 | 279 | 13 | 0.342
C1b13a | 15482 | 2 | 0.051
C1b13a | 16129 | 2 | 0.051
C1b13a | 16131 | 13 | 0.333
C1b13a | 16207 | 2 | 0.051
C1b13a | 195 | 2 | 0.051
C1b13a | 234 | 2 | 0.051
C1b13a1 | 10238 | 1 | 0.026
C1b13a1 | 10927 | 1 | 0.026
C1b13a1 | 14587 | 2 | 0.053
C1b13a1 | 15071 | 1 | 0.026

C1b13a1 | 15781 | 1 | 0.026
C1b13a1 | 15884 | 1 | 0.026
C1b13a1 | 16189 | 1 | 0.026
C1b13a1 | 16263 | 1 | 0.026
C1b13a1 | 16298G | 1 | 0.026
C1b13a1 | 16362 | 1 | 0.026
C1b13a1 | 16448 | 1 | 0.026
C1b13a1 | 16471 | 14 | 0.368
C1b13a1 | 214 | 15 | 0.395
C1b13a1 | 4375 | 1 | 0.026
C1b13a1 | 5985 | 1 | 0.026
C1b13a1 | 7859 | 1 | 0.026
C1b13a1 | 7897 | 1 | 0.026
C1b13a1 | 8537 | 1 | 0.026
C1b13a1 | 8825 | 1 | 0.026
C1b13b | 103 | 1 | 0.027
C1b13b | 150 | 2 | 0.054
C1b13b | 15262 | 1 | 0.027
C1b13b | 16131 | 1 | 0.027
C1b13b | 16137 | 1 | 0.027
C1b13b | 16219 | 13 | 0.351
C1b13b | 16301 | 13 | 0.351
C1b13b | 16311 | 1 | 0.027
C1b13b | 4452 | 1 | 0.027
C1b13b | 5414 | 1 | 0.027
C1b13b | 5894 | 1 | 0.027
C1b13b | 6248 | 1 | 0.027
C1b13b | 7621 | 1 | 0.027
C1b13c | 1346 | 1 | 0.028
C1b13c | 16131 | 13 | 0.361
C1b13c | 194 | 2 | 0.056
C1b13c | 5775 | 2 | 0.056
C1b13c1 | 10742 | 1 | 0.024
C1b13c1 | 11963 | 1 | 0.024
C1b13c1 | 16075 | 1 | 0.024
C1b13c1 | 16221 | 1 | 0.024
C1b13c1 | 16234 | 1 | 0.024
C1b13c1 | 16235 | 1 | 0.024
C1b13c1 | 16266 | 2 | 0.049
C1b13c1 | 16294 | 15 | 0.366
C1b13c1 | 194 | 1 | 0.024
C1b13d | 11117 | 1 | 0.077
C1b13d | 13674 | 1 | 0.077
C1b13d | 16166 | 1 | 0.077
C1b13d | 16189 | 1 | 0.077
C1b13d | 16294 | 2 | 0.154
C1b13d | 16319 | 1 | 0.077
C1b13d | 16335 | 2 | 0.154
C1b13d | 498.1C | 1 | 0.077
C1b13e | 103 | 1 | 0.022
C1b13e | 12175 | 1 | 0.022
C1b13e | 14693 | 1 | 0.022
C1b13e | 150 | 1 | 0.022
C1b13e | 16075 | 1 | 0.022
C1b13e | 16093 | 1 | 0.022
C1b13e | 16119 | 1 | 0.022
C1b13e | 16136 | 2 | 0.044
C1b13e | 16221 | 1 | 0.022
C1b13e | 16234 | 1 | 0.022
C1b13e | 16235 | 1 | 0.022
C1b13e | 16293 | 1 | 0.022
C1b13e | 16359G | 13 | 0.289
C1b13e | 189 | 1 | 0.022
C1b13e | 7269 | 1 | 0.022
C1b14 | 10040 | 1 | 0.1
C1b14 | 13813 | 1 | 0.1
C1b14 | 13953 | 2 | 0.2
C1b14 | 14215 | 1 | 0.1
C1b14 | 153 | 5 | 0.5
C1b14 | 16172 | 2 | 0.2
C1b14 | 16192 | 5 | 0.5
C1b14 | 16336 | 1 | 0.1
C1b14 | 16348 | 1 | 0.1
C1b14 | 195 | 1 | 0.1
C1b14 | 215 | 5 | 0.5
C1b14 | 228 | 1 | 0.1
C1b14 | 241 | 1 | 0.1
C1b14 | 310 | 1 | 0.1
C1b14 | 3204 | 1 | 0.1
C1b14 | 6261 | 1 | 0.1
C1b14 | 6872 | 2 | 0.2
C1b14 | 709 | 2 | 0.2
C1b14 | 7985G | 1 | 0.1
C1b14 | 8047 | 1 | 0.1
C1b2 | 11866 | 5 | 0.066
C1b2 | 12574 | 1 | 0.013
C1b2 | 12651 | 4 | 0.053
C1b2 | 13135 | 1 | 0.013
C1b2 | 14053 | 1 | 0.013
C1b2 | 14067 | 1 | 0.013
C1b2 | 146 | 5 | 0.066
C1b2 | 14883 | 1 | 0.013
C1b2 | 152 | 1 | 0.013
C1b2 | 16154 | 4 | 0.053
C1b2 | 16172 | 1 | 0.013
C1b2 | 16176 | 2 | 0.026
C1b2 | 16239 | 3 | 0.039
C1b2 | 16319 | 5 | 0.066
C1b2 | 215 | 1 | 0.013
C1b2 | 228 | 1 | 0.013
C1b2 | 263 | 11 | 0.145
C1b2 | 3579 | 1 | 0.013
C1b2 | 6260 | 1 | 0.013
C1b2 | 6528 | 5 | 0.066
C1b2 | 7805 | 5 | 0.066
C1b2 | 7841T | 4 | 0.053
C1b2 | 8038 | 1 | 0.013
C1b2 | 8289.1CCCCCTCTA | 1 | 0.013
C1b2 | 9165 | 1 | 0.013
C1b2 | 9914 | 1 | 0.013
C1b3 | 10192 | 2 | 0.023

C1b3 | 10310 | 1 | 0.011
C1b3 | 10454 | 1 | 0.011
C1b3 | 106-111d | 1 | 0.011
C1b3 | 12346 | 1 | 0.011
C1b3 | 14016 | 1 | 0.011
C1b3 | 14364 | 2 | 0.023
C1b3 | 152 | 1 | 0.011
C1b3 | 15265 | 1 | 0.011
C1b3 | 16086 | 1 | 0.011
C1b3 | 16093 | 17 | 0.193
C1b3 | 16126 | 13 | 0.148
C1b3 | 16147A | 1 | 0.011
C1b3 | 16172 | 2 | 0.023
C1b3 | 16173 | 5 | 0.057
C1b3 | 16176 | 1 | 0.011
C1b3 | 16189 | 1 | 0.011
C1b3 | 16192 | 24 | 0.273
C1b3 | 16209 | 1 | 0.011
C1b3 | 16234 | 1 | 0.011
C1b3 | 16265 | 1 | 0.011
C1b3 | 16266 | 1 | 0.011
C1b3 | 16270 | 3 | 0.034
C1b3 | 16311 | 1 | 0.011
C1b3 | 16344 | 1 | 0.011
C1b3 | 16346 | 4 | 0.045
C1b3 | 16362 | 1 | 0.011
C1b3 | 16385 | 11 | 0.125
C1b3 | 16390 | 1 | 0.011
C1b3 | 16438 | 1 | 0.011
C1b3 | 16497 | 1 | 0.011
C1b3 | 16526 | 1 | 0.011
C1b3 | 185 | 1 | 0.011
C1b3 | 188 | 1 | 0.011
C1b3 | 195 | 1 | 0.011
C1b3 | 201 | 7 | 0.08
C1b3 | 203 | 1 | 0.011
C1b3 | 204 | 1 | 0.011
C1b3 | 3204 | 3 | 0.034
C1b3 | 3468 | 2 | 0.023
C1b3 | 385 | 1 | 0.011
C1b3 | 460 | 1 | 0.011
C1b3 | 463.1C | 1 | 0.011
C1b3 | 523d | 1 | 0.011
C1b3 | 5664 | 1 | 0.011
C1b3 | 6293 | 1 | 0.011
C1b3 | 72 | 5 | 0.057
C1b3 | 8567 | 1 | 0.011
C1b3 | 9115 | 3 | 0.034
C1b3 | 9644 | 3 | 0.034
C1b4 | 152 | 1 | 0.071
C1b4 | 16093 | 1 | 0.071
C1b4 | 204 | 1 | 0.071
C1b4 | 9530 | 1 | 0.071
C1b5 | 100 | 1 | 0.007
C1b5 | 103 | 1 | 0.007
C1b5 | 143 | 1 | 0.007
C1b5 | 146 | 2 | 0.015
C1b5 | 150 | 24 | 0.176
C1b5 | 152 | 2 | 0.015
C1b5 | 16086 | 2 | 0.015
C1b5 | 16092 | 18 | 0.132
C1b5 | 16093 | 5 | 0.037
C1b5 | 16096 | 1 | 0.007
C1b5 | 161 | 1 | 0.007
C1b5 | 16126 | 1 | 0.007
C1b5 | 16127 | 2 | 0.015
C1b5 | 16129 | 4 | 0.029
C1b5 | 16146 | 1 | 0.007
C1b5 | 16157 | 2 | 0.015
C1b5 | 16163 | 1 | 0.007
C1b5 | 16166 | 1 | 0.007
C1b5 | 16185 | 2 | 0.015
C1b5 | 16189 | 19 | 0.14
C1b5 | 16193 | 2 | 0.015
C1b5 | 16197G | 1 | 0.007
C1b5 | 16199 | 1 | 0.007
C1b5 | 16209 | 3 | 0.022
C1b5 | 16214 | 1 | 0.007
C1b5 | 16230 | 1 | 0.007
C1b5 | 16234 | 6 | 0.044
C1b5 | 16254 | 2 | 0.015
C1b5 | 16259 | 1 | 0.007
C1b5 | 16266 | 1 | 0.007
C1b5 | 16270 | 5 | 0.037
C1b5 | 16274 | 3 | 0.022
C1b5 | 16278 | 1 | 0.007
C1b5 | 16299 | 1 | 0.007
C1b5 | 16300 | 2 | 0.015
C1b5 | 16343 | 5 | 0.037
C1b5 | 16344 | 1 | 0.007
C1b5 | 16357 | 1 | 0.007
C1b5 | 16456 | 1 | 0.007
C1b5 | 16540A | 2 | 0.015
C1b5 | 188 | 1 | 0.007
C1b5 | 194 | 33 | 0.243
C1b5 | 199 | 1 | 0.007
C1b5 | 201 | 1 | 0.007
C1b5 | 215 | 2 | 0.015
C1b5 | 225 | 2 | 0.015
C1b5 | 228 | 1 | 0.007
C1b5 | 237 | 4 | 0.029
C1b5 | 325 | 1 | 0.007
C1b5 | 377 | 1 | 0.007
C1b5 | 485 | 3 | 0.022
C1b5 | 62 | 1 | 0.007
C1b5 | 68 | 1 | 0.007
C1b5 | 72 | 31 | 0.228
C1b5a | 146 | 30 | 0.411
C1b5a | 150 | 1 | 0.014
C1b5a | 15790 | 1 | 0.014
C1b5a | 16093 | 1 | 0.014

C1b5a | 16124 | 1 | 0.014
C1b5a | 16146 | 1 | 0.014
C1b5a | 16189 | 2 | 0.027
C1b5a | 16192 | 1 | 0.014
C1b5a | 16212 | 1 | 0.014
C1b5a | 16227 | 1 | 0.014
C1b5a | 16294 | 1 | 0.014
C1b5a | 16309 | 1 | 0.014
C1b5a | 16320 | 1 | 0.014
C1b5a | 16428 | 10 | 0.137
C1b5a | 16474C | 1 | 0.014
C1b5a | 16526 | 1 | 0.014
C1b5a | 195 | 1 | 0.014
C1b5a | 207 | 1 | 0.014
C1b5a | 214 | 1 | 0.014
C1b5a | 235 | 2 | 0.027
C1b5a | 3357T | 1 | 0.014
C1b5a | 3531 | 1 | 0.014
C1b5a | 471 | 10 | 0.137
C1b5a | 512 | 1 | 0.014
C1b5a | 523 | 10 | 0.137
C1b5a | 5605 | 1 | 0.014
C1b5a | 5821 | 1 | 0.014
C1b5a | 7628A | 1 | 0.014
C1b5a | 7747 | 1 | 0.014
C1b5a | 79T | 1 | 0.014
C1b5a | 8572 | 1 | 0.014
C1b5b | 11755 | 1 | 0.077
C1b5b | 14061 | 2 | 0.154
C1b5b | 14070 | 2 | 0.154
C1b5b | 15586 | 2 | 0.154
C1b5b | 16092 | 7 | 0.538
C1b5b | 16166 | 1 | 0.077
C1b5b | 16193 | 1 | 0.077
C1b5b | 16354 | 1 | 0.077
C1b5b | 16400 | 7 | 0.538
C1b5b | 16463 | 7 | 0.538
C1b5b | 195 | 6 | 0.462
C1b5b | 255 | 2 | 0.154
C1b5b | 331 | 2 | 0.154
C1b5b | 46G | 7 | 0.538
C1b5b | 59A | 7 | 0.538
C1b5b | 61 | 7 | 0.538
C1b5b | 62T | 7 | 0.538
C1b5b | 9449 | 1 | 0.077
C1b6 | 10310 | 1 | 0.333
C1b6 | 441G | 1 | 0.333
C1b7 | 12361 | 1 | 0.02
C1b7 | 12603 | 1 | 0.02
C1b7 | 143 | 4 | 0.082
C1b7 | 14389 | 1 | 0.02
C1b7 | 146 | 8 | 0.163
C1b7 | 152 | 8 | 0.163
C1b7 | 15300 | 1 | 0.02
C1b7 | 16058C | 1 | 0.02
C1b7 | 16078 | 2 | 0.041
C1b7 | 16092 | 1 | 0.02
C1b7 | 16124 | 1 | 0.02
C1b7 | 16129 | 7 | 0.143
C1b7 | 16140 | 3 | 0.061
C1b7 | 16146T | 2 | 0.041
C1b7 | 16172 | 18 | 0.367
C1b7 | 16175 | 2 | 0.041
C1b7 | 16179 | 2 | 0.041
C1b7 | 16185 | 3 | 0.061
C1b7 | 16221 | 2 | 0.041
C1b7 | 16261 | 1 | 0.02
C1b7 | 16468 | 1 | 0.02
C1b7 | 194 | 1 | 0.02
C1b7 | 195 | 6 | 0.122
C1b7 | 200 | 1 | 0.02
C1b7 | 204 | 13 | 0.265
C1b7 | 207 | 5 | 0.102
C1b7 | 228 | 4 | 0.082
C1b7 | 234 | 2 | 0.041
C1b7 | 335 | 1 | 0.02
C1b7 | 33G | 1 | 0.02
C1b7 | 385 | 1 | 0.02
C1b7 | 513 | 3 | 0.061
C1b7 | 5279 | 1 | 0.02
C1b7 | 709 | 1 | 0.02
C1b7 | 72 | 1 | 0.02
C1b7 | 7606 | 2 | 0.041
C1b7a | 1117 | 2 | 0.118
C1b7a | 11800 | 1 | 0.059
C1b7a | 1310 | 5 | 0.294
C1b7a | 16145 | 3 | 0.176
C1b7a | 16157 | 1 | 0.059
C1b7a | 16157A | 1 | 0.059
C1b7a | 16189 | 7 | 0.412
C1b7a | 16390 | 2 | 0.118
C1b7a | 16463 | 2 | 0.118
C1b7a | 195 | 1 | 0.059
C1b7a | 204 | 2 | 0.118
C1b7a | 207 | 2 | 0.118
C1b7a | 333 | 3 | 0.176
C1b7a | 4695 | 1 | 0.059
C1b7a | 512C | 1 | 0.059
C1b7a | 54 | 2 | 0.118
C1b7a | 5671 | 1 | 0.059
C1b7a | 60 | 2 | 0.118
C1b7a | 60.1T | 2 | 0.118
C1b7a | 64 | 1 | 0.059
C1b7a | 71d | 3 | 0.176
C1b7a | 8251 | 2 | 0.118
C1b8 | 10601 | 2 | 0.053
C1b8 | 10993 | 1 | 0.026
C1b8 | 11797 | 1 | 0.026
C1b8 | 12136 | 1 | 0.026
C1b8 | 12631G | 2 | 0.053
C1b8 | 13602 | 1 | 0.026

C1b8 | 143 | 1 | 0.026
C1b8 | 146 | 5 | 0.132
C1b8 | 152 | 1 | 0.026
C1b8 | 15244 | 1 | 0.026
C1b8 | 16000 | 2 | 0.053
C1b8 | 16094 | 2 | 0.053
C1b8 | 16104 | 3 | 0.079
C1b8 | 16146 | 1 | 0.026
C1b8 | 16172 | 2 | 0.053
C1b8 | 16188 | 2 | 0.053
C1b8 | 16195 | 2 | 0.053
C1b8 | 16292 | 20 | 0.526
C1b8 | 16343 | 4 | 0.105
C1b8 | 16357 | 4 | 0.105
C1b8 | 1692C | 1 | 0.026
C1b8 | 195 | 2 | 0.053
C1b8 | 204 | 1 | 0.026
C1b8 | 285 | 1 | 0.026
C1b8 | 3442 | 1 | 0.026
C1b8 | 3764 | 1 | 0.026
C1b8 | 4496 | 2 | 0.053
C1b8 | 4924 | 1 | 0.026
C1b8 | 525.1AC | 1 | 0.026
C1b8 | 5563 | 2 | 0.053
C1b8 | 5821 | 4 | 0.105
C1b8 | 5894C | 2 | 0.053
C1b8 | 636 | 3 | 0.079
C1b8 | 6605 | 1 | 0.026
C1b8 | 7076 | 1 | 0.026
C1b8 | 8078 | 1 | 0.026
C1b8 | 8155 | 1 | 0.026
C1b8 | 8389 | 2 | 0.053
C1b8 | 9025 | 1 | 0.026
C1b8 | 960.1C | 1 | 0.026
C1b8a | 14813 | 1 | 0.1
C1b8a | 150 | 1 | 0.1
C1b8a | 16265C | 1 | 0.1
C1b8a | 16335 | 1 | 0.1
C1b8a | 6260 | 1 | 0.1
C1b8a | 6473 | 2 | 0.2
C1b9 | 11440 | 1 | 0.017
C1b9 | 11944 | 1 | 0.017
C1b9 | 13434 | 1 | 0.017
C1b9 | 14226 | 1 | 0.017
C1b9 | 14562G | 1 | 0.017
C1b9 | 146 | 1 | 0.017
C1b9 | 16172 | 1 | 0.017
C1b9 | 16179 | 1 | 0.017
C1b9 | 16400 | 10 | 0.167
C1b9 | 1719 | 1 | 0.017
C1b9 | 195 | 1 | 0.017
C1b9 | 198 | 9 | 0.15
C1b9 | 210 | 1 | 0.017
C1b9 | 247 | 1 | 0.017
C1b9 | 5237 | 1 | 0.017
C1b9 | 5887 | 1 | 0.017
C1c | 10053 | 1 | 0.004
C1c | 10084 | 2 | 0.008
C1c | 1018 | 8 | 0.032
C1c | 1019 | 3 | 0.012
C1c | 10352 | 1 | 0.004
C1c | 10373 | 5 | 0.02
C1c | 10394A | 1 | 0.004
C1c | 10604 | 1 | 0.004
C1c | 10688 | 1 | 0.004
C1c | 10744 | 2 | 0.008
C1c | 11009 | 2 | 0.008
C1c | 11087 | 1 | 0.004
C1c | 11170 | 1 | 0.004
C1c | 11242 | 1 | 0.004
C1c | 11278 | 1 | 0.004
C1c | 11299 | 1 | 0.004
C1c | 11464 | 1 | 0.004
C1c | 115 | 22 | 0.088
C1c | 115d | 18 | 0.072
C1c | 116d | 22 | 0.088
C1c | 11731 | 5 | 0.02
C1c | 11887 | 1 | 0.004
C1c | 1189 | 1 | 0.004
C1c | 11905 | 3 | 0.012
C1c | 12071 | 2 | 0.008
C1c | 12193 | 1 | 0.004
C1c | 12248 | 1 | 0.004
C1c | 12265 | 2 | 0.008
C1c | 12342 | 1 | 0.004
C1c | 12397 | 1 | 0.004
C1c | 12696 | 2 | 0.008
C1c | 12774 | 1 | 0.004
C1c | 12940 | 1 | 0.004
C1c | 131 | 3 | 0.012
C1c | 13135 | 1 | 0.004
C1c | 13153 | 1 | 0.004
C1c | 13362 | 22 | 0.088
C1c | 13395 | 22 | 0.088
C1c | 13500 | 1 | 0.004
C1c | 13650 | 1 | 0.004
C1c | 13759 | 1 | 0.004
C1c | 13866 | 1 | 0.004
C1c | 13879 | 1 | 0.004
C1c | 13934 | 1 | 0.004
C1c | 14025 | 1 | 0.004
C1c | 14118 | 3 | 0.012
C1c | 14129 | 2 | 0.008
C1c | 14207 | 1 | 0.004
C1c | 14226 | 1 | 0.004
C1c | 143 | 3 | 0.012
C1c | 14305 | 3 | 0.012
C1c | 14384 | 2 | 0.008
C1c | 14494 | 1 | 0.004
C1c | 14515 | 1 | 0.004
C1c | 14581 | 4 | 0.016

C1c | 146 | 17 | 0.068
C1c | 1462 | 1 | 0.004
C1c | 14830A | 2 | 0.008
C1c | 14861 | 1 | 0.004
C1c | 150 | 11 | 0.044
C1c | 15007 | 8 | 0.032
C1c | 15064 | 1 | 0.004
C1c | 15077 | 12 | 0.048
C1c | 151 | 2 | 0.008
C1c | 15106 | 1 | 0.004
C1c | 152 | 44 | 0.175
C1c | 15244 | 5 | 0.02
C1c | 15262 | 1 | 0.004
C1c | 15263 | 1 | 0.004
C1c | 153 | 3 | 0.012
C1c | 15323 | 2 | 0.008
C1c | 15596 | 1 | 0.004
C1c | 15706 | 1 | 0.004
C1c | 15740 | 2 | 0.008
C1c | 15849 | 3 | 0.012
C1c | 15924 | 1 | 0.004
C1c | 15954 | 7 | 0.028
C1c | 16051 | 2 | 0.008
C1c | 16075 | 1 | 0.004
C1c | 16076 | 1 | 0.004
C1c | 16086 | 2 | 0.008
C1c | 16091 | 1 | 0.004
C1c | 16092 | 5 | 0.02
C1c | 16093 | 7 | 0.028
C1c | 16111 | 1 | 0.004
C1c | 16126 | 1 | 0.004
C1c | 16129 | 5 | 0.02
C1c | 16145 | 1 | 0.004
C1c | 16148 | 1 | 0.004
C1c | 16163.1T | 1 | 0.004
C1c | 16169 | 5 | 0.02
C1c | 16172 | 2 | 0.008
C1c | 16187 | 1 | 0.004
C1c | 16189 | 5 | 0.02
C1c | 16192A | 4 | 0.016
C1c | 16207 | 5 | 0.02
C1c | 16209 | 4 | 0.016
C1c | 16212 | 1 | 0.004
C1c | 16234 | 3 | 0.012
C1c | 16242 | 2 | 0.008
C1c | 16243 | 2 | 0.008
C1c | 16249 | 9 | 0.036
C1c | 16260 | 2 | 0.008
C1c | 16270 | 2 | 0.008
C1c | 16274 | 1 | 0.004
C1c | 16278 | 3 | 0.012
C1c | 16295 | 2 | 0.008
C1c | 16297 | 1 | 0.004
C1c | 16301 | 1 | 0.004
C1c | 16311 | 3 | 0.012
C1c | 16316 | 6 | 0.024
C1c | 16319 | 5 | 0.02
C1c | 16343 | 5 | 0.02
C1c | 16357 | 2 | 0.008
C1c | 16362 | 21 | 0.084
C1c | 16381 | 1 | 0.004
C1c | 16390 | 7 | 0.028
C1c | 16456 | 1 | 0.004
C1c | 16468 | 1 | 0.004
C1c | 16485 | 2 | 0.008
C1c | 16490 | 2 | 0.008
C1c | 16491 | 2 | 0.008
C1c | 16496 | 2 | 0.008
C1c | 16526 | 12 | 0.048
C1c | 1694 | 1 | 0.004
C1c | 179 | 3 | 0.012
C1c | 18 | 1 | 0.004
C1c | 182 | 1 | 0.004
C1c | 183 | 1 | 0.004
C1c | 185 | 1 | 0.004
C1c | 188 | 2 | 0.008
C1c | 196 | 1 | 0.004
C1c | 199 | 1 | 0.004
C1c | 200 | 1 | 0.004
C1c | 201 | 1 | 0.004
C1c | 204 | 2 | 0.008
C1c | 207 | 2 | 0.008
C1c | 2071 | 1 | 0.004
C1c | 214 | 1 | 0.004
C1c | 228 | 2 | 0.008
C1c | 2294 | 3 | 0.012
C1c | 2362 | 1 | 0.004
C1c | 2366 | 1 | 0.004
C1c | 237 | 2 | 0.008
C1c | 2404 | 4 | 0.016
C1c | 2707 | 1 | 0.004
C1c | 2833 | 4 | 0.016
C1c | 293 | 3 | 0.012
C1c | 3083 | 1 | 0.004
C1c | 310 | 1 | 0.004
C1c | 3158.1T | 1 | 0.004
C1c | 3316 | 1 | 0.004
C1c | 3394 | 1 | 0.004
C1c | 3398 | 1 | 0.004
C1c | 3531 | 1 | 0.004
C1c | 3801 | 1 | 0.004
C1c | 3948 | 2 | 0.008
C1c | 3999 | 1 | 0.004
C1c | 4052 | 1 | 0.004
C1c | 4117 | 1 | 0.004
C1c | 4216 | 1 | 0.004
C1c | 4233 | 1 | 0.004
C1c | 4353 | 2 | 0.008
C1c | 4394 | 22 | 0.088
C1c | 4479 | 1 | 0.004
C1c | 4506 | 1 | 0.004

C1c | 4654 | 1 | 0.004
C1c | 4702 | 8 | 0.032
C1c | 4784 | 2 | 0.008
C1c | 4805 | 5 | 0.02
C1c | 4892 | 7 | 0.028
C1c | 4965 | 1 | 0.004
C1c | 5046 | 3 | 0.012
C1c | 5147 | 1 | 0.004
C1c | 5162 | 1 | 0.004
C1c | 5201 | 1 | 0.004
C1c | 523 | 5 | 0.02
C1c | 5294 | 1 | 0.004
C1c | 5333 | 2 | 0.008
C1c | 538C | 1 | 0.004
C1c | 5493 | 2 | 0.008
C1c | 55 | 2 | 0.008
C1c | 5554G | 1 | 0.004
C1c | 5581 | 8 | 0.032
C1c | 56 | 2 | 0.008
C1c | 5628 | 1 | 0.004
C1c | 5899.1C | 1 | 0.004
C1c | 5899.3C | 1 | 0.004
C1c | 6260 | 4 | 0.016
C1c | 6480 | 4 | 0.016
C1c | 6554 | 1 | 0.004
C1c | 6575 | 2 | 0.008
C1c | 6707 | 1 | 0.004
C1c | 6947 | 3 | 0.012
C1c | 7055 | 1 | 0.004
C1c | 709 | 4 | 0.016
C1c | 71.1G | 1 | 0.004
C1c | 710 | 1 | 0.004
C1c | 7118 | 1 | 0.004
C1c | 7124 | 1 | 0.004
C1c | 7150 | 2 | 0.008
C1c | 7253 | 1 | 0.004
C1c | 7278 | 2 | 0.008
C1c | 7444 | 1 | 0.004
C1c | 7474d | 1 | 0.004
C1c | 749 | 1 | 0.004
C1c | 7766 | 1 | 0.004
C1c | 7853 | 2 | 0.008
C1c | 7948 | 2 | 0.008
C1c | 8143 | 1 | 0.004
C1c | 8251 | 1 | 0.004
C1c | 8269 | 1 | 0.004
C1c | 8308 | 1 | 0.004
C1c | 8310 | 2 | 0.008
C1c | 8380 | 1 | 0.004
C1c | 8450 | 1 | 0.004
C1c | 8477 | 1 | 0.004
C1c | 85 | 1 | 0.004
C1c | 8565 | 1 | 0.004
C1c | 8567 | 1 | 0.004
C1c | 8618 | 22 | 0.088
C1c | 8668 | 5 | 0.02
C1c | 8700T | 2 | 0.008
C1c | 8973 | 2 | 0.008
C1c | 8994 | 1 | 0.004
C1c | 9055 | 4 | 0.016
C1c | 9091T | 1 | 0.004
C1c | 914 | 1 | 0.004
C1c | 9204 | 1 | 0.004
C1c | 930 | 1 | 0.004
C1c | 94 | 2 | 0.008
C1c | 9554 | 2 | 0.008
C1c | 961 | 2 | 0.008
C1c | 9689 | 1 | 0.004
C1c | 9854 | 1 | 0.004
C1c | 9947 | 8 | 0.032
C1c+195 | 10 | 1 | 0.007
C1c+195 | 10915 | 1 | 0.007
C1c+195 | 115d | 1 | 0.007
C1c+195 | 12372 | 1 | 0.007
C1c+195 | 12378 | 1 | 0.007
C1c+195 | 12425 | 1 | 0.007
C1c+195 | 1243 | 1 | 0.007
C1c+195 | 12687 | 1 | 0.007
C1c+195 | 13153 | 1 | 0.007
C1c+195 | 14180 | 1 | 0.007
C1c+195 | 143 | 2 | 0.015
C1c+195 | 14581 | 1 | 0.007
C1c+195 | 146 | 1 | 0.007
C1c+195 | 152 | 1 | 0.007
C1c+195 | 15466 | 1 | 0.007
C1c+195 | 16129 | 1 | 0.007
C1c+195 | 16189 | 2 | 0.015
C1c+195 | 16192 | 1 | 0.007
C1c+195 | 16209 | 1 | 0.007
C1c+195 | 16221 | 1 | 0.007
C1c+195 | 16261 | 1 | 0.007
C1c+195 | 16291 | 2 | 0.015
C1c+195 | 16294 | 4 | 0.03
C1c+195 | 16311 | 6 | 0.044
C1c+195 | 16463 | 4 | 0.03
C1c+195 | 16526 | 1 | 0.007
C1c+195 | 185 | 1 | 0.007
C1c+195 | 199 | 1 | 0.007
C1c+195 | 204 | 1 | 0.007
C1c+195 | 214 | 4 | 0.03
C1c+195 | 2731G | 1 | 0.007
C1c+195 | 3221 | 1 | 0.007
C1c+195 | 3316 | 1 | 0.007
C1c+195 | 3333 | 1 | 0.007
C1c+195 | 335 | 1 | 0.007
C1c+195 | 3744 | 1 | 0.007
C1c+195 | 3808 | 1 | 0.007
C1c+195 | 3897 | 1 | 0.007
C1c+195 | 4092 | 1 | 0.007
C1c+195 | 493 | 4 | 0.03
C1c+195 | 5054 | 1 | 0.007

C1c+195 | 5581 | 2 | 0.015
C1c+195 | 6059 | 1 | 0.007
C1c+195 | 6503 | 1 | 0.007
C1c+195 | 6531 | 1 | 0.007
C1c+195 | 7598 | 1 | 0.007
C1c+195 | 7837 | 1 | 0.007
C1c1 | 11377 | 1 | 0.007
C1c1 | 146 | 14 | 0.093
C1c1 | 16129 | 1 | 0.007
C1c1 | 16189 | 1 | 0.007
C1c1 | 16317 | 1 | 0.007
C1c1 | 204 | 2 | 0.013
C1c1 | 2833 | 2 | 0.013
C1c1 | 5075 | 1 | 0.007
C1c1 | 5471 | 1 | 0.007
C1c1 | 573d | 2 | 0.013
C1c1 | 8251 | 1 | 0.007
C1c1a | 12642 | 2 | 0.014
C1c1a | 2735 | 1 | 0.007
C1c1b | 10927 | 1 | 0.007
C1c1b | 11866 | 2 | 0.014
C1c1b | 14178 | 1 | 0.007
C1c1b | 14690 | 1 | 0.007
C1c1b | 14830A | 1 | 0.007
C1c1b | 16189 | 1 | 0.007
C1c1b | 16266 | 1 | 0.007
C1c1b | 16362 | 2 | 0.014
C1c1b | 7149 | 1 | 0.007
C1c1b | 8540 | 1 | 0.007
C1c1b | 9380 | 1 | 0.007
C1c2 | 1055G | 1 | 0.007
C1c2 | 11455 | 1 | 0.007
C1c2 | 12473 | 1 | 0.007
C1c2 | 13717 | 1 | 0.007
C1c2 | 143 | 1 | 0.007
C1c2 | 152 | 1 | 0.007
C1c2 | 15235 | 1 | 0.007
C1c2 | 16368 | 2 | 0.014
C1c2 | 310 | 1 | 0.007
C1c2 | 315.2C | 1 | 0.007
C1c2 | 3678 | 1 | 0.007
C1c2 | 3832 | 1 | 0.007
C1c2 | 450 | 1 | 0.007
C1c2 | 5196 | 1 | 0.007
C1c2 | 5471 | 1 | 0.007
C1c2 | 64 | 1 | 0.007
C1c3 | 11011 | 1 | 0.009
C1c3 | 12545 | 1 | 0.009
C1c3 | 15140 | 1 | 0.009
C1c3 | 15479 | 3 | 0.028
C1c3 | 15497 | 3 | 0.028
C1c3 | 16184 | 1 | 0.009
C1c3 | 16362 | 1 | 0.009
C1c3 | 310 | 1 | 0.009
C1c3 | 3202 | 1 | 0.009
C1c3 | 4316 | 1 | 0.009
C1c3 | 4435 | 2 | 0.019
C1c4 | 12372 | 1 | 0.009
C1c4 | 12618 | 1 | 0.009
C1c4 | 13321 | 1 | 0.009
C1c4 | 13879 | 1 | 0.009
C1c4 | 14581 | 1 | 0.009
C1c4 | 14769 | 1 | 0.009
C1c4 | 15077 | 1 | 0.009
C1c4 | 15119 | 1 | 0.009
C1c4 | 152 | 3 | 0.028
C1c4 | 15313 | 1 | 0.009
C1c4 | 15715 | 2 | 0.019
C1c4 | 16104 | 2 | 0.019
C1c4 | 16256 | 1 | 0.009
C1c4 | 16319 | 1 | 0.009
C1c4 | 200 | 1 | 0.009
C1c4 | 204 | 1 | 0.009
C1c4 | 2486 | 1 | 0.009
C1c4 | 328 | 1 | 0.009
C1c4 | 4315T | 1 | 0.009
C1c4 | 5118 | 1 | 0.009
C1c4 | 5813 | 1 | 0.009
C1c4 | 709 | 1 | 0.009
C1c4 | 9156 | 1 | 0.009
C1c4 | 9617 | 2 | 0.019
C1c4 | 9657 | 1 | 0.009
C1c5 | 10 | 1 | 0.008
C1c5 | 10197 | 1 | 0.008
C1c5 | 115d | 1 | 0.008
C1c5 | 13359 | 1 | 0.008
C1c5 | 146 | 1 | 0.008
C1c5 | 16127T | 10 | 0.078
C1c5 | 16189 | 1 | 0.008
C1c5 | 16309 | 10 | 0.078
C1c5 | 16319 | 9 | 0.07
C1c5 | 16354 | 5 | 0.039
C1c5 | 195 | 11 | 0.085
C1c5 | 2690 | 1 | 0.008
C1c5 | 309d | 1 | 0.008
C1c5 | 408A | 1 | 0.008
C1c5 | 6366 | 6 | 0.047
C1c5 | 6392 | 6 | 0.047
C1c6 | 10853 | 1 | 0.009
C1c6 | 114 | 2 | 0.018
C1c6 | 12092 | 3 | 0.027
C1c6 | 13105 | 1 | 0.009
C1c6 | 13707 | 1 | 0.009
C1c6 | 152 | 1 | 0.009
C1c6 | 15670 | 2 | 0.018
C1c6 | 16320 | 1 | 0.009
C1c6 | 3693 | 1 | 0.009
C1c6 | 3766 | 1 | 0.009
C1c6 | 6272 | 1 | 0.009
C1c6 | 9230 | 1 | 0.009
C1c7 | 14979 | 2 | 0.019

C1c7 | 16213 | 2 | 0.019
C1c7 | 16362 | 2 | 0.019
C1c8 | 13839 | 1 | 0.01
C1c8 | 15613 | 1 | 0.01
C1c8 | 16357 | 1 | 0.01
C1c8 | 16512 | 1 | 0.01
C1d | 10 | 1 | 0.006
C1d | 106 | 2 | 0.013
C1d | 10619 | 1 | 0.006
C1d | 10646 | 1 | 0.006
C1d | 10750 | 1 | 0.006
C1d | 10978 | 1 | 0.006
C1d | 1100 | 1 | 0.006
C1d | 11020 | 1 | 0.006
C1d | 11025 | 2 | 0.013
C1d | 11087 | 1 | 0.006
C1d | 11090T | 10 | 0.064
C1d | 11150 | 2 | 0.013
C1d | 11437 | 2 | 0.013
C1d | 11482 | 1 | 0.006
C1d | 11935 | 1 | 0.006
C1d | 1211 | 1 | 0.006
C1d | 12372 | 1 | 0.006
C1d | 12490 | 2 | 0.013
C1d | 12501 | 11 | 0.07
C1d | 12561 | 2 | 0.013
C1d | 12717 | 2 | 0.013
C1d | 1282 | 2 | 0.013
C1d | 12879 | 10 | 0.064
C1d | 12937 | 2 | 0.013
C1d | 13471 | 1 | 0.006
C1d | 13604C | 1 | 0.006
C1d | 13748 | 1 | 0.006
C1d | 14020 | 1 | 0.006
C1d | 14053 | 1 | 0.006
C1d | 14122 | 3 | 0.019
C1d | 14128 | 2 | 0.013
C1d | 14207 | 1 | 0.006
C1d | 143 | 4 | 0.025
C1d | 14530 | 2 | 0.013
C1d | 146 | 6 | 0.038
C1d | 14668 | 2 | 0.013
C1d | 14827 | 2 | 0.013
C1d | 14872A | 2 | 0.013
C1d | 150 | 1 | 0.006
C1d | 15004 | 2 | 0.013
C1d | 151 | 2 | 0.013
C1d | 15164 | 1 | 0.006
C1d | 15172 | 1 | 0.006
C1d | 152 | 1 | 0.006
C1d | 153 | 1 | 0.006
C1d | 15346 | 1 | 0.006
C1d | 15355 | 1 | 0.006
C1d | 15497 | 1 | 0.006
C1d | 15758 | 1 | 0.006
C1d | 15884 | 2 | 0.013
C1d | 16017 | 2 | 0.013
C1d | 16086 | 4 | 0.025
C1d | 16093 | 14 | 0.089
C1d | 16129 | 4 | 0.025
C1d | 16147 | 2 | 0.013
C1d | 16166 | 1 | 0.006
C1d | 16184 | 2 | 0.013
C1d | 16188 | 1 | 0.006
C1d | 16189 | 1 | 0.006
C1d | 16192 | 2 | 0.013
C1d | 16234 | 5 | 0.032
C1d | 16270 | 11 | 0.07
C1d | 16274 | 3 | 0.019
C1d | 16294 | 2 | 0.013
C1d | 16297 | 1 | 0.006
C1d | 16311 | 8 | 0.051
C1d | 16319 | 2 | 0.013
C1d | 16360 | 1 | 0.006
C1d | 16362 | 1 | 0.006
C1d | 16390 | 1 | 0.006
C1d | 16437 | 1 | 0.006
C1d | 16533 | 3 | 0.019
C1d | 1719 | 1 | 0.006
C1d | 185 | 2 | 0.013
C1d | 189 | 4 | 0.025
C1d | 195 | 11 | 0.07
C1d | 198 | 1 | 0.006
C1d | 199 | 1 | 0.006
C1d | 226 | 2 | 0.013
C1d | 235 | 1 | 0.006
C1d | 271 | 1 | 0.006
C1d | 310 | 2 | 0.013
C1d | 311 | 1 | 0.006
C1d | 315.2C | 2 | 0.013
C1d | 3338 | 1 | 0.006
C1d | 3439 | 1 | 0.006
C1d | 3586 | 1 | 0.006
C1d | 3606 | 1 | 0.006
C1d | 3644 | 2 | 0.013
C1d | 3831A | 2 | 0.013
C1d | 4706 | 1 | 0.006
C1d | 4907 | 2 | 0.013
C1d | 493 | 3 | 0.019
C1d | 5231 | 1 | 0.006
C1d | 5249 | 1 | 0.006
C1d | 5294 | 1 | 0.006
C1d | 5460 | 1 | 0.006
C1d | 5553 | 1 | 0.006
C1d | 573.1C | 2 | 0.013
C1d | 573.2C | 1 | 0.006
C1d | 574 | 1 | 0.006
C1d | 5806 | 1 | 0.006
C1d | 5876 | 4 | 0.025
C1d | 5899.1C | 1 | 0.006
C1d | 5988 | 4 | 0.025

C1d | 60.1T | 1 | 0.006
C1d | 6252 | 1 | 0.006
C1d | 6253 | 1 | 0.006
C1d | 629 | 1 | 0.006
C1d | 6297 | 1 | 0.006
C1d | 6340 | 1 | 0.006
C1d | 6536 | 2 | 0.013
C1d | 6569 | 1 | 0.006
C1d | 7269 | 1 | 0.006
C1d | 7618 | 1 | 0.006
C1d | 7697 | 32 | 0.204
C1d | 8463T | 1 | 0.006
C1d | 8487 | 2 | 0.013
C1d | 8520 | 1 | 0.006
C1d | 8556 | 1 | 0.006
C1d | 8670 | 2 | 0.013
C1d | 8745 | 2 | 0.013
C1d | 8756 | 1 | 0.006
C1d | 9053 | 1 | 0.006
C1d | 9056 | 4 | 0.025
C1d | 9115 | 3 | 0.019
C1d | 9132 | 2 | 0.013
C1d | 9722 | 4 | 0.025
C1d | 9758 | 2 | 0.013
C1d | 9854 | 2 | 0.013
C1d+194 | 10274 | 1 | 0.009
C1d+194 | 106-111d | 2 | 0.017
C1d+194 | 12885 | 2 | 0.017
C1d+194 | 14020 | 1 | 0.009
C1d+194 | 15440 | 2 | 0.017
C1d+194 | 16247 | 1 | 0.009
C1d+194 | 16291 | 1 | 0.009
C1d+194 | 16352 | 3 | 0.026
C1d+194 | 16362 | 3 | 0.026
C1d+194 | 195 | 1 | 0.009
C1d+194 | 200 | 3 | 0.026
C1d+194 | 315.2C | 2 | 0.017
C1d+194 | 3753 | 1 | 0.009
C1d+194 | 3849 | 2 | 0.017
C1d+194 | 4688 | 1 | 0.009
C1d+194 | 4973 | 2 | 0.017
C1d+194 | 5656 | 1 | 0.009
C1d+194 | 64 | 3 | 0.026
C1d+194 | 7828 | 1 | 0.009
C1d+194 | 9380 | 1 | 0.009
C1d1 | 10 | 1 | 0.006
C1d1 | 10211 | 1 | 0.006
C1d1 | 10295 | 1 | 0.006
C1d1 | 10688 | 2 | 0.011
C1d1 | 10991 | 1 | 0.006
C1d1 | 10995 | 1 | 0.006
C1d1 | 11015 | 1 | 0.006
C1d1 | 11016 | 2 | 0.011
C1d1 | 11087 | 2 | 0.011
C1d1 | 11090T | 19 | 0.105
C1d1 | 11167 | 2 | 0.011
C1d1 | 11347 | 2 | 0.011
C1d1 | 115 | 3 | 0.017
C1d1 | 12188A | 1 | 0.006
C1d1 | 12372 | 1 | 0.006
C1d1 | 12384 | 1 | 0.006
C1d1 | 12501 | 25 | 0.138
C1d1 | 12667 | 1 | 0.006
C1d1 | 12727 | 1 | 0.006
C1d1 | 12771 | 1 | 0.006
C1d1 | 12773 | 1 | 0.006
C1d1 | 12879 | 3 | 0.017
C1d1 | 12892 | 1 | 0.006
C1d1 | 12930 | 1 | 0.006
C1d1 | 12973 | 1 | 0.006
C1d1 | 13077A | 1 | 0.006
C1d1 | 13350 | 1 | 0.006
C1d1 | 13470 | 2 | 0.011
C1d1 | 13500 | 2 | 0.011
C1d1 | 13593 | 2 | 0.011
C1d1 | 13728 | 1 | 0.006
C1d1 | 13928C | 1 | 0.006
C1d1 | 1393 | 1 | 0.006
C1d1 | 140 | 1 | 0.006
C1d1 | 14002 | 1 | 0.006
C1d1 | 14133 | 1 | 0.006
C1d1 | 14199G | 1 | 0.006
C1d1 | 14272G | 1 | 0.006
C1d1 | 14305 | 2 | 0.011
C1d1 | 14365G | 1 | 0.006
C1d1 | 14368G | 2 | 0.011
C1d1 | 14443 | 1 | 0.006
C1d1 | 146 | 10 | 0.055
C1d1 | 14962 | 2 | 0.011
C1d1 | 15034 | 2 | 0.011
C1d1 | 151 | 2 | 0.011
C1d1 | 15106 | 6 | 0.033
C1d1 | 152 | 3 | 0.017
C1d1 | 15529A | 1 | 0.006
C1d1 | 15628 | 2 | 0.011
C1d1 | 15645 | 1 | 0.006
C1d1 | 15673 | 1 | 0.006
C1d1 | 15728 | 1 | 0.006
C1d1 | 15730 | 1 | 0.006
C1d1 | 15743A | 1 | 0.006
C1d1 | 15805 | 1 | 0.006
C1d1 | 15924 | 4 | 0.022
C1d1 | 15929 | 3 | 0.017
C1d1 | 16086 | 3 | 0.017
C1d1 | 16092 | 2 | 0.011
C1d1 | 16093 | 4 | 0.022
C1d1 | 16094 | 1 | 0.006
C1d1 | 16129 | 2 | 0.011
C1d1 | 16145 | 1 | 0.006
C1d1 | 16170 | 1 | 0.006
C1d1 | 16184 | 2 | 0.011

C1d1 | 16185 | 18 | 0.099
C1d1 | 16186 | 1 | 0.006
C1d1 | 16188 | 1 | 0.006
C1d1 | 16189 | 3 | 0.017
C1d1 | 16193d | 1 | 0.006
C1d1 | 16201 | 1 | 0.006
C1d1 | 16209 | 2 | 0.011
C1d1 | 1625 | 18 | 0.099
C1d1 | 16264 | 1 | 0.006
C1d1 | 16270 | 1 | 0.006
C1d1 | 16287 | 4 | 0.022
C1d1 | 16292 | 2 | 0.011
C1d1 | 16295 | 1 | 0.006
C1d1 | 16301 | 2 | 0.011
C1d1 | 16310 | 7 | 0.039
C1d1 | 16311 | 9 | 0.05
C1d1 | 16356 | 7 | 0.039
C1d1 | 16362 | 3 | 0.017
C1d1 | 16438 | 2 | 0.011
C1d1 | 16512 | 1 | 0.006
C1d1 | 16526 | 1 | 0.006
C1d1 | 16534C | 1 | 0.006
C1d1 | 1719 | 6 | 0.033
C1d1 | 1790 | 1 | 0.006
C1d1 | 18 | 1 | 0.006
C1d1 | 1842 | 1 | 0.006
C1d1 | 189 | 2 | 0.011
C1d1 | 194.1A | 1 | 0.006
C1d1 | 195 | 20 | 0.11
C1d1 | 199 | 7 | 0.039
C1d1 | 200 | 2 | 0.011
C1d1 | 204 | 1 | 0.006
C1d1 | 207 | 6 | 0.033
C1d1 | 215 | 1 | 0.006
C1d1 | 228 | 1 | 0.006
C1d1 | 26 | 1 | 0.006
C1d1 | 271 | 6 | 0.033
C1d1 | 2763 | 1 | 0.006
C1d1 | 2835A | 1 | 0.006
C1d1 | 3010 | 2 | 0.011
C1d1 | 311 | 1 | 0.006
C1d1 | 315.2C | 1 | 0.006
C1d1 | 327 | 2 | 0.011
C1d1 | 3338 | 4 | 0.022
C1d1 | 3366 | 1 | 0.006
C1d1 | 3438 | 2 | 0.011
C1d1 | 35 | 1 | 0.006
C1d1 | 3511 | 1 | 0.006
C1d1 | 3598 | 1 | 0.006
C1d1 | 3720 | 1 | 0.006
C1d1 | 3984 | 4 | 0.022
C1d1 | 4011 | 2 | 0.011
C1d1 | 4048 | 1 | 0.006
C1d1 | 4084 | 1 | 0.006
C1d1 | 41 | 1 | 0.006
C1d1 | 4102 | 1 | 0.006
C1d1 | 456 | 1 | 0.006
C1d1 | 4562 | 1 | 0.006
C1d1 | 4652 | 1 | 0.006
C1d1 | 4667 | 1 | 0.006
C1d1 | 47 | 1 | 0.006
C1d1 | 4928 | 2 | 0.011
C1d1 | 4947 | 18 | 0.099
C1d1 | 4955 | 2 | 0.011
C1d1 | 4991 | 2 | 0.011
C1d1 | 501 | 2 | 0.011
C1d1 | 5060 | 1 | 0.006
C1d1 | 5277 | 2 | 0.011
C1d1 | 5460 | 4 | 0.022
C1d1 | 548 | 1 | 0.006
C1d1 | 55.1T | 2 | 0.011
C1d1 | 57 | 3 | 0.017
C1d1 | 5806 | 9 | 0.05
C1d1 | 59 | 3 | 0.017
C1d1 | 5C | 1 | 0.006
C1d1 | 60 | 1 | 0.006
C1d1 | 60.1T | 1 | 0.006
C1d1 | 6260 | 1 | 0.006
C1d1 | 6332 | 1 | 0.006
C1d1 | 64 | 1 | 0.006
C1d1 | 6665 | 1 | 0.006
C1d1 | 6770 | 1 | 0.006
C1d1 | 6815 | 5 | 0.028
C1d1 | 6872 | 1 | 0.006
C1d1 | 6923 | 1 | 0.006
C1d1 | 72 | 1 | 0.006
C1d1 | 721 | 18 | 0.099
C1d1 | 7245 | 1 | 0.006
C1d1 | 7269 | 1 | 0.006
C1d1 | 7302 | 1 | 0.006
C1d1 | 7354 | 2 | 0.011
C1d1 | 7723 | 2 | 0.011
C1d1 | 8393 | 1 | 0.006
C1d1 | 8467 | 1 | 0.006
C1d1 | 8503 | 1 | 0.006
C1d1 | 8555 | 1 | 0.006
C1d1 | 8557 | 1 | 0.006
C1d1 | 8588 | 2 | 0.011
C1d1 | 8680 | 1 | 0.006
C1d1 | 8928 | 2 | 0.011
C1d1 | 9006 | 1 | 0.006
C1d1 | 9316A | 18 | 0.099
C1d1 | 9398 | 2 | 0.011
C1d1 | 94 | 1 | 0.006
C1d1 | 9559G | 2 | 0.011
C1d1 | 9591 | 2 | 0.011
C1d1 | 9722 | 1 | 0.006
C1d1a | 10978 | 1 | 0.009
C1d1a | 16209 | 1 | 0.009
C1d1a | 16357 | 1 | 0.009
C1d1a | 200 | 1 | 0.009

C1d1a | 3591 | 1 | 0.009
C1d1a | 93 | 1 | 0.009
C1d1a1 | 12820 | 1 | 0.009
C1d1a1 | 16311 | 1 | 0.009
C1d1a1 | 16357 | 1 | 0.009
C1d1a1 | 200 | 1 | 0.009
C1d1b | 14122 | 2 | 0.118
C1d1b | 14341 | 2 | 0.118
C1d1b | 16093 | 2 | 0.118
C1d1b | 16153 | 1 | 0.059
C1d1b | 16259 | 4 | 0.235
C1d1b | 16264 | 2 | 0.118
C1d1b | 16271 | 4 | 0.235
C1d1b | 16287 | 2 | 0.118
C1d1b | 16311 | 7 | 0.412
C1d1b | 2689.1C | 1 | 0.059
C1d1b | 3438 | 2 | 0.118
C1d1b | 573.2C | 2 | 0.118
C1d1b | 6252 | 1 | 0.059
C1d1b | 9449 | 2 | 0.118
C1d1b1 | 10202 | 1 | 0.059
C1d1b1 | 13689 | 2 | 0.118
C1d1b1 | 15014 | 2 | 0.118
C1d1b1 | 16266 | 2 | 0.118
C1d1b1 | 16270 | 2 | 0.118
C1d1b1 | 204 | 2 | 0.118
C1d1b1 | 6245 | 1 | 0.059
C1d1b1 | 64 | 1 | 0.059
C1d1b1 | 65 | 1 | 0.059
C1d1b1 | 8691 | 1 | 0.059
C1d1c | 143 | 4 | 0.038
C1d1c | 15670 | 1 | 0.01
C1d1c | 3504 | 1 | 0.01
C1d1c1 | 16093 | 1 | 0.009
C1d1c1 | 16204 | 11 | 0.095
C1d1c1 | 16242 | 3 | 0.026
C1d1c1 | 16300 | 1 | 0.009
C1d1c1 | 16527 | 11 | 0.095
C1d1c1 | 1656d | 1 | 0.009
C1d1c1 | 3582 | 1 | 0.009
C1d1c1 | 6018 | 1 | 0.009
C1d1c1 | 9196 | 1 | 0.009
C1d1d | 1246 | 1 | 0.008
C1d1d | 13215 | 1 | 0.008
C1d1d | 13604 | 1 | 0.008
C1d1d | 13770 | 2 | 0.017
C1d1d | 13934 | 1 | 0.008
C1d1d | 1416 | 1 | 0.008
C1d1d | 1452 | 7 | 0.059
C1d1d | 14569 | 1 | 0.008
C1d1d | 15244 | 1 | 0.008
C1d1d | 15628 | 1 | 0.008
C1d1d | 15852 | 7 | 0.059
C1d1d | 15884 | 2 | 0.017
C1d1d | 15905G | 1 | 0.008
C1d1d | 16209 | 1 | 0.008
C1d1d | 16293 | 1 | 0.008
C1d1d | 16295 | 2 | 0.017
C1d1d | 16311 | 1 | 0.008
C1d1d | 16335 | 2 | 0.017
C1d1d | 195 | 1 | 0.008
C1d1d | 197 | 1 | 0.008
C1d1d | 2262 | 1 | 0.008
C1d1d | 235 | 1 | 0.008
C1d1d | 310 | 1 | 0.008
C1d1d | 493 | 1 | 0.008
C1d1d | 5252 | 1 | 0.008
C1d1d | 5836 | 7 | 0.059
C1d1d | 6221 | 1 | 0.008
C1d1d | 6272 | 2 | 0.017
C1d1d | 6347A | 1 | 0.008
C1d1d | 6545 | 1 | 0.008
C1d1d | 709 | 4 | 0.034
C1d1d | 7269 | 1 | 0.008
C1d1d | 7397 | 2 | 0.017
C1d1d | 7609 | 1 | 0.008
C1d1d | 8425 | 7 | 0.059
C1d1d | 8749 | 1 | 0.008
C1d1d | 9007 | 1 | 0.008
C1d1d | 9055 | 2 | 0.017
C1d1d | 9148 | 1 | 0.008
C1d1d | 9921 | 1 | 0.008
C1d2 | 12396 | 1 | 0.009
C1d2 | 16069 | 2 | 0.018
C1d2 | 16104 | 1 | 0.009
C1d2 | 16174 | 1 | 0.009
C1d2 | 16291 | 1 | 0.009
C1d2 | 7129 | 1 | 0.009
C1d2 | 7151 | 1 | 0.009
C1d2a | 12997 | 1 | 0.25
C1d2a | 13966 | 1 | 0.25
C1d2a | 14040 | 1 | 0.25
C1d2a | 15784 | 1 | 0.25
C1d2a | 15955 | 1 | 0.25
C1d2a | 16300 | 1 | 0.25
C1d2a | 16357 | 1 | 0.25
C1d2a | 5417 | 1 | 0.25
C1d3 | 10365 | 1 | 0.01
C1d3 | 14992 | 2 | 0.019
C1d3 | 15313 | 2 | 0.019
C1d3 | 15662 | 2 | 0.019
C1d3 | 16209 | 2 | 0.019
C1d3 | 16400 | 2 | 0.019
C1d3 | 16422 | 4 | 0.038
C1d3 | 507 | 2 | 0.019
C1d3 | 8474 | 1 | 0.01
C1e | 151 | 2 | 0.333
C1e | 16287 | 1 | 0.167
C1e | 16368 | 3 | 0.5
C1e | 16390 | 3 | 0.5
C1e | 247 | 1 | 0.167

C1e | 379 | 2 | 0.333
C1f | 11887 | 1 | 0.008
C1f | 12984 | 1 | 0.008
C1f | 13980 | 1 | 0.008
C1f | 152 | 1 | 0.008
C1f | 16093 | 1 | 0.008
C1f | 16320 | 1 | 0.008
C1f | 16362 | 2 | 0.016
C1f | 16508 | 1 | 0.008
C1f | 4216 | 2 | 0.016
C1f | 5892.1TTTTCCC | 1 | 0.008
C1f | 5894C | 2 | 0.016
C1f | 8251 | 1 | 0.008
C4 | 14004 | 1 | 0.004
C4 | 15052 | 2 | 0.008
C4 | 16147 | 1 | 0.004
C4 | 16243 | 1 | 0.004
C4 | 16287 | 2 | 0.008
C4 | 16325 | 3 | 0.012
C4 | 16399 | 5 | 0.02
C4 | 16566 | 1 | 0.004
C4 | 189 | 1 | 0.004
C4 | 195 | 3 | 0.012
C4 | 200 | 2 | 0.008
C4 | 204 | 2 | 0.008
C4 | 310 | 2 | 0.008
C4 | 3192 | 2 | 0.008
C4 | 447 | 1 | 0.004
C4 | 513 | 1 | 0.004
C4 | 5706 | 1 | 0.004
C4 | 5707 | 1 | 0.004
C4 | 574 | 1 | 0.004
C4 | 8473 | 2 | 0.008
C4+152 | 11176 | 1 | 0.005
C4+152 | 12780 | 1 | 0.005
C4+152 | 143 | 1 | 0.005
C4+152 | 14384 | 4 | 0.019
C4+152 | 151 | 1 | 0.005
C4+152 | 15236 | 1 | 0.005
C4+152 | 16129 | 10 | 0.048
C4+152 | 16148 | 10 | 0.048
C4+152 | 16189 | 1 | 0.005
C4+152 | 16242 | 1 | 0.005
C4+152 | 16243 | 1 | 0.005
C4+152 | 16248 | 1 | 0.005
C4+152 | 16297 | 11 | 0.052
C4+152 | 16301 | 5 | 0.024
C4+152 | 16304 | 1 | 0.005
C4+152 | 16311 | 1 | 0.005
C4+152 | 16319 | 3 | 0.014
C4+152 | 16324 | 1 | 0.005
C4+152 | 16357 | 2 | 0.01
C4+152 | 16368 | 1 | 0.005
C4+152 | 16390 | 1 | 0.005
C4+152 | 195 | 1 | 0.005
C4+152 | 200 | 1 | 0.005
C4+152 | 207 | 1 | 0.005
C4+152 | 214 | 1 | 0.005
C4+152 | 225 | 1 | 0.005
C4+152 | 315.2C | 6 | 0.029
C4+152 | 316C | 1 | 0.005
C4+152 | 3801 | 1 | 0.005
C4+152 | 449 | 8 | 0.038
C4+152 | 456 | 1 | 0.005
C4+152 | 4742 | 5 | 0.024
C4+152 | 721A | 1 | 0.005
C4+152 | 745 | 1 | 0.005
C4+152 | 8152 | 1 | 0.005
C4+152 | 8602 | 4 | 0.019
C4+152 | 8682 | 1 | 0.005
C4+152 | 9102A | 4 | 0.019
C4+152 | 9739 | 1 | 0.005
C4+152 | 9966 | 4 | 0.019
C4+152+16093 | 10314 | 1 | 0.008
C4+152+16093 | 146 | 1 | 0.008
C4+152+16093 | 16129 | 2 | 0.016
C4+152+16093 | 16184 | 1 | 0.008
C4+152+16093 | 16193 | 2 | 0.016
C4+152+16093 | 16218 | 1 | 0.008
C4+152+16093 | 16242 | 1 | 0.008
C4+152+16093 | 16266 | 2 | 0.016
C4+152+16093 | 16290 | 1 | 0.008
C4+152+16093 | 16292A | 1 | 0.008
C4+152+16093 | 16297 | 13 | 0.102
C4+152+16093 | 16311 | 3 | 0.024
C4+152+16093 | 207 | 1 | 0.008
C4+152+16093 | 310 | 2 | 0.016
C4+152+16093 | 315.2C | 7 | 0.055
C4+152+16093 | 316C | 2 | 0.016
C4+152+16093 | 318.1CT | 1 | 0.008
C4+152+16093 | 368 | 3 | 0.024
C4+152+16093 | 385 | 1 | 0.008
C4+152+16093 | 4742 | 3 | 0.024
C4+152+16093 | 7106 | 2 | 0.016
C4+152+16093 | 8152 | 3 | 0.024
C4a | 16086 | 6 | 0.024
C4a | 16104 | 1 | 0.004
C4a | 16126 | 2 | 0.008
C4a | 16147 | 1 | 0.004
C4a | 16243 | 1 | 0.004
C4a | 16319 | 1 | 0.004
C4a | 16325 | 3 | 0.012
C4a | 16359 | 1 | 0.004
C4a | 16399 | 5 | 0.02
C4a | 513 | 1 | 0.004
C4a | 574 | 1 | 0.004
C4a1 | 11008 | 2 | 0.005
C4a1 | 11179 | 3 | 0.008
C4a1 | 11899 | 2 | 0.005
C4a1 | 13420 | 1 | 0.003
C4a1 | 13674 | 6 | 0.015

C4a1 | 146 | 1 | 0.003
C4a1 | 152 | 1 | 0.003
C4a1 | 15734 | 3 | 0.008
C4a1 | 16075A | 2 | 0.005
C4a1 | 16076d | 1 | 0.003
C4a1 | 16085G | 1 | 0.003
C4a1 | 16086 | 2 | 0.005
C4a1 | 16103 | 1 | 0.003
C4a1 | 16121G | 1 | 0.003
C4a1 | 16148 | 7 | 0.018
C4a1 | 16158 | 1 | 0.003
C4a1 | 16172 | 1 | 0.003
C4a1 | 16189 | 20 | 0.051
C4a1 | 16209 | 1 | 0.003
C4a1 | 16234 | 1 | 0.003
C4a1 | 16242 | 1 | 0.003
C4a1 | 16289 | 1 | 0.003
C4a1 | 16299 | 1 | 0.003
C4a1 | 16311 | 2 | 0.005
C4a1 | 16314T | 2 | 0.005
C4a1 | 16319 | 7 | 0.018
C4a1 | 16350C | 1 | 0.003
C4a1 | 16362 | 3 | 0.008
C4a1 | 16390 | 2 | 0.005
C4a1 | 16463 | 1 | 0.003
C4a1 | 16527 | 3 | 0.008
C4a1 | 199 | 1 | 0.003
C4a1 | 310 | 1 | 0.003
C4a1 | 315.2C | 2 | 0.005
C4a1 | 315d | 1 | 0.003
C4a1 | 3290 | 1 | 0.003
C4a1 | 44.1C | 1 | 0.003
C4a1 | 456 | 2 | 0.005
C4a1 | 8078 | 6 | 0.015
C4a1 | 9722 | 1 | 0.003
C4a1a | 146 | 5 | 0.014
C4a1a | 16221A | 1 | 0.003
C4a1a | 16224.1T | 1 | 0.003
C4a1a | 16297 | 5 | 0.014
C4a1a | 16319 | 1 | 0.003
C4a1a | 16362 | 2 | 0.006
C4a1a | 16399 | 2 | 0.006
C4a1a | 209A | 1 | 0.003
C4a1a | 315.2C | 2 | 0.006
C4a1a | 316C | 1 | 0.003
C4a1a | 3714 | 2 | 0.006
C4a1a | 4021 | 1 | 0.003
C4a1a | 449 | 1 | 0.003
C4a1a | 456 | 2 | 0.006
C4a1a | 64 | 2 | 0.006
C4a1a | 709 | 1 | 0.003
C4a1a | 719 | 1 | 0.003
C4a1a | 7675 | 1 | 0.003
C4a1a | 9129 | 1 | 0.003
C4a1a+195 | 10322 | 1 | 0.003
C4a1a+195 | 10484 | 2 | 0.006
C4a1a+195 | 11020 | 2 | 0.006
C4a1a+195 | 1193 | 2 | 0.006
C4a1a+195 | 11963 | 3 | 0.008
C4a1a+195 | 12098 | 3 | 0.008
C4a1a+195 | 12245 | 1 | 0.003
C4a1a+195 | 1243 | 1 | 0.003
C4a1a+195 | 12432 | 4 | 0.011
C4a1a+195 | 13470 | 3 | 0.008
C4a1a+195 | 13583 | 3 | 0.008
C4a1a+195 | 13635 | 4 | 0.011
C4a1a+195 | 1410 | 1 | 0.003
C4a1a+195 | 14207 | 2 | 0.006
C4a1a+195 | 14515 | 1 | 0.003
C4a1a+195 | 146 | 2 | 0.006
C4a1a+195 | 14878 | 5 | 0.014
C4a1a+195 | 14971 | 1 | 0.003
C4a1a+195 | 151 | 5 | 0.014
C4a1a+195 | 152 | 1 | 0.003
C4a1a+195 | 15773 | 1 | 0.003
C4a1a+195 | 16037 | 2 | 0.006
C4a1a+195 | 16077 | 1 | 0.003
C4a1a+195 | 16111 | 1 | 0.003
C4a1a+195 | 16114A | 4 | 0.011
C4a1a+195 | 16148 | 1 | 0.003
C4a1a+195 | 16168 | 1 | 0.003
C4a1a+195 | 16189 | 5 | 0.014
C4a1a+195 | 16213 | 14 | 0.039
C4a1a+195 | 16234 | 3 | 0.008
C4a1a+195 | 16249 | 1 | 0.003
C4a1a+195 | 16260 | 8 | 0.022
C4a1a+195 | 16261 | 1 | 0.003
C4a1a+195 | 16263 | 8 | 0.022
C4a1a+195 | 16272 | 2 | 0.006
C4a1a+195 | 16291 | 2 | 0.006
C4a1a+195 | 16524C | 4 | 0.011
C4a1a+195 | 207 | 1 | 0.003
C4a1a+195 | 248 | 1 | 0.003
C4a1a+195 | 310 | 3 | 0.008
C4a1a+195 | 3106A | 1 | 0.003
C4a1a+195 | 316C | 1 | 0.003
C4a1a+195 | 3212 | 2 | 0.006
C4a1a+195 | 3548 | 2 | 0.006
C4a1a+195 | 3593 | 1 | 0.003
C4a1a+195 | 495 | 1 | 0.003
C4a1a+195 | 507 | 1 | 0.003
C4a1a+195 | 513 | 1 | 0.003
C4a1a+195 | 5201 | 1 | 0.003
C4a1a+195 | 5222 | 1 | 0.003
C4a1a+195 | 5883 | 1 | 0.003
C4a1a+195 | 5957 | 1 | 0.003
C4a1a+195 | 6071 | 2 | 0.006
C4a1a+195 | 6221 | 3 | 0.008
C4a1a+195 | 6563 | 1 | 0.003
C4a1a+195 | 709 | 1 | 0.003
C4a1a+195 | 7310 | 3 | 0.008

C4a1a+195 | 7424 | 2 | 0.006
C4a1a+195 | 7444 | 1 | 0.003
C4a1a+195 | 8281-8289d | 1 | 0.003
C4a1a+195 | 8865 | 1 | 0.003
C4a1a+195 | 9025 | 1 | 0.003
C4a1a+195 | 980 | 1 | 0.003
C4a1a+195 | 990 | 1 | 0.003
C4a1a1 | 11023C | 1 | 0.003
C4a1a1 | 11732 | 2 | 0.006
C4a1a1 | 11900 | 1 | 0.003
C4a1a1 | 1201 | 1 | 0.003
C4a1a1 | 13392 | 2 | 0.006
C4a1a1 | 14693 | 2 | 0.006
C4a1a1 | 152 | 2 | 0.006
C4a1a1 | 16297 | 5 | 0.014
C4a1a1 | 16362 | 2 | 0.006
C4a1a1 | 16399 | 2 | 0.006
C4a1a1 | 194 | 6 | 0.017
C4a1a1 | 209A | 1 | 0.003
C4a1a1 | 315.2C | 2 | 0.006
C4a1a1 | 3906 | 2 | 0.006
C4a1a1 | 4216 | 1 | 0.003
C4a1a1 | 4254 | 1 | 0.003
C4a1a1 | 456 | 2 | 0.006
C4a1a1 | 6497 | 2 | 0.006
C4a1a1 | 6951 | 2 | 0.006
C4a1a1 | 7581 | 1 | 0.003
C4a1a1 | 8206 | 2 | 0.006
C4a1a1a | 10410 | 2 | 0.005
C4a1a1a | 10775 | 2 | 0.005
C4a1a1a | 13488 | 2 | 0.005
C4a1a1a | 14978 | 1 | 0.003
C4a1a1a | 16117 | 2 | 0.005
C4a1a1a | 16224 | 41 | 0.104
C4a1a1a | 16325 | 1 | 0.003
C4a1a1a | 16362 | 3 | 0.008
C4a1a1a | 16399 | 2 | 0.005
C4a1a1a | 315.2C | 2 | 0.005
C4a1a1a | 316C | 1 | 0.003
C4a1a1a | 456 | 2 | 0.005
C4a1a1a | 5493 | 2 | 0.005
C4a1a1a | 5843 | 2 | 0.005
C4a1a1a | 8110 | 1 | 0.003
C4a1a2 | 10352C | 5 | 0.015
C4a1a2 | 11893 | 7 | 0.02
C4a1a2 | 12084 | 1 | 0.003
C4a1a2 | 14233 | 2 | 0.006
C4a1a2 | 151 | 1 | 0.003
C4a1a2 | 153 | 1 | 0.003
C4a1a2 | 15907 | 5 | 0.015
C4a1a2 | 16051 | 2 | 0.006
C4a1a2 | 16092 | 2 | 0.006
C4a1a2 | 16117 | 1 | 0.003
C4a1a2 | 16179 | 2 | 0.006
C4a1a2 | 16209 | 4 | 0.012
C4a1a2 | 16221A | 1 | 0.003
C4a1a2 | 16224.1T | 1 | 0.003
C4a1a2 | 16248 | 1 | 0.003
C4a1a2 | 16249 | 1 | 0.003
C4a1a2 | 16261 | 1 | 0.003
C4a1a2 | 16272 | 1 | 0.003
C4a1a2 | 16284 | 1 | 0.003
C4a1a2 | 16384 | 2 | 0.006
C4a1a2 | 198 | 2 | 0.006
C4a1a2 | 207 | 1 | 0.003
C4a1a2 | 228T | 11 | 0.032
C4a1a2 | 310 | 1 | 0.003
C4a1a2 | 513 | 1 | 0.003
C4a1a2 | 513.1AC | 1 | 0.003
C4a1a2 | 535 | 1 | 0.003
C4a1a2 | 709 | 1 | 0.003
C4a1a2 | 8119 | 1 | 0.003
C4a1a2 | 8137 | 2 | 0.006
C4a1a2 | 9452 | 1 | 0.003
C4a1a2a | 11176 | 2 | 0.006
C4a1a2a | 15511 | 1 | 0.003
C4a1a2a | 16051 | 2 | 0.006
C4a1a2a | 16092 | 2 | 0.006
C4a1a2a | 16179 | 2 | 0.006
C4a1a2a | 16189 | 2 | 0.006
C4a1a2a | 16248 | 1 | 0.003
C4a1a2a | 16249 | 1 | 0.003
C4a1a2a | 16261 | 1 | 0.003
C4a1a2a | 16272 | 1 | 0.003
C4a1a2a | 16384 | 2 | 0.006
C4a1a2a | 198 | 2 | 0.006
C4a1a2a | 207 | 1 | 0.003
C4a1a2a | 4248 | 1 | 0.003
C4a1a2a | 513 | 1 | 0.003
C4a1a2a | 709 | 1 | 0.003
C4a1a2a | 7302 | 1 | 0.003
C4a1a2a | 8188 | 2 | 0.006
C4a1a2a | 8573 | 1 | 0.003
C4a1a3 | 13708 | 1 | 0.003
C4a1a3 | 13857 | 4 | 0.011
C4a1a3 | 14129 | 1 | 0.003
C4a1a3 | 152 | 11 | 0.03
C4a1a3 | 15944d | 1 | 0.003
C4a1a3 | 16037 | 2 | 0.005
C4a1a3 | 16172 | 1 | 0.003
C4a1a3 | 16249 | 1 | 0.003
C4a1a3 | 16261 | 1 | 0.003
C4a1a3 | 16272 | 1 | 0.003
C4a1a3 | 16311 | 4 | 0.011
C4a1a3 | 16354 | 2 | 0.005
C4a1a3 | 16362 | 15 | 0.041
C4a1a3 | 16434 | 3 | 0.008
C4a1a3 | 16555 | 1 | 0.003
C4a1a3 | 198 | 1 | 0.003
C4a1a3 | 200 | 2 | 0.005
C4a1a3 | 207 | 1 | 0.003

C4a1a3 | 255 | 8 | 0.022
C4a1a3 | 310 | 1 | 0.003
C4a1a3 | 3720 | 2 | 0.005
C4a1a3 | 513 | 1 | 0.003
C4a1a3 | 573.1C | 1 | 0.003
C4a1a3 | 573.4C | 2 | 0.005
C4a1a3 | 6852 | 5 | 0.014
C4a1a3 | 709 | 1 | 0.003
C4a1a3 | 7422 | 1 | 0.003
C4a1a3 | 7424 | 1 | 0.003
C4a1a3 | 7954 | 5 | 0.014
C4a1a3a | 125 | 1 | 0.003
C4a1a3a | 12662 | 1 | 0.003
C4a1a3a | 127 | 1 | 0.003
C4a1a3a | 150 | 4 | 0.012
C4a1a3a | 15280 | 1 | 0.003
C4a1a3a | 16051 | 2 | 0.006
C4a1a3a | 16092 | 2 | 0.006
C4a1a3a | 16179 | 2 | 0.006
C4a1a3a | 16248 | 1 | 0.003
C4a1a3a | 16249 | 1 | 0.003
C4a1a3a | 16261 | 1 | 0.003
C4a1a3a | 16266 | 1 | 0.003
C4a1a3a | 16272 | 1 | 0.003
C4a1a3a | 16384 | 2 | 0.006
C4a1a3a | 16399 | 1 | 0.003
C4a1a3a | 198 | 2 | 0.006
C4a1a3a | 207 | 1 | 0.003
C4a1a3a | 310 | 5 | 0.015
C4a1a3a | 333 | 2 | 0.006
C4a1a3a | 4739 | 1 | 0.003
C4a1a3a | 513 | 1 | 0.003
C4a1a3a | 709 | 1 | 0.003
C4a1a3a | 9371 | 1 | 0.003
C4a1a3a1 | 16051 | 2 | 0.006
C4a1a3a1 | 16092 | 2 | 0.006
C4a1a3a1 | 16179 | 2 | 0.006
C4a1a3a1 | 16235 | 1 | 0.003
C4a1a3a1 | 16248 | 1 | 0.003
C4a1a3a1 | 16249 | 1 | 0.003
C4a1a3a1 | 16261 | 1 | 0.003
C4a1a3a1 | 16272 | 1 | 0.003
C4a1a3a1 | 16384 | 2 | 0.006
C4a1a3a1 | 16390 | 1 | 0.003
C4a1a3a1 | 198 | 2 | 0.006
C4a1a3a1 | 207 | 1 | 0.003
C4a1a3a1 | 234 | 1 | 0.003
C4a1a3a1 | 310 | 1 | 0.003
C4a1a3a1 | 3644 | 3 | 0.009
C4a1a3a1 | 513 | 1 | 0.003
C4a1a3a1 | 5606 | 1 | 0.003
C4a1a3a1 | 709 | 1 | 0.003
C4a1a3b | 14110 | 1 | 0.003
C4a1a3b | 14180 | 1 | 0.003
C4a1a3b | 14225 | 2 | 0.006
C4a1a3b | 15355 | 1 | 0.003
C4a1a3b | 16051 | 2 | 0.006
C4a1a3b | 16092 | 2 | 0.006
C4a1a3b | 16179 | 2 | 0.006
C4a1a3b | 16248 | 1 | 0.003
C4a1a3b | 16249 | 1 | 0.003
C4a1a3b | 16261 | 1 | 0.003
C4a1a3b | 16272 | 1 | 0.003
C4a1a3b | 16293 | 1 | 0.003
C4a1a3b | 16384 | 2 | 0.006
C4a1a3b | 198 | 2 | 0.006
C4a1a3b | 207 | 1 | 0.003
C4a1a3b | 430 | 2 | 0.006
C4a1a3b | 513 | 1 | 0.003
C4a1a3b | 573.3C | 1 | 0.003
C4a1a3b | 709 | 1 | 0.003
C4a1a3b | 7258 | 1 | 0.003
C4a1a3c | 11447 | 9 | 0.027
C4a1a3c | 11875 | 8 | 0.024
C4a1a3c | 16092 | 2 | 0.006
C4a1a3c | 16179 | 2 | 0.006
C4a1a3c | 16249 | 1 | 0.003
C4a1a3c | 16261 | 1 | 0.003
C4a1a3c | 16272 | 1 | 0.003
C4a1a3c | 16384 | 2 | 0.006
C4a1a3c | 16428 | 1 | 0.003
C4a1a3c | 207 | 1 | 0.003
C4a1a3c | 310 | 2 | 0.006
C4a1a3c | 513 | 1 | 0.003
C4a1a3c | 5752.1A | 2 | 0.006
C4a1a3c | 597.1T | 1 | 0.003
C4a1a3c | 709 | 1 | 0.003
C4a1a3c | 8281-8289d | 4 | 0.012
C4a1a3d | 10885 | 3 | 0.021
C4a1a3d | 15110 | 2 | 0.014
C4a1a3d | 16311 | 1 | 0.007
C4a1a3d | 204 | 1 | 0.007
C4a1a3d | 207 | 2 | 0.014
C4a1a3d | 310 | 2 | 0.014
C4a1a3d | 5444 | 1 | 0.007
C4a1a3d | 6563 | 3 | 0.021
C4a1a3d | 7888 | 2 | 0.014
C4a1a4 | 1008 | 1 | 0.003
C4a1a4 | 1047 | 3 | 0.009
C4a1a4 | 11992 | 1 | 0.003
C4a1a4 | 12599 | 3 | 0.009
C4a1a4 | 16051 | 2 | 0.006
C4a1a4 | 16092 | 2 | 0.006
C4a1a4 | 16179 | 2 | 0.006
C4a1a4 | 16248 | 1 | 0.003
C4a1a4 | 16249 | 1 | 0.003
C4a1a4 | 16261 | 1 | 0.003
C4a1a4 | 16272 | 1 | 0.003
C4a1a4 | 16319 | 12 | 0.036
C4a1a4 | 16384 | 2 | 0.006
C4a1a4 | 198 | 2 | 0.006

C4a1a4 | 207 | 1 | 0.003
C4a1a4 | 4491 | 2 | 0.006
C4a1a4 | 4688 | 1 | 0.003
C4a1a4 | 502d | 2 | 0.006
C4a1a4 | 513 | 1 | 0.003
C4a1a4 | 709 | 1 | 0.003
C4a1a4 | 8276.1C | 1 | 0.003
C4a1a4a | 10908 | 2 | 0.012
C4a1a4a | 11002 | 1 | 0.006
C4a1a4a | 12372 | 4 | 0.025
C4a1a4a | 14757 | 2 | 0.012
C4a1a4a | 16037 | 1 | 0.006
C4a1a4a | 16086 | 2 | 0.012
C4a1a4a | 16189 | 2 | 0.012
C4a1a4a | 16209 | 1 | 0.006
C4a1a4a | 16235 | 1 | 0.006
C4a1a4a | 16249 | 1 | 0.006
C4a1a4a | 16309 | 1 | 0.006
C4a1a4a | 16311 | 1 | 0.006
C4a1a4a | 16318 | 1 | 0.006
C4a1a4a | 16319 | 2 | 0.012
C4a1a4a | 16354 | 1 | 0.006
C4a1a4a | 16357 | 2 | 0.012
C4a1a4a | 16362 | 1 | 0.006
C4a1a4a | 16418 | 1 | 0.006
C4a1a4a | 207 | 1 | 0.006
C4a1a4a | 2363 | 1 | 0.006
C4a1a4a | 316C | 1 | 0.006
C4a1a4a | 333 | 2 | 0.012
C4a1a4a | 3338 | 1 | 0.006
C4a1a4a | 3472 | 1 | 0.006
C4a1a4a | 347d | 1 | 0.006
C4a1a4a | 4688 | 2 | 0.012
C4a1a4a | 502d | 1 | 0.006
C4a1a4a | 5120 | 1 | 0.006
C4a1a4a | 549 | 1 | 0.006
C4a1a4a | 634 | 1 | 0.006
C4a1a4a | 7196 | 1 | 0.006
C4a1a4a | 7773 | 1 | 0.006
C4a1a4a | 8276.1C | 2 | 0.012
C4a1a4a | 8639 | 2 | 0.012
C4a1a4a | 8856 | 1 | 0.006
C4a1a4a | 928 | 1 | 0.006
C4a1a5 | 16234 | 1 | 0.003
C4a1a5 | 16297 | 5 | 0.014
C4a1a5 | 16362 | 2 | 0.006
C4a1a5 | 16399 | 2 | 0.006
C4a1a5 | 209A | 1 | 0.003
C4a1a5 | 315.2C | 2 | 0.006
C4a1a5 | 456 | 2 | 0.006
C4a1a5 | 4830 | 1 | 0.003
C4a1a5 | 8623 | 2 | 0.006
C4a1a6 | 11253 | 5 | 0.014
C4a1a6 | 16051 | 1 | 0.003
C4a1a6 | 16213 | 1 | 0.003
C4a1a6 | 16297 | 5 | 0.014
C4a1a6 | 16362 | 2 | 0.006
C4a1a6 | 16399 | 2 | 0.006
C4a1a6 | 209A | 1 | 0.003
C4a1a6 | 2281 | 1 | 0.003
C4a1a6 | 3027 | 1 | 0.003
C4a1a6 | 315.2C | 2 | 0.006
C4a1a6 | 456 | 2 | 0.006
C4a1b | 14484 | 1 | 0.007
C4a1b | 146 | 1 | 0.007
C4a1b | 16117 | 2 | 0.014
C4a1b | 16181 | 1 | 0.007
C4a1b | 16286 | 1 | 0.007
C4a1b | 16295 | 1 | 0.007
C4a1b | 16311 | 8 | 0.054
C4a1b | 16325 | 1 | 0.007
C4a1b | 16357 | 3 | 0.02
C4a1b | 195 | 1 | 0.007
C4a1b | 315.2C | 1 | 0.007
C4a1b | 316C | 1 | 0.007
C4a1b | 5082 | 1 | 0.007
C4a1b | 573.4C | 1 | 0.007
C4a1b | 9137 | 1 | 0.007
C4a1b | 9148 | 5 | 0.034
C4a2 | 16057 | 1 | 0.005
C4a2 | 16086 | 4 | 0.021
C4a2 | 16092 | 1 | 0.005
C4a2 | 16214 | 10 | 0.052
C4a2 | 16218 | 1 | 0.005
C4a2 | 16310 | 1 | 0.005
C4a2 | 16318 | 3 | 0.016
C4a2 | 16354 | 2 | 0.01
C4a2 | 195 | 1 | 0.005
C4a2 | 204 | 1 | 0.005
C4a2 | 316C | 2 | 0.01
C4a2 | 634 | 1 | 0.005
C4a2 | 93 | 1 | 0.005
C4a2a | 16167 | 1 | 0.008
C4a2a | 16184 | 1 | 0.008
C4a2a | 16301 | 1 | 0.008
C4a2a | 47 | 1 | 0.008
C4a2a | 7080 | 1 | 0.008
C4a2a1 | 10463 | 1 | 0.005
C4a2a1 | 11147 | 6 | 0.029
C4a2a1 | 13677 | 1 | 0.005
C4a2a1 | 14053 | 2 | 0.01
C4a2a1 | 146 | 1 | 0.005
C4a2a1 | 150 | 3 | 0.015
C4a2a1 | 15191 | 1 | 0.005
C4a2a1 | 152 | 3 | 0.015
C4a2a1 | 15924 | 1 | 0.005
C4a2a1 | 15978 | 1 | 0.005
C4a2a1 | 16077 | 1 | 0.005
C4a2a1 | 16124 | 1 | 0.005
C4a2a1 | 16167 | 2 | 0.01
C4a2a1 | 16174 | 2 | 0.01

C4a2a1 | 16184 | 1 | 0.005
C4a2a1 | 16189 | 2 | 0.01
C4a2a1 | 16209 | 2 | 0.01
C4a2a1 | 16224 | 1 | 0.005
C4a2a1 | 16244 | 2 | 0.01
C4a2a1 | 16256 | 1 | 0.005
C4a2a1 | 16261 | 2 | 0.01
C4a2a1 | 16278 | 4 | 0.02
C4a2a1 | 16300 | 1 | 0.005
C4a2a1 | 16311 | 1 | 0.005
C4a2a1 | 183 | 1 | 0.005
C4a2a1 | 195 | 8 | 0.039
C4a2a1 | 207 | 5 | 0.025
C4a2a1 | 248 | 1 | 0.005
C4a2a1 | 310 | 1 | 0.005
C4a2a1 | 3355 | 1 | 0.005
C4a2a1 | 47 | 19 | 0.093
C4a2a1 | 503 | 2 | 0.01
C4a2a1 | 508 | 1 | 0.005
C4a2a1 | 5984 | 1 | 0.005
C4a2a1 | 64 | 1 | 0.005
C4a2a1 | 6663 | 2 | 0.01
C4a2a1 | 8146C | 4 | 0.02
C4a2a1 | 8337 | 1 | 0.005
C4a2a1 | 8577 | 1 | 0.005
C4a2a1a | 146 | 2 | 0.012
C4a2a1a | 15860 | 1 | 0.006
C4a2a1a | 16248 | 1 | 0.006
C4a2a1a | 310 | 1 | 0.006
C4a2a1b | 13818 | 9 | 0.055
C4a2a1b | 146 | 2 | 0.012
C4a2a1b | 16000 | 5 | 0.03
C4a2a1b | 16167 | 4 | 0.024
C4a2a1b | 16248 | 1 | 0.006
C4a2a1b | 16278 | 1 | 0.006
C4a2b | 13239 | 1 | 0.005
C4a2b | 15115 | 3 | 0.015
C4a2b | 16092 | 2 | 0.01
C4a2b | 16129 | 4 | 0.02
C4a2b | 16189 | 24 | 0.119
C4a2b | 16239 | 3 | 0.015
C4a2b | 16249 | 1 | 0.005
C4a2b | 16255 | 1 | 0.005
C4a2b | 16310 | 1 | 0.005
C4a2b | 16311 | 5 | 0.025
C4a2b | 16368 | 1 | 0.005
C4a2b | 1719 | 1 | 0.005
C4a2b | 1888 | 1 | 0.005
C4a2b | 200 | 6 | 0.03
C4a2b | 204 | 1 | 0.005
C4a2b | 26.1T | 1 | 0.005
C4a2b | 309d | 1 | 0.005
C4a2b | 310 | 1 | 0.005
C4a2b | 310d | 1 | 0.005
C4a2b | 3462 | 3 | 0.015
C4a2b | 372 | 3 | 0.015
C4a2b | 513 | 1 | 0.005
C4a2b | 5333 | 1 | 0.005
C4a2b | 6131 | 3 | 0.015
C4a2b | 6218 | 1 | 0.005
C4a2b | 8093 | 1 | 0.005
C4a2b | 8251 | 1 | 0.005
C4a2b1 | 13563 | 1 | 0.008
C4a2b1 | 15553 | 1 | 0.008
C4a2b1 | 2232.2A | 1 | 0.008
C4a2b1 | 2857 | 1 | 0.008
C4a2b1 | 315.2C | 1 | 0.008
C4a2b1 | 3753 | 1 | 0.008
C4a2b1 | 6297 | 1 | 0.008
C4a2b2 | 12954 | 1 | 0.004
C4a2b2 | 13173 | 2 | 0.008
C4a2b2 | 13269 | 1 | 0.004
C4a2b2 | 13635 | 2 | 0.008
C4a2b2 | 14384 | 2 | 0.008
C4a2b2 | 16053G | 3 | 0.013
C4a2b2 | 16093 | 2 | 0.008
C4a2b2 | 16188 | 1 | 0.004
C4a2b2 | 16189 | 9 | 0.038
C4a2b2 | 16230 | 1 | 0.004
C4a2b2 | 16234 | 1 | 0.004
C4a2b2 | 16243 | 1 | 0.004
C4a2b2 | 16249 | 3 | 0.013
C4a2b2 | 16272 | 2 | 0.008
C4a2b2 | 16278 | 2 | 0.008
C4a2b2 | 16288 | 2 | 0.008
C4a2b2 | 16289 | 1 | 0.004
C4a2b2 | 16320 | 3 | 0.013
C4a2b2 | 16325 | 3 | 0.013
C4a2b2 | 16354 | 1 | 0.004
C4a2b2 | 16504 | 1 | 0.004
C4a2b2 | 188 | 2 | 0.008
C4a2b2 | 189 | 3 | 0.013
C4a2b2 | 194 | 1 | 0.004
C4a2b2 | 199 | 3 | 0.013
C4a2b2 | 207 | 2 | 0.008
C4a2b2 | 315d | 2 | 0.008
C4a2b2 | 3766 | 2 | 0.008
C4a2b2 | 4136 | 2 | 0.008
C4a2b2 | 4767 | 2 | 0.008
C4a2b2 | 573.1C | 1 | 0.004
C4a2b2 | 5899.2C | 1 | 0.004
C4a2b2 | 64 | 1 | 0.004
C4a2b2 | 6480 | 1 | 0.004
C4a2b2 | 7196 | 1 | 0.004
C4a2b2 | 8269 | 2 | 0.008
C4a2b2 | 9629 | 2 | 0.008
C4a2b2 | 9752 | 1 | 0.004
C4a2b2a | 11398 | 2 | 0.008
C4a2b2a | 15658 | 4 | 0.015
C4a2b2a | 16037 | 3 | 0.011
C4a2b2a | 16093 | 2 | 0.008

C4a2b2a | 16234 | 2 | 0.008
C4a2b2a | 16272 | 2 | 0.008
C4a2b2a | 16354 | 1 | 0.004
C4a2b2a | 16524 | 1 | 0.004
C4a2b2a | 16527 | 1 | 0.004
C4a2b2a | 385 | 1 | 0.004
C4a2b2a | 709 | 1 | 0.004
C4a2c | 11654 | 3 | 0.016
C4a2c | 13759 | 1 | 0.005
C4a2c | 146 | 1 | 0.005
C4a2c | 152 | 2 | 0.01
C4a2c | 15499 | 1 | 0.005
C4a2c | 16037 | 1 | 0.005
C4a2c | 16126 | 1 | 0.005
C4a2c | 16172 | 1 | 0.005
C4a2c | 16189 | 3 | 0.016
C4a2c | 16214 | 10 | 0.052
C4a2c | 16218 | 1 | 0.005
C4a2c | 16239 | 5 | 0.026
C4a2c | 16247 | 1 | 0.005
C4a2c | 16259 | 3 | 0.016
C4a2c | 16269 | 1 | 0.005
C4a2c | 16310 | 1 | 0.005
C4a2c | 16354 | 24 | 0.125
C4a2c | 16362 | 6 | 0.031
C4a2c | 200 | 1 | 0.005
C4a2c | 207 | 2 | 0.01
C4a2c | 214 | 4 | 0.021
C4a2c | 215 | 1 | 0.005
C4a2c | 310 | 1 | 0.005
C4a2c | 316C | 2 | 0.01
C4a2c | 366 | 1 | 0.005
C4a2c | 411G | 1 | 0.005
C4a2c | 541A | 1 | 0.005
C4a2c | 64 | 1 | 0.005
C4a2c | 6665 | 1 | 0.005
C4a2c | 674A | 1 | 0.005
C4a2c | 681 | 1 | 0.005
C4a2c | 683 | 1 | 0.005
C4a2c | 689A | 1 | 0.005
C4a2c | 6929 | 1 | 0.005
C4a2c | 8443 | 3 | 0.016
C4a2c | 951 | 1 | 0.005
C4a2c1 | 16013C | 1 | 0.008
C4a2c1 | 16031d | 1 | 0.008
C4a2c1 | 16140 | 1 | 0.008
C4a2c1 | 16300 | 1 | 0.008
C4a2c1 | 16411d | 1 | 0.008
C4a2c1 | 3523T | 1 | 0.008
C4a2c1 | 4772 | 1 | 0.008
C4a2c1 | 482 | 1 | 0.008
C4a2c2 | 16192 | 1 | 0.007
C4a2c2 | 16234 | 7 | 0.052
C4a2c2 | 16255 | 2 | 0.015
C4a2c2 | 16354 | 2 | 0.015
C4a2c2 | 316C | 1 | 0.007
C4a2c2 | 709 | 2 | 0.015
C4a2c2 | 7765 | 1 | 0.007
C4a2c2a | 15300 | 1 | 0.008
C4a2c2a | 16241T | 1 | 0.008
C4a2c2a | 16263 | 1 | 0.008
C4a2c2a | 16344 | 1 | 0.008
C4a2c2a | 16451d | 1 | 0.008
C4a2c2a | 64 | 1 | 0.008
C4a2c2a | 8269 | 3 | 0.023
C4a'b'c | 16086 | 6 | 0.024
C4a'b'c | 16104 | 1 | 0.004
C4a'b'c | 16126 | 2 | 0.008
C4a'b'c | 16147 | 1 | 0.004
C4a'b'c | 16243 | 1 | 0.004
C4a'b'c | 16319 | 1 | 0.004
C4a'b'c | 16325 | 3 | 0.012
C4a'b'c | 16359 | 1 | 0.004
C4a'b'c | 16399 | 5 | 0.02
C4a'b'c | 513 | 1 | 0.004
C4a'b'c | 574 | 1 | 0.004
C4b | 11928 | 8 | 0.028
C4b | 13212 | 1 | 0.004
C4b | 152 | 1 | 0.004
C4b | 15268 | 1 | 0.004
C4b | 16093 | 1 | 0.004
C4b | 16242 | 1 | 0.004
C4b | 16287 | 1 | 0.004
C4b | 16293 | 1 | 0.004
C4b | 16299 | 1 | 0.004
C4b | 16311 | 24 | 0.085
C4b | 16497 | 2 | 0.007
C4b | 189 | 19 | 0.068
C4b | 199 | 8 | 0.028
C4b | 228 | 1 | 0.004
C4b | 310 | 9 | 0.032
C4b | 3306 | 8 | 0.028
C4b | 3319 | 8 | 0.028
C4b | 4125 | 1 | 0.004
C4b | 4353 | 1 | 0.004
C4b | 449 | 1 | 0.004
C4b | 6485 | 2 | 0.007
C4b | 709 | 2 | 0.007
C4b | 8093 | 1 | 0.004
C4b | 9802 | 1 | 0.004
C4b1 | 10792 | 1 | 0.004
C4b1 | 12311 | 10 | 0.037
C4b1 | 12870 | 3 | 0.011
C4b1 | 14153 | 3 | 0.011
C4b1 | 14502A | 1 | 0.004
C4b1 | 14502T | 1 | 0.004
C4b1 | 152 | 2 | 0.007
C4b1 | 15325 | 2 | 0.007
C4b1 | 16086 | 1 | 0.004
C4b1 | 16090 | 1 | 0.004
C4b1 | 16189 | 17 | 0.062

C4b1 | 16192 | 1 | 0.004
C4b1 | 16243 | 1 | 0.004
C4b1 | 16261 | 1 | 0.004
C4b1 | 16329 | 1 | 0.004
C4b1 | 16344 | 3 | 0.011
C4b1 | 189 | 3 | 0.011
C4b1 | 199 | 1 | 0.004
C4b1 | 204 | 1 | 0.004
C4b1 | 214 | 1 | 0.004
C4b1 | 234 | 4 | 0.015
C4b1 | 310 | 10 | 0.037
C4b1 | 4129 | 3 | 0.011
C4b1 | 466 | 1 | 0.004
C4b1 | 6596 | 2 | 0.007
C4b1 | 709 | 3 | 0.011
C4b1 | 8336 | 1 | 0.004
C4b1 | 8419 | 2 | 0.007
C4b1 | 8764 | 1 | 0.004
C4b1a | 12757 | 1 | 0.05
C4b1a | 13470 | 1 | 0.05
C4b1a | 13928C | 1 | 0.05
C4b1a | 14793 | 1 | 0.05
C4b1a | 15930 | 3 | 0.15
C4b1a | 15974T | 1 | 0.05
C4b1a | 16294 | 4 | 0.2
C4b1a | 16311 | 1 | 0.05
C4b1a | 4231 | 1 | 0.05
C4b1a | 6221 | 1 | 0.05
C4b1b | 151 | 1 | 0.004
C4b1b | 16086 | 1 | 0.004
C4b1b | 16090 | 1 | 0.004
C4b1b | 16129 | 6 | 0.025
C4b1b | 16230 | 2 | 0.008
C4b1b | 16231 | 2 | 0.008
C4b1b | 16242 | 2 | 0.008
C4b1b | 16243 | 1 | 0.004
C4b1b | 16266 | 1 | 0.004
C4b1b | 16270 | 9 | 0.037
C4b1b | 16290 | 1 | 0.004
C4b1b | 16329 | 1 | 0.004
C4b1b | 16390 | 3 | 0.012
C4b1b | 199 | 1 | 0.004
C4b1b | 204 | 1 | 0.004
C4b1b | 214 | 1 | 0.004
C4b1b | 215 | 6 | 0.025
C4b1b | 315.2C | 1 | 0.004
C4b1b | 316C | 3 | 0.012
C4b2 | 146 | 1 | 0.007
C4b2 | 195 | 3 | 0.022
C4b2 | 310 | 3 | 0.022
C4b2a | 16311 | 2 | 0.013
C4b2a | 16319 | 1 | 0.007
C4b2a | 8632 | 1 | 0.007
C4b3 | 10843 | 1 | 0.007
C4b3 | 13401 | 1 | 0.007
C4b3 | 16051 | 1 | 0.007
C4b3 | 16072 | 3 | 0.02
C4b3 | 16322T | 1 | 0.007
C4b3 | 16326T | 1 | 0.007
C4b3 | 310 | 1 | 0.007
C4b3 | 4109 | 1 | 0.007
C4b3 | 7702 | 3 | 0.02
C4b3a | 10704 | 3 | 0.019
C4b3a | 11150 | 2 | 0.013
C4b3a | 13395 | 5 | 0.032
C4b3a | 152 | 4 | 0.026
C4b3a | 15865 | 5 | 0.032
C4b3a | 310 | 4 | 0.026
C4b3a1 | 1119 | 1 | 0.008
C4b3a1 | 16311 | 3 | 0.023
C4b3a1 | 310 | 3 | 0.023
C4b3b | 12171 | 3 | 0.023
C4b3b | 16295 | 3 | 0.023
C4b3b | 310 | 1 | 0.008
C4b3b | 5894 | 3 | 0.023
C4b5 | 16086 | 6 | 0.024
C4b5 | 16104 | 1 | 0.004
C4b5 | 16126 | 2 | 0.008
C4b5 | 16147 | 1 | 0.004
C4b5 | 16243 | 1 | 0.004
C4b5 | 16319 | 1 | 0.004
C4b5 | 16325 | 3 | 0.012
C4b5 | 16359 | 1 | 0.004
C4b5 | 16399 | 5 | 0.02
C4b5 | 310 | 1 | 0.004
C4b5 | 513 | 1 | 0.004
C4b5 | 574 | 1 | 0.004
C4b6 | 10867 | 1 | 0.004
C4b6 | 12612 | 1 | 0.004
C4b6 | 13765 | 1 | 0.004
C4b6 | 14890 | 6 | 0.024
C4b6 | 16067 | 1 | 0.004
C4b6 | 16145 | 7 | 0.028
C4b6 | 16147 | 2 | 0.008
C4b6 | 16243 | 2 | 0.008
C4b6 | 16294 | 2 | 0.008
C4b6 | 513 | 2 | 0.008
C4b6 | 574 | 2 | 0.008
C4b6 | 5973 | 1 | 0.004
C4b6 | 983 | 4 | 0.016
C4b7 | 12007 | 1 | 0.004
C4b7 | 13260 | 2 | 0.008
C4b7 | 16147 | 1 | 0.004
C4b7 | 16243 | 1 | 0.004
C4b7 | 16325 | 3 | 0.012
C4b7 | 16399 | 5 | 0.02
C4b7 | 310 | 3 | 0.012
C4b7 | 513 | 1 | 0.004
C4b7 | 574 | 1 | 0.004
C4b8 | 13896 | 1 | 0.004
C4b8 | 16086 | 6 | 0.024

C4b8 | 16104 | 1 | 0.004
C4b8 | 16126 | 2 | 0.008
C4b8 | 16147 | 1 | 0.004
C4b8 | 16243 | 1 | 0.004
C4b8 | 16319 | 1 | 0.004
C4b8 | 16325 | 3 | 0.012
C4b8 | 16359 | 1 | 0.004
C4b8 | 16399 | 5 | 0.02
C4b8 | 513 | 1 | 0.004
C4b8 | 574 | 1 | 0.004
C4b8a | 12254 | 1 | 0.005
C4b8a | 16030d | 1 | 0.005
C4b8a | 16067 | 3 | 0.016
C4b8a | 16075A | 1 | 0.005
C4b8a | 16076d | 2 | 0.011
C4b8a | 16145 | 4 | 0.021
C4b8a | 16172 | 32 | 0.17
C4b8a | 16189 | 1 | 0.005
C4b8a | 16193 | 1 | 0.005
C4b8a | 16218 | 2 | 0.011
C4b8a | 16266 | 5 | 0.027
C4b8a | 16284 | 6 | 0.032
C4b8a | 16294 | 3 | 0.016
C4b8a | 16311 | 10 | 0.053
C4b8a | 16317 | 1 | 0.005
C4b8a | 16422 | 1 | 0.005
C4b8a | 16450T | 1 | 0.005
C4b8a | 195 | 1 | 0.005
C4b8a | 466 | 1 | 0.005
C4c | 11440 | 1 | 0.004
C4c | 13368 | 1 | 0.004
C4c | 16086 | 6 | 0.024
C4c | 16104 | 1 | 0.004
C4c | 16126 | 2 | 0.008
C4c | 16147 | 1 | 0.004
C4c | 16243 | 1 | 0.004
C4c | 16245 | 1 | 0.004
C4c | 16319 | 1 | 0.004
C4c | 16325 | 3 | 0.012
C4c | 16359 | 1 | 0.004
C4c | 16399 | 5 | 0.02
C4c | 513 | 1 | 0.004
C4c | 574 | 1 | 0.004
C4c1 | 1007 | 4 | 0.015
C4c1 | 11431 | 1 | 0.004
C4c1 | 13674 | 1 | 0.004
C4c1 | 13781 | 1 | 0.004
C4c1 | 1413 | 1 | 0.004
C4c1 | 14180 | 1 | 0.004
C4c1 | 14208 | 1 | 0.004
C4c1 | 143 | 8 | 0.03
C4c1 | 14311 | 1 | 0.004
C4c1 | 150 | 2 | 0.008
C4c1 | 16051 | 1 | 0.004
C4c1 | 16147 | 1 | 0.004
C4c1 | 16203 | 1 | 0.004
C4c1 | 16243 | 1 | 0.004
C4c1 | 16265C | 1 | 0.004
C4c1 | 16362 | 8 | 0.03
C4c1 | 1719 | 1 | 0.004
C4c1 | 195 | 1 | 0.004
C4c1 | 207 | 1 | 0.004
C4c1 | 310 | 3 | 0.011
C4c1 | 513 | 1 | 0.004
C4c1 | 5460 | 3 | 0.011
C4c1 | 574 | 1 | 0.004
C4c1 | 6911 | 3 | 0.011
C4c1 | 7043 | 11 | 0.042
C4c1 | 7064 | 11 | 0.042
C4c1 | 7084 | 1 | 0.004
C4c1 | 7444 | 1 | 0.004
C4c1 | 8027 | 1 | 0.004
C4c1 | 8269 | 1 | 0.004
C4c1 | 980 | 1 | 0.004
C4c1a | 11821 | 1 | 0.008
C4c1a | 12397 | 1 | 0.008
C4c1b | 1007 | 2 | 0.009
C4c1b | 14208 | 1 | 0.004
C4c1b | 16032d | 1 | 0.004
C4c1b | 16038d | 1 | 0.004
C4c1b | 16042d | 1 | 0.004
C4c1b | 16075A | 2 | 0.009
C4c1b | 16086 | 1 | 0.004
C4c1b | 16092 | 8 | 0.036
C4c1b | 16110d | 1 | 0.004
C4c1b | 16126 | 1 | 0.004
C4c1b | 16129 | 2 | 0.009
C4c1b | 16140 | 1 | 0.004
C4c1b | 16145 | 1 | 0.004
C4c1b | 16172 | 1 | 0.004
C4c1b | 16186 | 1 | 0.004
C4c1b | 16203 | 1 | 0.004
C4c1b | 16237C | 1 | 0.004
C4c1b | 16287 | 1 | 0.004
C4c1b | 16288 | 1 | 0.004
C4c1b | 16293 | 1 | 0.004
C4c1b | 16293C | 1 | 0.004
C4c1b | 16311 | 4 | 0.018
C4c1b | 16354 | 1 | 0.004
C4c1b | 16355 | 10 | 0.045
C4c1b | 16362 | 1 | 0.004
C4c1b | 16368A | 1 | 0.004
C4c1b | 16384 | 5 | 0.022
C4c1b | 16405T | 1 | 0.004
C4c1b | 16527 | 4 | 0.018
C4c1b | 415 | 1 | 0.004
C4c1b | 44.1C | 1 | 0.004
C4c1b | 513 | 2 | 0.009
C4c1b | 529 | 1 | 0.004
C4c2 | 13153 | 1 | 0.013
C4c2 | 15996 | 1 | 0.013

C4c2 | 16061 | 1 | 0.013
C4c2 | 16111 | 1 | 0.013
C4c2 | 16278 | 1 | 0.013
C4c2 | 16295 | 1 | 0.013
C4c2 | 16368 | 3 | 0.038
C4d | 10248 | 1 | 0.005
C4d | 12996T | 1 | 0.005
C4d | 13581 | 1 | 0.005
C4d | 16090 | 1 | 0.005
C4d | 16111A | 2 | 0.01
C4d | 16126 | 5 | 0.024
C4d | 16140 | 1 | 0.005
C4d | 16172 | 1 | 0.005
C4d | 16266 | 1 | 0.005
C4d | 16301 | 1 | 0.005
C4d | 16311 | 4 | 0.02
C4d | 195 | 4 | 0.02
C4d | 316C | 2 | 0.01
C4d | 5785 | 1 | 0.005
C4d | 5978 | 1 | 0.005
C4d | 6185 | 1 | 0.005
C4d | 628 | 1 | 0.005
C4d | 6927 | 1 | 0.005
C4e | 13722 | 1 | 0.005
C4e | 14392 | 1 | 0.005
C4e | 146 | 2 | 0.011
C4e | 16051 | 1 | 0.005
C4e | 16258 | 4 | 0.022
C4e | 204 | 1 | 0.005
C4e | 207 | 5 | 0.027
C4e | 5752d | 1 | 0.005
C4e | 922 | 1 | 0.005
C5 | 11092 | 5 | 0.109
C5 | 13722 | 5 | 0.109
C5 | 15080 | 5 | 0.109
C5 | 15884 | 5 | 0.109
C5 | 16177 | 1 | 0.022
C5 | 16189 | 1 | 0.022
C5 | 16214 | 1 | 0.022
C5 | 16300 | 13 | 0.283
C5 | 16390 | 2 | 0.043
C5 | 18 | 1 | 0.022
C5 | 1839 | 1 | 0.022
C5 | 248 | 1 | 0.022
C5 | 308 | 1 | 0.022
C5 | 310 | 1 | 0.022
C5 | 6191 | 1 | 0.022
C5 | 7326 | 1 | 0.022
C5 | 8407 | 1 | 0.022
C5 | 8409 | 1 | 0.022
C5+16093 | 1118T | 1 | 0.027
C5+16093 | 11827 | 1 | 0.027
C5+16093 | 12161 | 1 | 0.027
C5+16093 | 13708 | 1 | 0.027
C5+16093 | 14315 | 1 | 0.027
C5+16093 | 16166C | 1 | 0.027
C5+16093 | 16179A | 7 | 0.189
C5+16093 | 16189 | 1 | 0.027
C5+16093 | 16290 | 1 | 0.027
C5+16093 | 16362 | 1 | 0.027
C5+16093 | 16518T | 1 | 0.027
C5+16093 | 195 | 7 | 0.189
C5+16093 | 198 | 1 | 0.027
C5+16093 | 207 | 1 | 0.027
C5+16093 | 234 | 2 | 0.054
C5+16093 | 295 | 1 | 0.027
C5+16093 | 4562 | 1 | 0.027
C5+16093 | 573.6C | 1 | 0.027
C5+16093 | 6113 | 1 | 0.027
C5+16093 | 650 | 1 | 0.027
C5+16093 | 650G | 1 | 0.027
C5+16093 | 9489 | 1 | 0.027
C5a | 16016.1G | 1 | 0.019
C5a | 16019.1C | 1 | 0.019
C5a | 16031d | 1 | 0.019
C5a | 16411d | 1 | 0.019
C5a1 | 10828 | 2 | 0.027
C5a1 | 1211 | 2 | 0.027
C5a1 | 14384 | 1 | 0.014
C5a1 | 152 | 2 | 0.027
C5a1 | 16093 | 11 | 0.151
C5a1 | 3423 | 1 | 0.014
C5a1 | 345 | 2 | 0.027
C5a1 | 4216 | 3 | 0.041
C5a1 | 5319 | 3 | 0.041
C5a1 | 57 | 4 | 0.055
C5a1 | 596 | 1 | 0.014
C5a1 | 60.1T | 1 | 0.014
C5a1 | 9007 | 7 | 0.096
C5a1 | 9374 | 3 | 0.041
C5a2 | 152 | 1 | 0.016
C5a2 | 15996G | 1 | 0.016
C5a2 | 16129 | 1 | 0.016
C5a2 | 16259 | 1 | 0.016
C5a2 | 16260 | 1 | 0.016
C5a2 | 16429A | 1 | 0.016
C5a2 | 16430C | 1 | 0.016
C5a2 | 345 | 1 | 0.016
C5a2 | 8440 | 1 | 0.016
C5a2a | 16214 | 5 | 0.077
C5a2a | 310 | 1 | 0.015
C5a2b | 12351 | 1 | 0.015
C5a2b | 14845 | 4 | 0.062
C5a2b | 16299 | 1 | 0.015
C5a2b | 16309 | 4 | 0.062
C5a2b | 189 | 1 | 0.015
C5a2b | 345 | 1 | 0.015
C5a2b | 4892 | 3 | 0.046
C5a2b1 | 15151 | 1 | 0.017
C5a2b1 | 16127 | 2 | 0.034
C5a2b1 | 16243 | 1 | 0.017

C5a2b1 | 9541 | 1 | 0.017
C5b | 10031 | 1 | 0.026
C5b | 114 | 1 | 0.026
C5b | 12235 | 2 | 0.053
C5b | 152 | 3 | 0.079
C5b | 16086 | 2 | 0.053
C5b | 16172 | 1 | 0.026
C5b | 16174 | 2 | 0.053
C5b | 16189 | 2 | 0.053
C5b | 16218 | 1 | 0.026
C5b | 16230 | 1 | 0.026
C5b | 16274 | 1 | 0.026
C5b | 16305T | 1 | 0.026
C5b | 16357 | 1 | 0.026
C5b | 16497 | 1 | 0.026
C5b | 310 | 2 | 0.053
C5b | 315d | 1 | 0.026
C5b | 4705 | 2 | 0.053
C5b | 6040 | 1 | 0.026
C5b | 7775 | 2 | 0.053
C5b1 | 10927 | 1 | 0.02
C5b1 | 12151 | 1 | 0.02
C5b1 | 12295 | 1 | 0.02
C5b1 | 12530 | 1 | 0.02
C5b1 | 15217 | 1 | 0.02
C5b1 | 15799 | 1 | 0.02
C5b1 | 16126 | 2 | 0.04
C5b1 | 16192 | 2 | 0.04
C5b1 | 16274 | 1 | 0.02
C5b1 | 16278 | 2 | 0.04
C5b1 | 16319 | 4 | 0.08
C5b1 | 195 | 1 | 0.02
C5b1 | 207 | 2 | 0.04
C5b1 | 4637 | 1 | 0.02
C5b1 | 573.2C | 1 | 0.02
C5b1 | 597.1C | 1 | 0.02
C5b1 | 93 | 2 | 0.04
C5b1 | 95C | 2 | 0.04
C5b1a | 12855 | 1 | 0.022
C5b1a | 14142 | 1 | 0.022
C5b1a | 15848 | 1 | 0.022
C5b1a | 16142 | 1 | 0.022
C5b1a | 16257 | 1 | 0.022
C5b1a | 16274 | 1 | 0.022
C5b1a | 16343 | 1 | 0.022
C5b1a | 573.2C | 1 | 0.022
C5b1a | 64 | 4 | 0.089
C5b1a | 7187 | 1 | 0.022
C5b1a1 | 16094 | 1 | 0.02
C5b1a1 | 16294 | 1 | 0.02
C5b1a1 | 16297A | 2 | 0.04
C5b1a1 | 16311 | 1 | 0.02
C5b1a1 | 7568 | 1 | 0.02
C5b1a1 | 8289.1CCCCCTCTA | 2 | 0.04
C5b1b | 16325 | 1 | 0.026
C5b1b | 282 | 3 | 0.079
C5b1b1 | 12696 | 1 | 0.022
C5b1b1 | 14476 | 1 | 0.022
C5b1b1 | 310 | 1 | 0.022
C5b1b1 | 7498 | 9 | 0.2
C5c | 10967 | 1 | 0.034
C5c | 12241.1C | 1 | 0.034
C5c | 152 | 4 | 0.138
C5c | 16291 | 2 | 0.069
C5c | 3754 | 1 | 0.034
C5c+16234 | 11465 | 1 | 0.038
C5c+16234 | 146 | 1 | 0.038
C5c+16234 | 15323 | 1 | 0.038
C5c+16234 | 16311 | 1 | 0.038
C5c+16234 | 47.2G | 1 | 0.038
C5c+16234 | 475C | 1 | 0.038
C5c+16234 | 485-486d | 1 | 0.038
C5c+16234 | 49.1AATC | 1 | 0.038
C5c+16234 | 491 | 1 | 0.038
C5c+16234 | 7046 | 1 | 0.038
C5c+16234 | 71d | 1 | 0.038
C5c+16234 | 7521 | 1 | 0.038
C5c+16234 | 7694 | 1 | 0.038
C5c+16234 | 88.1G | 1 | 0.038
C5c+16234 | 9431A | 1 | 0.038
C5c1 | 16293 | 1 | 0.036
C5c1 | 249T | 2 | 0.071
C5c1 | 48G | 1 | 0.036
C5c1 | 54 | 4 | 0.143
C5c1 | 574C | 1 | 0.036
C5c1 | 576C | 1 | 0.036
C5c1 | 577C | 1 | 0.036
C5c1 | 578 | 1 | 0.036
C5c1 | 579 | 1 | 0.036
C5c1 | 580 | 1 | 0.036
C5c1a | 10042 | 1 | 0.026
C5c1a | 10927 | 1 | 0.026
C5c1a | 11150 | 1 | 0.026
C5c1a | 12130 | 1 | 0.026
C5c1a | 152 | 7 | 0.184
C5c1a | 15314 | 1 | 0.026
C5c1a | 15355 | 1 | 0.026
C5c1a | 16129 | 1 | 0.026
C5c1a | 16169 | 1 | 0.026
C5c1a | 16187 | 1 | 0.026
C5c1a | 16293 | 1 | 0.026
C5c1a | 195 | 1 | 0.026
C5c1a | 200 | 1 | 0.026
C5c1a | 5436 | 1 | 0.026
C5c1a | 597.1C | 1 | 0.026
C5c1a | 71d | 1 | 0.026
C5c1a | 7749G | 1 | 0.026
C5c1a | 9902 | 1 | 0.026
C5c1a | 9947 | 1 | 0.026
C5c1a | 9983 | 1 | 0.026
C5d | 16234 | 1 | 0.034

C5d1 | 14133 | 2 | 0.032
C5d1 | 14450 | 1 | 0.016
C5d1 | 16145 | 1 | 0.016
C5d1 | 16169 | 1 | 0.016
C5d1 | 16294 | 1 | 0.016
C5d1 | 2515 | 1 | 0.016
C5d1 | 3460 | 1 | 0.016
C5d2 | 10790 | 2 | 0.061
C5d2 | 11902 | 1 | 0.03
C5d2 | 12810 | 2 | 0.061
C5d2 | 13956 | 1 | 0.03
C5d2 | 14067 | 1 | 0.03
C5d2 | 15924 | 2 | 0.061
C5d2 | 15930 | 1 | 0.03
C5d2 | 16148 | 2 | 0.061
C5d2 | 16284 | 1 | 0.03
C5d2 | 16311 | 1 | 0.03
C5d2 | 195 | 2 | 0.061
C5d2 | 207 | 1 | 0.03
C5d2 | 3797G | 1 | 0.03
C5d2 | 4219 | 2 | 0.061
C5d2 | 5189 | 2 | 0.061
C5d2 | 6755 | 1 | 0.03
C5d2 | 7861 | 1 | 0.03
C5d2 | 8293 | 2 | 0.061
C7 | 10370 | 4 | 0.015
C7 | 10640 | 1 | 0.004
C7 | 12732 | 1 | 0.004
C7 | 13722C | 1 | 0.004
C7 | 1393 | 1 | 0.004
C7 | 13986 | 1 | 0.004
C7 | 14124 | 1 | 0.004
C7 | 143 | 1 | 0.004
C7 | 14569 | 1 | 0.004
C7 | 152 | 5 | 0.019
C7 | 15299 | 1 | 0.004
C7 | 16093 | 10 | 0.038
C7 | 16129 | 4 | 0.015
C7 | 16147 | 1 | 0.004
C7 | 16148 | 4 | 0.015
C7 | 16172 | 12 | 0.045
C7 | 16183 | 1 | 0.004
C7 | 16189 | 1 | 0.004
C7 | 16218 | 1 | 0.004
C7 | 16239 | 2 | 0.008
C7 | 16243 | 1 | 0.004
C7 | 16248 | 1 | 0.004
C7 | 16249 | 1 | 0.004
C7 | 16250 | 1 | 0.004
C7 | 16266 | 3 | 0.011
C7 | 16284 | 1 | 0.004
C7 | 16291 | 1 | 0.004
C7 | 16293 | 1 | 0.004
C7 | 16311 | 1 | 0.004
C7 | 16390 | 5 | 0.019
C7 | 1711 | 1 | 0.004
C7 | 1719 | 1 | 0.004
C7 | 195 | 3 | 0.011
C7 | 207 | 1 | 0.004
C7 | 225 | 1 | 0.004
C7 | 3394 | 1 | 0.004
C7 | 3398 | 1 | 0.004
C7 | 4216 | 1 | 0.004
C7 | 449 | 3 | 0.011
C7 | 513 | 1 | 0.004
C7 | 5177 | 1 | 0.004
C7 | 574 | 1 | 0.004
C7 | 6515 | 1 | 0.004
C7 | 7424 | 1 | 0.004
C7 | 745 | 1 | 0.004
C7 | 7861 | 1 | 0.004
C7 | 8856 | 4 | 0.015
C7 | 9 | 1 | 0.004
C7 | 9641 | 1 | 0.004
C7+16051 | 11447 | 1 | 0.007
C7+16051 | 152 | 1 | 0.007
C7+16051 | 16093 | 2 | 0.015
C7+16051 | 16145 | 1 | 0.007
C7+16051 | 16166 | 1 | 0.007
C7+16051 | 16291 | 1 | 0.007
C7+16051 | 16311 | 1 | 0.007
C7+16051 | 16326T | 1 | 0.007
C7+16051 | 16330 | 1 | 0.007
C7+16051 | 249 | 1 | 0.007
C7+16051 | 250d | 1 | 0.007
C7+16051 | 4401 | 1 | 0.007
C7+16051 | 5262 | 1 | 0.007
C7+16051 | 5264 | 1 | 0.007
C7+16051 | 593 | 1 | 0.007
C7+16051 | 6386 | 1 | 0.007
C7+16051 | 9389 | 1 | 0.007
C7a | 10310 | 3 | 0.009
C7a | 10750 | 9 | 0.028
C7a | 11255 | 2 | 0.006
C7a | 11935 | 2 | 0.006
C7a | 12672 | 5 | 0.015
C7a | 12888A | 1 | 0.003
C7a | 13926 | 1 | 0.003
C7a | 13928 | 1 | 0.003
C7a | 13934 | 1 | 0.003
C7a | 14030 | 7 | 0.022
C7a | 14180A | 1 | 0.003
C7a | 14194 | 1 | 0.003
C7a | 14463 | 1 | 0.003
C7a | 14514 | 1 | 0.003
C7a | 146 | 1 | 0.003
C7a | 1462 | 2 | 0.006
C7a | 150 | 1 | 0.003
C7a | 1503 | 1 | 0.003
C7a | 152 | 3 | 0.009
C7a | 15244 | 3 | 0.009

C7a | 15314 | 1 | 0.003
C7a | 15467 | 2 | 0.006
C7a | 15924 | 4 | 0.012
C7a | 15930 | 1 | 0.003
C7a | 16092 | 3 | 0.009
C7a | 16129 | 5 | 0.015
C7a | 16153 | 1 | 0.003
C7a | 16172 | 1 | 0.003
C7a | 16173 | 1 | 0.003
C7a | 16187 | 9 | 0.028
C7a | 16189 | 12 | 0.037
C7a | 16266 | 1 | 0.003
C7a | 16293 | 1 | 0.003
C7a | 16311 | 3 | 0.009
C7a | 16325 | 1 | 0.003
C7a | 16355 | 1 | 0.003
C7a | 16359 | 1 | 0.003
C7a | 16362 | 1 | 0.003
C7a | 16457 | 7 | 0.022
C7a | 1719 | 1 | 0.003
C7a | 1787 | 1 | 0.003
C7a | 207 | 1 | 0.003
C7a | 234 | 8 | 0.025
C7a | 310 | 1 | 0.003
C7a | 3338 | 9 | 0.028
C7a | 4062 | 7 | 0.022
C7a | 41 | 9 | 0.028
C7a | 4232 | 6 | 0.019
C7a | 4233 | 5 | 0.015
C7a | 4254 | 1 | 0.003
C7a | 44.1C | 19 | 0.059
C7a | 460 | 5 | 0.015
C7a | 463.1C | 2 | 0.006
C7a | 4977 | 1 | 0.003
C7a | 52 | 1 | 0.003
C7a | 5311 | 3 | 0.009
C7a | 574C | 1 | 0.003
C7a | 5773 | 1 | 0.003
C7a | 6218 | 1 | 0.003
C7a | 6485 | 1 | 0.003
C7a | 6533 | 1 | 0.003
C7a | 6722 | 2 | 0.006
C7a | 6890 | 1 | 0.003
C7a | 709 | 2 | 0.006
C7a | 7196 | 1 | 0.003
C7a | 7801 | 2 | 0.006
C7a | 7990 | 1 | 0.003
C7a | 8093 | 1 | 0.003
C7a | 814 | 2 | 0.006
C7a | 8152 | 1 | 0.003
C7a | 8602 | 3 | 0.009
C7a | 8832 | 1 | 0.003
C7a | 8988 | 1 | 0.003
C7a1 | 10750 | 4 | 0.015
C7a1 | 114 | 1 | 0.004
C7a1 | 11582 | 1 | 0.004
C7a1 | 12175G | 1 | 0.004
C7a1 | 12770 | 1 | 0.004
C7a1 | 13602 | 2 | 0.007
C7a1 | 143 | 5 | 0.018
C7a1 | 14755 | 1 | 0.004
C7a1 | 151 | 3 | 0.011
C7a1 | 152 | 4 | 0.015
C7a1 | 15346 | 1 | 0.004
C7a1 | 15758 | 1 | 0.004
C7a1 | 15773 | 1 | 0.004
C7a1 | 15924 | 1 | 0.004
C7a1 | 15928 | 19 | 0.07
C7a1 | 16086 | 1 | 0.004
C7a1 | 16090 | 1 | 0.004
C7a1 | 16093 | 1 | 0.004
C7a1 | 16126 | 1 | 0.004
C7a1 | 16150 | 1 | 0.004
C7a1 | 16164 | 1 | 0.004
C7a1 | 16231 | 2 | 0.007
C7a1 | 16242 | 1 | 0.004
C7a1 | 16243 | 1 | 0.004
C7a1 | 16245 | 5 | 0.018
C7a1 | 16263 | 11 | 0.04
C7a1 | 16290 | 3 | 0.011
C7a1 | 16311 | 1 | 0.004
C7a1 | 16329 | 1 | 0.004
C7a1 | 16355 | 1 | 0.004
C7a1 | 16357 | 1 | 0.004
C7a1 | 16399 | 4 | 0.015
C7a1 | 16400 | 1 | 0.004
C7a1 | 16497 | 1 | 0.004
C7a1 | 1664 | 4 | 0.015
C7a1 | 195 | 3 | 0.011
C7a1 | 199 | 1 | 0.004
C7a1 | 204 | 1 | 0.004
C7a1 | 214 | 1 | 0.004
C7a1 | 228T | 7 | 0.026
C7a1 | 319 | 38 | 0.139
C7a1 | 3537 | 4 | 0.015
C7a1 | 3786 | 1 | 0.004
C7a1 | 3795 | 2 | 0.007
C7a1 | 5174 | 19 | 0.07
C7a1 | 5628 | 1 | 0.004
C7a1 | 6206 | 1 | 0.004
C7a1 | 6250 | 1 | 0.004
C7a1 | 7085 | 10 | 0.037
C7a1 | 8296 | 1 | 0.004
C7a1 | 8515 | 1 | 0.004
C7a1 | 870 | 19 | 0.07
C7a1 | 9077 | 1 | 0.004
C7a1 | 9084 | 1 | 0.004
C7a1 | 922 | 1 | 0.004
C7a1 | 9828 | 1 | 0.004
C7a1 | 9957 | 3 | 0.011
C7a1a | 151 | 1 | 0.006

C7a1a | 16362 | 1 | 0.006
C7a1a | 401d | 1 | 0.006
C7a1a | 408G | 1 | 0.006
C7a1a | 460 | 1 | 0.006
C7a1a | 6791 | 1 | 0.006
C7a1a | 6923 | 1 | 0.006
C7a1a2 | 12612 | 1 | 0.009
C7a1a2 | 15926 | 1 | 0.009
C7a1a2 | 16217 | 1 | 0.009
C7a1a2 | 16294 | 1 | 0.009
C7a1a2 | 16318T | 1 | 0.009
C7a1a2 | 3167.1T | 3 | 0.026
C7a1a2 | 5899.1C | 1 | 0.009
C7a1a2 | 709 | 1 | 0.009
C7a1c | 13681 | 2 | 0.008
C7a1c | 1503 | 1 | 0.004
C7a1c | 15670 | 4 | 0.016
C7a1c | 159 | 3 | 0.012
C7a1c | 15930 | 1 | 0.004
C7a1c | 15951 | 3 | 0.012
C7a1c | 16086 | 1 | 0.004
C7a1c | 16090 | 1 | 0.004
C7a1c | 16172 | 4 | 0.016
C7a1c | 16175 | 4 | 0.016
C7a1c | 16217 | 1 | 0.004
C7a1c | 16219 | 1 | 0.004
C7a1c | 16243 | 1 | 0.004
C7a1c | 16266 | 5 | 0.021
C7a1c | 16309 | 4 | 0.016
C7a1c | 16311 | 4 | 0.016
C7a1c | 16316 | 1 | 0.004
C7a1c | 16326 | 3 | 0.012
C7a1c | 16329 | 1 | 0.004
C7a1c | 16362 | 4 | 0.016
C7a1c | 16527 | 1 | 0.004
C7a1c | 16540 | 3 | 0.012
C7a1c | 1719 | 1 | 0.004
C7a1c | 199 | 1 | 0.004
C7a1c | 204 | 1 | 0.004
C7a1c | 214 | 1 | 0.004
C7a1c | 338 | 4 | 0.016
C7a1c | 3736 | 1 | 0.004
C7a1c | 7982 | 1 | 0.004
C7a1c | 8270 | 1 | 0.004
C7a1c | 8281-8289d | 1 | 0.004
C7a1c | 8648 | 1 | 0.004
C7a1c | 8679 | 3 | 0.012
C7a1d | 16086 | 6 | 0.055
C7a1d | 16209 | 1 | 0.009
C7a1d | 16390 | 3 | 0.027
C7a1d | 720 | 1 | 0.009
C7a2 | 10084 | 1 | 0.004
C7a2 | 10086 | 3 | 0.012
C7a2 | 10754 | 5 | 0.021
C7a2 | 11023C | 1 | 0.004
C7a2 | 11778 | 1 | 0.004
C7a2 | 11839 | 5 | 0.021
C7a2 | 12541 | 4 | 0.017
C7a2 | 12672 | 6 | 0.025
C7a2 | 13681 | 1 | 0.004
C7a2 | 13737 | 1 | 0.004
C7a2 | 14287 | 1 | 0.004
C7a2 | 1503 | 1 | 0.004
C7a2 | 16032d | 1 | 0.004
C7a2 | 16038d | 1 | 0.004
C7a2 | 16042d | 1 | 0.004
C7a2 | 16086 | 1 | 0.004
C7a2 | 16110d | 1 | 0.004
C7a2 | 16140 | 1 | 0.004
C7a2 | 16153 | 1 | 0.004
C7a2 | 16172 | 1 | 0.004
C7a2 | 16213 | 2 | 0.008
C7a2 | 16237C | 1 | 0.004
C7a2 | 16239 | 6 | 0.025
C7a2 | 16287 | 1 | 0.004
C7a2 | 16293 | 1 | 0.004
C7a2 | 16293C | 1 | 0.004
C7a2 | 16319 | 11 | 0.046
C7a2 | 16384 | 5 | 0.021
C7a2 | 1711 | 3 | 0.012
C7a2 | 189 | 1 | 0.004
C7a2 | 200 | 4 | 0.017
C7a2 | 207 | 9 | 0.038
C7a2 | 214 | 1 | 0.004
C7a2 | 310 | 2 | 0.008
C7a2 | 315.3C | 1 | 0.004
C7a2 | 372 | 1 | 0.004
C7a2 | 4062 | 7 | 0.029
C7a2 | 4254 | 1 | 0.004
C7a2 | 44.1C | 1 | 0.004
C7a2 | 508 | 1 | 0.004
C7a2 | 5918 | 1 | 0.004
C7a2 | 593 | 2 | 0.008
C7a2 | 6152 | 1 | 0.004
C7a2 | 6249 | 1 | 0.004
C7a2 | 7007 | 1 | 0.004
C7a2 | 7828 | 1 | 0.004
C7a2 | 7990 | 1 | 0.004
C7a2 | 8289.1CCCCCTCTA | 1 | 0.004
C7a2 | 8473 | 1 | 0.004
C7a2a | 12634 | 1 | 0.004
C7a2a | 13858 | 1 | 0.004
C7a2a | 14226 | 3 | 0.013
C7a2a | 14769 | 1 | 0.004
C7a2a | 16032d | 1 | 0.004
C7a2a | 16038d | 1 | 0.004
C7a2a | 16042d | 1 | 0.004
C7a2a | 16086 | 1 | 0.004
C7a2a | 16104G | 3 | 0.013
C7a2a | 16110d | 1 | 0.004
C7a2a | 16129 | 1 | 0.004

C7a2a | 16140 | 1 | 0.004
C7a2a | 16172 | 1 | 0.004
C7a2a | 16237C | 1 | 0.004
C7a2a | 16261 | 16 | 0.072
C7a2a | 16287 | 1 | 0.004
C7a2a | 16293 | 1 | 0.004
C7a2a | 16293C | 1 | 0.004
C7a2a | 16384 | 1 | 0.004
C7a2a | 16391 | 5 | 0.022
C7a2a | 195 | 1 | 0.004
C7a2a | 378 | 1 | 0.004
C7a2a | 44.1C | 1 | 0.004
C7a2a | 7007 | 1 | 0.004
C7b | 11923 | 1 | 0.006
C7b | 13770 | 2 | 0.013
C7b | 146 | 3 | 0.019
C7b | 15256 | 1 | 0.006
C7b | 16093 | 1 | 0.006
C7b | 16111 | 1 | 0.006
C7b | 16126 | 1 | 0.006
C7b | 16144A | 1 | 0.006
C7b | 16164 | 2 | 0.013
C7b | 16189 | 12 | 0.077
C7b | 16209 | 3 | 0.019
C7b | 16278 | 7 | 0.045
C7b | 16311 | 1 | 0.006
C7b | 16319 | 3 | 0.019
C7b | 16325 | 3 | 0.019
C7b | 16362 | 1 | 0.006
C7b | 188 | 3 | 0.019
C7b | 195 | 4 | 0.026
C7b | 200 | 1 | 0.006
C7b | 204 | 3 | 0.019
C7b | 2762 | 1 | 0.006
C7b | 316C | 1 | 0.006
C7b | 5573 | 1 | 0.006
C7b | 8233 | 1 | 0.006
C7b | 9449 | 2 | 0.013
CZ | 152 | 1 | 0.007
CZ | 16051 | 3 | 0.02
CZ | 16075 | 1 | 0.007
CZ | 16095 | 1 | 0.007
CZ | 16129 | 1 | 0.007
CZ | 16136 | 2 | 0.014
CZ | 16148 | 1 | 0.007
CZ | 16189 | 2 | 0.014
CZ | 16218 | 2 | 0.014
CZ | 16234 | 2 | 0.014
CZ | 16260 | 4 | 0.027
CZ | 16261 | 3 | 0.02
CZ | 16311 | 2 | 0.014
CZ | 16326T | 1 | 0.007
CZ | 16357 | 2 | 0.014
CZ | 16527 | 1 | 0.007
CZ | 200 | 1 | 0.007
CZ | 204 | 3 | 0.02
CZ | 207 | 3 | 0.02
CZ | 210 | 1 | 0.007
CZ | 316C | 2 | 0.014
CZ | 544A | 1 | 0.007
CZ | 93 | 3 | 0.02
D | 16165 | 1 | 0.001
D | 16179 | 1 | 0.001
D | 16218 | 4 | 0.006
D | 446 | 1 | 0.001
D | 648 | 1 | 0.001
D+16189 | 137 | 1 | 0.005
D+16189 | 142 | 1 | 0.005
D+16189 | 143 | 1 | 0.005
D+16189 | 152 | 1 | 0.005
D+16189 | 153 | 1 | 0.005
D+16189 | 16037 | 2 | 0.01
D+16189 | 16051 | 2 | 0.01
D+16189 | 16074 | 1 | 0.005
D+16189 | 16090 | 1 | 0.005
D+16189 | 16093 | 1 | 0.005
D+16189 | 16095 | 1 | 0.005
D+16189 | 16130 | 1 | 0.005
D+16189 | 16167 | 2 | 0.01
D+16189 | 16172 | 1 | 0.005
D+16189 | 16188 | 2 | 0.01
D+16189 | 16192 | 1 | 0.005
D+16189 | 16193.4C | 1 | 0.005
D+16189 | 16193d | 3 | 0.016
D+16189 | 16218 | 22 | 0.115
D+16189 | 16259A | 1 | 0.005
D+16189 | 16277T | 1 | 0.005
D+16189 | 16288 | 1 | 0.005
D+16189 | 16291 | 1 | 0.005
D+16189 | 16295 | 2 | 0.01
D+16189 | 16316 | 2 | 0.01
D+16189 | 16377 | 1 | 0.005
D+16189 | 16391 | 1 | 0.005
D+16189 | 16421 | 1 | 0.005
D+16189 | 16497 | 1 | 0.005
D+16189 | 194 | 4 | 0.021
D+16189 | 195 | 6 | 0.031
D+16189 | 200 | 1 | 0.005
D+16189 | 205 | 1 | 0.005
D+16189 | 21 | 1 | 0.005
D+16189 | 257 | 1 | 0.005
D+16189 | 310 | 1 | 0.005
D+16189 | 314-315d | 1 | 0.005
D+16189 | 329 | 1 | 0.005
D+16189 | 366 | 1 | 0.005
D+16189 | 408 | 1 | 0.005
D+16189 | 416 | 1 | 0.005
D+16189 | 438 | 1 | 0.005
D+16189 | 461 | 1 | 0.005
D+16189 | 484 | 1 | 0.005
D+16189 | 488 | 2 | 0.01

D+16189 | 499 | 1 | 0.005
D+16189 | 512T | 1 | 0.005
D+16189 | 519 | 1 | 0.005
D+16189 | 556d | 1 | 0.005
D+16189 | 64 | 1 | 0.005
D+16189 | 770 | 1 | 0.005
D+16189 | 850 | 1 | 0.005
D+16189 | 93 | 1 | 0.005
D1 | 10007 | 1 | 0.002
D1 | 10030 | 4 | 0.009
D1 | 10042 | 1 | 0.002
D1 | 10097 | 1 | 0.002
D1 | 10118 | 21 | 0.048
D1 | 10163 | 1 | 0.002
D1 | 10188 | 1 | 0.002
D1 | 10208 | 1 | 0.002
D1 | 103 | 1 | 0.002
D1 | 10448 | 1 | 0.002
D1 | 10524 | 1 | 0.002
D1 | 10601 | 1 | 0.002
D1 | 10604 | 2 | 0.005
D1 | 10739 | 2 | 0.005
D1 | 10793 | 1 | 0.002
D1 | 10822G | 7 | 0.016
D1 | 10993 | 2 | 0.005
D1 | 11002 | 2 | 0.005
D1 | 11087 | 7 | 0.016
D1 | 11092 | 1 | 0.002
D1 | 11150 | 3 | 0.007
D1 | 11152 | 5 | 0.011
D1 | 11176 | 1 | 0.002
D1 | 11204 | 1 | 0.002
D1 | 11252 | 1 | 0.002
D1 | 11253 | 1 | 0.002
D1 | 11302 | 1 | 0.002
D1 | 11318 | 1 | 0.002
D1 | 11339 | 1 | 0.002
D1 | 11353 | 1 | 0.002
D1 | 11365 | 2 | 0.005
D1 | 11368 | 1 | 0.002
D1 | 11410 | 6 | 0.014
D1 | 11455 | 2 | 0.005
D1 | 11545 | 1 | 0.002
D1 | 11608 | 1 | 0.002
D1 | 11665 | 3 | 0.007
D1 | 11797 | 1 | 0.002
D1 | 11830 | 3 | 0.007
D1 | 11884 | 1 | 0.002
D1 | 11906 | 2 | 0.005
D1 | 11928 | 22 | 0.05
D1 | 11959 | 8 | 0.018
D1 | 12011 | 1 | 0.002
D1 | 12063 | 1 | 0.002
D1 | 12127 | 7 | 0.016
D1 | 12215 | 1 | 0.002
D1 | 12280 | 1 | 0.002
D1 | 12358 | 1 | 0.002
D1 | 12361 | 1 | 0.002
D1 | 12366 | 1 | 0.002
D1 | 12372 | 1 | 0.002
D1 | 12381 | 1 | 0.002
D1 | 12397 | 1 | 0.002
D1 | 12403 | 2 | 0.005
D1 | 12481 | 2 | 0.005
D1 | 12557 | 2 | 0.005
D1 | 12568 | 1 | 0.002
D1 | 12618 | 4 | 0.009
D1 | 12630 | 2 | 0.005
D1 | 12732 | 21 | 0.048
D1 | 12771 | 7 | 0.016
D1 | 12793 | 1 | 0.002
D1 | 12795 | 1 | 0.002
D1 | 12930 | 1 | 0.002
D1 | 131 | 1 | 0.002
D1 | 13105 | 26 | 0.059
D1 | 13227 | 1 | 0.002
D1 | 13287 | 3 | 0.007
D1 | 133 | 1 | 0.002
D1 | 13308 | 1 | 0.002
D1 | 13317 | 1 | 0.002
D1 | 13326 | 1 | 0.002
D1 | 13395 | 1 | 0.002
D1 | 13404 | 3 | 0.007
D1 | 13419 | 1 | 0.002
D1 | 13431 | 5 | 0.011
D1 | 13488 | 1 | 0.002
D1 | 13514A | 1 | 0.002
D1 | 13514T | 1 | 0.002
D1 | 13528 | 1 | 0.002
D1 | 13581 | 1 | 0.002
D1 | 13597 | 1 | 0.002
D1 | 13604C | 1 | 0.002
D1 | 13608 | 1 | 0.002
D1 | 13681 | 1 | 0.002
D1 | 13719 | 1 | 0.002
D1 | 13734 | 3 | 0.007
D1 | 13743 | 1 | 0.002
D1 | 13781 | 1 | 0.002
D1 | 13809A | 1 | 0.002
D1 | 13812 | 15 | 0.034
D1 | 13827 | 3 | 0.007
D1 | 139 | 2 | 0.005
D1 | 13908 | 1 | 0.002
D1 | 13934 | 3 | 0.007
D1 | 140 | 1 | 0.002
D1 | 14020 | 1 | 0.002
D1 | 14025 | 1 | 0.002
D1 | 14034 | 1 | 0.002
D1 | 14055 | 1 | 0.002
D1 | 14071 | 1 | 0.002
D1 | 14097 | 1 | 0.002

D1 | 14110 | 3 | 0.007
D1 | 14129 | 1 | 0.002
D1 | 14197A | 3 | 0.007
D1 | 14215 | 6 | 0.014
D1 | 14249 | 1 | 0.002
D1 | 14256 | 21 | 0.048
D1 | 14272G | 1 | 0.002
D1 | 14275 | 1 | 0.002
D1 | 14280 | 1 | 0.002
D1 | 143 | 41 | 0.094
D1 | 14334 | 1 | 0.002
D1 | 14365G | 1 | 0.002
D1 | 14368G | 1 | 0.002
D1 | 14384 | 1 | 0.002
D1 | 14443 | 1 | 0.002
D1 | 14459 | 1 | 0.002
D1 | 14470 | 1 | 0.002
D1 | 14484 | 1 | 0.002
D1 | 14494 | 1 | 0.002
D1 | 14569 | 1 | 0.002
D1 | 14593 | 1 | 0.002
D1 | 146 | 30 | 0.069
D1 | 14605 | 1 | 0.002
D1 | 14632 | 3 | 0.007
D1 | 14665 | 2 | 0.005
D1 | 14690 | 2 | 0.005
D1 | 14693 | 1 | 0.002
D1 | 14776 | 1 | 0.002
D1 | 14803 | 3 | 0.007
D1 | 14861 | 2 | 0.005
D1 | 14914 | 1 | 0.002
D1 | 150 | 13 | 0.03
D1 | 15034 | 1 | 0.002
D1 | 1508 | 1 | 0.002
D1 | 15106 | 9 | 0.021
D1 | 15172 | 2 | 0.005
D1 | 15193 | 2 | 0.005
D1 | 152 | 10 | 0.023
D1 | 15221 | 1 | 0.002
D1 | 15229 | 1 | 0.002
D1 | 15244 | 5 | 0.011
D1 | 15287 | 3 | 0.007
D1 | 15289 | 1 | 0.002
D1 | 153 | 4 | 0.009
D1 | 15316 | 4 | 0.009
D1 | 15346 | 1 | 0.002
D1 | 15388 | 1 | 0.002
D1 | 154 | 1 | 0.002
D1 | 15402 | 6 | 0.014
D1 | 15440 | 1 | 0.002
D1 | 15452 | 1 | 0.002
D1 | 15497 | 5 | 0.011
D1 | 15514 | 5 | 0.011
D1 | 15519 | 1 | 0.002
D1 | 15530 | 5 | 0.011
D1 | 15565 | 2 | 0.005
D1 | 15672 | 1 | 0.002
D1 | 15697 | 1 | 0.002
D1 | 15727 | 1 | 0.002
D1 | 15760 | 11 | 0.025
D1 | 15777 | 1 | 0.002
D1 | 15784 | 2 | 0.005
D1 | 15799 | 1 | 0.002
D1 | 15813 | 3 | 0.007
D1 | 15884 | 2 | 0.005
D1 | 15903 | 1 | 0.002
D1 | 15924 | 3 | 0.007
D1 | 15928 | 1 | 0.002
D1 | 15930 | 1 | 0.002
D1 | 15938 | 1 | 0.002
D1 | 1598 | 2 | 0.005
D1 | 15984 | 1 | 0.002
D1 | 16051 | 3 | 0.007
D1 | 16092 | 2 | 0.005
D1 | 16093 | 23 | 0.053
D1 | 16104 | 1 | 0.002
D1 | 16114 | 1 | 0.002
D1 | 16124 | 1 | 0.002
D1 | 16126 | 1 | 0.002
D1 | 16129 | 12 | 0.027
D1 | 16140 | 1 | 0.002
D1 | 16147 | 1 | 0.002
D1 | 16154 | 2 | 0.005
D1 | 16157 | 2 | 0.005
D1 | 16166 | 1 | 0.002
D1 | 16168 | 1 | 0.002
D1 | 16172 | 2 | 0.005
D1 | 16174 | 27 | 0.062
D1 | 16175 | 1 | 0.002
D1 | 16179 | 2 | 0.005
D1 | 16184 | 3 | 0.007
D1 | 16186 | 1 | 0.002
D1 | 16187A | 1 | 0.002
D1 | 16188 | 2 | 0.005
D1 | 16189 | 17 | 0.039
D1 | 16190 | 2 | 0.005
D1 | 16192 | 1 | 0.002
D1 | 16193 | 3 | 0.007
D1 | 16193d | 1 | 0.002
D1 | 16194C | 1 | 0.002
D1 | 16195 | 1 | 0.002
D1 | 16209 | 22 | 0.05
D1 | 16217 | 1 | 0.002
D1 | 16221 | 1 | 0.002
D1 | 16222 | 1 | 0.002
D1 | 16239 | 6 | 0.014
D1 | 16241 | 1 | 0.002
D1 | 16248 | 1 | 0.002
D1 | 16256 | 2 | 0.005
D1 | 16261 | 1 | 0.002
D1 | 16263 | 25 | 0.057

D1 | 16264.1C | 1 | 0.002
D1 | 16265C | 1 | 0.002
D1 | 16270 | 1 | 0.002
D1 | 16274 | 2 | 0.005
D1 | 16278 | 1 | 0.002
D1 | 1627A | 1 | 0.002
D1 | 16286 | 4 | 0.009
D1 | 16288 | 1 | 0.002
D1 | 16291 | 13 | 0.03
D1 | 16292 | 2 | 0.005
D1 | 16294 | 1 | 0.002
D1 | 16298 | 1 | 0.002
D1 | 16299 | 1 | 0.002
D1 | 16300 | 1 | 0.002
D1 | 16301 | 2 | 0.005
D1 | 16304 | 3 | 0.007
D1 | 16311 | 6 | 0.014
D1 | 16317 | 1 | 0.002
D1 | 16319 | 6 | 0.014
D1 | 16320 | 1 | 0.002
D1 | 16324 | 1 | 0.002
D1 | 16325A | 1 | 0.002
D1 | 16342 | 3 | 0.007
D1 | 16352 | 1 | 0.002
D1 | 16354 | 1 | 0.002
D1 | 16355 | 1 | 0.002
D1 | 16356 | 5 | 0.011
D1 | 16357 | 2 | 0.005
D1 | 16361 | 25 | 0.057
D1 | 16362d | 1 | 0.002
D1 | 16365 | 2 | 0.005
D1 | 16368 | 2 | 0.005
D1 | 16371 | 1 | 0.002
D1 | 16380 | 2 | 0.005
D1 | 16381 | 1 | 0.002
D1 | 16390 | 33 | 0.076
D1 | 16399 | 1 | 0.002
D1 | 16422 | 9 | 0.021
D1 | 16428 | 1 | 0.002
D1 | 16483 | 5 | 0.011
D1 | 16524C | 1 | 0.002
D1 | 16526 | 2 | 0.005
D1 | 16527 | 6 | 0.014
D1 | 1686 | 4 | 0.009
D1 | 1719 | 6 | 0.014
D1 | 179 | 1 | 0.002
D1 | 1821 | 21 | 0.048
D1 | 183 | 6 | 0.014
D1 | 1848 | 1 | 0.002
D1 | 185 | 2 | 0.005
D1 | 189 | 3 | 0.007
D1 | 195 | 7 | 0.016
D1 | 196 | 9 | 0.021
D1 | 199 | 3 | 0.007
D1 | 200 | 2 | 0.005
D1 | 204 | 7 | 0.016
D1 | 207 | 27 | 0.062
D1 | 210 | 4 | 0.009
D1 | 213 | 1 | 0.002
D1 | 214 | 6 | 0.014
D1 | 2156.1A | 1 | 0.002
D1 | 222 | 1 | 0.002
D1 | 2251 | 1 | 0.002
D1 | 226 | 2 | 0.005
D1 | 2263A | 4 | 0.009
D1 | 228 | 4 | 0.009
D1 | 2387 | 1 | 0.002
D1 | 240 | 1 | 0.002
D1 | 2404 | 1 | 0.002
D1 | 2417 | 2 | 0.005
D1 | 2757 | 1 | 0.002
D1 | 285 | 8 | 0.018
D1 | 289 | 1 | 0.002
D1 | 292 | 1 | 0.002
D1 | 297 | 1 | 0.002
D1 | 309.3C | 2 | 0.005
D1 | 310 | 6 | 0.014
D1 | 315.2C | 2 | 0.005
D1 | 318 | 1 | 0.002
D1 | 3200 | 1 | 0.002
D1 | 327 | 1 | 0.002
D1 | 3363 | 1 | 0.002
D1 | 3397 | 4 | 0.009
D1 | 3421 | 1 | 0.002
D1 | 3438 | 1 | 0.002
D1 | 3531 | 1 | 0.002
D1 | 3584 | 4 | 0.009
D1 | 3591 | 21 | 0.048
D1 | 3639 | 1 | 0.002
D1 | 3699G | 1 | 0.002
D1 | 3736 | 1 | 0.002
D1 | 3764 | 1 | 0.002
D1 | 3805 | 1 | 0.002
D1 | 3834 | 1 | 0.002
D1 | 3921 | 1 | 0.002
D1 | 4131 | 26 | 0.059
D1 | 4204 | 1 | 0.002
D1 | 4218 | 1 | 0.002
D1 | 4219 | 28 | 0.064
D1 | 4550 | 1 | 0.002
D1 | 456 | 4 | 0.009
D1 | 4583 | 1 | 0.002
D1 | 460 | 1 | 0.002
D1 | 4658 | 1 | 0.002
D1 | 466 | 1 | 0.002
D1 | 4688 | 2 | 0.005
D1 | 4704 | 1 | 0.002
D1 | 4814 | 1 | 0.002
D1 | 482 | 1 | 0.002
D1 | 4853 | 7 | 0.016
D1 | 487 | 1 | 0.002

D1 | 4904 | 1 | 0.002
D1 | 4907 | 3 | 0.007
D1 | 4973 | 1 | 0.002
D1 | 502 | 5 | 0.011
D1 | 5095 | 4 | 0.009
D1 | 513 | 1 | 0.002
D1 | 5205 | 1 | 0.002
D1 | 5208 | 2 | 0.005
D1 | 524.1ACCA | 1 | 0.002
D1 | 525.1AC | 1 | 0.002
D1 | 5324 | 2 | 0.005
D1 | 5417 | 4 | 0.009
D1 | 544 | 6 | 0.014
D1 | 5451 | 1 | 0.002
D1 | 5460 | 2 | 0.005
D1 | 5465 | 2 | 0.005
D1 | 55 | 1 | 0.002
D1 | 55.1T | 5 | 0.011
D1 | 5585 | 1 | 0.002
D1 | 56 | 1 | 0.002
D1 | 5655 | 4 | 0.009
D1 | 57 | 6 | 0.014
D1 | 57.1G | 1 | 0.002
D1 | 5752.1A | 1 | 0.002
D1 | 5786 | 1 | 0.002
D1 | 5839 | 11 | 0.025
D1 | 5894C | 1 | 0.002
D1 | 59 | 5 | 0.011
D1 | 593 | 1 | 0.002
D1 | 60 | 2 | 0.005
D1 | 6053 | 1 | 0.002
D1 | 61 | 2 | 0.005
D1 | 6113 | 2 | 0.005
D1 | 6218 | 2 | 0.005
D1 | 6254 | 1 | 0.002
D1 | 6261 | 2 | 0.005
D1 | 6267 | 1 | 0.002
D1 | 6272 | 1 | 0.002
D1 | 629 | 5 | 0.011
D1 | 62T | 4 | 0.009
D1 | 63 | 1 | 0.002
D1 | 630 | 3 | 0.007
D1 | 6340 | 1 | 0.002
D1 | 6359 | 3 | 0.007
D1 | 6366 | 1 | 0.002
D1 | 6395 | 1 | 0.002
D1 | 64 | 2 | 0.005
D1 | 6446 | 1 | 0.002
D1 | 6465 | 2 | 0.005
D1 | 6485 | 1 | 0.002
D1 | 64A | 3 | 0.007
D1 | 6515 | 1 | 0.002
D1 | 6528 | 1 | 0.002
D1 | 65G | 2 | 0.005
D1 | 66 | 4 | 0.009
D1 | 6602 | 1 | 0.002
D1 | 6635 | 6 | 0.014
D1 | 6680 | 1 | 0.002
D1 | 66T | 1 | 0.002
D1 | 6719G | 1 | 0.002
D1 | 6752 | 1 | 0.002
D1 | 6779 | 2 | 0.005
D1 | 6859T | 1 | 0.002
D1 | 6872 | 2 | 0.005
D1 | 7055 | 2 | 0.005
D1 | 7073 | 1 | 0.002
D1 | 7080 | 1 | 0.002
D1 | 709 | 1 | 0.002
D1 | 71.1G | 6 | 0.014
D1 | 7175 | 1 | 0.002
D1 | 71d | 2 | 0.005
D1 | 72 | 3 | 0.007
D1 | 7278 | 1 | 0.002
D1 | 729 | 1 | 0.002
D1 | 735 | 2 | 0.005
D1 | 7352 | 4 | 0.009
D1 | 7361 | 1 | 0.002
D1 | 7376 | 1 | 0.002
D1 | 7389 | 3 | 0.007
D1 | 7407 | 1 | 0.002
D1 | 7427 | 1 | 0.002
D1 | 745.1T | 3 | 0.007
D1 | 748 | 1 | 0.002
D1 | 75 | 7 | 0.016
D1 | 752A | 3 | 0.007
D1 | 769 | 2 | 0.005
D1 | 7747 | 12 | 0.027
D1 | 7805 | 1 | 0.002
D1 | 7859 | 1 | 0.002
D1 | 8022 | 8 | 0.018
D1 | 8038 | 3 | 0.007
D1 | 8093 | 2 | 0.005
D1 | 8113 | 3 | 0.007
D1 | 8125 | 1 | 0.002
D1 | 813 | 1 | 0.002
D1 | 8191 | 1 | 0.002
D1 | 8222 | 3 | 0.007
D1 | 8251 | 1 | 0.002
D1 | 827 | 1 | 0.002
D1 | 8348 | 2 | 0.005
D1 | 8387 | 1 | 0.002
D1 | 8392 | 1 | 0.002
D1 | 8477 | 1 | 0.002
D1 | 85 | 5 | 0.011
D1 | 8541 | 1 | 0.002
D1 | 8557 | 2 | 0.005
D1 | 8592 | 1 | 0.002
D1 | 8659 | 1 | 0.002
D1 | 8705 | 1 | 0.002
D1 | 8843 | 1 | 0.002
D1 | 8848 | 1 | 0.002

D1 | 8860T | 1 | 0.002
D1 | 8871 | 1 | 0.002
D1 | 8959 | 2 | 0.005
D1 | 9 | 2 | 0.005
D1 | 9055 | 1 | 0.002
D1 | 9098 | 1 | 0.002
D1 | 9098G | 1 | 0.002
D1 | 9111 | 3 | 0.007
D1 | 9198 | 1 | 0.002
D1 | 9254 | 1 | 0.002
D1 | 9287C | 2 | 0.005
D1 | 93 | 2 | 0.005
D1 | 931 | 2 | 0.005
D1 | 9316 | 1 | 0.002
D1 | 9355 | 1 | 0.002
D1 | 9389 | 1 | 0.002
D1 | 94 | 1 | 0.002
D1 | 9424 | 7 | 0.016
D1 | 9469 | 1 | 0.002
D1 | 9490 | 1 | 0.002
D1 | 9536 | 3 | 0.007
D1 | 9559G | 1 | 0.002
D1 | 9591 | 1 | 0.002
D1 | 960.1C | 20 | 0.046
D1 | 961 | 2 | 0.005
D1 | 9612 | 1 | 0.002
D1 | 965.2C | 1 | 0.002
D1 | 965.3C | 1 | 0.002
D1 | 9667 | 2 | 0.005
D1 | 9668 | 1 | 0.002
D1 | 9682 | 1 | 0.002
D1 | 9701 | 5 | 0.011
D1 | 9716 | 1 | 0.002
D1 | 9739 | 7 | 0.016
D1 | 9758 | 1 | 0.002
D1 | 9801 | 2 | 0.005
D1 | 9804 | 1 | 0.002
D1 | 9881 | 1 | 0.002
D1 | 9965 | 1 | 0.002
D1 | 9966 | 2 | 0.005
D1a | 152 | 1 | 0.004
D1a | 15765 | 1 | 0.004
D1a | 16173 | 1 | 0.004
D1a | 16192 | 1 | 0.004
D1a | 16217 | 1 | 0.004
D1a | 16268 | 1 | 0.004
D1a | 16311 | 1 | 0.004
D1a | 16380 | 1 | 0.004
D1a | 16505 | 1 | 0.004
D1a | 16527 | 1 | 0.004
D1a | 194 | 1 | 0.004
D1a | 195 | 11 | 0.049
D1a | 205 | 1 | 0.004
D1a | 244 | 4 | 0.018
D1a | 4626 | 1 | 0.004
D1a | 5460 | 1 | 0.004
D1a | 63 | 4 | 0.018
D1a | 64 | 4 | 0.018
D1a | 66 | 4 | 0.018
D1a | 7930 | 1 | 0.004
D1a1 | 16131G | 1 | 0.006
D1a1 | 310 | 8 | 0.05
D1a1 | 315.2C | 1 | 0.006
D1a1 | 5471 | 40 | 0.248
D1a1 | 70 | 55 | 0.342
D1a2 | 10505 | 1 | 0.004
D1a2 | 106-111d | 3 | 0.012
D1a2 | 14016 | 2 | 0.008
D1a2 | 14094 | 2 | 0.008
D1a2 | 152 | 4 | 0.016
D1a2 | 153 | 1 | 0.004
D1a2 | 154 | 1 | 0.004
D1a2 | 16140 | 2 | 0.008
D1a2 | 16153 | 1 | 0.004
D1a2 | 16172 | 1 | 0.004
D1a2 | 16176 | 1 | 0.004
D1a2 | 16188 | 3 | 0.012
D1a2 | 16189 | 1 | 0.004
D1a2 | 16192 | 4 | 0.016
D1a2 | 16217 | 1 | 0.004
D1a2 | 16221 | 1 | 0.004
D1a2 | 16235 | 1 | 0.004
D1a2 | 16239 | 1 | 0.004
D1a2 | 16243 | 2 | 0.008
D1a2 | 16260 | 1 | 0.004
D1a2 | 16261 | 1 | 0.004
D1a2 | 16270 | 1 | 0.004
D1a2 | 16290 | 1 | 0.004
D1a2 | 16292 | 1 | 0.004
D1a2 | 16293 | 2 | 0.008
D1a2 | 16303 | 7 | 0.027
D1a2 | 16327 | 1 | 0.004
D1a2 | 16356 | 1 | 0.004
D1a2 | 16482T | 1 | 0.004
D1a2 | 16526 | 1 | 0.004
D1a2 | 182 | 4 | 0.016
D1a2 | 189 | 1 | 0.004
D1a2 | 194 | 1 | 0.004
D1a2 | 199 | 2 | 0.008
D1a2 | 204 | 2 | 0.008
D1a2 | 205 | 1 | 0.004
D1a2 | 227 | 4 | 0.016
D1a2 | 279 | 1 | 0.004
D1a2 | 315.2C | 1 | 0.004
D1a2 | 316 | 1 | 0.004
D1a2 | 326 | 2 | 0.008
D1a2 | 453 | 1 | 0.004
D1a2 | 518 | 7 | 0.027
D1a2 | 55.1T | 1 | 0.004
D1a2 | 57 | 1 | 0.004
D1a2 | 93 | 1 | 0.004

D1a2 | 97 | 2 | 0.008
D1b | 16217 | 1 | 0.005
D1b | 16266 | 3 | 0.014
D1b | 16311 | 1 | 0.005
D1b | 194 | 1 | 0.005
D1b | 205 | 1 | 0.005
D1b | 6182 | 1 | 0.005
D1c | 12468 | 1 | 0.005
D1c | 12474 | 1 | 0.005
D1c | 13681 | 2 | 0.009
D1c | 13920 | 2 | 0.009
D1c | 16217 | 1 | 0.005
D1c | 194 | 1 | 0.005
D1c | 205 | 1 | 0.005
D1c | 8380 | 2 | 0.009
D1d | 146 | 1 | 0.005
D1d | 16172 | 1 | 0.005
D1d | 16217 | 1 | 0.005
D1d | 16249 | 2 | 0.009
D1d | 16266 | 3 | 0.014
D1d | 16311 | 1 | 0.005
D1d | 194 | 1 | 0.005
D1d | 205 | 1 | 0.005
D1d | 5147 | 1 | 0.005
D1d | 5752d | 1 | 0.005
D1d1 | 11630 | 1 | 0.005
D1d1 | 13105 | 2 | 0.009
D1d1 | 13821A | 2 | 0.009
D1d1 | 16145 | 3 | 0.014
D1d1 | 16217 | 1 | 0.005
D1d1 | 194 | 1 | 0.005
D1d1 | 200 | 1 | 0.005
D1d1 | 205 | 1 | 0.005
D1d1 | 7142 | 1 | 0.005
D1d2 | 10915 | 1 | 0.006
D1d2 | 143 | 10 | 0.055
D1d2 | 146 | 1 | 0.006
D1d2 | 158 | 1 | 0.006
D1d2 | 16126 | 1 | 0.006
D1d2 | 16398 | 1 | 0.006
D1d2 | 195 | 2 | 0.011
D1d2 | 321 | 1 | 0.006
D1d2 | 5752d | 1 | 0.006
D1e | 109 | 12 | 0.054
D1e | 11482 | 1 | 0.004
D1e | 12490 | 2 | 0.009
D1e | 12892 | 1 | 0.004
D1e | 143 | 4 | 0.018
D1e | 151 | 2 | 0.009
D1e | 152 | 4 | 0.018
D1e | 16051 | 1 | 0.004
D1e | 16095 | 1 | 0.004
D1e | 16172 | 1 | 0.004
D1e | 16174 | 1 | 0.004
D1e | 16189 | 4 | 0.018
D1e | 16209 | 1 | 0.004
D1e | 16217 | 3 | 0.013
D1e | 16221 | 2 | 0.009
D1e | 16236A | 1 | 0.004
D1e | 16249 | 1 | 0.004
D1e | 16261 | 46 | 0.206
D1e | 16263 | 1 | 0.004
D1e | 16289 | 9 | 0.04
D1e | 16291 | 1 | 0.004
D1e | 16304 | 2 | 0.009
D1e | 16343 | 4 | 0.018
D1e | 16357 | 5 | 0.022
D1e | 16361 | 1 | 0.004
D1e | 16390 | 3 | 0.013
D1e | 182 | 4 | 0.018
D1e | 183 | 2 | 0.009
D1e | 195 | 1 | 0.004
D1e | 196 | 4 | 0.018
D1e | 207 | 1 | 0.004
D1e | 309d | 1 | 0.004
D1e | 310 | 5 | 0.022
D1e | 3663 | 27 | 0.121
D1e | 390 | 1 | 0.004
D1e | 9753 | 1 | 0.004
D1f | 10 | 1 | 0.005
D1f | 10084 | 2 | 0.01
D1f | 10172 | 1 | 0.005
D1f | 10328 | 2 | 0.01
D1f | 10454 | 1 | 0.005
D1f | 105-110d | 1 | 0.005
D1f | 10652 | 3 | 0.015
D1f | 10688 | 4 | 0.02
D1f | 10799 | 1 | 0.005
D1f | 10900 | 1 | 0.005
D1f | 11101 | 1 | 0.005
D1f | 11116 | 1 | 0.005
D1f | 1193 | 4 | 0.02
D1f | 12121 | 1 | 0.005
D1f | 12346 | 1 | 0.005
D1f | 12358 | 2 | 0.01
D1f | 12477 | 1 | 0.005
D1f | 12530 | 7 | 0.035
D1f | 12561 | 1 | 0.005
D1f | 13500 | 4 | 0.02
D1f | 14034 | 1 | 0.005
D1f | 14569 | 3 | 0.015
D1f | 14587 | 1 | 0.005
D1f | 146 | 1 | 0.005
D1f | 14798 | 1 | 0.005
D1f | 150 | 1 | 0.005
D1f | 151 | 2 | 0.01
D1f | 15199 | 4 | 0.02
D1f | 152 | 17 | 0.084
D1f | 15212 | 1 | 0.005
D1f | 153 | 1 | 0.005
D1f | 15776 | 2 | 0.01

D1f | 16086 | 2 | 0.01
D1f | 16092 | 1 | 0.005
D1f | 16093 | 4 | 0.02
D1f | 16148 | 1 | 0.005
D1f | 16168 | 2 | 0.01
D1f | 16189 | 1 | 0.005
D1f | 16207 | 1 | 0.005
D1f | 16213 | 1 | 0.005
D1f | 16231 | 1 | 0.005
D1f | 16272 | 1 | 0.005
D1f | 16274 | 1 | 0.005
D1f | 16278 | 2 | 0.01
D1f | 16290 | 2 | 0.01
D1f | 16292 | 1 | 0.005
D1f | 16293 | 3 | 0.015
D1f | 16298 | 2 | 0.01
D1f | 16356 | 2 | 0.01
D1f | 16400 | 2 | 0.01
D1f | 16456 | 2 | 0.01
D1f | 195 | 8 | 0.04
D1f | 204 | 2 | 0.01
D1f | 224 | 5 | 0.025
D1f | 228 | 1 | 0.005
D1f | 2281C | 1 | 0.005
D1f | 234 | 3 | 0.015
D1f | 239 | 1 | 0.005
D1f | 26 | 3 | 0.015
D1f | 289 | 3 | 0.015
D1f | 309d | 1 | 0.005
D1f | 3531 | 2 | 0.01
D1f | 3666 | 1 | 0.005
D1f | 39 | 3 | 0.015
D1f | 4056A | 1 | 0.005
D1f | 4137 | 1 | 0.005
D1f | 4491 | 1 | 0.005
D1f | 4562 | 2 | 0.01
D1f | 4626 | 4 | 0.02
D1f | 4823 | 1 | 0.005
D1f | 4886 | 2 | 0.01
D1f | 4935T | 1 | 0.005
D1f | 4937 | 1 | 0.005
D1f | 513 | 1 | 0.005
D1f | 5201 | 1 | 0.005
D1f | 5237 | 1 | 0.005
D1f | 5252 | 3 | 0.015
D1f | 5460 | 9 | 0.045
D1f | 57 | 1 | 0.005
D1f | 5821 | 9 | 0.045
D1f | 5964 | 2 | 0.01
D1f | 6221 | 4 | 0.02
D1f | 6253 | 2 | 0.01
D1f | 6320 | 4 | 0.02
D1f | 6351 | 2 | 0.01
D1f | 6378 | 5 | 0.025
D1f | 64 | 5 | 0.025
D1f | 6827 | 4 | 0.02
D1f | 709 | 1 | 0.005
D1f | 7196A | 1 | 0.005
D1f | 723 | 1 | 0.005
D1f | 789 | 2 | 0.01
D1f | 8265 | 1 | 0.005
D1f | 8473 | 1 | 0.005
D1f | 9196 | 2 | 0.01
D1f | 9554 | 1 | 0.005
D1f | 9612 | 1 | 0.005
D1f | 9621 | 1 | 0.005
D1f | 964A | 1 | 0.005
D1f | 9667 | 2 | 0.01
D1f | 97 | 1 | 0.005
D1f | 9804 | 1 | 0.005
D1f | 989 | 3 | 0.015
D1f+16189 | 10598 | 1 | 0.006
D1f+16189 | 12166 | 1 | 0.006
D1f+16189 | 12381 | 1 | 0.006
D1f+16189 | 12804 | 1 | 0.006
D1f+16189 | 12840 | 1 | 0.006
D1f+16189 | 13174 | 1 | 0.006
D1f+16189 | 13928C | 1 | 0.006
D1f+16189 | 14122 | 1 | 0.006
D1f+16189 | 14278 | 1 | 0.006
D1f+16189 | 14494 | 1 | 0.006
D1f+16189 | 14687 | 1 | 0.006
D1f+16189 | 14693 | 1 | 0.006
D1f+16189 | 151 | 1 | 0.006
D1f+16189 | 15148 | 1 | 0.006
D1f+16189 | 15266 | 1 | 0.006
D1f+16189 | 16092 | 1 | 0.006
D1f+16189 | 16124 | 1 | 0.006
D1f+16189 | 16242 | 1 | 0.006
D1f+16189 | 16398 | 1 | 0.006
D1f+16189 | 16400 | 1 | 0.006
D1f+16189 | 1811 | 1 | 0.006
D1f+16189 | 189 | 1 | 0.006
D1f+16189 | 309d | 1 | 0.006
D1f+16189 | 3390 | 1 | 0.006
D1f+16189 | 4435 | 1 | 0.006
D1f+16189 | 4796A | 1 | 0.006
D1f+16189 | 5824 | 1 | 0.006
D1f+16189 | 593 | 1 | 0.006
D1f+16189 | 6261 | 1 | 0.006
D1f+16189 | 6951 | 1 | 0.006
D1f+16189 | 7040 | 1 | 0.006
D1f+16189 | 729 | 1 | 0.006
D1f+16189 | 8155 | 1 | 0.006
D1f+16189 | 8251 | 1 | 0.006
D1f+16189 | 8503 | 1 | 0.006
D1f+16189 | 8680 | 1 | 0.006
D1f1 | 10159G | 1 | 0.006
D1f1 | 12406 | 2 | 0.011
D1f1 | 12810 | 2 | 0.011
D1f1 | 15323 | 1 | 0.006

D1f1 | 16153 | 1 | 0.006
D1f1 | 16192 | 2 | 0.011
D1f1 | 16296 | 1 | 0.006
D1f1 | 16344 | 3 | 0.017
D1f1 | 16360 | 3 | 0.017
D1f1 | 16399 | 3 | 0.017
D1f1 | 228 | 2 | 0.011
D1f1 | 3483 | 3 | 0.017
D1f1 | 525.1AC | 1 | 0.006
D1f1 | 6446 | 3 | 0.017
D1f1 | 7112 | 3 | 0.017
D1f1 | 7134C | 1 | 0.006
D1f2 | 133 | 1 | 0.008
D1f2 | 14106A | 1 | 0.008
D1f2 | 1420 | 1 | 0.008
D1f2 | 14280 | 1 | 0.008
D1f2 | 15221 | 1 | 0.008
D1f2 | 16092 | 1 | 0.008
D1f2 | 16256 | 1 | 0.008
D1f2 | 16291 | 1 | 0.008
D1f2 | 16311 | 1 | 0.008
D1f2 | 16456 | 1 | 0.008
D1f2 | 2177 | 1 | 0.008
D1f2 | 2445 | 1 | 0.008
D1f2 | 3203 | 2 | 0.016
D1f2 | 3396 | 1 | 0.008
D1f2 | 477 | 1 | 0.008
D1f2 | 9615 | 1 | 0.008
D1f3 | 146 | 5 | 0.029
D1f3 | 151 | 1 | 0.006
D1f3 | 16124 | 1 | 0.006
D1f3 | 16129 | 1 | 0.006
D1f3 | 16184A | 5 | 0.029
D1f3 | 16242 | 1 | 0.006
D1f3 | 16448 | 4 | 0.023
D1f3 | 186 | 1 | 0.006
D1f3 | 272 | 1 | 0.006
D1f3 | 309d | 1 | 0.006
D1f3 | 3666 | 2 | 0.012
D1f3 | 3766 | 1 | 0.006
D1f3 | 5140 | 1 | 0.006
D1f3 | 7149 | 2 | 0.012
D1f3 | 8519C | 1 | 0.006
D1g | 11914 | 1 | 0.006
D1g | 1393 | 7 | 0.04
D1g | 140 | 7 | 0.04
D1g | 14148 | 1 | 0.006
D1g | 152 | 3 | 0.017
D1g | 16093 | 1 | 0.006
D1g | 16249 | 1 | 0.006
D1g | 16311 | 7 | 0.04
D1g | 199 | 1 | 0.006
D1g | 207 | 1 | 0.006
D1g | 234 | 7 | 0.04
D1g | 5252 | 7 | 0.04
D1g | 5492 | 7 | 0.04
D1g | 5899.1C | 7 | 0.04
D1g | 6713 | 7 | 0.04
D1g | 70 | 1 | 0.006
D1g | 7598 | 1 | 0.006
D1g+16189 | 11809 | 1 | 0.006
D1g+16189 | 16086 | 7 | 0.039
D1g+16189 | 16209 | 6 | 0.033
D1g+16189 | 16234 | 2 | 0.011
D1g+16189 | 16286 | 7 | 0.039
D1g+16189 | 16311 | 2 | 0.011
D1g+16189 | 16445 | 2 | 0.011
D1g+16189 | 185 | 2 | 0.011
D1g+16189 | 195 | 2 | 0.011
D1g+16189 | 199A | 1 | 0.006
D1g+16189 | 3381 | 1 | 0.006
D1g+16189 | 64 | 6 | 0.033
D1g1 | 16352 | 3 | 0.028
D1g1 | 16399 | 3 | 0.028
D1g1a | 14129 | 1 | 0.009
D1g1a | 16342 | 1 | 0.009
D1g1a | 16352 | 3 | 0.027
D1g1a | 16399 | 3 | 0.027
D1g1b | 13392 | 1 | 0.009
D1g1b | 16126 | 1 | 0.009
D1g1b | 16153 | 1 | 0.009
D1g1b | 16218 | 1 | 0.009
D1g1b | 16270 | 7 | 0.061
D1g1b | 3316 | 1 | 0.009
D1g1b | 4452 | 1 | 0.009
D1g1b | 63 | 7 | 0.061
D1g1b | 8589 | 1 | 0.009
D1g1b | 90 | 1 | 0.009
D1g1b | 98 | 1 | 0.009
D1g2 | 14169 | 1 | 0.01
D1g2 | 14319 | 2 | 0.019
D1g2 | 15148 | 1 | 0.01
D1g2 | 16304 | 3 | 0.029
D1g2a | 10595 | 1 | 0.062
D1g2a | 11914 | 1 | 0.062
D1g2a | 16092 | 12 | 0.75
D1g2a | 16129 | 3 | 0.188
D1g2a | 16274 | 2 | 0.125
D1g2a | 16294 | 2 | 0.125
D1g2a | 16319 | 2 | 0.125
D1g2a | 16320 | 2 | 0.125
D1g2a | 16518C | 1 | 0.062
D1g2a | 195 | 2 | 0.125
D1g2a | 204 | 1 | 0.062
D1g2a | 3421 | 1 | 0.062
D1g2a | 593 | 1 | 0.062
D1g2a | 8348 | 1 | 0.062
D1g2a | 9438 | 1 | 0.062
D1g3 | 10646 | 1 | 0.006
D1g3 | 12618 | 1 | 0.006
D1g3 | 152 | 3 | 0.017

D1g3 | 16304 | 10 | 0.056
D1g3 | 16399 | 10 | 0.056
D1g3 | 207 | 1 | 0.006
D1g3 | 7521 | 1 | 0.006
D1g4 | 14097 | 1 | 0.01
D1g4 | 15119 | 1 | 0.01
D1g4 | 152 | 1 | 0.01
D1g4 | 4823 | 1 | 0.01
D1g5 | 16092 | 5 | 0.833
D1g5 | 64 | 3 | 0.5
D1g6 | 13590 | 1 | 0.01
D1g6 | 204 | 1 | 0.01
D1h | 10598 | 1 | 0.006
D1h | 11593T | 1 | 0.006
D1h | 12166 | 1 | 0.006
D1h | 12381 | 1 | 0.006
D1h | 13928C | 1 | 0.006
D1h | 14122 | 1 | 0.006
D1h | 14278 | 1 | 0.006
D1h | 14494 | 1 | 0.006
D1h | 14687 | 1 | 0.006
D1h | 14693 | 1 | 0.006
D1h | 15266 | 1 | 0.006
D1h | 267 | 1 | 0.006
D1h | 4435 | 1 | 0.006
D1h | 5824 | 1 | 0.006
D1h | 593 | 1 | 0.006
D1h | 6260 | 1 | 0.006
D1h | 6261 | 1 | 0.006
D1h | 6951 | 1 | 0.006
D1h | 729 | 1 | 0.006
D1h | 8251 | 1 | 0.006
D1h | 8680 | 1 | 0.006
D1h1 | 12594 | 1 | 0.009
D1h1 | 13327 | 1 | 0.009
D1h1 | 13651 | 1 | 0.009
D1h1 | 16092 | 1 | 0.009
D1h1 | 16189 | 2 | 0.019
D1h1 | 16256 | 1 | 0.009
D1h1 | 203 | 2 | 0.019
D1h1 | 211 | 3 | 0.028
D1h1 | 286 | 1 | 0.009
D1h1 | 9095 | 1 | 0.009
D1h2 | 15940 | 1 | 0.006
D1h2 | 1735 | 1 | 0.006
D1h2 | 2558 | 1 | 0.006
D1h2 | 5972 | 1 | 0.006
D1h2 | 9716 | 1 | 0.006
D1i | 152 | 4 | 0.039
D1i | 16051 | 1 | 0.01
D1i | 16093 | 1 | 0.01
D1i | 16111 | 1 | 0.01
D1i | 16188 | 1 | 0.01
D1i | 16189 | 1 | 0.01
D1i | 16209 | 2 | 0.02
D1i | 16256 | 1 | 0.01
D1i | 16291 | 1 | 0.01
D1i | 16352 | 9 | 0.088
D1i | 179 | 2 | 0.02
D1i | 196 | 5 | 0.049
D1i | 200 | 3 | 0.029
D1i | 204 | 1 | 0.01
D1i | 214 | 1 | 0.01
D1i | 3438 | 3 | 0.029
D1i | 5237 | 3 | 0.029
D1i | 85 | 9 | 0.088
D1i | 94 | 9 | 0.088
D1i1 | 13602 | 1 | 0.013
D1i1 | 16176 | 2 | 0.025
D1i1 | 16224 | 1 | 0.013
D1i1 | 195 | 1 | 0.013
D1i1 | 517-524d | 1 | 0.013
D1i1 | 7084 | 1 | 0.013
D1i1 | 8278.3C | 1 | 0.013
D1i2 | 15001 | 2 | 0.042
D1i2 | 16274 | 2 | 0.042
D1i2 | 16532d | 9 | 0.188
D1i2 | 16T | 1 | 0.021
D1i2 | 188 | 7 | 0.146
D1i2 | 286 | 1 | 0.021
D1i2 | 3316 | 1 | 0.021
D1i2 | 5462 | 1 | 0.021
D1i2 | 7642 | 1 | 0.021
D1j | 10598 | 1 | 0.006
D1j | 11227G | 1 | 0.006
D1j | 11229 | 1 | 0.006
D1j | 11593T | 2 | 0.013
D1j | 11746 | 1 | 0.006
D1j | 12166 | 1 | 0.006
D1j | 12317G | 1 | 0.006
D1j | 12381 | 1 | 0.006
D1j | 12622 | 1 | 0.006
D1j | 13928C | 1 | 0.006
D1j | 13933 | 1 | 0.006
D1j | 14116 | 1 | 0.006
D1j | 14122 | 1 | 0.006
D1j | 14278 | 1 | 0.006
D1j | 143 | 1 | 0.006
D1j | 14460G | 1 | 0.006
D1j | 14494 | 1 | 0.006
D1j | 146 | 1 | 0.006
D1j | 14687 | 1 | 0.006
D1j | 14693 | 1 | 0.006
D1j | 15266 | 1 | 0.006
D1j | 15314 | 1 | 0.006
D1j | 15468 | 1 | 0.006
D1j | 16083 | 2 | 0.013
D1j | 16086 | 1 | 0.006
D1j | 16157 | 3 | 0.019
D1j | 16167 | 1 | 0.006
D1j | 16189 | 4 | 0.026

D1j | 16327 | 1 | 0.006
D1j | 16391 | 2 | 0.013
D1j | 16465 | 2 | 0.013
D1j | 182 | 1 | 0.006
D1j | 200 | 1 | 0.006
D1j | 2056 | 1 | 0.006
D1j | 207 | 1 | 0.006
D1j | 2080 | 1 | 0.006
D1j | 2404 | 1 | 0.006
D1j | 3579 | 1 | 0.006
D1j | 417 | 1 | 0.006
D1j | 4435 | 1 | 0.006
D1j | 5147 | 1 | 0.006
D1j | 54.1C | 1 | 0.006
D1j | 5824 | 1 | 0.006
D1j | 593 | 1 | 0.006
D1j | 59-60d | 1 | 0.006
D1j | 6260 | 3 | 0.019
D1j | 6261 | 1 | 0.006
D1j | 6951 | 1 | 0.006
D1j | 71.1G | 1 | 0.006
D1j | 729 | 1 | 0.006
D1j | 7705 | 1 | 0.006
D1j | 7853 | 1 | 0.006
D1j | 8134G | 1 | 0.006
D1j | 8251 | 1 | 0.006
D1j | 8680 | 1 | 0.006
D1j1 | 10352 | 1 | 0.007
D1j1 | 11149 | 1 | 0.007
D1j1 | 12715 | 1 | 0.007
D1j1 | 14544 | 1 | 0.007
D1j1 | 16148 | 1 | 0.007
D1j1 | 16172 | 5 | 0.034
D1j1 | 16214 | 1 | 0.007
D1j1 | 183 | 3 | 0.021
D1j1 | 3834 | 1 | 0.007
D1j1 | 3873 | 1 | 0.007
D1j1 | 3927 | 1 | 0.007
D1j1 | 455.1T | 1 | 0.007
D1j1 | 8222 | 1 | 0.007
D1j1a | 109 | 1 | 0.007
D1j1a | 1309 | 2 | 0.013
D1j1a | 13263 | 1 | 0.007
D1j1a | 146 | 1 | 0.007
D1j1a | 15380T | 1 | 0.007
D1j1a | 16148 | 3 | 0.02
D1j1a | 16272 | 1 | 0.007
D1j1a | 4080 | 1 | 0.007
D1j1a | 455.1T | 1 | 0.007
D1j1a | 8649T | 1 | 0.007
D1j1a1 | 15530 | 1 | 0.007
D1j1a1 | 16148 | 3 | 0.02
D1j1a1 | 235 | 4 | 0.027
D1j1a1 | 455.1T | 1 | 0.007
D1j1a1 | 538C | 2 | 0.014
D1j1a1 | 6164 | 1 | 0.007
D1j1a2 | 12134 | 1 | 0.008
D1j1a2 | 16294 | 1 | 0.008
D1j1a2 | 16298 | 1 | 0.008
D1j1a2 | 16326T | 2 | 0.016
D1j1a2 | 16327 | 5 | 0.041
D1j1a2 | 16356 | 1 | 0.008
D1j1a2 | 5715 | 1 | 0.008
D1j1a2 | 96 | 1 | 0.008
D1k | 11026 | 2 | 0.009
D1k | 12882 | 2 | 0.009
D1k | 143 | 2 | 0.009
D1k | 16189 | 2 | 0.009
D1k | 16217 | 1 | 0.004
D1k | 16292 | 4 | 0.018
D1k | 16527 | 7 | 0.031
D1k | 189 | 2 | 0.009
D1k | 194 | 1 | 0.004
D1k | 205 | 1 | 0.004
D1k | 279 | 1 | 0.004
D1k | 3348 | 2 | 0.009
D1k | 5460 | 2 | 0.009
D1k | 56.1A | 2 | 0.009
D1k | 5655 | 2 | 0.009
D1k | 64d | 2 | 0.009
D1k | 66C | 2 | 0.009
D1k | 7046 | 1 | 0.004
D1k | 9266 | 1 | 0.004
D1m | 1007 | 1 | 0.006
D1m | 12474 | 1 | 0.006
D1m | 16278 | 1 | 0.006
D1m | 195 | 1 | 0.006
D1n | 10238 | 1 | 0.005
D1n | 12223 | 1 | 0.005
D1n | 12346 | 1 | 0.005
D1n | 1284 | 1 | 0.005
D1n | 15097 | 1 | 0.005
D1n | 16217 | 1 | 0.005
D1n | 16266 | 3 | 0.014
D1n | 16311 | 1 | 0.005
D1n | 194 | 1 | 0.005
D1n | 205 | 1 | 0.005
D1n | 4580 | 1 | 0.005
D2 | 12089C | 5 | 0.034
D2 | 12603 | 2 | 0.014
D2 | 13105 | 2 | 0.014
D2 | 143 | 4 | 0.027
D2 | 146 | 2 | 0.014
D2 | 150 | 1 | 0.007
D2 | 152 | 4 | 0.027
D2 | 15745 | 1 | 0.007
D2 | 16076A | 1 | 0.007
D2 | 16093 | 3 | 0.021
D2 | 16134 | 1 | 0.007
D2 | 16140 | 2 | 0.014
D2 | 16188 | 1 | 0.007

D2 | 16209 | 1 | 0.007
D2 | 16214 | 1 | 0.007
D2 | 16240 | 1 | 0.007
D2 | 16278 | 5 | 0.034
D2 | 16298 | 1 | 0.007
D2 | 1719 | 2 | 0.014
D2 | 194 | 1 | 0.007
D2 | 215 | 1 | 0.007
D2 | 461 | 1 | 0.007
D2 | 593 | 1 | 0.007
D2 | 9095 | 2 | 0.014
D2a1 | 11176 | 2 | 0.025
D2a1 | 11234 | 1 | 0.013
D2a1 | 13011 | 1 | 0.013
D2a1 | 13037 | 1 | 0.013
D2a1 | 14226 | 1 | 0.013
D2a1 | 15049 | 1 | 0.013
D2a1 | 15050 | 1 | 0.013
D2a1 | 152 | 1 | 0.013
D2a1 | 15390 | 1 | 0.013
D2a1 | 15403 | 1 | 0.013
D2a1 | 16128 | 1 | 0.013
D2a1 | 3380 | 1 | 0.013
D2a1 | 4107 | 1 | 0.013
D2a1 | 525 | 2 | 0.025
D2a1 | 664 | 1 | 0.013
D2a1 | 7174 | 1 | 0.013
D2a1 | 7320 | 1 | 0.013
D2a1 | 7424 | 2 | 0.025
D2a1a | 10685 | 1 | 0.009
D2a1a | 10695 | 1 | 0.009
D2a1a | 11062 | 3 | 0.028
D2a1a | 11113 | 1 | 0.009
D2a1a | 153 | 1 | 0.009
D2a1a | 16311 | 5 | 0.046
D2a1a | 5081 | 1 | 0.009
D2a1a | 6554 | 4 | 0.037
D2a1a | 8460 | 1 | 0.009
D2a1a | 8639 | 4 | 0.037
D2a1b | 10381 | 1 | 0.014
D2a1b | 16294 | 1 | 0.014
D2a1b | 7403 | 4 | 0.055
D2a1b | 795 | 1 | 0.014
D2a1b | 8895 | 1 | 0.014
D2a2 | 11065 | 2 | 0.022
D2a2 | 16111 | 1 | 0.011
D2a2 | 5147 | 1 | 0.011
D2a2 | 5436 | 2 | 0.022
D2a'b | 106-111d | 1 | 0.013
D2a'b | 11963 | 1 | 0.013
D2a'b | 16086 | 2 | 0.025
D2a'b | 310 | 1 | 0.013
D2a'b | 316C | 1 | 0.013
D2b | 153 | 1 | 0.042
D2b | 16079G | 1 | 0.042
D2b | 16092A | 1 | 0.042
D2b | 16093 | 1 | 0.042
D2b | 16111A | 1 | 0.042
D2b | 16234 | 1 | 0.042
D2b | 16308 | 1 | 0.042
D2b | 16368 | 1 | 0.042
D2b | 16399 | 1 | 0.042
D2b1 | 16111G | 1 | 0.033
D2b1 | 16192 | 1 | 0.033
D2b1 | 16245 | 6 | 0.2
D2b1 | 16359 | 1 | 0.033
D2b1 | 574 | 2 | 0.067
D2b1 | 801 | 1 | 0.033
D2b1a | 16148 | 5 | 0.179
D2b1a | 198 | 1 | 0.036
D2b1a | 291T | 4 | 0.143
D2b1a | 310 | 1 | 0.036
D2b2 | 14380 | 1 | 0.042
D2b2 | 16092A | 1 | 0.042
D2b2 | 16296 | 1 | 0.042
D2b2 | 16308 | 1 | 0.042
D2b2 | 16368 | 1 | 0.042
D2b2 | 16399 | 1 | 0.042
D2c | 15937C | 1 | 0.016
D2c | 16051 | 1 | 0.016
D2c | 16164 | 1 | 0.016
D2c | 16187 | 1 | 0.016
D2c | 16231A | 1 | 0.016
D2c | 16254 | 5 | 0.078
D2c | 16325 | 1 | 0.016
D2c | 16355A | 1 | 0.016
D2c | 16428C | 1 | 0.016
D2c | 16429G | 1 | 0.016
D2c | 16446 | 1 | 0.016
D2c | 16453 | 1 | 0.016
D2c | 16468 | 1 | 0.016
D2c | 16470T | 1 | 0.016
D2c | 16527 | 1 | 0.016
D3 | 10042 | 6 | 0.086
D3 | 10160A | 1 | 0.014
D3 | 11353 | 6 | 0.086
D3 | 13515 | 1 | 0.014
D3 | 13834 | 1 | 0.014
D3 | 13879 | 2 | 0.029
D3 | 14034 | 1 | 0.014
D3 | 152 | 2 | 0.029
D3 | 15928 | 1 | 0.014
D3 | 16093 | 1 | 0.014
D3 | 3079 | 1 | 0.014
D3 | 310 | 1 | 0.014
D3 | 5054 | 2 | 0.029
D3 | 523d | 1 | 0.014
D3 | 6040 | 1 | 0.014
D3 | 6515G | 1 | 0.014
D3 | 9007 | 1 | 0.014
D4 | 10101 | 1 | 0.001

D4 | 10356A | 1 | 0.001
D4 | 10751 | 2 | 0.002
D4 | 10843 | 1 | 0.001
D4 | 11287 | 1 | 0.001
D4 | 11746 | 1 | 0.001
D4 | 120 | 1 | 0.001
D4 | 12236 | 2 | 0.002
D4 | 12280 | 3 | 0.004
D4 | 12351 | 3 | 0.004
D4 | 12384 | 2 | 0.002
D4 | 12396 | 1 | 0.001
D4 | 12432 | 6 | 0.007
D4 | 12912 | 2 | 0.002
D4 | 13071 | 2 | 0.002
D4 | 13153 | 3 | 0.004
D4 | 13461 | 2 | 0.002
D4 | 13474 | 1 | 0.001
D4 | 13713 | 1 | 0.001
D4 | 13926 | 1 | 0.001
D4 | 13983 | 1 | 0.001
D4 | 14203 | 1 | 0.001
D4 | 143 | 1 | 0.001
D4 | 14438C | 1 | 0.001
D4 | 146 | 4 | 0.005
D4 | 14882 | 2 | 0.002
D4 | 14883 | 1 | 0.001
D4 | 150 | 5 | 0.006
D4 | 15110 | 1 | 0.001
D4 | 15115 | 1 | 0.001
D4 | 15130 | 1 | 0.001
D4 | 152 | 10 | 0.012
D4 | 15400 | 3 | 0.004
D4 | 15431 | 1 | 0.001
D4 | 15586 | 1 | 0.001
D4 | 15721 | 3 | 0.004
D4 | 15758 | 1 | 0.001
D4 | 15839 | 1 | 0.001
D4 | 15884 | 4 | 0.005
D4 | 16086 | 1 | 0.001
D4 | 16093 | 10 | 0.012
D4 | 16111 | 1 | 0.001
D4 | 16129 | 1 | 0.001
D4 | 16147 | 1 | 0.001
D4 | 16150 | 4 | 0.005
D4 | 16153 | 1 | 0.001
D4 | 16158 | 2 | 0.002
D4 | 16165 | 1 | 0.001
D4 | 16169 | 5 | 0.006
D4 | 16172 | 1 | 0.001
D4 | 16176 | 2 | 0.002
D4 | 16185 | 1 | 0.001
D4 | 16186 | 1 | 0.001
D4 | 16187 | 1 | 0.001
D4 | 16189 | 4 | 0.005
D4 | 16192 | 29 | 0.035
D4 | 16218 | 1 | 0.001
D4 | 16234 | 1 | 0.001
D4 | 16243 | 1 | 0.001
D4 | 16249 | 48 | 0.057
D4 | 16250 | 1 | 0.001
D4 | 16260 | 1 | 0.001
D4 | 16261 | 1 | 0.001
D4 | 16271 | 1 | 0.001
D4 | 16274 | 3 | 0.004
D4 | 16278 | 1 | 0.001
D4 | 16291 | 1 | 0.001
D4 | 16309 | 1 | 0.001
D4 | 16311 | 1 | 0.001
D4 | 16316 | 35 | 0.042
D4 | 16319 | 1 | 0.001
D4 | 16356 | 7 | 0.008
D4 | 16360 | 1 | 0.001
D4 | 163C | 1 | 0.001
D4 | 16491A | 1 | 0.001
D4 | 16526T | 1 | 0.001
D4 | 183 | 23 | 0.028
D4 | 184 | 19 | 0.023
D4 | 199 | 2 | 0.002
D4 | 200 | 1 | 0.001
D4 | 2010 | 1 | 0.001
D4 | 217 | 1 | 0.001
D4 | 227T | 1 | 0.001
D4 | 228 | 5 | 0.006
D4 | 236 | 1 | 0.001
D4 | 2483 | 1 | 0.001
D4 | 2789A | 1 | 0.001
D4 | 310 | 2 | 0.002
D4 | 316C | 1 | 0.001
D4 | 3316 | 1 | 0.001
D4 | 3522 | 1 | 0.001
D4 | 3523 | 1 | 0.001
D4 | 3540 | 2 | 0.002
D4 | 3583 | 1 | 0.001
D4 | 3603 | 4 | 0.005
D4 | 3606 | 1 | 0.001
D4 | 374 | 1 | 0.001
D4 | 3764 | 3 | 0.004
D4 | 385 | 1 | 0.001
D4 | 4038 | 1 | 0.001
D4 | 4182 | 1 | 0.001
D4 | 43 | 1 | 0.001
D4 | 4335 | 2 | 0.002
D4 | 4633 | 1 | 0.001
D4 | 466 | 1 | 0.001
D4 | 4928 | 1 | 0.001
D4 | 508 | 4 | 0.005
D4 | 5211 | 1 | 0.001
D4 | 5262 | 1 | 0.001
D4 | 5460 | 1 | 0.001
D4 | 57 | 3 | 0.004
D4 | 570A | 2 | 0.002

D4 | 59 | 3 | 0.004
D4 | 60.1T | 3 | 0.004
D4 | 6260 | 1 | 0.001
D4 | 6383 | 1 | 0.001
D4 | 648 | 1 | 0.001
D4 | 7337 | 1 | 0.001
D4 | 735 | 1 | 0.001
D4 | 7382 | 1 | 0.001
D4 | 7581 | 1 | 0.001
D4 | 7673 | 1 | 0.001
D4 | 7706 | 1 | 0.001
D4 | 7709A | 1 | 0.001
D4 | 7754 | 1 | 0.001
D4 | 7762 | 3 | 0.004
D4 | 7783 | 1 | 0.001
D4 | 8251 | 1 | 0.001
D4 | 8588 | 1 | 0.001
D4 | 869 | 2 | 0.002
D4 | 8745 | 1 | 0.001
D4 | 9531 | 4 | 0.005
D4 | 9696 | 2 | 0.002
D4 | 9725 | 7 | 0.008
D4 | 9758 | 3 | 0.004
D4 | 9812 | 3 | 0.004
D4 | 9856 | 10 | 0.012
D4 | 9896 | 1 | 0.001
D4+195 | 10649 | 3 | 0.006
D4+195 | 11696 | 3 | 0.006
D4+195 | 11984 | 2 | 0.004
D4+195 | 13681 | 1 | 0.002
D4+195 | 13788A | 2 | 0.004
D4+195 | 152 | 10 | 0.021
D4+195 | 153 | 1 | 0.002
D4+195 | 16092 | 1 | 0.002
D4+195 | 16095 | 5 | 0.011
D4+195 | 16108 | 1 | 0.002
D4+195 | 16111A | 1 | 0.002
D4+195 | 16126 | 1 | 0.002
D4+195 | 16131 | 1 | 0.002
D4+195 | 16168 | 1 | 0.002
D4+195 | 16172 | 3 | 0.006
D4+195 | 16192 | 3 | 0.006
D4+195 | 16193 | 1 | 0.002
D4+195 | 16209 | 2 | 0.004
D4+195 | 16222 | 2 | 0.004
D4+195 | 16232 | 7 | 0.015
D4+195 | 16249 | 4 | 0.008
D4+195 | 16250A | 1 | 0.002
D4+195 | 16255 | 1 | 0.002
D4+195 | 16260 | 2 | 0.004
D4+195 | 16262 | 1 | 0.002
D4+195 | 16271 | 3 | 0.006
D4+195 | 16288 | 1 | 0.002
D4+195 | 16293T | 3 | 0.006
D4+195 | 16316 | 2 | 0.004
D4+195 | 16318C | 1 | 0.002
D4+195 | 16324 | 1 | 0.002
D4+195 | 16355 | 3 | 0.006
D4+195 | 16356 | 3 | 0.006
D4+195 | 183 | 1 | 0.002
D4+195 | 184 | 2 | 0.004
D4+195 | 189 | 1 | 0.002
D4+195 | 199 | 1 | 0.002
D4+195 | 207 | 1 | 0.002
D4+195 | 234 | 1 | 0.002
D4+195 | 29 | 1 | 0.002
D4+195 | 298 | 1 | 0.002
D4+195 | 3083 | 1 | 0.002
D4+195 | 41 | 1 | 0.002
D4+195 | 4254 | 1 | 0.002
D4+195 | 507 | 1 | 0.002
D4+195 | 55.1T | 1 | 0.002
D4+195 | 64 | 1 | 0.002
D4+195 | 721A | 1 | 0.002
D4+195 | 7356 | 1 | 0.002
D4+195 | 9130 | 1 | 0.002
D4+195 | 9856 | 1 | 0.002
D4a | 1 | 1 | 0.003
D4a | 10993 | 2 | 0.007
D4a | 11425 | 1 | 0.003
D4a | 11800T | 4 | 0.014
D4a | 12034 | 1 | 0.003
D4a | 13602 | 3 | 0.01
D4a | 13650G | 1 | 0.003
D4a | 13708 | 1 | 0.003
D4a | 13767 | 1 | 0.003
D4a | 143 | 1 | 0.003
D4a | 14443 | 1 | 0.003
D4a | 14494 | 1 | 0.003
D4a | 14560 | 1 | 0.003
D4a | 150 | 2 | 0.007
D4a | 151 | 4 | 0.014
D4a | 15596 | 8 | 0.028
D4a | 15715 | 4 | 0.014
D4a | 16097 | 1 | 0.003
D4a | 16105 | 1 | 0.003
D4a | 16111 | 1 | 0.003
D4a | 16111G | 2 | 0.007
D4a | 16124 | 1 | 0.003
D4a | 16147 | 1 | 0.003
D4a | 16148 | 3 | 0.01
D4a | 16153 | 1 | 0.003
D4a | 16162 | 3 | 0.01
D4a | 16168 | 1 | 0.003
D4a | 16180 | 1 | 0.003
D4a | 16185 | 4 | 0.014
D4a | 16203T | 1 | 0.003
D4a | 16242 | 1 | 0.003
D4a | 16255 | 1 | 0.003
D4a | 16256 | 1 | 0.003
D4a | 16260 | 1 | 0.003

D4a | 16290A | 1 | 0.003
D4a | 16300 | 1 | 0.003
D4a | 16318d | 1 | 0.003
D4a | 16390 | 1 | 0.003
D4a | 16399 | 4 | 0.014
D4a | 16465 | 1 | 0.003
D4a | 16474 | 1 | 0.003
D4a | 16512 | 1 | 0.003
D4a | 185 | 1 | 0.003
D4a | 189 | 1 | 0.003
D4a | 194 | 1 | 0.003
D4a | 195 | 1 | 0.003
D4a | 199 | 1 | 0.003
D4a | 215 | 1 | 0.003
D4a | 222 | 1 | 0.003
D4a | 232 | 2 | 0.007
D4a | 239 | 1 | 0.003
D4a | 2833 | 1 | 0.003
D4a | 309.3C | 2 | 0.007
D4a | 337 | 1 | 0.003
D4a | 403 | 1 | 0.003
D4a | 426 | 1 | 0.003
D4a | 446 | 1 | 0.003
D4a | 450 | 1 | 0.003
D4a | 4745 | 1 | 0.003
D4a | 496 | 1 | 0.003
D4a | 518 | 1 | 0.003
D4a | 519 | 1 | 0.003
D4a | 529 | 1 | 0.003
D4a | 5582 | 12 | 0.041
D4a | 562 | 1 | 0.003
D4a | 5899.1C | 1 | 0.003
D4a | 6278 | 2 | 0.007
D4a | 650 | 1 | 0.003
D4a | 6524 | 1 | 0.003
D4a | 6975 | 1 | 0.003
D4a | 709 | 1 | 0.003
D4a | 71.1G | 2 | 0.007
D4a | 7789 | 1 | 0.003
D4a | 8683 | 1 | 0.003
D4a | 94 | 1 | 0.003
D4a | 9518 | 2 | 0.007
D4a+16294 | 16249 | 1 | 0.008
D4a+16294 | 5744 | 1 | 0.008
D4a+16294 | 6551 | 1 | 0.008
D4a+16294 | 9197 | 1 | 0.008
D4a1 | 1 | 1 | 0.003
D4a1 | 10006d | 1 | 0.003
D4a1 | 10365 | 1 | 0.003
D4a1 | 1053T | 1 | 0.003
D4a1 | 106-111d | 1 | 0.003
D4a1 | 10646 | 1 | 0.003
D4a1 | 11809 | 1 | 0.003
D4a1 | 11908 | 1 | 0.003
D4a1 | 11969 | 1 | 0.003
D4a1 | 14016 | 1 | 0.003
D4a1 | 143 | 1 | 0.003
D4a1 | 146 | 4 | 0.013
D4a1 | 150 | 2 | 0.007
D4a1 | 153 | 1 | 0.003
D4a1 | 15468 | 1 | 0.003
D4a1 | 15530 | 1 | 0.003
D4a1 | 16086 | 1 | 0.003
D4a1 | 16097 | 1 | 0.003
D4a1 | 16111 | 1 | 0.003
D4a1 | 16111G | 2 | 0.007
D4a1 | 16124 | 1 | 0.003
D4a1 | 16140 | 1 | 0.003
D4a1 | 16147 | 1 | 0.003
D4a1 | 16148 | 3 | 0.01
D4a1 | 16153 | 1 | 0.003
D4a1 | 16162 | 3 | 0.01
D4a1 | 16168 | 1 | 0.003
D4a1 | 16180 | 1 | 0.003
D4a1 | 16192 | 4 | 0.013
D4a1 | 16203T | 1 | 0.003
D4a1 | 16215 | 1 | 0.003
D4a1 | 16242 | 1 | 0.003
D4a1 | 16247 | 2 | 0.007
D4a1 | 16255 | 1 | 0.003
D4a1 | 16272 | 1 | 0.003
D4a1 | 16290A | 1 | 0.003
D4a1 | 16300 | 1 | 0.003
D4a1 | 16311 | 1 | 0.003
D4a1 | 16318d | 1 | 0.003
D4a1 | 16320 | 1 | 0.003
D4a1 | 16399 | 4 | 0.013
D4a1 | 16465 | 1 | 0.003
D4a1 | 16474 | 1 | 0.003
D4a1 | 16512 | 1 | 0.003
D4a1 | 194 | 1 | 0.003
D4a1 | 195 | 1 | 0.003
D4a1 | 199 | 1 | 0.003
D4a1 | 211T | 1 | 0.003
D4a1 | 2140 | 1 | 0.003
D4a1 | 215 | 1 | 0.003
D4a1 | 222 | 1 | 0.003
D4a1 | 232 | 2 | 0.007
D4a1 | 239 | 1 | 0.003
D4a1 | 309.3C | 2 | 0.007
D4a1 | 3317 | 1 | 0.003
D4a1 | 337 | 1 | 0.003
D4a1 | 403 | 1 | 0.003
D4a1 | 446 | 1 | 0.003
D4a1 | 450 | 1 | 0.003
D4a1 | 481 | 1 | 0.003
D4a1 | 496 | 1 | 0.003
D4a1 | 519 | 1 | 0.003
D4a1 | 5268 | 1 | 0.003
D4a1 | 529 | 1 | 0.003
D4a1 | 562 | 1 | 0.003

D4a1 | 63 | 1 | 0.003
D4a1 | 6485 | 1 | 0.003
D4a1 | 709 | 1 | 0.003
D4a1 | 71.1G | 2 | 0.007
D4a1 | 7146 | 1 | 0.003
D4a1 | 735 | 2 | 0.007
D4a1 | 7789 | 1 | 0.003
D4a1 | 8292 | 1 | 0.003
D4a1 | 8725 | 1 | 0.003
D4a1 | 9374 | 1 | 0.003
D4a1 | 9631 | 1 | 0.003
D4a1 | 9804 | 1 | 0.003
D4a1a | 1 | 1 | 0.003
D4a1a | 143 | 1 | 0.003
D4a1a | 150 | 2 | 0.007
D4a1a | 16019G | 1 | 0.003
D4a1a | 16028A | 1 | 0.003
D4a1a | 16029G | 1 | 0.003
D4a1a | 16037 | 13 | 0.045
D4a1a | 16097 | 1 | 0.003
D4a1a | 16111 | 1 | 0.003
D4a1a | 16111G | 2 | 0.007
D4a1a | 16124 | 1 | 0.003
D4a1a | 16147 | 1 | 0.003
D4a1a | 16148 | 3 | 0.01
D4a1a | 16153 | 1 | 0.003
D4a1a | 16162 | 3 | 0.01
D4a1a | 16168 | 1 | 0.003
D4a1a | 16180 | 1 | 0.003
D4a1a | 16192 | 1 | 0.003
D4a1a | 16203T | 1 | 0.003
D4a1a | 16217 | 5 | 0.017
D4a1a | 16217A | 1 | 0.003
D4a1a | 16242 | 1 | 0.003
D4a1a | 16255 | 1 | 0.003
D4a1a | 16261 | 13 | 0.045
D4a1a | 16290A | 1 | 0.003
D4a1a | 16300 | 1 | 0.003
D4a1a | 16318d | 1 | 0.003
D4a1a | 16342G | 1 | 0.003
D4a1a | 16351d | 2 | 0.007
D4a1a | 16381 | 2 | 0.007
D4a1a | 16399 | 4 | 0.014
D4a1a | 16465 | 1 | 0.003
D4a1a | 16474 | 1 | 0.003
D4a1a | 16512 | 1 | 0.003
D4a1a | 194 | 1 | 0.003
D4a1a | 199 | 1 | 0.003
D4a1a | 200 | 1 | 0.003
D4a1a | 207 | 4 | 0.014
D4a1a | 215 | 1 | 0.003
D4a1a | 222 | 1 | 0.003
D4a1a | 232 | 2 | 0.007
D4a1a | 235 | 6 | 0.021
D4a1a | 239 | 1 | 0.003
D4a1a | 309.3C | 2 | 0.007
D4a1a | 315.2C | 1 | 0.003
D4a1a | 337 | 1 | 0.003
D4a1a | 403 | 1 | 0.003
D4a1a | 446 | 1 | 0.003
D4a1a | 450 | 1 | 0.003
D4a1a | 496 | 1 | 0.003
D4a1a | 519 | 1 | 0.003
D4a1a | 529 | 1 | 0.003
D4a1a | 562 | 1 | 0.003
D4a1a | 709 | 1 | 0.003
D4a1a | 71.1G | 2 | 0.007
D4a1a | 9588 | 1 | 0.003
D4a1a1 | 1 | 1 | 0.003
D4a1a1 | 12235 | 1 | 0.003
D4a1a1 | 143 | 1 | 0.003
D4a1a1 | 150 | 2 | 0.007
D4a1a1 | 15119 | 1 | 0.003
D4a1a1 | 15803 | 1 | 0.003
D4a1a1 | 16079A | 1 | 0.003
D4a1a1 | 16085G | 1 | 0.003
D4a1a1 | 16097 | 1 | 0.003
D4a1a1 | 16111 | 1 | 0.003
D4a1a1 | 16111G | 2 | 0.007
D4a1a1 | 16122T | 1 | 0.003
D4a1a1 | 16124 | 1 | 0.003
D4a1a1 | 16147 | 1 | 0.003
D4a1a1 | 16148 | 3 | 0.01
D4a1a1 | 16153 | 1 | 0.003
D4a1a1 | 16162 | 3 | 0.01
D4a1a1 | 16168 | 1 | 0.003
D4a1a1 | 16180 | 1 | 0.003
D4a1a1 | 16186 | 1 | 0.003
D4a1a1 | 16189 | 2 | 0.007
D4a1a1 | 16193d | 1 | 0.003
D4a1a1 | 16203T | 1 | 0.003
D4a1a1 | 16209 | 6 | 0.021
D4a1a1 | 16242 | 1 | 0.003
D4a1a1 | 16255 | 1 | 0.003
D4a1a1 | 16290A | 1 | 0.003
D4a1a1 | 16300 | 1 | 0.003
D4a1a1 | 16318d | 1 | 0.003
D4a1a1 | 16335 | 2 | 0.007
D4a1a1 | 16399 | 4 | 0.014
D4a1a1 | 16465 | 1 | 0.003
D4a1a1 | 16474 | 1 | 0.003
D4a1a1 | 16512 | 1 | 0.003
D4a1a1 | 185 | 1 | 0.003
D4a1a1 | 194 | 1 | 0.003
D4a1a1 | 199 | 1 | 0.003
D4a1a1 | 215 | 1 | 0.003
D4a1a1 | 222 | 1 | 0.003
D4a1a1 | 232 | 2 | 0.007
D4a1a1 | 239 | 1 | 0.003
D4a1a1 | 309.3C | 2 | 0.007
D4a1a1 | 310 | 1 | 0.003

D4a1a1 | 337 | 1 | 0.003
D4a1a1 | 403 | 1 | 0.003
D4a1a1 | 4435 | 1 | 0.003
D4a1a1 | 446 | 1 | 0.003
D4a1a1 | 450 | 1 | 0.003
D4a1a1 | 496 | 1 | 0.003
D4a1a1 | 515 | 1 | 0.003
D4a1a1 | 519 | 1 | 0.003
D4a1a1 | 5201 | 1 | 0.003
D4a1a1 | 529 | 1 | 0.003
D4a1a1 | 5582 | 1 | 0.003
D4a1a1 | 562 | 1 | 0.003
D4a1a1 | 6494 | 1 | 0.003
D4a1a1 | 709 | 1 | 0.003
D4a1a1 | 71.1G | 2 | 0.007
D4a1a1 | 7418 | 1 | 0.003
D4a1a1a | 1 | 1 | 0.004
D4a1a1a | 14226 | 2 | 0.007
D4a1a1a | 143 | 1 | 0.004
D4a1a1a | 1462 | 1 | 0.004
D4a1a1a | 150 | 2 | 0.007
D4a1a1a | 15355 | 2 | 0.007
D4a1a1a | 15927 | 1 | 0.004
D4a1a1a | 16097 | 1 | 0.004
D4a1a1a | 16111 | 1 | 0.004
D4a1a1a | 16111G | 2 | 0.007
D4a1a1a | 16124 | 1 | 0.004
D4a1a1a | 16147 | 1 | 0.004
D4a1a1a | 16148 | 3 | 0.011
D4a1a1a | 16153 | 1 | 0.004
D4a1a1a | 16162 | 3 | 0.011
D4a1a1a | 16168 | 1 | 0.004
D4a1a1a | 16180 | 1 | 0.004
D4a1a1a | 16203T | 1 | 0.004
D4a1a1a | 16234 | 3 | 0.011
D4a1a1a | 16242 | 1 | 0.004
D4a1a1a | 16255 | 1 | 0.004
D4a1a1a | 16290A | 1 | 0.004
D4a1a1a | 16300 | 1 | 0.004
D4a1a1a | 16318d | 1 | 0.004
D4a1a1a | 16399 | 4 | 0.014
D4a1a1a | 16465 | 1 | 0.004
D4a1a1a | 16474 | 1 | 0.004
D4a1a1a | 16512 | 1 | 0.004
D4a1a1a | 194 | 1 | 0.004
D4a1a1a | 199 | 1 | 0.004
D4a1a1a | 215 | 1 | 0.004
D4a1a1a | 222 | 1 | 0.004
D4a1a1a | 232 | 2 | 0.007
D4a1a1a | 239 | 1 | 0.004
D4a1a1a | 309.3C | 2 | 0.007
D4a1a1a | 337 | 1 | 0.004
D4a1a1a | 403 | 1 | 0.004
D4a1a1a | 446 | 1 | 0.004
D4a1a1a | 450 | 1 | 0.004
D4a1a1a | 496 | 1 | 0.004
D4a1a1a | 519 | 1 | 0.004
D4a1a1a | 529 | 1 | 0.004
D4a1a1a | 562 | 1 | 0.004
D4a1a1a | 6899 | 2 | 0.007
D4a1a1a | 6908 | 1 | 0.004
D4a1a1a | 709 | 1 | 0.004
D4a1a1a | 71.1G | 2 | 0.007
D4a1b | 1 | 1 | 0.003
D4a1b | 10084 | 1 | 0.003
D4a1b | 14180 | 1 | 0.003
D4a1b | 143 | 1 | 0.003
D4a1b | 14332 | 1 | 0.003
D4a1b | 14755 | 1 | 0.003
D4a1b | 150 | 2 | 0.007
D4a1b | 15403 | 1 | 0.003
D4a1b | 15645 | 1 | 0.003
D4a1b | 15731 | 1 | 0.003
D4a1b | 16093 | 1 | 0.003
D4a1b | 16097 | 1 | 0.003
D4a1b | 16111 | 1 | 0.003
D4a1b | 16111G | 2 | 0.007
D4a1b | 16124 | 1 | 0.003
D4a1b | 16147 | 1 | 0.003
D4a1b | 16148 | 3 | 0.01
D4a1b | 16153 | 1 | 0.003
D4a1b | 16162 | 3 | 0.01
D4a1b | 16168 | 1 | 0.003
D4a1b | 16172 | 4 | 0.014
D4a1b | 16180 | 1 | 0.003
D4a1b | 16203T | 1 | 0.003
D4a1b | 16242 | 1 | 0.003
D4a1b | 16255 | 1 | 0.003
D4a1b | 16270 | 3 | 0.01
D4a1b | 16290A | 1 | 0.003
D4a1b | 16300 | 1 | 0.003
D4a1b | 16318d | 1 | 0.003
D4a1b | 16399 | 4 | 0.014
D4a1b | 16465 | 1 | 0.003
D4a1b | 16474 | 1 | 0.003
D4a1b | 16512 | 1 | 0.003
D4a1b | 194 | 1 | 0.003
D4a1b | 195 | 1 | 0.003
D4a1b | 196 | 1 | 0.003
D4a1b | 199 | 1 | 0.003
D4a1b | 214 | 1 | 0.003
D4a1b | 215 | 1 | 0.003
D4a1b | 222 | 1 | 0.003
D4a1b | 228 | 3 | 0.01
D4a1b | 232 | 2 | 0.007
D4a1b | 239 | 1 | 0.003
D4a1b | 309.3C | 2 | 0.007
D4a1b | 337 | 1 | 0.003
D4a1b | 403 | 1 | 0.003
D4a1b | 446 | 1 | 0.003
D4a1b | 450 | 1 | 0.003

D4a1b | 4655 | 1 | 0.003
D4a1b | 496 | 1 | 0.003
D4a1b | 5147 | 1 | 0.003
D4a1b | 519 | 1 | 0.003
D4a1b | 529 | 1 | 0.003
D4a1b | 562 | 1 | 0.003
D4a1b | 7043A | 1 | 0.003
D4a1b | 7085 | 1 | 0.003
D4a1b | 709 | 1 | 0.003
D4a1b | 71.1G | 2 | 0.007
D4a1b | 8285.1C | 1 | 0.003
D4a1b | 9341T | 1 | 0.003
D4a1b1 | 150 | 1 | 0.008
D4a1b1 | 16249 | 1 | 0.008
D4a1b1 | 533 | 2 | 0.015
D4a1b1 | 6524 | 1 | 0.008
D4a1c | 1 | 1 | 0.004
D4a1c | 11902 | 1 | 0.004
D4a1c | 143 | 1 | 0.004
D4a1c | 146 | 4 | 0.014
D4a1c | 150 | 2 | 0.007
D4a1c | 16092 | 1 | 0.004
D4a1c | 16097 | 1 | 0.004
D4a1c | 16111 | 1 | 0.004
D4a1c | 16111G | 2 | 0.007
D4a1c | 16124 | 1 | 0.004
D4a1c | 16147 | 1 | 0.004
D4a1c | 16148 | 3 | 0.011
D4a1c | 16153 | 1 | 0.004
D4a1c | 16162 | 3 | 0.011
D4a1c | 16168 | 1 | 0.004
D4a1c | 16180 | 1 | 0.004
D4a1c | 16189 | 2 | 0.007
D4a1c | 16193d | 1 | 0.004
D4a1c | 16203T | 1 | 0.004
D4a1c | 16242 | 1 | 0.004
D4a1c | 16255 | 1 | 0.004
D4a1c | 16290A | 1 | 0.004
D4a1c | 16300 | 1 | 0.004
D4a1c | 16318d | 1 | 0.004
D4a1c | 16399 | 4 | 0.014
D4a1c | 16465 | 1 | 0.004
D4a1c | 16474 | 1 | 0.004
D4a1c | 16512 | 1 | 0.004
D4a1c | 194 | 1 | 0.004
D4a1c | 199 | 1 | 0.004
D4a1c | 215 | 1 | 0.004
D4a1c | 222 | 1 | 0.004
D4a1c | 232 | 2 | 0.007
D4a1c | 239 | 1 | 0.004
D4a1c | 309.3C | 2 | 0.007
D4a1c | 337 | 1 | 0.004
D4a1c | 3398 | 1 | 0.004
D4a1c | 403 | 1 | 0.004
D4a1c | 446 | 1 | 0.004
D4a1c | 450 | 1 | 0.004
D4a1c | 496 | 1 | 0.004
D4a1c | 519 | 1 | 0.004
D4a1c | 529 | 1 | 0.004
D4a1c | 544.1C | 1 | 0.004
D4a1c | 562 | 1 | 0.004
D4a1c | 6039 | 1 | 0.004
D4a1c | 709 | 1 | 0.004
D4a1c | 71.1G | 2 | 0.007
D4a1c | 9123 | 1 | 0.004
D4a1c | 9801 | 1 | 0.004
D4a1d | 143 | 1 | 0.008
D4a1d | 151 | 1 | 0.008
D4a1d | 15697 | 1 | 0.008
D4a1d | 16304 | 1 | 0.008
D4a1d | 16325 | 1 | 0.008
D4a1d | 1709 | 1 | 0.008
D4a1d | 195 | 1 | 0.008
D4a1d | 2356 | 1 | 0.008
D4a1e | 12900 | 2 | 0.014
D4a1e | 13140 | 1 | 0.007
D4a1e | 13371 | 1 | 0.007
D4a1e | 13590 | 1 | 0.007
D4a1e | 15839 | 1 | 0.007
D4a1e | 16254d | 1 | 0.007
D4a1e | 16286 | 3 | 0.021
D4a1e | 16299 | 8 | 0.055
D4a1e | 1888 | 1 | 0.007
D4a1e | 199 | 1 | 0.007
D4a1e | 242 | 2 | 0.014
D4a1e | 3705 | 1 | 0.007
D4a1e | 4580 | 1 | 0.007
D4a1e | 6575 | 1 | 0.007
D4a1e | 72 | 2 | 0.014
D4a1e | 723 | 4 | 0.028
D4a1e | 745 | 2 | 0.014
D4a1e | 8014 | 1 | 0.007
D4a1e | 8614 | 2 | 0.014
D4a1e | 9389 | 2 | 0.014
D4a1e1 | 151 | 1 | 0.007
D4a1e1 | 16093 | 1 | 0.007
D4a1e1 | 16126 | 1 | 0.007
D4a1e1 | 16132 | 2 | 0.014
D4a1e1 | 16189 | 1 | 0.007
D4a1e1 | 16193 | 1 | 0.007
D4a1e1 | 16261 | 2 | 0.014
D4a1e1 | 16278 | 2 | 0.014
D4a1e1 | 291d | 1 | 0.007
D4a1e1 | 411A | 1 | 0.007
D4a1e1 | 6755 | 1 | 0.007
D4a1e1 | 7334 | 1 | 0.007
D4a1e1 | 738C | 1 | 0.007
D4a1f | 1 | 1 | 0.003
D4a1f | 143 | 1 | 0.003
D4a1f | 150 | 2 | 0.007
D4a1f | 16019G | 1 | 0.003

D4a1f | 16028A | 1 | 0.003
D4a1f | 16029G | 1 | 0.003
D4a1f | 16037 | 13 | 0.045
D4a1f | 16097 | 1 | 0.003
D4a1f | 16111 | 1 | 0.003
D4a1f | 16111G | 2 | 0.007
D4a1f | 16124 | 1 | 0.003
D4a1f | 16147 | 1 | 0.003
D4a1f | 16148 | 3 | 0.01
D4a1f | 16153 | 1 | 0.003
D4a1f | 16162 | 3 | 0.01
D4a1f | 16168 | 1 | 0.003
D4a1f | 16180 | 1 | 0.003
D4a1f | 16203T | 1 | 0.003
D4a1f | 16217 | 5 | 0.017
D4a1f | 16217A | 1 | 0.003
D4a1f | 16242 | 1 | 0.003
D4a1f | 16255 | 1 | 0.003
D4a1f | 16261 | 13 | 0.045
D4a1f | 16290A | 1 | 0.003
D4a1f | 16300 | 1 | 0.003
D4a1f | 16318d | 1 | 0.003
D4a1f | 16342G | 1 | 0.003
D4a1f | 16351d | 2 | 0.007
D4a1f | 16381 | 2 | 0.007
D4a1f | 16399 | 4 | 0.014
D4a1f | 16465 | 1 | 0.003
D4a1f | 16474 | 1 | 0.003
D4a1f | 16512 | 1 | 0.003
D4a1f | 194 | 1 | 0.003
D4a1f | 199 | 1 | 0.003
D4a1f | 200 | 1 | 0.003
D4a1f | 207 | 4 | 0.014
D4a1f | 215 | 1 | 0.003
D4a1f | 222 | 1 | 0.003
D4a1f | 232 | 2 | 0.007
D4a1f | 235 | 6 | 0.021
D4a1f | 239 | 1 | 0.003
D4a1f | 309.3C | 2 | 0.007
D4a1f | 315.2C | 1 | 0.003
D4a1f | 337 | 1 | 0.003
D4a1f | 403 | 1 | 0.003
D4a1f | 446 | 1 | 0.003
D4a1f | 450 | 1 | 0.003
D4a1f | 496 | 1 | 0.003
D4a1f | 519 | 1 | 0.003
D4a1f | 529 | 1 | 0.003
D4a1f | 562 | 1 | 0.003
D4a1f | 709 | 1 | 0.003
D4a1f | 71.1G | 2 | 0.007
D4a1f | 9545 | 1 | 0.003
D4a1f1 | 1 | 1 | 0.004
D4a1f1 | 143 | 1 | 0.004
D4a1f1 | 146 | 1 | 0.004
D4a1f1 | 14978 | 2 | 0.007
D4a1f1 | 150 | 2 | 0.007
D4a1f1 | 16019G | 1 | 0.004
D4a1f1 | 16037 | 9 | 0.032
D4a1f1 | 16086 | 1 | 0.004
D4a1f1 | 16097 | 1 | 0.004
D4a1f1 | 16111 | 1 | 0.004
D4a1f1 | 16111G | 2 | 0.007
D4a1f1 | 16124 | 1 | 0.004
D4a1f1 | 16147 | 1 | 0.004
D4a1f1 | 16148 | 3 | 0.011
D4a1f1 | 16153 | 1 | 0.004
D4a1f1 | 16162 | 3 | 0.011
D4a1f1 | 16168 | 1 | 0.004
D4a1f1 | 16180 | 1 | 0.004
D4a1f1 | 16203T | 1 | 0.004
D4a1f1 | 16217A | 1 | 0.004
D4a1f1 | 16242 | 1 | 0.004
D4a1f1 | 16255 | 1 | 0.004
D4a1f1 | 16261 | 8 | 0.028
D4a1f1 | 16290A | 1 | 0.004
D4a1f1 | 16300 | 1 | 0.004
D4a1f1 | 16318d | 1 | 0.004
D4a1f1 | 16381 | 1 | 0.004
D4a1f1 | 16399 | 4 | 0.014
D4a1f1 | 16465 | 1 | 0.004
D4a1f1 | 16474 | 1 | 0.004
D4a1f1 | 16512 | 1 | 0.004
D4a1f1 | 194 | 1 | 0.004
D4a1f1 | 199 | 1 | 0.004
D4a1f1 | 215 | 1 | 0.004
D4a1f1 | 222 | 1 | 0.004
D4a1f1 | 232 | 2 | 0.007
D4a1f1 | 235 | 5 | 0.018
D4a1f1 | 239 | 1 | 0.004
D4a1f1 | 309.3C | 2 | 0.007
D4a1f1 | 315.2C | 1 | 0.004
D4a1f1 | 337 | 1 | 0.004
D4a1f1 | 403 | 1 | 0.004
D4a1f1 | 446 | 1 | 0.004
D4a1f1 | 450 | 1 | 0.004
D4a1f1 | 4959 | 1 | 0.004
D4a1f1 | 496 | 1 | 0.004
D4a1f1 | 519 | 1 | 0.004
D4a1f1 | 529 | 1 | 0.004
D4a1f1 | 562 | 1 | 0.004
D4a1f1 | 709 | 1 | 0.004
D4a1f1 | 71.1G | 2 | 0.007
D4a1f1 | 7202 | 1 | 0.004
D4a1g | 1 | 1 | 0.004
D4a1g | 13285 | 1 | 0.004
D4a1g | 143 | 1 | 0.004
D4a1g | 150 | 2 | 0.007
D4a1g | 16019G | 1 | 0.004
D4a1g | 16037 | 9 | 0.032
D4a1g | 16097 | 1 | 0.004
D4a1g | 16111 | 1 | 0.004

D4a1g | 16111G | 2 | 0.007
D4a1g | 16124 | 1 | 0.004
D4a1g | 16147 | 1 | 0.004
D4a1g | 16148 | 3 | 0.011
D4a1g | 16153 | 1 | 0.004
D4a1g | 16162 | 3 | 0.011
D4a1g | 16168 | 1 | 0.004
D4a1g | 16180 | 1 | 0.004
D4a1g | 16203T | 1 | 0.004
D4a1g | 16217A | 1 | 0.004
D4a1g | 16242 | 1 | 0.004
D4a1g | 16255 | 1 | 0.004
D4a1g | 16261 | 8 | 0.028
D4a1g | 16290A | 1 | 0.004
D4a1g | 16300 | 1 | 0.004
D4a1g | 16318d | 1 | 0.004
D4a1g | 16356 | 1 | 0.004
D4a1g | 16381 | 1 | 0.004
D4a1g | 16399 | 4 | 0.014
D4a1g | 16465 | 1 | 0.004
D4a1g | 16474 | 1 | 0.004
D4a1g | 16512 | 1 | 0.004
D4a1g | 194 | 1 | 0.004
D4a1g | 199 | 1 | 0.004
D4a1g | 215 | 1 | 0.004
D4a1g | 222 | 1 | 0.004
D4a1g | 232 | 2 | 0.007
D4a1g | 235 | 5 | 0.018
D4a1g | 239 | 1 | 0.004
D4a1g | 309.3C | 2 | 0.007
D4a1g | 315.2C | 1 | 0.004
D4a1g | 337 | 1 | 0.004
D4a1g | 403 | 1 | 0.004
D4a1g | 446 | 1 | 0.004
D4a1g | 450 | 1 | 0.004
D4a1g | 496 | 1 | 0.004
D4a1g | 519 | 1 | 0.004
D4a1g | 529 | 1 | 0.004
D4a1g | 562 | 1 | 0.004
D4a1g | 5809 | 1 | 0.004
D4a1g | 709 | 1 | 0.004
D4a1g | 71.1G | 2 | 0.007
D4a1g | 9064 | 1 | 0.004
D4a1h | 1 | 1 | 0.004
D4a1h | 13635A | 1 | 0.004
D4a1h | 143 | 1 | 0.004
D4a1h | 150 | 2 | 0.007
D4a1h | 16093 | 7 | 0.025
D4a1h | 16097 | 1 | 0.004
D4a1h | 16111 | 1 | 0.004
D4a1h | 16111G | 2 | 0.007
D4a1h | 16124 | 1 | 0.004
D4a1h | 16147 | 1 | 0.004
D4a1h | 16148 | 3 | 0.011
D4a1h | 16153 | 1 | 0.004
D4a1h | 16162 | 3 | 0.011
D4a1h | 16168 | 1 | 0.004
D4a1h | 16180 | 1 | 0.004
D4a1h | 16203T | 1 | 0.004
D4a1h | 16242 | 1 | 0.004
D4a1h | 16247 | 3 | 0.011
D4a1h | 16255 | 1 | 0.004
D4a1h | 16270 | 1 | 0.004
D4a1h | 16274 | 1 | 0.004
D4a1h | 16290A | 1 | 0.004
D4a1h | 16300 | 1 | 0.004
D4a1h | 16311 | 4 | 0.014
D4a1h | 16318d | 1 | 0.004
D4a1h | 16325 | 1 | 0.004
D4a1h | 16399 | 4 | 0.014
D4a1h | 16465 | 1 | 0.004
D4a1h | 16474 | 1 | 0.004
D4a1h | 16512 | 1 | 0.004
D4a1h | 194 | 1 | 0.004
D4a1h | 196 | 2 | 0.007
D4a1h | 199 | 1 | 0.004
D4a1h | 215 | 1 | 0.004
D4a1h | 222 | 1 | 0.004
D4a1h | 232 | 2 | 0.007
D4a1h | 239 | 1 | 0.004
D4a1h | 309.3C | 2 | 0.007
D4a1h | 337 | 1 | 0.004
D4a1h | 403 | 1 | 0.004
D4a1h | 446 | 1 | 0.004
D4a1h | 450 | 1 | 0.004
D4a1h | 496 | 1 | 0.004
D4a1h | 519 | 1 | 0.004
D4a1h | 529 | 1 | 0.004
D4a1h | 562 | 1 | 0.004
D4a1h | 709 | 1 | 0.004
D4a1h | 71.1G | 2 | 0.007
D4a2 | 1 | 1 | 0.003
D4a2 | 143 | 1 | 0.003
D4a2 | 150 | 2 | 0.007
D4a2 | 15052 | 1 | 0.003
D4a2 | 15622 | 1 | 0.003
D4a2 | 16019G | 1 | 0.003
D4a2 | 16028A | 1 | 0.003
D4a2 | 16029G | 1 | 0.003
D4a2 | 16037 | 13 | 0.045
D4a2 | 16097 | 1 | 0.003
D4a2 | 16111 | 1 | 0.003
D4a2 | 16111G | 2 | 0.007
D4a2 | 16124 | 1 | 0.003
D4a2 | 16147 | 1 | 0.003
D4a2 | 16148 | 3 | 0.01
D4a2 | 16153 | 1 | 0.003
D4a2 | 16162 | 3 | 0.01
D4a2 | 16168 | 1 | 0.003
D4a2 | 16180 | 1 | 0.003
D4a2 | 16203T | 1 | 0.003

D4a2 | 16217 | 5 | 0.017
D4a2 | 16217A | 1 | 0.003
D4a2 | 16242 | 1 | 0.003
D4a2 | 16255 | 1 | 0.003
D4a2 | 16261 | 13 | 0.045
D4a2 | 16290A | 1 | 0.003
D4a2 | 16300 | 1 | 0.003
D4a2 | 16318d | 1 | 0.003
D4a2 | 16342G | 1 | 0.003
D4a2 | 16351d | 2 | 0.007
D4a2 | 16381 | 2 | 0.007
D4a2 | 16399 | 4 | 0.014
D4a2 | 16465 | 1 | 0.003
D4a2 | 16474 | 1 | 0.003
D4a2 | 16512 | 1 | 0.003
D4a2 | 194 | 1 | 0.003
D4a2 | 199 | 1 | 0.003
D4a2 | 200 | 1 | 0.003
D4a2 | 207 | 4 | 0.014
D4a2 | 215 | 1 | 0.003
D4a2 | 222 | 1 | 0.003
D4a2 | 232 | 2 | 0.007
D4a2 | 235 | 6 | 0.021
D4a2 | 239 | 1 | 0.003
D4a2 | 309.3C | 2 | 0.007
D4a2 | 315.2C | 1 | 0.003
D4a2 | 337 | 1 | 0.003
D4a2 | 403 | 1 | 0.003
D4a2 | 446 | 1 | 0.003
D4a2 | 450 | 1 | 0.003
D4a2 | 496 | 1 | 0.003
D4a2 | 519 | 1 | 0.003
D4a2 | 529 | 1 | 0.003
D4a2 | 562 | 1 | 0.003
D4a2 | 6998 | 1 | 0.003
D4a2 | 709 | 1 | 0.003
D4a2 | 71.1G | 2 | 0.007
D4a2a | 1 | 1 | 0.004
D4a2a | 143 | 1 | 0.004
D4a2a | 150 | 2 | 0.007
D4a2a | 151 | 4 | 0.014
D4a2a | 15613 | 1 | 0.004
D4a2a | 15868 | 1 | 0.004
D4a2a | 16097 | 1 | 0.004
D4a2a | 16105 | 1 | 0.004
D4a2a | 16111 | 1 | 0.004
D4a2a | 16111G | 2 | 0.007
D4a2a | 16124 | 1 | 0.004
D4a2a | 16147 | 1 | 0.004
D4a2a | 16148 | 3 | 0.011
D4a2a | 16153 | 1 | 0.004
D4a2a | 16162 | 3 | 0.011
D4a2a | 16168 | 1 | 0.004
D4a2a | 16180 | 1 | 0.004
D4a2a | 16203T | 1 | 0.004
D4a2a | 16242 | 1 | 0.004
D4a2a | 16247 | 3 | 0.011
D4a2a | 16255 | 1 | 0.004
D4a2a | 16290A | 1 | 0.004
D4a2a | 16300 | 1 | 0.004
D4a2a | 16311 | 3 | 0.011
D4a2a | 16318d | 1 | 0.004
D4a2a | 16390 | 1 | 0.004
D4a2a | 16399 | 4 | 0.014
D4a2a | 16465 | 1 | 0.004
D4a2a | 16474 | 1 | 0.004
D4a2a | 16512 | 1 | 0.004
D4a2a | 185 | 1 | 0.004
D4a2a | 189 | 1 | 0.004
D4a2a | 194 | 1 | 0.004
D4a2a | 195 | 1 | 0.004
D4a2a | 196 | 1 | 0.004
D4a2a | 199 | 1 | 0.004
D4a2a | 215 | 1 | 0.004
D4a2a | 222 | 1 | 0.004
D4a2a | 232 | 2 | 0.007
D4a2a | 239 | 1 | 0.004
D4a2a | 241 | 1 | 0.004
D4a2a | 309.3C | 2 | 0.007
D4a2a | 337 | 1 | 0.004
D4a2a | 403 | 1 | 0.004
D4a2a | 426 | 1 | 0.004
D4a2a | 446 | 1 | 0.004
D4a2a | 450 | 1 | 0.004
D4a2a | 496 | 1 | 0.004
D4a2a | 518 | 1 | 0.004
D4a2a | 519 | 1 | 0.004
D4a2a | 529 | 1 | 0.004
D4a2a | 562 | 1 | 0.004
D4a2a | 709 | 1 | 0.004
D4a2a | 71.1G | 2 | 0.007
D4a2b | 1 | 1 | 0.003
D4a2b | 143 | 1 | 0.003
D4a2b | 150 | 2 | 0.007
D4a2b | 16019G | 1 | 0.003
D4a2b | 16028A | 1 | 0.003
D4a2b | 16029G | 1 | 0.003
D4a2b | 16037 | 13 | 0.045
D4a2b | 16097 | 1 | 0.003
D4a2b | 16111 | 1 | 0.003
D4a2b | 16111G | 2 | 0.007
D4a2b | 16124 | 1 | 0.003
D4a2b | 16147 | 1 | 0.003
D4a2b | 16148 | 3 | 0.01
D4a2b | 16153 | 1 | 0.003
D4a2b | 16162 | 3 | 0.01
D4a2b | 16168 | 1 | 0.003
D4a2b | 16180 | 1 | 0.003
D4a2b | 16203T | 1 | 0.003
D4a2b | 16217 | 5 | 0.017
D4a2b | 16217A | 1 | 0.003

D4a2b | 16242 | 1 | 0.003
D4a2b | 16255 | 1 | 0.003
D4a2b | 16261 | 13 | 0.045
D4a2b | 16290A | 1 | 0.003
D4a2b | 16298 | 1 | 0.003
D4a2b | 16300 | 1 | 0.003
D4a2b | 16318d | 1 | 0.003
D4a2b | 16342G | 1 | 0.003
D4a2b | 16351d | 2 | 0.007
D4a2b | 16381 | 2 | 0.007
D4a2b | 16399 | 4 | 0.014
D4a2b | 16465 | 1 | 0.003
D4a2b | 16474 | 1 | 0.003
D4a2b | 16512 | 1 | 0.003
D4a2b | 194 | 1 | 0.003
D4a2b | 199 | 1 | 0.003
D4a2b | 200 | 1 | 0.003
D4a2b | 207 | 4 | 0.014
D4a2b | 215 | 1 | 0.003
D4a2b | 222 | 1 | 0.003
D4a2b | 232 | 2 | 0.007
D4a2b | 235 | 6 | 0.021
D4a2b | 239 | 1 | 0.003
D4a2b | 309.3C | 2 | 0.007
D4a2b | 315.2C | 1 | 0.003
D4a2b | 337 | 1 | 0.003
D4a2b | 403 | 1 | 0.003
D4a2b | 446 | 1 | 0.003
D4a2b | 450 | 1 | 0.003
D4a2b | 496 | 1 | 0.003
D4a2b | 519 | 1 | 0.003
D4a2b | 5196 | 1 | 0.003
D4a2b | 529 | 1 | 0.003
D4a2b | 562 | 1 | 0.003
D4a2b | 709 | 1 | 0.003
D4a2b | 71.1G | 2 | 0.007
D4a3 | 146 | 1 | 0.007
D4a3 | 16051 | 2 | 0.015
D4a3 | 16189 | 1 | 0.007
D4a3 | 16192 | 1 | 0.007
D4a3 | 16193 | 1 | 0.007
D4a3 | 16263 | 1 | 0.007
D4a3 | 16269 | 2 | 0.015
D4a3 | 196 | 1 | 0.007
D4a3 | 215 | 1 | 0.007
D4a3 | 237 | 1 | 0.007
D4a3 | 290-291d | 1 | 0.007
D4a3 | 291d | 2 | 0.015
D4a3a | 146 | 1 | 0.007
D4a3a | 16051 | 2 | 0.015
D4a3a | 16189 | 1 | 0.007
D4a3a | 16192 | 1 | 0.007
D4a3a | 16193 | 1 | 0.007
D4a3a | 16263 | 1 | 0.007
D4a3a | 16269 | 2 | 0.015
D4a3a | 196 | 1 | 0.007
D4a3a | 215 | 1 | 0.007
D4a3a | 237 | 1 | 0.007
D4a3a | 290-291d | 1 | 0.007
D4a3a | 291d | 2 | 0.015
D4a3a1 | 13401 | 2 | 0.018
D4a3a1 | 16093 | 6 | 0.055
D4a3a1 | 29 | 1 | 0.009
D4a3a1 | 3338 | 1 | 0.009
D4a3a1 | 3483 | 2 | 0.018
D4a3a1 | 6515 | 1 | 0.009
D4a3a1 | 7257 | 1 | 0.009
D4a3a1 | 7854 | 1 | 0.009
D4a3a1 | 9662 | 1 | 0.009
D4a3a1 | 9725 | 2 | 0.018
D4a3a2 | 143 | 1 | 0.006
D4a3a2 | 146 | 3 | 0.019
D4a3a2 | 15776 | 1 | 0.006
D4a3a2 | 16189 | 1 | 0.006
D4a3a2 | 16192 | 2 | 0.012
D4a3a2 | 16221 | 1 | 0.006
D4a3a2 | 16264 | 1 | 0.006
D4a3a2 | 16371 | 2 | 0.012
D4a3a2 | 16399 | 1 | 0.006
D4a3a2 | 195A | 1 | 0.006
D4a3a2 | 196 | 3 | 0.019
D4a3a2 | 237 | 7 | 0.044
D4a3a2 | 455.1T | 1 | 0.006
D4a3a2 | 459.1C | 1 | 0.006
D4a3a2 | 460 | 2 | 0.012
D4a3a2 | 5237 | 1 | 0.006
D4a3a2 | 63 | 2 | 0.012
D4a3a2 | 64 | 1 | 0.006
D4a3a2 | 9545 | 1 | 0.006
D4a3b | 13659 | 1 | 0.007
D4a3b | 146 | 1 | 0.007
D4a3b | 16051 | 2 | 0.014
D4a3b | 16093 | 3 | 0.022
D4a3b | 16189 | 1 | 0.007
D4a3b | 16192 | 1 | 0.007
D4a3b | 16193 | 1 | 0.007
D4a3b | 16263 | 1 | 0.007
D4a3b | 16269 | 2 | 0.014
D4a3b | 16311 | 1 | 0.007
D4a3b | 195 | 1 | 0.007
D4a3b | 196 | 1 | 0.007
D4a3b | 215 | 1 | 0.007
D4a3b | 237 | 1 | 0.007
D4a3b | 290-291d | 1 | 0.007
D4a3b | 291d | 2 | 0.014
D4a3b1 | 13418 | 1 | 0.008
D4a3b1 | 146 | 1 | 0.008
D4a3b1 | 16243 | 1 | 0.008
D4a3b1 | 16309 | 1 | 0.008
D4a3b1 | 195 | 1 | 0.008
D4a3b1 | 6366 | 1 | 0.008

D4a3b1 | 9705 | 1 | 0.008
D4a3b2 | 12892 | 6 | 0.029
D4a3b2 | 13302T | 2 | 0.01
D4a3b2 | 146 | 3 | 0.014
D4a3b2 | 147 | 1 | 0.005
D4a3b2 | 15470 | 2 | 0.01
D4a3b2 | 15481 | 7 | 0.034
D4a3b2 | 15799 | 1 | 0.005
D4a3b2 | 16086 | 1 | 0.005
D4a3b2 | 16093 | 1 | 0.005
D4a3b2 | 16094 | 1 | 0.005
D4a3b2 | 16111 | 3 | 0.014
D4a3b2 | 16177 | 1 | 0.005
D4a3b2 | 16189 | 1 | 0.005
D4a3b2 | 16192 | 2 | 0.01
D4a3b2 | 16231 | 2 | 0.01
D4a3b2 | 16233 | 1 | 0.005
D4a3b2 | 16234 | 3 | 0.014
D4a3b2 | 16244 | 1 | 0.005
D4a3b2 | 16265 | 1 | 0.005
D4a3b2 | 16274 | 1 | 0.005
D4a3b2 | 16278 | 41 | 0.198
D4a3b2 | 16284 | 2 | 0.01
D4a3b2 | 16342 | 1 | 0.005
D4a3b2 | 16376G | 2 | 0.01
D4a3b2 | 16385 | 1 | 0.005
D4a3b2 | 16387 | 2 | 0.01
D4a3b2 | 16411 | 1 | 0.005
D4a3b2 | 16415 | 1 | 0.005
D4a3b2 | 16426 | 1 | 0.005
D4a3b2 | 16464 | 2 | 0.01
D4a3b2 | 16478 | 2 | 0.01
D4a3b2 | 16481 | 1 | 0.005
D4a3b2 | 16484-16489d | 1 | 0.005
D4a3b2 | 16486 | 2 | 0.01
D4a3b2 | 16518 | 1 | 0.005
D4a3b2 | 16541T | 1 | 0.005
D4a3b2 | 16550 | 1 | 0.005
D4a3b2 | 177 | 1 | 0.005
D4a3b2 | 210 | 1 | 0.005
D4a3b2 | 248T | 1 | 0.005
D4a3b2 | 249d | 2 | 0.01
D4a3b2 | 284.1A | 1 | 0.005
D4a3b2 | 284d | 1 | 0.005
D4a3b2 | 309d | 1 | 0.005
D4a3b2 | 310 | 1 | 0.005
D4a3b2 | 323.1G | 1 | 0.005
D4a3b2 | 325d | 1 | 0.005
D4a3b2 | 330 | 1 | 0.005
D4a3b2 | 361d | 2 | 0.01
D4a3b2 | 438.1C | 1 | 0.005
D4a3b2 | 44 | 1 | 0.005
D4a3b2 | 445.1C | 1 | 0.005
D4a3b2 | 455.1T | 1 | 0.005
D4a3b2 | 459.1C | 1 | 0.005
D4a3b2 | 465.1C | 1 | 0.005
D4a3b2 | 470.1A | 1 | 0.005
D4a3b2 | 472.1A | 1 | 0.005
D4a3b2 | 479.1A | 1 | 0.005
D4a3b2 | 492 | 1 | 0.005
D4a3b2 | 508C | 1 | 0.005
D4a3b2 | 514 | 1 | 0.005
D4a3b2 | 533 | 1 | 0.005
D4a3b2 | 5460 | 1 | 0.005
D4a3b2 | 559 | 2 | 0.01
D4a3b2 | 6022 | 1 | 0.005
D4a3b2 | 63 | 1 | 0.005
D4a3b2 | 71.1G | 4 | 0.019
D4a3b2 | 7572 | 2 | 0.01
D4a3b2 | 7823 | 1 | 0.005
D4a3b2 | 95 | 1 | 0.005
D4a4 | 16214 | 1 | 0.042
D4a4 | 16234 | 2 | 0.083
D4a4 | 16296 | 1 | 0.042
D4a4 | 16316 | 2 | 0.083
D4a4 | 9569 | 4 | 0.167
D4a5 | 1 | 1 | 0.004
D4a5 | 12865 | 1 | 0.004
D4a5 | 143 | 1 | 0.004
D4a5 | 146 | 1 | 0.004
D4a5 | 150 | 2 | 0.007
D4a5 | 16097 | 1 | 0.004
D4a5 | 16111 | 1 | 0.004
D4a5 | 16111G | 2 | 0.007
D4a5 | 16124 | 1 | 0.004
D4a5 | 16147 | 1 | 0.004
D4a5 | 16148 | 3 | 0.011
D4a5 | 16153 | 1 | 0.004
D4a5 | 16162 | 3 | 0.011
D4a5 | 16168 | 1 | 0.004
D4a5 | 16180 | 1 | 0.004
D4a5 | 16201 | 1 | 0.004
D4a5 | 16203T | 1 | 0.004
D4a5 | 16234 | 3 | 0.011
D4a5 | 16242 | 1 | 0.004
D4a5 | 16255 | 1 | 0.004
D4a5 | 16290A | 1 | 0.004
D4a5 | 16300 | 1 | 0.004
D4a5 | 16318d | 1 | 0.004
D4a5 | 16390 | 1 | 0.004
D4a5 | 16399 | 4 | 0.015
D4a5 | 16465 | 1 | 0.004
D4a5 | 16474 | 1 | 0.004
D4a5 | 16512 | 1 | 0.004
D4a5 | 194 | 1 | 0.004
D4a5 | 199 | 1 | 0.004
D4a5 | 215 | 1 | 0.004
D4a5 | 222 | 1 | 0.004
D4a5 | 232 | 2 | 0.007
D4a5 | 239 | 1 | 0.004
D4a5 | 309.3C | 2 | 0.007

D4a5 | 337 | 1 | 0.004
D4a5 | 403 | 1 | 0.004
D4a5 | 4053 | 1 | 0.004
D4a5 | 446 | 1 | 0.004
D4a5 | 450 | 1 | 0.004
D4a5 | 4913 | 1 | 0.004
D4a5 | 496 | 1 | 0.004
D4a5 | 519 | 1 | 0.004
D4a5 | 529 | 1 | 0.004
D4a5 | 5558 | 1 | 0.004
D4a5 | 562 | 1 | 0.004
D4a5 | 709 | 1 | 0.004
D4a5 | 71.1G | 2 | 0.007
D4a6 | 10003 | 1 | 0.006
D4a6 | 146 | 1 | 0.006
D4a6 | 16093 | 1 | 0.006
D4a6 | 16188 | 1 | 0.006
D4a6 | 16297 | 3 | 0.018
D4a6 | 447 | 1 | 0.006
D4a6 | 709 | 1 | 0.006
D4a6 | 7367 | 1 | 0.006
D4a6 | 7921 | 1 | 0.006
D4a6 | 8120G | 1 | 0.006
D4a6 | 9377 | 1 | 0.006
D4a7 | 12216 | 1 | 0.005
D4a7 | 146 | 1 | 0.005
D4a7 | 14664 | 1 | 0.005
D4a7 | 15066A | 3 | 0.014
D4a7 | 151 | 1 | 0.005
D4a7 | 15883 | 6 | 0.027
D4a7 | 16051 | 1 | 0.005
D4a7 | 16073d | 1 | 0.005
D4a7 | 16075A | 1 | 0.005
D4a7 | 16084 | 1 | 0.005
D4a7 | 16088A | 1 | 0.005
D4a7 | 16093 | 11 | 0.05
D4a7 | 16111 | 1 | 0.005
D4a7 | 16113C | 1 | 0.005
D4a7 | 16116 | 1 | 0.005
D4a7 | 16116C | 1 | 0.005
D4a7 | 16118T | 1 | 0.005
D4a7 | 16126A | 1 | 0.005
D4a7 | 16148 | 2 | 0.009
D4a7 | 16177 | 1 | 0.005
D4a7 | 16194 | 1 | 0.005
D4a7 | 16207 | 1 | 0.005
D4a7 | 16209 | 1 | 0.005
D4a7 | 16244 | 1 | 0.005
D4a7 | 16255 | 1 | 0.005
D4a7 | 16260 | 3 | 0.014
D4a7 | 16271 | 1 | 0.005
D4a7 | 16274 | 1 | 0.005
D4a7 | 16283C | 1 | 0.005
D4a7 | 16299 | 1 | 0.005
D4a7 | 16311 | 6 | 0.027
D4a7 | 16350T | 1 | 0.005
D4a7 | 16390 | 2 | 0.009
D4a7 | 16413 | 1 | 0.005
D4a7 | 16451d | 1 | 0.005
D4a7 | 16462G | 4 | 0.018
D4a7 | 16465 | 1 | 0.005
D4a7 | 16527 | 2 | 0.009
D4a7 | 183 | 1 | 0.005
D4a7 | 195 | 1 | 0.005
D4a7 | 199 | 34 | 0.153
D4a7 | 204 | 1 | 0.005
D4a7 | 207 | 1 | 0.005
D4a7 | 213.1T | 1 | 0.005
D4a7 | 214 | 1 | 0.005
D4a7 | 237T | 1 | 0.005
D4a7 | 246 | 1 | 0.005
D4a7 | 260T | 1 | 0.005
D4a7 | 276 | 1 | 0.005
D4a7 | 279 | 1 | 0.005
D4a7 | 290 | 1 | 0.005
D4a7 | 3 | 1 | 0.005
D4a7 | 309.3C | 4 | 0.018
D4a7 | 310 | 1 | 0.005
D4a7 | 315.3C | 1 | 0.005
D4a7 | 316C | 1 | 0.005
D4a7 | 333 | 1 | 0.005
D4a7 | 352 | 1 | 0.005
D4a7 | 363 | 1 | 0.005
D4a7 | 385 | 2 | 0.009
D4a7 | 40 | 1 | 0.005
D4a7 | 401A | 1 | 0.005
D4a7 | 4491 | 2 | 0.009
D4a7 | 4622 | 1 | 0.005
D4a7 | 475d | 1 | 0.005
D4a7 | 507 | 20 | 0.09
D4a7 | 513 | 1 | 0.005
D4a7 | 515 | 1 | 0.005
D4a7 | 538 | 1 | 0.005
D4a7 | 550C | 1 | 0.005
D4a7 | 567 | 1 | 0.005
D4a7 | 5673 | 5 | 0.023
D4a7 | 6039 | 1 | 0.005
D4a7 | 64 | 1 | 0.005
D4a7 | 67 | 1 | 0.005
D4a7 | 751 | 1 | 0.005
D4a7 | 792 | 2 | 0.009
D4a7 | 81 | 1 | 0.005
D4a7 | 8410 | 5 | 0.023
D4a7 | 91 | 1 | 0.005
D4a7 | 95 | 1 | 0.005
D4a8 | 10680 | 1 | 0.008
D4a8 | 16189 | 1 | 0.008
D4a8 | 16265 | 6 | 0.048
D4a8 | 16311 | 1 | 0.008
D4a8 | 5216A | 1 | 0.008
D4a8 | 7859 | 1 | 0.008

D4b | 11287 | 1 | 0.001
D4b | 150 | 1 | 0.001
D4b | 16165 | 1 | 0.001
D4b | 16179 | 1 | 0.001
D4b | 16218 | 4 | 0.006
D4b | 16249 | 1 | 0.001
D4b | 446 | 1 | 0.001
D4b | 648 | 1 | 0.001
D4b | 7852 | 1 | 0.001
D4b | 8292 | 1 | 0.001
D4b1 | 13928C | 1 | 0.005
D4b1 | 14180 | 3 | 0.015
D4b1 | 152 | 3 | 0.015
D4b1 | 15211 | 3 | 0.015
D4b1 | 16093 | 1 | 0.005
D4b1 | 16239 | 3 | 0.015
D4b1 | 16243 | 3 | 0.015
D4b1 | 195 | 2 | 0.01
D4b1 | 4136 | 3 | 0.015
D4b1 | 5319 | 3 | 0.015
D4b1 | 5824 | 1 | 0.005
D4b1 | 6146 | 3 | 0.015
D4b1 | 6647 | 1 | 0.005
D4b1 | 6881 | 3 | 0.015
D4b1a | 10502 | 1 | 0.005
D4b1a | 12231 | 1 | 0.005
D4b1a | 14180 | 1 | 0.005
D4b1a | 146 | 1 | 0.005
D4b1a | 16129 | 3 | 0.015
D4b1a | 7270 | 1 | 0.005
D4b1a1 | 10365 | 1 | 0.006
D4b1a1 | 11015 | 1 | 0.006
D4b1a1 | 12429 | 1 | 0.006
D4b1a1 | 13928C | 1 | 0.006
D4b1a1 | 146 | 1 | 0.006
D4b1a1 | 150 | 1 | 0.006
D4b1a1 | 151 | 3 | 0.017
D4b1a1 | 16184 | 1 | 0.006
D4b1a1 | 16224 | 1 | 0.006
D4b1a1 | 16290 | 1 | 0.006
D4b1a1 | 16293C | 1 | 0.006
D4b1a1 | 16297 | 1 | 0.006
D4b1a1 | 16298d | 1 | 0.006
D4b1a1 | 16320A | 3 | 0.017
D4b1a1 | 16355 | 1 | 0.006
D4b1a1 | 4113 | 2 | 0.011
D4b1a1 | 4538 | 1 | 0.006
D4b1a1 | 5471 | 1 | 0.006
D4b1a1 | 7673 | 1 | 0.006
D4b1a1 | 7979 | 1 | 0.006
D4b1a1a | 146 | 1 | 0.005
D4b1a1a | 150 | 1 | 0.005
D4b1a1a | 16093 | 7 | 0.036
D4b1a1a | 16184 | 1 | 0.005
D4b1a1a | 16201 | 6 | 0.031
D4b1a1a | 16239 | 14 | 0.072
D4b1a1a | 16242 | 2 | 0.01
D4b1a1a | 16243 | 14 | 0.072
D4b1a1a | 16293C | 1 | 0.005
D4b1a1a | 16297 | 1 | 0.005
D4b1a1a | 16298d | 1 | 0.005
D4b1a1a | 195 | 2 | 0.01
D4b1a1a | 234 | 1 | 0.005
D4b1a1a | 316C | 2 | 0.01
D4b1a1a | 5821 | 1 | 0.005
D4b1a2 | 12957 | 1 | 0.005
D4b1a2 | 13759 | 2 | 0.01
D4b1a2 | 13815 | 1 | 0.005
D4b1a2 | 146 | 1 | 0.005
D4b1a2 | 16129 | 3 | 0.015
D4b1a2 | 16320 | 1 | 0.005
D4b1a2 | 310 | 1 | 0.005
D4b1a2 | 3397 | 1 | 0.005
D4b1a2 | 6842 | 2 | 0.01
D4b1a2 | 7581 | 1 | 0.005
D4b1a2a | 1001A | 1 | 0.005
D4b1a2a | 11107 | 1 | 0.005
D4b1a2a | 13656 | 1 | 0.005
D4b1a2a | 146 | 1 | 0.005
D4b1a2a | 16129 | 3 | 0.015
D4b1a2a | 16189 | 1 | 0.005
D4b1a2a | 16311 | 1 | 0.005
D4b1a2a | 3834C | 1 | 0.005
D4b1a2a | 6620 | 1 | 0.005
D4b1a2a | 9142 | 1 | 0.005
D4b1a2a | 9431 | 1 | 0.005
D4b1a2a1 | 11383 | 10 | 0.042
D4b1a2a1 | 11617 | 1 | 0.004
D4b1a2a1 | 11914 | 2 | 0.008
D4b1a2a1 | 14122C | 10 | 0.042
D4b1a2a1 | 14305 | 3 | 0.013
D4b1a2a1 | 15172 | 2 | 0.008
D4b1a2a1 | 15370 | 4 | 0.017
D4b1a2a1 | 15448A | 3 | 0.013
D4b1a2a1 | 15909 | 1 | 0.004
D4b1a2a1 | 16093 | 52 | 0.219
D4b1a2a1 | 16129 | 21 | 0.089
D4b1a2a1 | 16150 | 1 | 0.004
D4b1a2a1 | 16172 | 10 | 0.042
D4b1a2a1 | 16215 | 9 | 0.038
D4b1a2a1 | 16234 | 1 | 0.004
D4b1a2a1 | 16255 | 2 | 0.008
D4b1a2a1 | 16265C | 1 | 0.004
D4b1a2a1 | 16266 | 1 | 0.004
D4b1a2a1 | 16311 | 1 | 0.004
D4b1a2a1 | 16352 | 1 | 0.004
D4b1a2a1 | 16362G | 1 | 0.004
D4b1a2a1 | 183 | 8 | 0.034
D4b1a2a1 | 183C | 1 | 0.004
D4b1a2a1 | 185 | 1 | 0.004
D4b1a2a1 | 195 | 1 | 0.004

D4b1a2a1 | 310 | 1 | 0.004
D4b1a2a1 | 4841 | 1 | 0.004
D4b1a2a1 | 508 | 1 | 0.004
D4b1a2a1 | 523d | 3 | 0.013
D4b1a2a1 | 650 | 2 | 0.008
D4b1a2a1 | 7076 | 1 | 0.004
D4b1a2a1 | 7762 | 8 | 0.034
D4b1a2a1 | 8945 | 1 | 0.004
D4b1a2a2 | 10754 | 2 | 0.01
D4b1a2a2 | 14207 | 2 | 0.01
D4b1a2a2 | 14798 | 1 | 0.005
D4b1a2a2 | 16260 | 2 | 0.01
D4b1b | 10172 | 3 | 0.048
D4b1b | 12188 | 1 | 0.016
D4b1b | 12372 | 3 | 0.048
D4b1b | 146 | 1 | 0.016
D4b1b | 15635 | 1 | 0.016
D4b1b | 15731 | 1 | 0.016
D4b1b | 16172 | 4 | 0.065
D4b1b | 16176 | 1 | 0.016
D4b1b | 16177 | 1 | 0.016
D4b1b | 16271 | 6 | 0.097
D4b1b | 16348 | 1 | 0.016
D4b1b | 16356 | 4 | 0.065
D4b1b | 16381 | 1 | 0.016
D4b1b | 16390 | 9 | 0.145
D4b1b | 16468 | 1 | 0.016
D4b1b | 420 | 2 | 0.032
D4b1b | 4310 | 2 | 0.032
D4b1b | 5048 | 1 | 0.016
D4b1b | 6218 | 3 | 0.048
D4b1b | 8281-8289d | 1 | 0.016
D4b1b | 8374 | 3 | 0.048
D4b1b | 8532 | 2 | 0.032
D4b1b | 8920 | 1 | 0.016
D4b1b | 9150 | 2 | 0.032
D4b1b | 979 | 1 | 0.016
D4b1b | 9894 | 2 | 0.032
D4b1b1 | 10801 | 1 | 0.02
D4b1b1 | 16111 | 1 | 0.02
D4b1b1 | 16145 | 1 | 0.02
D4b1b1 | 16209 | 3 | 0.059
D4b1b1 | 16259A | 1 | 0.02
D4b1b1 | 16310 | 1 | 0.02
D4b1b1 | 16352 | 1 | 0.02
D4b1b1 | 16529 | 1 | 0.02
D4b1b1 | 1719 | 1 | 0.02
D4b1b1 | 194 | 1 | 0.02
D4b1b1 | 204 | 1 | 0.02
D4b1b1 | 259 | 1 | 0.02
D4b1b1 | 504 | 1 | 0.02
D4b1b1 | 9129 | 1 | 0.02
D4b1b1a | 146 | 1 | 0.023
D4b1b1a | 16354 | 1 | 0.023
D4b1b1a | 310 | 1 | 0.023
D4b1b1a | 5128 | 1 | 0.023
D4b1b1a1 | 10223 | 1 | 0.017
D4b1b1a1 | 11257 | 2 | 0.033
D4b1b1a1 | 11668 | 1 | 0.017
D4b1b1a1 | 1393 | 1 | 0.017
D4b1b1a1 | 14635 | 1 | 0.017
D4b1b1a1 | 15900 | 1 | 0.017
D4b1b1a1 | 16077 | 3 | 0.05
D4b1b1a1 | 16192 | 1 | 0.017
D4b1b1a1 | 16227 | 3 | 0.05
D4b1b1a1 | 16274 | 1 | 0.017
D4b1b1a1 | 16325 | 1 | 0.017
D4b1b1a1 | 16400 | 1 | 0.017
D4b1b1a1 | 195 | 4 | 0.067
D4b1b1a1 | 291d | 1 | 0.017
D4b1b1a1 | 309.3C | 1 | 0.017
D4b1b1a1 | 3472 | 1 | 0.017
D4b1b1a1 | 5899.1C | 1 | 0.017
D4b1b1a1 | 8730 | 2 | 0.033
D4b1b1a1 | 8776 | 1 | 0.017
D4b1b2 | 12360 | 1 | 0.017
D4b1b2 | 14584 | 2 | 0.034
D4b1b2 | 16093 | 13 | 0.224
D4b1b2 | 16380 | 11 | 0.19
D4b1b2 | 209 | 2 | 0.034
D4b1b2 | 5460 | 1 | 0.017
D4b1b2 | 7043 | 2 | 0.034
D4b1b2 | 921 | 1 | 0.017
D4b1c | 15110 | 1 | 0.007
D4b1c | 16243 | 1 | 0.007
D4b1c | 200 | 2 | 0.014
D4b1c | 310 | 2 | 0.014
D4b1c | 3576 | 1 | 0.007
D4b1c | 4023 | 2 | 0.014
D4b1c | 6808 | 1 | 0.007
D4b1c | 7115 | 1 | 0.007
D4b1c | 9469 | 3 | 0.021
D4b1c | 9604 | 3 | 0.021
D4b1c | 9785 | 2 | 0.014
D4b1d | 10049 | 1 | 0.008
D4b1d | 16293 | 4 | 0.03
D4b1d | 204 | 4 | 0.03
D4b1d | 207 | 1 | 0.008
D4b1d | 389 | 1 | 0.008
D4b1d | 5097 | 1 | 0.008
D4b1d | 8823 | 1 | 0.008
D4b2 | 10454 | 1 | 0.001
D4b2 | 10692 | 1 | 0.001
D4b2 | 10858 | 1 | 0.001
D4b2 | 12172 | 1 | 0.001
D4b2 | 12549 | 1 | 0.001
D4b2 | 12795 | 1 | 0.001
D4b2 | 13135 | 1 | 0.001
D4b2 | 13152 | 1 | 0.001
D4b2 | 13392 | 1 | 0.001
D4b2 | 13928C | 1 | 0.001

D4b2 | 15040 | 1 | 0.001
D4b2 | 1555 | 1 | 0.001
D4b2 | 16093 | 1 | 0.001
D4b2 | 16129 | 1 | 0.001
D4b2 | 16165 | 1 | 0.001
D4b2 | 16172 | 1 | 0.001
D4b2 | 16179 | 1 | 0.001
D4b2 | 16218 | 4 | 0.006
D4b2 | 319 | 1 | 0.001
D4b2 | 3460 | 1 | 0.001
D4b2 | 3866 | 1 | 0.001
D4b2 | 4140 | 1 | 0.001
D4b2 | 446 | 1 | 0.001
D4b2 | 4655 | 1 | 0.001
D4b2 | 5600 | 1 | 0.001
D4b2 | 648 | 1 | 0.001
D4b2a | 1211 | 1 | 0.001
D4b2a | 13105 | 1 | 0.001
D4b2a | 15265 | 1 | 0.001
D4b2a | 15271 | 1 | 0.001
D4b2a | 16165 | 1 | 0.001
D4b2a | 16179 | 1 | 0.001
D4b2a | 16218 | 4 | 0.006
D4b2a | 16254 | 1 | 0.001
D4b2a | 1719 | 1 | 0.001
D4b2a | 1888 | 1 | 0.001
D4b2a | 207 | 1 | 0.001
D4b2a | 3327 | 1 | 0.001
D4b2a | 446 | 1 | 0.001
D4b2a | 648 | 1 | 0.001
D4b2a1 | 16216 | 1 | 0.009
D4b2a1 | 16527 | 1 | 0.009
D4b2a1 | 1662 | 1 | 0.009
D4b2a1 | 7403 | 1 | 0.009
D4b2a1 | 9058 | 1 | 0.009
D4b2a2 | 11878 | 1 | 0.001
D4b2a2 | 16165 | 1 | 0.001
D4b2a2 | 16179 | 1 | 0.001
D4b2a2 | 16187 | 1 | 0.001
D4b2a2 | 16218 | 4 | 0.006
D4b2a2 | 16227 | 1 | 0.001
D4b2a2 | 16278 | 1 | 0.001
D4b2a2 | 16290 | 1 | 0.001
D4b2a2 | 16311 | 1 | 0.001
D4b2a2 | 199 | 2 | 0.003
D4b2a2 | 446 | 1 | 0.001
D4b2a2 | 4824 | 2 | 0.003
D4b2a2 | 648 | 1 | 0.001
D4b2a2a | 11431 | 1 | 0.004
D4b2a2a | 13759 | 1 | 0.004
D4b2a2a | 14002 | 1 | 0.004
D4b2a2a | 15217 | 1 | 0.004
D4b2a2a | 16209 | 1 | 0.004
D4b2a2a | 16278 | 1 | 0.004
D4b2a2a | 4646 | 1 | 0.004
D4b2a2a | 6239 | 1 | 0.004
D4b2a2a | 6698 | 1 | 0.004
D4b2a2a1 | 10352 | 1 | 0.005
D4b2a2a1 | 11447 | 1 | 0.005
D4b2a2a1 | 11581 | 1 | 0.005
D4b2a2a1 | 12630 | 1 | 0.005
D4b2a2a1 | 16111 | 1 | 0.005
D4b2a2a1 | 16178G | 1 | 0.005
D4b2a2a1 | 16190 | 3 | 0.014
D4b2a2a1 | 16191 | 1 | 0.005
D4b2a2a1 | 16192 | 30 | 0.136
D4b2a2a1 | 16311 | 1 | 0.005
D4b2a2a1 | 16316 | 31 | 0.141
D4b2a2a1 | 183 | 1 | 0.005
D4b2a2a1 | 184 | 4 | 0.018
D4b2a2a1 | 204 | 5 | 0.023
D4b2a2a1 | 207 | 1 | 0.005
D4b2a2a1 | 309.3C | 1 | 0.005
D4b2a2a1 | 3423 | 1 | 0.005
D4b2a2a1 | 495 | 4 | 0.018
D4b2a2a1 | 6286 | 1 | 0.005
D4b2a2a1 | 7214 | 2 | 0.009
D4b2a2a1 | 8149 | 2 | 0.009
D4b2a2a1 | 8595 | 1 | 0.005
D4b2a2a1 | 8901 | 1 | 0.005
D4b2a2a1 | 9041 | 2 | 0.009
D4b2a2a2 | 14470 | 1 | 0.004
D4b2a2a2 | 191 | 1 | 0.004
D4b2a2b | 10 | 2 | 0.003
D4b2a2b | 11884 | 1 | 0.001
D4b2a2b | 13105 | 1 | 0.001
D4b2a2b | 13855 | 1 | 0.001
D4b2a2b | 15804 | 1 | 0.001
D4b2a2b | 16165 | 1 | 0.001
D4b2a2b | 16179 | 1 | 0.001
D4b2a2b | 16218 | 4 | 0.006
D4b2a2b | 3395 | 1 | 0.001
D4b2a2b | 446 | 1 | 0.001
D4b2a2b | 648 | 1 | 0.001
D4b2a2b | 6713 | 2 | 0.003
D4b2b | 10310 | 2 | 0.002
D4b2b | 10373 | 1 | 0.001
D4b2b | 10742G | 2 | 0.002
D4b2b | 11476 | 1 | 0.001
D4b2b | 11506 | 2 | 0.002
D4b2b | 11887 | 1 | 0.001
D4b2b | 13722 | 3 | 0.004
D4b2b | 13959 | 2 | 0.002
D4b2b | 14002 | 2 | 0.002
D4b2b | 14065 | 2 | 0.002
D4b2b | 14290 | 1 | 0.001
D4b2b | 143 | 1 | 0.001
D4b2b | 14384 | 3 | 0.004
D4b2b | 146 | 1 | 0.001
D4b2b | 14751 | 2 | 0.002
D4b2b | 152 | 7 | 0.008

D4b2b | 15422 | 1 | 0.001
D4b2b | 15511 | 2 | 0.002
D4b2b | 15613 | 1 | 0.001
D4b2b | 15883 | 1 | 0.001
D4b2b | 15930 | 2 | 0.002
D4b2b | 16037 | 2 | 0.002
D4b2b | 16046d | 1 | 0.001
D4b2b | 16051 | 1 | 0.001
D4b2b | 16076d | 1 | 0.001
D4b2b | 16077 | 1 | 0.001
D4b2b | 16086 | 1 | 0.001
D4b2b | 16093 | 21 | 0.025
D4b2b | 16111 | 2 | 0.002
D4b2b | 16124 | 7 | 0.008
D4b2b | 16139.1A | 1 | 0.001
D4b2b | 16165 | 1 | 0.001
D4b2b | 16172 | 1 | 0.001
D4b2b | 16185 | 9 | 0.011
D4b2b | 16189 | 3 | 0.004
D4b2b | 16215 | 3 | 0.004
D4b2b | 16218 | 1 | 0.001
D4b2b | 16224 | 7 | 0.008
D4b2b | 16231G | 1 | 0.001
D4b2b | 16258 | 1 | 0.001
D4b2b | 16283T | 3 | 0.004
D4b2b | 16288 | 1 | 0.001
D4b2b | 16289 | 1 | 0.001
D4b2b | 16290 | 1 | 0.001
D4b2b | 16291 | 3 | 0.004
D4b2b | 16293 | 1 | 0.001
D4b2b | 16295 | 1 | 0.001
D4b2b | 16304 | 1 | 0.001
D4b2b | 16319 | 4 | 0.005
D4b2b | 16320 | 1 | 0.001
D4b2b | 16465 | 4 | 0.005
D4b2b | 16512 | 1 | 0.001
D4b2b | 16526 | 1 | 0.001
D4b2b | 187 | 1 | 0.001
D4b2b | 189 | 1 | 0.001
D4b2b | 191.1A | 4 | 0.005
D4b2b | 195 | 2 | 0.002
D4b2b | 200 | 3 | 0.004
D4b2b | 205 | 1 | 0.001
D4b2b | 207 | 1 | 0.001
D4b2b | 2352 | 1 | 0.001
D4b2b | 246 | 1 | 0.001
D4b2b | 251 | 1 | 0.001
D4b2b | 257 | 1 | 0.001
D4b2b | 310 | 1 | 0.001
D4b2b | 3144 | 2 | 0.002
D4b2b | 316C | 9 | 0.011
D4b2b | 3338 | 2 | 0.002
D4b2b | 3398 | 2 | 0.002
D4b2b | 3608 | 1 | 0.001
D4b2b | 374 | 1 | 0.001
D4b2b | 390 | 1 | 0.001
D4b2b | 41 | 1 | 0.001
D4b2b | 4123 | 2 | 0.002
D4b2b | 4695 | 3 | 0.004
D4b2b | 4799 | 1 | 0.001
D4b2b | 499 | 2 | 0.002
D4b2b | 573 | 2 | 0.002
D4b2b | 573.3C | 1 | 0.001
D4b2b | 5894 | 1 | 0.001
D4b2b | 629 | 3 | 0.004
D4b2b | 636 | 1 | 0.001
D4b2b | 6383 | 1 | 0.001
D4b2b | 648 | 1 | 0.001
D4b2b | 6731 | 1 | 0.001
D4b2b | 723 | 1 | 0.001
D4b2b | 747 | 1 | 0.001
D4b2b | 7933 | 1 | 0.001
D4b2b | 8212 | 2 | 0.002
D4b2b | 8420 | 2 | 0.002
D4b2b | 8545 | 2 | 0.002
D4b2b | 8551 | 1 | 0.001
D4b2b | 8901 | 1 | 0.001
D4b2b | 9260 | 3 | 0.004
D4b2b | 9477 | 1 | 0.001
D4b2b | 9676 | 1 | 0.001
D4b2b | 9804 | 2 | 0.002
D4b2b1 | 11386 | 1 | 0.001
D4b2b1 | 11854 | 1 | 0.001
D4b2b1 | 13461 | 1 | 0.001
D4b2b1 | 13708 | 1 | 0.001
D4b2b1 | 1402C | 1 | 0.001
D4b2b1 | 143 | 1 | 0.001
D4b2b1 | 1452 | 1 | 0.001
D4b2b1 | 14862 | 1 | 0.001
D4b2b1 | 14992 | 1 | 0.001
D4b2b1 | 150 | 2 | 0.002
D4b2b1 | 152 | 1 | 0.001
D4b2b1 | 15924 | 1 | 0.001
D4b2b1 | 16046d | 1 | 0.001
D4b2b1 | 16051 | 1 | 0.001
D4b2b1 | 16086 | 1 | 0.001
D4b2b1 | 16111 | 1 | 0.001
D4b2b1 | 16124 | 7 | 0.009
D4b2b1 | 16126 | 1 | 0.001
D4b2b1 | 16139.1A | 1 | 0.001
D4b2b1 | 16165 | 1 | 0.001
D4b2b1 | 16169 | 1 | 0.001
D4b2b1 | 16175 | 1 | 0.001
D4b2b1 | 16188 | 2 | 0.002
D4b2b1 | 16218 | 1 | 0.001
D4b2b1 | 16231G | 1 | 0.001
D4b2b1 | 16258 | 1 | 0.001
D4b2b1 | 16293 | 1 | 0.001
D4b2b1 | 16294 | 1 | 0.001
D4b2b1 | 16295 | 1 | 0.001
D4b2b1 | 16320 | 4 | 0.005

D4b2b1 | 16355 | 1 | 0.001
D4b2b1 | 16527 | 1 | 0.001
D4b2b1 | 189 | 1 | 0.001
D4b2b1 | 195 | 2 | 0.002
D4b2b1 | 199 | 1 | 0.001
D4b2b1 | 205 | 1 | 0.001
D4b2b1 | 251 | 4 | 0.005
D4b2b1 | 2617 | 1 | 0.001
D4b2b1 | 310 | 1 | 0.001
D4b2b1 | 3505 | 1 | 0.001
D4b2b1 | 3866 | 1 | 0.001
D4b2b1 | 3975 | 1 | 0.001
D4b2b1 | 4098 | 1 | 0.001
D4b2b1 | 4655 | 3 | 0.004
D4b2b1 | 5894 | 1 | 0.001
D4b2b1 | 6112 | 1 | 0.001
D4b2b1 | 648 | 1 | 0.001
D4b2b1 | 709 | 1 | 0.001
D4b2b1 | 722 | 1 | 0.001
D4b2b1 | 723 | 1 | 0.001
D4b2b1 | 7244 | 1 | 0.001
D4b2b1 | 8227 | 1 | 0.001
D4b2b1 | 8485C | 1 | 0.001
D4b2b1 | 8911 | 1 | 0.001
D4b2b1 | 9163 | 1 | 0.001
D4b2b1 | 9355 | 1 | 0.001
D4b2b1 | 9824 | 1 | 0.001
D4b2b1 | 9843 | 1 | 0.001
D4b2b1+146 | 10031 | 1 | 0.002
D4b2b1+146 | 11260 | 1 | 0.002
D4b2b1+146 | 14185 | 1 | 0.002
D4b2b1+146 | 14608 | 1 | 0.002
D4b2b1+146 | 16108 | 1 | 0.002
D4b2b1+146 | 16111A | 1 | 0.002
D4b2b1+146 | 16172 | 1 | 0.002
D4b2b1+146 | 16176 | 1 | 0.002
D4b2b1+146 | 16218 | 1 | 0.002
D4b2b1+146 | 16250A | 1 | 0.002
D4b2b1+146 | 16269 | 1 | 0.002
D4b2b1+146 | 16290 | 1 | 0.002
D4b2b1+146 | 16318C | 1 | 0.002
D4b2b1+146 | 183 | 1 | 0.002
D4b2b1+146 | 3720 | 1 | 0.002
D4b2b1+146 | 3796 | 1 | 0.002
D4b2b1+146 | 383 | 1 | 0.002
D4b2b1+146 | 3873 | 1 | 0.002
D4b2b1+146 | 5894 | 1 | 0.002
D4b2b1+146 | 60A | 1 | 0.002
D4b2b1+146 | 6719 | 1 | 0.002
D4b2b1+146 | 6761 | 1 | 0.002
D4b2b1+146 | 8227 | 1 | 0.002
D4b2b1+146 | 8610 | 1 | 0.002
D4b2b1a | 11009 | 1 | 0.001
D4b2b1a | 12727 | 1 | 0.001
D4b2b1a | 14148 | 1 | 0.001
D4b2b1a | 143 | 5 | 0.006
D4b2b1a | 15455 | 1 | 0.001
D4b2b1a | 1555 | 1 | 0.001
D4b2b1a | 15930 | 1 | 0.001
D4b2b1a | 16046d | 1 | 0.001
D4b2b1a | 16051 | 1 | 0.001
D4b2b1a | 16086 | 1 | 0.001
D4b2b1a | 16124 | 7 | 0.009
D4b2b1a | 16139.1A | 1 | 0.001
D4b2b1a | 16145 | 1 | 0.001
D4b2b1a | 16165 | 1 | 0.001
D4b2b1a | 16189 | 1 | 0.001
D4b2b1a | 16209 | 1 | 0.001
D4b2b1a | 16218 | 1 | 0.001
D4b2b1a | 16231G | 1 | 0.001
D4b2b1a | 16258 | 1 | 0.001
D4b2b1a | 16291 | 6 | 0.008
D4b2b1a | 16293 | 1 | 0.001
D4b2b1a | 16295 | 1 | 0.001
D4b2b1a | 16320 | 1 | 0.001
D4b2b1a | 16360 | 1 | 0.001
D4b2b1a | 16399 | 7 | 0.009
D4b2b1a | 189 | 1 | 0.001
D4b2b1a | 1995 | 1 | 0.001
D4b2b1a | 204 | 2 | 0.002
D4b2b1a | 205 | 1 | 0.001
D4b2b1a | 215 | 1 | 0.001
D4b2b1a | 251 | 1 | 0.001
D4b2b1a | 335 | 1 | 0.001
D4b2b1a | 3639 | 1 | 0.001
D4b2b1a | 390 | 4 | 0.005
D4b2b1a | 6297 | 1 | 0.001
D4b2b1a | 648 | 1 | 0.001
D4b2b1a | 723 | 1 | 0.001
D4b2b1a | 8434 | 1 | 0.001
D4b2b1a | 93 | 1 | 0.001
D4b2b1b | 143 | 5 | 0.006
D4b2b1b | 16046d | 1 | 0.001
D4b2b1b | 16051 | 1 | 0.001
D4b2b1b | 16086 | 1 | 0.001
D4b2b1b | 16124 | 7 | 0.009
D4b2b1b | 16139.1A | 1 | 0.001
D4b2b1b | 16145 | 1 | 0.001
D4b2b1b | 16165 | 1 | 0.001
D4b2b1b | 16209 | 1 | 0.001
D4b2b1b | 16218 | 1 | 0.001
D4b2b1b | 16231G | 1 | 0.001
D4b2b1b | 16258 | 1 | 0.001
D4b2b1b | 16293 | 1 | 0.001
D4b2b1b | 16295 | 1 | 0.001
D4b2b1b | 16320 | 1 | 0.001
D4b2b1b | 16360 | 1 | 0.001
D4b2b1b | 16399 | 7 | 0.009
D4b2b1b | 189 | 1 | 0.001
D4b2b1b | 204 | 2 | 0.003
D4b2b1b | 205 | 1 | 0.001

D4b2b1b | 251 | 1 | 0.001
D4b2b1b | 335 | 1 | 0.001
D4b2b1b | 390 | 4 | 0.005
D4b2b1b | 4659 | 1 | 0.001
D4b2b1b | 648 | 1 | 0.001
D4b2b1b | 723 | 1 | 0.001
D4b2b1c | 13225 | 1 | 0.001
D4b2b1c | 143 | 5 | 0.006
D4b2b1c | 16046d | 1 | 0.001
D4b2b1c | 16051 | 1 | 0.001
D4b2b1c | 16086 | 1 | 0.001
D4b2b1c | 16124 | 7 | 0.009
D4b2b1c | 16139.1A | 1 | 0.001
D4b2b1c | 16145 | 1 | 0.001
D4b2b1c | 16165 | 1 | 0.001
D4b2b1c | 16209 | 1 | 0.001
D4b2b1c | 16218 | 1 | 0.001
D4b2b1c | 16231G | 1 | 0.001
D4b2b1c | 16258 | 1 | 0.001
D4b2b1c | 16293 | 1 | 0.001
D4b2b1c | 16295 | 1 | 0.001
D4b2b1c | 16320 | 1 | 0.001
D4b2b1c | 16360 | 1 | 0.001
D4b2b1c | 16399 | 7 | 0.009
D4b2b1c | 189 | 1 | 0.001
D4b2b1c | 204 | 2 | 0.003
D4b2b1c | 205 | 1 | 0.001
D4b2b1c | 251 | 1 | 0.001
D4b2b1c | 335 | 1 | 0.001
D4b2b1c | 390 | 4 | 0.005
D4b2b1c | 648 | 1 | 0.001
D4b2b1c | 723 | 1 | 0.001
D4b2b1d | 13680 | 1 | 0.002
D4b2b1d | 16108 | 1 | 0.002
D4b2b1d | 16111A | 1 | 0.002
D4b2b1d | 16172 | 1 | 0.002
D4b2b1d | 16218 | 1 | 0.002
D4b2b1d | 16250A | 1 | 0.002
D4b2b1d | 16269 | 1 | 0.002
D4b2b1d | 16290 | 1 | 0.002
D4b2b1d | 16292 | 2 | 0.004
D4b2b1d | 16318C | 1 | 0.002
D4b2b1d | 183 | 1 | 0.002
D4b2b1d | 310 | 1 | 0.002
D4b2b1d | 383 | 1 | 0.002
D4b2b1d | 4002 | 1 | 0.002
D4b2b2 | 11499 | 1 | 0.001
D4b2b2 | 143 | 2 | 0.002
D4b2b2 | 14867 | 1 | 0.001
D4b2b2 | 16046d | 1 | 0.001
D4b2b2 | 16051 | 1 | 0.001
D4b2b2 | 16086 | 1 | 0.001
D4b2b2 | 16124 | 7 | 0.009
D4b2b2 | 16139.1A | 1 | 0.001
D4b2b2 | 16145 | 1 | 0.001
D4b2b2 | 16165 | 1 | 0.001
D4b2b2 | 16209 | 1 | 0.001
D4b2b2 | 16218 | 1 | 0.001
D4b2b2 | 16231G | 1 | 0.001
D4b2b2 | 16234 | 1 | 0.001
D4b2b2 | 16258 | 1 | 0.001
D4b2b2 | 16293 | 1 | 0.001
D4b2b2 | 16295 | 1 | 0.001
D4b2b2 | 16320 | 1 | 0.001
D4b2b2 | 16360 | 1 | 0.001
D4b2b2 | 16399 | 4 | 0.005
D4b2b2 | 16465 | 4 | 0.005
D4b2b2 | 189 | 1 | 0.001
D4b2b2 | 191.1A | 5 | 0.006
D4b2b2 | 199 | 1 | 0.001
D4b2b2 | 204 | 2 | 0.002
D4b2b2 | 205 | 1 | 0.001
D4b2b2 | 207 | 5 | 0.006
D4b2b2 | 224.1T | 1 | 0.001
D4b2b2 | 251 | 1 | 0.001
D4b2b2 | 2581 | 1 | 0.001
D4b2b2 | 316C | 4 | 0.005
D4b2b2 | 3334 | 1 | 0.001
D4b2b2 | 335 | 1 | 0.001
D4b2b2 | 390 | 4 | 0.005
D4b2b2 | 41 | 1 | 0.001
D4b2b2 | 513 | 1 | 0.001
D4b2b2 | 620.1T | 1 | 0.001
D4b2b2 | 648 | 1 | 0.001
D4b2b2 | 723 | 1 | 0.001
D4b2b2 | 8270 | 1 | 0.001
D4b2b2 | 8281-8289d | 1 | 0.001
D4b2b2 | 922 | 1 | 0.001
D4b2b2 | 9861 | 1 | 0.001
D4b2b2a | 11098 | 2 | 0.004
D4b2b2a | 152 | 1 | 0.002
D4b2b2a | 15754 | 3 | 0.005
D4b2b2a | 16108 | 1 | 0.002
D4b2b2a | 16111A | 1 | 0.002
D4b2b2a | 16172 | 3 | 0.005
D4b2b2a | 16221 | 1 | 0.002
D4b2b2a | 16249 | 1 | 0.002
D4b2b2a | 16250A | 1 | 0.002
D4b2b2a | 16286 | 4 | 0.007
D4b2b2a | 16290 | 1 | 0.002
D4b2b2a | 16318C | 1 | 0.002
D4b2b2a | 16390 | 1 | 0.002
D4b2b2a | 16526T | 2 | 0.004
D4b2b2a | 298 | 1 | 0.002
D4b2b2a | 3666 | 3 | 0.005
D4b2b2a | 374 | 1 | 0.002
D4b2b2a | 6317 | 2 | 0.004
D4b2b2a | 711.1T | 1 | 0.002
D4b2b2a | 8152 | 2 | 0.004
D4b2b2a1 | 152 | 1 | 0.002
D4b2b2a1 | 16108 | 1 | 0.002

D4b2b2a1 | 16111A | 1 | 0.002
D4b2b2a1 | 16150 | 1 | 0.002
D4b2b2a1 | 16172 | 3 | 0.005
D4b2b2a1 | 16221 | 1 | 0.002
D4b2b2a1 | 16249 | 1 | 0.002
D4b2b2a1 | 16250A | 1 | 0.002
D4b2b2a1 | 16286 | 4 | 0.007
D4b2b2a1 | 16290 | 1 | 0.002
D4b2b2a1 | 16318C | 1 | 0.002
D4b2b2a1 | 16344 | 1 | 0.002
D4b2b2a1 | 16526T | 2 | 0.004
D4b2b2a1 | 205 | 6 | 0.011
D4b2b2a1 | 298 | 1 | 0.002
D4b2b2a1 | 374 | 1 | 0.002
D4b2b2a1 | 711.1T | 1 | 0.002
D4b2b2b | 11887 | 1 | 0.005
D4b2b2b | 12408 | 2 | 0.009
D4b2b2b | 1393 | 1 | 0.005
D4b2b2b | 152 | 7 | 0.033
D4b2b2b | 16042 | 1 | 0.005
D4b2b2b | 16066 | 1 | 0.005
D4b2b2b | 16076d | 2 | 0.009
D4b2b2b | 16093 | 1 | 0.005
D4b2b2b | 16093A | 1 | 0.005
D4b2b2b | 16093T | 1 | 0.005
D4b2b2b | 16104 | 3 | 0.014
D4b2b2b | 16111 | 1 | 0.005
D4b2b2b | 16168 | 2 | 0.009
D4b2b2b | 16174 | 1 | 0.005
D4b2b2b | 16183T | 2 | 0.009
D4b2b2b | 16189 | 3 | 0.014
D4b2b2b | 16197 | 2 | 0.009
D4b2b2b | 16220 | 1 | 0.005
D4b2b2b | 16222 | 2 | 0.009
D4b2b2b | 16223 | 4 | 0.019
D4b2b2b | 16224.1T | 2 | 0.009
D4b2b2b | 16234 | 5 | 0.024
D4b2b2b | 16270G | 2 | 0.009
D4b2b2b | 16293 | 1 | 0.005
D4b2b2b | 16294 | 1 | 0.005
D4b2b2b | 16304 | 1 | 0.005
D4b2b2b | 16316 | 3 | 0.014
D4b2b2b | 16328 | 2 | 0.009
D4b2b2b | 16342 | 1 | 0.005
D4b2b2b | 16391 | 1 | 0.005
D4b2b2b | 16519G | 1 | 0.005
D4b2b2b | 205 | 1 | 0.005
D4b2b2b | 3460 | 1 | 0.005
D4b2b2b | 5674 | 1 | 0.005
D4b2b2b | 629 | 3 | 0.014
D4b2b2b | 722 | 1 | 0.005
D4b2b2b | 93 | 1 | 0.005
D4b2b2c | 10646 | 1 | 0.005
D4b2b2c | 15959d | 1 | 0.005
D4b2b2c | 16093 | 7 | 0.038
D4b2b2c | 16134 | 1 | 0.005
D4b2b2c | 16145 | 1 | 0.005
D4b2b2c | 16184A | 2 | 0.011
D4b2b2c | 16231 | 1 | 0.005
D4b2b2c | 16243 | 1 | 0.005
D4b2b2c | 16266 | 1 | 0.005
D4b2b2c | 16311 | 2 | 0.011
D4b2b2c | 16325 | 3 | 0.016
D4b2b2c | 310 | 1 | 0.005
D4b2b2c | 315d | 1 | 0.005
D4b2b2c | 316 | 1 | 0.005
D4b2b2c | 316C | 1 | 0.005
D4b2b2c | 3744 | 3 | 0.016
D4b2b2c | 64 | 1 | 0.005
D4b2b2c | 6566 | 3 | 0.016
D4b2b3 | 13395 | 1 | 0.001
D4b2b3 | 143 | 5 | 0.006
D4b2b3 | 15299 | 1 | 0.001
D4b2b3 | 16046d | 1 | 0.001
D4b2b3 | 16051 | 1 | 0.001
D4b2b3 | 16086 | 1 | 0.001
D4b2b3 | 16092 | 2 | 0.002
D4b2b3 | 16124 | 7 | 0.009
D4b2b3 | 16139.1A | 1 | 0.001
D4b2b3 | 16145 | 1 | 0.001
D4b2b3 | 16165 | 1 | 0.001
D4b2b3 | 16187 | 1 | 0.001
D4b2b3 | 16209 | 1 | 0.001
D4b2b3 | 16218 | 1 | 0.001
D4b2b3 | 16231G | 1 | 0.001
D4b2b3 | 16258 | 1 | 0.001
D4b2b3 | 16293 | 1 | 0.001
D4b2b3 | 16295 | 1 | 0.001
D4b2b3 | 16301 | 2 | 0.002
D4b2b3 | 16320 | 1 | 0.001
D4b2b3 | 16360 | 1 | 0.001
D4b2b3 | 16399 | 7 | 0.009
D4b2b3 | 16565 | 1 | 0.001
D4b2b3 | 189 | 1 | 0.001
D4b2b3 | 204 | 2 | 0.002
D4b2b3 | 205 | 1 | 0.001
D4b2b3 | 226 | 1 | 0.001
D4b2b3 | 251 | 1 | 0.001
D4b2b3 | 335 | 1 | 0.001
D4b2b3 | 390 | 4 | 0.005
D4b2b3 | 41 | 9 | 0.011
D4b2b3 | 425 | 2 | 0.002
D4b2b3 | 648 | 1 | 0.001
D4b2b3 | 71.1G | 5 | 0.006
D4b2b3 | 723 | 1 | 0.001
D4b2b3 | 75 | 1 | 0.001
D4b2b3 | 8075 | 1 | 0.001
D4b2b4 | 1014 | 2 | 0.004
D4b2b4 | 10640 | 2 | 0.004
D4b2b4 | 1284 | 1 | 0.002
D4b2b4 | 14335 | 2 | 0.004

D4b2b4 | 152 | 1 | 0.002
D4b2b4 | 16108 | 1 | 0.002
D4b2b4 | 16111A | 1 | 0.002
D4b2b4 | 16172 | 3 | 0.005
D4b2b4 | 16189 | 2 | 0.004
D4b2b4 | 16221 | 1 | 0.002
D4b2b4 | 16249 | 1 | 0.002
D4b2b4 | 16250A | 1 | 0.002
D4b2b4 | 16286 | 4 | 0.007
D4b2b4 | 16290 | 1 | 0.002
D4b2b4 | 16291A | 1 | 0.002
D4b2b4 | 16311 | 1 | 0.002
D4b2b4 | 16318C | 1 | 0.002
D4b2b4 | 16526T | 2 | 0.004
D4b2b4 | 1719 | 2 | 0.004
D4b2b4 | 195 | 3 | 0.005
D4b2b4 | 199 | 2 | 0.004
D4b2b4 | 298 | 1 | 0.002
D4b2b4 | 3391 | 2 | 0.004
D4b2b4 | 374 | 1 | 0.002
D4b2b4 | 4824 | 2 | 0.004
D4b2b4 | 5492 | 2 | 0.004
D4b2b4 | 629 | 1 | 0.002
D4b2b4 | 711.1T | 1 | 0.002
D4b2b4 | 8743 | 2 | 0.004
D4b2b4 | 960.1C | 1 | 0.002
D4b2b5 | 11734 | 2 | 0.004
D4b2b5 | 14182 | 2 | 0.004
D4b2b5 | 14218 | 5 | 0.009
D4b2b5 | 151 | 1 | 0.002
D4b2b5 | 15511 | 4 | 0.007
D4b2b5 | 15525 | 1 | 0.002
D4b2b5 | 16108 | 1 | 0.002
D4b2b5 | 16111A | 1 | 0.002
D4b2b5 | 16126 | 1 | 0.002
D4b2b5 | 16158 | 2 | 0.004
D4b2b5 | 16172 | 1 | 0.002
D4b2b5 | 16248 | 1 | 0.002
D4b2b5 | 16250A | 1 | 0.002
D4b2b5 | 16259 | 1 | 0.002
D4b2b5 | 16318C | 1 | 0.002
D4b2b5 | 16335 | 2 | 0.004
D4b2b5 | 239 | 2 | 0.004
D4b2b5 | 315.2C | 1 | 0.002
D4b2b5 | 3537 | 1 | 0.002
D4b2b5 | 3763 | 2 | 0.004
D4b2b5 | 5774 | 6 | 0.011
D4b2b5 | 7372 | 1 | 0.002
D4b2b5 | 7999 | 6 | 0.011
D4b2b6 | 12950 | 1 | 0.001
D4b2b6 | 143 | 5 | 0.006
D4b2b6 | 16046d | 1 | 0.001
D4b2b6 | 16051 | 1 | 0.001
D4b2b6 | 16093 | 1 | 0.001
D4b2b6 | 16139.1A | 1 | 0.001
D4b2b6 | 16145 | 1 | 0.001
D4b2b6 | 16165 | 1 | 0.001
D4b2b6 | 16209 | 1 | 0.001
D4b2b6 | 16218 | 1 | 0.001
D4b2b6 | 16231G | 1 | 0.001
D4b2b6 | 16243 | 3 | 0.004
D4b2b6 | 16258 | 1 | 0.001
D4b2b6 | 16278 | 1 | 0.001
D4b2b6 | 16293 | 1 | 0.001
D4b2b6 | 16295 | 1 | 0.001
D4b2b6 | 16320 | 1 | 0.001
D4b2b6 | 16360 | 1 | 0.001
D4b2b6 | 16399 | 7 | 0.009
D4b2b6 | 189 | 1 | 0.001
D4b2b6 | 204 | 2 | 0.003
D4b2b6 | 205 | 1 | 0.001
D4b2b6 | 251 | 1 | 0.001
D4b2b6 | 335 | 1 | 0.001
D4b2b6 | 3796 | 1 | 0.001
D4b2b6 | 390 | 4 | 0.005
D4b2b6 | 5291 | 3 | 0.004
D4b2b6 | 573.1C | 1 | 0.001
D4b2b6 | 648 | 1 | 0.001
D4b2b6 | 723 | 1 | 0.001
D4b2b6 | 8485 | 1 | 0.001
D4b2b7 | 13500 | 1 | 0.006
D4b2b7 | 13590 | 2 | 0.012
D4b2b7 | 2904 | 1 | 0.006
D4b2d | 16111 | 5 | 0.027
D4b2d | 16189 | 4 | 0.021
D4b2d | 16243 | 1 | 0.005
D4b2d | 16259A | 1 | 0.005
D4b2d | 16291A | 2 | 0.011
D4b2d | 16295 | 9 | 0.048
D4b2d | 16317 | 8 | 0.043
D4b2d | 16325 | 1 | 0.005
D4b2d | 297 | 4 | 0.021
D4b2d | 709 | 2 | 0.011
D4c | 16178 | 1 | 0.013
D4c | 16297 | 1 | 0.013
D4c | 194 | 1 | 0.013
D4c1 | 13105 | 6 | 0.08
D4c1 | 14693 | 6 | 0.08
D4c1 | 16223 | 6 | 0.08
D4c1 | 16224 | 2 | 0.027
D4c1 | 4502 | 6 | 0.08
D4c1a | 11087 | 1 | 0.037
D4c1a | 11257 | 1 | 0.037
D4c1a | 11482 | 1 | 0.037
D4c1a | 11914 | 1 | 0.037
D4c1a | 12509 | 1 | 0.037
D4c1a | 13768 | 1 | 0.037
D4c1a | 159 | 1 | 0.037
D4c1a | 16093 | 1 | 0.037
D4c1a | 16186 | 1 | 0.037
D4c1a | 16189 | 1 | 0.037

D4c1a | 16201 | 1 | 0.037
D4c1a | 16352 | 1 | 0.037
D4c1a | 16368 | 1 | 0.037
D4c1a | 188 | 1 | 0.037
D4c1a | 204 | 1 | 0.037
D4c1a | 315.1T | 1 | 0.037
D4c1a | 325 | 1 | 0.037
D4c1a | 394 | 1 | 0.037
D4c1a | 573.6C | 1 | 0.037
D4c1a | 629 | 1 | 0.037
D4c1a | 7912 | 1 | 0.037
D4c1a | 801 | 1 | 0.037
D4c1a | 8264 | 1 | 0.037
D4c1a | 9084 | 2 | 0.074
D4c1a | 9337 | 1 | 0.037
D4c1a1 | 152 | 4 | 0.148
D4c1a1 | 16075 | 1 | 0.037
D4c1a1 | 16093 | 1 | 0.037
D4c1a1 | 16169 | 1 | 0.037
D4c1a1 | 16172 | 1 | 0.037
D4c1a1 | 16186 | 1 | 0.037
D4c1a1 | 16223 | 6 | 0.222
D4c1a1 | 16243 | 1 | 0.037
D4c1a1 | 16244 | 1 | 0.037
D4c1a1 | 16258T | 1 | 0.037
D4c1a1 | 16261 | 1 | 0.037
D4c1a1 | 16265 | 1 | 0.037
D4c1a1 | 16278 | 2 | 0.074
D4c1a1 | 16286 | 1 | 0.037
D4c1a1 | 16352 | 1 | 0.037
D4c1a1 | 16360 | 1 | 0.037
D4c1a1 | 16511 | 1 | 0.037
D4c1a1 | 16512 | 1 | 0.037
D4c1a1 | 16516 | 1 | 0.037
D4c1a1 | 1719 | 1 | 0.037
D4c1b | 11113 | 1 | 0.014
D4c1b | 13928C | 2 | 0.028
D4c1b | 16156 | 3 | 0.042
D4c1b | 9738 | 2 | 0.028
D4c1b1 | 10819 | 1 | 0.033
D4c1b1 | 11080 | 1 | 0.033
D4c1b1 | 11569 | 1 | 0.033
D4c1b1 | 152 | 1 | 0.033
D4c1b1 | 15244 | 1 | 0.033
D4c1b1 | 15924 | 3 | 0.1
D4c1b1 | 16092 | 1 | 0.033
D4c1b1 | 16126 | 3 | 0.1
D4c1b1 | 16177 | 1 | 0.033
D4c1b1 | 16184 | 1 | 0.033
D4c1b1 | 16187 | 1 | 0.033
D4c1b1 | 16189 | 3 | 0.1
D4c1b1 | 16193 | 1 | 0.033
D4c1b1 | 16287 | 1 | 0.033
D4c1b1 | 16311 | 5 | 0.167
D4c1b1 | 185 | 1 | 0.033
D4c1b1 | 195 | 7 | 0.233
D4c1b1 | 319.1T | 1 | 0.033
D4c1b1 | 3504 | 1 | 0.033
D4c1b1 | 5460 | 1 | 0.033
D4c1b1 | 7665 | 1 | 0.033
D4c1b1 | 9254 | 1 | 0.033
D4c1b2 | 11253 | 1 | 0.014
D4c2 | 10307 | 2 | 0.018
D4c2 | 1211 | 1 | 0.009
D4c2 | 13105 | 3 | 0.027
D4c2 | 151 | 1 | 0.009
D4c2 | 152 | 7 | 0.063
D4c2 | 16176 | 3 | 0.027
D4c2 | 16269 | 1 | 0.009
D4c2 | 16368 | 34 | 0.306
D4c2 | 195 | 4 | 0.036
D4c2 | 250 | 1 | 0.009
D4c2 | 385 | 7 | 0.063
D4c2 | 4233 | 6 | 0.054
D4c2 | 7854 | 1 | 0.009
D4c2a | 11152 | 5 | 0.069
D4c2a | 3226T | 1 | 0.014
D4c2b | 10920 | 3 | 0.035
D4c2b | 152 | 2 | 0.023
D4c2b | 15836 | 2 | 0.023
D4c2b | 16093 | 5 | 0.058
D4c2b | 16111 | 1 | 0.012
D4c2b | 16142G | 1 | 0.012
D4c2b | 16164 | 2 | 0.023
D4c2b | 16172 | 1 | 0.012
D4c2b | 16178 | 1 | 0.012
D4c2b | 16188 | 1 | 0.012
D4c2b | 16189 | 10 | 0.116
D4c2b | 16197G | 1 | 0.012
D4c2b | 16266 | 3 | 0.035
D4c2b | 16278 | 2 | 0.023
D4c2b | 16335 | 1 | 0.012
D4c2b | 1958 | 3 | 0.035
D4c2b | 208 | 5 | 0.058
D4c2b | 310 | 2 | 0.023
D4c2b | 4004 | 1 | 0.012
D4c2b | 545 | 1 | 0.012
D4c2b | 5777 | 1 | 0.012
D4c2b | 5939 | 2 | 0.023
D4c2b | 6914 | 3 | 0.035
D4c2b | 7741 | 1 | 0.012
D4c2b | 8251 | 1 | 0.012
D4c2b | 8289.1CCCCCTCTA | 2 | 0.023
D4c2b | 8485 | 1 | 0.012
D4c2b | 8867 | 1 | 0.012
D4c2b | 9438 | 2 | 0.023
D4c2c | 10410 | 1 | 0.011
D4c2c | 12245 | 1 | 0.011
D4c2c | 16311 | 4 | 0.046
D4c2c | 1719 | 1 | 0.011
D4c2c | 5319 | 2 | 0.023

D4c2c | 603 | 1 | 0.011
D4d | 143 | 1 | 0.005
D4d | 145d | 1 | 0.005
D4d | 146 | 9 | 0.041
D4d | 150 | 1 | 0.005
D4d | 153 | 1 | 0.005
D4d | 16051 | 1 | 0.005
D4d | 16070 | 2 | 0.009
D4d | 16092 | 6 | 0.027
D4d | 16093 | 33 | 0.149
D4d | 16104 | 1 | 0.005
D4d | 16126 | 5 | 0.023
D4d | 16129 | 12 | 0.054
D4d | 16138 | 1 | 0.005
D4d | 16145 | 1 | 0.005
D4d | 16150 | 1 | 0.005
D4d | 16172 | 13 | 0.059
D4d | 16174 | 1 | 0.005
D4d | 16185 | 18 | 0.081
D4d | 16186 | 1 | 0.005
D4d | 16189 | 28 | 0.127
D4d | 16192 | 2 | 0.009
D4d | 16193d | 10 | 0.045
D4d | 16224 | 8 | 0.036
D4d | 16232A | 15 | 0.068
D4d | 16234 | 1 | 0.005
D4d | 16241 | 1 | 0.005
D4d | 16243 | 1 | 0.005
D4d | 16261 | 1 | 0.005
D4d | 16269 | 1 | 0.005
D4d | 16274 | 1 | 0.005
D4d | 16291 | 1 | 0.005
D4d | 16292 | 1 | 0.005
D4d | 16293 | 5 | 0.023
D4d | 16298 | 2 | 0.009
D4d | 16303 | 3 | 0.014
D4d | 16304 | 1 | 0.005
D4d | 16305C | 1 | 0.005
D4d | 16305T | 3 | 0.014
D4d | 16311 | 15 | 0.068
D4d | 16316 | 2 | 0.009
D4d | 16326 | 1 | 0.005
D4d | 16327 | 2 | 0.009
D4d | 16356 | 3 | 0.014
D4d | 16380 | 7 | 0.032
D4d | 16390 | 1 | 0.005
D4d | 16398 | 1 | 0.005
D4d | 16399 | 1 | 0.005
D4d | 183 | 2 | 0.009
D4d | 184 | 2 | 0.009
D4d | 185 | 5 | 0.023
D4d | 189 | 1 | 0.005
D4d | 194 | 1 | 0.005
D4d | 195 | 4 | 0.018
D4d | 199 | 32 | 0.145
D4d | 200 | 2 | 0.009
D4d | 207 | 2 | 0.009
D4d | 228 | 1 | 0.005
D4d | 239 | 1 | 0.005
D4d | 309 | 13 | 0.059
D4d | 316C | 19 | 0.086
D4d | 374 | 1 | 0.005
D4d | 431 | 1 | 0.005
D4d | 573.2C | 1 | 0.005
D4d | 64 | 1 | 0.005
D4d | 709 | 1 | 0.005
D4d | 94 | 1 | 0.005
D4e | 13654 | 1 | 0.002
D4e | 14905 | 1 | 0.002
D4e | 150A | 4 | 0.006
D4e | 16114 | 6 | 0.01
D4e | 16165 | 1 | 0.002
D4e | 16167 | 1 | 0.002
D4e | 16179 | 1 | 0.002
D4e | 16218 | 1 | 0.002
D4e | 16274 | 1 | 0.002
D4e | 16335 | 4 | 0.006
D4e | 3483 | 1 | 0.002
D4e | 4021 | 1 | 0.002
D4e | 446 | 1 | 0.002
D4e | 573.3C | 1 | 0.002
D4e | 64 | 2 | 0.003
D4e | 648 | 1 | 0.002
D4e | 789 | 5 | 0.008
D4e | 8573 | 1 | 0.002
D4e | 9804 | 1 | 0.002
D4e1 | 15217 | 1 | 0.001
D4e1 | 15927 | 1 | 0.001
D4e1 | 16145 | 2 | 0.003
D4e1 | 16165 | 1 | 0.001
D4e1 | 16179 | 1 | 0.001
D4e1 | 16188 | 12 | 0.018
D4e1 | 16214 | 1 | 0.001
D4e1 | 16218 | 1 | 0.001
D4e1 | 16270 | 2 | 0.003
D4e1 | 16298 | 1 | 0.001
D4e1 | 16474d | 6 | 0.009
D4e1 | 183 | 1 | 0.001
D4e1 | 184 | 1 | 0.001
D4e1 | 197 | 5 | 0.007
D4e1 | 200 | 1 | 0.001
D4e1 | 214 | 1 | 0.001
D4e1 | 230T | 6 | 0.009
D4e1 | 4385 | 2 | 0.003
D4e1 | 446 | 1 | 0.001
D4e1 | 51A | 6 | 0.009
D4e1 | 629 | 1 | 0.001
D4e1 | 6366 | 2 | 0.003
D4e1 | 648 | 1 | 0.001
D4e1 | 7696 | 1 | 0.001
D4e1'3 | 150A | 4 | 0.006

D4e1'3 | 16114 | 6 | 0.01
D4e1'3 | 16165 | 1 | 0.002
D4e1'3 | 16167 | 1 | 0.002
D4e1'3 | 16179 | 1 | 0.002
D4e1'3 | 16218 | 1 | 0.002
D4e1'3 | 16335 | 4 | 0.006
D4e1'3 | 446 | 1 | 0.002
D4e1'3 | 64 | 2 | 0.003
D4e1'3 | 648 | 1 | 0.002
D4e1'3 | 789 | 5 | 0.008
D4e1a | 11963 | 6 | 0.011
D4e1a | 12232 | 1 | 0.002
D4e1a | 12771 | 1 | 0.002
D4e1a | 13203 | 1 | 0.002
D4e1a | 13651 | 2 | 0.004
D4e1a | 14566 | 1 | 0.002
D4e1a | 151 | 1 | 0.002
D4e1a | 15654 | 1 | 0.002
D4e1a | 16051 | 1 | 0.002
D4e1a | 16051T | 1 | 0.002
D4e1a | 16059C | 1 | 0.002
D4e1a | 16071A | 1 | 0.002
D4e1a | 16075A | 2 | 0.004
D4e1a | 16086 | 2 | 0.004
D4e1a | 16097 | 1 | 0.002
D4e1a | 16108 | 1 | 0.002
D4e1a | 16111A | 1 | 0.002
D4e1a | 16126 | 2 | 0.004
D4e1a | 16147 | 1 | 0.002
D4e1a | 16148 | 3 | 0.006
D4e1a | 16167 | 8 | 0.015
D4e1a | 16176 | 1 | 0.002
D4e1a | 16189 | 2 | 0.004
D4e1a | 16249 | 1 | 0.002
D4e1a | 16250A | 1 | 0.002
D4e1a | 16261 | 1 | 0.002
D4e1a | 16278 | 2 | 0.004
D4e1a | 16291 | 2 | 0.004
D4e1a | 16311 | 8 | 0.015
D4e1a | 16318C | 1 | 0.002
D4e1a | 16320 | 4 | 0.008
D4e1a | 16325 | 3 | 0.006
D4e1a | 16368 | 1 | 0.002
D4e1a | 16399 | 2 | 0.004
D4e1a | 16497 | 1 | 0.002
D4e1a | 1864 | 2 | 0.004
D4e1a | 195 | 4 | 0.008
D4e1a | 200 | 2 | 0.004
D4e1a | 204 | 4 | 0.008
D4e1a | 207 | 3 | 0.006
D4e1a | 240 | 1 | 0.002
D4e1a | 262 | 6 | 0.011
D4e1a | 272 | 1 | 0.002
D4e1a | 279 | 1 | 0.002
D4e1a | 3849 | 6 | 0.011
D4e1a | 508 | 3 | 0.006
D4e1a | 573.1C | 1 | 0.002
D4e1a | 8545 | 6 | 0.011
D4e1a | 8740 | 2 | 0.004
D4e1a | 9187 | 1 | 0.002
D4e1a | 9380 | 6 | 0.011
D4e1a | 9803 | 1 | 0.002
D4e1a1 | 16071 | 1 | 0.033
D4e1a1 | 16129 | 5 | 0.167
D4e1a1 | 16291A | 1 | 0.033
D4e1a1 | 16311 | 1 | 0.033
D4e1a1 | 16357 | 1 | 0.033
D4e1a1 | 16446 | 1 | 0.033
D4e1a1 | 16454d | 1 | 0.033
D4e1a1 | 189 | 1 | 0.033
D4e1a1 | 3395 | 1 | 0.033
D4e1a1 | 5585 | 1 | 0.033
D4e1a1 | 64 | 1 | 0.033
D4e1a2 | 10915 | 1 | 0.002
D4e1a2 | 12879 | 1 | 0.002
D4e1a2 | 146 | 2 | 0.004
D4e1a2 | 16042 | 1 | 0.002
D4e1a2 | 16051T | 1 | 0.002
D4e1a2 | 16059C | 1 | 0.002
D4e1a2 | 16071A | 1 | 0.002
D4e1a2 | 16075A | 2 | 0.004
D4e1a2 | 16097 | 1 | 0.002
D4e1a2 | 16108 | 1 | 0.002
D4e1a2 | 16111A | 1 | 0.002
D4e1a2 | 16153 | 1 | 0.002
D4e1a2 | 16188 | 4 | 0.008
D4e1a2 | 16234 | 1 | 0.002
D4e1a2 | 16240T | 1 | 0.002
D4e1a2 | 16250A | 1 | 0.002
D4e1a2 | 16261 | 1 | 0.002
D4e1a2 | 16266 | 1 | 0.002
D4e1a2 | 16291 | 1 | 0.002
D4e1a2 | 16311 | 23 | 0.048
D4e1a2 | 16318C | 1 | 0.002
D4e1a2 | 16325 | 1 | 0.002
D4e1a2 | 16399 | 2 | 0.004
D4e1a2 | 183 | 1 | 0.002
D4e1a2 | 189 | 8 | 0.017
D4e1a2 | 195 | 3 | 0.006
D4e1a2 | 204 | 1 | 0.002
D4e1a2 | 207 | 3 | 0.006
D4e1a2 | 262 | 1 | 0.002
D4e1a2 | 279 | 1 | 0.002
D4e1a2 | 315.2C | 2 | 0.004
D4e1a2 | 401d | 1 | 0.002
D4e1a2 | 417 | 1 | 0.002
D4e1a2 | 455.1T | 1 | 0.002
D4e1a2 | 5480 | 1 | 0.002
D4e1a2 | 55.1T | 1 | 0.002
D4e1a2 | 59-60d | 1 | 0.002
D4e1a2 | 71.1G | 1 | 0.002

D4e1a2a | 11824 | 1 | 0.002
D4e1a2a | 16032d | 8 | 0.017
D4e1a2a | 16042d | 8 | 0.017
D4e1a2a | 16051T | 1 | 0.002
D4e1a2a | 16059C | 1 | 0.002
D4e1a2a | 16071A | 1 | 0.002
D4e1a2a | 16075A | 2 | 0.004
D4e1a2a | 16097 | 1 | 0.002
D4e1a2a | 16100T | 1 | 0.002
D4e1a2a | 16108 | 1 | 0.002
D4e1a2a | 16110d | 7 | 0.015
D4e1a2a | 16111A | 1 | 0.002
D4e1a2a | 16153 | 1 | 0.002
D4e1a2a | 16234 | 1 | 0.002
D4e1a2a | 16250A | 1 | 0.002
D4e1a2a | 16261 | 1 | 0.002
D4e1a2a | 16311 | 1 | 0.002
D4e1a2a | 16318C | 1 | 0.002
D4e1a2a | 189 | 2 | 0.004
D4e1a2a | 195 | 1 | 0.002
D4e1a2a | 204 | 1 | 0.002
D4e1a2a | 297 | 1 | 0.002
D4e1a2a | 9620A | 1 | 0.002
D4e1a3 | 13934 | 1 | 0.002
D4e1a3 | 146 | 6 | 0.012
D4e1a3 | 14860 | 2 | 0.004
D4e1a3 | 152 | 6 | 0.012
D4e1a3 | 16051 | 1 | 0.002
D4e1a3 | 16051T | 1 | 0.002
D4e1a3 | 16059C | 1 | 0.002
D4e1a3 | 16071A | 1 | 0.002
D4e1a3 | 16075A | 2 | 0.004
D4e1a3 | 16086 | 2 | 0.004
D4e1a3 | 16097 | 1 | 0.002
D4e1a3 | 16108 | 1 | 0.002
D4e1a3 | 16111A | 1 | 0.002
D4e1a3 | 16126 | 2 | 0.004
D4e1a3 | 16147 | 1 | 0.002
D4e1a3 | 16148 | 3 | 0.006
D4e1a3 | 16172 | 1 | 0.002
D4e1a3 | 16176 | 1 | 0.002
D4e1a3 | 16189 | 2 | 0.004
D4e1a3 | 16249 | 1 | 0.002
D4e1a3 | 16250A | 1 | 0.002
D4e1a3 | 16261 | 1 | 0.002
D4e1a3 | 16278 | 2 | 0.004
D4e1a3 | 16318C | 1 | 0.002
D4e1a3 | 16368 | 1 | 0.002
D4e1a3 | 183 | 1 | 0.002
D4e1a3 | 200 | 2 | 0.004
D4e1a3 | 478 | 1 | 0.002
D4e1a3 | 517 | 10 | 0.019
D4e1a3 | 573.1C | 1 | 0.002
D4e1a3 | 63 | 1 | 0.002
D4e1a3 | 644 | 1 | 0.002
D4e1a3 | 6692 | 1 | 0.002
D4e1a3 | 6962 | 1 | 0.002
D4e1a3 | 709 | 3 | 0.006
D4e1a3 | 72 | 1 | 0.002
D4e1a3 | 771T | 1 | 0.002
D4e1a3 | 9097 | 1 | 0.002
D4e1c | 16145 | 2 | 0.003
D4e1c | 16165 | 1 | 0.001
D4e1c | 16172 | 2 | 0.003
D4e1c | 16179 | 1 | 0.001
D4e1c | 16188 | 1 | 0.001
D4e1c | 16218 | 1 | 0.001
D4e1c | 16270 | 2 | 0.003
D4e1c | 16298 | 1 | 0.001
D4e1c | 16474d | 6 | 0.009
D4e1c | 183 | 1 | 0.001
D4e1c | 184 | 1 | 0.001
D4e1c | 200 | 1 | 0.001
D4e1c | 214 | 1 | 0.001
D4e1c | 230T | 6 | 0.009
D4e1c | 446 | 1 | 0.001
D4e1c | 51A | 6 | 0.009
D4e1c | 629 | 1 | 0.001
D4e1c | 6366 | 1 | 0.001
D4e1c | 648 | 1 | 0.001
D4e2 | 10238 | 1 | 0.002
D4e2 | 10355 | 1 | 0.002
D4e2 | 11013A | 1 | 0.002
D4e2 | 11461 | 1 | 0.002
D4e2 | 11530 | 1 | 0.002
D4e2 | 12684 | 2 | 0.003
D4e2 | 13651 | 1 | 0.002
D4e2 | 14133 | 1 | 0.002
D4e2 | 14142 | 1 | 0.002
D4e2 | 14560 | 1 | 0.002
D4e2 | 14968 | 1 | 0.002
D4e2 | 150A | 1 | 0.002
D4e2 | 152 | 2 | 0.003
D4e2 | 15257 | 1 | 0.002
D4e2 | 1598 | 1 | 0.002
D4e2 | 16093 | 4 | 0.006
D4e2 | 16129 | 9 | 0.014
D4e2 | 16165 | 1 | 0.002
D4e2 | 16177 | 1 | 0.002
D4e2 | 16179 | 1 | 0.002
D4e2 | 16218 | 1 | 0.002
D4e2 | 16304 | 1 | 0.002
D4e2 | 16311 | 4 | 0.006
D4e2 | 16319 | 1 | 0.002
D4e2 | 16320 | 1 | 0.002
D4e2 | 16335 | 1 | 0.002
D4e2 | 16354 | 5 | 0.008
D4e2 | 16497 | 1 | 0.002
D4e2 | 195 | 1 | 0.002
D4e2 | 214 | 4 | 0.006
D4e2 | 256 | 1 | 0.002

D4e2 | 316d | 1 | 0.002
D4e2 | 3434 | 1 | 0.002
D4e2 | 3593 | 1 | 0.002
D4e2 | 4232 | 1 | 0.002
D4e2 | 446 | 1 | 0.002
D4e2 | 5773 | 1 | 0.002
D4e2 | 5822 | 1 | 0.002
D4e2 | 6261 | 2 | 0.003
D4e2 | 6267 | 1 | 0.002
D4e2 | 6305 | 1 | 0.002
D4e2 | 64 | 2 | 0.003
D4e2 | 648 | 1 | 0.002
D4e2 | 7444 | 1 | 0.002
D4e2 | 7460 | 1 | 0.002
D4e2 | 7702 | 1 | 0.002
D4e2 | 7783 | 1 | 0.002
D4e2 | 789 | 2 | 0.003
D4e2 | 827 | 1 | 0.002
D4e2 | 8566 | 1 | 0.002
D4e2 | 9112 | 1 | 0.002
D4e2 | 93 | 7 | 0.011
D4e2 | 9755 | 1 | 0.002
D4e2 | 9804 | 1 | 0.002
D4e2 | 9935 | 1 | 0.002
D4e2a | 14129 | 1 | 0.002
D4e2a | 150 | 1 | 0.002
D4e2a | 152G | 1 | 0.002
D4e2a | 16093 | 1 | 0.002
D4e2a | 16108 | 1 | 0.002
D4e2a | 16111A | 1 | 0.002
D4e2a | 16140 | 1 | 0.002
D4e2a | 16150 | 1 | 0.002
D4e2a | 16157 | 1 | 0.002
D4e2a | 16169 | 1 | 0.002
D4e2a | 16177 | 1 | 0.002
D4e2a | 16185 | 1 | 0.002
D4e2a | 16240C | 2 | 0.004
D4e2a | 16241 | 1 | 0.002
D4e2a | 16242 | 1 | 0.002
D4e2a | 16250A | 1 | 0.002
D4e2a | 16256 | 15 | 0.029
D4e2a | 16311 | 1 | 0.002
D4e2a | 16318C | 1 | 0.002
D4e2a | 16471 | 4 | 0.008
D4e2a | 171 | 1 | 0.002
D4e2a | 200 | 1 | 0.002
D4e2a | 316C | 15 | 0.029
D4e2a | 629 | 1 | 0.002
D4e2a | 64 | 4 | 0.008
D4e2a | 8945 | 1 | 0.002
D4e2b | 150A | 1 | 0.002
D4e2b | 15314 | 1 | 0.002
D4e2b | 16114 | 6 | 0.01
D4e2b | 16165 | 1 | 0.002
D4e2b | 16167 | 1 | 0.002
D4e2b | 16179 | 1 | 0.002
D4e2b | 16218 | 1 | 0.002
D4e2b | 16335 | 1 | 0.002
D4e2b | 16360 | 5 | 0.008
D4e2b | 260 | 1 | 0.002
D4e2b | 446 | 1 | 0.002
D4e2b | 64 | 2 | 0.003
D4e2b | 648 | 1 | 0.002
D4e2b | 789 | 2 | 0.003
D4e2c | 128 | 1 | 0.002
D4e2c | 14858 | 1 | 0.002
D4e2c | 150A | 4 | 0.006
D4e2c | 16093 | 1 | 0.002
D4e2c | 16114 | 6 | 0.01
D4e2c | 16144 | 1 | 0.002
D4e2c | 16165 | 1 | 0.002
D4e2c | 16167 | 1 | 0.002
D4e2c | 16179 | 1 | 0.002
D4e2c | 16218 | 1 | 0.002
D4e2c | 16335 | 4 | 0.006
D4e2c | 323.1G | 1 | 0.002
D4e2c | 361d | 1 | 0.002
D4e2c | 397d | 1 | 0.002
D4e2c | 414 | 1 | 0.002
D4e2c | 42 | 2 | 0.003
D4e2c | 446 | 1 | 0.002
D4e2c | 4674 | 1 | 0.002
D4e2c | 64 | 2 | 0.003
D4e2c | 648 | 1 | 0.002
D4e2c | 7444 | 1 | 0.002
D4e2c | 789 | 5 | 0.008
D4e2c | 9425 | 1 | 0.002
D4e2d | 11287 | 1 | 0.002
D4e2d | 13737 | 1 | 0.002
D4e2d | 150A | 4 | 0.006
D4e2d | 152 | 1 | 0.002
D4e2d | 16114 | 6 | 0.01
D4e2d | 16165 | 1 | 0.002
D4e2d | 16167 | 1 | 0.002
D4e2d | 16179 | 1 | 0.002
D4e2d | 16218 | 1 | 0.002
D4e2d | 16335 | 4 | 0.006
D4e2d | 446 | 1 | 0.002
D4e2d | 64 | 2 | 0.003
D4e2d | 648 | 1 | 0.002
D4e2d | 789 | 5 | 0.008
D4e3 | 11299 | 1 | 0.002
D4e3 | 113 | 2 | 0.003
D4e3 | 13443 | 3 | 0.005
D4e3 | 146 | 2 | 0.003
D4e3 | 150A | 4 | 0.006
D4e3 | 153 | 2 | 0.003
D4e3 | 15827T | 1 | 0.002
D4e3 | 15835 | 1 | 0.002
D4e3 | 15836 | 1 | 0.002
D4e3 | 15841 | 1 | 0.002

D4e3 | 15848T | 1 | 0.002
D4e3 | 15875G | 1 | 0.002
D4e3 | 15893 | 1 | 0.002
D4e3 | 15911T | 1 | 0.002
D4e3 | 15921 | 1 | 0.002
D4e3 | 15924C | 1 | 0.002
D4e3 | 15927T | 1 | 0.002
D4e3 | 16059 | 1 | 0.002
D4e3 | 16165 | 1 | 0.002
D4e3 | 16179 | 1 | 0.002
D4e3 | 16190 | 1 | 0.002
D4e3 | 16194 | 1 | 0.002
D4e3 | 16218 | 1 | 0.002
D4e3 | 16256 | 9 | 0.014
D4e3 | 16335 | 4 | 0.006
D4e3 | 16348 | 2 | 0.003
D4e3 | 16475G | 1 | 0.002
D4e3 | 247 | 8 | 0.013
D4e3 | 310 | 1 | 0.002
D4e3 | 446 | 1 | 0.002
D4e3 | 5783 | 7 | 0.011
D4e3 | 64 | 2 | 0.003
D4e3 | 648 | 1 | 0.002
D4e3 | 789 | 5 | 0.008
D4e4 | 10667 | 1 | 0.002
D4e4 | 10746 | 1 | 0.002
D4e4 | 13105 | 1 | 0.002
D4e4 | 150A | 4 | 0.006
D4e4 | 16114 | 6 | 0.01
D4e4 | 16165 | 1 | 0.002
D4e4 | 16167 | 1 | 0.002
D4e4 | 16179 | 1 | 0.002
D4e4 | 16218 | 1 | 0.002
D4e4 | 16263 | 1 | 0.002
D4e4 | 16291 | 1 | 0.002
D4e4 | 16335 | 4 | 0.006
D4e4 | 195 | 1 | 0.002
D4e4 | 2623 | 1 | 0.002
D4e4 | 310 | 1 | 0.002
D4e4 | 446 | 1 | 0.002
D4e4 | 603 | 1 | 0.002
D4e4 | 64 | 2 | 0.003
D4e4 | 648 | 1 | 0.002
D4e4 | 7624 | 1 | 0.002
D4e4 | 7805 | 1 | 0.002
D4e4 | 789 | 5 | 0.008
D4e4 | 8743 | 1 | 0.002
D4e4a | 10352 | 1 | 0.002
D4e4a | 150A | 4 | 0.006
D4e4a | 15930 | 1 | 0.002
D4e4a | 16114 | 6 | 0.009
D4e4a | 16165 | 1 | 0.002
D4e4a | 16167 | 1 | 0.002
D4e4a | 16179 | 1 | 0.002
D4e4a | 16218 | 1 | 0.002
D4e4a | 16291 | 13 | 0.02
D4e4a | 16335 | 4 | 0.006
D4e4a | 204 | 3 | 0.005
D4e4a | 207 | 1 | 0.002
D4e4a | 310 | 7 | 0.011
D4e4a | 314-315d | 1 | 0.002
D4e4a | 446 | 1 | 0.002
D4e4a | 4676 | 1 | 0.002
D4e4a | 64 | 2 | 0.003
D4e4a | 648 | 1 | 0.002
D4e4a | 789 | 6 | 0.009
D4e4a | 8348 | 12 | 0.019
D4e4a | 8812 | 12 | 0.019
D4e4a1 | 16129 | 3 | 0.036
D4e4a1 | 16140 | 1 | 0.012
D4e4a1 | 16163 | 1 | 0.012
D4e4a1 | 16193 | 1 | 0.012
D4e4a1 | 16293T | 1 | 0.012
D4e4a1 | 16310 | 1 | 0.012
D4e4a1 | 16348G | 1 | 0.012
D4e4a1 | 16414T | 1 | 0.012
D4e4a1 | 16526 | 1 | 0.012
D4e4a1 | 309.3C | 1 | 0.012
D4e4a1 | 312 | 1 | 0.012
D4e4a1 | 315.2C | 1 | 0.012
D4e4a1 | 488.7G | 1 | 0.012
D4e4b | 150A | 4 | 0.006
D4e4b | 16114 | 6 | 0.01
D4e4b | 16138 | 1 | 0.002
D4e4b | 16165 | 1 | 0.002
D4e4b | 16167 | 1 | 0.002
D4e4b | 16179 | 1 | 0.002
D4e4b | 16218 | 1 | 0.002
D4e4b | 16335 | 4 | 0.006
D4e4b | 196.1T | 1 | 0.002
D4e4b | 446 | 1 | 0.002
D4e4b | 64 | 2 | 0.003
D4e4b | 648 | 1 | 0.002
D4e4b | 789 | 5 | 0.008
D4e5 | 16092 | 1 | 0.007
D4e5 | 461 | 1 | 0.007
D4e5 | 513 | 1 | 0.007
D4e5 | 549 | 1 | 0.007
D4e5a | 12280 | 1 | 0.009
D4e5a | 14569 | 1 | 0.009
D4e5a | 16086 | 1 | 0.009
D4e5a | 16126 | 1 | 0.009
D4e5a | 16140 | 1 | 0.009
D4e5a | 16150 | 1 | 0.009
D4e5a | 16173 | 9 | 0.079
D4e5a | 16209 | 3 | 0.026
D4e5a | 16249 | 9 | 0.079
D4e5a | 16291 | 10 | 0.088
D4e5a | 16301 | 1 | 0.009
D4e5a | 16311 | 1 | 0.009
D4e5a | 185 | 1 | 0.009

D4e5a | 189 | 1 | 0.009
D4e5a | 195 | 1 | 0.009
D4e5a | 207 | 1 | 0.009
D4e5a | 279 | 1 | 0.009
D4e5a | 298 | 1 | 0.009
D4e5a | 316C | 1 | 0.009
D4e5a | 4678 | 1 | 0.009
D4e5a | 497 | 1 | 0.009
D4e5a | 6465 | 1 | 0.009
D4e5a | 7807 | 1 | 0.009
D4e5a | 8718 | 1 | 0.009
D4e5b | 16086 | 8 | 0.096
D4e5b | 16129 | 1 | 0.012
D4e5b | 16173 | 6 | 0.072
D4e5b | 16234 | 1 | 0.012
D4e5b | 16278 | 1 | 0.012
D4e5b | 16311 | 1 | 0.012
D4e5b | 296d | 1 | 0.012
D4e5b | 4395 | 1 | 0.012
D4e5b | 4637 | 1 | 0.012
D4e5b | 6827 | 1 | 0.012
D4f | 150A | 4 | 0.006
D4f | 16114 | 6 | 0.01
D4f | 16165 | 1 | 0.002
D4f | 16167 | 1 | 0.002
D4f | 16179 | 1 | 0.002
D4f | 16218 | 1 | 0.002
D4f | 16335 | 4 | 0.006
D4f | 207 | 1 | 0.002
D4f | 446 | 1 | 0.002
D4f | 64 | 2 | 0.003
D4f | 648 | 1 | 0.002
D4f | 7746 | 1 | 0.002
D4f | 789 | 5 | 0.008
D4f1 | 11536 | 1 | 0.002
D4f1 | 12667 | 1 | 0.002
D4f1 | 150A | 4 | 0.006
D4f1 | 152 | 2 | 0.003
D4f1 | 16037 | 2 | 0.003
D4f1 | 16067 | 1 | 0.002
D4f1 | 16114 | 6 | 0.009
D4f1 | 16165 | 1 | 0.002
D4f1 | 16167 | 1 | 0.002
D4f1 | 16179 | 1 | 0.002
D4f1 | 16218 | 1 | 0.002
D4f1 | 16335 | 4 | 0.006
D4f1 | 16399 | 2 | 0.003
D4f1 | 182 | 1 | 0.002
D4f1 | 446 | 1 | 0.002
D4f1 | 5384 | 1 | 0.002
D4f1 | 5438 | 1 | 0.002
D4f1 | 5471 | 1 | 0.002
D4f1 | 6018 | 1 | 0.002
D4f1 | 6227 | 1 | 0.002
D4f1 | 6266 | 1 | 0.002
D4f1 | 64 | 2 | 0.003
D4f1 | 648 | 1 | 0.002
D4f1 | 789 | 5 | 0.008
D4f1 | 7897 | 1 | 0.002
D4f1 | 8281-8289d | 1 | 0.002
D4f1 | 9039 | 1 | 0.002
D4f1 | 961 | 1 | 0.002
D4f1 | 965.4C | 1 | 0.002
D4g | 150A | 4 | 0.006
D4g | 16114 | 6 | 0.01
D4g | 16165 | 1 | 0.002
D4g | 16167 | 1 | 0.002
D4g | 16179 | 1 | 0.002
D4g | 16218 | 1 | 0.002
D4g | 16335 | 4 | 0.006
D4g | 446 | 1 | 0.002
D4g | 64 | 2 | 0.003
D4g | 648 | 1 | 0.002
D4g | 789 | 5 | 0.008
D4g1 | 11068 | 1 | 0.009
D4g1 | 12123 | 1 | 0.009
D4g1 | 13512 | 1 | 0.009
D4g1 | 13886 | 1 | 0.009
D4g1 | 14020 | 1 | 0.009
D4g1 | 14180 | 1 | 0.009
D4g1 | 15172 | 1 | 0.009
D4g1 | 16189 | 1 | 0.009
D4g1 | 16290 | 1 | 0.009
D4g1 | 16292 | 1 | 0.009
D4g1 | 1888 | 1 | 0.009
D4g1 | 2283 | 1 | 0.009
D4g1 | 4541 | 2 | 0.019
D4g1 | 6962 | 1 | 0.009
D4g1 | 7805 | 1 | 0.009
D4g1 | 9144A | 1 | 0.009
D4g1a | 11809 | 2 | 0.021
D4g1a | 13695A | 1 | 0.01
D4g1a | 14200 | 1 | 0.01
D4g1a | 14544 | 1 | 0.01
D4g1a | 16261 | 1 | 0.01
D4g1a | 16342 | 1 | 0.01
D4g1a | 183 | 1 | 0.01
D4g1a | 310 | 1 | 0.01
D4g1b | 10463 | 2 | 0.02
D4g1b | 12684 | 1 | 0.01
D4g1b | 14305 | 1 | 0.01
D4g1b | 146 | 1 | 0.01
D4g1b | 152 | 1 | 0.01
D4g1b | 15823 | 1 | 0.01
D4g1b | 15977 | 1 | 0.01
D4g1b | 16145 | 3 | 0.029
D4g1b | 16261 | 1 | 0.01
D4g1b | 16342 | 1 | 0.01
D4g1b | 183 | 1 | 0.01
D4g1b | 3202 | 1 | 0.01
D4g1b | 3349 | 1 | 0.01

D4g1b | 4313 | 2 | 0.02
D4g1b | 4820 | 1 | 0.01
D4g1b | 5764 | 1 | 0.01
D4g1b | 7543 | 1 | 0.01
D4g1b | 850d | 1 | 0.01
D4g1c | 152 | 3 | 0.029
D4g1c | 16260 | 1 | 0.01
D4g1c | 16261 | 1 | 0.01
D4g1c | 16269 | 2 | 0.02
D4g1c | 16342 | 1 | 0.01
D4g1c | 183 | 1 | 0.01
D4g1c | 2885 | 1 | 0.01
D4g1c | 8929 | 2 | 0.02
D4g1c | 961 | 1 | 0.01
D4g1c | 9851 | 1 | 0.01
D4g2 | 10750 | 1 | 0.002
D4g2 | 10969 | 1 | 0.002
D4g2 | 11778 | 1 | 0.002
D4g2 | 11935 | 1 | 0.002
D4g2 | 12972 | 1 | 0.002
D4g2 | 150 | 1 | 0.002
D4g2 | 16093 | 1 | 0.002
D4g2 | 16108 | 1 | 0.002
D4g2 | 16111A | 1 | 0.002
D4g2 | 16180 | 1 | 0.002
D4g2 | 16185 | 1 | 0.002
D4g2 | 16189 | 1 | 0.002
D4g2 | 16214 | 1 | 0.002
D4g2 | 16218 | 1 | 0.002
D4g2 | 16221 | 1 | 0.002
D4g2 | 16234 | 1 | 0.002
D4g2 | 16250A | 1 | 0.002
D4g2 | 16271 | 6 | 0.014
D4g2 | 16304 | 1 | 0.002
D4g2 | 16311 | 1 | 0.002
D4g2 | 16318C | 1 | 0.002
D4g2 | 16365 | 1 | 0.002
D4g2 | 16526 | 9 | 0.02
D4g2 | 3504 | 1 | 0.002
D4g2 | 4959 | 1 | 0.002
D4g2 | 5615 | 1 | 0.002
D4g2 | 574C | 1 | 0.002
D4g2 | 576C | 1 | 0.002
D4g2 | 577C | 1 | 0.002
D4g2 | 5894 | 1 | 0.002
D4g2 | 593 | 5 | 0.011
D4g2 | 709 | 1 | 0.002
D4g2 | 9575T | 1 | 0.002
D4g2a | 150 | 3 | 0.027
D4g2a | 152 | 2 | 0.018
D4g2a | 15496 | 1 | 0.009
D4g2a | 16093 | 6 | 0.054
D4g2a | 16129 | 3 | 0.027
D4g2a | 16167 | 1 | 0.009
D4g2a | 16189 | 1 | 0.009
D4g2a | 16209 | 1 | 0.009
D4g2a | 16217 | 2 | 0.018
D4g2a | 16239 | 2 | 0.018
D4g2a | 16240 | 1 | 0.009
D4g2a | 16278 | 3 | 0.027
D4g2a | 16295 | 1 | 0.009
D4g2a | 16325 | 1 | 0.009
D4g2a | 189 | 1 | 0.009
D4g2a | 195 | 2 | 0.018
D4g2a | 200 | 1 | 0.009
D4g2a | 203 | 1 | 0.009
D4g2a | 204 | 1 | 0.009
D4g2a | 228 | 1 | 0.009
D4g2a | 2772 | 1 | 0.009
D4g2a | 315.2C | 1 | 0.009
D4g2a | 325 | 1 | 0.009
D4g2a | 455d | 1 | 0.009
D4g2a | 499 | 1 | 0.009
D4g2a | 546 | 8 | 0.072
D4g2a | 549 | 1 | 0.009
D4g2a | 551 | 1 | 0.009
D4g2a | 709 | 2 | 0.018
D4g2a | 9128 | 1 | 0.009
D4g2a | 93 | 1 | 0.009
D4g2a1 | 10694 | 3 | 0.023
D4g2a1 | 11914 | 2 | 0.016
D4g2a1 | 12346 | 1 | 0.008
D4g2a1 | 12372 | 4 | 0.031
D4g2a1 | 12432 | 1 | 0.008
D4g2a1 | 13022G | 1 | 0.008
D4g2a1 | 13054G | 1 | 0.008
D4g2a1 | 13105 | 2 | 0.016
D4g2a1 | 13269 | 1 | 0.008
D4g2a1 | 146 | 3 | 0.023
D4g2a1 | 14650 | 2 | 0.016
D4g2a1 | 150 | 3 | 0.023
D4g2a1 | 15085 | 2 | 0.016
D4g2a1 | 152 | 1 | 0.008
D4g2a1 | 16093 | 6 | 0.047
D4g2a1 | 16095 | 2 | 0.016
D4g2a1 | 16129 | 2 | 0.016
D4g2a1 | 16169 | 19 | 0.147
D4g2a1 | 16179G | 1 | 0.008
D4g2a1 | 16189 | 1 | 0.008
D4g2a1 | 16266 | 14 | 0.109
D4g2a1 | 16271 | 1 | 0.008
D4g2a1 | 16297 | 1 | 0.008
D4g2a1 | 16311 | 8 | 0.062
D4g2a1 | 16319 | 2 | 0.016
D4g2a1 | 16327 | 1 | 0.008
D4g2a1 | 16465 | 1 | 0.008
D4g2a1 | 200 | 1 | 0.008
D4g2a1 | 310 | 1 | 0.008
D4g2a1 | 315.2C | 1 | 0.008
D4g2a1 | 3150 | 2 | 0.016
D4g2a1 | 315d | 1 | 0.008

D4g2a1 | 316 | 1 | 0.008
D4g2a1 | 316C | 1 | 0.008
D4g2a1 | 4135 | 1 | 0.008
D4g2a1 | 499 | 1 | 0.008
D4g2a1 | 5306 | 1 | 0.008
D4g2a1 | 5585 | 1 | 0.008
D4g2a1 | 5655 | 4 | 0.031
D4g2a1 | 5656 | 1 | 0.008
D4g2a1 | 7850 | 1 | 0.008
D4g2a1 | 8462 | 2 | 0.016
D4g2a1 | 9007 | 1 | 0.008
D4g2a1 | 9156 | 2 | 0.016
D4g2a1 | 93 | 1 | 0.008
D4g2a1a | 12622 | 1 | 0.091
D4g2a1a | 16145 | 1 | 0.091
D4g2a1a | 16189 | 1 | 0.091
D4g2a1a | 16249 | 4 | 0.364
D4g2a1a | 16288 | 1 | 0.091
D4g2a1a | 16291G | 1 | 0.091
D4g2a1a | 16343 | 1 | 0.091
D4g2a1a | 16365 | 1 | 0.091
D4g2a1a | 16390 | 1 | 0.091
D4g2a1a | 189 | 1 | 0.091
D4g2a1b | 11287 | 1 | 0.008
D4g2a1b | 12804 | 1 | 0.008
D4g2a1b | 152 | 1 | 0.008
D4g2a1b | 15497 | 3 | 0.024
D4g2a1b | 16086 | 1 | 0.008
D4g2a1b | 16172 | 1 | 0.008
D4g2a1b | 16174 | 1 | 0.008
D4g2a1b | 16189 | 1 | 0.008
D4g2a1b | 16213 | 1 | 0.008
D4g2a1b | 16240 | 26 | 0.21
D4g2a1b | 16264 | 9 | 0.073
D4g2a1b | 16292 | 1 | 0.008
D4g2a1b | 16294 | 8 | 0.065
D4g2a1b | 16304 | 3 | 0.024
D4g2a1b | 182 | 1 | 0.008
D4g2a1b | 195 | 1 | 0.008
D4g2a1b | 196 | 2 | 0.016
D4g2a1b | 310 | 2 | 0.016
D4g2a1b | 316C | 6 | 0.048
D4g2a1b | 3391 | 1 | 0.008
D4g2a1b | 466 | 10 | 0.081
D4g2a1c | 11025 | 1 | 0.009
D4g2a1c | 11041 | 1 | 0.009
D4g2a1c | 11875 | 1 | 0.009
D4g2a1c | 12127 | 1 | 0.009
D4g2a1c | 14302 | 1 | 0.009
D4g2a1c | 14368 | 1 | 0.009
D4g2a1c | 15156 | 1 | 0.009
D4g2a1c | 15773 | 1 | 0.009
D4g2a1c | 16086 | 1 | 0.009
D4g2a1c | 16094 | 1 | 0.009
D4g2a1c | 16164 | 1 | 0.009
D4g2a1c | 16172 | 4 | 0.034
D4g2a1c | 16189 | 3 | 0.026
D4g2a1c | 16213 | 1 | 0.009
D4g2a1c | 16232A | 2 | 0.017
D4g2a1c | 16254 | 1 | 0.009
D4g2a1c | 16259A | 3 | 0.026
D4g2a1c | 16295 | 1 | 0.009
D4g2a1c | 16303 | 1 | 0.009
D4g2a1c | 16309 | 1 | 0.009
D4g2a1c | 185 | 4 | 0.034
D4g2a1c | 204 | 4 | 0.034
D4g2a1c | 207 | 1 | 0.009
D4g2a1c | 225 | 1 | 0.009
D4g2a1c | 226 | 1 | 0.009
D4g2a1c | 310 | 4 | 0.034
D4g2a1c | 3106A | 1 | 0.009
D4g2a1c | 314-315d | 1 | 0.009
D4g2a1c | 315.2C | 2 | 0.017
D4g2a1c | 315d | 1 | 0.009
D4g2a1c | 316 | 3 | 0.026
D4g2a1c | 347d | 1 | 0.009
D4g2a1c | 392 | 1 | 0.009
D4g2a1c | 4165 | 1 | 0.009
D4g2a1c | 573.2C | 2 | 0.017
D4g2a1c | 573.3C | 1 | 0.009
D4g2a1c | 710 | 1 | 0.009
D4g2a1c | 7248 | 1 | 0.009
D4g2a1c | 8167 | 1 | 0.009
D4g2a1c | 983 | 1 | 0.009
D4g2b | 13287 | 1 | 0.002
D4g2b | 14221 | 1 | 0.002
D4g2b | 150 | 1 | 0.002
D4g2b | 152 | 7 | 0.016
D4g2b | 15893 | 1 | 0.002
D4g2b | 16108 | 1 | 0.002
D4g2b | 16111A | 1 | 0.002
D4g2b | 16140 | 7 | 0.016
D4g2b | 16188 | 1 | 0.002
D4g2b | 16189 | 1 | 0.002
D4g2b | 16214 | 1 | 0.002
D4g2b | 16218 | 1 | 0.002
D4g2b | 16221 | 1 | 0.002
D4g2b | 16250A | 1 | 0.002
D4g2b | 16290 | 1 | 0.002
D4g2b | 16318C | 1 | 0.002
D4g2b | 203 | 1 | 0.002
D4g2b | 204 | 2 | 0.005
D4g2b | 316 | 1 | 0.002
D4g2b | 3421 | 1 | 0.002
D4g2b | 3635 | 1 | 0.002
D4g2b | 574C | 1 | 0.002
D4g2b | 576C | 1 | 0.002
D4g2b | 577C | 1 | 0.002
D4g2b | 709 | 1 | 0.002
D4g2b | 8793 | 1 | 0.002
D4g2b1 | 150 | 1 | 0.002

D4g2b1 | 16076d | 6 | 0.014
D4g2b1 | 16086 | 1 | 0.002
D4g2b1 | 16108 | 1 | 0.002
D4g2b1 | 16111A | 1 | 0.002
D4g2b1 | 16184 | 3 | 0.007
D4g2b1 | 16214 | 1 | 0.002
D4g2b1 | 16218 | 1 | 0.002
D4g2b1 | 16221 | 1 | 0.002
D4g2b1 | 16250A | 1 | 0.002
D4g2b1 | 16318C | 1 | 0.002
D4g2b1 | 573.2C | 4 | 0.009
D4g2b1 | 574C | 1 | 0.002
D4g2b1 | 576C | 1 | 0.002
D4g2b1 | 577C | 1 | 0.002
D4g2b1 | 709 | 1 | 0.002
D4g2b1 | 9052 | 3 | 0.007
D4g2b1a | 10376 | 1 | 0.002
D4g2b1a | 10667 | 1 | 0.002
D4g2b1a | 16076d | 6 | 0.014
D4g2b1a | 16108 | 1 | 0.002
D4g2b1a | 16111A | 1 | 0.002
D4g2b1a | 16250A | 1 | 0.002
D4g2b1a | 16318C | 1 | 0.002
D4g2b1a | 235 | 1 | 0.002
D4g2b1a | 310 | 1 | 0.002
D4h | 10192 | 5 | 0.008
D4h | 13879 | 5 | 0.008
D4h | 150A | 4 | 0.006
D4h | 15323 | 2 | 0.003
D4h | 16114 | 6 | 0.01
D4h | 16165 | 1 | 0.002
D4h | 16167 | 1 | 0.002
D4h | 16179 | 1 | 0.002
D4h | 16218 | 1 | 0.002
D4h | 16248 | 3 | 0.005
D4h | 16263 | 1 | 0.002
D4h | 16264.1C | 1 | 0.002
D4h | 16335 | 4 | 0.006
D4h | 446 | 1 | 0.002
D4h | 4580 | 5 | 0.008
D4h | 516 | 2 | 0.003
D4h | 571G | 5 | 0.008
D4h | 64 | 2 | 0.003
D4h | 648 | 1 | 0.002
D4h | 6663 | 5 | 0.008
D4h | 789 | 5 | 0.008
D4h | 9424 | 5 | 0.008
D4h1 | 13095G | 2 | 0.015
D4h1 | 131 | 3 | 0.022
D4h1 | 13677 | 2 | 0.015
D4h1 | 150 | 1 | 0.007
D4h1 | 152 | 1 | 0.007
D4h1 | 16048 | 1 | 0.007
D4h1 | 16076d | 1 | 0.007
D4h1 | 16093 | 7 | 0.051
D4h1 | 16126 | 13 | 0.096
D4h1 | 16166 | 2 | 0.015
D4h1 | 16169 | 1 | 0.007
D4h1 | 16172 | 12 | 0.088
D4h1 | 16177 | 2 | 0.015
D4h1 | 16179 | 4 | 0.029
D4h1 | 16189 | 1 | 0.007
D4h1 | 16192 | 2 | 0.015
D4h1 | 16209 | 2 | 0.015
D4h1 | 16216 | 1 | 0.007
D4h1 | 16239 | 5 | 0.037
D4h1 | 16243 | 1 | 0.007
D4h1 | 16254 | 1 | 0.007
D4h1 | 16286 | 4 | 0.029
D4h1 | 16311 | 3 | 0.022
D4h1 | 16325 | 1 | 0.007
D4h1 | 16343 | 19 | 0.14
D4h1 | 16365 | 1 | 0.007
D4h1 | 16468 | 1 | 0.007
D4h1 | 16526 | 2 | 0.015
D4h1 | 189 | 1 | 0.007
D4h1 | 196 | 2 | 0.015
D4h1 | 199 | 1 | 0.007
D4h1 | 207 | 1 | 0.007
D4h1 | 214 | 5 | 0.037
D4h1 | 228 | 1 | 0.007
D4h1 | 259 | 2 | 0.015
D4h1 | 315.2C | 1 | 0.007
D4h1 | 3308 | 2 | 0.015
D4h1 | 356.1C | 6 | 0.044
D4h1 | 4176 | 2 | 0.015
D4h1 | 499 | 1 | 0.007
D4h1 | 513 | 2 | 0.015
D4h1 | 573.2C | 3 | 0.022
D4h1 | 573.3C | 4 | 0.029
D4h1 | 573.4C | 1 | 0.007
D4h1 | 573.5C | 3 | 0.022
D4h1 | 593 | 1 | 0.007
D4h1 | 63 | 1 | 0.007
D4h1 | 7181 | 2 | 0.015
D4h1 | 7673 | 2 | 0.015
D4h1 | 9300 | 2 | 0.015
D4h1a | 16114 | 1 | 0.067
D4h1a | 16468 | 1 | 0.067
D4h1a | 16533d | 1 | 0.067
D4h1a | 16542.1A | 1 | 0.067
D4h1a | 32.1G | 1 | 0.067
D4h1a1 | 152 | 1 | 0.083
D4h1a1 | 16021G | 1 | 0.083
D4h1a1 | 16172 | 1 | 0.083
D4h1a1 | 16259 | 1 | 0.083
D4h1a1 | 3666 | 1 | 0.083
D4h1a1 | 4541T | 2 | 0.167
D4h1a1 | 8669C | 1 | 0.083
D4h1a1 | 9038 | 1 | 0.083
D4h1a2 | 11654 | 1 | 0.062

D4h1a2	16172	1	0.062
D4h1a2	2074d	1	0.062
D4h1a2	6581	1	0.062
D4h1a2	9922	1	0.062
D4h1b	1007	1	0.011
D4h1b	11257	1	0.011
D4h1b	16111	1	0.011
D4h1b	16169A	1	0.011
D4h1b	16241	2	0.023
D4h1b	16288	1	0.011
D4h1b	16352	2	0.023
D4h1b	3915	2	0.023
D4h1b	6791T	1	0.011
D4h1b	7196A	1	0.011
D4h1b	7859	1	0.011
D4h1c	12477	2	0.022
D4h1c	14178	2	0.022
D4h1c	15236	6	0.065
D4h1c	16147	1	0.011
D4h1c	16172	4	0.043
D4h1c	16189	1	0.011
D4h1c	16192	7	0.076
D4h1c	16194T	2	0.022
D4h1c	16213	8	0.087
D4h1c	16257	1	0.011
D4h1c	16262.1C	1	0.011
D4h1c	16278	1	0.011
D4h1c	16290G	1	0.011
D4h1c	16293	2	0.022
D4h1c	16319	6	0.065
D4h1c	16320	4	0.043
D4h1c	16325	3	0.033
D4h1c	16343	1	0.011
D4h1c	178	1	0.011
D4h1c	195	1	0.011
D4h1c	2702	1	0.011
D4h1c	4182	8	0.087
D4h1c	4310	1	0.011
D4h1c	507	1	0.011
D4h1c	513	1	0.011
D4h1c	5936	8	0.087
D4h1c	709	9	0.098
D4h1c	8179	10	0.109
D4h1c1	12842	1	0.029
D4h1c1	14569	1	0.029
D4h1c1	15916	1	0.029
D4h1c1	16265	1	0.029
D4h1c1	195	1	0.029
D4h1d	8152	1	0.015
D4h2	146	3	0.045
D4h2	16142	3	0.045
D4h2	16148	3	0.045
D4h2	16356	3	0.045
D4h2	228	3	0.045
D4h3	11150	2	0.065
D4h3	12373	2	0.065
D4h3	15346	2	0.065
D4h3	16178	2	0.065
D4h3	4418	2	0.065
D4h3	4562	2	0.065
D4h3	5147	1	0.032
D4h3	6620	1	0.032
D4h3a	10256	1	0.025
D4h3a	106-111d	1	0.025
D4h3a	10652	1	0.025
D4h3a	11016	1	0.025
D4h3a	11053	1	0.025
D4h3a	11465	1	0.025
D4h3a	11482	1	0.025
D4h3a	11890	1	0.025
D4h3a	12188	1	0.025
D4h3a	12338	1	0.025
D4h3a	12528	2	0.05
D4h3a	12717	1	0.025
D4h3a	12771	2	0.05
D4h3a	12906A	1	0.025
D4h3a	1309	1	0.025
D4h3a	13404	1	0.025
D4h3a	13748	1	0.025
D4h3a	14136	1	0.025
D4h3a	14203	1	0.025
D4h3a	14287	1	0.025
D4h3a	151	1	0.025
D4h3a	15257	1	0.025
D4h3a	15791	1	0.025
D4h3a	15944d	1	0.025
D4h3a	16051	2	0.05
D4h3a	16092	1	0.025
D4h3a	16111	1	0.025
D4h3a	16129	2	0.05
D4h3a	16187	1	0.025
D4h3a	16189	2	0.05
D4h3a	16203	1	0.025
D4h3a	16235	1	0.025
D4h3a	16245	1	0.025
D4h3a	16255	3	0.075
D4h3a	16256	1	0.025
D4h3a	16278	1	0.025
D4h3a	16291	1	0.025
D4h3a	16311	3	0.075
D4h3a	16319	1	0.025
D4h3a	16390	1	0.025
D4h3a	16468	1	0.025
D4h3a	16533	1	0.025
D4h3a	1664	1	0.025
D4h3a	1719	1	0.025
D4h3a	185	1	0.025
D4h3a	2056	1	0.025
D4h3a	2065	1	0.025
D4h3a	294	1	0.025
D4h3a	3594	1	0.025

D4h3a | 408 | 1 | 0.025
D4h3a | 4820 | 1 | 0.025
D4h3a | 4958 | 1 | 0.025
D4h3a | 507 | 1 | 0.025
D4h3a | 5147 | 1 | 0.025
D4h3a | 5192 | 2 | 0.05
D4h3a | 573d | 1 | 0.025
D4h3a | 5745 | 1 | 0.025
D4h3a | 6092 | 1 | 0.025
D4h3a | 6287 | 1 | 0.025
D4h3a | 6480 | 1 | 0.025
D4h3a | 6572 | 1 | 0.025
D4h3a | 709 | 1 | 0.025
D4h3a | 72 | 1 | 0.025
D4h3a | 722 | 1 | 0.025
D4h3a | 742 | 1 | 0.025
D4h3a | 7805 | 1 | 0.025
D4h3a | 7990 | 1 | 0.025
D4h3a | 8014 | 1 | 0.025
D4h3a | 8296 | 1 | 0.025
D4h3a | 8718 | 2 | 0.05
D4h3a | 9163 | 1 | 0.025
D4h3a | 9299 | 2 | 0.05
D4h3a | 9413 | 1 | 0.025
D4h3a | 9770 | 2 | 0.05
D4h3a | 9821 | 2 | 0.05
D4h3a | 9965 | 1 | 0.025
D4h3a+@152 | 11569A | 1 | 0.015
D4h3a+@152 | 12393G | 1 | 0.015
D4h3a+@152 | 14218 | 1 | 0.015
D4h3a+@152 | 14335 | 1 | 0.015
D4h3a+@152 | 15734 | 2 | 0.03
D4h3a+@152 | 16162 | 1 | 0.015
D4h3a+@152 | 16263 | 2 | 0.03
D4h3a+@152 | 16319 | 1 | 0.015
D4h3a+@152 | 195 | 1 | 0.015
D4h3a+@152 | 3927 | 2 | 0.03
D4h3a+@152 | 4092 | 2 | 0.03
D4h3a+@152 | 4646 | 1 | 0.015
D4h3a+@152 | 5480 | 2 | 0.03
D4h3a+@152 | 709 | 1 | 0.015
D4h3a+@152 | 7269 | 1 | 0.015
D4h3a+@152 | 8713 | 2 | 0.03
D4h3a1 | 12681 | 2 | 0.077
D4h3a1 | 14384 | 2 | 0.077
D4h3a1 | 15804 | 2 | 0.077
D4h3a1 | 16265C | 2 | 0.077
D4h3a1 | 203 | 3 | 0.115
D4h3a1 | 204 | 3 | 0.115
D4h3a1 | 9533 | 2 | 0.077
D4h3a1a1 | 16092 | 1 | 0.1
D4h3a1a1 | 16532d | 2 | 0.2
D4h3a1a1 | 7609 | 1 | 0.1
D4h3a1a1 | 9616 | 1 | 0.1
D4h3a1a2 | 15928 | 3 | 0.103
D4h3a1a2 | 16292 | 1 | 0.034
D4h3a1a2 | 16527 | 1 | 0.034
D4h3a1a2 | 54 | 1 | 0.034
D4h3a1a2 | 709 | 1 | 0.034
D4h3a1a2 | 7849 | 2 | 0.069
D4h3a1a2 | 960.1C | 1 | 0.034
D4h3a1a2 | 9848 | 1 | 0.034
D4h3a2 | 10172 | 2 | 0.08
D4h3a2 | 11102G | 1 | 0.04
D4h3a2 | 11782 | 1 | 0.04
D4h3a2 | 12673 | 1 | 0.04
D4h3a2 | 13809 | 3 | 0.12
D4h3a2 | 146 | 1 | 0.04
D4h3a2 | 16051 | 5 | 0.2
D4h3a2 | 16092 | 1 | 0.04
D4h3a2 | 16189 | 2 | 0.08
D4h3a2 | 16298 | 2 | 0.08
D4h3a2 | 16368 | 1 | 0.04
D4h3a2 | 195 | 2 | 0.08
D4h3a2 | 208A | 2 | 0.08
D4h3a2 | 498 | 1 | 0.04
D4h3a2 | 5237 | 2 | 0.08
D4h3a2 | 75 | 2 | 0.08
D4h3a2 | 8270 | 2 | 0.08
D4h3a2 | 8281-8289d | 2 | 0.08
D4h3a3 | 10616 | 1 | 0.25
D4h3a3 | 11407A | 1 | 0.25
D4h3a3 | 15556A | 1 | 0.25
D4h3a3 | 16114A | 1 | 0.25
D4h3a3 | 16234 | 1 | 0.25
D4h3a3 | 16256G | 1 | 0.25
D4h3a3 | 16288 | 1 | 0.25
D4h3a3 | 16320 | 1 | 0.25
D4h3a3 | 16342G | 1 | 0.25
D4h3a3 | 16526 | 1 | 0.25
D4h3a3 | 199 | 1 | 0.25
D4h3a3 | 2260 | 1 | 0.25
D4h3a3 | 55.1T | 1 | 0.25
D4h3a3 | 57 | 1 | 0.25
D4h3a3 | 59 | 1 | 0.25
D4h3a3 | 60.1T | 1 | 0.25
D4h3a3a | 11002 | 1 | 0.111
D4h3a3a | 14861 | 4 | 0.444
D4h3a3a | 153 | 1 | 0.111
D4h3a3a | 16092 | 2 | 0.222
D4h3a3a | 16209 | 2 | 0.222
D4h3a3a | 178 | 1 | 0.111
D4h3a3a | 3565 | 4 | 0.444
D4h3a3a | 4778 | 1 | 0.111
D4h3a3a | 547 | 1 | 0.111
D4h3a3a | 7278 | 1 | 0.111
D4h3a3a | 8496 | 1 | 0.111
D4h3a4 | 125 | 1 | 0.036
D4h3a4 | 127 | 1 | 0.036
D4h3a4 | 16075 | 1 | 0.036
D4h3a4 | 16260 | 1 | 0.036

D4h3a4 | 16292 | 1 | 0.036
D4h3a4 | 16462 | 1 | 0.036
D4h3a4 | 16533 | 1 | 0.036
D4h3a4 | 195 | 1 | 0.036
D4h3a4 | 279 | 1 | 0.036
D4h3a4 | 4413 | 1 | 0.036
D4h3a4 | 8998 | 1 | 0.036
D4h3a4 | 94 | 1 | 0.036
D4h3a5 | 11482 | 1 | 0.027
D4h3a5 | 12040 | 3 | 0.081
D4h3a5 | 12528 | 3 | 0.081
D4h3a5 | 15383 | 1 | 0.027
D4h3a5 | 16051 | 14 | 0.378
D4h3a5 | 16234 | 2 | 0.054
D4h3a5 | 16311 | 7 | 0.189
D4h3a5 | 16445 | 1 | 0.027
D4h3a5 | 2065 | 1 | 0.027
D4h3a5 | 207 | 2 | 0.054
D4h3a5 | 3434 | 1 | 0.027
D4h3a5 | 4948 | 2 | 0.054
D4h3a5 | 573d | 1 | 0.027
D4h3a5 | 7802 | 1 | 0.027
D4h3a5 | 7990 | 1 | 0.027
D4h3a5 | 8281-8289d | 1 | 0.027
D4h3a5 | 9770 | 3 | 0.081
D4h3a5 | 9821 | 3 | 0.081
D4h3a5 | 9956 | 1 | 0.027
D4h3a6 | 15930 | 1 | 0.167
D4h3a6 | 16129 | 2 | 0.333
D4h3a6 | 16172 | 1 | 0.167
D4h3a6 | 16192 | 4 | 0.667
D4h3a6 | 16311 | 2 | 0.333
D4h3a6 | 195 | 1 | 0.167
D4h3a6 | 2308 | 1 | 0.167
D4h3a6 | 249d | 1 | 0.167
D4h3a6 | 6293 | 2 | 0.333
D4h3a6 | 7348 | 2 | 0.333
D4h3a6 | 8854 | 1 | 0.167
D4h3a6 | 8989 | 1 | 0.167
D4h3a6 | 9110 | 1 | 0.167
D4h3a7 | 765G | 1 | 0.015
D4h3a8 | 119 | 1 | 0.014
D4h3a8 | 121 | 1 | 0.014
D4h3a8 | 14016 | 1 | 0.014
D4h3a8 | 14233 | 1 | 0.014
D4h3a8 | 16093 | 1 | 0.014
D4h3a8 | 16189 | 1 | 0.014
D4h3a8 | 16293C | 1 | 0.014
D4h3a8 | 292A | 1 | 0.014
D4h3a8 | 324 | 1 | 0.014
D4h3a8 | 508 | 1 | 0.014
D4h3a8 | 562 | 1 | 0.014
D4h3a8 | 580 | 1 | 0.014
D4h3a8 | 645.1A | 1 | 0.014
D4h3a8 | 676C | 1 | 0.014
D4h3a9 | 13851 | 2 | 0.029
D4h3a9 | 13986 | 2 | 0.029
D4h3a9 | 14065 | 2 | 0.029
D4h3a9 | 146 | 2 | 0.029
D4h3a9 | 15253 | 1 | 0.015
D4h3a9 | 15944d | 3 | 0.044
D4h3a9 | 16249 | 2 | 0.029
D4h3a9 | 16293C | 2 | 0.029
D4h3a9 | 16296.1C | 2 | 0.029
D4h3a9 | 16311 | 2 | 0.029
D4h3a9 | 1971 | 2 | 0.029
D4h3a9 | 6326 | 1 | 0.015
D4h3a9 | 7879 | 3 | 0.044
D4h4 | 10463 | 1 | 0.002
D4h4 | 10646 | 1 | 0.002
D4h4 | 13681 | 1 | 0.002
D4h4 | 13965 | 2 | 0.003
D4h4 | 14155 | 2 | 0.003
D4h4 | 150A | 4 | 0.006
D4h4 | 152 | 2 | 0.003
D4h4 | 16093 | 2 | 0.003
D4h4 | 16114 | 6 | 0.01
D4h4 | 16146 | 1 | 0.002
D4h4 | 16165 | 1 | 0.002
D4h4 | 16167 | 1 | 0.002
D4h4 | 16179 | 1 | 0.002
D4h4 | 16218 | 1 | 0.002
D4h4 | 16256 | 1 | 0.002
D4h4 | 16309 | 1 | 0.002
D4h4 | 16311 | 1 | 0.002
D4h4 | 16335 | 4 | 0.006
D4h4 | 195 | 1 | 0.002
D4h4 | 446 | 1 | 0.002
D4h4 | 5074 | 1 | 0.002
D4h4 | 64 | 2 | 0.003
D4h4 | 648 | 1 | 0.002
D4h4 | 709 | 2 | 0.003
D4h4 | 7502 | 1 | 0.002
D4h4 | 789 | 5 | 0.008
D4h4a | 136 | 1 | 0.006
D4h4a | 146 | 3 | 0.018
D4h4a | 151 | 7 | 0.043
D4h4a | 153 | 3 | 0.018
D4h4a | 159d | 1 | 0.006
D4h4a | 16086 | 4 | 0.024
D4h4a | 16093 | 5 | 0.03
D4h4a | 16129 | 1 | 0.006
D4h4a | 16131 | 1 | 0.006
D4h4a | 16172 | 1 | 0.006
D4h4a | 16185 | 2 | 0.012
D4h4a | 16209 | 6 | 0.037
D4h4a | 16234 | 2 | 0.012
D4h4a | 16235 | 1 | 0.006
D4h4a | 16249 | 2 | 0.012
D4h4a | 16258 | 1 | 0.006
D4h4a | 16355 | 41 | 0.25

D4h4a | 16406 | 1 | 0.006
D4h4a | 249d | 1 | 0.006
D4h4a | 268 | 1 | 0.006
D4h4a | 284.1A | 1 | 0.006
D4h4a | 315.2C | 1 | 0.006
D4h4a | 5186 | 3 | 0.018
D4h4a | 5363 | 3 | 0.018
D4h4a | 71.1G | 1 | 0.006
D4h4a | 7642 | 1 | 0.006
D4i | 11150 | 1 | 0.007
D4i | 1187 | 1 | 0.007
D4i | 12432 | 4 | 0.026
D4i | 13105 | 1 | 0.007
D4i | 13711 | 2 | 0.013
D4i | 14070 | 3 | 0.02
D4i | 146 | 4 | 0.026
D4i | 150 | 4 | 0.026
D4i | 151 | 2 | 0.013
D4i | 15734 | 3 | 0.02
D4i | 16033 | 1 | 0.007
D4i | 16051 | 3 | 0.02
D4i | 16052 | 1 | 0.007
D4i | 16103T | 1 | 0.007
D4i | 16107A | 1 | 0.007
D4i | 16111 | 6 | 0.04
D4i | 16114 | 1 | 0.007
D4i | 16128G | 1 | 0.007
D4i | 16129 | 18 | 0.119
D4i | 16131 | 1 | 0.007
D4i | 16133A | 1 | 0.007
D4i | 16147 | 1 | 0.007
D4i | 16166C | 1 | 0.007
D4i | 16174 | 1 | 0.007
D4i | 16185 | 1 | 0.007
D4i | 16192 | 1 | 0.007
D4i | 16214G | 2 | 0.013
D4i | 16229 | 2 | 0.013
D4i | 16241T | 1 | 0.007
D4i | 16256A | 1 | 0.007
D4i | 16265C | 1 | 0.007
D4i | 16278G | 1 | 0.007
D4i | 16281 | 1 | 0.007
D4i | 16299 | 1 | 0.007
D4i | 16304 | 1 | 0.007
D4i | 16309 | 1 | 0.007
D4i | 16311 | 3 | 0.02
D4i | 16319 | 4 | 0.026
D4i | 16344 | 1 | 0.007
D4i | 16356 | 6 | 0.04
D4i | 16390 | 1 | 0.007
D4i | 183 | 23 | 0.152
D4i | 185 | 1 | 0.007
D4i | 186 | 4 | 0.026
D4i | 189 | 1 | 0.007
D4i | 193 | 1 | 0.007
D4i | 195 | 3 | 0.02
D4i | 199 | 2 | 0.013
D4i | 215 | 1 | 0.007
D4i | 228T | 4 | 0.026
D4i | 234 | 3 | 0.02
D4i | 298 | 1 | 0.007
D4i | 310 | 2 | 0.013
D4i | 3196C | 1 | 0.007
D4i | 485 | 1 | 0.007
D4i | 573.1C | 1 | 0.007
D4i | 6680 | 1 | 0.007
D4i | 709 | 16 | 0.106
D4i | 770 | 1 | 0.007
D4i | 8080 | 1 | 0.007
D4i | 8279 | 2 | 0.013
D4i | 85 | 1 | 0.007
D4i | 8682 | 1 | 0.007
D4i | 9725 | 4 | 0.026
D4i | 9861 | 2 | 0.013
D4i1 | 15257 | 1 | 0.014
D4i1 | 16126 | 1 | 0.014
D4i1 | 16184 | 3 | 0.043
D4i1 | 16311 | 1 | 0.014
D4i1 | 16356 | 1 | 0.014
D4i1 | 204 | 3 | 0.043
D4i1 | 207 | 3 | 0.043
D4i1 | 6345 | 1 | 0.014
D4i2 | 12192 | 1 | 0.006
D4i2 | 14182 | 1 | 0.006
D4i2 | 146 | 38 | 0.236
D4i2 | 150 | 1 | 0.006
D4i2 | 16086 | 1 | 0.006
D4i2 | 16093 | 16 | 0.099
D4i2 | 16095 | 1 | 0.006
D4i2 | 16129 | 2 | 0.012
D4i2 | 16172 | 3 | 0.019
D4i2 | 16180 | 1 | 0.006
D4i2 | 16189 | 2 | 0.012
D4i2 | 16209 | 1 | 0.006
D4i2 | 16274 | 1 | 0.006
D4i2 | 16278 | 1 | 0.006
D4i2 | 16299 | 1 | 0.006
D4i2 | 16311 | 2 | 0.012
D4i2 | 195 | 44 | 0.273
D4i2 | 200 | 16 | 0.099
D4i2 | 204 | 1 | 0.006
D4i2 | 207 | 1 | 0.006
D4i2 | 237 | 11 | 0.068
D4i2 | 258 | 1 | 0.006
D4i2 | 310 | 6 | 0.037
D4i2 | 324G | 1 | 0.006
D4i2 | 350C | 1 | 0.006
D4i2 | 4775 | 1 | 0.006
D4i2 | 501 | 11 | 0.068
D4i2 | 512.1A | 1 | 0.006
D4i2 | 64 | 6 | 0.037

D4i2 | 65A | 11 | 0.068
D4i2 | 7440 | 1 | 0.006
D4i3 | 10652 | 1 | 0.056
D4i3 | 11899 | 1 | 0.056
D4i3 | 132 | 1 | 0.056
D4i3 | 141 | 1 | 0.056
D4i3 | 14178 | 1 | 0.056
D4i3 | 150 | 1 | 0.056
D4i3 | 15856 | 1 | 0.056
D4i3 | 16093 | 3 | 0.167
D4i3 | 16376 | 1 | 0.056
D4i3 | 16378 | 1 | 0.056
D4i3 | 16380 | 1 | 0.056
D4i3 | 207 | 1 | 0.056
D4i3 | 3184 | 1 | 0.056
D4i3 | 3798 | 2 | 0.111
D4i3 | 4736 | 1 | 0.056
D4i3 | 64G | 1 | 0.056
D4i3 | 7444 | 1 | 0.056
D4j | 10724 | 1 | 0.002
D4j | 11422 | 1 | 0.002
D4j | 11509 | 5 | 0.008
D4j | 11617 | 5 | 0.008
D4j | 11935 | 1 | 0.002
D4j | 12127 | 1 | 0.002
D4j | 12501 | 5 | 0.008
D4j | 12662 | 1 | 0.002
D4j | 12732 | 1 | 0.002
D4j | 13020 | 3 | 0.005
D4j | 13071A | 1 | 0.002
D4j | 14128 | 3 | 0.005
D4j | 14393 | 1 | 0.002
D4j | 1442 | 1 | 0.002
D4j | 14693 | 5 | 0.008
D4j | 150A | 1 | 0.002
D4j | 153 | 1 | 0.002
D4j | 16051 | 5 | 0.008
D4j | 16075 | 1 | 0.002
D4j | 16165 | 1 | 0.002
D4j | 16172 | 1 | 0.002
D4j | 16179 | 1 | 0.002
D4j | 16218 | 1 | 0.002
D4j | 16265C | 2 | 0.003
D4j | 16288 | 7 | 0.011
D4j | 16299 | 5 | 0.008
D4j | 16327 | 1 | 0.002
D4j | 16335 | 1 | 0.002
D4j | 16359 | 6 | 0.009
D4j | 234 | 1 | 0.002
D4j | 243 | 3 | 0.005
D4j | 309 | 1 | 0.002
D4j | 341C | 1 | 0.002
D4j | 3918 | 1 | 0.002
D4j | 4048 | 4 | 0.006
D4j | 4315.1T | 1 | 0.002
D4j | 4353 | 1 | 0.002
D4j | 446 | 1 | 0.002
D4j | 4820 | 1 | 0.002
D4j | 5131 | 1 | 0.002
D4j | 5483 | 1 | 0.002
D4j | 60 | 1 | 0.002
D4j | 6216 | 1 | 0.002
D4j | 6261 | 1 | 0.002
D4j | 64 | 2 | 0.003
D4j | 648 | 1 | 0.002
D4j | 6503T | 2 | 0.003
D4j | 7501A | 1 | 0.002
D4j | 7543 | 2 | 0.003
D4j | 789 | 2 | 0.003
D4j | 8277 | 1 | 0.002
D4j | 8278.4C | 1 | 0.002
D4j | 8285.1C | 1 | 0.002
D4j | 8572 | 1 | 0.002
D4j | 9007 | 1 | 0.002
D4j | 9128 | 1 | 0.002
D4j | 9389 | 1 | 0.002
D4j | 9612 | 1 | 0.002
D4j | 9909 | 1 | 0.002
D4j+(16286) | 10158A | 1 | 0.002
D4j+(16286) | 10724 | 1 | 0.002
D4j+(16286) | 10915 | 5 | 0.008
D4j+(16286) | 11422 | 1 | 0.002
D4j+(16286) | 11509 | 5 | 0.008
D4j+(16286) | 11617 | 5 | 0.008
D4j+(16286) | 11935 | 1 | 0.002
D4j+(16286) | 12501 | 5 | 0.008
D4j+(16286) | 12732 | 1 | 0.002
D4j+(16286) | 14393 | 1 | 0.002
D4j+(16286) | 1442 | 1 | 0.002
D4j+(16286) | 146 | 1 | 0.002
D4j+(16286) | 14693 | 5 | 0.008
D4j+(16286) | 153 | 1 | 0.002
D4j+(16286) | 16051 | 5 | 0.008
D4j+(16286) | 16075 | 1 | 0.002
D4j+(16286) | 16165 | 1 | 0.002
D4j+(16286) | 16172 | 1 | 0.002
D4j+(16286) | 16179 | 1 | 0.002
D4j+(16286) | 16192 | 2 | 0.003
D4j+(16286) | 16218 | 1 | 0.002
D4j+(16286) | 16265C | 2 | 0.003
D4j+(16286) | 16286 | 1 | 0.002
D4j+(16286) | 16288 | 7 | 0.011
D4j+(16286) | 16290 | 1 | 0.002
D4j+(16286) | 16294 | 1 | 0.002
D4j+(16286) | 16299 | 8 | 0.012
D4j+(16286) | 16327 | 1 | 0.002
D4j+(16286) | 341C | 1 | 0.002
D4j+(16286) | 3438 | 3 | 0.005
D4j+(16286) | 3510 | 2 | 0.003
D4j+(16286) | 4048 | 4 | 0.006
D4j+(16286) | 4315.1T | 1 | 0.002

D4j+(16286) | 446 | 1 | 0.002
D4j+(16286) | 5131 | 1 | 0.002
D4j+(16286) | 60 | 1 | 0.002
D4j+(16286) | 6216 | 1 | 0.002
D4j+(16286) | 6261 | 1 | 0.002
D4j+(16286) | 6340 | 2 | 0.003
D4j+(16286) | 648 | 1 | 0.002
D4j+(16286) | 7501A | 1 | 0.002
D4j+(16286) | 8077 | 3 | 0.005
D4j+(16286) | 8277 | 1 | 0.002
D4j+(16286) | 8278.4C | 1 | 0.002
D4j+(16286) | 8572 | 1 | 0.002
D4j+(16286) | 9007 | 1 | 0.002
D4j+(16286) | 9128 | 1 | 0.002
D4j+(16286) | 9612 | 1 | 0.002
D4j+(16286) | 9909 | 1 | 0.002
D4j+146 | 10361 | 2 | 0.004
D4j+146 | 11239 | 3 | 0.006
D4j+146 | 11377 | 2 | 0.004
D4j+146 | 11878 | 2 | 0.004
D4j+146 | 13020 | 3 | 0.006
D4j+146 | 13477 | 3 | 0.006
D4j+146 | 14989 | 2 | 0.004
D4j+146 | 150 | 1 | 0.002
D4j+146 | 151 | 1 | 0.002
D4j+146 | 153 | 1 | 0.002
D4j+146 | 15529A | 5 | 0.011
D4j+146 | 16093 | 1 | 0.002
D4j+146 | 16108 | 1 | 0.002
D4j+146 | 16111A | 1 | 0.002
D4j+146 | 16140 | 1 | 0.002
D4j+146 | 16188 | 22 | 0.048
D4j+146 | 16192 | 1 | 0.002
D4j+146 | 16209 | 2 | 0.004
D4j+146 | 16214 | 22 | 0.048
D4j+146 | 16218 | 1 | 0.002
D4j+146 | 16225 | 1 | 0.002
D4j+146 | 16249 | 1 | 0.002
D4j+146 | 16250A | 1 | 0.002
D4j+146 | 16261 | 1 | 0.002
D4j+146 | 16269 | 1 | 0.002
D4j+146 | 16290 | 1 | 0.002
D4j+146 | 16293 | 2 | 0.004
D4j+146 | 16318C | 1 | 0.002
D4j+146 | 16356 | 17 | 0.037
D4j+146 | 16365 | 1 | 0.002
D4j+146 | 183 | 1 | 0.002
D4j+146 | 185 | 3 | 0.006
D4j+146 | 296 | 1 | 0.002
D4j+146 | 371 | 2 | 0.004
D4j+146 | 3746 | 3 | 0.006
D4j+146 | 383 | 1 | 0.002
D4j+146 | 4315.1T | 1 | 0.002
D4j+146 | 6125 | 1 | 0.002
D4j+146 | 6176 | 2 | 0.004
D4j+146 | 7912 | 1 | 0.002
D4j+16311 | 12580 | 1 | 0.005
D4j+16311 | 13105 | 2 | 0.009
D4j+16311 | 13377 | 1 | 0.005
D4j+16311 | 146 | 7 | 0.033
D4j+16311 | 14883 | 2 | 0.009
D4j+16311 | 1494 | 2 | 0.009
D4j+16311 | 150 | 4 | 0.019
D4j+16311 | 15130 | 2 | 0.009
D4j+16311 | 15672 | 6 | 0.028
D4j+16311 | 16051 | 1 | 0.005
D4j+16311 | 16066 | 1 | 0.005
D4j+16311 | 16092 | 2 | 0.009
D4j+16311 | 16093 | 1 | 0.005
D4j+16311 | 16129 | 1 | 0.005
D4j+16311 | 16158 | 1 | 0.005
D4j+16311 | 16171 | 25 | 0.117
D4j+16311 | 16172 | 1 | 0.005
D4j+16311 | 16185 | 1 | 0.005
D4j+16311 | 16224 | 1 | 0.005
D4j+16311 | 16234 | 2 | 0.009
D4j+16311 | 16243 | 1 | 0.005
D4j+16311 | 16248 | 1 | 0.005
D4j+16311 | 16260 | 4 | 0.019
D4j+16311 | 16291 | 3 | 0.014
D4j+16311 | 16292 | 4 | 0.019
D4j+16311 | 16381 | 1 | 0.005
D4j+16311 | 16390T | 1 | 0.005
D4j+16311 | 16453 | 1 | 0.005
D4j+16311 | 16497 | 1 | 0.005
D4j+16311 | 16526T | 1 | 0.005
D4j+16311 | 189 | 1 | 0.005
D4j+16311 | 194 | 3 | 0.014
D4j+16311 | 196 | 1 | 0.005
D4j+16311 | 200 | 1 | 0.005
D4j+16311 | 215 | 3 | 0.014
D4j+16311 | 243 | 1 | 0.005
D4j+16311 | 298 | 1 | 0.005
D4j+16311 | 3316 | 2 | 0.009
D4j+16311 | 3606 | 2 | 0.009
D4j+16311 | 3714 | 1 | 0.005
D4j+16311 | 374 | 1 | 0.005
D4j+16311 | 3744 | 1 | 0.005
D4j+16311 | 385 | 1 | 0.005
D4j+16311 | 4107 | 2 | 0.009
D4j+16311 | 4316 | 1 | 0.005
D4j+16311 | 458A | 1 | 0.005
D4j+16311 | 4659T | 1 | 0.005
D4j+16311 | 5021 | 1 | 0.005
D4j+16311 | 573.1C | 4 | 0.019
D4j+16311 | 574C | 1 | 0.005
D4j+16311 | 593 | 1 | 0.005
D4j+16311 | 618G | 1 | 0.005
D4j+16311 | 63d | 1 | 0.005
D4j+16311 | 66T | 1 | 0.005
D4j+16311 | 67 | 1 | 0.005

D4j+16311 | 676 | 1 | 0.005
D4j+16311 | 683 | 1 | 0.005
D4j+16311 | 7687 | 1 | 0.005
D4j+16311 | 8538 | 1 | 0.005
D4j+16311 | 94 | 1 | 0.005
D4j+16311 | 9621 | 1 | 0.005
D4j+16311 | 9896 | 2 | 0.009
D4j1 | 10005 | 2 | 0.003
D4j1 | 11404 | 1 | 0.002
D4j1 | 14130 | 1 | 0.002
D4j1 | 14305 | 1 | 0.002
D4j1 | 14769 | 1 | 0.002
D4j1 | 150A | 4 | 0.006
D4j1 | 152 | 1 | 0.002
D4j1 | 15314 | 4 | 0.006
D4j1 | 16114 | 6 | 0.01
D4j1 | 16165 | 1 | 0.002
D4j1 | 16167 | 1 | 0.002
D4j1 | 16179 | 1 | 0.002
D4j1 | 16218 | 1 | 0.002
D4j1 | 16260 | 2 | 0.003
D4j1 | 16335 | 4 | 0.006
D4j1 | 16526 | 4 | 0.006
D4j1 | 310 | 1 | 0.002
D4j1 | 4263 | 2 | 0.003
D4j1 | 446 | 1 | 0.002
D4j1 | 5196 | 4 | 0.006
D4j1 | 5250 | 4 | 0.006
D4j1 | 64 | 2 | 0.003
D4j1 | 648 | 1 | 0.002
D4j1 | 7058 | 1 | 0.002
D4j1 | 789 | 5 | 0.008
D4j1 | 8281-8289d | 1 | 0.002
D4j1 | 8904 | 1 | 0.002
D4j1 | 8950 | 1 | 0.002
D4j1 | 9948 | 1 | 0.002
D4j10 | 12957 | 3 | 0.005
D4j10 | 150A | 4 | 0.006
D4j10 | 16114 | 6 | 0.01
D4j10 | 16165 | 1 | 0.002
D4j10 | 16167 | 1 | 0.002
D4j10 | 16179 | 1 | 0.002
D4j10 | 16218 | 1 | 0.002
D4j10 | 16335 | 4 | 0.006
D4j10 | 310 | 1 | 0.002
D4j10 | 446 | 1 | 0.002
D4j10 | 64 | 2 | 0.003
D4j10 | 648 | 1 | 0.002
D4j10 | 7677 | 1 | 0.002
D4j10 | 789 | 5 | 0.008
D4j10 | 9996 | 1 | 0.002
D4j11 | 13356 | 1 | 0.005
D4j11 | 14905 | 2 | 0.01
D4j11 | 16051 | 1 | 0.005
D4j11 | 16066 | 1 | 0.005
D4j11 | 16075 | 1 | 0.005
D4j11 | 16077 | 1 | 0.005
D4j11 | 16185 | 1 | 0.005
D4j11 | 16224 | 1 | 0.005
D4j11 | 16248 | 1 | 0.005
D4j11 | 16256 | 3 | 0.016
D4j11 | 16293 | 1 | 0.005
D4j11 | 16398 | 9 | 0.047
D4j11 | 16446 | 1 | 0.005
D4j11 | 16526T | 1 | 0.005
D4j11 | 189 | 1 | 0.005
D4j11 | 243 | 1 | 0.005
D4j11 | 298 | 1 | 0.005
D4j11 | 328T | 1 | 0.005
D4j11 | 333A | 1 | 0.005
D4j11 | 374 | 1 | 0.005
D4j11 | 417 | 2 | 0.01
D4j11 | 425T | 1 | 0.005
D4j11 | 449 | 1 | 0.005
D4j11 | 4916 | 1 | 0.005
D4j11 | 573.1C | 2 | 0.01
D4j11 | 574C | 1 | 0.005
D4j11 | 618G | 1 | 0.005
D4j11 | 676 | 1 | 0.005
D4j11 | 683 | 1 | 0.005
D4j12 | 13708 | 1 | 0.002
D4j12 | 150A | 4 | 0.006
D4j12 | 15272 | 2 | 0.003
D4j12 | 16114 | 6 | 0.01
D4j12 | 16165 | 1 | 0.002
D4j12 | 16167 | 1 | 0.002
D4j12 | 16179 | 1 | 0.002
D4j12 | 16218 | 1 | 0.002
D4j12 | 16335 | 4 | 0.006
D4j12 | 446 | 1 | 0.002
D4j12 | 5899.1C | 1 | 0.002
D4j12 | 5973 | 1 | 0.002
D4j12 | 64 | 2 | 0.003
D4j12 | 648 | 1 | 0.002
D4j12 | 6821 | 1 | 0.002
D4j12 | 7073 | 1 | 0.002
D4j12 | 7119 | 1 | 0.002
D4j12 | 7681 | 6 | 0.01
D4j12 | 789 | 5 | 0.008
D4j13 | 10658 | 1 | 0.002
D4j13 | 14582 | 1 | 0.002
D4j13 | 16108 | 1 | 0.002
D4j13 | 16111A | 1 | 0.002
D4j13 | 16172 | 1 | 0.002
D4j13 | 16176 | 1 | 0.002
D4j13 | 16243 | 3 | 0.007
D4j13 | 16250A | 1 | 0.002
D4j13 | 16290 | 1 | 0.002
D4j13 | 16318C | 1 | 0.002
D4j13 | 183 | 1 | 0.002
D4j13 | 383 | 1 | 0.002

D4j13 | 44.1C | 1 | 0.002
D4j13 | 9269 | 1 | 0.002
D4j14 | 16066C | 1 | 0.014
D4j14 | 16129 | 1 | 0.014
D4j14 | 16138C | 1 | 0.014
D4j14 | 16203C | 1 | 0.014
D4j14 | 16356 | 1 | 0.014
D4j14 | 16513G | 1 | 0.014
D4j14 | 16551 | 1 | 0.014
D4j14 | 195 | 1 | 0.014
D4j14 | 207 | 1 | 0.014
D4j14 | 542 | 1 | 0.014
D4j14 | 6392 | 1 | 0.014
D4j15 | 15079 | 1 | 0.002
D4j15 | 150A | 4 | 0.006
D4j15 | 16114 | 6 | 0.01
D4j15 | 16165 | 1 | 0.002
D4j15 | 16167 | 1 | 0.002
D4j15 | 16179 | 1 | 0.002
D4j15 | 16218 | 1 | 0.002
D4j15 | 16274 | 1 | 0.002
D4j15 | 16293 | 1 | 0.002
D4j15 | 16335 | 4 | 0.006
D4j15 | 185 | 1 | 0.002
D4j15 | 195 | 1 | 0.002
D4j15 | 446 | 1 | 0.002
D4j15 | 64 | 2 | 0.003
D4j15 | 648 | 1 | 0.002
D4j15 | 6842 | 1 | 0.002
D4j15 | 789 | 5 | 0.008
D4j16 | 13759 | 1 | 0.002
D4j16 | 146 | 1 | 0.002
D4j16 | 150A | 4 | 0.006
D4j16 | 15851 | 1 | 0.002
D4j16 | 16114 | 6 | 0.01
D4j16 | 16165 | 1 | 0.002
D4j16 | 16167 | 1 | 0.002
D4j16 | 16179 | 1 | 0.002
D4j16 | 16218 | 1 | 0.002
D4j16 | 16325 | 1 | 0.002
D4j16 | 16335 | 4 | 0.006
D4j16 | 446 | 1 | 0.002
D4j16 | 64 | 2 | 0.003
D4j16 | 648 | 1 | 0.002
D4j16 | 789 | 5 | 0.008
D4j1a | 16069 | 1 | 0.005
D4j1a | 16111 | 1 | 0.005
D4j1a | 16129 | 5 | 0.026
D4j1a | 16148 | 1 | 0.005
D4j1a | 16168 | 1 | 0.005
D4j1a | 16184 | 1 | 0.005
D4j1a | 16184A | 1 | 0.005
D4j1a | 16189 | 6 | 0.032
D4j1a | 16192 | 1 | 0.005
D4j1a | 16234 | 2 | 0.011
D4j1a | 16289 | 1 | 0.005
D4j1a | 16301 | 1 | 0.005
D4j1a | 16304 | 1 | 0.005
D4j1a | 16316 | 3 | 0.016
D4j1a | 16327 | 1 | 0.005
D4j1a | 16360 | 1 | 0.005
D4j1a | 16361 | 1 | 0.005
D4j1a | 16384 | 1 | 0.005
D4j1a | 16399 | 24 | 0.127
D4j1a | 195 | 1 | 0.005
D4j1a | 227 | 1 | 0.005
D4j1a | 430 | 1 | 0.005
D4j1a | 94 | 1 | 0.005
D4j1a1 | 11611 | 1 | 0.004
D4j1a1 | 12557 | 1 | 0.004
D4j1a1 | 12717 | 1 | 0.004
D4j1a1 | 13830 | 2 | 0.009
D4j1a1 | 14587 | 8 | 0.034
D4j1a1 | 152 | 4 | 0.017
D4j1a1 | 16051 | 2 | 0.009
D4j1a1 | 16104 | 1 | 0.004
D4j1a1 | 16150 | 8 | 0.034
D4j1a1 | 16187 | 1 | 0.004
D4j1a1 | 16188 | 1 | 0.004
D4j1a1 | 16218 | 2 | 0.009
D4j1a1 | 16270 | 1 | 0.004
D4j1a1 | 16274 | 56 | 0.239
D4j1a1 | 16291 | 5 | 0.021
D4j1a1 | 16311 | 9 | 0.038
D4j1a1 | 1888 | 1 | 0.004
D4j1a1 | 199 | 1 | 0.004
D4j1a1 | 200 | 2 | 0.009
D4j1a1 | 204 | 1 | 0.004
D4j1a1 | 259 | 1 | 0.004
D4j1a1 | 2857 | 1 | 0.004
D4j1a1 | 310 | 1 | 0.004
D4j1a1 | 315.2C | 1 | 0.004
D4j1a1 | 3203 | 1 | 0.004
D4j1a1 | 338 | 1 | 0.004
D4j1a1 | 3397 | 1 | 0.004
D4j1a1 | 3918 | 2 | 0.009
D4j1a1 | 4135 | 1 | 0.004
D4j1a1 | 4435 | 4 | 0.017
D4j1a1 | 4898 | 1 | 0.004
D4j1a1 | 55 | 1 | 0.004
D4j1a1 | 5563 | 1 | 0.004
D4j1a1 | 56 | 1 | 0.004
D4j1a1 | 5628 | 1 | 0.004
D4j1a1 | 574C | 1 | 0.004
D4j1a1 | 6293 | 4 | 0.017
D4j1a1 | 6722 | 1 | 0.004
D4j1a1 | 7419 | 1 | 0.004
D4j1a1 | 8671 | 10 | 0.043
D4j1a1 | 8711 | 1 | 0.004
D4j1a1 | 8877 | 1 | 0.004
D4j1a1 | 8896 | 1 | 0.004

D4j1a1 | 9053 | 3 | 0.013
D4j1a1a | 10589 | 1 | 0.005
D4j1a1a | 14569 | 1 | 0.005
D4j1a1a | 16069 | 1 | 0.005
D4j1a1a | 16111 | 1 | 0.005
D4j1a1a | 16148 | 1 | 0.005
D4j1a1a | 16168 | 1 | 0.005
D4j1a1a | 16184 | 1 | 0.005
D4j1a1a | 16188 | 2 | 0.011
D4j1a1a | 16192 | 1 | 0.005
D4j1a1a | 16234 | 2 | 0.011
D4j1a1a | 16289 | 1 | 0.005
D4j1a1a | 16301 | 1 | 0.005
D4j1a1a | 16304 | 1 | 0.005
D4j1a1a | 16316 | 3 | 0.016
D4j1a1a | 16327 | 1 | 0.005
D4j1a1a | 16360 | 1 | 0.005
D4j1a1a | 16361 | 1 | 0.005
D4j1a1a | 16384 | 1 | 0.005
D4j1a1a | 16399 | 24 | 0.126
D4j1a1a | 195 | 1 | 0.005
D4j1a1a | 199 | 1 | 0.005
D4j1a1a | 2281 | 8 | 0.042
D4j1a1a | 234 | 4 | 0.021
D4j1a1a | 430 | 1 | 0.005
D4j1a1a | 5237 | 2 | 0.011
D4j1a1a | 94 | 1 | 0.005
D4j1a1b | 14189.1A | 1 | 0.005
D4j1a1b | 15839 | 6 | 0.032
D4j1a1b | 16069 | 1 | 0.005
D4j1a1b | 16111 | 1 | 0.005
D4j1a1b | 16148 | 1 | 0.005
D4j1a1b | 16168 | 1 | 0.005
D4j1a1b | 16184 | 1 | 0.005
D4j1a1b | 16192 | 1 | 0.005
D4j1a1b | 16234 | 2 | 0.011
D4j1a1b | 16289 | 1 | 0.005
D4j1a1b | 16301 | 1 | 0.005
D4j1a1b | 16304 | 1 | 0.005
D4j1a1b | 16316 | 3 | 0.016
D4j1a1b | 16319 | 9 | 0.048
D4j1a1b | 16327 | 1 | 0.005
D4j1a1b | 16360 | 1 | 0.005
D4j1a1b | 16361 | 1 | 0.005
D4j1a1b | 16384 | 1 | 0.005
D4j1a1b | 16399 | 24 | 0.127
D4j1a1b | 195 | 1 | 0.005
D4j1a1b | 430 | 1 | 0.005
D4j1a1b | 8774 | 2 | 0.011
D4j1a1b | 94 | 1 | 0.005
D4j1a2 | 14088 | 1 | 0.01
D4j1a2 | 16111 | 2 | 0.02
D4j1a2 | 16172 | 1 | 0.01
D4j1a2 | 16188 | 2 | 0.02
D4j1a2 | 16189 | 1 | 0.01
D4j1a2 | 16192 | 1 | 0.01
D4j1a2 | 16239 | 1 | 0.01
D4j1a2 | 16264 | 1 | 0.01
D4j1a2 | 16266 | 2 | 0.02
D4j1a2 | 16316 | 1 | 0.01
D4j1a2 | 184 | 1 | 0.01
D4j1a2 | 376 | 1 | 0.01
D4j1a2 | 7702 | 1 | 0.01
D4j1b | 11009 | 2 | 0.003
D4j1b | 11347 | 1 | 0.002
D4j1b | 11749 | 1 | 0.002
D4j1b | 12618 | 1 | 0.002
D4j1b | 12623G | 1 | 0.002
D4j1b | 12633 | 1 | 0.002
D4j1b | 12749A | 1 | 0.002
D4j1b | 13469A | 1 | 0.002
D4j1b | 13543G | 1 | 0.002
D4j1b | 13648A | 1 | 0.002
D4j1b | 13708 | 2 | 0.003
D4j1b | 14097 | 2 | 0.003
D4j1b | 14696 | 2 | 0.003
D4j1b | 150A | 4 | 0.006
D4j1b | 15237 | 2 | 0.003
D4j1b | 15380 | 2 | 0.003
D4j1b | 15924 | 2 | 0.003
D4j1b | 15930 | 2 | 0.003
D4j1b | 16000 | 1 | 0.002
D4j1b | 16093 | 1 | 0.002
D4j1b | 16114 | 6 | 0.009
D4j1b | 16124 | 1 | 0.002
D4j1b | 16165 | 1 | 0.002
D4j1b | 16167 | 1 | 0.002
D4j1b | 16172 | 2 | 0.003
D4j1b | 16179 | 1 | 0.002
D4j1b | 16218 | 1 | 0.002
D4j1b | 16261 | 2 | 0.003
D4j1b | 16311 | 3 | 0.005
D4j1b | 16319 | 2 | 0.003
D4j1b | 16320 | 1 | 0.002
D4j1b | 16335 | 4 | 0.006
D4j1b | 200 | 1 | 0.002
D4j1b | 2080 | 1 | 0.002
D4j1b | 446 | 1 | 0.002
D4j1b | 4639 | 3 | 0.005
D4j1b | 4775 | 1 | 0.002
D4j1b | 636 | 1 | 0.002
D4j1b | 64 | 2 | 0.003
D4j1b | 648 | 1 | 0.002
D4j1b | 6722 | 1 | 0.002
D4j1b | 789 | 5 | 0.008
D4j1b | 8292 | 2 | 0.003
D4j1b | 8296 | 1 | 0.002
D4j1b | 9096 | 1 | 0.002
D4j1b | 9125 | 3 | 0.005
D4j1b | 9145 | 1 | 0.002
D4j1b | 9428 | 1 | 0.002

D4j1b2 | 16076d | 7 | 0.016
D4j1b2 | 16093 | 1 | 0.002
D4j1b2 | 16104 | 1 | 0.002
D4j1b2 | 16108 | 1 | 0.002
D4j1b2 | 16111A | 1 | 0.002
D4j1b2 | 16172 | 2 | 0.005
D4j1b2 | 16192 | 1 | 0.002
D4j1b2 | 16224 | 2 | 0.005
D4j1b2 | 16250A | 1 | 0.002
D4j1b2 | 16278 | 1 | 0.002
D4j1b2 | 16316 | 1 | 0.002
D4j1b2 | 16318C | 1 | 0.002
D4j1b2 | 184 | 1 | 0.002
D4j1b2 | 189 | 1 | 0.002
D4j1b2 | 326C | 1 | 0.002
D4j1b2 | 331 | 1 | 0.002
D4j1b2 | 333G | 1 | 0.002
D4j1b2 | 60 | 1 | 0.002
D4j1b2 | 729d | 1 | 0.002
D4j1b2 | 735.1TA | 1 | 0.002
D4j1b2 | 736.1A | 1 | 0.002
D4j1b2 | 739.1A | 1 | 0.002
D4j1b2 | 742G | 1 | 0.002
D4j1b2 | 743d | 1 | 0.002
D4j1b2 | 746 | 1 | 0.002
D4j1b2 | 747 | 2 | 0.005
D4j1b2 | 751 | 1 | 0.002
D4j1b2 | 752G | 1 | 0.002
D4j1b2 | 754 | 1 | 0.002
D4j1b2 | 9682 | 1 | 0.002
D4j2 | 150 | 1 | 0.013
D4j2 | 16032 | 1 | 0.013
D4j2 | 16046.1C | 1 | 0.013
D4j2 | 16051 | 2 | 0.025
D4j2 | 16064.1A | 1 | 0.013
D4j2 | 16069 | 1 | 0.013
D4j2 | 16129 | 1 | 0.013
D4j2 | 16179 | 1 | 0.013
D4j2 | 16209 | 2 | 0.025
D4j2 | 16224 | 1 | 0.013
D4j2 | 16234 | 1 | 0.013
D4j2 | 16249 | 21 | 0.266
D4j2 | 16256 | 2 | 0.025
D4j2 | 16259 | 23 | 0.291
D4j2 | 16261 | 1 | 0.013
D4j2 | 16269 | 1 | 0.013
D4j2 | 16271 | 1 | 0.013
D4j2 | 16274 | 1 | 0.013
D4j2 | 16278 | 23 | 0.291
D4j2 | 16300T | 1 | 0.013
D4j2 | 16304A | 1 | 0.013
D4j2 | 16316 | 3 | 0.038
D4j2 | 16317T | 1 | 0.013
D4j2 | 16318 | 1 | 0.013
D4j2 | 16325 | 2 | 0.025
D4j2 | 16355 | 1 | 0.013
D4j2 | 16356 | 2 | 0.025
D4j2 | 16357 | 1 | 0.013
D4j2 | 16361T | 1 | 0.013
D4j2 | 16390 | 1 | 0.013
D4j2 | 16442 | 1 | 0.013
D4j2 | 16462 | 2 | 0.025
D4j2 | 16463 | 1 | 0.013
D4j2 | 194 | 1 | 0.013
D4j2 | 249d | 3 | 0.038
D4j2 | 574C | 1 | 0.013
D4j2 | 576C | 1 | 0.013
D4j2 | 789 | 1 | 0.013
D4j2a | 10804 | 1 | 0.022
D4j2a | 11908 | 1 | 0.022
D4j2a | 151 | 1 | 0.022
D4j2a | 15530 | 1 | 0.022
D4j2a | 16192 | 1 | 0.022
D4j2a | 16311 | 3 | 0.065
D4j2a | 709 | 1 | 0.022
D4j3 | 10601 | 1 | 0.004
D4j3 | 11611 | 2 | 0.007
D4j3 | 12A | 1 | 0.004
D4j3 | 13632 | 1 | 0.004
D4j3 | 13711 | 2 | 0.007
D4j3 | 13967 | 2 | 0.007
D4j3 | 143 | 2 | 0.007
D4j3 | 146 | 2 | 0.007
D4j3 | 151 | 3 | 0.011
D4j3 | 152 | 1 | 0.004
D4j3 | 15300 | 2 | 0.007
D4j3 | 15734 | 1 | 0.004
D4j3 | 16027 | 1 | 0.004
D4j3 | 16044 | 1 | 0.004
D4j3 | 16045 | 3 | 0.011
D4j3 | 16045.1C | 1 | 0.004
D4j3 | 16057d | 1 | 0.004
D4j3 | 16093 | 7 | 0.026
D4j3 | 16098 | 1 | 0.004
D4j3 | 16101 | 1 | 0.004
D4j3 | 16129 | 9 | 0.033
D4j3 | 16157 | 2 | 0.007
D4j3 | 16164 | 1 | 0.004
D4j3 | 16166 | 1 | 0.004
D4j3 | 16168 | 1 | 0.004
D4j3 | 16172 | 6 | 0.022
D4j3 | 16177T | 1 | 0.004
D4j3 | 16182 | 1 | 0.004
D4j3 | 16188 | 1 | 0.004
D4j3 | 16189 | 3 | 0.011
D4j3 | 16192 | 1 | 0.004
D4j3 | 16202 | 1 | 0.004
D4j3 | 16213 | 24 | 0.089
D4j3 | 16233 | 1 | 0.004
D4j3 | 16234 | 1 | 0.004
D4j3 | 16237 | 1 | 0.004

D4j3 | 16255 | 5 | 0.018
D4j3 | 16260 | 1 | 0.004
D4j3 | 16266 | 4 | 0.015
D4j3 | 16274 | 6 | 0.022
D4j3 | 16288 | 1 | 0.004
D4j3 | 16290 | 1 | 0.004
D4j3 | 16292 | 1 | 0.004
D4j3 | 16293 | 2 | 0.007
D4j3 | 16294 | 2 | 0.007
D4j3 | 16295A | 1 | 0.004
D4j3 | 16296 | 1 | 0.004
D4j3 | 16312 | 1 | 0.004
D4j3 | 16335 | 1 | 0.004
D4j3 | 16338 | 1 | 0.004
D4j3 | 16346 | 1 | 0.004
D4j3 | 16348 | 1 | 0.004
D4j3 | 16352 | 8 | 0.03
D4j3 | 16374 | 1 | 0.004
D4j3 | 16386 | 1 | 0.004
D4j3 | 16390 | 3 | 0.011
D4j3 | 16400 | 1 | 0.004
D4j3 | 16424 | 1 | 0.004
D4j3 | 16454 | 1 | 0.004
D4j3 | 16471 | 1 | 0.004
D4j3 | 16486 | 1 | 0.004
D4j3 | 16526 | 1 | 0.004
D4j3 | 16546 | 1 | 0.004
D4j3 | 16559 | 1 | 0.004
D4j3 | 172 | 1 | 0.004
D4j3 | 194 | 2 | 0.007
D4j3 | 199 | 2 | 0.007
D4j3 | 200 | 1 | 0.004
D4j3 | 212 | 1 | 0.004
D4j3 | 224A | 1 | 0.004
D4j3 | 246 | 1 | 0.004
D4j3 | 247T | 1 | 0.004
D4j3 | 266 | 1 | 0.004
D4j3 | 284 | 1 | 0.004
D4j3 | 288T | 1 | 0.004
D4j3 | 310 | 1 | 0.004
D4j3 | 315.2C | 1 | 0.004
D4j3 | 316C | 3 | 0.011
D4j3 | 325A | 1 | 0.004
D4j3 | 338 | 10 | 0.037
D4j3 | 3753 | 1 | 0.004
D4j3 | 376 | 1 | 0.004
D4j3 | 401 | 1 | 0.004
D4j3 | 441A | 1 | 0.004
D4j3 | 4466 | 1 | 0.004
D4j3 | 46 | 2 | 0.007
D4j3 | 515 | 1 | 0.004
D4j3 | 550 | 1 | 0.004
D4j3 | 5578 | 1 | 0.004
D4j3 | 5580 | 1 | 0.004
D4j3 | 564 | 1 | 0.004
D4j3 | 569 | 1 | 0.004
D4j3 | 593 | 1 | 0.004
D4j3 | 722 | 1 | 0.004
D4j3 | 7761 | 1 | 0.004
D4j3 | 7805 | 2 | 0.007
D4j3 | 87 | 1 | 0.004
D4j3 | 89 | 1 | 0.004
D4j3 | 9055 | 2 | 0.007
D4j3 | 9192 | 2 | 0.007
D4j3 | 990 | 2 | 0.007
D4j3 | 9983 | 1 | 0.004
D4j3a | 12A | 1 | 0.004
D4j3a | 151 | 3 | 0.012
D4j3a | 152 | 8 | 0.032
D4j3a | 158 | 1 | 0.004
D4j3a | 16027 | 1 | 0.004
D4j3a | 16043C | 1 | 0.004
D4j3a | 16044 | 1 | 0.004
D4j3a | 16045 | 4 | 0.016
D4j3a | 16045.1C | 1 | 0.004
D4j3a | 16057d | 1 | 0.004
D4j3a | 16078T | 1 | 0.004
D4j3a | 16080 | 1 | 0.004
D4j3a | 16093 | 7 | 0.028
D4j3a | 16101 | 1 | 0.004
D4j3a | 16112 | 1 | 0.004
D4j3a | 16114A | 1 | 0.004
D4j3a | 16118 | 1 | 0.004
D4j3a | 16129 | 1 | 0.004
D4j3a | 16131 | 1 | 0.004
D4j3a | 16150 | 1 | 0.004
D4j3a | 16152A | 1 | 0.004
D4j3a | 16166 | 1 | 0.004
D4j3a | 16168 | 1 | 0.004
D4j3a | 16177T | 1 | 0.004
D4j3a | 16182 | 1 | 0.004
D4j3a | 16188 | 1 | 0.004
D4j3a | 16189 | 1 | 0.004
D4j3a | 16192 | 2 | 0.008
D4j3a | 16199d | 1 | 0.004
D4j3a | 16202 | 1 | 0.004
D4j3a | 16233 | 1 | 0.004
D4j3a | 16237 | 1 | 0.004
D4j3a | 16246T | 1 | 0.004
D4j3a | 16256 | 2 | 0.008
D4j3a | 16264 | 24 | 0.095
D4j3a | 16288 | 1 | 0.004
D4j3a | 16294 | 3 | 0.012
D4j3a | 16295A | 1 | 0.004
D4j3a | 16296 | 1 | 0.004
D4j3a | 16312 | 1 | 0.004
D4j3a | 16338 | 1 | 0.004
D4j3a | 16346 | 1 | 0.004
D4j3a | 16348 | 1 | 0.004
D4j3a | 16351 | 1 | 0.004
D4j3a | 16360 | 2 | 0.008

D4j3a | 16374 | 1 | 0.004
D4j3a | 16386 | 1 | 0.004
D4j3a | 16397 | 1 | 0.004
D4j3a | 16399 | 2 | 0.008
D4j3a | 16424 | 1 | 0.004
D4j3a | 16454 | 1 | 0.004
D4j3a | 16471 | 1 | 0.004
D4j3a | 16476 | 1 | 0.004
D4j3a | 16486 | 1 | 0.004
D4j3a | 16510 | 1 | 0.004
D4j3a | 16542 | 1 | 0.004
D4j3a | 16546 | 1 | 0.004
D4j3a | 16559 | 1 | 0.004
D4j3a | 16564 | 1 | 0.004
D4j3a | 172 | 1 | 0.004
D4j3a | 195 | 3 | 0.012
D4j3a | 206 | 1 | 0.004
D4j3a | 207 | 1 | 0.004
D4j3a | 212 | 1 | 0.004
D4j3a | 224A | 1 | 0.004
D4j3a | 226 | 1 | 0.004
D4j3a | 237 | 1 | 0.004
D4j3a | 241 | 1 | 0.004
D4j3a | 246 | 1 | 0.004
D4j3a | 247T | 1 | 0.004
D4j3a | 282 | 1 | 0.004
D4j3a | 288T | 1 | 0.004
D4j3a | 309 | 2 | 0.008
D4j3a | 310.1T | 1 | 0.004
D4j3a | 316C | 1 | 0.004
D4j3a | 325A | 1 | 0.004
D4j3a | 338 | 1 | 0.004
D4j3a | 368 | 1 | 0.004
D4j3a | 37 | 2 | 0.008
D4j3a | 374 | 1 | 0.004
D4j3a | 376 | 1 | 0.004
D4j3a | 387 | 1 | 0.004
D4j3a | 390 | 1 | 0.004
D4j3a | 401 | 1 | 0.004
D4j3a | 417 | 3 | 0.012
D4j3a | 4500 | 3 | 0.012
D4j3a | 512 | 1 | 0.004
D4j3a | 515 | 1 | 0.004
D4j3a | 564 | 1 | 0.004
D4j3a | 568 | 1 | 0.004
D4j3a | 569 | 1 | 0.004
D4j3a | 722 | 1 | 0.004
D4j3a | 7220 | 1 | 0.004
D4j3a | 78 | 1 | 0.004
D4j3a | 8149 | 1 | 0.004
D4j3a | 8601 | 2 | 0.008
D4j3a | 87 | 1 | 0.004
D4j3a | 8702 | 3 | 0.012
D4j3a | 89 | 1 | 0.004
D4j3a | 94 | 24 | 0.095
D4j3a1 | 13410 | 1 | 0.008
D4j3a1 | 152 | 1 | 0.008
D4j3a1 | 16086 | 2 | 0.015
D4j3a1 | 16092 | 1 | 0.008
D4j3a1 | 16111 | 1 | 0.008
D4j3a1 | 16181 | 3 | 0.023
D4j3a1 | 16203 | 1 | 0.008
D4j3a1 | 16218 | 1 | 0.008
D4j3a1 | 16269 | 1 | 0.008
D4j3a1 | 5824 | 1 | 0.008
D4j4 | 14470 | 3 | 0.005
D4j4 | 150A | 4 | 0.006
D4j4 | 16114 | 6 | 0.01
D4j4 | 16165 | 1 | 0.002
D4j4 | 16167 | 1 | 0.002
D4j4 | 16179 | 1 | 0.002
D4j4 | 16218 | 1 | 0.002
D4j4 | 16335 | 4 | 0.006
D4j4 | 310 | 1 | 0.002
D4j4 | 446 | 1 | 0.002
D4j4 | 64 | 2 | 0.003
D4j4 | 648 | 1 | 0.002
D4j4 | 789 | 5 | 0.008
D4j4a | 152 | 3 | 0.042
D4j4a | 16148 | 2 | 0.028
D4j4a | 16455 | 3 | 0.042
D4j4a | 199 | 3 | 0.042
D4j4a | 246A | 3 | 0.042
D4j4a | 253A | 3 | 0.042
D4j4a | 254G | 3 | 0.042
D4j4a | 343G | 3 | 0.042
D4j4a | 7861 | 1 | 0.014
D4j5 | 150A | 4 | 0.006
D4j5 | 15260C | 1 | 0.002
D4j5 | 16114 | 6 | 0.01
D4j5 | 16165 | 1 | 0.002
D4j5 | 16167 | 1 | 0.002
D4j5 | 16174 | 1 | 0.002
D4j5 | 16179 | 1 | 0.002
D4j5 | 16218 | 1 | 0.002
D4j5 | 16325 | 1 | 0.002
D4j5 | 16335 | 4 | 0.006
D4j5 | 2064 | 1 | 0.002
D4j5 | 446 | 1 | 0.002
D4j5 | 61A | 1 | 0.002
D4j5 | 62C | 1 | 0.002
D4j5 | 64 | 2 | 0.003
D4j5 | 648 | 1 | 0.002
D4j5 | 789 | 5 | 0.008
D4j5a | 12855 | 1 | 0.002
D4j5a | 146 | 2 | 0.003
D4j5a | 150A | 4 | 0.006
D4j5a | 15773 | 7 | 0.011
D4j5a | 1598 | 5 | 0.008
D4j5a | 16114 | 6 | 0.009
D4j5a | 16165 | 1 | 0.002

D4j5a | 16167 | 1 | 0.002
D4j5a | 16179 | 1 | 0.002
D4j5a | 16218 | 1 | 0.002
D4j5a | 16302 | 1 | 0.002
D4j5a | 16319 | 5 | 0.008
D4j5a | 16335 | 4 | 0.006
D4j5a | 310 | 4 | 0.006
D4j5a | 3540 | 1 | 0.002
D4j5a | 446 | 1 | 0.002
D4j5a | 5843 | 1 | 0.002
D4j5a | 64 | 2 | 0.003
D4j5a | 648 | 1 | 0.002
D4j5a | 7620 | 1 | 0.002
D4j5a | 789 | 5 | 0.008
D4j5a | 7986 | 1 | 0.002
D4j6 | 11023C | 1 | 0.002
D4j6 | 12106 | 1 | 0.002
D4j6 | 14218 | 1 | 0.002
D4j6 | 150 | 1 | 0.002
D4j6 | 16093 | 2 | 0.004
D4j6 | 16108 | 1 | 0.002
D4j6 | 16111A | 1 | 0.002
D4j6 | 16182 | 2 | 0.004
D4j6 | 16192 | 1 | 0.002
D4j6 | 16218 | 1 | 0.002
D4j6 | 16225 | 1 | 0.002
D4j6 | 16243 | 3 | 0.007
D4j6 | 16249 | 1 | 0.002
D4j6 | 16250A | 1 | 0.002
D4j6 | 16261 | 1 | 0.002
D4j6 | 16269 | 1 | 0.002
D4j6 | 16290 | 1 | 0.002
D4j6 | 16293 | 2 | 0.004
D4j6 | 16293T | 1 | 0.002
D4j6 | 16318C | 1 | 0.002
D4j6 | 16526 | 3 | 0.007
D4j6 | 183 | 1 | 0.002
D4j6 | 200 | 1 | 0.002
D4j6 | 214 | 1 | 0.002
D4j6 | 248 | 1 | 0.002
D4j6 | 316C | 2 | 0.004
D4j6 | 383 | 1 | 0.002
D4j6 | 6040 | 1 | 0.002
D4j7 | 12406 | 1 | 0.014
D4j7 | 16147A | 1 | 0.014
D4j7a | 16145 | 2 | 0.028
D4j7a | 16172 | 2 | 0.028
D4j7a | 16189 | 2 | 0.028
D4j7a | 16311 | 2 | 0.028
D4j7a | 16399 | 1 | 0.014
D4j7a | 194 | 2 | 0.028
D4j8 | 11061 | 3 | 0.028
D4j8 | 12609 | 1 | 0.009
D4j8 | 12795 | 2 | 0.019
D4j8 | 16111A | 1 | 0.009
D4j8 | 16129 | 3 | 0.028
D4j8 | 16136 | 1 | 0.009
D4j8 | 16259 | 1 | 0.009
D4j8 | 16274 | 3 | 0.028
D4j8 | 16355 | 3 | 0.028
D4j8 | 16399 | 4 | 0.037
D4j8 | 16566 | 1 | 0.009
D4j8 | 200 | 4 | 0.037
D4j8 | 217 | 1 | 0.009
D4j8 | 4204 | 1 | 0.009
D4j8 | 5656 | 1 | 0.009
D4j8 | 6869 | 2 | 0.019
D4j8 | 7205 | 4 | 0.037
D4j8 | 7386 | 1 | 0.009
D4j8 | 7859 | 1 | 0.009
D4j8 | 8619A | 1 | 0.009
D4j8 | 9548 | 1 | 0.009
D4j8 | 9661 | 1 | 0.009
D4j9 | 12186 | 1 | 0.002
D4j9 | 146 | 1 | 0.002
D4j9 | 15265 | 1 | 0.002
D4j9 | 16114 | 6 | 0.009
D4j9 | 16165 | 1 | 0.002
D4j9 | 16167 | 1 | 0.002
D4j9 | 16179 | 1 | 0.002
D4j9 | 16218 | 1 | 0.002
D4j9 | 16270 | 1 | 0.002
D4j9 | 16286 | 1 | 0.002
D4j9 | 16290 | 1 | 0.002
D4j9 | 16474 | 4 | 0.006
D4j9 | 1692 | 2 | 0.003
D4j9 | 1766G | 3 | 0.005
D4j9 | 1977 | 1 | 0.002
D4j9 | 2242 | 1 | 0.002
D4j9 | 446 | 1 | 0.002
D4j9 | 648 | 1 | 0.002
D4k | 131 | 1 | 0.006
D4k | 13380 | 1 | 0.006
D4k | 13965 | 1 | 0.006
D4k | 143 | 3 | 0.017
D4k | 150 | 1 | 0.006
D4k | 15110 | 1 | 0.006
D4k | 15236 | 3 | 0.017
D4k | 153 | 1 | 0.006
D4k | 15737 | 1 | 0.006
D4k | 16076d | 1 | 0.006
D4k | 16094 | 1 | 0.006
D4k | 16111A | 1 | 0.006
D4k | 16157 | 1 | 0.006
D4k | 16172 | 1 | 0.006
D4k | 16174 | 8 | 0.045
D4k | 16189 | 1 | 0.006
D4k | 16192 | 36 | 0.203
D4k | 16230 | 1 | 0.006
D4k | 16234 | 1 | 0.006
D4k | 16249 | 1 | 0.006

D4k | 16274 | 1 | 0.006
D4k | 16297 | 1 | 0.006
D4k | 16298 | 1 | 0.006
D4k | 16320 | 1 | 0.006
D4k | 16354 | 2 | 0.011
D4k | 16357 | 1 | 0.006
D4k | 16381 | 2 | 0.011
D4k | 1766 | 2 | 0.011
D4k | 185 | 3 | 0.017
D4k | 204 | 1 | 0.006
D4k | 315.2C | 1 | 0.006
D4k | 4086 | 1 | 0.006
D4k | 482 | 1 | 0.006
D4k | 4904 | 3 | 0.017
D4k | 4993 | 1 | 0.006
D4k | 6040 | 1 | 0.006
D4k | 709 | 1 | 0.006
D4k | 7964 | 1 | 0.006
D4k | 8905A | 1 | 0.006
D4k | 8937 | 1 | 0.006
D4k | 9407 | 3 | 0.017
D4k | 9449 | 1 | 0.006
D4l | 16036 | 1 | 0.013
D4l | 173 | 1 | 0.013
D4l | 204 | 1 | 0.013
D4l | 499 | 1 | 0.013
D4l | 593 | 1 | 0.013
D4l1 | 12786 | 1 | 0.012
D4l1 | 1308 | 1 | 0.012
D4l1 | 146 | 2 | 0.025
D4l1 | 15315 | 1 | 0.012
D4l1 | 16176G | 1 | 0.012
D4l1 | 16325 | 3 | 0.037
D4l1 | 185 | 2 | 0.025
D4l1 | 373 | 7 | 0.086
D4l1 | 3746 | 1 | 0.012
D4l1 | 3808 | 1 | 0.012
D4l1 | 4336 | 1 | 0.012
D4l1 | 5704 | 1 | 0.012
D4l1 | 93 | 7 | 0.086
D4l1a | 13830 | 1 | 0.015
D4l1a1 | 152 | 1 | 0.014
D4l1a1 | 16129 | 1 | 0.014
D4l1a1 | 16526 | 1 | 0.014
D4l2 | 310 | 3 | 0.115
D4l2 | 6260 | 1 | 0.038
D4l2a | 128 | 1 | 0.045
D4l2a | 150 | 2 | 0.091
D4l2a | 16166 | 2 | 0.091
D4l2a | 16213 | 1 | 0.045
D4l2a | 16325 | 2 | 0.091
D4l2a | 310 | 1 | 0.045
D4l2a1 | 195A | 1 | 0.111
D4l2a1 | 310 | 2 | 0.222
D4l2a1 | 4917 | 1 | 0.111
D4l2a1 | 8843 | 5 | 0.556
D4l2a2 | 15749 | 5 | 0.455
D4l2a2 | 310 | 5 | 0.455
D4l2a2 | 4682 | 1 | 0.091
D4l2b | 15090 | 1 | 0.05
D4l2b | 16092 | 1 | 0.05
D4l2b | 16129 | 1 | 0.05
D4l2b | 16209 | 2 | 0.1
D4l2b | 16241 | 2 | 0.1
D4l2b | 16304 | 1 | 0.05
D4l2b | 16399 | 1 | 0.05
D4l2b | 195 | 2 | 0.1
D4l2b | 6852 | 1 | 0.05
D4l2b | 73 | 1 | 0.05
D4l2b | 9725 | 1 | 0.05
D4m | 11782 | 3 | 0.005
D4m | 14305 | 3 | 0.005
D4m | 14484 | 1 | 0.002
D4m | 150A | 4 | 0.006
D4m | 15283 | 1 | 0.002
D4m | 16114 | 6 | 0.01
D4m | 16165 | 1 | 0.002
D4m | 16167 | 1 | 0.002
D4m | 16179 | 1 | 0.002
D4m | 16218 | 1 | 0.002
D4m | 16335 | 4 | 0.006
D4m | 3492 | 3 | 0.005
D4m | 446 | 1 | 0.002
D4m | 499 | 9 | 0.014
D4m | 64 | 2 | 0.003
D4m | 648 | 1 | 0.002
D4m | 789 | 5 | 0.008
D4m | 8089 | 3 | 0.005
D4m | 8730 | 3 | 0.005
D4m | 8978 | 3 | 0.005
D4m1 | 1118 | 1 | 0.014
D4m1 | 16129 | 3 | 0.042
D4m1 | 16311 | 1 | 0.014
D4m1 | 204 | 3 | 0.042
D4m1 | 310 | 1 | 0.014
D4m1 | 4310 | 1 | 0.014
D4m1 | 593 | 2 | 0.028
D4m1 | 7150 | 2 | 0.028
D4m1 | 8730 | 1 | 0.014
D4m1 | 9938 | 1 | 0.014
D4m2 | 11353 | 1 | 0.006
D4m2 | 152 | 3 | 0.019
D4m2 | 15229 | 5 | 0.032
D4m2 | 15927 | 1 | 0.006
D4m2 | 16022.1C | 1 | 0.006
D4m2 | 16032G | 1 | 0.006
D4m2 | 16079G | 1 | 0.006
D4m2 | 16092 | 1 | 0.006
D4m2 | 16093 | 3 | 0.019
D4m2 | 16102 | 1 | 0.006
D4m2 | 16110d | 1 | 0.006

D4m2 | 16124 | 2 | 0.013
D4m2 | 16172 | 11 | 0.07
D4m2 | 16219 | 1 | 0.006
D4m2 | 16244C | 2 | 0.013
D4m2 | 16246T | 2 | 0.013
D4m2 | 16292 | 1 | 0.006
D4m2 | 16295 | 3 | 0.019
D4m2 | 16326T | 1 | 0.006
D4m2 | 16335 | 2 | 0.013
D4m2 | 16354 | 1 | 0.006
D4m2 | 16356 | 3 | 0.019
D4m2 | 16399 | 1 | 0.006
D4m2 | 16488 | 1 | 0.006
D4m2 | 16497 | 1 | 0.006
D4m2 | 200 | 5 | 0.032
D4m2 | 243 | 1 | 0.006
D4m2 | 247 | 2 | 0.013
D4m2 | 309.3C | 1 | 0.006
D4m2 | 315.2C | 2 | 0.013
D4m2 | 468 | 2 | 0.013
D4m2 | 521 | 2 | 0.013
D4m2 | 595.1A | 1 | 0.006
D4m2 | 629 | 1 | 0.006
D4m2 | 789 | 5 | 0.032
D4m2 | 8868 | 5 | 0.032
D4m2a | 13881 | 5 | 0.06
D4m2a | 14180 | 1 | 0.012
D4m2a | 15847 | 2 | 0.024
D4m2a | 16192A | 4 | 0.048
D4m2a | 16311 | 1 | 0.012
D4m2a | 189 | 1 | 0.012
D4m2a | 194 | 1 | 0.012
D4m2a | 310 | 4 | 0.048
D4m2a | 4676 | 1 | 0.012
D4m2a | 4767 | 1 | 0.012
D4m2a | 7013 | 5 | 0.06
D4m2a | 7430 | 1 | 0.012
D4m2a | 9755 | 1 | 0.012
D4m2a1 | 16093 | 1 | 0.014
D4m2a1 | 16316 | 1 | 0.014
D4m2a1 | 195 | 2 | 0.028
D4m2a1 | 709 | 1 | 0.014
D4m2a1a | 12 | 2 | 0.026
D4m2a1a | 16093 | 7 | 0.092
D4m2a1a | 16150 | 1 | 0.013
D4m2a1a | 16256 | 4 | 0.053
D4m2a1a | 310 | 1 | 0.013
D4m2a1a | 3388A | 1 | 0.013
D4n | 152 | 1 | 0.01
D4n | 16185 | 2 | 0.02
D4n | 16189 | 1 | 0.01
D4n | 16193d | 1 | 0.01
D4n | 16217 | 1 | 0.01
D4n | 16357 | 2 | 0.02
D4n | 456 | 1 | 0.01
D4n | 7805 | 1 | 0.01
D4n | 8404 | 1 | 0.01
D4n | 93 | 1 | 0.01
D4n1 | 150 | 1 | 0.011
D4n1 | 154 | 1 | 0.011
D4n1a | 10187 | 1 | 0.01
D4n1a | 9708A | 1 | 0.01
D4n2 | 10166 | 1 | 0.016
D4n2 | 11086 | 2 | 0.033
D4n2 | 14514 | 1 | 0.016
D4n2 | 151 | 2 | 0.033
D4n2 | 15625 | 2 | 0.033
D4n2 | 16042 | 1 | 0.016
D4n2 | 16051 | 1 | 0.016
D4n2 | 16092 | 2 | 0.033
D4n2 | 16102 | 1 | 0.016
D4n2 | 16129 | 1 | 0.016
D4n2 | 16171T | 13 | 0.213
D4n2 | 16189 | 2 | 0.033
D4n2 | 16266 | 1 | 0.016
D4n2 | 16325 | 1 | 0.016
D4n2 | 16352 | 1 | 0.016
D4n2 | 1643 | 1 | 0.016
D4n2 | 198 | 2 | 0.033
D4n2 | 291.2A | 4 | 0.066
D4n2 | 316C | 2 | 0.033
D4n2 | 9181 | 1 | 0.016
D4o | 11809 | 2 | 0.074
D4o | 11944 | 1 | 0.037
D4o | 12612 | 2 | 0.074
D4o | 146 | 1 | 0.037
D4o | 16168 | 1 | 0.037
D4o | 16172 | 2 | 0.074
D4o | 16187 | 2 | 0.074
D4o | 16232 | 12 | 0.444
D4o | 16294 | 1 | 0.037
D4o | 16311 | 8 | 0.296
D4o | 16325 | 3 | 0.111
D4o | 183 | 2 | 0.074
D4o | 573.3C | 1 | 0.037
D4o | 573.4C | 1 | 0.037
D4o | 5899.1C | 2 | 0.074
D4o | 8658 | 1 | 0.037
D4o | 9196 | 2 | 0.074
D4o1 | 13269 | 1 | 0.007
D4o1 | 16051 | 1 | 0.007
D4o1 | 16086 | 2 | 0.014
D4o1 | 16092 | 1 | 0.007
D4o1 | 16129 | 1 | 0.007
D4o1 | 16150 | 1 | 0.007
D4o1 | 16176 | 8 | 0.055
D4o1 | 16182 | 1 | 0.007
D4o1 | 16188 | 1 | 0.007
D4o1 | 16189 | 4 | 0.027
D4o1 | 16209 | 1 | 0.007
D4o1 | 16248 | 3 | 0.021

D4o1 | 16291 | 2 | 0.014
D4o1 | 16301 | 1 | 0.007
D4o1 | 16325 | 1 | 0.007
D4o1 | 16327A | 5 | 0.034
D4o1 | 16342 | 8 | 0.055
D4o1 | 16400 | 1 | 0.007
D4o1 | 189 | 1 | 0.007
D4o1 | 310 | 1 | 0.007
D4o1 | 4048 | 4 | 0.027
D4o1 | 6917 | 6 | 0.041
D4o1 | 8065 | 1 | 0.007
D4o1 | 8478 | 2 | 0.014
D4o1 | 8631 | 1 | 0.007
D4o1a | 11084 | 1 | 0.034
D4o1a | 12084 | 1 | 0.034
D4o1a | 13928 | 1 | 0.034
D4o1a | 15380 | 1 | 0.034
D4o1a | 16173 | 1 | 0.034
D4o1a | 16249 | 1 | 0.034
D4o1a | 16260 | 1 | 0.034
D4o1a | 3213 | 1 | 0.034
D4o1a | 8854 | 2 | 0.069
D4o1a | 8901 | 1 | 0.034
D4o2 | 16192 | 1 | 0.062
D4o2 | 16249 | 1 | 0.062
D4o2 | 16261 | 1 | 0.062
D4o2 | 16365 | 1 | 0.062
D4o2 | 194 | 1 | 0.062
D4o2 | 310 | 1 | 0.062
D4o2 | 374C | 1 | 0.062
D4o2 | 573d | 1 | 0.062
D4o2 | 8897 | 1 | 0.062
D4o2a | 11914 | 2 | 0.036
D4o2a | 12013T | 1 | 0.018
D4o2a | 12705A | 1 | 0.018
D4o2a | 14016 | 1 | 0.018
D4o2a | 14883 | 1 | 0.018
D4o2a | 150 | 9 | 0.164
D4o2a | 16192 | 1 | 0.018
D4o2a | 16261 | 9 | 0.164
D4o2a | 16356 | 4 | 0.073
D4o2a | 16471 | 1 | 0.018
D4o2a | 198 | 3 | 0.055
D4o2a | 2487C | 1 | 0.018
D4o2a | 310 | 10 | 0.182
D4o2a | 311 | 1 | 0.018
D4o2a | 316C | 1 | 0.018
D4o2a | 3338 | 4 | 0.073
D4o2a | 3789 | 2 | 0.036
D4o2a | 4907 | 1 | 0.018
D4o2a | 5004 | 1 | 0.018
D4o2a | 533 | 5 | 0.091
D4o2a | 6260 | 2 | 0.036
D4o2a | 64 | 1 | 0.018
D4o2a | 66T | 1 | 0.018
D4o2a | 8730 | 1 | 0.018
D4o2a | 8897 | 5 | 0.091
D4o2a | 9042 | 2 | 0.036
D4o2a | 930C | 9 | 0.164
D4o2a1 | 14062 | 1 | 0.032
D4o2a1 | 16261 | 11 | 0.355
D4o2a1 | 16294 | 2 | 0.065
D4o2a1 | 16471 | 1 | 0.032
D4o2a1 | 4682 | 1 | 0.032
D4o2a1 | 7080 | 2 | 0.065
D4o2a1 | 9078 | 2 | 0.065
D4p | 153 | 1 | 0.002
D4p | 15470 | 2 | 0.005
D4p | 16076d | 6 | 0.014
D4p | 16095 | 1 | 0.002
D4p | 16108 | 1 | 0.002
D4p | 16111A | 1 | 0.002
D4p | 16189 | 1 | 0.002
D4p | 16209 | 1 | 0.002
D4p | 16250A | 1 | 0.002
D4p | 16318C | 1 | 0.002
D4p | 16325 | 1 | 0.002
D4p | 16360 | 1 | 0.002
D4p | 207 | 1 | 0.002
D4p | 217 | 1 | 0.002
D4p | 3460 | 1 | 0.002
D4p | 548 | 1 | 0.002
D4p1 | 11020 | 1 | 0.002
D4p1 | 153 | 1 | 0.002
D4p1 | 16076d | 6 | 0.014
D4p1 | 16095 | 1 | 0.002
D4p1 | 16108 | 1 | 0.002
D4p1 | 16111A | 1 | 0.002
D4p1 | 16189 | 1 | 0.002
D4p1 | 16209 | 1 | 0.002
D4p1 | 16250A | 1 | 0.002
D4p1 | 16318C | 1 | 0.002
D4p1 | 16325 | 1 | 0.002
D4p1 | 16360 | 1 | 0.002
D4p1 | 207 | 1 | 0.002
D4p1 | 217 | 1 | 0.002
D4p1 | 548 | 1 | 0.002
D4p1 | 8014 | 1 | 0.002
D4q | 10373 | 1 | 0.008
D4q | 11150 | 1 | 0.008
D4q | 11732 | 1 | 0.008
D4q | 12 | 2 | 0.017
D4q | 125G | 1 | 0.008
D4q | 131 | 1 | 0.008
D4q | 13236 | 1 | 0.008
D4q | 13899 | 2 | 0.017
D4q | 13956 | 1 | 0.008
D4q | 146 | 8 | 0.067
D4q | 14827 | 2 | 0.017
D4q | 15118 | 1 | 0.008
D4q | 152 | 3 | 0.025

D4q | 153 | 2 | 0.017
D4q | 1555 | 1 | 0.008
D4q | 16042 | 1 | 0.008
D4q | 16080 | 2 | 0.017
D4q | 16081 | 1 | 0.008
D4q | 16086 | 1 | 0.008
D4q | 16093 | 1 | 0.008
D4q | 16150 | 1 | 0.008
D4q | 16163T | 1 | 0.008
D4q | 16167 | 1 | 0.008
D4q | 16172G | 1 | 0.008
D4q | 16177 | 1 | 0.008
D4q | 16192 | 4 | 0.033
D4q | 16193A | 1 | 0.008
D4q | 16212 | 1 | 0.008
D4q | 16214 | 1 | 0.008
D4q | 16215T | 1 | 0.008
D4q | 16218 | 2 | 0.017
D4q | 16229 | 1 | 0.008
D4q | 16242 | 1 | 0.008
D4q | 16243A | 1 | 0.008
D4q | 16261 | 8 | 0.067
D4q | 16274 | 2 | 0.017
D4q | 16281 | 2 | 0.017
D4q | 16281T | 1 | 0.008
D4q | 16284 | 1 | 0.008
D4q | 16304 | 1 | 0.008
D4q | 16312 | 1 | 0.008
D4q | 16316 | 1 | 0.008
D4q | 16325 | 1 | 0.008
D4q | 16332 | 1 | 0.008
D4q | 16333 | 1 | 0.008
D4q | 16343 | 1 | 0.008
D4q | 16346T | 1 | 0.008
D4q | 16350T | 1 | 0.008
D4q | 16355 | 1 | 0.008
D4q | 16356 | 5 | 0.042
D4q | 16373 | 1 | 0.008
D4q | 16416T | 1 | 0.008
D4q | 16422 | 2 | 0.017
D4q | 16528 | 1 | 0.008
D4q | 189 | 1 | 0.008
D4q | 193 | 1 | 0.008
D4q | 195 | 8 | 0.067
D4q | 198 | 1 | 0.008
D4q | 20 | 1 | 0.008
D4q | 228 | 4 | 0.033
D4q | 230 | 1 | 0.008
D4q | 234 | 1 | 0.008
D4q | 310 | 1 | 0.008
D4q | 3106A | 1 | 0.008
D4q | 315.2C | 1 | 0.008
D4q | 316C | 5 | 0.042
D4q | 321 | 1 | 0.008
D4q | 3505 | 1 | 0.008
D4q | 376 | 1 | 0.008
D4q | 3765 | 1 | 0.008
D4q | 3918 | 1 | 0.008
D4q | 4086 | 1 | 0.008
D4q | 469d | 1 | 0.008
D4q | 4959 | 6 | 0.05
D4q | 5033 | 1 | 0.008
D4q | 574 | 1 | 0.008
D4q | 5824 | 4 | 0.033
D4q | 593 | 1 | 0.008
D4q | 6297 | 1 | 0.008
D4q | 87 | 1 | 0.008
D4q | 8794 | 2 | 0.017
D4q | 9371 | 1 | 0.008
D4q1 | 15791 | 1 | 0.333
D4q1 | 15839 | 1 | 0.333
D4q1 | 15854A | 1 | 0.333
D4q1 | 5220 | 1 | 0.333
D4q1 | 9861 | 1 | 0.333
D4q1a | 14842 | 1 | 0.125
D4q1a | 15839 | 3 | 0.375
D4q1a | 8705 | 1 | 0.125
D4s | 10364 | 1 | 0.002
D4s | 13269 | 2 | 0.004
D4s | 14665 | 1 | 0.002
D4s | 152 | 6 | 0.013
D4s | 15731 | 1 | 0.002
D4s | 15734 | 12 | 0.025
D4s | 16072.1A | 2 | 0.004
D4s | 16093 | 3 | 0.006
D4s | 16108 | 1 | 0.002
D4s | 16111A | 1 | 0.002
D4s | 16124 | 1 | 0.002
D4s | 16145 | 15 | 0.032
D4s | 16187 | 2 | 0.004
D4s | 16193 | 1 | 0.002
D4s | 16243 | 1 | 0.002
D4s | 16249 | 1 | 0.002
D4s | 16250A | 1 | 0.002
D4s | 16271 | 2 | 0.004
D4s | 16286 | 1 | 0.002
D4s | 16295 | 3 | 0.006
D4s | 16316 | 1 | 0.002
D4s | 16318C | 1 | 0.002
D4s | 16319 | 1 | 0.002
D4s | 16320 | 1 | 0.002
D4s | 173 | 2 | 0.004
D4s | 194 | 2 | 0.004
D4s | 207 | 1 | 0.002
D4s | 217 | 1 | 0.002
D4s | 279 | 6 | 0.013
D4s | 315.2C | 3 | 0.006
D4s | 356.1C | 1 | 0.002
D4s | 6902 | 1 | 0.002
D4s | 8239 | 8 | 0.017
D4t | 105.1C | 1 | 0.002

D4t | 110 | 1 | 0.002
D4t | 13356 | 1 | 0.002
D4t | 135 | 1 | 0.002
D4t | 13928 | 1 | 0.002
D4t | 15323 | 2 | 0.004
D4t | 16086 | 1 | 0.002
D4t | 16093 | 1 | 0.002
D4t | 16108 | 1 | 0.002
D4t | 16111A | 1 | 0.002
D4t | 16115 | 1 | 0.002
D4t | 16189 | 1 | 0.002
D4t | 16202 | 1 | 0.002
D4t | 16250A | 1 | 0.002
D4t | 16263 | 1 | 0.002
D4t | 16311 | 2 | 0.004
D4t | 16318C | 1 | 0.002
D4t | 16321 | 1 | 0.002
D4t | 16339 | 1 | 0.002
D4t | 16455 | 1 | 0.002
D4t | 16505 | 1 | 0.002
D4t | 16564C | 1 | 0.002
D4t | 200 | 1 | 0.002
D4t | 263C | 1 | 0.002
D4t | 306 | 1 | 0.002
D4t | 326 | 1 | 0.002
D4t | 393 | 1 | 0.002
D4t | 446 | 1 | 0.002
D4t | 448 | 1 | 0.002
D4t | 573d | 1 | 0.002
D5 | 152 | 2 | 0.02
D5 | 16051 | 1 | 0.01
D5 | 16129 | 2 | 0.02
D5 | 16244 | 1 | 0.01
D5 | 16467 | 4 | 0.04
D5 | 195 | 1 | 0.01
D5 | 310 | 1 | 0.01
D5 | 459d | 2 | 0.02
D5 | 681 | 2 | 0.02
D5a | 146 | 2 | 0.018
D5a | 16102 | 1 | 0.009
D5a | 16129 | 1 | 0.009
D5a | 16164 | 2 | 0.018
D5a | 16167 | 1 | 0.009
D5a | 16178 | 1 | 0.009
D5a | 16239 | 1 | 0.009
D5a | 16243 | 3 | 0.027
D5a | 16245 | 1 | 0.009
D5a | 16266 | 16 | 0.142
D5a | 16288 | 1 | 0.009
D5a | 16302 | 1 | 0.009
D5a | 16363 | 1 | 0.009
D5a | 16399 | 1 | 0.009
D5a | 195 | 1 | 0.009
D5a | 214 | 4 | 0.035
D5a | 263C | 4 | 0.035
D5a | 309.3C | 1 | 0.009
D5a | 309d | 1 | 0.009
D5a | 459d | 1 | 0.009
D5a1 | 12040 | 1 | 0.062
D5a1 | 146 | 2 | 0.125
D5a1 | 15817 | 1 | 0.062
D5a1 | 195 | 1 | 0.062
D5a1a | 16093 | 2 | 0.053
D5a1a | 16108 | 1 | 0.026
D5a1a | 16124 | 1 | 0.026
D5a1a | 16209 | 4 | 0.105
D5a1a | 16227 | 3 | 0.079
D5a1a | 16260 | 3 | 0.079
D5a1a | 16265 | 1 | 0.026
D5a1a | 16291 | 25 | 0.658
D5a1a | 16292 | 5 | 0.132
D5a1a | 16309 | 1 | 0.026
D5a1a | 16399 | 3 | 0.079
D5a1a1 | 14161 | 1 | 0.05
D5a1a1 | 1442 | 1 | 0.05
D5a1a1 | 16192-16193d | 1 | 0.05
D5a1a1 | 16240 | 1 | 0.05
D5a1a1 | 16256 | 1 | 0.05
D5a1a1 | 3397 | 1 | 0.05
D5a1a1 | 3666 | 1 | 0.05
D5a1a2 | 12613 | 1 | 0.059
D5a1a2 | 13527 | 1 | 0.059
D5a1a2 | 16169 | 1 | 0.059
D5a1a2 | 16359 | 1 | 0.059
D5a1a2 | 5899.1C | 1 | 0.059
D5a1a2 | 9377 | 1 | 0.059
D5a2 | 10790 | 1 | 0.009
D5a2 | 11233 | 1 | 0.009
D5a2 | 146 | 1 | 0.009
D5a2 | 14668 | 1 | 0.009
D5a2 | 152 | 1 | 0.009
D5a2 | 16051 | 1 | 0.009
D5a2 | 16056 | 1 | 0.009
D5a2 | 16066 | 1 | 0.009
D5a2 | 16093 | 1 | 0.009
D5a2 | 16105 | 1 | 0.009
D5a2 | 16111 | 2 | 0.019
D5a2 | 16153 | 1 | 0.009
D5a2 | 16158 | 3 | 0.028
D5a2 | 16164 | 34 | 0.315
D5a2 | 16179 | 1 | 0.009
D5a2 | 16184 | 1 | 0.009
D5a2 | 16186 | 1 | 0.009
D5a2 | 16192-16193d | 1 | 0.009
D5a2 | 16193d | 4 | 0.037
D5a2 | 16203 | 1 | 0.009
D5a2 | 16209 | 1 | 0.009
D5a2 | 16220 | 1 | 0.009
D5a2 | 16234 | 3 | 0.028
D5a2 | 16243 | 1 | 0.009
D5a2 | 16256 | 2 | 0.019

D5a2 | 16259 | 13 | 0.12
D5a2 | 16278 | 1 | 0.009
D5a2 | 16294 | 19 | 0.176
D5a2 | 16311 | 2 | 0.019
D5a2 | 16315 | 1 | 0.009
D5a2 | 16350T | 1 | 0.009
D5a2 | 16468 | 1 | 0.009
D5a2 | 16526T | 4 | 0.037
D5a2 | 195 | 2 | 0.019
D5a2 | 196A | 1 | 0.009
D5a2 | 199 | 1 | 0.009
D5a2 | 200 | 1 | 0.009
D5a2 | 204 | 1 | 0.009
D5a2 | 243 | 1 | 0.009
D5a2 | 2469d | 1 | 0.009
D5a2 | 263C | 2 | 0.019
D5a2 | 312 | 1 | 0.009
D5a2 | 315.2C | 1 | 0.009
D5a2 | 316C | 8 | 0.074
D5a2 | 322 | 1 | 0.009
D5a2 | 3355 | 1 | 0.009
D5a2 | 3528 | 2 | 0.019
D5a2 | 367 | 1 | 0.009
D5a2 | 3936G | 1 | 0.009
D5a2 | 409.1TTGTTG | 1 | 0.009
D5a2 | 435 | 1 | 0.009
D5a2 | 484 | 1 | 0.009
D5a2 | 4973 | 1 | 0.009
D5a2 | 564 | 1 | 0.009
D5a2 | 61 | 1 | 0.009
D5a2 | 6267 | 1 | 0.009
D5a2 | 6797 | 1 | 0.009
D5a2 | 756 | 1 | 0.009
D5a2 | 7609 | 1 | 0.009
D5a2 | 7975 | 1 | 0.009
D5a2 | 7978A | 1 | 0.009
D5a2 | 8843 | 1 | 0.009
D5a2 | 9304 | 1 | 0.009
D5a2a | 10143 | 1 | 0.005
D5a2a | 10237 | 1 | 0.005
D5a2a | 10399 | 1 | 0.005
D5a2a | 11017 | 1 | 0.005
D5a2a | 11150 | 1 | 0.005
D5a2a | 11152 | 4 | 0.018
D5a2a | 11447 | 1 | 0.005
D5a2a | 121 | 1 | 0.005
D5a2a | 12528 | 1 | 0.005
D5a2a | 13098 | 1 | 0.005
D5a2a | 13356 | 1 | 0.005
D5a2a | 13431 | 2 | 0.009
D5a2a | 13708 | 1 | 0.005
D5a2a | 13926 | 1 | 0.005
D5a2a | 14109 | 1 | 0.005
D5a2a | 14308 | 1 | 0.005
D5a2a | 14484 | 1 | 0.005
D5a2a | 146 | 9 | 0.041
D5a2a | 15191 | 1 | 0.005
D5a2a | 152 | 5 | 0.023
D5a2a | 15262 | 1 | 0.005
D5a2a | 15295 | 1 | 0.005
D5a2a | 15458 | 1 | 0.005
D5a2a | 1555 | 2 | 0.009
D5a2a | 15968 | 1 | 0.005
D5a2a | 160 | 1 | 0.005
D5a2a | 16051 | 3 | 0.014
D5a2a | 16076d | 1 | 0.005
D5a2a | 16086 | 5 | 0.023
D5a2a | 16093 | 6 | 0.028
D5a2a | 16102d | 1 | 0.005
D5a2a | 16103 | 1 | 0.005
D5a2a | 16111 | 1 | 0.005
D5a2a | 16129 | 1 | 0.005
D5a2a | 16131A | 1 | 0.005
D5a2a | 16148 | 1 | 0.005
D5a2a | 16158 | 2 | 0.009
D5a2a | 16160 | 1 | 0.005
D5a2a | 16164 | 166 | 0.761
D5a2a | 16168 | 1 | 0.005
D5a2a | 16169 | 1 | 0.005
D5a2a | 16171T | 1 | 0.005
D5a2a | 16173 | 1 | 0.005
D5a2a | 16178 | 1 | 0.005
D5a2a | 16179 | 1 | 0.005
D5a2a | 16182T | 7 | 0.032
D5a2a | 16185 | 2 | 0.009
D5a2a | 16187 | 2 | 0.009
D5a2a | 16192-16193d | 1 | 0.005
D5a2a | 16193d | 6 | 0.028
D5a2a | 16209 | 7 | 0.032
D5a2a | 16234 | 3 | 0.014
D5a2a | 16235 | 2 | 0.009
D5a2a | 16243 | 19 | 0.087
D5a2a | 16249 | 4 | 0.018
D5a2a | 16260 | 4 | 0.018
D5a2a | 16261 | 2 | 0.009
D5a2a | 16267 | 1 | 0.005
D5a2a | 16269C | 1 | 0.005
D5a2a | 16271 | 3 | 0.014
D5a2a | 16274 | 3 | 0.014
D5a2a | 16278 | 1 | 0.005
D5a2a | 16290 | 5 | 0.023
D5a2a | 16291 | 1 | 0.005
D5a2a | 16293T | 4 | 0.018
D5a2a | 16299 | 2 | 0.009
D5a2a | 16300 | 1 | 0.005
D5a2a | 16311 | 8 | 0.037
D5a2a | 16319 | 5 | 0.023
D5a2a | 16324 | 1 | 0.005
D5a2a | 16325 | 1 | 0.005
D5a2a | 16343 | 1 | 0.005
D5a2a | 16348 | 1 | 0.005

D5a2a | 16390 | 1 | 0.005
D5a2a | 16425G | 1 | 0.005
D5a2a | 16464 | 1 | 0.005
D5a2a | 16491G | 1 | 0.005
D5a2a | 16497 | 2 | 0.009
D5a2a | 16T | 9 | 0.041
D5a2a | 170 | 1 | 0.005
D5a2a | 1719 | 1 | 0.005
D5a2a | 180 | 1 | 0.005
D5a2a | 183 | 2 | 0.009
D5a2a | 194 | 2 | 0.009
D5a2a | 195 | 6 | 0.028
D5a2a | 199 | 1 | 0.005
D5a2a | 200 | 1 | 0.005
D5a2a | 20G | 1 | 0.005
D5a2a | 217 | 1 | 0.005
D5a2a | 224.1T | 1 | 0.005
D5a2a | 263C | 3 | 0.014
D5a2a | 282 | 1 | 0.005
D5a2a | 309.3C | 2 | 0.009
D5a2a | 315.2C | 1 | 0.005
D5a2a | 316C | 3 | 0.014
D5a2a | 321 | 1 | 0.005
D5a2a | 3394 | 1 | 0.005
D5a2a | 3402 | 3 | 0.014
D5a2a | 3714 | 1 | 0.005
D5a2a | 373 | 1 | 0.005
D5a2a | 389 | 2 | 0.009
D5a2a | 3918 | 1 | 0.005
D5a2a | 411 | 1 | 0.005
D5a2a | 4164 | 1 | 0.005
D5a2a | 4232 | 1 | 0.005
D5a2a | 439T | 1 | 0.005
D5a2a | 456 | 1 | 0.005
D5a2a | 458A | 1 | 0.005
D5a2a | 474 | 1 | 0.005
D5a2a | 487 | 1 | 0.005
D5a2a | 494A | 1 | 0.005
D5a2a | 4973 | 1 | 0.005
D5a2a | 518 | 1 | 0.005
D5a2a | 523C | 1 | 0.005
D5a2a | 5263 | 1 | 0.005
D5a2a | 5291 | 1 | 0.005
D5a2a | 538C | 2 | 0.009
D5a2a | 5563 | 1 | 0.005
D5a2a | 5698 | 1 | 0.005
D5a2a | 573d | 1 | 0.005
D5a2a | 574C | 1 | 0.005
D5a2a | 576C | 1 | 0.005
D5a2a | 577C | 1 | 0.005
D5a2a | 5899d | 1 | 0.005
D5a2a | 6272 | 1 | 0.005
D5a2a | 6671 | 1 | 0.005
D5a2a | 709 | 2 | 0.009
D5a2a | 71d | 1 | 0.005
D5a2a | 7250 | 1 | 0.005
D5a2a | 7340 | 1 | 0.005
D5a2a | 7513.1T | 1 | 0.005
D5a2a | 77 | 1 | 0.005
D5a2a | 8322 | 1 | 0.005
D5a2a | 8410 | 1 | 0.005
D5a2a | 8479 | 3 | 0.014
D5a2a | 8516 | 1 | 0.005
D5a2a | 870 | 1 | 0.005
D5a2a | 9182 | 1 | 0.005
D5a2a | 9608 | 2 | 0.009
D5a2a | 9755 | 1 | 0.005
D5a2a | 9977 | 1 | 0.005
D5a2a+16092 | 1 | 1 | 0.011
D5a2a+16092 | 105 | 1 | 0.011
D5a2a+16092 | 146 | 1 | 0.011
D5a2a+16092 | 149 | 2 | 0.022
D5a2a+16092 | 151d | 1 | 0.011
D5a2a+16092 | 152 | 1 | 0.011
D5a2a+16092 | 16025 | 1 | 0.011
D5a2a+16092 | 16043 | 1 | 0.011
D5a2a+16092 | 16069 | 1 | 0.011
D5a2a+16092 | 16076d | 1 | 0.011
D5a2a+16092 | 16086 | 3 | 0.032
D5a2a+16092 | 16098 | 1 | 0.011
D5a2a+16092 | 16134d | 1 | 0.011
D5a2a+16092 | 16164T | 1 | 0.011
D5a2a+16092 | 16167 | 2 | 0.022
D5a2a+16092 | 16173 | 1 | 0.011
D5a2a+16092 | 16185 | 5 | 0.054
D5a2a+16092 | 16188 | 1 | 0.011
D5a2a+16092 | 16193 | 1 | 0.011
D5a2a+16092 | 16193.3C | 1 | 0.011
D5a2a+16092 | 16193d | 8 | 0.086
D5a2a+16092 | 16213 | 3 | 0.032
D5a2a+16092 | 16231A | 1 | 0.011
D5a2a+16092 | 16243 | 1 | 0.011
D5a2a+16092 | 16260 | 2 | 0.022
D5a2a+16092 | 16271 | 1 | 0.011
D5a2a+16092 | 16280C | 1 | 0.011
D5a2a+16092 | 16289 | 1 | 0.011
D5a2a+16092 | 16290 | 24 | 0.258
D5a2a+16092 | 16317 | 1 | 0.011
D5a2a+16092 | 16366 | 1 | 0.011
D5a2a+16092 | 16367 | 1 | 0.011
D5a2a+16092 | 16466T | 1 | 0.011
D5a2a+16092 | 16475 | 1 | 0.011
D5a2a+16092 | 16526 | 1 | 0.011
D5a2a+16092 | 16530 | 1 | 0.011
D5a2a+16092 | 172 | 1 | 0.011
D5a2a+16092 | 183 | 1 | 0.011
D5a2a+16092 | 189 | 2 | 0.022
D5a2a+16092 | 195 | 1 | 0.011
D5a2a+16092 | 214 | 1 | 0.011
D5a2a+16092 | 235 | 1 | 0.011
D5a2a+16092 | 243 | 1 | 0.011

D5a2a+16092 | 247 | 1 | 0.011
D5a2a+16092 | 259T | 1 | 0.011
D5a2a+16092 | 309d | 1 | 0.011
D5a2a+16092 | 315 | 1 | 0.011
D5a2a+16092 | 323d | 1 | 0.011
D5a2a+16092 | 323T | 1 | 0.011
D5a2a+16092 | 325 | 1 | 0.011
D5a2a+16092 | 347 | 1 | 0.011
D5a2a+16092 | 379 | 1 | 0.011
D5a2a+16092 | 385d | 1 | 0.011
D5a2a+16092 | 438.1C | 1 | 0.011
D5a2a+16092 | 453 | 1 | 0.011
D5a2a+16092 | 463d | 1 | 0.011
D5a2a+16092 | 500 | 1 | 0.011
D5a2a+16092 | 503 | 1 | 0.011
D5a2a+16092 | 506 | 1 | 0.011
D5a2a+16092 | 533T | 1 | 0.011
D5a2a+16092 | 573.5C | 2 | 0.022
D5a2a+16092 | 574C | 2 | 0.022
D5a2a+16092 | 576C | 2 | 0.022
D5a2a+16092 | 6596 | 1 | 0.011
D5a2a+16092 | 709 | 1 | 0.011
D5a2a+16092 | 732T | 1 | 0.011
D5a2a+16092 | 735.1A | 1 | 0.011
D5a2a+16092 | 738C | 1 | 0.011
D5a2a+16092 | 7673 | 1 | 0.011
D5a2a1 | 10403 | 1 | 0.009
D5a2a1 | 11929 | 1 | 0.009
D5a2a1 | 12810 | 1 | 0.009
D5a2a1 | 12954 | 1 | 0.009
D5a2a1 | 13966 | 1 | 0.009
D5a2a1 | 14484 | 1 | 0.009
D5a2a1 | 14502 | 1 | 0.009
D5a2a1 | 146 | 4 | 0.036
D5a2a1 | 14920 | 1 | 0.009
D5a2a1 | 151 | 1 | 0.009
D5a2a1 | 16103 | 1 | 0.009
D5a2a1 | 16111A | 1 | 0.009
D5a2a1 | 16129 | 2 | 0.018
D5a2a1 | 16153 | 4 | 0.036
D5a2a1 | 16193d | 1 | 0.009
D5a2a1 | 16243 | 40 | 0.364
D5a2a1 | 16247 | 1 | 0.009
D5a2a1 | 16249 | 2 | 0.018
D5a2a1 | 16261 | 2 | 0.018
D5a2a1 | 16287 | 2 | 0.018
D5a2a1 | 16311 | 2 | 0.018
D5a2a1 | 16319 | 1 | 0.009
D5a2a1 | 16360 | 1 | 0.009
D5a2a1 | 16368 | 5 | 0.045
D5a2a1 | 195 | 1 | 0.009
D5a2a1 | 1957 | 1 | 0.009
D5a2a1 | 204 | 1 | 0.009
D5a2a1 | 215 | 1 | 0.009
D5a2a1 | 252 | 1 | 0.009
D5a2a1 | 2581 | 1 | 0.009
D5a2a1 | 316C | 8 | 0.073
D5a2a1 | 4491 | 1 | 0.009
D5a2a1 | 455d | 1 | 0.009
D5a2a1 | 4947 | 1 | 0.009
D5a2a1 | 8251 | 1 | 0.009
D5a2a1 | 8838 | 1 | 0.009
D5a2a1 | 8911 | 1 | 0.009
D5a2a1+@16172 | 10885 | 1 | 0.006
D5a2a1+@16172 | 10972 | 1 | 0.006
D5a2a1+@16172 | 11233 | 1 | 0.006
D5a2a1+@16172 | 12414 | 3 | 0.017
D5a2a1+@16172 | 12696 | 2 | 0.011
D5a2a1+@16172 | 12753 | 1 | 0.006
D5a2a1+@16172 | 12940 | 1 | 0.006
D5a2a1+@16172 | 13278 | 5 | 0.028
D5a2a1+@16172 | 14007 | 3 | 0.017
D5a2a1+@16172 | 14215G | 2 | 0.011
D5a2a1+@16172 | 14274T | 1 | 0.006
D5a2a1+@16172 | 1438 | 5 | 0.028
D5a2a1+@16172 | 146 | 9 | 0.05
D5a2a1+@16172 | 14668 | 1 | 0.006
D5a2a1+@16172 | 152 | 13 | 0.072
D5a2a1+@16172 | 153 | 1 | 0.006
D5a2a1+@16172 | 15334 | 2 | 0.011
D5a2a1+@16172 | 15479 | 1 | 0.006
D5a2a1+@16172 | 15562 | 4 | 0.022
D5a2a1+@16172 | 15766 | 4 | 0.022
D5a2a1+@16172 | 16066 | 1 | 0.006
D5a2a1+@16172 | 16073 | 1 | 0.006
D5a2a1+@16172 | 16086 | 3 | 0.017
D5a2a1+@16172 | 16092A | 1 | 0.006
D5a2a1+@16172 | 16093 | 4 | 0.022
D5a2a1+@16172 | 16097 | 1 | 0.006
D5a2a1+@16172 | 16104 | 1 | 0.006
D5a2a1+@16172 | 16111 | 2 | 0.011
D5a2a1+@16172 | 16126 | 1 | 0.006
D5a2a1+@16172 | 16129 | 47 | 0.26
D5a2a1+@16172 | 16145 | 1 | 0.006
D5a2a1+@16172 | 16148 | 2 | 0.011
D5a2a1+@16172 | 16153 | 2 | 0.011
D5a2a1+@16172 | 16167 | 6 | 0.033
D5a2a1+@16172 | 16170 | 1 | 0.006
D5a2a1+@16172 | 16173 | 1 | 0.006
D5a2a1+@16172 | 16176 | 1 | 0.006
D5a2a1+@16172 | 16181C | 1 | 0.006
D5a2a1+@16172 | 16185 | 14 | 0.077
D5a2a1+@16172 | 16186 | 1 | 0.006
D5a2a1+@16172 | 16187A | 2 | 0.011
D5a2a1+@16172 | 16192-16193d | 1 | 0.006
D5a2a1+@16172 | 16193d | 17 | 0.094
D5a2a1+@16172 | 16209 | 1 | 0.006
D5a2a1+@16172 | 16213C | 1 | 0.006
D5a2a1+@16172 | 16224 | 1 | 0.006
D5a2a1+@16172 | 16234 | 1 | 0.006
D5a2a1+@16172 | 16242 | 2 | 0.011

D5a2a1+@16172 | 16243 | 3 | 0.017
D5a2a1+@16172 | 16264 | 1 | 0.006
D5a2a1+@16172 | 16274 | 2 | 0.011
D5a2a1+@16172 | 16278 | 2 | 0.011
D5a2a1+@16172 | 16291 | 1 | 0.006
D5a2a1+@16172 | 16292 | 1 | 0.006
D5a2a1+@16172 | 16294 | 6 | 0.033
D5a2a1+@16172 | 16311 | 7 | 0.039
D5a2a1+@16172 | 16319 | 2 | 0.011
D5a2a1+@16172 | 16325 | 1 | 0.006
D5a2a1+@16172 | 16343 | 1 | 0.006
D5a2a1+@16172 | 16354 | 1 | 0.006
D5a2a1+@16172 | 16356 | 3 | 0.017
D5a2a1+@16172 | 183 | 1 | 0.006
D5a2a1+@16172 | 189 | 2 | 0.011
D5a2a1+@16172 | 189T | 1 | 0.006
D5a2a1+@16172 | 1927 | 1 | 0.006
D5a2a1+@16172 | 194 | 2 | 0.011
D5a2a1+@16172 | 195 | 6 | 0.033
D5a2a1+@16172 | 199 | 7 | 0.039
D5a2a1+@16172 | 204 | 3 | 0.017
D5a2a1+@16172 | 207 | 9 | 0.05
D5a2a1+@16172 | 214 | 2 | 0.011
D5a2a1+@16172 | 228 | 2 | 0.011
D5a2a1+@16172 | 2315 | 4 | 0.022
D5a2a1+@16172 | 243 | 1 | 0.006
D5a2a1+@16172 | 2581 | 3 | 0.017
D5a2a1+@16172 | 263C | 6 | 0.033
D5a2a1+@16172 | 309.3C | 2 | 0.011
D5a2a1+@16172 | 309d | 1 | 0.006
D5a2a1+@16172 | 310 | 2 | 0.011
D5a2a1+@16172 | 3150 | 3 | 0.017
D5a2a1+@16172 | 316 | 1 | 0.006
D5a2a1+@16172 | 316C | 3 | 0.017
D5a2a1+@16172 | 320 | 1 | 0.006
D5a2a1+@16172 | 324G | 1 | 0.006
D5a2a1+@16172 | 3355 | 1 | 0.006
D5a2a1+@16172 | 3565 | 2 | 0.011
D5a2a1+@16172 | 3591 | 4 | 0.022
D5a2a1+@16172 | 366 | 1 | 0.006
D5a2a1+@16172 | 3903 | 3 | 0.017
D5a2a1+@16172 | 444 | 4 | 0.022
D5a2a1+@16172 | 455d | 1 | 0.006
D5a2a1+@16172 | 4632 | 1 | 0.006
D5a2a1+@16172 | 4637 | 1 | 0.006
D5a2a1+@16172 | 4907 | 1 | 0.006
D5a2a1+@16172 | 493 | 1 | 0.006
D5a2a1+@16172 | 4959 | 1 | 0.006
D5a2a1+@16172 | 513 | 1 | 0.006
D5a2a1+@16172 | 518 | 1 | 0.006
D5a2a1+@16172 | 54 | 1 | 0.006
D5a2a1+@16172 | 549 | 2 | 0.011
D5a2a1+@16172 | 57 | 1 | 0.006
D5a2a1+@16172 | 573.1C | 5 | 0.028
D5a2a1+@16172 | 59 | 1 | 0.006
D5a2a1+@16172 | 597.1T | 1 | 0.006
D5a2a1+@16172 | 60.1T | 1 | 0.006
D5a2a1+@16172 | 6112 | 1 | 0.006
D5a2a1+@16172 | 6326 | 2 | 0.011
D5a2a1+@16172 | 6671 | 1 | 0.006
D5a2a1+@16172 | 6755 | 1 | 0.006
D5a2a1+@16172 | 6779 | 3 | 0.017
D5a2a1+@16172 | 6908 | 1 | 0.006
D5a2a1+@16172 | 739 | 2 | 0.011
D5a2a1+@16172 | 7757 | 1 | 0.006
D5a2a1+@16172 | 8116 | 1 | 0.006
D5a2a1+@16172 | 8271 | 1 | 0.006
D5a2a1+@16172 | 8479 | 1 | 0.006
D5a2a1+@16172 | 8793 | 1 | 0.006
D5a2a1+@16172 | 9033 | 3 | 0.017
D5a2a1+@16172 | 9077 | 1 | 0.006
D5a2a1+@16172 | 9117 | 4 | 0.022
D5a2a1+@16172 | 9181 | 1 | 0.006
D5a2a1+@16172 | 9215 | 1 | 0.006
D5a2a1+@16172 | 9554 | 1 | 0.006
D5a2a1+@16172 | 9770 | 1 | 0.006
D5a2a1+@16172 | 9966 | 2 | 0.011
D5a2a1a | 12398 | 2 | 0.118
D5a2a1a | 15731 | 1 | 0.059
D5a2a1a | 15924 | 1 | 0.059
D5a2a1a | 16181C | 1 | 0.059
D5a2a1a | 16356 | 2 | 0.118
D5a2a1a | 16394 | 1 | 0.059
D5a2a1a | 4435 | 1 | 0.059
D5a2a1a | 6617 | 1 | 0.059
D5a2a1a1 | 10373 | 1 | 0.077
D5a2a1a1 | 11746 | 1 | 0.077
D5a2a1a1 | 13896 | 1 | 0.077
D5a2a1a1 | 14003 | 1 | 0.077
D5a2a1a1 | 14028 | 1 | 0.077
D5a2a1a1 | 1438 | 1 | 0.077
D5a2a1a1 | 146 | 2 | 0.154
D5a2a1a1 | 14872 | 1 | 0.077
D5a2a1a1 | 15218 | 5 | 0.385
D5a2a1a1 | 15412 | 1 | 0.077
D5a2a1a1 | 15905 | 3 | 0.231
D5a2a1a1 | 16147 | 1 | 0.077
D5a2a1a1 | 16148 | 1 | 0.077
D5a2a1a1 | 16256 | 1 | 0.077
D5a2a1a1 | 16319 | 1 | 0.077
D5a2a1a1 | 189 | 1 | 0.077
D5a2a1a1 | 193 | 1 | 0.077
D5a2a1a1 | 194 | 1 | 0.077
D5a2a1a1 | 2259 | 1 | 0.077
D5a2a1a1 | 249 | 1 | 0.077
D5a2a1a1 | 310 | 1 | 0.077
D5a2a1a1 | 316 | 1 | 0.077
D5a2a1a1 | 3391 | 2 | 0.154
D5a2a1a1 | 4577 | 1 | 0.077
D5a2a1a1 | 6126 | 1 | 0.077
D5a2a1a1 | 6827 | 1 | 0.077

D5a2a1a1 | 709 | 1 | 0.077
D5a2a1a1 | 9012 | 1 | 0.077
D5a2a1a1a | 10007 | 1 | 0.077
D5a2a1a1a | 11150 | 1 | 0.077
D5a2a1a1a | 16193.3C | 1 | 0.077
D5a2a1a1a | 16252 | 1 | 0.077
D5a2a1a1a | 16260 | 1 | 0.077
D5a2a1a1a | 16365 | 2 | 0.154
D5a2a1a1a | 16525 | 1 | 0.077
D5a2a1a1a | 279 | 1 | 0.077
D5a2a1a1a | 309.4C | 1 | 0.077
D5a2a1a1a | 4594 | 1 | 0.077
D5a2a1a1a | 573.1C | 1 | 0.077
D5a2a1a1a | 5775 | 1 | 0.077
D5a2a1a1a | 615.1A | 1 | 0.077
D5a2a1a1a | 642A | 1 | 0.077
D5a2a1a1a | 676 | 1 | 0.077
D5a2a1a1a | 683T | 1 | 0.077
D5a2a1a2 | 12007 | 2 | 0.1
D5a2a1a2 | 13057 | 1 | 0.05
D5a2a1a2 | 16076d | 1 | 0.05
D5a2a1a2 | 16179 | 1 | 0.05
D5a2a1a2 | 16186 | 1 | 0.05
D5a2a1a2 | 16187 | 3 | 0.15
D5a2a1a2 | 16227T | 1 | 0.05
D5a2a1a2 | 16259 | 1 | 0.05
D5a2a1a2 | 16292 | 1 | 0.05
D5a2a1a2 | 1810T | 1 | 0.05
D5a2a1a2 | 8027 | 1 | 0.05
D5a2a1b | 10364 | 1 | 0.01
D5a2a1b | 11365 | 1 | 0.01
D5a2a1b | 11914 | 8 | 0.083
D5a2a1b | 12361 | 3 | 0.031
D5a2a1b | 14040 | 1 | 0.01
D5a2a1b | 146 | 2 | 0.021
D5a2a1b | 15077 | 3 | 0.031
D5a2a1b | 152 | 1 | 0.01
D5a2a1b | 15226T | 3 | 0.031
D5a2a1b | 15250 | 2 | 0.021
D5a2a1b | 15553 | 3 | 0.031
D5a2a1b | 15885 | 1 | 0.01
D5a2a1b | 15968 | 3 | 0.031
D5a2a1b | 16025d | 1 | 0.01
D5a2a1b | 16051 | 3 | 0.031
D5a2a1b | 16072.1A | 1 | 0.01
D5a2a1b | 16076d | 1 | 0.01
D5a2a1b | 16093 | 1 | 0.01
D5a2a1b | 16181C | 1 | 0.01
D5a2a1b | 16188 | 1 | 0.01
D5a2a1b | 16192-16193d | 1 | 0.01
D5a2a1b | 16193d | 1 | 0.01
D5a2a1b | 16240 | 1 | 0.01
D5a2a1b | 16260 | 1 | 0.01
D5a2a1b | 16311 | 1 | 0.01
D5a2a1b | 16317 | 1 | 0.01
D5a2a1b | 16325 | 8 | 0.083
D5a2a1b | 16390 | 5 | 0.052
D5a2a1b | 16482 | 4 | 0.042
D5a2a1b | 2222 | 1 | 0.01
D5a2a1b | 263C | 1 | 0.01
D5a2a1b | 315.2C | 1 | 0.01
D5a2a1b | 4082 | 1 | 0.01
D5a2a1b | 5093 | 1 | 0.01
D5a2a1b | 513 | 1 | 0.01
D5a2a1b | 52 | 1 | 0.01
D5a2a1b | 5582 | 2 | 0.021
D5a2a1b | 573d | 7 | 0.073
D5a2a1b | 6975 | 2 | 0.021
D5a2a1b | 7025 | 1 | 0.01
D5a2a1b | 8185 | 3 | 0.031
D5a2a1b | 8479 | 1 | 0.01
D5a2a1b | 8628 | 5 | 0.052
D5a2a1b | 870 | 1 | 0.01
D5a2a1b | 9355 | 1 | 0.01
D5a2a1b1 | 11974T | 1 | 0.022
D5a2a1b1 | 146 | 2 | 0.044
D5a2a1b1 | 15254 | 1 | 0.022
D5a2a1b1 | 16076d | 1 | 0.022
D5a2a1b1 | 16114A | 1 | 0.022
D5a2a1b1 | 16148 | 1 | 0.022
D5a2a1b1 | 16179 | 2 | 0.044
D5a2a1b1 | 16224 | 1 | 0.022
D5a2a1b1 | 16265C | 2 | 0.044
D5a2a1b1 | 16274 | 1 | 0.022
D5a2a1b1 | 16293 | 17 | 0.378
D5a2a1b1 | 16311 | 2 | 0.044
D5a2a1b1 | 185 | 1 | 0.022
D5a2a1b1 | 22 | 1 | 0.022
D5a2a1b1 | 2404 | 1 | 0.022
D5a2a1b1 | 493 | 2 | 0.044
D5a2a2 | 13584 | 4 | 0.04
D5a2a2 | 14207 | 1 | 0.01
D5a2a2 | 14389G | 1 | 0.01
D5a2a2 | 14530 | 3 | 0.03
D5a2a2 | 15388 | 2 | 0.02
D5a2a2 | 16111A | 1 | 0.01
D5a2a2 | 16192 | 2 | 0.02
D5a2a2 | 16243 | 1 | 0.01
D5a2a2 | 16274 | 3 | 0.03
D5a2a2 | 16311 | 1 | 0.01
D5a2a2 | 204 | 1 | 0.01
D5a2a2 | 2399 | 1 | 0.01
D5a2a2 | 310 | 18 | 0.178
D5a2a2 | 3460 | 1 | 0.01
D5a2a2 | 385 | 2 | 0.02
D5a2a2 | 4089 | 1 | 0.01
D5a2a2 | 4880 | 8 | 0.079
D5a2a2 | 573.5C | 1 | 0.01
D5a2a2 | 7389 | 1 | 0.01
D5a2a2 | 8348 | 1 | 0.01
D5a2b | 10861 | 1 | 0.017

D5a2b | 12528 | 2 | 0.034
D5a2b | 13759 | 1 | 0.017
D5a2b | 14305 | 2 | 0.034
D5a2b | 15166 | 4 | 0.068
D5a2b | 153 | 1 | 0.017
D5a2b | 15323 | 2 | 0.034
D5a2b | 1555 | 2 | 0.034
D5a2b | 15928 | 1 | 0.017
D5a2b | 16140A | 1 | 0.017
D5a2b | 16142 | 1 | 0.017
D5a2b | 16149C | 1 | 0.017
D5a2b | 16152 | 1 | 0.017
D5a2b | 16152.1C | 1 | 0.017
D5a2b | 16154G | 1 | 0.017
D5a2b | 16155 | 1 | 0.017
D5a2b | 16157G | 1 | 0.017
D5a2b | 16159A | 1 | 0.017
D5a2b | 16161A | 1 | 0.017
D5a2b | 16175C | 1 | 0.017
D5a2b | 16179 | 1 | 0.017
D5a2b | 16192 | 3 | 0.051
D5a2b | 16311 | 1 | 0.017
D5a2b | 16399 | 8 | 0.136
D5a2b | 16483 | 6 | 0.102
D5a2b | 1806 | 1 | 0.017
D5a2b | 207 | 6 | 0.102
D5a2b | 3337 | 1 | 0.017
D5a2b | 347d | 1 | 0.017
D5a2b | 3866 | 4 | 0.068
D5a2b | 4742 | 2 | 0.034
D5a2b | 5211 | 3 | 0.051
D5a2b | 5894C | 1 | 0.017
D5a2b | 6018 | 1 | 0.017
D5a2b | 6645 | 1 | 0.017
D5a2b | 735 | 1 | 0.017
D5a2b | 8020 | 1 | 0.017
D5a2b | 8295d | 2 | 0.034
D5a2b | 8454 | 2 | 0.034
D5a2b | 8857 | 2 | 0.034
D5a3 | 11 | 1 | 0.014
D5a3 | 114 | 1 | 0.014
D5a3 | 125 | 1 | 0.014
D5a3 | 13135 | 1 | 0.014
D5a3 | 14020 | 1 | 0.014
D5a3 | 146 | 6 | 0.087
D5a3 | 148 | 1 | 0.014
D5a3 | 151G | 1 | 0.014
D5a3 | 153 | 1 | 0.014
D5a3 | 15948d | 1 | 0.014
D5a3 | 16045 | 1 | 0.014
D5a3 | 16051 | 1 | 0.014
D5a3 | 16092 | 3 | 0.043
D5a3 | 16131 | 2 | 0.029
D5a3 | 16172 | 1 | 0.014
D5a3 | 16178 | 20 | 0.29
D5a3 | 16179 | 1 | 0.014
D5a3 | 16192-16193d | 4 | 0.058
D5a3 | 16193d | 3 | 0.043
D5a3 | 16194C | 3 | 0.043
D5a3 | 16195 | 3 | 0.043
D5a3 | 16219 | 1 | 0.014
D5a3 | 16221 | 1 | 0.014
D5a3 | 16260 | 3 | 0.043
D5a3 | 16263 | 1 | 0.014
D5a3 | 16267 | 1 | 0.014
D5a3 | 16280 | 1 | 0.014
D5a3 | 16311 | 4 | 0.058
D5a3 | 16318 | 1 | 0.014
D5a3 | 16323 | 1 | 0.014
D5a3 | 16359A | 1 | 0.014
D5a3 | 16384 | 1 | 0.014
D5a3 | 16390 | 1 | 0.014
D5a3 | 16405T | 1 | 0.014
D5a3 | 16426A | 1 | 0.014
D5a3 | 16447 | 1 | 0.014
D5a3 | 16457 | 1 | 0.014
D5a3 | 187 | 1 | 0.014
D5a3 | 195 | 1 | 0.014
D5a3 | 200 | 19 | 0.275
D5a3 | 202T | 1 | 0.014
D5a3 | 204 | 2 | 0.029
D5a3 | 205 | 1 | 0.014
D5a3 | 207 | 3 | 0.043
D5a3 | 263C | 2 | 0.029
D5a3 | 288T | 1 | 0.014
D5a3 | 309.3C | 1 | 0.014
D5a3 | 316C | 1 | 0.014
D5a3 | 391 | 1 | 0.014
D5a3 | 400 | 1 | 0.014
D5a3 | 405 | 1 | 0.014
D5a3 | 438d | 1 | 0.014
D5a3 | 455d | 1 | 0.014
D5a3 | 457 | 1 | 0.014
D5a3 | 486 | 1 | 0.014
D5a3 | 4924 | 1 | 0.014
D5a3 | 518 | 1 | 0.014
D5a3 | 55A | 1 | 0.014
D5a3 | 563 | 1 | 0.014
D5a3 | 569 | 1 | 0.014
D5a3 | 573.3C | 1 | 0.014
D5a3 | 5911 | 1 | 0.014
D5a3 | 71T | 1 | 0.014
D5a3 | 78 | 1 | 0.014
D5a3 | 86 | 1 | 0.014
D5a3 | 93 | 2 | 0.029
D5a3a | 11215 | 3 | 0.057
D5a3a | 11257 | 1 | 0.019
D5a3a | 11722 | 1 | 0.019
D5a3a | 15172 | 3 | 0.057
D5a3a | 15287 | 1 | 0.019
D5a3a | 16093 | 12 | 0.226

D5a3a | 16111 | 26 | 0.491
D5a3a | 16129 | 5 | 0.094
D5a3a | 16145 | 1 | 0.019
D5a3a | 16173 | 1 | 0.019
D5a3a | 16181C | 5 | 0.094
D5a3a | 16256 | 3 | 0.057
D5a3a | 16293C | 1 | 0.019
D5a3a | 16296.1C | 1 | 0.019
D5a3a | 16301 | 1 | 0.019
D5a3a | 16399 | 6 | 0.113
D5a3a | 308-309d | 1 | 0.019
D5a3a | 309d | 1 | 0.019
D5a3a | 3212 | 1 | 0.019
D5a3a | 471 | 1 | 0.019
D5a3a | 4823 | 1 | 0.019
D5a3a | 573.1C | 1 | 0.019
D5a3a | 6253 | 3 | 0.057
D5a3a | 727 | 1 | 0.019
D5a3a | 7797 | 3 | 0.057
D5a3a | 9116 | 1 | 0.019
D5a3a1 | 1291A | 2 | 0.067
D5a3a1 | 152 | 2 | 0.067
D5a3a1 | 15530 | 2 | 0.067
D5a3a1 | 16126 | 1 | 0.033
D5a3a1 | 16129 | 2 | 0.067
D5a3a1 | 16169 | 2 | 0.067
D5a3a1 | 16209 | 2 | 0.067
D5a3a1 | 16270 | 2 | 0.067
D5a3a1 | 16295 | 1 | 0.033
D5a3a1 | 6264 | 1 | 0.033
D5a3a1 | 8119 | 2 | 0.067
D5a3a1 | 9738 | 1 | 0.033
D5a3a1a | 10685 | 1 | 0.033
D5a3a1a | 12811 | 1 | 0.033
D5a3a1a | 146 | 2 | 0.067
D5a3a1a | 16194C | 1 | 0.033
D5a3a1a | 16195 | 1 | 0.033
D5a3a1a | 16327 | 2 | 0.067
D5a3a1a | 315d | 1 | 0.033
D5a'b | 152 | 2 | 0.02
D5a'b | 16051 | 1 | 0.01
D5a'b | 16129 | 2 | 0.02
D5a'b | 16244 | 1 | 0.01
D5a'b | 16467 | 4 | 0.04
D5a'b | 195 | 1 | 0.01
D5a'b | 459d | 2 | 0.02
D5a'b | 681 | 2 | 0.02
D5b | 11023 | 4 | 0.029
D5b | 12224 | 4 | 0.029
D5b | 13145 | 1 | 0.007
D5b | 1462 | 4 | 0.029
D5b | 16076d | 1 | 0.007
D5b | 16160 | 1 | 0.007
D5b | 16166 | 1 | 0.007
D5b | 16172 | 3 | 0.022
D5b | 16184A | 1 | 0.007
D5b | 16194C | 2 | 0.014
D5b | 16195 | 2 | 0.014
D5b | 16212 | 1 | 0.007
D5b | 16217 | 1 | 0.007
D5b | 16242 | 1 | 0.007
D5b | 16258T | 2 | 0.014
D5b | 16260.1G | 4 | 0.029
D5b | 16265C | 1 | 0.007
D5b | 16266 | 1 | 0.007
D5b | 1822 | 4 | 0.029
D5b | 204 | 2 | 0.014
D5b | 207 | 1 | 0.007
D5b | 309d | 1 | 0.007
D5b | 3744 | 4 | 0.029
D5b | 4724 | 1 | 0.007
D5b | 511 | 1 | 0.007
D5b | 740 | 1 | 0.007
D5b | 7964 | 4 | 0.029
D5b | 8412 | 4 | 0.029
D5b | 8530 | 4 | 0.029
D5b | 8602A | 1 | 0.007
D5b | 865 | 1 | 0.007
D5b | 94 | 1 | 0.007
D5b | 9764 | 1 | 0.007
D5b1 | 11128 | 2 | 0.014
D5b1 | 13269 | 2 | 0.014
D5b1 | 152 | 21 | 0.151
D5b1 | 16014 | 1 | 0.007
D5b1 | 16104 | 2 | 0.014
D5b1 | 16145 | 1 | 0.007
D5b1 | 16167 | 2 | 0.014
D5b1 | 16172 | 2 | 0.014
D5b1 | 16214 | 1 | 0.007
D5b1 | 16273 | 4 | 0.029
D5b1 | 194 | 1 | 0.007
D5b1 | 457 | 1 | 0.007
D5b1 | 4943 | 2 | 0.014
D5b1 | 7280 | 2 | 0.014
D5b1 | 9554 | 2 | 0.014
D5b1a | 16051 | 2 | 0.014
D5b1a | 16076d | 1 | 0.007
D5b1a | 16150 | 2 | 0.014
D5b1a | 16153 | 1 | 0.007
D5b1a | 16160 | 1 | 0.007
D5b1a | 16166 | 1 | 0.007
D5b1a | 16184A | 1 | 0.007
D5b1a | 16192 | 2 | 0.014
D5b1a | 16194C | 2 | 0.014
D5b1a | 16195 | 2 | 0.014
D5b1a | 16212 | 1 | 0.007
D5b1a | 16242 | 1 | 0.007
D5b1a | 16256 | 2 | 0.014
D5b1a | 16258T | 2 | 0.014
D5b1a | 16260.1G | 1 | 0.007
D5b1a | 16264 | 2 | 0.014

D5b1a | 16265C | 1 | 0.007
D5b1a | 16266 | 1 | 0.007
D5b1a | 16288 | 6 | 0.043
D5b1a | 16299 | 1 | 0.007
D5b1a | 16362A | 1 | 0.007
D5b1a | 189 | 1 | 0.007
D5b1a | 207 | 1 | 0.007
D5b1a | 228 | 2 | 0.014
D5b1a | 309.3C | 2 | 0.014
D5b1a | 309d | 1 | 0.007
D5b1a | 511 | 1 | 0.007
D5b1a | 545 | 5 | 0.036
D5b1a | 865 | 1 | 0.007
D5b1a | 94 | 1 | 0.007
D5b1a1 | 13029 | 1 | 0.029
D5b1a1 | 152 | 2 | 0.057
D5b1a1 | 16091 | 4 | 0.114
D5b1a1 | 16132 | 1 | 0.029
D5b1a1 | 16179 | 1 | 0.029
D5b1a1 | 16213 | 1 | 0.029
D5b1a1 | 16270 | 1 | 0.029
D5b1a1 | 16478G | 1 | 0.029
D5b1a1 | 1842 | 2 | 0.057
D5b1a1 | 189 | 2 | 0.057
D5b1a1 | 316C | 1 | 0.029
D5b1a1 | 61 | 1 | 0.029
D5b1a1 | 6749 | 1 | 0.029
D5b1a1 | 7076 | 1 | 0.029
D5b1a1 | 9590 | 1 | 0.029
D5b1a2 | 16325 | 1 | 0.05
D5b1a2 | 183 | 1 | 0.05
D5b1a2 | 225 | 2 | 0.1
D5b1a2 | 236 | 1 | 0.05
D5b1a2 | 4186 | 1 | 0.05
D5b1b | 14082 | 2 | 0.014
D5b1b | 146 | 2 | 0.014
D5b1b | 1598 | 2 | 0.014
D5b1b | 16076d | 1 | 0.007
D5b1b | 16093 | 2 | 0.014
D5b1b | 16160 | 1 | 0.007
D5b1b | 16166 | 1 | 0.007
D5b1b | 16184A | 1 | 0.007
D5b1b | 16194C | 2 | 0.014
D5b1b | 16195 | 2 | 0.014
D5b1b | 16212 | 1 | 0.007
D5b1b | 16242 | 1 | 0.007
D5b1b | 16258T | 2 | 0.014
D5b1b | 16260.1G | 4 | 0.029
D5b1b | 16265C | 1 | 0.007
D5b1b | 16266 | 1 | 0.007
D5b1b | 195 | 3 | 0.022
D5b1b | 207 | 1 | 0.007
D5b1b | 252 | 2 | 0.014
D5b1b | 309d | 1 | 0.007
D5b1b | 511 | 1 | 0.007
D5b1b | 8063 | 1 | 0.007
D5b1b | 865 | 1 | 0.007
D5b1b | 8793 | 2 | 0.014
D5b1b | 94 | 1 | 0.007
D5b1b1 | 103 | 1 | 0.036
D5b1b1 | 13224 | 1 | 0.036
D5b1b1 | 146 | 1 | 0.036
D5b1b1 | 16058C | 1 | 0.036
D5b1b1 | 16086 | 1 | 0.036
D5b1b1 | 16093 | 1 | 0.036
D5b1b1 | 16252C | 1 | 0.036
D5b1b1 | 4695 | 1 | 0.036
D5b1b2 | 10320 | 1 | 0.018
D5b1b2 | 11869 | 2 | 0.036
D5b1b2 | 13764 | 1 | 0.018
D5b1b2 | 146 | 4 | 0.071
D5b1b2 | 152 | 4 | 0.071
D5b1b2 | 16076d | 1 | 0.018
D5b1b2 | 16093 | 2 | 0.036
D5b1b2 | 16129 | 1 | 0.018
D5b1b2 | 16193d | 1 | 0.018
D5b1b2 | 16249 | 1 | 0.018
D5b1b2 | 16259 | 2 | 0.036
D5b1b2 | 16326 | 3 | 0.054
D5b1b2 | 200 | 1 | 0.018
D5b1b2 | 207 | 3 | 0.054
D5b1b2 | 309.3C | 1 | 0.018
D5b1b2 | 309d | 1 | 0.018
D5b1b2 | 499 | 1 | 0.018
D5b1b2 | 5206G | 2 | 0.036
D5b1b2 | 520A | 1 | 0.018
D5b1b2 | 6465 | 2 | 0.036
D5b1b2 | 8290 | 1 | 0.018
D5b1b2 | 8886 | 2 | 0.036
D5b1b2 | 9168 | 3 | 0.054
D5b1b2 | 93 | 1 | 0.018
D5b1b2 | 94 | 3 | 0.054
D5b1b2 | 9531 | 2 | 0.036
D5b1c | 11497 | 4 | 0.027
D5b1c | 12297 | 1 | 0.007
D5b1c | 12501 | 1 | 0.007
D5b1c | 15400 | 4 | 0.027
D5b1c | 16092 | 4 | 0.027
D5b1c | 16093 | 1 | 0.007
D5b1c | 16160 | 1 | 0.007
D5b1c | 16166 | 1 | 0.007
D5b1c | 16178 | 3 | 0.02
D5b1c | 16184A | 1 | 0.007
D5b1c | 16212 | 1 | 0.007
D5b1c | 16217 | 10 | 0.068
D5b1c | 16260.1G | 1 | 0.007
D5b1c | 16261 | 4 | 0.027
D5b1c | 16265C | 1 | 0.007
D5b1c | 16266 | 1 | 0.007
D5b1c | 16311 | 15 | 0.102
D5b1c | 16317 | 1 | 0.007

D5b1c | 16319 | 10 | 0.068
D5b1c | 185 | 8 | 0.054
D5b1c | 189 | 2 | 0.014
D5b1c | 207 | 1 | 0.007
D5b1c | 237 | 8 | 0.054
D5b1c | 309d | 1 | 0.007
D5b1c | 3338 | 1 | 0.007
D5b1c | 3510 | 1 | 0.007
D5b1c | 4092 | 4 | 0.027
D5b1c | 511 | 1 | 0.007
D5b1c | 8485 | 1 | 0.007
D5b1c | 865 | 1 | 0.007
D5b1c | 94 | 1 | 0.007
D5b1c1 | 10034 | 2 | 0.039
D5b1c1 | 13681 | 1 | 0.02
D5b1c1 | 16076d | 1 | 0.02
D5b1c1 | 16129 | 2 | 0.039
D5b1c1 | 16179 | 3 | 0.059
D5b1c1 | 16195 | 1 | 0.02
D5b1c1 | 16233 | 1 | 0.02
D5b1c1 | 16262 | 1 | 0.02
D5b1c1 | 16264 | 1 | 0.02
D5b1c1 | 16287 | 2 | 0.039
D5b1c1 | 16295 | 1 | 0.02
D5b1c1 | 16368 | 1 | 0.02
D5b1c1 | 16399 | 11 | 0.216
D5b1c1 | 188 | 2 | 0.039
D5b1c1 | 189 | 2 | 0.039
D5b1c1 | 195 | 3 | 0.059
D5b1c1 | 3111T | 6 | 0.118
D5b1c1 | 5147 | 2 | 0.039
D5b1c1 | 6626 | 1 | 0.02
D5b1c1 | 9128 | 6 | 0.118
D5b1c1a | 10202 | 3 | 0.022
D5b1c1a | 10548C | 3 | 0.022
D5b1c1a | 114 | 1 | 0.007
D5b1c1a | 12501 | 9 | 0.066
D5b1c1a | 13681 | 1 | 0.007
D5b1c1a | 14305 | 1 | 0.007
D5b1c1a | 15287 | 2 | 0.015
D5b1c1a | 16037 | 1 | 0.007
D5b1c1a | 16051 | 1 | 0.007
D5b1c1a | 16076d | 1 | 0.007
D5b1c1a | 16086 | 9 | 0.066
D5b1c1a | 16092 | 112 | 0.824
D5b1c1a | 16095 | 1 | 0.007
D5b1c1a | 16124 | 2 | 0.015
D5b1c1a | 16126 | 1 | 0.007
D5b1c1a | 16129 | 7 | 0.051
D5b1c1a | 16172 | 1 | 0.007
D5b1c1a | 16184 | 14 | 0.103
D5b1c1a | 16239 | 3 | 0.022
D5b1c1a | 16242G | 2 | 0.015
D5b1c1a | 16256 | 1 | 0.007
D5b1c1a | 16291 | 2 | 0.015
D5b1c1a | 16293C | 1 | 0.007
D5b1c1a | 16297 | 1 | 0.007
D5b1c1a | 16311 | 2 | 0.015
D5b1c1a | 16319 | 3 | 0.022
D5b1c1a | 16335 | 1 | 0.007
D5b1c1a | 16355 | 1 | 0.007
D5b1c1a | 16356 | 1 | 0.007
D5b1c1a | 16390 | 1 | 0.007
D5b1c1a | 16398 | 1 | 0.007
D5b1c1a | 16399 | 1 | 0.007
D5b1c1a | 16439A | 2 | 0.015
D5b1c1a | 16526 | 1 | 0.007
D5b1c1a | 183 | 1 | 0.007
D5b1c1a | 189 | 2 | 0.015
D5b1c1a | 195 | 2 | 0.015
D5b1c1a | 225 | 1 | 0.007
D5b1c1a | 309d | 1 | 0.007
D5b1c1a | 345G | 1 | 0.007
D5b1c1a | 420G | 1 | 0.007
D5b1c1a | 4216 | 1 | 0.007
D5b1c1a | 459d | 2 | 0.015
D5b1c1a | 5303G | 1 | 0.007
D5b1c1a | 5899.2C | 1 | 0.007
D5b1c1a | 5899.3C | 1 | 0.007
D5b1c1a | 6260 | 1 | 0.007
D5b1c1a | 7403 | 1 | 0.007
D5b1c1a | 9545 | 1 | 0.007
D5b1d | 11252 | 2 | 0.017
D5b1d | 13563 | 2 | 0.017
D5b1d | 14230 | 2 | 0.017
D5b1d | 14821 | 1 | 0.009
D5b1d | 152 | 1 | 0.009
D5b1d | 15286 | 1 | 0.009
D5b1d | 15451 | 1 | 0.009
D5b1d | 15496 | 1 | 0.009
D5b1d | 15902 | 2 | 0.017
D5b1d | 16086 | 1 | 0.009
D5b1d | 16093 | 10 | 0.087
D5b1d | 16109 | 1 | 0.009
D5b1d | 16129 | 11 | 0.096
D5b1d | 16173 | 3 | 0.026
D5b1d | 16194C | 9 | 0.078
D5b1d | 16195 | 7 | 0.061
D5b1d | 16292 | 1 | 0.009
D5b1d | 16293 | 2 | 0.017
D5b1d | 16295 | 1 | 0.009
D5b1d | 16311 | 1 | 0.009
D5b1d | 16532d | 2 | 0.017
D5b1d | 247 | 1 | 0.009
D5b1d | 252 | 2 | 0.017
D5b1d | 310 | 1 | 0.009
D5b1d | 311 | 1 | 0.009
D5b1d | 391 | 1 | 0.009
D5b1d | 593 | 1 | 0.009
D5b1d | 60 | 1 | 0.009
D5b1d | 8979 | 1 | 0.009

D5b1d | 9316 | 1 | 0.009
D5b2 | 16126 | 1 | 0.25
D5b2 | 16193.3C | 1 | 0.25
D5b2 | 16311 | 1 | 0.25
D5b2 | 309.3C | 2 | 0.5
D5b2 | 69 | 1 | 0.25
D5b2 | 9554 | 1 | 0.25
D5b3 | 11288 | 1 | 0.007
D5b3 | 11293 | 3 | 0.021
D5b3 | 13386 | 1 | 0.007
D5b3 | 14094 | 4 | 0.028
D5b3 | 14276A | 1 | 0.007
D5b3 | 14798 | 2 | 0.014
D5b3 | 14821G | 3 | 0.021
D5b3 | 152 | 1 | 0.007
D5b3 | 15616 | 2 | 0.014
D5b3 | 16111 | 2 | 0.014
D5b3 | 16129 | 1 | 0.007
D5b3 | 16160 | 1 | 0.007
D5b3 | 16166 | 1 | 0.007
D5b3 | 16184A | 1 | 0.007
D5b3 | 16212 | 1 | 0.007
D5b3 | 16234 | 1 | 0.007
D5b3 | 16260.1G | 1 | 0.007
D5b3 | 16265C | 1 | 0.007
D5b3 | 16266 | 1 | 0.007
D5b3 | 16273 | 7 | 0.049
D5b3 | 16287 | 2 | 0.014
D5b3 | 194 | 5 | 0.035
D5b3 | 195 | 1 | 0.007
D5b3 | 207 | 1 | 0.007
D5b3 | 309.3C | 1 | 0.007
D5b3 | 309d | 1 | 0.007
D5b3 | 3335 | 1 | 0.007
D5b3 | 3892 | 1 | 0.007
D5b3 | 4643 | 1 | 0.007
D5b3 | 511 | 1 | 0.007
D5b3 | 759 | 1 | 0.007
D5b3 | 8158 | 3 | 0.021
D5b3 | 865 | 1 | 0.007
D5b3 | 8968 | 1 | 0.007
D5b3 | 94 | 1 | 0.007
D5b3 | 9548 | 1 | 0.007
D5b3a | 12361 | 8 | 0.055
D5b3a | 12528 | 1 | 0.007
D5b3a | 13434 | 7 | 0.048
D5b3a | 14905 | 2 | 0.014
D5b3a | 16017 | 1 | 0.007
D5b3a | 16129 | 1 | 0.007
D5b3a | 16160 | 1 | 0.007
D5b3a | 16166 | 1 | 0.007
D5b3a | 16184A | 1 | 0.007
D5b3a | 16212 | 1 | 0.007
D5b3a | 16260.1G | 4 | 0.028
D5b3a | 16265C | 1 | 0.007
D5b3a | 16266 | 1 | 0.007
D5b3a | 207 | 1 | 0.007
D5b3a | 2206A | 1 | 0.007
D5b3a | 309d | 1 | 0.007
D5b3a | 314-315d | 1 | 0.007
D5b3a | 318 | 1 | 0.007
D5b3a | 4452 | 1 | 0.007
D5b3a | 511 | 1 | 0.007
D5b3a | 865 | 1 | 0.007
D5b3a | 94 | 1 | 0.007
D5b3a1 | 14296 | 1 | 0.023
D5b3a1 | 143 | 18 | 0.409
D5b3a1 | 16051 | 1 | 0.023
D5b3a1 | 16092 | 1 | 0.023
D5b3a1 | 16213 | 1 | 0.023
D5b3a1 | 16292 | 1 | 0.023
D5b3a1 | 4491 | 3 | 0.068
D5b4 | 10192 | 1 | 0.01
D5b4 | 11536 | 1 | 0.01
D5b4 | 13477 | 1 | 0.01
D5b4 | 13812 | 3 | 0.029
D5b4 | 14094 | 2 | 0.019
D5b4 | 14797 | 1 | 0.01
D5b4 | 14798 | 1 | 0.01
D5b4 | 15110 | 3 | 0.029
D5b4 | 152 | 1 | 0.01
D5b4 | 15617 | 1 | 0.01
D5b4 | 15734 | 4 | 0.038
D5b4 | 16129 | 1 | 0.01
D5b4 | 16166d | 2 | 0.019
D5b4 | 16188 | 1 | 0.01
D5b4 | 16213 | 1 | 0.01
D5b4 | 16214 | 4 | 0.038
D5b4 | 16291 | 4 | 0.038
D5b4 | 16311 | 1 | 0.01
D5b4 | 16326T | 1 | 0.01
D5b4 | 185 | 3 | 0.029
D5b4 | 1868T | 1 | 0.01
D5b4 | 189 | 1 | 0.01
D5b4 | 195 | 3 | 0.029
D5b4 | 2028 | 1 | 0.01
D5b4 | 204 | 1 | 0.01
D5b4 | 237 | 2 | 0.019
D5b4 | 239 | 1 | 0.01
D5b4 | 310 | 1 | 0.01
D5b4 | 3826 | 1 | 0.01
D5b4 | 711 | 1 | 0.01
D5b4 | 7598 | 1 | 0.01
D5b4 | 769 | 1 | 0.01
D5b4 | 8464 | 1 | 0.01
D5b4 | 8602 | 1 | 0.01
D5b4 | 8701 | 6 | 0.057
D5b4 | 9175A | 2 | 0.019
D5c | 146 | 1 | 0.333
D5c | 16079 | 1 | 0.333
D5c | 16093 | 1 | 0.333

D5c | 16145 | 1 | 0.333
D5c | 16241 | 1 | 0.333
D5c | 16294 | 1 | 0.333
D5c | 709 | 1 | 0.333
D5c+16311 | 1120 | 1 | 0.071
D5c+16311 | 12945 | 1 | 0.071
D5c+16311 | 14470 | 1 | 0.071
D5c+16311 | 1524 | 1 | 0.071
D5c+16311 | 15737 | 1 | 0.071
D5c+16311 | 16086 | 4 | 0.286
D5c+16311 | 16126 | 4 | 0.286
D5c+16311 | 16136 | 1 | 0.071
D5c+16311 | 16234 | 9 | 0.643
D5c+16311 | 16295 | 4 | 0.286
D5c+16311 | 16297 | 4 | 0.286
D5c+16311 | 16299 | 4 | 0.286
D5c+16311 | 16316 | 1 | 0.071
D5c+16311 | 195 | 1 | 0.071
D5c+16311 | 310 | 4 | 0.286
D5c+16311 | 315d | 2 | 0.143
D5c+16311 | 3359 | 1 | 0.071
D5c+16311 | 3396 | 1 | 0.071
D5c+16311 | 3720 | 1 | 0.071
D5c+16311 | 4080 | 1 | 0.071
D5c+16311 | 521 | 1 | 0.071
D5c+16311 | 7269 | 1 | 0.071
D5c+16311 | 7408 | 1 | 0.071
D5c+16311 | 9845 | 1 | 0.071
D5c+16311 | 9970 | 1 | 0.071
D5c1 | 10289 | 1 | 0.167
D5c1 | 16051 | 6 | 1
D5c1 | 16240 | 1 | 0.167
D5c1 | 16319 | 1 | 0.167
D5c1 | 16320 | 5 | 0.833
D5c1 | 4025 | 1 | 0.167
D5c1 | 94 | 1 | 0.167
D5c1a | 10352 | 1 | 0.028
D5c1a | 11914 | 1 | 0.028
D5c1a | 12236 | 1 | 0.028
D5c1a | 12444 | 1 | 0.028
D5c1a | 13104 | 1 | 0.028
D5c1a | 14696 | 1 | 0.028
D5c1a | 15128 | 1 | 0.028
D5c1a | 15529 | 1 | 0.028
D5c1a | 1555 | 1 | 0.028
D5c1a | 15928 | 1 | 0.028
D5c1a | 16093 | 7 | 0.194
D5c1a | 16129 | 4 | 0.111
D5c1a | 16257 | 1 | 0.028
D5c1a | 16290 | 12 | 0.333
D5c1a | 16293C | 3 | 0.083
D5c1a | 16296.1C | 3 | 0.083
D5c1a | 16304 | 1 | 0.028
D5c1a | 16311 | 1 | 0.028
D5c1a | 16325 | 1 | 0.028
D5c1a | 16343 | 1 | 0.028
D5c1a | 16465 | 1 | 0.028
D5c1a | 1802 | 1 | 0.028
D5c1a | 185 | 5 | 0.139
D5c1a | 195 | 12 | 0.333
D5c1a | 3221 | 1 | 0.028
D5c1a | 3395 | 1 | 0.028
D5c1a | 3768 | 1 | 0.028
D5c1a | 4659 | 1 | 0.028
D5c1a | 5153 | 1 | 0.028
D5c1a | 5261 | 2 | 0.056
D5c1a | 5585 | 1 | 0.028
D5c1a | 6614 | 1 | 0.028
D5c1a | 7133A | 1 | 0.028
D5c1a | 8764 | 1 | 0.028
D5c1a | 9139 | 1 | 0.028
D5c1a | 9554 | 1 | 0.028
D5c2 | 12696 | 1 | 0.059
D5c2 | 13260 | 1 | 0.059
D5c2 | 14484 | 1 | 0.059
D5c2 | 15562 | 1 | 0.059
D5c2 | 16079 | 1 | 0.059
D5c2 | 16169 | 1 | 0.059
D5c2 | 16184 | 1 | 0.059
D5c2 | 16188 | 2 | 0.118
D5c2 | 16210 | 1 | 0.059
D5c2 | 16269 | 1 | 0.059
D5c2 | 16356 | 1 | 0.059
D5c2 | 185 | 1 | 0.059
D5c2 | 200 | 1 | 0.059
D5c2 | 295A | 2 | 0.118
D5c2 | 3316 | 1 | 0.059
D5c2 | 4317 | 1 | 0.059
D5c2 | 5442 | 1 | 0.059
D5c2 | 5554 | 1 | 0.059
D5c2 | 7643 | 1 | 0.059
D5c2 | 9545 | 1 | 0.059
D6 | 146 | 1 | 0.011
D6 | 16086 | 2 | 0.022
D6 | 16092 | 2 | 0.022
D6 | 16140 | 1 | 0.011
D6 | 16171 | 2 | 0.022
D6 | 16184 | 3 | 0.032
D6 | 16185 | 1 | 0.011
D6 | 16232A | 1 | 0.011
D6 | 16242 | 1 | 0.011
D6 | 16243 | 1 | 0.011
D6 | 16291 | 1 | 0.011
D6 | 16319 | 1 | 0.011
D6 | 16355 | 1 | 0.011
D6 | 16356 | 1 | 0.011
D6 | 16390 | 1 | 0.011
D6 | 309.3C | 1 | 0.011
D6 | 94 | 2 | 0.022
D6a | 11017 | 1 | 0.014
D6a | 119 | 1 | 0.014

D6a | 121 | 1 | 0.014
D6a | 14551 | 1 | 0.014
D6a | 16192 | 1 | 0.014
D6a | 292A | 1 | 0.014
D6a | 324 | 1 | 0.014
D6a | 508 | 1 | 0.014
D6a | 562 | 1 | 0.014
D6a | 580 | 1 | 0.014
D6a | 645.1A | 1 | 0.014
D6a | 676C | 1 | 0.014
D6a | 8473 | 1 | 0.014
D6a1 | 10680 | 1 | 0.01
D6a1 | 11809 | 1 | 0.01
D6a1 | 13327 | 1 | 0.01
D6a1 | 14344 | 1 | 0.01
D6a1 | 146 | 3 | 0.031
D6a1 | 15688 | 1 | 0.01
D6a1 | 16192 | 12 | 0.122
D6a1 | 16316 | 1 | 0.01
D6a1 | 200 | 1 | 0.01
D6a1 | 3745 | 1 | 0.01
D6a1 | 4670 | 1 | 0.01
D6a1 | 8251 | 1 | 0.01
D6a1a | 12882 | 1 | 0.01
D6a1a | 152 | 2 | 0.02
D6a1a | 16166 | 1 | 0.01
D6a1a | 16172 | 1 | 0.01
D6a1a | 16186 | 1 | 0.01
D6a1a | 16209 | 1 | 0.01
D6a1a | 16224 | 1 | 0.01
D6a1a | 16242 | 1 | 0.01
D6a1a | 16269 | 3 | 0.03
D6a1a | 16278 | 2 | 0.02
D6a1a | 16319 | 3 | 0.03
D6a1a | 16327 | 1 | 0.01
D6a1a | 3808 | 1 | 0.01
D6a1a | 4048 | 1 | 0.01
D6a1a | 4884 | 2 | 0.02
D6a1a | 6494 | 1 | 0.01
D6a1a | 71d | 1 | 0.01
D6a1a | 7598 | 2 | 0.02
D6a1a | 8618 | 1 | 0.01
D6a1a | 8895 | 1 | 0.01
D6a1a | 9910 | 1 | 0.01
D6a2 | 11167 | 2 | 0.024
D6a2 | 14869 | 1 | 0.012
D6a2 | 16093 | 1 | 0.012
D6a2 | 16187 | 2 | 0.024
D6a2 | 16234 | 1 | 0.012
D6a2 | 16247 | 2 | 0.024
D6a2 | 16292 | 1 | 0.012
D6a2 | 16297 | 1 | 0.012
D6a2 | 16316 | 1 | 0.012
D6a2 | 204 | 2 | 0.024
D6a2 | 207 | 2 | 0.024
D6a2 | 310 | 2 | 0.024
D6a2 | 315d | 2 | 0.024
D6a2 | 5319 | 3 | 0.037
D6a2 | 537 | 2 | 0.024
D6a2 | 573.1C | 1 | 0.012
D6a2 | 6480 | 2 | 0.024
D6a2 | 8459 | 1 | 0.012
D6a2 | 9377 | 1 | 0.012
D6a2 | 961 | 4 | 0.049
D6c | 10978 | 1 | 0.015
D6c | 12471 | 1 | 0.015
D6c | 13962 | 1 | 0.015
D6c | 146 | 8 | 0.119
D6c | 14832 | 1 | 0.015
D6c | 16184 | 6 | 0.09
D6c | 16214A | 1 | 0.015
D6c | 16218 | 1 | 0.015
D6c | 16256G | 1 | 0.015
D6c | 16294 | 2 | 0.03
D6c | 204 | 8 | 0.119
D6c | 207 | 2 | 0.03
D6c | 228T | 1 | 0.015
D6c | 3510 | 1 | 0.015
D6c | 3882 | 2 | 0.03
D6c | 4652 | 1 | 0.015
D6c | 8730 | 1 | 0.015
D6c | 8952 | 1 | 0.015
D6c1 | 12984 | 1 | 0.012
D6c1 | 14 | 1 | 0.012
D6c1 | 151 | 2 | 0.024
D6c1 | 16035 | 1 | 0.012
D6c1 | 16046 | 1 | 0.012
D6c1 | 16067 | 1 | 0.012
D6c1 | 16075 | 1 | 0.012
D6c1 | 16079 | 1 | 0.012
D6c1 | 16093 | 7 | 0.084
D6c1 | 16123 | 1 | 0.012
D6c1 | 16129 | 1 | 0.012
D6c1 | 16145 | 5 | 0.06
D6c1 | 16172 | 4 | 0.048
D6c1 | 16191 | 1 | 0.012
D6c1 | 16193.1T | 1 | 0.012
D6c1 | 16193.3C | 1 | 0.012
D6c1 | 16240C | 4 | 0.048
D6c1 | 16243 | 2 | 0.024
D6c1 | 16243A | 1 | 0.012
D6c1 | 16252 | 1 | 0.012
D6c1 | 16254 | 1 | 0.012
D6c1 | 16257A | 1 | 0.012
D6c1 | 16258C | 1 | 0.012
D6c1 | 16259 | 1 | 0.012
D6c1 | 16261 | 1 | 0.012
D6c1 | 16277T | 1 | 0.012
D6c1 | 16334A | 1 | 0.012
D6c1 | 16380d | 1 | 0.012
D6c1 | 16497 | 1 | 0.012

D6c1 | 16526 | 1 | 0.012
D6c1 | 16T | 3 | 0.036
D6c1 | 195 | 1 | 0.012
D6c1 | 208 | 1 | 0.012
D6c1 | 235 | 1 | 0.012
D6c1 | 239 | 3 | 0.036
D6c1 | 251 | 1 | 0.012
D6c1 | 267 | 1 | 0.012
D6c1 | 338 | 1 | 0.012
D6c1 | 34 | 1 | 0.012
D6c1 | 383 | 1 | 0.012
D6c1 | 495 | 1 | 0.012
D6c1 | 5357 | 2 | 0.024
D6c1 | 6260 | 2 | 0.024
D6c1 | 65G | 1 | 0.012
D6c1 | 68 | 1 | 0.012
D6c1 | 79 | 1 | 0.012
D6c1 | 94 | 1 | 0.012
D6c1a | 11950 | 1 | 0.012
D6c1a | 13227 | 2 | 0.025
D6c1a | 14 | 1 | 0.012
D6c1a | 151 | 2 | 0.025
D6c1a | 16035 | 1 | 0.012
D6c1a | 16046 | 1 | 0.012
D6c1a | 16067 | 1 | 0.012
D6c1a | 16075 | 1 | 0.012
D6c1a | 16079 | 1 | 0.012
D6c1a | 16093 | 7 | 0.086
D6c1a | 16123 | 1 | 0.012
D6c1a | 16129 | 1 | 0.012
D6c1a | 16145 | 5 | 0.062
D6c1a | 16172 | 4 | 0.049
D6c1a | 16191 | 1 | 0.012
D6c1a | 16193.1T | 1 | 0.012
D6c1a | 16193.3C | 1 | 0.012
D6c1a | 16240C | 4 | 0.049
D6c1a | 16243A | 1 | 0.012
D6c1a | 16252 | 1 | 0.012
D6c1a | 16254 | 1 | 0.012
D6c1a | 16257A | 1 | 0.012
D6c1a | 16258C | 1 | 0.012
D6c1a | 16259 | 1 | 0.012
D6c1a | 16261 | 1 | 0.012
D6c1a | 16277T | 1 | 0.012
D6c1a | 16290 | 3 | 0.037
D6c1a | 16334A | 1 | 0.012
D6c1a | 16380d | 1 | 0.012
D6c1a | 16497 | 1 | 0.012
D6c1a | 16526 | 1 | 0.012
D6c1a | 16T | 3 | 0.037
D6c1a | 195 | 1 | 0.012
D6c1a | 208 | 1 | 0.012
D6c1a | 235 | 1 | 0.012
D6c1a | 2371 | 1 | 0.012
D6c1a | 251 | 1 | 0.012
D6c1a | 267 | 1 | 0.012
D6c1a | 338 | 1 | 0.012
D6c1a | 34 | 1 | 0.012
D6c1a | 383 | 1 | 0.012
D6c1a | 495 | 1 | 0.012
D6c1a | 65G | 1 | 0.012
D6c1a | 68 | 1 | 0.012
D6c1a | 79 | 1 | 0.012
D6c1a | 94 | 1 | 0.012
E | 103 | 2 | 0.009
E | 16114 | 1 | 0.004
E | 16311 | 6 | 0.027
E1 | 103 | 2 | 0.009
E1 | 13434 | 1 | 0.004
E1 | 14443 | 1 | 0.004
E1 | 16114 | 1 | 0.004
E1 | 16311 | 6 | 0.027
E1 | 310 | 1 | 0.004
E1 | 372 | 1 | 0.004
E1 | 4311T | 1 | 0.004
E1 | 6620 | 1 | 0.004
E1a | 103 | 2 | 0.009
E1a | 16114 | 1 | 0.004
E1a | 16311 | 6 | 0.027
E1a1 | 103 | 2 | 0.009
E1a1 | 16114 | 1 | 0.004
E1a1 | 16129 | 1 | 0.004
E1a1 | 16172 | 1 | 0.004
E1a1 | 16311 | 6 | 0.026
E1a1 | 316 | 1 | 0.004
E1a1 | 3310 | 1 | 0.004
E1a1 | 7798A | 1 | 0.004
E1a1a | 103 | 2 | 0.009
E1a1a | 14443 | 2 | 0.009
E1a1a | 16114 | 1 | 0.004
E1a1a | 16221 | 2 | 0.009
E1a1a | 16311 | 6 | 0.026
E1a1a | 372 | 2 | 0.009
E1a1a | 5306 | 1 | 0.004
E1a1a | 7897 | 1 | 0.004
E1a1a1 | 10775 | 1 | 0.002
E1a1a1 | 10931 | 1 | 0.002
E1a1a1 | 11107 | 5 | 0.008
E1a1a1 | 11732 | 4 | 0.007
E1a1a1 | 11836 | 1 | 0.002
E1a1a1 | 11893 | 1 | 0.002
E1a1a1 | 11935 | 1 | 0.002
E1a1a1 | 12133 | 6 | 0.01
E1a1a1 | 12196 | 1 | 0.002
E1a1a1 | 12237 | 1 | 0.002
E1a1a1 | 1231T | 1 | 0.002
E1a1a1 | 12358 | 1 | 0.002
E1a1a1 | 12522 | 1 | 0.002
E1a1a1 | 12651 | 1 | 0.002
E1a1a1 | 12663A | 1 | 0.002
E1a1a1 | 12714 | 1 | 0.002

E1a1a1 | 12738 | 1 | 0.002
E1a1a1 | 12984G | 1 | 0.002
E1a1a1 | 13326 | 2 | 0.003
E1a1a1 | 13395 | 1 | 0.002
E1a1a1 | 13759 | 1 | 0.002
E1a1a1 | 13827 | 1 | 0.002
E1a1a1 | 14227 | 1 | 0.002
E1a1a1 | 14256 | 1 | 0.002
E1a1a1 | 143 | 1 | 0.002
E1a1a1 | 14364 | 2 | 0.003
E1a1a1 | 14405 | 2 | 0.003
E1a1a1 | 14443 | 180 | 0.3
E1a1a1 | 14560 | 1 | 0.002
E1a1a1 | 14566T | 1 | 0.002
E1a1a1 | 146 | 6 | 0.01
E1a1a1 | 14790 | 1 | 0.002
E1a1a1 | 1508 | 1 | 0.002
E1a1a1 | 152 | 5 | 0.008
E1a1a1 | 153 | 1 | 0.002
E1a1a1 | 15313 | 2 | 0.003
E1a1a1 | 15433 | 1 | 0.002
E1a1a1 | 1555 | 3 | 0.005
E1a1a1 | 15596 | 1 | 0.002
E1a1a1 | 15722A | 1 | 0.002
E1a1a1 | 15784 | 1 | 0.002
E1a1a1 | 15851 | 2 | 0.003
E1a1a1 | 15896 | 1 | 0.002
E1a1a1 | 15966d | 1 | 0.002
E1a1a1 | 16072G | 1 | 0.002
E1a1a1 | 16086 | 1 | 0.002
E1a1a1 | 16093 | 2 | 0.003
E1a1a1 | 16134 | 1 | 0.002
E1a1a1 | 16140 | 5 | 0.008
E1a1a1 | 16166 | 1 | 0.002
E1a1a1 | 16167 | 3 | 0.005
E1a1a1 | 16172 | 2 | 0.003
E1a1a1 | 16174 | 3 | 0.005
E1a1a1 | 16178 | 2 | 0.003
E1a1a1 | 16185 | 5 | 0.008
E1a1a1 | 16189 | 3 | 0.005
E1a1a1 | 16192 | 1 | 0.002
E1a1a1 | 16209 | 1 | 0.002
E1a1a1 | 16221 | 227 | 0.378
E1a1a1 | 16224 | 2 | 0.003
E1a1a1 | 16248 | 2 | 0.003
E1a1a1 | 16251d | 1 | 0.002
E1a1a1 | 16263 | 1 | 0.002
E1a1a1 | 16265T | 1 | 0.002
E1a1a1 | 16270 | 1 | 0.002
E1a1a1 | 16278 | 2 | 0.003
E1a1a1 | 16294 | 2 | 0.003
E1a1a1 | 16295 | 1 | 0.002
E1a1a1 | 16311 | 1 | 0.002
E1a1a1 | 16325 | 1 | 0.002
E1a1a1 | 16354 | 3 | 0.005
E1a1a1 | 182 | 1 | 0.002
E1a1a1 | 186 | 1 | 0.002
E1a1a1 | 195 | 4 | 0.007
E1a1a1 | 204 | 1 | 0.002
E1a1a1 | 2220 | 1 | 0.002
E1a1a1 | 2244 | 3 | 0.005
E1a1a1 | 2523 | 1 | 0.002
E1a1a1 | 2618 | 1 | 0.002
E1a1a1 | 264 | 1 | 0.002
E1a1a1 | 279 | 1 | 0.002
E1a1a1 | 2997 | 1 | 0.002
E1a1a1 | 310 | 5 | 0.008
E1a1a1 | 315d | 1 | 0.002
E1a1a1 | 316 | 2 | 0.003
E1a1a1 | 3395 | 1 | 0.002
E1a1a1 | 3594 | 1 | 0.002
E1a1a1 | 3693 | 1 | 0.002
E1a1a1 | 372 | 182 | 0.303
E1a1a1 | 372A | 1 | 0.002
E1a1a1 | 3849 | 2 | 0.003
E1a1a1 | 3995 | 1 | 0.002
E1a1a1 | 4109 | 1 | 0.002
E1a1a1 | 4487 | 1 | 0.002
E1a1a1 | 5082 | 1 | 0.002
E1a1a1 | 5201 | 1 | 0.002
E1a1a1 | 533 | 1 | 0.002
E1a1a1 | 5417 | 1 | 0.002
E1a1a1 | 55 | 2 | 0.003
E1a1a1 | 5581 | 1 | 0.002
E1a1a1 | 56C | 2 | 0.003
E1a1a1 | 5741T | 1 | 0.002
E1a1a1 | 5770G | 1 | 0.002
E1a1a1 | 5774 | 2 | 0.003
E1a1a1 | 5777 | 1 | 0.002
E1a1a1 | 6179 | 2 | 0.003
E1a1a1 | 6465 | 3 | 0.005
E1a1a1 | 6605 | 4 | 0.007
E1a1a1 | 6852 | 1 | 0.002
E1a1a1 | 6871T | 1 | 0.002
E1a1a1 | 709 | 6 | 0.01
E1a1a1 | 7265C | 1 | 0.002
E1a1a1 | 7389 | 1 | 0.002
E1a1a1 | 7444 | 1 | 0.002
E1a1a1 | 7498 | 2 | 0.003
E1a1a1 | 7789 | 1 | 0.002
E1a1a1 | 7897 | 6 | 0.01
E1a1a1 | 7967G | 1 | 0.002
E1a1a1 | 7970 | 1 | 0.002
E1a1a1 | 8020 | 1 | 0.002
E1a1a1 | 8080A | 2 | 0.003
E1a1a1 | 8093 | 1 | 0.002
E1a1a1 | 8251 | 1 | 0.002
E1a1a1 | 8281-8289d | 1 | 0.002
E1a1a1 | 8392 | 1 | 0.002
E1a1a1 | 8650 | 1 | 0.002
E1a1a1 | 9007 | 5 | 0.008

E1a1a1 | 9025 | 1 | 0.002
E1a1a1 | 9037 | 1 | 0.002
E1a1a1 | 9055 | 3 | 0.005
E1a1a1 | 9129 | 1 | 0.002
E1a1a1 | 9182 | 1 | 0.002
E1a1a1 | 93 | 4 | 0.007
E1a1a1 | 9512 | 2 | 0.003
E1a1a1 | 961 | 1 | 0.002
E1a1a1 | 965.2C | 1 | 0.002
E1a1a1 | 9661 | 1 | 0.002
E1a1a1 | 9699 | 1 | 0.002
E1a1a1 | 9983 | 4 | 0.007
E1a1a1a | 10410 | 1 | 0.003
E1a1a1a | 11075 | 4 | 0.011
E1a1a1a | 11311 | 4 | 0.011
E1a1a1a | 125 | 1 | 0.003
E1a1a1a | 127 | 1 | 0.003
E1a1a1a | 12723 | 1 | 0.003
E1a1a1a | 128 | 1 | 0.003
E1a1a1a | 12850 | 2 | 0.006
E1a1a1a | 13934 | 7 | 0.02
E1a1a1a | 14016 | 1 | 0.003
E1a1a1a | 146 | 1 | 0.003
E1a1a1a | 152 | 2 | 0.006
E1a1a1a | 15221 | 1 | 0.003
E1a1a1a | 15848 | 1 | 0.003
E1a1a1a | 16086 | 3 | 0.009
E1a1a1a | 16172 | 1 | 0.003
E1a1a1a | 16176 | 1 | 0.003
E1a1a1a | 16189 | 6 | 0.017
E1a1a1a | 16218 | 1 | 0.003
E1a1a1a | 16266 | 2 | 0.006
E1a1a1a | 16297 | 1 | 0.003
E1a1a1a | 16304 | 1 | 0.003
E1a1a1a | 16311 | 5 | 0.014
E1a1a1a | 1780 | 1 | 0.003
E1a1a1a | 1948d | 1 | 0.003
E1a1a1a | 195 | 1 | 0.003
E1a1a1a | 2595d | 1 | 0.003
E1a1a1a | 291d | 1 | 0.003
E1a1a1a | 310 | 15 | 0.043
E1a1a1a | 315.2C | 1 | 0.003
E1a1a1a | 315d | 14 | 0.04
E1a1a1a | 3488A | 1 | 0.003
E1a1a1a | 3736 | 2 | 0.006
E1a1a1a | 3866 | 1 | 0.003
E1a1a1a | 5471 | 1 | 0.003
E1a1a1a | 5777 | 5 | 0.014
E1a1a1a | 712A | 1 | 0.003
E1a1a1a | 7418 | 1 | 0.003
E1a1a1a | 8149 | 1 | 0.003
E1a1a1a | 8281-8289d | 1 | 0.003
E1a1a1a | 961 | 5 | 0.014
E1a1a1a | 965.2C | 5 | 0.014
E1a1a1b | 15034 | 1 | 0.004
E1a1a1b | 16114 | 1 | 0.004
E1a1a1b | 16117 | 1 | 0.004
E1a1a1b | 16129 | 4 | 0.016
E1a1a1b | 16145 | 1 | 0.004
E1a1a1b | 16153 | 1 | 0.004
E1a1a1b | 16169 | 1 | 0.004
E1a1a1b | 16193 | 1 | 0.004
E1a1a1b | 16218 | 1 | 0.004
E1a1a1b | 16243 | 1 | 0.004
E1a1a1b | 16265 | 1 | 0.004
E1a1a1b | 16271 | 1 | 0.004
E1a1a1b | 16304 | 2 | 0.008
E1a1a1b | 16319 | 1 | 0.004
E1a1a1b | 16320 | 1 | 0.004
E1a1a1b | 16325 | 1 | 0.004
E1a1a1b | 16356 | 2 | 0.008
E1a1a1b | 16523C | 1 | 0.004
E1a1a1b | 5319 | 1 | 0.004
E1a1a1b | 5393 | 1 | 0.004
E1a1a1b1 | 16114 | 1 | 0.004
E1a1a1b1 | 16117 | 1 | 0.004
E1a1a1b1 | 16129 | 4 | 0.016
E1a1a1b1 | 16145 | 1 | 0.004
E1a1a1b1 | 16153 | 1 | 0.004
E1a1a1b1 | 16169 | 1 | 0.004
E1a1a1b1 | 16193 | 1 | 0.004
E1a1a1b1 | 16218 | 1 | 0.004
E1a1a1b1 | 16243 | 1 | 0.004
E1a1a1b1 | 16248 | 1 | 0.004
E1a1a1b1 | 16265 | 1 | 0.004
E1a1a1b1 | 16271 | 1 | 0.004
E1a1a1b1 | 16296 | 1 | 0.004
E1a1a1b1 | 16304 | 2 | 0.008
E1a1a1b1 | 16319 | 1 | 0.004
E1a1a1b1 | 16320 | 1 | 0.004
E1a1a1b1 | 16325 | 1 | 0.004
E1a1a1b1 | 16356 | 2 | 0.008
E1a1a1b1 | 16523C | 1 | 0.004
E1a1a1b1 | 5277 | 1 | 0.004
E1a1a1b1 | 8953 | 1 | 0.004
E1a1a1b2 | 16114 | 1 | 0.004
E1a1a1b2 | 16117 | 1 | 0.004
E1a1a1b2 | 16129 | 4 | 0.016
E1a1a1b2 | 16145 | 1 | 0.004
E1a1a1b2 | 16153 | 1 | 0.004
E1a1a1b2 | 16169 | 1 | 0.004
E1a1a1b2 | 16193 | 1 | 0.004
E1a1a1b2 | 16243 | 1 | 0.004
E1a1a1b2 | 16265 | 1 | 0.004
E1a1a1b2 | 16271 | 1 | 0.004
E1a1a1b2 | 16319 | 1 | 0.004
E1a1a1b2 | 16320 | 1 | 0.004
E1a1a1b2 | 16325 | 1 | 0.004
E1a1a1b2 | 16523C | 1 | 0.004
E1a1a1b2 | 3345 | 7 | 0.028
E1a1a1c | 125 | 6 | 0.024

E1a1a1c | 127 | 6 | 0.024
E1a1a1c | 150 | 1 | 0.004
E1a1a1c | 15001 | 1 | 0.004
E1a1a1c | 153 | 1 | 0.004
E1a1a1c | 16093 | 7 | 0.028
E1a1a1c | 16111 | 1 | 0.004
E1a1a1c | 16114 | 2 | 0.008
E1a1a1c | 16117 | 1 | 0.004
E1a1a1c | 16145 | 1 | 0.004
E1a1a1c | 16189 | 16 | 0.063
E1a1a1c | 16193 | 1 | 0.004
E1a1a1c | 16271 | 1 | 0.004
E1a1a1c | 16274 | 3 | 0.012
E1a1a1c | 16301 | 1 | 0.004
E1a1a1c | 16311 | 2 | 0.008
E1a1a1c | 16319 | 1 | 0.004
E1a1a1c | 16320 | 1 | 0.004
E1a1a1c | 16352 | 1 | 0.004
E1a1a1c | 16465 | 1 | 0.004
E1a1a1c | 16497 | 1 | 0.004
E1a1a1c | 16527 | 1 | 0.004
E1a1a1c | 185 | 1 | 0.004
E1a1a1c | 193 | 1 | 0.004
E1a1a1c | 195 | 13 | 0.051
E1a1a1c | 235 | 5 | 0.02
E1a1a1c | 257 | 9 | 0.035
E1a1a1c | 292 | 3 | 0.012
E1a1a1c | 366 | 1 | 0.004
E1a1a1c | 513 | 1 | 0.004
E1a1a1c | 574C | 1 | 0.004
E1a1b | 14767 | 1 | 0.006
E1a1b | 15235 | 1 | 0.006
E1a1b | 16069 | 1 | 0.006
E1a1b | 16174 | 1 | 0.006
E1a1b | 16179 | 1 | 0.006
E1a1b | 16256 | 1 | 0.006
E1a1b | 16261 | 1 | 0.006
E1a1b | 16316 | 1 | 0.006
E1a1b | 16321 | 4 | 0.023
E1a1b | 16343 | 1 | 0.006
E1a1b | 1821 | 1 | 0.006
E1a1b | 1901 | 1 | 0.006
E1a1b | 198 | 1 | 0.006
E1a1b | 207 | 1 | 0.006
E1a1b | 2833 | 1 | 0.006
E1a1b | 3254 | 1 | 0.006
E1a1b | 3254A | 1 | 0.006
E1a1b | 3866 | 1 | 0.006
E1a1b | 4232 | 2 | 0.012
E1a1b | 6261 | 1 | 0.006
E1a1b | 7364 | 1 | 0.006
E1a1b | 9288 | 1 | 0.006
E1a1b | 961 | 1 | 0.006
E1a1b1 | 11016 | 1 | 0.006
E1a1b1 | 13813 | 1 | 0.006
E1a1b1 | 16069 | 1 | 0.006
E1a1b1 | 16179 | 1 | 0.006
E1a1b1 | 16256 | 1 | 0.006
E1a1b1 | 16261 | 1 | 0.006
E1a1b1 | 16305 | 5 | 0.029
E1a1b1 | 16316 | 1 | 0.006
E1a1b1 | 16321 | 4 | 0.023
E1a1b1 | 16343 | 1 | 0.006
E1a1b1 | 3411 | 1 | 0.006
E1a1b2 | 13933 | 1 | 0.01
E1a1b2 | 16256 | 1 | 0.01
E1a1b2 | 5662 | 1 | 0.01
E1a1b2 | 8551 | 1 | 0.01
E1a1b3 | 12892 | 1 | 0.01
E1a1b3 | 1452 | 1 | 0.01
E1a1b3 | 16124 | 1 | 0.01
E1a1b3 | 16148 | 1 | 0.01
E1a1b3 | 16304 | 1 | 0.01
E1a1b3 | 5124G | 1 | 0.01
E1a1b4 | 16189 | 1 | 0.01
E1a1b4 | 16291 | 1 | 0.01
E1a1b4 | 16311 | 1 | 0.01
E1a1b4 | 195 | 4 | 0.039
E1a1b4 | 709 | 4 | 0.039
E1a1b4 | 9554 | 4 | 0.039
E1a1c | 3338 | 1 | 0.004
E1a1c | 7706 | 2 | 0.009
E1a1c | 8155 | 1 | 0.004
E1a1c | 9653 | 2 | 0.009
E1a1c | 9861 | 3 | 0.013
E1a2 | 13494 | 1 | 0.01
E1a2 | 151 | 4 | 0.04
E1a2 | 15317 | 1 | 0.01
E1a2 | 15974T | 1 | 0.01
E1a2 | 15984.1T | 1 | 0.01
E1a2 | 16037 | 1 | 0.01
E1a2 | 16080 | 1 | 0.01
E1a2 | 16093 | 1 | 0.01
E1a2 | 16129 | 9 | 0.089
E1a2 | 16171 | 1 | 0.01
E1a2 | 16180 | 1 | 0.01
E1a2 | 16188 | 3 | 0.03
E1a2 | 16196T | 2 | 0.02
E1a2 | 16214 | 3 | 0.03
E1a2 | 16234 | 1 | 0.01
E1a2 | 16286 | 1 | 0.01
E1a2 | 16295 | 2 | 0.02
E1a2 | 16304 | 1 | 0.01
E1a2 | 16356 | 3 | 0.03
E1a2 | 16524 | 1 | 0.01
E1a2 | 249d | 1 | 0.01
E1a2+(16261) | 12599 | 1 | 0.005
E1a2+(16261) | 12606 | 1 | 0.005
E1a2+(16261) | 13269 | 1 | 0.005
E1a2+(16261) | 13494 | 1 | 0.005
E1a2+(16261) | 13722 | 1 | 0.005

E1a2+(16261) | 151 | 1 | 0.005
E1a2+(16261) | 16093 | 2 | 0.011
E1a2+(16261) | 16234 | 1 | 0.005
E1a2+(16261) | 16284 | 2 | 0.011
E1a2+(16261) | 16288 | 3 | 0.016
E1a2+(16261) | 16311 | 1 | 0.005
E1a2+(16261) | 195 | 1 | 0.005
E1a2+(16261) | 207 | 2 | 0.011
E1a2+(16261) | 374T | 1 | 0.005
E1a2+(16261) | 4176 | 1 | 0.005
E1a2+(16261) | 742 | 1 | 0.005
E1a2+(16261) | 7747 | 1 | 0.005
E1a2+(16261) | 8191 | 1 | 0.005
E1a2+(16261) | 8494 | 1 | 0.005
E1a2a | 11963 | 2 | 0.011
E1a2a | 16093 | 2 | 0.011
E1a2a | 16183 | 1 | 0.005
E1a2a | 16234 | 1 | 0.005
E1a2a | 16284 | 2 | 0.011
E1a2a | 16291 | 6 | 0.032
E1a2a | 16311 | 1 | 0.005
E1a2a | 16391 | 1 | 0.005
E1a2a | 310 | 1 | 0.005
E1a2a | 6908 | 1 | 0.005
E1a2a | 8414 | 1 | 0.005
E1a2a | 8730 | 1 | 0.005
E1a2a1 | 16093 | 2 | 0.011
E1a2a1 | 16156 | 1 | 0.005
E1a2a1 | 16192 | 1 | 0.005
E1a2a1 | 16234 | 2 | 0.011
E1a2a1 | 16257 | 1 | 0.005
E1a2a1 | 16275C | 1 | 0.005
E1a2a1 | 16284 | 2 | 0.011
E1a2a1 | 16291 | 8 | 0.043
E1a2a1 | 16311 | 2 | 0.011
E1a2a1 | 185 | 1 | 0.005
E1a2a1 | 199 | 1 | 0.005
E1a2a1 | 249d | 1 | 0.005
E1a2a1 | 279 | 1 | 0.005
E1a2a1 | 527 | 1 | 0.005
E1a2a1 | 573.2C | 1 | 0.005
E1a2a1 | 573.3C | 1 | 0.005
E1a2a1 | 9018 | 1 | 0.005
E1a2a2 | 153 | 1 | 0.042
E1a2a2 | 215 | 1 | 0.042
E1a2a3 | 16093 | 2 | 0.011
E1a2a3 | 16234 | 1 | 0.005
E1a2a3 | 16284 | 2 | 0.011
E1a2a3 | 16311 | 1 | 0.005
E1a2a3 | 2080 | 4 | 0.021
E1a2a4 | 10700 | 1 | 0.006
E1a2a4 | 16069 | 6 | 0.037
E1a2a4 | 16092 | 1 | 0.006
E1a2a4 | 16104 | 1 | 0.006
E1a2a4 | 16193 | 2 | 0.012
E1a2a4 | 16203 | 1 | 0.006
E1a2a4 | 16215 | 2 | 0.012
E1a2a4 | 16240C | 2 | 0.012
E1a2a4 | 16250 | 1 | 0.006
E1a2a4 | 16264 | 1 | 0.006
E1a2a4 | 16294 | 1 | 0.006
E1a2a4 | 16295 | 1 | 0.006
E1a2a4 | 16524C | 1 | 0.006
E1a2a4 | 6620 | 1 | 0.006
E2 | 10685 | 2 | 0.011
E2 | 15974T | 1 | 0.005
E2 | 15982.1A | 1 | 0.005
E2 | 16129 | 4 | 0.022
E2 | 16131 | 1 | 0.005
E2 | 16291 | 2 | 0.011
E2 | 16302 | 2 | 0.011
E2 | 16305 | 1 | 0.005
E2 | 16343 | 1 | 0.005
E2 | 16411G | 3 | 0.016
E2 | 16497 | 1 | 0.005
E2 | 16524 | 3 | 0.016
E2a | 10721 | 1 | 0.004
E2a | 10909 | 1 | 0.004
E2a | 12346 | 1 | 0.004
E2a | 12723 | 1 | 0.004
E2a | 13279 | 1 | 0.004
E2a | 13708 | 1 | 0.004
E2a | 14100 | 1 | 0.004
E2a | 15287 | 1 | 0.004
E2a | 16093 | 9 | 0.038
E2a | 16153 | 1 | 0.004
E2a | 16189 | 1 | 0.004
E2a | 16193d | 1 | 0.004
E2a | 16215 | 33 | 0.138
E2a | 16244 | 1 | 0.004
E2a | 16258C | 1 | 0.004
E2a | 16304 | 3 | 0.013
E2a | 16309 | 1 | 0.004
E2a | 16311 | 3 | 0.013
E2a | 16356 | 1 | 0.004
E2a | 16368 | 1 | 0.004
E2a | 16385 | 6 | 0.025
E2a | 194 | 1 | 0.004
E2a | 198 | 1 | 0.004
E2a | 2222 | 1 | 0.004
E2a | 309d | 1 | 0.004
E2a | 384 | 2 | 0.008
E2a | 4136 | 1 | 0.004
E2a | 523T | 1 | 0.004
E2a | 524-525d | 1 | 0.004
E2a | 5460 | 2 | 0.008
E2a | 5558 | 1 | 0.004
E2a | 5775 | 1 | 0.004
E2a | 63 | 1 | 0.004
E2a | 6734 | 1 | 0.004
E2a | 7022 | 1 | 0.004

E2a | 7340 | 1 | 0.004
E2a | 7364 | 2 | 0.008
E2a | 8476 | 1 | 0.004
E2a | 9233 | 1 | 0.004
E2a | 9293 | 1 | 0.004
E2a | 9776 | 1 | 0.004
E2a | 9947 | 1 | 0.004
E2a | 9966 | 1 | 0.004
E2a1 | 15974T | 1 | 0.006
E2a1 | 15982.1A | 1 | 0.006
E2a1 | 16131 | 1 | 0.006
E2a1 | 16189 | 1 | 0.006
E2a1 | 16291 | 2 | 0.011
E2a1 | 16302 | 2 | 0.011
E2a1 | 16305 | 1 | 0.006
E2a1 | 16343 | 1 | 0.006
E2a1 | 16411G | 3 | 0.017
E2a1 | 16497 | 1 | 0.006
E2a1 | 16524 | 3 | 0.017
E2a1a | 15974T | 1 | 0.005
E2a1a | 15982.1A | 1 | 0.005
E2a1a | 16131 | 1 | 0.005
E2a1a | 16189 | 3 | 0.016
E2a1a | 16291 | 2 | 0.011
E2a1a | 16302 | 2 | 0.011
E2a1a | 16305 | 1 | 0.005
E2a1a | 16343 | 1 | 0.005
E2a1a | 16411G | 3 | 0.016
E2a1a | 16497 | 1 | 0.005
E2a1a | 16524 | 3 | 0.016
E2a1a | 9143 | 1 | 0.005
E2a2 | 15777 | 1 | 0.006
E2a2 | 15974T | 1 | 0.006
E2a2 | 15982.1A | 1 | 0.006
E2a2 | 16131 | 1 | 0.006
E2a2 | 16291 | 2 | 0.011
E2a2 | 16302 | 2 | 0.011
E2a2 | 16305 | 1 | 0.006
E2a2 | 16343 | 1 | 0.006
E2a2 | 16411G | 3 | 0.017
E2a2 | 16497 | 1 | 0.006
E2a2 | 16524 | 3 | 0.017
E2a2 | 485 | 1 | 0.006
E2a2 | 573.1C | 1 | 0.006
E2b | 10208 | 3 | 0.021
E2b | 12397 | 4 | 0.028
E2b | 13942 | 1 | 0.007
E2b | 15510C | 1 | 0.007
E2b | 16233 | 4 | 0.028
E2b | 16287 | 4 | 0.028
E2b | 16319 | 4 | 0.028
E2b | 16445 | 4 | 0.028
E2b | 310 | 3 | 0.021
E2b | 315d | 3 | 0.021
E2b | 55 | 1 | 0.007
E2b | 6929 | 5 | 0.035
E2b | 709 | 5 | 0.035
E2b | 8730 | 1 | 0.007
E2b | 9220 | 1 | 0.007
E2b | 9722 | 3 | 0.021
E2b1 | 12952 | 1 | 0.008
E2b1 | 593 | 1 | 0.008
E2b1 | 9248 | 1 | 0.008
E2b2 | 10631 | 1 | 0.008
E2b2 | 11710 | 1 | 0.008
E2b2 | 12280 | 7 | 0.058
E2b2 | 12745A | 1 | 0.008
E2b2 | 16249 | 1 | 0.008
E2b2 | 16408A | 1 | 0.008
E2b2 | 450 | 1 | 0.008
F | 125G | 1 | 0.005
F | 146G | 1 | 0.005
F | 147G | 1 | 0.005
F | 16086 | 1 | 0.005
F | 16134 | 1 | 0.005
F | 16179 | 1 | 0.005
F | 16184 | 2 | 0.01
F | 16192 | 1 | 0.005
F | 16193A | 1 | 0.005
F | 16209 | 2 | 0.01
F | 16218 | 1 | 0.005
F | 16222 | 1 | 0.005
F | 16224 | 1 | 0.005
F | 16274 | 1 | 0.005
F | 16294 | 1 | 0.005
F | 16299 | 4 | 0.021
F | 16327 | 1 | 0.005
F | 16335 | 4 | 0.021
F | 16360 | 1 | 0.005
F | 16399 | 1 | 0.005
F | 189 | 1 | 0.005
F | 199 | 1 | 0.005
F | 200 | 1 | 0.005
F | 207 | 1 | 0.005
F | 208 | 1 | 0.005
F | 309.3C | 1 | 0.005
F | 318 | 1 | 0.005
F | 509 | 1 | 0.005
F | 548 | 2 | 0.01
F | 574C | 1 | 0.005
F | 576C | 1 | 0.005
F | 606 | 1 | 0.005
F | 709 | 1 | 0.005
F | 929 | 1 | 0.005
F | 930 | 1 | 0.005
F1 | 125G | 1 | 0.005
F1 | 146G | 1 | 0.005
F1 | 147G | 1 | 0.005
F1 | 16086 | 1 | 0.005
F1 | 16134 | 1 | 0.005
F1 | 16179 | 1 | 0.005

F1 | 16184 | 2 | 0.01
F1 | 16192 | 1 | 0.005
F1 | 16193A | 1 | 0.005
F1 | 16209 | 2 | 0.01
F1 | 16218 | 1 | 0.005
F1 | 16222 | 1 | 0.005
F1 | 16224 | 1 | 0.005
F1 | 16274 | 1 | 0.005
F1 | 16294 | 1 | 0.005
F1 | 16299 | 4 | 0.021
F1 | 16327 | 1 | 0.005
F1 | 16335 | 4 | 0.021
F1 | 16360 | 1 | 0.005
F1 | 16399 | 1 | 0.005
F1 | 189 | 1 | 0.005
F1 | 199 | 1 | 0.005
F1 | 200 | 1 | 0.005
F1 | 207 | 1 | 0.005
F1 | 208 | 1 | 0.005
F1 | 309.3C | 1 | 0.005
F1 | 318 | 1 | 0.005
F1 | 509 | 1 | 0.005
F1 | 548 | 2 | 0.01
F1 | 574C | 1 | 0.005
F1 | 576C | 1 | 0.005
F1 | 606 | 1 | 0.005
F1 | 709 | 1 | 0.005
F1 | 929 | 1 | 0.005
F1 | 930 | 1 | 0.005
F1+16189 | 11887 | 1 | 0.003
F1+16189 | 14563 | 1 | 0.003
F1+16189 | 14566C | 2 | 0.005
F1+16189 | 15047 | 1 | 0.003
F1+16189 | 152 | 6 | 0.016
F1+16189 | 15850 | 1 | 0.003
F1+16189 | 16048 | 1 | 0.003
F1+16189 | 16067A | 2 | 0.005
F1+16189 | 16079 | 6 | 0.016
F1+16189 | 16086 | 5 | 0.013
F1+16189 | 16126 | 1 | 0.003
F1+16189 | 16146 | 1 | 0.003
F1+16189 | 16147 | 5 | 0.013
F1+16189 | 16166C | 6 | 0.016
F1+16189 | 16168 | 2 | 0.005
F1+16189 | 16185 | 2 | 0.005
F1+16189 | 16186 | 2 | 0.005
F1+16189 | 16188G | 1 | 0.003
F1+16189 | 16207 | 1 | 0.003
F1+16189 | 16209 | 2 | 0.005
F1+16189 | 16213 | 3 | 0.008
F1+16189 | 16214 | 2 | 0.005
F1+16189 | 16221 | 1 | 0.003
F1+16189 | 16223 | 1 | 0.003
F1+16189 | 16232A | 1 | 0.003
F1+16189 | 16242 | 2 | 0.005
F1+16189 | 16256 | 2 | 0.005
F1+16189 | 16260 | 1 | 0.003
F1+16189 | 16261 | 1 | 0.003
F1+16189 | 16271 | 3 | 0.008
F1+16189 | 16274 | 6 | 0.016
F1+16189 | 16287 | 4 | 0.01
F1+16189 | 16288 | 1 | 0.003
F1+16189 | 16291A | 1 | 0.003
F1+16189 | 16292 | 1 | 0.003
F1+16189 | 16293 | 1 | 0.003
F1+16189 | 16295 | 1 | 0.003
F1+16189 | 16301 | 1 | 0.003
F1+16189 | 16309 | 1 | 0.003
F1+16189 | 16311 | 3 | 0.008
F1+16189 | 16318 | 2 | 0.005
F1+16189 | 16356 | 2 | 0.005
F1+16189 | 16362 | 1 | 0.003
F1+16189 | 16371 | 1 | 0.003
F1+16189 | 16381 | 1 | 0.003
F1+16189 | 199 | 3 | 0.008
F1+16189 | 208 | 1 | 0.003
F1+16189 | 237 | 3 | 0.008
F1+16189 | 309.3C | 2 | 0.005
F1+16189 | 316C | 2 | 0.005
F1+16189 | 464 | 1 | 0.003
F1+16189 | 573.4C | 1 | 0.003
F1+16189 | 709 | 3 | 0.008
F1+16189 | 735 | 2 | 0.005
F1+16189 | 745.1T | 1 | 0.003
F1+16189 | 761 | 1 | 0.003
F1+16189 | 7852 | 2 | 0.005
F1+16189 | 8563 | 2 | 0.005
F1+16189 | 874.1G | 1 | 0.003
F1+16189 | 8870 | 2 | 0.005
F1+16189 | 93 | 1 | 0.003
F1+16189 | 9509 | 1 | 0.003
F1a | 10327 | 1 | 0.003
F1a | 10604 | 12 | 0.033
F1a | 109 | 2 | 0.006
F1a | 10920 | 1 | 0.003
F1a | 11928 | 1 | 0.003
F1a | 12398 | 1 | 0.003
F1a | 12771 | 2 | 0.006
F1a | 12811 | 2 | 0.006
F1a | 13477C | 1 | 0.003
F1a | 13681 | 1 | 0.003
F1a | 13888 | 1 | 0.003
F1a | 14053 | 1 | 0.003
F1a | 14063 | 6 | 0.017
F1a | 146 | 5 | 0.014
F1a | 14668 | 1 | 0.003
F1a | 15175 | 1 | 0.003
F1a | 15237 | 2 | 0.006
F1a | 15262 | 1 | 0.003
F1a | 16037 | 1 | 0.003
F1a | 16051 | 1 | 0.003

F1a | 16111G | 1 | 0.003
F1a | 16141 | 2 | 0.006
F1a | 16154 | 1 | 0.003
F1a | 16179 | 4 | 0.011
F1a | 16188 | 1 | 0.003
F1a | 16190 | 1 | 0.003
F1a | 16193 | 1 | 0.003
F1a | 16207 | 1 | 0.003
F1a | 16209 | 1 | 0.003
F1a | 16218 | 2 | 0.006
F1a | 16223 | 1 | 0.003
F1a | 16224 | 1 | 0.003
F1a | 16239 | 1 | 0.003
F1a | 16274 | 4 | 0.011
F1a | 16278 | 2 | 0.006
F1a | 16284 | 1 | 0.003
F1a | 16291 | 1 | 0.003
F1a | 16311 | 2 | 0.006
F1a | 16319 | 2 | 0.006
F1a | 16320 | 3 | 0.008
F1a | 16354 | 11 | 0.03
F1a | 16527 | 2 | 0.006
F1a | 189 | 16 | 0.044
F1a | 195 | 13 | 0.036
F1a | 200 | 2 | 0.006
F1a | 204 | 7 | 0.019
F1a | 207 | 13 | 0.036
F1a | 215 | 1 | 0.003
F1a | 234 | 1 | 0.003
F1a | 255 | 3 | 0.008
F1a | 260 | 2 | 0.006
F1a | 275 | 1 | 0.003
F1a | 279 | 1 | 0.003
F1a | 316C | 1 | 0.003
F1a | 3417 | 1 | 0.003
F1a | 3535 | 1 | 0.003
F1a | 3635 | 2 | 0.006
F1a | 3736 | 1 | 0.003
F1a | 4204 | 2 | 0.006
F1a | 4316 | 2 | 0.006
F1a | 4695 | 1 | 0.003
F1a | 513 | 1 | 0.003
F1a | 5301 | 2 | 0.006
F1a | 5655 | 1 | 0.003
F1a | 573.1C | 1 | 0.003
F1a | 573.3C | 1 | 0.003
F1a | 601 | 1 | 0.003
F1a | 6378 | 2 | 0.006
F1a | 6448A | 1 | 0.003
F1a | 7007 | 2 | 0.006
F1a | 7372 | 1 | 0.003
F1a | 8281-8289d | 1 | 0.003
F1a | 8285.1C | 1 | 0.003
F1a | 93 | 4 | 0.011
F1a | 9770 | 2 | 0.006
F1a1 | 10043 | 1 | 0.003
F1a1 | 10211 | 1 | 0.003
F1a1 | 10410 | 1 | 0.003
F1a1 | 10420 | 1 | 0.003
F1a1 | 10463 | 1 | 0.003
F1a1 | 10685 | 2 | 0.006
F1a1 | 10972 | 2 | 0.006
F1a1 | 11002 | 7 | 0.021
F1a1 | 11176 | 1 | 0.003
F1a1 | 11253 | 2 | 0.006
F1a1 | 11255 | 1 | 0.003
F1a1 | 11381 | 1 | 0.003
F1a1 | 11518 | 1 | 0.003
F1a1 | 11778 | 1 | 0.003
F1a1 | 12561 | 1 | 0.003
F1a1 | 12624 | 1 | 0.003
F1a1 | 12630 | 1 | 0.003
F1a1 | 12715 | 7 | 0.021
F1a1 | 12963 | 1 | 0.003
F1a1 | 1310 | 1 | 0.003
F1a1 | 13260 | 2 | 0.006
F1a1 | 13590 | 1 | 0.003
F1a1 | 13760 | 1 | 0.003
F1a1 | 14002 | 1 | 0.003
F1a1 | 14258 | 3 | 0.009
F1a1 | 146 | 4 | 0.012
F1a1 | 14791G | 1 | 0.003
F1a1 | 14879 | 1 | 0.003
F1a1 | 14953 | 1 | 0.003
F1a1 | 150 | 2 | 0.006
F1a1 | 151 | 13 | 0.04
F1a1 | 15189C | 1 | 0.003
F1a1 | 152 | 29 | 0.088
F1a1 | 153 | 4 | 0.012
F1a1 | 15380 | 3 | 0.009
F1a1 | 15884 | 2 | 0.006
F1a1 | 15930 | 1 | 0.003
F1a1 | 15967T | 1 | 0.003
F1a1 | 15968 | 1 | 0.003
F1a1 | 1598 | 1 | 0.003
F1a1 | 16032d | 1 | 0.003
F1a1 | 16042d | 1 | 0.003
F1a1 | 16076d | 1 | 0.003
F1a1 | 16086 | 3 | 0.009
F1a1 | 16092A | 1 | 0.003
F1a1 | 16093 | 2 | 0.006
F1a1 | 16108 | 1 | 0.003
F1a1 | 16110d | 1 | 0.003
F1a1 | 16140 | 1 | 0.003
F1a1 | 16148 | 3 | 0.009
F1a1 | 16150 | 1 | 0.003
F1a1 | 16167 | 5 | 0.015
F1a1 | 16169 | 1 | 0.003
F1a1 | 16180 | 2 | 0.006
F1a1 | 16184 | 1 | 0.003
F1a1 | 16186 | 4 | 0.012

F1a1 | 16189 | 19 | 0.058
F1a1 | 16192 | 3 | 0.009
F1a1 | 16193 | 4 | 0.012
F1a1 | 16209 | 2 | 0.006
F1a1 | 16222 | 1 | 0.003
F1a1 | 16224 | 1 | 0.003
F1a1 | 16235 | 1 | 0.003
F1a1 | 16242 | 4 | 0.012
F1a1 | 16243 | 23 | 0.07
F1a1 | 16244 | 3 | 0.009
F1a1 | 16261 | 6 | 0.018
F1a1 | 16263 | 1 | 0.003
F1a1 | 16266 | 4 | 0.012
F1a1 | 16266G | 2 | 0.006
F1a1 | 16270 | 1 | 0.003
F1a1 | 16272 | 1 | 0.003
F1a1 | 16274 | 2 | 0.006
F1a1 | 16288 | 1 | 0.003
F1a1 | 16289 | 1 | 0.003
F1a1 | 16291 | 2 | 0.006
F1a1 | 16292 | 18 | 0.055
F1a1 | 16293 | 2 | 0.006
F1a1 | 16293C | 1 | 0.003
F1a1 | 16294 | 1 | 0.003
F1a1 | 16303 | 4 | 0.012
F1a1 | 16311 | 42 | 0.128
F1a1 | 16335 | 17 | 0.052
F1a1 | 16352 | 1 | 0.003
F1a1 | 16354 | 3 | 0.009
F1a1 | 16362 | 1 | 0.003
F1a1 | 16368 | 3 | 0.009
F1a1 | 16371 | 1 | 0.003
F1a1 | 16390 | 2 | 0.006
F1a1 | 16392 | 2 | 0.006
F1a1 | 16399 | 1 | 0.003
F1a1 | 16488 | 1 | 0.003
F1a1 | 16497 | 30 | 0.091
F1a1 | 16506 | 1 | 0.003
F1a1 | 16527 | 4 | 0.012
F1a1 | 1709 | 3 | 0.009
F1a1 | 1772 | 1 | 0.003
F1a1 | 1860 | 1 | 0.003
F1a1 | 187T | 2 | 0.006
F1a1 | 189 | 1 | 0.003
F1a1 | 1896 | 4 | 0.012
F1a1 | 195 | 11 | 0.034
F1a1 | 198 | 1 | 0.003
F1a1 | 199 | 1 | 0.003
F1a1 | 200 | 9 | 0.027
F1a1 | 203 | 1 | 0.003
F1a1 | 204 | 1 | 0.003
F1a1 | 207 | 1 | 0.003
F1a1 | 2248 | 1 | 0.003
F1a1 | 228 | 1 | 0.003
F1a1 | 234 | 4 | 0.012
F1a1 | 248 | 1 | 0.003
F1a1 | 251 | 9 | 0.027
F1a1 | 268 | 1 | 0.003
F1a1 | 269G | 1 | 0.003
F1a1 | 315.2C | 1 | 0.003
F1a1 | 316C | 1 | 0.003
F1a1 | 321 | 1 | 0.003
F1a1 | 3540 | 2 | 0.006
F1a1 | 3579 | 1 | 0.003
F1a1 | 3676C | 1 | 0.003
F1a1 | 372 | 1 | 0.003
F1a1 | 3768 | 1 | 0.003
F1a1 | 417 | 1 | 0.003
F1a1 | 4216 | 1 | 0.003
F1a1 | 4313 | 1 | 0.003
F1a1 | 437 | 1 | 0.003
F1a1 | 460 | 1 | 0.003
F1a1 | 4622 | 1 | 0.003
F1a1 | 464 | 1 | 0.003
F1a1 | 4646 | 1 | 0.003
F1a1 | 477 | 4 | 0.012
F1a1 | 4772 | 1 | 0.003
F1a1 | 489 | 3 | 0.009
F1a1 | 490 | 1 | 0.003
F1a1 | 4907 | 1 | 0.003
F1a1 | 4967 | 1 | 0.003
F1a1 | 509 | 4 | 0.012
F1a1 | 513 | 1 | 0.003
F1a1 | 5147 | 1 | 0.003
F1a1 | 5147C | 3 | 0.009
F1a1 | 515 | 2 | 0.006
F1a1 | 5183 | 1 | 0.003
F1a1 | 5322C | 2 | 0.006
F1a1 | 5460 | 1 | 0.003
F1a1 | 5483 | 1 | 0.003
F1a1 | 5654 | 3 | 0.009
F1a1 | 57 | 1 | 0.003
F1a1 | 573.1C | 1 | 0.003
F1a1 | 5899.1C | 1 | 0.003
F1a1 | 593 | 4 | 0.012
F1a1 | 60.1T | 1 | 0.003
F1a1 | 6182 | 1 | 0.003
F1a1 | 6254 | 1 | 0.003
F1a1 | 6263 | 1 | 0.003
F1a1 | 6293 | 2 | 0.006
F1a1 | 63d | 1 | 0.003
F1a1 | 6527 | 1 | 0.003
F1a1 | 66T | 1 | 0.003
F1a1 | 6791 | 1 | 0.003
F1a1 | 6961 | 1 | 0.003
F1a1 | 709 | 4 | 0.012
F1a1 | 7296 | 1 | 0.003
F1a1 | 7681A | 1 | 0.003
F1a1 | 7859 | 2 | 0.006
F1a1 | 7976 | 2 | 0.006
F1a1 | 7990 | 1 | 0.003

F1a1 | 8069 | 1 | 0.003
F1a1 | 8146 | 1 | 0.003
F1a1 | 8149 | 7 | 0.021
F1a1 | 8222 | 1 | 0.003
F1a1 | 8251 | 1 | 0.003
F1a1 | 8281-8289d | 1 | 0.003
F1a1 | 8282A | 1 | 0.003
F1a1 | 8301 | 1 | 0.003
F1a1 | 8379 | 1 | 0.003
F1a1 | 8409G | 1 | 0.003
F1a1 | 8433 | 7 | 0.021
F1a1 | 8507 | 1 | 0.003
F1a1 | 8545 | 1 | 0.003
F1a1 | 8589 | 1 | 0.003
F1a1 | 8592 | 1 | 0.003
F1a1 | 8631 | 1 | 0.003
F1a1 | 8697 | 3 | 0.009
F1a1 | 8839 | 1 | 0.003
F1a1 | 8944 | 2 | 0.006
F1a1 | 9141 | 4 | 0.012
F1a1 | 9217 | 1 | 0.003
F1a1 | 94 | 23 | 0.07
F1a1 | 9527 | 1 | 0.003
F1a1 | 961 | 1 | 0.003
F1a1 | 9632 | 1 | 0.003
F1a1 | 9987A | 1 | 0.003
F1a1'4 | 11914 | 3 | 0.008
F1a1'4 | 12630 | 10 | 0.027
F1a1'4 | 14386 | 3 | 0.008
F1a1'4 | 152 | 4 | 0.011
F1a1'4 | 15220 | 10 | 0.027
F1a1'4 | 15373 | 1 | 0.003
F1a1'4 | 15784 | 10 | 0.027
F1a1'4 | 15787 | 10 | 0.027
F1a1'4 | 16051 | 1 | 0.003
F1a1'4 | 16058T | 1 | 0.003
F1a1'4 | 16104 | 2 | 0.005
F1a1'4 | 16108 | 17 | 0.045
F1a1'4 | 16145 | 1 | 0.003
F1a1'4 | 16154 | 1 | 0.003
F1a1'4 | 16163 | 3 | 0.008
F1a1'4 | 16169 | 5 | 0.013
F1a1'4 | 16174 | 1 | 0.003
F1a1'4 | 16184 | 1 | 0.003
F1a1'4 | 16187 | 4 | 0.011
F1a1'4 | 16188 | 1 | 0.003
F1a1'4 | 16190 | 1 | 0.003
F1a1'4 | 16193 | 1 | 0.003
F1a1'4 | 16194T | 1 | 0.003
F1a1'4 | 16207 | 1 | 0.003
F1a1'4 | 16223 | 1 | 0.003
F1a1'4 | 16224 | 1 | 0.003
F1a1'4 | 16261 | 2 | 0.005
F1a1'4 | 16278 | 2 | 0.005
F1a1'4 | 16293 | 1 | 0.003
F1a1'4 | 16294 | 1 | 0.003
F1a1'4 | 16304G | 1 | 0.003
F1a1'4 | 16319 | 2 | 0.005
F1a1'4 | 16320 | 3 | 0.008
F1a1'4 | 16362 | 5 | 0.013
F1a1'4 | 16390 | 8 | 0.021
F1a1'4 | 16399 | 9 | 0.024
F1a1'4 | 16497 | 2 | 0.005
F1a1'4 | 16527 | 2 | 0.005
F1a1'4 | 1719 | 9 | 0.024
F1a1'4 | 185 | 2 | 0.005
F1a1'4 | 215 | 1 | 0.003
F1a1'4 | 234 | 1 | 0.003
F1a1'4 | 275 | 1 | 0.003
F1a1'4 | 310 | 2 | 0.005
F1a1'4 | 315.2C | 1 | 0.003
F1a1'4 | 3396 | 1 | 0.003
F1a1'4 | 4733 | 1 | 0.003
F1a1'4 | 573.1C | 3 | 0.008
F1a1'4 | 573.3C | 1 | 0.003
F1a1'4 | 601 | 1 | 0.003
F1a1'4 | 6680 | 2 | 0.005
F1a1'4 | 7348 | 1 | 0.003
F1a1'4 | 9468 | 10 | 0.027
F1a1a | 10184 | 1 | 0.002
F1a1a | 10454 | 1 | 0.002
F1a1a | 10589 | 2 | 0.003
F1a1a | 10637G | 1 | 0.002
F1a1a | 10654 | 1 | 0.002
F1a1a | 10685 | 1 | 0.002
F1a1a | 10915 | 1 | 0.002
F1a1a | 11025 | 1 | 0.002
F1a1a | 11350 | 1 | 0.002
F1a1a | 11353 | 1 | 0.002
F1a1a | 11365 | 1 | 0.002
F1a1a | 11422 | 1 | 0.002
F1a1a | 11638 | 8 | 0.013
F1a1a | 12373 | 1 | 0.002
F1a1a | 12501C | 2 | 0.003
F1a1a | 12561 | 6 | 0.009
F1a1a | 12663 | 2 | 0.003
F1a1a | 12732 | 1 | 0.002
F1a1a | 12810 | 2 | 0.003
F1a1a | 12820 | 1 | 0.002
F1a1a | 12906A | 1 | 0.002
F1a1a | 12957 | 1 | 0.002
F1a1a | 12972 | 1 | 0.002
F1a1a | 13005 | 1 | 0.002
F1a1a | 13020 | 18 | 0.028
F1a1a | 13174 | 1 | 0.002
F1a1a | 13353 | 8 | 0.013
F1a1a | 13368 | 1 | 0.002
F1a1a | 13659 | 23 | 0.036
F1a1a | 13662 | 1 | 0.002
F1a1a | 13677 | 1 | 0.002
F1a1a | 13768 | 6 | 0.009

F1a1a | 14121 | 1 | 0.002
F1a1a | 14134 | 1 | 0.002
F1a1a | 14142G | 18 | 0.028
F1a1a | 14180 | 1 | 0.002
F1a1a | 14226 | 1 | 0.002
F1a1a | 143 | 1 | 0.002
F1a1a | 14311 | 1 | 0.002
F1a1a | 14364 | 1 | 0.002
F1a1a | 14383A | 4 | 0.006
F1a1a | 14384 | 1 | 0.002
F1a1a | 14419 | 1 | 0.002
F1a1a | 146 | 5 | 0.008
F1a1a | 14790 | 2 | 0.003
F1a1a | 14841 | 7 | 0.011
F1a1a | 14953 | 1 | 0.002
F1a1a | 150 | 95 | 0.149
F1a1a | 1503 | 19 | 0.03
F1a1a | 15043 | 18 | 0.028
F1a1a | 15071 | 1 | 0.002
F1a1a | 15110 | 1 | 0.002
F1a1a | 152 | 9 | 0.014
F1a1a | 15244 | 1 | 0.002
F1a1a | 15314 | 10 | 0.016
F1a1a | 15340 | 2 | 0.003
F1a1a | 15596 | 6 | 0.009
F1a1a | 15638T | 1 | 0.002
F1a1a | 15643 | 2 | 0.003
F1a1a | 15658 | 4 | 0.006
F1a1a | 15937 | 1 | 0.002
F1a1a | 16037 | 1 | 0.002
F1a1a | 16075 | 1 | 0.002
F1a1a | 16076 | 1 | 0.002
F1a1a | 16086 | 1 | 0.002
F1a1a | 16092 | 1 | 0.002
F1a1a | 16093 | 5 | 0.008
F1a1a | 16111 | 15 | 0.024
F1a1a | 16124 | 7 | 0.011
F1a1a | 16140 | 1 | 0.002
F1a1a | 16145 | 3 | 0.005
F1a1a | 16150 | 1 | 0.002
F1a1a | 16153 | 1 | 0.002
F1a1a | 16154 | 1 | 0.002
F1a1a | 16169 | 2 | 0.003
F1a1a | 16169A | 1 | 0.002
F1a1a | 16170 | 2 | 0.003
F1a1a | 16174 | 1 | 0.002
F1a1a | 16179 | 3 | 0.005
F1a1a | 16183 | 2 | 0.003
F1a1a | 16187 | 2 | 0.003
F1a1a | 16189 | 31 | 0.049
F1a1a | 16201 | 4 | 0.006
F1a1a | 16209 | 7 | 0.011
F1a1a | 16214 | 19 | 0.03
F1a1a | 16216 | 1 | 0.002
F1a1a | 16218 | 2 | 0.003
F1a1a | 16227.1A | 2 | 0.003
F1a1a | 16234 | 2 | 0.003
F1a1a | 16239 | 1 | 0.002
F1a1a | 16240 | 2 | 0.003
F1a1a | 16243 | 3 | 0.005
F1a1a | 16247 | 1 | 0.002
F1a1a | 16249 | 1 | 0.002
F1a1a | 16256 | 12 | 0.019
F1a1a | 16259A | 4 | 0.006
F1a1a | 16261 | 1 | 0.002
F1a1a | 16263.1A | 1 | 0.002
F1a1a | 16266 | 2 | 0.003
F1a1a | 16270 | 10 | 0.016
F1a1a | 16274 | 18 | 0.028
F1a1a | 16284 | 3 | 0.005
F1a1a | 16291 | 1 | 0.002
F1a1a | 16292 | 2 | 0.003
F1a1a | 16293 | 3 | 0.005
F1a1a | 16294 | 7 | 0.011
F1a1a | 16295 | 3 | 0.005
F1a1a | 16303 | 1 | 0.002
F1a1a | 16311 | 41 | 0.064
F1a1a | 16325 | 1 | 0.002
F1a1a | 16326 | 2 | 0.003
F1a1a | 16343T | 2 | 0.003
F1a1a | 16354 | 10 | 0.016
F1a1a | 16355 | 1 | 0.002
F1a1a | 16356 | 2 | 0.003
F1a1a | 16362 | 6 | 0.009
F1a1a | 16368 | 25 | 0.039
F1a1a | 16372A | 11 | 0.017
F1a1a | 16384 | 1 | 0.002
F1a1a | 16390 | 4 | 0.006
F1a1a | 16434 | 1 | 0.002
F1a1a | 16465 | 2 | 0.003
F1a1a | 16519G | 1 | 0.002
F1a1a | 16525.1A | 1 | 0.002
F1a1a | 16527 | 1 | 0.002
F1a1a | 1719 | 1 | 0.002
F1a1a | 183 | 1 | 0.002
F1a1a | 186 | 6 | 0.009
F1a1a | 189 | 5 | 0.008
F1a1a | 195 | 71 | 0.111
F1a1a | 198 | 1 | 0.002
F1a1a | 199 | 5 | 0.008
F1a1a | 207 | 5 | 0.008
F1a1a | 2140 | 4 | 0.006
F1a1a | 215 | 1 | 0.002
F1a1a | 2388 | 3 | 0.005
F1a1a | 257.1T | 1 | 0.002
F1a1a | 279 | 3 | 0.005
F1a1a | 2818 | 1 | 0.002
F1a1a | 2968 | 1 | 0.002
F1a1a | 3275G | 1 | 0.002
F1a1a | 3395 | 1 | 0.002
F1a1a | 3396 | 3 | 0.005

F1a1a | 3531 | 1 | 0.002
F1a1a | 3540 | 5 | 0.008
F1a1a | 3915 | 4 | 0.006
F1a1a | 4029 | 1 | 0.002
F1a1a | 4315 | 3 | 0.005
F1a1a | 4592 | 5 | 0.008
F1a1a | 4768 | 2 | 0.003
F1a1a | 482 | 4 | 0.006
F1a1a | 4820 | 2 | 0.003
F1a1a | 4826 | 1 | 0.002
F1a1a | 492 | 2 | 0.003
F1a1a | 494 | 3 | 0.005
F1a1a | 513 | 3 | 0.005
F1a1a | 5147 | 5 | 0.008
F1a1a | 515d | 1 | 0.002
F1a1a | 5171 | 1 | 0.002
F1a1a | 5177 | 1 | 0.002
F1a1a | 5196 | 4 | 0.006
F1a1a | 5486A | 1 | 0.002
F1a1a | 5492 | 1 | 0.002
F1a1a | 57 | 1 | 0.002
F1a1a | 573.3C | 1 | 0.002
F1a1a | 5774 | 1 | 0.002
F1a1a | 59 | 1 | 0.002
F1a1a | 594A | 1 | 0.002
F1a1a | 60.1T | 1 | 0.002
F1a1a | 6040 | 3 | 0.005
F1a1a | 6253 | 1 | 0.002
F1a1a | 6261 | 1 | 0.002
F1a1a | 63 | 1 | 0.002
F1a1a | 6345 | 1 | 0.002
F1a1a | 6378 | 1 | 0.002
F1a1a | 6891 | 1 | 0.002
F1a1a | 6932 | 1 | 0.002
F1a1a | 6951 | 2 | 0.003
F1a1a | 6956G | 5 | 0.008
F1a1a | 709 | 1 | 0.002
F1a1a | 7258 | 8 | 0.013
F1a1a | 7366 | 2 | 0.003
F1a1a | 7609 | 2 | 0.003
F1a1a | 7774 | 1 | 0.002
F1a1a | 7925 | 1 | 0.002
F1a1a | 7934 | 6 | 0.009
F1a1a | 794 | 1 | 0.002
F1a1a | 8167 | 1 | 0.002
F1a1a | 8280d | 1 | 0.002
F1a1a | 8292 | 1 | 0.002
F1a1a | 8389 | 1 | 0.002
F1a1a | 8419 | 7 | 0.011
F1a1a | 8488 | 1 | 0.002
F1a1a | 8566 | 1 | 0.002
F1a1a | 8584 | 3 | 0.005
F1a1a | 8654 | 1 | 0.002
F1a1a | 8723 | 1 | 0.002
F1a1a | 89 | 1 | 0.002
F1a1a | 9145 | 1 | 0.002
F1a1a | 918 | 3 | 0.005
F1a1a | 9181 | 1 | 0.002
F1a1a | 9214 | 1 | 0.002
F1a1a | 93 | 2 | 0.003
F1a1a | 930 | 20 | 0.031
F1a1a | 9425 | 2 | 0.003
F1a1a | 9449 | 8 | 0.013
F1a1a | 9612 | 2 | 0.003
F1a1a | 9777 | 1 | 0.002
F1a1a | 9845 | 2 | 0.003
F1a1a | 9854 | 1 | 0.002
F1a1a1 | 10128 | 1 | 0.002
F1a1a1 | 10253 | 1 | 0.002
F1a1a1 | 10321 | 4 | 0.007
F1a1a1 | 10326 | 2 | 0.003
F1a1a1 | 10523 | 1 | 0.002
F1a1a1 | 10547 | 1 | 0.002
F1a1a1 | 10653 | 2 | 0.003
F1a1a1 | 10900 | 1 | 0.002
F1a1a1 | 10975 | 8 | 0.014
F1a1a1 | 11204 | 1 | 0.002
F1a1a1 | 11383 | 2 | 0.003
F1a1a1 | 11467 | 1 | 0.002
F1a1a1 | 11476 | 1 | 0.002
F1a1a1 | 11809 | 1 | 0.002
F1a1a1 | 12007 | 1 | 0.002
F1a1a1 | 12064A | 2 | 0.003
F1a1a1 | 12136 | 4 | 0.007
F1a1a1 | 12192 | 1 | 0.002
F1a1a1 | 12193 | 5 | 0.009
F1a1a1 | 12308 | 1 | 0.002
F1a1a1 | 12372 | 7 | 0.012
F1a1a1 | 12437 | 1 | 0.002
F1a1a1 | 12651 | 1 | 0.002
F1a1a1 | 12738 | 1 | 0.002
F1a1a1 | 12820 | 7 | 0.012
F1a1a1 | 12879 | 1 | 0.002
F1a1a1 | 12975 | 1 | 0.002
F1a1a1 | 13062T | 1 | 0.002
F1a1a1 | 13104 | 1 | 0.002
F1a1a1 | 13263 | 1 | 0.002
F1a1a1 | 13350 | 2 | 0.003
F1a1a1 | 13722 | 2 | 0.003
F1a1a1 | 13909C | 2 | 0.003
F1a1a1 | 14037 | 1 | 0.002
F1a1a1 | 14047 | 1 | 0.002
F1a1a1 | 14070 | 1 | 0.002
F1a1a1 | 14152 | 3 | 0.005
F1a1a1 | 14182 | 1 | 0.002
F1a1a1 | 14209 | 5 | 0.009
F1a1a1 | 14212 | 1 | 0.002
F1a1a1 | 143 | 3 | 0.005
F1a1a1 | 14325 | 3 | 0.005
F1a1a1 | 14350 | 2 | 0.003
F1a1a1 | 14364 | 1 | 0.002

F1a1a1 | 14512 | 1 | 0.002
F1a1a1 | 14530 | 1 | 0.002
F1a1a1 | 14553G | 1 | 0.002
F1a1a1 | 14561 | 1 | 0.002
F1a1a1 | 146 | 10 | 0.017
F1a1a1 | 15001 | 1 | 0.002
F1a1a1 | 1503 | 2 | 0.003
F1a1a1 | 15043 | 1 | 0.002
F1a1a1 | 15100 | 1 | 0.002
F1a1a1 | 15115 | 1 | 0.002
F1a1a1 | 15148 | 1 | 0.002
F1a1a1 | 152 | 14 | 0.024
F1a1a1 | 15217 | 1 | 0.002
F1a1a1 | 15236 | 6 | 0.01
F1a1a1 | 15249T | 8 | 0.014
F1a1a1 | 15262 | 1 | 0.002
F1a1a1 | 15301 | 1 | 0.002
F1a1a1 | 15328 | 1 | 0.002
F1a1a1 | 15394 | 1 | 0.002
F1a1a1 | 15777 | 1 | 0.002
F1a1a1 | 15850 | 1 | 0.002
F1a1a1 | 15913 | 1 | 0.002
F1a1a1 | 15954C | 1 | 0.002
F1a1a1 | 16032d | 21 | 0.036
F1a1a1 | 16042 | 1 | 0.002
F1a1a1 | 16042d | 21 | 0.036
F1a1a1 | 16070C | 1 | 0.002
F1a1a1 | 16073A | 1 | 0.002
F1a1a1 | 16075 | 3 | 0.005
F1a1a1 | 16075A | 1 | 0.002
F1a1a1 | 16076A | 1 | 0.002
F1a1a1 | 16079 | 1 | 0.002
F1a1a1 | 16083 | 1 | 0.002
F1a1a1 | 16093 | 1 | 0.002
F1a1a1 | 16109 | 1 | 0.002
F1a1a1 | 16110d | 21 | 0.036
F1a1a1 | 16111 | 2 | 0.003
F1a1a1 | 16126 | 2 | 0.003
F1a1a1 | 16139T | 1 | 0.002
F1a1a1 | 16145 | 3 | 0.005
F1a1a1 | 16147 | 4 | 0.007
F1a1a1 | 16150 | 2 | 0.003
F1a1a1 | 16154 | 1 | 0.002
F1a1a1 | 16158 | 1 | 0.002
F1a1a1 | 16169 | 3 | 0.005
F1a1a1 | 16178 | 25 | 0.043
F1a1a1 | 16184 | 6 | 0.01
F1a1a1 | 16189 | 9 | 0.016
F1a1a1 | 1619 | 1 | 0.002
F1a1a1 | 16191 | 1 | 0.002
F1a1a1 | 16192 | 1 | 0.002
F1a1a1 | 16209 | 1 | 0.002
F1a1a1 | 16218 | 2 | 0.003
F1a1a1 | 16220 | 1 | 0.002
F1a1a1 | 16223 | 1 | 0.002
F1a1a1 | 16234 | 2 | 0.003
F1a1a1 | 16239 | 7 | 0.012
F1a1a1 | 16241 | 12 | 0.021
F1a1a1 | 16241C | 1 | 0.002
F1a1a1 | 16241T | 1 | 0.002
F1a1a1 | 16242 | 5 | 0.009
F1a1a1 | 16243 | 1 | 0.002
F1a1a1 | 16245 | 1 | 0.002
F1a1a1 | 16248 | 11 | 0.019
F1a1a1 | 16249 | 1 | 0.002
F1a1a1 | 16250A | 1 | 0.002
F1a1a1 | 16256 | 50 | 0.086
F1a1a1 | 16261 | 3 | 0.005
F1a1a1 | 16265C | 2 | 0.003
F1a1a1 | 16266 | 2 | 0.003
F1a1a1 | 16278 | 6 | 0.01
F1a1a1 | 16284 | 14 | 0.024
F1a1a1 | 16286 | 1 | 0.002
F1a1a1 | 16291 | 1 | 0.002
F1a1a1 | 16292 | 3 | 0.005
F1a1a1 | 16293 | 34 | 0.059
F1a1a1 | 16293C | 1 | 0.002
F1a1a1 | 16295 | 6 | 0.01
F1a1a1 | 16299 | 2 | 0.003
F1a1a1 | 16300 | 3 | 0.005
F1a1a1 | 16302 | 1 | 0.002
F1a1a1 | 16304G | 15 | 0.026
F1a1a1 | 16311 | 23 | 0.04
F1a1a1 | 16317 | 1 | 0.002
F1a1a1 | 16320 | 4 | 0.007
F1a1a1 | 16325 | 2 | 0.003
F1a1a1 | 16326 | 1 | 0.002
F1a1a1 | 16327 | 19 | 0.033
F1a1a1 | 16343T | 2 | 0.003
F1a1a1 | 16350 | 1 | 0.002
F1a1a1 | 16355 | 3 | 0.005
F1a1a1 | 16357 | 1 | 0.002
F1a1a1 | 16362 | 7 | 0.012
F1a1a1 | 16367C | 1 | 0.002
F1a1a1 | 16368 | 6 | 0.01
F1a1a1 | 16371 | 1 | 0.002
F1a1a1 | 16390 | 1 | 0.002
F1a1a1 | 16391 | 16 | 0.028
F1a1a1 | 16398 | 1 | 0.002
F1a1a1 | 16399 | 3 | 0.005
F1a1a1 | 16405C | 1 | 0.002
F1a1a1 | 16413d | 1 | 0.002
F1a1a1 | 16434d | 2 | 0.003
F1a1a1 | 16441T | 1 | 0.002
F1a1a1 | 16527 | 4 | 0.007
F1a1a1 | 16558d | 1 | 0.002
F1a1a1 | 1685 | 1 | 0.002
F1a1a1 | 194 | 1 | 0.002
F1a1a1 | 195 | 7 | 0.012
F1a1a1 | 198 | 1 | 0.002
F1a1a1 | 199 | 3 | 0.005

F1a1a1 | 1d | 1 | 0.002
F1a1a1 | 207 | 1 | 0.002
F1a1a1 | 211 | 1 | 0.002
F1a1a1 | 2120 | 3 | 0.005
F1a1a1 | 214 | 1 | 0.002
F1a1a1 | 2218 | 1 | 0.002
F1a1a1 | 222 | 4 | 0.007
F1a1a1 | 2251 | 3 | 0.005
F1a1a1 | 227 | 2 | 0.003
F1a1a1 | 234 | 1 | 0.002
F1a1a1 | 2626 | 1 | 0.002
F1a1a1 | 2886d | 2 | 0.003
F1a1a1 | 293 | 13 | 0.022
F1a1a1 | 3027 | 1 | 0.002
F1a1a1 | 310 | 1 | 0.002
F1a1a1 | 3144 | 1 | 0.002
F1a1a1 | 3202 | 1 | 0.002
F1a1a1 | 3398 | 1 | 0.002
F1a1a1 | 3414 | 1 | 0.002
F1a1a1 | 3753 | 1 | 0.002
F1a1a1 | 385 | 4 | 0.007
F1a1a1 | 3882 | 2 | 0.003
F1a1a1 | 3995 | 2 | 0.003
F1a1a1 | 408A | 1 | 0.002
F1a1a1 | 4113 | 7 | 0.012
F1a1a1 | 4221 | 1 | 0.002
F1a1a1 | 4343 | 2 | 0.003
F1a1a1 | 4386 | 1 | 0.002
F1a1a1 | 4435 | 1 | 0.002
F1a1a1 | 4596 | 1 | 0.002
F1a1a1 | 462 | 1 | 0.002
F1a1a1 | 477 | 3 | 0.005
F1a1a1 | 480 | 1 | 0.002
F1a1a1 | 485 | 1 | 0.002
F1a1a1 | 4965 | 2 | 0.003
F1a1a1 | 4991 | 1 | 0.002
F1a1a1 | 5082 | 1 | 0.002
F1a1a1 | 513-516d | 1 | 0.002
F1a1a1 | 5231 | 3 | 0.005
F1a1a1 | 5234 | 2 | 0.003
F1a1a1 | 5369 | 1 | 0.002
F1a1a1 | 5528 | 2 | 0.003
F1a1a1 | 5567 | 1 | 0.002
F1a1a1 | 5605C | 1 | 0.002
F1a1a1 | 5671 | 2 | 0.003
F1a1a1 | 573.1C | 1 | 0.002
F1a1a1 | 6026 | 1 | 0.002
F1a1a1 | 6029 | 1 | 0.002
F1a1a1 | 6302 | 1 | 0.002
F1a1a1 | 6329A | 4 | 0.007
F1a1a1 | 6340 | 1 | 0.002
F1a1a1 | 6359 | 1 | 0.002
F1a1a1 | 6893 | 1 | 0.002
F1a1a1 | 6911 | 1 | 0.002
F1a1a1 | 709 | 6 | 0.01
F1a1a1 | 7299 | 1 | 0.002
F1a1a1 | 7310 | 1 | 0.002
F1a1a1 | 7352 | 1 | 0.002
F1a1a1 | 7581 | 2 | 0.003
F1a1a1 | 7606 | 4 | 0.007
F1a1a1 | 765A | 1 | 0.002
F1a1a1 | 7673 | 1 | 0.002
F1a1a1 | 7747 | 1 | 0.002
F1a1a1 | 7775 | 1 | 0.002
F1a1a1 | 7805 | 1 | 0.002
F1a1a1 | 786 | 1 | 0.002
F1a1a1 | 789 | 1 | 0.002
F1a1a1 | 7934 | 1 | 0.002
F1a1a1 | 804 | 1 | 0.002
F1a1a1 | 806 | 1 | 0.002
F1a1a1 | 8286 | 1 | 0.002
F1a1a1 | 8477 | 3 | 0.005
F1a1a1 | 8572 | 1 | 0.002
F1a1a1 | 8838 | 3 | 0.005
F1a1a1 | 9009 | 3 | 0.005
F1a1a1 | 9097 | 2 | 0.003
F1a1a1 | 9276 | 1 | 0.002
F1a1a1 | 93 | 5 | 0.009
F1a1a1 | 9300 | 1 | 0.002
F1a1a1 | 9540 | 1 | 0.002
F1a1a1 | 961 | 1 | 0.002
F1a1a1 | 9667 | 1 | 0.002
F1a1a1 | 9812 | 1 | 0.002
F1a1a1 | 9869 | 1 | 0.002
F1a1a1 | 9926 | 6 | 0.01
F1a1a1 | 9948 | 4 | 0.007
F1a1b | 14890 | 1 | 0.013
F1a1b | 152 | 2 | 0.027
F1a1b | 15618 | 1 | 0.013
F1a1b | 15774 | 1 | 0.013
F1a1b | 15929 | 2 | 0.027
F1a1b | 16037 | 1 | 0.013
F1a1b | 16213 | 1 | 0.013
F1a1b | 16218 | 4 | 0.053
F1a1b | 16220 | 2 | 0.027
F1a1b | 16233 | 2 | 0.027
F1a1b | 16234 | 1 | 0.013
F1a1b | 16250A | 1 | 0.013
F1a1b | 16309 | 1 | 0.013
F1a1b | 16319 | 1 | 0.013
F1a1b | 16390 | 1 | 0.013
F1a1b | 195 | 1 | 0.013
F1a1b | 200 | 1 | 0.013
F1a1b | 2882 | 1 | 0.013
F1a1b | 310 | 2 | 0.027
F1a1b | 315d | 1 | 0.013
F1a1b | 3705 | 1 | 0.013
F1a1b | 5788 | 1 | 0.013
F1a1b | 63 | 9 | 0.12
F1a1b | 66 | 5 | 0.067
F1a1b | 9099 | 1 | 0.013

F1a1b | 9254 | 1 | 0.013
F1a1c | 10208 | 4 | 0.024
F1a1c | 10394G | 1 | 0.006
F1a1c | 10876 | 1 | 0.006
F1a1c | 11200 | 2 | 0.012
F1a1c | 11482 | 1 | 0.006
F1a1c | 11653 | 4 | 0.024
F1a1c | 11965A | 1 | 0.006
F1a1c | 11969 | 1 | 0.006
F1a1c | 12026 | 1 | 0.006
F1a1c | 12372 | 1 | 0.006
F1a1c | 12373 | 1 | 0.006
F1a1c | 12465 | 1 | 0.006
F1a1c | 12528 | 1 | 0.006
F1a1c | 12940 | 2 | 0.012
F1a1c | 13149 | 4 | 0.024
F1a1c | 13269 | 3 | 0.018
F1a1c | 13431 | 1 | 0.006
F1a1c | 13638 | 1 | 0.006
F1a1c | 13681 | 1 | 0.006
F1a1c | 14325 | 1 | 0.006
F1a1c | 14530 | 1 | 0.006
F1a1c | 14560 | 1 | 0.006
F1a1c | 146 | 3 | 0.018
F1a1c | 14870 | 2 | 0.012
F1a1c | 14983A | 1 | 0.006
F1a1c | 15099 | 1 | 0.006
F1a1c | 15145 | 1 | 0.006
F1a1c | 152 | 12 | 0.073
F1a1c | 15314 | 5 | 0.03
F1a1c | 15466 | 1 | 0.006
F1a1c | 15574 | 1 | 0.006
F1a1c | 15747 | 2 | 0.012
F1a1c | 15758 | 1 | 0.006
F1a1c | 15777 | 1 | 0.006
F1a1c | 16066 | 1 | 0.006
F1a1c | 16091 | 1 | 0.006
F1a1c | 16145 | 1 | 0.006
F1a1c | 16148 | 1 | 0.006
F1a1c | 16167 | 1 | 0.006
F1a1c | 16169 | 1 | 0.006
F1a1c | 16174 | 4 | 0.024
F1a1c | 16212 | 1 | 0.006
F1a1c | 16215C | 1 | 0.006
F1a1c | 16217 | 1 | 0.006
F1a1c | 16221 | 1 | 0.006
F1a1c | 16223 | 1 | 0.006
F1a1c | 16260 | 2 | 0.012
F1a1c | 16264 | 6 | 0.037
F1a1c | 16274 | 4 | 0.024
F1a1c | 16311 | 8 | 0.049
F1a1c | 16320 | 2 | 0.012
F1a1c | 16342 | 1 | 0.006
F1a1c | 16346 | 1 | 0.006
F1a1c | 16355 | 2 | 0.012
F1a1c | 16362 | 6 | 0.037
F1a1c | 16399 | 1 | 0.006
F1a1c | 16529 | 1 | 0.006
F1a1c | 1719 | 2 | 0.012
F1a1c | 185 | 4 | 0.024
F1a1c | 195 | 2 | 0.012
F1a1c | 199 | 1 | 0.006
F1a1c | 200 | 1 | 0.006
F1a1c | 2416 | 3 | 0.018
F1a1c | 3640 | 1 | 0.006
F1a1c | 3645 | 5 | 0.03
F1a1c | 4343 | 1 | 0.006
F1a1c | 466G | 7 | 0.043
F1a1c | 4856 | 1 | 0.006
F1a1c | 5417 | 1 | 0.006
F1a1c | 5471 | 1 | 0.006
F1a1c | 549 | 3 | 0.018
F1a1c | 5561 | 1 | 0.006
F1a1c | 573.3C | 1 | 0.006
F1a1c | 5894 | 2 | 0.012
F1a1c | 5984 | 1 | 0.006
F1a1c | 6011 | 3 | 0.018
F1a1c | 6287 | 1 | 0.006
F1a1c | 6366 | 2 | 0.012
F1a1c | 6899 | 1 | 0.006
F1a1c | 6995 | 1 | 0.006
F1a1c | 6996 | 1 | 0.006
F1a1c | 7250C | 1 | 0.006
F1a1c | 7270 | 1 | 0.006
F1a1c | 7521 | 1 | 0.006
F1a1c | 7804 | 1 | 0.006
F1a1c | 8167 | 1 | 0.006
F1a1c | 8471G | 1 | 0.006
F1a1c | 8519 | 1 | 0.006
F1a1c | 8537 | 1 | 0.006
F1a1c | 8701 | 1 | 0.006
F1a1c | 8743 | 1 | 0.006
F1a1c | 8875 | 1 | 0.006
F1a1c | 9111 | 1 | 0.006
F1a1c | 9120 | 1 | 0.006
F1a1c | 9181 | 1 | 0.006
F1a1c | 9300 | 1 | 0.006
F1a1c | 9554 | 1 | 0.006
F1a1c | 9584 | 1 | 0.006
F1a1c | 980 | 1 | 0.006
F1a1c | 9824 | 1 | 0.006
F1a1c | 9948 | 1 | 0.006
F1a1c1 | 14148 | 2 | 0.111
F1a1c1 | 275 | 1 | 0.056
F1a1c1 | 42.1T | 1 | 0.056
F1a1c1 | 8787G | 1 | 0.056
F1a1c1 | 8838 | 1 | 0.056
F1a1c2 | 151 | 1 | 0.008
F1a1c2 | 16042 | 1 | 0.008
F1a1c2 | 16056 | 1 | 0.008
F1a1c2 | 16112 | 1 | 0.008

F1a1c2 | 16117G | 1 | 0.008
F1a1c2 | 16167 | 15 | 0.123
F1a1c2 | 16181 | 1 | 0.008
F1a1c2 | 16207 | 3 | 0.025
F1a1c2 | 16293 | 1 | 0.008
F1a1c2 | 16299 | 1 | 0.008
F1a1c2 | 16386 | 1 | 0.008
F1a1c2 | 16469G | 1 | 0.008
F1a1c2 | 16486 | 1 | 0.008
F1a1c2 | 16551 | 1 | 0.008
F1a1c2 | 219 | 1 | 0.008
F1a1c2 | 24 | 1 | 0.008
F1a1c2 | 315.2C | 4 | 0.033
F1a1c2 | 32 | 1 | 0.008
F1a1c2 | 379 | 1 | 0.008
F1a1c2 | 3915 | 1 | 0.008
F1a1c2 | 4 | 1 | 0.008
F1a1c2 | 400 | 1 | 0.008
F1a1c2 | 425T | 1 | 0.008
F1a1c2 | 446 | 1 | 0.008
F1a1c2 | 456 | 1 | 0.008
F1a1c2 | 502 | 1 | 0.008
F1a1c2 | 508 | 1 | 0.008
F1a1c2 | 573.3C | 1 | 0.008
F1a1c2 | 6254 | 1 | 0.008
F1a1c3 | 12414 | 1 | 0.008
F1a1c3 | 13713 | 1 | 0.008
F1a1c3 | 14198 | 1 | 0.008
F1a1c3 | 14683 | 2 | 0.017
F1a1c3 | 150 | 5 | 0.042
F1a1c3 | 15534 | 1 | 0.008
F1a1c3 | 16086 | 1 | 0.008
F1a1c3 | 16189 | 1 | 0.008
F1a1c3 | 16327 | 2 | 0.017
F1a1c3 | 16451d | 2 | 0.017
F1a1c3 | 16497 | 2 | 0.017
F1a1c3 | 16527 | 1 | 0.008
F1a1c3 | 227 | 1 | 0.008
F1a1c3 | 2706 | 1 | 0.008
F1a1c3 | 3565 | 1 | 0.008
F1a1c3 | 385 | 2 | 0.017
F1a1c3 | 3867 | 1 | 0.008
F1a1c3 | 5277 | 1 | 0.008
F1a1c3 | 5553 | 1 | 0.008
F1a1c3 | 573.3C | 1 | 0.008
F1a1c3 | 7269 | 1 | 0.008
F1a1d | 1007 | 1 | 0.005
F1a1d | 11377 | 2 | 0.01
F1a1d | 11381 | 1 | 0.005
F1a1d | 13753 | 1 | 0.005
F1a1d | 13980 | 5 | 0.024
F1a1d | 14226 | 4 | 0.019
F1a1d | 14417 | 1 | 0.005
F1a1d | 146 | 12 | 0.057
F1a1d | 14783 | 1 | 0.005
F1a1d | 14797C | 1 | 0.005
F1a1d | 150 | 4 | 0.019
F1a1d | 15013 | 1 | 0.005
F1a1d | 151 | 1 | 0.005
F1a1d | 152 | 30 | 0.144
F1a1d | 15236 | 1 | 0.005
F1a1d | 15343 | 2 | 0.01
F1a1d | 16051 | 1 | 0.005
F1a1d | 16076A | 1 | 0.005
F1a1d | 16086 | 4 | 0.019
F1a1d | 16093 | 16 | 0.077
F1a1d | 16094 | 1 | 0.005
F1a1d | 16108 | 1 | 0.005
F1a1d | 16168 | 13 | 0.062
F1a1d | 16186 | 1 | 0.005
F1a1d | 16189 | 13 | 0.062
F1a1d | 16192 | 2 | 0.01
F1a1d | 16209 | 1 | 0.005
F1a1d | 16218G | 1 | 0.005
F1a1d | 16234 | 1 | 0.005
F1a1d | 16248A | 1 | 0.005
F1a1d | 16255 | 1 | 0.005
F1a1d | 16270A | 1 | 0.005
F1a1d | 16278 | 4 | 0.019
F1a1d | 16287 | 1 | 0.005
F1a1d | 16292 | 1 | 0.005
F1a1d | 16295 | 1 | 0.005
F1a1d | 16311 | 1 | 0.005
F1a1d | 16355 | 1 | 0.005
F1a1d | 16356 | 1 | 0.005
F1a1d | 16362 | 2 | 0.01
F1a1d | 199 | 1 | 0.005
F1a1d | 200 | 5 | 0.024
F1a1d | 295 | 1 | 0.005
F1a1d | 3144 | 1 | 0.005
F1a1d | 3585 | 1 | 0.005
F1a1d | 372 | 1 | 0.005
F1a1d | 4722 | 1 | 0.005
F1a1d | 5773 | 2 | 0.01
F1a1d | 6351 | 1 | 0.005
F1a1d | 6398 | 2 | 0.01
F1a1d | 710 | 2 | 0.01
F1a1d | 7711 | 5 | 0.024
F1a1d | 801 | 1 | 0.005
F1a1d | 8158T | 3 | 0.014
F1a1d | 8277 | 1 | 0.005
F1a1d | 8387 | 1 | 0.005
F1a1d | 8994 | 1 | 0.005
F1a1d | 9092 | 3 | 0.014
F1a1d | 9844 | 1 | 0.005
F1a1d1 | 16076A | 1 | 0.005
F1a1d1 | 16094 | 1 | 0.005
F1a1d1 | 16108 | 1 | 0.005
F1a1d1 | 16168 | 1 | 0.005
F1a1d1 | 16186 | 1 | 0.005
F1a1d1 | 16218G | 1 | 0.005

F1a1d1 | 16223 | 2 | 0.011
F1a1d1 | 16234 | 1 | 0.005
F1a1d1 | 16258 | 2 | 0.011
F1a1d1 | 16260 | 1 | 0.005
F1a1d1 | 16265C | 3 | 0.016
F1a1d1 | 16274 | 3 | 0.016
F1a1d1 | 16287 | 5 | 0.027
F1a1d1 | 16294 | 1 | 0.005
F1a1d1 | 16311 | 6 | 0.032
F1a1d1 | 16355 | 1 | 0.005
F1a1d1 | 16360 | 1 | 0.005
F1a1d1 | 16362 | 16 | 0.086
F1a1d1 | 16446 | 3 | 0.016
F1a1d1 | 16453 | 2 | 0.011
F1a1d1 | 16455C | 2 | 0.011
F1a1d1 | 16497 | 1 | 0.005
F1a1d1 | 195 | 8 | 0.043
F1a1d1 | 199 | 1 | 0.005
F1a1d1 | 372 | 2 | 0.011
F1a1d1 | 709 | 1 | 0.005
F1a1d1 | 801 | 1 | 0.005
F1a2 | 103 | 1 | 0.007
F1a2 | 12501 | 3 | 0.02
F1a2 | 13599 | 2 | 0.014
F1a2 | 13827 | 1 | 0.007
F1a2 | 14259 | 1 | 0.007
F1a2 | 146 | 1 | 0.007
F1a2 | 14905 | 1 | 0.007
F1a2 | 151 | 1 | 0.007
F1a2 | 152 | 25 | 0.169
F1a2 | 153 | 2 | 0.014
F1a2 | 16029 | 1 | 0.007
F1a2 | 16038d | 1 | 0.007
F1a2 | 16045 | 2 | 0.014
F1a2 | 16045.1C | 1 | 0.007
F1a2 | 16048T | 1 | 0.007
F1a2 | 16049T | 1 | 0.007
F1a2 | 16054 | 1 | 0.007
F1a2 | 16069 | 1 | 0.007
F1a2 | 16075 | 3 | 0.02
F1a2 | 16092 | 1 | 0.007
F1a2 | 16094 | 1 | 0.007
F1a2 | 16148A | 1 | 0.007
F1a2 | 16153 | 1 | 0.007
F1a2 | 16162 | 44 | 0.297
F1a2 | 16168 | 3 | 0.02
F1a2 | 16171 | 1 | 0.007
F1a2 | 16174 | 1 | 0.007
F1a2 | 16186 | 1 | 0.007
F1a2 | 16189 | 7 | 0.047
F1a2 | 16201 | 1 | 0.007
F1a2 | 16209 | 4 | 0.027
F1a2 | 16223 | 1 | 0.007
F1a2 | 16224 | 2 | 0.014
F1a2 | 16245 | 3 | 0.02
F1a2 | 16247 | 1 | 0.007
F1a2 | 16260 | 1 | 0.007
F1a2 | 16261 | 3 | 0.02
F1a2 | 16266 | 2 | 0.014
F1a2 | 16269 | 1 | 0.007
F1a2 | 16274 | 10 | 0.068
F1a2 | 16278 | 1 | 0.007
F1a2 | 16291 | 1 | 0.007
F1a2 | 16293 | 1 | 0.007
F1a2 | 16311 | 2 | 0.014
F1a2 | 16338 | 1 | 0.007
F1a2 | 16340 | 1 | 0.007
F1a2 | 16362 | 5 | 0.034
F1a2 | 16373 | 1 | 0.007
F1a2 | 16417 | 1 | 0.007
F1a2 | 16477 | 1 | 0.007
F1a2 | 16554 | 1 | 0.007
F1a2 | 189 | 2 | 0.014
F1a2 | 195 | 1 | 0.007
F1a2 | 200 | 10 | 0.068
F1a2 | 204 | 1 | 0.007
F1a2 | 207 | 2 | 0.014
F1a2 | 214 | 1 | 0.007
F1a2 | 215 | 1 | 0.007
F1a2 | 4025 | 9 | 0.061
F1a2 | 447 | 1 | 0.007
F1a2 | 472T | 1 | 0.007
F1a2 | 479 | 1 | 0.007
F1a2 | 482 | 1 | 0.007
F1a2 | 5191 | 9 | 0.061
F1a2 | 564 | 1 | 0.007
F1a2 | 7853 | 1 | 0.007
F1a2 | 8506 | 2 | 0.014
F1a2 | 9139 | 1 | 0.007
F1a2 | 9655 | 1 | 0.007
F1a2a | 11437 | 1 | 0.014
F1a2a | 13326 | 1 | 0.014
F1a2a | 13748 | 1 | 0.014
F1a2a | 14110 | 1 | 0.014
F1a2a | 14178 | 1 | 0.014
F1a2a | 15097 | 1 | 0.014
F1a2a | 152 | 1 | 0.014
F1a2a | 16080 | 1 | 0.014
F1a2a | 16081 | 1 | 0.014
F1a2a | 16114A | 1 | 0.014
F1a2a | 16115A | 1 | 0.014
F1a2a | 16180 | 3 | 0.043
F1a2a | 16189 | 4 | 0.058
F1a2a | 194 | 1 | 0.014
F1a2a | 217 | 2 | 0.029
F1a2a | 4224 | 1 | 0.014
F1a2a | 5567 | 1 | 0.014
F1a2a | 6852 | 1 | 0.014
F1a2a | 7444 | 4 | 0.058
F1a2a | 8659 | 3 | 0.043
F1a3 | 10223 | 19 | 0.048

F1a3 | 1037 | 2 | 0.005
F1a3 | 10427 | 1 | 0.003
F1a3 | 10688 | 5 | 0.013
F1a3 | 11860 | 6 | 0.015
F1a3 | 150 | 3 | 0.008
F1a3 | 152 | 1 | 0.003
F1a3 | 15670 | 1 | 0.003
F1a3 | 16000 | 6 | 0.015
F1a3 | 16051 | 1 | 0.003
F1a3 | 16058T | 1 | 0.003
F1a3 | 16086 | 30 | 0.075
F1a3 | 16108 | 17 | 0.043
F1a3 | 16145 | 1 | 0.003
F1a3 | 16154 | 1 | 0.003
F1a3 | 16163 | 3 | 0.008
F1a3 | 16169 | 5 | 0.013
F1a3 | 16169.1C | 1 | 0.003
F1a3 | 16184 | 1 | 0.003
F1a3 | 16185 | 1 | 0.003
F1a3 | 16187 | 4 | 0.01
F1a3 | 16188 | 1 | 0.003
F1a3 | 16189 | 36 | 0.09
F1a3 | 16190 | 1 | 0.003
F1a3 | 16193 | 1 | 0.003
F1a3 | 16194T | 1 | 0.003
F1a3 | 16207 | 1 | 0.003
F1a3 | 16220 | 1 | 0.003
F1a3 | 16221 | 4 | 0.01
F1a3 | 16223 | 1 | 0.003
F1a3 | 16224 | 1 | 0.003
F1a3 | 16243 | 1 | 0.003
F1a3 | 16258C | 1 | 0.003
F1a3 | 16261 | 2 | 0.005
F1a3 | 16278 | 2 | 0.005
F1a3 | 16294 | 1 | 0.003
F1a3 | 16303 | 1 | 0.003
F1a3 | 16304G | 1 | 0.003
F1a3 | 16320 | 3 | 0.008
F1a3 | 16360 | 1 | 0.003
F1a3 | 16375G | 1 | 0.003
F1a3 | 16376G | 1 | 0.003
F1a3 | 16399 | 9 | 0.023
F1a3 | 16414T | 1 | 0.003
F1a3 | 16438 | 4 | 0.01
F1a3 | 16520.1C | 1 | 0.003
F1a3 | 185 | 2 | 0.005
F1a3 | 195 | 1 | 0.003
F1a3 | 215 | 1 | 0.003
F1a3 | 234 | 1 | 0.003
F1a3 | 235 | 3 | 0.008
F1a3 | 3116 | 3 | 0.008
F1a3 | 316 | 1 | 0.003
F1a3 | 333 | 1 | 0.003
F1a3 | 5252 | 5 | 0.013
F1a3 | 573.1C | 1 | 0.003
F1a3 | 573.3C | 1 | 0.003
F1a3 | 601 | 1 | 0.003
F1a3 | 8618 | 3 | 0.008
F1a3 | 868 | 5 | 0.013
F1a3 | 8866 | 2 | 0.005
F1a3 | 9142 | 1 | 0.003
F1a3 | 9533 | 1 | 0.003
F1a3+16311 | 11287 | 1 | 0.005
F1a3+16311 | 14560 | 1 | 0.005
F1a3+16311 | 152 | 4 | 0.022
F1a3+16311 | 16051 | 4 | 0.022
F1a3+16311 | 16076d | 1 | 0.005
F1a3+16311 | 16154 | 2 | 0.011
F1a3+16311 | 16169 | 15 | 0.082
F1a3+16311 | 16235 | 1 | 0.005
F1a3+16311 | 16265 | 3 | 0.016
F1a3+16311 | 16295 | 2 | 0.011
F1a3+16311 | 16301 | 1 | 0.005
F1a3+16311 | 16320 | 2 | 0.011
F1a3+16311 | 16360 | 3 | 0.016
F1a3+16311 | 16465 | 5 | 0.027
F1a3+16311 | 16493 | 10 | 0.054
F1a3+16311 | 16497 | 3 | 0.016
F1a3+16311 | 198 | 1 | 0.005
F1a3+16311 | 573.1C | 1 | 0.005
F1a3a | 10463 | 1 | 0.006
F1a3a | 12654 | 1 | 0.006
F1a3a | 13434 | 1 | 0.006
F1a3a | 13812 | 1 | 0.006
F1a3a | 13886 | 1 | 0.006
F1a3a | 14040 | 1 | 0.006
F1a3a | 15148 | 1 | 0.006
F1a3a | 15218 | 1 | 0.006
F1a3a | 153 | 1 | 0.006
F1a3a | 16037 | 2 | 0.012
F1a3a | 16076d | 1 | 0.006
F1a3a | 16174 | 6 | 0.037
F1a3a | 16179 | 10 | 0.061
F1a3a | 16235 | 1 | 0.006
F1a3a | 16258T | 10 | 0.061
F1a3a | 16301 | 3 | 0.018
F1a3a | 195 | 6 | 0.037
F1a3a | 198 | 1 | 0.006
F1a3a | 3394 | 1 | 0.006
F1a3a | 385 | 1 | 0.006
F1a3a | 444 | 2 | 0.012
F1a3a | 4880 | 7 | 0.043
F1a3a | 573.1C | 1 | 0.006
F1a3a | 6376 | 1 | 0.006
F1a3a | 6899 | 1 | 0.006
F1a3a | 8985 | 7 | 0.043
F1a3a | 9138A | 1 | 0.006
F1a3a1 | 14905 | 1 | 0.022
F1a3a1 | 16189 | 2 | 0.043
F1a3a1 | 324G | 1 | 0.022
F1a3a1 | 3531 | 5 | 0.109

F1a3a1a | 11869 | 2 | 0.051
F1a3a1a | 146 | 1 | 0.026
F1a3a1a | 152 | 3 | 0.077
F1a3a1a | 214 | 1 | 0.026
F1a3a1a | 2589 | 1 | 0.026
F1a3a1a | 385 | 1 | 0.026
F1a3a1a | 6842 | 1 | 0.026
F1a3a1a | 9128 | 1 | 0.026
F1a3a2 | 13502A | 1 | 0.083
F1a3a2 | 16086 | 3 | 0.25
F1a3a2 | 16111 | 1 | 0.083
F1a3a2 | 16213 | 1 | 0.083
F1a3a2 | 16344 | 1 | 0.083
F1a3a2 | 194 | 1 | 0.083
F1a3a2 | 310 | 1 | 0.083
F1a3a2 | 9377 | 1 | 0.083
F1a3a3 | 146 | 5 | 0.03
F1a3a3 | 1462 | 1 | 0.006
F1a3a3 | 16076d | 1 | 0.006
F1a3a3 | 16093 | 1 | 0.006
F1a3a3 | 16169 | 15 | 0.089
F1a3a3 | 16235 | 1 | 0.006
F1a3a3 | 16256 | 1 | 0.006
F1a3a3 | 16265 | 3 | 0.018
F1a3a3 | 16301 | 1 | 0.006
F1a3a3 | 16362 | 3 | 0.018
F1a3a3 | 16465 | 5 | 0.03
F1a3a3 | 198 | 1 | 0.006
F1a3a3 | 5302 | 1 | 0.006
F1a3a3 | 573.1C | 1 | 0.006
F1a3a3a | 10724 | 1 | 0.006
F1a3a3a | 14883 | 3 | 0.019
F1a3a3a | 16076d | 1 | 0.006
F1a3a3a | 16235 | 1 | 0.006
F1a3a3a | 16301 | 3 | 0.019
F1a3a3a | 16465 | 5 | 0.032
F1a3a3a | 198 | 1 | 0.006
F1a3a3a | 5432 | 3 | 0.019
F1a3a3a | 573.1C | 1 | 0.006
F1a3a3a | 6261 | 3 | 0.019
F1a3a3a | 8093 | 1 | 0.006
F1a3b | 12717 | 1 | 0.005
F1a3b | 146 | 1 | 0.005
F1a3b | 1462 | 1 | 0.005
F1a3b | 16010.1T | 1 | 0.005
F1a3b | 16059T | 1 | 0.005
F1a3b | 16066 | 2 | 0.01
F1a3b | 16066C | 1 | 0.005
F1a3b | 16076d | 1 | 0.005
F1a3b | 16092 | 2 | 0.01
F1a3b | 16095 | 2 | 0.01
F1a3b | 16164 | 1 | 0.005
F1a3b | 16184 | 1 | 0.005
F1a3b | 16189 | 1 | 0.005
F1a3b | 16209 | 3 | 0.015
F1a3b | 16218A | 2 | 0.01
F1a3b | 16242 | 13 | 0.065
F1a3b | 16243 | 1 | 0.005
F1a3b | 16257A | 1 | 0.005
F1a3b | 16265 | 1 | 0.005
F1a3b | 16293 | 18 | 0.09
F1a3b | 16294 | 6 | 0.03
F1a3b | 16355 | 1 | 0.005
F1a3b | 16361T | 1 | 0.005
F1a3b | 16399C | 1 | 0.005
F1a3b | 16446 | 1 | 0.005
F1a3b | 16463T | 2 | 0.01
F1a3b | 385 | 4 | 0.02
F1a3b | 4709 | 1 | 0.005
F1a3b | 4732 | 1 | 0.005
F1a3b | 52 | 17 | 0.085
F1a3b | 54T | 7 | 0.035
F1a3b | 5899.1C | 1 | 0.005
F1a3b | 629 | 1 | 0.005
F1a3b | 71d | 22 | 0.11
F1a4 | 146 | 7 | 0.018
F1a4 | 150 | 1 | 0.003
F1a4 | 16051 | 1 | 0.003
F1a4 | 16058T | 1 | 0.003
F1a4 | 16108 | 18 | 0.047
F1a4 | 16145 | 1 | 0.003
F1a4 | 16154 | 1 | 0.003
F1a4 | 16163 | 3 | 0.008
F1a4 | 16169 | 5 | 0.013
F1a4 | 16184 | 1 | 0.003
F1a4 | 16187 | 4 | 0.01
F1a4 | 16188 | 1 | 0.003
F1a4 | 16190 | 1 | 0.003
F1a4 | 16193 | 1 | 0.003
F1a4 | 16194T | 1 | 0.003
F1a4 | 16207 | 1 | 0.003
F1a4 | 16223 | 1 | 0.003
F1a4 | 16224 | 1 | 0.003
F1a4 | 16260 | 2 | 0.005
F1a4 | 16261 | 2 | 0.005
F1a4 | 16265 | 1 | 0.003
F1a4 | 16278 | 2 | 0.005
F1a4 | 16287 | 2 | 0.005
F1a4 | 16293 | 1 | 0.003
F1a4 | 16294 | 1 | 0.003
F1a4 | 16295 | 20 | 0.052
F1a4 | 16304G | 1 | 0.003
F1a4 | 16319 | 2 | 0.005
F1a4 | 16320 | 3 | 0.008
F1a4 | 16365 | 5 | 0.013
F1a4 | 16399 | 9 | 0.024
F1a4 | 16527 | 2 | 0.005
F1a4 | 185 | 2 | 0.005
F1a4 | 200 | 3 | 0.008
F1a4 | 215 | 1 | 0.003
F1a4 | 234 | 1 | 0.003

F1a4 | 275 | 1 | 0.003
F1a4 | 573.1C | 1 | 0.003
F1a4 | 573.3C | 1 | 0.003
F1a4 | 601 | 1 | 0.003
F1a4a | 11549 | 1 | 0.019
F1a4a | 146 | 4 | 0.077
F1a4a | 153 | 1 | 0.019
F1a4a | 16093 | 1 | 0.019
F1a4a | 16108 | 1 | 0.019
F1a4a | 16162 | 2 | 0.038
F1a4a | 16168 | 1 | 0.019
F1a4a | 16223 | 1 | 0.019
F1a4a | 16224 | 1 | 0.019
F1a4a | 16249 | 1 | 0.019
F1a4a | 16295 | 1 | 0.019
F1a4a | 16311 | 40 | 0.769
F1a4a | 16360 | 1 | 0.019
F1a4a | 195 | 3 | 0.058
F1a4a | 200 | 1 | 0.019
F1a4a | 234 | 2 | 0.038
F1a4a | 5773 | 3 | 0.058
F1a4a | 5978 | 5 | 0.096
F1a4a | 681 | 1 | 0.019
F1a4a1 | 11075 | 1 | 0.003
F1a4a1 | 12810 | 1 | 0.003
F1a4a1 | 13105 | 2 | 0.007
F1a4a1 | 13434 | 1 | 0.003
F1a4a1 | 14319 | 1 | 0.003
F1a4a1 | 146 | 3 | 0.01
F1a4a1 | 151 | 1 | 0.003
F1a4a1 | 15646 | 1 | 0.003
F1a4a1 | 15924 | 3 | 0.01
F1a4a1 | 16092 | 1 | 0.003
F1a4a1 | 16093 | 6 | 0.02
F1a4a1 | 16111A | 1 | 0.003
F1a4a1 | 16147 | 1 | 0.003
F1a4a1 | 16173 | 4 | 0.013
F1a4a1 | 16186 | 1 | 0.003
F1a4a1 | 16189 | 12 | 0.04
F1a4a1 | 16192 | 2 | 0.007
F1a4a1 | 16220 | 1 | 0.003
F1a4a1 | 16239 | 2 | 0.007
F1a4a1 | 16240T | 2 | 0.007
F1a4a1 | 16242G | 1 | 0.003
F1a4a1 | 16243 | 1 | 0.003
F1a4a1 | 16271 | 2 | 0.007
F1a4a1 | 16274 | 3 | 0.01
F1a4a1 | 16311 | 6 | 0.02
F1a4a1 | 16318 | 1 | 0.003
F1a4a1 | 16319 | 7 | 0.024
F1a4a1 | 16320 | 11 | 0.037
F1a4a1 | 16356 | 1 | 0.003
F1a4a1 | 16361T | 3 | 0.01
F1a4a1 | 16489 | 1 | 0.003
F1a4a1 | 195 | 1 | 0.003
F1a4a1 | 214 | 1 | 0.003
F1a4a1 | 234 | 1 | 0.003
F1a4a1 | 2626 | 1 | 0.003
F1a4a1 | 295 | 1 | 0.003
F1a4a1 | 309d | 1 | 0.003
F1a4a1 | 310 | 2 | 0.007
F1a4a1 | 315d | 1 | 0.003
F1a4a1 | 324 | 7 | 0.024
F1a4a1 | 3456 | 1 | 0.003
F1a4a1 | 5102 | 1 | 0.003
F1a4a1 | 534 | 1 | 0.003
F1a4a1 | 573.1C | 1 | 0.003
F1a4a1 | 573.4C | 1 | 0.003
F1a4a1 | 6719 | 2 | 0.007
F1a4a1 | 6956 | 1 | 0.003
F1a4a1 | 7241 | 1 | 0.003
F1a4a1 | 7337 | 1 | 0.003
F1a4a1 | 8129 | 1 | 0.003
F1a4a1 | 8252G | 1 | 0.003
F1a4a1 | 8276.1C | 1 | 0.003
F1a4a1 | 8278.2C | 1 | 0.003
F1a4a1 | 8278.3C | 2 | 0.007
F1a4a1 | 8757 | 4 | 0.013
F1a4a1 | 8760 | 1 | 0.003
F1a4a1 | 8981 | 1 | 0.003
F1a4a1 | 8998 | 1 | 0.003
F1a4a1 | 9010 | 1 | 0.003
F1a4a1 | 9329 | 3 | 0.01
F1a4a1 | 9604 | 2 | 0.007
F1a4b | 1149 | 1 | 0.005
F1a4b | 151 | 2 | 0.009
F1a4b | 15301 | 1 | 0.005
F1a4b | 15992T | 1 | 0.005
F1a4b | 16038d | 1 | 0.005
F1a4b | 16093 | 2 | 0.009
F1a4b | 16171C | 1 | 0.005
F1a4b | 16176 | 1 | 0.005
F1a4b | 16242 | 2 | 0.009
F1a4b | 16248 | 1 | 0.005
F1a4b | 16256 | 2 | 0.009
F1a4b | 16266 | 14 | 0.065
F1a4b | 16301 | 17 | 0.079
F1a4b | 16400 | 2 | 0.009
F1a4b | 16474 | 6 | 0.028
F1a4b | 185 | 3 | 0.014
F1a4b | 275 | 1 | 0.005
F1a4b | 316C | 1 | 0.005
F1a4b | 318 | 3 | 0.014
F1a4b | 388 | 1 | 0.005
F1a4b | 54C | 1 | 0.005
F1a4b | 64 | 1 | 0.005
F1a4b | 71d | 2 | 0.009
F1a4b | 7419 | 1 | 0.005
F1a4b | 784d | 1 | 0.005
F1a'c'f | 11002 | 1 | 0.011
F1a'c'f | 12372 | 1 | 0.011

F1a'c'f | 13488 | 1 | 0.011
F1a'c'f | 14047 | 5 | 0.054
F1a'c'f | 14384 | 5 | 0.054
F1a'c'f | 14553 | 1 | 0.011
F1a'c'f | 146 | 5 | 0.054
F1a'c'f | 15061 | 1 | 0.011
F1a'c'f | 152 | 1 | 0.011
F1a'c'f | 15553 | 5 | 0.054
F1a'c'f | 16051 | 1 | 0.011
F1a'c'f | 16075A | 1 | 0.011
F1a'c'f | 16082G | 1 | 0.011
F1a'c'f | 16083G | 1 | 0.011
F1a'c'f | 16093 | 1 | 0.011
F1a'c'f | 16145 | 11 | 0.118
F1a'c'f | 16148 | 1 | 0.011
F1a'c'f | 16156T | 1 | 0.011
F1a'c'f | 16159 | 1 | 0.011
F1a'c'f | 16162 | 1 | 0.011
F1a'c'f | 16172 | 10 | 0.108
F1a'c'f | 16189 | 51 | 0.548
F1a'c'f | 16203 | 7 | 0.075
F1a'c'f | 16221 | 2 | 0.022
F1a'c'f | 16255 | 4 | 0.043
F1a'c'f | 16284 | 11 | 0.118
F1a'c'f | 16289 | 2 | 0.022
F1a'c'f | 16294 | 1 | 0.011
F1a'c'f | 16295 | 1 | 0.011
F1a'c'f | 16297 | 1 | 0.011
F1a'c'f | 185 | 3 | 0.032
F1a'c'f | 204 | 1 | 0.011
F1a'c'f | 236 | 1 | 0.011
F1a'c'f | 2581 | 1 | 0.011
F1a'c'f | 316C | 1 | 0.011
F1a'c'f | 7130 | 1 | 0.011
F1a'c'f | 7436 | 1 | 0.011
F1a'c'f | 8251 | 5 | 0.054
F1a'c'f | 8572 | 1 | 0.011
F1a'c'f | 8706 | 1 | 0.011
F1a'c'f | 961 | 1 | 0.011
F1b | 16399 | 2 | 0.024
F1b | 204 | 4 | 0.048
F1b1 | 16399 | 2 | 0.024
F1b1 | 204 | 4 | 0.048
F1b1+@152 | 10688 | 1 | 0.005
F1b1+@152 | 1189 | 1 | 0.005
F1b1+@152 | 11928 | 1 | 0.005
F1b1+@152 | 12505 | 1 | 0.005
F1b1+@152 | 12795 | 1 | 0.005
F1b1+@152 | 12876 | 2 | 0.01
F1b1+@152 | 13482 | 1 | 0.005
F1b1+@152 | 13759 | 4 | 0.021
F1b1+@152 | 1377 | 1 | 0.005
F1b1+@152 | 13889 | 1 | 0.005
F1b1+@152 | 146 | 9 | 0.046
F1b1+@152 | 15398 | 4 | 0.021
F1b1+@152 | 15617 | 1 | 0.005
F1b1+@152 | 15707 | 1 | 0.005
F1b1+@152 | 15924 | 2 | 0.01
F1b1+@152 | 16026d | 1 | 0.005
F1b1+@152 | 16111 | 5 | 0.026
F1b1+@152 | 16129 | 2 | 0.01
F1b1+@152 | 16132 | 3 | 0.015
F1b1+@152 | 16148 | 3 | 0.015
F1b1+@152 | 16184 | 1 | 0.005
F1b1+@152 | 16217 | 1 | 0.005
F1b1+@152 | 16278 | 1 | 0.005
F1b1+@152 | 16297A | 1 | 0.005
F1b1+@152 | 16335 | 1 | 0.005
F1b1+@152 | 16399 | 1 | 0.005
F1b1+@152 | 16427 | 3 | 0.015
F1b1+@152 | 183 | 1 | 0.005
F1b1+@152 | 195 | 1 | 0.005
F1b1+@152 | 199 | 5 | 0.026
F1b1+@152 | 203 | 2 | 0.01
F1b1+@152 | 204 | 49 | 0.251
F1b1+@152 | 207 | 2 | 0.01
F1b1+@152 | 2706C | 1 | 0.005
F1b1+@152 | 309.3C | 1 | 0.005
F1b1+@152 | 310 | 4 | 0.021
F1b1+@152 | 3531 | 5 | 0.026
F1b1+@152 | 3533 | 4 | 0.021
F1b1+@152 | 3591 | 1 | 0.005
F1b1+@152 | 4131 | 1 | 0.005
F1b1+@152 | 4218 | 1 | 0.005
F1b1+@152 | 4795 | 1 | 0.005
F1b1+@152 | 5237 | 1 | 0.005
F1b1+@152 | 5460 | 1 | 0.005
F1b1+@152 | 573.1C | 1 | 0.005
F1b1+@152 | 5899.1C | 1 | 0.005
F1b1+@152 | 6018 | 1 | 0.005
F1b1+@152 | 6040 | 2 | 0.01
F1b1+@152 | 6581 | 3 | 0.015
F1b1+@152 | 6629 | 2 | 0.01
F1b1+@152 | 6710 | 3 | 0.015
F1b1+@152 | 709 | 1 | 0.005
F1b1+@152 | 7624 | 1 | 0.005
F1b1+@152 | 8521 | 1 | 0.005
F1b1+@152 | 8537 | 1 | 0.005
F1b1+@152 | 8718 | 1 | 0.005
F1b1+@152 | 8774 | 2 | 0.01
F1b1+@152 | 9548 | 1 | 0.005
F1b1a | 11404 | 1 | 0.036
F1b1a | 13470 | 1 | 0.036
F1b1a | 15924 | 1 | 0.036
F1b1a | 16187 | 1 | 0.036
F1b1a | 188 | 1 | 0.036
F1b1a | 4314 | 1 | 0.036
F1b1a | 4456 | 1 | 0.036
F1b1a | 9092 | 1 | 0.036
F1b1a | 9449 | 1 | 0.036
F1b1a1 | 15884 | 1 | 0.032

F1b1a1 | 189 | 1 | 0.032
F1b1a1 | 207 | 3 | 0.097
F1b1a1 | 2079 | 1 | 0.032
F1b1a1 | 309.3C | 4 | 0.129
F1b1a1 | 385 | 2 | 0.065
F1b1a1a | 16178 | 1 | 0.028
F1b1a1a | 16193d | 3 | 0.083
F1b1a1a | 16223 | 1 | 0.028
F1b1a1a | 16284 | 1 | 0.028
F1b1a1a | 308-309d | 2 | 0.056
F1b1a1a | 4123 | 1 | 0.028
F1b1a1a | 8994 | 1 | 0.028
F1b1a1a1 | 10658 | 1 | 0.026
F1b1a1a1 | 13759 | 1 | 0.026
F1b1a1a1 | 13800 | 1 | 0.026
F1b1a1a1 | 13878 | 1 | 0.026
F1b1a1a1 | 14312 | 1 | 0.026
F1b1a1a1 | 151 | 5 | 0.132
F1b1a1a1 | 1738 | 1 | 0.026
F1b1a1a1 | 309.3C | 1 | 0.026
F1b1a1a1 | 310 | 1 | 0.026
F1b1a1a1 | 3745 | 1 | 0.026
F1b1a1a1 | 377.1T | 1 | 0.026
F1b1a1a1 | 5809 | 1 | 0.026
F1b1a1a1 | 8888 | 3 | 0.079
F1b1a1a1 | 8902 | 1 | 0.026
F1b1a1a1a | 15475 | 1 | 0.027
F1b1a1a1a | 16093 | 3 | 0.081
F1b1a1a1a | 309.3C | 1 | 0.027
F1b1a1a1a | 309.4C | 2 | 0.054
F1b1a1a1a | 3897 | 1 | 0.027
F1b1a1a1a | 408A | 1 | 0.027
F1b1a1a1a | 4296 | 1 | 0.027
F1b1a1a1a | 4820 | 1 | 0.027
F1b1a1a1a | 8725 | 1 | 0.027
F1b1a1a2 | 15649 | 1 | 0.031
F1b1a1a2 | 16524 | 1 | 0.031
F1b1a1a2 | 3696 | 1 | 0.031
F1b1a1a2 | 509 | 2 | 0.062
F1b1a1a2 | 53 | 1 | 0.031
F1b1a1a2 | 5836 | 1 | 0.031
F1b1a1a3 | 14118 | 1 | 0.034
F1b1a1a3 | 16145 | 2 | 0.069
F1b1a1a3 | 16220C | 1 | 0.034
F1b1a1a3 | 16355 | 1 | 0.034
F1b1a1a3 | 302.1A | 1 | 0.034
F1b1a1a3 | 339 | 1 | 0.034
F1b1a2 | 16145 | 2 | 0.069
F1b1a2 | 16220C | 1 | 0.034
F1b1a2 | 302.1A | 1 | 0.034
F1b1a2 | 339 | 1 | 0.034
F1b1a2 | 465 | 1 | 0.034
F1b1a2 | 5899 | 1 | 0.034
F1b1a2 | 8251 | 1 | 0.034
F1b1b | 10227 | 3 | 0.06
F1b1b | 11710 | 1 | 0.02
F1b1b | 13122 | 2 | 0.04
F1b1b | 13590 | 1 | 0.02
F1b1b | 146 | 3 | 0.06
F1b1b | 15924 | 8 | 0.16
F1b1b | 16108 | 1 | 0.02
F1b1b | 16169 | 1 | 0.02
F1b1b | 16179 | 8 | 0.16
F1b1b | 16193d | 1 | 0.02
F1b1b | 16342 | 1 | 0.02
F1b1b | 199 | 1 | 0.02
F1b1b | 200 | 1 | 0.02
F1b1b | 215 | 3 | 0.06
F1b1b | 3027 | 1 | 0.02
F1b1b | 310 | 4 | 0.08
F1b1b | 3392C | 1 | 0.02
F1b1b | 5262 | 1 | 0.02
F1b1b | 6357A | 1 | 0.02
F1b1b | 678 | 1 | 0.02
F1b1b | 7966 | 1 | 0.02
F1b1b | 8988 | 1 | 0.02
F1b1b | 9179 | 1 | 0.02
F1b1b | 9180 | 11 | 0.22
F1b1c | 10223 | 1 | 0.01
F1b1c | 10907 | 2 | 0.02
F1b1c | 10915 | 1 | 0.01
F1b1c | 11084 | 1 | 0.01
F1b1c | 11778 | 1 | 0.01
F1b1c | 11860 | 13 | 0.129
F1b1c | 12136 | 1 | 0.01
F1b1c | 13521 | 1 | 0.01
F1b1c | 13708 | 13 | 0.129
F1b1c | 14133 | 1 | 0.01
F1b1c | 14305 | 1 | 0.01
F1b1c | 146 | 1 | 0.01
F1b1c | 150 | 4 | 0.04
F1b1c | 15119 | 16 | 0.158
F1b1c | 153 | 2 | 0.02
F1b1c | 15301 | 1 | 0.01
F1b1c | 15734 | 3 | 0.03
F1b1c | 15948d | 1 | 0.01
F1b1c | 16086 | 2 | 0.02
F1b1c | 16093 | 1 | 0.01
F1b1c | 16124 | 1 | 0.01
F1b1c | 16129 | 5 | 0.05
F1b1c | 16134 | 10 | 0.099
F1b1c | 16136 | 1 | 0.01
F1b1c | 16145 | 1 | 0.01
F1b1c | 16162 | 1 | 0.01
F1b1c | 16174 | 1 | 0.01
F1b1c | 16181 | 1 | 0.01
F1b1c | 16181C | 2 | 0.02
F1b1c | 16188 | 2 | 0.02
F1b1c | 16194C | 1 | 0.01
F1b1c | 16195 | 1 | 0.01
F1b1c | 16209 | 1 | 0.01

F1b1c | 16242 | 2 | 0.02
F1b1c | 16243G | 1 | 0.01
F1b1c | 16254 | 1 | 0.01
F1b1c | 16266 | 22 | 0.218
F1b1c | 16271 | 1 | 0.01
F1b1c | 16278 | 2 | 0.02
F1b1c | 16295 | 1 | 0.01
F1b1c | 16302 | 1 | 0.01
F1b1c | 16344 | 1 | 0.01
F1b1c | 16357 | 7 | 0.069
F1b1c | 16360 | 1 | 0.01
F1b1c | 16362 | 3 | 0.03
F1b1c | 1719 | 1 | 0.01
F1b1c | 189 | 1 | 0.01
F1b1c | 234 | 1 | 0.01
F1b1c | 308-309d | 1 | 0.01
F1b1c | 309 | 1 | 0.01
F1b1c | 309.3C | 1 | 0.01
F1b1c | 309d | 4 | 0.04
F1b1c | 316C | 9 | 0.089
F1b1c | 318 | 2 | 0.02
F1b1c | 373 | 1 | 0.01
F1b1c | 379 | 1 | 0.01
F1b1c | 3834 | 2 | 0.02
F1b1c | 455d | 1 | 0.01
F1b1c | 573 | 1 | 0.01
F1b1c | 573.1C | 2 | 0.02
F1b1c | 573.2C | 1 | 0.01
F1b1c | 574C | 2 | 0.02
F1b1c | 576C | 1 | 0.01
F1b1c | 577C | 1 | 0.01
F1b1c | 578 | 1 | 0.01
F1b1c | 579 | 1 | 0.01
F1b1c | 6150 | 1 | 0.01
F1b1c | 9615 | 1 | 0.01
F1b1c | 9899 | 1 | 0.01
F1b1d | 14368 | 1 | 0.011
F1b1d | 146 | 1 | 0.011
F1b1d | 151 | 1 | 0.011
F1b1d | 16256 | 1 | 0.011
F1b1d | 16264 | 1 | 0.011
F1b1d | 16284 | 1 | 0.011
F1b1d | 16362 | 1 | 0.011
F1b1d | 16399 | 2 | 0.022
F1b1d | 16456 | 7 | 0.076
F1b1d | 194.1C | 1 | 0.011
F1b1d | 195 | 1 | 0.011
F1b1d | 200 | 1 | 0.011
F1b1d | 309.3C | 1 | 0.011
F1b1d | 326C | 1 | 0.011
F1b1d | 366 | 1 | 0.011
F1b1d | 676 | 1 | 0.011
F1b1e | 134A | 1 | 0.005
F1b1e | 14587 | 2 | 0.01
F1b1e | 146 | 1 | 0.005
F1b1e | 152 | 1 | 0.005
F1b1e | 15747 | 1 | 0.005
F1b1e | 16046G | 1 | 0.005
F1b1e | 16050G | 1 | 0.005
F1b1e | 16117 | 1 | 0.005
F1b1e | 16129 | 1 | 0.005
F1b1e | 16140 | 1 | 0.005
F1b1e | 16188 | 1 | 0.005
F1b1e | 16192-16193d | 2 | 0.01
F1b1e | 16193d | 1 | 0.005
F1b1e | 16209 | 1 | 0.005
F1b1e | 16217 | 1 | 0.005
F1b1e | 16219 | 1 | 0.005
F1b1e | 16261 | 1 | 0.005
F1b1e | 16274 | 1 | 0.005
F1b1e | 16331 | 1 | 0.005
F1b1e | 16340 | 1 | 0.005
F1b1e | 16343 | 1 | 0.005
F1b1e | 16356 | 2 | 0.01
F1b1e | 16360 | 13 | 0.068
F1b1e | 16374 | 1 | 0.005
F1b1e | 16374d | 1 | 0.005
F1b1e | 16390 | 1 | 0.005
F1b1e | 16396 | 1 | 0.005
F1b1e | 16419.1C | 1 | 0.005
F1b1e | 16421T | 1 | 0.005
F1b1e | 16432 | 1 | 0.005
F1b1e | 16485 | 2 | 0.01
F1b1e | 16496 | 2 | 0.01
F1b1e | 16502 | 1 | 0.005
F1b1e | 16531 | 1 | 0.005
F1b1e | 16532.1A | 1 | 0.005
F1b1e | 16544 | 1 | 0.005
F1b1e | 1717 | 1 | 0.005
F1b1e | 189 | 1 | 0.005
F1b1e | 195 | 3 | 0.016
F1b1e | 199 | 1 | 0.005
F1b1e | 220G | 1 | 0.005
F1b1e | 224 | 1 | 0.005
F1b1e | 234 | 1 | 0.005
F1b1e | 247 | 1 | 0.005
F1b1e | 252 | 4 | 0.021
F1b1e | 265 | 1 | 0.005
F1b1e | 283C | 1 | 0.005
F1b1e | 284.1A | 1 | 0.005
F1b1e | 309.3C | 1 | 0.005
F1b1e | 316C | 4 | 0.021
F1b1e | 328 | 1 | 0.005
F1b1e | 338 | 1 | 0.005
F1b1e | 365 | 1 | 0.005
F1b1e | 379 | 1 | 0.005
F1b1e | 395 | 1 | 0.005
F1b1e | 438d | 1 | 0.005
F1b1e | 44.1C | 1 | 0.005
F1b1e | 444C | 1 | 0.005
F1b1e | 489 | 1 | 0.005

F1b1e | 498d | 1 | 0.005
F1b1e | 573d | 3 | 0.016
F1b1e | 5794 | 2 | 0.01
F1b1e | 616 | 1 | 0.005
F1b1e | 6707 | 1 | 0.005
F1b1e | 6722 | 2 | 0.01
F1b1e | 71.1G | 2 | 0.01
F1b1e | 7547 | 2 | 0.01
F1b1e | 7572 | 1 | 0.005
F1b1e | 7964 | 1 | 0.005
F1b1e | 824 | 1 | 0.005
F1b1e | 827 | 2 | 0.01
F1b1e1 | 13245 | 1 | 0.006
F1b1e1 | 134A | 1 | 0.006
F1b1e1 | 16046G | 1 | 0.006
F1b1e1 | 16050G | 1 | 0.006
F1b1e1 | 16086 | 6 | 0.034
F1b1e1 | 16117 | 1 | 0.006
F1b1e1 | 16129 | 1 | 0.006
F1b1e1 | 16140 | 1 | 0.006
F1b1e1 | 16188 | 1 | 0.006
F1b1e1 | 16192-16193d | 2 | 0.011
F1b1e1 | 16193d | 1 | 0.006
F1b1e1 | 16209 | 1 | 0.006
F1b1e1 | 16215 | 9 | 0.052
F1b1e1 | 16217 | 1 | 0.006
F1b1e1 | 16219 | 1 | 0.006
F1b1e1 | 16240T | 1 | 0.006
F1b1e1 | 16331 | 1 | 0.006
F1b1e1 | 16340 | 1 | 0.006
F1b1e1 | 16343 | 1 | 0.006
F1b1e1 | 16374 | 1 | 0.006
F1b1e1 | 16390 | 1 | 0.006
F1b1e1 | 16396 | 1 | 0.006
F1b1e1 | 16421T | 1 | 0.006
F1b1e1 | 16432 | 1 | 0.006
F1b1e1 | 16485 | 2 | 0.011
F1b1e1 | 16496 | 2 | 0.011
F1b1e1 | 16502 | 1 | 0.006
F1b1e1 | 16531 | 1 | 0.006
F1b1e1 | 16532.1A | 1 | 0.006
F1b1e1 | 16544 | 1 | 0.006
F1b1e1 | 199 | 1 | 0.006
F1b1e1 | 220G | 1 | 0.006
F1b1e1 | 224 | 1 | 0.006
F1b1e1 | 234 | 1 | 0.006
F1b1e1 | 247 | 1 | 0.006
F1b1e1 | 265 | 1 | 0.006
F1b1e1 | 283C | 1 | 0.006
F1b1e1 | 284.1A | 1 | 0.006
F1b1e1 | 309.3C | 1 | 0.006
F1b1e1 | 328 | 1 | 0.006
F1b1e1 | 338 | 1 | 0.006
F1b1e1 | 365 | 1 | 0.006
F1b1e1 | 379 | 1 | 0.006
F1b1e1 | 395 | 1 | 0.006
F1b1e1 | 438d | 1 | 0.006
F1b1e1 | 444C | 1 | 0.006
F1b1e1 | 489 | 1 | 0.006
F1b1e1 | 498d | 1 | 0.006
F1b1e1 | 616 | 1 | 0.006
F1b1e1 | 71.1G | 2 | 0.011
F1b1f | 13470 | 2 | 0.012
F1b1f | 134A | 1 | 0.006
F1b1f | 16046G | 1 | 0.006
F1b1f | 16050G | 1 | 0.006
F1b1f | 16114A | 2 | 0.012
F1b1f | 16117 | 1 | 0.006
F1b1f | 16140 | 1 | 0.006
F1b1f | 16188 | 1 | 0.006
F1b1f | 16192-16193d | 2 | 0.012
F1b1f | 16193d | 1 | 0.006
F1b1f | 16209 | 1 | 0.006
F1b1f | 16217 | 1 | 0.006
F1b1f | 16219 | 1 | 0.006
F1b1f | 16331 | 1 | 0.006
F1b1f | 16340 | 1 | 0.006
F1b1f | 16343 | 1 | 0.006
F1b1f | 16374 | 1 | 0.006
F1b1f | 16390 | 1 | 0.006
F1b1f | 16396 | 1 | 0.006
F1b1f | 16421T | 1 | 0.006
F1b1f | 16432 | 1 | 0.006
F1b1f | 16485 | 2 | 0.012
F1b1f | 16496 | 2 | 0.012
F1b1f | 16502 | 1 | 0.006
F1b1f | 16531 | 1 | 0.006
F1b1f | 16532.1A | 1 | 0.006
F1b1f | 16544 | 1 | 0.006
F1b1f | 199 | 1 | 0.006
F1b1f | 220G | 1 | 0.006
F1b1f | 224 | 1 | 0.006
F1b1f | 234 | 1 | 0.006
F1b1f | 247 | 1 | 0.006
F1b1f | 265 | 1 | 0.006
F1b1f | 283C | 1 | 0.006
F1b1f | 284.1A | 1 | 0.006
F1b1f | 309.3C | 1 | 0.006
F1b1f | 310 | 1 | 0.006
F1b1f | 328 | 1 | 0.006
F1b1f | 338 | 1 | 0.006
F1b1f | 365 | 1 | 0.006
F1b1f | 379 | 1 | 0.006
F1b1f | 395 | 1 | 0.006
F1b1f | 438d | 1 | 0.006
F1b1f | 444C | 1 | 0.006
F1b1f | 489 | 1 | 0.006
F1b1f | 498d | 1 | 0.006
F1b1f | 616 | 1 | 0.006
F1b1f | 71.1G | 2 | 0.012
F1c | 10256 | 1 | 0.016

F1c | 10649 | 1 | 0.016
F1c | 142A | 1 | 0.016
F1c | 14364 | 1 | 0.016
F1c | 16035T | 1 | 0.016
F1c | 16048 | 21 | 0.339
F1c | 16068 | 1 | 0.016
F1c | 16093 | 1 | 0.016
F1c | 16108 | 1 | 0.016
F1c | 16116T | 1 | 0.016
F1c | 16166C | 1 | 0.016
F1c | 16172 | 6 | 0.097
F1c | 16189 | 21 | 0.339
F1c | 16191-16193d | 1 | 0.016
F1c | 16193.3C | 1 | 0.016
F1c | 16193d | 5 | 0.081
F1c | 16203 | 1 | 0.016
F1c | 16219 | 1 | 0.016
F1c | 16288 | 1 | 0.016
F1c | 16309 | 21 | 0.339
F1c | 16325 | 1 | 0.016
F1c | 16330 | 1 | 0.016
F1c | 16459 | 1 | 0.016
F1c | 16487 | 1 | 0.016
F1c | 183 | 1 | 0.016
F1c | 195 | 22 | 0.355
F1c | 1T | 1 | 0.016
F1c | 2308 | 1 | 0.016
F1c | 376 | 1 | 0.016
F1c | 398 | 1 | 0.016
F1c | 4232 | 1 | 0.016
F1c | 460 | 1 | 0.016
F1c | 515 | 1 | 0.016
F1c | 556 | 1 | 0.016
F1c | 82 | 1 | 0.016
F1c | 95 | 1 | 0.016
F1c1 | 16051 | 1 | 0.038
F1c1 | 16076d | 1 | 0.038
F1c1 | 16077 | 1 | 0.038
F1c1 | 16079G | 1 | 0.038
F1c1 | 16086 | 1 | 0.038
F1c1 | 16109 | 1 | 0.038
F1c1 | 16162 | 3 | 0.115
F1c1 | 16163 | 1 | 0.038
F1c1 | 16168 | 1 | 0.038
F1c1 | 16172 | 6 | 0.231
F1c1 | 16189 | 1 | 0.038
F1c1 | 16228G | 1 | 0.038
F1c1 | 16265 | 1 | 0.038
F1c1 | 16265C | 1 | 0.038
F1c1 | 16266 | 5 | 0.192
F1c1 | 16311 | 2 | 0.077
F1c1 | 16497 | 1 | 0.038
F1c1 | 1766 | 1 | 0.038
F1c1 | 1978 | 1 | 0.038
F1c1 | 234 | 3 | 0.115
F1c1 | 279 | 1 | 0.038
F1c1 | 2887 | 1 | 0.038
F1c1 | 3816 | 1 | 0.038
F1c1 | 432C | 1 | 0.038
F1c1 | 43G | 1 | 0.038
F1c1 | 46 | 1 | 0.038
F1c1 | 474d | 1 | 0.038
F1c1 | 477.1T | 1 | 0.038
F1c1 | 485-486d | 1 | 0.038
F1c1 | 490C | 1 | 0.038
F1c1 | 492T | 1 | 0.038
F1c1 | 5178 | 1 | 0.038
F1c1 | 573.1C | 1 | 0.038
F1c1a | 16051 | 1 | 0.032
F1c1a | 16086 | 13 | 0.419
F1c1a | 16172 | 1 | 0.032
F1c1a | 16189 | 2 | 0.065
F1c1a | 16294 | 1 | 0.032
F1c1a | 16526 | 1 | 0.032
F1c1a | 234 | 13 | 0.419
F1c1a | 316C | 1 | 0.032
F1c1a1 | 10118 | 1 | 0.006
F1c1a1 | 10192 | 1 | 0.006
F1c1a1 | 10479 | 1 | 0.006
F1c1a1 | 10790 | 1 | 0.006
F1c1a1 | 11425 | 1 | 0.006
F1c1a1 | 12192 | 1 | 0.006
F1c1a1 | 12853 | 1 | 0.006
F1c1a1 | 13050 | 2 | 0.012
F1c1a1 | 13105 | 1 | 0.006
F1c1a1 | 13194 | 2 | 0.012
F1c1a1 | 13708 | 1 | 0.006
F1c1a1 | 13887 | 2 | 0.012
F1c1a1 | 14059 | 1 | 0.006
F1c1a1 | 14587 | 2 | 0.012
F1c1a1 | 1511.1T | 1 | 0.006
F1c1a1 | 15394 | 1 | 0.006
F1c1a1 | 16017 | 1 | 0.006
F1c1a1 | 16029.1A | 1 | 0.006
F1c1a1 | 16033 | 1 | 0.006
F1c1a1 | 16037 | 1 | 0.006
F1c1a1 | 16076d | 1 | 0.006
F1c1a1 | 16092 | 1 | 0.006
F1c1a1 | 16093 | 1 | 0.006
F1c1a1 | 16102 | 1 | 0.006
F1c1a1 | 16172 | 1 | 0.006
F1c1a1 | 16215 | 3 | 0.018
F1c1a1 | 16223 | 2 | 0.012
F1c1a1 | 16245 | 1 | 0.006
F1c1a1 | 16250A | 1 | 0.006
F1c1a1 | 16251A | 1 | 0.006
F1c1a1 | 16259 | 8 | 0.047
F1c1a1 | 16271 | 1 | 0.006
F1c1a1 | 16311 | 4 | 0.023
F1c1a1 | 16356 | 2 | 0.012
F1c1a1 | 16357 | 5 | 0.029

F1c1a1 | 16514d | 1 | 0.006
F1c1a1 | 195 | 1 | 0.006
F1c1a1 | 2746 | 1 | 0.006
F1c1a1 | 279 | 1 | 0.006
F1c1a1 | 310 | 1 | 0.006
F1c1a1 | 315.2C | 1 | 0.006
F1c1a1 | 4435 | 1 | 0.006
F1c1a1 | 5363 | 1 | 0.006
F1c1a1 | 6863 | 1 | 0.006
F1c1a1 | 7175 | 1 | 0.006
F1c1a1 | 8567 | 6 | 0.035
F1c1a1 | 9357 | 1 | 0.006
F1c1a1 | 9815 | 1 | 0.006
F1c1a1 | 9965 | 1 | 0.006
F1c1a1a | 10324 | 2 | 0.013
F1c1a1a | 10739 | 1 | 0.007
F1c1a1a | 10887 | 2 | 0.013
F1c1a1a | 12771 | 2 | 0.013
F1c1a1a | 143 | 5 | 0.033
F1c1a1a | 16051 | 3 | 0.02
F1c1a1a | 16189 | 3 | 0.02
F1c1a1a | 16224 | 3 | 0.02
F1c1a1a | 16256 | 1 | 0.007
F1c1a1a | 16311 | 1 | 0.007
F1c1a1a | 16362 | 30 | 0.2
F1c1a1a | 200 | 1 | 0.007
F1c1a1a | 215 | 1 | 0.007
F1c1a1a | 309.3C | 1 | 0.007
F1c1a1a | 316C | 7 | 0.047
F1c1a1a | 4123 | 1 | 0.007
F1c1a1a | 8825 | 1 | 0.007
F1c1a1a | 9389 | 1 | 0.007
F1c1a1b | 12360 | 1 | 0.006
F1c1a1b | 12585 | 1 | 0.006
F1c1a1b | 151 | 2 | 0.012
F1c1a1b | 16092A | 1 | 0.006
F1c1a1b | 16189 | 40 | 0.248
F1c1a1b | 16247 | 2 | 0.012
F1c1a1b | 16295 | 1 | 0.006
F1c1a1b | 16356 | 2 | 0.012
F1c1a1b | 16357 | 4 | 0.025
F1c1a1b | 204 | 5 | 0.031
F1c1a1b | 234 | 1 | 0.006
F1c1a1b | 316C | 9 | 0.056
F1c1a1b | 3338 | 1 | 0.006
F1c1a1b | 6218 | 1 | 0.006
F1c1a1b | 7805 | 2 | 0.012
F1c1a2 | 1018 | 1 | 0.009
F1c1a2 | 10398 | 1 | 0.009
F1c1a2 | 103T | 1 | 0.009
F1c1a2 | 11365 | 1 | 0.009
F1c1a2 | 12469 | 1 | 0.009
F1c1a2 | 14180 | 1 | 0.009
F1c1a2 | 14484 | 1 | 0.009
F1c1a2 | 14502 | 1 | 0.009
F1c1a2 | 14526 | 1 | 0.009
F1c1a2 | 14776 | 1 | 0.009
F1c1a2 | 14783 | 1 | 0.009
F1c1a2 | 14954 | 1 | 0.009
F1c1a2 | 150 | 1 | 0.009
F1c1a2 | 151 | 2 | 0.018
F1c1a2 | 15217 | 1 | 0.009
F1c1a2 | 15412 | 1 | 0.009
F1c1a2 | 15580 | 7 | 0.064
F1c1a2 | 16041 | 1 | 0.009
F1c1a2 | 16051 | 1 | 0.009
F1c1a2 | 16058C | 11 | 0.101
F1c1a2 | 16072.1A | 1 | 0.009
F1c1a2 | 16086 | 3 | 0.028
F1c1a2 | 16092 | 4 | 0.037
F1c1a2 | 16093 | 1 | 0.009
F1c1a2 | 16094 | 1 | 0.009
F1c1a2 | 16107 | 1 | 0.009
F1c1a2 | 16145 | 9 | 0.083
F1c1a2 | 16189 | 5 | 0.046
F1c1a2 | 16213 | 1 | 0.009
F1c1a2 | 16220 | 1 | 0.009
F1c1a2 | 16223 | 1 | 0.009
F1c1a2 | 16243 | 1 | 0.009
F1c1a2 | 16248 | 1 | 0.009
F1c1a2 | 16261 | 1 | 0.009
F1c1a2 | 16268 | 2 | 0.018
F1c1a2 | 16311 | 4 | 0.037
F1c1a2 | 16320 | 1 | 0.009
F1c1a2 | 16368 | 1 | 0.009
F1c1a2 | 16399 | 1 | 0.009
F1c1a2 | 16527 | 1 | 0.009
F1c1a2 | 1849 | 1 | 0.009
F1c1a2 | 185 | 4 | 0.037
F1c1a2 | 195 | 1 | 0.009
F1c1a2 | 207 | 1 | 0.009
F1c1a2 | 2140 | 6 | 0.055
F1c1a2 | 228 | 1 | 0.009
F1c1a2 | 234 | 94 | 0.862
F1c1a2 | 310 | 2 | 0.018
F1c1a2 | 3106A | 2 | 0.018
F1c1a2 | 315.2C | 6 | 0.055
F1c1a2 | 317G | 1 | 0.009
F1c1a2 | 3258 | 1 | 0.009
F1c1a2 | 327G | 1 | 0.009
F1c1a2 | 3398 | 1 | 0.009
F1c1a2 | 3588 | 6 | 0.055
F1c1a2 | 4659 | 1 | 0.009
F1c1a2 | 4820 | 1 | 0.009
F1c1a2 | 489 | 1 | 0.009
F1c1a2 | 513 | 1 | 0.009
F1c1a2 | 515 | 2 | 0.018
F1c1a2 | 5420 | 1 | 0.009
F1c1a2 | 5429 | 1 | 0.009
F1c1a2 | 6047 | 1 | 0.009
F1c1a2 | 6150 | 1 | 0.009

F1c1a2 | 6377 | 1 | 0.009
F1c1a2 | 7226T | 1 | 0.009
F1c1a2 | 8152 | 1 | 0.009
F1d | 10095 | 1 | 0.003
F1d | 10192 | 2 | 0.005
F1d | 10653 | 1 | 0.003
F1d | 10828 | 2 | 0.005
F1d | 11092 | 5 | 0.014
F1d | 1187 | 1 | 0.003
F1d | 11935 | 1 | 0.003
F1d | 11998C | 1 | 0.003
F1d | 12375 | 4 | 0.011
F1d | 12545 | 1 | 0.003
F1d | 12696 | 2 | 0.005
F1d | 12858 | 1 | 0.003
F1d | 12940 | 1 | 0.003
F1d | 13287 | 1 | 0.003
F1d | 13299 | 1 | 0.003
F1d | 13350 | 1 | 0.003
F1d | 13388 | 1 | 0.003
F1d | 13635 | 1 | 0.003
F1d | 13708 | 1 | 0.003
F1d | 13759 | 1 | 0.003
F1d | 13899 | 1 | 0.003
F1d | 14259 | 1 | 0.003
F1d | 143 | 7 | 0.019
F1d | 14305 | 1 | 0.003
F1d | 14512 | 1 | 0.003
F1d | 14564 | 1 | 0.003
F1d | 14954 | 1 | 0.003
F1d | 150 | 4 | 0.011
F1d | 151 | 2 | 0.005
F1d | 15172 | 1 | 0.003
F1d | 152 | 7 | 0.019
F1d | 15244 | 1 | 0.003
F1d | 16023d | 1 | 0.003
F1d | 16026 | 1 | 0.003
F1d | 16037 | 1 | 0.003
F1d | 16067A | 2 | 0.005
F1d | 16093 | 2 | 0.005
F1d | 16124 | 1 | 0.003
F1d | 16148 | 1 | 0.003
F1d | 16166 | 1 | 0.003
F1d | 16172 | 10 | 0.027
F1d | 16176 | 1 | 0.003
F1d | 16185 | 2 | 0.005
F1d | 16187 | 2 | 0.005
F1d | 16188 | 1 | 0.003
F1d | 16191 | 1 | 0.003
F1d | 16194C | 1 | 0.003
F1d | 16207 | 2 | 0.005
F1d | 16214 | 2 | 0.005
F1d | 16223 | 1 | 0.003
F1d | 16224 | 1 | 0.003
F1d | 16260 | 1 | 0.003
F1d | 16261 | 1 | 0.003
F1d | 16278 | 1 | 0.003
F1d | 16294 | 4 | 0.011
F1d | 16295 | 2 | 0.005
F1d | 16309 | 7 | 0.019
F1d | 16310 | 1 | 0.003
F1d | 16311 | 45 | 0.124
F1d | 16318 | 2 | 0.005
F1d | 16343 | 2 | 0.005
F1d | 16357 | 1 | 0.003
F1d | 16390 | 1 | 0.003
F1d | 16400 | 3 | 0.008
F1d | 16465 | 1 | 0.003
F1d | 16C | 1 | 0.003
F1d | 186G | 1 | 0.003
F1d | 199 | 2 | 0.005
F1d | 200 | 1 | 0.003
F1d | 204 | 2 | 0.005
F1d | 207 | 1 | 0.003
F1d | 208 | 1 | 0.003
F1d | 2135d | 2 | 0.005
F1d | 217 | 6 | 0.016
F1d | 228 | 4 | 0.011
F1d | 234 | 1 | 0.003
F1d | 236 | 4 | 0.011
F1d | 247 | 1 | 0.003
F1d | 2755 | 2 | 0.005
F1d | 291d | 1 | 0.003
F1d | 315.3C | 1 | 0.003
F1d | 316C | 1 | 0.003
F1d | 390 | 1 | 0.003
F1d | 4722 | 1 | 0.003
F1d | 486 | 3 | 0.008
F1d | 4907 | 1 | 0.003
F1d | 490T | 1 | 0.003
F1d | 504 | 1 | 0.003
F1d | 546 | 1 | 0.003
F1d | 5460 | 7 | 0.019
F1d | 549 | 2 | 0.005
F1d | 5563 | 2 | 0.005
F1d | 574d | 1 | 0.003
F1d | 576C | 2 | 0.005
F1d | 592.1C | 1 | 0.003
F1d | 595.1C | 1 | 0.003
F1d | 597 | 1 | 0.003
F1d | 7076 | 1 | 0.003
F1d | 709 | 1 | 0.003
F1d | 7598 | 1 | 0.003
F1d | 7702 | 2 | 0.005
F1d | 7861 | 2 | 0.005
F1d | 8448 | 1 | 0.003
F1d | 8521 | 2 | 0.005
F1d | 8625 | 1 | 0.003
F1d | 8705 | 1 | 0.003
F1d | 8757 | 1 | 0.003
F1d | 8854 | 1 | 0.003

F1d | 8913 | 1 | 0.003
F1d | 9063 | 2 | 0.005
F1d | 9104 | 1 | 0.003
F1d | 929 | 1 | 0.003
F1d | 9423 | 1 | 0.003
F1d | 9950 | 6 | 0.016
F1d1 | 13251 | 1 | 0.002
F1d1 | 147.1C | 1 | 0.002
F1d1 | 150 | 1 | 0.002
F1d1 | 151 | 1 | 0.002
F1d1 | 152 | 4 | 0.009
F1d1 | 15924 | 1 | 0.002
F1d1 | 16072.1A | 1 | 0.002
F1d1 | 16086 | 1 | 0.002
F1d1 | 16093 | 9 | 0.021
F1d1 | 16103 | 1 | 0.002
F1d1 | 16129 | 1 | 0.002
F1d1 | 16145 | 96 | 0.223
F1d1 | 16154 | 2 | 0.005
F1d1 | 16171 | 1 | 0.002
F1d1 | 16172 | 1 | 0.002
F1d1 | 16179 | 7 | 0.016
F1d1 | 16223 | 1 | 0.002
F1d1 | 16234 | 1 | 0.002
F1d1 | 16245 | 1 | 0.002
F1d1 | 16255 | 47 | 0.109
F1d1 | 16261 | 3 | 0.007
F1d1 | 16265C | 2 | 0.005
F1d1 | 16266 | 1 | 0.002
F1d1 | 16269 | 1 | 0.002
F1d1 | 16271 | 1 | 0.002
F1d1 | 16274 | 1 | 0.002
F1d1 | 16278 | 2 | 0.005
F1d1 | 16284 | 148 | 0.344
F1d1 | 16291 | 2 | 0.005
F1d1 | 16293T | 1 | 0.002
F1d1 | 16311 | 3 | 0.007
F1d1 | 16319 | 10 | 0.023
F1d1 | 16362 | 14 | 0.033
F1d1 | 1814 | 1 | 0.002
F1d1 | 195 | 4 | 0.009
F1d1 | 200 | 1 | 0.002
F1d1 | 2124 | 1 | 0.002
F1d1 | 228 | 1 | 0.002
F1d1 | 315.2C | 4 | 0.009
F1d1 | 316C | 10 | 0.023
F1d1 | 377.1T | 1 | 0.002
F1d1 | 415T | 1 | 0.002
F1d1 | 420 | 1 | 0.002
F1d1 | 420G | 1 | 0.002
F1d1 | 424.2T | 2 | 0.005
F1d1 | 430A | 2 | 0.005
F1d1 | 431A | 1 | 0.002
F1d1 | 447 | 1 | 0.002
F1d1 | 573.1C | 1 | 0.002
F1d1 | 61A | 1 | 0.002
F1d1 | 650 | 1 | 0.002
F1d1 | 7191 | 1 | 0.002
F1d1 | 8896 | 1 | 0.002
F1d1 | 93 | 1 | 0.002
F1e | 11950 | 3 | 0.008
F1e | 13305 | 3 | 0.008
F1e | 152 | 1 | 0.003
F1e | 15657 | 1 | 0.003
F1e | 15812 | 3 | 0.008
F1e | 16093 | 1 | 0.003
F1e | 16146 | 1 | 0.003
F1e | 16221 | 1 | 0.003
F1e | 16232A | 1 | 0.003
F1e | 16243 | 27 | 0.076
F1e | 16260 | 1 | 0.003
F1e | 16261 | 1 | 0.003
F1e | 16287 | 3 | 0.008
F1e | 16292 | 1 | 0.003
F1e | 16301 | 1 | 0.003
F1e | 16305 | 3 | 0.008
F1e | 16311 | 2 | 0.006
F1e | 16362 | 1 | 0.003
F1e | 16371 | 1 | 0.003
F1e | 185 | 5 | 0.014
F1e | 185C | 4 | 0.011
F1e | 189 | 5 | 0.014
F1e | 204 | 1 | 0.003
F1e | 228 | 1 | 0.003
F1e | 237 | 3 | 0.008
F1e | 309.4C | 1 | 0.003
F1e | 312A | 1 | 0.003
F1e | 315.2C | 1 | 0.003
F1e | 3531 | 3 | 0.008
F1e | 372 | 3 | 0.008
F1e | 381 | 1 | 0.003
F1e | 464 | 1 | 0.003
F1e | 5192T | 3 | 0.008
F1e | 573.1C | 2 | 0.006
F1e | 573.2C | 1 | 0.003
F1e | 573.4C | 1 | 0.003
F1e | 573.5C | 1 | 0.003
F1e | 574C | 1 | 0.003
F1e | 5990 | 1 | 0.003
F1e | 70 | 3 | 0.008
F1e | 745.1T | 1 | 0.003
F1e | 761 | 1 | 0.003
F1e | 7805 | 1 | 0.003
F1e | 8164 | 3 | 0.008
F1e | 8248 | 3 | 0.008
F1e | 850d | 1 | 0.003
F1e | 874.1G | 1 | 0.003
F1e | 8784 | 3 | 0.008
F1e | 9115 | 3 | 0.008
F1e | 93 | 1 | 0.003
F1e | 9386 | 3 | 0.008

F1e1 | 10223 | 1 | 0.002
F1e1 | 10963 | 1 | 0.002
F1e1 | 16048 | 12 | 0.03
F1e1 | 16067A | 2 | 0.005
F1e1 | 16079 | 6 | 0.015
F1e1 | 16086 | 5 | 0.012
F1e1 | 16146 | 1 | 0.002
F1e1 | 16147 | 6 | 0.015
F1e1 | 16166C | 6 | 0.015
F1e1 | 16168 | 2 | 0.005
F1e1 | 16169 | 1 | 0.002
F1e1 | 16172 | 4 | 0.01
F1e1 | 16185 | 2 | 0.005
F1e1 | 16188G | 1 | 0.002
F1e1 | 16207 | 2 | 0.005
F1e1 | 16210 | 13 | 0.032
F1e1 | 16213 | 3 | 0.007
F1e1 | 16214 | 2 | 0.005
F1e1 | 16221 | 1 | 0.002
F1e1 | 16223 | 1 | 0.002
F1e1 | 16232A | 1 | 0.002
F1e1 | 16239 | 1 | 0.002
F1e1 | 16256 | 2 | 0.005
F1e1 | 16260 | 1 | 0.002
F1e1 | 16261 | 1 | 0.002
F1e1 | 16263 | 5 | 0.012
F1e1 | 16271 | 3 | 0.007
F1e1 | 16274 | 6 | 0.015
F1e1 | 16287 | 4 | 0.01
F1e1 | 16288 | 2 | 0.005
F1e1 | 16291 | 1 | 0.002
F1e1 | 16291A | 1 | 0.002
F1e1 | 16292 | 1 | 0.002
F1e1 | 16293 | 1 | 0.002
F1e1 | 16295 | 1 | 0.002
F1e1 | 16301 | 1 | 0.002
F1e1 | 16309 | 19 | 0.047
F1e1 | 16318 | 2 | 0.005
F1e1 | 16356 | 3 | 0.007
F1e1 | 16362 | 1 | 0.002
F1e1 | 16371 | 1 | 0.002
F1e1 | 16381 | 1 | 0.002
F1e1 | 199 | 3 | 0.007
F1e1 | 208 | 1 | 0.002
F1e1 | 237 | 3 | 0.007
F1e1 | 309.3C | 2 | 0.005
F1e1 | 3777 | 1 | 0.002
F1e1 | 464 | 1 | 0.002
F1e1 | 494A | 5 | 0.012
F1e1 | 5585 | 1 | 0.002
F1e1 | 573.4C | 1 | 0.002
F1e1 | 709 | 3 | 0.007
F1e1 | 735 | 2 | 0.005
F1e1 | 7357 | 1 | 0.002
F1e1 | 745.1T | 1 | 0.002
F1e1 | 761 | 1 | 0.002
F1e1 | 7765 | 1 | 0.002
F1e1 | 874.1G | 1 | 0.002
F1e1 | 93 | 1 | 0.002
F1e1a | 1227 | 1 | 0.012
F1e1a | 146 | 1 | 0.012
F1e1a | 151 | 1 | 0.012
F1e1a | 16046d | 1 | 0.012
F1e1a | 16051 | 1 | 0.012
F1e1a | 16059 | 1 | 0.012
F1e1a | 16066 | 1 | 0.012
F1e1a | 16092 | 1 | 0.012
F1e1a | 16103 | 2 | 0.025
F1e1a | 16129 | 1 | 0.012
F1e1a | 16145 | 1 | 0.012
F1e1a | 16160 | 1 | 0.012
F1e1a | 16172 | 5 | 0.062
F1e1a | 16180 | 1 | 0.012
F1e1a | 16192-16193d | 1 | 0.012
F1e1a | 16193d | 4 | 0.049
F1e1a | 16209 | 1 | 0.012
F1e1a | 16210C | 1 | 0.012
F1e1a | 16232 | 1 | 0.012
F1e1a | 16262 | 1 | 0.012
F1e1a | 16262A | 1 | 0.012
F1e1a | 16272 | 1 | 0.012
F1e1a | 16291 | 2 | 0.025
F1e1a | 16344 | 1 | 0.012
F1e1a | 16359 | 1 | 0.012
F1e1a | 16381 | 1 | 0.012
F1e1a | 16445 | 1 | 0.012
F1e1a | 16474 | 1 | 0.012
F1e1a | 16517 | 1 | 0.012
F1e1a | 16518 | 1 | 0.012
F1e1a | 202 | 1 | 0.012
F1e1a | 204 | 2 | 0.025
F1e1a | 215 | 1 | 0.012
F1e1a | 223A | 1 | 0.012
F1e1a | 238 | 1 | 0.012
F1e1a | 258 | 1 | 0.012
F1e1a | 265 | 21 | 0.259
F1e1a | 283T | 1 | 0.012
F1e1a | 302 | 1 | 0.012
F1e1a | 336 | 1 | 0.012
F1e1a | 3519 | 1 | 0.012
F1e1a | 364T | 1 | 0.012
F1e1a | 385 | 1 | 0.012
F1e1a | 408d | 1 | 0.012
F1e1a | 413 | 1 | 0.012
F1e1a | 413.1G | 1 | 0.012
F1e1a | 429 | 1 | 0.012
F1e1a | 451.1A | 1 | 0.012
F1e1a | 513 | 2 | 0.025
F1e1a | 530 | 1 | 0.012
F1e1a | 544 | 1 | 0.012
F1e1a | 573 | 1 | 0.012

F1e1a | 574 | 1 | 0.012
F1e1a | 9983 | 1 | 0.012
F1e2 | 10653 | 1 | 0.071
F1e2 | 13590 | 1 | 0.071
F1e2 | 16093 | 5 | 0.357
F1e2 | 16186 | 7 | 0.5
F1e2 | 16209 | 7 | 0.5
F1e2 | 16256 | 1 | 0.071
F1e2 | 16271 | 9 | 0.643
F1e2 | 16311 | 8 | 0.571
F1e2 | 4314d | 3 | 0.214
F1e2 | 6734 | 3 | 0.214
F1e2 | 9095 | 3 | 0.214
F1e3 | 10364 | 2 | 0.029
F1e3 | 1053 | 1 | 0.014
F1e3 | 10882 | 1 | 0.014
F1e3 | 10966 | 1 | 0.014
F1e3 | 11935 | 3 | 0.043
F1e3 | 131 | 1 | 0.014
F1e3 | 13359 | 1 | 0.014
F1e3 | 14040 | 1 | 0.014
F1e3 | 146 | 9 | 0.129
F1e3 | 152 | 5 | 0.071
F1e3 | 15924 | 1 | 0.014
F1e3 | 16042 | 12 | 0.171
F1e3 | 16051 | 17 | 0.243
F1e3 | 16086 | 2 | 0.029
F1e3 | 16093 | 2 | 0.029
F1e3 | 16131 | 2 | 0.029
F1e3 | 16137 | 1 | 0.014
F1e3 | 16145 | 1 | 0.014
F1e3 | 16167 | 1 | 0.014
F1e3 | 16172 | 1 | 0.014
F1e3 | 16212 | 1 | 0.014
F1e3 | 16239 | 1 | 0.014
F1e3 | 16249 | 7 | 0.1
F1e3 | 16269 | 11 | 0.157
F1e3 | 16274 | 9 | 0.129
F1e3 | 16284 | 10 | 0.143
F1e3 | 16287 | 1 | 0.014
F1e3 | 16292 | 15 | 0.214
F1e3 | 16294 | 3 | 0.043
F1e3 | 16305 | 4 | 0.057
F1e3 | 16311 | 25 | 0.357
F1e3 | 16327 | 1 | 0.014
F1e3 | 16342 | 2 | 0.029
F1e3 | 16355 | 1 | 0.014
F1e3 | 16356 | 1 | 0.014
F1e3 | 16357 | 1 | 0.014
F1e3 | 189 | 3 | 0.043
F1e3 | 195 | 51 | 0.729
F1e3 | 198 | 3 | 0.043
F1e3 | 204 | 1 | 0.014
F1e3 | 214 | 1 | 0.014
F1e3 | 215 | 3 | 0.043
F1e3 | 234 | 1 | 0.014
F1e3 | 235 | 1 | 0.014
F1e3 | 237 | 1 | 0.014
F1e3 | 291d | 1 | 0.014
F1e3 | 310 | 1 | 0.014
F1e3 | 315d | 1 | 0.014
F1e3 | 3434 | 1 | 0.014
F1e3 | 3834 | 2 | 0.029
F1e3 | 4435 | 2 | 0.029
F1e3 | 4649 | 2 | 0.029
F1e3 | 489 | 8 | 0.114
F1e3 | 5147 | 2 | 0.029
F1e3 | 5263 | 1 | 0.014
F1e3 | 5492 | 1 | 0.014
F1e3 | 5961A | 1 | 0.014
F1e3 | 6620 | 1 | 0.014
F1e3 | 6842 | 1 | 0.014
F1e3 | 7675 | 1 | 0.014
F1e3 | 7888 | 2 | 0.029
F1e3 | 7909 | 1 | 0.014
F1e3 | 8008 | 1 | 0.014
F1f | 10101 | 8 | 0.049
F1f | 1018 | 1 | 0.006
F1f | 10192A | 1 | 0.006
F1f | 10265 | 1 | 0.006
F1f | 10685 | 1 | 0.006
F1f | 10687G | 1 | 0.006
F1f | 10969 | 1 | 0.006
F1f | 10972 | 1 | 0.006
F1f | 11087 | 4 | 0.024
F1f | 11287 | 2 | 0.012
F1f | 11581 | 2 | 0.012
F1f | 12055 | 1 | 0.006
F1f | 12361 | 1 | 0.006
F1f | 12372 | 1 | 0.006
F1f | 12441 | 2 | 0.012
F1f | 12495 | 4 | 0.024
F1f | 13236 | 1 | 0.006
F1f | 13275 | 4 | 0.024
F1f | 1348 | 1 | 0.006
F1f | 13708 | 5 | 0.03
F1f | 13888 | 1 | 0.006
F1f | 13932 | 1 | 0.006
F1f | 14131 | 4 | 0.024
F1f | 14180 | 1 | 0.006
F1f | 14344 | 1 | 0.006
F1f | 14370 | 1 | 0.006
F1f | 146 | 8 | 0.049
F1f | 1462 | 1 | 0.006
F1f | 14696 | 2 | 0.012
F1f | 14839 | 2 | 0.012
F1f | 14905 | 1 | 0.006
F1f | 15077 | 1 | 0.006
F1f | 15115 | 1 | 0.006
F1f | 152 | 21 | 0.128
F1f | 15217 | 2 | 0.012

F1f | 15226 | 1 | 0.006
F1f | 15317 | 2 | 0.012
F1f | 15355 | 1 | 0.006
F1f | 15499 | 5 | 0.03
F1f | 15604 | 1 | 0.006
F1f | 15706 | 1 | 0.006
F1f | 15718 | 1 | 0.006
F1f | 15924 | 1 | 0.006
F1f | 15930 | 5 | 0.03
F1f | 15939 | 4 | 0.024
F1f | 16039 | 2 | 0.012
F1f | 16086 | 3 | 0.018
F1f | 16095 | 1 | 0.006
F1f | 16164 | 1 | 0.006
F1f | 16172 | 122 | 0.744
F1f | 16189 | 4 | 0.024
F1f | 16192 | 3 | 0.018
F1f | 16207 | 1 | 0.006
F1f | 16209 | 1 | 0.006
F1f | 16245 | 4 | 0.024
F1f | 16256 | 1 | 0.006
F1f | 16258T | 7 | 0.043
F1f | 16293 | 1 | 0.006
F1f | 16294 | 5 | 0.03
F1f | 16295 | 5 | 0.03
F1f | 16311 | 5 | 0.03
F1f | 16320 | 3 | 0.018
F1f | 1824 | 1 | 0.006
F1f | 195 | 3 | 0.018
F1f | 204 | 1 | 0.006
F1f | 2218 | 4 | 0.024
F1f | 225T | 1 | 0.006
F1f | 234 | 1 | 0.006
F1f | 2352 | 8 | 0.049
F1f | 243 | 4 | 0.024
F1f | 251 | 8 | 0.049
F1f | 266 | 1 | 0.006
F1f | 2916 | 1 | 0.006
F1f | 310 | 1 | 0.006
F1f | 3394 | 2 | 0.012
F1f | 3397 | 1 | 0.006
F1f | 3432 | 9 | 0.055
F1f | 3509 | 4 | 0.024
F1f | 3645 | 2 | 0.012
F1f | 365d | 1 | 0.006
F1f | 4227 | 1 | 0.006
F1f | 456 | 1 | 0.006
F1f | 4659 | 2 | 0.012
F1f | 4688 | 1 | 0.006
F1f | 4705 | 7 | 0.043
F1f | 4823 | 1 | 0.006
F1f | 4907 | 4 | 0.024
F1f | 4976 | 2 | 0.012
F1f | 504 | 1 | 0.006
F1f | 5054 | 2 | 0.012
F1f | 5057 | 1 | 0.006
F1f | 5147 | 13 | 0.079
F1f | 5442 | 1 | 0.006
F1f | 5460 | 1 | 0.006
F1f | 5585 | 1 | 0.006
F1f | 5964 | 1 | 0.006
F1f | 6109 | 1 | 0.006
F1f | 6176 | 1 | 0.006
F1f | 6323 | 5 | 0.03
F1f | 6353 | 1 | 0.006
F1f | 7055 | 1 | 0.006
F1f | 709 | 1 | 0.006
F1f | 7298 | 1 | 0.006
F1f | 7337 | 1 | 0.006
F1f | 7830 | 3 | 0.018
F1f | 7849 | 1 | 0.006
F1f | 7961 | 1 | 0.006
F1f | 8119 | 2 | 0.012
F1f | 8144 | 1 | 0.006
F1f | 8490 | 3 | 0.018
F1f | 8572 | 5 | 0.03
F1f | 8618 | 2 | 0.012
F1f | 8872 | 1 | 0.006
F1f | 8929 | 7 | 0.043
F1f | 8937 | 1 | 0.006
F1f | 8970 | 3 | 0.018
F1f | 9021 | 1 | 0.006
F1f | 90C | 1 | 0.006
F1f | 9104 | 1 | 0.006
F1f | 9119 | 1 | 0.006
F1f | 9142 | 1 | 0.006
F1f | 9266 | 9 | 0.055
F1f | 9438 | 1 | 0.006
F1f | 9452 | 1 | 0.006
F1f | 9575 | 1 | 0.006
F1f | 961 | 1 | 0.006
F1f | 9692 | 8 | 0.049
F1f | 9966 | 1 | 0.006
F1f | 9968 | 4 | 0.024
F1g | 1018 | 1 | 0.003
F1g | 106-111d | 1 | 0.003
F1g | 11335 | 2 | 0.005
F1g | 11992 | 1 | 0.003
F1g | 12166 | 5 | 0.013
F1g | 12699 | 1 | 0.003
F1g | 13398 | 1 | 0.003
F1g | 13879 | 1 | 0.003
F1g | 13907 | 1 | 0.003
F1g | 14484 | 1 | 0.003
F1g | 146 | 1 | 0.003
F1g | 1462 | 1 | 0.003
F1g | 14861 | 2 | 0.005
F1g | 14894 | 1 | 0.003
F1g | 15013 | 1 | 0.003
F1g | 151 | 4 | 0.01
F1g | 15119 | 1 | 0.003

F1g | 152 | 1 | 0.003
F1g | 15247 | 1 | 0.003
F1g | 15442 | 1 | 0.003
F1g | 15688 | 1 | 0.003
F1g | 15908 | 3 | 0.008
F1g | 15924 | 1 | 0.003
F1g | 15927 | 1 | 0.003
F1g | 16092 | 2 | 0.005
F1g | 16093 | 1 | 0.003
F1g | 16126 | 1 | 0.003
F1g | 16129 | 1 | 0.003
F1g | 16146 | 1 | 0.003
F1g | 16153 | 7 | 0.018
F1g | 16167 | 2 | 0.005
F1g | 16188G | 1 | 0.003
F1g | 16190 | 1 | 0.003
F1g | 16209 | 1 | 0.003
F1g | 16213 | 3 | 0.008
F1g | 16221 | 1 | 0.003
F1g | 16223 | 1 | 0.003
F1g | 16228 | 1 | 0.003
F1g | 16232A | 1 | 0.003
F1g | 16260 | 1 | 0.003
F1g | 16261 | 1 | 0.003
F1g | 16278 | 9 | 0.024
F1g | 16287 | 3 | 0.008
F1g | 16292 | 1 | 0.003
F1g | 16300 | 7 | 0.018
F1g | 16301 | 1 | 0.003
F1g | 16316 | 2 | 0.005
F1g | 16319 | 2 | 0.005
F1g | 16352 | 1 | 0.003
F1g | 16356 | 2 | 0.005
F1g | 16357 | 10 | 0.026
F1g | 16362 | 1 | 0.003
F1g | 16368 | 1 | 0.003
F1g | 16371 | 1 | 0.003
F1g | 16374C | 4 | 0.01
F1g | 16390 | 8 | 0.021
F1g | 16398 | 1 | 0.003
F1g | 186 | 2 | 0.005
F1g | 195 | 5 | 0.013
F1g | 199 | 3 | 0.008
F1g | 204 | 1 | 0.003
F1g | 2581 | 1 | 0.003
F1g | 2857 | 1 | 0.003
F1g | 310 | 2 | 0.005
F1g | 3421 | 1 | 0.003
F1g | 3483 | 1 | 0.003
F1g | 3592 | 1 | 0.003
F1g | 3702 | 2 | 0.005
F1g | 373 | 1 | 0.003
F1g | 374d | 1 | 0.003
F1g | 4048 | 2 | 0.005
F1g | 464 | 1 | 0.003
F1g | 4853 | 1 | 0.003
F1g | 4924C | 1 | 0.003
F1g | 4967 | 5 | 0.013
F1g | 4985 | 1 | 0.003
F1g | 509 | 1 | 0.003
F1g | 573.4C | 1 | 0.003
F1g | 6260 | 1 | 0.003
F1g | 709 | 2 | 0.005
F1g | 745.1T | 1 | 0.003
F1g | 761 | 1 | 0.003
F1g | 8167 | 1 | 0.003
F1g | 8308 | 5 | 0.013
F1g | 8637 | 1 | 0.003
F1g | 8696 | 1 | 0.003
F1g | 8784 | 1 | 0.003
F1g | 8854 | 1 | 0.003
F1g | 93 | 1 | 0.003
F1g | 9377 | 5 | 0.013
F1g1 | 10653 | 1 | 0.002
F1g1 | 12171 | 1 | 0.002
F1g1 | 12892 | 3 | 0.007
F1g1 | 13759 | 1 | 0.002
F1g1 | 14311 | 1 | 0.002
F1g1 | 14977 | 1 | 0.002
F1g1 | 152 | 2 | 0.005
F1g1 | 15261 | 1 | 0.002
F1g1 | 153 | 2 | 0.005
F1g1 | 15317 | 1 | 0.002
F1g1 | 15758 | 1 | 0.002
F1g1 | 16067A | 2 | 0.005
F1g1 | 16079 | 6 | 0.015
F1g1 | 16086 | 5 | 0.012
F1g1 | 16093 | 11 | 0.027
F1g1 | 16114 | 1 | 0.002
F1g1 | 16124 | 1 | 0.002
F1g1 | 16129 | 4 | 0.01
F1g1 | 16136 | 1 | 0.002
F1g1 | 16140 | 4 | 0.01
F1g1 | 16146 | 1 | 0.002
F1g1 | 16147 | 1 | 0.002
F1g1 | 16166C | 6 | 0.015
F1g1 | 16168 | 3 | 0.007
F1g1 | 16185 | 2 | 0.005
F1g1 | 16188G | 1 | 0.002
F1g1 | 16213 | 3 | 0.007
F1g1 | 16214 | 2 | 0.005
F1g1 | 16221 | 1 | 0.002
F1g1 | 16223 | 1 | 0.002
F1g1 | 16232A | 1 | 0.002
F1g1 | 16256 | 2 | 0.005
F1g1 | 16260 | 1 | 0.002
F1g1 | 16261 | 4 | 0.01
F1g1 | 16263 | 4 | 0.01
F1g1 | 16271 | 2 | 0.005
F1g1 | 16274 | 6 | 0.015
F1g1 | 16287 | 4 | 0.01

F1g1 | 16291A | 1 | 0.002
F1g1 | 16292 | 1 | 0.002
F1g1 | 16295 | 1 | 0.002
F1g1 | 16301 | 1 | 0.002
F1g1 | 16318 | 2 | 0.005
F1g1 | 16325 | 2 | 0.005
F1g1 | 16356 | 2 | 0.005
F1g1 | 16359 | 1 | 0.002
F1g1 | 16362 | 1 | 0.002
F1g1 | 16371 | 1 | 0.002
F1g1 | 16381 | 1 | 0.002
F1g1 | 16390 | 1 | 0.002
F1g1 | 16527 | 2 | 0.005
F1g1 | 199 | 3 | 0.007
F1g1 | 204 | 4 | 0.01
F1g1 | 207 | 3 | 0.007
F1g1 | 208 | 1 | 0.002
F1g1 | 215 | 3 | 0.007
F1g1 | 237 | 3 | 0.007
F1g1 | 2889 | 4 | 0.01
F1g1 | 3010 | 1 | 0.002
F1g1 | 310 | 1 | 0.002
F1g1 | 315.2C | 1 | 0.002
F1g1 | 315d | 1 | 0.002
F1g1 | 316C | 11 | 0.027
F1g1 | 3736 | 1 | 0.002
F1g1 | 4086 | 1 | 0.002
F1g1 | 44.1C | 1 | 0.002
F1g1 | 464 | 1 | 0.002
F1g1 | 5300 | 3 | 0.007
F1g1 | 5663 | 3 | 0.007
F1g1 | 573.4C | 1 | 0.002
F1g1 | 6887 | 1 | 0.002
F1g1 | 709 | 3 | 0.007
F1g1 | 722 | 1 | 0.002
F1g1 | 735 | 2 | 0.005
F1g1 | 745.1T | 1 | 0.002
F1g1 | 761 | 1 | 0.002
F1g1 | 7813 | 1 | 0.002
F1g1 | 7859 | 1 | 0.002
F1g1 | 7935 | 1 | 0.002
F1g1 | 874.1G | 1 | 0.002
F1g1 | 9254 | 1 | 0.002
F1g1 | 93 | 1 | 0.002
F2 | 11016 | 1 | 0.005
F2 | 11893 | 1 | 0.005
F2 | 14002 | 1 | 0.005
F2 | 146G | 1 | 0.005
F2 | 147G | 1 | 0.005
F2 | 15403A | 1 | 0.005
F2 | 15803 | 1 | 0.005
F2 | 16129 | 1 | 0.005
F2 | 16169 | 1 | 0.005
F2 | 16179 | 1 | 0.005
F2 | 16184 | 1 | 0.005
F2 | 16189 | 1 | 0.005
F2 | 16193A | 1 | 0.005
F2 | 16209 | 2 | 0.011
F2 | 16218 | 1 | 0.005
F2 | 16221 | 1 | 0.005
F2 | 16224 | 1 | 0.005
F2 | 16272 | 3 | 0.016
F2 | 16360 | 1 | 0.005
F2 | 16390 | 3 | 0.016
F2 | 16399 | 1 | 0.005
F2 | 207 | 1 | 0.005
F2 | 3010 | 2 | 0.011
F2 | 318 | 1 | 0.005
F2 | 574C | 1 | 0.005
F2 | 576C | 1 | 0.005
F2 | 5894C | 1 | 0.005
F2 | 6020 | 1 | 0.005
F2 | 606 | 1 | 0.005
F2 | 6464 | 1 | 0.005
F2 | 7861 | 1 | 0.005
F2 | 8764 | 1 | 0.005
F2 | 8964 | 1 | 0.005
F2 | 8998 | 1 | 0.005
F2 | 9096 | 1 | 0.005
F2 | 9497 | 1 | 0.005
F2+16291 | 10927 | 1 | 0.013
F2+16291 | 147 | 1 | 0.013
F2+16291 | 16037 | 1 | 0.013
F2+16291 | 16086 | 1 | 0.013
F2+16291 | 16092A | 1 | 0.013
F2+16291 | 16092T | 1 | 0.013
F2+16291 | 16111A | 1 | 0.013
F2+16291 | 16185 | 4 | 0.051
F2+16291 | 16189 | 1 | 0.013
F2+16291 | 16198A | 1 | 0.013
F2+16291 | 16206 | 1 | 0.013
F2+16291 | 16221 | 1 | 0.013
F2+16291 | 16252 | 1 | 0.013
F2+16291 | 16266G | 16 | 0.205
F2+16291 | 16287 | 1 | 0.013
F2+16291 | 16311 | 27 | 0.346
F2+16291 | 16325 | 1 | 0.013
F2+16291 | 16335 | 26 | 0.333
F2+16291 | 16368 | 1 | 0.013
F2+16291 | 16399 | 1 | 0.013
F2+16291 | 16548 | 1 | 0.013
F2+16291 | 266 | 1 | 0.013
F2+16291 | 278 | 1 | 0.013
F2+16291 | 289 | 1 | 0.013
F2+16291 | 309.3C | 1 | 0.013
F2+16291 | 316C | 1 | 0.013
F2+16291 | 350 | 1 | 0.013
F2+16291 | 383 | 1 | 0.013
F2+16291 | 456 | 1 | 0.013
F2+16291 | 462 | 1 | 0.013
F2+16291 | 464C | 1 | 0.013

F2+16291 | 489 | 1 | 0.013
F2+16291 | 507 | 1 | 0.013
F2+16291 | 526 | 1 | 0.013
F2+16291 | 537 | 1 | 0.013
F2+16291 | 537d | 1 | 0.013
F2+16291 | 69 | 4 | 0.051
F2+195 | 11339 | 2 | 0.013
F2+195 | 11569 | 1 | 0.006
F2+195 | 13275 | 1 | 0.006
F2+195 | 14002 | 1 | 0.006
F2+195 | 16086 | 1 | 0.006
F2+195 | 16093 | 2 | 0.013
F2+195 | 16233 | 3 | 0.019
F2+195 | 279 | 1 | 0.006
F2+195 | 3010 | 1 | 0.006
F2+195 | 5177 | 2 | 0.013
F2+195 | 5258 | 1 | 0.006
F2+195 | 606 | 1 | 0.006
F2+195 | 6248 | 2 | 0.013
F2+195 | 709 | 1 | 0.006
F2+195 | 8269 | 1 | 0.006
F2+195 | 8998 | 1 | 0.006
F2a | 10325 | 3 | 0.022
F2a | 10927 | 9 | 0.065
F2a | 11009 | 1 | 0.007
F2a | 114 | 1 | 0.007
F2a | 11485 | 1 | 0.007
F2a | 11614 | 1 | 0.007
F2a | 11778 | 1 | 0.007
F2a | 11969 | 1 | 0.007
F2a | 12172 | 1 | 0.007
F2a | 12372 | 4 | 0.029
F2a | 12477 | 1 | 0.007
F2a | 12603 | 1 | 0.007
F2a | 12696 | 1 | 0.007
F2a | 13077 | 1 | 0.007
F2a | 13563 | 1 | 0.007
F2a | 13860 | 1 | 0.007
F2a | 14037 | 1 | 0.007
F2a | 14049 | 1 | 0.007
F2a | 14308 | 1 | 0.007
F2a | 146 | 4 | 0.029
F2a | 14687 | 1 | 0.007
F2a | 14790 | 1 | 0.007
F2a | 14831 | 3 | 0.022
F2a | 152 | 4 | 0.029
F2a | 15586 | 3 | 0.022
F2a | 15597 | 1 | 0.007
F2a | 15670 | 1 | 0.007
F2a | 15852 | 1 | 0.007
F2a | 15884 | 2 | 0.014
F2a | 16066 | 1 | 0.007
F2a | 16076d | 1 | 0.007
F2a | 16086 | 2 | 0.014
F2a | 16093 | 14 | 0.101
F2a | 16111A | 11 | 0.08
F2a | 16126 | 8 | 0.058
F2a | 16129 | 4 | 0.029
F2a | 16140 | 1 | 0.007
F2a | 16193 | 1 | 0.007
F2a | 16218 | 1 | 0.007
F2a | 16223 | 1 | 0.007
F2a | 16231 | 6 | 0.043
F2a | 16234 | 1 | 0.007
F2a | 16239 | 1 | 0.007
F2a | 16241 | 1 | 0.007
F2a | 16254d | 1 | 0.007
F2a | 16256 | 3 | 0.022
F2a | 16258 | 1 | 0.007
F2a | 16259 | 6 | 0.043
F2a | 16262.1C | 1 | 0.007
F2a | 16274 | 1 | 0.007
F2a | 16278 | 1 | 0.007
F2a | 16295 | 1 | 0.007
F2a | 16311 | 24 | 0.174
F2a | 16335 | 16 | 0.116
F2a | 16352 | 3 | 0.022
F2a | 16356 | 1 | 0.007
F2a | 16362 | 1 | 0.007
F2a | 16390 | 2 | 0.014
F2a | 185 | 1 | 0.007
F2a | 186 | 2 | 0.014
F2a | 189 | 1 | 0.007
F2a | 195 | 7 | 0.051
F2a | 198 | 1 | 0.007
F2a | 200 | 3 | 0.022
F2a | 204 | 1 | 0.007
F2a | 2149.1AG | 1 | 0.007
F2a | 228 | 2 | 0.014
F2a | 246 | 1 | 0.007
F2a | 2581 | 1 | 0.007
F2a | 260 | 1 | 0.007
F2a | 2746 | 2 | 0.014
F2a | 2833d | 1 | 0.007
F2a | 296 | 1 | 0.007
F2a | 310 | 1 | 0.007
F2a | 315.3C | 1 | 0.007
F2a | 3290 | 1 | 0.007
F2a | 3342 | 1 | 0.007
F2a | 3547 | 1 | 0.007
F2a | 3828 | 1 | 0.007
F2a | 385 | 2 | 0.014
F2a | 3852 | 1 | 0.007
F2a | 3957T | 1 | 0.007
F2a | 4084 | 1 | 0.007
F2a | 4452 | 1 | 0.007
F2a | 4554 | 1 | 0.007
F2a | 455d | 1 | 0.007
F2a | 499 | 1 | 0.007
F2a | 5147 | 1 | 0.007
F2a | 57A | 1 | 0.007

F2a | 5894C | 1 | 0.007
F2a | 59 | 2 | 0.014
F2a | 60.1T | 2 | 0.014
F2a | 6216 | 1 | 0.007
F2a | 6566 | 2 | 0.014
F2a | 6710 | 1 | 0.007
F2a | 6722 | 3 | 0.022
F2a | 7298 | 1 | 0.007
F2a | 7419 | 1 | 0.007
F2a | 7646 | 1 | 0.007
F2a | 8292 | 1 | 0.007
F2a | 8448 | 1 | 0.007
F2a | 8473 | 1 | 0.007
F2a | 8479 | 1 | 0.007
F2a | 8538 | 1 | 0.007
F2a | 9128 | 4 | 0.029
F2a | 93 | 1 | 0.007
F2a | 9324 | 1 | 0.007
F2a | 9377 | 1 | 0.007
F2a | 9455 | 1 | 0.007
F2a | 9540 | 1 | 0.007
F2a | 9722 | 1 | 0.007
F2a+@16291 | 12696 | 1 | 0.018
F2a+@16291 | 14831 | 1 | 0.018
F2a+@16291 | 152 | 2 | 0.036
F2a+@16291 | 16093 | 1 | 0.018
F2a+@16291 | 16094 | 1 | 0.018
F2a+@16291 | 16129 | 5 | 0.091
F2a+@16291 | 16166 | 1 | 0.018
F2a+@16291 | 16189 | 1 | 0.018
F2a+@16291 | 16234 | 1 | 0.018
F2a+@16291 | 16272 | 1 | 0.018
F2a+@16291 | 16293 | 1 | 0.018
F2a+@16291 | 16446 | 1 | 0.018
F2a+@16291 | 214 | 1 | 0.018
F2a+@16291 | 5191 | 1 | 0.018
F2a+@16291 | 6566 | 1 | 0.018
F2a+@16291 | 7080 | 1 | 0.018
F2a+@16291 | 8448 | 1 | 0.018
F2a1 | 12366 | 2 | 0.017
F2a1 | 12609G | 1 | 0.009
F2a1 | 14100 | 10 | 0.085
F2a1 | 14560 | 13 | 0.111
F2a1 | 14587 | 1 | 0.009
F2a1 | 14884G | 1 | 0.009
F2a1 | 150 | 4 | 0.034
F2a1 | 15151 | 14 | 0.12
F2a1 | 15203 | 14 | 0.12
F2a1 | 15884 | 1 | 0.009
F2a1 | 16066 | 1 | 0.009
F2a1 | 16086 | 36 | 0.308
F2a1 | 16124 | 4 | 0.034
F2a1 | 16145 | 3 | 0.026
F2a1 | 16167 | 58 | 0.496
F2a1 | 16176 | 1 | 0.009
F2a1 | 16234 | 1 | 0.009
F2a1 | 16256 | 1 | 0.009
F2a1 | 16260 | 3 | 0.026
F2a1 | 16311 | 2 | 0.017
F2a1 | 16318 | 57 | 0.487
F2a1 | 16399 | 1 | 0.009
F2a1 | 16422 | 3 | 0.026
F2a1 | 16474.1G | 1 | 0.009
F2a1 | 182 | 12 | 0.103
F2a1 | 199 | 2 | 0.017
F2a1 | 2179 | 1 | 0.009
F2a1 | 3420 | 2 | 0.017
F2a1 | 408A | 1 | 0.009
F2a1 | 4353 | 1 | 0.009
F2a1 | 5153 | 1 | 0.009
F2a1 | 5752d | 13 | 0.111
F2a1 | 6320 | 1 | 0.009
F2a1 | 7076 | 2 | 0.017
F2a1 | 710 | 2 | 0.017
F2a1 | 7129 | 1 | 0.009
F2a1 | 7740 | 1 | 0.009
F2a1 | 8281-8289d | 10 | 0.085
F2a1 | 8552 | 2 | 0.017
F2a1 | 8583 | 2 | 0.017
F2a1 | 8856 | 3 | 0.026
F2a1 | 9089 | 2 | 0.017
F2a1 | 9297 | 1 | 0.009
F2b | 10801 | 1 | 0.022
F2b | 12609 | 1 | 0.022
F2b | 13500 | 1 | 0.022
F2b | 14016 | 1 | 0.022
F2b | 146 | 9 | 0.2
F2b | 16086 | 1 | 0.022
F2b | 16092 | 2 | 0.044
F2b | 16093 | 3 | 0.067
F2b | 16264 | 2 | 0.044
F2b | 16266 | 6 | 0.133
F2b | 16294 | 1 | 0.022
F2b | 1709 | 1 | 0.022
F2b | 3290 | 1 | 0.022
F2b | 5417 | 1 | 0.022
F2b | 5846 | 1 | 0.022
F2b | 709 | 1 | 0.022
F2b | 8636 | 1 | 0.022
F2b | 9137 | 1 | 0.022
F2b1 | 11918G | 2 | 0.011
F2b1 | 12715 | 1 | 0.005
F2b1 | 12810 | 1 | 0.005
F2b1 | 13050 | 2 | 0.011
F2b1 | 13191 | 2 | 0.011
F2b1 | 13641 | 1 | 0.005
F2b1 | 13879 | 2 | 0.011
F2b1 | 143 | 1 | 0.005
F2b1 | 146 | 2 | 0.011
F2b1 | 15052 | 1 | 0.005
F2b1 | 152 | 1 | 0.005

F2b1 | 15211 | 1 | 0.005
F2b1 | 15218 | 1 | 0.005
F2b1 | 153 | 1 | 0.005
F2b1 | 15316 | 1 | 0.005
F2b1 | 15900 | 1 | 0.005
F2b1 | 16086 | 5 | 0.027
F2b1 | 16093 | 4 | 0.022
F2b1 | 16095 | 1 | 0.005
F2b1 | 16108 | 1 | 0.005
F2b1 | 16124 | 2 | 0.011
F2b1 | 16136 | 3 | 0.016
F2b1 | 16137T | 1 | 0.005
F2b1 | 16154 | 1 | 0.005
F2b1 | 16166 | 1 | 0.005
F2b1 | 16167 | 1 | 0.005
F2b1 | 16168 | 1 | 0.005
F2b1 | 16170C | 1 | 0.005
F2b1 | 16170T | 11 | 0.059
F2b1 | 16172 | 3 | 0.016
F2b1 | 16183T | 7 | 0.038
F2b1 | 16184 | 1 | 0.005
F2b1 | 16186 | 1 | 0.005
F2b1 | 16189 | 34 | 0.183
F2b1 | 16192 | 2 | 0.011
F2b1 | 16193d | 1 | 0.005
F2b1 | 16243 | 2 | 0.011
F2b1 | 16245 | 3 | 0.016
F2b1 | 16249 | 1 | 0.005
F2b1 | 16274 | 2 | 0.011
F2b1 | 16278 | 1 | 0.005
F2b1 | 16289 | 5 | 0.027
F2b1 | 16294 | 2 | 0.011
F2b1 | 16297 | 2 | 0.011
F2b1 | 16298 | 1 | 0.005
F2b1 | 16299 | 7 | 0.038
F2b1 | 16311 | 19 | 0.102
F2b1 | 16318 | 1 | 0.005
F2b1 | 16359 | 5 | 0.027
F2b1 | 16362 | 1 | 0.005
F2b1 | 16400 | 1 | 0.005
F2b1 | 16526T | 1 | 0.005
F2b1 | 16T | 1 | 0.005
F2b1 | 185 | 1 | 0.005
F2b1 | 195 | 13 | 0.07
F2b1 | 198 | 1 | 0.005
F2b1 | 199 | 1 | 0.005
F2b1 | 207 | 2 | 0.011
F2b1 | 210 | 2 | 0.011
F2b1 | 236 | 1 | 0.005
F2b1 | 2707 | 1 | 0.005
F2b1 | 3010 | 2 | 0.011
F2b1 | 3169 | 2 | 0.011
F2b1 | 3199A | 1 | 0.005
F2b1 | 3278 | 1 | 0.005
F2b1 | 337 | 1 | 0.005
F2b1 | 3579 | 1 | 0.005
F2b1 | 4065 | 1 | 0.005
F2b1 | 4643 | 9 | 0.048
F2b1 | 5048 | 1 | 0.005
F2b1 | 5090 | 3 | 0.016
F2b1 | 512 | 1 | 0.005
F2b1 | 513C | 1 | 0.005
F2b1 | 515d | 1 | 0.005
F2b1 | 517 | 3 | 0.016
F2b1 | 533 | 1 | 0.005
F2b1 | 5442 | 1 | 0.005
F2b1 | 5800 | 2 | 0.011
F2b1 | 613 | 1 | 0.005
F2b1 | 6164 | 1 | 0.005
F2b1 | 6710 | 2 | 0.011
F2b1 | 6782 | 3 | 0.016
F2b1 | 6806C | 1 | 0.005
F2b1 | 6935 | 1 | 0.005
F2b1 | 736 | 1 | 0.005
F2b1 | 7961 | 2 | 0.011
F2b1 | 8010 | 1 | 0.005
F2b1 | 8697 | 11 | 0.059
F2b1 | 8865 | 2 | 0.011
F2b1 | 9 | 2 | 0.011
F2b1 | 9210 | 1 | 0.005
F2c | 14133 | 1 | 0.005
F2c | 146 | 3 | 0.016
F2c | 146G | 1 | 0.005
F2c | 147G | 1 | 0.005
F2c | 152 | 5 | 0.026
F2c | 15950 | 1 | 0.005
F2c | 16179 | 1 | 0.005
F2c | 16184 | 1 | 0.005
F2c | 16193A | 1 | 0.005
F2c | 16209 | 2 | 0.01
F2c | 16218 | 1 | 0.005
F2c | 16224 | 1 | 0.005
F2c | 16235 | 1 | 0.005
F2c | 16311 | 7 | 0.036
F2c | 16360 | 1 | 0.005
F2c | 16399 | 1 | 0.005
F2c | 16463T | 2 | 0.01
F2c | 200 | 1 | 0.005
F2c | 207 | 1 | 0.005
F2c | 2833 | 1 | 0.005
F2c | 318 | 1 | 0.005
F2c | 4117 | 1 | 0.005
F2c | 5147 | 1 | 0.005
F2c | 5460 | 1 | 0.005
F2c | 573.4C | 2 | 0.01
F2c | 574C | 1 | 0.005
F2c | 576C | 1 | 0.005
F2c | 606 | 1 | 0.005
F2c | 761 | 3 | 0.016
F2c | 9632 | 1 | 0.005
F2c | 9833 | 1 | 0.005

F2c1	1048	1	0.003
F2c1	15714	3	0.008
F2c1	15721	2	0.005
F2c1	15843	1	0.003
F2c1	15884	2	0.005
F2c1	16066	3	0.008
F2c1	16067A	2	0.005
F2c1	16079	6	0.016
F2c1	16086	5	0.013
F2c1	16146	1	0.003
F2c1	16147	5	0.013
F2c1	16166C	6	0.016
F2c1	16168	2	0.005
F2c1	16185	2	0.005
F2c1	16188G	1	0.003
F2c1	16213	3	0.008
F2c1	16214	2	0.005
F2c1	16221	1	0.003
F2c1	16223	1	0.003
F2c1	16232A	1	0.003
F2c1	16256	2	0.005
F2c1	16258	1	0.003
F2c1	16260	1	0.003
F2c1	16261	1	0.003
F2c1	16271	3	0.008
F2c1	16274	6	0.016
F2c1	16287	4	0.01
F2c1	16288	1	0.003
F2c1	16291A	1	0.003
F2c1	16292	1	0.003
F2c1	16293	1	0.003
F2c1	16295	1	0.003
F2c1	16301	1	0.003
F2c1	16318	2	0.005
F2c1	16355	5	0.013
F2c1	16356	2	0.005
F2c1	16362	1	0.003
F2c1	16371	1	0.003
F2c1	16381	1	0.003
F2c1	199	3	0.008
F2c1	208	1	0.003
F2c1	237	3	0.008
F2c1	309.3C	1	0.003
F2c1	464	1	0.003
F2c1	573.4C	1	0.003
F2c1	5824	2	0.005
F2c1	6221	4	0.01
F2c1	709	3	0.008
F2c1	71d	1	0.003
F2c1	735	2	0.005
F2c1	745.1T	1	0.003
F2c1	761	1	0.003
F2c1	8706	2	0.005
F2c1	874.1G	1	0.003
F2c1	93	1	0.003
F2c2	10978	1	0.027
F2c2	13470	1	0.027
F2c2	16192	1	0.027
F2c2	16311	1	0.027
F2c2	16547	1	0.027
F2c2	16552T	1	0.027
F2c2	16556T	1	0.027
F2c2	307G	1	0.027
F2c2	318	1	0.027
F2c2	4823	1	0.027
F2c2	5318	1	0.027
F2c2	589A	1	0.027
F2c2	595G	1	0.027
F2c2	596A	1	0.027
F2c2	597G	1	0.027
F2d	10527	8	0.136
F2d	10795	2	0.034
F2d	11527	1	0.017
F2d	12406	1	0.017
F2d	12821	2	0.034
F2d	13161	1	0.017
F2d	13290	1	0.017
F2d	14509	2	0.034
F2d	146	3	0.051
F2d	14755C	1	0.017
F2d	15040	1	0.017
F2d	152	10	0.169
F2d	15388	5	0.085
F2d	15529	8	0.136
F2d	16017	1	0.017
F2d	16051	1	0.017
F2d	16093	5	0.085
F2d	16158	4	0.068
F2d	16172	1	0.017
F2d	16189	3	0.051
F2d	16274	1	0.017
F2d	16286	1	0.017
F2d	16291	2	0.034
F2d	16295	4	0.068
F2d	16311	6	0.102
F2d	16319	5	0.085
F2d	16362	2	0.034
F2d	1717	2	0.034
F2d	195	3	0.051
F2d	200	1	0.017
F2d	204	1	0.017
F2d	214	1	0.017
F2d	228	2	0.034
F2d	259	1	0.017
F2d	2875	3	0.051
F2d	297	2	0.034
F2d	4538	3	0.051
F2d	5705	3	0.051
F2d	574	6	0.102
F2d	5790A	3	0.051
F2d	709	5	0.085

F2d | 7403 | 1 | 0.017
F2d | 7492 | 4 | 0.068
F2d | 9099 | 4 | 0.068
F2d | 912d | 1 | 0.017
F2e | 10265 | 7 | 0.034
F2e | 10325 | 4 | 0.02
F2e | 10640 | 1 | 0.005
F2e | 11696 | 3 | 0.015
F2e | 125G | 1 | 0.005
F2e | 14173 | 1 | 0.005
F2e | 143 | 1 | 0.005
F2e | 146G | 1 | 0.005
F2e | 147G | 1 | 0.005
F2e | 15010 | 2 | 0.01
F2e | 151 | 1 | 0.005
F2e | 16086 | 3 | 0.015
F2e | 16093 | 1 | 0.005
F2e | 16167 | 1 | 0.005
F2e | 16176 | 1 | 0.005
F2e | 16179 | 1 | 0.005
F2e | 16192 | 2 | 0.01
F2e | 16193A | 1 | 0.005
F2e | 16218 | 1 | 0.005
F2e | 16274 | 1 | 0.005
F2e | 16304G | 2 | 0.01
F2e | 16311 | 1 | 0.005
F2e | 16318 | 1 | 0.005
F2e | 16342 | 2 | 0.01
F2e | 16360 | 1 | 0.005
F2e | 16399 | 1 | 0.005
F2e | 16527 | 25 | 0.123
F2e | 189 | 1 | 0.005
F2e | 207 | 1 | 0.005
F2e | 318 | 1 | 0.005
F2e | 3645 | 1 | 0.005
F2e | 5460 | 4 | 0.02
F2e | 5461 | 1 | 0.005
F2e | 5493 | 2 | 0.01
F2e | 574C | 1 | 0.005
F2e | 576C | 1 | 0.005
F2e | 606 | 1 | 0.005
F2e | 6260 | 1 | 0.005
F2e | 709 | 6 | 0.029
F2e | 7109 | 1 | 0.005
F2e | 930 | 1 | 0.005
F2e1 | 16311 | 1 | 0.5
F2e1 | 6927 | 1 | 0.5
F2f | 10754 | 3 | 0.016
F2f | 14587 | 1 | 0.005
F2f | 14687 | 1 | 0.005
F2f | 146G | 1 | 0.005
F2f | 14798 | 1 | 0.005
F2f | 147G | 1 | 0.005
F2f | 15438C | 1 | 0.005
F2f | 16179 | 1 | 0.005
F2f | 16184 | 1 | 0.005
F2f | 16193A | 1 | 0.005
F2f | 16209 | 2 | 0.011
F2f | 16218 | 1 | 0.005
F2f | 16224 | 2 | 0.011
F2f | 16360 | 1 | 0.005
F2f | 16399 | 1 | 0.005
F2f | 185 | 6 | 0.032
F2f | 194 | 2 | 0.011
F2f | 207 | 1 | 0.005
F2f | 2619 | 3 | 0.016
F2f | 318 | 1 | 0.005
F2f | 5153 | 1 | 0.005
F2f | 574C | 1 | 0.005
F2f | 576C | 1 | 0.005
F2f | 606 | 1 | 0.005
F2f | 8185 | 1 | 0.005
F2f | 8461 | 2 | 0.011
F2g | 10031 | 2 | 0.023
F2g | 13191 | 2 | 0.023
F2g | 16051 | 1 | 0.012
F2g | 16062T | 1 | 0.012
F2g | 16075 | 1 | 0.012
F2g | 16092 | 2 | 0.023
F2g | 16093 | 4 | 0.047
F2g | 16145 | 3 | 0.035
F2g | 16185 | 38 | 0.442
F2g | 16189 | 4 | 0.047
F2g | 16231 | 1 | 0.012
F2g | 16254.1A | 4 | 0.047
F2g | 16258 | 4 | 0.047
F2g | 16266 | 1 | 0.012
F2g | 16266A | 27 | 0.314
F2g | 16266G | 10 | 0.116
F2g | 16317T | 1 | 0.012
F2g | 16318 | 1 | 0.012
F2g | 16390 | 4 | 0.047
F2g | 199 | 1 | 0.012
F2g | 2226 | 2 | 0.023
F2g | 316C | 1 | 0.012
F2g | 57 | 1 | 0.012
F2g | 59 | 1 | 0.012
F2g | 60.1T | 1 | 0.012
F2g | 6683 | 1 | 0.012
F2g | 6951 | 1 | 0.012
F2g | 7238 | 1 | 0.012
F2g | 8185 | 1 | 0.012
F2h | 15171 | 1 | 0.006
F2h | 16048 | 1 | 0.006
F2h | 16092 | 3 | 0.018
F2h | 16126 | 3 | 0.018
F2h | 16126G | 1 | 0.006
F2h | 16169 | 3 | 0.018
F2h | 16182 | 1 | 0.006
F2h | 16189 | 12 | 0.073
F2h | 16309 | 2 | 0.012

F2h | 16357 | 1 | 0.006
F2h | 16390 | 2 | 0.012
F2h | 16488 | 1 | 0.006
F2h | 279 | 1 | 0.006
F2h | 291d | 1 | 0.006
F2h | 316C | 4 | 0.024
F2h | 3460 | 1 | 0.006
F2h | 4580 | 2 | 0.012
F2h | 5744 | 1 | 0.006
F2h | 7976 | 1 | 0.006
F2i | 13710 | 1 | 0.031
F2i | 14230 | 1 | 0.031
F2i | 16051 | 1 | 0.031
F2i | 16093 | 1 | 0.031
F2i | 16140 | 1 | 0.031
F2i | 16167 | 1 | 0.031
F2i | 16187 | 1 | 0.031
F2i | 16189 | 1 | 0.031
F2i | 16217 | 2 | 0.062
F2i | 16291 | 1 | 0.031
F2i | 16311 | 1 | 0.031
F2i | 16352 | 2 | 0.062
F2i | 16365 | 1 | 0.031
F2i | 207 | 1 | 0.031
F2i | 272 | 1 | 0.031
F2i | 316C | 1 | 0.031
F2i | 471 | 2 | 0.062
F2i | 5821 | 1 | 0.031
F2i | 6345 | 1 | 0.031
F2i | 6465 | 1 | 0.031
F2i | 7076 | 1 | 0.031
F2i | 9452 | 1 | 0.031
F3 | 291d | 1 | 0.03
F3a | 12237 | 2 | 0.033
F3a | 12477 | 1 | 0.016
F3a | 13105 | 1 | 0.016
F3a | 13681 | 1 | 0.016
F3a | 14356 | 1 | 0.016
F3a | 15837.1G | 1 | 0.016
F3a | 15924 | 2 | 0.033
F3a | 16093 | 9 | 0.148
F3a | 16111 | 4 | 0.066
F3a | 16117 | 4 | 0.066
F3a | 16129 | 1 | 0.016
F3a | 16145 | 1 | 0.016
F3a | 16189 | 6 | 0.098
F3a | 16192 | 4 | 0.066
F3a | 16209 | 6 | 0.098
F3a | 16221 | 5 | 0.082
F3a | 16235 | 1 | 0.016
F3a | 16249 | 5 | 0.082
F3a | 16260 | 8 | 0.131
F3a | 16263 | 4 | 0.066
F3a | 16311 | 3 | 0.049
F3a | 16390 | 4 | 0.066
F3a | 195 | 7 | 0.115
F3a | 251 | 2 | 0.033
F3a | 267d | 1 | 0.016
F3a | 3390 | 4 | 0.066
F3a | 3510 | 1 | 0.016
F3a | 37d | 1 | 0.016
F3a | 39-44d | 1 | 0.016
F3a | 4047 | 1 | 0.016
F3a | 4136 | 2 | 0.033
F3a | 432C | 1 | 0.016
F3a | 455d | 1 | 0.016
F3a | 47.1G | 1 | 0.016
F3a | 475C | 1 | 0.016
F3a | 479d | 1 | 0.016
F3a | 485A | 1 | 0.016
F3a | 486A | 1 | 0.016
F3a | 487.1T | 1 | 0.016
F3a | 489 | 4 | 0.066
F3a | 489G | 1 | 0.016
F3a | 490C | 1 | 0.016
F3a | 491G | 2 | 0.033
F3a | 492 | 1 | 0.016
F3a | 492T | 1 | 0.016
F3a | 512d | 1 | 0.016
F3a | 516d | 1 | 0.016
F3a | 5894 | 2 | 0.033
F3a | 709 | 1 | 0.016
F3a | 7094 | 4 | 0.066
F3a | 7258 | 2 | 0.033
F3a | 7741 | 1 | 0.016
F3a | 88.1G | 1 | 0.016
F3a | 9301 | 2 | 0.033
F3a | 9438 | 1 | 0.016
F3a | 9813 | 2 | 0.033
F3a+207 | 105-110d | 1 | 0.02
F3a+207 | 114 | 2 | 0.039
F3a+207 | 12237 | 1 | 0.02
F3a+207 | 12810 | 1 | 0.02
F3a+207 | 150 | 1 | 0.02
F3a+207 | 152 | 2 | 0.039
F3a+207 | 15586 | 2 | 0.039
F3a+207 | 15854G | 4 | 0.078
F3a+207 | 16069 | 1 | 0.02
F3a+207 | 16093 | 38 | 0.745
F3a+207 | 16111 | 23 | 0.451
F3a+207 | 16126 | 1 | 0.02
F3a+207 | 16136 | 2 | 0.039
F3a+207 | 16187 | 5 | 0.098
F3a+207 | 16189 | 7 | 0.137
F3a+207 | 16192 | 24 | 0.471
F3a+207 | 16209 | 7 | 0.137
F3a+207 | 16221 | 7 | 0.137
F3a+207 | 16249 | 29 | 0.569
F3a+207 | 16263 | 14 | 0.275
F3a+207 | 16264.1C | 2 | 0.039
F3a+207 | 16311 | 2 | 0.039

F3a+207 | 16390 | 31 | 0.608
F3a+207 | 1719 | 2 | 0.039
F3a+207 | 195 | 1 | 0.02
F3a+207 | 204 | 2 | 0.039
F3a+207 | 295 | 1 | 0.02
F3a+207 | 3390 | 1 | 0.02
F3a+207 | 4907 | 2 | 0.039
F3a+207 | 622 | 1 | 0.02
F3a+207 | 6480 | 1 | 0.02
F3a+207 | 709 | 7 | 0.137
F3a+207 | 7094 | 1 | 0.02
F3a+207 | 9182 | 2 | 0.039
F3a+207 | 961 | 2 | 0.039
F3a+207 | 97 | 1 | 0.02
F3a+207 | 9962 | 1 | 0.02
F3a1 | 10499 | 9 | 0.043
F3a1 | 10646C | 4 | 0.019
F3a1 | 11053 | 1 | 0.005
F3a1 | 11485 | 4 | 0.019
F3a1 | 11617 | 4 | 0.019
F3a1 | 11620 | 1 | 0.005
F3a1 | 11989 | 1 | 0.005
F3a1 | 12189 | 1 | 0.005
F3a1 | 126 | 1 | 0.005
F3a1 | 12696 | 7 | 0.033
F3a1 | 13095 | 1 | 0.005
F3a1 | 1310 | 2 | 0.01
F3a1 | 13145 | 4 | 0.019
F3a1 | 13656 | 1 | 0.005
F3a1 | 14178 | 5 | 0.024
F3a1 | 14263 | 2 | 0.01
F3a1 | 14530 | 1 | 0.005
F3a1 | 14560 | 1 | 0.005
F3a1 | 146 | 3 | 0.014
F3a1 | 14783 | 4 | 0.019
F3a1 | 14956 | 1 | 0.005
F3a1 | 15160C | 1 | 0.005
F3a1 | 152 | 5 | 0.024
F3a1 | 15412G | 29 | 0.139
F3a1 | 15483 | 6 | 0.029
F3a1 | 15493 | 5 | 0.024
F3a1 | 15808 | 3 | 0.014
F3a1 | 15943 | 9 | 0.043
F3a1 | 16037 | 1 | 0.005
F3a1 | 16042T | 1 | 0.005
F3a1 | 16069 | 1 | 0.005
F3a1 | 16075A | 1 | 0.005
F3a1 | 16076 | 1 | 0.005
F3a1 | 16076d | 2 | 0.01
F3a1 | 16092 | 2 | 0.01
F3a1 | 16093 | 40 | 0.191
F3a1 | 16103 | 1 | 0.005
F3a1 | 16111 | 1 | 0.005
F3a1 | 16114A | 2 | 0.01
F3a1 | 16140 | 1 | 0.005
F3a1 | 16174 | 1 | 0.005
F3a1 | 16180 | 1 | 0.005
F3a1 | 16184 | 1 | 0.005
F3a1 | 16189 | 2 | 0.01
F3a1 | 16190 | 1 | 0.005
F3a1 | 16192 | 1 | 0.005
F3a1 | 16194T | 1 | 0.005
F3a1 | 16234 | 2 | 0.01
F3a1 | 16239 | 6 | 0.029
F3a1 | 16243 | 1 | 0.005
F3a1 | 16249 | 6 | 0.029
F3a1 | 16261 | 1 | 0.005
F3a1 | 16266 | 1 | 0.005
F3a1 | 16274 | 5 | 0.024
F3a1 | 16278 | 5 | 0.024
F3a1 | 16288G | 3 | 0.014
F3a1 | 16309 | 2 | 0.01
F3a1 | 16311 | 13 | 0.062
F3a1 | 16327 | 1 | 0.005
F3a1 | 16343T | 1 | 0.005
F3a1 | 16356 | 2 | 0.01
F3a1 | 16390 | 6 | 0.029
F3a1 | 16402C | 1 | 0.005
F3a1 | 16405T | 1 | 0.005
F3a1 | 16526 | 9 | 0.043
F3a1 | 182 | 1 | 0.005
F3a1 | 195 | 1 | 0.005
F3a1 | 204 | 25 | 0.12
F3a1 | 2045 | 1 | 0.005
F3a1 | 2120 | 1 | 0.005
F3a1 | 215 | 3 | 0.014
F3a1 | 246 | 1 | 0.005
F3a1 | 25d | 1 | 0.005
F3a1 | 310 | 1 | 0.005
F3a1 | 315.2C | 1 | 0.005
F3a1 | 385 | 1 | 0.005
F3a1 | 4053 | 1 | 0.005
F3a1 | 41 | 1 | 0.005
F3a1 | 4652 | 1 | 0.005
F3a1 | 4695 | 1 | 0.005
F3a1 | 4824 | 29 | 0.139
F3a1 | 4991 | 29 | 0.139
F3a1 | 5096 | 2 | 0.01
F3a1 | 515d | 2 | 0.01
F3a1 | 516d | 1 | 0.005
F3a1 | 5237 | 1 | 0.005
F3a1 | 5302 | 1 | 0.005
F3a1 | 5423 | 3 | 0.014
F3a1 | 5474 | 1 | 0.005
F3a1 | 573.1C | 1 | 0.005
F3a1 | 5823 | 1 | 0.005
F3a1 | 6365 | 1 | 0.005
F3a1 | 6736 | 1 | 0.005
F3a1 | 6962 | 2 | 0.01
F3a1 | 7151 | 6 | 0.029
F3a1 | 7444 | 1 | 0.005

F3a1 | 8614 | 1 | 0.005
F3a1 | 8638 | 1 | 0.005
F3a1 | 8689 | 2 | 0.01
F3a1 | 9152 | 1 | 0.005
F3a1 | 9550C | 1 | 0.005
F3a1 | 9851A | 3 | 0.014
F3a1 | 9854 | 19 | 0.091
F3b | 10849 | 1 | 0.023
F3b | 10873 | 1 | 0.023
F3b | 10922 | 1 | 0.023
F3b | 11914 | 2 | 0.047
F3b | 1193 | 1 | 0.023
F3b | 12354 | 1 | 0.023
F3b | 12405 | 1 | 0.023
F3b | 12705 | 1 | 0.023
F3b | 12950 | 1 | 0.023
F3b | 13722 | 2 | 0.047
F3b | 14305 | 1 | 0.023
F3b | 14464 | 1 | 0.023
F3b | 14560 | 1 | 0.023
F3b | 14861 | 1 | 0.023
F3b | 150 | 1 | 0.023
F3b | 15043 | 1 | 0.023
F3b | 15301 | 1 | 0.023
F3b | 15440 | 3 | 0.07
F3b | 16075 | 2 | 0.047
F3b | 16093 | 10 | 0.233
F3b | 16129 | 1 | 0.023
F3b | 16189 | 5 | 0.116
F3b | 16223 | 1 | 0.023
F3b | 16254 | 8 | 0.186
F3b | 16311 | 1 | 0.023
F3b | 195 | 2 | 0.047
F3b | 3150 | 3 | 0.07
F3b | 3210 | 3 | 0.07
F3b | 5261 | 3 | 0.07
F3b | 5262 | 1 | 0.023
F3b | 5460 | 1 | 0.023
F3b | 5999 | 1 | 0.023
F3b | 64 | 1 | 0.023
F3b | 6455 | 1 | 0.023
F3b | 6719 | 3 | 0.07
F3b | 7271 | 1 | 0.023
F3b | 7861 | 3 | 0.07
F3b | 8701 | 2 | 0.047
F3b | 9438 | 1 | 0.023
F3b | 9452 | 1 | 0.023
F3b | 9540 | 1 | 0.023
F3b | 9824 | 1 | 0.023
F3b+152 | 10398 | 1 | 0.036
F3b+152 | 11914 | 1 | 0.036
F3b+152 | 12014 | 1 | 0.036
F3b+152 | 13044 | 5 | 0.179
F3b+152 | 151 | 1 | 0.036
F3b+152 | 15910 | 6 | 0.214
F3b+152 | 16126 | 2 | 0.071
F3b+152 | 16227 | 25 | 0.893
F3b+152 | 1811 | 2 | 0.071
F3b+152 | 2392 | 5 | 0.179
F3b+152 | 310 | 1 | 0.036
F3b+152 | 315.2C | 1 | 0.036
F3b+152 | 3411 | 2 | 0.071
F3b+152 | 3504 | 1 | 0.036
F3b+152 | 3535 | 5 | 0.179
F3b+152 | 3918 | 2 | 0.071
F3b+152 | 4802 | 5 | 0.179
F3b+152 | 8281-8289d | 2 | 0.071
F3b+152 | 8292 | 2 | 0.071
F3b+152 | 9456 | 1 | 0.036
F3b1 | 10894 | 1 | 0.037
F3b1 | 15154 | 1 | 0.037
F3b1 | 16093 | 4 | 0.148
F3b1 | 16213 | 3 | 0.111
F3b1 | 16224 | 1 | 0.037
F3b1 | 16335 | 2 | 0.074
F3b1 | 16355 | 1 | 0.037
F3b1a | 11209 | 1 | 0.2
F3b1a | 11914 | 1 | 0.2
F3b1a | 16051 | 1 | 0.2
F3b1a | 16189 | 1 | 0.2
F3b1a | 2157 | 1 | 0.2
F3b1a | 913 | 1 | 0.2
F3b1a+16093 | 10382 | 3 | 0.07
F3b1a+16093 | 11020 | 1 | 0.023
F3b1a+16093 | 14488 | 1 | 0.023
F3b1a+16093 | 15229 | 1 | 0.023
F3b1a+16093 | 15300 | 4 | 0.093
F3b1a+16093 | 16095 | 2 | 0.047
F3b1a+16093 | 16136 | 1 | 0.023
F3b1a+16093 | 16166 | 4 | 0.093
F3b1a+16093 | 16189 | 1 | 0.023
F3b1a+16093 | 16192 | 2 | 0.047
F3b1a+16093 | 251 | 1 | 0.023
F3b1a+16093 | 4733 | 4 | 0.093
F3b1a+16093 | 4907 | 4 | 0.093
F3b1a+16093 | 71d | 1 | 0.023
F3b1a+16093 | 7354 | 4 | 0.093
F3b1a+16093 | 8285 | 2 | 0.047
F3b1a+16093 | 8757 | 1 | 0.023
F3b1a1 | 10143 | 1 | 0.25
F3b1a1 | 10335 | 1 | 0.25
F3b1a1 | 151 | 1 | 0.25
F3b1a1 | 15927 | 1 | 0.25
F3b1a1 | 16129T | 1 | 0.25
F3b1a1 | 16344 | 1 | 0.25
F3b1a1 | 310 | 1 | 0.25
F3b1a1 | 5773 | 1 | 0.25
F3b1a1 | 5824 | 1 | 0.25
F3b1a1 | 6278 | 1 | 0.25
F3b1a1 | 8281-8289d | 1 | 0.25
F3b1a2 | 11299 | 1 | 0.017

F3b1a2 | 14212 | 1 | 0.017
F3b1a2 | 15454 | 5 | 0.083
F3b1a2 | 16271 | 1 | 0.017
F3b1a2 | 5774 | 2 | 0.033
F3b1a2 | 8591 | 1 | 0.017
F3b1a2 | 9469 | 1 | 0.017
F3b1a2 | 980 | 2 | 0.033
F3b1b | 10172 | 2 | 0.012
F3b1b | 10511A | 1 | 0.006
F3b1b | 11809 | 1 | 0.006
F3b1b | 11887 | 1 | 0.006
F3b1b | 12105 | 1 | 0.006
F3b1b | 12113 | 1 | 0.006
F3b1b | 12148 | 1 | 0.006
F3b1b | 13225 | 1 | 0.006
F3b1b | 13254 | 1 | 0.006
F3b1b | 13392 | 4 | 0.025
F3b1b | 13455 | 3 | 0.019
F3b1b | 13677 | 1 | 0.006
F3b1b | 14129 | 1 | 0.006
F3b1b | 14203 | 1 | 0.006
F3b1b | 14410 | 1 | 0.006
F3b1b | 14693 | 1 | 0.006
F3b1b | 152d | 27 | 0.168
F3b1b | 15670 | 1 | 0.006
F3b1b | 15848 | 4 | 0.025
F3b1b | 15859 | 2 | 0.012
F3b1b | 16093 | 2 | 0.012
F3b1b | 16172 | 1 | 0.006
F3b1b | 16213 | 1 | 0.006
F3b1b | 16240 | 1 | 0.006
F3b1b | 16256 | 2 | 0.012
F3b1b | 16258C | 1 | 0.006
F3b1b | 16265C | 2 | 0.012
F3b1b | 16309 | 3 | 0.019
F3b1b | 16335 | 1 | 0.006
F3b1b | 16422 | 1 | 0.006
F3b1b | 16456 | 1 | 0.006
F3b1b | 200 | 49 | 0.304
F3b1b | 204 | 1 | 0.006
F3b1b | 234 | 1 | 0.006
F3b1b | 3421 | 1 | 0.006
F3b1b | 3441 | 1 | 0.006
F3b1b | 3930 | 7 | 0.043
F3b1b | 4386 | 1 | 0.006
F3b1b | 4991 | 1 | 0.006
F3b1b | 5063 | 2 | 0.012
F3b1b | 513 | 1 | 0.006
F3b1b | 5655 | 1 | 0.006
F3b1b | 573.1C | 2 | 0.012
F3b1b | 60 | 4 | 0.025
F3b1b | 6374 | 1 | 0.006
F3b1b | 6482 | 1 | 0.006
F3b1b | 7157 | 1 | 0.006
F3b1b | 7496 | 1 | 0.006
F3b1b | 8155 | 2 | 0.012
F3b1b | 8557 | 2 | 0.012
F3b1b | 8654 | 1 | 0.006
F3b1b | 8705 | 1 | 0.006
F3b1b | 8854 | 4 | 0.025
F3b1b | 9210 | 2 | 0.012
F3b1b | 9300 | 1 | 0.006
F3b1b | 9716 | 132 | 0.82
F3b1b1 | 1189 | 1 | 0.037
F3b1b1 | 15889 | 1 | 0.037
F3b1b1 | 15905 | 1 | 0.037
F3b1b1 | 16168 | 2 | 0.074
F3b1b1 | 16184 | 1 | 0.037
F3b1b1 | 2746 | 1 | 0.037
F3b1b1 | 310 | 1 | 0.037
F4 | 125G | 1 | 0.005
F4 | 146G | 1 | 0.005
F4 | 147G | 1 | 0.005
F4 | 16086 | 1 | 0.005
F4 | 16134 | 1 | 0.005
F4 | 16179 | 1 | 0.005
F4 | 16184 | 2 | 0.01
F4 | 16192 | 1 | 0.005
F4 | 16193A | 1 | 0.005
F4 | 16209 | 2 | 0.01
F4 | 16218 | 1 | 0.005
F4 | 16222 | 1 | 0.005
F4 | 16224 | 1 | 0.005
F4 | 16274 | 1 | 0.005
F4 | 16294 | 1 | 0.005
F4 | 16299 | 4 | 0.021
F4 | 16327 | 1 | 0.005
F4 | 16335 | 4 | 0.021
F4 | 16360 | 1 | 0.005
F4 | 16399 | 1 | 0.005
F4 | 189 | 1 | 0.005
F4 | 199 | 1 | 0.005
F4 | 200 | 1 | 0.005
F4 | 207 | 1 | 0.005
F4 | 208 | 1 | 0.005
F4 | 309.3C | 1 | 0.005
F4 | 318 | 1 | 0.005
F4 | 509 | 1 | 0.005
F4 | 548 | 2 | 0.01
F4 | 574C | 1 | 0.005
F4 | 576C | 1 | 0.005
F4 | 606 | 1 | 0.005
F4 | 709 | 1 | 0.005
F4 | 929 | 1 | 0.005
F4 | 930 | 1 | 0.005
F4a | 10208 | 1 | 0.083
F4a | 11116 | 2 | 0.167
F4a | 11227A | 1 | 0.083
F4a | 13923 | 1 | 0.083
F4a | 16032d | 1 | 0.083
F4a | 16042d | 1 | 0.083

F4a | 16110d | 1 | 0.083
F4a | 16179 | 3 | 0.25
F4a | 16187 | 1 | 0.083
F4a | 16216 | 1 | 0.083
F4a | 16256 | 1 | 0.083
F4a | 16291 | 1 | 0.083
F4a | 16357 | 1 | 0.083
F4a | 207 | 1 | 0.083
F4a | 291d | 1 | 0.083
F4a | 317A | 2 | 0.167
F4a | 3705 | 1 | 0.083
F4a | 644 | 1 | 0.083
F4a | 8023 | 1 | 0.083
F4a | 9725 | 1 | 0.083
F4a1 | 151 | 1 | 0.077
F4a1 | 16076d | 1 | 0.077
F4a1 | 16360 | 1 | 0.077
F4a1 | 16497 | 3 | 0.231
F4a1 | 182 | 1 | 0.077
F4a1 | 207 | 3 | 0.231
F4a1 | 281 | 2 | 0.154
F4a1a | 14693 | 1 | 0.043
F4a1a | 14831 | 1 | 0.043
F4a1a | 1520 | 5 | 0.217
F4a1a | 16093 | 2 | 0.087
F4a1a | 16312 | 1 | 0.043
F4a1a | 16360 | 4 | 0.174
F4a1a | 16494G | 1 | 0.043
F4a1a | 338 | 1 | 0.043
F4a1a | 3699G | 1 | 0.043
F4a1a | 3858 | 1 | 0.043
F4a1a | 5563 | 1 | 0.043
F4a1a | 5664 | 1 | 0.043
F4a1a | 709 | 5 | 0.217
F4a1b | 12134 | 1 | 0.042
F4a1b | 12882 | 1 | 0.042
F4a1b | 1391 | 1 | 0.042
F4a1b | 13926 | 1 | 0.042
F4a1b | 14020 | 1 | 0.042
F4a1b | 150 | 1 | 0.042
F4a1b | 15110 | 1 | 0.042
F4a1b | 15803 | 1 | 0.042
F4a1b | 16082 | 1 | 0.042
F4a1b | 16083 | 1 | 0.042
F4a1b | 16086 | 3 | 0.125
F4a1b | 16093 | 1 | 0.042
F4a1b | 16140 | 10 | 0.417
F4a1b | 16145 | 1 | 0.042
F4a1b | 16181 | 1 | 0.042
F4a1b | 16187 | 1 | 0.042
F4a1b | 16189 | 3 | 0.125
F4a1b | 16274 | 1 | 0.042
F4a1b | 16343 | 1 | 0.042
F4a1b | 16456 | 3 | 0.125
F4a1b | 195 | 1 | 0.042
F4a1b | 200 | 1 | 0.042
F4a1b | 215 | 1 | 0.042
F4a1b | 251 | 1 | 0.042
F4a1b | 2763 | 1 | 0.042
F4a1b | 316C | 1 | 0.042
F4a1b | 4512 | 1 | 0.042
F4a1b | 4820 | 1 | 0.042
F4a1b | 544 | 1 | 0.042
F4a1b | 9804 | 1 | 0.042
F4a2 | 10909 | 5 | 0.147
F4a2 | 11512 | 1 | 0.029
F4a2 | 11825 | 1 | 0.029
F4a2 | 11914 | 1 | 0.029
F4a2 | 12007 | 1 | 0.029
F4a2 | 12406 | 1 | 0.029
F4a2 | 13359 | 2 | 0.059
F4a2 | 13368 | 5 | 0.147
F4a2 | 13533 | 1 | 0.029
F4a2 | 13759 | 1 | 0.029
F4a2 | 14328 | 1 | 0.029
F4a2 | 150 | 2 | 0.059
F4a2 | 15314 | 2 | 0.059
F4a2 | 15440 | 1 | 0.029
F4a2 | 1555 | 1 | 0.029
F4a2 | 16086 | 1 | 0.029
F4a2 | 16129 | 10 | 0.294
F4a2 | 16179 | 1 | 0.029
F4a2 | 16185 | 9 | 0.265
F4a2 | 16187 | 3 | 0.088
F4a2 | 16189 | 1 | 0.029
F4a2 | 16192 | 1 | 0.029
F4a2 | 16223 | 1 | 0.029
F4a2 | 16243 | 4 | 0.118
F4a2 | 185C | 2 | 0.059
F4a2 | 204 | 2 | 0.059
F4a2 | 207 | 2 | 0.059
F4a2 | 2445 | 2 | 0.059
F4a2 | 3010 | 2 | 0.059
F4a2 | 310 | 1 | 0.029
F4a2 | 374 | 1 | 0.029
F4a2 | 4194 | 2 | 0.059
F4a2 | 4703 | 8 | 0.235
F4a2 | 5069 | 5 | 0.147
F4a2 | 5231 | 2 | 0.059
F4a2 | 5354 | 1 | 0.029
F4a2 | 538T | 2 | 0.059
F4a2 | 5585 | 1 | 0.029
F4a2 | 573.1C | 2 | 0.059
F4a2 | 573.5C | 2 | 0.059
F4a2 | 574C | 1 | 0.029
F4a2 | 576C | 1 | 0.029
F4a2 | 577C | 1 | 0.029
F4a2 | 578 | 1 | 0.029
F4a2 | 6827 | 1 | 0.029
F4a2 | 709 | 1 | 0.029
F4a2 | 869 | 1 | 0.029

F4a2 | 8839 | 1 | 0.029
F4a2 | 9950 | 2 | 0.059
F4b | 10084 | 3 | 0.038
F4b | 12091 | 1 | 0.012
F4b | 12354 | 1 | 0.012
F4b | 12879 | 2 | 0.025
F4b | 14552 | 3 | 0.038
F4b | 152 | 2 | 0.025
F4b | 15944d | 1 | 0.012
F4b | 16093 | 1 | 0.012
F4b | 16129 | 7 | 0.088
F4b | 16161A | 1 | 0.012
F4b | 16172 | 7 | 0.088
F4b | 16213 | 2 | 0.025
F4b | 16230 | 1 | 0.012
F4b | 16241 | 1 | 0.012
F4b | 16241T | 2 | 0.025
F4b | 16255 | 1 | 0.012
F4b | 16264 | 1 | 0.012
F4b | 16265 | 4 | 0.05
F4b | 16287 | 1 | 0.012
F4b | 16355 | 2 | 0.025
F4b | 16497 | 4 | 0.05
F4b | 185 | 3 | 0.038
F4b | 200 | 1 | 0.012
F4b | 2158 | 3 | 0.038
F4b | 2308 | 1 | 0.012
F4b | 2833 | 1 | 0.012
F4b | 310 | 1 | 0.012
F4b | 3640 | 3 | 0.038
F4b | 4991 | 1 | 0.012
F4b | 5006 | 3 | 0.038
F4b | 5843 | 1 | 0.012
F4b | 5930 | 1 | 0.012
F4b | 7445T | 3 | 0.038
F4b | 7741 | 2 | 0.025
F4b | 8986 | 3 | 0.038
F4b | 8989 | 1 | 0.012
F4b | 9266 | 2 | 0.025
F4b | 9327 | 1 | 0.012
F4b | 9764 | 2 | 0.025
F4b | 9827 | 3 | 0.038
F4b1 | 10688 | 1 | 0.007
F4b1 | 11027 | 1 | 0.007
F4b1 | 12477 | 1 | 0.007
F4b1 | 12618 | 2 | 0.014
F4b1 | 13145 | 1 | 0.007
F4b1 | 13812 | 4 | 0.028
F4b1 | 14215 | 4 | 0.028
F4b1 | 14233 | 2 | 0.014
F4b1 | 14551 | 1 | 0.007
F4b1 | 146 | 1 | 0.007
F4b1 | 14927 | 2 | 0.014
F4b1 | 15148 | 1 | 0.007
F4b1 | 152 | 23 | 0.163
F4b1 | 15924 | 4 | 0.028
F4b1 | 15930 | 3 | 0.021
F4b1 | 16129 | 4 | 0.028
F4b1 | 16172 | 1 | 0.007
F4b1 | 16207 | 1 | 0.007
F4b1 | 16213 | 1 | 0.007
F4b1 | 16241 | 23 | 0.163
F4b1 | 16255 | 18 | 0.128
F4b1 | 16287 | 1 | 0.007
F4b1 | 16319 | 1 | 0.007
F4b1 | 16343C | 2 | 0.014
F4b1 | 185 | 1 | 0.007
F4b1 | 198 | 1 | 0.007
F4b1 | 200 | 1 | 0.007
F4b1 | 2010 | 1 | 0.007
F4b1 | 310 | 1 | 0.007
F4b1 | 368 | 1 | 0.007
F4b1 | 41 | 1 | 0.007
F4b1 | 4580 | 1 | 0.007
F4b1 | 489 | 1 | 0.007
F4b1 | 513 | 27 | 0.191
F4b1 | 5460 | 1 | 0.007
F4b1 | 567C | 1 | 0.007
F4b1 | 574C | 4 | 0.028
F4b1 | 576C | 4 | 0.028
F4b1 | 577C | 3 | 0.021
F4b1 | 578 | 1 | 0.007
F4b1 | 6261 | 1 | 0.007
F4b1 | 7129 | 1 | 0.007
F4b1 | 7270 | 1 | 0.007
F4b1 | 7298C | 1 | 0.007
F4b1 | 7681 | 8 | 0.057
F4b1 | 8548 | 5 | 0.035
F4b1 | 8989 | 18 | 0.128
F4b1 | 9286 | 1 | 0.007
F4b1 | 9350 | 1 | 0.007
F4b1 | 9899 | 1 | 0.007
G | 10286 | 6 | 0.009
G | 11410 | 6 | 0.009
G | 115A | 1 | 0.002
G | 12397 | 6 | 0.009
G | 14530 | 6 | 0.009
G | 14905 | 6 | 0.009
G | 153 | 7 | 0.011
G | 16032.1A | 1 | 0.002
G | 16044.1C | 1 | 0.002
G | 16045 | 1 | 0.002
G | 16047T | 1 | 0.002
G | 16048T | 2 | 0.003
G | 16051 | 1 | 0.002
G | 16086 | 6 | 0.009
G | 16092 | 7 | 0.011
G | 16095 | 1 | 0.002
G | 16108 | 1 | 0.002
G | 16111A | 1 | 0.002
G | 16126 | 1 | 0.002

G | 16136 | 1 | 0.002
G | 16179 | 2 | 0.003
G | 16234 | 1 | 0.002
G | 16241 | 1 | 0.002
G | 16242 | 1 | 0.002
G | 16249 | 1 | 0.002
G | 16250A | 1 | 0.002
G | 16293 | 2 | 0.003
G | 16293T | 1 | 0.002
G | 16318C | 1 | 0.002
G | 16326C | 1 | 0.002
G | 16355 | 1 | 0.002
G | 16357 | 1 | 0.002
G | 16396A | 1 | 0.002
G | 16434 | 1 | 0.002
G | 16443 | 1 | 0.002
G | 16447 | 1 | 0.002
G | 16452 | 4 | 0.006
G | 16507 | 1 | 0.002
G | 16522 | 1 | 0.002
G | 16525 | 1 | 0.002
G | 171 | 1 | 0.002
G | 183 | 6 | 0.009
G | 1959 | 6 | 0.009
G | 217 | 4 | 0.006
G | 249 | 1 | 0.002
G | 274T | 1 | 0.002
G | 2772 | 6 | 0.009
G | 290.1T | 1 | 0.002
G | 297 | 1 | 0.002
G | 298 | 1 | 0.002
G | 309.3C | 1 | 0.002
G | 323.1G | 1 | 0.002
G | 324 | 1 | 0.002
G | 325d | 1 | 0.002
G | 3342 | 6 | 0.009
G | 337d | 1 | 0.002
G | 346 | 1 | 0.002
G | 384A | 1 | 0.002
G | 384T | 1 | 0.002
G | 401.1T | 1 | 0.002
G | 403.1T | 1 | 0.002
G | 408.1T | 1 | 0.002
G | 439 | 1 | 0.002
G | 446 | 2 | 0.003
G | 446T | 1 | 0.002
G | 47 | 1 | 0.002
G | 472 | 1 | 0.002
G | 48 | 1 | 0.002
G | 488 | 1 | 0.002
G | 517 | 1 | 0.002
G | 5222 | 6 | 0.009
G | 529 | 1 | 0.002
G | 530 | 1 | 0.002
G | 553 | 1 | 0.002
G | 574 | 1 | 0.002
G | 574d | 3 | 0.005
G | 72 | 1 | 0.002
G | 75 | 1 | 0.002
G | 82 | 1 | 0.002
G | 8289.1CCCCCTCTA | 5 | 0.008
G | 97C | 1 | 0.002
G1 | 11152 | 3 | 0.005
G1 | 11253 | 1 | 0.002
G1 | 115A | 1 | 0.002
G1 | 16032.1A | 1 | 0.002
G1 | 16044.1C | 1 | 0.002
G1 | 16045 | 1 | 0.002
G1 | 16047T | 1 | 0.002
G1 | 16048T | 2 | 0.003
G1 | 16051 | 1 | 0.002
G1 | 16093 | 3 | 0.005
G1 | 16095 | 1 | 0.002
G1 | 16108 | 1 | 0.002
G1 | 16111A | 1 | 0.002
G1 | 16136 | 1 | 0.002
G1 | 16179 | 2 | 0.003
G1 | 16234 | 1 | 0.002
G1 | 16241 | 1 | 0.002
G1 | 16242 | 1 | 0.002
G1 | 16250A | 1 | 0.002
G1 | 16293 | 2 | 0.003
G1 | 16293T | 1 | 0.002
G1 | 16318C | 1 | 0.002
G1 | 16326C | 1 | 0.002
G1 | 16355 | 1 | 0.002
G1 | 16357 | 1 | 0.002
G1 | 16396A | 1 | 0.002
G1 | 16434 | 1 | 0.002
G1 | 16443 | 1 | 0.002
G1 | 16447 | 1 | 0.002
G1 | 16452 | 4 | 0.006
G1 | 16507 | 1 | 0.002
G1 | 16522 | 1 | 0.002
G1 | 16525 | 1 | 0.002
G1 | 171 | 1 | 0.002
G1 | 217 | 4 | 0.006
G1 | 249 | 1 | 0.002
G1 | 274T | 1 | 0.002
G1 | 290.1T | 1 | 0.002
G1 | 297 | 1 | 0.002
G1 | 298 | 1 | 0.002
G1 | 309.3C | 1 | 0.002
G1 | 323.1G | 1 | 0.002
G1 | 324 | 1 | 0.002
G1 | 325d | 1 | 0.002
G1 | 3278 | 1 | 0.002
G1 | 337d | 1 | 0.002
G1 | 346 | 1 | 0.002
G1 | 384A | 1 | 0.002
G1 | 384T | 1 | 0.002

G1 | 401.1T | 1 | 0.002
G1 | 403.1T | 1 | 0.002
G1 | 408.1T | 1 | 0.002
G1 | 439 | 1 | 0.002
G1 | 446 | 2 | 0.003
G1 | 446T | 1 | 0.002
G1 | 47 | 1 | 0.002
G1 | 472 | 1 | 0.002
G1 | 48 | 1 | 0.002
G1 | 488 | 1 | 0.002
G1 | 517 | 1 | 0.002
G1 | 529 | 1 | 0.002
G1 | 530 | 1 | 0.002
G1 | 553 | 1 | 0.002
G1 | 574 | 1 | 0.002
G1 | 574d | 3 | 0.005
G1 | 72 | 1 | 0.002
G1 | 75 | 1 | 0.002
G1 | 7521 | 1 | 0.002
G1 | 7867 | 3 | 0.005
G1 | 82 | 1 | 0.002
G1 | 8572 | 1 | 0.002
G1 | 9300 | 1 | 0.002
G1 | 97C | 1 | 0.002
G1a | 16092 | 1 | 0.002
G1a | 16108 | 1 | 0.002
G1a | 16111A | 1 | 0.002
G1a | 16124 | 1 | 0.002
G1a | 16250A | 1 | 0.002
G1a | 16318C | 1 | 0.002
G1a | 16T | 1 | 0.002
G1a | 183 | 1 | 0.002
G1a | 258 | 1 | 0.002
G1a | 467 | 1 | 0.002
G1a1 | 10658 | 3 | 0.029
G1a1 | 12867 | 1 | 0.01
G1a1 | 13145 | 5 | 0.049
G1a1 | 13763A | 3 | 0.029
G1a1 | 14370 | 1 | 0.01
G1a1 | 14484 | 1 | 0.01
G1a1 | 152 | 1 | 0.01
G1a1 | 15442 | 1 | 0.01
G1a1 | 15462 | 1 | 0.01
G1a1 | 15769 | 1 | 0.01
G1a1 | 15968 | 2 | 0.019
G1a1 | 16086 | 1 | 0.01
G1a1 | 16104 | 1 | 0.01
G1a1 | 16129T | 1 | 0.01
G1a1 | 16145 | 1 | 0.01
G1a1 | 16172 | 4 | 0.039
G1a1 | 16213 | 1 | 0.01
G1a1 | 16221 | 1 | 0.01
G1a1 | 16239 | 1 | 0.01
G1a1 | 16319 | 1 | 0.01
G1a1 | 16320 | 1 | 0.01
G1a1 | 16355 | 4 | 0.039
G1a1 | 16365 | 1 | 0.01
G1a1 | 16422 | 1 | 0.01
G1a1 | 16463 | 2 | 0.019
G1a1 | 1694 | 3 | 0.029
G1a1 | 185 | 1 | 0.01
G1a1 | 200 | 4 | 0.039
G1a1 | 3336 | 1 | 0.01
G1a1 | 3447 | 1 | 0.01
G1a1 | 3579 | 1 | 0.01
G1a1 | 3834 | 1 | 0.01
G1a1 | 4694A | 3 | 0.029
G1a1 | 4793 | 1 | 0.01
G1a1 | 5806 | 1 | 0.01
G1a1 | 6668 | 3 | 0.029
G1a1 | 6842 | 1 | 0.01
G1a1 | 7581 | 2 | 0.019
G1a1 | 7750 | 4 | 0.039
G1a1 | 7772 | 1 | 0.01
G1a1 | 8654 | 1 | 0.01
G1a1 | 9041 | 5 | 0.049
G1a1a | 11204 | 1 | 0.009
G1a1a | 11549 | 1 | 0.009
G1a1a | 1393 | 1 | 0.009
G1a1a | 152 | 1 | 0.009
G1a1a | 15813 | 1 | 0.009
G1a1a | 16086 | 1 | 0.009
G1a1a | 16102 | 1 | 0.009
G1a1a | 16158 | 2 | 0.018
G1a1a | 16166 | 1 | 0.009
G1a1a | 16184A | 1 | 0.009
G1a1a | 16189 | 6 | 0.054
G1a1a | 16221 | 2 | 0.018
G1a1a | 16275 | 1 | 0.009
G1a1a | 16294 | 1 | 0.009
G1a1a | 16304 | 1 | 0.009
G1a1a | 194 | 1 | 0.009
G1a1a | 195 | 5 | 0.045
G1a1a | 279 | 1 | 0.009
G1a1a | 316C | 1 | 0.009
G1a1a | 3338 | 2 | 0.018
G1a1a | 3954 | 1 | 0.009
G1a1a | 5655 | 1 | 0.009
G1a1a | 592 | 1 | 0.009
G1a1a | 6533 | 3 | 0.027
G1a1a | 6983 | 1 | 0.009
G1a1a | 7124 | 1 | 0.009
G1a1a | 7646 | 1 | 0.009
G1a1a | 8227 | 1 | 0.009
G1a1a | 8563 | 1 | 0.009
G1a1a | 8610 | 1 | 0.009
G1a1a | 9494 | 1 | 0.009
G1a1a | 9753 | 1 | 0.009
G1a1a1 | 14386 | 1 | 0.009
G1a1a1 | 16075 | 13 | 0.123
G1a1a1 | 16082 | 1 | 0.009

G1a1a1 | 16093 | 1 | 0.009
G1a1a1 | 16126 | 1 | 0.009
G1a1a1 | 16129 | 1 | 0.009
G1a1a1 | 16153 | 1 | 0.009
G1a1a1 | 16175 | 1 | 0.009
G1a1a1 | 16189 | 2 | 0.019
G1a1a1 | 16234 | 1 | 0.009
G1a1a1 | 16274 | 3 | 0.028
G1a1a1 | 16311 | 1 | 0.009
G1a1a1 | 16327 | 2 | 0.019
G1a1a1 | 16365 | 1 | 0.009
G1a1a1 | 1822 | 2 | 0.019
G1a1a1 | 183 | 5 | 0.047
G1a1a1 | 189 | 1 | 0.009
G1a1a1 | 2708 | 1 | 0.009
G1a1a1 | 316 | 1 | 0.009
G1a1a1 | 3644 | 1 | 0.009
G1a1a1 | 438 | 1 | 0.009
G1a1a2 | 10 | 2 | 0.022
G1a1a2 | 10550 | 1 | 0.011
G1a1a2 | 12144 | 1 | 0.011
G1a1a2 | 14016 | 1 | 0.011
G1a1a2 | 152 | 5 | 0.056
G1a1a2 | 16176 | 1 | 0.011
G1a1a2 | 16324 | 1 | 0.011
G1a1a2 | 16327 | 1 | 0.011
G1a1a2 | 16390 | 1 | 0.011
G1a1a2 | 16400 | 1 | 0.011
G1a1a2 | 5344 | 1 | 0.011
G1a1a3 | 15941 | 1 | 0.04
G1a1a3 | 16254 | 2 | 0.08
G1a1a3 | 16291 | 2 | 0.08
G1a1a3 | 195 | 2 | 0.08
G1a1a3 | 207 | 2 | 0.08
G1a1a3 | 5951 | 1 | 0.04
G1a1a3 | 7909 | 1 | 0.04
G1a1a4 | 14470 | 1 | 0.012
G1a1a4 | 14575 | 2 | 0.023
G1a1a4 | 16176 | 1 | 0.012
G1a1a4 | 1625 | 2 | 0.023
G1a1a4 | 16256 | 2 | 0.023
G1a1a4 | 16390 | 1 | 0.012
G1a1a4 | 16422 | 1 | 0.012
G1a1a4 | 4612 | 1 | 0.012
G1a1a4 | 482 | 2 | 0.023
G1a1a4 | 5147 | 2 | 0.023
G1a1a4 | 6164 | 2 | 0.023
G1a1a4 | 8485 | 2 | 0.023
G1a1a4 | 9380 | 2 | 0.023
G1a1b | 106 | 1 | 0.011
G1a1b | 145 | 1 | 0.011
G1a1b | 146 | 12 | 0.128
G1a1b | 149 | 1 | 0.011
G1a1b | 15077 | 1 | 0.011
G1a1b | 151G | 1 | 0.011
G1a1b | 152 | 2 | 0.021
G1a1b | 152A | 2 | 0.021
G1a1b | 16145 | 1 | 0.011
G1a1b | 16176 | 1 | 0.011
G1a1b | 16209 | 2 | 0.021
G1a1b | 16274 | 1 | 0.011
G1a1b | 16390 | 1 | 0.011
G1a1b | 16553 | 1 | 0.011
G1a1b | 16564 | 1 | 0.011
G1a1b | 193 | 1 | 0.011
G1a1b | 195 | 1 | 0.011
G1a1b | 291 | 1 | 0.011
G1a1b | 3221 | 1 | 0.011
G1a1b | 37 | 1 | 0.011
G1a1b | 4257 | 1 | 0.011
G1a1b | 548 | 1 | 0.011
G1a1b | 576 | 1 | 0.011
G1a1b | 64G | 1 | 0.011
G1a1b | 68-76d | 1 | 0.011
G1a2 | 11887 | 1 | 0.023
G1a2 | 14905 | 1 | 0.023
G1a2 | 14950 | 1 | 0.023
G1a2 | 152 | 1 | 0.023
G1a2 | 16051 | 1 | 0.023
G1a2 | 16093 | 1 | 0.023
G1a2 | 16126 | 2 | 0.047
G1a2 | 16145 | 2 | 0.047
G1a2 | 16300 | 5 | 0.116
G1a2 | 16311 | 1 | 0.023
G1a2 | 238 | 1 | 0.023
G1a2 | 247 | 1 | 0.023
G1a2 | 2760 | 1 | 0.023
G1a2 | 316C | 1 | 0.023
G1a2 | 389 | 1 | 0.023
G1a2 | 513 | 1 | 0.023
G1a2 | 5378 | 1 | 0.023
G1a2 | 596 | 1 | 0.023
G1a2 | 8520 | 1 | 0.023
G1a2'3 | 146 | 1 | 0.036
G1a2'3 | 16069 | 1 | 0.036
G1a2'3 | 16161G | 1 | 0.036
G1a2'3 | 16163 | 1 | 0.036
G1a2'3 | 16185 | 1 | 0.036
G1a2'3 | 16192 | 1 | 0.036
G1a2'3 | 16291 | 7 | 0.25
G1a2'3 | 16311 | 1 | 0.036
G1a2'3 | 16325 | 7 | 0.25
G1a2'3 | 16475 | 1 | 0.036
G1a2'3 | 195 | 1 | 0.036
G1a2'3 | 208A | 1 | 0.036
G1a2'3 | 325 | 1 | 0.036
G1a3 | 14990 | 1 | 0.014
G1a3 | 152 | 1 | 0.014
G1a3 | 153 | 2 | 0.029
G1a3 | 200 | 3 | 0.043
G1a3 | 316.1T | 1 | 0.014

G1a3 | 316C | 1 | 0.014
G1a3 | 573.1C | 1 | 0.014
G1a3 | 6260 | 1 | 0.014
G1a3 | 7220 | 2 | 0.029
G1a3 | 9527 | 2 | 0.029
G1b | 146 | 6 | 0.037
G1b | 150 | 1 | 0.006
G1b | 152 | 1 | 0.006
G1b | 16051 | 3 | 0.018
G1b | 16062T | 1 | 0.006
G1b | 16124 | 1 | 0.006
G1b | 16126 | 4 | 0.024
G1b | 16145 | 2 | 0.012
G1b | 16147 | 2 | 0.012
G1b | 16155 | 1 | 0.006
G1b | 16156 | 1 | 0.006
G1b | 16189 | 1 | 0.006
G1b | 16192 | 2 | 0.012
G1b | 16194T | 1 | 0.006
G1b | 16196 | 1 | 0.006
G1b | 16214A | 1 | 0.006
G1b | 16217 | 1 | 0.006
G1b | 16217A | 1 | 0.006
G1b | 16218 | 3 | 0.018
G1b | 16220T | 3 | 0.018
G1b | 16243 | 1 | 0.006
G1b | 16258 | 1 | 0.006
G1b | 16258d | 2 | 0.012
G1b | 16266 | 1 | 0.006
G1b | 16271 | 1 | 0.006
G1b | 16272 | 2 | 0.012
G1b | 16274 | 1 | 0.006
G1b | 16278 | 1 | 0.006
G1b | 16289 | 1 | 0.006
G1b | 16291 | 1 | 0.006
G1b | 16292 | 1 | 0.006
G1b | 16295 | 1 | 0.006
G1b | 16320 | 6 | 0.037
G1b | 16325 | 1 | 0.006
G1b | 16344 | 1 | 0.006
G1b | 16353 | 1 | 0.006
G1b | 16356 | 1 | 0.006
G1b | 16362d | 1 | 0.006
G1b | 16381 | 2 | 0.012
G1b | 16391 | 1 | 0.006
G1b | 16399 | 8 | 0.049
G1b | 16452 | 2 | 0.012
G1b | 16488 | 4 | 0.024
G1b | 16497 | 4 | 0.024
G1b | 16502 | 1 | 0.006
G1b | 16513G | 2 | 0.012
G1b | 16543C | 2 | 0.012
G1b | 16547G | 1 | 0.006
G1b | 185 | 3 | 0.018
G1b | 188 | 1 | 0.006
G1b | 195 | 1 | 0.006
G1b | 199 | 2 | 0.012
G1b | 204 | 1 | 0.006
G1b | 214 | 6 | 0.037
G1b | 242-244d | 1 | 0.006
G1b | 315.2C | 5 | 0.03
G1b | 356.1C | 4 | 0.024
G1b | 374 | 1 | 0.006
G1b | 461 | 1 | 0.006
G1b | 482 | 1 | 0.006
G1b | 521 | 1 | 0.006
G1b | 55.1T | 1 | 0.006
G1b | 57 | 1 | 0.006
G1b | 59 | 1 | 0.006
G1b | 60.1T | 1 | 0.006
G1b | 71d | 1 | 0.006
G1b+16129 | 11440C | 1 | 0.007
G1b+16129 | 152 | 9 | 0.059
G1b+16129 | 15706 | 1 | 0.007
G1b+16129 | 16042 | 1 | 0.007
G1b+16129 | 16111 | 1 | 0.007
G1b+16129 | 16144 | 1 | 0.007
G1b+16129 | 16147 | 1 | 0.007
G1b+16129 | 16148 | 2 | 0.013
G1b+16129 | 16155 | 1 | 0.007
G1b+16129 | 16166 | 1 | 0.007
G1b+16129 | 16176 | 2 | 0.013
G1b+16129 | 16184 | 1 | 0.007
G1b+16129 | 16192 | 1 | 0.007
G1b+16129 | 16213 | 1 | 0.007
G1b+16129 | 16220T | 1 | 0.007
G1b+16129 | 16263 | 2 | 0.013
G1b+16129 | 16263d | 1 | 0.007
G1b+16129 | 16264.1C | 2 | 0.013
G1b+16129 | 16270 | 1 | 0.007
G1b+16129 | 16271 | 1 | 0.007
G1b+16129 | 16274 | 2 | 0.013
G1b+16129 | 16278 | 1 | 0.007
G1b+16129 | 16291 | 1 | 0.007
G1b+16129 | 16292 | 1 | 0.007
G1b+16129 | 16310 | 1 | 0.007
G1b+16129 | 16311 | 2 | 0.013
G1b+16129 | 16390 | 1 | 0.007
G1b+16129 | 16497 | 1 | 0.007
G1b+16129 | 16526 | 2 | 0.013
G1b+16129 | 16566 | 1 | 0.007
G1b+16129 | 1719 | 1 | 0.007
G1b+16129 | 195A | 1 | 0.007
G1b+16129 | 204 | 1 | 0.007
G1b+16129 | 309d | 1 | 0.007
G1b+16129 | 310 | 1 | 0.007
G1b+16129 | 5276 | 1 | 0.007
G1b+16129 | 5704 | 2 | 0.013
G1b+16129 | 571-573d | 1 | 0.007
G1b+16129 | 7389 | 1 | 0.007
G1b1 | 10586C | 9 | 0.094

G1b1 | 13646 | 1 | 0.01
G1b1 | 15383 | 6 | 0.062
G1b1 | 16188 | 2 | 0.021
G1b1 | 16244 | 4 | 0.042
G1b1 | 16295 | 1 | 0.01
G1b1 | 16399 | 11 | 0.115
G1b1 | 204 | 3 | 0.031
G1b1 | 3398 | 2 | 0.021
G1b1 | 4923 | 6 | 0.062
G1b1 | 8865 | 9 | 0.094
G1b1 | 9068 | 1 | 0.01
G1b2 | 12408 | 2 | 0.012
G1b2 | 150 | 3 | 0.018
G1b2 | 16042 | 1 | 0.006
G1b2 | 16051 | 1 | 0.006
G1b2 | 16086 | 1 | 0.006
G1b2 | 16111 | 1 | 0.006
G1b2 | 16117A | 1 | 0.006
G1b2 | 16155 | 1 | 0.006
G1b2 | 16189 | 1 | 0.006
G1b2 | 16220T | 1 | 0.006
G1b2 | 16224 | 1 | 0.006
G1b2 | 16256 | 24 | 0.146
G1b2 | 16262 | 2 | 0.012
G1b2 | 16263d | 1 | 0.006
G1b2 | 16271 | 1 | 0.006
G1b2 | 16278 | 1 | 0.006
G1b2 | 16292 | 1 | 0.006
G1b2 | 16296 | 4 | 0.024
G1b2 | 16297 | 1 | 0.006
G1b2 | 16309 | 1 | 0.006
G1b2 | 16310 | 1 | 0.006
G1b2 | 16390 | 1 | 0.006
G1b2 | 16526 | 6 | 0.037
G1b2 | 16566 | 1 | 0.006
G1b2 | 195A | 1 | 0.006
G1b2 | 246 | 1 | 0.006
G1b2 | 315.2C | 1 | 0.006
G1b2 | 4695 | 1 | 0.006
G1b3 | 10685 | 1 | 0.012
G1b3 | 11361 | 1 | 0.012
G1b3 | 16212 | 1 | 0.012
G1b3 | 7521 | 1 | 0.012
G1b4 | 11569 | 1 | 0.006
G1b4 | 150 | 3 | 0.018
G1b4 | 16042 | 1 | 0.006
G1b4 | 16051 | 1 | 0.006
G1b4 | 16086 | 1 | 0.006
G1b4 | 16111 | 1 | 0.006
G1b4 | 16117A | 1 | 0.006
G1b4 | 16153 | 1 | 0.006
G1b4 | 16155 | 1 | 0.006
G1b4 | 16189 | 1 | 0.006
G1b4 | 16220T | 1 | 0.006
G1b4 | 16224 | 1 | 0.006
G1b4 | 16256 | 24 | 0.146
G1b4 | 16262 | 2 | 0.012
G1b4 | 16263d | 1 | 0.006
G1b4 | 16271 | 1 | 0.006
G1b4 | 16278 | 1 | 0.006
G1b4 | 16292 | 1 | 0.006
G1b4 | 16296 | 4 | 0.024
G1b4 | 16297 | 1 | 0.006
G1b4 | 16309 | 1 | 0.006
G1b4 | 16310 | 1 | 0.006
G1b4 | 16390 | 1 | 0.006
G1b4 | 16526 | 6 | 0.037
G1b4 | 16566 | 1 | 0.006
G1b4 | 195A | 1 | 0.006
G1b4 | 246 | 1 | 0.006
G1b4 | 315.2C | 1 | 0.006
G1c | 11470 | 1 | 0.002
G1c | 12361 | 1 | 0.002
G1c | 12519 | 1 | 0.002
G1c | 14956 | 1 | 0.002
G1c | 152 | 1 | 0.002
G1c | 153 | 1 | 0.002
G1c | 16051 | 9 | 0.015
G1c | 16092 | 1 | 0.002
G1c | 16093 | 4 | 0.007
G1c | 16103 | 5 | 0.008
G1c | 16114 | 6 | 0.01
G1c | 16172A | 1 | 0.002
G1c | 16179 | 1 | 0.002
G1c | 16189 | 8 | 0.013
G1c | 16192 | 2 | 0.003
G1c | 16213 | 1 | 0.002
G1c | 16253 | 1 | 0.002
G1c | 16254 | 1 | 0.002
G1c | 16255 | 2 | 0.003
G1c | 16261 | 2 | 0.003
G1c | 16264 | 1 | 0.002
G1c | 16319 | 3 | 0.005
G1c | 16474C | 4 | 0.007
G1c | 1719 | 2 | 0.003
G1c | 185 | 1 | 0.002
G1c | 188 | 1 | 0.002
G1c | 199 | 1 | 0.002
G1c | 200 | 1 | 0.002
G1c | 215 | 1 | 0.002
G1c | 236 | 1 | 0.002
G1c | 2757 | 1 | 0.002
G1c | 3277 | 1 | 0.002
G1c | 374 | 1 | 0.002
G1c | 408A | 2 | 0.003
G1c | 4353 | 1 | 0.002
G1c | 446 | 1 | 0.002
G1c | 455.1T | 4 | 0.007
G1c | 527 | 1 | 0.002
G1c | 63 | 1 | 0.002
G1c | 636 | 1 | 0.002

G1c | 723 | 2 | 0.003
G1c | 8694 | 1 | 0.002
G1c1 | 14726 | 1 | 0.011
G1c1 | 16157 | 3 | 0.033
G1c1 | 16291 | 1 | 0.011
G1c1 | 16295 | 25 | 0.275
G1c1 | 341T | 1 | 0.011
G1c1 | 350T | 1 | 0.011
G1c1 | 3593 | 1 | 0.011
G1c1 | 372 | 1 | 0.011
G1c1 | 780.1C | 1 | 0.011
G1c1 | 9165 | 1 | 0.011
G1c2 | 11778 | 1 | 0.002
G1c2 | 125G | 1 | 0.002
G1c2 | 152 | 1 | 0.002
G1c2 | 153 | 1 | 0.002
G1c2 | 16051 | 9 | 0.015
G1c2 | 16092 | 1 | 0.002
G1c2 | 16103 | 5 | 0.008
G1c2 | 16114 | 6 | 0.01
G1c2 | 16126 | 12 | 0.019
G1c2 | 16179 | 1 | 0.002
G1c2 | 16189 | 8 | 0.013
G1c2 | 16192 | 2 | 0.003
G1c2 | 16213 | 1 | 0.002
G1c2 | 16253 | 1 | 0.002
G1c2 | 16254 | 1 | 0.002
G1c2 | 16261 | 2 | 0.003
G1c2 | 16264 | 1 | 0.002
G1c2 | 16319 | 3 | 0.005
G1c2 | 16474C | 4 | 0.006
G1c2 | 185 | 1 | 0.002
G1c2 | 188 | 1 | 0.002
G1c2 | 200 | 1 | 0.002
G1c2 | 215 | 1 | 0.002
G1c2 | 236 | 1 | 0.002
G1c2 | 374 | 1 | 0.002
G1c2 | 408A | 2 | 0.003
G1c2 | 446 | 1 | 0.002
G1c2 | 455.1T | 4 | 0.006
G1c2 | 527 | 1 | 0.002
G1c2 | 5333 | 1 | 0.002
G1c2 | 547 | 1 | 0.002
G1c2 | 63 | 2 | 0.003
G2 | 11443 | 3 | 0.005
G2 | 115A | 1 | 0.002
G2 | 12067 | 3 | 0.005
G2 | 146 | 4 | 0.006
G2 | 16032.1A | 1 | 0.002
G2 | 16044.1C | 1 | 0.002
G2 | 16045 | 1 | 0.002
G2 | 16047T | 1 | 0.002
G2 | 16048T | 2 | 0.003
G2 | 16051 | 1 | 0.002
G2 | 16095 | 1 | 0.002
G2 | 16108 | 1 | 0.002
G2 | 16111A | 1 | 0.002
G2 | 16136 | 1 | 0.002
G2 | 16179 | 2 | 0.003
G2 | 16234 | 1 | 0.002
G2 | 16241 | 1 | 0.002
G2 | 16242 | 1 | 0.002
G2 | 16250A | 1 | 0.002
G2 | 16293 | 2 | 0.003
G2 | 16293T | 1 | 0.002
G2 | 16318C | 1 | 0.002
G2 | 16326C | 1 | 0.002
G2 | 16355 | 1 | 0.002
G2 | 16357 | 1 | 0.002
G2 | 16396A | 1 | 0.002
G2 | 16434 | 1 | 0.002
G2 | 16443 | 1 | 0.002
G2 | 16447 | 1 | 0.002
G2 | 16452 | 4 | 0.006
G2 | 16507 | 1 | 0.002
G2 | 16522 | 1 | 0.002
G2 | 16525 | 1 | 0.002
G2 | 171 | 1 | 0.002
G2 | 210 | 3 | 0.005
G2 | 217 | 4 | 0.006
G2 | 249 | 1 | 0.002
G2 | 274T | 1 | 0.002
G2 | 290.1T | 1 | 0.002
G2 | 297 | 1 | 0.002
G2 | 298 | 1 | 0.002
G2 | 309.3C | 1 | 0.002
G2 | 323.1G | 1 | 0.002
G2 | 324 | 1 | 0.002
G2 | 325d | 1 | 0.002
G2 | 328 | 1 | 0.002
G2 | 337d | 1 | 0.002
G2 | 346 | 1 | 0.002
G2 | 384A | 1 | 0.002
G2 | 384T | 1 | 0.002
G2 | 401.1T | 1 | 0.002
G2 | 403.1T | 1 | 0.002
G2 | 408.1T | 1 | 0.002
G2 | 439 | 1 | 0.002
G2 | 446 | 2 | 0.003
G2 | 446T | 1 | 0.002
G2 | 47 | 1 | 0.002
G2 | 472 | 1 | 0.002
G2 | 48 | 1 | 0.002
G2 | 488 | 1 | 0.002
G2 | 517 | 1 | 0.002
G2 | 529 | 1 | 0.002
G2 | 530 | 1 | 0.002
G2 | 553 | 1 | 0.002
G2 | 5581 | 3 | 0.005
G2 | 574 | 1 | 0.002
G2 | 574d | 3 | 0.005

G2 | 5960 | 3 | 0.005
G2 | 72 | 1 | 0.002
G2 | 75 | 1 | 0.002
G2 | 82 | 1 | 0.002
G2 | 97C | 1 | 0.002
G2a | 146 | 21 | 0.087
G2a | 153 | 1 | 0.004
G2a | 15955.1A | 1 | 0.004
G2a | 16051 | 5 | 0.021
G2a | 16056G | 1 | 0.004
G2a | 16063A | 1 | 0.004
G2a | 16075A | 2 | 0.008
G2a | 16093 | 21 | 0.087
G2a | 16111A | 1 | 0.004
G2a | 16112A | 1 | 0.004
G2a | 16114A | 1 | 0.004
G2a | 16121A | 1 | 0.004
G2a | 16126 | 3 | 0.012
G2a | 16136 | 9 | 0.037
G2a | 16144 | 1 | 0.004
G2a | 16150 | 1 | 0.004
G2a | 16169 | 1 | 0.004
G2a | 16187 | 4 | 0.017
G2a | 16193 | 1 | 0.004
G2a | 16209 | 2 | 0.008
G2a | 16221 | 1 | 0.004
G2a | 16224 | 8 | 0.033
G2a | 16233 | 1 | 0.004
G2a | 16249 | 1 | 0.004
G2a | 16261 | 1 | 0.004
G2a | 16274 | 6 | 0.025
G2a | 16287 | 1 | 0.004
G2a | 16288 | 2 | 0.008
G2a | 16304 | 9 | 0.037
G2a | 16348 | 1 | 0.004
G2a | 16448G | 1 | 0.004
G2a | 16465 | 3 | 0.012
G2a | 16527 | 15 | 0.062
G2a | 189 | 1 | 0.004
G2a | 195 | 7 | 0.029
G2a | 198 | 1 | 0.004
G2a | 199 | 1 | 0.004
G2a | 228 | 1 | 0.004
G2a | 235 | 2 | 0.008
G2a | 283 | 17 | 0.071
G2a | 316C | 5 | 0.021
G2a | 466 | 5 | 0.021
G2a | 507 | 2 | 0.008
G2a | 524.1GC | 1 | 0.004
G2a | 544A | 1 | 0.004
G2a | 562 | 1 | 0.004
G2a | 574C | 14 | 0.058
G2a | 576C | 14 | 0.058
G2a | 577C | 5 | 0.021
G2a | 578 | 3 | 0.012
G2a | 579 | 1 | 0.004
G2a+152 | 10043 | 2 | 0.011
G2a+152 | 10804 | 2 | 0.011
G2a+152 | 11696 | 4 | 0.023
G2a+152 | 11778 | 1 | 0.006
G2a+152 | 1189 | 7 | 0.04
G2a+152 | 12372 | 7 | 0.04
G2a+152 | 14200 | 2 | 0.011
G2a+152 | 14587 | 2 | 0.011
G2a+152 | 146 | 4 | 0.023
G2a+152 | 150 | 2 | 0.011
G2a+152 | 151 | 21 | 0.12
G2a+152 | 15670 | 1 | 0.006
G2a+152 | 15731 | 1 | 0.006
G2a+152 | 16092 | 6 | 0.034
G2a+152 | 16093 | 7 | 0.04
G2a+152 | 16126 | 1 | 0.006
G2a+152 | 16129 | 3 | 0.017
G2a+152 | 16167 | 1 | 0.006
G2a+152 | 16192 | 1 | 0.006
G2a+152 | 16203 | 2 | 0.011
G2a+152 | 16204 | 2 | 0.011
G2a+152 | 16207 | 2 | 0.011
G2a+152 | 16219 | 1 | 0.006
G2a+152 | 16255 | 1 | 0.006
G2a+152 | 16262 | 4 | 0.023
G2a+152 | 16263 | 1 | 0.006
G2a+152 | 16271 | 1 | 0.006
G2a+152 | 16274 | 2 | 0.011
G2a+152 | 16287 | 7 | 0.04
G2a+152 | 16304 | 11 | 0.063
G2a+152 | 16319 | 1 | 0.006
G2a+152 | 16325 | 2 | 0.011
G2a+152 | 16362d | 2 | 0.011
G2a+152 | 16399 | 1 | 0.006
G2a+152 | 199 | 1 | 0.006
G2a+152 | 204 | 1 | 0.006
G2a+152 | 222G | 1 | 0.006
G2a+152 | 228 | 1 | 0.006
G2a+152 | 2357A | 1 | 0.006
G2a+152 | 237 | 2 | 0.011
G2a+152 | 249d | 1 | 0.006
G2a+152 | 283 | 1 | 0.006
G2a+152 | 325 | 5 | 0.029
G2a+152 | 3351 | 2 | 0.011
G2a+152 | 3915 | 1 | 0.006
G2a+152 | 4189 | 1 | 0.006
G2a+152 | 451 | 2 | 0.011
G2a+152 | 4721 | 2 | 0.011
G2a+152 | 551 | 1 | 0.006
G2a+152 | 573.2C | 1 | 0.006
G2a+152 | 573.3C | 1 | 0.006
G2a+152 | 573.4C | 2 | 0.011
G2a+152 | 573.5C | 2 | 0.011
G2a+152 | 573.7C | 1 | 0.006
G2a+152 | 574C | 2 | 0.011

G2a+152 | 576C | 2 | 0.011
G2a+152 | 5899.1C | 3 | 0.017
G2a+152 | 595 | 1 | 0.006
G2a+152 | 6101 | 4 | 0.023
G2a+152 | 6340 | 1 | 0.006
G2a+152 | 7604 | 2 | 0.011
G2a+152 | 8251 | 4 | 0.023
G2a+152 | 8547 | 2 | 0.011
G2a+152 | 87 | 1 | 0.006
G2a+152 | 9932 | 2 | 0.011
G2a1 | 10 | 1 | 0.003
G2a1 | 10742 | 1 | 0.003
G2a1 | 11087 | 2 | 0.006
G2a1 | 11778 | 3 | 0.009
G2a1 | 11809 | 1 | 0.003
G2a1 | 1192 | 1 | 0.003
G2a1 | 12525 | 2 | 0.006
G2a1 | 12557 | 1 | 0.003
G2a1 | 12662 | 3 | 0.009
G2a1 | 12753 | 6 | 0.018
G2a1 | 12825 | 8 | 0.023
G2a1 | 13194 | 1 | 0.003
G2a1 | 13299 | 1 | 0.003
G2a1 | 13395 | 1 | 0.003
G2a1 | 13648A | 1 | 0.003
G2a1 | 13708 | 2 | 0.006
G2a1 | 13926 | 2 | 0.006
G2a1 | 13928C | 1 | 0.003
G2a1 | 14071 | 1 | 0.003
G2a1 | 14155 | 2 | 0.006
G2a1 | 14180 | 2 | 0.006
G2a1 | 14484 | 1 | 0.003
G2a1 | 146 | 2 | 0.006
G2a1 | 14839 | 2 | 0.006
G2a1 | 14861 | 3 | 0.009
G2a1 | 150 | 4 | 0.012
G2a1 | 15172 | 1 | 0.003
G2a1 | 15236 | 1 | 0.003
G2a1 | 15262 | 1 | 0.003
G2a1 | 15930 | 2 | 0.006
G2a1 | 16037 | 1 | 0.003
G2a1 | 16072.1A | 2 | 0.006
G2a1 | 16086 | 9 | 0.026
G2a1 | 16093 | 5 | 0.015
G2a1 | 16109C | 10 | 0.029
G2a1 | 16117 | 1 | 0.003
G2a1 | 16126 | 4 | 0.012
G2a1 | 16129 | 146 | 0.427
G2a1 | 16145 | 2 | 0.006
G2a1 | 16150 | 2 | 0.006
G2a1 | 16172 | 10 | 0.029
G2a1 | 16185 | 3 | 0.009
G2a1 | 16192 | 1 | 0.003
G2a1 | 16193 | 1 | 0.003
G2a1 | 16209 | 1 | 0.003
G2a1 | 16233 | 1 | 0.003
G2a1 | 16234 | 16 | 0.047
G2a1 | 16269 | 2 | 0.006
G2a1 | 16271 | 1 | 0.003
G2a1 | 16274 | 3 | 0.009
G2a1 | 16292 | 8 | 0.023
G2a1 | 16293C | 3 | 0.009
G2a1 | 16295 | 3 | 0.009
G2a1 | 16316 | 1 | 0.003
G2a1 | 16319 | 6 | 0.018
G2a1 | 16360 | 1 | 0.003
G2a1 | 16368 | 2 | 0.006
G2a1 | 16390 | 1 | 0.003
G2a1 | 16419d | 1 | 0.003
G2a1 | 16463 | 1 | 0.003
G2a1 | 16526 | 2 | 0.006
G2a1 | 16527 | 1 | 0.003
G2a1 | 173 | 2 | 0.006
G2a1 | 1808 | 8 | 0.023
G2a1 | 1811 | 1 | 0.003
G2a1 | 184 | 1 | 0.003
G2a1 | 1888 | 1 | 0.003
G2a1 | 189 | 1 | 0.003
G2a1 | 194 | 2 | 0.006
G2a1 | 195 | 1 | 0.003
G2a1 | 198 | 2 | 0.006
G2a1 | 199 | 2 | 0.006
G2a1 | 278 | 1 | 0.003
G2a1 | 283 | 2 | 0.006
G2a1 | 291T | 2 | 0.006
G2a1 | 315.2C | 13 | 0.038
G2a1 | 315.3C | 1 | 0.003
G2a1 | 316C | 1 | 0.003
G2a1 | 34 | 3 | 0.009
G2a1 | 3417 | 2 | 0.006
G2a1 | 3438 | 1 | 0.003
G2a1 | 3546 | 1 | 0.003
G2a1 | 374 | 2 | 0.006
G2a1 | 3943 | 1 | 0.003
G2a1 | 4102 | 2 | 0.006
G2a1 | 4158 | 1 | 0.003
G2a1 | 4225C | 1 | 0.003
G2a1 | 4229A | 1 | 0.003
G2a1 | 4277 | 2 | 0.006
G2a1 | 4395 | 2 | 0.006
G2a1 | 52 | 4 | 0.012
G2a1 | 5261 | 1 | 0.003
G2a1 | 5581T | 1 | 0.003
G2a1 | 5634 | 1 | 0.003
G2a1 | 5840 | 3 | 0.009
G2a1 | 5892 | 1 | 0.003
G2a1 | 6266 | 1 | 0.003
G2a1 | 6293 | 2 | 0.006
G2a1 | 6333 | 1 | 0.003
G2a1 | 64 | 1 | 0.003
G2a1 | 7148 | 1 | 0.003

G2a1 | 7229 | 7 | 0.02
G2a1 | 7606 | 3 | 0.009
G2a1 | 8087 | 1 | 0.003
G2a1 | 8289.1CCCCCTCTA | 1 | 0.003
G2a1 | 8521 | 2 | 0.006
G2a1 | 8766 | 8 | 0.023
G2a1 | 8832C | 2 | 0.006
G2a1 | 9007 | 2 | 0.006
G2a1 | 9163 | 1 | 0.003
G2a1+16189 | 10310 | 1 | 0.007
G2a1+16189 | 11377 | 2 | 0.014
G2a1+16189 | 11399 | 1 | 0.007
G2a1+16189 | 12406 | 2 | 0.014
G2a1+16189 | 12525 | 2 | 0.014
G2a1+16189 | 12540 | 1 | 0.007
G2a1+16189 | 13203 | 1 | 0.007
G2a1+16189 | 13926 | 1 | 0.007
G2a1+16189 | 14155 | 1 | 0.007
G2a1+16189 | 14374 | 1 | 0.007
G2a1+16189 | 146 | 1 | 0.007
G2a1+16189 | 14839 | 1 | 0.007
G2a1+16189 | 150 | 2 | 0.014
G2a1+16189 | 152 | 6 | 0.041
G2a1+16189 | 153 | 2 | 0.014
G2a1+16189 | 15784 | 2 | 0.014
G2a1+16189 | 16092 | 6 | 0.041
G2a1+16189 | 16093 | 5 | 0.034
G2a1+16189 | 16124 | 1 | 0.007
G2a1+16189 | 16126 | 1 | 0.007
G2a1+16189 | 16129 | 4 | 0.027
G2a1+16189 | 16147 | 6 | 0.041
G2a1+16189 | 16167 | 1 | 0.007
G2a1+16189 | 16168 | 1 | 0.007
G2a1+16189 | 16190 | 3 | 0.021
G2a1+16189 | 16194C | 3 | 0.021
G2a1+16189 | 16195.1C | 3 | 0.021
G2a1+16189 | 16195G | 3 | 0.021
G2a1+16189 | 16224 | 1 | 0.007
G2a1+16189 | 16234 | 22 | 0.151
G2a1+16189 | 16243 | 7 | 0.048
G2a1+16189 | 16256 | 5 | 0.034
G2a1+16189 | 16261 | 8 | 0.055
G2a1+16189 | 16274 | 4 | 0.027
G2a1+16189 | 16294 | 2 | 0.014
G2a1+16189 | 16309 | 2 | 0.014
G2a1+16189 | 16311 | 1 | 0.007
G2a1+16189 | 16352 | 1 | 0.007
G2a1+16189 | 16356 | 3 | 0.021
G2a1+16189 | 16474T | 1 | 0.007
G2a1+16189 | 16526 | 5 | 0.034
G2a1+16189 | 1692 | 1 | 0.007
G2a1+16189 | 1719 | 1 | 0.007
G2a1+16189 | 185 | 1 | 0.007
G2a1+16189 | 195 | 3 | 0.021
G2a1+16189 | 200 | 3 | 0.021
G2a1+16189 | 204 | 1 | 0.007
G2a1+16189 | 207 | 6 | 0.041
G2a1+16189 | 234 | 7 | 0.048
G2a1+16189 | 2483 | 1 | 0.007
G2a1+16189 | 249 | 2 | 0.014
G2a1+16189 | 259 | 7 | 0.048
G2a1+16189 | 2831 | 1 | 0.007
G2a1+16189 | 2871 | 1 | 0.007
G2a1+16189 | 296 | 7 | 0.048
G2a1+16189 | 309d | 1 | 0.007
G2a1+16189 | 310 | 1 | 0.007
G2a1+16189 | 315.1T | 1 | 0.007
G2a1+16189 | 316C | 4 | 0.027
G2a1+16189 | 3970 | 1 | 0.007
G2a1+16189 | 4102 | 1 | 0.007
G2a1+16189 | 4277 | 1 | 0.007
G2a1+16189 | 455.1T | 1 | 0.007
G2a1+16189 | 455.2T | 1 | 0.007
G2a1+16189 | 466 | 1 | 0.007
G2a1+16189 | 5082 | 1 | 0.007
G2a1+16189 | 571-573d | 1 | 0.007
G2a1+16189 | 574C | 2 | 0.014
G2a1+16189 | 5752d | 1 | 0.007
G2a1+16189 | 576C | 2 | 0.014
G2a1+16189 | 577C | 2 | 0.014
G2a1+16189 | 578 | 1 | 0.007
G2a1+16189 | 7298 | 1 | 0.007
G2a1+16189 | 7533 | 1 | 0.007
G2a1+16189 | 7598 | 1 | 0.007
G2a1+16189 | 7609 | 2 | 0.014
G2a1+16189 | 93 | 1 | 0.007
G2a1+16189+16194 | 13968 | 1 | 0.015
G2a1+16189+16194 | 16093 | 1 | 0.015
G2a1+16189+16194 | 16311 | 1 | 0.015
G2a1+16189+16194 | 9182 | 1 | 0.015
G2a1b | 10018 | 1 | 0.011
G2a1b | 16030d | 1 | 0.011
G2a1b | 16075A | 2 | 0.021
G2a1b | 16080 | 1 | 0.011
G2a1b | 16085A | 1 | 0.011
G2a1b | 16093 | 1 | 0.011
G2a1b | 16166 | 1 | 0.011
G2a1b | 16171 | 1 | 0.011
G2a1b | 16235 | 2 | 0.021
G2a1b | 16242G | 1 | 0.011
G2a1b | 16265C | 2 | 0.021
G2a1b | 16289 | 1 | 0.011
G2a1b | 16293 | 1 | 0.011
G2a1b | 16311 | 1 | 0.011
G2a1b | 16319 | 1 | 0.011
G2a1b | 16320 | 1 | 0.011
G2a1b | 16332A | 1 | 0.011
G2a1b | 16335 | 1 | 0.011
G2a1b | 16359 | 1 | 0.011
G2a1b | 16370 | 1 | 0.011
G2a1b | 16372G | 1 | 0.011

G2a1b | 16373 | 1 | 0.011
G2a1b | 16427d | 1 | 0.011
G2a1b | 195 | 1 | 0.011
G2a1b | 337 | 2 | 0.021
G2a1b | 3591 | 1 | 0.011
G2a1c | 14158 | 1 | 0.012
G2a1c | 15754A | 1 | 0.012
G2a1c | 15914 | 1 | 0.012
G2a1c | 16093 | 2 | 0.025
G2a1c | 16183T | 1 | 0.012
G2a1c | 16274 | 1 | 0.012
G2a1c | 4977 | 1 | 0.012
G2a1c | 6734 | 1 | 0.012
G2a1c | 6962 | 1 | 0.012
G2a1c | 8289.1CCCCCTCTA | 1 | 0.012
G2a1c1 | 16093 | 1 | 0.014
G2a1c1 | 16094 | 1 | 0.014
G2a1c1 | 16124 | 1 | 0.014
G2a1c1 | 16173 | 1 | 0.014
G2a1c1 | 16260 | 1 | 0.014
G2a1c1 | 16293C | 1 | 0.014
G2a1c1 | 3278 | 2 | 0.028
G2a1c1 | 5970 | 1 | 0.014
G2a1c1 | 6383 | 1 | 0.014
G2a1c2 | 1313 | 1 | 0.014
G2a1c2 | 13827 | 1 | 0.014
G2a1c2 | 16190A | 1 | 0.014
G2a1c2 | 16311 | 2 | 0.028
G2a1c2 | 214 | 1 | 0.014
G2a1c2 | 961 | 1 | 0.014
G2a1c2 | 965.6C | 1 | 0.014
G2a1d | 16086 | 4 | 0.091
G2a1d | 16093 | 2 | 0.045
G2a1d | 16129 | 4 | 0.091
G2a1d | 16172 | 11 | 0.25
G2a1d | 16304 | 2 | 0.045
G2a1d | 16524 | 1 | 0.023
G2a1d | 16527 | 1 | 0.023
G2a1d1 | 15670 | 1 | 0.031
G2a1d1 | 16148 | 1 | 0.031
G2a1d1 | 16241 | 1 | 0.031
G2a1d1 | 16249 | 1 | 0.031
G2a1d1 | 16301 | 1 | 0.031
G2a1d1 | 16316 | 1 | 0.031
G2a1d1 | 16325 | 1 | 0.031
G2a1d1 | 16384 | 1 | 0.031
G2a1d1 | 16434 | 1 | 0.031
G2a1d1 | 2851 | 1 | 0.031
G2a1d1 | 8470 | 1 | 0.031
G2a1d1a | 10655 | 3 | 0.067
G2a1d1a | 11362 | 2 | 0.044
G2a1d1a | 12172 | 1 | 0.022
G2a1d1a | 13606 | 1 | 0.022
G2a1d1a | 13948 | 1 | 0.022
G2a1d1a | 14198 | 1 | 0.022
G2a1d1a | 14389 | 1 | 0.022
G2a1d1a | 14562G | 1 | 0.022
G2a1d1a | 15758 | 1 | 0.022
G2a1d1a | 16269 | 17 | 0.378
G2a1d1a | 16311 | 1 | 0.022
G2a1d1a | 16319 | 1 | 0.022
G2a1d1a | 16344 | 1 | 0.022
G2a1d1a | 185 | 1 | 0.022
G2a1d1a | 189 | 1 | 0.022
G2a1d1a | 200 | 1 | 0.022
G2a1d1a | 2360-2361d | 1 | 0.022
G2a1d1a | 284 | 1 | 0.022
G2a1d1a | 6221 | 1 | 0.022
G2a1d1a | 8188 | 2 | 0.044
G2a1d1a | 8343 | 1 | 0.022
G2a1d1a | 8489 | 1 | 0.022
G2a1d1a | 8572 | 1 | 0.022
G2a1d1a | 9494 | 2 | 0.044
G2a1d2 | 15706 | 1 | 0.011
G2a1d2 | 15930 | 1 | 0.011
G2a1d2 | 16051 | 3 | 0.032
G2a1d2 | 16192 | 3 | 0.032
G2a1d2 | 16193 | 6 | 0.063
G2a1d2 | 16260 | 1 | 0.011
G2a1d2 | 16274 | 1 | 0.011
G2a1d2 | 16291 | 1 | 0.011
G2a1d2 | 16294 | 2 | 0.021
G2a1d2 | 16300 | 1 | 0.011
G2a1d2 | 16311 | 1 | 0.011
G2a1d2 | 16316 | 1 | 0.011
G2a1d2 | 16429A | 2 | 0.021
G2a1d2 | 16430C | 2 | 0.021
G2a1d2 | 194 | 1 | 0.011
G2a1d2 | 2223 | 1 | 0.011
G2a1d2 | 389 | 1 | 0.011
G2a1d2 | 4833 | 2 | 0.021
G2a1d2 | 516A | 1 | 0.011
G2a1d2 | 622 | 1 | 0.011
G2a1d2 | 6719 | 1 | 0.011
G2a1d2 | 683T | 1 | 0.011
G2a1d2 | 8688T | 1 | 0.011
G2a1d2 | 8964 | 1 | 0.011
G2a1d2a | 10172 | 1 | 0.011
G2a1d2a | 16051 | 3 | 0.032
G2a1d2a | 16192 | 3 | 0.032
G2a1d2a | 16193 | 6 | 0.063
G2a1d2a | 16260 | 1 | 0.011
G2a1d2a | 16291 | 1 | 0.011
G2a1d2a | 16294 | 2 | 0.021
G2a1d2a | 16300 | 1 | 0.011
G2a1d2a | 16311 | 1 | 0.011
G2a1d2a | 16316 | 1 | 0.011
G2a1d2a | 16429A | 2 | 0.021
G2a1d2a | 16430C | 2 | 0.021
G2a1d2a | 194 | 1 | 0.011
G2a1d2a | 389 | 1 | 0.011

G2a1d2a | 516A | 1 | 0.011
G2a1d2a | 622 | 1 | 0.011
G2a1d2a | 683T | 1 | 0.011
G2a1d2a | 7754 | 1 | 0.011
G2a1d2a | 9105 | 1 | 0.011
G2a1d2a | 9935 | 1 | 0.011
G2a1e | 151 | 1 | 0.014
G2a1e | 16086 | 1 | 0.014
G2a1e | 2392 | 1 | 0.014
G2a1e | 8393 | 1 | 0.014
G2a1e | 8955 | 1 | 0.014
G2a1f | 13884 | 1 | 0.012
G2a1f | 14443 | 1 | 0.012
G2a1f | 16051 | 20 | 0.233
G2a1f | 16093 | 1 | 0.012
G2a1f | 16124 | 1 | 0.012
G2a1f | 16143 | 1 | 0.012
G2a1f | 16239 | 3 | 0.035
G2a1f | 16291 | 19 | 0.221
G2a1f | 316C | 1 | 0.012
G2a1f | 9659 | 1 | 0.012
G2a1f1 | 10107 | 1 | 0.125
G2a1f1 | 1393 | 2 | 0.25
G2a1f1 | 146 | 2 | 0.25
G2a1f1 | 153 | 2 | 0.25
G2a1f1 | 217 | 1 | 0.125
G2a1f1 | 308-309d | 1 | 0.125
G2a1f1 | 3290 | 2 | 0.25
G2a1f1 | 7245 | 1 | 0.125
G2a1g | 12070 | 1 | 0.2
G2a1g | 14484 | 1 | 0.2
G2a1g | 16111 | 1 | 0.2
G2a1g | 16176 | 1 | 0.2
G2a1g | 16243G | 1 | 0.2
G2a1g | 16294 | 1 | 0.2
G2a1h | 14668 | 1 | 0.01
G2a1h | 14696 | 1 | 0.01
G2a1h | 15553 | 1 | 0.01
G2a1h | 16051 | 2 | 0.02
G2a1h | 16086 | 1 | 0.01
G2a1h | 16093 | 3 | 0.031
G2a1h | 16104 | 1 | 0.01
G2a1h | 16126 | 1 | 0.01
G2a1h | 16129 | 1 | 0.01
G2a1h | 16172 | 1 | 0.01
G2a1h | 16184 | 7 | 0.071
G2a1h | 16231 | 1 | 0.01
G2a1h | 16249 | 3 | 0.031
G2a1h | 16259 | 1 | 0.01
G2a1h | 16291 | 1 | 0.01
G2a1h | 16303 | 1 | 0.01
G2a1h | 16316 | 1 | 0.01
G2a1h | 16485-16488d | 1 | 0.01
G2a1h | 215 | 1 | 0.01
G2a1h | 573.3C | 1 | 0.01
G2a1h | 573.6C | 1 | 0.01
G2a1h | 574C | 1 | 0.01
G2a1h | 576C | 1 | 0.01
G2a1h | 63 | 1 | 0.01
G2a1h | 64 | 3 | 0.031
G2a1h | 792 | 1 | 0.01
G2a2 | 11152 | 1 | 0.006
G2a2 | 12007 | 2 | 0.011
G2a2 | 12397 | 13 | 0.074
G2a2 | 12501 | 1 | 0.006
G2a2 | 16018 | 2 | 0.011
G2a2 | 16035C | 2 | 0.011
G2a2 | 16043C | 2 | 0.011
G2a2 | 16111 | 2 | 0.011
G2a2 | 16126 | 2 | 0.011
G2a2 | 16136 | 3 | 0.017
G2a2 | 16169 | 1 | 0.006
G2a2 | 16187 | 1 | 0.006
G2a2 | 16193G | 2 | 0.011
G2a2 | 16207 | 2 | 0.011
G2a2 | 16209 | 2 | 0.011
G2a2 | 16274 | 17 | 0.097
G2a2 | 16304 | 1 | 0.006
G2a2 | 16311 | 1 | 0.006
G2a2 | 16326 | 1 | 0.006
G2a2 | 16390 | 2 | 0.011
G2a2 | 189 | 1 | 0.006
G2a2 | 195 | 7 | 0.04
G2a2 | 2363 | 1 | 0.006
G2a2 | 310 | 1 | 0.006
G2a2 | 315.2C | 1 | 0.006
G2a2 | 316C | 1 | 0.006
G2a2 | 3736 | 1 | 0.006
G2a2 | 4772 | 2 | 0.011
G2a2 | 523d | 1 | 0.006
G2a2 | 5456 | 13 | 0.074
G2a2 | 574C | 2 | 0.011
G2a2 | 576C | 2 | 0.011
G2a2 | 6104 | 2 | 0.011
G2a2 | 8978 | 4 | 0.023
G2a2 | 9650 | 1 | 0.006
G2a2a | 153 | 1 | 0.011
G2a2a | 16051 | 1 | 0.011
G2a2a | 16093 | 1 | 0.011
G2a2a | 16094 | 1 | 0.011
G2a2a | 16172 | 1 | 0.011
G2a2a | 16189 | 1 | 0.011
G2a2a | 16239 | 2 | 0.023
G2a2a | 16356 | 2 | 0.023
G2a2a | 194.1A | 1 | 0.011
G2a2a | 198 | 1 | 0.011
G2a2a | 204 | 1 | 0.011
G2a2a | 2109 | 1 | 0.011
G2a2a | 316C | 1 | 0.011
G2a2a | 4493 | 1 | 0.011
G2a2a | 7364 | 1 | 0.011

G2a2a | 7859 | 1 | 0.011
G2a3 | 14155 | 1 | 0.5
G2a3 | 14470 | 1 | 0.5
G2a3 | 16356 | 1 | 0.5
G2a3 | 7805 | 1 | 0.5
G2a3 | 9986 | 1 | 0.5
G2a3a | 11764 | 1 | 0.25
G2a3a | 1833 | 1 | 0.25
G2a3a | 6152 | 1 | 0.25
G2a4 | 103 | 1 | 0.02
G2a4 | 11914 | 1 | 0.02
G2a4 | 1389 | 1 | 0.02
G2a4 | 14053 | 1 | 0.02
G2a4 | 143 | 1 | 0.02
G2a4 | 16051 | 1 | 0.02
G2a4 | 16076d | 1 | 0.02
G2a4 | 16086 | 1 | 0.02
G2a4 | 16129 | 1 | 0.02
G2a4 | 16172 | 7 | 0.137
G2a4 | 16176 | 1 | 0.02
G2a4 | 16189 | 1 | 0.02
G2a4 | 16242 | 2 | 0.039
G2a4 | 16274 | 1 | 0.02
G2a4 | 16301 | 2 | 0.039
G2a4 | 16311 | 3 | 0.059
G2a4 | 16320 | 1 | 0.02
G2a4 | 16365 | 1 | 0.02
G2a4 | 189 | 8 | 0.157
G2a4 | 195A | 1 | 0.02
G2a4 | 198 | 2 | 0.039
G2a4 | 215 | 1 | 0.02
G2a4 | 2383 | 1 | 0.02
G2a4 | 282 | 2 | 0.039
G2a4 | 3531 | 1 | 0.02
G2a4 | 55.1T | 2 | 0.039
G2a4 | 57 | 2 | 0.039
G2a4 | 5899.1C | 1 | 0.02
G2a4 | 59 | 2 | 0.039
G2a4 | 710 | 1 | 0.02
G2a4 | 8763 | 1 | 0.02
G2a5 | 146 | 1 | 0.013
G2a5 | 16092 | 2 | 0.027
G2a5 | 16093 | 17 | 0.227
G2a5 | 16147 | 3 | 0.04
G2a5 | 16158 | 2 | 0.027
G2a5 | 16189 | 2 | 0.027
G2a5 | 16209 | 3 | 0.04
G2a5 | 16213 | 1 | 0.013
G2a5 | 16274 | 3 | 0.04
G2a5 | 16289 | 1 | 0.013
G2a5 | 16295 | 1 | 0.013
G2a5 | 16309 | 25 | 0.333
G2a5 | 16311 | 2 | 0.027
G2a5 | 16354A | 1 | 0.013
G2a5 | 16463 | 1 | 0.013
G2a5 | 316 | 1 | 0.013
G2a5 | 390 | 1 | 0.013
G2a5 | 4021 | 2 | 0.027
G2a5 | 573.1C | 2 | 0.027
G2a5 | 573.3C | 1 | 0.013
G2a5 | 573.4C | 1 | 0.013
G2a5 | 573.6C | 1 | 0.013
G2a5 | 9168 | 1 | 0.013
G2a5 | 998 | 1 | 0.013
G2a'c | 10609 | 6 | 0.01
G2a'c | 11084 | 1 | 0.002
G2a'c | 11836 | 2 | 0.003
G2a'c | 11963 | 1 | 0.002
G2a'c | 12771 | 2 | 0.003
G2a'c | 14126 | 1 | 0.002
G2a'c | 14683 | 6 | 0.01
G2a'c | 151 | 1 | 0.002
G2a'c | 152 | 12 | 0.02
G2a'c | 15262 | 4 | 0.007
G2a'c | 15313 | 1 | 0.002
G2a'c | 15670 | 6 | 0.01
G2a'c | 16093 | 6 | 0.01
G2a'c | 16179 | 1 | 0.002
G2a'c | 16192 | 1 | 0.002
G2a'c | 16214 | 3 | 0.005
G2a'c | 16224 | 1 | 0.002
G2a'c | 16291 | 1 | 0.002
G2a'c | 16293 | 1 | 0.002
G2a'c | 16311 | 14 | 0.023
G2a'c | 3397 | 8 | 0.013
G2a'c | 446 | 1 | 0.002
G2a'c | 4659 | 1 | 0.002
G2a'c | 5493 | 1 | 0.002
G2a'c | 5783 | 1 | 0.002
G2a'c | 6071 | 6 | 0.01
G2a'c | 6125 | 6 | 0.01
G2a'c | 6398 | 6 | 0.01
G2a'c | 6623 | 6 | 0.01
G2a'c | 8839 | 6 | 0.01
G2a'c | 8856 | 6 | 0.01
G2a'c | 9230 | 1 | 0.002
G2a'c | 9752 | 2 | 0.003
G2b | 115A | 1 | 0.002
G2b | 16032.1A | 1 | 0.002
G2b | 16044.1C | 1 | 0.002
G2b | 16045 | 1 | 0.002
G2b | 16047T | 1 | 0.002
G2b | 16048T | 2 | 0.003
G2b | 16051 | 1 | 0.002
G2b | 16095 | 1 | 0.002
G2b | 16108 | 1 | 0.002
G2b | 16111A | 1 | 0.002
G2b | 16124 | 2 | 0.003
G2b | 16126 | 2 | 0.003
G2b | 16136 | 1 | 0.002
G2b | 16179 | 2 | 0.003

G2b | 16234 | 1 | 0.002
G2b | 16240C | 1 | 0.002
G2b | 16241 | 1 | 0.002
G2b | 16242 | 1 | 0.002
G2b | 16244C | 2 | 0.003
G2b | 16246T | 2 | 0.003
G2b | 16250A | 1 | 0.002
G2b | 16293 | 2 | 0.003
G2b | 16293T | 1 | 0.002
G2b | 16295 | 2 | 0.003
G2b | 16318C | 1 | 0.002
G2b | 16326C | 1 | 0.002
G2b | 16355 | 1 | 0.002
G2b | 16356 | 2 | 0.003
G2b | 16357 | 1 | 0.002
G2b | 16396A | 1 | 0.002
G2b | 16434 | 1 | 0.002
G2b | 16443 | 1 | 0.002
G2b | 16447 | 1 | 0.002
G2b | 16452 | 4 | 0.006
G2b | 16507 | 1 | 0.002
G2b | 16522 | 1 | 0.002
G2b | 16525 | 1 | 0.002
G2b | 171 | 1 | 0.002
G2b | 200 | 7 | 0.011
G2b | 217 | 4 | 0.006
G2b | 249 | 1 | 0.002
G2b | 274T | 1 | 0.002
G2b | 290.1T | 1 | 0.002
G2b | 297 | 1 | 0.002
G2b | 298 | 1 | 0.002
G2b | 309.3C | 1 | 0.002
G2b | 315.2C | 2 | 0.003
G2b | 323.1G | 1 | 0.002
G2b | 324 | 1 | 0.002
G2b | 325d | 1 | 0.002
G2b | 337d | 1 | 0.002
G2b | 346 | 1 | 0.002
G2b | 384A | 1 | 0.002
G2b | 384T | 1 | 0.002
G2b | 401.1T | 1 | 0.002
G2b | 403.1T | 1 | 0.002
G2b | 408.1T | 1 | 0.002
G2b | 439 | 1 | 0.002
G2b | 446 | 2 | 0.003
G2b | 446T | 1 | 0.002
G2b | 468 | 1 | 0.002
G2b | 47 | 1 | 0.002
G2b | 472 | 1 | 0.002
G2b | 48 | 1 | 0.002
G2b | 488 | 1 | 0.002
G2b | 517 | 1 | 0.002
G2b | 521 | 2 | 0.003
G2b | 529 | 1 | 0.002
G2b | 530 | 1 | 0.002
G2b | 553 | 1 | 0.002
G2b | 574 | 1 | 0.002
G2b | 574d | 3 | 0.005
G2b | 72 | 1 | 0.002
G2b | 739 | 1 | 0.002
G2b | 75 | 1 | 0.002
G2b | 82 | 1 | 0.002
G2b | 97C | 1 | 0.002
G2b1 | 115A | 1 | 0.002
G2b1 | 12406 | 1 | 0.002
G2b1 | 13928C | 1 | 0.002
G2b1 | 14782 | 1 | 0.002
G2b1 | 15402 | 1 | 0.002
G2b1 | 16032.1A | 1 | 0.002
G2b1 | 16044.1C | 1 | 0.002
G2b1 | 16045 | 1 | 0.002
G2b1 | 16047T | 1 | 0.002
G2b1 | 16048T | 2 | 0.003
G2b1 | 16051 | 1 | 0.002
G2b1 | 16095 | 1 | 0.002
G2b1 | 16108 | 1 | 0.002
G2b1 | 16111A | 1 | 0.002
G2b1 | 16136 | 1 | 0.002
G2b1 | 16179 | 2 | 0.003
G2b1 | 16234 | 1 | 0.002
G2b1 | 16241 | 1 | 0.002
G2b1 | 16242 | 1 | 0.002
G2b1 | 16250A | 1 | 0.002
G2b1 | 16269 | 1 | 0.002
G2b1 | 16293 | 2 | 0.003
G2b1 | 16293T | 1 | 0.002
G2b1 | 16318C | 1 | 0.002
G2b1 | 16326C | 1 | 0.002
G2b1 | 16355 | 1 | 0.002
G2b1 | 16357 | 1 | 0.002
G2b1 | 16396A | 1 | 0.002
G2b1 | 16434 | 1 | 0.002
G2b1 | 16443 | 1 | 0.002
G2b1 | 16447 | 1 | 0.002
G2b1 | 16452 | 4 | 0.006
G2b1 | 16507 | 1 | 0.002
G2b1 | 16522 | 1 | 0.002
G2b1 | 16525 | 1 | 0.002
G2b1 | 171 | 1 | 0.002
G2b1 | 217 | 4 | 0.006
G2b1 | 249 | 1 | 0.002
G2b1 | 274T | 1 | 0.002
G2b1 | 290.1T | 1 | 0.002
G2b1 | 297 | 1 | 0.002
G2b1 | 298 | 1 | 0.002
G2b1 | 309.3C | 1 | 0.002
G2b1 | 323.1G | 1 | 0.002
G2b1 | 324 | 1 | 0.002
G2b1 | 325d | 1 | 0.002
G2b1 | 337d | 1 | 0.002
G2b1 | 346 | 1 | 0.002

G2b1 | 384A | 1 | 0.002
G2b1 | 384T | 1 | 0.002
G2b1 | 3970 | 1 | 0.002
G2b1 | 401.1T | 1 | 0.002
G2b1 | 403.1T | 1 | 0.002
G2b1 | 408.1T | 1 | 0.002
G2b1 | 439 | 1 | 0.002
G2b1 | 446 | 2 | 0.003
G2b1 | 446T | 1 | 0.002
G2b1 | 47 | 1 | 0.002
G2b1 | 472 | 1 | 0.002
G2b1 | 48 | 1 | 0.002
G2b1 | 488 | 1 | 0.002
G2b1 | 517 | 1 | 0.002
G2b1 | 529 | 1 | 0.002
G2b1 | 530 | 1 | 0.002
G2b1 | 553 | 1 | 0.002
G2b1 | 574 | 1 | 0.002
G2b1 | 574d | 3 | 0.005
G2b1 | 72 | 1 | 0.002
G2b1 | 75 | 1 | 0.002
G2b1 | 7738 | 1 | 0.002
G2b1 | 82 | 1 | 0.002
G2b1 | 97C | 1 | 0.002
G2b1a | 10978 | 1 | 0.002
G2b1a | 11353 | 1 | 0.002
G2b1a | 11778 | 1 | 0.002
G2b1a | 11887 | 1 | 0.002
G2b1a | 12236 | 1 | 0.002
G2b1a | 13975T | 2 | 0.003
G2b1a | 14924A | 1 | 0.002
G2b1a | 15433 | 1 | 0.002
G2b1a | 1555 | 1 | 0.002
G2b1a | 16179 | 1 | 0.002
G2b1a | 16265 | 11 | 0.018
G2b1a | 16293 | 1 | 0.002
G2b1a | 16319 | 1 | 0.002
G2b1a | 16326 | 1 | 0.002
G2b1a | 16463 | 6 | 0.01
G2b1a | 243 | 2 | 0.003
G2b1a | 4048 | 1 | 0.002
G2b1a | 446 | 1 | 0.002
G2b1a | 4562T | 2 | 0.003
G2b1a | 4599 | 5 | 0.008
G2b1a | 6112 | 1 | 0.002
G2b1a | 8573 | 5 | 0.008
G2b1a | 9003A | 1 | 0.002
G2b1a1 | 14167 | 1 | 0.002
G2b1a1 | 146 | 2 | 0.004
G2b1a1 | 15148 | 1 | 0.002
G2b1a1 | 152 | 1 | 0.002
G2b1a1 | 16108 | 1 | 0.002
G2b1a1 | 16111A | 1 | 0.002
G2b1a1 | 16239 | 4 | 0.009
G2b1a1 | 16250A | 1 | 0.002
G2b1a1 | 16284 | 2 | 0.004
G2b1a1 | 16295 | 2 | 0.004
G2b1a1 | 16297 | 2 | 0.004
G2b1a1 | 16316 | 1 | 0.002
G2b1a1 | 16318C | 1 | 0.002
G2b1a1 | 16355 | 1 | 0.002
G2b1a1 | 199 | 1 | 0.002
G2b1a1 | 204 | 3 | 0.007
G2b1a1 | 356.1C | 1 | 0.002
G2b1a1 | 408A | 2 | 0.004
G2b1a1 | 593 | 2 | 0.004
G2b1a2 | 12076 | 1 | 0.021
G2b1a2 | 14129 | 1 | 0.021
G2b1a2 | 146 | 1 | 0.021
G2b1a2 | 16093 | 1 | 0.021
G2b1a2 | 16193 | 2 | 0.042
G2b1a2 | 16209 | 2 | 0.042
G2b1a2 | 16555 | 1 | 0.021
G2b1a2 | 209 | 1 | 0.021
G2b1a2 | 2120 | 5 | 0.104
G2b1a2 | 263 | 6 | 0.125
G2b1a2 | 319 | 1 | 0.021
G2b1b | 15916 | 1 | 0.008
G2b1b | 16086 | 5 | 0.042
G2b1b | 16090 | 1 | 0.008
G2b1b | 16110 | 1 | 0.008
G2b1b | 16153 | 1 | 0.008
G2b1b | 16154 | 1 | 0.008
G2b1b | 16185 | 1 | 0.008
G2b1b | 16241 | 10 | 0.083
G2b1b | 16286 | 1 | 0.008
G2b1b | 16286G | 1 | 0.008
G2b1b | 16318C | 2 | 0.017
G2b1b | 16356 | 8 | 0.067
G2b1b | 16363 | 1 | 0.008
G2b1b | 16390 | 3 | 0.025
G2b1b | 195 | 1 | 0.008
G2b1b | 2361 | 1 | 0.008
G2b1b | 316C | 3 | 0.025
G2b1b | 460 | 1 | 0.008
G2b1b | 573.1C | 1 | 0.008
G2b1b | 64 | 1 | 0.008
G2b1b | 6422 | 3 | 0.025
G2b1b | 7852 | 1 | 0.008
G2b1b | 8896 | 2 | 0.017
G2b1b | 9585 | 3 | 0.025
G2b2 | 11002 | 1 | 0.002
G2b2 | 115A | 1 | 0.002
G2b2 | 11650 | 1 | 0.002
G2b2 | 11809 | 1 | 0.002
G2b2 | 11984 | 6 | 0.009
G2b2 | 13895 | 1 | 0.002
G2b2 | 13926 | 1 | 0.002
G2b2 | 14956 | 2 | 0.003
G2b2 | 152 | 2 | 0.003
G2b2 | 16032.1A | 1 | 0.002

G2b2 | 16044.1C | 1 | 0.002
G2b2 | 16045 | 1 | 0.002
G2b2 | 16047T | 1 | 0.002
G2b2 | 16048T | 2 | 0.003
G2b2 | 16051 | 1 | 0.002
G2b2 | 16093 | 1 | 0.002
G2b2 | 16095 | 1 | 0.002
G2b2 | 16108 | 1 | 0.002
G2b2 | 16111A | 1 | 0.002
G2b2 | 16136 | 1 | 0.002
G2b2 | 16179 | 2 | 0.003
G2b2 | 16184 | 2 | 0.003
G2b2 | 16213 | 1 | 0.002
G2b2 | 16234 | 1 | 0.002
G2b2 | 16241 | 1 | 0.002
G2b2 | 16242 | 1 | 0.002
G2b2 | 16250A | 1 | 0.002
G2b2 | 16293 | 2 | 0.003
G2b2 | 16293T | 1 | 0.002
G2b2 | 16300 | 2 | 0.003
G2b2 | 16318C | 1 | 0.002
G2b2 | 16326C | 1 | 0.002
G2b2 | 16355 | 1 | 0.002
G2b2 | 16357 | 1 | 0.002
G2b2 | 16396A | 1 | 0.002
G2b2 | 16434 | 1 | 0.002
G2b2 | 16443 | 1 | 0.002
G2b2 | 16447 | 1 | 0.002
G2b2 | 16452 | 4 | 0.006
G2b2 | 16507 | 1 | 0.002
G2b2 | 16522 | 1 | 0.002
G2b2 | 16525 | 1 | 0.002
G2b2 | 1709 | 2 | 0.003
G2b2 | 171 | 1 | 0.002
G2b2 | 217 | 4 | 0.006
G2b2 | 249 | 1 | 0.002
G2b2 | 257 | 1 | 0.002
G2b2 | 274T | 1 | 0.002
G2b2 | 290.1T | 1 | 0.002
G2b2 | 297 | 1 | 0.002
G2b2 | 298 | 1 | 0.002
G2b2 | 309.3C | 1 | 0.002
G2b2 | 323.1G | 1 | 0.002
G2b2 | 324 | 1 | 0.002
G2b2 | 325d | 1 | 0.002
G2b2 | 337d | 1 | 0.002
G2b2 | 346 | 1 | 0.002
G2b2 | 3593 | 1 | 0.002
G2b2 | 384A | 1 | 0.002
G2b2 | 384T | 1 | 0.002
G2b2 | 401.1T | 1 | 0.002
G2b2 | 403.1T | 1 | 0.002
G2b2 | 408.1T | 1 | 0.002
G2b2 | 439 | 1 | 0.002
G2b2 | 446 | 2 | 0.003
G2b2 | 446T | 1 | 0.002
G2b2 | 47 | 1 | 0.002
G2b2 | 4703 | 1 | 0.002
G2b2 | 472 | 1 | 0.002
G2b2 | 48 | 1 | 0.002
G2b2 | 488 | 1 | 0.002
G2b2 | 517 | 1 | 0.002
G2b2 | 529 | 1 | 0.002
G2b2 | 530 | 1 | 0.002
G2b2 | 5460 | 1 | 0.002
G2b2 | 553 | 1 | 0.002
G2b2 | 5641 | 6 | 0.009
G2b2 | 574 | 1 | 0.002
G2b2 | 574d | 3 | 0.005
G2b2 | 6647T | 2 | 0.003
G2b2 | 72 | 1 | 0.002
G2b2 | 75 | 1 | 0.002
G2b2 | 7705 | 1 | 0.002
G2b2 | 82 | 1 | 0.002
G2b2 | 8467 | 1 | 0.002
G2b2 | 8896 | 1 | 0.002
G2b2 | 97C | 1 | 0.002
G2b2a | 115A | 1 | 0.002
G2b2a | 1198 | 1 | 0.002
G2b2a | 12130 | 1 | 0.002
G2b2a | 12358 | 1 | 0.002
G2b2a | 12912 | 1 | 0.002
G2b2a | 16032.1A | 1 | 0.002
G2b2a | 16044.1C | 1 | 0.002
G2b2a | 16045 | 1 | 0.002
G2b2a | 16047T | 1 | 0.002
G2b2a | 16048T | 2 | 0.003
G2b2a | 16051 | 1 | 0.002
G2b2a | 16095 | 1 | 0.002
G2b2a | 16108 | 1 | 0.002
G2b2a | 16111A | 1 | 0.002
G2b2a | 16136 | 1 | 0.002
G2b2a | 16179 | 2 | 0.003
G2b2a | 16234 | 1 | 0.002
G2b2a | 16241 | 1 | 0.002
G2b2a | 16242 | 1 | 0.002
G2b2a | 16250A | 1 | 0.002
G2b2a | 16293 | 2 | 0.003
G2b2a | 16293T | 1 | 0.002
G2b2a | 16294 | 1 | 0.002
G2b2a | 16318C | 1 | 0.002
G2b2a | 16326C | 1 | 0.002
G2b2a | 16355 | 1 | 0.002
G2b2a | 16357 | 1 | 0.002
G2b2a | 16396A | 1 | 0.002
G2b2a | 16434 | 1 | 0.002
G2b2a | 16443 | 1 | 0.002
G2b2a | 16447 | 1 | 0.002
G2b2a | 16452 | 4 | 0.006
G2b2a | 16507 | 1 | 0.002
G2b2a | 16522 | 1 | 0.002

G2b2a | 16525 | 1 | 0.002
G2b2a | 171 | 1 | 0.002
G2b2a | 183 | 1 | 0.002
G2b2a | 195 | 1 | 0.002
G2b2a | 217 | 4 | 0.006
G2b2a | 249 | 1 | 0.002
G2b2a | 274T | 1 | 0.002
G2b2a | 290.1T | 1 | 0.002
G2b2a | 297 | 1 | 0.002
G2b2a | 298 | 1 | 0.002
G2b2a | 309.3C | 1 | 0.002
G2b2a | 323.1G | 1 | 0.002
G2b2a | 324 | 1 | 0.002
G2b2a | 325d | 1 | 0.002
G2b2a | 337d | 1 | 0.002
G2b2a | 346 | 1 | 0.002
G2b2a | 384A | 1 | 0.002
G2b2a | 384T | 1 | 0.002
G2b2a | 401.1T | 1 | 0.002
G2b2a | 403.1T | 1 | 0.002
G2b2a | 408.1T | 1 | 0.002
G2b2a | 4216 | 1 | 0.002
G2b2a | 439 | 1 | 0.002
G2b2a | 446 | 2 | 0.003
G2b2a | 446T | 1 | 0.002
G2b2a | 47 | 1 | 0.002
G2b2a | 472 | 1 | 0.002
G2b2a | 48 | 1 | 0.002
G2b2a | 488 | 1 | 0.002
G2b2a | 517 | 1 | 0.002
G2b2a | 529 | 1 | 0.002
G2b2a | 530 | 1 | 0.002
G2b2a | 553 | 1 | 0.002
G2b2a | 574 | 1 | 0.002
G2b2a | 574d | 3 | 0.005
G2b2a | 6116 | 1 | 0.002
G2b2a | 72 | 1 | 0.002
G2b2a | 75 | 1 | 0.002
G2b2a | 82 | 1 | 0.002
G2b2a | 9305 | 1 | 0.002
G2b2a | 97C | 1 | 0.002
G2b2b | 12678 | 1 | 0.011
G2b2b | 12892 | 1 | 0.011
G2b2b | 13434 | 1 | 0.011
G2b2b | 13590 | 1 | 0.011
G2b2b | 13708 | 1 | 0.011
G2b2b | 143 | 2 | 0.022
G2b2b | 14484 | 1 | 0.011
G2b2b | 146 | 2 | 0.022
G2b2b | 15034 | 1 | 0.011
G2b2b | 15346 | 1 | 0.011
G2b2b | 15525 | 1 | 0.011
G2b2b | 15852 | 1 | 0.011
G2b2b | 16086 | 1 | 0.011
G2b2b | 16093 | 1 | 0.011
G2b2b | 16189 | 1 | 0.011
G2b2b | 16192A | 2 | 0.022
G2b2b | 16193 | 2 | 0.022
G2b2b | 16234 | 1 | 0.011
G2b2b | 16286 | 1 | 0.011
G2b2b | 16291 | 3 | 0.034
G2b2b | 16292 | 1 | 0.011
G2b2b | 16390 | 1 | 0.011
G2b2b | 16527 | 1 | 0.011
G2b2b | 195 | 2 | 0.022
G2b2b | 204 | 2 | 0.022
G2b2b | 315.2C | 1 | 0.011
G2b2b | 4388 | 1 | 0.011
G2b2b | 9168 | 1 | 0.011
G2b2c | 148 | 1 | 0.014
G2b2c | 16086 | 2 | 0.027
G2b2c | 16129 | 1 | 0.014
G2b2c | 16209 | 2 | 0.027
G2b2c | 314 | 1 | 0.014
G2b2c | 488 | 1 | 0.014
G2b2c | 513 | 1 | 0.014
G2b2c | 9110 | 2 | 0.027
G2c | 10335 | 2 | 0.004
G2c | 12438 | 1 | 0.002
G2c | 13928 | 2 | 0.004
G2c | 150 | 1 | 0.002
G2c | 15508 | 1 | 0.002
G2c | 16086 | 1 | 0.002
G2c | 16108 | 1 | 0.002
G2c | 16111A | 1 | 0.002
G2c | 16133 | 1 | 0.002
G2c | 16189 | 1 | 0.002
G2c | 16242 | 1 | 0.002
G2c | 16250A | 1 | 0.002
G2c | 16311 | 1 | 0.002
G2c | 16318 | 1 | 0.002
G2c | 16318C | 1 | 0.002
G2c | 16325 | 2 | 0.004
G2c | 16381 | 6 | 0.013
G2c | 1736 | 1 | 0.002
G2c | 198 | 1 | 0.002
G2c | 207 | 2 | 0.004
G2c | 482 | 1 | 0.002
G2c | 5351 | 1 | 0.002
G2c | 5474 | 1 | 0.002
G2c | 5498 | 1 | 0.002
G2c | 62.1G | 1 | 0.002
G2c | 663 | 1 | 0.002
G2c | 6647 | 1 | 0.002
G2c | 735 | 1 | 0.002
G2c | 8626 | 6 | 0.013
G3 | 16229 | 2 | 0.014
G3 | 16234 | 1 | 0.007
G3 | 461 | 1 | 0.007
G3 | 513 | 1 | 0.007
G3 | 549 | 1 | 0.007

G3a | 16229 | 2 | 0.014
G3a | 16234 | 1 | 0.007
G3a | 461 | 1 | 0.007
G3a | 513 | 1 | 0.007
G3a | 549 | 1 | 0.007
G3a1 | 13326 | 1 | 0.009
G3a1 | 16075 | 5 | 0.044
G3a1 | 16086 | 1 | 0.009
G3a1 | 16093 | 1 | 0.009
G3a1 | 16153 | 3 | 0.027
G3a1 | 16173 | 2 | 0.018
G3a1 | 16183 | 1 | 0.009
G3a1 | 16214 | 1 | 0.009
G3a1 | 16225 | 35 | 0.31
G3a1 | 16278 | 3 | 0.027
G3a1 | 16294 | 1 | 0.009
G3a1 | 16298 | 4 | 0.035
G3a1 | 16311 | 5 | 0.044
G3a1 | 16356 | 1 | 0.009
G3a1 | 316C | 1 | 0.009
G3a1'2 | 10248 | 2 | 0.017
G3a1'2 | 12661 | 2 | 0.017
G3a1'2 | 13681 | 1 | 0.009
G3a1'2 | 15106 | 1 | 0.009
G3a1'2 | 15244 | 1 | 0.009
G3a1'2 | 15996 | 1 | 0.009
G3a1'2 | 16019A | 1 | 0.009
G3a1'2 | 16021 | 1 | 0.009
G3a1'2 | 16039C | 1 | 0.009
G3a1'2 | 16042 | 1 | 0.009
G3a1'2 | 16081 | 3 | 0.026
G3a1'2 | 16092A | 14 | 0.12
G3a1'2 | 16093 | 2 | 0.017
G3a1'2 | 16095 | 1 | 0.009
G3a1'2 | 16095G | 1 | 0.009
G3a1'2 | 16103T | 1 | 0.009
G3a1'2 | 16104 | 1 | 0.009
G3a1'2 | 16107A | 1 | 0.009
G3a1'2 | 16112 | 1 | 0.009
G3a1'2 | 16129 | 16 | 0.137
G3a1'2 | 16147 | 1 | 0.009
G3a1'2 | 16150 | 1 | 0.009
G3a1'2 | 16172 | 3 | 0.026
G3a1'2 | 16177 | 2 | 0.017
G3a1'2 | 16209 | 3 | 0.026
G3a1'2 | 16261 | 16 | 0.137
G3a1'2 | 16263 | 1 | 0.009
G3a1'2 | 16265 | 2 | 0.017
G3a1'2 | 16292 | 2 | 0.017
G3a1'2 | 16295 | 3 | 0.026
G3a1'2 | 16311 | 34 | 0.291
G3a1'2 | 16342G | 1 | 0.009
G3a1'2 | 16364 | 1 | 0.009
G3a1'2 | 16365 | 1 | 0.009
G3a1'2 | 16370C | 1 | 0.009
G3a1'2 | 16374T | 1 | 0.009
G3a1'2 | 16375A | 1 | 0.009
G3a1'2 | 16376A | 1 | 0.009
G3a1'2 | 195 | 3 | 0.026
G3a1'2 | 198 | 2 | 0.017
G3a1'2 | 227 | 2 | 0.017
G3a1'2 | 234 | 1 | 0.009
G3a1'2 | 4113 | 2 | 0.017
G3a1'2 | 7382 | 1 | 0.009
G3a1'2 | 84 | 1 | 0.009
G3a1'2 | 9 | 2 | 0.017
G3a1a | 146 | 1 | 0.009
G3a1a | 153 | 1 | 0.009
G3a1a | 16093 | 1 | 0.009
G3a1a | 16148 | 23 | 0.205
G3a1a | 16153 | 23 | 0.205
G3a1a | 16176 | 3 | 0.027
G3a1a | 16183 | 1 | 0.009
G3a1a | 16214 | 1 | 0.009
G3a1a | 16260 | 1 | 0.009
G3a1a | 16294 | 1 | 0.009
G3a1a | 16356 | 1 | 0.009
G3a1a | 16390 | 1 | 0.009
G3a1a | 16399 | 17 | 0.152
G3a1a | 16512 | 1 | 0.009
G3a1a | 182 | 2 | 0.018
G3a1a | 195 | 3 | 0.027
G3a1a | 310 | 1 | 0.009
G3a1a | 316C | 11 | 0.098
G3a1a | 8887 | 1 | 0.009
G3a2 | 15104 | 1 | 0.01
G3a2 | 16076d | 1 | 0.01
G3a2 | 16085G | 1 | 0.01
G3a2 | 16086 | 2 | 0.021
G3a2 | 16124 | 1 | 0.01
G3a2 | 16172 | 4 | 0.041
G3a2 | 16184 | 1 | 0.01
G3a2 | 16242 | 1 | 0.01
G3a2 | 16259 | 1 | 0.01
G3a2 | 16262.1C | 1 | 0.01
G3a2 | 16266 | 1 | 0.01
G3a2 | 16305 | 1 | 0.01
G3a2 | 16309 | 1 | 0.01
G3a2 | 16325 | 2 | 0.021
G3a2 | 16354 | 7 | 0.072
G3a2 | 16356 | 1 | 0.01
G3a2 | 16357 | 2 | 0.021
G3a2 | 16360 | 2 | 0.021
G3a2 | 16380 | 1 | 0.01
G3a2 | 234 | 3 | 0.031
G3a2 | 484T | 1 | 0.01
G3a2 | 675 | 1 | 0.01
G3a2 | 732.1A | 1 | 0.01
G3a2+152 | 12842 | 2 | 0.02
G3a2+152 | 13209 | 4 | 0.04
G3a2+152 | 13350 | 4 | 0.04

G3a2+152 | 13718C | 2 | 0.02
G3a2+152 | 14110 | 2 | 0.02
G3a2+152 | 146 | 2 | 0.02
G3a2+152 | 153 | 1 | 0.01
G3a2+152 | 16092 | 2 | 0.02
G3a2+152 | 16093 | 2 | 0.02
G3a2+152 | 16186 | 10 | 0.099
G3a2+152 | 16189 | 2 | 0.02
G3a2+152 | 16201 | 4 | 0.04
G3a2+152 | 16240 | 2 | 0.02
G3a2+152 | 16291 | 2 | 0.02
G3a2+152 | 16311 | 1 | 0.01
G3a2+152 | 16311G | 2 | 0.02
G3a2+152 | 16319 | 2 | 0.02
G3a2+152 | 16335 | 1 | 0.01
G3a2+152 | 195 | 6 | 0.059
G3a2+152 | 204 | 1 | 0.01
G3a2+152 | 226 | 1 | 0.01
G3a2+152 | 227 | 1 | 0.01
G3a2+152 | 573.1C | 1 | 0.01
G3a2+152 | 9054 | 4 | 0.04
G3a2a | 16093 | 1 | 0.062
G3a2a | 16126 | 1 | 0.062
G3a2a | 16176 | 1 | 0.062
G3a2a | 16184A | 1 | 0.062
G3a2a | 16214 | 1 | 0.062
G3a2a | 195 | 1 | 0.062
G3a2a | 199 | 1 | 0.062
G3a2a | 574C | 6 | 0.375
G3a2a | 576C | 6 | 0.375
G3a2a | 577C | 1 | 0.062
G3a3 | 10492 | 1 | 0.011
G3a3 | 11050 | 3 | 0.033
G3a3 | 14218 | 3 | 0.033
G3a3 | 16093 | 1 | 0.011
G3a3 | 16156 | 7 | 0.078
G3a3 | 16174 | 1 | 0.011
G3a3 | 16189 | 1 | 0.011
G3a3 | 16209 | 2 | 0.022
G3a3 | 16299 | 1 | 0.011
G3a3 | 16320 | 1 | 0.011
G3a3 | 16381 | 1 | 0.011
G3a3 | 214 | 1 | 0.011
G3a3 | 2263A | 1 | 0.011
G3a3 | 390 | 4 | 0.044
G3a3 | 5041 | 3 | 0.033
G3a3 | 574 | 1 | 0.011
G3a3 | 574C | 2 | 0.022
G3a3 | 576C | 2 | 0.022
G3a3 | 8680 | 1 | 0.011
G3b | 152 | 7 | 0.037
G3b | 16092 | 1 | 0.005
G3b | 16093 | 2 | 0.011
G3b | 16172 | 3 | 0.016
G3b | 16180 | 1 | 0.005
G3b | 16180C | 1 | 0.005
G3b | 16192 | 23 | 0.122
G3b | 16209 | 24 | 0.127
G3b | 16249 | 1 | 0.005
G3b | 16261 | 1 | 0.005
G3b | 16278 | 2 | 0.011
G3b | 16294 | 1 | 0.005
G3b | 16311 | 8 | 0.042
G3b | 16365 | 1 | 0.005
G3b | 16381 | 1 | 0.005
G3b | 16503-16504d | 1 | 0.005
G3b | 16507-16511d | 1 | 0.005
G3b | 16515-16518d | 1 | 0.005
G3b | 189 | 3 | 0.016
G3b | 1d | 1 | 0.005
G3b | 204 | 1 | 0.005
G3b | 3394 | 3 | 0.016
G3b | 4113 | 2 | 0.011
G3b | 63d | 1 | 0.005
G3b | 66T | 1 | 0.005
G3b | 67 | 1 | 0.005
G3b | 9966 | 2 | 0.011
G3b1 | 10427 | 1 | 0.008
G3b1 | 12121 | 1 | 0.008
G3b1 | 13194 | 1 | 0.008
G3b1 | 13281 | 1 | 0.008
G3b1 | 137 | 1 | 0.008
G3b1 | 142 | 1 | 0.008
G3b1 | 143 | 1 | 0.008
G3b1 | 150 | 1 | 0.008
G3b1 | 15236 | 1 | 0.008
G3b1 | 16086 | 1 | 0.008
G3b1 | 16093 | 30 | 0.236
G3b1 | 16104 | 9 | 0.071
G3b1 | 16129 | 2 | 0.016
G3b1 | 16153 | 2 | 0.016
G3b1 | 16168 | 2 | 0.016
G3b1 | 16184A | 1 | 0.008
G3b1 | 16233 | 1 | 0.008
G3b1 | 16249 | 1 | 0.008
G3b1 | 16259 | 1 | 0.008
G3b1 | 16260 | 5 | 0.039
G3b1 | 16286 | 1 | 0.008
G3b1 | 16291 | 2 | 0.016
G3b1 | 16293 | 1 | 0.008
G3b1 | 16297 | 1 | 0.008
G3b1 | 16319 | 7 | 0.055
G3b1 | 16320 | 1 | 0.008
G3b1 | 16527 | 2 | 0.016
G3b1 | 198 | 2 | 0.016
G3b1 | 279 | 4 | 0.031
G3b1 | 316C | 3 | 0.024
G3b1 | 337 | 1 | 0.008
G3b1 | 3546 | 1 | 0.008
G3b1 | 4883 | 1 | 0.008
G3b1 | 5063 | 1 | 0.008

G3b1 | 573.1C | 1 | 0.008
G3b1 | 64 | 1 | 0.008
G3b1 | 7076 | 1 | 0.008
G3b2 | 146 | 6 | 0.039
G3b2 | 15758 | 1 | 0.007
G3b2 | 16093 | 5 | 0.033
G3b2 | 16150 | 1 | 0.007
G3b2 | 16166 | 8 | 0.052
G3b2 | 16209 | 2 | 0.013
G3b2 | 16294 | 1 | 0.007
G3b2 | 16309 | 2 | 0.013
G3b2 | 1658 | 1 | 0.007
G3b2 | 437 | 1 | 0.007
G3b2 | 5460 | 2 | 0.013
G3b2 | 6515G | 1 | 0.007
G4 | 12684 | 2 | 0.067
G4 | 1393 | 1 | 0.033
G4 | 1413 | 2 | 0.067
G4 | 16051 | 1 | 0.033
G4 | 16145 | 2 | 0.067
G4 | 16189 | 2 | 0.067
G4 | 16213 | 1 | 0.033
G4 | 16215 | 3 | 0.1
G4 | 16243 | 6 | 0.2
G4 | 16261 | 1 | 0.033
G4 | 16395 | 1 | 0.033
G4 | 198 | 1 | 0.033
G4 | 203 | 4 | 0.133
G4 | 204 | 9 | 0.3
G4 | 2109T | 2 | 0.067
G4 | 3434 | 2 | 0.067
G4 | 3483 | 2 | 0.067
G4 | 4541 | 2 | 0.067
G4 | 456 | 1 | 0.033
G4 | 485 | 1 | 0.033
G4 | 9011 | 2 | 0.067
H | 10007 | 1 | 0.001
H | 1007 | 1 | 0.001
H | 10227 | 1 | 0.001
H | 10238 | 1 | 0.001
H | 10248 | 2 | 0.002
H | 1038 | 1 | 0.001
H | 10398 | 1 | 0.001
H | 10410 | 1 | 0.001
H | 10410A | 1 | 0.001
H | 10454 | 1 | 0.001
H | 1053 | 1 | 0.001
H | 10532 | 2 | 0.002
H | 10550 | 1 | 0.001
H | 10586 | 1 | 0.001
H | 10610 | 1 | 0.001
H | 10685 | 1 | 0.001
H | 10697 | 1 | 0.001
H | 10855 | 1 | 0.001
H | 10999 | 1 | 0.001
H | 11084 | 1 | 0.001
H | 11085-11086d | 1 | 0.001
H | 111 | 1 | 0.001
H | 11113 | 1 | 0.001
H | 11134 | 1 | 0.001
H | 11167 | 2 | 0.002
H | 11172 | 1 | 0.001
H | 11176 | 1 | 0.001
H | 11209T | 2 | 0.002
H | 113 | 1 | 0.001
H | 11359 | 1 | 0.001
H | 11362 | 1 | 0.001
H | 11461 | 1 | 0.001
H | 11465 | 1 | 0.001
H | 11506 | 1 | 0.001
H | 11509 | 1 | 0.001
H | 11516 | 1 | 0.001
H | 11536 | 2 | 0.002
H | 11563G | 1 | 0.001
H | 11587 | 1 | 0.001
H | 11635 | 1 | 0.001
H | 11665 | 4 | 0.004
H | 11719 | 1 | 0.001
H | 11770 | 1 | 0.001
H | 11778 | 1 | 0.001
H | 11807 | 1 | 0.001
H | 11899 | 1 | 0.001
H | 11914 | 4 | 0.004
H | 11962 | 1 | 0.001
H | 11963 | 1 | 0.001
H | 12127 | 1 | 0.001
H | 12172 | 1 | 0.001
H | 12235 | 1 | 0.001
H | 12236 | 1 | 0.001
H | 12285 | 1 | 0.001
H | 12346 | 1 | 0.001
H | 12362 | 1 | 0.001
H | 12369A | 1 | 0.001
H | 12373 | 1 | 0.001
H | 12501 | 6 | 0.006
H | 12528 | 1 | 0.001
H | 12557 | 2 | 0.002
H | 12599 | 2 | 0.002
H | 12630 | 1 | 0.001
H | 12662 | 8 | 0.007
H | 12705 | 1 | 0.001
H | 12712 | 2 | 0.002
H | 12727 | 1 | 0.001
H | 12730 | 1 | 0.001
H | 12732 | 2 | 0.002
H | 12741 | 1 | 0.001
H | 12777 | 3 | 0.003
H | 12804 | 1 | 0.001
H | 12822 | 1 | 0.001
H | 12843 | 2 | 0.002
H | 12894 | 1 | 0.001

H | 1290 | 1 | 0.001
H | 12900 | 3 | 0.003
H | 12981 | 2 | 0.002
H | 13056 | 1 | 0.001
H | 13077A | 1 | 0.001
H | 131 | 2 | 0.002
H | 13105 | 3 | 0.003
H | 13191 | 1 | 0.001
H | 13401 | 1 | 0.001
H | 13431 | 3 | 0.003
H | 13477 | 1 | 0.001
H | 13488 | 2 | 0.002
H | 13590 | 1 | 0.001
H | 13638 | 1 | 0.001
H | 13641 | 1 | 0.001
H | 13651 | 4 | 0.004
H | 13677 | 1 | 0.001
H | 13707 | 1 | 0.001
H | 13748 | 4 | 0.004
H | 13759 | 5 | 0.005
H | 13819 | 1 | 0.001
H | 13830 | 1 | 0.001
H | 13884 | 1 | 0.001
H | 13885 | 1 | 0.001
H | 13889 | 1 | 0.001
H | 13928 | 1 | 0.001
H | 13943 | 1 | 0.001
H | 13956 | 1 | 0.001
H | 13983G | 1 | 0.001
H | 14016 | 2 | 0.002
H | 14180 | 1 | 0.001
H | 14180G | 1 | 0.001
H | 14215 | 1 | 0.001
H | 14218 | 1 | 0.001
H | 14235G | 1 | 0.001
H | 14249 | 1 | 0.001
H | 14281 | 1 | 0.001
H | 14299 | 2 | 0.002
H | 143 | 3 | 0.003
H | 14339 | 2 | 0.002
H | 14388 | 2 | 0.002
H | 14514 | 2 | 0.002
H | 1452 | 6 | 0.006
H | 14560T | 2 | 0.002
H | 146 | 12 | 0.011
H | 14605 | 1 | 0.001
H | 14693 | 1 | 0.001
H | 14766 | 1 | 0.001
H | 14797 | 1 | 0.001
H | 14851 | 1 | 0.001
H | 14869 | 8 | 0.007
H | 14870 | 1 | 0.001
H | 14956 | 1 | 0.001
H | 14968 | 1 | 0.001
H | 14971 | 1 | 0.001
H | 150 | 16 | 0.015
H | 15031 | 1 | 0.001
H | 15037 | 3 | 0.003
H | 151 | 12 | 0.011
H | 15106 | 6 | 0.006
H | 15109 | 1 | 0.001
H | 15173 | 1 | 0.001
H | 1520 | 1 | 0.001
H | 15217 | 2 | 0.002
H | 15244 | 1 | 0.001
H | 15266 | 1 | 0.001
H | 15289 | 1 | 0.001
H | 1530 | 1 | 0.001
H | 15313 | 2 | 0.002
H | 15319 | 1 | 0.001
H | 15323 | 1 | 0.001
H | 15386 | 1 | 0.001
H | 15439 | 6 | 0.006
H | 15466 | 1 | 0.001
H | 15497 | 3 | 0.003
H | 15514 | 1 | 0.001
H | 1555 | 1 | 0.001
H | 15565 | 5 | 0.005
H | 15586 | 1 | 0.001
H | 15620 | 2 | 0.002
H | 15670 | 1 | 0.001
H | 15679 | 1 | 0.001
H | 15724 | 1 | 0.001
H | 15751T | 1 | 0.001
H | 1578 | 1 | 0.001
H | 15805 | 1 | 0.001
H | 15850 | 1 | 0.001
H | 15884 | 1 | 0.001
H | 15885 | 2 | 0.002
H | 15908 | 1 | 0.001
H | 15930 | 1 | 0.001
H | 16051 | 7 | 0.007
H | 16075 | 2 | 0.002
H | 16076A | 1 | 0.001
H | 16093 | 12 | 0.011
H | 16104A | 1 | 0.001
H | 16111 | 3 | 0.003
H | 16126 | 5 | 0.005
H | 16136 | 1 | 0.001
H | 16145 | 1 | 0.001
H | 16157 | 1 | 0.001
H | 16162 | 5 | 0.005
H | 16167 | 1 | 0.001
H | 16168 | 3 | 0.003
H | 16169 | 1 | 0.001
H | 16169G | 1 | 0.001
H | 16172 | 9 | 0.008
H | 16174 | 2 | 0.002
H | 16176 | 3 | 0.003
H | 16179 | 2 | 0.002
H | 16186 | 1 | 0.001

H | 16188 | 4 | 0.004
H | 16189 | 7 | 0.007
H | 16192 | 12 | 0.011
H | 16209 | 1 | 0.001
H | 16213 | 1 | 0.001
H | 16214 | 1 | 0.001
H | 16217 | 1 | 0.001
H | 16219 | 6 | 0.006
H | 16221 | 2 | 0.002
H | 16222 | 4 | 0.004
H | 16223 | 6 | 0.006
H | 16224 | 1 | 0.001
H | 16234 | 4 | 0.004
H | 16235 | 1 | 0.001
H | 16241 | 1 | 0.001
H | 16243 | 1 | 0.001
H | 16249 | 1 | 0.001
H | 16256 | 1 | 0.001
H | 16261 | 5 | 0.005
H | 16263 | 1 | 0.001
H | 16266 | 2 | 0.002
H | 16269 | 8 | 0.007
H | 16271 | 1 | 0.001
H | 16272 | 1 | 0.001
H | 16274 | 4 | 0.004
H | 16288 | 2 | 0.002
H | 16289 | 9 | 0.008
H | 16292 | 2 | 0.002
H | 16293C | 1 | 0.001
H | 16294 | 7 | 0.007
H | 16295 | 1 | 0.001
H | 16298 | 3 | 0.003
H | 16300 | 2 | 0.002
H | 16301 | 1 | 0.001
H | 16304 | 1 | 0.001
H | 16311 | 25 | 0.023
H | 16317 | 3 | 0.003
H | 16319 | 6 | 0.006
H | 16324 | 3 | 0.003
H | 16325 | 1 | 0.001
H | 16335 | 1 | 0.001
H | 16352 | 1 | 0.001
H | 16354 | 5 | 0.005
H | 16355 | 1 | 0.001
H | 16362 | 131 | 0.122
H | 16368 | 1 | 0.001
H | 16390 | 1 | 0.001
H | 16399 | 3 | 0.003
H | 16422 | 2 | 0.002
H | 16438 | 2 | 0.002
H | 16465 | 1 | 0.001
H | 16524C | 8 | 0.007
H | 16527 | 2 | 0.002
H | 168 | 1 | 0.001
H | 1692 | 2 | 0.002
H | 1716 | 2 | 0.002
H | 1717 | 1 | 0.001
H | 1719 | 1 | 0.001
H | 1809 | 1 | 0.001
H | 1810 | 1 | 0.001
H | 1811 | 1 | 0.001
H | 185 | 1 | 0.001
H | 188 | 1 | 0.001
H | 189 | 1 | 0.001
H | 191.1A | 1 | 0.001
H | 195 | 1 | 0.001
H | 1978C | 1 | 0.001
H | 198 | 1 | 0.001
H | 199 | 1 | 0.001
H | 200 | 17 | 0.016
H | 204 | 1 | 0.001
H | 2065 | 1 | 0.001
H | 208 | 1 | 0.001
H | 2158 | 1 | 0.001
H | 2222 | 2 | 0.002
H | 225 | 1 | 0.001
H | 226 | 1 | 0.001
H | 227 | 1 | 0.001
H | 228 | 2 | 0.002
H | 235 | 3 | 0.003
H | 236 | 1 | 0.001
H | 2360 | 1 | 0.001
H | 237 | 1 | 0.001
H | 238 | 1 | 0.001
H | 259 | 1 | 0.001
H | 261 | 1 | 0.001
H | 2648 | 1 | 0.001
H | 270 | 1 | 0.001
H | 2746 | 1 | 0.001
H | 2772 | 1 | 0.001
H | 281C | 1 | 0.001
H | 2885 | 1 | 0.001
H | 291d | 4 | 0.004
H | 292 | 1 | 0.001
H | 292.1AT | 2 | 0.002
H | 309d | 2 | 0.002
H | 310 | 22 | 0.02
H | 312 | 2 | 0.002
H | 315.2C | 6 | 0.006
H | 315.3C | 2 | 0.002
H | 315d | 1 | 0.001
H | 316d | 1 | 0.001
H | 3172 | 1 | 0.001
H | 319 | 3 | 0.003
H | 3203 | 1 | 0.001
H | 3221 | 4 | 0.004
H | 3254 | 1 | 0.001
H | 3290 | 4 | 0.004
H | 3338 | 1 | 0.001
H | 338 | 1 | 0.001
H | 3456 | 1 | 0.001

H | 3460 | 2 | 0.002
H | 3505 | 6 | 0.006
H | 3507 | 1 | 0.001
H | 3531 | 3 | 0.003
H | 3537 | 1 | 0.001
H | 3588A | 1 | 0.001
H | 3621 | 2 | 0.002
H | 3627 | 1 | 0.001
H | 3666 | 1 | 0.001
H | 368 | 1 | 0.001
H | 374 | 9 | 0.008
H | 3769 | 2 | 0.002
H | 3786 | 2 | 0.002
H | 3798 | 2 | 0.002
H | 3826 | 2 | 0.002
H | 385 | 2 | 0.002
H | 3870 | 1 | 0.001
H | 3924 | 1 | 0.001
H | 4012 | 2 | 0.002
H | 4080 | 1 | 0.001
H | 4086 | 1 | 0.001
H | 4117 | 1 | 0.001
H | 4135 | 1 | 0.001
H | 417 | 1 | 0.001
H | 4215 | 1 | 0.001
H | 4216 | 2 | 0.002
H | 4277 | 2 | 0.002
H | 4317d | 1 | 0.001
H | 4491 | 2 | 0.002
H | 4502 | 7 | 0.007
H | 4505 | 1 | 0.001
H | 451 | 1 | 0.001
H | 460.1T | 1 | 0.001
H | 4612 | 1 | 0.001
H | 4639 | 1 | 0.001
H | 4659 | 2 | 0.002
H | 4679 | 1 | 0.001
H | 4703 | 1 | 0.001
H | 4715 | 1 | 0.001
H | 4859 | 1 | 0.001
H | 4883 | 2 | 0.002
H | 491 | 1 | 0.001
H | 4919 | 2 | 0.002
H | 499 | 1 | 0.001
H | 5019 | 5 | 0.005
H | 502 | 1 | 0.001
H | 5081 | 1 | 0.001
H | 5120 | 1 | 0.001
H | 5147 | 2 | 0.002
H | 5162 | 1 | 0.001
H | 5201 | 1 | 0.001
H | 5216 | 1 | 0.001
H | 5231 | 1 | 0.001
H | 5277 | 2 | 0.002
H | 5420G | 1 | 0.001
H | 5456 | 1 | 0.001
H | 5460 | 5 | 0.005
H | 5461 | 1 | 0.001
H | 5483 | 1 | 0.001
H | 5501 | 3 | 0.003
H | 5516 | 1 | 0.001
H | 5553 | 1 | 0.001
H | 5554 | 3 | 0.003
H | 573.1C | 4 | 0.004
H | 573.2C | 5 | 0.005
H | 573.5C | 1 | 0.001
H | 5746 | 1 | 0.001
H | 5752d | 1 | 0.001
H | 5840 | 1 | 0.001
H | 5899d | 1 | 0.001
H | 5913 | 2 | 0.002
H | 5964 | 1 | 0.001
H | 6002 | 1 | 0.001
H | 6173 | 2 | 0.002
H | 6215A | 1 | 0.001
H | 6285 | 2 | 0.002
H | 629 | 1 | 0.001
H | 63 | 1 | 0.001
H | 6311 | 1 | 0.001
H | 6392 | 3 | 0.003
H | 6425 | 1 | 0.001
H | 6434 | 1 | 0.001
H | 6458 | 1 | 0.001
H | 6465 | 8 | 0.007
H | 6476 | 2 | 0.002
H | 6503 | 2 | 0.002
H | 6524 | 1 | 0.001
H | 6524G | 1 | 0.001
H | 6542 | 1 | 0.001
H | 6620 | 2 | 0.002
H | 6650 | 1 | 0.001
H | 6671 | 2 | 0.002
H | 6677 | 1 | 0.001
H | 6722 | 1 | 0.001
H | 6752C | 1 | 0.001
H | 6776 | 1 | 0.001
H | 6866 | 1 | 0.001
H | 6881 | 1 | 0.001
H | 6902 | 1 | 0.001
H | 6905 | 1 | 0.001
H | 6917 | 1 | 0.001
H | 6932 | 2 | 0.002
H | 7025 | 1 | 0.001
H | 7083 | 8 | 0.007
H | 709 | 5 | 0.005
H | 7098 | 1 | 0.001
H | 7109A | 1 | 0.001
H | 7114 | 9 | 0.008
H | 7118 | 1 | 0.001
H | 714 | 1 | 0.001
H | 72 | 1 | 0.001

H | 7245 | 1 | 0.001
H | 7268 | 1 | 0.001
H | 7269 | 2 | 0.002
H | 7270 | 2 | 0.002
H | 73 | 11 | 0.01
H | 7325 | 1 | 0.001
H | 7379 | 1 | 0.001
H | 7424 | 1 | 0.001
H | 745 | 1 | 0.001
H | 7604 | 1 | 0.001
H | 7630 | 1 | 0.001
H | 7795 | 1 | 0.001
H | 7796 | 2 | 0.002
H | 7805 | 1 | 0.001
H | 7813 | 8 | 0.007
H | 7819G | 1 | 0.001
H | 7897 | 2 | 0.002
H | 7904 | 1 | 0.001
H | 8130 | 2 | 0.002
H | 8185 | 1 | 0.001
H | 8269 | 1 | 0.001
H | 8282 | 1 | 0.001
H | 8289.1CCCCCTCTA | 2 | 0.002
H | 8400 | 3 | 0.003
H | 8411C | 1 | 0.001
H | 8490 | 8 | 0.007
H | 8516 | 3 | 0.003
H | 8542 | 1 | 0.001
H | 8545 | 1 | 0.001
H | 8574 | 1 | 0.001
H | 8584 | 3 | 0.003
H | 8618 | 1 | 0.001
H | 8635A | 1 | 0.001
H | 8636 | 1 | 0.001
H | 868 | 1 | 0.001
H | 870 | 2 | 0.002
H | 8701 | 1 | 0.001
H | 8704 | 1 | 0.001
H | 8718 | 1 | 0.001
H | 8772 | 1 | 0.001
H | 8830 | 1 | 0.001
H | 8838 | 1 | 0.001
H | 8864 | 1 | 0.001
H | 8865 | 2 | 0.002
H | 8877 | 1 | 0.001
H | 8902 | 1 | 0.001
H | 8911 | 1 | 0.001
H | 8937 | 2 | 0.002
H | 8961 | 2 | 0.002
H | 8962 | 2 | 0.002
H | 8981 | 1 | 0.001
H | 9027 | 3 | 0.003
H | 9053 | 1 | 0.001
H | 9056 | 1 | 0.001
H | 9071 | 1 | 0.001
H | 9074 | 1 | 0.001
H | 9078 | 2 | 0.002
H | 9101 | 1 | 0.001
H | 9103 | 1 | 0.001
H | 9110 | 1 | 0.001
H | 9126A | 1 | 0.001
H | 9152 | 1 | 0.001
H | 9157 | 3 | 0.003
H | 9178 | 2 | 0.002
H | 9182 | 2 | 0.002
H | 9299 | 4 | 0.004
H | 93 | 13 | 0.012
H | 930 | 1 | 0.001
H | 9310 | 1 | 0.001
H | 9311 | 2 | 0.002
H | 9338 | 1 | 0.001
H | 9371 | 1 | 0.001
H | 9377 | 2 | 0.002
H | 9431 | 3 | 0.003
H | 9449 | 2 | 0.002
H | 9488 | 2 | 0.002
H | 9494 | 2 | 0.002
H | 951 | 1 | 0.001
H | 9521 | 1 | 0.001
H | 9525 | 1 | 0.001
H | 9548 | 3 | 0.003
H | 9550 | 1 | 0.001
H | 9575 | 1 | 0.001
H | 9591 | 1 | 0.001
H | 9593 | 1 | 0.001
H | 95C | 1 | 0.001
H | 9632 | 1 | 0.001
H | 9644 | 1 | 0.001
H | 9698 | 8 | 0.007
H | 9699 | 1 | 0.001
H | 9708 | 2 | 0.002
H | 9742 | 1 | 0.001
H | 9755 | 2 | 0.002
H | 980 | 1 | 0.001
H | 9809 | 1 | 0.001
H | 9851 | 3 | 0.003
H | 9944 | 2 | 0.002
H | 9957 | 1 | 0.001
H | 9995 | 1 | 0.001
H+13708 | 10876 | 1 | 0.001
H+13708 | 113 | 1 | 0.001
H+13708 | 11318 | 1 | 0.001
H+13708 | 12172 | 1 | 0.001
H+13708 | 12612 | 1 | 0.001
H+13708 | 14790 | 1 | 0.001
H+13708 | 15370 | 1 | 0.001
H+13708 | 159 | 1 | 0.001
H+13708 | 16076A | 123 | 0.156
H+13708 | 16287 | 1 | 0.001
H+13708 | 16311 | 1 | 0.001
H+13708 | 200 | 1 | 0.001

H+13708 | 208 | 1 | 0.001
H+13708 | 261 | 1 | 0.001
H+13708 | 281C | 1 | 0.001
H+13708 | 316d | 1 | 0.001
H+13708 | 338 | 1 | 0.001
H+13708 | 456 | 1 | 0.001
H+13708 | 468 | 3 | 0.004
H+13708 | 502 | 1 | 0.001
H+13708 | 573.1C | 3 | 0.004
H+13708 | 573.2C | 5 | 0.006
H+13708 | 573.5C | 1 | 0.001
H+13708 | 8393 | 1 | 0.001
H+13708 | 9230 | 1 | 0.001
H+13708 | 93 | 1 | 0.001
H+152 | 10006 | 2 | 0.009
H+152 | 10211 | 2 | 0.009
H+152 | 10248 | 1 | 0.004
H+152 | 10310 | 1 | 0.004
H+152 | 10313C | 1 | 0.004
H+152 | 10313T | 1 | 0.004
H+152 | 10389 | 1 | 0.004
H+152 | 10398 | 1 | 0.004
H+152 | 10398T | 1 | 0.004
H+152 | 10410 | 1 | 0.004
H+152 | 10493 | 2 | 0.009
H+152 | 1053 | 1 | 0.004
H+152 | 10550 | 1 | 0.004
H+152 | 1063 | 2 | 0.009
H+152 | 10654 | 1 | 0.004
H+152 | 10682A | 1 | 0.004
H+152 | 10682T | 1 | 0.004
H+152 | 10685 | 1 | 0.004
H+152 | 10808 | 1 | 0.004
H+152 | 10993 | 1 | 0.004
H+152 | 11137 | 7 | 0.03
H+152 | 11151 | 1 | 0.004
H+152 | 11167 | 3 | 0.013
H+152 | 11245 | 1 | 0.004
H+152 | 11253 | 1 | 0.004
H+152 | 11361 | 1 | 0.004
H+152 | 11467 | 1 | 0.004
H+152 | 11497 | 1 | 0.004
H+152 | 11719 | 1 | 0.004
H+152 | 11743 | 1 | 0.004
H+152 | 11788 | 2 | 0.009
H+152 | 11827 | 1 | 0.004
H+152 | 11854 | 1 | 0.004
H+152 | 11890 | 1 | 0.004
H+152 | 11914 | 1 | 0.004
H+152 | 11944 | 1 | 0.004
H+152 | 12070 | 1 | 0.004
H+152 | 12192 | 1 | 0.004
H+152 | 12469 | 1 | 0.004
H+152 | 12579 | 4 | 0.017
H+152 | 12599 | 1 | 0.004
H+152 | 12616 | 1 | 0.004
H+152 | 12624 | 1 | 0.004
H+152 | 12651 | 1 | 0.004
H+152 | 12705 | 1 | 0.004
H+152 | 13104 | 1 | 0.004
H+152 | 13547 | 1 | 0.004
H+152 | 13705 | 1 | 0.004
H+152 | 13752 | 2 | 0.009
H+152 | 13759 | 3 | 0.013
H+152 | 13768 | 1 | 0.004
H+152 | 13773 | 1 | 0.004
H+152 | 13893 | 1 | 0.004
H+152 | 14128 | 1 | 0.004
H+152 | 14148 | 1 | 0.004
H+152 | 14162 | 1 | 0.004
H+152 | 14180 | 1 | 0.004
H+152 | 1420 | 1 | 0.004
H+152 | 143 | 1 | 0.004
H+152 | 14323 | 1 | 0.004
H+152 | 14334 | 1 | 0.004
H+152 | 14344 | 1 | 0.004
H+152 | 14364 | 1 | 0.004
H+152 | 14512 | 2 | 0.009
H+152 | 14560 | 2 | 0.009
H+152 | 14569 | 1 | 0.004
H+152 | 146 | 2 | 0.009
H+152 | 14750 | 1 | 0.004
H+152 | 14956 | 1 | 0.004
H+152 | 14971 | 1 | 0.004
H+152 | 150 | 2 | 0.009
H+152 | 15043 | 8 | 0.034
H+152 | 15089 | 1 | 0.004
H+152 | 151 | 4 | 0.017
H+152 | 15106 | 1 | 0.004
H+152 | 15163 | 1 | 0.004
H+152 | 15214 | 2 | 0.009
H+152 | 15217 | 1 | 0.004
H+152 | 15229 | 1 | 0.004
H+152 | 15257 | 1 | 0.004
H+152 | 15430 | 2 | 0.009
H+152 | 15454 | 1 | 0.004
H+152 | 15484 | 1 | 0.004
H+152 | 15497 | 1 | 0.004
H+152 | 15519 | 3 | 0.013
H+152 | 15746 | 7 | 0.03
H+152 | 15784 | 1 | 0.004
H+152 | 15930 | 1 | 0.004
H+152 | 15940 | 1 | 0.004
H+152 | 15941 | 3 | 0.013
H+152 | 16092 | 1 | 0.004
H+152 | 16093 | 3 | 0.013
H+152 | 16126 | 1 | 0.004
H+152 | 16145 | 1 | 0.004
H+152 | 16162 | 1 | 0.004
H+152 | 16168 | 1 | 0.004
H+152 | 16169 | 1 | 0.004

H+152 | 16170C | 1 | 0.004
H+152 | 16185 | 1 | 0.004
H+152 | 16189 | 2 | 0.009
H+152 | 16192 | 4 | 0.017
H+152 | 16209 | 41 | 0.174
H+152 | 16219 | 1 | 0.004
H+152 | 16223 | 2 | 0.009
H+152 | 16249 | 1 | 0.004
H+152 | 16261 | 3 | 0.013
H+152 | 16265 | 1 | 0.004
H+152 | 16271 | 1 | 0.004
H+152 | 16289 | 1 | 0.004
H+152 | 16319 | 1 | 0.004
H+152 | 16320 | 2 | 0.009
H+152 | 16323 | 2 | 0.009
H+152 | 16325 | 3 | 0.013
H+152 | 16327 | 1 | 0.004
H+152 | 16354 | 22 | 0.094
H+152 | 16362 | 1 | 0.004
H+152 | 16390 | 1 | 0.004
H+152 | 16525 | 2 | 0.009
H+152 | 1700 | 10 | 0.043
H+152 | 1719 | 1 | 0.004
H+152 | 182 | 1 | 0.004
H+152 | 195 | 1 | 0.004
H+152 | 198 | 1 | 0.004
H+152 | 200 | 2 | 0.009
H+152 | 2070 | 1 | 0.004
H+152 | 242 | 1 | 0.004
H+152 | 2444 | 1 | 0.004
H+152 | 309d | 1 | 0.004
H+152 | 310 | 4 | 0.017
H+152 | 315.3C | 1 | 0.004
H+152 | 3277 | 4 | 0.017
H+152 | 3316 | 1 | 0.004
H+152 | 3378 | 1 | 0.004
H+152 | 3397 | 1 | 0.004
H+152 | 3511 | 1 | 0.004
H+152 | 3594 | 1 | 0.004
H+152 | 3700 | 1 | 0.004
H+152 | 3945 | 1 | 0.004
H+152 | 4185 | 1 | 0.004
H+152 | 4277 | 1 | 0.004
H+152 | 4452 | 1 | 0.004
H+152 | 4502 | 1 | 0.004
H+152 | 4655 | 1 | 0.004
H+152 | 4659 | 1 | 0.004
H+152 | 4793 | 16 | 0.068
H+152 | 4833 | 1 | 0.004
H+152 | 5108 | 1 | 0.004
H+152 | 5128 | 1 | 0.004
H+152 | 5201 | 1 | 0.004
H+152 | 5460 | 5 | 0.021
H+152 | 5561 | 1 | 0.004
H+152 | 573.1C | 2 | 0.009
H+152 | 573.2C | 10 | 0.043
H+152 | 573.4C | 1 | 0.004
H+152 | 5855 | 1 | 0.004
H+152 | 5913 | 1 | 0.004
H+152 | 593 | 2 | 0.009
H+152 | 5C | 1 | 0.004
H+152 | 6261 | 1 | 0.004
H+152 | 6269T | 2 | 0.009
H+152 | 6632 | 1 | 0.004
H+152 | 6719 | 2 | 0.009
H+152 | 6899 | 1 | 0.004
H+152 | 6975 | 1 | 0.004
H+152 | 7028 | 3 | 0.013
H+152 | 709 | 1 | 0.004
H+152 | 7153 | 1 | 0.004
H+152 | 73 | 3 | 0.013
H+152 | 7337 | 1 | 0.004
H+152 | 738 | 1 | 0.004
H+152 | 7681 | 1 | 0.004
H+152 | 7852 | 1 | 0.004
H+152 | 7861 | 1 | 0.004
H+152 | 8020 | 1 | 0.004
H+152 | 8027 | 1 | 0.004
H+152 | 8170 | 7 | 0.03
H+152 | 824 | 1 | 0.004
H+152 | 8269 | 1 | 0.004
H+152 | 8519C | 1 | 0.004
H+152 | 8573 | 1 | 0.004
H+152 | 8614 | 1 | 0.004
H+152 | 8618 | 1 | 0.004
H+152 | 8812 | 1 | 0.004
H+152 | 8850 | 1 | 0.004
H+152 | 8856 | 1 | 0.004
H+152 | 8869 | 2 | 0.009
H+152 | 8939 | 1 | 0.004
H+152 | 9055 | 1 | 0.004
H+152 | 9066 | 2 | 0.009
H+152 | 9103 | 1 | 0.004
H+152 | 93 | 2 | 0.009
H+152 | 9324 | 1 | 0.004
H+152 | 9380 | 3 | 0.013
H+152 | 9431 | 1 | 0.004
H+152 | 9494 | 2 | 0.009
H+152 | 9548 | 2 | 0.009
H+152 | 9554 | 1 | 0.004
H+152 | 9604 | 1 | 0.004
H+152 | 9948 | 1 | 0.004
H+16129 | 1018 | 1 | 0.004
H+16129 | 10843 | 1 | 0.004
H+16129 | 11263 | 1 | 0.004
H+16129 | 11518 | 1 | 0.004
H+16129 | 11761 | 1 | 0.004
H+16129 | 12042 | 1 | 0.004
H+16129 | 12136 | 1 | 0.004
H+16129 | 12843 | 1 | 0.004
H+16129 | 13326 | 1 | 0.004

H+16129 | 14339 | 1 | 0.004
H+16129 | 14495 | 1 | 0.004
H+16129 | 15449 | 1 | 0.004
H+16129 | 16066 | 2 | 0.007
H+16129 | 16093 | 1 | 0.004
H+16129 | 16176 | 1 | 0.004
H+16129 | 16240 | 1 | 0.004
H+16129 | 16248 | 2 | 0.007
H+16129 | 16249 | 1 | 0.004
H+16129 | 16257 | 7 | 0.026
H+16129 | 16311 | 1 | 0.004
H+16129 | 16316 | 2 | 0.007
H+16129 | 16468 | 1 | 0.004
H+16129 | 195 | 2 | 0.007
H+16129 | 2060 | 2 | 0.007
H+16129 | 2087 | 1 | 0.004
H+16129 | 2262 | 1 | 0.004
H+16129 | 2377 | 1 | 0.004
H+16129 | 2483 | 1 | 0.004
H+16129 | 2755T | 1 | 0.004
H+16129 | 281C | 1 | 0.004
H+16129 | 3010 | 3 | 0.011
H+16129 | 3308 | 1 | 0.004
H+16129 | 3316 | 1 | 0.004
H+16129 | 3757 | 1 | 0.004
H+16129 | 518 | 1 | 0.004
H+16129 | 6221 | 1 | 0.004
H+16129 | 6283 | 1 | 0.004
H+16129 | 6456 | 1 | 0.004
H+16129 | 6600 | 1 | 0.004
H+16129 | 6776 | 1 | 0.004
H+16129 | 709 | 1 | 0.004
H+16129 | 7283 | 2 | 0.007
H+16129 | 7337 | 1 | 0.004
H+16129 | 8113 | 1 | 0.004
H+16129 | 8619 | 1 | 0.004
H+16129 | 8720 | 1 | 0.004
H+16129 | 9015 | 1 | 0.004
H+16129 | 9264 | 1 | 0.004
H+16129 | 9265 | 1 | 0.004
H+16129 | 9391 | 1 | 0.004
H+16129 | 9497 | 1 | 0.004
H+16291 | 10203 | 1 | 0.006
H+16291 | 10394 | 2 | 0.012
H+16291 | 11788 | 1 | 0.006
H+16291 | 11935 | 1 | 0.006
H+16291 | 13708 | 1 | 0.006
H+16291 | 14791 | 2 | 0.012
H+16291 | 15043 | 1 | 0.006
H+16291 | 152 | 1 | 0.006
H+16291 | 15458G | 1 | 0.006
H+16291 | 15650 | 1 | 0.006
H+16291 | 15884 | 1 | 0.006
H+16291 | 15889 | 1 | 0.006
H+16291 | 16067 | 26 | 0.159
H+16291 | 16093 | 3 | 0.018
H+16291 | 16189 | 4 | 0.024
H+16291 | 16249 | 1 | 0.006
H+16291 | 16256 | 1 | 0.006
H+16291 | 16311 | 1 | 0.006
H+16291 | 16324 | 23 | 0.14
H+16291 | 16390 | 2 | 0.012
H+16291 | 1664 | 1 | 0.006
H+16291 | 195 | 1 | 0.006
H+16291 | 3738 | 1 | 0.006
H+16291 | 3866 | 1 | 0.006
H+16291 | 408A | 1 | 0.006
H+16291 | 4511 | 1 | 0.006
H+16291 | 4793 | 1 | 0.006
H+16291 | 4820 | 2 | 0.012
H+16291 | 5673 | 1 | 0.006
H+16291 | 573.1C | 1 | 0.006
H+16291 | 5773 | 1 | 0.006
H+16291 | 6040 | 1 | 0.006
H+16291 | 6221 | 1 | 0.006
H+16291 | 6293 | 1 | 0.006
H+16291 | 6446 | 1 | 0.006
H+16291 | 6776 | 26 | 0.159
H+16291 | 6821 | 2 | 0.012
H+16291 | 6975 | 1 | 0.006
H+16291 | 73 | 1 | 0.006
H+16291 | 8033 | 2 | 0.012
H+16291 | 8790 | 1 | 0.006
H+16291 | 8939 | 1 | 0.006
H+16291 | 9096 | 1 | 0.006
H+16291 | 93 | 2 | 0.012
H+16291 | 9320 | 1 | 0.006
H+16291 | 9380 | 1 | 0.006
H+16291 | 940 | 1 | 0.006
H+16291 | 9438 | 1 | 0.006
H+16291 | 9596 | 1 | 0.006
H+16291 | 9770 | 1 | 0.006
H+16291 | 980 | 1 | 0.006
H+195 | 10211 | 1 | 0.013
H+195 | 10256 | 2 | 0.026
H+195 | 10361 | 1 | 0.013
H+195 | 10463 | 1 | 0.013
H+195 | 10658 | 2 | 0.026
H+195 | 11204 | 1 | 0.013
H+195 | 11377 | 1 | 0.013
H+195 | 11404 | 1 | 0.013
H+195 | 11696 | 1 | 0.013
H+195 | 11719 | 2 | 0.026
H+195 | 11776 | 1 | 0.013
H+195 | 11914 | 2 | 0.026
H+195 | 11950 | 1 | 0.013
H+195 | 12630 | 1 | 0.013
H+195 | 12674 | 1 | 0.013
H+195 | 12705 | 2 | 0.026
H+195 | 12990 | 2 | 0.026
H+195 | 13254 | 2 | 0.026

H+195 | 13359 | 1 | 0.013
H+195 | 13681 | 1 | 0.013
H+195 | 13708 | 1 | 0.013
H+195 | 13759 | 2 | 0.026
H+195 | 13899 | 1 | 0.013
H+195 | 13927T | 1 | 0.013
H+195 | 14195 | 1 | 0.013
H+195 | 14241 | 1 | 0.013
H+195 | 143 | 1 | 0.013
H+195 | 14329 | 1 | 0.013
H+195 | 14339T | 2 | 0.026
H+195 | 14428 | 1 | 0.013
H+195 | 1453 | 2 | 0.026
H+195 | 14587 | 1 | 0.013
H+195 | 14596T | 1 | 0.013
H+195 | 14869 | 1 | 0.013
H+195 | 150 | 1 | 0.013
H+195 | 152 | 7 | 0.09
H+195 | 15465 | 1 | 0.013
H+195 | 15544 | 1 | 0.013
H+195 | 15933.1G | 1 | 0.013
H+195 | 16093 | 4 | 0.051
H+195 | 16129 | 1 | 0.013
H+195 | 16148 | 2 | 0.026
H+195 | 16172 | 5 | 0.064
H+195 | 16192 | 2 | 0.026
H+195 | 16193 | 6 | 0.077
H+195 | 16219 | 2 | 0.026
H+195 | 16230 | 1 | 0.013
H+195 | 16260 | 1 | 0.013
H+195 | 16261 | 2 | 0.026
H+195 | 16293C | 1 | 0.013
H+195 | 16311 | 2 | 0.026
H+195 | 16319 | 1 | 0.013
H+195 | 16320 | 1 | 0.013
H+195 | 16368 | 1 | 0.013
H+195 | 1780 | 2 | 0.026
H+195 | 182 | 3 | 0.038
H+195 | 1915A | 1 | 0.013
H+195 | 1917 | 1 | 0.013
H+195 | 2308 | 1 | 0.013
H+195 | 235 | 1 | 0.013
H+195 | 252 | 1 | 0.013
H+195 | 2563A | 1 | 0.013
H+195 | 2626 | 2 | 0.026
H+195 | 2831T | 2 | 0.026
H+195 | 292 | 2 | 0.026
H+195 | 294 | 1 | 0.013
H+195 | 295 | 1 | 0.013
H+195 | 3010 | 14 | 0.179
H+195 | 309d | 1 | 0.013
H+195 | 310 | 1 | 0.013
H+195 | 3116 | 1 | 0.013
H+195 | 3460 | 1 | 0.013
H+195 | 3548 | 1 | 0.013
H+195 | 3630 | 2 | 0.026
H+195 | 447G | 2 | 0.026
H+195 | 459d | 2 | 0.026
H+195 | 4667 | 1 | 0.013
H+195 | 477 | 1 | 0.013
H+195 | 489 | 2 | 0.026
H+195 | 504 | 1 | 0.013
H+195 | 5183 | 2 | 0.026
H+195 | 5263 | 2 | 0.026
H+195 | 5279 | 1 | 0.013
H+195 | 5465 | 2 | 0.026
H+195 | 5498 | 2 | 0.026
H+195 | 55 | 1 | 0.013
H+195 | 57 | 1 | 0.013
H+195 | 573.2C | 1 | 0.013
H+195 | 5744 | 2 | 0.026
H+195 | 5775 | 2 | 0.026
H+195 | 6128 | 1 | 0.013
H+195 | 6293 | 1 | 0.013
H+195 | 6570T | 2 | 0.026
H+195 | 6776 | 3 | 0.038
H+195 | 6842 | 2 | 0.026
H+195 | 7028 | 2 | 0.026
H+195 | 73 | 2 | 0.026
H+195 | 7543 | 6 | 0.077
H+195 | 7762 | 2 | 0.026
H+195 | 789 | 2 | 0.026
H+195 | 8155 | 1 | 0.013
H+195 | 8200 | 1 | 0.013
H+195 | 8448 | 1 | 0.013
H+195 | 8536 | 1 | 0.013
H+195 | 8638 | 1 | 0.013
H+195 | 8817 | 1 | 0.013
H+195 | 9109 | 1 | 0.013
H+195 | 9233 | 2 | 0.026
H+195 | 9575C | 1 | 0.013
H+195+146 | 10042T | 11 | 0.268
H+195+146 | 10045 | 1 | 0.024
H+195+146 | 10166 | 1 | 0.024
H+195+146 | 10771 | 2 | 0.049
H+195+146 | 11986 | 1 | 0.024
H+195+146 | 12059C | 11 | 0.268
H+195+146 | 12633 | 3 | 0.073
H+195+146 | 13708 | 1 | 0.024
H+195+146 | 13830 | 1 | 0.024
H+195+146 | 13966 | 1 | 0.024
H+195+146 | 14463 | 1 | 0.024
H+195+146 | 152 | 1 | 0.024
H+195+146 | 15607 | 1 | 0.024
H+195+146 | 16051 | 3 | 0.073
H+195+146 | 16129 | 12 | 0.293
H+195+146 | 16144 | 2 | 0.049
H+195+146 | 16153 | 3 | 0.073
H+195+146 | 16189 | 2 | 0.049
H+195+146 | 16192 | 1 | 0.024
H+195+146 | 16209 | 1 | 0.024

H+195+146 | 16248 | 1 | 0.024
H+195+146 | 16270 | 1 | 0.024
H+195+146 | 16271 | 1 | 0.024
H+195+146 | 16278 | 1 | 0.024
H+195+146 | 16293T | 2 | 0.049
H+195+146 | 16311 | 13 | 0.317
H+195+146 | 16355 | 3 | 0.073
H+195+146 | 16356 | 1 | 0.024
H+195+146 | 16399 | 4 | 0.098
H+195+146 | 16400 | 1 | 0.024
H+195+146 | 1719 | 1 | 0.024
H+195+146 | 236 | 2 | 0.049
H+195+146 | 2392 | 3 | 0.073
H+195+146 | 2746 | 1 | 0.024
H+195+146 | 3010 | 13 | 0.317
H+195+146 | 315.2C | 1 | 0.024
H+195+146 | 3394 | 1 | 0.024
H+195+146 | 4188 | 1 | 0.024
H+195+146 | 4732 | 1 | 0.024
H+195+146 | 4967 | 1 | 0.024
H+195+146 | 6249 | 2 | 0.049
H+195+146 | 6962 | 3 | 0.073
H+195+146 | 7040 | 1 | 0.024
H+195+146 | 73 | 1 | 0.024
H+195+146 | 7521 | 1 | 0.024
H+195+146 | 7772 | 2 | 0.049
H+195+146 | 7930T | 1 | 0.024
H+195+146 | 8014 | 1 | 0.024
H+195+146 | 9254 | 2 | 0.049
H+195+146 | 93 | 1 | 0.024
H+195+146 | 9530 | 3 | 0.073
H+195+146 | 9804 | 1 | 0.024
H+195+146 | 9938 | 11 | 0.268
H1 | 10007 | 2 | 0.002
H1 | 10049 | 2 | 0.002
H1 | 10124 | 1 | 0.001
H1 | 10130 | 1 | 0.001
H1 | 10211 | 3 | 0.002
H1 | 10237 | 1 | 0.001
H1 | 10245 | 2 | 0.002
H1 | 10257 | 25 | 0.021
H1 | 10310 | 1 | 0.001
H1 | 10370 | 3 | 0.002
H1 | 10410 | 2 | 0.002
H1 | 10463 | 1 | 0.001
H1 | 10524 | 1 | 0.001
H1 | 10609 | 1 | 0.001
H1 | 10658 | 1 | 0.001
H1 | 10685 | 2 | 0.002
H1 | 10784 | 1 | 0.001
H1 | 10790 | 1 | 0.001
H1 | 10978 | 1 | 0.001
H1 | 11017 | 1 | 0.001
H1 | 11053 | 1 | 0.001
H1 | 11113 | 6 | 0.005
H1 | 11150 | 1 | 0.001
H1 | 11170A | 1 | 0.001
H1 | 11215 | 1 | 0.001
H1 | 11293T | 1 | 0.001
H1 | 11299 | 1 | 0.001
H1 | 113 | 1 | 0.001
H1 | 11447 | 1 | 0.001
H1 | 11479 | 1 | 0.001
H1 | 11800 | 1 | 0.001
H1 | 11809 | 1 | 0.001
H1 | 1187 | 1 | 0.001
H1 | 11914 | 3 | 0.002
H1 | 11929 | 1 | 0.001
H1 | 11930 | 1 | 0.001
H1 | 11944 | 4 | 0.003
H1 | 11950 | 38 | 0.032
H1 | 11956 | 1 | 0.001
H1 | 12236 | 13 | 0.011
H1 | 12245 | 1 | 0.001
H1 | 12280 | 1 | 0.001
H1 | 12358 | 13 | 0.011
H1 | 12372 | 1 | 0.001
H1 | 12390 | 1 | 0.001
H1 | 12465 | 1 | 0.001
H1 | 12507 | 1 | 0.001
H1 | 12804 | 1 | 0.001
H1 | 12810 | 1 | 0.001
H1 | 13094 | 1 | 0.001
H1 | 13095 | 1 | 0.001
H1 | 13191 | 1 | 0.001
H1 | 13194 | 1 | 0.001
H1 | 13356 | 1 | 0.001
H1 | 13359 | 1 | 0.001
H1 | 13401 | 1 | 0.001
H1 | 1342 | 1 | 0.001
H1 | 13485 | 1 | 0.001
H1 | 13518 | 2 | 0.002
H1 | 13563 | 1 | 0.001
H1 | 13604C | 4 | 0.003
H1 | 13626 | 1 | 0.001
H1 | 13627 | 1 | 0.001
H1 | 13656 | 1 | 0.001
H1 | 1368 | 1 | 0.001
H1 | 13681 | 1 | 0.001
H1 | 13708 | 1 | 0.001
H1 | 13711 | 1 | 0.001
H1 | 13746 | 1 | 0.001
H1 | 13759 | 1 | 0.001
H1 | 13836 | 1 | 0.001
H1 | 13879 | 1 | 0.001
H1 | 13907 | 7 | 0.006
H1 | 13928 | 1 | 0.001
H1 | 1393 | 1 | 0.001
H1 | 13943 | 3 | 0.002
H1 | 14004 | 1 | 0.001
H1 | 14025 | 1 | 0.001

H1 | 14128 | 1 | 0.001
H1 | 14148 | 1 | 0.001
H1 | 14180 | 6 | 0.005
H1 | 14212 | 1 | 0.001
H1 | 14220T | 1 | 0.001
H1 | 14226 | 3 | 0.002
H1 | 14233 | 1 | 0.001
H1 | 14279 | 1 | 0.001
H1 | 14287 | 1 | 0.001
H1 | 14318 | 1 | 0.001
H1 | 14329 | 53 | 0.044
H1 | 14370 | 1 | 0.001
H1 | 14380 | 1 | 0.001
H1 | 14392 | 1 | 0.001
H1 | 14410 | 1 | 0.001
H1 | 14470 | 2 | 0.002
H1 | 14502 | 2 | 0.002
H1 | 14530 | 8 | 0.007
H1 | 14560 | 1 | 0.001
H1 | 14581 | 2 | 0.002
H1 | 14766 | 2 | 0.002
H1 | 14839 | 1 | 0.001
H1 | 14867A | 1 | 0.001
H1 | 14905 | 3 | 0.002
H1 | 14927 | 1 | 0.001
H1 | 14964 | 1 | 0.001
H1 | 14978 | 1 | 0.001
H1 | 150 | 6 | 0.005
H1 | 1503 | 1 | 0.001
H1 | 15043 | 1 | 0.001
H1 | 15107A | 1 | 0.001
H1 | 15110 | 2 | 0.002
H1 | 15115 | 1 | 0.001
H1 | 152 | 2 | 0.002
H1 | 15217 | 23 | 0.019
H1 | 15261 | 1 | 0.001
H1 | 15262 | 4 | 0.003
H1 | 15297 | 1 | 0.001
H1 | 15301 | 1 | 0.001
H1 | 15314 | 2 | 0.002
H1 | 15380C | 1 | 0.001
H1 | 15465 | 2 | 0.002
H1 | 15470 | 3 | 0.002
H1 | 15497 | 2 | 0.002
H1 | 15527 | 1 | 0.001
H1 | 15530 | 1 | 0.001
H1 | 1555 | 1 | 0.001
H1 | 15618 | 7 | 0.006
H1 | 15731 | 1 | 0.001
H1 | 15734 | 2 | 0.002
H1 | 15836 | 1 | 0.001
H1 | 15874 | 1 | 0.001
H1 | 15884 | 1 | 0.001
H1 | 15904 | 1 | 0.001
H1 | 15912 | 1 | 0.001
H1 | 15924 | 2 | 0.002
H1 | 15944 | 2 | 0.002
H1 | 16037 | 2 | 0.002
H1 | 16041 | 1 | 0.001
H1 | 16042 | 24 | 0.02
H1 | 16051 | 1 | 0.001
H1 | 16076A | 1 | 0.001
H1 | 16092 | 1 | 0.001
H1 | 16093 | 9 | 0.008
H1 | 16124 | 1 | 0.001
H1 | 16126 | 1 | 0.001
H1 | 16134 | 4 | 0.003
H1 | 16136 | 2 | 0.002
H1 | 16145 | 34 | 0.028
H1 | 16147 | 6 | 0.005
H1 | 16153 | 2 | 0.002
H1 | 16167 | 2 | 0.002
H1 | 16169 | 1 | 0.001
H1 | 16172 | 4 | 0.003
H1 | 16179 | 2 | 0.002
H1 | 16181 | 1 | 0.001
H1 | 16182 | 2 | 0.002
H1 | 16184 | 1 | 0.001
H1 | 16187 | 1 | 0.001
H1 | 16188 | 1 | 0.001
H1 | 16192 | 1 | 0.001
H1 | 16193 | 24 | 0.02
H1 | 16209 | 8 | 0.007
H1 | 16209G | 1 | 0.001
H1 | 16213 | 1 | 0.001
H1 | 16221 | 1 | 0.001
H1 | 16222 | 26 | 0.022
H1 | 16233 | 1 | 0.001
H1 | 16234 | 1 | 0.001
H1 | 16248 | 3 | 0.002
H1 | 16256 | 2 | 0.002
H1 | 16258C | 1 | 0.001
H1 | 16259 | 1 | 0.001
H1 | 16264 | 1 | 0.001
H1 | 16267 | 1 | 0.001
H1 | 16274 | 3 | 0.002
H1 | 16284 | 1 | 0.001
H1 | 16287 | 1 | 0.001
H1 | 16288 | 1 | 0.001
H1 | 16290 | 2 | 0.002
H1 | 16292 | 1 | 0.001
H1 | 16293C | 3 | 0.002
H1 | 16294 | 7 | 0.006
H1 | 16295 | 2 | 0.002
H1 | 16296 | 1 | 0.001
H1 | 16298 | 1 | 0.001
H1 | 16302 | 1 | 0.001
H1 | 16304 | 1 | 0.001
H1 | 16309 | 1 | 0.001
H1 | 16316 | 1 | 0.001
H1 | 16319 | 25 | 0.021

H1 | 16320 | 11 | 0.009
H1 | 16320A | 1 | 0.001
H1 | 16325 | 1 | 0.001
H1 | 16344 | 1 | 0.001
H1 | 16354 | 1 | 0.001
H1 | 16356 | 163 | 0.136
H1 | 16368 | 1 | 0.001
H1 | 16456 | 1 | 0.001
H1 | 1692 | 1 | 0.001
H1 | 1717 | 1 | 0.001
H1 | 1719 | 1 | 0.001
H1 | 1811 | 1 | 0.001
H1 | 1817 | 1 | 0.001
H1 | 185 | 1 | 0.001
H1 | 1888 | 2 | 0.002
H1 | 189 | 1 | 0.001
H1 | 1900 | 1 | 0.001
H1 | 1900C | 2 | 0.002
H1 | 1923 | 1 | 0.001
H1 | 1927 | 1 | 0.001
H1 | 1938.1A | 1 | 0.001
H1 | 1943A | 1 | 0.001
H1 | 1943T | 1 | 0.001
H1 | 200 | 2 | 0.002
H1 | 2010 | 1 | 0.001
H1 | 208 | 1 | 0.001
H1 | 2120 | 1 | 0.001
H1 | 2220 | 1 | 0.001
H1 | 2244 | 1 | 0.001
H1 | 2272 | 1 | 0.001
H1 | 228 | 1 | 0.001
H1 | 2284 | 1 | 0.001
H1 | 236 | 1 | 0.001
H1 | 2392 | 1 | 0.001
H1 | 246G | 1 | 0.001
H1 | 2589 | 1 | 0.001
H1 | 261 | 1 | 0.001
H1 | 2626A | 1 | 0.001
H1 | 2706 | 1 | 0.001
H1 | 2710 | 2 | 0.002
H1 | 2746 | 1 | 0.001
H1 | 279 | 2 | 0.002
H1 | 281C | 1 | 0.001
H1 | 2851 | 1 | 0.001
H1 | 2889 | 1 | 0.001
H1 | 2891 | 1 | 0.001
H1 | 291C | 1 | 0.001
H1 | 292 | 2 | 0.002
H1 | 2931 | 1 | 0.001
H1 | 2955 | 1 | 0.001
H1 | 3083 | 1 | 0.001
H1 | 310 | 4 | 0.003
H1 | 3123d | 1 | 0.001
H1 | 316d | 1 | 0.001
H1 | 317.1C | 1 | 0.001
H1 | 318 | 1 | 0.001
H1 | 3306 | 1 | 0.001
H1 | 338 | 1 | 0.001
H1 | 3421 | 1 | 0.001
H1 | 3496 | 1 | 0.001
H1 | 3531 | 1 | 0.001
H1 | 3556 | 3 | 0.002
H1 | 3579 | 3 | 0.002
H1 | 3612 | 1 | 0.001
H1 | 3777 | 1 | 0.001
H1 | 3816 | 1 | 0.001
H1 | 3826 | 1 | 0.001
H1 | 3842 | 1 | 0.001
H1 | 3849 | 1 | 0.001
H1 | 385 | 2 | 0.002
H1 | 3874 | 1 | 0.001
H1 | 3890 | 1 | 0.001
H1 | 3972 | 1 | 0.001
H1 | 4047 | 1 | 0.001
H1 | 4062 | 1 | 0.001
H1 | 414G | 2 | 0.002
H1 | 4197 | 1 | 0.001
H1 | 4219 | 1 | 0.001
H1 | 4388 | 1 | 0.001
H1 | 4454 | 1 | 0.001
H1 | 453 | 1 | 0.001
H1 | 4655 | 1 | 0.001
H1 | 4659 | 1 | 0.001
H1 | 4707A | 1 | 0.001
H1 | 4772 | 2 | 0.002
H1 | 4793 | 2 | 0.002
H1 | 482 | 1 | 0.001
H1 | 4961C | 1 | 0.001
H1 | 4967 | 1 | 0.001
H1 | 4973G | 1 | 0.001
H1 | 4976 | 1 | 0.001
H1 | 498d | 1 | 0.001
H1 | 5004 | 4 | 0.003
H1 | 502 | 1 | 0.001
H1 | 5075 | 8 | 0.007
H1 | 5111 | 24 | 0.02
H1 | 513 | 1 | 0.001
H1 | 5147 | 2 | 0.002
H1 | 5189 | 1 | 0.001
H1 | 5196 | 1 | 0.001
H1 | 5237 | 1 | 0.001
H1 | 5263 | 1 | 0.001
H1 | 527G | 1 | 0.001
H1 | 535 | 1 | 0.001
H1 | 5492 | 1 | 0.001
H1 | 5581 | 3 | 0.002
H1 | 573.1C | 3 | 0.002
H1 | 573.2C | 5 | 0.004
H1 | 573.3C | 1 | 0.001
H1 | 573.5C | 1 | 0.001
H1 | 5774 | 1 | 0.001

H1 | 5899.1C | 1 | 0.001
H1 | 5913 | 1 | 0.001
H1 | 6040 | 1 | 0.001
H1 | 6182 | 1 | 0.001
H1 | 6249 | 1 | 0.001
H1 | 6253 | 1 | 0.001
H1 | 6267 | 3 | 0.002
H1 | 6284 | 1 | 0.001
H1 | 629 | 1 | 0.001
H1 | 634 | 1 | 0.001
H1 | 6366C | 3 | 0.002
H1 | 6367 | 1 | 0.001
H1 | 6383 | 1 | 0.001
H1 | 6480 | 1 | 0.001
H1 | 6581 | 2 | 0.002
H1 | 6599 | 1 | 0.001
H1 | 6620 | 1 | 0.001
H1 | 6776 | 1 | 0.001
H1 | 6815 | 5 | 0.004
H1 | 6824 | 1 | 0.001
H1 | 6848 | 1 | 0.001
H1 | 6881 | 1 | 0.001
H1 | 6941 | 1 | 0.001
H1 | 7022 | 1 | 0.001
H1 | 709 | 8 | 0.007
H1 | 7191 | 1 | 0.001
H1 | 72 | 1 | 0.001
H1 | 7220 | 2 | 0.002
H1 | 7269 | 2 | 0.002
H1 | 73 | 4 | 0.003
H1 | 7358 | 5 | 0.004
H1 | 7543 | 17 | 0.014
H1 | 7598 | 1 | 0.001
H1 | 7657 | 1 | 0.001
H1 | 7660 | 1 | 0.001
H1 | 7669 | 1 | 0.001
H1 | 7761 | 1 | 0.001
H1 | 7765 | 1 | 0.001
H1 | 7805 | 1 | 0.001
H1 | 7853 | 1 | 0.001
H1 | 7861 | 1 | 0.001
H1 | 7885 | 1 | 0.001
H1 | 789 | 3 | 0.002
H1 | 7948 | 1 | 0.001
H1 | 795 | 1 | 0.001
H1 | 7954 | 1 | 0.001
H1 | 7976 | 1 | 0.001
H1 | 8014 | 1 | 0.001
H1 | 8075 | 1 | 0.001
H1 | 8080 | 1 | 0.001
H1 | 8108 | 2 | 0.002
H1 | 8119 | 1 | 0.001
H1 | 8167 | 2 | 0.002
H1 | 8224 | 2 | 0.002
H1 | 8251 | 1 | 0.001
H1 | 8260A | 1 | 0.001
H1 | 8281-8289d | 1 | 0.001
H1 | 8308 | 1 | 0.001
H1 | 8347 | 2 | 0.002
H1 | 8380 | 1 | 0.001
H1 | 8392 | 1 | 0.001
H1 | 8461 | 1 | 0.001
H1 | 8477 | 1 | 0.001
H1 | 8483 | 1 | 0.001
H1 | 8536 | 4 | 0.003
H1 | 8551 | 2 | 0.002
H1 | 8603 | 2 | 0.002
H1 | 8619 | 1 | 0.001
H1 | 8623 | 1 | 0.001
H1 | 8634 | 1 | 0.001
H1 | 8701 | 1 | 0.001
H1 | 8702 | 1 | 0.001
H1 | 8713 | 1 | 0.001
H1 | 8718 | 1 | 0.001
H1 | 8749 | 2 | 0.002
H1 | 8764 | 3 | 0.002
H1 | 8793 | 1 | 0.001
H1 | 8839 | 1 | 0.001
H1 | 884 | 1 | 0.001
H1 | 8859 | 1 | 0.001
H1 | 8864 | 1 | 0.001
H1 | 8865 | 2 | 0.002
H1 | 8930 | 1 | 0.001
H1 | 8939 | 1 | 0.001
H1 | 8945 | 1 | 0.001
H1 | 8974 | 1 | 0.001
H1 | 8989 | 1 | 0.001
H1 | 9025 | 1 | 0.001
H1 | 9026C | 1 | 0.001
H1 | 9033 | 1 | 0.001
H1 | 9168 | 1 | 0.001
H1 | 9180 | 1 | 0.001
H1 | 9181 | 1 | 0.001
H1 | 9185G | 1 | 0.001
H1 | 93 | 15 | 0.012
H1 | 9347 | 1 | 0.001
H1 | 9391 | 1 | 0.001
H1 | 9419 | 1 | 0.001
H1 | 9477 | 1 | 0.001
H1 | 9592 | 1 | 0.001
H1 | 961 | 1 | 0.001
H1 | 9630 | 1 | 0.001
H1 | 9632 | 1 | 0.001
H1 | 965d | 1 | 0.001
H1 | 9660 | 1 | 0.001
H1 | 9756G | 1 | 0.001
H1 | 9777 | 1 | 0.001
H1 | 978 | 1 | 0.001
H1 | 980 | 2 | 0.002
H1 | 9845 | 1 | 0.001
H1 | 9932 | 1 | 0.001

H1+152 | 10187 | 1 | 0.003
H1+152 | 10211 | 57 | 0.189
H1+152 | 10427 | 1 | 0.003
H1+152 | 10643 | 1 | 0.003
H1+152 | 11002 | 1 | 0.003
H1+152 | 11944 | 1 | 0.003
H1+152 | 12092A | 1 | 0.003
H1+152 | 12362 | 4 | 0.013
H1+152 | 12397 | 2 | 0.007
H1+152 | 12399 | 1 | 0.003
H1+152 | 12477 | 1 | 0.003
H1+152 | 12562G | 1 | 0.003
H1+152 | 12687 | 1 | 0.003
H1+152 | 12807 | 1 | 0.003
H1+152 | 12952 | 1 | 0.003
H1+152 | 131 | 1 | 0.003
H1+152 | 13206 | 1 | 0.003
H1+152 | 13386 | 1 | 0.003
H1+152 | 13470 | 1 | 0.003
H1+152 | 13525 | 1 | 0.003
H1+152 | 13620 | 1 | 0.003
H1+152 | 13818 | 1 | 0.003
H1+152 | 1393 | 1 | 0.003
H1+152 | 13932 | 2 | 0.007
H1+152 | 13966 | 1 | 0.003
H1+152 | 14085 | 2 | 0.007
H1+152 | 14118T | 1 | 0.003
H1+152 | 14123 | 1 | 0.003
H1+152 | 14180 | 1 | 0.003
H1+152 | 14290 | 1 | 0.003
H1+152 | 14384 | 1 | 0.003
H1+152 | 14470 | 1 | 0.003
H1+152 | 146 | 1 | 0.003
H1+152 | 14668 | 1 | 0.003
H1+152 | 14743C | 1 | 0.003
H1+152 | 14869 | 1 | 0.003
H1+152 | 151 | 2 | 0.007
H1+152 | 15238A | 1 | 0.003
H1+152 | 15323 | 1 | 0.003
H1+152 | 15346 | 8 | 0.026
H1+152 | 15404 | 1 | 0.003
H1+152 | 15603 | 1 | 0.003
H1+152 | 15632 | 1 | 0.003
H1+152 | 15672 | 1 | 0.003
H1+152 | 15904 | 1 | 0.003
H1+152 | 15908 | 3 | 0.01
H1+152 | 15961 | 1 | 0.003
H1+152 | 16023T | 1 | 0.003
H1+152 | 16069 | 1 | 0.003
H1+152 | 16104 | 1 | 0.003
H1+152 | 16126 | 1 | 0.003
H1+152 | 16129 | 1 | 0.003
H1+152 | 16140 | 1 | 0.003
H1+152 | 16147 | 1 | 0.003
H1+152 | 16161 | 1 | 0.003
H1+152 | 16174 | 3 | 0.01
H1+152 | 16179 | 1 | 0.003
H1+152 | 16192 | 1 | 0.003
H1+152 | 16207 | 6 | 0.02
H1+152 | 16209 | 2 | 0.007
H1+152 | 16224 | 8 | 0.026
H1+152 | 16233T | 21 | 0.07
H1+152 | 16256 | 1 | 0.003
H1+152 | 16291 | 1 | 0.003
H1+152 | 16300 | 3 | 0.01
H1+152 | 16301 | 2 | 0.007
H1+152 | 16305 | 1 | 0.003
H1+152 | 16317 | 1 | 0.003
H1+152 | 16319 | 1 | 0.003
H1+152 | 16356 | 2 | 0.007
H1+152 | 16360 | 1 | 0.003
H1+152 | 16362 | 3 | 0.01
H1+152 | 16484-16489d | 1 | 0.003
H1+152 | 16526 | 1 | 0.003
H1+152 | 194 | 1 | 0.003
H1+152 | 195 | 1 | 0.003
H1+152 | 1977 | 1 | 0.003
H1+152 | 204 | 1 | 0.003
H1+152 | 2098 | 1 | 0.003
H1+152 | 238 | 4 | 0.013
H1+152 | 242 | 2 | 0.007
H1+152 | 2486 | 1 | 0.003
H1+152 | 2593 | 1 | 0.003
H1+152 | 2760 | 2 | 0.007
H1+152 | 2831 | 1 | 0.003
H1+152 | 291A | 1 | 0.003
H1+152 | 291T | 1 | 0.003
H1+152 | 3 | 1 | 0.003
H1+152 | 310 | 2 | 0.007
H1+152 | 337 | 7 | 0.023
H1+152 | 3434 | 1 | 0.003
H1+152 | 3892 | 7 | 0.023
H1+152 | 4204 | 1 | 0.003
H1+152 | 4216 | 1 | 0.003
H1+152 | 4384-4389d | 1 | 0.003
H1+152 | 4562 | 1 | 0.003
H1+152 | 4580 | 1 | 0.003
H1+152 | 4733 | 3 | 0.01
H1+152 | 4895 | 4 | 0.013
H1+152 | 5067 | 1 | 0.003
H1+152 | 5147 | 1 | 0.003
H1+152 | 5390 | 2 | 0.007
H1+152 | 5471 | 24 | 0.079
H1+152 | 5492 | 1 | 0.003
H1+152 | 5601 | 2 | 0.007
H1+152 | 5655 | 4 | 0.013
H1+152 | 573.1C | 12 | 0.04
H1+152 | 573.2C | 1 | 0.003
H1+152 | 5752d | 1 | 0.003
H1+152 | 5753 | 1 | 0.003
H1+152 | 6023 | 1 | 0.003

H1+152 | 6113 | 2 | 0.007
H1+152 | 6164 | 8 | 0.026
H1+152 | 6221 | 1 | 0.003
H1+152 | 6260 | 1 | 0.003
H1+152 | 64 | 1 | 0.003
H1+152 | 6456 | 1 | 0.003
H1+152 | 6481 | 2 | 0.007
H1+152 | 709 | 1 | 0.003
H1+152 | 7226 | 1 | 0.003
H1+152 | 7471 | 1 | 0.003
H1+152 | 7570 | 24 | 0.079
H1+152 | 7612 | 1 | 0.003
H1+152 | 7691 | 1 | 0.003
H1+152 | 789 | 1 | 0.003
H1+152 | 7927 | 1 | 0.003
H1+152 | 7941 | 1 | 0.003
H1+152 | 8027 | 1 | 0.003
H1+152 | 8245 | 1 | 0.003
H1+152 | 8260A | 57 | 0.189
H1+152 | 8346 | 3 | 0.01
H1+152 | 8503 | 1 | 0.003
H1+152 | 8524 | 1 | 0.003
H1+152 | 8566 | 2 | 0.007
H1+152 | 8603 | 57 | 0.189
H1+152 | 8676 | 1 | 0.003
H1+152 | 8730 | 1 | 0.003
H1+152 | 8805 | 1 | 0.003
H1+152 | 9041 | 1 | 0.003
H1+152 | 9356 | 2 | 0.007
H1+152 | 9615 | 1 | 0.003
H1+152 | 9647 | 1 | 0.003
H1+152 | 9722 | 1 | 0.003
H1+152 | 9833 | 2 | 0.007
H1+152 | 9861 | 2 | 0.007
H1+152 | 9923 | 6 | 0.02
H1+152 | 9935 | 1 | 0.003
H1+16189 | 10143 | 1 | 0.003
H1+16189 | 10262 | 1 | 0.003
H1+16189 | 10303 | 1 | 0.003
H1+16189 | 10410A | 1 | 0.003
H1+16189 | 10616A | 1 | 0.003
H1+16189 | 10646 | 1 | 0.003
H1+16189 | 10680 | 1 | 0.003
H1+16189 | 11016 | 4 | 0.013
H1+16189 | 11149 | 1 | 0.003
H1+16189 | 11335 | 1 | 0.003
H1+16189 | 11617 | 1 | 0.003
H1+16189 | 1189 | 1 | 0.003
H1+16189 | 12130 | 2 | 0.007
H1+16189 | 12172 | 3 | 0.01
H1+16189 | 12354 | 2 | 0.007
H1+16189 | 12406 | 1 | 0.003
H1+16189 | 12630 | 2 | 0.007
H1+16189 | 13051 | 1 | 0.003
H1+16189 | 13671 | 1 | 0.003
H1+16189 | 13681 | 1 | 0.003
H1+16189 | 13702G | 1 | 0.003
H1+16189 | 13708 | 3 | 0.01
H1+16189 | 13722 | 1 | 0.003
H1+16189 | 13746 | 2 | 0.007
H1+16189 | 13759 | 1 | 0.003
H1+16189 | 13896 | 2 | 0.007
H1+16189 | 14016 | 1 | 0.003
H1+16189 | 14034 | 1 | 0.003
H1+16189 | 14178 | 1 | 0.003
H1+16189 | 14199G | 1 | 0.003
H1+16189 | 14272G | 1 | 0.003
H1+16189 | 14278 | 1 | 0.003
H1+16189 | 14319 | 1 | 0.003
H1+16189 | 14365G | 1 | 0.003
H1+16189 | 14368G | 1 | 0.003
H1+16189 | 14467 | 1 | 0.003
H1+16189 | 14484 | 1 | 0.003
H1+16189 | 14577 | 1 | 0.003
H1+16189 | 14587 | 1 | 0.003
H1+16189 | 146 | 3 | 0.01
H1+16189 | 1462 | 3 | 0.01
H1+16189 | 14650 | 1 | 0.003
H1+16189 | 14887 | 2 | 0.007
H1+16189 | 14971 | 1 | 0.003
H1+16189 | 14981 | 1 | 0.003
H1+16189 | 152 | 16 | 0.052
H1+16189 | 15261 | 2 | 0.007
H1+16189 | 15265 | 2 | 0.007
H1+16189 | 15383 | 1 | 0.003
H1+16189 | 15497 | 1 | 0.003
H1+16189 | 1555 | 1 | 0.003
H1+16189 | 15637 | 1 | 0.003
H1+16189 | 15670 | 1 | 0.003
H1+16189 | 15703T | 1 | 0.003
H1+16189 | 15735 | 1 | 0.003
H1+16189 | 15757 | 1 | 0.003
H1+16189 | 15892 | 1 | 0.003
H1+16189 | 15924 | 2 | 0.007
H1+16189 | 15930 | 1 | 0.003
H1+16189 | 16075 | 1 | 0.003
H1+16189 | 16084 | 1 | 0.003
H1+16189 | 16093 | 4 | 0.013
H1+16189 | 16095 | 1 | 0.003
H1+16189 | 16117 | 1 | 0.003
H1+16189 | 16126 | 2 | 0.007
H1+16189 | 16129 | 1 | 0.003
H1+16189 | 16147 | 1 | 0.003
H1+16189 | 16184 | 1 | 0.003
H1+16189 | 16185 | 1 | 0.003
H1+16189 | 16188 | 1 | 0.003
H1+16189 | 16193d | 2 | 0.007
H1+16189 | 16211 | 1 | 0.003
H1+16189 | 16213 | 1 | 0.003
H1+16189 | 16224 | 1 | 0.003
H1+16189 | 16264 | 1 | 0.003

H1+16189 | 16293C | 3 | 0.01
H1+16189 | 16298 | 1 | 0.003
H1+16189 | 16311 | 1 | 0.003
H1+16189 | 16319 | 20 | 0.065
H1+16189 | 16324 | 1 | 0.003
H1+16189 | 16344 | 1 | 0.003
H1+16189 | 16368 | 2 | 0.007
H1+16189 | 16390 | 1 | 0.003
H1+16189 | 16400 | 3 | 0.01
H1+16189 | 182 | 1 | 0.003
H1+16189 | 1834 | 2 | 0.007
H1+16189 | 1888 | 1 | 0.003
H1+16189 | 189 | 1 | 0.003
H1+16189 | 194 | 1 | 0.003
H1+16189 | 207 | 2 | 0.007
H1+16189 | 214 | 1 | 0.003
H1+16189 | 227 | 1 | 0.003
H1+16189 | 267 | 1 | 0.003
H1+16189 | 2746 | 2 | 0.007
H1+16189 | 301C | 1 | 0.003
H1+16189 | 302C | 2 | 0.007
H1+16189 | 305G | 1 | 0.003
H1+16189 | 308 | 1 | 0.003
H1+16189 | 309d | 2 | 0.007
H1+16189 | 310 | 10 | 0.033
H1+16189 | 312 | 2 | 0.007
H1+16189 | 3144 | 3 | 0.01
H1+16189 | 315.2C | 1 | 0.003
H1+16189 | 316d | 1 | 0.003
H1+16189 | 319 | 1 | 0.003
H1+16189 | 3316 | 1 | 0.003
H1+16189 | 3398 | 1 | 0.003
H1+16189 | 3423G | 1 | 0.003
H1+16189 | 3528 | 1 | 0.003
H1+16189 | 3591 | 1 | 0.003
H1+16189 | 3796 | 5 | 0.016
H1+16189 | 3858 | 2 | 0.007
H1+16189 | 3903 | 2 | 0.007
H1+16189 | 4219 | 1 | 0.003
H1+16189 | 4336 | 1 | 0.003
H1+16189 | 44.1C | 2 | 0.007
H1+16189 | 4454 | 1 | 0.003
H1+16189 | 4655 | 1 | 0.003
H1+16189 | 4732 | 1 | 0.003
H1+16189 | 4742 | 1 | 0.003
H1+16189 | 477 | 2 | 0.007
H1+16189 | 4907 | 1 | 0.003
H1+16189 | 494 | 2 | 0.007
H1+16189 | 4985 | 1 | 0.003
H1+16189 | 5054 | 1 | 0.003
H1+16189 | 5460 | 2 | 0.007
H1+16189 | 55 | 1 | 0.003
H1+16189 | 56 | 1 | 0.003
H1+16189 | 5911 | 1 | 0.003
H1+16189 | 6284 | 1 | 0.003
H1+16189 | 6852 | 1 | 0.003
H1+16189 | 709 | 2 | 0.007
H1+16189 | 714 | 6 | 0.02
H1+16189 | 723 | 1 | 0.003
H1+16189 | 7471d | 1 | 0.003
H1+16189 | 7642 | 1 | 0.003
H1+16189 | 7747 | 1 | 0.003
H1+16189 | 7762 | 4 | 0.013
H1+16189 | 8107T | 1 | 0.003
H1+16189 | 8119 | 6 | 0.02
H1+16189 | 8149 | 1 | 0.003
H1+16189 | 8281-8289d | 1 | 0.003
H1+16189 | 8538 | 1 | 0.003
H1+16189 | 8566 | 1 | 0.003
H1+16189 | 8730 | 3 | 0.01
H1+16189 | 8864 | 1 | 0.003
H1+16189 | 8865 | 2 | 0.007
H1+16189 | 9017 | 2 | 0.007
H1+16189 | 9101 | 1 | 0.003
H1+16189 | 9139 | 1 | 0.003
H1+16189 | 9149 | 2 | 0.007
H1+16189 | 9185 | 1 | 0.003
H1+16189 | 93 | 2 | 0.007
H1+16189 | 9335 | 1 | 0.003
H1+16189 | 9356 | 1 | 0.003
H1+16189 | 9377 | 1 | 0.003
H1+16189 | 9467 | 1 | 0.003
H1+16189 | 9545 | 1 | 0.003
H1+16189 | 9559G | 1 | 0.003
H1+16189 | 9612 | 2 | 0.007
H1+16189 | 9676 | 2 | 0.007
H1+16189 | 9708 | 1 | 0.003
H1+16189 | 9779 | 1 | 0.003
H1+16189 | 9932 | 1 | 0.003
H1+16189 | 9966 | 2 | 0.007
H1+16189 | 9994 | 1 | 0.003
H1+16239 | 10098 | 1 | 0.008
H1+16239 | 11914 | 6 | 0.047
H1+16239 | 12366 | 2 | 0.016
H1+16239 | 12651 | 1 | 0.008
H1+16239 | 12879 | 1 | 0.008
H1+16239 | 13759 | 1 | 0.008
H1+16239 | 14180 | 1 | 0.008
H1+16239 | 14189 | 1 | 0.008
H1+16239 | 146 | 4 | 0.031
H1+16239 | 150 | 1 | 0.008
H1+16239 | 15196 | 1 | 0.008
H1+16239 | 152 | 9 | 0.071
H1+16239 | 15301 | 1 | 0.008
H1+16239 | 15313 | 3 | 0.024
H1+16239 | 15529 | 1 | 0.008
H1+16239 | 15785 | 1 | 0.008
H1+16239 | 1598 | 1 | 0.008
H1+16239 | 16066 | 2 | 0.016
H1+16239 | 16127C | 1 | 0.008
H1+16239 | 16129 | 1 | 0.008

H1+16239 | 16153 | 1 | 0.008
H1+16239 | 16184 | 1 | 0.008
H1+16239 | 16220C | 1 | 0.008
H1+16239 | 16259 | 3 | 0.024
H1+16239 | 16261 | 1 | 0.008
H1+16239 | 183 | 1 | 0.008
H1+16239 | 1888C | 6 | 0.047
H1+16239 | 2263 | 1 | 0.008
H1+16239 | 2352 | 1 | 0.008
H1+16239 | 2626 | 2 | 0.016
H1+16239 | 2792C | 1 | 0.008
H1+16239 | 5006 | 1 | 0.008
H1+16239 | 513 | 1 | 0.008
H1+16239 | 5460 | 1 | 0.008
H1+16239 | 5516 | 1 | 0.008
H1+16239 | 5978 | 1 | 0.008
H1+16239 | 709 | 2 | 0.016
H1+16239 | 72 | 1 | 0.008
H1+16239 | 73 | 1 | 0.008
H1+16239 | 8149 | 1 | 0.008
H1+16239 | 8281-8289d | 1 | 0.008
H1+16239 | 8521C | 1 | 0.008
H1+16239 | 8994 | 1 | 0.008
H1+16239 | 93 | 3 | 0.024
H1+16239 | 9708 | 1 | 0.008
H1+16239 | 9804 | 1 | 0.008
H1+16239 | 9861 | 1 | 0.008
H1+16239 | 9911 | 1 | 0.008
H1+16239 | 9938 | 1 | 0.008
H1+16278 | 1290 | 7 | 0.065
H1+16278 | 13455 | 1 | 0.009
H1+16278 | 13581 | 1 | 0.009
H1+16278 | 14329 | 1 | 0.009
H1+16278 | 14384 | 1 | 0.009
H1+16278 | 16129 | 1 | 0.009
H1+16278 | 16189 | 1 | 0.009
H1+16278 | 16193 | 1 | 0.009
H1+16278 | 16230 | 1 | 0.009
H1+16278 | 16311 | 7 | 0.065
H1+16278 | 16324 | 1 | 0.009
H1+16278 | 183 | 1 | 0.009
H1+16278 | 4227 | 1 | 0.009
H1+16278 | 4991 | 1 | 0.009
H1+16278 | 73 | 2 | 0.019
H1+16278 | 7543 | 1 | 0.009
H1+16278 | 7859 | 1 | 0.009
H1+16278 | 921 | 1 | 0.009
H1+16311 | 10007 | 1 | 0.002
H1+16311 | 10042T | 2 | 0.005
H1+16311 | 10493 | 1 | 0.002
H1+16311 | 10553 | 1 | 0.002
H1+16311 | 11278C | 8 | 0.02
H1+16311 | 11549 | 1 | 0.002
H1+16311 | 12059C | 3 | 0.007
H1+16311 | 12170T | 1 | 0.002
H1+16311 | 12194 | 1 | 0.002
H1+16311 | 12236 | 1 | 0.002
H1+16311 | 12630 | 1 | 0.002
H1+16311 | 12662 | 1 | 0.002
H1+16311 | 1290 | 8 | 0.02
H1+16311 | 1374 | 1 | 0.002
H1+16311 | 14467 | 1 | 0.002
H1+16311 | 146 | 2 | 0.005
H1+16311 | 150 | 1 | 0.002
H1+16311 | 151 | 5 | 0.012
H1+16311 | 152 | 50 | 0.122
H1+16311 | 15301 | 1 | 0.002
H1+16311 | 15314 | 1 | 0.002
H1+16311 | 15519 | 1 | 0.002
H1+16311 | 15574 | 1 | 0.002
H1+16311 | 15924 | 1 | 0.002
H1+16311 | 15927 | 1 | 0.002
H1+16311 | 16092 | 1 | 0.002
H1+16311 | 16114 | 1 | 0.002
H1+16311 | 16126 | 1 | 0.002
H1+16311 | 16129 | 7 | 0.017
H1+16311 | 16172 | 1 | 0.002
H1+16311 | 16188G | 2 | 0.005
H1+16311 | 16209 | 1 | 0.002
H1+16311 | 16215 | 1 | 0.002
H1+16311 | 16222 | 1 | 0.002
H1+16311 | 16235 | 1 | 0.002
H1+16311 | 16298 | 1 | 0.002
H1+16311 | 16301 | 1 | 0.002
H1+16311 | 16356 | 6 | 0.015
H1+16311 | 16391 | 37 | 0.09
H1+16311 | 1888 | 8 | 0.02
H1+16311 | 189 | 1 | 0.002
H1+16311 | 195 | 3 | 0.007
H1+16311 | 200 | 1 | 0.002
H1+16311 | 204 | 1 | 0.002
H1+16311 | 215 | 1 | 0.002
H1+16311 | 260T | 1 | 0.002
H1+16311 | 3144 | 1 | 0.002
H1+16311 | 3403 | 1 | 0.002
H1+16311 | 3438 | 1 | 0.002
H1+16311 | 368.1AGAA | 1 | 0.002
H1+16311 | 4166 | 1 | 0.002
H1+16311 | 4204 | 1 | 0.002
H1+16311 | 449 | 3 | 0.007
H1+16311 | 456 | 36 | 0.088
H1+16311 | 4648 | 1 | 0.002
H1+16311 | 477 | 2 | 0.005
H1+16311 | 4859 | 3 | 0.007
H1+16311 | 4997 | 1 | 0.002
H1+16311 | 550 | 1 | 0.002
H1+16311 | 573.1C | 3 | 0.007
H1+16311 | 5964 | 1 | 0.002
H1+16311 | 6040 | 1 | 0.002
H1+16311 | 6287 | 1 | 0.002
H1+16311 | 6887 | 1 | 0.002

H1+16311 | 6911 | 1 | 0.002
H1+16311 | 6914 | 1 | 0.002
H1+16311 | 723 | 1 | 0.002
H1+16311 | 7271 | 1 | 0.002
H1+16311 | 73 | 1 | 0.002
H1+16311 | 7424 | 1 | 0.002
H1+16311 | 7761 | 1 | 0.002
H1+16311 | 789 | 1 | 0.002
H1+16311 | 8110 | 1 | 0.002
H1+16311 | 8200 | 1 | 0.002
H1+16311 | 8282 | 8 | 0.02
H1+16311 | 8426 | 1 | 0.002
H1+16311 | 8952 | 1 | 0.002
H1+16311 | 9644 | 1 | 0.002
H1+16311 | 9923 | 4 | 0.01
H1+16311 | 9938 | 2 | 0.005
H1+16355 | 12594 | 1 | 0.011
H1+16355 | 13194 | 1 | 0.011
H1+16355 | 15110 | 1 | 0.011
H1+16355 | 16263 | 1 | 0.011
H1+16355 | 16311 | 1 | 0.011
H1+16355 | 1888 | 1 | 0.011
H1+16355 | 44.1C | 1 | 0.011
H1+16355 | 6365 | 1 | 0.011
H10 | 10172 | 1 | 0.001
H10 | 10643 | 1 | 0.001
H10 | 10914 | 1 | 0.001
H10 | 113 | 1 | 0.001
H10 | 15643 | 1 | 0.001
H10 | 16076A | 123 | 0.155
H10 | 1692 | 2 | 0.003
H10 | 208 | 1 | 0.001
H10 | 261 | 1 | 0.001
H10 | 281C | 1 | 0.001
H10 | 316d | 1 | 0.001
H10 | 338 | 1 | 0.001
H10 | 3705 | 1 | 0.001
H10 | 3810 | 2 | 0.003
H10 | 468 | 3 | 0.004
H10 | 4977 | 1 | 0.001
H10 | 502 | 1 | 0.001
H10 | 5201 | 1 | 0.001
H10 | 573.1C | 3 | 0.004
H10 | 573.2C | 5 | 0.006
H10 | 573.5C | 1 | 0.001
H10 | 7771 | 1 | 0.001
H10 | 8020 | 1 | 0.001
H10+(16093) | 10172 | 1 | 0.001
H10+(16093) | 10643 | 1 | 0.001
H10+(16093) | 10914 | 1 | 0.001
H10+(16093) | 113 | 1 | 0.001
H10+(16093) | 12084 | 1 | 0.001
H10+(16093) | 13503 | 1 | 0.001
H10+(16093) | 14569 | 1 | 0.001
H10+(16093) | 146 | 1 | 0.001
H10+(16093) | 150 | 2 | 0.002
H10+(16093) | 152 | 1 | 0.001
H10+(16093) | 155 | 3 | 0.003
H10+(16093) | 15643 | 1 | 0.001
H10+(16093) | 16042 | 1 | 0.001
H10+(16093) | 16076A | 1 | 0.001
H10+(16093) | 16111A | 1 | 0.001
H10+(16093) | 16189 | 2 | 0.002
H10+(16093) | 16209 | 3 | 0.003
H10+(16093) | 16265C | 1 | 0.001
H10+(16093) | 16265T | 2 | 0.002
H10+(16093) | 16292 | 4 | 0.005
H10+(16093) | 16327 | 126 | 0.143
H10+(16093) | 16328 | 126 | 0.143
H10+(16093) | 1692 | 2 | 0.002
H10+(16093) | 188 | 1 | 0.001
H10+(16093) | 189 | 2 | 0.002
H10+(16093) | 197 | 1 | 0.001
H10+(16093) | 204 | 1 | 0.001
H10+(16093) | 207 | 2 | 0.002
H10+(16093) | 208 | 1 | 0.001
H10+(16093) | 261 | 1 | 0.001
H10+(16093) | 281C | 1 | 0.001
H10+(16093) | 310 | 2 | 0.002
H10+(16093) | 315.2C | 1 | 0.001
H10+(16093) | 316d | 1 | 0.001
H10+(16093) | 338 | 1 | 0.001
H10+(16093) | 3705 | 1 | 0.001
H10+(16093) | 3810 | 2 | 0.002
H10+(16093) | 483 | 1 | 0.001
H10+(16093) | 4977 | 1 | 0.001
H10+(16093) | 502 | 1 | 0.001
H10+(16093) | 5201 | 1 | 0.001
H10+(16093) | 573.1C | 3 | 0.003
H10+(16093) | 573.2C | 5 | 0.006
H10+(16093) | 573.5C | 1 | 0.001
H10+(16093) | 66T | 2 | 0.002
H10+(16093) | 7771 | 1 | 0.001
H10+(16093) | 8020 | 1 | 0.001
H10+(16093) | 9022 | 1 | 0.001
H10+(16093) | 93 | 1 | 0.001
H100 | 1041 | 1 | 0.001
H100 | 113 | 1 | 0.001
H100 | 13656 | 1 | 0.001
H100 | 1391 | 2 | 0.003
H100 | 15110 | 2 | 0.003
H100 | 16076A | 123 | 0.158
H100 | 16129 | 1 | 0.001
H100 | 16189 | 1 | 0.001
H100 | 16192 | 1 | 0.001
H100 | 200 | 2 | 0.003
H100 | 208 | 1 | 0.001
H100 | 261 | 1 | 0.001
H100 | 281C | 1 | 0.001
H100 | 316d | 1 | 0.001
H100 | 338 | 1 | 0.001

H100 | 468 | 3 | 0.004
H100 | 502 | 1 | 0.001
H100 | 573.1C | 3 | 0.004
H100 | 573.2C | 5 | 0.006
H100 | 573.5C | 1 | 0.001
H100 | 93 | 2 | 0.003
H100 | 9410 | 1 | 0.001
H101 | 10237 | 1 | 0.01
H101 | 10274 | 6 | 0.06
H101 | 10598 | 1 | 0.01
H101 | 11011 | 1 | 0.01
H101 | 11623 | 1 | 0.01
H101 | 11914 | 4 | 0.04
H101 | 12134A | 1 | 0.01
H101 | 12417 | 1 | 0.01
H101 | 12618 | 1 | 0.01
H101 | 13020 | 1 | 0.01
H101 | 13759 | 1 | 0.01
H101 | 13879A | 4 | 0.04
H101 | 14211 | 1 | 0.01
H101 | 146 | 3 | 0.03
H101 | 152 | 5 | 0.05
H101 | 15221 | 1 | 0.01
H101 | 15514 | 4 | 0.04
H101 | 16124 | 1 | 0.01
H101 | 16169 | 2 | 0.02
H101 | 16288 | 1 | 0.01
H101 | 16311 | 8 | 0.08
H101 | 16352 | 1 | 0.01
H101 | 16360 | 2 | 0.02
H101 | 16526 | 1 | 0.01
H101 | 2189 | 1 | 0.01
H101 | 2528 | 1 | 0.01
H101 | 3097 | 1 | 0.01
H101 | 310 | 1 | 0.01
H101 | 315.2C | 1 | 0.01
H101 | 3397 | 2 | 0.02
H101 | 402T | 1 | 0.01
H101 | 5399 | 1 | 0.01
H101 | 55A | 3 | 0.03
H101 | 56T | 1 | 0.01
H101 | 5779 | 1 | 0.01
H101 | 5824 | 2 | 0.02
H101 | 6962 | 1 | 0.01
H101 | 73 | 1 | 0.01
H101 | 8683 | 1 | 0.01
H102 | 113 | 1 | 0.001
H102 | 16076A | 1 | 0.001
H102 | 16218 | 123 | 0.159
H102 | 16278 | 123 | 0.159
H102 | 208 | 1 | 0.001
H102 | 261 | 1 | 0.001
H102 | 281C | 1 | 0.001
H102 | 316d | 1 | 0.001
H102 | 338 | 1 | 0.001
H102 | 4171A | 1 | 0.001
H102 | 468 | 3 | 0.004
H102 | 4705 | 1 | 0.001
H102 | 502 | 1 | 0.001
H102 | 5263 | 1 | 0.001
H102 | 573.1C | 3 | 0.004
H102 | 573.2C | 5 | 0.006
H102 | 573.5C | 1 | 0.001
H103 | 11752G | 1 | 0.042
H103 | 12841 | 1 | 0.042
H103 | 15745 | 1 | 0.042
H103 | 16092 | 1 | 0.042
H103 | 16111 | 1 | 0.042
H103 | 16187 | 1 | 0.042
H103 | 16189 | 4 | 0.167
H103 | 16234 | 1 | 0.042
H103 | 16287 | 1 | 0.042
H103 | 16291 | 3 | 0.125
H103 | 16311 | 4 | 0.167
H103 | 4264 | 1 | 0.042
H104 | 113 | 1 | 0.001
H104 | 16076A | 123 | 0.159
H104 | 208 | 1 | 0.001
H104 | 261 | 1 | 0.001
H104 | 281C | 1 | 0.001
H104 | 316d | 1 | 0.001
H104 | 338 | 1 | 0.001
H104 | 468 | 3 | 0.004
H104 | 502 | 1 | 0.001
H104 | 573.1C | 3 | 0.004
H104 | 573.2C | 5 | 0.006
H104 | 573.5C | 1 | 0.001
H104 | 5794 | 1 | 0.001
H104 | 8485 | 1 | 0.001
H104a | 11353 | 1 | 0.02
H104a | 1780 | 1 | 0.02
H104a | 5899.1C | 6 | 0.12
H105 | 113 | 1 | 0.001
H105 | 12378 | 1 | 0.001
H105 | 14133 | 1 | 0.001
H105 | 16041 | 1 | 0.001
H105 | 16076A | 21 | 0.027
H105 | 16189 | 1 | 0.001
H105 | 16287 | 1 | 0.001
H105 | 16491 | 103 | 0.133
H105 | 16526 | 1 | 0.001
H105 | 208 | 1 | 0.001
H105 | 261 | 1 | 0.001
H105 | 281C | 1 | 0.001
H105 | 316d | 1 | 0.001
H105 | 338 | 1 | 0.001
H105 | 4002 | 1 | 0.001
H105 | 4029 | 1 | 0.001
H105 | 4232 | 1 | 0.001
H105 | 468 | 3 | 0.004
H105 | 4907 | 1 | 0.001

H105 | 502 | 1 | 0.001
H105 | 573.1C | 3 | 0.004
H105 | 573.2C | 5 | 0.006
H105 | 573.5C | 1 | 0.001
H105 | 6975 | 1 | 0.001
H105a | 11084 | 1 | 0.001
H105a | 113 | 1 | 0.001
H105a | 12141 | 1 | 0.001
H105a | 1415 | 1 | 0.001
H105a | 14683 | 1 | 0.001
H105a | 16076A | 123 | 0.163
H105a | 16111 | 1 | 0.001
H105a | 208 | 1 | 0.001
H105a | 261 | 1 | 0.001
H105a | 281C | 1 | 0.001
H105a | 316 | 2 | 0.003
H105a | 316d | 1 | 0.001
H105a | 3316 | 1 | 0.001
H105a | 338 | 1 | 0.001
H105a | 468 | 3 | 0.004
H105a | 502 | 1 | 0.001
H105a | 573.1C | 3 | 0.004
H105a | 573.2C | 5 | 0.007
H105a | 573.5C | 1 | 0.001
H106 | 146 | 1 | 0.033
H106 | 152 | 2 | 0.067
H106 | 16069 | 1 | 0.033
H106 | 16092 | 1 | 0.033
H106 | 16124 | 1 | 0.033
H106 | 16129 | 1 | 0.033
H106 | 16189 | 1 | 0.033
H106 | 16190 | 1 | 0.033
H106 | 16311 | 1 | 0.033
H106 | 1766 | 1 | 0.033
H106 | 182.1T | 1 | 0.033
H106 | 235 | 1 | 0.033
H106 | 324G | 1 | 0.033
H106 | 378 | 1 | 0.033
H106 | 389 | 1 | 0.033
H106 | 42G | 1 | 0.033
H106 | 432C | 1 | 0.033
H106 | 43G | 1 | 0.033
H106 | 451T | 1 | 0.033
H106 | 475C | 1 | 0.033
H106 | 477.1T | 1 | 0.033
H106 | 485-486d | 1 | 0.033
H106 | 7385 | 1 | 0.033
H106 | 763A | 1 | 0.033
H106 | 9284 | 1 | 0.033
H106 | 9328 | 1 | 0.033
H107 | 111 | 1 | 0.04
H107 | 14198 | 1 | 0.04
H107 | 16399 | 1 | 0.04
H107 | 195 | 4 | 0.16
H107 | 3396 | 1 | 0.04
H107 | 4581G | 1 | 0.04
H107 | 5480 | 2 | 0.08
H107 | 960d | 2 | 0.08
H108 | 14020 | 1 | 0.031
H108 | 16189 | 1 | 0.031
H108 | 16292 | 4 | 0.125
H108 | 16319 | 8 | 0.25
H108 | 2763 | 1 | 0.031
H108 | 6392 | 1 | 0.031
H10a | 10340 | 1 | 0.001
H10a | 113 | 1 | 0.001
H10a | 14831 | 1 | 0.001
H10a | 16076A | 123 | 0.155
H10a | 208 | 1 | 0.001
H10a | 261 | 1 | 0.001
H10a | 281C | 1 | 0.001
H10a | 310 | 1 | 0.001
H10a | 315.3C | 1 | 0.001
H10a | 316d | 1 | 0.001
H10a | 338 | 1 | 0.001
H10a | 468 | 3 | 0.004
H10a | 502 | 1 | 0.001
H10a | 573.1C | 3 | 0.004
H10a | 573.2C | 5 | 0.006
H10a | 573.5C | 1 | 0.001
H10a | 8704 | 1 | 0.001
H10a1 | 10238 | 1 | 0.012
H10a1 | 11087 | 1 | 0.012
H10a1 | 12810 | 2 | 0.025
H10a1 | 13708 | 2 | 0.025
H10a1 | 13983 | 1 | 0.012
H10a1 | 14207 | 1 | 0.012
H10a1 | 146 | 2 | 0.025
H10a1 | 150 | 1 | 0.012
H10a1 | 152 | 1 | 0.012
H10a1 | 15497 | 1 | 0.012
H10a1 | 15927 | 2 | 0.025
H10a1 | 16270 | 1 | 0.012
H10a1 | 16304 | 1 | 0.012
H10a1 | 16319 | 3 | 0.038
H10a1 | 198 | 1 | 0.012
H10a1 | 199 | 1 | 0.012
H10a1 | 309d | 1 | 0.012
H10a1 | 310 | 5 | 0.062
H10a1 | 3209 | 1 | 0.012
H10a1 | 3290 | 1 | 0.012
H10a1 | 3308 | 1 | 0.012
H10a1 | 5105 | 1 | 0.012
H10a1 | 5585 | 1 | 0.012
H10a1 | 616 | 1 | 0.012
H10a1 | 7419 | 1 | 0.012
H10a1 | 7533 | 1 | 0.012
H10a1 | 9151 | 1 | 0.012
H10a1a | 16129 | 2 | 0.029
H10a1a | 16179 | 1 | 0.014
H10a1a | 16287 | 1 | 0.014

H10a1a | 374 | 1 | 0.014
H10a1a | 5378 | 1 | 0.014
H10a1a1 | 10915 | 1 | 0.014
H10a1a1 | 16129 | 2 | 0.028
H10a1a1 | 16179 | 1 | 0.014
H10a1a1 | 16287 | 1 | 0.014
H10a1a1 | 374 | 1 | 0.014
H10a1a1 | 6340 | 2 | 0.028
H10a1b | 223 | 1 | 0.025
H10a1b | 286T | 1 | 0.025
H10a1b | 342 | 1 | 0.025
H10a1b | 376C | 2 | 0.05
H10a1b | 376T | 1 | 0.025
H10a1b | 395 | 1 | 0.025
H10a1b | 397 | 1 | 0.025
H10a1b | 40 | 1 | 0.025
H10a1b | 7356 | 1 | 0.025
H10b | 10828 | 1 | 0.001
H10b | 113 | 1 | 0.001
H10b | 13494 | 1 | 0.001
H10b | 152 | 1 | 0.001
H10b | 15889 | 1 | 0.001
H10b | 16076A | 1 | 0.001
H10b | 16301 | 5 | 0.006
H10b | 16355 | 1 | 0.001
H10b | 16399 | 124 | 0.156
H10b | 208 | 1 | 0.001
H10b | 261 | 1 | 0.001
H10b | 281C | 1 | 0.001
H10b | 310 | 1 | 0.001
H10b | 316d | 1 | 0.001
H10b | 338 | 1 | 0.001
H10b | 3733 | 1 | 0.001
H10b | 3918 | 1 | 0.001
H10b | 468 | 3 | 0.004
H10b | 502 | 1 | 0.001
H10b | 573.1C | 3 | 0.004
H10b | 573.2C | 5 | 0.006
H10b | 573.5C | 1 | 0.001
H10b1 | 113 | 1 | 0.001
H10b1 | 13658 | 1 | 0.001
H10b1 | 16076A | 123 | 0.156
H10b1 | 208 | 1 | 0.001
H10b1 | 261 | 1 | 0.001
H10b1 | 281C | 1 | 0.001
H10b1 | 3169 | 1 | 0.001
H10b1 | 316d | 1 | 0.001
H10b1 | 338 | 1 | 0.001
H10b1 | 468 | 3 | 0.004
H10b1 | 502 | 1 | 0.001
H10b1 | 573.1C | 3 | 0.004
H10b1 | 573.2C | 5 | 0.006
H10b1 | 573.5C | 1 | 0.001
H10b1 | 6575 | 1 | 0.001
H10c | 113 | 1 | 0.001
H10c | 16076A | 123 | 0.158
H10c | 208 | 1 | 0.001
H10c | 261 | 1 | 0.001
H10c | 281C | 1 | 0.001
H10c | 316d | 1 | 0.001
H10c | 338 | 1 | 0.001
H10c | 366 | 1 | 0.001
H10c | 389 | 1 | 0.001
H10c | 468 | 3 | 0.004
H10c | 502 | 1 | 0.001
H10c | 573.1C | 3 | 0.004
H10c | 573.2C | 5 | 0.006
H10c | 573.5C | 1 | 0.001
H10c | 622 | 1 | 0.001
H10c1 | 113 | 1 | 0.001
H10c1 | 1193 | 1 | 0.001
H10c1 | 14370 | 1 | 0.001
H10c1 | 16042 | 1 | 0.001
H10c1 | 16076A | 123 | 0.157
H10c1 | 16215 | 1 | 0.001
H10c1 | 16243 | 3 | 0.004
H10c1 | 16266 | 1 | 0.001
H10c1 | 16362 | 1 | 0.001
H10c1 | 208 | 1 | 0.001
H10c1 | 249 | 1 | 0.001
H10c1 | 261 | 1 | 0.001
H10c1 | 281C | 1 | 0.001
H10c1 | 310 | 1 | 0.001
H10c1 | 316d | 1 | 0.001
H10c1 | 338 | 1 | 0.001
H10c1 | 3645 | 3 | 0.004
H10c1 | 366 | 1 | 0.001
H10c1 | 389 | 1 | 0.001
H10c1 | 468 | 3 | 0.004
H10c1 | 502 | 1 | 0.001
H10c1 | 573.1C | 3 | 0.004
H10c1 | 573.2C | 5 | 0.006
H10c1 | 573.5C | 1 | 0.001
H10c1 | 622 | 1 | 0.001
H10c1 | 73 | 3 | 0.004
H10c1 | 8395 | 1 | 0.001
H10c1 | 8596C | 1 | 0.001
H10c1 | 9266 | 1 | 0.001
H10d | 1462 | 1 | 0.003
H10d | 15884 | 1 | 0.003
H10d | 16042C | 1 | 0.003
H10d | 16129T | 1 | 0.003
H10d | 16150 | 1 | 0.003
H10d | 16188A | 2 | 0.005
H10d | 16209 | 16 | 0.042
H10d | 16231 | 1 | 0.003
H10d | 16293C | 2 | 0.005
H10d | 16327 | 125 | 0.326
H10d | 176C | 2 | 0.005
H10d | 252 | 3 | 0.008
H10d | 261.1T | 1 | 0.003

H10d | 264 | 3 | 0.008
H10d | 299d | 1 | 0.003
H10d | 332G | 1 | 0.003
H10d | 49T | 1 | 0.003
H10d | 73 | 1 | 0.003
H10e | 10172 | 1 | 0.008
H10e | 10187 | 2 | 0.016
H10e | 11362 | 1 | 0.008
H10e | 11778 | 1 | 0.008
H10e | 12358 | 1 | 0.008
H10e | 12362 | 1 | 0.008
H10e | 12636 | 3 | 0.024
H10e | 13767 | 1 | 0.008
H10e | 14587 | 1 | 0.008
H10e | 14770 | 1 | 0.008
H10e | 150 | 2 | 0.016
H10e | 15367 | 1 | 0.008
H10e | 15924 | 2 | 0.016
H10e | 16093 | 4 | 0.032
H10e | 16230 | 3 | 0.024
H10e | 16311 | 3 | 0.024
H10e | 16316 | 1 | 0.008
H10e | 16362 | 3 | 0.024
H10e | 207 | 3 | 0.024
H10e | 2483 | 1 | 0.008
H10e | 309d | 1 | 0.008
H10e | 310 | 1 | 0.008
H10e | 3290 | 1 | 0.008
H10e | 3363 | 2 | 0.016
H10e | 3584 | 1 | 0.008
H10e | 4295 | 1 | 0.008
H10e | 4454 | 1 | 0.008
H10e | 4638 | 1 | 0.008
H10e | 5135 | 1 | 0.008
H10e | 533 | 1 | 0.008
H10e | 5348 | 1 | 0.008
H10e | 5381 | 1 | 0.008
H10e | 5747T | 1 | 0.008
H10e | 5780 | 1 | 0.008
H10e | 6260 | 1 | 0.008
H10e | 6480 | 4 | 0.032
H10e | 709 | 1 | 0.008
H10e | 7598 | 1 | 0.008
H10e | 7797 | 1 | 0.008
H10e | 7849 | 1 | 0.008
H10e | 8270 | 1 | 0.008
H10e | 8485 | 1 | 0.008
H10e | 9055 | 4 | 0.032
H10e | 9902 | 1 | 0.008
H10e1 | 11383 | 1 | 0.01
H10e1 | 12040 | 1 | 0.01
H10e1 | 14180 | 1 | 0.01
H10e1 | 15289 | 1 | 0.01
H10e1 | 16093 | 3 | 0.031
H10e1 | 16124 | 4 | 0.041
H10e1 | 16298 | 1 | 0.01
H10e1 | 16362 | 1 | 0.01
H10e1 | 310 | 1 | 0.01
H10e1 | 315.2C | 1 | 0.01
H10e1 | 4020 | 1 | 0.01
H10e1 | 5045A | 1 | 0.01
H10e1 | 5045T | 1 | 0.01
H10e1 | 6575 | 1 | 0.01
H10e1 | 72 | 1 | 0.01
H10e1 | 7645 | 1 | 0.01
H10e1 | 9251 | 1 | 0.01
H10e1 | 960.1C | 2 | 0.02
H10e1a | 10214 | 1 | 0.016
H10e1a | 10410 | 1 | 0.016
H10e1a | 11314 | 1 | 0.016
H10e1a | 12284A | 1 | 0.016
H10e1a | 152 | 1 | 0.016
H10e1a | 16093 | 1 | 0.016
H10e1a | 16399 | 2 | 0.031
H10e1a | 279 | 1 | 0.016
H10e2 | 13105 | 1 | 0.011
H10e2 | 15301 | 1 | 0.011
H10e2 | 16093 | 3 | 0.033
H10e2 | 310 | 1 | 0.011
H10e2 | 315.3C | 1 | 0.011
H10e2 | 4823 | 4 | 0.044
H10e3 | 16270 | 2 | 0.024
H10e3 | 200 | 1 | 0.012
H10e3 | 6351 | 1 | 0.012
H10e3 | 64 | 2 | 0.024
H10e3a | 152 | 1 | 0.011
H10e3a | 15317 | 1 | 0.011
H10e3a | 16093 | 2 | 0.023
H10e3a | 16270 | 2 | 0.023
H10e3a | 310 | 1 | 0.011
H10e3a | 5530 | 1 | 0.011
H10e3a | 6032 | 1 | 0.011
H10e3a | 64 | 2 | 0.023
H10f | 113 | 1 | 0.001
H10f | 12028 | 2 | 0.002
H10f | 13209 | 2 | 0.002
H10f | 146 | 1 | 0.001
H10f | 15194 | 1 | 0.001
H10f | 16042 | 1 | 0.001
H10f | 16076A | 1 | 0.001
H10f | 16093 | 1 | 0.001
H10f | 16111A | 1 | 0.001
H10f | 16188G | 1 | 0.001
H10f | 16209 | 3 | 0.003
H10f | 16216 | 6 | 0.007
H10f | 16292 | 4 | 0.005
H10f | 16327 | 126 | 0.144
H10f | 16328 | 126 | 0.144
H10f | 16337G | 2 | 0.002
H10f | 16392 | 1 | 0.001
H10f | 16405 | 2 | 0.002

H10f | 189 | 2 | 0.002
H10f | 207 | 2 | 0.002
H10f | 208 | 1 | 0.001
H10f | 261 | 1 | 0.001
H10f | 281C | 1 | 0.001
H10f | 310 | 1 | 0.001
H10f | 311 | 1 | 0.001
H10f | 3145 | 2 | 0.002
H10f | 316d | 1 | 0.001
H10f | 338 | 1 | 0.001
H10f | 379C | 1 | 0.001
H10f | 380T | 2 | 0.002
H10f | 404 | 1 | 0.001
H10f | 483 | 1 | 0.001
H10f | 502 | 1 | 0.001
H10f | 573.1C | 3 | 0.003
H10f | 573.2C | 5 | 0.006
H10f | 573.5C | 1 | 0.001
H10f | 6150 | 1 | 0.001
H10f | 8657 | 1 | 0.001
H10f | 9966 | 1 | 0.001
H10g | 12084 | 1 | 0.015
H10g | 12358 | 1 | 0.015
H10g | 15265 | 1 | 0.015
H10g | 317.1C | 1 | 0.015
H10g | 748 | 1 | 0.015
H10g | 9022 | 1 | 0.015
H10g | 9115 | 2 | 0.031
H10g | 9591 | 1 | 0.015
H10h | 113 | 1 | 0.001
H10h | 16076A | 123 | 0.156
H10h | 208 | 1 | 0.001
H10h | 261 | 1 | 0.001
H10h | 281C | 1 | 0.001
H10h | 310 | 1 | 0.001
H10h | 316d | 1 | 0.001
H10h | 338 | 1 | 0.001
H10h | 468 | 3 | 0.004
H10h | 502 | 1 | 0.001
H10h | 573.1C | 3 | 0.004
H10h | 573.2C | 5 | 0.006
H10h | 573.5C | 1 | 0.001
H10h | 5843 | 1 | 0.001
H11 | 11395 | 1 | 0.007
H11 | 14377 | 1 | 0.007
H11 | 14386 | 1 | 0.007
H11 | 14502 | 1 | 0.007
H11 | 152 | 2 | 0.014
H11 | 15883 | 1 | 0.007
H11 | 15913 | 2 | 0.014
H11 | 16126 | 1 | 0.007
H11 | 16172 | 2 | 0.014
H11 | 16189 | 1 | 0.007
H11 | 16223 | 1 | 0.007
H11 | 16293 | 3 | 0.021
H11 | 16301 | 1 | 0.007
H11 | 16354 | 2 | 0.014
H11 | 16526 | 1 | 0.007
H11 | 438 | 1 | 0.007
H11 | 499 | 3 | 0.021
H11 | 6018 | 1 | 0.007
H11 | 6531 | 1 | 0.007
H11 | 6723 | 2 | 0.014
H11 | 7444 | 2 | 0.014
H11 | 9000 | 2 | 0.014
H11 | 9285 | 2 | 0.014
H11 | 93 | 2 | 0.014
H11 | 961A | 1 | 0.007
H11 | 961G | 3 | 0.021
H11a | 11206 | 2 | 0.027
H11a | 12215 | 1 | 0.014
H11a | 12486 | 1 | 0.014
H11a | 12771 | 1 | 0.014
H11a | 143 | 5 | 0.068
H11a | 146 | 2 | 0.027
H11a | 14668 | 1 | 0.014
H11a | 14922 | 1 | 0.014
H11a | 1531 | 1 | 0.014
H11a | 15940 | 1 | 0.014
H11a | 16093 | 2 | 0.027
H11a | 16129 | 1 | 0.014
H11a | 16187 | 1 | 0.014
H11a | 16189 | 1 | 0.014
H11a | 16189A | 1 | 0.014
H11a | 16220 | 1 | 0.014
H11a | 16244 | 1 | 0.014
H11a | 16266 | 1 | 0.014
H11a | 16304 | 1 | 0.014
H11a | 16344 | 1 | 0.014
H11a | 16354 | 1 | 0.014
H11a | 16355 | 1 | 0.014
H11a | 16368 | 4 | 0.054
H11a | 185 | 1 | 0.014
H11a | 204 | 1 | 0.014
H11a | 207 | 1 | 0.014
H11a | 226 | 1 | 0.014
H11a | 228 | 2 | 0.027
H11a | 2851 | 1 | 0.014
H11a | 310 | 10 | 0.135
H11a | 312 | 2 | 0.027
H11a | 315.2C | 1 | 0.014
H11a | 315d | 1 | 0.014
H11a | 316C | 1 | 0.014
H11a | 373T | 1 | 0.014
H11a | 385 | 1 | 0.014
H11a | 3865 | 1 | 0.014
H11a | 4056 | 1 | 0.014
H11a | 480 | 1 | 0.014
H11a | 5162 | 1 | 0.014
H11a | 5821 | 1 | 0.014
H11a | 7278 | 1 | 0.014

H11a | 8227 | 1 | 0.014
H11a | 8618 | 1 | 0.014
H11a | 8865 | 1 | 0.014
H11a | 9033 | 1 | 0.014
H11a | 9116 | 2 | 0.027
H11a | 9794 | 1 | 0.014
H11a | 9932 | 2 | 0.027
H11a | 9966 | 1 | 0.014
H11a+152 | 11062 | 1 | 0.048
H11a+152 | 12188 | 1 | 0.048
H11a+152 | 14560 | 1 | 0.048
H11a+152 | 14587 | 1 | 0.048
H11a+152 | 151 | 1 | 0.048
H11a+152 | 16129 | 1 | 0.048
H11a+152 | 16291 | 1 | 0.048
H11a+152 | 16305 | 1 | 0.048
H11a+152 | 310 | 1 | 0.048
H11a+152 | 3736 | 2 | 0.095
H11a+152 | 4820 | 2 | 0.095
H11a+152 | 5054 | 2 | 0.095
H11a+152 | 5515 | 2 | 0.095
H11a+152 | 6510 | 1 | 0.048
H11a+152 | 6854 | 1 | 0.048
H11a+152 | 6950 | 1 | 0.048
H11a+152 | 93 | 1 | 0.048
H11a1 | 10235 | 1 | 0.012
H11a1 | 10376 | 1 | 0.012
H11a1 | 10785 | 1 | 0.012
H11a1 | 11503A | 4 | 0.048
H11a1 | 11914 | 1 | 0.012
H11a1 | 12678 | 2 | 0.024
H11a1 | 13434 | 7 | 0.083
H11a1 | 13470C | 1 | 0.012
H11a1 | 13635 | 1 | 0.012
H11a1 | 13708 | 1 | 0.012
H11a1 | 13971 | 1 | 0.012
H11a1 | 14097 | 1 | 0.012
H11a1 | 143 | 3 | 0.036
H11a1 | 14386 | 1 | 0.012
H11a1 | 14544 | 1 | 0.012
H11a1 | 146 | 13 | 0.155
H11a1 | 15001 | 1 | 0.012
H11a1 | 15077 | 2 | 0.024
H11a1 | 15355 | 2 | 0.024
H11a1 | 1542 | 1 | 0.012
H11a1 | 15487T | 1 | 0.012
H11a1 | 15639 | 1 | 0.012
H11a1 | 15924 | 1 | 0.012
H11a1 | 16086 | 1 | 0.012
H11a1 | 16094 | 1 | 0.012
H11a1 | 16134 | 1 | 0.012
H11a1 | 16189 | 8 | 0.095
H11a1 | 16209 | 3 | 0.036
H11a1 | 16224 | 7 | 0.083
H11a1 | 16234 | 2 | 0.024
H11a1 | 16261 | 2 | 0.024
H11a1 | 16263 | 1 | 0.012
H11a1 | 16299 | 1 | 0.012
H11a1 | 16301 | 1 | 0.012
H11a1 | 16319 | 1 | 0.012
H11a1 | 16362 | 1 | 0.012
H11a1 | 16390 | 3 | 0.036
H11a1 | 185 | 2 | 0.024
H11a1 | 214C | 1 | 0.012
H11a1 | 214T | 1 | 0.012
H11a1 | 215 | 3 | 0.036
H11a1 | 2430 | 1 | 0.012
H11a1 | 249d | 1 | 0.012
H11a1 | 2784 | 1 | 0.012
H11a1 | 310 | 5 | 0.06
H11a1 | 315.2C | 1 | 0.012
H11a1 | 4188 | 1 | 0.012
H11a1 | 42.1G | 1 | 0.012
H11a1 | 4215 | 2 | 0.024
H11a1 | 4898 | 1 | 0.012
H11a1 | 5021 | 4 | 0.048
H11a1 | 513 | 1 | 0.012
H11a1 | 570 | 1 | 0.012
H11a1 | 573.1C | 1 | 0.012
H11a1 | 6848 | 1 | 0.012
H11a1 | 7076 | 1 | 0.012
H11a1 | 72 | 1 | 0.012
H11a1 | 7389 | 1 | 0.012
H11a1 | 7598 | 2 | 0.024
H11a1 | 7674 | 1 | 0.012
H11a1 | 7712 | 1 | 0.012
H11a1 | 7906 | 1 | 0.012
H11a1 | 8152 | 1 | 0.012
H11a1 | 8530 | 1 | 0.012
H11a1 | 93 | 1 | 0.012
H11a1 | 9316 | 1 | 0.012
H11a1 | 961 | 1 | 0.012
H11a1 | 961A | 2 | 0.024
H11a1 | 963.1GCC | 1 | 0.012
H11a2 | 10454 | 2 | 0.034
H11a2 | 12651 | 1 | 0.017
H11a2 | 143 | 1 | 0.017
H11a2 | 14476C | 1 | 0.017
H11a2 | 146 | 14 | 0.241
H11a2 | 151 | 4 | 0.069
H11a2 | 15139 | 2 | 0.034
H11a2 | 15424 | 1 | 0.017
H11a2 | 15765T | 1 | 0.017
H11a2 | 15943 | 1 | 0.017
H11a2 | 16129 | 1 | 0.017
H11a2 | 16169 | 2 | 0.034
H11a2 | 16183 | 1 | 0.017
H11a2 | 16189 | 4 | 0.069
H11a2 | 16207 | 1 | 0.017
H11a2 | 16212 | 1 | 0.017
H11a2 | 16259 | 1 | 0.017

H11a2 | 16261 | 4 | 0.069
H11a2 | 16301 | 3 | 0.052
H11a2 | 16343 | 4 | 0.069
H11a2 | 310 | 1 | 0.017
H11a2 | 459 | 1 | 0.017
H11a2 | 513 | 1 | 0.017
H11a2 | 6261 | 1 | 0.017
H11a2 | 709 | 1 | 0.017
H11a2 | 8618 | 2 | 0.034
H11a2 | 8793 | 1 | 0.017
H11a2 | 9038 | 1 | 0.017
H11a2 | 9150 | 1 | 0.017
H11a2 | 9254 | 2 | 0.034
H11a2a | 11778 | 1 | 0.043
H11a2a | 12014 | 1 | 0.043
H11a2a | 152 | 1 | 0.043
H11a2a | 16092 | 2 | 0.087
H11a2a | 16254 | 1 | 0.043
H11a2a | 3591 | 1 | 0.043
H11a2a | 3834C | 1 | 0.043
H11a2a | 5102 | 1 | 0.043
H11a2a | 5252 | 1 | 0.043
H11a2a | 5471 | 1 | 0.043
H11a2a | 7313 | 1 | 0.043
H11a2a | 7939 | 1 | 0.043
H11a2a | 7996 | 1 | 0.043
H11a2a | 8898 | 1 | 0.043
H11a2a | 8979 | 1 | 0.043
H11a2a | 9709 | 1 | 0.043
H11a2a | 982 | 1 | 0.043
H11a2a | 9903 | 1 | 0.043
H11a2a1 | 7521 | 1 | 0.077
H11a2a1 | 9204 | 1 | 0.077
H11a2a1 | 9230 | 1 | 0.077
H11a2a2 | 10070 | 1 | 0.043
H11a2a2 | 12235 | 3 | 0.13
H11a2a2 | 13080 | 1 | 0.043
H11a2a2 | 13752 | 1 | 0.043
H11a2a2 | 14566 | 1 | 0.043
H11a2a2 | 146 | 1 | 0.043
H11a2a2 | 152 | 1 | 0.043
H11a2a2 | 16129 | 2 | 0.087
H11a2a2 | 16239 | 2 | 0.087
H11a2a2 | 183 | 1 | 0.043
H11a2a2 | 198 | 1 | 0.043
H11a2a2 | 207 | 1 | 0.043
H11a2a2 | 6386 | 2 | 0.087
H11a2a2 | 7805 | 1 | 0.043
H11a2a3 | 146 | 1 | 0.083
H11a2a3 | 16129 | 1 | 0.083
H11a2a3 | 16380 | 1 | 0.083
H11a2a3 | 3447 | 1 | 0.083
H11a2a3 | 4612 | 1 | 0.083
H11a2a3 | 8557 | 1 | 0.083
H11a3 | 11260 | 1 | 0.091
H11a3 | 146 | 1 | 0.091
H11a3 | 16532d | 2 | 0.182
H11a3 | 186 | 1 | 0.091
H11a4 | 151 | 2 | 0.2
H11a4 | 298 | 1 | 0.1
H11a4 | 489 | 2 | 0.2
H11a4 | 5806 | 1 | 0.1
H11a4 | 96A | 1 | 0.1
H11a5 | 11101 | 1 | 0.026
H11a5 | 14569 | 1 | 0.026
H11a5 | 15623 | 2 | 0.053
H11a5 | 16093 | 9 | 0.237
H11a5 | 16114 | 2 | 0.053
H11a5 | 16320 | 2 | 0.053
H11a5 | 16354 | 2 | 0.053
H11a5 | 16391 | 1 | 0.026
H11a5 | 16497 | 2 | 0.053
H11a6 | 152 | 1 | 0.091
H11a6 | 16256 | 1 | 0.091
H11a6 | 16532d | 2 | 0.182
H11a6 | 186 | 1 | 0.091
H11a7 | 15034 | 2 | 0.143
H11a7 | 16092 | 1 | 0.071
H11a7 | 16266 | 1 | 0.071
H11a7 | 16355 | 2 | 0.143
H11a7 | 198 | 1 | 0.071
H11a7 | 4820 | 1 | 0.071
H11a7 | 5675 | 1 | 0.071
H11a7 | 7190 | 1 | 0.071
H11a7 | 72 | 1 | 0.071
H11a7 | 8269 | 1 | 0.071
H11a7 | 9530 | 1 | 0.071
H11a8 | 1393 | 1 | 0.026
H11a8 | 14581 | 1 | 0.026
H11a8 | 15617 | 1 | 0.026
H11a8 | 16090 | 1 | 0.026
H11a8 | 16093 | 9 | 0.237
H11a8 | 16114 | 2 | 0.053
H11a8 | 16320 | 2 | 0.053
H11a8 | 16354 | 2 | 0.053
H11a8 | 16497 | 2 | 0.053
H11a8 | 8604A | 1 | 0.026
H11b | 150 | 4 | 0.028
H11b | 16079 | 1 | 0.007
H11b | 16093 | 2 | 0.014
H11b | 16252 | 1 | 0.007
H11b | 16254 | 4 | 0.028
H11b | 16291 | 3 | 0.021
H11b | 16355 | 2 | 0.014
H11b | 16368 | 4 | 0.028
H11b | 64 | 1 | 0.007
H11b | 66T | 1 | 0.007
H11b1 | 10088 | 2 | 0.014
H11b1 | 1187 | 1 | 0.007
H11b1 | 131 | 1 | 0.007
H11b1 | 14093 | 1 | 0.007

H11b1 | 146 | 3 | 0.021
H11b1 | 15097 | 1 | 0.007
H11b1 | 152 | 3 | 0.021
H11b1 | 15943 | 1 | 0.007
H11b1 | 16051 | 2 | 0.014
H11b1 | 16179 | 1 | 0.007
H11b1 | 16261 | 2 | 0.014
H11b1 | 16319 | 1 | 0.007
H11b1 | 16356 | 1 | 0.007
H11b1 | 16357 | 1 | 0.007
H11b1 | 16390 | 3 | 0.021
H11b1 | 1681 | 1 | 0.007
H11b1 | 1850 | 1 | 0.007
H11b1 | 214 | 1 | 0.007
H11b1 | 310 | 3 | 0.021
H11b1 | 315.2C | 1 | 0.007
H11b1 | 315.3C | 2 | 0.014
H11b1 | 4050 | 1 | 0.007
H11b1 | 4135 | 1 | 0.007
H11b1 | 42.1G | 2 | 0.014
H11b1 | 4859 | 1 | 0.007
H11b1 | 499 | 1 | 0.007
H11b1 | 6260 | 2 | 0.014
H11b1 | 7861 | 1 | 0.007
H11b1 | 9138A | 1 | 0.007
H11b1 | 93 | 2 | 0.014
H11b1 | 9315 | 1 | 0.007
H12 | 146 | 4 | 0.129
H12 | 16274 | 3 | 0.097
H12 | 3460 | 3 | 0.097
H12a | 152 | 1 | 0.033
H12a | 16093 | 1 | 0.033
H12a | 16189 | 1 | 0.033
H12a | 16304 | 4 | 0.133
H12a | 73 | 2 | 0.067
H12a | 93 | 4 | 0.133
H13 | 113 | 1 | 0.001
H13 | 11650 | 2 | 0.003
H13 | 14830 | 1 | 0.001
H13 | 16076A | 123 | 0.156
H13 | 208 | 1 | 0.001
H13 | 261 | 1 | 0.001
H13 | 281C | 1 | 0.001
H13 | 316d | 1 | 0.001
H13 | 338 | 1 | 0.001
H13 | 466 | 4 | 0.005
H13 | 468 | 3 | 0.004
H13 | 502 | 1 | 0.001
H13 | 573.1C | 3 | 0.004
H13 | 573.2C | 5 | 0.006
H13 | 573.5C | 1 | 0.001
H13a | 1008 | 1 | 0.001
H13a | 113 | 1 | 0.001
H13a | 12662 | 1 | 0.001
H13a | 13105 | 1 | 0.001
H13a | 16076A | 123 | 0.156
H13a | 16172 | 1 | 0.001
H13a | 16282A | 1 | 0.001
H13a | 16283C | 1 | 0.001
H13a | 208 | 1 | 0.001
H13a | 261 | 1 | 0.001
H13a | 281C | 1 | 0.001
H13a | 316d | 1 | 0.001
H13a | 338 | 1 | 0.001
H13a | 3571.1C | 1 | 0.001
H13a | 4134 | 1 | 0.001
H13a | 468 | 3 | 0.004
H13a | 502 | 1 | 0.001
H13a | 5656 | 1 | 0.001
H13a | 573.1C | 3 | 0.004
H13a | 573.2C | 5 | 0.006
H13a | 573.5C | 1 | 0.001
H13a | 9575 | 1 | 0.001
H13a1 | 113 | 1 | 0.001
H13a1 | 16076A | 123 | 0.157
H13a1 | 208 | 1 | 0.001
H13a1 | 261 | 1 | 0.001
H13a1 | 281C | 1 | 0.001
H13a1 | 316d | 1 | 0.001
H13a1 | 338 | 1 | 0.001
H13a1 | 468 | 3 | 0.004
H13a1 | 502 | 1 | 0.001
H13a1 | 573.1C | 3 | 0.004
H13a1 | 573.2C | 5 | 0.006
H13a1 | 573.5C | 1 | 0.001
H13a1+152 | 10457 | 1 | 0.007
H13a1+152 | 1189 | 1 | 0.007
H13a1+152 | 12471 | 1 | 0.007
H13a1+152 | 13135 | 1 | 0.007
H13a1+152 | 13965 | 1 | 0.007
H13a1+152 | 14245 | 1 | 0.007
H13a1+152 | 146 | 3 | 0.021
H13a1+152 | 15109 | 1 | 0.007
H13a1+152 | 16111 | 21 | 0.147
H13a1+152 | 16311 | 3 | 0.021
H13a1+152 | 2387 | 1 | 0.007
H13a1+152 | 3435 | 1 | 0.007
H13a1+152 | 573.1C | 1 | 0.007
H13a1+152 | 7083 | 1 | 0.007
H13a1+152 | 93 | 1 | 0.007
H13a1a | 113 | 1 | 0.001
H13a1a | 12061 | 1 | 0.001
H13a1a | 12771 | 1 | 0.001
H13a1a | 12930 | 3 | 0.004
H13a1a | 13011 | 1 | 0.001
H13a1a | 13708 | 1 | 0.001
H13a1a | 14484 | 1 | 0.001
H13a1a | 14560 | 1 | 0.001
H13a1a | 146 | 2 | 0.002
H13a1a | 14770 | 1 | 0.001
H13a1a | 14794 | 1 | 0.001

H13a1a | 153 | 1 | 0.001
H13a1a | 15355 | 1 | 0.001
H13a1a | 15721 | 1 | 0.001
H13a1a | 15784 | 1 | 0.001
H13a1a | 15913 | 1 | 0.001
H13a1a | 15927 | 1 | 0.001
H13a1a | 16076A | 1 | 0.001
H13a1a | 16129 | 1 | 0.001
H13a1a | 16153 | 2 | 0.002
H13a1a | 16180 | 1 | 0.001
H13a1a | 16230 | 1 | 0.001
H13a1a | 16269 | 124 | 0.152
H13a1a | 16304 | 1 | 0.001
H13a1a | 16311 | 1 | 0.001
H13a1a | 200 | 1 | 0.001
H13a1a | 2059 | 1 | 0.001
H13a1a | 2074d | 1 | 0.001
H13a1a | 208 | 1 | 0.001
H13a1a | 2326 | 1 | 0.001
H13a1a | 261 | 1 | 0.001
H13a1a | 2803 | 1 | 0.001
H13a1a | 281C | 1 | 0.001
H13a1a | 301C | 1 | 0.001
H13a1a | 302d | 1 | 0.001
H13a1a | 310 | 1 | 0.001
H13a1a | 3150 | 1 | 0.001
H13a1a | 316d | 1 | 0.001
H13a1a | 338 | 1 | 0.001
H13a1a | 3637 | 1 | 0.001
H13a1a | 4201C | 1 | 0.001
H13a1a | 468 | 3 | 0.004
H13a1a | 4939G | 1 | 0.001
H13a1a | 502 | 1 | 0.001
H13a1a | 5582 | 1 | 0.001
H13a1a | 5618 | 1 | 0.001
H13a1a | 573.1C | 5 | 0.006
H13a1a | 573.2C | 6 | 0.007
H13a1a | 573.5C | 1 | 0.001
H13a1a | 6480 | 1 | 0.001
H13a1a | 6951 | 1 | 0.001
H13a1a | 7146 | 1 | 0.001
H13a1a | 7313 | 1 | 0.001
H13a1a | 7849 | 1 | 0.001
H13a1a | 811 | 1 | 0.001
H13a1a | 8256 | 1 | 0.001
H13a1a | 858 | 1 | 0.001
H13a1a | 8764 | 2 | 0.002
H13a1a | 8790 | 1 | 0.001
H13a1a | 9233 | 1 | 0.001
H13a1a | 9287 | 1 | 0.001
H13a1a | 9575T | 1 | 0.001
H13a1a | 9755 | 1 | 0.001
H13a1a | 9827 | 2 | 0.002
H13a1a1 | 10688 | 1 | 0.001
H13a1a1 | 10792 | 1 | 0.001
H13a1a1 | 11025 | 1 | 0.001
H13a1a1 | 11204 | 2 | 0.002
H13a1a1 | 113 | 1 | 0.001
H13a1a1 | 11485 | 1 | 0.001
H13a1a1 | 12235 | 2 | 0.002
H13a1a1 | 12397 | 2 | 0.002
H13a1a1 | 13042 | 1 | 0.001
H13a1a1 | 13485 | 1 | 0.001
H13a1a1 | 1352A | 8 | 0.01
H13a1a1 | 14053 | 1 | 0.001
H13a1a1 | 14221 | 1 | 0.001
H13a1a1 | 14280 | 2 | 0.002
H13a1a1 | 146 | 4 | 0.005
H13a1a1 | 14783 | 1 | 0.001
H13a1a1 | 14790 | 1 | 0.001
H13a1a1 | 150 | 5 | 0.006
H13a1a1 | 15043 | 1 | 0.001
H13a1a1 | 152 | 2 | 0.002
H13a1a1 | 15900 | 8 | 0.01
H13a1a1 | 16076A | 1 | 0.001
H13a1a1 | 16188 | 1 | 0.001
H13a1a1 | 16257 | 1 | 0.001
H13a1a1 | 16261 | 124 | 0.151
H13a1a1 | 16297 | 2 | 0.002
H13a1a1 | 16311 | 1 | 0.001
H13a1a1 | 16527 | 1 | 0.001
H13a1a1 | 1709 | 1 | 0.001
H13a1a1 | 195 | 1 | 0.001
H13a1a1 | 208 | 1 | 0.001
H13a1a1 | 236 | 1 | 0.001
H13a1a1 | 2524T | 1 | 0.001
H13a1a1 | 261 | 1 | 0.001
H13a1a1 | 281C | 1 | 0.001
H13a1a1 | 310 | 3 | 0.004
H13a1a1 | 316d | 1 | 0.001
H13a1a1 | 338 | 1 | 0.001
H13a1a1 | 3921 | 1 | 0.001
H13a1a1 | 4117 | 1 | 0.001
H13a1a1 | 459.1C | 1 | 0.001
H13a1a1 | 502 | 1 | 0.001
H13a1a1 | 5616 | 1 | 0.001
H13a1a1 | 573.1C | 3 | 0.004
H13a1a1 | 573.2C | 9 | 0.011
H13a1a1 | 573.5C | 1 | 0.001
H13a1a1 | 5899.1C | 1 | 0.001
H13a1a1 | 6221 | 1 | 0.001
H13a1a1 | 6480 | 1 | 0.001
H13a1a1 | 679A | 2 | 0.002
H13a1a1 | 709 | 2 | 0.002
H13a1a1 | 7125 | 1 | 0.001
H13a1a1 | 73 | 4 | 0.005
H13a1a1 | 7521 | 1 | 0.001
H13a1a1 | 7804 | 1 | 0.001
H13a1a1 | 9007 | 1 | 0.001
H13a1a1 | 93 | 1 | 0.001
H13a1a1 | 9336 | 1 | 0.001

H13a1a1 | 960.1C | 1 | 0.001
H13a1a1 | 9612 | 1 | 0.001
H13a1a1 | 9708 | 4 | 0.005
H13a1a1a | 10421 | 1 | 0.001
H13a1a1a | 113 | 1 | 0.001
H13a1a1a | 11778 | 1 | 0.001
H13a1a1a | 13851 | 2 | 0.002
H13a1a1a | 152 | 25 | 0.031
H13a1a1a | 15603 | 1 | 0.001
H13a1a1a | 15779 | 1 | 0.001
H13a1a1a | 16037 | 1 | 0.001
H13a1a1a | 16076A | 1 | 0.001
H13a1a1a | 16111 | 1 | 0.001
H13a1a1a | 16129 | 1 | 0.001
H13a1a1a | 16181 | 4 | 0.005
H13a1a1a | 16189 | 1 | 0.001
H13a1a1a | 16209 | 1 | 0.001
H13a1a1a | 16218 | 1 | 0.001
H13a1a1a | 16224 | 1 | 0.001
H13a1a1a | 16271 | 1 | 0.001
H13a1a1a | 16327 | 123 | 0.151
H13a1a1a | 208 | 1 | 0.001
H13a1a1a | 234 | 1 | 0.001
H13a1a1a | 2557 | 2 | 0.002
H13a1a1a | 261 | 1 | 0.001
H13a1a1a | 281C | 1 | 0.001
H13a1a1a | 309d | 1 | 0.001
H13a1a1a | 310 | 2 | 0.002
H13a1a1a | 316d | 1 | 0.001
H13a1a1a | 338 | 1 | 0.001
H13a1a1a | 468 | 3 | 0.004
H13a1a1a | 502 | 1 | 0.001
H13a1a1a | 5070 | 1 | 0.001
H13a1a1a | 5460 | 1 | 0.001
H13a1a1a | 573.1C | 3 | 0.004
H13a1a1a | 573.2C | 5 | 0.006
H13a1a1a | 573.5C | 1 | 0.001
H13a1a1a | 5899d | 1 | 0.001
H13a1a1a | 6605 | 1 | 0.001
H13a1a1a | 6755 | 1 | 0.001
H13a1a1a | 7049 | 1 | 0.001
H13a1a1a | 709 | 2 | 0.002
H13a1a1a | 7813 | 1 | 0.001
H13a1a1a | 7964 | 1 | 0.001
H13a1a1a | 8281-8289d | 1 | 0.001
H13a1a1a | 8614 | 1 | 0.001
H13a1a1a | 9055 | 1 | 0.001
H13a1a1b | 113 | 1 | 0.001
H13a1a1b | 16076A | 1 | 0.001
H13a1a1b | 16129 | 1 | 0.001
H13a1a1b | 16153 | 124 | 0.157
H13a1a1b | 208 | 1 | 0.001
H13a1a1b | 214 | 1 | 0.001
H13a1a1b | 261 | 1 | 0.001
H13a1a1b | 281C | 1 | 0.001
H13a1a1b | 316d | 1 | 0.001
H13a1a1b | 338 | 1 | 0.001
H13a1a1b | 468 | 3 | 0.004
H13a1a1b | 502 | 1 | 0.001
H13a1a1b | 573.1C | 3 | 0.004
H13a1a1b | 573.2C | 5 | 0.006
H13a1a1b | 573.5C | 1 | 0.001
H13a1a1c | 113 | 1 | 0.001
H13a1a1c | 13820 | 3 | 0.004
H13a1a1c | 15927 | 1 | 0.001
H13a1a1c | 16076A | 1 | 0.001
H13a1a1c | 16093 | 124 | 0.157
H13a1a1c | 182 | 1 | 0.001
H13a1a1c | 208 | 1 | 0.001
H13a1a1c | 2443 | 1 | 0.001
H13a1a1c | 261 | 1 | 0.001
H13a1a1c | 281C | 1 | 0.001
H13a1a1c | 316d | 1 | 0.001
H13a1a1c | 338 | 1 | 0.001
H13a1a1c | 468 | 3 | 0.004
H13a1a1c | 502 | 1 | 0.001
H13a1a1c | 573.1C | 3 | 0.004
H13a1a1c | 573.2C | 5 | 0.006
H13a1a1c | 573.5C | 1 | 0.001
H13a1a1c | 7365 | 1 | 0.001
H13a1a1d | 152 | 2 | 0.029
H13a1a1d | 16093 | 1 | 0.015
H13a1a1d | 16192 | 1 | 0.015
H13a1a1d | 16249 | 3 | 0.044
H13a1a1d | 16292 | 3 | 0.044
H13a1a1d | 16301 | 1 | 0.015
H13a1a1d | 16320 | 3 | 0.044
H13a1a1d | 195 | 5 | 0.074
H13a1a1d | 3918 | 1 | 0.015
H13a1a1d | 6927 | 1 | 0.015
H13a1a1d | 8764 | 1 | 0.015
H13a1a1d | 9083 | 1 | 0.015
H13a1a1d1 | 11549 | 1 | 0.014
H13a1a1d1 | 12811 | 1 | 0.014
H13a1a1d1 | 13617 | 1 | 0.014
H13a1a1d1 | 13759 | 1 | 0.014
H13a1a1d1 | 152 | 1 | 0.014
H13a1a1d1 | 16301 | 1 | 0.014
H13a1a1d1 | 3010 | 1 | 0.014
H13a1a1d1 | 309.3C | 1 | 0.014
H13a1a1d1 | 3549 | 1 | 0.014
H13a1a1d1 | 373 | 1 | 0.014
H13a1a1d1 | 8281-8289d | 3 | 0.043
H13a1a1d1 | 8290d | 1 | 0.014
H13a1a1d1 | 8588A | 1 | 0.014
H13a1a1d1 | 8740 | 1 | 0.014
H13a1a1e | 113 | 1 | 0.001
H13a1a1e | 131 | 1 | 0.001
H13a1a1e | 14040 | 1 | 0.001
H13a1a1e | 15601 | 1 | 0.001
H13a1a1e | 16076A | 1 | 0.001

H13a1a1e | 16129 | 1 | 0.001
H13a1a1e | 16209 | 123 | 0.155
H13a1a1e | 16261 | 123 | 0.155
H13a1a1e | 208 | 1 | 0.001
H13a1a1e | 261 | 1 | 0.001
H13a1a1e | 281C | 1 | 0.001
H13a1a1e | 310 | 1 | 0.001
H13a1a1e | 316d | 1 | 0.001
H13a1a1e | 338 | 1 | 0.001
H13a1a1e | 468 | 3 | 0.004
H13a1a1e | 502 | 1 | 0.001
H13a1a1e | 573.1C | 3 | 0.004
H13a1a1e | 573.2C | 5 | 0.006
H13a1a1e | 573.5C | 1 | 0.001
H13a1a1e | 5812 | 1 | 0.001
H13a1a1e | 9755 | 1 | 0.001
H13a1a2 | 113 | 1 | 0.001
H13a1a2 | 16076A | 123 | 0.156
H13a1a2 | 16193 | 1 | 0.001
H13a1a2 | 16305 | 1 | 0.001
H13a1a2 | 195 | 1 | 0.001
H13a1a2 | 208 | 1 | 0.001
H13a1a2 | 261 | 1 | 0.001
H13a1a2 | 281C | 1 | 0.001
H13a1a2 | 316d | 1 | 0.001
H13a1a2 | 338 | 1 | 0.001
H13a1a2 | 468 | 3 | 0.004
H13a1a2 | 502 | 1 | 0.001
H13a1a2 | 573.1C | 3 | 0.004
H13a1a2 | 573.2C | 5 | 0.006
H13a1a2 | 573.5C | 1 | 0.001
H13a1a2 | 709 | 1 | 0.001
H13a1a2 | 9025 | 1 | 0.001
H13a1a2 | 9449 | 2 | 0.003
H13a1a2+16311 | 16150 | 1 | 0.003
H13a1a2+16311 | 16159 | 1 | 0.003
H13a1a2+16311 | 16177 | 1 | 0.003
H13a1a2+16311 | 16209 | 3 | 0.009
H13a1a2+16311 | 16284 | 35 | 0.108
H13a1a2+16311 | 16566 | 1 | 0.003
H13a1a2+16311 | 237 | 1 | 0.003
H13a1a2+16311 | 246 | 1 | 0.003
H13a1a2+16311 | 288 | 1 | 0.003
H13a1a2+16311 | 484 | 1 | 0.003
H13a1a2a | 11563 | 2 | 0.019
H13a1a2a | 12738 | 1 | 0.01
H13a1a2a | 13980 | 1 | 0.01
H13a1a2a | 152 | 11 | 0.107
H13a1a2a | 16086 | 2 | 0.019
H13a1a2a | 16129 | 3 | 0.029
H13a1a2a | 385 | 1 | 0.01
H13a1a2a | 497 | 18 | 0.175
H13a1a2a | 73 | 23 | 0.223
H13a1a2a | 7471.1C | 1 | 0.01
H13a1a2b | 16187 | 16 | 0.333
H13a1a2b | 189 | 4 | 0.083
H13a1a2b | 573.3C | 1 | 0.021
H13a1a2b | 93 | 1 | 0.021
H13a1a3 | 10373 | 1 | 0.001
H13a1a3 | 113 | 1 | 0.001
H13a1a3 | 11368 | 1 | 0.001
H13a1a3 | 1187 | 1 | 0.001
H13a1a3 | 14281 | 1 | 0.001
H13a1a3 | 14683 | 1 | 0.001
H13a1a3 | 14992A | 1 | 0.001
H13a1a3 | 16076A | 123 | 0.156
H13a1a3 | 208 | 1 | 0.001
H13a1a3 | 261 | 1 | 0.001
H13a1a3 | 281C | 1 | 0.001
H13a1a3 | 310 | 1 | 0.001
H13a1a3 | 315.2C | 1 | 0.001
H13a1a3 | 316d | 1 | 0.001
H13a1a3 | 338 | 1 | 0.001
H13a1a3 | 468 | 3 | 0.004
H13a1a3 | 4775 | 1 | 0.001
H13a1a3 | 502 | 1 | 0.001
H13a1a3 | 573.1C | 3 | 0.004
H13a1a3 | 573.2C | 5 | 0.006
H13a1a3 | 573.5C | 1 | 0.001
H13a1a3 | 8865 | 1 | 0.001
H13a1a4 | 113 | 1 | 0.001
H13a1a4 | 16076A | 123 | 0.156
H13a1a4 | 208 | 1 | 0.001
H13a1a4 | 261 | 1 | 0.001
H13a1a4 | 281C | 1 | 0.001
H13a1a4 | 310 | 1 | 0.001
H13a1a4 | 315.2C | 1 | 0.001
H13a1a4 | 316d | 1 | 0.001
H13a1a4 | 338 | 1 | 0.001
H13a1a4 | 468 | 3 | 0.004
H13a1a4 | 502 | 1 | 0.001
H13a1a4 | 573.1C | 3 | 0.004
H13a1a4 | 573.2C | 5 | 0.006
H13a1a4 | 573.5C | 1 | 0.001
H13a1a4 | 6716 | 1 | 0.001
H13a1a5 | 113 | 1 | 0.001
H13a1a5 | 15758 | 1 | 0.001
H13a1a5 | 16076A | 123 | 0.156
H13a1a5 | 182 | 1 | 0.001
H13a1a5 | 208 | 1 | 0.001
H13a1a5 | 261 | 1 | 0.001
H13a1a5 | 281C | 1 | 0.001
H13a1a5 | 316d | 1 | 0.001
H13a1a5 | 338 | 1 | 0.001
H13a1a5 | 468 | 3 | 0.004
H13a1a5 | 502 | 1 | 0.001
H13a1a5 | 573.1C | 3 | 0.004
H13a1a5 | 573.2C | 5 | 0.006
H13a1a5 | 573.5C | 1 | 0.001
H13a1a6 | 108.1A | 1 | 0.077
H13a1a6 | 152 | 2 | 0.154

H13a1a6 | 15323 | 1 | 0.077
H13a1a6 | 16071 | 1 | 0.077
H13a1a6 | 16172 | 1 | 0.077
H13a1a6 | 16192A | 1 | 0.077
H13a1a6 | 16304 | 2 | 0.154
H13a1a6 | 16325 | 1 | 0.077
H13a1a6 | 16375A | 1 | 0.077
H13a1a6 | 16419A | 1 | 0.077
H13a1a6 | 234 | 1 | 0.077
H13a1a6 | 291d | 1 | 0.077
H13a1a6 | 315.2C | 1 | 0.077
H13a1a6 | 85C | 1 | 0.077
H13a1a6 | 9755 | 1 | 0.077
H13a1b | 13813 | 1 | 0.033
H13a1b | 15998C | 1 | 0.033
H13a1b | 16022A | 1 | 0.033
H13a1b | 16059 | 1 | 0.033
H13a1b | 16061 | 1 | 0.033
H13a1b | 16064 | 1 | 0.033
H13a1b | 16084T | 1 | 0.033
H13a1b | 16087 | 2 | 0.067
H13a1b | 16089 | 1 | 0.033
H13a1b | 16143 | 1 | 0.033
H13a1b | 16149 | 1 | 0.033
H13a1b | 16173G | 1 | 0.033
H13a1b | 16177T | 1 | 0.033
H13a1b | 16189G | 1 | 0.033
H13a1b | 16194T | 1 | 0.033
H13a1b | 16199A | 1 | 0.033
H13a1b | 16200 | 1 | 0.033
H13a1b | 16220T | 1 | 0.033
H13a1b | 16226 | 1 | 0.033
H13a1b | 16277 | 1 | 0.033
H13a1b | 16291 | 1 | 0.033
H13a1b | 16311 | 1 | 0.033
H13a1b | 16311G | 1 | 0.033
H13a1b | 16342G | 1 | 0.033
H13a1b | 16347 | 2 | 0.067
H13a1b | 16350 | 1 | 0.033
H13a1b | 16354A | 1 | 0.033
H13a1b | 16374 | 2 | 0.067
H13a1b | 16396 | 1 | 0.033
H13a1b | 7080 | 3 | 0.1
H13a1c | 1007 | 2 | 0.027
H13a1c | 11242 | 2 | 0.027
H13a1c | 11930 | 1 | 0.014
H13a1c | 13416 | 1 | 0.014
H13a1c | 152 | 2 | 0.027
H13a1c | 16092 | 2 | 0.027
H13a1c | 16169 | 1 | 0.014
H13a1c | 16189 | 4 | 0.055
H13a1c | 16214 | 1 | 0.014
H13a1c | 16278 | 3 | 0.041
H13a1c | 16311 | 1 | 0.014
H13a1c | 16384 | 3 | 0.041
H13a1c | 16399 | 6 | 0.082
H13a1c | 207 | 6 | 0.082
H13a1c | 5366 | 2 | 0.027
H13a1c | 789 | 1 | 0.014
H13a1c | 8251 | 1 | 0.014
H13a1c | 8293 | 2 | 0.027
H13a1c | 8388 | 2 | 0.027
H13a1c | 9135 | 1 | 0.014
H13a1d | 14858 | 1 | 0.02
H13a1d | 150 | 1 | 0.02
H13a1d | 16085d | 1 | 0.02
H13a1d | 16093 | 1 | 0.02
H13a1d | 16129 | 1 | 0.02
H13a1d | 16145 | 8 | 0.16
H13a1d | 16209 | 2 | 0.04
H13a1d | 16220 | 1 | 0.02
H13a1d | 16254 | 1 | 0.02
H13a1d | 16260 | 1 | 0.02
H13a1d | 16270 | 1 | 0.02
H13a1d | 16284 | 3 | 0.06
H13a1d | 16296 | 1 | 0.02
H13a1d | 16304 | 1 | 0.02
H13a1d | 16362 | 5 | 0.1
H13a1d | 16T | 1 | 0.02
H13a1d | 1888 | 1 | 0.02
H13a1d | 195 | 1 | 0.02
H13a1d | 235 | 1 | 0.02
H13a1d | 513 | 1 | 0.02
H13a1d | 6.1C | 1 | 0.02
H13a1d | 73 | 1 | 0.02
H13a2 | 113 | 1 | 0.001
H13a2 | 14857 | 1 | 0.001
H13a2 | 16076A | 123 | 0.158
H13a2 | 16129 | 1 | 0.001
H13a2 | 16256 | 1 | 0.001
H13a2 | 193 | 1 | 0.001
H13a2 | 208 | 1 | 0.001
H13a2 | 249 | 1 | 0.001
H13a2 | 261 | 1 | 0.001
H13a2 | 281C | 1 | 0.001
H13a2 | 316d | 1 | 0.001
H13a2 | 338 | 1 | 0.001
H13a2 | 366 | 1 | 0.001
H13a2 | 389 | 1 | 0.001
H13a2 | 468 | 3 | 0.004
H13a2 | 502 | 1 | 0.001
H13a2 | 573.1C | 3 | 0.004
H13a2 | 573.2C | 5 | 0.006
H13a2 | 573.5C | 1 | 0.001
H13a2 | 622 | 1 | 0.001
H13a2a | 10680 | 1 | 0.001
H13a2a | 10751 | 1 | 0.001
H13a2a | 11047A | 1 | 0.001
H13a2a | 11151 | 1 | 0.001
H13a2a | 113 | 1 | 0.001
H13a2a | 11350 | 2 | 0.002

H13a2a | 12076 | 9 | 0.011
H13a2a | 14323 | 1 | 0.001
H13a2a | 15043 | 2 | 0.002
H13a2a | 151 | 2 | 0.002
H13a2a | 15110 | 1 | 0.001
H13a2a | 15118 | 2 | 0.002
H13a2a | 152 | 2 | 0.002
H13a2a | 153 | 9 | 0.011
H13a2a | 16076A | 1 | 0.001
H13a2a | 16243 | 9 | 0.011
H13a2a | 16256G | 125 | 0.156
H13a2a | 16311 | 1 | 0.001
H13a2a | 16465 | 1 | 0.001
H13a2a | 1768 | 1 | 0.001
H13a2a | 183 | 1 | 0.001
H13a2a | 200 | 9 | 0.011
H13a2a | 208 | 1 | 0.001
H13a2a | 2232.1A | 1 | 0.001
H13a2a | 234 | 1 | 0.001
H13a2a | 261 | 1 | 0.001
H13a2a | 281C | 1 | 0.001
H13a2a | 3106A | 1 | 0.001
H13a2a | 3156 | 3 | 0.004
H13a2a | 316d | 1 | 0.001
H13a2a | 338 | 1 | 0.001
H13a2a | 366 | 1 | 0.001
H13a2a | 3834 | 3 | 0.004
H13a2a | 389 | 1 | 0.001
H13a2a | 468 | 3 | 0.004
H13a2a | 4820 | 3 | 0.004
H13a2a | 4947 | 1 | 0.001
H13a2a | 502 | 1 | 0.001
H13a2a | 5442 | 1 | 0.001
H13a2a | 573.1C | 3 | 0.004
H13a2a | 573.2C | 5 | 0.006
H13a2a | 573.5C | 1 | 0.001
H13a2a | 5773 | 3 | 0.004
H13a2a | 5794 | 2 | 0.002
H13a2a | 5894 | 1 | 0.001
H13a2a | 622 | 1 | 0.001
H13a2a | 7184 | 1 | 0.001
H13a2a | 8555 | 2 | 0.002
H13a2a | 8632 | 1 | 0.001
H13a2a | 8790 | 1 | 0.001
H13a2a | 9269 | 2 | 0.002
H13a2a | 9824 | 1 | 0.001
H13a2a1 | 11017 | 1 | 0.001
H13a2a1 | 113 | 1 | 0.001
H13a2a1 | 11914 | 1 | 0.001
H13a2a1 | 12082 | 1 | 0.001
H13a2a1 | 131 | 1 | 0.001
H13a2a1 | 13695 | 1 | 0.001
H13a2a1 | 15154 | 1 | 0.001
H13a2a1 | 16076A | 123 | 0.157
H13a2a1 | 16290 | 1 | 0.001
H13a2a1 | 16362 | 1 | 0.001
H13a2a1 | 195 | 1 | 0.001
H13a2a1 | 208 | 1 | 0.001
H13a2a1 | 261 | 1 | 0.001
H13a2a1 | 281C | 1 | 0.001
H13a2a1 | 309d | 1 | 0.001
H13a2a1 | 316d | 1 | 0.001
H13a2a1 | 338 | 1 | 0.001
H13a2a1 | 366 | 1 | 0.001
H13a2a1 | 389 | 1 | 0.001
H13a2a1 | 468 | 3 | 0.004
H13a2a1 | 4977 | 1 | 0.001
H13a2a1 | 502 | 1 | 0.001
H13a2a1 | 573.1C | 3 | 0.004
H13a2a1 | 573.2C | 5 | 0.006
H13a2a1 | 573.5C | 1 | 0.001
H13a2a1 | 622 | 1 | 0.001
H13a2a1 | 6366 | 1 | 0.001
H13a2a1 | 8648 | 1 | 0.001
H13a2b | 113 | 1 | 0.001
H13a2b | 13152T | 2 | 0.003
H13a2b | 14857 | 2 | 0.003
H13a2b | 16076A | 123 | 0.158
H13a2b | 208 | 1 | 0.001
H13a2b | 261 | 1 | 0.001
H13a2b | 281C | 1 | 0.001
H13a2b | 316d | 1 | 0.001
H13a2b | 338 | 1 | 0.001
H13a2b | 366 | 1 | 0.001
H13a2b | 389 | 1 | 0.001
H13a2b | 468 | 3 | 0.004
H13a2b | 502 | 1 | 0.001
H13a2b | 573.1C | 3 | 0.004
H13a2b | 573.2C | 5 | 0.006
H13a2b | 573.5C | 1 | 0.001
H13a2b | 622 | 1 | 0.001
H13a2b1 | 13889 | 1 | 0.003
H13a2b1 | 143 | 2 | 0.006
H13a2b1 | 16150 | 1 | 0.003
H13a2b1 | 16159 | 1 | 0.003
H13a2b1 | 16177 | 1 | 0.003
H13a2b1 | 16209 | 3 | 0.009
H13a2b1 | 16214 | 1 | 0.003
H13a2b1 | 16258 | 3 | 0.009
H13a2b1 | 16284 | 35 | 0.106
H13a2b1 | 16293 | 1 | 0.003
H13a2b1 | 16335 | 1 | 0.003
H13a2b1 | 16527 | 4 | 0.012
H13a2b1 | 16566 | 1 | 0.003
H13a2b1 | 237 | 1 | 0.003
H13a2b1 | 246 | 1 | 0.003
H13a2b1 | 288 | 1 | 0.003
H13a2b1 | 456 | 1 | 0.003
H13a2b1 | 484 | 1 | 0.003
H13a2b1 | 5899.1C | 3 | 0.009
H13a2b1 | 5901.1C | 1 | 0.003

H13a2b1 | 7322 | 1 | 0.003
H13a2b2 | 113 | 1 | 0.001
H13a2b2 | 14696 | 1 | 0.001
H13a2b2 | 14956 | 1 | 0.001
H13a2b2 | 15299 | 1 | 0.001
H13a2b2 | 16075 | 1 | 0.001
H13a2b2 | 16076A | 123 | 0.157
H13a2b2 | 16093 | 1 | 0.001
H13a2b2 | 16129 | 3 | 0.004
H13a2b2 | 16311 | 1 | 0.001
H13a2b2 | 208 | 1 | 0.001
H13a2b2 | 2413 | 1 | 0.001
H13a2b2 | 261 | 1 | 0.001
H13a2b2 | 2757 | 6 | 0.008
H13a2b2 | 281C | 1 | 0.001
H13a2b2 | 316d | 1 | 0.001
H13a2b2 | 338 | 1 | 0.001
H13a2b2 | 356.1C | 1 | 0.001
H13a2b2 | 366 | 1 | 0.001
H13a2b2 | 389 | 1 | 0.001
H13a2b2 | 468 | 3 | 0.004
H13a2b2 | 502 | 1 | 0.001
H13a2b2 | 573.1C | 3 | 0.004
H13a2b2 | 573.2C | 5 | 0.006
H13a2b2 | 573.5C | 1 | 0.001
H13a2b2 | 622 | 1 | 0.001
H13a2b2 | 8697 | 1 | 0.001
H13a2b2a | 10993 | 1 | 0.001
H13a2b2a | 113 | 1 | 0.001
H13a2b2a | 13812 | 1 | 0.001
H13a2b2a | 14476 | 1 | 0.001
H13a2b2a | 14663 | 1 | 0.001
H13a2b2a | 16076A | 123 | 0.156
H13a2b2a | 208 | 1 | 0.001
H13a2b2a | 261 | 1 | 0.001
H13a2b2a | 281C | 1 | 0.001
H13a2b2a | 310 | 4 | 0.005
H13a2b2a | 315.2C | 1 | 0.001
H13a2b2a | 316d | 1 | 0.001
H13a2b2a | 338 | 1 | 0.001
H13a2b2a | 366 | 1 | 0.001
H13a2b2a | 389 | 1 | 0.001
H13a2b2a | 468 | 3 | 0.004
H13a2b2a | 502 | 1 | 0.001
H13a2b2a | 573.1C | 3 | 0.004
H13a2b2a | 573.2C | 5 | 0.006
H13a2b2a | 573.5C | 1 | 0.001
H13a2b2a | 5933 | 1 | 0.001
H13a2b2a | 622 | 1 | 0.001
H13a2b2a | 6929 | 1 | 0.001
H13a2b2a | 72 | 2 | 0.003
H13a2b2a | 7340 | 6 | 0.008
H13a2b2a | 8093 | 1 | 0.001
H13a2b2a | 8700 | 1 | 0.001
H13a2b3 | 103 | 1 | 0.111
H13a2b3 | 11641 | 1 | 0.111
H13a2b3 | 16188 | 1 | 0.111
H13a2b3 | 16192 | 1 | 0.111
H13a2b3 | 16278 | 2 | 0.222
H13a2b3 | 16357 | 1 | 0.111
H13a2b3 | 207 | 1 | 0.111
H13a2b3 | 2264 | 1 | 0.111
H13a2b3 | 573.2C | 1 | 0.111
H13a2b4 | 113 | 1 | 0.001
H13a2b4 | 152 | 1 | 0.001
H13a2b4 | 15662 | 3 | 0.004
H13a2b4 | 16076A | 123 | 0.157
H13a2b4 | 16145 | 1 | 0.001
H13a2b4 | 208 | 1 | 0.001
H13a2b4 | 261 | 1 | 0.001
H13a2b4 | 281C | 1 | 0.001
H13a2b4 | 316d | 1 | 0.001
H13a2b4 | 338 | 1 | 0.001
H13a2b4 | 366 | 1 | 0.001
H13a2b4 | 389 | 1 | 0.001
H13a2b4 | 468 | 3 | 0.004
H13a2b4 | 502 | 1 | 0.001
H13a2b4 | 5196 | 3 | 0.004
H13a2b4 | 573.1C | 3 | 0.004
H13a2b4 | 573.2C | 5 | 0.006
H13a2b4 | 573.5C | 1 | 0.001
H13a2b4 | 622 | 1 | 0.001
H13a2b4 | 93 | 1 | 0.001
H13a2b4 | 9335 | 1 | 0.001
H13a2b5 | 12954 | 1 | 0.011
H13a2b5 | 12A | 2 | 0.022
H13a2b5 | 143 | 2 | 0.022
H13a2b5 | 150.1T | 1 | 0.011
H13a2b5 | 151 | 1 | 0.011
H13a2b5 | 16129 | 1 | 0.011
H13a2b5 | 16192 | 1 | 0.011
H13a2b5 | 16218 | 11 | 0.12
H13a2b5 | 16222 | 1 | 0.011
H13a2b5 | 16223 | 1 | 0.011
H13a2b5 | 16294 | 1 | 0.011
H13a2b5 | 16304 | 2 | 0.022
H13a2b5 | 16309 | 1 | 0.011
H13a2b5 | 16318 | 1 | 0.011
H13a2b5 | 16325 | 1 | 0.011
H13a2b5 | 16356 | 1 | 0.011
H13a2b5 | 16422 | 1 | 0.011
H13a2b5 | 16484-16489d | 1 | 0.011
H13a2b5 | 16524C | 1 | 0.011
H13a2b5 | 183 | 1 | 0.011
H13a2b5 | 189 | 1 | 0.011
H13a2b5 | 203 | 1 | 0.011
H13a2b5 | 204 | 1 | 0.011
H13a2b5 | 214 | 1 | 0.011
H13a2b5 | 249d | 2 | 0.022
H13a2b5 | 309 | 1 | 0.011
H13a2b5 | 341d | 2 | 0.022

H13a2b5 | 3736 | 1 | 0.011
H13a2b5 | 73 | 4 | 0.043
H13a2b5 | 93 | 11 | 0.12
H13a2c | 16145 | 1 | 0.019
H13a2c | 16189 | 1 | 0.019
H13a2c | 16201 | 1 | 0.019
H13a2c | 16362 | 2 | 0.037
H13a2c | 195A | 1 | 0.019
H13a2c | 1978C | 1 | 0.019
H13a2c | 2755 | 1 | 0.019
H13a2c | 324G | 1 | 0.019
H13a2c | 338 | 1 | 0.019
H13a2c | 73 | 1 | 0.019
H13a2c | 93 | 1 | 0.019
H13a2c1 | 10007 | 1 | 0.048
H13a2c1 | 10237 | 6 | 0.286
H13a2c1 | 10373 | 1 | 0.048
H13a2c1 | 11084 | 1 | 0.048
H13a2c1 | 12819 | 1 | 0.048
H13a2c1 | 14028 | 1 | 0.048
H13a2c1 | 143 | 1 | 0.048
H13a2c1 | 14308 | 1 | 0.048
H13a2c1 | 146 | 4 | 0.19
H13a2c1 | 152 | 1 | 0.048
H13a2c1 | 15412 | 13 | 0.619
H13a2c1 | 16129 | 7 | 0.333
H13a2c1 | 16147 | 1 | 0.048
H13a2c1 | 16193 | 3 | 0.143
H13a2c1 | 16311 | 1 | 0.048
H13a2c1 | 16390 | 1 | 0.048
H13a2c1 | 193 | 17 | 0.81
H13a2c1 | 194 | 1 | 0.048
H13a2c1 | 214 | 1 | 0.048
H13a2c1 | 215 | 1 | 0.048
H13a2c1 | 249 | 14 | 0.667
H13a2c1 | 309d | 1 | 0.048
H13a2c1 | 310 | 4 | 0.19
H13a2c1 | 316 | 1 | 0.048
H13a2c1 | 334A | 1 | 0.048
H13a2c1 | 342 | 1 | 0.048
H13a2c1 | 4577 | 14 | 0.667
H13a2c1 | 4820 | 2 | 0.095
H13a2c1 | 5772 | 1 | 0.048
H13a2c1 | 8084 | 1 | 0.048
H13a2c1 | 8993G | 1 | 0.048
H13a2c1 | 93 | 1 | 0.048
H13a2c1 | 9489 | 1 | 0.048
H13b | 113 | 1 | 0.001
H13b | 13635 | 1 | 0.001
H13b | 16016 | 1 | 0.001
H13b | 16076A | 1 | 0.001
H13b | 16134 | 1 | 0.001
H13b | 16263 | 1 | 0.001
H13b | 16335 | 129 | 0.151
H13b | 16446 | 129 | 0.151
H13b | 173 | 1 | 0.001
H13b | 199 | 1 | 0.001
H13b | 208 | 1 | 0.001
H13b | 2280 | 1 | 0.001
H13b | 261 | 1 | 0.001
H13b | 2730 | 1 | 0.001
H13b | 281C | 1 | 0.001
H13b | 316d | 1 | 0.001
H13b | 338 | 1 | 0.001
H13b | 502 | 1 | 0.001
H13b | 573.1C | 3 | 0.004
H13b | 573.2C | 5 | 0.006
H13b | 573.5C | 1 | 0.001
H13b | 6383 | 1 | 0.001
H13b | 8485 | 1 | 0.001
H13b1 | 10370 | 3 | 0.031
H13b1 | 10511A | 1 | 0.01
H13b1 | 11778 | 1 | 0.01
H13b1 | 13620 | 2 | 0.02
H13b1 | 14182 | 1 | 0.01
H13b1 | 152 | 7 | 0.071
H13b1 | 16058T | 1 | 0.01
H13b1 | 16129 | 1 | 0.01
H13b1 | 16145 | 1 | 0.01
H13b1 | 16192 | 4 | 0.041
H13b1 | 16219 | 1 | 0.01
H13b1 | 16239 | 1 | 0.01
H13b1 | 16242 | 3 | 0.031
H13b1 | 16257 | 1 | 0.01
H13b1 | 16289 | 2 | 0.02
H13b1 | 16311 | 3 | 0.031
H13b1 | 16325 | 1 | 0.01
H13b1 | 16344 | 1 | 0.01
H13b1 | 16516 | 1 | 0.01
H13b1 | 195 | 3 | 0.031
H13b1 | 310 | 1 | 0.01
H13b1 | 73 | 1 | 0.01
H13b1 | 8236 | 3 | 0.031
H13b1 | 9225 | 1 | 0.01
H13b1 | 9398 | 3 | 0.031
H13b1+200 | 10274 | 2 | 0.067
H13b1+200 | 10550 | 1 | 0.033
H13b1+200 | 10790 | 1 | 0.033
H13b1+200 | 12015 | 2 | 0.067
H13b1+200 | 13327 | 2 | 0.067
H13b1+200 | 13515 | 1 | 0.033
H13b1+200 | 14070 | 1 | 0.033
H13b1+200 | 14757 | 2 | 0.067
H13b1+200 | 152 | 2 | 0.067
H13b1+200 | 15247 | 1 | 0.033
H13b1+200 | 15617 | 1 | 0.033
H13b1+200 | 16047 | 1 | 0.033
H13b1+200 | 16048 | 3 | 0.1
H13b1+200 | 16076 | 1 | 0.033
H13b1+200 | 16150 | 1 | 0.033
H13b1+200 | 16189 | 6 | 0.2

H13b1+200 | 16247C | 1 | 0.033
H13b1+200 | 16270 | 1 | 0.033
H13b1+200 | 16271 | 1 | 0.033
H13b1+200 | 16291 | 1 | 0.033
H13b1+200 | 16335 | 1 | 0.033
H13b1+200 | 16343 | 1 | 0.033
H13b1+200 | 310 | 1 | 0.033
H13b1+200 | 466 | 1 | 0.033
H13b1+200 | 499 | 1 | 0.033
H13b1+200 | 720 | 2 | 0.067
H13b1+200 | 9083 | 1 | 0.033
H13b1+200 | 960d | 1 | 0.033
H13b1a | 16111 | 3 | 0.081
H13b1a | 16150 | 1 | 0.027
H13b1a | 16192 | 4 | 0.108
H13b1a | 16223 | 5 | 0.135
H13b1a | 16227 | 1 | 0.027
H13b1a | 16242 | 2 | 0.054
H13b1a | 16352 | 1 | 0.027
H13b1a | 16516 | 1 | 0.027
H13b1a | 195 | 1 | 0.027
H13b1a | 204 | 1 | 0.027
H13b1a | 2887 | 1 | 0.027
H13b1a | 513 | 1 | 0.027
H13b1a | 5752.1A | 1 | 0.027
H13b1a | 73 | 1 | 0.027
H13b1a | 9554 | 1 | 0.027
H13b1b | 14118 | 1 | 0.111
H13b1b | 15804 | 1 | 0.111
H13b1b | 16147 | 1 | 0.111
H13b1b | 16189 | 1 | 0.111
H13b1b | 16311 | 3 | 0.333
H13b1b | 16320 | 1 | 0.111
H13b1b | 16343 | 1 | 0.111
H13b1b | 3462 | 1 | 0.111
H13b1b | 4086 | 1 | 0.111
H13b2 | 10909 | 2 | 0.002
H13b2 | 11050G | 1 | 0.001
H13b2 | 113 | 1 | 0.001
H13b2 | 16016 | 1 | 0.001
H13b2 | 16076A | 1 | 0.001
H13b2 | 16223 | 1 | 0.001
H13b2 | 16335 | 129 | 0.151
H13b2 | 16446 | 129 | 0.151
H13b2 | 208 | 1 | 0.001
H13b2 | 234 | 1 | 0.001
H13b2 | 261 | 1 | 0.001
H13b2 | 281C | 1 | 0.001
H13b2 | 316d | 1 | 0.001
H13b2 | 338 | 1 | 0.001
H13b2 | 502 | 1 | 0.001
H13b2 | 573.1C | 3 | 0.004
H13b2 | 573.2C | 5 | 0.006
H13b2 | 573.5C | 1 | 0.001
H13c | 113 | 1 | 0.001
H13c | 16076A | 123 | 0.157
H13c | 208 | 1 | 0.001
H13c | 261 | 1 | 0.001
H13c | 281C | 1 | 0.001
H13c | 316d | 1 | 0.001
H13c | 338 | 1 | 0.001
H13c | 468 | 3 | 0.004
H13c | 502 | 1 | 0.001
H13c | 573.1C | 3 | 0.004
H13c | 573.2C | 5 | 0.006
H13c | 573.5C | 1 | 0.001
H13c1 | 10202 | 1 | 0.001
H13c1 | 10601 | 1 | 0.001
H13c1 | 10604 | 1 | 0.001
H13c1 | 113 | 1 | 0.001
H13c1 | 14189 | 1 | 0.001
H13c1 | 16076A | 123 | 0.156
H13c1 | 16188 | 1 | 0.001
H13c1 | 16192 | 1 | 0.001
H13c1 | 16201 | 1 | 0.001
H13c1 | 16263 | 1 | 0.001
H13c1 | 16264.1C | 1 | 0.001
H13c1 | 1842 | 1 | 0.001
H13c1 | 195 | 1 | 0.001
H13c1 | 200 | 1 | 0.001
H13c1 | 208 | 1 | 0.001
H13c1 | 261 | 1 | 0.001
H13c1 | 281C | 1 | 0.001
H13c1 | 316d | 1 | 0.001
H13c1 | 338 | 1 | 0.001
H13c1 | 3622 | 1 | 0.001
H13c1 | 468 | 3 | 0.004
H13c1 | 502 | 1 | 0.001
H13c1 | 573.1C | 3 | 0.004
H13c1 | 573.2C | 5 | 0.006
H13c1 | 573.5C | 1 | 0.001
H13c1 | 6947 | 1 | 0.001
H13c1 | 7245 | 1 | 0.001
H13c1 | 8870 | 1 | 0.001
H13c1 | 9389 | 1 | 0.001
H13c1 | 971 | 1 | 0.001
H13c1a | 113 | 1 | 0.001
H13c1a | 11866 | 1 | 0.001
H13c1a | 12236 | 2 | 0.003
H13c1a | 14152 | 2 | 0.003
H13c1a | 16076A | 123 | 0.156
H13c1a | 16233 | 1 | 0.001
H13c1a | 16355 | 1 | 0.001
H13c1a | 208 | 1 | 0.001
H13c1a | 261 | 1 | 0.001
H13c1a | 281C | 1 | 0.001
H13c1a | 316d | 1 | 0.001
H13c1a | 338 | 1 | 0.001
H13c1a | 468 | 3 | 0.004
H13c1a | 502 | 1 | 0.001
H13c1a | 573.1C | 3 | 0.004

H13c1a | 573.2C | 5 | 0.006
H13c1a | 573.5C | 1 | 0.001
H13c1a | 6116 | 2 | 0.003
H13c1a | 6179 | 1 | 0.001
H13c1a | 8280 | 2 | 0.003
H13c2 | 10427 | 1 | 0.091
H13c2 | 11188 | 1 | 0.091
H13c2 | 14818 | 1 | 0.091
H13c2 | 152 | 3 | 0.273
H13c2 | 153 | 1 | 0.091
H13c2 | 15406 | 1 | 0.091
H13c2 | 16270 | 1 | 0.091
H13c2 | 16297 | 1 | 0.091
H13c2 | 16298 | 1 | 0.091
H13c2 | 16299 | 1 | 0.091
H13c2 | 16301 | 1 | 0.091
H13c2 | 16497 | 1 | 0.091
H13c2 | 249d | 3 | 0.273
H13c2 | 299d | 1 | 0.091
H13c2 | 324 | 1 | 0.091
H13c2 | 373 | 1 | 0.091
H13c2 | 489 | 2 | 0.182
H13c2 | 8557 | 1 | 0.091
H13c2 | 96d | 1 | 0.091
H14 | 113 | 1 | 0.001
H14 | 11761 | 5 | 0.006
H14 | 15449 | 5 | 0.006
H14 | 16076A | 1 | 0.001
H14 | 16092 | 130 | 0.164
H14 | 16111 | 1 | 0.001
H14 | 16189 | 5 | 0.006
H14 | 185C | 2 | 0.003
H14 | 208 | 1 | 0.001
H14 | 261 | 1 | 0.001
H14 | 281C | 1 | 0.001
H14 | 310 | 4 | 0.005
H14 | 314-315d | 1 | 0.001
H14 | 315d | 3 | 0.004
H14 | 316d | 1 | 0.001
H14 | 338 | 1 | 0.001
H14 | 468 | 3 | 0.004
H14 | 475 | 123 | 0.155
H14 | 502 | 1 | 0.001
H14 | 5508 | 4 | 0.005
H14 | 573.1C | 3 | 0.004
H14 | 573.2C | 5 | 0.006
H14 | 573.5C | 1 | 0.001
H14 | 6340 | 1 | 0.001
H14 | 8857 | 4 | 0.005
H14a | 10398 | 1 | 0.009
H14a | 10573 | 1 | 0.009
H14a | 11084 | 1 | 0.009
H14a | 11377 | 1 | 0.009
H14a | 11914 | 1 | 0.009
H14a | 11969 | 2 | 0.018
H14a | 12172 | 1 | 0.009
H14a | 12501 | 1 | 0.009
H14a | 12810 | 1 | 0.009
H14a | 12879 | 1 | 0.009
H14a | 13194 | 1 | 0.009
H14a | 13470 | 1 | 0.009
H14a | 13500 | 1 | 0.009
H14a | 14043 | 2 | 0.018
H14a | 14470 | 1 | 0.009
H14a | 1462 | 1 | 0.009
H14a | 14766 | 1 | 0.009
H14a | 15132 | 1 | 0.009
H14a | 152 | 3 | 0.028
H14a | 15456G | 1 | 0.009
H14a | 15457G | 1 | 0.009
H14a | 15460A | 1 | 0.009
H14a | 15606 | 1 | 0.009
H14a | 15607T | 1 | 0.009
H14a | 16048 | 1 | 0.009
H14a | 16114A | 1 | 0.009
H14a | 16124 | 1 | 0.009
H14a | 16172 | 1 | 0.009
H14a | 16189 | 1 | 0.009
H14a | 16192 | 2 | 0.018
H14a | 16209G | 1 | 0.009
H14a | 16249 | 5 | 0.046
H14a | 16286 | 1 | 0.009
H14a | 16295 | 9 | 0.083
H14a | 16304 | 1 | 0.009
H14a | 16311 | 16 | 0.147
H14a | 16456 | 3 | 0.028
H14a | 16526 | 3 | 0.028
H14a | 179 | 2 | 0.018
H14a | 183 | 1 | 0.009
H14a | 189 | 1 | 0.009
H14a | 200 | 1 | 0.009
H14a | 204 | 1 | 0.009
H14a | 207 | 2 | 0.018
H14a | 2614 | 1 | 0.009
H14a | 309d | 1 | 0.009
H14a | 310 | 3 | 0.028
H14a | 3106A | 1 | 0.009
H14a | 316C | 1 | 0.009
H14a | 3579 | 1 | 0.009
H14a | 3589 | 1 | 0.009
H14a | 3621 | 1 | 0.009
H14a | 3666 | 1 | 0.009
H14a | 4113 | 1 | 0.009
H14a | 417 | 3 | 0.028
H14a | 4336 | 1 | 0.009
H14a | 4541 | 6 | 0.055
H14a | 573.1C | 1 | 0.009
H14a | 574C | 1 | 0.009
H14a | 6035 | 1 | 0.009
H14a | 6152 | 1 | 0.009
H14a | 7861 | 1 | 0.009

H14a | 8027 | 6 | 0.055
H14a | 8080 | 1 | 0.009
H14a | 8152 | 1 | 0.009
H14a | 8460 | 1 | 0.009
H14a | 8562 | 1 | 0.009
H14a | 9174 | 1 | 0.009
H14a | 9290 | 1 | 0.009
H14a | 93 | 1 | 0.009
H14a | 9438 | 1 | 0.009
H14a | 980 | 1 | 0.009
H14a+146 | 10101 | 1 | 0.043
H14a+146 | 12972 | 1 | 0.043
H14a+146 | 14945 | 1 | 0.043
H14a+146 | 15097 | 5 | 0.217
H14a+146 | 152 | 2 | 0.087
H14a+146 | 15530 | 1 | 0.043
H14a+146 | 16184 | 1 | 0.043
H14a+146 | 16203 | 5 | 0.217
H14a+146 | 16223 | 1 | 0.043
H14a+146 | 16346T | 1 | 0.043
H14a+146 | 16399 | 3 | 0.13
H14a+146 | 200 | 1 | 0.043
H14a+146 | 2881 | 1 | 0.043
H14a+146 | 3541 | 5 | 0.217
H14a+146 | 4248 | 5 | 0.217
H14a+146 | 6983 | 1 | 0.043
H14a+146 | 7094 | 1 | 0.043
H14a+146 | 7777 | 5 | 0.217
H14a+146 | 8238 | 1 | 0.043
H14a+146 | 8610 | 1 | 0.043
H14a1 | 11002 | 1 | 0.033
H14a1 | 15835 | 1 | 0.033
H14a1 | 15836 | 1 | 0.033
H14a1 | 16092 | 1 | 0.033
H14a1 | 16093 | 1 | 0.033
H14a1 | 16114 | 1 | 0.033
H14a1 | 16176 | 1 | 0.033
H14a1 | 16189 | 1 | 0.033
H14a1 | 16209 | 1 | 0.033
H14a1 | 16248 | 1 | 0.033
H14a1 | 16249 | 5 | 0.167
H14a1 | 16278 | 1 | 0.033
H14a1 | 16311 | 8 | 0.267
H14a1 | 16362 | 1 | 0.033
H14a1 | 16456 | 1 | 0.033
H14a1 | 16503C | 1 | 0.033
H14a1 | 7864 | 1 | 0.033
H14a2 | 11499 | 1 | 0.012
H14a2 | 11893 | 1 | 0.012
H14a2 | 13404 | 1 | 0.012
H14a2 | 152 | 8 | 0.098
H14a2 | 15262 | 1 | 0.012
H14a2 | 15596 | 1 | 0.012
H14a2 | 15758 | 1 | 0.012
H14a2 | 15886 | 1 | 0.012
H14a2 | 15891 | 1 | 0.012
H14a2 | 16114A | 1 | 0.012
H14a2 | 16189 | 1 | 0.012
H14a2 | 16217 | 1 | 0.012
H14a2 | 16239 | 1 | 0.012
H14a2 | 16271 | 1 | 0.012
H14a2 | 195 | 1 | 0.012
H14a2 | 3803 | 1 | 0.012
H14a2 | 396T | 2 | 0.024
H14a2 | 4727 | 1 | 0.012
H14a2 | 5774 | 1 | 0.012
H14a2 | 7389 | 1 | 0.012
H14a2 | 8296 | 2 | 0.024
H14a2 | 8812 | 1 | 0.012
H14a2 | 9554 | 1 | 0.012
H14a2 | 95C | 2 | 0.024
H14a2 | 9716 | 1 | 0.012
H14a2 | 9758 | 1 | 0.012
H14a2 | 9966 | 1 | 0.012
H14a2a | 11194 | 1 | 0.017
H14a2a | 16399 | 1 | 0.017
H14a2a | 3010 | 1 | 0.017
H14a2a | 6570T | 1 | 0.017
H14a2a | 8790 | 1 | 0.017
H14a2b | 16145 | 1 | 0.012
H14a2b | 16209 | 1 | 0.012
H14a2b | 16230 | 1 | 0.012
H14a2b | 16258C | 1 | 0.012
H14a2b | 16362 | 2 | 0.025
H14a2b | 214 | 1 | 0.012
H14a2b | 4136 | 1 | 0.012
H14a2c | 12738 | 1 | 0.012
H14a2c | 13710 | 4 | 0.049
H14a2c | 14220 | 4 | 0.049
H14a2c | 150 | 1 | 0.012
H14a2c | 15172 | 1 | 0.012
H14a2c | 16145 | 1 | 0.012
H14a2c | 16209 | 1 | 0.012
H14a2c | 16230 | 1 | 0.012
H14a2c | 16258C | 1 | 0.012
H14a2c | 16293C | 1 | 0.012
H14a2c | 16295 | 1 | 0.012
H14a2c | 16362 | 2 | 0.024
H14a2c | 309.3C | 1 | 0.012
H14a2c | 3496 | 1 | 0.012
H14a2c | 3552 | 1 | 0.012
H14a2c | 4065 | 1 | 0.012
H14a2c | 6995 | 1 | 0.012
H14a2c | 9018 | 1 | 0.012
H14b | 1038 | 1 | 0.001
H14b | 113 | 1 | 0.001
H14b | 13617 | 2 | 0.002
H14b | 13788A | 1 | 0.001
H14b | 13863 | 1 | 0.001
H14b | 14053 | 1 | 0.001
H14b | 143 | 5 | 0.005

H14b | 14887 | 1 | 0.001
H14b | 15553 | 2 | 0.002
H14b | 15924 | 2 | 0.002
H14b | 16076A | 1 | 0.001
H14b | 16093 | 1 | 0.001
H14b | 16129 | 1 | 0.001
H14b | 16146 | 1 | 0.001
H14b | 16172 | 1 | 0.001
H14b | 16192 | 1 | 0.001
H14b | 16239A | 1 | 0.001
H14b | 16270 | 2 | 0.002
H14b | 16311 | 142 | 0.153
H14b | 16319 | 142 | 0.153
H14b | 16354 | 2 | 0.002
H14b | 16391 | 1 | 0.001
H14b | 1888 | 2 | 0.002
H14b | 208 | 1 | 0.001
H14b | 215 | 1 | 0.001
H14b | 234 | 1 | 0.001
H14b | 261 | 1 | 0.001
H14b | 281C | 1 | 0.001
H14b | 316d | 1 | 0.001
H14b | 338 | 1 | 0.001
H14b | 4012 | 1 | 0.001
H14b | 4248 | 2 | 0.002
H14b | 4775 | 1 | 0.001
H14b | 502 | 1 | 0.001
H14b | 54.1C | 1 | 0.001
H14b | 56C | 1 | 0.001
H14b | 573.1C | 4 | 0.004
H14b | 573.2C | 5 | 0.005
H14b | 573.5C | 1 | 0.001
H14b | 7403 | 1 | 0.001
H14b | 7610 | 1 | 0.001
H14b | 7702 | 1 | 0.001
H14b1 | 150 | 1 | 0.01
H14b1 | 16081 | 1 | 0.01
H14b1 | 16180 | 1 | 0.01
H14b1 | 16192 | 4 | 0.041
H14b1 | 16247 | 1 | 0.01
H14b1 | 16311 | 5 | 0.051
H14b1 | 16343 | 1 | 0.01
H14b1 | 16362 | 4 | 0.041
H14b1 | 16484-16489d | 2 | 0.02
H14b1 | 16526 | 1 | 0.01
H14b1 | 195 | 1 | 0.01
H14b1 | 198 | 1 | 0.01
H14b1 | 204 | 1 | 0.01
H14b1 | 226 | 1 | 0.01
H14b1 | 234 | 1 | 0.01
H14b1 | 301 | 1 | 0.01
H14b1 | 319 | 1 | 0.01
H14b2 | 113 | 1 | 0.001
H14b2 | 16076A | 142 | 0.154
H14b2 | 16093 | 1 | 0.001
H14b2 | 16129 | 2 | 0.002
H14b2 | 16189 | 1 | 0.001
H14b2 | 208 | 1 | 0.001
H14b2 | 215 | 1 | 0.001
H14b2 | 261 | 1 | 0.001
H14b2 | 281C | 1 | 0.001
H14b2 | 309.3C | 1 | 0.001
H14b2 | 316d | 1 | 0.001
H14b2 | 338 | 1 | 0.001
H14b2 | 502 | 1 | 0.001
H14b2 | 54.1C | 1 | 0.001
H14b2 | 56C | 1 | 0.001
H14b2 | 573.1C | 4 | 0.004
H14b2 | 573.2C | 5 | 0.005
H14b2 | 573.5C | 1 | 0.001
H14b2 | 6267 | 3 | 0.003
H14b2 | 93 | 1 | 0.001
H14b2a | 14016 | 1 | 0.125
H14b2a | 16093 | 1 | 0.125
H14b2a | 16185 | 2 | 0.25
H14b2a | 16300 | 2 | 0.25
H14b2a | 16309 | 1 | 0.125
H14b2a | 499 | 1 | 0.125
H14b2a | 765G | 1 | 0.125
H14b3 | 14305 | 1 | 0.01
H14b3 | 150 | 1 | 0.01
H14b3 | 15287 | 1 | 0.01
H14b3 | 16093 | 3 | 0.031
H14b3 | 16126 | 3 | 0.031
H14b3 | 16176 | 2 | 0.02
H14b3 | 16189 | 1 | 0.01
H14b3 | 16223 | 1 | 0.01
H14b3 | 16232A | 1 | 0.01
H14b3 | 16262 | 3 | 0.031
H14b3 | 16266 | 1 | 0.01
H14b3 | 16319 | 1 | 0.01
H14b3 | 16357 | 2 | 0.02
H14b3 | 16540 | 1 | 0.01
H14b3 | 185 | 1 | 0.01
H14b3 | 189 | 8 | 0.082
H14b3 | 225 | 1 | 0.01
H14b3 | 228 | 1 | 0.01
H14b3 | 3531 | 1 | 0.01
H14b3 | 5460 | 1 | 0.01
H14b3 | 552 | 1 | 0.01
H14b3 | 6722 | 1 | 0.01
H14b3 | 8020 | 2 | 0.02
H14b4 | 10876 | 1 | 0.001
H14b4 | 113 | 1 | 0.001
H14b4 | 13135 | 2 | 0.002
H14b4 | 151 | 1 | 0.001
H14b4 | 16076A | 142 | 0.154
H14b4 | 16456 | 1 | 0.001
H14b4 | 208 | 1 | 0.001
H14b4 | 215 | 1 | 0.001
H14b4 | 261 | 1 | 0.001

H14b4 | 281C | 1 | 0.001
H14b4 | 316d | 1 | 0.001
H14b4 | 338 | 1 | 0.001
H14b4 | 502 | 1 | 0.001
H14b4 | 54.1C | 1 | 0.001
H14b4 | 56C | 1 | 0.001
H14b4 | 573.1C | 4 | 0.004
H14b4 | 573.2C | 5 | 0.005
H14b4 | 573.5C | 1 | 0.001
H14b4 | 5773 | 1 | 0.001
H15 | 131 | 1 | 0.029
H15 | 14384 | 1 | 0.029
H15 | 152 | 2 | 0.057
H15 | 16092 | 3 | 0.086
H15 | 16224 | 1 | 0.029
H15 | 16241 | 1 | 0.029
H15 | 16295 | 1 | 0.029
H15 | 16357 | 1 | 0.029
H15 | 185 | 1 | 0.029
H15 | 26 | 1 | 0.029
H15 | 309.3C | 2 | 0.057
H15 | 3693 | 1 | 0.029
H15 | 4122 | 2 | 0.057
H15 | 56T | 1 | 0.029
H15 | 7609 | 1 | 0.029
H15 | 8448 | 1 | 0.029
H15 | 9731 | 1 | 0.029
H15a | 11050 | 1 | 0.029
H15a | 146 | 1 | 0.029
H15a | 14959T | 1 | 0.029
H15a | 16129 | 1 | 0.029
H15a | 16153 | 4 | 0.114
H15a | 16224 | 1 | 0.029
H15a | 16241 | 1 | 0.029
H15a | 26 | 1 | 0.029
H15a | 324G | 1 | 0.029
H15a | 326C | 1 | 0.029
H15a | 329C | 1 | 0.029
H15a | 345 | 2 | 0.057
H15a | 366 | 4 | 0.114
H15a | 389 | 3 | 0.086
H15a | 414G | 1 | 0.029
H15a | 415T | 1 | 0.029
H15a | 427A | 1 | 0.029
H15a | 429 | 1 | 0.029
H15a | 515-524d | 4 | 0.114
H15a | 529T | 1 | 0.029
H15a | 564 | 1 | 0.029
H15a1 | 10283 | 2 | 0.032
H15a1 | 10335 | 1 | 0.016
H15a1 | 10604 | 1 | 0.016
H15a1 | 10937 | 1 | 0.016
H15a1 | 11722 | 1 | 0.016
H15a1 | 11821 | 2 | 0.032
H15a1 | 11914 | 1 | 0.016
H15a1 | 12187 | 1 | 0.016
H15a1 | 13182 | 1 | 0.016
H15a1 | 13209 | 2 | 0.032
H15a1 | 13227 | 1 | 0.016
H15a1 | 13590 | 1 | 0.016
H15a1 | 14384 | 2 | 0.032
H15a1 | 146 | 1 | 0.016
H15a1 | 152 | 5 | 0.081
H15a1 | 15316 | 2 | 0.032
H15a1 | 15929 | 1 | 0.016
H15a1 | 16093 | 3 | 0.048
H15a1 | 16209 | 3 | 0.048
H15a1 | 16245 | 1 | 0.016
H15a1 | 16292 | 1 | 0.016
H15a1 | 16319 | 1 | 0.016
H15a1 | 16356 | 1 | 0.016
H15a1 | 1888 | 1 | 0.016
H15a1 | 193 | 2 | 0.032
H15a1 | 194 | 1 | 0.016
H15a1 | 204 | 2 | 0.032
H15a1 | 2736 | 2 | 0.032
H15a1 | 302d | 1 | 0.016
H15a1 | 309d | 2 | 0.032
H15a1 | 310 | 1 | 0.016
H15a1 | 315.2C | 1 | 0.016
H15a1 | 3865 | 1 | 0.016
H15a1 | 4276 | 1 | 0.016
H15a1 | 44.1C | 1 | 0.016
H15a1 | 4596 | 1 | 0.016
H15a1 | 4695 | 1 | 0.016
H15a1 | 5051 | 1 | 0.016
H15a1 | 5147 | 1 | 0.016
H15a1 | 56 | 1 | 0.016
H15a1 | 56.1G | 1 | 0.016
H15a1 | 56T | 1 | 0.016
H15a1 | 57.1G | 2 | 0.032
H15a1 | 57G | 2 | 0.032
H15a1 | 60.1T | 2 | 0.032
H15a1 | 6515 | 1 | 0.016
H15a1 | 6748 | 1 | 0.016
H15a1 | 709 | 3 | 0.048
H15a1 | 71d | 1 | 0.016
H15a1 | 723 | 2 | 0.032
H15a1 | 748 | 1 | 0.016
H15a1 | 8857 | 1 | 0.016
H15a1 | 9037 | 1 | 0.016
H15a1 | 9188 | 2 | 0.032
H15a1 | 93 | 1 | 0.016
H15a1 | 9599A | 1 | 0.016
H15a1a | 16171 | 2 | 0.054
H15a1a | 16189 | 3 | 0.081
H15a1a | 16224 | 1 | 0.027
H15a1a | 16234 | 3 | 0.081
H15a1a | 16241 | 1 | 0.027
H15a1a | 26 | 1 | 0.027
H15a1a | 5495 | 1 | 0.027

H15a1a | 60.1T | 1 | 0.027
H15a1a1 | 11152 | 3 | 0.073
H15a1a1 | 12702 | 1 | 0.024
H15a1a1 | 14180 | 1 | 0.024
H15a1a1 | 14364 | 1 | 0.024
H15a1a1 | 14548 | 1 | 0.024
H15a1a1 | 146 | 3 | 0.073
H15a1a1 | 152 | 1 | 0.024
H15a1a1 | 15217 | 1 | 0.024
H15a1a1 | 15886 | 1 | 0.024
H15a1a1 | 16184A | 1 | 0.024
H15a1a1 | 16187 | 1 | 0.024
H15a1a1 | 16189 | 13 | 0.317
H15a1a1 | 16325 | 12 | 0.293
H15a1a1 | 16525 | 1 | 0.024
H15a1a1 | 16527 | 1 | 0.024
H15a1a1 | 204 | 2 | 0.049
H15a1a1 | 253 | 1 | 0.024
H15a1a1 | 310 | 1 | 0.024
H15a1a1 | 315.2C | 1 | 0.024
H15a1a1 | 3565 | 3 | 0.073
H15a1a1 | 374 | 1 | 0.024
H15a1a1 | 467 | 1 | 0.024
H15a1a1 | 476A | 1 | 0.024
H15a1a1 | 478T | 1 | 0.024
H15a1a1 | 544.1C | 2 | 0.049
H15a1a1 | 5442 | 1 | 0.024
H15a1a1 | 544d | 1 | 0.024
H15a1a1 | 5590 | 1 | 0.024
H15a1a1 | 56T | 1 | 0.024
H15a1a1 | 57G | 2 | 0.049
H15a1a1 | 60.1T | 8 | 0.195
H15a1a1 | 709 | 1 | 0.024
H15a1a1 | 7440G | 1 | 0.024
H15a1a1 | 8289.1CCCCCTCTA | 1 | 0.024
H15a1a1 | 9261 | 1 | 0.024
H15a1b | 10653 | 3 | 0.055
H15a1b | 1243 | 3 | 0.055
H15a1b | 13469A | 1 | 0.018
H15a1b | 14070 | 3 | 0.055
H15a1b | 150 | 1 | 0.018
H15a1b | 15930 | 1 | 0.018
H15a1b | 16072 | 4 | 0.073
H15a1b | 16084 | 4 | 0.073
H15a1b | 16086 | 2 | 0.036
H15a1b | 16093 | 1 | 0.018
H15a1b | 16124 | 21 | 0.382
H15a1b | 16129 | 1 | 0.018
H15a1b | 16157 | 2 | 0.036
H15a1b | 16179 | 3 | 0.055
H15a1b | 16291 | 1 | 0.018
H15a1b | 16292 | 1 | 0.018
H15a1b | 16311 | 6 | 0.109
H15a1b | 16318T | 1 | 0.018
H15a1b | 16565d | 1 | 0.018
H15a1b | 324G | 1 | 0.018
H15a1b | 5082 | 1 | 0.018
H15a1b | 5294 | 1 | 0.018
H15a1b | 5301C | 1 | 0.018
H15a1b | 5460 | 1 | 0.018
H15a1b | 56.1A | 1 | 0.018
H15a1b | 573.1C | 1 | 0.018
H15a1b | 57G | 1 | 0.018
H15a1b | 5999 | 1 | 0.018
H15a1b | 60.1T | 6 | 0.109
H15a1b | 709 | 1 | 0.018
H15a1b | 756 | 2 | 0.036
H15a1b | 7598 | 4 | 0.073
H15a1b | 9181 | 3 | 0.055
H15b | 10819 | 1 | 0.02
H15b | 10993 | 1 | 0.02
H15b | 1117 | 1 | 0.02
H15b | 13145 | 1 | 0.02
H15b | 146 | 2 | 0.039
H15b | 16086 | 1 | 0.02
H15b | 16093 | 1 | 0.02
H15b | 16178 | 2 | 0.039
H15b | 16189 | 1 | 0.02
H15b | 16234 | 1 | 0.02
H15b | 16274 | 1 | 0.02
H15b | 16323 | 1 | 0.02
H15b | 16527 | 2 | 0.039
H15b | 185 | 7 | 0.137
H15b | 200 | 1 | 0.02
H15b | 309.3C | 1 | 0.02
H15b | 309d | 2 | 0.039
H15b | 310 | 4 | 0.078
H15b | 315.3C | 1 | 0.02
H15b | 316C | 2 | 0.039
H15b | 3552 | 1 | 0.02
H15b | 5075 | 3 | 0.059
H15b | 54C | 1 | 0.02
H15b | 56.1A | 1 | 0.02
H15b | 59 | 1 | 0.02
H15b | 60.1T | 12 | 0.235
H15b | 61 | 1 | 0.02
H15b | 62 | 1 | 0.02
H15b | 9336 | 1 | 0.02
H15b | 9612 | 1 | 0.02
H15b | 9644 | 1 | 0.02
H15b1 | 15181 | 1 | 0.024
H15b1 | 15617 | 1 | 0.024
H15b1 | 16256 | 6 | 0.146
H15b1 | 16311 | 2 | 0.049
H15b1 | 16390 | 1 | 0.024
H15b1 | 2416 | 1 | 0.024
H15b1 | 246 | 6 | 0.146
H15b1 | 2673 | 1 | 0.024
H15b1 | 310 | 3 | 0.073
H15b1 | 315.2C | 3 | 0.073
H15b1 | 3864 | 1 | 0.024

H15b1 | 4025 | 1 | 0.024
H15b1 | 55.1T | 7 | 0.171
H15b1 | 56.1A | 2 | 0.049
H15b1 | 573.1C | 4 | 0.098
H15b1 | 573.2C | 1 | 0.024
H15b1 | 57G | 1 | 0.024
H15b1 | 59 | 7 | 0.171
H15b1 | 6746 | 1 | 0.024
H15b1 | 709 | 5 | 0.122
H15b1 | 7572 | 1 | 0.024
H15b1 | 93 | 1 | 0.024
H15b1 | 9921 | 6 | 0.146
H15b2 | 13371 | 1 | 0.027
H15b2 | 15115 | 1 | 0.027
H15b2 | 16171 | 2 | 0.054
H15b2 | 16189 | 3 | 0.081
H15b2 | 16224 | 1 | 0.027
H15b2 | 16234 | 3 | 0.081
H15b2 | 16241 | 1 | 0.027
H15b2 | 26 | 1 | 0.027
H15b2 | 56.1A | 1 | 0.027
H15b2 | 56T | 1 | 0.027
H16 | 10493 | 2 | 0.002
H16 | 113 | 1 | 0.001
H16 | 146 | 3 | 0.004
H16 | 16071 | 2 | 0.002
H16 | 16076A | 1 | 0.001
H16 | 16086 | 3 | 0.004
H16 | 16119 | 1 | 0.001
H16 | 16189 | 130 | 0.162
H16 | 16248 | 123 | 0.153
H16 | 208 | 1 | 0.001
H16 | 261 | 1 | 0.001
H16 | 281C | 1 | 0.001
H16 | 296 | 1 | 0.001
H16 | 310 | 2 | 0.002
H16 | 315.2C | 1 | 0.001
H16 | 316d | 1 | 0.001
H16 | 338 | 1 | 0.001
H16 | 468 | 3 | 0.004
H16 | 4916 | 1 | 0.001
H16 | 502 | 1 | 0.001
H16 | 5662 | 1 | 0.001
H16 | 573.1C | 3 | 0.004
H16 | 573.2C | 5 | 0.006
H16 | 573.5C | 1 | 0.001
H16 | 5789 | 1 | 0.001
H16 | 6071 | 3 | 0.004
H16 | 6340 | 2 | 0.002
H16 | 72 | 1 | 0.001
H16 | 769 | 1 | 0.001
H16 | 7830 | 1 | 0.001
H16 | 8251 | 2 | 0.002
H16 | 8563 | 1 | 0.001
H16 | 9344 | 1 | 0.001
H16+152 | 11020 | 1 | 0.006
H16+152 | 12454 | 1 | 0.006
H16+152 | 1328 | 1 | 0.006
H16+152 | 1442 | 1 | 0.006
H16+152 | 15110 | 1 | 0.006
H16+152 | 16319 | 21 | 0.134
H16+152 | 204 | 1 | 0.006
H16+152 | 2156.1A | 5 | 0.032
H16+152 | 310 | 1 | 0.006
H16+152 | 389 | 1 | 0.006
H16+152 | 4452 | 1 | 0.006
H16+152 | 5492 | 1 | 0.006
H16+152 | 573.1C | 1 | 0.006
H16+152 | 6071 | 3 | 0.019
H16+152 | 73 | 1 | 0.006
H16+152 | 8022 | 1 | 0.006
H16+152 | 8308 | 2 | 0.013
H16+152 | 9129 | 1 | 0.006
H16+152 | 93 | 1 | 0.006
H16+152 | 9438 | 1 | 0.006
H16+152 | 9632 | 1 | 0.006
H16a | 14587C | 1 | 0.007
H16a | 16214 | 3 | 0.02
H16a | 16233T | 1 | 0.007
H16a | 16247 | 20 | 0.132
H16a | 16258T | 1 | 0.007
H16a | 200T | 1 | 0.007
H16a | 207 | 1 | 0.007
H16a | 456 | 1 | 0.007
H16a | 463 | 1 | 0.007
H16a | 515-524d | 1 | 0.007
H16a | 529T | 1 | 0.007
H16a | 573.1C | 1 | 0.007
H16a | 8681 | 1 | 0.007
H16a1 | 12167A | 20 | 0.139
H16a1 | 15671 | 1 | 0.007
H16a1 | 16294 | 1 | 0.007
H16a1 | 16318 | 20 | 0.139
H16a1 | 573.1C | 1 | 0.007
H16a1 | 739 | 4 | 0.028
H16a1 | 7501 | 1 | 0.007
H16b | 113 | 1 | 0.001
H16b | 11654 | 1 | 0.001
H16b | 12879 | 1 | 0.001
H16b | 12945 | 1 | 0.001
H16b | 12996 | 1 | 0.001
H16b | 13149 | 1 | 0.001
H16b | 1406 | 2 | 0.002
H16b | 15811 | 1 | 0.001
H16b | 16076A | 1 | 0.001
H16b | 16136 | 2 | 0.002
H16b | 16145 | 1 | 0.001
H16b | 16172 | 1 | 0.001
H16b | 16223 | 1 | 0.001
H16b | 16274 | 1 | 0.001
H16b | 16278 | 124 | 0.153

H16b | 16293 | 1 | 0.001
H16b | 16295 | 1 | 0.001
H16b | 208 | 1 | 0.001
H16b | 2483 | 2 | 0.002
H16b | 261 | 1 | 0.001
H16b | 281C | 1 | 0.001
H16b | 310 | 2 | 0.002
H16b | 316d | 1 | 0.001
H16b | 3335 | 1 | 0.001
H16b | 338 | 1 | 0.001
H16b | 468 | 3 | 0.004
H16b | 502 | 1 | 0.001
H16b | 573.1C | 3 | 0.004
H16b | 573.2C | 5 | 0.006
H16b | 573.5C | 1 | 0.001
H16b | 6261 | 2 | 0.002
H16b | 64 | 1 | 0.001
H16b | 6891 | 1 | 0.001
H16b | 8764 | 3 | 0.004
H16b | 9707 | 1 | 0.001
H16b | 9833 | 1 | 0.001
H16b | 9932 | 1 | 0.001
H16b | 9947 | 1 | 0.001
H16c | 10130T | 1 | 0.007
H16c | 16172 | 1 | 0.007
H16c | 16214 | 3 | 0.02
H16c | 16233T | 1 | 0.007
H16c | 16247 | 20 | 0.133
H16c | 16258T | 1 | 0.007
H16c | 195 | 1 | 0.007
H16c | 200T | 1 | 0.007
H16c | 207 | 1 | 0.007
H16c | 456 | 1 | 0.007
H16c | 463 | 1 | 0.007
H16c | 515-524d | 1 | 0.007
H16c | 529T | 1 | 0.007
H16c | 573.1C | 1 | 0.007
H16c | 709 | 1 | 0.007
H16d | 13168 | 20 | 0.135
H16d | 14577 | 20 | 0.135
H16d | 16163 | 3 | 0.02
H16d | 16278 | 2 | 0.014
H16d | 16527 | 1 | 0.007
H16d | 200 | 4 | 0.027
H16d | 237 | 2 | 0.014
H16d | 310 | 1 | 0.007
H16d | 5208 | 1 | 0.007
H16d | 573.1C | 1 | 0.007
H16d | 8051 | 1 | 0.007
H16e | 113 | 1 | 0.001
H16e | 13818 | 1 | 0.001
H16e | 152 | 2 | 0.003
H16e | 15616 | 1 | 0.001
H16e | 16076A | 1 | 0.001
H16e | 16278 | 1 | 0.001
H16e | 16287 | 123 | 0.155
H16e | 16311 | 1 | 0.001
H16e | 208 | 1 | 0.001
H16e | 261 | 1 | 0.001
H16e | 281C | 1 | 0.001
H16e | 316d | 1 | 0.001
H16e | 338 | 1 | 0.001
H16e | 468 | 3 | 0.004
H16e | 502 | 1 | 0.001
H16e | 5021 | 1 | 0.001
H16e | 573.1C | 3 | 0.004
H16e | 573.2C | 5 | 0.006
H16e | 573.5C | 1 | 0.001
H16e | 7598 | 1 | 0.001
H16e | 9845 | 1 | 0.001
H17 | 10292 | 1 | 0.004
H17 | 11864 | 2 | 0.008
H17 | 12774 | 2 | 0.008
H17 | 13168 | 1 | 0.004
H17 | 15071 | 1 | 0.004
H17 | 151 | 1 | 0.004
H17 | 152 | 2 | 0.008
H17 | 15323 | 1 | 0.004
H17 | 15951 | 1 | 0.004
H17 | 16093 | 1 | 0.004
H17 | 16172 | 2 | 0.008
H17 | 16189 | 1 | 0.004
H17 | 16257 | 7 | 0.026
H17 | 16266 | 1 | 0.004
H17 | 16311 | 1 | 0.004
H17 | 16319 | 1 | 0.004
H17 | 256 | 2 | 0.008
H17 | 281C | 1 | 0.004
H17 | 325 | 1 | 0.004
H17 | 3657A | 2 | 0.008
H17 | 437 | 1 | 0.004
H17 | 518 | 1 | 0.004
H17 | 6121 | 1 | 0.004
H17 | 7785 | 1 | 0.004
H17 | 7844 | 1 | 0.004
H17 | 93 | 2 | 0.008
H17a | 11779 | 1 | 0.004
H17a | 14276 | 2 | 0.007
H17a | 14812 | 1 | 0.004
H17a | 16093 | 25 | 0.091
H17a | 16240T | 1 | 0.004
H17a | 16266 | 1 | 0.004
H17a | 16357 | 7 | 0.026
H17a | 1709T | 1 | 0.004
H17a | 1762 | 1 | 0.004
H17a | 185 | 2 | 0.007
H17a | 2607 | 1 | 0.004
H17a | 310 | 4 | 0.015
H17a | 315.2C | 1 | 0.004
H17a | 3358 | 1 | 0.004
H17a | 491 | 1 | 0.004

H17a | 709 | 5 | 0.018
H17a | 7353 | 1 | 0.004
H17a | 7761 | 1 | 0.004
H17a | 8393 | 1 | 0.004
H17a1 | 11778 | 1 | 0.015
H17a1 | 13528 | 2 | 0.03
H17a1 | 146 | 2 | 0.03
H17a1 | 16093 | 3 | 0.045
H17a1 | 16177 | 1 | 0.015
H17a1 | 16189 | 2 | 0.03
H17a1 | 16206C | 1 | 0.015
H17a1 | 16301 | 1 | 0.015
H17a1 | 193 | 2 | 0.03
H17a1 | 198 | 2 | 0.03
H17a1 | 200 | 1 | 0.015
H17a1 | 394 | 1 | 0.015
H17a1 | 44.1C | 1 | 0.015
H17a1 | 480 | 2 | 0.03
H17a1 | 5662 | 1 | 0.015
H17a1 | 73 | 1 | 0.015
H17a2 | 14869 | 1 | 0.004
H17a2 | 152 | 12 | 0.047
H17a2 | 16150 | 6 | 0.024
H17a2 | 16209 | 1 | 0.004
H17a2 | 16215 | 1 | 0.004
H17a2 | 16242 | 4 | 0.016
H17a2 | 16257 | 7 | 0.028
H17a2 | 16356 | 4 | 0.016
H17a2 | 207 | 1 | 0.004
H17a2 | 281C | 1 | 0.004
H17a2 | 518 | 1 | 0.004
H17b | 1000A | 1 | 0.004
H17b | 12191 | 1 | 0.004
H17b | 12193 | 1 | 0.004
H17b | 146 | 2 | 0.008
H17b | 150 | 1 | 0.004
H17b | 1547.1T | 1 | 0.004
H17b | 16257 | 7 | 0.027
H17b | 195 | 1 | 0.004
H17b | 281C | 1 | 0.004
H17b | 518 | 1 | 0.004
H17b | 6152 | 1 | 0.004
H17b | 9990 | 1 | 0.004
H17c | 152 | 1 | 0.014
H17c | 16066 | 3 | 0.041
H17c | 16223 | 3 | 0.041
H17c | 16325 | 3 | 0.041
H18 | 10136 | 1 | 0.001
H18 | 10190 | 1 | 0.001
H18 | 10870 | 1 | 0.001
H18 | 113 | 1 | 0.001
H18 | 11719 | 1 | 0.001
H18 | 11778 | 1 | 0.001
H18 | 11864 | 1 | 0.001
H18 | 12964A | 1 | 0.001
H18 | 131 | 2 | 0.002
H18 | 13515 | 1 | 0.001
H18 | 14198 | 1 | 0.001
H18 | 14574 | 1 | 0.001
H18 | 1462 | 2 | 0.002
H18 | 14858 | 2 | 0.002
H18 | 14992 | 1 | 0.001
H18 | 150 | 2 | 0.002
H18 | 152 | 2 | 0.002
H18 | 15209 | 1 | 0.001
H18 | 15287 | 1 | 0.001
H18 | 16076A | 1 | 0.001
H18 | 16146 | 1 | 0.001
H18 | 16209 | 123 | 0.153
H18 | 16278 | 1 | 0.001
H18 | 16311 | 2 | 0.002
H18 | 1717 | 1 | 0.001
H18 | 208 | 1 | 0.001
H18 | 2393 | 1 | 0.001
H18 | 261 | 1 | 0.001
H18 | 281C | 1 | 0.001
H18 | 309d | 1 | 0.001
H18 | 316d | 1 | 0.001
H18 | 3197 | 1 | 0.001
H18 | 338 | 1 | 0.001
H18 | 3771 | 1 | 0.001
H18 | 4048 | 1 | 0.001
H18 | 468 | 3 | 0.004
H18 | 502 | 1 | 0.001
H18 | 5585 | 1 | 0.001
H18 | 573.1C | 3 | 0.004
H18 | 573.2C | 5 | 0.006
H18 | 573.5C | 1 | 0.001
H18 | 6152 | 1 | 0.001
H18 | 709 | 2 | 0.002
H18 | 7278 | 1 | 0.001
H18 | 8839 | 2 | 0.002
H18 | 93 | 1 | 0.001
H18b | 10586 | 2 | 0.002
H18b | 113 | 1 | 0.001
H18b | 11588 | 1 | 0.001
H18b | 152 | 2 | 0.002
H18b | 16076A | 1 | 0.001
H18b | 16209 | 1 | 0.001
H18b | 16294 | 1 | 0.001
H18b | 16309 | 124 | 0.155
H18b | 208 | 1 | 0.001
H18b | 261 | 1 | 0.001
H18b | 2638 | 7 | 0.009
H18b | 266 | 2 | 0.002
H18b | 281C | 1 | 0.001
H18b | 310 | 2 | 0.002
H18b | 316d | 1 | 0.001
H18b | 338 | 1 | 0.001
H18b | 468 | 3 | 0.004
H18b | 4823 | 1 | 0.001

H18b | 502 | 1 | 0.001
H18b | 573.1C | 3 | 0.004
H18b | 573.2C | 5 | 0.006
H18b | 573.5C | 1 | 0.001
H18b | 9947 | 1 | 0.001
H19 | 60 | 3 | 0.231
H19 | 60A | 1 | 0.077
H19 | 64 | 3 | 0.231
H19 | 6713 | 1 | 0.077
H1a | 10172 | 1 | 0.005
H1a | 1018 | 1 | 0.005
H1a | 10448 | 1 | 0.005
H1a | 10828 | 1 | 0.005
H1a | 11017 | 2 | 0.01
H1a | 11257 | 3 | 0.015
H1a | 11374 | 1 | 0.005
H1a | 11719 | 1 | 0.005
H1a | 119 | 1 | 0.005
H1a | 12030 | 1 | 0.005
H1a | 12588 | 2 | 0.01
H1a | 12630 | 1 | 0.005
H1a | 1290 | 1 | 0.005
H1a | 13627 | 4 | 0.02
H1a | 13708 | 2 | 0.01
H1a | 13942 | 1 | 0.005
H1a | 143 | 3 | 0.015
H1a | 14502 | 1 | 0.005
H1a | 146 | 8 | 0.04
H1a | 14769 | 1 | 0.005
H1a | 14983 | 2 | 0.01
H1a | 152 | 7 | 0.035
H1a | 153 | 1 | 0.005
H1a | 15301 | 5 | 0.025
H1a | 15815 | 2 | 0.01
H1a | 16093 | 1 | 0.005
H1a | 16111 | 3 | 0.015
H1a | 16129 | 4 | 0.02
H1a | 16140 | 1 | 0.005
H1a | 16172 | 2 | 0.01
H1a | 16183 | 1 | 0.005
H1a | 16234 | 1 | 0.005
H1a | 16258C | 2 | 0.01
H1a | 16260 | 1 | 0.005
H1a | 16263 | 1 | 0.005
H1a | 16288 | 2 | 0.01
H1a | 16290 | 2 | 0.01
H1a | 16290G | 1 | 0.005
H1a | 16294 | 1 | 0.005
H1a | 16301 | 1 | 0.005
H1a | 16304 | 1 | 0.005
H1a | 16311 | 1 | 0.005
H1a | 16368 | 6 | 0.03
H1a | 16497 | 1 | 0.005
H1a | 185 | 1 | 0.005
H1a | 2689 | 1 | 0.005
H1a | 275 | 1 | 0.005
H1a | 310 | 10 | 0.05
H1a | 315.2C | 2 | 0.01
H1a | 3150 | 1 | 0.005
H1a | 316C | 1 | 0.005
H1a | 3398 | 3 | 0.015
H1a | 3483 | 1 | 0.005
H1a | 3504 | 1 | 0.005
H1a | 3777 | 1 | 0.005
H1a | 447 | 1 | 0.005
H1a | 4561 | 1 | 0.005
H1a | 4676 | 1 | 0.005
H1a | 480 | 1 | 0.005
H1a | 4891 | 1 | 0.005
H1a | 4958 | 1 | 0.005
H1a | 5093 | 1 | 0.005
H1a | 513 | 5 | 0.025
H1a | 545 | 1 | 0.005
H1a | 5460 | 3 | 0.015
H1a | 5498 | 1 | 0.005
H1a | 5788 | 1 | 0.005
H1a | 6040 | 1 | 0.005
H1a | 6620 | 1 | 0.005
H1a | 7160 | 1 | 0.005
H1a | 7269 | 1 | 0.005
H1a | 7576 | 1 | 0.005
H1a | 7702 | 1 | 0.005
H1a | 7772 | 1 | 0.005
H1a | 8224 | 1 | 0.005
H1a | 8289.1CCCCCTCTA | 1 | 0.005
H1a | 8601 | 1 | 0.005
H1a | 8610 | 2 | 0.01
H1a | 9055 | 1 | 0.005
H1a | 9449 | 2 | 0.01
H1a | 9525 | 1 | 0.005
H1a | 9615 | 1 | 0.005
H1a | 9632 | 1 | 0.005
H1a1 | 10356 | 1 | 0.006
H1a1 | 1051 | 1 | 0.006
H1a1 | 10604 | 1 | 0.006
H1a1 | 10801 | 1 | 0.006
H1a1 | 11167 | 1 | 0.006
H1a1 | 11188 | 1 | 0.006
H1a1 | 12248 | 1 | 0.006
H1a1 | 12651 | 1 | 0.006
H1a1 | 14189 | 1 | 0.006
H1a1 | 150 | 2 | 0.012
H1a1 | 15154 | 2 | 0.012
H1a1 | 15172 | 1 | 0.006
H1a1 | 15457 | 1 | 0.006
H1a1 | 15661 | 2 | 0.012
H1a1 | 16093 | 4 | 0.025
H1a1 | 16136 | 1 | 0.006
H1a1 | 16178 | 2 | 0.012
H1a1 | 16239 | 1 | 0.006
H1a1 | 16311 | 3 | 0.018

H1a1 | 16320 | 1 | 0.006
H1a1 | 16368 | 1 | 0.006
H1a1 | 16390 | 1 | 0.006
H1a1 | 171.1G | 1 | 0.006
H1a1 | 1822 | 1 | 0.006
H1a1 | 195 | 2 | 0.012
H1a1 | 2350 | 1 | 0.006
H1a1 | 2628 | 1 | 0.006
H1a1 | 2882 | 1 | 0.006
H1a1 | 310 | 3 | 0.018
H1a1 | 315d | 1 | 0.006
H1a1 | 3175 | 1 | 0.006
H1a1 | 3460 | 1 | 0.006
H1a1 | 4500A | 1 | 0.006
H1a1 | 4843 | 1 | 0.006
H1a1 | 5147 | 1 | 0.006
H1a1 | 5250 | 1 | 0.006
H1a1 | 5460 | 1 | 0.006
H1a1 | 5471 | 1 | 0.006
H1a1 | 5603 | 2 | 0.012
H1a1 | 5773 | 1 | 0.006
H1a1 | 5922 | 1 | 0.006
H1a1 | 64 | 1 | 0.006
H1a1 | 6480 | 1 | 0.006
H1a1 | 709 | 2 | 0.012
H1a1 | 7262 | 1 | 0.006
H1a1 | 7331 | 1 | 0.006
H1a1 | 7980 | 1 | 0.006
H1a1 | 8399 | 1 | 0.006
H1a1 | 8961T | 1 | 0.006
H1a1 | 930 | 1 | 0.006
H1a1a | 11084 | 1 | 0.01
H1a1a | 11253 | 2 | 0.02
H1a1a | 152 | 1 | 0.01
H1a1a | 153 | 1 | 0.01
H1a1a | 16129 | 2 | 0.02
H1a1a | 16278 | 1 | 0.01
H1a1a | 16325 | 1 | 0.01
H1a1a | 210 | 1 | 0.01
H1a1a | 217 | 1 | 0.01
H1a1a | 315.2C | 1 | 0.01
H1a1a | 519 | 1 | 0.01
H1a1a | 5477 | 2 | 0.02
H1a1a | 8821 | 1 | 0.01
H1a1a1 | 12771 | 1 | 0.01
H1a1a1 | 153 | 1 | 0.01
H1a1a1 | 1555 | 1 | 0.01
H1a1a1 | 16067 | 1 | 0.01
H1a1a1 | 16166 | 1 | 0.01
H1a1a1 | 16231 | 3 | 0.029
H1a1a1 | 16278 | 1 | 0.01
H1a1a1 | 16325 | 1 | 0.01
H1a1a1 | 16327 | 1 | 0.01
H1a1a1 | 16343 | 7 | 0.067
H1a1a1 | 16436 | 1 | 0.01
H1a1a1 | 210 | 1 | 0.01
H1a1a1 | 217 | 1 | 0.01
H1a1a1 | 4679 | 1 | 0.01
H1a1a1 | 519 | 1 | 0.01
H1a1a1 | 8989 | 1 | 0.01
H1a1b | 10478 | 1 | 0.01
H1a1b | 1116 | 1 | 0.01
H1a1b | 13886 | 1 | 0.01
H1a1b | 14020 | 1 | 0.01
H1a1b | 16311 | 1 | 0.01
H1a1b | 195 | 1 | 0.01
H1a1b | 310 | 1 | 0.01
H1a1b | 3396 | 2 | 0.019
H1a1b | 5964 | 1 | 0.01
H1a1b | 7292 | 1 | 0.01
H1a1b | 9790 | 1 | 0.01
H1a1c | 16266 | 8 | 0.088
H1a1c | 16283 | 4 | 0.044
H1a1c | 200 | 1 | 0.011
H1a1c | 2361 | 1 | 0.011
H1a1c | 3505 | 1 | 0.011
H1a1c | 5460 | 1 | 0.011
H1a1c | 5806 | 1 | 0.011
H1a1c | 7692 | 1 | 0.011
H1a1c | 8602 | 1 | 0.011
H1a2 | 1007 | 1 | 0.008
H1a2 | 11231 | 1 | 0.008
H1a2 | 1187 | 1 | 0.008
H1a2 | 15071 | 1 | 0.008
H1a2 | 152 | 9 | 0.068
H1a2 | 15226 | 1 | 0.008
H1a2 | 15454 | 1 | 0.008
H1a2 | 15929 | 1 | 0.008
H1a2 | 16093 | 1 | 0.008
H1a2 | 16172 | 1 | 0.008
H1a2 | 16294 | 1 | 0.008
H1a2 | 16296 | 1 | 0.008
H1a2 | 16311 | 6 | 0.045
H1a2 | 1712 | 1 | 0.008
H1a2 | 195 | 1 | 0.008
H1a2 | 241.1TAA | 1 | 0.008
H1a2 | 310 | 1 | 0.008
H1a2 | 4452 | 2 | 0.015
H1a2 | 537.1C | 1 | 0.008
H1a2 | 7309 | 2 | 0.015
H1a2 | 9 | 1 | 0.008
H1a2 | 9012 | 1 | 0.008
H1a2 | 9066 | 2 | 0.015
H1a3 | 10320 | 1 | 0.008
H1a3 | 10376 | 1 | 0.008
H1a3 | 11251 | 1 | 0.008
H1a3 | 11812 | 1 | 0.008
H1a3 | 12618 | 1 | 0.008
H1a3 | 13708 | 1 | 0.008
H1a3 | 14130 | 1 | 0.008
H1a3 | 146 | 1 | 0.008

H1a3 | 14980A | 1 | 0.008
H1a3 | 150 | 2 | 0.016
H1a3 | 15052 | 1 | 0.008
H1a3 | 15100 | 2 | 0.016
H1a3 | 152 | 2 | 0.016
H1a3 | 15204 | 1 | 0.008
H1a3 | 15244 | 1 | 0.008
H1a3 | 1555 | 1 | 0.008
H1a3 | 16075 | 4 | 0.033
H1a3 | 16126 | 1 | 0.008
H1a3 | 16129 | 1 | 0.008
H1a3 | 16172 | 4 | 0.033
H1a3 | 16174 | 1 | 0.008
H1a3 | 16257 | 1 | 0.008
H1a3 | 16291 | 2 | 0.016
H1a3 | 16294 | 1 | 0.008
H1a3 | 16296 | 1 | 0.008
H1a3 | 16304 | 4 | 0.033
H1a3 | 1835 | 1 | 0.008
H1a3 | 1837G | 1 | 0.008
H1a3 | 195 | 1 | 0.008
H1a3 | 2098 | 1 | 0.008
H1a3 | 214 | 1 | 0.008
H1a3 | 2709 | 1 | 0.008
H1a3 | 309d | 1 | 0.008
H1a3 | 310 | 2 | 0.016
H1a3 | 315.2C | 1 | 0.008
H1a3 | 3421 | 1 | 0.008
H1a3 | 3746 | 4 | 0.033
H1a3 | 4318A | 1 | 0.008
H1a3 | 5054 | 1 | 0.008
H1a3 | 55.1T | 1 | 0.008
H1a3 | 5628 | 3 | 0.024
H1a3 | 57 | 1 | 0.008
H1a3 | 59 | 1 | 0.008
H1a3 | 6026 | 1 | 0.008
H1a3 | 63A | 1 | 0.008
H1a3 | 64 | 1 | 0.008
H1a3 | 6570T | 1 | 0.008
H1a3 | 7211 | 1 | 0.008
H1a3 | 7775 | 1 | 0.008
H1a3 | 8222 | 1 | 0.008
H1a3 | 8989 | 1 | 0.008
H1a3 | 9275 | 2 | 0.016
H1a3a | 10416A | 1 | 0.011
H1a3a | 12354 | 1 | 0.011
H1a3a | 14484 | 1 | 0.011
H1a3a | 146 | 2 | 0.022
H1a3a | 15244 | 1 | 0.011
H1a3a | 16111 | 1 | 0.011
H1a3a | 16153 | 1 | 0.011
H1a3a | 16292 | 1 | 0.011
H1a3a | 16303 | 1 | 0.011
H1a3a | 16527 | 1 | 0.011
H1a3a | 302C | 1 | 0.011
H1a3a | 5744 | 1 | 0.011
H1a3a | 6260 | 1 | 0.011
H1a3a | 7317 | 1 | 0.011
H1a3a | 8468 | 1 | 0.011
H1a3a | 9275 | 2 | 0.022
H1a3a | 9951 | 1 | 0.011
H1a3a1 | 4011 | 1 | 0.012
H1a3a2 | 3399C | 1 | 0.033
H1a3a2 | 6962 | 1 | 0.033
H1a3a3 | 150 | 1 | 0.013
H1a3a3 | 16136 | 1 | 0.013
H1a3a3 | 16209 | 3 | 0.038
H1a3a3 | 16527 | 2 | 0.026
H1a3a3 | 217 | 1 | 0.013
H1a3a3 | 6320 | 1 | 0.013
H1a3a3 | 9921 | 1 | 0.013
H1a3a4 | 10238 | 1 | 0.022
H1a3a4 | 12738 | 1 | 0.022
H1a3a4 | 16092 | 1 | 0.022
H1a3a4 | 16159 | 1 | 0.022
H1a3a4 | 3531 | 1 | 0.022
H1a3b | 11270 | 1 | 0.01
H1a3b | 11935 | 2 | 0.019
H1a3b | 13804 | 1 | 0.01
H1a3b | 14025 | 1 | 0.01
H1a3b | 14530 | 1 | 0.01
H1a3b | 14569 | 1 | 0.01
H1a3b | 16172 | 1 | 0.01
H1a3b | 16223 | 1 | 0.01
H1a3b | 16241C | 2 | 0.019
H1a3b | 16259 | 1 | 0.01
H1a3b | 195 | 8 | 0.077
H1a3b | 3803 | 1 | 0.01
H1a3b | 709 | 1 | 0.01
H1a3b | 7269 | 2 | 0.019
H1a3b | 7754C | 1 | 0.01
H1a3b | 9966 | 1 | 0.01
H1a3b1 | 13830 | 1 | 0.012
H1a3b1 | 152 | 1 | 0.012
H1a3b1 | 15204 | 1 | 0.012
H1a3b1 | 16124 | 1 | 0.012
H1a3b1 | 16189 | 2 | 0.024
H1a3b1 | 16264 | 3 | 0.036
H1a3b1 | 16311 | 2 | 0.024
H1a3b1 | 16343 | 1 | 0.012
H1a3c | 12279 | 1 | 0.011
H1a3c | 13269 | 2 | 0.022
H1a3c | 1406 | 1 | 0.011
H1a3c | 3438 | 1 | 0.011
H1a3c | 6188 | 1 | 0.011
H1a3c | 8435 | 1 | 0.011
H1a3c | 93 | 2 | 0.022
H1a3c1 | 10336 | 7 | 0.064
H1a3c1 | 10535 | 1 | 0.009
H1a3c1 | 10604 | 1 | 0.009
H1a3c1 | 1125 | 1 | 0.009

H1a3c1 | 12372 | 7 | 0.064
H1a3c1 | 14020 | 1 | 0.009
H1a3c1 | 146 | 1 | 0.009
H1a3c1 | 150 | 7 | 0.064
H1a3c1 | 15355 | 1 | 0.009
H1a3c1 | 15511 | 7 | 0.064
H1a3c1 | 15977 | 1 | 0.009
H1a3c1 | 16123 | 1 | 0.009
H1a3c1 | 16232 | 3 | 0.028
H1a3c1 | 16287 | 1 | 0.009
H1a3c1 | 16304 | 2 | 0.018
H1a3c1 | 310 | 2 | 0.018
H1a3c1 | 4924 | 1 | 0.009
H1a3c1 | 7372 | 2 | 0.018
H1a3c1 | 8557 | 1 | 0.009
H1a3c1 | 9026 | 1 | 0.009
H1a3c1 | 9392 | 6 | 0.055
H1a3d | 8020 | 1 | 0.012
H1a4 | 14530 | 1 | 0.009
H1a4 | 15655 | 2 | 0.019
H1a4 | 16219 | 1 | 0.009
H1a4 | 16230 | 1 | 0.009
H1a4 | 16465 | 8 | 0.075
H1a4 | 189 | 1 | 0.009
H1a4 | 204 | 1 | 0.009
H1a5 | 10411 | 1 | 0.01
H1a5 | 13098 | 2 | 0.019
H1a5 | 152 | 1 | 0.01
H1a5 | 15314 | 1 | 0.01
H1a5 | 16093 | 7 | 0.067
H1a5 | 16258 | 1 | 0.01
H1a5 | 3468 | 1 | 0.01
H1a6 | 152 | 1 | 0.05
H1a6 | 16127 | 2 | 0.1
H1a6 | 16286 | 1 | 0.05
H1a6 | 295 | 1 | 0.05
H1a6 | 310 | 1 | 0.05
H1a6 | 6052 | 1 | 0.05
H1a6 | 7927G | 1 | 0.05
H1a6 | 7978G | 1 | 0.05
H1a6 | 96A | 1 | 0.05
H1a6 | 9756G | 1 | 0.05
H1a7 | 12162A | 1 | 0.009
H1a7 | 12361 | 1 | 0.009
H1a7 | 13933 | 1 | 0.009
H1a7 | 15314 | 1 | 0.009
H1a7 | 16219 | 1 | 0.009
H1a7 | 16220C | 1 | 0.009
H1a7 | 16230 | 6 | 0.057
H1a7 | 189 | 1 | 0.009
H1a7 | 204 | 1 | 0.009
H1a7 | 9593 | 1 | 0.009
H1a8 | 10289 | 1 | 0.009
H1a8 | 16219 | 1 | 0.009
H1a8 | 16362 | 6 | 0.057
H1a8 | 189 | 1 | 0.009
H1a8 | 194 | 1 | 0.009
H1a8 | 204 | 1 | 0.009
H1a8 | 9758 | 1 | 0.009
H1a8a | 10084 | 1 | 0.009
H1a8a | 10142 | 1 | 0.009
H1a8a | 16219 | 1 | 0.009
H1a8a | 16230 | 6 | 0.057
H1a8a | 189 | 1 | 0.009
H1a8a | 204 | 1 | 0.009
H1a8a | 6855 | 1 | 0.009
H1a9 | 11239 | 1 | 0.014
H1a9 | 214 | 1 | 0.014
H1a9 | 2519T | 1 | 0.014
H1aa | 103 | 3 | 0.011
H1aa | 109 | 1 | 0.004
H1aa | 121C | 1 | 0.004
H1aa | 143 | 4 | 0.015
H1aa | 150 | 23 | 0.085
H1aa | 16037 | 1 | 0.004
H1aa | 16038 | 1 | 0.004
H1aa | 16051 | 15 | 0.055
H1aa | 16086 | 1 | 0.004
H1aa | 16111 | 1 | 0.004
H1aa | 16124 | 2 | 0.007
H1aa | 16126 | 1 | 0.004
H1aa | 16129 | 9 | 0.033
H1aa | 16150 | 2 | 0.007
H1aa | 16168 | 3 | 0.011
H1aa | 16172 | 2 | 0.007
H1aa | 16179 | 1 | 0.004
H1aa | 16209 | 1 | 0.004
H1aa | 16217 | 55 | 0.203
H1aa | 16233 | 1 | 0.004
H1aa | 16234G | 4 | 0.015
H1aa | 16243 | 1 | 0.004
H1aa | 16249 | 1 | 0.004
H1aa | 16254 | 1 | 0.004
H1aa | 16266 | 2 | 0.007
H1aa | 16270A | 2 | 0.007
H1aa | 16290 | 1 | 0.004
H1aa | 16293 | 1 | 0.004
H1aa | 16295 | 1 | 0.004
H1aa | 16298 | 3 | 0.011
H1aa | 16324 | 2 | 0.007
H1aa | 16327 | 1 | 0.004
H1aa | 16357 | 1 | 0.004
H1aa | 16362 | 1 | 0.004
H1aa | 16484-16489d | 20 | 0.074
H1aa | 189 | 5 | 0.018
H1aa | 195 | 1 | 0.004
H1aa | 200 | 2 | 0.007
H1aa | 204 | 28 | 0.103
H1aa | 214 | 3 | 0.011
H1aa | 226 | 1 | 0.004
H1aa | 240 | 4 | 0.015

H1aa | 249d | 6 | 0.022
H1aa | 297 | 1 | 0.004
H1aa | 302C | 1 | 0.004
H1aa | 315.2C | 1 | 0.004
H1aa | 316 | 1 | 0.004
H1aa | 319 | 1 | 0.004
H1aa | 319.1T | 1 | 0.004
H1aa | 480 | 1 | 0.004
H1aa | 499 | 1 | 0.004
H1aa | 554T | 1 | 0.004
H1aa | 567T | 1 | 0.004
H1aa1 | 11719 | 2 | 0.009
H1aa1 | 13143 | 1 | 0.005
H1aa1 | 14517 | 1 | 0.005
H1aa1 | 152 | 1 | 0.005
H1aa1 | 16038.1A | 1 | 0.005
H1aa1 | 16111 | 1 | 0.005
H1aa1 | 16168 | 1 | 0.005
H1aa1 | 16233 | 1 | 0.005
H1aa1 | 16243 | 19 | 0.09
H1aa1 | 16255 | 1 | 0.005
H1aa1 | 16316 | 1 | 0.005
H1aa1 | 199 | 1 | 0.005
H1aa1 | 297 | 19 | 0.09
H1aa1 | 3099 | 1 | 0.005
H1aa1 | 310 | 2 | 0.009
H1aa1 | 315.2C | 1 | 0.005
H1aa1 | 319.1T | 1 | 0.005
H1aa1 | 480 | 1 | 0.005
H1aa1 | 499 | 19 | 0.09
H1aa1 | 554T | 1 | 0.005
H1aa1 | 567T | 1 | 0.005
H1aa1 | 596 | 1 | 0.005
H1aa1 | 6960 | 1 | 0.005
H1aa1 | 8738 | 3 | 0.014
H1aa1 | 8928 | 1 | 0.005
H1aa1 | 9095 | 1 | 0.005
H1ab | 150 | 22 | 0.103
H1ab | 16111 | 1 | 0.005
H1ab | 16126 | 1 | 0.005
H1ab | 16129 | 4 | 0.019
H1ab | 16233 | 1 | 0.005
H1ab | 16243 | 1 | 0.005
H1ab | 16295 | 1 | 0.005
H1ab | 16298 | 1 | 0.005
H1ab | 16362 | 1 | 0.005
H1ab | 16484-16489d | 19 | 0.089
H1ab | 1896 | 1 | 0.005
H1ab | 195 | 19 | 0.089
H1ab | 214 | 1 | 0.005
H1ab | 297 | 1 | 0.005
H1ab | 315.2C | 1 | 0.005
H1ab | 316 | 1 | 0.005
H1ab | 319.1T | 1 | 0.005
H1ab | 3795 | 1 | 0.005
H1ab | 480 | 1 | 0.005
H1ab | 499 | 1 | 0.005
H1ab | 554T | 1 | 0.005
H1ab | 567T | 1 | 0.005
H1ab1 | 12326 | 1 | 0.011
H1ab1 | 12358 | 1 | 0.011
H1ab1 | 143 | 1 | 0.011
H1ab1 | 15758 | 1 | 0.011
H1ab1 | 16114A | 4 | 0.043
H1ab1 | 16172 | 1 | 0.011
H1ab1 | 16209 | 1 | 0.011
H1ab1 | 16223 | 1 | 0.011
H1ab1 | 16243 | 1 | 0.011
H1ab1 | 16317 | 3 | 0.032
H1ab1 | 16319 | 2 | 0.022
H1ab1 | 16320 | 1 | 0.011
H1ab1 | 214 | 1 | 0.011
H1ab1 | 2792C | 1 | 0.011
H1ab1 | 489 | 1 | 0.011
H1ab1 | 73 | 1 | 0.011
H1ac | 11447 | 1 | 0.005
H1ac | 14979G | 1 | 0.005
H1ac | 16129 | 3 | 0.015
H1ac | 16298 | 19 | 0.095
H1ac | 316 | 1 | 0.005
H1ac | 6560 | 1 | 0.005
H1ac | 73 | 1 | 0.005
H1ad | 16129 | 3 | 0.015
H1ad | 16298 | 19 | 0.094
H1ad | 316 | 1 | 0.005
H1ad | 5899.2C | 1 | 0.005
H1ad | 8945 | 1 | 0.005
H1ae | 113 | 1 | 0.001
H1ae | 13452 | 2 | 0.003
H1ae | 15226 | 1 | 0.001
H1ae | 16038 | 2 | 0.003
H1ae | 16076A | 123 | 0.154
H1ae | 16316 | 2 | 0.003
H1ae | 208 | 1 | 0.001
H1ae | 2483 | 1 | 0.001
H1ae | 261 | 1 | 0.001
H1ae | 281C | 1 | 0.001
H1ae | 316d | 1 | 0.001
H1ae | 338 | 1 | 0.001
H1ae | 468 | 3 | 0.004
H1ae | 502 | 1 | 0.001
H1ae | 573.1C | 3 | 0.004
H1ae | 573.2C | 5 | 0.006
H1ae | 573.5C | 1 | 0.001
H1ae | 6620 | 1 | 0.001
H1ae | 9316 | 2 | 0.003
H1ae1 | 11016 | 2 | 0.002
H1ae1 | 113 | 1 | 0.001
H1ae1 | 11722 | 2 | 0.002
H1ae1 | 13111 | 2 | 0.002
H1ae1 | 15848 | 1 | 0.001

H1ae1 | 16076A | 123 | 0.154
H1ae1 | 208 | 1 | 0.001
H1ae1 | 261 | 1 | 0.001
H1ae1 | 281C | 1 | 0.001
H1ae1 | 316d | 1 | 0.001
H1ae1 | 338 | 1 | 0.001
H1ae1 | 468 | 3 | 0.004
H1ae1 | 502 | 1 | 0.001
H1ae1 | 573.1C | 3 | 0.004
H1ae1 | 573.2C | 5 | 0.006
H1ae1 | 573.5C | 1 | 0.001
H1ae1 | 8176 | 1 | 0.001
H1ae2 | 1053 | 2 | 0.003
H1ae2 | 10586 | 1 | 0.001
H1ae2 | 113 | 1 | 0.001
H1ae2 | 13395 | 1 | 0.001
H1ae2 | 16076A | 123 | 0.154
H1ae2 | 16189 | 1 | 0.001
H1ae2 | 16483 | 2 | 0.003
H1ae2 | 208 | 1 | 0.001
H1ae2 | 261 | 1 | 0.001
H1ae2 | 281C | 1 | 0.001
H1ae2 | 316d | 1 | 0.001
H1ae2 | 338 | 1 | 0.001
H1ae2 | 3483 | 1 | 0.001
H1ae2 | 468 | 3 | 0.004
H1ae2 | 502 | 1 | 0.001
H1ae2 | 573.1C | 3 | 0.004
H1ae2 | 573.2C | 5 | 0.006
H1ae2 | 573.5C | 1 | 0.001
H1ae2 | 7909 | 2 | 0.003
H1ae2a | 113 | 1 | 0.001
H1ae2a | 15355 | 2 | 0.003
H1ae2a | 16076A | 123 | 0.154
H1ae2a | 16192 | 2 | 0.003
H1ae2a | 16311 | 1 | 0.001
H1ae2a | 208 | 1 | 0.001
H1ae2a | 215 | 1 | 0.001
H1ae2a | 261 | 1 | 0.001
H1ae2a | 281C | 1 | 0.001
H1ae2a | 316d | 1 | 0.001
H1ae2a | 338 | 1 | 0.001
H1ae2a | 4012 | 2 | 0.003
H1ae2a | 468 | 3 | 0.004
H1ae2a | 502 | 1 | 0.001
H1ae2a | 573.1C | 3 | 0.004
H1ae2a | 573.2C | 5 | 0.006
H1ae2a | 573.5C | 1 | 0.001
H1ae3 | 113 | 1 | 0.001
H1ae3 | 15217 | 3 | 0.004
H1ae3 | 16076A | 123 | 0.154
H1ae3 | 16274 | 3 | 0.004
H1ae3 | 208 | 1 | 0.001
H1ae3 | 261 | 1 | 0.001
H1ae3 | 281C | 1 | 0.001
H1ae3 | 309.3C | 2 | 0.003
H1ae3 | 316d | 1 | 0.001
H1ae3 | 338 | 1 | 0.001
H1ae3 | 465 | 2 | 0.003
H1ae3 | 468 | 3 | 0.004
H1ae3 | 502 | 1 | 0.001
H1ae3 | 573.1C | 3 | 0.004
H1ae3 | 573.2C | 5 | 0.006
H1ae3 | 573.5C | 1 | 0.001
H1ae3 | 8993G | 1 | 0.001
H1ae3a | 10130 | 1 | 0.001
H1ae3a | 10306C | 1 | 0.001
H1ae3a | 113 | 1 | 0.001
H1ae3a | 16076A | 123 | 0.154
H1ae3a | 208 | 1 | 0.001
H1ae3a | 2487C | 1 | 0.001
H1ae3a | 261 | 1 | 0.001
H1ae3a | 2697 | 1 | 0.001
H1ae3a | 281C | 1 | 0.001
H1ae3a | 316d | 1 | 0.001
H1ae3a | 338 | 1 | 0.001
H1ae3a | 468 | 3 | 0.004
H1ae3a | 502 | 1 | 0.001
H1ae3a | 5063 | 1 | 0.001
H1ae3a | 5252 | 1 | 0.001
H1ae3a | 573.1C | 3 | 0.004
H1ae3a | 573.2C | 5 | 0.006
H1ae3a | 573.5C | 1 | 0.001
H1af | 143 | 1 | 0.011
H1af | 16239 | 1 | 0.011
H1af | 309d | 1 | 0.011
H1af | 374 | 3 | 0.032
H1af | 513 | 1 | 0.011
H1af | 9592 | 1 | 0.011
H1af1 | 15827T | 1 | 0.012
H1af1 | 15835 | 1 | 0.012
H1af1 | 15836 | 1 | 0.012
H1af1 | 15841 | 1 | 0.012
H1af1 | 15848T | 1 | 0.012
H1af1 | 16140 | 1 | 0.012
H1af1 | 16193 | 3 | 0.036
H1af1 | 16194 | 1 | 0.012
H1af1 | 16209G | 1 | 0.012
H1af1 | 16232 | 1 | 0.012
H1af1 | 16343 | 1 | 0.012
H1af1 | 16358 | 1 | 0.012
H1af1 | 16363 | 1 | 0.012
H1af1 | 16475G | 1 | 0.012
H1af1 | 16539C | 1 | 0.012
H1af1 | 3666 | 1 | 0.012
H1af1 | 709 | 1 | 0.012
H1af1 | 9644 | 1 | 0.012
H1af1a | 11084 | 1 | 0.012
H1af1a | 1373 | 1 | 0.012
H1af1a | 15106 | 1 | 0.012
H1af1a | 152 | 1 | 0.012

H1af1a | 15942 | 1 | 0.012
H1af1a | 8286 | 1 | 0.012
H1af1b | 131 | 4 | 0.047
H1af1b | 146 | 1 | 0.012
H1af1b | 16093 | 5 | 0.058
H1af1b | 16184 | 2 | 0.023
H1af1b | 16189 | 2 | 0.023
H1af1b | 16256 | 5 | 0.058
H1af1b | 16274 | 1 | 0.012
H1af1b | 16304 | 5 | 0.058
H1af1b | 16399 | 4 | 0.047
H1af1b | 199 | 2 | 0.023
H1af1b | 204 | 1 | 0.012
H1af1b | 234 | 1 | 0.012
H1af1b | 2581 | 4 | 0.047
H1af1b | 292 | 1 | 0.012
H1af1b | 310 | 1 | 0.012
H1af1b | 73 | 3 | 0.035
H1af1b | 9581 | 1 | 0.012
H1af1b | 9729 | 1 | 0.012
H1af2 | 16111 | 4 | 0.051
H1ag | 113 | 1 | 0.001
H1ag | 12810 | 1 | 0.001
H1ag | 14180 | 1 | 0.001
H1ag | 15140 | 1 | 0.001
H1ag | 15670 | 1 | 0.001
H1ag | 16076A | 123 | 0.154
H1ag | 208 | 1 | 0.001
H1ag | 261 | 1 | 0.001
H1ag | 281C | 1 | 0.001
H1ag | 316d | 1 | 0.001
H1ag | 338 | 1 | 0.001
H1ag | 4659 | 1 | 0.001
H1ag | 468 | 3 | 0.004
H1ag | 502 | 1 | 0.001
H1ag | 573.1C | 3 | 0.004
H1ag | 573.2C | 5 | 0.006
H1ag | 573.5C | 1 | 0.001
H1ag | 5899.1C | 2 | 0.003
H1ag | 6086 | 1 | 0.001
H1ag | 8207A | 1 | 0.001
H1ag | 9210 | 2 | 0.003
H1ag | 93 | 2 | 0.003
H1ag1 | 10259 | 1 | 0.001
H1ag1 | 10448 | 1 | 0.001
H1ag1 | 10978C | 1 | 0.001
H1ag1 | 113 | 1 | 0.001
H1ag1 | 11440 | 1 | 0.001
H1ag1 | 11800 | 1 | 0.001
H1ag1 | 12011 | 1 | 0.001
H1ag1 | 13029 | 1 | 0.001
H1ag1 | 13708 | 1 | 0.001
H1ag1 | 14803 | 1 | 0.001
H1ag1 | 152 | 1 | 0.001
H1ag1 | 15256 | 1 | 0.001
H1ag1 | 16076A | 1 | 0.001
H1ag1 | 16092 | 1 | 0.001
H1ag1 | 16189 | 123 | 0.152
H1ag1 | 16267 | 1 | 0.001
H1ag1 | 16311 | 1 | 0.001
H1ag1 | 16362 | 1 | 0.001
H1ag1 | 189 | 1 | 0.001
H1ag1 | 208 | 1 | 0.001
H1ag1 | 261 | 1 | 0.001
H1ag1 | 281C | 1 | 0.001
H1ag1 | 2885 | 2 | 0.002
H1ag1 | 291.1A | 1 | 0.001
H1ag1 | 316d | 1 | 0.001
H1ag1 | 338 | 1 | 0.001
H1ag1 | 468 | 3 | 0.004
H1ag1 | 502 | 1 | 0.001
H1ag1 | 5483 | 1 | 0.001
H1ag1 | 573.1C | 3 | 0.004
H1ag1 | 573.2C | 5 | 0.006
H1ag1 | 573.5C | 1 | 0.001
H1ag1 | 7153 | 3 | 0.004
H1ag1 | 8380 | 1 | 0.001
H1ag1 | 8764 | 1 | 0.001
H1ag1a | 16179 | 1 | 0.012
H1ag1a | 16219 | 1 | 0.012
H1ag1a | 16220 | 1 | 0.012
H1ag1a | 16266 | 1 | 0.012
H1ag1a | 267 | 1 | 0.012
H1ag1a | 334 | 1 | 0.012
H1ag1a | 5460 | 1 | 0.012
H1ag1b | 113 | 1 | 0.001
H1ag1b | 16076A | 1 | 0.001
H1ag1b | 16114 | 123 | 0.154
H1ag1b | 208 | 1 | 0.001
H1ag1b | 235 | 1 | 0.001
H1ag1b | 261 | 1 | 0.001
H1ag1b | 281C | 1 | 0.001
H1ag1b | 316d | 1 | 0.001
H1ag1b | 338 | 1 | 0.001
H1ag1b | 468 | 3 | 0.004
H1ag1b | 502 | 1 | 0.001
H1ag1b | 573.1C | 3 | 0.004
H1ag1b | 573.2C | 5 | 0.006
H1ag1b | 573.5C | 1 | 0.001
H1ag1b | 8238 | 1 | 0.001
H1ah | 113 | 1 | 0.001
H1ah | 12501 | 1 | 0.001
H1ah | 13662 | 1 | 0.001
H1ah | 16076A | 123 | 0.155
H1ah | 208 | 1 | 0.001
H1ah | 261 | 1 | 0.001
H1ah | 281C | 1 | 0.001
H1ah | 316d | 1 | 0.001
H1ah | 338 | 1 | 0.001
H1ah | 468 | 3 | 0.004
H1ah | 502 | 1 | 0.001

H1ah | 573.1C | 3 | 0.004
H1ah | 573.2C | 5 | 0.006
H1ah | 573.5C | 1 | 0.001
H1ah1 | 11260 | 3 | 0.033
H1ah1 | 150 | 2 | 0.022
H1ah1 | 16213 | 1 | 0.011
H1ah1 | 16311 | 5 | 0.056
H1ah1 | 16319 | 1 | 0.011
H1ah1 | 55 | 1 | 0.011
H1ah1 | 57G | 1 | 0.011
H1ah1 | 64 | 1 | 0.011
H1ah1 | 709 | 1 | 0.011
H1ah1 | 7444 | 1 | 0.011
H1ah1 | 93 | 1 | 0.011
H1ah2 | 15254 | 1 | 0.014
H1ah2 | 15656 | 1 | 0.014
H1ah2 | 16278 | 1 | 0.014
H1ah2 | 310 | 1 | 0.014
H1ah2 | 4380 | 1 | 0.014
H1ah2 | 6566 | 1 | 0.014
H1ai | 113 | 1 | 0.001
H1ai | 16076A | 123 | 0.155
H1ai | 208 | 1 | 0.001
H1ai | 261 | 1 | 0.001
H1ai | 281C | 1 | 0.001
H1ai | 316d | 1 | 0.001
H1ai | 338 | 1 | 0.001
H1ai | 468 | 3 | 0.004
H1ai | 502 | 1 | 0.001
H1ai | 573.1C | 3 | 0.004
H1ai | 573.2C | 5 | 0.006
H1ai | 573.5C | 1 | 0.001
H1ai1 | 10364 | 1 | 0.001
H1ai1 | 10370 | 2 | 0.003
H1ai1 | 113 | 1 | 0.001
H1ai1 | 146 | 1 | 0.001
H1ai1 | 16076A | 123 | 0.154
H1ai1 | 16359 | 1 | 0.001
H1ai1 | 208 | 1 | 0.001
H1ai1 | 261 | 1 | 0.001
H1ai1 | 281C | 1 | 0.001
H1ai1 | 310 | 1 | 0.001
H1ai1 | 312 | 1 | 0.001
H1ai1 | 316d | 1 | 0.001
H1ai1 | 338 | 1 | 0.001
H1ai1 | 356.1C | 1 | 0.001
H1ai1 | 4005 | 1 | 0.001
H1ai1 | 468 | 3 | 0.004
H1ai1 | 502 | 1 | 0.001
H1ai1 | 573.1C | 3 | 0.004
H1ai1 | 573.2C | 5 | 0.006
H1ai1 | 573.5C | 1 | 0.001
H1aj | 113 | 1 | 0.001
H1aj | 12414 | 1 | 0.001
H1aj | 12714 | 1 | 0.001
H1aj | 13263 | 2 | 0.002
H1aj | 13326 | 1 | 0.001
H1aj | 13533T | 1 | 0.001
H1aj | 14259 | 1 | 0.001
H1aj | 152 | 1 | 0.001
H1aj | 16076A | 1 | 0.001
H1aj | 16111G | 1 | 0.001
H1aj | 16189 | 1 | 0.001
H1aj | 16249 | 1 | 0.001
H1aj | 16263 | 125 | 0.156
H1aj | 16296 | 1 | 0.001
H1aj | 16316 | 1 | 0.001
H1aj | 1719 | 1 | 0.001
H1aj | 208 | 1 | 0.001
H1aj | 261 | 1 | 0.001
H1aj | 281C | 1 | 0.001
H1aj | 316d | 1 | 0.001
H1aj | 3316 | 1 | 0.001
H1aj | 338 | 1 | 0.001
H1aj | 468 | 3 | 0.004
H1aj | 499C | 123 | 0.153
H1aj | 502 | 1 | 0.001
H1aj | 5147 | 1 | 0.001
H1aj | 5426G | 1 | 0.001
H1aj | 573.1C | 3 | 0.004
H1aj | 573.2C | 5 | 0.006
H1aj | 573.5C | 1 | 0.001
H1aj | 6716 | 2 | 0.002
H1aj | 7124 | 1 | 0.001
H1aj | 7202 | 1 | 0.001
H1aj | 73 | 1 | 0.001
H1aj | 7984 | 5 | 0.006
H1aj | 8673 | 1 | 0.001
H1aj | 8859 | 1 | 0.001
H1aj | 9149 | 1 | 0.001
H1aj1 | 16129 | 1 | 0.012
H1aj1 | 16186 | 1 | 0.012
H1aj1 | 16215 | 1 | 0.012
H1aj1 | 93 | 1 | 0.012
H1aj1a | 10006 | 1 | 0.2
H1aj1a | 14798 | 1 | 0.2
H1aj1a | 15951 | 1 | 0.2
H1aj1a | 16234 | 1 | 0.2
H1aj1a | 204 | 1 | 0.2
H1ak | 113 | 1 | 0.001
H1ak | 11719 | 1 | 0.001
H1ak | 13966 | 1 | 0.001
H1ak | 14287 | 1 | 0.001
H1ak | 14326 | 1 | 0.001
H1ak | 15258 | 1 | 0.001
H1ak | 15778 | 1 | 0.001
H1ak | 16076A | 123 | 0.154
H1ak | 16311 | 1 | 0.001
H1ak | 16356 | 1 | 0.001
H1ak | 195 | 2 | 0.002
H1ak | 208 | 1 | 0.001

H1ak | 2244 | 1 | 0.001
H1ak | 261 | 1 | 0.001
H1ak | 281C | 1 | 0.001
H1ak | 316d | 1 | 0.001
H1ak | 338 | 1 | 0.001
H1ak | 468 | 3 | 0.004
H1ak | 502 | 1 | 0.001
H1ak | 573.1C | 3 | 0.004
H1ak | 573.2C | 5 | 0.006
H1ak | 573.5C | 1 | 0.001
H1ak1 | 113 | 1 | 0.001
H1ak1 | 12094 | 1 | 0.001
H1ak1 | 12142 | 1 | 0.001
H1ak1 | 146 | 1 | 0.001
H1ak1 | 15257 | 1 | 0.001
H1ak1 | 16076A | 123 | 0.154
H1ak1 | 16311 | 1 | 0.001
H1ak1 | 195 | 1 | 0.001
H1ak1 | 208 | 1 | 0.001
H1ak1 | 261 | 1 | 0.001
H1ak1 | 281C | 1 | 0.001
H1ak1 | 316d | 1 | 0.001
H1ak1 | 338 | 1 | 0.001
H1ak1 | 468 | 3 | 0.004
H1ak1 | 502 | 1 | 0.001
H1ak1 | 573.1C | 3 | 0.004
H1ak1 | 573.2C | 5 | 0.006
H1ak1 | 573.5C | 1 | 0.001
H1ak1 | 8634 | 1 | 0.001
H1ak2 | 10187G | 2 | 0.002
H1ak2 | 113 | 1 | 0.001
H1ak2 | 12561 | 2 | 0.002
H1ak2 | 16076A | 123 | 0.154
H1ak2 | 16129 | 1 | 0.001
H1ak2 | 208 | 1 | 0.001
H1ak2 | 261 | 1 | 0.001
H1ak2 | 281C | 1 | 0.001
H1ak2 | 316d | 1 | 0.001
H1ak2 | 338 | 1 | 0.001
H1ak2 | 468 | 3 | 0.004
H1ak2 | 502 | 1 | 0.001
H1ak2 | 573.1C | 3 | 0.004
H1ak2 | 573.2C | 5 | 0.006
H1ak2 | 573.5C | 1 | 0.001
H1ak2 | 934 | 1 | 0.001
H1am | 113 | 1 | 0.001
H1am | 16076A | 123 | 0.155
H1am | 208 | 1 | 0.001
H1am | 261 | 1 | 0.001
H1am | 281C | 1 | 0.001
H1am | 316d | 1 | 0.001
H1am | 338 | 1 | 0.001
H1am | 468 | 3 | 0.004
H1am | 502 | 1 | 0.001
H1am | 573.1C | 3 | 0.004
H1am | 573.2C | 5 | 0.006
H1am | 573.5C | 1 | 0.001
H1am1 | 113 | 1 | 0.001
H1am1 | 16076A | 123 | 0.154
H1am1 | 208 | 1 | 0.001
H1am1 | 261 | 1 | 0.001
H1am1 | 281C | 1 | 0.001
H1am1 | 316d | 1 | 0.001
H1am1 | 338 | 1 | 0.001
H1am1 | 468 | 3 | 0.004
H1am1 | 502 | 1 | 0.001
H1am1 | 573.1C | 3 | 0.004
H1am1 | 573.2C | 5 | 0.006
H1am1 | 573.5C | 1 | 0.001
H1am1 | 9145 | 21 | 0.026
H1an | 573.1C | 20 | 0.138
H1an1 | 16188 | 1 | 0.007
H1an1 | 573.1C | 1 | 0.007
H1an1 | 7930 | 1 | 0.007
H1an1a | 13365 | 1 | 0.007
H1an1a | 16207 | 1 | 0.007
H1an1a | 16286 | 22 | 0.144
H1an1a | 237 | 2 | 0.013
H1an1a | 3796 | 1 | 0.007
H1an1a | 573.1C | 1 | 0.007
H1an2 | 11632 | 1 | 0.05
H1an2 | 11638 | 1 | 0.05
H1an2 | 16023.1G | 1 | 0.05
H1an2 | 16029d | 1 | 0.05
H1an2 | 16068 | 1 | 0.05
H1an2 | 16126 | 1 | 0.05
H1an2 | 16179 | 1 | 0.05
H1an2 | 16188 | 1 | 0.05
H1an2 | 16189 | 1 | 0.05
H1an2 | 16193d | 1 | 0.05
H1an2 | 16209 | 1 | 0.05
H1an2 | 16226 | 1 | 0.05
H1an2 | 16230T | 1 | 0.05
H1an2 | 16231A | 1 | 0.05
H1an2 | 16256 | 1 | 0.05
H1an2 | 16266 | 1 | 0.05
H1an2 | 2854G | 3 | 0.15
H1an2 | 310 | 1 | 0.05
H1an2 | 3861 | 1 | 0.05
H1an2 | 6713 | 1 | 0.05
H1an2 | 8712 | 1 | 0.05
H1an2 | 8998 | 1 | 0.05
H1an2 | 9117 | 1 | 0.05
H1ao | 13899 | 1 | 0.033
H1ao | 152 | 1 | 0.033
H1ao | 16311 | 1 | 0.033
H1ao | 199 | 3 | 0.1
H1ao | 2889A | 1 | 0.033
H1ao | 310 | 2 | 0.067
H1ao | 5028 | 4 | 0.133
H1ao | 519 | 1 | 0.033

H1ao | 8632 | 1 | 0.033
H1ao | 9299 | 1 | 0.033
H1ao1 | 16129 | 2 | 0.083
H1ao1 | 16162 | 1 | 0.042
H1ao1 | 16174 | 1 | 0.042
H1ao1 | 16223 | 1 | 0.042
H1ao1 | 16262 | 1 | 0.042
H1ao1 | 16274 | 1 | 0.042
H1ao1 | 246 | 1 | 0.042
H1ao1 | 438 | 1 | 0.042
H1ao1 | 73 | 1 | 0.042
H1ao1 | 95C | 1 | 0.042
H1ap | 10398 | 1 | 0.001
H1ap | 113 | 1 | 0.001
H1ap | 11465 | 1 | 0.001
H1ap | 13194 | 1 | 0.001
H1ap | 16076A | 123 | 0.155
H1ap | 16174 | 1 | 0.001
H1ap | 208 | 1 | 0.001
H1ap | 261 | 1 | 0.001
H1ap | 281C | 1 | 0.001
H1ap | 310 | 1 | 0.001
H1ap | 316d | 1 | 0.001
H1ap | 338 | 1 | 0.001
H1ap | 3612 | 1 | 0.001
H1ap | 3870 | 1 | 0.001
H1ap | 468 | 3 | 0.004
H1ap | 502 | 1 | 0.001
H1ap | 573.1C | 3 | 0.004
H1ap | 573.2C | 5 | 0.006
H1ap | 573.5C | 1 | 0.001
H1ap | 6150 | 1 | 0.001
H1ap1 | 11002 | 1 | 0.009
H1ap1 | 14386 | 1 | 0.009
H1ap1 | 14560 | 1 | 0.009
H1ap1 | 14894 | 1 | 0.009
H1ap1 | 151 | 2 | 0.019
H1ap1 | 16093 | 2 | 0.019
H1ap1 | 16172 | 2 | 0.019
H1ap1 | 16185 | 1 | 0.009
H1ap1 | 16186A | 1 | 0.009
H1ap1 | 16213 | 1 | 0.009
H1ap1 | 16214 | 1 | 0.009
H1ap1 | 16265C | 2 | 0.019
H1ap1 | 16270 | 1 | 0.009
H1ap1 | 16278 | 1 | 0.009
H1ap1 | 16291 | 2 | 0.019
H1ap1 | 16293 | 2 | 0.019
H1ap1 | 16304 | 1 | 0.009
H1ap1 | 16354 | 1 | 0.009
H1ap1 | 16363 | 4 | 0.038
H1ap1 | 16524 | 1 | 0.009
H1ap1 | 191.1A | 3 | 0.028
H1ap1 | 200 | 2 | 0.019
H1ap1 | 207 | 1 | 0.009
H1ap1 | 309d | 1 | 0.009
H1ap1 | 310 | 1 | 0.009
H1ap1 | 408G | 2 | 0.019
H1ap1 | 4092 | 1 | 0.009
H1ap1 | 7217 | 1 | 0.009
H1ap1 | 7521 | 1 | 0.009
H1ap1 | 7801 | 1 | 0.009
H1ap1 | 94 | 1 | 0.009
H1aq | 113 | 1 | 0.001
H1aq | 11314 | 3 | 0.004
H1aq | 13590 | 1 | 0.001
H1aq | 15067 | 1 | 0.001
H1aq | 16076A | 123 | 0.154
H1aq | 208 | 1 | 0.001
H1aq | 261 | 1 | 0.001
H1aq | 281C | 1 | 0.001
H1aq | 316d | 1 | 0.001
H1aq | 338 | 1 | 0.001
H1aq | 468 | 3 | 0.004
H1aq | 502 | 1 | 0.001
H1aq | 5498 | 1 | 0.001
H1aq | 573.1C | 3 | 0.004
H1aq | 573.2C | 5 | 0.006
H1aq | 573.5C | 1 | 0.001
H1aq | 7894 | 3 | 0.004
H1aq | 93 | 3 | 0.004
H1aq1 | 16179 | 1 | 0.012
H1aq1 | 16219 | 1 | 0.012
H1aq1 | 16220 | 1 | 0.012
H1aq1 | 16266 | 1 | 0.012
H1aq1 | 267 | 1 | 0.012
H1aq1 | 334 | 1 | 0.012
H1aq1 | 3666 | 2 | 0.025
H1ar | 10654 | 1 | 0.025
H1ar | 10695 | 1 | 0.025
H1ar | 12311.1A | 1 | 0.025
H1ar | 12453 | 1 | 0.025
H1ar | 13781 | 1 | 0.025
H1ar | 14180 | 3 | 0.075
H1ar | 14344 | 2 | 0.05
H1ar | 14974G | 1 | 0.025
H1ar | 1508 | 1 | 0.025
H1ar | 15153 | 1 | 0.025
H1ar | 15172 | 1 | 0.025
H1ar | 15589A | 2 | 0.05
H1ar | 16311 | 3 | 0.075
H1ar | 182 | 3 | 0.075
H1ar | 2281 | 1 | 0.025
H1ar | 2330 | 1 | 0.025
H1ar | 3396 | 1 | 0.025
H1ar | 4772 | 2 | 0.05
H1ar | 5788 | 1 | 0.025
H1ar | 6253 | 1 | 0.025
H1ar | 6456 | 1 | 0.025
H1ar | 6480 | 1 | 0.025
H1ar | 6755 | 1 | 0.025

H1ar | 735 | 1 | 0.025
H1ar | 7510 | 1 | 0.025
H1ar | 7830 | 1 | 0.025
H1ar | 7891 | 1 | 0.025
H1ar | 7984 | 1 | 0.025
H1ar | 8277 | 1 | 0.025
H1ar | 8371 | 1 | 0.025
H1ar | 8461 | 1 | 0.025
H1ar | 8736 | 1 | 0.025
H1ar | 8921 | 1 | 0.025
H1ar | 8997A | 3 | 0.075
H1ar | 9058 | 1 | 0.025
H1ar1 | 152 | 1 | 0.333
H1as | 113 | 1 | 0.001
H1as | 16076A | 123 | 0.154
H1as | 208 | 1 | 0.001
H1as | 225 | 1 | 0.001
H1as | 261 | 1 | 0.001
H1as | 281C | 1 | 0.001
H1as | 316d | 1 | 0.001
H1as | 338 | 1 | 0.001
H1as | 4674 | 2 | 0.003
H1as | 468 | 3 | 0.004
H1as | 502 | 1 | 0.001
H1as | 573.1C | 3 | 0.004
H1as | 573.2C | 5 | 0.006
H1as | 573.5C | 1 | 0.001
H1as | 72 | 2 | 0.003
H1as | 8603 | 1 | 0.001
H1as1 | 114 | 5 | 0.065
H1as1 | 15924 | 1 | 0.013
H1as1 | 16078 | 2 | 0.026
H1as1 | 16086 | 1 | 0.013
H1as1 | 16163 | 1 | 0.013
H1as1 | 16174 | 1 | 0.013
H1as1 | 16192A | 1 | 0.013
H1as1 | 16243 | 1 | 0.013
H1as1 | 16263 | 5 | 0.065
H1as1 | 16297 | 1 | 0.013
H1as1 | 16309 | 1 | 0.013
H1as1 | 16318T | 1 | 0.013
H1as1 | 16362 | 2 | 0.026
H1as1 | 16484-16489d | 1 | 0.013
H1as1 | 189 | 2 | 0.026
H1as1 | 227T | 5 | 0.065
H1as1 | 248 | 1 | 0.013
H1as1 | 315.2C | 1 | 0.013
H1as1 | 316C | 1 | 0.013
H1as1 | 477 | 2 | 0.026
H1as1 | 573.1C | 5 | 0.065
H1as1a | 16126 | 1 | 0.032
H1as1a | 2385A | 1 | 0.032
H1as1a | 2836 | 1 | 0.032
H1as1a | 417 | 2 | 0.065
H1as1a | 5978 | 1 | 0.032
H1as1a | 73 | 1 | 0.032
H1as2 | 113 | 1 | 0.001
H1as2 | 13145 | 1 | 0.001
H1as2 | 14025 | 1 | 0.001
H1as2 | 16076A | 123 | 0.154
H1as2 | 208 | 1 | 0.001
H1as2 | 261 | 1 | 0.001
H1as2 | 281C | 1 | 0.001
H1as2 | 316d | 1 | 0.001
H1as2 | 338 | 1 | 0.001
H1as2 | 468 | 3 | 0.004
H1as2 | 502 | 1 | 0.001
H1as2 | 573.1C | 3 | 0.004
H1as2 | 573.2C | 5 | 0.006
H1as2 | 573.5C | 1 | 0.001
H1at | 10790 | 1 | 0.001
H1at | 11172 | 1 | 0.001
H1at | 113 | 1 | 0.001
H1at | 12507 | 1 | 0.001
H1at | 16076A | 123 | 0.155
H1at | 208 | 1 | 0.001
H1at | 239 | 1 | 0.001
H1at | 2501 | 1 | 0.001
H1at | 261 | 1 | 0.001
H1at | 281C | 1 | 0.001
H1at | 316d | 1 | 0.001
H1at | 338 | 1 | 0.001
H1at | 468 | 3 | 0.004
H1at | 502 | 1 | 0.001
H1at | 573.1C | 3 | 0.004
H1at | 573.2C | 5 | 0.006
H1at | 573.5C | 1 | 0.001
H1at | 8764 | 1 | 0.001
H1at1 | 113 | 1 | 0.001
H1at1 | 16076A | 123 | 0.154
H1at1 | 16311 | 1 | 0.001
H1at1 | 1719 | 1 | 0.001
H1at1 | 208 | 1 | 0.001
H1at1 | 261 | 1 | 0.001
H1at1 | 281C | 1 | 0.001
H1at1 | 316d | 1 | 0.001
H1at1 | 338 | 1 | 0.001
H1at1 | 3472 | 1 | 0.001
H1at1 | 468 | 3 | 0.004
H1at1 | 502 | 1 | 0.001
H1at1 | 573.1C | 3 | 0.004
H1at1 | 573.2C | 5 | 0.006
H1at1 | 573.5C | 1 | 0.001
H1at1 | 5814 | 1 | 0.001
H1at1 | 6305 | 1 | 0.001
H1at1 | 9311 | 3 | 0.004
H1at1a | 113 | 1 | 0.001
H1at1a | 1393 | 2 | 0.002
H1at1a | 152 | 1 | 0.001
H1at1a | 16076A | 1 | 0.001
H1at1a | 16259 | 123 | 0.154

H1at1a | 16260 | 1 | 0.001
H1at1a | 16311 | 123 | 0.154
H1at1a | 208 | 1 | 0.001
H1at1a | 261 | 1 | 0.001
H1at1a | 281C | 1 | 0.001
H1at1a | 316d | 1 | 0.001
H1at1a | 3196 | 1 | 0.001
H1at1a | 338 | 1 | 0.001
H1at1a | 468 | 3 | 0.004
H1at1a | 502 | 1 | 0.001
H1at1a | 573.1C | 3 | 0.004
H1at1a | 573.2C | 5 | 0.006
H1at1a | 573.5C | 1 | 0.001
H1at1a | 6593 | 1 | 0.001
H1au | 13404 | 2 | 0.022
H1au | 15619 | 2 | 0.022
H1au | 15936 | 1 | 0.011
H1au | 16093 | 1 | 0.011
H1au | 16172 | 1 | 0.011
H1au | 16174 | 1 | 0.011
H1au | 16201 | 1 | 0.011
H1au | 16247 | 1 | 0.011
H1au | 3826 | 1 | 0.011
H1au | 5460 | 1 | 0.011
H1au | 8251 | 1 | 0.011
H1au1 | 152 | 2 | 0.026
H1au1 | 16129 | 1 | 0.013
H1au1 | 16145 | 1 | 0.013
H1au1 | 16381d | 1 | 0.013
H1au1 | 16383T | 1 | 0.013
H1au1 | 16385.1G | 1 | 0.013
H1au1 | 207 | 1 | 0.013
H1au1a | 14305 | 1 | 0.013
H1au1a | 16129 | 1 | 0.013
H1au1a | 16145 | 1 | 0.013
H1au1a | 16189 | 2 | 0.025
H1au1a | 16304 | 1 | 0.013
H1au1a | 16354 | 1 | 0.013
H1au1a | 16381d | 1 | 0.013
H1au1a | 16383T | 1 | 0.013
H1au1a | 16385.1G | 1 | 0.013
H1au1a | 5580 | 1 | 0.013
H1au1a | 8393 | 1 | 0.013
H1au1a | 93 | 1 | 0.013
H1au1a | 9359 | 1 | 0.013
H1au1b | 16129 | 1 | 0.012
H1au1b | 16145 | 1 | 0.012
H1au1b | 16356 | 2 | 0.025
H1au1b | 16381d | 1 | 0.012
H1au1b | 16383T | 1 | 0.012
H1au1b | 16385.1G | 1 | 0.012
H1au1b | 4456 | 1 | 0.012
H1au1b | 7789 | 1 | 0.012
H1av | 113 | 1 | 0.001
H1av | 14587 | 1 | 0.001
H1av | 16076A | 123 | 0.155
H1av | 16311 | 1 | 0.001
H1av | 208 | 1 | 0.001
H1av | 261 | 1 | 0.001
H1av | 281C | 1 | 0.001
H1av | 310 | 1 | 0.001
H1av | 312 | 1 | 0.001
H1av | 316d | 1 | 0.001
H1av | 338 | 1 | 0.001
H1av | 4232 | 1 | 0.001
H1av | 468 | 3 | 0.004
H1av | 502 | 1 | 0.001
H1av | 573.1C | 3 | 0.004
H1av | 573.2C | 5 | 0.006
H1av | 573.5C | 1 | 0.001
H1av | 9845 | 1 | 0.001
H1av1 | 10448 | 6 | 0.071
H1av1 | 12437 | 1 | 0.012
H1av1 | 14207 | 1 | 0.012
H1av1 | 152 | 13 | 0.155
H1av1 | 16239 | 5 | 0.06
H1av1 | 215 | 1 | 0.012
H1av1 | 3903 | 1 | 0.012
H1av1 | 4219 | 1 | 0.012
H1av1 | 714 | 1 | 0.012
H1av1 | 8065 | 6 | 0.071
H1av1 | 8119 | 1 | 0.012
H1av1 | 8572 | 5 | 0.06
H1av1a | 4353 | 1 | 0.014
H1aw | 12493 | 1 | 0.007
H1aw | 15546 | 2 | 0.014
H1aw | 16311 | 7 | 0.049
H1aw | 3621 | 1 | 0.007
H1aw | 5564 | 2 | 0.014
H1aw | 7951 | 1 | 0.007
H1aw | 8152 | 2 | 0.014
H1aw1 | 103 | 1 | 0.007
H1aw1 | 10909 | 1 | 0.007
H1aw1 | 16061 | 1 | 0.007
H1aw1 | 16264 | 2 | 0.013
H1aw1 | 16327 | 7 | 0.047
H1aw1 | 292 | 1 | 0.007
H1aw1 | 299A | 1 | 0.007
H1aw1 | 368 | 1 | 0.007
H1aw1 | 455.1T | 1 | 0.007
H1aw1 | 459.1C | 1 | 0.007
H1aw1 | 463.1C | 8 | 0.053
H1aw1 | 4920 | 1 | 0.007
H1aw1 | 9948 | 1 | 0.007
H1ax | 10670 | 2 | 0.002
H1ax | 113 | 1 | 0.001
H1ax | 13602 | 1 | 0.001
H1ax | 14061A | 1 | 0.001
H1ax | 15236 | 1 | 0.001
H1ax | 16076A | 1 | 0.001
H1ax | 16140 | 123 | 0.154

H1ax | 16187 | 123 | 0.154
H1ax | 16248 | 1 | 0.001
H1ax | 16249 | 2 | 0.002
H1ax | 208 | 1 | 0.001
H1ax | 261 | 1 | 0.001
H1ax | 281C | 1 | 0.001
H1ax | 316d | 1 | 0.001
H1ax | 338 | 1 | 0.001
H1ax | 3630 | 1 | 0.001
H1ax | 468 | 3 | 0.004
H1ax | 502 | 1 | 0.001
H1ax | 513 | 1 | 0.001
H1ax | 5460 | 1 | 0.001
H1ax | 573.1C | 3 | 0.004
H1ax | 573.2C | 5 | 0.006
H1ax | 573.5C | 1 | 0.001
H1ax | 73 | 1 | 0.001
H1ax1 | 11101 | 3 | 0.004
H1ax1 | 113 | 1 | 0.001
H1ax1 | 150 | 3 | 0.004
H1ax1 | 16076A | 123 | 0.154
H1ax1 | 16150 | 1 | 0.001
H1ax1 | 16189 | 1 | 0.001
H1ax1 | 200 | 1 | 0.001
H1ax1 | 208 | 1 | 0.001
H1ax1 | 261 | 1 | 0.001
H1ax1 | 281C | 1 | 0.001
H1ax1 | 316d | 1 | 0.001
H1ax1 | 338 | 1 | 0.001
H1ax1 | 468 | 3 | 0.004
H1ax1 | 502 | 1 | 0.001
H1ax1 | 573.1C | 3 | 0.004
H1ax1 | 573.2C | 5 | 0.006
H1ax1 | 573.5C | 1 | 0.001
H1ax1 | 7499 | 1 | 0.001
H1ax1 | 8583 | 1 | 0.001
H1ay | 113 | 1 | 0.001
H1ay | 14971 | 1 | 0.001
H1ay | 16076A | 123 | 0.155
H1ay | 208 | 1 | 0.001
H1ay | 2352 | 1 | 0.001
H1ay | 261 | 1 | 0.001
H1ay | 281C | 1 | 0.001
H1ay | 316d | 1 | 0.001
H1ay | 338 | 1 | 0.001
H1ay | 468 | 3 | 0.004
H1ay | 502 | 1 | 0.001
H1ay | 5516 | 1 | 0.001
H1ay | 573.1C | 3 | 0.004
H1ay | 573.2C | 5 | 0.006
H1ay | 573.5C | 1 | 0.001
H1ay | 8149 | 1 | 0.001
H1ay | 9804 | 1 | 0.001
H1ay | 9861 | 1 | 0.001
H1az | 113 | 1 | 0.001
H1az | 12387 | 1 | 0.001
H1az | 12810 | 1 | 0.001
H1az | 16076A | 1 | 0.001
H1az | 16176 | 1 | 0.001
H1az | 16214 | 1 | 0.001
H1az | 16288A | 126 | 0.154
H1az | 16390 | 1 | 0.001
H1az | 208 | 1 | 0.001
H1az | 261 | 1 | 0.001
H1az | 281C | 1 | 0.001
H1az | 310 | 3 | 0.004
H1az | 316d | 1 | 0.001
H1az | 334 | 1 | 0.001
H1az | 338 | 1 | 0.001
H1az | 468 | 3 | 0.004
H1az | 502 | 1 | 0.001
H1az | 573.1C | 3 | 0.004
H1az | 573.2C | 5 | 0.006
H1az | 573.5C | 1 | 0.001
H1az | 73 | 2 | 0.002
H1az | 7444 | 4 | 0.005
H1az | 8812 | 1 | 0.001
H1az | 9518 | 2 | 0.002
H1b | 10680 | 1 | 0.006
H1b | 10700 | 1 | 0.006
H1b | 1118 | 2 | 0.011
H1b | 11708 | 1 | 0.006
H1b | 11819 | 1 | 0.006
H1b | 12192 | 1 | 0.006
H1b | 12630 | 1 | 0.006
H1b | 131 | 3 | 0.017
H1b | 13105 | 1 | 0.006
H1b | 13389 | 1 | 0.006
H1b | 13731 | 1 | 0.006
H1b | 13781 | 1 | 0.006
H1b | 13830 | 1 | 0.006
H1b | 140 | 1 | 0.006
H1b | 14118C | 1 | 0.006
H1b | 14208 | 1 | 0.006
H1b | 146 | 1 | 0.006
H1b | 14696C | 1 | 0.006
H1b | 1508 | 1 | 0.006
H1b | 15148 | 1 | 0.006
H1b | 15172 | 1 | 0.006
H1b | 152 | 5 | 0.029
H1b | 15617 | 1 | 0.006
H1b | 15977 | 1 | 0.006
H1b | 16086 | 1 | 0.006
H1b | 16093 | 2 | 0.011
H1b | 16129 | 2 | 0.011
H1b | 16137 | 1 | 0.006
H1b | 16172 | 1 | 0.006
H1b | 16173 | 1 | 0.006
H1b | 16207 | 1 | 0.006
H1b | 16221 | 1 | 0.006
H1b | 16223 | 1 | 0.006

H1b | 16234 | 1 | 0.006
H1b | 16240 | 1 | 0.006
H1b | 16248 | 1 | 0.006
H1b | 16249 | 3 | 0.017
H1b | 16263 | 1 | 0.006
H1b | 16291 | 1 | 0.006
H1b | 16318 | 1 | 0.006
H1b | 16354 | 1 | 0.006
H1b | 16360 | 3 | 0.017
H1b | 16362 | 1 | 0.006
H1b | 1836 | 1 | 0.006
H1b | 199 | 2 | 0.011
H1b | 2140 | 1 | 0.006
H1b | 2248 | 1 | 0.006
H1b | 271 | 1 | 0.006
H1b | 310 | 2 | 0.011
H1b | 315.2C | 1 | 0.006
H1b | 316 | 2 | 0.011
H1b | 345 | 1 | 0.006
H1b | 3537 | 1 | 0.006
H1b | 3849 | 1 | 0.006
H1b | 3912 | 1 | 0.006
H1b | 3969 | 1 | 0.006
H1b | 4065 | 1 | 0.006
H1b | 4117 | 1 | 0.006
H1b | 4317 | 1 | 0.006
H1b | 4649 | 1 | 0.006
H1b | 4742 | 1 | 0.006
H1b | 4823 | 2 | 0.011
H1b | 4853 | 2 | 0.011
H1b | 60 | 2 | 0.011
H1b | 61 | 2 | 0.011
H1b | 621 | 1 | 0.006
H1b | 6216 | 2 | 0.011
H1b | 6755 | 1 | 0.006
H1b | 721 | 1 | 0.006
H1b | 735 | 1 | 0.006
H1b | 7751G | 1 | 0.006
H1b | 7789 | 1 | 0.006
H1b | 7830 | 1 | 0.006
H1b | 7984 | 1 | 0.006
H1b | 8406 | 2 | 0.011
H1b | 8461 | 1 | 0.006
H1b | 8581 | 1 | 0.006
H1b | 8603 | 2 | 0.011
H1b | 8715 | 1 | 0.006
H1b | 9163 | 1 | 0.006
H1b | 93 | 3 | 0.017
H1b1 | 10364 | 1 | 0.007
H1b1 | 1189 | 1 | 0.007
H1b1 | 11923 | 2 | 0.015
H1b1 | 12717 | 1 | 0.007
H1b1 | 12957 | 1 | 0.007
H1b1 | 13552 | 2 | 0.015
H1b1 | 146 | 2 | 0.015
H1b1 | 15043 | 1 | 0.007
H1b1 | 15152 | 1 | 0.007
H1b1 | 152 | 12 | 0.088
H1b1 | 15519A | 4 | 0.029
H1b1 | 15757 | 1 | 0.007
H1b1 | 15777 | 1 | 0.007
H1b1 | 15914C | 1 | 0.007
H1b1 | 15932 | 1 | 0.007
H1b1 | 16111 | 1 | 0.007
H1b1 | 16172 | 1 | 0.007
H1b1 | 16192 | 1 | 0.007
H1b1 | 16261 | 1 | 0.007
H1b1 | 16274 | 1 | 0.007
H1b1 | 16287 | 1 | 0.007
H1b1 | 16311 | 2 | 0.015
H1b1 | 16362 | 1 | 0.007
H1b1 | 1809 | 1 | 0.007
H1b1 | 198 | 1 | 0.007
H1b1 | 2486A | 1 | 0.007
H1b1 | 2833 | 1 | 0.007
H1b1 | 2870 | 1 | 0.007
H1b1 | 304 | 1 | 0.007
H1b1 | 305 | 1 | 0.007
H1b1 | 310 | 4 | 0.029
H1b1 | 3826 | 1 | 0.007
H1b1 | 408A | 1 | 0.007
H1b1 | 4491 | 1 | 0.007
H1b1 | 489 | 1 | 0.007
H1b1 | 513 | 2 | 0.015
H1b1 | 5387 | 1 | 0.007
H1b1 | 58 | 3 | 0.022
H1b1 | 60.1T | 2 | 0.015
H1b1 | 6137 | 1 | 0.007
H1b1 | 6272 | 1 | 0.007
H1b1 | 6293 | 1 | 0.007
H1b1 | 6353 | 1 | 0.007
H1b1 | 6366 | 1 | 0.007
H1b1 | 72 | 1 | 0.007
H1b1 | 738 | 1 | 0.007
H1b1 | 8545 | 1 | 0.007
H1b1 | 8706 | 1 | 0.007
H1b1 | 8723 | 1 | 0.007
H1b1 | 93 | 1 | 0.007
H1b1 | 9540 | 1 | 0.007
H1b1 | 9947 | 4 | 0.029
H1b1+16362 | 10253 | 1 | 0.007
H1b1+16362 | 10289 | 1 | 0.007
H1b1+16362 | 10822 | 2 | 0.014
H1b1+16362 | 11719 | 1 | 0.007
H1b1+16362 | 1193 | 1 | 0.007
H1b1+16362 | 12103 | 1 | 0.007
H1b1+16362 | 12285 | 1 | 0.007
H1b1+16362 | 12768 | 1 | 0.007
H1b1+16362 | 13928C | 1 | 0.007
H1b1+16362 | 14602 | 1 | 0.007
H1b1+16362 | 150 | 6 | 0.043

H1b1+16362 | 151 | 3 | 0.022
H1b1+16362 | 152 | 3 | 0.022
H1b1+16362 | 15221 | 1 | 0.007
H1b1+16362 | 15244 | 1 | 0.007
H1b1+16362 | 15254 | 1 | 0.007
H1b1+16362 | 15468 | 1 | 0.007
H1b1+16362 | 15519 | 1 | 0.007
H1b1+16362 | 15924 | 1 | 0.007
H1b1+16362 | 16093 | 1 | 0.007
H1b1+16362 | 16153 | 1 | 0.007
H1b1+16362 | 16172 | 3 | 0.022
H1b1+16362 | 16243 | 1 | 0.007
H1b1+16362 | 16271 | 1 | 0.007
H1b1+16362 | 16291 | 1 | 0.007
H1b1+16362 | 16300 | 1 | 0.007
H1b1+16362 | 16311 | 2 | 0.014
H1b1+16362 | 16374C | 1 | 0.007
H1b1+16362 | 16463 | 3 | 0.022
H1b1+16362 | 1822 | 1 | 0.007
H1b1+16362 | 186 | 1 | 0.007
H1b1+16362 | 195 | 2 | 0.014
H1b1+16362 | 309d | 2 | 0.014
H1b1+16362 | 320 | 1 | 0.007
H1b1+16362 | 3472 | 1 | 0.007
H1b1+16362 | 3736 | 1 | 0.007
H1b1+16362 | 4021 | 1 | 0.007
H1b1+16362 | 482 | 1 | 0.007
H1b1+16362 | 5746 | 1 | 0.007
H1b1+16362 | 6635 | 1 | 0.007
H1b1+16362 | 6770C | 1 | 0.007
H1b1+16362 | 709 | 1 | 0.007
H1b1+16362 | 72G | 1 | 0.007
H1b1+16362 | 7598 | 1 | 0.007
H1b1+16362 | 8027 | 1 | 0.007
H1b1+16362 | 8298 | 1 | 0.007
H1b1+16362 | 8346A | 1 | 0.007
H1b1+16362 | 8348 | 4 | 0.029
H1b1+16362 | 9182 | 1 | 0.007
H1b1+16362 | 9831 | 1 | 0.007
H1b1+16362 | 9861 | 1 | 0.007
H1b1a | 10978 | 1 | 0.009
H1b1a | 12561 | 1 | 0.009
H1b1a | 13608 | 2 | 0.017
H1b1a | 13822 | 1 | 0.009
H1b1a | 14751 | 2 | 0.017
H1b1a | 14824 | 1 | 0.009
H1b1a | 152 | 1 | 0.009
H1b1a | 15346 | 1 | 0.009
H1b1a | 16129 | 1 | 0.009
H1b1a | 16185A | 4 | 0.034
H1b1a | 16193d | 3 | 0.026
H1b1a | 16274 | 3 | 0.026
H1b1a | 1957 | 1 | 0.009
H1b1a | 200 | 2 | 0.017
H1b1a | 215 | 1 | 0.009
H1b1a | 2483 | 1 | 0.009
H1b1a | 249 | 3 | 0.026
H1b1a | 3243 | 1 | 0.009
H1b1a | 3338 | 1 | 0.009
H1b1a | 374 | 4 | 0.034
H1b1a | 3952 | 1 | 0.009
H1b1a | 4226 | 1 | 0.009
H1b1a | 5046 | 1 | 0.009
H1b1a | 5252 | 1 | 0.009
H1b1a | 7870 | 1 | 0.009
H1b1a | 9022 | 1 | 0.009
H1b1a | 9061 | 1 | 0.009
H1b1a | 93 | 1 | 0.009
H1b1b | 13536 | 1 | 0.012
H1b1b | 309d | 1 | 0.012
H1b1b | 310 | 2 | 0.025
H1b1b | 315.2C | 1 | 0.012
H1b1b | 517 | 1 | 0.012
H1b1b | 6040 | 1 | 0.012
H1b1c | 10172 | 1 | 0.01
H1b1c | 12408 | 1 | 0.01
H1b1c | 15468 | 1 | 0.01
H1b1c | 16162 | 1 | 0.01
H1b1c | 16294 | 1 | 0.01
H1b1c | 16299 | 1 | 0.01
H1b1c | 16469G | 3 | 0.03
H1b1c | 204 | 1 | 0.01
H1b1c | 310 | 1 | 0.01
H1b1c | 315.2C | 1 | 0.01
H1b1c | 483 | 3 | 0.03
H1b1c | 64 | 1 | 0.01
H1b1c | 73 | 1 | 0.01
H1b1c | 9110 | 1 | 0.01
H1b1d | 13926 | 1 | 0.024
H1b1d | 14209T | 1 | 0.024
H1b1d | 15787 | 1 | 0.024
H1b1d | 15924 | 1 | 0.024
H1b1d | 16265C | 1 | 0.024
H1b1d | 16280 | 1 | 0.024
H1b1d | 6182 | 1 | 0.024
H1b1d | 723 | 1 | 0.024
H1b1d | 8994 | 1 | 0.024
H1b1d | 9007 | 1 | 0.024
H1b1d | 9212 | 4 | 0.098
H1b1e | 12338 | 1 | 0.007
H1b1e | 143 | 1 | 0.007
H1b1e | 16051 | 1 | 0.007
H1b1e | 16086 | 3 | 0.022
H1b1e | 16104 | 1 | 0.007
H1b1e | 16129 | 7 | 0.052
H1b1e | 16145 | 1 | 0.007
H1b1e | 16153 | 1 | 0.007
H1b1e | 16192 | 3 | 0.022
H1b1e | 16209 | 3 | 0.022
H1b1e | 16226C | 1 | 0.007
H1b1e | 16243 | 1 | 0.007

H1b1e | 16263 | 2 | 0.015
H1b1e | 16265C | 1 | 0.007
H1b1e | 16292 | 2 | 0.015
H1b1e | 282 | 1 | 0.007
H1b1e | 309.3C | 4 | 0.03
H1b1e | 316 | 1 | 0.007
H1b1e | 335 | 3 | 0.022
H1b1e | 417 | 1 | 0.007
H1b1e | 813 | 1 | 0.007
H1b1e | 8649 | 1 | 0.007
H1b1e1 | 143 | 1 | 0.007
H1b1e1 | 16051 | 1 | 0.007
H1b1e1 | 16086 | 3 | 0.022
H1b1e1 | 16104 | 1 | 0.007
H1b1e1 | 16129 | 7 | 0.052
H1b1e1 | 16145 | 1 | 0.007
H1b1e1 | 16153 | 1 | 0.007
H1b1e1 | 16192 | 3 | 0.022
H1b1e1 | 16209 | 3 | 0.022
H1b1e1 | 16222 | 1 | 0.007
H1b1e1 | 16226C | 1 | 0.007
H1b1e1 | 16243 | 1 | 0.007
H1b1e1 | 16263 | 2 | 0.015
H1b1e1 | 16265C | 1 | 0.007
H1b1e1 | 16292 | 2 | 0.015
H1b1e1 | 282 | 1 | 0.007
H1b1e1 | 309.3C | 4 | 0.03
H1b1e1 | 316 | 1 | 0.007
H1b1e1 | 335 | 3 | 0.022
H1b1e1 | 417 | 1 | 0.007
H1b1e1 | 4973 | 1 | 0.007
H1b1e1 | 5237 | 1 | 0.007
H1b1f | 770 | 1 | 0.012
H1b1g | 16179 | 5 | 0.192
H1b1g | 16223 | 2 | 0.077
H1b1g | 16255 | 1 | 0.038
H1b1g | 16256 | 1 | 0.038
H1b1g | 16319 | 1 | 0.038
H1b1g | 16361C | 1 | 0.038
H1b1g | 709 | 1 | 0.038
H1b1h | 15199 | 1 | 0.01
H1b1h | 16162 | 1 | 0.01
H1b1h | 16261 | 2 | 0.02
H1b1h | 16299 | 1 | 0.01
H1b1h | 16469G | 3 | 0.03
H1b1h | 199 | 2 | 0.02
H1b1h | 204 | 1 | 0.01
H1b1h | 64 | 1 | 0.01
H1b1h | 73 | 1 | 0.01
H1b1i | 11719 | 1 | 0.007
H1b1i | 11890 | 1 | 0.007
H1b1i | 143 | 1 | 0.007
H1b1i | 16051 | 1 | 0.007
H1b1i | 16086 | 3 | 0.022
H1b1i | 16104 | 1 | 0.007
H1b1i | 16129 | 7 | 0.052
H1b1i | 16145 | 1 | 0.007
H1b1i | 16153 | 1 | 0.007
H1b1i | 16192 | 3 | 0.022
H1b1i | 16209 | 3 | 0.022
H1b1i | 16226C | 1 | 0.007
H1b1i | 16243 | 1 | 0.007
H1b1i | 16263 | 2 | 0.015
H1b1i | 16265C | 1 | 0.007
H1b1i | 16292 | 2 | 0.015
H1b1i | 282 | 1 | 0.007
H1b1i | 309.3C | 4 | 0.03
H1b1i | 316 | 1 | 0.007
H1b1i | 335 | 3 | 0.022
H1b1i | 417 | 1 | 0.007
H1b1i | 9782 | 1 | 0.007
H1b1i | 9788G | 1 | 0.007
H1b2 | 10003 | 1 | 0.01
H1b2 | 10527 | 1 | 0.01
H1b2 | 11152 | 5 | 0.048
H1b2 | 11361 | 1 | 0.01
H1b2 | 12501 | 1 | 0.01
H1b2 | 12510 | 1 | 0.01
H1b2 | 12731 | 1 | 0.01
H1b2 | 13579 | 1 | 0.01
H1b2 | 14052 | 1 | 0.01
H1b2 | 14207 | 1 | 0.01
H1b2 | 146 | 4 | 0.038
H1b2 | 151 | 5 | 0.048
H1b2 | 16092 | 1 | 0.01
H1b2 | 16111 | 1 | 0.01
H1b2 | 16126 | 1 | 0.01
H1b2 | 16129 | 3 | 0.029
H1b2 | 16223 | 4 | 0.038
H1b2 | 16265 | 1 | 0.01
H1b2 | 16309 | 1 | 0.01
H1b2 | 16360 | 1 | 0.01
H1b2 | 16371d | 2 | 0.019
H1b2 | 16383d | 2 | 0.019
H1b2 | 309.3C | 3 | 0.029
H1b2 | 310 | 4 | 0.038
H1b2 | 315.2C | 1 | 0.01
H1b2 | 4971 | 1 | 0.01
H1b2 | 5516 | 2 | 0.019
H1b2 | 5555T | 1 | 0.01
H1b2 | 5921 | 1 | 0.01
H1b2 | 7211 | 1 | 0.01
H1b2 | 73 | 1 | 0.01
H1b2 | 7337 | 2 | 0.019
H1b2 | 8419 | 2 | 0.019
H1b2 | 8625A | 1 | 0.01
H1b2 | 990 | 1 | 0.01
H1b2a | 1896 | 1 | 0.333
H1b2a1 | 8287.2C | 1 | 0.333
H1b3 | 14978 | 1 | 0.011
H1b3 | 16037 | 1 | 0.011

H1b3 | 16111 | 3 | 0.034
H1b3 | 16209 | 4 | 0.046
H1b3 | 16218 | 2 | 0.023
H1b3 | 4907G | 1 | 0.011
H1b3 | 7922 | 1 | 0.011
H1b3 | 8485 | 3 | 0.034
H1b3 | 8950 | 3 | 0.034
H1b3 | 9004 | 3 | 0.034
H1b4 | 11377 | 1 | 0.007
H1b4 | 143 | 1 | 0.007
H1b4 | 16051 | 1 | 0.007
H1b4 | 16086 | 3 | 0.022
H1b4 | 16104 | 1 | 0.007
H1b4 | 16129 | 7 | 0.052
H1b4 | 16145 | 1 | 0.007
H1b4 | 16153 | 1 | 0.007
H1b4 | 16192 | 3 | 0.022
H1b4 | 16209 | 3 | 0.022
H1b4 | 16226C | 1 | 0.007
H1b4 | 16243 | 1 | 0.007
H1b4 | 16263 | 2 | 0.015
H1b4 | 16265C | 1 | 0.007
H1b4 | 16292 | 2 | 0.015
H1b4 | 1778 | 1 | 0.007
H1b4 | 282 | 1 | 0.007
H1b4 | 309.3C | 4 | 0.03
H1b4 | 316 | 1 | 0.007
H1b4 | 335 | 3 | 0.022
H1b4 | 417 | 1 | 0.007
H1b5 | 10325 | 1 | 0.008
H1b5 | 10463 | 2 | 0.016
H1b5 | 11253 | 1 | 0.008
H1b5 | 143 | 1 | 0.008
H1b5 | 1461 | 1 | 0.008
H1b5 | 150 | 1 | 0.008
H1b5 | 15924 | 2 | 0.016
H1b5 | 16051 | 1 | 0.008
H1b5 | 16104 | 1 | 0.008
H1b5 | 16153 | 1 | 0.008
H1b5 | 16188 | 3 | 0.024
H1b5 | 16193d | 2 | 0.016
H1b5 | 16243 | 1 | 0.008
H1b5 | 16265C | 1 | 0.008
H1b5 | 16292 | 2 | 0.016
H1b5 | 16294 | 2 | 0.016
H1b5 | 16298 | 1 | 0.008
H1b5 | 282 | 1 | 0.008
H1b5 | 309d | 1 | 0.008
H1b5 | 310 | 1 | 0.008
H1b5 | 3163 | 1 | 0.008
H1b5 | 417 | 1 | 0.008
H1b5 | 5147 | 1 | 0.008
H1b5 | 7543 | 1 | 0.008
H1b5 | 8084 | 1 | 0.008
H1b5 | 827 | 1 | 0.008
H1ba | 10101 | 3 | 0.018
H1ba | 10790 | 1 | 0.006
H1ba | 11335 | 1 | 0.006
H1ba | 11453 | 2 | 0.012
H1ba | 11620 | 1 | 0.006
H1ba | 11728 | 2 | 0.012
H1ba | 12346 | 2 | 0.012
H1ba | 13015 | 2 | 0.012
H1ba | 13602 | 1 | 0.006
H1ba | 13702G | 1 | 0.006
H1ba | 14050G | 1 | 0.006
H1ba | 14199G | 1 | 0.006
H1ba | 14272G | 1 | 0.006
H1ba | 14365G | 1 | 0.006
H1ba | 14368G | 1 | 0.006
H1ba | 146 | 1 | 0.006
H1ba | 14687 | 2 | 0.012
H1ba | 14831 | 1 | 0.006
H1ba | 150 | 3 | 0.018
H1ba | 152 | 3 | 0.018
H1ba | 16051 | 1 | 0.006
H1ba | 16093 | 8 | 0.047
H1ba | 16145 | 1 | 0.006
H1ba | 16189 | 1 | 0.006
H1ba | 16190 | 1 | 0.006
H1ba | 16192 | 4 | 0.024
H1ba | 16220T | 2 | 0.012
H1ba | 16223 | 1 | 0.006
H1ba | 16234 | 1 | 0.006
H1ba | 16262 | 1 | 0.006
H1ba | 16269 | 2 | 0.012
H1ba | 16311 | 3 | 0.018
H1ba | 16320 | 1 | 0.006
H1ba | 16343T | 1 | 0.006
H1ba | 16375 | 2 | 0.012
H1ba | 16527 | 1 | 0.006
H1ba | 183 | 1 | 0.006
H1ba | 189 | 1 | 0.006
H1ba | 207 | 1 | 0.006
H1ba | 251 | 1 | 0.006
H1ba | 261 | 1 | 0.006
H1ba | 2833 | 1 | 0.006
H1ba | 315.1T | 1 | 0.006
H1ba | 3423G | 1 | 0.006
H1ba | 482 | 1 | 0.006
H1ba | 489 | 1 | 0.006
H1ba | 4985 | 1 | 0.006
H1ba | 5333 | 1 | 0.006
H1ba | 6569 | 1 | 0.006
H1ba | 709 | 1 | 0.006
H1ba | 76 | 1 | 0.006
H1ba | 8119 | 1 | 0.006
H1ba | 8631 | 1 | 0.006
H1ba | 9083 | 4 | 0.024
H1ba | 9185 | 1 | 0.006
H1ba | 93 | 3 | 0.018

H1ba | 9335 | 1 | 0.006
H1ba | 9559G | 1 | 0.006
H1ba1 | 11736 | 1 | 0.012
H1ba1 | 12618 | 1 | 0.012
H1ba1 | 13886 | 1 | 0.012
H1ba1 | 16566 | 2 | 0.024
H1ba1 | 3882 | 1 | 0.012
H1ba1 | 684A | 1 | 0.012
H1ba1 | 684T | 1 | 0.012
H1bb | 11695 | 1 | 0.006
H1bb | 11824 | 1 | 0.006
H1bb | 11872 | 1 | 0.006
H1bb | 11930 | 1 | 0.006
H1bb | 12888A | 1 | 0.006
H1bb | 12905G | 1 | 0.006
H1bb | 13404 | 1 | 0.006
H1bb | 14375 | 1 | 0.006
H1bb | 146 | 1 | 0.006
H1bb | 15106 | 1 | 0.006
H1bb | 15497 | 2 | 0.011
H1bb | 1555 | 2 | 0.011
H1bb | 16086 | 22 | 0.124
H1bb | 16129 | 1 | 0.006
H1bb | 16288 | 1 | 0.006
H1bb | 16311 | 1 | 0.006
H1bb | 194 | 1 | 0.006
H1bb | 195 | 1 | 0.006
H1bb | 199 | 10 | 0.056
H1bb | 310 | 1 | 0.006
H1bb | 41 | 20 | 0.113
H1bb | 480 | 1 | 0.006
H1bb | 5351 | 1 | 0.006
H1bb | 573.1C | 1 | 0.006
H1bb | 593 | 1 | 0.006
H1bb | 64 | 1 | 0.006
H1bb | 73 | 1 | 0.006
H1bb | 8485 | 1 | 0.006
H1bb | 8508 | 1 | 0.006
H1bb | 9615 | 1 | 0.006
H1bb | 998 | 1 | 0.006
H1bc | 10640 | 1 | 0.007
H1bc | 12880 | 20 | 0.132
H1bc | 13695 | 2 | 0.013
H1bc | 13768 | 1 | 0.007
H1bc | 15927 | 1 | 0.007
H1bc | 16362 | 1 | 0.007
H1bc | 4739 | 2 | 0.013
H1bc | 573.1C | 1 | 0.007
H1bc | 732 | 1 | 0.007
H1bc | 9039 | 2 | 0.013
H1bd | 113 | 1 | 0.001
H1bd | 1555 | 1 | 0.001
H1bd | 16076A | 123 | 0.154
H1bd | 16222 | 1 | 0.001
H1bd | 16318C | 1 | 0.001
H1bd | 200 | 1 | 0.001
H1bd | 204 | 3 | 0.004
H1bd | 208 | 1 | 0.001
H1bd | 261 | 1 | 0.001
H1bd | 281C | 1 | 0.001
H1bd | 316d | 1 | 0.001
H1bd | 3308 | 1 | 0.001
H1bd | 338 | 1 | 0.001
H1bd | 468 | 3 | 0.004
H1bd | 502 | 1 | 0.001
H1bd | 573.1C | 3 | 0.004
H1bd | 573.2C | 5 | 0.006
H1bd | 573.5C | 1 | 0.001
H1bd | 73 | 1 | 0.001
H1be | 113 | 1 | 0.001
H1be | 13260 | 2 | 0.003
H1be | 16076A | 1 | 0.001
H1be | 16214 | 1 | 0.001
H1be | 16362 | 123 | 0.154
H1be | 208 | 1 | 0.001
H1be | 261 | 1 | 0.001
H1be | 281C | 1 | 0.001
H1be | 316d | 1 | 0.001
H1be | 338 | 1 | 0.001
H1be | 468 | 3 | 0.004
H1be | 502 | 1 | 0.001
H1be | 573.1C | 3 | 0.004
H1be | 573.2C | 5 | 0.006
H1be | 573.5C | 1 | 0.001
H1bf | 11596 | 2 | 0.023
H1bf | 13230 | 2 | 0.023
H1bf | 152 | 2 | 0.023
H1bf | 16129 | 11 | 0.125
H1bf | 16189 | 2 | 0.023
H1bf | 5474 | 1 | 0.011
H1bf1 | 12672T | 1 | 0.014
H1bg | 150 | 2 | 0.025
H1bg | 1555 | 3 | 0.038
H1bg | 16092 | 1 | 0.012
H1bg | 4691 | 1 | 0.012
H1bh | 13716 | 1 | 0.011
H1bh | 152A | 2 | 0.022
H1bh | 16093 | 1 | 0.011
H1bh | 16136 | 2 | 0.022
H1bh | 16192 | 1 | 0.011
H1bh | 16223 | 1 | 0.011
H1bh | 16278 | 1 | 0.011
H1bh | 16311 | 3 | 0.034
H1bh | 16390 | 3 | 0.034
H1bh | 1737 | 1 | 0.011
H1bh | 6268 | 1 | 0.011
H1bh | 8981 | 1 | 0.011
H1bi | 113 | 1 | 0.001
H1bi | 12083G | 1 | 0.001
H1bi | 16076A | 1 | 0.001
H1bi | 16093 | 1 | 0.001

H1bi | 16311 | 123 | 0.154
H1bi | 208 | 1 | 0.001
H1bi | 261 | 1 | 0.001
H1bi | 281C | 1 | 0.001
H1bi | 316d | 1 | 0.001
H1bi | 338 | 1 | 0.001
H1bi | 468 | 3 | 0.004
H1bi | 502 | 1 | 0.001
H1bi | 573.1C | 3 | 0.004
H1bi | 573.2C | 5 | 0.006
H1bi | 573.5C | 1 | 0.001
H1bj | 113 | 1 | 0.001
H1bj | 14693 | 1 | 0.001
H1bj | 16076A | 123 | 0.155
H1bj | 208 | 1 | 0.001
H1bj | 261 | 1 | 0.001
H1bj | 281C | 1 | 0.001
H1bj | 316d | 1 | 0.001
H1bj | 338 | 1 | 0.001
H1bj | 468 | 3 | 0.004
H1bj | 502 | 1 | 0.001
H1bj | 573.1C | 3 | 0.004
H1bj | 573.2C | 5 | 0.006
H1bj | 573.5C | 1 | 0.001
H1bk | 113 | 1 | 0.001
H1bk | 16076A | 123 | 0.153
H1bk | 16311 | 1 | 0.001
H1bk | 208 | 1 | 0.001
H1bk | 261 | 1 | 0.001
H1bk | 281C | 1 | 0.001
H1bk | 310 | 2 | 0.002
H1bk | 316d | 1 | 0.001
H1bk | 338 | 1 | 0.001
H1bk | 468 | 3 | 0.004
H1bk | 502 | 1 | 0.001
H1bk | 573.1C | 3 | 0.004
H1bk | 573.2C | 5 | 0.006
H1bk | 573.5C | 1 | 0.001
H1bk | 93 | 1 | 0.001
H1bk | 95C | 2 | 0.002
H1bk | 9921 | 1 | 0.001
H1bm | 113 | 1 | 0.001
H1bm | 11614 | 1 | 0.001
H1bm | 11941 | 1 | 0.001
H1bm | 16076A | 123 | 0.154
H1bm | 16185 | 1 | 0.001
H1bm | 183 | 1 | 0.001
H1bm | 208 | 1 | 0.001
H1bm | 261 | 1 | 0.001
H1bm | 281C | 1 | 0.001
H1bm | 316d | 1 | 0.001
H1bm | 338 | 1 | 0.001
H1bm | 4011 | 1 | 0.001
H1bm | 468 | 3 | 0.004
H1bm | 4733 | 1 | 0.001
H1bm | 502 | 1 | 0.001
H1bm | 573.1C | 3 | 0.004
H1bm | 573.2C | 5 | 0.006
H1bm | 573.5C | 1 | 0.001
H1bm | 8450 | 1 | 0.001
H1bn | 113 | 1 | 0.001
H1bn | 12618 | 3 | 0.004
H1bn | 13191G | 1 | 0.001
H1bn | 16076A | 123 | 0.154
H1bn | 208 | 1 | 0.001
H1bn | 261 | 1 | 0.001
H1bn | 281C | 1 | 0.001
H1bn | 316d | 1 | 0.001
H1bn | 338 | 1 | 0.001
H1bn | 356.1C | 1 | 0.001
H1bn | 468 | 3 | 0.004
H1bn | 502 | 1 | 0.001
H1bn | 573.1C | 3 | 0.004
H1bn | 573.2C | 5 | 0.006
H1bn | 573.5C | 1 | 0.001
H1bn | 6340 | 1 | 0.001
H1bn | 6719 | 3 | 0.004
H1bn | 6896 | 1 | 0.001
H1bn | 7698 | 1 | 0.001
H1bn | 869 | 1 | 0.001
H1bo | 14053 | 1 | 0.011
H1bo | 16104 | 1 | 0.011
H1bo | 16111 | 1 | 0.011
H1bo | 16124 | 1 | 0.011
H1bo | 16189 | 3 | 0.033
H1bo | 16209 | 1 | 0.011
H1bo | 16319 | 1 | 0.011
H1bo | 165 | 1 | 0.011
H1bo | 6483 | 2 | 0.022
H1bo | 6512 | 2 | 0.022
H1bo | 6542 | 2 | 0.022
H1bo | 6569A | 2 | 0.022
H1bo | 6569G | 1 | 0.011
H1bo | 6641 | 2 | 0.022
H1bp | 113 | 1 | 0.001
H1bp | 14329 | 1 | 0.001
H1bp | 150 | 1 | 0.001
H1bp | 16076A | 123 | 0.154
H1bp | 16291 | 1 | 0.001
H1bp | 16294 | 1 | 0.001
H1bp | 16325 | 1 | 0.001
H1bp | 208 | 1 | 0.001
H1bp | 261 | 1 | 0.001
H1bp | 281C | 1 | 0.001
H1bp | 316d | 1 | 0.001
H1bp | 338 | 1 | 0.001
H1bp | 368 | 1 | 0.001
H1bp | 468 | 3 | 0.004
H1bp | 502 | 1 | 0.001
H1bp | 573.1C | 3 | 0.004
H1bp | 573.2C | 5 | 0.006

H1bp | 573.5C | 1 | 0.001
H1bp | 9599 | 1 | 0.001
H1bq | 113 | 1 | 0.001
H1bq | 16076A | 123 | 0.155
H1bq | 208 | 1 | 0.001
H1bq | 261 | 1 | 0.001
H1bq | 281C | 1 | 0.001
H1bq | 316d | 1 | 0.001
H1bq | 338 | 1 | 0.001
H1bq | 468 | 3 | 0.004
H1bq | 502 | 1 | 0.001
H1bq | 573.1C | 3 | 0.004
H1bq | 573.2C | 5 | 0.006
H1bq | 573.5C | 1 | 0.001
H1br | 10583 | 1 | 0.001
H1br | 113 | 1 | 0.001
H1br | 11914 | 1 | 0.001
H1br | 12007 | 1 | 0.001
H1br | 12771 | 1 | 0.001
H1br | 16076A | 123 | 0.154
H1br | 16093 | 1 | 0.001
H1br | 208 | 1 | 0.001
H1br | 261 | 1 | 0.001
H1br | 281C | 1 | 0.001
H1br | 316d | 1 | 0.001
H1br | 338 | 1 | 0.001
H1br | 468 | 3 | 0.004
H1br | 502 | 1 | 0.001
H1br | 573.1C | 3 | 0.004
H1br | 573.2C | 5 | 0.006
H1br | 573.5C | 1 | 0.001
H1br | 5960 | 1 | 0.001
H1br | 73 | 1 | 0.001
H1br | 7870 | 2 | 0.003
H1br | 9947 | 1 | 0.001
H1bs | 11204 | 3 | 0.027
H1bs | 11536 | 1 | 0.009
H1bs | 14239 | 1 | 0.009
H1bs | 14560 | 1 | 0.009
H1bs | 146 | 2 | 0.018
H1bs | 14861 | 5 | 0.045
H1bs | 150 | 1 | 0.009
H1bs | 15110 | 1 | 0.009
H1bs | 15122 | 5 | 0.045
H1bs | 152 | 1 | 0.009
H1bs | 15773 | 2 | 0.018
H1bs | 15924 | 6 | 0.054
H1bs | 16033T | 1 | 0.009
H1bs | 16035 | 1 | 0.009
H1bs | 16129 | 3 | 0.027
H1bs | 16189 | 2 | 0.018
H1bs | 16247 | 3 | 0.027
H1bs | 16274 | 3 | 0.027
H1bs | 16278 | 1 | 0.009
H1bs | 16311 | 2 | 0.018
H1bs | 16318 | 3 | 0.027
H1bs | 16362 | 2 | 0.018
H1bs | 2222 | 1 | 0.009
H1bs | 309d | 1 | 0.009
H1bs | 4688 | 2 | 0.018
H1bs | 477 | 1 | 0.009
H1bs | 5466 | 1 | 0.009
H1bs | 7080 | 2 | 0.018
H1bs | 709 | 2 | 0.018
H1bs | 721 | 1 | 0.009
H1bs | 7269 | 2 | 0.018
H1bs | 7278 | 2 | 0.018
H1bs | 8093 | 2 | 0.018
H1bs | 8152 | 1 | 0.009
H1bs | 93 | 1 | 0.009
H1bs | 980 | 1 | 0.009
H1bs | 9804 | 2 | 0.018
H1bt | 16518T | 1 | 0.007
H1bt | 310 | 1 | 0.007
H1bt | 315.2C | 1 | 0.007
H1bt | 477 | 1 | 0.007
H1bt | 54 | 1 | 0.007
H1bt | 5875 | 1 | 0.007
H1bt | 73 | 1 | 0.007
H1bt1 | 16188G | 3 | 0.021
H1bt1 | 16311 | 2 | 0.014
H1bt1 | 4395 | 1 | 0.007
H1bt1 | 5262 | 1 | 0.007
H1bu | 10432 | 1 | 0.001
H1bu | 113 | 1 | 0.001
H1bu | 16076A | 123 | 0.155
H1bu | 208 | 1 | 0.001
H1bu | 261 | 1 | 0.001
H1bu | 281C | 1 | 0.001
H1bu | 316d | 1 | 0.001
H1bu | 338 | 1 | 0.001
H1bu | 468 | 3 | 0.004
H1bu | 502 | 1 | 0.001
H1bu | 573.1C | 3 | 0.004
H1bu | 573.2C | 5 | 0.006
H1bu | 573.5C | 1 | 0.001
H1bv | 113 | 1 | 0.001
H1bv | 16076A | 123 | 0.155
H1bv | 16319 | 1 | 0.001
H1bv | 208 | 1 | 0.001
H1bv | 261 | 1 | 0.001
H1bv | 281C | 1 | 0.001
H1bv | 316d | 1 | 0.001
H1bv | 338 | 1 | 0.001
H1bv | 468 | 3 | 0.004
H1bv | 502 | 1 | 0.001
H1bv | 573.1C | 3 | 0.004
H1bv | 573.2C | 5 | 0.006
H1bv | 573.5C | 1 | 0.001
H1bv | 6755 | 1 | 0.001
H1bv1 | 146 | 1 | 0.006

H1bv1 | 150 | 4 | 0.023
H1bv1 | 152 | 11 | 0.064
H1bv1 | 16104 | 1 | 0.006
H1bv1 | 16129 | 1 | 0.006
H1bv1 | 16140 | 2 | 0.012
H1bv1 | 16145 | 3 | 0.017
H1bv1 | 16153 | 1 | 0.006
H1bv1 | 16209 | 20 | 0.116
H1bv1 | 16217 | 3 | 0.017
H1bv1 | 16250G | 1 | 0.006
H1bv1 | 16256 | 1 | 0.006
H1bv1 | 16256G | 1 | 0.006
H1bv1 | 16286 | 1 | 0.006
H1bv1 | 16294 | 3 | 0.017
H1bv1 | 16304 | 1 | 0.006
H1bv1 | 16312 | 3 | 0.017
H1bv1 | 16325 | 23 | 0.133
H1bv1 | 16360 | 3 | 0.017
H1bv1 | 16368 | 1 | 0.006
H1bv1 | 16390 | 7 | 0.04
H1bv1 | 16399 | 1 | 0.006
H1bv1 | 182 | 1 | 0.006
H1bv1 | 183 | 8 | 0.046
H1bv1 | 214 | 4 | 0.023
H1bv1 | 315.2C | 1 | 0.006
H1bv1 | 456 | 1 | 0.006
H1bv1 | 8050 | 2 | 0.012
H1bv1 | 93 | 1 | 0.006
H1bv1 | 986C | 1 | 0.006
H1bv1 | 9932 | 1 | 0.006
H1bw | 113 | 1 | 0.001
H1bw | 11407A | 1 | 0.001
H1bw | 14251 | 2 | 0.003
H1bw | 14259 | 1 | 0.001
H1bw | 16076A | 123 | 0.154
H1bw | 16290 | 2 | 0.003
H1bw | 16362 | 2 | 0.003
H1bw | 208 | 1 | 0.001
H1bw | 261 | 1 | 0.001
H1bw | 281C | 1 | 0.001
H1bw | 316d | 1 | 0.001
H1bw | 3290 | 1 | 0.001
H1bw | 335 | 2 | 0.003
H1bw | 338 | 1 | 0.001
H1bw | 468 | 3 | 0.004
H1bw | 502 | 1 | 0.001
H1bw | 573.1C | 3 | 0.004
H1bw | 573.2C | 5 | 0.006
H1bw | 573.5C | 1 | 0.001
H1bw | 8129 | 1 | 0.001
H1bw | 9007 | 2 | 0.003
H1bx | 10325 | 1 | 0.001
H1bx | 113 | 1 | 0.001
H1bx | 16076A | 1 | 0.001
H1bx | 16192 | 123 | 0.157
H1bx | 16362 | 1 | 0.001
H1bx | 208 | 1 | 0.001
H1bx | 261 | 1 | 0.001
H1bx | 281C | 1 | 0.001
H1bx | 316d | 1 | 0.001
H1bx | 338 | 1 | 0.001
H1bx | 468 | 3 | 0.004
H1bx | 502 | 1 | 0.001
H1bx | 573.1C | 3 | 0.004
H1bx | 573.2C | 5 | 0.006
H1bx | 573.5C | 1 | 0.001
H1bx | 9053 | 1 | 0.001
H1bz | 146 | 1 | 0.013
H1bz | 16093 | 1 | 0.013
H1bz | 16111 | 1 | 0.013
H1bz | 16186 | 1 | 0.013
H1bz | 16309 | 4 | 0.051
H1bz | 200 | 1 | 0.013
H1bz | 72 | 1 | 0.013
H1bz | 72G | 1 | 0.013
H1bz | 8720C | 1 | 0.013
H1c | 10397 | 2 | 0.004
H1c | 1053 | 1 | 0.002
H1c | 10653C | 1 | 0.002
H1c | 11884 | 1 | 0.002
H1c | 11944 | 1 | 0.002
H1c | 11950 | 1 | 0.002
H1c | 12406 | 1 | 0.002
H1c | 12583 | 1 | 0.002
H1c | 12684 | 1 | 0.002
H1c | 12732 | 1 | 0.002
H1c | 12757 | 1 | 0.002
H1c | 12952 | 1 | 0.002
H1c | 13437 | 1 | 0.002
H1c | 14091 | 1 | 0.002
H1c | 14118 | 1 | 0.002
H1c | 14128 | 1 | 0.002
H1c | 14233 | 1 | 0.002
H1c | 14263 | 1 | 0.002
H1c | 14305 | 1 | 0.002
H1c | 14423C | 1 | 0.002
H1c | 146 | 4 | 0.008
H1c | 150 | 1 | 0.002
H1c | 15043 | 1 | 0.002
H1c | 15152 | 1 | 0.002
H1c | 15496 | 1 | 0.002
H1c | 15655 | 4 | 0.008
H1c | 15905 | 1 | 0.002
H1c | 15928 | 1 | 0.002
H1c | 15930 | 2 | 0.004
H1c | 15937 | 2 | 0.004
H1c | 1595 | 1 | 0.002
H1c | 16042 | 8 | 0.016
H1c | 16042C | 1 | 0.002
H1c | 16092 | 1 | 0.002
H1c | 16126 | 1 | 0.002

H1c | 16129 | 8 | 0.016
H1c | 16132 | 3 | 0.006
H1c | 16136 | 1 | 0.002
H1c | 16168 | 1 | 0.002
H1c | 16172 | 1 | 0.002
H1c | 16173 | 1 | 0.002
H1c | 16188 | 3 | 0.006
H1c | 16254 | 1 | 0.002
H1c | 16259 | 1 | 0.002
H1c | 16271 | 1 | 0.002
H1c | 16274 | 1 | 0.002
H1c | 16288 | 9 | 0.018
H1c | 16290 | 4 | 0.008
H1c | 16294 | 1 | 0.002
H1c | 16301 | 5 | 0.01
H1c | 16311 | 1 | 0.002
H1c | 16316 | 1 | 0.002
H1c | 16390 | 120 | 0.244
H1c | 189 | 1 | 0.002
H1c | 195 | 3 | 0.006
H1c | 214 | 1 | 0.002
H1c | 292 | 2 | 0.004
H1c | 310 | 10 | 0.02
H1c | 312 | 1 | 0.002
H1c | 315.2C | 1 | 0.002
H1c | 315.3C | 2 | 0.004
H1c | 3321 | 1 | 0.002
H1c | 332G | 1 | 0.002
H1c | 343 | 2 | 0.004
H1c | 3606 | 3 | 0.006
H1c | 3639 | 2 | 0.004
H1c | 4052 | 1 | 0.002
H1c | 411A | 1 | 0.002
H1c | 4454 | 3 | 0.006
H1c | 45C | 2 | 0.004
H1c | 4688 | 1 | 0.002
H1c | 5048 | 1 | 0.002
H1c | 513 | 1 | 0.002
H1c | 5585 | 1 | 0.002
H1c | 5752.1A | 1 | 0.002
H1c | 5899.1C | 2 | 0.004
H1c | 5899.3C | 1 | 0.002
H1c | 6293 | 1 | 0.002
H1c | 6671 | 9 | 0.018
H1c | 6710 | 1 | 0.002
H1c | 709 | 2 | 0.004
H1c | 7436 | 1 | 0.002
H1c | 7685 | 1 | 0.002
H1c | 7861 | 1 | 0.002
H1c | 8027 | 1 | 0.002
H1c | 8060 | 1 | 0.002
H1c | 8139 | 1 | 0.002
H1c | 824 | 1 | 0.002
H1c | 8338 | 2 | 0.004
H1c | 866d | 1 | 0.002
H1c | 8764 | 1 | 0.002
H1c | 8772 | 2 | 0.004
H1c | 8843 | 2 | 0.004
H1c | 8994 | 1 | 0.002
H1c | 9196 | 1 | 0.002
H1c | 93 | 3 | 0.006
H1c | 980G | 1 | 0.002
H1c | 9881 | 1 | 0.002
H1c+152 | 10397 | 1 | 0.011
H1c+152 | 11087 | 1 | 0.011
H1c+152 | 11516 | 3 | 0.033
H1c+152 | 12310.1A | 1 | 0.011
H1c+152 | 12354 | 1 | 0.011
H1c+152 | 14233 | 1 | 0.011
H1c+152 | 15724 | 1 | 0.011
H1c+152 | 15930 | 1 | 0.011
H1c+152 | 16093 | 1 | 0.011
H1c+152 | 16129 | 1 | 0.011
H1c+152 | 16145 | 1 | 0.011
H1c+152 | 16265 | 20 | 0.217
H1c+152 | 16267 | 1 | 0.011
H1c+152 | 16271 | 1 | 0.011
H1c+152 | 16274 | 20 | 0.217
H1c+152 | 189 | 23 | 0.25
H1c+152 | 214 | 1 | 0.011
H1c+152 | 309.3C | 1 | 0.011
H1c+152 | 310 | 1 | 0.011
H1c+152 | 5654 | 1 | 0.011
H1c+152 | 6267 | 1 | 0.011
H1c+152 | 8271T | 1 | 0.011
H1c+152 | 8645 | 1 | 0.011
H1c+152 | 8723 | 1 | 0.011
H1c+152 | 9025 | 1 | 0.011
H1c+152 | 9192 | 1 | 0.011
H1c+152 | 9478 | 1 | 0.011
H1c1 | 10197 | 1 | 0.007
H1c1 | 10232 | 1 | 0.007
H1c1 | 10568C | 1 | 0.007
H1c1 | 10653 | 2 | 0.014
H1c1 | 10981 | 1 | 0.007
H1c1 | 11778 | 4 | 0.028
H1c1 | 1193 | 1 | 0.007
H1c1 | 11983A | 1 | 0.007
H1c1 | 12070 | 1 | 0.007
H1c1 | 12372 | 1 | 0.007
H1c1 | 12775 | 3 | 0.021
H1c1 | 14048 | 1 | 0.007
H1c1 | 14485 | 1 | 0.007
H1c1 | 14568 | 1 | 0.007
H1c1 | 14831 | 1 | 0.007
H1c1 | 15055 | 1 | 0.007
H1c1 | 151 | 1 | 0.007
H1c1 | 152 | 3 | 0.021
H1c1 | 15498 | 1 | 0.007
H1c1 | 15884 | 1 | 0.007
H1c1 | 15951 | 1 | 0.007

H1c1 | 16051 | 1 | 0.007
H1c1 | 16093 | 4 | 0.028
H1c1 | 16129 | 1 | 0.007
H1c1 | 16147 | 1 | 0.007
H1c1 | 16167 | 1 | 0.007
H1c1 | 16189 | 1 | 0.007
H1c1 | 16193 | 2 | 0.014
H1c1 | 16239 | 1 | 0.007
H1c1 | 16265C | 1 | 0.007
H1c1 | 16269 | 1 | 0.007
H1c1 | 16291 | 3 | 0.021
H1c1 | 16295 | 3 | 0.021
H1c1 | 16311 | 1 | 0.007
H1c1 | 1661 | 1 | 0.007
H1c1 | 195 | 1 | 0.007
H1c1 | 200 | 1 | 0.007
H1c1 | 215 | 1 | 0.007
H1c1 | 234 | 1 | 0.007
H1c1 | 2539 | 1 | 0.007
H1c1 | 309d | 1 | 0.007
H1c1 | 310 | 3 | 0.021
H1c1 | 3109d | 1 | 0.007
H1c1 | 318 | 3 | 0.021
H1c1 | 3398 | 1 | 0.007
H1c1 | 3600A | 1 | 0.007
H1c1 | 3744 | 1 | 0.007
H1c1 | 3826 | 1 | 0.007
H1c1 | 3918 | 2 | 0.014
H1c1 | 4188 | 1 | 0.007
H1c1 | 4317 | 1 | 0.007
H1c1 | 4429 | 1 | 0.007
H1c1 | 472 | 1 | 0.007
H1c1 | 5147 | 2 | 0.014
H1c1 | 5204 | 1 | 0.007
H1c1 | 5460 | 1 | 0.007
H1c1 | 5794 | 1 | 0.007
H1c1 | 5821 | 2 | 0.014
H1c1 | 5945 | 3 | 0.021
H1c1 | 6116 | 1 | 0.007
H1c1 | 6293 | 1 | 0.007
H1c1 | 6531 | 1 | 0.007
H1c1 | 6719 | 1 | 0.007
H1c1 | 6956 | 2 | 0.014
H1c1 | 73 | 1 | 0.007
H1c1 | 7337 | 1 | 0.007
H1c1 | 7468 | 1 | 0.007
H1c1 | 7598 | 1 | 0.007
H1c1 | 7830 | 2 | 0.014
H1c1 | 8280 | 1 | 0.007
H1c1 | 845 | 1 | 0.007
H1c1 | 8531 | 1 | 0.007
H1c1 | 9055 | 1 | 0.007
H1c1 | 9180 | 1 | 0.007
H1c1 | 9194 | 1 | 0.007
H1c1 | 961 | 1 | 0.007
H1c1 | 965.2C | 1 | 0.007
H1c1 | 9750 | 3 | 0.021
H1c1 | 983 | 1 | 0.007
H1c1 | 9983 | 1 | 0.007
H1c1+16093 | 16209 | 1 | 0.02
H1c1+16093 | 1632 | 1 | 0.02
H1c1+16093 | 310 | 2 | 0.041
H1c1+16093 | 315.2C | 1 | 0.02
H1c1+16093 | 3826 | 1 | 0.02
H1c1+16093 | 6374 | 2 | 0.041
H1c1+16093 | 7533 | 1 | 0.02
H1c10 | 14198 | 1 | 0.003
H1c10 | 16042C | 1 | 0.003
H1c10 | 16129T | 1 | 0.003
H1c10 | 16150 | 1 | 0.003
H1c10 | 16188A | 2 | 0.005
H1c10 | 16231 | 1 | 0.003
H1c10 | 16287 | 1 | 0.003
H1c10 | 16293C | 2 | 0.005
H1c10 | 16300 | 1 | 0.003
H1c10 | 16327 | 125 | 0.314
H1c10 | 189 | 1 | 0.003
H1c10 | 214 | 1 | 0.003
H1c10 | 261.1T | 1 | 0.003
H1c10 | 332G | 1 | 0.003
H1c10 | 411A | 1 | 0.003
H1c10 | 49T | 1 | 0.003
H1c11 | 1193 | 1 | 0.003
H1c11 | 14112 | 1 | 0.003
H1c11 | 16042C | 1 | 0.003
H1c11 | 16150 | 1 | 0.003
H1c11 | 16193 | 1 | 0.003
H1c11 | 16231 | 1 | 0.003
H1c11 | 16261 | 1 | 0.003
H1c11 | 16287 | 1 | 0.003
H1c11 | 16293C | 2 | 0.005
H1c11 | 16300 | 1 | 0.003
H1c11 | 16327 | 125 | 0.313
H1c11 | 189 | 1 | 0.003
H1c11 | 214 | 1 | 0.003
H1c11 | 261.1T | 1 | 0.003
H1c11 | 310 | 1 | 0.003
H1c11 | 332G | 1 | 0.003
H1c11 | 411A | 1 | 0.003
H1c11 | 8388 | 1 | 0.003
H1c11 | 960d | 1 | 0.003
H1c12 | 1192 | 1 | 0.003
H1c12 | 16042C | 1 | 0.003
H1c12 | 16129T | 1 | 0.003
H1c12 | 16150 | 1 | 0.003
H1c12 | 16188A | 2 | 0.005
H1c12 | 16231 | 1 | 0.003
H1c12 | 16287 | 1 | 0.003
H1c12 | 16293C | 2 | 0.005
H1c12 | 16300 | 1 | 0.003
H1c12 | 16327 | 125 | 0.314

H1c12 | 189 | 1 | 0.003
H1c12 | 214 | 1 | 0.003
H1c12 | 261.1T | 1 | 0.003
H1c12 | 332G | 1 | 0.003
H1c12 | 411A | 1 | 0.003
H1c12 | 49T | 1 | 0.003
H1c13 | 146 | 1 | 0.002
H1c13 | 16042C | 1 | 0.002
H1c13 | 16150 | 1 | 0.002
H1c13 | 16231 | 1 | 0.002
H1c13 | 16287 | 1 | 0.002
H1c13 | 16293C | 2 | 0.005
H1c13 | 16300 | 1 | 0.002
H1c13 | 16325 | 2 | 0.005
H1c13 | 16327 | 3 | 0.007
H1c13 | 16362 | 4 | 0.01
H1c13 | 16368 | 1 | 0.002
H1c13 | 16390 | 120 | 0.298
H1c13 | 1719 | 1 | 0.002
H1c13 | 189 | 1 | 0.002
H1c13 | 214 | 1 | 0.002
H1c13 | 261.1T | 1 | 0.002
H1c13 | 2706 | 1 | 0.002
H1c13 | 309d | 1 | 0.002
H1c13 | 332G | 1 | 0.002
H1c13 | 411A | 1 | 0.002
H1c13 | 8488 | 1 | 0.002
H1c14 | 10801 | 1 | 0.003
H1c14 | 14180 | 1 | 0.003
H1c14 | 16042C | 1 | 0.003
H1c14 | 16129T | 1 | 0.003
H1c14 | 16150 | 1 | 0.003
H1c14 | 16188A | 2 | 0.005
H1c14 | 16231 | 1 | 0.003
H1c14 | 16287 | 1 | 0.003
H1c14 | 16293C | 2 | 0.005
H1c14 | 16300 | 1 | 0.003
H1c14 | 16327 | 125 | 0.315
H1c14 | 189 | 1 | 0.003
H1c14 | 214 | 1 | 0.003
H1c14 | 261.1T | 1 | 0.003
H1c14 | 332G | 1 | 0.003
H1c14 | 411A | 1 | 0.003
H1c14 | 49T | 1 | 0.003
H1c15 | 10463 | 1 | 0.003
H1c15 | 16042C | 1 | 0.003
H1c15 | 16150 | 1 | 0.003
H1c15 | 16231 | 1 | 0.003
H1c15 | 16287 | 1 | 0.003
H1c15 | 16293C | 2 | 0.005
H1c15 | 16300 | 1 | 0.003
H1c15 | 16327 | 125 | 0.316
H1c15 | 189 | 1 | 0.003
H1c15 | 214 | 1 | 0.003
H1c15 | 261.1T | 1 | 0.003
H1c15 | 332G | 1 | 0.003
H1c15 | 411A | 1 | 0.003
H1c15 | 4430 | 1 | 0.003
H1c15 | 49T | 1 | 0.003
H1c16 | 11839 | 1 | 0.003
H1c16 | 12858 | 1 | 0.003
H1c16 | 14050A | 1 | 0.003
H1c16 | 16042C | 1 | 0.003
H1c16 | 16129T | 1 | 0.003
H1c16 | 16150 | 1 | 0.003
H1c16 | 16188A | 2 | 0.005
H1c16 | 16231 | 1 | 0.003
H1c16 | 16287 | 1 | 0.003
H1c16 | 16293C | 2 | 0.005
H1c16 | 16300 | 1 | 0.003
H1c16 | 16327 | 125 | 0.314
H1c16 | 189 | 1 | 0.003
H1c16 | 214 | 1 | 0.003
H1c16 | 261.1T | 1 | 0.003
H1c16 | 332G | 1 | 0.003
H1c16 | 411A | 1 | 0.003
H1c16 | 49T | 1 | 0.003
H1c17 | 16042C | 1 | 0.003
H1c17 | 16150 | 1 | 0.003
H1c17 | 16231 | 1 | 0.003
H1c17 | 16287 | 1 | 0.003
H1c17 | 16293C | 2 | 0.005
H1c17 | 16300 | 1 | 0.003
H1c17 | 16327 | 125 | 0.315
H1c17 | 189 | 1 | 0.003
H1c17 | 214 | 1 | 0.003
H1c17 | 261.1T | 1 | 0.003
H1c17 | 332G | 1 | 0.003
H1c17 | 411A | 1 | 0.003
H1c17 | 46 | 1 | 0.003
H1c17 | 49T | 1 | 0.003
H1c17 | 73 | 1 | 0.003
H1c18 | 152 | 1 | 0.003
H1c18 | 16042C | 1 | 0.003
H1c18 | 16129T | 1 | 0.003
H1c18 | 16150 | 1 | 0.003
H1c18 | 16188A | 2 | 0.005
H1c18 | 16231 | 1 | 0.003
H1c18 | 16287 | 1 | 0.003
H1c18 | 16293C | 2 | 0.005
H1c18 | 16300 | 1 | 0.003
H1c18 | 16327 | 125 | 0.314
H1c18 | 189 | 1 | 0.003
H1c18 | 214 | 1 | 0.003
H1c18 | 261.1T | 1 | 0.003
H1c18 | 332G | 1 | 0.003
H1c18 | 411A | 1 | 0.003
H1c18 | 49T | 1 | 0.003
H1c19 | 12950 | 2 | 0.005
H1c19 | 16042C | 1 | 0.003
H1c19 | 16129T | 1 | 0.003

H1c19 | 16150 | 1 | 0.003
H1c19 | 16188A | 2 | 0.005
H1c19 | 16231 | 1 | 0.003
H1c19 | 16287 | 1 | 0.003
H1c19 | 16293C | 2 | 0.005
H1c19 | 16300 | 1 | 0.003
H1c19 | 16327 | 125 | 0.313
H1c19 | 189 | 1 | 0.003
H1c19 | 214 | 1 | 0.003
H1c19 | 261.1T | 1 | 0.003
H1c19 | 332G | 1 | 0.003
H1c19 | 411A | 1 | 0.003
H1c19 | 49T | 1 | 0.003
H1c19 | 9947 | 1 | 0.003
H1c1a | 11151 | 1 | 0.02
H1c1a | 15734 | 1 | 0.02
H1c1a | 16092 | 1 | 0.02
H1c1a | 16092A | 1 | 0.02
H1c1a | 16189 | 2 | 0.039
H1c1a | 16249 | 2 | 0.039
H1c1a | 16304G | 1 | 0.02
H1c1a | 2581 | 1 | 0.02
H1c1a | 310 | 1 | 0.02
H1c1a | 315.2C | 1 | 0.02
H1c1a | 6293 | 1 | 0.02
H1c1a1 | 152 | 1 | 0.02
H1c1a1 | 16092 | 1 | 0.02
H1c1a1 | 16145 | 1 | 0.02
H1c1a1 | 16259 | 1 | 0.02
H1c1a1 | 1824 | 1 | 0.02
H1c1a1 | 196 | 2 | 0.039
H1c1a1 | 3391 | 1 | 0.02
H1c1a1 | 3784 | 1 | 0.02
H1c1a1 | 513 | 1 | 0.02
H1c1a1 | 5231 | 3 | 0.059
H1c1b | 11347 | 1 | 0.026
H1c1b | 14458A | 1 | 0.026
H1c1b | 5821 | 1 | 0.026
H1c1b | 5892 | 1 | 0.026
H1c1b | 6032 | 1 | 0.026
H1c1b | 8696 | 1 | 0.026
H1c1c | 1031 | 1 | 0.024
H1c1c | 13326 | 1 | 0.024
H1c1c | 16258C | 1 | 0.024
H1c1c | 16362 | 1 | 0.024
H1c1c | 16384 | 1 | 0.024
H1c1c | 16390 | 1 | 0.024
H1c1c | 309d | 1 | 0.024
H1c1c | 310 | 3 | 0.073
H1c1d | 16075A | 1 | 0.02
H1c1d | 16111 | 3 | 0.06
H1c1d | 16192 | 3 | 0.06
H1c1d | 16249 | 3 | 0.06
H1c1d | 16298 | 3 | 0.06
H1c1d | 16311 | 1 | 0.02
H1c1d | 16355 | 3 | 0.06
H1c1d | 16390 | 2 | 0.04
H1c1d | 199 | 2 | 0.04
H1c1d | 385 | 1 | 0.02
H1c1d | 471 | 1 | 0.02
H1c1d | 93 | 1 | 0.02
H1c2 | 11857 | 1 | 0.002
H1c2 | 1193 | 1 | 0.002
H1c2 | 12172 | 1 | 0.002
H1c2 | 16042C | 1 | 0.002
H1c2 | 16231 | 1 | 0.002
H1c2 | 16287 | 1 | 0.002
H1c2 | 16300 | 1 | 0.002
H1c2 | 16325 | 2 | 0.005
H1c2 | 16327 | 125 | 0.309
H1c2 | 16390 | 1 | 0.002
H1c2 | 189 | 1 | 0.002
H1c2 | 195 | 3 | 0.007
H1c2 | 214 | 1 | 0.002
H1c2 | 261.1T | 1 | 0.002
H1c2 | 3083 | 1 | 0.002
H1c2 | 332G | 1 | 0.002
H1c2 | 411A | 1 | 0.002
H1c2 | 44.1C | 1 | 0.002
H1c2 | 5004 | 1 | 0.002
H1c2 | 5249 | 2 | 0.005
H1c2 | 6267 | 1 | 0.002
H1c2 | 6284 | 1 | 0.002
H1c2 | 8520 | 1 | 0.002
H1c2 | 8762 | 1 | 0.002
H1c2 | 9104 | 1 | 0.002
H1c2 | 9591 | 1 | 0.002
H1c2 | 9716 | 1 | 0.002
H1c20 | 10232 | 1 | 0.003
H1c20 | 1038 | 1 | 0.003
H1c20 | 143 | 2 | 0.005
H1c20 | 16042C | 1 | 0.003
H1c20 | 16150 | 1 | 0.003
H1c20 | 16231 | 1 | 0.003
H1c20 | 16287 | 1 | 0.003
H1c20 | 16293C | 2 | 0.005
H1c20 | 16300 | 1 | 0.003
H1c20 | 16327 | 125 | 0.315
H1c20 | 189 | 1 | 0.003
H1c20 | 214 | 1 | 0.003
H1c20 | 261.1T | 1 | 0.003
H1c20 | 332G | 1 | 0.003
H1c20 | 411A | 1 | 0.003
H1c20 | 4659 | 1 | 0.003
H1c20 | 49T | 1 | 0.003
H1c20 | 8108 | 1 | 0.003
H1c21 | 16042C | 1 | 0.003
H1c21 | 16129T | 1 | 0.003
H1c21 | 16150 | 1 | 0.003
H1c21 | 16188A | 2 | 0.005
H1c21 | 16189 | 1 | 0.003

H1c21 | 16231 | 1 | 0.003
H1c21 | 16287 | 1 | 0.003
H1c21 | 16293C | 2 | 0.005
H1c21 | 16300 | 1 | 0.003
H1c21 | 16327 | 125 | 0.315
H1c21 | 189 | 1 | 0.003
H1c21 | 214 | 1 | 0.003
H1c21 | 261.1T | 1 | 0.003
H1c21 | 332G | 1 | 0.003
H1c21 | 411A | 1 | 0.003
H1c21 | 4638 | 1 | 0.003
H1c21 | 49T | 1 | 0.003
H1c22 | 10335 | 1 | 0.003
H1c22 | 13803 | 1 | 0.003
H1c22 | 14364 | 1 | 0.003
H1c22 | 15470 | 1 | 0.003
H1c22 | 16042C | 1 | 0.003
H1c22 | 16150 | 1 | 0.003
H1c22 | 16231 | 1 | 0.003
H1c22 | 16287 | 1 | 0.003
H1c22 | 16293C | 2 | 0.005
H1c22 | 16300 | 1 | 0.003
H1c22 | 16327 | 125 | 0.313
H1c22 | 1850 | 1 | 0.003
H1c22 | 189 | 1 | 0.003
H1c22 | 204 | 1 | 0.003
H1c22 | 214 | 1 | 0.003
H1c22 | 261.1T | 1 | 0.003
H1c22 | 2835 | 1 | 0.003
H1c22 | 332G | 1 | 0.003
H1c22 | 411A | 1 | 0.003
H1c22 | 5530 | 1 | 0.003
H1c22 | 7765T | 1 | 0.003
H1c22 | 7805 | 1 | 0.003
H1c2a | 13167T | 1 | 0.003
H1c2a | 13879A | 2 | 0.005
H1c2a | 152 | 2 | 0.005
H1c2a | 15466 | 1 | 0.003
H1c2a | 16042C | 1 | 0.003
H1c2a | 16150 | 1 | 0.003
H1c2a | 16231 | 1 | 0.003
H1c2a | 16287 | 1 | 0.003
H1c2a | 16293C | 2 | 0.005
H1c2a | 16300 | 1 | 0.003
H1c2a | 16311 | 1 | 0.003
H1c2a | 16327 | 125 | 0.314
H1c2a | 189 | 1 | 0.003
H1c2a | 214 | 1 | 0.003
H1c2a | 261.1T | 1 | 0.003
H1c2a | 2851 | 2 | 0.005
H1c2a | 332G | 1 | 0.003
H1c2a | 411A | 1 | 0.003
H1c2a | 4363 | 1 | 0.003
H1c2a | 5237 | 2 | 0.005
H1c2a | 6297 | 1 | 0.003
H1c3 | 10370 | 1 | 0.017
H1c3 | 12172 | 1 | 0.017
H1c3 | 12280 | 1 | 0.017
H1c3 | 12406 | 1 | 0.017
H1c3 | 12966 | 3 | 0.051
H1c3 | 131 | 1 | 0.017
H1c3 | 13215 | 3 | 0.051
H1c3 | 14116 | 1 | 0.017
H1c3 | 152 | 3 | 0.051
H1c3 | 15261 | 1 | 0.017
H1c3 | 15662 | 1 | 0.017
H1c3 | 15937d | 1 | 0.017
H1c3 | 16093 | 1 | 0.017
H1c3 | 16148 | 1 | 0.017
H1c3 | 16186 | 5 | 0.085
H1c3 | 16189 | 1 | 0.017
H1c3 | 16234 | 1 | 0.017
H1c3 | 16265 | 1 | 0.017
H1c3 | 16288 | 1 | 0.017
H1c3 | 16311 | 7 | 0.119
H1c3 | 16356 | 1 | 0.017
H1c3 | 16361 | 1 | 0.017
H1c3 | 16362 | 1 | 0.017
H1c3 | 185 | 1 | 0.017
H1c3 | 195 | 36 | 0.61
H1c3 | 234 | 1 | 0.017
H1c3 | 309d | 2 | 0.034
H1c3 | 310 | 4 | 0.068
H1c3 | 315.2C | 1 | 0.017
H1c3 | 3278 | 5 | 0.085
H1c3 | 3316 | 1 | 0.017
H1c3 | 3338 | 1 | 0.017
H1c3 | 4464 | 1 | 0.017
H1c3 | 5177 | 1 | 0.017
H1c3 | 573A | 1 | 0.017
H1c3 | 6671 | 1 | 0.017
H1c3 | 7274 | 1 | 0.017
H1c3 | 73 | 1 | 0.017
H1c3 | 7595 | 1 | 0.017
H1c3 | 7598 | 1 | 0.017
H1c3 | 8504 | 1 | 0.017
H1c3 | 8931 | 1 | 0.017
H1c3 | 93 | 2 | 0.034
H1c3a | 10667 | 1 | 0.056
H1c3a | 12501 | 1 | 0.056
H1c3a | 12966 | 1 | 0.056
H1c3a | 13296 | 1 | 0.056
H1c3a | 13896 | 1 | 0.056
H1c3a | 1969 | 1 | 0.056
H1c3a | 2389 | 1 | 0.056
H1c3a | 3801 | 1 | 0.056
H1c3a | 7270 | 1 | 0.056
H1c3a | 93 | 1 | 0.056
H1c3b | 107 | 1 | 0.011
H1c3b | 12585 | 1 | 0.011
H1c3b | 12795 | 5 | 0.057

H1c3b | 13161 | 2 | 0.023
H1c3b | 13933 | 4 | 0.046
H1c3b | 1462 | 1 | 0.011
H1c3b | 14981 | 1 | 0.011
H1c3b | 16000 | 2 | 0.023
H1c3b | 16069 | 2 | 0.023
H1c3b | 16108 | 1 | 0.011
H1c3b | 16126 | 55 | 0.632
H1c3b | 16129 | 1 | 0.011
H1c3b | 16145 | 2 | 0.023
H1c3b | 16150 | 1 | 0.011
H1c3b | 16173A | 1 | 0.011
H1c3b | 16174 | 4 | 0.046
H1c3b | 16178 | 1 | 0.011
H1c3b | 16179 | 2 | 0.023
H1c3b | 16213T | 1 | 0.011
H1c3b | 16232A | 23 | 0.264
H1c3b | 16240 | 1 | 0.011
H1c3b | 16243 | 3 | 0.034
H1c3b | 16249 | 4 | 0.046
H1c3b | 16266A | 1 | 0.011
H1c3b | 16272 | 1 | 0.011
H1c3b | 16290 | 1 | 0.011
H1c3b | 16309 | 1 | 0.011
H1c3b | 16318C | 1 | 0.011
H1c3b | 16353 | 1 | 0.011
H1c3b | 16360 | 3 | 0.034
H1c3b | 16399T | 1 | 0.011
H1c3b | 16400 | 1 | 0.011
H1c3b | 16526 | 2 | 0.023
H1c3b | 16527 | 2 | 0.023
H1c3b | 307-309d | 1 | 0.011
H1c3b | 309d | 1 | 0.011
H1c3b | 310 | 2 | 0.023
H1c3b | 5997 | 1 | 0.011
H1c3b | 7169 | 1 | 0.011
H1c3b | 8870 | 1 | 0.011
H1c3b | 8981 | 1 | 0.011
H1c3b | 9254 | 2 | 0.023
H1c3b | 9899 | 1 | 0.011
H1c4 | 16042C | 1 | 0.003
H1c4 | 16150 | 1 | 0.003
H1c4 | 16188 | 2 | 0.005
H1c4 | 16189 | 1 | 0.003
H1c4 | 16231 | 1 | 0.003
H1c4 | 16287 | 1 | 0.003
H1c4 | 16293C | 2 | 0.005
H1c4 | 16300 | 1 | 0.003
H1c4 | 16327 | 125 | 0.315
H1c4 | 189 | 1 | 0.003
H1c4 | 199 | 2 | 0.005
H1c4 | 214 | 1 | 0.003
H1c4 | 261.1T | 1 | 0.003
H1c4 | 332G | 1 | 0.003
H1c4 | 411A | 1 | 0.003
H1c4 | 49T | 1 | 0.003
H1c4 | 7253 | 1 | 0.003
H1c4 | 8567 | 1 | 0.003
H1c4a | 16042C | 1 | 0.003
H1c4a | 16129T | 1 | 0.003
H1c4a | 16150 | 1 | 0.003
H1c4a | 16188A | 2 | 0.005
H1c4a | 16231 | 1 | 0.003
H1c4a | 16287 | 1 | 0.003
H1c4a | 16293C | 2 | 0.005
H1c4a | 16300 | 1 | 0.003
H1c4a | 16327 | 125 | 0.315
H1c4a | 189 | 1 | 0.003
H1c4a | 195 | 1 | 0.003
H1c4a | 214 | 1 | 0.003
H1c4a | 261.1T | 1 | 0.003
H1c4a | 332G | 1 | 0.003
H1c4a | 411A | 1 | 0.003
H1c4a | 49T | 1 | 0.003
H1c4a1 | 1598 | 1 | 0.002
H1c4a1 | 16042C | 1 | 0.002
H1c4a1 | 16150 | 1 | 0.002
H1c4a1 | 16187 | 2 | 0.005
H1c4a1 | 16231 | 1 | 0.002
H1c4a1 | 16287 | 1 | 0.002
H1c4a1 | 16293C | 2 | 0.005
H1c4a1 | 16300 | 1 | 0.002
H1c4a1 | 16327 | 125 | 0.312
H1c4a1 | 189 | 1 | 0.002
H1c4a1 | 214 | 1 | 0.002
H1c4a1 | 261.1T | 1 | 0.002
H1c4a1 | 332G | 1 | 0.002
H1c4a1 | 411A | 1 | 0.002
H1c4a1 | 5222 | 1 | 0.002
H1c4b | 12366 | 1 | 0.025
H1c4b | 12401 | 1 | 0.025
H1c4b | 152 | 2 | 0.05
H1c4b | 16093 | 1 | 0.025
H1c4b | 16311 | 1 | 0.025
H1c4b | 16400 | 1 | 0.025
H1c4b | 9007 | 1 | 0.025
H1c4b | 9380 | 1 | 0.025
H1c4b1 | 15323 | 1 | 0.026
H1c4b1 | 8851 | 1 | 0.026
H1c5 | 16042C | 1 | 0.003
H1c5 | 16129T | 1 | 0.003
H1c5 | 16150 | 1 | 0.003
H1c5 | 16188A | 2 | 0.005
H1c5 | 16231 | 1 | 0.003
H1c5 | 16287 | 1 | 0.003
H1c5 | 16293C | 2 | 0.005
H1c5 | 16300 | 1 | 0.003
H1c5 | 16327 | 125 | 0.314
H1c5 | 189 | 1 | 0.003
H1c5 | 214 | 1 | 0.003
H1c5 | 261.1T | 1 | 0.003

H1c5 | 332G | 1 | 0.003
H1c5 | 411A | 1 | 0.003
H1c5 | 49T | 1 | 0.003
H1c5a | 14407G | 1 | 0.038
H1c5a | 16188G | 1 | 0.038
H1c5a | 16290 | 2 | 0.077
H1c5a | 16297 | 1 | 0.038
H1c5a | 5093 | 1 | 0.038
H1c5a | 60A | 4 | 0.154
H1c5a | 8864 | 3 | 0.115
H1c6 | 10454 | 1 | 0.003
H1c6 | 12123 | 1 | 0.003
H1c6 | 15226 | 1 | 0.003
H1c6 | 16042C | 1 | 0.003
H1c6 | 16150 | 1 | 0.003
H1c6 | 16231 | 1 | 0.003
H1c6 | 16287 | 1 | 0.003
H1c6 | 16293C | 2 | 0.005
H1c6 | 16300 | 1 | 0.003
H1c6 | 16327 | 125 | 0.313
H1c6 | 189 | 1 | 0.003
H1c6 | 214 | 1 | 0.003
H1c6 | 261.1T | 1 | 0.003
H1c6 | 332G | 1 | 0.003
H1c6 | 41 | 1 | 0.003
H1c6 | 411A | 1 | 0.003
H1c6 | 8383 | 1 | 0.003
H1c7 | 13359 | 1 | 0.002
H1c7 | 16042C | 1 | 0.002
H1c7 | 16150 | 1 | 0.002
H1c7 | 16231 | 1 | 0.002
H1c7 | 16287 | 1 | 0.002
H1c7 | 16293C | 2 | 0.005
H1c7 | 16300 | 1 | 0.002
H1c7 | 16311 | 1 | 0.002
H1c7 | 16327 | 125 | 0.312
H1c7 | 189 | 1 | 0.002
H1c7 | 214 | 1 | 0.002
H1c7 | 261.1T | 1 | 0.002
H1c7 | 332G | 1 | 0.002
H1c7 | 3699G | 1 | 0.002
H1c7 | 411A | 1 | 0.002
H1c7 | 709 | 1 | 0.002
H1c8 | 10337 | 1 | 0.003
H1c8 | 12528C | 1 | 0.003
H1c8 | 152 | 1 | 0.003
H1c8 | 16042C | 1 | 0.003
H1c8 | 16129T | 1 | 0.003
H1c8 | 16150 | 1 | 0.003
H1c8 | 16188A | 2 | 0.005
H1c8 | 16189 | 1 | 0.003
H1c8 | 16231 | 1 | 0.003
H1c8 | 16287 | 1 | 0.003
H1c8 | 16293C | 2 | 0.005
H1c8 | 16300 | 1 | 0.003
H1c8 | 16327 | 125 | 0.313
H1c8 | 189 | 1 | 0.003
H1c8 | 214 | 1 | 0.003
H1c8 | 261.1T | 1 | 0.003
H1c8 | 332G | 1 | 0.003
H1c8 | 3511 | 1 | 0.003
H1c8 | 411A | 1 | 0.003
H1c8 | 49T | 1 | 0.003
H1c9 | 15355T | 1 | 0.014
H1c9 | 16039 | 1 | 0.014
H1c9 | 16169 | 1 | 0.014
H1c9 | 16269 | 1 | 0.014
H1c9 | 16316 | 6 | 0.083
H1c9 | 16484d | 16 | 0.222
H1c9 | 16486-16488d | 16 | 0.222
H1c9 | 316C | 1 | 0.014
H1c9 | 8723 | 7 | 0.097
H1c9a | 143 | 7 | 0.088
H1c9a | 15692 | 1 | 0.012
H1c9a | 16039 | 1 | 0.012
H1c9a | 16063 | 1 | 0.012
H1c9a | 16069 | 1 | 0.012
H1c9a | 16080 | 3 | 0.038
H1c9a | 16269 | 1 | 0.012
H1c9a | 16300 | 1 | 0.012
H1c9a | 16319 | 5 | 0.062
H1c9a | 16484d | 16 | 0.2
H1c9a | 16486-16488d | 16 | 0.2
H1c9a | 195 | 1 | 0.012
H1c9a | 242 | 1 | 0.012
H1c9a | 309d | 2 | 0.025
H1c9a | 310 | 1 | 0.012
H1c9a | 316C | 1 | 0.012
H1c9a | 380T | 2 | 0.025
H1c9a | 5276 | 1 | 0.012
H1c9a | 532 | 1 | 0.012
H1c9a | 6734 | 1 | 0.012
H1c9a | 9320 | 1 | 0.012
H1ca | 113 | 1 | 0.001
H1ca | 14153 | 2 | 0.002
H1ca | 14162 | 2 | 0.002
H1ca | 16076A | 1 | 0.001
H1ca | 16189 | 125 | 0.155
H1ca | 208 | 1 | 0.001
H1ca | 2416 | 9 | 0.011
H1ca | 261 | 1 | 0.001
H1ca | 281C | 1 | 0.001
H1ca | 316d | 1 | 0.001
H1ca | 338 | 1 | 0.001
H1ca | 468 | 3 | 0.004
H1ca | 502 | 1 | 0.001
H1ca | 573.1C | 3 | 0.004
H1ca | 573.2C | 5 | 0.006
H1ca | 573.5C | 1 | 0.001
H1ca | 93 | 1 | 0.001
H1cc | 16249 | 2 | 0.08

H1cc | 16260 | 2 | 0.08
H1cc | 16265 | 1 | 0.04
H1cc | 63 | 1 | 0.04
H1cc | 64 | 1 | 0.04
H1cc | 66 | 1 | 0.04
H1cc | 67T | 1 | 0.04
H1cc | 89 | 1 | 0.04
H1cc | 9914 | 1 | 0.04
H1cd | 143 | 1 | 0.011
H1cd | 146 | 3 | 0.033
H1cd | 152 | 2 | 0.022
H1cd | 16189 | 19 | 0.211
H1cd | 16293 | 1 | 0.011
H1cd | 195 | 3 | 0.033
H1cd | 6048 | 1 | 0.011
H1cf | 10044 | 1 | 0.01
H1cf | 10454 | 1 | 0.01
H1cf | 10795 | 1 | 0.01
H1cf | 13452 | 1 | 0.01
H1cf | 14207 | 1 | 0.01
H1cf | 15553 | 1 | 0.01
H1cf | 16038 | 1 | 0.01
H1cf | 16042 | 1 | 0.01
H1cf | 16145 | 1 | 0.01
H1cf | 16189 | 3 | 0.031
H1cf | 16221 | 1 | 0.01
H1cf | 16235 | 1 | 0.01
H1cf | 16263 | 1 | 0.01
H1cf | 16316 | 1 | 0.01
H1cf | 204 | 1 | 0.01
H1cf | 3687 | 1 | 0.01
H1cf | 4733 | 1 | 0.01
H1cf | 5293 | 1 | 0.01
H1cf | 73 | 1 | 0.01
H1cf | 8435 | 1 | 0.01
H1cf | 9075 | 1 | 0.01
H1cf | 9316 | 1 | 0.01
H1cf | 9722 | 1 | 0.01
H1cg | 15323 | 1 | 0.012
H1cg | 16362 | 2 | 0.023
H1cg | 8750 | 1 | 0.012
H1cg | 9438 | 1 | 0.012
H1cg | 960 | 1 | 0.012
H1ch | 152A | 2 | 0.023
H1ch | 15799 | 1 | 0.011
H1ch | 16136 | 2 | 0.023
H1ch | 16192 | 1 | 0.011
H1ch | 16215 | 1 | 0.011
H1ch | 16223 | 1 | 0.011
H1ch | 16245 | 1 | 0.011
H1ch | 16278 | 1 | 0.011
H1ch | 16311 | 3 | 0.034
H1ch | 16390 | 3 | 0.034
H1ch | 567 | 1 | 0.011
H1ch | 8027 | 1 | 0.011
H1ci | 10188 | 2 | 0.003
H1ci | 113 | 1 | 0.001
H1ci | 15928 | 2 | 0.003
H1ci | 16076A | 123 | 0.155
H1ci | 208 | 1 | 0.001
H1ci | 261 | 1 | 0.001
H1ci | 281C | 1 | 0.001
H1ci | 316d | 1 | 0.001
H1ci | 338 | 1 | 0.001
H1ci | 468 | 3 | 0.004
H1ci | 502 | 1 | 0.001
H1ci | 573.1C | 3 | 0.004
H1ci | 573.2C | 5 | 0.006
H1ci | 573.5C | 1 | 0.001
H1cj | 16189 | 1 | 0.004
H1cj | 16257 | 7 | 0.026
H1cj | 16355 | 2 | 0.008
H1cj | 2352 | 1 | 0.004
H1cj | 281C | 1 | 0.004
H1cj | 310 | 1 | 0.004
H1cj | 518 | 1 | 0.004
H1cj | 6014 | 1 | 0.004
H1ck | 132 | 1 | 0.013
H1ck | 16036 | 1 | 0.013
H1ck | 186G | 1 | 0.013
H1ck | 195 | 2 | 0.026
H1ck | 204 | 1 | 0.013
H1ck | 217 | 1 | 0.013
H1ck | 253 | 1 | 0.013
H1ck | 489 | 1 | 0.013
H1ck | 64 | 1 | 0.013
H1ck | 73 | 1 | 0.013
H1e | 10042 | 1 | 0.001
H1e | 113 | 1 | 0.001
H1e | 11914 | 1 | 0.001
H1e | 12121 | 1 | 0.001
H1e | 12172 | 1 | 0.001
H1e | 12358 | 1 | 0.001
H1e | 12972 | 1 | 0.001
H1e | 13359 | 1 | 0.001
H1e | 14233 | 1 | 0.001
H1e | 14992 | 1 | 0.001
H1e | 150 | 1 | 0.001
H1e | 15071 | 8 | 0.01
H1e | 15217 | 1 | 0.001
H1e | 15220 | 1 | 0.001
H1e | 15346 | 1 | 0.001
H1e | 15401 | 1 | 0.001
H1e | 15518 | 1 | 0.001
H1e | 15747 | 1 | 0.001
H1e | 16076A | 1 | 0.001
H1e | 16092 | 1 | 0.001
H1e | 16093 | 2 | 0.002
H1e | 16218 | 1 | 0.001
H1e | 16240 | 1 | 0.001
H1e | 16264 | 1 | 0.001

H1e | 16293 | 1 | 0.001
H1e | 16318T | 124 | 0.151
H1e | 16319 | 1 | 0.001
H1e | 16497 | 1 | 0.001
H1e | 16525 | 1 | 0.001
H1e | 1766 | 1 | 0.001
H1e | 185 | 1 | 0.001
H1e | 199 | 1 | 0.001
H1e | 207 | 1 | 0.001
H1e | 208 | 1 | 0.001
H1e | 2395 | 1 | 0.001
H1e | 261 | 1 | 0.001
H1e | 2772 | 1 | 0.001
H1e | 281 | 1 | 0.001
H1e | 281C | 1 | 0.001
H1e | 2863 | 1 | 0.001
H1e | 2885 | 1 | 0.001
H1e | 2887 | 1 | 0.001
H1e | 300-302d | 1 | 0.001
H1e | 310 | 1 | 0.001
H1e | 316d | 1 | 0.001
H1e | 338 | 1 | 0.001
H1e | 3771 | 1 | 0.001
H1e | 3796 | 1 | 0.001
H1e | 468 | 3 | 0.004
H1e | 502 | 1 | 0.001
H1e | 573.1C | 3 | 0.004
H1e | 573.2C | 5 | 0.006
H1e | 573.5C | 1 | 0.001
H1e | 6150 | 1 | 0.001
H1e | 709 | 1 | 0.001
H1e | 8302 | 1 | 0.001
H1e | 8986 | 1 | 0.001
H1e | 93 | 2 | 0.002
H1e | 960.1C | 1 | 0.001
H1e | 9948 | 1 | 0.001
H1e+16129 | 16257 | 7 | 0.027
H1e+16129 | 256 | 1 | 0.004
H1e+16129 | 281C | 1 | 0.004
H1e+16129 | 518 | 1 | 0.004
H1e+16129 | 6365 | 1 | 0.004
H1e1 | 10583 | 2 | 0.002
H1e1 | 10978 | 1 | 0.001
H1e1 | 113 | 1 | 0.001
H1e1 | 11914 | 11 | 0.014
H1e1 | 13488 | 2 | 0.002
H1e1 | 13500 | 2 | 0.002
H1e1 | 13576 | 1 | 0.001
H1e1 | 13938 | 11 | 0.014
H1e1 | 14180 | 7 | 0.009
H1e1 | 146 | 2 | 0.002
H1e1 | 1555 | 1 | 0.001
H1e1 | 15813G | 1 | 0.001
H1e1 | 15930 | 10 | 0.012
H1e1 | 16076A | 1 | 0.001
H1e1 | 16174 | 1 | 0.001
H1e1 | 16185 | 126 | 0.155
H1e1 | 16189 | 1 | 0.001
H1e1 | 16291 | 1 | 0.001
H1e1 | 16400 | 1 | 0.001
H1e1 | 16497 | 1 | 0.001
H1e1 | 208 | 1 | 0.001
H1e1 | 261 | 1 | 0.001
H1e1 | 281C | 1 | 0.001
H1e1 | 316d | 1 | 0.001
H1e1 | 338 | 1 | 0.001
H1e1 | 3520 | 1 | 0.001
H1e1 | 4078 | 1 | 0.001
H1e1 | 4560C | 1 | 0.001
H1e1 | 468 | 3 | 0.004
H1e1 | 502 | 1 | 0.001
H1e1 | 5105 | 2 | 0.002
H1e1 | 573.1C | 4 | 0.005
H1e1 | 573.2C | 5 | 0.006
H1e1 | 573.5C | 1 | 0.001
H1e1 | 6071 | 1 | 0.001
H1e1 | 789 | 2 | 0.002
H1e1 | 8227 | 2 | 0.002
H1e1 | 8251 | 2 | 0.002
H1e1 | 8705 | 1 | 0.001
H1e1 | 9082 | 1 | 0.001
H1e1a | 113 | 1 | 0.001
H1e1a | 11778 | 1 | 0.001
H1e1a | 11830 | 1 | 0.001
H1e1a | 12007 | 1 | 0.001
H1e1a | 13183 | 1 | 0.001
H1e1a | 13474 | 1 | 0.001
H1e1a | 13934 | 1 | 0.001
H1e1a | 14257 | 2 | 0.002
H1e1a | 14271 | 2 | 0.002
H1e1a | 14362 | 1 | 0.001
H1e1a | 146 | 2 | 0.002
H1e1a | 14921 | 1 | 0.001
H1e1a | 152 | 2 | 0.002
H1e1a | 15445 | 1 | 0.001
H1e1a | 15748 | 1 | 0.001
H1e1a | 15758 | 1 | 0.001
H1e1a | 16076A | 1 | 0.001
H1e1a | 16093 | 8 | 0.01
H1e1a | 16129 | 5 | 0.006
H1e1a | 16172 | 1 | 0.001
H1e1a | 16192 | 1 | 0.001
H1e1a | 16239 | 1 | 0.001
H1e1a | 16259 | 3 | 0.004
H1e1a | 16266 | 5 | 0.006
H1e1a | 16287 | 5 | 0.006
H1e1a | 16304 | 1 | 0.001
H1e1a | 16311 | 2 | 0.002
H1e1a | 16312 | 1 | 0.001
H1e1a | 16325 | 1 | 0.001
H1e1a | 16342 | 1 | 0.001

H1e1a | 16343 | 123 | 0.147
H1e1a | 16523 | 1 | 0.001
H1e1a | 16527 | 1 | 0.001
H1e1a | 182 | 2 | 0.002
H1e1a | 195 | 2 | 0.002
H1e1a | 204 | 2 | 0.002
H1e1a | 208 | 1 | 0.001
H1e1a | 2581 | 1 | 0.001
H1e1a | 261 | 1 | 0.001
H1e1a | 281C | 1 | 0.001
H1e1a | 309d | 1 | 0.001
H1e1a | 310 | 3 | 0.004
H1e1a | 3106d | 1 | 0.001
H1e1a | 3109 | 1 | 0.001
H1e1a | 315.2C | 2 | 0.002
H1e1a | 316d | 1 | 0.001
H1e1a | 3172 | 1 | 0.001
H1e1a | 3202 | 2 | 0.002
H1e1a | 3308 | 1 | 0.001
H1e1a | 338 | 1 | 0.001
H1e1a | 3397 | 1 | 0.001
H1e1a | 3555 | 1 | 0.001
H1e1a | 408A | 1 | 0.001
H1e1a | 4913 | 1 | 0.001
H1e1a | 502 | 1 | 0.001
H1e1a | 520 | 1 | 0.001
H1e1a | 525.1AC | 1 | 0.001
H1e1a | 5301 | 1 | 0.001
H1e1a | 55.1T | 1 | 0.001
H1e1a | 5600C | 1 | 0.001
H1e1a | 57 | 2 | 0.002
H1e1a | 573.1C | 4 | 0.005
H1e1a | 573.2C | 5 | 0.006
H1e1a | 573.5C | 1 | 0.001
H1e1a | 59 | 1 | 0.001
H1e1a | 5960 | 1 | 0.001
H1e1a | 5979 | 1 | 0.001
H1e1a | 60.1T | 1 | 0.001
H1e1a | 6263A | 1 | 0.001
H1e1a | 6367 | 5 | 0.006
H1e1a | 6587 | 1 | 0.001
H1e1a | 6659 | 1 | 0.001
H1e1a | 678 | 1 | 0.001
H1e1a | 6956 | 1 | 0.001
H1e1a | 7151 | 1 | 0.001
H1e1a | 7572 | 1 | 0.001
H1e1a | 8271 | 1 | 0.001
H1e1a | 8273 | 1 | 0.001
H1e1a | 8460 | 1 | 0.001
H1e1a | 8503 | 1 | 0.001
H1e1a | 8790 | 2 | 0.002
H1e1a | 8812 | 2 | 0.002
H1e1a | 8839 | 2 | 0.002
H1e1a | 8865 | 1 | 0.001
H1e1a | 9327 | 1 | 0.001
H1e1a | 9531 | 1 | 0.001
H1e1a | 960d | 1 | 0.001
H1e1a | 9655 | 1 | 0.001
H1e1a | 9909 | 1 | 0.001
H1e1a+16278 | 11290 | 1 | 0.012
H1e1a+16278 | 4826A | 1 | 0.012
H1e1a+16278 | 7280A | 1 | 0.012
H1e1a1 | 13830 | 1 | 0.012
H1e1a1 | 16093 | 30 | 0.357
H1e1a1 | 16129 | 1 | 0.012
H1e1a1 | 16249 | 2 | 0.024
H1e1a1 | 16274 | 3 | 0.036
H1e1a1 | 16354 | 5 | 0.06
H1e1a1 | 183 | 3 | 0.036
H1e1a1 | 207 | 2 | 0.024
H1e1a1 | 57 | 3 | 0.036
H1e1a1 | 60.1T | 3 | 0.036
H1e1a1 | 7754 | 1 | 0.012
H1e1a1 | 7960 | 1 | 0.012
H1e1a2 | 10323 | 1 | 0.001
H1e1a2 | 113 | 1 | 0.001
H1e1a2 | 11623 | 1 | 0.001
H1e1a2 | 15061 | 1 | 0.001
H1e1a2 | 152 | 1 | 0.001
H1e1a2 | 16076A | 123 | 0.154
H1e1a2 | 16129 | 1 | 0.001
H1e1a2 | 16188 | 1 | 0.001
H1e1a2 | 16250 | 2 | 0.003
H1e1a2 | 16311 | 1 | 0.001
H1e1a2 | 16463 | 1 | 0.001
H1e1a2 | 208 | 1 | 0.001
H1e1a2 | 260 | 1 | 0.001
H1e1a2 | 261 | 1 | 0.001
H1e1a2 | 281C | 1 | 0.001
H1e1a2 | 316d | 1 | 0.001
H1e1a2 | 338 | 1 | 0.001
H1e1a2 | 3736 | 1 | 0.001
H1e1a2 | 468 | 3 | 0.004
H1e1a2 | 502 | 1 | 0.001
H1e1a2 | 573.1C | 3 | 0.004
H1e1a2 | 573.2C | 5 | 0.006
H1e1a2 | 573.5C | 1 | 0.001
H1e1a2 | 7244 | 1 | 0.001
H1e1a2 | 9438 | 1 | 0.001
H1e1a3 | 13434 | 1 | 0.012
H1e1a3 | 16183 | 1 | 0.012
H1e1a3 | 16189 | 1 | 0.012
H1e1a3 | 16222 | 2 | 0.024
H1e1a3 | 195 | 2 | 0.024
H1e1a3 | 3290 | 1 | 0.012
H1e1a3 | 4092 | 1 | 0.012
H1e1a3 | 573.1C | 2 | 0.024
H1e1a4 | 143 | 2 | 0.006
H1e1a4 | 16150 | 1 | 0.003
H1e1a4 | 16159 | 1 | 0.003
H1e1a4 | 16177 | 1 | 0.003

H1e1a4 | 16209 | 3 | 0.009
H1e1a4 | 16214 | 1 | 0.003
H1e1a4 | 16243 | 1 | 0.003
H1e1a4 | 16258 | 3 | 0.009
H1e1a4 | 16284 | 35 | 0.107
H1e1a4 | 16319 | 1 | 0.003
H1e1a4 | 16566 | 1 | 0.003
H1e1a4 | 237 | 1 | 0.003
H1e1a4 | 246 | 1 | 0.003
H1e1a4 | 2707 | 1 | 0.003
H1e1a4 | 288 | 1 | 0.003
H1e1a4 | 484 | 1 | 0.003
H1e1a4 | 5319 | 1 | 0.003
H1e1a4 | 8952 | 1 | 0.003
H1e1a5 | 14207 | 1 | 0.012
H1e1a5 | 16067G | 1 | 0.012
H1e1a5 | 16126 | 1 | 0.012
H1e1a5 | 16160 | 1 | 0.012
H1e1a5 | 16166 | 2 | 0.024
H1e1a5 | 16172 | 1 | 0.012
H1e1a5 | 16209 | 1 | 0.012
H1e1a5 | 16223 | 10 | 0.12
H1e1a5 | 489 | 1 | 0.012
H1e1a5 | 55 | 1 | 0.012
H1e1a5 | 55A | 1 | 0.012
H1e1a5 | 56 | 2 | 0.024
H1e1a5 | 7270 | 1 | 0.012
H1e1a5 | 9266 | 1 | 0.012
H1e1a6 | 1117 | 1 | 0.012
H1e1a6 | 114 | 5 | 0.061
H1e1a6 | 11590 | 1 | 0.012
H1e1a6 | 14398 | 1 | 0.012
H1e1a6 | 14861 | 1 | 0.012
H1e1a6 | 16066 | 2 | 0.024
H1e1a6 | 16163 | 1 | 0.012
H1e1a6 | 16174 | 1 | 0.012
H1e1a6 | 16243 | 1 | 0.012
H1e1a6 | 16254 | 3 | 0.037
H1e1a6 | 16256 | 1 | 0.012
H1e1a6 | 16263 | 5 | 0.061
H1e1a6 | 182 | 1 | 0.012
H1e1a6 | 227T | 5 | 0.061
H1e1a6 | 2320 | 2 | 0.024
H1e1a6 | 248 | 1 | 0.012
H1e1a6 | 315.2C | 1 | 0.012
H1e1a6 | 316C | 1 | 0.012
H1e1a6 | 3308 | 1 | 0.012
H1e1a6 | 408A | 1 | 0.012
H1e1a6 | 4823 | 2 | 0.024
H1e1a6 | 5201 | 1 | 0.012
H1e1a6 | 573.1C | 5 | 0.061
H1e1a6 | 5964 | 1 | 0.012
H1e1a6 | 6216 | 2 | 0.024
H1e1a6 | 7598 | 1 | 0.012
H1e1a6 | 7702 | 1 | 0.012
H1e1a6 | 8772 | 1 | 0.012
H1e1a6 | 8861 | 1 | 0.012
H1e1a7 | 113 | 1 | 0.001
H1e1a7 | 12373 | 3 | 0.004
H1e1a7 | 16076A | 123 | 0.154
H1e1a7 | 208 | 1 | 0.001
H1e1a7 | 261 | 1 | 0.001
H1e1a7 | 281C | 1 | 0.001
H1e1a7 | 316d | 1 | 0.001
H1e1a7 | 338 | 1 | 0.001
H1e1a7 | 468 | 3 | 0.004
H1e1a7 | 502 | 1 | 0.001
H1e1a7 | 573.1C | 3 | 0.004
H1e1a7 | 573.2C | 5 | 0.006
H1e1a7 | 573.5C | 1 | 0.001
H1e1a8 | 13020 | 2 | 0.08
H1e1a8 | 16041 | 2 | 0.08
H1e1a8 | 16189 | 1 | 0.04
H1e1a8 | 16223 | 2 | 0.08
H1e1a8 | 16356 | 2 | 0.08
H1e1a8 | 16484-16489d | 2 | 0.08
H1e1a8 | 310 | 2 | 0.08
H1e1a8 | 606 | 2 | 0.08
H1e1a8 | 8975 | 1 | 0.04
H1e1b | 10274 | 2 | 0.005
H1e1b | 16042C | 1 | 0.003
H1e1b | 16129T | 1 | 0.003
H1e1b | 16150 | 1 | 0.003
H1e1b | 16188A | 2 | 0.005
H1e1b | 16209 | 16 | 0.042
H1e1b | 16231 | 1 | 0.003
H1e1b | 16293C | 2 | 0.005
H1e1b | 16327 | 125 | 0.326
H1e1b | 176C | 2 | 0.005
H1e1b | 252 | 3 | 0.008
H1e1b | 261.1T | 1 | 0.003
H1e1b | 264 | 3 | 0.008
H1e1b | 310 | 1 | 0.003
H1e1b | 332G | 1 | 0.003
H1e1b | 385 | 1 | 0.003
H1e1b | 49T | 1 | 0.003
H1e1b1 | 12640 | 1 | 0.003
H1e1b1 | 12754 | 1 | 0.003
H1e1b1 | 13437 | 2 | 0.005
H1e1b1 | 13911 | 2 | 0.005
H1e1b1 | 13923 | 8 | 0.021
H1e1b1 | 14275 | 1 | 0.003
H1e1b1 | 143 | 6 | 0.015
H1e1b1 | 146 | 1 | 0.003
H1e1b1 | 16042C | 1 | 0.003
H1e1b1 | 16129T | 1 | 0.003
H1e1b1 | 16150 | 1 | 0.003
H1e1b1 | 16188A | 2 | 0.005
H1e1b1 | 16231 | 1 | 0.003
H1e1b1 | 16263 | 2 | 0.005
H1e1b1 | 16291 | 1 | 0.003

H1e1b1 | 16293C | 2 | 0.005
H1e1b1 | 16327 | 125 | 0.321
H1e1b1 | 16362 | 1 | 0.003
H1e1b1 | 176C | 2 | 0.005
H1e1b1 | 182 | 1 | 0.003
H1e1b1 | 195 | 1 | 0.003
H1e1b1 | 252 | 3 | 0.008
H1e1b1 | 261.1T | 1 | 0.003
H1e1b1 | 264 | 3 | 0.008
H1e1b1 | 309d | 1 | 0.003
H1e1b1 | 310 | 2 | 0.005
H1e1b1 | 332G | 1 | 0.003
H1e1b1 | 3397 | 1 | 0.003
H1e1b1 | 3438 | 1 | 0.003
H1e1b1 | 3592 | 1 | 0.003
H1e1b1 | 366 | 1 | 0.003
H1e1b1 | 385 | 1 | 0.003
H1e1b1 | 389 | 1 | 0.003
H1e1b1 | 463 | 1 | 0.003
H1e1b1 | 49T | 1 | 0.003
H1e1b1 | 729 | 1 | 0.003
H1e1b1 | 73 | 4 | 0.01
H1e1b1 | 861 | 1 | 0.003
H1e1b1 | 9357 | 1 | 0.003
H1e1b1 | 9545 | 9 | 0.023
H1e1b1a | 15454 | 2 | 0.005
H1e1b1a | 15692 | 1 | 0.003
H1e1b1a | 16042C | 1 | 0.003
H1e1b1a | 16129T | 1 | 0.003
H1e1b1a | 16150 | 1 | 0.003
H1e1b1a | 16188A | 2 | 0.005
H1e1b1a | 16209 | 16 | 0.041
H1e1b1a | 16231 | 1 | 0.003
H1e1b1a | 16293C | 2 | 0.005
H1e1b1a | 16309 | 2 | 0.005
H1e1b1a | 16327 | 125 | 0.324
H1e1b1a | 176C | 2 | 0.005
H1e1b1a | 252 | 3 | 0.008
H1e1b1a | 261.1T | 1 | 0.003
H1e1b1a | 264 | 3 | 0.008
H1e1b1a | 332G | 1 | 0.003
H1e1b1a | 385 | 1 | 0.003
H1e1b1a | 49T | 1 | 0.003
H1e1b1a | 7436 | 2 | 0.005
H1e1b1a | 75 | 2 | 0.005
H1e1b1a | 9148 | 2 | 0.005
H1e1b1b | 11080 | 1 | 0.003
H1e1b1b | 152 | 1 | 0.003
H1e1b1b | 16042C | 1 | 0.003
H1e1b1b | 16129T | 1 | 0.003
H1e1b1b | 16150 | 1 | 0.003
H1e1b1b | 16188A | 2 | 0.005
H1e1b1b | 16231 | 1 | 0.003
H1e1b1b | 16257 | 5 | 0.013
H1e1b1b | 16293C | 2 | 0.005
H1e1b1b | 16327 | 125 | 0.335
H1e1b1b | 176C | 2 | 0.005
H1e1b1b | 252 | 3 | 0.008
H1e1b1b | 261.1T | 1 | 0.003
H1e1b1b | 264 | 3 | 0.008
H1e1b1b | 332G | 1 | 0.003
H1e1b1b | 385 | 1 | 0.003
H1e1b1b | 49T | 1 | 0.003
H1e1c | 11204 | 1 | 0.001
H1e1c | 113 | 1 | 0.001
H1e1c | 11914 | 1 | 0.001
H1e1c | 12397 | 9 | 0.011
H1e1c | 152 | 1 | 0.001
H1e1c | 16076A | 1 | 0.001
H1e1c | 16271 | 131 | 0.162
H1e1c | 16288 | 1 | 0.001
H1e1c | 208 | 1 | 0.001
H1e1c | 261 | 1 | 0.001
H1e1c | 281C | 1 | 0.001
H1e1c | 316d | 1 | 0.001
H1e1c | 338 | 1 | 0.001
H1e1c | 427A | 123 | 0.152
H1e1c | 468 | 3 | 0.004
H1e1c | 502 | 1 | 0.001
H1e1c | 573.1C | 3 | 0.004
H1e1c | 573.2C | 5 | 0.006
H1e1c | 573.5C | 1 | 0.001
H1e2 | 113 | 1 | 0.001
H1e2 | 11404 | 1 | 0.001
H1e2 | 13020 | 1 | 0.001
H1e2 | 14477 | 1 | 0.001
H1e2 | 146 | 1 | 0.001
H1e2 | 14634 | 1 | 0.001
H1e2 | 150 | 1 | 0.001
H1e2 | 152 | 2 | 0.002
H1e2 | 15245 | 1 | 0.001
H1e2 | 15670 | 1 | 0.001
H1e2 | 16076A | 1 | 0.001
H1e2 | 16215 | 2 | 0.002
H1e2 | 16362 | 14 | 0.017
H1e2 | 16399 | 1 | 0.001
H1e2 | 16472 | 111 | 0.136
H1e2 | 208 | 1 | 0.001
H1e2 | 261 | 1 | 0.001
H1e2 | 2626 | 1 | 0.001
H1e2 | 281C | 1 | 0.001
H1e2 | 310 | 2 | 0.002
H1e2 | 316d | 1 | 0.001
H1e2 | 3238 | 1 | 0.001
H1e2 | 338 | 1 | 0.001
H1e2 | 468 | 3 | 0.004
H1e2 | 502 | 1 | 0.001
H1e2 | 5345 | 1 | 0.001
H1e2 | 573.1C | 3 | 0.004
H1e2 | 573.2C | 5 | 0.006
H1e2 | 573.5C | 1 | 0.001

H1e2 | 5896A | 1 | 0.001
H1e2 | 7258 | 1 | 0.001
H1e2 | 7771 | 1 | 0.001
H1e2 | 8210 | 2 | 0.002
H1e2 | 9338 | 1 | 0.001
H1e2 | 9489 | 2 | 0.002
H1e2a | 113 | 1 | 0.001
H1e2a | 11914 | 2 | 0.002
H1e2a | 13326 | 3 | 0.004
H1e2a | 13368 | 5 | 0.006
H1e2a | 14431 | 1 | 0.001
H1e2a | 146 | 1 | 0.001
H1e2a | 15889 | 1 | 0.001
H1e2a | 16051 | 1 | 0.001
H1e2a | 16076A | 123 | 0.153
H1e2a | 208 | 1 | 0.001
H1e2a | 261 | 1 | 0.001
H1e2a | 281C | 1 | 0.001
H1e2a | 316d | 1 | 0.001
H1e2a | 338 | 1 | 0.001
H1e2a | 468 | 3 | 0.004
H1e2a | 502 | 1 | 0.001
H1e2a | 573.1C | 3 | 0.004
H1e2a | 573.2C | 5 | 0.006
H1e2a | 573.5C | 1 | 0.001
H1e2b | 113 | 1 | 0.001
H1e2b | 16076A | 1 | 0.001
H1e2b | 16175 | 123 | 0.155
H1e2b | 208 | 1 | 0.001
H1e2b | 261 | 1 | 0.001
H1e2b | 281C | 1 | 0.001
H1e2b | 316d | 1 | 0.001
H1e2b | 338 | 1 | 0.001
H1e2b | 468 | 3 | 0.004
H1e2b | 502 | 1 | 0.001
H1e2b | 573.1C | 3 | 0.004
H1e2b | 573.2C | 5 | 0.006
H1e2b | 573.5C | 1 | 0.001
H1e2c | 13782 | 1 | 0.007
H1e2c | 15944d | 1 | 0.007
H1e2c | 16153 | 4 | 0.029
H1e2c | 16318 | 1 | 0.007
H1e2c | 16362 | 1 | 0.007
H1e2c | 1808 | 1 | 0.007
H1e2c | 200 | 1 | 0.007
H1e2c | 310 | 1 | 0.007
H1e2c | 513 | 1 | 0.007
H1e2c | 5899.1C | 7 | 0.05
H1e2c | 5899.2C | 1 | 0.007
H1e2c | 8119 | 1 | 0.007
H1e2c | 9006 | 1 | 0.007
H1e2d | 113 | 1 | 0.001
H1e2d | 14180 | 1 | 0.001
H1e2d | 16076A | 123 | 0.155
H1e2d | 16244 | 1 | 0.001
H1e2d | 189 | 1 | 0.001
H1e2d | 208 | 1 | 0.001
H1e2d | 261 | 1 | 0.001
H1e2d | 281C | 1 | 0.001
H1e2d | 316d | 1 | 0.001
H1e2d | 338 | 1 | 0.001
H1e2d | 468 | 3 | 0.004
H1e2d | 502 | 1 | 0.001
H1e2d | 573.1C | 3 | 0.004
H1e2d | 573.2C | 5 | 0.006
H1e2d | 573.5C | 1 | 0.001
H1e3 | 11778 | 1 | 0.009
H1e3 | 152 | 4 | 0.034
H1e3 | 16028A | 1 | 0.009
H1e3 | 16029G | 1 | 0.009
H1e3 | 16037 | 1 | 0.009
H1e3 | 16085 | 2 | 0.017
H1e3 | 16093 | 1 | 0.009
H1e3 | 16131 | 1 | 0.009
H1e3 | 16170T | 1 | 0.009
H1e3 | 16179 | 1 | 0.009
H1e3 | 16189 | 1 | 0.009
H1e3 | 16285.1A | 3 | 0.026
H1e3 | 16309 | 3 | 0.026
H1e3 | 185 | 11 | 0.095
H1e3 | 6164 | 1 | 0.009
H1e3 | 6314 | 1 | 0.009
H1e3 | 7191 | 1 | 0.009
H1e4 | 113 | 1 | 0.001
H1e4 | 146 | 1 | 0.001
H1e4 | 16076A | 1 | 0.001
H1e4 | 16189 | 123 | 0.154
H1e4 | 208 | 1 | 0.001
H1e4 | 261 | 1 | 0.001
H1e4 | 281C | 1 | 0.001
H1e4 | 316d | 1 | 0.001
H1e4 | 338 | 1 | 0.001
H1e4 | 468 | 3 | 0.004
H1e4 | 502 | 1 | 0.001
H1e4 | 573.1C | 3 | 0.004
H1e4 | 573.2C | 5 | 0.006
H1e4 | 573.5C | 1 | 0.001
H1e4a | 14259 | 1 | 0.013
H1e4a | 16126 | 1 | 0.013
H1e4a | 16311 | 1 | 0.013
H1e4a | 8951 | 1 | 0.013
H1e5 | 11253 | 2 | 0.021
H1e5 | 16072 | 1 | 0.01
H1e5 | 16209 | 1 | 0.01
H1e5 | 16233 | 1 | 0.01
H1e5 | 16261 | 1 | 0.01
H1e5 | 16274 | 1 | 0.01
H1e5 | 16311 | 1 | 0.01
H1e5 | 16354 | 1 | 0.01
H1e5 | 16512 | 1 | 0.01
H1e5 | 316C | 1 | 0.01

H1e5 | 3511 | 2 | 0.021
H1e5 | 372 | 1 | 0.01
H1e5 | 443C | 1 | 0.01
H1e5 | 573.2C | 1 | 0.01
H1e5 | 72A | 1 | 0.01
H1e5 | 75 | 1 | 0.01
H1e5 | 9389 | 2 | 0.021
H1e5a | 11404 | 1 | 0.01
H1e5a | 12070 | 1 | 0.01
H1e5a | 12127 | 1 | 0.01
H1e5a | 12714 | 1 | 0.01
H1e5a | 1346 | 3 | 0.029
H1e5a | 13948 | 2 | 0.019
H1e5a | 14287 | 1 | 0.01
H1e5a | 14897 | 1 | 0.01
H1e5a | 152 | 1 | 0.01
H1e5a | 15265 | 1 | 0.01
H1e5a | 16072 | 1 | 0.01
H1e5a | 16092 | 1 | 0.01
H1e5a | 16209 | 1 | 0.01
H1e5a | 16233 | 1 | 0.01
H1e5a | 16255 | 1 | 0.01
H1e5a | 16261 | 1 | 0.01
H1e5a | 16274 | 1 | 0.01
H1e5a | 16287 | 1 | 0.01
H1e5a | 16311 | 1 | 0.01
H1e5a | 16354 | 1 | 0.01
H1e5a | 16362 | 4 | 0.039
H1e5a | 16512 | 1 | 0.01
H1e5a | 294 | 3 | 0.029
H1e5a | 309.3C | 1 | 0.01
H1e5a | 316C | 1 | 0.01
H1e5a | 372 | 1 | 0.01
H1e5a | 4084 | 3 | 0.029
H1e5a | 443C | 1 | 0.01
H1e5a | 449 | 1 | 0.01
H1e5a | 480 | 3 | 0.029
H1e5a | 499 | 1 | 0.01
H1e5a | 573.2C | 1 | 0.01
H1e5a | 6044 | 3 | 0.029
H1e5a | 72A | 1 | 0.01
H1e5a | 75 | 1 | 0.01
H1e5b | 15670 | 1 | 0.01
H1e5b | 16072 | 1 | 0.01
H1e5b | 16185 | 1 | 0.01
H1e5b | 16187 | 1 | 0.01
H1e5b | 16209 | 1 | 0.01
H1e5b | 16233 | 1 | 0.01
H1e5b | 16261 | 1 | 0.01
H1e5b | 16274 | 1 | 0.01
H1e5b | 16311 | 1 | 0.01
H1e5b | 16354 | 1 | 0.01
H1e5b | 16512 | 1 | 0.01
H1e5b | 316C | 1 | 0.01
H1e5b | 372 | 1 | 0.01
H1e5b | 443C | 1 | 0.01
H1e5b | 573.2C | 1 | 0.01
H1e5b | 72A | 1 | 0.01
H1e5b | 75 | 1 | 0.01
H1e6 | 113 | 1 | 0.001
H1e6 | 146 | 1 | 0.001
H1e6 | 16076A | 123 | 0.155
H1e6 | 16290 | 1 | 0.001
H1e6 | 208 | 1 | 0.001
H1e6 | 261 | 1 | 0.001
H1e6 | 281C | 1 | 0.001
H1e6 | 316d | 1 | 0.001
H1e6 | 338 | 1 | 0.001
H1e6 | 468 | 3 | 0.004
H1e6 | 502 | 1 | 0.001
H1e6 | 573.1C | 3 | 0.004
H1e6 | 573.2C | 5 | 0.006
H1e6 | 573.5C | 1 | 0.001
H1e6 | 9007 | 1 | 0.001
H1e7 | 113 | 1 | 0.001
H1e7 | 16076A | 123 | 0.155
H1e7 | 208 | 1 | 0.001
H1e7 | 261 | 1 | 0.001
H1e7 | 281C | 1 | 0.001
H1e7 | 316d | 1 | 0.001
H1e7 | 3203 | 1 | 0.001
H1e7 | 338 | 1 | 0.001
H1e7 | 468 | 3 | 0.004
H1e7 | 502 | 1 | 0.001
H1e7 | 573.1C | 3 | 0.004
H1e7 | 573.2C | 6 | 0.008
H1e7 | 573.5C | 1 | 0.001
H1e8 | 113 | 1 | 0.001
H1e8 | 14831 | 1 | 0.001
H1e8 | 16076A | 123 | 0.155
H1e8 | 16271 | 1 | 0.001
H1e8 | 208 | 1 | 0.001
H1e8 | 261 | 1 | 0.001
H1e8 | 281C | 1 | 0.001
H1e8 | 316d | 1 | 0.001
H1e8 | 338 | 1 | 0.001
H1e8 | 468 | 3 | 0.004
H1e8 | 502 | 1 | 0.001
H1e8 | 573.1C | 3 | 0.004
H1e8 | 573.2C | 5 | 0.006
H1e8 | 573.5C | 1 | 0.001
H1e8a | 113 | 1 | 0.001
H1e8a | 150 | 5 | 0.006
H1e8a | 16076A | 123 | 0.154
H1e8a | 198 | 4 | 0.005
H1e8a | 208 | 1 | 0.001
H1e8a | 228 | 1 | 0.001
H1e8a | 261 | 1 | 0.001
H1e8a | 281C | 1 | 0.001
H1e8a | 310 | 2 | 0.002
H1e8a | 316d | 1 | 0.001

H1e8a | 338 | 1 | 0.001
H1e8a | 468 | 3 | 0.004
H1e8a | 4745 | 1 | 0.001
H1e8a | 502 | 1 | 0.001
H1e8a | 573.1C | 3 | 0.004
H1e8a | 573.2C | 5 | 0.006
H1e8a | 573.5C | 1 | 0.001
H1e8a | 73 | 5 | 0.006
H1e8a | 9413 | 1 | 0.001
H1f | 1189 | 1 | 0.005
H1f | 13680 | 1 | 0.005
H1f | 14872 | 1 | 0.005
H1f | 152 | 1 | 0.005
H1f | 16111 | 1 | 0.005
H1f | 16193 | 1 | 0.005
H1f | 16233 | 1 | 0.005
H1f | 16243 | 1 | 0.005
H1f | 16319 | 1 | 0.005
H1f | 16390 | 19 | 0.091
H1f | 16517 | 1 | 0.005
H1f | 297 | 1 | 0.005
H1f | 315.2C | 1 | 0.005
H1f | 319.1T | 1 | 0.005
H1f | 385 | 1 | 0.005
H1f | 480 | 1 | 0.005
H1f | 499 | 1 | 0.005
H1f | 554T | 1 | 0.005
H1f | 567T | 1 | 0.005
H1f | 72 | 1 | 0.005
H1f | 7309 | 5 | 0.024
H1f | 7585.1C | 1 | 0.005
H1f+16093 | 103C | 1 | 0.011
H1f+16093 | 104G | 1 | 0.011
H1f+16093 | 152 | 1 | 0.011
H1f+16093 | 16075A | 1 | 0.011
H1f+16093 | 16178 | 10 | 0.111
H1f+16093 | 16209 | 1 | 0.011
H1f+16093 | 16219 | 3 | 0.033
H1f+16093 | 16311 | 1 | 0.011
H1f+16093 | 16405C | 1 | 0.011
H1f+16093 | 182 | 1 | 0.011
H1f+16093 | 1957 | 1 | 0.011
H1f+16093 | 234 | 3 | 0.033
H1f+16093 | 249d | 1 | 0.011
H1f+16093 | 384 | 1 | 0.011
H1f+16093 | 64 | 1 | 0.011
H1f+16093 | 650 | 1 | 0.011
H1f+16093 | 73 | 10 | 0.111
H1f+16093 | 93 | 1 | 0.011
H1f1 | 10343 | 1 | 0.01
H1f1 | 10578 | 1 | 0.01
H1f1 | 11778 | 1 | 0.01
H1f1 | 12164 | 1 | 0.01
H1f1 | 13356 | 2 | 0.019
H1f1 | 152 | 1 | 0.01
H1f1 | 16172 | 3 | 0.029
H1f1 | 16193d | 1 | 0.01
H1f1 | 1842 | 1 | 0.01
H1f1 | 195 | 1 | 0.01
H1f1 | 573.1C | 1 | 0.01
H1f1 | 5772 | 2 | 0.019
H1f1 | 729 | 1 | 0.01
H1f1 | 8411 | 1 | 0.01
H1f1 | 8765 | 1 | 0.01
H1f1a | 13145 | 1 | 0.015
H1f1a | 146 | 7 | 0.106
H1f1a | 16051 | 1 | 0.015
H1f1a | 16075 | 1 | 0.015
H1f1a | 16079G | 1 | 0.015
H1f1a | 16092A | 1 | 0.015
H1f1a | 16115 | 1 | 0.015
H1f1a | 16129 | 2 | 0.03
H1f1a | 16140 | 1 | 0.015
H1f1a | 16173 | 2 | 0.03
H1f1a | 16183 | 1 | 0.015
H1f1a | 16184A | 1 | 0.015
H1f1a | 16188 | 1 | 0.015
H1f1a | 16193d | 3 | 0.045
H1f1a | 16213C | 1 | 0.015
H1f1a | 16217 | 10 | 0.152
H1f1a | 16239 | 1 | 0.015
H1f1a | 16265 | 1 | 0.015
H1f1a | 16266 | 5 | 0.076
H1f1a | 16270A | 1 | 0.015
H1f1a | 16271 | 1 | 0.015
H1f1a | 16295 | 1 | 0.015
H1f1a | 16405T | 1 | 0.015
H1f1a | 16465 | 1 | 0.015
H1f1a | 309d | 1 | 0.015
H1f1a | 8269 | 1 | 0.015
H1g | 103 | 3 | 0.011
H1g | 109 | 1 | 0.004
H1g | 121C | 1 | 0.004
H1g | 143 | 4 | 0.015
H1g | 150 | 23 | 0.085
H1g | 16037 | 1 | 0.004
H1g | 16038 | 1 | 0.004
H1g | 16051 | 15 | 0.055
H1g | 16086 | 1 | 0.004
H1g | 16111 | 1 | 0.004
H1g | 16124 | 2 | 0.007
H1g | 16126 | 1 | 0.004
H1g | 16129 | 9 | 0.033
H1g | 16150 | 2 | 0.007
H1g | 16168 | 3 | 0.011
H1g | 16172 | 2 | 0.007
H1g | 16179 | 1 | 0.004
H1g | 16209 | 1 | 0.004
H1g | 16217 | 55 | 0.203
H1g | 16233 | 1 | 0.004
H1g | 16234G | 4 | 0.015

H1g | 16243 | 1 | 0.004
H1g | 16249 | 1 | 0.004
H1g | 16254 | 1 | 0.004
H1g | 16266 | 2 | 0.007
H1g | 16270A | 2 | 0.007
H1g | 16290 | 1 | 0.004
H1g | 16293 | 1 | 0.004
H1g | 16295 | 1 | 0.004
H1g | 16298 | 3 | 0.011
H1g | 16324 | 2 | 0.007
H1g | 16327 | 1 | 0.004
H1g | 16357 | 1 | 0.004
H1g | 16362 | 1 | 0.004
H1g | 16484-16489d | 20 | 0.074
H1g | 189 | 5 | 0.018
H1g | 195 | 1 | 0.004
H1g | 200 | 2 | 0.007
H1g | 204 | 28 | 0.103
H1g | 214 | 3 | 0.011
H1g | 226 | 1 | 0.004
H1g | 240 | 4 | 0.015
H1g | 249d | 6 | 0.022
H1g | 297 | 1 | 0.004
H1g | 302C | 1 | 0.004
H1g | 315.2C | 1 | 0.004
H1g | 316 | 1 | 0.004
H1g | 319 | 1 | 0.004
H1g | 319.1T | 1 | 0.004
H1g | 480 | 1 | 0.004
H1g | 499 | 1 | 0.004
H1g | 554T | 1 | 0.004
H1g | 567T | 1 | 0.004
H1g1 | 10310 | 1 | 0.004
H1g1 | 10565 | 1 | 0.004
H1g1 | 10739 | 1 | 0.004
H1g1 | 12657 | 1 | 0.004
H1g1 | 13272 | 1 | 0.004
H1g1 | 13635 | 1 | 0.004
H1g1 | 13708 | 1 | 0.004
H1g1 | 14097 | 1 | 0.004
H1g1 | 14311 | 1 | 0.004
H1g1 | 14319 | 8 | 0.034
H1g1 | 14980 | 1 | 0.004
H1g1 | 152 | 3 | 0.013
H1g1 | 15262 | 1 | 0.004
H1g1 | 15301 | 1 | 0.004
H1g1 | 15684 | 2 | 0.008
H1g1 | 15884 | 1 | 0.004
H1g1 | 16092 | 2 | 0.008
H1g1 | 16111 | 1 | 0.004
H1g1 | 16233 | 1 | 0.004
H1g1 | 16265C | 19 | 0.08
H1g1 | 200 | 1 | 0.004
H1g1 | 2294 | 1 | 0.004
H1g1 | 309.3C | 2 | 0.008
H1g1 | 310 | 2 | 0.008
H1g1 | 3394 | 1 | 0.004
H1g1 | 3418 | 1 | 0.004
H1g1 | 3421 | 1 | 0.004
H1g1 | 3991 | 1 | 0.004
H1g1 | 4775 | 1 | 0.004
H1g1 | 480 | 1 | 0.004
H1g1 | 5250 | 1 | 0.004
H1g1 | 554T | 1 | 0.004
H1g1 | 5582 | 1 | 0.004
H1g1 | 567T | 1 | 0.004
H1g1 | 6249 | 1 | 0.004
H1g1 | 7149 | 1 | 0.004
H1g1 | 8020 | 1 | 0.004
H1g1 | 8485 | 1 | 0.004
H1g1 | 895A | 1 | 0.004
H1g1 | 9630 | 1 | 0.004
H1g1 | 9812 | 8 | 0.034
H1g1 | 9944 | 1 | 0.004
H1g2 | 150 | 22 | 0.103
H1g2 | 16111 | 1 | 0.005
H1g2 | 16126 | 1 | 0.005
H1g2 | 16129 | 4 | 0.019
H1g2 | 16233 | 1 | 0.005
H1g2 | 16243 | 1 | 0.005
H1g2 | 16295 | 1 | 0.005
H1g2 | 16298 | 1 | 0.005
H1g2 | 16362 | 1 | 0.005
H1g2 | 16484-16489d | 19 | 0.089
H1g2 | 195 | 19 | 0.089
H1g2 | 214 | 1 | 0.005
H1g2 | 297 | 1 | 0.005
H1g2 | 315.2C | 1 | 0.005
H1g2 | 316 | 1 | 0.005
H1g2 | 319.1T | 1 | 0.005
H1g2 | 480 | 1 | 0.005
H1g2 | 499 | 1 | 0.005
H1g2 | 554T | 1 | 0.005
H1g2 | 567T | 1 | 0.005
H1g2 | 73 | 1 | 0.005
H1h | 113 | 1 | 0.001
H1h | 16076A | 123 | 0.155
H1h | 208 | 1 | 0.001
H1h | 261 | 1 | 0.001
H1h | 281C | 1 | 0.001
H1h | 316d | 1 | 0.001
H1h | 338 | 1 | 0.001
H1h | 468 | 3 | 0.004
H1h | 502 | 1 | 0.001
H1h | 573.1C | 3 | 0.004
H1h | 573.2C | 5 | 0.006
H1h | 573.5C | 1 | 0.001
H1h1 | 10907 | 1 | 0.001
H1h1 | 11167 | 1 | 0.001
H1h1 | 113 | 1 | 0.001
H1h1 | 11432 | 2 | 0.002

H1h1 | 11935G | 1 | 0.001
H1h1 | 12882 | 1 | 0.001
H1h1 | 13089 | 1 | 0.001
H1h1 | 13889 | 1 | 0.001
H1h1 | 14482 | 3 | 0.004
H1h1 | 14564 | 3 | 0.004
H1h1 | 146 | 4 | 0.005
H1h1 | 14815 | 2 | 0.002
H1h1 | 14887 | 1 | 0.001
H1h1 | 152 | 4 | 0.005
H1h1 | 16072 | 1 | 0.001
H1h1 | 16076A | 1 | 0.001
H1h1 | 16111A | 124 | 0.153
H1h1 | 16129 | 2 | 0.002
H1h1 | 16189 | 1 | 0.001
H1h1 | 16194C | 1 | 0.001
H1h1 | 16243 | 1 | 0.001
H1h1 | 16260 | 1 | 0.001
H1h1 | 16261 | 1 | 0.001
H1h1 | 16265 | 1 | 0.001
H1h1 | 16297 | 1 | 0.001
H1h1 | 16343 | 1 | 0.001
H1h1 | 195 | 1 | 0.001
H1h1 | 208 | 1 | 0.001
H1h1 | 2231 | 1 | 0.001
H1h1 | 234 | 1 | 0.001
H1h1 | 261 | 1 | 0.001
H1h1 | 281C | 1 | 0.001
H1h1 | 2850 | 1 | 0.001
H1h1 | 316d | 1 | 0.001
H1h1 | 338 | 1 | 0.001
H1h1 | 3394 | 1 | 0.001
H1h1 | 468 | 3 | 0.004
H1h1 | 4740 | 1 | 0.001
H1h1 | 502 | 1 | 0.001
H1h1 | 5417 | 1 | 0.001
H1h1 | 573.1C | 3 | 0.004
H1h1 | 573.2C | 5 | 0.006
H1h1 | 573.5C | 1 | 0.001
H1h1 | 5899.1C | 2 | 0.002
H1h1 | 7818A | 1 | 0.001
H1h1 | 7818G | 1 | 0.001
H1h1 | 8331 | 1 | 0.001
H1h1 | 9046 | 1 | 0.001
H1h1 | 9656 | 1 | 0.001
H1h2 | 113 | 1 | 0.001
H1h2 | 152 | 1 | 0.001
H1h2 | 16076A | 123 | 0.155
H1h2 | 208 | 1 | 0.001
H1h2 | 261 | 1 | 0.001
H1h2 | 281C | 1 | 0.001
H1h2 | 316d | 1 | 0.001
H1h2 | 338 | 1 | 0.001
H1h2 | 468 | 3 | 0.004
H1h2 | 502 | 1 | 0.001
H1h2 | 573.1C | 3 | 0.004
H1h2 | 573.2C | 5 | 0.006
H1h2 | 573.5C | 1 | 0.001
H1i | 11204 | 1 | 0.007
H1i | 14470 | 3 | 0.02
H1i | 146 | 1 | 0.007
H1i | 16051 | 23 | 0.151
H1i | 16311 | 1 | 0.007
H1i | 252 | 1 | 0.007
H1i | 310 | 1 | 0.007
H1i | 3652 | 1 | 0.007
H1i | 5585 | 1 | 0.007
H1i | 573.1C | 1 | 0.007
H1i1 | 11611 | 1 | 0.006
H1i1 | 143 | 1 | 0.006
H1i1 | 14311 | 1 | 0.006
H1i1 | 14470 | 1 | 0.006
H1i1 | 151 | 10 | 0.062
H1i1 | 15110 | 1 | 0.006
H1i1 | 16051 | 1 | 0.006
H1i1 | 16071 | 1 | 0.006
H1i1 | 16111 | 1 | 0.006
H1i1 | 16177 | 2 | 0.012
H1i1 | 16223 | 1 | 0.006
H1i1 | 16300 | 1 | 0.006
H1i1 | 16318C | 21 | 0.13
H1i1 | 189 | 1 | 0.006
H1i1 | 2784 | 1 | 0.006
H1i1 | 2872 | 1 | 0.006
H1i1 | 573.1C | 1 | 0.006
H1i1 | 8420 | 1 | 0.006
H1i1 | 8507T | 1 | 0.006
H1i1 | 954 | 1 | 0.006
H1i2 | 15718 | 1 | 0.007
H1i2 | 16300 | 20 | 0.136
H1i2 | 16344 | 20 | 0.136
H1i2 | 573.1C | 1 | 0.007
H1i2a | 14323 | 21 | 0.141
H1i2a | 3144 | 1 | 0.007
H1i2a | 573.1C | 1 | 0.007
H1i2a | 6951 | 1 | 0.007
H1i2a | 9966 | 1 | 0.007
H1j | 113 | 1 | 0.001
H1j | 11614 | 1 | 0.001
H1j | 11941 | 1 | 0.001
H1j | 12879 | 1 | 0.001
H1j | 13500 | 1 | 0.001
H1j | 13911 | 2 | 0.002
H1j | 13966 | 1 | 0.001
H1j | 14025 | 2 | 0.002
H1j | 14180 | 1 | 0.001
H1j | 14182 | 1 | 0.001
H1j | 14512 | 1 | 0.001
H1j | 15052 | 1 | 0.001
H1j | 15320 | 1 | 0.001
H1j | 15440 | 1 | 0.001

H1j | 15465 | 1 | 0.001
H1j | 15924 | 1 | 0.001
H1j | 15929 | 1 | 0.001
H1j | 16076A | 1 | 0.001
H1j | 16093 | 1 | 0.001
H1j | 16185 | 1 | 0.001
H1j | 16257 | 1 | 0.001
H1j | 16303 | 124 | 0.153
H1j | 16311 | 1 | 0.001
H1j | 16362 | 1 | 0.001
H1j | 183 | 1 | 0.001
H1j | 208 | 1 | 0.001
H1j | 2445 | 1 | 0.001
H1j | 261 | 1 | 0.001
H1j | 281C | 1 | 0.001
H1j | 310 | 1 | 0.001
H1j | 316d | 1 | 0.001
H1j | 338 | 1 | 0.001
H1j | 4216 | 1 | 0.001
H1j | 468 | 3 | 0.004
H1j | 480 | 1 | 0.001
H1j | 502 | 1 | 0.001
H1j | 513 | 1 | 0.001
H1j | 573.1C | 3 | 0.004
H1j | 573.2C | 5 | 0.006
H1j | 573.5C | 1 | 0.001
H1j | 7394 | 1 | 0.001
H1j | 8348 | 1 | 0.001
H1j | 9 | 1 | 0.001
H1j | 9254 | 1 | 0.001
H1j | 93 | 1 | 0.001
H1j | 9500 | 1 | 0.001
H1j | 9530 | 1 | 0.001
H1j | 9615 | 1 | 0.001
H1j1 | 12241d | 1 | 0.004
H1j1 | 13731 | 1 | 0.004
H1j1 | 152 | 4 | 0.014
H1j1 | 16241 | 2 | 0.007
H1j1 | 16311 | 7 | 0.025
H1j1 | 16488 | 2 | 0.007
H1j1 | 3645 | 1 | 0.004
H1j1 | 4317 | 2 | 0.007
H1j1 | 5237 | 1 | 0.004
H1j1 | 5426 | 3 | 0.011
H1j1 | 592A | 1 | 0.004
H1j1 | 6791 | 1 | 0.004
H1j1a | 12957 | 1 | 0.004
H1j1a | 13314 | 7 | 0.026
H1j1a | 13490A | 1 | 0.004
H1j1a | 14502 | 1 | 0.004
H1j1a | 14831 | 1 | 0.004
H1j1a | 15257 | 1 | 0.004
H1j1a | 1555 | 1 | 0.004
H1j1a | 15942 | 1 | 0.004
H1j1a | 16390 | 9 | 0.033
H1j1a | 195 | 1 | 0.004
H1j1a | 3593 | 1 | 0.004
H1j1a | 5843 | 1 | 0.004
H1j1a | 6465 | 1 | 0.004
H1j1a | 73 | 2 | 0.007
H1j1a | 93 | 1 | 0.004
H1j1a | 930 | 7 | 0.026
H1j1a | 961 | 7 | 0.026
H1j1a1 | 8772 | 1 | 0.007
H1j1a2 | 15244 | 1 | 0.004
H1j1a2 | 16257 | 7 | 0.026
H1j1a2 | 281C | 1 | 0.004
H1j1a2 | 518 | 1 | 0.004
H1j1a2 | 6815 | 2 | 0.008
H1j1b | 10646 | 1 | 0.004
H1j1b | 12477 | 1 | 0.004
H1j1b | 150 | 3 | 0.011
H1j1b | 15430 | 1 | 0.004
H1j1b | 16093 | 1 | 0.004
H1j1b | 16298 | 1 | 0.004
H1j1b | 16390 | 9 | 0.033
H1j1b | 5978 | 1 | 0.004
H1j1b | 8176 | 1 | 0.004
H1j1b | 9069 | 1 | 0.004
H1j1b | 9983 | 1 | 0.004
H1j1c | 16154 | 4 | 0.015
H1j1c | 16248 | 1 | 0.004
H1j1c | 16257 | 7 | 0.026
H1j1c | 234 | 2 | 0.007
H1j1c | 281C | 1 | 0.004
H1j1c | 513 | 2 | 0.007
H1j1c | 518 | 1 | 0.004
H1j2 | 13474 | 1 | 0.007
H1j2 | 15071 | 1 | 0.007
H1j2 | 16221 | 1 | 0.007
H1j2 | 16270 | 1 | 0.007
H1j2 | 16298 | 3 | 0.022
H1j2 | 204 | 1 | 0.007
H1j2a | 12771 | 2 | 0.069
H1j2a | 15052 | 2 | 0.069
H1j2a | 152 | 1 | 0.034
H1j2a | 16071 | 1 | 0.034
H1j2a | 16145 | 1 | 0.034
H1j2a | 16169 | 1 | 0.034
H1j2a | 16234 | 6 | 0.207
H1j2a | 16240 | 1 | 0.034
H1j2a | 16301 | 1 | 0.034
H1j2a | 16312C | 1 | 0.034
H1j2a | 16356 | 5 | 0.172
H1j2a | 308-309d | 1 | 0.034
H1j3 | 113 | 1 | 0.001
H1j3 | 16076A | 123 | 0.154
H1j3 | 208 | 1 | 0.001
H1j3 | 261 | 1 | 0.001
H1j3 | 281C | 1 | 0.001
H1j3 | 316d | 1 | 0.001

H1j3 | 338 | 1 | 0.001
H1j3 | 468 | 3 | 0.004
H1j3 | 502 | 1 | 0.001
H1j3 | 573.1C | 3 | 0.004
H1j3 | 573.2C | 5 | 0.006
H1j3 | 573.5C | 1 | 0.001
H1j3 | 5752.1A | 1 | 0.001
H1j3 | 7678 | 1 | 0.001
H1j3 | 9948 | 1 | 0.001
H1j4 | 10325 | 2 | 0.024
H1j4 | 10892T | 1 | 0.012
H1j4 | 12136 | 1 | 0.012
H1j4 | 13623 | 1 | 0.012
H1j4 | 13731 | 1 | 0.012
H1j4 | 15932 | 1 | 0.012
H1j4 | 16051 | 11 | 0.133
H1j4 | 16093 | 1 | 0.012
H1j4 | 16172 | 1 | 0.012
H1j4 | 16179 | 1 | 0.012
H1j4 | 16216 | 2 | 0.024
H1j4 | 16317 | 1 | 0.012
H1j4 | 195 | 11 | 0.133
H1j4 | 204 | 1 | 0.012
H1j4 | 5270 | 1 | 0.012
H1j4 | 6755 | 1 | 0.012
H1j4 | 7598 | 1 | 0.012
H1j4 | 93 | 1 | 0.012
H1j5 | 113 | 1 | 0.001
H1j5 | 16076A | 123 | 0.155
H1j5 | 208 | 1 | 0.001
H1j5 | 261 | 1 | 0.001
H1j5 | 281C | 1 | 0.001
H1j5 | 316d | 1 | 0.001
H1j5 | 338 | 1 | 0.001
H1j5 | 468 | 3 | 0.004
H1j5 | 502 | 1 | 0.001
H1j5 | 573.1C | 3 | 0.004
H1j5 | 573.2C | 5 | 0.006
H1j5 | 573.5C | 1 | 0.001
H1j5 | 7375 | 1 | 0.001
H1j5 | 9668 | 1 | 0.001
H1j6 | 113 | 1 | 0.001
H1j6 | 15629 | 1 | 0.001
H1j6 | 15937d | 1 | 0.001
H1j6 | 16076A | 123 | 0.157
H1j6 | 208 | 1 | 0.001
H1j6 | 261 | 1 | 0.001
H1j6 | 281C | 1 | 0.001
H1j6 | 316d | 1 | 0.001
H1j6 | 338 | 1 | 0.001
H1j6 | 366 | 1 | 0.001
H1j6 | 389 | 1 | 0.001
H1j6 | 468 | 3 | 0.004
H1j6 | 502 | 1 | 0.001
H1j6 | 573.1C | 3 | 0.004
H1j6 | 573.2C | 5 | 0.006
H1j6 | 573.5C | 1 | 0.001
H1j6 | 622 | 1 | 0.001
H1j6 | 884 | 1 | 0.001
H1j7 | 113 | 1 | 0.001
H1j7 | 16076A | 123 | 0.155
H1j7 | 208 | 1 | 0.001
H1j7 | 261 | 1 | 0.001
H1j7 | 281C | 1 | 0.001
H1j7 | 316d | 1 | 0.001
H1j7 | 338 | 1 | 0.001
H1j7 | 3696 | 1 | 0.001
H1j7 | 468 | 3 | 0.004
H1j7 | 502 | 1 | 0.001
H1j7 | 573.1C | 3 | 0.004
H1j7 | 573.2C | 5 | 0.006
H1j7 | 573.5C | 1 | 0.001
H1j7 | 7674 | 1 | 0.001
H1j8 | 11440 | 1 | 0.013
H1j8 | 11626 | 1 | 0.013
H1j8 | 12654 | 1 | 0.013
H1j8 | 152 | 3 | 0.039
H1j8 | 16239 | 1 | 0.013
H1j8 | 5355 | 1 | 0.013
H1j8 | 5471 | 2 | 0.026
H1j8 | 7419 | 1 | 0.013
H1j8 | 8047 | 1 | 0.013
H1j9 | 10455 | 2 | 0.002
H1j9 | 113 | 1 | 0.001
H1j9 | 12696 | 1 | 0.001
H1j9 | 143 | 1 | 0.001
H1j9 | 16076A | 1 | 0.001
H1j9 | 16104 | 1 | 0.001
H1j9 | 16111 | 2 | 0.002
H1j9 | 16131 | 1 | 0.001
H1j9 | 16188G | 1 | 0.001
H1j9 | 16284 | 3 | 0.003
H1j9 | 16316 | 18 | 0.019
H1j9 | 16468 | 130 | 0.138
H1j9 | 204 | 5 | 0.005
H1j9 | 207 | 13 | 0.014
H1j9 | 208 | 1 | 0.001
H1j9 | 261 | 1 | 0.001
H1j9 | 281C | 1 | 0.001
H1j9 | 316d | 1 | 0.001
H1j9 | 338 | 1 | 0.001
H1j9 | 502 | 1 | 0.001
H1j9 | 573.1C | 3 | 0.003
H1j9 | 573.2C | 5 | 0.005
H1j9 | 573.5C | 1 | 0.001
H1j9 | 593G | 2 | 0.002
H1k1 | 14298 | 1 | 0.014
H1k1 | 16129 | 3 | 0.041
H1k1 | 16217 | 4 | 0.054
H1k1 | 16233 | 1 | 0.014
H1k1 | 16384 | 1 | 0.014

H1k1a | 146 | 1 | 0.013
H1k1a | 15106 | 1 | 0.013
H1k1a | 152 | 1 | 0.013
H1k1a | 16042T | 1 | 0.013
H1k1a | 16304 | 1 | 0.013
H1k1a | 16384 | 2 | 0.026
H1k1a | 8062 | 1 | 0.013
H1m | 10527 | 1 | 0.001
H1m | 113 | 1 | 0.001
H1m | 11878 | 4 | 0.005
H1m | 12341 | 2 | 0.002
H1m | 14097 | 1 | 0.001
H1m | 14136 | 4 | 0.005
H1m | 146 | 2 | 0.002
H1m | 150 | 7 | 0.009
H1m | 16076A | 1 | 0.001
H1m | 16129 | 1 | 0.001
H1m | 16169 | 124 | 0.154
H1m | 16192 | 2 | 0.002
H1m | 16241 | 124 | 0.154
H1m | 16299 | 4 | 0.005
H1m | 208 | 1 | 0.001
H1m | 2294 | 1 | 0.001
H1m | 261 | 1 | 0.001
H1m | 281C | 1 | 0.001
H1m | 316d | 1 | 0.001
H1m | 338 | 1 | 0.001
H1m | 4136 | 1 | 0.001
H1m | 4204 | 1 | 0.001
H1m | 468 | 3 | 0.004
H1m | 471 | 1 | 0.001
H1m | 4820 | 1 | 0.001
H1m | 502 | 1 | 0.001
H1m | 573.1C | 3 | 0.004
H1m | 573.2C | 5 | 0.006
H1m | 573.5C | 1 | 0.001
H1m | 8281-8289d | 1 | 0.001
H1m | 8645 | 2 | 0.002
H1m | 9604 | 1 | 0.001
H1m1 | 16124 | 1 | 0.016
H1m1 | 16142 | 2 | 0.032
H1m1 | 16153 | 1 | 0.016
H1m1 | 16215 | 2 | 0.032
H1m1 | 16219 | 1 | 0.016
H1m1 | 16234 | 1 | 0.016
H1m1 | 16263A | 1 | 0.016
H1m1 | 16266A | 1 | 0.016
H1m1 | 16290 | 2 | 0.032
H1m1 | 16319 | 1 | 0.016
H1m1 | 16325 | 3 | 0.048
H1m1 | 16348 | 2 | 0.032
H1m1 | 16399 | 4 | 0.063
H1m1 | 16527 | 2 | 0.032
H1m1 | 217 | 1 | 0.016
H1m1 | 460.1T | 1 | 0.016
H1m1 | 515-524d | 1 | 0.016
H1m1 | 5483 | 1 | 0.016
H1m1 | 57 | 1 | 0.016
H1m1 | 573.3C | 1 | 0.016
H1m1 | 9058 | 1 | 0.016
H1m1 | 9655 | 1 | 0.016
H1n | 113 | 1 | 0.001
H1n | 16076A | 123 | 0.155
H1n | 208 | 1 | 0.001
H1n | 261 | 1 | 0.001
H1n | 281C | 1 | 0.001
H1n | 316d | 1 | 0.001
H1n | 338 | 1 | 0.001
H1n | 468 | 3 | 0.004
H1n | 502 | 1 | 0.001
H1n | 573.1C | 3 | 0.004
H1n | 573.2C | 5 | 0.006
H1n | 573.5C | 1 | 0.001
H1n+146 | 10007 | 1 | 0.015
H1n+146 | 10169 | 1 | 0.015
H1n+146 | 14384 | 1 | 0.015
H1n+146 | 153 | 1 | 0.015
H1n+146 | 15436 | 1 | 0.015
H1n+146 | 16124 | 1 | 0.015
H1n+146 | 16153 | 1 | 0.015
H1n+146 | 16172 | 1 | 0.015
H1n+146 | 16209G | 1 | 0.015
H1n+146 | 16215 | 2 | 0.03
H1n+146 | 16219 | 1 | 0.015
H1n+146 | 16234 | 1 | 0.015
H1n+146 | 16263A | 1 | 0.015
H1n+146 | 16266A | 1 | 0.015
H1n+146 | 16290 | 2 | 0.03
H1n+146 | 16319 | 1 | 0.015
H1n+146 | 16348 | 5 | 0.076
H1n+146 | 16527 | 5 | 0.076
H1n+146 | 217 | 1 | 0.015
H1n+146 | 3745 | 1 | 0.015
H1n+146 | 460.1T | 1 | 0.015
H1n+146 | 515-524d | 1 | 0.015
H1n+146 | 57 | 1 | 0.015
H1n+146 | 573.3C | 1 | 0.015
H1n+146 | 9329 | 1 | 0.015
H1n+146 | 9695C | 1 | 0.015
H1n+146+195 | 10169 | 1 | 0.1
H1n+146+195 | 12338 | 1 | 0.1
H1n+146+195 | 14274 | 1 | 0.1
H1n+146+195 | 152 | 2 | 0.2
H1n+146+195 | 15217 | 1 | 0.1
H1n+146+195 | 15804 | 1 | 0.1
H1n+146+195 | 16153 | 1 | 0.1
H1n+146+195 | 16173 | 1 | 0.1
H1n1 | 13980 | 1 | 0.016
H1n1 | 152 | 1 | 0.016
H1n1 | 16086 | 1 | 0.016
H1n1 | 16124 | 1 | 0.016

H1n1 | 16153 | 1 | 0.016
H1n1 | 16215 | 2 | 0.032
H1n1 | 16219 | 1 | 0.016
H1n1 | 16234 | 1 | 0.016
H1n1 | 16263A | 1 | 0.016
H1n1 | 16266A | 1 | 0.016
H1n1 | 16290 | 2 | 0.032
H1n1 | 16319 | 1 | 0.016
H1n1 | 16348 | 2 | 0.032
H1n1 | 16399 | 4 | 0.063
H1n1 | 16527 | 2 | 0.032
H1n1 | 215 | 1 | 0.016
H1n1 | 217 | 1 | 0.016
H1n1 | 310 | 1 | 0.016
H1n1 | 3526 | 1 | 0.016
H1n1 | 460.1T | 1 | 0.016
H1n1 | 515-524d | 1 | 0.016
H1n1 | 5196 | 1 | 0.016
H1n1 | 57 | 1 | 0.016
H1n1 | 573.3C | 1 | 0.016
H1n1 | 6050 | 3 | 0.048
H1n1 | 8634 | 3 | 0.048
H1n1a | 13722 | 1 | 0.016
H1n1a | 16124 | 1 | 0.016
H1n1a | 16153 | 1 | 0.016
H1n1a | 16215 | 2 | 0.031
H1n1a | 16219 | 1 | 0.016
H1n1a | 16234 | 1 | 0.016
H1n1a | 16263A | 1 | 0.016
H1n1a | 16266A | 1 | 0.016
H1n1a | 16270 | 1 | 0.016
H1n1a | 16274 | 2 | 0.031
H1n1a | 16290 | 2 | 0.031
H1n1a | 16319 | 1 | 0.016
H1n1a | 16348 | 2 | 0.031
H1n1a | 16399 | 4 | 0.062
H1n1a | 16527 | 2 | 0.031
H1n1a | 217 | 1 | 0.016
H1n1a | 310 | 1 | 0.016
H1n1a | 3394 | 1 | 0.016
H1n1a | 460.1T | 1 | 0.016
H1n1a | 4736 | 1 | 0.016
H1n1a | 515-524d | 1 | 0.016
H1n1a | 57 | 1 | 0.016
H1n1a | 573.3C | 1 | 0.016
H1n1a | 6297 | 1 | 0.016
H1n1a | 7245 | 1 | 0.016
H1n1a | 8632 | 1 | 0.016
H1n1a | 9899 | 2 | 0.031
H1n1b | 14199 | 1 | 0.028
H1n1b | 16077 | 1 | 0.028
H1n1b | 16142 | 4 | 0.111
H1n1b | 16215 | 3 | 0.083
H1n1b | 16216 | 3 | 0.083
H1n1b | 16223 | 1 | 0.028
H1n1b | 16248 | 2 | 0.056
H1n1b | 16290 | 4 | 0.111
H1n1b | 16325 | 4 | 0.111
H1n1b | 16380.1C | 4 | 0.111
H1n1b | 16387 | 1 | 0.028
H1n1b | 16455C | 1 | 0.028
H1n1b | 16484-16489d | 2 | 0.056
H1n1b | 525.1AC | 1 | 0.028
H1n1b | 8772 | 1 | 0.028
H1n1b | 9932 | 1 | 0.028
H1n2 | 11227 | 2 | 0.036
H1n2 | 152 | 2 | 0.036
H1n2 | 15244 | 1 | 0.018
H1n2 | 16189 | 8 | 0.145
H1n2 | 16270 | 1 | 0.018
H1n2 | 16497 | 4 | 0.073
H1n2 | 200 | 2 | 0.036
H1n2 | 3316 | 1 | 0.018
H1n2 | 573.3C | 1 | 0.018
H1n2 | 64 | 1 | 0.018
H1n2 | 8277 | 2 | 0.036
H1n2 | 8278.1C | 1 | 0.018
H1n2 | 8380 | 5 | 0.091
H1n2 | 8865 | 1 | 0.018
H1n3 | 15650 | 1 | 0.016
H1n3 | 16124 | 1 | 0.016
H1n3 | 16142 | 2 | 0.032
H1n3 | 16153 | 1 | 0.016
H1n3 | 16215 | 2 | 0.032
H1n3 | 16219 | 1 | 0.016
H1n3 | 16234 | 1 | 0.016
H1n3 | 16263A | 1 | 0.016
H1n3 | 16266A | 1 | 0.016
H1n3 | 16290 | 2 | 0.032
H1n3 | 16319 | 1 | 0.016
H1n3 | 16325 | 3 | 0.048
H1n3 | 16348 | 2 | 0.032
H1n3 | 16399 | 4 | 0.065
H1n3 | 16527 | 2 | 0.032
H1n3 | 217 | 1 | 0.016
H1n3 | 460.1T | 1 | 0.016
H1n3 | 515-524d | 1 | 0.016
H1n3 | 57 | 1 | 0.016
H1n3 | 573.3C | 1 | 0.016
H1n3 | 6040 | 1 | 0.016
H1n4 | 1189 | 1 | 0.015
H1n4 | 12188 | 1 | 0.015
H1n4 | 12906 | 1 | 0.015
H1n4 | 13246 | 1 | 0.015
H1n4 | 1413 | 1 | 0.015
H1n4 | 14482 | 1 | 0.015
H1n4 | 14564 | 1 | 0.015
H1n4 | 152 | 1 | 0.015
H1n4 | 1607 | 1 | 0.015
H1n4 | 16111 | 5 | 0.076
H1n4 | 16126 | 1 | 0.015

H1n4 | 16207 | 1 | 0.015
H1n4 | 16362 | 1 | 0.015
H1n4 | 204 | 1 | 0.015
H1n4 | 291.1A | 1 | 0.015
H1n4 | 310 | 1 | 0.015
H1n4 | 312 | 1 | 0.015
H1n4 | 3278 | 1 | 0.015
H1n4 | 366C | 1 | 0.015
H1n4 | 366T | 1 | 0.015
H1n4 | 513 | 1 | 0.015
H1n4 | 5461 | 1 | 0.015
H1n4 | 573.3C | 1 | 0.015
H1n4 | 6518 | 1 | 0.015
H1n4 | 7389 | 1 | 0.015
H1n4 | 7859 | 1 | 0.015
H1n5 | 1040 | 2 | 0.286
H1n5 | 12100 | 2 | 0.286
H1n5 | 7744 | 1 | 0.143
H1n6 | 16042C | 1 | 0.003
H1n6 | 16129T | 1 | 0.003
H1n6 | 16150 | 1 | 0.003
H1n6 | 16188A | 2 | 0.005
H1n6 | 16209 | 16 | 0.042
H1n6 | 16231 | 1 | 0.003
H1n6 | 16293C | 2 | 0.005
H1n6 | 16327 | 3 | 0.008
H1n6 | 16336 | 21 | 0.055
H1n6 | 16505 | 103 | 0.269
H1n6 | 176C | 2 | 0.005
H1n6 | 195 | 1 | 0.003
H1n6 | 252 | 3 | 0.008
H1n6 | 261.1T | 1 | 0.003
H1n6 | 264 | 3 | 0.008
H1n6 | 332G | 1 | 0.003
H1n6 | 456 | 1 | 0.003
H1n6 | 49T | 1 | 0.003
H1n6 | 5029 | 1 | 0.003
H1n6 | 73 | 1 | 0.003
H1n6 | 8084 | 1 | 0.003
H1n6 | 8888 | 1 | 0.003
H1o | 12615 | 1 | 0.023
H1o | 16129 | 2 | 0.047
H1o | 16144 | 1 | 0.023
H1o | 16145 | 7 | 0.163
H1o | 16148 | 1 | 0.023
H1o | 16169 | 1 | 0.023
H1o | 16172 | 1 | 0.023
H1o | 16189 | 1 | 0.023
H1o | 16209 | 1 | 0.023
H1o | 16213 | 1 | 0.023
H1o | 16217 | 1 | 0.023
H1o | 16256 | 1 | 0.023
H1o | 16261 | 1 | 0.023
H1o | 16265 | 1 | 0.023
H1o | 16284 | 2 | 0.047
H1o | 16292 | 1 | 0.023
H1o | 16311 | 11 | 0.256
H1o | 16343 | 7 | 0.163
H1o | 16362 | 1 | 0.023
H1o | 6081T | 1 | 0.023
H1o | 6185 | 1 | 0.023
H1o | 7337T | 1 | 0.023
H1o | 8349 | 2 | 0.047
H1p | 113 | 1 | 0.001
H1p | 15773 | 1 | 0.001
H1p | 16076A | 123 | 0.155
H1p | 208 | 1 | 0.001
H1p | 261 | 1 | 0.001
H1p | 281C | 1 | 0.001
H1p | 316d | 1 | 0.001
H1p | 338 | 1 | 0.001
H1p | 468 | 3 | 0.004
H1p | 502 | 1 | 0.001
H1p | 573.1C | 3 | 0.004
H1p | 573.2C | 5 | 0.006
H1p | 573.5C | 1 | 0.001
H1p | 9545 | 1 | 0.001
H1q | 10043 | 1 | 0.001
H1q | 10373 | 1 | 0.001
H1q | 10493 | 1 | 0.001
H1q | 10586 | 1 | 0.001
H1q | 10657G | 1 | 0.001
H1q | 10658T | 1 | 0.001
H1q | 11087 | 1 | 0.001
H1q | 113 | 1 | 0.001
H1q | 12070 | 2 | 0.002
H1q | 13235.1T | 1 | 0.001
H1q | 1345 | 1 | 0.001
H1q | 14016 | 1 | 0.001
H1q | 14569 | 1 | 0.001
H1q | 152 | 1 | 0.001
H1q | 15301 | 1 | 0.001
H1q | 15314 | 1 | 0.001
H1q | 1555 | 1 | 0.001
H1q | 15954 | 1 | 0.001
H1q | 16023T | 1 | 0.001
H1q | 16076A | 1 | 0.001
H1q | 16093 | 1 | 0.001
H1q | 16129 | 2 | 0.002
H1q | 16172 | 1 | 0.001
H1q | 16173 | 3 | 0.004
H1q | 16188A | 6 | 0.007
H1q | 16188G | 15 | 0.018
H1q | 16189 | 3 | 0.004
H1q | 16209 | 124 | 0.151
H1q | 16311 | 124 | 0.151
H1q | 16319 | 1 | 0.001
H1q | 16356 | 3 | 0.004
H1q | 1780 | 1 | 0.001
H1q | 201 | 1 | 0.001
H1q | 204 | 1 | 0.001

H1q | 208 | 1 | 0.001
H1q | 249d | 1 | 0.001
H1q | 261 | 1 | 0.001
H1q | 281C | 1 | 0.001
H1q | 3144 | 1 | 0.001
H1q | 316d | 1 | 0.001
H1q | 3244 | 1 | 0.001
H1q | 338 | 1 | 0.001
H1q | 3447 | 1 | 0.001
H1q | 3483 | 1 | 0.001
H1q | 4148 | 1 | 0.001
H1q | 4688 | 1 | 0.001
H1q | 4973 | 1 | 0.001
H1q | 502 | 1 | 0.001
H1q | 5263 | 1 | 0.001
H1q | 573.1C | 3 | 0.004
H1q | 573.2C | 5 | 0.006
H1q | 573.5C | 1 | 0.001
H1q | 593 | 1 | 0.001
H1q | 6101 | 1 | 0.001
H1q | 6287 | 2 | 0.002
H1q | 6366C | 3 | 0.004
H1q | 6481 | 1 | 0.001
H1q | 7163 | 1 | 0.001
H1q | 8835 | 2 | 0.002
H1q | 8839 | 1 | 0.001
H1q | 9055 | 1 | 0.001
H1q | 9299 | 1 | 0.001
H1q | 9438 | 1 | 0.001
H1q | 9948 | 1 | 0.001
H1q1 | 113 | 1 | 0.001
H1q1 | 16076A | 1 | 0.001
H1q1 | 16209 | 9 | 0.011
H1q1 | 16311 | 123 | 0.153
H1q1 | 208 | 1 | 0.001
H1q1 | 261 | 1 | 0.001
H1q1 | 281C | 1 | 0.001
H1q1 | 316d | 1 | 0.001
H1q1 | 338 | 1 | 0.001
H1q1 | 468 | 3 | 0.004
H1q1 | 4985 | 1 | 0.001
H1q1 | 502 | 1 | 0.001
H1q1 | 573.1C | 3 | 0.004
H1q1 | 573.2C | 5 | 0.006
H1q1 | 573.5C | 1 | 0.001
H1q1 | 8289.1CCCCCTCTA | 1 | 0.001
H1q1a | 13752 | 1 | 0.01
H1q1a | 16072 | 1 | 0.01
H1q1a | 16209 | 1 | 0.01
H1q1a | 16233 | 1 | 0.01
H1q1a | 16261 | 1 | 0.01
H1q1a | 16274 | 1 | 0.01
H1q1a | 16311 | 1 | 0.01
H1q1a | 16354 | 1 | 0.01
H1q1a | 16512 | 1 | 0.01
H1q1a | 2119 | 1 | 0.01
H1q1a | 316C | 1 | 0.01
H1q1a | 372 | 1 | 0.01
H1q1a | 443C | 1 | 0.01
H1q1a | 573.2C | 1 | 0.01
H1q1a | 72A | 1 | 0.01
H1q1a | 75 | 1 | 0.01
H1q1a | 8290.1G | 1 | 0.01
H1q2 | 11288 | 1 | 0.001
H1q2 | 113 | 1 | 0.001
H1q2 | 16076A | 123 | 0.162
H1q2 | 16129 | 2 | 0.003
H1q2 | 208 | 1 | 0.001
H1q2 | 261 | 1 | 0.001
H1q2 | 281C | 1 | 0.001
H1q2 | 316 | 2 | 0.003
H1q2 | 316d | 1 | 0.001
H1q2 | 3290G | 1 | 0.001
H1q2 | 338 | 1 | 0.001
H1q2 | 468 | 3 | 0.004
H1q2 | 502 | 1 | 0.001
H1q2 | 573.1C | 2 | 0.003
H1q2 | 573.2C | 4 | 0.005
H1q2 | 573.5C | 1 | 0.001
H1q2 | 8462 | 1 | 0.001
H1q2 | 9856 | 1 | 0.001
H1q3 | 1009 | 1 | 0.009
H1q3 | 11266 | 1 | 0.009
H1q3 | 146 | 1 | 0.009
H1q3 | 16014G | 1 | 0.009
H1q3 | 16025.1T | 1 | 0.009
H1q3 | 16067 | 1 | 0.009
H1q3 | 16075 | 1 | 0.009
H1q3 | 16077 | 1 | 0.009
H1q3 | 16140 | 2 | 0.019
H1q3 | 16166d | 1 | 0.009
H1q3 | 16189 | 2 | 0.019
H1q3 | 16211 | 1 | 0.009
H1q3 | 16283 | 1 | 0.009
H1q3 | 16286 | 2 | 0.019
H1q3 | 16287 | 3 | 0.028
H1q3 | 16304G | 2 | 0.019
H1q3 | 16357 | 1 | 0.009
H1q3 | 16508 | 1 | 0.009
H1q3 | 16509 | 1 | 0.009
H1q3 | 185 | 1 | 0.009
H1q3 | 270C | 1 | 0.009
H1q3 | 309d | 1 | 0.009
H1q3 | 3511 | 3 | 0.028
H1q3 | 390 | 1 | 0.009
H1q3 | 4084 | 1 | 0.009
H1q3 | 5237 | 1 | 0.009
H1q3 | 56.1A | 1 | 0.009
H1q3 | 9948 | 1 | 0.009
H1r | 10187 | 1 | 0.001
H1r | 113 | 1 | 0.001

H1r | 16076A | 123 | 0.155
H1r | 208 | 1 | 0.001
H1r | 261 | 1 | 0.001
H1r | 281C | 1 | 0.001
H1r | 316d | 1 | 0.001
H1r | 338 | 1 | 0.001
H1r | 468 | 3 | 0.004
H1r | 502 | 1 | 0.001
H1r | 573.1C | 3 | 0.004
H1r | 573.2C | 5 | 0.006
H1r | 573.5C | 1 | 0.001
H1r | 678 | 1 | 0.001
H1r1 | 10187 | 1 | 0.029
H1r1 | 10885 | 2 | 0.057
H1r1 | 16170T | 2 | 0.057
H1r1 | 16189 | 1 | 0.029
H1r1 | 16245 | 3 | 0.086
H1r1 | 16256 | 2 | 0.057
H1r1 | 16311 | 2 | 0.057
H1s | 113 | 1 | 0.001
H1s | 16076A | 1 | 0.001
H1s | 16243 | 123 | 0.154
H1s | 208 | 1 | 0.001
H1s | 261 | 1 | 0.001
H1s | 281C | 1 | 0.001
H1s | 310 | 1 | 0.001
H1s | 316d | 1 | 0.001
H1s | 338 | 1 | 0.001
H1s | 468 | 3 | 0.004
H1s | 502 | 1 | 0.001
H1s | 573.1C | 3 | 0.004
H1s | 573.2C | 5 | 0.006
H1s | 573.5C | 1 | 0.001
H1s1 | 113 | 1 | 0.001
H1s1 | 16076A | 123 | 0.154
H1s1 | 208 | 1 | 0.001
H1s1 | 261 | 1 | 0.001
H1s1 | 281C | 1 | 0.001
H1s1 | 310 | 1 | 0.001
H1s1 | 316d | 1 | 0.001
H1s1 | 338 | 1 | 0.001
H1s1 | 468 | 3 | 0.004
H1s1 | 502 | 1 | 0.001
H1s1 | 5563 | 4 | 0.005
H1s1 | 573.1C | 3 | 0.004
H1s1 | 573.2C | 5 | 0.006
H1s1 | 573.5C | 1 | 0.001
H1s1 | 709 | 3 | 0.004
H1s1 | 8574 | 1 | 0.001
H1t | 10680 | 1 | 0.001
H1t | 113 | 1 | 0.001
H1t | 12645 | 1 | 0.001
H1t | 1414 | 1 | 0.001
H1t | 15613 | 1 | 0.001
H1t | 16076A | 1 | 0.001
H1t | 16222 | 124 | 0.155
H1t | 195 | 1 | 0.001
H1t | 208 | 1 | 0.001
H1t | 2141 | 1 | 0.001
H1t | 261 | 1 | 0.001
H1t | 281C | 1 | 0.001
H1t | 316d | 1 | 0.001
H1t | 338 | 1 | 0.001
H1t | 3447 | 1 | 0.001
H1t | 468 | 3 | 0.004
H1t | 502 | 1 | 0.001
H1t | 573.1C | 3 | 0.004
H1t | 573.2C | 5 | 0.006
H1t | 573.5C | 1 | 0.001
H1t | 6167 | 1 | 0.001
H1t | 6293 | 1 | 0.001
H1t | 8093 | 1 | 0.001
H1t | 95C | 1 | 0.001
H1t | 9911A | 1 | 0.001
H1t1 | 113 | 1 | 0.001
H1t1 | 16076A | 123 | 0.154
H1t1 | 208 | 1 | 0.001
H1t1 | 261 | 1 | 0.001
H1t1 | 281C | 1 | 0.001
H1t1 | 316d | 1 | 0.001
H1t1 | 338 | 1 | 0.001
H1t1 | 468 | 3 | 0.004
H1t1 | 502 | 1 | 0.001
H1t1 | 573.1C | 3 | 0.004
H1t1 | 573.2C | 5 | 0.006
H1t1 | 573.5C | 1 | 0.001
H1t1a | 113 | 1 | 0.001
H1t1a | 12358 | 1 | 0.001
H1t1a | 12372 | 2 | 0.002
H1t1a | 12831 | 1 | 0.001
H1t1a | 13145 | 1 | 0.001
H1t1a | 14130 | 2 | 0.002
H1t1a | 152 | 1 | 0.001
H1t1a | 16076A | 1 | 0.001
H1t1a | 16148 | 1 | 0.001
H1t1a | 16311 | 127 | 0.154
H1t1a | 1700 | 1 | 0.001
H1t1a | 1819 | 1 | 0.001
H1t1a | 208 | 1 | 0.001
H1t1a | 261 | 1 | 0.001
H1t1a | 281C | 1 | 0.001
H1t1a | 316d | 1 | 0.001
H1t1a | 338 | 1 | 0.001
H1t1a | 3897A | 1 | 0.001
H1t1a | 41 | 2 | 0.002
H1t1a | 444 | 1 | 0.001
H1t1a | 502 | 1 | 0.001
H1t1a | 5238 | 3 | 0.004
H1t1a | 573.1C | 3 | 0.004
H1t1a | 573.2C | 5 | 0.006
H1t1a | 573.5C | 1 | 0.001

H1t1a | 5930 | 1 | 0.001
H1t1a | 6023 | 1 | 0.001
H1t1a | 7805 | 3 | 0.004
H1t1a | 7853 | 1 | 0.001
H1t1a | 9025 | 1 | 0.001
H1t1a1 | 146 | 1 | 0.01
H1t1a1 | 15930 | 1 | 0.01
H1t1a1 | 16032d | 2 | 0.021
H1t1a1 | 16032G | 1 | 0.01
H1t1a1 | 16042 | 1 | 0.01
H1t1a1 | 16042d | 2 | 0.021
H1t1a1 | 16056 | 1 | 0.01
H1t1a1 | 16070C | 1 | 0.01
H1t1a1 | 16110d | 3 | 0.031
H1t1a1 | 16140 | 2 | 0.021
H1t1a1 | 16189 | 3 | 0.031
H1t1a1 | 16267 | 2 | 0.021
H1t1a1 | 16268 | 2 | 0.021
H1t1a1 | 9398 | 1 | 0.01
H1t1a1 | 9482 | 7 | 0.073
H1t2 | 113 | 1 | 0.001
H1t2 | 16076A | 123 | 0.154
H1t2 | 16291 | 1 | 0.001
H1t2 | 208 | 1 | 0.001
H1t2 | 261 | 1 | 0.001
H1t2 | 281C | 1 | 0.001
H1t2 | 316d | 1 | 0.001
H1t2 | 338 | 1 | 0.001
H1t2 | 468 | 3 | 0.004
H1t2 | 502 | 1 | 0.001
H1t2 | 569 | 1 | 0.001
H1t2 | 573.1C | 3 | 0.004
H1t2 | 573.2C | 5 | 0.006
H1t2 | 573.5C | 1 | 0.001
H1t2 | 8393 | 1 | 0.001
H1u | 10535 | 5 | 0.006
H1u | 10667 | 1 | 0.001
H1u | 113 | 1 | 0.001
H1u | 11656 | 1 | 0.001
H1u | 12189 | 1 | 0.001
H1u | 12241d | 1 | 0.001
H1u | 12984 | 5 | 0.006
H1u | 14226 | 1 | 0.001
H1u | 15082 | 1 | 0.001
H1u | 16076A | 1 | 0.001
H1u | 16166 | 1 | 0.001
H1u | 16300 | 123 | 0.153
H1u | 1819 | 5 | 0.006
H1u | 185 | 1 | 0.001
H1u | 188 | 1 | 0.001
H1u | 208 | 1 | 0.001
H1u | 261 | 1 | 0.001
H1u | 281C | 1 | 0.001
H1u | 316d | 1 | 0.001
H1u | 3337 | 1 | 0.001
H1u | 338 | 1 | 0.001
H1u | 3535 | 1 | 0.001
H1u | 468 | 3 | 0.004
H1u | 502 | 1 | 0.001
H1u | 5162 | 1 | 0.001
H1u | 573.1C | 3 | 0.004
H1u | 573.2C | 5 | 0.006
H1u | 573.5C | 1 | 0.001
H1u | 6425 | 1 | 0.001
H1u | 7119 | 1 | 0.001
H1u | 7897 | 6 | 0.007
H1u | 8014 | 1 | 0.001
H1u | 8311 | 1 | 0.001
H1u | 9531 | 1 | 0.001
H1u | 980 | 6 | 0.007
H1u | 9950 | 1 | 0.001
H1u1 | 11113 | 1 | 0.001
H1u1 | 11260 | 1 | 0.001
H1u1 | 113 | 1 | 0.001
H1u1 | 146 | 1 | 0.001
H1u1 | 15916 | 1 | 0.001
H1u1 | 16068 | 3 | 0.004
H1u1 | 16076A | 123 | 0.152
H1u1 | 16311 | 1 | 0.001
H1u1 | 16362 | 1 | 0.001
H1u1 | 208 | 1 | 0.001
H1u1 | 261 | 1 | 0.001
H1u1 | 281C | 1 | 0.001
H1u1 | 316d | 1 | 0.001
H1u1 | 338 | 1 | 0.001
H1u1 | 468 | 3 | 0.004
H1u1 | 502 | 1 | 0.001
H1u1 | 573.1C | 3 | 0.004
H1u1 | 573.2C | 5 | 0.006
H1u1 | 573.5C | 1 | 0.001
H1u1 | 8566 | 1 | 0.001
H1u1 | 8589 | 1 | 0.001
H1u2 | 10586 | 1 | 0.012
H1u2 | 131 | 1 | 0.012
H1u2 | 13708 | 1 | 0.012
H1u2 | 14766 | 1 | 0.012
H1u2 | 14767 | 1 | 0.012
H1u2 | 15519G | 1 | 0.012
H1u2 | 16104 | 1 | 0.012
H1u2 | 16216 | 1 | 0.012
H1u2 | 16266 | 1 | 0.012
H1u2 | 16337G | 1 | 0.012
H1u2 | 1643 | 1 | 0.012
H1u2 | 1764 | 1 | 0.012
H1u2 | 456 | 1 | 0.012
H1u2 | 5093 | 1 | 0.012
H1u2 | 8573 | 2 | 0.023
H1u2 | 8634 | 1 | 0.012
H1u2 | 9563 | 1 | 0.012
H1v | 10101 | 8 | 0.01
H1v | 113 | 1 | 0.001

H1v | 146 | 8 | 0.01
H1v | 16076A | 123 | 0.153
H1v | 208 | 1 | 0.001
H1v | 261 | 1 | 0.001
H1v | 281C | 1 | 0.001
H1v | 310 | 1 | 0.001
H1v | 316d | 1 | 0.001
H1v | 338 | 1 | 0.001
H1v | 4092 | 1 | 0.001
H1v | 468 | 3 | 0.004
H1v | 502 | 1 | 0.001
H1v | 573.1C | 3 | 0.004
H1v | 573.2C | 5 | 0.006
H1v | 573.5C | 1 | 0.001
H1v | 745 | 1 | 0.001
H1v1 | 113 | 1 | 0.001
H1v1 | 16076A | 123 | 0.155
H1v1 | 208 | 1 | 0.001
H1v1 | 261 | 1 | 0.001
H1v1 | 281C | 1 | 0.001
H1v1 | 316d | 1 | 0.001
H1v1 | 338 | 1 | 0.001
H1v1 | 468 | 3 | 0.004
H1v1 | 502 | 1 | 0.001
H1v1 | 573.1C | 3 | 0.004
H1v1 | 573.2C | 5 | 0.006
H1v1 | 573.5C | 1 | 0.001
H1v1a | 113 | 1 | 0.001
H1v1a | 16076A | 123 | 0.154
H1v1a | 208 | 1 | 0.001
H1v1a | 261 | 1 | 0.001
H1v1a | 281C | 1 | 0.001
H1v1a | 316d | 1 | 0.001
H1v1a | 338 | 1 | 0.001
H1v1a | 468 | 3 | 0.004
H1v1a | 502 | 1 | 0.001
H1v1a | 573.1C | 3 | 0.004
H1v1a | 573.2C | 5 | 0.006
H1v1a | 573.5C | 1 | 0.001
H1v1b | 113 | 1 | 0.001
H1v1b | 16076A | 123 | 0.155
H1v1b | 208 | 1 | 0.001
H1v1b | 261 | 1 | 0.001
H1v1b | 281C | 1 | 0.001
H1v1b | 316d | 1 | 0.001
H1v1b | 338 | 1 | 0.001
H1v1b | 468 | 3 | 0.004
H1v1b | 502 | 1 | 0.001
H1v1b | 573.1C | 3 | 0.004
H1v1b | 573.2C | 5 | 0.006
H1v1b | 573.5C | 1 | 0.001
H1w | 113 | 1 | 0.001
H1w | 16076A | 123 | 0.154
H1w | 208 | 1 | 0.001
H1w | 261 | 1 | 0.001
H1w | 281C | 1 | 0.001
H1w | 316d | 1 | 0.001
H1w | 338 | 1 | 0.001
H1w | 468 | 3 | 0.004
H1w | 502 | 1 | 0.001
H1w | 573.1C | 3 | 0.004
H1w | 573.2C | 5 | 0.006
H1w | 573.5C | 1 | 0.001
H1w | 748 | 1 | 0.001
H1x | 13759 | 1 | 0.014
H1x | 16213 | 1 | 0.014
H1x | 9 | 1 | 0.014
H1y | 11204 | 1 | 0.005
H1y | 12354 | 2 | 0.01
H1y | 13905 | 1 | 0.005
H1y | 15153 | 1 | 0.005
H1y | 16086 | 1 | 0.005
H1y | 16111 | 1 | 0.005
H1y | 16150 | 1 | 0.005
H1y | 16193 | 1 | 0.005
H1y | 16233 | 1 | 0.005
H1y | 16243 | 19 | 0.09
H1y | 16319 | 1 | 0.005
H1y | 195 | 2 | 0.01
H1y | 2619 | 1 | 0.005
H1y | 297 | 19 | 0.09
H1y | 310 | 1 | 0.005
H1y | 315.2C | 1 | 0.005
H1y | 319.1T | 1 | 0.005
H1y | 480 | 1 | 0.005
H1y | 499 | 19 | 0.09
H1y | 554T | 1 | 0.005
H1y | 567T | 1 | 0.005
H1y | 569A | 1 | 0.005
H1y | 5960 | 1 | 0.005
H1y | 6587 | 2 | 0.01
H1y | 7055 | 2 | 0.01
H1y | 73 | 1 | 0.005
H1y | 7930 | 2 | 0.01
H1z | 14110 | 1 | 0.011
H1z | 16104 | 1 | 0.011
H1z | 16131 | 1 | 0.011
H1z | 16215 | 1 | 0.011
H1z | 16218.1T | 1 | 0.011
H1z | 16266 | 1 | 0.011
H1z | 16284 | 7 | 0.079
H1z | 16286 | 7 | 0.079
H1z | 16325 | 1 | 0.011
H1z | 16365 | 1 | 0.011
H1z | 207 | 6 | 0.067
H1z | 3009 | 1 | 0.011
H1z | 6620 | 2 | 0.022
H1z1 | 10115 | 7 | 0.219
H1z1 | 15930 | 1 | 0.031
H1z1 | 16075 | 1 | 0.031
H1z1 | 16218 | 2 | 0.062

H1z1 | 16240 | 1 | 0.031
H1z1 | 16249 | 1 | 0.031
H1z1 | 180 | 1 | 0.031
H1z1 | 227 | 1 | 0.031
H1z1 | 2380 | 2 | 0.062
H1z1 | 308-309d | 1 | 0.031
H1z1 | 309d | 1 | 0.031
H1z1 | 310 | 4 | 0.125
H1z1 | 312 | 1 | 0.031
H1z1 | 315d | 1 | 0.031
H1z1 | 5595 | 1 | 0.031
H1z1 | 7685 | 1 | 0.031
H1z1 | 93 | 1 | 0.031
H2 | 10325 | 1 | 0.001
H2 | 113 | 1 | 0.001
H2 | 11362 | 1 | 0.001
H2 | 12127 | 1 | 0.001
H2 | 12397 | 1 | 0.001
H2 | 12561 | 1 | 0.001
H2 | 14470A | 1 | 0.001
H2 | 15319 | 1 | 0.001
H2 | 16076A | 123 | 0.154
H2 | 16189 | 1 | 0.001
H2 | 16270 | 1 | 0.001
H2 | 208 | 1 | 0.001
H2 | 261 | 1 | 0.001
H2 | 2772 | 1 | 0.001
H2 | 281C | 1 | 0.001
H2 | 316d | 1 | 0.001
H2 | 338 | 1 | 0.001
H2 | 468 | 3 | 0.004
H2 | 477 | 1 | 0.001
H2 | 502 | 1 | 0.001
H2 | 573.1C | 3 | 0.004
H2 | 573.2C | 5 | 0.006
H2 | 573.5C | 1 | 0.001
H2 | 6311 | 1 | 0.001
H2 | 8129 | 1 | 0.001
H2 | 8485 | 1 | 0.001
H2+152_16311 | 11017 | 2 | 0.011
H2+152_16311 | 12017 | 1 | 0.006
H2+152_16311 | 1290 | 2 | 0.011
H2+152_16311 | 1438 | 6 | 0.034
H2+152_16311 | 146 | 4 | 0.023
H2+152_16311 | 151 | 7 | 0.04
H2+152_16311 | 16051 | 2 | 0.011
H2+152_16311 | 16082 | 2 | 0.011
H2+152_16311 | 16093 | 1 | 0.006
H2+152_16311 | 16145 | 1 | 0.006
H2+152_16311 | 16154 | 1 | 0.006
H2+152_16311 | 16256 | 1 | 0.006
H2+152_16311 | 16266 | 1 | 0.006
H2+152_16311 | 16286 | 2 | 0.011
H2+152_16311 | 16343 | 1 | 0.006
H2+152_16311 | 16356 | 2 | 0.011
H2+152_16311 | 16399 | 1 | 0.006
H2+152_16311 | 16497 | 1 | 0.006
H2+152_16311 | 1719 | 1 | 0.006
H2+152_16311 | 198 | 1 | 0.006
H2+152_16311 | 2083 | 1 | 0.006
H2+152_16311 | 280 | 2 | 0.011
H2+152_16311 | 310 | 1 | 0.006
H2+152_16311 | 320 | 1 | 0.006
H2+152_16311 | 343 | 1 | 0.006
H2+152_16311 | 3627 | 2 | 0.011
H2+152_16311 | 5105 | 1 | 0.006
H2+152_16311 | 573d | 1 | 0.006
H2+152_16311 | 6045 | 1 | 0.006
H2+152_16311 | 7118 | 1 | 0.006
H2+152_16311 | 73 | 1 | 0.006
H2+152_16311 | 7521 | 1 | 0.006
H2+152_16311 | 8269 | 1 | 0.006
H20 | 13781 | 1 | 0.012
H20 | 13816 | 1 | 0.012
H20 | 14207 | 1 | 0.012
H20 | 15226 | 1 | 0.012
H20 | 15244 | 2 | 0.025
H20 | 15265 | 1 | 0.012
H20 | 15908 | 1 | 0.012
H20 | 16093 | 3 | 0.038
H20 | 16179 | 1 | 0.012
H20 | 16209 | 1 | 0.012
H20 | 16219 | 1 | 0.012
H20 | 16220 | 1 | 0.012
H20 | 16242A | 1 | 0.012
H20 | 16309 | 5 | 0.062
H20 | 16318T | 1 | 0.012
H20 | 16390 | 1 | 0.012
H20 | 1717 | 1 | 0.012
H20 | 200 | 1 | 0.012
H20 | 236 | 1 | 0.012
H20 | 309.3C | 1 | 0.012
H20 | 310 | 1 | 0.012
H20 | 315d | 1 | 0.012
H20 | 3423 | 1 | 0.012
H20 | 4703 | 1 | 0.012
H20 | 6002 | 2 | 0.025
H20 | 6039 | 1 | 0.012
H20 | 6221 | 1 | 0.012
H20 | 6261 | 1 | 0.012
H20 | 6299 | 1 | 0.012
H20 | 6366 | 1 | 0.012
H20 | 6852 | 1 | 0.012
H20 | 8347 | 1 | 0.012
H20 | 8705 | 1 | 0.012
H20 | 8723 | 1 | 0.012
H20 | 93 | 1 | 0.012
H20 | 9449 | 1 | 0.012
H20a | 10978 | 2 | 0.026
H20a | 11016 | 3 | 0.039
H20a | 15490 | 2 | 0.026

H20a | 16000 | 3 | 0.039
H20a | 16066 | 3 | 0.039
H20a | 16150 | 2 | 0.026
H20a | 16172 | 3 | 0.039
H20a | 16181 | 2 | 0.026
H20a | 16318C | 3 | 0.039
H20a | 1763 | 3 | 0.039
H20a | 195 | 3 | 0.039
H20a | 198 | 3 | 0.039
H20a | 3308 | 1 | 0.013
H20a | 73 | 2 | 0.026
H20a | 7819A | 3 | 0.039
H20a1 | 103 | 1 | 0.048
H20a1 | 11084 | 1 | 0.048
H20a1 | 13105 | 1 | 0.048
H20a1 | 13344 | 1 | 0.048
H20a1 | 13547 | 1 | 0.048
H20a1 | 14831 | 1 | 0.048
H20a1 | 152 | 2 | 0.095
H20a1 | 15852 | 1 | 0.048
H20a1 | 15894 | 1 | 0.048
H20a1 | 15939 | 1 | 0.048
H20a1 | 16037 | 1 | 0.048
H20a1 | 16150 | 1 | 0.048
H20a1 | 16243 | 1 | 0.048
H20a1 | 16256 | 1 | 0.048
H20a1 | 16260 | 2 | 0.095
H20a1 | 16284 | 1 | 0.048
H20a1 | 235 | 1 | 0.048
H20a1 | 292 | 1 | 0.048
H20a1 | 4395 | 1 | 0.048
H20a1 | 4655 | 1 | 0.048
H20a1 | 489 | 1 | 0.048
H20a1 | 5108 | 1 | 0.048
H20a1 | 5471 | 1 | 0.048
H20a1 | 9300 | 1 | 0.048
H20a1 | 9656 | 1 | 0.048
H20a1a | 10955 | 1 | 0.033
H20a1a | 11017 | 1 | 0.033
H20a1a | 11290 | 1 | 0.033
H20a1a | 11353 | 1 | 0.033
H20a1a | 12172 | 1 | 0.033
H20a1a | 12579 | 2 | 0.067
H20a1a | 14553 | 1 | 0.033
H20a1a | 14978 | 1 | 0.033
H20a1a | 15115 | 1 | 0.033
H20a1a | 15172 | 1 | 0.033
H20a1a | 15221 | 1 | 0.033
H20a1a | 15833 | 1 | 0.033
H20a1a | 15924 | 1 | 0.033
H20a1a | 2386 | 1 | 0.033
H20a1a | 2757 | 1 | 0.033
H20a1a | 3277 | 2 | 0.067
H20a1a | 3666 | 1 | 0.033
H20a1a | 4012 | 1 | 0.033
H20a1a | 4562 | 1 | 0.033
H20a1a | 5618 | 1 | 0.033
H20a1a | 573.1C | 1 | 0.033
H20a1a | 5899.1C | 1 | 0.033
H20a1a | 6367 | 1 | 0.033
H20a1a | 6671 | 1 | 0.033
H20a1a | 6869 | 1 | 0.033
H20a1a | 7025 | 1 | 0.033
H20a1a | 73 | 1 | 0.033
H20a1a | 7999 | 1 | 0.033
H20a1a | 8618 | 1 | 0.033
H20a1a | 8715 | 1 | 0.033
H20a1a | 8940 | 1 | 0.033
H20a1a | 9305 | 1 | 0.033
H20a1a | 9391 | 1 | 0.033
H20a2 | 11518 | 1 | 0.013
H20a2 | 12562G | 1 | 0.013
H20a2 | 12906 | 3 | 0.039
H20a2 | 14750 | 2 | 0.026
H20a2 | 152 | 2 | 0.026
H20a2 | 15448A | 2 | 0.026
H20a2 | 16192 | 1 | 0.013
H20a2 | 16362 | 1 | 0.013
H20a2 | 16390 | 2 | 0.026
H20a2 | 251 | 1 | 0.013
H20a2 | 4216 | 1 | 0.013
H20a2 | 6791 | 2 | 0.026
H20a2 | 7388 | 1 | 0.013
H20a2 | 7961 | 2 | 0.026
H20a2 | 8167 | 1 | 0.013
H20a2 | 8842T | 1 | 0.013
H20a2 | 9300 | 2 | 0.026
H20a2 | 9891 | 1 | 0.013
H20b | 143 | 2 | 0.023
H20b | 152 | 3 | 0.035
H20b | 15924 | 1 | 0.012
H20b | 16145 | 3 | 0.035
H20b | 16150 | 3 | 0.035
H20b | 16187A | 1 | 0.012
H20b | 16189 | 3 | 0.035
H20b | 16291 | 5 | 0.058
H20b | 16303 | 1 | 0.012
H20b | 16311 | 4 | 0.047
H20b | 16354 | 1 | 0.012
H20b | 16545A | 1 | 0.012
H20b | 188 | 1 | 0.012
H20b | 203 | 1 | 0.012
H20b | 204 | 1 | 0.012
H20b | 8289.1CCCCCTCTA | 1 | 0.012
H20b | 93 | 2 | 0.023
H20c | 10248 | 8 | 0.096
H20c | 16179 | 1 | 0.012
H20c | 16217 | 6 | 0.072
H20c | 16219 | 1 | 0.012
H20c | 16220 | 1 | 0.012
H20c | 16266 | 1 | 0.012

H20c | 195 | 2 | 0.024
H20c | 267 | 1 | 0.012
H20c | 334 | 1 | 0.012
H20c | 4135 | 2 | 0.024
H20c | 4204 | 2 | 0.024
H20c | 6891 | 2 | 0.024
H20c | 8300 | 2 | 0.024
H21 | 16022.1T | 1 | 0.038
H21 | 16025.1T | 1 | 0.038
H21 | 16031d | 1 | 0.038
H21 | 16038d | 1 | 0.038
H21 | 16047.1T | 1 | 0.038
H21 | 16162 | 4 | 0.154
H21 | 16201 | 2 | 0.077
H21 | 16209 | 1 | 0.038
H21 | 16292 | 2 | 0.077
H21 | 16295 | 1 | 0.038
H21 | 16304G | 1 | 0.038
H21 | 16356 | 3 | 0.115
H21 | 16422A | 1 | 0.038
H21 | 709 | 1 | 0.038
H22 | 93 | 1 | 0.015
H23 | 10101 | 1 | 0.001
H23 | 113 | 1 | 0.001
H23 | 11962 | 1 | 0.001
H23 | 12033 | 2 | 0.002
H23 | 13886 | 1 | 0.001
H23 | 14180 | 1 | 0.001
H23 | 146 | 1 | 0.001
H23 | 1555 | 1 | 0.001
H23 | 16076A | 1 | 0.001
H23 | 16318 | 125 | 0.154
H23 | 208 | 1 | 0.001
H23 | 214 | 1 | 0.001
H23 | 261 | 1 | 0.001
H23 | 281C | 1 | 0.001
H23 | 310 | 2 | 0.002
H23 | 316d | 1 | 0.001
H23 | 338 | 1 | 0.001
H23 | 3736 | 1 | 0.001
H23 | 468 | 3 | 0.004
H23 | 502 | 1 | 0.001
H23 | 573.1C | 3 | 0.004
H23 | 573.2C | 5 | 0.006
H23 | 573.5C | 1 | 0.001
H23 | 5999 | 1 | 0.001
H23 | 72 | 1 | 0.001
H23 | 742 | 1 | 0.001
H24 | 1007 | 1 | 0.009
H24 | 11194 | 1 | 0.009
H24 | 12192 | 1 | 0.009
H24 | 13281 | 1 | 0.009
H24 | 13392 | 1 | 0.009
H24 | 14374 | 3 | 0.027
H24 | 146 | 3 | 0.027
H24 | 14766 | 2 | 0.018
H24 | 16148 | 1 | 0.009
H24 | 16213 | 5 | 0.045
H24 | 16255 | 1 | 0.009
H24 | 16274 | 3 | 0.027
H24 | 189 | 1 | 0.009
H24 | 2418 | 1 | 0.009
H24 | 2757 | 1 | 0.009
H24 | 3355 | 1 | 0.009
H24 | 573.1C | 1 | 0.009
H24 | 73 | 1 | 0.009
H24 | 8242 | 1 | 0.009
H24 | 9371 | 7 | 0.062
H24a | 10289 | 1 | 0.007
H24a | 10398 | 1 | 0.007
H24a | 10550 | 1 | 0.007
H24a | 11778 | 2 | 0.015
H24a | 11800 | 1 | 0.007
H24a | 13104 | 1 | 0.007
H24a | 13105 | 1 | 0.007
H24a | 13473 | 1 | 0.007
H24a | 13635 | 1 | 0.007
H24a | 13967 | 1 | 0.007
H24a | 14167 | 1 | 0.007
H24a | 14553 | 2 | 0.015
H24a | 14560 | 1 | 0.007
H24a | 14574 | 1 | 0.007
H24a | 146 | 8 | 0.058
H24a | 14798 | 1 | 0.007
H24a | 150 | 4 | 0.029
H24a | 15133 | 1 | 0.007
H24a | 152 | 9 | 0.066
H24a | 15257 | 1 | 0.007
H24a | 15394 | 2 | 0.015
H24a | 1555 | 5 | 0.036
H24a | 15670 | 1 | 0.007
H24a | 15727 | 1 | 0.007
H24a | 15766 | 3 | 0.022
H24a | 16077 | 1 | 0.007
H24a | 16079G | 1 | 0.007
H24a | 16093 | 4 | 0.029
H24a | 16148 | 2 | 0.015
H24a | 16180 | 1 | 0.007
H24a | 16209 | 1 | 0.007
H24a | 16261 | 2 | 0.015
H24a | 16270 | 1 | 0.007
H24a | 16272 | 1 | 0.007
H24a | 16309 | 3 | 0.022
H24a | 1700 | 1 | 0.007
H24a | 236 | 2 | 0.015
H24a | 2626 | 5 | 0.036
H24a | 310 | 2 | 0.015
H24a | 315.2C | 2 | 0.015
H24a | 3335 | 1 | 0.007
H24a | 3609 | 1 | 0.007
H24a | 373 | 1 | 0.007

H24a | 44.1C | 1 | 0.007
H24a | 480 | 1 | 0.007
H24a | 5302 | 1 | 0.007
H24a | 6398 | 5 | 0.036
H24a | 64 | 1 | 0.007
H24a | 6446 | 1 | 0.007
H24a | 6926 | 1 | 0.007
H24a | 7083 | 3 | 0.022
H24a | 7598 | 4 | 0.029
H24a | 7830 | 1 | 0.007
H24a | 8104 | 1 | 0.007
H24a | 9027 | 1 | 0.007
H24a | 9314 | 2 | 0.015
H24a | 9962 | 1 | 0.007
H24a1 | 12907 | 1 | 0.015
H24a1 | 146 | 1 | 0.015
H24a1 | 16129 | 1 | 0.015
H24a1 | 16180 | 1 | 0.015
H24a1 | 16188 | 1 | 0.015
H24a1 | 186A | 1 | 0.015
H24a1 | 214 | 1 | 0.015
H24a1 | 315.2C | 6 | 0.092
H24a1 | 7510 | 1 | 0.015
H24a1 | 9947 | 1 | 0.015
H24a2 | 151 | 1 | 0.011
H24a2 | 16078T | 1 | 0.011
H24a2 | 16079G | 1 | 0.011
H24a2 | 16145 | 1 | 0.011
H24a2 | 16257 | 1 | 0.011
H24a2 | 16304 | 1 | 0.011
H24a2 | 16326 | 1 | 0.011
H24a2 | 16362 | 3 | 0.032
H24a2 | 93 | 3 | 0.032
H24b | 16077 | 1 | 0.011
H24b | 16189 | 1 | 0.011
H24b | 16261 | 3 | 0.033
H25 | 113 | 1 | 0.001
H25 | 16076A | 123 | 0.156
H25 | 208 | 1 | 0.001
H25 | 261 | 1 | 0.001
H25 | 281C | 1 | 0.001
H25 | 316d | 1 | 0.001
H25 | 338 | 1 | 0.001
H25 | 468 | 3 | 0.004
H25 | 502 | 1 | 0.001
H25 | 573.1C | 3 | 0.004
H25 | 573.2C | 5 | 0.006
H25 | 573.5C | 1 | 0.001
H26 | 10079 | 1 | 0.001
H26 | 10685 | 1 | 0.001
H26 | 113 | 1 | 0.001
H26 | 16076A | 123 | 0.156
H26 | 16222 | 1 | 0.001
H26 | 208 | 1 | 0.001
H26 | 261 | 1 | 0.001
H26 | 281C | 1 | 0.001
H26 | 316d | 1 | 0.001
H26 | 338 | 1 | 0.001
H26 | 3508 | 1 | 0.001
H26 | 468 | 3 | 0.004
H26 | 502 | 1 | 0.001
H26 | 573.1C | 3 | 0.004
H26 | 573.2C | 5 | 0.006
H26 | 573.5C | 1 | 0.001
H26 | 8234 | 1 | 0.001
H26 | 9053 | 1 | 0.001
H26a | 113 | 1 | 0.001
H26a | 14374 | 1 | 0.001
H26a | 16076A | 123 | 0.156
H26a | 208 | 1 | 0.001
H26a | 210 | 2 | 0.003
H26a | 261 | 1 | 0.001
H26a | 281C | 1 | 0.001
H26a | 316d | 1 | 0.001
H26a | 3305 | 1 | 0.001
H26a | 338 | 1 | 0.001
H26a | 3700 | 1 | 0.001
H26a | 468 | 3 | 0.004
H26a | 502 | 1 | 0.001
H26a | 573.1C | 3 | 0.004
H26a | 573.2C | 5 | 0.006
H26a | 573.5C | 1 | 0.001
H26a | 5843 | 1 | 0.001
H26a | 721 | 1 | 0.001
H26a | 73 | 1 | 0.001
H26a1 | 10077 | 1 | 0.001
H26a1 | 113 | 1 | 0.001
H26a1 | 11314 | 1 | 0.001
H26a1 | 1291A | 1 | 0.001
H26a1 | 13191 | 1 | 0.001
H26a1 | 14162 | 2 | 0.002
H26a1 | 152 | 7 | 0.009
H26a1 | 15731 | 3 | 0.004
H26a1 | 15790 | 2 | 0.002
H26a1 | 15909 | 1 | 0.001
H26a1 | 16076A | 1 | 0.001
H26a1 | 16189 | 123 | 0.151
H26a1 | 16311 | 1 | 0.001
H26a1 | 1719 | 1 | 0.001
H26a1 | 194 | 2 | 0.002
H26a1 | 1999 | 1 | 0.001
H26a1 | 208 | 1 | 0.001
H26a1 | 2557 | 1 | 0.001
H26a1 | 261 | 1 | 0.001
H26a1 | 281C | 1 | 0.001
H26a1 | 310 | 12 | 0.015
H26a1 | 316d | 1 | 0.001
H26a1 | 338 | 1 | 0.001
H26a1 | 3398 | 1 | 0.001
H26a1 | 3866 | 1 | 0.001
H26a1 | 4059 | 2 | 0.002

H26a1 | 4218 | 1 | 0.001
H26a1 | 468 | 3 | 0.004
H26a1 | 502 | 1 | 0.001
H26a1 | 573.1C | 3 | 0.004
H26a1 | 573.2C | 5 | 0.006
H26a1 | 573.5C | 1 | 0.001
H26a1 | 5899.1C | 3 | 0.004
H26a1 | 5899.2C | 1 | 0.001
H26a1 | 6023 | 1 | 0.001
H26a1 | 7271 | 1 | 0.001
H26a1a | 113 | 1 | 0.001
H26a1a | 16076A | 123 | 0.156
H26a1a | 208 | 1 | 0.001
H26a1a | 261 | 1 | 0.001
H26a1a | 281C | 1 | 0.001
H26a1a | 316d | 1 | 0.001
H26a1a | 338 | 1 | 0.001
H26a1a | 468 | 3 | 0.004
H26a1a | 502 | 1 | 0.001
H26a1a | 5471 | 1 | 0.001
H26a1a | 573.1C | 3 | 0.004
H26a1a | 573.2C | 5 | 0.006
H26a1a | 573.5C | 1 | 0.001
H26a1a | 709 | 1 | 0.001
H26a1a1 | 12241d | 1 | 0.056
H26a1a1 | 13752 | 1 | 0.056
H26a1a1 | 14013 | 1 | 0.056
H26a1a1 | 15110 | 1 | 0.056
H26a1a1 | 16124 | 1 | 0.056
H26a1a1 | 16166 | 2 | 0.111
H26a1a1 | 16290 | 1 | 0.056
H26a1a1 | 16362 | 1 | 0.056
H26a1a1 | 309d | 1 | 0.056
H26a1a1 | 310 | 5 | 0.278
H26a1a1 | 315.2C | 1 | 0.056
H26a1a1 | 315d | 1 | 0.056
H26a1a1 | 8412 | 1 | 0.056
H26a1a1 | 9909 | 1 | 0.056
H26a1b | 11914 | 1 | 0.016
H26a1b | 2071 | 1 | 0.016
H26b | 13656 | 1 | 0.011
H26b | 13899 | 9 | 0.103
H26b | 14198 | 9 | 0.103
H26b | 14398 | 1 | 0.011
H26b | 152 | 4 | 0.046
H26b | 16216 | 1 | 0.011
H26b | 16270 | 1 | 0.011
H26b | 16323 | 2 | 0.023
H26b | 16325 | 2 | 0.023
H26b | 16337G | 1 | 0.011
H26b | 16465A | 1 | 0.011
H26b | 4336 | 1 | 0.011
H26b | 456 | 1 | 0.011
H26b | 6221 | 9 | 0.103
H26b | 73 | 1 | 0.011
H26b | 7419 | 1 | 0.011
H26b | 8119 | 1 | 0.011
H26b | 8251 | 9 | 0.103
H26c | 103 | 1 | 0.014
H26c | 114 | 5 | 0.068
H26c | 143 | 2 | 0.027
H26c | 14745 | 1 | 0.014
H26c | 14935 | 1 | 0.014
H26c | 152 | 21 | 0.288
H26c | 16041 | 1 | 0.014
H26c | 16082 | 1 | 0.014
H26c | 16129 | 7 | 0.096
H26c | 16174 | 2 | 0.027
H26c | 16182 | 1 | 0.014
H26c | 16209 | 1 | 0.014
H26c | 16223 | 2 | 0.027
H26c | 16235T | 3 | 0.041
H26c | 16261 | 2 | 0.027
H26c | 16286 | 5 | 0.068
H26c | 16293 | 1 | 0.014
H26c | 16311 | 4 | 0.055
H26c | 16327 | 1 | 0.014
H26c | 16327A | 4 | 0.055
H26c | 165 | 4 | 0.055
H26c | 199 | 2 | 0.027
H26c | 235 | 1 | 0.014
H26c | 4622 | 1 | 0.014
H26c | 513 | 1 | 0.014
H26c | 5262 | 1 | 0.014
H26c | 573.3C | 1 | 0.014
H26c | 709 | 1 | 0.014
H26c | 74G | 1 | 0.014
H26c | 9545 | 1 | 0.014
H27 | 11255 | 1 | 0.01
H27 | 11353 | 1 | 0.01
H27 | 12034 | 5 | 0.049
H27 | 12657 | 1 | 0.01
H27 | 12855 | 1 | 0.01
H27 | 13008 | 2 | 0.019
H27 | 13353 | 1 | 0.01
H27 | 13926 | 1 | 0.01
H27 | 13945 | 1 | 0.01
H27 | 146 | 1 | 0.01
H27 | 14640 | 1 | 0.01
H27 | 14668 | 1 | 0.01
H27 | 14793 | 1 | 0.01
H27 | 150 | 6 | 0.058
H27 | 15131C | 1 | 0.01
H27 | 15301 | 1 | 0.01
H27 | 16093 | 4 | 0.039
H27 | 16108 | 1 | 0.01
H27 | 16169 | 1 | 0.01
H27 | 16189 | 1 | 0.01
H27 | 16278 | 2 | 0.019
H27 | 1675 | 1 | 0.01
H27 | 195 | 1 | 0.01

H27 | 2141 | 1 | 0.01
H27 | 215 | 1 | 0.01
H27 | 2246 | 1 | 0.01
H27 | 234 | 1 | 0.01
H27 | 262 | 1 | 0.01
H27 | 2649 | 1 | 0.01
H27 | 3565 | 1 | 0.01
H27 | 4386 | 1 | 0.01
H27 | 4531 | 1 | 0.01
H27 | 521 | 1 | 0.01
H27 | 5460 | 2 | 0.019
H27 | 63 | 2 | 0.019
H27 | 64 | 2 | 0.019
H27 | 66 | 2 | 0.019
H27 | 73 | 8 | 0.078
H27 | 8281G | 1 | 0.01
H27 | 8282A | 1 | 0.01
H27 | 8283G | 1 | 0.01
H27 | 8764 | 1 | 0.01
H27 | 8843 | 1 | 0.01
H27 | 9066 | 1 | 0.01
H27 | 9391 | 2 | 0.019
H27 | 961 | 1 | 0.01
H27 | 9682 | 1 | 0.01
H27 | 980 | 1 | 0.01
H27+16093 | 12366 | 1 | 0.012
H27+16093 | 13008 | 1 | 0.012
H27+16093 | 14208 | 1 | 0.012
H27+16093 | 14831 | 1 | 0.012
H27+16093 | 15301 | 1 | 0.012
H27+16093 | 15519 | 1 | 0.012
H27+16093 | 15784 | 1 | 0.012
H27+16093 | 16147 | 1 | 0.012
H27+16093 | 16189 | 1 | 0.012
H27+16093 | 16234 | 1 | 0.012
H27+16093 | 16239 | 2 | 0.025
H27+16093 | 16311 | 1 | 0.012
H27+16093 | 1719 | 1 | 0.012
H27+16093 | 185 | 4 | 0.05
H27+16093 | 195 | 2 | 0.025
H27+16093 | 215 | 1 | 0.012
H27+16093 | 2386 | 1 | 0.012
H27+16093 | 3547C | 1 | 0.012
H27+16093 | 3593 | 1 | 0.012
H27+16093 | 4386 | 1 | 0.012
H27+16093 | 573d | 1 | 0.012
H27+16093 | 635 | 4 | 0.05
H27+16093 | 6776 | 1 | 0.012
H27+16093 | 72 | 1 | 0.012
H27+16093 | 73 | 2 | 0.025
H27+16093 | 93 | 4 | 0.05
H27+16093 | 9512 | 1 | 0.012
H27a | 11809 | 1 | 0.012
H27a | 11950 | 1 | 0.012
H27a | 13569 | 4 | 0.048
H27a | 14199 | 1 | 0.012
H27a | 14857 | 1 | 0.012
H27a | 152 | 4 | 0.048
H27a | 16184 | 1 | 0.012
H27a | 16189 | 1 | 0.012
H27a | 16234 | 1 | 0.012
H27a | 16274 | 1 | 0.012
H27a | 16278 | 1 | 0.012
H27a | 16311 | 1 | 0.012
H27a | 16368 | 2 | 0.024
H27a | 1717 | 1 | 0.012
H27a | 260 | 1 | 0.012
H27a | 2746 | 1 | 0.012
H27a | 4236 | 1 | 0.012
H27a | 72 | 1 | 0.012
H27a | 9380 | 1 | 0.012
H27b | 15758 | 1 | 0.016
H27b | 1677 | 1 | 0.016
H27c | 310 | 1 | 0.015
H27d | 11353 | 2 | 0.111
H27d | 14110 | 1 | 0.056
H27d | 146 | 1 | 0.056
H27d | 14803G | 1 | 0.056
H27d | 150 | 1 | 0.056
H27d | 152 | 3 | 0.167
H27d | 16051 | 1 | 0.056
H27d | 16140 | 2 | 0.111
H27d | 16145 | 1 | 0.056
H27d | 16189 | 3 | 0.167
H27d | 16217 | 1 | 0.056
H27d | 16227 | 1 | 0.056
H27d | 16242 | 1 | 0.056
H27d | 16311 | 2 | 0.111
H27d | 16356 | 1 | 0.056
H27d | 16360 | 1 | 0.056
H27d | 195 | 1 | 0.056
H27d | 3573 | 1 | 0.056
H27d | 4531 | 2 | 0.111
H27d | 4619 | 1 | 0.056
H27d | 63 | 1 | 0.056
H27d | 64 | 1 | 0.056
H27d | 66 | 1 | 0.056
H27d | 9266 | 1 | 0.056
H27e | 2141 | 1 | 0.015
H27e | 751T | 2 | 0.031
H27e | 7624 | 1 | 0.015
H27f | 234 | 1 | 0.016
H27f | 4820 | 1 | 0.016
H28 | 11611 | 1 | 0.048
H28 | 152 | 2 | 0.095
H28 | 16129 | 1 | 0.048
H28 | 16189 | 2 | 0.095
H28 | 16192 | 12 | 0.571
H28 | 16213 | 1 | 0.048
H28 | 182 | 1 | 0.048
H28 | 2789 | 2 | 0.095

H28 | 3398 | 1 | 0.048
H28 | 4491 | 1 | 0.048
H28 | 4823 | 3 | 0.143
H28 | 8634 | 4 | 0.19
H28a | 10463 | 3 | 0.056
H28a | 11038d | 6 | 0.111
H28a | 12085 | 1 | 0.019
H28a | 12634 | 1 | 0.019
H28a | 13327 | 2 | 0.037
H28a | 13790 | 1 | 0.019
H28a | 13886 | 1 | 0.019
H28a | 14560 | 1 | 0.019
H28a | 146 | 4 | 0.074
H28a | 15758 | 1 | 0.019
H28a | 15961 | 1 | 0.019
H28a | 16092 | 13 | 0.241
H28a | 16093 | 7 | 0.13
H28a | 16188 | 4 | 0.074
H28a | 16189 | 1 | 0.019
H28a | 16218 | 1 | 0.019
H28a | 16303 | 1 | 0.019
H28a | 16311 | 8 | 0.148
H28a | 16536G | 1 | 0.019
H28a | 16539C | 1 | 0.019
H28a | 182 | 1 | 0.019
H28a | 186 | 1 | 0.019
H28a | 188 | 1 | 0.019
H28a | 189 | 1 | 0.019
H28a | 195 | 1 | 0.019
H28a | 2484 | 1 | 0.019
H28a | 310 | 2 | 0.037
H28a | 315.2C | 2 | 0.037
H28a | 3414 | 6 | 0.111
H28a | 3666 | 1 | 0.019
H28a | 42.1G | 3 | 0.056
H28a | 4505 | 1 | 0.019
H28a | 4688 | 1 | 0.019
H28a | 4820 | 4 | 0.074
H28a | 573.1C | 1 | 0.019
H28a | 6635 | 1 | 0.019
H28a | 6647 | 2 | 0.037
H28a | 72 | 6 | 0.111
H28a | 7309 | 1 | 0.019
H28a | 7472 | 1 | 0.019
H28a | 7681A | 1 | 0.019
H28a | 7830 | 2 | 0.037
H28a | 8251 | 1 | 0.019
H28a | 9055 | 3 | 0.056
H28a | 9329 | 1 | 0.019
H28a | 9545 | 1 | 0.019
H28a1 | 11889 | 1 | 0.125
H28a1 | 182 | 1 | 0.125
H28a1 | 6722 | 1 | 0.125
H28a1 | 7495 | 1 | 0.125
H28a2 | 152 | 3 | 0.429
H28a2 | 3398 | 3 | 0.429
H28a2 | 6426 | 3 | 0.429
H29 | 10400 | 1 | 0.017
H29 | 11914 | 1 | 0.017
H29 | 12133 | 1 | 0.017
H29 | 14118 | 1 | 0.017
H29 | 15110 | 1 | 0.017
H29 | 153 | 1 | 0.017
H29 | 15930 | 1 | 0.017
H29 | 16079 | 3 | 0.051
H29 | 16145 | 1 | 0.017
H29 | 16300 | 1 | 0.017
H29 | 16319 | 1 | 0.017
H29 | 16356 | 1 | 0.017
H29 | 16399 | 29 | 0.492
H29 | 16400 | 1 | 0.017
H29 | 194 | 1 | 0.017
H29 | 215 | 2 | 0.034
H29 | 234 | 1 | 0.017
H29 | 315.2C | 1 | 0.017
H29 | 3398 | 1 | 0.017
H29 | 4496 | 1 | 0.017
H29 | 73 | 1 | 0.017
H29 | 8551 | 1 | 0.017
H29 | 8793 | 1 | 0.017
H29 | 95C | 20 | 0.339
H29a | 12115 | 2 | 0.05
H29a | 16216 | 3 | 0.075
H29a | 16224 | 2 | 0.05
H29a | 16240 | 2 | 0.05
H29a | 16248 | 4 | 0.1
H29a | 16266 | 2 | 0.05
H29a | 16284 | 1 | 0.025
H29a | 195 | 3 | 0.075
H29a | 200 | 1 | 0.025
H29a | 291.1A | 1 | 0.025
H29a | 316d | 1 | 0.025
H29a | 54.1C | 1 | 0.025
H29a | 56C | 1 | 0.025
H29a | 6131 | 2 | 0.05
H29a | 87 | 4 | 0.1
H29a | 88A | 4 | 0.1
H29a | 9103 | 2 | 0.05
H29b | 12397 | 1 | 0.036
H29b | 13618A | 1 | 0.036
H29b | 16189 | 1 | 0.036
H29b | 9077 | 1 | 0.036
H29b | 95T | 1 | 0.036
H2a | 10237 | 2 | 0.002
H2a | 10376 | 1 | 0.001
H2a | 10398 | 1 | 0.001
H2a | 10586 | 1 | 0.001
H2a | 10685 | 1 | 0.001
H2a | 113 | 1 | 0.001
H2a | 11351 | 1 | 0.001
H2a | 12131 | 1 | 0.001

H2a | 12362 | 1 | 0.001
H2a | 12406 | 1 | 0.001
H2a | 13575 | 1 | 0.001
H2a | 13968 | 2 | 0.002
H2a | 14002 | 1 | 0.001
H2a | 146 | 1 | 0.001
H2a | 152 | 2 | 0.002
H2a | 1555 | 1 | 0.001
H2a | 15784 | 1 | 0.001
H2a | 15787 | 1 | 0.001
H2a | 16076A | 1 | 0.001
H2a | 16114 | 1 | 0.001
H2a | 16189 | 125 | 0.153
H2a | 16203 | 1 | 0.001
H2a | 16260 | 1 | 0.001
H2a | 16266 | 125 | 0.153
H2a | 16271 | 1 | 0.001
H2a | 16294 | 1 | 0.001
H2a | 16563 | 1 | 0.001
H2a | 1677 | 1 | 0.001
H2a | 186 | 1 | 0.001
H2a | 208 | 1 | 0.001
H2a | 261 | 1 | 0.001
H2a | 281C | 1 | 0.001
H2a | 310 | 1 | 0.001
H2a | 3106A | 1 | 0.001
H2a | 316d | 1 | 0.001
H2a | 3357 | 1 | 0.001
H2a | 338 | 1 | 0.001
H2a | 3394 | 2 | 0.002
H2a | 4136 | 1 | 0.001
H2a | 4350 | 5 | 0.006
H2a | 4491 | 1 | 0.001
H2a | 468 | 3 | 0.004
H2a | 4793 | 1 | 0.001
H2a | 502 | 1 | 0.001
H2a | 5206 | 2 | 0.002
H2a | 5460 | 5 | 0.006
H2a | 573.1C | 3 | 0.004
H2a | 573.2C | 5 | 0.006
H2a | 573.5C | 1 | 0.001
H2a | 6038 | 1 | 0.001
H2a | 6257 | 1 | 0.001
H2a | 72 | 1 | 0.001
H2a | 73 | 5 | 0.006
H2a | 7650G | 2 | 0.002
H2a | 7762 | 1 | 0.001
H2a | 7819 | 1 | 0.001
H2a | 7840 | 1 | 0.001
H2a | 8027 | 2 | 0.002
H2a | 8654 | 1 | 0.001
H2a | 8709 | 5 | 0.006
H2a | 8856 | 4 | 0.005
H2a | 951 | 3 | 0.004
H2a1 | 10172 | 2 | 0.009
H2a1 | 10235 | 1 | 0.004
H2a1 | 10310 | 3 | 0.013
H2a1 | 10352 | 1 | 0.004
H2a1 | 10952 | 1 | 0.004
H2a1 | 10954 | 1 | 0.004
H2a1 | 10955 | 1 | 0.004
H2a1 | 11443 | 1 | 0.004
H2a1 | 11719 | 1 | 0.004
H2a1 | 11866 | 1 | 0.004
H2a1 | 11899 | 2 | 0.009
H2a1 | 11914 | 3 | 0.013
H2a1 | 12418C | 1 | 0.004
H2a1 | 12940 | 1 | 0.004
H2a1 | 13326 | 3 | 0.013
H2a1 | 13395 | 2 | 0.009
H2a1 | 13434 | 1 | 0.004
H2a1 | 13993 | 1 | 0.004
H2a1 | 14133 | 1 | 0.004
H2a1 | 14266 | 1 | 0.004
H2a1 | 14305 | 1 | 0.004
H2a1 | 1438 | 1 | 0.004
H2a1 | 14403 | 1 | 0.004
H2a1 | 14494 | 1 | 0.004
H2a1 | 14587 | 2 | 0.009
H2a1 | 14674 | 2 | 0.009
H2a1 | 14806 | 1 | 0.004
H2a1 | 14860 | 1 | 0.004
H2a1 | 150 | 1 | 0.004
H2a1 | 151 | 1 | 0.004
H2a1 | 152 | 9 | 0.038
H2a1 | 15313 | 1 | 0.004
H2a1 | 1532 | 1 | 0.004
H2a1 | 16108 | 1 | 0.004
H2a1 | 16129 | 7 | 0.03
H2a1 | 16150 | 1 | 0.004
H2a1 | 16188 | 1 | 0.004
H2a1 | 16189 | 8 | 0.034
H2a1 | 16192 | 5 | 0.021
H2a1 | 16193d | 1 | 0.004
H2a1 | 16243 | 1 | 0.004
H2a1 | 16248 | 1 | 0.004
H2a1 | 16291 | 2 | 0.009
H2a1 | 16311 | 12 | 0.051
H2a1 | 16527 | 2 | 0.009
H2a1 | 182 | 1 | 0.004
H2a1 | 1833 | 3 | 0.013
H2a1 | 194 | 6 | 0.026
H2a1 | 236 | 1 | 0.004
H2a1 | 2887 | 1 | 0.004
H2a1 | 3082 | 1 | 0.004
H2a1 | 3090 | 1 | 0.004
H2a1 | 310 | 4 | 0.017
H2a1 | 3106A | 1 | 0.004
H2a1 | 315.3C | 1 | 0.004
H2a1 | 3150 | 3 | 0.013
H2a1 | 3514 | 1 | 0.004

H2a1 | 3516 | 1 | 0.004
H2a1 | 3930 | 2 | 0.009
H2a1 | 4012 | 1 | 0.004
H2a1 | 4025 | 1 | 0.004
H2a1 | 4048 | 1 | 0.004
H2a1 | 4074 | 2 | 0.009
H2a1 | 451 | 1 | 0.004
H2a1 | 5063 | 1 | 0.004
H2a1 | 5297A | 5 | 0.021
H2a1 | 5384 | 1 | 0.004
H2a1 | 5420 | 3 | 0.013
H2a1 | 5486 | 1 | 0.004
H2a1 | 5493 | 1 | 0.004
H2a1 | 6071 | 1 | 0.004
H2a1 | 6261 | 1 | 0.004
H2a1 | 6680 | 1 | 0.004
H2a1 | 6714C | 1 | 0.004
H2a1 | 6890 | 2 | 0.009
H2a1 | 709 | 1 | 0.004
H2a1 | 73 | 1 | 0.004
H2a1 | 7308C | 1 | 0.004
H2a1 | 7492 | 1 | 0.004
H2a1 | 7665 | 1 | 0.004
H2a1 | 8027 | 1 | 0.004
H2a1 | 8251 | 1 | 0.004
H2a1 | 8406 | 1 | 0.004
H2a1 | 8857 | 1 | 0.004
H2a1 | 8918 | 1 | 0.004
H2a1 | 9043 | 1 | 0.004
H2a1 | 9089 | 1 | 0.004
H2a1 | 9145 | 2 | 0.009
H2a1 | 9182 | 1 | 0.004
H2a1 | 93 | 1 | 0.004
H2a1 | 9360 | 1 | 0.004
H2a1 | 9364 | 1 | 0.004
H2a1 | 9365 | 2 | 0.009
H2a1 | 9438 | 5 | 0.021
H2a1 | 9548 | 1 | 0.004
H2a1 | 960d | 1 | 0.004
H2a1 | 9739 | 1 | 0.004
H2a1 | 9758 | 1 | 0.004
H2a1+146 | 1555 | 1 | 0.015
H2a1+146 | 15884 | 1 | 0.015
H2a1+146 | 185T | 1 | 0.015
H2a1+146 | 204 | 1 | 0.015
H2a1+146 | 207 | 1 | 0.015
H2a1+146 | 269 | 3 | 0.046
H2a1+146 | 4722 | 1 | 0.015
H2a1+146 | 8433 | 1 | 0.015
H2a1+146 | 8648 | 1 | 0.015
H2a1+146 | 8886 | 1 | 0.015
H2a1+146 | 93 | 2 | 0.031
H2a1a | 10293 | 1 | 0.005
H2a1a | 11902 | 1 | 0.005
H2a1a | 11914 | 8 | 0.036
H2a1a | 12474 | 1 | 0.005
H2a1a | 14410 | 1 | 0.005
H2a1a | 14488 | 1 | 0.005
H2a1a | 150 | 5 | 0.023
H2a1a | 15067 | 1 | 0.005
H2a1a | 151 | 1 | 0.005
H2a1a | 152 | 24 | 0.109
H2a1a | 15497 | 1 | 0.005
H2a1a | 15629 | 1 | 0.005
H2a1a | 15670 | 1 | 0.005
H2a1a | 15932 | 2 | 0.009
H2a1a | 16093 | 1 | 0.005
H2a1a | 16157 | 2 | 0.009
H2a1a | 16178 | 1 | 0.005
H2a1a | 16189 | 1 | 0.005
H2a1a | 16209 | 3 | 0.014
H2a1a | 16240T | 1 | 0.005
H2a1a | 16274 | 10 | 0.045
H2a1a | 16291 | 1 | 0.005
H2a1a | 16292 | 3 | 0.014
H2a1a | 16311 | 2 | 0.009
H2a1a | 183 | 2 | 0.009
H2a1a | 2483 | 1 | 0.005
H2a1a | 26 | 10 | 0.045
H2a1a | 293 | 1 | 0.005
H2a1a | 310 | 2 | 0.009
H2a1a | 3335 | 3 | 0.014
H2a1a | 3591 | 2 | 0.009
H2a1a | 36 | 10 | 0.045
H2a1a | 4475 | 1 | 0.005
H2a1a | 5964 | 8 | 0.036
H2a1a | 64 | 1 | 0.005
H2a1a | 6854 | 4 | 0.018
H2a1a | 6866 | 1 | 0.005
H2a1a | 6975 | 1 | 0.005
H2a1a | 7001 | 1 | 0.005
H2a1a | 709 | 1 | 0.005
H2a1a | 8697 | 1 | 0.005
H2a1a | 9055 | 4 | 0.018
H2a1a | 9077A | 1 | 0.005
H2a1a | 9126 | 1 | 0.005
H2a1a | 9182 | 8 | 0.036
H2a1a | 94 | 1 | 0.005
H2a1a | 9804 | 1 | 0.005
H2a1a1 | 16145 | 11 | 0.064
H2a1a1 | 16526 | 3 | 0.018
H2a1a1 | 199 | 2 | 0.012
H2a1a1 | 9461 | 1 | 0.006
H2a1a2 | 16145 | 2 | 0.012
H2a1a2 | 16215 | 11 | 0.065
H2a1a2 | 195 | 1 | 0.006
H2a1a2 | 199 | 2 | 0.012
H2a1b | 16145 | 11 | 0.066
H2a1b | 199 | 2 | 0.012
H2a1b1 | 16093 | 1 | 0.02
H2a1b1 | 16111 | 1 | 0.02

H2a1b1 | 16126 | 1 | 0.02
H2a1b1 | 16148 | 1 | 0.02
H2a1b1 | 16218 | 1 | 0.02
H2a1b1 | 8538 | 1 | 0.02
H2a1b1 | 9309 | 1 | 0.02
H2a1b2 | 14871 | 1 | 0.03
H2a1b2 | 16311 | 1 | 0.03
H2a1b2 | 195 | 1 | 0.03
H2a1b2 | 310 | 2 | 0.061
H2a1b2 | 3395C | 1 | 0.03
H2a1b2 | 3540 | 1 | 0.03
H2a1b2 | 6053 | 1 | 0.03
H2a1b2 | 6890 | 1 | 0.03
H2a1c | 10724 | 1 | 0.005
H2a1c | 11158 | 3 | 0.016
H2a1c | 11899 | 1 | 0.005
H2a1c | 12351 | 4 | 0.022
H2a1c | 152 | 3 | 0.016
H2a1c | 15831 | 1 | 0.005
H2a1c | 15936 | 1 | 0.005
H2a1c | 15968 | 1 | 0.005
H2a1c | 16287 | 1 | 0.005
H2a1c | 16320 | 10 | 0.054
H2a1c | 1662 | 1 | 0.005
H2a1c | 228 | 1 | 0.005
H2a1c | 310 | 2 | 0.011
H2a1c | 315.2C | 1 | 0.005
H2a1c | 5177 | 3 | 0.016
H2a1c | 6353 | 1 | 0.005
H2a1c | 7289 | 1 | 0.005
H2a1c | 7775 | 1 | 0.005
H2a1c | 8470 | 1 | 0.005
H2a1c | 9323A | 1 | 0.005
H2a1c | 9677 | 1 | 0.005
H2a1d | 16145 | 11 | 0.064
H2a1d | 199 | 2 | 0.012
H2a1d | 73 | 2 | 0.012
H2a1e | 10850 | 1 | 0.009
H2a1e | 13359 | 2 | 0.018
H2a1e | 150 | 10 | 0.092
H2a1e | 16249 | 10 | 0.092
H2a1e | 228 | 10 | 0.092
H2a1e | 2483 | 2 | 0.018
H2a1e | 309d | 1 | 0.009
H2a1e | 310 | 1 | 0.009
H2a1e | 3397 | 1 | 0.009
H2a1e | 4733 | 1 | 0.009
H2a1e | 5963C | 1 | 0.009
H2a1e | 8902 | 2 | 0.018
H2a1e | 9754 | 1 | 0.009
H2a1e1 | 150 | 10 | 0.097
H2a1e1 | 16176 | 2 | 0.019
H2a1e1 | 16249 | 10 | 0.097
H2a1e1 | 228 | 10 | 0.097
H2a1e1 | 310 | 1 | 0.01
H2a1e1 | 8701 | 1 | 0.01
H2a1e1a | 10192 | 1 | 0.022
H2a1e1a | 310 | 1 | 0.022
H2a1e1a | 6339 | 1 | 0.022
H2a1e1a | 9052 | 2 | 0.044
H2a1e1a1 | 223 | 1 | 0.025
H2a1e1a1 | 286T | 1 | 0.025
H2a1e1a1 | 342 | 1 | 0.025
H2a1e1a1 | 376C | 2 | 0.05
H2a1e1a1 | 376T | 1 | 0.025
H2a1e1a1 | 395 | 1 | 0.025
H2a1e1a1 | 397 | 1 | 0.025
H2a1e1a1 | 40 | 1 | 0.025
H2a1e1a1 | 8281-8289d | 1 | 0.025
H2a1e1b | 10855 | 1 | 0.009
H2a1e1b | 150 | 1 | 0.009
H2a1e1b | 16075 | 1 | 0.009
H2a1e1b | 16230 | 1 | 0.009
H2a1e1b | 16249 | 1 | 0.009
H2a1e1b | 16356 | 14 | 0.131
H2a1e1b | 228 | 1 | 0.009
H2a1f | 10256 | 2 | 0.024
H2a1f | 13759 | 1 | 0.012
H2a1f | 14484 | 1 | 0.012
H2a1f | 15133 | 1 | 0.012
H2a1f | 152 | 4 | 0.049
H2a1f | 153 | 1 | 0.012
H2a1f | 16086 | 1 | 0.012
H2a1f | 16189 | 1 | 0.012
H2a1f | 16311 | 2 | 0.024
H2a1f | 310 | 2 | 0.024
H2a1f | 311 | 1 | 0.012
H2a1f | 4312 | 1 | 0.012
H2a1f1 | 10586 | 1 | 0.053
H2a1f1 | 152 | 1 | 0.053
H2a1f1 | 16285T | 1 | 0.053
H2a1f1 | 16344 | 1 | 0.053
H2a1f2 | 10274 | 1 | 0.048
H2a1f2 | 12501 | 1 | 0.048
H2a1f2 | 16218G | 1 | 0.048
H2a1f2 | 368 | 1 | 0.048
H2a1f2 | 45 | 1 | 0.048
H2a1f2 | 489 | 1 | 0.048
H2a1g | 10172 | 1 | 0.006
H2a1g | 11299 | 1 | 0.006
H2a1g | 1168C | 1 | 0.006
H2a1g | 16145 | 11 | 0.064
H2a1g | 195 | 1 | 0.006
H2a1g | 199 | 2 | 0.012
H2a1g | 310 | 1 | 0.006
H2a1g | 3693 | 1 | 0.006
H2a1i | 10389 | 1 | 0.059
H2a1i | 11719 | 1 | 0.059
H2a1i | 12441 | 1 | 0.059
H2a1i | 146 | 2 | 0.118
H2a1i | 16126 | 2 | 0.118

H2a1i | 3312 | 1 | 0.059
H2a1i | 5460 | 1 | 0.059
H2a1i | 9145 | 1 | 0.059
H2a1j | 12055 | 1 | 0.009
H2a1j | 16018 | 1 | 0.009
H2a1j | 16170 | 1 | 0.009
H2a1j | 16230 | 1 | 0.009
H2a1j | 16356 | 14 | 0.132
H2a1j | 8659 | 1 | 0.009
H2a1k | 16145 | 11 | 0.065
H2a1k | 16173 | 1 | 0.006
H2a1k | 199 | 2 | 0.012
H2a1k | 3951 | 1 | 0.006
H2a1k | 41 | 1 | 0.006
H2a1k | 573.1C | 1 | 0.006
H2a1k | 8251 | 1 | 0.006
H2a1m | 16174 | 1 | 0.02
H2a1m | 16311 | 1 | 0.02
H2a1m | 204 | 1 | 0.02
H2a1m | 482 | 1 | 0.02
H2a1m | 489 | 1 | 0.02
H2a1m | 73 | 1 | 0.02
H2a1n | 14226 | 1 | 0.015
H2a1n | 16497 | 1 | 0.015
H2a1n | 185T | 1 | 0.015
H2a1n | 6042 | 1 | 0.015
H2a1n | 8084 | 1 | 0.015
H2a2 | 1007 | 1 | 0.001
H2a2 | 113 | 1 | 0.001
H2a2 | 14769 | 1 | 0.001
H2a2 | 14798 | 1 | 0.001
H2a2 | 16076A | 1 | 0.001
H2a2 | 16172 | 1 | 0.001
H2a2 | 16235 | 123 | 0.158
H2a2 | 208 | 1 | 0.001
H2a2 | 228 | 1 | 0.001
H2a2 | 234 | 1 | 0.001
H2a2 | 261 | 1 | 0.001
H2a2 | 2772 | 1 | 0.001
H2a2 | 281C | 1 | 0.001
H2a2 | 316d | 1 | 0.001
H2a2 | 338 | 1 | 0.001
H2a2 | 468 | 3 | 0.004
H2a2 | 502 | 1 | 0.001
H2a2 | 5054 | 1 | 0.001
H2a2 | 573.1C | 3 | 0.004
H2a2 | 573.2C | 5 | 0.006
H2a2 | 573.5C | 1 | 0.001
H2a2+(16235) | 1007 | 1 | 0.001
H2a2+(16235) | 113 | 1 | 0.001
H2a2+(16235) | 13801 | 1 | 0.001
H2a2+(16235) | 14769 | 1 | 0.001
H2a2+(16235) | 14798 | 1 | 0.001
H2a2+(16235) | 16076A | 1 | 0.001
H2a2+(16235) | 16111 | 1 | 0.001
H2a2+(16235) | 16172 | 1 | 0.001
H2a2+(16235) | 16235 | 1 | 0.001
H2a2+(16235) | 16290 | 123 | 0.156
H2a2+(16235) | 16362 | 2 | 0.003
H2a2+(16235) | 208 | 1 | 0.001
H2a2+(16235) | 228 | 1 | 0.001
H2a2+(16235) | 234 | 1 | 0.001
H2a2+(16235) | 261 | 1 | 0.001
H2a2+(16235) | 2772 | 1 | 0.001
H2a2+(16235) | 281C | 1 | 0.001
H2a2+(16235) | 310 | 1 | 0.001
H2a2+(16235) | 315.2C | 1 | 0.001
H2a2+(16235) | 316d | 1 | 0.001
H2a2+(16235) | 338 | 1 | 0.001
H2a2+(16235) | 468 | 3 | 0.004
H2a2+(16235) | 502 | 1 | 0.001
H2a2+(16235) | 5054 | 1 | 0.001
H2a2+(16235) | 5563 | 2 | 0.003
H2a2+(16235) | 573.1C | 3 | 0.004
H2a2+(16235) | 573.2C | 5 | 0.006
H2a2+(16235) | 573.5C | 1 | 0.001
H2a2a | 108C | 1 | 0.001
H2a2a | 109T | 1 | 0.001
H2a2a | 113 | 1 | 0.001
H2a2a | 11929 | 2 | 0.002
H2a2a | 121C | 1 | 0.001
H2a2a | 14058 | 2 | 0.002
H2a2a | 143 | 1 | 0.001
H2a2a | 146 | 7 | 0.007
H2a2a | 150 | 21 | 0.022
H2a2a | 152 | 22 | 0.023
H2a2a | 153 | 2 | 0.002
H2a2a | 16051 | 2 | 0.002
H2a2a | 16069 | 12 | 0.012
H2a2a | 16076A | 1 | 0.001
H2a2a | 16086 | 2 | 0.002
H2a2a | 16093 | 4 | 0.004
H2a2a | 16111 | 4 | 0.004
H2a2a | 16114A | 1 | 0.001
H2a2a | 16126 | 18 | 0.019
H2a2a | 16129 | 17 | 0.018
H2a2a | 16134 | 1 | 0.001
H2a2a | 16136 | 2 | 0.002
H2a2a | 16142 | 1 | 0.001
H2a2a | 16145 | 3 | 0.003
H2a2a | 16146 | 1 | 0.001
H2a2a | 16162 | 4 | 0.004
H2a2a | 16170 | 1 | 0.001
H2a2a | 16176 | 4 | 0.004
H2a2a | 16179A | 1 | 0.001
H2a2a | 16181 | 1 | 0.001
H2a2a | 16188G | 1 | 0.001
H2a2a | 16192 | 10 | 0.01
H2a2a | 16193 | 5 | 0.005
H2a2a | 16196 | 1 | 0.001
H2a2a | 16209 | 6 | 0.006

H2a2a | 16210 | 3 | 0.003
H2a2a | 16213 | 2 | 0.002
H2a2a | 16218 | 2 | 0.002
H2a2a | 16219 | 4 | 0.004
H2a2a | 16220C | 1 | 0.001
H2a2a | 16223 | 36 | 0.037
H2a2a | 16224 | 8 | 0.008
H2a2a | 16231 | 1 | 0.001
H2a2a | 16235 | 1 | 0.001
H2a2a | 16239 | 2 | 0.002
H2a2a | 16242 | 1 | 0.001
H2a2a | 16248 | 2 | 0.002
H2a2a | 16249A | 2 | 0.002
H2a2a | 16256 | 6 | 0.006
H2a2a | 16260 | 4 | 0.004
H2a2a | 16261 | 2 | 0.002
H2a2a | 16262 | 1 | 0.001
H2a2a | 16265 | 1 | 0.001
H2a2a | 16266 | 2 | 0.002
H2a2a | 16270 | 13 | 0.013
H2a2a | 16274 | 1 | 0.001
H2a2a | 16278 | 10 | 0.01
H2a2a | 16290 | 1 | 0.001
H2a2a | 16291 | 11 | 0.011
H2a2a | 16292 | 3 | 0.003
H2a2a | 16293 | 2 | 0.002
H2a2a | 16293T | 1 | 0.001
H2a2a | 16294 | 10 | 0.01
H2a2a | 16295 | 1 | 0.001
H2a2a | 16296 | 4 | 0.004
H2a2a | 16298 | 14 | 0.014
H2a2a | 16304 | 7 | 0.007
H2a2a | 16309 | 1 | 0.001
H2a2a | 16311 | 17 | 0.018
H2a2a | 16312 | 1 | 0.001
H2a2a | 16313A | 1 | 0.001
H2a2a | 16316 | 1 | 0.001
H2a2a | 16319 | 3 | 0.003
H2a2a | 16325 | 4 | 0.004
H2a2a | 16327 | 1 | 0.001
H2a2a | 16328A | 2 | 0.002
H2a2a | 16335 | 2 | 0.002
H2a2a | 16343 | 6 | 0.006
H2a2a | 16346 | 1 | 0.001
H2a2a | 16354 | 4 | 0.004
H2a2a | 16356 | 6 | 0.006
H2a2a | 16362 | 15 | 0.015
H2a2a | 16366 | 2 | 0.002
H2a2a | 16368 | 1 | 0.001
H2a2a | 16390 | 123 | 0.127
H2a2a | 16391 | 2 | 0.002
H2a2a | 16399 | 2 | 0.002
H2a2a | 16422 | 1 | 0.001
H2a2a | 16482 | 1 | 0.001
H2a2a | 16496 | 1 | 0.001
H2a2a | 16526 | 2 | 0.002
H2a2a | 182 | 1 | 0.001
H2a2a | 185 | 6 | 0.006
H2a2a | 188 | 2 | 0.002
H2a2a | 189 | 5 | 0.005
H2a2a | 194 | 1 | 0.001
H2a2a | 195 | 21 | 0.022
H2a2a | 198 | 2 | 0.002
H2a2a | 199 | 4 | 0.004
H2a2a | 200 | 2 | 0.002
H2a2a | 202 | 1 | 0.001
H2a2a | 204 | 9 | 0.009
H2a2a | 207 | 6 | 0.006
H2a2a | 208 | 1 | 0.001
H2a2a | 215 | 1 | 0.001
H2a2a | 228 | 5 | 0.005
H2a2a | 235 | 1 | 0.001
H2a2a | 237 | 1 | 0.001
H2a2a | 239 | 5 | 0.005
H2a2a | 246 | 1 | 0.001
H2a2a | 247 | 1 | 0.001
H2a2a | 249d | 3 | 0.003
H2a2a | 250 | 4 | 0.004
H2a2a | 257 | 2 | 0.002
H2a2a | 261 | 1 | 0.001
H2a2a | 281C | 1 | 0.001
H2a2a | 292 | 2 | 0.002
H2a2a | 293 | 2 | 0.002
H2a2a | 295 | 14 | 0.014
H2a2a | 310 | 4 | 0.004
H2a2a | 310.1T | 1 | 0.001
H2a2a | 3116 | 3 | 0.003
H2a2a | 315.2C | 1 | 0.001
H2a2a | 316d | 1 | 0.001
H2a2a | 319 | 1 | 0.001
H2a2a | 324 | 1 | 0.001
H2a2a | 338 | 1 | 0.001
H2a2a | 3693 | 3 | 0.003
H2a2a | 373 | 1 | 0.001
H2a2a | 390 | 1 | 0.001
H2a2a | 408A | 1 | 0.001
H2a2a | 44.1C | 1 | 0.001
H2a2a | 456 | 1 | 0.001
H2a2a | 462 | 4 | 0.004
H2a2a | 474A | 1 | 0.001
H2a2a | 482 | 1 | 0.001
H2a2a | 489 | 8 | 0.008
H2a2a | 497 | 5 | 0.005
H2a2a | 499 | 6 | 0.006
H2a2a | 502 | 1 | 0.001
H2a2a | 507A | 1 | 0.001
H2a2a | 513 | 1 | 0.001
H2a2a | 515-524d | 3 | 0.003
H2a2a | 517T | 1 | 0.001
H2a2a | 518 | 1 | 0.001
H2a2a | 573.1C | 3 | 0.003

H2a2a | 573.2C | 6 | 0.006
H2a2a | 573.4C | 1 | 0.001
H2a2a | 573.5C | 1 | 0.001
H2a2a | 5988 | 1 | 0.001
H2a2a | 63 | 1 | 0.001
H2a2a | 64 | 3 | 0.003
H2a2a | 66 | 1 | 0.001
H2a2a | 72 | 5 | 0.005
H2a2a | 73 | 64 | 0.066
H2a2a | 7521 | 1 | 0.001
H2a2a | 8736 | 3 | 0.003
H2a2a1 | 10208 | 4 | 0.027
H2a2a1 | 10336A | 2 | 0.014
H2a2a1 | 10397 | 1 | 0.007
H2a2a1 | 10398 | 5 | 0.034
H2a2a1 | 10400 | 3 | 0.02
H2a2a1 | 10446 | 1 | 0.007
H2a2a1 | 10535 | 1 | 0.007
H2a2a1 | 10577 | 1 | 0.007
H2a2a1 | 10586 | 1 | 0.007
H2a2a1 | 10652 | 1 | 0.007
H2a2a1 | 10803 | 1 | 0.007
H2a2a1 | 10861 | 1 | 0.007
H2a2a1 | 10873 | 1 | 0.007
H2a2a1 | 10951 | 1 | 0.007
H2a2a1 | 11137 | 1 | 0.007
H2a2a1 | 1119 | 1 | 0.007
H2a2a1 | 11335 | 1 | 0.007
H2a2a1 | 11447C | 1 | 0.007
H2a2a1 | 11555 | 1 | 0.007
H2a2a1 | 11719 | 1 | 0.007
H2a2a1 | 11740 | 1 | 0.007
H2a2a1 | 11778 | 1 | 0.007
H2a2a1 | 11970 | 1 | 0.007
H2a2a1 | 11986 | 1 | 0.007
H2a2a1 | 12348 | 1 | 0.007
H2a2a1 | 12349 | 1 | 0.007
H2a2a1 | 12379 | 1 | 0.007
H2a2a1 | 12393 | 1 | 0.007
H2a2a1 | 12399 | 1 | 0.007
H2a2a1 | 12498 | 1 | 0.007
H2a2a1 | 12507 | 1 | 0.007
H2a2a1 | 12588 | 1 | 0.007
H2a2a1 | 12705 | 1 | 0.007
H2a2a1 | 12711 | 1 | 0.007
H2a2a1 | 12782 | 1 | 0.007
H2a2a1 | 12783 | 1 | 0.007
H2a2a1 | 12858 | 1 | 0.007
H2a2a1 | 12873 | 1 | 0.007
H2a2a1 | 13105 | 1 | 0.007
H2a2a1 | 13113 | 1 | 0.007
H2a2a1 | 13150 | 1 | 0.007
H2a2a1 | 13157 | 1 | 0.007
H2a2a1 | 13204 | 1 | 0.007
H2a2a1 | 13254 | 1 | 0.007
H2a2a1 | 13269 | 1 | 0.007
H2a2a1 | 13449 | 1 | 0.007
H2a2a1 | 13461 | 1 | 0.007
H2a2a1 | 13506 | 1 | 0.007
H2a2a1 | 13539 | 1 | 0.007
H2a2a1 | 13702G | 1 | 0.007
H2a2a1 | 13774 | 1 | 0.007
H2a2a1 | 13777 | 1 | 0.007
H2a2a1 | 13820 | 1 | 0.007
H2a2a1 | 13821 | 1 | 0.007
H2a2a1 | 13889 | 1 | 0.007
H2a2a1 | 14047 | 1 | 0.007
H2a2a1 | 14182 | 1 | 0.007
H2a2a1 | 14199G | 1 | 0.007
H2a2a1 | 14203 | 1 | 0.007
H2a2a1 | 14209 | 1 | 0.007
H2a2a1 | 14218 | 1 | 0.007
H2a2a1 | 14272G | 1 | 0.007
H2a2a1 | 14302 | 1 | 0.007
H2a2a1 | 14357 | 1 | 0.007
H2a2a1 | 14365G | 1 | 0.007
H2a2a1 | 14368G | 1 | 0.007
H2a2a1 | 14375 | 1 | 0.007
H2a2a1 | 14410 | 1 | 0.007
H2a2a1 | 14431 | 1 | 0.007
H2a2a1 | 14470 | 2 | 0.014
H2a2a1 | 14536 | 1 | 0.007
H2a2a1 | 14554 | 1 | 0.007
H2a2a1 | 14566 | 3 | 0.02
H2a2a1 | 14690 | 1 | 0.007
H2a2a1 | 14766 | 2 | 0.014
H2a2a1 | 14793 | 1 | 0.007
H2a2a1 | 14827 | 1 | 0.007
H2a2a1 | 14839 | 1 | 0.007
H2a2a1 | 14854 | 1 | 0.007
H2a2a1 | 14881 | 1 | 0.007
H2a2a1 | 150 | 1 | 0.007
H2a2a1 | 15013 | 1 | 0.007
H2a2a1 | 15016 | 1 | 0.007
H2a2a1 | 15042 | 1 | 0.007
H2a2a1 | 15047 | 1 | 0.007
H2a2a1 | 15094 | 1 | 0.007
H2a2a1 | 15124 | 2 | 0.014
H2a2a1 | 152 | 8 | 0.054
H2a2a1 | 15256 | 1 | 0.007
H2a2a1 | 15346 | 1 | 0.007
H2a2a1 | 15349 | 1 | 0.007
H2a2a1 | 15388 | 1 | 0.007
H2a2a1 | 15410 | 1 | 0.007
H2a2a1 | 15457A | 1 | 0.007
H2a2a1 | 15493 | 1 | 0.007
H2a2a1 | 1555 | 1 | 0.007
H2a2a1 | 15784 | 1 | 0.007
H2a2a1 | 15832 | 1 | 0.007
H2a2a1 | 15883 | 2 | 0.014
H2a2a1 | 16129 | 2 | 0.014

H2a2a1 | 16147 | 1 | 0.007
H2a2a1 | 16149C | 1 | 0.007
H2a2a1 | 16153 | 1 | 0.007
H2a2a1 | 16169 | 3 | 0.02
H2a2a1 | 16192 | 1 | 0.007
H2a2a1 | 16193 | 3 | 0.02
H2a2a1 | 16207 | 1 | 0.007
H2a2a1 | 16215 | 1 | 0.007
H2a2a1 | 16217 | 1 | 0.007
H2a2a1 | 16219 | 2 | 0.014
H2a2a1 | 16235 | 1 | 0.007
H2a2a1 | 16255 | 1 | 0.007
H2a2a1 | 16263 | 1 | 0.007
H2a2a1 | 16266 | 1 | 0.007
H2a2a1 | 16270 | 3 | 0.02
H2a2a1 | 16289 | 1 | 0.007
H2a2a1 | 16293 | 1 | 0.007
H2a2a1 | 16294 | 1 | 0.007
H2a2a1 | 16295 | 2 | 0.014
H2a2a1 | 16305 | 1 | 0.007
H2a2a1 | 16311 | 2 | 0.014
H2a2a1 | 16335 | 1 | 0.007
H2a2a1 | 16362 | 3 | 0.02
H2a2a1 | 16368 | 1 | 0.007
H2a2a1 | 1719 | 1 | 0.007
H2a2a1 | 1809 | 1 | 0.007
H2a2a1 | 183 | 2 | 0.014
H2a2a1 | 195 | 1 | 0.007
H2a2a1 | 200 | 1 | 0.007
H2a2a1 | 204 | 1 | 0.007
H2a2a1 | 2120 | 1 | 0.007
H2a2a1 | 2124 | 3 | 0.02
H2a2a1 | 215 | 1 | 0.007
H2a2a1 | 2162 | 1 | 0.007
H2a2a1 | 2220 | 1 | 0.007
H2a2a1 | 2225 | 1 | 0.007
H2a2a1 | 2232.1A | 1 | 0.007
H2a2a1 | 2246T | 1 | 0.007
H2a2a1 | 236G | 1 | 0.007
H2a2a1 | 2378 | 1 | 0.007
H2a2a1 | 2388 | 1 | 0.007
H2a2a1 | 249 | 1 | 0.007
H2a2a1 | 2616 | 1 | 0.007
H2a2a1 | 2672 | 1 | 0.007
H2a2a1 | 2707 | 1 | 0.007
H2a2a1 | 2872 | 1 | 0.007
H2a2a1 | 2878T | 1 | 0.007
H2a2a1 | 3010 | 1 | 0.007
H2a2a1 | 310 | 5 | 0.034
H2a2a1 | 3144 | 1 | 0.007
H2a2a1 | 315.2C | 1 | 0.007
H2a2a1 | 3221 | 1 | 0.007
H2a2a1 | 3315 | 1 | 0.007
H2a2a1 | 3411 | 1 | 0.007
H2a2a1 | 3423G | 1 | 0.007
H2a2a1 | 3486 | 1 | 0.007
H2a2a1 | 3519 | 1 | 0.007
H2a2a1 | 3523T | 1 | 0.007
H2a2a1 | 3630 | 1 | 0.007
H2a2a1 | 3644 | 3 | 0.02
H2a2a1 | 3645 | 1 | 0.007
H2a2a1 | 3667G | 1 | 0.007
H2a2a1 | 3769 | 1 | 0.007
H2a2a1 | 3918 | 1 | 0.007
H2a2a1 | 3978 | 1 | 0.007
H2a2a1 | 4122 | 1 | 0.007
H2a2a1 | 4216 | 1 | 0.007
H2a2a1 | 4248 | 1 | 0.007
H2a2a1 | 4290 | 1 | 0.007
H2a2a1 | 43 | 1 | 0.007
H2a2a1 | 4353 | 1 | 0.007
H2a2a1 | 4638 | 1 | 0.007
H2a2a1 | 4655 | 1 | 0.007
H2a2a1 | 4688 | 1 | 0.007
H2a2a1 | 4838 | 1 | 0.007
H2a2a1 | 4851 | 1 | 0.007
H2a2a1 | 4985 | 1 | 0.007
H2a2a1 | 5009 | 1 | 0.007
H2a2a1 | 5082 | 1 | 0.007
H2a2a1 | 5108 | 1 | 0.007
H2a2a1 | 5118 | 1 | 0.007
H2a2a1 | 5147 | 1 | 0.007
H2a2a1 | 5180 | 1 | 0.007
H2a2a1 | 5252 | 1 | 0.007
H2a2a1 | 567 | 1 | 0.007
H2a2a1 | 5899.1C | 1 | 0.007
H2a2a1 | 598.1T | 2 | 0.014
H2a2a1 | 6020 | 1 | 0.007
H2a2a1 | 6120 | 1 | 0.007
H2a2a1 | 6347 | 1 | 0.007
H2a2a1 | 6374 | 1 | 0.007
H2a2a1 | 6461 | 1 | 0.007
H2a2a1 | 6480 | 1 | 0.007
H2a2a1 | 6915 | 1 | 0.007
H2a2a1 | 7028 | 1 | 0.007
H2a2a1 | 7041 | 1 | 0.007
H2a2a1 | 709 | 1 | 0.007
H2a2a1 | 7119 | 1 | 0.007
H2a2a1 | 7280 | 1 | 0.007
H2a2a1 | 73 | 5 | 0.034
H2a2a1 | 7318A | 1 | 0.007
H2a2a1 | 7430 | 4 | 0.027
H2a2a1 | 750 | 2 | 0.014
H2a2a1 | 7521 | 1 | 0.007
H2a2a1 | 7598 | 1 | 0.007
H2a2a1 | 7633 | 1 | 0.007
H2a2a1 | 769 | 1 | 0.007
H2a2a1 | 7786 | 1 | 0.007
H2a2a1 | 7805 | 1 | 0.007
H2a2a1 | 7922 | 1 | 0.007
H2a2a1 | 8041 | 1 | 0.007

H2a2a1 | 8065 | 1 | 0.007
H2a2a1 | 806d | 1 | 0.007
H2a2a1 | 8125 | 3 | 0.02
H2a2a1 | 8251 | 1 | 0.007
H2a2a1 | 8276.1C | 1 | 0.007
H2a2a1 | 8281-8289d | 7 | 0.047
H2a2a1 | 8375 | 1 | 0.007
H2a2a1 | 8458 | 1 | 0.007
H2a2a1 | 8490 | 1 | 0.007
H2a2a1 | 8597 | 1 | 0.007
H2a2a1 | 8631 | 1 | 0.007
H2a2a1 | 8634 | 1 | 0.007
H2a2a1 | 8640 | 1 | 0.007
H2a2a1 | 8667 | 1 | 0.007
H2a2a1 | 8679 | 1 | 0.007
H2a2a1 | 8699 | 1 | 0.007
H2a2a1 | 8701 | 1 | 0.007
H2a2a1 | 8802 | 1 | 0.007
H2a2a1 | 8862 | 1 | 0.007
H2a2a1 | 8966 | 1 | 0.007
H2a2a1 | 9018 | 1 | 0.007
H2a2a1 | 9045T | 1 | 0.007
H2a2a1 | 9061 | 1 | 0.007
H2a2a1 | 9126 | 1 | 0.007
H2a2a1 | 9194 | 1 | 0.007
H2a2a1 | 9245G | 1 | 0.007
H2a2a1 | 9275 | 1 | 0.007
H2a2a1 | 9473 | 1 | 0.007
H2a2a1 | 9494 | 1 | 0.007
H2a2a1 | 9559G | 1 | 0.007
H2a2a1 | 955C | 1 | 0.007
H2a2a1 | 9592 | 1 | 0.007
H2a2a1 | 9773 | 1 | 0.007
H2a2a1 | 9848 | 1 | 0.007
H2a2a1 | 9877A | 1 | 0.007
H2a2a1 | 9893 | 1 | 0.007
H2a2a1 | 9947 | 2 | 0.014
H2a2a1a | 14279 | 1 | 0.083
H2a2a1a | 15386 | 1 | 0.083
H2a2a1a | 16195 | 2 | 0.167
H2a2a1a | 16259 | 5 | 0.417
H2a2a1a | 189 | 2 | 0.167
H2a2a1a | 310 | 1 | 0.083
H2a2a1a | 7220 | 1 | 0.083
H2a2a1a | 9126A | 2 | 0.167
H2a2a1b | 12366 | 1 | 0.077
H2a2a1b | 13299 | 1 | 0.077
H2a2a1b | 15130 | 1 | 0.077
H2a2a1b | 16145 | 3 | 0.231
H2a2a1b | 5046 | 1 | 0.077
H2a2a1b | 6719 | 1 | 0.077
H2a2a1c | 151 | 1 | 0.05
H2a2a1c | 152 | 1 | 0.05
H2a2a1c | 15827T | 2 | 0.1
H2a2a1c | 15835 | 2 | 0.1
H2a2a1c | 15836 | 2 | 0.1
H2a2a1c | 15841 | 2 | 0.1
H2a2a1c | 16131 | 3 | 0.15
H2a2a1c | 16217 | 1 | 0.05
H2a2a1c | 16221 | 1 | 0.05
H2a2a1c | 16264 | 1 | 0.05
H2a2a1c | 16274 | 1 | 0.05
H2a2a1c | 16309 | 1 | 0.05
H2a2a1c | 16311 | 1 | 0.05
H2a2a1c | 16465 | 1 | 0.05
H2a2a1c | 16475G | 2 | 0.1
H2a2a1c | 16502 | 1 | 0.05
H2a2a1c | 16513G | 1 | 0.05
H2a2a1c | 226 | 1 | 0.05
H2a2a1c | 263 | 3 | 0.15
H2a2a1d | 10373C | 3 | 0.071
H2a2a1d | 11335 | 3 | 0.071
H2a2a1d | 11440 | 3 | 0.071
H2a2a1d | 11719 | 3 | 0.071
H2a2a1d | 12135A | 1 | 0.024
H2a2a1d | 12654 | 3 | 0.071
H2a2a1d | 13474 | 3 | 0.071
H2a2a1d | 13702G | 3 | 0.071
H2a2a1d | 14199G | 3 | 0.071
H2a2a1d | 14272G | 3 | 0.071
H2a2a1d | 14357 | 2 | 0.048
H2a2a1d | 14365G | 3 | 0.071
H2a2a1d | 14368G | 3 | 0.071
H2a2a1d | 146 | 1 | 0.024
H2a2a1d | 14766 | 3 | 0.071
H2a2a1d | 150 | 3 | 0.071
H2a2a1d | 152 | 5 | 0.119
H2a2a1d | 15211 | 3 | 0.071
H2a2a1d | 15326 | 3 | 0.071
H2a2a1d | 16069 | 1 | 0.024
H2a2a1d | 16086 | 1 | 0.024
H2a2a1d | 16092 | 1 | 0.024
H2a2a1d | 16111 | 4 | 0.095
H2a2a1d | 16126 | 1 | 0.024
H2a2a1d | 16145 | 1 | 0.024
H2a2a1d | 16164 | 1 | 0.024
H2a2a1d | 16168 | 3 | 0.071
H2a2a1d | 16169 | 1 | 0.024
H2a2a1d | 16186 | 4 | 0.095
H2a2a1d | 16189 | 13 | 0.31
H2a2a1d | 16193 | 1 | 0.024
H2a2a1d | 16194C | 3 | 0.071
H2a2a1d | 16213 | 2 | 0.048
H2a2a1d | 16219 | 4 | 0.095
H2a2a1d | 16222 | 1 | 0.024
H2a2a1d | 16223 | 1 | 0.024
H2a2a1d | 16261 | 1 | 0.024
H2a2a1d | 16270 | 1 | 0.024
H2a2a1d | 16274 | 2 | 0.048
H2a2a1d | 16278 | 4 | 0.095
H2a2a1d | 16290 | 1 | 0.024

H2a2a1d | 16293C | 1 | 0.024
H2a2a1d | 16311 | 2 | 0.048
H2a2a1d | 16319 | 2 | 0.048
H2a2a1d | 16325 | 1 | 0.024
H2a2a1d | 16327 | 1 | 0.024
H2a2a1d | 16357A | 1 | 0.024
H2a2a1d | 16376 | 1 | 0.024
H2a2a1d | 16377 | 1 | 0.024
H2a2a1d | 16378 | 1 | 0.024
H2a2a1d | 16379A | 1 | 0.024
H2a2a1d | 16465 | 1 | 0.024
H2a2a1d | 16468 | 1 | 0.024
H2a2a1d | 185 | 1 | 0.024
H2a2a1d | 188 | 1 | 0.024
H2a2a1d | 195 | 1 | 0.024
H2a2a1d | 196 | 1 | 0.024
H2a2a1d | 207 | 6 | 0.143
H2a2a1d | 242 | 1 | 0.024
H2a2a1d | 263 | 6 | 0.143
H2a2a1d | 2706 | 3 | 0.071
H2a2a1d | 295 | 1 | 0.024
H2a2a1d | 3423G | 3 | 0.071
H2a2a1d | 44.1C | 4 | 0.095
H2a2a1d | 4985 | 3 | 0.071
H2a2a1d | 5213G | 3 | 0.071
H2a2a1d | 5581 | 3 | 0.071
H2a2a1d | 650 | 3 | 0.071
H2a2a1d | 73 | 7 | 0.167
H2a2a1d | 7894 | 3 | 0.071
H2a2a1d | 8652 | 3 | 0.071
H2a2a1d | 93 | 1 | 0.024
H2a2a1d | 9531 | 3 | 0.071
H2a2a1d | 9559G | 3 | 0.071
H2a2a1e | 146 | 1 | 0.071
H2a2a1e | 16111G | 4 | 0.286
H2a2a1e | 16129 | 1 | 0.071
H2a2a1e | 16291 | 3 | 0.214
H2a2a1e | 64 | 1 | 0.071
H2a2a1f | 11778 | 1 | 0.034
H2a2a1f | 146 | 2 | 0.069
H2a2a1f | 14883 | 1 | 0.034
H2a2a1f | 152 | 1 | 0.034
H2a2a1f | 15314 | 1 | 0.034
H2a2a1f | 16126 | 1 | 0.034
H2a2a1f | 16169 | 1 | 0.034
H2a2a1f | 16188G | 3 | 0.103
H2a2a1f | 16223 | 1 | 0.034
H2a2a1f | 16260 | 1 | 0.034
H2a2a1f | 16278 | 3 | 0.103
H2a2a1f | 16294 | 1 | 0.034
H2a2a1f | 16296 | 2 | 0.069
H2a2a1f | 16304 | 2 | 0.069
H2a2a1f | 16311 | 1 | 0.034
H2a2a1f | 16318T | 1 | 0.034
H2a2a1f | 16324 | 1 | 0.034
H2a2a1f | 16356 | 1 | 0.034
H2a2a1f | 16357 | 3 | 0.103
H2a2a1f | 1709 | 1 | 0.034
H2a2a1f | 194 | 1 | 0.034
H2a2a1f | 200 | 1 | 0.034
H2a2a1f | 204 | 2 | 0.069
H2a2a1f | 246 | 1 | 0.034
H2a2a1f | 263 | 8 | 0.276
H2a2a1f | 279 | 1 | 0.034
H2a2a1f | 6480 | 1 | 0.034
H2a2a1f | 73 | 1 | 0.034
H2a2a1f | 9299 | 1 | 0.034
H2a2a1f | 95 | 1 | 0.034
H2a2a1g | 146 | 2 | 0.021
H2a2a1g | 14798 | 1 | 0.011
H2a2a1g | 150 | 3 | 0.032
H2a2a1g | 152 | 3 | 0.032
H2a2a1g | 153 | 2 | 0.021
H2a2a1g | 16075 | 1 | 0.011
H2a2a1g | 16093 | 1 | 0.011
H2a2a1g | 16126 | 4 | 0.043
H2a2a1g | 16129 | 1 | 0.011
H2a2a1g | 16153 | 1 | 0.011
H2a2a1g | 16163 | 3 | 0.032
H2a2a1g | 16178 | 1 | 0.011
H2a2a1g | 16180 | 1 | 0.011
H2a2a1g | 16181C | 1 | 0.011
H2a2a1g | 16186 | 3 | 0.032
H2a2a1g | 16187 | 1 | 0.011
H2a2a1g | 16192 | 1 | 0.011
H2a2a1g | 16193d | 1 | 0.011
H2a2a1g | 16223 | 3 | 0.032
H2a2a1g | 16234 | 1 | 0.011
H2a2a1g | 16234d | 1 | 0.011
H2a2a1g | 16249 | 1 | 0.011
H2a2a1g | 16264 | 1 | 0.011
H2a2a1g | 16265C | 1 | 0.011
H2a2a1g | 16270 | 3 | 0.032
H2a2a1g | 16274 | 1 | 0.011
H2a2a1g | 16278 | 4 | 0.043
H2a2a1g | 16292 | 1 | 0.011
H2a2a1g | 16293 | 1 | 0.011
H2a2a1g | 16293C | 1 | 0.011
H2a2a1g | 16294 | 5 | 0.053
H2a2a1g | 16298 | 4 | 0.043
H2a2a1g | 16309 | 4 | 0.043
H2a2a1g | 16311 | 3 | 0.032
H2a2a1g | 16356 | 3 | 0.032
H2a2a1g | 16362 | 4 | 0.043
H2a2a1g | 16390 | 1 | 0.011
H2a2a1g | 16526 | 1 | 0.011
H2a2a1g | 182 | 1 | 0.011
H2a2a1g | 185T | 1 | 0.011
H2a2a1g | 189 | 1 | 0.011
H2a2a1g | 195 | 4 | 0.043
H2a2a1g | 200 | 1 | 0.011

H2a2a1g | 225 | 1 | 0.011
H2a2a1g | 226 | 1 | 0.011
H2a2a1g | 247 | 1 | 0.011
H2a2a1g | 256 | 1 | 0.011
H2a2a1g | 263 | 25 | 0.266
H2a2a1g | 327 | 1 | 0.011
H2a2a1g | 456 | 1 | 0.011
H2a2a1g | 512 | 1 | 0.011
H2a2a1g | 72 | 1 | 0.011
H2a2a1g | 73 | 12 | 0.128
H2a2a1g | 93 | 1 | 0.011
H2a2a1h | 10245 | 1 | 0.03
H2a2a1h | 10700 | 1 | 0.03
H2a2a1h | 12684 | 1 | 0.03
H2a2a1h | 12810 | 1 | 0.03
H2a2a1h | 13017 | 1 | 0.03
H2a2a1h | 13477 | 1 | 0.03
H2a2a1h | 13637 | 1 | 0.03
H2a2a1h | 13958C | 1 | 0.03
H2a2a1h | 1438 | 2 | 0.061
H2a2a1h | 146 | 2 | 0.061
H2a2a1h | 152 | 3 | 0.091
H2a2a1h | 15340 | 1 | 0.03
H2a2a1h | 15884 | 1 | 0.03
H2a2a1h | 16069 | 1 | 0.03
H2a2a1h | 16150 | 1 | 0.03
H2a2a1h | 16179 | 1 | 0.03
H2a2a1h | 16180 | 1 | 0.03
H2a2a1h | 16189 | 3 | 0.091
H2a2a1h | 16192 | 1 | 0.03
H2a2a1h | 16223 | 1 | 0.03
H2a2a1h | 16227 | 1 | 0.03
H2a2a1h | 16243 | 1 | 0.03
H2a2a1h | 16256 | 2 | 0.061
H2a2a1h | 16256A | 1 | 0.03
H2a2a1h | 16261 | 1 | 0.03
H2a2a1h | 16270 | 2 | 0.061
H2a2a1h | 16284 | 1 | 0.03
H2a2a1h | 16298 | 1 | 0.03
H2a2a1h | 16311 | 1 | 0.03
H2a2a1h | 16316 | 1 | 0.03
H2a2a1h | 16368 | 1 | 0.03
H2a2a1h | 1721 | 1 | 0.03
H2a2a1h | 189C | 1 | 0.03
H2a2a1h | 194 | 1 | 0.03
H2a2a1h | 195 | 1 | 0.03
H2a2a1h | 204 | 2 | 0.061
H2a2a1h | 263 | 3 | 0.091
H2a2a1h | 263C | 2 | 0.061
H2a2a1h | 310 | 1 | 0.03
H2a2a1h | 315d | 1 | 0.03
H2a2a1h | 3546 | 1 | 0.03
H2a2a1h | 4080 | 1 | 0.03
H2a2a1h | 4580 | 1 | 0.03
H2a2a1h | 4718 | 1 | 0.03
H2a2a1h | 4769 | 1 | 0.03
H2a2a1h | 4985 | 1 | 0.03
H2a2a1h | 5420G | 1 | 0.03
H2a2a1h | 6231 | 2 | 0.061
H2a2a1h | 6998 | 1 | 0.03
H2a2a1h | 7028 | 1 | 0.03
H2a2a1h | 721 | 1 | 0.03
H2a2a1h | 73 | 3 | 0.091
H2a2a1h | 7444 | 1 | 0.03
H2a2a1h | 750 | 1 | 0.03
H2a2a1h | 7561 | 1 | 0.03
H2a2a1h | 7954 | 1 | 0.03
H2a2a1h | 8654 | 1 | 0.03
H2a2a1h | 960.1C | 1 | 0.03
H2a2a2 | 1133 | 1 | 0.007
H2a2a2 | 12903 | 1 | 0.007
H2a2a2 | 13816 | 1 | 0.007
H2a2a2 | 14162 | 1 | 0.007
H2a2a2 | 14311 | 1 | 0.007
H2a2a2 | 14872 | 1 | 0.007
H2a2a2 | 16093 | 1 | 0.007
H2a2a2 | 16129 | 7 | 0.046
H2a2a2 | 16214 | 3 | 0.02
H2a2a2 | 16231 | 1 | 0.007
H2a2a2 | 16233T | 1 | 0.007
H2a2a2 | 16247 | 20 | 0.132
H2a2a2 | 16258T | 1 | 0.007
H2a2a2 | 16285.1A | 2 | 0.013
H2a2a2 | 182 | 1 | 0.007
H2a2a2 | 200 | 1 | 0.007
H2a2a2 | 200T | 1 | 0.007
H2a2a2 | 207 | 1 | 0.007
H2a2a2 | 408A | 1 | 0.007
H2a2a2 | 456 | 1 | 0.007
H2a2a2 | 463 | 1 | 0.007
H2a2a2 | 515-524d | 1 | 0.007
H2a2a2 | 529T | 1 | 0.007
H2a2a2 | 573.1C | 1 | 0.007
H2a2b | 10172 | 1 | 0.007
H2a2b | 10235 | 1 | 0.007
H2a2b | 10310 | 1 | 0.007
H2a2b | 10373 | 2 | 0.013
H2a2b | 10609 | 1 | 0.007
H2a2b | 12622 | 2 | 0.013
H2a2b | 12941 | 3 | 0.02
H2a2b | 13105 | 1 | 0.007
H2a2b | 13708 | 3 | 0.02
H2a2b | 1393 | 3 | 0.02
H2a2b | 14384 | 2 | 0.013
H2a2b | 152 | 1 | 0.007
H2a2b | 15916 | 1 | 0.007
H2a2b | 16051 | 1 | 0.007
H2a2b | 16093 | 1 | 0.007
H2a2b | 16150 | 1 | 0.007
H2a2b | 16154 | 3 | 0.02
H2a2b | 16274 | 1 | 0.007

H2a2b | 16311 | 2 | 0.013
H2a2b | 16319 | 1 | 0.007
H2a2b | 16357 | 10 | 0.066
H2a2b | 16390 | 1 | 0.007
H2a2b | 193 | 5 | 0.033
H2a2b | 200 | 1 | 0.007
H2a2b | 207 | 1 | 0.007
H2a2b | 3388A | 1 | 0.007
H2a2b | 391 | 2 | 0.013
H2a2b | 4655 | 1 | 0.007
H2a2b | 497 | 1 | 0.007
H2a2b | 573.1C | 1 | 0.007
H2a2b | 573.2C | 1 | 0.007
H2a2b | 6261 | 1 | 0.007
H2a2b | 6527 | 1 | 0.007
H2a2b | 6674 | 2 | 0.013
H2a2b | 750 | 4 | 0.026
H2a2b | 7853 | 2 | 0.013
H2a2b | 8020 | 1 | 0.007
H2a2b | 8286 | 2 | 0.013
H2a2b | 8906 | 3 | 0.02
H2a2b | 9085 | 1 | 0.007
H2a2b | 9182 | 1 | 0.007
H2a2b | 9467 | 1 | 0.007
H2a2b1 | 10410 | 1 | 0.018
H2a2b1 | 11548 | 1 | 0.018
H2a2b1 | 12151 | 1 | 0.018
H2a2b1 | 182 | 1 | 0.018
H2a2b1 | 5893 | 1 | 0.018
H2a2b1 | 7080 | 1 | 0.018
H2a2b1a | 146 | 1 | 0.019
H2a2b1a | 1555 | 2 | 0.038
H2a2b1a | 16256 | 1 | 0.019
H2a2b1a | 16284 | 1 | 0.019
H2a2b1a1 | 10142 | 1 | 0.017
H2a2b1a1 | 10698 | 2 | 0.034
H2a2b1a1 | 12280 | 1 | 0.017
H2a2b1a1 | 16086 | 1 | 0.017
H2a2b1a1 | 16284 | 1 | 0.017
H2a2b1a1 | 16346 | 1 | 0.017
H2a2b1a1 | 16471 | 1 | 0.017
H2a2b2 | 146 | 1 | 0.008
H2a2b2 | 16221 | 1 | 0.008
H2a2b2 | 16263 | 1 | 0.008
H2a2b2 | 16270 | 1 | 0.008
H2a2b2 | 16298 | 1 | 0.008
H2a2b2 | 16311 | 1 | 0.008
H2a2b2 | 16330 | 4 | 0.031
H2a2b2 | 204 | 1 | 0.008
H2a2b2 | 384 | 1 | 0.008
H2a2b3 | 12188 | 4 | 0.03
H2a2b3 | 12811 | 1 | 0.007
H2a2b3 | 16220C | 2 | 0.015
H2a2b3 | 16311 | 3 | 0.022
H2a2b3 | 309d | 1 | 0.007
H2a2b3 | 310 | 2 | 0.015
H2a2b3 | 311 | 1 | 0.007
H2a2b3 | 3261 | 2 | 0.015
H2a2b3 | 486 | 1 | 0.007
H2a2b3 | 532C | 1 | 0.007
H2a2b3 | 593 | 1 | 0.007
H2a2b3 | 9157 | 1 | 0.007
H2a2b3 | 9180 | 4 | 0.03
H2a2b4 | 13749 | 2 | 0.016
H2a2b4 | 16221 | 1 | 0.008
H2a2b4 | 16222 | 1 | 0.008
H2a2b4 | 16263 | 1 | 0.008
H2a2b4 | 16270 | 1 | 0.008
H2a2b4 | 16298 | 1 | 0.008
H2a2b4 | 16330 | 4 | 0.031
H2a2b4 | 204 | 1 | 0.008
H2a2b4 | 384 | 1 | 0.008
H2a2b4 | 3882 | 2 | 0.016
H2a2b4 | 73 | 1 | 0.008
H2a2b5 | 16221 | 1 | 0.007
H2a2b5 | 16235 | 1 | 0.007
H2a2b5 | 16263 | 1 | 0.007
H2a2b5 | 16270 | 1 | 0.007
H2a2b5 | 16298 | 3 | 0.022
H2a2b5 | 204 | 1 | 0.007
H2a2b5 | 384 | 1 | 0.007
H2a2b5 | 5528 | 1 | 0.007
H2a2b5 | 9287 | 1 | 0.007
H2a2b5 | 93 | 10 | 0.075
H2a2b5a | 13708 | 1 | 0.008
H2a2b5a | 14581 | 1 | 0.008
H2a2b5a | 15941 | 1 | 0.008
H2a2b5a | 16221 | 1 | 0.008
H2a2b5a | 16263 | 1 | 0.008
H2a2b5a | 16270 | 1 | 0.008
H2a2b5a | 16292 | 3 | 0.023
H2a2b5a | 16298 | 1 | 0.008
H2a2b5a | 16319 | 3 | 0.023
H2a2b5a | 16330 | 4 | 0.031
H2a2b5a | 204 | 1 | 0.008
H2a2b5a | 310 | 1 | 0.008
H2a2b5a | 3360 | 1 | 0.008
H2a2b5a | 384 | 1 | 0.008
H2a2b5a | 9142 | 1 | 0.008
H2a2b5a | 9300 | 1 | 0.008
H2a3 | 10373 | 1 | 0.009
H2a3 | 10899 | 1 | 0.009
H2a3 | 13911 | 1 | 0.009
H2a3 | 14470 | 1 | 0.009
H2a3 | 152 | 5 | 0.046
H2a3 | 15299 | 1 | 0.009
H2a3 | 16121 | 1 | 0.009
H2a3 | 16158 | 1 | 0.009
H2a3 | 16167 | 1 | 0.009
H2a3 | 16215 | 7 | 0.065
H2a3 | 16223d | 1 | 0.009

H2a3 | 16225.1T | 1 | 0.009
H2a3 | 16294G | 1 | 0.009
H2a3 | 16304 | 1 | 0.009
H2a3 | 16311 | 2 | 0.019
H2a3 | 16355 | 1 | 0.009
H2a3 | 16488 | 1 | 0.009
H2a3 | 182 | 1 | 0.009
H2a3 | 200 | 1 | 0.009
H2a3 | 204 | 1 | 0.009
H2a3 | 262 | 2 | 0.019
H2a3 | 310 | 2 | 0.019
H2a3 | 3396 | 2 | 0.019
H2a3 | 3438 | 1 | 0.009
H2a3 | 506 | 1 | 0.009
H2a3 | 5821 | 1 | 0.009
H2a3 | 9682 | 2 | 0.019
H2a3a | 14290 | 2 | 0.019
H2a3a | 146 | 2 | 0.019
H2a3a | 16121 | 1 | 0.01
H2a3a | 16126 | 4 | 0.039
H2a3a | 16158 | 1 | 0.01
H2a3a | 16167 | 3 | 0.029
H2a3a | 16214 | 2 | 0.019
H2a3a | 16223d | 1 | 0.01
H2a3a | 16225.1T | 1 | 0.01
H2a3a | 16304 | 1 | 0.01
H2a3a | 16311 | 2 | 0.019
H2a3a | 16488 | 1 | 0.01
H2a3a | 204 | 1 | 0.01
H2a3a | 2259 | 1 | 0.01
H2a3a | 506 | 1 | 0.01
H2a3a | 824 | 1 | 0.01
H2a3a1 | 11719 | 1 | 0.01
H2a3a1 | 12903 | 1 | 0.01
H2a3a1 | 146 | 1 | 0.01
H2a3a1 | 16121 | 1 | 0.01
H2a3a1 | 16158 | 1 | 0.01
H2a3a1 | 16223d | 1 | 0.01
H2a3a1 | 16225.1T | 1 | 0.01
H2a3a1 | 16253T | 1 | 0.01
H2a3a1 | 16311 | 2 | 0.019
H2a3a1 | 200 | 1 | 0.01
H2a3a1 | 204 | 1 | 0.01
H2a3a1 | 309.3C | 1 | 0.01
H2a3a1 | 310 | 2 | 0.019
H2a3a1 | 506 | 1 | 0.01
H2a3a1 | 5806 | 1 | 0.01
H2a3a1 | 824 | 11 | 0.107
H2a3a1 | 9579 | 1 | 0.01
H2a3b | 14927 | 1 | 0.01
H2a3b | 16121 | 1 | 0.01
H2a3b | 16158 | 1 | 0.01
H2a3b | 16223d | 1 | 0.01
H2a3b | 16225.1T | 1 | 0.01
H2a3b | 16287 | 6 | 0.059
H2a3b | 16304 | 1 | 0.01
H2a3b | 16311 | 2 | 0.02
H2a3b | 199 | 2 | 0.02
H2a3b | 204 | 1 | 0.01
H2a3b | 506 | 1 | 0.01
H2a3b | 73 | 1 | 0.01
H2a4 | 113 | 1 | 0.001
H2a4 | 16076A | 1 | 0.001
H2a4 | 16187 | 1 | 0.001
H2a4 | 16227 | 124 | 0.156
H2a4 | 16362 | 1 | 0.001
H2a4 | 208 | 1 | 0.001
H2a4 | 261 | 1 | 0.001
H2a4 | 281C | 1 | 0.001
H2a4 | 316d | 1 | 0.001
H2a4 | 338 | 1 | 0.001
H2a4 | 468 | 3 | 0.004
H2a4 | 502 | 1 | 0.001
H2a4 | 5557 | 1 | 0.001
H2a4 | 573.1C | 3 | 0.004
H2a4 | 573.2C | 5 | 0.006
H2a4 | 573.5C | 1 | 0.001
H2a4 | 6221 | 1 | 0.001
H2a5 | 10993 | 1 | 0.001
H2a5 | 113 | 1 | 0.001
H2a5 | 13962A | 2 | 0.002
H2a5 | 14180 | 1 | 0.001
H2a5 | 15266 | 1 | 0.001
H2a5 | 16076A | 123 | 0.153
H2a5 | 16362 | 1 | 0.001
H2a5 | 208 | 1 | 0.001
H2a5 | 2352 | 1 | 0.001
H2a5 | 261 | 1 | 0.001
H2a5 | 281C | 1 | 0.001
H2a5 | 310 | 1 | 0.001
H2a5 | 316d | 1 | 0.001
H2a5 | 338 | 1 | 0.001
H2a5 | 468 | 3 | 0.004
H2a5 | 4976 | 1 | 0.001
H2a5 | 502 | 1 | 0.001
H2a5 | 573.1C | 3 | 0.004
H2a5 | 573.2C | 5 | 0.006
H2a5 | 573.5C | 1 | 0.001
H2a5 | 6179 | 1 | 0.001
H2a5a | 113 | 1 | 0.001
H2a5a | 16076A | 123 | 0.155
H2a5a | 208 | 1 | 0.001
H2a5a | 261 | 1 | 0.001
H2a5a | 281C | 1 | 0.001
H2a5a | 316d | 1 | 0.001
H2a5a | 338 | 1 | 0.001
H2a5a | 468 | 3 | 0.004
H2a5a | 502 | 1 | 0.001
H2a5a | 573.1C | 3 | 0.004
H2a5a | 573.2C | 5 | 0.006
H2a5a | 573.5C | 1 | 0.001

H2a5a1 | 11368 | 1 | 0.007
H2a5a1 | 11530 | 1 | 0.007
H2a5a1 | 146 | 4 | 0.027
H2a5a1 | 16178 | 1 | 0.007
H2a5a1 | 16304 | 9 | 0.062
H2a5a1 | 225 | 1 | 0.007
H2a5a1 | 227 | 1 | 0.007
H2a5a1 | 5899.1C | 3 | 0.021
H2a5a1a | 10391 | 3 | 0.019
H2a5a1a | 4354 | 2 | 0.013
H2a5a1a | 7051 | 1 | 0.006
H2a5a1a | 8285.1C | 1 | 0.006
H2a5a1a | 8916 | 1 | 0.006
H2a5a1a | 9033 | 5 | 0.032
H2a5a1b | 14323 | 1 | 0.007
H2a5a1b | 16221 | 1 | 0.007
H2a5a1b | 16270 | 1 | 0.007
H2a5a1b | 16298 | 3 | 0.022
H2a5a1b | 204 | 1 | 0.007
H2a5b | 113 | 1 | 0.001
H2a5b | 11914 | 1 | 0.001
H2a5b | 12052 | 1 | 0.001
H2a5b | 146 | 1 | 0.001
H2a5b | 14971 | 1 | 0.001
H2a5b | 15315 | 1 | 0.001
H2a5b | 16076A | 123 | 0.153
H2a5b | 16189 | 1 | 0.001
H2a5b | 16209 | 1 | 0.001
H2a5b | 208 | 1 | 0.001
H2a5b | 261 | 1 | 0.001
H2a5b | 281C | 1 | 0.001
H2a5b | 316d | 1 | 0.001
H2a5b | 338 | 1 | 0.001
H2a5b | 3488 | 1 | 0.001
H2a5b | 4227 | 1 | 0.001
H2a5b | 468 | 3 | 0.004
H2a5b | 502 | 1 | 0.001
H2a5b | 512C | 2 | 0.002
H2a5b | 573.1C | 3 | 0.004
H2a5b | 573.2C | 5 | 0.006
H2a5b | 573.5C | 1 | 0.001
H2a5b | 9596 | 1 | 0.001
H2a5b1 | 11260 | 1 | 0.056
H2a5b1 | 12052 | 1 | 0.056
H2a5b1 | 12136 | 1 | 0.056
H2a5b1 | 16209 | 2 | 0.111
H2a5b1 | 16274 | 1 | 0.056
H2a5b1 | 16311 | 1 | 0.056
H2a5b1 | 518 | 2 | 0.111
H2a5b1 | 5819A | 2 | 0.111
H2a5b1 | 73 | 2 | 0.111
H2a5b1 | 93 | 1 | 0.056
H2a5b2 | 152 | 1 | 0.013
H2a5b2 | 16067 | 1 | 0.013
H2a5b2 | 16147 | 1 | 0.013
H2a5b2 | 16189 | 1 | 0.013
H2a5b2 | 16311 | 2 | 0.027
H2a5b2 | 16335 | 1 | 0.013
H2a5b2 | 16390 | 1 | 0.013
H2a5b2 | 225 | 1 | 0.013
H2a5b2 | 226 | 1 | 0.013
H2a5b2 | 237 | 1 | 0.013
H2b | 11440 | 1 | 0.005
H2b | 11524 | 1 | 0.005
H2b | 12007 | 1 | 0.005
H2b | 13281 | 1 | 0.005
H2b | 13928 | 1 | 0.005
H2b | 13942 | 1 | 0.005
H2b | 146 | 2 | 0.01
H2b | 15077 | 1 | 0.005
H2b | 15373 | 3 | 0.015
H2b | 15386A | 1 | 0.005
H2b | 1555 | 1 | 0.005
H2b | 15929 | 1 | 0.005
H2b | 16086 | 8 | 0.04
H2b | 16093 | 1 | 0.005
H2b | 16111 | 1 | 0.005
H2b | 16184 | 1 | 0.005
H2b | 16189 | 1 | 0.005
H2b | 16209 | 1 | 0.005
H2b | 16266 | 1 | 0.005
H2b | 16362 | 4 | 0.02
H2b | 16399 | 1 | 0.005
H2b | 199 | 4 | 0.02
H2b | 2244 | 1 | 0.005
H2b | 310 | 5 | 0.025
H2b | 3106A | 2 | 0.01
H2b | 311 | 2 | 0.01
H2b | 316C | 1 | 0.005
H2b | 3173 | 1 | 0.005
H2b | 319 | 1 | 0.005
H2b | 3394 | 2 | 0.01
H2b | 408A | 3 | 0.015
H2b | 5460 | 3 | 0.015
H2b | 6215 | 2 | 0.01
H2b | 6512 | 1 | 0.005
H2b | 8065 | 1 | 0.005
H2b | 8101 | 2 | 0.01
H2b | 8277 | 1 | 0.005
H2b | 8577 | 1 | 0.005
H2b | 93 | 1 | 0.005
H2b | 9448 | 1 | 0.005
H2b | 9596 | 1 | 0.005
H2b | 9801 | 1 | 0.005
H2c | 14249 | 1 | 0.006
H2c | 14560 | 1 | 0.006
H2c | 16093 | 1 | 0.006
H2c | 16209 | 3 | 0.018
H2c | 16465 | 2 | 0.012
H2c | 4385 | 1 | 0.006
H2c | 709 | 1 | 0.006

H2c | 9090 | 1 | 0.006
H2c | 9386 | 1 | 0.006
H2c1 | 15930 | 1 | 0.009
H2c1 | 16249 | 2 | 0.017
H2c1 | 573.1C | 1 | 0.009
H2c1 | 6018 | 1 | 0.009
H3 | 1019 | 12 | 0.01
H3 | 10245 | 1 | 0.001
H3 | 10316 | 1 | 0.001
H3 | 10658 | 1 | 0.001
H3 | 10727 | 1 | 0.001
H3 | 10745 | 1 | 0.001
H3 | 10837 | 1 | 0.001
H3 | 10963 | 1 | 0.001
H3 | 10978 | 1 | 0.001
H3 | 11086 | 2 | 0.002
H3 | 1119 | 2 | 0.002
H3 | 11200 | 22 | 0.019
H3 | 11230A | 1 | 0.001
H3 | 11233 | 1 | 0.001
H3 | 11290 | 2 | 0.002
H3 | 113 | 1 | 0.001
H3 | 11447 | 1 | 0.001
H3 | 11458 | 2 | 0.002
H3 | 11731 | 1 | 0.001
H3 | 11863 | 2 | 0.002
H3 | 11893 | 2 | 0.002
H3 | 11936 | 1 | 0.001
H3 | 12033 | 11 | 0.009
H3 | 12113 | 1 | 0.001
H3 | 12133 | 1 | 0.001
H3 | 12136 | 1 | 0.001
H3 | 12165 | 1 | 0.001
H3 | 12285 | 1 | 0.001
H3 | 1243 | 1 | 0.001
H3 | 12450 | 2 | 0.002
H3 | 12480 | 1 | 0.001
H3 | 12630 | 2 | 0.002
H3 | 12684 | 2 | 0.002
H3 | 12705 | 4 | 0.003
H3 | 12738 | 2 | 0.002
H3 | 12771 | 1 | 0.001
H3 | 12804 | 3 | 0.003
H3 | 12950 | 2 | 0.002
H3 | 13105 | 1 | 0.001
H3 | 1313 | 16 | 0.014
H3 | 13145 | 1 | 0.001
H3 | 13350 | 2 | 0.002
H3 | 13359 | 4 | 0.003
H3 | 13368 | 1 | 0.001
H3 | 13371 | 1 | 0.001
H3 | 13419 | 1 | 0.001
H3 | 13431 | 1 | 0.001
H3 | 13535 | 2 | 0.002
H3 | 13575 | 1 | 0.001
H3 | 13635 | 1 | 0.001
H3 | 13698 | 4 | 0.003
H3 | 13708 | 1 | 0.001
H3 | 13759 | 2 | 0.002
H3 | 13768 | 1 | 0.001
H3 | 13928C | 13 | 0.011
H3 | 13950A | 1 | 0.001
H3 | 14002 | 2 | 0.002
H3 | 14016 | 1 | 0.001
H3 | 14180 | 1 | 0.001
H3 | 14259 | 1 | 0.001
H3 | 143 | 2 | 0.002
H3 | 14353 | 9 | 0.008
H3 | 14428 | 4 | 0.003
H3 | 14470 | 11 | 0.009
H3 | 14530 | 1 | 0.001
H3 | 14569 | 1 | 0.001
H3 | 14577 | 1 | 0.001
H3 | 146 | 6 | 0.005
H3 | 14798 | 30 | 0.026
H3 | 14831 | 1 | 0.001
H3 | 14858 | 1 | 0.001
H3 | 14900 | 3 | 0.003
H3 | 150 | 6 | 0.005
H3 | 15071 | 1 | 0.001
H3 | 15074 | 1 | 0.001
H3 | 15115G | 3 | 0.003
H3 | 15133 | 1 | 0.001
H3 | 15184 | 1 | 0.001
H3 | 152 | 1 | 0.001
H3 | 15238A | 1 | 0.001
H3 | 15289 | 25 | 0.021
H3 | 15301 | 3 | 0.003
H3 | 15315 | 144 | 0.123
H3 | 15346 | 1 | 0.001
H3 | 15412 | 2 | 0.002
H3 | 15434 | 1 | 0.001
H3 | 15440 | 1 | 0.001
H3 | 15519 | 3 | 0.003
H3 | 15525 | 1 | 0.001
H3 | 15530 | 4 | 0.003
H3 | 1555 | 2 | 0.002
H3 | 15562 | 1 | 0.001
H3 | 15667 | 1 | 0.001
H3 | 15670 | 1 | 0.001
H3 | 15772T | 1 | 0.001
H3 | 15790 | 1 | 0.001
H3 | 15803 | 1 | 0.001
H3 | 15902 | 1 | 0.001
H3 | 15930 | 1 | 0.001
H3 | 16000 | 1 | 0.001
H3 | 16038 | 1 | 0.001
H3 | 16051 | 1 | 0.001
H3 | 16076A | 1 | 0.001
H3 | 16093 | 1 | 0.001
H3 | 16126 | 2 | 0.002

H3 | 16129 | 1 | 0.001
H3 | 16140 | 4 | 0.003
H3 | 16145 | 3 | 0.003
H3 | 16166 | 1 | 0.001
H3 | 16172 | 5 | 0.004
H3 | 16180 | 1 | 0.001
H3 | 16181 | 5 | 0.004
H3 | 16188 | 1 | 0.001
H3 | 16192 | 1 | 0.001
H3 | 16209 | 3 | 0.003
H3 | 16213 | 1 | 0.001
H3 | 16234 | 2 | 0.002
H3 | 16242 | 1 | 0.001
H3 | 16245 | 1 | 0.001
H3 | 16248 | 3 | 0.003
H3 | 16257 | 11 | 0.009
H3 | 16260 | 1 | 0.001
H3 | 16261 | 1 | 0.001
H3 | 16264 | 2 | 0.002
H3 | 16269 | 1 | 0.001
H3 | 16274 | 2 | 0.002
H3 | 16278 | 1 | 0.001
H3 | 16289 | 2 | 0.002
H3 | 16290 | 1 | 0.001
H3 | 16291 | 1 | 0.001
H3 | 16292 | 1 | 0.001
H3 | 16294 | 18 | 0.015
H3 | 16298 | 1 | 0.001
H3 | 16301 | 3 | 0.003
H3 | 16311 | 123 | 0.105
H3 | 16319 | 1 | 0.001
H3 | 16325 | 1 | 0.001
H3 | 16335 | 5 | 0.004
H3 | 16362 | 1 | 0.001
H3 | 16390 | 1 | 0.001
H3 | 16468 | 1 | 0.001
H3 | 1717 | 1 | 0.001
H3 | 1719 | 1 | 0.001
H3 | 1721 | 1 | 0.001
H3 | 1725 | 1 | 0.001
H3 | 189 | 1 | 0.001
H3 | 195A | 1 | 0.001
H3 | 199 | 12 | 0.01
H3 | 2069 | 1 | 0.001
H3 | 207 | 1 | 0.001
H3 | 208 | 1 | 0.001
H3 | 2251 | 1 | 0.001
H3 | 2393 | 1 | 0.001
H3 | 248 | 2 | 0.002
H3 | 260 | 1 | 0.001
H3 | 261 | 1 | 0.001
H3 | 269 | 2 | 0.002
H3 | 2709 | 1 | 0.001
H3 | 2755 | 1 | 0.001
H3 | 281C | 1 | 0.001
H3 | 2851 | 20 | 0.017
H3 | 295 | 2 | 0.002
H3 | 3010 | 1 | 0.001
H3 | 310 | 3 | 0.003
H3 | 315.2C | 1 | 0.001
H3 | 316d | 1 | 0.001
H3 | 3197 | 1 | 0.001
H3 | 3254 | 1 | 0.001
H3 | 33 | 1 | 0.001
H3 | 3306 | 1 | 0.001
H3 | 3311 | 3 | 0.003
H3 | 338 | 1 | 0.001
H3 | 3511 | 1 | 0.001
H3 | 3519 | 1 | 0.001
H3 | 3523 | 1 | 0.001
H3 | 3618 | 1 | 0.001
H3 | 3666 | 1 | 0.001
H3 | 3766 | 1 | 0.001
H3 | 3786 | 1 | 0.001
H3 | 3816 | 1 | 0.001
H3 | 3826 | 1 | 0.001
H3 | 385 | 2 | 0.002
H3 | 3915 | 1 | 0.001
H3 | 3918 | 2 | 0.002
H3 | 4047 | 1 | 0.001
H3 | 4092 | 1 | 0.001
H3 | 4137 | 1 | 0.001
H3 | 4185 | 1 | 0.001
H3 | 4363 | 1 | 0.001
H3 | 438 | 1 | 0.001
H3 | 4674 | 1 | 0.001
H3 | 4924 | 1 | 0.001
H3 | 4959 | 2 | 0.002
H3 | 502 | 1 | 0.001
H3 | 503 | 1 | 0.001
H3 | 5054 | 2 | 0.002
H3 | 515C | 4 | 0.003
H3 | 5162 | 1 | 0.001
H3 | 5205 | 1 | 0.001
H3 | 524A | 4 | 0.003
H3 | 5252 | 1 | 0.001
H3 | 5360 | 1 | 0.001
H3 | 5450A | 1 | 0.001
H3 | 5451C | 1 | 0.001
H3 | 5460 | 5 | 0.004
H3 | 5471 | 29 | 0.025
H3 | 5586 | 3 | 0.003
H3 | 573.1C | 7 | 0.006
H3 | 573.2C | 5 | 0.004
H3 | 573.5C | 1 | 0.001
H3 | 5773 | 1 | 0.001
H3 | 58 | 8 | 0.007
H3 | 5819A | 1 | 0.001
H3 | 5899.1C | 1 | 0.001
H3 | 59 | 9 | 0.008
H3 | 60.1T | 2 | 0.002

H3 | 6040 | 1 | 0.001
H3 | 6185 | 1 | 0.001
H3 | 6206G | 1 | 0.001
H3 | 6217A | 1 | 0.001
H3 | 6217T | 1 | 0.001
H3 | 6247 | 1 | 0.001
H3 | 6267 | 1 | 0.001
H3 | 629 | 1 | 0.001
H3 | 62T | 1 | 0.001
H3 | 6344 | 1 | 0.001
H3 | 6377 | 1 | 0.001
H3 | 6392 | 1 | 0.001
H3 | 64 | 4 | 0.003
H3 | 6419 | 1 | 0.001
H3 | 6570T | 1 | 0.001
H3 | 66T | 2 | 0.002
H3 | 6719 | 1 | 0.001
H3 | 6827 | 5 | 0.004
H3 | 709 | 1 | 0.001
H3 | 7117 | 1 | 0.001
H3 | 7158 | 1 | 0.001
H3 | 7159 | 1 | 0.001
H3 | 7184 | 1 | 0.001
H3 | 72 | 1 | 0.001
H3 | 72G | 1 | 0.001
H3 | 7421 | 1 | 0.001
H3 | 7445C | 1 | 0.001
H3 | 7598 | 1 | 0.001
H3 | 7720 | 1 | 0.001
H3 | 7805 | 1 | 0.001
H3 | 7861 | 1 | 0.001
H3 | 7897 | 1 | 0.001
H3 | 7980 | 1 | 0.001
H3 | 8084T | 3 | 0.003
H3 | 8108 | 4 | 0.003
H3 | 8251 | 2 | 0.002
H3 | 8258 | 1 | 0.001
H3 | 8290 | 12 | 0.01
H3 | 8359 | 3 | 0.003
H3 | 8518 | 1 | 0.001
H3 | 8520 | 1 | 0.001
H3 | 8522 | 3 | 0.003
H3 | 8580A | 12 | 0.01
H3 | 8705 | 3 | 0.003
H3 | 8851 | 1 | 0.001
H3 | 8854 | 1 | 0.001
H3 | 8932 | 1 | 0.001
H3 | 9025 | 2 | 0.002
H3 | 9033 | 12 | 0.01
H3 | 9053 | 1 | 0.001
H3 | 9055 | 1 | 0.001
H3 | 9128 | 1 | 0.001
H3 | 9151 | 12 | 0.01
H3 | 9156 | 1 | 0.001
H3 | 9201 | 1 | 0.001
H3 | 93 | 195 | 0.167
H3 | 9425 | 1 | 0.001
H3 | 9456 | 3 | 0.003
H3 | 9545 | 3 | 0.003
H3 | 9548 | 1 | 0.001
H3 | 9554 | 1 | 0.001
H3 | 95C | 21 | 0.018
H3 | 95T | 69 | 0.059
H3 | 960.1C | 1 | 0.001
H3 | 961 | 1 | 0.001
H3 | 9804 | 1 | 0.001
H3 | 9804C | 1 | 0.001
H3+152 | 1019 | 5 | 0.02
H3+152 | 10247 | 2 | 0.008
H3+152 | 10679 | 1 | 0.004
H3+152 | 10912 | 2 | 0.008
H3+152 | 11002 | 1 | 0.004
H3+152 | 11182 | 3 | 0.012
H3+152 | 11386 | 2 | 0.008
H3+152 | 11560 | 1 | 0.004
H3+152 | 11893 | 5 | 0.02
H3+152 | 12372 | 1 | 0.004
H3+152 | 12696 | 1 | 0.004
H3+152 | 12940 | 1 | 0.004
H3+152 | 13494 | 1 | 0.004
H3+152 | 13759 | 3 | 0.012
H3+152 | 13980C | 1 | 0.004
H3+152 | 14016 | 1 | 0.004
H3+152 | 14198 | 1 | 0.004
H3+152 | 14278 | 5 | 0.02
H3+152 | 146 | 1 | 0.004
H3+152 | 1462 | 1 | 0.004
H3+152 | 14693 | 2 | 0.008
H3+152 | 150 | 1 | 0.004
H3+152 | 15090 | 1 | 0.004
H3+152 | 151 | 1 | 0.004
H3+152 | 15113 | 1 | 0.004
H3+152 | 15148 | 1 | 0.004
H3+152 | 15202 | 1 | 0.004
H3+152 | 15224 | 1 | 0.004
H3+152 | 15289 | 9 | 0.036
H3+152 | 15315 | 1 | 0.004
H3+152 | 15758 | 3 | 0.012
H3+152 | 15924 | 1 | 0.004
H3+152 | 16067 | 1 | 0.004
H3+152 | 16093 | 1 | 0.004
H3+152 | 16111A | 1 | 0.004
H3+152 | 16111G | 1 | 0.004
H3+152 | 16129 | 1 | 0.004
H3+152 | 16150 | 1 | 0.004
H3+152 | 16172 | 1 | 0.004
H3+152 | 16184 | 1 | 0.004
H3+152 | 16189 | 1 | 0.004
H3+152 | 16239A | 1 | 0.004
H3+152 | 16245 | 1 | 0.004
H3+152 | 16249 | 6 | 0.024

H3+152 | 16274 | 1 | 0.004
H3+152 | 16283 | 1 | 0.004
H3+152 | 16288 | 1 | 0.004
H3+152 | 16291 | 1 | 0.004
H3+152 | 16294 | 6 | 0.024
H3+152 | 16298 | 20 | 0.079
H3+152 | 16311 | 1 | 0.004
H3+152 | 16342 | 1 | 0.004
H3+152 | 16354 | 1 | 0.004
H3+152 | 16357 | 1 | 0.004
H3+152 | 16399 | 1 | 0.004
H3+152 | 195 | 1 | 0.004
H3+152 | 235 | 1 | 0.004
H3+152 | 2416 | 1 | 0.004
H3+152 | 2706 | 1 | 0.004
H3+152 | 279 | 2 | 0.008
H3+152 | 293 | 2 | 0.008
H3+152 | 3014 | 1 | 0.004
H3+152 | 310 | 5 | 0.02
H3+152 | 315.2C | 1 | 0.004
H3+152 | 3243 | 1 | 0.004
H3+152 | 3502G | 1 | 0.004
H3+152 | 3970 | 3 | 0.012
H3+152 | 4025 | 4 | 0.016
H3+152 | 4135 | 1 | 0.004
H3+152 | 4688 | 1 | 0.004
H3+152 | 4812C | 2 | 0.008
H3+152 | 486 | 1 | 0.004
H3+152 | 5132 | 1 | 0.004
H3+152 | 5206 | 1 | 0.004
H3+152 | 5387 | 1 | 0.004
H3+152 | 5460 | 1 | 0.004
H3+152 | 5492 | 1 | 0.004
H3+152 | 5496 | 1 | 0.004
H3+152 | 571 | 38 | 0.15
H3+152 | 573.1C | 1 | 0.004
H3+152 | 5773 | 1 | 0.004
H3+152 | 6125 | 12 | 0.047
H3+152 | 64 | 26 | 0.103
H3+152 | 6599 | 1 | 0.004
H3+152 | 6959 | 1 | 0.004
H3+152 | 7302 | 2 | 0.008
H3+152 | 7805 | 4 | 0.016
H3+152 | 8251 | 2 | 0.008
H3+152 | 8290 | 5 | 0.02
H3+152 | 8485 | 1 | 0.004
H3+152 | 8556 | 1 | 0.004
H3+152 | 8679 | 1 | 0.004
H3+152 | 8745 | 1 | 0.004
H3+152 | 8763 | 1 | 0.004
H3+152 | 8871 | 1 | 0.004
H3+152 | 9017 | 3 | 0.012
H3+152 | 9033 | 5 | 0.02
H3+152 | 9041 | 1 | 0.004
H3+152 | 9151 | 5 | 0.02
H3+152 | 9156 | 1 | 0.004
H3+152 | 9195 | 1 | 0.004
H3+152 | 93 | 8 | 0.032
H3+152 | 9456 | 1 | 0.004
H3+152 | 9515 | 1 | 0.004
H3+152 | 9525 | 1 | 0.004
H3+152 | 978 | 1 | 0.004
H3+152 | 990 | 1 | 0.004
H3+16189 | 10410 | 2 | 0.009
H3+16189 | 10535 | 1 | 0.004
H3+16189 | 12033 | 1 | 0.004
H3+16189 | 13928C | 1 | 0.004
H3+16189 | 14470 | 2 | 0.009
H3+16189 | 152 | 1 | 0.004
H3+16189 | 15289 | 1 | 0.004
H3+16189 | 15315 | 4 | 0.017
H3+16189 | 16041 | 1 | 0.004
H3+16189 | 16051 | 1 | 0.004
H3+16189 | 16111 | 1 | 0.004
H3+16189 | 16139T | 1 | 0.004
H3+16189 | 16157 | 1 | 0.004
H3+16189 | 16188 | 1 | 0.004
H3+16189 | 16194C | 4 | 0.017
H3+16189 | 16195 | 4 | 0.017
H3+16189 | 16212 | 2 | 0.009
H3+16189 | 16233 | 1 | 0.004
H3+16189 | 16243 | 1 | 0.004
H3+16189 | 16284 | 4 | 0.017
H3+16189 | 16286 | 5 | 0.022
H3+16189 | 16288 | 1 | 0.004
H3+16189 | 16311 | 2 | 0.009
H3+16189 | 16320 | 1 | 0.004
H3+16189 | 16342 | 1 | 0.004
H3+16189 | 16362 | 1 | 0.004
H3+16189 | 195 | 1 | 0.004
H3+16189 | 199 | 1 | 0.004
H3+16189 | 242 | 1 | 0.004
H3+16189 | 297 | 1 | 0.004
H3+16189 | 315.2C | 1 | 0.004
H3+16189 | 319.1T | 1 | 0.004
H3+16189 | 3618 | 1 | 0.004
H3+16189 | 390 | 3 | 0.013
H3+16189 | 4767 | 1 | 0.004
H3+16189 | 477 | 3 | 0.013
H3+16189 | 480 | 1 | 0.004
H3+16189 | 499 | 1 | 0.004
H3+16189 | 554T | 1 | 0.004
H3+16189 | 567T | 1 | 0.004
H3+16189 | 5899.1C | 1 | 0.004
H3+16189 | 6488 | 1 | 0.004
H3+16189 | 8986 | 1 | 0.004
H3+16189 | 93 | 29 | 0.126
H3+16189 | 9530A | 1 | 0.004
H3+16189 | 9693 | 1 | 0.004
H3+16189 | 9966 | 3 | 0.013
H3+16311 | 11147 | 1 | 0.003

H3+16311 | 11200 | 1 | 0.003
H3+16311 | 12127 | 1 | 0.003
H3+16311 | 13866 | 1 | 0.003
H3+16311 | 146 | 1 | 0.003
H3+16311 | 15115G | 1 | 0.003
H3+16311 | 152 | 2 | 0.006
H3+16311 | 15355 | 1 | 0.003
H3+16311 | 16150 | 1 | 0.003
H3+16311 | 16159 | 1 | 0.003
H3+16311 | 16177 | 1 | 0.003
H3+16311 | 16284 | 35 | 0.102
H3+16311 | 16566 | 1 | 0.003
H3+16311 | 194 | 1 | 0.003
H3+16311 | 237 | 1 | 0.003
H3+16311 | 246 | 1 | 0.003
H3+16311 | 2851 | 1 | 0.003
H3+16311 | 288 | 1 | 0.003
H3+16311 | 310 | 1 | 0.003
H3+16311 | 311 | 1 | 0.003
H3+16311 | 3645 | 1 | 0.003
H3+16311 | 3714C | 1 | 0.003
H3+16311 | 484 | 1 | 0.003
H3+16311 | 6293 | 1 | 0.003
H3+16311 | 73 | 1 | 0.003
H3+16311 | 7521 | 1 | 0.003
H3+16311 | 8258 | 1 | 0.003
H3+16311 | 9254 | 1 | 0.003
H3+73 | 10310 | 1 | 0.007
H3+73 | 11200 | 2 | 0.013
H3+73 | 11914 | 1 | 0.007
H3+73 | 12A | 2 | 0.013
H3+73 | 13896 | 1 | 0.007
H3+73 | 15315 | 10 | 0.066
H3+73 | 15317 | 2 | 0.013
H3+73 | 16192 | 9 | 0.059
H3+73 | 195 | 2 | 0.013
H3+73 | 207 | 1 | 0.007
H3+73 | 2851 | 2 | 0.013
H3+73 | 3200 | 1 | 0.007
H3+73 | 3714 | 2 | 0.013
H3+73 | 4248 | 1 | 0.007
H3+73 | 7148 | 1 | 0.007
H3+73 | 8334 | 5 | 0.033
H3+73 | 93 | 13 | 0.086
H30 | 113 | 1 | 0.001
H30 | 16076A | 123 | 0.157
H30 | 208 | 1 | 0.001
H30 | 261 | 1 | 0.001
H30 | 281C | 1 | 0.001
H30 | 316d | 1 | 0.001
H30 | 338 | 1 | 0.001
H30 | 468 | 3 | 0.004
H30 | 502 | 1 | 0.001
H30 | 573.1C | 3 | 0.004
H30 | 573.2C | 5 | 0.006
H30 | 573.5C | 1 | 0.001
H30a | 11084 | 1 | 0.001
H30a | 113 | 1 | 0.001
H30a | 14221 | 1 | 0.001
H30a | 16076A | 123 | 0.156
H30a | 16148 | 1 | 0.001
H30a | 208 | 1 | 0.001
H30a | 2280 | 1 | 0.001
H30a | 261 | 1 | 0.001
H30a | 281C | 1 | 0.001
H30a | 316d | 1 | 0.001
H30a | 338 | 1 | 0.001
H30a | 468 | 3 | 0.004
H30a | 502 | 1 | 0.001
H30a | 521 | 1 | 0.001
H30a | 530G | 1 | 0.001
H30a | 573.1C | 3 | 0.004
H30a | 573.2C | 5 | 0.006
H30a | 573.5C | 1 | 0.001
H30a | 616G | 1 | 0.001
H30a | 6221 | 1 | 0.001
H30a | 8004 | 1 | 0.001
H30a | 8281-8289d | 1 | 0.001
H30b | 113 | 1 | 0.001
H30b | 16076A | 123 | 0.157
H30b | 208 | 1 | 0.001
H30b | 261 | 1 | 0.001
H30b | 281C | 1 | 0.001
H30b | 316d | 1 | 0.001
H30b | 338 | 1 | 0.001
H30b | 468 | 3 | 0.004
H30b | 502 | 1 | 0.001
H30b | 573.1C | 3 | 0.004
H30b | 573.2C | 5 | 0.006
H30b | 573.5C | 1 | 0.001
H30b1 | 16214 | 3 | 0.02
H30b1 | 16233T | 1 | 0.007
H30b1 | 16247 | 20 | 0.135
H30b1 | 16258T | 1 | 0.007
H30b1 | 200T | 1 | 0.007
H30b1 | 207 | 1 | 0.007
H30b1 | 3150 | 2 | 0.014
H30b1 | 456 | 1 | 0.007
H30b1 | 463 | 1 | 0.007
H30b1 | 4673 | 1 | 0.007
H30b1 | 515-524d | 1 | 0.007
H30b1 | 529T | 1 | 0.007
H30b1 | 573.1C | 1 | 0.007
H30b1 | 6284 | 1 | 0.007
H30b1 | 8276 | 2 | 0.014
H31 | 1007 | 1 | 0.028
H31 | 10562 | 1 | 0.028
H31 | 12649 | 1 | 0.028
H31 | 13638 | 1 | 0.028
H31 | 14990 | 1 | 0.028
H31 | 151 | 3 | 0.083

H31 | 15217C | 1 | 0.028
H31 | 16104 | 1 | 0.028
H31 | 16257A | 1 | 0.028
H31 | 16265 | 1 | 0.028
H31 | 16294 | 1 | 0.028
H31 | 16295 | 1 | 0.028
H31 | 16360 | 1 | 0.028
H31 | 1686T | 1 | 0.028
H31 | 198 | 4 | 0.111
H31 | 2220 | 1 | 0.028
H31 | 259 | 1 | 0.028
H31 | 2789 | 4 | 0.111
H31 | 284 | 1 | 0.028
H31 | 309d | 3 | 0.083
H31 | 310 | 1 | 0.028
H31 | 315.2C | 1 | 0.028
H31 | 4560 | 1 | 0.028
H31 | 4755 | 1 | 0.028
H31 | 6413 | 1 | 0.028
H31 | 73 | 3 | 0.083
H31 | 7927 | 1 | 0.028
H31 | 7996 | 1 | 0.028
H31 | 8219 | 1 | 0.028
H31 | 827 | 1 | 0.028
H31 | 8946 | 1 | 0.028
H31 | 9744 | 1 | 0.028
H31a | 10192 | 2 | 0.105
H31a | 11254 | 1 | 0.053
H31a | 11893 | 1 | 0.053
H31a | 12561 | 1 | 0.053
H31a | 16213 | 1 | 0.053
H31a | 16286 | 1 | 0.053
H31a | 16319 | 15 | 0.789
H31a | 16390 | 1 | 0.053
H31a | 309d | 1 | 0.053
H31a | 4316 | 1 | 0.053
H31a | 5821 | 1 | 0.053
H31b | 10903 | 1 | 0.111
H31b | 234 | 1 | 0.111
H31b | 374 | 1 | 0.111
H31b | 7859 | 1 | 0.111
H31b | 9753C | 1 | 0.111
H32 | 12358 | 4 | 0.068
H32 | 14956 | 1 | 0.017
H32 | 15314 | 2 | 0.034
H32 | 16025 | 1 | 0.017
H32 | 16026 | 1 | 0.017
H32 | 16035 | 1 | 0.017
H32 | 16145 | 11 | 0.186
H32 | 16179 | 1 | 0.017
H32 | 16184 | 1 | 0.017
H32 | 16318C | 4 | 0.068
H32 | 16390 | 1 | 0.017
H32 | 16497 | 1 | 0.017
H32 | 1943 | 1 | 0.017
H32 | 195 | 1 | 0.017
H32 | 480 | 1 | 0.017
H32 | 5302 | 1 | 0.017
H32 | 9368 | 1 | 0.017
H32 | 9496 | 1 | 0.017
H33 | 10915 | 1 | 0.001
H33 | 113 | 1 | 0.001
H33 | 12852 | 1 | 0.001
H33 | 146 | 1 | 0.001
H33 | 16076A | 123 | 0.155
H33 | 207 | 1 | 0.001
H33 | 208 | 1 | 0.001
H33 | 261 | 1 | 0.001
H33 | 281C | 1 | 0.001
H33 | 3010 | 1 | 0.001
H33 | 316d | 1 | 0.001
H33 | 338 | 1 | 0.001
H33 | 468 | 3 | 0.004
H33 | 4970 | 1 | 0.001
H33 | 502 | 1 | 0.001
H33 | 573.1C | 3 | 0.004
H33 | 573.2C | 5 | 0.006
H33 | 573.5C | 1 | 0.001
H33 | 8702 | 1 | 0.001
H33 | 9064 | 1 | 0.001
H33 | 9115 | 1 | 0.001
H33 | 93 | 1 | 0.001
H33a | 113 | 1 | 0.001
H33a | 1374T | 3 | 0.004
H33a | 16076A | 123 | 0.156
H33a | 2056 | 1 | 0.001
H33a | 208 | 1 | 0.001
H33a | 261 | 1 | 0.001
H33a | 281C | 1 | 0.001
H33a | 316d | 1 | 0.001
H33a | 338 | 1 | 0.001
H33a | 468 | 3 | 0.004
H33a | 502 | 1 | 0.001
H33a | 573.1C | 3 | 0.004
H33a | 573.2C | 5 | 0.006
H33a | 573.5C | 1 | 0.001
H33a | 5973 | 1 | 0.001
H33b | 14121 | 4 | 0.056
H33b | 16104 | 2 | 0.028
H33b | 16355 | 1 | 0.014
H33c | 10885 | 1 | 0.012
H33c | 11050 | 1 | 0.012
H33c | 14569 | 1 | 0.012
H33c | 14831 | 1 | 0.012
H33c | 150 | 3 | 0.035
H33c | 16180T | 1 | 0.012
H33c | 16189 | 7 | 0.082
H33c | 16189A | 4 | 0.047
H33c | 16193d | 3 | 0.035
H33c | 16212 | 1 | 0.012
H33c | 16235 | 1 | 0.012

H33c | 16249 | 1 | 0.012
H33c | 16266A | 1 | 0.012
H33c | 16298 | 1 | 0.012
H33c | 16304 | 1 | 0.012
H33c | 16311 | 1 | 0.012
H33c | 16327 | 1 | 0.012
H33c | 195 | 1 | 0.012
H33c | 456 | 1 | 0.012
H33c | 477 | 1 | 0.012
H33c | 573.2C | 1 | 0.012
H33c | 709 | 1 | 0.012
H33c | 93 | 2 | 0.024
H34 | 10750 | 1 | 0.007
H34 | 11800 | 1 | 0.007
H34 | 14502 | 1 | 0.007
H34 | 151 | 2 | 0.014
H34 | 152 | 24 | 0.163
H34 | 16093 | 15 | 0.102
H34 | 16167 | 3 | 0.02
H34 | 16218 | 1 | 0.007
H34 | 16319 | 1 | 0.007
H34 | 1866 | 1 | 0.007
H34 | 215 | 1 | 0.007
H34 | 2368 | 1 | 0.007
H34 | 3618 | 1 | 0.007
H34 | 3633 | 1 | 0.007
H34 | 3745 | 1 | 0.007
H34 | 460 | 1 | 0.007
H34 | 5288 | 1 | 0.007
H34 | 6908 | 1 | 0.007
H34 | 723 | 1 | 0.007
H34 | 7711 | 1 | 0.007
H34 | 8277 | 1 | 0.007
H34 | 8496 | 1 | 0.007
H34 | 8718 | 2 | 0.014
H35 | 1009 | 1 | 0.001
H35 | 10810 | 1 | 0.001
H35 | 11086 | 1 | 0.001
H35 | 113 | 1 | 0.001
H35 | 11590 | 1 | 0.001
H35 | 11698 | 1 | 0.001
H35 | 12618 | 1 | 0.001
H35 | 13271 | 1 | 0.001
H35 | 13414 | 1 | 0.001
H35 | 13752 | 1 | 0.001
H35 | 13818 | 1 | 0.001
H35 | 14573 | 1 | 0.001
H35 | 146 | 3 | 0.004
H35 | 14790 | 1 | 0.001
H35 | 15187 | 1 | 0.001
H35 | 152 | 1 | 0.001
H35 | 16076A | 123 | 0.153
H35 | 16176 | 1 | 0.001
H35 | 16311 | 1 | 0.001
H35 | 208 | 1 | 0.001
H35 | 261 | 1 | 0.001
H35 | 281C | 1 | 0.001
H35 | 310 | 1 | 0.001
H35 | 311 | 1 | 0.001
H35 | 316d | 1 | 0.001
H35 | 3221 | 1 | 0.001
H35 | 338 | 1 | 0.001
H35 | 3394 | 1 | 0.001
H35 | 3439 | 1 | 0.001
H35 | 468 | 3 | 0.004
H35 | 502 | 1 | 0.001
H35 | 5600 | 1 | 0.001
H35 | 573.1C | 3 | 0.004
H35 | 573.2C | 5 | 0.006
H35 | 573.5C | 1 | 0.001
H35 | 6603 | 1 | 0.001
H35 | 7585.1T | 6 | 0.007
H35 | 8047 | 1 | 0.001
H35 | 8934 | 1 | 0.001
H35 | 8940 | 1 | 0.001
H35 | 9383 | 1 | 0.001
H35a | 10481 | 1 | 0.001
H35a | 113 | 1 | 0.001
H35a | 15169 | 1 | 0.001
H35a | 15244 | 1 | 0.001
H35a | 15924 | 1 | 0.001
H35a | 16076A | 123 | 0.155
H35a | 16298 | 2 | 0.003
H35a | 208 | 1 | 0.001
H35a | 261 | 1 | 0.001
H35a | 281C | 1 | 0.001
H35a | 316d | 1 | 0.001
H35a | 338 | 1 | 0.001
H35a | 468 | 3 | 0.004
H35a | 502 | 1 | 0.001
H35a | 513 | 6 | 0.008
H35a | 573.1C | 5 | 0.006
H35a | 573.2C | 5 | 0.006
H35a | 573.5C | 1 | 0.001
H36 | 13065G | 1 | 0.022
H36 | 13260 | 7 | 0.152
H36 | 13746 | 1 | 0.022
H36 | 15191 | 1 | 0.022
H36 | 16051 | 1 | 0.022
H36 | 16081 | 1 | 0.022
H36 | 16093 | 1 | 0.022
H36 | 16129 | 1 | 0.022
H36 | 16230 | 1 | 0.022
H36 | 16286 | 1 | 0.022
H36 | 16295 | 1 | 0.022
H36 | 16304 | 17 | 0.37
H36 | 16311 | 1 | 0.022
H36 | 16315G | 1 | 0.022
H36 | 16343T | 2 | 0.043
H36 | 1787 | 1 | 0.022
H36 | 215 | 7 | 0.152

H36 | 2706 | 2 | 0.043
H36 | 2765 | 15 | 0.326
H36 | 3010 | 2 | 0.043
H36 | 309d | 1 | 0.022
H36 | 310 | 6 | 0.13
H36 | 315.3C | 2 | 0.043
H36 | 4387 | 1 | 0.022
H36 | 4841 | 1 | 0.022
H36 | 5147 | 1 | 0.022
H36 | 5773 | 1 | 0.022
H36 | 8179 | 1 | 0.022
H36 | 8485 | 1 | 0.022
H36 | 8865 | 1 | 0.022
H36 | 9370 | 1 | 0.022
H39 | 12145 | 1 | 0.014
H39 | 12622 | 1 | 0.014
H39 | 16093 | 1 | 0.014
H39 | 16189 | 2 | 0.027
H39 | 16311 | 1 | 0.014
H39 | 16422 | 2 | 0.027
H39 | 239 | 1 | 0.014
H39 | 2396 | 1 | 0.014
H39 | 4890 | 1 | 0.014
H39 | 7424 | 1 | 0.014
H39a | 16111 | 2 | 0.032
H39a | 16158 | 1 | 0.016
H39a | 6831 | 1 | 0.016
H39a1 | 16111 | 2 | 0.032
H39a1 | 16158 | 1 | 0.016
H39a1 | 310 | 1 | 0.016
H39a1 | 315d | 1 | 0.016
H39a1 | 5899.1C | 1 | 0.016
H39b | 10463 | 1 | 0.014
H39b | 150 | 2 | 0.028
H39b | 16111 | 3 | 0.042
H39b | 16167 | 1 | 0.014
H39b | 16189 | 5 | 0.07
H39b | 16286 | 2 | 0.028
H39b | 16298 | 1 | 0.014
H39b | 408A | 1 | 0.014
H39b | 8477 | 1 | 0.014
H39c | 10057 | 1 | 0.014
H39c | 15191 | 1 | 0.014
H39c | 15257 | 1 | 0.014
H39c | 15452A | 1 | 0.014
H39c | 16167 | 1 | 0.014
H39c | 16298 | 1 | 0.014
H39c | 195 | 3 | 0.043
H3a | 10352 | 1 | 0.018
H3a | 10410 | 1 | 0.018
H3a | 10837 | 1 | 0.018
H3a | 11233 | 1 | 0.018
H3a | 12014 | 2 | 0.036
H3a | 12630 | 1 | 0.018
H3a | 12720 | 1 | 0.018
H3a | 12771 | 1 | 0.018
H3a | 13293 | 1 | 0.018
H3a | 13768 | 1 | 0.018
H3a | 15885 | 1 | 0.018
H3a | 16086 | 1 | 0.018
H3a | 16092 | 1 | 0.018
H3a | 4233 | 1 | 0.018
H3a | 4386 | 1 | 0.018
H3a | 7159 | 1 | 0.018
H3a | 7424 | 1 | 0.018
H3a | 8993G | 1 | 0.018
H3a | 9053 | 1 | 0.018
H3a | 9196 | 1 | 0.018
H3a | 9764 | 1 | 0.018
H3a | 9948 | 1 | 0.018
H3a1 | 10328 | 2 | 0.036
H3a1 | 10972 | 2 | 0.036
H3a1 | 11778 | 1 | 0.018
H3a1 | 11953 | 1 | 0.018
H3a1 | 12258A | 1 | 0.018
H3a1 | 12522 | 1 | 0.018
H3a1 | 12879 | 1 | 0.018
H3a1 | 13722 | 2 | 0.036
H3a1 | 14152 | 1 | 0.018
H3a1 | 16092 | 1 | 0.018
H3a1 | 16126 | 1 | 0.018
H3a1 | 16189 | 1 | 0.018
H3a1 | 16214 | 1 | 0.018
H3a1 | 16239A | 1 | 0.018
H3a1 | 16242A | 2 | 0.036
H3a1 | 16257 | 1 | 0.018
H3a1 | 16263 | 2 | 0.036
H3a1 | 16270 | 1 | 0.018
H3a1 | 16311 | 4 | 0.071
H3a1 | 195 | 1 | 0.018
H3a1 | 3866 | 1 | 0.018
H3a1 | 4679 | 1 | 0.018
H3a1 | 5450 | 1 | 0.018
H3a1 | 64 | 2 | 0.036
H3a1 | 7007 | 2 | 0.036
H3a1 | 8994 | 1 | 0.018
H3a1 | 9478 | 1 | 0.018
H3a1 | 9818 | 2 | 0.036
H3a1a | 10124 | 1 | 0.018
H3a1a | 12358 | 1 | 0.018
H3a1a | 13464 | 2 | 0.036
H3a1a | 16092 | 1 | 0.018
H3a1a | 228 | 5 | 0.091
H3a1a | 4065 | 4 | 0.073
H3a1a | 6261 | 1 | 0.018
H3a1a | 7080 | 1 | 0.018
H3a1a | 8134 | 1 | 0.018
H3a1a | 8170 | 1 | 0.018
H3aa | 113 | 1 | 0.001
H3aa | 16076A | 123 | 0.155
H3aa | 16212 | 1 | 0.001

H3aa | 208 | 1 | 0.001
H3aa | 261 | 1 | 0.001
H3aa | 281C | 1 | 0.001
H3aa | 316d | 1 | 0.001
H3aa | 338 | 1 | 0.001
H3aa | 468 | 3 | 0.004
H3aa | 502 | 1 | 0.001
H3aa | 573.1C | 3 | 0.004
H3aa | 573.2C | 5 | 0.006
H3aa | 573.5C | 1 | 0.001
H3aa | 9101 | 1 | 0.001
H3ab | 11009 | 1 | 0.001
H3ab | 113 | 1 | 0.001
H3ab | 12693 | 1 | 0.001
H3ab | 14040 | 1 | 0.001
H3ab | 14207 | 1 | 0.001
H3ab | 16076A | 123 | 0.154
H3ab | 16266 | 1 | 0.001
H3ab | 208 | 1 | 0.001
H3ab | 2392 | 1 | 0.001
H3ab | 261 | 1 | 0.001
H3ab | 281C | 1 | 0.001
H3ab | 316d | 1 | 0.001
H3ab | 338 | 1 | 0.001
H3ab | 468 | 3 | 0.004
H3ab | 502 | 1 | 0.001
H3ab | 573.1C | 3 | 0.004
H3ab | 573.2C | 5 | 0.006
H3ab | 573.5C | 1 | 0.001
H3ab | 8993G | 1 | 0.001
H3ab | 9194 | 1 | 0.001
H3ac | 113 | 1 | 0.001
H3ac | 16076A | 123 | 0.154
H3ac | 208 | 1 | 0.001
H3ac | 261 | 1 | 0.001
H3ac | 281C | 1 | 0.001
H3ac | 316d | 1 | 0.001
H3ac | 338 | 1 | 0.001
H3ac | 468 | 3 | 0.004
H3ac | 472 | 1 | 0.001
H3ac | 502 | 1 | 0.001
H3ac | 573.1C | 3 | 0.004
H3ac | 573.2C | 5 | 0.006
H3ac | 573.5C | 1 | 0.001
H3ac | 8410 | 1 | 0.001
H3ac | 9145 | 1 | 0.001
H3ad | 113 | 1 | 0.001
H3ad | 16076A | 1 | 0.001
H3ad | 16145 | 123 | 0.155
H3ad | 208 | 1 | 0.001
H3ad | 261 | 1 | 0.001
H3ad | 281C | 1 | 0.001
H3ad | 316d | 1 | 0.001
H3ad | 338 | 1 | 0.001
H3ad | 468 | 3 | 0.004
H3ad | 4924 | 1 | 0.001
H3ad | 502 | 1 | 0.001
H3ad | 573.1C | 3 | 0.004
H3ad | 573.2C | 5 | 0.006
H3ad | 573.5C | 1 | 0.001
H3ae | 113 | 1 | 0.001
H3ae | 12438 | 1 | 0.001
H3ae | 15972 | 1 | 0.001
H3ae | 16076A | 123 | 0.155
H3ae | 208 | 1 | 0.001
H3ae | 261 | 1 | 0.001
H3ae | 281C | 1 | 0.001
H3ae | 316d | 1 | 0.001
H3ae | 338 | 1 | 0.001
H3ae | 3975 | 1 | 0.001
H3ae | 468 | 3 | 0.004
H3ae | 502 | 1 | 0.001
H3ae | 573.1C | 3 | 0.004
H3ae | 573.2C | 5 | 0.006
H3ae | 573.5C | 1 | 0.001
H3af | 10 | 7 | 0.026
H3af | 10343 | 1 | 0.004
H3af | 11176 | 1 | 0.004
H3af | 11314 | 2 | 0.007
H3af | 11437 | 1 | 0.004
H3af | 11887 | 1 | 0.004
H3af | 12241d | 7 | 0.026
H3af | 14180 | 1 | 0.004
H3af | 15317 | 1 | 0.004
H3af | 189 | 1 | 0.004
H3af | 198 | 1 | 0.004
H3af | 414G | 1 | 0.004
H3af | 9747 | 2 | 0.007
H3ag | 1031 | 1 | 0.001
H3ag | 10322 | 1 | 0.001
H3ag | 10915 | 1 | 0.001
H3ag | 113 | 1 | 0.001
H3ag | 114 | 1 | 0.001
H3ag | 1393 | 1 | 0.001
H3ag | 14692 | 1 | 0.001
H3ag | 16076A | 123 | 0.155
H3ag | 16214 | 1 | 0.001
H3ag | 208 | 1 | 0.001
H3ag | 2302 | 1 | 0.001
H3ag | 261 | 1 | 0.001
H3ag | 281C | 1 | 0.001
H3ag | 316d | 1 | 0.001
H3ag | 338 | 1 | 0.001
H3ag | 4050 | 1 | 0.001
H3ag | 468 | 3 | 0.004
H3ag | 502 | 1 | 0.001
H3ag | 573.1C | 3 | 0.004
H3ag | 573.2C | 5 | 0.006
H3ag | 573.5C | 1 | 0.001
H3ag1 | 113 | 1 | 0.001
H3ag1 | 11956 | 1 | 0.001

H3ag1 | 16076A | 123 | 0.155
H3ag1 | 208 | 1 | 0.001
H3ag1 | 261 | 1 | 0.001
H3ag1 | 281C | 1 | 0.001
H3ag1 | 316d | 1 | 0.001
H3ag1 | 338 | 1 | 0.001
H3ag1 | 468 | 3 | 0.004
H3ag1 | 502 | 1 | 0.001
H3ag1 | 510 | 1 | 0.001
H3ag1 | 573.1C | 3 | 0.004
H3ag1 | 573.2C | 5 | 0.006
H3ag1 | 573.5C | 1 | 0.001
H3ah | 113 | 1 | 0.001
H3ah | 146 | 1 | 0.001
H3ah | 16076A | 123 | 0.155
H3ah | 208 | 1 | 0.001
H3ah | 261 | 1 | 0.001
H3ah | 281C | 1 | 0.001
H3ah | 316d | 1 | 0.001
H3ah | 338 | 1 | 0.001
H3ah | 468 | 3 | 0.004
H3ah | 502 | 1 | 0.001
H3ah | 573.1C | 3 | 0.004
H3ah | 573.2C | 5 | 0.006
H3ah | 573.5C | 1 | 0.001
H3ai | 113 | 1 | 0.001
H3ai | 16076A | 123 | 0.155
H3ai | 1720 | 1 | 0.001
H3ai | 208 | 1 | 0.001
H3ai | 261 | 1 | 0.001
H3ai | 281C | 1 | 0.001
H3ai | 316d | 1 | 0.001
H3ai | 338 | 1 | 0.001
H3ai | 4612 | 1 | 0.001
H3ai | 468 | 3 | 0.004
H3ai | 502 | 1 | 0.001
H3ai | 573.1C | 3 | 0.004
H3ai | 573.2C | 5 | 0.006
H3ai | 573.5C | 1 | 0.001
H3aj | 113 | 1 | 0.001
H3aj | 15859 | 1 | 0.001
H3aj | 16076A | 123 | 0.155
H3aj | 208 | 1 | 0.001
H3aj | 261 | 1 | 0.001
H3aj | 281C | 1 | 0.001
H3aj | 316d | 1 | 0.001
H3aj | 338 | 1 | 0.001
H3aj | 468 | 3 | 0.004
H3aj | 502 | 1 | 0.001
H3aj | 573.1C | 4 | 0.005
H3aj | 573.2C | 5 | 0.006
H3aj | 573.5C | 1 | 0.001
H3ak | 10659 | 1 | 0.015
H3ak | 11204 | 3 | 0.045
H3ak | 152 | 1 | 0.015
H3ak | 16025 | 1 | 0.015
H3ak | 16026 | 1 | 0.015
H3ak | 16067d | 1 | 0.015
H3ak | 16083G | 1 | 0.015
H3ak | 16095d | 1 | 0.015
H3ak | 16102G | 1 | 0.015
H3ak | 16104A | 1 | 0.015
H3ak | 16128d | 1 | 0.015
H3ak | 16172 | 3 | 0.045
H3ak | 16181 | 1 | 0.015
H3ak | 16188 | 1 | 0.015
H3ak | 16212 | 1 | 0.015
H3ak | 16213 | 2 | 0.03
H3ak | 16258C | 1 | 0.015
H3ak | 16260 | 3 | 0.045
H3ak | 16299 | 1 | 0.015
H3ak | 16301G | 1 | 0.015
H3ak | 16339A | 1 | 0.015
H3ak | 16399T | 1 | 0.015
H3ak | 16488 | 1 | 0.015
H3ak | 228 | 2 | 0.03
H3ak | 297 | 1 | 0.015
H3ak | 310 | 1 | 0.015
H3ak | 315.2C | 1 | 0.015
H3ak | 32.1G | 1 | 0.015
H3ak | 3202 | 2 | 0.03
H3ak | 7193 | 1 | 0.015
H3ak | 7194 | 1 | 0.015
H3ak | 73 | 5 | 0.076
H3ak | 8084T | 1 | 0.015
H3am | 11324A | 1 | 0.03
H3am | 11344 | 1 | 0.03
H3am | 14194 | 1 | 0.03
H3am | 152 | 4 | 0.121
H3am | 16093 | 3 | 0.091
H3am | 16292 | 1 | 0.03
H3am | 16486 | 3 | 0.091
H3am | 16491 | 3 | 0.091
H3am | 16524C | 1 | 0.03
H3am | 16527 | 1 | 0.03
H3am | 200 | 12 | 0.364
H3am | 2396 | 1 | 0.03
H3am | 326 | 1 | 0.03
H3am | 448 | 1 | 0.03
H3am | 6915 | 1 | 0.03
H3am | 8656 | 4 | 0.121
H3am | 9053 | 1 | 0.03
H3am | 93 | 6 | 0.182
H3an | 146 | 1 | 0.067
H3an | 16154 | 1 | 0.067
H3an | 16166 | 1 | 0.067
H3an | 16227 | 1 | 0.067
H3an | 16311 | 1 | 0.067
H3an | 16553 | 1 | 0.067
H3an | 7976 | 2 | 0.133
H3ao | 10915 | 1 | 0.01

H3ao | 1334 | 1 | 0.01
H3ao | 15257 | 1 | 0.01
H3ao | 15758 | 1 | 0.01
H3ao | 16172 | 1 | 0.01
H3ao | 16189 | 1 | 0.01
H3ao | 1719 | 1 | 0.01
H3ao | 4577 | 1 | 0.01
H3ao | 6293 | 1 | 0.01
H3ao | 8659 | 1 | 0.01
H3ao | 93 | 1 | 0.01
H3ao1 | 12171 | 1 | 0.012
H3ao1 | 13111 | 1 | 0.012
H3ao1 | 152 | 1 | 0.012
H3ao1 | 16124 | 5 | 0.062
H3ao1 | 195 | 4 | 0.049
H3ao1 | 513 | 1 | 0.012
H3ap | 10199 | 1 | 0.001
H3ap | 1031 | 1 | 0.001
H3ap | 113 | 1 | 0.001
H3ap | 114 | 1 | 0.001
H3ap | 11527 | 1 | 0.001
H3ap | 12351 | 1 | 0.001
H3ap | 12480 | 1 | 0.001
H3ap | 12942 | 1 | 0.001
H3ap | 12972 | 1 | 0.001
H3ap | 13934 | 1 | 0.001
H3ap | 146 | 2 | 0.002
H3ap | 14692 | 1 | 0.001
H3ap | 150 | 1 | 0.001
H3ap | 15314 | 2 | 0.002
H3ap | 15930 | 1 | 0.001
H3ap | 16042 | 1 | 0.001
H3ap | 16076A | 123 | 0.152
H3ap | 16114 | 2 | 0.002
H3ap | 16169 | 1 | 0.001
H3ap | 16189 | 1 | 0.001
H3ap | 16213 | 1 | 0.001
H3ap | 199 | 2 | 0.002
H3ap | 208 | 1 | 0.001
H3ap | 2302 | 1 | 0.001
H3ap | 261 | 1 | 0.001
H3ap | 281C | 1 | 0.001
H3ap | 306-309d | 1 | 0.001
H3ap | 316d | 1 | 0.001
H3ap | 338 | 1 | 0.001
H3ap | 4023 | 1 | 0.001
H3ap | 4050 | 1 | 0.001
H3ap | 468 | 3 | 0.004
H3ap | 4682 | 1 | 0.001
H3ap | 4960 | 1 | 0.001
H3ap | 502 | 1 | 0.001
H3ap | 573.1C | 4 | 0.005
H3ap | 573.2C | 5 | 0.006
H3ap | 573.5C | 1 | 0.001
H3ap | 5785 | 1 | 0.001
H3ap | 6293 | 1 | 0.001
H3ap | 6447 | 1 | 0.001
H3ap | 6465 | 1 | 0.001
H3ap | 6590 | 1 | 0.001
H3ap | 6821 | 1 | 0.001
H3ap | 73 | 1 | 0.001
H3ap | 8464 | 1 | 0.001
H3ap | 8723 | 1 | 0.001
H3ap | 9103 | 1 | 0.001
H3ap | 9575 | 1 | 0.001
H3aq | 113 | 1 | 0.001
H3aq | 14305 | 1 | 0.001
H3aq | 16076A | 123 | 0.155
H3aq | 208 | 1 | 0.001
H3aq | 261 | 1 | 0.001
H3aq | 281C | 1 | 0.001
H3aq | 316d | 1 | 0.001
H3aq | 338 | 1 | 0.001
H3aq | 468 | 3 | 0.004
H3aq | 502 | 1 | 0.001
H3aq | 573.1C | 3 | 0.004
H3aq | 573.2C | 5 | 0.006
H3aq | 573.5C | 1 | 0.001
H3ar | 11017 | 1 | 0.001
H3ar | 113 | 1 | 0.001
H3ar | 13368 | 1 | 0.001
H3ar | 13581 | 1 | 0.001
H3ar | 14034 | 1 | 0.001
H3ar | 16076A | 123 | 0.154
H3ar | 208 | 1 | 0.001
H3ar | 261 | 1 | 0.001
H3ar | 281C | 1 | 0.001
H3ar | 316d | 1 | 0.001
H3ar | 338 | 1 | 0.001
H3ar | 3745 | 1 | 0.001
H3ar | 468 | 3 | 0.004
H3ar | 502 | 1 | 0.001
H3ar | 573.1C | 3 | 0.004
H3ar | 573.2C | 5 | 0.006
H3ar | 573.5C | 1 | 0.001
H3as | 113 | 1 | 0.001
H3as | 16039 | 1 | 0.001
H3as | 16076A | 1 | 0.001
H3as | 16325 | 123 | 0.154
H3as | 208 | 1 | 0.001
H3as | 261 | 1 | 0.001
H3as | 281C | 1 | 0.001
H3as | 316d | 1 | 0.001
H3as | 338 | 1 | 0.001
H3as | 468 | 3 | 0.004
H3as | 502 | 1 | 0.001
H3as | 573.1C | 3 | 0.004
H3as | 573.2C | 5 | 0.006
H3as | 573.5C | 1 | 0.001
H3as | 8721 | 1 | 0.001
H3as | 8762 | 1 | 0.001

H3at | 11167 | 1 | 0.001
H3at | 113 | 1 | 0.001
H3at | 16076A | 123 | 0.155
H3at | 208 | 1 | 0.001
H3at | 261 | 1 | 0.001
H3at | 281C | 1 | 0.001
H3at | 316d | 1 | 0.001
H3at | 338 | 1 | 0.001
H3at | 468 | 3 | 0.004
H3at | 502 | 1 | 0.001
H3at | 573.1C | 3 | 0.004
H3at | 573.2C | 5 | 0.006
H3at | 573.5C | 1 | 0.001
H3at1 | 113 | 1 | 0.001
H3at1 | 12187 | 1 | 0.001
H3at1 | 16076A | 123 | 0.154
H3at1 | 16179 | 1 | 0.001
H3at1 | 208 | 1 | 0.001
H3at1 | 261 | 1 | 0.001
H3at1 | 281C | 1 | 0.001
H3at1 | 2875 | 3 | 0.004
H3at1 | 316d | 1 | 0.001
H3at1 | 338 | 1 | 0.001
H3at1 | 3426 | 1 | 0.001
H3at1 | 468 | 3 | 0.004
H3at1 | 502 | 1 | 0.001
H3at1 | 573.1C | 3 | 0.004
H3at1 | 573.2C | 5 | 0.006
H3at1 | 573.5C | 1 | 0.001
H3au | 113 | 1 | 0.001
H3au | 16076A | 123 | 0.155
H3au | 208 | 1 | 0.001
H3au | 261 | 1 | 0.001
H3au | 281C | 1 | 0.001
H3au | 2887 | 1 | 0.001
H3au | 316d | 1 | 0.001
H3au | 338 | 1 | 0.001
H3au | 468 | 3 | 0.004
H3au | 502 | 1 | 0.001
H3au | 573.1C | 3 | 0.004
H3au | 573.2C | 5 | 0.006
H3au | 573.5C | 1 | 0.001
H3av | 10581 | 1 | 0.008
H3av | 146 | 4 | 0.034
H3av | 15244 | 1 | 0.008
H3av | 16051 | 14 | 0.119
H3av | 16126 | 2 | 0.017
H3av | 16129 | 1 | 0.008
H3av | 16156 | 1 | 0.008
H3av | 16162 | 1 | 0.008
H3av | 16190 | 3 | 0.025
H3av | 16217 | 20 | 0.169
H3av | 16302 | 1 | 0.008
H3av | 16311 | 4 | 0.034
H3av | 16350 | 1 | 0.008
H3av | 16360 | 13 | 0.11
H3av | 183 | 6 | 0.051
H3av | 195 | 9 | 0.076
H3av | 307 | 1 | 0.008
H3av | 310 | 1 | 0.008
H3av | 314-315d | 1 | 0.008
H3av | 389T | 1 | 0.008
H3av | 5566 | 1 | 0.008
H3b | 113 | 1 | 0.001
H3b | 11662 | 1 | 0.001
H3b | 11914 | 3 | 0.004
H3b | 12972 | 3 | 0.004
H3b | 14769 | 1 | 0.001
H3b | 16076A | 123 | 0.154
H3b | 16148 | 3 | 0.004
H3b | 16362 | 1 | 0.001
H3b | 183 | 1 | 0.001
H3b | 195 | 1 | 0.001
H3b | 208 | 1 | 0.001
H3b | 261 | 1 | 0.001
H3b | 281C | 1 | 0.001
H3b | 316d | 1 | 0.001
H3b | 338 | 1 | 0.001
H3b | 3387G | 1 | 0.001
H3b | 468 | 3 | 0.004
H3b | 502 | 1 | 0.001
H3b | 573.1C | 3 | 0.004
H3b | 573.2C | 5 | 0.006
H3b | 573.5C | 1 | 0.001
H3b | 5911 | 1 | 0.001
H3b | 709 | 1 | 0.001
H3b | 8562 | 1 | 0.001
H3b+16129 | 11914 | 1 | 0.004
H3b+16129 | 12679 | 1 | 0.004
H3b+16129 | 12879 | 1 | 0.004
H3b+16129 | 13590C | 2 | 0.007
H3b+16129 | 13708 | 4 | 0.014
H3b+16129 | 14470 | 3 | 0.011
H3b+16129 | 16266 | 4 | 0.014
H3b+16129 | 16300 | 7 | 0.025
H3b+16129 | 1767 | 1 | 0.004
H3b+16129 | 310 | 1 | 0.004
H3b+16129 | 3547 | 1 | 0.004
H3b+16129 | 6735 | 1 | 0.004
H3b+16129 | 7853 | 2 | 0.007
H3b+16129 | 8359 | 1 | 0.004
H3b1 | 16352 | 1 | 0.014
H3b1a | 10203 | 1 | 0.014
H3b1a | 12373 | 1 | 0.014
H3b1a | 15218C | 2 | 0.027
H3b1a | 310 | 1 | 0.014
H3b1a | 9105 | 2 | 0.027
H3b1b | 13984 | 1 | 0.091
H3b1b | 14380 | 1 | 0.091
H3b1b | 152 | 1 | 0.091
H3b1b | 8854 | 1 | 0.091

H3b1b1 | 12397 | 1 | 0.067
H3b1b1 | 13722 | 1 | 0.067
H3b1b1 | 146 | 4 | 0.267
H3b1b1 | 15323 | 2 | 0.133
H3b1b1 | 16243 | 1 | 0.067
H3b1b1 | 16320 | 1 | 0.067
H3b1b1 | 310 | 1 | 0.067
H3b1b1 | 6167 | 2 | 0.133
H3b1b1 | 7403 | 1 | 0.067
H3b1b1 | 765G | 1 | 0.067
H3b1b1 | 8863 | 1 | 0.067
H3b1b1 | 9148 | 1 | 0.067
H3b1b1a | 152 | 1 | 0.077
H3b1b1a | 411G | 1 | 0.077
H3b1b1a | 502A | 1 | 0.077
H3b1b1a | 503T | 1 | 0.077
H3b1b1a | 6395 | 1 | 0.077
H3b1b1a | 684 | 1 | 0.077
H3b2 | 16257 | 7 | 0.027
H3b2 | 281C | 1 | 0.004
H3b2 | 481 | 1 | 0.004
H3b2 | 518 | 1 | 0.004
H3b2 | 5460 | 1 | 0.004
H3b3 | 12397 | 1 | 0.004
H3b3 | 12561 | 1 | 0.004
H3b3 | 15431 | 3 | 0.011
H3b3 | 16257 | 7 | 0.026
H3b3 | 2626 | 1 | 0.004
H3b3 | 281C | 1 | 0.004
H3b3 | 309d | 1 | 0.004
H3b3 | 310 | 2 | 0.008
H3b3 | 4850 | 1 | 0.004
H3b3 | 518 | 1 | 0.004
H3b3 | 5387 | 3 | 0.011
H3b3 | 9852 | 1 | 0.004
H3b4 | 103 | 1 | 0.007
H3b4 | 152 | 21 | 0.138
H3b4 | 16051 | 1 | 0.007
H3b4 | 16061 | 1 | 0.007
H3b4 | 16108 | 1 | 0.007
H3b4 | 16172 | 1 | 0.007
H3b4 | 16189 | 1 | 0.007
H3b4 | 16209 | 1 | 0.007
H3b4 | 16210 | 3 | 0.02
H3b4 | 16213 | 1 | 0.007
H3b4 | 16297 | 4 | 0.026
H3b4 | 16311 | 2 | 0.013
H3b4 | 16398 | 10 | 0.066
H3b4 | 185 | 2 | 0.013
H3b4 | 207 | 3 | 0.02
H3b4 | 225 | 1 | 0.007
H3b4 | 299A | 1 | 0.007
H3b4 | 309.3C | 8 | 0.053
H3b4 | 316C | 2 | 0.013
H3b4 | 368 | 1 | 0.007
H3b4a | 103 | 1 | 0.008
H3b4a | 16051 | 1 | 0.008
H3b4a | 16061 | 1 | 0.008
H3b4a | 16108 | 1 | 0.008
H3b4a | 16189 | 1 | 0.008
H3b4a | 16209 | 1 | 0.008
H3b4a | 16260 | 1 | 0.008
H3b4a | 16311 | 2 | 0.015
H3b4a | 16398 | 10 | 0.076
H3b4a | 207 | 3 | 0.023
H3b4a | 299A | 1 | 0.008
H3b4a | 309.3C | 8 | 0.061
H3b4a | 316C | 1 | 0.008
H3b4a | 73 | 1 | 0.008
H3b5 | 13116 | 1 | 0.004
H3b5 | 14002 | 1 | 0.004
H3b5 | 16184 | 1 | 0.004
H3b5 | 16257 | 7 | 0.027
H3b5 | 16260 | 1 | 0.004
H3b5 | 16302 | 1 | 0.004
H3b5 | 281C | 1 | 0.004
H3b5 | 4922 | 1 | 0.004
H3b5 | 5144 | 1 | 0.004
H3b5 | 518 | 1 | 0.004
H3b6 | 1462 | 1 | 0.007
H3b6 | 153 | 1 | 0.007
H3b6 | 16189 | 1 | 0.007
H3b6 | 16213 | 1 | 0.007
H3b6 | 16249 | 1 | 0.007
H3b6 | 16270 | 1 | 0.007
H3b6 | 16293d | 1 | 0.007
H3b6 | 16311 | 2 | 0.013
H3b6 | 16318T | 1 | 0.007
H3b6 | 16352G | 2 | 0.013
H3b6 | 199 | 2 | 0.013
H3b6 | 200 | 1 | 0.007
H3b6 | 204 | 1 | 0.007
H3b6 | 250 | 1 | 0.007
H3b6 | 285 | 1 | 0.007
H3b6 | 3338 | 2 | 0.013
H3b6 | 9018 | 2 | 0.013
H3b6a | 13047 | 1 | 0.007
H3b6a | 153 | 1 | 0.007
H3b6a | 16189 | 1 | 0.007
H3b6a | 16213 | 1 | 0.007
H3b6a | 16249 | 1 | 0.007
H3b6a | 16270 | 1 | 0.007
H3b6a | 16293d | 1 | 0.007
H3b6a | 16311 | 2 | 0.013
H3b6a | 16318T | 1 | 0.007
H3b6a | 16352G | 2 | 0.013
H3b6a | 199 | 2 | 0.013
H3b6a | 200 | 1 | 0.007
H3b6a | 204 | 1 | 0.007
H3b6a | 250 | 1 | 0.007
H3b6a | 285 | 1 | 0.007

H3b7 | 12070 | 1 | 0.004
H3b7 | 16257 | 7 | 0.026
H3b7 | 281C | 1 | 0.004
H3b7 | 310 | 1 | 0.004
H3b7 | 518 | 1 | 0.004
H3b7 | 5460 | 1 | 0.004
H3b7 | 6635 | 1 | 0.004
H3b7 | 93 | 1 | 0.004
H3c | 113 | 1 | 0.001
H3c | 12193 | 1 | 0.001
H3c | 14152 | 1 | 0.001
H3c | 16076A | 123 | 0.153
H3c | 16290 | 1 | 0.001
H3c | 208 | 1 | 0.001
H3c | 2232.1A | 1 | 0.001
H3c | 228 | 1 | 0.001
H3c | 261 | 1 | 0.001
H3c | 2706 | 1 | 0.001
H3c | 281C | 1 | 0.001
H3c | 316d | 1 | 0.001
H3c | 338 | 1 | 0.001
H3c | 3705 | 1 | 0.001
H3c | 468 | 3 | 0.004
H3c | 502 | 1 | 0.001
H3c | 5063 | 1 | 0.001
H3c | 5581 | 3 | 0.004
H3c | 573.1C | 3 | 0.004
H3c | 573.2C | 7 | 0.009
H3c | 573.5C | 1 | 0.001
H3c | 6261 | 1 | 0.001
H3c | 6465 | 3 | 0.004
H3c | 789 | 3 | 0.004
H3c | 8879 | 1 | 0.001
H3c | 9854 | 3 | 0.004
H3c1 | 10667 | 1 | 0.001
H3c1 | 113 | 1 | 0.001
H3c1 | 11453 | 1 | 0.001
H3c1 | 12981 | 1 | 0.001
H3c1 | 13143 | 1 | 0.001
H3c1 | 14467 | 1 | 0.001
H3c1 | 150 | 1 | 0.001
H3c1 | 15632 | 1 | 0.001
H3c1 | 16076A | 123 | 0.153
H3c1 | 16189 | 2 | 0.002
H3c1 | 16260 | 7 | 0.009
H3c1 | 189 | 5 | 0.006
H3c1 | 191 | 1 | 0.001
H3c1 | 208 | 1 | 0.001
H3c1 | 261 | 1 | 0.001
H3c1 | 281C | 1 | 0.001
H3c1 | 310 | 1 | 0.001
H3c1 | 315.3C | 1 | 0.001
H3c1 | 316d | 1 | 0.001
H3c1 | 338 | 1 | 0.001
H3c1 | 3969 | 1 | 0.001
H3c1 | 468 | 3 | 0.004
H3c1 | 502 | 1 | 0.001
H3c1 | 5153 | 1 | 0.001
H3c1 | 5237 | 1 | 0.001
H3c1 | 573.1C | 3 | 0.004
H3c1 | 573.2C | 5 | 0.006
H3c1 | 573.5C | 1 | 0.001
H3c1 | 72 | 1 | 0.001
H3c1 | 7980 | 1 | 0.001
H3c1 | 8845 | 1 | 0.001
H3c1 | 9175A | 1 | 0.001
H3c1 | 9379 | 1 | 0.001
H3c2 | 14259 | 1 | 0.021
H3c2 | 15172 | 1 | 0.021
H3c2 | 16126 | 1 | 0.021
H3c2 | 16178 | 1 | 0.021
H3c2 | 16390 | 1 | 0.021
H3c2 | 198 | 1 | 0.021
H3c2 | 3027 | 1 | 0.021
H3c2 | 4025 | 1 | 0.021
H3c2 | 8865 | 1 | 0.021
H3c2a | 10192A | 1 | 0.017
H3c2a | 12408 | 5 | 0.083
H3c2a | 14404 | 1 | 0.017
H3c2a | 152 | 1 | 0.017
H3c2a | 1555 | 1 | 0.017
H3c2a | 16093 | 2 | 0.033
H3c2a | 16311 | 1 | 0.017
H3c2a | 2709 | 1 | 0.017
H3c2a1 | 146 | 2 | 0.04
H3c2a1 | 152 | 3 | 0.06
H3c2a1 | 15894 | 1 | 0.02
H3c2a1 | 1926 | 1 | 0.02
H3c2a1 | 5195 | 1 | 0.02
H3c2a1 | 6761 | 1 | 0.02
H3c2b | 14287 | 1 | 0.021
H3c2b | 16126 | 1 | 0.021
H3c2b | 16178 | 1 | 0.021
H3c2b | 16184 | 1 | 0.021
H3c2b | 16390 | 1 | 0.021
H3c2b | 198 | 1 | 0.021
H3c2b1 | 14484 | 2 | 0.042
H3c2b1 | 16311 | 2 | 0.042
H3c2b1 | 6261 | 2 | 0.042
H3c2b1 | 7444 | 2 | 0.042
H3c2c | 16294 | 2 | 0.041
H3c3 | 11272 | 1 | 0.045
H3c3 | 16045A | 1 | 0.045
H3c3 | 16067A | 1 | 0.045
H3c3 | 16069 | 1 | 0.045
H3c3 | 16072G | 1 | 0.045
H3c3 | 16074T | 1 | 0.045
H3c3 | 16075 | 1 | 0.045
H3c3 | 16075A | 1 | 0.045
H3c3 | 16192 | 5 | 0.227
H3c3 | 16233 | 4 | 0.182

H3c3 | 16325 | 1 | 0.045
H3c3 | 16362 | 8 | 0.364
H3c3 | 6053 | 1 | 0.045
H3c3 | 8632 | 1 | 0.045
H3d | 14325 | 4 | 0.035
H3e | 113 | 1 | 0.001
H3e | 15145 | 1 | 0.001
H3e | 16076A | 1 | 0.001
H3e | 16181 | 123 | 0.154
H3e | 200 | 1 | 0.001
H3e | 208 | 1 | 0.001
H3e | 261 | 1 | 0.001
H3e | 281C | 1 | 0.001
H3e | 316d | 1 | 0.001
H3e | 338 | 1 | 0.001
H3e | 468 | 3 | 0.004
H3e | 502 | 1 | 0.001
H3e | 573.1C | 3 | 0.004
H3e | 573.2C | 5 | 0.006
H3e | 573.5C | 1 | 0.001
H3g | 12040 | 1 | 0.006
H3g | 12362 | 2 | 0.013
H3g | 13422 | 1 | 0.006
H3g | 15244 | 1 | 0.006
H3g | 15376 | 1 | 0.006
H3g | 16320 | 1 | 0.006
H3g | 16362 | 20 | 0.127
H3g | 1934 | 1 | 0.006
H3g | 310 | 1 | 0.006
H3g | 315.2C | 1 | 0.006
H3g | 4823 | 1 | 0.006
H3g | 573.1C | 1 | 0.006
H3g1 | 10256 | 1 | 0.006
H3g1 | 12142 | 1 | 0.006
H3g1 | 13710 | 1 | 0.006
H3g1 | 13711 | 1 | 0.006
H3g1 | 14016 | 2 | 0.011
H3g1 | 14560 | 1 | 0.006
H3g1 | 16093 | 2 | 0.011
H3g1 | 16129 | 3 | 0.017
H3g1 | 16157 | 1 | 0.006
H3g1 | 16189 | 2 | 0.011
H3g1 | 16223 | 26 | 0.149
H3g1 | 16287 | 2 | 0.011
H3g1 | 16311 | 1 | 0.006
H3g1 | 16399 | 1 | 0.006
H3g1 | 194 | 1 | 0.006
H3g1 | 195 | 2 | 0.011
H3g1 | 215 | 20 | 0.115
H3g1 | 310 | 3 | 0.017
H3g1 | 4164 | 1 | 0.006
H3g1 | 5249 | 1 | 0.006
H3g1 | 573.1C | 2 | 0.011
H3g1 | 5772 | 1 | 0.006
H3g1 | 6755 | 1 | 0.006
H3g1 | 7854 | 2 | 0.011
H3g1 | 9132 | 1 | 0.006
H3g1 | 960.1C | 1 | 0.006
H3g1 | 9668 | 1 | 0.006
H3g1a | 12965 | 1 | 0.007
H3g1a | 151 | 20 | 0.136
H3g1a | 573.1C | 1 | 0.007
H3g1a | 9029 | 20 | 0.136
H3g1b | 10652 | 20 | 0.133
H3g1b | 573.1C | 1 | 0.007
H3g1b | 8251 | 1 | 0.007
H3g1b | 9025 | 1 | 0.007
H3g2 | 16188G | 1 | 0.007
H3g2 | 16214 | 3 | 0.02
H3g2 | 16233T | 1 | 0.007
H3g2 | 16247 | 1 | 0.007
H3g2 | 16258T | 1 | 0.007
H3g2 | 16309 | 20 | 0.132
H3g2 | 200T | 1 | 0.007
H3g2 | 204 | 2 | 0.013
H3g2 | 207 | 1 | 0.007
H3g2 | 215 | 1 | 0.007
H3g2 | 456 | 1 | 0.007
H3g2 | 463 | 1 | 0.007
H3g2 | 480 | 2 | 0.013
H3g2 | 515-524d | 1 | 0.007
H3g2 | 529T | 1 | 0.007
H3g2 | 573.1C | 1 | 0.007
H3g2 | 7001 | 2 | 0.013
H3g3 | 10646 | 1 | 0.007
H3g3 | 11581A | 1 | 0.007
H3g3 | 12127 | 1 | 0.007
H3g3 | 14180 | 1 | 0.007
H3g3 | 15221 | 1 | 0.007
H3g3 | 15928 | 1 | 0.007
H3g3 | 16126 | 1 | 0.007
H3g3 | 16184 | 2 | 0.013
H3g3 | 16278 | 1 | 0.007
H3g3 | 16390 | 20 | 0.132
H3g3 | 3394 | 1 | 0.007
H3g3 | 3398 | 2 | 0.013
H3g3 | 573.1C | 1 | 0.007
H3g4 | 10685 | 1 | 0.007
H3g4 | 12236 | 1 | 0.007
H3g4 | 13933 | 1 | 0.007
H3g4 | 15496 | 1 | 0.007
H3g4 | 16242 | 26 | 0.17
H3g4 | 3144 | 1 | 0.007
H3g4 | 5102 | 1 | 0.007
H3g4 | 5483 | 1 | 0.007
H3g4 | 573.1C | 1 | 0.007
H3h | 14182 | 1 | 0.003
H3h | 146 | 1 | 0.003
H3h | 152 | 1 | 0.003
H3h | 16150 | 1 | 0.003
H3h | 16159 | 1 | 0.003

H3h | 16177 | 1 | 0.003
H3h | 16284 | 35 | 0.103
H3h | 16355 | 1 | 0.003
H3h | 16566 | 1 | 0.003
H3h | 237 | 1 | 0.003
H3h | 246 | 1 | 0.003
H3h | 288 | 1 | 0.003
H3h | 310 | 1 | 0.003
H3h | 311 | 1 | 0.003
H3h | 3350 | 1 | 0.003
H3h | 484 | 1 | 0.003
H3h | 5354 | 1 | 0.003
H3h | 6563 | 1 | 0.003
H3h | 7002 | 1 | 0.003
H3h | 73 | 1 | 0.003
H3h | 8594 | 2 | 0.006
H3h | 9977 | 1 | 0.003
H3h1 | 10427 | 1 | 0.003
H3h1 | 12519 | 1 | 0.003
H3h1 | 13434 | 1 | 0.003
H3h1 | 152 | 1 | 0.003
H3h1 | 16150 | 1 | 0.003
H3h1 | 16159 | 1 | 0.003
H3h1 | 16177 | 1 | 0.003
H3h1 | 16261 | 1 | 0.003
H3h1 | 16284 | 35 | 0.099
H3h1 | 16566 | 1 | 0.003
H3h1 | 237 | 1 | 0.003
H3h1 | 246 | 1 | 0.003
H3h1 | 288 | 1 | 0.003
H3h1 | 4452 | 1 | 0.003
H3h1 | 484 | 1 | 0.003
H3h1 | 5824 | 8 | 0.023
H3h1 | 7064 | 2 | 0.006
H3h1 | 9542 | 1 | 0.003
H3h2 | 146 | 1 | 0.003
H3h2 | 150 | 2 | 0.006
H3h2 | 16067 | 2 | 0.006
H3h2 | 16145 | 1 | 0.003
H3h2 | 16150 | 1 | 0.003
H3h2 | 16159 | 1 | 0.003
H3h2 | 16177 | 1 | 0.003
H3h2 | 16189 | 7 | 0.02
H3h2 | 16223 | 1 | 0.003
H3h2 | 16235 | 1 | 0.003
H3h2 | 16260 | 1 | 0.003
H3h2 | 16265 | 1 | 0.003
H3h2 | 16284 | 1 | 0.003
H3h2 | 16304G | 36 | 0.101
H3h2 | 16357A | 1 | 0.003
H3h2 | 16381G | 1 | 0.003
H3h2 | 16382G | 1 | 0.003
H3h2 | 16566 | 1 | 0.003
H3h2 | 197 | 1 | 0.003
H3h2 | 200 | 1 | 0.003
H3h2 | 212 | 1 | 0.003
H3h2 | 237 | 1 | 0.003
H3h2 | 246 | 1 | 0.003
H3h2 | 288 | 1 | 0.003
H3h2 | 456 | 36 | 0.101
H3h2 | 484 | 1 | 0.003
H3h2a | 13474 | 1 | 0.014
H3h2a | 14530 | 1 | 0.014
H3h2a | 15804 | 1 | 0.014
H3h2a | 15823 | 1 | 0.014
H3h2a | 15860 | 1 | 0.014
H3h2a | 16176 | 2 | 0.027
H3h2a | 16224 | 1 | 0.014
H3h2a | 16270 | 2 | 0.027
H3h2a | 16399 | 3 | 0.041
H3h2a | 411A | 1 | 0.014
H3h2a | 4222 | 1 | 0.014
H3h3 | 12795 | 3 | 0.009
H3h3 | 15178 | 3 | 0.009
H3h3 | 16150 | 1 | 0.003
H3h3 | 16159 | 1 | 0.003
H3h3 | 16177 | 1 | 0.003
H3h3 | 16284 | 35 | 0.105
H3h3 | 16566 | 1 | 0.003
H3h3 | 237 | 1 | 0.003
H3h3 | 246 | 1 | 0.003
H3h3 | 288 | 1 | 0.003
H3h3 | 484 | 1 | 0.003
H3h3 | 5423 | 3 | 0.009
H3h3 | 9214 | 1 | 0.003
H3h3a | 12528 | 1 | 0.003
H3h3a | 15061 | 1 | 0.003
H3h3a | 16150 | 1 | 0.003
H3h3a | 16159 | 1 | 0.003
H3h3a | 16177 | 1 | 0.003
H3h3a | 16284 | 35 | 0.105
H3h3a | 16566 | 1 | 0.003
H3h3a | 237 | 1 | 0.003
H3h3a | 246 | 1 | 0.003
H3h3a | 288 | 1 | 0.003
H3h3a | 484 | 1 | 0.003
H3h3b | 16150 | 1 | 0.003
H3h3b | 16159 | 1 | 0.003
H3h3b | 16172 | 1 | 0.003
H3h3b | 16177 | 1 | 0.003
H3h3b | 16284 | 35 | 0.105
H3h3b | 16566 | 1 | 0.003
H3h3b | 237 | 1 | 0.003
H3h3b | 246 | 1 | 0.003
H3h3b | 288 | 1 | 0.003
H3h3b | 484 | 1 | 0.003
H3h3b | 5899.1C | 1 | 0.003
H3h4 | 13743 | 1 | 0.008
H3h4 | 16180 | 2 | 0.017
H3h4 | 16271 | 3 | 0.025
H3h5 | 11778 | 1 | 0.003

H3h5 | 12603 | 1 | 0.003
H3h5 | 15184 | 2 | 0.006
H3h5 | 15299 | 1 | 0.003
H3h5 | 16129 | 1 | 0.003
H3h5 | 16150 | 1 | 0.003
H3h5 | 16157 | 2 | 0.006
H3h5 | 16159 | 1 | 0.003
H3h5 | 16162 | 1 | 0.003
H3h5 | 16177 | 1 | 0.003
H3h5 | 16284 | 35 | 0.104
H3h5 | 16543 | 1 | 0.003
H3h5 | 16566 | 1 | 0.003
H3h5 | 237 | 1 | 0.003
H3h5 | 246 | 1 | 0.003
H3h5 | 288 | 1 | 0.003
H3h5 | 484 | 1 | 0.003
H3h5 | 5566 | 1 | 0.003
H3h5 | 6261 | 1 | 0.003
H3h5 | 6406 | 1 | 0.003
H3h5 | 73 | 2 | 0.006
H3h5 | 8651 | 1 | 0.003
H3h5 | 9114T | 1 | 0.003
H3h6 | 12172 | 1 | 0.003
H3h6 | 12397 | 1 | 0.003
H3h6 | 16150 | 1 | 0.003
H3h6 | 16159 | 1 | 0.003
H3h6 | 16177 | 1 | 0.003
H3h6 | 16284 | 35 | 0.105
H3h6 | 16566 | 1 | 0.003
H3h6 | 237 | 1 | 0.003
H3h6 | 246 | 1 | 0.003
H3h6 | 288 | 1 | 0.003
H3h6 | 484 | 1 | 0.003
H3h6 | 6710 | 1 | 0.003
H3h6 | 8251 | 1 | 0.003
H3h7 | 11002 | 1 | 0.008
H3h7 | 11778 | 1 | 0.008
H3h7 | 13299 | 1 | 0.008
H3h7 | 14162 | 1 | 0.008
H3h7 | 14325 | 1 | 0.008
H3h7 | 146 | 1 | 0.008
H3h7 | 150 | 1 | 0.008
H3h7 | 152 | 2 | 0.016
H3h7 | 15418 | 1 | 0.008
H3h7 | 16223 | 1 | 0.008
H3h7 | 16264 | 1 | 0.008
H3h7 | 16292 | 1 | 0.008
H3h7 | 16359 | 1 | 0.008
H3h7 | 164 | 1 | 0.008
H3h7 | 246 | 1 | 0.008
H3h7 | 249d | 1 | 0.008
H3h7 | 3460 | 1 | 0.008
H3h7 | 4092 | 1 | 0.008
H3h7 | 4109 | 1 | 0.008
H3h7 | 5585 | 1 | 0.008
H3h7 | 6026 | 1 | 0.008
H3h7 | 7356 | 1 | 0.008
H3h7 | 95C | 1 | 0.008
H3i | 12595 | 20 | 0.135
H3i | 573.1C | 1 | 0.007
H3i | 8222 | 1 | 0.007
H3i | 8928 | 1 | 0.007
H3i | 9554 | 1 | 0.007
H3i1 | 14776 | 1 | 0.007
H3i1 | 16179 | 20 | 0.133
H3i1 | 3531T | 1 | 0.007
H3i1 | 3640 | 2 | 0.013
H3i1 | 573.1C | 1 | 0.007
H3j | 1044 | 1 | 0.007
H3j | 16189 | 1 | 0.007
H3j | 16311 | 21 | 0.141
H3j | 4435 | 1 | 0.007
H3j | 573.1C | 1 | 0.007
H3k | 11518 | 2 | 0.013
H3k | 1193 | 1 | 0.006
H3k | 1282 | 1 | 0.006
H3k | 1452 | 1 | 0.006
H3k | 151 | 1 | 0.006
H3k | 15355 | 1 | 0.006
H3k | 15742 | 1 | 0.006
H3k | 16145 | 20 | 0.13
H3k | 16162 | 1 | 0.006
H3k | 16192 | 2 | 0.013
H3k | 16311 | 1 | 0.006
H3k | 195 | 2 | 0.013
H3k | 3395 | 1 | 0.006
H3k | 4336 | 1 | 0.006
H3k | 479 | 1 | 0.006
H3k | 5030 | 1 | 0.006
H3k | 573.1C | 1 | 0.006
H3k | 573.2C | 1 | 0.006
H3k | 7229 | 1 | 0.006
H3k | 7258 | 1 | 0.006
H3k | 8602 | 1 | 0.006
H3k | 9801 | 1 | 0.006
H3k1 | 10343 | 1 | 0.007
H3k1 | 14587 | 20 | 0.136
H3k1 | 16189 | 20 | 0.136
H3k1 | 2707 | 1 | 0.007
H3k1 | 3398 | 1 | 0.007
H3k1 | 573.1C | 20 | 0.136
H3k1a | 11151 | 1 | 0.007
H3k1a | 12804 | 1 | 0.007
H3k1a | 3591 | 4 | 0.026
H3k1a | 3645 | 2 | 0.013
H3k1a | 4561 | 1 | 0.007
H3k1a | 573.1C | 1 | 0.007
H3m | 10927 | 1 | 0.003
H3m | 11995A | 1 | 0.003
H3m | 13656 | 1 | 0.003
H3m | 16150 | 1 | 0.003

H3m | 16159 | 1 | 0.003
H3m | 16177 | 1 | 0.003
H3m | 16284 | 35 | 0.104
H3m | 16566 | 1 | 0.003
H3m | 195 | 1 | 0.003
H3m | 237 | 1 | 0.003
H3m | 246 | 1 | 0.003
H3m | 2848 | 1 | 0.003
H3m | 288 | 1 | 0.003
H3m | 4263 | 1 | 0.003
H3m | 463 | 1 | 0.003
H3m | 484 | 1 | 0.003
H3m | 6150 | 1 | 0.003
H3m | 7223 | 1 | 0.003
H3m | 73 | 1 | 0.003
H3m | 979 | 2 | 0.006
H3m | 9797 | 1 | 0.003
H3n | 13542C | 1 | 0.012
H3n | 146 | 1 | 0.012
H3n | 15924 | 1 | 0.012
H3n | 16093 | 3 | 0.038
H3n | 16362 | 2 | 0.025
H3n | 16399 | 1 | 0.012
H3n | 200 | 2 | 0.025
H3n | 2033 | 1 | 0.012
H3n | 4206 | 1 | 0.012
H3n | 8790 | 2 | 0.025
H3n | 9168 | 1 | 0.012
H3p | 146 | 2 | 0.022
H3p | 15885 | 2 | 0.022
H3p | 16129 | 1 | 0.011
H3p | 16145 | 10 | 0.108
H3p | 16242 | 1 | 0.011
H3p | 16273 | 1 | 0.011
H3p | 16299 | 2 | 0.022
H3p | 16311 | 1 | 0.011
H3p | 16318T | 1 | 0.011
H3p | 16320 | 4 | 0.043
H3p | 16356 | 1 | 0.011
H3p | 200 | 1 | 0.011
H3p | 324G | 1 | 0.011
H3p | 357C | 1 | 0.011
H3p | 363C | 1 | 0.011
H3p | 383 | 1 | 0.011
H3p | 388C | 1 | 0.011
H3p | 391A | 1 | 0.011
H3p | 414G | 1 | 0.011
H3p | 498d | 1 | 0.011
H3p | 515-524d | 6 | 0.065
H3p | 529T | 1 | 0.011
H3p | 8152 | 1 | 0.011
H3p | 8245 | 2 | 0.022
H3p | 9007 | 2 | 0.022
H3q | 113 | 1 | 0.001
H3q | 11963 | 1 | 0.001
H3q | 13203 | 1 | 0.001
H3q | 16076A | 123 | 0.154
H3q | 16079 | 2 | 0.003
H3q | 16278 | 2 | 0.003
H3q | 208 | 1 | 0.001
H3q | 261 | 1 | 0.001
H3q | 281C | 1 | 0.001
H3q | 316d | 1 | 0.001
H3q | 338 | 1 | 0.001
H3q | 356.1C | 1 | 0.001
H3q | 4592 | 1 | 0.001
H3q | 468 | 3 | 0.004
H3q | 502 | 1 | 0.001
H3q | 573.1C | 3 | 0.004
H3q | 573.2C | 5 | 0.006
H3q | 573.5C | 1 | 0.001
H3q | 6221 | 1 | 0.001
H3q | 7897 | 1 | 0.001
H3q1 | 113 | 1 | 0.001
H3q1 | 13434 | 2 | 0.003
H3q1 | 152 | 3 | 0.004
H3q1 | 16076A | 123 | 0.154
H3q1 | 16390 | 1 | 0.001
H3q1 | 208 | 1 | 0.001
H3q1 | 261 | 1 | 0.001
H3q1 | 281C | 1 | 0.001
H3q1 | 316d | 1 | 0.001
H3q1 | 338 | 1 | 0.001
H3q1 | 468 | 3 | 0.004
H3q1 | 502 | 1 | 0.001
H3q1 | 573.1C | 3 | 0.004
H3q1 | 573.2C | 5 | 0.006
H3q1 | 573.5C | 1 | 0.001
H3r | 113 | 1 | 0.001
H3r | 12046 | 1 | 0.001
H3r | 1342 | 1 | 0.001
H3r | 16076A | 123 | 0.154
H3r | 16092 | 2 | 0.003
H3r | 208 | 1 | 0.001
H3r | 261 | 1 | 0.001
H3r | 281C | 1 | 0.001
H3r | 310 | 1 | 0.001
H3r | 311 | 1 | 0.001
H3r | 316d | 1 | 0.001
H3r | 338 | 1 | 0.001
H3r | 468 | 3 | 0.004
H3r | 502 | 1 | 0.001
H3r | 573.1C | 3 | 0.004
H3r | 573.2C | 5 | 0.006
H3r | 573.5C | 1 | 0.001
H3r | 6506 | 2 | 0.003
H3r | 8155 | 1 | 0.001
H3r | 8648C | 2 | 0.003
H3r | 9716 | 1 | 0.001
H3r1 | 113 | 1 | 0.001
H3r1 | 15944d | 1 | 0.001

H3r1 | 16076A | 123 | 0.155
H3r1 | 208 | 1 | 0.001
H3r1 | 261 | 1 | 0.001
H3r1 | 281C | 1 | 0.001
H3r1 | 316d | 1 | 0.001
H3r1 | 338 | 1 | 0.001
H3r1 | 468 | 3 | 0.004
H3r1 | 502 | 1 | 0.001
H3r1 | 573.1C | 3 | 0.004
H3r1 | 573.2C | 5 | 0.006
H3r1 | 573.5C | 1 | 0.001
H3r1 | 8251 | 1 | 0.001
H3s | 10849 | 1 | 0.111
H3s | 11150 | 1 | 0.111
H3s | 15043 | 1 | 0.111
H3s | 152 | 4 | 0.444
H3s | 16270 | 1 | 0.111
H3s | 489 | 1 | 0.111
H3s | 709 | 2 | 0.222
H3t | 113 | 1 | 0.001
H3t | 15383 | 1 | 0.001
H3t | 15519 | 1 | 0.001
H3t | 15955 | 1 | 0.001
H3t | 16076A | 123 | 0.154
H3t | 16301 | 1 | 0.001
H3t | 208 | 1 | 0.001
H3t | 261 | 1 | 0.001
H3t | 281C | 1 | 0.001
H3t | 316d | 1 | 0.001
H3t | 338 | 1 | 0.001
H3t | 372 | 1 | 0.001
H3t | 468 | 3 | 0.004
H3t | 502 | 1 | 0.001
H3t | 573.1C | 3 | 0.004
H3t | 573.2C | 5 | 0.006
H3t | 573.5C | 1 | 0.001
H3t | 8152 | 1 | 0.001
H3u | 10084 | 8 | 0.01
H3u | 113 | 1 | 0.001
H3u | 13641 | 1 | 0.001
H3u | 16076A | 1 | 0.001
H3u | 16213 | 1 | 0.001
H3u | 16240 | 142 | 0.174
H3u | 16244 | 7 | 0.009
H3u | 16261 | 2 | 0.002
H3u | 16269 | 10 | 0.012
H3u | 16298 | 2 | 0.002
H3u | 199 | 1 | 0.001
H3u | 208 | 1 | 0.001
H3u | 249d | 6 | 0.007
H3u | 261 | 1 | 0.001
H3u | 281C | 1 | 0.001
H3u | 3010 | 2 | 0.002
H3u | 316d | 1 | 0.001
H3u | 338 | 1 | 0.001
H3u | 3951 | 1 | 0.001
H3u | 468 | 3 | 0.004
H3u | 502 | 1 | 0.001
H3u | 573.1C | 3 | 0.004
H3u | 573.2C | 5 | 0.006
H3u | 573.5C | 1 | 0.001
H3u | 7571C | 14 | 0.017
H3u | 8485 | 2 | 0.002
H3u | 9452 | 1 | 0.001
H3u1 | 10241 | 1 | 0.013
H3u1 | 13260 | 1 | 0.013
H3u1 | 151 | 1 | 0.013
H3u1 | 16129 | 1 | 0.013
H3u1 | 16145 | 1 | 0.013
H3u1 | 16381d | 1 | 0.013
H3u1 | 16383T | 1 | 0.013
H3u1 | 16385.1G | 1 | 0.013
H3v | 140 | 1 | 0.005
H3v | 16093 | 1 | 0.005
H3v | 16174 | 1 | 0.005
H3v | 16179 | 1 | 0.005
H3v | 16215 | 1 | 0.005
H3v | 16247 | 1 | 0.005
H3v | 16259 | 2 | 0.01
H3v | 16263A | 1 | 0.005
H3v | 16283 | 21 | 0.101
H3v | 16484-16489d | 1 | 0.005
H3v | 246 | 1 | 0.005
H3v | 316 | 1 | 0.005
H3v | 316C | 1 | 0.005
H3v+16093 | 11137 | 1 | 0.014
H3v+16093 | 11824 | 1 | 0.014
H3v+16093 | 12092A | 1 | 0.014
H3v+16093 | 13986 | 1 | 0.014
H3v+16093 | 14148 | 1 | 0.014
H3v+16093 | 14440 | 1 | 0.014
H3v+16093 | 150 | 2 | 0.028
H3v+16093 | 16129 | 1 | 0.014
H3v+16093 | 16153 | 2 | 0.028
H3v+16093 | 16189 | 2 | 0.028
H3v+16093 | 16192 | 1 | 0.014
H3v+16093 | 16256 | 1 | 0.014
H3v+16093 | 16260 | 1 | 0.014
H3v+16093 | 16274 | 3 | 0.042
H3v+16093 | 16278 | 1 | 0.014
H3v+16093 | 207 | 1 | 0.014
H3v+16093 | 242 | 2 | 0.028
H3v+16093 | 5042 | 1 | 0.014
H3v+16093 | 5423 | 1 | 0.014
H3v+16093 | 8200 | 1 | 0.014
H3v+16093 | 9554 | 1 | 0.014
H3v+16093 | 9965 | 1 | 0.014
H3v1 | 13105 | 1 | 0.005
H3v1 | 140 | 1 | 0.005
H3v1 | 15884 | 1 | 0.005
H3v1 | 16092 | 1 | 0.005

H3v1 | 16174 | 1 | 0.005
H3v1 | 16179 | 1 | 0.005
H3v1 | 16215 | 1 | 0.005
H3v1 | 16247 | 1 | 0.005
H3v1 | 16259 | 2 | 0.01
H3v1 | 16263A | 1 | 0.005
H3v1 | 16283 | 1 | 0.005
H3v1 | 16380d | 21 | 0.101
H3v1 | 16484-16489d | 1 | 0.005
H3v1 | 199 | 2 | 0.01
H3v1 | 246 | 1 | 0.005
H3v1 | 295.1A | 1 | 0.005
H3v1 | 316 | 1 | 0.005
H3v1 | 316C | 1 | 0.005
H3v2 | 16463 | 1 | 0.016
H3v2 | 16465 | 2 | 0.032
H3v2 | 198.1C | 1 | 0.016
H3w | 152 | 3 | 0.034
H3w | 15927 | 1 | 0.011
H3w | 16129 | 4 | 0.045
H3w | 16154 | 1 | 0.011
H3w | 16172 | 1 | 0.011
H3w | 16319 | 1 | 0.011
H3w | 3360 | 1 | 0.011
H3w | 55 | 1 | 0.011
H3w | 57G | 1 | 0.011
H3w | 64 | 1 | 0.011
H3w | 7979 | 3 | 0.034
H3w | 9081 | 1 | 0.011
H3w | 93 | 1 | 0.011
H3x | 150 | 8 | 0.074
H3x | 16037 | 2 | 0.019
H3x | 16093 | 5 | 0.046
H3x | 16129 | 1 | 0.009
H3x | 16162 | 1 | 0.009
H3x | 16254 | 1 | 0.009
H3x | 16261 | 1 | 0.009
H3x | 16271 | 1 | 0.009
H3x | 16311 | 1 | 0.009
H3x | 16319 | 1 | 0.009
H3x | 16343 | 10 | 0.093
H3x | 195 | 8 | 0.074
H3x | 259 | 1 | 0.009
H3x | 37d | 1 | 0.009
H3x | 40A | 1 | 0.009
H3x | 41-44d | 1 | 0.009
H3x | 42.1T | 1 | 0.009
H3x | 432C | 1 | 0.009
H3x | 467 | 1 | 0.009
H3x | 475C | 1 | 0.009
H3x | 479.1A | 1 | 0.009
H3x | 485A | 1 | 0.009
H3x | 486A | 1 | 0.009
H3x | 489G | 1 | 0.009
H3x | 6827 | 1 | 0.009
H3x1 | 12921 | 1 | 0.012
H3x1 | 16352 | 1 | 0.012
H3x1 | 183 | 1 | 0.012
H3x1 | 195 | 2 | 0.025
H3x1 | 239 | 1 | 0.012
H3x1 | 337C | 10 | 0.123
H3x1 | 480 | 13 | 0.16
H3x1 | 4802 | 1 | 0.012
H3x1 | 6750 | 1 | 0.012
H3x1 | 9063 | 1 | 0.012
H3y | 113 | 1 | 0.001
H3y | 11935 | 1 | 0.001
H3y | 13708 | 1 | 0.001
H3y | 16076A | 1 | 0.001
H3y | 16189 | 1 | 0.001
H3y | 16192 | 125 | 0.156
H3y | 195 | 1 | 0.001
H3y | 208 | 1 | 0.001
H3y | 261 | 1 | 0.001
H3y | 281C | 1 | 0.001
H3y | 316d | 1 | 0.001
H3y | 338 | 1 | 0.001
H3y | 373 | 1 | 0.001
H3y | 468 | 3 | 0.004
H3y | 502 | 1 | 0.001
H3y | 573.1C | 3 | 0.004
H3y | 573.2C | 5 | 0.006
H3y | 573.5C | 1 | 0.001
H3y | 8167 | 1 | 0.001
H3z | 151 | 1 | 0.011
H3z | 152 | 4 | 0.043
H3z | 16104 | 1 | 0.011
H3z | 16124 | 2 | 0.022
H3z | 16209 | 1 | 0.011
H3z | 165 | 1 | 0.011
H3z | 189 | 1 | 0.011
H3z | 215 | 1 | 0.011
H3z | 8572 | 1 | 0.011
H3z | 8954 | 1 | 0.011
H3z1 | 11719 | 1 | 0.011
H3z1 | 15777 | 1 | 0.011
H3z1 | 16104 | 1 | 0.011
H3z1 | 16111 | 1 | 0.011
H3z1 | 16124 | 1 | 0.011
H3z1 | 16209 | 3 | 0.033
H3z1 | 16295 | 2 | 0.022
H3z1 | 16305T | 2 | 0.022
H3z1 | 165 | 1 | 0.011
H3z1 | 3203 | 1 | 0.011
H3z1 | 3396 | 2 | 0.022
H3z1 | 709 | 4 | 0.043
H3z1 | 7157 | 2 | 0.022
H3z1 | 73 | 1 | 0.011
H3z1 | 8886 | 2 | 0.022
H3z1 | 8937 | 1 | 0.011
H3z1 | 93 | 2 | 0.022

H3z2 | 16104 | 1 | 0.011
H3z2 | 16111 | 1 | 0.011
H3z2 | 16124 | 1 | 0.011
H3z2 | 16129 | 1 | 0.011
H3z2 | 16188A | 8 | 0.088
H3z2 | 16213 | 2 | 0.022
H3z2 | 165 | 1 | 0.011
H4 | 113 | 1 | 0.001
H4 | 114 | 1 | 0.001
H4 | 13350 | 1 | 0.001
H4 | 13879 | 1 | 0.001
H4 | 14208 | 1 | 0.001
H4 | 143 | 1 | 0.001
H4 | 146 | 2 | 0.002
H4 | 14766 | 1 | 0.001
H4 | 152 | 1 | 0.001
H4 | 15286 | 3 | 0.004
H4 | 15457 | 2 | 0.002
H4 | 16076A | 123 | 0.154
H4 | 16093 | 1 | 0.001
H4 | 16145 | 1 | 0.001
H4 | 16231 | 1 | 0.001
H4 | 1719 | 1 | 0.001
H4 | 195 | 2 | 0.002
H4 | 204 | 1 | 0.001
H4 | 208 | 1 | 0.001
H4 | 234 | 1 | 0.001
H4 | 261 | 1 | 0.001
H4 | 281C | 1 | 0.001
H4 | 315.2C | 1 | 0.001
H4 | 316d | 1 | 0.001
H4 | 338 | 1 | 0.001
H4 | 4024 | 1 | 0.001
H4 | 4315 | 1 | 0.001
H4 | 4418 | 1 | 0.001
H4 | 4639 | 3 | 0.004
H4 | 468 | 3 | 0.004
H4 | 4733 | 1 | 0.001
H4 | 502 | 1 | 0.001
H4 | 5051T | 1 | 0.001
H4 | 573.1C | 3 | 0.004
H4 | 573.2C | 5 | 0.006
H4 | 573.5C | 1 | 0.001
H4 | 689 | 1 | 0.001
H4 | 93 | 2 | 0.002
H40 | 113 | 1 | 0.001
H40 | 13306 | 1 | 0.001
H40 | 13857 | 1 | 0.001
H40 | 15331 | 1 | 0.001
H40 | 16076A | 1 | 0.001
H40 | 16153 | 124 | 0.157
H40 | 16239 | 1 | 0.001
H40 | 16356 | 1 | 0.001
H40 | 208 | 1 | 0.001
H40 | 261 | 1 | 0.001
H40 | 281C | 1 | 0.001
H40 | 295 | 1 | 0.001
H40 | 310 | 1 | 0.001
H40 | 316d | 1 | 0.001
H40 | 338 | 1 | 0.001
H40 | 468 | 3 | 0.004
H40 | 499 | 1 | 0.001
H40 | 502 | 1 | 0.001
H40 | 5156T | 2 | 0.003
H40 | 573.1C | 3 | 0.004
H40 | 573.2C | 5 | 0.006
H40 | 573.5C | 1 | 0.001
H40 | 7600 | 1 | 0.001
H40a | 113 | 1 | 0.001
H40a | 11740 | 1 | 0.001
H40a | 11914 | 1 | 0.001
H40a | 13224 | 1 | 0.001
H40a | 13879 | 1 | 0.001
H40a | 16076A | 123 | 0.156
H40a | 208 | 1 | 0.001
H40a | 261 | 1 | 0.001
H40a | 281C | 1 | 0.001
H40a | 3144 | 1 | 0.001
H40a | 316d | 1 | 0.001
H40a | 338 | 1 | 0.001
H40a | 3714C | 1 | 0.001
H40a | 4386 | 1 | 0.001
H40a | 468 | 3 | 0.004
H40a | 502 | 1 | 0.001
H40a | 573.1C | 3 | 0.004
H40a | 573.2C | 5 | 0.006
H40a | 573.5C | 1 | 0.001
H40b | 113 | 1 | 0.001
H40b | 152 | 1 | 0.001
H40b | 16076A | 123 | 0.156
H40b | 208 | 1 | 0.001
H40b | 261 | 1 | 0.001
H40b | 281C | 1 | 0.001
H40b | 316d | 1 | 0.001
H40b | 338 | 1 | 0.001
H40b | 4561 | 1 | 0.001
H40b | 468 | 3 | 0.004
H40b | 502 | 1 | 0.001
H40b | 573.1C | 3 | 0.004
H40b | 573.2C | 5 | 0.006
H40b | 573.5C | 1 | 0.001
H41 | 113 | 1 | 0.001
H41 | 16076A | 123 | 0.157
H41 | 208 | 1 | 0.001
H41 | 261 | 1 | 0.001
H41 | 281C | 1 | 0.001
H41 | 316d | 1 | 0.001
H41 | 338 | 1 | 0.001
H41 | 468 | 3 | 0.004
H41 | 502 | 1 | 0.001
H41 | 573.1C | 3 | 0.004

H41 | 573.2C | 5 | 0.006
H41 | 573.5C | 1 | 0.001
H41a | 10685 | 1 | 0.02
H41a | 11899 | 2 | 0.039
H41a | 12121 | 1 | 0.02
H41a | 12630 | 2 | 0.039
H41a | 12696 | 1 | 0.02
H41a | 14055 | 2 | 0.039
H41a | 14142 | 1 | 0.02
H41a | 14433 | 1 | 0.02
H41a | 146 | 1 | 0.02
H41a | 152 | 2 | 0.039
H41a | 16072 | 4 | 0.078
H41a | 16189 | 2 | 0.039
H41a | 16246C | 1 | 0.02
H41a | 16261 | 5 | 0.098
H41a | 16264A | 1 | 0.02
H41a | 16274 | 1 | 0.02
H41a | 16289 | 1 | 0.02
H41a | 16293C | 11 | 0.216
H41a | 16328 | 1 | 0.02
H41a | 16356 | 1 | 0.02
H41a | 3039 | 1 | 0.02
H41a | 309d | 1 | 0.02
H41a | 310 | 2 | 0.039
H41a | 3106A | 1 | 0.02
H41a | 315.2C | 1 | 0.02
H41a | 3208 | 1 | 0.02
H41a | 3792 | 1 | 0.02
H41a | 4838 | 1 | 0.02
H41a | 573.1C | 1 | 0.02
H41a | 6284 | 2 | 0.039
H41a | 6392 | 2 | 0.039
H41a | 7674 | 1 | 0.02
H41a | 8251 | 2 | 0.039
H41a | 9218 | 1 | 0.02
H41a | 9548 | 1 | 0.02
H41a | 9554 | 2 | 0.039
H41a | 9804 | 1 | 0.02
H42 | 113 | 1 | 0.001
H42 | 12028 | 1 | 0.001
H42 | 16076A | 123 | 0.156
H42 | 208 | 1 | 0.001
H42 | 261 | 1 | 0.001
H42 | 281C | 1 | 0.001
H42 | 316d | 1 | 0.001
H42 | 338 | 1 | 0.001
H42 | 468 | 3 | 0.004
H42 | 502 | 1 | 0.001
H42 | 573.1C | 3 | 0.004
H42 | 573.2C | 5 | 0.006
H42 | 573.5C | 1 | 0.001
H42 | 7283 | 1 | 0.001
H42a | 152 | 3 | 0.035
H42a | 16092 | 1 | 0.012
H42a | 16153 | 1 | 0.012
H42a | 16172 | 2 | 0.023
H42a | 16188 | 3 | 0.035
H42a | 16189 | 1 | 0.012
H42a | 16241 | 1 | 0.012
H42a | 16243 | 1 | 0.012
H42a | 16300 | 3 | 0.035
H42a | 16319 | 1 | 0.012
H42a | 16362 | 3 | 0.035
H42a | 16390 | 2 | 0.023
H42a | 16484-16489d | 1 | 0.012
H42a | 293 | 1 | 0.012
H42a | 477 | 1 | 0.012
H42a | 8584 | 1 | 0.012
H42a1 | 10237 | 2 | 0.023
H42a1 | 146 | 5 | 0.057
H42a1 | 15978 | 1 | 0.011
H42a1 | 16092 | 1 | 0.011
H42a1 | 16111 | 2 | 0.023
H42a1 | 16153 | 1 | 0.011
H42a1 | 16187A | 1 | 0.011
H42a1 | 16241 | 1 | 0.011
H42a1 | 16243 | 1 | 0.011
H42a1 | 16271 | 1 | 0.011
H42a1 | 16311 | 4 | 0.045
H42a1 | 16319 | 1 | 0.011
H42a1 | 16484-16489d | 1 | 0.011
H42a2 | 11116 | 1 | 0.012
H42a2 | 11914 | 1 | 0.012
H42a2 | 14659 | 1 | 0.012
H42a2 | 15299 | 1 | 0.012
H42a2 | 16092 | 1 | 0.012
H42a2 | 16153 | 1 | 0.012
H42a2 | 16172 | 2 | 0.023
H42a2 | 16241 | 1 | 0.012
H42a2 | 16243 | 1 | 0.012
H42a2 | 16256 | 1 | 0.012
H42a2 | 16319 | 1 | 0.012
H42a2 | 16325 | 2 | 0.023
H42a2 | 16484-16489d | 1 | 0.012
H42a2 | 1842 | 1 | 0.012
H42a2 | 310 | 1 | 0.012
H42a2 | 477 | 1 | 0.012
H42a2 | 9531 | 1 | 0.012
H43 | 10846 | 1 | 0.001
H43 | 113 | 1 | 0.001
H43 | 15924 | 1 | 0.001
H43 | 16076A | 123 | 0.156
H43 | 208 | 1 | 0.001
H43 | 261 | 1 | 0.001
H43 | 281C | 1 | 0.001
H43 | 316d | 1 | 0.001
H43 | 338 | 1 | 0.001
H43 | 468 | 3 | 0.004
H43 | 502 | 1 | 0.001
H43 | 573.1C | 3 | 0.004

H43 | 573.2C | 5 | 0.006
H43 | 573.5C | 1 | 0.001
H43 | 6956 | 1 | 0.001
H44 | 113 | 1 | 0.001
H44 | 16076A | 123 | 0.156
H44 | 208 | 1 | 0.001
H44 | 261 | 1 | 0.001
H44 | 281C | 1 | 0.001
H44 | 316d | 1 | 0.001
H44 | 338 | 1 | 0.001
H44 | 468 | 3 | 0.004
H44 | 502 | 1 | 0.001
H44 | 573.1C | 3 | 0.004
H44 | 573.2C | 5 | 0.006
H44 | 573.5C | 1 | 0.001
H44 | 6215A | 1 | 0.001
H44 | 8618 | 1 | 0.001
H44a | 113 | 1 | 0.001
H44a | 12562G | 1 | 0.001
H44a | 14187 | 1 | 0.001
H44a | 16076A | 1 | 0.001
H44a | 16093 | 123 | 0.155
H44a | 208 | 1 | 0.001
H44a | 261 | 1 | 0.001
H44a | 281C | 1 | 0.001
H44a | 310 | 1 | 0.001
H44a | 316d | 1 | 0.001
H44a | 338 | 1 | 0.001
H44a | 468 | 3 | 0.004
H44a | 502 | 1 | 0.001
H44a | 573.1C | 3 | 0.004
H44a | 573.2C | 5 | 0.006
H44a | 573.5C | 1 | 0.001
H44a1 | 113 | 1 | 0.001
H44a1 | 16076A | 1 | 0.001
H44a1 | 16234 | 123 | 0.156
H44a1 | 208 | 1 | 0.001
H44a1 | 261 | 1 | 0.001
H44a1 | 281C | 1 | 0.001
H44a1 | 316d | 1 | 0.001
H44a1 | 338 | 1 | 0.001
H44a1 | 468 | 3 | 0.004
H44a1 | 502 | 1 | 0.001
H44a1 | 573.1C | 3 | 0.004
H44a1 | 573.2C | 5 | 0.006
H44a1 | 573.5C | 1 | 0.001
H44b | 12512T | 1 | 0.023
H44b | 12612 | 8 | 0.186
H44b | 146 | 2 | 0.047
H44b | 150 | 2 | 0.047
H44b | 15221 | 1 | 0.023
H44b | 16051 | 15 | 0.349
H44b | 16093 | 2 | 0.047
H44b | 16189 | 1 | 0.023
H44b | 16256 | 1 | 0.023
H44b | 16312 | 13 | 0.302
H44b | 16355 | 4 | 0.093
H44b | 204 | 5 | 0.116
H44b | 309d | 3 | 0.07
H44b | 310 | 1 | 0.023
H44b | 5605 | 1 | 0.023
H44b | 6305 | 2 | 0.047
H44b | 8596 | 1 | 0.023
H44b | 8632 | 2 | 0.047
H44b | 9127 | 1 | 0.023
H45 | 113 | 1 | 0.001
H45 | 12192 | 1 | 0.001
H45 | 16076A | 123 | 0.156
H45 | 208 | 1 | 0.001
H45 | 261 | 1 | 0.001
H45 | 281C | 1 | 0.001
H45 | 311 | 1 | 0.001
H45 | 316d | 1 | 0.001
H45 | 338 | 1 | 0.001
H45 | 468 | 3 | 0.004
H45 | 502 | 1 | 0.001
H45 | 573.1C | 3 | 0.004
H45 | 573.2C | 5 | 0.006
H45 | 573.5C | 1 | 0.001
H45a | 113 | 1 | 0.001
H45a | 152 | 1 | 0.001
H45a | 16076A | 123 | 0.154
H45a | 199 | 6 | 0.008
H45a | 208 | 1 | 0.001
H45a | 234 | 1 | 0.001
H45a | 261 | 1 | 0.001
H45a | 281C | 1 | 0.001
H45a | 316d | 1 | 0.001
H45a | 338 | 1 | 0.001
H45a | 4025 | 1 | 0.001
H45a | 468 | 3 | 0.004
H45a | 502 | 1 | 0.001
H45a | 573.1C | 3 | 0.004
H45a | 573.2C | 5 | 0.006
H45a | 573.5C | 1 | 0.001
H45a | 6467 | 1 | 0.001
H45a | 9377 | 1 | 0.001
H45a | 980 | 1 | 0.001
H45b | 113 | 1 | 0.001
H45b | 13708 | 3 | 0.004
H45b | 16076A | 123 | 0.156
H45b | 16316 | 1 | 0.001
H45b | 208 | 1 | 0.001
H45b | 261 | 1 | 0.001
H45b | 281C | 1 | 0.001
H45b | 316d | 1 | 0.001
H45b | 338 | 1 | 0.001
H45b | 468 | 3 | 0.004
H45b | 502 | 1 | 0.001
H45b | 5408 | 1 | 0.001
H45b | 573.1C | 3 | 0.004

H45b | 573.2C | 5 | 0.006
H45b | 573.5C | 1 | 0.001
H45b | 9932 | 1 | 0.001
H46 | 12406 | 3 | 0.02
H46 | 16178 | 4 | 0.026
H46 | 16214 | 3 | 0.02
H46 | 16233T | 1 | 0.007
H46 | 16247 | 20 | 0.132
H46 | 16258T | 1 | 0.007
H46 | 200T | 1 | 0.007
H46 | 207 | 1 | 0.007
H46 | 310 | 1 | 0.007
H46 | 456 | 1 | 0.007
H46 | 463 | 1 | 0.007
H46 | 515-524d | 1 | 0.007
H46 | 529T | 1 | 0.007
H46 | 573.1C | 1 | 0.007
H46 | 8865 | 1 | 0.007
H46 | 93 | 4 | 0.026
H46a | 14198 | 1 | 0.007
H46a | 16214 | 3 | 0.02
H46a | 16233T | 1 | 0.007
H46a | 16247 | 20 | 0.132
H46a | 16258T | 1 | 0.007
H46a | 200T | 1 | 0.007
H46a | 207 | 1 | 0.007
H46a | 430A | 1 | 0.007
H46a | 4435 | 1 | 0.007
H46a | 456 | 1 | 0.007
H46a | 463 | 1 | 0.007
H46a | 515-524d | 1 | 0.007
H46a | 529T | 1 | 0.007
H46a | 573.1C | 1 | 0.007
H46a | 6040 | 3 | 0.02
H46a | 6881T | 2 | 0.013
H46b | 16092 | 1 | 0.007
H46b | 16214 | 3 | 0.02
H46b | 16233T | 1 | 0.007
H46b | 16247 | 20 | 0.134
H46b | 16258T | 1 | 0.007
H46b | 200T | 1 | 0.007
H46b | 207 | 1 | 0.007
H46b | 456 | 1 | 0.007
H46b | 463 | 1 | 0.007
H46b | 515-524d | 1 | 0.007
H46b | 529T | 1 | 0.007
H46b | 573.1C | 1 | 0.007
H46b | 57A | 1 | 0.007
H46b | 58 | 1 | 0.007
H46b | 60.1T | 2 | 0.013
H47 | 113 | 1 | 0.001
H47 | 11499 | 1 | 0.001
H47 | 12561 | 1 | 0.001
H47 | 131 | 1 | 0.001
H47 | 13203 | 1 | 0.001
H47 | 13260 | 1 | 0.001
H47 | 15514 | 1 | 0.001
H47 | 16076A | 1 | 0.001
H47 | 16153 | 123 | 0.155
H47 | 16209 | 1 | 0.001
H47 | 16230 | 1 | 0.001
H47 | 16246T | 1 | 0.001
H47 | 16362 | 1 | 0.001
H47 | 16399 | 1 | 0.001
H47 | 195 | 1 | 0.001
H47 | 208 | 1 | 0.001
H47 | 2392 | 1 | 0.001
H47 | 261 | 1 | 0.001
H47 | 2804 | 1 | 0.001
H47 | 281C | 1 | 0.001
H47 | 296A | 2 | 0.003
H47 | 316d | 1 | 0.001
H47 | 338 | 1 | 0.001
H47 | 468 | 3 | 0.004
H47 | 502 | 1 | 0.001
H47 | 573.1C | 3 | 0.004
H47 | 573.2C | 5 | 0.006
H47 | 573.5C | 1 | 0.001
H47 | 6719 | 1 | 0.001
H47 | 709 | 1 | 0.001
H47 | 8659 | 1 | 0.001
H47a | 12453 | 1 | 0.007
H47a | 13676 | 1 | 0.007
H47a | 16214 | 3 | 0.02
H47a | 16233T | 1 | 0.007
H47a | 16247 | 20 | 0.132
H47a | 16258T | 1 | 0.007
H47a | 200T | 1 | 0.007
H47a | 207 | 1 | 0.007
H47a | 456 | 1 | 0.007
H47a | 463 | 1 | 0.007
H47a | 515-524d | 1 | 0.007
H47a | 529T | 1 | 0.007
H47a | 573.1C | 1 | 0.007
H47a | 8986 | 3 | 0.02
H47a | 93 | 1 | 0.007
H48 | 113 | 1 | 0.001
H48 | 16076A | 1 | 0.001
H48 | 16129 | 1 | 0.001
H48 | 16325 | 2 | 0.003
H48 | 16356 | 123 | 0.154
H48 | 208 | 1 | 0.001
H48 | 235 | 1 | 0.001
H48 | 261 | 1 | 0.001
H48 | 264 | 3 | 0.004
H48 | 281C | 1 | 0.001
H48 | 310 | 2 | 0.003
H48 | 315.2C | 1 | 0.001
H48 | 316d | 1 | 0.001
H48 | 338 | 1 | 0.001
H48 | 468 | 3 | 0.004

H48 | 502 | 1 | 0.001
H48 | 573.1C | 3 | 0.004
H48 | 573.2C | 5 | 0.006
H48 | 573.5C | 1 | 0.001
H48 | 6173 | 1 | 0.001
H48 | 8011 | 1 | 0.001
H48 | 9230 | 2 | 0.003
H49 | 113 | 1 | 0.001
H49 | 12416 | 7 | 0.009
H49 | 15115 | 1 | 0.001
H49 | 15213 | 1 | 0.001
H49 | 16076A | 123 | 0.153
H49 | 16176 | 1 | 0.001
H49 | 16356 | 1 | 0.001
H49 | 16390 | 1 | 0.001
H49 | 16399 | 1 | 0.001
H49 | 208 | 1 | 0.001
H49 | 261 | 1 | 0.001
H49 | 281C | 1 | 0.001
H49 | 310 | 3 | 0.004
H49 | 315.3C | 2 | 0.002
H49 | 316d | 1 | 0.001
H49 | 3338 | 1 | 0.001
H49 | 338 | 1 | 0.001
H49 | 3882 | 3 | 0.004
H49 | 468 | 3 | 0.004
H49 | 502 | 1 | 0.001
H49 | 5553 | 1 | 0.001
H49 | 573.1C | 3 | 0.004
H49 | 573.2C | 5 | 0.006
H49 | 573.5C | 1 | 0.001
H49 | 6827 | 1 | 0.001
H49 | 723 | 1 | 0.001
H49 | 732 | 1 | 0.001
H49 | 8251 | 1 | 0.001
H49 | 8565 | 1 | 0.001
H49 | 9054 | 1 | 0.001
H49 | 93 | 1 | 0.001
H49 | 9368 | 1 | 0.001
H49 | 9422T | 1 | 0.001
H49a | 113 | 1 | 0.001
H49a | 12130 | 1 | 0.001
H49a | 15077 | 2 | 0.003
H49a | 15191 | 1 | 0.001
H49a | 16076A | 1 | 0.001
H49a | 16177 | 4 | 0.005
H49a | 16319 | 123 | 0.154
H49a | 16399 | 124 | 0.156
H49a | 195 | 1 | 0.001
H49a | 208 | 1 | 0.001
H49a | 261 | 1 | 0.001
H49a | 281C | 1 | 0.001
H49a | 310 | 1 | 0.001
H49a | 316d | 1 | 0.001
H49a | 338 | 1 | 0.001
H49a | 4093 | 1 | 0.001
H49a | 4164 | 1 | 0.001
H49a | 468 | 3 | 0.004
H49a | 502 | 1 | 0.001
H49a | 573.1C | 3 | 0.004
H49a | 573.2C | 8 | 0.01
H49a | 573.3C | 4 | 0.005
H49a | 573.4C | 1 | 0.001
H49a | 573.5C | 1 | 0.001
H49a | 574C | 1 | 0.001
H49a | 8404 | 1 | 0.001
H49a1 | 113 | 1 | 0.001
H49a1 | 11872 | 1 | 0.001
H49a1 | 16076A | 123 | 0.156
H49a1 | 208 | 1 | 0.001
H49a1 | 261 | 1 | 0.001
H49a1 | 281C | 1 | 0.001
H49a1 | 316d | 1 | 0.001
H49a1 | 338 | 1 | 0.001
H49a1 | 468 | 3 | 0.004
H49a1 | 502 | 1 | 0.001
H49a1 | 573.1C | 3 | 0.004
H49a1 | 573.2C | 8 | 0.01
H49a1 | 573.5C | 1 | 0.001
H49a1 | 6299 | 1 | 0.001
H49a2 | 14893 | 1 | 0.018
H49a2 | 151 | 5 | 0.091
H49a2 | 152 | 2 | 0.036
H49a2 | 16167 | 1 | 0.018
H49a2 | 16174 | 1 | 0.018
H49a2 | 16181 | 1 | 0.018
H49a2 | 16240T | 2 | 0.036
H49a2 | 295 | 1 | 0.018
H49a2 | 408A | 1 | 0.018
H49a2 | 444 | 1 | 0.018
H49a2 | 513 | 3 | 0.055
H49a2 | 515-524d | 2 | 0.036
H49a2 | 55 | 1 | 0.018
H49a2 | 552A | 3 | 0.055
H49a2 | 57G | 1 | 0.018
H49a2 | 72 | 1 | 0.018
H49a2 | 95T | 1 | 0.018
H49b | 113 | 1 | 0.001
H49b | 13184 | 1 | 0.001
H49b | 15055 | 1 | 0.001
H49b | 16076A | 123 | 0.156
H49b | 208 | 1 | 0.001
H49b | 2118 | 1 | 0.001
H49b | 261 | 1 | 0.001
H49b | 281C | 1 | 0.001
H49b | 310 | 1 | 0.001
H49b | 3110d | 1 | 0.001
H49b | 315.2C | 1 | 0.001
H49b | 316d | 1 | 0.001
H49b | 338 | 1 | 0.001
H49b | 372 | 2 | 0.003

H49b | 468 | 3 | 0.004
H49b | 502 | 1 | 0.001
H49b | 573.1C | 4 | 0.005
H49b | 573.2C | 5 | 0.006
H49b | 573.5C | 1 | 0.001
H49b | 63 | 2 | 0.003
H49b | 64 | 3 | 0.004
H49b | 66 | 1 | 0.001
H49b | 8281-8289d | 1 | 0.001
H4a | 113 | 1 | 0.001
H4a | 12834 | 1 | 0.001
H4a | 16076A | 123 | 0.155
H4a | 16235 | 1 | 0.001
H4a | 16362 | 1 | 0.001
H4a | 208 | 1 | 0.001
H4a | 261 | 1 | 0.001
H4a | 281C | 1 | 0.001
H4a | 316d | 1 | 0.001
H4a | 338 | 1 | 0.001
H4a | 4248 | 1 | 0.001
H4a | 465 | 1 | 0.001
H4a | 468 | 3 | 0.004
H4a | 502 | 1 | 0.001
H4a | 5093 | 1 | 0.001
H4a | 573.1C | 3 | 0.004
H4a | 573.2C | 5 | 0.006
H4a | 573.5C | 1 | 0.001
H4a | 6750 | 1 | 0.001
H4a | 6956G | 1 | 0.001
H4a | 93 | 1 | 0.001
H4a1 | 10095A | 1 | 0.001
H4a1 | 10448 | 1 | 0.001
H4a1 | 10535 | 1 | 0.001
H4a1 | 10620 | 6 | 0.007
H4a1 | 113 | 1 | 0.001
H4a1 | 11371 | 1 | 0.001
H4a1 | 11957 | 1 | 0.001
H4a1 | 12171 | 1 | 0.001
H4a1 | 12705 | 1 | 0.001
H4a1 | 13104 | 1 | 0.001
H4a1 | 14218 | 1 | 0.001
H4a1 | 14305 | 2 | 0.002
H4a1 | 146 | 2 | 0.002
H4a1 | 14767 | 1 | 0.001
H4a1 | 15071 | 1 | 0.001
H4a1 | 152 | 7 | 0.009
H4a1 | 15211 | 1 | 0.001
H4a1 | 1536 | 1 | 0.001
H4a1 | 15394 | 1 | 0.001
H4a1 | 15910 | 1 | 0.001
H4a1 | 16037 | 1 | 0.001
H4a1 | 16076A | 1 | 0.001
H4a1 | 16162 | 1 | 0.001
H4a1 | 16176G | 1 | 0.001
H4a1 | 16189 | 2 | 0.002
H4a1 | 16209 | 2 | 0.002
H4a1 | 16235T | 2 | 0.002
H4a1 | 16239 | 1 | 0.001
H4a1 | 16259 | 1 | 0.001
H4a1 | 16271 | 1 | 0.001
H4a1 | 16286 | 10 | 0.012
H4a1 | 16290 | 129 | 0.157
H4a1 | 16304 | 1 | 0.001
H4a1 | 16311 | 4 | 0.005
H4a1 | 16356 | 1 | 0.001
H4a1 | 16362 | 1 | 0.001
H4a1 | 182 | 2 | 0.002
H4a1 | 194 | 1 | 0.001
H4a1 | 208 | 1 | 0.001
H4a1 | 215 | 1 | 0.001
H4a1 | 261 | 1 | 0.001
H4a1 | 281C | 1 | 0.001
H4a1 | 3083 | 2 | 0.002
H4a1 | 310 | 1 | 0.001
H4a1 | 316d | 1 | 0.001
H4a1 | 3203 | 2 | 0.002
H4a1 | 338 | 1 | 0.001
H4a1 | 3459 | 1 | 0.001
H4a1 | 4232 | 1 | 0.001
H4a1 | 456 | 3 | 0.004
H4a1 | 460 | 1 | 0.001
H4a1 | 502 | 1 | 0.001
H4a1 | 5147 | 2 | 0.002
H4a1 | 5267 | 1 | 0.001
H4a1 | 5471 | 1 | 0.001
H4a1 | 5585 | 1 | 0.001
H4a1 | 573.1C | 3 | 0.004
H4a1 | 573.2C | 5 | 0.006
H4a1 | 573.3C | 123 | 0.15
H4a1 | 573.5C | 1 | 0.001
H4a1 | 7079 | 1 | 0.001
H4a1 | 73 | 1 | 0.001
H4a1 | 7741 | 1 | 0.001
H4a1 | 7853 | 3 | 0.004
H4a1 | 8093 | 1 | 0.001
H4a1 | 8433 | 1 | 0.001
H4a1 | 8730 | 2 | 0.002
H4a1 | 8954 | 1 | 0.001
H4a1 | 8975 | 1 | 0.001
H4a1 | 9007 | 10 | 0.012
H4a1 | 9110 | 1 | 0.001
H4a1 | 93 | 1 | 0.001
H4a1 | 960d | 1 | 0.001
H4a1a | 1027 | 1 | 0.001
H4a1a | 10718 | 1 | 0.001
H4a1a | 113 | 1 | 0.001
H4a1a | 11347 | 1 | 0.001
H4a1a | 11377 | 2 | 0.002
H4a1a | 13578 | 1 | 0.001
H4a1a | 14502 | 1 | 0.001
H4a1a | 152 | 1 | 0.001

H4a1a | 15924 | 2 | 0.002
H4a1a | 16076A | 1 | 0.001
H4a1a | 16093 | 1 | 0.001
H4a1a | 16104 | 1 | 0.001
H4a1a | 16209 | 1 | 0.001
H4a1a | 16260 | 1 | 0.001
H4a1a | 16265C | 2 | 0.002
H4a1a | 16311 | 124 | 0.154
H4a1a | 208 | 1 | 0.001
H4a1a | 261 | 1 | 0.001
H4a1a | 281C | 1 | 0.001
H4a1a | 316d | 1 | 0.001
H4a1a | 338 | 1 | 0.001
H4a1a | 468 | 3 | 0.004
H4a1a | 502 | 1 | 0.001
H4a1a | 573.1C | 4 | 0.005
H4a1a | 573.2C | 5 | 0.006
H4a1a | 573.5C | 1 | 0.001
H4a1a | 7894 | 1 | 0.001
H4a1a+195 | 10589 | 1 | 0.03
H4a1a+195 | 15071 | 1 | 0.03
H4a1a+195 | 15884 | 1 | 0.03
H4a1a+195 | 15924 | 1 | 0.03
H4a1a+195 | 16093 | 1 | 0.03
H4a1a+195 | 16189 | 8 | 0.242
H4a1a+195 | 16193 | 1 | 0.03
H4a1a+195 | 4113C | 1 | 0.03
H4a1a+195 | 6095 | 1 | 0.03
H4a1a+195 | 6293 | 1 | 0.03
H4a1a+195 | 73 | 1 | 0.03
H4a1a+195 | 7354 | 1 | 0.03
H4a1a+195 | 9591 | 1 | 0.03
H4a1a1 | 10364 | 1 | 0.001
H4a1a1 | 113 | 1 | 0.001
H4a1a1 | 11632 | 1 | 0.001
H4a1a1 | 152 | 1 | 0.001
H4a1a1 | 15323 | 2 | 0.002
H4a1a1 | 16076A | 123 | 0.151
H4a1a1 | 16162 | 2 | 0.002
H4a1a1 | 16287 | 1 | 0.001
H4a1a1 | 16390 | 1 | 0.001
H4a1a1 | 208 | 1 | 0.001
H4a1a1 | 2416 | 2 | 0.002
H4a1a1 | 261 | 1 | 0.001
H4a1a1 | 281C | 1 | 0.001
H4a1a1 | 316d | 1 | 0.001
H4a1a1 | 338 | 1 | 0.001
H4a1a1 | 3644 | 1 | 0.001
H4a1a1 | 468 | 3 | 0.004
H4a1a1 | 502 | 1 | 0.001
H4a1a1 | 573.1C | 3 | 0.004
H4a1a1 | 573.2C | 5 | 0.006
H4a1a1 | 573.5C | 1 | 0.001
H4a1a1 | 5814 | 4 | 0.005
H4a1a1 | 5899.1C | 1 | 0.001
H4a1a1 | 8281-8289d | 2 | 0.002
H4a1a1 | 8290C | 2 | 0.002
H4a1a1 | 8681G | 1 | 0.001
H4a1a1 | 990 | 1 | 0.001
H4a1a1a | 10601 | 1 | 0.005
H4a1a1a | 11710 | 1 | 0.005
H4a1a1a | 12687 | 1 | 0.005
H4a1a1a | 12702 | 1 | 0.005
H4a1a1a | 13161 | 1 | 0.005
H4a1a1a | 13545 | 2 | 0.011
H4a1a1a | 14318 | 1 | 0.005
H4a1a1a | 14527 | 1 | 0.005
H4a1a1a | 146 | 6 | 0.032
H4a1a1a | 15077 | 1 | 0.005
H4a1a1a | 15148 | 1 | 0.005
H4a1a1a | 15172 | 3 | 0.016
H4a1a1a | 152 | 1 | 0.005
H4a1a1a | 15244 | 1 | 0.005
H4a1a1a | 15808 | 3 | 0.016
H4a1a1a | 15955 | 1 | 0.005
H4a1a1a | 16082 | 1 | 0.005
H4a1a1a | 16129 | 5 | 0.027
H4a1a1a | 16176 | 1 | 0.005
H4a1a1a | 16180 | 2 | 0.011
H4a1a1a | 16192 | 3 | 0.016
H4a1a1a | 16234 | 1 | 0.005
H4a1a1a | 16256 | 1 | 0.005
H4a1a1a | 16266 | 1 | 0.005
H4a1a1a | 16274 | 1 | 0.005
H4a1a1a | 16320 | 4 | 0.022
H4a1a1a | 16465A | 4 | 0.022
H4a1a1a | 16497 | 1 | 0.005
H4a1a1a | 182 | 1 | 0.005
H4a1a1a | 1822 | 1 | 0.005
H4a1a1a | 2361 | 1 | 0.005
H4a1a1a | 239 | 1 | 0.005
H4a1a1a | 2416 | 1 | 0.005
H4a1a1a | 3010 | 1 | 0.005
H4a1a1a | 310 | 2 | 0.011
H4a1a1a | 3157A | 1 | 0.005
H4a1a1a | 4659 | 1 | 0.005
H4a1a1a | 5220 | 1 | 0.005
H4a1a1a | 5276 | 1 | 0.005
H4a1a1a | 5513 | 1 | 0.005
H4a1a1a | 560 | 1 | 0.005
H4a1a1a | 593 | 1 | 0.005
H4a1a1a | 629 | 1 | 0.005
H4a1a1a | 64 | 1 | 0.005
H4a1a1a | 6719 | 1 | 0.005
H4a1a1a | 7058 | 1 | 0.005
H4a1a1a | 709 | 3 | 0.016
H4a1a1a | 710A | 1 | 0.005
H4a1a1a | 7834 | 1 | 0.005
H4a1a1a | 8077 | 1 | 0.005
H4a1a1a | 8582 | 1 | 0.005
H4a1a1a | 8626A | 1 | 0.005

H4a1a1a | 8832 | 1 | 0.005
H4a1a1a | 8841 | 1 | 0.005
H4a1a1a | 8989 | 1 | 0.005
H4a1a1a | 9997 | 4 | 0.022
H4a1a1a1a | 12406 | 1 | 0.008
H4a1a1a1a | 150 | 4 | 0.031
H4a1a1a1a | 16317 | 1 | 0.008
H4a1a1a1a | 16362 | 1 | 0.008
H4a1a1a1a | 3305 | 1 | 0.008
H4a1a1a1a | 5318 | 1 | 0.008
H4a1a1a1a | 7831 | 4 | 0.031
H4a1a1a1a | 960d | 4 | 0.031
H4a1a1a1a1 | 12651 | 2 | 0.015
H4a1a1a1a1 | 152 | 2 | 0.015
H4a1a1a1a1 | 16080 | 1 | 0.007
H4a1a1a1a1 | 16184 | 5 | 0.037
H4a1a1a1a1 | 16311 | 1 | 0.007
H4a1a1a1a1 | 4353 | 1 | 0.007
H4a1a1a1a1 | 5460 | 1 | 0.007
H4a1a1a1a1 | 6164 | 1 | 0.007
H4a1a1a1a1 | 9218 | 1 | 0.007
H4a1a1a2 | 11173 | 1 | 0.008
H4a1a1a2 | 11629 | 1 | 0.008
H4a1a1a2 | 11914 | 2 | 0.015
H4a1a1a2 | 12508 | 1 | 0.008
H4a1a1a2 | 13545 | 7 | 0.053
H4a1a1a2 | 228T | 1 | 0.008
H4a1a1a2 | 292.1AT | 2 | 0.015
H4a1a1a2 | 4688 | 1 | 0.008
H4a1a1a2 | 5618 | 1 | 0.008
H4a1a1a2 | 8185 | 1 | 0.008
H4a1a1a2 | 8348 | 1 | 0.008
H4a1a1a3 | 10166 | 1 | 0.013
H4a1a1a3 | 146 | 5 | 0.064
H4a1a1a3 | 15154A | 1 | 0.013
H4a1a1a3 | 16147 | 1 | 0.013
H4a1a1a3 | 16234 | 1 | 0.013
H4a1a1a3 | 3398 | 1 | 0.013
H4a1a1a3 | 385 | 1 | 0.013
H4a1a1a3 | 9422 | 1 | 0.013
H4a1a1a4 | 11377 | 4 | 0.031
H4a1a1a4 | 7648 | 1 | 0.008
H4a1a1a4 | 9053 | 1 | 0.008
H4a1a2 | 113 | 1 | 0.001
H4a1a2 | 16076A | 123 | 0.155
H4a1a2 | 208 | 1 | 0.001
H4a1a2 | 261 | 1 | 0.001
H4a1a2 | 281C | 1 | 0.001
H4a1a2 | 316d | 1 | 0.001
H4a1a2 | 338 | 1 | 0.001
H4a1a2 | 468 | 3 | 0.004
H4a1a2 | 502 | 1 | 0.001
H4a1a2 | 573.1C | 3 | 0.004
H4a1a2 | 573.2C | 5 | 0.006
H4a1a2 | 573.5C | 1 | 0.001
H4a1a2 | 73 | 1 | 0.001
H4a1a2 | 8566 | 1 | 0.001
H4a1a2a | 113 | 1 | 0.001
H4a1a2a | 11875 | 1 | 0.001
H4a1a2a | 146 | 1 | 0.001
H4a1a2a | 16076A | 123 | 0.154
H4a1a2a | 208 | 1 | 0.001
H4a1a2a | 261 | 1 | 0.001
H4a1a2a | 281C | 1 | 0.001
H4a1a2a | 316d | 1 | 0.001
H4a1a2a | 338 | 1 | 0.001
H4a1a2a | 468 | 3 | 0.004
H4a1a2a | 502 | 1 | 0.001
H4a1a2a | 573.1C | 3 | 0.004
H4a1a2a | 573.2C | 5 | 0.006
H4a1a2a | 573.5C | 1 | 0.001
H4a1a2a1 | 113 | 1 | 0.001
H4a1a2a1 | 13368 | 1 | 0.001
H4a1a2a1 | 16076A | 123 | 0.155
H4a1a2a1 | 16189 | 1 | 0.001
H4a1a2a1 | 16325 | 1 | 0.001
H4a1a2a1 | 208 | 1 | 0.001
H4a1a2a1 | 261 | 1 | 0.001
H4a1a2a1 | 281C | 1 | 0.001
H4a1a2a1 | 316d | 1 | 0.001
H4a1a2a1 | 338 | 1 | 0.001
H4a1a2a1 | 3816 | 1 | 0.001
H4a1a2a1 | 468 | 3 | 0.004
H4a1a2a1 | 502 | 1 | 0.001
H4a1a2a1 | 573.1C | 3 | 0.004
H4a1a2a1 | 573.2C | 5 | 0.006
H4a1a2a1 | 573.5C | 1 | 0.001
H4a1a3 | 150 | 7 | 0.233
H4a1a3 | 310 | 1 | 0.033
H4a1a3 | 6951 | 5 | 0.167
H4a1a3a | 12118 | 1 | 0.032
H4a1a3a | 14133 | 6 | 0.194
H4a1a3a | 16526 | 1 | 0.032
H4a1a3a | 513 | 2 | 0.065
H4a1a3a | 6293 | 1 | 0.032
H4a1a4 | 15941 | 1 | 0.03
H4a1a4 | 16153 | 2 | 0.061
H4a1a4 | 16485-16488d | 3 | 0.091
H4a1a4 | 16490-16501d | 3 | 0.091
H4a1a4 | 16507-16509d | 3 | 0.091
H4a1a4 | 16524C | 1 | 0.03
H4a1a4 | 16527 | 1 | 0.03
H4a1a4 | 198 | 2 | 0.061
H4a1a4 | 280 | 1 | 0.03
H4a1a4 | 477 | 1 | 0.03
H4a1a4a | 152 | 4 | 0.075
H4a1a4a | 16086 | 3 | 0.057
H4a1a4a | 16148 | 1 | 0.019
H4a1a4a | 16233 | 25 | 0.472
H4a1a4a | 16356 | 4 | 0.075
H4a1a4a | 4232 | 1 | 0.019

H4a1a4b | 10972 | 2 | 0.033
H4a1a4b | 12741 | 1 | 0.016
H4a1a4b | 13471 | 1 | 0.016
H4a1a4b | 14484 | 1 | 0.016
H4a1a4b | 15172 | 1 | 0.016
H4a1a4b | 16145 | 1 | 0.016
H4a1a4b | 16311 | 4 | 0.066
H4a1a4b | 200 | 1 | 0.016
H4a1a4b | 310 | 3 | 0.049
H4a1a4b | 3591 | 2 | 0.033
H4a1a4b | 573.1C | 3 | 0.049
H4a1a4b | 629 | 1 | 0.016
H4a1a4b | 6374 | 2 | 0.033
H4a1a4b | 7080 | 1 | 0.016
H4a1a4b | 7091 | 1 | 0.016
H4a1a4b | 7397 | 1 | 0.016
H4a1a4b | 7984 | 1 | 0.016
H4a1a4b | 8400 | 1 | 0.016
H4a1a4b | 8583 | 2 | 0.033
H4a1a4b | 9033 | 2 | 0.033
H4a1a4b | 93 | 1 | 0.016
H4a1a4b | 9300 | 1 | 0.016
H4a1a4b | 9647 | 1 | 0.016
H4a1a4b | 9977 | 1 | 0.016
H4a1a4b1 | 12397 | 1 | 0.077
H4a1a4b1 | 12406 | 1 | 0.077
H4a1a4b1 | 152 | 1 | 0.077
H4a1a4b1 | 15613 | 1 | 0.077
H4a1a4b1 | 16093 | 2 | 0.154
H4a1a4b1 | 16172 | 1 | 0.077
H4a1a4b1 | 16189 | 2 | 0.154
H4a1a4b1 | 316C | 1 | 0.077
H4a1a4b1 | 64A | 2 | 0.154
H4a1a4b1 | 65A | 1 | 0.077
H4a1a4b1 | 8485 | 1 | 0.077
H4a1a4b2 | 13015 | 1 | 0.029
H4a1a4b2 | 15941 | 2 | 0.057
H4a1a4b2 | 16485-16488d | 3 | 0.086
H4a1a4b2 | 16490-16501d | 3 | 0.086
H4a1a4b2 | 16507-16509d | 3 | 0.086
H4a1a4b2 | 16524C | 1 | 0.029
H4a1a4b2 | 16527 | 1 | 0.029
H4a1a4b2 | 280 | 1 | 0.029
H4a1a4b2 | 477 | 1 | 0.029
H4a1a5 | 113 | 1 | 0.001
H4a1a5 | 16076A | 123 | 0.155
H4a1a5 | 208 | 1 | 0.001
H4a1a5 | 261 | 1 | 0.001
H4a1a5 | 2789 | 1 | 0.001
H4a1a5 | 281C | 1 | 0.001
H4a1a5 | 316d | 1 | 0.001
H4a1a5 | 338 | 1 | 0.001
H4a1a5 | 3394 | 1 | 0.001
H4a1a5 | 468 | 3 | 0.004
H4a1a5 | 502 | 1 | 0.001
H4a1a5 | 573.1C | 3 | 0.004
H4a1a5 | 573.2C | 5 | 0.006
H4a1a5 | 573.5C | 1 | 0.001
H4a1c | 113 | 1 | 0.001
H4a1c | 152 | 1 | 0.001
H4a1c | 16076A | 123 | 0.156
H4a1c | 16445 | 1 | 0.001
H4a1c | 208 | 1 | 0.001
H4a1c | 261 | 1 | 0.001
H4a1c | 281C | 1 | 0.001
H4a1c | 316d | 1 | 0.001
H4a1c | 338 | 1 | 0.001
H4a1c | 468 | 3 | 0.004
H4a1c | 502 | 1 | 0.001
H4a1c | 573.1C | 3 | 0.004
H4a1c | 573.2C | 5 | 0.006
H4a1c | 573.5C | 1 | 0.001
H4a1c1 | 113 | 1 | 0.001
H4a1c1 | 13477 | 1 | 0.001
H4a1c1 | 14861 | 1 | 0.001
H4a1c1 | 16076A | 123 | 0.156
H4a1c1 | 16189 | 1 | 0.001
H4a1c1 | 208 | 1 | 0.001
H4a1c1 | 261 | 1 | 0.001
H4a1c1 | 281C | 1 | 0.001
H4a1c1 | 316d | 1 | 0.001
H4a1c1 | 3316 | 1 | 0.001
H4a1c1 | 338 | 1 | 0.001
H4a1c1 | 468 | 3 | 0.004
H4a1c1 | 502 | 1 | 0.001
H4a1c1 | 573.1C | 3 | 0.004
H4a1c1 | 573.2C | 5 | 0.006
H4a1c1 | 573.5C | 1 | 0.001
H4a1c1a | 113 | 1 | 0.001
H4a1c1a | 15314 | 1 | 0.001
H4a1c1a | 15498d | 1 | 0.001
H4a1c1a | 16076A | 1 | 0.001
H4a1c1a | 16164 | 123 | 0.155
H4a1c1a | 208 | 1 | 0.001
H4a1c1a | 261 | 1 | 0.001
H4a1c1a | 281C | 1 | 0.001
H4a1c1a | 316d | 1 | 0.001
H4a1c1a | 338 | 1 | 0.001
H4a1c1a | 468 | 3 | 0.004
H4a1c1a | 4934 | 1 | 0.001
H4a1c1a | 498d | 1 | 0.001
H4a1c1a | 502 | 1 | 0.001
H4a1c1a | 573.1C | 3 | 0.004
H4a1c1a | 573.2C | 5 | 0.006
H4a1c1a | 573.5C | 1 | 0.001
H4a1c1a | 6845 | 2 | 0.003
H4a1c1a | 9106 | 2 | 0.003
H4a1c2 | 4991 | 1 | 0.015
H4a1d | 113 | 1 | 0.001
H4a1d | 14818 | 6 | 0.007
H4a1d | 15148 | 2 | 0.002

H4a1d | 16051 | 1 | 0.001
H4a1d | 16076A | 123 | 0.153
H4a1d | 16287 | 1 | 0.001
H4a1d | 16354 | 2 | 0.002
H4a1d | 208 | 1 | 0.001
H4a1d | 261 | 1 | 0.001
H4a1d | 281C | 1 | 0.001
H4a1d | 316d | 1 | 0.001
H4a1d | 338 | 1 | 0.001
H4a1d | 468 | 3 | 0.004
H4a1d | 502 | 1 | 0.001
H4a1d | 573.1C | 3 | 0.004
H4a1d | 573.2C | 5 | 0.006
H4a1d | 573.5C | 1 | 0.001
H4a1d | 6719 | 1 | 0.001
H4a1d | 7419 | 1 | 0.001
H4a2 | 10376 | 1 | 0.014
H4a2 | 152 | 1 | 0.014
H4a2 | 16167 | 1 | 0.014
H4a2 | 16232 | 1 | 0.014
H4a2 | 16271 | 1 | 0.014
H4a2 | 16311 | 1 | 0.014
H4a2 | 453 | 1 | 0.014
H4b | 10523T | 1 | 0.001
H4b | 113 | 1 | 0.001
H4b | 152 | 2 | 0.003
H4b | 16076A | 123 | 0.155
H4b | 16092 | 1 | 0.001
H4b | 16189 | 1 | 0.001
H4b | 16526 | 1 | 0.001
H4b | 208 | 1 | 0.001
H4b | 261 | 1 | 0.001
H4b | 281C | 1 | 0.001
H4b | 316d | 1 | 0.001
H4b | 338 | 1 | 0.001
H4b | 468 | 3 | 0.004
H4b | 502 | 1 | 0.001
H4b | 573.1C | 3 | 0.004
H4b | 573.2C | 5 | 0.006
H4b | 573.5C | 1 | 0.001
H4b1 | 16117 | 1 | 0.019
H4b1 | 16145 | 1 | 0.019
H4b1 | 16188 | 1 | 0.019
H4b1 | 16189 | 1 | 0.019
H4b1 | 16193d | 1 | 0.019
H4b1 | 16223 | 1 | 0.019
H4b1 | 16298 | 1 | 0.019
H4b1 | 16301A | 1 | 0.019
H4b1 | 16390 | 9 | 0.167
H4b1 | 16524C | 1 | 0.019
H4b1 | 183d | 1 | 0.019
H4b1 | 267 | 4 | 0.074
H4b1 | 533T | 5 | 0.093
H4b1 | 73 | 2 | 0.037
H4b1 | 8901 | 1 | 0.019
H4c | 113 | 1 | 0.001
H4c | 14539 | 1 | 0.001
H4c | 16076A | 123 | 0.155
H4c | 208 | 1 | 0.001
H4c | 261 | 1 | 0.001
H4c | 281C | 1 | 0.001
H4c | 316d | 1 | 0.001
H4c | 338 | 1 | 0.001
H4c | 468 | 3 | 0.004
H4c | 502 | 1 | 0.001
H4c | 573.1C | 4 | 0.005
H4c | 573.2C | 5 | 0.006
H4c | 573.5C | 1 | 0.001
H4c | 9629 | 1 | 0.001
H4c1 | 11410 | 2 | 0.026
H4c1 | 152 | 1 | 0.013
H4c1 | 16189 | 1 | 0.013
H4c1 | 16302 | 1 | 0.013
H4c1 | 16354 | 1 | 0.013
H4c1 | 16398 | 1 | 0.013
H4c1 | 16473 | 1 | 0.013
H4c1 | 8584 | 1 | 0.013
H4c1 | 9947 | 1 | 0.013
H4d | 16042C | 1 | 0.003
H4d | 16129T | 1 | 0.003
H4d | 16150 | 1 | 0.003
H4d | 16188A | 2 | 0.005
H4d | 16231 | 1 | 0.003
H4d | 16249 | 8 | 0.021
H4d | 16293C | 2 | 0.005
H4d | 16327 | 125 | 0.333
H4d | 176C | 2 | 0.005
H4d | 239 | 1 | 0.003
H4d | 252 | 3 | 0.008
H4d | 261.1T | 1 | 0.003
H4d | 264 | 3 | 0.008
H4d | 3027 | 1 | 0.003
H4d | 332G | 1 | 0.003
H4d | 4248 | 2 | 0.005
H4d | 49T | 1 | 0.003
H4d | 523-525d | 1 | 0.003
H4d | 7562 | 2 | 0.005
H4d | 9540 | 1 | 0.003
H5 | 10256 | 1 | 0.003
H5 | 10265 | 1 | 0.003
H5 | 10289 | 2 | 0.005
H5 | 10310 | 7 | 0.019
H5 | 10313 | 1 | 0.003
H5 | 10376 | 1 | 0.003
H5 | 10554 | 1 | 0.003
H5 | 10646 | 1 | 0.003
H5 | 10664A | 1 | 0.003
H5 | 10727 | 1 | 0.003
H5 | 10751 | 1 | 0.003
H5 | 10864 | 3 | 0.008
H5 | 10907 | 1 | 0.003

H5 | 10963 | 1 | 0.003
H5 | 11147 | 1 | 0.003
H5 | 11152 | 2 | 0.005
H5 | 11172 | 7 | 0.019
H5 | 11719 | 4 | 0.011
H5 | 11914 | 1 | 0.003
H5 | 12236 | 1 | 0.003
H5 | 12696 | 1 | 0.003
H5 | 13105 | 1 | 0.003
H5 | 13145 | 2 | 0.005
H5 | 13470 | 1 | 0.003
H5 | 13651C | 1 | 0.003
H5 | 13665 | 1 | 0.003
H5 | 13674 | 1 | 0.003
H5 | 13708 | 1 | 0.003
H5 | 1412.1G | 1 | 0.003
H5 | 14180 | 1 | 0.003
H5 | 14198 | 1 | 0.003
H5 | 14287 | 1 | 0.003
H5 | 143 | 1 | 0.003
H5 | 14308 | 2 | 0.005
H5 | 14323 | 1 | 0.003
H5 | 14560 | 1 | 0.003
H5 | 14566 | 1 | 0.003
H5 | 146 | 22 | 0.06
H5 | 150 | 7 | 0.019
H5 | 15172 | 1 | 0.003
H5 | 152 | 4 | 0.011
H5 | 15217 | 1 | 0.003
H5 | 15219 | 7 | 0.019
H5 | 15257 | 1 | 0.003
H5 | 152A | 1 | 0.003
H5 | 153 | 3 | 0.008
H5 | 15314 | 1 | 0.003
H5 | 15511 | 1 | 0.003
H5 | 15601 | 1 | 0.003
H5 | 15884 | 1 | 0.003
H5 | 15889 | 2 | 0.005
H5 | 15928 | 1 | 0.003
H5 | 16000 | 1 | 0.003
H5 | 16037 | 1 | 0.003
H5 | 16114A | 1 | 0.003
H5 | 16129 | 4 | 0.011
H5 | 16138 | 1 | 0.003
H5 | 16148 | 1 | 0.003
H5 | 16162 | 1 | 0.003
H5 | 16166 | 2 | 0.005
H5 | 16167 | 2 | 0.005
H5 | 16176 | 1 | 0.003
H5 | 16178 | 3 | 0.008
H5 | 16181 | 1 | 0.003
H5 | 16185 | 1 | 0.003
H5 | 16189 | 3 | 0.008
H5 | 16221 | 1 | 0.003
H5 | 16222 | 5 | 0.014
H5 | 16241C | 2 | 0.005
H5 | 16243 | 1 | 0.003
H5 | 16247 | 1 | 0.003
H5 | 16249 | 1 | 0.003
H5 | 16256 | 2 | 0.005
H5 | 16259 | 1 | 0.003
H5 | 16265C | 1 | 0.003
H5 | 16269 | 1 | 0.003
H5 | 16274 | 2 | 0.005
H5 | 16291 | 5 | 0.014
H5 | 16293 | 1 | 0.003
H5 | 16305 | 3 | 0.008
H5 | 16311 | 23 | 0.063
H5 | 16342 | 1 | 0.003
H5 | 16362 | 2 | 0.005
H5 | 16390 | 1 | 0.003
H5 | 16497 | 1 | 0.003
H5 | 16529A | 1 | 0.003
H5 | 1842 | 1 | 0.003
H5 | 194 | 1 | 0.003
H5 | 195A | 1 | 0.003
H5 | 199 | 1 | 0.003
H5 | 204 | 8 | 0.022
H5 | 2149 | 2 | 0.005
H5 | 215 | 1 | 0.003
H5 | 225 | 2 | 0.005
H5 | 227 | 2 | 0.005
H5 | 252 | 1 | 0.003
H5 | 2757 | 1 | 0.003
H5 | 310 | 3 | 0.008
H5 | 316 | 2 | 0.005
H5 | 3197 | 1 | 0.003
H5 | 3200 | 1 | 0.003
H5 | 3204 | 1 | 0.003
H5 | 3396 | 1 | 0.003
H5 | 3434 | 1 | 0.003
H5 | 3438 | 1 | 0.003
H5 | 365 | 1 | 0.003
H5 | 3826 | 1 | 0.003
H5 | 4098 | 1 | 0.003
H5 | 4216 | 1 | 0.003
H5 | 4432.1C | 1 | 0.003
H5 | 459d | 1 | 0.003
H5 | 4790 | 1 | 0.003
H5 | 4820 | 2 | 0.005
H5 | 506 | 1 | 0.003
H5 | 5238 | 2 | 0.005
H5 | 5277 | 1 | 0.003
H5 | 5460 | 2 | 0.005
H5 | 573.6C | 1 | 0.003
H5 | 5784 | 1 | 0.003
H5 | 593 | 1 | 0.003
H5 | 5993 | 1 | 0.003
H5 | 61 | 3 | 0.008
H5 | 62 | 3 | 0.008
H5 | 6365 | 1 | 0.003

H5 | 6497 | 1 | 0.003
H5 | 7106 | 1 | 0.003
H5 | 7120 | 1 | 0.003
H5 | 7153 | 1 | 0.003
H5 | 7211 | 1 | 0.003
H5 | 7278 | 2 | 0.005
H5 | 73 | 8 | 0.022
H5 | 7519d | 1 | 0.003
H5 | 7741 | 1 | 0.003
H5 | 7746 | 1 | 0.003
H5 | 7999 | 5 | 0.014
H5 | 8149 | 2 | 0.005
H5 | 8176 | 1 | 0.003
H5 | 8348 | 1 | 0.003
H5 | 8400 | 1 | 0.003
H5 | 8481 | 3 | 0.008
H5 | 8947 | 2 | 0.005
H5 | 9070 | 1 | 0.003
H5 | 9110 | 7 | 0.019
H5 | 9128 | 1 | 0.003
H5 | 93 | 1 | 0.003
H5 | 961 | 4 | 0.011
H5 | 965.2C | 1 | 0.003
H5 | 9804 | 3 | 0.008
H5+16192 | 11233 | 1 | 0.02
H5+16192 | 11593 | 1 | 0.02
H5+16192 | 15257 | 1 | 0.02
H5+16192 | 16086G | 1 | 0.02
H5+16192 | 16093 | 1 | 0.02
H5+16192 | 16121G | 1 | 0.02
H5+16192 | 16187 | 1 | 0.02
H5+16192 | 16207 | 1 | 0.02
H5+16192 | 16228G | 1 | 0.02
H5+16192 | 16241 | 1 | 0.02
H5+16192 | 16266 | 2 | 0.041
H5+16192 | 16272C | 1 | 0.02
H5+16192 | 16274 | 1 | 0.02
H5+16192 | 16280 | 1 | 0.02
H5+16192 | 16282G | 1 | 0.02
H5+16192 | 16287 | 1 | 0.02
H5+16192 | 16290G | 1 | 0.02
H5+16192 | 16310 | 1 | 0.02
H5+16192 | 16311 | 1 | 0.02
H5+16192 | 16318 | 1 | 0.02
H5+16192 | 16322T | 1 | 0.02
H5+16192 | 16470T | 1 | 0.02
H5+16192 | 3197 | 1 | 0.02
H5+16192 | 4314A | 1 | 0.02
H5+16192 | 4435 | 1 | 0.02
H5+16192 | 444 | 1 | 0.02
H5+16192 | 5460 | 1 | 0.02
H5+16192 | 6359 | 1 | 0.02
H5+16192 | 6425 | 2 | 0.041
H5+16192 | 6779 | 1 | 0.02
H5+16192 | 9509 | 1 | 0.02
H5+16311 | 10172 | 2 | 0.024
H5+16311 | 12236 | 1 | 0.012
H5+16311 | 125A | 1 | 0.012
H5+16311 | 131d | 1 | 0.012
H5+16311 | 13788 | 1 | 0.012
H5+16311 | 150 | 8 | 0.098
H5+16311 | 152 | 3 | 0.037
H5+16311 | 16051T | 1 | 0.012
H5+16311 | 16052 | 1 | 0.012
H5+16311 | 16189 | 1 | 0.012
H5+16311 | 16234 | 1 | 0.012
H5+16311 | 16266 | 4 | 0.049
H5+16311 | 16319 | 3 | 0.037
H5+16311 | 16362 | 1 | 0.012
H5+16311 | 16526 | 1 | 0.012
H5+16311 | 189 | 1 | 0.012
H5+16311 | 195 | 1 | 0.012
H5+16311 | 204G | 1 | 0.012
H5+16311 | 206G | 1 | 0.012
H5+16311 | 207 | 1 | 0.012
H5+16311 | 209A | 1 | 0.012
H5+16311 | 217d | 1 | 0.012
H5+16311 | 224G | 1 | 0.012
H5+16311 | 226A | 1 | 0.012
H5+16311 | 3316 | 1 | 0.012
H5+16311 | 373 | 2 | 0.024
H5+16311 | 4336 | 1 | 0.012
H5+16311 | 489 | 1 | 0.012
H5+16311 | 500 | 1 | 0.012
H5+16311 | 515-524d | 1 | 0.012
H5+16311 | 5492 | 1 | 0.012
H5+16311 | 5894 | 1 | 0.012
H5+16311 | 6425 | 1 | 0.012
H5+16311 | 8857 | 1 | 0.012
H5+16311 | 9111 | 1 | 0.012
H5+16311 | 9948 | 1 | 0.012
H5+709 | 14311G | 1 | 0.003
H5+709 | 15019 | 1 | 0.003
H5+709 | 15498 | 1 | 0.003
H5+709 | 16068 | 1 | 0.003
H5+709 | 1608 | 1 | 0.003
H5+709 | 16111A | 2 | 0.007
H5+709 | 16114A | 1 | 0.003
H5+709 | 16181 | 1 | 0.003
H5+709 | 16185 | 1 | 0.003
H5+709 | 16189 | 2 | 0.007
H5+709 | 16241C | 22 | 0.075
H5+709 | 16247 | 1 | 0.003
H5+709 | 16257 | 1 | 0.003
H5+709 | 16342 | 1 | 0.003
H5+709 | 16527 | 1 | 0.003
H5+709 | 506 | 1 | 0.003
H5+709 | 5471 | 1 | 0.003
H5+709 | 573.6C | 1 | 0.003
H5+709 | 73 | 1 | 0.003
H5+709 | 8251 | 2 | 0.007

H5+709 | 9530 | 2 | 0.007
H5+709 | 960.1C | 1 | 0.003
H50 | 11182 | 1 | 0.001
H50 | 113 | 1 | 0.001
H50 | 146 | 3 | 0.004
H50 | 16076A | 1 | 0.001
H50 | 16111A | 4 | 0.005
H50 | 16172 | 125 | 0.157
H50 | 16189 | 4 | 0.005
H50 | 16256 | 1 | 0.001
H50 | 208 | 1 | 0.001
H50 | 261 | 1 | 0.001
H50 | 281C | 1 | 0.001
H50 | 316d | 1 | 0.001
H50 | 338 | 1 | 0.001
H50 | 4317d | 1 | 0.001
H50 | 4639 | 1 | 0.001
H50 | 468 | 3 | 0.004
H50 | 502 | 1 | 0.001
H50 | 573.1C | 3 | 0.004
H50 | 573.2C | 5 | 0.006
H50 | 573.5C | 1 | 0.001
H50 | 9018 | 1 | 0.001
H50 | 9337 | 1 | 0.001
H50 | 9852 | 4 | 0.005
H51 | 11016 | 2 | 0.003
H51 | 113 | 1 | 0.001
H51 | 12640 | 1 | 0.001
H51 | 15439 | 3 | 0.004
H51 | 16076A | 123 | 0.155
H51 | 16189 | 1 | 0.001
H51 | 204 | 1 | 0.001
H51 | 208 | 1 | 0.001
H51 | 261 | 1 | 0.001
H51 | 281C | 1 | 0.001
H51 | 316d | 1 | 0.001
H51 | 3333 | 1 | 0.001
H51 | 338 | 1 | 0.001
H51 | 3394 | 1 | 0.001
H51 | 468 | 3 | 0.004
H51 | 502 | 1 | 0.001
H51 | 513 | 1 | 0.001
H51 | 573.1C | 3 | 0.004
H51 | 573.2C | 5 | 0.006
H51 | 573.5C | 1 | 0.001
H51 | 6458 | 3 | 0.004
H51 | 8251 | 1 | 0.001
H51 | 9531 | 1 | 0.001
H51a | 113 | 1 | 0.001
H51a | 16076A | 1 | 0.001
H51a | 16320 | 123 | 0.156
H51a | 208 | 1 | 0.001
H51a | 261 | 1 | 0.001
H51a | 281C | 1 | 0.001
H51a | 316d | 1 | 0.001
H51a | 338 | 1 | 0.001
H51a | 468 | 3 | 0.004
H51a | 502 | 1 | 0.001
H51a | 573.1C | 3 | 0.004
H51a | 573.2C | 5 | 0.006
H51a | 573.5C | 1 | 0.001
H51a | 7948 | 1 | 0.001
H51a | 8119 | 1 | 0.001
H52 | 12173 | 1 | 0.006
H52 | 12397 | 2 | 0.013
H52 | 14945 | 1 | 0.006
H52 | 15314 | 1 | 0.006
H52 | 16093 | 19 | 0.12
H52 | 16167 | 1 | 0.006
H52 | 16255 | 20 | 0.127
H52 | 16261 | 1 | 0.006
H52 | 16324 | 1 | 0.006
H52 | 16325 | 2 | 0.013
H52 | 16468 | 2 | 0.013
H52 | 16524C | 1 | 0.006
H52 | 2361 | 1 | 0.006
H52 | 4084 | 1 | 0.006
H52 | 4856 | 1 | 0.006
H52 | 532 | 2 | 0.013
H52 | 57 | 1 | 0.006
H52 | 573.1C | 1 | 0.006
H52 | 5899.1C | 1 | 0.006
H52 | 59 | 1 | 0.006
H52 | 60.1T | 1 | 0.006
H53 | 11253 | 1 | 0.001
H53 | 113 | 1 | 0.001
H53 | 13938 | 4 | 0.005
H53 | 14323 | 4 | 0.005
H53 | 16076A | 1 | 0.001
H53 | 16092 | 123 | 0.155
H53 | 208 | 1 | 0.001
H53 | 261 | 1 | 0.001
H53 | 281C | 1 | 0.001
H53 | 316d | 1 | 0.001
H53 | 3333 | 1 | 0.001
H53 | 338 | 1 | 0.001
H53 | 4216 | 1 | 0.001
H53 | 468 | 3 | 0.004
H53 | 502 | 1 | 0.001
H53 | 573.1C | 3 | 0.004
H53 | 573.2C | 5 | 0.006
H53 | 573.5C | 1 | 0.001
H53 | 7859 | 4 | 0.005
H53 | 9612 | 4 | 0.005
H5'36 | 10398 | 1 | 0.003
H5'36 | 11719 | 1 | 0.003
H5'36 | 11965 | 1 | 0.003
H5'36 | 11974 | 1 | 0.003
H5'36 | 13327T | 1 | 0.003
H5'36 | 14180 | 1 | 0.003
H5'36 | 14560 | 1 | 0.003

H5'36 | 146 | 1 | 0.003
H5'36 | 15139 | 1 | 0.003
H5'36 | 16042C | 1 | 0.003
H5'36 | 16093 | 1 | 0.003
H5'36 | 16129T | 1 | 0.003
H5'36 | 16148 | 1 | 0.003
H5'36 | 16150 | 1 | 0.003
H5'36 | 16188A | 2 | 0.005
H5'36 | 16231 | 1 | 0.003
H5'36 | 16233 | 2 | 0.005
H5'36 | 16239 | 1 | 0.003
H5'36 | 16249 | 1 | 0.003
H5'36 | 16293C | 2 | 0.005
H5'36 | 16304G | 2 | 0.005
H5'36 | 16311 | 2 | 0.005
H5'36 | 16327 | 3 | 0.008
H5'36 | 16362 | 5 | 0.013
H5'36 | 16390 | 25 | 0.065
H5'36 | 16505 | 103 | 0.268
H5'36 | 1926T | 1 | 0.003
H5'36 | 195A | 1 | 0.003
H5'36 | 207 | 1 | 0.003
H5'36 | 252 | 3 | 0.008
H5'36 | 261.1T | 1 | 0.003
H5'36 | 2857 | 1 | 0.003
H5'36 | 332G | 1 | 0.003
H5'36 | 49T | 1 | 0.003
H5'36 | 5471 | 2 | 0.005
H5'36 | 7028 | 1 | 0.003
H5'36 | 7364 | 1 | 0.003
H5'36 | 7642 | 1 | 0.003
H5'36 | 7897 | 2 | 0.005
H5'36 | 7976 | 1 | 0.003
H5'36 | 8291 | 1 | 0.003
H5'36 | 9698 | 1 | 0.003
H54 | 113 | 1 | 0.001
H54 | 15311 | 1 | 0.001
H54 | 15394 | 1 | 0.001
H54 | 16076A | 123 | 0.156
H54 | 182 | 2 | 0.003
H54 | 208 | 1 | 0.001
H54 | 2325.1T | 1 | 0.001
H54 | 261 | 1 | 0.001
H54 | 281C | 1 | 0.001
H54 | 316d | 1 | 0.001
H54 | 338 | 1 | 0.001
H54 | 468 | 3 | 0.004
H54 | 502 | 1 | 0.001
H54 | 5252 | 1 | 0.001
H54 | 573.1C | 3 | 0.004
H54 | 573.2C | 5 | 0.006
H54 | 573.5C | 1 | 0.001
H54 | 6215A | 1 | 0.001
H54 | 6267 | 1 | 0.001
H54 | 709 | 2 | 0.003
H54 | 72 | 1 | 0.001
H54 | 8618 | 1 | 0.001
H54 | 8788 | 1 | 0.001
H55 | 113 | 1 | 0.001
H55 | 16076A | 123 | 0.157
H55 | 208 | 1 | 0.001
H55 | 261 | 1 | 0.001
H55 | 281C | 1 | 0.001
H55 | 316d | 1 | 0.001
H55 | 338 | 1 | 0.001
H55 | 468 | 3 | 0.004
H55 | 502 | 1 | 0.001
H55 | 573.1C | 3 | 0.004
H55 | 573.2C | 5 | 0.006
H55 | 573.5C | 1 | 0.001
H55+153 | 146 | 3 | 0.273
H55+153 | 152 | 3 | 0.273
H55+153 | 16240 | 1 | 0.091
H55+153 | 16261 | 1 | 0.091
H55+153 | 16319 | 3 | 0.273
H55+153 | 16360 | 1 | 0.091
H55+153 | 16380 | 2 | 0.182
H55+153 | 204 | 1 | 0.091
H55+153 | 356.1C | 2 | 0.182
H55+153 | 573.1C | 1 | 0.091
H55+153 | 6719 | 1 | 0.091
H55+153 | 9482 | 1 | 0.091
H55a | 113 | 1 | 0.001
H55a | 12217 | 1 | 0.001
H55a | 12613T | 1 | 0.001
H55a | 12630 | 1 | 0.001
H55a | 13145 | 1 | 0.001
H55a | 13713G | 1 | 0.001
H55a | 14932 | 1 | 0.001
H55a | 16076A | 1 | 0.001
H55a | 16145 | 124 | 0.157
H55a | 16189 | 1 | 0.001
H55a | 208 | 1 | 0.001
H55a | 261 | 1 | 0.001
H55a | 281C | 1 | 0.001
H55a | 310 | 1 | 0.001
H55a | 316d | 1 | 0.001
H55a | 338 | 1 | 0.001
H55a | 468 | 3 | 0.004
H55a | 502 | 1 | 0.001
H55a | 573.1C | 3 | 0.004
H55a | 573.2C | 5 | 0.006
H55a | 573.5C | 1 | 0.001
H55a | 742 | 1 | 0.001
H55b | 108 | 1 | 0.011
H55b | 1126 | 1 | 0.011
H55b | 12501 | 1 | 0.011
H55b | 13899 | 1 | 0.011
H55b | 146 | 6 | 0.065
H55b | 14884 | 2 | 0.022
H55b | 152 | 3 | 0.032

H55b | 1603 | 1 | 0.011
H55b | 16124 | 3 | 0.032
H55b | 16191 | 1 | 0.011
H55b | 16248 | 1 | 0.011
H55b | 16320 | 3 | 0.032
H55b | 16368 | 3 | 0.032
H55b | 195 | 1 | 0.011
H55b | 199 | 1 | 0.011
H55b | 203 | 4 | 0.043
H55b | 204 | 34 | 0.366
H55b | 204G | 2 | 0.022
H55b | 207 | 6 | 0.065
H55b | 310 | 1 | 0.011
H55b | 315.2C | 1 | 0.011
H55b | 3531 | 1 | 0.011
H55b | 485 | 1 | 0.011
H55b | 6503 | 2 | 0.022
H55b | 7028 | 1 | 0.011
H55b | 71 | 2 | 0.022
H55b | 7861 | 2 | 0.022
H55b | 8185 | 1 | 0.011
H55b | 9966 | 1 | 0.011
H56 | 10685 | 1 | 0.001
H56 | 113 | 1 | 0.001
H56 | 12236 | 1 | 0.001
H56 | 12441 | 1 | 0.001
H56 | 1555 | 1 | 0.001
H56 | 16076A | 1 | 0.001
H56 | 16093 | 2 | 0.002
H56 | 16172 | 1 | 0.001
H56 | 16240 | 123 | 0.154
H56 | 16274 | 1 | 0.001
H56 | 16362 | 1 | 0.001
H56 | 208 | 1 | 0.001
H56 | 239 | 2 | 0.002
H56 | 261 | 1 | 0.001
H56 | 281C | 1 | 0.001
H56 | 310 | 2 | 0.002
H56 | 316d | 1 | 0.001
H56 | 338 | 1 | 0.001
H56 | 468 | 3 | 0.004
H56 | 502 | 1 | 0.001
H56 | 5447 | 1 | 0.001
H56 | 5581 | 3 | 0.004
H56 | 573.1C | 3 | 0.004
H56 | 573.2C | 5 | 0.006
H56 | 573.5C | 1 | 0.001
H56 | 6293 | 1 | 0.001
H56 | 6626 | 2 | 0.002
H56 | 6635 | 3 | 0.004
H56 | 7747 | 1 | 0.001
H56 | 7810 | 1 | 0.001
H56 | 8674 | 1 | 0.001
H56 | 8850 | 1 | 0.001
H56 | 9027 | 1 | 0.001
H56 | 9163 | 1 | 0.001
H56 | 9548 | 1 | 0.001
H56 | 9650 | 1 | 0.001
H56a | 113 | 1 | 0.001
H56a | 13327 | 1 | 0.001
H56a | 13708 | 1 | 0.001
H56a | 16076A | 123 | 0.156
H56a | 208 | 1 | 0.001
H56a | 261 | 1 | 0.001
H56a | 281C | 1 | 0.001
H56a | 316d | 1 | 0.001
H56a | 338 | 1 | 0.001
H56a | 468 | 3 | 0.004
H56a | 502 | 1 | 0.001
H56a | 573.1C | 3 | 0.004
H56a | 573.2C | 5 | 0.006
H56a | 573.5C | 1 | 0.001
H56a | 9380 | 1 | 0.001
H56a1 | 113 | 1 | 0.001
H56a1 | 15258 | 1 | 0.001
H56a1 | 16076A | 123 | 0.156
H56a1 | 195 | 1 | 0.001
H56a1 | 208 | 1 | 0.001
H56a1 | 261 | 1 | 0.001
H56a1 | 281C | 1 | 0.001
H56a1 | 316d | 1 | 0.001
H56a1 | 338 | 1 | 0.001
H56a1 | 468 | 3 | 0.004
H56a1 | 502 | 1 | 0.001
H56a1 | 573.1C | 3 | 0.004
H56a1 | 573.2C | 5 | 0.006
H56a1 | 573.5C | 1 | 0.001
H56b | 113 | 1 | 0.001
H56b | 15412 | 1 | 0.001
H56b | 16076A | 123 | 0.156
H56b | 16255 | 1 | 0.001
H56b | 208 | 1 | 0.001
H56b | 261 | 1 | 0.001
H56b | 281C | 1 | 0.001
H56b | 316d | 1 | 0.001
H56b | 338 | 1 | 0.001
H56b | 468 | 3 | 0.004
H56b | 502 | 1 | 0.001
H56b | 573.1C | 3 | 0.004
H56b | 573.2C | 5 | 0.006
H56b | 573.5C | 1 | 0.001
H56c | 113 | 1 | 0.001
H56c | 16076A | 1 | 0.001
H56c | 16129 | 123 | 0.156
H56c | 208 | 1 | 0.001
H56c | 261 | 1 | 0.001
H56c | 281C | 1 | 0.001
H56c | 316d | 1 | 0.001
H56c | 338 | 1 | 0.001
H56c | 4622 | 1 | 0.001
H56c | 468 | 3 | 0.004

H56c | 502 | 1 | 0.001
H56c | 573.1C | 3 | 0.004
H56c | 573.2C | 5 | 0.006
H56c | 573.5C | 1 | 0.001
H56d | 11778 | 1 | 0.048
H56d | 146 | 2 | 0.095
H56d | 15317 | 1 | 0.048
H56d | 15431 | 1 | 0.048
H56d | 16243 | 17 | 0.81
H56d | 16278 | 1 | 0.048
H56d | 200 | 15 | 0.714
H56d | 4626 | 1 | 0.048
H56d | 6251 | 1 | 0.048
H56d | 709 | 1 | 0.048
H57 | 11972 | 1 | 0.024
H57 | 13488 | 1 | 0.024
H57 | 14539 | 1 | 0.024
H57 | 15077 | 1 | 0.024
H57 | 15721 | 1 | 0.024
H57 | 16145 | 3 | 0.071
H57 | 16269 | 2 | 0.048
H57 | 16271 | 1 | 0.024
H57 | 16316 | 1 | 0.024
H57 | 1658 | 1 | 0.024
H57 | 519 | 2 | 0.048
H57 | 5835 | 1 | 0.024
H57 | 709 | 1 | 0.024
H57 | 72 | 1 | 0.024
H57 | 8251 | 1 | 0.024
H57 | 8855 | 1 | 0.024
H57 | 93 | 1 | 0.024
H58 | 10283 | 1 | 0.001
H58 | 10907 | 1 | 0.001
H58 | 113 | 1 | 0.001
H58 | 146 | 1 | 0.001
H58 | 16076A | 123 | 0.156
H58 | 208 | 1 | 0.001
H58 | 261 | 1 | 0.001
H58 | 281C | 1 | 0.001
H58 | 316d | 1 | 0.001
H58 | 338 | 1 | 0.001
H58 | 3837 | 1 | 0.001
H58 | 468 | 3 | 0.004
H58 | 502 | 1 | 0.001
H58 | 573.1C | 3 | 0.004
H58 | 573.2C | 5 | 0.006
H58 | 573.5C | 1 | 0.001
H58 | 5807 | 1 | 0.001
H58 | 8251 | 1 | 0.001
H58 | 8252A | 1 | 0.001
H58 | 9461 | 1 | 0.001
H58a | 113 | 1 | 0.001
H58a | 11908 | 4 | 0.005
H58a | 13145 | 2 | 0.003
H58a | 152 | 2 | 0.003
H58a | 16076A | 123 | 0.155
H58a | 16192 | 1 | 0.001
H58a | 208 | 1 | 0.001
H58a | 261 | 1 | 0.001
H58a | 281C | 1 | 0.001
H58a | 310 | 1 | 0.001
H58a | 316d | 1 | 0.001
H58a | 338 | 1 | 0.001
H58a | 468 | 3 | 0.004
H58a | 502 | 1 | 0.001
H58a | 5318 | 2 | 0.003
H58a | 573.1C | 3 | 0.004
H58a | 573.2C | 5 | 0.006
H58a | 573.5C | 1 | 0.001
H58a | 9389 | 1 | 0.001
H58a | 9922 | 1 | 0.001
H58a | 9950 | 2 | 0.003
H59 | 113 | 1 | 0.001
H59 | 146 | 4 | 0.005
H59 | 15544 | 2 | 0.003
H59 | 16076A | 123 | 0.155
H59 | 16189 | 1 | 0.001
H59 | 16192 | 1 | 0.001
H59 | 208 | 1 | 0.001
H59 | 261 | 1 | 0.001
H59 | 2760 | 1 | 0.001
H59 | 281C | 1 | 0.001
H59 | 310 | 2 | 0.003
H59 | 315.2C | 1 | 0.001
H59 | 316d | 1 | 0.001
H59 | 338 | 1 | 0.001
H59 | 468 | 3 | 0.004
H59 | 502 | 1 | 0.001
H59 | 573.1C | 4 | 0.005
H59 | 573.2C | 6 | 0.008
H59 | 573.5C | 1 | 0.001
H59 | 73 | 1 | 0.001
H59a | 10608 | 1 | 0.001
H59a | 113 | 1 | 0.001
H59a | 15934 | 2 | 0.003
H59a | 1607 | 1 | 0.001
H59a | 16076A | 1 | 0.001
H59a | 16311 | 123 | 0.156
H59a | 208 | 1 | 0.001
H59a | 261 | 1 | 0.001
H59a | 281C | 1 | 0.001
H59a | 316d | 1 | 0.001
H59a | 338 | 1 | 0.001
H59a | 3398 | 1 | 0.001
H59a | 3434 | 1 | 0.001
H59a | 468 | 3 | 0.004
H59a | 502 | 1 | 0.001
H59a | 573.1C | 3 | 0.004
H59a | 573.2C | 5 | 0.006
H59a | 573.5C | 1 | 0.001
H5a | 10172 | 2 | 0.007

H5a | 10211 | 1 | 0.003
H5a | 10643 | 1 | 0.003
H5a | 11087 | 2 | 0.007
H5a | 12246 | 1 | 0.003
H5a | 12364 | 1 | 0.003
H5a | 13105 | 1 | 0.003
H5a | 13419 | 1 | 0.003
H5a | 13708 | 1 | 0.003
H5a | 13768 | 1 | 0.003
H5a | 14179 | 1 | 0.003
H5a | 14305 | 1 | 0.003
H5a | 146 | 1 | 0.003
H5a | 14979 | 1 | 0.003
H5a | 15479 | 1 | 0.003
H5a | 15930 | 1 | 0.003
H5a | 16114A | 1 | 0.003
H5a | 16174 | 1 | 0.003
H5a | 16181 | 1 | 0.003
H5a | 16185 | 1 | 0.003
H5a | 16241C | 22 | 0.073
H5a | 16247 | 1 | 0.003
H5a | 16254 | 1 | 0.003
H5a | 16259 | 1 | 0.003
H5a | 16302 | 1 | 0.003
H5a | 16342 | 1 | 0.003
H5a | 16357 | 1 | 0.003
H5a | 16527 | 1 | 0.003
H5a | 2125 | 1 | 0.003
H5a | 259 | 1 | 0.003
H5a | 3645 | 1 | 0.003
H5a | 3744 | 2 | 0.007
H5a | 4659 | 1 | 0.003
H5a | 506 | 1 | 0.003
H5a | 522d | 1 | 0.003
H5a | 573.6C | 1 | 0.003
H5a | 6076G | 1 | 0.003
H5a | 721 | 1 | 0.003
H5a | 73 | 1 | 0.003
H5a | 7444 | 1 | 0.003
H5a | 8588A | 1 | 0.003
H5a | 8670 | 1 | 0.003
H5a | 9912 | 1 | 0.003
H5a+152 | 10275 | 1 | 0.006
H5a+152 | 11047 | 1 | 0.006
H5a+152 | 11251 | 1 | 0.006
H5a+152 | 1438C | 1 | 0.006
H5a+152 | 146 | 13 | 0.072
H5a+152 | 15613 | 1 | 0.006
H5a+152 | 15910 | 1 | 0.006
H5a+152 | 16093 | 1 | 0.006
H5a+152 | 16186 | 1 | 0.006
H5a+152 | 16399 | 4 | 0.022
H5a+152 | 207 | 1 | 0.006
H5a+152 | 249d | 1 | 0.006
H5a+152 | 3368 | 1 | 0.006
H5a+152 | 373 | 2 | 0.011
H5a+152 | 393d | 1 | 0.006
H5a+152 | 4020 | 2 | 0.011
H5a+152 | 473 | 1 | 0.006
H5a+152 | 5095 | 1 | 0.006
H5a+152 | 573.3C | 1 | 0.006
H5a+152 | 5821 | 1 | 0.006
H5a+152 | 5839 | 1 | 0.006
H5a+152 | 7028 | 1 | 0.006
H5a+152 | 9254 | 1 | 0.006
H5a+152 | 9297 | 1 | 0.006
H5a1 | 10238 | 6 | 0.015
H5a1 | 10634 | 6 | 0.015
H5a1 | 10688 | 1 | 0.003
H5a1 | 10822 | 2 | 0.005
H5a1 | 11050 | 1 | 0.003
H5a1 | 11151 | 2 | 0.005
H5a1 | 11386 | 1 | 0.003
H5a1 | 11404 | 1 | 0.003
H5a1 | 11830 | 1 | 0.003
H5a1 | 11884 | 1 | 0.003
H5a1 | 11887 | 1 | 0.003
H5a1 | 12094 | 2 | 0.005
H5a1 | 12354 | 5 | 0.013
H5a1 | 12362 | 1 | 0.003
H5a1 | 12414 | 2 | 0.005
H5a1 | 12603 | 1 | 0.003
H5a1 | 12630 | 1 | 0.003
H5a1 | 12634 | 2 | 0.005
H5a1 | 12678 | 1 | 0.003
H5a1 | 12948 | 1 | 0.003
H5a1 | 13023 | 2 | 0.005
H5a1 | 13527 | 1 | 0.003
H5a1 | 13674 | 1 | 0.003
H5a1 | 13928 | 1 | 0.003
H5a1 | 14530G | 1 | 0.003
H5a1 | 14552 | 1 | 0.003
H5a1 | 146 | 2 | 0.005
H5a1 | 14905 | 1 | 0.003
H5a1 | 150 | 2 | 0.005
H5a1 | 15076 | 1 | 0.003
H5a1 | 15119 | 1 | 0.003
H5a1 | 152 | 3 | 0.008
H5a1 | 15249 | 1 | 0.003
H5a1 | 15454 | 1 | 0.003
H5a1 | 15466 | 1 | 0.003
H5a1 | 15498 | 2 | 0.005
H5a1 | 15511 | 1 | 0.003
H5a1 | 15514 | 1 | 0.003
H5a1 | 15530 | 1 | 0.003
H5a1 | 15596 | 1 | 0.003
H5a1 | 15650 | 1 | 0.003
H5a1 | 15658 | 1 | 0.003
H5a1 | 15772 | 1 | 0.003
H5a1 | 15812 | 1 | 0.003
H5a1 | 15930 | 1 | 0.003

H5a1 | 16069 | 2 | 0.005
H5a1 | 16075 | 1 | 0.003
H5a1 | 16129 | 1 | 0.003
H5a1 | 16179 | 2 | 0.005
H5a1 | 16184 | 1 | 0.003
H5a1 | 16189 | 6 | 0.015
H5a1 | 16192 | 2 | 0.005
H5a1 | 16243 | 2 | 0.005
H5a1 | 16256 | 1 | 0.003
H5a1 | 16278 | 1 | 0.003
H5a1 | 16294A | 3 | 0.008
H5a1 | 16311 | 4 | 0.01
H5a1 | 16343T | 22 | 0.057
H5a1 | 16362 | 1 | 0.003
H5a1 | 16465 | 1 | 0.003
H5a1 | 186 | 2 | 0.005
H5a1 | 1888 | 1 | 0.003
H5a1 | 195 | 2 | 0.005
H5a1 | 199 | 1 | 0.003
H5a1 | 2294 | 1 | 0.003
H5a1 | 235 | 5 | 0.013
H5a1 | 240 | 2 | 0.005
H5a1 | 2803 | 1 | 0.003
H5a1 | 310 | 6 | 0.015
H5a1 | 315.2C | 2 | 0.005
H5a1 | 3337 | 1 | 0.003
H5a1 | 3338 | 1 | 0.003
H5a1 | 3609 | 1 | 0.003
H5a1 | 4011 | 1 | 0.003
H5a1 | 452A | 1 | 0.003
H5a1 | 452T | 1 | 0.003
H5a1 | 4688 | 1 | 0.003
H5a1 | 4703 | 1 | 0.003
H5a1 | 4947 | 1 | 0.003
H5a1 | 5067 | 1 | 0.003
H5a1 | 513 | 4 | 0.01
H5a1 | 514 | 1 | 0.003
H5a1 | 5147 | 1 | 0.003
H5a1 | 5195 | 1 | 0.003
H5a1 | 5213 | 1 | 0.003
H5a1 | 522d | 1 | 0.003
H5a1 | 531 | 1 | 0.003
H5a1 | 5310 | 1 | 0.003
H5a1 | 5477 | 1 | 0.003
H5a1 | 5582 | 1 | 0.003
H5a1 | 573.2C | 1 | 0.003
H5a1 | 573.4C | 1 | 0.003
H5a1 | 573.6C | 1 | 0.003
H5a1 | 5788 | 3 | 0.008
H5a1 | 6603 | 4 | 0.01
H5a1 | 66T | 1 | 0.003
H5a1 | 6710 | 1 | 0.003
H5a1 | 6722 | 2 | 0.005
H5a1 | 7299 | 1 | 0.003
H5a1 | 73 | 2 | 0.005
H5a1 | 7642 | 2 | 0.005
H5a1 | 7691 | 3 | 0.008
H5a1 | 7732 | 1 | 0.003
H5a1 | 7961 | 1 | 0.003
H5a1 | 8277 | 1 | 0.003
H5a1 | 8389 | 2 | 0.005
H5a1 | 8433 | 1 | 0.003
H5a1 | 8462 | 4 | 0.01
H5a1 | 8556 | 1 | 0.003
H5a1 | 9020 | 1 | 0.003
H5a1 | 9053 | 2 | 0.005
H5a1 | 9103 | 2 | 0.005
H5a1 | 9254 | 2 | 0.005
H5a1 | 93 | 1 | 0.003
H5a1 | 9392T | 2 | 0.005
H5a1 | 9425C | 1 | 0.003
H5a1 | 9531 | 2 | 0.005
H5a1 | 9722 | 1 | 0.003
H5a1 | 9902A | 1 | 0.003
H5a1+152 | 10391 | 1 | 0.006
H5a1+152 | 10634 | 2 | 0.011
H5a1+152 | 12757 | 3 | 0.017
H5a1+152 | 12867 | 3 | 0.017
H5a1+152 | 13668 | 3 | 0.017
H5a1+152 | 13708 | 1 | 0.006
H5a1+152 | 14180 | 1 | 0.006
H5a1+152 | 16230 | 3 | 0.017
H5a1+152 | 16311 | 2 | 0.011
H5a1+152 | 310 | 1 | 0.006
H5a1+152 | 459d | 1 | 0.006
H5a1+152 | 573.3C | 1 | 0.006
H5a1+152 | 7124 | 1 | 0.006
H5a1+152 | 721 | 1 | 0.006
H5a1+152 | 7502 | 1 | 0.006
H5a1+152 | 794 | 1 | 0.006
H5a1+152 | 8928 | 1 | 0.006
H5a1+16093 | 11329 | 1 | 0.02
H5a1+16093 | 12633 | 1 | 0.02
H5a1+16093 | 13468A | 1 | 0.02
H5a1+16093 | 14142A | 1 | 0.02
H5a1+16093 | 16167 | 2 | 0.041
H5a1+16093 | 16192 | 1 | 0.02
H5a1+16093 | 16274 | 2 | 0.041
H5a1+16093 | 16305 | 2 | 0.041
H5a1+16093 | 200 | 1 | 0.02
H5a1+16093 | 225 | 1 | 0.02
H5a1+16093 | 373 | 1 | 0.02
H5a1+16093 | 448T | 1 | 0.02
H5a1+16093 | 721 | 1 | 0.02
H5a1+16093 | 961 | 1 | 0.02
H5a1a | 11134 | 1 | 0.003
H5a1a | 12397 | 1 | 0.003
H5a1a | 13194 | 1 | 0.003
H5a1a | 15014 | 2 | 0.006
H5a1a | 15034 | 2 | 0.006
H5a1a | 15440 | 2 | 0.006

H5a1a | 15940 | 1 | 0.003
H5a1a | 15994 | 1 | 0.003
H5a1a | 16114A | 1 | 0.003
H5a1a | 16181 | 1 | 0.003
H5a1a | 16184 | 1 | 0.003
H5a1a | 16185 | 1 | 0.003
H5a1a | 16213 | 2 | 0.006
H5a1a | 16223 | 1 | 0.003
H5a1a | 16241C | 1 | 0.003
H5a1a | 16242 | 1 | 0.003
H5a1a | 16247 | 1 | 0.003
H5a1a | 16278 | 1 | 0.003
H5a1a | 16286 | 2 | 0.006
H5a1a | 16311 | 1 | 0.003
H5a1a | 16342 | 1 | 0.003
H5a1a | 16370 | 1 | 0.003
H5a1a | 16483 | 26 | 0.081
H5a1a | 16527 | 1 | 0.003
H5a1a | 1798 | 1 | 0.003
H5a1a | 1819 | 1 | 0.003
H5a1a | 309d | 1 | 0.003
H5a1a | 310 | 1 | 0.003
H5a1a | 3849 | 1 | 0.003
H5a1a | 4048 | 1 | 0.003
H5a1a | 506 | 1 | 0.003
H5a1a | 523d | 1 | 0.003
H5a1a | 533 | 1 | 0.003
H5a1a | 549 | 1 | 0.003
H5a1a | 573.6C | 1 | 0.003
H5a1a | 709 | 1 | 0.003
H5a1a | 7283 | 1 | 0.003
H5a1a | 7697 | 3 | 0.009
H5a1a | 8020 | 1 | 0.003
H5a1a | 8167 | 1 | 0.003
H5a1a | 8251 | 1 | 0.003
H5a1a | 8271 | 2 | 0.006
H5a1a | 93 | 27 | 0.084
H5a1b | 11731 | 1 | 0.003
H5a1b | 16114A | 1 | 0.003
H5a1b | 16162 | 1 | 0.003
H5a1b | 16181 | 1 | 0.003
H5a1b | 16185 | 1 | 0.003
H5a1b | 16241C | 22 | 0.075
H5a1b | 16247 | 1 | 0.003
H5a1b | 16342 | 1 | 0.003
H5a1b | 16527 | 1 | 0.003
H5a1b | 1719 | 1 | 0.003
H5a1b | 506 | 1 | 0.003
H5a1b | 573.6C | 1 | 0.003
H5a1c | 16114A | 1 | 0.003
H5a1c | 16181 | 1 | 0.003
H5a1c | 16185 | 1 | 0.003
H5a1c | 16241C | 1 | 0.003
H5a1c | 16247 | 1 | 0.003
H5a1c | 16265 | 1 | 0.003
H5a1c | 16320 | 24 | 0.082
H5a1c | 16342 | 1 | 0.003
H5a1c | 16527 | 1 | 0.003
H5a1c | 506 | 1 | 0.003
H5a1c | 573.6C | 1 | 0.003
H5a1c1 | 16114A | 1 | 0.003
H5a1c1 | 16181 | 1 | 0.003
H5a1c1 | 16185 | 1 | 0.003
H5a1c1 | 16241C | 1 | 0.003
H5a1c1 | 16247 | 1 | 0.003
H5a1c1 | 16265 | 1 | 0.003
H5a1c1 | 16320 | 24 | 0.082
H5a1c1 | 16342 | 1 | 0.003
H5a1c1 | 16527 | 1 | 0.003
H5a1c1 | 506 | 1 | 0.003
H5a1c1 | 573.6C | 1 | 0.003
H5a1c1a | 14110 | 1 | 0.003
H5a1c1a | 16114A | 1 | 0.003
H5a1c1a | 16144 | 1 | 0.003
H5a1c1a | 16181 | 1 | 0.003
H5a1c1a | 16185 | 1 | 0.003
H5a1c1a | 16241C | 1 | 0.003
H5a1c1a | 16247 | 1 | 0.003
H5a1c1a | 16316 | 22 | 0.075
H5a1c1a | 16342 | 1 | 0.003
H5a1c1a | 16527 | 1 | 0.003
H5a1c1a | 310 | 1 | 0.003
H5a1c1a | 3447 | 1 | 0.003
H5a1c1a | 506 | 1 | 0.003
H5a1c1a | 573.6C | 1 | 0.003
H5a1c1a | 6480 | 1 | 0.003
H5a1c1a | 650 | 1 | 0.003
H5a1c2 | 16304G | 1 | 0.028
H5a1c2 | 16319 | 1 | 0.028
H5a1c2 | 16346 | 1 | 0.028
H5a1c2 | 593 | 1 | 0.028
H5a1c2 | 7642 | 1 | 0.028
H5a1d | 1120 | 1 | 0.003
H5a1d | 12561 | 1 | 0.003
H5a1d | 12723 | 1 | 0.003
H5a1d | 14668 | 1 | 0.003
H5a1d | 150 | 1 | 0.003
H5a1d | 16114A | 1 | 0.003
H5a1d | 16181 | 1 | 0.003
H5a1d | 16185 | 1 | 0.003
H5a1d | 16241C | 22 | 0.075
H5a1d | 16247 | 1 | 0.003
H5a1d | 16342 | 1 | 0.003
H5a1d | 16527 | 1 | 0.003
H5a1d | 334 | 1 | 0.003
H5a1d | 3460 | 1 | 0.003
H5a1d | 506 | 1 | 0.003
H5a1d | 573.6C | 1 | 0.003
H5a1d | 8027 | 1 | 0.003
H5a1d | 8557 | 1 | 0.003
H5a1e | 15880 | 1 | 0.025

H5a1e | 16344 | 2 | 0.05
H5a1e | 7471.1C | 1 | 0.025
H5a1e | 8265 | 1 | 0.025
H5a1f | 114A | 1 | 0.003
H5a1f | 12535 | 1 | 0.003
H5a1f | 146 | 1 | 0.003
H5a1f | 15808 | 1 | 0.003
H5a1f | 16114A | 1 | 0.003
H5a1f | 16181 | 1 | 0.003
H5a1f | 16185 | 1 | 0.003
H5a1f | 16218 | 2 | 0.007
H5a1f | 16223 | 1 | 0.003
H5a1f | 16241C | 22 | 0.074
H5a1f | 16247 | 1 | 0.003
H5a1f | 16342 | 1 | 0.003
H5a1f | 16527 | 1 | 0.003
H5a1f | 225 | 1 | 0.003
H5a1f | 310 | 1 | 0.003
H5a1f | 4736 | 4 | 0.013
H5a1f | 506 | 1 | 0.003
H5a1f | 573.6C | 1 | 0.003
H5a1f | 6422 | 1 | 0.003
H5a1f | 965.1C | 1 | 0.003
H5a1g | 150 | 1 | 0.021
H5a1g1 | 1248 | 1 | 0.023
H5a1g1 | 146 | 1 | 0.023
H5a1g1 | 14755 | 1 | 0.023
H5a1g1 | 152 | 1 | 0.023
H5a1g1 | 16114 | 2 | 0.047
H5a1g1 | 16189 | 1 | 0.023
H5a1g1 | 16192 | 1 | 0.023
H5a1g1 | 16193d | 1 | 0.023
H5a1g1 | 3460 | 1 | 0.023
H5a1g1 | 5082 | 1 | 0.023
H5a1g1 | 5132 | 1 | 0.023
H5a1g1 | 7517 | 1 | 0.023
H5a1g1 | 7689C | 1 | 0.023
H5a1g1a | 11914 | 1 | 0.021
H5a1g1a | 14203 | 1 | 0.021
H5a1g1a | 14412 | 1 | 0.021
H5a1g1a | 14755 | 1 | 0.021
H5a1g1a | 152 | 3 | 0.062
H5a1g1a | 195 | 1 | 0.021
H5a1g1a | 200 | 1 | 0.021
H5a1g1a | 3483 | 1 | 0.021
H5a1g1a | 64 | 2 | 0.042
H5a1g2 | 11800 | 1 | 0.026
H5a1g2 | 14335 | 1 | 0.026
H5a1g2 | 15064 | 1 | 0.026
H5a1g2 | 870 | 1 | 0.026
H5a1g2 | 9214 | 1 | 0.026
H5a1h | 16114A | 1 | 0.003
H5a1h | 16181 | 1 | 0.003
H5a1h | 16185 | 1 | 0.003
H5a1h | 16241C | 1 | 0.003
H5a1h | 16247 | 1 | 0.003
H5a1h | 16265 | 1 | 0.003
H5a1h | 16320 | 24 | 0.082
H5a1h | 16342 | 1 | 0.003
H5a1h | 16527 | 1 | 0.003
H5a1h | 506 | 1 | 0.003
H5a1h | 573.6C | 1 | 0.003
H5a1i | 13542 | 1 | 0.025
H5a1i | 152 | 1 | 0.025
H5a1i | 16218 | 1 | 0.025
H5a1i | 16263 | 1 | 0.025
H5a1i | 16390 | 1 | 0.025
H5a1i | 199 | 1 | 0.025
H5a1i | 3540 | 1 | 0.025
H5a1i | 42.1G | 1 | 0.025
H5a1j | 11506 | 1 | 0.018
H5a1j | 13966 | 1 | 0.018
H5a1j | 146 | 1 | 0.018
H5a1j | 152 | 2 | 0.036
H5a1j | 16148 | 1 | 0.018
H5a1j | 16189 | 1 | 0.018
H5a1j | 16261 | 1 | 0.018
H5a1j | 16278 | 1 | 0.018
H5a1j | 16305 | 3 | 0.055
H5a1j | 256 | 1 | 0.018
H5a1j | 257 | 1 | 0.018
H5a1j | 310 | 3 | 0.055
H5a1j | 3397 | 2 | 0.036
H5a1j | 4173 | 2 | 0.036
H5a1j | 513 | 1 | 0.018
H5a1j | 523d | 1 | 0.018
H5a1j | 5460 | 2 | 0.036
H5a1j | 593 | 1 | 0.018
H5a1j | 8149 | 1 | 0.018
H5a1j | 8282 | 1 | 0.018
H5a1j | 8727A | 2 | 0.036
H5a1j | 8856 | 1 | 0.018
H5a1k | 10084 | 1 | 0.003
H5a1k | 10250 | 1 | 0.003
H5a1k | 10310 | 1 | 0.003
H5a1k | 14200 | 1 | 0.003
H5a1k | 16114A | 1 | 0.003
H5a1k | 16181 | 1 | 0.003
H5a1k | 16185 | 1 | 0.003
H5a1k | 16241C | 1 | 0.003
H5a1k | 16247 | 1 | 0.003
H5a1k | 16265 | 1 | 0.003
H5a1k | 16320 | 24 | 0.082
H5a1k | 16342 | 1 | 0.003
H5a1k | 16527 | 1 | 0.003
H5a1k | 309d | 1 | 0.003
H5a1k | 506 | 1 | 0.003
H5a1k | 573.6C | 1 | 0.003
H5a1k | 6779 | 1 | 0.003
H5a1m | 16111 | 1 | 0.006
H5a1m | 16124 | 1 | 0.006

H5a1m | 16179 | 1 | 0.006
H5a1m | 16257 | 3 | 0.018
H5a1m | 3397 | 1 | 0.006
H5a1m | 4116 | 1 | 0.006
H5a1m | 573.3C | 1 | 0.006
H5a1m | 8167 | 1 | 0.006
H5a1n | 16111 | 1 | 0.006
H5a1n | 16124 | 1 | 0.006
H5a1n | 16257 | 3 | 0.018
H5a1n | 573.3C | 1 | 0.006
H5a1p | 15436 | 1 | 0.015
H5a1p | 16189 | 24 | 0.369
H5a1p | 16217 | 2 | 0.031
H5a1p | 16299 | 3 | 0.046
H5a1p | 16311 | 1 | 0.015
H5a1p | 199 | 2 | 0.031
H5a1p | 226 | 2 | 0.031
H5a1p | 324A | 1 | 0.015
H5a1p | 8573 | 1 | 0.015
H5a1q | 12151 | 1 | 0.003
H5a1q | 13879 | 1 | 0.003
H5a1q | 14771A | 1 | 0.003
H5a1q | 16114A | 1 | 0.003
H5a1q | 16181 | 1 | 0.003
H5a1q | 16185 | 1 | 0.003
H5a1q | 16241C | 1 | 0.003
H5a1q | 16247 | 1 | 0.003
H5a1q | 16249 | 1 | 0.003
H5a1q | 16265 | 1 | 0.003
H5a1q | 16320 | 24 | 0.081
H5a1q | 16342 | 1 | 0.003
H5a1q | 16527 | 1 | 0.003
H5a1q | 235 | 1 | 0.003
H5a1q | 506 | 1 | 0.003
H5a1q | 523d | 1 | 0.003
H5a1q | 573.6C | 1 | 0.003
H5a2 | 10344C | 1 | 0.003
H5a2 | 10707G | 3 | 0.009
H5a2 | 11281 | 1 | 0.003
H5a2 | 12160 | 1 | 0.003
H5a2 | 12406 | 1 | 0.003
H5a2 | 12976 | 1 | 0.003
H5a2 | 13135 | 3 | 0.009
H5a2 | 13708 | 1 | 0.003
H5a2 | 13866 | 1 | 0.003
H5a2 | 14180 | 3 | 0.009
H5a2 | 143 | 1 | 0.003
H5a2 | 14515 | 1 | 0.003
H5a2 | 14569 | 1 | 0.003
H5a2 | 146 | 1 | 0.003
H5a2 | 150 | 1 | 0.003
H5a2 | 15043C | 1 | 0.003
H5a2 | 151 | 3 | 0.009
H5a2 | 152 | 1 | 0.003
H5a2 | 15261 | 1 | 0.003
H5a2 | 15287 | 1 | 0.003
H5a2 | 15355 | 1 | 0.003
H5a2 | 15521 | 1 | 0.003
H5a2 | 16093 | 1 | 0.003
H5a2 | 16114A | 1 | 0.003
H5a2 | 16129 | 5 | 0.015
H5a2 | 16148 | 2 | 0.006
H5a2 | 16181 | 1 | 0.003
H5a2 | 16185 | 1 | 0.003
H5a2 | 16189 | 4 | 0.012
H5a2 | 16213 | 1 | 0.003
H5a2 | 16223 | 1 | 0.003
H5a2 | 16240 | 1 | 0.003
H5a2 | 16244 | 1 | 0.003
H5a2 | 16247 | 1 | 0.003
H5a2 | 16255 | 1 | 0.003
H5a2 | 16256 | 1 | 0.003
H5a2 | 16303 | 1 | 0.003
H5a2 | 16311 | 2 | 0.006
H5a2 | 16342 | 1 | 0.003
H5a2 | 16343 | 1 | 0.003
H5a2 | 16362 | 2 | 0.006
H5a2 | 16390 | 31 | 0.094
H5a2 | 16491 | 1 | 0.003
H5a2 | 16494 | 1 | 0.003
H5a2 | 16527 | 1 | 0.003
H5a2 | 195 | 1 | 0.003
H5a2 | 200 | 1 | 0.003
H5a2 | 207 | 1 | 0.003
H5a2 | 2564T | 1 | 0.003
H5a2 | 291.1A | 1 | 0.003
H5a2 | 310 | 3 | 0.009
H5a2 | 3746 | 1 | 0.003
H5a2 | 4005 | 1 | 0.003
H5a2 | 459 | 1 | 0.003
H5a2 | 506 | 1 | 0.003
H5a2 | 515-524d | 1 | 0.003
H5a2 | 573.6C | 1 | 0.003
H5a2 | 5823 | 1 | 0.003
H5a2 | 72G | 2 | 0.006
H5a2 | 73 | 5 | 0.015
H5a2 | 7472C | 1 | 0.003
H5a2 | 7844 | 1 | 0.003
H5a2 | 8032A | 1 | 0.003
H5a2 | 8348 | 1 | 0.003
H5a2 | 8508 | 1 | 0.003
H5a2 | 93 | 1 | 0.003
H5a2 | 9983 | 1 | 0.003
H5a2a | 14530 | 1 | 0.003
H5a2a | 16114A | 1 | 0.003
H5a2a | 16181 | 1 | 0.003
H5a2a | 16185 | 1 | 0.003
H5a2a | 16241C | 1 | 0.003
H5a2a | 16247 | 1 | 0.003
H5a2a | 16265 | 1 | 0.003
H5a2a | 16320 | 24 | 0.082

H5a2a | 16342 | 1 | 0.003
H5a2a | 16527 | 1 | 0.003
H5a2a | 1719 | 1 | 0.003
H5a2a | 506 | 1 | 0.003
H5a2a | 573.6C | 1 | 0.003
H5a3 | 11147 | 1 | 0.004
H5a3 | 14491A | 1 | 0.004
H5a3 | 16124 | 1 | 0.004
H5a3 | 16145 | 5 | 0.02
H5a3 | 16209 | 1 | 0.004
H5a3 | 16241C | 22 | 0.089
H5a3 | 16312 | 1 | 0.004
H5a3 | 16438 | 1 | 0.004
H5a3 | 182 | 1 | 0.004
H5a3 | 262 | 1 | 0.004
H5a3 | 9948 | 1 | 0.004
H5a3a | 14040 | 1 | 0.004
H5a3a | 15519 | 1 | 0.004
H5a3a | 15924 | 1 | 0.004
H5a3a | 16124 | 1 | 0.004
H5a3a | 16241C | 1 | 0.004
H5a3a | 16305 | 32 | 0.126
H5a3a | 182 | 1 | 0.004
H5a3a | 262 | 2 | 0.008
H5a3a | 310 | 1 | 0.004
H5a3a | 315.2C | 1 | 0.004
H5a3a | 4092 | 1 | 0.004
H5a3a | 5012 | 1 | 0.004
H5a3a | 5369 | 1 | 0.004
H5a3a | 7962 | 1 | 0.004
H5a3a | 9055 | 1 | 0.004
H5a3a+152 | 16362 | 4 | 0.025
H5a3a+152 | 327 | 1 | 0.006
H5a3a1 | 11809 | 3 | 0.018
H5a3a1 | 14674 | 1 | 0.006
H5a3a1 | 15734 | 1 | 0.006
H5a3a1 | 16311 | 4 | 0.024
H5a3a1 | 3396 | 1 | 0.006
H5a3a1 | 4084 | 1 | 0.006
H5a3a1 | 64 | 2 | 0.012
H5a3a1 | 7362 | 1 | 0.006
H5a3a1 | 7444 | 1 | 0.006
H5a3a1 | 8119 | 1 | 0.006
H5a3a2 | 13473 | 1 | 0.006
H5a3a2 | 13651 | 1 | 0.006
H5a3a2 | 14319 | 1 | 0.006
H5a3a2 | 16362 | 4 | 0.025
H5a3a2 | 327 | 1 | 0.006
H5a3a2 | 8563 | 1 | 0.006
H5a3a3 | 13350C | 1 | 0.004
H5a3a3 | 15456G | 1 | 0.004
H5a3a3 | 16124 | 1 | 0.004
H5a3a3 | 16241C | 1 | 0.004
H5a3a3 | 16305 | 11 | 0.044
H5a3a3 | 16343 | 22 | 0.087
H5a3a3 | 16527 | 1 | 0.004
H5a3a3 | 182 | 1 | 0.004
H5a3a3 | 262 | 1 | 0.004
H5a3a3 | 5817 | 1 | 0.004
H5a3a3 | 9725 | 1 | 0.004
H5a3b | 15773 | 1 | 0.004
H5a3b | 16093 | 1 | 0.004
H5a3b | 16124 | 1 | 0.004
H5a3b | 16241C | 1 | 0.004
H5a3b | 16305 | 32 | 0.126
H5a3b | 182 | 1 | 0.004
H5a3b | 199 | 1 | 0.004
H5a3b | 262 | 1 | 0.004
H5a3b | 7142 | 1 | 0.004
H5a4 | 12397 | 1 | 0.014
H5a4 | 14569 | 3 | 0.043
H5a4 | 15792 | 2 | 0.029
H5a4 | 16126 | 2 | 0.029
H5a4 | 16189 | 1 | 0.014
H5a4 | 16300 | 20 | 0.29
H5a4 | 16362 | 20 | 0.29
H5a4 | 195 | 1 | 0.014
H5a4 | 4616 | 1 | 0.014
H5a4 | 7076 | 1 | 0.014
H5a4 | 742 | 3 | 0.043
H5a4 | 8870 | 1 | 0.014
H5a4 | 93 | 1 | 0.014
H5a4a | 146 | 1 | 0.024
H5a4a | 159 | 1 | 0.024
H5a4a | 16278 | 1 | 0.024
H5a4a | 16311 | 1 | 0.024
H5a4a | 4164 | 1 | 0.024
H5a4a1 | 11914 | 1 | 0.023
H5a4a1 | 12471 | 1 | 0.023
H5a4a1 | 13194 | 2 | 0.047
H5a4a1 | 152 | 4 | 0.093
H5a4a1 | 16093 | 3 | 0.07
H5a4a1 | 16335 | 1 | 0.023
H5a4a1 | 385 | 1 | 0.023
H5a4a1 | 4832 | 2 | 0.047
H5a4a1a | 12188 | 1 | 0.024
H5a4a1a | 146 | 1 | 0.024
H5a4a1a | 159 | 1 | 0.024
H5a4a1a | 16278 | 1 | 0.024
H5a4a1a | 16311 | 1 | 0.024
H5a4a1a | 282 | 2 | 0.049
H5a4a1a | 3351 | 1 | 0.024
H5a4a1a | 8521 | 2 | 0.049
H5a5 | 150 | 2 | 0.013
H5a5 | 16172 | 1 | 0.006
H5a5 | 16179 | 1 | 0.006
H5a5 | 16302 | 1 | 0.006
H5a5 | 16311 | 1 | 0.006
H5a5 | 194 | 1 | 0.006
H5a5 | 4143C | 1 | 0.006
H5a5 | 573.3C | 1 | 0.006

H5a5 | 7805 | 1 | 0.006
H5a6 | 1300 | 1 | 0.006
H5a6 | 13434 | 2 | 0.011
H5a6 | 13708 | 1 | 0.006
H5a6 | 13710 | 1 | 0.006
H5a6 | 15773 | 1 | 0.006
H5a6 | 16148 | 1 | 0.006
H5a6 | 16260 | 3 | 0.017
H5a6 | 16261 | 4 | 0.023
H5a6 | 16291 | 2 | 0.011
H5a6 | 16497 | 1 | 0.006
H5a6 | 214 | 1 | 0.006
H5a6 | 3027 | 3 | 0.017
H5a6 | 310 | 2 | 0.011
H5a6 | 315.2C | 2 | 0.011
H5a6 | 3229.1A | 2 | 0.011
H5a6 | 3396 | 1 | 0.006
H5a6 | 340 | 3 | 0.017
H5a6 | 526 | 1 | 0.006
H5a6 | 573.3C | 1 | 0.006
H5a6 | 5899.1C | 1 | 0.006
H5a6 | 6408 | 1 | 0.006
H5a6 | 7388A | 1 | 0.006
H5a6 | 7388T | 1 | 0.006
H5a6 | 9010 | 1 | 0.006
H5a6 | 9881 | 1 | 0.006
H5a6a | 12007 | 2 | 0.012
H5a6a | 15883 | 1 | 0.006
H5a6a | 16111 | 1 | 0.006
H5a6a | 16124 | 1 | 0.006
H5a6a | 16257 | 3 | 0.018
H5a6a | 573.3C | 1 | 0.006
H5a7 | 10915 | 1 | 0.003
H5a7 | 11150 | 2 | 0.007
H5a7 | 12088 | 2 | 0.007
H5a7 | 12930 | 1 | 0.003
H5a7 | 14826 | 1 | 0.003
H5a7 | 152 | 2 | 0.007
H5a7 | 16114A | 1 | 0.003
H5a7 | 16181 | 1 | 0.003
H5a7 | 16185 | 1 | 0.003
H5a7 | 16193 | 1 | 0.003
H5a7 | 16213 | 1 | 0.003
H5a7 | 16241C | 22 | 0.074
H5a7 | 16247 | 1 | 0.003
H5a7 | 16342 | 1 | 0.003
H5a7 | 16527 | 1 | 0.003
H5a7 | 1717 | 2 | 0.007
H5a7 | 185 | 2 | 0.007
H5a7 | 316.1G | 1 | 0.003
H5a7 | 506 | 1 | 0.003
H5a7 | 533 | 2 | 0.007
H5a7 | 573.6C | 1 | 0.003
H5a7 | 6800 | 1 | 0.003
H5a7 | 8041 | 1 | 0.003
H5a7 | 8281-8289d | 1 | 0.003
H5a7 | 9010 | 1 | 0.003
H5a8 | 11807 | 1 | 0.003
H5a8 | 14325 | 2 | 0.007
H5a8 | 16114A | 1 | 0.003
H5a8 | 16181 | 1 | 0.003
H5a8 | 16185 | 1 | 0.003
H5a8 | 16241C | 1 | 0.003
H5a8 | 16247 | 1 | 0.003
H5a8 | 16265 | 1 | 0.003
H5a8 | 16320 | 24 | 0.081
H5a8 | 16342 | 1 | 0.003
H5a8 | 16527 | 1 | 0.003
H5a8 | 506 | 1 | 0.003
H5a8 | 573.6C | 1 | 0.003
H5a9 | 14040 | 1 | 0.003
H5a9 | 16114A | 1 | 0.003
H5a9 | 16181 | 1 | 0.003
H5a9 | 16185 | 1 | 0.003
H5a9 | 16241C | 22 | 0.075
H5a9 | 16247 | 1 | 0.003
H5a9 | 16342 | 1 | 0.003
H5a9 | 16527 | 1 | 0.003
H5a9 | 195 | 4 | 0.014
H5a9 | 366 | 1 | 0.003
H5a9 | 506 | 1 | 0.003
H5a9 | 564 | 1 | 0.003
H5a9 | 573.6C | 1 | 0.003
H5a9 | 769 | 1 | 0.003
H5b | 10238 | 5 | 0.015
H5b | 10248 | 1 | 0.003
H5b | 10680 | 1 | 0.003
H5b | 10972 | 1 | 0.003
H5b | 11113 | 1 | 0.003
H5b | 11329 | 1 | 0.003
H5b | 11914 | 1 | 0.003
H5b | 12033 | 1 | 0.003
H5b | 12127 | 1 | 0.003
H5b | 12401 | 1 | 0.003
H5b | 12726 | 1 | 0.003
H5b | 12864 | 1 | 0.003
H5b | 12879 | 1 | 0.003
H5b | 13285 | 1 | 0.003
H5b | 13516 | 1 | 0.003
H5b | 13608 | 1 | 0.003
H5b | 13704 | 1 | 0.003
H5b | 13734 | 1 | 0.003
H5b | 13908 | 2 | 0.006
H5b | 14007 | 5 | 0.015
H5b | 14258 | 1 | 0.003
H5b | 14311G | 1 | 0.003
H5b | 14319 | 1 | 0.003
H5b | 14384 | 1 | 0.003
H5b | 145 | 1 | 0.003
H5b | 146 | 3 | 0.009
H5b | 14773 | 1 | 0.003

H5b | 14798 | 1 | 0.003
H5b | 150 | 1 | 0.003
H5b | 15190 | 1 | 0.003
H5b | 152 | 7 | 0.021
H5b | 15709 | 1 | 0.003
H5b | 15929 | 1 | 0.003
H5b | 15940 | 1 | 0.003
H5b | 16051 | 2 | 0.006
H5b | 16114A | 1 | 0.003
H5b | 16129 | 4 | 0.012
H5b | 16181 | 1 | 0.003
H5b | 16185 | 1 | 0.003
H5b | 16224 | 1 | 0.003
H5b | 16241C | 1 | 0.003
H5b | 16247 | 1 | 0.003
H5b | 16255 | 1 | 0.003
H5b | 16260 | 2 | 0.006
H5b | 16261 | 5 | 0.015
H5b | 16271 | 3 | 0.009
H5b | 16278 | 1 | 0.003
H5b | 16309 | 1 | 0.003
H5b | 16318 | 25 | 0.076
H5b | 16319 | 4 | 0.012
H5b | 16327 | 2 | 0.006
H5b | 16342 | 1 | 0.003
H5b | 16355 | 1 | 0.003
H5b | 16497 | 1 | 0.003
H5b | 16527 | 1 | 0.003
H5b | 183 | 1 | 0.003
H5b | 227 | 1 | 0.003
H5b | 228 | 1 | 0.003
H5b | 2706 | 1 | 0.003
H5b | 310 | 3 | 0.009
H5b | 4343 | 1 | 0.003
H5b | 4607 | 1 | 0.003
H5b | 4694A | 1 | 0.003
H5b | 497 | 1 | 0.003
H5b | 506 | 1 | 0.003
H5b | 508 | 1 | 0.003
H5b | 5270 | 1 | 0.003
H5b | 533 | 1 | 0.003
H5b | 573.6C | 1 | 0.003
H5b | 6710 | 1 | 0.003
H5b | 6755 | 1 | 0.003
H5b | 709 | 1 | 0.003
H5b | 721 | 1 | 0.003
H5b | 7258 | 2 | 0.006
H5b | 7269 | 2 | 0.006
H5b | 73 | 1 | 0.003
H5b | 7853 | 2 | 0.006
H5b | 7861 | 1 | 0.003
H5b | 7897 | 2 | 0.006
H5b | 7941 | 2 | 0.006
H5b | 801 | 1 | 0.003
H5b | 8416 | 1 | 0.003
H5b | 8701 | 1 | 0.003
H5b | 8854 | 1 | 0.003
H5b | 93 | 1 | 0.003
H5b | 9327 | 1 | 0.003
H5b | 9966 | 1 | 0.003
H5b1 | 10589 | 1 | 0.006
H5b1 | 13581 | 2 | 0.012
H5b1 | 150 | 1 | 0.006
H5b1 | 1503 | 1 | 0.006
H5b1 | 152 | 2 | 0.012
H5b1 | 15596 | 1 | 0.006
H5b1 | 16311 | 1 | 0.006
H5b1 | 16399 | 5 | 0.03
H5b1 | 1822 | 1 | 0.006
H5b1 | 198 | 1 | 0.006
H5b1 | 249 | 1 | 0.006
H5b1 | 310 | 2 | 0.012
H5b1 | 315.2C | 1 | 0.006
H5b1 | 4002 | 1 | 0.006
H5b1 | 5492 | 4 | 0.024
H5b1 | 6662 | 1 | 0.006
H5b1 | 9452 | 3 | 0.018
H5b2 | 15493 | 2 | 0.01
H5b2 | 16111G | 1 | 0.005
H5b2 | 16177 | 1 | 0.005
H5b2 | 16290 | 2 | 0.01
H5b2 | 16304G | 1 | 0.005
H5b2 | 195 | 1 | 0.005
H5b2 | 309d | 1 | 0.005
H5b2 | 310 | 1 | 0.005
H5b2 | 316C | 1 | 0.005
H5b2 | 5147 | 1 | 0.005
H5b2 | 573.1C | 2 | 0.01
H5b2 | 573.2C | 1 | 0.005
H5b2 | 573.3C | 3 | 0.015
H5b2 | 7058 | 1 | 0.005
H5b2 | 709 | 1 | 0.005
H5b2 | 9948 | 2 | 0.01
H5b3 | 10490 | 1 | 0.003
H5b3 | 152 | 1 | 0.003
H5b3 | 16114A | 1 | 0.003
H5b3 | 16171 | 1 | 0.003
H5b3 | 16181 | 1 | 0.003
H5b3 | 16185 | 1 | 0.003
H5b3 | 16241C | 1 | 0.003
H5b3 | 16247 | 1 | 0.003
H5b3 | 16265 | 1 | 0.003
H5b3 | 16320 | 24 | 0.082
H5b3 | 16342 | 1 | 0.003
H5b3 | 16527 | 1 | 0.003
H5b3 | 199 | 1 | 0.003
H5b3 | 391 | 1 | 0.003
H5b3 | 506 | 1 | 0.003
H5b3 | 573.6C | 1 | 0.003
H5b3 | 7245 | 1 | 0.003
H5b3 | 7609 | 1 | 0.003

H5b3 | 81 | 1 | 0.003
H5b3 | 827 | 1 | 0.003
H5b4 | 12501 | 1 | 0.006
H5b4 | 16278 | 14 | 0.083
H5b4 | 16T | 2 | 0.012
H5b4 | 194 | 1 | 0.006
H5b4 | 200 | 3 | 0.018
H5b4 | 309d | 1 | 0.006
H5b4 | 7768 | 1 | 0.006
H5b5 | 146 | 1 | 0.003
H5b5 | 152 | 1 | 0.003
H5b5 | 16075 | 1 | 0.003
H5b5 | 16114A | 1 | 0.003
H5b5 | 16181 | 1 | 0.003
H5b5 | 16185 | 1 | 0.003
H5b5 | 16241C | 1 | 0.003
H5b5 | 16247 | 1 | 0.003
H5b5 | 16265 | 1 | 0.003
H5b5 | 16294 | 1 | 0.003
H5b5 | 16320 | 24 | 0.082
H5b5 | 16342 | 1 | 0.003
H5b5 | 16527 | 1 | 0.003
H5b5 | 4002 | 1 | 0.003
H5b5 | 506 | 1 | 0.003
H5b5 | 573.6C | 1 | 0.003
H5b5 | 7891 | 1 | 0.003
H5c | 16114A | 1 | 0.003
H5c | 16181 | 1 | 0.003
H5c | 16185 | 1 | 0.003
H5c | 16223 | 1 | 0.003
H5c | 16241C | 1 | 0.003
H5c | 16247 | 1 | 0.003
H5c | 16258T | 1 | 0.003
H5c | 16265 | 1 | 0.003
H5c | 16320 | 24 | 0.082
H5c | 16342 | 1 | 0.003
H5c | 16527 | 1 | 0.003
H5c | 506 | 1 | 0.003
H5c | 5580 | 1 | 0.003
H5c | 573.6C | 1 | 0.003
H5c | 8188 | 1 | 0.003
H5c | 9377 | 1 | 0.003
H5c1 | 14071 | 1 | 0.003
H5c1 | 150 | 1 | 0.003
H5c1 | 16114A | 1 | 0.003
H5c1 | 16181 | 1 | 0.003
H5c1 | 16185 | 1 | 0.003
H5c1 | 16241C | 22 | 0.072
H5c1 | 16247 | 1 | 0.003
H5c1 | 16311 | 1 | 0.003
H5c1 | 16342 | 1 | 0.003
H5c1 | 16527 | 1 | 0.003
H5c1 | 316 | 1 | 0.003
H5c1 | 347 | 1 | 0.003
H5c1 | 377 | 1 | 0.003
H5c1 | 506 | 1 | 0.003
H5c1 | 5400 | 1 | 0.003
H5c1 | 573.6C | 1 | 0.003
H5c1 | 6917 | 9 | 0.03
H5c1 | 72 | 6 | 0.02
H5c1a | 12126 | 1 | 0.003
H5c1a | 15902 | 1 | 0.003
H5c1a | 16114A | 1 | 0.003
H5c1a | 16181 | 1 | 0.003
H5c1a | 16185 | 1 | 0.003
H5c1a | 16189 | 1 | 0.003
H5c1a | 16241C | 1 | 0.003
H5c1a | 16247 | 1 | 0.003
H5c1a | 16265 | 1 | 0.003
H5c1a | 16320 | 24 | 0.082
H5c1a | 16342 | 1 | 0.003
H5c1a | 16527 | 1 | 0.003
H5c1a | 506 | 1 | 0.003
H5c1a | 573.6C | 1 | 0.003
H5c1a | 8762 | 1 | 0.003
H5c2 | 16037 | 1 | 0.028
H5c2 | 16207 | 1 | 0.028
H5c2 | 16216 | 3 | 0.083
H5d | 131 | 2 | 0.007
H5d | 152 | 1 | 0.003
H5d | 16093 | 1 | 0.003
H5d | 16114A | 1 | 0.003
H5d | 16181 | 1 | 0.003
H5d | 16185 | 1 | 0.003
H5d | 16232 | 1 | 0.003
H5d | 16241C | 22 | 0.072
H5d | 16247 | 1 | 0.003
H5d | 16342 | 1 | 0.003
H5d | 16362 | 1 | 0.003
H5d | 16527 | 1 | 0.003
H5d | 234 | 1 | 0.003
H5d | 310 | 1 | 0.003
H5d | 315.3C | 1 | 0.003
H5d | 506 | 1 | 0.003
H5d | 515d | 1 | 0.003
H5d | 573.6C | 1 | 0.003
H5d | 5984T | 11 | 0.036
H5d | 8434G | 1 | 0.003
H5d | 9099 | 1 | 0.003
H5e | 15287 | 1 | 0.015
H5e | 154 | 21 | 0.313
H5e | 15805 | 1 | 0.015
H5e1 | 12193 | 2 | 0.027
H5e1 | 13967 | 1 | 0.014
H5e1 | 143 | 1 | 0.014
H5e1 | 14687 | 3 | 0.041
H5e1 | 14956 | 1 | 0.014
H5e1 | 16189 | 24 | 0.329
H5e1 | 16527 | 23 | 0.315
H5e1 | 189 | 1 | 0.014
H5e1 | 226 | 1 | 0.014

H5e1 | 3421 | 3 | 0.041
H5e1 | 390 | 2 | 0.027
H5e1 | 5899.1C | 3 | 0.041
H5e1 | 8931 | 1 | 0.014
H5e1 | 93 | 2 | 0.027
H5e1a | 12376A | 1 | 0.014
H5e1a | 12376T | 1 | 0.014
H5e1a | 14476 | 1 | 0.014
H5e1a | 152 | 24 | 0.338
H5e1a | 16114 | 3 | 0.042
H5e1a | 16304G | 20 | 0.282
H5e1a | 2124 | 1 | 0.014
H5e1a | 8344 | 1 | 0.014
H5e1a1 | 11630 | 1 | 0.013
H5e1a1 | 13731 | 1 | 0.013
H5e1a1 | 16092 | 1 | 0.013
H5e1a1 | 16222A | 6 | 0.077
H5e1a1 | 16271 | 3 | 0.038
H5e1a1 | 16301 | 20 | 0.256
H5e1a1 | 16311 | 26 | 0.333
H5e1a1 | 200 | 4 | 0.051
H5e1a1 | 3438 | 1 | 0.013
H5e1a1 | 8552 | 1 | 0.013
H5e1a1 | 9656 | 1 | 0.013
H5e1b | 146 | 2 | 0.026
H5e1b | 16086 | 1 | 0.013
H5e1b | 16093 | 1 | 0.013
H5e1b | 16153 | 1 | 0.013
H5e1b | 16167 | 1 | 0.013
H5e1b | 16192 | 1 | 0.013
H5e1b | 16223 | 2 | 0.026
H5e1b | 16260 | 21 | 0.276
H5e1b | 198 | 2 | 0.026
H5e1b | 363C | 2 | 0.026
H5e1b | 5566 | 1 | 0.013
H5e1b | 573.5C | 1 | 0.013
H5e1b | 73 | 4 | 0.053
H5f | 11089 | 1 | 0.003
H5f | 11686 | 1 | 0.003
H5f | 11959 | 1 | 0.003
H5f | 16114A | 1 | 0.003
H5f | 16145 | 1 | 0.003
H5f | 16181 | 1 | 0.003
H5f | 16185 | 1 | 0.003
H5f | 16241C | 1 | 0.003
H5f | 16247 | 1 | 0.003
H5f | 16265 | 1 | 0.003
H5f | 16320 | 24 | 0.081
H5f | 16342 | 1 | 0.003
H5f | 16527 | 1 | 0.003
H5f | 456.1T | 1 | 0.003
H5f | 506 | 1 | 0.003
H5f | 573.6C | 1 | 0.003
H5f | 7444 | 1 | 0.003
H5g | 146 | 3 | 0.071
H5g | 152 | 2 | 0.048
H5g | 16292 | 3 | 0.071
H5g | 310 | 1 | 0.024
H5g | 5460 | 1 | 0.024
H5g | 6032 | 1 | 0.024
H5g | 9947 | 1 | 0.024
H5h | 16311 | 2 | 0.05
H5h | 223 | 1 | 0.025
H5h | 286T | 1 | 0.025
H5h | 342 | 1 | 0.025
H5h | 376T | 1 | 0.025
H5h | 395 | 1 | 0.025
H5h | 397 | 1 | 0.025
H5h | 40 | 1 | 0.025
H5h | 6261 | 1 | 0.025
H5j | 12408 | 2 | 0.007
H5j | 14218 | 1 | 0.003
H5j | 15043 | 1 | 0.003
H5j | 16114A | 1 | 0.003
H5j | 16181 | 1 | 0.003
H5j | 16185 | 1 | 0.003
H5j | 16241C | 1 | 0.003
H5j | 16247 | 1 | 0.003
H5j | 16265C | 1 | 0.003
H5j | 16284 | 1 | 0.003
H5j | 16342 | 1 | 0.003
H5j | 16343T | 22 | 0.074
H5j | 16527 | 1 | 0.003
H5j | 1957 | 2 | 0.007
H5j | 309d | 1 | 0.003
H5j | 3768 | 1 | 0.003
H5j | 506 | 1 | 0.003
H5j | 526 | 2 | 0.007
H5j | 573.1C | 23 | 0.077
H5j | 573.2C | 3 | 0.01
H5j | 573.6C | 1 | 0.003
H5j | 7270 | 1 | 0.003
H5k | 11204 | 1 | 0.003
H5k | 13650 | 1 | 0.003
H5k | 16114A | 1 | 0.003
H5k | 16181 | 1 | 0.003
H5k | 16185 | 1 | 0.003
H5k | 16241C | 22 | 0.075
H5k | 16247 | 1 | 0.003
H5k | 16342 | 1 | 0.003
H5k | 16527 | 1 | 0.003
H5k | 2887 | 1 | 0.003
H5k | 506 | 1 | 0.003
H5k | 573.6C | 1 | 0.003
H5m | 10915 | 5 | 0.028
H5m | 11016 | 1 | 0.006
H5m | 14153 | 2 | 0.011
H5m | 14502 | 2 | 0.011
H5m | 14569 | 1 | 0.006
H5m | 14750 | 2 | 0.011
H5m | 15047 | 2 | 0.011

H5m | 15482G | 1 | 0.006
H5m | 15766 | 1 | 0.006
H5m | 16086 | 8 | 0.045
H5m | 16186 | 1 | 0.006
H5m | 16189 | 9 | 0.051
H5m | 16192 | 1 | 0.006
H5m | 16214 | 5 | 0.028
H5m | 16291 | 1 | 0.006
H5m | 16390 | 1 | 0.006
H5m | 189 | 1 | 0.006
H5m | 2755 | 1 | 0.006
H5m | 3355 | 2 | 0.011
H5m | 3776 | 1 | 0.006
H5m | 573.2C | 4 | 0.023
H5m | 573.3C | 4 | 0.023
H5m | 573.4C | 3 | 0.017
H5m | 574C | 1 | 0.006
H5m | 6095 | 1 | 0.006
H5m | 7070G | 1 | 0.006
H5m | 7148 | 2 | 0.011
H5m | 8206 | 1 | 0.006
H5n | 10454 | 1 | 0.005
H5n | 13215A | 1 | 0.005
H5n | 13638 | 1 | 0.005
H5n | 14659 | 1 | 0.005
H5n | 14893 | 2 | 0.01
H5n | 152 | 1 | 0.005
H5n | 15646 | 1 | 0.005
H5n | 15734 | 1 | 0.005
H5n | 16092 | 1 | 0.005
H5n | 16093 | 2 | 0.01
H5n | 16111G | 1 | 0.005
H5n | 16145 | 1 | 0.005
H5n | 16166C | 1 | 0.005
H5n | 16177 | 1 | 0.005
H5n | 16189 | 1 | 0.005
H5n | 16201 | 1 | 0.005
H5n | 16261 | 1 | 0.005
H5n | 16269 | 1 | 0.005
H5n | 16271 | 4 | 0.02
H5n | 16274 | 1 | 0.005
H5n | 16290 | 2 | 0.01
H5n | 16327 | 9 | 0.045
H5n | 16342 | 1 | 0.005
H5n | 228 | 1 | 0.005
H5n | 316C | 1 | 0.005
H5n | 507 | 1 | 0.005
H5n | 6755 | 1 | 0.005
H5n | 7004 | 1 | 0.005
H5n | 73 | 1 | 0.005
H5n | 7598 | 1 | 0.005
H5n | 7720 | 1 | 0.005
H5n | 8489C | 1 | 0.005
H5n | 9240 | 1 | 0.005
H5p | 16114A | 1 | 0.003
H5p | 16181 | 1 | 0.003
H5p | 16185 | 1 | 0.003
H5p | 16241C | 1 | 0.003
H5p | 16247 | 1 | 0.003
H5p | 16265 | 1 | 0.003
H5p | 16320 | 24 | 0.082
H5p | 16342 | 1 | 0.003
H5p | 16527 | 1 | 0.003
H5p | 506 | 1 | 0.003
H5p | 573.6C | 1 | 0.003
H5p | 5899.1C | 1 | 0.003
H5p | 9145 | 1 | 0.003
H5q | 150 | 1 | 0.019
H5q | 16167 | 1 | 0.019
H5q | 16239 | 2 | 0.038
H5q | 16264 | 1 | 0.019
H5q | 16294 | 1 | 0.019
H5q | 16311 | 6 | 0.115
H5q | 16318 | 1 | 0.019
H5q | 236 | 1 | 0.019
H5q | 239 | 1 | 0.019
H5q | 485 | 1 | 0.019
H5r | 152 | 1 | 0.029
H5r | 310 | 1 | 0.029
H5r | 4596 | 1 | 0.029
H5r | 5268 | 1 | 0.029
H5r | 9966 | 2 | 0.059
H5r1 | 15988 | 1 | 0.026
H5r1 | 15989G | 1 | 0.026
H5r1 | 15994-15995d | 1 | 0.026
H5r1 | 15999d | 1 | 0.026
H5r1 | 16006 | 1 | 0.026
H5r1 | 16007 | 1 | 0.026
H5r1 | 16024 | 1 | 0.026
H5r1 | 16032d | 3 | 0.079
H5r1 | 16032G | 1 | 0.026
H5r1 | 16042d | 3 | 0.079
H5r1 | 16052 | 1 | 0.026
H5r1 | 16053d | 1 | 0.026
H5r1 | 16054C | 1 | 0.026
H5r1 | 16092 | 1 | 0.026
H5r1 | 16110d | 3 | 0.079
H5r1 | 16145 | 3 | 0.079
H5r1 | 16223 | 3 | 0.079
H5r1 | 16273 | 2 | 0.053
H5r1 | 16274.1G | 1 | 0.026
H5r1 | 16288 | 2 | 0.053
H5r1 | 16320 | 1 | 0.026
H5r1 | 16359 | 1 | 0.026
H5r1 | 16370.1G | 1 | 0.026
H5r1 | 16390 | 2 | 0.053
H5r1 | 16434.1G | 1 | 0.026
H5r1 | 19.1C | 1 | 0.026
H5r1 | 310 | 1 | 0.026
H5r1 | 36.1G | 1 | 0.026
H5r1 | 4991 | 1 | 0.026

H5r1 | 55G | 1 | 0.026
H5r1 | 71.1G | 1 | 0.026
H5r2 | 14831 | 1 | 0.026
H5r2 | 151 | 1 | 0.026
H5r2 | 15988 | 1 | 0.026
H5r2 | 15989G | 1 | 0.026
H5r2 | 15994-15995d | 1 | 0.026
H5r2 | 15999d | 1 | 0.026
H5r2 | 16006 | 1 | 0.026
H5r2 | 16007 | 1 | 0.026
H5r2 | 16024 | 1 | 0.026
H5r2 | 16032d | 3 | 0.079
H5r2 | 16032G | 1 | 0.026
H5r2 | 16042d | 3 | 0.079
H5r2 | 16052 | 1 | 0.026
H5r2 | 16053d | 1 | 0.026
H5r2 | 16054C | 1 | 0.026
H5r2 | 16092 | 1 | 0.026
H5r2 | 16110d | 3 | 0.079
H5r2 | 16145 | 3 | 0.079
H5r2 | 16223 | 3 | 0.079
H5r2 | 16273 | 2 | 0.053
H5r2 | 16274.1G | 1 | 0.026
H5r2 | 16288 | 2 | 0.053
H5r2 | 16320 | 1 | 0.026
H5r2 | 16359 | 1 | 0.026
H5r2 | 16370.1G | 1 | 0.026
H5r2 | 16390 | 2 | 0.053
H5r2 | 16434.1G | 1 | 0.026
H5r2 | 1717 | 1 | 0.026
H5r2 | 19.1C | 1 | 0.026
H5r2 | 309d | 1 | 0.026
H5r2 | 36.1G | 1 | 0.026
H5r2 | 55G | 1 | 0.026
H5r2 | 71.1G | 1 | 0.026
H5s | 12972 | 1 | 0.019
H5s | 15001 | 1 | 0.019
H5s | 15213 | 1 | 0.019
H5s | 16259 | 3 | 0.057
H5s | 16362 | 1 | 0.019
H5s | 204 | 1 | 0.019
H5s | 310 | 1 | 0.019
H5s | 311 | 1 | 0.019
H5s | 315.2C | 1 | 0.019
H5s | 316C | 1 | 0.019
H5s | 3666 | 3 | 0.057
H5s | 4140 | 1 | 0.019
H5s | 4576 | 1 | 0.019
H5s | 573.1C | 1 | 0.019
H5s | 6448A | 1 | 0.019
H5s | 72 | 1 | 0.019
H5s | 73 | 1 | 0.019
H5t | 13609G | 3 | 0.083
H5t | 152 | 4 | 0.111
H5t | 8053 | 3 | 0.083
H5u | 11475 | 1 | 0.024
H5u | 11581 | 1 | 0.024
H5u | 16311 | 1 | 0.024
H5u | 185 | 4 | 0.095
H5u | 188 | 1 | 0.024
H5u | 189 | 2 | 0.048
H5u | 5447 | 3 | 0.071
H5u | 709 | 1 | 0.024
H5u | 72G | 1 | 0.024
H5u1 | 16261 | 1 | 0.018
H5u1 | 16287 | 3 | 0.054
H5u1 | 310 | 3 | 0.054
H5u1 | 5582 | 1 | 0.018
H5u1 | 5899.1C | 16 | 0.286
H5u1 | 709 | 1 | 0.018
H5u1 | 73 | 1 | 0.018
H5u1 | 93 | 1 | 0.018
H5u1 | 9923 | 1 | 0.018
H5v | 152 | 1 | 0.003
H5v | 16042 | 1 | 0.003
H5v | 16114A | 1 | 0.003
H5v | 16181 | 1 | 0.003
H5v | 16185 | 1 | 0.003
H5v | 16189 | 1 | 0.003
H5v | 16190 | 1 | 0.003
H5v | 16241C | 1 | 0.003
H5v | 16247 | 1 | 0.003
H5v | 16335 | 1 | 0.003
H5v | 16342 | 1 | 0.003
H5v | 16343C | 1 | 0.003
H5v | 16355 | 3 | 0.01
H5v | 16362 | 29 | 0.097
H5v | 16527 | 1 | 0.003
H5v | 310 | 1 | 0.003
H5v | 3244 | 1 | 0.003
H5v | 506 | 1 | 0.003
H5v | 573.6C | 1 | 0.003
H5v | 73 | 2 | 0.007
H5v | 7521 | 1 | 0.003
H5v | 7978 | 2 | 0.007
H5v | 8521 | 1 | 0.003
H6 | 10589 | 1 | 0.007
H6 | 11467 | 1 | 0.007
H6 | 12308 | 1 | 0.007
H6 | 12372 | 1 | 0.007
H6 | 12438 | 3 | 0.021
H6 | 146 | 2 | 0.014
H6 | 14620 | 1 | 0.007
H6 | 15693 | 1 | 0.007
H6 | 15804 | 1 | 0.007
H6 | 16111 | 1 | 0.007
H6 | 16120 | 1 | 0.007
H6 | 16124 | 9 | 0.063
H6 | 16189 | 2 | 0.014
H6 | 16243 | 1 | 0.007
H6 | 1811 | 1 | 0.007

H6 | 2159 | 1 | 0.007
H6 | 3349 | 2 | 0.014
H6 | 499 | 1 | 0.007
H6 | 6164 | 1 | 0.007
H6 | 6293 | 1 | 0.007
H6 | 6351 | 1 | 0.007
H6 | 6852 | 2 | 0.014
H6 | 7028 | 1 | 0.007
H6 | 709 | 1 | 0.007
H6 | 73 | 3 | 0.021
H6 | 7775 | 1 | 0.007
H6 | 7897 | 2 | 0.014
H6 | 839 | 1 | 0.007
H6 | 873 | 1 | 0.007
H6 | 8818 | 1 | 0.007
H6 | 9254 | 1 | 0.007
H60 | 113 | 1 | 0.001
H60 | 16076A | 123 | 0.157
H60 | 208 | 1 | 0.001
H60 | 261 | 1 | 0.001
H60 | 281C | 1 | 0.001
H60 | 316d | 1 | 0.001
H60 | 338 | 1 | 0.001
H60 | 468 | 3 | 0.004
H60 | 502 | 1 | 0.001
H60 | 573.1C | 3 | 0.004
H60 | 573.2C | 5 | 0.006
H60 | 573.5C | 1 | 0.001
H60a | 113 | 1 | 0.001
H60a | 12654 | 1 | 0.001
H60a | 150 | 1 | 0.001
H60a | 15747 | 1 | 0.001
H60a | 15928T | 1 | 0.001
H60a | 16076A | 123 | 0.155
H60a | 16093 | 4 | 0.005
H60a | 16175 | 1 | 0.001
H60a | 16189 | 2 | 0.003
H60a | 16234 | 1 | 0.001
H60a | 16311 | 2 | 0.003
H60a | 185 | 2 | 0.003
H60a | 195 | 2 | 0.003
H60a | 208 | 1 | 0.001
H60a | 261 | 1 | 0.001
H60a | 281C | 1 | 0.001
H60a | 316d | 1 | 0.001
H60a | 338 | 1 | 0.001
H60a | 466 | 4 | 0.005
H60a | 468 | 3 | 0.004
H60a | 502 | 1 | 0.001
H60a | 513 | 1 | 0.001
H60a | 573.1C | 3 | 0.004
H60a | 573.2C | 5 | 0.006
H60a | 573.5C | 1 | 0.001
H60a | 6137 | 1 | 0.001
H60a | 73 | 1 | 0.001
H60a | 9102A | 2 | 0.003
H61 | 113 | 1 | 0.001
H61 | 13743 | 1 | 0.001
H61 | 16076A | 123 | 0.156
H61 | 16192 | 1 | 0.001
H61 | 16216 | 1 | 0.001
H61 | 16297 | 1 | 0.001
H61 | 208 | 1 | 0.001
H61 | 261 | 1 | 0.001
H61 | 281C | 1 | 0.001
H61 | 316d | 1 | 0.001
H61 | 338 | 1 | 0.001
H61 | 468 | 3 | 0.004
H61 | 502 | 1 | 0.001
H61 | 573.1C | 3 | 0.004
H61 | 573.2C | 5 | 0.006
H61 | 573.5C | 1 | 0.001
H61 | 5964 | 1 | 0.001
H61 | 73 | 2 | 0.003
H61a | 14687 | 1 | 0.011
H61a | 151 | 3 | 0.033
H61a | 152 | 4 | 0.044
H61a | 16126 | 2 | 0.022
H61a | 16189 | 1 | 0.011
H61a | 16201 | 1 | 0.011
H61a | 16209 | 1 | 0.011
H61a | 16311 | 2 | 0.022
H61a | 16354 | 4 | 0.044
H61a | 195 | 1 | 0.011
H61a | 200 | 2 | 0.022
H61a | 207 | 2 | 0.022
H61a | 235 | 1 | 0.011
H61a | 449A | 1 | 0.011
H61a | 5786 | 1 | 0.011
H61a | 73 | 3 | 0.033
H62 | 113 | 1 | 0.001
H62 | 16076A | 123 | 0.156
H62 | 194 | 1 | 0.001
H62 | 208 | 1 | 0.001
H62 | 261 | 1 | 0.001
H62 | 281C | 1 | 0.001
H62 | 316d | 1 | 0.001
H62 | 338 | 1 | 0.001
H62 | 468 | 3 | 0.004
H62 | 502 | 1 | 0.001
H62 | 573.1C | 3 | 0.004
H62 | 573.2C | 5 | 0.006
H62 | 573.5C | 1 | 0.001
H63 | 11016 | 1 | 0.001
H63 | 113 | 1 | 0.001
H63 | 14569 | 1 | 0.001
H63 | 14905 | 1 | 0.001
H63 | 16076A | 1 | 0.001
H63 | 16093 | 2 | 0.003
H63 | 16265C | 1 | 0.001
H63 | 16357 | 123 | 0.155

H63 | 208 | 1 | 0.001
H63 | 2322 | 1 | 0.001
H63 | 261 | 1 | 0.001
H63 | 281C | 1 | 0.001
H63 | 316d | 1 | 0.001
H63 | 338 | 1 | 0.001
H63 | 468 | 3 | 0.004
H63 | 502 | 1 | 0.001
H63 | 573.1C | 3 | 0.004
H63 | 573.2C | 5 | 0.006
H63 | 573.5C | 1 | 0.001
H63 | 5752d | 1 | 0.001
H63a | 16257 | 7 | 0.028
H63a | 281C | 1 | 0.004
H63a | 518 | 1 | 0.004
H63a | 64 | 1 | 0.004
H64 | 14410 | 2 | 0.015
H64 | 16185 | 1 | 0.008
H64 | 16195 | 1 | 0.008
H64 | 16221 | 1 | 0.008
H64 | 16270 | 1 | 0.008
H64 | 16298 | 1 | 0.008
H64 | 16399 | 3 | 0.023
H64 | 204 | 1 | 0.008
H64 | 499 | 3 | 0.023
H64 | 8065 | 1 | 0.008
H64 | 8661 | 1 | 0.008
H64 | 9211 | 1 | 0.008
H65 | 113 | 1 | 0.001
H65 | 14727 | 1 | 0.001
H65 | 16076A | 123 | 0.156
H65 | 16172 | 1 | 0.001
H65 | 16209 | 1 | 0.001
H65 | 16304 | 1 | 0.001
H65 | 208 | 1 | 0.001
H65 | 2099 | 1 | 0.001
H65 | 261 | 1 | 0.001
H65 | 281C | 1 | 0.001
H65 | 316d | 1 | 0.001
H65 | 338 | 1 | 0.001
H65 | 4491 | 1 | 0.001
H65 | 4502 | 1 | 0.001
H65 | 468 | 3 | 0.004
H65 | 502 | 1 | 0.001
H65 | 573.1C | 3 | 0.004
H65 | 573.2C | 5 | 0.006
H65 | 573.5C | 1 | 0.001
H65 | 7861 | 1 | 0.001
H65 | 7918A | 1 | 0.001
H65 | 8648 | 1 | 0.001
H65 | 9842 | 1 | 0.001
H65 | 9948 | 1 | 0.001
H65a | 113 | 1 | 0.001
H65a | 15341 | 1 | 0.001
H65a | 16076A | 123 | 0.156
H65a | 16189 | 1 | 0.001
H65a | 208 | 1 | 0.001
H65a | 261 | 1 | 0.001
H65a | 281C | 1 | 0.001
H65a | 316d | 1 | 0.001
H65a | 338 | 1 | 0.001
H65a | 468 | 3 | 0.004
H65a | 502 | 1 | 0.001
H65a | 573.1C | 3 | 0.004
H65a | 573.2C | 5 | 0.006
H65a | 573.5C | 1 | 0.001
H66 | 113 | 1 | 0.001
H66 | 16076A | 123 | 0.157
H66 | 208 | 1 | 0.001
H66 | 261 | 1 | 0.001
H66 | 281C | 1 | 0.001
H66 | 316d | 1 | 0.001
H66 | 338 | 1 | 0.001
H66 | 468 | 3 | 0.004
H66 | 502 | 1 | 0.001
H66 | 573.1C | 3 | 0.004
H66 | 573.2C | 5 | 0.006
H66 | 573.5C | 1 | 0.001
H66a | 12609 | 2 | 0.019
H66a | 13314 | 1 | 0.01
H66a | 14022 | 1 | 0.01
H66a | 14443 | 1 | 0.01
H66a | 146 | 1 | 0.01
H66a | 16147 | 1 | 0.01
H66a | 16162 | 2 | 0.019
H66a | 16173 | 14 | 0.135
H66a | 16174 | 1 | 0.01
H66a | 16186 | 1 | 0.01
H66a | 16272 | 1 | 0.01
H66a | 16343 | 1 | 0.01
H66a | 16354 | 3 | 0.029
H66a | 16362 | 3 | 0.029
H66a | 207 | 2 | 0.019
H66a | 228 | 1 | 0.01
H66a | 315.2C | 2 | 0.019
H66a | 3943 | 2 | 0.019
H66a | 4002 | 1 | 0.01
H66a | 44.1C | 1 | 0.01
H66a | 5561 | 2 | 0.019
H66a | 93 | 1 | 0.01
H66a1 | 12950 | 1 | 0.016
H66a1 | 146 | 1 | 0.016
H66a1 | 16129 | 6 | 0.098
H66a1 | 16167 | 1 | 0.016
H66a1 | 16318T | 1 | 0.016
H66a1 | 195A | 1 | 0.016
H66a1 | 343 | 1 | 0.016
H66a1 | 404 | 1 | 0.016
H66a1 | 489 | 1 | 0.016
H66a1 | 73 | 1 | 0.016
H67 | 11233 | 1 | 0.001

H67 | 113 | 1 | 0.001
H67 | 16076A | 123 | 0.158
H67 | 16093 | 1 | 0.001
H67 | 208 | 1 | 0.001
H67 | 261 | 1 | 0.001
H67 | 281C | 1 | 0.001
H67 | 316d | 1 | 0.001
H67 | 338 | 1 | 0.001
H67 | 4481 | 1 | 0.001
H67 | 468 | 3 | 0.004
H67 | 502 | 1 | 0.001
H67 | 573.1C | 3 | 0.004
H67 | 573.2C | 5 | 0.006
H67 | 573.5C | 1 | 0.001
H67a | 1018 | 1 | 0.001
H67a | 113 | 1 | 0.001
H67a | 11989 | 1 | 0.001
H67a | 12549 | 2 | 0.003
H67a | 16076A | 1 | 0.001
H67a | 16129 | 123 | 0.157
H67a | 208 | 1 | 0.001
H67a | 261 | 1 | 0.001
H67a | 281C | 1 | 0.001
H67a | 316d | 1 | 0.001
H67a | 338 | 1 | 0.001
H67a | 4353 | 1 | 0.001
H67a | 468 | 3 | 0.004
H67a | 502 | 1 | 0.001
H67a | 573.1C | 3 | 0.004
H67a | 573.2C | 5 | 0.006
H67a | 573.5C | 1 | 0.001
H69 | 14460G | 1 | 0.007
H69 | 15884C | 1 | 0.007
H69 | 16214 | 3 | 0.02
H69 | 16233T | 1 | 0.007
H69 | 16247 | 20 | 0.132
H69 | 16258T | 1 | 0.007
H69 | 200T | 1 | 0.007
H69 | 207 | 1 | 0.007
H69 | 3316 | 1 | 0.007
H69 | 4080 | 1 | 0.007
H69 | 456 | 1 | 0.007
H69 | 463 | 1 | 0.007
H69 | 515-524d | 1 | 0.007
H69 | 529T | 1 | 0.007
H69 | 573.1C | 1 | 0.007
H6a | 11969 | 1 | 0.007
H6a | 14896 | 1 | 0.007
H6a | 16051 | 1 | 0.007
H6a | 16093 | 5 | 0.034
H6a | 16120 | 1 | 0.007
H6a | 16124 | 1 | 0.007
H6a | 16157 | 1 | 0.007
H6a | 16158 | 1 | 0.007
H6a | 16173 | 2 | 0.014
H6a | 16192 | 1 | 0.007
H6a | 16278 | 10 | 0.069
H6a | 16342 | 10 | 0.069
H6a | 16363 | 4 | 0.028
H6a | 16364 | 4 | 0.028
H6a | 1719 | 1 | 0.007
H6a | 199 | 1 | 0.007
H6a | 6755 | 1 | 0.007
H6a1 | 10915 | 1 | 0.007
H6a1 | 11969 | 1 | 0.007
H6a1 | 14896 | 1 | 0.007
H6a1 | 16051 | 1 | 0.007
H6a1 | 16092 | 1 | 0.007
H6a1 | 16120 | 1 | 0.007
H6a1 | 16124 | 1 | 0.007
H6a1 | 16245 | 1 | 0.007
H6a1 | 16278 | 10 | 0.072
H6a1 | 16342 | 10 | 0.072
H6a1 | 199 | 1 | 0.007
H6a1 | 7270 | 1 | 0.007
H6a1 | 9024 | 1 | 0.007
H6a1a | 10187 | 1 | 0.004
H6a1a | 10370 | 2 | 0.008
H6a1a | 10586 | 1 | 0.004
H6a1a | 10685 | 1 | 0.004
H6a1a | 10742 | 1 | 0.004
H6a1a | 11083 | 1 | 0.004
H6a1a | 11311 | 2 | 0.008
H6a1a | 11914 | 1 | 0.004
H6a1a | 11978A | 2 | 0.008
H6a1a | 12097 | 1 | 0.004
H6a1a | 12280 | 1 | 0.004
H6a1a | 12369 | 3 | 0.012
H6a1a | 12372 | 1 | 0.004
H6a1a | 12501 | 1 | 0.004
H6a1a | 12557 | 2 | 0.008
H6a1a | 12865 | 1 | 0.004
H6a1a | 13020 | 1 | 0.004
H6a1a | 13104 | 2 | 0.008
H6a1a | 13174 | 1 | 0.004
H6a1a | 13191 | 1 | 0.004
H6a1a | 13602 | 2 | 0.008
H6a1a | 13710 | 1 | 0.004
H6a1a | 13958C | 1 | 0.004
H6a1a | 14029 | 1 | 0.004
H6a1a | 14094 | 1 | 0.004
H6a1a | 14476 | 2 | 0.008
H6a1a | 14527 | 3 | 0.012
H6a1a | 14560 | 3 | 0.012
H6a1a | 14587 | 1 | 0.004
H6a1a | 146 | 7 | 0.028
H6a1a | 14944 | 1 | 0.004
H6a1a | 152 | 44 | 0.176
H6a1a | 15226C | 1 | 0.004
H6a1a | 15298A | 1 | 0.004
H6a1a | 15944d | 1 | 0.004

H6a1a | 16092 | 6 | 0.024
H6a1a | 16126 | 7 | 0.028
H6a1a | 16134 | 1 | 0.004
H6a1a | 16168 | 1 | 0.004
H6a1a | 16172 | 1 | 0.004
H6a1a | 16174 | 1 | 0.004
H6a1a | 16189 | 6 | 0.024
H6a1a | 16192 | 2 | 0.008
H6a1a | 16213 | 2 | 0.008
H6a1a | 16218 | 1 | 0.004
H6a1a | 16223 | 1 | 0.004
H6a1a | 16224 | 1 | 0.004
H6a1a | 16245 | 1 | 0.004
H6a1a | 16278 | 1 | 0.004
H6a1a | 16295 | 2 | 0.008
H6a1a | 16311 | 5 | 0.02
H6a1a | 16317 | 1 | 0.004
H6a1a | 16325 | 4 | 0.016
H6a1a | 16356 | 15 | 0.06
H6a1a | 16468 | 1 | 0.004
H6a1a | 16526 | 7 | 0.028
H6a1a | 16527 | 1 | 0.004
H6a1a | 199 | 1 | 0.004
H6a1a | 214 | 1 | 0.004
H6a1a | 2352 | 1 | 0.004
H6a1a | 249d | 1 | 0.004
H6a1a | 2581 | 2 | 0.008
H6a1a | 279 | 1 | 0.004
H6a1a | 309d | 2 | 0.008
H6a1a | 310 | 7 | 0.028
H6a1a | 315.2C | 1 | 0.004
H6a1a | 315.3C | 1 | 0.004
H6a1a | 3402 | 1 | 0.004
H6a1a | 3600 | 1 | 0.004
H6a1a | 3992 | 1 | 0.004
H6a1a | 41 | 2 | 0.008
H6a1a | 4340 | 1 | 0.004
H6a1a | 4580 | 1 | 0.004
H6a1a | 4823 | 2 | 0.008
H6a1a | 4829 | 1 | 0.004
H6a1a | 4988 | 1 | 0.004
H6a1a | 513 | 2 | 0.008
H6a1a | 5237 | 1 | 0.004
H6a1a | 5562 | 1 | 0.004
H6a1a | 568 | 1 | 0.004
H6a1a | 573.1C | 1 | 0.004
H6a1a | 573.2C | 1 | 0.004
H6a1a | 5894C | 1 | 0.004
H6a1a | 60 | 14 | 0.056
H6a1a | 6156 | 1 | 0.004
H6a1a | 6179 | 1 | 0.004
H6a1a | 6260 | 1 | 0.004
H6a1a | 64 | 3 | 0.012
H6a1a | 650 | 3 | 0.012
H6a1a | 6548 | 1 | 0.004
H6a1a | 6635 | 2 | 0.008
H6a1a | 7080 | 1 | 0.004
H6a1a | 7280 | 1 | 0.004
H6a1a | 7316C | 2 | 0.008
H6a1a | 7609 | 1 | 0.004
H6a1a | 7706 | 1 | 0.004
H6a1a | 7775 | 3 | 0.012
H6a1a | 7789 | 1 | 0.004
H6a1a | 7813 | 1 | 0.004
H6a1a | 7925 | 1 | 0.004
H6a1a | 8047 | 1 | 0.004
H6a1a | 8383 | 1 | 0.004
H6a1a | 869 | 1 | 0.004
H6a1a | 8723 | 1 | 0.004
H6a1a | 8978 | 1 | 0.004
H6a1a | 9022 | 1 | 0.004
H6a1a | 9053 | 1 | 0.004
H6a1a | 9055 | 2 | 0.008
H6a1a | 9116 | 1 | 0.004
H6a1a | 93 | 2 | 0.008
H6a1a | 9545 | 1 | 0.004
H6a1a | 9804C | 1 | 0.004
H6a1a | 9804T | 1 | 0.004
H6a1a | 9905 | 1 | 0.004
H6a1a | 9926 | 1 | 0.004
H6a1a | 9938A | 1 | 0.004
H6a1a | 9948 | 2 | 0.008
H6a1a1 | 10237 | 1 | 0.007
H6a1a1 | 11923 | 1 | 0.007
H6a1a1 | 16120 | 1 | 0.007
H6a1a1 | 16124 | 1 | 0.007
H6a1a1 | 16218 | 1 | 0.007
H6a1a1 | 16278 | 10 | 0.072
H6a1a1 | 16297 | 1 | 0.007
H6a1a1 | 16342 | 10 | 0.072
H6a1a1 | 199 | 1 | 0.007
H6a1a1 | 4164 | 1 | 0.007
H6a1a1 | 573.1C | 1 | 0.007
H6a1a1 | 6681 | 1 | 0.007
H6a1a10 | 10166 | 2 | 0.014
H6a1a10 | 10250 | 1 | 0.007
H6a1a10 | 14211 | 1 | 0.007
H6a1a10 | 16120 | 1 | 0.007
H6a1a10 | 16124 | 1 | 0.007
H6a1a10 | 16192 | 1 | 0.007
H6a1a10 | 16278 | 2 | 0.014
H6a1a10 | 1633 | 9 | 0.064
H6a1a10 | 16342 | 2 | 0.014
H6a1a10 | 16445 | 1 | 0.007
H6a1a10 | 5048 | 2 | 0.014
H6a1a1a | 16114A | 5 | 0.227
H6a1a1a | 16156 | 1 | 0.045
H6a1a1a | 16250 | 3 | 0.136
H6a1a1a | 16290 | 1 | 0.045
H6a1a1a | 16381 | 1 | 0.045
H6a1a1a | 16T | 1 | 0.045

H6a1a1a | 4991 | 2 | 0.091
H6a1a1a | 5228G | 1 | 0.045
H6a1a2 | 16120 | 1 | 0.007
H6a1a2 | 16124 | 1 | 0.007
H6a1a2 | 16278 | 10 | 0.074
H6a1a2 | 16342 | 10 | 0.074
H6a1a2 | 199 | 1 | 0.007
H6a1a2a | 1193 | 1 | 0.014
H6a1a2a | 13802 | 1 | 0.014
H6a1a2a | 14970 | 6 | 0.087
H6a1a2a | 150 | 2 | 0.029
H6a1a2a | 1598 | 1 | 0.014
H6a1a2a | 297 | 6 | 0.087
H6a1a2a | 310 | 1 | 0.014
H6a1a2a | 3766 | 1 | 0.014
H6a1a2a | 4386 | 1 | 0.014
H6a1a2a | 537G | 3 | 0.043
H6a1a2a | 6339 | 1 | 0.014
H6a1a2a | 7001 | 1 | 0.014
H6a1a2a | 7364 | 1 | 0.014
H6a1a2a | 980 | 3 | 0.043
H6a1a2b | 146 | 1 | 0.007
H6a1a2b | 16120 | 1 | 0.007
H6a1a2b | 16124 | 1 | 0.007
H6a1a2b | 16278 | 10 | 0.072
H6a1a2b | 16342 | 10 | 0.072
H6a1a2b | 16360 | 1 | 0.007
H6a1a2b | 199 | 1 | 0.007
H6a1a2b | 9145 | 1 | 0.007
H6a1a2b1 | 15287 | 1 | 0.007
H6a1a2b1 | 16120 | 1 | 0.007
H6a1a2b1 | 16124 | 1 | 0.007
H6a1a2b1 | 16209 | 10 | 0.071
H6a1a2b1 | 2361 | 1 | 0.007
H6a1a2b1 | 269 | 9 | 0.064
H6a1a2b1 | 5979 | 1 | 0.007
H6a1a2b1 | 9025 | 1 | 0.007
H6a1a2b1 | 9818 | 1 | 0.007
H6a1a3 | 11852 | 1 | 0.007
H6a1a3 | 16120 | 1 | 0.007
H6a1a3 | 16124 | 1 | 0.007
H6a1a3 | 16311 | 9 | 0.062
H6a1a3 | 310 | 1 | 0.007
H6a1a3 | 7094 | 4 | 0.027
H6a1a3 | 7269 | 1 | 0.007
H6a1a3 | 8436 | 1 | 0.007
H6a1a3 | 8455 | 1 | 0.007
H6a1a3 | 8470 | 1 | 0.007
H6a1a3a | 15099 | 1 | 0.007
H6a1a3a | 16120 | 1 | 0.007
H6a1a3a | 16124 | 9 | 0.066
H6a1a3a | 195 | 2 | 0.015
H6a1a3a | 199 | 1 | 0.007
H6a1a4 | 10724 | 1 | 0.007
H6a1a4 | 11204 | 1 | 0.007
H6a1a4 | 12810 | 1 | 0.007
H6a1a4 | 13105 | 1 | 0.007
H6a1a4 | 13218 | 1 | 0.007
H6a1a4 | 13404 | 1 | 0.007
H6a1a4 | 1389 | 1 | 0.007
H6a1a4 | 14226 | 1 | 0.007
H6a1a4 | 14326 | 1 | 0.007
H6a1a4 | 146 | 1 | 0.007
H6a1a4 | 152 | 2 | 0.014
H6a1a4 | 15222 | 1 | 0.007
H6a1a4 | 15930 | 1 | 0.007
H6a1a4 | 16148 | 9 | 0.061
H6a1a4 | 189 | 2 | 0.014
H6a1a4 | 195 | 1 | 0.007
H6a1a4 | 2263A | 1 | 0.007
H6a1a4 | 310 | 2 | 0.014
H6a1a4 | 315.2C | 2 | 0.014
H6a1a4 | 3591 | 1 | 0.007
H6a1a4 | 3944 | 1 | 0.007
H6a1a4 | 6468 | 1 | 0.007
H6a1a4 | 7588 | 1 | 0.007
H6a1a4 | 7855A | 1 | 0.007
H6a1a4 | 9055 | 1 | 0.007
H6a1a4 | 93 | 1 | 0.007
H6a1a5 | 16093 | 1 | 0.007
H6a1a5 | 16120 | 1 | 0.007
H6a1a5 | 16124 | 1 | 0.007
H6a1a5 | 16278 | 10 | 0.071
H6a1a5 | 16342 | 10 | 0.071
H6a1a5 | 2010 | 1 | 0.007
H6a1a5 | 4216 | 1 | 0.007
H6a1a5 | 4947 | 1 | 0.007
H6a1a5 | 5302 | 2 | 0.014
H6a1a5 | 748 | 1 | 0.007
H6a1a6 | 16140 | 1 | 0.011
H6a1a6 | 16145 | 2 | 0.022
H6a1a6 | 16154 | 2 | 0.022
H6a1a6 | 16280 | 1 | 0.011
H6a1a6 | 709 | 1 | 0.011
H6a1a7 | 1005 | 1 | 0.01
H6a1a7 | 13215 | 1 | 0.01
H6a1a7 | 14182A | 1 | 0.01
H6a1a7 | 1578 | 1 | 0.01
H6a1a7 | 16179 | 1 | 0.01
H6a1a7 | 16184 | 6 | 0.062
H6a1a7 | 16319 | 2 | 0.021
H6a1a7 | 200 | 1 | 0.01
H6a1a7 | 515-524d | 1 | 0.01
H6a1a8 | 10936 | 1 | 0.05
H6a1a8 | 16075A | 1 | 0.05
H6a1a8 | 16124 | 5 | 0.25
H6a1a8 | 16162 | 1 | 0.05
H6a1a8 | 16260 | 3 | 0.15
H6a1a8 | 16265 | 1 | 0.05
H6a1a8 | 16291 | 1 | 0.05
H6a1a8 | 16294A | 1 | 0.05

H6a1a8 | 16356 | 1 | 0.05
H6a1a8 | 3523 | 1 | 0.05
H6a1a8a | 16287 | 1 | 0.111
H6a1a8a | 3397 | 1 | 0.111
H6a1a8a | 432C | 1 | 0.111
H6a1a8a | 43G | 1 | 0.111
H6a1a8a | 451T | 1 | 0.111
H6a1a8a | 45d | 1 | 0.111
H6a1a8a | 473 | 1 | 0.111
H6a1a8a | 474 | 1 | 0.111
H6a1a8a | 476d | 1 | 0.111
H6a1a8a | 480A | 1 | 0.111
H6a1a8a | 481A | 1 | 0.111
H6a1a8a | 484d | 1 | 0.111
H6a1a8a | 489A | 1 | 0.111
H6a1a8a | 52d | 1 | 0.111
H6a1a8a | 9068 | 1 | 0.111
H6a1a9 | 15884 | 1 | 0.007
H6a1a9 | 16120 | 1 | 0.007
H6a1a9 | 16124 | 1 | 0.007
H6a1a9 | 16278 | 10 | 0.072
H6a1a9 | 16342 | 10 | 0.072
H6a1a9 | 204 | 1 | 0.007
H6a1a9 | 310 | 1 | 0.007
H6a1a9 | 6182 | 1 | 0.007
H6a1a9 | 9055 | 1 | 0.007
H6a1a9 | 942 | 1 | 0.007
H6a1b | 10373 | 3 | 0.018
H6a1b | 10490 | 3 | 0.018
H6a1b | 11255 | 1 | 0.006
H6a1b | 11440 | 3 | 0.018
H6a1b | 11914 | 1 | 0.006
H6a1b | 12241d | 1 | 0.006
H6a1b | 12519 | 4 | 0.024
H6a1b | 146 | 1 | 0.006
H6a1b | 14696 | 1 | 0.006
H6a1b | 14790 | 1 | 0.006
H6a1b | 15055 | 2 | 0.012
H6a1b | 152 | 1 | 0.006
H6a1b | 15804 | 1 | 0.006
H6a1b | 16111 | 19 | 0.112
H6a1b | 16129 | 2 | 0.012
H6a1b | 16147 | 3 | 0.018
H6a1b | 16172 | 5 | 0.03
H6a1b | 16176 | 1 | 0.006
H6a1b | 16189 | 2 | 0.012
H6a1b | 16287 | 1 | 0.006
H6a1b | 16355 | 9 | 0.053
H6a1b | 16390 | 2 | 0.012
H6a1b | 1692 | 1 | 0.006
H6a1b | 200 | 1 | 0.006
H6a1b | 207 | 1 | 0.006
H6a1b | 2246 | 1 | 0.006
H6a1b | 2283 | 1 | 0.006
H6a1b | 310 | 3 | 0.018
H6a1b | 3106A | 1 | 0.006
H6a1b | 315.2C | 1 | 0.006
H6a1b | 4092 | 1 | 0.006
H6a1b | 477 | 1 | 0.006
H6a1b | 478 | 1 | 0.006
H6a1b | 513 | 1 | 0.006
H6a1b | 5147 | 1 | 0.006
H6a1b | 5423 | 1 | 0.006
H6a1b | 6191 | 1 | 0.006
H6a1b | 7269 | 1 | 0.006
H6a1b | 73 | 14 | 0.083
H6a1b | 8047 | 1 | 0.006
H6a1b | 8238 | 1 | 0.006
H6a1b | 8686 | 1 | 0.006
H6a1b | 9016 | 1 | 0.006
H6a1b | 9554 | 1 | 0.006
H6a1b1 | 16233 | 1 | 0.071
H6a1b2 | 10310 | 1 | 0.006
H6a1b2 | 10365 | 1 | 0.006
H6a1b2 | 10422 | 1 | 0.006
H6a1b2 | 10882 | 1 | 0.006
H6a1b2 | 11047 | 1 | 0.006
H6a1b2 | 11260 | 1 | 0.006
H6a1b2 | 12397 | 1 | 0.006
H6a1b2 | 12789A | 1 | 0.006
H6a1b2 | 12810 | 1 | 0.006
H6a1b2 | 14305 | 1 | 0.006
H6a1b2 | 146 | 1 | 0.006
H6a1b2 | 152 | 4 | 0.023
H6a1b2 | 15924 | 1 | 0.006
H6a1b2 | 15930 | 1 | 0.006
H6a1b2 | 16093 | 1 | 0.006
H6a1b2 | 16126 | 1 | 0.006
H6a1b2 | 16129 | 6 | 0.034
H6a1b2 | 16153 | 1 | 0.006
H6a1b2 | 16169 | 2 | 0.011
H6a1b2 | 16223 | 2 | 0.011
H6a1b2 | 16286 | 2 | 0.011
H6a1b2 | 16290 | 1 | 0.006
H6a1b2 | 16291 | 4 | 0.023
H6a1b2 | 16311 | 2 | 0.011
H6a1b2 | 16390 | 8 | 0.045
H6a1b2 | 16526 | 1 | 0.006
H6a1b2 | 1878 | 1 | 0.006
H6a1b2 | 194 | 1 | 0.006
H6a1b2 | 195 | 1 | 0.006
H6a1b2 | 207 | 2 | 0.011
H6a1b2 | 2251 | 1 | 0.006
H6a1b2 | 310 | 3 | 0.017
H6a1b2 | 318 | 1 | 0.006
H6a1b2 | 3398 | 1 | 0.006
H6a1b2 | 3897 | 1 | 0.006
H6a1b2 | 4353 | 1 | 0.006
H6a1b2 | 44.1C | 9 | 0.051
H6a1b2 | 4562 | 1 | 0.006
H6a1b2 | 5634 | 1 | 0.006

H6a1b2 | 573.2C | 1 | 0.006
H6a1b2 | 6359 | 1 | 0.006
H6a1b2 | 6422 | 1 | 0.006
H6a1b2 | 7031 | 1 | 0.006
H6a1b2 | 7371 | 1 | 0.006
H6a1b2 | 827 | 1 | 0.006
H6a1b2 | 8435 | 1 | 0.006
H6a1b2 | 8521 | 1 | 0.006
H6a1b2 | 8605 | 1 | 0.006
H6a1b2 | 8989 | 1 | 0.006
H6a1b2 | 8994 | 1 | 0.006
H6a1b2 | 9391 | 1 | 0.006
H6a1b2 | 9478 | 1 | 0.006
H6a1b2 | 9758 | 1 | 0.006
H6a1b2 | 979 | 2 | 0.011
H6a1b2a | 14163 | 1 | 0.007
H6a1b2a | 15650 | 1 | 0.007
H6a1b2a | 16075 | 1 | 0.007
H6a1b2a | 16120 | 1 | 0.007
H6a1b2a | 16124 | 1 | 0.007
H6a1b2a | 16278 | 10 | 0.072
H6a1b2a | 16342 | 10 | 0.072
H6a1b2a | 199 | 1 | 0.007
H6a1b2a | 513 | 1 | 0.007
H6a1b2b | 16120 | 1 | 0.007
H6a1b2b | 16124 | 1 | 0.007
H6a1b2b | 16278 | 10 | 0.073
H6a1b2b | 16342 | 10 | 0.073
H6a1b2b | 199 | 1 | 0.007
H6a1b2c | 16093 | 5 | 0.035
H6a1b2c | 16120 | 1 | 0.007
H6a1b2c | 16124 | 1 | 0.007
H6a1b2c | 16157 | 1 | 0.007
H6a1b2c | 16158 | 1 | 0.007
H6a1b2c | 16173 | 2 | 0.014
H6a1b2c | 16278 | 10 | 0.069
H6a1b2c | 16342 | 10 | 0.069
H6a1b2c | 16363 | 4 | 0.028
H6a1b2c | 16364 | 4 | 0.028
H6a1b2c | 199 | 1 | 0.007
H6a1b2c | 709 | 1 | 0.007
H6a1b2c | 8939 | 1 | 0.007
H6a1b2d | 14016 | 1 | 0.071
H6a1b2d | 16188 | 2 | 0.143
H6a1b2d | 16212 | 1 | 0.071
H6a1b2d | 5634 | 1 | 0.071
H6a1b2e | 12864 | 1 | 0.007
H6a1b2e | 146 | 8 | 0.059
H6a1b2e | 16120 | 1 | 0.007
H6a1b2e | 16124 | 9 | 0.066
H6a1b2e | 16189 | 1 | 0.007
H6a1b2e | 16469 | 1 | 0.007
H6a1b2e | 302C | 1 | 0.007
H6a1b2e | 310 | 3 | 0.022
H6a1b2e | 315.2C | 1 | 0.007
H6a1b3 | 10130C | 2 | 0.074
H6a1b3 | 10754 | 1 | 0.037
H6a1b3 | 11969 | 1 | 0.037
H6a1b3 | 12030 | 2 | 0.074
H6a1b3 | 12972 | 1 | 0.037
H6a1b3 | 13683 | 1 | 0.037
H6a1b3 | 13926 | 1 | 0.037
H6a1b3 | 13941G | 1 | 0.037
H6a1b3 | 146 | 2 | 0.074
H6a1b3 | 152 | 4 | 0.148
H6a1b3 | 15618 | 1 | 0.037
H6a1b3 | 15679 | 1 | 0.037
H6a1b3 | 16195 | 1 | 0.037
H6a1b3 | 16260 | 2 | 0.074
H6a1b3 | 16274 | 1 | 0.037
H6a1b3 | 16319 | 2 | 0.074
H6a1b3 | 16356 | 1 | 0.037
H6a1b3 | 174.1C | 1 | 0.037
H6a1b3 | 198 | 1 | 0.037
H6a1b3 | 203 | 1 | 0.037
H6a1b3 | 23 | 1 | 0.037
H6a1b3 | 26 | 2 | 0.074
H6a1b3 | 2619 | 2 | 0.074
H6a1b3 | 309d | 1 | 0.037
H6a1b3 | 310 | 3 | 0.111
H6a1b3 | 4014 | 1 | 0.037
H6a1b3 | 5785 | 1 | 0.037
H6a1b3 | 619 | 2 | 0.074
H6a1b3 | 6272 | 1 | 0.037
H6a1b3 | 709 | 3 | 0.111
H6a1b3 | 7151 | 1 | 0.037
H6a1b3 | 7985G | 1 | 0.037
H6a1b3 | 8483 | 1 | 0.037
H6a1b3 | 8685 | 2 | 0.074
H6a1b3 | 93 | 5 | 0.185
H6a1b3 | 960 | 1 | 0.037
H6a1b3a | 15055 | 1 | 0.091
H6a1b3a | 15788 | 1 | 0.091
H6a1b3a | 1842 | 1 | 0.091
H6a1b3a | 1888 | 1 | 0.091
H6a1b3a | 525.1AC | 1 | 0.091
H6a1b3a | 8216 | 1 | 0.091
H6a1b3b | 146 | 2 | 0.2
H6a1b3b | 16093 | 1 | 0.1
H6a1b3b | 16482 | 1 | 0.1
H6a1b3b | 207 | 2 | 0.2
H6a1b3b | 23 | 2 | 0.2
H6a1b3b | 26 | 2 | 0.2
H6a1b3b | 4691 | 1 | 0.1
H6a1b3b | 5417 | 2 | 0.2
H6a1b3b | 93 | 2 | 0.2
H6a1b4 | 10697 | 1 | 0.02
H6a1b4 | 10908A | 1 | 0.02
H6a1b4 | 11515 | 1 | 0.02
H6a1b4 | 11779 | 1 | 0.02
H6a1b4 | 12414 | 1 | 0.02

H6a1b4 | 12843 | 6 | 0.12
H6a1b4 | 13653 | 1 | 0.02
H6a1b4 | 13947 | 1 | 0.02
H6a1b4 | 14053 | 1 | 0.02
H6a1b4 | 14180 | 1 | 0.02
H6a1b4 | 1420 | 1 | 0.02
H6a1b4 | 14488 | 1 | 0.02
H6a1b4 | 146 | 1 | 0.02
H6a1b4 | 152 | 5 | 0.1
H6a1b4 | 15565 | 1 | 0.02
H6a1b4 | 15804 | 8 | 0.16
H6a1b4 | 16129 | 2 | 0.04
H6a1b4 | 16136 | 2 | 0.04
H6a1b4 | 16189 | 2 | 0.04
H6a1b4 | 16209 | 1 | 0.02
H6a1b4 | 16296 | 2 | 0.04
H6a1b4 | 16335 | 7 | 0.14
H6a1b4 | 16390 | 1 | 0.02
H6a1b4 | 1811 | 1 | 0.02
H6a1b4 | 1888 | 1 | 0.02
H6a1b4 | 310 | 2 | 0.04
H6a1b4 | 4136 | 1 | 0.02
H6a1b4 | 479 | 1 | 0.02
H6a1b4 | 5567 | 1 | 0.02
H6a1b4 | 5665 | 1 | 0.02
H6a1b4 | 6249 | 1 | 0.02
H6a1b4 | 63 | 1 | 0.02
H6a1b4 | 64 | 1 | 0.02
H6a1b4 | 66 | 1 | 0.02
H6a1b4 | 709 | 1 | 0.02
H6a1b4 | 769 | 1 | 0.02
H6a1b4 | 8251 | 1 | 0.02
H6a1b4 | 9282A | 1 | 0.02
H6a2 | 10307 | 1 | 0.007
H6a2 | 11719 | 1 | 0.007
H6a2 | 11974 | 2 | 0.014
H6a2 | 14662 | 1 | 0.007
H6a2 | 150 | 1 | 0.007
H6a2 | 152 | 1 | 0.007
H6a2 | 15784 | 1 | 0.007
H6a2 | 16120 | 1 | 0.007
H6a2 | 16124 | 9 | 0.062
H6a2 | 5821 | 1 | 0.007
H6a2 | 8521 | 1 | 0.007
H6a2a | 152 | 1 | 0.008
H6a2a | 16124 | 1 | 0.008
H6a2a | 16221 | 1 | 0.008
H6a2a | 16224 | 7 | 0.053
H6a2a | 16357 | 9 | 0.068
H6a2a | 93 | 7 | 0.053
H6b | 12411 | 2 | 0.067
H6b | 12609 | 1 | 0.033
H6b | 1406 | 1 | 0.033
H6b | 152 | 1 | 0.033
H6b | 16093 | 3 | 0.1
H6b | 16104 | 1 | 0.033
H6b | 16145 | 1 | 0.033
H6b | 16167 | 2 | 0.067
H6b | 16172 | 4 | 0.133
H6b | 16189 | 1 | 0.033
H6b | 16234 | 2 | 0.067
H6b | 16311 | 1 | 0.033
H6b | 210 | 1 | 0.033
H6b | 324G | 1 | 0.033
H6b | 345 | 1 | 0.033
H6b | 366 | 2 | 0.067
H6b | 373 | 1 | 0.033
H6b | 3750 | 1 | 0.033
H6b | 386A | 1 | 0.033
H6b | 411G | 1 | 0.033
H6b | 414G | 1 | 0.033
H6b | 429 | 2 | 0.067
H6b | 445A | 1 | 0.033
H6b | 452A | 1 | 0.033
H6b | 476A | 1 | 0.033
H6b | 505 | 1 | 0.033
H6b | 509A | 1 | 0.033
H6b | 514-522d | 1 | 0.033
H6b | 515-524d | 1 | 0.033
H6b | 525G | 1 | 0.033
H6b | 527 | 1 | 0.033
H6b | 529d | 1 | 0.033
H6b | 534A | 1 | 0.033
H6b | 8167 | 1 | 0.033
H6b | 9587 | 2 | 0.067
H6b1 | 11353 | 1 | 0.042
H6b1 | 13155 | 2 | 0.083
H6b1 | 13933 | 2 | 0.083
H6b1 | 14180 | 1 | 0.042
H6b1 | 143 | 1 | 0.042
H6b1 | 146 | 2 | 0.083
H6b1 | 16189 | 1 | 0.042
H6b1 | 16223 | 1 | 0.042
H6b1 | 16274 | 1 | 0.042
H6b1 | 16287 | 1 | 0.042
H6b1 | 16294 | 3 | 0.125
H6b1 | 16352 | 3 | 0.125
H6b1 | 189 | 1 | 0.042
H6b1 | 204 | 3 | 0.125
H6b1 | 214 | 6 | 0.25
H6b1 | 533 | 4 | 0.167
H6b1 | 6263 | 1 | 0.042
H6b1 | 7202 | 1 | 0.042
H6b1 | 8668 | 1 | 0.042
H6b2 | 13154 | 1 | 0.02
H6b2 | 14364 | 1 | 0.02
H6b2 | 152 | 7 | 0.14
H6b2 | 15758 | 1 | 0.02
H6b2 | 16093 | 2 | 0.04
H6b2 | 16104 | 1 | 0.02
H6b2 | 16129 | 1 | 0.02

H6b2 | 16180 | 1 | 0.02
H6b2 | 16186 | 12 | 0.24
H6b2 | 16233 | 1 | 0.02
H6b2 | 16248 | 1 | 0.02
H6b2 | 16261 | 15 | 0.3
H6b2 | 16474T | 1 | 0.02
H6b2 | 189 | 2 | 0.04
H6b2 | 260 | 1 | 0.02
H6b2 | 2626 | 1 | 0.02
H6b2 | 309d | 1 | 0.02
H6b2 | 3766 | 1 | 0.02
H6b2 | 5459 | 1 | 0.02
H6b2 | 6467 | 3 | 0.06
H6b2 | 7749 | 1 | 0.02
H6b2 | 9100 | 2 | 0.04
H6b2 | 93 | 1 | 0.02
H6c | 10373 | 3 | 0.02
H6c | 10646 | 1 | 0.007
H6c | 10870 | 1 | 0.007
H6c | 11151 | 1 | 0.007
H6c | 12771 | 1 | 0.007
H6c | 12909 | 1 | 0.007
H6c | 13105 | 1 | 0.007
H6c | 14185T | 1 | 0.007
H6c | 152 | 1 | 0.007
H6c | 16134 | 9 | 0.06
H6c | 16527 | 1 | 0.007
H6c | 300C | 1 | 0.007
H6c | 310 | 4 | 0.027
H6c | 3902 | 1 | 0.007
H6c | 4940 | 1 | 0.007
H6c | 8464 | 1 | 0.007
H6c | 930 | 1 | 0.007
H6c | 9305 | 1 | 0.007
H6c1 | 16129 | 1 | 0.059
H6c1 | 16222 | 3 | 0.176
H6c1 | 2294 | 1 | 0.059
H6c1 | 9233 | 2 | 0.118
H7 | 10398 | 2 | 0.002
H7 | 10586 | 1 | 0.001
H7 | 11204 | 2 | 0.002
H7 | 113 | 1 | 0.001
H7 | 11440 | 1 | 0.001
H7 | 11497 | 1 | 0.001
H7 | 11824 | 1 | 0.001
H7 | 11875 | 1 | 0.001
H7 | 12280 | 1 | 0.001
H7 | 12603 | 1 | 0.001
H7 | 12630 | 1 | 0.001
H7 | 12843 | 1 | 0.001
H7 | 131 | 1 | 0.001
H7 | 13299 | 1 | 0.001
H7 | 13401 | 1 | 0.001
H7 | 14041 | 1 | 0.001
H7 | 14128 | 1 | 0.001
H7 | 14180 | 1 | 0.001
H7 | 1420 | 1 | 0.001
H7 | 14272G | 1 | 0.001
H7 | 14422 | 1 | 0.001
H7 | 146 | 1 | 0.001
H7 | 14890 | 2 | 0.002
H7 | 15051 | 1 | 0.001
H7 | 15601 | 1 | 0.001
H7 | 15928 | 1 | 0.001
H7 | 16076A | 1 | 0.001
H7 | 16140 | 1 | 0.001
H7 | 16169 | 1 | 0.001
H7 | 16224 | 2 | 0.002
H7 | 16249 | 1 | 0.001
H7 | 16260 | 1 | 0.001
H7 | 16272 | 1 | 0.001
H7 | 16290 | 1 | 0.001
H7 | 16311 | 3 | 0.004
H7 | 16354 | 1 | 0.001
H7 | 16355 | 124 | 0.151
H7 | 16362 | 1 | 0.001
H7 | 1693 | 1 | 0.001
H7 | 1709 | 2 | 0.002
H7 | 191C | 1 | 0.001
H7 | 208 | 1 | 0.001
H7 | 2244 | 1 | 0.001
H7 | 2581 | 1 | 0.001
H7 | 261 | 1 | 0.001
H7 | 281C | 1 | 0.001
H7 | 2831 | 1 | 0.001
H7 | 309d | 1 | 0.001
H7 | 310 | 1 | 0.001
H7 | 316d | 1 | 0.001
H7 | 3338 | 2 | 0.002
H7 | 338 | 1 | 0.001
H7 | 4742 | 1 | 0.001
H7 | 502 | 1 | 0.001
H7 | 5060 | 1 | 0.001
H7 | 5417 | 1 | 0.001
H7 | 573.1C | 4 | 0.005
H7 | 573.2C | 5 | 0.006
H7 | 573.5C | 1 | 0.001
H7 | 593 | 2 | 0.002
H7 | 6125 | 1 | 0.001
H7 | 6177 | 1 | 0.001
H7 | 6239C | 1 | 0.001
H7 | 6710 | 4 | 0.005
H7 | 6737 | 1 | 0.001
H7 | 6827 | 1 | 0.001
H7 | 6890 | 4 | 0.005
H7 | 6915 | 1 | 0.001
H7 | 72 | 2 | 0.002
H7 | 73 | 1 | 0.001
H7 | 7365 | 1 | 0.001
H7 | 8089 | 1 | 0.001
H7 | 8251 | 1 | 0.001

H7 | 8587 | 1 | 0.001
H7 | 8973 | 1 | 0.001
H7 | 9559G | 1 | 0.001
H7 | 961 | 1 | 0.001
H7 | 9629 | 1 | 0.001
H7 | 9935 | 1 | 0.001
H70 | 10775 | 1 | 0.001
H70 | 113 | 1 | 0.001
H70 | 13513 | 1 | 0.001
H70 | 14560 | 1 | 0.001
H70 | 16076A | 123 | 0.157
H70 | 16150 | 1 | 0.001
H70 | 16209 | 3 | 0.004
H70 | 16215T | 1 | 0.001
H70 | 189 | 2 | 0.003
H70 | 208 | 1 | 0.001
H70 | 261 | 1 | 0.001
H70 | 281C | 1 | 0.001
H70 | 3010 | 1 | 0.001
H70 | 3083 | 1 | 0.001
H70 | 316d | 1 | 0.001
H70 | 338 | 1 | 0.001
H70 | 468 | 3 | 0.004
H70 | 502 | 1 | 0.001
H70 | 573.1C | 3 | 0.004
H70 | 573.2C | 5 | 0.006
H70 | 573.5C | 1 | 0.001
H70 | 73 | 1 | 0.001
H70 | 9299 | 2 | 0.003
H71 | 10084 | 1 | 0.001
H71 | 10532 | 1 | 0.001
H71 | 113 | 1 | 0.001
H71 | 15885 | 1 | 0.001
H71 | 16076A | 123 | 0.158
H71 | 16086 | 1 | 0.001
H71 | 16311 | 1 | 0.001
H71 | 1692 | 1 | 0.001
H71 | 208 | 1 | 0.001
H71 | 261 | 1 | 0.001
H71 | 281C | 1 | 0.001
H71 | 316d | 1 | 0.001
H71 | 338 | 1 | 0.001
H71 | 468 | 3 | 0.004
H71 | 502 | 1 | 0.001
H71 | 573.1C | 3 | 0.004
H71 | 573.2C | 5 | 0.006
H71 | 573.5C | 1 | 0.001
H71 | 5913 | 1 | 0.001
H72 | 10750 | 1 | 0.001
H72 | 113 | 1 | 0.001
H72 | 16076A | 1 | 0.001
H72 | 16093 | 2 | 0.003
H72 | 16248 | 1 | 0.001
H72 | 16311 | 125 | 0.16
H72 | 208 | 1 | 0.001
H72 | 261 | 1 | 0.001
H72 | 281C | 1 | 0.001
H72 | 316d | 1 | 0.001
H72 | 338 | 1 | 0.001
H72 | 3834 | 1 | 0.001
H72 | 468 | 3 | 0.004
H72 | 502 | 1 | 0.001
H72 | 573.1C | 3 | 0.004
H72 | 573.2C | 5 | 0.006
H72 | 573.5C | 1 | 0.001
H73 | 113 | 1 | 0.001
H73 | 16076A | 1 | 0.001
H73 | 16213 | 123 | 0.157
H73 | 198 | 1 | 0.001
H73 | 208 | 1 | 0.001
H73 | 261 | 1 | 0.001
H73 | 281C | 1 | 0.001
H73 | 316d | 1 | 0.001
H73 | 3221 | 1 | 0.001
H73 | 338 | 1 | 0.001
H73 | 468 | 3 | 0.004
H73 | 502 | 1 | 0.001
H73 | 5147 | 1 | 0.001
H73 | 573.1C | 3 | 0.004
H73 | 573.2C | 5 | 0.006
H73 | 573.5C | 1 | 0.001
H73 | 93 | 1 | 0.001
H73 | 9857 | 1 | 0.001
H73a | 113 | 1 | 0.001
H73a | 16076A | 123 | 0.157
H73a | 208 | 1 | 0.001
H73a | 261 | 1 | 0.001
H73a | 281C | 1 | 0.001
H73a | 316d | 1 | 0.001
H73a | 338 | 1 | 0.001
H73a | 468 | 3 | 0.004
H73a | 502 | 1 | 0.001
H73a | 573.1C | 3 | 0.004
H73a | 573.2C | 5 | 0.006
H73a | 573.5C | 1 | 0.001
H73a | 960.1C | 2 | 0.003
H73a1 | 113 | 1 | 0.001
H73a1 | 11824 | 1 | 0.001
H73a1 | 16076A | 1 | 0.001
H73a1 | 16153 | 124 | 0.158
H73a1 | 208 | 1 | 0.001
H73a1 | 261 | 1 | 0.001
H73a1 | 281C | 1 | 0.001
H73a1 | 316d | 1 | 0.001
H73a1 | 338 | 1 | 0.001
H73a1 | 468 | 3 | 0.004
H73a1 | 502 | 1 | 0.001
H73a1 | 573.1C | 3 | 0.004
H73a1 | 573.2C | 5 | 0.006
H73a1 | 573.5C | 1 | 0.001
H73a1 | 7762 | 1 | 0.001

H74 | 11119 | 1 | 0.077
H74 | 13299 | 1 | 0.077
H74 | 152 | 2 | 0.154
H74 | 15891 | 1 | 0.077
H74 | 16192 | 1 | 0.077
H74 | 249 | 5 | 0.385
H74 | 6080 | 1 | 0.077
H74 | 6962 | 1 | 0.077
H74 | 8277 | 2 | 0.154
H74 | 8281-8289d | 1 | 0.077
H74 | 93 | 2 | 0.154
H74 | 9932 | 1 | 0.077
H75 | 113 | 1 | 0.001
H75 | 12286 | 1 | 0.001
H75 | 152 | 2 | 0.003
H75 | 16076A | 123 | 0.157
H75 | 208 | 1 | 0.001
H75 | 261 | 1 | 0.001
H75 | 281C | 1 | 0.001
H75 | 310 | 1 | 0.001
H75 | 315.2C | 1 | 0.001
H75 | 316d | 1 | 0.001
H75 | 338 | 1 | 0.001
H75 | 4454 | 1 | 0.001
H75 | 468 | 3 | 0.004
H75 | 4973 | 1 | 0.001
H75 | 502 | 1 | 0.001
H75 | 573.1C | 3 | 0.004
H75 | 573.2C | 5 | 0.006
H75 | 573.5C | 1 | 0.001
H75 | 6722 | 1 | 0.001
H75 | 961 | 1 | 0.001
H76 | 152 | 1 | 0.003
H76 | 16093 | 2 | 0.006
H76 | 16150 | 1 | 0.003
H76 | 16159 | 1 | 0.003
H76 | 16169 | 1 | 0.003
H76 | 16177 | 1 | 0.003
H76 | 16209 | 3 | 0.009
H76 | 16243 | 15 | 0.044
H76 | 16278 | 2 | 0.006
H76 | 16284 | 35 | 0.103
H76 | 16290 | 2 | 0.006
H76 | 16319 | 1 | 0.003
H76 | 16566 | 1 | 0.003
H76 | 195 | 1 | 0.003
H76 | 204 | 11 | 0.032
H76 | 237 | 1 | 0.003
H76 | 246 | 1 | 0.003
H76 | 288 | 1 | 0.003
H76 | 484 | 1 | 0.003
H76 | 6060 | 1 | 0.003
H76 | 8940 | 2 | 0.006
H76a | 12714 | 1 | 0.042
H76a | 15827T | 2 | 0.083
H76a | 15835 | 2 | 0.083
H76a | 15836 | 2 | 0.083
H76a | 15841 | 2 | 0.083
H76a | 15848T | 1 | 0.042
H76a | 16016 | 1 | 0.042
H76a | 16169 | 1 | 0.042
H76a | 16223 | 4 | 0.167
H76a | 16224 | 1 | 0.042
H76a | 16475G | 1 | 0.042
H76a | 16484-16489d | 1 | 0.042
H76a | 16505 | 2 | 0.083
H76a | 257 | 1 | 0.042
H76a | 341 | 1 | 0.042
H76a | 573.1C | 1 | 0.042
H76a | 8152 | 1 | 0.042
H76a | 8276G | 1 | 0.042
H76a | 9738 | 1 | 0.042
H77 | 16104 | 1 | 0.011
H77 | 16111 | 1 | 0.011
H77 | 16124 | 1 | 0.011
H77 | 16319 | 1 | 0.011
H77 | 165 | 1 | 0.011
H77 | 199 | 7 | 0.079
H78 | 113 | 1 | 0.001
H78 | 12115 | 1 | 0.001
H78 | 146 | 2 | 0.003
H78 | 16076A | 123 | 0.157
H78 | 16240 | 2 | 0.003
H78 | 16311 | 1 | 0.001
H78 | 208 | 1 | 0.001
H78 | 261 | 1 | 0.001
H78 | 281C | 1 | 0.001
H78 | 316d | 1 | 0.001
H78 | 338 | 1 | 0.001
H78 | 3411 | 2 | 0.003
H78 | 4243 | 1 | 0.001
H78 | 468 | 3 | 0.004
H78 | 502 | 1 | 0.001
H78 | 5291 | 1 | 0.001
H78 | 573.1C | 3 | 0.004
H78 | 573.2C | 5 | 0.006
H78 | 573.5C | 1 | 0.001
H78 | 711 | 1 | 0.001
H78 | 9077 | 1 | 0.001
H79 | 113 | 1 | 0.001
H79 | 12123 | 1 | 0.001
H79 | 16076A | 123 | 0.157
H79 | 16286 | 1 | 0.001
H79 | 208 | 1 | 0.001
H79 | 261 | 1 | 0.001
H79 | 281C | 1 | 0.001
H79 | 316d | 1 | 0.001
H79 | 338 | 1 | 0.001
H79 | 468 | 3 | 0.004
H79 | 502 | 1 | 0.001
H79 | 5147 | 1 | 0.001

H79 | 573.1C | 3 | 0.004
H79 | 573.2C | 5 | 0.006
H79 | 573.5C | 1 | 0.001
H79 | 5899.1C | 1 | 0.001
H79 | 6347 | 4 | 0.005
H79 | 6960 | 1 | 0.001
H79 | 9325 | 1 | 0.001
H79a | 113 | 1 | 0.001
H79a | 14668 | 1 | 0.001
H79a | 16076A | 123 | 0.158
H79a | 208 | 1 | 0.001
H79a | 261 | 1 | 0.001
H79a | 281C | 1 | 0.001
H79a | 316d | 1 | 0.001
H79a | 338 | 1 | 0.001
H79a | 468 | 3 | 0.004
H79a | 502 | 1 | 0.001
H79a | 573.1C | 3 | 0.004
H79a | 573.2C | 5 | 0.006
H79a | 573.5C | 1 | 0.001
H79a | 8425 | 1 | 0.001
H7a | 113 | 1 | 0.001
H7a | 11377 | 1 | 0.001
H7a | 146 | 1 | 0.001
H7a | 15884 | 1 | 0.001
H7a | 16076A | 123 | 0.154
H7a | 16093 | 1 | 0.001
H7a | 16124 | 1 | 0.001
H7a | 16189 | 1 | 0.001
H7a | 16223 | 1 | 0.001
H7a | 16255 | 1 | 0.001
H7a | 16278 | 1 | 0.001
H7a | 199 | 1 | 0.001
H7a | 208 | 1 | 0.001
H7a | 261 | 1 | 0.001
H7a | 281C | 1 | 0.001
H7a | 316d | 1 | 0.001
H7a | 338 | 1 | 0.001
H7a | 468 | 3 | 0.004
H7a | 502 | 1 | 0.001
H7a | 573.1C | 3 | 0.004
H7a | 573.2C | 5 | 0.006
H7a | 573.5C | 1 | 0.001
H7a | 6038 | 1 | 0.001
H7a | 6296A | 1 | 0.001
H7a | 9878 | 1 | 0.001
H7a1 | 106-111d | 1 | 0.009
H7a1 | 10736 | 1 | 0.009
H7a1 | 11914 | 1 | 0.009
H7a1 | 12063 | 1 | 0.009
H7a1 | 12654 | 1 | 0.009
H7a1 | 12966A | 1 | 0.009
H7a1 | 13651 | 1 | 0.009
H7a1 | 14163 | 1 | 0.009
H7a1 | 14180 | 1 | 0.009
H7a1 | 15939 | 1 | 0.009
H7a1 | 1607 | 1 | 0.009
H7a1 | 16093 | 2 | 0.017
H7a1 | 16126 | 2 | 0.017
H7a1 | 16189 | 5 | 0.043
H7a1 | 16220C | 1 | 0.009
H7a1 | 16234 | 1 | 0.009
H7a1 | 16286 | 2 | 0.017
H7a1 | 16311 | 1 | 0.009
H7a1 | 16312 | 1 | 0.009
H7a1 | 16325 | 5 | 0.043
H7a1 | 16365 | 1 | 0.009
H7a1 | 1824 | 1 | 0.009
H7a1 | 1861 | 1 | 0.009
H7a1 | 188 | 1 | 0.009
H7a1 | 194 | 1 | 0.009
H7a1 | 204 | 3 | 0.026
H7a1 | 2558 | 1 | 0.009
H7a1 | 2581 | 1 | 0.009
H7a1 | 2757 | 1 | 0.009
H7a1 | 309.3C | 1 | 0.009
H7a1 | 310 | 4 | 0.035
H7a1 | 315.2C | 2 | 0.017
H7a1 | 3244 | 1 | 0.009
H7a1 | 3531 | 1 | 0.009
H7a1 | 5460 | 1 | 0.009
H7a1 | 6285 | 1 | 0.009
H7a1 | 709 | 1 | 0.009
H7a1 | 8344 | 1 | 0.009
H7a1 | 8762 | 1 | 0.009
H7a1 | 8864 | 1 | 0.009
H7a1 | 9053 | 1 | 0.009
H7a1 | 9329 | 1 | 0.009
H7a1a | 11016 | 3 | 0.064
H7a1a | 13466 | 1 | 0.021
H7a1a | 13896 | 3 | 0.064
H7a1a | 15930 | 1 | 0.021
H7a1a | 16249 | 1 | 0.021
H7a1a | 16270 | 2 | 0.043
H7a1a | 16295 | 1 | 0.021
H7a1a | 16304 | 10 | 0.213
H7a1a | 16390 | 7 | 0.149
H7a1a | 3613 | 2 | 0.043
H7a1a | 5417 | 3 | 0.064
H7a1a | 742 | 1 | 0.021
H7a1a | 8022 | 3 | 0.064
H7a1a | 8110 | 1 | 0.021
H7a1b | 10931 | 1 | 0.01
H7a1b | 11151 | 1 | 0.01
H7a1b | 11318A | 2 | 0.019
H7a1b | 11377 | 2 | 0.019
H7a1b | 12223 | 1 | 0.01
H7a1b | 13835 | 1 | 0.01
H7a1b | 152 | 1 | 0.01
H7a1b | 16189 | 1 | 0.01
H7a1b | 16266 | 3 | 0.029

H7a1b | 16294 | 2 | 0.019
H7a1b | 16380 | 1 | 0.01
H7a1b | 3058 | 1 | 0.01
H7a1b | 310 | 1 | 0.01
H7a1b | 60 | 1 | 0.01
H7a1b | 73 | 1 | 0.01
H7a1b | 8002 | 1 | 0.01
H7a1b | 8395 | 2 | 0.019
H7a1b | 8752 | 3 | 0.029
H7a1b | 89 | 1 | 0.01
H7a1b | 9055 | 1 | 0.01
H7a1b | 93 | 2 | 0.019
H7a1c | 16129 | 1 | 0.011
H7a1c | 16145 | 2 | 0.021
H7a1c | 16209 | 1 | 0.011
H7a1c | 16219 | 1 | 0.011
H7a1c | 16311 | 1 | 0.011
H7a1c | 16344 | 1 | 0.011
H7a1c | 6407 | 1 | 0.011
H7a1c | 6950 | 1 | 0.011
H7a1c | 7859 | 1 | 0.011
H7a1d | 151 | 2 | 0.021
H7a1d | 16048 | 1 | 0.01
H7a1d | 16129 | 1 | 0.01
H7a1d | 16219 | 1 | 0.01
H7a1d | 16249 | 1 | 0.01
H7a1d | 16270 | 1 | 0.01
H7a1d | 16318T | 2 | 0.021
H7a1d | 16344 | 1 | 0.01
H7a1d | 16516 | 1 | 0.01
H7a1d | 326 | 1 | 0.01
H7a1d | 5557 | 1 | 0.01
H7a1d | 5580 | 1 | 0.01
H7a1d | 8287.1C | 1 | 0.01
H7a2 | 1007 | 1 | 0.013
H7a2 | 150 | 1 | 0.013
H7a2 | 152 | 2 | 0.027
H7a2 | 16169 | 1 | 0.013
H7a2 | 16357 | 1 | 0.013
H7a2 | 200 | 1 | 0.013
H7a2 | 249d | 1 | 0.013
H7a2 | 6947 | 1 | 0.013
H7a2 | 8289.1CCCCCTCTA | 1 | 0.013
H7b | 113 | 1 | 0.001
H7b | 11884 | 1 | 0.001
H7b | 11914 | 1 | 0.001
H7b | 12250 | 2 | 0.002
H7b | 12471 | 1 | 0.001
H7b | 12501 | 1 | 0.001
H7b | 12946 | 1 | 0.001
H7b | 13708 | 2 | 0.002
H7b | 13935T | 1 | 0.001
H7b | 14119 | 1 | 0.001
H7b | 143 | 1 | 0.001
H7b | 14514 | 2 | 0.002
H7b | 14783 | 1 | 0.001
H7b | 14905 | 1 | 0.001
H7b | 1503 | 2 | 0.002
H7b | 152 | 3 | 0.004
H7b | 15607 | 1 | 0.001
H7b | 16076A | 123 | 0.15
H7b | 16193 | 1 | 0.001
H7b | 16217 | 1 | 0.001
H7b | 16223 | 2 | 0.002
H7b | 16295 | 1 | 0.001
H7b | 16311 | 1 | 0.001
H7b | 16356 | 2 | 0.002
H7b | 195 | 2 | 0.002
H7b | 208 | 1 | 0.001
H7b | 261 | 1 | 0.001
H7b | 2626 | 2 | 0.002
H7b | 281C | 1 | 0.001
H7b | 291.2A | 5 | 0.006
H7b | 309d | 1 | 0.001
H7b | 310 | 1 | 0.001
H7b | 316d | 1 | 0.001
H7b | 338 | 1 | 0.001
H7b | 4245 | 2 | 0.002
H7b | 468 | 3 | 0.004
H7b | 502 | 1 | 0.001
H7b | 5196 | 1 | 0.001
H7b | 5263 | 1 | 0.001
H7b | 5585 | 1 | 0.001
H7b | 573.1C | 7 | 0.009
H7b | 573.2C | 5 | 0.006
H7b | 573.5C | 1 | 0.001
H7b | 7034 | 1 | 0.001
H7b | 73 | 5 | 0.006
H7b | 7471d | 2 | 0.002
H7b | 8022 | 2 | 0.002
H7b | 8023 | 1 | 0.001
H7b | 8251 | 1 | 0.001
H7b | 8388 | 1 | 0.001
H7b | 8697 | 1 | 0.001
H7b | 8877 | 2 | 0.002
H7b | 9661 | 1 | 0.001
H7b | 9722 | 2 | 0.002
H7b1 | 10245 | 1 | 0.001
H7b1 | 10365 | 1 | 0.001
H7b1 | 10969 | 1 | 0.001
H7b1 | 113 | 1 | 0.001
H7b1 | 12019 | 1 | 0.001
H7b1 | 12630 | 1 | 0.001
H7b1 | 13266C | 1 | 0.001
H7b1 | 13347 | 1 | 0.001
H7b1 | 14053 | 1 | 0.001
H7b1 | 152 | 1 | 0.001
H7b1 | 15297 | 1 | 0.001
H7b1 | 15467 | 1 | 0.001
H7b1 | 16042 | 2 | 0.002
H7b1 | 16076A | 123 | 0.153

H7b1 | 16093 | 1 | 0.001
H7b1 | 16189 | 4 | 0.005
H7b1 | 16221 | 1 | 0.001
H7b1 | 16257 | 1 | 0.001
H7b1 | 16295 | 3 | 0.004
H7b1 | 208 | 1 | 0.001
H7b1 | 261 | 1 | 0.001
H7b1 | 2756A | 1 | 0.001
H7b1 | 281C | 1 | 0.001
H7b1 | 310 | 2 | 0.002
H7b1 | 316d | 1 | 0.001
H7b1 | 3278 | 1 | 0.001
H7b1 | 338 | 1 | 0.001
H7b1 | 468 | 3 | 0.004
H7b1 | 4802 | 1 | 0.001
H7b1 | 502 | 1 | 0.001
H7b1 | 573.1C | 3 | 0.004
H7b1 | 573.2C | 5 | 0.006
H7b1 | 573.5C | 1 | 0.001
H7b1 | 6464 | 1 | 0.001
H7b1 | 7757 | 1 | 0.001
H7b1 | 7979 | 1 | 0.001
H7b1 | 8958 | 1 | 0.001
H7b1 | 9152 | 3 | 0.004
H7b2 | 10007 | 1 | 0.001
H7b2 | 10876 | 1 | 0.001
H7b2 | 113 | 1 | 0.001
H7b2 | 14007 | 1 | 0.001
H7b2 | 14180 | 1 | 0.001
H7b2 | 1555 | 1 | 0.001
H7b2 | 16076A | 1 | 0.001
H7b2 | 16189 | 1 | 0.001
H7b2 | 16209 | 123 | 0.154
H7b2 | 16320 | 1 | 0.001
H7b2 | 199 | 1 | 0.001
H7b2 | 208 | 1 | 0.001
H7b2 | 261 | 1 | 0.001
H7b2 | 281C | 1 | 0.001
H7b2 | 316d | 1 | 0.001
H7b2 | 338 | 1 | 0.001
H7b2 | 468 | 3 | 0.004
H7b2 | 502 | 1 | 0.001
H7b2 | 5580 | 1 | 0.001
H7b2 | 570 | 1 | 0.001
H7b2 | 573.1C | 3 | 0.004
H7b2 | 573.2C | 5 | 0.006
H7b2 | 573.5C | 1 | 0.001
H7b2 | 6671 | 1 | 0.001
H7b2 | 8603 | 1 | 0.001
H7b2 | 8623 | 1 | 0.001
H7b2a | 113 | 1 | 0.001
H7b2a | 16076A | 123 | 0.156
H7b2a | 16278 | 1 | 0.001
H7b2a | 189 | 2 | 0.003
H7b2a | 194 | 1 | 0.001
H7b2a | 208 | 1 | 0.001
H7b2a | 261 | 1 | 0.001
H7b2a | 281C | 1 | 0.001
H7b2a | 316d | 1 | 0.001
H7b2a | 338 | 1 | 0.001
H7b2a | 468 | 3 | 0.004
H7b2a | 502 | 1 | 0.001
H7b2a | 573.1C | 3 | 0.004
H7b2a | 573.2C | 5 | 0.006
H7b2a | 573.5C | 1 | 0.001
H7b2a | 8817 | 1 | 0.001
H7b3 | 113 | 1 | 0.001
H7b3 | 13707 | 1 | 0.001
H7b3 | 16076A | 123 | 0.156
H7b3 | 208 | 1 | 0.001
H7b3 | 261 | 1 | 0.001
H7b3 | 281C | 1 | 0.001
H7b3 | 316d | 1 | 0.001
H7b3 | 338 | 1 | 0.001
H7b3 | 468 | 3 | 0.004
H7b3 | 502 | 1 | 0.001
H7b3 | 573.1C | 3 | 0.004
H7b3 | 573.2C | 5 | 0.006
H7b3 | 573.5C | 1 | 0.001
H7b3 | 7184 | 1 | 0.001
H7b4 | 113 | 1 | 0.001
H7b4 | 16076A | 123 | 0.155
H7b4 | 16311 | 1 | 0.001
H7b4 | 208 | 1 | 0.001
H7b4 | 261 | 1 | 0.001
H7b4 | 281C | 1 | 0.001
H7b4 | 316d | 1 | 0.001
H7b4 | 338 | 1 | 0.001
H7b4 | 468 | 3 | 0.004
H7b4 | 502 | 1 | 0.001
H7b4 | 573.1C | 4 | 0.005
H7b4 | 573.2C | 5 | 0.006
H7b4 | 573.5C | 1 | 0.001
H7b4 | 6185 | 1 | 0.001
H7b4 | 7205 | 1 | 0.001
H7b5 | 113 | 1 | 0.001
H7b5 | 16076A | 1 | 0.001
H7b5 | 16311 | 123 | 0.156
H7b5 | 208 | 1 | 0.001
H7b5 | 261 | 1 | 0.001
H7b5 | 281C | 1 | 0.001
H7b5 | 316d | 1 | 0.001
H7b5 | 338 | 1 | 0.001
H7b5 | 468 | 3 | 0.004
H7b5 | 502 | 1 | 0.001
H7b5 | 573.1C | 3 | 0.004
H7b5 | 573.2C | 5 | 0.006
H7b5 | 573.5C | 1 | 0.001
H7b6 | 113 | 1 | 0.001
H7b6 | 114 | 123 | 0.162
H7b6 | 14527 | 1 | 0.001

H7b6 | 16076A | 1 | 0.001
H7b6 | 16174 | 1 | 0.001
H7b6 | 16318C | 123 | 0.162
H7b6 | 203C | 6 | 0.008
H7b6 | 208 | 1 | 0.001
H7b6 | 214 | 1 | 0.001
H7b6 | 228T | 2 | 0.003
H7b6 | 252 | 1 | 0.001
H7b6 | 261 | 1 | 0.001
H7b6 | 281C | 1 | 0.001
H7b6 | 316d | 1 | 0.001
H7b6 | 338 | 1 | 0.001
H7b6 | 41 | 2 | 0.003
H7b6 | 414G | 124 | 0.164
H7b6 | 468 | 3 | 0.004
H7b6 | 502 | 1 | 0.001
H7b6 | 5072 | 1 | 0.001
H7b6 | 573.1C | 2 | 0.003
H7b6 | 573.2C | 4 | 0.005
H7b6 | 573.5C | 1 | 0.001
H7b6 | 8882 | 1 | 0.001
H7c | 113 | 1 | 0.001
H7c | 146 | 1 | 0.001
H7c | 15115 | 1 | 0.001
H7c | 15884 | 1 | 0.001
H7c | 16076A | 1 | 0.001
H7c | 16093 | 123 | 0.156
H7c | 16124 | 1 | 0.001
H7c | 16319 | 123 | 0.156
H7c | 1719 | 1 | 0.001
H7c | 208 | 1 | 0.001
H7c | 261 | 1 | 0.001
H7c | 281C | 1 | 0.001
H7c | 316d | 1 | 0.001
H7c | 338 | 1 | 0.001
H7c | 468 | 3 | 0.004
H7c | 502 | 1 | 0.001
H7c | 573.1C | 3 | 0.004
H7c | 573.2C | 5 | 0.006
H7c | 573.5C | 1 | 0.001
H7c | 8516 | 1 | 0.001
H7c1 | 11299 | 2 | 0.017
H7c1 | 11499 | 1 | 0.009
H7c1 | 11914 | 1 | 0.009
H7c1 | 13389 | 1 | 0.009
H7c1 | 14831 | 1 | 0.009
H7c1 | 14869C | 1 | 0.009
H7c1 | 150 | 2 | 0.017
H7c1 | 151 | 3 | 0.026
H7c1 | 152 | 5 | 0.043
H7c1 | 15758 | 1 | 0.009
H7c1 | 1598 | 1 | 0.009
H7c1 | 16067 | 2 | 0.017
H7c1 | 16093 | 31 | 0.267
H7c1 | 16169 | 1 | 0.009
H7c1 | 16179 | 1 | 0.009
H7c1 | 16181 | 1 | 0.009
H7c1 | 16184 | 3 | 0.026
H7c1 | 16263 | 3 | 0.026
H7c1 | 16271 | 1 | 0.009
H7c1 | 16298 | 4 | 0.034
H7c1 | 16304 | 1 | 0.009
H7c1 | 16311 | 3 | 0.026
H7c1 | 16333d | 1 | 0.009
H7c1 | 1909 | 1 | 0.009
H7c1 | 198 | 1 | 0.009
H7c1 | 207 | 2 | 0.017
H7c1 | 4224 | 2 | 0.017
H7c1 | 5210 | 1 | 0.009
H7c1 | 5601 | 1 | 0.009
H7c1 | 573.2C | 1 | 0.009
H7c1 | 5899.1C | 1 | 0.009
H7c1 | 73 | 1 | 0.009
H7c1 | 7822 | 1 | 0.009
H7c1 | 8395 | 1 | 0.009
H7c1 | 8503 | 1 | 0.009
H7c1 | 8702A | 1 | 0.009
H7c2 | 113 | 1 | 0.001
H7c2 | 16076A | 1 | 0.001
H7c2 | 16189 | 1 | 0.001
H7c2 | 16311 | 123 | 0.155
H7c2 | 208 | 1 | 0.001
H7c2 | 261 | 1 | 0.001
H7c2 | 281C | 1 | 0.001
H7c2 | 316d | 1 | 0.001
H7c2 | 338 | 1 | 0.001
H7c2 | 468 | 3 | 0.004
H7c2 | 5001 | 1 | 0.001
H7c2 | 502 | 1 | 0.001
H7c2 | 573.1C | 3 | 0.004
H7c2 | 573.2C | 5 | 0.006
H7c2 | 573.5C | 1 | 0.001
H7c2 | 8227 | 1 | 0.001
H7c3 | 10915 | 1 | 0.001
H7c3 | 113 | 1 | 0.001
H7c3 | 16076A | 1 | 0.001
H7c3 | 16093 | 125 | 0.158
H7c3 | 195 | 1 | 0.001
H7c3 | 208 | 1 | 0.001
H7c3 | 261 | 1 | 0.001
H7c3 | 281C | 1 | 0.001
H7c3 | 310 | 1 | 0.001
H7c3 | 315.2C | 1 | 0.001
H7c3 | 316d | 1 | 0.001
H7c3 | 338 | 1 | 0.001
H7c3 | 468 | 3 | 0.004
H7c3 | 502 | 1 | 0.001
H7c3 | 573.1C | 3 | 0.004
H7c3 | 573.2C | 5 | 0.006
H7c3 | 573.5C | 1 | 0.001
H7c3 | 6249 | 1 | 0.001

H7c4 | 11176 | 1 | 0.014
H7c4 | 12141 | 1 | 0.014
H7c4 | 150 | 1 | 0.014
H7c4 | 152 | 2 | 0.029
H7c4 | 16086 | 1 | 0.014
H7c4 | 16129 | 1 | 0.014
H7c4 | 16189 | 1 | 0.014
H7c4 | 16291 | 1 | 0.014
H7c4 | 16311 | 2 | 0.029
H7c4 | 316 | 2 | 0.029
H7c4 | 3705 | 1 | 0.014
H7c4 | 72 | 2 | 0.029
H7c4 | 75 | 1 | 0.014
H7c4 | 8020 | 1 | 0.014
H7c4 | 94 | 1 | 0.014
H7c4 | 9770 | 1 | 0.014
H7c5 | 10978 | 1 | 0.001
H7c5 | 11204 | 1 | 0.001
H7c5 | 113 | 1 | 0.001
H7c5 | 13105 | 1 | 0.001
H7c5 | 16076A | 123 | 0.156
H7c5 | 208 | 1 | 0.001
H7c5 | 261 | 1 | 0.001
H7c5 | 281C | 1 | 0.001
H7c5 | 316d | 1 | 0.001
H7c5 | 338 | 1 | 0.001
H7c5 | 468 | 3 | 0.004
H7c5 | 502 | 1 | 0.001
H7c5 | 573.1C | 3 | 0.004
H7c5 | 573.2C | 5 | 0.006
H7c5 | 573.5C | 1 | 0.001
H7c6 | 10907 | 1 | 0.001
H7c6 | 113 | 1 | 0.001
H7c6 | 11347 | 3 | 0.004
H7c6 | 146 | 1 | 0.001
H7c6 | 152 | 1 | 0.001
H7c6 | 16076A | 123 | 0.156
H7c6 | 16266 | 3 | 0.004
H7c6 | 208 | 1 | 0.001
H7c6 | 261 | 1 | 0.001
H7c6 | 281C | 1 | 0.001
H7c6 | 310 | 1 | 0.001
H7c6 | 316d | 1 | 0.001
H7c6 | 338 | 1 | 0.001
H7c6 | 468 | 3 | 0.004
H7c6 | 502 | 1 | 0.001
H7c6 | 573.1C | 3 | 0.004
H7c6 | 573.2C | 5 | 0.006
H7c6 | 573.5C | 1 | 0.001
H7d | 113 | 1 | 0.001
H7d | 11778 | 1 | 0.001
H7d | 13359 | 1 | 0.001
H7d | 13477 | 1 | 0.001
H7d | 14207 | 1 | 0.001
H7d | 152 | 1 | 0.001
H7d | 16076A | 1 | 0.001
H7d | 16080 | 24 | 0.03
H7d | 16169 | 1 | 0.001
H7d | 16388 | 123 | 0.151
H7d | 208 | 1 | 0.001
H7d | 261 | 1 | 0.001
H7d | 281C | 1 | 0.001
H7d | 310 | 3 | 0.004
H7d | 316d | 1 | 0.001
H7d | 338 | 1 | 0.001
H7d | 468 | 3 | 0.004
H7d | 502 | 1 | 0.001
H7d | 573.1C | 3 | 0.004
H7d | 573.2C | 5 | 0.006
H7d | 573.5C | 1 | 0.001
H7d | 596 | 1 | 0.001
H7d1 | 113 | 1 | 0.001
H7d1 | 12441 | 1 | 0.001
H7d1 | 13005 | 1 | 0.001
H7d1 | 13681 | 1 | 0.001
H7d1 | 16076A | 1 | 0.001
H7d1 | 16145 | 123 | 0.156
H7d1 | 208 | 1 | 0.001
H7d1 | 261 | 1 | 0.001
H7d1 | 281C | 1 | 0.001
H7d1 | 316d | 1 | 0.001
H7d1 | 338 | 1 | 0.001
H7d1 | 4655 | 1 | 0.001
H7d1 | 468 | 3 | 0.004
H7d1 | 502 | 1 | 0.001
H7d1 | 573.1C | 3 | 0.004
H7d1 | 573.2C | 5 | 0.006
H7d1 | 573.5C | 1 | 0.001
H7d1 | 8255 | 2 | 0.003
H7d1 | 9656 | 1 | 0.001
H7d2 | 15866 | 2 | 0.022
H7d2 | 16104 | 1 | 0.011
H7d2 | 16111 | 1 | 0.011
H7d2 | 16124 | 1 | 0.011
H7d2 | 16179 | 3 | 0.033
H7d2 | 16294 | 1 | 0.011
H7d2 | 16335 | 4 | 0.044
H7d2 | 165 | 1 | 0.011
H7d2 | 237 | 3 | 0.033
H7d2 | 6719 | 1 | 0.011
H7d2a | 16104 | 1 | 0.011
H7d2a | 16111 | 1 | 0.011
H7d2a | 16124 | 1 | 0.011
H7d2a | 16179 | 3 | 0.033
H7d2a | 16311 | 1 | 0.011
H7d2a | 16335 | 4 | 0.044
H7d2a | 165 | 1 | 0.011
H7d2a | 237 | 3 | 0.033
H7d2a | 8972G | 1 | 0.011
H7d3 | 113 | 1 | 0.001
H7d3 | 16076A | 1 | 0.001

H7d3 | 16153 | 124 | 0.158
H7d3 | 208 | 1 | 0.001
H7d3 | 261 | 1 | 0.001
H7d3 | 281C | 1 | 0.001
H7d3 | 316d | 1 | 0.001
H7d3 | 338 | 1 | 0.001
H7d3 | 468 | 3 | 0.004
H7d3 | 502 | 1 | 0.001
H7d3 | 573.1C | 3 | 0.004
H7d3 | 573.2C | 5 | 0.006
H7d3 | 573.5C | 1 | 0.001
H7d3a | 113 | 1 | 0.001
H7d3a | 11878 | 1 | 0.001
H7d3a | 150 | 1 | 0.001
H7d3a | 16076A | 123 | 0.156
H7d3a | 208 | 1 | 0.001
H7d3a | 261 | 1 | 0.001
H7d3a | 281C | 1 | 0.001
H7d3a | 316d | 1 | 0.001
H7d3a | 338 | 1 | 0.001
H7d3a | 468 | 3 | 0.004
H7d3a | 502 | 1 | 0.001
H7d3a | 573.1C | 3 | 0.004
H7d3a | 573.2C | 5 | 0.006
H7d3a | 573.5C | 1 | 0.001
H7d3a | 6216 | 1 | 0.001
H7d3a | 8784 | 1 | 0.001
H7d4 | 113 | 1 | 0.001
H7d4 | 12007 | 1 | 0.001
H7d4 | 16076A | 1 | 0.001
H7d4 | 16234 | 123 | 0.156
H7d4 | 208 | 1 | 0.001
H7d4 | 261 | 1 | 0.001
H7d4 | 281C | 1 | 0.001
H7d4 | 316d | 1 | 0.001
H7d4 | 338 | 1 | 0.001
H7d4 | 468 | 3 | 0.004
H7d4 | 502 | 1 | 0.001
H7d4 | 573.1C | 3 | 0.004
H7d4 | 573.2C | 5 | 0.006
H7d4 | 573.5C | 1 | 0.001
H7d5 | 16189 | 1 | 0.016
H7d5 | 3396 | 1 | 0.016
H7d5 | 502 | 1 | 0.016
H7e | 11260 | 1 | 0.001
H7e | 113 | 1 | 0.001
H7e | 11776 | 1 | 0.001
H7e | 11890 | 2 | 0.002
H7e | 1192 | 1 | 0.001
H7e | 12651 | 4 | 0.005
H7e | 13828 | 1 | 0.001
H7e | 14129 | 1 | 0.001
H7e | 1536 | 1 | 0.001
H7e | 16076A | 1 | 0.001
H7e | 16218 | 1 | 0.001
H7e | 16230 | 123 | 0.152
H7e | 16305 | 1 | 0.001
H7e | 208 | 1 | 0.001
H7e | 2222 | 1 | 0.001
H7e | 261 | 1 | 0.001
H7e | 281C | 1 | 0.001
H7e | 292.1AT | 1 | 0.001
H7e | 316d | 1 | 0.001
H7e | 338 | 1 | 0.001
H7e | 4093 | 1 | 0.001
H7e | 468 | 3 | 0.004
H7e | 502 | 1 | 0.001
H7e | 573.1C | 3 | 0.004
H7e | 573.2C | 5 | 0.006
H7e | 573.5C | 1 | 0.001
H7e | 8027 | 2 | 0.002
H7e | 8994 | 3 | 0.004
H7e | 9596 | 1 | 0.001
H7e | 9932 | 1 | 0.001
H7f | 10398T | 1 | 0.014
H7f | 12737G | 1 | 0.014
H7f | 13708 | 1 | 0.014
H7f | 15043 | 1 | 0.014
H7f | 15758 | 1 | 0.014
H7f | 16176 | 3 | 0.041
H7f | 16189 | 1 | 0.014
H7f | 16195 | 1 | 0.014
H7f | 16265C | 1 | 0.014
H7f | 16325 | 1 | 0.014
H7f | 16362 | 1 | 0.014
H7f | 1809 | 1 | 0.014
H7f | 4185 | 1 | 0.014
H7f | 573.2C | 1 | 0.014
H7f | 73 | 2 | 0.027
H7f | 7861 | 1 | 0.014
H7f | 9041 | 1 | 0.014
H7f | 9324 | 1 | 0.014
H7g | 113 | 1 | 0.001
H7g | 16076A | 123 | 0.155
H7g | 208 | 1 | 0.001
H7g | 261 | 1 | 0.001
H7g | 281C | 1 | 0.001
H7g | 309d | 5 | 0.006
H7g | 316d | 1 | 0.001
H7g | 338 | 1 | 0.001
H7g | 468 | 3 | 0.004
H7g | 502 | 1 | 0.001
H7g | 573.1C | 3 | 0.004
H7g | 573.2C | 5 | 0.006
H7g | 573.5C | 1 | 0.001
H7h | 10057 | 1 | 0.012
H7h | 11025 | 1 | 0.012
H7h | 13005 | 1 | 0.012
H7h | 146 | 1 | 0.012
H7h | 15301 | 1 | 0.012
H7h | 16037 | 1 | 0.012

H7h | 16172 | 1 | 0.012
H7h | 16175 | 1 | 0.012
H7h | 16189 | 1 | 0.012
H7h | 16240 | 1 | 0.012
H7h | 16294 | 1 | 0.012
H7h | 16354 | 1 | 0.012
H7h | 200 | 1 | 0.012
H7h | 299d | 1 | 0.012
H7h | 8247 | 1 | 0.012
H7h | 93 | 3 | 0.038
H7h1 | 13105 | 1 | 0.012
H7h1 | 14560 | 1 | 0.012
H7h1 | 16037 | 1 | 0.012
H7h1 | 16172 | 1 | 0.012
H7h1 | 16175 | 1 | 0.012
H7h1 | 16189 | 1 | 0.012
H7h1 | 16240 | 1 | 0.012
H7h1 | 16294 | 1 | 0.012
H7h1 | 16354 | 1 | 0.012
H7h1 | 16362 | 2 | 0.025
H7h1 | 1719 | 1 | 0.012
H7h1 | 185 | 1 | 0.012
H7h1 | 195 | 1 | 0.012
H7h1 | 93 | 3 | 0.037
H7i | 16086 | 3 | 0.103
H7i | 16092 | 1 | 0.034
H7i | 16129 | 1 | 0.034
H7i | 16158 | 6 | 0.207
H7i | 16188G | 7 | 0.241
H7i | 16193 | 6 | 0.207
H7i | 16264 | 1 | 0.034
H7i | 16298 | 1 | 0.034
H7i | 16311 | 1 | 0.034
H7i | 16319 | 1 | 0.034
H7i | 185 | 1 | 0.034
H7i | 3873 | 1 | 0.034
H7i1 | 15910 | 2 | 0.154
H7i1 | 16209 | 1 | 0.077
H7i1 | 16213 | 1 | 0.077
H7i1 | 16311 | 3 | 0.231
H7i1 | 309d | 1 | 0.077
H7i1 | 310 | 1 | 0.077
H7i1 | 315.2C | 1 | 0.077
H7i1 | 4435 | 1 | 0.077
H7i1 | 64 | 1 | 0.077
H7i1 | 65 | 1 | 0.077
H7i1 | 709 | 1 | 0.077
H7i1 | 9337 | 1 | 0.077
H7i1 | 988T | 1 | 0.077
H8 | 16068 | 4 | 0.333
H8 | 225 | 1 | 0.083
H8 | 4820 | 1 | 0.083
H8 | 9196 | 1 | 0.083
H8+(114) | 114 | 1 | 0.111
H8+(114) | 16093 | 2 | 0.222
H8+(114) | 16263 | 2 | 0.222
H8+(114)+152 | 14198 | 1 | 0.083
H8+(114)+152 | 143 | 1 | 0.083
H8+(114)+152 | 16145 | 1 | 0.083
H8+(114)+152 | 16221 | 1 | 0.083
H8+(114)+152 | 16265 | 1 | 0.083
H8+(114)+152 | 16317 | 1 | 0.083
H8+(114)+152 | 319 | 2 | 0.167
H80 | 12788G | 1 | 0.003
H80 | 13943 | 2 | 0.006
H80 | 16150 | 1 | 0.003
H80 | 16159 | 1 | 0.003
H80 | 16177 | 1 | 0.003
H80 | 16209 | 3 | 0.009
H80 | 16243 | 15 | 0.044
H80 | 16254 | 1 | 0.003
H80 | 16284 | 35 | 0.104
H80 | 16319 | 1 | 0.003
H80 | 16566 | 1 | 0.003
H80 | 204 | 11 | 0.033
H80 | 237 | 1 | 0.003
H80 | 246 | 1 | 0.003
H80 | 288 | 1 | 0.003
H80 | 484 | 1 | 0.003
H80 | 5558 | 2 | 0.006
H80 | 6851 | 2 | 0.006
H80 | 8265-8274d | 1 | 0.003
H80 | 8940 | 2 | 0.006
H81 | 113 | 1 | 0.001
H81 | 15479 | 1 | 0.001
H81 | 16076A | 123 | 0.158
H81 | 16295 | 1 | 0.001
H81 | 208 | 1 | 0.001
H81 | 261 | 1 | 0.001
H81 | 281C | 1 | 0.001
H81 | 316d | 1 | 0.001
H81 | 338 | 1 | 0.001
H81 | 468 | 3 | 0.004
H81 | 502 | 1 | 0.001
H81 | 573.1C | 3 | 0.004
H81 | 573.2C | 5 | 0.006
H81 | 573.5C | 1 | 0.001
H81 | 7519d | 1 | 0.001
H81a | 113 | 1 | 0.001
H81a | 16076A | 123 | 0.158
H81a | 208 | 1 | 0.001
H81a | 261 | 1 | 0.001
H81a | 281C | 1 | 0.001
H81a | 316d | 1 | 0.001
H81a | 338 | 1 | 0.001
H81a | 468 | 3 | 0.004
H81a | 502 | 1 | 0.001
H81a | 573.1C | 3 | 0.004
H81a | 573.2C | 5 | 0.006
H81a | 573.5C | 1 | 0.001
H82 | 12561 | 1 | 0.012

H82 | 14869 | 1 | 0.012
H82 | 152 | 2 | 0.025
H82 | 15529 | 1 | 0.012
H82 | 16086 | 2 | 0.025
H82 | 16218 | 1 | 0.012
H82 | 16219 | 1 | 0.012
H82 | 16288 | 1 | 0.012
H82 | 16296 | 2 | 0.025
H82 | 195 | 13 | 0.16
H82 | 204 | 1 | 0.012
H82 | 374 | 1 | 0.012
H82 | 7118 | 1 | 0.012
H82 | 9065 | 1 | 0.012
H82 | 9088 | 1 | 0.012
H83 | 13827 | 1 | 0.017
H83 | 150 | 1 | 0.017
H83 | 159 | 2 | 0.034
H83 | 16093 | 3 | 0.051
H83 | 16129 | 1 | 0.017
H83 | 16189 | 1 | 0.017
H83 | 16390 | 1 | 0.017
H83 | 16438 | 3 | 0.051
H83 | 243 | 1 | 0.017
H84 | 113 | 1 | 0.001
H84 | 12127 | 1 | 0.001
H84 | 14514 | 1 | 0.001
H84 | 15319 | 1 | 0.001
H84 | 16076A | 123 | 0.158
H84 | 208 | 1 | 0.001
H84 | 261 | 1 | 0.001
H84 | 281C | 1 | 0.001
H84 | 316d | 1 | 0.001
H84 | 338 | 1 | 0.001
H84 | 468 | 3 | 0.004
H84 | 502 | 1 | 0.001
H84 | 573.1C | 3 | 0.004
H84 | 573.2C | 5 | 0.006
H84 | 573.5C | 1 | 0.001
H84 | 6311 | 1 | 0.001
H84 | 8946 | 1 | 0.001
H85 | 13857 | 1 | 0.012
H85 | 16086 | 1 | 0.012
H85 | 16114 | 1 | 0.012
H85 | 16124 | 3 | 0.036
H85 | 16129 | 1 | 0.012
H85 | 16166 | 1 | 0.012
H85 | 16172 | 1 | 0.012
H85 | 16188G | 1 | 0.012
H85 | 16223 | 2 | 0.024
H85 | 16270 | 1 | 0.012
H85 | 16274 | 1 | 0.012
H85 | 16287 | 3 | 0.036
H85 | 16290 | 1 | 0.012
H85 | 16311 | 1 | 0.012
H85 | 16319 | 1 | 0.012
H85 | 16343 | 11 | 0.133
H85 | 16352 | 1 | 0.012
H85 | 16362 | 2 | 0.024
H85 | 16391 | 1 | 0.012
H85 | 189 | 1 | 0.012
H85 | 199 | 1 | 0.012
H85 | 310 | 1 | 0.012
H85 | 315.2C | 1 | 0.012
H85 | 338 | 1 | 0.012
H85 | 456 | 2 | 0.024
H85 | 573.1C | 1 | 0.012
H85 | 8155 | 1 | 0.012
H86 | 113 | 1 | 0.001
H86 | 16076A | 123 | 0.157
H86 | 200 | 1 | 0.001
H86 | 208 | 1 | 0.001
H86 | 261 | 1 | 0.001
H86 | 281C | 1 | 0.001
H86 | 310 | 1 | 0.001
H86 | 316d | 1 | 0.001
H86 | 338 | 1 | 0.001
H86 | 4561 | 1 | 0.001
H86 | 4639 | 1 | 0.001
H86 | 468 | 3 | 0.004
H86 | 502 | 1 | 0.001
H86 | 573.1C | 3 | 0.004
H86 | 573.2C | 5 | 0.006
H86 | 573.5C | 1 | 0.001
H86 | 8857 | 2 | 0.003
H86 | 8958 | 1 | 0.001
H86 | 9004 | 1 | 0.001
H86 | 9425 | 1 | 0.001
H87 | 10172 | 2 | 0.003
H87 | 113 | 1 | 0.001
H87 | 13781 | 2 | 0.003
H87 | 14869 | 1 | 0.001
H87 | 150 | 1 | 0.001
H87 | 1555 | 1 | 0.001
H87 | 16076A | 123 | 0.157
H87 | 16311 | 2 | 0.003
H87 | 16474C | 1 | 0.001
H87 | 208 | 1 | 0.001
H87 | 261 | 1 | 0.001
H87 | 281C | 1 | 0.001
H87 | 316d | 1 | 0.001
H87 | 3335 | 1 | 0.001
H87 | 338 | 1 | 0.001
H87 | 3637 | 1 | 0.001
H87 | 3714 | 1 | 0.001
H87 | 4295 | 1 | 0.001
H87 | 468 | 3 | 0.004
H87 | 502 | 1 | 0.001
H87 | 573.1C | 3 | 0.004
H87 | 573.2C | 5 | 0.006
H87 | 573.5C | 1 | 0.001
H87 | 8706 | 1 | 0.001

H87 | 9647 | 1 | 0.001
H88 | 113 | 1 | 0.001
H88 | 16076A | 123 | 0.158
H88 | 208 | 1 | 0.001
H88 | 261 | 1 | 0.001
H88 | 281C | 1 | 0.001
H88 | 316d | 1 | 0.001
H88 | 338 | 1 | 0.001
H88 | 468 | 3 | 0.004
H88 | 502 | 1 | 0.001
H88 | 573.1C | 3 | 0.004
H88 | 573.2C | 5 | 0.006
H88 | 573.5C | 1 | 0.001
H89 | 10057 | 1 | 0.001
H89 | 113 | 1 | 0.001
H89 | 16076A | 123 | 0.158
H89 | 208 | 1 | 0.001
H89 | 261 | 1 | 0.001
H89 | 281C | 1 | 0.001
H89 | 316d | 1 | 0.001
H89 | 338 | 1 | 0.001
H89 | 468 | 3 | 0.004
H89 | 502 | 1 | 0.001
H89 | 573.1C | 3 | 0.004
H89 | 573.2C | 5 | 0.006
H89 | 573.5C | 1 | 0.001
H89 | 6014 | 1 | 0.001
H89 | 709 | 1 | 0.001
H8a | 14500 | 1 | 0.2
H8a | 16292 | 1 | 0.2
H8a | 16311 | 1 | 0.2
H8a | 16325 | 1 | 0.2
H8a | 246 | 2 | 0.4
H8a | 8788 | 1 | 0.2
H8a1 | 12672 | 1 | 0.091
H8a1 | 15498 | 2 | 0.182
H8a1 | 15928 | 1 | 0.091
H8a1 | 3999 | 1 | 0.091
H8a1 | 4385 | 1 | 0.091
H8a1 | 444 | 1 | 0.091
H8a1 | 8381 | 2 | 0.182
H8b | 13933 | 1 | 0.071
H8b | 16189 | 1 | 0.071
H8b | 16311 | 5 | 0.357
H8b | 225 | 1 | 0.071
H8b | 310 | 1 | 0.071
H8b | 315.2C | 1 | 0.071
H8b | 4820 | 1 | 0.071
H8b | 486A | 2 | 0.143
H8b | 6267 | 1 | 0.071
H8b | 8167 | 1 | 0.071
H8b1 | 12236.1G | 1 | 0.048
H8b1 | 14311 | 1 | 0.048
H8b1 | 15221 | 7 | 0.333
H8b1 | 15301 | 2 | 0.095
H8b1 | 16093 | 2 | 0.095
H8b1 | 16344 | 7 | 0.333
H8b1 | 203C | 7 | 0.333
H8b1 | 252 | 2 | 0.095
H8b1 | 310 | 2 | 0.095
H8b1 | 456 | 1 | 0.048
H8b1 | 7624 | 2 | 0.095
H8b1 | 961 | 4 | 0.19
H8b1 | 965.1C | 2 | 0.095
H8c | 10027d | 1 | 0.04
H8c | 10265 | 1 | 0.04
H8c | 10784 | 1 | 0.04
H8c | 11368 | 1 | 0.04
H8c | 11623 | 1 | 0.04
H8c | 12765 | 1 | 0.04
H8c | 13368 | 1 | 0.04
H8c | 13752 | 1 | 0.04
H8c | 13768 | 1 | 0.04
H8c | 13928C | 1 | 0.04
H8c | 15092 | 1 | 0.04
H8c | 15440A | 1 | 0.04
H8c | 15924 | 1 | 0.04
H8c | 16093 | 1 | 0.04
H8c | 16095A | 1 | 0.04
H8c | 16224 | 1 | 0.04
H8c | 16249 | 1 | 0.04
H8c | 16258 | 1 | 0.04
H8c | 16263 | 1 | 0.04
H8c | 16269 | 1 | 0.04
H8c | 16294 | 2 | 0.08
H8c | 196 | 2 | 0.08
H8c | 220 | 1 | 0.04
H8c | 2706 | 1 | 0.04
H8c | 310 | 1 | 0.04
H8c | 3473 | 1 | 0.04
H8c | 3593 | 1 | 0.04
H8c | 4722 | 2 | 0.08
H8c | 5093 | 1 | 0.04
H8c | 533 | 1 | 0.04
H8c | 6060C | 1 | 0.04
H8c | 6712 | 1 | 0.04
H8c | 7705 | 1 | 0.04
H8c | 8289.1CCCCCTCTA | 1 | 0.04
H8c | 8331 | 2 | 0.08
H8c | 8864 | 2 | 0.08
H8c | 9193A | 1 | 0.04
H8c | 93 | 4 | 0.16
H8c | 9404 | 1 | 0.04
H8c | 9575 | 2 | 0.08
H8c1 | 11494 | 1 | 0.125
H8c1 | 12879 | 1 | 0.125
H8c1 | 13204 | 1 | 0.125
H8c1 | 14854A | 1 | 0.125
H8c1 | 9052 | 3 | 0.375
H8c2 | 12373 | 1 | 0.333
H8c2 | 198 | 1 | 0.333

H9 | 10166 | 2 | 0.013
H9 | 146 | 2 | 0.013
H9 | 16092 | 1 | 0.007
H9 | 16214 | 3 | 0.02
H9 | 16233T | 1 | 0.007
H9 | 16247 | 20 | 0.133
H9 | 16256 | 1 | 0.007
H9 | 16258T | 1 | 0.007
H9 | 200T | 1 | 0.007
H9 | 207 | 1 | 0.007
H9 | 456 | 1 | 0.007
H9 | 463 | 1 | 0.007
H9 | 515-524d | 1 | 0.007
H9 | 529T | 1 | 0.007
H9 | 573.1C | 1 | 0.007
H9 | 57A | 1 | 0.007
H9 | 58 | 1 | 0.007
H9 | 60.1T | 2 | 0.013
H90 | 113 | 1 | 0.001
H90 | 16076A | 123 | 0.158
H90 | 208 | 1 | 0.001
H90 | 261 | 1 | 0.001
H90 | 281C | 1 | 0.001
H90 | 316d | 1 | 0.001
H90 | 338 | 1 | 0.001
H90 | 468 | 3 | 0.004
H90 | 502 | 1 | 0.001
H90 | 573.1C | 3 | 0.004
H90 | 573.2C | 5 | 0.006
H90 | 573.5C | 1 | 0.001
H91 | 11038d | 1 | 0.042
H91 | 13602 | 1 | 0.042
H91 | 15047 | 1 | 0.042
H91 | 151 | 3 | 0.125
H91 | 152 | 3 | 0.125
H91 | 16086 | 2 | 0.083
H91 | 16188G | 2 | 0.083
H91 | 16261 | 1 | 0.042
H91 | 16264 | 1 | 0.042
H91 | 16265C | 2 | 0.083
H91 | 16301 | 1 | 0.042
H91 | 478 | 5 | 0.208
H91 | 4831 | 1 | 0.042
H92 | 113 | 1 | 0.001
H92 | 12236C | 1 | 0.001
H92 | 16076A | 123 | 0.158
H92 | 16368 | 1 | 0.001
H92 | 208 | 1 | 0.001
H92 | 2483 | 1 | 0.001
H92 | 261 | 1 | 0.001
H92 | 281C | 1 | 0.001
H92 | 316d | 1 | 0.001
H92 | 338 | 1 | 0.001
H92 | 468 | 3 | 0.004
H92 | 502 | 1 | 0.001
H92 | 573.1C | 3 | 0.004
H92 | 573.2C | 5 | 0.006
H92 | 573.5C | 1 | 0.001
H92 | 5785 | 1 | 0.001
H92 | 709 | 1 | 0.001
H92 | 9554 | 1 | 0.001
H93 | 113 | 1 | 0.001
H93 | 11344 | 1 | 0.001
H93 | 16076A | 123 | 0.158
H93 | 16524C | 1 | 0.001
H93 | 208 | 1 | 0.001
H93 | 2205 | 1 | 0.001
H93 | 261 | 1 | 0.001
H93 | 281C | 1 | 0.001
H93 | 316d | 1 | 0.001
H93 | 338 | 1 | 0.001
H93 | 468 | 3 | 0.004
H93 | 502 | 1 | 0.001
H93 | 573.1C | 3 | 0.004
H93 | 573.2C | 5 | 0.006
H93 | 573.5C | 1 | 0.001
H93 | 7270 | 1 | 0.001
H93 | 942 | 1 | 0.001
H94 | 15511 | 1 | 0.015
H94 | 16093 | 4 | 0.06
H94 | 16189 | 2 | 0.03
H94 | 16316 | 2 | 0.03
H94 | 16357 | 1 | 0.015
H94 | 200 | 1 | 0.015
H94 | 291.1A | 1 | 0.015
H94 | 9374 | 2 | 0.03
H95 | 113 | 1 | 0.001
H95 | 11353 | 2 | 0.003
H95 | 151 | 2 | 0.003
H95 | 16076A | 123 | 0.158
H95 | 16249 | 2 | 0.003
H95 | 208 | 1 | 0.001
H95 | 261 | 1 | 0.001
H95 | 281C | 1 | 0.001
H95 | 316d | 1 | 0.001
H95 | 338 | 1 | 0.001
H95 | 468 | 3 | 0.004
H95 | 502 | 1 | 0.001
H95 | 573.1C | 3 | 0.004
H95 | 573.2C | 5 | 0.006
H95 | 573.5C | 1 | 0.001
H95 | 6423G | 1 | 0.001
H95a | 113 | 1 | 0.001
H95a | 11944 | 2 | 0.003
H95a | 16076A | 123 | 0.158
H95a | 16319 | 2 | 0.003
H95a | 208 | 1 | 0.001
H95a | 261 | 1 | 0.001
H95a | 2707 | 1 | 0.001
H95a | 281C | 1 | 0.001
H95a | 316d | 1 | 0.001

H95a | 338 | 1 | 0.001
H95a | 468 | 3 | 0.004
H95a | 502 | 1 | 0.001
H95a | 573.1C | 3 | 0.004
H95a | 573.2C | 5 | 0.006
H95a | 573.5C | 1 | 0.001
H95a | 6340 | 1 | 0.001
H96 | 11854 | 3 | 0.041
H96 | 150 | 9 | 0.122
H96 | 152 | 9 | 0.122
H96 | 15381 | 2 | 0.027
H96 | 15774 | 1 | 0.014
H96 | 16129 | 11 | 0.149
H96 | 16136 | 8 | 0.108
H96 | 16234d | 1 | 0.014
H96 | 16248 | 1 | 0.014
H96 | 16254 | 2 | 0.027
H96 | 16257 | 1 | 0.014
H96 | 16266 | 2 | 0.027
H96 | 16266A | 1 | 0.014
H96 | 16298 | 1 | 0.014
H96 | 16300 | 2 | 0.027
H96 | 16352 | 9 | 0.122
H96 | 16354 | 2 | 0.027
H96 | 16355 | 11 | 0.149
H96 | 16390 | 1 | 0.014
H96 | 16399 | 1 | 0.014
H96 | 16527 | 1 | 0.014
H96 | 194 | 1 | 0.014
H96 | 204 | 1 | 0.014
H96 | 236 | 2 | 0.027
H96 | 308-309d | 1 | 0.014
H96 | 309.3C | 2 | 0.027
H96 | 573.2C | 1 | 0.014
H96 | 573.4C | 1 | 0.014
H96 | 574C | 2 | 0.027
H96 | 576C | 1 | 0.014
H96 | 577C | 1 | 0.014
H96 | 578 | 1 | 0.014
H96 | 73 | 10 | 0.135
H96 | 8334 | 2 | 0.027
H96 | 9801 | 1 | 0.014
H96 | 9980 | 2 | 0.027
H9a | 12358 | 1 | 0.022
H9a | 13161 | 1 | 0.022
H9a | 13734 | 1 | 0.022
H9a | 14593 | 1 | 0.022
H9a | 15064 | 1 | 0.022
H9a | 16111 | 2 | 0.044
H9a | 16129 | 1 | 0.022
H9a | 16172 | 1 | 0.022
H9a | 16193 | 1 | 0.022
H9a | 16209 | 1 | 0.022
H9a | 16218 | 1 | 0.022
H9a | 16254 | 2 | 0.044
H9a | 16261 | 1 | 0.022
H9a | 16269 | 1 | 0.022
H9a | 16325 | 1 | 0.022
H9a | 16355 | 6 | 0.133
H9a | 195 | 1 | 0.022
H9a | 292 | 1 | 0.022
H9a | 294.1T | 1 | 0.022
H9a | 310 | 1 | 0.022
H9a | 338 | 2 | 0.044
H9a | 4491 | 1 | 0.022
H9a | 513 | 2 | 0.044
H9a | 5755G | 1 | 0.022
H9a | 709 | 2 | 0.044
H9a | 7471d | 1 | 0.022
H9a | 8108 | 2 | 0.044
H9a | 8289.1CCCCCTCTA | 1 | 0.022
H9a | 9148 | 1 | 0.022
H9a | 93 | 2 | 0.044
HV | 103 | 4 | 0.005
HV | 10556 | 3 | 0.004
HV | 11050 | 2 | 0.002
HV | 11137 | 1 | 0.001
HV | 11155 | 1 | 0.001
HV | 11245 | 2 | 0.002
HV | 113 | 1 | 0.001
HV | 11770 | 1 | 0.001
HV | 11800 | 3 | 0.004
HV | 11903 | 2 | 0.002
HV | 12425d | 1 | 0.001
HV | 12468 | 1 | 0.001
HV | 12477 | 1 | 0.001
HV | 12777 | 1 | 0.001
HV | 13461 | 2 | 0.002
HV | 13635 | 1 | 0.001
HV | 13768 | 1 | 0.001
HV | 14263 | 1 | 0.001
HV | 143 | 2 | 0.002
HV | 14323 | 2 | 0.002
HV | 1452 | 1 | 0.001
HV | 14580 | 1 | 0.001
HV | 146 | 8 | 0.01
HV | 14750 | 1 | 0.001
HV | 14764 | 2 | 0.002
HV | 14831 | 2 | 0.002
HV | 14968 | 1 | 0.001
HV | 15172 | 1 | 0.001
HV | 152 | 10 | 0.012
HV | 15217 | 1 | 0.001
HV | 15272 | 1 | 0.001
HV | 15924 | 2 | 0.002
HV | 16037 | 1 | 0.001
HV | 16076A | 1 | 0.001
HV | 16079 | 1 | 0.001
HV | 16086 | 1 | 0.001
HV | 16092 | 3 | 0.004
HV | 16129 | 1 | 0.001

HV | 16154 | 1 | 0.001
HV | 16162 | 1 | 0.001
HV | 16177 | 1 | 0.001
HV | 16189 | 2 | 0.002
HV | 16220 | 1 | 0.001
HV | 16223 | 4 | 0.005
HV | 16234 | 1 | 0.001
HV | 16243 | 1 | 0.001
HV | 16248 | 3 | 0.004
HV | 16261 | 1 | 0.001
HV | 16266 | 1 | 0.001
HV | 16267 | 1 | 0.001
HV | 16294 | 2 | 0.002
HV | 16295 | 1 | 0.001
HV | 16296 | 3 | 0.004
HV | 16309 | 1 | 0.001
HV | 16319 | 2 | 0.002
HV | 16327 | 125 | 0.15
HV | 16327A | 1 | 0.001
HV | 16356 | 1 | 0.001
HV | 16362 | 2 | 0.002
HV | 1664 | 2 | 0.002
HV | 195 | 5 | 0.006
HV | 199 | 2 | 0.002
HV | 200 | 1 | 0.001
HV | 208 | 1 | 0.001
HV | 2280 | 1 | 0.001
HV | 235 | 6 | 0.007
HV | 249d | 3 | 0.004
HV | 261 | 1 | 0.001
HV | 281C | 1 | 0.001
HV | 310 | 3 | 0.004
HV | 3106A | 1 | 0.001
HV | 316d | 1 | 0.001
HV | 3202 | 1 | 0.001
HV | 338 | 1 | 0.001
HV | 3397 | 4 | 0.005
HV | 3398 | 1 | 0.001
HV | 385 | 1 | 0.001
HV | 4435 | 2 | 0.002
HV | 4907 | 2 | 0.002
HV | 499 | 1 | 0.001
HV | 502 | 1 | 0.001
HV | 5087 | 1 | 0.001
HV | 5121 | 1 | 0.001
HV | 515 | 1 | 0.001
HV | 5191 | 2 | 0.002
HV | 5295 | 2 | 0.002
HV | 5460 | 7 | 0.008
HV | 5471 | 3 | 0.004
HV | 5673 | 2 | 0.002
HV | 573.1C | 3 | 0.004
HV | 573.2C | 5 | 0.006
HV | 573.5C | 1 | 0.001
HV | 5782 | 1 | 0.001
HV | 593 | 3 | 0.004
HV | 6216 | 2 | 0.002
HV | 6248 | 1 | 0.001
HV | 6365 | 1 | 0.001
HV | 7521 | 1 | 0.001
HV | 7598 | 1 | 0.001
HV | 7664 | 2 | 0.002
HV | 8348 | 1 | 0.001
HV | 8636 | 3 | 0.004
HV | 8706 | 2 | 0.002
HV | 8842 | 1 | 0.001
HV | 8862T | 1 | 0.001
HV | 9065 | 1 | 0.001
HV | 9152 | 1 | 0.001
HV | 9548 | 2 | 0.002
HV | 9738 | 1 | 0.001
HV+16311 | 10007 | 1 | 0.003
HV+16311 | 10205 | 3 | 0.008
HV+16311 | 10237 | 2 | 0.005
HV+16311 | 10321 | 1 | 0.003
HV+16311 | 10331 | 1 | 0.003
HV+16311 | 10389 | 1 | 0.003
HV+16311 | 10508 | 1 | 0.003
HV+16311 | 10680 | 1 | 0.003
HV+16311 | 10750 | 1 | 0.003
HV+16311 | 10819 | 1 | 0.003
HV+16311 | 10920 | 4 | 0.01
HV+16311 | 11176 | 1 | 0.003
HV+16311 | 11302 | 1 | 0.003
HV+16311 | 11566 | 5 | 0.013
HV+16311 | 12007 | 1 | 0.003
HV+16311 | 12278 | 1 | 0.003
HV+16311 | 12397 | 1 | 0.003
HV+16311 | 12468 | 1 | 0.003
HV+16311 | 12562G | 1 | 0.003
HV+16311 | 12630 | 2 | 0.005
HV+16311 | 12771 | 1 | 0.003
HV+16311 | 13111 | 1 | 0.003
HV+16311 | 13347 | 1 | 0.003
HV+16311 | 13359 | 1 | 0.003
HV+16311 | 13401 | 1 | 0.003
HV+16311 | 13419 | 1 | 0.003
HV+16311 | 13437 | 2 | 0.005
HV+16311 | 13470 | 1 | 0.003
HV+16311 | 13650 | 1 | 0.003
HV+16311 | 13708 | 1 | 0.003
HV+16311 | 13941 | 1 | 0.003
HV+16311 | 14071 | 1 | 0.003
HV+16311 | 14162 | 1 | 0.003
HV+16311 | 14386 | 1 | 0.003
HV+16311 | 14500 | 1 | 0.003
HV+16311 | 1453 | 1 | 0.003
HV+16311 | 14577 | 1 | 0.003
HV+16311 | 146 | 2 | 0.005
HV+16311 | 14830 | 1 | 0.003
HV+16311 | 150 | 2 | 0.005

HV+16311 | 15004 | 1 | 0.003
HV+16311 | 1503 | 1 | 0.003
HV+16311 | 15213 | 1 | 0.003
HV+16311 | 15223 | 1 | 0.003
HV+16311 | 15301 | 2 | 0.005
HV+16311 | 15314 | 1 | 0.003
HV+16311 | 15514 | 4 | 0.01
HV+16311 | 15574 | 2 | 0.005
HV+16311 | 15773 | 1 | 0.003
HV+16311 | 15784 | 1 | 0.003
HV+16311 | 15902 | 1 | 0.003
HV+16311 | 15930 | 2 | 0.005
HV+16311 | 15937d | 1 | 0.003
HV+16311 | 16038 | 1 | 0.003
HV+16311 | 16079 | 1 | 0.003
HV+16311 | 16092 | 2 | 0.005
HV+16311 | 16093 | 2 | 0.005
HV+16311 | 16129 | 2 | 0.005
HV+16311 | 16145 | 1 | 0.003
HV+16311 | 16147 | 4 | 0.01
HV+16311 | 16148 | 1 | 0.003
HV+16311 | 16153 | 2 | 0.005
HV+16311 | 16154 | 1 | 0.003
HV+16311 | 16158C | 2 | 0.005
HV+16311 | 16158T | 5 | 0.013
HV+16311 | 16172 | 4 | 0.01
HV+16311 | 16179 | 1 | 0.003
HV+16311 | 16187 | 1 | 0.003
HV+16311 | 16188 | 1 | 0.003
HV+16311 | 16189 | 1 | 0.003
HV+16311 | 16192 | 1 | 0.003
HV+16311 | 16206C | 1 | 0.003
HV+16311 | 16234 | 3 | 0.008
HV+16311 | 16239 | 1 | 0.003
HV+16311 | 16256 | 1 | 0.003
HV+16311 | 16266 | 1 | 0.003
HV+16311 | 16271 | 1 | 0.003
HV+16311 | 16286 | 36 | 0.092
HV+16311 | 16293 | 1 | 0.003
HV+16311 | 16298 | 1 | 0.003
HV+16311 | 16299 | 1 | 0.003
HV+16311 | 16362 | 2 | 0.005
HV+16311 | 1692 | 3 | 0.008
HV+16311 | 1746 | 1 | 0.003
HV+16311 | 183 | 1 | 0.003
HV+16311 | 185 | 1 | 0.003
HV+16311 | 195 | 6 | 0.015
HV+16311 | 207 | 2 | 0.005
HV+16311 | 228 | 1 | 0.003
HV+16311 | 2486 | 1 | 0.003
HV+16311 | 2628 | 1 | 0.003
HV+16311 | 2667 | 1 | 0.003
HV+16311 | 2885 | 1 | 0.003
HV+16311 | 310 | 6 | 0.015
HV+16311 | 3204 | 1 | 0.003
HV+16311 | 332A | 1 | 0.003
HV+16311 | 3335 | 1 | 0.003
HV+16311 | 3337 | 1 | 0.003
HV+16311 | 3351 | 2 | 0.005
HV+16311 | 3368 | 1 | 0.003
HV+16311 | 3394 | 1 | 0.003
HV+16311 | 3744 | 4 | 0.01
HV+16311 | 3903 | 1 | 0.003
HV+16311 | 4226 | 1 | 0.003
HV+16311 | 4314A | 1 | 0.003
HV+16311 | 455.1T | 1 | 0.003
HV+16311 | 4561 | 1 | 0.003
HV+16311 | 4562 | 1 | 0.003
HV+16311 | 4706 | 1 | 0.003
HV+16311 | 471 | 1 | 0.003
HV+16311 | 4829 | 2 | 0.005
HV+16311 | 498.1C | 1 | 0.003
HV+16311 | 5029 | 4 | 0.01
HV+16311 | 5147 | 1 | 0.003
HV+16311 | 5263 | 1 | 0.003
HV+16311 | 5295 | 2 | 0.005
HV+16311 | 5460 | 2 | 0.005
HV+16311 | 5528 | 4 | 0.01
HV+16311 | 5592 | 1 | 0.003
HV+16311 | 5655 | 1 | 0.003
HV+16311 | 5746 | 1 | 0.003
HV+16311 | 5774 | 1 | 0.003
HV+16311 | 5821 | 1 | 0.003
HV+16311 | 5910 | 1 | 0.003
HV+16311 | 5913 | 1 | 0.003
HV+16311 | 5984 | 1 | 0.003
HV+16311 | 6054T | 1 | 0.003
HV+16311 | 6890 | 1 | 0.003
HV+16311 | 6956G | 1 | 0.003
HV+16311 | 6983 | 1 | 0.003
HV+16311 | 709 | 1 | 0.003
HV+16311 | 7270 | 1 | 0.003
HV+16311 | 7472C | 1 | 0.003
HV+16311 | 7521 | 1 | 0.003
HV+16311 | 7717 | 1 | 0.003
HV+16311 | 7853 | 1 | 0.003
HV+16311 | 8075 | 1 | 0.003
HV+16311 | 8376 | 1 | 0.003
HV+16311 | 8485 | 1 | 0.003
HV+16311 | 8573 | 4 | 0.01
HV+16311 | 8628 | 1 | 0.003
HV+16311 | 8703 | 1 | 0.003
HV+16311 | 8842T | 1 | 0.003
HV+16311 | 8963 | 4 | 0.01
HV+16311 | 9010 | 1 | 0.003
HV+16311 | 9151 | 1 | 0.003
HV+16311 | 9182 | 1 | 0.003
HV+16311 | 93 | 4 | 0.01
HV+16311 | 9575 | 1 | 0.003
HV+16311 | 95C | 1 | 0.003
HV+16311 | 961 | 1 | 0.003

HV+16311 | 9801 | 4 | 0.01
HV+16311 | 9804 | 1 | 0.003
HV+16311 | 9833 | 4 | 0.01
HV+16311 | 9911 | 3 | 0.008
HV+16311 | 996 | 1 | 0.003
HV+16311 | 998 | 4 | 0.01
HV+73 | 10398 | 1 | 0.006
HV+73 | 11075 | 1 | 0.006
HV+73 | 11719 | 2 | 0.012
HV+73 | 12133 | 1 | 0.006
HV+73 | 12523 | 1 | 0.006
HV+73 | 12662 | 33 | 0.195
HV+73 | 12882 | 1 | 0.006
HV+73 | 14178 | 1 | 0.006
HV+73 | 14325 | 2 | 0.012
HV+73 | 14668 | 1 | 0.006
HV+73 | 14693 | 1 | 0.006
HV+73 | 14766 | 1 | 0.006
HV+73 | 152 | 1 | 0.006
HV+73 | 15202 | 1 | 0.006
HV+73 | 15301 | 1 | 0.006
HV+73 | 15511 | 1 | 0.006
HV+73 | 1555 | 1 | 0.006
HV+73 | 15574 | 1 | 0.006
HV+73 | 15904 | 33 | 0.195
HV+73 | 15914 | 1 | 0.006
HV+73 | 16304G | 1 | 0.006
HV+73 | 16311 | 1 | 0.006
HV+73 | 16362 | 1 | 0.006
HV+73 | 195 | 1 | 0.006
HV+73 | 246 | 1 | 0.006
HV+73 | 249d | 1 | 0.006
HV+73 | 3106A | 1 | 0.006
HV+73 | 455.1T | 1 | 0.006
HV+73 | 5441 | 1 | 0.006
HV+73 | 5634 | 1 | 0.006
HV+73 | 5894 | 1 | 0.006
HV+73 | 6786 | 1 | 0.006
HV+73 | 7140 | 1 | 0.006
HV+73 | 72 | 32 | 0.189
HV+73 | 7733 | 1 | 0.006
HV+73 | 7737 | 1 | 0.006
HV+73 | 7754 | 1 | 0.006
HV+73 | 7757 | 1 | 0.006
HV+73 | 7763 | 1 | 0.006
HV+73 | 7768 | 1 | 0.006
HV+73 | 7897 | 1 | 0.006
HV+73 | 8430 | 1 | 0.006
HV+73 | 8574 | 1 | 0.006
HV+73 | 9064 | 27 | 0.16
HV0 | 10039 | 1 | 0.005
HV0 | 11518 | 2 | 0.01
HV0 | 12425 | 4 | 0.02
HV0 | 13980 | 1 | 0.005
HV0 | 14836 | 1 | 0.005
HV0 | 150 | 1 | 0.005
HV0 | 151 | 1 | 0.005
HV0 | 15317 | 1 | 0.005
HV0 | 15924 | 1 | 0.005
HV0 | 16172 | 4 | 0.02
HV0 | 16189 | 1 | 0.005
HV0 | 16193 | 1 | 0.005
HV0 | 16240 | 14 | 0.07
HV0 | 16249 | 1 | 0.005
HV0 | 16278 | 1 | 0.005
HV0 | 16317 | 1 | 0.005
HV0 | 16368 | 1 | 0.005
HV0 | 16527 | 2 | 0.01
HV0 | 182 | 1 | 0.005
HV0 | 183 | 1 | 0.005
HV0 | 1842 | 2 | 0.01
HV0 | 186 | 3 | 0.015
HV0 | 239 | 1 | 0.005
HV0 | 249 | 1 | 0.005
HV0 | 310 | 1 | 0.005
HV0 | 334 | 1 | 0.005
HV0 | 338 | 1 | 0.005
HV0 | 3531 | 1 | 0.005
HV0 | 3565 | 1 | 0.005
HV0 | 3576 | 1 | 0.005
HV0 | 3745 | 1 | 0.005
HV0 | 4715 | 1 | 0.005
HV0 | 5366 | 1 | 0.005
HV0 | 5493 | 1 | 0.005
HV0 | 705 | 1 | 0.005
HV0 | 7958 | 1 | 0.005
HV0 | 8058 | 1 | 0.005
HV0 | 8473 | 2 | 0.01
HV0 | 8787A | 1 | 0.005
HV0 | 8843 | 1 | 0.005
HV0 | 9145 | 1 | 0.005
HV0+195 | 10685 | 2 | 0.012
HV0+195 | 10882 | 1 | 0.006
HV0+195 | 10894 | 1 | 0.006
HV0+195 | 11335 | 1 | 0.006
HV0+195 | 11337 | 2 | 0.012
HV0+195 | 11719 | 1 | 0.006
HV0+195 | 12354 | 1 | 0.006
HV0+195 | 12370 | 1 | 0.006
HV0+195 | 12630 | 1 | 0.006
HV0+195 | 12635 | 1 | 0.006
HV0+195 | 13145 | 1 | 0.006
HV0+195 | 13503 | 1 | 0.006
HV0+195 | 13535 | 2 | 0.012
HV0+195 | 13804 | 1 | 0.006
HV0+195 | 13933 | 1 | 0.006
HV0+195 | 14560 | 2 | 0.012
HV0+195 | 14581 | 1 | 0.006
HV0+195 | 14582 | 1 | 0.006
HV0+195 | 14693 | 1 | 0.006
HV0+195 | 14860 | 1 | 0.006

HV0+195 | 14978 | 3 | 0.018
HV0+195 | 150 | 2 | 0.012
HV0+195 | 15145 | 2 | 0.012
HV0+195 | 152 | 7 | 0.043
HV0+195 | 15217 | 2 | 0.012
HV0+195 | 15315 | 1 | 0.006
HV0+195 | 15317 | 2 | 0.012
HV0+195 | 15324 | 1 | 0.006
HV0+195 | 15617 | 3 | 0.018
HV0+195 | 15734 | 1 | 0.006
HV0+195 | 15951 | 1 | 0.006
HV0+195 | 16037 | 2 | 0.012
HV0+195 | 16070C | 2 | 0.012
HV0+195 | 16075 | 3 | 0.018
HV0+195 | 16092 | 3 | 0.018
HV0+195 | 16111 | 7 | 0.043
HV0+195 | 16129 | 1 | 0.006
HV0+195 | 16153 | 4 | 0.024
HV0+195 | 16166 | 2 | 0.012
HV0+195 | 16168 | 2 | 0.012
HV0+195 | 16169 | 2 | 0.012
HV0+195 | 16189 | 17 | 0.104
HV0+195 | 16201 | 1 | 0.006
HV0+195 | 16239 | 1 | 0.006
HV0+195 | 16260 | 1 | 0.006
HV0+195 | 16261 | 1 | 0.006
HV0+195 | 16267 | 2 | 0.012
HV0+195 | 16284 | 1 | 0.006
HV0+195 | 16291 | 3 | 0.018
HV0+195 | 16298G | 2 | 0.012
HV0+195 | 16301 | 1 | 0.006
HV0+195 | 16311 | 4 | 0.024
HV0+195 | 16326 | 1 | 0.006
HV0+195 | 16463 | 2 | 0.012
HV0+195 | 1706 | 1 | 0.006
HV0+195 | 200 | 3 | 0.018
HV0+195 | 228 | 1 | 0.006
HV0+195 | 2280 | 4 | 0.024
HV0+195 | 309.3C | 1 | 0.006
HV0+195 | 310 | 4 | 0.024
HV0+195 | 315.3C | 1 | 0.006
HV0+195 | 3316 | 1 | 0.006
HV0+195 | 3337 | 1 | 0.006
HV0+195 | 3565 | 2 | 0.012
HV0+195 | 3618 | 1 | 0.006
HV0+195 | 3892 | 1 | 0.006
HV0+195 | 417C | 1 | 0.006
HV0+195 | 4248 | 2 | 0.012
HV0+195 | 4560 | 4 | 0.024
HV0+195 | 4688 | 4 | 0.024
HV0+195 | 5093 | 1 | 0.006
HV0+195 | 518 | 1 | 0.006
HV0+195 | 573.4C | 1 | 0.006
HV0+195 | 5985 | 1 | 0.006
HV0+195 | 6059 | 1 | 0.006
HV0+195 | 6392 | 1 | 0.006
HV0+195 | 64 | 4 | 0.024
HV0+195 | 6776 | 1 | 0.006
HV0+195 | 7004 | 1 | 0.006
HV0+195 | 7055 | 2 | 0.012
HV0+195 | 709 | 1 | 0.006
HV0+195 | 7684 | 1 | 0.006
HV0+195 | 769 | 1 | 0.006
HV0+195 | 7762 | 1 | 0.006
HV0+195 | 7774 | 1 | 0.006
HV0+195 | 7805 | 2 | 0.012
HV0+195 | 7930 | 1 | 0.006
HV0+195 | 8014 | 2 | 0.012
HV0+195 | 8251 | 1 | 0.006
HV0+195 | 8273 | 2 | 0.012
HV0+195 | 8302 | 1 | 0.006
HV0+195 | 8477 | 2 | 0.012
HV0+195 | 8572 | 3 | 0.018
HV0+195 | 8674 | 1 | 0.006
HV0+195 | 8715 | 2 | 0.012
HV0+195 | 8853 | 1 | 0.006
HV0+195 | 8862T | 6 | 0.037
HV0+195 | 8922 | 2 | 0.012
HV0+195 | 9007 | 1 | 0.006
HV0+195 | 9086 | 1 | 0.006
HV0+195 | 93 | 1 | 0.006
HV0+195 | 9987 | 1 | 0.006
HV0a | 10313 | 1 | 0.005
HV0a | 11150 | 1 | 0.005
HV0a | 12372 | 2 | 0.009
HV0a | 12669 | 1 | 0.005
HV0a | 13862 | 1 | 0.005
HV0a | 14470 | 25 | 0.115
HV0a | 14963 | 1 | 0.005
HV0a | 15221 | 1 | 0.005
HV0a | 15262 | 2 | 0.009
HV0a | 15731 | 1 | 0.005
HV0a | 16189 | 2 | 0.009
HV0a | 16222 | 15 | 0.069
HV0a | 2833 | 1 | 0.005
HV0a | 295 | 7 | 0.032
HV0a | 3459 | 1 | 0.005
HV0a | 3540 | 3 | 0.014
HV0a | 368 | 1 | 0.005
HV0a | 3995 | 1 | 0.005
HV0a | 480 | 2 | 0.009
HV0a | 5581 | 1 | 0.005
HV0a | 6635 | 25 | 0.115
HV0a | 7930 | 1 | 0.005
HV0a | 8020 | 1 | 0.005
HV0a | 8488 | 1 | 0.005
HV0a | 8557 | 1 | 0.005
HV0a1 | 10885 | 2 | 0.056
HV0a1 | 15315 | 1 | 0.028
HV0a1 | 15596 | 1 | 0.028
HV0a1 | 15992 | 1 | 0.028

HV0a1 | 16189 | 1 | 0.028
HV0a1 | 16214A | 2 | 0.056
HV0a1 | 16223 | 4 | 0.111
HV0a1 | 16260 | 2 | 0.056
HV0a1 | 16271 | 2 | 0.056
HV0a1 | 16278 | 2 | 0.056
HV0a1 | 204 | 2 | 0.056
HV0a1 | 2626 | 1 | 0.028
HV0a1 | 310 | 2 | 0.056
HV0a1 | 311 | 1 | 0.028
HV0a1 | 4179 | 1 | 0.028
HV0a1 | 573.1C | 1 | 0.028
HV0a1 | 9500 | 2 | 0.056
HV0a1a | 10084 | 2 | 0.25
HV0a1a | 12530 | 1 | 0.125
HV0a1a | 189 | 1 | 0.125
HV0a1a | 207 | 2 | 0.25
HV0a1a | 3368 | 1 | 0.125
HV0a1a | 5027 | 2 | 0.25
HV0a1a | 5277 | 1 | 0.125
HV0a1a | 596 | 2 | 0.25
HV0b | 11613 | 1 | 0.011
HV0b | 14582 | 2 | 0.023
HV0b | 15001 | 1 | 0.011
HV0b | 15119 | 1 | 0.011
HV0b | 16093 | 3 | 0.034
HV0b | 16129 | 1 | 0.011
HV0b | 16153 | 2 | 0.023
HV0b | 16269 | 1 | 0.011
HV0b | 16293 | 1 | 0.011
HV0b | 1719 | 1 | 0.011
HV0b | 1888 | 1 | 0.011
HV0b | 2620C | 1 | 0.011
HV0b | 2833 | 1 | 0.011
HV0b | 291.1A | 1 | 0.011
HV0b | 2940d | 1 | 0.011
HV0b | 3618 | 1 | 0.011
HV0b | 4688 | 1 | 0.011
HV0b | 518 | 2 | 0.023
HV0b | 6266 | 1 | 0.011
HV0b | 6446 | 1 | 0.011
HV0b | 6457 | 1 | 0.011
HV0b | 6824 | 1 | 0.011
HV0b | 8520 | 1 | 0.011
HV0b | 8628 | 1 | 0.011
HV0b | 9181 | 1 | 0.011
HV0b | 93 | 2 | 0.023
HV0b | 9467 | 1 | 0.011
HV0b | 986T | 1 | 0.011
HV0c | 12164 | 1 | 0.008
HV0c | 13398 | 5 | 0.039
HV0c | 13980 | 5 | 0.039
HV0c | 150 | 1 | 0.008
HV0c | 15747 | 2 | 0.016
HV0c | 16153 | 16 | 0.126
HV0c | 16186 | 1 | 0.008
HV0c | 16258C | 2 | 0.016
HV0c | 16311 | 1 | 0.008
HV0c | 16362 | 2 | 0.016
HV0c | 2779 | 1 | 0.008
HV0c | 4226 | 2 | 0.016
HV0c | 489 | 2 | 0.016
HV0c | 522G | 2 | 0.016
HV0c | 573.4C | 1 | 0.008
HV0c | 6284 | 2 | 0.016
HV0c | 64 | 1 | 0.008
HV0c | 73 | 2 | 0.016
HV0c | 9214 | 1 | 0.008
HV0c | 9533 | 1 | 0.008
HV0c | 9923 | 2 | 0.016
HV0d | 10463 | 2 | 0.015
HV0d | 10700 | 1 | 0.008
HV0d | 11260 | 3 | 0.023
HV0d | 11651 | 1 | 0.008
HV0d | 12397 | 1 | 0.008
HV0d | 146 | 1 | 0.008
HV0d | 15314 | 1 | 0.008
HV0d | 15758 | 1 | 0.008
HV0d | 15968 | 1 | 0.008
HV0d | 16169 | 1 | 0.008
HV0d | 16172 | 5 | 0.038
HV0d | 16192 | 1 | 0.008
HV0d | 16258C | 2 | 0.015
HV0d | 16266 | 2 | 0.015
HV0d | 16292 | 1 | 0.008
HV0d | 16293T | 1 | 0.008
HV0d | 16309 | 1 | 0.008
HV0d | 16311 | 1 | 0.008
HV0d | 16355 | 8 | 0.061
HV0d | 16362 | 1 | 0.008
HV0d | 200 | 3 | 0.023
HV0d | 204 | 1 | 0.008
HV0d | 215 | 1 | 0.008
HV0d | 2332A | 1 | 0.008
HV0d | 234 | 1 | 0.008
HV0d | 2535 | 1 | 0.008
HV0d | 3010 | 1 | 0.008
HV0d | 310 | 1 | 0.008
HV0d | 340 | 1 | 0.008
HV0d | 489 | 2 | 0.015
HV0d | 4904A | 1 | 0.008
HV0d | 5046 | 1 | 0.008
HV0d | 508 | 1 | 0.008
HV0d | 5692 | 1 | 0.008
HV0d | 573.1C | 1 | 0.008
HV0d | 573.4C | 1 | 0.008
HV0d | 5768 | 3 | 0.023
HV0d | 5814 | 1 | 0.008
HV0d | 5821 | 1 | 0.008
HV0d | 6095 | 1 | 0.008
HV0d | 64 | 2 | 0.015

HV0d | 7083 | 1 | 0.008
HV0d | 709 | 1 | 0.008
HV0d | 7543 | 1 | 0.008
HV0d | 7910 | 1 | 0.008
HV0d | 8736 | 1 | 0.008
HV0e | 12373 | 2 | 0.043
HV0e | 13105 | 1 | 0.022
HV0e | 143 | 2 | 0.043
HV0e | 14587 | 1 | 0.022
HV0e | 15289 | 1 | 0.022
HV0e | 16051 | 2 | 0.043
HV0e | 16092 | 3 | 0.065
HV0e | 16093 | 1 | 0.022
HV0e | 16124A | 1 | 0.022
HV0e | 16126A | 1 | 0.022
HV0e | 16129 | 3 | 0.065
HV0e | 16130C | 1 | 0.022
HV0e | 16187 | 4 | 0.087
HV0e | 16188 | 1 | 0.022
HV0e | 16189 | 2 | 0.043
HV0e | 16234 | 1 | 0.022
HV0e | 16257 | 1 | 0.022
HV0e | 16291 | 1 | 0.022
HV0e | 16327 | 1 | 0.022
HV0e | 198 | 2 | 0.043
HV0e | 200 | 3 | 0.065
HV0e | 204 | 1 | 0.022
HV0e | 2443 | 1 | 0.022
HV0e | 2905 | 1 | 0.022
HV0e | 309d | 1 | 0.022
HV0e | 310 | 2 | 0.043
HV0e | 3221 | 2 | 0.043
HV0e | 3335 | 1 | 0.022
HV0e | 8260 | 1 | 0.022
HV0e | 8347 | 1 | 0.022
HV0e | 894A | 1 | 0.022
HV0e | 895G | 1 | 0.022
HV0e | 897 | 1 | 0.022
HV0e | 9192 | 3 | 0.065
HV0e | 9525 | 1 | 0.022
HV0e | 9750 | 2 | 0.043
HV0f | 10873 | 1 | 0.011
HV0f | 115A | 1 | 0.011
HV0f | 11944 | 1 | 0.011
HV0f | 12026 | 1 | 0.011
HV0f | 12705 | 1 | 0.011
HV0f | 1275 | 3 | 0.033
HV0f | 13278 | 1 | 0.011
HV0f | 13708 | 1 | 0.011
HV0f | 14053 | 1 | 0.011
HV0f | 14323 | 1 | 0.011
HV0f | 14766 | 1 | 0.011
HV0f | 14783 | 1 | 0.011
HV0f | 150 | 1 | 0.011
HV0f | 152 | 2 | 0.022
HV0f | 15218 | 1 | 0.011
HV0f | 15301 | 1 | 0.011
HV0f | 15324 | 1 | 0.011
HV0f | 15459 | 1 | 0.011
HV0f | 15617 | 1 | 0.011
HV0f | 15905 | 1 | 0.011
HV0f | 16093 | 1 | 0.011
HV0f | 16212 | 1 | 0.011
HV0f | 16261 | 1 | 0.011
HV0f | 16274 | 1 | 0.011
HV0f | 16291 | 2 | 0.022
HV0f | 16311 | 7 | 0.077
HV0f | 16355 | 2 | 0.022
HV0f | 16362 | 3 | 0.033
HV0f | 16484-16489d | 4 | 0.044
HV0f | 228 | 1 | 0.011
HV0f | 307 | 1 | 0.011
HV0f | 310 | 2 | 0.022
HV0f | 315.2C | 1 | 0.011
HV0f | 315.3C | 1 | 0.011
HV0f | 316C | 1 | 0.011
HV0f | 3431 | 1 | 0.011
HV0f | 357C | 1 | 0.011
HV0f | 44.1C | 1 | 0.011
HV0f | 4655 | 1 | 0.011
HV0f | 4793 | 1 | 0.011
HV0f | 5004 | 1 | 0.011
HV0f | 515-524d | 3 | 0.033
HV0f | 6029 | 1 | 0.011
HV0f | 6281 | 1 | 0.011
HV0f | 6296A | 1 | 0.011
HV0f | 64 | 1 | 0.011
HV0f | 6776 | 1 | 0.011
HV0f | 6962 | 1 | 0.011
HV0f | 709 | 1 | 0.011
HV0f | 72 | 4 | 0.044
HV0f | 73 | 5 | 0.055
HV0f | 7419 | 1 | 0.011
HV0f | 7521 | 1 | 0.011
HV0f | 7813 | 1 | 0.011
HV0f | 8014 | 1 | 0.011
HV0f | 93 | 4 | 0.044
HV0f | 9329 | 1 | 0.011
HV0f | 9540 | 1 | 0.011
HV0g | 131 | 1 | 0.05
HV0g | 15154A | 1 | 0.05
HV0g | 16025 | 1 | 0.05
HV0g | 16026 | 1 | 0.05
HV0g | 16166 | 1 | 0.05
HV0g | 16172 | 4 | 0.2
HV0g | 16271A | 2 | 0.1
HV0g | 310 | 2 | 0.1
HV0g | 7646 | 1 | 0.05
HV0g | 93 | 1 | 0.05
HV1 | 114 | 1 | 0.011
HV1 | 16176 | 1 | 0.011

HV1 | 16354 | 1 | 0.011
HV1 | 16356 | 2 | 0.023
HV1 | 195 | 3 | 0.034
HV1 | 199 | 1 | 0.011
HV1 | 316 | 3 | 0.034
HV1 | 389T | 1 | 0.011
HV1 | 4766 | 1 | 0.011
HV1 | 9548 | 1 | 0.011
HV10 | 16129 | 1 | 0.003
HV10 | 16150 | 1 | 0.003
HV10 | 16159 | 1 | 0.003
HV10 | 16177 | 1 | 0.003
HV10 | 16284 | 35 | 0.104
HV10 | 16566 | 1 | 0.003
HV10 | 200 | 1 | 0.003
HV10 | 227 | 1 | 0.003
HV10 | 237 | 1 | 0.003
HV10 | 246 | 1 | 0.003
HV10 | 288 | 1 | 0.003
HV10 | 310 | 1 | 0.003
HV10 | 315.2C | 1 | 0.003
HV10 | 3882 | 1 | 0.003
HV10 | 484 | 1 | 0.003
HV10 | 499 | 1 | 0.003
HV10 | 7754 | 1 | 0.003
HV10 | 8289.1CCCCCTCTA | 4 | 0.012
HV11 | 146 | 1 | 0.003
HV11 | 150 | 38 | 0.113
HV11 | 16093 | 5 | 0.015
HV11 | 16150 | 1 | 0.003
HV11 | 16159 | 1 | 0.003
HV11 | 16177 | 1 | 0.003
HV11 | 16284 | 1 | 0.003
HV11 | 16319 | 35 | 0.104
HV11 | 16566 | 1 | 0.003
HV11 | 237 | 1 | 0.003
HV11 | 246 | 1 | 0.003
HV11 | 288 | 1 | 0.003
HV11 | 4336 | 1 | 0.003
HV11 | 484 | 1 | 0.003
HV11 | 499 | 35 | 0.104
HV11a | 12803 | 1 | 0.003
HV11a | 15110 | 1 | 0.003
HV11a | 15775 | 1 | 0.003
HV11a | 16150 | 1 | 0.003
HV11a | 16159 | 1 | 0.003
HV11a | 16177 | 1 | 0.003
HV11a | 16284 | 35 | 0.105
HV11a | 16566 | 1 | 0.003
HV11a | 215 | 1 | 0.003
HV11a | 237 | 1 | 0.003
HV11a | 246 | 1 | 0.003
HV11a | 288 | 1 | 0.003
HV11a | 310 | 1 | 0.003
HV11a | 316C | 1 | 0.003
HV11a | 484 | 1 | 0.003
HV12 | 113 | 1 | 0.001
HV12 | 14497 | 1 | 0.001
HV12 | 15718 | 1 | 0.001
HV12 | 16076A | 123 | 0.155
HV12 | 208 | 1 | 0.001
HV12 | 261 | 1 | 0.001
HV12 | 281C | 1 | 0.001
HV12 | 316d | 1 | 0.001
HV12 | 338 | 1 | 0.001
HV12 | 468 | 3 | 0.004
HV12 | 502 | 1 | 0.001
HV12 | 573.1C | 3 | 0.004
HV12 | 573.2C | 5 | 0.006
HV12 | 573.5C | 1 | 0.001
HV12 | 7934 | 1 | 0.001
HV12a | 146 | 6 | 0.072
HV12a | 16093 | 5 | 0.06
HV12a | 16172 | 1 | 0.012
HV12a | 16234G | 5 | 0.06
HV12a | 16264 | 1 | 0.012
HV12a | 16278 | 1 | 0.012
HV12a | 16294 | 1 | 0.012
HV12a | 16342 | 1 | 0.012
HV12a | 16355 | 6 | 0.072
HV12a | 1766 | 1 | 0.012
HV12a | 1888C | 5 | 0.06
HV12a | 310 | 4 | 0.048
HV12a | 456 | 1 | 0.012
HV12a | 9944 | 5 | 0.06
HV12a1 | 13708 | 1 | 0.034
HV12a1 | 1393 | 1 | 0.034
HV12a1 | 15402G | 1 | 0.034
HV12a1 | 16261 | 1 | 0.034
HV12a1 | 16362 | 2 | 0.069
HV12a1 | 234 | 1 | 0.034
HV12a1 | 235 | 2 | 0.069
HV12a1 | 457A | 1 | 0.034
HV12a1 | 489 | 2 | 0.069
HV12b | 114 | 1 | 0.012
HV12b | 16078 | 2 | 0.025
HV12b | 16086 | 1 | 0.012
HV12b | 16129 | 1 | 0.012
HV12b | 16163 | 1 | 0.012
HV12b | 16174 | 1 | 0.012
HV12b | 16192A | 1 | 0.012
HV12b | 16243 | 1 | 0.012
HV12b | 16263 | 1 | 0.012
HV12b | 16298 | 7 | 0.088
HV12b | 16309 | 1 | 0.012
HV12b | 16318T | 1 | 0.012
HV12b | 16362 | 2 | 0.025
HV12b | 16484-16489d | 1 | 0.012
HV12b | 189 | 2 | 0.025
HV12b | 227T | 1 | 0.012
HV12b | 248 | 1 | 0.012

HV12b | 309.3C | 5 | 0.062
HV12b | 315.2C | 1 | 0.012
HV12b | 316C | 1 | 0.012
HV12b | 477 | 2 | 0.025
HV12b | 573.1C | 1 | 0.012
HV12b1 | 11204 | 1 | 0.01
HV12b1 | 1284 | 1 | 0.01
HV12b1 | 15725 | 1 | 0.01
HV12b1 | 15924 | 1 | 0.01
HV12b1 | 16124 | 3 | 0.029
HV12b1 | 16129 | 2 | 0.019
HV12b1 | 16209 | 6 | 0.058
HV12b1 | 16218 | 7 | 0.068
HV12b1 | 16222 | 1 | 0.01
HV12b1 | 16223 | 1 | 0.01
HV12b1 | 16242 | 6 | 0.058
HV12b1 | 16265 | 1 | 0.01
HV12b1 | 16273 | 1 | 0.01
HV12b1 | 16311 | 1 | 0.01
HV12b1 | 16325 | 1 | 0.01
HV12b1 | 16335 | 1 | 0.01
HV12b1 | 16353 | 1 | 0.01
HV12b1 | 16362 | 1 | 0.01
HV12b1 | 16399 | 1 | 0.01
HV12b1 | 236 | 1 | 0.01
HV12b1 | 310 | 1 | 0.01
HV12b1 | 4353 | 2 | 0.019
HV12b1 | 4883A | 1 | 0.01
HV12b1 | 5252 | 1 | 0.01
HV12b1 | 73 | 8 | 0.078
HV12b1 | 7473 | 1 | 0.01
HV12b1 | 7498 | 1 | 0.01
HV12b1 | 7568 | 1 | 0.01
HV12b1 | 7852 | 1 | 0.01
HV12b1 | 8251 | 1 | 0.01
HV12b1 | 9452 | 1 | 0.01
HV12b1 | 961 | 1 | 0.01
HV12b1a | 15884 | 1 | 0.015
HV12b1a | 16069 | 1 | 0.015
HV12b1a | 16096 | 1 | 0.015
HV12b1a | 189 | 2 | 0.029
HV12b1a | 298 | 2 | 0.029
HV12b1a | 4171 | 1 | 0.015
HV12b1a | 7645 | 1 | 0.015
HV13 | 12879 | 1 | 0.012
HV13 | 14569 | 1 | 0.012
HV13 | 16129 | 1 | 0.012
HV13 | 16173 | 1 | 0.012
HV13 | 16189 | 3 | 0.037
HV13 | 16266A | 1 | 0.012
HV13 | 310 | 1 | 0.012
HV13 | 5460 | 1 | 0.012
HV13 | 7664 | 1 | 0.012
HV13a | 146 | 1 | 0.011
HV13a | 16092 | 1 | 0.011
HV13a | 16093 | 4 | 0.045
HV13a | 16145 | 1 | 0.011
HV13a | 16187 | 1 | 0.011
HV13a | 16233 | 1 | 0.011
HV13a | 16243 | 1 | 0.011
HV13a | 16526 | 1 | 0.011
HV13a | 16527 | 1 | 0.011
HV13a | 308 | 1 | 0.011
HV13a | 310 | 1 | 0.011
HV13a | 480 | 2 | 0.022
HV13a | 73 | 3 | 0.034
HV13b | 11002 | 1 | 0.011
HV13b | 11152 | 2 | 0.022
HV13b | 12879 | 1 | 0.011
HV13b | 14052 | 3 | 0.032
HV13b | 146 | 1 | 0.011
HV13b | 152 | 1 | 0.011
HV13b | 16092 | 2 | 0.022
HV13b | 16093 | 1 | 0.011
HV13b | 16129 | 3 | 0.032
HV13b | 16176 | 1 | 0.011
HV13b | 16184 | 17 | 0.183
HV13b | 16188 | 2 | 0.022
HV13b | 16209 | 1 | 0.011
HV13b | 16263 | 1 | 0.011
HV13b | 16291 | 4 | 0.043
HV13b | 16304 | 1 | 0.011
HV13b | 16355 | 1 | 0.011
HV13b | 16497 | 1 | 0.011
HV13b | 1654 | 2 | 0.022
HV13b | 199 | 1 | 0.011
HV13b | 200 | 1 | 0.011
HV13b | 7308 | 1 | 0.011
HV13b | 9377 | 2 | 0.022
HV13b | 9989 | 3 | 0.032
HV14 | 10188 | 1 | 0.004
HV14 | 124T | 1 | 0.004
HV14 | 14d | 2 | 0.007
HV14 | 16040 | 1 | 0.004
HV14 | 16105G | 2 | 0.007
HV14 | 16124A | 2 | 0.007
HV14 | 16144A | 2 | 0.007
HV14 | 16145 | 2 | 0.007
HV14 | 16148 | 1 | 0.004
HV14 | 16150 | 1 | 0.004
HV14 | 16159 | 1 | 0.004
HV14 | 16180 | 1 | 0.004
HV14 | 16216 | 2 | 0.007
HV14 | 16241 | 1 | 0.004
HV14 | 16248 | 1 | 0.004
HV14 | 16256 | 1 | 0.004
HV14 | 16312 | 2 | 0.007
HV14 | 16313 | 1 | 0.004
HV14 | 16362 | 1 | 0.004
HV14 | 16399 | 3 | 0.011
HV14 | 16488 | 34 | 0.123

HV14 | 16504 | 1 | 0.004
HV14 | 215 | 2 | 0.007
HV14 | 288 | 1 | 0.004
HV14 | 310 | 1 | 0.004
HV14 | 372 | 1 | 0.004
HV14 | 411G | 1 | 0.004
HV14 | 414G | 1 | 0.004
HV14 | 459d | 33 | 0.12
HV14 | 683 | 1 | 0.004
HV14 | 701C | 1 | 0.004
HV14 | 784d | 1 | 0.004
HV14 | 8289.1CCCCCTCTA | 1 | 0.004
HV14 | 9.1C | 2 | 0.007
HV14 | 905C | 1 | 0.004
HV14a | 10084 | 5 | 0.015
HV14a | 10172 | 3 | 0.009
HV14a | 11440 | 1 | 0.003
HV14a | 11719 | 1 | 0.003
HV14a | 11778 | 1 | 0.003
HV14a | 11830 | 2 | 0.006
HV14a | 12097A | 1 | 0.003
HV14a | 12098G | 1 | 0.003
HV14a | 14110 | 1 | 0.003
HV14a | 146 | 46 | 0.142
HV14a | 150 | 45 | 0.139
HV14a | 153 | 1 | 0.003
HV14a | 15679 | 3 | 0.009
HV14a | 16093 | 3 | 0.009
HV14a | 16131 | 1 | 0.003
HV14a | 16142 | 1 | 0.003
HV14a | 16150 | 1 | 0.003
HV14a | 16159 | 1 | 0.003
HV14a | 16168 | 3 | 0.009
HV14a | 16193 | 5 | 0.015
HV14a | 16219 | 1 | 0.003
HV14a | 16220T | 1 | 0.003
HV14a | 16224 | 2 | 0.006
HV14a | 16249A | 1 | 0.003
HV14a | 16274 | 2 | 0.006
HV14a | 16291 | 1 | 0.003
HV14a | 16304 | 44 | 0.136
HV14a | 16308 | 1 | 0.003
HV14a | 16324A | 35 | 0.108
HV14a | 16357 | 5 | 0.015
HV14a | 1842 | 1 | 0.003
HV14a | 194 | 1 | 0.003
HV14a | 288 | 1 | 0.003
HV14a | 310 | 1 | 0.003
HV14a | 3106A | 1 | 0.003
HV14a | 372 | 1 | 0.003
HV14a | 3834 | 38 | 0.117
HV14a | 3858 | 2 | 0.006
HV14a | 411G | 1 | 0.003
HV14a | 4204 | 1 | 0.003
HV14a | 4512 | 2 | 0.006
HV14a | 5642.1T | 1 | 0.003
HV14a | 5823 | 3 | 0.009
HV14a | 6221 | 1 | 0.003
HV14a | 6569 | 1 | 0.003
HV14a | 6671 | 2 | 0.006
HV14a | 683 | 1 | 0.003
HV14a | 701C | 1 | 0.003
HV14a | 7148 | 1 | 0.003
HV14a | 7269 | 3 | 0.009
HV14a | 7754 | 1 | 0.003
HV14a | 784d | 1 | 0.003
HV14a | 8251 | 1 | 0.003
HV14a | 905C | 1 | 0.003
HV15 | 10581 | 1 | 0.011
HV15 | 13368 | 1 | 0.011
HV15 | 14061 | 1 | 0.011
HV15 | 14608 | 1 | 0.011
HV15 | 14627 | 1 | 0.011
HV15 | 150 | 1 | 0.011
HV15 | 152 | 2 | 0.023
HV15 | 16158C | 2 | 0.023
HV15 | 16274 | 1 | 0.011
HV15 | 16298 | 1 | 0.011
HV15 | 16362 | 3 | 0.034
HV15 | 1835 | 1 | 0.011
HV15 | 195 | 3 | 0.034
HV15 | 200 | 7 | 0.08
HV15 | 310 | 2 | 0.023
HV15 | 4561 | 1 | 0.011
HV15 | 513 | 1 | 0.011
HV15 | 6267 | 1 | 0.011
HV15 | 8821 | 1 | 0.011
HV15 | 9350 | 1 | 0.011
HV15 | 9758 | 1 | 0.011
HV16 | 1193 | 1 | 0.003
HV16 | 12599 | 1 | 0.003
HV16 | 12654 | 2 | 0.006
HV16 | 146 | 3 | 0.009
HV16 | 150 | 1 | 0.003
HV16 | 15213 | 1 | 0.003
HV16 | 15784 | 1 | 0.003
HV16 | 15949 | 1 | 0.003
HV16 | 16051 | 1 | 0.003
HV16 | 16150 | 1 | 0.003
HV16 | 16159 | 1 | 0.003
HV16 | 16177 | 1 | 0.003
HV16 | 16239 | 2 | 0.006
HV16 | 16284 | 1 | 0.003
HV16 | 16286 | 36 | 0.104
HV16 | 16566 | 1 | 0.003
HV16 | 237 | 1 | 0.003
HV16 | 246 | 1 | 0.003
HV16 | 288 | 1 | 0.003
HV16 | 310 | 1 | 0.003
HV16 | 4204 | 1 | 0.003
HV16 | 472-482d | 1 | 0.003

HV16 | 4787 | 1 | 0.003
HV16 | 484 | 1 | 0.003
HV16 | 5581 | 9 | 0.026
HV16 | 5595 | 1 | 0.003
HV16 | 8059 | 1 | 0.003
HV16 | 9123 | 2 | 0.006
HV16 | 9425C | 1 | 0.003
HV16 | 9792 | 1 | 0.003
HV17 | 146 | 1 | 0.013
HV17 | 150 | 5 | 0.064
HV17 | 152 | 1 | 0.013
HV17 | 16071 | 5 | 0.064
HV17 | 16147 | 5 | 0.064
HV17 | 16203 | 5 | 0.064
HV17 | 73 | 5 | 0.064
HV17a | 152 | 2 | 0.05
HV17a | 16189 | 1 | 0.025
HV17a | 16274 | 1 | 0.025
HV17a | 16291 | 1 | 0.025
HV17a | 195 | 2 | 0.05
HV17a | 2905 | 1 | 0.025
HV17a | 4100 | 1 | 0.025
HV17a | 5208 | 1 | 0.025
HV17a | 7424 | 1 | 0.025
HV17a | 7925 | 1 | 0.025
HV18 | 12127 | 2 | 0.009
HV18 | 13818 | 1 | 0.005
HV18 | 143 | 4 | 0.018
HV18 | 1462 | 1 | 0.005
HV18 | 150 | 1 | 0.005
HV18 | 152 | 1 | 0.005
HV18 | 15261 | 2 | 0.009
HV18 | 16111 | 1 | 0.005
HV18 | 16168 | 1 | 0.005
HV18 | 16233 | 1 | 0.005
HV18 | 16243 | 1 | 0.005
HV18 | 16258T | 1 | 0.005
HV18 | 16309 | 1 | 0.005
HV18 | 16316 | 24 | 0.11
HV18 | 16362 | 19 | 0.087
HV18 | 189 | 1 | 0.005
HV18 | 207 | 1 | 0.005
HV18 | 297 | 1 | 0.005
HV18 | 298 | 3 | 0.014
HV18 | 310 | 2 | 0.009
HV18 | 315.2C | 1 | 0.005
HV18 | 319.1T | 1 | 0.005
HV18 | 3350 | 2 | 0.009
HV18 | 3394 | 1 | 0.005
HV18 | 3849 | 1 | 0.005
HV18 | 4136 | 1 | 0.005
HV18 | 4322.1C | 1 | 0.005
HV18 | 4562 | 1 | 0.005
HV18 | 480 | 1 | 0.005
HV18 | 4832 | 1 | 0.005
HV18 | 4892 | 2 | 0.009
HV18 | 499 | 1 | 0.005
HV18 | 554T | 1 | 0.005
HV18 | 567T | 1 | 0.005
HV18 | 6248 | 1 | 0.005
HV18 | 6285 | 1 | 0.005
HV18 | 6632 | 3 | 0.014
HV18 | 7624 | 1 | 0.005
HV18 | 7775 | 2 | 0.009
HV18 | 960.1C | 1 | 0.005
HV19 | 13966 | 1 | 0.012
HV19 | 146 | 3 | 0.037
HV19 | 152 | 2 | 0.024
HV19 | 16093 | 1 | 0.012
HV19 | 16213 | 3 | 0.037
HV19 | 16264 | 2 | 0.024
HV19 | 16290 | 1 | 0.012
HV19 | 316C | 1 | 0.012
HV19 | 5585 | 1 | 0.012
HV19 | 73 | 3 | 0.037
HV19 | 8311 | 1 | 0.012
HV19 | 9716 | 1 | 0.012
HV1a | 114 | 1 | 0.011
HV1a | 13801 | 1 | 0.011
HV1a | 151 | 1 | 0.011
HV1a | 152 | 1 | 0.011
HV1a | 15287 | 1 | 0.011
HV1a | 16176 | 1 | 0.011
HV1a | 16354 | 1 | 0.011
HV1a | 16356 | 2 | 0.023
HV1a | 16400 | 1 | 0.011
HV1a | 1692 | 1 | 0.011
HV1a | 195 | 3 | 0.034
HV1a | 199 | 1 | 0.011
HV1a | 310 | 1 | 0.011
HV1a | 315.2C | 1 | 0.011
HV1a | 316 | 3 | 0.034
HV1a | 389T | 1 | 0.011
HV1a | 990 | 1 | 0.011
HV1a1 | 10172 | 1 | 0.03
HV1a1 | 11549 | 3 | 0.091
HV1a1 | 15692-15694d | 2 | 0.061
HV1a1 | 15697 | 1 | 0.03
HV1a1 | 15721 | 1 | 0.03
HV1a1 | 16172 | 2 | 0.061
HV1a1 | 16209 | 1 | 0.03
HV1a1 | 16263 | 1 | 0.03
HV1a1 | 204 | 1 | 0.03
HV1a1 | 257 | 1 | 0.03
HV1a1 | 310 | 1 | 0.03
HV1a1 | 311 | 1 | 0.03
HV1a1 | 3531 | 2 | 0.061
HV1a1 | 3666 | 1 | 0.03
HV1a1 | 4227 | 2 | 0.061
HV1a1 | 6150 | 3 | 0.091
HV1a1 | 8020 | 1 | 0.03

HV1a1 | 8278.2C | 4 | 0.121
HV1a1 | 8278.3C | 1 | 0.03
HV1a1 | 8278.4C | 1 | 0.03
HV1a1 | 8279 | 1 | 0.03
HV1a1 | 8868 | 1 | 0.03
HV1a1 | 9725 | 2 | 0.061
HV1a1a | 10203 | 1 | 0.024
HV1a1a | 13392 | 2 | 0.049
HV1a1a | 13650 | 1 | 0.024
HV1a1a | 15004 | 1 | 0.024
HV1a1a | 15110 | 1 | 0.024
HV1a1a | 15610 | 1 | 0.024
HV1a1a | 15617 | 1 | 0.024
HV1a1a | 16092 | 3 | 0.073
HV1a1a | 16260 | 4 | 0.098
HV1a1a | 16311 | 1 | 0.024
HV1a1a | 16354 | 1 | 0.024
HV1a1a | 249d | 5 | 0.122
HV1a1a | 2831 | 1 | 0.024
HV1a1a | 309 | 1 | 0.024
HV1a1a | 3110d | 5 | 0.122
HV1a1a | 385 | 4 | 0.098
HV1a1a | 4011 | 1 | 0.024
HV1a1a | 4257 | 1 | 0.024
HV1a1a | 4688 | 2 | 0.049
HV1a1a | 6779 | 4 | 0.098
HV1a1a | 73 | 1 | 0.024
HV1a1a | 8278.2C | 2 | 0.049
HV1a1a | 8278.3C | 1 | 0.024
HV1a1a | 8278.4C | 3 | 0.073
HV1a1a | 8279 | 1 | 0.024
HV1a1a | 8281-8289d | 3 | 0.073
HV1a1a | 9007 | 3 | 0.073
HV1a1a | 9021 | 4 | 0.098
HV1a1a | 93 | 1 | 0.024
HV1a1b | 152 | 1 | 0.029
HV1a1b | 16071 | 2 | 0.059
HV1a1b | 16167 | 1 | 0.029
HV1a1b | 16239 | 1 | 0.029
HV1a1b | 16284 | 5 | 0.147
HV1a1b | 4823 | 1 | 0.029
HV1a1b | 6935 | 1 | 0.029
HV1a1b | 93 | 4 | 0.118
HV1a2 | 11080 | 1 | 0.011
HV1a2 | 11233 | 2 | 0.021
HV1a2 | 12361 | 1 | 0.011
HV1a2 | 12601 | 1 | 0.011
HV1a2 | 14022 | 1 | 0.011
HV1a2 | 15796 | 1 | 0.011
HV1a2 | 16240 | 1 | 0.011
HV1a2 | 16298 | 1 | 0.011
HV1a2 | 16362 | 2 | 0.021
HV1a2 | 1664C | 1 | 0.011
HV1a2 | 310 | 8 | 0.085
HV1a2 | 374 | 1 | 0.011
HV1a2 | 3801 | 1 | 0.011
HV1a2 | 73 | 3 | 0.032
HV1a2 | 7403 | 2 | 0.021
HV1a2 | 8279 | 1 | 0.011
HV1a2 | 8280C | 1 | 0.011
HV1a2 | 8994 | 1 | 0.011
HV1a2 | 9007 | 2 | 0.021
HV1a2a | 13317 | 2 | 0.024
HV1a2a | 16176 | 1 | 0.012
HV1a2a | 16192 | 2 | 0.024
HV1a2a | 16220C | 1 | 0.012
HV1a2a | 16354 | 1 | 0.012
HV1a2a | 199 | 1 | 0.012
HV1a2a | 6060C | 2 | 0.024
HV1a2a | 9438 | 1 | 0.012
HV1a2b | 16131 | 1 | 0.023
HV1a2b | 16147 | 2 | 0.047
HV1a2b | 16153 | 1 | 0.023
HV1a2b | 16172 | 1 | 0.023
HV1a2b | 16183 | 3 | 0.07
HV1a2b | 16249 | 1 | 0.023
HV1a2b | 16256 | 1 | 0.023
HV1a2b | 16278 | 1 | 0.023
HV1a2b | 16291 | 1 | 0.023
HV1a2b | 16292 | 11 | 0.256
HV1a2b | 16327A | 1 | 0.023
HV1a2b | 16354 | 1 | 0.023
HV1a2b | 2848 | 2 | 0.047
HV1a2b | 508 | 1 | 0.023
HV1a2b | 64 | 9 | 0.209
HV1a2b | 8278.2C | 1 | 0.023
HV1a2b | 8278-8279d | 1 | 0.023
HV1a3 | 13656 | 1 | 0.013
HV1a3 | 13980 | 1 | 0.013
HV1a3 | 152 | 1 | 0.013
HV1a3 | 15244 | 1 | 0.013
HV1a3 | 16147 | 2 | 0.026
HV1a3 | 16183 | 5 | 0.066
HV1a3 | 16294 | 1 | 0.013
HV1a3 | 227 | 1 | 0.013
HV1a3 | 4580 | 1 | 0.013
HV1a3 | 4655 | 1 | 0.013
HV1a3 | 513 | 1 | 0.013
HV1a3 | 8278.3C | 1 | 0.013
HV1a3 | 8618 | 1 | 0.013
HV1a3 | 9163 | 1 | 0.013
HV1a3 | 9770 | 1 | 0.013
HV1a3a | 15778 | 1 | 0.014
HV1a3a | 16183 | 1 | 0.014
HV1a3a | 16311 | 1 | 0.014
HV1a3a | 8278.4C | 1 | 0.014
HV1a3a | 8279 | 1 | 0.014
HV1a3a | 8837 | 1 | 0.014
HV1a3a | 94 | 1 | 0.014
HV1a'b'c | 10365 | 1 | 0.011
HV1a'b'c | 13535 | 1 | 0.011

HV1a'b'c | 146 | 1 | 0.011
HV1a'b'c | 15140 | 1 | 0.011
HV1a'b'c | 152 | 2 | 0.022
HV1a'b'c | 15805 | 1 | 0.011
HV1a'b'c | 16129 | 14 | 0.151
HV1a'b'c | 16242 | 3 | 0.032
HV1a'b'c | 16311 | 1 | 0.011
HV1a'b'c | 16317 | 1 | 0.011
HV1a'b'c | 16368 | 9 | 0.097
HV1a'b'c | 201 | 1 | 0.011
HV1a'b'c | 2403 | 1 | 0.011
HV1a'b'c | 2851 | 1 | 0.011
HV1a'b'c | 310 | 1 | 0.011
HV1a'b'c | 3906 | 3 | 0.032
HV1a'b'c | 4722 | 1 | 0.011
HV1a'b'c | 5483 | 2 | 0.022
HV1a'b'c | 6002 | 1 | 0.011
HV1a'b'c | 6956 | 2 | 0.022
HV1a'b'c | 709 | 5 | 0.054
HV1a'b'c | 729 | 1 | 0.011
HV1a'b'c | 7569 | 1 | 0.011
HV1a'b'c | 8376 | 1 | 0.011
HV1a'b'c | 9018 | 2 | 0.022
HV1a'b'c | 9692 | 4 | 0.043
HV1a'b'c | 9755 | 1 | 0.011
HV1b | 10801 | 1 | 0.012
HV1b | 12373 | 1 | 0.012
HV1b | 12477 | 1 | 0.012
HV1b | 13287 | 1 | 0.012
HV1b | 13368 | 1 | 0.012
HV1b | 14207 | 1 | 0.012
HV1b | 150 | 1 | 0.012
HV1b | 16176 | 1 | 0.012
HV1b | 16291 | 1 | 0.012
HV1b | 16354 | 1 | 0.012
HV1b | 199 | 1 | 0.012
HV1b | 2903 | 1 | 0.012
HV1b | 3107-3108d | 1 | 0.012
HV1b | 3290 | 1 | 0.012
HV1b | 3398 | 2 | 0.024
HV1b | 3547 | 1 | 0.012
HV1b | 5134 | 1 | 0.012
HV1b | 6023 | 1 | 0.012
HV1b | 6263 | 1 | 0.012
HV1b | 6266 | 1 | 0.012
HV1b | 7353 | 1 | 0.012
HV1b | 8251 | 1 | 0.012
HV1b | 8419 | 1 | 0.012
HV1b | 8545 | 1 | 0.012
HV1b | 9585 | 1 | 0.012
HV1b+152 | 14305 | 1 | 0.023
HV1b+152 | 16093 | 2 | 0.045
HV1b+152 | 16129 | 2 | 0.045
HV1b+152 | 16158 | 1 | 0.023
HV1b+152 | 16166 | 1 | 0.023
HV1b+152 | 16215 | 1 | 0.023
HV1b+152 | 16234 | 4 | 0.091
HV1b+152 | 16242 | 1 | 0.023
HV1b+152 | 16311 | 7 | 0.159
HV1b+152 | 16327 | 1 | 0.023
HV1b+152 | 16335 | 1 | 0.023
HV1b+152 | 498d | 1 | 0.023
HV1b+152 | 560 | 1 | 0.023
HV1b1 | 16235T | 1 | 0.013
HV1b1 | 16265 | 2 | 0.026
HV1b1 | 16304 | 1 | 0.013
HV1b1 | 200 | 1 | 0.013
HV1b1 | 3687 | 1 | 0.013
HV1b1 | 515-524d | 2 | 0.026
HV1b1a | 13708 | 2 | 0.026
HV1b1a | 152 | 1 | 0.013
HV1b1a | 16235T | 1 | 0.013
HV1b1a | 16304 | 1 | 0.013
HV1b1a | 200 | 1 | 0.013
HV1b1a | 2755 | 2 | 0.026
HV1b1a | 3591 | 1 | 0.013
HV1b1a | 7912 | 1 | 0.013
HV1b1a | 8027 | 1 | 0.013
HV1b1a | 9117 | 2 | 0.026
HV1b1b | 14464 | 1 | 0.013
HV1b1b | 16129 | 3 | 0.038
HV1b1b | 16158 | 2 | 0.025
HV1b1b | 16235T | 1 | 0.013
HV1b1b | 16304 | 1 | 0.013
HV1b1b | 16368 | 2 | 0.025
HV1b1b | 200 | 1 | 0.013
HV1b1b | 4856 | 1 | 0.013
HV1b1b | 5460 | 1 | 0.013
HV1b1b | 709 | 1 | 0.013
HV1b1b | 961 | 3 | 0.038
HV1b2 | 11081T | 1 | 0.024
HV1b2 | 131 | 1 | 0.024
HV1b2 | 13434 | 1 | 0.024
HV1b2 | 15463 | 1 | 0.024
HV1b2 | 16037 | 1 | 0.024
HV1b2 | 16129 | 1 | 0.024
HV1b2 | 16311 | 1 | 0.024
HV1b2 | 1694 | 1 | 0.024
HV1b2 | 5033 | 1 | 0.024
HV1b2 | 5327 | 1 | 0.024
HV1b2 | 6137 | 1 | 0.024
HV1b2 | 7888 | 1 | 0.024
HV1b2 | 9438 | 1 | 0.024
HV1b3 | 10044 | 3 | 0.2
HV1b3 | 151 | 4 | 0.267
HV1b3 | 16294 | 1 | 0.067
HV1b3 | 16304 | 1 | 0.067
HV1b3 | 16356 | 3 | 0.2
HV1b3 | 198 | 3 | 0.2
HV1b3 | 3531 | 1 | 0.067
HV1b3a | 16129 | 6 | 0.24

HV1b3a | 16166 | 3 | 0.12
HV1b3a | 16178 | 1 | 0.04
HV1b3a | 16215 | 4 | 0.16
HV1b3a | 16234 | 4 | 0.16
HV1b3a | 16258C | 1 | 0.04
HV1b3a | 16290 | 1 | 0.04
HV1b3a | 16327 | 7 | 0.28
HV1b3a | 16335 | 4 | 0.16
HV1b3a | 16362 | 2 | 0.08
HV1b3a | 16368 | 3 | 0.12
HV1b3a | 16399 | 1 | 0.04
HV1b3b | 10532 | 1 | 0.083
HV1b3b | 12879 | 1 | 0.083
HV1b3b | 14040 | 1 | 0.083
HV1b3b | 146 | 1 | 0.083
HV1b3b | 16188 | 2 | 0.167
HV1b3b | 16319 | 2 | 0.167
HV1b3b | 8020 | 1 | 0.083
HV1b3b | 817 | 1 | 0.083
HV1b3b | 8715 | 1 | 0.083
HV1b3b | 8754 | 1 | 0.083
HV1c | 12630 | 2 | 0.022
HV1c | 13713A | 1 | 0.011
HV1c | 13748 | 1 | 0.011
HV1c | 14305 | 2 | 0.022
HV1c | 15930 | 3 | 0.032
HV1c | 16093 | 1 | 0.011
HV1c | 16166 | 2 | 0.022
HV1c | 16172 | 9 | 0.097
HV1c | 16242A | 1 | 0.011
HV1c | 16243 | 2 | 0.022
HV1c | 16261 | 1 | 0.011
HV1c | 16311 | 5 | 0.054
HV1c | 16362 | 1 | 0.011
HV1c | 16366 | 2 | 0.022
HV1c | 182 | 2 | 0.022
HV1c | 207 | 1 | 0.011
HV1c | 228 | 1 | 0.011
HV1c | 4561 | 2 | 0.022
HV1c | 65 | 1 | 0.011
HV1c | 7349A | 2 | 0.022
HV1c | 7930 | 1 | 0.011
HV1c | 8020 | 1 | 0.011
HV1c | 9055 | 2 | 0.022
HV1d | 12396 | 1 | 0.5
HV1d | 6054T | 1 | 0.5
HV1d | 6284 | 1 | 0.5
HV1d | 709 | 1 | 0.5
HV1d | 72G | 1 | 0.5
HV1d | 8584 | 1 | 0.5
HV2 | 11167 | 1 | 0.013
HV2 | 11719 | 1 | 0.013
HV2 | 12408 | 4 | 0.053
HV2 | 12477 | 1 | 0.013
HV2 | 12630 | 1 | 0.013
HV2 | 12681 | 12 | 0.16
HV2 | 12705 | 1 | 0.013
HV2 | 13269 | 1 | 0.013
HV2 | 13469 | 1 | 0.013
HV2 | 13543G | 1 | 0.013
HV2 | 13603C | 1 | 0.013
HV2 | 13708 | 11 | 0.147
HV2 | 13768 | 7 | 0.093
HV2 | 14016 | 1 | 0.013
HV2 | 14176T | 1 | 0.013
HV2 | 14766 | 1 | 0.013
HV2 | 14767 | 2 | 0.027
HV2 | 15262 | 1 | 0.013
HV2 | 15930 | 1 | 0.013
HV2 | 16000 | 1 | 0.013
HV2 | 16173 | 4 | 0.053
HV2 | 16256 | 4 | 0.053
HV2 | 16311 | 1 | 0.013
HV2 | 16325 | 2 | 0.027
HV2 | 200 | 1 | 0.013
HV2 | 204 | 1 | 0.013
HV2 | 207 | 1 | 0.013
HV2 | 2080 | 1 | 0.013
HV2 | 2248 | 1 | 0.013
HV2 | 247 | 1 | 0.013
HV2 | 3311 | 12 | 0.16
HV2 | 455.1T | 16 | 0.213
HV2 | 4562 | 4 | 0.053
HV2 | 4615 | 12 | 0.16
HV2 | 499 | 4 | 0.053
HV2 | 5319 | 1 | 0.013
HV2 | 5471 | 1 | 0.013
HV2 | 5899.1C | 6 | 0.08
HV2 | 5899.2C | 2 | 0.027
HV2 | 794 | 2 | 0.027
HV2 | 8289.1CCCCCTCTA | 1 | 0.013
HV2 | 8823 | 1 | 0.013
HV2 | 8843 | 12 | 0.16
HV2 | 9064 | 2 | 0.027
HV2 | 9096 | 2 | 0.027
HV2 | 9554 | 1 | 0.013
HV20 | 10685 | 1 | 0.008
HV20 | 12453 | 1 | 0.008
HV20 | 14256 | 3 | 0.023
HV20 | 16189 | 3 | 0.023
HV20 | 16390 | 3 | 0.023
HV20 | 1733 | 3 | 0.023
HV20 | 3606 | 3 | 0.023
HV20 | 7444 | 1 | 0.008
HV20 | 8345 | 1 | 0.008
HV20 | 8772 | 3 | 0.023
HV20 | 9704T | 1 | 0.008
HV21 | 11167 | 1 | 0.009
HV21 | 135.1T | 1 | 0.009
HV21 | 146 | 1 | 0.009
HV21 | 152 | 5 | 0.046

HV21 | 16111 | 1 | 0.009
HV21 | 16147 | 2 | 0.019
HV21 | 16189 | 1 | 0.009
HV21 | 16193 | 1 | 0.009
HV21 | 16219 | 1 | 0.009
HV21 | 16222 | 1 | 0.009
HV21 | 16241 | 9 | 0.083
HV21 | 16274 | 1 | 0.009
HV21 | 16305 | 2 | 0.019
HV21 | 16311 | 2 | 0.019
HV21 | 16355 | 2 | 0.019
HV21 | 16362 | 8 | 0.074
HV21 | 16527 | 1 | 0.009
HV21 | 195 | 2 | 0.019
HV21 | 200 | 7 | 0.065
HV21 | 262 | 1 | 0.009
HV21 | 3316 | 1 | 0.009
HV21 | 338 | 1 | 0.009
HV21 | 3397 | 3 | 0.028
HV21 | 455.1T | 1 | 0.009
HV21 | 60.1T | 1 | 0.009
HV21 | 644 | 3 | 0.028
HV21 | 72A | 1 | 0.009
HV21 | 7436 | 3 | 0.028
HV21 | 7706 | 1 | 0.009
HV21 | 8715 | 1 | 0.009
HV21 | 9007 | 1 | 0.009
HV21 | 93 | 8 | 0.074
HV22 | 13870 | 1 | 0.056
HV22 | 15172 | 1 | 0.056
HV22 | 16243 | 1 | 0.056
HV22 | 6366 | 1 | 0.056
HV22 | 8614 | 1 | 0.056
HV23 | 12624 | 1 | 0.003
HV23 | 14323 | 1 | 0.003
HV23 | 16150 | 1 | 0.003
HV23 | 16159 | 1 | 0.003
HV23 | 16177 | 1 | 0.003
HV23 | 16284 | 3 | 0.009
HV23 | 16391 | 1 | 0.003
HV23 | 16566 | 1 | 0.003
HV23 | 199 | 34 | 0.102
HV23 | 237 | 1 | 0.003
HV23 | 246 | 1 | 0.003
HV23 | 288 | 1 | 0.003
HV23 | 484 | 1 | 0.003
HV23 | 593 | 1 | 0.003
HV24 | 12940 | 2 | 0.006
HV24 | 15530 | 1 | 0.003
HV24 | 16150 | 1 | 0.003
HV24 | 16159 | 1 | 0.003
HV24 | 16177 | 1 | 0.003
HV24 | 16284 | 35 | 0.105
HV24 | 16566 | 1 | 0.003
HV24 | 237 | 1 | 0.003
HV24 | 246 | 1 | 0.003
HV24 | 288 | 1 | 0.003
HV24 | 484 | 1 | 0.003
HV24 | 5417 | 1 | 0.003
HV24 | 8143 | 2 | 0.006
HV2a | 11353 | 1 | 0.031
HV2a | 11614 | 1 | 0.031
HV2a | 13581 | 1 | 0.031
HV2a | 16126 | 1 | 0.031
HV2a | 16189 | 1 | 0.031
HV2a | 16240 | 1 | 0.031
HV2a | 16290 | 1 | 0.031
HV2a | 310 | 1 | 0.031
HV2a | 3106A | 1 | 0.031
HV2a | 6182 | 1 | 0.031
HV2a | 739 | 2 | 0.062
HV2a | 8277G | 1 | 0.031
HV2a | 8381 | 1 | 0.031
HV2a | 8691 | 1 | 0.031
HV2a | 9539 | 1 | 0.031
HV2a1 | 10304 | 1 | 0.023
HV2a1 | 10457G | 1 | 0.023
HV2a1 | 10969 | 3 | 0.068
HV2a1 | 11002 | 1 | 0.023
HV2a1 | 11254 | 1 | 0.023
HV2a1 | 11881 | 1 | 0.023
HV2a1 | 12192 | 5 | 0.114
HV2a1 | 12280 | 1 | 0.023
HV2a1 | 12939 | 1 | 0.023
HV2a1 | 13204 | 1 | 0.023
HV2a1 | 13651 | 1 | 0.023
HV2a1 | 13708 | 1 | 0.023
HV2a1 | 14208 | 1 | 0.023
HV2a1 | 146 | 1 | 0.023
HV2a1 | 151 | 7 | 0.159
HV2a1 | 15164 | 2 | 0.045
HV2a1 | 15287 | 1 | 0.023
HV2a1 | 15613 | 1 | 0.023
HV2a1 | 15850G | 5 | 0.114
HV2a1 | 15930 | 1 | 0.023
HV2a1 | 16093 | 1 | 0.023
HV2a1 | 16140 | 1 | 0.023
HV2a1 | 16223 | 3 | 0.068
HV2a1 | 16260 | 1 | 0.023
HV2a1 | 16274 | 2 | 0.045
HV2a1 | 16284 | 1 | 0.023
HV2a1 | 16311 | 2 | 0.045
HV2a1 | 16325 | 1 | 0.023
HV2a1 | 16398 | 1 | 0.023
HV2a1 | 198 | 1 | 0.023
HV2a1 | 207 | 1 | 0.023
HV2a1 | 246 | 28 | 0.636
HV2a1 | 252 | 1 | 0.023
HV2a1 | 279 | 1 | 0.023
HV2a1 | 310 | 2 | 0.045
HV2a1 | 315.2C | 1 | 0.023

HV2a1 | 4491 | 1 | 0.023
HV2a1 | 4580 | 1 | 0.023
HV2a1 | 4706C | 1 | 0.023
HV2a1 | 4946 | 5 | 0.114
HV2a1 | 573.1C | 1 | 0.023
HV2a1 | 573.2C | 2 | 0.045
HV2a1 | 573.3C | 5 | 0.114
HV2a1 | 573.4C | 2 | 0.045
HV2a1 | 573.5C | 1 | 0.023
HV2a1 | 574C | 4 | 0.091
HV2a1 | 576C | 1 | 0.023
HV2a1 | 5773 | 1 | 0.023
HV2a1 | 5821 | 1 | 0.023
HV2a1 | 5824 | 5 | 0.114
HV2a1 | 5899.1C | 2 | 0.045
HV2a1 | 596 | 1 | 0.023
HV2a1 | 629 | 5 | 0.114
HV2a1 | 6491 | 7 | 0.159
HV2a1 | 6632 | 2 | 0.045
HV2a1 | 709 | 1 | 0.023
HV2a1 | 7684 | 1 | 0.023
HV2a1 | 7927 | 1 | 0.023
HV2a1 | 9591 | 1 | 0.023
HV2a1 | 9617 | 4 | 0.091
HV2a1 | 9804 | 1 | 0.023
HV2a2 | 10306 | 7 | 0.137
HV2a2 | 11084 | 1 | 0.02
HV2a2 | 13184 | 1 | 0.02
HV2a2 | 14258 | 2 | 0.039
HV2a2 | 14319 | 1 | 0.02
HV2a2 | 15244 | 1 | 0.02
HV2a2 | 15792 | 1 | 0.02
HV2a2 | 16167 | 4 | 0.078
HV2a2 | 16168 | 1 | 0.02
HV2a2 | 16184A | 5 | 0.098
HV2a2 | 16189 | 2 | 0.039
HV2a2 | 16243 | 3 | 0.059
HV2a2 | 16256 | 8 | 0.157
HV2a2 | 16261 | 6 | 0.118
HV2a2 | 16287 | 1 | 0.02
HV2a2 | 16309 | 1 | 0.02
HV2a2 | 16325 | 2 | 0.039
HV2a2 | 16362 | 2 | 0.039
HV2a2 | 266 | 1 | 0.02
HV2a2 | 310 | 1 | 0.02
HV2a2 | 3742 | 1 | 0.02
HV2a2 | 392 | 1 | 0.02
HV2a2 | 6563 | 2 | 0.039
HV2a2 | 9055 | 7 | 0.137
HV2a3 | 12372 | 1 | 0.022
HV2a3 | 16051 | 1 | 0.022
HV2a3 | 16093 | 2 | 0.043
HV2a3 | 16129 | 1 | 0.022
HV2a3 | 16172 | 5 | 0.109
HV2a3 | 16189 | 1 | 0.022
HV2a3 | 16234 | 2 | 0.043
HV2a3 | 16243 | 6 | 0.13
HV2a3 | 16311 | 1 | 0.022
HV2a3 | 16325 | 1 | 0.022
HV2a3 | 16336 | 2 | 0.043
HV2a3 | 16352 | 1 | 0.022
HV2a3 | 16359 | 1 | 0.022
HV2a3 | 16456 | 2 | 0.043
HV2a3 | 198 | 1 | 0.022
HV2a3 | 246 | 1 | 0.022
HV2a3 | 263C | 1 | 0.022
HV2a3 | 266 | 1 | 0.022
HV2a3 | 310 | 1 | 0.022
HV2a3 | 5414 | 1 | 0.022
HV4 | 10314 | 1 | 0.001
HV4 | 10356 | 3 | 0.004
HV4 | 10790 | 1 | 0.001
HV4 | 11014 | 1 | 0.001
HV4 | 11087 | 1 | 0.001
HV4 | 11129T | 1 | 0.001
HV4 | 11290T | 1 | 0.001
HV4 | 113 | 1 | 0.001
HV4 | 11722 | 1 | 0.001
HV4 | 11914 | 1 | 0.001
HV4 | 12061 | 2 | 0.002
HV4 | 13389 | 1 | 0.001
HV4 | 13656 | 1 | 0.001
HV4 | 13866 | 1 | 0.001
HV4 | 14180 | 1 | 0.001
HV4 | 14470 | 1 | 0.001
HV4 | 150 | 1 | 0.001
HV4 | 15071 | 1 | 0.001
HV4 | 152 | 1 | 0.001
HV4 | 15244 | 1 | 0.001
HV4 | 15514 | 5 | 0.006
HV4 | 15924 | 1 | 0.001
HV4 | 16076A | 1 | 0.001
HV4 | 16129 | 1 | 0.001
HV4 | 16176 | 1 | 0.001
HV4 | 16192A | 129 | 0.159
HV4 | 16265C | 1 | 0.001
HV4 | 16295 | 1 | 0.001
HV4 | 199 | 1 | 0.001
HV4 | 208 | 1 | 0.001
HV4 | 2220C | 1 | 0.001
HV4 | 2392 | 1 | 0.001
HV4 | 261 | 1 | 0.001
HV4 | 281C | 1 | 0.001
HV4 | 310 | 1 | 0.001
HV4 | 316d | 1 | 0.001
HV4 | 338 | 1 | 0.001
HV4 | 3398 | 1 | 0.001
HV4 | 3808 | 1 | 0.001
HV4 | 390 | 124 | 0.153
HV4 | 468 | 3 | 0.004
HV4 | 489 | 1 | 0.001

HV4 | 502 | 1 | 0.001
HV4 | 524A | 1 | 0.001
HV4 | 573.1C | 3 | 0.004
HV4 | 573.2C | 5 | 0.006
HV4 | 573.5C | 1 | 0.001
HV4 | 5902 | 1 | 0.001
HV4 | 63 | 1 | 0.001
HV4 | 6971 | 1 | 0.001
HV4 | 7701 | 1 | 0.001
HV4 | 8703 | 1 | 0.001
HV4 | 9096 | 1 | 0.001
HV4 | 9139 | 1 | 0.001
HV4 | 9355 | 4 | 0.005
HV4a | 16083 | 1 | 0.012
HV4a | 16092 | 1 | 0.012
HV4a | 16126 | 1 | 0.012
HV4a | 16222A | 1 | 0.012
HV4a | 16256 | 1 | 0.012
HV4a1 | 10493 | 1 | 0.01
HV4a1 | 11389 | 2 | 0.021
HV4a1 | 11440 | 4 | 0.042
HV4a1 | 11944 | 2 | 0.021
HV4a1 | 12397 | 2 | 0.021
HV4a1 | 12399 | 1 | 0.01
HV4a1 | 12781 | 2 | 0.021
HV4a1 | 13527 | 1 | 0.01
HV4a1 | 14518 | 2 | 0.021
HV4a1 | 146 | 3 | 0.031
HV4a1 | 152 | 14 | 0.146
HV4a1 | 15442 | 1 | 0.01
HV4a1 | 16126 | 1 | 0.01
HV4a1 | 16147 | 1 | 0.01
HV4a1 | 16260 | 1 | 0.01
HV4a1 | 16319 | 3 | 0.031
HV4a1 | 3254 | 2 | 0.021
HV4a1 | 3645 | 1 | 0.01
HV4a1 | 4212 | 2 | 0.021
HV4a1 | 4277 | 2 | 0.021
HV4a1 | 455.1T | 1 | 0.01
HV4a1 | 4768 | 1 | 0.01
HV4a1 | 5978 | 2 | 0.021
HV4a1 | 6680 | 1 | 0.01
HV4a1 | 8260 | 4 | 0.042
HV4a1 | 8639 | 1 | 0.01
HV4a1 | 8648 | 1 | 0.01
HV4a1 | 8794 | 1 | 0.01
HV4a1+16291 | 12972 | 1 | 0.012
HV4a1+16291 | 204 | 1 | 0.012
HV4a1+16291 | 345 | 1 | 0.012
HV4a1+16291 | 64 | 1 | 0.012
HV4a1+16291 | 709 | 1 | 0.012
HV4a1+16291 | 7843 | 1 | 0.012
HV4a1+16291 | 93 | 1 | 0.012
HV4a1+16291 | 9977 | 1 | 0.012
HV4a1a | 10325 | 1 | 0.011
HV4a1a | 11641 | 1 | 0.011
HV4a1a | 12477 | 1 | 0.011
HV4a1a | 12909 | 1 | 0.011
HV4a1a | 13135 | 1 | 0.011
HV4a1a | 13327 | 1 | 0.011
HV4a1a | 13455 | 1 | 0.011
HV4a1a | 13928C | 4 | 0.043
HV4a1a | 14325 | 1 | 0.011
HV4a1a | 16172 | 4 | 0.043
HV4a1a | 16189 | 2 | 0.021
HV4a1a | 16241 | 1 | 0.011
HV4a1a | 16300 | 4 | 0.043
HV4a1a | 16399 | 1 | 0.011
HV4a1a | 195 | 1 | 0.011
HV4a1a | 6340 | 1 | 0.011
HV4a1a | 6575 | 1 | 0.011
HV4a1a | 7258 | 1 | 0.011
HV4a1a | 7769 | 1 | 0.011
HV4a1a | 7915 | 1 | 0.011
HV4a1a | 9755 | 4 | 0.043
HV4a1a | 9959 | 1 | 0.011
HV4a1a1 | 16302 | 1 | 0.038
HV4a1a1 | 207 | 2 | 0.077
HV4a1a2 | 10192 | 2 | 0.026
HV4a1a2 | 11914 | 1 | 0.013
HV4a1a2 | 14118 | 1 | 0.013
HV4a1a2 | 146 | 1 | 0.013
HV4a1a2 | 152 | 1 | 0.013
HV4a1a2 | 15934 | 1 | 0.013
HV4a1a2 | 16213 | 2 | 0.026
HV4a1a2 | 1717 | 1 | 0.013
HV4a1a2 | 3630 | 2 | 0.026
HV4a1a2 | 4314 | 1 | 0.013
HV4a1a3 | 309.3C | 1 | 0.013
HV4a1a4 | 12043A | 3 | 0.034
HV4a1a4 | 152 | 7 | 0.08
HV4a1a4 | 15301 | 1 | 0.011
HV4a1a4 | 16311 | 1 | 0.011
HV4a1a4 | 16355 | 3 | 0.034
HV4a1a4 | 251 | 1 | 0.011
HV4a1a4 | 6230 | 3 | 0.034
HV4a1a4 | 9091 | 1 | 0.011
HV4a2 | 10599 | 1 | 0.014
HV4a2 | 146 | 1 | 0.014
HV4a2 | 152 | 3 | 0.041
HV4a2 | 16301 | 1 | 0.014
HV4a2 | 16309 | 1 | 0.014
HV4a2 | 2380 | 3 | 0.041
HV4a2 | 709 | 1 | 0.014
HV4a2 | 7114 | 1 | 0.014
HV4a2 | 8035 | 3 | 0.041
HV4a2 | 93 | 1 | 0.014
HV4a2 | 9797 | 3 | 0.041
HV4a2a | 152 | 1 | 0.014
HV4a2a | 16189 | 1 | 0.014
HV4a2a | 16209 | 1 | 0.014

HV4a2a | 204 | 1 | 0.014
HV4a2b | 16301 | 1 | 0.014
HV4a2b | 236 | 1 | 0.014
HV4a2b | 5298 | 2 | 0.027
HV4a2b | 7808 | 2 | 0.027
HV4a2b | 8251 | 1 | 0.014
HV4a2b | 9055 | 1 | 0.014
HV4a2b | 93 | 2 | 0.027
HV4b | 12557 | 1 | 0.009
HV4b | 12950 | 1 | 0.009
HV4b | 12952 | 1 | 0.009
HV4b | 13735A | 1 | 0.009
HV4b | 146 | 9 | 0.08
HV4b | 1530 | 1 | 0.009
HV4b | 15777 | 3 | 0.027
HV4b | 15910 | 3 | 0.027
HV4b | 15943 | 1 | 0.009
HV4b | 16126 | 1 | 0.009
HV4b | 16193 | 1 | 0.009
HV4b | 16227 | 1 | 0.009
HV4b | 16318C | 1 | 0.009
HV4b | 16319 | 1 | 0.009
HV4b | 16354 | 1 | 0.009
HV4b | 16355 | 5 | 0.044
HV4b | 16359 | 1 | 0.009
HV4b | 1760 | 1 | 0.009
HV4b | 207 | 2 | 0.018
HV4b | 310 | 2 | 0.018
HV4b | 4375 | 1 | 0.009
HV4b | 709 | 4 | 0.035
HV4b | 7278 | 3 | 0.027
HV4b | 8618 | 1 | 0.009
HV4b | 9053 | 1 | 0.009
HV4b | 9182 | 1 | 0.009
HV4b | 9278 | 1 | 0.009
HV4c | 11252 | 1 | 0.001
HV4c | 113 | 1 | 0.001
HV4c | 16076A | 123 | 0.155
HV4c | 16168 | 1 | 0.001
HV4c | 208 | 1 | 0.001
HV4c | 261 | 1 | 0.001
HV4c | 281C | 1 | 0.001
HV4c | 316d | 1 | 0.001
HV4c | 338 | 1 | 0.001
HV4c | 468 | 3 | 0.004
HV4c | 502 | 1 | 0.001
HV4c | 573.1C | 3 | 0.004
HV4c | 573.2C | 5 | 0.006
HV4c | 573.5C | 1 | 0.001
HV4c | 6317 | 1 | 0.001
HV5 | 10993 | 2 | 0.003
HV5 | 113 | 1 | 0.001
HV5 | 11800 | 2 | 0.003
HV5 | 13020 | 2 | 0.003
HV5 | 14323 | 2 | 0.003
HV5 | 152 | 2 | 0.003
HV5 | 153 | 2 | 0.003
HV5 | 16076A | 123 | 0.154
HV5 | 16319 | 2 | 0.003
HV5 | 194 | 2 | 0.003
HV5 | 208 | 1 | 0.001
HV5 | 261 | 1 | 0.001
HV5 | 281C | 1 | 0.001
HV5 | 316d | 1 | 0.001
HV5 | 338 | 1 | 0.001
HV5 | 468 | 3 | 0.004
HV5 | 502 | 1 | 0.001
HV5 | 573.1C | 3 | 0.004
HV5 | 573.2C | 5 | 0.006
HV5 | 573.5C | 1 | 0.001
HV5 | 6216 | 2 | 0.003
HV5 | 6230 | 2 | 0.003
HV5 | 6293 | 2 | 0.003
HV5 | 8636 | 2 | 0.003
HV5a | 113 | 1 | 0.001
HV5a | 14259 | 1 | 0.001
HV5a | 16076A | 123 | 0.154
HV5a | 208 | 1 | 0.001
HV5a | 261 | 1 | 0.001
HV5a | 281C | 1 | 0.001
HV5a | 316d | 1 | 0.001
HV5a | 338 | 1 | 0.001
HV5a | 3397 | 2 | 0.002
HV5a | 468 | 3 | 0.004
HV5a | 502 | 1 | 0.001
HV5a | 573.1C | 3 | 0.004
HV5a | 573.2C | 5 | 0.006
HV5a | 573.5C | 1 | 0.001
HV5a | 8119 | 1 | 0.001
HV5b | 114 | 1 | 0.013
HV5b | 12759 | 1 | 0.013
HV5b | 13812 | 1 | 0.013
HV5b | 14803 | 1 | 0.013
HV5b | 15132 | 1 | 0.013
HV5b | 15556 | 1 | 0.013
HV5b | 16042 | 4 | 0.052
HV5b | 16163 | 1 | 0.013
HV5b | 16174 | 1 | 0.013
HV5b | 16212 | 1 | 0.013
HV5b | 16243 | 1 | 0.013
HV5b | 16263 | 1 | 0.013
HV5b | 16269 | 5 | 0.065
HV5b | 16288 | 2 | 0.026
HV5b | 16290 | 2 | 0.026
HV5b | 200 | 6 | 0.078
HV5b | 227T | 1 | 0.013
HV5b | 248 | 1 | 0.013
HV5b | 315.2C | 1 | 0.013
HV5b | 316C | 1 | 0.013
HV5b | 3513 | 2 | 0.026
HV5b | 374 | 5 | 0.065

HV5b | 4580 | 1 | 0.013
HV5b | 4958 | 2 | 0.026
HV5b | 573.1C | 1 | 0.013
HV6 | 11152 | 1 | 0.009
HV6 | 11969 | 1 | 0.009
HV6 | 143 | 4 | 0.035
HV6 | 146 | 1 | 0.009
HV6 | 14696 | 1 | 0.009
HV6 | 152 | 1 | 0.009
HV6 | 15586 | 1 | 0.009
HV6 | 16114 | 1 | 0.009
HV6 | 16188 | 1 | 0.009
HV6 | 16189 | 5 | 0.044
HV6 | 16209 | 1 | 0.009
HV6 | 16259 | 2 | 0.018
HV6 | 16266 | 1 | 0.009
HV6 | 16300 | 1 | 0.009
HV6 | 16327 | 1 | 0.009
HV6 | 16352 | 3 | 0.027
HV6 | 16362 | 1 | 0.009
HV6 | 16526 | 1 | 0.009
HV6 | 1719 | 1 | 0.009
HV6 | 200 | 1 | 0.009
HV6 | 214 | 1 | 0.009
HV6 | 3360 | 5 | 0.044
HV6 | 573.4C | 1 | 0.009
HV6 | 593 | 1 | 0.009
HV6 | 711 | 2 | 0.018
HV6 | 75 | 1 | 0.009
HV6 | 8119 | 1 | 0.009
HV6 | 8772 | 4 | 0.035
HV6a | 12612 | 1 | 0.014
HV6a | 316d | 1 | 0.014
HV6a | 9630 | 1 | 0.014
HV7 | 12133 | 1 | 0.013
HV7 | 12684 | 1 | 0.013
HV7 | 12918 | 1 | 0.013
HV7 | 13191 | 1 | 0.013
HV7 | 146 | 5 | 0.063
HV7 | 14965 | 1 | 0.013
HV7 | 16160 | 1 | 0.013
HV7 | 16170T | 2 | 0.025
HV7 | 16212 | 1 | 0.013
HV7 | 16238 | 1 | 0.013
HV7 | 16256 | 2 | 0.025
HV7 | 16274 | 2 | 0.025
HV7 | 16304 | 1 | 0.013
HV7 | 248 | 1 | 0.013
HV7 | 7948 | 1 | 0.013
HV7 | 8167 | 1 | 0.013
HV7 | 8380 | 2 | 0.025
HV7 | 93 | 2 | 0.025
HV7 | 930 | 1 | 0.013
HV7 | 9344 | 1 | 0.013
HV7 | 95C | 2 | 0.025
HV8 | 10978 | 1 | 0.011
HV8 | 152 | 7 | 0.08
HV8 | 15904A | 1 | 0.011
HV8 | 16309 | 1 | 0.011
HV8 | 2850 | 1 | 0.011
HV8 | 4580 | 1 | 0.011
HV8 | 466 | 1 | 0.011
HV8 | 482 | 1 | 0.011
HV8 | 6060 | 1 | 0.011
HV8 | 709 | 1 | 0.011
HV9 | 14145 | 3 | 0.009
HV9 | 14605 | 1 | 0.003
HV9 | 15406 | 1 | 0.003
HV9 | 15734 | 1 | 0.003
HV9 | 16150 | 1 | 0.003
HV9 | 16159 | 1 | 0.003
HV9 | 16167 | 2 | 0.006
HV9 | 16177 | 1 | 0.003
HV9 | 16284 | 1 | 0.003
HV9 | 16362 | 37 | 0.107
HV9 | 16566 | 1 | 0.003
HV9 | 237 | 1 | 0.003
HV9 | 246 | 1 | 0.003
HV9 | 288 | 1 | 0.003
HV9 | 310 | 4 | 0.012
HV9 | 484 | 1 | 0.003
HV9 | 4958 | 1 | 0.003
HV9 | 5009 | 3 | 0.009
HV9 | 633T | 1 | 0.003
HV9 | 6366 | 1 | 0.003
HV9 | 7340 | 1 | 0.003
HV9 | 9056 | 1 | 0.003
HV9 | 9368 | 1 | 0.003
HV9+152 | 13449 | 1 | 0.006
HV9+152 | 14142 | 1 | 0.006
HV9+152 | 15169 | 1 | 0.006
HV9+152 | 15461 | 1 | 0.006
HV9+152 | 16093 | 1 | 0.006
HV9+152 | 16104 | 1 | 0.006
HV9+152 | 16129 | 3 | 0.018
HV9+152 | 16186 | 1 | 0.006
HV9+152 | 16189 | 1 | 0.006
HV9+152 | 16217 | 1 | 0.006
HV9+152 | 16293 | 2 | 0.012
HV9+152 | 234 | 1 | 0.006
HV9+152 | 249 | 1 | 0.006
HV9+152 | 3915 | 1 | 0.006
HV9a | 16390 | 1 | 0.008
HV9a1 | 12892 | 1 | 0.008
HV9a1 | 146 | 1 | 0.008
HV9a1 | 16254 | 1 | 0.008
HV9a1 | 16255 | 1 | 0.008
HV9a1 | 309d | 1 | 0.008
HV9a1 | 310 | 1 | 0.008
HV9a1 | 315.2C | 1 | 0.008
HV9a1a | 10364 | 1 | 0.008

HV9a1a | 14182G | 2 | 0.016
HV9a1a | 195 | 2 | 0.016
HV9a1a | 4080 | 1 | 0.008
HV9a1a | 9947 | 2 | 0.016
HV9b | 12406 | 2 | 0.019
HV9b | 13651 | 4 | 0.038
HV9b | 146 | 1 | 0.01
HV9b | 150 | 1 | 0.01
HV9b | 16086 | 1 | 0.01
HV9b | 16172 | 1 | 0.01
HV9b | 16261 | 2 | 0.019
HV9b | 310 | 2 | 0.019
HV9b | 315.2C | 2 | 0.019
HV9b | 408A | 1 | 0.01
HV9b | 7990 | 1 | 0.01
HV9c | 113 | 1 | 0.001
HV9c | 14440 | 1 | 0.001
HV9c | 16076A | 123 | 0.154
HV9c | 16286 | 1 | 0.001
HV9c | 208 | 1 | 0.001
HV9c | 261 | 1 | 0.001
HV9c | 281C | 1 | 0.001
HV9c | 316d | 1 | 0.001
HV9c | 338 | 1 | 0.001
HV9c | 468 | 3 | 0.004
HV9c | 502 | 1 | 0.001
HV9c | 5372 | 1 | 0.001
HV9c | 573.1C | 4 | 0.005
HV9c | 573.2C | 5 | 0.006
HV9c | 573.5C | 1 | 0.001
HV9c | 8419 | 1 | 0.001
HV9c | 9096 | 1 | 0.001
HV9c | 9449 | 1 | 0.001
HV9c | 9554 | 1 | 0.001
I | 10124 | 1 | 0.014
I | 10405G | 1 | 0.014
I | 10407T | 1 | 0.014
I | 1120 | 2 | 0.029
I | 1183 | 2 | 0.029
I | 11929 | 1 | 0.014
I | 13572A | 1 | 0.014
I | 13813 | 2 | 0.029
I | 1415 | 1 | 0.014
I | 14552 | 1 | 0.014
I | 14839 | 1 | 0.014
I | 14969 | 1 | 0.014
I | 152 | 5 | 0.071
I | 15204 | 2 | 0.029
I | 15244 | 1 | 0.014
I | 15439 | 1 | 0.014
I | 15511 | 1 | 0.014
I | 15553 | 2 | 0.029
I | 15951 | 1 | 0.014
I | 16093 | 1 | 0.014
I | 16148 | 1 | 0.014
I | 16166d | 3 | 0.043
I | 16168 | 3 | 0.043
I | 16172 | 3 | 0.043
I | 16173 | 3 | 0.043
I | 16184 | 3 | 0.043
I | 16311 | 4 | 0.057
I | 16320 | 1 | 0.014
I | 1822 | 2 | 0.029
I | 1900 | 2 | 0.029
I | 194 | 3 | 0.043
I | 2898 | 1 | 0.014
I | 291.1A | 6 | 0.086
I | 310 | 1 | 0.014
I | 315.2C | 1 | 0.014
I | 324 | 1 | 0.014
I | 3316 | 2 | 0.029
I | 3438 | 1 | 0.014
I | 3447 | 1 | 0.014
I | 3565 | 2 | 0.029
I | 4083 | 2 | 0.029
I | 4772 | 2 | 0.029
I | 4976 | 1 | 0.014
I | 5147 | 1 | 0.014
I | 5174 | 1 | 0.014
I | 5319 | 1 | 0.014
I | 5460 | 2 | 0.029
I | 5480 | 1 | 0.014
I | 5483 | 1 | 0.014
I | 573.1CCCCCACAGTTTATGTAGCC | 1 | 0.014
I | 573.1TCCC | 2 | 0.029
I | 574C | 1 | 0.014
I | 5821 | 1 | 0.014
I | 6221 | 3 | 0.043
I | 6251 | 1 | 0.014
I | 6854 | 1 | 0.014
I | 6956 | 1 | 0.014
I | 709 | 2 | 0.029
I | 7299 | 1 | 0.014
I | 7337 | 1 | 0.014
I | 7362 | 3 | 0.043
I | 7609 | 1 | 0.014
I | 8089 | 2 | 0.029
I | 8547 | 2 | 0.029
I | 8603 | 1 | 0.014
I | 8882 | 1 | 0.014
I | 9716 | 1 | 0.014
I | 9801 | 1 | 0.014
I | 9804 | 1 | 0.014
I1 | 10589 | 1 | 0.03
I1 | 11016 | 2 | 0.061
I1 | 1120 | 1 | 0.03
I1 | 11392C | 1 | 0.03
I1 | 11887 | 2 | 0.061
I1 | 11914 | 1 | 0.03
I1 | 13194 | 3 | 0.091
I1 | 13254 | 1 | 0.03

I1 | 13314 | 1 | 0.03
I1 | 13708 | 1 | 0.03
I1 | 14596 | 1 | 0.03
I1 | 14757 | 2 | 0.061
I1 | 152 | 5 | 0.152
I1 | 15221C | 1 | 0.03
I1 | 16093 | 1 | 0.03
I1 | 16140 | 1 | 0.03
I1 | 16188 | 1 | 0.03
I1 | 16272 | 1 | 0.03
I1 | 16274 | 1 | 0.03
I1 | 16278 | 8 | 0.242
I1 | 16357 | 2 | 0.061
I1 | 189 | 3 | 0.091
I1 | 211 | 3 | 0.091
I1 | 2244 | 1 | 0.03
I1 | 291.1AT | 1 | 0.03
I1 | 292.1AT | 1 | 0.03
I1 | 3010 | 1 | 0.03
I1 | 310 | 1 | 0.03
I1 | 4050 | 1 | 0.03
I1 | 5783 | 2 | 0.061
I1 | 5786 | 1 | 0.03
I1 | 5836 | 2 | 0.061
I1 | 5894C | 1 | 0.03
I1 | 5899.4C | 1 | 0.03
I1 | 64 | 1 | 0.03
I1 | 6524 | 1 | 0.03
I1 | 6578 | 1 | 0.03
I1 | 7013 | 1 | 0.03
I1 | 7705 | 1 | 0.03
I1 | 8050 | 1 | 0.03
I1 | 8440 | 2 | 0.061
I1 | 8642C | 1 | 0.03
I1 | 9233 | 3 | 0.091
I1 | 9377 | 2 | 0.061
I1 | 9767A | 2 | 0.061
I1a | 10327 | 2 | 0.074
I1a | 10609 | 1 | 0.037
I1a | 11149 | 2 | 0.074
I1a | 12864 | 1 | 0.037
I1a | 13557 | 1 | 0.037
I1a | 14182 | 1 | 0.037
I1a | 14395 | 2 | 0.074
I1a | 1531 | 1 | 0.037
I1a | 15589 | 1 | 0.037
I1a | 15601 | 1 | 0.037
I1a | 16234 | 1 | 0.037
I1a | 16255 | 1 | 0.037
I1a | 16390 | 1 | 0.037
I1a | 185C | 1 | 0.037
I1a | 207 | 1 | 0.037
I1a | 310 | 1 | 0.037
I1a | 316 | 1 | 0.037
I1a | 3316 | 2 | 0.074
I1a | 3744 | 2 | 0.074
I1a | 4964 | 1 | 0.037
I1a | 8260 | 2 | 0.074
I1a | 8260G | 1 | 0.037
I1a1 | 10310 | 2 | 0.026
I1a1 | 10454 | 1 | 0.013
I1a1 | 10685 | 1 | 0.013
I1a1 | 10718 | 1 | 0.013
I1a1 | 12535 | 1 | 0.013
I1a1 | 13064 | 1 | 0.013
I1a1 | 13386 | 1 | 0.013
I1a1 | 13629 | 1 | 0.013
I1a1 | 13681T | 1 | 0.013
I1a1 | 13790 | 1 | 0.013
I1a1 | 14180 | 1 | 0.013
I1a1 | 14392 | 1 | 0.013
I1a1 | 14769 | 1 | 0.013
I1a1 | 150 | 1 | 0.013
I1a1 | 15337 | 1 | 0.013
I1a1 | 15498 | 1 | 0.013
I1a1 | 15617 | 1 | 0.013
I1a1 | 15670 | 1 | 0.013
I1a1 | 15916 | 1 | 0.013
I1a1 | 15928 | 1 | 0.013
I1a1 | 15955 | 1 | 0.013
I1a1 | 16037 | 1 | 0.013
I1a1 | 16093 | 1 | 0.013
I1a1 | 16189 | 1 | 0.013
I1a1 | 16259 | 1 | 0.013
I1a1 | 16298 | 3 | 0.038
I1a1 | 16390 | 9 | 0.115
I1a1 | 189 | 4 | 0.051
I1a1 | 202 | 14 | 0.179
I1a1 | 2779 | 2 | 0.026
I1a1 | 2971 | 1 | 0.013
I1a1 | 302C | 1 | 0.013
I1a1 | 310.1T | 1 | 0.013
I1a1 | 3338 | 1 | 0.013
I1a1 | 3398 | 3 | 0.038
I1a1 | 375 | 1 | 0.013
I1a1 | 3866 | 1 | 0.013
I1a1 | 456 | 2 | 0.026
I1a1 | 4561 | 1 | 0.013
I1a1 | 498d | 1 | 0.013
I1a1 | 5263 | 1 | 0.013
I1a1 | 5351 | 1 | 0.013
I1a1 | 5460 | 4 | 0.051
I1a1 | 5504T | 1 | 0.013
I1a1 | 574C | 2 | 0.026
I1a1 | 6023 | 1 | 0.013
I1a1 | 6383 | 1 | 0.013
I1a1 | 6563 | 5 | 0.064
I1a1 | 7364 | 3 | 0.038
I1a1 | 7690 | 1 | 0.013
I1a1 | 8107 | 4 | 0.051
I1a1 | 8277 | 1 | 0.013

I1a1 | 8278.3C | 1 | 0.013
I1a1 | 8292 | 1 | 0.013
I1a1 | 8563 | 1 | 0.013
I1a1 | 9110 | 1 | 0.013
I1a1 | 9180 | 1 | 0.013
I1a1 | 93 | 9 | 0.115
I1a1 | 9380 | 1 | 0.013
I1a1 | 9614 | 1 | 0.013
I1a1 | 9617 | 1 | 0.013
I1a1 | 9840A | 1 | 0.013
I1a1a | 10463 | 2 | 0.026
I1a1a | 11425 | 1 | 0.013
I1a1a | 1193 | 1 | 0.013
I1a1a | 12792 | 1 | 0.013
I1a1a | 12904 | 1 | 0.013
I1a1a | 13617 | 1 | 0.013
I1a1a | 15184 | 3 | 0.038
I1a1a | 153 | 1 | 0.013
I1a1a | 15884 | 1 | 0.013
I1a1a | 15944d | 1 | 0.013
I1a1a | 16093 | 1 | 0.013
I1a1a | 1625 | 1 | 0.013
I1a1a | 16260 | 1 | 0.013
I1a1a | 16266 | 2 | 0.026
I1a1a | 16293T | 9 | 0.115
I1a1a | 16318 | 1 | 0.013
I1a1a | 16355 | 1 | 0.013
I1a1a | 189 | 1 | 0.013
I1a1a | 291.1A | 1 | 0.013
I1a1a | 309d | 1 | 0.013
I1a1a | 310 | 3 | 0.038
I1a1a | 315.2C | 2 | 0.026
I1a1a | 317.1C | 1 | 0.013
I1a1a | 322 | 1 | 0.013
I1a1a | 3229.1T | 1 | 0.013
I1a1a | 3486 | 2 | 0.026
I1a1a | 3513 | 1 | 0.013
I1a1a | 3627 | 1 | 0.013
I1a1a | 3666 | 1 | 0.013
I1a1a | 4033A | 1 | 0.013
I1a1a | 456.1T | 1 | 0.013
I1a1a | 465 | 1 | 0.013
I1a1a | 481 | 1 | 0.013
I1a1a | 5213 | 1 | 0.013
I1a1a | 5587 | 1 | 0.013
I1a1a | 5663 | 1 | 0.013
I1a1a | 567C | 1 | 0.013
I1a1a | 574C | 2 | 0.026
I1a1a | 576C | 1 | 0.013
I1a1a | 577C | 1 | 0.013
I1a1a | 593 | 1 | 0.013
I1a1a | 64 | 1 | 0.013
I1a1a | 6680 | 3 | 0.038
I1a1a | 7471.1C | 1 | 0.013
I1a1a | 7609 | 1 | 0.013
I1a1a | 7618 | 1 | 0.013
I1a1a | 8567 | 1 | 0.013
I1a1a | 9180 | 1 | 0.013
I1a1a | 9254 | 1 | 0.013
I1a1a | 9300 | 1 | 0.013
I1a1a | 9477 | 1 | 0.013
I1a1a1 | 15152 | 1 | 0.021
I1a1a1 | 16189 | 9 | 0.188
I1a1a1 | 7332 | 1 | 0.021
I1a1a1 | 8455 | 1 | 0.021
I1a1a2 | 16292 | 9 | 0.196
I1a1a2 | 295 | 1 | 0.022
I1a1a2 | 9531 | 1 | 0.022
I1a1a3 | 10256 | 1 | 0.111
I1a1a3 | 12084 | 1 | 0.111
I1a1a3 | 13443 | 1 | 0.111
I1a1a3 | 14182 | 1 | 0.111
I1a1a3 | 15731 | 2 | 0.222
I1a1a3 | 15884 | 1 | 0.111
I1a1a3 | 16274 | 1 | 0.111
I1a1a3 | 16290 | 1 | 0.111
I1a1a3 | 16352 | 2 | 0.222
I1a1a3 | 16362 | 2 | 0.222
I1a1a3 | 189 | 1 | 0.111
I1a1a3 | 243 | 1 | 0.111
I1a1a3 | 327 | 1 | 0.111
I1a1a3 | 6267 | 1 | 0.111
I1a1a3 | 6465 | 1 | 0.111
I1a1a3 | 794 | 2 | 0.222
I1a1a3a | 15758 | 1 | 0.143
I1a1a3a | 3834 | 1 | 0.143
I1a1a3a | 7075C | 1 | 0.143
I1a1a3a | 8588 | 1 | 0.143
I1a1a3a | 9853 | 1 | 0.143
I1a1a3a | 9966 | 1 | 0.143
I1a1b | 10373 | 3 | 0.044
I1a1b | 10410 | 1 | 0.015
I1a1b | 10463 | 1 | 0.015
I1a1b | 10493 | 1 | 0.015
I1a1b | 11233 | 1 | 0.015
I1a1b | 12011 | 1 | 0.015
I1a1b | 14180 | 1 | 0.015
I1a1b | 14502 | 3 | 0.044
I1a1b | 14580 | 1 | 0.015
I1a1b | 1462 | 1 | 0.015
I1a1b | 15344 | 1 | 0.015
I1a1b | 16082 | 1 | 0.015
I1a1b | 16111 | 1 | 0.015
I1a1b | 16136 | 3 | 0.044
I1a1b | 16209 | 1 | 0.015
I1a1b | 16362 | 12 | 0.176
I1a1b | 16390 | 3 | 0.044
I1a1b | 182 | 1 | 0.015
I1a1b | 1973 | 2 | 0.029
I1a1b | 4021 | 1 | 0.015
I1a1b | 455.2T | 1 | 0.015

I1a1b | 5417 | 1 | 0.015
I1a1b | 709 | 3 | 0.044
I1a1b | 7941 | 2 | 0.029
I1a1b | 7984C | 1 | 0.015
I1a1b | 8632 | 1 | 0.015
I1a1b | 8676 | 1 | 0.015
I1a1b | 9010 | 1 | 0.015
I1a1b | 9554 | 1 | 0.015
I1a1b | 9591 | 1 | 0.015
I1a1c | 10427 | 1 | 0.019
I1a1c | 10454 | 1 | 0.019
I1a1c | 146 | 11 | 0.204
I1a1c | 15322 | 1 | 0.019
I1a1c | 16093 | 1 | 0.019
I1a1c | 16355 | 1 | 0.019
I1a1c | 16368 | 1 | 0.019
I1a1c | 16390 | 9 | 0.167
I1a1c | 189 | 20 | 0.37
I1a1c | 2285 | 1 | 0.019
I1a1c | 4991 | 1 | 0.019
I1a1c | 5147 | 1 | 0.019
I1a1c | 574C | 1 | 0.019
I1a1c | 6221 | 1 | 0.019
I1a1c | 8615 | 9 | 0.167
I1a1c | 8701 | 9 | 0.167
I1a1d | 10310 | 1 | 0.111
I1a1d | 12966 | 1 | 0.111
I1a1d | 14890 | 3 | 0.333
I1a1d | 16173 | 1 | 0.111
I1a1d | 16265C | 3 | 0.333
I1a1d | 16362 | 1 | 0.111
I1a1d | 8281-8289d | 1 | 0.111
I1a1e | 11337 | 1 | 0.021
I1a1e | 13954 | 1 | 0.021
I1a1e | 14178 | 1 | 0.021
I1a1e | 146 | 1 | 0.021
I1a1e | 15789G | 1 | 0.021
I1a1e | 16271 | 1 | 0.021
I1a1e | 16320 | 9 | 0.191
I1a1e | 16362 | 1 | 0.021
I1a1e | 5585 | 1 | 0.021
I1a1e | 574C | 1 | 0.021
I1a1e | 576.2C | 1 | 0.021
I1a1e | 576C | 1 | 0.021
I1a1e | 5951 | 1 | 0.021
I1a1e | 7897 | 1 | 0.021
I1a1e | 9518 | 1 | 0.021
I1b | 10283 | 2 | 0.032
I1b | 10410 | 2 | 0.032
I1b | 10463 | 1 | 0.016
I1b | 10595 | 1 | 0.016
I1b | 11020 | 2 | 0.032
I1b | 11113 | 2 | 0.032
I1b | 11299 | 2 | 0.032
I1b | 11305 | 3 | 0.048
I1b | 11314 | 2 | 0.032
I1b | 11380 | 1 | 0.016
I1b | 11440 | 1 | 0.016
I1b | 11479 | 1 | 0.016
I1b | 11941 | 1 | 0.016
I1b | 12104A | 1 | 0.016
I1b | 12172 | 1 | 0.016
I1b | 12375 | 2 | 0.032
I1b | 12684 | 3 | 0.048
I1b | 13359 | 1 | 0.016
I1b | 13368 | 1 | 0.016
I1b | 13590 | 1 | 0.016
I1b | 13635 | 1 | 0.016
I1b | 13890 | 1 | 0.016
I1b | 143 | 1 | 0.016
I1b | 14484 | 1 | 0.016
I1b | 14569 | 1 | 0.016
I1b | 146 | 2 | 0.032
I1b | 14769 | 1 | 0.016
I1b | 14830 | 1 | 0.016
I1b | 150 | 3 | 0.048
I1b | 15045 | 1 | 0.016
I1b | 152 | 17 | 0.274
I1b | 15514 | 2 | 0.032
I1b | 15670 | 1 | 0.016
I1b | 15731 | 2 | 0.032
I1b | 15951 | 1 | 0.016
I1b | 16093 | 1 | 0.016
I1b | 16145 | 1 | 0.016
I1b | 16174 | 1 | 0.016
I1b | 16188 | 1 | 0.016
I1b | 16188A | 1 | 0.016
I1b | 16189 | 18 | 0.29
I1b | 16209 | 1 | 0.016
I1b | 16218 | 1 | 0.016
I1b | 16242 | 4 | 0.065
I1b | 16249 | 1 | 0.016
I1b | 16256 | 2 | 0.032
I1b | 16293d | 1 | 0.016
I1b | 16295 | 1 | 0.016
I1b | 16319 | 1 | 0.016
I1b | 16320 | 1 | 0.016
I1b | 16366 | 3 | 0.048
I1b | 16368 | 1 | 0.016
I1b | 16400 | 1 | 0.016
I1b | 183 | 1 | 0.016
I1b | 189 | 1 | 0.016
I1b | 203 | 1 | 0.016
I1b | 207 | 3 | 0.048
I1b | 2222 | 2 | 0.032
I1b | 291.1A | 1 | 0.016
I1b | 3010 | 2 | 0.032
I1b | 316 | 1 | 0.016
I1b | 3350 | 1 | 0.016
I1b | 3531 | 2 | 0.032
I1b | 3540 | 2 | 0.032

I1b | 3777 | 1 | 0.016
I1b | 3847 | 2 | 0.032
I1b | 4227 | 1 | 0.016
I1b | 4317 | 1 | 0.016
I1b | 437 | 1 | 0.016
I1b | 4560 | 2 | 0.032
I1b | 4562 | 1 | 0.016
I1b | 471 | 3 | 0.048
I1b | 4790 | 1 | 0.016
I1b | 5009 | 1 | 0.016
I1b | 5054 | 2 | 0.032
I1b | 5319 | 1 | 0.016
I1b | 5530 | 1 | 0.016
I1b | 574C | 1 | 0.016
I1b | 5913 | 1 | 0.016
I1b | 5951 | 1 | 0.016
I1b | 6083 | 1 | 0.016
I1b | 6230 | 2 | 0.032
I1b | 7073 | 1 | 0.016
I1b | 7424 | 1 | 0.016
I1b | 7471.1C | 1 | 0.016
I1b | 7598 | 1 | 0.016
I1b | 789 | 2 | 0.032
I1b | 7948 | 1 | 0.016
I1b | 8020 | 1 | 0.016
I1b | 8078 | 1 | 0.016
I1b | 8093 | 1 | 0.016
I1b | 8191 | 1 | 0.016
I1b | 8485 | 1 | 0.016
I1b | 8563 | 1 | 0.016
I1b | 8638 | 3 | 0.048
I1b | 8711 | 2 | 0.032
I1b | 8756 | 3 | 0.048
I1b | 9181 | 1 | 0.016
I1b | 9286 | 2 | 0.032
I1b | 9456 | 2 | 0.032
I1b | 959 | 2 | 0.032
I1b | 965d | 1 | 0.016
I1b | 9944 | 2 | 0.032
I1c | 10463 | 1 | 0.143
I1c | 10530 | 3 | 0.429
I1c | 15001 | 3 | 0.429
I1c | 16270 | 1 | 0.143
I1c | 2524C | 3 | 0.429
I1c | 3144 | 1 | 0.143
I1c | 3398 | 1 | 0.143
I1c1 | 12432 | 2 | 0.182
I1c1 | 12681 | 1 | 0.091
I1c1 | 16048 | 2 | 0.182
I1c1 | 16074 | 1 | 0.091
I1c1 | 16309 | 1 | 0.091
I1c1 | 16390 | 1 | 0.091
I1c1 | 207 | 3 | 0.273
I1c1 | 310 | 1 | 0.091
I1c1 | 312 | 1 | 0.091
I1c1 | 315.2C | 1 | 0.091
I1c1 | 315.3C | 1 | 0.091
I1c1 | 6359 | 1 | 0.091
I1c1 | 6722 | 1 | 0.091
I1c1 | 7774 | 1 | 0.091
I1c1a | 13191 | 1 | 0.077
I1c1a | 152 | 1 | 0.077
I1c1a | 15940 | 1 | 0.077
I1c1a | 16390 | 1 | 0.077
I1c1a | 16482 | 1 | 0.077
I1c1a | 207 | 1 | 0.077
I1c1a | 7483 | 1 | 0.077
I1c1a | 9845 | 1 | 0.077
I1d | 152 | 5 | 0.172
I1d | 16069 | 1 | 0.034
I1d | 16076d | 1 | 0.034
I1d | 16145 | 2 | 0.069
I1d | 16153 | 1 | 0.034
I1d | 16168 | 1 | 0.034
I1d | 16172 | 1 | 0.034
I1d | 16193 | 1 | 0.034
I1d | 16234 | 1 | 0.034
I1d | 16242 | 6 | 0.207
I1d | 16249 | 1 | 0.034
I1d | 16272 | 1 | 0.034
I1d | 16296 | 1 | 0.034
I1d | 16353 | 1 | 0.034
I1d | 16391T | 1 | 0.034
I1d | 183 | 4 | 0.138
I1d | 198 | 1 | 0.034
I1d | 254 | 1 | 0.034
I1d | 485 | 1 | 0.034
I1d | 8825 | 1 | 0.034
I1d | 9302 | 1 | 0.034
I1e | 103 | 1 | 0.029
I1e | 11778 | 1 | 0.029
I1e | 12007 | 1 | 0.029
I1e | 12076 | 1 | 0.029
I1e | 12861 | 1 | 0.029
I1e | 14542 | 1 | 0.029
I1e | 15312 | 1 | 0.029
I1e | 16086 | 1 | 0.029
I1e | 16093 | 3 | 0.086
I1e | 16167 | 1 | 0.029
I1e | 16270 | 1 | 0.029
I1e | 16283T | 1 | 0.029
I1e | 16293C | 2 | 0.057
I1e | 16294 | 1 | 0.029
I1e | 16319 | 3 | 0.086
I1e | 16321 | 1 | 0.029
I1e | 16355 | 3 | 0.086
I1e | 16362 | 4 | 0.114
I1e | 1809 | 1 | 0.029
I1e | 195 | 2 | 0.057
I1e | 203 | 7 | 0.2
I1e | 207 | 2 | 0.057

I1e | 228 | 1 | 0.029
I1e | 2831 | 1 | 0.029
I1e | 501G | 1 | 0.029
I1e | 5277 | 1 | 0.029
I1e | 61 | 1 | 0.029
I1e | 6185 | 3 | 0.086
I1e | 64 | 3 | 0.086
I1e | 7771 | 1 | 0.029
I1e | 8793 | 1 | 0.029
I1e | 9263 | 1 | 0.029
I1f | 13272 | 1 | 0.045
I1f | 14405 | 9 | 0.409
I1f | 152 | 4 | 0.182
I1f | 16148 | 1 | 0.045
I1f | 16184 | 1 | 0.045
I1f | 16186 | 1 | 0.045
I1f | 16218G | 1 | 0.045
I1f | 309d | 1 | 0.045
I1f | 310 | 5 | 0.227
I1f | 3746 | 1 | 0.045
I1f | 3849 | 5 | 0.227
I1f | 4532 | 1 | 0.045
I1f | 567C | 1 | 0.045
I1f | 574C | 1 | 0.045
I1f | 576C | 1 | 0.045
I1f | 7474d | 1 | 0.045
I1f | 8567 | 13 | 0.591
I2 | 10289 | 1 | 0.008
I2 | 10586 | 1 | 0.008
I2 | 10927 | 1 | 0.008
I2 | 11150 | 1 | 0.008
I2 | 11353 | 3 | 0.024
I2 | 11368 | 1 | 0.008
I2 | 11479A | 1 | 0.008
I2 | 11485 | 1 | 0.008
I2 | 11696 | 4 | 0.032
I2 | 11899 | 1 | 0.008
I2 | 12070 | 1 | 0.008
I2 | 12136 | 2 | 0.016
I2 | 12285 | 1 | 0.008
I2 | 12303 | 3 | 0.024
I2 | 12372 | 1 | 0.008
I2 | 12469 | 1 | 0.008
I2 | 12477 | 1 | 0.008
I2 | 12603 | 3 | 0.024
I2 | 12618 | 1 | 0.008
I2 | 12996 | 1 | 0.008
I2 | 13131 | 1 | 0.008
I2 | 13590 | 1 | 0.008
I2 | 13681 | 1 | 0.008
I2 | 13980C | 1 | 0.008
I2 | 14121 | 1 | 0.008
I2 | 1420 | 2 | 0.016
I2 | 14290 | 2 | 0.016
I2 | 14374 | 7 | 0.056
I2 | 14488 | 3 | 0.024
I2 | 14560 | 2 | 0.016
I2 | 146 | 4 | 0.032
I2 | 14869 | 1 | 0.008
I2 | 14908 | 1 | 0.008
I2 | 150 | 3 | 0.024
I2 | 151 | 3 | 0.024
I2 | 15136 | 1 | 0.008
I2 | 15153 | 2 | 0.016
I2 | 15355 | 1 | 0.008
I2 | 15394 | 2 | 0.016
I2 | 15601 | 3 | 0.024
I2 | 15655 | 1 | 0.008
I2 | 15731 | 2 | 0.016
I2 | 15734 | 2 | 0.016
I2 | 15784 | 1 | 0.008
I2 | 15950 | 1 | 0.008
I2 | 16038 | 6 | 0.048
I2 | 16086 | 1 | 0.008
I2 | 16093 | 7 | 0.056
I2 | 16148 | 1 | 0.008
I2 | 16172 | 1 | 0.008
I2 | 16185 | 2 | 0.016
I2 | 16189 | 2 | 0.016
I2 | 16193d | 1 | 0.008
I2 | 16215 | 1 | 0.008
I2 | 16234 | 1 | 0.008
I2 | 16239 | 1 | 0.008
I2 | 16256 | 1 | 0.008
I2 | 16259G | 1 | 0.008
I2 | 16260 | 1 | 0.008
I2 | 16261 | 3 | 0.024
I2 | 16266 | 1 | 0.008
I2 | 16302 | 1 | 0.008
I2 | 16311 | 3 | 0.024
I2 | 16318 | 1 | 0.008
I2 | 16362 | 5 | 0.04
I2 | 16390 | 3 | 0.024
I2 | 16474T | 1 | 0.008
I2 | 182 | 2 | 0.016
I2 | 188 | 2 | 0.016
I2 | 2040 | 1 | 0.008
I2 | 2581 | 1 | 0.008
I2 | 2769 | 2 | 0.016
I2 | 2772 | 1 | 0.008
I2 | 3027 | 1 | 0.008
I2 | 309d | 1 | 0.008
I2 | 310 | 2 | 0.016
I2 | 315.2C | 2 | 0.016
I2 | 3196T | 1 | 0.008
I2 | 4532 | 1 | 0.008
I2 | 4561 | 2 | 0.016
I2 | 460 | 1 | 0.008
I2 | 460d | 1 | 0.008
I2 | 463.1C | 1 | 0.008
I2 | 4703 | 1 | 0.008

I2 | 499 | 3 | 0.024
I2 | 503 | 3 | 0.024
I2 | 5054 | 2 | 0.016
I2 | 513 | 1 | 0.008
I2 | 535 | 1 | 0.008
I2 | 5440 | 2 | 0.016
I2 | 5492 | 3 | 0.024
I2 | 5516 | 1 | 0.008
I2 | 5553 | 1 | 0.008
I2 | 5654 | 1 | 0.008
I2 | 5664 | 2 | 0.016
I2 | 567C | 1 | 0.008
I2 | 5715 | 1 | 0.008
I2 | 574C | 1 | 0.008
I2 | 5823 | 1 | 0.008
I2 | 5846 | 2 | 0.016
I2 | 5973 | 2 | 0.016
I2 | 6123C | 1 | 0.008
I2 | 6167 | 1 | 0.008
I2 | 6216 | 2 | 0.016
I2 | 6227 | 3 | 0.024
I2 | 6488 | 1 | 0.008
I2 | 6965 | 1 | 0.008
I2 | 702 | 1 | 0.008
I2 | 7340 | 1 | 0.008
I2 | 746 | 1 | 0.008
I2 | 7476 | 1 | 0.008
I2 | 7561 | 1 | 0.008
I2 | 7822 | 2 | 0.016
I2 | 8104 | 1 | 0.008
I2 | 8110 | 1 | 0.008
I2 | 8119 | 10 | 0.08
I2 | 8281-8289d | 2 | 0.016
I2 | 8557 | 9 | 0.072
I2 | 8635 | 1 | 0.008
I2 | 8802 | 2 | 0.016
I2 | 9055 | 1 | 0.008
I2 | 9163 | 1 | 0.008
I2 | 9438 | 1 | 0.008
I2 | 9604 | 2 | 0.016
I2 | 9653 | 1 | 0.008
I2'3 | 16051 | 1 | 0.021
I2'3 | 16168 | 1 | 0.021
I2'3 | 16261 | 8 | 0.17
I2'3 | 16390 | 5 | 0.106
I2a | 11146 | 1 | 0.091
I2a | 15803 | 1 | 0.091
I2a | 4216 | 1 | 0.091
I2a | 533 | 1 | 0.091
I2a | 6164 | 1 | 0.091
I2a | 8155 | 1 | 0.091
I2a | 8281-8289d | 1 | 0.091
I2a | 960d | 1 | 0.091
I2a1 | 12134 | 1 | 0.143
I2a1 | 14226 | 1 | 0.143
I2a1a | 16504 | 1 | 0.143
I2a1a | 3447 | 1 | 0.143
I2a1a | 7604 | 2 | 0.286
I2a2 | 10754 | 1 | 0.083
I2a2 | 11518 | 1 | 0.083
I2a2 | 12477 | 1 | 0.083
I2a2 | 15772T | 1 | 0.083
I2a2 | 15937 | 1 | 0.083
I2a2 | 16189 | 1 | 0.083
I2a2 | 16504 | 1 | 0.083
I2a2 | 189 | 1 | 0.083
I2a2 | 5147 | 1 | 0.083
I2a2 | 5558 | 1 | 0.083
I2a2 | 6182 | 2 | 0.167
I2a2 | 7055 | 1 | 0.083
I2a2 | 7813 | 1 | 0.083
I2a2 | 7912 | 1 | 0.083
I2a3 | 10667 | 1 | 0.25
I2a3 | 15530 | 1 | 0.25
I2a3 | 16189 | 1 | 0.25
I2a3 | 3951 | 1 | 0.25
I2a3 | 491 | 1 | 0.25
I2a3 | 9096G | 1 | 0.25
I2b | 143 | 1 | 0.25
I2b | 309d | 1 | 0.25
I2b | 311 | 1 | 0.25
I2c | 13056 | 1 | 0.022
I2c | 14207 | 1 | 0.022
I2c | 146 | 1 | 0.022
I2c | 16148 | 1 | 0.022
I2c | 16218 | 5 | 0.109
I2c | 16263 | 3 | 0.065
I2c | 16278 | 2 | 0.043
I2c | 16318 | 1 | 0.022
I2c | 335 | 3 | 0.065
I2c | 455.1T | 4 | 0.087
I2c | 455.1TCC | 1 | 0.022
I2c | 455.2T | 2 | 0.043
I2c | 456 | 1 | 0.022
I2c | 459.1C | 2 | 0.043
I2c | 4712 | 1 | 0.022
I2c | 5457 | 1 | 0.022
I2c | 6293 | 1 | 0.022
I2d | 11930 | 3 | 0.056
I2d | 13224 | 1 | 0.019
I2d | 14182 | 1 | 0.019
I2d | 14552 | 1 | 0.019
I2d | 15110 | 1 | 0.019
I2d | 15497 | 1 | 0.019
I2d | 15871 | 1 | 0.019
I2d | 16209 | 2 | 0.037
I2d | 16309 | 1 | 0.019
I2d | 16311 | 3 | 0.056
I2d | 16325 | 3 | 0.056
I2d | 16356 | 2 | 0.037
I2d | 16380 | 1 | 0.019

I2d | 189 | 1 | 0.019
I2d | 310 | 3 | 0.056
I2d | 315.3C | 1 | 0.019
I2d | 3531 | 1 | 0.019
I2d | 3757 | 1 | 0.019
I2d | 4380 | 1 | 0.019
I2d | 567C | 1 | 0.019
I2d | 5876 | 1 | 0.019
I2d | 719 | 1 | 0.019
I2d | 7260 | 1 | 0.019
I2d | 7861 | 1 | 0.019
I2d | 8264 | 1 | 0.019
I2d | 8583 | 1 | 0.019
I2d | 9214 | 1 | 0.019
I2d | 9338 | 1 | 0.019
I2e | 16289 | 2 | 0.054
I2e | 16474T | 1 | 0.027
I2e | 709 | 1 | 0.027
I2f | 15226 | 1 | 0.027
I2f | 16290 | 2 | 0.054
I2f | 16474T | 1 | 0.027
I2f | 3633 | 1 | 0.027
I3 | 14511 | 1 | 0.025
I3 | 150 | 2 | 0.05
I3 | 16235 | 2 | 0.05
I3 | 16260 | 3 | 0.075
I3 | 16278 | 1 | 0.025
I3 | 16485 | 1 | 0.025
I3 | 16485.1T | 1 | 0.025
I3 | 18 | 1 | 0.025
I3 | 2111 | 1 | 0.025
I3 | 567C | 1 | 0.025
I3 | 6260 | 1 | 0.025
I3a | 10 | 3 | 0.077
I3a | 10535 | 1 | 0.026
I3a | 10894 | 1 | 0.026
I3a | 11386 | 1 | 0.026
I3a | 11488 | 1 | 0.026
I3a | 1173 | 1 | 0.026
I3a | 12346 | 1 | 0.026
I3a | 12471 | 1 | 0.026
I3a | 12708 | 1 | 0.026
I3a | 13064 | 1 | 0.026
I3a | 13194 | 2 | 0.051
I3a | 13692 | 1 | 0.026
I3a | 13928C | 1 | 0.026
I3a | 14836 | 1 | 0.026
I3a | 151 | 1 | 0.026
I3a | 15169 | 1 | 0.026
I3a | 15565 | 2 | 0.051
I3a | 16051 | 3 | 0.077
I3a | 16134 | 1 | 0.026
I3a | 16148G | 2 | 0.051
I3a | 16179 | 1 | 0.026
I3a | 16189 | 1 | 0.026
I3a | 16214 | 1 | 0.026
I3a | 16233 | 1 | 0.026
I3a | 16235 | 3 | 0.077
I3a | 16239 | 1 | 0.026
I3a | 16287 | 3 | 0.077
I3a | 16319 | 6 | 0.154
I3a | 16491 | 3 | 0.077
I3a | 16497 | 3 | 0.077
I3a | 1807 | 1 | 0.026
I3a | 189 | 2 | 0.051
I3a | 246 | 2 | 0.051
I3a | 310 | 1 | 0.026
I3a | 3487A | 1 | 0.026
I3a | 385 | 1 | 0.026
I3a | 4300 | 1 | 0.026
I3a | 499 | 1 | 0.026
I3a | 501 | 1 | 0.026
I3a | 525.1ACAC | 1 | 0.026
I3a | 525.1ACACACAC | 1 | 0.026
I3a | 6218 | 1 | 0.026
I3a | 6323 | 1 | 0.026
I3a | 6359 | 1 | 0.026
I3a | 7720 | 1 | 0.026
I3a | 8987 | 1 | 0.026
I3a | 9581 | 1 | 0.026
I3a1 | 11284 | 1 | 0.043
I3a1 | 12396 | 1 | 0.043
I3a1 | 13395 | 4 | 0.174
I3a1 | 16172 | 1 | 0.043
I3a1 | 16174 | 1 | 0.043
I3a1 | 16209 | 1 | 0.043
I3a1 | 16239 | 1 | 0.043
I3a1 | 16261 | 1 | 0.043
I3a1 | 16399 | 1 | 0.043
I3a1 | 258 | 1 | 0.043
I3a1 | 310 | 1 | 0.043
I3a1 | 315.2C | 1 | 0.043
I3a1 | 4577 | 1 | 0.043
I3a1 | 5484 | 1 | 0.043
I3a1 | 709 | 1 | 0.043
I3a1 | 8962 | 1 | 0.043
I3a1 | 9010 | 1 | 0.043
I3a1 | 9053 | 1 | 0.043
I3b | 13194 | 1 | 0.111
I3b | 14122 | 1 | 0.111
I3b | 15431 | 3 | 0.333
I3b | 16288 | 1 | 0.111
I3b | 16300 | 1 | 0.111
I3b | 16497 | 1 | 0.111
I3b | 7521 | 1 | 0.111
I3c | 15530 | 1 | 0.029
I3c | 8882 | 1 | 0.029
I3d | 13105 | 1 | 0.027
I3d | 13974T | 2 | 0.054
I3d | 15559 | 5 | 0.135
I3d | 15628 | 1 | 0.027

I3d | 16086 | 8 | 0.216
I3d | 16189 | 3 | 0.081
I3d | 16233 | 2 | 0.054
I3d | 183 | 1 | 0.027
I3d | 1900 | 1 | 0.027
I3d | 3736 | 5 | 0.135
I3d | 3882 | 2 | 0.054
I3d | 456 | 5 | 0.135
I3d | 4813 | 2 | 0.054
I3d | 486A | 2 | 0.054
I3d | 6040 | 2 | 0.054
I3d | 7444 | 1 | 0.027
I3d | 9039 | 5 | 0.135
I3d1 | 16166 | 1 | 0.029
I3d1 | 16180 | 1 | 0.029
I3d1 | 16256A | 1 | 0.029
I3d1 | 16265C | 1 | 0.029
I3d1 | 16271 | 1 | 0.029
I3d1 | 16294 | 1 | 0.029
I3d1 | 16320 | 2 | 0.057
I3d1 | 9148 | 1 | 0.029
I3d1 | 960.1C | 1 | 0.029
I4 | 10410 | 2 | 0.042
I4 | 106-111d | 1 | 0.021
I4 | 12693 | 2 | 0.042
I4 | 146 | 2 | 0.042
I4 | 15237 | 1 | 0.021
I4 | 188 | 1 | 0.021
I4 | 260 | 1 | 0.021
I4 | 455.1T | 1 | 0.021
I4 | 467 | 1 | 0.021
I4a | 10083 | 1 | 0.008
I4a | 10742 | 2 | 0.017
I4a | 10900 | 2 | 0.017
I4a | 11016 | 2 | 0.017
I4a | 11068 | 1 | 0.008
I4a | 11075 | 1 | 0.008
I4a | 11137 | 1 | 0.008
I4a | 11563A | 1 | 0.008
I4a | 119 | 1 | 0.008
I4a | 11944 | 1 | 0.008
I4a | 121 | 1 | 0.008
I4a | 12495 | 1 | 0.008
I4a | 12879 | 1 | 0.008
I4a | 13708 | 2 | 0.017
I4a | 13710 | 1 | 0.008
I4a | 14180 | 1 | 0.008
I4a | 146 | 1 | 0.008
I4a | 14839 | 2 | 0.017
I4a | 14935 | 1 | 0.008
I4a | 14978 | 1 | 0.008
I4a | 150 | 2 | 0.017
I4a | 152 | 5 | 0.042
I4a | 15323 | 1 | 0.008
I4a | 16093 | 6 | 0.051
I4a | 16093.1GA | 1 | 0.008
I4a | 16093A | 1 | 0.008
I4a | 16111G | 1 | 0.008
I4a | 16125 | 1 | 0.008
I4a | 16128-16129d | 1 | 0.008
I4a | 16172 | 1 | 0.008
I4a | 16179 | 3 | 0.025
I4a | 16189 | 2 | 0.017
I4a | 16192 | 1 | 0.008
I4a | 16218 | 2 | 0.017
I4a | 16261 | 2 | 0.017
I4a | 16271 | 1 | 0.008
I4a | 16278 | 1 | 0.008
I4a | 16290 | 1 | 0.008
I4a | 16292 | 2 | 0.017
I4a | 16294 | 1 | 0.008
I4a | 16311 | 1 | 0.008
I4a | 16325 | 1 | 0.008
I4a | 16362 | 3 | 0.025
I4a | 16390 | 1 | 0.008
I4a | 16454 | 1 | 0.008
I4a | 16456 | 1 | 0.008
I4a | 16513 | 1 | 0.008
I4a | 1850 | 1 | 0.008
I4a | 188 | 2 | 0.017
I4a | 1888 | 2 | 0.017
I4a | 207 | 11 | 0.093
I4a | 2238 | 1 | 0.008
I4a | 2399 | 1 | 0.008
I4a | 2626 | 1 | 0.008
I4a | 2833 | 2 | 0.017
I4a | 310 | 5 | 0.042
I4a | 3157.1T | 1 | 0.008
I4a | 3199 | 2 | 0.017
I4a | 328 | 1 | 0.008
I4a | 3327 | 1 | 0.008
I4a | 3396 | 3 | 0.025
I4a | 3438 | 1 | 0.008
I4a | 3834 | 1 | 0.008
I4a | 4056 | 1 | 0.008
I4a | 4160 | 1 | 0.008
I4a | 4219 | 1 | 0.008
I4a | 467 | 1 | 0.008
I4a | 4949 | 1 | 0.008
I4a | 5231 | 1 | 0.008
I4a | 5237 | 1 | 0.008
I4a | 5441C | 4 | 0.034
I4a | 5465 | 2 | 0.017
I4a | 5562 | 1 | 0.008
I4a | 572.1TCCCC | 1 | 0.008
I4a | 574C | 4 | 0.034
I4a | 5821 | 1 | 0.008
I4a | 6221 | 9 | 0.076
I4a | 6227 | 1 | 0.008
I4a | 6635 | 2 | 0.017
I4a | 7092 | 1 | 0.008

I4a | 7170 | 1 | 0.008
I4a | 7269 | 1 | 0.008
I4a | 7874 | 1 | 0.008
I4a | 7934 | 1 | 0.008
I4a | 8258 | 1 | 0.008
I4a | 8354 | 1 | 0.008
I4a | 8886 | 2 | 0.017
I4a | 9188 | 1 | 0.008
I4a | 93 | 1 | 0.008
I4a | 9540 | 6 | 0.051
I4a | 9575 | 1 | 0.008
I4a1 | 11050 | 1 | 0.023
I4a1 | 11063 | 1 | 0.023
I4a1 | 11253 | 1 | 0.023
I4a1 | 11255 | 2 | 0.047
I4a1 | 12083G | 3 | 0.07
I4a1 | 14040 | 1 | 0.023
I4a1 | 14305 | 1 | 0.023
I4a1 | 14821 | 1 | 0.023
I4a1 | 14861 | 5 | 0.116
I4a1 | 15773 | 1 | 0.023
I4a1 | 16032.1T | 1 | 0.023
I4a1 | 16189 | 1 | 0.023
I4a1 | 16249 | 4 | 0.093
I4a1 | 16265C | 1 | 0.023
I4a1 | 16320 | 1 | 0.023
I4a1 | 16390 | 2 | 0.047
I4a1 | 194 | 1 | 0.023
I4a1 | 236 | 1 | 0.023
I4a1 | 2833 | 2 | 0.047
I4a1 | 294 | 1 | 0.023
I4a1 | 310 | 4 | 0.093
I4a1 | 315.2C | 1 | 0.023
I4a1 | 3504 | 2 | 0.047
I4a1 | 3528 | 1 | 0.023
I4a1 | 3666 | 2 | 0.047
I4a1 | 4025 | 10 | 0.233
I4a1 | 5656 | 3 | 0.07
I4a1 | 567C | 1 | 0.023
I4a1 | 575G | 1 | 0.023
I4a1 | 5786 | 1 | 0.023
I4a1 | 6413 | 2 | 0.047
I4a1 | 7762 | 4 | 0.093
I4a1 | 8087 | 1 | 0.023
I4a1 | 8697 | 3 | 0.07
I4a1 | 9053 | 2 | 0.047
I4a1 | 9380 | 1 | 0.023
I4a1 | 9467 | 1 | 0.023
I4a1 | 9804 | 1 | 0.023
I4a2 | 15499 | 1 | 0.021
I4a2 | 260 | 1 | 0.021
I4a2 | 4047 | 1 | 0.021
I4a2 | 455.1T | 1 | 0.021
I4a2 | 4561 | 1 | 0.021
I4a2 | 467 | 1 | 0.021
I4a2 | 523C | 1 | 0.021
I4b | 11530 | 4 | 0.068
I4b | 14353 | 1 | 0.017
I4b | 16092 | 1 | 0.017
I4b | 16093 | 1 | 0.017
I4b | 16271 | 2 | 0.034
I4b | 16311 | 1 | 0.017
I4b | 16400 | 1 | 0.017
I4b | 189 | 1 | 0.017
I4b | 207 | 3 | 0.051
I4b | 2124 | 2 | 0.034
I4b | 310 | 2 | 0.034
I4b | 3106A | 1 | 0.017
I4b | 316C | 2 | 0.034
I4b | 3483 | 1 | 0.017
I4b | 4021 | 2 | 0.034
I4b | 491.1T | 3 | 0.051
I4b | 5078 | 1 | 0.017
I4b | 509 | 1 | 0.017
I4b | 6014 | 1 | 0.017
I4b | 7717 | 1 | 0.017
I4b | 7853 | 2 | 0.034
I4b | 8468 | 2 | 0.034
I4b | 8768 | 4 | 0.068
I5 | 10679 | 1 | 0.02
I5 | 12134 | 1 | 0.02
I5 | 12880 | 2 | 0.04
I5 | 13368 | 4 | 0.08
I5 | 146 | 2 | 0.04
I5 | 150 | 3 | 0.06
I5 | 15074 | 2 | 0.04
I5 | 16093 | 1 | 0.02
I5 | 16148 | 5 | 0.1
I5 | 16257 | 1 | 0.02
I5 | 16311 | 1 | 0.02
I5 | 16354 | 4 | 0.08
I5 | 16497 | 1 | 0.02
I5 | 1888 | 1 | 0.02
I5 | 4065 | 2 | 0.04
I5 | 4532 | 4 | 0.08
I5 | 4890 | 1 | 0.02
I5 | 7211 | 2 | 0.04
I5 | 7783 | 1 | 0.02
I5 | 8547 | 2 | 0.04
I5 | 8581 | 1 | 0.02
I5 | 9115 | 1 | 0.02
I5 | 9156 | 4 | 0.08
I5 | 9554 | 1 | 0.02
I5 | 961 | 1 | 0.02
I5 | 965.1C | 1 | 0.02
I5a | 11024 | 3 | 0.176
I5a | 11914 | 1 | 0.059
I5a | 12073 | 1 | 0.059
I5a | 12397 | 2 | 0.118
I5a | 12774A | 3 | 0.176
I5a | 15052 | 1 | 0.059

I5a | 15244 | 1 | 0.059
I5a | 16188 | 3 | 0.176
I5a | 16284 | 1 | 0.059
I5a | 16292 | 6 | 0.353
I5a | 16294 | 1 | 0.059
I5a | 16295 | 1 | 0.059
I5a | 2392 | 3 | 0.176
I5a | 5231 | 1 | 0.059
I5a | 537 | 1 | 0.059
I5a | 574C | 1 | 0.059
I5a | 575.2C | 1 | 0.059
I5a | 576.5C | 1 | 0.059
I5a | 576C | 1 | 0.059
I5a | 7805 | 3 | 0.176
I5a | 7843 | 1 | 0.059
I5a | 8281-8289d | 3 | 0.176
I5a | 9287 | 1 | 0.059
I5a1 | 12771 | 1 | 0.05
I5a1 | 16189 | 1 | 0.05
I5a1 | 16274 | 1 | 0.05
I5a1 | 16298 | 1 | 0.05
I5a1 | 16301 | 1 | 0.05
I5a1 | 16465 | 5 | 0.25
I5a1 | 6593 | 1 | 0.05
I5a1a | 11305 | 1 | 0.053
I5a1a | 11898 | 1 | 0.053
I5a1a | 152 | 3 | 0.158
I5a1a | 15315 | 7 | 0.368
I5a1a | 1536 | 1 | 0.053
I5a1a | 16189 | 1 | 0.053
I5a1a | 16217 | 1 | 0.053
I5a1a | 16260 | 1 | 0.053
I5a1a | 16274 | 1 | 0.053
I5a1a | 16294 | 3 | 0.158
I5a1a | 4924 | 1 | 0.053
I5a1a | 8519 | 1 | 0.053
I5a1a | 961 | 1 | 0.053
I5a1a | 965.2C | 1 | 0.053
I5a1b | 152 | 1 | 0.077
I5a1b | 15452A | 1 | 0.077
I5a1b | 16192 | 1 | 0.077
I5a1b | 16230 | 1 | 0.077
I5a1b | 16258C | 1 | 0.077
I5a1b | 16292 | 1 | 0.077
I5a1b | 16311 | 1 | 0.077
I5a1b | 16354 | 1 | 0.077
I5a1b | 16400 | 1 | 0.077
I5a1b | 204A | 1 | 0.077
I5a1b | 455.1T | 1 | 0.077
I5a1b | 494A | 1 | 0.077
I5a1b | 8188 | 1 | 0.077
I5a1c | 10550 | 2 | 0.133
I5a1c | 13889 | 4 | 0.267
I5a1c | 16292 | 2 | 0.133
I5a1c | 813 | 1 | 0.067
I5a1c | 8706T | 4 | 0.267
I5a2 | 10098 | 1 | 0.029
I5a2 | 11447 | 19 | 0.543
I5a2 | 12810 | 19 | 0.543
I5a2 | 14088 | 2 | 0.057
I5a2 | 15002 | 2 | 0.057
I5a2 | 16092 | 3 | 0.086
I5a2 | 16289 | 1 | 0.029
I5a2 | 16305 | 20 | 0.571
I5a2 | 16311 | 1 | 0.029
I5a2 | 16354 | 3 | 0.086
I5a2 | 1664 | 1 | 0.029
I5a2 | 3335 | 1 | 0.029
I5a2 | 385 | 21 | 0.6
I5a2 | 3918 | 3 | 0.086
I5a2 | 5054 | 1 | 0.029
I5a2 | 5096 | 3 | 0.086
I5a2 | 6227 | 1 | 0.029
I5a2 | 9254 | 2 | 0.057
I5a2 | 9377 | 1 | 0.029
I5a2 | 9621 | 1 | 0.029
I5a2+16086 | 152 | 1 | 0.143
I5a2+16086 | 15314 | 1 | 0.143
I5a2+16086 | 1842 | 1 | 0.143
I5a2+16086 | 3335 | 1 | 0.143
I5a2+16086 | 492 | 1 | 0.143
I5a2+16086 | 622 | 2 | 0.286
I5a2+16086 | 684A | 1 | 0.143
I5a2+16086 | 687 | 1 | 0.143
I5a2a | 14007 | 1 | 0.091
I5a2a | 1793 | 1 | 0.091
I5a2a | 4688 | 1 | 0.091
I5a2a | 4742 | 1 | 0.091
I5a2a | 489 | 1 | 0.091
I5a3 | 13269 | 1 | 0.5
I5a3 | 14482A | 1 | 0.5
I5a3 | 152 | 1 | 0.5
I5a3 | 15883 | 1 | 0.5
I5a3 | 16311 | 1 | 0.5
I5a4 | 153 | 1 | 0.036
I5a4 | 16193 | 1 | 0.036
I5a4 | 16295 | 3 | 0.107
I5a4 | 16319 | 1 | 0.036
I5a4 | 16327A | 1 | 0.036
I5a4 | 16374C | 9 | 0.321
I5a4 | 16527 | 3 | 0.107
I5a4 | 310 | 1 | 0.036
I5a4 | 3200 | 1 | 0.036
I5a4 | 537 | 2 | 0.071
I5a4 | 576C | 2 | 0.071
I5a4 | 64 | 1 | 0.036
I5a4 | 8705 | 1 | 0.036
I5b | 12621 | 1 | 0.022
I5b | 12684 | 1 | 0.022
I5b | 13368 | 1 | 0.022
I5b | 16111G | 3 | 0.065

I5b | 2792C | 1 | 0.022
I5b | 54C | 1 | 0.022
I5b | 9717 | 1 | 0.022
I5b1 | 10502C | 1 | 0.048
I5b1 | 11002 | 1 | 0.048
I5b1 | 11778 | 1 | 0.048
I5b1 | 14259 | 1 | 0.048
I5b1 | 150 | 1 | 0.048
I5b1 | 15331A | 1 | 0.048
I5b1 | 15777 | 1 | 0.048
I5b1 | 16086 | 1 | 0.048
I5b1 | 16111 | 1 | 0.048
I5b1 | 16271 | 1 | 0.048
I5b1 | 16288 | 1 | 0.048
I5b1 | 16399 | 1 | 0.048
I5b1 | 188 | 1 | 0.048
I5b1 | 199 | 2 | 0.095
I5b1 | 199A | 1 | 0.048
I5b1 | 310 | 1 | 0.048
I5b1 | 6590 | 1 | 0.048
I5b1 | 9010 | 1 | 0.048
I5c | 10389 | 1 | 0.042
I5c | 11914 | 1 | 0.042
I5c | 12438 | 2 | 0.083
I5c | 12627 | 1 | 0.042
I5c | 12684 | 1 | 0.042
I5c | 12987 | 2 | 0.083
I5c | 13105 | 1 | 0.042
I5c | 13968 | 1 | 0.042
I5c | 15119 | 4 | 0.167
I5c | 16114 | 10 | 0.417
I5c | 16154 | 1 | 0.042
I5c | 16230 | 1 | 0.042
I5c | 16292 | 1 | 0.042
I5c | 16354 | 1 | 0.042
I5c | 16390T | 1 | 0.042
I5c | 16400 | 1 | 0.042
I5c | 2044 | 4 | 0.167
I5c | 3546 | 1 | 0.042
I5c | 3567 | 1 | 0.042
I5c | 4113 | 1 | 0.042
I5c | 5471 | 4 | 0.167
I5c | 574C | 1 | 0.042
I5c | 709 | 1 | 0.042
I5c | 8176 | 1 | 0.042
I5c | 8255C | 1 | 0.042
I5c | 8269 | 4 | 0.167
I5c | 9025 | 1 | 0.042
I5c | 960.1C | 1 | 0.042
I5c1 | 13371 | 3 | 0.176
I5c1 | 14574 | 5 | 0.294
I5c1 | 14798 | 3 | 0.176
I5c1 | 16167 | 1 | 0.059
I5c1 | 16172 | 2 | 0.118
I5c1 | 16189 | 1 | 0.059
I5c1 | 16298 | 1 | 0.059
I5c1 | 4695 | 1 | 0.059
I5c1 | 5585 | 1 | 0.059
I5c1 | 7389 | 1 | 0.059
I5c1 | 8027 | 3 | 0.176
I5c1 | 9007 | 5 | 0.294
I6 | 13943 | 1 | 0.022
I6 | 16111G | 3 | 0.065
I6 | 4892 | 1 | 0.022
I6 | 729 | 1 | 0.022
I6 | 7804 | 1 | 0.022
I6a | 13527 | 1 | 0.02
I6a | 13779C | 1 | 0.02
I6a | 15760 | 1 | 0.02
I6a | 16254 | 1 | 0.02
I6a | 16261 | 1 | 0.02
I6a | 16296.1C | 1 | 0.02
I6a | 16311 | 1 | 0.02
I6a | 16325 | 1 | 0.02
I6a | 188 | 1 | 0.02
I6a | 3828 | 1 | 0.02
I6a | 7051 | 1 | 0.02
I6a | 8286 | 4 | 0.08
I6a | 8287.4C | 1 | 0.02
I6a | 8288 | 2 | 0.04
I6a | 8288.2C | 1 | 0.02
I6b | 11365 | 1 | 0.125
I6b | 13327 | 1 | 0.125
I6b | 15916 | 1 | 0.125
I6b | 16186 | 1 | 0.125
I6b | 16188 | 1 | 0.125
I6b | 16327 | 4 | 0.5
I6b | 195 | 1 | 0.125
I6b | 310 | 1 | 0.125
I6b | 5460 | 1 | 0.125
I6b | 9200 | 1 | 0.125
I7 | 12477 | 1 | 0.067
I7 | 152 | 1 | 0.067
I7 | 15422 | 1 | 0.067
I7 | 16240 | 2 | 0.133
I7 | 188 | 1 | 0.067
I7 | 5460 | 1 | 0.067
I7 | 6722 | 1 | 0.067
I7 | 9182 | 1 | 0.067
J | 16145 | 1 | 0.008
J | 16216 | 5 | 0.038
J | 16263.1A | 1 | 0.008
J | 271 | 1 | 0.008
J | 507G | 1 | 0.008
J | 516A | 1 | 0.008
J | 564 | 1 | 0.008
J1 | 16093 | 5 | 0.033
J1 | 16142 | 2 | 0.013
J1 | 16153 | 1 | 0.007
J1 | 16187 | 3 | 0.02
J1 | 16189 | 1 | 0.007

J1 | 16192 | 5 | 0.033
J1 | 16223 | 5 | 0.033
J1 | 16266 | 3 | 0.02
J1 | 16494 | 1 | 0.007
J1 | 235 | 1 | 0.007
J1 | 242 | 5 | 0.033
J1+16193 | 10933 | 1 | 0.014
J1+16193 | 11260 | 1 | 0.014
J1+16193 | 16093 | 5 | 0.069
J1+16193 | 16169 | 1 | 0.014
J1+16193 | 16188 | 1 | 0.014
J1+16193 | 16274 | 1 | 0.014
J1+16193 | 16288 | 4 | 0.056
J1+16193 | 16293 | 1 | 0.014
J1+16193 | 16304 | 4 | 0.056
J1+16193 | 16305T | 2 | 0.028
J1+16193 | 16355A | 2 | 0.028
J1+16193 | 16356 | 2 | 0.028
J1+16193 | 200 | 2 | 0.028
J1+16193 | 294 | 1 | 0.014
J1+16193 | 5423 | 1 | 0.014
J1b | 10685 | 1 | 0.005
J1b | 10873 | 7 | 0.038
J1b | 12127 | 1 | 0.005
J1b | 12172 | 1 | 0.005
J1b | 12591 | 2 | 0.011
J1b | 12757 | 1 | 0.005
J1b | 12779 | 1 | 0.005
J1b | 13105 | 1 | 0.005
J1b | 13132 | 1 | 0.005
J1b | 13329 | 4 | 0.022
J1b | 13488 | 1 | 0.005
J1b | 13692 | 1 | 0.005
J1b | 138 | 1 | 0.005
J1b | 13933 | 7 | 0.038
J1b | 14020 | 5 | 0.027
J1b | 14353 | 1 | 0.005
J1b | 14569 | 1 | 0.005
J1b | 146 | 2 | 0.011
J1b | 14629 | 1 | 0.005
J1b | 14893 | 1 | 0.005
J1b | 15043 | 1 | 0.005
J1b | 151 | 5 | 0.027
J1b | 15193 | 4 | 0.022
J1b | 152 | 12 | 0.066
J1b | 1555 | 1 | 0.005
J1b | 15663 | 1 | 0.005
J1b | 15712 | 1 | 0.005
J1b | 15773 | 1 | 0.005
J1b | 16124 | 1 | 0.005
J1b | 16172 | 6 | 0.033
J1b | 16189 | 1 | 0.005
J1b | 16224 | 1 | 0.005
J1b | 16256 | 2 | 0.011
J1b | 16274 | 4 | 0.022
J1b | 16278 | 1 | 0.005
J1b | 16294 | 1 | 0.005
J1b | 16311 | 1 | 0.005
J1b | 16319 | 1 | 0.005
J1b | 16357 | 1 | 0.005
J1b | 16362 | 1 | 0.005
J1b | 195 | 1 | 0.005
J1b | 199 | 1 | 0.005
J1b | 285 | 4 | 0.022
J1b | 316 | 1 | 0.005
J1b | 3338 | 1 | 0.005
J1b | 3593 | 1 | 0.005
J1b | 4025 | 1 | 0.005
J1b | 4080 | 4 | 0.022
J1b | 4354 | 5 | 0.027
J1b | 4755 | 1 | 0.005
J1b | 5237 | 1 | 0.005
J1b | 523d | 1 | 0.005
J1b | 5432 | 1 | 0.005
J1b | 5471 | 4 | 0.022
J1b | 6962 | 7 | 0.038
J1b | 7080 | 1 | 0.005
J1b | 7364 | 5 | 0.027
J1b | 7521 | 1 | 0.005
J1b | 8110 | 1 | 0.005
J1b | 8994 | 1 | 0.005
J1b | 980 | 1 | 0.005
J1b1 | 10410A | 11 | 0.062
J1b1 | 13581 | 1 | 0.006
J1b1 | 14727 | 2 | 0.011
J1b1 | 15301 | 2 | 0.011
J1b1 | 15773 | 2 | 0.011
J1b1 | 16169 | 1 | 0.006
J1b1 | 16248 | 1 | 0.006
J1b1 | 16311 | 1 | 0.006
J1b1 | 3337 | 2 | 0.011
J1b1 | 3349 | 2 | 0.011
J1b1 | 3403 | 2 | 0.011
J1b1 | 3732 | 4 | 0.022
J1b1 | 4742 | 2 | 0.011
J1b1 | 515-524d | 4 | 0.022
J1b1 | 518G | 1 | 0.006
J1b1 | 5646 | 2 | 0.011
J1b1 | 6249 | 1 | 0.006
J1b1a | 16184 | 1 | 0.008
J1b1a | 16234 | 1 | 0.008
J1b1a | 16242 | 1 | 0.008
J1b1a | 16399 | 2 | 0.017
J1b1a1 | 10237 | 4 | 0.033
J1b1a1 | 10410 | 3 | 0.024
J1b1a1 | 10454 | 1 | 0.008
J1b1a1 | 10654 | 4 | 0.033
J1b1a1 | 10908 | 1 | 0.008
J1b1a1 | 11359C | 1 | 0.008
J1b1a1 | 11386 | 1 | 0.008
J1b1a1 | 11821T | 1 | 0.008

J1b1a1 | 11953 | 3 | 0.024
J1b1a1 | 12361 | 1 | 0.008
J1b1a1 | 12622 | 1 | 0.008
J1b1a1 | 12810 | 1 | 0.008
J1b1a1 | 12867A | 1 | 0.008
J1b1a1 | 13056 | 1 | 0.008
J1b1a1 | 13185 | 2 | 0.016
J1b1a1 | 13215 | 3 | 0.024
J1b1a1 | 13269 | 1 | 0.008
J1b1a1 | 13605 | 1 | 0.008
J1b1a1 | 13632 | 5 | 0.041
J1b1a1 | 13641 | 1 | 0.008
J1b1a1 | 13722 | 1 | 0.008
J1b1a1 | 13935 | 1 | 0.008
J1b1a1 | 13943 | 1 | 0.008
J1b1a1 | 14022 | 1 | 0.008
J1b1a1 | 14028 | 1 | 0.008
J1b1a1 | 14484 | 2 | 0.016
J1b1a1 | 14560 | 3 | 0.024
J1b1a1 | 14563 | 1 | 0.008
J1b1a1 | 14569 | 1 | 0.008
J1b1a1 | 14581 | 1 | 0.008
J1b1a1 | 14582 | 1 | 0.008
J1b1a1 | 150 | 1 | 0.008
J1b1a1 | 152 | 1 | 0.008
J1b1a1 | 15283 | 1 | 0.008
J1b1a1 | 153 | 1 | 0.008
J1b1a1 | 15740 | 3 | 0.024
J1b1a1 | 16093 | 8 | 0.065
J1b1a1 | 16192 | 1 | 0.008
J1b1a1 | 16215 | 2 | 0.016
J1b1a1 | 16222 | 1 | 0.008
J1b1a1 | 16248 | 1 | 0.008
J1b1a1 | 16254 | 2 | 0.016
J1b1a1 | 16305 | 1 | 0.008
J1b1a1 | 16311 | 1 | 0.008
J1b1a1 | 16474T | 2 | 0.016
J1b1a1 | 185 | 2 | 0.016
J1b1a1 | 1913 | 1 | 0.008
J1b1a1 | 195 | 2 | 0.016
J1b1a1 | 199 | 3 | 0.024
J1b1a1 | 2239 | 1 | 0.008
J1b1a1 | 228 | 8 | 0.065
J1b1a1 | 2322 | 1 | 0.008
J1b1a1 | 2639A | 1 | 0.008
J1b1a1 | 2695 | 1 | 0.008
J1b1a1 | 309d | 1 | 0.008
J1b1a1 | 3254 | 4 | 0.033
J1b1a1 | 3324 | 2 | 0.016
J1b1a1 | 356.1C | 4 | 0.033
J1b1a1 | 3690 | 1 | 0.008
J1b1a1 | 4204 | 4 | 0.033
J1b1a1 | 4679 | 1 | 0.008
J1b1a1 | 503C | 1 | 0.008
J1b1a1 | 5201 | 2 | 0.016
J1b1a1 | 5319 | 1 | 0.008
J1b1a1 | 573.3C | 1 | 0.008
J1b1a1 | 6179 | 1 | 0.008
J1b1a1 | 6254 | 4 | 0.033
J1b1a1 | 6359 | 1 | 0.008
J1b1a1 | 6528 | 1 | 0.008
J1b1a1 | 6599 | 1 | 0.008
J1b1a1 | 6620 | 1 | 0.008
J1b1a1 | 6681 | 1 | 0.008
J1b1a1 | 721 | 1 | 0.008
J1b1a1 | 7444 | 2 | 0.016
J1b1a1 | 7754 | 1 | 0.008
J1b1a1 | 7805 | 4 | 0.033
J1b1a1 | 8020 | 1 | 0.008
J1b1a1 | 808 | 1 | 0.008
J1b1a1 | 8167 | 1 | 0.008
J1b1a1 | 8203 | 1 | 0.008
J1b1a1 | 8557C | 1 | 0.008
J1b1a1 | 8752 | 5 | 0.041
J1b1a1 | 8764 | 1 | 0.008
J1b1a1 | 9221 | 2 | 0.016
J1b1a1 | 9308 | 3 | 0.024
J1b1a1 | 9438 | 1 | 0.008
J1b1a1 | 9612 | 1 | 0.008
J1b1a1 | 9755 | 1 | 0.008
J1b1a1 | 9938 | 1 | 0.008
J1b1a1+146 | 10322 | 1 | 0.016
J1b1a1+146 | 10427 | 1 | 0.016
J1b1a1+146 | 10972 | 1 | 0.016
J1b1a1+146 | 11935 | 2 | 0.032
J1b1a1+146 | 12172 | 1 | 0.016
J1b1a1+146 | 13743 | 1 | 0.016
J1b1a1+146 | 14434 | 3 | 0.048
J1b1a1+146 | 14484 | 1 | 0.016
J1b1a1+146 | 14761 | 1 | 0.016
J1b1a1+146 | 15119 | 2 | 0.032
J1b1a1+146 | 152 | 6 | 0.097
J1b1a1+146 | 15883 | 1 | 0.016
J1b1a1+146 | 16092 | 6 | 0.097
J1b1a1+146 | 16271 | 3 | 0.048
J1b1a1+146 | 16274 | 1 | 0.016
J1b1a1+146 | 16304G | 1 | 0.016
J1b1a1+146 | 16311 | 1 | 0.016
J1b1a1+146 | 16319 | 4 | 0.065
J1b1a1+146 | 16325 | 3 | 0.048
J1b1a1+146 | 189C | 1 | 0.016
J1b1a1+146 | 2025 | 1 | 0.016
J1b1a1+146 | 203 | 2 | 0.032
J1b1a1+146 | 204 | 2 | 0.032
J1b1a1+146 | 2352 | 1 | 0.016
J1b1a1+146 | 310 | 1 | 0.016
J1b1a1+146 | 333 | 1 | 0.016
J1b1a1+146 | 3840 | 1 | 0.016
J1b1a1+146 | 456 | 1 | 0.016
J1b1a1+146 | 4917 | 1 | 0.016
J1b1a1+146 | 523d | 1 | 0.016

J1b1a1+146 | 6836 | 1 | 0.016
J1b1a1+146 | 789 | 1 | 0.016
J1b1a1+146 | 8027 | 1 | 0.016
J1b1a1+146 | 827 | 1 | 0.016
J1b1a1+146 | 8658A | 2 | 0.032
J1b1a1+146 | 8835 | 2 | 0.032
J1b1a1+146 | 8938 | 2 | 0.032
J1b1a1a | 10289 | 1 | 0.012
J1b1a1a | 11969 | 1 | 0.012
J1b1a1a | 12074C | 1 | 0.012
J1b1a1a | 12561 | 1 | 0.012
J1b1a1a | 14368 | 1 | 0.012
J1b1a1a | 15153 | 1 | 0.012
J1b1a1a | 152 | 1 | 0.012
J1b1a1a | 1555 | 1 | 0.012
J1b1a1a | 15643 | 1 | 0.012
J1b1a1a | 15670 | 2 | 0.024
J1b1a1a | 16000d | 1 | 0.012
J1b1a1a | 16173 | 1 | 0.012
J1b1a1a | 16184 | 7 | 0.084
J1b1a1a | 16188 | 1 | 0.012
J1b1a1a | 16189 | 8 | 0.096
J1b1a1a | 16222 | 1 | 0.012
J1b1a1a | 16271 | 1 | 0.012
J1b1a1a | 16311 | 2 | 0.024
J1b1a1a | 16362 | 17 | 0.205
J1b1a1a | 16390 | 1 | 0.012
J1b1a1a | 195 | 1 | 0.012
J1b1a1a | 215 | 1 | 0.012
J1b1a1a | 2581 | 1 | 0.012
J1b1a1a | 3254 | 1 | 0.012
J1b1a1a | 3618 | 1 | 0.012
J1b1a1a | 3981 | 1 | 0.012
J1b1a1a | 4892 | 1 | 0.012
J1b1a1a | 573.1C | 1 | 0.012
J1b1a1a | 5794 | 1 | 0.012
J1b1a1a | 6080 | 1 | 0.012
J1b1a1a | 6770 | 1 | 0.012
J1b1a1a | 6842 | 1 | 0.012
J1b1a1a | 8573 | 1 | 0.012
J1b1a1a | 929 | 1 | 0.012
J1b1a1a | 94 | 1 | 0.012
J1b1a1b | 10352 | 1 | 0.012
J1b1a1b | 10410 | 2 | 0.024
J1b1a1b | 12311 | 1 | 0.012
J1b1a1b | 125G | 1 | 0.012
J1b1a1b | 12618 | 1 | 0.012
J1b1a1b | 13980 | 1 | 0.012
J1b1a1b | 14602 | 1 | 0.012
J1b1a1b | 14947A | 1 | 0.012
J1b1a1b | 151 | 1 | 0.012
J1b1a1b | 152 | 5 | 0.06
J1b1a1b | 153 | 1 | 0.012
J1b1a1b | 15467 | 1 | 0.012
J1b1a1b | 15735 | 2 | 0.024
J1b1a1b | 16111 | 1 | 0.012
J1b1a1b | 16150 | 1 | 0.012
J1b1a1b | 16179 | 1 | 0.012
J1b1a1b | 16186 | 1 | 0.012
J1b1a1b | 16189 | 3 | 0.036
J1b1a1b | 16192 | 1 | 0.012
J1b1a1b | 16219 | 1 | 0.012
J1b1a1b | 16224 | 1 | 0.012
J1b1a1b | 16247 | 1 | 0.012
J1b1a1b | 16260 | 5 | 0.06
J1b1a1b | 16274 | 2 | 0.024
J1b1a1b | 16311 | 2 | 0.024
J1b1a1b | 16316 | 5 | 0.06
J1b1a1b | 16320 | 1 | 0.012
J1b1a1b | 16355 | 1 | 0.012
J1b1a1b | 16399 | 7 | 0.084
J1b1a1b | 2157 | 2 | 0.024
J1b1a1b | 264 | 2 | 0.024
J1b1a1b | 327 | 1 | 0.012
J1b1a1b | 5298 | 1 | 0.012
J1b1a1b | 5705 | 2 | 0.024
J1b1a1b | 6221 | 1 | 0.012
J1b1a1b | 8286 | 2 | 0.024
J1b1a1b | 8287.1C | 2 | 0.024
J1b1a1b | 91d | 1 | 0.012
J1b1a1c | 10978 | 1 | 0.031
J1b1a1c | 10993 | 1 | 0.031
J1b1a1c | 12346 | 1 | 0.031
J1b1a1c | 14016 | 1 | 0.031
J1b1a1c | 14034 | 1 | 0.031
J1b1a1c | 146 | 1 | 0.031
J1b1a1c | 15439 | 1 | 0.031
J1b1a1c | 16129 | 1 | 0.031
J1b1a1c | 16218 | 1 | 0.031
J1b1a1c | 16249 | 1 | 0.031
J1b1a1c | 16255 | 1 | 0.031
J1b1a1c | 16278 | 2 | 0.062
J1b1a1c | 16293 | 1 | 0.031
J1b1a1c | 16295 | 1 | 0.031
J1b1a1c | 16302 | 2 | 0.062
J1b1a1c | 16355 | 1 | 0.031
J1b1a1c | 327 | 1 | 0.031
J1b1a1c | 3543 | 1 | 0.031
J1b1a1c | 5465 | 1 | 0.031
J1b1a1c | 9T | 2 | 0.062
J1b1a1d | 13576 | 1 | 0.034
J1b1a1d | 13815 | 1 | 0.034
J1b1a1d | 1462 | 1 | 0.034
J1b1a1d | 16136 | 1 | 0.034
J1b1a1d | 16223d | 1 | 0.034
J1b1a1d | 1943 | 1 | 0.034
J1b1a1d | 228 | 1 | 0.034
J1b1a1d | 310 | 1 | 0.034
J1b1a1d | 735 | 1 | 0.034
J1b1a1d | 9007 | 1 | 0.034
J1b1a1e | 13753 | 1 | 0.021

J1b1a1e | 14706 | 2 | 0.042
J1b1a1e | 150 | 1 | 0.021
J1b1a1e | 15910 | 1 | 0.021
J1b1a1e | 16093 | 5 | 0.104
J1b1a1e | 16129 | 1 | 0.021
J1b1a1e | 16192 | 1 | 0.021
J1b1a1e | 16242 | 1 | 0.021
J1b1a1e | 16259 | 1 | 0.021
J1b1a1e | 16311 | 3 | 0.062
J1b1a1e | 16362 | 2 | 0.042
J1b1a1e | 189 | 3 | 0.062
J1b1a1e | 2356 | 1 | 0.021
J1b1a1e | 456 | 1 | 0.021
J1b1a1e | 4562 | 1 | 0.021
J1b1a1e | 6890 | 1 | 0.021
J1b1a1e | 8167 | 1 | 0.021
J1b1a1e | 8520 | 1 | 0.021
J1b1a2 | 16111G | 1 | 0.125
J1b1a2 | 16189 | 6 | 0.75
J1b1a2 | 16362 | 5 | 0.625
J1b1a2a | 13056 | 1 | 0.333
J1b1a2a | 16189 | 1 | 0.333
J1b1a2a | 16362 | 1 | 0.333
J1b1a2a | 310 | 1 | 0.333
J1b1a2a | 315d | 1 | 0.333
J1b1a2a | 4984 | 1 | 0.333
J1b1a2a | 9716 | 1 | 0.333
J1b1a2a | 9744 | 1 | 0.333
J1b1a2b | 16468 | 1 | 0.333
J1b1a2b | 16497 | 1 | 0.333
J1b1a2b | 7407 | 1 | 0.333
J1b1a2b | 8412 | 1 | 0.333
J1b1a2b | 9968 | 1 | 0.333
J1b1a3 | 10667 | 2 | 0.015
J1b1a3 | 10899 | 1 | 0.008
J1b1a3 | 11914 | 1 | 0.008
J1b1a3 | 11959 | 1 | 0.008
J1b1a3 | 12117 | 1 | 0.008
J1b1a3 | 12795 | 2 | 0.015
J1b1a3 | 146 | 5 | 0.038
J1b1a3 | 15314 | 5 | 0.038
J1b1a3 | 16079 | 1 | 0.008
J1b1a3 | 16189 | 3 | 0.023
J1b1a3 | 16209 | 2 | 0.015
J1b1a3 | 16215 | 6 | 0.046
J1b1a3 | 194 | 4 | 0.031
J1b1a3 | 195A | 2 | 0.015
J1b1a3 | 204 | 1 | 0.008
J1b1a3 | 4234 | 1 | 0.008
J1b1a3 | 5191 | 5 | 0.038
J1b1a3 | 6261 | 1 | 0.008
J1b1a3 | 6497 | 1 | 0.008
J1b1a3 | 6899 | 1 | 0.008
J1b1a3 | 7112 | 1 | 0.008
J1b1a3 | 7211 | 5 | 0.038
J1b1a3 | 735 | 5 | 0.038
J1b1a3 | 8152 | 1 | 0.008
J1b1a3 | 8331 | 5 | 0.038
J1b1a3 | 8567 | 1 | 0.008
J1b1a3 | 9055 | 1 | 0.008
J1b1a3 | 9380 | 1 | 0.008
J1b1b | 16260 | 2 | 0.017
J1b1b | 16263.1A | 2 | 0.017
J1b1b1 | 10 | 1 | 0.007
J1b1b1 | 10245 | 2 | 0.014
J1b1b1 | 10411 | 1 | 0.007
J1b1b1 | 10924 | 2 | 0.014
J1b1b1 | 11353 | 1 | 0.007
J1b1b1 | 12046 | 1 | 0.007
J1b1b1 | 12246 | 1 | 0.007
J1b1b1 | 12501 | 2 | 0.014
J1b1b1 | 12714 | 1 | 0.007
J1b1b1 | 12780 | 1 | 0.007
J1b1b1 | 13032 | 1 | 0.007
J1b1b1 | 13760 | 1 | 0.007
J1b1b1 | 14470 | 1 | 0.007
J1b1b1 | 146 | 1 | 0.007
J1b1b1 | 14668 | 2 | 0.014
J1b1b1 | 15103 | 1 | 0.007
J1b1b1 | 15110 | 1 | 0.007
J1b1b1 | 152 | 1 | 0.007
J1b1b1 | 15718 | 2 | 0.014
J1b1b1 | 15884 | 2 | 0.014
J1b1b1 | 16136 | 2 | 0.014
J1b1b1 | 16218 | 3 | 0.021
J1b1b1 | 16248 | 1 | 0.007
J1b1b1 | 16255 | 1 | 0.007
J1b1b1 | 16258C | 3 | 0.021
J1b1b1 | 16262 | 1 | 0.007
J1b1b1 | 16263 | 4 | 0.028
J1b1b1 | 16269 | 1 | 0.007
J1b1b1 | 16295 | 1 | 0.007
J1b1b1 | 16311 | 5 | 0.035
J1b1b1 | 16344 | 1 | 0.007
J1b1b1 | 16362 | 3 | 0.021
J1b1b1 | 16399 | 1 | 0.007
J1b1b1 | 182 | 1 | 0.007
J1b1b1 | 185 | 3 | 0.021
J1b1b1 | 195 | 1 | 0.007
J1b1b1 | 204 | 1 | 0.007
J1b1b1 | 310 | 3 | 0.021
J1b1b1 | 315d | 1 | 0.007
J1b1b1 | 3912 | 1 | 0.007
J1b1b1 | 4386 | 1 | 0.007
J1b1b1 | 4880 | 1 | 0.007
J1b1b1 | 4913C | 2 | 0.014
J1b1b1 | 513 | 3 | 0.021
J1b1b1 | 523d | 1 | 0.007
J1b1b1 | 547 | 1 | 0.007
J1b1b1 | 5483 | 1 | 0.007
J1b1b1 | 709 | 1 | 0.007

J1b1b1 | 7257 | 1 | 0.007
J1b1b1 | 7270 | 2 | 0.014
J1b1b1 | 7647 | 1 | 0.007
J1b1b1 | 7805 | 1 | 0.007
J1b1b1 | 7861 | 1 | 0.007
J1b1b1 | 8251 | 1 | 0.007
J1b1b1 | 8790 | 1 | 0.007
J1b1b1 | 9055 | 3 | 0.021
J1b1b1 | 980 | 1 | 0.007
J1b1b1a | 10682 | 1 | 0.059
J1b1b1a | 11893 | 1 | 0.059
J1b1b1a | 12082 | 1 | 0.059
J1b1b1a | 13204 | 2 | 0.118
J1b1b1a | 146 | 3 | 0.176
J1b1b1a | 150 | 1 | 0.059
J1b1b1a | 152 | 1 | 0.059
J1b1b1a | 16295 | 1 | 0.059
J1b1b1a | 195 | 3 | 0.176
J1b1b1a | 3591 | 3 | 0.176
J1b1b1a | 4561 | 1 | 0.059
J1b1b1a | 513 | 1 | 0.059
J1b1b1a | 522d | 1 | 0.059
J1b1b1a | 5592 | 1 | 0.059
J1b1b1a | 5899.1C | 2 | 0.118
J1b1b1a | 709 | 2 | 0.118
J1b1b1a | 7372 | 2 | 0.118
J1b1b1a | 8997 | 1 | 0.059
J1b1b1b | 114G | 3 | 0.025
J1b1b1b | 12389 | 1 | 0.008
J1b1b1b | 12880 | 2 | 0.017
J1b1b1b | 146 | 3 | 0.025
J1b1b1b | 9530 | 1 | 0.008
J1b1b1c | 12661 | 1 | 0.125
J1b1b1c | 12952 | 1 | 0.125
J1b1b1c | 143 | 1 | 0.125
J1b1b1c | 16178 | 1 | 0.125
J1b1b1c | 8462 | 1 | 0.125
J1b1b2 | 10676 | 1 | 0.1
J1b1b2 | 15511 | 1 | 0.1
J1b1b2 | 16147G | 1 | 0.1
J1b1b2 | 16186 | 1 | 0.1
J1b1b2 | 16224 | 1 | 0.1
J1b1b2 | 16291 | 1 | 0.1
J1b1b2 | 16311 | 1 | 0.1
J1b1b2 | 401.1T | 1 | 0.1
J1b1b2 | 4231 | 1 | 0.1
J1b1b2 | 5231 | 1 | 0.1
J1b1b2 | 573.3C | 1 | 0.1
J1b1b2 | 574C | 1 | 0.1
J1b1b2 | 6071 | 1 | 0.1
J1b1b2 | 64 | 3 | 0.3
J1b1b2 | 7964 | 1 | 0.1
J1b1b2 | 8251 | 1 | 0.1
J1b1b2 | 9192 | 1 | 0.1
J1b1b3 | 10343 | 1 | 0.014
J1b1b3 | 13326 | 1 | 0.014
J1b1b3 | 13656 | 2 | 0.027
J1b1b3 | 14002 | 1 | 0.014
J1b1b3 | 152 | 1 | 0.014
J1b1b3 | 16064d | 1 | 0.014
J1b1b3 | 16179 | 1 | 0.014
J1b1b3 | 16185 | 3 | 0.041
J1b1b3 | 16189 | 9 | 0.122
J1b1b3 | 16218 | 4 | 0.054
J1b1b3 | 16235 | 2 | 0.027
J1b1b3 | 16235T | 1 | 0.014
J1b1b3 | 16236 | 1 | 0.014
J1b1b3 | 16241C | 1 | 0.014
J1b1b3 | 16247C | 1 | 0.014
J1b1b3 | 16258C | 4 | 0.054
J1b1b3 | 16263.1A | 1 | 0.014
J1b1b3 | 16274 | 1 | 0.014
J1b1b3 | 16278 | 1 | 0.014
J1b1b3 | 16291 | 1 | 0.014
J1b1b3 | 16295 | 4 | 0.054
J1b1b3 | 16301 | 1 | 0.014
J1b1b3 | 16305 | 1 | 0.014
J1b1b3 | 16311 | 10 | 0.135
J1b1b3 | 16319 | 2 | 0.027
J1b1b3 | 16325 | 1 | 0.014
J1b1b3 | 16344 | 4 | 0.054
J1b1b3 | 16362 | 1 | 0.014
J1b1b3 | 16368 | 2 | 0.027
J1b1b3 | 16516 | 2 | 0.027
J1b1b3 | 189 | 2 | 0.027
J1b1b3 | 194 | 1 | 0.014
J1b1b3 | 195 | 1 | 0.014
J1b1b3 | 199 | 1 | 0.014
J1b1b3 | 214 | 2 | 0.027
J1b1b3 | 246 | 1 | 0.014
J1b1b3 | 4562C | 1 | 0.014
J1b1b3 | 459d | 1 | 0.014
J1b1b3 | 508 | 1 | 0.014
J1b1b3 | 512 | 1 | 0.014
J1b1b3 | 513 | 2 | 0.027
J1b1b3 | 573.5C | 1 | 0.014
J1b1b3 | 64 | 1 | 0.014
J1b1b3 | 8494 | 2 | 0.027
J1b2 | 11204 | 1 | 0.005
J1b2 | 11377 | 1 | 0.005
J1b2 | 12164 | 1 | 0.005
J1b2 | 12406 | 1 | 0.005
J1b2 | 12585 | 2 | 0.01
J1b2 | 13768 | 1 | 0.005
J1b2 | 14180 | 1 | 0.005
J1b2 | 146 | 1 | 0.005
J1b2 | 14927 | 7 | 0.034
J1b2 | 150 | 2 | 0.01
J1b2 | 152 | 16 | 0.078
J1b2 | 15700 | 1 | 0.005
J1b2 | 15978 | 9 | 0.044

J1b2 | 16093 | 8 | 0.039
J1b2 | 16129 | 3 | 0.015
J1b2 | 16292 | 1 | 0.005
J1b2 | 16300 | 1 | 0.005
J1b2 | 16311 | 3 | 0.015
J1b2 | 16355 | 3 | 0.015
J1b2 | 183 | 1 | 0.005
J1b2 | 188 | 2 | 0.01
J1b2 | 234 | 1 | 0.005
J1b2 | 262 | 1 | 0.005
J1b2 | 2628 | 1 | 0.005
J1b2 | 262G | 1 | 0.005
J1b2 | 4975 | 1 | 0.005
J1b2 | 4991 | 4 | 0.02
J1b2 | 5008G | 1 | 0.005
J1b2 | 5009 | 1 | 0.005
J1b2 | 522d | 1 | 0.005
J1b2 | 5237C | 1 | 0.005
J1b2 | 545 | 2 | 0.01
J1b2 | 5773 | 1 | 0.005
J1b2 | 6340 | 1 | 0.005
J1b2 | 6701 | 2 | 0.01
J1b2 | 6719 | 7 | 0.034
J1b2 | 709 | 1 | 0.005
J1b2 | 7270 | 1 | 0.005
J1b2 | 7521 | 1 | 0.005
J1b2 | 7738 | 1 | 0.005
J1b2 | 9767 | 2 | 0.01
J1b2 | 9948 | 1 | 0.005
J1b2a | 16137 | 1 | 0.026
J1b2a | 16218 | 1 | 0.026
J1b2a | 16221 | 10 | 0.256
J1b2a | 16239 | 1 | 0.026
J1b2a | 16249 | 1 | 0.026
J1b2a | 195 | 3 | 0.077
J1b2a | 3735 | 1 | 0.026
J1b2a | 3918 | 1 | 0.026
J1b2a | 5465G | 1 | 0.026
J1b2a | 565C | 1 | 0.026
J1b2a | 567C | 1 | 0.026
J1b2a | 6309G | 1 | 0.026
J1b2a | 6331G | 1 | 0.026
J1b2a | 7 | 1 | 0.026
J1b2a | 7148 | 1 | 0.026
J1b2a | 8 | 1 | 0.026
J1b2a | 8962 | 1 | 0.026
J1b2a | 9120 | 2 | 0.051
J1b2a | 93 | 1 | 0.026
J1b3 | 11710 | 2 | 0.012
J1b3 | 13681 | 1 | 0.006
J1b3 | 13782 | 2 | 0.012
J1b3 | 14180 | 1 | 0.006
J1b3 | 146 | 2 | 0.012
J1b3 | 16092 | 1 | 0.006
J1b3 | 16185 | 1 | 0.006
J1b3 | 16187 | 1 | 0.006
J1b3 | 16294 | 1 | 0.006
J1b3 | 16303 | 1 | 0.006
J1b3 | 16311 | 1 | 0.006
J1b3 | 16362 | 1 | 0.006
J1b3 | 16364 | 1 | 0.006
J1b3 | 16516 | 1 | 0.006
J1b3 | 228 | 1 | 0.006
J1b3 | 3714 | 1 | 0.006
J1b3 | 498 | 1 | 0.006
J1b3 | 8231 | 1 | 0.006
J1b3 | 8642 | 1 | 0.006
J1b3 | 8659 | 1 | 0.006
J1b3 | 9180 | 1 | 0.006
J1b3a | 12079 | 1 | 0.056
J1b3a | 16234A | 1 | 0.056
J1b3a | 16239 | 2 | 0.111
J1b3a | 16290 | 2 | 0.111
J1b3a | 6137 | 1 | 0.056
J1b3b | 15758 | 1 | 0.005
J1b3b | 16031d | 1 | 0.005
J1b3b | 16172 | 3 | 0.016
J1b3b | 16189 | 18 | 0.097
J1b3b | 16249 | 3 | 0.016
J1b3b | 16256 | 1 | 0.005
J1b3b | 16278 | 1 | 0.005
J1b3b | 16311 | 5 | 0.027
J1b3b | 189 | 2 | 0.011
J1b3b | 199 | 1 | 0.005
J1b3b | 310 | 1 | 0.005
J1b3b | 8107 | 1 | 0.005
J1b3b1 | 10685 | 8 | 0.045
J1b3b1 | 13962 | 1 | 0.006
J1b3b1 | 152 | 13 | 0.073
J1b3b1 | 185 | 1 | 0.006
J1b3b1 | 2833 | 1 | 0.006
J1b3b1 | 6527 | 1 | 0.006
J1b4 | 12406 | 2 | 0.011
J1b4 | 14757 | 1 | 0.006
J1b4 | 15479 | 1 | 0.006
J1b4 | 15649 | 4 | 0.022
J1b4 | 16187 | 1 | 0.006
J1b4 | 16248 | 2 | 0.011
J1b4 | 16303 | 1 | 0.006
J1b4 | 16364 | 1 | 0.006
J1b4 | 16368 | 1 | 0.006
J1b4 | 16566 | 1 | 0.006
J1b4 | 228 | 1 | 0.006
J1b4 | 2757 | 2 | 0.011
J1b4 | 318 | 1 | 0.006
J1b4 | 33 | 1 | 0.006
J1b4 | 447 | 2 | 0.011
J1b4 | 4677 | 1 | 0.006
J1b4 | 498 | 1 | 0.006
J1b4 | 515-524d | 4 | 0.022
J1b4 | 5336 | 4 | 0.022

J1b4 | 9103 | 1 | 0.006
J1b4 | 9739 | 1 | 0.006
J1b4a | 16325 | 1 | 0.111
J1b4a | 765G | 1 | 0.111
J1b4a1 | 13149 | 1 | 0.062
J1b4a1 | 14364 | 1 | 0.062
J1b4a1 | 15328 | 1 | 0.062
J1b4a1 | 16278 | 4 | 0.25
J1b4a1 | 310 | 1 | 0.062
J1b4a1 | 6503 | 1 | 0.062
J1b4a2 | 16234 | 1 | 0.111
J1b5 | 11695 | 3 | 0.143
J1b5 | 11875 | 1 | 0.048
J1b5 | 12372 | 5 | 0.238
J1b5 | 152 | 3 | 0.143
J1b5 | 16186 | 1 | 0.048
J1b5 | 16286 | 1 | 0.048
J1b5 | 16287 | 2 | 0.095
J1b5 | 16311 | 2 | 0.095
J1b5 | 16319 | 1 | 0.048
J1b5 | 16355 | 1 | 0.048
J1b5 | 16362 | 4 | 0.19
J1b5 | 16535 | 1 | 0.048
J1b5 | 200 | 2 | 0.095
J1b5 | 227 | 1 | 0.048
J1b5 | 242 | 1 | 0.048
J1b5 | 3196 | 1 | 0.048
J1b5 | 4464 | 1 | 0.048
J1b5 | 567 | 1 | 0.048
J1b5 | 6253 | 1 | 0.048
J1b5 | 71.1G | 1 | 0.048
J1b5 | 7158 | 1 | 0.048
J1b5 | 94 | 1 | 0.048
J1b5 | 9591 | 1 | 0.048
J1b5a | 6182 | 1 | 0.2
J1b5a1 | 16093 | 1 | 0.143
J1b5a1 | 16223 | 2 | 0.286
J1b5a1 | 9389 | 1 | 0.143
J1b6 | 143 | 3 | 0.017
J1b6 | 146 | 1 | 0.006
J1b6 | 14769 | 1 | 0.006
J1b6 | 14769C | 2 | 0.011
J1b6 | 15314 | 1 | 0.006
J1b6 | 15883 | 1 | 0.006
J1b6 | 1607 | 1 | 0.006
J1b6 | 16163 | 2 | 0.011
J1b6 | 16187 | 1 | 0.006
J1b6 | 16192 | 1 | 0.006
J1b6 | 16248 | 2 | 0.011
J1b6 | 16291 | 1 | 0.006
J1b6 | 16296 | 2 | 0.011
J1b6 | 16303 | 1 | 0.006
J1b6 | 16364 | 1 | 0.006
J1b6 | 16368 | 1 | 0.006
J1b6 | 16526 | 2 | 0.011
J1b6 | 228 | 1 | 0.006
J1b6 | 318 | 1 | 0.006
J1b6 | 498 | 1 | 0.006
J1b6 | 515-524d | 4 | 0.022
J1b6 | 6218 | 1 | 0.006
J1b6 | 9587 | 2 | 0.011
J1b6a | 13590 | 1 | 0.006
J1b6a | 16114A | 1 | 0.006
J1b6a | 16187 | 1 | 0.006
J1b6a | 16248 | 2 | 0.011
J1b6a | 16288 | 1 | 0.006
J1b6a | 16303 | 1 | 0.006
J1b6a | 16362 | 1 | 0.006
J1b6a | 16364 | 1 | 0.006
J1b6a | 16368 | 1 | 0.006
J1b6a | 195 | 3 | 0.017
J1b6a | 228 | 1 | 0.006
J1b6a | 318 | 1 | 0.006
J1b6a | 3504 | 1 | 0.006
J1b6a | 498 | 1 | 0.006
J1b6a | 515-524d | 4 | 0.022
J1b6a | 6902 | 2 | 0.011
J1b6a | 9438 | 1 | 0.006
J1b6b | 14858 | 1 | 0.333
J1b6b | 189 | 1 | 0.333
J1b6b | 195 | 1 | 0.333
J1b7 | 10043 | 2 | 0.017
J1b7 | 152 | 1 | 0.008
J1b7 | 16189 | 1 | 0.008
J1b7 | 16234 | 1 | 0.008
J1b7 | 16242 | 1 | 0.008
J1b7 | 709 | 2 | 0.017
J1b7a | 10634 | 1 | 0.25
J1b7a | 11383 | 1 | 0.25
J1b7a | 16390 | 2 | 0.5
J1b7a | 573.1C | 2 | 0.5
J1b8 | 11499 | 1 | 0.006
J1b8 | 146 | 1 | 0.006
J1b8 | 151 | 6 | 0.034
J1b8 | 16066 | 1 | 0.006
J1b8 | 16093 | 3 | 0.017
J1b8 | 16124 | 1 | 0.006
J1b8 | 16129 | 14 | 0.08
J1b8 | 16173 | 1 | 0.006
J1b8 | 16189 | 5 | 0.029
J1b8 | 16201 | 1 | 0.006
J1b8 | 16224 | 1 | 0.006
J1b8 | 16241C | 1 | 0.006
J1b8 | 16242A | 1 | 0.006
J1b8 | 16247C | 1 | 0.006
J1b8 | 16260 | 6 | 0.034
J1b8 | 16286 | 2 | 0.011
J1b8 | 16355 | 7 | 0.04
J1b8 | 16362 | 3 | 0.017
J1b8 | 195 | 2 | 0.011
J1b8 | 226 | 1 | 0.006

J1b8	245	2	0.011
J1b8	311	1	0.006
J1b8	4092	1	0.006
J1b8	4136	1	0.006
J1b8	4491	1	0.006
J1b8	515-524d	6	0.034
J1b8	64	3	0.017
J1b8	9138A	1	0.006
J1b9	153	3	0.273
J1b9	15673	2	0.182
J1b9	16193	1	0.091
J1b9	16362	1	0.091
J1b9	16526	2	0.182
J1b9	310	2	0.182
J1b9	6116	1	0.091
J1b9	6212	1	0.091
J1b9	8853	1	0.091
J1b9	9288	1	0.091
J1c	10040	1	0.004
J1c	10205	1	0.004
J1c	10706	1	0.004
J1c	10775	1	0.004
J1c	10870	2	0.009
J1c	11140	1	0.004
J1c	11204	1	0.004
J1c	11611	2	0.009
J1c	11887	1	0.004
J1c	12133	4	0.017
J1c	12501	1	0.004
J1c	12568	1	0.004
J1c	1284	1	0.004
J1c	13350	1	0.004
J1c	13650	1	0.004
J1c	13768	1	0.004
J1c	13840	1	0.004
J1c	13962	1	0.004
J1c	14384	1	0.004
J1c	14394	1	0.004
J1c	14401	1	0.004
J1c	14494	1	0.004
J1c	14582	2	0.009
J1c	14653	5	0.021
J1c	14745	1	0.004
J1c	150	1	0.004
J1c	15110	1	0.004
J1c	15301	1	0.004
J1c	15394	1	0.004
J1c	15497	1	0.004
J1c	15520	1	0.004
J1c	15653A	1	0.004
J1c	15653T	1	0.004
J1c	15747	1	0.004
J1c	16075	1	0.004
J1c	16093	23	0.098
J1c	16114	1	0.004
J1c	16117	1	0.004
J1c	16145	1	0.004
J1c	16183	1	0.004
J1c	16185	1	0.004
J1c	16189	2	0.009
J1c	16207	1	0.004
J1c	16248	3	0.013
J1c	16265T	1	0.004
J1c	16298	1	0.004
J1c	16309	1	0.004
J1c	16311	2	0.009
J1c	16319	5	0.021
J1c	16343	1	0.004
J1c	16362	3	0.013
J1c	189	5	0.021
J1c	2308	1	0.004
J1c	2861	1	0.004
J1c	3394	1	0.004
J1c	3434	1	0.004
J1c	3657A	1	0.004
J1c	4017	1	0.004
J1c	4219	1	0.004
J1c	4454	1	0.004
J1c	5054	1	0.004
J1c	5105	1	0.004
J1c	513	1	0.004
J1c	5465	1	0.004
J1c	573.1C	1	0.004
J1c	573.2C	1	0.004
J1c	5788	1	0.004
J1c	5913	4	0.017
J1c	592	1	0.004
J1c	6299	1	0.004
J1c	709	1	0.004
J1c	729	1	0.004
J1c	7521	2	0.009
J1c	7772	1	0.004
J1c	7859	1	0.004
J1c	8087	2	0.009
J1c	8497	2	0.009
J1c	8656	2	0.009
J1c	8730	2	0.009
J1c	8764	1	0.004
J1c	8838	2	0.009
J1c	8839	1	0.004
J1c	8865	1	0.004
J1c	9425	1	0.004
J1c+16261	11864	1	0.012
J1c+16261	11992	1	0.012
J1c+16261	12218	1	0.012
J1c+16261	12290	1	0.012
J1c+16261	12501	1	0.012
J1c+16261	13278	1	0.012
J1c+16261	13942	1	0.012
J1c+16261	14401	1	0.012
J1c+16261	150	1	0.012

J1c+16261 | 15034C | 1 | 0.012
J1c+16261 | 16114 | 1 | 0.012
J1c+16261 | 16114G | 1 | 0.012
J1c+16261 | 16145 | 5 | 0.062
J1c+16261 | 16187 | 1 | 0.012
J1c+16261 | 16189 | 3 | 0.037
J1c+16261 | 16190 | 3 | 0.037
J1c+16261 | 16241 | 1 | 0.012
J1c+16261 | 16260 | 1 | 0.012
J1c+16261 | 16274 | 1 | 0.012
J1c+16261 | 16286 | 1 | 0.012
J1c+16261 | 16311 | 2 | 0.025
J1c+16261 | 16474 | 20 | 0.247
J1c+16261 | 188 | 4 | 0.049
J1c+16261 | 234 | 2 | 0.025
J1c+16261 | 2851 | 2 | 0.025
J1c+16261 | 310 | 1 | 0.012
J1c+16261 | 316 | 1 | 0.012
J1c+16261 | 3606 | 1 | 0.012
J1c+16261 | 5054 | 1 | 0.012
J1c+16261 | 5308 | 2 | 0.025
J1c+16261 | 64 | 1 | 0.012
J1c+16261 | 709 | 2 | 0.025
J1c+16261 | 7154 | 1 | 0.012
J1c+16261 | 8222 | 1 | 0.012
J1c+16261 | 8380 | 1 | 0.012
J1c+16261 | 9117 | 1 | 0.012
J1c+16261 | 9488 | 1 | 0.012
J1c+16261+189 | 12405A | 1 | 0.062
J1c+16261+189 | 13651 | 1 | 0.062
J1c+16261+189 | 13766A | 1 | 0.062
J1c+16261+189 | 14482A | 1 | 0.062
J1c+16261+189 | 200 | 1 | 0.062
J1c+16261+189 | 204 | 1 | 0.062
J1c+16261+189 | 3981 | 1 | 0.062
J1c+16261+189 | 6570T | 1 | 0.062
J1c+16261+189 | 7154 | 1 | 0.062
J1c+16261+189 | 7547 | 1 | 0.062
J1c+16261+189 | 8760 | 1 | 0.062
J1c1 | 10143 | 1 | 0.004
J1c1 | 10321 | 1 | 0.004
J1c1 | 10454 | 1 | 0.004
J1c1 | 11176 | 1 | 0.004
J1c1 | 11434 | 1 | 0.004
J1c1 | 11437 | 1 | 0.004
J1c1 | 11778 | 1 | 0.004
J1c1 | 11810 | 1 | 0.004
J1c1 | 11908 | 1 | 0.004
J1c1 | 1193 | 1 | 0.004
J1c1 | 11998 | 1 | 0.004
J1c1 | 12007 | 1 | 0.004
J1c1 | 12599 | 1 | 0.004
J1c1 | 12873 | 1 | 0.004
J1c1 | 13062C | 1 | 0.004
J1c1 | 13287 | 1 | 0.004
J1c1 | 13590 | 1 | 0.004
J1c1 | 14189 | 1 | 0.004
J1c1 | 14198 | 3 | 0.012
J1c1 | 14332 | 3 | 0.012
J1c1 | 14443 | 1 | 0.004
J1c1 | 146 | 1 | 0.004
J1c1 | 151 | 26 | 0.108
J1c1 | 15119 | 1 | 0.004
J1c1 | 15557 | 1 | 0.004
J1c1 | 15731 | 1 | 0.004
J1c1 | 15894 | 1 | 0.004
J1c1 | 15903 | 3 | 0.012
J1c1 | 15968 | 4 | 0.017
J1c1 | 16048 | 1 | 0.004
J1c1 | 16093 | 5 | 0.021
J1c1 | 16111 | 2 | 0.008
J1c1 | 16114 | 1 | 0.004
J1c1 | 16189 | 5 | 0.021
J1c1 | 16192 | 4 | 0.017
J1c1 | 16215 | 4 | 0.017
J1c1 | 16218A | 1 | 0.004
J1c1 | 16249 | 5 | 0.021
J1c1 | 16256 | 25 | 0.104
J1c1 | 16265 | 1 | 0.004
J1c1 | 16311 | 4 | 0.017
J1c1 | 16355 | 2 | 0.008
J1c1 | 16422 | 1 | 0.004
J1c1 | 16431A | 1 | 0.004
J1c1 | 1790 | 1 | 0.004
J1c1 | 186 | 1 | 0.004
J1c1 | 189 | 3 | 0.012
J1c1 | 198 | 1 | 0.004
J1c1 | 234 | 1 | 0.004
J1c1 | 241 | 1 | 0.004
J1c1 | 291T | 1 | 0.004
J1c1 | 296d | 1 | 0.004
J1c1 | 297 | 1 | 0.004
J1c1 | 309d | 1 | 0.004
J1c1 | 310 | 2 | 0.008
J1c1 | 3110d | 1 | 0.004
J1c1 | 3483 | 1 | 0.004
J1c1 | 4316 | 1 | 0.004
J1c1 | 4435 | 2 | 0.008
J1c1 | 4646 | 1 | 0.004
J1c1 | 4862A | 1 | 0.004
J1c1 | 524A | 2 | 0.008
J1c1 | 573.2C | 1 | 0.004
J1c1 | 5773 | 1 | 0.004
J1c1 | 6698 | 2 | 0.008
J1c1 | 709 | 1 | 0.004
J1c1 | 7244 | 1 | 0.004
J1c1 | 7533 | 1 | 0.004
J1c1 | 7735 | 3 | 0.012
J1c1 | 7844 | 1 | 0.004
J1c1 | 789 | 1 | 0.004
J1c1 | 7966 | 3 | 0.012

J1c1 | 8038 | 1 | 0.004
J1c1 | 8152 | 1 | 0.004
J1c1 | 8206 | 1 | 0.004
J1c1 | 8222 | 1 | 0.004
J1c1 | 8387 | 1 | 0.004
J1c1 | 8676 | 2 | 0.008
J1c1 | 870d | 1 | 0.004
J1c1 | 8793 | 1 | 0.004
J1c1 | 8848 | 3 | 0.012
J1c1 | 9205 | 1 | 0.004
J1c1 | 9389 | 1 | 0.004
J1c1 | 9497 | 3 | 0.012
J1c1 | 9708 | 1 | 0.004
J1c1 | 9854 | 1 | 0.004
J1c10 | 10616 | 1 | 0.005
J1c10 | 11335 | 1 | 0.005
J1c10 | 13702G | 1 | 0.005
J1c10 | 14199G | 1 | 0.005
J1c10 | 14272G | 1 | 0.005
J1c10 | 14365G | 1 | 0.005
J1c10 | 14368G | 1 | 0.005
J1c10 | 14386 | 1 | 0.005
J1c10 | 14569 | 1 | 0.005
J1c10 | 15907 | 1 | 0.005
J1c10 | 15992T | 1 | 0.005
J1c10 | 16093 | 1 | 0.005
J1c10 | 16168 | 2 | 0.009
J1c10 | 16176 | 23 | 0.108
J1c10 | 3423G | 1 | 0.005
J1c10 | 4721 | 1 | 0.005
J1c10 | 4985 | 1 | 0.005
J1c10 | 7098 | 2 | 0.009
J1c10 | 794 | 1 | 0.005
J1c10 | 8572 | 1 | 0.005
J1c10 | 9559G | 1 | 0.005
J1c10a | 12063 | 1 | 0.005
J1c10a | 143 | 21 | 0.097
J1c10a | 16319 | 21 | 0.097
J1c10a | 1978C | 1 | 0.005
J1c10a | 2065 | 1 | 0.005
J1c10a | 3397 | 7 | 0.032
J1c10a | 4316 | 2 | 0.009
J1c10a | 5563 | 2 | 0.009
J1c10a | 6253 | 1 | 0.005
J1c10a | 709 | 2 | 0.009
J1c10a | 8188 | 2 | 0.009
J1c10a | 9554 | 2 | 0.009
J1c10a | 9744 | 21 | 0.097
J1c11 | 117G | 1 | 0.056
J1c11 | 16189 | 1 | 0.056
J1c11 | 16287 | 3 | 0.167
J1c11 | 189 | 6 | 0.333
J1c11 | 2085-2088d | 1 | 0.056
J1c11 | 2805d | 1 | 0.056
J1c11 | 9777 | 1 | 0.056
J1c11 | 9782d | 1 | 0.056
J1c11 | 9785.1C | 1 | 0.056
J1c11 | 9788G | 1 | 0.056
J1c11 | 9790 | 1 | 0.056
J1c11 | 9791T | 1 | 0.056
J1c11 | 9850G | 1 | 0.056
J1c11 | 9852 | 1 | 0.056
J1c11a | 10899 | 1 | 0.067
J1c11a | 1132 | 1 | 0.067
J1c11a | 14696 | 1 | 0.067
J1c11a | 152 | 1 | 0.067
J1c11a | 15434 | 1 | 0.067
J1c11a | 1578 | 1 | 0.067
J1c11a | 15902 | 3 | 0.2
J1c11a | 194 | 1 | 0.067
J1c11a | 3335 | 1 | 0.067
J1c11a | 4002 | 2 | 0.133
J1c11a | 6812 | 1 | 0.067
J1c11a | 824 | 1 | 0.067
J1c11a | 9386 | 1 | 0.067
J1c11a | 9497 | 1 | 0.067
J1c12 | 10586 | 1 | 0.077
J1c12 | 152 | 1 | 0.077
J1c12a | 226 | 1 | 0.067
J1c12a | 2905 | 2 | 0.133
J1c12a | 3592 | 2 | 0.133
J1c12a | 573.1C | 1 | 0.067
J1c12a | 6224 | 2 | 0.133
J1c12a | 9932 | 1 | 0.067
J1c12b | 152 | 1 | 0.077
J1c12b | 15462 | 1 | 0.077
J1c12b | 6481 | 1 | 0.077
J1c12b | 8027 | 1 | 0.077
J1c12b | 8557 | 1 | 0.077
J1c13 | 153 | 1 | 0.067
J1c13 | 16209 | 1 | 0.067
J1c13 | 16249 | 1 | 0.067
J1c13 | 16295 | 1 | 0.067
J1c13 | 16311 | 2 | 0.133
J1c13 | 207 | 1 | 0.067
J1c13 | 316C | 1 | 0.067
J1c14 | 11016 | 1 | 0.125
J1c14 | 13966 | 1 | 0.125
J1c14 | 16093 | 5 | 0.625
J1c15 | 10427 | 2 | 0.009
J1c15 | 11821 | 1 | 0.005
J1c15 | 12241.1C | 1 | 0.005
J1c15 | 12346 | 1 | 0.005
J1c15 | 12780 | 1 | 0.005
J1c15 | 15466 | 2 | 0.009
J1c15 | 16136 | 1 | 0.005
J1c15 | 16243 | 1 | 0.005
J1c15 | 16291 | 22 | 0.103
J1c15 | 16293 | 22 | 0.103
J1c15 | 16294G | 1 | 0.005
J1c15 | 189 | 2 | 0.009

J1c15 | 195 | 1 | 0.005
J1c15 | 198 | 1 | 0.005
J1c15 | 3553 | 1 | 0.005
J1c15 | 3915 | 1 | 0.005
J1c15 | 4767 | 1 | 0.005
J1c15 | 5414 | 1 | 0.005
J1c15 | 5814 | 1 | 0.005
J1c15 | 7208 | 1 | 0.005
J1c15 | 7912 | 2 | 0.009
J1c15 | 8857 | 1 | 0.005
J1c15a | 14260 | 1 | 0.005
J1c15a | 152 | 1 | 0.005
J1c15a | 15985 | 1 | 0.005
J1c15a | 16092 | 1 | 0.005
J1c15a | 16187 | 3 | 0.014
J1c15a | 16189 | 1 | 0.005
J1c15a | 16245 | 24 | 0.112
J1c15a | 16325 | 25 | 0.117
J1c15a | 1888 | 2 | 0.009
J1c15a | 235 | 1 | 0.005
J1c15a | 870 | 1 | 0.005
J1c15a1 | 11204 | 1 | 0.005
J1c15a1 | 15244 | 1 | 0.005
J1c15a1 | 16148 | 2 | 0.009
J1c15a1 | 16201 | 1 | 0.005
J1c15a1 | 16259.1A | 23 | 0.107
J1c15a1 | 16299 | 23 | 0.107
J1c15a1 | 183 | 1 | 0.005
J1c15a1 | 310 | 1 | 0.005
J1c15a1 | 4176 | 1 | 0.005
J1c15b | 11792A | 1 | 0.01
J1c15b | 16031d | 2 | 0.02
J1c15b | 16075 | 3 | 0.03
J1c15b | 16290 | 2 | 0.02
J1c15b | 1719 | 4 | 0.04
J1c15b | 189 | 5 | 0.051
J1c15b | 7775 | 4 | 0.04
J1c15b | 9286 | 4 | 0.04
J1c16 | 16090 | 1 | 0.062
J1c16 | 16187 | 1 | 0.062
J1c16 | 16267 | 1 | 0.062
J1c16 | 16294 | 4 | 0.25
J1c16 | 182 | 1 | 0.062
J1c17 | 16111A | 25 | 0.117
J1c17 | 16292 | 1 | 0.005
J1c17 | 16311 | 1 | 0.005
J1c17 | 316 | 1 | 0.005
J1c17 | 5836 | 3 | 0.014
J1c17 | 8567 | 3 | 0.014
J1c17a | 16051 | 1 | 0.071
J1c17a | 16145 | 1 | 0.071
J1c17a | 16188 | 1 | 0.071
J1c17a | 16189 | 1 | 0.071
J1c1a | 11116 | 1 | 0.005
J1c1a | 11830 | 1 | 0.005
J1c1a | 150 | 1 | 0.005
J1c1a | 15371 | 1 | 0.005
J1c1a | 16178 | 1 | 0.005
J1c1a | 16192 | 1 | 0.005
J1c1a | 16265 | 1 | 0.005
J1c1a | 16271 | 1 | 0.005
J1c1a | 16311 | 1 | 0.005
J1c1a | 16362 | 21 | 0.101
J1c1a | 183 | 1 | 0.005
J1c1a | 2095 | 1 | 0.005
J1c1a | 210 | 1 | 0.005
J1c1a | 264 | 1 | 0.005
J1c1a | 3612 | 1 | 0.005
J1c1a | 3705 | 1 | 0.005
J1c1a | 5843 | 1 | 0.005
J1c1a | 6260 | 1 | 0.005
J1c1a | 6G | 2 | 0.01
J1c1a | 7444 | 1 | 0.005
J1c1a | 7762 | 1 | 0.005
J1c1a | 93 | 1 | 0.005
J1c1b | 11440 | 1 | 0.004
J1c1b | 12112 | 1 | 0.004
J1c1b | 12696 | 3 | 0.013
J1c1b | 14323 | 1 | 0.004
J1c1b | 14552 | 1 | 0.004
J1c1b | 15484 | 1 | 0.004
J1c1b | 15740 | 1 | 0.004
J1c1b | 16093 | 1 | 0.004
J1c1b | 16172 | 1 | 0.004
J1c1b | 16192 | 4 | 0.017
J1c1b | 16193 | 14 | 0.06
J1c1b | 16256 | 10 | 0.043
J1c1b | 16286 | 1 | 0.004
J1c1b | 16291 | 24 | 0.103
J1c1b | 16304 | 1 | 0.004
J1c1b | 195 | 1 | 0.004
J1c1b | 234 | 1 | 0.004
J1c1b | 315.2C | 1 | 0.004
J1c1b | 3548 | 1 | 0.004
J1c1b | 3834 | 1 | 0.004
J1c1b | 508 | 3 | 0.013
J1c1b | 5320 | 1 | 0.004
J1c1b | 735 | 1 | 0.004
J1c1b | 7975 | 1 | 0.004
J1c1b | 8558 | 1 | 0.004
J1c1b | 8581 | 5 | 0.022
J1c1b | 8706 | 1 | 0.004
J1c1b | 9077 | 1 | 0.004
J1c1b | 930 | 1 | 0.004
J1c1b | 9554 | 1 | 0.004
J1c1b1 | 12535 | 1 | 0.005
J1c1b1 | 13368 | 2 | 0.01
J1c1b1 | 14200 | 2 | 0.01
J1c1b1 | 14971 | 1 | 0.005
J1c1b1 | 15244 | 1 | 0.005
J1c1b1 | 16147A | 3 | 0.014

J1c1b1 | 16178 | 1 | 0.005
J1c1b1 | 16189 | 1 | 0.005
J1c1b1 | 16192 | 1 | 0.005
J1c1b1 | 16249 | 1 | 0.005
J1c1b1 | 16271 | 2 | 0.01
J1c1b1 | 16357 | 22 | 0.106
J1c1b1 | 16422 | 1 | 0.005
J1c1b1 | 174A | 1 | 0.005
J1c1b1 | 182 | 23 | 0.111
J1c1b1 | 183 | 1 | 0.005
J1c1b1 | 188 | 1 | 0.005
J1c1b1 | 225 | 1 | 0.005
J1c1b1 | 264 | 1 | 0.005
J1c1b1 | 3796 | 1 | 0.005
J1c1b1 | 5411 | 2 | 0.01
J1c1b1 | 5450 | 1 | 0.005
J1c1b1 | 5563 | 1 | 0.005
J1c1b1 | 64 | 1 | 0.005
J1c1b1 | 6G | 2 | 0.01
J1c1b1 | 7444 | 1 | 0.005
J1c1b1 | 789 | 1 | 0.005
J1c1b1a | 11878 | 2 | 0.008
J1c1b1a | 12192 | 9 | 0.037
J1c1b1a | 12963 | 1 | 0.004
J1c1b1a | 13020 | 3 | 0.012
J1c1b1a | 14016 | 1 | 0.004
J1c1b1a | 146 | 1 | 0.004
J1c1b1a | 150 | 3 | 0.012
J1c1b1a | 15047 | 1 | 0.004
J1c1b1a | 1555 | 1 | 0.004
J1c1b1a | 15937d | 3 | 0.012
J1c1b1a | 16086 | 1 | 0.004
J1c1b1a | 16093 | 5 | 0.02
J1c1b1a | 16111 | 1 | 0.004
J1c1b1a | 16145 | 3 | 0.012
J1c1b1a | 16172 | 2 | 0.008
J1c1b1a | 16185 | 1 | 0.004
J1c1b1a | 16187 | 2 | 0.008
J1c1b1a | 16189 | 30 | 0.122
J1c1b1a | 16192 | 3 | 0.012
J1c1b1a | 16193d | 1 | 0.004
J1c1b1a | 16235 | 7 | 0.029
J1c1b1a | 16261 | 1 | 0.004
J1c1b1a | 16272 | 3 | 0.012
J1c1b1a | 16274 | 22 | 0.09
J1c1b1a | 16304 | 1 | 0.004
J1c1b1a | 16311 | 1 | 0.004
J1c1b1a | 16319 | 1 | 0.004
J1c1b1a | 188 | 11 | 0.045
J1c1b1a | 210 | 4 | 0.016
J1c1b1a | 234 | 2 | 0.008
J1c1b1a | 235 | 1 | 0.004
J1c1b1a | 2396 | 3 | 0.012
J1c1b1a | 3197 | 2 | 0.008
J1c1b1a | 4204 | 3 | 0.012
J1c1b1a | 44.1C | 1 | 0.004
J1c1b1a | 4820 | 1 | 0.004
J1c1b1a | 5078 | 1 | 0.004
J1c1b1a | 5418G | 1 | 0.004
J1c1b1a | 5461G | 1 | 0.004
J1c1b1a | 5539C | 1 | 0.004
J1c1b1a | 5972 | 1 | 0.004
J1c1b1a | 5987 | 1 | 0.004
J1c1b1a | 6040 | 1 | 0.004
J1c1b1a | 650 | 1 | 0.004
J1c1b1a | 6912C | 2 | 0.008
J1c1b1a | 7055 | 3 | 0.012
J1c1b1a | 7581 | 1 | 0.004
J1c1b1a | 870 | 1 | 0.004
J1c1b1a | 9548 | 3 | 0.012
J1c1b1a1 | 11150 | 1 | 0.005
J1c1b1a1 | 12534 | 1 | 0.005
J1c1b1a1 | 13707 | 1 | 0.005
J1c1b1a1 | 152 | 1 | 0.005
J1c1b1a1 | 16114 | 3 | 0.015
J1c1b1a1 | 16147A | 3 | 0.015
J1c1b1a1 | 16178 | 21 | 0.103
J1c1b1a1 | 16259A | 2 | 0.01
J1c1b1a1 | 174A | 1 | 0.005
J1c1b1a1 | 183 | 1 | 0.005
J1c1b1a1 | 195 | 1 | 0.005
J1c1b1a1 | 264 | 1 | 0.005
J1c1b1a1 | 6G | 2 | 0.01
J1c1b1a1 | 8104 | 1 | 0.005
J1c1b2 | 10203 | 3 | 0.014
J1c1b2 | 152 | 1 | 0.005
J1c1b2 | 15784 | 1 | 0.005
J1c1b2 | 16114 | 3 | 0.014
J1c1b2 | 16147A | 3 | 0.014
J1c1b2 | 16178 | 1 | 0.005
J1c1b2 | 16234 | 22 | 0.106
J1c1b2 | 16259A | 2 | 0.01
J1c1b2 | 16527 | 1 | 0.005
J1c1b2 | 174A | 1 | 0.005
J1c1b2 | 183 | 1 | 0.005
J1c1b2 | 264 | 1 | 0.005
J1c1b2 | 6G | 2 | 0.01
J1c1b2a | 152 | 1 | 0.005
J1c1b2a | 15902 | 1 | 0.005
J1c1b2a | 16114 | 3 | 0.015
J1c1b2a | 16147A | 3 | 0.015
J1c1b2a | 16178 | 21 | 0.102
J1c1b2a | 16259A | 2 | 0.01
J1c1b2a | 174A | 1 | 0.005
J1c1b2a | 183 | 1 | 0.005
J1c1b2a | 264 | 1 | 0.005
J1c1b2a | 297 | 1 | 0.005
J1c1b2a | 4718 | 2 | 0.01
J1c1b2a | 6G | 2 | 0.01
J1c1b2a | 8426 | 1 | 0.005
J1c1c | 10370 | 1 | 0.059

J1c1c | 13224 | 1 | 0.059
J1c1c | 14128 | 1 | 0.059
J1c1c | 14634 | 1 | 0.059
J1c1c | 15977 | 1 | 0.059
J1c1c | 16111A | 1 | 0.059
J1c1c | 16189 | 10 | 0.588
J1c1c | 16243 | 10 | 0.588
J1c1c | 16290 | 1 | 0.059
J1c1c | 16325 | 1 | 0.059
J1c1c | 16366 | 1 | 0.059
J1c1c | 5460 | 1 | 0.059
J1c1c | 6539 | 1 | 0.059
J1c1c | 9377 | 1 | 0.059
J1c1d | 11884 | 7 | 0.241
J1c1d | 14484 | 8 | 0.276
J1c1d | 15323 | 1 | 0.034
J1c1d | 15514 | 1 | 0.034
J1c1d | 16189 | 1 | 0.034
J1c1d | 16193 | 1 | 0.034
J1c1d | 16291 | 1 | 0.034
J1c1d | 195 | 1 | 0.034
J1c1d | 309d | 1 | 0.034
J1c1d | 310 | 1 | 0.034
J1c1d | 4080 | 1 | 0.034
J1c1d | 438 | 2 | 0.069
J1c1d | 4435 | 1 | 0.034
J1c1d | 5255 | 1 | 0.034
J1c1d | 5387A | 1 | 0.034
J1c1d | 7297G | 1 | 0.034
J1c1d | 9053 | 3 | 0.103
J1c1d | 9682 | 2 | 0.069
J1c1e | 10073T | 1 | 0.111
J1c1e | 15769 | 1 | 0.111
J1c1e | 16189 | 3 | 0.333
J1c1e | 16532d | 1 | 0.111
J1c1e | 1836 | 1 | 0.111
J1c1e | 8468 | 1 | 0.111
J1c1f | 13647 | 2 | 0.01
J1c1f | 152 | 1 | 0.005
J1c1f | 15337 | 1 | 0.005
J1c1f | 16114 | 3 | 0.014
J1c1f | 16147A | 3 | 0.014
J1c1f | 16172 | 2 | 0.01
J1c1f | 16178 | 1 | 0.005
J1c1f | 16259A | 2 | 0.01
J1c1f | 16357 | 22 | 0.106
J1c1f | 174A | 1 | 0.005
J1c1f | 182 | 23 | 0.111
J1c1f | 183 | 1 | 0.005
J1c1f | 264 | 1 | 0.005
J1c1f | 5315 | 1 | 0.005
J1c1f | 6G | 2 | 0.01
J1c1g | 152 | 1 | 0.005
J1c1g | 16114 | 3 | 0.015
J1c1g | 16145 | 1 | 0.005
J1c1g | 16147A | 3 | 0.015
J1c1g | 16178 | 1 | 0.005
J1c1g | 16259A | 2 | 0.01
J1c1g | 16357 | 22 | 0.107
J1c1g | 16362 | 1 | 0.005
J1c1g | 174A | 1 | 0.005
J1c1g | 182 | 23 | 0.112
J1c1g | 183 | 1 | 0.005
J1c1g | 264 | 1 | 0.005
J1c1g | 310 | 1 | 0.005
J1c1g | 6G | 2 | 0.01
J1c1g1 | 11778 | 1 | 0.005
J1c1g1 | 12471 | 1 | 0.005
J1c1g1 | 152 | 1 | 0.005
J1c1g1 | 16114 | 3 | 0.014
J1c1g1 | 16147A | 3 | 0.014
J1c1g1 | 16178 | 1 | 0.005
J1c1g1 | 16259A | 2 | 0.01
J1c1g1 | 16292 | 1 | 0.005
J1c1g1 | 16357 | 22 | 0.106
J1c1g1 | 174A | 1 | 0.005
J1c1g1 | 182 | 23 | 0.111
J1c1g1 | 183 | 1 | 0.005
J1c1g1 | 264 | 1 | 0.005
J1c1g1 | 6G | 2 | 0.01
J1c1g1 | 7910 | 1 | 0.005
J1c1h | 11177 | 1 | 0.111
J1c1h | 15928 | 1 | 0.111
J1c1h | 16213 | 1 | 0.111
J1c1h | 16355 | 1 | 0.111
J1c1h | 16532d | 1 | 0.111
J1c1h | 6951 | 1 | 0.111
J1c2 | 10042 | 6 | 0.021
J1c2 | 10118 | 1 | 0.003
J1c2 | 10389 | 1 | 0.003
J1c2 | 1040 | 22 | 0.075
J1c2 | 10463 | 1 | 0.003
J1c2 | 10801 | 7 | 0.024
J1c2 | 10876 | 1 | 0.003
J1c2 | 11014 | 1 | 0.003
J1c2 | 11204 | 2 | 0.007
J1c2 | 11365 | 1 | 0.003
J1c2 | 11422A | 1 | 0.003
J1c2 | 11654 | 2 | 0.007
J1c2 | 11914 | 1 | 0.003
J1c2 | 12054T | 1 | 0.003
J1c2 | 12339 | 1 | 0.003
J1c2 | 12397 | 1 | 0.003
J1c2 | 12501 | 1 | 0.003
J1c2 | 12561 | 1 | 0.003
J1c2 | 12870 | 1 | 0.003
J1c2 | 12879 | 2 | 0.007
J1c2 | 12975T | 1 | 0.003
J1c2 | 13111 | 1 | 0.003
J1c2 | 13204 | 1 | 0.003
J1c2 | 13368 | 1 | 0.003

J1c2 | 13590 | 2 | 0.007
J1c2 | 13617 | 1 | 0.003
J1c2 | 13651C | 1 | 0.003
J1c2 | 13711 | 1 | 0.003
J1c2 | 13824 | 1 | 0.003
J1c2 | 13827 | 2 | 0.007
J1c2 | 13934 | 36 | 0.123
J1c2 | 13995 | 3 | 0.01
J1c2 | 14025 | 1 | 0.003
J1c2 | 14091 | 1 | 0.003
J1c2 | 14130 | 2 | 0.007
J1c2 | 14149 | 1 | 0.003
J1c2 | 14302 | 1 | 0.003
J1c2 | 14482 | 2 | 0.007
J1c2 | 14560 | 1 | 0.003
J1c2 | 14569 | 1 | 0.003
J1c2 | 14598 | 1 | 0.003
J1c2 | 14783 | 2 | 0.007
J1c2 | 150 | 1 | 0.003
J1c2 | 15013 | 1 | 0.003
J1c2 | 1503 | 1 | 0.003
J1c2 | 151 | 1 | 0.003
J1c2 | 15110 | 1 | 0.003
J1c2 | 152 | 3 | 0.01
J1c2 | 15650 | 1 | 0.003
J1c2 | 15748 | 1 | 0.003
J1c2 | 15884 | 1 | 0.003
J1c2 | 16086 | 1 | 0.003
J1c2 | 16093 | 1 | 0.003
J1c2 | 16104 | 1 | 0.003
J1c2 | 16111 | 1 | 0.003
J1c2 | 16187 | 1 | 0.003
J1c2 | 16189 | 2 | 0.007
J1c2 | 16192 | 1 | 0.003
J1c2 | 16197 | 1 | 0.003
J1c2 | 16239 | 2 | 0.007
J1c2 | 16260 | 2 | 0.007
J1c2 | 16290 | 26 | 0.089
J1c2 | 16292 | 1 | 0.003
J1c2 | 16517 | 1 | 0.003
J1c2 | 194 | 2 | 0.007
J1c2 | 204 | 1 | 0.003
J1c2 | 228 | 1 | 0.003
J1c2 | 234 | 2 | 0.007
J1c2 | 310 | 4 | 0.014
J1c2 | 3337 | 1 | 0.003
J1c2 | 3417 | 1 | 0.003
J1c2 | 3608 | 1 | 0.003
J1c2 | 3666 | 1 | 0.003
J1c2 | 3705 | 1 | 0.003
J1c2 | 3705C | 1 | 0.003
J1c2 | 3796 | 2 | 0.007
J1c2 | 426 | 1 | 0.003
J1c2 | 438d | 1 | 0.003
J1c2 | 4703 | 1 | 0.003
J1c2 | 4812 | 9 | 0.031
J1c2 | 4853 | 22 | 0.075
J1c2 | 5460 | 1 | 0.003
J1c2 | 5505 | 1 | 0.003
J1c2 | 5769 | 1 | 0.003
J1c2 | 5774 | 2 | 0.007
J1c2 | 5899.1C | 1 | 0.003
J1c2 | 5945 | 1 | 0.003
J1c2 | 6563 | 1 | 0.003
J1c2 | 6620 | 2 | 0.007
J1c2 | 6875 | 1 | 0.003
J1c2 | 7098 | 2 | 0.007
J1c2 | 7245 | 2 | 0.007
J1c2 | 7270 | 1 | 0.003
J1c2 | 7604 | 1 | 0.003
J1c2 | 7785 | 1 | 0.003
J1c2 | 8027 | 1 | 0.003
J1c2 | 8155 | 1 | 0.003
J1c2 | 8156C | 1 | 0.003
J1c2 | 8251 | 9 | 0.031
J1c2 | 8270 | 22 | 0.075
J1c2 | 8281-8289d | 21 | 0.072
J1c2 | 8285 | 1 | 0.003
J1c2 | 8450 | 1 | 0.003
J1c2 | 8476 | 2 | 0.007
J1c2 | 8538 | 1 | 0.003
J1c2 | 8558 | 1 | 0.003
J1c2 | 8701 | 1 | 0.003
J1c2 | 8712 | 6 | 0.021
J1c2 | 8843 | 1 | 0.003
J1c2 | 8922 | 1 | 0.003
J1c2 | 8989 | 2 | 0.007
J1c2 | 9025 | 1 | 0.003
J1c2 | 9070 | 1 | 0.003
J1c2 | 930 | 1 | 0.003
J1c2 | 9591 | 1 | 0.003
J1c2 | 960d | 1 | 0.003
J1c2 | 9670 | 1 | 0.003
J1c2a | 16168 | 3 | 0.017
J1c2a | 16178 | 1 | 0.006
J1c2a | 16193 | 1 | 0.006
J1c2a | 16266 | 16 | 0.089
J1c2a1 | 11413 | 1 | 0.006
J1c2a1 | 13734 | 2 | 0.011
J1c2a1 | 146 | 1 | 0.006
J1c2a1 | 15047 | 2 | 0.011
J1c2a1 | 16108 | 1 | 0.006
J1c2a1 | 16298 | 1 | 0.006
J1c2a1 | 16301 | 16 | 0.09
J1c2a1 | 3351 | 2 | 0.011
J1c2a1a | 146 | 1 | 0.005
J1c2a1a | 150 | 1 | 0.005
J1c2a1a | 151 | 16 | 0.087
J1c2a1a | 152 | 22 | 0.12
J1c2a1a | 15450 | 1 | 0.005
J1c2a1a | 15909 | 2 | 0.011

J1c2a1a | 16193 | 2 | 0.011
J1c2a1a | 16255 | 16 | 0.087
J1c2a1a | 16362 | 1 | 0.005
J1c2a1a | 16399 | 1 | 0.005
J1c2a1a | 485 | 1 | 0.005
J1c2a2 | 16005.1T | 1 | 0.083
J1c2a2 | 16136 | 1 | 0.083
J1c2a2 | 16231 | 1 | 0.083
J1c2a2 | 16291 | 1 | 0.083
J1c2a2 | 9548 | 16 | 1.333
J1c2a3 | 14278 | 1 | 0.006
J1c2a3 | 195 | 1 | 0.006
J1c2a3 | 310 | 1 | 0.006
J1c2a3 | 6260 | 1 | 0.006
J1c2a3 | 8276A | 1 | 0.006
J1c2b | 10559 | 1 | 0.006
J1c2b | 152 | 2 | 0.011
J1c2b | 15604 | 16 | 0.089
J1c2b | 16093 | 1 | 0.006
J1c2b | 16299 | 16 | 0.089
J1c2b | 3666 | 1 | 0.006
J1c2b | 5033 | 1 | 0.006
J1c2b | 7674 | 1 | 0.006
J1c2b | 8989 | 1 | 0.006
J1c2b1 | 16108 | 1 | 0.006
J1c2b1 | 16179 | 16 | 0.09
J1c2b1 | 16298 | 1 | 0.006
J1c2b1 | 9341 | 1 | 0.006
J1c2b2 | 12311 | 1 | 0.006
J1c2b2 | 16108 | 16 | 0.09
J1c2b2 | 16298 | 16 | 0.09
J1c2b2 | 7424 | 1 | 0.006
J1c2b3 | 59 | 1 | 0.143
J1c2b3 | 9761 | 1 | 0.143
J1c2b4 | 13194 | 1 | 0.077
J1c2b4 | 15924 | 1 | 0.077
J1c2b4 | 16193 | 1 | 0.077
J1c2b4 | 16288 | 2 | 0.154
J1c2b4 | 16309 | 1 | 0.077
J1c2b4 | 4370 | 1 | 0.077
J1c2b4 | 6383 | 3 | 0.231
J1c2b4 | 8065 | 1 | 0.077
J1c2b5 | 12810 | 1 | 0.006
J1c2b5 | 13851 | 1 | 0.006
J1c2b5 | 1442 | 16 | 0.089
J1c2b5 | 16093 | 16 | 0.089
J1c2b5 | 16243 | 1 | 0.006
J1c2b5 | 16265 | 1 | 0.006
J1c2b5 | 310 | 2 | 0.011
J1c2c | 12810 | 1 | 0.005
J1c2c | 14769 | 2 | 0.011
J1c2c | 16213 | 17 | 0.09
J1c2c | 16291 | 1 | 0.005
J1c2c | 198 | 1 | 0.005
J1c2c | 222 | 1 | 0.005
J1c2c | 235 | 1 | 0.005
J1c2c | 5021 | 1 | 0.005
J1c2c | 7374 | 1 | 0.005
J1c2c | 8027 | 2 | 0.011
J1c2c | 8392 | 1 | 0.005
J1c2c1 | 10927 | 1 | 0.007
J1c2c1 | 11158 | 1 | 0.007
J1c2c1 | 114.1C | 1 | 0.007
J1c2c1 | 11437 | 1 | 0.007
J1c2c1 | 11701 | 1 | 0.007
J1c2c1 | 12771 | 1 | 0.007
J1c2c1 | 13617 | 1 | 0.007
J1c2c1 | 13656 | 1 | 0.007
J1c2c1 | 13980 | 1 | 0.007
J1c2c1 | 14793 | 1 | 0.007
J1c2c1 | 15310 | 1 | 0.007
J1c2c1 | 15457 | 1 | 0.007
J1c2c1 | 1555 | 1 | 0.007
J1c2c1 | 16167 | 1 | 0.007
J1c2c1 | 16192 | 2 | 0.013
J1c2c1 | 16213 | 1 | 0.007
J1c2c1 | 16289 | 1 | 0.007
J1c2c1 | 16294 | 1 | 0.007
J1c2c1 | 16309 | 1 | 0.007
J1c2c1 | 16311 | 1 | 0.007
J1c2c1 | 16362 | 2 | 0.013
J1c2c1 | 198 | 1 | 0.007
J1c2c1 | 203 | 1 | 0.007
J1c2c1 | 23A | 1 | 0.007
J1c2c1 | 2702 | 1 | 0.007
J1c2c1 | 2707 | 1 | 0.007
J1c2c1 | 291T | 1 | 0.007
J1c2c1 | 296d | 1 | 0.007
J1c2c1 | 31.1C | 1 | 0.007
J1c2c1 | 3420 | 1 | 0.007
J1c2c1 | 71.1G | 1 | 0.007
J1c2c1 | 7441A | 1 | 0.007
J1c2c1 | 8023 | 1 | 0.007
J1c2c1 | 8280 | 1 | 0.007
J1c2c1 | 8610 | 1 | 0.007
J1c2c1 | 8697 | 1 | 0.007
J1c2c1 | 8764 | 1 | 0.007
J1c2c1 | 9025 | 1 | 0.007
J1c2c1 | 9647A | 1 | 0.007
J1c2c1a | 10454 | 1 | 0.167
J1c2c1a | 11778 | 1 | 0.167
J1c2c1a | 8260 | 1 | 0.167
J1c2c2 | 10373 | 1 | 0.005
J1c2c2 | 12880 | 1 | 0.005
J1c2c2 | 13020 | 1 | 0.005
J1c2c2 | 14544 | 1 | 0.005
J1c2c2 | 15449 | 16 | 0.083
J1c2c2 | 16176 | 1 | 0.005
J1c2c2 | 16274 | 1 | 0.005
J1c2c2 | 16526 | 3 | 0.016
J1c2c2 | 198 | 1 | 0.005

J1c2c2 | 310 | 2 | 0.01
J1c2c2 | 8961 | 1 | 0.005
J1c2c2 | 9621 | 1 | 0.005
J1c2c2a | 10256 | 3 | 0.016
J1c2c2a | 12358 | 1 | 0.005
J1c2c2a | 12875 | 1 | 0.005
J1c2c2a | 14016 | 1 | 0.005
J1c2c2a | 14128 | 1 | 0.005
J1c2c2a | 151 | 2 | 0.01
J1c2c2a | 152 | 1 | 0.005
J1c2c2a | 15523 | 1 | 0.005
J1c2c2a | 15715 | 1 | 0.005
J1c2c2a | 16209 | 16 | 0.083
J1c2c2a | 16220C | 1 | 0.005
J1c2c2a | 198 | 2 | 0.01
J1c2c2a | 215 | 1 | 0.005
J1c2c2a | 5082 | 3 | 0.016
J1c2c2a | 8101 | 1 | 0.005
J1c2c2a | 9644 | 1 | 0.005
J1c2c3 | 1494 | 1 | 0.005
J1c2c3 | 16209 | 16 | 0.086
J1c2c3 | 198 | 1 | 0.005
J1c2c3 | 215 | 1 | 0.005
J1c2c3 | 8994 | 1 | 0.005
J1c2d | 150 | 1 | 0.006
J1c2d | 16108 | 1 | 0.006
J1c2d | 16298 | 1 | 0.006
J1c2d | 16301 | 16 | 0.09
J1c2e | 10237 | 1 | 0.023
J1c2e | 10463 | 1 | 0.023
J1c2e | 11204 | 1 | 0.023
J1c2e | 11482 | 1 | 0.023
J1c2e | 13192 | 1 | 0.023
J1c2e | 13635 | 1 | 0.023
J1c2e | 13681C | 1 | 0.023
J1c2e | 14568 | 2 | 0.045
J1c2e | 14926 | 1 | 0.023
J1c2e | 150 | 1 | 0.023
J1c2e | 152 | 1 | 0.023
J1c2e | 15951 | 1 | 0.023
J1c2e | 16051 | 1 | 0.023
J1c2e | 16093 | 1 | 0.023
J1c2e | 16136 | 1 | 0.023
J1c2e | 16189 | 1 | 0.023
J1c2e | 16233 | 1 | 0.023
J1c2e | 16239 | 1 | 0.023
J1c2e | 16261 | 1 | 0.023
J1c2e | 16292 | 1 | 0.023
J1c2e | 16299 | 1 | 0.023
J1c2e | 16325 | 1 | 0.023
J1c2e | 16337A | 6 | 0.136
J1c2e | 16344A | 6 | 0.136
J1c2e | 16399 | 2 | 0.045
J1c2e | 4452 | 1 | 0.023
J1c2e | 522d | 2 | 0.045
J1c2e | 523d | 1 | 0.023
J1c2e | 6620 | 1 | 0.023
J1c2e | 6638 | 1 | 0.023
J1c2e | 6806 | 1 | 0.023
J1c2e | 6842A | 1 | 0.023
J1c2e | 8308 | 1 | 0.023
J1c2e | 8697 | 1 | 0.023
J1c2e | 93 | 1 | 0.023
J1c2e1 | 11557 | 2 | 0.083
J1c2e1 | 12007 | 1 | 0.042
J1c2e1 | 12092A | 2 | 0.083
J1c2e1 | 14502 | 1 | 0.042
J1c2e1 | 1503 | 5 | 0.208
J1c2e1 | 15314 | 5 | 0.208
J1c2e1 | 1555 | 2 | 0.083
J1c2e1 | 16131 | 1 | 0.042
J1c2e1 | 16324 | 9 | 0.375
J1c2e1 | 234 | 2 | 0.083
J1c2e1 | 309d | 2 | 0.083
J1c2e1 | 310 | 4 | 0.167
J1c2e1 | 315.2C | 2 | 0.083
J1c2e1 | 522d | 1 | 0.042
J1c2e1 | 729 | 1 | 0.042
J1c2e1 | 7830 | 1 | 0.042
J1c2e1 | 8618 | 1 | 0.042
J1c2e1 | 9386 | 3 | 0.125
J1c2e2 | 14198 | 1 | 0.023
J1c2e2 | 150 | 1 | 0.023
J1c2e2 | 16093 | 6 | 0.14
J1c2e2 | 16189 | 5 | 0.116
J1c2e2 | 16256 | 1 | 0.023
J1c2e2 | 16291 | 4 | 0.093
J1c2e2 | 16368 | 1 | 0.023
J1c2e2 | 234 | 1 | 0.023
J1c2e2 | 310 | 1 | 0.023
J1c2e2 | 3460 | 1 | 0.023
J1c2e2 | 522d | 1 | 0.023
J1c2e2 | 527G | 1 | 0.023
J1c2e2 | 5492 | 1 | 0.023
J1c2e2 | 7521 | 1 | 0.023
J1c2e2 | 8943G | 1 | 0.023
J1c2e2 | 93 | 2 | 0.047
J1c2e2 | 9490 | 1 | 0.023
J1c2f | 15574 | 1 | 0.006
J1c2f | 16239 | 1 | 0.006
J1c2f | 16318 | 16 | 0.089
J1c2f | 3915 | 1 | 0.006
J1c2g | 16108 | 1 | 0.006
J1c2g | 16256 | 1 | 0.006
J1c2g | 16298 | 1 | 0.006
J1c2g | 16301 | 16 | 0.09
J1c2g | 9974 | 1 | 0.006
J1c2h | 10205 | 1 | 0.007
J1c2h | 10416 | 1 | 0.007
J1c2h | 16075 | 1 | 0.007
J1c2h | 16189 | 1 | 0.007

J1c2h | 16192G | 1 | 0.007
J1c2h | 16234 | 2 | 0.014
J1c2h | 16235 | 2 | 0.014
J1c2h | 16290 | 5 | 0.036
J1c2h | 16311 | 1 | 0.007
J1c2h | 16527 | 1 | 0.007
J1c2h | 3531 | 2 | 0.014
J1c2h | 5786.1T | 2 | 0.014
J1c2h | 592 | 1 | 0.007
J1c2i | 11122A | 1 | 0.067
J1c2i | 12873 | 1 | 0.067
J1c2i | 13563 | 1 | 0.067
J1c2i | 14207 | 1 | 0.067
J1c2i | 146 | 1 | 0.067
J1c2i | 152 | 1 | 0.067
J1c2i | 16104 | 3 | 0.2
J1c2i | 16244 | 1 | 0.067
J1c2i | 16311 | 3 | 0.2
J1c2i | 2887 | 1 | 0.067
J1c2i | 5021 | 1 | 0.067
J1c2j | 14279 | 1 | 0.143
J1c2j | 14484 | 1 | 0.143
J1c2j | 7042 | 1 | 0.143
J1c2j | 723 | 1 | 0.143
J1c2j | 8433 | 16 | 2.286
J1c2k | 15649 | 1 | 0.006
J1c2k | 15742 | 1 | 0.006
J1c2k | 3849 | 1 | 0.006
J1c2k | 522d | 1 | 0.006
J1c2k | 573.2C | 1 | 0.006
J1c2k | 8513 | 1 | 0.006
J1c2l | 16041 | 2 | 0.011
J1c2l | 16117 | 1 | 0.006
J1c2l | 16168 | 3 | 0.017
J1c2l | 16178 | 1 | 0.006
J1c2l | 16189 | 2 | 0.011
J1c2l | 16193 | 1 | 0.006
J1c2l | 16266 | 1 | 0.006
J1c2l | 16343 | 17 | 0.094
J1c2l | 16353A | 1 | 0.006
J1c2l | 200 | 2 | 0.011
J1c2l | 210 | 1 | 0.006
J1c2l | 4598 | 1 | 0.006
J1c2l | 9055 | 1 | 0.006
J1c2m | 16086 | 2 | 0.133
J1c2m | 16221 | 1 | 0.067
J1c2m | 16324 | 1 | 0.067
J1c2m | 3918 | 1 | 0.067
J1c2m | 5471 | 1 | 0.067
J1c2m | 6753 | 1 | 0.067
J1c2m | 7835 | 1 | 0.067
J1c2m | 9030 | 1 | 0.067
J1c2m1 | 10423 | 1 | 0.1
J1c2m1 | 11204 | 1 | 0.1
J1c2m1 | 13651C | 1 | 0.1
J1c2m1 | 195 | 1 | 0.1
J1c2m1 | 215 | 1 | 0.1
J1c2m1 | 5585 | 1 | 0.1
J1c2m1 | 6620 | 1 | 0.1
J1c2m1 | 7604 | 1 | 0.1
J1c2m1 | 7772 | 1 | 0.1
J1c2m1 | 8558 | 1 | 0.1
J1c2m1 | 9670 | 1 | 0.1
J1c2m1 | 9950 | 1 | 0.1
J1c2n | 10086 | 1 | 0.006
J1c2n | 13617 | 16 | 0.089
J1c2n | 14793 | 16 | 0.089
J1c2n | 14831 | 1 | 0.006
J1c2n | 16223 | 1 | 0.006
J1c2n | 16292 | 1 | 0.006
J1c2n | 463 | 1 | 0.006
J1c2n | 8023 | 2 | 0.011
J1c2n | 8705 | 1 | 0.006
J1c2n | 9084 | 1 | 0.006
J1c2n | 9300 | 2 | 0.011
J1c2n1 | 152 | 1 | 0.006
J1c2n1 | 16261 | 19 | 0.105
J1c2n1 | 2109 | 1 | 0.006
J1c2n1 | 9025 | 1 | 0.006
J1c2o | 12771 | 1 | 0.031
J1c2o | 1462 | 1 | 0.031
J1c2o | 15622 | 1 | 0.031
J1c2o | 16093 | 1 | 0.031
J1c2o | 16266 | 1 | 0.031
J1c2o | 16311 | 1 | 0.031
J1c2o | 1861 | 1 | 0.031
J1c2o | 2581 | 1 | 0.031
J1c2o | 309d | 2 | 0.062
J1c2o | 310 | 3 | 0.094
J1c2o | 3217 | 1 | 0.031
J1c2o | 5000 | 1 | 0.031
J1c2o | 8839 | 1 | 0.031
J1c2o | 9329 | 1 | 0.031
J1c2p | 12906A | 1 | 0.006
J1c2p | 146 | 16 | 0.09
J1c2p | 3834 | 1 | 0.006
J1c2p | 9210 | 1 | 0.006
J1c2q | 11083 | 1 | 0.006
J1c2q | 12557 | 1 | 0.006
J1c2q | 16108 | 1 | 0.006
J1c2q | 16298 | 1 | 0.006
J1c2q | 16301 | 16 | 0.09
J1c2q | 198 | 1 | 0.006
J1c2q1 | 13158 | 2 | 0.011
J1c2q1 | 15159 | 2 | 0.011
J1c2q1 | 15719 | 1 | 0.005
J1c2q1 | 16086 | 2 | 0.011
J1c2q1 | 16093 | 19 | 0.104
J1c2q1 | 309d | 1 | 0.005
J1c2q1 | 7754 | 1 | 0.005
J1c2q1 | 9123 | 1 | 0.005

J1c2r | 14568 | 1 | 0.067
J1c2r | 152 | 1 | 0.067
J1c2r | 16209 | 1 | 0.067
J1c2r | 16362 | 1 | 0.067
J1c2r | 310 | 1 | 0.067
J1c2r | 4917 | 1 | 0.067
J1c2s | 14290 | 1 | 0.006
J1c2s | 16168 | 3 | 0.017
J1c2s | 16178 | 1 | 0.006
J1c2s | 16193 | 1 | 0.006
J1c2s | 16266 | 16 | 0.089
J1c2s1 | 16178 | 2 | 0.017
J1c2s1 | 16242 | 1 | 0.008
J1c2s1 | 16258C | 1 | 0.008
J1c2s1 | 16272 | 1 | 0.008
J1c2s1 | 7211 | 1 | 0.008
J1c2t | 152 | 1 | 0.005
J1c2t | 16138C | 2 | 0.011
J1c2t | 16168 | 3 | 0.016
J1c2t | 16178 | 1 | 0.005
J1c2t | 16193 | 1 | 0.005
J1c2t | 16240 | 1 | 0.005
J1c2t | 16266 | 16 | 0.087
J1c2t | 204 | 2 | 0.011
J1c2t | 5585 | 4 | 0.022
J1c2t | 789 | 1 | 0.005
J1c2t | 9615 | 4 | 0.022
J1c3 | 10143 | 1 | 0.003
J1c3 | 10256 | 1 | 0.003
J1c3 | 10370 | 1 | 0.003
J1c3 | 11309A | 1 | 0.003
J1c3 | 11740 | 1 | 0.003
J1c3 | 11884 | 1 | 0.003
J1c3 | 11908 | 2 | 0.007
J1c3 | 11933T | 1 | 0.003
J1c3 | 12245 | 1 | 0.003
J1c3 | 12306 | 1 | 0.003
J1c3 | 12340 | 1 | 0.003
J1c3 | 12358 | 4 | 0.013
J1c3 | 12397 | 1 | 0.003
J1c3 | 12477 | 1 | 0.003
J1c3 | 12565A | 2 | 0.007
J1c3 | 12630 | 1 | 0.003
J1c3 | 12819 | 1 | 0.003
J1c3 | 12903 | 3 | 0.01
J1c3 | 12950 | 1 | 0.003
J1c3 | 13065 | 1 | 0.003
J1c3 | 13528 | 1 | 0.003
J1c3 | 13641 | 1 | 0.003
J1c3 | 13651 | 1 | 0.003
J1c3 | 14002 | 1 | 0.003
J1c3 | 14088 | 1 | 0.003
J1c3 | 14180 | 5 | 0.017
J1c3 | 14544 | 2 | 0.007
J1c3 | 146 | 2 | 0.007
J1c3 | 150 | 1 | 0.003
J1c3 | 15047 | 1 | 0.003
J1c3 | 152 | 3 | 0.01
J1c3 | 15217 | 1 | 0.003
J1c3 | 15394 | 1 | 0.003
J1c3 | 15431 | 1 | 0.003
J1c3 | 15542 | 5 | 0.017
J1c3 | 15697 | 1 | 0.003
J1c3 | 15848 | 1 | 0.003
J1c3 | 15851 | 1 | 0.003
J1c3 | 16093 | 3 | 0.01
J1c3 | 16150 | 1 | 0.003
J1c3 | 16153 | 1 | 0.003
J1c3 | 16158 | 1 | 0.003
J1c3 | 16187 | 2 | 0.007
J1c3 | 16189 | 1 | 0.003
J1c3 | 16245 | 1 | 0.003
J1c3 | 16248 | 3 | 0.01
J1c3 | 16271 | 2 | 0.007
J1c3 | 16278 | 3 | 0.01
J1c3 | 16289T | 1 | 0.003
J1c3 | 16290 | 1 | 0.003
J1c3 | 16291 | 1 | 0.003
J1c3 | 16292 | 1 | 0.003
J1c3 | 16293C | 1 | 0.003
J1c3 | 16294 | 1 | 0.003
J1c3 | 16304 | 1 | 0.003
J1c3 | 16311 | 1 | 0.003
J1c3 | 16320 | 3 | 0.01
J1c3 | 16362 | 59 | 0.195
J1c3 | 182 | 3 | 0.01
J1c3 | 188 | 2 | 0.007
J1c3 | 189 | 1 | 0.003
J1c3 | 1891 | 2 | 0.007
J1c3 | 195 | 1 | 0.003
J1c3 | 199 | 3 | 0.01
J1c3 | 207 | 1 | 0.003
J1c3 | 234 | 1 | 0.003
J1c3 | 2619 | 1 | 0.003
J1c3 | 2709 | 1 | 0.003
J1c3 | 272 | 46 | 0.152
J1c3 | 310 | 3 | 0.01
J1c3 | 326 | 1 | 0.003
J1c3 | 3456 | 2 | 0.007
J1c3 | 3496 | 4 | 0.013
J1c3 | 3543 | 1 | 0.003
J1c3 | 3897 | 1 | 0.003
J1c3 | 4017 | 2 | 0.007
J1c3 | 4316 | 1 | 0.003
J1c3 | 4325 | 1 | 0.003
J1c3 | 4550 | 1 | 0.003
J1c3 | 498.1C | 1 | 0.003
J1c3 | 5228 | 2 | 0.007
J1c3 | 524A | 2 | 0.007
J1c3 | 5351 | 1 | 0.003
J1c3 | 538 | 5 | 0.017

J1c3 | 573.5C | 1 | 0.003
J1c3 | 5821 | 1 | 0.003
J1c3 | 6221 | 2 | 0.007
J1c3 | 6261 | 2 | 0.007
J1c3 | 6480 | 3 | 0.01
J1c3 | 6713 | 1 | 0.003
J1c3 | 6896 | 1 | 0.003
J1c3 | 709 | 2 | 0.007
J1c3 | 7160 | 1 | 0.003
J1c3 | 7356 | 1 | 0.003
J1c3 | 8387 | 4 | 0.013
J1c3 | 8614 | 3 | 0.01
J1c3 | 8790 | 24 | 0.079
J1c3 | 9181 | 3 | 0.01
J1c3 | 9214 | 1 | 0.003
J1c3 | 9389 | 1 | 0.003
J1c3 | 9591 | 1 | 0.003
J1c3 | 9830A | 1 | 0.003
J1c3 | 9935 | 1 | 0.003
J1c3+189 | 14287 | 1 | 0.007
J1c3+189 | 16093 | 2 | 0.013
J1c3+189 | 16131 | 1 | 0.007
J1c3+189 | 16172 | 1 | 0.007
J1c3+189 | 16189 | 1 | 0.007
J1c3+189 | 16192 | 1 | 0.007
J1c3+189 | 16216 | 2 | 0.013
J1c3+189 | 16256 | 3 | 0.02
J1c3+189 | 227 | 1 | 0.007
J1c3+189 | 234 | 1 | 0.007
J1c3+189 | 3918 | 1 | 0.007
J1c3+189 | 5393 | 1 | 0.007
J1c3+189 | 5460 | 1 | 0.007
J1c3+189 | 7444 | 1 | 0.007
J1c3a | 16201 | 1 | 0.005
J1c3a | 16259.1A | 23 | 0.108
J1c3a | 16299 | 23 | 0.108
J1c3a | 183 | 1 | 0.005
J1c3a1 | 11143 | 1 | 0.004
J1c3a1 | 12558 | 1 | 0.004
J1c3a1 | 14560 | 1 | 0.004
J1c3a1 | 15436A | 1 | 0.004
J1c3a1 | 16219 | 21 | 0.093
J1c3a1 | 1780 | 1 | 0.004
J1c3a1 | 194 | 1 | 0.004
J1c3a1 | 309d | 1 | 0.004
J1c3a1 | 310 | 3 | 0.013
J1c3a1 | 315.2C | 1 | 0.004
J1c3a1 | 325 | 1 | 0.004
J1c3a1 | 3380 | 1 | 0.004
J1c3a1 | 438G | 1 | 0.004
J1c3a1 | 4454 | 1 | 0.004
J1c3a1 | 5600 | 1 | 0.004
J1c3a1 | 593 | 1 | 0.004
J1c3a1 | 709 | 1 | 0.004
J1c3a1 | 7691 | 3 | 0.013
J1c3a1 | 8289.1CCCCCTCTA | 2 | 0.009
J1c3a1 | 9482 | 1 | 0.004
J1c3a1 | 9797 | 1 | 0.004
J1c3a2 | 14323 | 2 | 0.009
J1c3a2 | 15355 | 2 | 0.009
J1c3a2 | 15799 | 1 | 0.005
J1c3a2 | 16189 | 1 | 0.005
J1c3a2 | 16288 | 1 | 0.005
J1c3a2 | 16290 | 1 | 0.005
J1c3a2 | 16311 | 21 | 0.097
J1c3a2 | 186 | 1 | 0.005
J1c3a2 | 309d | 1 | 0.005
J1c3a2 | 593 | 21 | 0.097
J1c3a2 | 5979 | 1 | 0.005
J1c3b | 13194 | 1 | 0.005
J1c3b | 1711 | 1 | 0.005
J1c3b | 1888 | 1 | 0.005
J1c3b | 3105 | 1 | 0.005
J1c3b | 8905 | 1 | 0.005
J1c3b | 9301 | 1 | 0.005
J1c3b1 | 15924 | 2 | 0.009
J1c3b1 | 16189 | 21 | 0.099
J1c3b1 | 3508 | 2 | 0.009
J1c3b1 | 9007 | 1 | 0.005
J1c3b1a | 16179 | 21 | 0.099
J1c3b1a | 8557 | 1 | 0.005
J1c3b2 | 152 | 1 | 0.005
J1c3b2 | 16245 | 25 | 0.117
J1c3b2 | 16325 | 24 | 0.112
J1c3b2 | 16390 | 1 | 0.005
J1c3b2 | 189 | 1 | 0.005
J1c3b2 | 4491 | 1 | 0.005
J1c3c | 10553 | 1 | 0.048
J1c3c | 1120 | 1 | 0.048
J1c3c | 11929 | 1 | 0.048
J1c3c | 12382 | 1 | 0.048
J1c3c | 14831 | 1 | 0.048
J1c3c | 14872 | 1 | 0.048
J1c3c | 16160 | 1 | 0.048
J1c3c | 16161 | 1 | 0.048
J1c3c | 16172 | 1 | 0.048
J1c3c | 16235 | 1 | 0.048
J1c3c | 16304 | 1 | 0.048
J1c3c | 3592 | 1 | 0.048
J1c3c | 4086 | 3 | 0.143
J1c3c | 6956 | 1 | 0.048
J1c3c | 6990 | 1 | 0.048
J1c3c | 8701 | 1 | 0.048
J1c3c | 962A | 1 | 0.048
J1c3c | 9932 | 1 | 0.048
J1c3c | 9948 | 1 | 0.048
J1c3c1 | 146 | 1 | 0.059
J1c3c1 | 152 | 1 | 0.059
J1c3c1 | 16145 | 2 | 0.118
J1c3c1 | 16274 | 3 | 0.176
J1c3c1 | 16319 | 1 | 0.059

J1c3c1 | 182 | 1 | 0.059
J1c3c1 | 195 | 1 | 0.059
J1c3c1 | 7301 | 1 | 0.059
J1c3c2 | 146 | 1 | 0.059
J1c3c2 | 152 | 1 | 0.059
J1c3c2 | 16145 | 2 | 0.118
J1c3c2 | 16274 | 3 | 0.176
J1c3c2 | 16319 | 1 | 0.059
J1c3c2 | 182 | 1 | 0.059
J1c3c2 | 195 | 1 | 0.059
J1c3c2 | 793 | 1 | 0.059
J1c3c2 | 8108 | 1 | 0.059
J1c3d | 11410G | 21 | 0.097
J1c3d | 2772 | 1 | 0.005
J1c3d | 309d | 1 | 0.005
J1c3d | 3203 | 1 | 0.005
J1c3d | 3540 | 1 | 0.005
J1c3d | 7711 | 2 | 0.009
J1c3e | 13020 | 1 | 0.042
J1c3e | 14047 | 1 | 0.042
J1c3e | 150 | 5 | 0.208
J1c3e | 16311 | 1 | 0.042
J1c3e | 16319 | 1 | 0.042
J1c3e | 16484-16489d | 3 | 0.125
J1c3e | 188 | 2 | 0.083
J1c3e | 189 | 1 | 0.042
J1c3e | 8563 | 1 | 0.042
J1c3e1 | 11233 | 2 | 0.08
J1c3e1 | 11254 | 1 | 0.04
J1c3e1 | 11440 | 1 | 0.04
J1c3e1 | 11653 | 1 | 0.04
J1c3e1 | 12070 | 1 | 0.04
J1c3e1 | 13488 | 5 | 0.2
J1c3e1 | 14502 | 1 | 0.04
J1c3e1 | 14927 | 1 | 0.04
J1c3e1 | 150 | 5 | 0.2
J1c3e1 | 15737 | 1 | 0.04
J1c3e1 | 16153 | 5 | 0.2
J1c3e1 | 195 | 9 | 0.36
J1c3e1 | 210 | 1 | 0.04
J1c3e1 | 2755 | 1 | 0.04
J1c3e1 | 3733C | 1 | 0.04
J1c3e1 | 4679 | 2 | 0.08
J1c3e1 | 4805 | 1 | 0.04
J1c3e1 | 6293 | 1 | 0.04
J1c3e2 | 10454 | 1 | 0.037
J1c3e2 | 11914 | 1 | 0.037
J1c3e2 | 13395 | 1 | 0.037
J1c3e2 | 14281 | 1 | 0.037
J1c3e2 | 152 | 1 | 0.037
J1c3e2 | 15883 | 1 | 0.037
J1c3e2 | 16249 | 3 | 0.111
J1c3e2 | 16256 | 1 | 0.037
J1c3e2 | 16261 | 2 | 0.074
J1c3e2 | 16362 | 5 | 0.185
J1c3e2 | 4243 | 1 | 0.037
J1c3e2 | 4955 | 2 | 0.074
J1c3e2 | 522d | 1 | 0.037
J1c3e2 | 6287A | 2 | 0.074
J1c3e2 | 7984 | 2 | 0.074
J1c3e2 | 9957 | 1 | 0.037
J1c3f | 10172 | 1 | 0.027
J1c3f | 10845 | 3 | 0.081
J1c3f | 11368 | 2 | 0.054
J1c3f | 12696 | 1 | 0.027
J1c3f | 14809 | 1 | 0.027
J1c3f | 14960 | 1 | 0.027
J1c3f | 15344 | 1 | 0.027
J1c3f | 16093 | 4 | 0.108
J1c3f | 16167 | 2 | 0.054
J1c3f | 16294 | 2 | 0.054
J1c3f | 16303 | 3 | 0.081
J1c3f | 16311 | 1 | 0.027
J1c3f | 16342 | 2 | 0.054
J1c3f | 16348 | 3 | 0.081
J1c3f | 189 | 1 | 0.027
J1c3f | 195 | 2 | 0.054
J1c3f | 210 | 1 | 0.027
J1c3f | 234 | 1 | 0.027
J1c3f | 310 | 3 | 0.081
J1c3f | 315.2C | 2 | 0.054
J1c3f | 3438 | 1 | 0.027
J1c3f | 6040 | 1 | 0.027
J1c3f | 709 | 4 | 0.108
J1c3f | 745 | 1 | 0.027
J1c3f | 7912 | 2 | 0.054
J1c3f | 8227 | 1 | 0.027
J1c3f | 8251 | 1 | 0.027
J1c3f | 9300 | 1 | 0.027
J1c3f | 9829 | 1 | 0.027
J1c3g | 10000 | 1 | 0.004
J1c3g | 10208 | 1 | 0.004
J1c3g | 11005 | 1 | 0.004
J1c3g | 11014 | 1 | 0.004
J1c3g | 11093 | 1 | 0.004
J1c3g | 11485 | 1 | 0.004
J1c3g | 11590 | 1 | 0.004
J1c3g | 11914 | 1 | 0.004
J1c3g | 12067 | 1 | 0.004
J1c3g | 12976 | 1 | 0.004
J1c3g | 13131 | 1 | 0.004
J1c3g | 13150 | 1 | 0.004
J1c3g | 13530 | 1 | 0.004
J1c3g | 13662 | 1 | 0.004
J1c3g | 14022 | 1 | 0.004
J1c3g | 14130 | 1 | 0.004
J1c3g | 14327 | 1 | 0.004
J1c3g | 150 | 1 | 0.004
J1c3g | 15337 | 1 | 0.004
J1c3g | 16145 | 1 | 0.004
J1c3g | 16243 | 2 | 0.009

J1c3g | 16260 | 25 | 0.108
J1c3g | 188 | 1 | 0.004
J1c3g | 189 | 2 | 0.009
J1c3g | 195 | 2 | 0.009
J1c3g | 3077 | 1 | 0.004
J1c3g | 309d | 1 | 0.004
J1c3g | 3298 | 1 | 0.004
J1c3g | 3308 | 1 | 0.004
J1c3g | 356.1C | 2 | 0.009
J1c3g | 4092 | 2 | 0.009
J1c3g | 4435 | 1 | 0.004
J1c3g | 4585 | 1 | 0.004
J1c3g | 5585 | 1 | 0.004
J1c3g | 5899.1C | 1 | 0.004
J1c3g | 6261 | 1 | 0.004
J1c3g | 6808 | 1 | 0.004
J1c3g | 6810 | 1 | 0.004
J1c3g | 7402 | 1 | 0.004
J1c3g | 7404 | 1 | 0.004
J1c3g | 7406 | 1 | 0.004
J1c3g | 7546 | 1 | 0.004
J1c3g | 8999 | 1 | 0.004
J1c3g | 9055 | 1 | 0.004
J1c3g | 9071 | 1 | 0.004
J1c3g | 9209 | 1 | 0.004
J1c3h | 10455 | 4 | 0.016
J1c3h | 10667 | 21 | 0.084
J1c3h | 10700T | 1 | 0.004
J1c3h | 10907 | 6 | 0.024
J1c3h | 11455 | 1 | 0.004
J1c3h | 14307A | 1 | 0.004
J1c3h | 14307T | 1 | 0.004
J1c3h | 14587 | 30 | 0.12
J1c3h | 146 | 1 | 0.004
J1c3h | 14629 | 1 | 0.004
J1c3h | 15254 | 1 | 0.004
J1c3h | 15313 | 1 | 0.004
J1c3h | 1578 | 21 | 0.084
J1c3h | 16043 | 1 | 0.004
J1c3h | 16093 | 2 | 0.008
J1c3h | 16270 | 1 | 0.004
J1c3h | 188 | 1 | 0.004
J1c3h | 189 | 1 | 0.004
J1c3h | 3591 | 1 | 0.004
J1c3h | 4688 | 10 | 0.04
J1c3h | 7205 | 2 | 0.008
J1c3h | 7797 | 1 | 0.004
J1c3h | 8463 | 29 | 0.116
J1c3h | 9221 | 1 | 0.004
J1c3i | 12858 | 3 | 0.014
J1c3i | 13326 | 21 | 0.099
J1c3i | 42.1G | 1 | 0.005
J1c3j | 11506 | 1 | 0.029
J1c3j | 150 | 1 | 0.029
J1c3j | 152 | 3 | 0.088
J1c3j | 153 | 1 | 0.029
J1c3j | 16093 | 1 | 0.029
J1c3j | 16167 | 2 | 0.059
J1c3j | 16184 | 1 | 0.029
J1c3j | 16188 | 2 | 0.059
J1c3j | 16193 | 2 | 0.059
J1c3j | 16261 | 4 | 0.118
J1c3j | 16309 | 1 | 0.029
J1c3j | 16319 | 2 | 0.059
J1c3j | 1766 | 1 | 0.029
J1c3j | 188 | 2 | 0.059
J1c3j | 195 | 1 | 0.029
J1c3j | 225 | 1 | 0.029
J1c3j | 482 | 3 | 0.088
J1c3j | 9055 | 1 | 0.029
J1c3k | 10018 | 21 | 0.097
J1c3k | 12957 | 1 | 0.005
J1c3k | 310 | 1 | 0.005
J1c3k | 315.2C | 1 | 0.005
J1c3k | 573.1C | 1 | 0.005
J1c3k | 9938 | 1 | 0.005
J1c3m | 14459 | 2 | 0.4
J1c3m | 260 | 1 | 0.2
J1c3m | 9531 | 1 | 0.2
J1c4 | 10373 | 1 | 0.004
J1c4 | 10834 | 1 | 0.004
J1c4 | 11260 | 1 | 0.004
J1c4 | 11778 | 3 | 0.012
J1c4 | 11914 | 3 | 0.012
J1c4 | 12127 | 1 | 0.004
J1c4 | 12172 | 1 | 0.004
J1c4 | 12480 | 1 | 0.004
J1c4 | 12807 | 1 | 0.004
J1c4 | 13020 | 2 | 0.008
J1c4 | 13858 | 2 | 0.008
J1c4 | 13863 | 1 | 0.004
J1c4 | 13911 | 1 | 0.004
J1c4 | 14569 | 1 | 0.004
J1c4 | 14869 | 1 | 0.004
J1c4 | 15924 | 2 | 0.008
J1c4 | 16093 | 1 | 0.004
J1c4 | 16145 | 1 | 0.004
J1c4 | 16179 | 2 | 0.008
J1c4 | 16185 | 1 | 0.004
J1c4 | 16187 | 1 | 0.004
J1c4 | 16189 | 31 | 0.129
J1c4 | 16243 | 21 | 0.088
J1c4 | 16245 | 1 | 0.004
J1c4 | 16290 | 1 | 0.004
J1c4 | 16294 | 21 | 0.088
J1c4 | 16311 | 2 | 0.008
J1c4 | 189 | 1 | 0.004
J1c4 | 195 | 3 | 0.012
J1c4 | 204 | 1 | 0.004
J1c4 | 2195 | 2 | 0.008
J1c4 | 2772 | 1 | 0.004

J1c4 | 309d | 1 | 0.004
J1c4 | 310 | 2 | 0.008
J1c4 | 315.2C | 1 | 0.004
J1c4 | 324 | 22 | 0.092
J1c4 | 3663 | 2 | 0.008
J1c4 | 5746 | 1 | 0.004
J1c4 | 5978 | 1 | 0.004
J1c4 | 8696 | 1 | 0.004
J1c4 | 9132 | 4 | 0.017
J1c4 | 9181 | 1 | 0.004
J1c4b | 12276 | 1 | 0.005
J1c4b | 13565 | 1 | 0.005
J1c4b | 16189 | 22 | 0.101
J1c4b | 4934 | 1 | 0.005
J1c4b | 573.1C | 1 | 0.005
J1c4b | 7853 | 1 | 0.005
J1c4b | 7912 | 1 | 0.005
J1c4b | 8310 | 1 | 0.005
J1c4b | 9701 | 2 | 0.009
J1c4c | 12708A | 1 | 0.045
J1c4c | 16051 | 2 | 0.091
J1c4c | 16093 | 1 | 0.045
J1c4c | 16220T | 1 | 0.045
J1c4c | 16362 | 2 | 0.091
J1c4c | 188 | 2 | 0.091
J1c4c | 195 | 1 | 0.045
J1c4c | 5582 | 2 | 0.091
J1c5 | 10192 | 1 | 0.004
J1c5 | 10529 | 1 | 0.004
J1c5 | 10972 | 1 | 0.004
J1c5 | 11080 | 1 | 0.004
J1c5 | 11087 | 6 | 0.023
J1c5 | 11204 | 1 | 0.004
J1c5 | 11778 | 2 | 0.008
J1c5 | 11963 | 1 | 0.004
J1c5 | 12239 | 6 | 0.023
J1c5 | 12361 | 1 | 0.004
J1c5 | 12587T | 1 | 0.004
J1c5 | 12732 | 1 | 0.004
J1c5 | 12741 | 1 | 0.004
J1c5 | 12930C | 1 | 0.004
J1c5 | 13966 | 1 | 0.004
J1c5 | 14311 | 1 | 0.004
J1c5 | 14314 | 1 | 0.004
J1c5 | 14364 | 2 | 0.008
J1c5 | 14470 | 1 | 0.004
J1c5 | 14587 | 1 | 0.004
J1c5 | 146 | 1 | 0.004
J1c5 | 14769 | 1 | 0.004
J1c5 | 150 | 1 | 0.004
J1c5 | 151 | 2 | 0.008
J1c5 | 152 | 12 | 0.045
J1c5 | 15378 | 1 | 0.004
J1c5 | 15850 | 1 | 0.004
J1c5 | 16093 | 1 | 0.004
J1c5 | 16167 | 1 | 0.004
J1c5 | 16185 | 1 | 0.004
J1c5 | 16193 | 1 | 0.004
J1c5 | 16239 | 1 | 0.004
J1c5 | 16266 | 1 | 0.004
J1c5 | 16292 | 1 | 0.004
J1c5 | 16301 | 1 | 0.004
J1c5 | 16311 | 1 | 0.004
J1c5 | 16399 | 25 | 0.094
J1c5 | 1719 | 3 | 0.011
J1c5 | 188 | 1 | 0.004
J1c5 | 189 | 3 | 0.011
J1c5 | 195 | 1 | 0.004
J1c5 | 198 | 1 | 0.004
J1c5 | 204 | 3 | 0.011
J1c5 | 309d | 1 | 0.004
J1c5 | 310 | 5 | 0.019
J1c5 | 310.1AC | 1 | 0.004
J1c5 | 3203 | 1 | 0.004
J1c5 | 3318 | 1 | 0.004
J1c5 | 3460 | 2 | 0.008
J1c5 | 3592 | 1 | 0.004
J1c5 | 3666 | 3 | 0.011
J1c5 | 408A | 1 | 0.004
J1c5 | 4484 | 1 | 0.004
J1c5 | 4838 | 2 | 0.008
J1c5 | 4976 | 1 | 0.004
J1c5 | 5046 | 1 | 0.004
J1c5 | 522d | 1 | 0.004
J1c5 | 5237C | 1 | 0.004
J1c5 | 5460 | 1 | 0.004
J1c5 | 5495 | 1 | 0.004
J1c5 | 573.2C | 2 | 0.008
J1c5 | 573.4C | 1 | 0.004
J1c5 | 5821 | 1 | 0.004
J1c5 | 5830.1T | 1 | 0.004
J1c5 | 5836 | 1 | 0.004
J1c5 | 5843 | 1 | 0.004
J1c5 | 5899.1C | 1 | 0.004
J1c5 | 6272 | 4 | 0.015
J1c5 | 6285 | 1 | 0.004
J1c5 | 6681 | 6 | 0.023
J1c5 | 6852 | 1 | 0.004
J1c5 | 6898 | 1 | 0.004
J1c5 | 709 | 1 | 0.004
J1c5 | 7364 | 1 | 0.004
J1c5 | 7372 | 1 | 0.004
J1c5 | 7412 | 1 | 0.004
J1c5 | 7519.1A | 4 | 0.015
J1c5 | 7912 | 1 | 0.004
J1c5 | 8289.1CCCCCTCTA | 1 | 0.004
J1c5 | 8479 | 1 | 0.004
J1c5 | 870 | 1 | 0.004
J1c5 | 8701 | 4 | 0.015
J1c5 | 9100 | 2 | 0.008
J1c5 | 9445 | 1 | 0.004

J1c5 | 9861 | 1 | 0.004
J1c5 | 9985 | 1 | 0.004
J1c5a | 10320 | 1 | 0.004
J1c5a | 10364 | 1 | 0.004
J1c5a | 11087 | 1 | 0.004
J1c5a | 150 | 3 | 0.013
J1c5a | 15390G | 1 | 0.004
J1c5a | 15601 | 1 | 0.004
J1c5a | 16111 | 4 | 0.017
J1c5a | 16148 | 1 | 0.004
J1c5a | 16172 | 4 | 0.017
J1c5a | 16174 | 2 | 0.009
J1c5a | 16184 | 1 | 0.004
J1c5a | 16230 | 25 | 0.109
J1c5a | 16270 | 1 | 0.004
J1c5a | 16280C | 1 | 0.004
J1c5a | 16281C | 1 | 0.004
J1c5a | 16287 | 1 | 0.004
J1c5a | 16527 | 1 | 0.004
J1c5a | 195 | 1 | 0.004
J1c5a | 261 | 1 | 0.004
J1c5a | 368 | 1 | 0.004
J1c5a | 384 | 1 | 0.004
J1c5a | 7269 | 1 | 0.004
J1c5a | 9053 | 1 | 0.004
J1c5a | 9612 | 1 | 0.004
J1c5a1 | 11197 | 1 | 0.004
J1c5a1 | 11583 | 1 | 0.004
J1c5a1 | 11860 | 2 | 0.008
J1c5a1 | 12895 | 1 | 0.004
J1c5a1 | 13135 | 1 | 0.004
J1c5a1 | 14162 | 1 | 0.004
J1c5a1 | 14207 | 1 | 0.004
J1c5a1 | 1535d | 1 | 0.004
J1c5a1 | 15553 | 1 | 0.004
J1c5a1 | 16051 | 1 | 0.004
J1c5a1 | 16093 | 3 | 0.012
J1c5a1 | 16209 | 22 | 0.092
J1c5a1 | 16325 | 1 | 0.004
J1c5a1 | 16327 | 1 | 0.004
J1c5a1 | 1709 | 1 | 0.004
J1c5a1 | 189 | 1 | 0.004
J1c5a1 | 310 | 2 | 0.008
J1c5a1 | 316 | 1 | 0.004
J1c5a1 | 3338 | 2 | 0.008
J1c5a1 | 3918 | 1 | 0.004
J1c5a1 | 5460 | 1 | 0.004
J1c5a1 | 5779 | 1 | 0.004
J1c5a1 | 5843 | 1 | 0.004
J1c5a1 | 6413 | 3 | 0.012
J1c5a1 | 6497 | 1 | 0.004
J1c5a1 | 7270 | 1 | 0.004
J1c5a1 | 9007 | 1 | 0.004
J1c5a1 | 9449 | 1 | 0.004
J1c5a1 | 9861 | 1 | 0.004
J1c5b | 10237 | 1 | 0.005
J1c5b | 14106 | 2 | 0.009
J1c5b | 16093 | 1 | 0.005
J1c5b | 16148 | 22 | 0.104
J1c5b | 189 | 1 | 0.005
J1c5b | 2911 | 1 | 0.005
J1c5b | 4639 | 1 | 0.005
J1c5b | 573.2C | 1 | 0.005
J1c5b | 9630 | 1 | 0.005
J1c5c | 16201 | 1 | 0.005
J1c5c | 16259.1A | 23 | 0.108
J1c5c | 16299 | 23 | 0.108
J1c5c | 183 | 1 | 0.005
J1c5c1 | 11761 | 1 | 0.019
J1c5c1 | 15565 | 1 | 0.019
J1c5c1 | 15940G | 3 | 0.056
J1c5c1 | 16189 | 3 | 0.056
J1c5c1 | 16234 | 1 | 0.019
J1c5c1 | 16356 | 1 | 0.019
J1c5c1 | 188 | 1 | 0.019
J1c5c1 | 3745 | 1 | 0.019
J1c5c1 | 513 | 1 | 0.019
J1c5d | 10237 | 1 | 0.005
J1c5d | 11852 | 21 | 0.099
J1c5d | 146 | 1 | 0.005
J1c5d | 150 | 21 | 0.099
J1c5d | 16192 | 1 | 0.005
J1c5d | 188 | 2 | 0.009
J1c5d | 5978 | 1 | 0.005
J1c5d | 7340 | 1 | 0.005
J1c5d | 7888 | 1 | 0.005
J1c5d | 8255 | 2 | 0.009
J1c5e | 16201 | 1 | 0.005
J1c5e | 16259.1A | 3 | 0.014
J1c5e | 16299 | 3 | 0.014
J1c5e | 16318 | 21 | 0.1
J1c5e | 183 | 1 | 0.005
J1c5e | 408A | 1 | 0.005
J1c5e | 4452 | 2 | 0.009
J1c5e | 5339 | 1 | 0.005
J1c5f | 11206 | 1 | 0.005
J1c5f | 12239 | 1 | 0.005
J1c5f | 16185 | 3 | 0.014
J1c5f | 16298 | 22 | 0.103
J1c5f | 16360 | 1 | 0.005
J1c5f | 6681 | 1 | 0.005
J1c6 | 11255 | 1 | 0.005
J1c6 | 11413 | 2 | 0.009
J1c6 | 14502 | 1 | 0.005
J1c6 | 15172 | 1 | 0.005
J1c6 | 16111 | 1 | 0.005
J1c6 | 16145 | 24 | 0.111
J1c6 | 16162 | 1 | 0.005
J1c6 | 16168 | 1 | 0.005
J1c6 | 16311 | 4 | 0.018
J1c6 | 16362 | 1 | 0.005

J1c6 | 188 | 2 | 0.009
J1c6 | 195 | 2 | 0.009
J1c6 | 3167.1T | 1 | 0.005
J1c6 | 5234 | 2 | 0.009
J1c6 | 573.4C | 21 | 0.097
J1c6 | 5951 | 1 | 0.005
J1c6 | 8406 | 1 | 0.005
J1c6 | 9738 | 1 | 0.005
J1c6a | 11914 | 1 | 0.005
J1c6a | 12566A | 1 | 0.005
J1c6a | 15814 | 1 | 0.005
J1c6a | 16201 | 1 | 0.005
J1c6a | 16220C | 5 | 0.023
J1c6a | 16258C | 1 | 0.005
J1c6a | 16259.1A | 23 | 0.106
J1c6a | 16299 | 23 | 0.106
J1c6a | 183 | 1 | 0.005
J1c6a | 234 | 2 | 0.009
J1c6a | 6261 | 1 | 0.005
J1c6a | 796 | 1 | 0.005
J1c7 | 10325 | 1 | 0.012
J1c7 | 10728 | 1 | 0.012
J1c7 | 10927 | 1 | 0.012
J1c7 | 12192 | 1 | 0.012
J1c7 | 13954 | 1 | 0.012
J1c7 | 14452 | 1 | 0.012
J1c7 | 146 | 4 | 0.048
J1c7 | 15055 | 1 | 0.012
J1c7 | 15749 | 1 | 0.012
J1c7 | 16216 | 1 | 0.012
J1c7 | 16291 | 1 | 0.012
J1c7 | 16311 | 1 | 0.012
J1c7 | 16319 | 1 | 0.012
J1c7 | 16325 | 22 | 0.265
J1c7 | 182 | 2 | 0.024
J1c7 | 214.1G | 2 | 0.024
J1c7 | 215 | 2 | 0.024
J1c7 | 2414 | 1 | 0.012
J1c7 | 2416 | 1 | 0.012
J1c7 | 282 | 1 | 0.012
J1c7 | 2835 | 1 | 0.012
J1c7 | 294.1T | 2 | 0.024
J1c7 | 3290 | 1 | 0.012
J1c7 | 3915 | 2 | 0.024
J1c7 | 513 | 1 | 0.012
J1c7 | 5417 | 1 | 0.012
J1c7 | 6221 | 2 | 0.024
J1c7 | 6734 | 2 | 0.024
J1c7 | 7664 | 1 | 0.012
J1c7 | 7711 | 1 | 0.012
J1c7 | 8856 | 1 | 0.012
J1c7a | 1000 | 1 | 0.009
J1c7a | 10915 | 1 | 0.009
J1c7a | 11074 | 1 | 0.009
J1c7a | 11088 | 1 | 0.009
J1c7a | 11302 | 1 | 0.009
J1c7a | 11404 | 1 | 0.009
J1c7a | 12519 | 1 | 0.009
J1c7a | 12630 | 1 | 0.009
J1c7a | 12649 | 1 | 0.009
J1c7a | 13470 | 5 | 0.044
J1c7a | 14207 | 1 | 0.009
J1c7a | 14552 | 1 | 0.009
J1c7a | 146 | 26 | 0.228
J1c7a | 14861 | 1 | 0.009
J1c7a | 14894 | 1 | 0.009
J1c7a | 15103 | 1 | 0.009
J1c7a | 152 | 4 | 0.035
J1c7a | 15380C | 1 | 0.009
J1c7a | 16037 | 1 | 0.009
J1c7a | 16093 | 2 | 0.018
J1c7a | 16189 | 1 | 0.009
J1c7a | 16209 | 1 | 0.009
J1c7a | 16254 | 1 | 0.009
J1c7a | 16255 | 1 | 0.009
J1c7a | 16263 | 1 | 0.009
J1c7a | 16278 | 1 | 0.009
J1c7a | 16284 | 1 | 0.009
J1c7a | 16365 | 2 | 0.018
J1c7a | 178 | 1 | 0.009
J1c7a | 1824 | 3 | 0.026
J1c7a | 188 | 5 | 0.044
J1c7a | 189 | 4 | 0.035
J1c7a | 195 | 26 | 0.228
J1c7a | 204 | 1 | 0.009
J1c7a | 2392 | 3 | 0.026
J1c7a | 2483 | 1 | 0.009
J1c7a | 2765 | 1 | 0.009
J1c7a | 2851 | 1 | 0.009
J1c7a | 309d | 1 | 0.009
J1c7a | 310 | 1 | 0.009
J1c7a | 314-315d | 1 | 0.009
J1c7a | 316C | 1 | 0.009
J1c7a | 3434 | 2 | 0.018
J1c7a | 3900 | 1 | 0.009
J1c7a | 4113 | 1 | 0.009
J1c7a | 463 | 1 | 0.009
J1c7a | 490 | 1 | 0.009
J1c7a | 515-524d | 18 | 0.158
J1c7a | 5291 | 1 | 0.009
J1c7a | 573.1C | 1 | 0.009
J1c7a | 593 | 1 | 0.009
J1c7a | 6887 | 1 | 0.009
J1c7a | 7270 | 1 | 0.009
J1c7a | 7547 | 1 | 0.009
J1c7a | 7810 | 1 | 0.009
J1c7a | 8113A | 1 | 0.009
J1c7a | 9152 | 2 | 0.018
J1c7a | 9554 | 1 | 0.009
J1c7a | 9896 | 2 | 0.018
J1c7a | 9973 | 1 | 0.009

J1c8 | 16201 | 1 | 0.005
J1c8 | 16259.1A | 23 | 0.108
J1c8 | 16299 | 23 | 0.108
J1c8 | 183 | 1 | 0.005
J1c8 | 188 | 1 | 0.005
J1c8 | 317.1C | 1 | 0.005
J1c8a | 1040 | 1 | 0.013
J1c8a | 12373 | 1 | 0.013
J1c8a | 12768 | 1 | 0.013
J1c8a | 13349 | 1 | 0.013
J1c8a | 14103 | 1 | 0.013
J1c8a | 14189 | 1 | 0.013
J1c8a | 14544 | 1 | 0.013
J1c8a | 14914 | 2 | 0.027
J1c8a | 153 | 1 | 0.013
J1c8a | 15884 | 1 | 0.013
J1c8a | 16093 | 1 | 0.013
J1c8a | 16114A | 2 | 0.027
J1c8a | 16145 | 2 | 0.027
J1c8a | 16148 | 1 | 0.013
J1c8a | 16153 | 1 | 0.013
J1c8a | 16188 | 1 | 0.013
J1c8a | 16189 | 5 | 0.067
J1c8a | 16193d | 1 | 0.013
J1c8a | 16228A | 2 | 0.027
J1c8a | 16241 | 1 | 0.013
J1c8a | 16242G | 2 | 0.027
J1c8a | 16248A | 2 | 0.027
J1c8a | 16270 | 2 | 0.027
J1c8a | 16301 | 2 | 0.027
J1c8a | 16326C | 2 | 0.027
J1c8a | 16328G | 2 | 0.027
J1c8a | 16343 | 5 | 0.067
J1c8a | 183 | 1 | 0.013
J1c8a | 1842 | 1 | 0.013
J1c8a | 185 | 1 | 0.013
J1c8a | 188 | 2 | 0.027
J1c8a | 195 | 2 | 0.027
J1c8a | 19A | 2 | 0.027
J1c8a | 228 | 1 | 0.013
J1c8a | 248 | 2 | 0.027
J1c8a | 267d | 1 | 0.013
J1c8a | 2792d | 1 | 0.013
J1c8a | 310 | 2 | 0.027
J1c8a | 3360 | 5 | 0.067
J1c8a | 3387A | 1 | 0.013
J1c8a | 3591 | 1 | 0.013
J1c8a | 3666 | 1 | 0.013
J1c8a | 3918 | 2 | 0.027
J1c8a | 3981 | 1 | 0.013
J1c8a | 5201 | 1 | 0.013
J1c8a | 55 | 3 | 0.04
J1c8a | 56 | 1 | 0.013
J1c8a | 568 | 1 | 0.013
J1c8a | 57 | 1 | 0.013
J1c8a | 6481 | 1 | 0.013
J1c8a | 7521 | 1 | 0.013
J1c8a | 9176 | 1 | 0.013
J1c8a1 | 11569 | 1 | 0.077
J1c8a1 | 14115 | 1 | 0.077
J1c8a1 | 14751 | 1 | 0.077
J1c8a1 | 150 | 1 | 0.077
J1c8a1 | 16153 | 1 | 0.077
J1c8a1 | 16209 | 2 | 0.154
J1c8a1 | 16262 | 1 | 0.077
J1c8a1 | 189 | 1 | 0.077
J1c8a1 | 3460 | 1 | 0.077
J1c8a1 | 522.2C | 1 | 0.077
J1c8a1 | 6570T | 1 | 0.077
J1c8a1 | 720 | 1 | 0.077
J1c8a1 | 8666 | 1 | 0.077
J1c8a1 | 9181 | 1 | 0.077
J1c8a1a | 10398 | 1 | 0.083
J1c8a1a | 14751 | 3 | 0.25
J1c8a1a | 16311 | 1 | 0.083
J1c8a1a | 6685 | 1 | 0.083
J1c8a1a | 8666 | 3 | 0.25
J1c8a1a | 9828 | 1 | 0.083
J1c8a2 | 15315 | 1 | 0.083
J1c8a2 | 15766 | 1 | 0.083
J1c8a2 | 2707 | 1 | 0.083
J1c8a2 | 522.1C | 1 | 0.083
J1c8a2 | 7598 | 1 | 0.083
J1c8a2 | 960d | 1 | 0.083
J1c8b | 16201 | 1 | 0.005
J1c8b | 16259.1A | 23 | 0.107
J1c8b | 16299 | 23 | 0.107
J1c8b | 183 | 1 | 0.005
J1c8b | 5306 | 1 | 0.005
J1c9 | 10873 | 1 | 0.005
J1c9 | 11518 | 1 | 0.005
J1c9 | 12813 | 21 | 0.096
J1c9 | 14552 | 1 | 0.005
J1c9 | 15883 | 1 | 0.005
J1c9 | 15940 | 4 | 0.018
J1c9 | 16189 | 1 | 0.005
J1c9 | 1719 | 4 | 0.018
J1c9 | 210 | 1 | 0.005
J1c9 | 225 | 1 | 0.005
J1c9 | 2703G | 1 | 0.005
J1c9 | 2704d | 1 | 0.005
J1c9 | 3155 | 1 | 0.005
J1c9 | 3768 | 1 | 0.005
J1c9 | 3882 | 1 | 0.005
J1c9 | 417 | 1 | 0.005
J1c9 | 5157 | 1 | 0.005
J1c9 | 522d | 1 | 0.005
J1c9 | 5957 | 1 | 0.005
J1c9 | 6365 | 1 | 0.005
J1c9 | 6499 | 1 | 0.005
J1c9 | 769 | 1 | 0.005

J1c9 | 7925 | 1 | 0.005
J1c9 | 9234 | 1 | 0.005
J1c9 | 9236 | 1 | 0.005
J1c9 | 951 | 2 | 0.009
J1c9 | 9947 | 1 | 0.005
J1d | 10203 | 1 | 0.013
J1d | 10322 | 2 | 0.027
J1d | 10410A | 1 | 0.013
J1d | 11089 | 1 | 0.013
J1d | 11353 | 1 | 0.013
J1d | 12123 | 2 | 0.027
J1d | 12823 | 1 | 0.013
J1d | 14203 | 2 | 0.027
J1d | 146 | 3 | 0.04
J1d | 150 | 1 | 0.013
J1d | 15314 | 4 | 0.053
J1d | 15530 | 1 | 0.013
J1d | 15672 | 2 | 0.027
J1d | 15924 | 1 | 0.013
J1d | 16051 | 2 | 0.027
J1d | 16114 | 3 | 0.04
J1d | 16192 | 1 | 0.013
J1d | 16212 | 2 | 0.027
J1d | 16223 | 1 | 0.013
J1d | 16241 | 4 | 0.053
J1d | 16311 | 5 | 0.067
J1d | 16312 | 1 | 0.013
J1d | 16368 | 2 | 0.027
J1d | 16409 | 1 | 0.013
J1d | 182 | 2 | 0.027
J1d | 271 | 2 | 0.027
J1d | 3474 | 1 | 0.013
J1d | 3483 | 1 | 0.013
J1d | 3523 | 2 | 0.027
J1d | 44.1C | 1 | 0.013
J1d | 459d | 1 | 0.013
J1d | 4655 | 1 | 0.013
J1d | 523d | 1 | 0.013
J1d | 55.1T | 2 | 0.027
J1d | 57 | 2 | 0.027
J1d | 59 | 2 | 0.027
J1d | 6515 | 1 | 0.013
J1d | 71d | 2 | 0.027
J1d | 7269 | 1 | 0.013
J1d | 8101 | 1 | 0.013
J1d | 8143 | 2 | 0.027
J1d | 8392 | 1 | 0.013
J1d | 8473 | 2 | 0.027
J1d | 9123C | 1 | 0.013
J1d1 | 1007 | 1 | 0.059
J1d1 | 12528 | 1 | 0.059
J1d1 | 13135 | 1 | 0.059
J1d1 | 15043 | 1 | 0.059
J1d1 | 16186 | 1 | 0.059
J1d1 | 16221 | 1 | 0.059
J1d1 | 1636 | 1 | 0.059
J1d1 | 199 | 1 | 0.059
J1d1 | 214 | 1 | 0.059
J1d1a | 10900 | 1 | 0.013
J1d1a | 11527 | 2 | 0.026
J1d1a | 14560 | 1 | 0.013
J1d1a | 146 | 1 | 0.013
J1d1a | 14602 | 1 | 0.013
J1d1a | 14996 | 1 | 0.013
J1d1a | 150 | 1 | 0.013
J1d1a | 151 | 1 | 0.013
J1d1a | 15616 | 1 | 0.013
J1d1a | 16051 | 1 | 0.013
J1d1a | 16172 | 1 | 0.013
J1d1a | 16173 | 1 | 0.013
J1d1a | 16234 | 3 | 0.038
J1d1a | 16256 | 2 | 0.026
J1d1a | 16287 | 2 | 0.026
J1d1a | 16311 | 1 | 0.013
J1d1a | 16399 | 2 | 0.026
J1d1a | 16T | 2 | 0.026
J1d1a | 194 | 1 | 0.013
J1d1a | 199 | 1 | 0.013
J1d1a | 468 | 1 | 0.013
J1d1a | 513 | 1 | 0.013
J1d1a | 514d | 1 | 0.013
J1d1a | 516 | 1 | 0.013
J1d1a | 522d | 1 | 0.013
J1d1a | 8622 | 1 | 0.013
J1d1a1 | 10454 | 1 | 0.012
J1d1a1 | 10795 | 1 | 0.012
J1d1a1 | 11447 | 4 | 0.047
J1d1a1 | 11461 | 2 | 0.024
J1d1a1 | 13420 | 1 | 0.012
J1d1a1 | 13967 | 1 | 0.012
J1d1a1 | 14020 | 2 | 0.024
J1d1a1 | 14065 | 1 | 0.012
J1d1a1 | 150 | 5 | 0.059
J1d1a1 | 16086 | 2 | 0.024
J1d1a1 | 16093 | 1 | 0.012
J1d1a1 | 16145 | 1 | 0.012
J1d1a1 | 16256 | 1 | 0.012
J1d1a1 | 195 | 3 | 0.035
J1d1a1 | 316 | 2 | 0.024
J1d1a1 | 370 | 4 | 0.047
J1d1a1 | 3832A | 2 | 0.024
J1d1a1 | 4703 | 1 | 0.012
J1d1a1 | 513 | 2 | 0.024
J1d1a1 | 5147 | 3 | 0.035
J1d1a1 | 515-524d | 1 | 0.012
J1d1a1 | 5498 | 1 | 0.012
J1d1a1 | 5528 | 2 | 0.024
J1d1a1 | 5704 | 5 | 0.059
J1d1a1 | 593 | 1 | 0.012
J1d1a1 | 6366C | 1 | 0.012
J1d1a1 | 6546 | 1 | 0.012

J1d1a1 | 709 | 1 | 0.012
J1d1a1 | 7119 | 1 | 0.012
J1d1a1 | 7202 | 1 | 0.012
J1d1a1 | 7269 | 1 | 0.012
J1d1a1 | 8131 | 1 | 0.012
J1d1a1 | 8152 | 2 | 0.024
J1d1a1 | 8269 | 1 | 0.012
J1d1a1 | 9254 | 1 | 0.012
J1d1a1 | 9380 | 4 | 0.047
J1d1a1a | 15924 | 2 | 0.047
J1d1a1a | 16319 | 1 | 0.023
J1d1a1a | 2404 | 1 | 0.023
J1d1a1a | 7474d | 1 | 0.023
J1d1b | 12975 | 1 | 0.053
J1d1b | 15260 | 1 | 0.053
J1d1b | 15924 | 1 | 0.053
J1d1b | 16129 | 4 | 0.211
J1d1b | 16354 | 1 | 0.053
J1d1b | 249d | 1 | 0.053
J1d1b | 282 | 1 | 0.053
J1d1b | 324 | 1 | 0.053
J1d1b | 6737 | 1 | 0.053
J1d1b | 8764 | 1 | 0.053
J1d1b | 9380 | 1 | 0.053
J1d1b1 | 10373 | 1 | 0.043
J1d1b1 | 11485 | 3 | 0.13
J1d1b1 | 13500 | 1 | 0.043
J1d1b1 | 2261 | 2 | 0.087
J1d1b1 | 239 | 1 | 0.043
J1d1b1 | 310 | 1 | 0.043
J1d1b1 | 515-524d | 1 | 0.043
J1d1b1 | 5747 | 2 | 0.087
J1d1b1 | 6446 | 2 | 0.087
J1d1b1 | 745 | 1 | 0.043
J1d1b1 | 8820 | 3 | 0.13
J1d1b1 | 9128 | 2 | 0.087
J1d1b1 | 9287 | 2 | 0.087
J1d2 | 11778 | 2 | 0.182
J1d2 | 14040 | 2 | 0.182
J1d2 | 14280 | 2 | 0.182
J1d2 | 16092 | 2 | 0.182
J1d2 | 16129 | 1 | 0.091
J1d2 | 16311 | 2 | 0.182
J1d2 | 16354A | 1 | 0.091
J1d2 | 16362 | 2 | 0.182
J1d2 | 689 | 2 | 0.182
J1d2 | 745.1T | 1 | 0.091
J1d2 | 9123 | 2 | 0.182
J1d2a | 11137 | 1 | 0.091
J1d2a | 13368 | 1 | 0.091
J1d2a | 16234 | 1 | 0.091
J1d2a | 16311 | 1 | 0.091
J1d2a | 16354A | 1 | 0.091
J1d2a | 1888 | 1 | 0.091
J1d2a | 522.1C | 1 | 0.091
J1d2a | 593 | 1 | 0.091
J1d2a | 9833 | 1 | 0.091
J1d3 | 10463 | 1 | 0.016
J1d3 | 12477 | 1 | 0.016
J1d3 | 151 | 2 | 0.031
J1d3 | 16054C | 1 | 0.016
J1d3 | 16093 | 1 | 0.016
J1d3 | 16239 | 1 | 0.016
J1d3 | 16266 | 1 | 0.016
J1d3 | 171C | 1 | 0.016
J1d3 | 2416 | 1 | 0.016
J1d3 | 316 | 1 | 0.016
J1d3 | 324G | 1 | 0.016
J1d3 | 376C | 1 | 0.016
J1d3 | 7843 | 1 | 0.016
J1d3a | 11061G | 1 | 0.015
J1d3a | 14551 | 1 | 0.015
J1d3a | 1508 | 1 | 0.015
J1d3a | 15498 | 1 | 0.015
J1d3a | 15894 | 1 | 0.015
J1d3a | 16054C | 1 | 0.015
J1d3a | 16093 | 1 | 0.015
J1d3a | 16218 | 1 | 0.015
J1d3a | 16239 | 1 | 0.015
J1d3a | 16266 | 1 | 0.015
J1d3a | 16344 | 1 | 0.015
J1d3a | 171C | 1 | 0.015
J1d3a | 200 | 2 | 0.031
J1d3a | 2109 | 1 | 0.015
J1d3a | 316 | 1 | 0.015
J1d3a | 324G | 1 | 0.015
J1d3a | 376C | 1 | 0.015
J1d3a1 | 14110 | 1 | 0.143
J1d3a1 | 16188 | 1 | 0.143
J1d3a1 | 16255 | 1 | 0.143
J1d3a1 | 16256 | 4 | 0.571
J1d3a1 | 522.1C | 2 | 0.286
J1d3a1 | 523.1A | 1 | 0.143
J1d3a2 | 146 | 1 | 0.015
J1d3a2 | 14669 | 1 | 0.015
J1d3a2 | 16362 | 1 | 0.015
J1d3a2 | 2281C | 1 | 0.015
J1d3a2 | 356.1C | 2 | 0.03
J1d3a2 | 5964 | 1 | 0.015
J1d3a2 | 794 | 1 | 0.015
J1d4 | 14422 | 1 | 0.016
J1d4 | 146 | 1 | 0.016
J1d4 | 153 | 1 | 0.016
J1d4 | 16054C | 1 | 0.016
J1d4 | 16093 | 1 | 0.016
J1d4 | 16189 | 1 | 0.016
J1d4 | 16239 | 1 | 0.016
J1d4 | 16266 | 1 | 0.016
J1d4 | 16294 | 1 | 0.016
J1d4 | 171C | 1 | 0.016
J1d4 | 316 | 1 | 0.016

J1d4 | 324G | 1 | 0.016
J1d4 | 376C | 1 | 0.016
J1d4 | 4508 | 1 | 0.016
J1d4 | 8538 | 1 | 0.016
J1d5 | 12811 | 1 | 0.034
J1d5 | 146 | 4 | 0.138
J1d5 | 14974 | 1 | 0.034
J1d5 | 16093 | 2 | 0.069
J1d5 | 16111 | 1 | 0.034
J1d5 | 16189 | 1 | 0.034
J1d5 | 16260 | 1 | 0.034
J1d5 | 16343 | 1 | 0.034
J1d5 | 16365 | 1 | 0.034
J1d5 | 200 | 1 | 0.034
J1d5 | 207 | 1 | 0.034
J1d5 | 310 | 1 | 0.034
J1d5 | 4164 | 1 | 0.034
J1d5 | 4772 | 1 | 0.034
J1d5 | 5553 | 1 | 0.034
J1d5 | 709 | 2 | 0.069
J1d5a | 127 | 1 | 0.05
J1d5a | 13132 | 1 | 0.05
J1d5a | 150 | 1 | 0.05
J1d5a | 5075 | 1 | 0.05
J1d6 | 12123 | 1 | 0.015
J1d6 | 12681 | 1 | 0.015
J1d6 | 14106 | 1 | 0.015
J1d6 | 15218 | 1 | 0.015
J1d6 | 16111 | 1 | 0.015
J1d6 | 16129 | 1 | 0.015
J1d6 | 16148 | 1 | 0.015
J1d6 | 16153 | 2 | 0.03
J1d6 | 16311 | 1 | 0.015
J1d6 | 16320 | 1 | 0.015
J1d6 | 16400 | 1 | 0.015
J1d6 | 236 | 1 | 0.015
J1d6 | 310 | 1 | 0.015
J1d6 | 335 | 1 | 0.015
J1d6 | 3915 | 1 | 0.015
J1d6 | 4553 | 1 | 0.015
J1d6 | 5557 | 1 | 0.015
J1d6 | 573.1C | 1 | 0.015
J1d6 | 573.5C | 1 | 0.015
J1d6 | 6305 | 1 | 0.015
J1d6 | 6719 | 1 | 0.015
J1d6 | 7473 | 1 | 0.015
J1d6 | 8863 | 1 | 0.015
J1d6 | 8874 | 1 | 0.015
J1d6a | 16362 | 1 | 0.143
J1d6a | 260.1G | 1 | 0.143
J1d6a | 573.1C | 1 | 0.143
J1d6a | 763A | 1 | 0.143
J1d6a | 763d | 1 | 0.143
J1d6a | 764 | 1 | 0.143
J1d6a | 765G | 1 | 0.143
J1d6a | 81.1C | 1 | 0.143
J2 | 16145 | 1 | 0.008
J2 | 16189 | 1 | 0.008
J2 | 16243 | 1 | 0.008
J2 | 16274 | 1 | 0.008
J2 | 16278 | 1 | 0.008
J2 | 16366 | 1 | 0.008
J2 | 182 | 1 | 0.008
J2 | 195 | 1 | 0.008
J2 | 247 | 1 | 0.008
J2a | 16051 | 7 | 0.054
J2a | 16092 | 1 | 0.008
J2a | 16188 | 7 | 0.054
J2a | 16311 | 6 | 0.046
J2a | 16375 | 2 | 0.015
J2a | 235 | 1 | 0.008
J2a | 345 | 3 | 0.023
J2a | 385 | 1 | 0.008
J2a1 | 12127 | 1 | 0.008
J2a1 | 13956 | 1 | 0.008
J2a1 | 14280 | 1 | 0.008
J2a1 | 14929 | 1 | 0.008
J2a1 | 16263d | 1 | 0.008
J2a1 | 16362 | 1 | 0.008
J2a1 | 16375 | 2 | 0.016
J2a1 | 4688 | 2 | 0.016
J2a1 | 8843 | 2 | 0.016
J2a1 | 9025 | 1 | 0.008
J2a1a | 16129 | 1 | 0.333
J2a1a | 310.1T | 1 | 0.333
J2a1a1 | 10007 | 1 | 0.017
J2a1a1 | 10172 | 2 | 0.033
J2a1a1 | 10333 | 1 | 0.017
J2a1a1 | 10456 | 1 | 0.017
J2a1a1 | 11242 | 1 | 0.017
J2a1a1 | 12684 | 1 | 0.017
J2a1a1 | 13020 | 1 | 0.017
J2a1a1 | 13395 | 1 | 0.017
J2a1a1 | 146 | 1 | 0.017
J2a1a1 | 14908 | 1 | 0.017
J2a1a1 | 14968 | 1 | 0.017
J2a1a1 | 15848 | 1 | 0.017
J2a1a1 | 16172 | 1 | 0.017
J2a1a1 | 16186 | 1 | 0.017
J2a1a1 | 16207 | 1 | 0.017
J2a1a1 | 16222 | 1 | 0.017
J2a1a1 | 16235 | 1 | 0.017
J2a1a1 | 16239 | 1 | 0.017
J2a1a1 | 16260 | 1 | 0.017
J2a1a1 | 16269 | 1 | 0.017
J2a1a1 | 16311 | 1 | 0.017
J2a1a1 | 16324 | 3 | 0.05
J2a1a1 | 1844 | 1 | 0.017
J2a1a1 | 1850 | 3 | 0.05
J2a1a1 | 189 | 2 | 0.033
J2a1a1 | 203 | 1 | 0.017

J2a1a1 | 214 | 1 | 0.017
J2a1a1 | 2392 | 2 | 0.033
J2a1a1 | 246 | 1 | 0.017
J2a1a1 | 2880 | 1 | 0.017
J2a1a1 | 310 | 1 | 0.017
J2a1a1 | 311 | 1 | 0.017
J2a1a1 | 3375 | 16 | 0.267
J2a1a1 | 3438 | 1 | 0.017
J2a1a1 | 3571.1C | 1 | 0.017
J2a1a1 | 389 | 1 | 0.017
J2a1a1 | 4021 | 1 | 0.017
J2a1a1 | 41 | 1 | 0.017
J2a1a1 | 4561 | 1 | 0.017
J2a1a1 | 509 | 1 | 0.017
J2a1a1 | 6032 | 1 | 0.017
J2a1a1 | 7051 | 1 | 0.017
J2a1a1 | 709 | 1 | 0.017
J2a1a1 | 75 | 1 | 0.017
J2a1a1 | 8857 | 1 | 0.017
J2a1a1 | 8958 | 1 | 0.017
J2a1a1 | 980 | 1 | 0.017
J2a1a1a | 10565 | 1 | 0.023
J2a1a1a | 11900 | 1 | 0.023
J2a1a1a | 13635 | 1 | 0.023
J2a1a1a | 146 | 4 | 0.091
J2a1a1a | 15217 | 7 | 0.159
J2a1a1a | 15905 | 1 | 0.023
J2a1a1a | 1617 | 1 | 0.023
J2a1a1a | 16299 | 2 | 0.045
J2a1a1a | 16311 | 1 | 0.023
J2a1a1a | 200 | 2 | 0.045
J2a1a1a | 203 | 1 | 0.023
J2a1a1a | 211 | 1 | 0.023
J2a1a1a | 279 | 1 | 0.023
J2a1a1a | 5585 | 7 | 0.159
J2a1a1a | 573.1C | 3 | 0.068
J2a1a1a | 573.2C | 2 | 0.045
J2a1a1a | 573.3C | 1 | 0.023
J2a1a1a | 8245 | 1 | 0.023
J2a1a1a | 9477 | 1 | 0.023
J2a1a1a1 | 12477 | 1 | 0.1
J2a1a1a1 | 2098 | 2 | 0.2
J2a1a1a1 | 214 | 1 | 0.1
J2a1a1a1 | 311 | 1 | 0.1
J2a1a1a1 | 8745 | 1 | 0.1
J2a1a1a1 | 9861 | 1 | 0.1
J2a1a1a2 | 1007 | 1 | 0.013
J2a1a1a2 | 10685 | 1 | 0.013
J2a1a1a2 | 11339 | 2 | 0.026
J2a1a1a2 | 11530 | 1 | 0.013
J2a1a1a2 | 11696 | 1 | 0.013
J2a1a1a2 | 11818 | 2 | 0.026
J2a1a1a2 | 12373 | 1 | 0.013
J2a1a1a2 | 12528 | 1 | 0.013
J2a1a1a2 | 12618 | 2 | 0.026
J2a1a1a2 | 13161 | 1 | 0.013
J2a1a1a2 | 13308 | 1 | 0.013
J2a1a1a2 | 13656 | 2 | 0.026
J2a1a1a2 | 14282 | 1 | 0.013
J2a1a1a2 | 14470 | 1 | 0.013
J2a1a1a2 | 146 | 2 | 0.026
J2a1a1a2 | 15613 | 1 | 0.013
J2a1a1a2 | 15930 | 1 | 0.013
J2a1a1a2 | 16037 | 1 | 0.013
J2a1a1a2 | 16093 | 5 | 0.066
J2a1a1a2 | 16154 | 1 | 0.013
J2a1a1a2 | 16168 | 1 | 0.013
J2a1a1a2 | 16209 | 1 | 0.013
J2a1a1a2 | 16231A | 1 | 0.013
J2a1a1a2 | 16248 | 1 | 0.013
J2a1a1a2 | 16270 | 1 | 0.013
J2a1a1a2 | 16301 | 6 | 0.079
J2a1a1a2 | 16311 | 2 | 0.026
J2a1a1a2 | 16319 | 1 | 0.013
J2a1a1a2 | 16344 | 1 | 0.013
J2a1a1a2 | 16399 | 1 | 0.013
J2a1a1a2 | 198 | 1 | 0.013
J2a1a1a2 | 200 | 1 | 0.013
J2a1a1a2 | 215C | 1 | 0.013
J2a1a1a2 | 2580 | 2 | 0.026
J2a1a1a2 | 2960 | 1 | 0.013
J2a1a1a2 | 311 | 1 | 0.013
J2a1a1a2 | 315.2C | 2 | 0.026
J2a1a1a2 | 316 | 2 | 0.026
J2a1a1a2 | 3197 | 1 | 0.013
J2a1a1a2 | 3915 | 1 | 0.013
J2a1a1a2 | 4011 | 1 | 0.013
J2a1a1a2 | 4232 | 1 | 0.013
J2a1a1a2 | 4769C | 1 | 0.013
J2a1a1a2 | 5231 | 1 | 0.013
J2a1a1a2 | 5307 | 3 | 0.039
J2a1a1a2 | 5319 | 1 | 0.013
J2a1a1a2 | 569 | 1 | 0.013
J2a1a1a2 | 5711 | 1 | 0.013
J2a1a1a2 | 573.1C | 2 | 0.026
J2a1a1a2 | 6285 | 1 | 0.013
J2a1a1a2 | 6731 | 1 | 0.013
J2a1a1a2 | 6852 | 1 | 0.013
J2a1a1a2 | 709 | 1 | 0.013
J2a1a1a2 | 7258 | 1 | 0.013
J2a1a1a2 | 7661 | 1 | 0.013
J2a1a1a2 | 8557 | 1 | 0.013
J2a1a1a2 | 8715 | 1 | 0.013
J2a1a1a2 | 8856 | 1 | 0.013
J2a1a1a2 | 8904 | 3 | 0.039
J2a1a1a2 | 93 | 1 | 0.013
J2a1a1a2 | 9540 | 1 | 0.013
J2a1a1a2 | 9644 | 2 | 0.026
J2a1a1a2 | 9739 | 1 | 0.013
J2a1a1a2 | 9932 | 1 | 0.013
J2a1a1a2a | 10271 | 1 | 0.029

J2a1a1a2a | 10801 | 3 | 0.088
J2a1a1a2a | 14194 | 1 | 0.029
J2a1a1a2a | 16324 | 1 | 0.029
J2a1a1a3 | 16051 | 1 | 0.043
J2a1a1a3 | 16140 | 1 | 0.043
J2a1a1a3 | 16189 | 1 | 0.043
J2a1a1a3 | 16311 | 2 | 0.087
J2a1a1a3 | 16355 | 4 | 0.174
J2a1a1a3 | 189 | 1 | 0.043
J2a1a1a3 | 5004 | 1 | 0.043
J2a1a1a3 | 7775 | 1 | 0.043
J2a1a1a3 | 9377 | 1 | 0.043
J2a1a1a3 | 9380 | 1 | 0.043
J2a1a1b | 16172 | 1 | 0.037
J2a1a1b | 16266 | 1 | 0.037
J2a1a1b | 16399 | 5 | 0.185
J2a1a1c | 10320 | 1 | 0.038
J2a1a1c | 146 | 2 | 0.077
J2a1a1c | 15217 | 1 | 0.038
J2a1a1c | 1542 | 1 | 0.038
J2a1a1c | 16172 | 1 | 0.038
J2a1a1c | 16209 | 5 | 0.192
J2a1a1c | 4976 | 1 | 0.038
J2a1a1c | 7768 | 1 | 0.038
J2a1a1c | 8008 | 3 | 0.115
J2a1a1c | 9254 | 1 | 0.038
J2a1a1d | 10461 | 1 | 0.012
J2a1a1d | 10909 | 1 | 0.012
J2a1a1d | 11551 | 1 | 0.012
J2a1a1d | 12070 | 2 | 0.023
J2a1a1d | 12879 | 3 | 0.035
J2a1a1d | 14357 | 1 | 0.012
J2a1a1d | 146 | 2 | 0.023
J2a1a1d | 15470 | 1 | 0.012
J2a1a1d | 15568 | 3 | 0.035
J2a1a1d | 16065 | 23 | 0.267
J2a1a1d | 16153 | 1 | 0.012
J2a1a1d | 16260 | 8 | 0.093
J2a1a1d | 16271 | 1 | 0.012
J2a1a1d | 16304 | 7 | 0.081
J2a1a1d | 16311 | 1 | 0.012
J2a1a1d | 1673 | 1 | 0.012
J2a1a1d | 4674 | 1 | 0.012
J2a1a1d | 6173 | 22 | 0.256
J2a1a1d | 6659 | 3 | 0.035
J2a1a1d | 6860 | 1 | 0.012
J2a1a1d | 709 | 19 | 0.221
J2a1a1d | 7318 | 1 | 0.012
J2a1a1d | 7785 | 1 | 0.012
J2a1a1d | 7840 | 2 | 0.023
J2a1a1d | 8337 | 1 | 0.012
J2a1a1d | 8538 | 32 | 0.372
J2a1a1d | 9163 | 1 | 0.012
J2a1a1e | 10044 | 1 | 0.027
J2a1a1e | 10604 | 1 | 0.027
J2a1a1e | 11512 | 2 | 0.054
J2a1a1e | 11914 | 2 | 0.054
J2a1a1e | 12007 | 2 | 0.054
J2a1a1e | 12140 | 1 | 0.027
J2a1a1e | 12375 | 1 | 0.027
J2a1a1e | 13768 | 1 | 0.027
J2a1a1e | 14302 | 1 | 0.027
J2a1a1e | 14305 | 1 | 0.027
J2a1a1e | 14316 | 2 | 0.054
J2a1a1e | 14587 | 1 | 0.027
J2a1a1e | 15534 | 1 | 0.027
J2a1a1e | 16249 | 1 | 0.027
J2a1a1e | 16269 | 1 | 0.027
J2a1a1e | 16286 | 3 | 0.081
J2a1a1e | 198 | 7 | 0.189
J2a1a1e | 2848 | 1 | 0.027
J2a1a1e | 308-309d | 1 | 0.027
J2a1a1e | 310 | 2 | 0.054
J2a1a1e | 319 | 10 | 0.27
J2a1a1e | 3306 | 1 | 0.027
J2a1a1e | 44.1C | 1 | 0.027
J2a1a1e | 509 | 3 | 0.081
J2a1a1e | 567T | 1 | 0.027
J2a1a1e | 573.2C | 1 | 0.027
J2a1a1e | 5780C | 1 | 0.027
J2a1a1e | 6152 | 1 | 0.027
J2a1a1e | 7080 | 3 | 0.081
J2a1a1e | 8270 | 4 | 0.108
J2a1a1e | 8697 | 1 | 0.027
J2a1a1e | 8832 | 1 | 0.027
J2a1a1e | 9025 | 1 | 0.027
J2a1a1e | 9548C | 1 | 0.027
J2a1a1e | 9612 | 1 | 0.027
J2a1a2 | 11704 | 1 | 0.333
J2a1a2 | 12501 | 1 | 0.333
J2a1a2 | 12705 | 1 | 0.333
J2a1a2 | 15055 | 1 | 0.333
J2a1a2 | 4991 | 1 | 0.333
J2a1a2a | 249d | 1 | 0.25
J2a1a2a | 7828 | 1 | 0.25
J2a1a2a | 9127 | 1 | 0.25
J2a1a2a1 | 11680 | 1 | 0.333
J2a1a2a1a | 11287 | 1 | 0.25
J2a1a2a1a | 14745 | 1 | 0.25
J2a1a2a1a | 2281C | 1 | 0.25
J2a2 | 14577 | 1 | 0.007
J2a2 | 16239 | 8 | 0.058
J2a2 | 16366 | 4 | 0.029
J2a2 | 16399 | 4 | 0.029
J2a2 | 3110 | 1 | 0.007
J2a2 | 3110d | 1 | 0.007
J2a2 | 316 | 1 | 0.007
J2a2 | 3316 | 2 | 0.015
J2a2 | 3511 | 2 | 0.015
J2a2 | 573.2C | 1 | 0.007
J2a2 | 5899.1C | 1 | 0.007

J2a2 | 6908 | 1 | 0.007
J2a2 | 709 | 1 | 0.007
J2a2 | 7830 | 1 | 0.007
J2a2 | 9189 | 1 | 0.007
J2a2a | 10684G | 1 | 0.008
J2a2a | 11204 | 1 | 0.008
J2a2a | 12171 | 2 | 0.015
J2a2a | 13105 | 1 | 0.008
J2a2a | 13581 | 1 | 0.008
J2a2a | 14656 | 1 | 0.008
J2a2a | 16048 | 1 | 0.008
J2a2a | 16114 | 1 | 0.008
J2a2a | 16179 | 3 | 0.023
J2a2a | 16214 | 1 | 0.008
J2a2a | 16215 | 1 | 0.008
J2a2a | 16265T | 1 | 0.008
J2a2a | 16311 | 1 | 0.008
J2a2a | 16362 | 1 | 0.008
J2a2a | 1888 | 1 | 0.008
J2a2a | 2149.1AG | 1 | 0.008
J2a2a | 326 | 1 | 0.008
J2a2a | 3316 | 2 | 0.015
J2a2a | 564C | 1 | 0.008
J2a2a | 565C | 1 | 0.008
J2a2a | 567C | 1 | 0.008
J2a2a | 574C | 1 | 0.008
J2a2a | 7585 | 1 | 0.008
J2a2a | 8281-8289d | 1 | 0.008
J2a2a1 | 13368 | 3 | 0.023
J2a2a1 | 15496 | 3 | 0.023
J2a2a1 | 16192 | 3 | 0.023
J2a2a1 | 8725 | 3 | 0.023
J2a2a1+16311 | 10586 | 1 | 0.083
J2a2a1+16311 | 10736 | 1 | 0.083
J2a2a1+16311 | 13759 | 2 | 0.167
J2a2a1+16311 | 14494 | 1 | 0.083
J2a2a1+16311 | 189 | 2 | 0.167
J2a2a1+16311 | 573.2C | 1 | 0.083
J2a2a1+16311 | 573.5C | 1 | 0.083
J2a2a1+16311 | 7325 | 2 | 0.167
J2a2a1a | 152 | 2 | 0.143
J2a2a1a | 16185 | 1 | 0.071
J2a2a1a | 16189 | 2 | 0.143
J2a2a1a | 16192 | 1 | 0.071
J2a2a1a | 16290 | 1 | 0.071
J2a2a1a | 16298 | 1 | 0.071
J2a2a1a | 573.5C | 1 | 0.071
J2a2a1a1 | 146 | 2 | 0.029
J2a2a1a1 | 152 | 29 | 0.42
J2a2a1a1 | 16051 | 50 | 0.725
J2a2a1a1 | 16168 | 1 | 0.014
J2a2a1a1 | 16188 | 54 | 0.783
J2a2a1a1 | 16189 | 2 | 0.029
J2a2a1a1 | 16209 | 1 | 0.014
J2a2a1a1 | 16239 | 1 | 0.014
J2a2a1a1 | 16264 | 2 | 0.029
J2a2a1a1 | 16278 | 1 | 0.014
J2a2a1a1 | 16362 | 1 | 0.014
J2a2a1a1 | 16496 | 1 | 0.014
J2a2a1a1 | 16513G | 1 | 0.014
J2a2a1a1 | 204 | 2 | 0.029
J2a2a1a1 | 257 | 1 | 0.014
J2a2a1a1 | 4322.1C | 1 | 0.014
J2a2a1a1 | 573.1C | 2 | 0.029
J2a2a1a1 | 573.5C | 1 | 0.014
J2a2a1a1 | 8473 | 5 | 0.072
J2a2a1a1 | 9758 | 1 | 0.014
J2a2a2 | 10657G | 1 | 0.071
J2a2a2 | 10658T | 1 | 0.071
J2a2a2 | 11809 | 1 | 0.071
J2a2a2 | 13056 | 1 | 0.071
J2a2a2 | 152 | 2 | 0.143
J2a2a2 | 16152 | 2 | 0.143
J2a2a2 | 2463.1A | 1 | 0.071
J2a2a2 | 262 | 1 | 0.071
J2a2a2 | 2755 | 1 | 0.071
J2a2a2 | 4309 | 1 | 0.071
J2a2a2 | 4955 | 1 | 0.071
J2a2a2 | 5839 | 1 | 0.071
J2a2a2 | 8865 | 2 | 0.143
J2a2a2 | 9176 | 1 | 0.071
J2a2b | 12858 | 1 | 0.032
J2a2b | 14058 | 2 | 0.065
J2a2b | 14947 | 1 | 0.032
J2a2b | 15217 | 2 | 0.065
J2a2b | 15924 | 1 | 0.032
J2a2b | 16145 | 3 | 0.097
J2a2b | 16150 | 1 | 0.032
J2a2b | 16173 | 1 | 0.032
J2a2b | 16234 | 1 | 0.032
J2a2b | 16278 | 17 | 0.548
J2a2b | 1676 | 1 | 0.032
J2a2b | 200 | 9 | 0.29
J2a2b | 204 | 2 | 0.065
J2a2b | 2483 | 2 | 0.065
J2a2b | 3337 | 1 | 0.032
J2a2b | 4257 | 2 | 0.065
J2a2b | 5054 | 1 | 0.032
J2a2b | 515-524d | 1 | 0.032
J2a2b | 525.1AC | 1 | 0.032
J2a2b | 5291 | 1 | 0.032
J2a2b | 6749 | 2 | 0.065
J2a2b | 7741 | 1 | 0.032
J2a2b | 9323 | 1 | 0.032
J2a2b1 | 16084 | 1 | 0.067
J2a2b1 | 16362 | 3 | 0.2
J2a2b1 | 185 | 6 | 0.4
J2a2b1 | 215 | 1 | 0.067
J2a2b1 | 234 | 1 | 0.067
J2a2b1 | 522 | 2 | 0.133
J2a2b1 | 8292 | 2 | 0.133

J2a2b1 | 8712 | 1 | 0.067
J2a2b1 | 9214 | 1 | 0.067
J2a2b1a | 11147 | 3 | 0.2
J2a2b1a | 152 | 5 | 0.333
J2a2b1a | 15924 | 3 | 0.2
J2a2b1a | 16084 | 1 | 0.067
J2a2b1a | 207 | 1 | 0.067
J2a2b1a | 215 | 1 | 0.067
J2a2b1a | 3397 | 1 | 0.067
J2a2b1a | 3915 | 1 | 0.067
J2a2b1a | 5387 | 1 | 0.067
J2a2b1a | 6524 | 1 | 0.067
J2a2b1a | 721 | 1 | 0.067
J2a2b1a | 7269 | 1 | 0.067
J2a2b2 | 11698G | 2 | 0.125
J2a2b2 | 12441 | 1 | 0.062
J2a2b2 | 12622 | 1 | 0.062
J2a2b2 | 15543 | 1 | 0.062
J2a2b2 | 16039 | 3 | 0.188
J2a2b2 | 16084 | 1 | 0.062
J2a2b2 | 16288 | 1 | 0.062
J2a2b2 | 204 | 2 | 0.125
J2a2b2 | 207 | 1 | 0.062
J2a2b2 | 262 | 1 | 0.062
J2a2b2 | 573.4C | 1 | 0.062
J2a2b2 | 573.5C | 1 | 0.062
J2a2b2 | 574C | 1 | 0.062
J2a2b2 | 9947 | 2 | 0.125
J2a2b3 | 310 | 1 | 0.071
J2a2b3 | 311 | 1 | 0.071
J2a2b3 | 522.1C | 1 | 0.071
J2a2b3 | 523.1A | 1 | 0.071
J2a2b3 | 8939 | 1 | 0.071
J2a2c | 11149 | 1 | 0.05
J2a2c | 14484 | 1 | 0.05
J2a2c | 14569 | 2 | 0.1
J2a2c | 152 | 1 | 0.05
J2a2c | 16092 | 1 | 0.05
J2a2c | 16319 | 8 | 0.4
J2a2c | 16362 | 1 | 0.05
J2a2c | 310 | 1 | 0.05
J2a2c | 3106A | 1 | 0.05
J2a2c | 3337 | 1 | 0.05
J2a2c | 3572 | 1 | 0.05
J2a2c | 4435 | 1 | 0.05
J2a2c | 7269 | 2 | 0.1
J2a2c1 | 10346G | 2 | 0.2
J2a2c1 | 10750 | 4 | 0.4
J2a2c1 | 14502 | 4 | 0.4
J2a2d | 11149 | 3 | 0.02
J2a2d | 11253 | 3 | 0.02
J2a2d | 13134 | 5 | 0.034
J2a2d | 14470 | 1 | 0.007
J2a2d | 16147 | 6 | 0.041
J2a2d | 16295 | 1 | 0.007
J2a2d | 16299 | 1 | 0.007
J2a2d | 16311 | 3 | 0.02
J2a2d | 16526 | 1 | 0.007
J2a2d | 189 | 1 | 0.007
J2a2d | 198 | 3 | 0.02
J2a2d | 204 | 5 | 0.034
J2a2d | 207 | 15 | 0.101
J2a2d | 459d | 1 | 0.007
J2a2d | 522d | 2 | 0.014
J2a2d | 7269 | 1 | 0.007
J2a2d | 7337 | 2 | 0.014
J2a2d | 7669 | 2 | 0.014
J2a2d | 8812 | 1 | 0.007
J2a2d | 8999 | 1 | 0.007
J2a2d | 9148 | 2 | 0.014
J2a2e | 13500 | 1 | 0.007
J2a2e | 13681 | 1 | 0.007
J2a2e | 14016 | 1 | 0.007
J2a2e | 15959 | 1 | 0.007
J2a2e | 16141 | 1 | 0.007
J2a2e | 16172 | 1 | 0.007
J2a2e | 16247 | 1 | 0.007
J2a2e | 16259A | 1 | 0.007
J2a2e | 16261 | 1 | 0.007
J2a2e | 16278 | 2 | 0.014
J2a2e | 16301 | 1 | 0.007
J2a2e | 16311 | 2 | 0.014
J2a2e | 16362 | 7 | 0.049
J2a2e | 16456 | 1 | 0.007
J2a2e | 200 | 2 | 0.014
J2a2e | 247 | 1 | 0.007
J2a2e | 316 | 1 | 0.007
J2a2e | 366 | 1 | 0.007
J2a2e | 389 | 1 | 0.007
J2a2e | 515-524d | 3 | 0.021
J2a2e | 8496 | 1 | 0.007
J2a2e | 9276 | 1 | 0.007
J2a2e | 93 | 2 | 0.014
J2a2e | 95C | 1 | 0.007
J2b | 16291 | 4 | 0.06
J2b | 207 | 2 | 0.03
J2b | 237 | 1 | 0.015
J2b1 | 10957 | 2 | 0.02
J2b1 | 11500 | 4 | 0.04
J2b1 | 11944 | 1 | 0.01
J2b1 | 12510 | 1 | 0.01
J2b1 | 12622 | 1 | 0.01
J2b1 | 12883 | 1 | 0.01
J2b1 | 13089 | 3 | 0.03
J2b1 | 13437 | 2 | 0.02
J2b1 | 14121 | 1 | 0.01
J2b1 | 1442 | 1 | 0.01
J2b1 | 14484 | 1 | 0.01
J2b1 | 14560 | 1 | 0.01
J2b1 | 14587 | 1 | 0.01
J2b1 | 146 | 2 | 0.02

J2b1 | 14744 | 1 | 0.01
J2b1 | 1508 | 4 | 0.04
J2b1 | 15213 | 2 | 0.02
J2b1 | 15442 | 2 | 0.02
J2b1 | 15673 | 2 | 0.02
J2b1 | 15784 | 1 | 0.01
J2b1 | 15949 | 1 | 0.01
J2b1 | 16037 | 1 | 0.01
J2b1 | 16092 | 1 | 0.01
J2b1 | 16145 | 1 | 0.01
J2b1 | 16148 | 4 | 0.04
J2b1 | 16167 | 2 | 0.02
J2b1 | 16172 | 2 | 0.02
J2b1 | 16220C | 2 | 0.02
J2b1 | 16256 | 1 | 0.01
J2b1 | 16265T | 4 | 0.04
J2b1 | 16270 | 5 | 0.051
J2b1 | 16274 | 2 | 0.02
J2b1 | 16290 | 3 | 0.03
J2b1 | 16311 | 4 | 0.04
J2b1 | 16352 | 1 | 0.01
J2b1 | 16355 | 1 | 0.01
J2b1 | 16356 | 1 | 0.01
J2b1 | 16362 | 4 | 0.04
J2b1 | 189 | 5 | 0.051
J2b1 | 195 | 4 | 0.04
J2b1 | 207 | 2 | 0.02
J2b1 | 215 | 1 | 0.01
J2b1 | 310 | 1 | 0.01
J2b1 | 3654 | 2 | 0.02
J2b1 | 3826 | 1 | 0.01
J2b1 | 4012 | 1 | 0.01
J2b1 | 4092 | 4 | 0.04
J2b1 | 4171A | 2 | 0.02
J2b1 | 4491 | 1 | 0.01
J2b1 | 4703 | 3 | 0.03
J2b1 | 4824 | 2 | 0.02
J2b1 | 488 | 1 | 0.01
J2b1 | 4884 | 1 | 0.01
J2b1 | 522d | 3 | 0.03
J2b1 | 5465 | 4 | 0.04
J2b1 | 5558 | 1 | 0.01
J2b1 | 5993 | 1 | 0.01
J2b1 | 6053 | 2 | 0.02
J2b1 | 6570T | 1 | 0.01
J2b1 | 6959 | 5 | 0.051
J2b1 | 7109 | 1 | 0.01
J2b1 | 7632 | 2 | 0.02
J2b1 | 7690 | 2 | 0.02
J2b1 | 8266 | 3 | 0.03
J2b1 | 8388 | 1 | 0.01
J2b1 | 8848 | 1 | 0.01
J2b1 | 8896 | 3 | 0.03
J2b1 | 8911 | 2 | 0.02
J2b1 | 8953 | 5 | 0.051
J2b1 | 9012 | 1 | 0.01
J2b1 | 9071 | 2 | 0.02
J2b1 | 9128 | 4 | 0.04
J2b1 | 94 | 2 | 0.02
J2b1 | 9434 | 1 | 0.01
J2b1 | 9593 | 1 | 0.01
J2b1 | 9852 | 1 | 0.01
J2b1a | 10286 | 47 | 0.294
J2b1a | 10321 | 1 | 0.006
J2b1a | 10410 | 3 | 0.019
J2b1a | 10915 | 2 | 0.012
J2b1a | 10961 | 1 | 0.006
J2b1a | 10972 | 1 | 0.006
J2b1a | 11084 | 7 | 0.044
J2b1a | 11089 | 2 | 0.012
J2b1a | 11152 | 1 | 0.006
J2b1a | 11204 | 12 | 0.075
J2b1a | 11278 | 1 | 0.006
J2b1a | 11685 | 2 | 0.012
J2b1a | 11914 | 2 | 0.012
J2b1a | 12092 | 1 | 0.006
J2b1a | 12092A | 1 | 0.006
J2b1a | 12187 | 1 | 0.006
J2b1a | 12279T | 1 | 0.006
J2b1a | 12397 | 2 | 0.012
J2b1a | 12510 | 1 | 0.006
J2b1a | 12662 | 1 | 0.006
J2b1a | 12705 | 1 | 0.006
J2b1a | 12810 | 8 | 0.05
J2b1a | 12888 | 1 | 0.006
J2b1a | 13194C | 2 | 0.012
J2b1a | 13533 | 1 | 0.006
J2b1a | 13886 | 1 | 0.006
J2b1a | 14180 | 1 | 0.006
J2b1a | 14539 | 3 | 0.019
J2b1a | 146 | 2 | 0.012
J2b1a | 14774 | 1 | 0.006
J2b1a | 14790 | 1 | 0.006
J2b1a | 151 | 6 | 0.038
J2b1a | 15110 | 1 | 0.006
J2b1a | 153 | 1 | 0.006
J2b1a | 15319 | 10 | 0.062
J2b1a | 15454 | 1 | 0.006
J2b1a | 15470 | 1 | 0.006
J2b1a | 15511 | 1 | 0.006
J2b1a | 15601 | 1 | 0.006
J2b1a | 15662 | 2 | 0.012
J2b1a | 15691 | 1 | 0.006
J2b1a | 15731 | 1 | 0.006
J2b1a | 16145 | 3 | 0.019
J2b1a | 16167 | 2 | 0.012
J2b1a | 16168 | 1 | 0.006
J2b1a | 16169 | 2 | 0.012
J2b1a | 16173 | 1 | 0.006
J2b1a | 16195 | 1 | 0.006
J2b1a | 16213 | 10 | 0.062

J2b1a | 16220 | 1 | 0.006
J2b1a | 16220T | 1 | 0.006
J2b1a | 16234 | 1 | 0.006
J2b1a | 16235 | 1 | 0.006
J2b1a | 16274 | 43 | 0.269
J2b1a | 16291 | 2 | 0.012
J2b1a | 16294 | 1 | 0.006
J2b1a | 16297 | 1 | 0.006
J2b1a | 16327 | 2 | 0.012
J2b1a | 16344 | 1 | 0.006
J2b1a | 16355 | 2 | 0.012
J2b1a | 16356 | 1 | 0.006
J2b1a | 16360 | 1 | 0.006
J2b1a | 16399 | 1 | 0.006
J2b1a | 1694 | 2 | 0.012
J2b1a | 1706 | 1 | 0.006
J2b1a | 185 | 1 | 0.006
J2b1a | 189 | 1 | 0.006
J2b1a | 195 | 1 | 0.006
J2b1a | 196.1T | 1 | 0.006
J2b1a | 199 | 49 | 0.306
J2b1a | 3010 | 2 | 0.012
J2b1a | 310 | 3 | 0.019
J2b1a | 3308 | 1 | 0.006
J2b1a | 3394 | 1 | 0.006
J2b1a | 3398 | 1 | 0.006
J2b1a | 3579 | 1 | 0.006
J2b1a | 3834 | 1 | 0.006
J2b1a | 390 | 1 | 0.006
J2b1a | 3992 | 1 | 0.006
J2b1a | 4012 | 1 | 0.006
J2b1a | 4131 | 2 | 0.012
J2b1a | 4454 | 1 | 0.006
J2b1a | 4586 | 1 | 0.006
J2b1a | 4688 | 2 | 0.012
J2b1a | 4973 | 2 | 0.012
J2b1a | 5290 | 1 | 0.006
J2b1a | 5295 | 2 | 0.012
J2b1a | 5417 | 1 | 0.006
J2b1a | 5580 | 1 | 0.006
J2b1a | 5743 | 1 | 0.006
J2b1a | 5773 | 1 | 0.006
J2b1a | 6050 | 1 | 0.006
J2b1a | 6257 | 1 | 0.006
J2b1a | 6353 | 1 | 0.006
J2b1a | 6465 | 1 | 0.006
J2b1a | 709 | 5 | 0.031
J2b1a | 7647 | 1 | 0.006
J2b1a | 849 | 2 | 0.012
J2b1a | 8633 | 1 | 0.006
J2b1a | 8634 | 1 | 0.006
J2b1a | 8878 | 1 | 0.006
J2b1a | 9116 | 1 | 0.006
J2b1a | 9129 | 1 | 0.006
J2b1a | 9214 | 1 | 0.006
J2b1a | 9299 | 1 | 0.006
J2b1a | 9377 | 1 | 0.006
J2b1a | 960.1C | 1 | 0.006
J2b1a | 9806 | 1 | 0.006
J2b1a | 9909 | 2 | 0.012
J2b1a | 9921 | 1 | 0.006
J2b1a | 9948 | 1 | 0.006
J2b1a | 9950 | 1 | 0.006
J2b1a+16311 | 10410 | 2 | 0.182
J2b1a+16311 | 11089 | 2 | 0.182
J2b1a+16311 | 13036 | 1 | 0.091
J2b1a+16311 | 15662 | 2 | 0.182
J2b1a+16311 | 1719 | 1 | 0.091
J2b1a+16311 | 3010 | 2 | 0.182
J2b1a+16311 | 3930 | 1 | 0.091
J2b1a+16311 | 4131 | 2 | 0.182
J2b1a+16311 | 5605 | 1 | 0.091
J2b1a+16311 | 9344 | 1 | 0.091
J2b1a1 | 13527 | 1 | 0.03
J2b1a1 | 14110 | 1 | 0.03
J2b1a1 | 16168 | 1 | 0.03
J2b1a1 | 16269 | 1 | 0.03
J2b1a1 | 16271 | 1 | 0.03
J2b1a1 | 16297 | 1 | 0.03
J2b1a1 | 16360 | 1 | 0.03
J2b1a1 | 1719 | 1 | 0.03
J2b1a1 | 189 | 1 | 0.03
J2b1a1 | 195 | 1 | 0.03
J2b1a1 | 3395 | 1 | 0.03
J2b1a1 | 3637 | 1 | 0.03
J2b1a1 | 396 | 1 | 0.03
J2b1a1 | 4496 | 1 | 0.03
J2b1a1 | 4907 | 1 | 0.03
J2b1a1 | 6261 | 1 | 0.03
J2b1a1 | 6734 | 1 | 0.03
J2b1a1 | 7690 | 1 | 0.03
J2b1a1 | 8633 | 1 | 0.03
J2b1a1a | 14405 | 2 | 0.154
J2b1a1a | 16093 | 2 | 0.154
J2b1a1a | 16234G | 2 | 0.154
J2b1a1a | 16319 | 1 | 0.077
J2b1a1a | 1719 | 1 | 0.077
J2b1a1a | 185 | 2 | 0.154
J2b1a1a | 194 | 1 | 0.077
J2b1a1a | 496 | 1 | 0.077
J2b1a1a | 5228G | 2 | 0.154
J2b1a1a | 5471 | 1 | 0.077
J2b1a2 | 10410 | 7 | 0.104
J2b1a2 | 11089 | 7 | 0.104
J2b1a2 | 12215A | 1 | 0.015
J2b1a2 | 13095 | 1 | 0.015
J2b1a2 | 15394 | 8 | 0.119
J2b1a2 | 15601 | 1 | 0.015
J2b1a2 | 15662 | 7 | 0.104
J2b1a2 | 15758 | 1 | 0.015
J2b1a2 | 16093 | 5 | 0.075

J2b1a2 | 16148 | 20 | 0.299
J2b1a2 | 16186 | 1 | 0.015
J2b1a2 | 16255 | 1 | 0.015
J2b1a2 | 16256 | 1 | 0.015
J2b1a2 | 16261 | 2 | 0.03
J2b1a2 | 16292 | 1 | 0.015
J2b1a2 | 16294 | 1 | 0.015
J2b1a2 | 16486C | 4 | 0.06
J2b1a2 | 1821 | 1 | 0.015
J2b1a2 | 195 | 5 | 0.075
J2b1a2 | 2010 | 1 | 0.015
J2b1a2 | 215 | 1 | 0.015
J2b1a2 | 228 | 1 | 0.015
J2b1a2 | 279 | 1 | 0.015
J2b1a2 | 3010 | 7 | 0.104
J2b1a2 | 324 | 1 | 0.015
J2b1a2 | 3348 | 2 | 0.03
J2b1a2 | 3866 | 1 | 0.015
J2b1a2 | 3943 | 1 | 0.015
J2b1a2 | 4131 | 7 | 0.104
J2b1a2 | 4226 | 1 | 0.015
J2b1a2 | 4850 | 1 | 0.015
J2b1a2 | 6260 | 1 | 0.015
J2b1a2 | 6494 | 1 | 0.015
J2b1a2 | 869 | 1 | 0.015
J2b1a2 | 9531 | 1 | 0.015
J2b1a2a | 103 | 1 | 0.027
J2b1a2a | 11740 | 1 | 0.027
J2b1a2a | 13968 | 3 | 0.081
J2b1a2a | 15212 | 4 | 0.108
J2b1a2a | 16287 | 1 | 0.027
J2b1a2a | 16295 | 2 | 0.054
J2b1a2a | 235 | 9 | 0.243
J2b1a2a | 3408 | 1 | 0.027
J2b1a2a | 3460 | 1 | 0.027
J2b1a2a | 5460 | 2 | 0.054
J2b1a2a | 72 | 1 | 0.027
J2b1a2a | 7394 | 1 | 0.027
J2b1a2a | 8962 | 4 | 0.108
J2b1a3 | 13135 | 1 | 0.029
J2b1a3 | 13242 | 1 | 0.029
J2b1a3 | 13857 | 1 | 0.029
J2b1a3 | 14484 | 1 | 0.029
J2b1a3 | 14488 | 1 | 0.029
J2b1a3 | 14905 | 1 | 0.029
J2b1a3 | 15499 | 1 | 0.029
J2b1a3 | 16042 | 1 | 0.029
J2b1a3 | 16111 | 1 | 0.029
J2b1a3 | 16265C | 1 | 0.029
J2b1a3 | 522.1C | 1 | 0.029
J2b1a3 | 709 | 1 | 0.029
J2b1a3 | 7598 | 1 | 0.029
J2b1a4 | 10864 | 1 | 0.019
J2b1a4 | 15983 | 1 | 0.019
J2b1a4 | 16086 | 1 | 0.019
J2b1a4 | 16145 | 7 | 0.13
J2b1a4 | 16172 | 1 | 0.019
J2b1a4 | 16195 | 1 | 0.019
J2b1a4 | 16221 | 1 | 0.019
J2b1a4 | 16234G | 1 | 0.019
J2b1a4 | 16344 | 1 | 0.019
J2b1a4 | 16368 | 11 | 0.204
J2b1a4 | 16390 | 1 | 0.019
J2b1a4 | 185 | 1 | 0.019
J2b1a4 | 196.1T | 4 | 0.074
J2b1a4 | 3238 | 1 | 0.019
J2b1a4 | 3882 | 1 | 0.019
J2b1a4 | 6378 | 1 | 0.019
J2b1a4 | 7960 | 1 | 0.019
J2b1a4 | 9145 | 1 | 0.019
J2b1a4 | 9266 | 1 | 0.019
J2b1a5 | 1009 | 1 | 0.011
J2b1a5 | 13064 | 2 | 0.022
J2b1a5 | 14302 | 1 | 0.011
J2b1a5 | 146 | 3 | 0.033
J2b1a5 | 1503 | 1 | 0.011
J2b1a5 | 15106 | 1 | 0.011
J2b1a5 | 15191 | 1 | 0.011
J2b1a5 | 15328 | 1 | 0.011
J2b1a5 | 15386 | 1 | 0.011
J2b1a5 | 15538 | 2 | 0.022
J2b1a5 | 15657 | 1 | 0.011
J2b1a5 | 16051 | 1 | 0.011
J2b1a5 | 16111 | 2 | 0.022
J2b1a5 | 16145 | 2 | 0.022
J2b1a5 | 16172 | 1 | 0.011
J2b1a5 | 16209 | 3 | 0.033
J2b1a5 | 16360 | 1 | 0.011
J2b1a5 | 1766 | 1 | 0.011
J2b1a5 | 204 | 1 | 0.011
J2b1a5 | 325 | 22 | 0.242
J2b1a5 | 3591 | 42 | 0.462
J2b1a5 | 3736 | 3 | 0.033
J2b1a5 | 5249 | 40 | 0.44
J2b1a5 | 529 | 1 | 0.011
J2b1a5 | 5319 | 1 | 0.011
J2b1a5 | 6465 | 1 | 0.011
J2b1a5 | 7051 | 1 | 0.011
J2b1a5 | 7119 | 1 | 0.011
J2b1a5 | 7557 | 1 | 0.011
J2b1a5 | 8838 | 5 | 0.055
J2b1a5 | 9168 | 1 | 0.011
J2b1a5 | 9512 | 27 | 0.297
J2b1a6 | 11767 | 1 | 0.033
J2b1a6 | 11854 | 1 | 0.033
J2b1a6 | 14245A | 1 | 0.033
J2b1a6 | 14544 | 1 | 0.033
J2b1a6 | 146 | 1 | 0.033
J2b1a6 | 15380 | 1 | 0.033
J2b1a6 | 16265 | 1 | 0.033
J2b1a6 | 16271 | 1 | 0.033

J2b1a6 | 16309 | 3 | 0.1
J2b1a6 | 16566 | 1 | 0.033
J2b1a6 | 195 | 2 | 0.067
J2b1a6 | 310 | 2 | 0.067
J2b1a6 | 315.2C | 1 | 0.033
J2b1a6 | 456 | 1 | 0.033
J2b1a6 | 5237 | 1 | 0.033
J2b1a6 | 9139 | 1 | 0.033
J2b1a6 | 93 | 1 | 0.033
J2b1b | 16054C | 1 | 0.015
J2b1b | 16217 | 2 | 0.03
J2b1b | 16249 | 2 | 0.03
J2b1b1 | 146 | 7 | 0.097
J2b1b1 | 151 | 1 | 0.014
J2b1b1 | 16286 | 2 | 0.028
J2b1b1 | 195 | 1 | 0.014
J2b1b1 | 60A | 2 | 0.028
J2b1b1 | 704G | 1 | 0.014
J2b1b1 | 705 | 1 | 0.014
J2b1b1 | 8503 | 1 | 0.014
J2b1b1 | 8711 | 1 | 0.014
J2b1c | 10646 | 1 | 0.015
J2b1c | 15184 | 1 | 0.015
J2b1c | 16217 | 3 | 0.045
J2b1c | 508 | 1 | 0.015
J2b1c | 513 | 2 | 0.03
J2b1c | 525d | 1 | 0.015
J2b1c | 7471d | 1 | 0.015
J2b1c | 8387 | 1 | 0.015
J2b1c | 9254 | 2 | 0.03
J2b1c1 | 10362 | 1 | 0.143
J2b1c1 | 11899 | 1 | 0.143
J2b1c1 | 12010 | 2 | 0.286
J2b1c1 | 12070 | 1 | 0.143
J2b1c1 | 13135 | 1 | 0.143
J2b1c1 | 16286 | 1 | 0.143
J2b1c1 | 16360 | 2 | 0.286
J2b1c1 | 16362 | 1 | 0.143
J2b1c1 | 242 | 1 | 0.143
J2b1c1 | 3635 | 1 | 0.143
J2b1c1 | 462 | 1 | 0.143
J2b1c1 | 5322C | 1 | 0.143
J2b1c1 | 8551 | 1 | 0.143
J2b1d | 12239 | 1 | 0.014
J2b1d | 13683 | 1 | 0.014
J2b1d | 15712 | 3 | 0.042
J2b1d | 16048 | 3 | 0.042
J2b1d | 16148 | 4 | 0.056
J2b1d | 16224 | 2 | 0.028
J2b1d | 16234 | 1 | 0.014
J2b1d | 16261 | 3 | 0.042
J2b1d | 3434 | 1 | 0.014
J2b1d | 4655 | 1 | 0.014
J2b1d | 4702 | 1 | 0.014
J2b1d | 4823 | 1 | 0.014
J2b1d | 5231 | 1 | 0.014
J2b1d | 7226 | 3 | 0.042
J2b1d | 739 | 3 | 0.042
J2b1d | 8089 | 3 | 0.042
J2b1d | 8945 | 2 | 0.028
J2b1e | 14761 | 1 | 0.015
J2b1e | 16054C | 1 | 0.015
J2b1e | 16217 | 2 | 0.03
J2b1e | 16249 | 2 | 0.03
J2b1e | 16362 | 1 | 0.015
J2b1e | 8311 | 1 | 0.015
J2b1e1 | 10373 | 1 | 0.043
J2b1e1 | 10397 | 1 | 0.043
J2b1e1 | 12361 | 1 | 0.043
J2b1e1 | 13851 | 1 | 0.043
J2b1e1 | 16250G | 1 | 0.043
J2b1e1 | 16270 | 1 | 0.043
J2b1e1 | 16324 | 2 | 0.087
J2b1e1 | 2844 | 1 | 0.043
J2b1e1 | 498 | 1 | 0.043
J2b1e1 | 8557 | 1 | 0.043
J2b1e1 | 8619 | 1 | 0.043
J2b1f | 10223 | 2 | 0.029
J2b1f | 10245 | 2 | 0.029
J2b1f | 11410 | 1 | 0.014
J2b1f | 11914 | 2 | 0.029
J2b1f | 13617 | 1 | 0.014
J2b1f | 16318 | 1 | 0.014
J2b1f | 3390 | 1 | 0.014
J2b1f | 4080 | 1 | 0.014
J2b1f | 5899.1C | 3 | 0.043
J2b1f | 5899.2C | 2 | 0.029
J2b1f | 9309 | 1 | 0.014
J2b1g | 13821 | 1 | 0.015
J2b1g | 16217 | 3 | 0.045
J2b1g | 204 | 1 | 0.015
J2b1g | 2789 | 1 | 0.015
J2b1h | 11050 | 1 | 0.5
J2b1h | 11383 | 1 | 0.5
J2b1h | 12969G | 1 | 0.5
J2b1h | 16357 | 1 | 0.5
J2b1h | 16496 | 1 | 0.5
J2b1h | 3766 | 1 | 0.5
J2b1h | 8020 | 1 | 0.5
J2b2 | 11399 | 1 | 0.015
J2b2 | 12070 | 1 | 0.015
J2b2 | 13135 | 1 | 0.015
J2b2 | 15001 | 2 | 0.029
J2b2 | 16051 | 1 | 0.015
J2b2 | 16093 | 3 | 0.044
J2b2 | 16169 | 1 | 0.015
J2b2 | 16320 | 2 | 0.029
J2b2 | 16324 | 1 | 0.015
J2b2 | 16442 | 2 | 0.029
J2b2 | 16443 | 2 | 0.029
J2b2 | 1821 | 1 | 0.015

J2b2 | 207 | 2 | 0.029
J2b2 | 4254 | 1 | 0.015
J2b2 | 8251 | 1 | 0.015
J2b2 | 8348 | 1 | 0.015
J2b2 | 9899 | 1 | 0.015
JT | 16048 | 1 | 0.016
JT | 16129 | 1 | 0.016
JT | 16193 | 1 | 0.016
JT | 16239 | 1 | 0.016
JT | 16242 | 1 | 0.016
JT | 16247C | 1 | 0.016
JT | 16296 | 1 | 0.016
JT | 32.1G | 1 | 0.016
JT | 489 | 1 | 0.016
K | 131 | 1 | 0.005
K | 16263 | 2 | 0.01
K | 16355 | 19 | 0.099
K | 480 | 1 | 0.005
K1 | 10113 | 19 | 0.098
K1 | 10966 | 1 | 0.005
K1 | 13008 | 1 | 0.005
K1 | 13350 | 1 | 0.005
K1 | 13711 | 1 | 0.005
K1 | 13967 | 20 | 0.103
K1 | 146 | 21 | 0.108
K1 | 15058 | 1 | 0.005
K1 | 15848 | 1 | 0.005
K1 | 16166C | 1 | 0.005
K1 | 16171d | 1 | 0.005
K1 | 16249 | 1 | 0.005
K1 | 215 | 20 | 0.103
K1 | 3995C | 1 | 0.005
K1 | 5258 | 1 | 0.005
K1 | 6351 | 20 | 0.103
K1 | 6446 | 20 | 0.103
K1 | 7120 | 1 | 0.005
K1 | 7356 | 1 | 0.005
K1 | 7751G | 1 | 0.005
K1 | 8462 | 19 | 0.098
K1+16362 | 10084 | 47 | 0.635
K1+16362 | 11929 | 47 | 0.635
K1+16362 | 12904 | 1 | 0.014
K1+16362 | 13143 | 4 | 0.054
K1+16362 | 13145 | 46 | 0.622
K1+16362 | 13434 | 1 | 0.014
K1+16362 | 13602 | 1 | 0.014
K1+16362 | 15314 | 1 | 0.014
K1+16362 | 16086 | 46 | 0.622
K1+16362 | 16124 | 1 | 0.014
K1+16362 | 16189 | 1 | 0.014
K1+16362 | 16291 | 47 | 0.635
K1+16362 | 16292 | 1 | 0.014
K1+16362 | 194 | 1 | 0.014
K1+16362 | 246 | 1 | 0.014
K1+16362 | 4243 | 1 | 0.014
K1+16362 | 4655 | 47 | 0.635
K1+16362 | 4688 | 1 | 0.014
K1+16362 | 6285 | 46 | 0.622
K1+16362 | 6973 | 46 | 0.622
K1+16362 | 709 | 46 | 0.622
K1+16362 | 7235 | 47 | 0.635
K1+16362 | 7468 | 47 | 0.635
K1+16362 | 9950 | 1 | 0.014
K1a | 10163 | 1 | 0.003
K1a | 10410 | 1 | 0.003
K1a | 10750 | 1 | 0.003
K1a | 11038 | 1 | 0.003
K1a | 11149 | 1 | 0.003
K1a | 11176 | 1 | 0.003
K1a | 11350 | 2 | 0.006
K1a | 114 | 1 | 0.003
K1a | 11452 | 1 | 0.003
K1a | 11764 | 1 | 0.003
K1a | 11887 | 2 | 0.006
K1a | 12312 | 1 | 0.003
K1a | 12795 | 1 | 0.003
K1a | 12880 | 1 | 0.003
K1a | 12975 | 1 | 0.003
K1a | 13224 | 1 | 0.003
K1a | 13635 | 1 | 0.003
K1a | 13692 | 1 | 0.003
K1a | 13812 | 1 | 0.003
K1a | 14071 | 1 | 0.003
K1a | 14178 | 1 | 0.003
K1a | 14258 | 1 | 0.003
K1a | 14296 | 1 | 0.003
K1a | 14377 | 1 | 0.003
K1a | 14413 | 1 | 0.003
K1a | 14470 | 1 | 0.003
K1a | 14560 | 1 | 0.003
K1a | 14569 | 1 | 0.003
K1a | 14582 | 1 | 0.003
K1a | 146 | 24 | 0.076
K1a | 14693 | 1 | 0.003
K1a | 14857 | 1 | 0.003
K1a | 14866 | 1 | 0.003
K1a | 14890 | 1 | 0.003
K1a | 15043 | 1 | 0.003
K1a | 15175 | 2 | 0.006
K1a | 152 | 4 | 0.013
K1a | 15218 | 1 | 0.003
K1a | 15253 | 1 | 0.003
K1a | 15479 | 2 | 0.006
K1a | 15616 | 1 | 0.003
K1a | 16037 | 1 | 0.003
K1a | 16129 | 5 | 0.016
K1a | 16172 | 1 | 0.003
K1a | 16177 | 1 | 0.003
K1a | 16180 | 2 | 0.006
K1a | 16189 | 2 | 0.006
K1a | 16218 | 1 | 0.003

K1a | 16225 | 1 | 0.003
K1a | 16234 | 3 | 0.009
K1a | 16249 | 6 | 0.019
K1a | 16256 | 6 | 0.019
K1a | 16292 | 3 | 0.009
K1a | 16298 | 1 | 0.003
K1a | 16301 | 4 | 0.013
K1a | 16304 | 2 | 0.006
K1a | 16319 | 2 | 0.006
K1a | 16400 | 19 | 0.06
K1a | 16422 | 1 | 0.003
K1a | 16438 | 1 | 0.003
K1a | 16527 | 1 | 0.003
K1a | 1673 | 2 | 0.006
K1a | 173 | 2 | 0.006
K1a | 1746 | 2 | 0.006
K1a | 195 | 1 | 0.003
K1a | 199 | 2 | 0.006
K1a | 200 | 1 | 0.003
K1a | 2071 | 1 | 0.003
K1a | 214 | 1 | 0.003
K1a | 217 | 1 | 0.003
K1a | 228 | 1 | 0.003
K1a | 2281 | 1 | 0.003
K1a | 2887 | 1 | 0.003
K1a | 310 | 3 | 0.009
K1a | 3110d | 1 | 0.003
K1a | 3278 | 1 | 0.003
K1a | 3505 | 1 | 0.003
K1a | 3768 | 1 | 0.003
K1a | 408A | 1 | 0.003
K1a | 4639 | 1 | 0.003
K1a | 4667 | 1 | 0.003
K1a | 4898 | 1 | 0.003
K1a | 4961 | 2 | 0.006
K1a | 4967 | 1 | 0.003
K1a | 4973 | 1 | 0.003
K1a | 508 | 1 | 0.003
K1a | 5206 | 1 | 0.003
K1a | 5252 | 1 | 0.003
K1a | 5437 | 1 | 0.003
K1a | 5567 | 1 | 0.003
K1a | 5581 | 1 | 0.003
K1a | 573.1C | 2 | 0.006
K1a | 5743 | 1 | 0.003
K1a | 5984 | 1 | 0.003
K1a | 5999 | 1 | 0.003
K1a | 6053 | 1 | 0.003
K1a | 6137 | 1 | 0.003
K1a | 6329 | 1 | 0.003
K1a | 6752 | 1 | 0.003
K1a | 6830G | 1 | 0.003
K1a | 6917 | 1 | 0.003
K1a | 6956 | 1 | 0.003
K1a | 7022 | 1 | 0.003
K1a | 7041 | 1 | 0.003
K1a | 7337 | 2 | 0.006
K1a | 7521 | 1 | 0.003
K1a | 7961 | 1 | 0.003
K1a | 8289.1CCCCCTCTA | 1 | 0.003
K1a | 8311 | 1 | 0.003
K1a | 8334 | 1 | 0.003
K1a | 8346 | 1 | 0.003
K1a | 8433G | 1 | 0.003
K1a | 8577 | 1 | 0.003
K1a | 8584 | 1 | 0.003
K1a | 8599 | 1 | 0.003
K1a | 8994 | 1 | 0.003
K1a | 9006 | 1 | 0.003
K1a | 9116 | 1 | 0.003
K1a | 9142 | 1 | 0.003
K1a | 9300 | 1 | 0.003
K1a | 9413 | 1 | 0.003
K1a | 9554 | 1 | 0.003
K1a | 960.1C | 1 | 0.003
K1a | 961 | 3 | 0.009
K1a | 965.4C | 1 | 0.003
K1a+150 | 10373 | 1 | 0.005
K1a+150 | 11485 | 2 | 0.01
K1a+150 | 1187 | 1 | 0.005
K1a+150 | 11969 | 1 | 0.005
K1a+150 | 12338 | 1 | 0.005
K1a+150 | 12880 | 1 | 0.005
K1a+150 | 13477 | 1 | 0.005
K1a+150 | 13566 | 1 | 0.005
K1a+150 | 1393 | 1 | 0.005
K1a+150 | 146 | 1 | 0.005
K1a+150 | 152 | 7 | 0.034
K1a+150 | 15317 | 1 | 0.005
K1a+150 | 15653T | 3 | 0.015
K1a+150 | 16023T | 1 | 0.005
K1a+150 | 16086 | 1 | 0.005
K1a+150 | 16093 | 1 | 0.005
K1a+150 | 16129 | 5 | 0.024
K1a+150 | 16150 | 1 | 0.005
K1a+150 | 16192 | 1 | 0.005
K1a+150 | 16286 | 1 | 0.005
K1a+150 | 16304 | 4 | 0.02
K1a+150 | 16319 | 1 | 0.005
K1a+150 | 16322 | 1 | 0.005
K1a+150 | 16325 | 1 | 0.005
K1a+150 | 16386 | 1 | 0.005
K1a+150 | 16399 | 1 | 0.005
K1a+150 | 16484-16489d | 1 | 0.005
K1a+150 | 16T | 1 | 0.005
K1a+150 | 189 | 1 | 0.005
K1a+150 | 195 | 8 | 0.039
K1a+150 | 231d | 1 | 0.005
K1a+150 | 243 | 1 | 0.005
K1a+150 | 281 | 1 | 0.005
K1a+150 | 295 | 1 | 0.005

K1a+150 | 310 | 1 | 0.005
K1a+150 | 315d | 1 | 0.005
K1a+150 | 316C | 1 | 0.005
K1a+150 | 3364 | 1 | 0.005
K1a+150 | 3553 | 1 | 0.005
K1a+150 | 4029A | 1 | 0.005
K1a+150 | 489 | 2 | 0.01
K1a+150 | 492 | 1 | 0.005
K1a+150 | 493 | 1 | 0.005
K1a+150 | 5581 | 1 | 0.005
K1a+150 | 6530 | 1 | 0.005
K1a+150 | 705 | 1 | 0.005
K1a+150 | 709 | 1 | 0.005
K1a+150 | 7897 | 1 | 0.005
K1a+150 | 8334 | 1 | 0.005
K1a+150 | 8599 | 1 | 0.005
K1a+150 | 8874 | 1 | 0.005
K1a+150 | 9336 | 1 | 0.005
K1a+150 | 9531 | 1 | 0.005
K1a+195 | 10143 | 1 | 0.003
K1a+195 | 11291 | 1 | 0.003
K1a+195 | 11356 | 1 | 0.003
K1a+195 | 11377 | 1 | 0.003
K1a+195 | 11428 | 1 | 0.003
K1a+195 | 11549 | 1 | 0.003
K1a+195 | 1187 | 8 | 0.027
K1a+195 | 11950 | 1 | 0.003
K1a+195 | 11963 | 1 | 0.003
K1a+195 | 11989 | 1 | 0.003
K1a+195 | 12063 | 2 | 0.007
K1a+195 | 12172 | 1 | 0.003
K1a+195 | 12351 | 1 | 0.003
K1a+195 | 12403 | 2 | 0.007
K1a+195 | 12630 | 1 | 0.003
K1a+195 | 12651 | 4 | 0.013
K1a+195 | 12696 | 1 | 0.003
K1a+195 | 12711 | 1 | 0.003
K1a+195 | 12712 | 1 | 0.003
K1a+195 | 12948 | 1 | 0.003
K1a+195 | 13117 | 2 | 0.007
K1a+195 | 13135 | 2 | 0.007
K1a+195 | 13143 | 1 | 0.003
K1a+195 | 13326 | 1 | 0.003
K1a+195 | 13602 | 2 | 0.007
K1a+195 | 13651 | 3 | 0.01
K1a+195 | 13928C | 1 | 0.003
K1a+195 | 13943 | 2 | 0.007
K1a+195 | 140 | 1 | 0.003
K1a+195 | 14180G | 1 | 0.003
K1a+195 | 14182 | 1 | 0.003
K1a+195 | 14249 | 1 | 0.003
K1a+195 | 14256 | 1 | 0.003
K1a+195 | 14440 | 1 | 0.003
K1a+195 | 14484 | 1 | 0.003
K1a+195 | 1452 | 1 | 0.003
K1a+195 | 14564 | 2 | 0.007
K1a+195 | 14569 | 1 | 0.003
K1a+195 | 146 | 2 | 0.007
K1a+195 | 14669 | 1 | 0.003
K1a+195 | 14854 | 2 | 0.007
K1a+195 | 14947 | 32 | 0.107
K1a+195 | 14997 | 1 | 0.003
K1a+195 | 150 | 1 | 0.003
K1a+195 | 15064C | 1 | 0.003
K1a+195 | 15100 | 3 | 0.01
K1a+195 | 152 | 11 | 0.037
K1a+195 | 15204 | 1 | 0.003
K1a+195 | 15226 | 1 | 0.003
K1a+195 | 15262 | 1 | 0.003
K1a+195 | 15323 | 1 | 0.003
K1a+195 | 15355 | 1 | 0.003
K1a+195 | 15625A | 1 | 0.003
K1a+195 | 15650 | 1 | 0.003
K1a+195 | 15758 | 1 | 0.003
K1a+195 | 16093 | 1 | 0.003
K1a+195 | 16111 | 2 | 0.007
K1a+195 | 16124 | 1 | 0.003
K1a+195 | 16145 | 1 | 0.003
K1a+195 | 16153 | 2 | 0.007
K1a+195 | 16162 | 1 | 0.003
K1a+195 | 16167 | 1 | 0.003
K1a+195 | 16172 | 1 | 0.003
K1a+195 | 16187 | 1 | 0.003
K1a+195 | 16189 | 4 | 0.013
K1a+195 | 16190 | 1 | 0.003
K1a+195 | 16214A | 4 | 0.013
K1a+195 | 16223 | 4 | 0.013
K1a+195 | 16234 | 2 | 0.007
K1a+195 | 16242 | 2 | 0.007
K1a+195 | 16249 | 1 | 0.003
K1a+195 | 16256 | 1 | 0.003
K1a+195 | 16265C | 4 | 0.013
K1a+195 | 16266 | 1 | 0.003
K1a+195 | 16274 | 3 | 0.01
K1a+195 | 16286 | 2 | 0.007
K1a+195 | 16294 | 1 | 0.003
K1a+195 | 16300 | 3 | 0.01
K1a+195 | 16305 | 1 | 0.003
K1a+195 | 16311G | 1 | 0.003
K1a+195 | 16319 | 2 | 0.007
K1a+195 | 16343 | 1 | 0.003
K1a+195 | 16354 | 4 | 0.013
K1a+195 | 16359 | 1 | 0.003
K1a+195 | 16362 | 3 | 0.01
K1a+195 | 16390 | 5 | 0.017
K1a+195 | 1657 | 1 | 0.003
K1a+195 | 183 | 3 | 0.01
K1a+195 | 189 | 1 | 0.003
K1a+195 | 1958 | 1 | 0.003
K1a+195 | 204 | 2 | 0.007
K1a+195 | 239 | 3 | 0.01

K1a+195 | 2416 | 1 | 0.003
K1a+195 | 2483 | 1 | 0.003
K1a+195 | 296 | 1 | 0.003
K1a+195 | 310 | 3 | 0.01
K1a+195 | 315.2C | 1 | 0.003
K1a+195 | 3221 | 2 | 0.007
K1a+195 | 3398 | 1 | 0.003
K1a+195 | 3522 | 1 | 0.003
K1a+195 | 3535 | 2 | 0.007
K1a+195 | 3540 | 1 | 0.003
K1a+195 | 3579 | 2 | 0.007
K1a+195 | 3606 | 1 | 0.003
K1a+195 | 3949 | 1 | 0.003
K1a+195 | 4020 | 1 | 0.003
K1a+195 | 4137 | 1 | 0.003
K1a+195 | 4164 | 1 | 0.003
K1a+195 | 4452 | 1 | 0.003
K1a+195 | 4491 | 1 | 0.003
K1a+195 | 4646 | 2 | 0.007
K1a+195 | 4739 | 1 | 0.003
K1a+195 | 5054 | 2 | 0.007
K1a+195 | 5093 | 2 | 0.007
K1a+195 | 5130 | 1 | 0.003
K1a+195 | 515-524d | 1 | 0.003
K1a+195 | 5240 | 1 | 0.003
K1a+195 | 525.1AC | 1 | 0.003
K1a+195 | 544 | 2 | 0.007
K1a+195 | 5460 | 14 | 0.047
K1a+195 | 5563 | 1 | 0.003
K1a+195 | 5655 | 1 | 0.003
K1a+195 | 573.1C | 1 | 0.003
K1a+195 | 5936 | 1 | 0.003
K1a+195 | 5964 | 1 | 0.003
K1a+195 | 6182 | 4 | 0.013
K1a+195 | 6212 | 1 | 0.003
K1a+195 | 6227 | 1 | 0.003
K1a+195 | 6267 | 1 | 0.003
K1a+195 | 6335 | 1 | 0.003
K1a+195 | 6357 | 9 | 0.03
K1a+195 | 6359 | 2 | 0.007
K1a+195 | 6570T | 1 | 0.003
K1a+195 | 722 | 1 | 0.003
K1a+195 | 7468 | 1 | 0.003
K1a+195 | 7747 | 2 | 0.007
K1a+195 | 7759 | 1 | 0.003
K1a+195 | 7853 | 1 | 0.003
K1a+195 | 8400 | 4 | 0.013
K1a+195 | 8521 | 4 | 0.013
K1a+195 | 8555 | 1 | 0.003
K1a+195 | 8856 | 1 | 0.003
K1a+195 | 8938 | 1 | 0.003
K1a+195 | 9093 | 1 | 0.003
K1a+195 | 9174 | 2 | 0.007
K1a+195 | 9266 | 1 | 0.003
K1a+195 | 9332 | 2 | 0.007
K1a+195 | 9629 | 2 | 0.007
K1a+195 | 9713 | 1 | 0.003
K1a+195 | 9714 | 1 | 0.003
K1a1 | 10410 | 1 | 0.003
K1a1 | 11084 | 2 | 0.007
K1a1 | 11101 | 1 | 0.003
K1a1 | 11471 | 2 | 0.007
K1a1 | 11485 | 2 | 0.007
K1a1 | 12076 | 1 | 0.003
K1a1 | 12085 | 2 | 0.007
K1a1 | 12358 | 1 | 0.003
K1a1 | 12534 | 1 | 0.003
K1a1 | 12597 | 2 | 0.007
K1a1 | 12634 | 1 | 0.003
K1a1 | 13434 | 2 | 0.007
K1a1 | 13578 | 2 | 0.007
K1a1 | 14053 | 1 | 0.003
K1a1 | 14110 | 1 | 0.003
K1a1 | 152 | 1 | 0.003
K1a1 | 15340 | 2 | 0.007
K1a1 | 16126 | 1 | 0.003
K1a1 | 16187 | 1 | 0.003
K1a1 | 16221 | 1 | 0.003
K1a1 | 16249 | 1 | 0.003
K1a1 | 16290 | 1 | 0.003
K1a1 | 16344 | 22 | 0.074
K1a1 | 16390 | 23 | 0.077
K1a1 | 199 | 1 | 0.003
K1a1 | 2416 | 1 | 0.003
K1a1 | 251 | 1 | 0.003
K1a1 | 3483 | 1 | 0.003
K1a1 | 393A | 1 | 0.003
K1a1 | 4048 | 2 | 0.007
K1a1 | 471 | 2 | 0.007
K1a1 | 5581 | 1 | 0.003
K1a1 | 5664 | 1 | 0.003
K1a1 | 573.1C | 2 | 0.007
K1a1 | 593 | 1 | 0.003
K1a1 | 6293 | 1 | 0.003
K1a1 | 6620 | 1 | 0.003
K1a1 | 6929 | 2 | 0.007
K1a1 | 7462 | 2 | 0.007
K1a1 | 7699 | 2 | 0.007
K1a1 | 7825 | 1 | 0.003
K1a1 | 8430 | 1 | 0.003
K1a1 | 8865 | 1 | 0.003
K1a1 | 9077 | 1 | 0.003
K1a1 | 9554 | 1 | 0.003
K1a1 | 9575 | 1 | 0.003
K1a10 | 10143 | 1 | 0.045
K1a10 | 13943 | 1 | 0.045
K1a10 | 16047 | 3 | 0.136
K1a10 | 16192A | 1 | 0.045
K1a10 | 16278 | 1 | 0.045
K1a10 | 16319 | 1 | 0.045
K1a10 | 316 | 4 | 0.182

K1a10 | 3206 | 1 | 0.045
K1a10 | 417 | 1 | 0.045
K1a10 | 524.1GC | 1 | 0.045
K1a10 | 525.1ACAC | 1 | 0.045
K1a10 | 6293 | 1 | 0.045
K1a10 | 6528 | 1 | 0.045
K1a10 | 6827 | 1 | 0.045
K1a10 | 8400 | 1 | 0.045
K1a10 | 8521 | 1 | 0.045
K1a10 | 8764 | 3 | 0.136
K1a10a | 10907 | 1 | 0.034
K1a10a | 150 | 1 | 0.034
K1a10a | 15431 | 1 | 0.034
K1a10a | 16051 | 2 | 0.069
K1a10a | 16093 | 1 | 0.034
K1a10a | 16172 | 1 | 0.034
K1a10a | 16189 | 1 | 0.034
K1a10a | 16201 | 1 | 0.034
K1a10a | 16223 | 1 | 0.034
K1a10a | 16305 | 1 | 0.034
K1a10a | 16358 | 1 | 0.034
K1a10a | 16360.1A | 1 | 0.034
K1a10a | 230T | 2 | 0.069
K1a10a | 338 | 1 | 0.034
K1a10a | 525.1ACAC | 2 | 0.069
K1a10a | 5300 | 1 | 0.034
K1a10a | 9477 | 1 | 0.034
K1a10a | 9951 | 1 | 0.034
K1a11 | 15229 | 1 | 0.034
K1a11 | 16092 | 4 | 0.138
K1a11 | 16203 | 1 | 0.034
K1a11 | 5263 | 1 | 0.034
K1a11a | 11698 | 1 | 0.125
K1a11a | 16168 | 1 | 0.125
K1a11a | 189 | 1 | 0.125
K1a11a | 286 | 1 | 0.125
K1a11a | 9468 | 1 | 0.125
K1a11a1 | 11887 | 1 | 0.125
K1a11a1 | 16168 | 1 | 0.125
K1a11a1 | 286 | 1 | 0.125
K1a11a1 | 8155 | 1 | 0.125
K1a11b | 10882 | 2 | 0.071
K1a11b | 11914 | 1 | 0.036
K1a11b | 151 | 1 | 0.036
K1a11b | 16093 | 5 | 0.179
K1a11b | 16102 | 1 | 0.036
K1a11b | 16215 | 1 | 0.036
K1a11b | 281 | 1 | 0.036
K1a12 | 1050G | 1 | 0.004
K1a12 | 12366 | 1 | 0.004
K1a12 | 12738G | 1 | 0.004
K1a12 | 12771 | 1 | 0.004
K1a12 | 14182 | 1 | 0.004
K1a12 | 15391G | 1 | 0.004
K1a12 | 15799 | 1 | 0.004
K1a12 | 16172 | 1 | 0.004
K1a12 | 16243 | 20 | 0.072
K1a12 | 16301 | 1 | 0.004
K1a12 | 204 | 1 | 0.004
K1a12 | 207 | 1 | 0.004
K1a12 | 2220 | 1 | 0.004
K1a12 | 401.1T | 1 | 0.004
K1a12 | 5196 | 1 | 0.004
K1a12 | 573.1C | 2 | 0.007
K1a12 | 7229 | 1 | 0.004
K1a12a | 10172 | 1 | 0.003
K1a12a | 10237 | 1 | 0.003
K1a12a | 11024 | 1 | 0.003
K1a12a | 11170 | 2 | 0.006
K1a12a | 11204 | 2 | 0.006
K1a12a | 12031 | 2 | 0.006
K1a12a | 12414 | 1 | 0.003
K1a12a | 12438 | 1 | 0.003
K1a12a | 12599 | 2 | 0.006
K1a12a | 12813 | 9 | 0.029
K1a12a | 13105 | 1 | 0.003
K1a12a | 13368 | 1 | 0.003
K1a12a | 13707 | 3 | 0.01
K1a12a | 13899 | 1 | 0.003
K1a12a | 14182 | 4 | 0.013
K1a12a | 14410 | 1 | 0.003
K1a12a | 14569 | 1 | 0.003
K1a12a | 146 | 4 | 0.013
K1a12a | 14831 | 1 | 0.003
K1a12a | 15244 | 2 | 0.006
K1a12a | 15248 | 1 | 0.003
K1a12a | 15257 | 2 | 0.006
K1a12a | 15391G | 9 | 0.029
K1a12a | 15670 | 1 | 0.003
K1a12a | 15734 | 1 | 0.003
K1a12a | 15769 | 2 | 0.006
K1a12a | 15799 | 9 | 0.029
K1a12a | 15859C | 1 | 0.003
K1a12a | 15928 | 2 | 0.006
K1a12a | 16111 | 1 | 0.003
K1a12a | 16126 | 1 | 0.003
K1a12a | 16129 | 3 | 0.01
K1a12a | 16167 | 2 | 0.006
K1a12a | 16172 | 15 | 0.048
K1a12a | 16180 | 1 | 0.003
K1a12a | 16216 | 2 | 0.006
K1a12a | 16225 | 1 | 0.003
K1a12a | 16258T | 1 | 0.003
K1a12a | 16266 | 1 | 0.003
K1a12a | 16301 | 35 | 0.111
K1a12a | 16320 | 20 | 0.063
K1a12a | 16327 | 1 | 0.003
K1a12a | 16352 | 1 | 0.003
K1a12a | 16354 | 2 | 0.006
K1a12a | 16368 | 2 | 0.006
K1a12a | 16497 | 2 | 0.006

K1a12a | 16527 | 2 | 0.006
K1a12a | 185 | 4 | 0.013
K1a12a | 189 | 3 | 0.01
K1a12a | 195 | 3 | 0.01
K1a12a | 199 | 1 | 0.003
K1a12a | 200 | 3 | 0.01
K1a12a | 204 | 11 | 0.035
K1a12a | 207 | 10 | 0.032
K1a12a | 2220 | 9 | 0.029
K1a12a | 234 | 1 | 0.003
K1a12a | 2413 | 1 | 0.003
K1a12a | 2442 | 1 | 0.003
K1a12a | 310 | 1 | 0.003
K1a12a | 385 | 1 | 0.003
K1a12a | 4248 | 2 | 0.006
K1a12a | 44.1C | 1 | 0.003
K1a12a | 4964 | 2 | 0.006
K1a12a | 5042 | 1 | 0.003
K1a12a | 5123C | 2 | 0.006
K1a12a | 5196 | 2 | 0.006
K1a12a | 525.1AC | 1 | 0.003
K1a12a | 573.1C | 4 | 0.013
K1a12a | 5892 | 1 | 0.003
K1a12a | 593 | 1 | 0.003
K1a12a | 6122 | 1 | 0.003
K1a12a | 7229 | 10 | 0.032
K1a12a | 723 | 1 | 0.003
K1a12a | 7245 | 3 | 0.01
K1a12a | 7262 | 2 | 0.006
K1a12a | 7711 | 1 | 0.003
K1a12a | 7927 | 1 | 0.003
K1a12a | 795T | 1 | 0.003
K1a12a | 7979 | 1 | 0.003
K1a12a | 8206 | 2 | 0.006
K1a12a | 8412 | 1 | 0.003
K1a12a | 9129 | 1 | 0.003
K1a12a | 9196 | 2 | 0.006
K1a12a | 9276 | 2 | 0.006
K1a12a | 9545 | 1 | 0.003
K1a12a | 9575 | 2 | 0.006
K1a12a1 | 10248 | 2 | 0.007
K1a12a1 | 10653 | 1 | 0.004
K1a12a1 | 14905 | 2 | 0.007
K1a12a1 | 150 | 1 | 0.004
K1a12a1 | 152 | 1 | 0.004
K1a12a1 | 15884 | 2 | 0.007
K1a12a1 | 16243 | 20 | 0.072
K1a12a1 | 16261 | 1 | 0.004
K1a12a1 | 1824 | 1 | 0.004
K1a12a1 | 3394 | 2 | 0.007
K1a12a1 | 401.1T | 1 | 0.004
K1a12a1 | 573.1C | 2 | 0.007
K1a12a1a | 10343 | 2 | 0.007
K1a12a1a | 10506 | 2 | 0.007
K1a12a1a | 12234 | 1 | 0.004
K1a12a1a | 12361 | 1 | 0.004
K1a12a1a | 13215 | 1 | 0.004
K1a12a1a | 14364 | 2 | 0.007
K1a12a1a | 14970 | 1 | 0.004
K1a12a1a | 152 | 1 | 0.004
K1a12a1a | 16092 | 1 | 0.004
K1a12a1a | 16129 | 2 | 0.007
K1a12a1a | 16243 | 20 | 0.071
K1a12a1a | 16278 | 1 | 0.004
K1a12a1a | 1719 | 1 | 0.004
K1a12a1a | 195 | 1 | 0.004
K1a12a1a | 401.1T | 1 | 0.004
K1a12a1a | 573.1C | 2 | 0.007
K1a12a1a | 7270 | 2 | 0.007
K1a12a1a | 8347C | 1 | 0.004
K1a12a1a | 8557 | 1 | 0.004
K1a12a1a | 8856 | 1 | 0.004
K1a12a1a | 8937 | 1 | 0.004
K1a13 | 152 | 6 | 0.034
K1a13 | 15808C | 5 | 0.029
K1a13 | 15851 | 5 | 0.029
K1a13 | 16227 | 5 | 0.029
K1a13 | 198 | 1 | 0.006
K1a13 | 81 | 1 | 0.006
K1a13a | 10289 | 3 | 0.017
K1a13a | 11025 | 2 | 0.011
K1a13a | 11236 | 7 | 0.04
K1a13a | 14097 | 2 | 0.011
K1a13a | 152 | 1 | 0.006
K1a13a | 16093 | 1 | 0.006
K1a13a | 16213 | 6 | 0.034
K1a13a | 16291 | 5 | 0.028
K1a13a | 16325 | 2 | 0.011
K1a13a | 1719 | 2 | 0.011
K1a13a | 204 | 1 | 0.006
K1a13a | 574.1A | 1 | 0.006
K1a13a | 71d | 1 | 0.006
K1a13a | 8875 | 1 | 0.006
K1a13a | 9548 | 4 | 0.023
K1a14 | 14198 | 1 | 0.005
K1a14 | 16189 | 1 | 0.005
K1a14 | 16246T | 1 | 0.005
K1a14 | 16325 | 3 | 0.015
K1a14 | 279 | 1 | 0.005
K1a14 | 452 | 1 | 0.005
K1a14 | 6735 | 1 | 0.005
K1a14 | 8392 | 1 | 0.005
K1a14 | 9137 | 1 | 0.005
K1a14 | 94 | 1 | 0.005
K1a15 | 10101 | 1 | 0.005
K1a15 | 16246T | 1 | 0.005
K1a15 | 16325 | 3 | 0.015
K1a15 | 279 | 1 | 0.005
K1a15 | 452 | 1 | 0.005
K1a15 | 6367 | 1 | 0.005
K1a15 | 93 | 1 | 0.005

K1a15 | 94 | 1 | 0.005
K1a16 | 16246T | 1 | 0.005
K1a16 | 16325 | 3 | 0.015
K1a16 | 279 | 1 | 0.005
K1a16 | 452 | 1 | 0.005
K1a16 | 7376 | 1 | 0.005
K1a16 | 7621 | 1 | 0.005
K1a16 | 8155 | 1 | 0.005
K1a16 | 94 | 1 | 0.005
K1a17 | 10499 | 2 | 0.011
K1a17 | 11902 | 1 | 0.005
K1a17 | 12892 | 1 | 0.005
K1a17 | 13174 | 2 | 0.011
K1a17 | 13854 | 1 | 0.005
K1a17 | 14180 | 1 | 0.005
K1a17 | 16223 | 4 | 0.022
K1a17 | 16254 | 1 | 0.005
K1a17 | 16259 | 2 | 0.011
K1a17 | 16261 | 2 | 0.011
K1a17 | 16295 | 2 | 0.011
K1a17 | 16343T | 1 | 0.005
K1a17 | 16344 | 1 | 0.005
K1a17 | 234 | 1 | 0.005
K1a17 | 249d | 1 | 0.005
K1a17 | 6719 | 2 | 0.011
K1a17 | 7148 | 1 | 0.005
K1a17 | 7673 | 2 | 0.011
K1a17 | 8164 | 2 | 0.011
K1a17 | 8677C | 1 | 0.005
K1a17 | 8701 | 1 | 0.005
K1a17 | 8718 | 1 | 0.005
K1a17a | 12362 | 1 | 0.167
K1a17a | 12900 | 2 | 0.333
K1a17a | 15466 | 2 | 0.333
K1a17a | 16239 | 2 | 0.333
K1a17a | 16477 | 1 | 0.167
K1a17a | 16532d | 1 | 0.167
K1a17a | 200 | 1 | 0.167
K1a17a | 242 | 1 | 0.167
K1a17a | 44 | 1 | 0.167
K1a17a | 4454 | 1 | 0.167
K1a17a | 4733 | 1 | 0.167
K1a17a | 5030 | 1 | 0.167
K1a17a | 6357 | 1 | 0.167
K1a17a | 9948 | 1 | 0.167
K1a18 | 1154T | 1 | 0.004
K1a18 | 152 | 2 | 0.007
K1a18 | 16158 | 1 | 0.004
K1a18 | 16243 | 20 | 0.071
K1a18 | 16294 | 1 | 0.004
K1a18 | 3398 | 1 | 0.004
K1a18 | 401.1T | 1 | 0.004
K1a18 | 485 | 3 | 0.011
K1a18 | 524.1ACAA | 1 | 0.004
K1a18 | 573.1C | 2 | 0.007
K1a18 | 6272 | 1 | 0.004
K1a18 | 6467 | 1 | 0.004
K1a18 | 6755 | 1 | 0.004
K1a18 | 8950 | 2 | 0.007
K1a18 | 9063 | 1 | 0.004
K1a18 | 9196 | 1 | 0.004
K1a18 | 9983 | 1 | 0.004
K1a19 | 10172 | 2 | 0.007
K1a19 | 11524 | 1 | 0.004
K1a19 | 12187 | 1 | 0.004
K1a19 | 12618 | 1 | 0.004
K1a19 | 13167 | 1 | 0.004
K1a19 | 14106 | 1 | 0.004
K1a19 | 14180 | 1 | 0.004
K1a19 | 15265 | 1 | 0.004
K1a19 | 15791 | 1 | 0.004
K1a19 | 16111 | 1 | 0.004
K1a19 | 16187 | 1 | 0.004
K1a19 | 16243 | 20 | 0.07
K1a19 | 16465 | 1 | 0.004
K1a19 | 16488 | 1 | 0.004
K1a19 | 2857 | 1 | 0.004
K1a19 | 321 | 1 | 0.004
K1a19 | 401.1T | 1 | 0.004
K1a19 | 507 | 1 | 0.004
K1a19 | 5072 | 1 | 0.004
K1a19 | 5598 | 1 | 0.004
K1a19 | 573.1C | 2 | 0.007
K1a19 | 5811 | 1 | 0.004
K1a19 | 6329 | 1 | 0.004
K1a19 | 6656 | 1 | 0.004
K1a19 | 6734 | 1 | 0.004
K1a19 | 7245 | 1 | 0.004
K1a19 | 7624A | 1 | 0.004
K1a19 | 7830 | 1 | 0.004
K1a19 | 8683 | 1 | 0.004
K1a19 | 9053 | 1 | 0.004
K1a19 | 9063 | 1 | 0.004
K1a19 | 9266 | 1 | 0.004
K1a19 | 9494 | 1 | 0.004
K1a19a | 10586 | 1 | 0.007
K1a19a | 14467 | 1 | 0.007
K1a19a | 150 | 1 | 0.007
K1a19a | 189 | 1 | 0.007
K1a19a | 6719 | 1 | 0.007
K1a1a | 10428 | 1 | 0.003
K1a1a | 10440 | 1 | 0.003
K1a1a | 11095 | 1 | 0.003
K1a1a | 12103 | 1 | 0.003
K1a1a | 12406 | 1 | 0.003
K1a1a | 16093 | 20 | 0.069
K1a1a | 16129 | 1 | 0.003
K1a1a | 16192 | 1 | 0.003
K1a1a | 249d | 1 | 0.003
K1a1a | 310 | 2 | 0.007
K1a1a | 3336 | 1 | 0.003

K1a1a | 3745 | 1 | 0.003
K1a1a | 573.1C | 2 | 0.007
K1a1a1 | 13782A | 21 | 0.074
K1a1a1 | 14311 | 1 | 0.004
K1a1a1 | 146 | 1 | 0.004
K1a1a1 | 152 | 20 | 0.07
K1a1a1 | 16017 | 1 | 0.004
K1a1a1 | 16256 | 2 | 0.007
K1a1a1 | 16357 | 1 | 0.004
K1a1a1 | 573.1C | 2 | 0.007
K1a1a1 | 7927G | 1 | 0.004
K1a1a1 | 7985G | 1 | 0.004
K1a1a1 | 8065 | 20 | 0.07
K1a1a1 | 8251 | 1 | 0.004
K1a1a2 | 15328 | 1 | 0.004
K1a1a2 | 16167 | 1 | 0.004
K1a1a2 | 401.1T | 1 | 0.004
K1a1a2 | 4017A | 1 | 0.004
K1a1a2 | 573.1C | 2 | 0.007
K1a1a2a | 12771 | 2 | 0.007
K1a1a2a | 16086 | 1 | 0.003
K1a1a2a | 16129 | 28 | 0.097
K1a1a2a | 16145 | 2 | 0.007
K1a1a2a | 16390 | 1 | 0.003
K1a1a2a | 16484-16489d | 20 | 0.069
K1a1a2a | 5628 | 2 | 0.007
K1a1a2a | 573.1C | 2 | 0.007
K1a1a2a | 71.1G | 1 | 0.003
K1a1a2a1 | 14226 | 1 | 0.003
K1a1a2a1 | 15364 | 20 | 0.07
K1a1a2a1 | 573.1C | 2 | 0.007
K1a1b | 10697 | 1 | 0.004
K1a1b | 11827 | 1 | 0.004
K1a1b | 12816 | 1 | 0.004
K1a1b | 13135 | 1 | 0.004
K1a1b | 14200 | 1 | 0.004
K1a1b | 15194 | 1 | 0.004
K1a1b | 15323 | 1 | 0.004
K1a1b | 16192 | 2 | 0.007
K1a1b | 16293 | 2 | 0.007
K1a1b | 16295 | 1 | 0.004
K1a1b | 16362 | 1 | 0.004
K1a1b | 16400 | 19 | 0.069
K1a1b | 1656 | 1 | 0.004
K1a1b | 3487 | 1 | 0.004
K1a1b | 401.1T | 1 | 0.004
K1a1b | 456 | 2 | 0.007
K1a1b | 573.1C | 2 | 0.007
K1a1b | 593 | 1 | 0.004
K1a1b | 7051 | 1 | 0.004
K1a1b1 | 12537 | 5 | 0.017
K1a1b1 | 13443 | 1 | 0.003
K1a1b1 | 146 | 2 | 0.007
K1a1b1 | 14659 | 3 | 0.01
K1a1b1 | 14674 | 1 | 0.003
K1a1b1 | 15074 | 1 | 0.003
K1a1b1 | 152 | 23 | 0.079
K1a1b1 | 16048 | 1 | 0.003
K1a1b1 | 16092 | 1 | 0.003
K1a1b1 | 16184A | 1 | 0.003
K1a1b1 | 16234 | 21 | 0.072
K1a1b1 | 16269 | 21 | 0.072
K1a1b1 | 16278 | 4 | 0.014
K1a1b1 | 1717G | 1 | 0.003
K1a1b1 | 1720 | 1 | 0.003
K1a1b1 | 181T | 1 | 0.003
K1a1b1 | 200 | 2 | 0.007
K1a1b1 | 2628 | 1 | 0.003
K1a1b1 | 2963 | 1 | 0.003
K1a1b1 | 310 | 1 | 0.003
K1a1b1 | 315.2C | 1 | 0.003
K1a1b1 | 4080 | 1 | 0.003
K1a1b1 | 5583 | 3 | 0.01
K1a1b1 | 573.1C | 3 | 0.01
K1a1b1 | 5742 | 4 | 0.014
K1a1b1 | 6254 | 5 | 0.017
K1a1b1 | 6261 | 21 | 0.072
K1a1b1 | 709 | 21 | 0.072
K1a1b1 | 7158 | 21 | 0.072
K1a1b1 | 9233 | 5 | 0.017
K1a1b1 | 9644 | 1 | 0.003
K1a1b1a | 106.1A | 1 | 0.011
K1a1b1a | 11005 | 1 | 0.011
K1a1b1a | 11020 | 1 | 0.011
K1a1b1a | 11665 | 1 | 0.011
K1a1b1a | 11765A | 1 | 0.011
K1a1b1a | 12189 | 3 | 0.033
K1a1b1a | 13395 | 1 | 0.011
K1a1b1a | 13416 | 1 | 0.011
K1a1b1a | 1393 | 1 | 0.011
K1a1b1a | 14040 | 2 | 0.022
K1a1b1a | 14118 | 1 | 0.011
K1a1b1a | 14203C | 1 | 0.011
K1a1b1a | 14249 | 1 | 0.011
K1a1b1a | 14458A | 1 | 0.011
K1a1b1a | 14517 | 1 | 0.011
K1a1b1a | 1462 | 1 | 0.011
K1a1b1a | 14843 | 1 | 0.011
K1a1b1a | 152 | 3 | 0.033
K1a1b1a | 15355 | 1 | 0.011
K1a1b1a | 15978 | 1 | 0.011
K1a1b1a | 16066 | 1 | 0.011
K1a1b1a | 16159 | 1 | 0.011
K1a1b1a | 16189 | 1 | 0.011
K1a1b1a | 16223 | 40 | 0.435
K1a1b1a | 16278 | 1 | 0.011
K1a1b1a | 16355 | 1 | 0.011
K1a1b1a | 1709 | 2 | 0.022
K1a1b1a | 189 | 1 | 0.011
K1a1b1a | 195 | 1 | 0.011
K1a1b1a | 199 | 1 | 0.011

K1a1b1a | 215 | 2 | 0.022
K1a1b1a | 264 | 1 | 0.011
K1a1b1a | 310 | 1 | 0.011
K1a1b1a | 316 | 1 | 0.011
K1a1b1a | 316C | 1 | 0.011
K1a1b1a | 3316 | 1 | 0.011
K1a1b1a | 489 | 2 | 0.022
K1a1b1a | 5094 | 1 | 0.011
K1a1b1a | 513 | 2 | 0.022
K1a1b1a | 5147 | 1 | 0.011
K1a1b1a | 5460 | 1 | 0.011
K1a1b1a | 6366 | 1 | 0.011
K1a1b1a | 723 | 1 | 0.011
K1a1b1a | 7927G | 1 | 0.011
K1a1b1a | 8023 | 1 | 0.011
K1a1b1a | 8047 | 1 | 0.011
K1a1b1a | 8412 | 1 | 0.011
K1a1b1a | 8462 | 1 | 0.011
K1a1b1a | 8787 | 1 | 0.011
K1a1b1a | 961 | 1 | 0.011
K1a1b1a | 96d | 1 | 0.011
K1a1b1a | 9861 | 3 | 0.033
K1a1b1a | 9921 | 1 | 0.011
K1a1b1b | 11620 | 1 | 0.004
K1a1b1b | 13617 | 1 | 0.004
K1a1b1b | 13851 | 1 | 0.004
K1a1b1b | 14793 | 1 | 0.004
K1a1b1b | 16242 | 1 | 0.004
K1a1b1b | 16293 | 20 | 0.074
K1a1b1b | 189 | 2 | 0.007
K1a1b1b | 195 | 4 | 0.015
K1a1b1b | 401.1T | 1 | 0.004
K1a1b1b | 573.1C | 2 | 0.007
K1a1b1b | 5746 | 1 | 0.004
K1a1b1b | 5876 | 1 | 0.004
K1a1b1b1 | 16246T | 1 | 0.004
K1a1b1b1 | 16300 | 20 | 0.075
K1a1b1b1 | 401.1T | 1 | 0.004
K1a1b1b1 | 573.1C | 2 | 0.008
K1a1b1b1 | 5746 | 1 | 0.004
K1a1b1b1 | 7598 | 1 | 0.004
K1a1b1c | 146 | 3 | 0.176
K1a1b1c | 16188 | 2 | 0.118
K1a1b1c | 16189 | 3 | 0.176
K1a1b1c | 16260 | 2 | 0.118
K1a1b1c | 16327 | 4 | 0.235
K1a1b1c | 16362 | 4 | 0.235
K1a1b1c | 16390 | 1 | 0.059
K1a1b1c | 2145 | 1 | 0.059
K1a1b1c | 2852 | 1 | 0.059
K1a1b1c | 2998 | 1 | 0.059
K1a1b1c | 9138 | 1 | 0.059
K1a1b1d | 14569 | 3 | 0.333
K1a1b1e | 11204 | 1 | 0.004
K1a1b1e | 146 | 1 | 0.004
K1a1b1e | 15047 | 1 | 0.004
K1a1b1e | 16288 | 1 | 0.004
K1a1b1e | 16293 | 20 | 0.071
K1a1b1e | 189 | 1 | 0.004
K1a1b1e | 3705 | 1 | 0.004
K1a1b1e | 401.1T | 1 | 0.004
K1a1b1e | 477 | 2 | 0.007
K1a1b1e | 573.1C | 2 | 0.007
K1a1b1e | 8291 | 1 | 0.004
K1a1b1e | 8521 | 1 | 0.004
K1a1b1e | 9214 | 1 | 0.004
K1a1b1f | 10609 | 1 | 0.004
K1a1b1f | 13050 | 1 | 0.004
K1a1b1f | 16293 | 20 | 0.074
K1a1b1f | 3796 | 1 | 0.004
K1a1b1f | 401.1T | 1 | 0.004
K1a1b1f | 573.1C | 2 | 0.007
K1a1b1f | 6284 | 1 | 0.004
K1a1b1f | 827 | 1 | 0.004
K1a1b1g | 14279C | 1 | 0.004
K1a1b1g | 16293 | 20 | 0.074
K1a1b1g | 390 | 1 | 0.004
K1a1b1g | 401.1T | 1 | 0.004
K1a1b1g | 573.1C | 2 | 0.007
K1a1b2 | 16293 | 20 | 0.075
K1a1b2 | 401.1T | 1 | 0.004
K1a1b2 | 573.1C | 2 | 0.007
K1a1b2a | 11204 | 1 | 0.005
K1a1b2a | 15562 | 1 | 0.005
K1a1b2a | 15731 | 1 | 0.005
K1a1b2a | 16016T | 1 | 0.005
K1a1b2a | 16129 | 1 | 0.005
K1a1b2a | 16174 | 2 | 0.01
K1a1b2a | 16187 | 5 | 0.025
K1a1b2a | 16256 | 2 | 0.01
K1a1b2a | 16484-16489d | 5 | 0.025
K1a1b2a | 2352 | 1 | 0.005
K1a1b2a | 280G | 1 | 0.005
K1a1b2a | 332 | 5 | 0.025
K1a1b2a1 | 15071 | 1 | 0.005
K1a1b2a1 | 16016T | 1 | 0.005
K1a1b2a1 | 16129 | 1 | 0.005
K1a1b2a1 | 16174 | 2 | 0.01
K1a1b2a1 | 16187 | 5 | 0.025
K1a1b2a1 | 16213 | 1 | 0.005
K1a1b2a1 | 16256 | 2 | 0.01
K1a1b2a1 | 16484-16489d | 5 | 0.025
K1a1b2a1 | 280G | 1 | 0.005
K1a1b2a1 | 332 | 5 | 0.025
K1a1b2a1a | 10046 | 1 | 0.005
K1a1b2a1a | 11053 | 4 | 0.02
K1a1b2a1a | 12630 | 1 | 0.005
K1a1b2a1a | 13665 | 3 | 0.015
K1a1b2a1a | 14371 | 1 | 0.005
K1a1b2a1a | 15908 | 1 | 0.005
K1a1b2a1a | 16129 | 1 | 0.005

K1a1b2a1a | 16172 | 5 | 0.025
K1a1b2a1a | 16231 | 4 | 0.02
K1a1b2a1a | 195 | 1 | 0.005
K1a1b2a1a | 209 | 4 | 0.02
K1a1b2a1a | 280G | 1 | 0.005
K1a1b2a1a | 286 | 4 | 0.02
K1a1b2a1a | 4170 | 1 | 0.005
K1a1b2a1a | 770 | 4 | 0.02
K1a1b2a1a | 8604 | 1 | 0.005
K1a1b2a1a | 9545 | 1 | 0.005
K1a1b2b | 12756 | 1 | 0.007
K1a1b2b | 12950 | 1 | 0.007
K1a1b2b | 152 | 1 | 0.007
K1a1b2b | 15346 | 2 | 0.013
K1a1b2b | 16400 | 1 | 0.007
K1a1b2b | 185 | 1 | 0.007
K1a1b2b | 195 | 2 | 0.013
K1a1b2b | 199 | 1 | 0.007
K1a1b2b | 2156.1A | 2 | 0.013
K1a1b2b | 236 | 2 | 0.013
K1a1b2b | 279 | 1 | 0.007
K1a1b2b | 6032 | 1 | 0.007
K1a1b2b | 9329 | 2 | 0.013
K1a1c | 10654 | 1 | 0.045
K1a1c | 12795 | 2 | 0.091
K1a1c | 14484 | 2 | 0.091
K1a1c | 16192 | 2 | 0.091
K1a1c | 16293 | 1 | 0.045
K1a1c | 183 | 1 | 0.045
K1a1c | 2080 | 1 | 0.045
K1a1c | 2087 | 1 | 0.045
K1a1c | 310 | 1 | 0.045
K1a1c | 3398 | 1 | 0.045
K1a1c | 4387 | 1 | 0.045
K1a1c | 4949 | 1 | 0.045
K1a1c | 5918 | 1 | 0.045
K1a1c | 794 | 1 | 0.045
K1a2 | 10250 | 1 | 0.003
K1a2 | 11812 | 1 | 0.003
K1a2 | 11914 | 1 | 0.003
K1a2 | 12477 | 1 | 0.003
K1a2 | 13135 | 1 | 0.003
K1a2 | 13539 | 1 | 0.003
K1a2 | 13617 | 1 | 0.003
K1a2 | 13781 | 2 | 0.007
K1a2 | 13827 | 1 | 0.003
K1a2 | 13928C | 1 | 0.003
K1a2 | 14410 | 4 | 0.013
K1a2 | 14530 | 1 | 0.003
K1a2 | 146 | 3 | 0.01
K1a2 | 14674 | 1 | 0.003
K1a2 | 15024 | 1 | 0.003
K1a2 | 152 | 2 | 0.007
K1a2 | 153 | 1 | 0.003
K1a2 | 15310 | 5 | 0.017
K1a2 | 16051 | 2 | 0.007
K1a2 | 16126 | 1 | 0.003
K1a2 | 16209 | 1 | 0.003
K1a2 | 16243 | 20 | 0.067
K1a2 | 16264 | 1 | 0.003
K1a2 | 16399 | 3 | 0.01
K1a2 | 182 | 3 | 0.01
K1a2 | 195 | 1 | 0.003
K1a2 | 199 | 1 | 0.003
K1a2 | 316.1T | 1 | 0.003
K1a2 | 316C | 1 | 0.003
K1a2 | 3335 | 2 | 0.007
K1a2 | 3816 | 1 | 0.003
K1a2 | 3918 | 4 | 0.013
K1a2 | 4254 | 1 | 0.003
K1a2 | 459d | 2 | 0.007
K1a2 | 4677 | 1 | 0.003
K1a2 | 4913 | 1 | 0.003
K1a2 | 513 | 5 | 0.017
K1a2 | 5301 | 9 | 0.03
K1a2 | 532 | 2 | 0.007
K1a2 | 5474 | 4 | 0.013
K1a2 | 5480 | 1 | 0.003
K1a2 | 573.1C | 2 | 0.007
K1a2 | 6305C | 1 | 0.003
K1a2 | 6425 | 1 | 0.003
K1a2 | 7366 | 1 | 0.003
K1a2 | 745 | 3 | 0.01
K1a2 | 8557 | 4 | 0.013
K1a2 | 8725 | 4 | 0.013
K1a2 | 9156 | 5 | 0.017
K1a2 | 930 | 1 | 0.003
K1a2 | 9524 | 1 | 0.003
K1a23 | 10084 | 1 | 0.004
K1a23 | 12618 | 1 | 0.004
K1a23 | 12681 | 1 | 0.004
K1a23 | 13335 | 1 | 0.004
K1a23 | 14602 | 1 | 0.004
K1a23 | 16144 | 1 | 0.004
K1a23 | 16243 | 20 | 0.072
K1a23 | 1709 | 1 | 0.004
K1a23 | 249d | 1 | 0.004
K1a23 | 401.1T | 1 | 0.004
K1a23 | 5387 | 1 | 0.004
K1a23 | 573.1C | 2 | 0.007
K1a23 | 7372 | 1 | 0.004
K1a23 | 7846 | 1 | 0.004
K1a23 | 8281 | 1 | 0.004
K1a24 | 131 | 5 | 0.027
K1a24 | 15043 | 2 | 0.011
K1a24 | 151 | 5 | 0.027
K1a24 | 16218 | 4 | 0.022
K1a24 | 16263 | 4 | 0.022
K1a24 | 195 | 1 | 0.005
K1a24a | 16126 | 4 | 0.222
K1a24a | 16233 | 1 | 0.056

K1a24a | 16327 | 1 | 0.056
K1a24a | 16357 | 1 | 0.056
K1a24a | 16527 | 1 | 0.056
K1a24a | 16559 | 1 | 0.056
K1a24a | 5147 | 1 | 0.056
K1a24a | 59 | 1 | 0.056
K1a25 | 11524 | 1 | 0.005
K1a25 | 14464 | 1 | 0.005
K1a25 | 15892 | 1 | 0.005
K1a25 | 16129 | 1 | 0.005
K1a25 | 16158 | 4 | 0.02
K1a25 | 16390 | 1 | 0.005
K1a25 | 280G | 1 | 0.005
K1a25 | 4385 | 1 | 0.005
K1a25 | 485 | 8 | 0.039
K1a25 | 5060 | 1 | 0.005
K1a25 | 7830 | 1 | 0.005
K1a25 | 8184 | 1 | 0.005
K1a26 | 16192 | 7 | 0.035
K1a26 | 16239 | 1 | 0.005
K1a26 | 16246T | 1 | 0.005
K1a26 | 16325 | 3 | 0.015
K1a26 | 279 | 1 | 0.005
K1a26 | 452 | 1 | 0.005
K1a26 | 6725A | 2 | 0.01
K1a26 | 94 | 1 | 0.005
K1a27 | 103 | 1 | 0.053
K1a27 | 12630 | 2 | 0.105
K1a27 | 13759 | 1 | 0.053
K1a27 | 13980 | 2 | 0.105
K1a27 | 14016 | 4 | 0.211
K1a27 | 14568 | 1 | 0.053
K1a27 | 16193 | 1 | 0.053
K1a27 | 16209 | 3 | 0.158
K1a27 | 16242 | 1 | 0.053
K1a27 | 16293 | 1 | 0.053
K1a27 | 16T | 2 | 0.105
K1a27 | 4092 | 4 | 0.211
K1a27 | 5466 | 4 | 0.211
K1a27 | 5492 | 2 | 0.105
K1a27 | 7299 | 1 | 0.053
K1a27 | 8145 | 2 | 0.105
K1a27 | 8938 | 2 | 0.105
K1a27 | 9338 | 2 | 0.105
K1a28 | 13329 | 22 | 0.095
K1a28 | 15758 | 1 | 0.004
K1a28 | 195 | 1 | 0.004
K1a28 | 7789 | 1 | 0.004
K1a28 | 8664 | 1 | 0.004
K1a29 | 10861 | 1 | 0.004
K1a29 | 16176 | 3 | 0.011
K1a29 | 16290 | 21 | 0.075
K1a29 | 401.1T | 1 | 0.004
K1a29 | 573.1C | 2 | 0.007
K1a29 | 5899.1C | 1 | 0.004
K1a29 | 8295 | 1 | 0.004
K1a29 | 960d | 2 | 0.007
K1a29a | 13681 | 1 | 0.111
K1a29a | 150 | 1 | 0.111
K1a29a | 152 | 1 | 0.111
K1a29a | 16254 | 1 | 0.111
K1a29a | 310 | 1 | 0.111
K1a29a | 4216 | 1 | 0.111
K1a2a | 10786 | 1 | 0.003
K1a2a | 13434 | 3 | 0.01
K1a2a | 13708 | 1 | 0.003
K1a2a | 13710 | 2 | 0.007
K1a2a | 14587 | 1 | 0.003
K1a2a | 14767A | 1 | 0.003
K1a2a | 150 | 1 | 0.003
K1a2a | 152 | 3 | 0.01
K1a2a | 15249 | 1 | 0.003
K1a2a | 15784 | 3 | 0.01
K1a2a | 16119 | 3 | 0.01
K1a2a | 16145 | 6 | 0.02
K1a2a | 16147 | 2 | 0.007
K1a2a | 16188 | 1 | 0.003
K1a2a | 16189 | 1 | 0.003
K1a2a | 16193d | 1 | 0.003
K1a2a | 16215 | 1 | 0.003
K1a2a | 16223 | 1 | 0.003
K1a2a | 16243 | 20 | 0.068
K1a2a | 16497 | 1 | 0.003
K1a2a | 1719 | 1 | 0.003
K1a2a | 195 | 1 | 0.003
K1a2a | 214 | 1 | 0.003
K1a2a | 3483 | 2 | 0.007
K1a2a | 4024T | 1 | 0.003
K1a2a | 514.1GCAC | 1 | 0.003
K1a2a | 5231 | 1 | 0.003
K1a2a | 525.1AC | 1 | 0.003
K1a2a | 573.1C | 2 | 0.007
K1a2a | 8289.1CCCCCTCTA | 1 | 0.003
K1a2a | 9285 | 1 | 0.003
K1a2a | 930T | 1 | 0.003
K1a2a | 9438 | 1 | 0.003
K1a2a | 9725 | 1 | 0.003
K1a2a1 | 11084 | 1 | 0.029
K1a2a1 | 121C | 1 | 0.029
K1a2a1 | 143 | 2 | 0.059
K1a2a1 | 1466 | 1 | 0.029
K1a2a1 | 150 | 1 | 0.029
K1a2a1 | 152 | 2 | 0.059
K1a2a1 | 16124 | 1 | 0.029
K1a2a1 | 16180 | 1 | 0.029
K1a2a1 | 16184 | 2 | 0.059
K1a2a1 | 16186 | 1 | 0.029
K1a2a1 | 16188 | 4 | 0.118
K1a2a1 | 16190 | 1 | 0.029
K1a2a1 | 16192 | 1 | 0.029
K1a2a1 | 16193d | 3 | 0.088

K1a2a1 | 16215 | 2 | 0.059
K1a2a1 | 16233 | 1 | 0.029
K1a2a1 | 16265C | 1 | 0.029
K1a2a1 | 16286 | 1 | 0.029
K1a2a1 | 16319 | 1 | 0.029
K1a2a1 | 16354 | 1 | 0.029
K1a2a1 | 16368A | 1 | 0.029
K1a2a1 | 16384 | 1 | 0.029
K1a2a1 | 16390 | 1 | 0.029
K1a2a1 | 185 | 1 | 0.029
K1a2a1 | 195 | 10 | 0.294
K1a2a1 | 199 | 1 | 0.029
K1a2a1 | 280G | 1 | 0.029
K1a2a1 | 544 | 1 | 0.029
K1a2a1 | 7804 | 1 | 0.029
K1a2a1 | 8310 | 1 | 0.029
K1a2a1 | 8350 | 1 | 0.029
K1a2a1 | 8581 | 1 | 0.029
K1a2a2 | 11500 | 1 | 0.004
K1a2a2 | 13848 | 3 | 0.011
K1a2a2 | 16243 | 20 | 0.071
K1a2a2 | 195 | 1 | 0.004
K1a2a2 | 200 | 3 | 0.011
K1a2a2 | 401.1T | 1 | 0.004
K1a2a2 | 573.1C | 2 | 0.007
K1a2a2 | 6340 | 3 | 0.011
K1a2b | 10658 | 1 | 0.003
K1a2b | 1116 | 1 | 0.003
K1a2b | 13419 | 1 | 0.003
K1a2b | 152 | 3 | 0.01
K1a2b | 15787 | 7 | 0.024
K1a2b | 16086 | 1 | 0.003
K1a2b | 16093 | 2 | 0.007
K1a2b | 16148 | 3 | 0.01
K1a2b | 16175 | 2 | 0.007
K1a2b | 16201 | 2 | 0.007
K1a2b | 16278 | 33 | 0.113
K1a2b | 16357 | 2 | 0.007
K1a2b | 16399 | 1 | 0.003
K1a2b | 2834 | 1 | 0.003
K1a2b | 3318 | 1 | 0.003
K1a2b | 3471A | 1 | 0.003
K1a2b | 4316 | 1 | 0.003
K1a2b | 4691 | 1 | 0.003
K1a2b | 525.1ACAC | 1 | 0.003
K1a2b | 573.1C | 2 | 0.007
K1a2b | 6261 | 1 | 0.003
K1a2b | 7694 | 1 | 0.003
K1a2b | 8185 | 1 | 0.003
K1a2b | 8281-8289d | 5 | 0.017
K1a2b | 8802 | 1 | 0.003
K1a2b | 9293 | 1 | 0.003
K1a2c | 12633A | 1 | 0.004
K1a2c | 1292 | 1 | 0.004
K1a2c | 13886 | 2 | 0.007
K1a2c | 146 | 3 | 0.011
K1a2c | 152 | 3 | 0.011
K1a2c | 15944 | 2 | 0.007
K1a2c | 16192 | 1 | 0.004
K1a2c | 16241 | 1 | 0.004
K1a2c | 16243 | 20 | 0.071
K1a2c | 16261 | 2 | 0.007
K1a2c | 16288 | 2 | 0.007
K1a2c | 1692 | 2 | 0.007
K1a2c | 183 | 1 | 0.004
K1a2c | 401.1T | 1 | 0.004
K1a2c | 499 | 1 | 0.004
K1a2c | 54T | 2 | 0.007
K1a2c | 573.1C | 2 | 0.007
K1a2c | 6915 | 1 | 0.004
K1a2c | 8392 | 2 | 0.007
K1a2c | 942 | 2 | 0.007
K1a2c | 9491 | 2 | 0.007
K1a3 | 10310 | 1 | 0.003
K1a3 | 10972 | 1 | 0.003
K1a3 | 12192 | 1 | 0.003
K1a3 | 12786 | 1 | 0.003
K1a3 | 13065A | 2 | 0.007
K1a3 | 13194 | 1 | 0.003
K1a3 | 13708 | 15 | 0.05
K1a3 | 13928 | 1 | 0.003
K1a3 | 1503 | 2 | 0.007
K1a3 | 15064C | 2 | 0.007
K1a3 | 151 | 4 | 0.013
K1a3 | 152 | 14 | 0.047
K1a3 | 15708C | 1 | 0.003
K1a3 | 15884 | 1 | 0.003
K1a3 | 15893 | 1 | 0.003
K1a3 | 15928 | 1 | 0.003
K1a3 | 15939 | 6 | 0.02
K1a3 | 16037 | 1 | 0.003
K1a3 | 16225 | 1 | 0.003
K1a3 | 16265C | 1 | 0.003
K1a3 | 16278 | 21 | 0.07
K1a3 | 16291 | 1 | 0.003
K1a3 | 16327 | 1 | 0.003
K1a3 | 16360 | 1 | 0.003
K1a3 | 16362 | 1 | 0.003
K1a3 | 1809 | 1 | 0.003
K1a3 | 189 | 1 | 0.003
K1a3 | 3547 | 2 | 0.007
K1a3 | 3687 | 1 | 0.003
K1a3 | 3918 | 1 | 0.003
K1a3 | 4363 | 1 | 0.003
K1a3 | 485 | 1 | 0.003
K1a3 | 5033 | 1 | 0.003
K1a3 | 5249 | 1 | 0.003
K1a3 | 525.1AC | 1 | 0.003
K1a3 | 573.1C | 2 | 0.007
K1a3 | 6267 | 1 | 0.003
K1a3 | 6575 | 1 | 0.003

K1a3 | 7418 | 1 | 0.003
K1a3 | 7570 | 1 | 0.003
K1a3 | 7581 | 1 | 0.003
K1a3 | 8258 | 1 | 0.003
K1a3 | 9300 | 1 | 0.003
K1a3 | 9540 | 1 | 0.003
K1a30 | 10873 | 2 | 0.011
K1a30 | 131 | 5 | 0.027
K1a30 | 151 | 5 | 0.027
K1a30 | 16093 | 1 | 0.005
K1a30 | 16218 | 4 | 0.022
K1a30 | 16263 | 4 | 0.022
K1a30 | 16362 | 2 | 0.011
K1a30 | 249d | 1 | 0.005
K1a30 | 6290 | 3 | 0.016
K1a30 | 9456 | 1 | 0.005
K1a30a | 13191 | 1 | 0.005
K1a30a | 14121 | 1 | 0.005
K1a30a | 15119 | 1 | 0.005
K1a30a | 16086 | 3 | 0.016
K1a30a | 16114 | 1 | 0.005
K1a30a | 16256 | 2 | 0.011
K1a30a | 16497 | 1 | 0.005
K1a30a | 200 | 1 | 0.005
K1a30a | 6261 | 1 | 0.005
K1a30a | 7897 | 1 | 0.005
K1a30a | 961 | 1 | 0.005
K1a30a | 965.4C | 1 | 0.005
K1a31 | 131 | 5 | 0.027
K1a31 | 151 | 5 | 0.027
K1a31 | 152 | 1 | 0.005
K1a31 | 16218 | 4 | 0.022
K1a31 | 16263 | 4 | 0.022
K1a31 | 3144 | 1 | 0.005
K1a31 | 340 | 1 | 0.005
K1a31 | 6320 | 1 | 0.005
K1a3a | 10750 | 2 | 0.007
K1a3a | 10909 | 1 | 0.003
K1a3a | 11902 | 2 | 0.007
K1a3a | 12027 | 1 | 0.003
K1a3a | 12358 | 1 | 0.003
K1a3a | 12642 | 1 | 0.003
K1a3a | 12882 | 1 | 0.003
K1a3a | 13350 | 1 | 0.003
K1a3a | 13440 | 1 | 0.003
K1a3a | 13661 | 1 | 0.003
K1a3a | 13818 | 1 | 0.003
K1a3a | 13928T | 1 | 0.003
K1a3a | 14001 | 2 | 0.007
K1a3a | 14022 | 2 | 0.007
K1a3a | 150 | 1 | 0.003
K1a3a | 152 | 2 | 0.007
K1a3a | 15283 | 1 | 0.003
K1a3a | 15657 | 2 | 0.007
K1a3a | 16189 | 1 | 0.003
K1a3a | 16192 | 1 | 0.003
K1a3a | 16243 | 20 | 0.069
K1a3a | 16265C | 2 | 0.007
K1a3a | 199 | 2 | 0.007
K1a3a | 2056 | 1 | 0.003
K1a3a | 2251 | 1 | 0.003
K1a3a | 310 | 2 | 0.007
K1a3a | 315.2C | 1 | 0.003
K1a3a | 3305 | 1 | 0.003
K1a3a | 3316 | 3 | 0.01
K1a3a | 374 | 1 | 0.003
K1a3a | 4596 | 1 | 0.003
K1a3a | 5333 | 1 | 0.003
K1a3a | 573.1C | 2 | 0.007
K1a3a | 5773 | 1 | 0.003
K1a3a | 6164 | 1 | 0.003
K1a3a | 6719 | 1 | 0.003
K1a3a | 7771 | 2 | 0.007
K1a3a | 8506 | 1 | 0.003
K1a3a | 9300 | 1 | 0.003
K1a3a1 | 10468 | 1 | 0.004
K1a3a1 | 12397 | 1 | 0.004
K1a3a1 | 13212 | 1 | 0.004
K1a3a1 | 13722 | 1 | 0.004
K1a3a1 | 14339 | 1 | 0.004
K1a3a1 | 16167 | 3 | 0.011
K1a3a1 | 16243 | 20 | 0.07
K1a3a1 | 1764 | 1 | 0.004
K1a3a1 | 245 | 1 | 0.004
K1a3a1 | 3398 | 1 | 0.004
K1a3a1 | 401.1T | 1 | 0.004
K1a3a1 | 5102 | 1 | 0.004
K1a3a1 | 573.1C | 2 | 0.007
K1a3a1 | 7340 | 1 | 0.004
K1a3a1a | 16243 | 20 | 0.072
K1a3a1a | 401.1T | 1 | 0.004
K1a3a1a | 573.1C | 2 | 0.007
K1a3a1b | 11151 | 3 | 0.011
K1a3a1b | 16243 | 20 | 0.071
K1a3a1b | 16261 | 1 | 0.004
K1a3a1b | 401.1T | 1 | 0.004
K1a3a1b | 4772G | 1 | 0.004
K1a3a1b | 4973 | 1 | 0.004
K1a3a1b | 573.1C | 2 | 0.007
K1a3a2 | 11362 | 1 | 0.004
K1a3a2 | 12007 | 1 | 0.004
K1a3a2 | 16243 | 20 | 0.072
K1a3a2 | 16422 | 2 | 0.007
K1a3a2 | 401.1T | 1 | 0.004
K1a3a2 | 4216 | 1 | 0.004
K1a3a2 | 5324 | 1 | 0.004
K1a3a2 | 573.1C | 2 | 0.007
K1a3a2 | 6179 | 1 | 0.004
K1a3a2 | 6587 | 1 | 0.004
K1a3a2 | 7229 | 1 | 0.004
K1a3a2 | 8632 | 1 | 0.004

K1a3a3 | 10160 | 1 | 0.004
K1a3a3 | 12811 | 2 | 0.007
K1a3a3 | 13759 | 1 | 0.004
K1a3a3 | 14503 | 2 | 0.007
K1a3a3 | 146 | 2 | 0.007
K1a3a3 | 16234 | 2 | 0.007
K1a3a3 | 16243 | 20 | 0.071
K1a3a3 | 401.1T | 1 | 0.004
K1a3a3 | 4048 | 2 | 0.007
K1a3a3 | 4387 | 2 | 0.007
K1a3a3 | 573.1C | 2 | 0.007
K1a3a3 | 7829 | 1 | 0.004
K1a3a3 | 8742 | 1 | 0.004
K1a3a3 | 9210 | 2 | 0.007
K1a3a3 | 9983 | 1 | 0.004
K1a3a4 | 14312 | 1 | 0.004
K1a3a4 | 16243 | 20 | 0.072
K1a3a4 | 401.1T | 1 | 0.004
K1a3a4 | 573.1C | 2 | 0.007
K1a3a4 | 9921 | 1 | 0.004
K1a4 | 10560 | 1 | 0.003
K1a4 | 11566 | 1 | 0.003
K1a4 | 11914 | 2 | 0.007
K1a4 | 12273 | 1 | 0.003
K1a4 | 12358 | 3 | 0.01
K1a4 | 12362 | 1 | 0.003
K1a4 | 12486 | 1 | 0.003
K1a4 | 12499 | 1 | 0.003
K1a4 | 14287 | 1 | 0.003
K1a4 | 152 | 4 | 0.013
K1a4 | 15412 | 1 | 0.003
K1a4 | 15562 | 1 | 0.003
K1a4 | 15622 | 1 | 0.003
K1a4 | 16041 | 1 | 0.003
K1a4 | 16051 | 3 | 0.01
K1a4 | 1607 | 1 | 0.003
K1a4 | 16093 | 1 | 0.003
K1a4 | 16215 | 1 | 0.003
K1a4 | 16249 | 1 | 0.003
K1a4 | 16304 | 2 | 0.007
K1a4 | 16354 | 23 | 0.076
K1a4 | 2392 | 1 | 0.003
K1a4 | 251 | 1 | 0.003
K1a4 | 3290 | 1 | 0.003
K1a4 | 334 | 20 | 0.066
K1a4 | 3762C | 1 | 0.003
K1a4 | 3970 | 1 | 0.003
K1a4 | 4092 | 1 | 0.003
K1a4 | 4313 | 4 | 0.013
K1a4 | 4688 | 1 | 0.003
K1a4 | 4817 | 1 | 0.003
K1a4 | 525.1AC | 1 | 0.003
K1a4 | 573.1C | 2 | 0.007
K1a4 | 5899.1C | 1 | 0.003
K1a4 | 6437 | 1 | 0.003
K1a4 | 6593 | 1 | 0.003
K1a4 | 7358 | 1 | 0.003
K1a4 | 7844 | 1 | 0.003
K1a4 | 8350C | 2 | 0.007
K1a4 | 8383 | 1 | 0.003
K1a4 | 8588 | 1 | 0.003
K1a4 | 9064 | 4 | 0.013
K1a4 | 9254 | 1 | 0.003
K1a4 | 9266T | 1 | 0.003
K1a4 | 9377 | 1 | 0.003
K1a4 | 9635 | 1 | 0.003
K1a4 | 9667 | 2 | 0.007
K1a4 | 9926 | 1 | 0.003
K1a4+146 | 10203 | 1 | 0.005
K1a4+146 | 13185A | 1 | 0.005
K1a4+146 | 13407 | 1 | 0.005
K1a4+146 | 14079 | 1 | 0.005
K1a4+146 | 14417 | 1 | 0.005
K1a4+146 | 15631 | 1 | 0.005
K1a4+146 | 16168 | 1 | 0.005
K1a4+146 | 16186 | 1 | 0.005
K1a4+146 | 16189 | 1 | 0.005
K1a4+146 | 16209 | 3 | 0.016
K1a4+146 | 16223 | 1 | 0.005
K1a4+146 | 16355 | 1 | 0.005
K1a4+146 | 16399 | 1 | 0.005
K1a4+146 | 16484-16489d | 2 | 0.011
K1a4+146 | 195 | 1 | 0.005
K1a4+146 | 2388 | 1 | 0.005
K1a4+146 | 408A | 1 | 0.005
K1a4+146 | 4232 | 1 | 0.005
K1a4+146 | 5498 | 1 | 0.005
K1a4+146 | 54T | 2 | 0.011
K1a4+146 | 57 | 1 | 0.005
K1a4+146 | 59 | 1 | 0.005
K1a4+146 | 6260 | 2 | 0.011
K1a4+146 | 6863 | 1 | 0.005
K1a4a | 10080 | 2 | 0.007
K1a4a | 10376 | 2 | 0.007
K1a4a | 13566 | 1 | 0.004
K1a4a | 13752 | 1 | 0.004
K1a4a | 150 | 1 | 0.004
K1a4a | 15014 | 1 | 0.004
K1a4a | 15721 | 1 | 0.004
K1a4a | 15727 | 1 | 0.004
K1a4a | 15930 | 1 | 0.004
K1a4a | 16168 | 4 | 0.014
K1a4a | 16234 | 1 | 0.004
K1a4a | 16243 | 20 | 0.07
K1a4a | 195 | 3 | 0.011
K1a4a | 3027 | 1 | 0.004
K1a4a | 573.1C | 2 | 0.007
K1a4a | 575 | 2 | 0.007
K1a4a | 6134 | 3 | 0.011
K1a4a | 6480 | 3 | 0.011
K1a4a | 6510 | 1 | 0.004

K1a4a | 6605 | 2 | 0.007
K1a4a | 7375 | 2 | 0.007
K1a4a | 7859 | 2 | 0.007
K1a4a | 8937 | 1 | 0.004
K1a4a1 | 10143 | 1 | 0.003
K1a4a1 | 10235 | 1 | 0.003
K1a4a1 | 11016 | 1 | 0.003
K1a4a1 | 11419 | 1 | 0.003
K1a4a1 | 12364 | 2 | 0.006
K1a4a1 | 12618 | 1 | 0.003
K1a4a1 | 12684 | 2 | 0.006
K1a4a1 | 13135 | 1 | 0.003
K1a4a1 | 13153 | 3 | 0.009
K1a4a1 | 1342 | 1 | 0.003
K1a4a1 | 14053 | 1 | 0.003
K1a4a1 | 14064 | 1 | 0.003
K1a4a1 | 14305 | 2 | 0.006
K1a4a1 | 14470 | 1 | 0.003
K1a4a1 | 14577 | 1 | 0.003
K1a4a1 | 146 | 5 | 0.016
K1a4a1 | 14809 | 1 | 0.003
K1a4a1 | 14950G | 1 | 0.003
K1a4a1 | 150 | 2 | 0.006
K1a4a1 | 15154A | 2 | 0.006
K1a4a1 | 15166 | 1 | 0.003
K1a4a1 | 153 | 1 | 0.003
K1a4a1 | 15314 | 1 | 0.003
K1a4a1 | 15317 | 1 | 0.003
K1a4a1 | 15404 | 2 | 0.006
K1a4a1 | 15478 | 1 | 0.003
K1a4a1 | 15547 | 1 | 0.003
K1a4a1 | 15777 | 1 | 0.003
K1a4a1 | 15889 | 2 | 0.006
K1a4a1 | 16048 | 1 | 0.003
K1a4a1 | 16086 | 3 | 0.009
K1a4a1 | 16129 | 1 | 0.003
K1a4a1 | 16162C | 1 | 0.003
K1a4a1 | 16172 | 1 | 0.003
K1a4a1 | 16189 | 1 | 0.003
K1a4a1 | 16192A | 1 | 0.003
K1a4a1 | 16222 | 1 | 0.003
K1a4a1 | 16256 | 1 | 0.003
K1a4a1 | 16257 | 23 | 0.072
K1a4a1 | 16264 | 1 | 0.003
K1a4a1 | 16265C | 1 | 0.003
K1a4a1 | 1628 | 2 | 0.006
K1a4a1 | 16298 | 1 | 0.003
K1a4a1 | 16305 | 2 | 0.006
K1a4a1 | 16352 | 1 | 0.003
K1a4a1 | 16354 | 1 | 0.003
K1a4a1 | 189 | 2 | 0.006
K1a4a1 | 195 | 4 | 0.012
K1a4a1 | 200 | 2 | 0.006
K1a4a1 | 204 | 1 | 0.003
K1a4a1 | 235 | 1 | 0.003
K1a4a1 | 236 | 1 | 0.003
K1a4a1 | 2361 | 2 | 0.006
K1a4a1 | 2417A | 1 | 0.003
K1a4a1 | 257 | 2 | 0.006
K1a4a1 | 2630 | 1 | 0.003
K1a4a1 | 297 | 1 | 0.003
K1a4a1 | 310 | 1 | 0.003
K1a4a1 | 3547 | 2 | 0.006
K1a4a1 | 3796 | 2 | 0.006
K1a4a1 | 3990 | 1 | 0.003
K1a4a1 | 4076 | 1 | 0.003
K1a4a1 | 4546 | 1 | 0.003
K1a4a1 | 471 | 1 | 0.003
K1a4a1 | 4777 | 1 | 0.003
K1a4a1 | 4853 | 1 | 0.003
K1a4a1 | 4931 | 1 | 0.003
K1a4a1 | 5021 | 2 | 0.006
K1a4a1 | 5093 | 1 | 0.003
K1a4a1 | 511 | 1 | 0.003
K1a4a1 | 5153 | 1 | 0.003
K1a4a1 | 533 | 1 | 0.003
K1a4a1 | 5474 | 2 | 0.006
K1a4a1 | 548 | 1 | 0.003
K1a4a1 | 573.1C | 2 | 0.006
K1a4a1 | 6101 | 1 | 0.003
K1a4a1 | 6254 | 1 | 0.003
K1a4a1 | 7269 | 1 | 0.003
K1a4a1 | 736 | 1 | 0.003
K1a4a1 | 7409 | 1 | 0.003
K1a4a1 | 7948 | 1 | 0.003
K1a4a1 | 8032 | 1 | 0.003
K1a4a1 | 8047 | 1 | 0.003
K1a4a1 | 8104 | 1 | 0.003
K1a4a1 | 8155 | 1 | 0.003
K1a4a1 | 8521 | 1 | 0.003
K1a4a1 | 8857 | 1 | 0.003
K1a4a1 | 8994 | 1 | 0.003
K1a4a1 | 9099 | 1 | 0.003
K1a4a1 | 93 | 2 | 0.006
K1a4a1 | 9300 | 1 | 0.003
K1a4a1 | 95C | 1 | 0.003
K1a4a1 | 9633G | 1 | 0.003
K1a4a1 | 9957 | 1 | 0.003
K1a4a1a | 10586 | 1 | 0.004
K1a4a1a | 12642 | 1 | 0.004
K1a4a1a | 131 | 2 | 0.007
K1a4a1a | 13886 | 1 | 0.004
K1a4a1a | 14127 | 1 | 0.004
K1a4a1a | 14502 | 1 | 0.004
K1a4a1a | 16293 | 20 | 0.074
K1a4a1a | 16362 | 1 | 0.004
K1a4a1a | 401.1T | 1 | 0.004
K1a4a1a | 5711 | 1 | 0.004
K1a4a1a | 573.1C | 3 | 0.011
K1a4a1a+195 | 10007 | 1 | 0.005
K1a4a1a+195 | 11144T | 1 | 0.005

K1a4a1a+195 | 11152 | 2 | 0.009
K1a4a1a+195 | 12904 | 1 | 0.005
K1a4a1a+195 | 14325 | 1 | 0.005
K1a4a1a+195 | 146 | 1 | 0.005
K1a4a1a+195 | 14793 | 1 | 0.005
K1a4a1a+195 | 15849 | 1 | 0.005
K1a4a1a+195 | 16246T | 1 | 0.005
K1a4a1a+195 | 16261 | 1 | 0.005
K1a4a1a+195 | 16319 | 1 | 0.005
K1a4a1a+195 | 16325 | 3 | 0.014
K1a4a1a+195 | 279 | 1 | 0.005
K1a4a1a+195 | 309d | 1 | 0.005
K1a4a1a+195 | 310 | 1 | 0.005
K1a4a1a+195 | 452 | 1 | 0.005
K1a4a1a+195 | 513 | 1 | 0.005
K1a4a1a+195 | 9123 | 1 | 0.005
K1a4a1a+195 | 94 | 1 | 0.005
K1a4a1a1 | 15927 | 1 | 0.005
K1a4a1a1 | 16167 | 1 | 0.005
K1a4a1a1 | 16172 | 1 | 0.005
K1a4a1a1 | 16246T | 1 | 0.005
K1a4a1a1 | 16256 | 1 | 0.005
K1a4a1a1 | 16325 | 3 | 0.015
K1a4a1a1 | 279 | 1 | 0.005
K1a4a1a1 | 310 | 1 | 0.005
K1a4a1a1 | 3745 | 1 | 0.005
K1a4a1a1 | 452 | 1 | 0.005
K1a4a1a1 | 7711 | 1 | 0.005
K1a4a1a1 | 8901 | 1 | 0.005
K1a4a1a1 | 94 | 1 | 0.005
K1a4a1a1 | 9804 | 1 | 0.005
K1a4a1a2 | 10527 | 1 | 0.038
K1a4a1a2 | 12879 | 1 | 0.038
K1a4a1a2 | 13434 | 1 | 0.038
K1a4a1a2 | 13885G | 1 | 0.038
K1a4a1a2 | 13886 | 1 | 0.038
K1a4a1a2 | 146 | 1 | 0.038
K1a4a1a2 | 16140 | 1 | 0.038
K1a4a1a2 | 16266 | 1 | 0.038
K1a4a1a2 | 3553 | 1 | 0.038
K1a4a1a2 | 5147 | 1 | 0.038
K1a4a1a2 | 5508 | 1 | 0.038
K1a4a1a2 | 567C | 1 | 0.038
K1a4a1a2 | 5711 | 1 | 0.038
K1a4a1a2 | 573.1C | 1 | 0.038
K1a4a1a2 | 573.5C | 1 | 0.038
K1a4a1a2 | 722 | 1 | 0.038
K1a4a1a2 | 7609 | 1 | 0.038
K1a4a1a2 | 8697 | 1 | 0.038
K1a4a1a2 | 9938 | 1 | 0.038
K1a4a1a2a | 12616 | 1 | 0.028
K1a4a1a2a | 146 | 1 | 0.028
K1a4a1a2a | 16037 | 2 | 0.056
K1a4a1a2a | 16140 | 1 | 0.028
K1a4a1a2a | 16295 | 2 | 0.056
K1a4a1a2a | 16527 | 1 | 0.028
K1a4a1a2a | 2080 | 1 | 0.028
K1a4a1a2a | 310 | 5 | 0.139
K1a4a1a2a | 311 | 1 | 0.028
K1a4a1a2a | 315.2C | 2 | 0.056
K1a4a1a2a | 3505 | 1 | 0.028
K1a4a1a2a | 4171 | 1 | 0.028
K1a4a1a2a | 5147 | 1 | 0.028
K1a4a1a2a | 567C | 1 | 0.028
K1a4a1a2a | 573.1C | 1 | 0.028
K1a4a1a2a | 573.2C | 8 | 0.222
K1a4a1a2a | 573.4C | 1 | 0.028
K1a4a1a2a | 573.5C | 1 | 0.028
K1a4a1a2b | 10777 | 1 | 0.023
K1a4a1a2b | 153 | 1 | 0.023
K1a4a1a2b | 15773 | 1 | 0.023
K1a4a1a2b | 195 | 1 | 0.023
K1a4a1a2b | 310 | 3 | 0.07
K1a4a1a2b | 3646 | 1 | 0.023
K1a4a1a2b | 4655 | 1 | 0.023
K1a4a1a2b | 5460 | 1 | 0.023
K1a4a1a2b | 6629 | 1 | 0.023
K1a4a1a2b | 6752 | 1 | 0.023
K1a4a1a2b | 8521 | 1 | 0.023
K1a4a1a2b | 9214 | 2 | 0.047
K1a4a1a3 | 152 | 1 | 0.005
K1a4a1a3 | 16037 | 5 | 0.025
K1a4a1a3 | 16167 | 1 | 0.005
K1a4a1a3 | 16172 | 1 | 0.005
K1a4a1a3 | 16187 | 1 | 0.005
K1a4a1a3 | 16246T | 1 | 0.005
K1a4a1a3 | 16256 | 1 | 0.005
K1a4a1a3 | 16278 | 5 | 0.025
K1a4a1a3 | 16325 | 3 | 0.015
K1a4a1a3 | 207 | 5 | 0.025
K1a4a1a3 | 279 | 1 | 0.005
K1a4a1a3 | 452 | 1 | 0.005
K1a4a1a3 | 94 | 1 | 0.005
K1a4a1b | 13105 | 1 | 0.004
K1a4a1b | 13989 | 1 | 0.004
K1a4a1b | 146 | 1 | 0.004
K1a4a1b | 15099 | 1 | 0.004
K1a4a1b | 152 | 1 | 0.004
K1a4a1b | 15300 | 1 | 0.004
K1a4a1b | 16129 | 1 | 0.004
K1a4a1b | 16176 | 1 | 0.004
K1a4a1b | 16243 | 20 | 0.072
K1a4a1b | 401.1T | 1 | 0.004
K1a4a1b | 573.1C | 2 | 0.007
K1a4a1b | 5894 | 1 | 0.004
K1a4a1b | 9053 | 1 | 0.004
K1a4a1b | 9962 | 1 | 0.004
K1a4a1b1 | 12957 | 1 | 0.004
K1a4a1b1 | 14384 | 1 | 0.004
K1a4a1b1 | 15508 | 2 | 0.007
K1a4a1b1 | 16188 | 1 | 0.004

K1a4a1b1 | 16243 | 20 | 0.071
K1a4a1b1 | 195 | 1 | 0.004
K1a4a1b1 | 401.1T | 1 | 0.004
K1a4a1b1 | 573.1C | 2 | 0.007
K1a4a1b1 | 6951 | 2 | 0.007
K1a4a1b2 | 12477 | 1 | 0.077
K1a4a1b2 | 12528 | 2 | 0.154
K1a4a1b2 | 14317 | 1 | 0.077
K1a4a1b2 | 15175 | 2 | 0.154
K1a4a1b2 | 16278 | 2 | 0.154
K1a4a1b2 | 1842 | 1 | 0.077
K1a4a1b2 | 6465C | 1 | 0.077
K1a4a1b2 | 8095 | 1 | 0.077
K1a4a1b2 | 9438 | 2 | 0.154
K1a4a1b2 | 9804 | 2 | 0.154
K1a4a1b2 | 98G | 1 | 0.077
K1a4a1c | 16243 | 20 | 0.072
K1a4a1c | 401.1T | 1 | 0.004
K1a4a1c | 573.1C | 2 | 0.007
K1a4a1c | 6428 | 1 | 0.004
K1a4a1c | 7247 | 1 | 0.004
K1a4a1c1 | 12811 | 1 | 0.006
K1a4a1c1 | 152 | 3 | 0.017
K1a4a1c1 | 16075 | 1 | 0.006
K1a4a1c1 | 16077 | 1 | 0.006
K1a4a1c1 | 16172 | 3 | 0.017
K1a4a1c1 | 16180 | 5 | 0.029
K1a4a1c1 | 16231 | 2 | 0.012
K1a4a1c1 | 16263 | 1 | 0.006
K1a4a1c1 | 16354 | 1 | 0.006
K1a4a1c1 | 1836 | 3 | 0.017
K1a4a1c1 | 204 | 2 | 0.012
K1a4a1c1 | 286 | 2 | 0.012
K1a4a1c1 | 4113 | 3 | 0.017
K1a4a1c1 | 44.1C | 5 | 0.029
K1a4a1c1 | 4848C | 1 | 0.006
K1a4a1c1 | 5561 | 3 | 0.017
K1a4a1c1 | 7298T | 3 | 0.017
K1a4a1c1 | 7609 | 1 | 0.006
K1a4a1d | 10493 | 1 | 0.004
K1a4a1d | 14082 | 1 | 0.004
K1a4a1d | 16243 | 20 | 0.072
K1a4a1d | 401.1T | 1 | 0.004
K1a4a1d | 5093 | 2 | 0.007
K1a4a1d | 573.1C | 2 | 0.007
K1a4a1e | 14470 | 1 | 0.019
K1a4a1e | 15236 | 1 | 0.019
K1a4a1e | 16051 | 2 | 0.038
K1a4a1e | 16129 | 2 | 0.038
K1a4a1e | 16158 | 1 | 0.019
K1a4a1e | 16176 | 3 | 0.057
K1a4a1e | 16218 | 5 | 0.094
K1a4a1e | 16278 | 2 | 0.038
K1a4a1e | 16311 | 4 | 0.075
K1a4a1e | 195 | 1 | 0.019
K1a4a1e | 200 | 3 | 0.057
K1a4a1e | 203 | 1 | 0.019
K1a4a1e | 204 | 4 | 0.075
K1a4a1e | 310 | 1 | 0.019
K1a4a1e | 3866G | 1 | 0.019
K1a4a1e | 4098 | 1 | 0.019
K1a4a1e | 471 | 2 | 0.038
K1a4a1e | 5021 | 3 | 0.057
K1a4a1e | 6338 | 3 | 0.057
K1a4a1e | 7408C | 1 | 0.019
K1a4a1e | 7471d | 1 | 0.019
K1a4a1e | 7976 | 1 | 0.019
K1a4a1e | 8610 | 2 | 0.038
K1a4a1e | 8939 | 2 | 0.038
K1a4a1e | 9266 | 1 | 0.019
K1a4a1f | 10370 | 1 | 0.006
K1a4a1f | 15236 | 1 | 0.006
K1a4a1f | 16357 | 1 | 0.006
K1a4a1f1 | 13753 | 1 | 0.006
K1a4a1f1 | 3441 | 1 | 0.006
K1a4a1g | 152 | 1 | 0.111
K1a4a1g | 2361 | 1 | 0.111
K1a4a1g | 3388A | 1 | 0.111
K1a4a1g | 5090 | 1 | 0.111
K1a4a1g | 7736 | 1 | 0.111
K1a4a1g | 96A | 1 | 0.111
K1a4a1h | 11204 | 1 | 0.004
K1a4a1h | 11545 | 1 | 0.004
K1a4a1h | 11841 | 1 | 0.004
K1a4a1h | 13590 | 1 | 0.004
K1a4a1h | 14180 | 1 | 0.004
K1a4a1h | 152 | 2 | 0.007
K1a4a1h | 16243 | 20 | 0.071
K1a4a1h | 16294 | 1 | 0.004
K1a4a1h | 195 | 1 | 0.004
K1a4a1h | 310 | 1 | 0.004
K1a4a1h | 3834 | 1 | 0.004
K1a4a1h | 401.1T | 1 | 0.004
K1a4a1h | 573.1C | 2 | 0.007
K1a4a1h | 8461 | 1 | 0.004
K1a4a1h | 8557 | 2 | 0.007
K1a4a1i | 15954 | 2 | 0.008
K1a4a1i | 16261 | 1 | 0.004
K1a4a1i | 16265 | 1 | 0.004
K1a4a1i | 16293 | 20 | 0.075
K1a4a1i | 16479G | 2 | 0.008
K1a4a1i | 16482 | 2 | 0.008
K1a4a1i | 16511 | 2 | 0.008
K1a4a1i | 401.1T | 1 | 0.004
K1a4a1i | 515-524d | 1 | 0.004
K1a4a1i | 573.1C | 2 | 0.008
K1a4a1i | 60.1T | 1 | 0.004
K1a4a1i | 8343 | 1 | 0.004
K1a4b | 12693 | 1 | 0.005
K1a4b | 13105 | 2 | 0.01
K1a4b | 13281 | 1 | 0.005

K1a4b | 13590 | 1 | 0.005
K1a4b | 13993 | 1 | 0.005
K1a4b | 14470 | 1 | 0.005
K1a4b | 152 | 1 | 0.005
K1a4b | 16080 | 3 | 0.015
K1a4b | 16239 | 1 | 0.005
K1a4b | 16242 | 1 | 0.005
K1a4b | 16247 | 1 | 0.005
K1a4b | 16290 | 7 | 0.036
K1a4b | 16292 | 3 | 0.015
K1a4b | 195 | 3 | 0.015
K1a4b | 310 | 1 | 0.005
K1a4b | 315.2C | 1 | 0.005
K1a4b | 4314 | 1 | 0.005
K1a4b | 5231 | 1 | 0.005
K1a4b | 6173 | 2 | 0.01
K1a4b | 6569 | 1 | 0.005
K1a4b | 6899 | 1 | 0.005
K1a4b | 7394 | 1 | 0.005
K1a4b | 7648 | 1 | 0.005
K1a4b | 8029 | 2 | 0.01
K1a4b | 9058 | 1 | 0.005
K1a4b | 93 | 1 | 0.005
K1a4b | 9569 | 1 | 0.005
K1a4b1 | 11654 | 1 | 0.005
K1a4b1 | 11800 | 1 | 0.005
K1a4b1 | 121C | 5 | 0.025
K1a4b1 | 13395 | 6 | 0.03
K1a4b1 | 146 | 4 | 0.02
K1a4b1 | 150 | 1 | 0.005
K1a4b1 | 152 | 1 | 0.005
K1a4b1 | 16124 | 8 | 0.04
K1a4b1 | 16242 | 1 | 0.005
K1a4b1 | 16271 | 1 | 0.005
K1a4b1 | 16343 | 6 | 0.03
K1a4b1 | 524.2A | 1 | 0.005
K1a4b1 | 5747 | 6 | 0.03
K1a4b1 | 6023 | 7 | 0.035
K1a4c | 12030 | 1 | 0.005
K1a4c | 15097 | 1 | 0.005
K1a4c | 15214 | 1 | 0.005
K1a4c | 15892 | 1 | 0.005
K1a4c | 16129 | 1 | 0.005
K1a4c | 16158 | 4 | 0.02
K1a4c | 16234 | 2 | 0.01
K1a4c | 280G | 1 | 0.005
K1a4c | 4586 | 1 | 0.005
K1a4c | 485 | 8 | 0.039
K1a4c | 5628 | 1 | 0.005
K1a4c | 7924 | 1 | 0.005
K1a4c1 | 10101 | 1 | 0.023
K1a4c1 | 10410 | 2 | 0.047
K1a4c1 | 10834 | 1 | 0.023
K1a4c1 | 114 | 2 | 0.047
K1a4c1 | 11582 | 2 | 0.047
K1a4c1 | 11782 | 2 | 0.047
K1a4c1 | 12173 | 1 | 0.023
K1a4c1 | 1250 | 2 | 0.047
K1a4c1 | 13098 | 3 | 0.07
K1a4c1 | 13359 | 1 | 0.023
K1a4c1 | 13708 | 1 | 0.023
K1a4c1 | 143 | 1 | 0.023
K1a4c1 | 14581 | 1 | 0.023
K1a4c1 | 151 | 1 | 0.023
K1a4c1 | 158A | 1 | 0.023
K1a4c1 | 16169 | 4 | 0.093
K1a4c1 | 16261 | 3 | 0.07
K1a4c1 | 16265 | 3 | 0.07
K1a4c1 | 215 | 1 | 0.023
K1a4c1 | 6267 | 1 | 0.023
K1a4c1 | 7241 | 1 | 0.023
K1a4c1 | 7325 | 1 | 0.023
K1a4c1 | 9758 | 1 | 0.023
K1a4c1 | 9759 | 1 | 0.023
K1a4d | 11296 | 1 | 0.003
K1a4d | 11482 | 1 | 0.003
K1a4d | 11491 | 1 | 0.003
K1a4d | 12007 | 1 | 0.003
K1a4d | 152 | 2 | 0.007
K1a4d | 15202 | 1 | 0.003
K1a4d | 15226 | 1 | 0.003
K1a4d | 153 | 4 | 0.013
K1a4d | 16294 | 22 | 0.073
K1a4d | 195 | 1 | 0.003
K1a4d | 2789 | 1 | 0.003
K1a4d | 310 | 4 | 0.013
K1a4d | 315d | 1 | 0.003
K1a4d | 3203 | 1 | 0.003
K1a4d | 3699G | 1 | 0.003
K1a4d | 4381 | 1 | 0.003
K1a4d | 5063 | 2 | 0.007
K1a4d | 525.1AC | 1 | 0.003
K1a4d | 573.1C | 2 | 0.007
K1a4d | 64 | 1 | 0.003
K1a4d | 7151 | 6 | 0.02
K1a4d | 7270 | 2 | 0.007
K1a4d | 8152 | 2 | 0.007
K1a4d | 8238 | 2 | 0.007
K1a4d | 8292 | 1 | 0.003
K1a4e | 10560 | 1 | 0.004
K1a4e | 12358 | 3 | 0.011
K1a4e | 16169 | 6 | 0.021
K1a4e | 16243 | 20 | 0.071
K1a4e | 16438 | 3 | 0.011
K1a4e | 2270 | 2 | 0.007
K1a4e | 401.1T | 1 | 0.004
K1a4e | 573.1C | 2 | 0.007
K1a4e | 8588 | 1 | 0.004
K1a4e | 9667 | 1 | 0.004
K1a4f | 11447 | 2 | 0.007
K1a4f | 12630 | 1 | 0.003

K1a4f | 13651 | 1 | 0.003
K1a4f | 13708 | 1 | 0.003
K1a4f | 152 | 3 | 0.01
K1a4f | 15514 | 1 | 0.003
K1a4f | 16189 | 1 | 0.003
K1a4f | 16356 | 20 | 0.07
K1a4f | 279 | 28 | 0.098
K1a4f | 310 | 1 | 0.003
K1a4f | 3338 | 1 | 0.003
K1a4f | 4002 | 1 | 0.003
K1a4f | 411A | 1 | 0.003
K1a4f | 4959 | 4 | 0.014
K1a4f | 573.1C | 2 | 0.007
K1a4f | 5910 | 1 | 0.003
K1a4f | 6734 | 2 | 0.007
K1a4f1 | 10202 | 1 | 0.004
K1a4f1 | 12399 | 1 | 0.004
K1a4f1 | 13708 | 1 | 0.004
K1a4f1 | 1413d | 1 | 0.004
K1a4f1 | 1415 | 1 | 0.004
K1a4f1 | 14181 | 1 | 0.004
K1a4f1 | 14569 | 1 | 0.004
K1a4f1 | 1461C | 1 | 0.004
K1a4f1 | 15466 | 1 | 0.004
K1a4f1 | 15734 | 1 | 0.004
K1a4f1 | 16086 | 1 | 0.004
K1a4f1 | 16129 | 1 | 0.004
K1a4f1 | 16177 | 1 | 0.004
K1a4f1 | 16243 | 20 | 0.071
K1a4f1 | 16390 | 1 | 0.004
K1a4f1 | 2723.1A | 1 | 0.004
K1a4f1 | 2739.1T | 1 | 0.004
K1a4f1 | 401.1T | 1 | 0.004
K1a4f1 | 480 | 1 | 0.004
K1a4f1 | 4924 | 1 | 0.004
K1a4f1 | 524.1ACC | 1 | 0.004
K1a4f1 | 573.1C | 2 | 0.007
K1a4f1 | 6465 | 1 | 0.004
K1a4f1 | 951 | 1 | 0.004
K1a4g | 10961 | 2 | 0.007
K1a4g | 13681 | 2 | 0.007
K1a4g | 150 | 1 | 0.004
K1a4g | 15613 | 2 | 0.007
K1a4g | 16189 | 1 | 0.004
K1a4g | 16243 | 20 | 0.071
K1a4g | 264 | 5 | 0.018
K1a4g | 401.1T | 1 | 0.004
K1a4g | 4622 | 1 | 0.004
K1a4g | 573.1C | 2 | 0.007
K1a4g | 8347T | 1 | 0.004
K1a4g | 8715 | 1 | 0.004
K1a4h | 11167 | 1 | 0.004
K1a4h | 15497 | 1 | 0.004
K1a4h | 16243 | 20 | 0.072
K1a4h | 401.1T | 1 | 0.004
K1a4h | 573.1C | 2 | 0.007
K1a4h | 8642 | 1 | 0.004
K1a4h1 | 15930 | 2 | 0.007
K1a4h1 | 16166 | 1 | 0.004
K1a4h1 | 16243 | 20 | 0.072
K1a4h1 | 207 | 2 | 0.007
K1a4h1 | 3010 | 2 | 0.007
K1a4h1 | 401.1T | 1 | 0.004
K1a4h1 | 525.1AC | 1 | 0.004
K1a4h1 | 573.1C | 2 | 0.007
K1a4i | 11269 | 1 | 0.003
K1a4i | 13830 | 5 | 0.017
K1a4i | 153 | 6 | 0.021
K1a4i | 15884 | 1 | 0.003
K1a4i | 16189 | 1 | 0.003
K1a4i | 16243 | 20 | 0.069
K1a4i | 195 | 1 | 0.003
K1a4i | 310 | 1 | 0.003
K1a4i | 417 | 1 | 0.003
K1a4i | 524.1GC | 1 | 0.003
K1a4i | 573.1C | 2 | 0.007
K1a4i | 7169 | 1 | 0.003
K1a4i | 8790 | 1 | 0.003
K1a4j | 13145 | 1 | 0.006
K1a4j | 14470 | 1 | 0.006
K1a4j | 14569 | 1 | 0.006
K1a4j | 16189 | 1 | 0.006
K1a4j | 16209 | 3 | 0.017
K1a4j | 16222 | 1 | 0.006
K1a4j | 16223 | 1 | 0.006
K1a4j | 16390 | 1 | 0.006
K1a4j | 16484-16489d | 2 | 0.011
K1a4j | 54T | 2 | 0.011
K1a4j | 57 | 1 | 0.006
K1a4j | 59 | 1 | 0.006
K1a4j1 | 14025 | 2 | 0.01
K1a4j1 | 14122 | 1 | 0.005
K1a4j1 | 15317 | 1 | 0.005
K1a4j1 | 16093 | 1 | 0.005
K1a4j1 | 16129 | 1 | 0.005
K1a4j1 | 16360 | 9 | 0.044
K1a4j1 | 316C | 2 | 0.01
K1a4j1 | 3398 | 2 | 0.01
K1a4j1 | 5054 | 1 | 0.005
K1a4j1 | 5585 | 1 | 0.005
K1a4j1 | 5899.1C | 1 | 0.005
K1a4j1 | 8501 | 2 | 0.01
K1a4j1 | 8856 | 1 | 0.005
K1a4j1 | 9130 | 2 | 0.01
K1a4j1 | 990 | 1 | 0.005
K1a5 | 11917 | 2 | 0.007
K1a5 | 13500 | 1 | 0.004
K1a5 | 14767 | 2 | 0.007
K1a5 | 152 | 2 | 0.007
K1a5 | 16172 | 2 | 0.007
K1a5 | 16231 | 2 | 0.007

K1a5 | 16243 | 20 | 0.072
K1a5 | 195 | 1 | 0.004
K1a5 | 199 | 2 | 0.007
K1a5 | 286 | 2 | 0.007
K1a5 | 309d | 1 | 0.004
K1a5 | 401.1T | 1 | 0.004
K1a5 | 5074 | 2 | 0.007
K1a5 | 573.1C | 2 | 0.007
K1a5 | 8478 | 1 | 0.004
K1a5 | 9932 | 1 | 0.004
K1a5a | 13834 | 1 | 0.024
K1a5a | 146 | 1 | 0.024
K1a5a | 152 | 1 | 0.024
K1a5a | 16093 | 1 | 0.024
K1a5a | 16227 | 1 | 0.024
K1a5a | 16256 | 1 | 0.024
K1a5a | 16266 | 3 | 0.071
K1a5a | 195 | 1 | 0.024
K1a5a | 198 | 1 | 0.024
K1a5a | 295A | 2 | 0.048
K1a5a | 3200 | 1 | 0.024
K1a5a | 524.2A | 2 | 0.048
K1a5a | 5752d | 1 | 0.024
K1a5b | 152 | 1 | 0.006
K1a5b | 204 | 1 | 0.006
K1a5b | 408 | 1 | 0.006
K1a5b | 4083 | 1 | 0.006
K1a5b | 93 | 1 | 0.006
K1a6 | 143 | 1 | 0.008
K1a6 | 146 | 1 | 0.008
K1a6 | 16129 | 1 | 0.008
K1a6 | 1692 | 1 | 0.008
K1a6 | 290 | 1 | 0.008
K1a6 | 3591 | 1 | 0.008
K1a6 | 5325 | 1 | 0.008
K1a6 | 573.3C | 1 | 0.008
K1a6 | 6689 | 1 | 0.008
K1a6 | 7867 | 1 | 0.008
K1a6 | 8703 | 16 | 0.134
K1a7 | 12425 | 1 | 0.004
K1a7 | 12561 | 2 | 0.009
K1a7 | 13174 | 1 | 0.004
K1a7 | 14053 | 1 | 0.004
K1a7 | 16017 | 1 | 0.004
K1a7 | 16234 | 1 | 0.004
K1a7 | 16260 | 22 | 0.095
K1a7 | 4310 | 1 | 0.004
K1a7 | 4742 | 1 | 0.004
K1a7 | 4745 | 1 | 0.004
K1a8 | 14259 | 1 | 0.005
K1a8 | 15893 | 1 | 0.005
K1a8 | 16223 | 4 | 0.021
K1a8 | 3398 | 1 | 0.005
K1a8 | 5262 | 1 | 0.005
K1a8 | 9422 | 1 | 0.005
K1a8a | 13368 | 1 | 0.111
K1a8a | 146 | 1 | 0.111
K1a8a | 150 | 1 | 0.111
K1a8a | 16037 | 1 | 0.111
K1a8a | 16111 | 1 | 0.111
K1a8a1 | 12952 | 1 | 0.111
K1a8a1 | 5821 | 1 | 0.111
K1a8a1 | 8215 | 1 | 0.111
K1a8a1 | 9116 | 1 | 0.111
K1a8b | 10293 | 1 | 0.013
K1a8b | 10294 | 1 | 0.013
K1a8b | 10373 | 2 | 0.026
K1a8b | 11151 | 1 | 0.013
K1a8b | 13614 | 2 | 0.026
K1a8b | 152 | 3 | 0.038
K1a8b | 16145 | 1 | 0.013
K1a8b | 16172 | 1 | 0.013
K1a8b | 16214 | 1 | 0.013
K1a8b | 16239 | 1 | 0.013
K1a8b | 16266 | 1 | 0.013
K1a8b | 16297A | 1 | 0.013
K1a8b | 16354 | 1 | 0.013
K1a8b | 295 | 3 | 0.038
K1a8b | 5460 | 2 | 0.026
K1a8b | 5461 | 1 | 0.013
K1a8b | 7159 | 2 | 0.026
K1a8b | 7624 | 1 | 0.013
K1a8b | 8477 | 1 | 0.013
K1a8b | 9554 | 1 | 0.013
K1a9 | 10601 | 1 | 0.009
K1a9 | 11287 | 1 | 0.009
K1a9 | 11453 | 1 | 0.009
K1a9 | 13629 | 1 | 0.009
K1a9 | 14160 | 1 | 0.009
K1a9 | 14831 | 1 | 0.009
K1a9 | 152 | 4 | 0.035
K1a9 | 15758 | 1 | 0.009
K1a9 | 15924 | 1 | 0.009
K1a9 | 16093 | 3 | 0.026
K1a9 | 16266 | 1 | 0.009
K1a9 | 16270 | 1 | 0.009
K1a9 | 16526 | 1 | 0.009
K1a9 | 16527 | 1 | 0.009
K1a9 | 2258 | 3 | 0.026
K1a9 | 3338 | 1 | 0.009
K1a9 | 573.1C | 1 | 0.009
K1a9 | 6515 | 1 | 0.009
K1a9 | 7568 | 1 | 0.009
K1a9 | 8723 | 1 | 0.009
K1b | 131 | 1 | 0.005
K1b | 16263 | 2 | 0.01
K1b | 16355 | 19 | 0.099
K1b | 480 | 1 | 0.005
K1b1 | 16074 | 1 | 0.005
K1b1 | 16195 | 2 | 0.011
K1b1 | 16264 | 2 | 0.011

K1b1 | 16300 | 7 | 0.037
K1b1 | 16392d | 18 | 0.095
K1b1 | 198 | 2 | 0.011
K1b1 | 200 | 2 | 0.011
K1b1 | 240 | 1 | 0.005
K1b1 | 332d | 1 | 0.005
K1b1+(16093) | 16261 | 20 | 0.088
K1b1+(16093) | 195 | 20 | 0.088
K1b1a | 143 | 3 | 0.057
K1b1a | 146 | 3 | 0.057
K1b1a | 151 | 1 | 0.019
K1b1a | 15877 | 1 | 0.019
K1b1a | 16145 | 1 | 0.019
K1b1a | 16167 | 3 | 0.057
K1b1a | 16178 | 1 | 0.019
K1b1a | 16189 | 1 | 0.019
K1b1a | 16209 | 3 | 0.057
K1b1a | 3330 | 1 | 0.019
K1b1a | 3357 | 1 | 0.019
K1b1a | 390T | 1 | 0.019
K1b1a | 4763 | 1 | 0.019
K1b1a | 505 | 1 | 0.019
K1b1a | 6290 | 1 | 0.019
K1b1a | 9746 | 1 | 0.019
K1b1a1 | 10389 | 1 | 0.018
K1b1a1 | 1041 | 1 | 0.018
K1b1a1 | 13602 | 1 | 0.018
K1b1a1 | 16093 | 2 | 0.036
K1b1a1 | 16168 | 1 | 0.018
K1b1a1 | 16284 | 1 | 0.018
K1b1a1 | 1717 | 1 | 0.018
K1b1a1 | 2262 | 2 | 0.036
K1b1a1 | 310 | 1 | 0.018
K1b1a1 | 315.2C | 1 | 0.018
K1b1a1 | 3316 | 1 | 0.018
K1b1a1 | 4646 | 1 | 0.018
K1b1a1 | 513 | 1 | 0.018
K1b1a1 | 5267 | 1 | 0.018
K1b1a1 | 573.1C | 1 | 0.018
K1b1a1 | 61 | 1 | 0.018
K1b1a1 | 64 | 1 | 0.018
K1b1a1 | 8429 | 2 | 0.036
K1b1a1 | 8805 | 2 | 0.036
K1b1a1+199 | 10084 | 1 | 0.05
K1b1a1+199 | 12723 | 1 | 0.05
K1b1a1+199 | 14560 | 2 | 0.1
K1b1a1+199 | 15884 | 2 | 0.1
K1b1a1+199 | 16093 | 2 | 0.1
K1b1a1+199 | 16532d | 1 | 0.05
K1b1a1+199 | 3200A | 1 | 0.05
K1b1a1+199 | 3200T | 1 | 0.05
K1b1a1+199 | 6710 | 1 | 0.05
K1b1a1+199 | 8429 | 1 | 0.05
K1b1a1+199 | 8805 | 1 | 0.05
K1b1a1+199 | 9468 | 1 | 0.05
K1b1a1+199 | 9545 | 1 | 0.05
K1b1a1+199 | 9813A | 1 | 0.05
K1b1a1a | 11084 | 1 | 0.031
K1b1a1a | 11204 | 1 | 0.031
K1b1a1a | 13743 | 1 | 0.031
K1b1a1a | 14053 | 1 | 0.031
K1b1a1a | 14544 | 1 | 0.031
K1b1a1a | 146 | 1 | 0.031
K1b1a1a | 15314 | 1 | 0.031
K1b1a1a | 15458 | 1 | 0.031
K1b1a1a | 16248 | 1 | 0.031
K1b1a1a | 16270 | 2 | 0.062
K1b1a1a | 16362 | 1 | 0.031
K1b1a1a | 5390 | 1 | 0.031
K1b1a1a | 5426 | 1 | 0.031
K1b1a1a | 6465 | 1 | 0.031
K1b1a1a | 7364 | 1 | 0.031
K1b1a1a | 994 | 1 | 0.031
K1b1a1b | 10336 | 1 | 0.05
K1b1a1b | 16178 | 2 | 0.1
K1b1a1b | 709 | 1 | 0.05
K1b1a1b | 7985G | 1 | 0.05
K1b1a1b | 9804 | 1 | 0.05
K1b1a1c | 13161 | 1 | 0.02
K1b1a1c | 16129 | 5 | 0.1
K1b1a1c | 1842 | 1 | 0.02
K1b1a1c | 310 | 1 | 0.02
K1b1a1c1 | 13020G | 1 | 0.021
K1b1a1c1 | 16201 | 1 | 0.021
K1b1a1d | 113 | 1 | 0.021
K1b1a1d | 16172 | 1 | 0.021
K1b1a1d | 1719 | 1 | 0.021
K1b1a1d | 195 | 1 | 0.021
K1b1a1d1 | 12444 | 2 | 0.042
K1b1a1d1 | 14968 | 1 | 0.021
K1b1a1d1 | 16290 | 1 | 0.021
K1b1a2 | 146 | 1 | 0.071
K1b1a2 | 16102G | 1 | 0.071
K1b1a2 | 2023 | 1 | 0.071
K1b1a2 | 5899.1C | 2 | 0.143
K1b1b | 15381 | 1 | 0.004
K1b1b | 16261 | 20 | 0.088
K1b1b | 16399 | 1 | 0.004
K1b1b | 195 | 20 | 0.088
K1b1b | 721 | 1 | 0.004
K1b1b | 8270 | 1 | 0.004
K1b1b | 8281-8289d | 1 | 0.004
K1b1b1 | 13594 | 1 | 0.004
K1b1b1 | 13986 | 1 | 0.004
K1b1b1 | 152 | 1 | 0.004
K1b1b1 | 16092 | 1 | 0.004
K1b1b1 | 16195 | 1 | 0.004
K1b1b1 | 16261 | 20 | 0.087
K1b1b1 | 1719 | 1 | 0.004
K1b1b1 | 195 | 20 | 0.087
K1b1b1 | 2285 | 1 | 0.004

K1b1b1 | 2758 | 1 | 0.004
K1b1b1 | 2761 | 1 | 0.004
K1b1b1 | 5264 | 1 | 0.004
K1b1b1 | 5899.1C | 1 | 0.004
K1b1b1 | 6645 | 1 | 0.004
K1b1b1 | 7391 | 1 | 0.004
K1b1b1 | 9007 | 1 | 0.004
K1b1b1 | 9181 | 1 | 0.004
K1b1c | 10223 | 3 | 0.024
K1b1c | 12397 | 1 | 0.008
K1b1c | 12609 | 3 | 0.024
K1b1c | 13026 | 2 | 0.016
K1b1c | 13145 | 2 | 0.016
K1b1c | 13794 | 1 | 0.008
K1b1c | 14053 | 2 | 0.016
K1b1c | 14178 | 1 | 0.008
K1b1c | 146 | 1 | 0.008
K1b1c | 152 | 1 | 0.008
K1b1c | 15529 | 1 | 0.008
K1b1c | 16051 | 3 | 0.024
K1b1c | 16070 | 1 | 0.008
K1b1c | 16129 | 1 | 0.008
K1b1c | 16148 | 1 | 0.008
K1b1c | 16192 | 2 | 0.016
K1b1c | 16240T | 4 | 0.031
K1b1c | 16266 | 2 | 0.016
K1b1c | 16289 | 6 | 0.047
K1b1c | 16295 | 1 | 0.008
K1b1c | 16318 | 1 | 0.008
K1b1c | 16325 | 2 | 0.016
K1b1c | 16365 | 1 | 0.008
K1b1c | 195 | 2 | 0.016
K1b1c | 199 | 2 | 0.016
K1b1c | 2971 | 1 | 0.008
K1b1c | 310 | 2 | 0.016
K1b1c | 4802 | 2 | 0.016
K1b1c | 4970 | 1 | 0.008
K1b1c | 499 | 1 | 0.008
K1b1c | 5811 | 1 | 0.008
K1b1c | 5843 | 1 | 0.008
K1b1c | 6040 | 3 | 0.024
K1b1c | 650 | 1 | 0.008
K1b1c | 6617 | 3 | 0.024
K1b1c | 6935 | 2 | 0.016
K1b1c | 709 | 2 | 0.016
K1b1c | 8545 | 1 | 0.008
K1b1c | 9489 | 1 | 0.008
K1b2 | 10154 | 1 | 0.008
K1b2 | 10646 | 1 | 0.008
K1b2 | 11289 | 1 | 0.008
K1b2 | 12522 | 1 | 0.008
K1b2 | 13194 | 1 | 0.008
K1b2 | 15301 | 1 | 0.008
K1b2 | 16145 | 1 | 0.008
K1b2 | 16189 | 1 | 0.008
K1b2 | 16213 | 3 | 0.024
K1b2 | 16438 | 1 | 0.008
K1b2 | 5414 | 1 | 0.008
K1b2 | 7211 | 1 | 0.008
K1b2a | 10810 | 1 | 0.007
K1b2a | 12396 | 1 | 0.007
K1b2a | 13708 | 1 | 0.007
K1b2a | 14539 | 1 | 0.007
K1b2a | 14569 | 3 | 0.022
K1b2a | 152 | 1 | 0.007
K1b2a | 15327G | 1 | 0.007
K1b2a | 15760 | 3 | 0.022
K1b2a | 16093 | 1 | 0.007
K1b2a | 16224A | 1 | 0.007
K1b2a | 16260 | 1 | 0.007
K1b2a | 16320 | 6 | 0.045
K1b2a | 310 | 2 | 0.015
K1b2a | 3760G | 2 | 0.015
K1b2a | 525.1ACAC | 1 | 0.007
K1b2a | 629 | 1 | 0.007
K1b2a | 8251 | 2 | 0.015
K1b2a | 9058 | 1 | 0.007
K1b2a | 9932 | 1 | 0.007
K1b2a1 | 11539 | 1 | 0.008
K1b2a1 | 11914 | 2 | 0.015
K1b2a1 | 12984 | 1 | 0.008
K1b2a1 | 150 | 1 | 0.008
K1b2a1 | 16400 | 1 | 0.008
K1b2a1 | 2885 | 4 | 0.03
K1b2a1 | 3204 | 4 | 0.03
K1b2a1 | 3483 | 1 | 0.008
K1b2a1 | 3967C | 1 | 0.008
K1b2a1 | 3967T | 1 | 0.008
K1b2a1 | 8119 | 1 | 0.008
K1b2a1 | 9918C | 1 | 0.008
K1b2a1 | 9918T | 1 | 0.008
K1b2a1a | 13617 | 1 | 0.008
K1b2a1a | 13827 | 1 | 0.008
K1b2a1a | 13928C | 1 | 0.008
K1b2a1a | 14793 | 1 | 0.008
K1b2a1a | 15514 | 4 | 0.031
K1b2a1a | 16114A | 1 | 0.008
K1b2a1a | 16148 | 1 | 0.008
K1b2a1a | 16158 | 1 | 0.008
K1b2a1a | 16189 | 1 | 0.008
K1b2a1a | 16192 | 1 | 0.008
K1b2a1a | 16256 | 1 | 0.008
K1b2a1a | 16270 | 1 | 0.008
K1b2a1a | 16293 | 1 | 0.008
K1b2a1a | 16294 | 2 | 0.016
K1b2a1a | 16526 | 1 | 0.008
K1b2a1a | 210 | 1 | 0.008
K1b2a1a | 3197 | 1 | 0.008
K1b2a1a | 3277 | 1 | 0.008
K1b2a1a | 3337 | 6 | 0.047
K1b2a1a | 4224 | 1 | 0.008

K1b2a1a | 6356 | 1 | 0.008
K1b2a1a | 7112 | 1 | 0.008
K1b2a1a | 9015 | 2 | 0.016
K1b2a1a | 9477 | 1 | 0.008
K1b2a1a1 | 15850 | 1 | 0.015
K1b2a1a1 | 16108 | 1 | 0.015
K1b2a1a1 | 16218 | 7 | 0.103
K1b2a1a1 | 16263 | 7 | 0.103
K1b2a1a1 | 16278 | 2 | 0.029
K1b2a1a1 | 16320 | 1 | 0.015
K1b2a1a1 | 16355 | 2 | 0.029
K1b2a1a1 | 2179 | 1 | 0.015
K1b2a1a1 | 5194 | 1 | 0.015
K1b2a1a1 | 8251 | 1 | 0.015
K1b2a2 | 11815 | 1 | 0.007
K1b2a2 | 12215 | 1 | 0.007
K1b2a2 | 15045 | 1 | 0.007
K1b2a2 | 15172 | 1 | 0.007
K1b2a2 | 152 | 1 | 0.007
K1b2a2 | 153 | 1 | 0.007
K1b2a2 | 16094 | 1 | 0.007
K1b2a2 | 16169 | 2 | 0.014
K1b2a2 | 16189 | 5 | 0.036
K1b2a2 | 16260 | 1 | 0.007
K1b2a2 | 16270 | 2 | 0.014
K1b2a2 | 259 | 5 | 0.036
K1b2a2 | 310 | 2 | 0.014
K1b2a2 | 316.1G | 1 | 0.007
K1b2a2 | 3972 | 2 | 0.014
K1b2a2 | 5021 | 1 | 0.007
K1b2a2 | 524.1ACAG | 1 | 0.007
K1b2a2 | 5915 | 1 | 0.007
K1b2a2 | 6317 | 1 | 0.007
K1b2a2 | 6962 | 1 | 0.007
K1b2a2 | 8709 | 3 | 0.022
K1b2a2 | 8880 | 1 | 0.007
K1b2a2 | 9554 | 1 | 0.007
K1b2a2a | 515.1GACA | 1 | 0.008
K1b2a2a | 516.1GCAC | 2 | 0.016
K1b2a2a | 5966 | 1 | 0.008
K1b2a2a | 6040 | 1 | 0.008
K1b2a2a | 9350 | 1 | 0.008
K1b2a2a | 9495 | 3 | 0.024
K1b2a3 | 11266 | 1 | 0.008
K1b2a3 | 11654 | 1 | 0.008
K1b2a3 | 3290 | 1 | 0.008
K1b2a3 | 3935 | 1 | 0.008
K1b2a3 | 510A | 1 | 0.008
K1b2a3 | 525.1ACACAC | 1 | 0.008
K1b2b | 1187 | 2 | 0.015
K1b2b | 12088 | 1 | 0.008
K1b2b | 12501 | 2 | 0.015
K1b2b | 152 | 1 | 0.008
K1b2b | 16189 | 2 | 0.015
K1b2b | 16265 | 1 | 0.008
K1b2b | 16266 | 2 | 0.015
K1b2b | 16320 | 1 | 0.008
K1b2b | 3208 | 1 | 0.008
K1b2b | 4501 | 1 | 0.008
K1b2b | 525.1AC | 1 | 0.008
K1b2b | 6473 | 1 | 0.008
K1b2b | 7258 | 1 | 0.008
K1b2b | 8839 | 1 | 0.008
K1b2b | 9554 | 1 | 0.008
K1b2b | 9896 | 1 | 0.008
K1b2b1 | 16126 | 1 | 0.009
K1b2b1 | 310 | 1 | 0.009
K1b2b1 | 315.2C | 1 | 0.009
K1b2b1 | 5073 | 1 | 0.009
K1b2b1 | 9812 | 1 | 0.009
K1c | 16092 | 1 | 0.005
K1c | 16095 | 1 | 0.005
K1c | 16124 | 1 | 0.005
K1c | 16145 | 1 | 0.005
K1c | 16179 | 2 | 0.01
K1c | 16300 | 1 | 0.005
K1c | 16355 | 1 | 0.005
K1c | 16360 | 7 | 0.036
K1c | 456 | 1 | 0.005
K1c | 487 | 1 | 0.005
K1c1 | 10685 | 3 | 0.013
K1c1 | 11084 | 1 | 0.004
K1c1 | 11291 | 1 | 0.004
K1c1 | 11638 | 1 | 0.004
K1c1 | 12130 | 2 | 0.008
K1c1 | 12241d | 1 | 0.004
K1c1 | 12618 | 1 | 0.004
K1c1 | 12906 | 1 | 0.004
K1c1 | 13080 | 2 | 0.008
K1c1 | 13375 | 2 | 0.008
K1c1 | 13866 | 1 | 0.004
K1c1 | 14751 | 1 | 0.004
K1c1 | 14962 | 1 | 0.004
K1c1 | 15296 | 1 | 0.004
K1c1 | 15746C | 1 | 0.004
K1c1 | 15790 | 1 | 0.004
K1c1 | 15853 | 2 | 0.008
K1c1 | 15884 | 2 | 0.008
K1c1 | 15928 | 1 | 0.004
K1c1 | 16093 | 1 | 0.004
K1c1 | 16159 | 1 | 0.004
K1c1 | 16172 | 1 | 0.004
K1c1 | 16179 | 2 | 0.008
K1c1 | 16242A | 1 | 0.004
K1c1 | 16256 | 1 | 0.004
K1c1 | 16261 | 1 | 0.004
K1c1 | 16301 | 1 | 0.004
K1c1 | 16304 | 2 | 0.008
K1c1 | 16343 | 9 | 0.038
K1c1 | 16474C | 2 | 0.008
K1c1 | 16527 | 3 | 0.013

K1c1 | 195 | 3 | 0.013
K1c1 | 214 | 4 | 0.017
K1c1 | 230 | 1 | 0.004
K1c1 | 257 | 2 | 0.008
K1c1 | 310 | 2 | 0.008
K1c1 | 315.2C | 1 | 0.004
K1c1 | 3199A | 1 | 0.004
K1c1 | 356.1C | 1 | 0.004
K1c1 | 3591 | 1 | 0.004
K1c1 | 3936 | 1 | 0.004
K1c1 | 460 | 1 | 0.004
K1c1 | 4883 | 1 | 0.004
K1c1 | 5003 | 2 | 0.008
K1c1 | 508 | 1 | 0.004
K1c1 | 5220 | 1 | 0.004
K1c1 | 573.1C | 1 | 0.004
K1c1 | 573.2C | 1 | 0.004
K1c1 | 5786 | 1 | 0.004
K1c1 | 6734 | 1 | 0.004
K1c1 | 8020 | 1 | 0.004
K1c1 | 8083 | 1 | 0.004
K1c1 | 8713 | 2 | 0.008
K1c1 | 9058 | 1 | 0.004
K1c1 | 9248 | 1 | 0.004
K1c1 | 9380 | 1 | 0.004
K1c1a | 1393 | 1 | 0.005
K1c1a | 14757 | 1 | 0.005
K1c1a | 15635 | 1 | 0.005
K1c1a | 16092 | 1 | 0.005
K1c1a | 16095 | 1 | 0.005
K1c1a | 16124 | 1 | 0.005
K1c1a | 16145 | 1 | 0.005
K1c1a | 16179 | 2 | 0.01
K1c1a | 16300 | 1 | 0.005
K1c1a | 16301 | 1 | 0.005
K1c1a | 16355 | 1 | 0.005
K1c1a | 16360 | 7 | 0.036
K1c1a | 456 | 1 | 0.005
K1c1a | 487 | 1 | 0.005
K1c1a | 5301 | 1 | 0.005
K1c1a | 5539 | 1 | 0.005
K1c1a | 9210 | 1 | 0.005
K1c1b | 1053T | 1 | 0.005
K1c1b | 10691 | 1 | 0.005
K1c1b | 11728 | 1 | 0.005
K1c1b | 13708 | 1 | 0.005
K1c1b | 13719 | 1 | 0.005
K1c1b | 14518 | 1 | 0.005
K1c1b | 14769 | 1 | 0.005
K1c1b | 14905 | 2 | 0.009
K1c1b | 159 | 1 | 0.005
K1c1b | 15936T | 1 | 0.005
K1c1b | 16093 | 1 | 0.005
K1c1b | 16223 | 1 | 0.005
K1c1b | 16240 | 1 | 0.005
K1c1b | 16259 | 1 | 0.005
K1c1b | 16261 | 1 | 0.005
K1c1b | 16264 | 2 | 0.009
K1c1b | 16360 | 7 | 0.033
K1c1b | 1924 | 1 | 0.005
K1c1b | 310 | 1 | 0.005
K1c1b | 3736 | 1 | 0.005
K1c1b | 4622 | 2 | 0.009
K1c1b | 4851 | 1 | 0.005
K1c1b | 6086 | 1 | 0.005
K1c1b | 6389 | 5 | 0.024
K1c1b | 709 | 1 | 0.005
K1c1b | 7759 | 3 | 0.014
K1c1b | 8047 | 1 | 0.005
K1c1b | 8557 | 1 | 0.005
K1c1c | 10306C | 1 | 0.005
K1c1c | 11392 | 3 | 0.014
K1c1c | 14125 | 1 | 0.005
K1c1c | 14757 | 1 | 0.005
K1c1c | 16270 | 1 | 0.005
K1c1c | 16320 | 1 | 0.005
K1c1c | 16355 | 1 | 0.005
K1c1c | 16360 | 7 | 0.033
K1c1c | 16362 | 1 | 0.005
K1c1c | 195 | 1 | 0.005
K1c1c | 2487C | 1 | 0.005
K1c1c | 310 | 1 | 0.005
K1c1c | 498.1C | 2 | 0.01
K1c1c | 6419C | 1 | 0.005
K1c1c | 7853 | 1 | 0.005
K1c1c | 8817 | 1 | 0.005
K1c1c | 93 | 1 | 0.005
K1c1d | 13182 | 1 | 0.005
K1c1d | 13651 | 3 | 0.015
K1c1d | 16092 | 1 | 0.005
K1c1d | 16095 | 1 | 0.005
K1c1d | 16124 | 1 | 0.005
K1c1d | 16145 | 1 | 0.005
K1c1d | 16179 | 2 | 0.01
K1c1d | 16300 | 1 | 0.005
K1c1d | 16355 | 1 | 0.005
K1c1d | 16360 | 7 | 0.035
K1c1d | 3849 | 1 | 0.005
K1c1d | 456 | 1 | 0.005
K1c1d | 487 | 1 | 0.005
K1c1d | 8251 | 1 | 0.005
K1c1e | 11914 | 2 | 0.059
K1c1e | 12630 | 1 | 0.029
K1c1e | 13497 | 1 | 0.029
K1c1e | 14404 | 2 | 0.059
K1c1e | 14668 | 1 | 0.029
K1c1e | 15401 | 1 | 0.029
K1c1e | 15671 | 1 | 0.029
K1c1e | 15900 | 1 | 0.029
K1c1e | 15908 | 1 | 0.029
K1c1e | 16086 | 1 | 0.029

K1c1e | 16316 | 1 | 0.029
K1c1e | 16362 | 14 | 0.412
K1c1e | 195 | 2 | 0.059
K1c1e | 2789A | 1 | 0.029
K1c1e | 3398 | 1 | 0.029
K1c1e | 597 | 1 | 0.029
K1c1e | 6383 | 1 | 0.029
K1c1e | 6389 | 1 | 0.029
K1c1e | 7158 | 1 | 0.029
K1c1e | 7270 | 1 | 0.029
K1c1e | 7912 | 1 | 0.029
K1c1e | 8027 | 1 | 0.029
K1c1f | 11733 | 1 | 0.005
K1c1f | 12636 | 1 | 0.005
K1c1f | 13272 | 1 | 0.005
K1c1f | 13708 | 1 | 0.005
K1c1f | 1393 | 1 | 0.005
K1c1f | 13934 | 2 | 0.01
K1c1f | 14878 | 1 | 0.005
K1c1f | 15790 | 1 | 0.005
K1c1f | 16092 | 1 | 0.005
K1c1f | 16095 | 1 | 0.005
K1c1f | 16124 | 1 | 0.005
K1c1f | 16145 | 1 | 0.005
K1c1f | 16179 | 2 | 0.01
K1c1f | 16300 | 1 | 0.005
K1c1f | 16355 | 1 | 0.005
K1c1f | 16360 | 7 | 0.035
K1c1f | 195 | 1 | 0.005
K1c1f | 282 | 1 | 0.005
K1c1f | 456 | 1 | 0.005
K1c1f | 487 | 1 | 0.005
K1c1f | 6305 | 1 | 0.005
K1c1f | 6614 | 1 | 0.005
K1c1f | 6962 | 1 | 0.005
K1c1f | 8658 | 1 | 0.005
K1c1g | 15314 | 1 | 0.005
K1c1g | 16092 | 1 | 0.005
K1c1g | 16095 | 1 | 0.005
K1c1g | 16124 | 1 | 0.005
K1c1g | 16129 | 1 | 0.005
K1c1g | 16145 | 1 | 0.005
K1c1g | 16179 | 2 | 0.01
K1c1g | 16300 | 1 | 0.005
K1c1g | 16355 | 1 | 0.005
K1c1g | 16360 | 7 | 0.036
K1c1g | 456 | 1 | 0.005
K1c1g | 487 | 1 | 0.005
K1c1g | 5563 | 1 | 0.005
K1c1h | 12031A | 1 | 0.005
K1c1h | 12630 | 2 | 0.01
K1c1h | 12912 | 1 | 0.005
K1c1h | 16179 | 2 | 0.01
K1c1h | 16209 | 1 | 0.005
K1c1h | 16291 | 7 | 0.034
K1c1h | 16355 | 1 | 0.005
K1c1h | 16360 | 7 | 0.034
K1c1h | 310 | 2 | 0.01
K1c1h | 326 | 1 | 0.005
K1c1h | 5471 | 1 | 0.005
K1c1h | 723 | 2 | 0.01
K1c1h | 8020 | 1 | 0.005
K1c1h | 8280 | 1 | 0.005
K1c1h | 9077 | 1 | 0.005
K1c1h | 9767 | 1 | 0.005
K1c1i | 11392 | 1 | 0.005
K1c1i | 16092 | 1 | 0.005
K1c1i | 16095 | 1 | 0.005
K1c1i | 16124 | 1 | 0.005
K1c1i | 16145 | 1 | 0.005
K1c1i | 16179 | 2 | 0.01
K1c1i | 16300 | 1 | 0.005
K1c1i | 16355 | 1 | 0.005
K1c1i | 16360 | 7 | 0.036
K1c1i | 16362 | 1 | 0.005
K1c1i | 189 | 2 | 0.01
K1c1i | 456 | 1 | 0.005
K1c1i | 487 | 1 | 0.005
K1c1i | 6671 | 2 | 0.01
K1c1i | 8020 | 1 | 0.005
K1c2 | 10248 | 1 | 0.01
K1c2 | 106-111d | 1 | 0.01
K1c2 | 10646 | 1 | 0.01
K1c2 | 11253 | 1 | 0.01
K1c2 | 11362 | 3 | 0.029
K1c2 | 11383 | 1 | 0.01
K1c2 | 11453 | 1 | 0.01
K1c2 | 11882 | 1 | 0.01
K1c2 | 12074C | 1 | 0.01
K1c2 | 12373 | 1 | 0.01
K1c2 | 12720 | 1 | 0.01
K1c2 | 12759 | 1 | 0.01
K1c2 | 12804 | 1 | 0.01
K1c2 | 12834 | 1 | 0.01
K1c2 | 12990 | 1 | 0.01
K1c2 | 13008 | 1 | 0.01
K1c2 | 13105 | 1 | 0.01
K1c2 | 13158 | 1 | 0.01
K1c2 | 13542 | 1 | 0.01
K1c2 | 14197 | 2 | 0.02
K1c2 | 143 | 1 | 0.01
K1c2 | 14404 | 1 | 0.01
K1c2 | 14509 | 1 | 0.01
K1c2 | 14814A | 1 | 0.01
K1c2 | 150 | 1 | 0.01
K1c2 | 15193 | 1 | 0.01
K1c2 | 15244 | 1 | 0.01
K1c2 | 15301 | 1 | 0.01
K1c2 | 16093 | 1 | 0.01
K1c2 | 16114G | 1 | 0.01
K1c2 | 16126 | 1 | 0.01

K1c2 | 16189 | 6 | 0.059
K1c2 | 16214 | 1 | 0.01
K1c2 | 16362 | 4 | 0.039
K1c2 | 16474C | 1 | 0.01
K1c2 | 1692 | 1 | 0.01
K1c2 | 183 | 1 | 0.01
K1c2 | 1888 | 1 | 0.01
K1c2 | 2061 | 2 | 0.02
K1c2 | 2294 | 1 | 0.01
K1c2 | 2378A | 2 | 0.02
K1c2 | 310 | 3 | 0.029
K1c2 | 315.2C | 1 | 0.01
K1c2 | 3221 | 1 | 0.01
K1c2 | 3397 | 1 | 0.01
K1c2 | 3738 | 1 | 0.01
K1c2 | 379 | 1 | 0.01
K1c2 | 4015 | 1 | 0.01
K1c2 | 453 | 2 | 0.02
K1c2 | 5099 | 2 | 0.02
K1c2 | 5202 | 1 | 0.01
K1c2 | 5301 | 1 | 0.01
K1c2 | 533 | 1 | 0.01
K1c2 | 5442 | 1 | 0.01
K1c2 | 55 | 1 | 0.01
K1c2 | 5918 | 1 | 0.01
K1c2 | 5973 | 1 | 0.01
K1c2 | 5979 | 2 | 0.02
K1c2 | 6176 | 1 | 0.01
K1c2 | 6179 | 1 | 0.01
K1c2 | 6267 | 1 | 0.01
K1c2 | 6468 | 1 | 0.01
K1c2 | 6620 | 1 | 0.01
K1c2 | 7046 | 1 | 0.01
K1c2 | 8027 | 1 | 0.01
K1c2 | 8050 | 1 | 0.01
K1c2 | 8155 | 1 | 0.01
K1c2 | 8485 | 1 | 0.01
K1c2 | 8584 | 1 | 0.01
K1c2 | 8589 | 3 | 0.029
K1c2 | 8619A | 2 | 0.02
K1c2 | 869 | 1 | 0.01
K1c2 | 9038 | 1 | 0.01
K1c2 | 9058 | 2 | 0.02
K1c2 | 9186 | 1 | 0.01
K1c2 | 9204 | 1 | 0.01
K1c2 | 9266 | 1 | 0.01
K1c2 | 9437 | 2 | 0.02
K1c2 | 9524 | 1 | 0.01
K1c2 | 9545 | 1 | 0.01
K1c2 | 9677 | 1 | 0.01
K1c2a | 12358 | 3 | 0.056
K1c2a | 14506 | 2 | 0.037
K1c2a | 150 | 7 | 0.13
K1c2a | 15244 | 2 | 0.037
K1c2a | 16093 | 3 | 0.056
K1c2a | 16158 | 1 | 0.019
K1c2a | 16239 | 1 | 0.019
K1c2a | 573.1C | 2 | 0.037
K1c2a | 573.2C | 1 | 0.019
K1c2a | 81.1T | 1 | 0.019
K1c2a | 8300 | 1 | 0.019
K1c2a | 8705 | 1 | 0.019
K1d | 10172 | 1 | 0.043
K1d | 16147 | 1 | 0.043
K1d | 16269C | 1 | 0.043
K1d | 3460 | 1 | 0.043
K1d | 8078 | 1 | 0.043
K1d1 | 10088 | 2 | 0.077
K1d1 | 11914 | 1 | 0.038
K1d1 | 200 | 1 | 0.038
K1d1 | 310 | 2 | 0.077
K1d1 | 7852 | 2 | 0.077
K1e | 10166 | 2 | 0.045
K1e | 10757 | 2 | 0.045
K1e | 16140 | 2 | 0.045
K1e | 16223 | 2 | 0.045
K1e | 16317T | 1 | 0.023
K1e1 | 12810 | 2 | 0.222
K1e1 | 15244 | 1 | 0.111
K1e1 | 16093 | 2 | 0.222
K1e1 | 222G | 1 | 0.111
K1e1 | 253G | 1 | 0.111
K1e1 | 5090 | 1 | 0.111
K1e1 | 6249 | 1 | 0.111
K1f | 146 | 1 | 0.036
K1f | 16260 | 2 | 0.071
K2 | 150 | 3 | 0.023
K2 | 16114 | 1 | 0.008
K2 | 16120d | 1 | 0.008
K2 | 16174 | 1 | 0.008
K2 | 16234 | 4 | 0.031
K2 | 16243 | 1 | 0.008
K2 | 16399 | 3 | 0.023
K2 | 16527 | 1 | 0.008
K2 | 182 | 1 | 0.008
K2 | 294 | 1 | 0.008
K2 | 324 | 1 | 0.008
K2 | 383 | 1 | 0.008
K2a | 10128 | 2 | 0.01
K2a | 1041 | 1 | 0.005
K2a | 10476 | 1 | 0.005
K2a | 106-111d | 1 | 0.005
K2a | 11116 | 1 | 0.005
K2a | 11969 | 1 | 0.005
K2a | 12892 | 1 | 0.005
K2a | 13044 | 1 | 0.005
K2a | 13068 | 1 | 0.005
K2a | 13101C | 1 | 0.005
K2a | 14166 | 1 | 0.005
K2a | 14265 | 1 | 0.005
K2a | 14267 | 1 | 0.005

K2a | 14319 | 1 | 0.005
K2a | 15314 | 1 | 0.005
K2a | 15520 | 1 | 0.005
K2a | 15715 | 1 | 0.005
K2a | 15718 | 1 | 0.005
K2a | 15978 | 1 | 0.005
K2a | 16093 | 1 | 0.005
K2a | 16129 | 1 | 0.005
K2a | 16134 | 1 | 0.005
K2a | 16189 | 2 | 0.01
K2a | 16256 | 1 | 0.005
K2a | 16260 | 1 | 0.005
K2a | 16292 | 1 | 0.005
K2a | 16368 | 1 | 0.005
K2a | 16398 | 10 | 0.05
K2a | 16438 | 1 | 0.005
K2a | 185 | 1 | 0.005
K2a | 195 | 2 | 0.01
K2a | 199 | 2 | 0.01
K2a | 207 | 3 | 0.015
K2a | 310 | 1 | 0.005
K2a | 33 | 1 | 0.005
K2a | 3394 | 1 | 0.005
K2a | 3447 | 1 | 0.005
K2a | 3526 | 1 | 0.005
K2a | 4859 | 1 | 0.005
K2a | 504 | 2 | 0.01
K2a | 573.2C | 1 | 0.005
K2a | 5921 | 1 | 0.005
K2a | 64 | 1 | 0.005
K2a | 6647 | 1 | 0.005
K2a | 7368A | 1 | 0.005
K2a | 7440 | 1 | 0.005
K2a | 7753T | 1 | 0.005
K2a | 8260 | 1 | 0.005
K2a | 8618 | 1 | 0.005
K2a | 9256 | 1 | 0.005
K2a | 930 | 1 | 0.005
K2a | 9674 | 1 | 0.005
K2a | 97 | 1 | 0.005
K2a1 | 143 | 1 | 0.006
K2a1 | 16111 | 2 | 0.011
K2a1 | 16145 | 1 | 0.006
K2a1 | 16150 | 1 | 0.006
K2a1 | 16154 | 2 | 0.011
K2a1 | 16178 | 2 | 0.011
K2a1 | 16188 | 1 | 0.006
K2a1 | 16213 | 1 | 0.006
K2a1 | 16298 | 2 | 0.011
K2a1 | 16319 | 1 | 0.006
K2a1 | 16360 | 11 | 0.062
K2a1 | 207 | 2 | 0.011
K2a1 | 482 | 1 | 0.006
K2a1 | 573.2C | 2 | 0.011
K2a1 | 8293 | 2 | 0.011
K2a1 | 8905 | 2 | 0.011
K2a10 | 12612 | 1 | 0.006
K2a10 | 13434 | 1 | 0.006
K2a10 | 14605 | 1 | 0.006
K2a10 | 16093 | 1 | 0.006
K2a10 | 16145 | 1 | 0.006
K2a10 | 16150 | 1 | 0.006
K2a10 | 16188 | 1 | 0.006
K2a10 | 16213 | 7 | 0.04
K2a10 | 482 | 1 | 0.006
K2a10 | 573.2C | 1 | 0.006
K2a11 | 11150 | 1 | 0.062
K2a11 | 143 | 1 | 0.062
K2a11 | 14750 | 4 | 0.25
K2a11 | 14968 | 1 | 0.062
K2a11 | 15109 | 1 | 0.062
K2a11 | 16093 | 1 | 0.062
K2a11 | 16124 | 3 | 0.188
K2a11 | 16261 | 1 | 0.062
K2a11 | 16278 | 1 | 0.062
K2a11 | 4122 | 1 | 0.062
K2a11 | 4123 | 1 | 0.062
K2a11 | 5492 | 1 | 0.062
K2a11 | 7819 | 1 | 0.062
K2a11 | 8265 | 1 | 0.062
K2a1a | 16092 | 1 | 0.05
K2a1a | 16145 | 1 | 0.05
K2a1a | 16172 | 1 | 0.05
K2a1a | 16193 | 2 | 0.1
K2a1a | 16240 | 1 | 0.05
K2a1a | 16266 | 1 | 0.05
K2a1a | 16292 | 1 | 0.05
K2a1a | 16295 | 1 | 0.05
K2a1a | 16300 | 1 | 0.05
K2a1a | 16319 | 2 | 0.1
K2a1a | 16343 | 2 | 0.1
K2a1a | 16357 | 2 | 0.1
K2a1a | 16438 | 1 | 0.05
K2a1a | 279 | 1 | 0.05
K2a1a | 522 | 1 | 0.05
K2a2 | 143 | 1 | 0.006
K2a2 | 15520 | 1 | 0.006
K2a2 | 16111 | 2 | 0.011
K2a2 | 16145 | 1 | 0.006
K2a2 | 16150 | 1 | 0.006
K2a2 | 16154 | 2 | 0.011
K2a2 | 16178 | 2 | 0.011
K2a2 | 16188 | 1 | 0.006
K2a2 | 16213 | 1 | 0.006
K2a2 | 16298 | 2 | 0.011
K2a2 | 16319 | 1 | 0.006
K2a2 | 16360 | 11 | 0.062
K2a2 | 207 | 2 | 0.011
K2a2 | 482 | 1 | 0.006
K2a2 | 573.2C | 1 | 0.006
K2a2 | 63 | 1 | 0.006

K2a2 | 64 | 2 | 0.011
K2a2a | 143 | 1 | 0.006
K2a2a | 16111 | 2 | 0.011
K2a2a | 16145 | 1 | 0.006
K2a2a | 16150 | 1 | 0.006
K2a2a | 16154 | 2 | 0.011
K2a2a | 16178 | 2 | 0.011
K2a2a | 16188 | 1 | 0.006
K2a2a | 16213 | 1 | 0.006
K2a2a | 16298 | 2 | 0.011
K2a2a | 16319 | 1 | 0.006
K2a2a | 16360 | 11 | 0.063
K2a2a | 16390 | 1 | 0.006
K2a2a | 195 | 1 | 0.006
K2a2a | 207 | 2 | 0.011
K2a2a | 482 | 1 | 0.006
K2a2a | 573.2C | 1 | 0.006
K2a2a | 9263T | 1 | 0.006
K2a2a1 | 14599 | 1 | 0.006
K2a2a1 | 14863 | 1 | 0.006
K2a2a1 | 153 | 1 | 0.006
K2a2a1 | 15968 | 1 | 0.006
K2a2a1 | 16216 | 8 | 0.05
K2a2a1 | 16337G | 8 | 0.05
K2a2a1 | 16405 | 8 | 0.05
K2a2a1 | 310 | 1 | 0.006
K2a2a1 | 4325 | 1 | 0.006
K2a2a1 | 9214 | 1 | 0.006
K2a2a1 | 9461 | 1 | 0.006
K2a2a1 | 9615 | 1 | 0.006
K2a3 | 11794 | 1 | 0.005
K2a3 | 13572 | 1 | 0.005
K2a3 | 13902 | 1 | 0.005
K2a3 | 1462 | 1 | 0.005
K2a3 | 14857 | 1 | 0.005
K2a3 | 14921 | 1 | 0.005
K2a3 | 15031 | 1 | 0.005
K2a3 | 15344 | 1 | 0.005
K2a3 | 15896 | 2 | 0.011
K2a3 | 15927 | 1 | 0.005
K2a3 | 15992 | 1 | 0.005
K2a3 | 16134 | 1 | 0.005
K2a3 | 16222 | 1 | 0.005
K2a3 | 16233 | 2 | 0.011
K2a3 | 16291 | 7 | 0.038
K2a3 | 1656d | 1 | 0.005
K2a3 | 1709 | 1 | 0.005
K2a3 | 2164 | 1 | 0.005
K2a3 | 310 | 1 | 0.005
K2a3 | 4520 | 1 | 0.005
K2a3 | 4688 | 2 | 0.011
K2a3 | 4748 | 1 | 0.005
K2a3 | 5460 | 1 | 0.005
K2a3 | 573.2C | 1 | 0.005
K2a3 | 6750 | 1 | 0.005
K2a3 | 8161 | 1 | 0.005
K2a3 | 9801 | 2 | 0.011
K2a3a | 143 | 1 | 0.006
K2a3a | 14890 | 1 | 0.006
K2a3a | 16111 | 2 | 0.011
K2a3a | 16145 | 1 | 0.006
K2a3a | 16150 | 1 | 0.006
K2a3a | 16154 | 2 | 0.011
K2a3a | 16178 | 2 | 0.011
K2a3a | 16188 | 1 | 0.006
K2a3a | 16213 | 1 | 0.006
K2a3a | 16298 | 2 | 0.011
K2a3a | 16319 | 1 | 0.006
K2a3a | 16360 | 11 | 0.062
K2a3a | 207 | 2 | 0.011
K2a3a | 482 | 1 | 0.006
K2a3a | 573.2C | 1 | 0.006
K2a3a | 7202 | 3 | 0.017
K2a3a | 8410 | 1 | 0.006
K2a3a1 | 13143 | 3 | 0.018
K2a3a1 | 1443 | 1 | 0.006
K2a3a1 | 14950 | 1 | 0.006
K2a3a1 | 16092 | 9 | 0.053
K2a3a1 | 16184A | 7 | 0.041
K2a3a1 | 573.2C | 1 | 0.006
K2a3a1 | 5979 | 1 | 0.006
K2a4 | 13889C | 1 | 0.006
K2a4 | 14148 | 1 | 0.006
K2a4 | 16129 | 3 | 0.017
K2a4 | 16145 | 1 | 0.006
K2a4 | 16150 | 1 | 0.006
K2a4 | 16188 | 1 | 0.006
K2a4 | 16213 | 7 | 0.04
K2a4 | 248 | 1 | 0.006
K2a4 | 280 | 2 | 0.011
K2a4 | 482 | 1 | 0.006
K2a4 | 5194 | 2 | 0.011
K2a4 | 573.2C | 1 | 0.006
K2a5 | 13719 | 1 | 0.007
K2a5 | 14208 | 3 | 0.021
K2a5 | 15799 | 4 | 0.028
K2a5 | 16093 | 4 | 0.028
K2a5 | 16111 | 1 | 0.007
K2a5 | 16189 | 1 | 0.007
K2a5 | 1640 | 1 | 0.007
K2a5 | 195 | 4 | 0.028
K2a5 | 310 | 1 | 0.007
K2a5 | 3106A | 1 | 0.007
K2a5 | 3397 | 1 | 0.007
K2a5 | 4086 | 1 | 0.007
K2a5 | 430 | 3 | 0.021
K2a5 | 5021 | 1 | 0.007
K2a5 | 5147 | 1 | 0.007
K2a5 | 5231 | 1 | 0.007
K2a5 | 5480 | 2 | 0.014
K2a5 | 551 | 1 | 0.007

K2a5 | 5899.2C | 1 | 0.007
K2a5 | 6249 | 1 | 0.007
K2a5 | 6663 | 1 | 0.007
K2a5 | 7241T | 1 | 0.007
K2a5 | 8224 | 1 | 0.007
K2a5 | 8516 | 1 | 0.007
K2a5 | 8930 | 1 | 0.007
K2a5 | 9966 | 1 | 0.007
K2a5a | 132 | 1 | 0.008
K2a5a | 16075 | 1 | 0.008
K2a5a | 16129 | 1 | 0.008
K2a5a | 194 | 1 | 0.008
K2a5a | 321 | 1 | 0.008
K2a5a | 8291 | 1 | 0.008
K2a5a1 | 16148 | 1 | 0.2
K2a5a1 | 16248 | 1 | 0.2
K2a5a1 | 3693 | 1 | 0.2
K2a5a1 | 8838 | 1 | 0.2
K2a5b | 11151 | 3 | 0.022
K2a5b | 15259 | 1 | 0.007
K2a5b | 15461 | 1 | 0.007
K2a5b | 16150 | 1 | 0.007
K2a5b | 16243 | 4 | 0.03
K2a5b | 16274 | 1 | 0.007
K2a5b | 16300 | 1 | 0.007
K2a5b | 189 | 4 | 0.03
K2a5b | 280 | 7 | 0.052
K2a5b | 4959 | 2 | 0.015
K2a5b | 7424 | 2 | 0.015
K2a6 | 11404 | 1 | 0.005
K2a6 | 12037A | 1 | 0.005
K2a6 | 12037T | 1 | 0.005
K2a6 | 14118 | 2 | 0.01
K2a6 | 14189 | 1 | 0.005
K2a6 | 14212 | 1 | 0.005
K2a6 | 14437 | 7 | 0.033
K2a6 | 15258 | 1 | 0.005
K2a6 | 15479 | 1 | 0.005
K2a6 | 15493 | 1 | 0.005
K2a6 | 15525 | 2 | 0.01
K2a6 | 15928 | 1 | 0.005
K2a6 | 16086 | 4 | 0.019
K2a6 | 16093 | 2 | 0.01
K2a6 | 16114 | 1 | 0.005
K2a6 | 16166d | 1 | 0.005
K2a6 | 16245 | 1 | 0.005
K2a6 | 16327 | 1 | 0.005
K2a6 | 16399 | 7 | 0.033
K2a6 | 1764 | 1 | 0.005
K2a6 | 195 | 2 | 0.01
K2a6 | 207 | 1 | 0.005
K2a6 | 2308 | 1 | 0.005
K2a6 | 234 | 7 | 0.033
K2a6 | 294.1T | 1 | 0.005
K2a6 | 297 | 2 | 0.01
K2a6 | 310 | 4 | 0.019
K2a6 | 3915 | 1 | 0.005
K2a6 | 4574 | 1 | 0.005
K2a6 | 5228 | 1 | 0.005
K2a6 | 5582 | 1 | 0.005
K2a6 | 573.2C | 1 | 0.005
K2a6 | 5772 | 1 | 0.005
K2a6 | 6896 | 1 | 0.005
K2a6 | 7286 | 1 | 0.005
K2a6 | 7468 | 2 | 0.01
K2a6 | 7606 | 3 | 0.014
K2a6 | 7897 | 1 | 0.005
K2a6 | 8227 | 1 | 0.005
K2a6 | 8567 | 2 | 0.01
K2a6 | 8701T | 1 | 0.005
K2a6 | 9845 | 1 | 0.005
K2a7 | 143 | 7 | 0.04
K2a7 | 15266 | 1 | 0.006
K2a7 | 16066 | 2 | 0.011
K2a7 | 16145 | 1 | 0.006
K2a7 | 16150 | 1 | 0.006
K2a7 | 16188 | 1 | 0.006
K2a7 | 16213 | 1 | 0.006
K2a7 | 16278 | 2 | 0.011
K2a7 | 16319 | 7 | 0.04
K2a7 | 186A | 1 | 0.006
K2a7 | 482 | 1 | 0.006
K2a7 | 573.2C | 1 | 0.006
K2a7 | 73C | 1 | 0.006
K2a7 | 8292 | 2 | 0.011
K2a8 | 13941G | 1 | 0.037
K2a8 | 15853A | 1 | 0.037
K2a8 | 16086 | 1 | 0.037
K2a8 | 16304 | 1 | 0.037
K2a8 | 195 | 1 | 0.037
K2a8 | 2098 | 1 | 0.037
K2a8 | 8888 | 1 | 0.037
K2a9 | 10398 | 1 | 0.005
K2a9 | 10966 | 1 | 0.005
K2a9 | 11318 | 1 | 0.005
K2a9 | 11914 | 17 | 0.09
K2a9 | 12384 | 1 | 0.005
K2a9 | 12981 | 1 | 0.005
K2a9 | 14020 | 1 | 0.005
K2a9 | 14577 | 1 | 0.005
K2a9 | 16189 | 1 | 0.005
K2a9 | 16192 | 1 | 0.005
K2a9 | 16261 | 1 | 0.005
K2a9 | 16362 | 11 | 0.059
K2a9 | 16399 | 17 | 0.09
K2a9 | 2158 | 2 | 0.011
K2a9 | 2626 | 1 | 0.005
K2a9 | 2898 | 1 | 0.005
K2a9 | 296 | 3 | 0.016
K2a9 | 3348 | 4 | 0.021
K2a9 | 3462 | 2 | 0.011

K2a9 | 3777 | 7 | 0.037
K2a9 | 3801 | 1 | 0.005
K2a9 | 3866 | 1 | 0.005
K2a9 | 4395 | 1 | 0.005
K2a9 | 573.2C | 1 | 0.005
K2a9 | 6722 | 1 | 0.005
K2a9 | 6915 | 17 | 0.09
K2a9 | 7076 | 1 | 0.005
K2a9 | 7337 | 1 | 0.005
K2a9 | 7757 | 1 | 0.005
K2a9 | 8375 | 1 | 0.005
K2a9 | 8691 | 1 | 0.005
K2a9 | 9300 | 2 | 0.011
K2a9 | 9548 | 3 | 0.016
K2a9 | 9575 | 1 | 0.005
K2b | 16114 | 1 | 0.008
K2b | 16120d | 1 | 0.008
K2b | 16145 | 1 | 0.008
K2b | 16174 | 1 | 0.008
K2b | 16189 | 2 | 0.016
K2b | 16234 | 1 | 0.008
K2b | 16243 | 1 | 0.008
K2b | 16527 | 1 | 0.008
K2b | 182 | 1 | 0.008
K2b | 2060C | 1 | 0.008
K2b | 2887 | 1 | 0.008
K2b | 294 | 1 | 0.008
K2b | 324 | 1 | 0.008
K2b | 383 | 1 | 0.008
K2b | 4561 | 1 | 0.008
K2b1 | 10398 | 1 | 0.008
K2b1 | 10456 | 1 | 0.008
K2b1 | 11410 | 1 | 0.008
K2b1 | 11969 | 1 | 0.008
K2b1 | 12164 | 1 | 0.008
K2b1 | 13816 | 1 | 0.008
K2b1 | 14502 | 1 | 0.008
K2b1 | 151 | 1 | 0.008
K2b1 | 152 | 1 | 0.008
K2b1 | 16120d | 1 | 0.008
K2b1 | 16129 | 1 | 0.008
K2b1 | 16168 | 1 | 0.008
K2b1 | 16174 | 1 | 0.008
K2b1 | 16256 | 4 | 0.031
K2b1 | 16344 | 2 | 0.016
K2b1 | 16527 | 1 | 0.008
K2b1 | 182 | 1 | 0.008
K2b1 | 1842 | 1 | 0.008
K2b1 | 294 | 1 | 0.008
K2b1 | 321 | 1 | 0.008
K2b1 | 324 | 1 | 0.008
K2b1 | 3696 | 1 | 0.008
K2b1 | 383 | 1 | 0.008
K2b1 | 384 | 1 | 0.008
K2b1 | 44.1C | 1 | 0.008
K2b1 | 709 | 1 | 0.008
K2b1 | 71 | 1 | 0.008
K2b1 | 7885 | 1 | 0.008
K2b1 | 9413 | 1 | 0.008
K2b1a | 11539 | 1 | 0.008
K2b1a | 11854 | 1 | 0.008
K2b1a | 12127 | 1 | 0.008
K2b1a | 12714 | 1 | 0.008
K2b1a | 13191 | 1 | 0.008
K2b1a | 153 | 1 | 0.008
K2b1a | 16188 | 1 | 0.008
K2b1a | 16189 | 1 | 0.008
K2b1a | 16213 | 2 | 0.016
K2b1a | 295 | 2 | 0.016
K2b1a | 4029 | 1 | 0.008
K2b1a | 6267 | 1 | 0.008
K2b1a | 6518 | 2 | 0.016
K2b1a | 6915 | 2 | 0.016
K2b1a1 | 11708 | 1 | 0.034
K2b1a1 | 11854 | 1 | 0.034
K2b1a1 | 12192 | 1 | 0.034
K2b1a1 | 12507 | 1 | 0.034
K2b1a1 | 12771 | 1 | 0.034
K2b1a1 | 14053 | 2 | 0.069
K2b1a1 | 14198 | 1 | 0.034
K2b1a1 | 14560 | 1 | 0.034
K2b1a1 | 14869 | 1 | 0.034
K2b1a1 | 152 | 1 | 0.034
K2b1a1 | 15784 | 1 | 0.034
K2b1a1 | 16129 | 2 | 0.069
K2b1a1 | 16234 | 1 | 0.034
K2b1a1 | 16355 | 1 | 0.034
K2b1a1 | 189 | 1 | 0.034
K2b1a1 | 195 | 1 | 0.034
K2b1a1 | 199 | 1 | 0.034
K2b1a1 | 2626 | 1 | 0.034
K2b1a1 | 488 | 3 | 0.103
K2b1a1 | 5460 | 1 | 0.034
K2b1a1 | 5814 | 1 | 0.034
K2b1a1 | 5836 | 1 | 0.034
K2b1a1 | 7080 | 1 | 0.034
K2b1a1 | 7853 | 2 | 0.069
K2b1a1 | 8065 | 2 | 0.069
K2b1a1 | 8108 | 1 | 0.034
K2b1a1 | 8911 | 1 | 0.034
K2b1a1 | 9058 | 1 | 0.034
K2b1a1a | 10202 | 1 | 0.034
K2b1a1a | 11440 | 1 | 0.034
K2b1a1a | 11854 | 1 | 0.034
K2b1a1a | 11872 | 1 | 0.034
K2b1a1a | 12507 | 1 | 0.034
K2b1a1a | 14002 | 1 | 0.034
K2b1a1a | 14040 | 1 | 0.034
K2b1a1a | 14869 | 1 | 0.034
K2b1a1a | 15153 | 1 | 0.034
K2b1a1a | 152 | 2 | 0.069

K2b1a1a | 153 | 1 | 0.034
K2b1a1a | 15484 | 1 | 0.034
K2b1a1a | 15784 | 1 | 0.034
K2b1a1a | 16051 | 1 | 0.034
K2b1a1a | 16086 | 1 | 0.034
K2b1a1a | 16187 | 1 | 0.034
K2b1a1a | 16304 | 1 | 0.034
K2b1a1a | 16355 | 2 | 0.069
K2b1a1a | 199 | 2 | 0.069
K2b1a1a | 243 | 1 | 0.034
K2b1a1a | 4020 | 1 | 0.034
K2b1a1a | 4400 | 1 | 0.034
K2b1a1a | 6221 | 1 | 0.034
K2b1a1a | 7754 | 1 | 0.034
K2b1a1a | 8533 | 1 | 0.034
K2b1a1a | 9194 | 1 | 0.034
K2b1a2 | 16120d | 1 | 0.008
K2b1a2 | 16174 | 1 | 0.008
K2b1a2 | 16399 | 3 | 0.023
K2b1a2 | 16527 | 1 | 0.008
K2b1a2 | 182 | 1 | 0.008
K2b1a2 | 294 | 1 | 0.008
K2b1a2 | 324 | 1 | 0.008
K2b1a2 | 383 | 1 | 0.008
K2b1a2 | 5049 | 1 | 0.008
K2b1a2 | 8964 | 1 | 0.008
K2b1a3 | 11665 | 1 | 0.008
K2b1a3 | 15262 | 1 | 0.008
K2b1a3 | 15949 | 1 | 0.008
K2b1a3 | 16120d | 1 | 0.008
K2b1a3 | 16126 | 1 | 0.008
K2b1a3 | 16174 | 1 | 0.008
K2b1a3 | 16390 | 2 | 0.015
K2b1a3 | 16399 | 3 | 0.023
K2b1a3 | 16527 | 1 | 0.008
K2b1a3 | 182 | 1 | 0.008
K2b1a3 | 2882 | 1 | 0.008
K2b1a3 | 294 | 1 | 0.008
K2b1a3 | 295 | 2 | 0.015
K2b1a3 | 324 | 1 | 0.008
K2b1a3 | 383 | 1 | 0.008
K2b1a3 | 4452 | 1 | 0.008
K2b1a3 | 572 | 2 | 0.015
K2b1a4 | 12634 | 3 | 0.028
K2b1a4 | 13632 | 3 | 0.028
K2b1a4 | 14207 | 1 | 0.009
K2b1a4 | 16291 | 3 | 0.028
K2b1a4 | 16380d | 1 | 0.009
K2b1a4 | 16475d | 1 | 0.009
K2b1a4 | 16508 | 1 | 0.009
K2b1a4 | 16535 | 1 | 0.009
K2b1a4 | 16545 | 1 | 0.009
K2b1a4 | 204 | 1 | 0.009
K2b1a4 | 751 | 3 | 0.028
K2b1a4 | 7830 | 2 | 0.019
K2b1a4 | 8022 | 1 | 0.009
K2b1a4 | 9972C | 1 | 0.009
K2b1b | 12950 | 1 | 0.042
K2b1b | 13641 | 1 | 0.042
K2b1b | 14812 | 1 | 0.042
K2b1b | 152 | 1 | 0.042
K2b1b | 16007d | 1 | 0.042
K2b1b | 16013d | 1 | 0.042
K2b1b | 16093 | 1 | 0.042
K2b1b | 16103 | 1 | 0.042
K2b1b | 16189 | 1 | 0.042
K2b1b | 16259 | 1 | 0.042
K2b1b | 16312 | 1 | 0.042
K2b1b | 16319 | 2 | 0.083
K2b1b | 16355 | 1 | 0.042
K2b1b | 16399 | 5 | 0.208
K2b1b | 16421.1A | 1 | 0.042
K2b1b | 16424A | 1 | 0.042
K2b1b | 195 | 10 | 0.417
K2b1b | 234 | 1 | 0.042
K2b1b | 235 | 1 | 0.042
K2b1b | 2581 | 1 | 0.042
K2b1b | 5460 | 1 | 0.042
K2b1b | 5913 | 1 | 0.042
K2b1b | 6002 | 5 | 0.208
K2b1b | 7673 | 1 | 0.042
K2b1b | 9922 | 1 | 0.042
K2b2 | 12127 | 2 | 0.016
K2b2 | 14743 | 1 | 0.008
K2b2 | 16120d | 1 | 0.008
K2b2 | 16172 | 1 | 0.008
K2b2 | 16174 | 1 | 0.008
K2b2 | 16225 | 1 | 0.008
K2b2 | 16260 | 1 | 0.008
K2b2 | 16527 | 1 | 0.008
K2b2 | 1692 | 1 | 0.008
K2b2 | 182 | 1 | 0.008
K2b2 | 1901 | 1 | 0.008
K2b2 | 195 | 1 | 0.008
K2b2 | 294 | 1 | 0.008
K2b2 | 324 | 1 | 0.008
K2b2 | 383 | 1 | 0.008
K2b2 | 44.1C | 2 | 0.016
K2b2 | 5453 | 1 | 0.008
K2b2 | 596 | 1 | 0.008
K2b2 | 7702 | 3 | 0.024
K2b2 | 8279 | 2 | 0.016
K2b2 | 8895 | 3 | 0.024
K2b2 | 9098 | 2 | 0.016
K2b2 | 9554 | 1 | 0.008
K2c | 10262 | 1 | 0.008
K2c | 11015 | 1 | 0.008
K2c | 11041A | 1 | 0.008
K2c | 11647 | 2 | 0.015
K2c | 14326 | 2 | 0.015
K2c | 16120d | 1 | 0.008

K2c | 16172 | 2 | 0.015
K2c | 16174 | 1 | 0.008
K2c | 16192 | 2 | 0.015
K2c | 16210 | 2 | 0.015
K2c | 16261 | 2 | 0.015
K2c | 16385 | 2 | 0.015
K2c | 16399 | 3 | 0.023
K2c | 16527 | 1 | 0.008
K2c | 1719 | 2 | 0.015
K2c | 182 | 1 | 0.008
K2c | 185 | 3 | 0.023
K2c | 189 | 2 | 0.015
K2c | 207 | 2 | 0.015
K2c | 294 | 1 | 0.008
K2c | 324 | 1 | 0.008
K2c | 3531 | 1 | 0.008
K2c | 383 | 1 | 0.008
K2c | 4976 | 1 | 0.008
K2c | 5893 | 2 | 0.015
K2c | 7912 | 1 | 0.008
K2c | 9053 | 1 | 0.008
K2c | 9491 | 2 | 0.015
K3 | 10493 | 1 | 0.2
K3 | 15951 | 1 | 0.2
K3 | 16239 | 1 | 0.2
K3 | 16286 | 1 | 0.2
K3 | 16472C | 1 | 0.2
K3 | 1888 | 1 | 0.2
K3 | 189 | 1 | 0.2
K3 | 310 | 1 | 0.2
K3 | 408A | 1 | 0.2
K3 | 6935 | 3 | 0.6
K3 | 799 | 1 | 0.2
K3 | 9478 | 2 | 0.4
L0a | 10032 | 1 | 0.043
L0a | 10143 | 7 | 0.304
L0a | 10550 | 1 | 0.043
L0a | 10885 | 1 | 0.043
L0a | 10993 | 8 | 0.348
L0a | 11365 | 7 | 0.304
L0a | 1193 | 1 | 0.043
L0a | 12082 | 8 | 0.348
L0a | 12609 | 1 | 0.043
L0a | 12795 | 1 | 0.043
L0a | 12909 | 1 | 0.043
L0a | 13368 | 1 | 0.043
L0a | 13437 | 1 | 0.043
L0a | 13485 | 1 | 0.043
L0a | 14185 | 1 | 0.043
L0a | 14290 | 1 | 0.043
L0a | 146 | 1 | 0.043
L0a | 151 | 1 | 0.043
L0a | 15250 | 1 | 0.043
L0a | 15317 | 1 | 0.043
L0a | 15323 | 1 | 0.043
L0a | 15403 | 1 | 0.043
L0a | 15418 | 1 | 0.043
L0a | 15941 | 1 | 0.043
L0a | 15954 | 1 | 0.043
L0a | 16066 | 1 | 0.043
L0a | 16093 | 2 | 0.087
L0a | 16192 | 2 | 0.087
L0a | 16260 | 7 | 0.304
L0a | 16291 | 2 | 0.087
L0a | 16317 | 2 | 0.087
L0a | 16343 | 1 | 0.043
L0a | 16355 | 1 | 0.043
L0a | 16532d | 1 | 0.043
L0a | 204 | 9 | 0.391
L0a | 207 | 11 | 0.478
L0a | 2072 | 7 | 0.304
L0a | 249d | 1 | 0.043
L0a | 309d | 1 | 0.043
L0a | 316 | 1 | 0.043
L0a | 319d | 1 | 0.043
L0a | 374 | 1 | 0.043
L0a | 3866 | 1 | 0.043
L0a | 3915 | 1 | 0.043
L0a | 4580 | 1 | 0.043
L0a | 5090 | 1 | 0.043
L0a | 5096 | 1 | 0.043
L0a | 527G | 1 | 0.043
L0a | 5393 | 2 | 0.087
L0a | 5605 | 1 | 0.043
L0a | 567C | 1 | 0.043
L0a | 573.5C | 1 | 0.043
L0a | 5964 | 1 | 0.043
L0a | 6050 | 1 | 0.043
L0a | 6689A | 1 | 0.043
L0a | 6722 | 1 | 0.043
L0a | 709C | 1 | 0.043
L0a | 7379 | 1 | 0.043
L0a | 7808 | 1 | 0.043
L0a | 7843 | 1 | 0.043
L0a | 796 | 1 | 0.043
L0a | 8573 | 1 | 0.043
L0a | 8659 | 1 | 0.043
L0a | 8838 | 7 | 0.304
L0a | 8982 | 1 | 0.043
L0a | 9386 | 1 | 0.043
L0a | 9833 | 1 | 0.043
L0a1 | 11696 | 1 | 0.03
L0a1 | 12771 | 1 | 0.03
L0a1 | 13866 | 1 | 0.03
L0a1 | 14053 | 1 | 0.03
L0a1 | 14569 | 1 | 0.03
L0a1 | 151 | 1 | 0.03
L0a1 | 16114A | 3 | 0.091
L0a1 | 16184 | 5 | 0.152
L0a1 | 16209 | 1 | 0.03
L0a1 | 16218 | 1 | 0.03

L0a1 | 16266 | 2 | 0.061
L0a1 | 16278 | 1 | 0.03
L0a1 | 16327A | 1 | 0.03
L0a1 | 194 | 2 | 0.061
L0a1 | 195 | 2 | 0.061
L0a1 | 324G | 1 | 0.03
L0a1 | 363C | 1 | 0.03
L0a1 | 509A | 1 | 0.03
L0a1 | 515-524d | 4 | 0.121
L0a1 | 529T | 1 | 0.03
L0a1 | 548A | 1 | 0.03
L0a1 | 749 | 1 | 0.03
L0a1 | 8203 | 1 | 0.03
L0a1+16293 | 11950 | 5 | 0.714
L0a1+16293 | 14872 | 5 | 0.714
L0a1+16293 | 150 | 4 | 0.571
L0a1+16293 | 152d | 1 | 0.143
L0a1+16293 | 16188A | 4 | 0.571
L0a1+16293 | 16192-16193d | 1 | 0.143
L0a1+16293 | 309d | 2 | 0.286
L0a1+16293 | 4907 | 5 | 0.714
L0a1+16293 | 553 | 5 | 0.714
L0a1+16293 | 64 | 2 | 0.286
L0a1+16293 | 73 | 2 | 0.286
L0a1+16293 | 7610 | 1 | 0.143
L0a1+16293 | 7744 | 3 | 0.429
L0a1'4 | 11809 | 4 | 0.16
L0a1'4 | 12396 | 2 | 0.08
L0a1'4 | 13889 | 2 | 0.08
L0a1'4 | 14001 | 4 | 0.16
L0a1'4 | 14191 | 2 | 0.08
L0a1'4 | 16173 | 2 | 0.08
L0a1'4 | 16234 | 4 | 0.16
L0a1'4 | 16319 | 4 | 0.16
L0a1'4 | 3010 | 4 | 0.16
L0a1'4 | 5074 | 2 | 0.08
L0a1'4 | 573.1C | 1 | 0.04
L0a1'4 | 6248 | 2 | 0.08
L0a1'4 | 6261 | 2 | 0.08
L0a1'4 | 6480 | 2 | 0.08
L0a1'4 | 6485 | 2 | 0.08
L0a1'4 | 8277 | 2 | 0.08
L0a1'4 | 8278.1C | 1 | 0.04
L0a1'4 | 8545 | 4 | 0.16
L0a1'4 | 8870 | 4 | 0.16
L0a1a | 12771 | 1 | 0.021
L0a1a | 13359 | 3 | 0.064
L0a1a | 13819 | 1 | 0.021
L0a1a | 13966 | 1 | 0.021
L0a1a | 15061 | 1 | 0.021
L0a1a | 16154 | 5 | 0.106
L0a1a | 16261 | 5 | 0.106
L0a1a | 16287 | 5 | 0.106
L0a1a | 16355 | 3 | 0.064
L0a1a | 16356 | 3 | 0.064
L0a1a | 1822 | 1 | 0.021
L0a1a | 195 | 2 | 0.043
L0a1a | 2581 | 1 | 0.021
L0a1a | 2759 | 1 | 0.021
L0a1a | 515-524d | 2 | 0.043
L0a1a | 6018 | 1 | 0.021
L0a1a | 8998 | 3 | 0.064
L0a1a | 9305C | 1 | 0.021
L0a1a | 9932 | 1 | 0.021
L0a1a+200 | 10256 | 1 | 0.022
L0a1a+200 | 1053T | 2 | 0.043
L0a1a+200 | 12372 | 1 | 0.022
L0a1a+200 | 13135 | 1 | 0.022
L0a1a+200 | 13491 | 4 | 0.087
L0a1a+200 | 14161 | 2 | 0.043
L0a1a+200 | 14431 | 3 | 0.065
L0a1a+200 | 14455 | 3 | 0.065
L0a1a+200 | 14981C | 2 | 0.043
L0a1a+200 | 151 | 1 | 0.022
L0a1a+200 | 15172 | 1 | 0.022
L0a1a+200 | 15813 | 1 | 0.022
L0a1a+200 | 15850 | 1 | 0.022
L0a1a+200 | 16093 | 2 | 0.043
L0a1a+200 | 16165 | 1 | 0.022
L0a1a+200 | 16188A | 1 | 0.022
L0a1a+200 | 16189G | 1 | 0.022
L0a1a+200 | 16256 | 1 | 0.022
L0a1a+200 | 16271 | 1 | 0.022
L0a1a+200 | 16278 | 8 | 0.174
L0a1a+200 | 16284 | 2 | 0.043
L0a1a+200 | 16291 | 1 | 0.022
L0a1a+200 | 16293C | 1 | 0.022
L0a1a+200 | 16294 | 1 | 0.022
L0a1a+200 | 16301 | 1 | 0.022
L0a1a+200 | 16527 | 1 | 0.022
L0a1a+200 | 194 | 3 | 0.065
L0a1a+200 | 249d | 1 | 0.022
L0a1a+200 | 3010 | 1 | 0.022
L0a1a+200 | 310 | 1 | 0.022
L0a1a+200 | 3325 | 1 | 0.022
L0a1a+200 | 3498 | 1 | 0.022
L0a1a+200 | 368 | 3 | 0.065
L0a1a+200 | 3736 | 1 | 0.022
L0a1a+200 | 374 | 1 | 0.022
L0a1a+200 | 3834 | 1 | 0.022
L0a1a+200 | 5618 | 3 | 0.065
L0a1a+200 | 7022 | 1 | 0.022
L0a1a+200 | 7247 | 2 | 0.043
L0a1a+200 | 72G | 1 | 0.022
L0a1a+200 | 8302 | 2 | 0.043
L0a1a+200 | 8896 | 2 | 0.043
L0a1a+200 | 960.1C | 2 | 0.043
L0a1a1 | 10420 | 1 | 0.025
L0a1a1 | 11992 | 1 | 0.025
L0a1a1 | 12280 | 1 | 0.025
L0a1a1 | 12738 | 2 | 0.05

L0a1a1 | 14364 | 2 | 0.05
L0a1a1 | 14560 | 2 | 0.05
L0a1a1 | 14569 | 2 | 0.05
L0a1a1 | 150 | 2 | 0.05
L0a1a1 | 151 | 1 | 0.025
L0a1a1 | 15301 | 2 | 0.05
L0a1a1 | 15758 | 1 | 0.025
L0a1a1 | 16086 | 2 | 0.05
L0a1a1 | 16165 | 2 | 0.05
L0a1a1 | 16166 | 1 | 0.025
L0a1a1 | 16318 | 5 | 0.125
L0a1a1 | 16344 | 5 | 0.125
L0a1a1 | 16390 | 5 | 0.125
L0a1a1 | 16532d | 1 | 0.025
L0a1a1 | 38.1G | 1 | 0.025
L0a1a1 | 44.1C | 1 | 0.025
L0a1a1 | 5811T | 2 | 0.05
L0a1a1 | 5899.3C | 1 | 0.025
L0a1a1 | 8017 | 5 | 0.125
L0a1a1 | 8869 | 2 | 0.05
L0a1a1 | 9380 | 2 | 0.05
L0a1a2 | 10237 | 2 | 0.015
L0a1a2 | 10245 | 1 | 0.008
L0a1a2 | 10306C | 1 | 0.008
L0a1a2 | 10313 | 1 | 0.008
L0a1a2 | 10601 | 1 | 0.008
L0a1a2 | 10942 | 1 | 0.008
L0a1a2 | 11548 | 1 | 0.008
L0a1a2 | 11875 | 1 | 0.008
L0a1a2 | 12016 | 1 | 0.008
L0a1a2 | 12092A | 3 | 0.023
L0a1a2 | 12172 | 2 | 0.015
L0a1a2 | 12192 | 1 | 0.008
L0a1a2 | 12241d | 2 | 0.015
L0a1a2 | 12471 | 1 | 0.008
L0a1a2 | 12513 | 2 | 0.015
L0a1a2 | 12612C | 1 | 0.008
L0a1a2 | 12732 | 2 | 0.015
L0a1a2 | 13215A | 1 | 0.008
L0a1a2 | 13759 | 1 | 0.008
L0a1a2 | 13977 | 1 | 0.008
L0a1a2 | 14016 | 1 | 0.008
L0a1a2 | 14194 | 1 | 0.008
L0a1a2 | 14329 | 1 | 0.008
L0a1a2 | 14502 | 1 | 0.008
L0a1a2 | 14544 | 1 | 0.008
L0a1a2 | 14560 | 1 | 0.008
L0a1a2 | 14587 | 1 | 0.008
L0a1a2 | 146 | 1 | 0.008
L0a1a2 | 14632 | 1 | 0.008
L0a1a2 | 150 | 4 | 0.03
L0a1a2 | 15061 | 1 | 0.008
L0a1a2 | 15119 | 1 | 0.008
L0a1a2 | 15191 | 1 | 0.008
L0a1a2 | 152 | 2 | 0.015
L0a1a2 | 15314 | 1 | 0.008
L0a1a2 | 15758 | 1 | 0.008
L0a1a2 | 15773 | 1 | 0.008
L0a1a2 | 15777 | 1 | 0.008
L0a1a2 | 15779 | 1 | 0.008
L0a1a2 | 15924 | 1 | 0.008
L0a1a2 | 16076 | 1 | 0.008
L0a1a2 | 16092 | 1 | 0.008
L0a1a2 | 16093 | 1 | 0.008
L0a1a2 | 16107 | 1 | 0.008
L0a1a2 | 16111 | 1 | 0.008
L0a1a2 | 16114A | 1 | 0.008
L0a1a2 | 16144 | 1 | 0.008
L0a1a2 | 16188A | 7 | 0.053
L0a1a2 | 16190G | 1 | 0.008
L0a1a2 | 16192-16193d | 2 | 0.015
L0a1a2 | 16193d | 1 | 0.008
L0a1a2 | 16207 | 1 | 0.008
L0a1a2 | 16215 | 4 | 0.03
L0a1a2 | 16246 | 2 | 0.015
L0a1a2 | 16258C | 1 | 0.008
L0a1a2 | 16271 | 1 | 0.008
L0a1a2 | 16278 | 2 | 0.015
L0a1a2 | 16291 | 2 | 0.015
L0a1a2 | 16294 | 1 | 0.008
L0a1a2 | 16319 | 1 | 0.008
L0a1a2 | 16325 | 2 | 0.015
L0a1a2 | 16344 | 1 | 0.008
L0a1a2 | 16355 | 1 | 0.008
L0a1a2 | 16357 | 2 | 0.015
L0a1a2 | 16360 | 1 | 0.008
L0a1a2 | 16362 | 32 | 0.241
L0a1a2 | 16399 | 4 | 0.03
L0a1a2 | 16456 | 1 | 0.008
L0a1a2 | 16481 | 1 | 0.008
L0a1a2 | 16527 | 1 | 0.008
L0a1a2 | 195 | 1 | 0.008
L0a1a2 | 2487C | 1 | 0.008
L0a1a2 | 2755 | 1 | 0.008
L0a1a2 | 309d | 10 | 0.075
L0a1a2 | 310 | 4 | 0.03
L0a1a2 | 3116 | 2 | 0.015
L0a1a2 | 315.2C | 1 | 0.008
L0a1a2 | 3438 | 1 | 0.008
L0a1a2 | 3873 | 1 | 0.008
L0a1a2 | 394A | 1 | 0.008
L0a1a2 | 4316 | 1 | 0.008
L0a1a2 | 5033 | 1 | 0.008
L0a1a2 | 5112 | 1 | 0.008
L0a1a2 | 514 | 16 | 0.12
L0a1a2 | 515-524d | 5 | 0.038
L0a1a2 | 5186 | 1 | 0.008
L0a1a2 | 5681 | 1 | 0.008
L0a1a2 | 629 | 1 | 0.008
L0a1a2 | 6419C | 1 | 0.008
L0a1a2 | 6891 | 1 | 0.008

L0a1a2 | 73 | 2 | 0.015
L0a1a2 | 7785 | 1 | 0.008
L0a1a2 | 7805 | 1 | 0.008
L0a1a2 | 8185 | 1 | 0.008
L0a1a2 | 8388 | 1 | 0.008
L0a1a2 | 8516 | 1 | 0.008
L0a1a2 | 8521 | 1 | 0.008
L0a1a2 | 8552 | 1 | 0.008
L0a1a2 | 8839 | 1 | 0.008
L0a1a2 | 89 | 1 | 0.008
L0a1a2 | 9101 | 1 | 0.008
L0a1a2 | 9218 | 2 | 0.015
L0a1a2 | 95 | 1 | 0.008
L0a1a2 | 9509 | 1 | 0.008
L0a1a2 | 95T | 1 | 0.008
L0a1a2 | 9903 | 1 | 0.008
L0a1a3 | 10143 | 1 | 0.014
L0a1a3 | 14386 | 1 | 0.014
L0a1a3 | 146 | 2 | 0.029
L0a1a3 | 150 | 1 | 0.014
L0a1a3 | 151 | 2 | 0.029
L0a1a3 | 15839 | 1 | 0.014
L0a1a3 | 16025 | 1 | 0.014
L0a1a3 | 16086 | 1 | 0.014
L0a1a3 | 16093 | 1 | 0.014
L0a1a3 | 16111A | 3 | 0.043
L0a1a3 | 16165 | 1 | 0.014
L0a1a3 | 16188A | 1 | 0.014
L0a1a3 | 16190G | 1 | 0.014
L0a1a3 | 16193 | 1 | 0.014
L0a1a3 | 16214 | 2 | 0.029
L0a1a3 | 16215 | 2 | 0.029
L0a1a3 | 16234 | 1 | 0.014
L0a1a3 | 16239 | 1 | 0.014
L0a1a3 | 16261 | 1 | 0.014
L0a1a3 | 16271 | 1 | 0.014
L0a1a3 | 16278 | 2 | 0.029
L0a1a3 | 16355 | 2 | 0.029
L0a1a3 | 16362 | 1 | 0.014
L0a1a3 | 16390 | 3 | 0.043
L0a1a3 | 16511A | 2 | 0.029
L0a1a3 | 194 | 1 | 0.014
L0a1a3 | 195 | 2 | 0.029
L0a1a3 | 234 | 2 | 0.029
L0a1a3 | 249d | 1 | 0.014
L0a1a3 | 316 | 1 | 0.014
L0a1a3 | 317.1C | 1 | 0.014
L0a1a3 | 324G | 2 | 0.029
L0a1a3 | 356.1C | 1 | 0.014
L0a1a3 | 357.1C | 1 | 0.014
L0a1a3 | 3573 | 1 | 0.014
L0a1a3 | 366 | 4 | 0.057
L0a1a3 | 368 | 2 | 0.029
L0a1a3 | 380C | 1 | 0.014
L0a1a3 | 3847 | 2 | 0.029
L0a1a3 | 389 | 2 | 0.029
L0a1a3 | 411G | 1 | 0.014
L0a1a3 | 438 | 1 | 0.014
L0a1a3 | 441 | 1 | 0.014
L0a1a3 | 474A | 1 | 0.014
L0a1a3 | 5012 | 1 | 0.014
L0a1a3 | 507A | 2 | 0.029
L0a1a3 | 509A | 1 | 0.014
L0a1a3 | 515-524d | 35 | 0.5
L0a1a3 | 529T | 2 | 0.029
L0a1a3 | 548A | 5 | 0.071
L0a1a3 | 89 | 1 | 0.014
L0a1a3 | 9195A | 1 | 0.014
L0a1a3 | 9801 | 1 | 0.014
L0a1b | 11332 | 1 | 0.027
L0a1b | 12930 | 1 | 0.027
L0a1b | 15886 | 1 | 0.027
L0a1b | 16093 | 2 | 0.054
L0a1b | 16193 | 12 | 0.324
L0a1b | 16209 | 4 | 0.108
L0a1b | 16218 | 1 | 0.027
L0a1b | 16344 | 1 | 0.027
L0a1b | 16368 | 1 | 0.027
L0a1b | 3849 | 1 | 0.027
L0a1b | 4194 | 1 | 0.027
L0a1b | 515-524d | 2 | 0.054
L0a1b | 634 | 1 | 0.027
L0a1b | 6575 | 1 | 0.027
L0a1b | 6848 | 1 | 0.027
L0a1b | 8134 | 1 | 0.027
L0a1b | 8413 | 1 | 0.027
L0a1b1 | 12432 | 1 | 0.011
L0a1b1 | 150 | 1 | 0.011
L0a1b1 | 15244 | 1 | 0.011
L0a1b1 | 1539A | 2 | 0.023
L0a1b1 | 16093 | 28 | 0.318
L0a1b1 | 16188A | 3 | 0.034
L0a1b1 | 16190G | 1 | 0.011
L0a1b1 | 16192-16193d | 26 | 0.295
L0a1b1 | 199 | 1 | 0.011
L0a1b1 | 2377 | 1 | 0.011
L0a1b1 | 309d | 2 | 0.023
L0a1b1 | 315.2C | 2 | 0.023
L0a1b1 | 3644 | 2 | 0.023
L0a1b1 | 3745 | 1 | 0.011
L0a1b1 | 515-524d | 3 | 0.034
L0a1b1 | 5628 | 3 | 0.034
L0a1b1 | 593A | 1 | 0.011
L0a1b1 | 5951 | 1 | 0.011
L0a1b1 | 6446 | 1 | 0.011
L0a1b1 | 6752 | 1 | 0.011
L0a1b1 | 6905 | 2 | 0.023
L0a1b1 | 7041 | 1 | 0.011
L0a1b1 | 7257 | 1 | 0.011
L0a1b1 | 7258 | 1 | 0.011
L0a1b1 | 7552 | 1 | 0.011

L0a1b1 | 8986 | 1 | 0.011
L0a1b1 | 9110 | 1 | 0.011
L0a1b1a | 10680 | 2 | 0.03
L0a1b1a | 11890 | 2 | 0.03
L0a1b1a | 12164 | 1 | 0.015
L0a1b1a | 1291 | 1 | 0.015
L0a1b1a | 14251 | 1 | 0.015
L0a1b1a | 150 | 1 | 0.015
L0a1b1a | 16093 | 1 | 0.015
L0a1b1a | 16173 | 2 | 0.03
L0a1b1a | 16188A | 1 | 0.015
L0a1b1a | 16192-16193d | 1 | 0.015
L0a1b1a | 309d | 6 | 0.091
L0a1b1a | 3311 | 8 | 0.121
L0a1b1a | 3591 | 1 | 0.015
L0a1b1a | 3732 | 1 | 0.015
L0a1b1a | 3906A | 1 | 0.015
L0a1b1a | 5665 | 1 | 0.015
L0a1b1a | 593 | 4 | 0.061
L0a1b1a | 7686 | 1 | 0.015
L0a1b1a | 8242 | 2 | 0.03
L0a1b1a | 9286 | 1 | 0.015
L0a1b1a | 961d | 1 | 0.015
L0a1b1a1 | 1063 | 2 | 0.013
L0a1b1a1 | 10643 | 1 | 0.007
L0a1b1a1 | 10876 | 1 | 0.007
L0a1b1a1 | 11137 | 3 | 0.02
L0a1b1a1 | 11170 | 1 | 0.007
L0a1b1a1 | 11237 | 3 | 0.02
L0a1b1a1 | 11361 | 1 | 0.007
L0a1b1a1 | 11399 | 1 | 0.007
L0a1b1a1 | 11662 | 1 | 0.007
L0a1b1a1 | 11887 | 2 | 0.013
L0a1b1a1 | 11971 | 1 | 0.007
L0a1b1a1 | 12438 | 1 | 0.007
L0a1b1a1 | 12651 | 4 | 0.027
L0a1b1a1 | 13143 | 2 | 0.013
L0a1b1a1 | 13269 | 4 | 0.027
L0a1b1a1 | 14110 | 2 | 0.013
L0a1b1a1 | 14251 | 3 | 0.02
L0a1b1a1 | 14551 | 1 | 0.007
L0a1b1a1 | 146 | 1 | 0.007
L0a1b1a1 | 152 | 3 | 0.02
L0a1b1a1 | 15257 | 1 | 0.007
L0a1b1a1 | 15301 | 1 | 0.007
L0a1b1a1 | 15481 | 19 | 0.127
L0a1b1a1 | 15748 | 1 | 0.007
L0a1b1a1 | 15844 | 1 | 0.007
L0a1b1a1 | 16093 | 2 | 0.013
L0a1b1a1 | 16192 | 2 | 0.013
L0a1b1a1 | 16192-16193d | 10 | 0.067
L0a1b1a1 | 16214 | 2 | 0.013
L0a1b1a1 | 16249 | 2 | 0.013
L0a1b1a1 | 16344 | 3 | 0.02
L0a1b1a1 | 16368 | 1 | 0.007
L0a1b1a1 | 195 | 1 | 0.007
L0a1b1a1 | 2140 | 1 | 0.007
L0a1b1a1 | 279 | 1 | 0.007
L0a1b1a1 | 309d | 24 | 0.16
L0a1b1a1 | 310 | 6 | 0.04
L0a1b1a1 | 315.2C | 1 | 0.007
L0a1b1a1 | 316 | 1 | 0.007
L0a1b1a1 | 3338 | 1 | 0.007
L0a1b1a1 | 3736 | 1 | 0.007
L0a1b1a1 | 374 | 2 | 0.013
L0a1b1a1 | 4491 | 1 | 0.007
L0a1b1a1 | 455.1T | 1 | 0.007
L0a1b1a1 | 4649 | 1 | 0.007
L0a1b1a1 | 4674 | 1 | 0.007
L0a1b1a1 | 4706 | 1 | 0.007
L0a1b1a1 | 5147 | 2 | 0.013
L0a1b1a1 | 5665 | 3 | 0.02
L0a1b1a1 | 6060 | 2 | 0.013
L0a1b1a1 | 6293 | 1 | 0.007
L0a1b1a1 | 6307 | 1 | 0.007
L0a1b1a1 | 6599 | 1 | 0.007
L0a1b1a1 | 678 | 2 | 0.013
L0a1b1a1 | 6908A | 2 | 0.013
L0a1b1a1 | 709 | 3 | 0.02
L0a1b1a1 | 806 | 1 | 0.007
L0a1b1a1 | 8289.1CCCCCTCTA | 1 | 0.007
L0a1b1a1 | 8577 | 6 | 0.04
L0a1b1a1 | 8820 | 1 | 0.007
L0a1b1a1 | 89 | 3 | 0.02
L0a1b1a1 | 9025 | 1 | 0.007
L0a1b1a1 | 9230 | 1 | 0.007
L0a1b1a1 | 9288 | 1 | 0.007
L0a1b1a1 | 9355 | 1 | 0.007
L0a1b1a1 | 9356 | 1 | 0.007
L0a1b1a1 | 965.1C | 9 | 0.06
L0a1b1a1 | 965.2C | 3 | 0.02
L0a1b1a1 | 9758 | 1 | 0.007
L0a1b1a1a | 10187 | 1 | 0.013
L0a1b1a1a | 10301 | 1 | 0.013
L0a1b1a1a | 1290 | 1 | 0.013
L0a1b1a1a | 131 | 2 | 0.027
L0a1b1a1a | 14383 | 2 | 0.027
L0a1b1a1a | 15236 | 1 | 0.013
L0a1b1a1a | 153 | 1 | 0.013
L0a1b1a1a | 15927 | 1 | 0.013
L0a1b1a1a | 16111 | 1 | 0.013
L0a1b1a1a | 16126 | 1 | 0.013
L0a1b1a1a | 16129C | 1 | 0.013
L0a1b1a1a | 16214 | 2 | 0.027
L0a1b1a1a | 16256 | 1 | 0.013
L0a1b1a1a | 16266 | 1 | 0.013
L0a1b1a1a | 16291 | 1 | 0.013
L0a1b1a1a | 16292 | 1 | 0.013
L0a1b1a1a | 16365 | 1 | 0.013
L0a1b1a1a | 16526 | 2 | 0.027
L0a1b1a1a | 187 | 1 | 0.013

L0a1b1a1a | 1888 | 1 | 0.013
L0a1b1a1a | 194G | 1 | 0.013
L0a1b1a1a | 195 | 1 | 0.013
L0a1b1a1a | 200 | 1 | 0.013
L0a1b1a1a | 249d | 1 | 0.013
L0a1b1a1a | 330G | 1 | 0.013
L0a1b1a1a | 4113 | 1 | 0.013
L0a1b1a1a | 8767 | 1 | 0.013
L0a1b1a1a | 89 | 4 | 0.053
L0a1b1a1a | 961d | 1 | 0.013
L0a1b1a1a | 965.1C | 1 | 0.013
L0a1b2 | 131 | 17 | 0.354
L0a1b2 | 13767A | 1 | 0.021
L0a1b2 | 16192-16193d | 11 | 0.229
L0a1b2 | 16209 | 3 | 0.062
L0a1b2 | 16292 | 1 | 0.021
L0a1b2 | 194 | 1 | 0.021
L0a1b2 | 2281 | 17 | 0.354
L0a1b2 | 309d | 6 | 0.125
L0a1b2 | 515-524d | 4 | 0.083
L0a1b2 | 5258 | 5 | 0.104
L0a1b2 | 7283 | 1 | 0.021
L0a1b2 | 7711 | 20 | 0.417
L0a1b2 | 8512 | 1 | 0.021
L0a1b2 | 8950 | 1 | 0.021
L0a1b2 | 9804 | 20 | 0.417
L0a1b2a | 11812 | 2 | 0.1
L0a1b2a | 14198 | 2 | 0.1
L0a1b2a | 16209 | 2 | 0.1
L0a1b2a | 309d | 3 | 0.15
L0a1b2a | 317.1C | 1 | 0.05
L0a1c | 12031A | 1 | 0.091
L0a1c | 14251 | 1 | 0.091
L0a1c | 151 | 1 | 0.091
L0a1c | 16114A | 4 | 0.364
L0a1c | 16162 | 1 | 0.091
L0a1c | 16184 | 1 | 0.091
L0a1c | 16511A | 1 | 0.091
L0a1c | 16532d | 4 | 0.364
L0a1c | 1719 | 1 | 0.091
L0a1c | 5073 | 1 | 0.091
L0a1c | 5147 | 1 | 0.091
L0a1c | 515-524d | 2 | 0.182
L0a1c | 9288 | 1 | 0.091
L0a1c1 | 12657 | 1 | 0.056
L0a1c1 | 13020 | 1 | 0.056
L0a1c1 | 14347 | 1 | 0.056
L0a1c1 | 15941 | 4 | 0.222
L0a1c1 | 16025 | 1 | 0.056
L0a1c1 | 16069 | 1 | 0.056
L0a1c1 | 16093 | 1 | 0.056
L0a1c1 | 16230C | 1 | 0.056
L0a1c1 | 16257 | 1 | 0.056
L0a1c1 | 16266 | 1 | 0.056
L0a1c1 | 16269 | 1 | 0.056
L0a1c1 | 16278 | 3 | 0.167
L0a1c1 | 3534 | 1 | 0.056
L0a1c1 | 36.1G | 1 | 0.056
L0a1c1 | 44.1C | 1 | 0.056
L0a1c1 | 515-524d | 2 | 0.111
L0a1c1 | 5964 | 2 | 0.111
L0a1c1 | 7299 | 1 | 0.056
L0a1c1 | 7394 | 1 | 0.056
L0a1c1 | 747 | 1 | 0.056
L0a1c1 | 8251 | 1 | 0.056
L0a1c1 | 9055 | 1 | 0.056
L0a1d | 10538 | 2 | 0.074
L0a1d | 11611 | 1 | 0.037
L0a1d | 13563 | 1 | 0.037
L0a1d | 143 | 1 | 0.037
L0a1d | 146 | 1 | 0.037
L0a1d | 151 | 3 | 0.111
L0a1d | 153 | 1 | 0.037
L0a1d | 15924 | 2 | 0.074
L0a1d | 16024 | 2 | 0.074
L0a1d | 16025 | 2 | 0.074
L0a1d | 16114A | 2 | 0.074
L0a1d | 16184 | 2 | 0.074
L0a1d | 16261 | 2 | 0.074
L0a1d | 16287 | 3 | 0.111
L0a1d | 16294 | 3 | 0.111
L0a1d | 16350 | 2 | 0.074
L0a1d | 16390 | 1 | 0.037
L0a1d | 16399 | 1 | 0.037
L0a1d | 16511A | 1 | 0.037
L0a1d | 16532d | 1 | 0.037
L0a1d | 194 | 1 | 0.037
L0a1d | 195 | 5 | 0.185
L0a1d | 200 | 2 | 0.074
L0a1d | 203 | 1 | 0.037
L0a1d | 204 | 1 | 0.037
L0a1d | 249d | 1 | 0.037
L0a1d | 3534A | 1 | 0.037
L0a1d | 36.1G | 1 | 0.037
L0a1d | 4086 | 1 | 0.037
L0a1d | 515-524d | 4 | 0.148
L0a1d | 5301 | 1 | 0.037
L0a1d | 6446 | 1 | 0.037
L0a1d | 7278 | 1 | 0.037
L0a1d | 73 | 1 | 0.037
L0a1d | 8577 | 1 | 0.037
L0a1d | 89 | 3 | 0.111
L0a1e | 13020 | 1 | 0.045
L0a1e | 15853 | 2 | 0.091
L0a1e | 15942 | 2 | 0.091
L0a1e | 16051 | 4 | 0.182
L0a1e | 16168 | 4 | 0.182
L0a1e | 16184 | 1 | 0.045
L0a1e | 16266 | 2 | 0.091
L0a1e | 199 | 1 | 0.045
L0a1e | 325 | 1 | 0.045

L0a1e | 5631 | 1 | 0.045
L0a1e | 7076 | 1 | 0.045
L0a1e | 7861 | 1 | 0.045
L0a2 | 11269 | 2 | 0.026
L0a2 | 11452 | 1 | 0.013
L0a2 | 11870G | 1 | 0.013
L0a2 | 11887 | 3 | 0.039
L0a2 | 12172 | 5 | 0.066
L0a2 | 12362 | 1 | 0.013
L0a2 | 13419 | 5 | 0.066
L0a2 | 13582 | 1 | 0.013
L0a2 | 14281 | 3 | 0.039
L0a2 | 143 | 1 | 0.013
L0a2 | 14470 | 2 | 0.026
L0a2 | 14560 | 4 | 0.053
L0a2 | 146 | 22 | 0.289
L0a2 | 14798 | 1 | 0.013
L0a2 | 150 | 2 | 0.026
L0a2 | 15002 | 2 | 0.026
L0a2 | 15067 | 5 | 0.066
L0a2 | 15479 | 2 | 0.026
L0a2 | 15769 | 3 | 0.039
L0a2 | 15848 | 1 | 0.013
L0a2 | 16024 | 2 | 0.026
L0a2 | 16025 | 2 | 0.026
L0a2 | 16170 | 1 | 0.013
L0a2 | 16174 | 5 | 0.066
L0a2 | 16188A | 7 | 0.092
L0a2 | 16192 | 4 | 0.053
L0a2 | 16192-16193d | 1 | 0.013
L0a2 | 16212 | 9 | 0.118
L0a2 | 16213 | 2 | 0.026
L0a2 | 16214 | 3 | 0.039
L0a2 | 16221 | 3 | 0.039
L0a2 | 16234 | 5 | 0.066
L0a2 | 16283T | 1 | 0.013
L0a2 | 16286 | 1 | 0.013
L0a2 | 16362 | 1 | 0.013
L0a2 | 16437 | 1 | 0.013
L0a2 | 16465 | 1 | 0.013
L0a2 | 16532d | 9 | 0.118
L0a2 | 185 | 1 | 0.013
L0a2 | 195 | 2 | 0.026
L0a2 | 1986C | 5 | 0.066
L0a2 | 199 | 2 | 0.026
L0a2 | 200 | 2 | 0.026
L0a2 | 207 | 1 | 0.013
L0a2 | 226 | 1 | 0.013
L0a2 | 234 | 1 | 0.013
L0a2 | 259 | 1 | 0.013
L0a2 | 25C | 1 | 0.013
L0a2 | 27A | 1 | 0.013
L0a2 | 2857 | 2 | 0.026
L0a2 | 288 | 1 | 0.013
L0a2 | 308G | 1 | 0.013
L0a2 | 3398 | 5 | 0.066
L0a2 | 3421 | 3 | 0.039
L0a2 | 36.1G | 2 | 0.026
L0a2 | 3624 | 5 | 0.066
L0a2 | 3693 | 3 | 0.039
L0a2 | 3882 | 3 | 0.039
L0a2 | 3954 | 1 | 0.013
L0a2 | 4113 | 1 | 0.013
L0a2 | 42.1T | 3 | 0.039
L0a2 | 44.1C | 1 | 0.013
L0a2 | 4452 | 3 | 0.039
L0a2 | 4917 | 4 | 0.053
L0a2 | 523d | 1 | 0.013
L0a2 | 525G | 1 | 0.013
L0a2 | 527G | 1 | 0.013
L0a2 | 538C | 9 | 0.118
L0a2 | 573.2C | 1 | 0.013
L0a2 | 573.3C | 1 | 0.013
L0a2 | 5892 | 1 | 0.013
L0a2 | 6479 | 5 | 0.066
L0a2 | 6827 | 1 | 0.013
L0a2 | 709 | 1 | 0.013
L0a2 | 71d | 1 | 0.013
L0a2 | 723 | 1 | 0.013
L0a2 | 73 | 11 | 0.145
L0a2 | 8498 | 3 | 0.039
L0a2 | 8572 | 1 | 0.013
L0a2 | 89 | 10 | 0.132
L0a2 | 9077 | 5 | 0.066
L0a2 | 9821C | 2 | 0.026
L0a2 | 99 | 1 | 0.013
L0a2 | 9948 | 2 | 0.026
L0a2 | 9963G | 1 | 0.013
L0a2a | 12778T | 1 | 0.03
L0a2a | 13685A | 1 | 0.03
L0a2a | 16042 | 1 | 0.03
L0a2a | 16111 | 1 | 0.03
L0a2a | 207 | 1 | 0.03
L0a2a | 2653d | 1 | 0.03
L0a2a | 4427 | 1 | 0.03
L0a2a | 6328A | 1 | 0.03
L0a2a | 73 | 1 | 0.03
L0a2a | 8070G | 1 | 0.03
L0a2a | 8071T | 1 | 0.03
L0a2a | 8126G | 1 | 0.03
L0a2a | 95T | 1 | 0.03
L0a2a1 | 15927 | 11 | 0.268
L0a2a1 | 16093 | 10 | 0.244
L0a2a1 | 16129 | 3 | 0.073
L0a2a1 | 310 | 2 | 0.049
L0a2a1 | 6407 | 11 | 0.268
L0a2a1a | 10493 | 8 | 0.16
L0a2a1a | 13803 | 1 | 0.02
L0a2a1a | 14560 | 8 | 0.16
L0a2a1a | 15466 | 1 | 0.02
L0a2a1a | 15491 | 1 | 0.02

L0a2a1a | 16093 | 9 | 0.18
L0a2a1a | 16164 | 1 | 0.02
L0a2a1a | 16192-16193d | 3 | 0.06
L0a2a1a | 16214 | 4 | 0.08
L0a2a1a | 16289 | 4 | 0.08
L0a2a1a | 16390 | 1 | 0.02
L0a2a1a | 185 | 1 | 0.02
L0a2a1a | 207 | 8 | 0.16
L0a2a1a | 309d | 9 | 0.18
L0a2a1a | 3666 | 3 | 0.06
L0a2a1a | 5493 | 1 | 0.02
L0a2a1a | 5563 | 1 | 0.02
L0a2a1a | 575 | 2 | 0.04
L0a2a1a | 7424 | 1 | 0.02
L0a2a1a | 8264 | 1 | 0.02
L0a2a1a | 8954 | 1 | 0.02
L0a2a1a | 961 | 7 | 0.14
L0a2a1a | 9978G | 1 | 0.02
L0a2a1a1 | 11830 | 3 | 0.088
L0a2a1a1 | 4080 | 1 | 0.029
L0a2a1a2 | 13500 | 1 | 0.026
L0a2a1a2 | 13973T | 1 | 0.026
L0a2a1a2 | 14569 | 1 | 0.026
L0a2a1a2 | 15930 | 1 | 0.026
L0a2a1a2 | 16093 | 7 | 0.184
L0a2a1a2 | 16188A | 4 | 0.105
L0a2a1a2 | 16193 | 1 | 0.026
L0a2a1a2 | 309d | 1 | 0.026
L0a2a1b | 10978 | 1 | 0.01
L0a2a1b | 11797 | 1 | 0.01
L0a2a1b | 12414 | 27 | 0.257
L0a2a1b | 146 | 1 | 0.01
L0a2a1b | 14668 | 1 | 0.01
L0a2a1b | 15119 | 1 | 0.01
L0a2a1b | 15258 | 1 | 0.01
L0a2a1b | 15562 | 4 | 0.038
L0a2a1b | 16093 | 66 | 0.629
L0a2a1b | 16104 | 1 | 0.01
L0a2a1b | 16171 | 1 | 0.01
L0a2a1b | 16173 | 1 | 0.01
L0a2a1b | 16174 | 2 | 0.019
L0a2a1b | 16184 | 5 | 0.048
L0a2a1b | 16188A | 4 | 0.038
L0a2a1b | 16192-16193d | 18 | 0.171
L0a2a1b | 16214 | 1 | 0.01
L0a2a1b | 16238A | 1 | 0.01
L0a2a1b | 16287 | 4 | 0.038
L0a2a1b | 16289 | 1 | 0.01
L0a2a1b | 16399 | 1 | 0.01
L0a2a1b | 199 | 1 | 0.01
L0a2a1b | 207 | 1 | 0.01
L0a2a1b | 309d | 25 | 0.238
L0a2a1b | 310 | 1 | 0.01
L0a2a1b | 3396 | 1 | 0.01
L0a2a1b | 3870 | 1 | 0.01
L0a2a1b | 507A | 1 | 0.01
L0a2a1b | 515-524d | 1 | 0.01
L0a2a1b | 573.2C | 2 | 0.019
L0a2a1b | 6366 | 1 | 0.01
L0a2a1b | 73 | 2 | 0.019
L0a2a1b | 7609 | 1 | 0.01
L0a2a1b | 7859 | 1 | 0.01
L0a2a1b | 8348 | 27 | 0.257
L0a2a1b | 89 | 2 | 0.019
L0a2a1b | 8945 | 2 | 0.019
L0a2a2 | 14371 | 1 | 0.013
L0a2a2 | 146 | 1 | 0.013
L0a2a2 | 151 | 2 | 0.027
L0a2a2 | 15301 | 3 | 0.04
L0a2a2 | 16024 | 1 | 0.013
L0a2a2 | 16025 | 1 | 0.013
L0a2a2 | 16144 | 2 | 0.027
L0a2a2 | 16242 | 1 | 0.013
L0a2a2 | 16274 | 5 | 0.067
L0a2a2 | 16484 | 1 | 0.013
L0a2a2 | 16566C | 1 | 0.013
L0a2a2 | 2C | 1 | 0.013
L0a2a2 | 4598 | 3 | 0.04
L0a2a2 | 4916 | 1 | 0.013
L0a2a2 | 6607A | 3 | 0.04
L0a2a2 | 83 | 1 | 0.013
L0a2a2a | 10007 | 3 | 0.011
L0a2a2a | 10338 | 1 | 0.004
L0a2a2a | 10361 | 3 | 0.011
L0a2a2a | 10400 | 1 | 0.004
L0a2a2a | 10775 | 1 | 0.004
L0a2a2a | 10876 | 4 | 0.014
L0a2a2a | 11016 | 1 | 0.004
L0a2a2a | 11491 | 1 | 0.004
L0a2a2a | 11602 | 1 | 0.004
L0a2a2a | 11770 | 1 | 0.004
L0a2a2a | 11903A | 1 | 0.004
L0a2a2a | 1192 | 1 | 0.004
L0a2a2a | 11935 | 1 | 0.004
L0a2a2a | 11944 | 1 | 0.004
L0a2a2a | 11992 | 1 | 0.004
L0a2a2a | 12175 | 1 | 0.004
L0a2a2a | 12280 | 1 | 0.004
L0a2a2a | 12372 | 1 | 0.004
L0a2a2a | 12634 | 1 | 0.004
L0a2a2a | 12771 | 11 | 0.039
L0a2a2a | 12886A | 1 | 0.004
L0a2a2a | 13104 | 11 | 0.039
L0a2a2a | 1313 | 1 | 0.004
L0a2a2a | 13681 | 1 | 0.004
L0a2a2a | 14053 | 1 | 0.004
L0a2a2a | 1406 | 1 | 0.004
L0a2a2a | 14094 | 1 | 0.004
L0a2a2a | 14106 | 1 | 0.004
L0a2a2a | 14179 | 1 | 0.004
L0a2a2a | 14197 | 3 | 0.011

L0a2a2a | 14221 | 1 | 0.004
L0a2a2a | 14311 | 1 | 0.004
L0a2a2a | 14323 | 1 | 0.004
L0a2a2a | 14371 | 2 | 0.007
L0a2a2a | 14403 | 2 | 0.007
L0a2a2a | 14467 | 1 | 0.004
L0a2a2a | 14484 | 1 | 0.004
L0a2a2a | 146 | 1 | 0.004
L0a2a2a | 14643A | 1 | 0.004
L0a2a2a | 14881 | 1 | 0.004
L0a2a2a | 150 | 49 | 0.174
L0a2a2a | 151 | 3 | 0.011
L0a2a2a | 15217 | 1 | 0.004
L0a2a2a | 15236 | 1 | 0.004
L0a2a2a | 15244 | 2 | 0.007
L0a2a2a | 152d | 4 | 0.014
L0a2a2a | 15777 | 1 | 0.004
L0a2a2a | 15784 | 1 | 0.004
L0a2a2a | 15793 | 1 | 0.004
L0a2a2a | 15804 | 4 | 0.014
L0a2a2a | 15808 | 1 | 0.004
L0a2a2a | 15893 | 1 | 0.004
L0a2a2a | 15905 | 5 | 0.018
L0a2a2a | 15928 | 1 | 0.004
L0a2a2a | 16017 | 1 | 0.004
L0a2a2a | 16093 | 3 | 0.011
L0a2a2a | 16129 | 1 | 0.004
L0a2a2a | 16166d | 1 | 0.004
L0a2a2a | 16168 | 1 | 0.004
L0a2a2a | 16169 | 1 | 0.004
L0a2a2a | 16170 | 2 | 0.007
L0a2a2a | 16174 | 1 | 0.004
L0a2a2a | 16188 | 2 | 0.007
L0a2a2a | 16188A | 1 | 0.004
L0a2a2a | 16190G | 1 | 0.004
L0a2a2a | 16192-16193d | 27 | 0.096
L0a2a2a | 16193d | 2 | 0.007
L0a2a2a | 16209 | 1 | 0.004
L0a2a2a | 16214 | 1 | 0.004
L0a2a2a | 16247 | 1 | 0.004
L0a2a2a | 16254 | 1 | 0.004
L0a2a2a | 16260 | 1 | 0.004
L0a2a2a | 16261 | 2 | 0.007
L0a2a2a | 16264 | 1 | 0.004
L0a2a2a | 16274 | 8 | 0.028
L0a2a2a | 16278 | 1 | 0.004
L0a2a2a | 1629 | 1 | 0.004
L0a2a2a | 16290 | 1 | 0.004
L0a2a2a | 16294 | 1 | 0.004
L0a2a2a | 16295 | 1 | 0.004
L0a2a2a | 16309 | 1 | 0.004
L0a2a2a | 16355 | 1 | 0.004
L0a2a2a | 16390 | 2 | 0.007
L0a2a2a | 16399A | 1 | 0.004
L0a2a2a | 16399T | 1 | 0.004
L0a2a2a | 1719 | 2 | 0.007
L0a2a2a | 185 | 5 | 0.018
L0a2a2a | 195 | 9 | 0.032
L0a2a2a | 199 | 6 | 0.021
L0a2a2a | 217 | 1 | 0.004
L0a2a2a | 2626 | 1 | 0.004
L0a2a2a | 2679 | 2 | 0.007
L0a2a2a | 2924 | 1 | 0.004
L0a2a2a | 293 | 1 | 0.004
L0a2a2a | 3010 | 1 | 0.004
L0a2a2a | 3027 | 4 | 0.014
L0a2a2a | 3083 | 1 | 0.004
L0a2a2a | 309d | 15 | 0.053
L0a2a2a | 310 | 1 | 0.004
L0a2a2a | 317.1C | 1 | 0.004
L0a2a2a | 3211 | 1 | 0.004
L0a2a2a | 3397 | 1 | 0.004
L0a2a2a | 3531 | 1 | 0.004
L0a2a2a | 3585 | 3 | 0.011
L0a2a2a | 3766 | 1 | 0.004
L0a2a2a | 4284 | 1 | 0.004
L0a2a2a | 4395 | 1 | 0.004
L0a2a2a | 4615 | 26 | 0.092
L0a2a2a | 4916 | 2 | 0.007
L0a2a2a | 51 | 1 | 0.004
L0a2a2a | 5118 | 1 | 0.004
L0a2a2a | 5201 | 5 | 0.018
L0a2a2a | 5945 | 1 | 0.004
L0a2a2a | 6050 | 8 | 0.028
L0a2a2a | 6128 | 2 | 0.007
L0a2a2a | 6212 | 2 | 0.007
L0a2a2a | 6221 | 1 | 0.004
L0a2a2a | 6297 | 1 | 0.004
L0a2a2a | 6366 | 1 | 0.004
L0a2a2a | 6480 | 1 | 0.004
L0a2a2a | 6915 | 2 | 0.007
L0a2a2a | 6975 | 1 | 0.004
L0a2a2a | 7151G | 1 | 0.004
L0a2a2a | 723 | 1 | 0.004
L0a2a2a | 727 | 1 | 0.004
L0a2a2a | 7319 | 1 | 0.004
L0a2a2a | 7354 | 2 | 0.007
L0a2a2a | 7403 | 1 | 0.004
L0a2a2a | 7498 | 2 | 0.007
L0a2a2a | 7674 | 1 | 0.004
L0a2a2a | 7784 | 2 | 0.007
L0a2a2a | 7975 | 1 | 0.004
L0a2a2a | 8020 | 1 | 0.004
L0a2a2a | 8276d | 1 | 0.004
L0a2a2a | 8311 | 1 | 0.004
L0a2a2a | 8398A | 1 | 0.004
L0a2a2a | 8555 | 1 | 0.004
L0a2a2a | 8730 | 1 | 0.004
L0a2a2a | 8749 | 1 | 0.004
L0a2a2a | 8854 | 1 | 0.004
L0a2a2a | 8865 | 1 | 0.004

L0a2a2a | 9110 | 1 | 0.004
L0a2a2a | 9181 | 2 | 0.007
L0a2a2a | 9214 | 3 | 0.011
L0a2a2a | 9338 | 1 | 0.004
L0a2a2a | 9398 | 8 | 0.028
L0a2a2a | 9428A | 1 | 0.004
L0a2a2a | 9581 | 1 | 0.004
L0a2a2a | 95T | 2 | 0.007
L0a2a2a | 9682 | 2 | 0.007
L0a2a2a | 9947 | 1 | 0.004
L0a2a2a1 | 10114 | 1 | 0.014
L0a2a2a1 | 11071 | 1 | 0.014
L0a2a2a1 | 11884 | 1 | 0.014
L0a2a2a1 | 1273 | 1 | 0.014
L0a2a2a1 | 13317 | 1 | 0.014
L0a2a2a1 | 16093 | 2 | 0.027
L0a2a2a1 | 16173 | 2 | 0.027
L0a2a2a1 | 16264 | 1 | 0.014
L0a2a2a1 | 16283 | 1 | 0.014
L0a2a2a1 | 16527 | 1 | 0.014
L0a2a2a1 | 16532d | 1 | 0.014
L0a2a2a1 | 184 | 1 | 0.014
L0a2a2a1 | 2442 | 2 | 0.027
L0a2a2a1 | 309d | 8 | 0.108
L0a2a2a1 | 310 | 2 | 0.027
L0a2a2a1 | 3705 | 1 | 0.014
L0a2a2a1 | 3796 | 1 | 0.014
L0a2a2a1 | 4763 | 1 | 0.014
L0a2a2a1 | 73 | 1 | 0.014
L0a2a2a1 | 8939 | 2 | 0.027
L0a2a2a1 | 9142 | 2 | 0.027
L0a2a2a1 | 9214 | 1 | 0.014
L0a2a2a2 | 14598 | 1 | 0.013
L0a2a2a2 | 146 | 1 | 0.013
L0a2a2a2 | 1462 | 1 | 0.013
L0a2a2a2 | 15019 | 1 | 0.013
L0a2a2a2 | 151 | 2 | 0.027
L0a2a2a2 | 15172 | 1 | 0.013
L0a2a2a2 | 16024 | 1 | 0.013
L0a2a2a2 | 16025 | 1 | 0.013
L0a2a2a2 | 16144 | 2 | 0.027
L0a2a2a2 | 16242 | 1 | 0.013
L0a2a2a2 | 16274 | 5 | 0.067
L0a2a2a2 | 16484 | 1 | 0.013
L0a2a2a2 | 16566C | 1 | 0.013
L0a2a2a2 | 249d | 1 | 0.013
L0a2a2a2 | 2C | 1 | 0.013
L0a2a2a2 | 309d | 1 | 0.013
L0a2a2a2 | 73 | 1 | 0.013
L0a2a2a2 | 83 | 1 | 0.013
L0a2a2a2 | 9305 | 1 | 0.013
L0a2b | 10389 | 5 | 0.172
L0a2b | 10909 | 1 | 0.034
L0a2b | 10946 | 1 | 0.034
L0a2b | 13761 | 1 | 0.034
L0a2b | 14446 | 1 | 0.034
L0a2b | 152 | 1 | 0.034
L0a2b | 309d | 1 | 0.034
L0a2b | 310 | 2 | 0.069
L0a2b | 4314 | 1 | 0.034
L0a2b | 4682 | 1 | 0.034
L0a2b | 5216 | 1 | 0.034
L0a2b | 5741 | 1 | 0.034
L0a2b | 597 | 5 | 0.172
L0a2b | 7571 | 1 | 0.034
L0a2b1 | 132G | 1 | 0.048
L0a2b1 | 14131 | 4 | 0.19
L0a2b1 | 152 | 4 | 0.19
L0a2b1 | 16497 | 1 | 0.048
L0a2b1 | 16527 | 1 | 0.048
L0a2b1 | 1888 | 1 | 0.048
L0a2b1 | 195 | 5 | 0.238
L0a2b1 | 455.1T | 4 | 0.19
L0a2b1 | 7792 | 1 | 0.048
L0a2c | 12557 | 1 | 0.167
L0a2c | 146 | 1 | 0.167
L0a2c | 15148 | 1 | 0.167
L0a2c | 1521 | 1 | 0.167
L0a2c | 15386G | 1 | 0.167
L0a2c | 16114 | 1 | 0.167
L0a2c | 195 | 1 | 0.167
L0a2c | 466 | 1 | 0.167
L0a2c | 5237 | 1 | 0.167
L0a2c | 8811 | 1 | 0.167
L0a2d | 10984G | 1 | 0.026
L0a2d | 11134 | 1 | 0.026
L0a2d | 11296 | 1 | 0.026
L0a2d | 12861 | 1 | 0.026
L0a2d | 12967C | 1 | 0.026
L0a2d | 13260 | 1 | 0.026
L0a2d | 14377 | 1 | 0.026
L0a2d | 14547 | 2 | 0.051
L0a2d | 146 | 3 | 0.077
L0a2d | 15250 | 3 | 0.077
L0a2d | 16037 | 1 | 0.026
L0a2d | 16093 | 1 | 0.026
L0a2d | 16129 | 4 | 0.103
L0a2d | 16184 | 3 | 0.077
L0a2d | 16188A | 3 | 0.077
L0a2d | 16293 | 1 | 0.026
L0a2d | 16301 | 1 | 0.026
L0a2d | 16317T | 1 | 0.026
L0a2d | 16368 | 1 | 0.026
L0a2d | 171 | 1 | 0.026
L0a2d | 185 | 1 | 0.026
L0a2d | 207 | 1 | 0.026
L0a2d | 234 | 1 | 0.026
L0a2d | 2387 | 1 | 0.026
L0a2d | 2857 | 1 | 0.026
L0a2d | 309d | 2 | 0.051
L0a2d | 3110d | 1 | 0.026

L0a2d | 3565 | 1 | 0.026
L0a2d | 3808 | 1 | 0.026
L0a2d | 3987 | 1 | 0.026
L0a2d | 4596 | 1 | 0.026
L0a2d | 4841 | 1 | 0.026
L0a2d | 573.3C | 1 | 0.026
L0a2d | 5954 | 1 | 0.026
L0a2d | 6607 | 1 | 0.026
L0a2d | 7561 | 1 | 0.026
L0a2d | 7775 | 1 | 0.026
L0a2d | 8020 | 1 | 0.026
L0a2d | 8027 | 1 | 0.026
L0a2d | 8251 | 1 | 0.026
L0a2d | 8718 | 1 | 0.026
L0a2d | 89 | 2 | 0.051
L0a2d | 8998 | 1 | 0.026
L0a2d | 9261 | 1 | 0.026
L0a2d | 9368 | 1 | 0.026
L0a2d | 9424 | 2 | 0.051
L0a2d | 9860 | 3 | 0.077
L0a3 | 10993 | 1 | 0.056
L0a3 | 11410 | 2 | 0.111
L0a3 | 1193 | 1 | 0.056
L0a3 | 12082 | 1 | 0.056
L0a3 | 12618 | 2 | 0.111
L0a3 | 143 | 2 | 0.111
L0a3 | 146 | 3 | 0.167
L0a3 | 150 | 3 | 0.167
L0a3 | 15323 | 1 | 0.056
L0a3 | 15418 | 1 | 0.056
L0a3 | 15848 | 1 | 0.056
L0a3 | 15852 | 2 | 0.111
L0a3 | 15941 | 1 | 0.056
L0a3 | 16008 | 2 | 0.111
L0a3 | 16017 | 1 | 0.056
L0a3 | 16024 | 1 | 0.056
L0a3 | 16025 | 1 | 0.056
L0a3 | 16166d | 1 | 0.056
L0a3 | 16174 | 4 | 0.222
L0a3 | 16180 | 3 | 0.167
L0a3 | 16186 | 3 | 0.167
L0a3 | 16188A | 1 | 0.056
L0a3 | 16256 | 3 | 0.167
L0a3 | 16289 | 4 | 0.222
L0a3 | 16301 | 2 | 0.111
L0a3 | 16304 | 4 | 0.222
L0a3 | 16362 | 2 | 0.111
L0a3 | 195 | 2 | 0.111
L0a3 | 207 | 1 | 0.056
L0a3 | 249d | 2 | 0.111
L0a3 | 318 | 1 | 0.056
L0a3 | 328 | 2 | 0.111
L0a3 | 3447 | 2 | 0.111
L0a3 | 44.1C | 1 | 0.056
L0a3 | 4959 | 2 | 0.111
L0a3 | 511 | 2 | 0.111
L0a3 | 527G | 1 | 0.056
L0a3 | 6050 | 1 | 0.056
L0a3 | 6261 | 1 | 0.056
L0a3 | 6689A | 1 | 0.056
L0a3 | 73 | 6 | 0.333
L0a3 | 7462.1C | 1 | 0.056
L0a3 | 796 | 1 | 0.056
L0a3 | 8659 | 1 | 0.056
L0a3 | 89 | 3 | 0.167
L0a3 | 9055 | 2 | 0.111
L0a3 | 95T | 1 | 0.056
L0a4 | 16024 | 1 | 0.059
L0a4 | 16025 | 1 | 0.059
L0a4 | 16532d | 1 | 0.059
L0a4 | 195 | 1 | 0.059
L0a4 | 215 | 1 | 0.059
L0a4 | 311 | 1 | 0.059
L0a4 | 320 | 1 | 0.059
L0a4 | 324G | 1 | 0.059
L0a4 | 345 | 1 | 0.059
L0a4 | 348G | 1 | 0.059
L0a4 | 411G | 1 | 0.059
L0a4 | 489 | 1 | 0.059
L0a4 | 505 | 1 | 0.059
L0a4 | 507 | 1 | 0.059
L0a4 | 527G | 2 | 0.118
L0a4 | 530G | 1 | 0.059
L0a4 | 544A | 1 | 0.059
L0a4 | 6 | 1 | 0.059
L0a4 | 73 | 1 | 0.059
L0a4 | 89 | 2 | 0.118
L0a'b'g | 16180 | 1 | 0.333
L0a'b'g | 16289 | 1 | 0.333
L0a'b'g | 16320 | 1 | 0.333
L0a'b'g | 16532d | 2 | 0.667
L0a'b'g | 44.1C | 1 | 0.333
L0a'b'g | 73 | 1 | 0.333
L0a'g | 143 | 1 | 0.167
L0a'g | 16180 | 1 | 0.167
L0a'g | 16289 | 1 | 0.167
L0a'g | 16362 | 1 | 0.167
L0a'g | 16532d | 5 | 0.833
L0a'g | 195 | 5 | 0.833
L0a'g | 199 | 1 | 0.167
L0a'g | 249d | 2 | 0.333
L0a'g | 36.1G | 1 | 0.167
L0a'g | 380 | 1 | 0.167
L0a'g | 73 | 3 | 0.5
L0b | 11016 | 1 | 0.111
L0b | 11638 | 3 | 0.333
L0b | 11884 | 3 | 0.333
L0b | 13965 | 2 | 0.222
L0b | 14016 | 1 | 0.111
L0b | 14440 | 3 | 0.333
L0b | 15148 | 2 | 0.222

L0b | 15244 | 3 | 0.333
L0b | 15262 | 2 | 0.222
L0b | 1536 | 2 | 0.222
L0b | 15663 | 1 | 0.111
L0b | 15913 | 3 | 0.333
L0b | 16003 | 1 | 0.111
L0b | 16004 | 1 | 0.111
L0b | 16063 | 6 | 0.667
L0b | 16162 | 1 | 0.111
L0b | 16170C | 2 | 0.222
L0b | 16185 | 4 | 0.444
L0b | 16186 | 2 | 0.222
L0b | 16193d | 6 | 0.667
L0b | 16209 | 2 | 0.222
L0b | 16249 | 2 | 0.222
L0b | 16255 | 1 | 0.111
L0b | 16287 | 2 | 0.222
L0b | 16291 | 2 | 0.222
L0b | 16399 | 1 | 0.111
L0b | 1664 | 2 | 0.222
L0b | 1719 | 3 | 0.333
L0b | 195 | 4 | 0.444
L0b | 3699G | 1 | 0.111
L0b | 44.1C | 1 | 0.111
L0b | 4679 | 2 | 0.222
L0b | 5580 | 3 | 0.333
L0b | 6248 | 3 | 0.333
L0b | 64 | 2 | 0.222
L0b | 648 | 1 | 0.111
L0b | 723 | 3 | 0.333
L0b | 7241 | 2 | 0.222
L0b | 7302 | 3 | 0.333
L0b | 7337 | 3 | 0.333
L0b | 7424 | 2 | 0.222
L0b | 7874 | 2 | 0.222
L0b | 8027 | 2 | 0.222
L0b | 8158 | 3 | 0.333
L0b | 8632 | 1 | 0.111
L0b | 9068 | 1 | 0.111
L0d1 | 198 | 3 | 0.061
L0d1a | 10289 | 1 | 0.071
L0d1a | 11116 | 1 | 0.071
L0d1a | 11626 | 1 | 0.071
L0d1a | 14502 | 1 | 0.071
L0d1a | 15418 | 1 | 0.071
L0d1a | 16086 | 5 | 0.357
L0d1a | 16111 | 5 | 0.357
L0d1a | 16192 | 1 | 0.071
L0d1a | 16294 | 5 | 0.357
L0d1a | 5177 | 1 | 0.071
L0d1a | 573.1C | 2 | 0.143
L0d1a | 8814 | 1 | 0.071
L0d1a | 8860C | 1 | 0.071
L0d1a1 | 11260 | 1 | 0.048
L0d1a1 | 12348 | 1 | 0.048
L0d1a1 | 207 | 3 | 0.143
L0d1a1 | 524 | 2 | 0.095
L0d1a1 | 5553 | 1 | 0.048
L0d1a1 | 8793 | 1 | 0.048
L0d1a1a | 10907 | 1 | 0.05
L0d1a1a | 12957 | 1 | 0.05
L0d1a1a | 16524T | 1 | 0.05
L0d1a1a | 1654 | 1 | 0.05
L0d1a1a | 198 | 2 | 0.1
L0d1a1a | 202 | 1 | 0.05
L0d1a1a | 4353 | 1 | 0.05
L0d1a1a | 7241 | 1 | 0.05
L0d1a1a | 7283 | 1 | 0.05
L0d1a1a | 8277 | 1 | 0.05
L0d1a1a | 8392 | 1 | 0.05
L0d1a1a | 9758 | 1 | 0.05
L0d1a1a1 | 14405 | 1 | 0.036
L0d1a1a1 | 15650 | 1 | 0.036
L0d1a1a1 | 15758 | 5 | 0.179
L0d1a1a1 | 16086 | 5 | 0.179
L0d1a1a1 | 16111 | 4 | 0.143
L0d1a1a1 | 16301 | 2 | 0.071
L0d1a1a1 | 198 | 5 | 0.179
L0d1a1a1 | 310 | 1 | 0.036
L0d1a1a1 | 5715 | 5 | 0.179
L0d1a1a1 | 573.1C | 2 | 0.071
L0d1a1a1 | 8508 | 4 | 0.143
L0d1a1a1 | 8566 | 4 | 0.143
L0d1a1a2 | 12245 | 1 | 0.053
L0d1a1a2 | 16051 | 6 | 0.316
L0d1a1a2 | 16093 | 1 | 0.053
L0d1a1a2 | 16146 | 2 | 0.105
L0d1a1a2 | 16148 | 1 | 0.053
L0d1a1a2 | 16245 | 2 | 0.105
L0d1a1a2 | 16291 | 3 | 0.158
L0d1a1a2 | 16320 | 2 | 0.105
L0d1a1a2 | 16524T | 1 | 0.053
L0d1a1a2 | 2234 | 1 | 0.053
L0d1a1a2 | 5529 | 1 | 0.053
L0d1a1a2 | 866 | 1 | 0.053
L0d1a1a3 | 13532 | 2 | 0.105
L0d1a1a3 | 14053 | 2 | 0.105
L0d1a1a3 | 189 | 1 | 0.053
L0d1a1b | 12951 | 1 | 0.031
L0d1a1b | 152 | 12 | 0.375
L0d1a1b | 16093 | 9 | 0.281
L0d1a1b | 16214 | 3 | 0.094
L0d1a1b | 16260 | 2 | 0.062
L0d1a1b | 16264 | 1 | 0.031
L0d1a1b | 310 | 1 | 0.031
L0d1a1b | 5471 | 1 | 0.031
L0d1a1b1 | 16093 | 1 | 0.062
L0d1a1b1 | 189 | 1 | 0.062
L0d1a1b1a | 16256 | 3 | 0.062
L0d1a1b1a | 245 | 2 | 0.042
L0d1a1b1a | 3316 | 30 | 0.625

L0d1a1b1a | 385 | 1 | 0.021
L0d1a1b1a | 5557 | 1 | 0.021
L0d1a1b1a | 6617 | 1 | 0.021
L0d1a1b1a | 8161 | 1 | 0.021
L0d1a1b1b | 11087 | 1 | 0.056
L0d1a1b1b | 14790 | 1 | 0.056
L0d1a1b1b | 16362 | 5 | 0.278
L0d1a1c | 15443 | 1 | 0.053
L0d1a1c | 16264 | 1 | 0.053
L0d1a1c | 240 | 2 | 0.105
L0d1a1c | 5813 | 3 | 0.158
L0d1a1c | 5899.1C | 1 | 0.053
L0d1a1d | 189 | 1 | 0.053
L0d1a1d | 2706 | 1 | 0.053
L0d1a1d | 7055 | 1 | 0.053
L0d1a1d | 7205 | 1 | 0.053
L0d1a1d | 8577 | 1 | 0.053
L0d1a1d | 9477 | 1 | 0.053
L0d1a'c'd | 241 | 1 | 0.03
L0d1a'd | 189 | 4 | 0.333
L0d1a'd | 573.1C | 6 | 0.5
L0d1b1 | 305 | 1 | 0.083
L0d1b1 | 573.1C | 2 | 0.167
L0d1b1 | 593 | 1 | 0.083
L0d1b1+@152 | 11404 | 3 | 0.6
L0d1b1+@152 | 12136 | 3 | 0.6
L0d1b1+@152 | 16093 | 1 | 0.2
L0d1b1+@152 | 1842 | 3 | 0.6
L0d1b1+@152 | 200 | 3 | 0.6
L0d1b1a | 15799 | 1 | 0.077
L0d1b1a | 16093 | 1 | 0.077
L0d1b1a | 4277 | 1 | 0.077
L0d1b1a | 573.1C | 2 | 0.154
L0d1b1a | 7960 | 1 | 0.077
L0d1b1a | 8392 | 1 | 0.077
L0d1b1a1 | 15670 | 1 | 0.034
L0d1b1a1 | 16399 | 11 | 0.379
L0d1b1a1 | 228T | 1 | 0.034
L0d1b1a1 | 309d | 1 | 0.034
L0d1b1a1 | 310 | 3 | 0.103
L0d1b1a1 | 5074 | 1 | 0.034
L0d1b1a1 | 573.1C | 2 | 0.069
L0d1b1a1 | 573.2C | 6 | 0.207
L0d1b1a1 | 573.3C | 1 | 0.034
L0d1b1a1 | 5899.1C | 2 | 0.069
L0d1b1b | 12390 | 1 | 0.091
L0d1b1b | 16290 | 1 | 0.091
L0d1b1b | 183 | 1 | 0.091
L0d1b1b | 573.1C | 2 | 0.182
L0d1b1b | 6392 | 1 | 0.091
L0d1b1b | 8380 | 1 | 0.091
L0d1b1b1 | 10586 | 2 | 0.059
L0d1b1b1 | 12662 | 1 | 0.029
L0d1b1b1 | 13676 | 1 | 0.029
L0d1b1b1 | 1465 | 2 | 0.059
L0d1b1b1 | 15040 | 1 | 0.029
L0d1b1b1 | 15355 | 1 | 0.029
L0d1b1b1 | 15758 | 2 | 0.059
L0d1b1b1 | 2625 | 1 | 0.029
L0d1b1b1 | 573.2C | 2 | 0.059
L0d1b1b1 | 735 | 2 | 0.059
L0d1b1b1 | 7705 | 1 | 0.029
L0d1b1b1 | 7964 | 1 | 0.029
L0d1b1c | 13230 | 2 | 0.333
L0d1b1c | 13933 | 2 | 0.333
L0d1b1c | 1453 | 2 | 0.333
L0d1b1c | 16266 | 2 | 0.333
L0d1b1c | 16290 | 1 | 0.167
L0d1b1c | 199 | 1 | 0.167
L0d1b1c | 200 | 2 | 0.333
L0d1b1c | 2056 | 2 | 0.333
L0d1b1c | 206 | 1 | 0.167
L0d1b1c | 309d | 1 | 0.167
L0d1b1c | 3780 | 1 | 0.167
L0d1b1c | 4025 | 2 | 0.333
L0d1b1c | 469G | 2 | 0.333
L0d1b1c | 8658 | 2 | 0.333
L0d1b2 | 249d | 4 | 0.121
L0d1b2a1 | 14198 | 1 | 0.012
L0d1b2a1 | 15217 | 1 | 0.012
L0d1b2a1 | 15790 | 2 | 0.024
L0d1b2a1 | 16295 | 2 | 0.024
L0d1b2a1 | 16534T | 1 | 0.012
L0d1b2a1 | 1770C | 1 | 0.012
L0d1b2a1 | 1772T | 1 | 0.012
L0d1b2a1 | 188 | 1 | 0.012
L0d1b2a1 | 207 | 16 | 0.188
L0d1b2a1 | 310 | 1 | 0.012
L0d1b2a1 | 315.2C | 1 | 0.012
L0d1b2a1 | 3278 | 2 | 0.024
L0d1b2a1 | 3549 | 2 | 0.024
L0d1b2a1 | 376.1T | 1 | 0.012
L0d1b2a1 | 499 | 2 | 0.024
L0d1b2a1 | 5899.1C | 2 | 0.024
L0d1b2a1 | 593 | 1 | 0.012
L0d1b2a1 | 6146 | 2 | 0.024
L0d1b2a1 | 7571 | 1 | 0.012
L0d1b2a1 | 9037 | 10 | 0.118
L0d1b2a1 | 9947 | 1 | 0.012
L0d1b2a2 | 13488 | 7 | 0.127
L0d1b2a2 | 14861 | 3 | 0.055
L0d1b2a2 | 249d | 10 | 0.182
L0d1b2a2 | 3699G | 2 | 0.036
L0d1b2a2 | 7403 | 7 | 0.127
L0d1b2b | 10609 | 1 | 0.016
L0d1b2b | 13488 | 1 | 0.016
L0d1b2b | 13818 | 1 | 0.016
L0d1b2b | 3796 | 1 | 0.016
L0d1b2b | 5562 | 1 | 0.016
L0d1b2b | 597 | 1 | 0.016
L0d1b2b1 | 16266 | 1 | 0.016

L0d1b2b1a | 15196 | 2 | 0.024
L0d1b2b1a | 189 | 2 | 0.024
L0d1b2b1a | 4541 | 1 | 0.012
L0d1b2b1a | 4820 | 3 | 0.036
L0d1b2b1a | 5985 | 1 | 0.012
L0d1b2b1b | 10042 | 1 | 0.012
L0d1b2b1b | 10352 | 5 | 0.062
L0d1b2b1b | 11176 | 3 | 0.037
L0d1b2b1b | 13827 | 2 | 0.025
L0d1b2b1b | 15110 | 1 | 0.012
L0d1b2b1b | 15364 | 2 | 0.025
L0d1b2b1b | 15852 | 1 | 0.012
L0d1b2b1b | 16093 | 3 | 0.037
L0d1b2b1b | 16172 | 4 | 0.049
L0d1b2b1b | 16320 | 6 | 0.074
L0d1b2b1b | 16325 | 9 | 0.111
L0d1b2b1b | 1900 | 2 | 0.025
L0d1b2b1b | 204 | 1 | 0.012
L0d1b2b1b | 3510 | 5 | 0.062
L0d1b2b1b | 3578 | 5 | 0.062
L0d1b2b1b | 5563 | 1 | 0.012
L0d1b2b1b | 6917 | 5 | 0.062
L0d1b2b1b | 8440 | 5 | 0.062
L0d1b2b1b | 8489 | 1 | 0.012
L0d1b2b1b1 | 10646 | 2 | 0.031
L0d1b2b1b1 | 12373 | 1 | 0.016
L0d1b2b1b1 | 15773 | 1 | 0.016
L0d1b2b1b1 | 5262 | 1 | 0.016
L0d1b2b1b1 | 5291 | 1 | 0.016
L0d1b2b1b1 | 709 | 2 | 0.031
L0d1b2b2 | 11440 | 1 | 0.021
L0d1b2b2 | 16183d | 1 | 0.021
L0d1b2b2 | 16192 | 9 | 0.188
L0d1b2b2 | 16271 | 1 | 0.021
L0d1b2b2 | 1719 | 2 | 0.042
L0d1b2b2 | 188 | 2 | 0.042
L0d1b2b2a | 12775 | 1 | 0.016
L0d1b2b2a | 13359 | 12 | 0.194
L0d1b2b2a | 14198 | 1 | 0.016
L0d1b2b2a | 16140 | 7 | 0.113
L0d1b2b2a | 16148G | 2 | 0.032
L0d1b2b2a | 188 | 2 | 0.032
L0d1b2b2a | 3157 | 2 | 0.032
L0d1b2b2a | 3555 | 12 | 0.194
L0d1b2b2a | 4955 | 1 | 0.016
L0d1b2b2a | 8848 | 3 | 0.048
L0d1b2b2b | 1008 | 1 | 0.023
L0d1b2b2b | 11611 | 1 | 0.023
L0d1b2b2b | 12414 | 3 | 0.07
L0d1b2b2b | 14212 | 2 | 0.047
L0d1b2b2b | 16037 | 4 | 0.093
L0d1b2b2b | 188 | 2 | 0.047
L0d1b2b2b | 198 | 2 | 0.047
L0d1b2b2b | 310 | 2 | 0.047
L0d1b2b2b | 3660 | 3 | 0.07
L0d1b2b2b | 4080 | 2 | 0.047
L0d1b2b2b | 567C | 1 | 0.023
L0d1b2b2b | 8577 | 1 | 0.023
L0d1b2b2b1 | 13542 | 4 | 0.074
L0d1b2b2b1 | 16227 | 7 | 0.13
L0d1b2b2b1 | 188 | 2 | 0.037
L0d1b2b2b1 | 2395d | 1 | 0.019
L0d1b2b2b1 | 2486 | 3 | 0.056
L0d1b2b2c | 16271 | 1 | 0.024
L0d1b2b2c | 16292 | 1 | 0.024
L0d1b2b2c | 16527 | 1 | 0.024
L0d1b2b2c | 188 | 2 | 0.048
L0d1b2b2c1 | 188 | 2 | 0.041
L0d1b2b2c1 | 310 | 5 | 0.102
L0d1b2b2c1 | 9116 | 2 | 0.041
L0d1b2b2c2 | 13500 | 1 | 0.023
L0d1b2b2c2 | 188 | 2 | 0.045
L0d1b2b2c2 | 236 | 2 | 0.045
L0d1b2b2c2 | 8704 | 4 | 0.091
L0d1b2b2c2 | 8853 | 1 | 0.023
L0d1c | 10313 | 1 | 0.037
L0d1c | 10373 | 4 | 0.148
L0d1c | 10882 | 1 | 0.037
L0d1c | 11020 | 1 | 0.037
L0d1c | 11150 | 4 | 0.148
L0d1c | 11167 | 5 | 0.185
L0d1c | 11237 | 1 | 0.037
L0d1c | 11770 | 1 | 0.037
L0d1c | 11809 | 2 | 0.074
L0d1c | 11872 | 2 | 0.074
L0d1c | 12615 | 2 | 0.074
L0d1c | 12771 | 2 | 0.074
L0d1c | 12950 | 2 | 0.074
L0d1c | 12957 | 2 | 0.074
L0d1c | 13473 | 5 | 0.185
L0d1c | 13705 | 1 | 0.037
L0d1c | 13768 | 1 | 0.037
L0d1c | 1396 | 2 | 0.074
L0d1c | 14224 | 2 | 0.074
L0d1c | 14318 | 1 | 0.037
L0d1c | 14338 | 1 | 0.037
L0d1c | 14384 | 4 | 0.148
L0d1c | 14869 | 1 | 0.037
L0d1c | 15119 | 1 | 0.037
L0d1c | 15187 | 4 | 0.148
L0d1c | 1530 | 2 | 0.074
L0d1c | 15315 | 5 | 0.185
L0d1c | 15884 | 1 | 0.037
L0d1c | 16080 | 1 | 0.037
L0d1c | 16184 | 1 | 0.037
L0d1c | 16249 | 8 | 0.296
L0d1c | 16278 | 1 | 0.037
L0d1c | 16291 | 1 | 0.037
L0d1c | 16294A | 2 | 0.074
L0d1c | 16343 | 1 | 0.037
L0d1c | 16355 | 1 | 0.037

L0d1c | 198 | 2 | 0.074
L0d1c | 2000 | 4 | 0.148
L0d1c | 228 | 2 | 0.074
L0d1c | 2707 | 1 | 0.037
L0d1c | 2755 | 1 | 0.037
L0d1c | 3083 | 4 | 0.148
L0d1c | 3345 | 1 | 0.037
L0d1c | 3540 | 1 | 0.037
L0d1c | 3591 | 2 | 0.074
L0d1c | 3834 | 1 | 0.037
L0d1c | 4011 | 1 | 0.037
L0d1c | 4216 | 1 | 0.037
L0d1c | 4502 | 1 | 0.037
L0d1c | 4511 | 1 | 0.037
L0d1c | 4742 | 1 | 0.037
L0d1c | 520 | 1 | 0.037
L0d1c | 5252 | 1 | 0.037
L0d1c | 5263 | 4 | 0.148
L0d1c | 5460 | 1 | 0.037
L0d1c | 5773 | 2 | 0.074
L0d1c | 593 | 1 | 0.037
L0d1c | 6060C | 2 | 0.074
L0d1c | 7129 | 2 | 0.074
L0d1c | 7265 | 2 | 0.074
L0d1c | 7696 | 4 | 0.148
L0d1c | 7699 | 1 | 0.037
L0d1c | 8227 | 1 | 0.037
L0d1c | 8269 | 1 | 0.037
L0d1c | 8652 | 2 | 0.074
L0d1c | 8994 | 5 | 0.185
L0d1c | 9055 | 2 | 0.074
L0d1c | 9111 | 1 | 0.037
L0d1c | 9144 | 2 | 0.074
L0d1c | 9449 | 1 | 0.037
L0d1c | 980 | 4 | 0.148
L0d1c1 | 11150 | 1 | 0.048
L0d1c1 | 12477 | 1 | 0.048
L0d1c1 | 12972 | 1 | 0.048
L0d1c1 | 14395 | 1 | 0.048
L0d1c1 | 16148 | 5 | 0.238
L0d1c1 | 16192 | 1 | 0.048
L0d1c1 | 16214 | 1 | 0.048
L0d1c1 | 6371 | 1 | 0.048
L0d1c1 | 7142 | 1 | 0.048
L0d1c1 | 7299 | 1 | 0.048
L0d1c1a | 10007 | 1 | 0.059
L0d1c1a | 10490 | 1 | 0.059
L0d1c1a | 13659 | 1 | 0.059
L0d1c1a | 13928 | 1 | 0.059
L0d1c1a | 15110 | 1 | 0.059
L0d1c1a | 15272 | 1 | 0.059
L0d1c1a | 16114G | 1 | 0.059
L0d1c1a | 16132 | 1 | 0.059
L0d1c1a | 16162 | 1 | 0.059
L0d1c1a | 4991 | 1 | 0.059
L0d1c1a | 5655 | 1 | 0.059
L0d1c1a | 7365 | 1 | 0.059
L0d1c1a | 7397 | 1 | 0.059
L0d1c1a | 7762 | 1 | 0.059
L0d1c1a | 9077 | 1 | 0.059
L0d1c1a1 | 14064 | 1 | 0.009
L0d1c1a1 | 1462 | 1 | 0.009
L0d1c1a1 | 16240C | 2 | 0.017
L0d1c1a1 | 16497 | 14 | 0.121
L0d1c1a1 | 499 | 1 | 0.009
L0d1c1a1 | 5843 | 1 | 0.009
L0d1c1a1a | 11332 | 1 | 0.006
L0d1c1a1a | 11852 | 1 | 0.006
L0d1c1a1a | 11899 | 1 | 0.006
L0d1c1a1a | 12780 | 1 | 0.006
L0d1c1a1a | 13368 | 1 | 0.006
L0d1c1a1a | 13743 | 3 | 0.019
L0d1c1a1a | 14212 | 1 | 0.006
L0d1c1a1a | 15245 | 1 | 0.006
L0d1c1a1a | 15601 | 1 | 0.006
L0d1c1a1a | 16129 | 1 | 0.006
L0d1c1a1a | 16179 | 1 | 0.006
L0d1c1a1a | 2759 | 1 | 0.006
L0d1c1a1a | 3426 | 1 | 0.006
L0d1c1a1a | 3504 | 1 | 0.006
L0d1c1a1a | 3591 | 1 | 0.006
L0d1c1a1a | 3918 | 1 | 0.006
L0d1c1a1a | 438d | 1 | 0.006
L0d1c1a1a | 4615 | 2 | 0.012
L0d1c1a1a | 4633A | 1 | 0.006
L0d1c1a1a | 502 | 2 | 0.012
L0d1c1a1a | 5147 | 1 | 0.006
L0d1c1a1a | 593 | 22 | 0.136
L0d1c1a1a | 5936 | 1 | 0.006
L0d1c1a1a | 6218 | 1 | 0.006
L0d1c1a1a | 6620 | 3 | 0.019
L0d1c1a1a | 7158 | 1 | 0.006
L0d1c1a1a | 7285A | 1 | 0.006
L0d1c1a1a | 7444 | 2 | 0.012
L0d1c1a1a | 7961 | 3 | 0.019
L0d1c1a1a | 8518 | 1 | 0.006
L0d1c1a1a | 8648 | 1 | 0.006
L0d1c1a1a | 8901 | 3 | 0.019
L0d1c1a1a | 9287 | 3 | 0.019
L0d1c1a1a | 9480 | 1 | 0.006
L0d1c1a1a | 9948 | 1 | 0.006
L0d1c1a1a1 | 13431 | 1 | 0.018
L0d1c1a1a1 | 15530 | 1 | 0.018
L0d1c1a1a1 | 16129 | 1 | 0.018
L0d1c1a1a1 | 16287 | 1 | 0.018
L0d1c1a1a1 | 189 | 1 | 0.018
L0d1c1a1a1 | 3990 | 1 | 0.018
L0d1c1a1a1 | 7001T | 2 | 0.036
L0d1c1a1a2 | 11233 | 1 | 0.009
L0d1c1a1a2 | 12715 | 5 | 0.045
L0d1c1a1a2 | 499 | 1 | 0.009

L0d1c1a1a2 | 8276.1C | 1 | 0.009
L0d1c1a1a2 | 8289.1CCCCCTCTA | 1 | 0.009
L0d1c1a1a2 | 9025 | 2 | 0.018
L0d1c1a1b | 11536 | 5 | 0.038
L0d1c1a1b | 12752C | 1 | 0.008
L0d1c1a1b | 13260 | 2 | 0.015
L0d1c1a1b | 13590 | 3 | 0.023
L0d1c1a1b | 15519 | 1 | 0.008
L0d1c1a1b | 15861A | 1 | 0.008
L0d1c1a1b | 16497 | 2 | 0.015
L0d1c1a1b | 309d | 1 | 0.008
L0d1c1a1b | 310 | 4 | 0.031
L0d1c1a1b | 315.2C | 1 | 0.008
L0d1c1a1b | 8641C | 1 | 0.008
L0d1c1a2 | 16185 | 2 | 0.062
L0d1c1a2 | 4343 | 1 | 0.031
L0d1c1a2 | 4703 | 1 | 0.031
L0d1c1a2 | 8946 | 4 | 0.125
L0d1c1a2 | 9804 | 1 | 0.031
L0d1c2 | 10005 | 4 | 0.182
L0d1c2 | 10463 | 1 | 0.045
L0d1c2 | 11518 | 4 | 0.182
L0d1c2 | 13041 | 4 | 0.182
L0d1c2 | 13350 | 2 | 0.091
L0d1c2 | 14053 | 2 | 0.091
L0d1c2 | 14110 | 1 | 0.045
L0d1c2 | 14209 | 4 | 0.182
L0d1c2 | 16244 | 1 | 0.045
L0d1c2 | 16244C | 5 | 0.227
L0d1c2 | 16264 | 1 | 0.045
L0d1c2 | 16278 | 1 | 0.045
L0d1c2 | 16291 | 2 | 0.091
L0d1c2 | 16354 | 2 | 0.091
L0d1c2 | 16488 | 1 | 0.045
L0d1c2 | 4079 | 2 | 0.091
L0d1c2 | 4491 | 1 | 0.045
L0d1c2 | 471 | 1 | 0.045
L0d1c2 | 6350 | 1 | 0.045
L0d1c2 | 6566 | 4 | 0.182
L0d1c2 | 7299 | 1 | 0.045
L0d1c2 | 8014 | 1 | 0.045
L0d1c2 | 8618 | 1 | 0.045
L0d1c2 | 9196 | 2 | 0.091
L0d1c2a | 189 | 1 | 0.091
L0d1c2a1 | 14271 | 4 | 0.5
L0d1c2a1 | 6253 | 3 | 0.375
L0d1c3 | 10016d | 1 | 0.031
L0d1c3 | 10811A | 1 | 0.031
L0d1c3 | 12952 | 5 | 0.156
L0d1c3 | 13347 | 4 | 0.125
L0d1c3 | 14443 | 4 | 0.125
L0d1c3 | 4856 | 5 | 0.156
L0d1c3 | 9111 | 4 | 0.125
L0d1c3 | 9438 | 1 | 0.031
L0d1d | 189 | 4 | 0.333
L0d1d | 573.1C | 6 | 0.5
L0d2a | 16148 | 2 | 0.019
L0d2a | 16173 | 1 | 0.01
L0d2a | 16179 | 1 | 0.01
L0d2a | 16266 | 1 | 0.01
L0d2a | 16320 | 4 | 0.039
L0d2a | 16362 | 8 | 0.078
L0d2a1 | 10007 | 1 | 0.007
L0d2a1 | 10084 | 2 | 0.014
L0d2a1 | 10771 | 1 | 0.007
L0d2a1 | 11084 | 1 | 0.007
L0d2a1 | 14258 | 1 | 0.007
L0d2a1 | 15043 | 1 | 0.007
L0d2a1 | 15061 | 1 | 0.007
L0d2a1 | 16145 | 1 | 0.007
L0d2a1 | 16284 | 2 | 0.014
L0d2a1 | 16524 | 1 | 0.007
L0d2a1 | 310 | 1 | 0.007
L0d2a1 | 3552 | 2 | 0.014
L0d2a1 | 4479 | 1 | 0.007
L0d2a1 | 4944 | 1 | 0.007
L0d2a1 | 5106 | 1 | 0.007
L0d2a1 | 5460 | 3 | 0.021
L0d2a1 | 573.1C | 2 | 0.014
L0d2a1 | 5899.1C | 1 | 0.007
L0d2a1 | 6445 | 1 | 0.007
L0d2a1a | 10373 | 1 | 0.005
L0d2a1a | 10586 | 2 | 0.011
L0d2a1a | 11016 | 1 | 0.005
L0d2a1a | 11560 | 1 | 0.005
L0d2a1a | 12841 | 1 | 0.005
L0d2a1a | 14040 | 2 | 0.011
L0d2a1a | 14517 | 1 | 0.005
L0d2a1a | 15892 | 1 | 0.005
L0d2a1a | 16093 | 2 | 0.011
L0d2a1a | 16188 | 5 | 0.027
L0d2a1a | 16260 | 2 | 0.011
L0d2a1a | 16362 | 1 | 0.005
L0d2a1a | 189 | 2 | 0.011
L0d2a1a | 2775 | 1 | 0.005
L0d2a1a | 309d | 1 | 0.005
L0d2a1a | 310 | 2 | 0.011
L0d2a1a | 315.2C | 4 | 0.021
L0d2a1a | 3338 | 1 | 0.005
L0d2a1a | 3434 | 1 | 0.005
L0d2a1a | 390 | 1 | 0.005
L0d2a1a | 4086 | 1 | 0.005
L0d2a1a | 4301T | 1 | 0.005
L0d2a1a | 4464 | 1 | 0.005
L0d2a1a | 456 | 2 | 0.011
L0d2a1a | 4917 | 1 | 0.005
L0d2a1a | 4965 | 1 | 0.005
L0d2a1a | 5418 | 1 | 0.005
L0d2a1a | 5441 | 2 | 0.011
L0d2a1a | 573.1C | 1 | 0.005
L0d2a1a | 6179 | 1 | 0.005

L0d2a1a | 6278 | 1 | 0.005
L0d2a1a | 7822 | 1 | 0.005
L0d2a1a | 8026 | 1 | 0.005
L0d2a1a | 8027 | 1 | 0.005
L0d2a1a | 8093 | 1 | 0.005
L0d2a1a | 8270 | 1 | 0.005
L0d2a1a | 8281-8289d | 1 | 0.005
L0d2a1a | 8410 | 1 | 0.005
L0d2a1a | 8994T | 1 | 0.005
L0d2a1a1 | 13752 | 1 | 0.006
L0d2a1a1 | 15346 | 3 | 0.019
L0d2a1a1 | 16093 | 8 | 0.052
L0d2a1a1 | 16145 | 5 | 0.032
L0d2a1a1 | 16264 | 4 | 0.026
L0d2a1a1 | 16294 | 1 | 0.006
L0d2a1a1 | 16524 | 1 | 0.006
L0d2a1a1 | 204 | 2 | 0.013
L0d2a1a1 | 309d | 2 | 0.013
L0d2a1a1 | 310 | 2 | 0.013
L0d2a1a1 | 482 | 1 | 0.006
L0d2a1a1 | 573.1C | 1 | 0.006
L0d2a1a1 | 6716 | 1 | 0.006
L0d2a1a1 | 7830 | 1 | 0.006
L0d2a1a1 | 8337 | 1 | 0.006
L0d2a1a1a | 13153 | 1 | 0.012
L0d2a1a1a | 5297 | 2 | 0.023
L0d2a1a1a | 573.1C | 1 | 0.012
L0d2a1a1a | 8165C | 1 | 0.012
L0d2a1a1a | 9266 | 1 | 0.012
L0d2a1a2 | 13819 | 1 | 0.007
L0d2a1a2 | 16320 | 1 | 0.007
L0d2a1a2 | 514G | 1 | 0.007
L0d2a1a2 | 573.1C | 1 | 0.007
L0d2a1a2 | 7258 | 1 | 0.007
L0d2a1a3 | 10373 | 3 | 0.033
L0d2a1a3 | 14040 | 1 | 0.011
L0d2a1a3 | 14088 | 1 | 0.011
L0d2a1a3 | 14517 | 3 | 0.033
L0d2a1a3 | 183 | 1 | 0.011
L0d2a1a3 | 188 | 1 | 0.011
L0d2a1a3 | 249d | 1 | 0.011
L0d2a1a3 | 291.1A | 1 | 0.011
L0d2a1a3 | 309d | 1 | 0.011
L0d2a1a3 | 3786 | 1 | 0.011
L0d2a1a3 | 390 | 1 | 0.011
L0d2a1a3 | 456 | 2 | 0.022
L0d2a1a3 | 4965 | 3 | 0.033
L0d2a1a3 | 515 | 5 | 0.055
L0d2a1a3 | 573.1C | 1 | 0.011
L0d2a1a3 | 6179 | 1 | 0.011
L0d2a1a3 | 8093 | 3 | 0.033
L0d2a1a3 | 960.1C | 2 | 0.022
L0d2a1a3 | 960.3C | 1 | 0.011
L0d2a1b | 10673 | 1 | 0.062
L0d2a1b | 14189 | 3 | 0.188
L0d2a1b | 16260 | 1 | 0.062
L0d2a1b | 16318T | 1 | 0.062
L0d2a1b | 309d | 1 | 0.062
L0d2a1b | 310 | 1 | 0.062
L0d2a1b | 3325A | 1 | 0.062
L0d2a1b | 4904A | 1 | 0.062
L0d2a1c | 12471 | 2 | 0.015
L0d2a1c | 16300 | 2 | 0.015
L0d2a1c | 309d | 1 | 0.007
L0d2a1c | 310 | 3 | 0.022
L0d2a1c | 573.1C | 1 | 0.007
L0d2a2 | 11239 | 4 | 0.8
L0d2a2 | 11485 | 1 | 0.2
L0d2a2 | 1187 | 2 | 0.4
L0d2a2 | 11899 | 1 | 0.2
L0d2a2 | 13008 | 1 | 0.2
L0d2a2 | 151 | 1 | 0.2
L0d2a2 | 189 | 4 | 0.8
L0d2a2 | 310 | 1 | 0.2
L0d2a2 | 3150 | 4 | 0.8
L0d2a2 | 3200 | 1 | 0.2
L0d2a2 | 6620 | 1 | 0.2
L0d2a2 | 7498 | 1 | 0.2
L0d2a2 | 8865 | 4 | 0.8
L0d2a2 | 9461 | 1 | 0.2
L0d2a2 | 9509 | 1 | 0.2
L0d2a'b'd | 10978 | 1 | 0.029
L0d2a'b'd | 11150 | 1 | 0.029
L0d2a'b'd | 11260 | 1 | 0.029
L0d2a'b'd | 13389 | 1 | 0.029
L0d2a'b'd | 13635 | 1 | 0.029
L0d2a'b'd | 14371 | 1 | 0.029
L0d2a'b'd | 15172 | 1 | 0.029
L0d2a'b'd | 15346 | 1 | 0.029
L0d2a'b'd | 16166d | 1 | 0.029
L0d2a'b'd | 16172 | 1 | 0.029
L0d2a'b'd | 16265 | 1 | 0.029
L0d2a'b'd | 16291 | 1 | 0.029
L0d2a'b'd | 385 | 1 | 0.029
L0d2a'b'd | 5420 | 1 | 0.029
L0d2a'b'd | 6620 | 1 | 0.029
L0d2a'b'd | 7933 | 1 | 0.029
L0d2a'b'd | 8284 | 1 | 0.029
L0d2b | 573.1C | 2 | 0.222
L0d2b1 | 573.1C | 3 | 0.6
L0d2b1a | 15706 | 1 | 0.167
L0d2b1a | 309d | 1 | 0.167
L0d2b1a | 3203 | 1 | 0.167
L0d2b1a | 3918 | 1 | 0.167
L0d2b1a | 5147 | 1 | 0.167
L0d2b1a | 573.1C | 4 | 0.667
L0d2b1a | 573.3C | 1 | 0.167
L0d2b1a | 7091 | 1 | 0.167
L0d2b1a | 7853 | 1 | 0.167
L0d2b1a | 9263 | 1 | 0.167
L0d2b1a1 | 16193d | 3 | 0.231

L0d2b1a1 | 2010 | 3 | 0.231
L0d2b1a1 | 310 | 2 | 0.154
L0d2b1a1 | 573.1C | 4 | 0.308
L0d2b1a1a | 15668A | 13 | 0.65
L0d2b1a1a | 16193d | 12 | 0.6
L0d2b1a1a | 309d | 1 | 0.05
L0d2b1a1a | 310 | 5 | 0.25
L0d2b1a1a | 573.1C | 3 | 0.15
L0d2b1a1a | 573.3C | 2 | 0.1
L0d2b1a1a | 9962 | 4 | 0.2
L0d2b1b | 10921 | 1 | 0.056
L0d2b1b | 152 | 5 | 0.278
L0d2b1b | 15942 | 1 | 0.056
L0d2b1b | 16187 | 3 | 0.167
L0d2b1b | 16188 | 8 | 0.444
L0d2b1b | 16193d | 3 | 0.167
L0d2b1b | 16258d | 5 | 0.278
L0d2b1b | 308-309d | 2 | 0.111
L0d2b1b | 309d | 9 | 0.5
L0d2b1b | 573.1C | 4 | 0.222
L0d2b1b | 573.3C | 1 | 0.056
L0d2b1b | 573.5C | 1 | 0.056
L0d2b1b | 6323 | 2 | 0.111
L0d2b1b | 6761 | 1 | 0.056
L0d2b2 | 1007 | 1 | 0.077
L0d2b2 | 11101 | 3 | 0.231
L0d2b2 | 12696 | 1 | 0.077
L0d2b2 | 12798 | 1 | 0.077
L0d2b2 | 12960 | 1 | 0.077
L0d2b2 | 13224 | 1 | 0.077
L0d2b2 | 14605 | 1 | 0.077
L0d2b2 | 15194 | 1 | 0.077
L0d2b2 | 152 | 3 | 0.231
L0d2b2 | 15511 | 3 | 0.231
L0d2b2 | 15940 | 2 | 0.154
L0d2b2 | 16527 | 1 | 0.077
L0d2b2 | 1749 | 1 | 0.077
L0d2b2 | 188 | 1 | 0.077
L0d2b2 | 200 | 1 | 0.077
L0d2b2 | 310 | 1 | 0.077
L0d2b2 | 368.1AGAA | 1 | 0.077
L0d2b2 | 3816 | 1 | 0.077
L0d2b2 | 3918 | 1 | 0.077
L0d2b2 | 5396 | 1 | 0.077
L0d2b2 | 573.1C | 2 | 0.154
L0d2b2 | 9053 | 1 | 0.077
L0d2c | 16081 | 1 | 0.053
L0d2c | 16391 | 1 | 0.053
L0d2c1 | 10822A | 5 | 0.152
L0d2c1 | 11279 | 1 | 0.03
L0d2c1 | 11431 | 1 | 0.03
L0d2c1 | 11884 | 4 | 0.121
L0d2c1 | 11928 | 2 | 0.061
L0d2c1 | 12173 | 2 | 0.061
L0d2c1 | 15119 | 1 | 0.03
L0d2c1 | 15546 | 1 | 0.03
L0d2c1 | 15703 | 1 | 0.03
L0d2c1 | 16086 | 7 | 0.212
L0d2c1 | 16145 | 1 | 0.03
L0d2c1 | 16261 | 1 | 0.03
L0d2c1 | 16261G | 7 | 0.212
L0d2c1 | 16289T | 2 | 0.061
L0d2c1 | 16355 | 8 | 0.242
L0d2c1 | 16399C | 1 | 0.03
L0d2c1 | 16508.1A | 1 | 0.03
L0d2c1 | 189 | 1 | 0.03
L0d2c1 | 198 | 1 | 0.03
L0d2c1 | 225 | 1 | 0.03
L0d2c1 | 249d | 3 | 0.091
L0d2c1 | 2581 | 3 | 0.091
L0d2c1 | 3308 | 1 | 0.03
L0d2c1 | 3591 | 1 | 0.03
L0d2c1 | 408A | 7 | 0.212
L0d2c1 | 4655 | 1 | 0.03
L0d2c1 | 4824 | 1 | 0.03
L0d2c1 | 5892 | 2 | 0.061
L0d2c1 | 6497 | 1 | 0.03
L0d2c1 | 7473 | 4 | 0.121
L0d2c1 | 8420C | 1 | 0.03
L0d2c1a | 10250 | 3 | 0.136
L0d2c1a | 10327 | 1 | 0.045
L0d2c1a | 1235 | 3 | 0.136
L0d2c1a | 15496 | 1 | 0.045
L0d2c1a | 15916 | 1 | 0.045
L0d2c1a | 16165T | 1 | 0.045
L0d2c1a | 16234 | 3 | 0.136
L0d2c1a | 16242 | 1 | 0.045
L0d2c1a | 16319 | 1 | 0.045
L0d2c1a | 2707 | 1 | 0.045
L0d2c1a | 5004 | 1 | 0.045
L0d2c1a | 5277 | 1 | 0.045
L0d2c1a | 6008 | 1 | 0.045
L0d2c1a | 8504 | 3 | 0.136
L0d2c1a | 8877 | 2 | 0.091
L0d2c1a | 9152 | 1 | 0.045
L0d2c1a | 930 | 1 | 0.045
L0d2c1a | 9477 | 2 | 0.091
L0d2c1a | 9566 | 1 | 0.045
L0d2c1a1 | 10907 | 2 | 0.095
L0d2c1a1 | 5894C | 1 | 0.048
L0d2c1a1 | 7891 | 1 | 0.048
L0d2c1a1 | 8279 | 1 | 0.048
L0d2c1b | 15353 | 1 | 0.083
L0d2c1b | 16093 | 1 | 0.083
L0d2c1b | 16257 | 5 | 0.417
L0d2c1b | 16294 | 5 | 0.417
L0d2c1b | 16527 | 5 | 0.417
L0d2c2 | 11116 | 1 | 0.031
L0d2c2 | 12136 | 1 | 0.031
L0d2c2 | 12161 | 1 | 0.031
L0d2c2 | 12432 | 1 | 0.031

L0d2c2 | 12501 | 3 | 0.094
L0d2c2a | 140 | 1 | 0.033
L0d2c2a1 | 140 | 1 | 0.033
L0d2c2a1a | 10325 | 2 | 0.062
L0d2c2a1a | 16188G | 1 | 0.031
L0d2c2b | 3010 | 1 | 0.032
L0d2c2b | 5899.2C | 2 | 0.065
L0d2c2b | 7051 | 1 | 0.032
L0d2d | 11872 | 1 | 0.111
L0d2d | 16093 | 4 | 0.444
L0d2d | 573.1C | 2 | 0.222
L0d2d | 9512 | 2 | 0.222
L0d3 | 10532 | 1 | 0.143
L0d3 | 13135 | 1 | 0.143
L0d3 | 13651 | 2 | 0.286
L0d3 | 15530 | 2 | 0.286
L0d3 | 15586 | 2 | 0.286
L0d3 | 15884 | 1 | 0.143
L0d3 | 16145 | 1 | 0.143
L0d3 | 16148 | 1 | 0.143
L0d3 | 16169 | 1 | 0.143
L0d3 | 16234 | 1 | 0.143
L0d3 | 16274 | 2 | 0.286
L0d3 | 16289 | 1 | 0.143
L0d3 | 16293 | 1 | 0.143
L0d3 | 16352 | 2 | 0.286
L0d3 | 16355 | 1 | 0.143
L0d3 | 16362 | 1 | 0.143
L0d3 | 16399 | 3 | 0.429
L0d3 | 2080 | 2 | 0.286
L0d3 | 239 | 1 | 0.143
L0d3 | 249d | 1 | 0.143
L0d3 | 2528 | 2 | 0.286
L0d3 | 3666 | 2 | 0.286
L0d3 | 3819 | 1 | 0.143
L0d3 | 7119 | 2 | 0.286
L0d3 | 8027 | 2 | 0.286
L0d3 | 94.1G | 1 | 0.143
L0d3a | 10015G | 1 | 0.077
L0d3a | 10599 | 2 | 0.154
L0d3a | 10727G | 1 | 0.077
L0d3a | 10812G | 1 | 0.077
L0d3a | 10841 | 1 | 0.077
L0d3a | 11390C | 1 | 0.077
L0d3a | 12172 | 1 | 0.077
L0d3a | 12390 | 4 | 0.308
L0d3a | 12522 | 1 | 0.077
L0d3a | 13487 | 1 | 0.077
L0d3a | 13882G | 1 | 0.077
L0d3a | 13932 | 4 | 0.308
L0d3a | 14185C | 1 | 0.077
L0d3a | 14517 | 2 | 0.154
L0d3a | 15235 | 1 | 0.077
L0d3a | 15631C | 1 | 0.077
L0d3a | 16025G | 1 | 0.077
L0d3a | 16148 | 2 | 0.154
L0d3a | 16192A | 1 | 0.077
L0d3a | 16352 | 1 | 0.077
L0d3a | 16362 | 2 | 0.154
L0d3a | 16505G | 1 | 0.077
L0d3a | 16506G | 1 | 0.077
L0d3a | 16507G | 1 | 0.077
L0d3a | 16526C | 1 | 0.077
L0d3a | 16552C | 1 | 0.077
L0d3a | 198 | 9 | 0.692
L0d3a | 2178C | 1 | 0.077
L0d3a | 224G | 1 | 0.077
L0d3a | 236 | 7 | 0.538
L0d3a | 2586G | 1 | 0.077
L0d3a | 36.1G | 1 | 0.077
L0d3a | 4113 | 4 | 0.308
L0d3a | 4812 | 4 | 0.308
L0d3a | 525d | 1 | 0.077
L0d3a | 537d | 1 | 0.077
L0d3a | 551d | 1 | 0.077
L0d3a | 567C | 1 | 0.077
L0d3a | 6261 | 1 | 0.077
L0d3a | 636T | 1 | 0.077
L0d3a | 679 | 1 | 0.077
L0d3a | 8013G | 1 | 0.077
L0d3a | 9899 | 1 | 0.077
L0d3a | 98G | 1 | 0.077
L0d3b | 13635 | 1 | 0.042
L0d3b | 15758 | 1 | 0.042
L0d3b | 16214 | 2 | 0.083
L0d3b | 16256 | 1 | 0.042
L0d3b | 455.1T | 1 | 0.042
L0d3b1 | 10581 | 1 | 0.024
L0d3b1 | 14290 | 1 | 0.024
L0d3b1 | 15132 | 1 | 0.024
L0d3b1 | 16172 | 1 | 0.024
L0d3b1 | 16266 | 13 | 0.31
L0d3b1 | 16293 | 1 | 0.024
L0d3b1 | 2083 | 2 | 0.048
L0d3b1 | 338 | 1 | 0.024
L0d3b1 | 538 | 1 | 0.024
L0d3b1 | 6629 | 2 | 0.048
L0d3b1 | 8485 | 1 | 0.024
L0d3b1 | 9278 | 1 | 0.024
L0d3b2 | 13204 | 1 | 0.043
L0d3b2 | 13928 | 1 | 0.043
L0d3b2 | 310 | 1 | 0.043
L0f | 10007 | 2 | 0.061
L0f | 10101 | 3 | 0.091
L0f | 10205 | 3 | 0.091
L0f | 10358 | 1 | 0.03
L0f | 10586 | 2 | 0.061
L0f | 10867 | 2 | 0.061
L0f | 11204 | 3 | 0.091
L0f | 11253 | 2 | 0.061
L0f | 11782 | 12 | 0.364

L0f | 11887 | 1 | 0.03
L0f | 12092A | 8 | 0.242
L0f | 12151 | 1 | 0.03
L0f | 12501 | 3 | 0.091
L0f | 12606 | 1 | 0.03
L0f | 12630 | 6 | 0.182
L0f | 12699 | 6 | 0.182
L0f | 12850 | 3 | 0.091
L0f | 12903 | 3 | 0.091
L0f | 12961 | 1 | 0.03
L0f | 13038A | 2 | 0.061
L0f | 13395 | 4 | 0.121
L0f | 13524 | 4 | 0.121
L0f | 13590 | 1 | 0.03
L0f | 13821A | 3 | 0.091
L0f | 13928C | 3 | 0.091
L0f | 13984 | 2 | 0.061
L0f | 14124 | 3 | 0.091
L0f | 143 | 14 | 0.424
L0f | 14305 | 12 | 0.364
L0f | 14326 | 3 | 0.091
L0f | 14364 | 1 | 0.03
L0f | 14401 | 3 | 0.091
L0f | 14482 | 8 | 0.242
L0f | 14555G | 1 | 0.03
L0f | 14560 | 3 | 0.091
L0f | 14577 | 4 | 0.121
L0f | 14757 | 3 | 0.091
L0f | 150 | 1 | 0.03
L0f | 15212 | 1 | 0.03
L0f | 15289 | 4 | 0.121
L0f | 15300 | 3 | 0.091
L0f | 15607 | 12 | 0.364
L0f | 15672 | 8 | 0.242
L0f | 15815 | 4 | 0.121
L0f | 15900 | 3 | 0.091
L0f | 16051 | 1 | 0.03
L0f | 16052 | 22 | 0.667
L0f | 16067 | 3 | 0.091
L0f | 16093 | 1 | 0.03
L0f | 16111 | 1 | 0.03
L0f | 16114A | 1 | 0.03
L0f | 16188 | 4 | 0.121
L0f | 16192-16193d | 2 | 0.061
L0f | 16239 | 1 | 0.03
L0f | 16265 | 1 | 0.03
L0f | 16274 | 3 | 0.091
L0f | 16290 | 21 | 0.636
L0f | 16294 | 1 | 0.03
L0f | 16316 | 3 | 0.091
L0f | 16318C | 1 | 0.03
L0f | 16319 | 1 | 0.03
L0f | 16325 | 22 | 0.667
L0f | 16344 | 1 | 0.03
L0f | 16352 | 1 | 0.03
L0f | 16354 | 19 | 0.576
L0f | 16357 | 1 | 0.03
L0f | 16360 | 1 | 0.03
L0f | 16362 | 4 | 0.121
L0f | 16399 | 4 | 0.121
L0f | 16551.1T | 1 | 0.03
L0f | 16557d | 1 | 0.03
L0f | 1822 | 12 | 0.364
L0f | 194 | 3 | 0.091
L0f | 195 | 3 | 0.091
L0f | 199 | 1 | 0.03
L0f | 200 | 3 | 0.091
L0f | 204 | 3 | 0.091
L0f | 205 | 2 | 0.061
L0f | 236 | 2 | 0.061
L0f | 272 | 4 | 0.121
L0f | 2879 | 8 | 0.242
L0f | 3027 | 1 | 0.03
L0f | 309d | 3 | 0.091
L0f | 3591 | 4 | 0.121
L0f | 3732 | 4 | 0.121
L0f | 3766 | 1 | 0.03
L0f | 4134C | 3 | 0.091
L0f | 4161 | 3 | 0.091
L0f | 4200 | 1 | 0.03
L0f | 4688 | 6 | 0.182
L0f | 4695 | 3 | 0.091
L0f | 489 | 3 | 0.091
L0f | 499 | 4 | 0.121
L0f | 505 | 1 | 0.03
L0f | 5063 | 6 | 0.182
L0f | 51 | 1 | 0.03
L0f | 5147 | 8 | 0.242
L0f | 5205G | 4 | 0.121
L0f | 5276 | 3 | 0.091
L0f | 527G | 1 | 0.03
L0f | 5586 | 1 | 0.03
L0f | 573.3C | 1 | 0.03
L0f | 5913 | 1 | 0.03
L0f | 6458 | 3 | 0.091
L0f | 6570T | 2 | 0.061
L0f | 6710 | 3 | 0.091
L0f | 6962 | 2 | 0.061
L0f | 7061 | 6 | 0.182
L0f | 7232 | 1 | 0.03
L0f | 7268 | 3 | 0.091
L0f | 7283 | 3 | 0.091
L0f | 73 | 2 | 0.061
L0f | 7316 | 1 | 0.03
L0f | 7364 | 3 | 0.091
L0f | 7650 | 1 | 0.03
L0f | 7660 | 11 | 0.333
L0f | 7705 | 1 | 0.03
L0f | 7810 | 1 | 0.03
L0f | 7868 | 1 | 0.03
L0f | 7891 | 1 | 0.03

L0f | 7912 | 3 | 0.091
L0f | 8047 | 3 | 0.091
L0f | 8227 | 1 | 0.03
L0f | 8230 | 1 | 0.03
L0f | 8251 | 4 | 0.121
L0f | 8485 | 3 | 0.091
L0f | 8674 | 4 | 0.121
L0f | 8C | 1 | 0.03
L0f | 9075 | 3 | 0.091
L0f | 9210 | 1 | 0.03
L0f | 9251 | 3 | 0.091
L0f | 93 | 3 | 0.091
L0f | 9438 | 3 | 0.091
L0f | 95C | 1 | 0.03
L0f | 9746 | 3 | 0.091
L0f | 9755 | 1 | 0.03
L0f1 | 10310 | 3 | 0.429
L0f1 | 10580 | 1 | 0.143
L0f1 | 10692 | 3 | 0.429
L0f1 | 10694 | 3 | 0.429
L0f1 | 10978 | 2 | 0.286
L0f1 | 11084 | 3 | 0.429
L0f1 | 11314 | 3 | 0.429
L0f1 | 12118 | 3 | 0.429
L0f1 | 12346 | 3 | 0.429
L0f1 | 12903 | 3 | 0.429
L0f1 | 13104 | 1 | 0.143
L0f1 | 13145 | 3 | 0.429
L0f1 | 14091 | 3 | 0.429
L0f1 | 14178 | 3 | 0.429
L0f1 | 14364 | 3 | 0.429
L0f1 | 14848 | 1 | 0.143
L0f1 | 15236 | 3 | 0.429
L0f1 | 16174 | 3 | 0.429
L0f1 | 16178 | 1 | 0.143
L0f1 | 16187 | 3 | 0.429
L0f1 | 16265 | 4 | 0.571
L0f1 | 16293 | 1 | 0.143
L0f1 | 16319 | 2 | 0.286
L0f1 | 173 | 2 | 0.286
L0f1 | 3644 | 3 | 0.429
L0f1 | 3699G | 3 | 0.429
L0f1 | 4562 | 1 | 0.143
L0f1 | 4695 | 3 | 0.429
L0f1 | 4703 | 3 | 0.429
L0f1 | 482 | 3 | 0.429
L0f1 | 4926A | 3 | 0.429
L0f1 | 5093 | 2 | 0.286
L0f1 | 5108 | 3 | 0.429
L0f1 | 5276 | 3 | 0.429
L0f1 | 5492 | 3 | 0.429
L0f1 | 573.5C | 1 | 0.143
L0f1 | 5951 | 1 | 0.143
L0f1 | 5984 | 3 | 0.429
L0f1 | 6923 | 3 | 0.429
L0f1 | 7007 | 3 | 0.429
L0f1 | 7543 | 3 | 0.429
L0f1 | 8200 | 1 | 0.143
L0f1 | 8281-8289d | 3 | 0.429
L0f1 | 89 | 2 | 0.286
L0f1 | 8911 | 3 | 0.429
L0f1 | 93 | 4 | 0.571
L0f1 | 95C | 4 | 0.571
L0f2 | 16025 | 1 | 0.059
L0f2 | 16214A | 1 | 0.059
L0f2 | 16237 | 1 | 0.059
L0f2 | 16239G | 1 | 0.059
L0f2 | 16265C | 10 | 0.588
L0f2 | 16270 | 10 | 0.588
L0f2 | 16291 | 1 | 0.059
L0f2 | 16293 | 1 | 0.059
L0f2 | 16301 | 3 | 0.176
L0f2 | 16330G | 1 | 0.059
L0f2 | 16465 | 11 | 0.647
L0f2 | 214C | 1 | 0.059
L0f2 | 215 | 1 | 0.059
L0f2 | 224G | 2 | 0.118
L0f2 | 226G | 1 | 0.059
L0f2 | 301T | 1 | 0.059
L0f2 | 371d | 1 | 0.059
L0f2 | 391 | 10 | 0.588
L0f2 | 41A | 1 | 0.059
L0f2 | 522d | 1 | 0.059
L0f2 | 525d | 1 | 0.059
L0f2 | 527d | 2 | 0.118
L0f2 | 69T | 1 | 0.059
L0f2 | 73 | 14 | 0.824
L0f2 | 8 | 1 | 0.059
L0f2a | 10361 | 6 | 0.316
L0f2a | 10517 | 1 | 0.053
L0f2a | 10532 | 10 | 0.526
L0f2a | 10707G | 1 | 0.053
L0f2a | 10810A | 1 | 0.053
L0f2a | 11200C | 3 | 0.158
L0f2a | 11617 | 7 | 0.368
L0f2a | 12612 | 7 | 0.368
L0f2a | 12678 | 2 | 0.105
L0f2a | 12684 | 1 | 0.053
L0f2a | 13899 | 2 | 0.105
L0f2a | 13938 | 7 | 0.368
L0f2a | 143 | 1 | 0.053
L0f2a | 14305 | 3 | 0.158
L0f2a | 14587 | 1 | 0.053
L0f2a | 14978 | 3 | 0.158
L0f2a | 1503 | 1 | 0.053
L0f2a | 15221 | 7 | 0.368
L0f2a | 15616 | 2 | 0.105
L0f2a | 1598 | 2 | 0.105
L0f2a | 16086 | 1 | 0.053
L0f2a | 16147A | 1 | 0.053
L0f2a | 16209 | 1 | 0.053

L0f2a | 16274 | 4 | 0.211
L0f2a | 16290 | 2 | 0.105
L0f2a | 16325 | 7 | 0.368
L0f2a | 16352 | 4 | 0.211
L0f2a | 16354 | 7 | 0.368
L0f2a | 16390 | 1 | 0.053
L0f2a | 16399 | 1 | 0.053
L0f2a | 1664 | 1 | 0.053
L0f2a | 1824 | 3 | 0.158
L0f2a | 199 | 7 | 0.368
L0f2a | 204 | 7 | 0.368
L0f2a | 3345 | 1 | 0.053
L0f2a | 3552 | 7 | 0.368
L0f2a | 37.1A | 1 | 0.053
L0f2a | 3846 | 1 | 0.053
L0f2a | 4117 | 2 | 0.105
L0f2a | 4393 | 1 | 0.053
L0f2a | 44.1C | 3 | 0.158
L0f2a | 4655 | 7 | 0.368
L0f2a | 4688 | 1 | 0.053
L0f2a | 5004 | 2 | 0.105
L0f2a | 511 | 8 | 0.421
L0f2a | 5201 | 1 | 0.053
L0f2a | 5484 | 1 | 0.053
L0f2a | 6489A | 2 | 0.105
L0f2a | 728 | 1 | 0.053
L0f2a | 73 | 12 | 0.632
L0f2a | 8281 | 1 | 0.053
L0f2a | 8346d | 6 | 0.316
L0f2a | 8634 | 1 | 0.053
L0f2a | 9027G | 1 | 0.053
L0f2a | 9078 | 6 | 0.316
L0f2a | 9120 | 1 | 0.053
L0f2a | 9260 | 6 | 0.316
L0f2a | 93 | 1 | 0.053
L0f2a | 9554 | 7 | 0.368
L0f2a | 9670 | 2 | 0.105
L0f2a | 9962 | 6 | 0.316
L0f2a1 | 11212 | 1 | 0.067
L0f2a1 | 15796G | 1 | 0.067
L0f2a1 | 16359 | 1 | 0.067
L0f2a1 | 16532d | 4 | 0.267
L0f2a1 | 195 | 3 | 0.2
L0f2a1 | 236 | 1 | 0.067
L0f2a1 | 2792C | 2 | 0.133
L0f2a1 | 343G | 1 | 0.067
L0f2a1 | 366 | 1 | 0.067
L0f2a1 | 389 | 1 | 0.067
L0f2a1 | 3945G | 1 | 0.067
L0f2a1 | 3946 | 1 | 0.067
L0f2a1 | 515-524d | 1 | 0.067
L0f2a1 | 6359 | 1 | 0.067
L0f2a1 | 6446 | 2 | 0.133
L0f2a1 | 73 | 1 | 0.067
L0f2a1 | 93 | 1 | 0.067
L0f2a1 | 9300 | 2 | 0.133
L0f2a1 | 961 | 1 | 0.067
L0f2b | 13810 | 1 | 0.1
L0f2b | 152 | 1 | 0.1
L0f2b | 16129 | 1 | 0.1
L0f2b | 16293 | 1 | 0.1
L0f2b | 16399 | 1 | 0.1
L0f2b | 3693 | 1 | 0.1
L0f2b | 505 | 2 | 0.2
L0f2b | 515-524d | 4 | 0.4
L0f2b | 650 | 1 | 0.1
L0g | 11938 | 1 | 0.125
L0g | 12957 | 1 | 0.125
L0g | 13306 | 1 | 0.125
L0g | 13434 | 1 | 0.125
L0g | 13911 | 1 | 0.125
L0g | 143 | 1 | 0.125
L0g | 15301 | 1 | 0.125
L0g | 1598 | 1 | 0.125
L0g | 16111 | 1 | 0.125
L0g | 16167 | 1 | 0.125
L0g | 16171 | 1 | 0.125
L0g | 16174 | 1 | 0.125
L0g | 16183 | 1 | 0.125
L0g | 16209 | 1 | 0.125
L0g | 16214 | 1 | 0.125
L0g | 16320 | 2 | 0.25
L0g | 16359 | 1 | 0.125
L0g | 16527 | 1 | 0.125
L0g | 199 | 1 | 0.125
L0g | 2425 | 2 | 0.25
L0g | 309d | 1 | 0.125
L0g | 316 | 1 | 0.125
L0g | 3167.1T | 1 | 0.125
L0g | 374 | 1 | 0.125
L0g | 4562 | 1 | 0.125
L0g | 5252 | 1 | 0.125
L0g | 6113 | 1 | 0.125
L0g | 64 | 1 | 0.125
L0g | 7444 | 1 | 0.125
L0g | 7598 | 1 | 0.125
L0g | 7664 | 1 | 0.125
L0g | 7765 | 1 | 0.125
L0g | 8251 | 1 | 0.125
L0g | 8469 | 1 | 0.125
L0g | 8512 | 1 | 0.125
L0g | 8987 | 1 | 0.125
L0g | 9190 | 1 | 0.125
L0g | 9758 | 1 | 0.125
L0k1a1 | 12414 | 3 | 0.038
L0k1a1 | 1284 | 5 | 0.063
L0k1a1 | 13572 | 1 | 0.013
L0k1a1 | 16284 | 1 | 0.013
L0k1a1 | 16291A | 2 | 0.025
L0k1a1 | 1700A | 1 | 0.013
L0k1a1 | 2010 | 5 | 0.063

L0k1a1 | 204 | 2 | 0.025
L0k1a1 | 310 | 1 | 0.013
L0k1a1 | 4454 | 1 | 0.013
L0k1a1 | 7934 | 3 | 0.038
L0k1a1a | 10388 | 1 | 0.014
L0k1a1a | 184 | 1 | 0.014
L0k1a1a | 2650 | 1 | 0.014
L0k1a1a | 310 | 6 | 0.083
L0k1a1a | 315.2C | 1 | 0.014
L0k1a1a | 4661T | 1 | 0.014
L0k1a1a | 7673 | 2 | 0.028
L0k1a1a | 8711 | 4 | 0.056
L0k1a1a | 9025 | 1 | 0.014
L0k1a1b | 10727G | 1 | 0.015
L0k1a1b | 11988 | 1 | 0.015
L0k1a1b | 13759 | 5 | 0.077
L0k1a1b | 15479 | 1 | 0.015
L0k1a1b | 15788-15792d | 1 | 0.015
L0k1a1b | 16093 | 3 | 0.046
L0k1a1b | 16266A | 1 | 0.015
L0k1a1b | 2356 | 5 | 0.077
L0k1a1b | 2363 | 2 | 0.031
L0k1a1b | 4525 | 1 | 0.015
L0k1a1b | 9833 | 1 | 0.015
L0k1a1c | 16284 | 2 | 0.031
L0k1a1c | 16327 | 1 | 0.015
L0k1a1c | 310 | 1 | 0.015
L0k1a1c | 4814 | 1 | 0.015
L0k1a1c | 7347 | 2 | 0.031
L0k1a1c | 8292 | 1 | 0.015
L0k1a1d | 10550 | 3 | 0.042
L0k1a1d | 150 | 1 | 0.014
L0k1a1d | 16291 | 1 | 0.014
L0k1a1d | 16291A | 8 | 0.113
L0k1a1d | 309d | 1 | 0.014
L0k1a1d | 310 | 2 | 0.028
L0k1a1d | 315.2C | 4 | 0.056
L0k1a1d | 9731 | 3 | 0.042
L0k1a2 | 11963 | 1 | 0.02
L0k1a2 | 12545 | 2 | 0.039
L0k1a2 | 150 | 1 | 0.02
L0k1a2 | 16093 | 1 | 0.02
L0k1a2 | 16129 | 1 | 0.02
L0k1a2 | 16217 | 1 | 0.02
L0k1a2 | 16243 | 2 | 0.039
L0k1a2 | 174 | 3 | 0.059
L0k1a2 | 234 | 1 | 0.02
L0k1a2 | 7598 | 1 | 0.02
L0k1a2a | 10680 | 1 | 0.029
L0k1a2a | 15236 | 1 | 0.029
L0k1a2a | 16291A | 1 | 0.029
L0k1a2a | 3876 | 1 | 0.029
L0k1a3 | 10214 | 1 | 0.125
L0k1a3 | 15518 | 2 | 0.25
L0k1a3 | 310 | 2 | 0.25
L0k1a3 | 315.2C | 1 | 0.125
L0k1b | 11864 | 3 | 0.107
L0k1b | 12390 | 3 | 0.107
L0k1b | 13362 | 1 | 0.036
L0k1b | 14605 | 1 | 0.036
L0k1b | 16093 | 1 | 0.036
L0k1b | 16176 | 1 | 0.036
L0k1b | 16274 | 1 | 0.036
L0k1b | 204 | 3 | 0.107
L0k1b | 310 | 4 | 0.143
L0k2 | 16209 | 1 | 1
L0k2 | 16223 | 1 | 1
L0k2a | 16186 | 1 | 0.5
L0k2a | 2883 | 2 | 1
L0k2a | 309d | 1 | 0.5
L0k2a | 4025 | 1 | 0.5
L0k2a | 8976 | 1 | 0.5
L0k2a | 9347 | 1 | 0.5
L0k2a1 | 10420 | 1 | 0.5
L0k2a1 | 11809 | 1 | 0.5
L0k2a1 | 3083 | 1 | 0.5
L0k2a1 | 6852 | 1 | 0.5
L0k2a1 | 6891 | 1 | 0.5
L0k2a1 | 7954 | 1 | 0.5
L0k2a1 | 9347 | 1 | 0.5
L0k2a1 | 9380 | 1 | 0.5
L0k2a1a | 16201 | 3 | 0.5
L0k2a1a | 185T | 4 | 0.667
L0k2a1a | 203 | 2 | 0.333
L0k2a1a | 510 | 2 | 0.333
L0k2a1a | 6260 | 4 | 0.667
L0k2b | 10454 | 1 | 0.333
L0k2b | 11061 | 2 | 0.667
L0k2b | 11237 | 1 | 0.333
L0k2b | 12651 | 1 | 0.333
L0k2b | 13535 | 2 | 0.667
L0k2b | 14118 | 1 | 0.333
L0k2b | 14696 | 1 | 0.333
L0k2b | 15467 | 1 | 0.333
L0k2b | 16129 | 2 | 0.667
L0k2b | 16148 | 1 | 0.333
L0k2b | 215 | 1 | 0.333
L0k2b | 249d | 1 | 0.333
L0k2b | 294 | 1 | 0.333
L0k2b | 3882 | 2 | 0.667
L0k2b | 4767 | 2 | 0.667
L0k2b | 5441 | 1 | 0.333
L0k2b | 5705 | 1 | 0.333
L0k2b | 6053 | 2 | 0.667
L0k2b | 6221 | 1 | 0.333
L0k2b | 7119 | 1 | 0.333
L0k2b | 9347 | 2 | 0.667
L1 | 16532d | 9 | 1
L1 | 183 | 2 | 0.222
L1 | 189 | 1 | 0.111
L1 | 81 | 1 | 0.111

L1'2'3'4'5'6 | 10088 | 1 | 0.038
L1'2'3'4'5'6 | 1009 | 3 | 0.115
L1'2'3'4'5'6 | 10101 | 21 | 0.808
L1'2'3'4'5'6 | 10151 | 1 | 0.038
L1'2'3'4'5'6 | 10172 | 3 | 0.115
L1'2'3'4'5'6 | 10196 | 3 | 0.115
L1'2'3'4'5'6 | 10223 | 3 | 0.115
L1'2'3'4'5'6 | 10238 | 3 | 0.115
L1'2'3'4'5'6 | 10256 | 17 | 0.654
L1'2'3'4'5'6 | 10281 | 18 | 0.692
L1'2'3'4'5'6 | 10307 | 21 | 0.808
L1'2'3'4'5'6 | 10310 | 17 | 0.654
L1'2'3'4'5'6 | 10320 | 1 | 0.038
L1'2'3'4'5'6 | 10324 | 20 | 0.769
L1'2'3'4'5'6 | 10373 | 18 | 0.692
L1'2'3'4'5'6 | 10385 | 3 | 0.115
L1'2'3'4'5'6 | 10397 | 1 | 0.038
L1'2'3'4'5'6 | 1040 | 2 | 0.077
L1'2'3'4'5'6 | 1048 | 3 | 0.115
L1'2'3'4'5'6 | 10520 | 9 | 0.346
L1'2'3'4'5'6 | 10532 | 18 | 0.692
L1'2'3'4'5'6 | 10547 | 3 | 0.115
L1'2'3'4'5'6 | 10586 | 4 | 0.154
L1'2'3'4'5'6 | 10664 | 18 | 0.692
L1'2'3'4'5'6 | 10670 | 3 | 0.115
L1'2'3'4'5'6 | 10742 | 3 | 0.115
L1'2'3'4'5'6 | 10750 | 21 | 0.808
L1'2'3'4'5'6 | 10780 | 1 | 0.038
L1'2'3'4'5'6 | 10790 | 1 | 0.038
L1'2'3'4'5'6 | 10876 | 3 | 0.115
L1'2'3'4'5'6 | 10909 | 1 | 0.038
L1'2'3'4'5'6 | 10915 | 21 | 0.808
L1'2'3'4'5'6 | 10930 | 1 | 0.038
L1'2'3'4'5'6 | 10966G | 3 | 0.115
L1'2'3'4'5'6 | 11016 | 3 | 0.115
L1'2'3'4'5'6 | 11065 | 3 | 0.115
L1'2'3'4'5'6 | 11071 | 3 | 0.115
L1'2'3'4'5'6 | 11074 | 1 | 0.038
L1'2'3'4'5'6 | 11075 | 3 | 0.115
L1'2'3'4'5'6 | 11101 | 1 | 0.038
L1'2'3'4'5'6 | 11102 | 1 | 0.038
L1'2'3'4'5'6 | 11147 | 3 | 0.115
L1'2'3'4'5'6 | 11186 | 1 | 0.038
L1'2'3'4'5'6 | 11215 | 3 | 0.115
L1'2'3'4'5'6 | 11233 | 1 | 0.038
L1'2'3'4'5'6 | 11270 | 3 | 0.115
L1'2'3'4'5'6 | 11314 | 2 | 0.077
L1'2'3'4'5'6 | 11383 | 17 | 0.654
L1'2'3'4'5'6 | 11419 | 1 | 0.038
L1'2'3'4'5'6 | 11458T | 18 | 0.692
L1'2'3'4'5'6 | 11503 | 3 | 0.115
L1'2'3'4'5'6 | 11527 | 17 | 0.654
L1'2'3'4'5'6 | 11549 | 3 | 0.115
L1'2'3'4'5'6 | 11551 | 3 | 0.115
L1'2'3'4'5'6 | 11560 | 16 | 0.615
L1'2'3'4'5'6 | 11590 | 21 | 0.808
L1'2'3'4'5'6 | 11623 | 20 | 0.769
L1'2'3'4'5'6 | 11770 | 21 | 0.808
L1'2'3'4'5'6 | 11824 | 6 | 0.231
L1'2'3'4'5'6 | 11864 | 3 | 0.115
L1'2'3'4'5'6 | 11914 | 21 | 0.808
L1'2'3'4'5'6 | 11950 | 16 | 0.615
L1'2'3'4'5'6 | 11969 | 1 | 0.038
L1'2'3'4'5'6 | 11977 | 2 | 0.077
L1'2'3'4'5'6 | 11983 | 3 | 0.115
L1'2'3'4'5'6 | 12001 | 3 | 0.115
L1'2'3'4'5'6 | 12007 | 4 | 0.154
L1'2'3'4'5'6 | 12064 | 3 | 0.115
L1'2'3'4'5'6 | 12070 | 18 | 0.692
L1'2'3'4'5'6 | 12189 | 21 | 0.808
L1'2'3'4'5'6 | 12193T | 3 | 0.115
L1'2'3'4'5'6 | 12236 | 1 | 0.038
L1'2'3'4'5'6 | 12238 | 1 | 0.038
L1'2'3'4'5'6 | 12311 | 3 | 0.115
L1'2'3'4'5'6 | 12346 | 3 | 0.115
L1'2'3'4'5'6 | 12351 | 17 | 0.654
L1'2'3'4'5'6 | 12366 | 18 | 0.692
L1'2'3'4'5'6 | 12372 | 3 | 0.115
L1'2'3'4'5'6 | 12375 | 3 | 0.115
L1'2'3'4'5'6 | 12406 | 21 | 0.808
L1'2'3'4'5'6 | 12420 | 3 | 0.115
L1'2'3'4'5'6 | 12474 | 21 | 0.808
L1'2'3'4'5'6 | 12477 | 4 | 0.154
L1'2'3'4'5'6 | 1250 | 1 | 0.038
L1'2'3'4'5'6 | 12528 | 3 | 0.115
L1'2'3'4'5'6 | 12561 | 3 | 0.115
L1'2'3'4'5'6 | 12570 | 1 | 0.038
L1'2'3'4'5'6 | 12630 | 3 | 0.115
L1'2'3'4'5'6 | 12654 | 2 | 0.077
L1'2'3'4'5'6 | 12684 | 3 | 0.115
L1'2'3'4'5'6 | 12699 | 3 | 0.115
L1'2'3'4'5'6 | 12714 | 3 | 0.115
L1'2'3'4'5'6 | 12756 | 3 | 0.115
L1'2'3'4'5'6 | 12771 | 1 | 0.038
L1'2'3'4'5'6 | 12801 | 3 | 0.115
L1'2'3'4'5'6 | 12810 | 1 | 0.038
L1'2'3'4'5'6 | 12864 | 12 | 0.462
L1'2'3'4'5'6 | 12879 | 3 | 0.115
L1'2'3'4'5'6 | 12921 | 3 | 0.115
L1'2'3'4'5'6 | 13015 | 1 | 0.038
L1'2'3'4'5'6 | 13020 | 3 | 0.115
L1'2'3'4'5'6 | 13023 | 1 | 0.038
L1'2'3'4'5'6 | 13062 | 4 | 0.154
L1'2'3'4'5'6 | 13095 | 21 | 0.808
L1'2'3'4'5'6 | 13104 | 1 | 0.038
L1'2'3'4'5'6 | 13194 | 18 | 0.692
L1'2'3'4'5'6 | 13269 | 20 | 0.769
L1'2'3'4'5'6 | 13276 | 21 | 0.808
L1'2'3'4'5'6 | 13350 | 1 | 0.038
L1'2'3'4'5'6 | 13359 | 21 | 0.808
L1'2'3'4'5'6 | 13368 | 3 | 0.115

L1'2'3'4'5'6 | 13434 | 3 | 0.115
L1'2'3'4'5'6 | 13437 | 1 | 0.038
L1'2'3'4'5'6 | 13488 | 3 | 0.115
L1'2'3'4'5'6 | 13528 | 3 | 0.115
L1'2'3'4'5'6 | 13563 | 1 | 0.038
L1'2'3'4'5'6 | 13629 | 3 | 0.115
L1'2'3'4'5'6 | 13635 | 3 | 0.115
L1'2'3'4'5'6 | 13651 | 1 | 0.038
L1'2'3'4'5'6 | 13656 | 21 | 0.808
L1'2'3'4'5'6 | 13680 | 21 | 0.808
L1'2'3'4'5'6 | 13681 | 1 | 0.038
L1'2'3'4'5'6 | 13707 | 18 | 0.692
L1'2'3'4'5'6 | 13722 | 2 | 0.077
L1'2'3'4'5'6 | 13762G | 1 | 0.038
L1'2'3'4'5'6 | 13801 | 18 | 0.692
L1'2'3'4'5'6 | 13819 | 1 | 0.038
L1'2'3'4'5'6 | 13827 | 3 | 0.115
L1'2'3'4'5'6 | 13879 | 3 | 0.115
L1'2'3'4'5'6 | 13879A | 18 | 0.692
L1'2'3'4'5'6 | 13889 | 21 | 0.808
L1'2'3'4'5'6 | 13896 | 3 | 0.115
L1'2'3'4'5'6 | 13917 | 9 | 0.346
L1'2'3'4'5'6 | 13923 | 3 | 0.115
L1'2'3'4'5'6 | 13928 | 3 | 0.115
L1'2'3'4'5'6 | 1393 | 1 | 0.038
L1'2'3'4'5'6 | 13968 | 3 | 0.115
L1'2'3'4'5'6 | 14007 | 2 | 0.077
L1'2'3'4'5'6 | 14016 | 5 | 0.192
L1'2'3'4'5'6 | 14020 | 3 | 0.115
L1'2'3'4'5'6 | 14034 | 3 | 0.115
L1'2'3'4'5'6 | 14040 | 1 | 0.038
L1'2'3'4'5'6 | 1405 | 3 | 0.115
L1'2'3'4'5'6 | 14053 | 20 | 0.769
L1'2'3'4'5'6 | 1406 | 17 | 0.654
L1'2'3'4'5'6 | 14063 | 16 | 0.615
L1'2'3'4'5'6 | 14073 | 3 | 0.115
L1'2'3'4'5'6 | 14088 | 3 | 0.115
L1'2'3'4'5'6 | 14110 | 1 | 0.038
L1'2'3'4'5'6 | 14133 | 1 | 0.038
L1'2'3'4'5'6 | 14144 | 16 | 0.615
L1'2'3'4'5'6 | 14178 | 18 | 0.692
L1'2'3'4'5'6 | 14194 | 1 | 0.038
L1'2'3'4'5'6 | 14215 | 1 | 0.038
L1'2'3'4'5'6 | 14230 | 1 | 0.038
L1'2'3'4'5'6 | 14296 | 20 | 0.769
L1'2'3'4'5'6 | 14305 | 4 | 0.154
L1'2'3'4'5'6 | 14311 | 3 | 0.115
L1'2'3'4'5'6 | 14409 | 3 | 0.115
L1'2'3'4'5'6 | 1442 | 3 | 0.115
L1'2'3'4'5'6 | 14446 | 1 | 0.038
L1'2'3'4'5'6 | 14458A | 1 | 0.038
L1'2'3'4'5'6 | 14476 | 3 | 0.115
L1'2'3'4'5'6 | 14494 | 3 | 0.115
L1'2'3'4'5'6 | 14560 | 21 | 0.808
L1'2'3'4'5'6 | 14569 | 3 | 0.115
L1'2'3'4'5'6 | 146 | 19 | 0.731
L1'2'3'4'5'6 | 14605 | 3 | 0.115
L1'2'3'4'5'6 | 14614 | 1 | 0.038
L1'2'3'4'5'6 | 14662 | 3 | 0.115
L1'2'3'4'5'6 | 14693 | 4 | 0.154
L1'2'3'4'5'6 | 14757 | 3 | 0.115
L1'2'3'4'5'6 | 14800 | 3 | 0.115
L1'2'3'4'5'6 | 14839 | 3 | 0.115
L1'2'3'4'5'6 | 14869 | 1 | 0.038
L1'2'3'4'5'6 | 14926 | 9 | 0.346
L1'2'3'4'5'6 | 14926C | 1 | 0.038
L1'2'3'4'5'6 | 14950 | 3 | 0.115
L1'2'3'4'5'6 | 14954 | 3 | 0.115
L1'2'3'4'5'6 | 14956 | 3 | 0.115
L1'2'3'4'5'6 | 14971 | 3 | 0.115
L1'2'3'4'5'6 | 150 | 18 | 0.692
L1'2'3'4'5'6 | 15043 | 21 | 0.808
L1'2'3'4'5'6 | 15077 | 14 | 0.538
L1'2'3'4'5'6 | 151 | 1 | 0.038
L1'2'3'4'5'6 | 15100 | 3 | 0.115
L1'2'3'4'5'6 | 15110 | 4 | 0.154
L1'2'3'4'5'6 | 15148 | 21 | 0.808
L1'2'3'4'5'6 | 15172C | 3 | 0.115
L1'2'3'4'5'6 | 15191 | 20 | 0.769
L1'2'3'4'5'6 | 15191A | 1 | 0.038
L1'2'3'4'5'6 | 15226 | 18 | 0.692
L1'2'3'4'5'6 | 15232 | 16 | 0.615
L1'2'3'4'5'6 | 15244 | 2 | 0.077
L1'2'3'4'5'6 | 15250 | 3 | 0.115
L1'2'3'4'5'6 | 15295 | 18 | 0.692
L1'2'3'4'5'6 | 15301 | 18 | 0.692
L1'2'3'4'5'6 | 15314 | 1 | 0.038
L1'2'3'4'5'6 | 15346 | 1 | 0.038
L1'2'3'4'5'6 | 15355 | 21 | 0.808
L1'2'3'4'5'6 | 15358 | 2 | 0.077
L1'2'3'4'5'6 | 15403 | 3 | 0.115
L1'2'3'4'5'6 | 15443 | 21 | 0.808
L1'2'3'4'5'6 | 15479 | 18 | 0.692
L1'2'3'4'5'6 | 15490 | 3 | 0.115
L1'2'3'4'5'6 | 15562 | 3 | 0.115
L1'2'3'4'5'6 | 15629 | 18 | 0.692
L1'2'3'4'5'6 | 15643 | 3 | 0.115
L1'2'3'4'5'6 | 15649 | 21 | 0.808
L1'2'3'4'5'6 | 15667 | 21 | 0.808
L1'2'3'4'5'6 | 15671 | 17 | 0.654
L1'2'3'4'5'6 | 15742 | 3 | 0.115
L1'2'3'4'5'6 | 15778 | 1 | 0.038
L1'2'3'4'5'6 | 15789 | 17 | 0.654
L1'2'3'4'5'6 | 15850 | 18 | 0.692
L1'2'3'4'5'6 | 15857 | 1 | 0.038
L1'2'3'4'5'6 | 15883 | 3 | 0.115
L1'2'3'4'5'6 | 15892 | 1 | 0.038
L1'2'3'4'5'6 | 15924 | 2 | 0.077
L1'2'3'4'5'6 | 15941 | 3 | 0.115
L1'2'3'4'5'6 | 16017 | 1 | 0.038
L1'2'3'4'5'6 | 16037 | 18 | 0.692

L1'2'3'4'5'6 | 16051 | 1 | 0.038
L1'2'3'4'5'6 | 16070 | 3 | 0.115
L1'2'3'4'5'6 | 16078 | 13 | 0.5
L1'2'3'4'5'6 | 16081 | 3 | 0.115
L1'2'3'4'5'6 | 16083 | 1 | 0.038
L1'2'3'4'5'6 | 16093 | 6 | 0.231
L1'2'3'4'5'6 | 16111 | 1 | 0.038
L1'2'3'4'5'6 | 16134 | 2 | 0.077
L1'2'3'4'5'6 | 16139T | 17 | 0.654
L1'2'3'4'5'6 | 16140 | 2 | 0.077
L1'2'3'4'5'6 | 16148 | 20 | 0.769
L1'2'3'4'5'6 | 16154 | 14 | 0.538
L1'2'3'4'5'6 | 16156 | 3 | 0.115
L1'2'3'4'5'6 | 16157 | 1 | 0.038
L1'2'3'4'5'6 | 16166 | 4 | 0.154
L1'2'3'4'5'6 | 16169 | 19 | 0.731
L1'2'3'4'5'6 | 16171C | 1 | 0.038
L1'2'3'4'5'6 | 16172 | 7 | 0.269
L1'2'3'4'5'6 | 16181 | 1 | 0.038
L1'2'3'4'5'6 | 16185 | 1 | 0.038
L1'2'3'4'5'6 | 16186 | 3 | 0.115
L1'2'3'4'5'6 | 16188 | 2 | 0.077
L1'2'3'4'5'6 | 16193d | 4 | 0.154
L1'2'3'4'5'6 | 16209 | 25 | 0.962
L1'2'3'4'5'6 | 16213 | 8 | 0.308
L1'2'3'4'5'6 | 16215 | 1 | 0.038
L1'2'3'4'5'6 | 16221 | 1 | 0.038
L1'2'3'4'5'6 | 16230 | 17 | 0.654
L1'2'3'4'5'6 | 16234 | 17 | 0.654
L1'2'3'4'5'6 | 16239 | 1 | 0.038
L1'2'3'4'5'6 | 16242 | 1 | 0.038
L1'2'3'4'5'6 | 16243 | 4 | 0.154
L1'2'3'4'5'6 | 16244 | 17 | 0.654
L1'2'3'4'5'6 | 16245 | 3 | 0.115
L1'2'3'4'5'6 | 16247 | 1 | 0.038
L1'2'3'4'5'6 | 16249 | 1 | 0.038
L1'2'3'4'5'6 | 16256A | 17 | 0.654
L1'2'3'4'5'6 | 16258 | 13 | 0.5
L1'2'3'4'5'6 | 16259A | 3 | 0.115
L1'2'3'4'5'6 | 16261.1T | 1 | 0.038
L1'2'3'4'5'6 | 16262 | 16 | 0.615
L1'2'3'4'5'6 | 16263.1A | 16 | 0.615
L1'2'3'4'5'6 | 16263A | 1 | 0.038
L1'2'3'4'5'6 | 16266 | 1 | 0.038
L1'2'3'4'5'6 | 16274 | 3 | 0.115
L1'2'3'4'5'6 | 16294 | 2 | 0.077
L1'2'3'4'5'6 | 16298 | 1 | 0.038
L1'2'3'4'5'6 | 16299 | 17 | 0.654
L1'2'3'4'5'6 | 16304 | 2 | 0.077
L1'2'3'4'5'6 | 16317C | 1 | 0.038
L1'2'3'4'5'6 | 16320 | 19 | 0.731
L1'2'3'4'5'6 | 16327 | 1 | 0.038
L1'2'3'4'5'6 | 16344 | 1 | 0.038
L1'2'3'4'5'6 | 16355 | 3 | 0.115
L1'2'3'4'5'6 | 16356 | 3 | 0.115
L1'2'3'4'5'6 | 16362 | 21 | 0.808
L1'2'3'4'5'6 | 16368 | 3 | 0.115
L1'2'3'4'5'6 | 16390 | 1 | 0.038
L1'2'3'4'5'6 | 16391 | 1 | 0.038
L1'2'3'4'5'6 | 16399 | 1 | 0.038
L1'2'3'4'5'6 | 16400 | 17 | 0.654
L1'2'3'4'5'6 | 16487 | 3 | 0.115
L1'2'3'4'5'6 | 16497 | 4 | 0.154
L1'2'3'4'5'6 | 16527 | 3 | 0.115
L1'2'3'4'5'6 | 1673 | 1 | 0.038
L1'2'3'4'5'6 | 1709 | 21 | 0.808
L1'2'3'4'5'6 | 1719 | 3 | 0.115
L1'2'3'4'5'6 | 1761T | 3 | 0.115
L1'2'3'4'5'6 | 1779 | 16 | 0.615
L1'2'3'4'5'6 | 185 | 3 | 0.115
L1'2'3'4'5'6 | 1888 | 3 | 0.115
L1'2'3'4'5'6 | 189 | 18 | 0.692
L1'2'3'4'5'6 | 195A | 1 | 0.038
L1'2'3'4'5'6 | 196.1C | 1 | 0.038
L1'2'3'4'5'6 | 200 | 11 | 0.423
L1'2'3'4'5'6 | 2056 | 21 | 0.808
L1'2'3'4'5'6 | 2259 | 1 | 0.038
L1'2'3'4'5'6 | 2284 | 3 | 0.115
L1'2'3'4'5'6 | 2294 | 1 | 0.038
L1'2'3'4'5'6 | 240 | 1 | 0.038
L1'2'3'4'5'6 | 2402 | 3 | 0.115
L1'2'3'4'5'6 | 2404 | 3 | 0.115
L1'2'3'4'5'6 | 243 | 18 | 0.692
L1'2'3'4'5'6 | 245 | 16 | 0.615
L1'2'3'4'5'6 | 245A | 1 | 0.038
L1'2'3'4'5'6 | 247T | 1 | 0.038
L1'2'3'4'5'6 | 249d | 3 | 0.115
L1'2'3'4'5'6 | 252 | 3 | 0.115
L1'2'3'4'5'6 | 2523 | 21 | 0.808
L1'2'3'4'5'6 | 262 | 14 | 0.538
L1'2'3'4'5'6 | 2649 | 1 | 0.038
L1'2'3'4'5'6 | 2783 | 1 | 0.038
L1'2'3'4'5'6 | 282 | 3 | 0.115
L1'2'3'4'5'6 | 2831C | 20 | 0.769
L1'2'3'4'5'6 | 2863 | 15 | 0.577
L1'2'3'4'5'6 | 291d | 3 | 0.115
L1'2'3'4'5'6 | 2955 | 3 | 0.115
L1'2'3'4'5'6 | 299d | 3 | 0.115
L1'2'3'4'5'6 | 3010 | 18 | 0.692
L1'2'3'4'5'6 | 310 | 1 | 0.038
L1'2'3'4'5'6 | 3130d | 1 | 0.038
L1'2'3'4'5'6 | 315.2C | 1 | 0.038
L1'2'3'4'5'6 | 316 | 1 | 0.038
L1'2'3'4'5'6 | 3200 | 1 | 0.038
L1'2'3'4'5'6 | 3221 | 1 | 0.038
L1'2'3'4'5'6 | 3277 | 3 | 0.115
L1'2'3'4'5'6 | 3290 | 4 | 0.154
L1'2'3'4'5'6 | 3308 | 15 | 0.577
L1'2'3'4'5'6 | 3334 | 16 | 0.615
L1'2'3'4'5'6 | 3357 | 3 | 0.115
L1'2'3'4'5'6 | 3399 | 18 | 0.692

L1'2'3'4'5'6 | 340 | 2 | 0.077
L1'2'3'4'5'6 | 3414 | 18 | 0.692
L1'2'3'4'5'6 | 3421 | 1 | 0.038
L1'2'3'4'5'6 | 3483 | 11 | 0.423
L1'2'3'4'5'6 | 3496T | 3 | 0.115
L1'2'3'4'5'6 | 3504 | 14 | 0.538
L1'2'3'4'5'6 | 3504A | 3 | 0.115
L1'2'3'4'5'6 | 3591 | 3 | 0.115
L1'2'3'4'5'6 | 3600 | 2 | 0.077
L1'2'3'4'5'6 | 3603 | 2 | 0.077
L1'2'3'4'5'6 | 3636 | 3 | 0.115
L1'2'3'4'5'6 | 3640 | 1 | 0.038
L1'2'3'4'5'6 | 3705 | 1 | 0.038
L1'2'3'4'5'6 | 3714 | 3 | 0.115
L1'2'3'4'5'6 | 3747 | 3 | 0.115
L1'2'3'4'5'6 | 3753 | 3 | 0.115
L1'2'3'4'5'6 | 3764 | 1 | 0.038
L1'2'3'4'5'6 | 3777 | 1 | 0.038
L1'2'3'4'5'6 | 3808 | 17 | 0.654
L1'2'3'4'5'6 | 3816 | 3 | 0.115
L1'2'3'4'5'6 | 3832 | 3 | 0.115
L1'2'3'4'5'6 | 3834 | 1 | 0.038
L1'2'3'4'5'6 | 385 | 3 | 0.115
L1'2'3'4'5'6 | 3870 | 3 | 0.115
L1'2'3'4'5'6 | 3873 | 1 | 0.038
L1'2'3'4'5'6 | 3888 | 3 | 0.115
L1'2'3'4'5'6 | 390 | 1 | 0.038
L1'2'3'4'5'6 | 3909 | 20 | 0.769
L1'2'3'4'5'6 | 391 | 3 | 0.115
L1'2'3'4'5'6 | 3915 | 1 | 0.038
L1'2'3'4'5'6 | 3918 | 16 | 0.615
L1'2'3'4'5'6 | 3921 | 1 | 0.038
L1'2'3'4'5'6 | 3939 | 15 | 0.577
L1'2'3'4'5'6 | 3957 | 3 | 0.115
L1'2'3'4'5'6 | 3978 | 3 | 0.115
L1'2'3'4'5'6 | 4047 | 3 | 0.115
L1'2'3'4'5'6 | 4048 | 20 | 0.769
L1'2'3'4'5'6 | 4092 | 3 | 0.115
L1'2'3'4'5'6 | 417 | 18 | 0.692
L1'2'3'4'5'6 | 4204 | 18 | 0.692
L1'2'3'4'5'6 | 4312 | 20 | 0.769
L1'2'3'4'5'6 | 4318 | 3 | 0.115
L1'2'3'4'5'6 | 438 | 17 | 0.654
L1'2'3'4'5'6 | 4395 | 1 | 0.038
L1'2'3'4'5'6 | 4456 | 3 | 0.115
L1'2'3'4'5'6 | 4532 | 18 | 0.692
L1'2'3'4'5'6 | 456 | 3 | 0.115
L1'2'3'4'5'6 | 4562 | 20 | 0.769
L1'2'3'4'5'6 | 4580 | 1 | 0.038
L1'2'3'4'5'6 | 4586 | 3 | 0.115
L1'2'3'4'5'6 | 4622 | 3 | 0.115
L1'2'3'4'5'6 | 4654 | 1 | 0.038
L1'2'3'4'5'6 | 4659 | 3 | 0.115
L1'2'3'4'5'6 | 4688 | 3 | 0.115
L1'2'3'4'5'6 | 4856 | 20 | 0.769
L1'2'3'4'5'6 | 486 | 3 | 0.115
L1'2'3'4'5'6 | 4904 | 21 | 0.808
L1'2'3'4'5'6 | 4914 | 2 | 0.077
L1'2'3'4'5'6 | 4928 | 19 | 0.731
L1'2'3'4'5'6 | 4937 | 3 | 0.115
L1'2'3'4'5'6 | 4940 | 21 | 0.808
L1'2'3'4'5'6 | 4956 | 1 | 0.038
L1'2'3'4'5'6 | 4958 | 3 | 0.115
L1'2'3'4'5'6 | 4959 | 3 | 0.115
L1'2'3'4'5'6 | 498.1C | 1 | 0.038
L1'2'3'4'5'6 | 4991 | 3 | 0.115
L1'2'3'4'5'6 | 5021 | 3 | 0.115
L1'2'3'4'5'6 | 505 | 3 | 0.115
L1'2'3'4'5'6 | 5147 | 3 | 0.115
L1'2'3'4'5'6 | 5189T | 3 | 0.115
L1'2'3'4'5'6 | 523d | 1 | 0.038
L1'2'3'4'5'6 | 5262 | 2 | 0.077
L1'2'3'4'5'6 | 5270 | 3 | 0.115
L1'2'3'4'5'6 | 5320 | 2 | 0.077
L1'2'3'4'5'6 | 534 | 3 | 0.115
L1'2'3'4'5'6 | 5351 | 3 | 0.115
L1'2'3'4'5'6 | 5381 | 1 | 0.038
L1'2'3'4'5'6 | 5387 | 20 | 0.769
L1'2'3'4'5'6 | 5447 | 3 | 0.115
L1'2'3'4'5'6 | 5456 | 1 | 0.038
L1'2'3'4'5'6 | 5460 | 18 | 0.692
L1'2'3'4'5'6 | 547 | 18 | 0.692
L1'2'3'4'5'6 | 5471 | 20 | 0.769
L1'2'3'4'5'6 | 5474 | 3 | 0.115
L1'2'3'4'5'6 | 5505 | 14 | 0.538
L1'2'3'4'5'6 | 5508 | 3 | 0.115
L1'2'3'4'5'6 | 5516 | 3 | 0.115
L1'2'3'4'5'6 | 5539 | 3 | 0.115
L1'2'3'4'5'6 | 5580 | 21 | 0.808
L1'2'3'4'5'6 | 5585 | 1 | 0.038
L1'2'3'4'5'6 | 5600 | 1 | 0.038
L1'2'3'4'5'6 | 5673 | 17 | 0.654
L1'2'3'4'5'6 | 572 | 1 | 0.038
L1'2'3'4'5'6 | 575 | 2 | 0.077
L1'2'3'4'5'6 | 5813.1A | 1 | 0.038
L1'2'3'4'5'6 | 5821 | 21 | 0.808
L1'2'3'4'5'6 | 5840 | 21 | 0.808
L1'2'3'4'5'6 | 5894.1AACCCC | 1 | 0.038
L1'2'3'4'5'6 | 5894.1ACCCCC | 1 | 0.038
L1'2'3'4'5'6 | 5910 | 1 | 0.038
L1'2'3'4'5'6 | 596 | 3 | 0.115
L1'2'3'4'5'6 | 5964 | 3 | 0.115
L1'2'3'4'5'6 | 5984 | 1 | 0.038
L1'2'3'4'5'6 | 6023 | 20 | 0.769
L1'2'3'4'5'6 | 6040 | 1 | 0.038
L1'2'3'4'5'6 | 6131 | 13 | 0.5
L1'2'3'4'5'6 | 6150 | 1 | 0.038
L1'2'3'4'5'6 | 6152 | 3 | 0.115
L1'2'3'4'5'6 | 6156 | 17 | 0.654
L1'2'3'4'5'6 | 6200 | 17 | 0.654
L1'2'3'4'5'6 | 6216 | 1 | 0.038

L1'2'3'4'5'6 | 6221 | 3 | 0.115
L1'2'3'4'5'6 | 6242 | 3 | 0.115
L1'2'3'4'5'6 | 6260 | 18 | 0.692
L1'2'3'4'5'6 | 6266C | 21 | 0.808
L1'2'3'4'5'6 | 6351 | 1 | 0.038
L1'2'3'4'5'6 | 6366 | 3 | 0.115
L1'2'3'4'5'6 | 6410 | 18 | 0.692
L1'2'3'4'5'6 | 6452 | 21 | 0.808
L1'2'3'4'5'6 | 6483 | 3 | 0.115
L1'2'3'4'5'6 | 6485 | 3 | 0.115
L1'2'3'4'5'6 | 6509 | 3 | 0.115
L1'2'3'4'5'6 | 6512 | 3 | 0.115
L1'2'3'4'5'6 | 6530 | 1 | 0.038
L1'2'3'4'5'6 | 6542 | 3 | 0.115
L1'2'3'4'5'6 | 6563 | 5 | 0.192
L1'2'3'4'5'6 | 6566 | 3 | 0.115
L1'2'3'4'5'6 | 6569A | 3 | 0.115
L1'2'3'4'5'6 | 6617 | 1 | 0.038
L1'2'3'4'5'6 | 6620 | 18 | 0.692
L1'2'3'4'5'6 | 6641 | 20 | 0.769
L1'2'3'4'5'6 | 6719 | 3 | 0.115
L1'2'3'4'5'6 | 6917 | 3 | 0.115
L1'2'3'4'5'6 | 6935 | 3 | 0.115
L1'2'3'4'5'6 | 6938 | 3 | 0.115
L1'2'3'4'5'6 | 6956A | 2 | 0.077
L1'2'3'4'5'6 | 6956G | 1 | 0.038
L1'2'3'4'5'6 | 7076 | 1 | 0.038
L1'2'3'4'5'6 | 709 | 21 | 0.808
L1'2'3'4'5'6 | 7106C | 17 | 0.654
L1'2'3'4'5'6 | 7127 | 16 | 0.615
L1'2'3'4'5'6 | 7150 | 1 | 0.038
L1'2'3'4'5'6 | 7232 | 3 | 0.115
L1'2'3'4'5'6 | 7270 | 4 | 0.154
L1'2'3'4'5'6 | 7316 | 3 | 0.115
L1'2'3'4'5'6 | 7337 | 1 | 0.038
L1'2'3'4'5'6 | 736A | 3 | 0.115
L1'2'3'4'5'6 | 7403 | 1 | 0.038
L1'2'3'4'5'6 | 7424 | 16 | 0.615
L1'2'3'4'5'6 | 7474 | 1 | 0.038
L1'2'3'4'5'6 | 7490 | 1 | 0.038
L1'2'3'4'5'6 | 7609 | 3 | 0.115
L1'2'3'4'5'6 | 7621 | 3 | 0.115
L1'2'3'4'5'6 | 7650 | 21 | 0.808
L1'2'3'4'5'6 | 7664 | 3 | 0.115
L1'2'3'4'5'6 | 7702 | 1 | 0.038
L1'2'3'4'5'6 | 7705 | 3 | 0.115
L1'2'3'4'5'6 | 7741 | 3 | 0.115
L1'2'3'4'5'6 | 7746 | 16 | 0.615
L1'2'3'4'5'6 | 7789 | 3 | 0.115
L1'2'3'4'5'6 | 7810 | 3 | 0.115
L1'2'3'4'5'6 | 7852 | 1 | 0.038
L1'2'3'4'5'6 | 7861 | 16 | 0.615
L1'2'3'4'5'6 | 7868 | 21 | 0.808
L1'2'3'4'5'6 | 789 | 1 | 0.038
L1'2'3'4'5'6 | 7891 | 21 | 0.808
L1'2'3'4'5'6 | 7961 | 3 | 0.115
L1'2'3'4'5'6 | 8005 | 1 | 0.038
L1'2'3'4'5'6 | 8008 | 3 | 0.115
L1'2'3'4'5'6 | 8021 | 21 | 0.808
L1'2'3'4'5'6 | 8065 | 21 | 0.808
L1'2'3'4'5'6 | 8140 | 3 | 0.115
L1'2'3'4'5'6 | 8152 | 3 | 0.115
L1'2'3'4'5'6 | 8167 | 3 | 0.115
L1'2'3'4'5'6 | 8203 | 3 | 0.115
L1'2'3'4'5'6 | 827 | 21 | 0.808
L1'2'3'4'5'6 | 8284 | 1 | 0.038
L1'2'3'4'5'6 | 8348 | 14 | 0.538
L1'2'3'4'5'6 | 8365 | 17 | 0.654
L1'2'3'4'5'6 | 8386 | 17 | 0.654
L1'2'3'4'5'6 | 8406 | 21 | 0.808
L1'2'3'4'5'6 | 8455 | 19 | 0.731
L1'2'3'4'5'6 | 8461 | 21 | 0.808
L1'2'3'4'5'6 | 8488 | 3 | 0.115
L1'2'3'4'5'6 | 8503 | 21 | 0.808
L1'2'3'4'5'6 | 8521 | 3 | 0.115
L1'2'3'4'5'6 | 8545 | 3 | 0.115
L1'2'3'4'5'6 | 8659 | 2 | 0.077
L1'2'3'4'5'6 | 8677C | 3 | 0.115
L1'2'3'4'5'6 | 868 | 3 | 0.115
L1'2'3'4'5'6 | 8718 | 21 | 0.808
L1'2'3'4'5'6 | 8764 | 18 | 0.692
L1'2'3'4'5'6 | 8772 | 3 | 0.115
L1'2'3'4'5'6 | 8784 | 3 | 0.115
L1'2'3'4'5'6 | 8840 | 1 | 0.038
L1'2'3'4'5'6 | 8943 | 21 | 0.808
L1'2'3'4'5'6 | 8986 | 16 | 0.615
L1'2'3'4'5'6 | 8994 | 3 | 0.115
L1'2'3'4'5'6 | 9027 | 17 | 0.654
L1'2'3'4'5'6 | 9053 | 18 | 0.692
L1'2'3'4'5'6 | 9060A | 3 | 0.115
L1'2'3'4'5'6 | 9075 | 3 | 0.115
L1'2'3'4'5'6 | 9080 | 3 | 0.115
L1'2'3'4'5'6 | 9090 | 1 | 0.038
L1'2'3'4'5'6 | 9168 | 3 | 0.115
L1'2'3'4'5'6 | 9248 | 4 | 0.154
L1'2'3'4'5'6 | 9254 | 3 | 0.115
L1'2'3'4'5'6 | 928 | 3 | 0.115
L1'2'3'4'5'6 | 9300 | 1 | 0.038
L1'2'3'4'5'6 | 9325 | 21 | 0.808
L1'2'3'4'5'6 | 9329C | 21 | 0.808
L1'2'3'4'5'6 | 9345 | 18 | 0.692
L1'2'3'4'5'6 | 9355T | 1 | 0.038
L1'2'3'4'5'6 | 9380 | 1 | 0.038
L1'2'3'4'5'6 | 94 | 2 | 0.077
L1'2'3'4'5'6 | 9410 | 1 | 0.038
L1'2'3'4'5'6 | 9434 | 3 | 0.115
L1'2'3'4'5'6 | 9449 | 1 | 0.038
L1'2'3'4'5'6 | 9456 | 17 | 0.654
L1'2'3'4'5'6 | 9469 | 1 | 0.038
L1'2'3'4'5'6 | 9527 | 2 | 0.077
L1'2'3'4'5'6 | 9530 | 3 | 0.115

L1'2'3'4'5'6 | 9536 | 3 | 0.115
L1'2'3'4'5'6 | 9566 | 3 | 0.115
L1'2'3'4'5'6 | 9629 | 3 | 0.115
L1'2'3'4'5'6 | 9647 | 3 | 0.115
L1'2'3'4'5'6 | 9716 | 2 | 0.077
L1'2'3'4'5'6 | 9755 | 21 | 0.808
L1'2'3'4'5'6 | 9758 | 3 | 0.115
L1'2'3'4'5'6 | 9797 | 4 | 0.154
L1'2'3'4'5'6 | 9839A | 3 | 0.115
L1'2'3'4'5'6 | 9869 | 21 | 0.808
L1'2'3'4'5'6 | 9884 | 3 | 0.115
L1'2'3'4'5'6 | 9899 | 3 | 0.115
L1'2'3'4'5'6 | 9932 | 4 | 0.154
L1'2'3'4'5'6 | 9966 | 4 | 0.154
L1b | 16051 | 1 | 0.012
L1b | 16114G | 5 | 0.062
L1b | 16274 | 1 | 0.012
L1b | 16301 | 3 | 0.037
L1b | 16320 | 1 | 0.012
L1b | 16362 | 4 | 0.049
L1b | 265 | 1 | 0.012
L1b | 573.1C | 1 | 0.012
L1b | 573.3C | 1 | 0.012
L1b | 573.5C | 1 | 0.012
L1b | 573.6C | 1 | 0.012
L1b1 | 11020 | 1 | 0.012
L1b1 | 11452 | 1 | 0.012
L1b1 | 13880 | 1 | 0.012
L1b1 | 14180 | 1 | 0.012
L1b1 | 14759G | 1 | 0.012
L1b1 | 14812 | 1 | 0.012
L1b1 | 15301 | 1 | 0.012
L1b1 | 16070 | 1 | 0.012
L1b1 | 16184 | 1 | 0.012
L1b1 | 16192 | 1 | 0.012
L1b1 | 16193 | 1 | 0.012
L1b1 | 16195 | 1 | 0.012
L1b1 | 16213 | 1 | 0.012
L1b1 | 16224 | 1 | 0.012
L1b1 | 16266 | 1 | 0.012
L1b1 | 16317 | 2 | 0.025
L1b1 | 185 | 1 | 0.012
L1b1 | 189 | 1 | 0.012
L1b1 | 384 | 1 | 0.012
L1b1 | 4123 | 1 | 0.012
L1b1 | 573.1C | 1 | 0.012
L1b1 | 573.3C | 1 | 0.012
L1b1 | 573.5C | 1 | 0.012
L1b1 | 573.6C | 1 | 0.012
L1b1 | 7444 | 1 | 0.012
L1b1 | 825 | 1 | 0.012
L1b1 | 8329G | 1 | 0.012
L1b1 | 8937 | 1 | 0.012
L1b1a | 10045 | 3 | 0.022
L1b1a | 10157 | 1 | 0.007
L1b1a | 10187 | 2 | 0.015
L1b1a | 10247 | 1 | 0.007
L1b1a | 10446 | 1 | 0.007
L1b1a | 10454 | 1 | 0.007
L1b1a | 10810A | 1 | 0.007
L1b1a | 10930 | 1 | 0.007
L1b1a | 11083 | 2 | 0.015
L1b1a | 11329 | 1 | 0.007
L1b1a | 11569 | 1 | 0.007
L1b1a | 11890 | 1 | 0.007
L1b1a | 11939 | 1 | 0.007
L1b1a | 12091 | 1 | 0.007
L1b1a | 12358 | 1 | 0.007
L1b1a | 12480 | 3 | 0.022
L1b1a | 13111 | 2 | 0.015
L1b1a | 13563 | 1 | 0.007
L1b1a | 13708 | 3 | 0.022
L1b1a | 13827 | 1 | 0.007
L1b1a | 13848 | 5 | 0.036
L1b1a | 13858 | 1 | 0.007
L1b1a | 13879 | 4 | 0.029
L1b1a | 13965 | 1 | 0.007
L1b1a | 14110 | 1 | 0.007
L1b1a | 14233 | 3 | 0.022
L1b1a | 14305 | 3 | 0.022
L1b1a | 14484 | 1 | 0.007
L1b1a | 14552 | 2 | 0.015
L1b1a | 146 | 4 | 0.029
L1b1a | 150 | 1 | 0.007
L1b1a | 15412 | 1 | 0.007
L1b1a | 15511 | 3 | 0.022
L1b1a | 15586 | 1 | 0.007
L1b1a | 15692 | 1 | 0.007
L1b1a | 15924 | 1 | 0.007
L1b1a | 16093 | 6 | 0.044
L1b1a | 16114 | 1 | 0.007
L1b1a | 16114G | 1 | 0.007
L1b1a | 16145 | 13 | 0.095
L1b1a | 16168 | 2 | 0.015
L1b1a | 16172 | 2 | 0.015
L1b1a | 16193 | 1 | 0.007
L1b1a | 16213 | 1 | 0.007
L1b1a | 16215C | 1 | 0.007
L1b1a | 16234 | 1 | 0.007
L1b1a | 16247 | 1 | 0.007
L1b1a | 16258d | 1 | 0.007
L1b1a | 16274 | 1 | 0.007
L1b1a | 16294 | 2 | 0.015
L1b1a | 16312 | 1 | 0.007
L1b1a | 185C | 1 | 0.007
L1b1a | 198 | 1 | 0.007
L1b1a | 204 | 2 | 0.015
L1b1a | 249d | 1 | 0.007
L1b1a | 264 | 1 | 0.007
L1b1a | 2812 | 1 | 0.007
L1b1a | 309d | 4 | 0.029

L1b1a | 310 | 1 | 0.007
L1b1a | 317.1C | 2 | 0.015
L1b1a | 3396 | 1 | 0.007
L1b1a | 3505 | 1 | 0.007
L1b1a | 3510A | 4 | 0.029
L1b1a | 3849 | 1 | 0.007
L1b1a | 3966 | 1 | 0.007
L1b1a | 4076 | 1 | 0.007
L1b1a | 4117 | 1 | 0.007
L1b1a | 4216 | 3 | 0.022
L1b1a | 461 | 1 | 0.007
L1b1a | 5004 | 1 | 0.007
L1b1a | 5082 | 1 | 0.007
L1b1a | 5177 | 1 | 0.007
L1b1a | 5499 | 6 | 0.044
L1b1a | 5628 | 1 | 0.007
L1b1a | 573.1C | 2 | 0.015
L1b1a | 573.3C | 1 | 0.007
L1b1a | 573.5C | 1 | 0.007
L1b1a | 573.6C | 1 | 0.007
L1b1a | 5988 | 1 | 0.007
L1b1a | 6150 | 1 | 0.007
L1b1a | 6252 | 3 | 0.022
L1b1a | 6366 | 1 | 0.007
L1b1a | 6446 | 1 | 0.007
L1b1a | 6671 | 1 | 0.007
L1b1a | 6881 | 1 | 0.007
L1b1a | 7022 | 1 | 0.007
L1b1a | 7598 | 2 | 0.015
L1b1a | 7609 | 1 | 0.007
L1b1a | 7807A | 2 | 0.015
L1b1a | 8251 | 2 | 0.015
L1b1a | 8790 | 1 | 0.007
L1b1a | 8843 | 1 | 0.007
L1b1a | 9374 | 3 | 0.022
L1b1a | 9482 | 1 | 0.007
L1b1a | 9804 | 1 | 0.007
L1b1a | 9851 | 3 | 0.022
L1b1a | 9932 | 1 | 0.007
L1b1a | 9983 | 1 | 0.007
L1b1a+189 | 10807 | 1 | 0.01
L1b1a+189 | 1193 | 1 | 0.01
L1b1a+189 | 13111 | 2 | 0.02
L1b1a+189 | 14164 | 1 | 0.01
L1b1a+189 | 14915 | 1 | 0.01
L1b1a+189 | 15479 | 1 | 0.01
L1b1a+189 | 15693 | 1 | 0.01
L1b1a+189 | 16159 | 1 | 0.01
L1b1a+189 | 16166 | 2 | 0.02
L1b1a+189 | 16193 | 2 | 0.02
L1b1a+189 | 16274 | 2 | 0.02
L1b1a+189 | 16362 | 5 | 0.051
L1b1a+189 | 16400 | 1 | 0.01
L1b1a+189 | 198 | 3 | 0.031
L1b1a+189 | 226 | 1 | 0.01
L1b1a+189 | 260 | 1 | 0.01
L1b1a+189 | 309d | 2 | 0.02
L1b1a+189 | 310 | 1 | 0.01
L1b1a+189 | 3411 | 1 | 0.01
L1b1a+189 | 5291 | 1 | 0.01
L1b1a+189 | 573.1C | 2 | 0.02
L1b1a+189 | 573.3C | 1 | 0.01
L1b1a+189 | 573.4C | 2 | 0.02
L1b1a+189 | 573.5C | 1 | 0.01
L1b1a+189 | 9180 | 1 | 0.01
L1b1a+189 | 930 | 1 | 0.01
L1b1a+189 | 94 | 1 | 0.01
L1b1a+189 | 9755 | 1 | 0.01
L1b1a10 | 11017 | 3 | 0.051
L1b1a10 | 11605 | 1 | 0.017
L1b1a10 | 11864 | 1 | 0.017
L1b1a10 | 12358 | 1 | 0.017
L1b1a10 | 12507 | 1 | 0.017
L1b1a10 | 12609 | 1 | 0.017
L1b1a10 | 13644 | 1 | 0.017
L1b1a10 | 13781 | 2 | 0.034
L1b1a10 | 14460G | 1 | 0.017
L1b1a10 | 14873A | 1 | 0.017
L1b1a10 | 16290 | 1 | 0.017
L1b1a10 | 16362 | 1 | 0.017
L1b1a10 | 16400 | 1 | 0.017
L1b1a10 | 198 | 1 | 0.017
L1b1a10 | 309d | 4 | 0.068
L1b1a10 | 310 | 1 | 0.017
L1b1a10 | 316 | 1 | 0.017
L1b1a10 | 317.1C | 3 | 0.051
L1b1a10 | 4733 | 1 | 0.017
L1b1a10 | 4856 | 1 | 0.017
L1b1a10 | 5563 | 1 | 0.017
L1b1a10 | 573.1C | 2 | 0.034
L1b1a10 | 6680 | 1 | 0.017
L1b1a10 | 7609 | 2 | 0.034
L1b1a10 | 9156 | 1 | 0.017
L1b1a10 | 9716 | 1 | 0.017
L1b1a10 | 9827 | 1 | 0.017
L1b1a10a | 16093 | 1 | 0.02
L1b1a10a | 16114 | 1 | 0.02
L1b1a10a | 16168 | 1 | 0.02
L1b1a10a | 16192 | 1 | 0.02
L1b1a10a | 16232A | 1 | 0.02
L1b1a10a | 16234 | 1 | 0.02
L1b1a10a | 16257 | 1 | 0.02
L1b1a10a | 16274 | 1 | 0.02
L1b1a10a | 16317T | 1 | 0.02
L1b1a10a | 185C | 1 | 0.02
L1b1a10a | 189 | 5 | 0.1
L1b1a10a | 4655 | 1 | 0.02
L1b1a10a | 4958 | 1 | 0.02
L1b1a10a | 515-524d | 2 | 0.04
L1b1a10a | 5162 | 1 | 0.02
L1b1a10a | 573.1C | 2 | 0.04

L1b1a10a | 573.2C | 1 | 0.02
L1b1a10a | 573.4C | 1 | 0.02
L1b1a10a | 573.5C | 2 | 0.04
L1b1a10a | 5899.1C | 1 | 0.02
L1b1a10a | 7444 | 1 | 0.02
L1b1a10b | 13020 | 2 | 0.035
L1b1a10b | 16145 | 3 | 0.053
L1b1a10b | 198 | 3 | 0.053
L1b1a10b | 309d | 4 | 0.07
L1b1a10b | 310 | 2 | 0.035
L1b1a10b | 4048 | 2 | 0.035
L1b1a10b | 5250 | 2 | 0.035
L1b1a10b | 573.1C | 2 | 0.035
L1b1a12 | 146 | 1 | 0.062
L1b1a12 | 16085 | 1 | 0.062
L1b1a12 | 16093 | 1 | 0.062
L1b1a12 | 16145 | 1 | 0.062
L1b1a12 | 16184 | 1 | 0.062
L1b1a12 | 16188 | 1 | 0.062
L1b1a12 | 16260 | 3 | 0.188
L1b1a12 | 16274 | 2 | 0.125
L1b1a12 | 16362 | 8 | 0.5
L1b1a12 | 16394 | 1 | 0.062
L1b1a12 | 16484-16489d | 1 | 0.062
L1b1a12 | 188 | 1 | 0.062
L1b1a12 | 189 | 2 | 0.125
L1b1a12 | 198 | 2 | 0.125
L1b1a12 | 249d | 1 | 0.062
L1b1a12a | 13602 | 1 | 0.071
L1b1a12a | 16184 | 1 | 0.071
L1b1a12a | 198 | 6 | 0.429
L1b1a12a | 6227 | 1 | 0.071
L1b1a12b | 12172.1A | 1 | 0.016
L1b1a12b | 14212 | 2 | 0.032
L1b1a12b | 146 | 1 | 0.016
L1b1a12b | 15725 | 1 | 0.016
L1b1a12b | 16092 | 1 | 0.016
L1b1a12b | 16093 | 13 | 0.21
L1b1a12b | 16172 | 1 | 0.016
L1b1a12b | 16175 | 2 | 0.032
L1b1a12b | 16186 | 1 | 0.016
L1b1a12b | 16213 | 7 | 0.113
L1b1a12b | 16234G | 1 | 0.016
L1b1a12b | 16239 | 1 | 0.016
L1b1a12b | 16249A | 1 | 0.016
L1b1a12b | 16252C | 2 | 0.032
L1b1a12b | 16255 | 1 | 0.016
L1b1a12b | 16256 | 2 | 0.032
L1b1a12b | 16260 | 7 | 0.113
L1b1a12b | 16261 | 1 | 0.016
L1b1a12b | 16301 | 1 | 0.016
L1b1a12b | 16362 | 2 | 0.032
L1b1a12b | 16496 | 4 | 0.065
L1b1a12b | 16496T | 1 | 0.016
L1b1a12b | 189 | 1 | 0.016
L1b1a12b | 198 | 2 | 0.032
L1b1a12b | 200 | 1 | 0.016
L1b1a12b | 3915 | 1 | 0.016
L1b1a12b | 438 | 1 | 0.016
L1b1a12b | 474A | 1 | 0.016
L1b1a12b | 507 | 1 | 0.016
L1b1a12b | 507A | 2 | 0.032
L1b1a12b | 515-524d | 55 | 0.887
L1b1a12b | 522A | 1 | 0.016
L1b1a12b | 7389A | 1 | 0.016
L1b1a13 | 16070 | 1 | 0.012
L1b1a13 | 16184 | 1 | 0.012
L1b1a13 | 16192 | 1 | 0.012
L1b1a13 | 16193 | 1 | 0.012
L1b1a13 | 16195 | 1 | 0.012
L1b1a13 | 16213 | 3 | 0.037
L1b1a13 | 16224 | 1 | 0.012
L1b1a13 | 16260 | 3 | 0.037
L1b1a13 | 16266 | 1 | 0.012
L1b1a13 | 384 | 1 | 0.012
L1b1a13 | 573.1C | 1 | 0.012
L1b1a13 | 573.3C | 1 | 0.012
L1b1a13 | 573.5C | 1 | 0.012
L1b1a13 | 573.6C | 1 | 0.012
L1b1a13 | 9493C | 1 | 0.012
L1b1a13 | 9948 | 1 | 0.012
L1b1a14 | 14338 | 2 | 0.027
L1b1a14 | 14527 | 2 | 0.027
L1b1a14 | 1453 | 2 | 0.027
L1b1a14 | 15812 | 2 | 0.027
L1b1a14 | 16256 | 4 | 0.053
L1b1a14 | 3930 | 1 | 0.013
L1b1a14 | 5147 | 2 | 0.027
L1b1a1'4 | 5147 | 1 | 0.071
L1b1a14 | 573.1C | 1 | 0.013
L1b1a14 | 573.3C | 1 | 0.013
L1b1a14 | 573.5C | 1 | 0.013
L1b1a14 | 573.6C | 1 | 0.013
L1b1a1'4 | 5988 | 1 | 0.071
L1b1a1'4 | 7419 | 1 | 0.071
L1b1a1'4 | 7785 | 1 | 0.071
L1b1a14 | 8116 | 2 | 0.027
L1b1a15 | 12121 | 1 | 0.01
L1b1a15 | 12471 | 1 | 0.01
L1b1a15 | 13326 | 1 | 0.01
L1b1a15 | 16176 | 2 | 0.02
L1b1a15 | 16192 | 1 | 0.01
L1b1a15 | 16497 | 1 | 0.01
L1b1a15 | 199 | 3 | 0.031
L1b1a15 | 200 | 1 | 0.01
L1b1a15 | 309d | 2 | 0.02
L1b1a15 | 310 | 1 | 0.01
L1b1a15 | 3861 | 1 | 0.01
L1b1a15 | 4080 | 3 | 0.031
L1b1a15 | 5471 | 1 | 0.01
L1b1a15 | 573.1C | 2 | 0.02

L1b1a15 | 573.3C | 1 | 0.01
L1b1a15 | 573.4C | 2 | 0.02
L1b1a15 | 573.5C | 1 | 0.01
L1b1a15 | 7403 | 1 | 0.01
L1b1a15 | 7492 | 1 | 0.01
L1b1a15 | 8999 | 1 | 0.01
L1b1a15 | 9668 | 1 | 0.01
L1b1a15a | 146 | 3 | 0.5
L1b1a15a | 199 | 4 | 0.667
L1b1a15a | 37d | 1 | 0.167
L1b1a15a | 40A | 1 | 0.167
L1b1a15a | 41-44d | 1 | 0.167
L1b1a15a | 4589 | 1 | 0.167
L1b1a15a | 46G | 1 | 0.167
L1b1a15a | 475C | 1 | 0.167
L1b1a15a | 477.1T | 1 | 0.167
L1b1a15a | 485A | 1 | 0.167
L1b1a15a | 486A | 1 | 0.167
L1b1a15a | 489A | 1 | 0.167
L1b1a15a | 721 | 1 | 0.167
L1b1a15a | 8839 | 1 | 0.167
L1b1a16 | 11944 | 1 | 0.024
L1b1a16 | 146 | 3 | 0.073
L1b1a16 | 150 | 1 | 0.024
L1b1a16 | 151 | 1 | 0.024
L1b1a16 | 15310 | 1 | 0.024
L1b1a16 | 16086 | 1 | 0.024
L1b1a16 | 16240 | 1 | 0.024
L1b1a16 | 16256 | 1 | 0.024
L1b1a16 | 16261 | 1 | 0.024
L1b1a16 | 16284 | 2 | 0.049
L1b1a16 | 16294 | 2 | 0.049
L1b1a16 | 16360 | 10 | 0.244
L1b1a16 | 185 | 1 | 0.024
L1b1a16 | 188 | 1 | 0.024
L1b1a16 | 189 | 1 | 0.024
L1b1a16 | 236 | 1 | 0.024
L1b1a16 | 279 | 1 | 0.024
L1b1a16 | 515 | 1 | 0.024
L1b1a16 | 515-524d | 1 | 0.024
L1b1a16 | 5493 | 1 | 0.024
L1b1a16 | 7205 | 1 | 0.024
L1b1a16 | 7674 | 1 | 0.024
L1b1a16 | 8285.1CTCTACCCC | 1 | 0.024
L1b1a16 | 9548 | 1 | 0.024
L1b1a17 | 146 | 1 | 0.009
L1b1a17 | 151 | 1 | 0.009
L1b1a17 | 15526 | 1 | 0.009
L1b1a17 | 15940 | 1 | 0.009
L1b1a17 | 16093 | 4 | 0.035
L1b1a17 | 16163T | 1 | 0.009
L1b1a17 | 16172 | 3 | 0.026
L1b1a17 | 16242 | 1 | 0.009
L1b1a17 | 16245 | 1 | 0.009
L1b1a17 | 16249A | 2 | 0.018
L1b1a17 | 16258C | 1 | 0.009
L1b1a17 | 16274 | 1 | 0.009
L1b1a17 | 16301 | 1 | 0.009
L1b1a17 | 16362 | 1 | 0.009
L1b1a17 | 16496 | 1 | 0.009
L1b1a17 | 199 | 1 | 0.009
L1b1a17 | 234 | 1 | 0.009
L1b1a17 | 249d | 2 | 0.018
L1b1a17 | 2905 | 5 | 0.044
L1b1a17 | 3027 | 1 | 0.009
L1b1a17 | 373 | 2 | 0.018
L1b1a17 | 507A | 2 | 0.018
L1b1a17 | 515-524d | 19 | 0.167
L1b1a17 | 573.1C | 2 | 0.018
L1b1a17 | 573.2C | 1 | 0.009
L1b1a17 | 573.3C | 2 | 0.018
L1b1a17 | 573.4C | 2 | 0.018
L1b1a17 | 573.5C | 1 | 0.009
L1b1a17 | 6899 | 1 | 0.009
L1b1a17 | 7859 | 2 | 0.018
L1b1a17 | 8531 | 6 | 0.053
L1b1a18 | 1007 | 1 | 0.016
L1b1a18 | 151 | 1 | 0.016
L1b1a18 | 16114G | 1 | 0.016
L1b1a18 | 16166 | 2 | 0.032
L1b1a18 | 16172 | 1 | 0.016
L1b1a18 | 16184 | 2 | 0.032
L1b1a18 | 16193 | 1 | 0.016
L1b1a18 | 16235 | 1 | 0.016
L1b1a18 | 16260 | 3 | 0.048
L1b1a18 | 16274 | 1 | 0.016
L1b1a18 | 163 | 2 | 0.032
L1b1a18 | 16484-16489d | 1 | 0.016
L1b1a18 | 185 | 1 | 0.016
L1b1a18 | 2000 | 1 | 0.016
L1b1a18 | 204 | 1 | 0.016
L1b1a18 | 236 | 1 | 0.016
L1b1a18 | 455.1T | 4 | 0.065
L1b1a18 | 4580 | 1 | 0.016
L1b1a18 | 515-524d | 2 | 0.032
L1b1a18 | 573.3C | 1 | 0.016
L1b1a18 | 573.5C | 1 | 0.016
L1b1a2 | 10321 | 5 | 0.068
L1b1a2 | 11150 | 5 | 0.068
L1b1a2 | 1508 | 5 | 0.068
L1b1a2 | 189 | 5 | 0.068
L1b1a2 | 573.1C | 1 | 0.014
L1b1a2 | 573.3C | 1 | 0.014
L1b1a2 | 573.5C | 1 | 0.014
L1b1a2 | 573.6C | 1 | 0.014
L1b1a2a | 10247 | 1 | 0.043
L1b1a2a | 10248 | 1 | 0.043
L1b1a2a | 151 | 2 | 0.087
L1b1a2a | 16037 | 4 | 0.174
L1b1a2a | 16075 | 1 | 0.043
L1b1a2a | 16104 | 3 | 0.13

L1b1a2a | 16108 | 1 | 0.043
L1b1a2a | 16129 | 1 | 0.043
L1b1a2a | 16304 | 1 | 0.043
L1b1a2a | 16320 | 4 | 0.174
L1b1a2a | 16340T | 1 | 0.043
L1b1a2a | 16343T | 1 | 0.043
L1b1a2a | 1709 | 1 | 0.043
L1b1a2a | 1842 | 1 | 0.043
L1b1a2a | 189 | 2 | 0.087
L1b1a2a | 198 | 1 | 0.043
L1b1a2a | 3579 | 1 | 0.043
L1b1a2a | 515-524d | 6 | 0.261
L1b1a2a | 7208 | 1 | 0.043
L1b1a2a | 8116 | 1 | 0.043
L1b1a2a | 9667 | 1 | 0.043
L1b1a2a | 9767 | 1 | 0.043
L1b1a3 | 10685 | 1 | 0.008
L1b1a3 | 10810A | 1 | 0.008
L1b1a3 | 11020 | 9 | 0.073
L1b1a3 | 11488 | 1 | 0.008
L1b1a3 | 14034 | 1 | 0.008
L1b1a3 | 14768 | 1 | 0.008
L1b1a3 | 14926 | 1 | 0.008
L1b1a3 | 150 | 3 | 0.024
L1b1a3 | 15077 | 1 | 0.008
L1b1a3 | 15152 | 1 | 0.008
L1b1a3 | 15217 | 1 | 0.008
L1b1a3 | 15530 | 3 | 0.024
L1b1a3 | 15773 | 1 | 0.008
L1b1a3 | 16265 | 1 | 0.008
L1b1a3 | 185C | 1 | 0.008
L1b1a3 | 198 | 1 | 0.008
L1b1a3 | 204 | 1 | 0.008
L1b1a3 | 309d | 3 | 0.024
L1b1a3 | 310 | 3 | 0.024
L1b1a3 | 317.1C | 3 | 0.024
L1b1a3 | 3441 | 1 | 0.008
L1b1a3 | 3543 | 1 | 0.008
L1b1a3 | 3828 | 1 | 0.008
L1b1a3 | 3867 | 1 | 0.008
L1b1a3 | 4140 | 1 | 0.008
L1b1a3 | 573.1C | 2 | 0.016
L1b1a3 | 573.3C | 1 | 0.008
L1b1a3 | 573.4C | 2 | 0.016
L1b1a3 | 573.5C | 1 | 0.008
L1b1a3 | 711 | 1 | 0.008
L1b1a3 | 8490 | 1 | 0.008
L1b1a3 | 8637 | 1 | 0.008
L1b1a3 | 9098G | 2 | 0.016
L1b1a3 | 9174 | 3 | 0.024
L1b1a3 | 9300 | 1 | 0.008
L1b1a3 | 9550 | 1 | 0.008
L1b1a3a | 10084 | 1 | 0.01
L1b1a3a | 11016 | 1 | 0.01
L1b1a3a | 11659 | 3 | 0.03
L1b1a3a | 12657 | 1 | 0.01
L1b1a3a | 12721 | 1 | 0.01
L1b1a3a | 13594 | 2 | 0.02
L1b1a3a | 14094 | 1 | 0.01
L1b1a3a | 14503 | 3 | 0.03
L1b1a3a | 1462 | 1 | 0.01
L1b1a3a | 14756 | 3 | 0.03
L1b1a3a | 15607 | 1 | 0.01
L1b1a3a | 16261 | 4 | 0.04
L1b1a3a | 1992 | 1 | 0.01
L1b1a3a | 310 | 1 | 0.01
L1b1a3a | 3762 | 1 | 0.01
L1b1a3a | 4080 | 2 | 0.02
L1b1a3a | 4113 | 1 | 0.01
L1b1a3a | 573.1C | 2 | 0.02
L1b1a3a | 573.3C | 1 | 0.01
L1b1a3a | 573.4C | 2 | 0.02
L1b1a3a | 573.5C | 1 | 0.01
L1b1a3a | 7747 | 2 | 0.02
L1b1a3a | 7868 | 1 | 0.01
L1b1a3a | 9270 | 3 | 0.03
L1b1a3a1 | 12906A | 2 | 0.02
L1b1a3a1 | 16051 | 1 | 0.01
L1b1a3a1 | 16068 | 1 | 0.01
L1b1a3a1 | 16145 | 1 | 0.01
L1b1a3a1 | 16167 | 1 | 0.01
L1b1a3a1 | 16169 | 1 | 0.01
L1b1a3a1 | 16243 | 1 | 0.01
L1b1a3a1 | 16255 | 1 | 0.01
L1b1a3a1 | 16292 | 3 | 0.029
L1b1a3a1 | 16300 | 1 | 0.01
L1b1a3a1 | 16301 | 1 | 0.01
L1b1a3a1 | 16305 | 1 | 0.01
L1b1a3a1 | 16390 | 1 | 0.01
L1b1a3a1 | 1822 | 1 | 0.01
L1b1a3a1 | 262 | 1 | 0.01
L1b1a3a1 | 297 | 1 | 0.01
L1b1a3a1 | 310 | 1 | 0.01
L1b1a3a1 | 408A | 1 | 0.01
L1b1a3a1 | 5237 | 1 | 0.01
L1b1a3a1 | 573.1C | 2 | 0.02
L1b1a3a1 | 573.3C | 1 | 0.01
L1b1a3a1 | 573.4C | 2 | 0.02
L1b1a3a1 | 573.5C | 1 | 0.01
L1b1a3b | 11015 | 2 | 0.019
L1b1a3b | 146 | 7 | 0.067
L1b1a3b | 15643 | 1 | 0.01
L1b1a3b | 16172 | 2 | 0.019
L1b1a3b | 16259 | 6 | 0.058
L1b1a3b | 16261 | 2 | 0.019
L1b1a3b | 16354 | 1 | 0.01
L1b1a3b | 16527 | 2 | 0.019
L1b1a3b | 225 | 2 | 0.019
L1b1a3b | 280 | 2 | 0.019
L1b1a3b | 294 | 1 | 0.01
L1b1a3b | 4340 | 1 | 0.01

L1b1a3b | 5096 | 1 | 0.01
L1b1a3b | 573.1C | 2 | 0.019
L1b1a3b | 573.3C | 1 | 0.01
L1b1a3b | 573.4C | 2 | 0.019
L1b1a3b | 573.5C | 1 | 0.01
L1b1a3b | 8149 | 1 | 0.01
L1b1a3b | 8479 | 1 | 0.01
L1b1a4 | 10172 | 1 | 0.04
L1b1a4 | 11701 | 1 | 0.04
L1b1a4 | 12280 | 1 | 0.04
L1b1a4 | 12477 | 1 | 0.04
L1b1a4 | 15204 | 1 | 0.04
L1b1a4 | 15714 | 1 | 0.04
L1b1a4 | 16234 | 4 | 0.16
L1b1a4 | 16239 | 4 | 0.16
L1b1a4 | 16265 | 1 | 0.04
L1b1a4 | 16274 | 4 | 0.16
L1b1a4 | 16399 | 2 | 0.08
L1b1a4 | 189 | 3 | 0.12
L1b1a4 | 4216 | 1 | 0.04
L1b1a4 | 474A | 1 | 0.04
L1b1a4 | 515-524d | 2 | 0.08
L1b1a4 | 5894 | 1 | 0.04
L1b1a4 | 6260 | 1 | 0.04
L1b1a4 | 6267 | 1 | 0.04
L1b1a4 | 6480 | 1 | 0.04
L1b1a4 | 8270 | 1 | 0.04
L1b1a4 | 8563 | 1 | 0.04
L1b1a4 | 9596 | 1 | 0.04
L1b1a4a | 13626 | 1 | 0.05
L1b1a4a | 13759 | 1 | 0.05
L1b1a4a | 146 | 5 | 0.25
L1b1a4a | 15219 | 1 | 0.05
L1b1a4a | 16114G | 8 | 0.4
L1b1a4a | 16234 | 1 | 0.05
L1b1a4a | 16239 | 1 | 0.05
L1b1a4a | 1842 | 2 | 0.1
L1b1a4a | 228 | 1 | 0.05
L1b1a4a | 317.1C | 1 | 0.05
L1b1a4a | 4248 | 1 | 0.05
L1b1a4a | 5243 | 1 | 0.05
L1b1a4a | 8269 | 1 | 0.05
L1b1a4a | 8762 | 1 | 0.05
L1b1a5 | 13293 | 1 | 0.011
L1b1a5 | 13485 | 1 | 0.011
L1b1a5 | 1406 | 3 | 0.034
L1b1a5 | 1438 | 1 | 0.011
L1b1a5 | 16070 | 1 | 0.011
L1b1a5 | 16184 | 1 | 0.011
L1b1a5 | 16192 | 1 | 0.011
L1b1a5 | 16193 | 1 | 0.011
L1b1a5 | 16195 | 1 | 0.011
L1b1a5 | 16224 | 1 | 0.011
L1b1a5 | 16256 | 4 | 0.046
L1b1a5 | 16266 | 1 | 0.011
L1b1a5 | 204 | 1 | 0.011
L1b1a5 | 241 | 1 | 0.011
L1b1a5 | 294 | 3 | 0.034
L1b1a5 | 384 | 1 | 0.011
L1b1a5 | 573.1C | 1 | 0.011
L1b1a5 | 573.3C | 1 | 0.011
L1b1a5 | 573.5C | 1 | 0.011
L1b1a5 | 573.6C | 1 | 0.011
L1b1a6 | 13368 | 1 | 0.01
L1b1a6 | 14305 | 1 | 0.01
L1b1a6 | 146 | 9 | 0.091
L1b1a6 | 14730 | 1 | 0.01
L1b1a6 | 151 | 1 | 0.01
L1b1a6 | 15172 | 1 | 0.01
L1b1a6 | 15262 | 1 | 0.01
L1b1a6 | 15271 | 1 | 0.01
L1b1a6 | 15346 | 1 | 0.01
L1b1a6 | 15903 | 1 | 0.01
L1b1a6 | 16093 | 6 | 0.061
L1b1a6 | 16114G | 1 | 0.01
L1b1a6 | 16172 | 1 | 0.01
L1b1a6 | 16181 | 1 | 0.01
L1b1a6 | 16256 | 3 | 0.03
L1b1a6 | 16301 | 1 | 0.01
L1b1a6 | 16362 | 3 | 0.03
L1b1a6 | 188 | 4 | 0.04
L1b1a6 | 198 | 1 | 0.01
L1b1a6 | 262 | 2 | 0.02
L1b1a6 | 3385 | 2 | 0.02
L1b1a6 | 372 | 1 | 0.01
L1b1a6 | 385 | 1 | 0.01
L1b1a6 | 4017 | 1 | 0.01
L1b1a6 | 4164 | 1 | 0.01
L1b1a6 | 4512 | 1 | 0.01
L1b1a6 | 515 | 2 | 0.02
L1b1a6 | 573.1C | 1 | 0.01
L1b1a6 | 573.3C | 1 | 0.01
L1b1a6 | 573.5C | 1 | 0.01
L1b1a6 | 573.6C | 1 | 0.01
L1b1a6 | 5773 | 1 | 0.01
L1b1a6 | 6348 | 1 | 0.01
L1b1a6 | 7805 | 1 | 0.01
L1b1a6 | 8835 | 1 | 0.01
L1b1a6 | 9716 | 1 | 0.01
L1b1a7 | 11929 | 1 | 0.013
L1b1a7 | 12358 | 1 | 0.013
L1b1a7 | 13831A | 1 | 0.013
L1b1a7 | 14025 | 1 | 0.013
L1b1a7 | 14053 | 1 | 0.013
L1b1a7 | 15406A | 1 | 0.013
L1b1a7 | 15706 | 1 | 0.013
L1b1a7 | 16148 | 1 | 0.013
L1b1a7 | 16290 | 1 | 0.013
L1b1a7 | 16320 | 1 | 0.013
L1b1a7 | 16360 | 1 | 0.013
L1b1a7 | 198 | 4 | 0.051

L1b1a7 | 573.1C | 1 | 0.013
L1b1a7 | 573.3C | 2 | 0.026
L1b1a7 | 573.5C | 1 | 0.013
L1b1a7 | 573.6C | 1 | 0.013
L1b1a7 | 7543 | 1 | 0.013
L1b1a7 | 7854 | 1 | 0.013
L1b1a7 | 9300 | 1 | 0.013
L1b1a7 | 9855 | 1 | 0.013
L1b1a7a | 14969 | 1 | 0.029
L1b1a7a | 15224 | 4 | 0.114
L1b1a7a | 16114G | 1 | 0.029
L1b1a7a | 16290 | 1 | 0.029
L1b1a7a | 16301 | 1 | 0.029
L1b1a7a | 16355 | 1 | 0.029
L1b1a7a | 1766 | 2 | 0.057
L1b1a7a | 198 | 1 | 0.029
L1b1a7a | 315.2C | 1 | 0.029
L1b1a7a | 515-524d | 1 | 0.029
L1b1a8 | 10094 | 1 | 0.011
L1b1a8 | 146 | 1 | 0.011
L1b1a8 | 14950 | 2 | 0.021
L1b1a8 | 150 | 3 | 0.032
L1b1a8 | 16037 | 1 | 0.011
L1b1a8 | 16071 | 1 | 0.011
L1b1a8 | 16114 | 3 | 0.032
L1b1a8 | 16114G | 2 | 0.021
L1b1a8 | 16145 | 5 | 0.053
L1b1a8 | 16148 | 1 | 0.011
L1b1a8 | 16186 | 1 | 0.011
L1b1a8 | 16207 | 1 | 0.011
L1b1a8 | 16215 | 1 | 0.011
L1b1a8 | 16215C | 2 | 0.021
L1b1a8 | 16257 | 1 | 0.011
L1b1a8 | 16274 | 1 | 0.011
L1b1a8 | 16301 | 1 | 0.011
L1b1a8 | 16344 | 1 | 0.011
L1b1a8 | 16355 | 2 | 0.021
L1b1a8 | 16368 | 2 | 0.021
L1b1a8 | 198 | 1 | 0.011
L1b1a8 | 200 | 1 | 0.011
L1b1a8 | 228T | 1 | 0.011
L1b1a8 | 236 | 1 | 0.011
L1b1a8 | 241C | 1 | 0.011
L1b1a8 | 4011 | 2 | 0.021
L1b1a8 | 5147 | 2 | 0.021
L1b1a8 | 573.1C | 1 | 0.011
L1b1a8 | 573.3C | 1 | 0.011
L1b1a8 | 573.5C | 1 | 0.011
L1b1a8 | 573.6C | 1 | 0.011
L1b1a8 | 8551 | 2 | 0.021
L1b1a8 | 93.1A | 1 | 0.011
L1b1a9 | 15061 | 1 | 0.009
L1b1a9 | 16037 | 1 | 0.009
L1b1a9 | 16093 | 7 | 0.065
L1b1a9 | 16172 | 3 | 0.028
L1b1a9 | 16184 | 1 | 0.009
L1b1a9 | 16234 | 1 | 0.009
L1b1a9 | 16261 | 1 | 0.009
L1b1a9 | 16266G | 1 | 0.009
L1b1a9 | 16286 | 1 | 0.009
L1b1a9 | 16301 | 1 | 0.009
L1b1a9 | 16340T | 2 | 0.019
L1b1a9 | 204 | 1 | 0.009
L1b1a9 | 207 | 2 | 0.019
L1b1a9 | 249d | 2 | 0.019
L1b1a9 | 310 | 3 | 0.028
L1b1a9 | 4116 | 1 | 0.009
L1b1a9 | 573.1C | 3 | 0.028
L1b1a9 | 573.3C | 3 | 0.028
L1b1a9 | 573.4C | 4 | 0.037
L1b1a9 | 573.5C | 4 | 0.037
L1b1a9 | 573.6C | 1 | 0.009
L1b1a9 | 5985 | 1 | 0.009
L1b1a9 | 61 | 2 | 0.019
L1b1a9 | 8176 | 1 | 0.009
L1b1a9 | 9458 | 3 | 0.028
L1b1a9 | 9873 | 1 | 0.009
L1b1a9 | 9932 | 1 | 0.009
L1b2 | 11812 | 1 | 0.042
L1b2 | 11902C | 1 | 0.042
L1b2 | 13470 | 1 | 0.042
L1b2 | 14152 | 1 | 0.042
L1b2 | 14569 | 1 | 0.042
L1b2 | 14632 | 1 | 0.042
L1b2 | 150 | 1 | 0.042
L1b2 | 15734 | 2 | 0.083
L1b2 | 15900 | 3 | 0.125
L1b2 | 16042 | 1 | 0.042
L1b2 | 16145 | 3 | 0.125
L1b2 | 16166 | 1 | 0.042
L1b2 | 16252 | 1 | 0.042
L1b2 | 16254 | 1 | 0.042
L1b2 | 16261 | 2 | 0.083
L1b2 | 16292 | 2 | 0.083
L1b2 | 16294 | 3 | 0.125
L1b2 | 16354 | 1 | 0.042
L1b2 | 16496 | 2 | 0.083
L1b2 | 1673 | 1 | 0.042
L1b2 | 185C | 1 | 0.042
L1b2 | 195 | 2 | 0.083
L1b2 | 199 | 2 | 0.083
L1b2 | 204 | 6 | 0.25
L1b2 | 474A | 3 | 0.125
L1b2 | 507 | 2 | 0.083
L1b2 | 507A | 1 | 0.042
L1b2 | 515-524d | 12 | 0.5
L1b2 | 6040 | 1 | 0.042
L1b2 | 7775 | 1 | 0.042
L1b2 | 8605 | 2 | 0.083
L1b2 | 9305 | 1 | 0.042
L1b2 | 9650 | 1 | 0.042

L1b2'3 | 151 | 1 | 0.036
L1b2'3 | 16172 | 3 | 0.107
L1b2'3 | 16186 | 1 | 0.036
L1b2'3 | 16193 | 1 | 0.036
L1b2'3 | 16195 | 1 | 0.036
L1b2'3 | 16272 | 1 | 0.036
L1b2'3 | 16310C | 1 | 0.036
L1b2'3 | 16325 | 1 | 0.036
L1b2'3 | 16327 | 1 | 0.036
L1b2'3 | 16484-16489d | 1 | 0.036
L1b2a | 10381 | 1 | 0.029
L1b2a | 11025 | 1 | 0.029
L1b2a | 12358 | 1 | 0.029
L1b2a | 13602 | 4 | 0.114
L1b2a | 14861 | 1 | 0.029
L1b2a | 151 | 2 | 0.057
L1b2a | 16067 | 2 | 0.057
L1b2a | 16093 | 4 | 0.114
L1b2a | 16221 | 2 | 0.057
L1b2a | 16249A | 1 | 0.029
L1b2a | 16274 | 1 | 0.029
L1b2a | 16292 | 1 | 0.029
L1b2a | 1842 | 1 | 0.029
L1b2a | 204 | 3 | 0.086
L1b2a | 207 | 1 | 0.029
L1b2a | 269 | 2 | 0.057
L1b2a | 309d | 5 | 0.143
L1b2a | 3339 | 1 | 0.029
L1b2a | 4715 | 1 | 0.029
L1b2a | 4755 | 1 | 0.029
L1b2a | 515-524d | 3 | 0.086
L1b2a | 5319 | 1 | 0.029
L1b2a | 5581 | 1 | 0.029
L1b2a | 569 | 1 | 0.029
L1b2a | 6032 | 1 | 0.029
L1b2a | 6066 | 1 | 0.029
L1b2a | 7598 | 1 | 0.029
L1b2a | 7830 | 1 | 0.029
L1b2a | 8282 | 17 | 0.486
L1b3 | 10077 | 1 | 0.036
L1b3 | 10352 | 1 | 0.036
L1b3 | 13928C | 1 | 0.036
L1b3 | 14305 | 1 | 0.036
L1b3 | 150 | 1 | 0.036
L1b3 | 16154 | 1 | 0.036
L1b3 | 16217 | 1 | 0.036
L1b3 | 16293T | 2 | 0.071
L1b3 | 16390 | 1 | 0.036
L1b3 | 16484-16489d | 1 | 0.036
L1b3 | 16527 | 5 | 0.179
L1b3 | 188 | 1 | 0.036
L1b3 | 195 | 1 | 0.036
L1b3 | 310 | 1 | 0.036
L1b3 | 408A | 1 | 0.036
L1b3 | 515-524d | 1 | 0.036
L1b3 | 6164 | 1 | 0.036
L1c | 146 | 1 | 0.5
L1c | 16274 | 1 | 0.5
L1c | 16287 | 1 | 0.5
L1c | 236 | 1 | 0.5
L1c | 93 | 1 | 0.5
L1c1 | 10181 | 2 | 0.154
L1c1 | 12007 | 8 | 0.615
L1c1 | 12127 | 8 | 0.615
L1c1 | 12990 | 2 | 0.154
L1c1 | 13111 | 1 | 0.077
L1c1 | 13500 | 8 | 0.615
L1c1 | 13692 | 3 | 0.231
L1c1 | 14007 | 3 | 0.231
L1c1 | 14161 | 8 | 0.615
L1c1 | 14305 | 8 | 0.615
L1c1 | 14311 | 8 | 0.615
L1c1 | 14476T | 8 | 0.615
L1c1 | 15172 | 8 | 0.615
L1c1 | 15944d | 1 | 0.077
L1c1 | 15970 | 4 | 0.308
L1c1 | 16093 | 1 | 0.077
L1c1 | 16117 | 3 | 0.231
L1c1 | 16153 | 1 | 0.077
L1c1 | 16162 | 2 | 0.154
L1c1 | 16172 | 10 | 0.769
L1c1 | 16173 | 10 | 0.769
L1c1 | 16188A | 9 | 0.692
L1c1 | 16256 | 10 | 0.769
L1c1 | 16291 | 3 | 0.231
L1c1 | 16368 | 10 | 0.769
L1c1 | 16390 | 2 | 0.154
L1c1 | 189 | 10 | 0.769
L1c1 | 1977 | 2 | 0.154
L1c1 | 2394C | 1 | 0.077
L1c1 | 2395C | 1 | 0.077
L1c1 | 249d | 1 | 0.077
L1c1 | 2707C | 3 | 0.231
L1c1 | 291d | 1 | 0.077
L1c1 | 309d | 2 | 0.154
L1c1 | 3573A | 1 | 0.077
L1c1 | 4314 | 3 | 0.231
L1c1 | 4924 | 2 | 0.154
L1c1 | 5417 | 8 | 0.615
L1c1 | 5628 | 8 | 0.615
L1c1 | 5899.1C | 1 | 0.077
L1c1 | 6249 | 3 | 0.231
L1c1 | 64 | 1 | 0.077
L1c1 | 7609 | 8 | 0.615
L1c1'2'4'5'6 | 146 | 1 | 0.2
L1c1'2'4'5'6 | 16037 | 1 | 0.2
L1c1'2'4'5'6 | 16265C | 1 | 0.2
L1c1'2'4'5'6 | 16271 | 1 | 0.2
L1c1'2'4'5'6 | 16274 | 2 | 0.4
L1c1'2'4'5'6 | 16287 | 1 | 0.2
L1c1'2'4'5'6 | 16290G | 1 | 0.2

L1c1'2'4'5'6 | 16291 | 1 | 0.2
L1c1'2'4'5'6 | 16527 | 1 | 0.2
L1c1'2'4'5'6 | 204 | 2 | 0.4
L1c1'2'4'5'6 | 236 | 1 | 0.2
L1c1'2'4'5'6 | 459d | 1 | 0.2
L1c1'2'4'5'6 | 89 | 1 | 0.2
L1c1'2'4'5'6 | 93 | 2 | 0.4
L1c1'2'4'6 | 16032.1TCTCTGTTCTTTCAT | 1 | 0.25
L1c1'2'4'6 | 16103C | 1 | 0.25
L1c1'2'4'6 | 16197 | 1 | 0.25
L1c1'2'4'6 | 16199 | 1 | 0.25
L1c1'2'4'6 | 16233T | 1 | 0.25
L1c1'2'4'6 | 16275C | 1 | 0.25
L1c1'2'4'6 | 16296G | 1 | 0.25
L1c1'2'4'6 | 16370T | 1 | 0.25
L1c1'2'4'6 | 16529 | 1 | 0.25
L1c1'2'4'6 | 16537 | 1 | 0.25
L1c1'2'4'6 | 200 | 1 | 0.25
L1c1a | 10976 | 1 | 0.143
L1c1a | 11044 | 1 | 0.143
L1c1a | 12281 | 1 | 0.143
L1c1a | 13260 | 1 | 0.143
L1c1a | 16093 | 5 | 0.714
L1c1a | 16170 | 2 | 0.286
L1c1a | 16263 | 3 | 0.429
L1c1a | 16368 | 5 | 0.714
L1c1a | 1762 | 1 | 0.143
L1c1a | 249d | 2 | 0.286
L1c1a | 3969 | 1 | 0.143
L1c1a | 4219 | 1 | 0.143
L1c1a | 4225 | 1 | 0.143
L1c1a | 6578C | 1 | 0.143
L1c1a | 8841 | 1 | 0.143
L1c1a | 9830 | 1 | 0.143
L1c1a+@198 | 16093 | 2 | 1
L1c1a+@198 | 16170 | 1 | 0.5
L1c1a+@198 | 16263 | 1 | 0.5
L1c1a+@198 | 16368 | 2 | 1
L1c1a+@198 | 249d | 1 | 0.5
L1c1a1 | 16270 | 1 | 1
L1c1a1a1a | 12004 | 1 | 0.019
L1c1a1a1a | 13966 | 9 | 0.17
L1c1a1a1a | 14180 | 1 | 0.019
L1c1a1a1a | 15758 | 1 | 0.019
L1c1a1a1a | 16189A | 1 | 0.019
L1c1a1a1a | 16258 | 1 | 0.019
L1c1a1a1a | 16266 | 1 | 0.019
L1c1a1a1a | 16271 | 5 | 0.094
L1c1a1a1a | 183 | 6 | 0.113
L1c1a1a1a | 5826 | 5 | 0.094
L1c1a1a1a | 708A | 1 | 0.019
L1c1a1a1a | 749 | 3 | 0.057
L1c1a1a1a | 8420 | 4 | 0.075
L1c1a1a1b | 11150 | 1 | 0.026
L1c1a1a1b | 13225 | 1 | 0.026
L1c1a1a1b | 14016 | 1 | 0.026
L1c1a1a1b | 1413 | 3 | 0.077
L1c1a1a1b | 16086 | 3 | 0.077
L1c1a1a1b | 16181 | 1 | 0.026
L1c1a1a1b | 16435-16441d | 1 | 0.026
L1c1a1a1b | 16512 | 2 | 0.051
L1c1a1a1b | 16529 | 2 | 0.051
L1c1a1a1b | 189 | 1 | 0.026
L1c1a1a1b | 198 | 2 | 0.051
L1c1a1a1b | 3394 | 3 | 0.077
L1c1a1a1b | 394 | 1 | 0.026
L1c1a1a1b | 5423 | 1 | 0.026
L1c1a1a1b | 7258 | 3 | 0.077
L1c1a1a1b | 9096 | 1 | 0.026
L1c1a1a1b | 9098 | 1 | 0.026
L1c1a1a1b | 9912 | 1 | 0.026
L1c1a1a1b1 | 10192 | 1 | 0.03
L1c1a1a1b1 | 11204 | 3 | 0.091
L1c1a1a1b1 | 14180 | 1 | 0.03
L1c1a1a1b1 | 15930 | 1 | 0.03
L1c1a1a1b1 | 16265 | 1 | 0.03
L1c1a1a1b1 | 2109 | 1 | 0.03
L1c1a1a1b1 | 2133C | 1 | 0.03
L1c1a1a1b1 | 2702 | 1 | 0.03
L1c1a1a1b1 | 308-309d | 1 | 0.03
L1c1a1a1b1 | 3901 | 1 | 0.03
L1c1a1a1b1 | 7785 | 1 | 0.03
L1c1a1a1b1 | 9355 | 8 | 0.242
L1c1a1a1b1 | 9494 | 2 | 0.061
L1c1a1a2 | 16527 | 2 | 0.667
L1c1a1a2 | 2707 | 1 | 0.333
L1c1a1a2 | 5913 | 2 | 0.667
L1c1a2 | 12843 | 3 | 0.088
L1c1a2 | 13209 | 1 | 0.029
L1c1a2 | 13269 | 1 | 0.029
L1c1a2 | 13315 | 3 | 0.088
L1c1a2 | 13827 | 1 | 0.029
L1c1a2 | 13948 | 1 | 0.029
L1c1a2 | 13980 | 1 | 0.029
L1c1a2 | 146 | 1 | 0.029
L1c1a2 | 151 | 4 | 0.118
L1c1a2 | 15884 | 3 | 0.088
L1c1a2 | 16145 | 1 | 0.029
L1c1a2 | 16235 | 2 | 0.059
L1c1a2 | 16292 | 1 | 0.029
L1c1a2 | 16344 | 1 | 0.029
L1c1a2 | 16355 | 3 | 0.088
L1c1a2 | 16524T | 4 | 0.118
L1c1a2 | 1822 | 3 | 0.088
L1c1a2 | 309d | 3 | 0.088
L1c1a2 | 310 | 1 | 0.029
L1c1a2 | 5186 | 1 | 0.029
L1c1a2 | 5351 | 1 | 0.029
L1c1a2 | 5843 | 1 | 0.029
L1c1a2 | 76 | 7 | 0.206
L1c1a2 | 89 | 14 | 0.412

L1c1a2 | 9064 | 2 | 0.059
L1c1a2 | 9948 | 1 | 0.029
L1c1a2a | 16399 | 9 | 0.333
L1c1a2a1 | 10365 | 2 | 0.065
L1c1a2a1 | 12735 | 1 | 0.032
L1c1a2a1 | 13834 | 1 | 0.032
L1c1a2a1 | 16093 | 7 | 0.226
L1c1a2a1 | 16284 | 2 | 0.065
L1c1a2a1 | 16399 | 2 | 0.065
L1c1a2a1 | 317.1A | 1 | 0.032
L1c1a2a1 | 573.3C | 1 | 0.032
L1c1a2a1 | 8419 | 2 | 0.065
L1c1a2a1 | 8988 | 5 | 0.161
L1c1a2a2 | 10203 | 1 | 0.034
L1c1a2a2 | 1503 | 4 | 0.138
L1c1a2a2 | 2674 | 1 | 0.034
L1c1a2a2 | 4820 | 1 | 0.034
L1c1a2a2 | 5054C | 3 | 0.103
L1c1a2a2 | 5250 | 1 | 0.034
L1c1a2a2 | 7056 | 1 | 0.034
L1c1a2a2 | 76 | 1 | 0.034
L1c1a2a2 | 8413 | 1 | 0.034
L1c1a2a2 | 89 | 1 | 0.034
L1c1a2b | 12610C | 1 | 0.021
L1c1a2b | 12972 | 1 | 0.021
L1c1a2b | 13422C | 1 | 0.021
L1c1a2b | 13620 | 1 | 0.021
L1c1a2b | 14325 | 2 | 0.042
L1c1a2b | 152 | 8 | 0.167
L1c1a2b | 16093 | 1 | 0.021
L1c1a2b | 198 | 1 | 0.021
L1c1a2b | 3323A | 1 | 0.021
L1c1a2b | 3338A | 1 | 0.021
L1c1a2b | 3483 | 3 | 0.062
L1c1a2b | 755 | 2 | 0.042
L1c1a2b | 7571 | 1 | 0.021
L1c1a2b | 8723 | 5 | 0.104
L1c1a2b | 8939 | 1 | 0.021
L1c1a2c | 16258C | 3 | 0.143
L1c1a2c | 16264 | 2 | 0.095
L1c1a'b'd | 16038 | 2 | 0.286
L1c1a'b'd | 16234 | 2 | 0.286
L1c1a'b'd | 16355 | 1 | 0.143
L1c1a'b'd | 248 | 1 | 0.143
L1c1a'b'd | 249d | 2 | 0.286
L1c1a'b'd | 303A | 4 | 0.571
L1c1a'b'd | 515-524d | 4 | 0.571
L1c1a'b'd | 529T | 1 | 0.143
L1c1b | 10861 | 5 | 0.096
L1c1b | 11087 | 1 | 0.019
L1c1b | 11353 | 32 | 0.615
L1c1b | 11440 | 32 | 0.615
L1c1b | 11929 | 1 | 0.019
L1c1b | 12212T | 1 | 0.019
L1c1b | 13874 | 41 | 0.788
L1c1b | 14323 | 1 | 0.019
L1c1b | 14693 | 1 | 0.019
L1c1b | 14954 | 1 | 0.019
L1c1b | 15930 | 5 | 0.096
L1c1b | 16291 | 9 | 0.173
L1c1b | 16399 | 1 | 0.019
L1c1b | 1821 | 1 | 0.019
L1c1b | 2352 | 3 | 0.058
L1c1b | 2581 | 32 | 0.615
L1c1b | 309d | 12 | 0.231
L1c1b | 3834 | 41 | 0.788
L1c1b | 416A | 48 | 0.923
L1c1b | 4589 | 41 | 0.788
L1c1b | 4703 | 2 | 0.038
L1c1b | 508 | 1 | 0.019
L1c1b | 518 | 39 | 0.75
L1c1b | 520 | 1 | 0.019
L1c1b | 5480 | 41 | 0.788
L1c1b | 5876 | 1 | 0.019
L1c1b | 6179 | 32 | 0.615
L1c1b | 6182 | 1 | 0.019
L1c1b | 7196 | 41 | 0.788
L1c1b | 7660 | 41 | 0.788
L1c1b | 7714 | 32 | 0.615
L1c1b | 7854 | 9 | 0.173
L1c1b | 7921 | 1 | 0.019
L1c1b | 7979 | 5 | 0.096
L1c1b | 8155 | 1 | 0.019
L1c1b | 8618 | 1 | 0.019
L1c1b | 9025 | 1 | 0.019
L1c1b | 9090 | 1 | 0.019
L1c1b | 9467 | 1 | 0.019
L1c1b | 9682 | 1 | 0.019
L1c1b | 9923 | 1 | 0.019
L1c1b1 | 10589 | 1 | 0.042
L1c1b1 | 11611 | 2 | 0.083
L1c1b1 | 12957 | 1 | 0.042
L1c1b1 | 14319 | 1 | 0.042
L1c1b1 | 14338 | 4 | 0.167
L1c1b1 | 14460G | 1 | 0.042
L1c1b1 | 15160 | 4 | 0.167
L1c1b1 | 15301 | 2 | 0.083
L1c1b1 | 15784 | 2 | 0.083
L1c1b1 | 15806 | 2 | 0.083
L1c1b1 | 15934 | 1 | 0.042
L1c1b1 | 16079 | 2 | 0.083
L1c1b1 | 16166d | 1 | 0.042
L1c1b1 | 16192 | 2 | 0.083
L1c1b1 | 16274 | 4 | 0.167
L1c1b1 | 16293 | 2 | 0.083
L1c1b1 | 16391 | 1 | 0.042
L1c1b1 | 16394 | 1 | 0.042
L1c1b1 | 2057 | 1 | 0.042
L1c1b1 | 317.1C | 1 | 0.042
L1c1b1 | 3792 | 2 | 0.083
L1c1b1 | 4025 | 1 | 0.042

L1c1b1 | 4219 | 1 | 0.042
L1c1b1 | 4562 | 2 | 0.083
L1c1b1 | 5231 | 1 | 0.042
L1c1b1 | 5651 | 1 | 0.042
L1c1b1 | 5876 | 1 | 0.042
L1c1b1 | 64 | 1 | 0.042
L1c1b1 | 7498 | 1 | 0.042
L1c1b1 | 8237 | 4 | 0.167
L1c1b1 | 8271.1TC | 2 | 0.083
L1c1b1 | 8271.1TCCC | 1 | 0.042
L1c1b1 | 8272 | 1 | 0.042
L1c1b1 | 8276.1C | 1 | 0.042
L1c1b1 | 8278.2C | 1 | 0.042
L1c1b1 | 8278.4C | 1 | 0.042
L1c1b1 | 8279 | 2 | 0.083
L1c1b1 | 8279.1C | 2 | 0.083
L1c1b1 | 8281-8289d | 1 | 0.042
L1c1b1 | 8287 | 2 | 0.083
L1c1b1 | 9054 | 1 | 0.042
L1c1b1 | 9467 | 1 | 0.042
L1c1b1 | 9824 | 4 | 0.167
L1c1b'd | 16153 | 2 | 0.4
L1c1b'd | 16219 | 1 | 0.2
L1c1b'd | 16261 | 1 | 0.2
L1c1b'd | 16362 | 1 | 0.2
L1c1b'd | 16390 | 1 | 0.2
L1c1b'd | 183 | 1 | 0.2
L1c1b'd | 204 | 1 | 0.2
L1c1b'd | 207 | 1 | 0.2
L1c1b'd | 242 | 2 | 0.4
L1c1b'd | 249d | 1 | 0.2
L1c1c | 10595 | 2 | 0.062
L1c1c | 11914 | 1 | 0.031
L1c1c | 12026 | 1 | 0.031
L1c1c | 12545 | 1 | 0.031
L1c1c | 12961 | 1 | 0.031
L1c1c | 13134 | 1 | 0.031
L1c1c | 146 | 1 | 0.031
L1c1c | 150 | 4 | 0.125
L1c1c | 16038 | 2 | 0.062
L1c1c | 16086 | 12 | 0.375
L1c1c | 16093 | 4 | 0.125
L1c1c | 16111A | 3 | 0.094
L1c1c | 16117 | 1 | 0.031
L1c1c | 16148 | 7 | 0.219
L1c1c | 16153 | 1 | 0.031
L1c1c | 16169 | 11 | 0.344
L1c1c | 16173 | 4 | 0.125
L1c1c | 16174A | 2 | 0.062
L1c1c | 16184 | 13 | 0.406
L1c1c | 16186 | 1 | 0.031
L1c1c | 16187 | 13 | 0.406
L1c1c | 16188 | 1 | 0.031
L1c1c | 16188A | 4 | 0.125
L1c1c | 16192 | 6 | 0.188
L1c1c | 16193 | 3 | 0.094
L1c1c | 16209 | 1 | 0.031
L1c1c | 16213 | 1 | 0.031
L1c1c | 16234 | 1 | 0.031
L1c1c | 16235 | 2 | 0.062
L1c1c | 16239 | 1 | 0.031
L1c1c | 16243 | 3 | 0.094
L1c1c | 16245 | 1 | 0.031
L1c1c | 16256 | 7 | 0.219
L1c1c | 16259 | 1 | 0.031
L1c1c | 16261 | 23 | 0.719
L1c1c | 16264 | 1 | 0.031
L1c1c | 16287 | 1 | 0.031
L1c1c | 16290 | 5 | 0.156
L1c1c | 16291 | 1 | 0.031
L1c1c | 16294 | 4 | 0.125
L1c1c | 16319 | 1 | 0.031
L1c1c | 16354 | 1 | 0.031
L1c1c | 16355 | 1 | 0.031
L1c1c | 16359 | 1 | 0.031
L1c1c | 16368 | 6 | 0.188
L1c1c | 16390 | 2 | 0.062
L1c1c | 16398 | 3 | 0.094
L1c1c | 16399 | 1 | 0.031
L1c1c | 16527 | 6 | 0.188
L1c1c | 189T | 1 | 0.031
L1c1c | 198 | 4 | 0.125
L1c1c | 204 | 1 | 0.031
L1c1c | 2232.1A | 2 | 0.062
L1c1c | 2887-2888d | 2 | 0.062
L1c1c | 3208 | 2 | 0.062
L1c1c | 3705 | 1 | 0.031
L1c1c | 3834 | 1 | 0.031
L1c1c | 385 | 1 | 0.031
L1c1c | 417 | 1 | 0.031
L1c1c | 4712 | 2 | 0.062
L1c1c | 4767 | 1 | 0.031
L1c1c | 4991 | 1 | 0.031
L1c1c | 5054 | 1 | 0.031
L1c1c | 529 | 1 | 0.031
L1c1c | 534 | 9 | 0.281
L1c1c | 573.3C | 1 | 0.031
L1c1c | 5984 | 1 | 0.031
L1c1c | 6297 | 1 | 0.031
L1c1c | 6401T | 1 | 0.031
L1c1c | 9497 | 1 | 0.031
L1c1d | 12103 | 13 | 0.565
L1c1d | 13651 | 1 | 0.043
L1c1d | 14319 | 12 | 0.522
L1c1d | 14467 | 1 | 0.043
L1c1d | 14869 | 1 | 0.043
L1c1d | 14905 | 1 | 0.043
L1c1d | 15076 | 1 | 0.043
L1c1d | 151d | 4 | 0.174
L1c1d | 16093 | 14 | 0.609
L1c1d | 16271 | 13 | 0.565

L1c1d | 207 | 1 | 0.043
L1c1d | 2363 | 13 | 0.565
L1c1d | 2686 | 9 | 0.391
L1c1d | 309d | 2 | 0.087
L1c1d | 3900 | 2 | 0.087
L1c1d | 5112 | 1 | 0.043
L1c1d | 521 | 13 | 0.565
L1c1d | 5821 | 12 | 0.522
L1c1d | 5964 | 9 | 0.391
L1c1d | 6378 | 1 | 0.043
L1c1d | 723 | 1 | 0.043
L1c1d | 7407 | 1 | 0.043
L1c1d | 7419 | 3 | 0.13
L1c1d | 7805 | 13 | 0.565
L1c1d | 8291 | 1 | 0.043
L1c1d | 8937 | 13 | 0.565
L1c1d | 9010 | 1 | 0.043
L1c1d | 9287 | 1 | 0.043
L1c1d | 9932 | 7 | 0.304
L1c1d1 | 10804 | 1 | 0.038
L1c1d1 | 13965 | 1 | 0.038
L1c1d1 | 15470 | 1 | 0.038
L1c1d1 | 15944d | 1 | 0.038
L1c1d1 | 16114G | 4 | 0.154
L1c1d1 | 16224 | 1 | 0.038
L1c1d1 | 16256 | 1 | 0.038
L1c1d1 | 16284 | 6 | 0.231
L1c1d1 | 16291 | 3 | 0.115
L1c1d1 | 16355 | 1 | 0.038
L1c1d1 | 204 | 10 | 0.385
L1c1d1 | 4167 | 2 | 0.077
L1c1d1 | 459d | 4 | 0.154
L1c1d1 | 4688 | 1 | 0.038
L1c1d1 | 514 | 2 | 0.077
L1c1d1 | 5480 | 1 | 0.038
L1c1d1 | 573.1C | 1 | 0.038
L1c1d1 | 8657 | 4 | 0.154
L1c2 | 16006-16007d | 1 | 0.053
L1c2 | 16010-16013d | 1 | 0.053
L1c2 | 16015d | 1 | 0.053
L1c2 | 16086 | 1 | 0.053
L1c2 | 16163 | 1 | 0.053
L1c2 | 16192 | 2 | 0.105
L1c2 | 16288 | 5 | 0.263
L1c2 | 16343T | 1 | 0.053
L1c2 | 16359 | 3 | 0.158
L1c2 | 16399 | 1 | 0.053
L1c2 | 16497 | 2 | 0.105
L1c2 | 487 | 2 | 0.105
L1c2'4 | 16032.1TCTCTGTTCTTTCAT | 1 | 0.25
L1c2'4 | 16103C | 1 | 0.25
L1c2'4 | 16197 | 1 | 0.25
L1c2'4 | 16199 | 1 | 0.25
L1c2'4 | 16233T | 1 | 0.25
L1c2'4 | 16275C | 1 | 0.25
L1c2'4 | 16296G | 1 | 0.25
L1c2'4 | 16370T | 1 | 0.25
L1c2'4 | 16529 | 1 | 0.25
L1c2'4 | 16537 | 1 | 0.25
L1c2'4 | 200 | 1 | 0.25
L1c2a | 16234 | 1 | 0.143
L1c2a | 16274 | 1 | 0.143
L1c2a | 523 | 1 | 0.143
L1c2a1 | 11984 | 8 | 0.571
L1c2a1 | 12759 | 8 | 0.571
L1c2a1 | 15388 | 8 | 0.571
L1c2a1 | 16134 | 11 | 0.786
L1c2a1 | 16209 | 8 | 0.571
L1c2a1 | 16214 | 8 | 0.571
L1c2a1 | 16256 | 3 | 0.214
L1c2a1 | 16274 | 11 | 0.786
L1c2a1 | 7157 | 8 | 0.571
L1c2a1a | 11182 | 2 | 0.026
L1c2a1a | 12389 | 4 | 0.053
L1c2a1a | 13866 | 1 | 0.013
L1c2a1a | 13933 | 1 | 0.013
L1c2a1a | 14129 | 1 | 0.013
L1c2a1a | 14325 | 1 | 0.013
L1c2a1a | 14544 | 1 | 0.013
L1c2a1a | 14858 | 2 | 0.026
L1c2a1a | 151d | 2 | 0.026
L1c2a1a | 15383 | 1 | 0.013
L1c2a1a | 15731 | 1 | 0.013
L1c2a1a | 15908 | 1 | 0.013
L1c2a1a | 1594 | 1 | 0.013
L1c2a1a | 16093 | 4 | 0.053
L1c2a1a | 16207 | 1 | 0.013
L1c2a1a | 16248 | 1 | 0.013
L1c2a1a | 16258C | 2 | 0.026
L1c2a1a | 16264 | 1 | 0.013
L1c2a1a | 16286A | 1 | 0.013
L1c2a1a | 16291 | 1 | 0.013
L1c2a1a | 16320 | 4 | 0.053
L1c2a1a | 16365 | 5 | 0.066
L1c2a1a | 1949 | 2 | 0.026
L1c2a1a | 204 | 1 | 0.013
L1c2a1a | 2955A | 1 | 0.013
L1c2a1a | 2968 | 1 | 0.013
L1c2a1a | 3009 | 1 | 0.013
L1c2a1a | 3019 | 1 | 0.013
L1c2a1a | 3027 | 1 | 0.013
L1c2a1a | 309d | 17 | 0.224
L1c2a1a | 310 | 1 | 0.013
L1c2a1a | 3543 | 1 | 0.013
L1c2a1a | 3892 | 1 | 0.013
L1c2a1a | 3995 | 1 | 0.013
L1c2a1a | 4500 | 1 | 0.013
L1c2a1a | 5585 | 2 | 0.026
L1c2a1a | 5899.2C | 4 | 0.053
L1c2a1a | 709 | 6 | 0.079
L1c2a1a | 7502 | 1 | 0.013

L1c2a1a | 752G | 5 | 0.066
L1c2a1a | 7954 | 1 | 0.013
L1c2a1a | 8214 | 1 | 0.013
L1c2a1a | 8477 | 1 | 0.013
L1c2a1a | 9181 | 4 | 0.053
L1c2a1a | 9333 | 1 | 0.013
L1c2a1a | 9631 | 1 | 0.013
L1c2a1a | 9667 | 1 | 0.013
L1c2a1b | 11392C | 1 | 0.053
L1c2a1b | 11914 | 3 | 0.158
L1c2a1b | 12082 | 2 | 0.105
L1c2a1b | 12172 | 3 | 0.158
L1c2a1b | 13477 | 1 | 0.053
L1c2a1b | 13708 | 1 | 0.053
L1c2a1b | 14267 | 1 | 0.053
L1c2a1b | 146 | 3 | 0.158
L1c2a1b | 15061 | 1 | 0.053
L1c2a1b | 15491 | 1 | 0.053
L1c2a1b | 15530 | 1 | 0.053
L1c2a1b | 15758 | 3 | 0.158
L1c2a1b | 1600 | 1 | 0.053
L1c2a1b | 16071 | 2 | 0.105
L1c2a1b | 16093 | 1 | 0.053
L1c2a1b | 16188 | 1 | 0.053
L1c2a1b | 16259 | 1 | 0.053
L1c2a1b | 16271 | 1 | 0.053
L1c2a1b | 16320 | 1 | 0.053
L1c2a1b | 16362 | 1 | 0.053
L1c2a1b | 16399 | 5 | 0.263
L1c2a1b | 2109 | 1 | 0.053
L1c2a1b | 2133 | 1 | 0.053
L1c2a1b | 2157.1A | 4 | 0.211
L1c2a1b | 2780 | 1 | 0.053
L1c2a1b | 303 | 1 | 0.053
L1c2a1b | 309d | 1 | 0.053
L1c2a1b | 317.1A | 2 | 0.105
L1c2a1b | 3372 | 1 | 0.053
L1c2a1b | 4548 | 1 | 0.053
L1c2a1b | 4742 | 1 | 0.053
L1c2a1b | 4820 | 1 | 0.053
L1c2a1b | 5655 | 1 | 0.053
L1c2a1b | 573.6C | 1 | 0.053
L1c2a1b | 5901.1C | 1 | 0.053
L1c2a1b | 5901.2C | 3 | 0.158
L1c2a1b | 7066 | 1 | 0.053
L1c2a1b | 7680 | 1 | 0.053
L1c2a1b | 9052 | 3 | 0.158
L1c2a1b | 94 | 1 | 0.053
L1c2a1b | 9449 | 1 | 0.053
L1c2a2 | 10397 | 1 | 0.125
L1c2a2 | 10752 | 1 | 0.125
L1c2a2 | 11899 | 1 | 0.125
L1c2a2 | 12011 | 1 | 0.125
L1c2a2 | 12285 | 1 | 0.125
L1c2a2 | 12741 | 1 | 0.125
L1c2a2 | 14016 | 1 | 0.125
L1c2a2 | 14476 | 1 | 0.125
L1c2a2 | 14668 | 1 | 0.125
L1c2a2 | 1531 | 1 | 0.125
L1c2a2 | 16181 | 1 | 0.125
L1c2a2 | 16360 | 3 | 0.375
L1c2a2 | 2764 | 1 | 0.125
L1c2a2 | 309d | 2 | 0.25
L1c2a2 | 3483 | 1 | 0.125
L1c2a2 | 4186 | 1 | 0.125
L1c2a2 | 4688 | 1 | 0.125
L1c2a2 | 4853 | 1 | 0.125
L1c2a2 | 5471 | 1 | 0.125
L1c2a2 | 5899.7C | 1 | 0.125
L1c2a2 | 5899.9C | 1 | 0.125
L1c2a2 | 6990 | 2 | 0.25
L1c2a2 | 8506 | 1 | 0.125
L1c2a2 | 9017 | 2 | 0.25
L1c2a2 | 942 | 2 | 0.25
L1c2a3 | 12654 | 9 | 0.818
L1c2a3 | 14757 | 9 | 0.818
L1c2a3 | 15119 | 1 | 0.091
L1c2a3 | 16154 | 1 | 0.091
L1c2a3 | 16169 | 2 | 0.182
L1c2a3 | 16284 | 1 | 0.091
L1c2a3 | 16292 | 1 | 0.091
L1c2a3 | 204 | 8 | 0.727
L1c2a3 | 309d | 2 | 0.182
L1c2a3 | 4761 | 1 | 0.091
L1c2a3 | 5262 | 7 | 0.636
L1c2a3 | 5414 | 7 | 0.636
L1c2a3 | 5899.7C | 2 | 0.182
L1c2a3 | 7744 | 9 | 0.818
L1c2a3 | 7861 | 7 | 0.636
L1c2a3 | 9632 | 1 | 0.091
L1c2a3a | 12033 | 1 | 0.067
L1c2a3a | 125G | 1 | 0.067
L1c2a3a | 146 | 1 | 0.067
L1c2a3a | 15191 | 1 | 0.067
L1c2a3a | 16169 | 1 | 0.067
L1c2a3a | 16184 | 1 | 0.067
L1c2a3a | 16193d | 1 | 0.067
L1c2a3a | 16286A | 1 | 0.067
L1c2a3a | 1694 | 1 | 0.067
L1c2a3a | 1719 | 1 | 0.067
L1c2a3a | 1822 | 1 | 0.067
L1c2a3a | 1888 | 1 | 0.067
L1c2a3a | 2394C | 3 | 0.2
L1c2a3a | 2395C | 4 | 0.267
L1c2a3a | 4012 | 1 | 0.067
L1c2a3a | 6203 | 1 | 0.067
L1c2a3a | 64 | 1 | 0.067
L1c2a3a | 8473 | 1 | 0.067
L1c2b | 1016 | 1 | 0.091
L1c2b | 140 | 1 | 0.091
L1c2b | 141 | 1 | 0.091

L1c2b | 142 | 1 | 0.091
L1c2b | 14869 | 1 | 0.091
L1c2b | 16181 | 2 | 0.182
L1c2b | 16230 | 1 | 0.091
L1c2b | 16233 | 1 | 0.091
L1c2b | 16242 | 1 | 0.091
L1c2b | 16319 | 1 | 0.091
L1c2b | 16399 | 1 | 0.091
L1c2b | 2831 | 1 | 0.091
L1c2b | 299d | 1 | 0.091
L1c2b | 309d | 2 | 0.182
L1c2b | 8167 | 1 | 0.091
L1c2b | 8562 | 1 | 0.091
L1c2b1 | 150 | 8 | 0.444
L1c2b1 | 16086 | 1 | 0.056
L1c2b1 | 16145 | 1 | 0.056
L1c2b1 | 16148 | 1 | 0.056
L1c2b1 | 16213 | 3 | 0.167
L1c2b1 | 16230 | 8 | 0.444
L1c2b1 | 16327 | 1 | 0.056
L1c2b1a | 12771 | 1 | 0.062
L1c2b1a | 12865 | 1 | 0.062
L1c2b1a | 14687 | 1 | 0.062
L1c2b1a | 15514 | 4 | 0.25
L1c2b1a | 16108 | 1 | 0.062
L1c2b1a | 16260 | 6 | 0.375
L1c2b1a | 16320 | 1 | 0.062
L1c2b1a | 16362 | 1 | 0.062
L1c2b1a | 16524 | 1 | 0.062
L1c2b1a | 1709 | 1 | 0.062
L1c2b1a | 204 | 1 | 0.062
L1c2b1a | 309d | 2 | 0.125
L1c2b1a | 4221 | 1 | 0.062
L1c2b1a | 4598 | 1 | 0.062
L1c2b1a | 489 | 1 | 0.062
L1c2b1a | 508 | 1 | 0.062
L1c2b1a | 5899.8C | 1 | 0.062
L1c2b1a | 6125 | 4 | 0.25
L1c2b1a | 6767 | 1 | 0.062
L1c2b1a | 7010 | 4 | 0.25
L1c2b1a | 8296 | 1 | 0.062
L1c2b1a1 | 10978 | 1 | 0.083
L1c2b1a1 | 11350 | 1 | 0.083
L1c2b1a1 | 16162 | 2 | 0.167
L1c2b1a1 | 16217 | 1 | 0.083
L1c2b1a1 | 183 | 1 | 0.083
L1c2b1a1 | 2162 | 1 | 0.083
L1c2b1a1 | 2352 | 1 | 0.083
L1c2b1a1 | 2395-2399d | 1 | 0.083
L1c2b1a1 | 3912 | 1 | 0.083
L1c2b1a1 | 5899.2C | 1 | 0.083
L1c2b1a1 | 7854 | 1 | 0.083
L1c2b1a1 | 8573 | 1 | 0.083
L1c2b1a'b | 11252 | 8 | 0.444
L1c2b1a'b | 11452 | 1 | 0.056
L1c2b1a'b | 11959 | 8 | 0.444
L1c2b1a'b | 12477 | 8 | 0.444
L1c2b1a'b | 12540 | 8 | 0.444
L1c2b1a'b | 13260 | 1 | 0.056
L1c2b1a'b | 151d | 1 | 0.056
L1c2b1a'b | 15929 | 10 | 0.556
L1c2b1a'b | 1598 | 8 | 0.444
L1c2b1a'b | 16006-16007d | 1 | 0.056
L1c2b1a'b | 16010-16013d | 1 | 0.056
L1c2b1a'b | 16015d | 1 | 0.056
L1c2b1a'b | 16030.1TCTGTTCTTTC | 1 | 0.056
L1c2b1a'b | 16032.1TCTCTGTTCTTTCAT | 1 | 0.056
L1c2b1a'b | 16086 | 1 | 0.056
L1c2b1a'b | 16255 | 1 | 0.056
L1c2b1a'b | 16284 | 1 | 0.056
L1c2b1a'b | 16319 | 1 | 0.056
L1c2b1a'b | 189 | 1 | 0.056
L1c2b1a'b | 200 | 8 | 0.444
L1c2b1a'b | 309d | 2 | 0.111
L1c2b1a'b | 316C | 1 | 0.056
L1c2b1a'b | 317.1AC | 1 | 0.056
L1c2b1a'b | 513 | 3 | 0.167
L1c2b1a'b | 5162 | 3 | 0.167
L1c2b1a'b | 6962 | 5 | 0.278
L1c2b1a'b | 7703 | 1 | 0.056
L1c2b1a'b | 8029 | 1 | 0.056
L1c2b1b | 11963 | 11 | 0.423
L1c2b1b | 12630 | 1 | 0.038
L1c2b1b | 13708 | 1 | 0.038
L1c2b1b | 14580 | 1 | 0.038
L1c2b1b | 14587 | 11 | 0.423
L1c2b1b | 15924 | 11 | 0.423
L1c2b1b | 16214 | 15 | 0.577
L1c2b1b | 16291 | 15 | 0.577
L1c2b1b | 16524 | 2 | 0.077
L1c2b1b | 1977 | 1 | 0.038
L1c2b1b | 204 | 8 | 0.308
L1c2b1b | 309d | 7 | 0.269
L1c2b1b | 3202 | 1 | 0.038
L1c2b1b | 513 | 15 | 0.577
L1c2b1b | 5814 | 2 | 0.077
L1c2b1b | 5899.2C | 1 | 0.038
L1c2b1b | 5899.3C | 1 | 0.038
L1c2b1b | 7982 | 1 | 0.038
L1c2b1b1 | 10313C | 1 | 0.018
L1c2b1b1 | 11914 | 2 | 0.036
L1c2b1b1 | 12573 | 1 | 0.018
L1c2b1b1 | 13827 | 2 | 0.036
L1c2b1b1 | 16093 | 1 | 0.018
L1c2b1b1 | 16249 | 13 | 0.232
L1c2b1b1 | 16390 | 1 | 0.018
L1c2b1b1 | 16465 | 1 | 0.018
L1c2b1b1 | 200 | 1 | 0.018
L1c2b1b1 | 204 | 4 | 0.071
L1c2b1b1 | 2702 | 1 | 0.018
L1c2b1b1 | 2707C | 1 | 0.018

L1c2b1b1 | 2775 | 5 | 0.089
L1c2b1b1 | 309d | 37 | 0.661
L1c2b1b1 | 3243 | 1 | 0.018
L1c2b1b1 | 3540 | 1 | 0.018
L1c2b1b1 | 3786 | 1 | 0.018
L1c2b1b1 | 462 | 8 | 0.143
L1c2b1b1 | 4898 | 11 | 0.196
L1c2b1b1 | 4940 | 1 | 0.018
L1c2b1b1 | 5123 | 1 | 0.018
L1c2b1b1 | 5894C | 1 | 0.018
L1c2b1b1 | 5896A | 1 | 0.018
L1c2b1b1 | 6017 | 1 | 0.018
L1c2b1b1 | 6212 | 2 | 0.036
L1c2b1b1 | 7016 | 2 | 0.036
L1c2b1b1 | 8566 | 1 | 0.018
L1c2b1b1 | 9196 | 3 | 0.054
L1c2b1b1 | 9531 | 8 | 0.143
L1c2b1b1 | 9836 | 1 | 0.018
L1c2b1c | 10523 | 1 | 0.033
L1c2b1c | 10724 | 1 | 0.033
L1c2b1c | 13941G | 1 | 0.033
L1c2b1c | 14319 | 1 | 0.033
L1c2b1c | 14682G | 1 | 0.033
L1c2b1c | 15968 | 1 | 0.033
L1c2b1c | 16207 | 2 | 0.067
L1c2b1c | 16243 | 1 | 0.033
L1c2b1c | 16270 | 1 | 0.033
L1c2b1c | 16274 | 1 | 0.033
L1c2b1c | 16284 | 1 | 0.033
L1c2b1c | 16288 | 1 | 0.033
L1c2b1c | 16290 | 2 | 0.067
L1c2b1c | 16304 | 1 | 0.033
L1c2b1c | 16327 | 2 | 0.067
L1c2b1c | 16390 | 1 | 0.033
L1c2b1c | 189T | 1 | 0.033
L1c2b1c | 204 | 1 | 0.033
L1c2b1c | 239 | 1 | 0.033
L1c2b1c | 264 | 2 | 0.067
L1c2b1c | 267 | 1 | 0.033
L1c2b1c | 316C | 1 | 0.033
L1c2b1c | 317.1AC | 1 | 0.033
L1c2b1c | 513 | 1 | 0.033
L1c2b1c | 5205G | 1 | 0.033
L1c2b1c | 574 | 1 | 0.033
L1c2b1c | 5894C | 6 | 0.2
L1c2b1c | 5899.6C | 1 | 0.033
L1c2b1c | 7985G | 1 | 0.033
L1c2b2 | 10133 | 1 | 0.025
L1c2b2 | 11290 | 8 | 0.2
L1c2b2 | 11347 | 4 | 0.1
L1c2b2 | 12192 | 4 | 0.1
L1c2b2 | 12634 | 2 | 0.05
L1c2b2 | 14063 | 4 | 0.1
L1c2b2 | 141 | 1 | 0.025
L1c2b2 | 142 | 1 | 0.025
L1c2b2 | 14302 | 4 | 0.1
L1c2b2 | 14502 | 1 | 0.025
L1c2b2 | 151d | 1 | 0.025
L1c2b2 | 15553 | 1 | 0.025
L1c2b2 | 15692 | 1 | 0.025
L1c2b2 | 15734 | 6 | 0.15
L1c2b2 | 15886 | 8 | 0.2
L1c2b2 | 16078 | 18 | 0.45
L1c2b2 | 16162 | 2 | 0.05
L1c2b2 | 16176.1TCCAC | 2 | 0.05
L1c2b2 | 16183 | 7 | 0.175
L1c2b2 | 16192 | 1 | 0.025
L1c2b2 | 16290 | 1 | 0.025
L1c2b2 | 16368 | 1 | 0.025
L1c2b2 | 183 | 1 | 0.025
L1c2b2 | 196A | 1 | 0.025
L1c2b2 | 197T | 1 | 0.025
L1c2b2 | 2887-2888d | 14 | 0.35
L1c2b2 | 309d | 11 | 0.275
L1c2b2 | 316C | 1 | 0.025
L1c2b2 | 317.1C | 1 | 0.025
L1c2b2 | 4094 | 8 | 0.2
L1c2b2 | 4454 | 2 | 0.05
L1c2b2 | 4722 | 5 | 0.125
L1c2b2 | 4808 | 3 | 0.075
L1c2b2 | 5255 | 1 | 0.025
L1c2b2 | 5510 | 1 | 0.025
L1c2b2 | 573.4C | 1 | 0.025
L1c2b2 | 5843 | 2 | 0.05
L1c2b2 | 5899.0C | 2 | 0.05
L1c2b2 | 5899.6C | 1 | 0.025
L1c2b2 | 6500 | 1 | 0.025
L1c2b2 | 709 | 3 | 0.075
L1c2b2 | 7697 | 1 | 0.025
L1c2b2 | 7854 | 1 | 0.025
L1c2b2 | 794 | 4 | 0.1
L1c2b2 | 8261 | 14 | 0.35
L1c3 | 11984 | 1 | 0.1
L1c3 | 14669 | 2 | 0.2
L1c3 | 14926 | 2 | 0.2
L1c3 | 16214 | 1 | 0.1
L1c3 | 16218 | 1 | 0.1
L1c3 | 16292 | 1 | 0.1
L1c3 | 16293 | 1 | 0.1
L1c3 | 16368 | 2 | 0.2
L1c3 | 195 | 1 | 0.1
L1c3 | 308-309d | 1 | 0.1
L1c3 | 3525 | 2 | 0.2
L1c3 | 4853 | 1 | 0.1
L1c3 | 573.1C | 1 | 0.1
L1c3 | 6227 | 2 | 0.2
L1c3 | 7247 | 2 | 0.2
L1c3 | 8281-8289d | 1 | 0.1
L1c3a | 1007 | 1 | 0.019
L1c3a | 11253 | 5 | 0.093
L1c3a | 11396 | 1 | 0.019

L1c3a | 11440 | 1 | 0.019
L1c3a | 12375 | 1 | 0.019
L1c3a | 12616 | 1 | 0.019
L1c3a | 12906 | 8 | 0.148
L1c3a | 13020 | 1 | 0.019
L1c3a | 13098 | 2 | 0.037
L1c3a | 13293 | 1 | 0.019
L1c3a | 13365 | 1 | 0.019
L1c3a | 13651 | 1 | 0.019
L1c3a | 13665 | 1 | 0.019
L1c3a | 13915 | 1 | 0.019
L1c3a | 14016 | 2 | 0.037
L1c3a | 14305 | 2 | 0.037
L1c3a | 14314 | 1 | 0.019
L1c3a | 14767 | 1 | 0.019
L1c3a | 151d | 5 | 0.093
L1c3a | 15244 | 1 | 0.019
L1c3a | 15497 | 1 | 0.019
L1c3a | 15622 | 1 | 0.019
L1c3a | 15643G | 1 | 0.019
L1c3a | 15860 | 1 | 0.019
L1c3a | 15940 | 1 | 0.019
L1c3a | 16093 | 5 | 0.093
L1c3a | 16145 | 2 | 0.037
L1c3a | 16154 | 1 | 0.019
L1c3a | 16188A | 7 | 0.13
L1c3a | 16193d | 12 | 0.222
L1c3a | 16194 | 1 | 0.019
L1c3a | 16209 | 1 | 0.019
L1c3a | 16293 | 1 | 0.019
L1c3a | 16360-16367d | 1 | 0.019
L1c3a | 16368 | 10 | 0.185
L1c3a | 2010 | 1 | 0.019
L1c3a | 2395C | 1 | 0.019
L1c3a | 2619 | 1 | 0.019
L1c3a | 309d | 8 | 0.148
L1c3a | 310 | 1 | 0.019
L1c3a | 317.1C | 1 | 0.019
L1c3a | 3192 | 1 | 0.019
L1c3a | 3316 | 1 | 0.019
L1c3a | 3591 | 2 | 0.037
L1c3a | 408A | 18 | 0.333
L1c3a | 4639 | 1 | 0.019
L1c3a | 494 | 18 | 0.333
L1c3a | 4977 | 1 | 0.019
L1c3a | 5004 | 1 | 0.019
L1c3a | 5046 | 2 | 0.037
L1c3a | 6128 | 1 | 0.019
L1c3a | 6227 | 2 | 0.037
L1c3a | 6345 | 1 | 0.019
L1c3a | 64 | 2 | 0.037
L1c3a | 6791 | 9 | 0.167
L1c3a | 7022 | 14 | 0.259
L1c3a | 7924 | 4 | 0.074
L1c3a | 8654 | 1 | 0.019
L1c3a | 8762 | 1 | 0.019
L1c3a | 9370 | 1 | 0.019
L1c3a | 9377 | 1 | 0.019
L1c3a | 9494 | 1 | 0.019
L1c3a | 9554 | 2 | 0.037
L1c3a | 9752A | 2 | 0.037
L1c3a | 990 | 1 | 0.019
L1c3a | 9917A | 1 | 0.019
L1c3a1 | 10685 | 1 | 0.029
L1c3a1 | 15553 | 1 | 0.029
L1c3a1 | 15954 | 1 | 0.029
L1c3a1 | 16086 | 1 | 0.029
L1c3a1 | 16145 | 6 | 0.171
L1c3a1 | 16166 | 1 | 0.029
L1c3a1 | 16184 | 2 | 0.057
L1c3a1 | 16184A | 1 | 0.029
L1c3a1 | 16243 | 1 | 0.029
L1c3a1 | 16254 | 1 | 0.029
L1c3a1 | 16266 | 1 | 0.029
L1c3a1 | 16287 | 1 | 0.029
L1c3a1 | 16290 | 1 | 0.029
L1c3a1 | 16293 | 1 | 0.029
L1c3a1 | 16319 | 2 | 0.057
L1c3a1 | 195 | 2 | 0.057
L1c3a1 | 198 | 2 | 0.057
L1c3a1 | 303A | 11 | 0.314
L1c3a1 | 309d | 1 | 0.029
L1c3a1 | 316C | 1 | 0.029
L1c3a1 | 317.1AC | 1 | 0.029
L1c3a1 | 338 | 2 | 0.057
L1c3a1 | 345 | 1 | 0.029
L1c3a1 | 408A | 4 | 0.114
L1c3a1 | 4353 | 1 | 0.029
L1c3a1 | 4823 | 1 | 0.029
L1c3a1 | 494 | 4 | 0.114
L1c3a1 | 515-524d | 11 | 0.314
L1c3a1 | 529T | 1 | 0.029
L1c3a1 | 5899.1C | 1 | 0.029
L1c3a1 | 7471.1C | 1 | 0.029
L1c3a1 | 9254 | 1 | 0.029
L1c3a1 | 9554 | 1 | 0.029
L1c3a1a | 10629A | 1 | 0.036
L1c3a1a | 10993 | 1 | 0.036
L1c3a1a | 11995G | 2 | 0.071
L1c3a1a | 13204 | 1 | 0.036
L1c3a1a | 13593T | 1 | 0.036
L1c3a1a | 14129 | 2 | 0.071
L1c3a1a | 15038 | 1 | 0.036
L1c3a1a | 15203 | 1 | 0.036
L1c3a1a | 15784 | 1 | 0.036
L1c3a1a | 15949 | 7 | 0.25
L1c3a1a | 16093 | 8 | 0.286
L1c3a1a | 16124 | 1 | 0.036
L1c3a1a | 16180C | 1 | 0.036
L1c3a1a | 16192-16193d | 3 | 0.107
L1c3a1a | 16193d | 1 | 0.036

L1c3a1a | 16209 | 2 | 0.071
L1c3a1a | 16241 | 1 | 0.036
L1c3a1a | 16263 | 1 | 0.036
L1c3a1a | 16289 | 1 | 0.036
L1c3a1a | 16292 | 1 | 0.036
L1c3a1a | 1700 | 2 | 0.071
L1c3a1a | 198 | 1 | 0.036
L1c3a1a | 309d | 2 | 0.071
L1c3a1a | 3897A | 1 | 0.036
L1c3a1a | 5165 | 2 | 0.071
L1c3a1a | 5471 | 1 | 0.036
L1c3a1a | 573.2C | 2 | 0.071
L1c3a1a | 573.4C | 1 | 0.036
L1c3a1a | 6531 | 5 | 0.179
L1c3a1a | 7897 | 5 | 0.179
L1c3a1a | 9053 | 1 | 0.036
L1c3a1a | 9410 | 1 | 0.036
L1c3a1b | 10055 | 1 | 0.026
L1c3a1b | 10289 | 1 | 0.026
L1c3a1b | 11506 | 5 | 0.128
L1c3a1b | 11701 | 1 | 0.026
L1c3a1b | 12684 | 1 | 0.026
L1c3a1b | 13020 | 4 | 0.103
L1c3a1b | 13528T | 1 | 0.026
L1c3a1b | 13577 | 1 | 0.026
L1c3a1b | 13768 | 5 | 0.128
L1c3a1b | 13866 | 1 | 0.026
L1c3a1b | 14022 | 2 | 0.051
L1c3a1b | 14581 | 5 | 0.128
L1c3a1b | 151d | 1 | 0.026
L1c3a1b | 15907 | 1 | 0.026
L1c3a1b | 15916 | 1 | 0.026
L1c3a1b | 16093 | 14 | 0.359
L1c3a1b | 16180 | 1 | 0.026
L1c3a1b | 16192-16193d | 1 | 0.026
L1c3a1b | 16213 | 2 | 0.051
L1c3a1b | 16231 | 2 | 0.051
L1c3a1b | 16234 | 2 | 0.051
L1c3a1b | 16258C | 1 | 0.026
L1c3a1b | 16266 | 1 | 0.026
L1c3a1b | 16291 | 3 | 0.077
L1c3a1b | 16327 | 2 | 0.051
L1c3a1b | 16390 | 19 | 0.487
L1c3a1b | 183 | 14 | 0.359
L1c3a1b | 195 | 4 | 0.103
L1c3a1b | 198 | 1 | 0.026
L1c3a1b | 204 | 11 | 0.282
L1c3a1b | 2486 | 1 | 0.026
L1c3a1b | 257 | 10 | 0.256
L1c3a1b | 309d | 4 | 0.103
L1c3a1b | 3552 | 5 | 0.128
L1c3a1b | 4345 | 1 | 0.026
L1c3a1b | 4883A | 2 | 0.051
L1c3a1b | 5033 | 4 | 0.103
L1c3a1b | 5177 | 1 | 0.026
L1c3a1b | 5291 | 1 | 0.026
L1c3a1b | 573.1C | 2 | 0.051
L1c3a1b | 573.2C | 2 | 0.051
L1c3a1b | 5821 | 4 | 0.103
L1c3a1b | 6101 | 1 | 0.026
L1c3a1b | 6261 | 1 | 0.026
L1c3a1b | 6932 | 1 | 0.026
L1c3a1b | 7296 | 1 | 0.026
L1c3a1b | 783 | 9 | 0.231
L1c3a1b | 8555 | 1 | 0.026
L1c3a1b | 8680 | 2 | 0.051
L1c3a1b | 8987 | 1 | 0.026
L1c3b | 16111 | 2 | 0.133
L1c3b | 16148 | 1 | 0.067
L1c3b | 16266 | 2 | 0.133
L1c3b | 16292 | 1 | 0.067
L1c3b | 16355 | 2 | 0.133
L1c3b | 65G | 1 | 0.067
L1c3b | 89 | 1 | 0.067
L1c3b | 93 | 1 | 0.067
L1c3b1 | 10813 | 1 | 0.067
L1c3b1 | 11641 | 1 | 0.067
L1c3b1 | 16093 | 1 | 0.067
L1c3b1 | 16111G | 1 | 0.067
L1c3b1 | 16249 | 1 | 0.067
L1c3b1 | 16304 | 3 | 0.2
L1c3b1 | 1943 | 1 | 0.067
L1c3b1 | 195 | 4 | 0.267
L1c3b1 | 204 | 1 | 0.067
L1c3b1 | 236 | 1 | 0.067
L1c3b1 | 2581 | 1 | 0.067
L1c3b1 | 303A | 1 | 0.067
L1c3b1 | 385 | 1 | 0.067
L1c3b1 | 513 | 5 | 0.333
L1c3b1 | 515-524d | 1 | 0.067
L1c3b1 | 5291 | 1 | 0.067
L1c3b1 | 8348 | 1 | 0.067
L1c3b1 | 8521 | 1 | 0.067
L1c3b1 | 9088 | 1 | 0.067
L1c3b1 | 93 | 1 | 0.067
L1c3b1a | 10031 | 1 | 0.023
L1c3b1a | 10042T | 2 | 0.047
L1c3b1a | 10265 | 1 | 0.023
L1c3b1a | 10400 | 1 | 0.023
L1c3b1a | 10535 | 1 | 0.023
L1c3b1a | 11812 | 1 | 0.023
L1c3b1a | 11914 | 1 | 0.023
L1c3b1a | 12735 | 1 | 0.023
L1c3b1a | 12855 | 1 | 0.023
L1c3b1a | 13279 | 1 | 0.023
L1c3b1a | 13488 | 1 | 0.023
L1c3b1a | 13740 | 2 | 0.047
L1c3b1a | 14070 | 2 | 0.047
L1c3b1a | 15907 | 1 | 0.023
L1c3b1a | 16111A | 2 | 0.047
L1c3b1a | 16271 | 1 | 0.023

L1c3b1a | 16298 | 2 | 0.047
L1c3b1a | 183 | 1 | 0.023
L1c3b1a | 194 | 1 | 0.023
L1c3b1a | 198 | 1 | 0.023
L1c3b1a | 211 | 1 | 0.023
L1c3b1a | 2222 | 1 | 0.023
L1c3b1a | 249d | 1 | 0.023
L1c3b1a | 3090 | 1 | 0.023
L1c3b1a | 309d | 13 | 0.302
L1c3b1a | 3200 | 1 | 0.023
L1c3b1a | 4913 | 1 | 0.023
L1c3b1a | 499 | 1 | 0.023
L1c3b1a | 523-525d | 1 | 0.023
L1c3b1a | 5237 | 1 | 0.023
L1c3b1a | 5291 | 1 | 0.023
L1c3b1a | 593 | 1 | 0.023
L1c3b1a | 6179 | 1 | 0.023
L1c3b1a | 7376 | 2 | 0.047
L1c3b1a | 7897 | 1 | 0.023
L1c3b1a | 8277 | 1 | 0.023
L1c3b1a | 8911 | 2 | 0.047
L1c3b1a | 9352 | 1 | 0.023
L1c3b1a | 9581 | 2 | 0.047
L1c3b1b | 11015 | 1 | 0.067
L1c3b1b | 11077 | 1 | 0.067
L1c3b1b | 11491 | 1 | 0.067
L1c3b1b | 13182 | 1 | 0.067
L1c3b1b | 13785 | 1 | 0.067
L1c3b1b | 15061 | 2 | 0.133
L1c3b1b | 16150 | 1 | 0.067
L1c3b1b | 16158 | 1 | 0.067
L1c3b1b | 16266 | 2 | 0.133
L1c3b1b | 16274 | 1 | 0.067
L1c3b1b | 16527 | 1 | 0.067
L1c3b1b | 1664 | 1 | 0.067
L1c3b1b | 1888C | 1 | 0.067
L1c3b1b | 310 | 1 | 0.067
L1c3b1b | 4092 | 1 | 0.067
L1c3b1b | 5225 | 1 | 0.067
L1c3b1b | 5351 | 1 | 0.067
L1c3b1b | 5417 | 1 | 0.067
L1c3b1b | 5987A | 1 | 0.067
L1c3b1b | 6182 | 1 | 0.067
L1c3b1b | 8939 | 1 | 0.067
L1c3b2 | 11233G | 1 | 0.025
L1c3b2 | 13651 | 1 | 0.025
L1c3b2 | 14124 | 1 | 0.025
L1c3b2 | 14266 | 1 | 0.025
L1c3b2 | 14905 | 1 | 0.025
L1c3b2 | 14929 | 12 | 0.3
L1c3b2 | 15115 | 1 | 0.025
L1c3b2 | 16037 | 2 | 0.05
L1c3b2 | 16104 | 32 | 0.8
L1c3b2 | 16185 | 1 | 0.025
L1c3b2 | 16188 | 1 | 0.025
L1c3b2 | 16189A | 1 | 0.025
L1c3b2 | 16291 | 1 | 0.025
L1c3b2 | 16301G | 7 | 0.175
L1c3b2 | 16399 | 1 | 0.025
L1c3b2 | 2000 | 1 | 0.025
L1c3b2 | 2884G | 1 | 0.025
L1c3b2 | 310 | 1 | 0.025
L1c3b2 | 317.1A | 1 | 0.025
L1c3b2 | 385 | 1 | 0.025
L1c3b2 | 3882 | 11 | 0.275
L1c3b2 | 4823 | 1 | 0.025
L1c3b2 | 515d | 1 | 0.025
L1c3b2 | 5424 | 1 | 0.025
L1c3b2 | 7762 | 1 | 0.025
L1c3b2 | 7888 | 1 | 0.025
L1c3b2 | 9058 | 1 | 0.025
L1c3b2 | 9758 | 1 | 0.025
L1c3b'c | 16527 | 2 | 0.333
L1c3b'c | 16532d | 2 | 0.333
L1c3b'c | 265 | 3 | 0.5
L1c3c | 13698 | 1 | 0.031
L1c3c | 14926 | 19 | 0.594
L1c3c | 15976 | 2 | 0.062
L1c3c | 16150 | 1 | 0.031
L1c3c | 16194C | 1 | 0.031
L1c3c | 16224 | 1 | 0.031
L1c3c | 16260 | 1 | 0.031
L1c3c | 16368 | 2 | 0.062
L1c3c | 198 | 1 | 0.031
L1c3c | 2010 | 1 | 0.031
L1c3c | 309d | 1 | 0.031
L1c3c | 3204 | 1 | 0.031
L1c3c | 4448 | 1 | 0.031
L1c3c | 517d | 1 | 0.031
L1c3c | 520d | 1 | 0.031
L1c3c | 6227 | 19 | 0.594
L1c3c | 7247 | 19 | 0.594
L1c3c | 89 | 1 | 0.031
L1c3c | 95C | 1 | 0.031
L1c4 | 16093 | 2 | 1
L1c4 | 16259 | 1 | 0.5
L1c4 | 16527 | 1 | 0.5
L1c4 | 204 | 1 | 0.5
L1c4 | 575 | 1 | 0.5
L1c4 | 94.1G | 1 | 0.5
L1c4a | 12540 | 3 | 0.6
L1c4a | 12732 | 3 | 0.6
L1c4a | 14913 | 1 | 0.2
L1c4a | 15530 | 1 | 0.2
L1c4a | 16304 | 1 | 0.2
L1c4a | 16390 | 2 | 0.4
L1c4a | 456 | 1 | 0.2
L1c4a | 5553 | 1 | 0.2
L1c4a | 5899.10C | 2 | 0.4
L1c4a | 5899.5C | 2 | 0.4
L1c4a | 9944 | 1 | 0.2

L1c4b | 10454 | 12 | 0.245
L1c4b | 10927 | 30 | 0.612
L1c4b | 11878 | 1 | 0.02
L1c4b | 11928 | 30 | 0.612
L1c4b | 13691 | 1 | 0.02
L1c4b | 14452 | 1 | 0.02
L1c4b | 146 | 44 | 0.898
L1c4b | 15314 | 1 | 0.02
L1c4b | 15877 | 1 | 0.02
L1c4b | 16093 | 1 | 0.02
L1c4b | 16102 | 6 | 0.122
L1c4b | 16355 | 1 | 0.02
L1c4b | 16356 | 1 | 0.02
L1c4b | 16362 | 1 | 0.02
L1c4b | 16368 | 1 | 0.02
L1c4b | 16390 | 1 | 0.02
L1c4b | 16399 | 1 | 0.02
L1c4b | 16527 | 1 | 0.02
L1c4b | 1884 | 3 | 0.061
L1c4b | 194 | 1 | 0.02
L1c4b | 256 | 35 | 0.714
L1c4b | 291d | 44 | 0.898
L1c4b | 309d | 1 | 0.02
L1c4b | 4452 | 1 | 0.02
L1c4b | 4732 | 1 | 0.02
L1c4b | 575 | 1 | 0.02
L1c4b | 5894T | 1 | 0.02
L1c4b | 5899.0C | 2 | 0.041
L1c4b | 5899.10C | 8 | 0.163
L1c4b | 5899.4C | 1 | 0.02
L1c4b | 5899.5C | 2 | 0.041
L1c4b | 747.1A | 1 | 0.02
L1c4b | 8348 | 23 | 0.469
L1c4b | 8648 | 1 | 0.02
L1c4b | 9010 | 3 | 0.061
L1c4b | 9266C | 1 | 0.02
L1c4b | 94.1G | 1 | 0.02
L1c4b | 9494 | 30 | 0.612
L1c4b | 9650 | 1 | 0.02
L1c5 | 12454 | 1 | 0.032
L1c5 | 13981 | 1 | 0.032
L1c5 | 16114G | 7 | 0.226
L1c5 | 16172 | 15 | 0.484
L1c5 | 16184 | 15 | 0.484
L1c5 | 16266 | 1 | 0.032
L1c5 | 16355 | 1 | 0.032
L1c5 | 16359 | 14 | 0.452
L1c5 | 16527 | 1 | 0.032
L1c5 | 236 | 1 | 0.032
L1c5 | 249d | 13 | 0.419
L1c5 | 257 | 1 | 0.032
L1c5 | 376T | 2 | 0.065
L1c5 | 573.5C | 1 | 0.032
L1c6 | 10601 | 1 | 0.167
L1c6 | 10915 | 1 | 0.167
L1c6 | 11339 | 1 | 0.167
L1c6 | 12396 | 1 | 0.167
L1c6 | 12714 | 1 | 0.167
L1c6 | 13659 | 1 | 0.167
L1c6 | 13933 | 1 | 0.167
L1c6 | 14798 | 1 | 0.167
L1c6 | 15034 | 1 | 0.167
L1c6 | 15700 | 1 | 0.167
L1c6 | 16148 | 1 | 0.167
L1c6 | 16191 | 1 | 0.167
L1c6 | 16193d | 1 | 0.167
L1c6 | 16234 | 1 | 0.167
L1c6 | 16255 | 1 | 0.167
L1c6 | 16256 | 1 | 0.167
L1c6 | 16274 | 1 | 0.167
L1c6 | 16293 | 1 | 0.167
L1c6 | 16527 | 1 | 0.167
L1c6 | 2308 | 1 | 0.167
L1c6 | 2315 | 1 | 0.167
L1c6 | 3874 | 1 | 0.167
L1c6 | 455 | 1 | 0.167
L1c6 | 455.1T | 1 | 0.167
L1c6 | 4562 | 1 | 0.167
L1c6 | 5074 | 1 | 0.167
L1c6 | 514 | 1 | 0.167
L1c6 | 5460 | 1 | 0.167
L1c6 | 5465 | 1 | 0.167
L1c6 | 5492 | 1 | 0.167
L1c6 | 593 | 1 | 0.167
L1c6 | 6908 | 1 | 0.167
L1c6 | 8251 | 1 | 0.167
L1c6 | 8538 | 1 | 0.167
L1c6 | 8829 | 1 | 0.167
L1c6 | 9611 | 1 | 0.167
L1c6 | 9986 | 1 | 0.167
L2 | 16291 | 1 | 0.2
L2 | 185C | 3 | 0.6
L2 | 189 | 3 | 0.6
L2 | 325 | 3 | 0.6
L2 | 356.1C | 2 | 0.4
L2 | 515-524d | 1 | 0.2
L2 | 93 | 3 | 0.6
L2'3'4'5'6 | 10084 | 1 | 0.083
L2'3'4'5'6 | 10275 | 1 | 0.083
L2'3'4'5'6 | 10G | 1 | 0.083
L2'3'4'5'6 | 11809 | 6 | 0.5
L2'3'4'5'6 | 12178 | 5 | 0.417
L2'3'4'5'6 | 12432 | 6 | 0.5
L2'3'4'5'6 | 12651C | 6 | 0.5
L2'3'4'5'6 | 12681 | 1 | 0.083
L2'3'4'5'6 | 1377 | 4 | 0.333
L2'3'4'5'6 | 14348 | 1 | 0.083
L2'3'4'5'6 | 14431 | 4 | 0.333
L2'3'4'5'6 | 150 | 1 | 0.083
L2'3'4'5'6 | 152 | 5 | 0.417
L2'3'4'5'6 | 15226 | 4 | 0.333

L2'3'4'5'6 | 152G | 1 | 0.083
L2'3'4'5'6 | 15346 | 4 | 0.333
L2'3'4'5'6 | 15514 | 1 | 0.083
L2'3'4'5'6 | 15733A | 4 | 0.333
L2'3'4'5'6 | 15911 | 1 | 0.083
L2'3'4'5'6 | 15927 | 5 | 0.417
L2'3'4'5'6 | 15944d | 1 | 0.083
L2'3'4'5'6 | 16093 | 4 | 0.333
L2'3'4'5'6 | 16166 | 12 | 1
L2'3'4'5'6 | 16172 | 8 | 0.667
L2'3'4'5'6 | 16179 | 1 | 0.083
L2'3'4'5'6 | 16209 | 11 | 0.917
L2'3'4'5'6 | 16213 | 11 | 0.917
L2'3'4'5'6 | 16256 | 3 | 0.25
L2'3'4'5'6 | 16265C | 2 | 0.167
L2'3'4'5'6 | 16274 | 2 | 0.167
L2'3'4'5'6 | 16283 | 1 | 0.083
L2'3'4'5'6 | 16298 | 1 | 0.083
L2'3'4'5'6 | 16355 | 1 | 0.083
L2'3'4'5'6 | 16362 | 1 | 0.083
L2'3'4'5'6 | 200 | 1 | 0.083
L2'3'4'5'6 | 3027 | 1 | 0.083
L2'3'4'5'6 | 3204 | 5 | 0.417
L2'3'4'5'6 | 3316 | 1 | 0.083
L2'3'4'5'6 | 3423 | 6 | 0.5
L2'3'4'5'6 | 3592 | 6 | 0.5
L2'3'4'5'6 | 3633 | 1 | 0.083
L2'3'4'5'6 | 3705 | 6 | 0.5
L2'3'4'5'6 | 4012 | 1 | 0.083
L2'3'4'5'6 | 4219 | 1 | 0.083
L2'3'4'5'6 | 4853 | 1 | 0.083
L2'3'4'5'6 | 4871 | 1 | 0.083
L2'3'4'5'6 | 5237 | 1 | 0.083
L2'3'4'5'6 | 5530 | 6 | 0.5
L2'3'4'5'6 | 593 | 5 | 0.417
L2'3'4'5'6 | 6044 | 1 | 0.083
L2'3'4'5'6 | 6216 | 1 | 0.083
L2'3'4'5'6 | 6527 | 6 | 0.5
L2'3'4'5'6 | 6620 | 1 | 0.083
L2'3'4'5'6 | 6671 | 1 | 0.083
L2'3'4'5'6 | 709 | 4 | 0.333
L2'3'4'5'6 | 7389 | 6 | 0.5
L2'3'4'5'6 | 7711 | 5 | 0.417
L2'3'4'5'6 | 7711G | 1 | 0.083
L2'3'4'5'6 | 8027 | 1 | 0.083
L2'3'4'5'6 | 8059 | 1 | 0.083
L2'3'4'5'6 | 8152 | 6 | 0.5
L2'3'4'5'6 | 8176 | 1 | 0.083
L2'3'4'5'6 | 8270 | 5 | 0.417
L2'3'4'5'6 | 8281-8289d | 5 | 0.417
L2'3'4'5'6 | 8581 | 1 | 0.083
L2'3'4'5'6 | 8611A | 1 | 0.083
L2'3'4'5'6 | 8634 | 5 | 0.417
L2'3'4'5'6 | 8830 | 5 | 0.417
L2'3'4'5'6 | 8992 | 5 | 0.417
L2'3'4'5'6 | 9072 | 1 | 0.083
L2'3'4'5'6 | 9320 | 1 | 0.083
L2'3'4'5'6 | 9449 | 1 | 0.083
L2'3'4'5'6 | 9512 | 1 | 0.083
L2'3'4'5'6 | 9755 | 6 | 0.5
L2'3'4'5'6 | 9983 | 1 | 0.083
L2'3'4'6 | 146 | 1 | 0.034
L2'3'4'6 | 16024A | 1 | 0.034
L2'3'4'6 | 16025 | 1 | 0.034
L2'3'4'6 | 16093 | 2 | 0.069
L2'3'4'6 | 16172 | 18 | 0.621
L2'3'4'6 | 16173 | 18 | 0.621
L2'3'4'6 | 16189 | 1 | 0.034
L2'3'4'6 | 16239G | 1 | 0.034
L2'3'4'6 | 16287 | 5 | 0.172
L2'3'4'6 | 16309 | 1 | 0.034
L2'3'4'6 | 16319 | 2 | 0.069
L2'3'4'6 | 16330G | 1 | 0.034
L2'3'4'6 | 16359 | 1 | 0.034
L2'3'4'6 | 16362A | 1 | 0.034
L2'3'4'6 | 183 | 1 | 0.034
L2'3'4'6 | 518 | 1 | 0.034
L2a | 12693 | 1 | 0.2
L2a | 14566 | 1 | 0.2
L2a | 15244 | 1 | 0.2
L2a | 15784 | 1 | 0.2
L2a | 16093 | 1 | 0.2
L2a | 16172 | 1 | 0.2
L2a | 16294 | 1 | 0.2
L2a | 16311 | 2 | 0.4
L2a | 16318 | 2 | 0.4
L2a | 185T | 1 | 0.2
L2a | 247 | 1 | 0.2
L2a | 249d | 1 | 0.2
L2a | 259.1A | 1 | 0.2
L2a | 309d | 1 | 0.2
L2a | 357 | 1 | 0.2
L2a | 3918 | 1 | 0.2
L2a | 515-524d | 1 | 0.2
L2a1 | 14152 | 1 | 0.008
L2a1 | 14212 | 1 | 0.008
L2a1 | 14484 | 1 | 0.008
L2a1 | 150 | 2 | 0.016
L2a1 | 15629 | 1 | 0.008
L2a1 | 16042 | 1 | 0.008
L2a1 | 16092 | 5 | 0.04
L2a1 | 16172 | 1 | 0.008
L2a1 | 16179 | 1 | 0.008
L2a1 | 16185 | 1 | 0.008
L2a1 | 16186 | 1 | 0.008
L2a1 | 16230 | 1 | 0.008
L2a1 | 16259 | 1 | 0.008
L2a1 | 16261 | 1 | 0.008
L2a1 | 16270 | 1 | 0.008
L2a1 | 16274 | 1 | 0.008
L2a1 | 16286 | 2 | 0.016

L2a1 | 16290 | 1 | 0.008
L2a1 | 16292 | 1 | 0.008
L2a1 | 16319 | 6 | 0.048
L2a1 | 16346 | 1 | 0.008
L2a1 | 16356 | 1 | 0.008
L2a1 | 16360 | 3 | 0.024
L2a1 | 16398C | 2 | 0.016
L2a1 | 16399T | 1 | 0.008
L2a1 | 183 | 1 | 0.008
L2a1 | 185 | 1 | 0.008
L2a1 | 189 | 1 | 0.008
L2a1 | 198 | 4 | 0.032
L2a1 | 199G | 1 | 0.008
L2a1 | 200 | 1 | 0.008
L2a1 | 214 | 1 | 0.008
L2a1 | 217 | 1 | 0.008
L2a1 | 260 | 2 | 0.016
L2a1 | 345 | 1 | 0.008
L2a1 | 384 | 1 | 0.008
L2a1 | 385 | 1 | 0.008
L2a1 | 445A | 1 | 0.008
L2a1 | 515-524d | 13 | 0.105
L2a1 | 5285 | 1 | 0.008
L2a1 | 529T | 2 | 0.016
L2a1 | 534 | 1 | 0.008
L2a1 | 548A | 1 | 0.008
L2a1+143 | 10685 | 1 | 0.019
L2a1+143 | 13858 | 1 | 0.019
L2a1+143 | 15217 | 1 | 0.019
L2a1+143 | 16129 | 1 | 0.019
L2a1+143 | 16224 | 1 | 0.019
L2a1+143 | 16234 | 2 | 0.038
L2a1+143 | 16242A | 1 | 0.019
L2a1+143 | 16380 | 1 | 0.019
L2a1+143 | 204 | 1 | 0.019
L2a1+143 | 3423G | 1 | 0.019
L2a1+143 | 5147 | 1 | 0.019
L2a1+143 | 548 | 1 | 0.019
L2a1+143 | 573.3C | 1 | 0.019
L2a1+143 | 93 | 1 | 0.019
L2a1+143+@16309 | 16051 | 1 | 0.017
L2a1+143+@16309 | 16086 | 1 | 0.017
L2a1+143+@16309 | 16093 | 3 | 0.052
L2a1+143+@16309 | 16114 | 1 | 0.017
L2a1+143+@16309 | 16114A | 2 | 0.034
L2a1+143+@16309 | 16126 | 1 | 0.017
L2a1+143+@16309 | 16129 | 1 | 0.017
L2a1+143+@16309 | 16162 | 1 | 0.017
L2a1+143+@16309 | 16184 | 3 | 0.052
L2a1+143+@16309 | 16221 | 1 | 0.017
L2a1+143+@16309 | 16239 | 1 | 0.017
L2a1+143+@16309 | 16248A | 1 | 0.017
L2a1+143+@16309 | 16264 | 3 | 0.052
L2a1+143+@16309 | 16270 | 1 | 0.017
L2a1+143+@16309 | 16274 | 3 | 0.052
L2a1+143+@16309 | 16291 | 1 | 0.017
L2a1+143+@16309 | 16292 | 2 | 0.034
L2a1+143+@16309 | 16293 | 1 | 0.017
L2a1+143+@16309 | 16300 | 1 | 0.017
L2a1+143+@16309 | 16309 | 2 | 0.034
L2a1+143+@16309 | 16311 | 2 | 0.034
L2a1+143+@16309 | 16325 | 1 | 0.017
L2a1+143+@16309 | 16335 | 1 | 0.017
L2a1+143+@16309 | 16354 | 1 | 0.017
L2a1+143+@16309 | 16362 | 9 | 0.155
L2a1+143+@16309 | 16399 | 1 | 0.017
L2a1+143+@16309 | 16497 | 1 | 0.017
L2a1+143+@16309 | 16511A | 1 | 0.017
L2a1+143+@16309 | 189 | 1 | 0.017
L2a1+143+@16309 | 198 | 4 | 0.069
L2a1+143+@16309 | 200 | 1 | 0.017
L2a1+143+@16309 | 228 | 7 | 0.121
L2a1+143+@16309 | 247 | 2 | 0.034
L2a1+143+@16309 | 264 | 1 | 0.017
L2a1+143+@16309 | 321G | 1 | 0.017
L2a1+143+@16309 | 324G | 1 | 0.017
L2a1+143+@16309 | 329C | 1 | 0.017
L2a1+143+@16309 | 366 | 2 | 0.034
L2a1+143+@16309 | 369A | 1 | 0.017
L2a1+143+@16309 | 380C | 1 | 0.017
L2a1+143+@16309 | 389 | 1 | 0.017
L2a1+143+@16309 | 466 | 3 | 0.052
L2a1+143+@16309 | 507A | 3 | 0.052
L2a1+143+@16309 | 515-524d | 30 | 0.517
L2a1+143+@16309 | 527 | 1 | 0.017
L2a1+143+@16309 | 529T | 1 | 0.017
L2a1+143+@16309 | 530 | 1 | 0.017
L2a1+143+@16309 | 534 | 8 | 0.138
L2a1+143+@16309 | 564 | 1 | 0.017
L2a1+143+@16309 | 573.5C | 1 | 0.017
L2a1+143+@16309 | 573.6C | 1 | 0.017
L2a1+143+@16309 | 6216 | 1 | 0.017
L2a1+143+16189_(16192) | 10389 | 1 | 0.008
L2a1+143+16189_(16192) | 10497 | 2 | 0.017
L2a1+143+16189_(16192) | 10601 | 2 | 0.017
L2a1+143+16189_(16192) | 10924 | 1 | 0.008
L2a1+143+16189_(16192) | 11016 | 3 | 0.025
L2a1+143+16189_(16192) | 1120 | 2 | 0.017
L2a1+143+16189_(16192) | 12239 | 1 | 0.008
L2a1+143+16189_(16192) | 12612 | 1 | 0.008
L2a1+143+16189_(16192) | 12715 | 1 | 0.008
L2a1+143+16189_(16192) | 12720 | 1 | 0.008
L2a1+143+16189_(16192) | 13062 | 1 | 0.008
L2a1+143+16189_(16192) | 13275 | 1 | 0.008
L2a1+143+16189_(16192) | 13623 | 4 | 0.033
L2a1+143+16189_(16192) | 13752 | 3 | 0.025
L2a1+143+16189_(16192) | 13759 | 1 | 0.008
L2a1+143+16189_(16192) | 13819 | 1 | 0.008
L2a1+143+16189_(16192) | 13889 | 1 | 0.008
L2a1+143+16189_(16192) | 1391 | 2 | 0.017
L2a1+143+16189_(16192) | 13980 | 1 | 0.008

L2a1+143+16189_(16192) | 14034 | 2 | 0.017
L2a1+143+16189_(16192) | 14239 | 2 | 0.017
L2a1+143+16189_(16192) | 14401 | 1 | 0.008
L2a1+143+16189_(16192) | 14563A | 1 | 0.008
L2a1+143+16189_(16192) | 150 | 2 | 0.017
L2a1+143+16189_(16192) | 15064 | 1 | 0.008
L2a1+143+16189_(16192) | 15115 | 1 | 0.008
L2a1+143+16189_(16192) | 15218 | 1 | 0.008
L2a1+143+16189_(16192) | 15289 | 1 | 0.008
L2a1+143+16189_(16192) | 15310 | 1 | 0.008
L2a1+143+16189_(16192) | 15412A | 1 | 0.008
L2a1+143+16189_(16192) | 15431 | 1 | 0.008
L2a1+143+16189_(16192) | 15445 | 4 | 0.033
L2a1+143+16189_(16192) | 15449 | 1 | 0.008
L2a1+143+16189_(16192) | 15670 | 1 | 0.008
L2a1+143+16189_(16192) | 15747 | 1 | 0.008
L2a1+143+16189_(16192) | 15869 | 1 | 0.008
L2a1+143+16189_(16192) | 15955 | 1 | 0.008
L2a1+143+16189_(16192) | 16039 | 1 | 0.008
L2a1+143+16189_(16192) | 16041 | 9 | 0.074
L2a1+143+16189_(16192) | 1607 | 1 | 0.008
L2a1+143+16189_(16192) | 16086 | 1 | 0.008
L2a1+143+16189_(16192) | 16092 | 1 | 0.008
L2a1+143+16189_(16192) | 16093 | 20 | 0.165
L2a1+143+16189_(16192) | 16124 | 7 | 0.058
L2a1+143+16189_(16192) | 16129 | 5 | 0.041
L2a1+143+16189_(16192) | 16150 | 1 | 0.008
L2a1+143+16189_(16192) | 16172 | 1 | 0.008
L2a1+143+16189_(16192) | 16187 | 1 | 0.008
L2a1+143+16189_(16192) | 16193 | 1 | 0.008
L2a1+143+16189_(16192) | 16213 | 1 | 0.008
L2a1+143+16189_(16192) | 16249 | 1 | 0.008
L2a1+143+16189_(16192) | 16256 | 2 | 0.017
L2a1+143+16189_(16192) | 16260 | 2 | 0.017
L2a1+143+16189_(16192) | 16271 | 1 | 0.008
L2a1+143+16189_(16192) | 16274 | 1 | 0.008
L2a1+143+16189_(16192) | 1628 | 1 | 0.008
L2a1+143+16189_(16192) | 16284 | 5 | 0.041
L2a1+143+16189_(16192) | 16292 | 15 | 0.124
L2a1+143+16189_(16192) | 16294A | 1 | 0.008
L2a1+143+16189_(16192) | 16304 | 1 | 0.008
L2a1+143+16189_(16192) | 16311G | 1 | 0.008
L2a1+143+16189_(16192) | 16484-16489d | 1 | 0.008
L2a1+143+16189_(16192) | 1706 | 1 | 0.008
L2a1+143+16189_(16192) | 1824 | 1 | 0.008
L2a1+143+16189_(16192) | 189 | 1 | 0.008
L2a1+143+16189_(16192) | 200 | 1 | 0.008
L2a1+143+16189_(16192) | 2083 | 2 | 0.017
L2a1+143+16189_(16192) | 2855 | 2 | 0.017
L2a1+143+16189_(16192) | 2887 | 1 | 0.008
L2a1+143+16189_(16192) | 317.1C | 1 | 0.008
L2a1+143+16189_(16192) | 3209C | 1 | 0.008
L2a1+143+16189_(16192) | 3221 | 1 | 0.008
L2a1+143+16189_(16192) | 3394 | 4 | 0.033
L2a1+143+16189_(16192) | 4371 | 2 | 0.017
L2a1+143+16189_(16192) | 5135 | 2 | 0.017
L2a1+143+16189_(16192) | 518 | 7 | 0.058
L2a1+143+16189_(16192) | 5237 | 1 | 0.008
L2a1+143+16189_(16192) | 5460 | 4 | 0.033
L2a1+143+16189_(16192) | 573.1C | 1 | 0.008
L2a1+143+16189_(16192) | 5814 | 2 | 0.017
L2a1+143+16189_(16192) | 6351 | 1 | 0.008
L2a1+143+16189_(16192) | 6722 | 3 | 0.025
L2a1+143+16189_(16192) | 723 | 1 | 0.008
L2a1+143+16189_(16192) | 7438T | 1 | 0.008
L2a1+143+16189_(16192) | 7444 | 2 | 0.017
L2a1+143+16189_(16192) | 8108 | 1 | 0.008
L2a1+143+16189_(16192) | 8544 | 1 | 0.008
L2a1+143+16189_(16192) | 8566 | 1 | 0.008
L2a1+143+16189_(16192) | 8631 | 1 | 0.008
L2a1+143+16189_(16192) | 8793 | 1 | 0.008
L2a1+143+16189_(16192) | 8799 | 1 | 0.008
L2a1+143+16189_(16192) | 8952 | 1 | 0.008
L2a1+143+16189_(16192) | 9055 | 1 | 0.008
L2a1+143+16189_(16192) | 9088 | 2 | 0.017
L2a1+143+16189_(16192) | 9090 | 1 | 0.008
L2a1+143+16189_(16192) | 9153A | 3 | 0.025
L2a1+143+16189_(16192) | 9165 | 1 | 0.008
L2a1+143+16189_(16192) | 9196 | 2 | 0.017
L2a1+143+16189_(16192) | 9905 | 1 | 0.008
L2a1+143+16189_(16192) | 9947 | 1 | 0.008
L2a1+143+16189_(16192) | 9966 | 4 | 0.033
L2a1+143+16189_(16192) | 9986 | 2 | 0.017
L2a1+143+16189_(16192)+@16309 | 10508 | 5 | 0.053
L2a1+143+16189_(16192)+@16309 | 11404 | 6 | 0.064
L2a1+143+16189_(16192)+@16309 | 11963 | 2 | 0.021
L2a1+143+16189_(16192)+@16309 | 12771 | 1 | 0.011
L2a1+143+16189_(16192)+@16309 | 12810 | 1 | 0.011
L2a1+143+16189_(16192)+@16309 | 13095A | 1 | 0.011
L2a1+143+16189_(16192)+@16309 | 13317 | 1 | 0.011
L2a1+143+16189_(16192)+@16309 | 13813 | 1 | 0.011
L2a1+143+16189_(16192)+@16309 | 14569 | 6 | 0.064
L2a1+143+16189_(16192)+@16309 | 15047 | 1 | 0.011
L2a1+143+16189_(16192)+@16309 | 15203 | 1 | 0.011
L2a1+143+16189_(16192)+@16309 | 15258 | 2 | 0.021
L2a1+143+16189_(16192)+@16309 | 15283 | 1 | 0.011
L2a1+143+16189_(16192)+@16309 | 153 | 1 | 0.011
L2a1+143+16189_(16192)+@16309 | 15382 | 1 | 0.011
L2a1+143+16189_(16192)+@16309 | 15703 | 1 | 0.011
L2a1+143+16189_(16192)+@16309 | 15777 | 1 | 0.011
L2a1+143+16189_(16192)+@16309 | 15924 | 6 | 0.064
L2a1+143+16189_(16192)+@16309 | 15926 | 2 | 0.021
L2a1+143+16189_(16192)+@16309 | 16024 | 1 | 0.011
L2a1+143+16189_(16192)+@16309 | 16025 | 1 | 0.011
L2a1+143+16189_(16192)+@16309 | 16093 | 4 | 0.043
L2a1+143+16189_(16192)+@16309 | 16129 | 2 | 0.021
L2a1+143+16189_(16192)+@16309 | 16145 | 3 | 0.032
L2a1+143+16189_(16192)+@16309 | 16173 | 1 | 0.011
L2a1+143+16189_(16192)+@16309 | 16187 | 1 | 0.011
L2a1+143+16189_(16192)+@16309 | 16188 | 1 | 0.011
L2a1+143+16189_(16192)+@16309 | 16193 | 2 | 0.021

L2a1+143+16189_(16192)+@16309 | 16193d | 1 | 0.011
L2a1+143+16189_(16192)+@16309 | 16209 | 1 | 0.011
L2a1+143+16189_(16192)+@16309 | 16224 | 3 | 0.032
L2a1+143+16189_(16192)+@16309 | 16234 | 2 | 0.021
L2a1+143+16189_(16192)+@16309 | 16239 | 4 | 0.043
L2a1+143+16189_(16192)+@16309 | 16239A | 1 | 0.011
L2a1+143+16189_(16192)+@16309 | 16248 | 1 | 0.011
L2a1+143+16189_(16192)+@16309 | 16249 | 2 | 0.021
L2a1+143+16189_(16192)+@16309 | 16256 | 1 | 0.011
L2a1+143+16189_(16192)+@16309 | 16266 | 3 | 0.032
L2a1+143+16189_(16192)+@16309 | 16269 | 1 | 0.011
L2a1+143+16189_(16192)+@16309 | 16274 | 1 | 0.011
L2a1+143+16189_(16192)+@16309 | 16290 | 1 | 0.011
L2a1+143+16189_(16192)+@16309 | 16291 | 5 | 0.053
L2a1+143+16189_(16192)+@16309 | 16292 | 6 | 0.064
L2a1+143+16189_(16192)+@16309 | 16295 | 2 | 0.021
L2a1+143+16189_(16192)+@16309 | 16311 | 4 | 0.043
L2a1+143+16189_(16192)+@16309 | 16353 | 3 | 0.032
L2a1+143+16189_(16192)+@16309 | 16355 | 3 | 0.032
L2a1+143+16189_(16192)+@16309 | 16358 | 1 | 0.011
L2a1+143+16189_(16192)+@16309 | 16360 | 1 | 0.011
L2a1+143+16189_(16192)+@16309 | 16362 | 2 | 0.021
L2a1+143+16189_(16192)+@16309 | 16365 | 1 | 0.011
L2a1+143+16189_(16192)+@16309 | 16511A | 1 | 0.011
L2a1+143+16189_(16192)+@16309 | 189 | 3 | 0.032
L2a1+143+16189_(16192)+@16309 | 198 | 2 | 0.021
L2a1+143+16189_(16192)+@16309 | 199 | 3 | 0.032
L2a1+143+16189_(16192)+@16309 | 200 | 2 | 0.021
L2a1+143+16189_(16192)+@16309 | 203 | 1 | 0.011
L2a1+143+16189_(16192)+@16309 | 204 | 1 | 0.011
L2a1+143+16189_(16192)+@16309 | 2308 | 1 | 0.011
L2a1+143+16189_(16192)+@16309 | 2352 | 1 | 0.011
L2a1+143+16189_(16192)+@16309 | 239 | 1 | 0.011
L2a1+143+16189_(16192)+@16309 | 2762 | 1 | 0.011
L2a1+143+16189_(16192)+@16309 | 291T | 1 | 0.011
L2a1+143+16189_(16192)+@16309 | 3338 | 1 | 0.011
L2a1+143+16189_(16192)+@16309 | 3606 | 1 | 0.011
L2a1+143+16189_(16192)+@16309 | 3666 | 1 | 0.011
L2a1+143+16189_(16192)+@16309 | 373 | 2 | 0.021
L2a1+143+16189_(16192)+@16309 | 3834 | 1 | 0.011
L2a1+143+16189_(16192)+@16309 | 4316 | 1 | 0.011
L2a1+143+16189_(16192)+@16309 | 4353 | 1 | 0.011
L2a1+143+16189_(16192)+@16309 | 4688 | 2 | 0.021
L2a1+143+16189_(16192)+@16309 | 4877 | 1 | 0.011
L2a1+143+16189_(16192)+@16309 | 489 | 1 | 0.011
L2a1+143+16189_(16192)+@16309 | 499 | 1 | 0.011
L2a1+143+16189_(16192)+@16309 | 518 | 1 | 0.011
L2a1+143+16189_(16192)+@16309 | 534 | 4 | 0.043
L2a1+143+16189_(16192)+@16309 | 5442 | 1 | 0.011
L2a1+143+16189_(16192)+@16309 | 5451 | 1 | 0.011
L2a1+143+16189_(16192)+@16309 | 5471 | 1 | 0.011
L2a1+143+16189_(16192)+@16309 | 5664 | 1 | 0.011
L2a1+143+16189_(16192)+@16309 | 573.1C | 1 | 0.011
L2a1+143+16189_(16192)+@16309 | 573.5C | 1 | 0.011
L2a1+143+16189_(16192)+@16309 | 6125 | 1 | 0.011
L2a1+143+16189_(16192)+@16309 | 6713 | 2 | 0.021
L2a1+143+16189_(16192)+@16309 | 6743 | 6 | 0.064
L2a1+143+16189_(16192)+@16309 | 7570 | 1 | 0.011
L2a1+143+16189_(16192)+@16309 | 7711 | 5 | 0.053
L2a1+143+16189_(16192)+@16309 | 7801 | 1 | 0.011
L2a1+143+16189_(16192)+@16309 | 7859 | 1 | 0.011
L2a1+143+16189_(16192)+@16309 | 7925 | 1 | 0.011
L2a1+143+16189_(16192)+@16309 | 8788 | 6 | 0.064
L2a1+143+16189_(16192)+@16309 | 9033 | 1 | 0.011
L2a1+143+16189_(16192)+@16309 | 9305 | 1 | 0.011
L2a1+16189_(16192) | 12453 | 1 | 0.008
L2a1+16189_(16192) | 13098 | 1 | 0.008
L2a1+16189_(16192) | 15511 | 1 | 0.008
L2a1+16189_(16192) | 16153 | 1 | 0.008
L2a1+16189_(16192) | 16194C | 1 | 0.008
L2a1+16189_(16192) | 16195 | 1 | 0.008
L2a1+16189_(16192) | 16201 | 1 | 0.008
L2a1+16189_(16192) | 16269 | 1 | 0.008
L2a1+16189_(16192) | 16293 | 1 | 0.008
L2a1+16189_(16192) | 16311 | 1 | 0.008
L2a1+16189_(16192) | 16325 | 1 | 0.008
L2a1+16189_(16192) | 16465 | 1 | 0.008
L2a1+16189_(16192) | 2779 | 1 | 0.008
L2a1+16189_(16192) | 282 | 1 | 0.008
L2a1+16189_(16192) | 310 | 1 | 0.008
L2a1+16189_(16192) | 3113.1A | 1 | 0.008
L2a1+16189_(16192) | 317 | 1 | 0.008
L2a1+16189_(16192) | 320 | 1 | 0.008
L2a1+16189_(16192) | 343 | 1 | 0.008
L2a1+16189_(16192) | 345 | 1 | 0.008
L2a1+16189_(16192) | 5237 | 1 | 0.008
L2a1+16189_(16192) | 553A | 1 | 0.008
L2a1'2'3'4 | 16169 | 1 | 0.1
L2a1'2'3'4 | 16209 | 1 | 0.1
L2a1'2'3'4 | 16209G | 1 | 0.1
L2a1'2'3'4 | 16224 | 1 | 0.1
L2a1'2'3'4 | 16266 | 1 | 0.1
L2a1'2'3'4 | 16287 | 1 | 0.1
L2a1'2'3'4 | 16354 | 1 | 0.1
L2a1'2'3'4 | 189 | 1 | 0.1
L2a1'2'3'4 | 198 | 1 | 0.1
L2a1'2'3'4 | 199 | 1 | 0.1
L2a1'2'3'4 | 236 | 1 | 0.1
L2a1'2'3'4 | 310A | 1 | 0.1
L2a1'2'3'4 | 515-524d | 2 | 0.2
L2a1'2'3'4 | 573.4C | 1 | 0.1
L2a1a | 10676 | 1 | 0.007
L2a1a | 11368 | 1 | 0.007
L2a1a | 12242C | 1 | 0.007
L2a1a | 12372 | 2 | 0.015
L2a1a | 12684 | 2 | 0.015
L2a1a | 12771 | 1 | 0.007
L2a1a | 13455 | 1 | 0.007
L2a1a | 13708 | 2 | 0.015
L2a1a | 13768 | 2 | 0.015
L2a1a | 13805G | 1 | 0.007

L2a1a | 14200 | 1 | 0.007
L2a1a | 14524 | 1 | 0.007
L2a1a | 14923 | 1 | 0.007
L2a1a | 14974G | 1 | 0.007
L2a1a | 14992 | 2 | 0.015
L2a1a | 15211 | 2 | 0.015
L2a1a | 15219 | 1 | 0.007
L2a1a | 15407G | 1 | 0.007
L2a1a | 15479 | 1 | 0.007
L2a1a | 15544 | 1 | 0.007
L2a1a | 15889 | 1 | 0.007
L2a1a | 1598 | 1 | 0.007
L2a1a | 16093 | 6 | 0.044
L2a1a | 16129 | 6 | 0.044
L2a1a | 16140 | 2 | 0.015
L2a1a | 16147 | 1 | 0.007
L2a1a | 16162 | 1 | 0.007
L2a1a | 16189 | 2 | 0.015
L2a1a | 16209 | 2 | 0.015
L2a1a | 16287 | 1 | 0.007
L2a1a | 16289 | 1 | 0.007
L2a1a | 16311 | 1 | 0.007
L2a1a | 16325 | 1 | 0.007
L2a1a | 16399 | 1 | 0.007
L2a1a | 16497 | 1 | 0.007
L2a1a | 173A | 1 | 0.007
L2a1a | 183 | 1 | 0.007
L2a1a | 1888 | 1 | 0.007
L2a1a | 2442 | 2 | 0.015
L2a1a | 3003 | 1 | 0.007
L2a1a | 309d | 4 | 0.029
L2a1a | 310 | 8 | 0.058
L2a1a | 316 | 1 | 0.007
L2a1a | 317.1C | 3 | 0.022
L2a1a | 3254A | 1 | 0.007
L2a1a | 373 | 1 | 0.007
L2a1a | 3834 | 1 | 0.007
L2a1a | 4823 | 1 | 0.007
L2a1a | 5074 | 1 | 0.007
L2a1a | 5460 | 2 | 0.015
L2a1a | 5580 | 1 | 0.007
L2a1a | 5585 | 1 | 0.007
L2a1a | 573.2C | 1 | 0.007
L2a1a | 575 | 1 | 0.007
L2a1a | 60d | 1 | 0.007
L2a1a | 6143 | 1 | 0.007
L2a1a | 6446 | 1 | 0.007
L2a1a | 6503 | 1 | 0.007
L2a1a | 6626 | 1 | 0.007
L2a1a | 6956 | 1 | 0.007
L2a1a | 7337 | 2 | 0.015
L2a1a | 7595 | 2 | 0.015
L2a1a | 7961 | 1 | 0.007
L2a1a | 8104 | 1 | 0.007
L2a1a | 8222 | 1 | 0.007
L2a1a | 8227 | 2 | 0.015
L2a1a | 8285.1C | 1 | 0.007
L2a1a | 8480 | 2 | 0.015
L2a1a | 8557 | 1 | 0.007
L2a1a | 9452 | 4 | 0.029
L2a1a | 9921 | 1 | 0.007
L2a1a | 9989 | 1 | 0.007
L2a1a1 | 10768 | 1 | 0.01
L2a1a1 | 10819 | 1 | 0.01
L2a1a1 | 10828 | 1 | 0.01
L2a1a1 | 11248 | 1 | 0.01
L2a1a1 | 114 | 1 | 0.01
L2a1a1 | 11963 | 1 | 0.01
L2a1a1 | 12142 | 1 | 0.01
L2a1a1 | 12715 | 1 | 0.01
L2a1a1 | 13129 | 1 | 0.01
L2a1a1 | 13376 | 1 | 0.01
L2a1a1 | 14131 | 2 | 0.019
L2a1a1 | 14258 | 1 | 0.01
L2a1a1 | 14305 | 1 | 0.01
L2a1a1 | 14323 | 1 | 0.01
L2a1a1 | 14484 | 1 | 0.01
L2a1a1 | 14544 | 2 | 0.019
L2a1a1 | 15358 | 1 | 0.01
L2a1a1 | 16086 | 5 | 0.048
L2a1a1 | 16092 | 3 | 0.029
L2a1a1 | 16093 | 2 | 0.019
L2a1a1 | 16129 | 4 | 0.038
L2a1a1 | 16179 | 1 | 0.01
L2a1a1 | 16189 | 1 | 0.01
L2a1a1 | 16233 | 1 | 0.01
L2a1a1 | 16234 | 2 | 0.019
L2a1a1 | 16248 | 1 | 0.01
L2a1a1 | 16264 | 1 | 0.01
L2a1a1 | 16265C | 1 | 0.01
L2a1a1 | 16267 | 1 | 0.01
L2a1a1 | 16274 | 1 | 0.01
L2a1a1 | 16290 | 1 | 0.01
L2a1a1 | 16291 | 1 | 0.01
L2a1a1 | 16299 | 3 | 0.029
L2a1a1 | 16311 | 1 | 0.01
L2a1a1 | 16497 | 1 | 0.01
L2a1a1 | 189 | 1 | 0.01
L2a1a1 | 1901 | 1 | 0.01
L2a1a1 | 198 | 1 | 0.01
L2a1a1 | 2069 | 1 | 0.01
L2a1a1 | 292.1AT | 2 | 0.019
L2a1a1 | 293.1AT | 2 | 0.019
L2a1a1 | 309d | 3 | 0.029
L2a1a1 | 310 | 5 | 0.048
L2a1a1 | 315.2C | 2 | 0.019
L2a1a1 | 315.3C | 1 | 0.01
L2a1a1 | 3834 | 1 | 0.01
L2a1a1 | 4161 | 2 | 0.019
L2a1a1 | 467 | 2 | 0.019
L2a1a1 | 4772 | 1 | 0.01

L2a1a1 | 499 | 1 | 0.01
L2a1a1 | 515-524d | 2 | 0.019
L2a1a1 | 525.1ACAC | 2 | 0.019
L2a1a1 | 525.1ACCA | 1 | 0.01
L2a1a1 | 5263 | 1 | 0.01
L2a1a1 | 5273 | 1 | 0.01
L2a1a1 | 5774 | 1 | 0.01
L2a1a1 | 7391 | 1 | 0.01
L2a1a1 | 7598 | 1 | 0.01
L2a1a1 | 789 | 1 | 0.01
L2a1a1 | 8020 | 1 | 0.01
L2a1a1 | 8078 | 1 | 0.01
L2a1a1 | 8188 | 1 | 0.01
L2a1a1 | 8470 | 1 | 0.01
L2a1a1 | 8477G | 1 | 0.01
L2a1a1 | 8616 | 1 | 0.01
L2a1a1 | 9212 | 1 | 0.01
L2a1a1 | 9752 | 1 | 0.01
L2a1a1 | 980 | 1 | 0.01
L2a1a2 | 10017 | 1 | 0.005
L2a1a2 | 1007 | 3 | 0.016
L2a1a2 | 10345 | 1 | 0.005
L2a1a2 | 10870 | 1 | 0.005
L2a1a2 | 11233 | 1 | 0.005
L2a1a2 | 11485 | 1 | 0.005
L2a1a2 | 11644 | 1 | 0.005
L2a1a2 | 11696 | 1 | 0.005
L2a1a2 | 12007 | 4 | 0.022
L2a1a2 | 12177 | 1 | 0.005
L2a1a2 | 1231T | 1 | 0.005
L2a1a2 | 12351 | 1 | 0.005
L2a1a2 | 12372 | 1 | 0.005
L2a1a2 | 12435 | 1 | 0.005
L2a1a2 | 12469 | 1 | 0.005
L2a1a2 | 13135 | 1 | 0.005
L2a1a2 | 13145 | 1 | 0.005
L2a1a2 | 13581 | 1 | 0.005
L2a1a2 | 13659 | 1 | 0.005
L2a1a2 | 13753 | 1 | 0.005
L2a1a2 | 13786A | 1 | 0.005
L2a1a2 | 14180 | 1 | 0.005
L2a1a2 | 14769 | 1 | 0.005
L2a1a2 | 14971 | 1 | 0.005
L2a1a2 | 15103 | 1 | 0.005
L2a1a2 | 15191 | 1 | 0.005
L2a1a2 | 15346 | 1 | 0.005
L2a1a2 | 15436 | 1 | 0.005
L2a1a2 | 15454 | 1 | 0.005
L2a1a2 | 1555 | 1 | 0.005
L2a1a2 | 15617 | 1 | 0.005
L2a1a2 | 15748 | 1 | 0.005
L2a1a2 | 15769 | 1 | 0.005
L2a1a2 | 15884 | 3 | 0.016
L2a1a2 | 15894 | 1 | 0.005
L2a1a2 | 15927 | 1 | 0.005
L2a1a2 | 15944d | 2 | 0.011
L2a1a2 | 1598 | 1 | 0.005
L2a1a2 | 16066 | 1 | 0.005
L2a1a2 | 16093 | 3 | 0.016
L2a1a2 | 16172 | 6 | 0.032
L2a1a2 | 16181 | 1 | 0.005
L2a1a2 | 16185 | 1 | 0.005
L2a1a2 | 16214 | 1 | 0.005
L2a1a2 | 16256 | 1 | 0.005
L2a1a2 | 16260 | 6 | 0.032
L2a1a2 | 16264 | 1 | 0.005
L2a1a2 | 16265C | 1 | 0.005
L2a1a2 | 16274 | 1 | 0.005
L2a1a2 | 16355 | 1 | 0.005
L2a1a2 | 16360 | 1 | 0.005
L2a1a2 | 16362 | 1 | 0.005
L2a1a2 | 1707 | 1 | 0.005
L2a1a2 | 1855 | 1 | 0.005
L2a1a2 | 2232.1A | 1 | 0.005
L2a1a2 | 2259 | 1 | 0.005
L2a1a2 | 228 | 2 | 0.011
L2a1a2 | 2412 | 1 | 0.005
L2a1a2 | 309d | 9 | 0.048
L2a1a2 | 310 | 7 | 0.038
L2a1a2 | 315.2C | 2 | 0.011
L2a1a2 | 317.1C | 1 | 0.005
L2a1a2 | 3203T | 1 | 0.005
L2a1a2 | 3391 | 1 | 0.005
L2a1a2 | 3438 | 1 | 0.005
L2a1a2 | 357C | 1 | 0.005
L2a1a2 | 373 | 1 | 0.005
L2a1a2 | 3987 | 1 | 0.005
L2a1a2 | 4048 | 1 | 0.005
L2a1a2 | 4216 | 2 | 0.011
L2a1a2 | 4231 | 1 | 0.005
L2a1a2 | 4561 | 1 | 0.005
L2a1a2 | 4631 | 1 | 0.005
L2a1a2 | 4634 | 1 | 0.005
L2a1a2 | 4703 | 6 | 0.032
L2a1a2 | 4739 | 1 | 0.005
L2a1a2 | 499C | 1 | 0.005
L2a1a2 | 513 | 2 | 0.011
L2a1a2 | 5153 | 1 | 0.005
L2a1a2 | 5442 | 1 | 0.005
L2a1a2 | 5460 | 1 | 0.005
L2a1a2 | 5563 | 1 | 0.005
L2a1a2 | 5585 | 1 | 0.005
L2a1a2 | 5788 | 1 | 0.005
L2a1a2 | 5911 | 1 | 0.005
L2a1a2 | 5951 | 1 | 0.005
L2a1a2 | 5978 | 1 | 0.005
L2a1a2 | 6113 | 1 | 0.005
L2a1a2 | 6150 | 1 | 0.005
L2a1a2 | 6152 | 1 | 0.005
L2a1a2 | 6569A | 1 | 0.005
L2a1a2 | 6572 | 1 | 0.005

L2a1a2 | 6587A | 1 | 0.005
L2a1a2 | 6590 | 1 | 0.005
L2a1a2 | 6857 | 2 | 0.011
L2a1a2 | 6875A | 1 | 0.005
L2a1a2 | 7079A | 2 | 0.011
L2a1a2 | 7283 | 1 | 0.005
L2a1a2 | 7389 | 1 | 0.005
L2a1a2 | 7444 | 1 | 0.005
L2a1a2 | 7562 | 2 | 0.011
L2a1a2 | 7795 | 1 | 0.005
L2a1a2 | 8020 | 2 | 0.011
L2a1a2 | 8104 | 2 | 0.011
L2a1a2 | 8302 | 2 | 0.011
L2a1a2 | 8477 | 1 | 0.005
L2a1a2 | 8551 | 1 | 0.005
L2a1a2 | 8700 | 1 | 0.005
L2a1a2 | 9335 | 1 | 0.005
L2a1a2 | 9530A | 2 | 0.011
L2a1a2 | 9587 | 1 | 0.005
L2a1a2a | 14857 | 1 | 0.011
L2a1a2a | 16150 | 1 | 0.011
L2a1a2a | 16192 | 1 | 0.011
L2a1a2a | 16256 | 1 | 0.011
L2a1a2a | 16266 | 1 | 0.011
L2a1a2a | 16320 | 1 | 0.011
L2a1a2a | 16394 | 1 | 0.011
L2a1a2a | 189 | 2 | 0.023
L2a1a2a | 198 | 1 | 0.011
L2a1a2a | 8686 | 1 | 0.011
L2a1a2a1 | 16129 | 4 | 0.047
L2a1a2a1 | 16497 | 1 | 0.012
L2a1a2a1 | 267 | 3 | 0.035
L2a1a2a1 | 309d | 1 | 0.012
L2a1a2a1 | 515-524d | 7 | 0.082
L2a1a2a1 | 6041 | 1 | 0.012
L2a1a2a1a | 10373 | 1 | 0.009
L2a1a2a1a | 10966 | 1 | 0.009
L2a1a2a1a | 11150 | 2 | 0.018
L2a1a2a1a | 11923 | 1 | 0.009
L2a1a2a1a | 12406 | 1 | 0.009
L2a1a2a1a | 12544 | 1 | 0.009
L2a1a2a1a | 13434 | 1 | 0.009
L2a1a2a1a | 13763 | 2 | 0.018
L2a1a2a1a | 14221 | 1 | 0.009
L2a1a2a1a | 14867 | 1 | 0.009
L2a1a2a1a | 16051 | 1 | 0.009
L2a1a2a1a | 16092 | 24 | 0.211
L2a1a2a1a | 16189 | 2 | 0.018
L2a1a2a1a | 16248 | 2 | 0.018
L2a1a2a1a | 16274 | 1 | 0.009
L2a1a2a1a | 1943 | 1 | 0.009
L2a1a2a1a | 198 | 1 | 0.009
L2a1a2a1a | 279 | 2 | 0.018
L2a1a2a1a | 293 | 1 | 0.009
L2a1a2a1a | 2960 | 1 | 0.009
L2a1a2a1a | 309d | 8 | 0.07
L2a1a2a1a | 317.1C | 2 | 0.018
L2a1a2a1a | 3615 | 1 | 0.009
L2a1a2a1a | 3796 | 1 | 0.009
L2a1a2a1a | 4191T | 1 | 0.009
L2a1a2a1a | 4216 | 1 | 0.009
L2a1a2a1a | 4659 | 1 | 0.009
L2a1a2a1a | 4913 | 1 | 0.009
L2a1a2a1a | 4976 | 1 | 0.009
L2a1a2a1a | 5436C | 1 | 0.009
L2a1a2a1a | 7278 | 2 | 0.018
L2a1a2a1a | 7389 | 1 | 0.009
L2a1a2a1a | 8119 | 1 | 0.009
L2a1a2a1a | 8625 | 1 | 0.009
L2a1a2a1a | 8751 | 2 | 0.018
L2a1a2a1a | 9010 | 1 | 0.009
L2a1a2a1a | 9145 | 1 | 0.009
L2a1a2a1a | 9211 | 8 | 0.07
L2a1a2a1a | 9548C | 1 | 0.009
L2a1a2a1a | 9803 | 1 | 0.009
L2a1a2b | 11896 | 2 | 0.022
L2a1a2b | 13392 | 3 | 0.032
L2a1a2b | 14560 | 1 | 0.011
L2a1a2b | 16086 | 1 | 0.011
L2a1a2b | 16093 | 7 | 0.075
L2a1a2b | 16150 | 1 | 0.011
L2a1a2b | 16192 | 1 | 0.011
L2a1a2b | 16256 | 1 | 0.011
L2a1a2b | 16266 | 1 | 0.011
L2a1a2b | 16320 | 1 | 0.011
L2a1a2b | 16394 | 1 | 0.011
L2a1a2b | 183 | 4 | 0.043
L2a1a2b | 315.2C | 1 | 0.011
L2a1a2b | 3438 | 2 | 0.022
L2a1a2b | 3613 | 3 | 0.032
L2a1a2b | 5048 | 2 | 0.022
L2a1a2b | 9428 | 2 | 0.022
L2a1a2c | 13938 | 1 | 0.013
L2a1a2c | 207 | 4 | 0.051
L2a1a2c | 9470 | 1 | 0.013
L2a1a3 | 11399 | 1 | 0.017
L2a1a3 | 13416 | 1 | 0.017
L2a1a3 | 15747 | 1 | 0.017
L2a1a3 | 15788 | 1 | 0.017
L2a1a3 | 16187 | 1 | 0.017
L2a1a3 | 16192 | 3 | 0.052
L2a1a3 | 16213 | 1 | 0.017
L2a1a3 | 16286 | 1 | 0.017
L2a1a3 | 16311 | 1 | 0.017
L2a1a3 | 16320 | 1 | 0.017
L2a1a3 | 182 | 1 | 0.017
L2a1a3 | 310 | 2 | 0.034
L2a1a3 | 3510A | 1 | 0.017
L2a1a3 | 4823 | 1 | 0.017
L2a1a3 | 573.3C | 1 | 0.017
L2a1a3 | 6269 | 1 | 0.017

L2a1a3 | 7753T | 1 | 0.017
L2a1a3 | 8521 | 1 | 0.017
L2a1a3 | 9007 | 3 | 0.052
L2a1a3a | 1008 | 1 | 0.018
L2a1a3a | 12031 | 1 | 0.018
L2a1a3a | 16215 | 1 | 0.018
L2a1a3a | 204 | 1 | 0.018
L2a1a3a | 2222 | 2 | 0.036
L2a1a3a | 228 | 1 | 0.018
L2a1a3a | 3221 | 2 | 0.036
L2a1a3a | 5460 | 2 | 0.036
L2a1a3a | 573.1C | 1 | 0.018
L2a1a3a | 573.2C | 1 | 0.018
L2a1a3a | 573.3C | 1 | 0.018
L2a1a3a | 573.5C | 2 | 0.036
L2a1a3a | 650 | 2 | 0.036
L2a1a3a | 8301 | 1 | 0.018
L2a1a3b | 12771 | 1 | 0.015
L2a1a3b | 14088 | 1 | 0.015
L2a1a3b | 14502 | 1 | 0.015
L2a1a3b | 16104 | 1 | 0.015
L2a1a3b | 16274 | 3 | 0.046
L2a1a3b | 16291 | 2 | 0.031
L2a1a3b | 16325 | 3 | 0.046
L2a1a3b | 16496 | 1 | 0.015
L2a1a3b | 16527 | 2 | 0.031
L2a1a3b | 2857 | 1 | 0.015
L2a1a3b | 296 | 1 | 0.015
L2a1a3b | 309d | 1 | 0.015
L2a1a3b | 3826 | 1 | 0.015
L2a1a3b | 391A | 1 | 0.015
L2a1a3b | 474A | 1 | 0.015
L2a1a3b | 507 | 1 | 0.015
L2a1a3b | 507A | 1 | 0.015
L2a1a3b | 515-524d | 8 | 0.123
L2a1a3b | 529T | 1 | 0.015
L2a1a3b | 548A | 1 | 0.015
L2a1a3b | 573.3C | 1 | 0.015
L2a1a3b | 5814 | 1 | 0.015
L2a1a3b | 8270 | 1 | 0.015
L2a1a3b | 8281-8289d | 1 | 0.015
L2a1a3c | 15043 | 1 | 0.037
L2a1a3c | 15734 | 10 | 0.37
L2a1a3c | 16046 | 1 | 0.037
L2a1a3c | 16209 | 1 | 0.037
L2a1a3c | 16241 | 1 | 0.037
L2a1a3c | 16291 | 1 | 0.037
L2a1a3c | 16292 | 1 | 0.037
L2a1a3c | 16311 | 1 | 0.037
L2a1a3c | 309d | 2 | 0.074
L2a1a3c | 3197 | 1 | 0.037
L2a1a3c | 4435 | 1 | 0.037
L2a1a3c | 4976 | 2 | 0.074
L2a1a3c | 515-524d | 2 | 0.074
L2a1a3c | 534 | 1 | 0.037
L2a1a3c | 6524 | 1 | 0.037
L2a1a3c | 8086 | 3 | 0.111
L2a1a3c | 8520 | 1 | 0.037
L2a1a3c | 8598 | 1 | 0.037
L2a1b | 12026 | 1 | 0.007
L2a1b | 12241d | 1 | 0.007
L2a1b | 16081 | 1 | 0.007
L2a1b | 16095 | 2 | 0.015
L2a1b | 16168 | 1 | 0.007
L2a1b | 16179A | 1 | 0.007
L2a1b | 16187 | 1 | 0.007
L2a1b | 16192 | 1 | 0.007
L2a1b | 16231 | 1 | 0.007
L2a1b | 16234 | 2 | 0.015
L2a1b | 16270 | 1 | 0.007
L2a1b | 16274 | 1 | 0.007
L2a1b | 183 | 2 | 0.015
L2a1b | 198 | 1 | 0.007
L2a1b | 207 | 2 | 0.015
L2a1b | 260 | 1 | 0.007
L2a1b | 270d | 1 | 0.007
L2a1b | 309d | 1 | 0.007
L2a1b | 709 | 1 | 0.007
L2a1b | 8347 | 1 | 0.007
L2a1b+143 | 11650 | 1 | 0.015
L2a1b+143 | 12241d | 2 | 0.03
L2a1b+143 | 12406 | 1 | 0.015
L2a1b+143 | 16093 | 1 | 0.015
L2a1b+143 | 16129 | 2 | 0.03
L2a1b+143 | 16166d | 1 | 0.015
L2a1b+143 | 16172 | 1 | 0.015
L2a1b+143 | 16187 | 4 | 0.061
L2a1b+143 | 16188G | 1 | 0.015
L2a1b+143 | 16214 | 4 | 0.061
L2a1b+143 | 16293 | 1 | 0.015
L2a1b+143 | 16325 | 1 | 0.015
L2a1b+143 | 5628 | 2 | 0.03
L2a1b+143 | 8838 | 1 | 0.015
L2a1b+143 | 9422 | 1 | 0.015
L2a1b1 | 12130 | 3 | 0.024
L2a1b1 | 12357 | 2 | 0.016
L2a1b1 | 15258 | 1 | 0.008
L2a1b1 | 15883 | 1 | 0.008
L2a1b1 | 16192 | 3 | 0.024
L2a1b1 | 5090 | 1 | 0.008
L2a1b1 | 5583 | 1 | 0.008
L2a1b1 | 7830 | 2 | 0.016
L2a1b1a | 10634 | 1 | 0.003
L2a1b1a | 10671 | 1 | 0.003
L2a1b1a | 10672 | 1 | 0.003
L2a1b1a | 10757G | 1 | 0.003
L2a1b1a | 10943 | 1 | 0.003
L2a1b1a | 11002 | 2 | 0.006
L2a1b1a | 11017 | 1 | 0.003
L2a1b1a | 1120 | 1 | 0.003
L2a1b1a | 11732 | 2 | 0.006

L2a1b1a | 11887 | 1 | 0.003
L2a1b1a | 11992 | 1 | 0.003
L2a1b1a | 12234C | 1 | 0.003
L2a1b1a | 12241.1C | 2 | 0.006
L2a1b1a | 12397 | 1 | 0.003
L2a1b1a | 12399 | 1 | 0.003
L2a1b1a | 12471 | 1 | 0.003
L2a1b1a | 12522 | 1 | 0.003
L2a1b1a | 12557 | 1 | 0.003
L2a1b1a | 12634 | 1 | 0.003
L2a1b1a | 12651 | 1 | 0.003
L2a1b1a | 12843 | 1 | 0.003
L2a1b1a | 12882 | 1 | 0.003
L2a1b1a | 13195A | 1 | 0.003
L2a1b1a | 13225 | 2 | 0.006
L2a1b1a | 14053 | 1 | 0.003
L2a1b1a | 14176 | 4 | 0.012
L2a1b1a | 14494 | 1 | 0.003
L2a1b1a | 14502 | 1 | 0.003
L2a1b1a | 14518C | 1 | 0.003
L2a1b1a | 14560 | 1 | 0.003
L2a1b1a | 1469T | 1 | 0.003
L2a1b1a | 14992 | 1 | 0.003
L2a1b1a | 15151 | 1 | 0.003
L2a1b1a | 15172 | 1 | 0.003
L2a1b1a | 15514 | 1 | 0.003
L2a1b1a | 15638 | 1 | 0.003
L2a1b1a | 15850 | 1 | 0.003
L2a1b1a | 15907 | 3 | 0.009
L2a1b1a | 15930 | 2 | 0.006
L2a1b1a | 16051 | 2 | 0.006
L2a1b1a | 16086 | 1 | 0.003
L2a1b1a | 16093 | 1 | 0.003
L2a1b1a | 16102 | 1 | 0.003
L2a1b1a | 16111 | 4 | 0.012
L2a1b1a | 16126 | 1 | 0.003
L2a1b1a | 16129 | 1 | 0.003
L2a1b1a | 16155 | 1 | 0.003
L2a1b1a | 16167 | 1 | 0.003
L2a1b1a | 16173 | 1 | 0.003
L2a1b1a | 16187 | 1 | 0.003
L2a1b1a | 16192 | 5 | 0.015
L2a1b1a | 16192-16193d | 10 | 0.03
L2a1b1a | 16193d | 8 | 0.024
L2a1b1a | 16210C | 1 | 0.003
L2a1b1a | 16235 | 2 | 0.006
L2a1b1a | 16243 | 1 | 0.003
L2a1b1a | 16247 | 1 | 0.003
L2a1b1a | 16262 | 1 | 0.003
L2a1b1a | 16266 | 1 | 0.003
L2a1b1a | 16268d | 1 | 0.003
L2a1b1a | 16270 | 1 | 0.003
L2a1b1a | 16274 | 4 | 0.012
L2a1b1a | 16284 | 1 | 0.003
L2a1b1a | 16291 | 1 | 0.003
L2a1b1a | 16292 | 1 | 0.003
L2a1b1a | 16304 | 1 | 0.003
L2a1b1a | 16311 | 1 | 0.003
L2a1b1a | 16320 | 1 | 0.003
L2a1b1a | 16355 | 1 | 0.003
L2a1b1a | 16356 | 5 | 0.015
L2a1b1a | 16359 | 1 | 0.003
L2a1b1a | 16368 | 1 | 0.003
L2a1b1a | 16380 | 1 | 0.003
L2a1b1a | 16399 | 1 | 0.003
L2a1b1a | 16526 | 1 | 0.003
L2a1b1a | 189 | 1 | 0.003
L2a1b1a | 198 | 2 | 0.006
L2a1b1a | 1980 | 3 | 0.009
L2a1b1a | 204 | 5 | 0.015
L2a1b1a | 2304 | 1 | 0.003
L2a1b1a | 234 | 2 | 0.006
L2a1b1a | 257 | 1 | 0.003
L2a1b1a | 2762 | 12 | 0.036
L2a1b1a | 291.2A | 1 | 0.003
L2a1b1a | 292 | 3 | 0.009
L2a1b1a | 3010 | 1 | 0.003
L2a1b1a | 309.3C | 1 | 0.003
L2a1b1a | 309d | 11 | 0.033
L2a1b1a | 310 | 1 | 0.003
L2a1b1a | 3394 | 3 | 0.009
L2a1b1a | 3397 | 1 | 0.003
L2a1b1a | 3398 | 1 | 0.003
L2a1b1a | 3505 | 1 | 0.003
L2a1b1a | 3593 | 1 | 0.003
L2a1b1a | 3606 | 1 | 0.003
L2a1b1a | 3746 | 1 | 0.003
L2a1b1a | 4314 | 1 | 0.003
L2a1b1a | 4742 | 1 | 0.003
L2a1b1a | 489 | 1 | 0.003
L2a1b1a | 498.1C | 1 | 0.003
L2a1b1a | 513 | 3 | 0.009
L2a1b1a | 526.1G | 1 | 0.003
L2a1b1a | 5263 | 1 | 0.003
L2a1b1a | 5277 | 1 | 0.003
L2a1b1a | 5319 | 1 | 0.003
L2a1b1a | 5512 | 3 | 0.009
L2a1b1a | 5530 | 1 | 0.003
L2a1b1a | 5662 | 1 | 0.003
L2a1b1a | 573.1C | 2 | 0.006
L2a1b1a | 5814 | 2 | 0.006
L2a1b1a | 5899.1C | 2 | 0.006
L2a1b1a | 5981 | 2 | 0.006
L2a1b1a | 6233 | 1 | 0.003
L2a1b1a | 6240G | 1 | 0.003
L2a1b1a | 6267 | 1 | 0.003
L2a1b1a | 6521 | 2 | 0.006
L2a1b1a | 6608 | 1 | 0.003
L2a1b1a | 6707 | 1 | 0.003
L2a1b1a | 6734 | 1 | 0.003
L2a1b1a | 6893 | 1 | 0.003

L2a1b1a | 6960 | 1 | 0.003
L2a1b1a | 7245 | 1 | 0.003
L2a1b1a | 7511 | 1 | 0.003
L2a1b1a | 7515T | 1 | 0.003
L2a1b1a | 7578 | 1 | 0.003
L2a1b1a | 7663 | 3 | 0.009
L2a1b1a | 7705 | 1 | 0.003
L2a1b1a | 8077 | 1 | 0.003
L2a1b1a | 824 | 1 | 0.003
L2a1b1a | 8270 | 1 | 0.003
L2a1b1a | 8280 | 4 | 0.012
L2a1b1a | 8674 | 1 | 0.003
L2a1b1a | 8867 | 1 | 0.003
L2a1b1a | 9055 | 4 | 0.012
L2a1b1a | 9163 | 1 | 0.003
L2a1b1a | 9548C | 1 | 0.003
L2a1b1a | 9604 | 7 | 0.021
L2a1b1a | 9754 | 1 | 0.003
L2a1b1a | 9891A | 1 | 0.003
L2a1b2 | 14034 | 1 | 0.008
L2a1b2 | 16081 | 1 | 0.008
L2a1b2 | 16095 | 2 | 0.015
L2a1b2 | 16168 | 1 | 0.008
L2a1b2 | 16191 | 1 | 0.008
L2a1b2 | 16193d | 1 | 0.008
L2a1b2 | 16234 | 2 | 0.015
L2a1b2 | 16270 | 1 | 0.008
L2a1b2 | 16274 | 1 | 0.008
L2a1b2 | 16352 | 1 | 0.008
L2a1b2 | 183 | 2 | 0.015
L2a1b2 | 215 | 2 | 0.015
L2a1b2 | 260 | 1 | 0.008
L2a1b2 | 310 | 1 | 0.008
L2a1b2 | 9653 | 1 | 0.008
L2a1b2 | 9921T | 1 | 0.008
L2a1b3 | 16111A | 1 | 0.021
L2a1b3 | 16169 | 1 | 0.021
L2a1b3 | 16172 | 3 | 0.062
L2a1b3 | 16191 | 1 | 0.021
L2a1b3 | 16193d | 1 | 0.021
L2a1b3 | 16248 | 1 | 0.021
L2a1b3 | 16252C | 1 | 0.021
L2a1b3 | 16257 | 1 | 0.021
L2a1b3 | 16297 | 1 | 0.021
L2a1b3 | 16301 | 1 | 0.021
L2a1b3 | 16317T | 1 | 0.021
L2a1b3 | 16342 | 1 | 0.021
L2a1b3 | 16355 | 1 | 0.021
L2a1b3 | 188T | 1 | 0.021
L2a1b3 | 198 | 1 | 0.021
L2a1b3 | 3866 | 1 | 0.021
L2a1b3 | 5192 | 1 | 0.021
L2a1b3 | 5417 | 1 | 0.021
L2a1b3 | 549 | 2 | 0.042
L2a1c | 11020 | 1 | 0.014
L2a1c | 11197 | 1 | 0.014
L2a1c | 11299 | 1 | 0.014
L2a1c | 11404 | 1 | 0.014
L2a1c | 11614 | 1 | 0.014
L2a1c | 1183 | 1 | 0.014
L2a1c | 11887 | 1 | 0.014
L2a1c | 12727 | 1 | 0.014
L2a1c | 12941 | 1 | 0.014
L2a1c | 13105 | 1 | 0.014
L2a1c | 13488 | 1 | 0.014
L2a1c | 13708 | 2 | 0.027
L2a1c | 13933 | 1 | 0.014
L2a1c | 14002 | 1 | 0.014
L2a1c | 14764 | 1 | 0.014
L2a1c | 15297 | 1 | 0.014
L2a1c | 15317 | 1 | 0.014
L2a1c | 15476A | 1 | 0.014
L2a1c | 15721 | 1 | 0.014
L2a1c | 15884 | 1 | 0.014
L2a1c | 16071 | 2 | 0.027
L2a1c | 16140 | 2 | 0.027
L2a1c | 16259 | 2 | 0.027
L2a1c | 16293 | 2 | 0.027
L2a1c | 16399 | 2 | 0.027
L2a1c | 16497 | 1 | 0.014
L2a1c | 1719 | 1 | 0.014
L2a1c | 200 | 4 | 0.054
L2a1c | 236 | 1 | 0.014
L2a1c | 290-291d | 1 | 0.014
L2a1c | 309d | 1 | 0.014
L2a1c | 310 | 1 | 0.014
L2a1c | 325 | 1 | 0.014
L2a1c | 3398 | 1 | 0.014
L2a1c | 3693 | 1 | 0.014
L2a1c | 3852 | 1 | 0.014
L2a1c | 4435 | 1 | 0.014
L2a1c | 4561 | 1 | 0.014
L2a1c | 4722 | 1 | 0.014
L2a1c | 482 | 1 | 0.014
L2a1c | 5138 | 1 | 0.014
L2a1c | 5154A | 1 | 0.014
L2a1c | 5411 | 1 | 0.014
L2a1c | 5628 | 1 | 0.014
L2a1c | 573.3C | 1 | 0.014
L2a1c | 5774 | 1 | 0.014
L2a1c | 5899.1C | 1 | 0.014
L2a1c | 5912 | 1 | 0.014
L2a1c | 6044 | 1 | 0.014
L2a1c | 6164 | 1 | 0.014
L2a1c | 6267 | 1 | 0.014
L2a1c | 6359 | 1 | 0.014
L2a1c | 6464 | 1 | 0.014
L2a1c | 7679 | 1 | 0.014
L2a1c | 8065 | 1 | 0.014
L2a1c | 8155 | 1 | 0.014
L2a1c | 8281-8289d | 1 | 0.014

L2a1c | 9320 | 1 | 0.014
L2a1c | 9947 | 1 | 0.014
L2a1c+16086 | 10481 | 1 | 0.05
L2a1c+16086 | 14142 | 1 | 0.05
L2a1c+16086 | 15607 | 1 | 0.05
L2a1c+16086 | 15940 | 1 | 0.05
L2a1c+16086 | 16092 | 1 | 0.05
L2a1c+16086 | 16124 | 1 | 0.05
L2a1c+16086 | 16189 | 2 | 0.1
L2a1c+16086 | 16193 | 1 | 0.05
L2a1c+16086 | 16212 | 1 | 0.05
L2a1c+16086 | 16264 | 1 | 0.05
L2a1c+16086 | 16274 | 1 | 0.05
L2a1c+16086 | 16288 | 1 | 0.05
L2a1c+16086 | 16360 | 1 | 0.05
L2a1c+16086 | 2755 | 1 | 0.05
L2a1c+16086 | 309d | 1 | 0.05
L2a1c+16086 | 4646 | 1 | 0.05
L2a1c+16086 | 515-524d | 5 | 0.25
L2a1c+16086 | 534 | 1 | 0.05
L2a1c+16086 | 596 | 1 | 0.05
L2a1c+16086 | 8270 | 1 | 0.05
L2a1c+16086 | 9401 | 1 | 0.05
L2a1c+16086 | 9596 | 1 | 0.05
L2a1c+16086 | 9656 | 1 | 0.05
L2a1c+16129 | 10364 | 1 | 0.014
L2a1c+16129 | 11087 | 1 | 0.014
L2a1c+16129 | 11167 | 3 | 0.043
L2a1c+16129 | 12007 | 1 | 0.014
L2a1c+16129 | 14016 | 1 | 0.014
L2a1c+16129 | 14769 | 1 | 0.014
L2a1c+16129 | 15016 | 1 | 0.014
L2a1c+16129 | 16086 | 1 | 0.014
L2a1c+16129 | 16148 | 1 | 0.014
L2a1c+16129 | 16192 | 6 | 0.087
L2a1c+16129 | 16218 | 1 | 0.014
L2a1c+16129 | 16239 | 1 | 0.014
L2a1c+16129 | 16242A | 1 | 0.014
L2a1c+16129 | 16289 | 1 | 0.014
L2a1c+16129 | 16380 | 1 | 0.014
L2a1c+16129 | 198 | 4 | 0.058
L2a1c+16129 | 207 | 20 | 0.29
L2a1c+16129 | 253 | 1 | 0.014
L2a1c+16129 | 309d | 1 | 0.014
L2a1c+16129 | 3196 | 1 | 0.014
L2a1c+16129 | 4014 | 1 | 0.014
L2a1c+16129 | 4452 | 1 | 0.014
L2a1c+16129 | 5147 | 3 | 0.043
L2a1c+16129 | 515-524d | 1 | 0.014
L2a1c+16129 | 5899.1C | 1 | 0.014
L2a1c+16129 | 606 | 1 | 0.014
L2a1c+16129 | 6260 | 1 | 0.014
L2a1c+16129 | 710 | 1 | 0.014
L2a1c+16129 | 8065 | 1 | 0.014
L2a1c+16129 | 8222 | 2 | 0.029
L2a1c+16129 | 8246T | 1 | 0.014
L2a1c+16129 | 8772 | 3 | 0.043
L2a1c+16129 | 9461 | 3 | 0.043
L2a1c+16129 | 9575 | 1 | 0.014
L2a1c1 | 11708 | 3 | 0.12
L2a1c1 | 12189 | 3 | 0.12
L2a1c1 | 12361 | 3 | 0.12
L2a1c1 | 12406 | 3 | 0.12
L2a1c1 | 14001 | 1 | 0.04
L2a1c1 | 14790 | 1 | 0.04
L2a1c1 | 150 | 1 | 0.04
L2a1c1 | 15001 | 3 | 0.12
L2a1c1 | 15064 | 1 | 0.04
L2a1c1 | 15595A | 3 | 0.12
L2a1c1 | 16051 | 1 | 0.04
L2a1c1 | 16126 | 1 | 0.04
L2a1c1 | 16179 | 1 | 0.04
L2a1c1 | 189 | 1 | 0.04
L2a1c1 | 309d | 2 | 0.08
L2a1c1 | 310 | 2 | 0.08
L2a1c1 | 317.1C | 1 | 0.04
L2a1c1 | 3535 | 3 | 0.12
L2a1c1 | 4080 | 1 | 0.04
L2a1c1 | 5006 | 1 | 0.04
L2a1c1 | 5471 | 1 | 0.04
L2a1c1 | 5567 | 1 | 0.04
L2a1c1 | 6173 | 4 | 0.16
L2a1c1 | 7349 | 1 | 0.04
L2a1c1 | 7702 | 1 | 0.04
L2a1c1 | 8020 | 1 | 0.04
L2a1c1 | 9608 | 1 | 0.04
L2a1c1a | 16153 | 1 | 0.062
L2a1c1a | 16192 | 1 | 0.062
L2a1c1a | 16256 | 1 | 0.062
L2a1c1a | 16362 | 1 | 0.062
L2a1c1a | 199 | 2 | 0.125
L2a1c1a | 247 | 1 | 0.062
L2a1c1a | 249d | 2 | 0.125
L2a1c1a | 411A | 1 | 0.062
L2a1c1a | 438.1C | 1 | 0.062
L2a1c1a | 453 | 1 | 0.062
L2a1c1a1 | 10088 | 1 | 0.059
L2a1c1a1 | 12172 | 1 | 0.059
L2a1c1a1 | 14173 | 2 | 0.118
L2a1c1a1 | 14258 | 1 | 0.059
L2a1c1a1 | 15451 | 2 | 0.118
L2a1c1a1 | 16189 | 3 | 0.176
L2a1c1a1 | 16526 | 1 | 0.059
L2a1c1a1 | 1656d | 1 | 0.059
L2a1c1a1 | 2242 | 1 | 0.059
L2a1c1a1 | 317.1C | 1 | 0.059
L2a1c1a1 | 3936A | 2 | 0.118
L2a1c1a1 | 4052 | 1 | 0.059
L2a1c1a1 | 636 | 1 | 0.059
L2a1c1a1 | 7702 | 2 | 0.118
L2a1c1a1 | 8119 | 2 | 0.118

L2a1c1a1 | 8584 | 1 | 0.059
L2a1c1a2 | 11137 | 2 | 0.143
L2a1c1a2 | 14180 | 3 | 0.214
L2a1c1a2 | 14263 | 3 | 0.214
L2a1c1a2 | 8188 | 2 | 0.143
L2a1c1a2 | 9629 | 3 | 0.214
L2a1c2 | 11455 | 1 | 0.033
L2a1c2 | 11701 | 1 | 0.033
L2a1c2 | 13635 | 1 | 0.033
L2a1c2 | 14272 | 1 | 0.033
L2a1c2 | 15777 | 2 | 0.067
L2a1c2 | 16055A | 1 | 0.033
L2a1c2 | 16056A | 1 | 0.033
L2a1c2 | 16057A | 1 | 0.033
L2a1c2 | 16059.1A | 1 | 0.033
L2a1c2 | 16093 | 5 | 0.167
L2a1c2 | 16145 | 2 | 0.067
L2a1c2 | 16209 | 1 | 0.033
L2a1c2 | 16235 | 1 | 0.033
L2a1c2 | 16239 | 2 | 0.067
L2a1c2 | 16239d | 1 | 0.033
L2a1c2 | 16249A | 1 | 0.033
L2a1c2 | 16261 | 1 | 0.033
L2a1c2 | 16291 | 1 | 0.033
L2a1c2 | 16320 | 1 | 0.033
L2a1c2 | 171T | 1 | 0.033
L2a1c2 | 189 | 1 | 0.033
L2a1c2 | 198 | 1 | 0.033
L2a1c2 | 206G | 2 | 0.067
L2a1c2 | 310 | 4 | 0.133
L2a1c2 | 366 | 1 | 0.033
L2a1c2 | 3768 | 1 | 0.033
L2a1c2 | 3826 | 1 | 0.033
L2a1c2 | 389 | 1 | 0.033
L2a1c2 | 474A | 1 | 0.033
L2a1c2 | 507A | 1 | 0.033
L2a1c2 | 513-515d | 1 | 0.033
L2a1c2 | 513-522d | 3 | 0.1
L2a1c2 | 515-524d | 4 | 0.133
L2a1c2 | 5387 | 2 | 0.067
L2a1c2 | 5493 | 1 | 0.033
L2a1c2 | 564 | 1 | 0.033
L2a1c2 | 6113 | 1 | 0.033
L2a1c2 | 64 | 1 | 0.033
L2a1c2 | 644 | 5 | 0.167
L2a1c2 | 648 | 1 | 0.033
L2a1c2 | 6836 | 1 | 0.033
L2a1c2 | 6852 | 1 | 0.033
L2a1c2 | 7299 | 5 | 0.167
L2a1c2 | 7316 | 1 | 0.033
L2a1c2 | 8155 | 1 | 0.033
L2a1c2 | 8659 | 3 | 0.1
L2a1c2 | 8725 | 1 | 0.033
L2a1c2 | 8938 | 1 | 0.033
L2a1c2 | 9438 | 3 | 0.1
L2a1c2 | 9812A | 3 | 0.1
L2a1c2a | 10385 | 1 | 0.026
L2a1c2a | 12236 | 1 | 0.026
L2a1c2a | 12612 | 1 | 0.026
L2a1c2a | 12804 | 1 | 0.026
L2a1c2a | 13105 | 1 | 0.026
L2a1c2a | 13879 | 1 | 0.026
L2a1c2a | 14007 | 2 | 0.053
L2a1c2a | 14162 | 1 | 0.026
L2a1c2a | 14249 | 1 | 0.026
L2a1c2a | 14267 | 1 | 0.026
L2a1c2a | 14971 | 1 | 0.026
L2a1c2a | 151 | 1 | 0.026
L2a1c2a | 15203 | 1 | 0.026
L2a1c2a | 15496 | 1 | 0.026
L2a1c2a | 15901 | 1 | 0.026
L2a1c2a | 16124A | 1 | 0.026
L2a1c2a | 16203 | 1 | 0.026
L2a1c2a | 16244 | 1 | 0.026
L2a1c2a | 16265 | 1 | 0.026
L2a1c2a | 16301 | 1 | 0.026
L2a1c2a | 16319 | 1 | 0.026
L2a1c2a | 16327 | 1 | 0.026
L2a1c2a | 16362 | 1 | 0.026
L2a1c2a | 182 | 1 | 0.026
L2a1c2a | 189 | 1 | 0.026
L2a1c2a | 2083 | 2 | 0.053
L2a1c2a | 310 | 4 | 0.105
L2a1c2a | 316 | 1 | 0.026
L2a1c2a | 321 | 1 | 0.026
L2a1c2a | 385 | 1 | 0.026
L2a1c2a | 408A | 1 | 0.026
L2a1c2a | 4143 | 1 | 0.026
L2a1c2a | 5814A | 1 | 0.026
L2a1c2a | 6230A | 1 | 0.026
L2a1c2a | 6713 | 1 | 0.026
L2a1c2a | 6731 | 1 | 0.026
L2a1c2a | 678 | 1 | 0.026
L2a1c2a | 7642 | 1 | 0.026
L2a1c2a | 8419 | 1 | 0.026
L2a1c2a | 8733 | 1 | 0.026
L2a1c2a | 8944 | 1 | 0.026
L2a1c2a | 9128 | 1 | 0.026
L2a1c2a | 9163 | 1 | 0.026
L2a1c2a | 9968A | 2 | 0.053
L2a1c3 | 150 | 1 | 0.009
L2a1c3 | 15917 | 1 | 0.009
L2a1c3 | 15924 | 1 | 0.009
L2a1c3 | 16111A | 1 | 0.009
L2a1c3 | 16154 | 1 | 0.009
L2a1c3 | 16169 | 1 | 0.009
L2a1c3 | 16172 | 3 | 0.027
L2a1c3 | 16179 | 2 | 0.018
L2a1c3 | 16184 | 1 | 0.009
L2a1c3 | 16218G | 6 | 0.055
L2a1c3 | 16227 | 2 | 0.018

L2a1c3 | 16234 | 1 | 0.009
L2a1c3 | 16249 | 1 | 0.009
L2a1c3 | 16249A | 2 | 0.018
L2a1c3 | 16252C | 1 | 0.009
L2a1c3 | 16257 | 2 | 0.018
L2a1c3 | 16274 | 1 | 0.009
L2a1c3 | 16291 | 1 | 0.009
L2a1c3 | 16292 | 7 | 0.064
L2a1c3 | 16297 | 1 | 0.009
L2a1c3 | 16301 | 1 | 0.009
L2a1c3 | 16311 | 1 | 0.009
L2a1c3 | 16325 | 1 | 0.009
L2a1c3 | 16342 | 1 | 0.009
L2a1c3 | 16355 | 2 | 0.018
L2a1c3 | 16356 | 1 | 0.009
L2a1c3 | 16362 | 2 | 0.018
L2a1c3 | 16527 | 1 | 0.009
L2a1c3 | 16T | 1 | 0.009
L2a1c3 | 200 | 2 | 0.018
L2a1c3 | 204d | 1 | 0.009
L2a1c3 | 264 | 2 | 0.018
L2a1c3 | 324G | 1 | 0.009
L2a1c3 | 332A | 1 | 0.009
L2a1c3 | 345 | 1 | 0.009
L2a1c3 | 366 | 1 | 0.009
L2a1c3 | 388C | 2 | 0.018
L2a1c3 | 398 | 6 | 0.055
L2a1c3 | 452A | 1 | 0.009
L2a1c3 | 498 | 1 | 0.009
L2a1c3 | 507A | 1 | 0.009
L2a1c3 | 515-524d | 62 | 0.564
L2a1c3 | 529T | 4 | 0.036
L2a1c3 | 534 | 4 | 0.036
L2a1c3 | 548A | 3 | 0.027
L2a1c3 | 564 | 1 | 0.009
L2a1c3 | 573 | 1 | 0.009
L2a1c3 | 573.3C | 1 | 0.009
L2a1c3 | 63A | 1 | 0.009
L2a1c3 | 64 | 1 | 0.009
L2a1c3a | 14182 | 1 | 0.018
L2a1c3a | 15431 | 3 | 0.055
L2a1c3a | 16037 | 3 | 0.055
L2a1c3a | 16051 | 1 | 0.018
L2a1c3a | 16192 | 2 | 0.036
L2a1c3a | 16305 | 1 | 0.018
L2a1c3a | 16357 | 2 | 0.036
L2a1c3a | 237 | 1 | 0.018
L2a1c3a | 3966 | 1 | 0.018
L2a1c3a | 573.3C | 1 | 0.018
L2a1c3a | 6797 | 1 | 0.018
L2a1c3a | 7849 | 1 | 0.018
L2a1c3a | 8158 | 1 | 0.018
L2a1c3a1 | 11350 | 2 | 0.2
L2a1c3a1 | 13653 | 2 | 0.2
L2a1c3a1 | 14374 | 2 | 0.2
L2a1c3a1 | 15283 | 2 | 0.2
L2a1c3a1 | 15394 | 2 | 0.2
L2a1c3a1 | 16086 | 1 | 0.1
L2a1c3a1 | 204 | 1 | 0.1
L2a1c3a1 | 2702 | 2 | 0.2
L2a1c3a1 | 334 | 1 | 0.1
L2a1c3a1 | 8473 | 2 | 0.2
L2a1c3b | 10804 | 1 | 0.05
L2a1c3b | 12891 | 1 | 0.05
L2a1c3b | 12950 | 1 | 0.05
L2a1c3b | 12952 | 1 | 0.05
L2a1c3b | 14000A | 1 | 0.05
L2a1c3b | 1555 | 1 | 0.05
L2a1c3b | 16131 | 1 | 0.05
L2a1c3b | 16179 | 1 | 0.05
L2a1c3b | 16189 | 2 | 0.1
L2a1c3b | 16263 | 1 | 0.05
L2a1c3b | 16266 | 2 | 0.1
L2a1c3b | 16532d | 13 | 0.65
L2a1c3b | 16552 | 1 | 0.05
L2a1c3b | 214 | 1 | 0.05
L2a1c3b | 239 | 1 | 0.05
L2a1c3b | 2707 | 1 | 0.05
L2a1c3b | 310 | 1 | 0.05
L2a1c3b | 3394 | 1 | 0.05
L2a1c3b | 38-44d | 1 | 0.05
L2a1c3b | 455.1T | 1 | 0.05
L2a1c3b | 4688 | 1 | 0.05
L2a1c3b | 475C | 1 | 0.05
L2a1c3b | 479.1A | 1 | 0.05
L2a1c3b | 485A | 1 | 0.05
L2a1c3b | 486A | 1 | 0.05
L2a1c3b | 48A | 1 | 0.05
L2a1c3b | 515-524d | 1 | 0.05
L2a1c3b | 9096 | 1 | 0.05
L2a1c3b | 9545 | 1 | 0.05
L2a1c3b1 | 15924 | 1 | 0.059
L2a1c3b1 | 16093 | 3 | 0.176
L2a1c3b1 | 16148 | 15 | 0.882
L2a1c3b1 | 16150 | 12 | 0.706
L2a1c3b1 | 16309 | 1 | 0.059
L2a1c3b1 | 16496 | 1 | 0.059
L2a1c3b1 | 4206 | 9 | 0.529
L2a1c3b1 | 4883 | 3 | 0.176
L2a1c3b1 | 507A | 1 | 0.059
L2a1c3b1 | 515-524d | 1 | 0.059
L2a1c3b1 | 7762 | 9 | 0.529
L2a1c3b2 | 12079 | 1 | 0.111
L2a1c3b2 | 13827 | 1 | 0.111
L2a1c3b2 | 14180 | 1 | 0.111
L2a1c3b2 | 14259 | 1 | 0.111
L2a1c3b2 | 15496 | 2 | 0.222
L2a1c3b2 | 15519 | 1 | 0.111
L2a1c3b2 | 16086 | 1 | 0.111
L2a1c3b2 | 16111A | 1 | 0.111
L2a1c3b2 | 16169 | 1 | 0.111

L2a1c3b2 | 16172 | 1 | 0.111
L2a1c3b2 | 16293T | 1 | 0.111
L2a1c3b2 | 16356 | 1 | 0.111
L2a1c3b2 | 16399 | 1 | 0.111
L2a1c3b2 | 44.1C | 1 | 0.111
L2a1c3b2 | 489 | 1 | 0.111
L2a1c3b2 | 5201 | 1 | 0.111
L2a1c3b2 | 709 | 1 | 0.111
L2a1c3b2 | 7424 | 1 | 0.111
L2a1c3b2 | 7868A | 1 | 0.111
L2a1c3b2 | 9738 | 1 | 0.111
L2a1c4 | 13749 | 1 | 0.017
L2a1c4 | 15943 | 1 | 0.017
L2a1c4 | 16111 | 1 | 0.017
L2a1c4 | 16145 | 1 | 0.017
L2a1c4 | 16153 | 1 | 0.017
L2a1c4 | 16157 | 1 | 0.017
L2a1c4 | 16183 | 1 | 0.017
L2a1c4 | 16217 | 1 | 0.017
L2a1c4 | 16242 | 1 | 0.017
L2a1c4 | 16243 | 1 | 0.017
L2a1c4 | 16295 | 1 | 0.017
L2a1c4 | 183 | 1 | 0.017
L2a1c4 | 204 | 1 | 0.017
L2a1c4 | 228 | 2 | 0.034
L2a1c4 | 269.1C | 1 | 0.017
L2a1c4 | 316 | 1 | 0.017
L2a1c4 | 3699G | 1 | 0.017
L2a1c4 | 573.3C | 1 | 0.017
L2a1c4 | 573.5C | 1 | 0.017
L2a1c4 | 6299 | 1 | 0.017
L2a1c4 | 7436 | 1 | 0.017
L2a1c4a | 12192 | 1 | 0.017
L2a1c4a | 12311 | 1 | 0.017
L2a1c4a | 13617 | 1 | 0.017
L2a1c4a | 13948 | 1 | 0.017
L2a1c4a | 14912G | 1 | 0.017
L2a1c4a | 15061 | 1 | 0.017
L2a1c4a | 15154 | 1 | 0.017
L2a1c4a | 16274 | 1 | 0.017
L2a1c4a | 16365 | 1 | 0.017
L2a1c4a | 309d | 1 | 0.017
L2a1c4a | 310 | 2 | 0.033
L2a1c4a | 3948 | 1 | 0.017
L2a1c4a | 395.1T | 3 | 0.05
L2a1c4a | 4703 | 1 | 0.017
L2a1c4a | 514 | 1 | 0.017
L2a1c4a | 573.3C | 1 | 0.017
L2a1c4a | 7244 | 1 | 0.017
L2a1c4a | 7868 | 1 | 0.017
L2a1c4a | 813 | 1 | 0.017
L2a1c4a | 9218 | 1 | 0.017
L2a1c4a | 9266 | 1 | 0.017
L2a1c4a1 | 10208 | 1 | 0.018
L2a1c4a1 | 10700 | 1 | 0.018
L2a1c4a1 | 11708 | 1 | 0.018
L2a1c4a1 | 11923 | 1 | 0.018
L2a1c4a1 | 12007 | 2 | 0.035
L2a1c4a1 | 13818 | 1 | 0.018
L2a1c4a1 | 13952 | 1 | 0.018
L2a1c4a1 | 15790 | 1 | 0.018
L2a1c4a1 | 16284 | 1 | 0.018
L2a1c4a1 | 189 | 2 | 0.035
L2a1c4a1 | 309d | 2 | 0.035
L2a1c4a1 | 310 | 1 | 0.018
L2a1c4a1 | 4562 | 1 | 0.018
L2a1c4a1 | 573.3C | 1 | 0.018
L2a1c4a1 | 593 | 2 | 0.035
L2a1c4a1 | 6221 | 1 | 0.018
L2a1c4a1 | 9478 | 1 | 0.018
L2a1c4a1 | 9497 | 1 | 0.018
L2a1c4a1 | 9833 | 1 | 0.018
L2a1c5 | 10045 | 1 | 0.015
L2a1c5 | 11992 | 1 | 0.015
L2a1c5 | 12358 | 3 | 0.045
L2a1c5 | 12397 | 1 | 0.015
L2a1c5 | 12651 | 1 | 0.015
L2a1c5 | 13608 | 5 | 0.076
L2a1c5 | 13886 | 1 | 0.015
L2a1c5 | 15110 | 1 | 0.015
L2a1c5 | 16037 | 11 | 0.167
L2a1c5 | 16189 | 1 | 0.015
L2a1c5 | 16192 | 1 | 0.015
L2a1c5 | 16213 | 1 | 0.015
L2a1c5 | 1719 | 1 | 0.015
L2a1c5 | 1733 | 4 | 0.061
L2a1c5 | 236 | 1 | 0.015
L2a1c5 | 309d | 3 | 0.045
L2a1c5 | 317.1C | 1 | 0.015
L2a1c5 | 3334 | 1 | 0.015
L2a1c5 | 3834 | 1 | 0.015
L2a1c5 | 5263 | 1 | 0.015
L2a1c5 | 5747 | 1 | 0.015
L2a1c5 | 6713 | 1 | 0.015
L2a1c5 | 7853 | 2 | 0.03
L2a1c5 | 8289.1CCCCCTCTA | 1 | 0.015
L2a1c5 | 8538 | 1 | 0.015
L2a1c5 | 9887 | 1 | 0.015
L2a1c6 | 16239 | 5 | 0.625
L2a1c6 | 16265 | 1 | 0.125
L2a1c6 | 234 | 1 | 0.125
L2a1c6 | 9201 | 3 | 0.375
L2a1d | 16093 | 1 | 0.111
L2a1d | 16266A | 1 | 0.111
L2a1d | 16274 | 1 | 0.111
L2a1d | 16311 | 1 | 0.111
L2a1d | 16360 | 1 | 0.111
L2a1d | 16399 | 1 | 0.111
L2a1d1 | 10721 | 1 | 0.021
L2a1d1 | 10922 | 1 | 0.021
L2a1d1 | 12397 | 4 | 0.085

L2a1d1 | 13422 | 2 | 0.043
L2a1d1 | 14374 | 1 | 0.021
L2a1d1 | 15968 | 2 | 0.043
L2a1d1 | 16051 | 4 | 0.085
L2a1d1 | 16111 | 3 | 0.064
L2a1d1 | 16172 | 6 | 0.128
L2a1d1 | 16179 | 1 | 0.021
L2a1d1 | 16189 | 1 | 0.021
L2a1d1 | 16249A | 1 | 0.021
L2a1d1 | 16257 | 1 | 0.021
L2a1d1 | 16263 | 1 | 0.021
L2a1d1 | 16270 | 2 | 0.043
L2a1d1 | 16271 | 5 | 0.106
L2a1d1 | 16296 | 4 | 0.085
L2a1d1 | 16304 | 1 | 0.021
L2a1d1 | 16355 | 1 | 0.021
L2a1d1 | 16362 | 1 | 0.021
L2a1d1 | 16399 | 2 | 0.043
L2a1d1 | 16527 | 4 | 0.085
L2a1d1 | 1654 | 4 | 0.085
L2a1d1 | 185 | 3 | 0.064
L2a1d1 | 189 | 22 | 0.468
L2a1d1 | 207 | 1 | 0.021
L2a1d1 | 299d | 1 | 0.021
L2a1d1 | 303A | 1 | 0.021
L2a1d1 | 345 | 1 | 0.021
L2a1d1 | 366 | 1 | 0.021
L2a1d1 | 389 | 1 | 0.021
L2a1d1 | 429T | 1 | 0.021
L2a1d1 | 4491 | 2 | 0.043
L2a1d1 | 452A | 1 | 0.021
L2a1d1 | 452T | 1 | 0.021
L2a1d1 | 463 | 3 | 0.064
L2a1d1 | 4766 | 1 | 0.021
L2a1d1 | 5090 | 4 | 0.085
L2a1d1 | 515-524d | 19 | 0.404
L2a1d1 | 6480 | 1 | 0.021
L2a1d1 | 6635 | 2 | 0.043
L2a1d1 | 9377 | 2 | 0.043
L2a1d1 | 9380 | 2 | 0.043
L2a1d2 | 10790 | 1 | 0.028
L2a1d2 | 12007 | 1 | 0.028
L2a1d2 | 12091 | 1 | 0.028
L2a1d2 | 12297 | 1 | 0.028
L2a1d2 | 12696 | 1 | 0.028
L2a1d2 | 150 | 1 | 0.028
L2a1d2 | 151 | 1 | 0.028
L2a1d2 | 15596 | 28 | 0.778
L2a1d2 | 15629 | 1 | 0.028
L2a1d2 | 15927 | 1 | 0.028
L2a1d2 | 16129 | 29 | 0.806
L2a1d2 | 16192 | 1 | 0.028
L2a1d2 | 16218 | 1 | 0.028
L2a1d2 | 16264 | 1 | 0.028
L2a1d2 | 185C | 1 | 0.028
L2a1d2 | 2095 | 1 | 0.028
L2a1d2 | 2445 | 1 | 0.028
L2a1d2 | 2762 | 1 | 0.028
L2a1d2 | 291.1A | 1 | 0.028
L2a1d2 | 309d | 14 | 0.389
L2a1d2 | 310 | 7 | 0.194
L2a1d2 | 315.2C | 1 | 0.028
L2a1d2 | 3183 | 3 | 0.083
L2a1d2 | 3290 | 1 | 0.028
L2a1d2 | 4580 | 2 | 0.056
L2a1d2 | 4742 | 1 | 0.028
L2a1d2 | 4823 | 1 | 0.028
L2a1d2 | 5442 | 28 | 0.778
L2a1d2 | 5563 | 1 | 0.028
L2a1d2 | 5603 | 28 | 0.778
L2a1d2 | 6221 | 1 | 0.028
L2a1d2 | 6512 | 1 | 0.028
L2a1d2 | 745.1T | 6 | 0.167
L2a1d2 | 7741 | 26 | 0.722
L2a1d2 | 7749 | 1 | 0.028
L2a1d2 | 8251 | 1 | 0.028
L2a1d2 | 8254 | 1 | 0.028
L2a1d2 | 8280 | 1 | 0.028
L2a1d2 | 8531 | 1 | 0.028
L2a1d2 | 8681 | 1 | 0.028
L2a1d2 | 9180 | 1 | 0.028
L2a1d2 | 9338 | 1 | 0.028
L2a1d2 | 9932 | 1 | 0.028
L2a1e | 1007 | 1 | 0.018
L2a1e | 1282 | 2 | 0.036
L2a1e | 13810 | 2 | 0.036
L2a1e | 13980 | 1 | 0.018
L2a1e | 15601 | 1 | 0.018
L2a1e | 15758 | 1 | 0.018
L2a1e | 16051 | 4 | 0.073
L2a1e | 16484-16489d | 2 | 0.036
L2a1e | 198 | 1 | 0.018
L2a1e | 315.3C | 1 | 0.018
L2a1e | 573.3C | 1 | 0.018
L2a1e | 6431 | 1 | 0.018
L2a1e | 9230 | 1 | 0.018
L2a1e | 9833 | 1 | 0.018
L2a1e1 | 10604 | 2 | 0.02
L2a1e1 | 12173 | 1 | 0.01
L2a1e1 | 12603 | 2 | 0.02
L2a1e1 | 13194 | 2 | 0.02
L2a1e1 | 13644 | 2 | 0.02
L2a1e1 | 13827 | 1 | 0.01
L2a1e1 | 13953 | 1 | 0.01
L2a1e1 | 143 | 2 | 0.02
L2a1e1 | 15244 | 2 | 0.02
L2a1e1 | 15978 | 1 | 0.01
L2a1e1 | 16051 | 5 | 0.05
L2a1e1 | 16086 | 1 | 0.01
L2a1e1 | 16253 | 1 | 0.01
L2a1e1 | 16260 | 1 | 0.01

L2a1e1 | 16292 | 1 | 0.01
L2a1e1 | 16295 | 1 | 0.01
L2a1e1 | 16484-16489d | 2 | 0.02
L2a1e1 | 1709 | 1 | 0.01
L2a1e1 | 2442 | 2 | 0.02
L2a1e1 | 310 | 2 | 0.02
L2a1e1 | 315.2C | 2 | 0.02
L2a1e1 | 4216 | 1 | 0.01
L2a1e1 | 4316 | 1 | 0.01
L2a1e1 | 4655 | 2 | 0.02
L2a1e1 | 5231 | 1 | 0.01
L2a1e1 | 5237 | 4 | 0.04
L2a1e1 | 7424 | 1 | 0.01
L2a1e1 | 7808 | 2 | 0.02
L2a1e1 | 7978G | 1 | 0.01
L2a1e1 | 827 | 2 | 0.02
L2a1e1 | 9380 | 1 | 0.01
L2a1e1 | 9758 | 2 | 0.02
L2a1f | 1005 | 1 | 0.005
L2a1f | 10750 | 1 | 0.005
L2a1f | 10790 | 1 | 0.005
L2a1f | 10900 | 1 | 0.005
L2a1f | 10915 | 1 | 0.005
L2a1f | 11020 | 1 | 0.005
L2a1f | 11023 | 2 | 0.009
L2a1f | 11137 | 1 | 0.005
L2a1f | 11151 | 4 | 0.019
L2a1f | 11167 | 1 | 0.005
L2a1f | 1118 | 1 | 0.005
L2a1f | 1129 | 1 | 0.005
L2a1f | 11380 | 1 | 0.005
L2a1f | 11872 | 1 | 0.005
L2a1f | 11920 | 4 | 0.019
L2a1f | 12079 | 1 | 0.005
L2a1f | 12408 | 1 | 0.005
L2a1f | 12603 | 1 | 0.005
L2a1f | 12940 | 1 | 0.005
L2a1f | 13203 | 1 | 0.005
L2a1f | 13708 | 1 | 0.005
L2a1f | 13788A | 1 | 0.005
L2a1f | 13866 | 4 | 0.019
L2a1f | 13884 | 1 | 0.005
L2a1f | 14025 | 1 | 0.005
L2a1f | 14180 | 1 | 0.005
L2a1f | 14562 | 1 | 0.005
L2a1f | 15022A | 3 | 0.014
L2a1f | 15364 | 1 | 0.005
L2a1f | 15616 | 1 | 0.005
L2a1f | 15697 | 1 | 0.005
L2a1f | 15706 | 1 | 0.005
L2a1f | 15731 | 1 | 0.005
L2a1f | 15854G | 1 | 0.005
L2a1f | 16093 | 2 | 0.009
L2a1f | 16111G | 1 | 0.005
L2a1f | 16129 | 2 | 0.009
L2a1f | 16184 | 1 | 0.005
L2a1f | 16192 | 1 | 0.005
L2a1f | 16193 | 7 | 0.033
L2a1f | 16193d | 1 | 0.005
L2a1f | 16213 | 1 | 0.005
L2a1f | 16214 | 1 | 0.005
L2a1f | 16217 | 1 | 0.005
L2a1f | 16234 | 1 | 0.005
L2a1f | 16254 | 1 | 0.005
L2a1f | 16256 | 22 | 0.103
L2a1f | 16261 | 1 | 0.005
L2a1f | 16270 | 1 | 0.005
L2a1f | 16272 | 1 | 0.005
L2a1f | 16290 | 1 | 0.005
L2a1f | 16292 | 3 | 0.014
L2a1f | 16296 | 2 | 0.009
L2a1f | 16298 | 1 | 0.005
L2a1f | 16344 | 18 | 0.084
L2a1f | 1780 | 1 | 0.005
L2a1f | 198 | 1 | 0.005
L2a1f | 204 | 1 | 0.005
L2a1f | 207 | 1 | 0.005
L2a1f | 2220 | 1 | 0.005
L2a1f | 2244 | 1 | 0.005
L2a1f | 2483 | 1 | 0.005
L2a1f | 2760 | 2 | 0.009
L2a1f | 309d | 10 | 0.047
L2a1f | 310 | 10 | 0.047
L2a1f | 315.2C | 3 | 0.014
L2a1f | 317.1C | 2 | 0.009
L2a1f | 3308 | 2 | 0.009
L2a1f | 3396 | 1 | 0.005
L2a1f | 3397 | 1 | 0.005
L2a1f | 3666 | 1 | 0.005
L2a1f | 3744 | 1 | 0.005
L2a1f | 4363 | 1 | 0.005
L2a1f | 4435 | 1 | 0.005
L2a1f | 493 | 2 | 0.009
L2a1f | 513 | 1 | 0.005
L2a1f | 5417 | 1 | 0.005
L2a1f | 604T | 2 | 0.009
L2a1f | 6131 | 1 | 0.005
L2a1f | 6260 | 1 | 0.005
L2a1f | 6366 | 1 | 0.005
L2a1f | 723 | 1 | 0.005
L2a1f | 7400 | 1 | 0.005
L2a1f | 7419 | 2 | 0.009
L2a1f | 7516 | 1 | 0.005
L2a1f | 7630 | 1 | 0.005
L2a1f | 7702 | 1 | 0.005
L2a1f | 7740 | 1 | 0.005
L2a1f | 7837 | 1 | 0.005
L2a1f | 7853 | 1 | 0.005
L2a1f | 7854 | 1 | 0.005
L2a1f | 813 | 1 | 0.005
L2a1f | 8289.1CCCCCTCTACCCCCTCTA | 1 | 0.005

L2a1f | 8433 | 1 | 0.005
L2a1f | 8449 | 1 | 0.005
L2a1f | 8473 | 1 | 0.005
L2a1f | 8743 | 4 | 0.019
L2a1f | 8865 | 1 | 0.005
L2a1f | 9098 | 1 | 0.005
L2a1f | 9248 | 2 | 0.009
L2a1f | 930 | 1 | 0.005
L2a1f | 9337 | 1 | 0.005
L2a1f | 9456 | 1 | 0.005
L2a1f | 9632 | 1 | 0.005
L2a1f | 9932 | 1 | 0.005
L2a1f | 9986 | 4 | 0.019
L2a1f1 | 10685 | 1 | 0.007
L2a1f1 | 11204 | 3 | 0.022
L2a1f1 | 11305 | 1 | 0.007
L2a1f1 | 11536 | 3 | 0.022
L2a1f1 | 11903 | 1 | 0.007
L2a1f1 | 12753 | 7 | 0.051
L2a1f1 | 13146 | 1 | 0.007
L2a1f1 | 13420 | 1 | 0.007
L2a1f1 | 13677 | 3 | 0.022
L2a1f1 | 13759 | 1 | 0.007
L2a1f1 | 14002 | 3 | 0.022
L2a1f1 | 14040 | 1 | 0.007
L2a1f1 | 14434 | 2 | 0.014
L2a1f1 | 14869 | 3 | 0.022
L2a1f1 | 14927 | 2 | 0.014
L2a1f1 | 15497 | 1 | 0.007
L2a1f1 | 15749 | 4 | 0.029
L2a1f1 | 15903 | 1 | 0.007
L2a1f1 | 15944d | 1 | 0.007
L2a1f1 | 16145 | 2 | 0.014
L2a1f1 | 16193 | 1 | 0.007
L2a1f1 | 1836 | 3 | 0.022
L2a1f1 | 1957 | 1 | 0.007
L2a1f1 | 2169T | 1 | 0.007
L2a1f1 | 2649 | 3 | 0.022
L2a1f1 | 309d | 4 | 0.029
L2a1f1 | 310 | 1 | 0.007
L2a1f1 | 315.2C | 1 | 0.007
L2a1f1 | 3705 | 2 | 0.014
L2a1f1 | 5899.4C | 2 | 0.014
L2a1f1 | 6317 | 1 | 0.007
L2a1f1 | 6480 | 4 | 0.029
L2a1f1 | 7424 | 3 | 0.022
L2a1f1 | 8276.1C | 1 | 0.007
L2a1f1 | 8310 | 2 | 0.014
L2a1f1 | 8477 | 1 | 0.007
L2a1f1 | 9133 | 1 | 0.007
L2a1f1a | 13887 | 1 | 0.007
L2a1f1a | 15244 | 1 | 0.007
L2a1f1a | 15637 | 2 | 0.015
L2a1f1a | 16081 | 1 | 0.007
L2a1f1a | 16086 | 3 | 0.022
L2a1f1a | 16095 | 2 | 0.015
L2a1f1a | 16124 | 1 | 0.007
L2a1f1a | 16129 | 5 | 0.036
L2a1f1a | 16168 | 1 | 0.007
L2a1f1a | 16234 | 2 | 0.015
L2a1f1a | 16270 | 1 | 0.007
L2a1f1a | 16274 | 1 | 0.007
L2a1f1a | 16360 | 1 | 0.007
L2a1f1a | 183 | 2 | 0.015
L2a1f1a | 260 | 1 | 0.007
L2a1f1a | 291.1A | 2 | 0.015
L2a1f1a | 961 | 2 | 0.015
L2a1f1a | 965.1C | 1 | 0.007
L2a1f2 | 11252 | 3 | 0.018
L2a1f2 | 12022 | 3 | 0.018
L2a1f2 | 12346 | 3 | 0.018
L2a1f2 | 12732 | 1 | 0.006
L2a1f2 | 13888 | 1 | 0.006
L2a1f2 | 151 | 1 | 0.006
L2a1f2 | 16023C | 1 | 0.006
L2a1f2 | 16093 | 25 | 0.153
L2a1f2 | 16114 | 1 | 0.006
L2a1f2 | 16173 | 2 | 0.012
L2a1f2 | 16187 | 2 | 0.012
L2a1f2 | 16193 | 22 | 0.135
L2a1f2 | 16195G | 1 | 0.006
L2a1f2 | 16234A | 1 | 0.006
L2a1f2 | 16259 | 1 | 0.006
L2a1f2 | 16293 | 1 | 0.006
L2a1f2 | 16362 | 4 | 0.025
L2a1f2 | 16384 | 1 | 0.006
L2a1f2 | 16399 | 2 | 0.012
L2a1f2 | 16434 | 1 | 0.006
L2a1f2 | 204 | 6 | 0.037
L2a1f2 | 228 | 1 | 0.006
L2a1f2 | 260 | 1 | 0.006
L2a1f2 | 3589G | 1 | 0.006
L2a1f2 | 4322.1C | 1 | 0.006
L2a1f2 | 513 | 1 | 0.006
L2a1f2 | 5663 | 5 | 0.031
L2a1f2 | 7870 | 1 | 0.006
L2a1f2 | 8155 | 1 | 0.006
L2a1f2 | 9941 | 3 | 0.018
L2a1f3 | 10229 | 1 | 0.011
L2a1f3 | 10245 | 1 | 0.011
L2a1f3 | 11176 | 1 | 0.011
L2a1f3 | 11852 | 1 | 0.011
L2a1f3 | 13651 | 1 | 0.011
L2a1f3 | 13759 | 1 | 0.011
L2a1f3 | 13928C | 1 | 0.011
L2a1f3 | 143 | 1 | 0.011
L2a1f3 | 14544 | 1 | 0.011
L2a1f3 | 16093 | 15 | 0.167
L2a1f3 | 16150 | 1 | 0.011
L2a1f3 | 16184A | 1 | 0.011
L2a1f3 | 16187 | 3 | 0.033

L2a1f3 | 16231 | 2 | 0.022
L2a1f3 | 16232 | 1 | 0.011
L2a1f3 | 16258C | 2 | 0.022
L2a1f3 | 16262.1C | 1 | 0.011
L2a1f3 | 16297 | 1 | 0.011
L2a1f3 | 16304 | 2 | 0.022
L2a1f3 | 16325 | 1 | 0.011
L2a1f3 | 16497 | 1 | 0.011
L2a1f3 | 1719 | 3 | 0.033
L2a1f3 | 200 | 1 | 0.011
L2a1f3 | 2157A | 2 | 0.022
L2a1f3 | 252 | 1 | 0.011
L2a1f3 | 309.3C | 1 | 0.011
L2a1f3 | 309d | 4 | 0.044
L2a1f3 | 310 | 2 | 0.022
L2a1f3 | 3243 | 1 | 0.011
L2a1f3 | 390 | 1 | 0.011
L2a1f3 | 4083 | 1 | 0.011
L2a1f3 | 456 | 1 | 0.011
L2a1f3 | 5042 | 1 | 0.011
L2a1f3 | 545T | 1 | 0.011
L2a1f3 | 6413 | 1 | 0.011
L2a1f3 | 6710 | 1 | 0.011
L2a1f3 | 6722 | 1 | 0.011
L2a1f3 | 731 | 1 | 0.011
L2a1f3 | 8251 | 1 | 0.011
L2a1f3 | 9033 | 1 | 0.011
L2a1g | 16223d | 1 | 0.019
L2a1g | 16225.1T | 1 | 0.019
L2a1g | 16286G | 1 | 0.019
L2a1g | 16291 | 1 | 0.019
L2a1g | 309d | 2 | 0.038
L2a1g | 310 | 2 | 0.038
L2a1g | 315.2C | 1 | 0.019
L2a1g | 4277 | 2 | 0.038
L2a1g | 6023 | 2 | 0.038
L2a1g | 6040 | 5 | 0.096
L2a1g | 64 | 1 | 0.019
L2a1g | 8557 | 1 | 0.019
L2a1h | 10420 | 2 | 0.053
L2a1h | 11404 | 3 | 0.079
L2a1h | 1193 | 1 | 0.026
L2a1h | 13764 | 13 | 0.342
L2a1h | 13824 | 13 | 0.342
L2a1h | 14094 | 1 | 0.026
L2a1h | 15490 | 1 | 0.026
L2a1h | 16129 | 5 | 0.132
L2a1h | 16189 | 1 | 0.026
L2a1h | 16234 | 23 | 0.605
L2a1h | 16249 | 23 | 0.605
L2a1h | 16291 | 1 | 0.026
L2a1h | 16292 | 3 | 0.079
L2a1h | 16295 | 22 | 0.579
L2a1h | 309d | 1 | 0.026
L2a1h | 4703 | 10 | 0.263
L2a1h | 4958 | 1 | 0.026
L2a1h | 5312 | 1 | 0.026
L2a1h | 573.5C | 1 | 0.026
L2a1h | 573.6C | 1 | 0.026
L2a1h | 5822 | 1 | 0.026
L2a1h | 9948 | 2 | 0.053
L2a1i | 10538 | 1 | 0.015
L2a1i | 10891 | 1 | 0.015
L2a1i | 13674 | 1 | 0.015
L2a1i | 13716 | 1 | 0.015
L2a1i | 13759 | 1 | 0.015
L2a1i | 14569 | 1 | 0.015
L2a1i | 14978 | 1 | 0.015
L2a1i | 15011 | 1 | 0.015
L2a1i | 15812 | 1 | 0.015
L2a1i | 16093 | 4 | 0.061
L2a1i | 16114A | 3 | 0.045
L2a1i | 16126 | 2 | 0.03
L2a1i | 16129 | 4 | 0.061
L2a1i | 16145 | 1 | 0.015
L2a1i | 16171 | 1 | 0.015
L2a1i | 16183 | 1 | 0.015
L2a1i | 16187 | 1 | 0.015
L2a1i | 16188 | 2 | 0.03
L2a1i | 16193 | 11 | 0.167
L2a1i | 16203 | 3 | 0.045
L2a1i | 16213 | 2 | 0.03
L2a1i | 16214 | 1 | 0.015
L2a1i | 16290 | 1 | 0.015
L2a1i | 16291 | 1 | 0.015
L2a1i | 16292 | 1 | 0.015
L2a1i | 16309 | 1 | 0.015
L2a1i | 16311 | 2 | 0.03
L2a1i | 236 | 1 | 0.015
L2a1i | 310 | 1 | 0.015
L2a1i | 317.1C | 1 | 0.015
L2a1i | 3456 | 1 | 0.015
L2a1i | 4853 | 1 | 0.015
L2a1i | 520 | 1 | 0.015
L2a1i | 9150 | 1 | 0.015
L2a1i | 9192 | 1 | 0.015
L2a1i1 | 13708 | 2 | 0.047
L2a1i1 | 14311 | 1 | 0.023
L2a1i1 | 150 | 1 | 0.023
L2a1i1 | 15663 | 1 | 0.023
L2a1i1 | 16162 | 1 | 0.023
L2a1i1 | 16193 | 1 | 0.023
L2a1i1 | 16234 | 1 | 0.023
L2a1i1 | 309d | 4 | 0.093
L2a1i1 | 315.2C | 1 | 0.023
L2a1i1 | 8987 | 2 | 0.047
L2a1i1 | 9903 | 2 | 0.047
L2a1j | 11386 | 1 | 0.014
L2a1j | 12519 | 1 | 0.014
L2a1j | 16024 | 3 | 0.041
L2a1j | 16024A | 1 | 0.014

L2a1j | 16025 | 2 | 0.027
L2a1j | 16025A | 3 | 0.041
L2a1j | 16025d | 1 | 0.014
L2a1j | 16029.1T | 1 | 0.014
L2a1j | 16093 | 1 | 0.014
L2a1j | 16149C | 1 | 0.014
L2a1j | 16192 | 2 | 0.027
L2a1j | 16225 | 1 | 0.014
L2a1j | 16230T | 1 | 0.014
L2a1j | 16232 | 3 | 0.041
L2a1j | 16239 | 1 | 0.014
L2a1j | 16239A | 3 | 0.041
L2a1j | 16245G | 1 | 0.014
L2a1j | 16248 | 1 | 0.014
L2a1j | 16249 | 1 | 0.014
L2a1j | 16279 | 1 | 0.014
L2a1j | 16311 | 1 | 0.014
L2a1j | 16311G | 1 | 0.014
L2a1j | 16330G | 1 | 0.014
L2a1j | 16358 | 3 | 0.041
L2a1j | 16361C | 1 | 0.014
L2a1j | 16384 | 3 | 0.041
L2a1j | 200 | 1 | 0.014
L2a1j | 224G | 1 | 0.014
L2a1j | 226G | 2 | 0.027
L2a1j | 244 | 1 | 0.014
L2a1j | 2863 | 1 | 0.014
L2a1j | 518 | 10 | 0.135
L2a1j | 525d | 2 | 0.027
L2a1j | 66C | 1 | 0.014
L2a1j | 81 | 1 | 0.014
L2a1j | 8842 | 1 | 0.014
L2a1k | 11204 | 1 | 0.111
L2a1k | 14256 | 1 | 0.111
L2a1k | 16172 | 1 | 0.111
L2a1k | 16247 | 3 | 0.333
L2a1k | 16357 | 1 | 0.111
L2a1k | 489 | 1 | 0.111
L2a1k | 6116 | 3 | 0.333
L2a1k | 714 | 2 | 0.222
L2a1k | 7756 | 1 | 0.111
L2a1k | 93 | 1 | 0.111
L2a1k | 95C | 1 | 0.111
L2a1l | 16111G | 1 | 0.017
L2a1l | 16167 | 1 | 0.017
L2a1l | 16259A | 1 | 0.017
L2a1l | 16292 | 2 | 0.034
L2a1l | 16354 | 1 | 0.017
L2a1l | 16356 | 1 | 0.017
L2a1l | 204 | 1 | 0.017
L2a1l | 247 | 1 | 0.017
L2a1l | 264 | 1 | 0.017
L2a1l | 507 | 1 | 0.017
L2a1l | 509 | 1 | 0.017
L2a1l1 | 12684 | 1 | 0.018
L2a1l1 | 16114 | 1 | 0.018
L2a1l1 | 16172 | 1 | 0.018
L2a1l1 | 16175C | 1 | 0.018
L2a1l1 | 16249 | 1 | 0.018
L2a1l1 | 16305T | 1 | 0.018
L2a1l1 | 16344 | 2 | 0.036
L2a1l1 | 16355 | 3 | 0.054
L2a1l1 | 198 | 4 | 0.071
L2a1l1 | 309d | 1 | 0.018
L2a1l1 | 310 | 1 | 0.018
L2a1l1 | 333 | 1 | 0.018
L2a1l1a | 10978 | 1 | 0.018
L2a1l1a | 12188 | 1 | 0.018
L2a1l1a | 13184 | 1 | 0.018
L2a1l1a | 14016 | 1 | 0.018
L2a1l1a | 14307 | 1 | 0.018
L2a1l1a | 15928 | 1 | 0.018
L2a1l1a | 16192 | 1 | 0.018
L2a1l1a | 16266 | 1 | 0.018
L2a1l1a | 1719 | 1 | 0.018
L2a1l1a | 198 | 1 | 0.018
L2a1l1a | 310 | 1 | 0.018
L2a1l1a | 3423 | 1 | 0.018
L2a1l1a | 9104 | 1 | 0.018
L2a1l1a | 9695 | 1 | 0.018
L2a1l1a1 | 14690 | 1 | 0.017
L2a1l1a1 | 16093 | 1 | 0.017
L2a1l1a1 | 16111G | 1 | 0.017
L2a1l1a1 | 16167 | 1 | 0.017
L2a1l1a1 | 16259A | 1 | 0.017
L2a1l1a1 | 16354 | 1 | 0.017
L2a1l1a1 | 16356 | 1 | 0.017
L2a1l1a1 | 247 | 1 | 0.017
L2a1l1a1 | 264 | 1 | 0.017
L2a1l1a1 | 310 | 1 | 0.017
L2a1l1a1 | 507 | 1 | 0.017
L2a1l1a1 | 509 | 1 | 0.017
L2a1l1a1 | 536 | 1 | 0.017
L2a1l1a1 | 606 | 1 | 0.017
L2a1l1a2 | 10227 | 1 | 0.017
L2a1l1a2 | 15111 | 1 | 0.017
L2a1l1a2 | 16111G | 1 | 0.017
L2a1l1a2 | 16167 | 1 | 0.017
L2a1l1a2 | 16259A | 1 | 0.017
L2a1l1a2 | 16354 | 1 | 0.017
L2a1l1a2 | 16356 | 1 | 0.017
L2a1l1a2 | 247 | 1 | 0.017
L2a1l1a2 | 264 | 1 | 0.017
L2a1l1a2 | 310 | 1 | 0.017
L2a1l1a2 | 507 | 1 | 0.017
L2a1l1a2 | 509 | 1 | 0.017
L2a1l1a2 | 7828 | 1 | 0.017
L2a1l1a2 | 8973 | 1 | 0.017
L2a1l1a2 | 8975 | 1 | 0.017
L2a1l1b | 11465 | 1 | 0.016
L2a1l1b | 12726 | 1 | 0.016

L2a1l1b | 153 | 1 | 0.016
L2a1l1b | 16093 | 3 | 0.048
L2a1l1b | 16111G | 1 | 0.016
L2a1l1b | 16167 | 1 | 0.016
L2a1l1b | 16172 | 1 | 0.016
L2a1l1b | 16193 | 1 | 0.016
L2a1l1b | 16214 | 1 | 0.016
L2a1l1b | 16218 | 1 | 0.016
L2a1l1b | 16249 | 2 | 0.032
L2a1l1b | 16259A | 1 | 0.016
L2a1l1b | 16261 | 2 | 0.032
L2a1l1b | 16266 | 2 | 0.032
L2a1l1b | 16270 | 1 | 0.016
L2a1l1b | 16291 | 1 | 0.016
L2a1l1b | 16293 | 2 | 0.032
L2a1l1b | 16354 | 1 | 0.016
L2a1l1b | 16356 | 1 | 0.016
L2a1l1b | 16362 | 1 | 0.016
L2a1l1b | 247 | 1 | 0.016
L2a1l1b | 264 | 1 | 0.016
L2a1l1b | 3591 | 1 | 0.016
L2a1l1b | 4038 | 1 | 0.016
L2a1l1b | 507 | 1 | 0.016
L2a1l1b | 509 | 1 | 0.016
L2a1l1b | 573.4C | 1 | 0.016
L2a1l1b | 6216 | 1 | 0.016
L2a1l1b | 7388 | 2 | 0.032
L2a1l2 | 10328 | 1 | 0.018
L2a1l2 | 14110 | 1 | 0.018
L2a1l2 | 14969 | 1 | 0.018
L2a1l2 | 16086 | 2 | 0.036
L2a1l2 | 16093 | 1 | 0.018
L2a1l2 | 16129 | 1 | 0.018
L2a1l2 | 16213 | 1 | 0.018
L2a1l2 | 16264 | 1 | 0.018
L2a1l2 | 16274 | 1 | 0.018
L2a1l2 | 16291 | 1 | 0.018
L2a1l2 | 16362 | 3 | 0.054
L2a1l2 | 309d | 1 | 0.018
L2a1l2 | 310 | 1 | 0.018
L2a1l2 | 5054 | 1 | 0.018
L2a1l2 | 6480 | 1 | 0.018
L2a1l2 | 8222 | 1 | 0.018
L2a1l2 | 9873 | 1 | 0.018
L2a1l2a | 10240 | 1 | 0.018
L2a1l2a | 12171 | 2 | 0.036
L2a1l2a | 12451 | 2 | 0.036
L2a1l2a | 14182 | 1 | 0.018
L2a1l2a | 16193.1TC | 1 | 0.018
L2a1l2a | 315.2C | 2 | 0.036
L2a1l2a | 3325A | 1 | 0.018
L2a1l2a | 7965 | 1 | 0.018
L2a1l2a | 8283 | 1 | 0.018
L2a1l2a | 8833 | 1 | 0.018
L2a1l2a1 | 14325 | 1 | 0.016
L2a1l2a1 | 151 | 1 | 0.016
L2a1l2a1 | 153 | 4 | 0.065
L2a1l2a1 | 16071 | 2 | 0.032
L2a1l2a1 | 16129 | 5 | 0.081
L2a1l2a1 | 16187 | 1 | 0.016
L2a1l2a1 | 16287 | 1 | 0.016
L2a1l2a1 | 16343C | 1 | 0.016
L2a1l2a1 | 316C | 1 | 0.016
L2a1l3 | 10042 | 1 | 0.111
L2a1l3 | 15760 | 1 | 0.111
L2a1l3 | 16093 | 1 | 0.111
L2a1l3 | 16129 | 1 | 0.111
L2a1l3 | 16193 | 1 | 0.111
L2a1l3 | 16209 | 1 | 0.111
L2a1l3 | 16311 | 1 | 0.111
L2a1l3 | 198 | 1 | 0.111
L2a1l3 | 199 | 1 | 0.111
L2a1l3 | 310 | 1 | 0.111
L2a1l3 | 4895 | 1 | 0.111
L2a1l3 | 573.2C | 1 | 0.111
L2a1l3 | 573.5C | 2 | 0.222
L2a1l3 | 6563 | 1 | 0.111
L2a1l3 | 7389 | 1 | 0.111
L2a1m | 11623 | 1 | 0.014
L2a1m | 11902 | 1 | 0.014
L2a1m | 16024 | 3 | 0.041
L2a1m | 16024A | 1 | 0.014
L2a1m | 16025 | 2 | 0.027
L2a1m | 16025A | 3 | 0.041
L2a1m | 16025d | 1 | 0.014
L2a1m | 16029.1T | 1 | 0.014
L2a1m | 16149C | 1 | 0.014
L2a1m | 16192 | 2 | 0.027
L2a1m | 16225 | 1 | 0.014
L2a1m | 16230T | 1 | 0.014
L2a1m | 16232 | 3 | 0.041
L2a1m | 16239 | 1 | 0.014
L2a1m | 16239A | 3 | 0.041
L2a1m | 16245G | 1 | 0.014
L2a1m | 16248 | 1 | 0.014
L2a1m | 16249 | 1 | 0.014
L2a1m | 16279 | 1 | 0.014
L2a1m | 16311 | 1 | 0.014
L2a1m | 16311G | 1 | 0.014
L2a1m | 16330G | 1 | 0.014
L2a1m | 16358 | 3 | 0.041
L2a1m | 16361C | 1 | 0.014
L2a1m | 16384 | 3 | 0.041
L2a1m | 200 | 1 | 0.014
L2a1m | 224G | 1 | 0.014
L2a1m | 226G | 2 | 0.027
L2a1m | 244 | 1 | 0.014
L2a1m | 310 | 3 | 0.041
L2a1m | 518 | 10 | 0.135
L2a1m | 525d | 2 | 0.027
L2a1m | 66C | 1 | 0.014

L2a1m | 6899 | 2 | 0.027
L2a1m | 81 | 1 | 0.014
L2a1m1 | 12961 | 1 | 0.014
L2a1m1 | 13926 | 1 | 0.014
L2a1m1 | 14757 | 1 | 0.014
L2a1m1 | 16024 | 3 | 0.041
L2a1m1 | 16024A | 1 | 0.014
L2a1m1 | 16025 | 2 | 0.027
L2a1m1 | 16025A | 3 | 0.041
L2a1m1 | 16025d | 1 | 0.014
L2a1m1 | 16029.1T | 1 | 0.014
L2a1m1 | 16111 | 1 | 0.014
L2a1m1 | 16149C | 1 | 0.014
L2a1m1 | 16192 | 2 | 0.027
L2a1m1 | 16225 | 1 | 0.014
L2a1m1 | 16230T | 1 | 0.014
L2a1m1 | 16232 | 3 | 0.041
L2a1m1 | 16239 | 1 | 0.014
L2a1m1 | 16239A | 3 | 0.041
L2a1m1 | 16245G | 1 | 0.014
L2a1m1 | 16248 | 1 | 0.014
L2a1m1 | 16249 | 1 | 0.014
L2a1m1 | 16279 | 1 | 0.014
L2a1m1 | 16311 | 1 | 0.014
L2a1m1 | 16311G | 1 | 0.014
L2a1m1 | 16330G | 1 | 0.014
L2a1m1 | 16358 | 3 | 0.041
L2a1m1 | 16361C | 1 | 0.014
L2a1m1 | 16384 | 3 | 0.041
L2a1m1 | 200 | 1 | 0.014
L2a1m1 | 224G | 1 | 0.014
L2a1m1 | 226G | 2 | 0.027
L2a1m1 | 244 | 1 | 0.014
L2a1m1 | 315.2C | 1 | 0.014
L2a1m1 | 518 | 10 | 0.135
L2a1m1 | 525d | 2 | 0.027
L2a1m1 | 573 | 2 | 0.027
L2a1m1 | 66C | 1 | 0.014
L2a1m1 | 7681A | 1 | 0.014
L2a1m1 | 81 | 1 | 0.014
L2a1m1 | 8269-8270d | 1 | 0.014
L2a1m1a | 11167 | 1 | 0.014
L2a1m1a | 114A | 1 | 0.014
L2a1m1a | 13191 | 1 | 0.014
L2a1m1a | 13326 | 2 | 0.029
L2a1m1a | 13528 | 1 | 0.014
L2a1m1a | 14371 | 1 | 0.014
L2a1m1a | 153 | 3 | 0.043
L2a1m1a | 16129 | 1 | 0.014
L2a1m1a | 16150 | 2 | 0.029
L2a1m1a | 16190 | 1 | 0.014
L2a1m1a | 16209 | 1 | 0.014
L2a1m1a | 16287 | 1 | 0.014
L2a1m1a | 199 | 1 | 0.014
L2a1m1a | 309d | 1 | 0.014
L2a1m1a | 3421 | 1 | 0.014
L2a1m1a | 6572 | 1 | 0.014
L2a1m1a | 9254 | 1 | 0.014
L2a1n | 12408 | 1 | 0.014
L2a1n | 13768 | 1 | 0.014
L2a1n | 14272G | 1 | 0.014
L2a1n | 14971 | 1 | 0.014
L2a1n | 15217 | 1 | 0.014
L2a1n | 15924 | 1 | 0.014
L2a1n | 16086 | 4 | 0.056
L2a1n | 16114 | 1 | 0.014
L2a1n | 16129 | 1 | 0.014
L2a1n | 16153 | 1 | 0.014
L2a1n | 16224 | 2 | 0.028
L2a1n | 16234 | 1 | 0.014
L2a1n | 16265 | 2 | 0.028
L2a1n | 16265C | 1 | 0.014
L2a1n | 16270 | 2 | 0.028
L2a1n | 16288 | 3 | 0.042
L2a1n | 189 | 1 | 0.014
L2a1n | 204 | 1 | 0.014
L2a1n | 228 | 1 | 0.014
L2a1n | 2571 | 1 | 0.014
L2a1n | 309.4C | 1 | 0.014
L2a1n | 310 | 1 | 0.014
L2a1n | 315 | 1 | 0.014
L2a1n | 315.5C | 1 | 0.014
L2a1n | 574 | 1 | 0.014
L2a1n | 7762 | 1 | 0.014
L2a1n | 8562 | 1 | 0.014
L2a1n | 9554 | 1 | 0.014
L2a1n | 9966 | 2 | 0.028
L2a1o | 10663 | 1 | 0.014
L2a1o | 13848 | 1 | 0.014
L2a1o | 15848 | 1 | 0.014
L2a1o | 15930 | 1 | 0.014
L2a1o | 16024 | 3 | 0.041
L2a1o | 16024A | 1 | 0.014
L2a1o | 16025 | 2 | 0.027
L2a1o | 16025A | 3 | 0.041
L2a1o | 16025d | 1 | 0.014
L2a1o | 16029.1T | 1 | 0.014
L2a1o | 16149C | 1 | 0.014
L2a1o | 16192 | 2 | 0.027
L2a1o | 16225 | 1 | 0.014
L2a1o | 16230T | 1 | 0.014
L2a1o | 16232 | 3 | 0.041
L2a1o | 16239 | 1 | 0.014
L2a1o | 16239A | 3 | 0.041
L2a1o | 16245G | 1 | 0.014
L2a1o | 16248 | 1 | 0.014
L2a1o | 16249 | 1 | 0.014
L2a1o | 16279 | 1 | 0.014
L2a1o | 16311 | 1 | 0.014
L2a1o | 16311G | 1 | 0.014
L2a1o | 16330G | 1 | 0.014

L2a1o | 16358 | 3 | 0.041
L2a1o | 16361C | 1 | 0.014
L2a1o | 16384 | 3 | 0.041
L2a1o | 200 | 1 | 0.014
L2a1o | 224G | 1 | 0.014
L2a1o | 226G | 2 | 0.027
L2a1o | 244 | 1 | 0.014
L2a1o | 2863 | 1 | 0.014
L2a1o | 310 | 1 | 0.014
L2a1o | 3372 | 2 | 0.027
L2a1o | 518 | 10 | 0.135
L2a1o | 525d | 2 | 0.027
L2a1o | 6260 | 1 | 0.014
L2a1o | 66C | 1 | 0.014
L2a1o | 711 | 1 | 0.014
L2a1o | 81 | 1 | 0.014
L2a1o | 9053 | 2 | 0.027
L2a1p | 13145 | 1 | 0.014
L2a1p | 14560 | 1 | 0.014
L2a1p | 14831 | 1 | 0.014
L2a1p | 16024 | 3 | 0.041
L2a1p | 16024A | 1 | 0.014
L2a1p | 16025 | 2 | 0.027
L2a1p | 16025A | 3 | 0.041
L2a1p | 16025d | 1 | 0.014
L2a1p | 16029.1T | 1 | 0.014
L2a1p | 16149C | 1 | 0.014
L2a1p | 16192 | 2 | 0.027
L2a1p | 16225 | 1 | 0.014
L2a1p | 16230T | 1 | 0.014
L2a1p | 16232 | 3 | 0.041
L2a1p | 16239 | 1 | 0.014
L2a1p | 16239A | 3 | 0.041
L2a1p | 16245G | 1 | 0.014
L2a1p | 16248 | 1 | 0.014
L2a1p | 16249 | 1 | 0.014
L2a1p | 16279 | 1 | 0.014
L2a1p | 16311 | 1 | 0.014
L2a1p | 16311G | 1 | 0.014
L2a1p | 16330G | 1 | 0.014
L2a1p | 16358 | 3 | 0.041
L2a1p | 16361C | 1 | 0.014
L2a1p | 16384 | 3 | 0.041
L2a1p | 200 | 1 | 0.014
L2a1p | 224G | 1 | 0.014
L2a1p | 226G | 2 | 0.027
L2a1p | 244 | 1 | 0.014
L2a1p | 310 | 1 | 0.014
L2a1p | 4464 | 1 | 0.014
L2a1p | 4767 | 1 | 0.014
L2a1p | 518 | 10 | 0.135
L2a1p | 525d | 2 | 0.027
L2a1p | 66C | 1 | 0.014
L2a1p | 7300 | 1 | 0.014
L2a1p | 81 | 1 | 0.014
L2a1p | 8869 | 2 | 0.027
L2a1q | 10337 | 1 | 0.125
L2a1q | 15164 | 1 | 0.125
L2a1q | 16209 | 1 | 0.125
L2a1q | 9932 | 1 | 0.125
L2a2 | 16187d | 1 | 0.111
L2a2 | 16188 | 2 | 0.222
L2a2 | 16191 | 1 | 0.111
L2a2 | 16193d | 1 | 0.111
L2a2 | 16261 | 1 | 0.111
L2a2 | 16527 | 1 | 0.111
L2a2 | 257 | 1 | 0.111
L2a2 | 385 | 1 | 0.111
L2a2 | 515-524d | 2 | 0.222
L2a2'3 | 16187d | 1 | 0.111
L2a2'3 | 16188 | 2 | 0.222
L2a2'3 | 16191 | 1 | 0.111
L2a2'3 | 16193d | 1 | 0.111
L2a2'3 | 16261 | 1 | 0.111
L2a2'3 | 16527 | 1 | 0.111
L2a2'3 | 257 | 1 | 0.111
L2a2'3 | 385 | 1 | 0.111
L2a2'3 | 515-524d | 2 | 0.222
L2a2'3'4 | 16067 | 2 | 0.4
L2a2'3'4 | 16086 | 2 | 0.4
L2a2'3'4 | 16169 | 2 | 0.4
L2a2'3'4 | 16192-16193d | 1 | 0.2
L2a2'3'4 | 16291 | 2 | 0.4
L2a2'3'4 | 318 | 1 | 0.2
L2a2'3'4 | 515-524d | 1 | 0.2
L2a2a | 10268 | 1 | 0.125
L2a2a | 11387 | 1 | 0.125
L2a2a | 11647 | 1 | 0.125
L2a2a | 12810 | 1 | 0.125
L2a2a | 15894 | 1 | 0.125
L2a2a | 16129 | 1 | 0.125
L2a2a | 16270 | 1 | 0.125
L2a2a | 194 | 1 | 0.125
L2a2a1 | 13443 | 5 | 0.357
L2a2a1 | 14560C | 1 | 0.071
L2a2a1 | 146 | 4 | 0.286
L2a2a1 | 15814 | 1 | 0.071
L2a2a1 | 16119 | 1 | 0.071
L2a2a1 | 16188 | 1 | 0.071
L2a2a1 | 16193d | 1 | 0.071
L2a2a1 | 16274 | 5 | 0.357
L2a2a1 | 262 | 1 | 0.071
L2a2a1 | 3834 | 4 | 0.286
L2a2a1 | 6359 | 1 | 0.071
L2a2a1 | 6656 | 2 | 0.143
L2a2a1 | 8964 | 1 | 0.071
L2a2a1 | 9053 | 1 | 0.071
L2a2a1 | 9299 | 5 | 0.357
L2a2a1 | 9716 | 1 | 0.071
L2a2b | 11506 | 1 | 0.111
L2a2b | 151 | 1 | 0.111

L2a2b | 16086 | 1 | 0.111
L2a2b | 16149C | 1 | 0.111
L2a2b | 16172 | 1 | 0.111
L2a2b | 16186 | 1 | 0.111
L2a2b | 16256 | 1 | 0.111
L2a2b | 16287 | 1 | 0.111
L2a2b | 16320 | 2 | 0.222
L2a2b | 16399 | 1 | 0.111
L2a2b | 2388 | 1 | 0.111
L2a2b | 257 | 1 | 0.111
L2a2b | 309d | 1 | 0.111
L2a2b | 507A | 1 | 0.111
L2a2b | 515-524d | 3 | 0.333
L2a2b | 7775 | 1 | 0.111
L2a2b | 8215 | 1 | 0.111
L2a2b | 9377 | 1 | 0.111
L2a2b1 | 13434 | 3 | 0.6
L2a2b1 | 13579 | 1 | 0.2
L2a2b1 | 16111 | 3 | 0.6
L2a2b1 | 4562 | 3 | 0.6
L2a2b1 | 5045 | 3 | 0.6
L2a2b1a | 10790 | 6 | 0.102
L2a2b1a | 12507 | 6 | 0.102
L2a2b1a | 16095G | 1 | 0.017
L2a2b1a | 16096C | 1 | 0.017
L2a2b1a | 16186 | 1 | 0.017
L2a2b1a | 16193d | 3 | 0.051
L2a2b1a | 1719 | 4 | 0.068
L2a2b1a | 204 | 1 | 0.017
L2a2b1a | 207C | 1 | 0.017
L2a2b1a | 3010 | 1 | 0.017
L2a2b1a | 5563 | 5 | 0.085
L2a2b1a | 7076 | 4 | 0.068
L2a2b1a | 8872 | 4 | 0.068
L2a2b2 | 15530 | 1 | 0.111
L2a2b2 | 16192-16193d | 2 | 0.222
L2a2b2 | 16274 | 1 | 0.111
L2a2b2 | 16368 | 1 | 0.111
L2a2b2 | 309.3C | 2 | 0.222
L2a2b2 | 3337 | 1 | 0.111
L2a2b2 | 6431 | 4 | 0.444
L2a3 | 16532d | 2 | 1
L2a4 | 41 | 1 | 0.333
L2a4a | 14180 | 1 | 0.071
L2a4a | 15449 | 2 | 0.143
L2a4a | 16093 | 1 | 0.071
L2a4a | 16319 | 2 | 0.143
L2a4a | 3398 | 5 | 0.357
L2a4a | 3615 | 1 | 0.071
L2a4a | 516 | 1 | 0.071
L2a4a | 5312A | 4 | 0.286
L2a4a | 6254 | 2 | 0.143
L2a4b | 11016 | 1 | 0.143
L2a4b | 13651 | 1 | 0.143
L2a4b | 15718 | 1 | 0.143
L2a4b | 15799 | 1 | 0.143
L2a4b | 16169 | 1 | 0.143
L2a4b | 16193d | 1 | 0.143
L2a4b | 16532d | 5 | 0.714
L2a4b | 199 | 1 | 0.143
L2a4b | 2352 | 1 | 0.143
L2a4b | 6227 | 1 | 0.143
L2a4b | 7702 | 1 | 0.143
L2a5 | 10463 | 4 | 0.083
L2a5 | 10589 | 1 | 0.021
L2a5 | 10659 | 1 | 0.021
L2a5 | 11009 | 10 | 0.208
L2a5 | 11353 | 6 | 0.125
L2a5 | 11815A | 5 | 0.104
L2a5 | 11908 | 1 | 0.021
L2a5 | 11A | 1 | 0.021
L2a5 | 12172 | 5 | 0.104
L2a5 | 12285G | 7 | 0.146
L2a5 | 12406 | 1 | 0.021
L2a5 | 12425 | 3 | 0.062
L2a5 | 12630 | 2 | 0.042
L2a5 | 12967 | 28 | 0.583
L2a5 | 12972 | 5 | 0.104
L2a5 | 13149 | 1 | 0.021
L2a5 | 13470 | 5 | 0.104
L2a5 | 13810 | 34 | 0.708
L2a5 | 13888 | 1 | 0.021
L2a5 | 13928C | 1 | 0.021
L2a5 | 14088 | 18 | 0.375
L2a5 | 14118 | 1 | 0.021
L2a5 | 14280 | 1 | 0.021
L2a5 | 143 | 1 | 0.021
L2a5 | 14314 | 6 | 0.125
L2a5 | 14356 | 1 | 0.021
L2a5 | 14368 | 1 | 0.021
L2a5 | 14410 | 1 | 0.021
L2a5 | 14557 | 6 | 0.125
L2a5 | 151 | 8 | 0.167
L2a5 | 151d | 1 | 0.021
L2a5 | 15632 | 1 | 0.021
L2a5 | 15736 | 2 | 0.042
L2a5 | 15758 | 7 | 0.146
L2a5 | 15796 | 6 | 0.125
L2a5 | 15862 | 2 | 0.042
L2a5 | 15883 | 1 | 0.021
L2a5 | 15916 | 1 | 0.021
L2a5 | 16038 | 1 | 0.021
L2a5 | 16051 | 1 | 0.021
L2a5 | 16066 | 7 | 0.146
L2a5 | 16092 | 1 | 0.021
L2a5 | 16093 | 2 | 0.042
L2a5 | 16148 | 2 | 0.042
L2a5 | 16189 | 23 | 0.479
L2a5 | 16192 | 1 | 0.021
L2a5 | 16192-16193d | 5 | 0.104
L2a5 | 16209 | 1 | 0.021

L2a5 | 16263 | 7 | 0.146
L2a5 | 16265C | 2 | 0.042
L2a5 | 16291 | 9 | 0.188
L2a5 | 16295 | 8 | 0.167
L2a5 | 16309 | 21 | 0.438
L2a5 | 16311 | 2 | 0.042
L2a5 | 16319 | 1 | 0.021
L2a5 | 16357 | 1 | 0.021
L2a5 | 16399 | 6 | 0.125
L2a5 | 16527 | 1 | 0.021
L2a5 | 16532d | 3 | 0.062
L2a5 | 16559 | 1 | 0.021
L2a5 | 1719 | 7 | 0.146
L2a5 | 1737 | 1 | 0.021
L2a5 | 183 | 19 | 0.396
L2a5 | 185C | 1 | 0.021
L2a5 | 186 | 7 | 0.146
L2a5 | 1900 | 10 | 0.208
L2a5 | 195 | 10 | 0.208
L2a5 | 198 | 1 | 0.021
L2a5 | 200 | 1 | 0.021
L2a5 | 215 | 3 | 0.062
L2a5 | 2345 | 1 | 0.021
L2a5 | 249d | 1 | 0.021
L2a5 | 292.1AT | 1 | 0.021
L2a5 | 309d | 19 | 0.396
L2a5 | 3277 | 1 | 0.021
L2a5 | 3321 | 2 | 0.042
L2a5 | 373 | 3 | 0.062
L2a5 | 3834 | 1 | 0.021
L2a5 | 4032 | 2 | 0.042
L2a5 | 4053T | 2 | 0.042
L2a5 | 4135 | 1 | 0.021
L2a5 | 4380 | 6 | 0.125
L2a5 | 4562 | 7 | 0.146
L2a5 | 4659 | 5 | 0.104
L2a5 | 466 | 4 | 0.083
L2a5 | 4823 | 34 | 0.708
L2a5 | 511 | 37 | 0.771
L2a5 | 513 | 4 | 0.083
L2a5 | 5183 | 5 | 0.104
L2a5 | 5205 | 5 | 0.104
L2a5 | 5246A | 18 | 0.375
L2a5 | 5316 | 3 | 0.062
L2a5 | 537 | 10 | 0.208
L2a5 | 5602 | 2 | 0.042
L2a5 | 573.2C | 1 | 0.021
L2a5 | 5899.1C | 1 | 0.021
L2a5 | 6080 | 18 | 0.375
L2a5 | 6083 | 5 | 0.104
L2a5 | 6164 | 1 | 0.021
L2a5 | 6248 | 18 | 0.375
L2a5 | 6366 | 3 | 0.062
L2a5 | 6647 | 6 | 0.125
L2a5 | 6893 | 7 | 0.146
L2a5 | 6915 | 1 | 0.021
L2a5 | 6918 | 7 | 0.146
L2a5 | 709 | 5 | 0.104
L2a5 | 742 | 2 | 0.042
L2a5 | 7543 | 1 | 0.021
L2a5 | 7715 | 1 | 0.021
L2a5 | 7961 | 1 | 0.021
L2a5 | 8014 | 7 | 0.146
L2a5 | 8027 | 1 | 0.021
L2a5 | 8041 | 6 | 0.125
L2a5 | 811 | 1 | 0.021
L2a5 | 8152 | 1 | 0.021
L2a5 | 8251 | 6 | 0.125
L2a5 | 8467 | 5 | 0.104
L2a5 | 870 | 1 | 0.021
L2a5 | 8723 | 18 | 0.375
L2a5 | 9380 | 1 | 0.021
L2a5 | 9530 | 1 | 0.021
L2a'b'c'd | 16177 | 2 | 0.286
L2a'b'c'd | 16192 | 3 | 0.429
L2a'b'c'd | 16255 | 1 | 0.143
L2a'b'c'd | 16261 | 3 | 0.429
L2a'b'c'd | 16311 | 2 | 0.286
L2b | 14384 | 1 | 0.125
L2b | 16266 | 1 | 0.125
L2b | 16311 | 1 | 0.125
L2b | 194 | 1 | 0.125
L2b | 257 | 2 | 0.25
L2b | 2626 | 1 | 0.125
L2b | 310 | 1 | 0.125
L2b | 515-524d | 2 | 0.25
L2b | 5747 | 1 | 0.125
L2b | 9438 | 1 | 0.125
L2b1 | 1007C | 1 | 0.143
L2b1 | 10685 | 1 | 0.143
L2b1 | 10724 | 1 | 0.143
L2b1 | 11204 | 1 | 0.143
L2b1 | 11809 | 1 | 0.143
L2b1 | 13629 | 1 | 0.143
L2b1 | 15014 | 1 | 0.143
L2b1 | 15777 | 1 | 0.143
L2b1 | 16148 | 2 | 0.286
L2b1 | 16189 | 1 | 0.143
L2b1 | 16217 | 1 | 0.143
L2b1 | 16354 | 1 | 0.143
L2b1 | 16355 | 2 | 0.286
L2b1 | 207 | 1 | 0.143
L2b1 | 260 | 2 | 0.286
L2b1 | 3417 | 1 | 0.143
L2b1 | 3777 | 1 | 0.143
L2b1 | 5046 | 1 | 0.143
L2b1 | 515-524d | 2 | 0.286
L2b1 | 5261 | 1 | 0.143
L2b1 | 7985G | 1 | 0.143
L2b1a | 1007C | 1 | 0.029
L2b1a | 11253 | 1 | 0.029

L2b1a | 11654 | 1 | 0.029
L2b1a | 11887 | 1 | 0.029
L2b1a | 12164T | 1 | 0.029
L2b1a | 12654 | 1 | 0.029
L2b1a | 12793 | 1 | 0.029
L2b1a | 13015 | 1 | 0.029
L2b1a | 13629 | 1 | 0.029
L2b1a | 13813 | 1 | 0.029
L2b1a | 13814 | 1 | 0.029
L2b1a | 14003 | 1 | 0.029
L2b1a | 14569 | 1 | 0.029
L2b1a | 16093 | 3 | 0.086
L2b1a | 16148 | 3 | 0.086
L2b1a | 16162 | 1 | 0.029
L2b1a | 16209 | 1 | 0.029
L2b1a | 16217 | 1 | 0.029
L2b1a | 16290 | 1 | 0.029
L2b1a | 16294 | 2 | 0.057
L2b1a | 16311 | 1 | 0.029
L2b1a | 16319 | 1 | 0.029
L2b1a | 16368 | 3 | 0.086
L2b1a | 207 | 2 | 0.057
L2b1a | 235 | 1 | 0.029
L2b1a | 247 | 1 | 0.029
L2b1a | 260 | 2 | 0.057
L2b1a | 310 | 1 | 0.029
L2b1a | 317.1C | 1 | 0.029
L2b1a | 3777 | 1 | 0.029
L2b1a | 385 | 1 | 0.029
L2b1a | 5231 | 1 | 0.029
L2b1a | 5563 | 1 | 0.029
L2b1a | 6131 | 1 | 0.029
L2b1a | 6185 | 1 | 0.029
L2b1a | 64 | 1 | 0.029
L2b1a | 7119 | 1 | 0.029
L2b1a | 7702 | 1 | 0.029
L2b1a | 801 | 1 | 0.029
L2b1a | 8419 | 1 | 0.029
L2b1a | 8939 | 1 | 0.029
L2b1a | 9663 | 1 | 0.029
L2b1a | 9935 | 1 | 0.029
L2b1a2 | 10907 | 5 | 0.111
L2b1a2 | 11020 | 1 | 0.022
L2b1a2 | 1118 | 1 | 0.022
L2b1a2 | 11377 | 1 | 0.022
L2b1a2 | 11836 | 1 | 0.022
L2b1a2 | 11903 | 1 | 0.022
L2b1a2 | 12007 | 1 | 0.022
L2b1a2 | 12557 | 4 | 0.089
L2b1a2 | 12895 | 2 | 0.044
L2b1a2 | 13635 | 5 | 0.111
L2b1a2 | 14180 | 1 | 0.022
L2b1a2 | 15883 | 1 | 0.022
L2b1a2 | 16093 | 1 | 0.022
L2b1a2 | 16294 | 2 | 0.044
L2b1a2 | 16295 | 1 | 0.022
L2b1a2 | 16356 | 1 | 0.022
L2b1a2 | 16368 | 7 | 0.156
L2b1a2 | 16465 | 1 | 0.022
L2b1a2 | 183 | 3 | 0.067
L2b1a2 | 2226 | 1 | 0.022
L2b1a2 | 228 | 8 | 0.178
L2b1a2 | 242 | 1 | 0.022
L2b1a2 | 249d | 2 | 0.044
L2b1a2 | 310 | 2 | 0.044
L2b1a2 | 3253 | 3 | 0.067
L2b1a2 | 590.1A | 1 | 0.022
L2b1a2 | 6197 | 1 | 0.022
L2b1a2 | 633 | 5 | 0.111
L2b1a2 | 6407 | 1 | 0.022
L2b1a2 | 6932 | 1 | 0.022
L2b1a2 | 8928 | 3 | 0.067
L2b1a2 | 9168 | 4 | 0.089
L2b1a2 | 9438 | 5 | 0.111
L2b1a2 | 9991 | 5 | 0.111
L2b1a3 | 11149 | 1 | 0.014
L2b1a3 | 11842 | 4 | 0.056
L2b1a3 | 13370 | 1 | 0.014
L2b1a3 | 13488 | 1 | 0.014
L2b1a3 | 13928C | 1 | 0.014
L2b1a3 | 13986 | 1 | 0.014
L2b1a3 | 1406 | 1 | 0.014
L2b1a3 | 14693 | 1 | 0.014
L2b1a3 | 151 | 4 | 0.056
L2b1a3 | 152d | 3 | 0.042
L2b1a3 | 15388 | 1 | 0.014
L2b1a3 | 15506 | 2 | 0.028
L2b1a3 | 15535 | 1 | 0.014
L2b1a3 | 15731 | 1 | 0.014
L2b1a3 | 15735 | 2 | 0.028
L2b1a3 | 16037 | 1 | 0.014
L2b1a3 | 16093 | 2 | 0.028
L2b1a3 | 16166 | 1 | 0.014
L2b1a3 | 16189 | 6 | 0.085
L2b1a3 | 16263d | 1 | 0.014
L2b1a3 | 16274 | 2 | 0.028
L2b1a3 | 16284 | 7 | 0.099
L2b1a3 | 16309 | 1 | 0.014
L2b1a3 | 16399 | 1 | 0.014
L2b1a3 | 1692 | 1 | 0.014
L2b1a3 | 1824 | 1 | 0.014
L2b1a3 | 186A | 1 | 0.014
L2b1a3 | 207 | 3 | 0.042
L2b1a3 | 227 | 1 | 0.014
L2b1a3 | 309d | 10 | 0.141
L2b1a3 | 4238 | 1 | 0.014
L2b1a3 | 4502 | 1 | 0.014
L2b1a3 | 4841 | 8 | 0.113
L2b1a3 | 492C | 1 | 0.014
L2b1a3 | 4947A | 1 | 0.014
L2b1a3 | 5567 | 1 | 0.014

L2b1a3 | 634 | 1 | 0.014
L2b1a3 | 6855 | 1 | 0.014
L2b1a3 | 8461 | 1 | 0.014
L2b1a3 | 8555 | 1 | 0.014
L2b1a3 | 9056 | 1 | 0.014
L2b1a4 | 10652 | 1 | 0.015
L2b1a4 | 13768 | 2 | 0.03
L2b1a4 | 15872 | 2 | 0.03
L2b1a4 | 16086 | 2 | 0.03
L2b1a4 | 16145 | 1 | 0.015
L2b1a4 | 16185 | 1 | 0.015
L2b1a4 | 16189 | 2 | 0.03
L2b1a4 | 16256 | 2 | 0.03
L2b1a4 | 16274 | 1 | 0.015
L2b1a4 | 16290 | 1 | 0.015
L2b1a4 | 16294 | 13 | 0.194
L2b1a4 | 16327 | 2 | 0.03
L2b1a4 | 16339 | 1 | 0.015
L2b1a4 | 16340T | 1 | 0.015
L2b1a4 | 16343T | 1 | 0.015
L2b1a4 | 16356 | 3 | 0.045
L2b1a4 | 16359 | 2 | 0.03
L2b1a4 | 16365 | 1 | 0.015
L2b1a4 | 16496 | 1 | 0.015
L2b1a4 | 16527 | 9 | 0.134
L2b1a4 | 189 | 1 | 0.015
L2b1a4 | 203 | 1 | 0.015
L2b1a4 | 207 | 4 | 0.06
L2b1a4 | 228 | 1 | 0.015
L2b1a4 | 242 | 3 | 0.045
L2b1a4 | 285 | 1 | 0.015
L2b1a4 | 292A | 4 | 0.06
L2b1a4 | 317.1C | 1 | 0.015
L2b1a4 | 4062 | 2 | 0.03
L2b1a4 | 456 | 9 | 0.134
L2b1a4 | 507 | 1 | 0.015
L2b1a4 | 507A | 1 | 0.015
L2b1a4 | 515-524d | 18 | 0.269
L2b1a4 | 529T | 1 | 0.015
L2b1a4 | 544 | 9 | 0.134
L2b1a4 | 5483 | 1 | 0.015
L2b1a4 | 573.1C | 1 | 0.015
L2b1a4 | 6524 | 1 | 0.015
L2b1a4 | 93 | 1 | 0.015
L2b1a4 | 9986 | 1 | 0.015
L2b1b | 10586C | 2 | 0.049
L2b1b | 11151 | 1 | 0.024
L2b1b | 12732 | 1 | 0.024
L2b1b | 14305 | 1 | 0.024
L2b1b | 16075 | 1 | 0.024
L2b1b | 16153 | 16 | 0.39
L2b1b | 16176 | 1 | 0.024
L2b1b | 16209 | 1 | 0.024
L2b1b | 16256 | 2 | 0.049
L2b1b | 16274 | 3 | 0.073
L2b1b | 16294 | 2 | 0.049
L2b1b | 16311 | 17 | 0.415
L2b1b | 16325 | 6 | 0.146
L2b1b | 16354 | 1 | 0.024
L2b1b | 16502 | 1 | 0.024
L2b1b | 16527 | 1 | 0.024
L2b1b | 16532d | 1 | 0.024
L2b1b | 183 | 35 | 0.854
L2b1b | 309d | 1 | 0.024
L2b1b | 310 | 1 | 0.024
L2b1b | 315.2C | 1 | 0.024
L2b1b | 4011 | 1 | 0.024
L2b1b | 408A | 2 | 0.049
L2b1b | 4727 | 1 | 0.024
L2b1b | 487T | 2 | 0.049
L2b1b | 5147 | 1 | 0.024
L2b1b | 515-524d | 1 | 0.024
L2b1b | 5442 | 1 | 0.024
L2b1b | 5460 | 1 | 0.024
L2b1b | 6481 | 1 | 0.024
L2b1b | 8350 | 1 | 0.024
L2b1b | 95C | 1 | 0.024
L2b2 | 10097 | 1 | 0.019
L2b2 | 10338 | 1 | 0.019
L2b2 | 10616 | 1 | 0.019
L2b2 | 10963 | 1 | 0.019
L2b2 | 11116 | 1 | 0.019
L2b2 | 11119 | 1 | 0.019
L2b2 | 11314 | 1 | 0.019
L2b2 | 11485 | 1 | 0.019
L2b2 | 11914 | 1 | 0.019
L2b2 | 12454 | 1 | 0.019
L2b2 | 12754 | 1 | 0.019
L2b2 | 12804 | 1 | 0.019
L2b2 | 13195 | 1 | 0.019
L2b2 | 13552 | 1 | 0.019
L2b2 | 13630 | 1 | 0.019
L2b2 | 13980 | 1 | 0.019
L2b2 | 13986 | 2 | 0.038
L2b2 | 14020A | 1 | 0.019
L2b2 | 14831 | 1 | 0.019
L2b2 | 15318 | 2 | 0.038
L2b2 | 15394 | 1 | 0.019
L2b2 | 15467 | 1 | 0.019
L2b2 | 15604 | 1 | 0.019
L2b2 | 15844 | 1 | 0.019
L2b2 | 16148 | 1 | 0.019
L2b2 | 16174 | 2 | 0.038
L2b2 | 16189 | 1 | 0.019
L2b2 | 16193 | 1 | 0.019
L2b2 | 16234 | 1 | 0.019
L2b2 | 16249 | 1 | 0.019
L2b2 | 16261 | 1 | 0.019
L2b2 | 16264 | 1 | 0.019
L2b2 | 16265 | 6 | 0.113
L2b2 | 16274 | 18 | 0.34

L2b2 | 16311 | 8 | 0.151
L2b2 | 16325 | 1 | 0.019
L2b2 | 16342 | 1 | 0.019
L2b2 | 16354A | 1 | 0.019
L2b2 | 16362 | 1 | 0.019
L2b2 | 16368 | 6 | 0.113
L2b2 | 16484-16489d | 1 | 0.019
L2b2 | 16496 | 1 | 0.019
L2b2 | 16497 | 1 | 0.019
L2b2 | 16527 | 2 | 0.038
L2b2 | 1766 | 1 | 0.019
L2b2 | 183 | 40 | 0.755
L2b2 | 184 | 2 | 0.038
L2b2 | 185C | 1 | 0.019
L2b2 | 186 | 1 | 0.019
L2b2 | 200 | 1 | 0.019
L2b2 | 205 | 1 | 0.019
L2b2 | 2243 | 1 | 0.019
L2b2 | 2436 | 1 | 0.019
L2b2 | 309d | 2 | 0.038
L2b2 | 3196 | 3 | 0.057
L2b2 | 3552 | 1 | 0.019
L2b2 | 3816 | 1 | 0.019
L2b2 | 4218 | 2 | 0.038
L2b2 | 4823 | 3 | 0.057
L2b2 | 489 | 1 | 0.019
L2b2 | 5074 | 1 | 0.019
L2b2 | 513 | 2 | 0.038
L2b2 | 515-524d | 4 | 0.075
L2b2 | 5258 | 3 | 0.057
L2b2 | 547 | 1 | 0.019
L2b2 | 5705 | 1 | 0.019
L2b2 | 6061 | 1 | 0.019
L2b2 | 6563 | 1 | 0.019
L2b2 | 6681 | 3 | 0.057
L2b2 | 748 | 1 | 0.019
L2b2 | 750C | 1 | 0.019
L2b2 | 8020 | 1 | 0.019
L2b2 | 804 | 1 | 0.019
L2b2 | 8749 | 1 | 0.019
L2b2a | 10795 | 2 | 0.049
L2b2a | 11654 | 1 | 0.024
L2b2a | 11968 | 1 | 0.024
L2b2a | 13623 | 2 | 0.049
L2b2a | 13907 | 1 | 0.024
L2b2a | 15872 | 1 | 0.024
L2b2a | 16086 | 1 | 0.024
L2b2a | 16175 | 1 | 0.024
L2b2a | 16189 | 2 | 0.049
L2b2a | 16218 | 3 | 0.073
L2b2a | 16260 | 2 | 0.049
L2b2a | 16291 | 1 | 0.024
L2b2a | 16292 | 1 | 0.024
L2b2a | 188T | 1 | 0.024
L2b2a | 203 | 1 | 0.024
L2b2a | 309d | 3 | 0.073
L2b2a | 456 | 1 | 0.024
L2b2a | 4976 | 1 | 0.024
L2b2a | 5996 | 1 | 0.024
L2b2a | 6212 | 1 | 0.024
L2b2a | 6710 | 1 | 0.024
L2b2a | 8705 | 2 | 0.049
L2b3 | 16343T | 1 | 0.1
L2b3 | 16344 | 1 | 0.1
L2b3 | 16532d | 4 | 0.4
L2b3 | 186A | 1 | 0.1
L2b3a | 10013 | 1 | 0.024
L2b3a | 15511 | 1 | 0.024
L2b3a | 15562 | 2 | 0.049
L2b3a | 15928 | 2 | 0.049
L2b3a | 15945.1T | 4 | 0.098
L2b3a | 16093 | 3 | 0.073
L2b3a | 16169 | 1 | 0.024
L2b3a | 16185 | 1 | 0.024
L2b3a | 16212 | 22 | 0.537
L2b3a | 16218 | 1 | 0.024
L2b3a | 16240 | 1 | 0.024
L2b3a | 16271 | 3 | 0.073
L2b3a | 16291 | 1 | 0.024
L2b3a | 16295 | 1 | 0.024
L2b3a | 16311 | 1 | 0.024
L2b3a | 16359 | 2 | 0.049
L2b3a | 16433 | 1 | 0.024
L2b3a | 16526 | 1 | 0.024
L2b3a | 183 | 1 | 0.024
L2b3a | 185C | 5 | 0.122
L2b3a | 207 | 32 | 0.78
L2b3a | 310 | 1 | 0.024
L2b3a | 317.1C | 2 | 0.049
L2b3a | 374 | 2 | 0.049
L2b3a | 5029 | 1 | 0.024
L2b3a | 5074 | 1 | 0.024
L2b3a | 5237 | 3 | 0.073
L2b3a | 64 | 1 | 0.024
L2b3a | 9438 | 1 | 0.024
L2b3b | 13105 | 1 | 0.2
L2b3b | 15298A | 1 | 0.2
L2b3b | 16234 | 1 | 0.2
L2b3b | 183 | 1 | 0.2
L2b3b | 189 | 1 | 0.2
L2b3b | 2885 | 1 | 0.2
L2b3b | 6095 | 1 | 0.2
L2b3b | 8566 | 1 | 0.2
L2b3c | 11150 | 1 | 0.077
L2b3c | 11914 | 1 | 0.077
L2b3c | 12397 | 1 | 0.077
L2b3c | 13105 | 1 | 0.077
L2b3c | 15924 | 1 | 0.077
L2b3c | 16145 | 5 | 0.385
L2b3c | 16169.1C | 2 | 0.154
L2b3c | 16179 | 1 | 0.077

L2b3c | 16189 | 1 | 0.077
L2b3c | 16287 | 1 | 0.077
L2b3c | 16293 | 1 | 0.077
L2b3c | 16311 | 4 | 0.308
L2b3c | 185C | 4 | 0.308
L2b3c | 194 | 1 | 0.077
L2b3c | 199 | 5 | 0.385
L2b3c | 202 | 1 | 0.077
L2b3c | 2768 | 1 | 0.077
L2b3c | 385 | 1 | 0.077
L2b3c | 408A | 3 | 0.231
L2b3c | 483 | 2 | 0.154
L2b3c | 573.1C | 1 | 0.077
L2b3c | 573.3C | 1 | 0.077
L2b3c | 573.4C | 1 | 0.077
L2b3c | 6261 | 1 | 0.077
L2b3c | 6284 | 1 | 0.077
L2b3c | 6293 | 2 | 0.154
L2b3c | 634 | 1 | 0.077
L2b3c | 8507 | 1 | 0.077
L2b3c | 8896 | 1 | 0.077
L2b3c | 9025 | 1 | 0.077
L2b'c | 16093 | 1 | 0.25
L2b'c | 16104 | 1 | 0.25
L2b'c | 16207 | 1 | 0.25
L2b'c | 16311 | 1 | 0.25
L2b'c | 249d | 1 | 0.25
L2b'c | 325 | 1 | 0.25
L2b'c'd | 16104 | 1 | 0.25
L2b'c'd | 325 | 2 | 0.5
L2b'c'd | 93 | 1 | 0.25
L2b'c'd | 95C | 1 | 0.25
L2c | 1040 | 1 | 0.021
L2c | 11020 | 1 | 0.021
L2c | 11074 | 1 | 0.021
L2c | 11404 | 1 | 0.021
L2c | 11440 | 1 | 0.021
L2c | 11852 | 1 | 0.021
L2c | 12266 | 1 | 0.021
L2c | 12523 | 1 | 0.021
L2c | 12678 | 1 | 0.021
L2c | 12771 | 3 | 0.062
L2c | 12795 | 2 | 0.042
L2c | 12909 | 1 | 0.021
L2c | 13368 | 1 | 0.021
L2c | 13470 | 4 | 0.083
L2c | 13656 | 1 | 0.021
L2c | 13759 | 2 | 0.042
L2c | 14311 | 3 | 0.062
L2c | 14353 | 1 | 0.021
L2c | 14569 | 3 | 0.062
L2c | 14696 | 1 | 0.021
L2c | 14750T | 2 | 0.042
L2c | 153 | 1 | 0.021
L2c | 15310 | 1 | 0.021
L2c | 15317 | 3 | 0.062
L2c | 15367 | 2 | 0.042
L2c | 15497 | 5 | 0.104
L2c | 15883 | 1 | 0.021
L2c | 15978 | 3 | 0.062
L2c | 16086 | 1 | 0.021
L2c | 16093 | 7 | 0.146
L2c | 16111 | 2 | 0.042
L2c | 16129 | 2 | 0.042
L2c | 16162 | 1 | 0.021
L2c | 16173 | 1 | 0.021
L2c | 16176 | 1 | 0.021
L2c | 16177 | 3 | 0.062
L2c | 16180 | 1 | 0.021
L2c | 16184 | 1 | 0.021
L2c | 16189 | 4 | 0.083
L2c | 16192 | 1 | 0.021
L2c | 16234 | 1 | 0.021
L2c | 16254 | 2 | 0.042
L2c | 16261 | 3 | 0.062
L2c | 16263 | 1 | 0.021
L2c | 16266 | 3 | 0.062
L2c | 16294 | 4 | 0.083
L2c | 16311 | 1 | 0.021
L2c | 16320 | 5 | 0.104
L2c | 16344 | 1 | 0.021
L2c | 16355 | 1 | 0.021
L2c | 16360 | 1 | 0.021
L2c | 16362 | 11 | 0.229
L2c | 16368 | 2 | 0.042
L2c | 16399 | 2 | 0.042
L2c | 199 | 1 | 0.021
L2c | 204 | 1 | 0.021
L2c | 2755 | 1 | 0.021
L2c | 2833 | 1 | 0.021
L2c | 310 | 2 | 0.042
L2c | 3316 | 1 | 0.021
L2c | 3591 | 1 | 0.021
L2c | 3882 | 2 | 0.042
L2c | 4050 | 1 | 0.021
L2c | 455.1T | 2 | 0.042
L2c | 485 | 1 | 0.021
L2c | 4907 | 1 | 0.021
L2c | 499 | 1 | 0.021
L2c | 5074 | 1 | 0.021
L2c | 5082 | 1 | 0.021
L2c | 513-522d | 2 | 0.042
L2c | 5390 | 1 | 0.021
L2c | 5460 | 1 | 0.021
L2c | 6150 | 5 | 0.104
L2c | 6203 | 1 | 0.021
L2c | 6293 | 1 | 0.021
L2c | 64 | 7 | 0.146
L2c | 7160 | 2 | 0.042
L2c | 7754 | 1 | 0.021
L2c | 7856 | 1 | 0.021

L2c | 8020 | 1 | 0.021
L2c | 8027 | 1 | 0.021
L2c | 8269 | 1 | 0.021
L2c | 8274 | 1 | 0.021
L2c | 8425 | 3 | 0.062
L2c | 850 | 1 | 0.021
L2c | 8744 | 1 | 0.021
L2c | 9300 | 1 | 0.021
L2c | 9591 | 1 | 0.021
L2c | 95C | 9 | 0.188
L2c | 9660 | 1 | 0.021
L2c | 9692 | 1 | 0.021
L2c | 9746 | 1 | 0.021
L2c | 9935 | 1 | 0.021
L2c | 9947 | 2 | 0.042
L2c1 | 11813 | 1 | 0.03
L2c1 | 13329 | 1 | 0.03
L2c1 | 14101 | 1 | 0.03
L2c1 | 15499 | 1 | 0.03
L2c1 | 16092 | 2 | 0.061
L2c1 | 16093 | 2 | 0.061
L2c1 | 16145 | 1 | 0.03
L2c1 | 16189 | 2 | 0.061
L2c1 | 16249A | 1 | 0.03
L2c1 | 16293 | 1 | 0.03
L2c1 | 16343C | 1 | 0.03
L2c1 | 16355 | 1 | 0.03
L2c1 | 16526 | 2 | 0.061
L2c1 | 16T | 2 | 0.061
L2c1 | 204 | 1 | 0.03
L2c1 | 262 | 1 | 0.03
L2c1 | 282 | 1 | 0.03
L2c1 | 2857 | 1 | 0.03
L2c1 | 309d | 1 | 0.03
L2c1 | 310 | 2 | 0.061
L2c1 | 3745 | 1 | 0.03
L2c1 | 515-524d | 8 | 0.242
L2c1 | 5585 | 1 | 0.03
L2c1 | 95C | 1 | 0.03
L2c1a | 11914 | 1 | 0.03
L2c1a | 12026 | 5 | 0.152
L2c1a | 12940 | 4 | 0.121
L2c1a | 13708 | 3 | 0.091
L2c1a | 13886 | 6 | 0.182
L2c1a | 14061A | 5 | 0.152
L2c1a | 14476 | 1 | 0.03
L2c1a | 14979 | 6 | 0.182
L2c1a | 15236 | 1 | 0.03
L2c1a | 15427 | 1 | 0.03
L2c1a | 15850 | 3 | 0.091
L2c1a | 16086 | 4 | 0.121
L2c1a | 16172 | 1 | 0.03
L2c1a | 16189 | 2 | 0.061
L2c1a | 16224 | 3 | 0.091
L2c1a | 16261 | 13 | 0.394
L2c1a | 16311 | 7 | 0.212
L2c1a | 16320 | 1 | 0.03
L2c1a | 16354 | 2 | 0.061
L2c1a | 200 | 3 | 0.091
L2c1a | 2463d | 1 | 0.03
L2c1a | 310 | 2 | 0.061
L2c1a | 3645 | 2 | 0.061
L2c1a | 4204 | 1 | 0.03
L2c1a | 513 | 6 | 0.182
L2c1a | 5147 | 1 | 0.03
L2c1a | 515-524d | 2 | 0.061
L2c1a | 5988 | 5 | 0.152
L2c1a | 6221 | 6 | 0.182
L2c1a | 6260 | 1 | 0.03
L2c1a | 6437 | 1 | 0.03
L2c1a | 7444 | 1 | 0.03
L2c1a | 7830 | 6 | 0.182
L2c1a | 8655 | 6 | 0.182
L2c2 | 11626 | 1 | 0.023
L2c2 | 12362 | 1 | 0.023
L2c2 | 12867 | 1 | 0.023
L2c2 | 1356T | 1 | 0.023
L2c2 | 13606 | 1 | 0.023
L2c2 | 14180 | 1 | 0.023
L2c2 | 14182 | 1 | 0.023
L2c2 | 14530 | 1 | 0.023
L2c2 | 14968 | 1 | 0.023
L2c2 | 15409 | 1 | 0.023
L2c2 | 15482 | 1 | 0.023
L2c2 | 15497 | 1 | 0.023
L2c2 | 16093 | 3 | 0.068
L2c2 | 16126 | 1 | 0.023
L2c2 | 16129 | 3 | 0.068
L2c2 | 16134 | 1 | 0.023
L2c2 | 16145 | 1 | 0.023
L2c2 | 16187 | 1 | 0.023
L2c2 | 16203 | 1 | 0.023
L2c2 | 16270 | 1 | 0.023
L2c2 | 16274 | 1 | 0.023
L2c2 | 16291 | 2 | 0.045
L2c2 | 16293 | 1 | 0.023
L2c2 | 16304 | 1 | 0.023
L2c2 | 16311 | 2 | 0.045
L2c2 | 16355 | 1 | 0.023
L2c2 | 16362 | 3 | 0.068
L2c2 | 1664 | 1 | 0.023
L2c2 | 185 | 2 | 0.045
L2c2 | 188 | 1 | 0.023
L2c2 | 189 | 3 | 0.068
L2c2 | 199 | 1 | 0.023
L2c2 | 207 | 3 | 0.068
L2c2 | 228 | 1 | 0.023
L2c2 | 2707 | 1 | 0.023
L2c2 | 309d | 1 | 0.023
L2c2 | 310 | 2 | 0.045
L2c2 | 315.2C | 1 | 0.023

L2c2 | 316 | 1 | 0.023
L2c2 | 3196 | 1 | 0.023
L2c2 | 3861 | 1 | 0.023
L2c2 | 4048 | 1 | 0.023
L2c2 | 456 | 1 | 0.023
L2c2 | 4561 | 1 | 0.023
L2c2 | 4841 | 1 | 0.023
L2c2 | 515-524d | 4 | 0.091
L2c2 | 5250 | 1 | 0.023
L2c2 | 5502 | 1 | 0.023
L2c2 | 5899.1C | 1 | 0.023
L2c2 | 6125 | 1 | 0.023
L2c2 | 6221 | 1 | 0.023
L2c2 | 7082 | 1 | 0.023
L2c2 | 7918 | 1 | 0.023
L2c2 | 8348 | 1 | 0.023
L2c2 | 8557 | 1 | 0.023
L2c2 | 8967 | 1 | 0.023
L2c2 | 9548 | 1 | 0.023
L2c2 | 95C | 5 | 0.114
L2c2a | 13182 | 1 | 0.015
L2c2a | 13708 | 1 | 0.015
L2c2a | 14208 | 1 | 0.015
L2c2a | 154 | 1 | 0.015
L2c2a | 16093 | 21 | 0.323
L2c2a | 16126 | 1 | 0.015
L2c2a | 16142 | 1 | 0.015
L2c2a | 16145 | 1 | 0.015
L2c2a | 16172 | 1 | 0.015
L2c2a | 16188 | 1 | 0.015
L2c2a | 16189 | 10 | 0.154
L2c2a | 16193d | 1 | 0.015
L2c2a | 16207 | 1 | 0.015
L2c2a | 16214 | 2 | 0.031
L2c2a | 16217 | 1 | 0.015
L2c2a | 16224 | 1 | 0.015
L2c2a | 16234 | 1 | 0.015
L2c2a | 16249 | 1 | 0.015
L2c2a | 16274 | 2 | 0.031
L2c2a | 16294 | 1 | 0.015
L2c2a | 16298 | 2 | 0.031
L2c2a | 16484-16489d | 2 | 0.031
L2c2a | 249 | 2 | 0.031
L2c2a | 489 | 1 | 0.015
L2c2a | 511d | 2 | 0.031
L2c2a | 515-524d | 2 | 0.031
L2c2a | 7897 | 2 | 0.031
L2c2a | 8392 | 2 | 0.031
L2c2a | 8843 | 1 | 0.015
L2c2a | 8870 | 2 | 0.031
L2c2a | 89 | 3 | 0.046
L2c2a | 9293 | 1 | 0.015
L2c2a | 9462 | 1 | 0.015
L2c2a | 95C | 1 | 0.015
L2c2a1 | 12393 | 1 | 0.029
L2c2a1 | 13759 | 2 | 0.059
L2c2a1 | 15670 | 1 | 0.029
L2c2a1 | 204 | 1 | 0.029
L2c2a1 | 309d | 8 | 0.235
L2c2a1 | 310 | 1 | 0.029
L2c2a1 | 5563 | 1 | 0.029
L2c2a1 | 5894 | 11 | 0.324
L2c2a1 | 8393 | 11 | 0.324
L2c2a1 | 8994 | 1 | 0.029
L2c2b | 12142 | 1 | 0.143
L2c2b | 12183 | 1 | 0.143
L2c2b | 4048 | 1 | 0.143
L2c2b | 6445 | 1 | 0.143
L2c2b | 9509 | 1 | 0.143
L2c2b1 | 15930 | 1 | 0.043
L2c2b1 | 16066 | 2 | 0.087
L2c2b1 | 16093 | 10 | 0.435
L2c2b1 | 16094 | 1 | 0.043
L2c2b1 | 16192 | 1 | 0.043
L2c2b1 | 16245 | 1 | 0.043
L2c2b1 | 16266 | 4 | 0.174
L2c2b1 | 16270 | 4 | 0.174
L2c2b1 | 185 | 1 | 0.043
L2c2b1 | 207 | 2 | 0.087
L2c2b1 | 515-524d | 1 | 0.043
L2c2b1 | 89 | 1 | 0.043
L2c2b1 | 95C | 1 | 0.043
L2c2b1a | 13269 | 1 | 0.143
L2c2b1a | 14755 | 1 | 0.143
L2c2b1a | 15227 | 1 | 0.143
L2c2b1a | 5480 | 1 | 0.143
L2c2b1a | 6965 | 1 | 0.143
L2c2b1b | 11653 | 2 | 0.069
L2c2b1b | 13194 | 1 | 0.034
L2c2b1b | 15749 | 1 | 0.034
L2c2b1b | 15926 | 1 | 0.034
L2c2b1b | 15927 | 2 | 0.069
L2c2b1b | 15928 | 1 | 0.034
L2c2b1b | 16069 | 1 | 0.034
L2c2b1b | 16093 | 1 | 0.034
L2c2b1b | 16189 | 1 | 0.034
L2c2b1b | 16209 | 1 | 0.034
L2c2b1b | 16230 | 1 | 0.034
L2c2b1b | 16260 | 1 | 0.034
L2c2b1b | 16265 | 1 | 0.034
L2c2b1b | 16301 | 1 | 0.034
L2c2b1b | 16527 | 1 | 0.034
L2c2b1b | 204 | 1 | 0.034
L2c2b1b | 249d | 1 | 0.034
L2c2b1b | 279 | 1 | 0.034
L2c2b1b | 309d | 5 | 0.172
L2c2b1b | 3769 | 1 | 0.034
L2c2b1b | 499 | 2 | 0.069
L2c2b1b | 6713 | 1 | 0.034
L2c2b1b | 8014 | 2 | 0.069
L2c2b1b | 8116 | 1 | 0.034

L2c2b1b | 9139 | 2 | 0.069
L2c2b1b | 9165 | 3 | 0.103
L2c2b2 | 10399 | 1 | 0.143
L2c2b2 | 11842 | 1 | 0.143
L2c2b2 | 186 | 1 | 0.143
L2c2b2 | 3784 | 1 | 0.143
L2c2b2 | 5051 | 1 | 0.143
L2c2b2 | 5978 | 1 | 0.143
L2c2b2 | 8994 | 1 | 0.143
L2c3 | 11362 | 5 | 0.111
L2c3 | 148 | 1 | 0.022
L2c3 | 153 | 4 | 0.089
L2c3 | 16066 | 1 | 0.022
L2c3 | 16086 | 2 | 0.044
L2c3 | 16129 | 1 | 0.022
L2c3 | 16167 | 1 | 0.022
L2c3 | 16176 | 1 | 0.022
L2c3 | 16178 | 2 | 0.044
L2c3 | 16189 | 1 | 0.022
L2c3 | 16193 | 9 | 0.2
L2c3 | 16242 | 1 | 0.022
L2c3 | 16248.1A | 1 | 0.022
L2c3 | 16270 | 1 | 0.022
L2c3 | 16311 | 2 | 0.044
L2c3 | 1811 | 5 | 0.111
L2c3 | 189 | 1 | 0.022
L2c3 | 200 | 1 | 0.022
L2c3 | 204 | 1 | 0.022
L2c3 | 230 | 1 | 0.022
L2c3 | 309d | 2 | 0.044
L2c3 | 3105 | 1 | 0.022
L2c3 | 315.2C | 1 | 0.022
L2c3 | 317.1C | 1 | 0.022
L2c3 | 4561 | 2 | 0.044
L2c3 | 471 | 1 | 0.022
L2c3 | 4742 | 1 | 0.022
L2c3 | 482 | 1 | 0.022
L2c3 | 4976 | 1 | 0.022
L2c3 | 6248 | 1 | 0.022
L2c3 | 7214 | 1 | 0.022
L2c3 | 7403 | 1 | 0.022
L2c3 | 7785 | 1 | 0.022
L2c3 | 8125 | 1 | 0.022
L2c3 | 8764 | 2 | 0.044
L2c3 | 89 | 22 | 0.489
L2c3 | 9052 | 1 | 0.022
L2c3 | 9301 | 1 | 0.022
L2c3 | 95C | 3 | 0.067
L2c3 | 95T | 1 | 0.022
L2c3 | 9804 | 1 | 0.022
L2c3a | 11204 | 1 | 0.04
L2c3a | 11909 | 1 | 0.04
L2c3a | 12612 | 1 | 0.04
L2c3a | 14118 | 3 | 0.12
L2c3a | 15077 | 6 | 0.24
L2c3a | 16093 | 6 | 0.24
L2c3a | 16187 | 4 | 0.16
L2c3a | 16207 | 2 | 0.08
L2c3a | 16234 | 9 | 0.36
L2c3a | 16263 | 1 | 0.04
L2c3a | 16290 | 1 | 0.04
L2c3a | 16291 | 1 | 0.04
L2c3a | 16362 | 4 | 0.16
L2c3a | 204 | 1 | 0.04
L2c3a | 2589 | 6 | 0.24
L2c3a | 310 | 2 | 0.08
L2c3a | 6521 | 6 | 0.24
L2c4 | 10586 | 3 | 0.053
L2c4 | 11197 | 2 | 0.035
L2c4 | 12651 | 1 | 0.018
L2c4 | 13587 | 1 | 0.018
L2c4 | 15912 | 1 | 0.018
L2c4 | 15917 | 2 | 0.035
L2c4 | 15924 | 1 | 0.018
L2c4 | 15928 | 2 | 0.035
L2c4 | 16004 | 1 | 0.018
L2c4 | 16051 | 1 | 0.018
L2c4 | 16061A | 1 | 0.018
L2c4 | 16081 | 6 | 0.105
L2c4 | 16086 | 3 | 0.053
L2c4 | 16090G | 1 | 0.018
L2c4 | 16093 | 3 | 0.053
L2c4 | 16111 | 1 | 0.018
L2c4 | 16126 | 1 | 0.018
L2c4 | 16131G | 1 | 0.018
L2c4 | 16138 | 1 | 0.018
L2c4 | 16172 | 1 | 0.018
L2c4 | 16193 | 3 | 0.053
L2c4 | 16214 | 3 | 0.053
L2c4 | 16240T | 1 | 0.018
L2c4 | 16256 | 1 | 0.018
L2c4 | 16261 | 1 | 0.018
L2c4 | 16265C | 2 | 0.035
L2c4 | 16266 | 3 | 0.053
L2c4 | 16274 | 5 | 0.088
L2c4 | 16309 | 1 | 0.018
L2c4 | 16311 | 6 | 0.105
L2c4 | 16318C | 1 | 0.018
L2c4 | 16380 | 3 | 0.053
L2c4 | 16399 | 1 | 0.018
L2c4 | 16496 | 4 | 0.07
L2c4 | 185 | 3 | 0.053
L2c4 | 204 | 2 | 0.035
L2c4 | 207 | 1 | 0.018
L2c4 | 249d | 3 | 0.053
L2c4 | 266 | 3 | 0.053
L2c4 | 309d | 1 | 0.018
L2c4 | 3547 | 1 | 0.018
L2c4 | 474A | 2 | 0.035
L2c4 | 499 | 12 | 0.211
L2c4 | 507 | 1 | 0.018

L2c4 | 507A | 3 | 0.053
L2c4 | 515-524d | 28 | 0.491
L2c4 | 534 | 1 | 0.018
L2c4 | 5451 | 1 | 0.018
L2c4 | 5471 | 3 | 0.053
L2c4 | 6518 | 2 | 0.035
L2c4 | 8329G | 1 | 0.018
L2c4 | 89 | 9 | 0.158
L2c4 | 8901 | 1 | 0.018
L2c4 | 9055 | 1 | 0.018
L2c4 | 95C | 10 | 0.175
L2c5 | 10530 | 1 | 0.067
L2c5 | 11016 | 1 | 0.067
L2c5 | 12717 | 1 | 0.067
L2c5 | 13135 | 2 | 0.133
L2c5 | 13708 | 2 | 0.133
L2c5 | 13711 | 2 | 0.133
L2c5 | 14319 | 1 | 0.067
L2c5 | 14374 | 3 | 0.2
L2c5 | 15514 | 1 | 0.067
L2c5 | 16000 | 1 | 0.067
L2c5 | 16051 | 1 | 0.067
L2c5 | 16176 | 1 | 0.067
L2c5 | 16240T | 1 | 0.067
L2c5 | 16255 | 1 | 0.067
L2c5 | 16265C | 1 | 0.067
L2c5 | 16352 | 1 | 0.067
L2c5 | 171 | 1 | 0.067
L2c5 | 183 | 1 | 0.067
L2c5 | 292 | 1 | 0.067
L2c5 | 3221 | 3 | 0.2
L2c5 | 3760G | 1 | 0.067
L2c5 | 3882 | 1 | 0.067
L2c5 | 4259 | 1 | 0.067
L2c5 | 484C | 1 | 0.067
L2c5 | 513-522d | 2 | 0.133
L2c5 | 5186 | 1 | 0.067
L2c5 | 5262 | 1 | 0.067
L2c5 | 5351 | 1 | 0.067
L2c5 | 5999 | 1 | 0.067
L2c5 | 6752 | 1 | 0.067
L2c5 | 7424 | 1 | 0.067
L2d | 310 | 1 | 1
L2d | 9254 | 1 | 1
L2d+16129 | 10217 | 1 | 0.1
L2d+16129 | 11150 | 1 | 0.1
L2d+16129 | 11253 | 2 | 0.2
L2d+16129 | 11914 | 1 | 0.1
L2d+16129 | 14798 | 1 | 0.1
L2d+16129 | 14979 | 1 | 0.1
L2d+16129 | 152 | 1 | 0.1
L2d+16129 | 15443 | 2 | 0.2
L2d+16129 | 15461 | 1 | 0.1
L2d+16129 | 15553 | 1 | 0.1
L2d+16129 | 15769 | 1 | 0.1
L2d+16129 | 16093 | 1 | 0.1
L2d+16129 | 16224 | 1 | 0.1
L2d+16129 | 1762 | 1 | 0.1
L2d+16129 | 199 | 2 | 0.2
L2d+16129 | 2008 | 1 | 0.1
L2d+16129 | 2035 | 1 | 0.1
L2d+16129 | 2086 | 1 | 0.1
L2d+16129 | 2818 | 1 | 0.1
L2d+16129 | 310 | 1 | 0.1
L2d+16129 | 3145 | 1 | 0.1
L2d+16129 | 3154 | 1 | 0.1
L2d+16129 | 4068 | 1 | 0.1
L2d+16129 | 499 | 2 | 0.2
L2d+16129 | 513 | 1 | 0.1
L2d+16129 | 5365 | 1 | 0.1
L2d+16129 | 549 | 1 | 0.1
L2d+16129 | 6941 | 1 | 0.1
L2d+16129 | 6956A | 1 | 0.1
L2d+16129 | 7007 | 1 | 0.1
L2d+16129 | 7094 | 1 | 0.1
L2d1 | 144A | 1 | 0.034
L2d1 | 16086 | 4 | 0.138
L2d1 | 16093 | 16 | 0.552
L2d1 | 16148 | 4 | 0.138
L2d1 | 16172 | 3 | 0.103
L2d1 | 16207 | 1 | 0.034
L2d1 | 16254 | 1 | 0.034
L2d1 | 16259 | 1 | 0.034
L2d1 | 16261 | 2 | 0.069
L2d1 | 16265C | 1 | 0.034
L2d1 | 16271 | 1 | 0.034
L2d1 | 16295 | 1 | 0.034
L2d1 | 16320 | 2 | 0.069
L2d1 | 16356 | 1 | 0.034
L2d1 | 16357 | 1 | 0.034
L2d1 | 198 | 2 | 0.069
L2d1 | 310 | 1 | 0.034
L2d1 | 456.1T | 1 | 0.034
L2d1 | 515-524d | 2 | 0.069
L2d1 | 6366 | 2 | 0.069
L2d1 | 8839 | 2 | 0.069
L2d1a | 11732 | 1 | 0.048
L2d1a | 12630 | 2 | 0.095
L2d1a | 13239 | 2 | 0.095
L2d1a | 13651 | 1 | 0.048
L2d1a | 14158 | 1 | 0.048
L2d1a | 1462 | 1 | 0.048
L2d1a | 15553 | 1 | 0.048
L2d1a | 15691 | 1 | 0.048
L2d1a | 16078 | 1 | 0.048
L2d1a | 16093 | 10 | 0.476
L2d1a | 16167 | 1 | 0.048
L2d1a | 16192 | 2 | 0.095
L2d1a | 16293 | 1 | 0.048
L2d1a | 16311 | 12 | 0.571
L2d1a | 16352 | 1 | 0.048

L2d1a | 16462-16466d | 1 | 0.048
L2d1a | 309d | 1 | 0.048
L2d1a | 310 | 2 | 0.095
L2d1a | 317.1C | 1 | 0.048
L2d1a | 3197 | 2 | 0.095
L2d1a | 3591 | 1 | 0.048
L2d1a | 527d | 1 | 0.048
L2d1a | 9007 | 1 | 0.048
L2d1a | 9130A | 2 | 0.095
L2d1a | 9290 | 1 | 0.048
L2d1a | 9727 | 1 | 0.048
L2e | 11930 | 1 | 0.029
L2e | 12441 | 1 | 0.029
L2e | 12950 | 1 | 0.029
L2e | 13020 | 1 | 0.029
L2e | 13602 | 1 | 0.029
L2e | 14687 | 1 | 0.029
L2e | 151 | 6 | 0.176
L2e | 15103G | 1 | 0.029
L2e | 15289 | 1 | 0.029
L2e | 15497 | 1 | 0.029
L2e | 15758 | 1 | 0.029
L2e | 15803 | 1 | 0.029
L2e | 15930 | 1 | 0.029
L2e | 16086 | 2 | 0.059
L2e | 16093 | 5 | 0.147
L2e | 16114A | 1 | 0.029
L2e | 16189 | 7 | 0.206
L2e | 16213 | 4 | 0.118
L2e | 16218 | 4 | 0.118
L2e | 16234 | 4 | 0.118
L2e | 16249A | 1 | 0.029
L2e | 16256 | 1 | 0.029
L2e | 16259 | 1 | 0.029
L2e | 16274 | 2 | 0.059
L2e | 16295 | 1 | 0.029
L2e | 16311 | 10 | 0.294
L2e | 16317T | 1 | 0.029
L2e | 16320 | 1 | 0.029
L2e | 16362 | 1 | 0.029
L2e | 185 | 6 | 0.176
L2e | 189 | 6 | 0.176
L2e | 194 | 2 | 0.059
L2e | 199 | 1 | 0.029
L2e | 3591 | 1 | 0.029
L2e | 3693 | 1 | 0.029
L2e | 3825 | 1 | 0.029
L2e | 4205 | 2 | 0.059
L2e | 44.1C | 1 | 0.029
L2e | 4418 | 1 | 0.029
L2e | 4481 | 2 | 0.059
L2e | 4512 | 1 | 0.029
L2e | 4832 | 1 | 0.029
L2e | 513 | 1 | 0.029
L2e | 515-524d | 6 | 0.176
L2e | 5228 | 1 | 0.029
L2e | 544 | 1 | 0.029
L2e | 5585 | 1 | 0.029
L2e | 6261 | 1 | 0.029
L2e | 6338 | 1 | 0.029
L2e | 7885 | 1 | 0.029
L2e | 8276.6C | 1 | 0.029
L2e | 8413 | 1 | 0.029
L2e | 8522 | 2 | 0.059
L2e | 8802 | 1 | 0.029
L2e | 9139 | 1 | 0.029
L2e | 9380 | 2 | 0.059
L2e | 9635 | 1 | 0.029
L2e1 | 10646 | 1 | 0.067
L2e1 | 11016 | 1 | 0.067
L2e1 | 13356 | 7 | 0.467
L2e1 | 13638 | 7 | 0.467
L2e1 | 13689C | 1 | 0.067
L2e1 | 15592 | 5 | 0.333
L2e1 | 16189 | 1 | 0.067
L2e1 | 16293 | 1 | 0.067
L2e1 | 185C | 1 | 0.067
L2e1 | 195 | 2 | 0.133
L2e1 | 204 | 1 | 0.067
L2e1 | 2789 | 1 | 0.067
L2e1 | 3010 | 1 | 0.067
L2e1 | 310 | 1 | 0.067
L2e1 | 317.1C | 1 | 0.067
L2e1 | 3316 | 5 | 0.333
L2e1 | 3531 | 1 | 0.067
L2e1 | 472 | 1 | 0.067
L2e1 | 505 | 1 | 0.067
L2e1 | 568 | 5 | 0.333
L2e1 | 5846 | 1 | 0.067
L2e1 | 593 | 1 | 0.067
L2e1 | 6221 | 7 | 0.467
L2e1 | 6872 | 1 | 0.067
L2e1 | 7196 | 1 | 0.067
L2e1 | 8167 | 1 | 0.067
L2e1 | 8614 | 1 | 0.067
L2e1 | 8986 | 1 | 0.067
L2e1 | 9163 | 1 | 0.067
L2e1a | 11016 | 1 | 0.053
L2e1a | 1171 | 1 | 0.053
L2e1a | 11989 | 1 | 0.053
L2e1a | 12236 | 1 | 0.053
L2e1a | 12373 | 2 | 0.105
L2e1a | 12651 | 1 | 0.053
L2e1a | 13023 | 1 | 0.053
L2e1a | 14266 | 1 | 0.053
L2e1a | 14364 | 6 | 0.316
L2e1a | 14825 | 2 | 0.105
L2e1a | 152d | 1 | 0.053
L2e1a | 16093 | 1 | 0.053
L2e1a | 16111 | 1 | 0.053
L2e1a | 16189 | 1 | 0.053

L2e1a | 16201 | 1 | 0.053
L2e1a | 16212 | 1 | 0.053
L2e1a | 16291 | 2 | 0.105
L2e1a | 16311 | 1 | 0.053
L2e1a | 16356 | 1 | 0.053
L2e1a | 16362 | 9 | 0.474
L2e1a | 183 | 2 | 0.105
L2e1a | 2710 | 1 | 0.053
L2e1a | 309d | 5 | 0.263
L2e1a | 3633 | 1 | 0.053
L2e1a | 3666 | 6 | 0.316
L2e1a | 4086 | 1 | 0.053
L2e1a | 4703 | 6 | 0.316
L2e1a | 5585 | 6 | 0.316
L2e1a | 5813d | 6 | 0.316
L2e1a | 6617 | 1 | 0.053
L2e1a | 7673 | 1 | 0.053
L2e1a | 8821 | 1 | 0.053
L3 | 10238 | 1 | 0.005
L3 | 10595 | 1 | 0.005
L3 | 11002 | 1 | 0.005
L3 | 135A | 1 | 0.005
L3 | 13886 | 1 | 0.005
L3 | 14022 | 1 | 0.005
L3 | 146 | 6 | 0.027
L3 | 1473 | 1 | 0.005
L3 | 14769 | 1 | 0.005
L3 | 15058 | 1 | 0.005
L3 | 152 | 2 | 0.009
L3 | 15746 | 2 | 0.009
L3 | 16072.1A | 1 | 0.005
L3 | 16093 | 3 | 0.014
L3 | 16188 | 1 | 0.005
L3 | 16221 | 1 | 0.005
L3 | 16234 | 1 | 0.005
L3 | 16235 | 1 | 0.005
L3 | 16278 | 3 | 0.014
L3 | 16291 | 1 | 0.005
L3 | 16311 | 1 | 0.005
L3 | 16319 | 1 | 0.005
L3 | 16390 | 3 | 0.014
L3 | 167A | 1 | 0.005
L3 | 189 | 1 | 0.005
L3 | 195 | 1 | 0.005
L3 | 199 | 1 | 0.005
L3 | 200 | 1 | 0.005
L3 | 206 | 1 | 0.005
L3 | 266 | 1 | 0.005
L3 | 279 | 1 | 0.005
L3 | 310 | 1 | 0.005
L3 | 315.2C | 1 | 0.005
L3 | 3438 | 1 | 0.005
L3 | 372 | 3 | 0.014
L3 | 406A | 1 | 0.005
L3 | 4084 | 1 | 0.005
L3 | 455G | 1 | 0.005
L3 | 468 | 1 | 0.005
L3 | 470 | 1 | 0.005
L3 | 488 | 1 | 0.005
L3 | 541 | 1 | 0.005
L3 | 5465 | 1 | 0.005
L3 | 6680 | 1 | 0.005
L3 | 7424 | 1 | 0.005
L3 | 8618 | 1 | 0.005
L3 | 9123 | 1 | 0.005
L3 | 93 | 1 | 0.005
L3'4 | 151 | 1 | 0.008
L3'4 | 16092 | 2 | 0.016
L3'4 | 16126 | 1 | 0.008
L3'4 | 16129 | 2 | 0.016
L3'4 | 16153 | 1 | 0.008
L3'4 | 16162 | 1 | 0.008
L3'4 | 16172 | 2 | 0.016
L3'4 | 16189 | 9 | 0.071
L3'4 | 16190 | 1 | 0.008
L3'4 | 16234 | 2 | 0.016
L3'4 | 16249 | 1 | 0.008
L3'4 | 16293 | 6 | 0.047
L3'4 | 16327 | 1 | 0.008
L3'4 | 16384 | 1 | 0.008
L3'4 | 16390 | 1 | 0.008
L3'4 | 189 | 2 | 0.016
L3'4 | 195A | 1 | 0.008
L3'4 | 200 | 2 | 0.016
L3'4 | 207 | 1 | 0.008
L3'4 | 249d | 1 | 0.008
L3'4 | 455d | 5 | 0.039
L3'4 | 482 | 1 | 0.008
L3'4 | 49T | 1 | 0.008
L3'4 | 50A | 1 | 0.008
L3'4 | 515-524d | 2 | 0.016
L3'4 | 51A | 1 | 0.008
L3'4'6 | 146 | 1 | 0.034
L3'4'6 | 16024A | 1 | 0.034
L3'4'6 | 16025 | 1 | 0.034
L3'4'6 | 16093 | 2 | 0.069
L3'4'6 | 16172 | 18 | 0.621
L3'4'6 | 16173 | 18 | 0.621
L3'4'6 | 16189 | 1 | 0.034
L3'4'6 | 16239G | 1 | 0.034
L3'4'6 | 16287 | 5 | 0.172
L3'4'6 | 16309 | 1 | 0.034
L3'4'6 | 16319 | 2 | 0.069
L3'4'6 | 16330G | 1 | 0.034
L3'4'6 | 16359 | 1 | 0.034
L3'4'6 | 16362A | 1 | 0.034
L3'4'6 | 183 | 1 | 0.034
L3'4'6 | 518 | 1 | 0.034
L3a | 16184A | 1 | 0.067
L3a | 16297 | 1 | 0.067
L3a | 16357 | 1 | 0.067

L3a+709 | 14668 | 15 | 0.536
L3a+709 | 1520 | 1 | 0.036
L3a+709 | 15803 | 15 | 0.536
L3a+709 | 15835 | 15 | 0.536
L3a+709 | 15905 | 16 | 0.571
L3a+709 | 16148 | 1 | 0.036
L3a+709 | 16169 | 20 | 0.714
L3a+709 | 16213 | 21 | 0.75
L3a+709 | 16240C | 20 | 0.714
L3a+709 | 16293 | 2 | 0.071
L3a+709 | 16335 | 19 | 0.679
L3a+709 | 189 | 1 | 0.036
L3a+709 | 2416 | 13 | 0.464
L3a+709 | 5261 | 15 | 0.536
L3a+709 | 6023 | 14 | 0.5
L3a+709 | 6527 | 1 | 0.036
L3a+709 | 7948 | 15 | 0.536
L3a+709 | 9117 | 15 | 0.536
L3a+709 | 9399 | 1 | 0.036
L3a+709 | 9591 | 1 | 0.036
L3a+709 | 9932 | 15 | 0.536
L3a1 | 12904 | 1 | 0.111
L3a1 | 13708 | 2 | 0.222
L3a1 | 14566 | 1 | 0.111
L3a1 | 150 | 1 | 0.111
L3a1 | 16086 | 1 | 0.111
L3a1 | 16126 | 2 | 0.222
L3a1 | 16145 | 1 | 0.111
L3a1 | 16184 | 1 | 0.111
L3a1 | 16189 | 1 | 0.111
L3a1 | 16220 | 1 | 0.111
L3a1 | 16274 | 1 | 0.111
L3a1 | 16391 | 1 | 0.111
L3a1 | 16399 | 1 | 0.111
L3a1 | 16468 | 1 | 0.111
L3a1 | 195 | 1 | 0.111
L3a1 | 235 | 1 | 0.111
L3a1 | 270 | 1 | 0.111
L3a1 | 414d | 1 | 0.111
L3a1 | 44.1C | 1 | 0.111
L3a1 | 7418 | 1 | 0.111
L3a1 | 7740 | 1 | 0.111
L3a1 | 8170 | 1 | 0.111
L3a1 | 8602 | 1 | 0.111
L3a1 | 979 | 1 | 0.111
L3a1 | 9947 | 1 | 0.111
L3a1a | 11328C | 1 | 0.023
L3a1a | 12681 | 1 | 0.023
L3a1a | 14319 | 1 | 0.023
L3a1a | 14927 | 2 | 0.047
L3a1a | 15109 | 2 | 0.047
L3a1a | 16024 | 1 | 0.023
L3a1a | 16025 | 1 | 0.023
L3a1a | 16029.1T | 1 | 0.023
L3a1a | 16093 | 2 | 0.047
L3a1a | 16293 | 2 | 0.047
L3a1a | 16294 | 2 | 0.047
L3a1a | 16357 | 1 | 0.023
L3a1a | 195 | 9 | 0.209
L3a1a | 2156.1A | 2 | 0.047
L3a1a | 2702 | 2 | 0.047
L3a1a | 2961 | 1 | 0.023
L3a1a | 3346A | 1 | 0.023
L3a1a | 3483 | 1 | 0.023
L3a1a | 3591 | 1 | 0.023
L3a1a | 3796 | 3 | 0.07
L3a1a | 3918 | 1 | 0.023
L3a1a | 4733 | 3 | 0.07
L3a1a | 5054 | 1 | 0.023
L3a1a | 514 | 1 | 0.023
L3a1a | 5628 | 1 | 0.023
L3a1a | 6182 | 1 | 0.023
L3a1a | 6500 | 2 | 0.047
L3a1a | 7325 | 1 | 0.023
L3a1a | 7385 | 1 | 0.023
L3a1a | 7474 | 1 | 0.023
L3a1a | 8896 | 1 | 0.023
L3a1a | 9740 | 1 | 0.023
L3a1a | 9804 | 1 | 0.023
L3a1a | 9951G | 1 | 0.023
L3a1a | 9963G | 1 | 0.023
L3a1a | 9966 | 1 | 0.023
L3a1a | 9967G | 1 | 0.023
L3a1b | 1005 | 2 | 0.222
L3a1b | 11299 | 1 | 0.111
L3a1b | 13392 | 1 | 0.111
L3a1b | 13443 | 3 | 0.333
L3a1b | 151d | 2 | 0.222
L3a1b | 16086 | 1 | 0.111
L3a1b | 16178 | 3 | 0.333
L3a1b | 16189 | 1 | 0.111
L3a1b | 16192 | 2 | 0.222
L3a1b | 16234 | 1 | 0.111
L3a1b | 16362 | 5 | 0.556
L3a1b | 204 | 1 | 0.111
L3a1b | 5310 | 3 | 0.333
L3a2 | 10289 | 1 | 0.019
L3a2 | 11368 | 1 | 0.019
L3a2 | 14215 | 1 | 0.019
L3a2 | 15312 | 1 | 0.019
L3a2 | 15754 | 1 | 0.019
L3a2 | 15924 | 1 | 0.019
L3a2 | 16075 | 1 | 0.019
L3a2 | 16076d | 1 | 0.019
L3a2 | 16093 | 28 | 0.519
L3a2 | 16145 | 3 | 0.056
L3a2 | 16169 | 1 | 0.019
L3a2 | 16176 | 3 | 0.056
L3a2 | 16192 | 38 | 0.704
L3a2 | 16222 | 1 | 0.019
L3a2 | 16230 | 1 | 0.019

L3a2 | 16234 | 2 | 0.037
L3a2 | 16270 | 1 | 0.019
L3a2 | 16278 | 1 | 0.019
L3a2 | 16300 | 3 | 0.056
L3a2 | 16304 | 1 | 0.019
L3a2 | 16357 | 2 | 0.037
L3a2 | 16362 | 43 | 0.796
L3a2 | 16400 | 1 | 0.019
L3a2 | 247 | 1 | 0.019
L3a2 | 3834 | 1 | 0.019
L3a2 | 4491 | 1 | 0.019
L3a2 | 6182 | 1 | 0.019
L3a2 | 7472 | 1 | 0.019
L3a2 | 8188 | 1 | 0.019
L3a2 | 8344T | 1 | 0.019
L3a2 | 8745 | 1 | 0.019
L3a2 | 8784 | 1 | 0.019
L3a2 | 9161 | 1 | 0.019
L3a2 | 9325 | 1 | 0.019
L3a2 | 957 | 1 | 0.019
L3a2 | 9612 | 1 | 0.019
L3a2a | 1118 | 1 | 0.04
L3a2a | 15442 | 2 | 0.08
L3a2a | 16018 | 1 | 0.04
L3a2a | 16019A | 1 | 0.04
L3a2a | 16021 | 1 | 0.04
L3a2a | 16113C | 1 | 0.04
L3a2a | 16132 | 1 | 0.04
L3a2a | 16188 | 1 | 0.04
L3a2a | 16189 | 3 | 0.12
L3a2a | 16223 | 3 | 0.12
L3a2a | 16225A | 1 | 0.04
L3a2a | 16256 | 1 | 0.04
L3a2a | 16278 | 1 | 0.04
L3a2a | 16351d | 1 | 0.04
L3a2a | 16357.1T | 1 | 0.04
L3a2a | 16362G | 1 | 0.04
L3a2a | 1812 | 2 | 0.08
L3a2a | 189 | 2 | 0.08
L3a2a | 4823 | 1 | 0.04
L3a2a | 5585 | 1 | 0.04
L3a2a | 64 | 1 | 0.04
L3a2a | 65 | 1 | 0.04
L3a2a | 6895 | 1 | 0.04
L3a2a | 70 | 1 | 0.04
L3a2a | 9254 | 2 | 0.08
L3a2a | 9458 | 2 | 0.08
L3b | 146 | 4 | 0.03
L3b | 150 | 2 | 0.015
L3b | 16068 | 1 | 0.008
L3b | 199 | 2 | 0.015
L3b | 200 | 1 | 0.008
L3b | 294 | 1 | 0.008
L3b | 366 | 1 | 0.008
L3b | 445A | 1 | 0.008
L3b | 507A | 1 | 0.008
L3b | 515-524d | 8 | 0.06
L3b | 573.1C | 1 | 0.008
L3b1 | 11176 | 2 | 0.014
L3b1 | 11800 | 2 | 0.014
L3b1 | 11800C | 2 | 0.014
L3b1 | 14133 | 1 | 0.007
L3b1 | 15099 | 4 | 0.029
L3b1 | 15734 | 1 | 0.007
L3b1 | 16048 | 3 | 0.022
L3b1 | 16224 | 1 | 0.007
L3b1 | 16234 | 3 | 0.022
L3b1 | 16243 | 1 | 0.007
L3b1 | 16274 | 1 | 0.007
L3b1 | 16292 | 1 | 0.007
L3b1 | 16294 | 1 | 0.007
L3b1 | 16311 | 3 | 0.022
L3b1 | 16327 | 1 | 0.007
L3b1 | 235 | 3 | 0.022
L3b1 | 317.1C | 3 | 0.022
L3b1 | 3644 | 1 | 0.007
L3b1 | 5063 | 2 | 0.014
L3b1 | 518 | 1 | 0.007
L3b1 | 573.1C | 1 | 0.007
L3b1 | 7618 | 4 | 0.029
L3b1 | 7859 | 1 | 0.007
L3b1 | 8530 | 2 | 0.014
L3b1 | 8616 | 4 | 0.029
L3b1 | 8875 | 2 | 0.014
L3b1a | 10424T | 1 | 0.006
L3b1a | 10493 | 2 | 0.012
L3b1a | 10538 | 1 | 0.006
L3b1a | 10595 | 3 | 0.018
L3b1a | 10786 | 1 | 0.006
L3b1a | 10909 | 1 | 0.006
L3b1a | 12028 | 1 | 0.006
L3b1a | 12408 | 1 | 0.006
L3b1a | 13862 | 1 | 0.006
L3b1a | 1393 | 1 | 0.006
L3b1a | 14004 | 1 | 0.006
L3b1a | 14554 | 1 | 0.006
L3b1a | 14560 | 2 | 0.012
L3b1a | 14791 | 1 | 0.006
L3b1a | 150 | 2 | 0.012
L3b1a | 15172 | 1 | 0.006
L3b1a | 15314 | 1 | 0.006
L3b1a | 15386G | 1 | 0.006
L3b1a | 15434 | 2 | 0.012
L3b1a | 15758 | 1 | 0.006
L3b1a | 15851 | 1 | 0.006
L3b1a | 15940 | 1 | 0.006
L3b1a | 16178 | 1 | 0.006
L3b1a | 16186 | 5 | 0.03
L3b1a | 16189 | 1 | 0.006
L3b1a | 16209 | 3 | 0.018
L3b1a | 16213 | 1 | 0.006

L3b1a | 16234 | 3 | 0.018
L3b1a | 16249 | 1 | 0.006
L3b1a | 16256 | 7 | 0.042
L3b1a | 16261 | 5 | 0.03
L3b1a | 16266 | 1 | 0.006
L3b1a | 16291 | 5 | 0.03
L3b1a | 16295 | 1 | 0.006
L3b1a | 16301 | 1 | 0.006
L3b1a | 16311 | 2 | 0.012
L3b1a | 16343 | 1 | 0.006
L3b1a | 16360 | 1 | 0.006
L3b1a | 16399 | 2 | 0.012
L3b1a | 195 | 20 | 0.119
L3b1a | 2775 | 2 | 0.012
L3b1a | 294 | 1 | 0.006
L3b1a | 309d | 2 | 0.012
L3b1a | 310 | 2 | 0.012
L3b1a | 3492 | 1 | 0.006
L3b1a | 3609 | 1 | 0.006
L3b1a | 372 | 1 | 0.006
L3b1a | 3866 | 1 | 0.006
L3b1a | 408A | 1 | 0.006
L3b1a | 4164 | 1 | 0.006
L3b1a | 4452 | 1 | 0.006
L3b1a | 455.1T | 2 | 0.012
L3b1a | 4843 | 1 | 0.006
L3b1a | 4917 | 1 | 0.006
L3b1a | 5099 | 1 | 0.006
L3b1a | 514G | 1 | 0.006
L3b1a | 515-517d | 1 | 0.006
L3b1a | 515-524d | 2 | 0.012
L3b1a | 573.1C | 1 | 0.006
L3b1a | 5892 | 1 | 0.006
L3b1a | 6249 | 1 | 0.006
L3b1a | 6371 | 1 | 0.006
L3b1a | 6932 | 3 | 0.018
L3b1a | 7160 | 1 | 0.006
L3b1a | 7340 | 3 | 0.018
L3b1a | 7772 | 1 | 0.006
L3b1a | 8155 | 1 | 0.006
L3b1a | 8347 | 1 | 0.006
L3b1a | 8393 | 1 | 0.006
L3b1a | 8512 | 1 | 0.006
L3b1a | 8588 | 2 | 0.012
L3b1a | 8772 | 2 | 0.012
L3b1a | 8995 | 1 | 0.006
L3b1a | 9055 | 1 | 0.006
L3b1a | 9305 | 1 | 0.006
L3b1a+@16124 | 10646 | 1 | 0.005
L3b1a+@16124 | 11016 | 1 | 0.005
L3b1a+@16124 | 111C | 1 | 0.005
L3b1a+@16124 | 11257 | 1 | 0.005
L3b1a+@16124 | 11944 | 1 | 0.005
L3b1a+@16124 | 12123 | 1 | 0.005
L3b1a+@16124 | 12557 | 1 | 0.005
L3b1a+@16124 | 13662 | 1 | 0.005
L3b1a+@16124 | 13932 | 1 | 0.005
L3b1a+@16124 | 13986C | 1 | 0.005
L3b1a+@16124 | 14553 | 1 | 0.005
L3b1a+@16124 | 15172 | 1 | 0.005
L3b1a+@16124 | 152 | 7 | 0.035
L3b1a+@16124 | 15883 | 1 | 0.005
L3b1a+@16124 | 16086G | 1 | 0.005
L3b1a+@16124 | 16093 | 65 | 0.322
L3b1a+@16124 | 16121G | 1 | 0.005
L3b1a+@16124 | 16124G | 1 | 0.005
L3b1a+@16124 | 16126G | 1 | 0.005
L3b1a+@16124 | 16174G | 1 | 0.005
L3b1a+@16124 | 16184 | 1 | 0.005
L3b1a+@16124 | 16209 | 1 | 0.005
L3b1a+@16124 | 16221 | 2 | 0.01
L3b1a+@16124 | 16228G | 1 | 0.005
L3b1a+@16124 | 16240 | 1 | 0.005
L3b1a+@16124 | 16249A | 3 | 0.015
L3b1a+@16124 | 16256 | 3 | 0.015
L3b1a+@16124 | 16274 | 1 | 0.005
L3b1a+@16124 | 16291 | 1 | 0.005
L3b1a+@16124 | 16311 | 1 | 0.005
L3b1a+@16124 | 16322T | 1 | 0.005
L3b1a+@16124 | 16355 | 1 | 0.005
L3b1a+@16124 | 16356 | 1 | 0.005
L3b1a+@16124 | 16399 | 2 | 0.01
L3b1a+@16124 | 16496 | 1 | 0.005
L3b1a+@16124 | 16527 | 2 | 0.01
L3b1a+@16124 | 1804 | 1 | 0.005
L3b1a+@16124 | 182 | 1 | 0.005
L3b1a+@16124 | 185T | 1 | 0.005
L3b1a+@16124 | 195 | 1 | 0.005
L3b1a+@16124 | 199 | 1 | 0.005
L3b1a+@16124 | 2416 | 1 | 0.005
L3b1a+@16124 | 247 | 1 | 0.005
L3b1a+@16124 | 357 | 1 | 0.005
L3b1a+@16124 | 4113C | 1 | 0.005
L3b1a+@16124 | 474A | 1 | 0.005
L3b1a+@16124 | 507A | 1 | 0.005
L3b1a+@16124 | 5090 | 1 | 0.005
L3b1a+@16124 | 515-524d | 40 | 0.198
L3b1a+@16124 | 5930 | 1 | 0.005
L3b1a+@16124 | 8447 | 1 | 0.005
L3b1a+@16124 | 8492 | 1 | 0.005
L3b1a+@16124 | 8654 | 1 | 0.005
L3b1a+@16124 | 8697 | 1 | 0.005
L3b1a+@16124 | 8928 | 1 | 0.005
L3b1a+@16124 | 9168 | 1 | 0.005
L3b1a+@16124 | 9300 | 1 | 0.005
L3b1a+@16124 | 94 | 1 | 0.005
L3b1a+@16124 | 9604 | 1 | 0.005
L3b1a+@16124 | 9947 | 1 | 0.005
L3b1a+152 | 10424T | 1 | 0.01
L3b1a+152 | 10601 | 1 | 0.01
L3b1a+152 | 10700 | 1 | 0.01

L3b1a+152 | 11149 | 1 | 0.01
L3b1a+152 | 11380 | 1 | 0.01
L3b1a+152 | 12612 | 1 | 0.01
L3b1a+152 | 12804 | 2 | 0.021
L3b1a+152 | 13926 | 1 | 0.01
L3b1a+152 | 14364 | 1 | 0.01
L3b1a+152 | 151 | 1 | 0.01
L3b1a+152 | 15119 | 1 | 0.01
L3b1a+152 | 15314 | 3 | 0.031
L3b1a+152 | 15434 | 1 | 0.01
L3b1a+152 | 15672 | 1 | 0.01
L3b1a+152 | 15812 | 1 | 0.01
L3b1a+152 | 15900 | 2 | 0.021
L3b1a+152 | 16170 | 2 | 0.021
L3b1a+152 | 16234 | 1 | 0.01
L3b1a+152 | 16249 | 1 | 0.01
L3b1a+152 | 16274 | 3 | 0.031
L3b1a+152 | 16291 | 2 | 0.021
L3b1a+152 | 16293 | 2 | 0.021
L3b1a+152 | 16309 | 1 | 0.01
L3b1a+152 | 16311 | 15 | 0.156
L3b1a+152 | 16320 | 1 | 0.01
L3b1a+152 | 16409 | 1 | 0.01
L3b1a+152 | 16527 | 3 | 0.031
L3b1a+152 | 1717 | 1 | 0.01
L3b1a+152 | 1780 | 1 | 0.01
L3b1a+152 | 200 | 7 | 0.073
L3b1a+152 | 2071 | 1 | 0.01
L3b1a+152 | 249 | 1 | 0.01
L3b1a+152 | 3019 | 1 | 0.01
L3b1a+152 | 310 | 4 | 0.042
L3b1a+152 | 3492 | 1 | 0.01
L3b1a+152 | 372 | 2 | 0.021
L3b1a+152 | 408A | 1 | 0.01
L3b1a+152 | 4164 | 1 | 0.01
L3b1a+152 | 480 | 2 | 0.021
L3b1a+152 | 482 | 2 | 0.021
L3b1a+152 | 499 | 1 | 0.01
L3b1a+152 | 5063 | 1 | 0.01
L3b1a+152 | 515-524d | 2 | 0.021
L3b1a+152 | 5417 | 1 | 0.01
L3b1a+152 | 5605 | 1 | 0.01
L3b1a+152 | 5633 | 1 | 0.01
L3b1a+152 | 6722 | 1 | 0.01
L3b1a+152 | 7759 | 1 | 0.01
L3b1a+152 | 8347 | 2 | 0.021
L3b1a+152 | 8393 | 1 | 0.01
L3b1a+152 | 8537 | 2 | 0.021
L3b1a+152 | 8547 | 1 | 0.01
L3b1a+152 | 8772 | 1 | 0.01
L3b1a+152 | 8812 | 1 | 0.01
L3b1a+152 | 9055 | 1 | 0.01
L3b1a+152 | 9174 | 1 | 0.01
L3b1a+152 | 9300 | 2 | 0.021
L3b1a+152 | 9305 | 1 | 0.01
L3b1a+152 | 9615 | 1 | 0.01
L3b1a+152 | 9983 | 1 | 0.01
L3b1a1 | 11944 | 3 | 0.024
L3b1a1 | 13708 | 3 | 0.024
L3b1a1 | 14233 | 1 | 0.008
L3b1a1 | 16093 | 2 | 0.016
L3b1a1 | 16189 | 3 | 0.024
L3b1a1 | 199 | 1 | 0.008
L3b1a1 | 264 | 1 | 0.008
L3b1a1 | 309d | 1 | 0.008
L3b1a1 | 3202 | 1 | 0.008
L3b1a1 | 3866 | 1 | 0.008
L3b1a1 | 5402 | 1 | 0.008
L3b1a1 | 573.1C | 1 | 0.008
L3b1a1 | 8027 | 1 | 0.008
L3b1a1 | 851 | 1 | 0.008
L3b1a10 | 14182 | 2 | 0.014
L3b1a10 | 14323 | 1 | 0.007
L3b1a10 | 16048 | 3 | 0.021
L3b1a10 | 16079 | 1 | 0.007
L3b1a10 | 16189 | 2 | 0.014
L3b1a10 | 16221 | 1 | 0.007
L3b1a10 | 16224 | 1 | 0.007
L3b1a10 | 16243 | 1 | 0.007
L3b1a10 | 16271 | 1 | 0.007
L3b1a10 | 16274 | 1 | 0.007
L3b1a10 | 16292 | 1 | 0.007
L3b1a10 | 16327 | 1 | 0.007
L3b1a10 | 16484-16489d | 2 | 0.014
L3b1a10 | 16527 | 1 | 0.007
L3b1a10 | 189 | 4 | 0.028
L3b1a10 | 207 | 1 | 0.007
L3b1a10 | 235 | 3 | 0.021
L3b1a10 | 309d | 1 | 0.007
L3b1a10 | 310 | 1 | 0.007
L3b1a10 | 467 | 1 | 0.007
L3b1a10 | 518 | 1 | 0.007
L3b1a10 | 573.1C | 1 | 0.007
L3b1a10 | 5978 | 1 | 0.007
L3b1a10 | 6249 | 2 | 0.014
L3b1a10 | 6510 | 1 | 0.007
L3b1a10 | 8279 | 2 | 0.014
L3b1a10 | 8455 | 1 | 0.007
L3b1a10 | 9647 | 2 | 0.014
L3b1a11 | 11315 | 1 | 0.009
L3b1a11 | 11794 | 1 | 0.009
L3b1a11 | 11914 | 1 | 0.009
L3b1a11 | 15043 | 1 | 0.009
L3b1a11 | 152 | 3 | 0.028
L3b1a11 | 15940 | 1 | 0.009
L3b1a11 | 16024 | 1 | 0.009
L3b1a11 | 16234 | 2 | 0.019
L3b1a11 | 16255 | 1 | 0.009
L3b1a11 | 16293 | 2 | 0.019
L3b1a11 | 16294 | 2 | 0.019
L3b1a11 | 309d | 5 | 0.047

L3b1a11 | 513 | 3 | 0.028
L3b1a11 | 5147 | 3 | 0.028
L3b1a11 | 523-525d | 1 | 0.009
L3b1a11 | 537 | 1 | 0.009
L3b1a11 | 5582 | 1 | 0.009
L3b1a11 | 8473 | 1 | 0.009
L3b1a11 | 9654 | 1 | 0.009
L3b1a1a | 10006 | 1 | 0.002
L3b1a1a | 100-103d | 1 | 0.002
L3b1a1a | 10042 | 1 | 0.002
L3b1a1a | 1007 | 1 | 0.002
L3b1a1a | 10496 | 3 | 0.007
L3b1a1a | 105 | 1 | 0.002
L3b1a1a | 105-110d | 3 | 0.007
L3b1a1a | 10586 | 4 | 0.01
L3b1a1a | 11087 | 1 | 0.002
L3b1a1a | 11167 | 1 | 0.002
L3b1a1a | 11347 | 4 | 0.01
L3b1a1a | 1142 | 1 | 0.002
L3b1a1a | 1145 | 1 | 0.002
L3b1a1a | 11827 | 1 | 0.002
L3b1a1a | 11845 | 1 | 0.002
L3b1a1a | 11914 | 1 | 0.002
L3b1a1a | 11965A | 1 | 0.002
L3b1a1a | 12022 | 2 | 0.005
L3b1a1a | 12684 | 1 | 0.002
L3b1a1a | 12903 | 3 | 0.007
L3b1a1a | 13092d | 1 | 0.002
L3b1a1a | 13179G | 1 | 0.002
L3b1a1a | 13420 | 1 | 0.002
L3b1a1a | 13639A | 1 | 0.002
L3b1a1a | 1369A | 1 | 0.002
L3b1a1a | 1370A | 1 | 0.002
L3b1a1a | 13712 | 1 | 0.002
L3b1a1a | 13743 | 11 | 0.026
L3b1a1a | 13759 | 1 | 0.002
L3b1a1a | 13928C | 1 | 0.002
L3b1a1a | 14180 | 1 | 0.002
L3b1a1a | 14182 | 1 | 0.002
L3b1a1a | 14280 | 1 | 0.002
L3b1a1a | 14374 | 1 | 0.002
L3b1a1a | 14433 | 1 | 0.002
L3b1a1a | 14443 | 1 | 0.002
L3b1a1a | 14540 | 1 | 0.002
L3b1a1a | 14565 | 12 | 0.029
L3b1a1a | 14577 | 3 | 0.007
L3b1a1a | 1473 | 1 | 0.002
L3b1a1a | 14811 | 1 | 0.002
L3b1a1a | 14905 | 3 | 0.007
L3b1a1a | 15013 | 2 | 0.005
L3b1a1a | 152 | 4 | 0.01
L3b1a1a | 1541 | 1 | 0.002
L3b1a1a | 15484 | 1 | 0.002
L3b1a1a | 15670 | 3 | 0.007
L3b1a1a | 15758 | 1 | 0.002
L3b1a1a | 15813 | 1 | 0.002
L3b1a1a | 15902 | 1 | 0.002
L3b1a1a | 16024 | 2 | 0.005
L3b1a1a | 16025 | 2 | 0.005
L3b1a1a | 16042 | 1 | 0.002
L3b1a1a | 16075 | 2 | 0.005
L3b1a1a | 16093 | 238 | 0.572
L3b1a1a | 16111G | 1 | 0.002
L3b1a1a | 16114 | 7 | 0.017
L3b1a1a | 16126 | 1 | 0.002
L3b1a1a | 16129 | 2 | 0.005
L3b1a1a | 16137 | 1 | 0.002
L3b1a1a | 16148 | 1 | 0.002
L3b1a1a | 16184 | 1 | 0.002
L3b1a1a | 16189 | 5 | 0.012
L3b1a1a | 16217 | 6 | 0.014
L3b1a1a | 16293 | 1 | 0.002
L3b1a1a | 16293C | 1 | 0.002
L3b1a1a | 16311 | 4 | 0.01
L3b1a1a | 16320 | 1 | 0.002
L3b1a1a | 16390 | 1 | 0.002
L3b1a1a | 16399 | 1 | 0.002
L3b1a1a | 16527 | 1 | 0.002
L3b1a1a | 1657 | 1 | 0.002
L3b1a1a | 1808 | 1 | 0.002
L3b1a1a | 1894 | 1 | 0.002
L3b1a1a | 195 | 6 | 0.014
L3b1a1a | 200 | 1 | 0.002
L3b1a1a | 204 | 2 | 0.005
L3b1a1a | 2634 | 1 | 0.002
L3b1a1a | 2778 | 1 | 0.002
L3b1a1a | 2831 | 1 | 0.002
L3b1a1a | 286 | 1 | 0.002
L3b1a1a | 2947 | 1 | 0.002
L3b1a1a | 3016C | 1 | 0.002
L3b1a1a | 3017 | 1 | 0.002
L3b1a1a | 3018T | 1 | 0.002
L3b1a1a | 309d | 4 | 0.01
L3b1a1a | 3105 | 2 | 0.005
L3b1a1a | 315.2C | 1 | 0.002
L3b1a1a | 3158.1T | 1 | 0.002
L3b1a1a | 316 | 1 | 0.002
L3b1a1a | 3183 | 1 | 0.002
L3b1a1a | 3394 | 2 | 0.005
L3b1a1a | 3592 | 1 | 0.002
L3b1a1a | 3684 | 1 | 0.002
L3b1a1a | 3720 | 3 | 0.007
L3b1a1a | 373 | 1 | 0.002
L3b1a1a | 3834 | 1 | 0.002
L3b1a1a | 385 | 2 | 0.005
L3b1a1a | 3915 | 3 | 0.007
L3b1a1a | 4188 | 1 | 0.002
L3b1a1a | 4215 | 1 | 0.002
L3b1a1a | 4227 | 1 | 0.002
L3b1a1a | 4317 | 3 | 0.007
L3b1a1a | 4491 | 2 | 0.005

L3b1a1a | 4512 | 2 | 0.005
L3b1a1a | 456 | 1 | 0.002
L3b1a1a | 4703 | 1 | 0.002
L3b1a1a | 4907 | 1 | 0.002
L3b1a1a | 4991 | 1 | 0.002
L3b1a1a | 5123 | 2 | 0.005
L3b1a1a | 514d | 1 | 0.002
L3b1a1a | 517d | 1 | 0.002
L3b1a1a | 5300 | 1 | 0.002
L3b1a1a | 5301 | 1 | 0.002
L3b1a1a | 5330 | 1 | 0.002
L3b1a1a | 573.1C | 1 | 0.002
L3b1a1a | 593 | 1 | 0.002
L3b1a1a | 5979 | 1 | 0.002
L3b1a1a | 6052 | 1 | 0.002
L3b1a1a | 62 | 1 | 0.002
L3b1a1a | 6366 | 4 | 0.01
L3b1a1a | 6512 | 1 | 0.002
L3b1a1a | 6707 | 1 | 0.002
L3b1a1a | 6743 | 1 | 0.002
L3b1a1a | 6918 | 1 | 0.002
L3b1a1a | 6962 | 2 | 0.005
L3b1a1a | 6975 | 1 | 0.002
L3b1a1a | 7444 | 3 | 0.007
L3b1a1a | 7501 | 1 | 0.002
L3b1a1a | 7830 | 1 | 0.002
L3b1a1a | 7925 | 1 | 0.002
L3b1a1a | 794 | 1 | 0.002
L3b1a1a | 8251 | 2 | 0.005
L3b1a1a | 8254 | 1 | 0.002
L3b1a1a | 8269 | 2 | 0.005
L3b1a1a | 8281-8289d | 1 | 0.002
L3b1a1a | 8393 | 1 | 0.002
L3b1a1a | 8412 | 1 | 0.002
L3b1a1a | 847T | 1 | 0.002
L3b1a1a | 8764 | 1 | 0.002
L3b1a1a | 8839 | 2 | 0.005
L3b1a1a | 8888 | 1 | 0.002
L3b1a1a | 8978 | 1 | 0.002
L3b1a1a | 9137 | 1 | 0.002
L3b1a1a | 9480G | 1 | 0.002
L3b1a1a | 9575 | 1 | 0.002
L3b1a1a | 97 | 1 | 0.002
L3b1a1a | 9780T | 1 | 0.002
L3b1a1a | 9966 | 1 | 0.002
L3b1a2 | 10462 | 1 | 0.007
L3b1a2 | 10931 | 1 | 0.007
L3b1a2 | 11410 | 2 | 0.015
L3b1a2 | 11545 | 1 | 0.007
L3b1a2 | 12490 | 3 | 0.022
L3b1a2 | 12771 | 1 | 0.007
L3b1a2 | 13269 | 1 | 0.007
L3b1a2 | 13419 | 1 | 0.007
L3b1a2 | 13599 | 1 | 0.007
L3b1a2 | 14393 | 2 | 0.015
L3b1a2 | 15203 | 1 | 0.007
L3b1a2 | 16048 | 3 | 0.022
L3b1a2 | 16086 | 2 | 0.015
L3b1a2 | 16093 | 1 | 0.007
L3b1a2 | 16224 | 1 | 0.007
L3b1a2 | 16243 | 1 | 0.007
L3b1a2 | 16274 | 1 | 0.007
L3b1a2 | 16292 | 1 | 0.007
L3b1a2 | 16311 | 3 | 0.022
L3b1a2 | 16325 | 1 | 0.007
L3b1a2 | 16327 | 1 | 0.007
L3b1a2 | 195 | 3 | 0.022
L3b1a2 | 198 | 1 | 0.007
L3b1a2 | 235 | 3 | 0.022
L3b1a2 | 3221 | 1 | 0.007
L3b1a2 | 3504 | 1 | 0.007
L3b1a2 | 518 | 1 | 0.007
L3b1a2 | 573.1C | 1 | 0.007
L3b1a2 | 7570 | 1 | 0.007
L3b1a2 | 8065 | 1 | 0.007
L3b1a2 | 8289.1CCCCCTCTA | 1 | 0.007
L3b1a3 | 1193 | 1 | 0.009
L3b1a3 | 146 | 4 | 0.036
L3b1a3 | 150 | 2 | 0.018
L3b1a3 | 152 | 2 | 0.018
L3b1a3 | 16093 | 3 | 0.027
L3b1a3 | 16111 | 1 | 0.009
L3b1a3 | 16117 | 3 | 0.027
L3b1a3 | 16126 | 1 | 0.009
L3b1a3 | 16189 | 1 | 0.009
L3b1a3 | 16196 | 2 | 0.018
L3b1a3 | 16215 | 1 | 0.009
L3b1a3 | 16234 | 2 | 0.018
L3b1a3 | 16290 | 2 | 0.018
L3b1a3 | 16294 | 2 | 0.018
L3b1a3 | 16320 | 1 | 0.009
L3b1a3 | 195 | 5 | 0.045
L3b1a3 | 199 | 1 | 0.009
L3b1a3 | 200 | 1 | 0.009
L3b1a3 | 249d | 1 | 0.009
L3b1a3 | 3523 | 1 | 0.009
L3b1a3 | 503 | 1 | 0.009
L3b1a3 | 515-524d | 4 | 0.036
L3b1a3 | 7964 | 1 | 0.009
L3b1a3 | 8206 | 2 | 0.018
L3b1a3 | 8251 | 1 | 0.009
L3b1a3 | 9548 | 1 | 0.009
L3b1a3 | 960d | 2 | 0.018
L3b1a4 | 10535 | 1 | 0.008
L3b1a4 | 12557 | 1 | 0.008
L3b1a4 | 13050 | 1 | 0.008
L3b1a4 | 13443 | 1 | 0.008
L3b1a4 | 14212 | 1 | 0.008
L3b1a4 | 14755 | 1 | 0.008
L3b1a4 | 152 | 1 | 0.008
L3b1a4 | 15884 | 1 | 0.008

L3b1a4 | 16075 | 1 | 0.008
L3b1a4 | 16255 | 1 | 0.008
L3b1a4 | 16311 | 2 | 0.016
L3b1a4 | 189 | 1 | 0.008
L3b1a4 | 199 | 1 | 0.008
L3b1a4 | 1999 | 1 | 0.008
L3b1a4 | 3645 | 1 | 0.008
L3b1a4 | 4562 | 1 | 0.008
L3b1a4 | 513 | 1 | 0.008
L3b1a4 | 5460 | 1 | 0.008
L3b1a4 | 573.1C | 1 | 0.008
L3b1a4 | 6249 | 1 | 0.008
L3b1a4 | 678 | 1 | 0.008
L3b1a4 | 9374 | 1 | 0.008
L3b1a5 | 11176 | 1 | 0.008
L3b1a5 | 14106 | 1 | 0.008
L3b1a5 | 14476 | 1 | 0.008
L3b1a5 | 15061 | 1 | 0.008
L3b1a5 | 15119 | 1 | 0.008
L3b1a5 | 15658 | 1 | 0.008
L3b1a5 | 16294 | 1 | 0.008
L3b1a5 | 16311 | 1 | 0.008
L3b1a5 | 16483 | 1 | 0.008
L3b1a5 | 195 | 1 | 0.008
L3b1a5 | 199 | 1 | 0.008
L3b1a5 | 310 | 1 | 0.008
L3b1a5 | 480 | 1 | 0.008
L3b1a5 | 4907 | 1 | 0.008
L3b1a5 | 497 | 1 | 0.008
L3b1a5 | 499 | 1 | 0.008
L3b1a5 | 5196 | 1 | 0.008
L3b1a5 | 573.1C | 1 | 0.008
L3b1a5 | 8155 | 1 | 0.008
L3b1a5 | 8723 | 1 | 0.008
L3b1a5 | 8875 | 1 | 0.008
L3b1a5a | 11004 | 1 | 0.008
L3b1a5a | 15145 | 1 | 0.008
L3b1a5a | 152 | 1 | 0.008
L3b1a5a | 16048 | 3 | 0.023
L3b1a5a | 16093 | 1 | 0.008
L3b1a5a | 16224 | 1 | 0.008
L3b1a5a | 16243 | 1 | 0.008
L3b1a5a | 16274 | 1 | 0.008
L3b1a5a | 16292 | 1 | 0.008
L3b1a5a | 16327 | 1 | 0.008
L3b1a5a | 199 | 1 | 0.008
L3b1a5a | 235 | 3 | 0.023
L3b1a5a | 518 | 1 | 0.008
L3b1a5a | 5460 | 1 | 0.008
L3b1a5a | 573.1C | 1 | 0.008
L3b1a6 | 10306C | 1 | 0.013
L3b1a6 | 11914 | 1 | 0.013
L3b1a6 | 13658 | 3 | 0.039
L3b1a6 | 14839 | 1 | 0.013
L3b1a6 | 15217 | 1 | 0.013
L3b1a6 | 15314 | 1 | 0.013
L3b1a6 | 15323 | 1 | 0.013
L3b1a6 | 16039 | 1 | 0.013
L3b1a6 | 16172 | 1 | 0.013
L3b1a6 | 16189 | 2 | 0.026
L3b1a6 | 16270 | 1 | 0.013
L3b1a6 | 16271 | 1 | 0.013
L3b1a6 | 16274 | 1 | 0.013
L3b1a6 | 189 | 1 | 0.013
L3b1a6 | 195 | 3 | 0.039
L3b1a6 | 200 | 1 | 0.013
L3b1a6 | 2487C | 1 | 0.013
L3b1a6 | 3003 | 1 | 0.013
L3b1a6 | 5585 | 1 | 0.013
L3b1a6 | 5673 | 1 | 0.013
L3b1a6 | 593 | 1 | 0.013
L3b1a6 | 6040 | 1 | 0.013
L3b1a6 | 634 | 1 | 0.013
L3b1a6 | 7313 | 1 | 0.013
L3b1a7 | 10181 | 2 | 0.015
L3b1a7 | 10646 | 1 | 0.008
L3b1a7 | 13506 | 1 | 0.008
L3b1a7 | 150 | 1 | 0.008
L3b1a7 | 16111 | 1 | 0.008
L3b1a7 | 16140 | 1 | 0.008
L3b1a7 | 16180 | 1 | 0.008
L3b1a7 | 16209 | 1 | 0.008
L3b1a7 | 16245 | 1 | 0.008
L3b1a7 | 16356 | 2 | 0.015
L3b1a7 | 16527 | 1 | 0.008
L3b1a7 | 195 | 1 | 0.008
L3b1a7 | 204 | 1 | 0.008
L3b1a7 | 499 | 2 | 0.015
L3b1a7 | 5460 | 1 | 0.008
L3b1a7 | 8108 | 1 | 0.008
L3b1a7a | 10320 | 3 | 0.022
L3b1a7a | 11465 | 2 | 0.015
L3b1a7a | 1193 | 2 | 0.015
L3b1a7a | 12214A | 1 | 0.007
L3b1a7a | 12634 | 1 | 0.007
L3b1a7a | 13711 | 1 | 0.007
L3b1a7a | 150 | 1 | 0.007
L3b1a7a | 15097 | 1 | 0.007
L3b1a7a | 1607 | 1 | 0.007
L3b1a7a | 16111 | 1 | 0.007
L3b1a7a | 16140 | 1 | 0.007
L3b1a7a | 16148 | 1 | 0.007
L3b1a7a | 16180 | 1 | 0.007
L3b1a7a | 16245 | 1 | 0.007
L3b1a7a | 16290 | 2 | 0.015
L3b1a7a | 16291 | 1 | 0.007
L3b1a7a | 16292 | 1 | 0.007
L3b1a7a | 16348 | 1 | 0.007
L3b1a7a | 16355 | 7 | 0.051
L3b1a7a | 2010 | 1 | 0.007
L3b1a7a | 204 | 1 | 0.007

L3b1a7a | 4586 | 1 | 0.007
L3b1a7a | 499 | 2 | 0.015
L3b1a7a | 5495 | 1 | 0.007
L3b1a7a | 8478 | 1 | 0.007
L3b1a8 | 10117 | 2 | 0.019
L3b1a8 | 11401 | 2 | 0.019
L3b1a8 | 11843 | 1 | 0.009
L3b1a8 | 12561 | 2 | 0.019
L3b1a8 | 13437 | 1 | 0.009
L3b1a8 | 152 | 6 | 0.056
L3b1a8 | 16093 | 2 | 0.019
L3b1a8 | 16129 | 3 | 0.028
L3b1a8 | 16172 | 1 | 0.009
L3b1a8 | 16192 | 1 | 0.009
L3b1a8 | 16235 | 2 | 0.019
L3b1a8 | 16271 | 1 | 0.009
L3b1a8 | 16295 | 3 | 0.028
L3b1a8 | 195 | 8 | 0.074
L3b1a8 | 1977 | 2 | 0.019
L3b1a8 | 249d | 1 | 0.009
L3b1a8 | 279 | 23 | 0.213
L3b1a8 | 281 | 1 | 0.009
L3b1a8 | 316 | 1 | 0.009
L3b1a8 | 317.1C | 2 | 0.019
L3b1a8 | 3434 | 1 | 0.009
L3b1a8 | 5028 | 1 | 0.009
L3b1a8 | 5147 | 2 | 0.019
L3b1a8 | 5585 | 1 | 0.009
L3b1a8 | 573.2C | 2 | 0.019
L3b1a8 | 575.2C | 1 | 0.009
L3b1a8 | 6340 | 16 | 0.148
L3b1a8 | 9009 | 1 | 0.009
L3b1a8 | 9117 | 1 | 0.009
L3b1a8 | 9355 | 1 | 0.009
L3b1a8 | 9531 | 1 | 0.009
L3b1a8 | 9533 | 2 | 0.019
L3b1a9 | 10819 | 1 | 0.011
L3b1a9 | 11386 | 1 | 0.011
L3b1a9 | 1243 | 1 | 0.011
L3b1a9 | 14267 | 1 | 0.011
L3b1a9 | 14670 | 1 | 0.011
L3b1a9 | 150 | 1 | 0.011
L3b1a9 | 152 | 1 | 0.011
L3b1a9 | 16256 | 1 | 0.011
L3b1a9 | 16266 | 1 | 0.011
L3b1a9 | 16354 | 1 | 0.011
L3b1a9 | 195 | 3 | 0.034
L3b1a9 | 297 | 1 | 0.011
L3b1a9 | 310 | 2 | 0.023
L3b1a9 | 383 | 1 | 0.011
L3b1a9 | 4712 | 2 | 0.023
L3b1a9 | 474A | 1 | 0.011
L3b1a9 | 4886 | 2 | 0.023
L3b1a9 | 507 | 1 | 0.011
L3b1a9 | 515-524d | 2 | 0.023
L3b1a9 | 529T | 1 | 0.011
L3b1a9 | 7521 | 2 | 0.023
L3b1a9a | 1005 | 1 | 0.012
L3b1a9a | 11962 | 1 | 0.012
L3b1a9a | 12070 | 1 | 0.012
L3b1a9a | 13586 | 2 | 0.024
L3b1a9a | 13651 | 1 | 0.012
L3b1a9a | 152 | 3 | 0.036
L3b1a9a | 15758 | 1 | 0.012
L3b1a9a | 16111 | 1 | 0.012
L3b1a9a | 16148 | 1 | 0.012
L3b1a9a | 16221 | 1 | 0.012
L3b1a9a | 16265C | 1 | 0.012
L3b1a9a | 16274 | 2 | 0.024
L3b1a9a | 16284 | 1 | 0.012
L3b1a9a | 16301 | 1 | 0.012
L3b1a9a | 1719 | 1 | 0.012
L3b1a9a | 228 | 1 | 0.012
L3b1a9a | 310 | 1 | 0.012
L3b1a9a | 3957 | 1 | 0.012
L3b1a9a | 6260 | 1 | 0.012
L3b1a9a | 7738 | 1 | 0.012
L3b1a9a | 7897 | 1 | 0.012
L3b1a9a | 8557 | 2 | 0.024
L3b1a9a | 8619 | 1 | 0.012
L3b1a9a | 8725 | 1 | 0.012
L3b1a9a | 9300 | 1 | 0.012
L3b1b | 151 | 13 | 0.157
L3b1b | 16093 | 1 | 0.012
L3b1b | 16172 | 1 | 0.012
L3b1b | 16189 | 2 | 0.024
L3b1b | 16220 | 1 | 0.012
L3b1b | 16234 | 2 | 0.024
L3b1b | 16256 | 1 | 0.012
L3b1b | 16352 | 1 | 0.012
L3b1b | 16496 | 2 | 0.024
L3b1b | 16527 | 1 | 0.012
L3b1b | 309d | 1 | 0.012
L3b1b | 389 | 1 | 0.012
L3b1b | 4062 | 1 | 0.012
L3b1b | 494A | 1 | 0.012
L3b1b | 498.1C | 1 | 0.012
L3b1b | 515-524d | 12 | 0.145
L3b1b | 6891 | 2 | 0.024
L3b1b1 | 10084 | 1 | 0.048
L3b1b1 | 12441 | 2 | 0.095
L3b1b1 | 13434 | 2 | 0.095
L3b1b1 | 14180 | 1 | 0.048
L3b1b1 | 150 | 1 | 0.048
L3b1b1 | 15110 | 1 | 0.048
L3b1b1 | 15217 | 1 | 0.048
L3b1b1 | 15394 | 1 | 0.048
L3b1b1 | 16052A | 1 | 0.048
L3b1b1 | 16172 | 1 | 0.048
L3b1b1 | 16203 | 1 | 0.048
L3b1b1 | 16294 | 4 | 0.19

L3b1b1 | 16327 | 1 | 0.048
L3b1b1 | 16474T | 1 | 0.048
L3b1b1 | 16484-16489d | 1 | 0.048
L3b1b1 | 3394 | 1 | 0.048
L3b1b1 | 480 | 1 | 0.048
L3b1b1 | 5371G | 1 | 0.048
L3b1b1 | 59 | 1 | 0.048
L3b1b1 | 8584 | 1 | 0.048
L3b2 | 12732 | 3 | 0.035
L3b2 | 14788 | 1 | 0.012
L3b2 | 14950 | 2 | 0.023
L3b2 | 14953 | 3 | 0.035
L3b2 | 15927 | 3 | 0.035
L3b2 | 16086 | 1 | 0.012
L3b2 | 16093 | 1 | 0.012
L3b2 | 16108 | 1 | 0.012
L3b2 | 16111 | 1 | 0.012
L3b2 | 16189 | 3 | 0.035
L3b2 | 16192 | 1 | 0.012
L3b2 | 16214 | 1 | 0.012
L3b2 | 16218 | 1 | 0.012
L3b2 | 16256 | 2 | 0.023
L3b2 | 16271 | 9 | 0.105
L3b2 | 16292 | 1 | 0.012
L3b2 | 16300 | 1 | 0.012
L3b2 | 16390 | 2 | 0.023
L3b2 | 309d | 1 | 0.012
L3b2 | 3645 | 1 | 0.012
L3b2 | 5231 | 2 | 0.023
L3b2 | 6392 | 1 | 0.012
L3b2 | 6917 | 2 | 0.023
L3b2 | 8811 | 3 | 0.035
L3b2 | 9299 | 3 | 0.035
L3b2 | 9386 | 3 | 0.035
L3b2a | 10586 | 1 | 0.012
L3b2a | 11150 | 1 | 0.012
L3b2a | 11485 | 1 | 0.012
L3b2a | 11935 | 1 | 0.012
L3b2a | 12236 | 1 | 0.012
L3b2a | 13889 | 1 | 0.012
L3b2a | 14161 | 1 | 0.012
L3b2a | 143 | 2 | 0.024
L3b2a | 146 | 1 | 0.012
L3b2a | 14914T | 1 | 0.012
L3b2a | 14931 | 1 | 0.012
L3b2a | 152 | 1 | 0.012
L3b2a | 15654A | 1 | 0.012
L3b2a | 15766 | 1 | 0.012
L3b2a | 16093 | 2 | 0.024
L3b2a | 16117 | 1 | 0.012
L3b2a | 16145 | 8 | 0.096
L3b2a | 16150 | 1 | 0.012
L3b2a | 16193d | 2 | 0.024
L3b2a | 16214 | 2 | 0.024
L3b2a | 16227 | 1 | 0.012
L3b2a | 16255 | 2 | 0.024
L3b2a | 16271 | 1 | 0.012
L3b2a | 16294 | 1 | 0.012
L3b2a | 16311 | 2 | 0.024
L3b2a | 16368 | 1 | 0.012
L3b2a | 189 | 4 | 0.048
L3b2a | 195 | 3 | 0.036
L3b2a | 2093 | 1 | 0.012
L3b2a | 225T | 1 | 0.012
L3b2a | 227 | 1 | 0.012
L3b2a | 279 | 1 | 0.012
L3b2a | 4336 | 1 | 0.012
L3b2a | 6071 | 1 | 0.012
L3b2a | 7100 | 1 | 0.012
L3b2a | 7301 | 2 | 0.024
L3b2a | 8496 | 1 | 0.012
L3b2a | 9010 | 1 | 0.012
L3b2a | 9438 | 1 | 0.012
L3b2b | 11260 | 1 | 0.011
L3b2b | 11800 | 1 | 0.011
L3b2b | 146 | 1 | 0.011
L3b2b | 15804 | 1 | 0.011
L3b2b | 15940 | 1 | 0.011
L3b2b | 16086 | 1 | 0.011
L3b2b | 16093 | 1 | 0.011
L3b2b | 16108 | 1 | 0.011
L3b2b | 16111 | 1 | 0.011
L3b2b | 16114G | 1 | 0.011
L3b2b | 16192 | 1 | 0.011
L3b2b | 16274 | 1 | 0.011
L3b2b | 16311 | 3 | 0.033
L3b2b | 16355 | 6 | 0.066
L3b2b | 16399 | 1 | 0.011
L3b2b | 16526 | 1 | 0.011
L3b2b | 195 | 5 | 0.055
L3b2b | 199 | 3 | 0.033
L3b2b | 2141 | 1 | 0.011
L3b2b | 2149.1AG | 1 | 0.011
L3b2b | 310 | 1 | 0.011
L3b2b | 4200 | 2 | 0.022
L3b2b | 515-524d | 6 | 0.066
L3b2b | 546 | 1 | 0.011
L3b2b | 7598 | 1 | 0.011
L3b2b | 8027 | 1 | 0.011
L3b2b | 9098 | 1 | 0.011
L3b3 | 11016 | 1 | 0.04
L3b3 | 12187 | 1 | 0.04
L3b3 | 13935 | 1 | 0.04
L3b3 | 14066G | 1 | 0.04
L3b3 | 14320 | 1 | 0.04
L3b3 | 150 | 5 | 0.2
L3b3 | 16093 | 1 | 0.04
L3b3 | 16163 | 3 | 0.12
L3b3 | 16189 | 1 | 0.04
L3b3 | 16298 | 1 | 0.04
L3b3 | 249d | 3 | 0.12

L3b3 | 374 | 1 | 0.04
L3b3 | 385 | 1 | 0.04
L3b3 | 5201 | 1 | 0.04
L3b3 | 5450 | 1 | 0.04
L3b3 | 593 | 1 | 0.04
L3b3 | 6261 | 1 | 0.04
L3b3 | 6620 | 1 | 0.04
L3b3 | 751 | 1 | 0.04
L3b3 | 8251 | 1 | 0.04
L3b3 | 8349 | 1 | 0.04
L3b3 | 8537 | 1 | 0.04
L3b'f | 16072.1A | 1 | 0.005
L3b'f | 16188 | 1 | 0.005
L3b'f | 16234 | 1 | 0.005
L3b'f | 16318T | 1 | 0.005
L3b'f | 199 | 1 | 0.005
L3b'f | 200 | 1 | 0.005
L3b'f | 285 | 2 | 0.009
L3b'f | 315.2C | 1 | 0.005
L3b'f | 316C | 2 | 0.009
L3b'f | 488 | 1 | 0.005
L3b'f | 541 | 1 | 0.005
L3b'f | 93 | 1 | 0.005
L3c | 11944 | 1 | 0.007
L3c | 13404 | 1 | 0.007
L3c | 15833 | 1 | 0.007
L3c | 16032d | 13 | 0.092
L3c | 16042d | 13 | 0.092
L3c | 16062T | 1 | 0.007
L3c | 16070C | 1 | 0.007
L3c | 16074C | 1 | 0.007
L3c | 16110d | 13 | 0.092
L3c | 16162 | 1 | 0.007
L3c | 16172 | 3 | 0.021
L3c | 16241 | 1 | 0.007
L3c | 16243 | 1 | 0.007
L3c | 16293 | 2 | 0.014
L3c | 16295 | 32 | 0.227
L3c | 16360 | 1 | 0.007
L3c | 16526 | 1 | 0.007
L3c | 16529 | 1 | 0.007
L3c | 16533A | 1 | 0.007
L3c | 16539C | 1 | 0.007
L3c | 16568 | 1 | 0.007
L3c | 368.1AGAA | 1 | 0.007
L3c | 792 | 1 | 0.007
L3c | 8835 | 1 | 0.007
L3c'd | 127 | 1 | 0.005
L3c'd | 146 | 2 | 0.011
L3c'd | 151 | 2 | 0.011
L3c'd | 153 | 1 | 0.005
L3c'd | 16048 | 1 | 0.005
L3c'd | 16060 | 1 | 0.005
L3c'd | 16067A | 1 | 0.005
L3c'd | 16095 | 1 | 0.005
L3c'd | 16098T | 2 | 0.011
L3c'd | 16099G | 1 | 0.005
L3c'd | 16116C | 1 | 0.005
L3c'd | 16126 | 1 | 0.005
L3c'd | 16129 | 1 | 0.005
L3c'd | 16134G | 1 | 0.005
L3c'd | 16166d | 2 | 0.011
L3c'd | 16179 | 3 | 0.016
L3c'd | 16184 | 1 | 0.005
L3c'd | 16206C | 1 | 0.005
L3c'd | 16211G | 1 | 0.005
L3c'd | 16234 | 1 | 0.005
L3c'd | 16249 | 1 | 0.005
L3c'd | 16290 | 1 | 0.005
L3c'd | 16291 | 1 | 0.005
L3c'd | 16292 | 1 | 0.005
L3c'd | 16319 | 2 | 0.011
L3c'd | 16359 | 1 | 0.005
L3c'd | 16372 | 1 | 0.005
L3c'd | 16449G | 1 | 0.005
L3c'd | 16496T | 1 | 0.005
L3c'd | 16513G | 1 | 0.005
L3c'd | 195 | 2 | 0.011
L3c'd | 210 | 2 | 0.011
L3c'd | 215 | 1 | 0.005
L3c'd | 280A | 1 | 0.005
L3c'd | 315.2C | 2 | 0.011
L3c'd | 373 | 1 | 0.005
L3c'd | 451 | 1 | 0.005
L3c'd | 72 | 1 | 0.005
L3c'd | 89 | 1 | 0.005
L3c'd | 93 | 1 | 0.005
L3c'd | 95C | 1 | 0.005
L3d | 11503 | 1 | 0.011
L3d | 13368 | 1 | 0.011
L3d | 15434 | 1 | 0.011
L3d | 16154 | 2 | 0.022
L3d | 16165 | 1 | 0.011
L3d | 16169 | 2 | 0.022
L3d | 16219 | 2 | 0.022
L3d | 16318C | 2 | 0.022
L3d | 16355 | 1 | 0.011
L3d | 3504 | 1 | 0.011
L3d | 3729 | 1 | 0.011
L3d | 4619 | 1 | 0.011
L3d | 491 | 1 | 0.011
L3d1 | 14831 | 1 | 0.011
L3d1 | 15058 | 1 | 0.011
L3d1 | 15061 | 1 | 0.011
L3d1 | 16153 | 2 | 0.021
L3d1 | 16187 | 1 | 0.011
L3d1 | 16291 | 11 | 0.116
L3d1 | 16309 | 1 | 0.011
L3d1 | 16320 | 1 | 0.011
L3d1 | 327 | 1 | 0.011
L3d1 | 515-524d | 3 | 0.032

L3d1 | 5580 | 1 | 0.011
L3d1 | 8865 | 1 | 0.011
L3d1'2'3'4'5'6 | 14188 | 3 | 0.033
L3d1'2'3'4'5'6 | 151 | 6 | 0.066
L3d1'2'3'4'5'6 | 15949 | 3 | 0.033
L3d1'2'3'4'5'6 | 16148 | 2 | 0.022
L3d1'2'3'4'5'6 | 16165 | 1 | 0.011
L3d1'2'3'4'5'6 | 16174 | 1 | 0.011
L3d1'2'3'4'5'6 | 16189 | 1 | 0.011
L3d1'2'3'4'5'6 | 16257 | 5 | 0.055
L3d1'2'3'4'5'6 | 195 | 1 | 0.011
L3d1'2'3'4'5'6 | 5187 | 3 | 0.033
L3d1'2'3'4'5'6 | 7594 | 1 | 0.011
L3d1'2'3'4'5'6 | 8950 | 1 | 0.011
L3d1'2'3'4'5'6 | 93 | 1 | 0.011
L3d1'2'3'4'5'6 | 9554 | 2 | 0.022
L3d1a | 12530 | 3 | 0.033
L3d1a | 16165 | 1 | 0.011
L3d1a | 16311 | 4 | 0.044
L3d1a | 215 | 1 | 0.011
L3d1a | 2304 | 4 | 0.044
L3d1a | 339 | 1 | 0.011
L3d1a | 8684 | 1 | 0.011
L3d1a1 | 11440 | 1 | 0.015
L3d1a1 | 11932 | 1 | 0.015
L3d1a1 | 13716 | 1 | 0.015
L3d1a1 | 14025 | 1 | 0.015
L3d1a1 | 14461 | 1 | 0.015
L3d1a1 | 146 | 1 | 0.015
L3d1a1 | 15235 | 1 | 0.015
L3d1a1 | 15565 | 1 | 0.015
L3d1a1 | 16086 | 1 | 0.015
L3d1a1 | 189 | 1 | 0.015
L3d1a1 | 195 | 1 | 0.015
L3d1a1 | 309d | 1 | 0.015
L3d1a1 | 3305 | 1 | 0.015
L3d1a1 | 3337 | 1 | 0.015
L3d1a1 | 4047 | 1 | 0.015
L3d1a1 | 4176 | 1 | 0.015
L3d1a1 | 6975 | 1 | 0.015
L3d1a1 | 7269 | 1 | 0.015
L3d1a1 | 8047 | 1 | 0.015
L3d1a1'2 | 15941 | 2 | 0.028
L3d1a1'2 | 16192 | 1 | 0.014
L3d1a1'2 | 16257 | 5 | 0.07
L3d1a1'2 | 328 | 1 | 0.014
L3d1a1'2 | 515-524d | 2 | 0.028
L3d1a1'2 | 533 | 1 | 0.014
L3d1a1a | 10101 | 1 | 0.006
L3d1a1a | 10373 | 3 | 0.019
L3d1a1a | 11009 | 2 | 0.013
L3d1a1a | 11016 | 1 | 0.006
L3d1a1a | 11539 | 2 | 0.013
L3d1a1a | 11599 | 1 | 0.006
L3d1a1a | 12172 | 5 | 0.032
L3d1a1a | 12651 | 1 | 0.006
L3d1a1a | 13064 | 2 | 0.013
L3d1a1a | 13611 | 1 | 0.006
L3d1a1a | 1378.1C | 1 | 0.006
L3d1a1a | 14125 | 2 | 0.013
L3d1a1a | 14137 | 1 | 0.006
L3d1a1a | 14210 | 1 | 0.006
L3d1a1a | 1428T | 1 | 0.006
L3d1a1a | 14668 | 2 | 0.013
L3d1a1a | 14831 | 1 | 0.006
L3d1a1a | 14890 | 1 | 0.006
L3d1a1a | 14927 | 1 | 0.006
L3d1a1a | 15097 | 1 | 0.006
L3d1a1a | 15256 | 1 | 0.006
L3d1a1a | 152d | 10 | 0.064
L3d1a1a | 15314 | 2 | 0.013
L3d1a1a | 15412G | 1 | 0.006
L3d1a1a | 15803 | 1 | 0.006
L3d1a1a | 15866 | 1 | 0.006
L3d1a1a | 15927 | 1 | 0.006
L3d1a1a | 16086 | 1 | 0.006
L3d1a1a | 16111 | 1 | 0.006
L3d1a1a | 16189 | 1 | 0.006
L3d1a1a | 16215 | 1 | 0.006
L3d1a1a | 16259 | 1 | 0.006
L3d1a1a | 16287 | 1 | 0.006
L3d1a1a | 16294 | 4 | 0.025
L3d1a1a | 16311 | 6 | 0.038
L3d1a1a | 1684 | 1 | 0.006
L3d1a1a | 1887C | 2 | 0.013
L3d1a1a | 189 | 1 | 0.006
L3d1a1a | 1991C | 1 | 0.006
L3d1a1a | 214 | 1 | 0.006
L3d1a1a | 2833 | 1 | 0.006
L3d1a1a | 309d | 6 | 0.038
L3d1a1a | 310 | 2 | 0.013
L3d1a1a | 316 | 1 | 0.006
L3d1a1a | 3666 | 4 | 0.025
L3d1a1a | 3908G | 1 | 0.006
L3d1a1a | 4384 | 12 | 0.076
L3d1a1a | 465 | 1 | 0.006
L3d1a1a | 5038 | 1 | 0.006
L3d1a1a | 523d | 1 | 0.006
L3d1a1a | 5876 | 1 | 0.006
L3d1a1a | 5899.1C | 1 | 0.006
L3d1a1a | 6050 | 3 | 0.019
L3d1a1a | 6339 | 1 | 0.006
L3d1a1a | 6799A | 1 | 0.006
L3d1a1a | 6872 | 1 | 0.006
L3d1a1a | 7789 | 1 | 0.006
L3d1a1a | 7999 | 1 | 0.006
L3d1a1a | 8743 | 1 | 0.006
L3d1a1a | 8853 | 1 | 0.006
L3d1a1a | 8865 | 1 | 0.006
L3d1a1a | 8997A | 1 | 0.006
L3d1a1a | 9117G | 4 | 0.025

L3d1a1a | 9266 | 1 | 0.006
L3d1a1a1 | 10327 | 3 | 0.023
L3d1a1a1 | 10410 | 1 | 0.008
L3d1a1a1 | 11230 | 1 | 0.008
L3d1a1a1 | 12065 | 1 | 0.008
L3d1a1a1 | 12106A | 1 | 0.008
L3d1a1a1 | 12297 | 1 | 0.008
L3d1a1a1 | 13093 | 1 | 0.008
L3d1a1a1 | 13104 | 1 | 0.008
L3d1a1a1 | 13311 | 1 | 0.008
L3d1a1a1 | 13651 | 1 | 0.008
L3d1a1a1 | 13681 | 1 | 0.008
L3d1a1a1 | 14577 | 1 | 0.008
L3d1a1a1 | 146 | 1 | 0.008
L3d1a1a1 | 152d | 7 | 0.053
L3d1a1a1 | 15313 | 1 | 0.008
L3d1a1a1 | 15355 | 30 | 0.226
L3d1a1a1 | 16051 | 1 | 0.008
L3d1a1a1 | 16093 | 3 | 0.023
L3d1a1a1 | 16182 | 1 | 0.008
L3d1a1a1 | 16189 | 2 | 0.015
L3d1a1a1 | 16212 | 1 | 0.008
L3d1a1a1 | 16214 | 3 | 0.023
L3d1a1a1 | 16245 | 1 | 0.008
L3d1a1a1 | 16274 | 1 | 0.008
L3d1a1a1 | 16300 | 1 | 0.008
L3d1a1a1 | 16311 | 4 | 0.03
L3d1a1a1 | 16361 | 3 | 0.023
L3d1a1a1 | 16399 | 1 | 0.008
L3d1a1a1 | 16468 | 1 | 0.008
L3d1a1a1 | 179 | 2 | 0.015
L3d1a1a1 | 2607 | 1 | 0.008
L3d1a1a1 | 291d | 1 | 0.008
L3d1a1a1 | 320 | 1 | 0.008
L3d1a1a1 | 345 | 1 | 0.008
L3d1a1a1 | 3826 | 1 | 0.008
L3d1a1a1 | 417T | 1 | 0.008
L3d1a1a1 | 4703 | 1 | 0.008
L3d1a1a1 | 494 | 3 | 0.023
L3d1a1a1 | 6152 | 1 | 0.008
L3d1a1a1 | 7337 | 1 | 0.008
L3d1a1a1 | 7765 | 30 | 0.226
L3d1a1a1 | 7942 | 1 | 0.008
L3d1a1a1 | 8989 | 1 | 0.008
L3d1a1a1 | 9548C | 1 | 0.008
L3d1a1b | 12311 | 1 | 0.014
L3d1a1b | 16051 | 1 | 0.014
L3d1a1b | 16086 | 1 | 0.014
L3d1a1b | 16126 | 1 | 0.014
L3d1a1b | 16145 | 1 | 0.014
L3d1a1b | 16154 | 1 | 0.014
L3d1a1b | 16169 | 1 | 0.014
L3d1a1b | 16172 | 1 | 0.014
L3d1a1b | 16189 | 1 | 0.014
L3d1a1b | 16209 | 1 | 0.014
L3d1a1b | 16218 | 1 | 0.014
L3d1a1b | 16254 | 2 | 0.027
L3d1a1b | 16362 | 5 | 0.068
L3d1a1b | 16385 | 2 | 0.027
L3d1a1b | 207 | 1 | 0.014
L3d1a1b | 257 | 1 | 0.014
L3d1a1b | 32.1G | 1 | 0.014
L3d1a1b | 3398 | 1 | 0.014
L3d1a1b | 3474 | 1 | 0.014
L3d1a1b | 515-524d | 7 | 0.095
L3d1a1b | 8749 | 1 | 0.014
L3d1a2 | 10103 | 1 | 0.014
L3d1a2 | 10463 | 2 | 0.028
L3d1a2 | 12189 | 1 | 0.014
L3d1a2 | 14602 | 1 | 0.014
L3d1a2 | 1503 | 1 | 0.014
L3d1a2 | 15344 | 1 | 0.014
L3d1a2 | 16093 | 1 | 0.014
L3d1a2 | 16148 | 1 | 0.014
L3d1a2 | 16189 | 1 | 0.014
L3d1a2 | 16261 | 1 | 0.014
L3d1a2 | 16300 | 6 | 0.085
L3d1a2 | 195 | 1 | 0.014
L3d1a2 | 309d | 1 | 0.014
L3d1a2 | 3796 | 3 | 0.042
L3d1a2 | 4924 | 1 | 0.014
L3d1a2 | 5875 | 1 | 0.014
L3d1a2 | 709 | 1 | 0.014
L3d1a2 | 8567 | 1 | 0.014
L3d1a2 | 870 | 1 | 0.014
L3d1a2 | 9300 | 1 | 0.014
L3d1b | 11194 | 2 | 0.022
L3d1b | 13389 | 1 | 0.011
L3d1b | 13581 | 1 | 0.011
L3d1b | 13708 | 2 | 0.022
L3d1b | 13899 | 3 | 0.033
L3d1b | 14110 | 3 | 0.033
L3d1b | 14311 | 3 | 0.033
L3d1b | 151 | 2 | 0.022
L3d1b | 15530 | 1 | 0.011
L3d1b | 15748 | 1 | 0.011
L3d1b | 16165 | 1 | 0.011
L3d1b | 16390 | 4 | 0.043
L3d1b | 1746 | 1 | 0.011
L3d1b | 195 | 4 | 0.043
L3d1b | 2308 | 2 | 0.022
L3d1b | 3957 | 1 | 0.011
L3d1b | 5036 | 1 | 0.011
L3d1b | 5246 | 1 | 0.011
L3d1b | 5605 | 1 | 0.011
L3d1b | 5894C | 1 | 0.011
L3d1b | 5899.4C | 1 | 0.011
L3d1b | 6119 | 3 | 0.033
L3d1b | 6152 | 1 | 0.011
L3d1b | 6842 | 1 | 0.011
L3d1b | 6980 | 1 | 0.011

L3d1b1 | 10646 | 2 | 0.022
L3d1b1 | 150 | 4 | 0.043
L3d1b1 | 16086 | 1 | 0.011
L3d1b1 | 16093 | 1 | 0.011
L3d1b1 | 16165 | 1 | 0.011
L3d1b1 | 16256 | 2 | 0.022
L3d1b1 | 16294 | 2 | 0.022
L3d1b1 | 16311 | 2 | 0.022
L3d1b1 | 16399 | 2 | 0.022
L3d1b1 | 1900 | 2 | 0.022
L3d1b1 | 2903 | 2 | 0.022
L3d1b1 | 317.1C | 1 | 0.011
L3d1b1 | 3459 | 2 | 0.022
L3d1b1 | 3597 | 1 | 0.011
L3d1b1 | 4910 | 2 | 0.022
L3d1b1 | 5605 | 2 | 0.022
L3d1b1 | 6842 | 2 | 0.022
L3d1b1 | 9156 | 1 | 0.011
L3d1b1 | 9647 | 2 | 0.022
L3d1b1a | 12454 | 1 | 0.011
L3d1b1a | 14587 | 1 | 0.011
L3d1b1a | 15924 | 1 | 0.011
L3d1b1a | 16148 | 1 | 0.011
L3d1b1a | 16165 | 1 | 0.011
L3d1b1a | 5029 | 1 | 0.011
L3d1b1a | 7754 | 1 | 0.011
L3d1b1a | 8614 | 3 | 0.033
L3d1b1b | 15924 | 3 | 0.088
L3d1b1b | 3316 | 1 | 0.029
L3d1b1b | 3360 | 2 | 0.059
L3d1b1b | 3438 | 1 | 0.029
L3d1b1b | 5238 | 2 | 0.059
L3d1b1b | 5567 | 2 | 0.059
L3d1b1b | 7705 | 1 | 0.029
L3d1b2 | 10307 | 1 | 0.014
L3d1b2 | 10750 | 1 | 0.014
L3d1b2 | 12082 | 1 | 0.014
L3d1b2 | 12461 | 1 | 0.014
L3d1b2 | 13659 | 1 | 0.014
L3d1b2 | 13866 | 1 | 0.014
L3d1b2 | 15860 | 1 | 0.014
L3d1b2 | 16093 | 2 | 0.029
L3d1b2 | 16111 | 1 | 0.014
L3d1b2 | 16150 | 2 | 0.029
L3d1b2 | 16218 | 1 | 0.014
L3d1b2 | 16248 | 1 | 0.014
L3d1b2 | 16249 | 1 | 0.014
L3d1b2 | 16288 | 2 | 0.029
L3d1b2 | 16292 | 1 | 0.014
L3d1b2 | 16357 | 3 | 0.043
L3d1b2 | 16399 | 4 | 0.058
L3d1b2 | 16496 | 2 | 0.029
L3d1b2 | 16497 | 1 | 0.014
L3d1b2 | 16526 | 1 | 0.014
L3d1b2 | 189 | 2 | 0.029
L3d1b2 | 195 | 2 | 0.029
L3d1b2 | 198 | 1 | 0.014
L3d1b2 | 266 | 2 | 0.029
L3d1b2 | 310 | 2 | 0.029
L3d1b2 | 515-524d | 3 | 0.043
L3d1b2 | 5561 | 1 | 0.014
L3d1b2 | 6221 | 1 | 0.014
L3d1b2 | 6710 | 1 | 0.014
L3d1b2 | 7609 | 1 | 0.014
L3d1b2 | 8251 | 1 | 0.014
L3d1b2 | 8473 | 1 | 0.014
L3d1b2 | 9873 | 1 | 0.014
L3d1b3 | 10694T | 2 | 0.032
L3d1b3 | 11383 | 1 | 0.016
L3d1b3 | 11815 | 1 | 0.016
L3d1b3 | 13002 | 1 | 0.016
L3d1b3 | 14389 | 1 | 0.016
L3d1b3 | 14587 | 1 | 0.016
L3d1b3 | 14684 | 1 | 0.016
L3d1b3 | 15883 | 1 | 0.016
L3d1b3 | 15910 | 1 | 0.016
L3d1b3 | 16086 | 1 | 0.016
L3d1b3 | 16093 | 6 | 0.095
L3d1b3 | 16126 | 2 | 0.032
L3d1b3 | 16179A | 1 | 0.016
L3d1b3 | 16261 | 1 | 0.016
L3d1b3 | 16278 | 3 | 0.048
L3d1b3 | 16327 | 1 | 0.016
L3d1b3 | 16343 | 1 | 0.016
L3d1b3 | 215 | 1 | 0.016
L3d1b3 | 309d | 1 | 0.016
L3d1b3 | 4910 | 1 | 0.016
L3d1b3 | 711 | 1 | 0.016
L3d1b3 | 7256 | 1 | 0.016
L3d1b3 | 7269 | 1 | 0.016
L3d1b3 | 9368 | 1 | 0.016
L3d1b3 | 9881 | 1 | 0.016
L3d1b3a | 11239 | 1 | 0.018
L3d1b3a | 11383 | 1 | 0.018
L3d1b3a | 12188 | 1 | 0.018
L3d1b3a | 12616 | 1 | 0.018
L3d1b3a | 13681 | 1 | 0.018
L3d1b3a | 14034 | 1 | 0.018
L3d1b3a | 14040 | 4 | 0.071
L3d1b3a | 14569 | 1 | 0.018
L3d1b3a | 15113 | 1 | 0.018
L3d1b3a | 15596 | 1 | 0.018
L3d1b3a | 15748 | 3 | 0.054
L3d1b3a | 16086 | 1 | 0.018
L3d1b3a | 16153 | 1 | 0.018
L3d1b3a | 16171 | 1 | 0.018
L3d1b3a | 16188 | 1 | 0.018
L3d1b3a | 16189 | 4 | 0.071
L3d1b3a | 16201 | 2 | 0.036
L3d1b3a | 16240C | 2 | 0.036
L3d1b3a | 16290G | 1 | 0.018

L3d1b3a | 16294 | 1 | 0.018
L3d1b3a | 163 | 2 | 0.036
L3d1b3a | 16311 | 1 | 0.018
L3d1b3a | 16362 | 2 | 0.036
L3d1b3a | 1842 | 1 | 0.018
L3d1b3a | 195 | 2 | 0.036
L3d1b3a | 199 | 1 | 0.018
L3d1b3a | 2438 | 1 | 0.018
L3d1b3a | 294 | 1 | 0.018
L3d1b3a | 309d | 2 | 0.036
L3d1b3a | 310 | 1 | 0.018
L3d1b3a | 3236 | 1 | 0.018
L3d1b3a | 515-524d | 1 | 0.018
L3d1b3a | 573.1C | 1 | 0.018
L3d1b3a | 574 | 1 | 0.018
L3d1b3a | 6305 | 1 | 0.018
L3d1b3a | 6446 | 3 | 0.054
L3d1b3a | 6641 | 3 | 0.054
L3d1b3a | 7771 | 1 | 0.018
L3d1b3a | 8910 | 1 | 0.018
L3d1b3a | 9254 | 1 | 0.018
L3d1b3a | 9305 | 2 | 0.036
L3d1c | 10589 | 1 | 0.015
L3d1c | 12342 | 1 | 0.015
L3d1c | 13820 | 1 | 0.015
L3d1c | 151 | 2 | 0.03
L3d1c | 153 | 1 | 0.015
L3d1c | 15938 | 1 | 0.015
L3d1c | 16037 | 1 | 0.015
L3d1c | 16093 | 5 | 0.075
L3d1c | 16172 | 1 | 0.015
L3d1c | 16272 | 1 | 0.015
L3d1c | 16294 | 2 | 0.03
L3d1c | 16298 | 1 | 0.015
L3d1c | 16309 | 1 | 0.015
L3d1c | 16335 | 1 | 0.015
L3d1c | 16352 | 1 | 0.015
L3d1c | 16357 | 1 | 0.015
L3d1c | 257 | 1 | 0.015
L3d1c | 291.2A | 1 | 0.015
L3d1c | 309d | 1 | 0.015
L3d1c | 515-524d | 1 | 0.015
L3d1c | 547 | 1 | 0.015
L3d1c | 5563 | 1 | 0.015
L3d1c | 6852 | 1 | 0.015
L3d1c | 7647 | 1 | 0.015
L3d1c | 9708 | 1 | 0.015
L3d1c1 | 11254 | 1 | 0.016
L3d1c1 | 11593 | 1 | 0.016
L3d1c1 | 12397 | 1 | 0.016
L3d1c1 | 12940 | 1 | 0.016
L3d1c1 | 15496 | 3 | 0.049
L3d1c1 | 15517 | 1 | 0.016
L3d1c1 | 16093 | 1 | 0.016
L3d1c1 | 16399 | 1 | 0.016
L3d1c1 | 2903 | 3 | 0.049
L3d1c1 | 291.1A | 1 | 0.016
L3d1c1 | 2971 | 1 | 0.016
L3d1c1 | 309d | 2 | 0.033
L3d1c1 | 310 | 1 | 0.016
L3d1c1 | 317.1C | 1 | 0.016
L3d1c1 | 3537 | 1 | 0.016
L3d1c1 | 4418 | 1 | 0.016
L3d1c1 | 5141 | 1 | 0.016
L3d1c1 | 709 | 1 | 0.016
L3d1c1 | 7691 | 1 | 0.016
L3d1c1 | 7859 | 1 | 0.016
L3d1c1 | 8599 | 1 | 0.016
L3d1c1 | 9728 | 3 | 0.049
L3d1c1 | 9932 | 3 | 0.049
L3d1d | 10559 | 1 | 0.008
L3d1d | 11401 | 1 | 0.008
L3d1d | 12654 | 1 | 0.008
L3d1d | 13632 | 2 | 0.017
L3d1d | 14128 | 7 | 0.058
L3d1d | 1452 | 1 | 0.008
L3d1d | 14659 | 1 | 0.008
L3d1d | 151 | 1 | 0.008
L3d1d | 153 | 3 | 0.025
L3d1d | 15672 | 1 | 0.008
L3d1d | 16086 | 7 | 0.058
L3d1d | 16093 | 2 | 0.017
L3d1d | 16127A | 1 | 0.008
L3d1d | 16127T | 1 | 0.008
L3d1d | 16129 | 2 | 0.017
L3d1d | 16153 | 1 | 0.008
L3d1d | 16172 | 1 | 0.008
L3d1d | 16245 | 1 | 0.008
L3d1d | 16288 | 2 | 0.017
L3d1d | 16291 | 2 | 0.017
L3d1d | 16291G | 1 | 0.008
L3d1d | 16311 | 2 | 0.017
L3d1d | 16399 | 2 | 0.017
L3d1d | 189 | 1 | 0.008
L3d1d | 195 | 1 | 0.008
L3d1d | 200 | 2 | 0.017
L3d1d | 207 | 1 | 0.008
L3d1d | 247 | 1 | 0.008
L3d1d | 296 | 1 | 0.008
L3d1d | 4040 | 1 | 0.008
L3d1d | 5054C | 1 | 0.008
L3d1d | 515-524d | 9 | 0.074
L3d1d | 6164 | 1 | 0.008
L3d1d | 7385 | 1 | 0.008
L3d1d | 8485 | 1 | 0.008
L3d1d | 8709 | 7 | 0.058
L3d1d | 8945 | 1 | 0.008
L3d2 | 150 | 1 | 0.016
L3d2 | 198 | 1 | 0.016
L3d2a | 11518 | 2 | 0.028
L3d2a | 12873 | 2 | 0.028

L3d2a | 15865 | 1 | 0.014
L3d2a | 199 | 1 | 0.014
L3d2a | 3394 | 2 | 0.028
L3d2a | 9022 | 1 | 0.014
L3d2b | 1000 | 1 | 0.015
L3d2b | 10307 | 1 | 0.015
L3d2b | 10864 | 1 | 0.015
L3d2b | 11150 | 1 | 0.015
L3d2b | 11659A | 1 | 0.015
L3d2b | 11878 | 1 | 0.015
L3d2b | 13191 | 3 | 0.046
L3d2b | 13708 | 1 | 0.015
L3d2b | 13711 | 2 | 0.031
L3d2b | 14007 | 1 | 0.015
L3d2b | 14133 | 1 | 0.015
L3d2b | 14215 | 2 | 0.031
L3d2b | 146 | 1 | 0.015
L3d2b | 150 | 7 | 0.108
L3d2b | 151 | 8 | 0.123
L3d2b | 15217 | 1 | 0.015
L3d2b | 15416 | 1 | 0.015
L3d2b | 16051 | 1 | 0.015
L3d2b | 16086 | 1 | 0.015
L3d2b | 16093 | 1 | 0.015
L3d2b | 16189 | 3 | 0.046
L3d2b | 16234 | 1 | 0.015
L3d2b | 16287 | 1 | 0.015
L3d2b | 16309 | 3 | 0.046
L3d2b | 16311 | 1 | 0.015
L3d2b | 16400 | 1 | 0.015
L3d2b | 16497 | 2 | 0.031
L3d2b | 16524 | 1 | 0.015
L3d2b | 1760 | 1 | 0.015
L3d2b | 195 | 1 | 0.015
L3d2b | 202 | 1 | 0.015
L3d2b | 204 | 2 | 0.031
L3d2b | 207 | 1 | 0.015
L3d2b | 2163 | 3 | 0.046
L3d2b | 2222 | 1 | 0.015
L3d2b | 2887 | 1 | 0.015
L3d2b | 2963 | 1 | 0.015
L3d2b | 309d | 1 | 0.015
L3d2b | 310 | 1 | 0.015
L3d2b | 373 | 2 | 0.031
L3d2b | 3745 | 2 | 0.031
L3d2b | 391 | 1 | 0.015
L3d2b | 4688 | 3 | 0.046
L3d2b | 5004 | 1 | 0.015
L3d2b | 515-524d | 2 | 0.031
L3d2b | 5252 | 1 | 0.015
L3d2b | 5899.2C | 1 | 0.015
L3d2b | 6080 | 1 | 0.015
L3d2b | 6365 | 1 | 0.015
L3d2b | 6492 | 1 | 0.015
L3d2b | 850 | 1 | 0.015
L3d2b | 8567 | 1 | 0.015
L3d2b | 9266 | 1 | 0.015
L3d3 | 16154 | 2 | 0.022
L3d3 | 16165 | 1 | 0.011
L3d3 | 16169 | 2 | 0.022
L3d3 | 16219 | 2 | 0.022
L3d3 | 16318C | 2 | 0.022
L3d3 | 16355 | 1 | 0.011
L3d3 | 491 | 1 | 0.011
L3d3a | 15565 | 1 | 0.007
L3d3a | 15928 | 1 | 0.007
L3d3a | 15940 | 1 | 0.007
L3d3a | 16150 | 1 | 0.007
L3d3a | 16230 | 1 | 0.007
L3d3a | 16274 | 2 | 0.015
L3d3a | 16291 | 1 | 0.007
L3d3a | 16293 | 1 | 0.007
L3d3a | 16335 | 1 | 0.007
L3d3a | 16362 | 4 | 0.03
L3d3a | 199 | 1 | 0.007
L3d3a | 200 | 1 | 0.007
L3d3a | 4393A | 1 | 0.007
L3d3a | 515-524d | 1 | 0.007
L3d3a | 6431 | 1 | 0.007
L3d3a1 | 10237 | 1 | 0.006
L3d3a1 | 11087 | 2 | 0.013
L3d3a1 | 11266 | 1 | 0.006
L3d3a1 | 12631G | 1 | 0.006
L3d3a1 | 12681 | 1 | 0.006
L3d3a1 | 14306 | 1 | 0.006
L3d3a1 | 14560 | 1 | 0.006
L3d3a1 | 146 | 2 | 0.013
L3d3a1 | 14944 | 1 | 0.006
L3d3a1 | 151 | 1 | 0.006
L3d3a1 | 15200 | 1 | 0.006
L3d3a1 | 15910 | 1 | 0.006
L3d3a1 | 16193d | 1 | 0.006
L3d3a1 | 195 | 4 | 0.025
L3d3a1 | 199 | 1 | 0.006
L3d3a1 | 309d | 11 | 0.069
L3d3a1 | 318 | 1 | 0.006
L3d3a1 | 513 | 1 | 0.006
L3d3a1 | 5979 | 1 | 0.006
L3d3a1 | 6185 | 1 | 0.006
L3d3a1 | 6239 | 1 | 0.006
L3d3a1 | 7085 | 1 | 0.006
L3d3a1 | 709 | 1 | 0.006
L3d3a1 | 7328 | 1 | 0.006
L3d3a1 | 7471 | 1 | 0.006
L3d3a1 | 8260 | 1 | 0.006
L3d3a1 | 8979A | 1 | 0.006
L3d3a1 | 9686 | 1 | 0.006
L3d3a1a | 11089 | 1 | 0.005
L3d3a1a | 12285 | 1 | 0.005
L3d3a1a | 12561 | 1 | 0.005
L3d3a1a | 13098 | 1 | 0.005

L3d3a1a | 13116 | 1 | 0.005
L3d3a1a | 14110 | 4 | 0.019
L3d3a1a | 14133C | 1 | 0.005
L3d3a1a | 14858 | 1 | 0.005
L3d3a1a | 15749 | 1 | 0.005
L3d3a1a | 15784 | 1 | 0.005
L3d3a1a | 16178 | 1 | 0.005
L3d3a1a | 16192 | 6 | 0.029
L3d3a1a | 16193d | 1 | 0.005
L3d3a1a | 16327 | 3 | 0.014
L3d3a1a | 195 | 12 | 0.057
L3d3a1a | 2003 | 2 | 0.01
L3d3a1a | 240 | 1 | 0.005
L3d3a1a | 2592-2593d | 1 | 0.005
L3d3a1a | 309d | 2 | 0.01
L3d3a1a | 310 | 3 | 0.014
L3d3a1a | 3744 | 2 | 0.01
L3d3a1a | 6473 | 1 | 0.005
L3d3a1a | 7238 | 1 | 0.005
L3d3a1a | 7245 | 2 | 0.01
L3d3a1a | 7679 | 1 | 0.005
L3d3a1a | 8078 | 1 | 0.005
L3d3a1a | 8155 | 2 | 0.01
L3d3a1a | 8705 | 3 | 0.014
L3d3a1a | 8730 | 1 | 0.005
L3d3a1a | 8965 | 1 | 0.005
L3d3a1a | 9174 | 1 | 0.005
L3d3a1a | 9468 | 4 | 0.019
L3d3a1b | 11887 | 1 | 0.008
L3d3a1b | 13068T | 4 | 0.033
L3d3a1b | 14290 | 1 | 0.008
L3d3a1b | 146 | 1 | 0.008
L3d3a1b | 158G | 1 | 0.008
L3d3a1b | 16086 | 1 | 0.008
L3d3a1b | 16148 | 1 | 0.008
L3d3a1b | 16153 | 1 | 0.008
L3d3a1b | 16162 | 1 | 0.008
L3d3a1b | 16172 | 3 | 0.024
L3d3a1b | 16188 | 2 | 0.016
L3d3a1b | 16193d | 1 | 0.008
L3d3a1b | 16214 | 6 | 0.049
L3d3a1b | 16249 | 1 | 0.008
L3d3a1b | 16272 | 1 | 0.008
L3d3a1b | 16274 | 1 | 0.008
L3d3a1b | 16463 | 5 | 0.041
L3d3a1b | 16484-16489d | 1 | 0.008
L3d3a1b | 16527 | 1 | 0.008
L3d3a1b | 189 | 2 | 0.016
L3d3a1b | 193 | 1 | 0.008
L3d3a1b | 309d | 2 | 0.016
L3d3a1b | 310 | 1 | 0.008
L3d3b | 10020 | 1 | 0.011
L3d3b | 11779 | 1 | 0.011
L3d3b | 12046T | 3 | 0.033
L3d3b | 12236 | 1 | 0.011
L3d3b | 12342 | 2 | 0.022
L3d3b | 13708 | 2 | 0.022
L3d3b | 13752 | 4 | 0.043
L3d3b | 1393 | 1 | 0.011
L3d3b | 16093 | 1 | 0.011
L3d3b | 16165 | 1 | 0.011
L3d3b | 16256 | 1 | 0.011
L3d3b | 16264 | 2 | 0.022
L3d3b | 207 | 3 | 0.033
L3d3b | 458 | 1 | 0.011
L3d3b | 5144 | 1 | 0.011
L3d3b | 5773 | 1 | 0.011
L3d3b | 6722 | 3 | 0.033
L3d3b | 8277 | 1 | 0.011
L3d3b | 8279 | 1 | 0.011
L3d4 | 12246A | 1 | 0.017
L3d4 | 12906A | 1 | 0.017
L3d4 | 13263 | 1 | 0.017
L3d4 | 13326 | 2 | 0.034
L3d4 | 13980 | 1 | 0.017
L3d4 | 14094 | 2 | 0.034
L3d4 | 14587 | 1 | 0.017
L3d4 | 146 | 1 | 0.017
L3d4 | 150 | 1 | 0.017
L3d4 | 15110 | 2 | 0.034
L3d4 | 15440 | 1 | 0.017
L3d4 | 15941 | 2 | 0.034
L3d4 | 16086 | 4 | 0.068
L3d4 | 16189 | 1 | 0.017
L3d4 | 16192 | 2 | 0.034
L3d4 | 16256 | 3 | 0.051
L3d4 | 16260 | 10 | 0.169
L3d4 | 16311 | 6 | 0.102
L3d4 | 16390 | 1 | 0.017
L3d4 | 185 | 2 | 0.034
L3d4 | 198 | 1 | 0.017
L3d4 | 200 | 3 | 0.051
L3d4 | 3010 | 2 | 0.034
L3d4 | 309d | 1 | 0.017
L3d4 | 317.1C | 1 | 0.017
L3d4 | 3420 | 2 | 0.034
L3d4 | 4679 | 2 | 0.034
L3d4 | 485 | 1 | 0.017
L3d4 | 515-524d | 1 | 0.017
L3d4 | 5320 | 1 | 0.017
L3d4 | 6150 | 2 | 0.034
L3d4 | 9098 | 2 | 0.034
L3d4a | 12904 | 1 | 0.02
L3d4a | 16114 | 1 | 0.02
L3d4a | 16114A | 1 | 0.02
L3d4a | 16212 | 1 | 0.02
L3d4a | 16291 | 1 | 0.02
L3d4a | 16362 | 2 | 0.04
L3d4a | 16502 | 1 | 0.02
L3d4a | 240 | 1 | 0.02
L3d4a | 335 | 1 | 0.02

L3d4a | 515-524d | 7 | 0.14
L3d4a | 533 | 1 | 0.02
L3d4a | 71d | 1 | 0.02
L3d5 | 11503 | 1 | 0.016
L3d5 | 1193 | 1 | 0.016
L3d5 | 13368 | 1 | 0.016
L3d5 | 13752 | 1 | 0.016
L3d5 | 13966 | 1 | 0.016
L3d5 | 15434 | 1 | 0.016
L3d5 | 15434A | 1 | 0.016
L3d5 | 15928 | 1 | 0.016
L3d5 | 16148 | 1 | 0.016
L3d5 | 16209 | 1 | 0.016
L3d5 | 16240T | 3 | 0.048
L3d5 | 16256 | 1 | 0.016
L3d5 | 16260 | 1 | 0.016
L3d5 | 16311 | 1 | 0.016
L3d5 | 16316 | 1 | 0.016
L3d5 | 16360 | 1 | 0.016
L3d5 | 16527 | 1 | 0.016
L3d5 | 195 | 1 | 0.016
L3d5 | 200 | 2 | 0.032
L3d5 | 262 | 1 | 0.016
L3d5 | 3504 | 1 | 0.016
L3d5 | 4619 | 1 | 0.016
L3d5 | 489 | 1 | 0.016
L3d5 | 515-524d | 1 | 0.016
L3d5 | 6480 | 1 | 0.016
L3d5 | 7471 | 1 | 0.016
L3d5 | 769 | 1 | 0.016
L3d5a | 10587 | 1 | 0.015
L3d5a | 13266 | 3 | 0.045
L3d5a | 13434 | 3 | 0.045
L3d5a | 13889 | 3 | 0.045
L3d5a | 16148 | 5 | 0.076
L3d5a | 16189 | 1 | 0.015
L3d5a | 16293 | 5 | 0.076
L3d5a | 16311 | 4 | 0.061
L3d5a | 16318 | 1 | 0.015
L3d5a | 16320 | 1 | 0.015
L3d5a | 16337 | 1 | 0.015
L3d5a | 194 | 1 | 0.015
L3d5a | 195 | 6 | 0.091
L3d5a | 199 | 6 | 0.091
L3d6 | 14007 | 1 | 0.011
L3d6 | 16165 | 1 | 0.011
L3d6 | 16166 | 1 | 0.011
L3d6 | 16185 | 1 | 0.011
L3d6 | 16272 | 1 | 0.011
L3d6 | 3523 | 4 | 0.044
L3d6 | 373 | 1 | 0.011
L3d6 | 4371 | 3 | 0.033
L3d6 | 5477 | 1 | 0.011
L3d6 | 6261 | 1 | 0.011
L3d6 | 9211 | 3 | 0.033
L3d6 | 9554 | 1 | 0.011
L3e | 152 | 1 | 0.006
L3e | 16111 | 1 | 0.006
L3e | 16265C | 5 | 0.03
L3e | 16274 | 1 | 0.006
L3e | 16304 | 1 | 0.006
L3e | 16316 | 1 | 0.006
L3e | 16327 | 1 | 0.006
L3e | 16343T | 1 | 0.006
L3e | 195 | 5 | 0.03
L3e | 303G | 1 | 0.006
L3e | 515-524d | 1 | 0.006
L3e1 | 10101 | 1 | 0.015
L3e1 | 10235 | 1 | 0.015
L3e1 | 10310 | 1 | 0.015
L3e1 | 10874 | 1 | 0.015
L3e1 | 11963 | 1 | 0.015
L3e1 | 12046 | 1 | 0.015
L3e1 | 12127 | 4 | 0.06
L3e1 | 13535 | 3 | 0.045
L3e1 | 14016 | 3 | 0.045
L3e1 | 14053 | 1 | 0.015
L3e1 | 14206 | 4 | 0.06
L3e1 | 14364 | 1 | 0.015
L3e1 | 14569 | 1 | 0.015
L3e1 | 15160 | 1 | 0.015
L3e1 | 15172 | 2 | 0.03
L3e1 | 15221 | 1 | 0.015
L3e1 | 15497 | 1 | 0.015
L3e1 | 15884 | 4 | 0.06
L3e1 | 16086 | 1 | 0.015
L3e1 | 16111 | 7 | 0.104
L3e1 | 16124 | 1 | 0.015
L3e1 | 16129 | 2 | 0.03
L3e1 | 16140 | 6 | 0.09
L3e1 | 16145 | 1 | 0.015
L3e1 | 16153 | 1 | 0.015
L3e1 | 16189 | 1 | 0.015
L3e1 | 16242 | 1 | 0.015
L3e1 | 16255 | 1 | 0.015
L3e1 | 16266 | 1 | 0.015
L3e1 | 16293C | 5 | 0.075
L3e1 | 16311 | 10 | 0.149
L3e1 | 16316 | 3 | 0.045
L3e1 | 16319 | 1 | 0.015
L3e1 | 16343T | 4 | 0.06
L3e1 | 16362 | 1 | 0.015
L3e1 | 16398 | 2 | 0.03
L3e1 | 16526 | 1 | 0.015
L3e1 | 1692C | 3 | 0.045
L3e1 | 185 | 7 | 0.104
L3e1 | 195 | 7 | 0.104
L3e1 | 2416 | 1 | 0.015
L3e1 | 309d | 7 | 0.104
L3e1 | 310 | 3 | 0.045
L3e1 | 4047 | 1 | 0.015

L3e1 | 4048 | 1 | 0.015
L3e1 | 503 | 4 | 0.06
L3e1 | 5196 | 2 | 0.03
L3e1 | 5261 | 4 | 0.06
L3e1 | 593 | 1 | 0.015
L3e1 | 595.1A | 1 | 0.015
L3e1 | 6169 | 1 | 0.015
L3e1 | 6261 | 1 | 0.015
L3e1 | 6896 | 1 | 0.015
L3e1 | 7142 | 1 | 0.015
L3e1 | 7444 | 4 | 0.06
L3e1 | 7462 | 4 | 0.06
L3e1 | 8300 | 1 | 0.015
L3e1 | 8772 | 1 | 0.015
L3e1 | 9656 | 1 | 0.015
L3e1 | 985 | 1 | 0.015
L3e1 | 9932 | 1 | 0.015
L3e1a | 152 | 8 | 0.258
L3e1a | 16189 | 1 | 0.032
L3e1a | 16193d | 1 | 0.032
L3e1a | 16264 | 2 | 0.065
L3e1a | 16269 | 7 | 0.226
L3e1a | 16292 | 1 | 0.032
L3e1a | 207 | 2 | 0.065
L3e1a | 9828 | 1 | 0.032
L3e1a1 | 152 | 7 | 0.269
L3e1a1 | 16218 | 3 | 0.115
L3e1a1 | 16532d | 3 | 0.115
L3e1a1 | 317.1C | 1 | 0.038
L3e1a1 | 4733 | 2 | 0.077
L3e1a1 | 573.1C | 1 | 0.038
L3e1a1a | 10042 | 2 | 0.03
L3e1a1a | 10876 | 1 | 0.015
L3e1a1a | 10915 | 1 | 0.015
L3e1a1a | 11692 | 2 | 0.03
L3e1a1a | 11914 | 2 | 0.03
L3e1a1a | 12007 | 1 | 0.015
L3e1a1a | 12297 | 1 | 0.015
L3e1a1a | 12396 | 2 | 0.03
L3e1a1a | 14110 | 1 | 0.015
L3e1a1a | 14180 | 3 | 0.045
L3e1a1a | 14319 | 1 | 0.015
L3e1a1a | 14687 | 1 | 0.015
L3e1a1a | 15924 | 1 | 0.015
L3e1a1a | 16093 | 1 | 0.015
L3e1a1a | 16169 | 3 | 0.045
L3e1a1a | 16189 | 1 | 0.015
L3e1a1a | 16192 | 1 | 0.015
L3e1a1a | 16209 | 3 | 0.045
L3e1a1a | 16234 | 1 | 0.015
L3e1a1a | 16291 | 1 | 0.015
L3e1a1a | 16391.1G | 1 | 0.015
L3e1a1a | 195 | 1 | 0.015
L3e1a1a | 309d | 6 | 0.09
L3e1a1a | 317.1C | 3 | 0.045
L3e1a1a | 4392 | 5 | 0.075
L3e1a1a | 5417 | 3 | 0.045
L3e1a1a | 573.1C | 1 | 0.015
L3e1a1a | 6152 | 3 | 0.045
L3e1a1a | 6632 | 1 | 0.015
L3e1a1a | 709 | 2 | 0.03
L3e1a1a | 8407 | 3 | 0.045
L3e1a1a | 8745 | 1 | 0.015
L3e1a1a | 9070 | 1 | 0.015
L3e1a1a | 9287 | 1 | 0.015
L3e1a2 | 10370 | 1 | 0.013
L3e1a2 | 10643 | 3 | 0.04
L3e1a2 | 10688 | 1 | 0.013
L3e1a2 | 12358 | 1 | 0.013
L3e1a2 | 13822 | 1 | 0.013
L3e1a2 | 14484 | 1 | 0.013
L3e1a2 | 14693 | 2 | 0.027
L3e1a2 | 14875A | 1 | 0.013
L3e1a2 | 15774 | 1 | 0.013
L3e1a2 | 15848 | 2 | 0.027
L3e1a2 | 16093 | 1 | 0.013
L3e1a2 | 204 | 3 | 0.04
L3e1a2 | 215 | 1 | 0.013
L3e1a2 | 309d | 5 | 0.067
L3e1a2 | 310 | 5 | 0.067
L3e1a2 | 315.2C | 1 | 0.013
L3e1a2 | 316 | 1 | 0.013
L3e1a2 | 4002 | 1 | 0.013
L3e1a2 | 4173 | 2 | 0.027
L3e1a2 | 573.1C | 1 | 0.013
L3e1a2 | 7158 | 1 | 0.013
L3e1a2 | 7775 | 1 | 0.013
L3e1a2 | 9533 | 7 | 0.093
L3e1a3 | 146 | 3 | 0.088
L3e1a3 | 16148 | 1 | 0.029
L3e1a3 | 16243 | 1 | 0.029
L3e1a3 | 16256 | 1 | 0.029
L3e1a3 | 16484-16489d | 1 | 0.029
L3e1a3 | 204 | 3 | 0.088
L3e1a3a | 10188 | 1 | 0.007
L3e1a3a | 10933 | 1 | 0.007
L3e1a3a | 11150 | 3 | 0.021
L3e1a3a | 1243 | 1 | 0.007
L3e1a3a | 12651 | 1 | 0.007
L3e1a3a | 12774 | 1 | 0.007
L3e1a3a | 12897 | 1 | 0.007
L3e1a3a | 13145 | 15 | 0.106
L3e1a3a | 13215 | 4 | 0.028
L3e1a3a | 13934 | 1 | 0.007
L3e1a3a | 13995 | 1 | 0.007
L3e1a3a | 14180 | 1 | 0.007
L3e1a3a | 14209 | 1 | 0.007
L3e1a3a | 146 | 1 | 0.007
L3e1a3a | 14935 | 1 | 0.007
L3e1a3a | 15099 | 1 | 0.007
L3e1a3a | 152 | 2 | 0.014

L3e1a3a | 15218 | 1 | 0.007
L3e1a3a | 15766 | 62 | 0.437
L3e1a3a | 15784 | 1 | 0.007
L3e1a3a | 16148 | 1 | 0.007
L3e1a3a | 16188 | 1 | 0.007
L3e1a3a | 16189 | 4 | 0.028
L3e1a3a | 16193d | 2 | 0.014
L3e1a3a | 16311 | 1 | 0.007
L3e1a3a | 16335 | 1 | 0.007
L3e1a3a | 16399 | 1 | 0.007
L3e1a3a | 185 | 2 | 0.014
L3e1a3a | 195 | 4 | 0.028
L3e1a3a | 204 | 4 | 0.028
L3e1a3a | 207 | 2 | 0.014
L3e1a3a | 214 | 1 | 0.007
L3e1a3a | 2351 | 2 | 0.014
L3e1a3a | 2704 | 1 | 0.007
L3e1a3a | 2765 | 1 | 0.007
L3e1a3a | 309d | 36 | 0.254
L3e1a3a | 310 | 4 | 0.028
L3e1a3a | 3786 | 1 | 0.007
L3e1a3a | 385 | 1 | 0.007
L3e1a3a | 4025 | 1 | 0.007
L3e1a3a | 4047 | 1 | 0.007
L3e1a3a | 4725C | 2 | 0.014
L3e1a3a | 4973 | 1 | 0.007
L3e1a3a | 5028 | 1 | 0.007
L3e1a3a | 5231C | 1 | 0.007
L3e1a3a | 5423 | 1 | 0.007
L3e1a3a | 5899.1C | 1 | 0.007
L3e1a3a | 6267 | 1 | 0.007
L3e1a3a | 6481 | 1 | 0.007
L3e1a3a | 6494 | 1 | 0.007
L3e1a3a | 709 | 1 | 0.007
L3e1a3a | 7621 | 1 | 0.007
L3e1a3a | 8856 | 1 | 0.007
L3e1a3a | 9024 | 1 | 0.007
L3e1a3a | 9193 | 1 | 0.007
L3e1a3a | 9316 | 1 | 0.007
L3e1a3a | 9428 | 1 | 0.007
L3e1a3a | 980 | 1 | 0.007
L3e1a3b | 152 | 3 | 0.25
L3e1a3b | 16293T | 1 | 0.083
L3e1a3b | 16311 | 1 | 0.083
L3e1a3b | 214 | 1 | 0.083
L3e1a3b | 5585 | 1 | 0.083
L3e1a3b | 765G | 1 | 0.083
L3e1b | 13302 | 1 | 0.067
L3e1b | 16086 | 1 | 0.067
L3e1b | 207 | 1 | 0.067
L3e1b | 32.1G | 1 | 0.067
L3e1b1 | 1018 | 2 | 0.08
L3e1b1 | 10235 | 2 | 0.08
L3e1b1 | 11929 | 3 | 0.12
L3e1b1 | 13105 | 2 | 0.08
L3e1b1 | 14364 | 3 | 0.12
L3e1b1 | 14563 | 1 | 0.04
L3e1b1 | 146 | 1 | 0.04
L3e1b1 | 16092 | 1 | 0.04
L3e1b1 | 16134 | 1 | 0.04
L3e1b1 | 16145 | 8 | 0.32
L3e1b1 | 16330 | 8 | 0.32
L3e1b1 | 16362 | 1 | 0.04
L3e1b1 | 16484-16489d | 1 | 0.04
L3e1b1 | 195 | 4 | 0.16
L3e1b1 | 214 | 6 | 0.24
L3e1b1 | 2463d | 2 | 0.08
L3e1b1 | 3882 | 1 | 0.04
L3e1b1 | 7867 | 1 | 0.04
L3e1b2 | 10245 | 1 | 0.019
L3e1b2 | 10334A | 2 | 0.037
L3e1b2 | 11337 | 1 | 0.019
L3e1b2 | 11893 | 1 | 0.019
L3e1b2 | 12528 | 1 | 0.019
L3e1b2 | 14278G | 1 | 0.019
L3e1b2 | 14542 | 2 | 0.037
L3e1b2 | 15115 | 1 | 0.019
L3e1b2 | 15140 | 1 | 0.019
L3e1b2 | 152 | 1 | 0.019
L3e1b2 | 15406 | 1 | 0.019
L3e1b2 | 16075 | 1 | 0.019
L3e1b2 | 16093 | 1 | 0.019
L3e1b2 | 16172 | 1 | 0.019
L3e1b2 | 16188 | 1 | 0.019
L3e1b2 | 16209 | 1 | 0.019
L3e1b2 | 16239 | 17 | 0.315
L3e1b2 | 16291 | 1 | 0.019
L3e1b2 | 16400 | 2 | 0.037
L3e1b2 | 16488 | 2 | 0.037
L3e1b2 | 200 | 2 | 0.037
L3e1b2 | 207 | 2 | 0.037
L3e1b2 | 258 | 3 | 0.056
L3e1b2 | 309d | 1 | 0.019
L3e1b2 | 310 | 2 | 0.037
L3e1b2 | 316C | 1 | 0.019
L3e1b2 | 317G | 1 | 0.019
L3e1b2 | 321.1T | 1 | 0.019
L3e1b2 | 330A | 1 | 0.019
L3e1b2 | 343 | 1 | 0.019
L3e1b2 | 345 | 1 | 0.019
L3e1b2 | 36.1G | 1 | 0.019
L3e1b2 | 3705 | 1 | 0.019
L3e1b2 | 406 | 1 | 0.019
L3e1b2 | 483A | 1 | 0.019
L3e1b2 | 486A | 1 | 0.019
L3e1b2 | 49T | 1 | 0.019
L3e1b2 | 514A | 1 | 0.019
L3e1b2 | 525d | 1 | 0.019
L3e1b2 | 530 | 1 | 0.019
L3e1b2 | 568 | 1 | 0.019
L3e1b2 | 67C | 1 | 0.019

L3e1b2 | 9548C | 1 | 0.019
L3e1c | 14384 | 1 | 0.022
L3e1c | 14924 | 1 | 0.022
L3e1c | 16114 | 1 | 0.022
L3e1c | 16124 | 1 | 0.022
L3e1c | 16160 | 1 | 0.022
L3e1c | 16172 | 1 | 0.022
L3e1c | 16186 | 1 | 0.022
L3e1c | 16187 | 1 | 0.022
L3e1c | 16189 | 1 | 0.022
L3e1c | 16249 | 1 | 0.022
L3e1c | 16255 | 1 | 0.022
L3e1c | 16259 | 1 | 0.022
L3e1c | 16291 | 2 | 0.043
L3e1c | 16294 | 1 | 0.022
L3e1c | 16319 | 2 | 0.043
L3e1c | 194 | 1 | 0.022
L3e1c | 217 | 1 | 0.022
L3e1c | 228 | 1 | 0.022
L3e1c | 257 | 1 | 0.022
L3e1c | 334 | 1 | 0.022
L3e1c | 455.1T | 1 | 0.022
L3e1c | 507A | 1 | 0.022
L3e1c | 515-524d | 2 | 0.043
L3e1c | 574 | 1 | 0.022
L3e1c | 7163 | 1 | 0.022
L3e1d | 11518 | 1 | 0.048
L3e1d | 12876A | 1 | 0.048
L3e1d | 16075 | 2 | 0.095
L3e1d | 16148 | 3 | 0.143
L3e1d | 16234 | 5 | 0.238
L3e1d | 16287 | 5 | 0.238
L3e1d | 16291 | 5 | 0.238
L3e1d | 16311 | 7 | 0.333
L3e1d | 16319 | 1 | 0.048
L3e1d | 16390 | 1 | 0.048
L3e1d | 16465 | 1 | 0.048
L3e1d | 16561 | 1 | 0.048
L3e1d | 185 | 1 | 0.048
L3e1d | 195 | 2 | 0.095
L3e1d | 205 | 1 | 0.048
L3e1d | 207 | 2 | 0.095
L3e1d | 36.1G | 1 | 0.048
L3e1d | 573.1C | 1 | 0.048
L3e1d | 7232 | 1 | 0.048
L3e1d1 | 11167 | 2 | 0.044
L3e1d1 | 11176 | 1 | 0.022
L3e1d1 | 11253 | 1 | 0.022
L3e1d1 | 11353 | 1 | 0.022
L3e1d1 | 12720 | 2 | 0.044
L3e1d1 | 13368 | 1 | 0.022
L3e1d1 | 152d | 2 | 0.044
L3e1d1 | 15721 | 1 | 0.022
L3e1d1 | 15977 | 1 | 0.022
L3e1d1 | 16086 | 1 | 0.022
L3e1d1 | 16189 | 6 | 0.133
L3e1d1 | 16220 | 1 | 0.022
L3e1d1 | 16234 | 5 | 0.111
L3e1d1 | 16256 | 6 | 0.133
L3e1d1 | 16287 | 5 | 0.111
L3e1d1 | 16291 | 5 | 0.111
L3e1d1 | 16294G | 1 | 0.022
L3e1d1 | 16311 | 3 | 0.067
L3e1d1 | 16360 | 1 | 0.022
L3e1d1 | 195 | 2 | 0.044
L3e1d1 | 207 | 1 | 0.022
L3e1d1 | 214 | 1 | 0.022
L3e1d1 | 2756 | 4 | 0.089
L3e1d1 | 309d | 11 | 0.244
L3e1d1 | 310 | 2 | 0.044
L3e1d1 | 3720 | 1 | 0.022
L3e1d1 | 375 | 1 | 0.022
L3e1d1 | 394 | 2 | 0.044
L3e1d1 | 5492 | 3 | 0.067
L3e1d1 | 5628 | 4 | 0.089
L3e1d1 | 5911 | 1 | 0.022
L3e1d1 | 6367 | 1 | 0.022
L3e1d1 | 6941 | 2 | 0.044
L3e1d1 | 709 | 2 | 0.044
L3e1d1 | 7337 | 1 | 0.022
L3e1d1 | 7859T | 1 | 0.022
L3e1d1 | 7993 | 1 | 0.022
L3e1d1 | 8086 | 1 | 0.022
L3e1d1 | 8490 | 2 | 0.044
L3e1d1 | 8723 | 1 | 0.022
L3e1d1 | 8875 | 6 | 0.133
L3e1d1 | 9053 | 1 | 0.022
L3e1d1 | 9145 | 1 | 0.022
L3e1d1 | 9854 | 1 | 0.022
L3e1d1a | 16294 | 1 | 0.062
L3e1d1a | 204 | 2 | 0.125
L3e1d1a | 215 | 2 | 0.125
L3e1d1a | 309d | 6 | 0.375
L3e1e | 10376 | 2 | 0.029
L3e1e | 11887 | 2 | 0.029
L3e1e | 14753 | 1 | 0.014
L3e1e | 14857 | 1 | 0.014
L3e1e | 152 | 1 | 0.014
L3e1e | 15223 | 4 | 0.057
L3e1e | 15547 | 2 | 0.029
L3e1e | 15758 | 1 | 0.014
L3e1e | 15909 | 2 | 0.029
L3e1e | 16086 | 2 | 0.029
L3e1e | 16093 | 5 | 0.071
L3e1e | 16124 | 1 | 0.014
L3e1e | 16172 | 1 | 0.014
L3e1e | 16179 | 4 | 0.057
L3e1e | 16192 | 1 | 0.014
L3e1e | 16207 | 1 | 0.014
L3e1e | 16260 | 1 | 0.014
L3e1e | 16278 | 1 | 0.014

L3e1e | 16309 | 25 | 0.357
L3e1e | 16311 | 1 | 0.014
L3e1e | 16326 | 1 | 0.014
L3e1e | 16399 | 1 | 0.014
L3e1e | 183 | 1 | 0.014
L3e1e | 185 | 1 | 0.014
L3e1e | 199 | 1 | 0.014
L3e1e | 2353C | 1 | 0.014
L3e1e | 513 | 1 | 0.014
L3e1e | 515-524d | 1 | 0.014
L3e1e | 5987A | 1 | 0.014
L3e1e | 7270 | 4 | 0.057
L3e1e | 8573 | 3 | 0.043
L3e1e | 8817 | 1 | 0.014
L3e1e | 8865 | 2 | 0.029
L3e1e | 9233 | 1 | 0.014
L3e1e | 9389 | 1 | 0.014
L3e1e | 9644 | 1 | 0.014
L3e1e1 | 10390d | 1 | 0.014
L3e1e1 | 11452 | 18 | 0.261
L3e1e1 | 11914 | 8 | 0.116
L3e1e1 | 13111 | 1 | 0.014
L3e1e1 | 13681 | 2 | 0.029
L3e1e1 | 14245 | 1 | 0.014
L3e1e1 | 14249 | 1 | 0.014
L3e1e1 | 14959 | 1 | 0.014
L3e1e1 | 15496 | 1 | 0.014
L3e1e1 | 16278 | 1 | 0.014
L3e1e1 | 16290 | 1 | 0.014
L3e1e1 | 16390 | 3 | 0.043
L3e1e1 | 1719 | 1 | 0.014
L3e1e1 | 185 | 3 | 0.043
L3e1e1 | 195 | 1 | 0.014
L3e1e1 | 204 | 1 | 0.014
L3e1e1 | 2056 | 3 | 0.043
L3e1e1 | 207 | 9 | 0.13
L3e1e1 | 309 | 1 | 0.014
L3e1e1 | 309d | 18 | 0.261
L3e1e1 | 315.2C | 2 | 0.029
L3e1e1 | 316C | 1 | 0.014
L3e1e1 | 3204 | 1 | 0.014
L3e1e1 | 3600 | 1 | 0.014
L3e1e1 | 3687 | 18 | 0.261
L3e1e1 | 4820 | 1 | 0.014
L3e1e1 | 5910 | 1 | 0.014
L3e1e1 | 5973 | 1 | 0.014
L3e1e1 | 6293 | 7 | 0.101
L3e1e1 | 6524 | 4 | 0.058
L3e1e1 | 6719 | 1 | 0.014
L3e1e1 | 7258 | 4 | 0.058
L3e1e1 | 7270 | 4 | 0.058
L3e1e1 | 7785 | 2 | 0.029
L3e1e1 | 8723 | 1 | 0.014
L3e1e2 | 12164 | 1 | 0.053
L3e1e2 | 16193 | 1 | 0.053
L3e1e2 | 16400 | 1 | 0.053
L3e1e2 | 16431d | 1 | 0.053
L3e1e2 | 7444 | 1 | 0.053
L3e1e2 | 8389 | 1 | 0.053
L3e1f | 13191 | 1 | 0.019
L3e1f | 14002 | 1 | 0.019
L3e1f | 152 | 3 | 0.057
L3e1f | 16093 | 3 | 0.057
L3e1f | 16129 | 1 | 0.019
L3e1f | 16179 | 9 | 0.17
L3e1f | 16184 | 6 | 0.113
L3e1f | 16207 | 3 | 0.057
L3e1f | 16234 | 1 | 0.019
L3e1f | 16255 | 2 | 0.038
L3e1f | 16355 | 1 | 0.019
L3e1f | 183 | 3 | 0.057
L3e1f | 193 | 1 | 0.019
L3e1f | 204 | 2 | 0.038
L3e1f | 23 | 3 | 0.057
L3e1f | 236 | 4 | 0.075
L3e1f | 241 | 1 | 0.019
L3e1f | 309d | 1 | 0.019
L3e1f | 5021 | 1 | 0.019
L3e1f | 7825 | 1 | 0.019
L3e1f | 8839 | 1 | 0.019
L3e1f | 930C | 1 | 0.019
L3e1f1 | 1007 | 1 | 0.043
L3e1f1 | 13581G | 1 | 0.043
L3e1f1 | 152 | 2 | 0.087
L3e1f1 | 16093 | 1 | 0.043
L3e1f1 | 16129 | 2 | 0.087
L3e1f1 | 16184A | 1 | 0.043
L3e1f1 | 16185 | 4 | 0.174
L3e1f1 | 16188 | 1 | 0.043
L3e1f1 | 16193d | 4 | 0.174
L3e1f1 | 16213 | 1 | 0.043
L3e1f1 | 16217 | 2 | 0.087
L3e1f1 | 16234 | 2 | 0.087
L3e1f1 | 16264 | 2 | 0.087
L3e1f1 | 16291 | 1 | 0.043
L3e1f1 | 16311 | 4 | 0.174
L3e1f1 | 16325 | 1 | 0.043
L3e1f1 | 16343T | 1 | 0.043
L3e1f1 | 16356 | 1 | 0.043
L3e1f1 | 16362 | 1 | 0.043
L3e1f1 | 185 | 2 | 0.087
L3e1f1 | 204 | 4 | 0.174
L3e1f1 | 207 | 3 | 0.13
L3e1f1 | 2392 | 1 | 0.043
L3e1f1 | 372 | 1 | 0.043
L3e1f1 | 8469 | 1 | 0.043
L3e1f1a | 14470 | 1 | 0.048
L3e1f1a | 16129 | 2 | 0.095
L3e1f1a | 16169 | 5 | 0.238
L3e1f1a | 16189A | 1 | 0.048
L3e1f1a | 16190-16193d | 1 | 0.048

L3e1f1a | 16192-16193d | 1 | 0.048
L3e1f1a | 185 | 1 | 0.048
L3e1f1a | 309d | 2 | 0.095
L3e1f1a | 310 | 1 | 0.048
L3e1f1a | 315.2C | 1 | 0.048
L3e1f1a | 316C | 1 | 0.048
L3e1f1a | 318 | 1 | 0.048
L3e1f2 | 12771 | 1 | 0.059
L3e1f2 | 16145 | 1 | 0.059
L3e1f2 | 16242 | 1 | 0.059
L3e1f2 | 16297 | 5 | 0.294
L3e1f2 | 16311 | 2 | 0.118
L3e1g | 16311 | 1 | 0.062
L3e1g | 16497 | 1 | 0.062
L3e1g | 195 | 1 | 0.062
L3e1g | 207 | 5 | 0.312
L3e1g | 7283 | 1 | 0.062
L3e1g | 9438 | 1 | 0.062
L3e2 | 11389 | 1 | 0.028
L3e2 | 15253 | 1 | 0.028
L3e2 | 15885 | 1 | 0.028
L3e2 | 15927 | 1 | 0.028
L3e2 | 16124 | 2 | 0.056
L3e2 | 16131G | 1 | 0.028
L3e2 | 16132 | 1 | 0.028
L3e2 | 16141 | 1 | 0.028
L3e2 | 16147G | 1 | 0.028
L3e2 | 16172 | 11 | 0.306
L3e2 | 16182 | 1 | 0.028
L3e2 | 16256 | 4 | 0.111
L3e2 | 16274 | 2 | 0.056
L3e2 | 16295 | 1 | 0.028
L3e2 | 16311 | 1 | 0.028
L3e2 | 16317 | 1 | 0.028
L3e2 | 16360 | 2 | 0.056
L3e2 | 200 | 2 | 0.056
L3e2 | 292 | 1 | 0.028
L3e2 | 309d | 1 | 0.028
L3e2 | 310 | 1 | 0.028
L3e2 | 366 | 1 | 0.028
L3e2 | 474A | 2 | 0.056
L3e2 | 507A | 4 | 0.111
L3e2 | 509A | 1 | 0.028
L3e2 | 515-524d | 15 | 0.417
L3e2 | 5201 | 1 | 0.028
L3e2 | 529T | 2 | 0.056
L3e2 | 548A | 2 | 0.056
L3e2 | 6267 | 1 | 0.028
L3e2 | 7256 | 1 | 0.028
L3e2a | 10915 | 1 | 0.037
L3e2a | 11347 | 2 | 0.074
L3e2a | 11617 | 1 | 0.037
L3e2a | 13879 | 1 | 0.037
L3e2a | 13889 | 1 | 0.037
L3e2a | 14668 | 1 | 0.037
L3e2a | 14927 | 1 | 0.037
L3e2a | 15927 | 1 | 0.037
L3e2a | 16093 | 1 | 0.037
L3e2a | 16183 | 1 | 0.037
L3e2a | 16258T | 2 | 0.074
L3e2a | 16261 | 1 | 0.037
L3e2a | 16295 | 1 | 0.037
L3e2a | 16325 | 5 | 0.185
L3e2a | 1737 | 2 | 0.074
L3e2a | 189 | 2 | 0.074
L3e2a | 309d | 1 | 0.037
L3e2a | 4216 | 1 | 0.037
L3e2a | 5082 | 1 | 0.037
L3e2a | 5093 | 1 | 0.037
L3e2a | 6044 | 2 | 0.074
L3e2a | 6200 | 2 | 0.074
L3e2a | 7226 | 1 | 0.037
L3e2a | 9098 | 1 | 0.037
L3e2a | 9196 | 1 | 0.037
L3e2a | 93.1A | 1 | 0.037
L3e2a1 | 10084 | 1 | 0.029
L3e2a1 | 10181 | 3 | 0.088
L3e2a1 | 11167 | 1 | 0.029
L3e2a1 | 152 | 2 | 0.059
L3e2a1 | 16068 | 1 | 0.029
L3e2a1 | 16189 | 3 | 0.088
L3e2a1 | 16218 | 1 | 0.029
L3e2a1 | 16256 | 1 | 0.029
L3e2a1 | 16271 | 1 | 0.029
L3e2a1 | 16286 | 1 | 0.029
L3e2a1 | 16292 | 1 | 0.029
L3e2a1 | 16325 | 3 | 0.088
L3e2a1 | 16335 | 3 | 0.088
L3e2a1 | 16362 | 6 | 0.176
L3e2a1 | 1935 | 1 | 0.029
L3e2a1 | 2917C | 1 | 0.029
L3e2a1 | 3645 | 1 | 0.029
L3e2a1 | 4907 | 3 | 0.088
L3e2a1 | 769 | 1 | 0.029
L3e2a1 | 8855 | 1 | 0.029
L3e2a1a | 15218 | 1 | 0.042
L3e2a1a | 16146 | 2 | 0.083
L3e2a1a | 16249A | 1 | 0.042
L3e2a1a | 16311 | 1 | 0.042
L3e2a1a | 1719 | 1 | 0.042
L3e2a1a | 1799 | 1 | 0.042
L3e2a1a | 2124 | 1 | 0.042
L3e2a1a | 2262 | 1 | 0.042
L3e2a1a | 309d | 1 | 0.042
L3e2a1a | 315.2C | 1 | 0.042
L3e2a1a | 3633 | 1 | 0.042
L3e2a1a | 515-524d | 3 | 0.125
L3e2a1a | 5774G | 1 | 0.042
L3e2a1a | 5951 | 1 | 0.042
L3e2a1a | 6956 | 1 | 0.042
L3e2a1a | 8251 | 1 | 0.042

L3e2a1a | 9653 | 1 | 0.042
L3e2a1b | 15319 | 1 | 0.048
L3e2a1b | 16129 | 1 | 0.048
L3e2a1b | 16148 | 1 | 0.048
L3e2a1b | 16170 | 1 | 0.048
L3e2a1b | 16221 | 1 | 0.048
L3e2a1b | 16311 | 3 | 0.143
L3e2a1b | 460A | 1 | 0.048
L3e2a1b | 6167 | 2 | 0.095
L3e2a1b | 6647 | 1 | 0.048
L3e2a1b | 793 | 1 | 0.048
L3e2a1b | 8011T | 1 | 0.048
L3e2a1b | 942 | 1 | 0.048
L3e2a1b | 9667 | 1 | 0.048
L3e2a1b1 | 10042 | 1 | 0.013
L3e2a1b1 | 10400 | 2 | 0.026
L3e2a1b1 | 11353 | 1 | 0.013
L3e2a1b1 | 12545 | 1 | 0.013
L3e2a1b1 | 12771 | 1 | 0.013
L3e2a1b1 | 13958C | 1 | 0.013
L3e2a1b1 | 14180 | 1 | 0.013
L3e2a1b1 | 14323 | 1 | 0.013
L3e2a1b1 | 14364 | 1 | 0.013
L3e2a1b1 | 14560 | 1 | 0.013
L3e2a1b1 | 146 | 1 | 0.013
L3e2a1b1 | 14693 | 1 | 0.013
L3e2a1b1 | 152 | 6 | 0.078
L3e2a1b1 | 15310 | 1 | 0.013
L3e2a1b1 | 15442 | 1 | 0.013
L3e2a1b1 | 15734 | 1 | 0.013
L3e2a1b1 | 16086 | 3 | 0.039
L3e2a1b1 | 16093 | 4 | 0.052
L3e2a1b1 | 16129 | 1 | 0.013
L3e2a1b1 | 16145 | 2 | 0.026
L3e2a1b1 | 16154 | 1 | 0.013
L3e2a1b1 | 16163 | 3 | 0.039
L3e2a1b1 | 16187 | 2 | 0.026
L3e2a1b1 | 16189 | 4 | 0.052
L3e2a1b1 | 16248 | 1 | 0.013
L3e2a1b1 | 16249 | 1 | 0.013
L3e2a1b1 | 16256 | 1 | 0.013
L3e2a1b1 | 16271 | 1 | 0.013
L3e2a1b1 | 16278 | 2 | 0.026
L3e2a1b1 | 16286 | 2 | 0.026
L3e2a1b1 | 16288 | 1 | 0.013
L3e2a1b1 | 16290 | 1 | 0.013
L3e2a1b1 | 16291 | 1 | 0.013
L3e2a1b1 | 16293T | 1 | 0.013
L3e2a1b1 | 16294 | 3 | 0.039
L3e2a1b1 | 16301 | 2 | 0.026
L3e2a1b1 | 16311 | 3 | 0.039
L3e2a1b1 | 16362 | 1 | 0.013
L3e2a1b1 | 16390 | 1 | 0.013
L3e2a1b1 | 16527 | 2 | 0.026
L3e2a1b1 | 1713 | 1 | 0.013
L3e2a1b1 | 185 | 2 | 0.026
L3e2a1b1 | 200 | 1 | 0.013
L3e2a1b1 | 204 | 1 | 0.013
L3e2a1b1 | 251 | 2 | 0.026
L3e2a1b1 | 2768 | 1 | 0.013
L3e2a1b1 | 309d | 1 | 0.013
L3e2a1b1 | 3531 | 1 | 0.013
L3e2a1b1 | 391 | 2 | 0.026
L3e2a1b1 | 4164 | 1 | 0.013
L3e2a1b1 | 4452 | 1 | 0.013
L3e2a1b1 | 499 | 66 | 0.857
L3e2a1b1 | 506 | 1 | 0.013
L3e2a1b1 | 515-524d | 3 | 0.039
L3e2a1b1 | 529T | 1 | 0.013
L3e2a1b1 | 533 | 1 | 0.013
L3e2a1b1 | 5508 | 1 | 0.013
L3e2a1b1 | 573.1C | 2 | 0.026
L3e2a1b1 | 5921 | 1 | 0.013
L3e2a1b1 | 593 | 2 | 0.026
L3e2a1b1 | 6480 | 1 | 0.013
L3e2a1b1 | 6680 | 1 | 0.013
L3e2a1b1 | 6915 | 1 | 0.013
L3e2a1b1 | 709 | 1 | 0.013
L3e2a1b1 | 7170 | 1 | 0.013
L3e2a1b1 | 723 | 1 | 0.013
L3e2a1b1 | 7444 | 2 | 0.026
L3e2a1b1 | 7521 | 1 | 0.013
L3e2a1b1 | 7852 | 1 | 0.013
L3e2a1b1 | 8047 | 1 | 0.013
L3e2a1b1 | 8155 | 1 | 0.013
L3e2a1b1 | 8711 | 1 | 0.013
L3e2a1b1 | 8764 | 1 | 0.013
L3e2a1b1 | 8911 | 1 | 0.013
L3e2a1b1 | 9006 | 1 | 0.013
L3e2a1b1 | 9156 | 1 | 0.013
L3e2a1b1 | 9353 | 1 | 0.013
L3e2a1b2 | 14198 | 1 | 0.056
L3e2a1b2 | 146 | 1 | 0.056
L3e2a1b2 | 15172 | 1 | 0.056
L3e2a1b2 | 16066 | 2 | 0.111
L3e2a1b2 | 16111 | 1 | 0.056
L3e2a1b2 | 16145 | 1 | 0.056
L3e2a1b2 | 16148 | 1 | 0.056
L3e2a1b2 | 16162 | 1 | 0.056
L3e2a1b2 | 16192 | 1 | 0.056
L3e2a1b2 | 16243 | 1 | 0.056
L3e2a1b2 | 16294 | 1 | 0.056
L3e2a1b2 | 16399 | 1 | 0.056
L3e2a1b2 | 16484-16489d | 1 | 0.056
L3e2a1b2 | 16497 | 1 | 0.056
L3e2a1b2 | 200 | 1 | 0.056
L3e2a1b2 | 3172.1C | 1 | 0.056
L3e2a1b2 | 3945 | 1 | 0.056
L3e2a1b2 | 8614 | 1 | 0.056
L3e2a1b2 | 8618 | 1 | 0.056
L3e2a1b2 | 9650 | 1 | 0.056

L3e2a1b3 | 11699 | 1 | 0.024
L3e2a1b3 | 13889 | 1 | 0.024
L3e2a1b3 | 16086 | 2 | 0.049
L3e2a1b3 | 16093 | 5 | 0.122
L3e2a1b3 | 16114 | 1 | 0.024
L3e2a1b3 | 16129 | 1 | 0.024
L3e2a1b3 | 16148 | 20 | 0.488
L3e2a1b3 | 16209 | 1 | 0.024
L3e2a1b3 | 16221 | 1 | 0.024
L3e2a1b3 | 16294 | 3 | 0.073
L3e2a1b3 | 16497 | 1 | 0.024
L3e2a1b3 | 1888 | 1 | 0.024
L3e2a1b3 | 200 | 10 | 0.244
L3e2a1b3 | 310 | 1 | 0.024
L3e2a1b3 | 4310 | 1 | 0.024
L3e2a1b3 | 5492 | 1 | 0.024
L3e2a2 | 10281 | 1 | 0.038
L3e2a2 | 10556 | 4 | 0.154
L3e2a2 | 152 | 8 | 0.308
L3e2a2 | 16148 | 1 | 0.038
L3e2a2 | 16179 | 1 | 0.038
L3e2a2 | 16188 | 1 | 0.038
L3e2a2 | 16189 | 1 | 0.038
L3e2a2 | 16257 | 3 | 0.115
L3e2a2 | 16290A | 3 | 0.115
L3e2a2 | 16311 | 1 | 0.038
L3e2a2 | 182 | 1 | 0.038
L3e2a2 | 185T | 1 | 0.038
L3e2a2 | 198 | 1 | 0.038
L3e2a2 | 247 | 1 | 0.038
L3e2a2 | 357 | 1 | 0.038
L3e2a2 | 3582A | 1 | 0.038
L3e2a2 | 3796 | 4 | 0.154
L3e2a2 | 515-524d | 2 | 0.077
L3e2a2 | 5951 | 1 | 0.038
L3e2a2 | 709 | 1 | 0.038
L3e2a3 | 146 | 1 | 0.025
L3e2a3 | 14974G | 1 | 0.025
L3e2a3 | 152 | 1 | 0.025
L3e2a3 | 16069 | 2 | 0.05
L3e2a3 | 16093 | 1 | 0.025
L3e2a3 | 16124 | 1 | 0.025
L3e2a3 | 16189 | 1 | 0.025
L3e2a3 | 16193 | 1 | 0.025
L3e2a3 | 16221 | 2 | 0.05
L3e2a3 | 16256 | 1 | 0.025
L3e2a3 | 16258 | 1 | 0.025
L3e2a3 | 16258T | 11 | 0.275
L3e2a3 | 16288 | 1 | 0.025
L3e2a3 | 16324 | 3 | 0.075
L3e2a3 | 16357 | 1 | 0.025
L3e2a3 | 16359 | 1 | 0.025
L3e2a3 | 16399 | 2 | 0.05
L3e2a3 | 189 | 14 | 0.35
L3e2a3 | 194 | 2 | 0.05
L3e2a3 | 200 | 1 | 0.025
L3e2a3 | 204 | 1 | 0.025
L3e2a3 | 253 | 1 | 0.025
L3e2a3 | 315.2C | 1 | 0.025
L3e2a3 | 44.1C | 1 | 0.025
L3e2a3 | 456 | 1 | 0.025
L3e2a3 | 4655 | 2 | 0.05
L3e2a3 | 4688 | 2 | 0.05
L3e2a3 | 515-524d | 1 | 0.025
L3e2a3 | 548A | 1 | 0.025
L3e2b | 10018 | 1 | 0.007
L3e2b | 10205 | 2 | 0.015
L3e2b | 10237 | 4 | 0.03
L3e2b | 10655 | 1 | 0.007
L3e2b | 10918 | 1 | 0.007
L3e2b | 11284 | 1 | 0.007
L3e2b | 11410 | 1 | 0.007
L3e2b | 11431 | 1 | 0.007
L3e2b | 11696 | 1 | 0.007
L3e2b | 11963 | 1 | 0.007
L3e2b | 12127 | 1 | 0.007
L3e2b | 12950 | 1 | 0.007
L3e2b | 13145 | 1 | 0.007
L3e2b | 13661 | 1 | 0.007
L3e2b | 13863 | 1 | 0.007
L3e2b | 13942 | 1 | 0.007
L3e2b | 14121 | 1 | 0.007
L3e2b | 1415 | 1 | 0.007
L3e2b | 14364 | 1 | 0.007
L3e2b | 14514 | 1 | 0.007
L3e2b | 14569 | 1 | 0.007
L3e2b | 146 | 4 | 0.03
L3e2b | 15253 | 5 | 0.037
L3e2b | 15287 | 7 | 0.052
L3e2b | 152d | 6 | 0.045
L3e2b | 15419T | 1 | 0.007
L3e2b | 15497 | 1 | 0.007
L3e2b | 15511 | 1 | 0.007
L3e2b | 15530 | 1 | 0.007
L3e2b | 15775 | 1 | 0.007
L3e2b | 15784 | 2 | 0.015
L3e2b | 15930 | 1 | 0.007
L3e2b | 16093 | 1 | 0.007
L3e2b | 16126 | 4 | 0.03
L3e2b | 16145 | 1 | 0.007
L3e2b | 16173 | 1 | 0.007
L3e2b | 16182 | 2 | 0.015
L3e2b | 16187 | 3 | 0.022
L3e2b | 16192-16193d | 5 | 0.037
L3e2b | 16193d | 3 | 0.022
L3e2b | 16194.1C | 1 | 0.007
L3e2b | 16209 | 1 | 0.007
L3e2b | 16213 | 5 | 0.037
L3e2b | 16234 | 1 | 0.007
L3e2b | 16259 | 1 | 0.007
L3e2b | 16265 | 1 | 0.007

L3e2b | 16288 | 5 | 0.037
L3e2b | 16301 | 2 | 0.015
L3e2b | 16311 | 1 | 0.007
L3e2b | 16337G | 1 | 0.007
L3e2b | 16359 | 1 | 0.007
L3e2b | 16384 | 1 | 0.007
L3e2b | 16484-16489d | 1 | 0.007
L3e2b | 1719 | 1 | 0.007
L3e2b | 1888 | 1 | 0.007
L3e2b | 189 | 2 | 0.015
L3e2b | 2068 | 1 | 0.007
L3e2b | 207 | 2 | 0.015
L3e2b | 2220 | 3 | 0.022
L3e2b | 228 | 1 | 0.007
L3e2b | 236 | 1 | 0.007
L3e2b | 247 | 1 | 0.007
L3e2b | 260 | 1 | 0.007
L3e2b | 309d | 13 | 0.097
L3e2b | 310 | 4 | 0.03
L3e2b | 315.2C | 1 | 0.007
L3e2b | 315.3C | 1 | 0.007
L3e2b | 316 | 1 | 0.007
L3e2b | 335 | 1 | 0.007
L3e2b | 3483 | 1 | 0.007
L3e2b | 3591 | 1 | 0.007
L3e2b | 3714 | 1 | 0.007
L3e2b | 384 | 1 | 0.007
L3e2b | 4164 | 6 | 0.045
L3e2b | 4205 | 1 | 0.007
L3e2b | 4418 | 1 | 0.007
L3e2b | 456 | 5 | 0.037
L3e2b | 5144 | 1 | 0.007
L3e2b | 5147 | 1 | 0.007
L3e2b | 517 | 1 | 0.007
L3e2b | 5186 | 11 | 0.082
L3e2b | 5201 | 1 | 0.007
L3e2b | 537 | 5 | 0.037
L3e2b | 5471 | 1 | 0.007
L3e2b | 5747 | 4 | 0.03
L3e2b | 5752d | 1 | 0.007
L3e2b | 6140G | 1 | 0.007
L3e2b | 6253 | 4 | 0.03
L3e2b | 6267 | 1 | 0.007
L3e2b | 6383 | 1 | 0.007
L3e2b | 6663 | 1 | 0.007
L3e2b | 679 | 1 | 0.007
L3e2b | 709 | 1 | 0.007
L3e2b | 7337 | 1 | 0.007
L3e2b | 7598 | 1 | 0.007
L3e2b | 769 | 12 | 0.09
L3e2b | 7717T | 1 | 0.007
L3e2b | 7775 | 2 | 0.015
L3e2b | 7819A | 1 | 0.007
L3e2b | 7957 | 1 | 0.007
L3e2b | 8238 | 1 | 0.007
L3e2b | 8290 | 1 | 0.007
L3e2b | 8576A | 1 | 0.007
L3e2b | 8688 | 4 | 0.03
L3e2b | 8802 | 1 | 0.007
L3e2b | 8838 | 1 | 0.007
L3e2b | 9018G | 1 | 0.007
L3e2b | 930 | 1 | 0.007
L3e2b | 9302 | 1 | 0.007
L3e2b | 9380 | 1 | 0.007
L3e2b | 95d | 1 | 0.007
L3e2b | 988 | 1 | 0.007
L3e2b | 9947 | 5 | 0.037
L3e2b+152 | 10098T | 8 | 0.084
L3e2b+152 | 1117 | 1 | 0.011
L3e2b+152 | 11260 | 1 | 0.011
L3e2b+152 | 11998 | 1 | 0.011
L3e2b+152 | 12630 | 1 | 0.011
L3e2b+152 | 13263 | 1 | 0.011
L3e2b+152 | 13528 | 1 | 0.011
L3e2b+152 | 14020 | 1 | 0.011
L3e2b+152 | 14226 | 1 | 0.011
L3e2b+152 | 14319 | 1 | 0.011
L3e2b+152 | 14470 | 1 | 0.011
L3e2b+152 | 15314 | 1 | 0.011
L3e2b+152 | 15431 | 1 | 0.011
L3e2b+152 | 15777 | 1 | 0.011
L3e2b+152 | 16186 | 1 | 0.011
L3e2b+152 | 16193d | 1 | 0.011
L3e2b+152 | 16213 | 15 | 0.158
L3e2b+152 | 16245 | 3 | 0.032
L3e2b+152 | 16260 | 3 | 0.032
L3e2b+152 | 16266 | 3 | 0.032
L3e2b+152 | 16278 | 1 | 0.011
L3e2b+152 | 16311 | 1 | 0.011
L3e2b+152 | 1676 | 1 | 0.011
L3e2b+152 | 2010 | 1 | 0.011
L3e2b+152 | 2445 | 1 | 0.011
L3e2b+152 | 309d | 7 | 0.074
L3e2b+152 | 3606 | 9 | 0.095
L3e2b+152 | 3834 | 1 | 0.011
L3e2b+152 | 4092 | 8 | 0.084
L3e2b+152 | 456 | 12 | 0.126
L3e2b+152 | 4823 | 1 | 0.011
L3e2b+152 | 5063 | 1 | 0.011
L3e2b+152 | 537 | 9 | 0.095
L3e2b+152 | 5486 | 1 | 0.011
L3e2b+152 | 5894 | 1 | 0.011
L3e2b+152 | 5899.1C | 1 | 0.011
L3e2b+152 | 6116 | 1 | 0.011
L3e2b+152 | 6179 | 1 | 0.011
L3e2b+152 | 647 | 8 | 0.084
L3e2b+152 | 6956 | 1 | 0.011
L3e2b+152 | 6987G | 1 | 0.011
L3e2b+152 | 7702 | 1 | 0.011
L3e2b+152 | 7746 | 1 | 0.011
L3e2b+152 | 7853 | 1 | 0.011

L3e2b+152 | 8848 | 1 | 0.011
L3e2b+152 | 8950 | 1 | 0.011
L3e2b+152 | 9078 | 1 | 0.011
L3e2b+152 | 9531 | 1 | 0.011
L3e2b+152 | 9947 | 9 | 0.095
L3e2b1 | 12561 | 1 | 0.014
L3e2b1 | 146 | 6 | 0.083
L3e2b1 | 14992 | 2 | 0.028
L3e2b1 | 16093 | 2 | 0.028
L3e2b1 | 16164 | 4 | 0.056
L3e2b1 | 16173 | 3 | 0.042
L3e2b1 | 309d | 1 | 0.014
L3e2b1 | 3975 | 2 | 0.028
L3e2b1 | 4496 | 2 | 0.028
L3e2b1 | 4772 | 4 | 0.056
L3e2b1 | 5964 | 4 | 0.056
L3e2b1 | 7859 | 2 | 0.028
L3e2b1 | 8730 | 4 | 0.056
L3e2b1 | 9132 | 1 | 0.014
L3e2b1 | 961 | 3 | 0.042
L3e2b1 | 965.1C | 1 | 0.014
L3e2b1 | 9948 | 2 | 0.028
L3e2b1a | 15412 | 1 | 0.015
L3e2b1a | 16209 | 1 | 0.015
L3e2b1a | 16234 | 1 | 0.015
L3e2b1a | 16239 | 6 | 0.088
L3e2b1a | 249d | 1 | 0.015
L3e2b1a | 310 | 1 | 0.015
L3e2b1a | 4167 | 1 | 0.015
L3e2b1a | 4500 | 1 | 0.015
L3e2b1a | 4550 | 1 | 0.015
L3e2b1a | 5471 | 1 | 0.015
L3e2b1a | 6092 | 2 | 0.029
L3e2b1a | 7609 | 1 | 0.015
L3e2b1a | 8823 | 1 | 0.015
L3e2b1a | 8953 | 1 | 0.015
L3e2b1a | 9503 | 1 | 0.015
L3e2b1a1 | 10007 | 1 | 0.014
L3e2b1a1 | 11017 | 1 | 0.014
L3e2b1a1 | 11337 | 1 | 0.014
L3e2b1a1 | 11654 | 1 | 0.014
L3e2b1a1 | 11722 | 8 | 0.108
L3e2b1a1 | 12172 | 1 | 0.014
L3e2b1a1 | 12285 | 1 | 0.014
L3e2b1a1 | 12850 | 1 | 0.014
L3e2b1a1 | 14580 | 1 | 0.014
L3e2b1a1 | 15047 | 1 | 0.014
L3e2b1a1 | 152 | 1 | 0.014
L3e2b1a1 | 15930 | 1 | 0.014
L3e2b1a1 | 15932 | 1 | 0.014
L3e2b1a1 | 16093 | 1 | 0.014
L3e2b1a1 | 16145 | 4 | 0.054
L3e2b1a1 | 16221 | 1 | 0.014
L3e2b1a1 | 16274 | 1 | 0.014
L3e2b1a1 | 16278 | 3 | 0.041
L3e2b1a1 | 16484-16489d | 1 | 0.014
L3e2b1a1 | 185T | 1 | 0.014
L3e2b1a1 | 3882 | 3 | 0.041
L3e2b1a1 | 408A | 3 | 0.041
L3e2b1a1 | 4209 | 1 | 0.014
L3e2b1a1 | 5210 | 4 | 0.054
L3e2b1a1 | 5460 | 1 | 0.014
L3e2b1a1 | 6480 | 4 | 0.054
L3e2b1a2 | 10454 | 1 | 0.013
L3e2b1a2 | 12432 | 1 | 0.013
L3e2b1a2 | 12900 | 2 | 0.025
L3e2b1a2 | 13674 | 1 | 0.013
L3e2b1a2 | 1415 | 1 | 0.013
L3e2b1a2 | 14180 | 1 | 0.013
L3e2b1a2 | 152 | 1 | 0.013
L3e2b1a2 | 15300 | 3 | 0.038
L3e2b1a2 | 15344 | 1 | 0.013
L3e2b1a2 | 15355 | 1 | 0.013
L3e2b1a2 | 15603 | 1 | 0.013
L3e2b1a2 | 15721 | 3 | 0.038
L3e2b1a2 | 198 | 2 | 0.025
L3e2b1a2 | 282 | 2 | 0.025
L3e2b1a2 | 309d | 7 | 0.089
L3e2b1a2 | 3866 | 3 | 0.038
L3e2b1a2 | 4386 | 1 | 0.013
L3e2b1a2 | 4811 | 1 | 0.013
L3e2b1a2 | 5585 | 1 | 0.013
L3e2b1a2 | 5899d | 1 | 0.013
L3e2b1a2 | 6167 | 1 | 0.013
L3e2b1a2 | 709 | 1 | 0.013
L3e2b2 | 11116 | 1 | 0.014
L3e2b2 | 11386 | 1 | 0.014
L3e2b2 | 14831 | 2 | 0.029
L3e2b2 | 15924 | 1 | 0.014
L3e2b2 | 16188 | 1 | 0.014
L3e2b2 | 16304 | 1 | 0.014
L3e2b2 | 16311 | 7 | 0.1
L3e2b2 | 1694 | 3 | 0.043
L3e2b2 | 235 | 1 | 0.014
L3e2b2 | 3496 | 1 | 0.014
L3e2b2 | 3523 | 2 | 0.029
L3e2b2 | 4113 | 1 | 0.014
L3e2b2 | 4560 | 4 | 0.057
L3e2b2 | 5582 | 1 | 0.014
L3e2b2 | 8009 | 1 | 0.014
L3e2b3 | 11659 | 2 | 0.033
L3e2b3 | 13269 | 2 | 0.033
L3e2b3 | 14091 | 1 | 0.016
L3e2b3 | 15670 | 1 | 0.016
L3e2b3 | 15941 | 1 | 0.016
L3e2b3 | 16069 | 1 | 0.016
L3e2b3 | 16192 | 1 | 0.016
L3e2b3 | 16532d | 1 | 0.016
L3e2b3 | 189 | 2 | 0.033
L3e2b3 | 189C | 1 | 0.016
L3e2b3 | 198 | 2 | 0.033

L3e2b3 | 199 | 1 | 0.016
L3e2b3 | 309d | 4 | 0.066
L3e2b3 | 315.2C | 1 | 0.016
L3e2b3 | 4242 | 1 | 0.016
L3e2b3 | 5160 | 1 | 0.016
L3e2b3 | 5978 | 1 | 0.016
L3e2b3 | 7978G | 1 | 0.016
L3e2b3 | 7985G | 1 | 0.016
L3e2b3 | 8614 | 1 | 0.016
L3e2b3 | 8818 | 2 | 0.033
L3e2b3 | 9304 | 1 | 0.016
L3e2b4 | 11440 | 1 | 0.011
L3e2b4 | 16093 | 3 | 0.034
L3e2b4 | 16129 | 2 | 0.022
L3e2b4 | 16175 | 3 | 0.034
L3e2b4 | 16188 | 3 | 0.034
L3e2b4 | 16190 | 1 | 0.011
L3e2b4 | 16209 | 2 | 0.022
L3e2b4 | 16261 | 1 | 0.011
L3e2b4 | 16263 | 1 | 0.011
L3e2b4 | 16265C | 1 | 0.011
L3e2b4 | 16302 | 1 | 0.011
L3e2b4 | 16311 | 6 | 0.067
L3e2b4 | 16318 | 1 | 0.011
L3e2b4 | 16318A | 1 | 0.011
L3e2b4 | 16318T | 1 | 0.011
L3e2b4 | 16335 | 1 | 0.011
L3e2b4 | 16344 | 1 | 0.011
L3e2b4 | 16360 | 1 | 0.011
L3e2b4 | 16527 | 1 | 0.011
L3e2b4 | 200 | 2 | 0.022
L3e2b4 | 204 | 2 | 0.022
L3e2b4 | 207 | 1 | 0.011
L3e2b4 | 247 | 1 | 0.011
L3e2b4 | 262 | 1 | 0.011
L3e2b4 | 309 | 1 | 0.011
L3e2b4 | 3816 | 1 | 0.011
L3e2b4 | 455.1T | 1 | 0.011
L3e2b4 | 463 | 1 | 0.011
L3e2b4 | 466 | 1 | 0.011
L3e2b4 | 483 | 1 | 0.011
L3e2b4 | 489 | 1 | 0.011
L3e2b4 | 508 | 1 | 0.011
L3e2b4 | 513 | 1 | 0.011
L3e2b4 | 515-524d | 4 | 0.045
L3e2b4 | 64 | 1 | 0.011
L3e2b4 | 8206 | 1 | 0.011
L3e2b4 | 93 | 1 | 0.011
L3e2b4 | 960d | 1 | 0.011
L3e2b5 | 13015 | 1 | 0.013
L3e2b5 | 16093 | 3 | 0.039
L3e2b5 | 16129 | 2 | 0.026
L3e2b5 | 16175 | 3 | 0.039
L3e2b5 | 16188 | 3 | 0.039
L3e2b5 | 16190 | 1 | 0.013
L3e2b5 | 16209 | 2 | 0.026
L3e2b5 | 16263 | 1 | 0.013
L3e2b5 | 16265C | 1 | 0.013
L3e2b5 | 16293T | 1 | 0.013
L3e2b5 | 16302 | 1 | 0.013
L3e2b5 | 16318 | 1 | 0.013
L3e2b5 | 16318A | 1 | 0.013
L3e2b5 | 16318T | 1 | 0.013
L3e2b5 | 16360 | 1 | 0.013
L3e2b5 | 16390 | 2 | 0.026
L3e2b5 | 16527 | 1 | 0.013
L3e2b5 | 200 | 1 | 0.013
L3e2b5 | 204 | 2 | 0.026
L3e2b5 | 207 | 1 | 0.013
L3e2b5 | 247 | 1 | 0.013
L3e2b5 | 309 | 1 | 0.013
L3e2b5 | 310 | 1 | 0.013
L3e2b5 | 455.1T | 1 | 0.013
L3e2b5 | 463 | 1 | 0.013
L3e2b5 | 466 | 1 | 0.013
L3e2b5 | 4811 | 1 | 0.013
L3e2b5 | 489 | 1 | 0.013
L3e2b5 | 508 | 1 | 0.013
L3e2b5 | 513 | 1 | 0.013
L3e2b5 | 8999 | 1 | 0.013
L3e2b5 | 93 | 1 | 0.013
L3e2b6 | 15631C | 1 | 0.062
L3e2b6 | 15703 | 1 | 0.062
L3e2b6 | 16126 | 1 | 0.062
L3e2b6 | 16131G | 1 | 0.062
L3e2b6 | 16132 | 1 | 0.062
L3e2b6 | 16141 | 1 | 0.062
L3e2b6 | 16147G | 1 | 0.062
L3e2b6 | 16263 | 1 | 0.062
L3e2b6 | 16327A | 1 | 0.062
L3e2b7 | 13166 | 1 | 0.009
L3e2b7 | 15113 | 1 | 0.009
L3e2b7 | 15325 | 1 | 0.009
L3e2b7 | 15358 | 1 | 0.009
L3e2b7 | 15533 | 1 | 0.009
L3e2b7 | 16051 | 1 | 0.009
L3e2b7 | 16111 | 1 | 0.009
L3e2b7 | 16126 | 1 | 0.009
L3e2b7 | 16129 | 3 | 0.027
L3e2b7 | 16148 | 1 | 0.009
L3e2b7 | 16153 | 1 | 0.009
L3e2b7 | 16171 | 1 | 0.009
L3e2b7 | 16186 | 2 | 0.018
L3e2b7 | 16188 | 8 | 0.072
L3e2b7 | 16192 | 1 | 0.009
L3e2b7 | 16242 | 1 | 0.009
L3e2b7 | 16261 | 4 | 0.036
L3e2b7 | 16278 | 1 | 0.009
L3e2b7 | 16284 | 1 | 0.009
L3e2b7 | 16287 | 1 | 0.009
L3e2b7 | 16291 | 1 | 0.009

L3e2b7 | 16309 | 1 | 0.009
L3e2b7 | 16311 | 2 | 0.018
L3e2b7 | 16353 | 3 | 0.027
L3e2b7 | 16357 | 1 | 0.009
L3e2b7 | 16362 | 1 | 0.009
L3e2b7 | 16390 | 3 | 0.027
L3e2b7 | 189 | 1 | 0.009
L3e2b7 | 204 | 1 | 0.009
L3e2b7 | 215 | 1 | 0.009
L3e2b7 | 228T | 1 | 0.009
L3e2b7 | 241.1TAA | 1 | 0.009
L3e2b7 | 282 | 18 | 0.162
L3e2b7 | 291.1A | 1 | 0.009
L3e2b7 | 385 | 1 | 0.009
L3e2b7 | 456 | 1 | 0.009
L3e2b7 | 513 | 1 | 0.009
L3e2b7 | 8745 | 1 | 0.009
L3e2b7 | 93 | 2 | 0.018
L3e2b8 | 12781 | 1 | 0.015
L3e2b8 | 16169 | 2 | 0.029
L3e2b8 | 16193d | 1 | 0.015
L3e2b8 | 16209 | 1 | 0.015
L3e2b8 | 228 | 1 | 0.015
L3e2b8 | 310 | 1 | 0.015
L3e2b8 | 5585 | 1 | 0.015
L3e2b8 | 6019 | 1 | 0.015
L3e2b8 | 8730 | 4 | 0.059
L3e3 | 16145 | 1 | 0.021
L3e3 | 16172 | 1 | 0.021
L3e3 | 16176 | 1 | 0.021
L3e3 | 16266 | 1 | 0.021
L3e3 | 16295 | 1 | 0.021
L3e3 | 16299 | 1 | 0.021
L3e3 | 204 | 1 | 0.021
L3e3 | 334 | 1 | 0.021
L3e3 | 527G | 1 | 0.021
L3e3'4 | 16172 | 4 | 0.027
L3e3'4 | 16234 | 2 | 0.013
L3e3'4 | 16258C | 3 | 0.02
L3e3'4 | 16291A | 3 | 0.02
L3e3'4 | 16292 | 1 | 0.007
L3e3'4 | 16295 | 1 | 0.007
L3e3'4 | 16298 | 3 | 0.02
L3e3'4 | 199 | 3 | 0.02
L3e3'4 | 504 | 1 | 0.007
L3e3'4'5 | 10667 | 1 | 0.006
L3e3'4'5 | 10816 | 1 | 0.006
L3e3'4'5 | 12248 | 1 | 0.006
L3e3'4'5 | 13651 | 1 | 0.006
L3e3'4'5 | 16148 | 12 | 0.076
L3e3'4'5 | 16296 | 1 | 0.006
L3e3'4'5 | 189 | 1 | 0.006
L3e3'4'5 | 195 | 1 | 0.006
L3e3'4'5 | 9554 | 1 | 0.006
L3e3a | 10042C | 1 | 0.007
L3e3a | 10790 | 1 | 0.007
L3e3a | 10909 | 1 | 0.007
L3e3a | 10978 | 1 | 0.007
L3e3a | 11016 | 1 | 0.007
L3e3a | 12034 | 1 | 0.007
L3e3a | 12357 | 1 | 0.007
L3e3a | 13866 | 1 | 0.007
L3e3a | 14218 | 1 | 0.007
L3e3a | 14225 | 1 | 0.007
L3e3a | 14392 | 1 | 0.007
L3e3a | 14544 | 1 | 0.007
L3e3a | 14587 | 9 | 0.06
L3e3a | 146 | 1 | 0.007
L3e3a | 14756T | 2 | 0.013
L3e3a | 14935 | 1 | 0.007
L3e3a | 152 | 10 | 0.066
L3e3a | 152d | 1 | 0.007
L3e3a | 1555 | 1 | 0.007
L3e3a | 15941 | 1 | 0.007
L3e3a | 16082 | 1 | 0.007
L3e3a | 16093 | 1 | 0.007
L3e3a | 16111 | 1 | 0.007
L3e3a | 16129 | 1 | 0.007
L3e3a | 16145 | 1 | 0.007
L3e3a | 16189 | 1 | 0.007
L3e3a | 16234 | 1 | 0.007
L3e3a | 16265C | 1 | 0.007
L3e3a | 16278 | 1 | 0.007
L3e3a | 16286 | 1 | 0.007
L3e3a | 16292 | 28 | 0.185
L3e3a | 16311 | 4 | 0.026
L3e3a | 16318 | 1 | 0.007
L3e3a | 16327 | 1 | 0.007
L3e3a | 189 | 1 | 0.007
L3e3a | 198 | 3 | 0.02
L3e3a | 207 | 1 | 0.007
L3e3a | 239 | 9 | 0.06
L3e3a | 309d | 3 | 0.02
L3e3a | 310 | 2 | 0.013
L3e3a | 315.2C | 1 | 0.007
L3e3a | 320 | 1 | 0.007
L3e3a | 3394 | 1 | 0.007
L3e3a | 3423A | 1 | 0.007
L3e3a | 3483C | 1 | 0.007
L3e3a | 3593 | 2 | 0.013
L3e3a | 499 | 7 | 0.046
L3e3a | 515d | 1 | 0.007
L3e3a | 5418 | 1 | 0.007
L3e3a | 574C | 2 | 0.013
L3e3a | 576.2C | 1 | 0.007
L3e3a | 576C | 1 | 0.007
L3e3a | 6233T | 1 | 0.007
L3e3a | 6387 | 1 | 0.007
L3e3a | 6551 | 1 | 0.007
L3e3a | 7129 | 2 | 0.013
L3e3a | 7379 | 8 | 0.053

L3e3a | 750 | 1 | 0.007
L3e3a | 8285 | 3 | 0.02
L3e3a | 8289.1CCCCCTCTA | 2 | 0.013
L3e3a | 8479 | 1 | 0.007
L3e3a | 8520 | 3 | 0.02
L3e3a | 8521 | 1 | 0.007
L3e3a | 8673C | 2 | 0.013
L3e3a | 8733 | 1 | 0.007
L3e3a | 9141G | 1 | 0.007
L3e3a | 921 | 1 | 0.007
L3e3a | 9214 | 1 | 0.007
L3e3a | 9311 | 1 | 0.007
L3e3a | 9974A | 1 | 0.007
L3e3b | 11149 | 1 | 0.009
L3e3b | 11710 | 1 | 0.009
L3e3b | 11914 | 1 | 0.009
L3e3b | 12358 | 1 | 0.009
L3e3b | 12561 | 2 | 0.019
L3e3b | 12618 | 1 | 0.009
L3e3b | 13116 | 1 | 0.009
L3e3b | 13135 | 1 | 0.009
L3e3b | 14137 | 1 | 0.009
L3e3b | 144A | 1 | 0.009
L3e3b | 15245 | 1 | 0.009
L3e3b | 15617 | 2 | 0.019
L3e3b | 15837 | 1 | 0.009
L3e3b | 15850 | 1 | 0.009
L3e3b | 15884 | 1 | 0.009
L3e3b | 16093 | 47 | 0.443
L3e3b | 16124 | 1 | 0.009
L3e3b | 16129 | 1 | 0.009
L3e3b | 16148 | 17 | 0.16
L3e3b | 16214 | 1 | 0.009
L3e3b | 16249 | 4 | 0.038
L3e3b | 16263 | 1 | 0.009
L3e3b | 16278 | 1 | 0.009
L3e3b | 16283C | 1 | 0.009
L3e3b | 16291 | 1 | 0.009
L3e3b | 16311 | 2 | 0.019
L3e3b | 16316 | 2 | 0.019
L3e3b | 16318 | 1 | 0.009
L3e3b | 16320 | 1 | 0.009
L3e3b | 16399 | 1 | 0.009
L3e3b | 1694 | 1 | 0.009
L3e3b | 189 | 7 | 0.066
L3e3b | 198 | 1 | 0.009
L3e3b | 208 | 2 | 0.019
L3e3b | 209 | 2 | 0.019
L3e3b | 2109 | 1 | 0.009
L3e3b | 2133 | 1 | 0.009
L3e3b | 214 | 1 | 0.009
L3e3b | 309d | 6 | 0.057
L3e3b | 316 | 1 | 0.009
L3e3b | 3531 | 1 | 0.009
L3e3b | 357C | 1 | 0.009
L3e3b | 408A | 2 | 0.019
L3e3b | 4114 | 1 | 0.009
L3e3b | 4166 | 1 | 0.009
L3e3b | 4386 | 1 | 0.009
L3e3b | 5054 | 1 | 0.009
L3e3b | 573.3C | 1 | 0.009
L3e3b | 6296 | 1 | 0.009
L3e3b | 6941 | 1 | 0.009
L3e3b | 6956A | 1 | 0.009
L3e3b | 6975 | 1 | 0.009
L3e3b | 6980C | 1 | 0.009
L3e3b | 7007 | 1 | 0.009
L3e3b | 7016 | 1 | 0.009
L3e3b | 7076 | 1 | 0.009
L3e3b | 7094 | 1 | 0.009
L3e3b | 7146 | 1 | 0.009
L3e3b | 7337 | 1 | 0.009
L3e3b | 7521 | 1 | 0.009
L3e3b | 8901 | 1 | 0.009
L3e3b | 9180 | 1 | 0.009
L3e3b | 9228 | 1 | 0.009
L3e3b | 9440 | 1 | 0.009
L3e3b | 9449 | 1 | 0.009
L3e3b | 9524 | 1 | 0.009
L3e3b | 9863 | 1 | 0.009
L3e3b1 | 10978 | 1 | 0.01
L3e3b1 | 11347 | 4 | 0.038
L3e3b1 | 11348 | 22 | 0.212
L3e3b1 | 12450 | 1 | 0.01
L3e3b1 | 13145 | 1 | 0.01
L3e3b1 | 13242 | 1 | 0.01
L3e3b1 | 13564G | 21 | 0.202
L3e3b1 | 13708 | 1 | 0.01
L3e3b1 | 13737A | 1 | 0.01
L3e3b1 | 14020 | 11 | 0.106
L3e3b1 | 14040 | 1 | 0.01
L3e3b1 | 14122 | 2 | 0.019
L3e3b1 | 152 | 9 | 0.087
L3e3b1 | 15734 | 1 | 0.01
L3e3b1 | 15884C | 1 | 0.01
L3e3b1 | 16060 | 1 | 0.01
L3e3b1 | 16260 | 1 | 0.01
L3e3b1 | 16265C | 2 | 0.019
L3e3b1 | 16291 | 4 | 0.038
L3e3b1 | 16311 | 5 | 0.048
L3e3b1 | 1943 | 2 | 0.019
L3e3b1 | 198 | 1 | 0.01
L3e3b1 | 309d | 4 | 0.038
L3e3b1 | 315.2C | 1 | 0.01
L3e3b1 | 3396 | 1 | 0.01
L3e3b1 | 3487 | 22 | 0.212
L3e3b1 | 385 | 1 | 0.01
L3e3b1 | 4149 | 1 | 0.01
L3e3b1 | 5261 | 1 | 0.01
L3e3b1 | 5460 | 1 | 0.01
L3e3b1 | 575.2C | 1 | 0.01

L3e3b1 | 6962 | 1 | 0.01
L3e3b1 | 702 | 1 | 0.01
L3e3b1 | 769 | 1 | 0.01
L3e3b1 | 9041 | 22 | 0.212
L3e3b1 | 9254 | 1 | 0.01
L3e3b2 | 11025 | 1 | 0.036
L3e3b2 | 11299 | 2 | 0.071
L3e3b2 | 11827 | 1 | 0.036
L3e3b2 | 11914 | 1 | 0.036
L3e3b2 | 11963 | 1 | 0.036
L3e3b2 | 12164 | 1 | 0.036
L3e3b2 | 12820 | 1 | 0.036
L3e3b2 | 12879 | 1 | 0.036
L3e3b2 | 14953 | 4 | 0.143
L3e3b2 | 15029 | 1 | 0.036
L3e3b2 | 15924 | 1 | 0.036
L3e3b2 | 16167 | 1 | 0.036
L3e3b2 | 16209 | 1 | 0.036
L3e3b2 | 16497 | 1 | 0.036
L3e3b2 | 309d | 3 | 0.107
L3e3b2 | 7609 | 1 | 0.036
L3e3b2 | 9545 | 1 | 0.036
L3e3b3 | 11016 | 1 | 0.014
L3e3b3 | 11350 | 3 | 0.043
L3e3b3 | 14750 | 1 | 0.014
L3e3b3 | 15043 | 1 | 0.014
L3e3b3 | 16129 | 2 | 0.029
L3e3b3 | 16176 | 1 | 0.014
L3e3b3 | 16189 | 10 | 0.143
L3e3b3 | 16216 | 2 | 0.029
L3e3b3 | 16300 | 2 | 0.029
L3e3b3 | 16319 | 1 | 0.014
L3e3b3 | 185 | 2 | 0.029
L3e3b3 | 189 | 2 | 0.029
L3e3b3 | 200 | 1 | 0.014
L3e3b3 | 2145 | 1 | 0.014
L3e3b3 | 310 | 1 | 0.014
L3e3b3 | 489 | 50 | 0.714
L3e3b3 | 4907 | 1 | 0.014
L3e3b3 | 5276 | 1 | 0.014
L3e3b3 | 5492 | 1 | 0.014
L3e3b3 | 980 | 1 | 0.014
L3e4 | 11665 | 1 | 0.023
L3e4 | 11914 | 1 | 0.023
L3e4 | 11963 | 1 | 0.023
L3e4 | 12007 | 1 | 0.023
L3e4 | 13131 | 1 | 0.023
L3e4 | 15924 | 1 | 0.023
L3e4 | 16093 | 2 | 0.047
L3e4 | 16116T | 1 | 0.023
L3e4 | 16129 | 4 | 0.093
L3e4 | 16136 | 1 | 0.023
L3e4 | 16138C | 1 | 0.023
L3e4 | 16140 | 1 | 0.023
L3e4 | 16145 | 1 | 0.023
L3e4 | 16204 | 1 | 0.023
L3e4 | 16234 | 1 | 0.023
L3e4 | 16291 | 1 | 0.023
L3e4 | 16502 | 1 | 0.023
L3e4 | 16513G | 1 | 0.023
L3e4 | 16543C | 1 | 0.023
L3e4 | 189 | 1 | 0.023
L3e4 | 2881 | 1 | 0.023
L3e4 | 315.2C | 1 | 0.023
L3e4 | 3609 | 1 | 0.023
L3e4 | 489 | 1 | 0.023
L3e4 | 5461 | 1 | 0.023
L3e4 | 9490 | 1 | 0.023
L3e4a | 11149 | 2 | 0.033
L3e4a | 12690 | 1 | 0.016
L3e4a | 12771 | 2 | 0.033
L3e4a | 13368 | 1 | 0.016
L3e4a | 13620 | 1 | 0.016
L3e4a | 13638 | 1 | 0.016
L3e4a | 13886 | 1 | 0.016
L3e4a | 14173 | 2 | 0.033
L3e4a | 14662 | 1 | 0.016
L3e4a | 15777 | 1 | 0.016
L3e4a | 16093 | 1 | 0.016
L3e4a | 16169G | 1 | 0.016
L3e4a | 16172 | 1 | 0.016
L3e4a | 16174 | 1 | 0.016
L3e4a | 16209 | 2 | 0.033
L3e4a | 16265 | 1 | 0.016
L3e4a | 16266 | 1 | 0.016
L3e4a | 16291 | 2 | 0.033
L3e4a | 16299 | 1 | 0.016
L3e4a | 16311 | 4 | 0.066
L3e4a | 195 | 1 | 0.016
L3e4a | 2863 | 1 | 0.016
L3e4a | 309d | 9 | 0.148
L3e4a | 3316 | 2 | 0.033
L3e4a | 493 | 22 | 0.361
L3e4a | 5711 | 1 | 0.016
L3e4a | 5979 | 1 | 0.016
L3e4a | 6366 | 1 | 0.016
L3e4a | 6750A | 3 | 0.049
L3e4a | 6827 | 1 | 0.016
L3e4a | 709 | 4 | 0.066
L3e4a | 8289.1CCCCCTCTA | 2 | 0.033
L3e4a | 8688 | 1 | 0.016
L3e4a | 8688C | 1 | 0.016
L3e4a | 8896 | 1 | 0.016
L3e4a | 9055T | 1 | 0.016
L3e4a1 | 1018 | 1 | 0.018
L3e4a1 | 11592 | 2 | 0.036
L3e4a1 | 12354 | 1 | 0.018
L3e4a1 | 12438 | 1 | 0.018
L3e4a1 | 14267 | 1 | 0.018
L3e4a1 | 15639 | 1 | 0.018
L3e4a1 | 16032d | 1 | 0.018

L3e4a1 | 16042d | 1 | 0.018
L3e4a1 | 16086 | 1 | 0.018
L3e4a1 | 16110d | 1 | 0.018
L3e4a1 | 16129 | 1 | 0.018
L3e4a1 | 16153 | 2 | 0.036
L3e4a1 | 16188 | 2 | 0.036
L3e4a1 | 16234 | 1 | 0.018
L3e4a1 | 16254 | 1 | 0.018
L3e4a1 | 16270 | 1 | 0.018
L3e4a1 | 16278 | 1 | 0.018
L3e4a1 | 16299 | 1 | 0.018
L3e4a1 | 16324 | 2 | 0.036
L3e4a1 | 16362 | 1 | 0.018
L3e4a1 | 189 | 1 | 0.018
L3e4a1 | 200 | 1 | 0.018
L3e4a1 | 2702 | 2 | 0.036
L3e4a1 | 315.2C | 1 | 0.018
L3e4a1 | 316 | 8 | 0.143
L3e4a1 | 466 | 6 | 0.107
L3e4a1 | 498.1C | 1 | 0.018
L3e4a1 | 513 | 1 | 0.018
L3e4a1 | 515-524d | 3 | 0.054
L3e4a1 | 8334 | 1 | 0.018
L3e5 | 10089 | 1 | 0.019
L3e5 | 1018 | 1 | 0.019
L3e5 | 10381 | 1 | 0.019
L3e5 | 11440 | 1 | 0.019
L3e5 | 11909 | 1 | 0.019
L3e5 | 11963 | 1 | 0.019
L3e5 | 12530 | 1 | 0.019
L3e5 | 12906A | 1 | 0.019
L3e5 | 13934 | 1 | 0.019
L3e5 | 14267 | 1 | 0.019
L3e5 | 14314 | 1 | 0.019
L3e5 | 152 | 1 | 0.019
L3e5 | 16239 | 1 | 0.019
L3e5 | 16254 | 2 | 0.038
L3e5 | 16355 | 3 | 0.058
L3e5 | 16390 | 1 | 0.019
L3e5 | 2109 | 1 | 0.019
L3e5 | 2133C | 1 | 0.019
L3e5 | 2438 | 1 | 0.019
L3e5 | 3901 | 1 | 0.019
L3e5 | 5951 | 1 | 0.019
L3e5 | 6941 | 1 | 0.019
L3e5 | 6956A | 1 | 0.019
L3e5 | 711 | 2 | 0.038
L3e5 | 7280 | 1 | 0.019
L3e5 | 7606 | 1 | 0.019
L3e5 | 8557 | 1 | 0.019
L3e5 | 93 | 1 | 0.019
L3e5 | 9647 | 1 | 0.019
L3e5 | 9972 | 1 | 0.019
L3e5+195 | 12397 | 1 | 0.029
L3e5+195 | 12969A | 1 | 0.029
L3e5+195 | 14207 | 1 | 0.029
L3e5+195 | 16172 | 1 | 0.029
L3e5+195 | 16186 | 1 | 0.029
L3e5+195 | 16193 | 1 | 0.029
L3e5+195 | 16232 | 1 | 0.029
L3e5+195 | 16266 | 1 | 0.029
L3e5+195 | 16355 | 1 | 0.029
L3e5+195 | 16362 | 1 | 0.029
L3e5+195 | 189 | 1 | 0.029
L3e5+195 | 2880 | 1 | 0.029
L3e5+195 | 515-524d | 1 | 0.029
L3e5a | 10751 | 1 | 0.022
L3e5a | 12123 | 1 | 0.022
L3e5a | 12280 | 1 | 0.022
L3e5a | 13928C | 1 | 0.022
L3e5a | 13934 | 2 | 0.043
L3e5a | 14798 | 1 | 0.022
L3e5a | 152 | 2 | 0.043
L3e5a | 16124 | 1 | 0.022
L3e5a | 242 | 1 | 0.022
L3e5a | 5460 | 1 | 0.022
L3e5a | 8410 | 1 | 0.022
L3e5a | 9102 | 1 | 0.022
L3e5a1 | 14073 | 2 | 0.032
L3e5a1 | 152 | 7 | 0.113
L3e5a1 | 15924 | 1 | 0.016
L3e5a1 | 16051 | 1 | 0.016
L3e5a1 | 16079 | 1 | 0.016
L3e5a1 | 16188 | 2 | 0.032
L3e5a1 | 16249A | 1 | 0.016
L3e5a1 | 16259 | 1 | 0.016
L3e5a1 | 16261 | 1 | 0.016
L3e5a1 | 16278 | 2 | 0.032
L3e5a1 | 16301 | 1 | 0.016
L3e5a1 | 16304 | 2 | 0.032
L3e5a1 | 16318 | 2 | 0.032
L3e5a1 | 16355 | 1 | 0.016
L3e5a1 | 204 | 1 | 0.016
L3e5a1 | 215 | 1 | 0.016
L3e5a1 | 493 | 1 | 0.016
L3e5a1 | 497 | 2 | 0.032
L3e5a1 | 515-524d | 10 | 0.161
L3e5a1a | 16129 | 1 | 0.025
L3e5a1a | 16209 | 1 | 0.025
L3e5a1a | 16235T | 1 | 0.025
L3e5a1a | 16256A | 1 | 0.025
L3e5a1a | 16291 | 1 | 0.025
L3e5a1a | 16484-16489d | 2 | 0.05
L3e5a1a | 2772 | 1 | 0.025
L3e5a1a | 2809A | 1 | 0.025
L3e5a1a | 316 | 1 | 0.025
L3e5a1a | 769 | 1 | 0.025
L3e5b | 10352C | 1 | 0.019
L3e5b | 10463 | 2 | 0.038
L3e5b | 10864 | 1 | 0.019
L3e5b | 10954A | 1 | 0.019

L3e5b | 11016 | 1 | 0.019
L3e5b | 13135 | 1 | 0.019
L3e5b | 13493A | 1 | 0.019
L3e5b | 146 | 4 | 0.077
L3e5b | 14756 | 1 | 0.019
L3e5b | 152 | 1 | 0.019
L3e5b | 15924 | 1 | 0.019
L3e5b | 16093 | 1 | 0.019
L3e5b | 16192 | 5 | 0.096
L3e5b | 16234 | 2 | 0.038
L3e5b | 16260 | 1 | 0.019
L3e5b | 16261 | 2 | 0.038
L3e5b | 16291 | 1 | 0.019
L3e5b | 16298 | 1 | 0.019
L3e5b | 1676 | 2 | 0.038
L3e5b | 3768 | 1 | 0.019
L3e5b | 634 | 1 | 0.019
L3e5b | 794 | 1 | 0.019
L3e5b | 961 | 1 | 0.019
L3e5c | 12769 | 1 | 0.029
L3e5c | 13448A | 1 | 0.029
L3e5c | 146 | 1 | 0.029
L3e5c | 16086 | 1 | 0.029
L3e5c | 16172 | 1 | 0.029
L3e5c | 16193 | 1 | 0.029
L3e5c | 16232 | 1 | 0.029
L3e5c | 16355 | 1 | 0.029
L3e5c | 16362 | 1 | 0.029
L3e5c | 189 | 1 | 0.029
L3e5c | 294 | 1 | 0.029
L3e5c | 515-524d | 1 | 0.029
L3e5c | 5312 | 1 | 0.029
L3e5c | 573.1C | 1 | 0.029
L3e5c | 8901 | 1 | 0.029
L3e5d | 10370 | 1 | 0.02
L3e5d | 10478 | 1 | 0.02
L3e5d | 15884 | 4 | 0.082
L3e5d | 16148 | 1 | 0.02
L3e5d | 16221 | 1 | 0.02
L3e5d | 16260 | 3 | 0.061
L3e5d | 16293C | 2 | 0.041
L3e5d | 16311 | 1 | 0.02
L3e5d | 16325 | 2 | 0.041
L3e5d | 1842 | 1 | 0.02
L3e5d | 2709 | 1 | 0.02
L3e5d | 515-524d | 1 | 0.02
L3e5d | 6366 | 1 | 0.02
L3e5d | 6725 | 1 | 0.02
L3e5d | 7419 | 1 | 0.02
L3e5d | 8292 | 1 | 0.02
L3e5d | 8749 | 1 | 0.02
L3e5e | 124 | 1 | 0.029
L3e5e | 12561 | 1 | 0.029
L3e5e | 15932A | 1 | 0.029
L3e5e | 16059d | 1 | 0.029
L3e5e | 16064d | 1 | 0.029
L3e5e | 16085G | 1 | 0.029
L3e5e | 16213 | 1 | 0.029
L3e5e | 16234 | 1 | 0.029
L3e5e | 16249A | 1 | 0.029
L3e5e | 16295G | 1 | 0.029
L3e5e | 16311 | 2 | 0.057
L3e5e | 16565d | 1 | 0.029
L3e5e | 25.1A | 1 | 0.029
L3e5e | 4561G | 1 | 0.029
L3e5e | 4715 | 1 | 0.029
L3e5f | 14325 | 1 | 0.125
L3e5f | 16189 | 1 | 0.125
L3e5f | 16494 | 1 | 0.125
L3e'i'k'x | 152 | 1 | 0.006
L3e'i'k'x | 16111 | 1 | 0.006
L3e'i'k'x | 16265C | 5 | 0.03
L3e'i'k'x | 16274 | 1 | 0.006
L3e'i'k'x | 16304 | 1 | 0.006
L3e'i'k'x | 16316 | 1 | 0.006
L3e'i'k'x | 16327 | 1 | 0.006
L3e'i'k'x | 16343T | 1 | 0.006
L3e'i'k'x | 195 | 5 | 0.03
L3e'i'k'x | 303G | 1 | 0.006
L3e'i'k'x | 515-524d | 1 | 0.006
L3f | 10017 | 2 | 0.025
L3f | 11299 | 1 | 0.012
L3f | 11467 | 1 | 0.012
L3f | 12183 | 1 | 0.012
L3f | 12545 | 1 | 0.012
L3f | 13260 | 1 | 0.012
L3f | 13863 | 1 | 0.012
L3f | 14148 | 3 | 0.037
L3f | 14971 | 1 | 0.012
L3f | 15106 | 1 | 0.012
L3f | 152 | 7 | 0.086
L3f | 16171 | 1 | 0.012
L3f | 16178 | 1 | 0.012
L3f | 16224 | 7 | 0.086
L3f | 16295 | 1 | 0.012
L3f | 16309 | 2 | 0.025
L3f | 16325 | 1 | 0.012
L3f | 1692 | 3 | 0.037
L3f | 1888 | 1 | 0.012
L3f | 200 | 5 | 0.062
L3f | 235 | 1 | 0.012
L3f | 3693 | 1 | 0.012
L3f | 491 | 4 | 0.049
L3f | 4917 | 1 | 0.012
L3f | 6524 | 3 | 0.037
L3f | 709 | 1 | 0.012
L3f | 7094 | 3 | 0.037
L3f | 813 | 3 | 0.037
L3f | 930 | 1 | 0.012
L3f1 | 150 | 1 | 0.014
L3f1 | 16360 | 1 | 0.014

L3f1a | 12236 | 1 | 0.014
L3f1a | 12545 | 1 | 0.014
L3f1a | 14971 | 1 | 0.014
L3f1a | 150 | 1 | 0.014
L3f1a | 152 | 1 | 0.014
L3f1a | 16360 | 1 | 0.014
L3f1a | 1658 | 1 | 0.014
L3f1a | 3504 | 1 | 0.014
L3f1a | 5471 | 1 | 0.014
L3f1a | 990 | 1 | 0.014
L3f1a1 | 11969 | 1 | 0.012
L3f1a1 | 12441 | 1 | 0.012
L3f1a1 | 12567 | 1 | 0.012
L3f1a1 | 12634 | 2 | 0.024
L3f1a1 | 13967 | 1 | 0.012
L3f1a1 | 14554 | 1 | 0.012
L3f1a1 | 15151 | 1 | 0.012
L3f1a1 | 152 | 1 | 0.012
L3f1a1 | 15317 | 1 | 0.012
L3f1a1 | 15401 | 3 | 0.037
L3f1a1 | 15952 | 2 | 0.024
L3f1a1 | 16086 | 1 | 0.012
L3f1a1 | 16126 | 5 | 0.061
L3f1a1 | 16129 | 1 | 0.012
L3f1a1 | 16172 | 2 | 0.024
L3f1a1 | 16213 | 2 | 0.024
L3f1a1 | 16311 | 1 | 0.012
L3f1a1 | 16320 | 1 | 0.012
L3f1a1 | 16381 | 1 | 0.012
L3f1a1 | 1699 | 3 | 0.037
L3f1a1 | 195 | 3 | 0.037
L3f1a1 | 228 | 3 | 0.037
L3f1a1 | 328 | 2 | 0.024
L3f1a1 | 3591 | 1 | 0.012
L3f1a1 | 3834C | 3 | 0.037
L3f1a1 | 4048 | 2 | 0.024
L3f1a1 | 515-524d | 1 | 0.012
L3f1a1 | 5460 | 1 | 0.012
L3f1a1 | 5913 | 1 | 0.012
L3f1a1 | 7270 | 2 | 0.024
L3f1a1 | 736 | 1 | 0.012
L3f1a1 | 8392 | 1 | 0.012
L3f1a1 | 8781A | 1 | 0.012
L3f1a1 | 8853 | 1 | 0.012
L3f1a1 | 9386 | 1 | 0.012
L3f1b | 16086 | 1 | 0.043
L3f1b | 16093 | 2 | 0.087
L3f1b | 16187 | 1 | 0.043
L3f1b | 16234 | 1 | 0.043
L3f1b | 16355 | 1 | 0.043
L3f1b | 193 | 1 | 0.043
L3f1b | 2438 | 1 | 0.043
L3f1b | 5073 | 1 | 0.043
L3f1b | 515-524d | 1 | 0.043
L3f1b | 8994 | 6 | 0.261
L3f1b | 9123 | 1 | 0.043
L3f1b+16292 | 11087 | 1 | 0.019
L3f1b+16292 | 11293 | 1 | 0.019
L3f1b+16292 | 11386 | 1 | 0.019
L3f1b+16292 | 12236 | 1 | 0.019
L3f1b+16292 | 12351 | 1 | 0.019
L3f1b+16292 | 12954 | 1 | 0.019
L3f1b+16292 | 12966 | 1 | 0.019
L3f1b+16292 | 12975 | 3 | 0.058
L3f1b+16292 | 13647 | 3 | 0.058
L3f1b+16292 | 13768 | 1 | 0.019
L3f1b+16292 | 13858 | 1 | 0.019
L3f1b+16292 | 13879A | 1 | 0.019
L3f1b+16292 | 14122 | 1 | 0.019
L3f1b+16292 | 14362 | 1 | 0.019
L3f1b+16292 | 14370 | 1 | 0.019
L3f1b+16292 | 15004 | 3 | 0.058
L3f1b+16292 | 152 | 5 | 0.096
L3f1b+16292 | 15217 | 1 | 0.019
L3f1b+16292 | 15924 | 3 | 0.058
L3f1b+16292 | 16018 | 1 | 0.019
L3f1b+16292 | 16086 | 1 | 0.019
L3f1b+16292 | 16093 | 2 | 0.038
L3f1b+16292 | 16129 | 5 | 0.096
L3f1b+16292 | 16153 | 1 | 0.019
L3f1b+16292 | 16174 | 4 | 0.077
L3f1b+16292 | 16179 | 1 | 0.019
L3f1b+16292 | 16192 | 2 | 0.038
L3f1b+16292 | 16207 | 1 | 0.019
L3f1b+16292 | 16218 | 1 | 0.019
L3f1b+16292 | 16230 | 1 | 0.019
L3f1b+16292 | 16260 | 1 | 0.019
L3f1b+16292 | 16274 | 1 | 0.019
L3f1b+16292 | 16293 | 2 | 0.038
L3f1b+16292 | 16294 | 2 | 0.038
L3f1b+16292 | 16319 | 2 | 0.038
L3f1b+16292 | 185 | 1 | 0.019
L3f1b+16292 | 1850 | 1 | 0.019
L3f1b+16292 | 186 | 1 | 0.019
L3f1b+16292 | 1891 | 1 | 0.019
L3f1b+16292 | 193 | 1 | 0.019
L3f1b+16292 | 195 | 7 | 0.135
L3f1b+16292 | 2251 | 1 | 0.019
L3f1b+16292 | 2355 | 1 | 0.019
L3f1b+16292 | 2423 | 1 | 0.019
L3f1b+16292 | 2483 | 1 | 0.019
L3f1b+16292 | 2541 | 1 | 0.019
L3f1b+16292 | 2557 | 1 | 0.019
L3f1b+16292 | 2626 | 1 | 0.019
L3f1b+16292 | 2668 | 1 | 0.019
L3f1b+16292 | 2779 | 1 | 0.019
L3f1b+16292 | 3010 | 1 | 0.019
L3f1b+16292 | 310 | 1 | 0.019
L3f1b+16292 | 3116 | 1 | 0.019
L3f1b+16292 | 315.2C | 1 | 0.019
L3f1b+16292 | 3200 | 1 | 0.019

L3f1b+16292 | 3308 | 1 | 0.019
L3f1b+16292 | 335 | 2 | 0.038
L3f1b+16292 | 3483 | 1 | 0.019
L3f1b+16292 | 3666 | 1 | 0.019
L3f1b+16292 | 437 | 1 | 0.019
L3f1b+16292 | 466 | 1 | 0.019
L3f1b+16292 | 4697 | 1 | 0.019
L3f1b+16292 | 5420 | 1 | 0.019
L3f1b+16292 | 5460 | 1 | 0.019
L3f1b+16292 | 573.2C | 1 | 0.019
L3f1b+16292 | 5786 | 1 | 0.019
L3f1b+16292 | 5843 | 1 | 0.019
L3f1b+16292 | 6090 | 1 | 0.019
L3f1b+16292 | 6150 | 1 | 0.019
L3f1b+16292 | 6485 | 1 | 0.019
L3f1b+16292 | 6498A | 1 | 0.019
L3f1b+16292 | 6908 | 3 | 0.058
L3f1b+16292 | 6941 | 1 | 0.019
L3f1b+16292 | 7146 | 1 | 0.019
L3f1b+16292 | 7424 | 1 | 0.019
L3f1b+16292 | 7852 | 1 | 0.019
L3f1b+16292 | 8618 | 1 | 0.019
L3f1b+16292 | 8632 | 1 | 0.019
L3f1b+16292 | 8922 | 1 | 0.019
L3f1b+16292 | 9163 | 1 | 0.019
L3f1b+16292 | 9287 | 1 | 0.019
L3f1b+16292 | 9344 | 1 | 0.019
L3f1b+16292+150 | 10670 | 3 | 0.049
L3f1b+16292+150 | 13263 | 1 | 0.016
L3f1b+16292+150 | 13879 | 1 | 0.016
L3f1b+16292+150 | 1406 | 1 | 0.016
L3f1b+16292+150 | 14206 | 1 | 0.016
L3f1b+16292+150 | 14484 | 1 | 0.016
L3f1b+16292+150 | 146 | 1 | 0.016
L3f1b+16292+150 | 152 | 1 | 0.016
L3f1b+16292+150 | 15226 | 1 | 0.016
L3f1b+16292+150 | 15244 | 1 | 0.016
L3f1b+16292+150 | 16042 | 1 | 0.016
L3f1b+16292+150 | 16140 | 1 | 0.016
L3f1b+16292+150 | 16188 | 1 | 0.016
L3f1b+16292+150 | 16189 | 2 | 0.033
L3f1b+16292+150 | 16218 | 2 | 0.033
L3f1b+16292+150 | 16234 | 1 | 0.016
L3f1b+16292+150 | 16235 | 4 | 0.066
L3f1b+16292+150 | 16249A | 1 | 0.016
L3f1b+16292+150 | 16263 | 1 | 0.016
L3f1b+16292+150 | 16266 | 1 | 0.016
L3f1b+16292+150 | 16274 | 14 | 0.23
L3f1b+16292+150 | 16293 | 1 | 0.016
L3f1b+16292+150 | 16305 | 1 | 0.016
L3f1b+16292+150 | 1719 | 1 | 0.016
L3f1b+16292+150 | 1821 | 1 | 0.016
L3f1b+16292+150 | 185 | 1 | 0.016
L3f1b+16292+150 | 1958 | 1 | 0.016
L3f1b+16292+150 | 207 | 9 | 0.148
L3f1b+16292+150 | 215 | 1 | 0.016
L3f1b+16292+150 | 310 | 1 | 0.016
L3f1b+16292+150 | 315d | 1 | 0.016
L3f1b+16292+150 | 317.1C | 1 | 0.016
L3f1b+16292+150 | 3777 | 1 | 0.016
L3f1b+16292+150 | 4902 | 1 | 0.016
L3f1b+16292+150 | 6179 | 1 | 0.016
L3f1b+16292+150 | 6650T | 1 | 0.016
L3f1b+16292+150 | 7082 | 1 | 0.016
L3f1b+16292+150 | 7379 | 1 | 0.016
L3f1b+16292+150 | 7852 | 1 | 0.016
L3f1b+16292+150 | 8075 | 1 | 0.016
L3f1b+16292+150 | 8167 | 1 | 0.016
L3f1b+16292+150 | 8503 | 1 | 0.016
L3f1b+16292+150 | 8612 | 1 | 0.016
L3f1b1 | 150 | 1 | 0.036
L3f1b1 | 15110 | 1 | 0.036
L3f1b1 | 16093 | 1 | 0.036
L3f1b1 | 16145 | 1 | 0.036
L3f1b1 | 16153 | 1 | 0.036
L3f1b1 | 16167 | 1 | 0.036
L3f1b1 | 16278 | 1 | 0.036
L3f1b1 | 16293 | 1 | 0.036
L3f1b1 | 16362 | 1 | 0.036
L3f1b1 | 16385 | 1 | 0.036
L3f1b1 | 16527 | 1 | 0.036
L3f1b1 | 195 | 2 | 0.071
L3f1b1 | 199 | 1 | 0.036
L3f1b1 | 207 | 1 | 0.036
L3f1b1 | 310 | 2 | 0.071
L3f1b1 | 317.1C | 1 | 0.036
L3f1b1 | 3909 | 1 | 0.036
L3f1b1 | 4086 | 1 | 0.036
L3f1b1 | 515-524d | 2 | 0.071
L3f1b1 | 5777 | 1 | 0.036
L3f1b1a | 10235 | 1 | 0.01
L3f1b1a | 11101 | 1 | 0.01
L3f1b1a | 11722 | 1 | 0.01
L3f1b1a | 12341 | 1 | 0.01
L3f1b1a | 12678 | 1 | 0.01
L3f1b1a | 12732 | 1 | 0.01
L3f1b1a | 12870 | 1 | 0.01
L3f1b1a | 13174 | 1 | 0.01
L3f1b1a | 13464 | 1 | 0.01
L3f1b1a | 13542 | 1 | 0.01
L3f1b1a | 13954A | 1 | 0.01
L3f1b1a | 14016 | 1 | 0.01
L3f1b1a | 14142 | 1 | 0.01
L3f1b1a | 143 | 1 | 0.01
L3f1b1a | 14582 | 1 | 0.01
L3f1b1a | 146 | 2 | 0.021
L3f1b1a | 14690 | 1 | 0.01
L3f1b1a | 14774 | 1 | 0.01
L3f1b1a | 150 | 2 | 0.021
L3f1b1a | 152 | 4 | 0.042
L3f1b1a | 15461 | 1 | 0.01

L3f1b1a | 15466 | 1 | 0.01
L3f1b1a | 15508 | 1 | 0.01
L3f1b1a | 15519 | 1 | 0.01
L3f1b1a | 15607 | 1 | 0.01
L3f1b1a | 15651 | 3 | 0.031
L3f1b1a | 15883 | 3 | 0.031
L3f1b1a | 16051 | 3 | 0.031
L3f1b1a | 16086 | 2 | 0.021
L3f1b1a | 16093 | 7 | 0.073
L3f1b1a | 16126 | 1 | 0.01
L3f1b1a | 16145 | 2 | 0.021
L3f1b1a | 16153 | 3 | 0.031
L3f1b1a | 16189 | 6 | 0.062
L3f1b1a | 16234 | 1 | 0.01
L3f1b1a | 16260 | 1 | 0.01
L3f1b1a | 16286 | 2 | 0.021
L3f1b1a | 16293 | 1 | 0.01
L3f1b1a | 16320 | 4 | 0.042
L3f1b1a | 16343T | 2 | 0.021
L3f1b1a | 16352 | 1 | 0.01
L3f1b1a | 16355 | 1 | 0.01
L3f1b1a | 16368 | 2 | 0.021
L3f1b1a | 16497 | 1 | 0.01
L3f1b1a | 16527 | 1 | 0.01
L3f1b1a | 185 | 6 | 0.062
L3f1b1a | 195 | 4 | 0.042
L3f1b1a | 200 | 1 | 0.01
L3f1b1a | 204 | 4 | 0.042
L3f1b1a | 207 | 2 | 0.021
L3f1b1a | 214 | 1 | 0.01
L3f1b1a | 234 | 4 | 0.042
L3f1b1a | 309d | 1 | 0.01
L3f1b1a | 310 | 4 | 0.042
L3f1b1a | 315.2C | 2 | 0.021
L3f1b1a | 316 | 1 | 0.01
L3f1b1a | 3434 | 1 | 0.01
L3f1b1a | 3850 | 1 | 0.01
L3f1b1a | 4136 | 1 | 0.01
L3f1b1a | 466 | 1 | 0.01
L3f1b1a | 573.1C | 1 | 0.01
L3f1b1a | 5843 | 1 | 0.01
L3f1b1a | 5899.1C | 1 | 0.01
L3f1b1a | 629 | 1 | 0.01
L3f1b1a | 6512 | 1 | 0.01
L3f1b1a | 6581 | 1 | 0.01
L3f1b1a | 6620A | 1 | 0.01
L3f1b1a | 6722 | 1 | 0.01
L3f1b1a | 7191 | 1 | 0.01
L3f1b1a | 8441 | 1 | 0.01
L3f1b1a | 9007 | 1 | 0.01
L3f1b1a | 9461 | 1 | 0.01
L3f1b1a | 95C | 1 | 0.01
L3f1b1a | 9717 | 1 | 0.01
L3f1b1a1 | 150 | 1 | 0.037
L3f1b1a1 | 15080 | 1 | 0.037
L3f1b1a1 | 152 | 1 | 0.037
L3f1b1a1 | 15421 | 2 | 0.074
L3f1b1a1 | 16086 | 1 | 0.037
L3f1b1a1 | 16261 | 1 | 0.037
L3f1b1a1 | 16291 | 1 | 0.037
L3f1b1a1 | 16294 | 1 | 0.037
L3f1b1a1 | 16360 | 1 | 0.037
L3f1b1a1 | 234 | 1 | 0.037
L3f1b1a1 | 309d | 2 | 0.074
L3f1b1a1 | 310 | 2 | 0.074
L3f1b1a1 | 4743A | 1 | 0.037
L3f1b1a1 | 482 | 1 | 0.037
L3f1b1a1 | 4843 | 1 | 0.037
L3f1b1a1 | 573.1C | 1 | 0.037
L3f1b1a1 | 6899 | 1 | 0.037
L3f1b1a1 | 7080 | 1 | 0.037
L3f1b1a1 | 8383 | 1 | 0.037
L3f1b2 | 12828 | 1 | 0.014
L3f1b2 | 146 | 2 | 0.029
L3f1b2 | 152 | 2 | 0.029
L3f1b2 | 15883 | 3 | 0.043
L3f1b2 | 16069 | 1 | 0.014
L3f1b2 | 16093 | 3 | 0.043
L3f1b2 | 16111 | 2 | 0.029
L3f1b2 | 16153 | 1 | 0.014
L3f1b2 | 16167 | 2 | 0.029
L3f1b2 | 16172 | 1 | 0.014
L3f1b2 | 16174 | 1 | 0.014
L3f1b2 | 16213C | 2 | 0.029
L3f1b2 | 16214 | 1 | 0.014
L3f1b2 | 16215 | 6 | 0.086
L3f1b2 | 16218 | 3 | 0.043
L3f1b2 | 16249A | 1 | 0.014
L3f1b2 | 16263 | 1 | 0.014
L3f1b2 | 16293 | 1 | 0.014
L3f1b2 | 16301 | 1 | 0.014
L3f1b2 | 16305 | 1 | 0.014
L3f1b2 | 16319 | 1 | 0.014
L3f1b2 | 16362 | 1 | 0.014
L3f1b2 | 16484-16489d | 1 | 0.014
L3f1b2 | 16527 | 1 | 0.014
L3f1b2 | 185 | 7 | 0.1
L3f1b2 | 195 | 9 | 0.129
L3f1b2 | 204 | 2 | 0.029
L3f1b2 | 214 | 1 | 0.014
L3f1b2 | 281C | 1 | 0.014
L3f1b2 | 489 | 1 | 0.014
L3f1b2 | 507A | 2 | 0.029
L3f1b2 | 515-524d | 42 | 0.6
L3f1b2 | 7444 | 1 | 0.014
L3f1b2 | 9174 | 1 | 0.014
L3f1b2a | 10327 | 1 | 0.1
L3f1b2a | 14110 | 1 | 0.1
L3f1b2a | 16086 | 2 | 0.2
L3f1b2a | 16183 | 1 | 0.1
L3f1b2a | 16271 | 1 | 0.1

L3f1b2a | 16274 | 1 | 0.1
L3f1b2a | 16532d | 1 | 0.1
L3f1b2a | 195 | 2 | 0.2
L3f1b2a | 44G | 1 | 0.1
L3f1b2a | 46.1CG | 1 | 0.1
L3f1b2a | 46A | 1 | 0.1
L3f1b2a | 474d | 1 | 0.1
L3f1b2a | 477G | 1 | 0.1
L3f1b2a | 479.1A | 1 | 0.1
L3f1b2a | 485-486d | 1 | 0.1
L3f1b2a | 489d | 1 | 0.1
L3f1b2a | 48A | 1 | 0.1
L3f1b2a | 492 | 1 | 0.1
L3f1b2a | 56d | 1 | 0.1
L3f1b2a | 720 | 1 | 0.1
L3f1b3 | 10034 | 1 | 0.027
L3f1b3 | 10493 | 1 | 0.027
L3f1b3 | 11928 | 1 | 0.027
L3f1b3 | 14178 | 1 | 0.027
L3f1b3 | 14757 | 1 | 0.027
L3f1b3 | 16260 | 1 | 0.027
L3f1b3 | 2483 | 1 | 0.027
L3f1b3 | 315.2C | 1 | 0.027
L3f1b3 | 515-524d | 1 | 0.027
L3f1b3 | 7349 | 1 | 0.027
L3f1b3 | 8167 | 1 | 0.027
L3f1b4 | 15629 | 2 | 0.057
L3f1b4 | 8937A | 2 | 0.057
L3f1b4a | 11491 | 4 | 0.033
L3f1b4a | 11692A | 1 | 0.008
L3f1b4a | 12408 | 7 | 0.058
L3f1b4a | 12450 | 1 | 0.008
L3f1b4a | 12771 | 1 | 0.008
L3f1b4a | 1291 | 2 | 0.017
L3f1b4a | 13899 | 1 | 0.008
L3f1b4a | 1415 | 1 | 0.008
L3f1b4a | 14170 | 3 | 0.025
L3f1b4a | 14384 | 1 | 0.008
L3f1b4a | 14405 | 1 | 0.008
L3f1b4a | 14587 | 1 | 0.008
L3f1b4a | 15043 | 76 | 0.628
L3f1b4a | 15229 | 1 | 0.008
L3f1b4a | 15497 | 2 | 0.017
L3f1b4a | 15672 | 3 | 0.025
L3f1b4a | 16129 | 1 | 0.008
L3f1b4a | 16145 | 1 | 0.008
L3f1b4a | 16219 | 1 | 0.008
L3f1b4a | 16360 | 1 | 0.008
L3f1b4a | 195 | 2 | 0.017
L3f1b4a | 207 | 6 | 0.05
L3f1b4a | 309d | 11 | 0.091
L3f1b4a | 3211 | 1 | 0.008
L3f1b4a | 3483C | 1 | 0.008
L3f1b4a | 3488A | 1 | 0.008
L3f1b4a | 3492C | 1 | 0.008
L3f1b4a | 3576 | 1 | 0.008
L3f1b4a | 3666 | 2 | 0.017
L3f1b4a | 4511 | 1 | 0.008
L3f1b4a | 4722 | 76 | 0.628
L3f1b4a | 5726A | 1 | 0.008
L3f1b4a | 6018 | 2 | 0.017
L3f1b4a | 6089 | 1 | 0.008
L3f1b4a | 6221 | 11 | 0.091
L3f1b4a | 6719 | 21 | 0.174
L3f1b4a | 8389 | 2 | 0.017
L3f1b4a | 93 | 2 | 0.017
L3f1b4a1 | 10055 | 2 | 0.031
L3f1b4a1 | 12366 | 1 | 0.016
L3f1b4a1 | 12397 | 1 | 0.016
L3f1b4a1 | 146 | 7 | 0.109
L3f1b4a1 | 15043 | 3 | 0.047
L3f1b4a1 | 152 | 2 | 0.031
L3f1b4a1 | 1555 | 2 | 0.031
L3f1b4a1 | 15940 | 2 | 0.031
L3f1b4a1 | 16131 | 1 | 0.016
L3f1b4a1 | 16261 | 1 | 0.016
L3f1b4a1 | 16278 | 1 | 0.016
L3f1b4a1 | 16293 | 1 | 0.016
L3f1b4a1 | 16304 | 1 | 0.016
L3f1b4a1 | 16325 | 1 | 0.016
L3f1b4a1 | 16343 | 1 | 0.016
L3f1b4a1 | 16368 | 1 | 0.016
L3f1b4a1 | 185 | 1 | 0.016
L3f1b4a1 | 200 | 1 | 0.016
L3f1b4a1 | 207 | 2 | 0.031
L3f1b4a1 | 3200 | 1 | 0.016
L3f1b4a1 | 3705 | 1 | 0.016
L3f1b4a1 | 3736 | 1 | 0.016
L3f1b4a1 | 573.1C | 1 | 0.016
L3f1b4a1 | 573.3C | 1 | 0.016
L3f1b4a1 | 6701 | 1 | 0.016
L3f1b4a1 | 7278 | 2 | 0.031
L3f1b4a1 | 9139 | 1 | 0.016
L3f1b4b | 13708 | 3 | 0.075
L3f1b4b | 14053 | 3 | 0.075
L3f1b4b | 14062 | 3 | 0.075
L3f1b4b | 152 | 3 | 0.075
L3f1b4b | 16066 | 1 | 0.025
L3f1b4b | 16075 | 2 | 0.05
L3f1b4b | 16093 | 1 | 0.025
L3f1b4b | 16126 | 1 | 0.025
L3f1b4b | 16129 | 1 | 0.025
L3f1b4b | 16214 | 1 | 0.025
L3f1b4b | 16294 | 4 | 0.1
L3f1b4b | 195 | 2 | 0.05
L3f1b4b | 215 | 4 | 0.1
L3f1b4b | 3927 | 1 | 0.025
L3f1b4b | 7322 | 1 | 0.025
L3f1b4c | 13602 | 1 | 0.021
L3f1b4c | 152 | 1 | 0.021
L3f1b4c | 15421 | 1 | 0.021

L3f1b4c | 15565 | 1 | 0.021
L3f1b4c | 16169 | 2 | 0.042
L3f1b4c | 16256 | 9 | 0.188
L3f1b4c | 16291 | 1 | 0.021
L3f1b4c | 16293 | 3 | 0.062
L3f1b4c | 16295 | 3 | 0.062
L3f1b4c | 16352 | 2 | 0.042
L3f1b4c | 16484-16489d | 4 | 0.083
L3f1b4c | 185 | 3 | 0.062
L3f1b4c | 195 | 1 | 0.021
L3f1b4c | 207 | 1 | 0.021
L3f1b4c | 214 | 1 | 0.021
L3f1b4c | 217 | 1 | 0.021
L3f1b4c | 234 | 1 | 0.021
L3f1b4c | 309d | 2 | 0.042
L3f1b4c | 310 | 1 | 0.021
L3f1b4c | 4216 | 1 | 0.021
L3f1b4c | 4767 | 1 | 0.021
L3f1b4c | 634G | 1 | 0.021
L3f1b5 | 10649 | 1 | 0.034
L3f1b5 | 13863 | 1 | 0.034
L3f1b5 | 13879 | 1 | 0.034
L3f1b5 | 16172 | 3 | 0.103
L3f1b5 | 16189 | 5 | 0.172
L3f1b5 | 16270 | 1 | 0.034
L3f1b5 | 16278 | 1 | 0.034
L3f1b5 | 16290 | 1 | 0.034
L3f1b5 | 16390 | 2 | 0.069
L3f1b5 | 185 | 1 | 0.034
L3f1b5 | 207 | 1 | 0.034
L3f1b5 | 279 | 1 | 0.034
L3f1b5 | 3796 | 1 | 0.034
L3f1b5 | 6827 | 1 | 0.034
L3f2 | 152 | 1 | 0.009
L3f2 | 16162 | 1 | 0.009
L3f2 | 16186 | 1 | 0.009
L3f2 | 16213 | 33 | 0.284
L3f2 | 16235 | 1 | 0.009
L3f2 | 16309 | 1 | 0.009
L3f2 | 16310 | 1 | 0.009
L3f2 | 16327 | 1 | 0.009
L3f2 | 195 | 33 | 0.284
L3f2 | 200 | 1 | 0.009
L3f2 | 328 | 32 | 0.276
L3f2 | 515-524d | 32 | 0.276
L3f2 | 527 | 1 | 0.009
L3f2 | 529T | 3 | 0.026
L3f2a | 12285 | 2 | 0.035
L3f2a | 12530 | 1 | 0.018
L3f2a | 13896 | 2 | 0.035
L3f2a | 13984 | 2 | 0.035
L3f2a | 15119 | 2 | 0.035
L3f2a | 15511 | 2 | 0.035
L3f2a | 15934.1G | 2 | 0.035
L3f2a | 16111A | 2 | 0.035
L3f2a | 16129 | 1 | 0.018
L3f2a | 16145 | 2 | 0.035
L3f2a | 16150 | 1 | 0.018
L3f2a | 16292 | 1 | 0.018
L3f2a | 2438 | 2 | 0.035
L3f2a | 252 | 1 | 0.018
L3f2a | 3756 | 1 | 0.018
L3f2a | 37d | 1 | 0.018
L3f2a | 41-46d | 1 | 0.018
L3f2a | 474d | 1 | 0.018
L3f2a | 477.1T | 1 | 0.018
L3f2a | 484-485d | 1 | 0.018
L3f2a | 490T | 1 | 0.018
L3f2a | 491 | 1 | 0.018
L3f2a | 492T | 1 | 0.018
L3f2a | 6413 | 1 | 0.018
L3f2a | 6872 | 1 | 0.018
L3f2a | 709 | 2 | 0.035
L3f2a1 | 103 | 1 | 0.015
L3f2a1 | 10376 | 9 | 0.132
L3f2a1 | 10535 | 1 | 0.015
L3f2a1 | 11101 | 2 | 0.029
L3f2a1 | 11150 | 2 | 0.029
L3f2a1 | 12151 | 9 | 0.132
L3f2a1 | 12280 | 5 | 0.074
L3f2a1 | 12534 | 5 | 0.074
L3f2a1 | 13105 | 5 | 0.074
L3f2a1 | 14260 | 2 | 0.029
L3f2a1 | 14727 | 9 | 0.132
L3f2a1 | 16024 | 1 | 0.015
L3f2a1 | 16025 | 1 | 0.015
L3f2a1 | 16186 | 1 | 0.015
L3f2a1 | 16213 | 3 | 0.044
L3f2a1 | 16224 | 1 | 0.015
L3f2a1 | 16234 | 3 | 0.044
L3f2a1 | 16235 | 1 | 0.015
L3f2a1 | 16256 | 1 | 0.015
L3f2a1 | 16309 | 1 | 0.015
L3f2a1 | 16327 | 1 | 0.015
L3f2a1 | 185 | 2 | 0.029
L3f2a1 | 189 | 3 | 0.044
L3f2a1 | 200 | 10 | 0.147
L3f2a1 | 2702 | 9 | 0.132
L3f2a1 | 309d | 5 | 0.074
L3f2a1 | 310 | 1 | 0.015
L3f2a1 | 3828 | 1 | 0.015
L3f2a1 | 3927 | 1 | 0.015
L3f2a1 | 4227 | 1 | 0.015
L3f2a1 | 430G | 1 | 0.015
L3f2a1 | 4580 | 9 | 0.132
L3f2a1 | 4688 | 1 | 0.015
L3f2a1 | 486G | 1 | 0.015
L3f2a1 | 491 | 1 | 0.015
L3f2a1 | 530 | 1 | 0.015
L3f2a1 | 541 | 1 | 0.015
L3f2a1 | 547 | 1 | 0.015

L3f2a1 | 557G | 1 | 0.015
L3f2a1 | 5964 | 3 | 0.044
L3f2a1 | 6164 | 9 | 0.132
L3f2a1 | 6179 | 7 | 0.103
L3f2a1 | 6182 | 5 | 0.074
L3f2a1 | 6722 | 2 | 0.029
L3f2a1 | 6899 | 1 | 0.015
L3f2a1 | 7406A | 1 | 0.015
L3f2a1 | 8065 | 1 | 0.015
L3f2a1 | 8676 | 5 | 0.074
L3f2a1 | 9132 | 1 | 0.015
L3f2a1 | 9177 | 1 | 0.015
L3f2a1 | 9257 | 7 | 0.103
L3f2a1 | 9338C | 1 | 0.015
L3f2a1 | 9425 | 9 | 0.132
L3f2a1 | 9731A | 5 | 0.074
L3f2a1a | 11466 | 1 | 0.021
L3f2a1a | 13194 | 2 | 0.042
L3f2a1a | 13269 | 1 | 0.021
L3f2a1a | 14167 | 1 | 0.021
L3f2a1a | 15518 | 1 | 0.021
L3f2a1a | 16024 | 1 | 0.021
L3f2a1a | 16025 | 1 | 0.021
L3f2a1a | 16093 | 1 | 0.021
L3f2a1a | 16129 | 2 | 0.042
L3f2a1a | 16274 | 1 | 0.021
L3f2a1a | 16290 | 1 | 0.021
L3f2a1a | 16293 | 1 | 0.021
L3f2a1a | 1719 | 1 | 0.021
L3f2a1a | 200 | 3 | 0.062
L3f2a1a | 300C | 1 | 0.021
L3f2a1a | 414G | 1 | 0.021
L3f2a1a | 527G | 1 | 0.021
L3f2a1a | 8610 | 1 | 0.021
L3f2b | 10310 | 1 | 0.043
L3f2b | 10410 | 1 | 0.043
L3f2b | 11137 | 1 | 0.043
L3f2b | 11253 | 1 | 0.043
L3f2b | 11293 | 1 | 0.043
L3f2b | 13020 | 1 | 0.043
L3f2b | 14118 | 1 | 0.043
L3f2b | 146 | 2 | 0.087
L3f2b | 152 | 1 | 0.043
L3f2b | 15930 | 1 | 0.043
L3f2b | 16038 | 1 | 0.043
L3f2b | 16080 | 1 | 0.043
L3f2b | 16111 | 1 | 0.043
L3f2b | 16144 | 1 | 0.043
L3f2b | 16162 | 1 | 0.043
L3f2b | 16166d | 1 | 0.043
L3f2b | 16167 | 1 | 0.043
L3f2b | 16176 | 1 | 0.043
L3f2b | 16185 | 3 | 0.13
L3f2b | 16186 | 4 | 0.174
L3f2b | 16201 | 1 | 0.043
L3f2b | 16214 | 1 | 0.043
L3f2b | 16235 | 3 | 0.13
L3f2b | 16259G | 1 | 0.043
L3f2b | 16260 | 1 | 0.043
L3f2b | 16261 | 1 | 0.043
L3f2b | 16265 | 1 | 0.043
L3f2b | 16294 | 2 | 0.087
L3f2b | 16309 | 4 | 0.174
L3f2b | 16325 | 1 | 0.043
L3f2b | 16327 | 4 | 0.174
L3f2b | 16362 | 1 | 0.043
L3f2b | 200 | 2 | 0.087
L3f2b | 325 | 1 | 0.043
L3f2b | 3438 | 1 | 0.043
L3f2b | 3552 | 1 | 0.043
L3f2b | 4117 | 1 | 0.043
L3f2b | 4491 | 1 | 0.043
L3f2b | 518 | 1 | 0.043
L3f2b | 520 | 1 | 0.043
L3f2b | 9077 | 1 | 0.043
L3f3 | 12904 | 1 | 0.056
L3f3 | 13518 | 1 | 0.056
L3f3 | 15106 | 1 | 0.056
L3f3 | 152 | 3 | 0.167
L3f3 | 15502G | 1 | 0.056
L3f3 | 15910 | 1 | 0.056
L3f3 | 16139 | 1 | 0.056
L3f3 | 16218 | 1 | 0.056
L3f3 | 16256 | 1 | 0.056
L3f3 | 16292 | 1 | 0.056
L3f3 | 16294 | 1 | 0.056
L3f3 | 16327 | 1 | 0.056
L3f3 | 195 | 3 | 0.167
L3f3 | 214 | 1 | 0.056
L3f3 | 303G | 2 | 0.111
L3f3 | 4820 | 1 | 0.056
L3f3 | 515-524d | 2 | 0.111
L3f3 | 5492 | 1 | 0.056
L3f3 | 7258 | 1 | 0.056
L3f3 | 8865 | 1 | 0.056
L3f3a | 15458 | 1 | 0.2
L3f3a | 16189 | 1 | 0.2
L3f3a | 16532d | 1 | 0.2
L3f3a | 4976 | 1 | 0.2
L3f3a | 9857 | 2 | 0.4
L3f3b | 11176 | 1 | 0.1
L3f3b | 143 | 1 | 0.1
L3f3b | 14590 | 1 | 0.1
L3f3b | 15106 | 5 | 0.5
L3f3b | 16189 | 2 | 0.2
L3f3b | 16215 | 1 | 0.1
L3f3b | 16278 | 1 | 0.1
L3f3b | 16311 | 1 | 0.1
L3f3b | 16355 | 1 | 0.1
L3f3b | 1692 | 1 | 0.1
L3f3b | 479 | 1 | 0.1

L3f3b | 6077.1GTC | 1 | 0.1
L3f3b | 6293 | 1 | 0.1
L3f3b | 6480 | 1 | 0.1
L3f3b | 8400 | 1 | 0.1
L3f3b | 8506 | 1 | 0.1
L3f3b | 9377 | 2 | 0.2
L3h | 16072.1A | 1 | 0.005
L3h | 16188 | 1 | 0.005
L3h | 16234 | 1 | 0.005
L3h | 16318T | 1 | 0.005
L3h | 199 | 1 | 0.005
L3h | 200 | 1 | 0.005
L3h | 285 | 2 | 0.009
L3h | 315.2C | 1 | 0.005
L3h | 316C | 2 | 0.009
L3h | 488 | 1 | 0.005
L3h | 541 | 1 | 0.005
L3h | 93 | 1 | 0.005
L3h1 | 152 | 3 | 0.022
L3h1 | 16126 | 3 | 0.022
L3h1 | 16214 | 6 | 0.043
L3h1 | 16274 | 6 | 0.043
L3h1 | 16320 | 1 | 0.007
L3h1 | 16366G | 1 | 0.007
L3h1 | 217 | 1 | 0.007
L3h1 | 309d | 1 | 0.007
L3h1 | 394 | 1 | 0.007
L3h1 | 508 | 1 | 0.007
L3h1a | 152 | 3 | 0.022
L3h1a | 16126 | 3 | 0.022
L3h1a | 16214 | 6 | 0.043
L3h1a | 16274 | 6 | 0.043
L3h1a | 16320 | 1 | 0.007
L3h1a | 16366G | 1 | 0.007
L3h1a | 217 | 1 | 0.007
L3h1a | 309d | 1 | 0.007
L3h1a | 394 | 1 | 0.007
L3h1a | 508 | 1 | 0.007
L3h1a1 | 10343 | 1 | 0.012
L3h1a1 | 10358 | 1 | 0.012
L3h1a1 | 10463 | 1 | 0.012
L3h1a1 | 11029 | 1 | 0.012
L3h1a1 | 11084 | 1 | 0.012
L3h1a1 | 11152 | 1 | 0.012
L3h1a1 | 1242 | 2 | 0.024
L3h1a1 | 12651 | 3 | 0.036
L3h1a1 | 12741 | 1 | 0.012
L3h1a1 | 12753 | 3 | 0.036
L3h1a1 | 14278 | 1 | 0.012
L3h1a1 | 14370 | 1 | 0.012
L3h1a1 | 14494 | 6 | 0.071
L3h1a1 | 146 | 1 | 0.012
L3h1a1 | 14664 | 6 | 0.071
L3h1a1 | 15924 | 5 | 0.06
L3h1a1 | 16129 | 1 | 0.012
L3h1a1 | 16170 | 1 | 0.012
L3h1a1 | 16189 | 4 | 0.048
L3h1a1 | 16195 | 7 | 0.083
L3h1a1 | 16212 | 1 | 0.012
L3h1a1 | 16245 | 7 | 0.083
L3h1a1 | 16255 | 1 | 0.012
L3h1a1 | 16390 | 7 | 0.083
L3h1a1 | 1888 | 3 | 0.036
L3h1a1 | 195 | 2 | 0.024
L3h1a1 | 205 | 1 | 0.012
L3h1a1 | 235 | 1 | 0.012
L3h1a1 | 2833 | 1 | 0.012
L3h1a1 | 3221 | 1 | 0.012
L3h1a1 | 3588 | 1 | 0.012
L3h1a1 | 4048 | 1 | 0.012
L3h1a1 | 4117 | 2 | 0.024
L3h1a1 | 516 | 2 | 0.024
L3h1a1 | 518 | 1 | 0.012
L3h1a1 | 5316 | 2 | 0.024
L3h1a1 | 5899.1C | 1 | 0.012
L3h1a1 | 6281 | 1 | 0.012
L3h1a1 | 709 | 5 | 0.06
L3h1a1 | 7372 | 6 | 0.071
L3h1a1 | 7493 | 1 | 0.012
L3h1a1 | 8387 | 1 | 0.012
L3h1a1 | 9080 | 2 | 0.024
L3h1a1 | 9428 | 6 | 0.071
L3h1a1 | 9845 | 2 | 0.024
L3h1a2 | 10042 | 1 | 0.007
L3h1a2 | 10304 | 2 | 0.015
L3h1a2 | 11204 | 1 | 0.007
L3h1a2 | 11533 | 1 | 0.007
L3h1a2 | 11935 | 1 | 0.007
L3h1a2 | 14311 | 1 | 0.007
L3h1a2 | 146 | 3 | 0.022
L3h1a2 | 15002 | 3 | 0.022
L3h1a2 | 15466 | 2 | 0.015
L3h1a2 | 15562 | 1 | 0.007
L3h1a2 | 16189 | 1 | 0.007
L3h1a2 | 16192 | 2 | 0.015
L3h1a2 | 16359 | 2 | 0.015
L3h1a2 | 1652A | 3 | 0.022
L3h1a2 | 2442 | 1 | 0.007
L3h1a2 | 246 | 3 | 0.022
L3h1a2 | 3834 | 3 | 0.022
L3h1a2 | 4937 | 1 | 0.007
L3h1a2 | 5054 | 1 | 0.007
L3h1a2 | 6482 | 2 | 0.015
L3h1a2 | 7364 | 1 | 0.007
L3h1a2 | 89 | 1 | 0.007
L3h1a2 | 93 | 3 | 0.022
L3h1a2 | 95C | 3 | 0.022
L3h1a2 | 980 | 1 | 0.007
L3h1a2 | 9932 | 3 | 0.022
L3h1a2a | 11146 | 1 | 0.029
L3h1a2a | 11157 | 1 | 0.029

L3h1a2a | 11467 | 1 | 0.029
L3h1a2a | 12284 | 2 | 0.059
L3h1a2a | 13158 | 1 | 0.029
L3h1a2a | 14578 | 1 | 0.029
L3h1a2a | 14862 | 3 | 0.088
L3h1a2a | 14955 | 1 | 0.029
L3h1a2a | 1508 | 3 | 0.088
L3h1a2a | 15229 | 1 | 0.029
L3h1a2a | 153 | 10 | 0.294
L3h1a2a | 15346 | 1 | 0.029
L3h1a2a | 16036 | 1 | 0.029
L3h1a2a | 16066 | 3 | 0.088
L3h1a2a | 16086 | 1 | 0.029
L3h1a2a | 16092A | 1 | 0.029
L3h1a2a | 16145 | 1 | 0.029
L3h1a2a | 16178 | 1 | 0.029
L3h1a2a | 16189 | 2 | 0.059
L3h1a2a | 16209 | 1 | 0.029
L3h1a2a | 16256 | 1 | 0.029
L3h1a2a | 16261 | 2 | 0.059
L3h1a2a | 16274 | 2 | 0.059
L3h1a2a | 16293T | 1 | 0.029
L3h1a2a | 16300 | 1 | 0.029
L3h1a2a | 16354 | 6 | 0.176
L3h1a2a | 16355 | 2 | 0.059
L3h1a2a | 16362 | 1 | 0.029
L3h1a2a | 16474d | 1 | 0.029
L3h1a2a | 16507G | 1 | 0.029
L3h1a2a | 2092 | 1 | 0.029
L3h1a2a | 257 | 1 | 0.029
L3h1a2a | 3892 | 1 | 0.029
L3h1a2a | 466 | 1 | 0.029
L3h1a2a | 4965 | 1 | 0.029
L3h1a2a | 515-524d | 1 | 0.029
L3h1a2a | 525d | 2 | 0.059
L3h1a2a | 5760 | 1 | 0.029
L3h1a2a | 6506 | 1 | 0.029
L3h1a2a | 7055 | 3 | 0.088
L3h1a2a | 7705 | 1 | 0.029
L3h1a2a | 9839A | 1 | 0.029
L3h1a2a1 | 10197 | 1 | 0.019
L3h1a2a1 | 10586 | 1 | 0.019
L3h1a2a1 | 11377 | 1 | 0.019
L3h1a2a1 | 11466 | 1 | 0.019
L3h1a2a1 | 11512 | 1 | 0.019
L3h1a2a1 | 1173 | 1 | 0.019
L3h1a2a1 | 12014 | 1 | 0.019
L3h1a2a1 | 12076 | 1 | 0.019
L3h1a2a1 | 12999 | 1 | 0.019
L3h1a2a1 | 14203 | 1 | 0.019
L3h1a2a1 | 14908 | 1 | 0.019
L3h1a2a1 | 1503 | 1 | 0.019
L3h1a2a1 | 15043 | 1 | 0.019
L3h1a2a1 | 15119 | 1 | 0.019
L3h1a2a1 | 152 | 13 | 0.25
L3h1a2a1 | 15346 | 1 | 0.019
L3h1a2a1 | 1597 | 1 | 0.019
L3h1a2a1 | 16036 | 1 | 0.019
L3h1a2a1 | 16093 | 1 | 0.019
L3h1a2a1 | 16126 | 1 | 0.019
L3h1a2a1 | 16129 | 1 | 0.019
L3h1a2a1 | 16148 | 13 | 0.25
L3h1a2a1 | 16165 | 9 | 0.173
L3h1a2a1 | 16172 | 3 | 0.058
L3h1a2a1 | 16175 | 3 | 0.058
L3h1a2a1 | 16177 | 2 | 0.038
L3h1a2a1 | 16178 | 4 | 0.077
L3h1a2a1 | 16180 | 1 | 0.019
L3h1a2a1 | 16189 | 6 | 0.115
L3h1a2a1 | 16192 | 22 | 0.423
L3h1a2a1 | 16193 | 1 | 0.019
L3h1a2a1 | 16214 | 3 | 0.058
L3h1a2a1 | 16234 | 3 | 0.058
L3h1a2a1 | 16263 | 1 | 0.019
L3h1a2a1 | 16270 | 1 | 0.019
L3h1a2a1 | 16274 | 2 | 0.038
L3h1a2a1 | 16292 | 2 | 0.038
L3h1a2a1 | 16359 | 4 | 0.077
L3h1a2a1 | 189 | 3 | 0.058
L3h1a2a1 | 195 | 1 | 0.019
L3h1a2a1 | 207 | 1 | 0.019
L3h1a2a1 | 207C | 1 | 0.019
L3h1a2a1 | 234 | 1 | 0.019
L3h1a2a1 | 246 | 4 | 0.077
L3h1a2a1 | 291 | 1 | 0.019
L3h1a2a1 | 315.2C | 1 | 0.019
L3h1a2a1 | 3290 | 2 | 0.038
L3h1a2a1 | 338 | 1 | 0.019
L3h1a2a1 | 36.1G | 1 | 0.019
L3h1a2a1 | 3808 | 1 | 0.019
L3h1a2a1 | 3892 | 1 | 0.019
L3h1a2a1 | 5004 | 1 | 0.019
L3h1a2a1 | 5090 | 1 | 0.019
L3h1a2a1 | 5108 | 5 | 0.096
L3h1a2a1 | 5147 | 1 | 0.019
L3h1a2a1 | 515-524d | 2 | 0.038
L3h1a2a1 | 6023 | 1 | 0.019
L3h1a2a1 | 7055 | 1 | 0.019
L3h1a2a1 | 7058A | 1 | 0.019
L3h1a2a1 | 723 | 2 | 0.038
L3h1a2a1 | 7705 | 1 | 0.019
L3h1a2a1 | 7858 | 1 | 0.019
L3h1a2a1 | 8485 | 1 | 0.019
L3h1a2a1 | 9084G | 2 | 0.038
L3h1a2a1 | 93 | 6 | 0.115
L3h1a2a1 | 9327 | 1 | 0.019
L3h1a2a1 | 9554 | 1 | 0.019
L3h1a2a1 | 95C | 4 | 0.077
L3h1a2a1 | 98A | 1 | 0.019
L3h1a2b | 11317 | 1 | 0.013
L3h1a2b | 11440 | 1 | 0.013

L3h1a2b | 11466 | 1 | 0.013
L3h1a2b | 12351 | 1 | 0.013
L3h1a2b | 12627 | 1 | 0.013
L3h1a2b | 12696 | 1 | 0.013
L3h1a2b | 12774 | 1 | 0.013
L3h1a2b | 12952 | 1 | 0.013
L3h1a2b | 12957 | 1 | 0.013
L3h1a2b | 13153 | 3 | 0.038
L3h1a2b | 13803 | 1 | 0.013
L3h1a2b | 14167 | 1 | 0.013
L3h1a2b | 14407 | 4 | 0.051
L3h1a2b | 14530 | 1 | 0.013
L3h1a2b | 14664 | 1 | 0.013
L3h1a2b | 15109 | 1 | 0.013
L3h1a2b | 152 | 1 | 0.013
L3h1a2b | 15924 | 2 | 0.025
L3h1a2b | 16093 | 3 | 0.038
L3h1a2b | 16126 | 1 | 0.013
L3h1a2b | 16129 | 3 | 0.038
L3h1a2b | 16188G | 1 | 0.013
L3h1a2b | 16189 | 1 | 0.013
L3h1a2b | 16242 | 1 | 0.013
L3h1a2b | 16256 | 2 | 0.025
L3h1a2b | 16264 | 1 | 0.013
L3h1a2b | 16278 | 1 | 0.013
L3h1a2b | 16293 | 1 | 0.013
L3h1a2b | 16327 | 2 | 0.025
L3h1a2b | 16347G | 1 | 0.013
L3h1a2b | 204 | 1 | 0.013
L3h1a2b | 3396 | 1 | 0.013
L3h1a2b | 3483 | 2 | 0.025
L3h1a2b | 4209 | 1 | 0.013
L3h1a2b | 4218 | 1 | 0.013
L3h1a2b | 4248 | 1 | 0.013
L3h1a2b | 4856 | 1 | 0.013
L3h1a2b | 512 | 1 | 0.013
L3h1a2b | 515-524d | 1 | 0.013
L3h1a2b | 6351 | 1 | 0.013
L3h1a2b | 7269 | 2 | 0.025
L3h1a2b | 7310 | 4 | 0.051
L3h1a2b | 7690 | 2 | 0.025
L3h1a2b | 7853 | 1 | 0.013
L3h1a2b | 8634 | 1 | 0.013
L3h1a2b | 8727 | 1 | 0.013
L3h1a2b | 9824A | 3 | 0.038
L3h1a2b | 9938 | 1 | 0.013
L3h1b | 16179 | 1 | 1
L3h1b | 16289 | 1 | 1
L3h1b | 16354 | 1 | 1
L3h1b1 | 10289 | 1 | 0.143
L3h1b1 | 10586 | 1 | 0.143
L3h1b1 | 10595 | 1 | 0.143
L3h1b1 | 11299 | 1 | 0.143
L3h1b1 | 11563 | 1 | 0.143
L3h1b1 | 11963 | 1 | 0.143
L3h1b1 | 12403 | 1 | 0.143
L3h1b1 | 12855 | 1 | 0.143
L3h1b1 | 13041 | 1 | 0.143
L3h1b1 | 14569 | 1 | 0.143
L3h1b1 | 15205 | 1 | 0.143
L3h1b1 | 16092 | 2 | 0.286
L3h1b1 | 16126 | 4 | 0.571
L3h1b1 | 16140 | 1 | 0.143
L3h1b1 | 16154 | 1 | 0.143
L3h1b1 | 16186 | 3 | 0.429
L3h1b1 | 16189 | 3 | 0.429
L3h1b1 | 16192 | 3 | 0.429
L3h1b1 | 16215 | 2 | 0.286
L3h1b1 | 16266 | 1 | 0.143
L3h1b1 | 16297 | 1 | 0.143
L3h1b1 | 2831 | 1 | 0.143
L3h1b1 | 3777 | 1 | 0.143
L3h1b1 | 4859 | 1 | 0.143
L3h1b1 | 7055 | 1 | 0.143
L3h1b1 | 8743 | 1 | 0.143
L3h1b1 | 8767 | 1 | 0.143
L3h1b1 | 9057 | 1 | 0.143
L3h1b1 | 9518 | 1 | 0.143
L3h1b1 | 9827 | 1 | 0.143
L3h1b1a | 10289 | 3 | 0.081
L3h1b1a | 10310 | 1 | 0.027
L3h1b1a | 10966 | 1 | 0.027
L3h1b1a | 11440 | 1 | 0.027
L3h1b1a | 11563 | 3 | 0.081
L3h1b1a | 11963 | 6 | 0.162
L3h1b1a | 12441 | 1 | 0.027
L3h1b1a | 12507 | 2 | 0.054
L3h1b1a | 13135 | 1 | 0.027
L3h1b1a | 13215 | 1 | 0.027
L3h1b1a | 14188 | 1 | 0.027
L3h1b1a | 14577 | 1 | 0.027
L3h1b1a | 14920 | 1 | 0.027
L3h1b1a | 14950 | 7 | 0.189
L3h1b1a | 15040A | 2 | 0.054
L3h1b1a | 152 | 5 | 0.135
L3h1b1a | 15924 | 2 | 0.054
L3h1b1a | 15950 | 1 | 0.027
L3h1b1a | 16024 | 1 | 0.027
L3h1b1a | 16025 | 1 | 0.027
L3h1b1a | 16048 | 2 | 0.054
L3h1b1a | 16086 | 2 | 0.054
L3h1b1a | 16093 | 3 | 0.081
L3h1b1a | 16114 | 1 | 0.027
L3h1b1a | 16126 | 4 | 0.108
L3h1b1a | 16153 | 2 | 0.054
L3h1b1a | 16168 | 1 | 0.027
L3h1b1a | 16172 | 2 | 0.054
L3h1b1a | 16189 | 3 | 0.081
L3h1b1a | 16192 | 14 | 0.378
L3h1b1a | 16215 | 18 | 0.486
L3h1b1a | 16234 | 1 | 0.027

L3h1b1a | 16243 | 4 | 0.108
L3h1b1a | 16278 | 2 | 0.054
L3h1b1a | 16320 | 4 | 0.108
L3h1b1a | 16335 | 1 | 0.027
L3h1b1a | 16559 | 1 | 0.027
L3h1b1a | 194 | 5 | 0.135
L3h1b1a | 198 | 2 | 0.054
L3h1b1a | 2383 | 2 | 0.054
L3h1b1a | 2389 | 1 | 0.027
L3h1b1a | 249d | 2 | 0.054
L3h1b1a | 2831 | 6 | 0.162
L3h1b1a | 3027 | 2 | 0.054
L3h1b1a | 320 | 1 | 0.027
L3h1b1a | 356.1C | 1 | 0.027
L3h1b1a | 36.1G | 1 | 0.027
L3h1b1a | 3786 | 1 | 0.027
L3h1b1a | 437 | 1 | 0.027
L3h1b1a | 4859 | 1 | 0.027
L3h1b1a | 4892 | 1 | 0.027
L3h1b1a | 513 | 3 | 0.081
L3h1b1a | 515-524d | 3 | 0.081
L3h1b1a | 527G | 1 | 0.027
L3h1b1a | 529T | 1 | 0.027
L3h1b1a | 5423 | 1 | 0.027
L3h1b1a | 573.5C | 1 | 0.027
L3h1b1a | 5846 | 2 | 0.054
L3h1b1a | 6137 | 2 | 0.054
L3h1b1a | 6359 | 3 | 0.081
L3h1b1a | 6497A | 1 | 0.027
L3h1b1a | 6756 | 1 | 0.027
L3h1b1a | 6911 | 1 | 0.027
L3h1b1a | 7055 | 6 | 0.162
L3h1b1a | 7717 | 3 | 0.081
L3h1b1a | 7762 | 1 | 0.027
L3h1b1a | 8152 | 3 | 0.081
L3h1b1a | 8176 | 1 | 0.027
L3h1b1a | 8767 | 6 | 0.162
L3h1b1a | 8790 | 1 | 0.027
L3h1b1a | 9287 | 1 | 0.027
L3h1b1a | 93 | 1 | 0.027
L3h1b1a | 93.1A | 1 | 0.027
L3h1b1a | 9323 | 2 | 0.054
L3h1b1a | 9524 | 2 | 0.054
L3h1b1a | 9776 | 4 | 0.108
L3h1b1a | 9827 | 4 | 0.108
L3h1b2 | 10754 | 4 | 0.2
L3h1b2 | 11253 | 1 | 0.05
L3h1b2 | 11287 | 4 | 0.2
L3h1b2 | 11380 | 1 | 0.05
L3h1b2 | 12912 | 1 | 0.05
L3h1b2 | 13105 | 4 | 0.2
L3h1b2 | 13194 | 7 | 0.35
L3h1b2 | 13476 | 1 | 0.05
L3h1b2 | 13863 | 1 | 0.05
L3h1b2 | 13879A | 1 | 0.05
L3h1b2 | 14271 | 1 | 0.05
L3h1b2 | 14364 | 1 | 0.05
L3h1b2 | 14370 | 1 | 0.05
L3h1b2 | 146 | 1 | 0.05
L3h1b2 | 14793 | 6 | 0.3
L3h1b2 | 15355 | 1 | 0.05
L3h1b2 | 16093 | 1 | 0.05
L3h1b2 | 16172 | 9 | 0.45
L3h1b2 | 16174 | 10 | 0.5
L3h1b2 | 16192 | 11 | 0.55
L3h1b2 | 16218 | 14 | 0.7
L3h1b2 | 16249 | 1 | 0.05
L3h1b2 | 16278 | 2 | 0.1
L3h1b2 | 16325 | 2 | 0.1
L3h1b2 | 1664 | 4 | 0.2
L3h1b2 | 198 | 1 | 0.05
L3h1b2 | 309d | 1 | 0.05
L3h1b2 | 310 | 1 | 0.05
L3h1b2 | 337d | 1 | 0.05
L3h1b2 | 4025 | 1 | 0.05
L3h1b2 | 4218 | 1 | 0.05
L3h1b2 | 4688 | 1 | 0.05
L3h1b2 | 4722 | 2 | 0.1
L3h1b2 | 499 | 3 | 0.15
L3h1b2 | 515-524d | 1 | 0.05
L3h1b2 | 6267 | 1 | 0.05
L3h1b2 | 7064 | 1 | 0.05
L3h1b2 | 7278 | 3 | 0.15
L3h1b2 | 7424 | 3 | 0.15
L3h1b2 | 7496 | 1 | 0.05
L3h1b2 | 769 | 1 | 0.05
L3h1b2 | 7810A | 1 | 0.05
L3h1b2 | 7853 | 2 | 0.1
L3h2 | 10325 | 1 | 0.077
L3h2 | 10858 | 1 | 0.077
L3h2 | 12969 | 1 | 0.077
L3h2 | 13753 | 10 | 0.769
L3h2 | 13824 | 1 | 0.077
L3h2 | 1530 | 1 | 0.077
L3h2 | 15910 | 1 | 0.077
L3h2 | 16129 | 1 | 0.077
L3h2 | 16177 | 4 | 0.308
L3h2 | 16189 | 1 | 0.077
L3h2 | 16192 | 1 | 0.077
L3h2 | 16234 | 1 | 0.077
L3h2 | 16274 | 1 | 0.077
L3h2 | 16301 | 3 | 0.231
L3h2 | 2707 | 1 | 0.077
L3h2 | 4122 | 1 | 0.077
L3h2 | 513 | 2 | 0.154
L3h2 | 5147 | 1 | 0.077
L3h2 | 5567 | 1 | 0.077
L3h2 | 6018 | 1 | 0.077
L3h2 | 628 | 1 | 0.077
L3h2 | 7805 | 7 | 0.538
L3h2 | 8155 | 1 | 0.077

L3h2 | 8281 | 3 | 0.231
L3h2 | 859 | 1 | 0.077
L3h2 | 9163 | 1 | 0.077
L3h2 | 9254 | 1 | 0.077
L3h2 | 9909 | 2 | 0.154
L3i | 152 | 1 | 0.006
L3i | 16111 | 1 | 0.006
L3i | 16265C | 5 | 0.03
L3i | 16274 | 1 | 0.006
L3i | 16304 | 1 | 0.006
L3i | 16316 | 1 | 0.006
L3i | 16327 | 1 | 0.006
L3i | 16343T | 1 | 0.006
L3i | 195 | 5 | 0.03
L3i | 303G | 1 | 0.006
L3i | 515-524d | 1 | 0.006
L3i1 | 10130 | 1 | 0.023
L3i1 | 10509 | 1 | 0.023
L3i1 | 10670 | 1 | 0.023
L3i1 | 11254 | 2 | 0.045
L3i1 | 11812 | 1 | 0.023
L3i1 | 12127 | 1 | 0.023
L3i1 | 12406 | 9 | 0.205
L3i1 | 12720 | 1 | 0.023
L3i1 | 13590 | 15 | 0.341
L3i1 | 13827 | 2 | 0.045
L3i1 | 13905 | 2 | 0.045
L3i1 | 14422 | 1 | 0.023
L3i1 | 14587 | 2 | 0.045
L3i1 | 152 | 1 | 0.023
L3i1 | 15323 | 1 | 0.023
L3i1 | 15346 | 3 | 0.068
L3i1 | 15731 | 1 | 0.023
L3i1 | 15784 | 3 | 0.068
L3i1 | 15924 | 8 | 0.182
L3i1 | 16066 | 8 | 0.182
L3i1 | 16093 | 2 | 0.045
L3i1 | 16138T | 1 | 0.023
L3i1 | 16172 | 4 | 0.091
L3i1 | 16174 | 15 | 0.341
L3i1 | 16219 | 1 | 0.023
L3i1 | 16230 | 2 | 0.045
L3i1 | 16286 | 1 | 0.023
L3i1 | 16319 | 15 | 0.341
L3i1 | 16399 | 1 | 0.023
L3i1 | 1719 | 15 | 0.341
L3i1 | 257 | 1 | 0.023
L3i1 | 3095 | 1 | 0.023
L3i1 | 3378 | 2 | 0.045
L3i1 | 3398 | 1 | 0.023
L3i1 | 408A | 1 | 0.023
L3i1 | 4484 | 2 | 0.045
L3i1 | 465.1A | 1 | 0.023
L3i1 | 4949 | 2 | 0.045
L3i1 | 5027 | 15 | 0.341
L3i1 | 5553 | 2 | 0.045
L3i1 | 5823 | 1 | 0.023
L3i1 | 709 | 15 | 0.341
L3i1 | 7146 | 1 | 0.023
L3i1 | 7256 | 1 | 0.023
L3i1 | 7897 | 2 | 0.045
L3i1 | 8167 | 1 | 0.023
L3i1 | 9575 | 1 | 0.023
L3i1 | 990 | 1 | 0.023
L3i1a | 1018 | 2 | 0.044
L3i1a | 11914 | 3 | 0.067
L3i1a | 13650 | 2 | 0.044
L3i1a | 13967 | 2 | 0.044
L3i1a | 14060 | 2 | 0.044
L3i1a | 14311 | 1 | 0.022
L3i1a | 146 | 1 | 0.022
L3i1a | 152 | 1 | 0.022
L3i1a | 15734 | 3 | 0.067
L3i1a | 16145 | 1 | 0.022
L3i1a | 16192 | 1 | 0.022
L3i1a | 16223 | 1 | 0.022
L3i1a | 16311 | 2 | 0.044
L3i1a | 16318T | 4 | 0.089
L3i1a | 16362 | 3 | 0.067
L3i1a | 1842 | 1 | 0.022
L3i1a | 4092 | 1 | 0.022
L3i1a | 515-524d | 3 | 0.067
L3i1a | 6932 | 1 | 0.022
L3i1a | 769 | 2 | 0.044
L3i1a | 8279 | 1 | 0.022
L3i1b | 11204 | 1 | 0.028
L3i1b | 14040 | 1 | 0.028
L3i1b | 14395 | 1 | 0.028
L3i1b | 146 | 1 | 0.028
L3i1b | 15924 | 1 | 0.028
L3i1b | 16148 | 4 | 0.111
L3i1b | 16215 | 2 | 0.056
L3i1b | 16220 | 1 | 0.028
L3i1b | 16256 | 1 | 0.028
L3i1b | 16274 | 1 | 0.028
L3i1b | 200 | 1 | 0.028
L3i1b | 291.1A | 1 | 0.028
L3i1b | 8598 | 1 | 0.028
L3i2 | 11002 | 1 | 0.033
L3i2 | 12172 | 1 | 0.033
L3i2 | 12630 | 8 | 0.267
L3i2 | 12680 | 1 | 0.033
L3i2 | 12753 | 1 | 0.033
L3i2 | 13753 | 1 | 0.033
L3i2 | 14053 | 1 | 0.033
L3i2 | 14155 | 3 | 0.1
L3i2 | 14197 | 1 | 0.033
L3i2 | 14305 | 3 | 0.1
L3i2 | 146 | 2 | 0.067
L3i2 | 15217 | 1 | 0.033
L3i2 | 15457 | 1 | 0.033

L3i2 | 15466 | 1 | 0.033
L3i2 | 15924 | 1 | 0.033
L3i2 | 15944d | 6 | 0.2
L3i2 | 16051 | 1 | 0.033
L3i2 | 16093 | 6 | 0.2
L3i2 | 16126 | 1 | 0.033
L3i2 | 16148 | 1 | 0.033
L3i2 | 16172 | 1 | 0.033
L3i2 | 16180 | 1 | 0.033
L3i2 | 16184 | 1 | 0.033
L3i2 | 16185 | 1 | 0.033
L3i2 | 16192 | 2 | 0.067
L3i2 | 16264 | 1 | 0.033
L3i2 | 16270 | 1 | 0.033
L3i2 | 16278 | 4 | 0.133
L3i2 | 16293 | 1 | 0.033
L3i2 | 16294 | 1 | 0.033
L3i2 | 16295 | 2 | 0.067
L3i2 | 16298 | 1 | 0.033
L3i2 | 16390 | 1 | 0.033
L3i2 | 16532d | 1 | 0.033
L3i2 | 185 | 1 | 0.033
L3i2 | 200 | 4 | 0.133
L3i2 | 204 | 2 | 0.067
L3i2 | 207 | 2 | 0.067
L3i2 | 266 | 1 | 0.033
L3i2 | 3209 | 1 | 0.033
L3i2 | 4216 | 1 | 0.033
L3i2 | 455.3T | 1 | 0.033
L3i2 | 460 | 1 | 0.033
L3i2 | 4959 | 1 | 0.033
L3i2 | 504 | 10 | 0.333
L3i2 | 515-524d | 2 | 0.067
L3i2 | 5237 | 1 | 0.033
L3i2 | 529T | 1 | 0.033
L3i2 | 5366A | 1 | 0.033
L3i2 | 6120 | 1 | 0.033
L3i2 | 68 | 1 | 0.033
L3i2 | 7762 | 1 | 0.033
L3i2 | 7984 | 1 | 0.033
L3i2 | 8642C | 1 | 0.033
L3i2 | 8679 | 1 | 0.033
L3i2 | 8973T | 1 | 0.033
L3i2 | 9371 | 2 | 0.067
L3k | 10235 | 1 | 0.007
L3k | 10324 | 1 | 0.007
L3k | 12082 | 1 | 0.007
L3k | 12880 | 1 | 0.007
L3k | 13191 | 1 | 0.007
L3k | 14097 | 1 | 0.007
L3k | 14248 | 1 | 0.007
L3k | 152d | 1 | 0.007
L3k | 15758 | 1 | 0.007
L3k | 15924 | 1 | 0.007
L3k | 16129 | 1 | 0.007
L3k | 16193A | 1 | 0.007
L3k | 16214 | 5 | 0.037
L3k | 16311 | 1 | 0.007
L3k | 16319 | 1 | 0.007
L3k | 16344 | 1 | 0.007
L3k | 16361 | 1 | 0.007
L3k | 16528d | 1 | 0.007
L3k | 1888 | 1 | 0.007
L3k | 3426 | 1 | 0.007
L3k | 4164 | 1 | 0.007
L3k | 4967 | 1 | 0.007
L3k | 5263 | 1 | 0.007
L3k | 5360 | 1 | 0.007
L3k | 732d | 1 | 0.007
L3k | 8251 | 1 | 0.007
L3k | 8516 | 1 | 0.007
L3k1 | 10308 | 1 | 0.036
L3k1 | 11926 | 1 | 0.036
L3k1 | 13477 | 1 | 0.036
L3k1 | 14013 | 13 | 0.464
L3k1 | 15064C | 1 | 0.036
L3k1 | 152d | 4 | 0.143
L3k1 | 15454 | 2 | 0.071
L3k1 | 16075 | 3 | 0.107
L3k1 | 16093 | 15 | 0.536
L3k1 | 16111 | 1 | 0.036
L3k1 | 16129 | 3 | 0.107
L3k1 | 16209 | 1 | 0.036
L3k1 | 16286 | 1 | 0.036
L3k1 | 16311 | 1 | 0.036
L3k1 | 16357 | 2 | 0.071
L3k1 | 16390 | 1 | 0.036
L3k1 | 16391 | 1 | 0.036
L3k1 | 709 | 1 | 0.036
L3k1 | 7202 | 11 | 0.393
L3k1 | 7428 | 1 | 0.036
L3k1 | 7775 | 1 | 0.036
L3k1 | 8817T | 1 | 0.036
L3k1 | 9055 | 1 | 0.036
L3k1 | 9235 | 1 | 0.036
L3k1 | 961 | 1 | 0.036
L3x | 16086 | 4 | 0.082
L3x | 16150 | 3 | 0.061
L3x | 16255 | 1 | 0.02
L3x | 16260 | 15 | 0.306
L3x | 16265 | 1 | 0.02
L3x | 16311 | 1 | 0.02
L3x | 16362 | 5 | 0.102
L3x | 200 | 1 | 0.02
L3x1 | 12236 | 1 | 0.125
L3x1 | 12609 | 1 | 0.125
L3x1 | 15002T | 1 | 0.125
L3x1 | 15496 | 1 | 0.125
L3x1 | 15937 | 1 | 0.125
L3x1 | 16066 | 5 | 0.625
L3x1 | 16136 | 5 | 0.625

L3x1 | 16292 | 1 | 0.125
L3x1 | 16368 | 4 | 0.5
L3x1 | 513 | 1 | 0.125
L3x1 | 5773 | 1 | 0.125
L3x1 | 5999A | 1 | 0.125
L3x1+16311 | 10084 | 2 | 0.222
L3x1+16311 | 1018 | 1 | 0.111
L3x1+16311 | 12280 | 2 | 0.222
L3x1+16311 | 14344 | 2 | 0.222
L3x1+16311 | 15148 | 1 | 0.111
L3x1+16311 | 152 | 2 | 0.222
L3x1+16311 | 15346 | 1 | 0.111
L3x1+16311 | 15482 | 1 | 0.111
L3x1+16311 | 15900 | 1 | 0.111
L3x1+16311 | 16086 | 1 | 0.111
L3x1+16311 | 16171 | 1 | 0.111
L3x1+16311 | 16189 | 1 | 0.111
L3x1+16311 | 16231 | 1 | 0.111
L3x1+16311 | 16298 | 3 | 0.333
L3x1+16311 | 207 | 2 | 0.222
L3x1+16311 | 2232d | 1 | 0.111
L3x1+16311 | 3010 | 2 | 0.222
L3x1+16311 | 334 | 2 | 0.222
L3x1+16311 | 41 | 2 | 0.222
L3x1+16311 | 5663 | 2 | 0.222
L3x1+16311 | 6134 | 1 | 0.111
L3x1+16311 | 6788 | 2 | 0.222
L3x1+16311 | 709 | 2 | 0.222
L3x1+16311 | 735 | 2 | 0.222
L3x1+16311 | 7830 | 1 | 0.111
L3x1+16311 | 8699 | 1 | 0.111
L3x1+16311 | 9461 | 2 | 0.222
L3x1+16311 | 961 | 1 | 0.111
L3x1a | 16192 | 1 | 0.25
L3x1a1 | 10497 | 1 | 0.2
L3x1a1 | 12753 | 1 | 0.2
L3x1a1 | 14750 | 1 | 0.2
L3x1a1 | 14944 | 1 | 0.2
L3x1a1 | 15310 | 1 | 0.2
L3x1a1 | 15718 | 1 | 0.2
L3x1a1 | 15924 | 1 | 0.2
L3x1a1 | 16298 | 1 | 0.2
L3x1a1 | 16311 | 1 | 0.2
L3x1a1 | 204A | 1 | 0.2
L3x1a1 | 225 | 1 | 0.2
L3x1a1 | 2263 | 1 | 0.2
L3x1a1 | 4092 | 1 | 0.2
L3x1a1 | 4216 | 1 | 0.2
L3x1a1 | 4490 | 1 | 0.2
L3x1a1 | 487 | 1 | 0.2
L3x1a1 | 5775 | 1 | 0.2
L3x1a1 | 5899.1C | 1 | 0.2
L3x1a1 | 6962 | 1 | 0.2
L3x1a1 | 7372 | 1 | 0.2
L3x1a1 | 8119 | 1 | 0.2
L3x1a1 | 9966 | 1 | 0.2
L3x1a2 | 10G | 1 | 0.05
L3x1a2 | 1242 | 1 | 0.05
L3x1a2 | 12599 | 1 | 0.05
L3x1a2 | 13008 | 1 | 0.05
L3x1a2 | 1413 | 8 | 0.4
L3x1a2 | 15473 | 2 | 0.1
L3x1a2 | 16069 | 1 | 0.05
L3x1a2 | 16162 | 1 | 0.05
L3x1a2 | 16171 | 4 | 0.2
L3x1a2 | 207 | 15 | 0.75
L3x1a2 | 2120 | 1 | 0.05
L3x1a2 | 250G | 1 | 0.05
L3x1a2 | 2857 | 1 | 0.05
L3x1a2 | 36.1G | 1 | 0.05
L3x1a2 | 523-525d | 1 | 0.05
L3x1a2 | 573.1C | 1 | 0.05
L3x1a2 | 6 | 1 | 0.05
L3x1a2 | 7 | 1 | 0.05
L3x1a2 | 8251 | 3 | 0.15
L3x1b | 10646 | 1 | 0.111
L3x1b | 14070 | 2 | 0.222
L3x1b | 1420 | 1 | 0.111
L3x1b | 14750 | 1 | 0.111
L3x1b | 14935 | 1 | 0.111
L3x1b | 15080 | 1 | 0.111
L3x1b | 16086 | 1 | 0.111
L3x1b | 16093 | 1 | 0.111
L3x1b | 16111A | 1 | 0.111
L3x1b | 16185 | 1 | 0.111
L3x1b | 16189 | 1 | 0.111
L3x1b | 16320 | 1 | 0.111
L3x1b | 189 | 1 | 0.111
L3x1b | 195 | 1 | 0.111
L3x1b | 199 | 2 | 0.222
L3x1b | 200 | 1 | 0.111
L3x1b | 203 | 3 | 0.333
L3x1b | 207 | 1 | 0.111
L3x1b | 294 | 1 | 0.111
L3x1b | 3010 | 1 | 0.111
L3x1b | 490 | 1 | 0.111
L3x1b | 504 | 1 | 0.111
L3x1b | 5775 | 1 | 0.111
L3x1b | 6917 | 2 | 0.222
L3x1b | 709 | 1 | 0.111
L3x1b | 7673 | 1 | 0.111
L3x1b | 9554 | 2 | 0.222
L3x1b | 96A | 1 | 0.111
L3x2 | 146 | 1 | 0.333
L3x2 | 16532d | 2 | 0.667
L3x2 | 189 | 1 | 0.333
L3x2a | 10101 | 2 | 0.154
L3x2a | 10667 | 1 | 0.077
L3x2a | 10978 | 2 | 0.154
L3x2a | 11063 | 4 | 0.308
L3x2a | 12400 | 1 | 0.077

L3x2a | 14167 | 2 | 0.154
L3x2a | 14388 | 1 | 0.077
L3x2a | 152 | 1 | 0.077
L3x2a | 15637 | 1 | 0.077
L3x2a | 16173 | 1 | 0.077
L3x2a | 16243 | 1 | 0.077
L3x2a | 16264 | 1 | 0.077
L3x2a | 2239 | 1 | 0.077
L3x2a | 234 | 1 | 0.077
L3x2a | 3316 | 2 | 0.154
L3x2a | 3592 | 1 | 0.077
L3x2a | 4200 | 1 | 0.077
L3x2a | 4859 | 1 | 0.077
L3x2a | 5585 | 2 | 0.154
L3x2a | 6263 | 1 | 0.077
L3x2a | 6267 | 1 | 0.077
L3x2a | 8567 | 1 | 0.077
L3x2a | 8982 | 1 | 0.077
L3x2a | 9477 | 2 | 0.154
L3x2a1 | 12340 | 1 | 0.5
L3x2a1 | 14034 | 1 | 0.5
L3x2a1 | 16147 | 1 | 0.5
L3x2a1 | 16362 | 1 | 0.5
L3x2a1 | 249 | 1 | 0.5
L3x2a1 | 3621 | 1 | 0.5
L3x2a1 | 5894.1T | 1 | 0.5
L3x2a1 | 8041 | 1 | 0.5
L3x2a1 | 8197 | 1 | 0.5
L3x2a1 | 8928 | 1 | 0.5
L3x2a1a | 13174 | 1 | 0.2
L3x2a1a | 15314 | 1 | 0.2
L3x2a1a | 15338 | 1 | 0.2
L3x2a1a | 15928 | 1 | 0.2
L3x2a1a | 16354 | 1 | 0.2
L3x2a1a | 16494 | 1 | 0.2
L3x2a1a | 1822 | 1 | 0.2
L3x2a1a | 1842 | 2 | 0.4
L3x2a1a | 195 | 1 | 0.2
L3x2a1a | 4548 | 1 | 0.2
L3x2a1a | 5900C | 1 | 0.2
L3x2a1a | 5901.1C | 1 | 0.2
L3x2a1a | 6227 | 2 | 0.4
L3x2b | 125G | 1 | 0.091
L3x2b | 13368 | 1 | 0.091
L3x2b | 14569 | 1 | 0.091
L3x2b | 15519 | 4 | 0.364
L3x2b | 16093 | 1 | 0.091
L3x2b | 16189 | 2 | 0.182
L3x2b | 16194C | 1 | 0.091
L3x2b | 16213 | 2 | 0.182
L3x2b | 16235 | 2 | 0.182
L3x2b | 16261 | 5 | 0.455
L3x2b | 16309 | 2 | 0.182
L3x2b | 16311 | 1 | 0.091
L3x2b | 188 | 1 | 0.091
L3x2b | 189 | 1 | 0.091
L3x2b | 194 | 1 | 0.091
L3x2b | 235 | 1 | 0.091
L3x2b | 4104 | 1 | 0.091
L3x2b | 4230 | 1 | 0.091
L3x2b | 515-524d | 1 | 0.091
L3x2b | 5656 | 1 | 0.091
L3x2b | 7933 | 4 | 0.364
L3x2b | 813 | 1 | 0.091
L3x2b | 8158 | 4 | 0.364
L3x2b | 8476 | 1 | 0.091
L3x2b | 8584 | 1 | 0.091
L4 | 152 | 2 | 0.016
L4 | 16013C | 1 | 0.008
L4 | 16015d | 1 | 0.008
L4 | 16090 | 1 | 0.008
L4 | 16148 | 1 | 0.008
L4 | 16166 | 4 | 0.033
L4 | 16189 | 1 | 0.008
L4 | 16231 | 1 | 0.008
L4 | 16231G | 1 | 0.008
L4 | 16243 | 2 | 0.016
L4 | 16266A | 7 | 0.057
L4 | 16280C | 1 | 0.008
L4 | 16284 | 1 | 0.008
L4 | 16300 | 1 | 0.008
L4 | 16354 | 1 | 0.008
L4 | 16355 | 2 | 0.016
L4 | 16361C | 1 | 0.008
L4 | 16411d | 1 | 0.008
L4 | 385 | 1 | 0.008
L4 | 515-524d | 1 | 0.008
L4a | 16093 | 2 | 0.667
L4a | 16169 | 1 | 0.333
L4a | 16207C | 2 | 0.667
L4a | 325 | 1 | 0.333
L4a1 | 13043 | 1 | 0.059
L4a1 | 152 | 1 | 0.059
L4a1 | 15937d | 1 | 0.059
L4a1 | 16093 | 9 | 0.529
L4a1 | 16129 | 5 | 0.294
L4a1 | 16220 | 6 | 0.353
L4a1 | 16261 | 6 | 0.353
L4a1 | 16318 | 1 | 0.059
L4a1 | 16368 | 2 | 0.118
L4a1 | 6701 | 1 | 0.059
L4a1 | 8817 | 1 | 0.059
L4a1a | 10978 | 1 | 0.091
L4a1a | 11023 | 1 | 0.091
L4a1a | 11025 | 1 | 0.091
L4a1a | 11143 | 2 | 0.182
L4a1a | 11149 | 2 | 0.182
L4a1a | 11260 | 1 | 0.091
L4a1a | 1193 | 1 | 0.091
L4a1a | 12280 | 2 | 0.182
L4a1a | 12814 | 1 | 0.091

L4a1a | 12814T | 2 | 0.182
L4a1a | 13269 | 1 | 0.091
L4a1a | 13344 | 4 | 0.364
L4a1a | 13506 | 1 | 0.091
L4a1a | 15497 | 1 | 0.091
L4a1a | 15568 | 1 | 0.091
L4a1a | 16093 | 2 | 0.182
L4a1a | 16148 | 1 | 0.091
L4a1a | 16207C | 1 | 0.091
L4a1a | 16219 | 1 | 0.091
L4a1a | 16220 | 1 | 0.091
L4a1a | 16234 | 1 | 0.091
L4a1a | 16261 | 5 | 0.455
L4a1a | 16291 | 1 | 0.091
L4a1a | 16320 | 1 | 0.091
L4a1a | 16360 | 1 | 0.091
L4a1a | 185 | 1 | 0.091
L4a1a | 189 | 5 | 0.455
L4a1a | 210 | 1 | 0.091
L4a1a | 228 | 1 | 0.091
L4a1a | 2416 | 2 | 0.182
L4a1a | 3700 | 1 | 0.091
L4a1a | 3796 | 1 | 0.091
L4a1a | 4297 | 1 | 0.091
L4a1a | 4967 | 1 | 0.091
L4a1a | 6176 | 1 | 0.091
L4a1a | 6527 | 1 | 0.091
L4a1a | 8133 | 2 | 0.182
L4a1a | 8572 | 1 | 0.091
L4a1a | 8622 | 1 | 0.091
L4a1a | 8634 | 1 | 0.091
L4a1a | 8697 | 5 | 0.455
L4a1a | 921 | 1 | 0.091
L4a2 | 10032 | 4 | 0.571
L4a2 | 10320 | 1 | 0.143
L4a2 | 11365 | 1 | 0.143
L4a2 | 11593 | 1 | 0.143
L4a2 | 11851 | 1 | 0.143
L4a2 | 11969 | 1 | 0.143
L4a2 | 13194 | 1 | 0.143
L4a2 | 13392 | 4 | 0.571
L4a2 | 152 | 4 | 0.571
L4a2 | 15514 | 1 | 0.143
L4a2 | 15586 | 1 | 0.143
L4a2 | 15733A | 1 | 0.143
L4a2 | 15924 | 3 | 0.429
L4a2 | 16176 | 1 | 0.143
L4a2 | 16179 | 1 | 0.143
L4a2 | 16189 | 4 | 0.571
L4a2 | 182 | 1 | 0.143
L4a2 | 200 | 3 | 0.429
L4a2 | 2232.1A | 1 | 0.143
L4a2 | 247 | 1 | 0.143
L4a2 | 304 | 1 | 0.143
L4a2 | 3221 | 1 | 0.143
L4a2 | 4047 | 1 | 0.143
L4a2 | 5231 | 1 | 0.143
L4a2 | 5333 | 1 | 0.143
L4a2 | 5471 | 2 | 0.286
L4a2 | 5580 | 1 | 0.143
L4a2 | 5891.2C | 1 | 0.143
L4a2 | 6293 | 1 | 0.143
L4a2 | 64 | 1 | 0.143
L4a2 | 7789 | 1 | 0.143
L4a2 | 866 | 1 | 0.143
L4a2 | 8790 | 1 | 0.143
L4a2 | 8838 | 2 | 0.286
L4a2 | 9081 | 1 | 0.143
L4a2 | 9103 | 1 | 0.143
L4a2 | 9112 | 1 | 0.143
L4a2 | 9221 | 3 | 0.429
L4a2 | 9344 | 3 | 0.429
L4a2 | 9758 | 1 | 0.143
L4a2 | 9827 | 3 | 0.429
L4a2 | 9899 | 1 | 0.143
L4b | 152 | 1 | 0.01
L4b | 16166 | 4 | 0.038
L4b | 16243 | 1 | 0.01
L4b | 16355 | 1 | 0.01
L4b1 | 10754 | 1 | 0.01
L4b1 | 11176 | 1 | 0.01
L4b1 | 11466 | 1 | 0.01
L4b1 | 12028 | 1 | 0.01
L4b1 | 12855 | 1 | 0.01
L4b1 | 12988 | 7 | 0.069
L4b1 | 13928C | 1 | 0.01
L4b1 | 14128 | 1 | 0.01
L4b1 | 14494 | 1 | 0.01
L4b1 | 14778 | 1 | 0.01
L4b1 | 14793 | 7 | 0.069
L4b1 | 14971 | 1 | 0.01
L4b1 | 15007 | 1 | 0.01
L4b1 | 15924 | 1 | 0.01
L4b1 | 15946 | 1 | 0.01
L4b1 | 16093 | 1 | 0.01
L4b1 | 16129 | 1 | 0.01
L4b1 | 16166d | 1 | 0.01
L4b1 | 16192 | 1 | 0.01
L4b1 | 16249 | 1 | 0.01
L4b1 | 16256 | 1 | 0.01
L4b1 | 16274 | 1 | 0.01
L4b1 | 16278 | 1 | 0.01
L4b1 | 16293 | 2 | 0.02
L4b1 | 16381 | 1 | 0.01
L4b1 | 16400 | 1 | 0.01
L4b1 | 1914 | 7 | 0.069
L4b1 | 195 | 2 | 0.02
L4b1 | 199 | 1 | 0.01
L4b1 | 207 | 1 | 0.01
L4b1 | 279 | 1 | 0.01
L4b1 | 316C | 1 | 0.01

L4b1 | 328 | 1 | 0.01
L4b1 | 3316 | 1 | 0.01
L4b1 | 356.1C | 1 | 0.01
L4b1 | 3915 | 2 | 0.02
L4b1 | 4137 | 7 | 0.069
L4b1 | 4248 | 1 | 0.01
L4b1 | 4394 | 1 | 0.01
L4b1 | 488 | 1 | 0.01
L4b1 | 515-524d | 1 | 0.01
L4b1 | 5318 | 1 | 0.01
L4b1 | 5999 | 1 | 0.01
L4b1 | 71.1G | 1 | 0.01
L4b1 | 7389 | 7 | 0.069
L4b1 | 7849 | 7 | 0.069
L4b1 | 7909 | 1 | 0.01
L4b1 | 8289.1CCCCCTCTA | 1 | 0.01
L4b1 | 8392 | 7 | 0.069
L4b1 | 8519 | 1 | 0.01
L4b1 | 8577 | 1 | 0.01
L4b1 | 8587 | 1 | 0.01
L4b1 | 8928 | 7 | 0.069
L4b1 | 9168 | 7 | 0.069
L4b1a | 10328 | 1 | 0.04
L4b1a | 10400 | 1 | 0.04
L4b1a | 10688 | 2 | 0.08
L4b1a | 11914 | 1 | 0.04
L4b1a | 12780 | 1 | 0.04
L4b1a | 13203 | 1 | 0.04
L4b1a | 1341 | 1 | 0.04
L4b1a | 13518 | 8 | 0.32
L4b1a | 14259 | 1 | 0.04
L4b1a | 14629 | 1 | 0.04
L4b1a | 14791 | 2 | 0.08
L4b1a | 14935 | 12 | 0.48
L4b1a | 15043 | 1 | 0.04
L4b1a | 152 | 4 | 0.16
L4b1a | 15344 | 11 | 0.44
L4b1a | 16093 | 1 | 0.04
L4b1a | 16111 | 1 | 0.04
L4b1a | 16111A | 1 | 0.04
L4b1a | 16153 | 2 | 0.08
L4b1a | 16207 | 1 | 0.04
L4b1a | 16295 | 1 | 0.04
L4b1a | 16365 | 1 | 0.04
L4b1a | 204A | 1 | 0.04
L4b1a | 207 | 2 | 0.08
L4b1a | 309d | 1 | 0.04
L4b1a | 310 | 1 | 0.04
L4b1a | 3693C | 8 | 0.32
L4b1a | 496 | 2 | 0.08
L4b1a | 507 | 1 | 0.04
L4b1a | 513-516d | 1 | 0.04
L4b1a | 513-522d | 1 | 0.04
L4b1a | 5894 | 1 | 0.04
L4b1a | 593 | 1 | 0.04
L4b1a | 6710 | 1 | 0.04
L4b1a | 7166 | 3 | 0.12
L4b1a | 789 | 1 | 0.04
L4b1a | 789A | 10 | 0.4
L4b1a | 8206 | 7 | 0.28
L4b1a | 8289.1CCCCCTCTA | 1 | 0.04
L4b1a | 8588 | 1 | 0.04
L4b1a | 8937 | 2 | 0.08
L4b1a | 9010 | 1 | 0.04
L4b1a | 980 | 1 | 0.04
L4b2 | 16025 | 1 | 0.056
L4b2 | 16093 | 2 | 0.111
L4b2 | 16146 | 1 | 0.056
L4b2 | 16147 | 1 | 0.056
L4b2 | 16172 | 1 | 0.056
L4b2 | 16189 | 9 | 0.5
L4b2 | 16192 | 5 | 0.278
L4b2 | 16193 | 1 | 0.056
L4b2 | 16230 | 1 | 0.056
L4b2 | 16261 | 1 | 0.056
L4b2 | 16294 | 4 | 0.222
L4b2 | 16300 | 1 | 0.056
L4b2 | 16344 | 9 | 0.5
L4b2 | 16507G | 1 | 0.056
L4b2 | 182 | 4 | 0.222
L4b2 | 183 | 4 | 0.222
L4b2 | 247 | 1 | 0.056
L4b2 | 249d | 3 | 0.167
L4b2 | 257 | 3 | 0.167
L4b2 | 459.1C | 1 | 0.056
L4b2 | 497 | 1 | 0.056
L4b2 | 513 | 4 | 0.222
L4b2 | 515-524d | 1 | 0.056
L4b2 | 527d | 1 | 0.056
L4b2 | 535 | 1 | 0.056
L4b2 | 93 | 1 | 0.056
L4b2a | 10834G | 1 | 0.048
L4b2a | 11431 | 1 | 0.048
L4b2a | 11653T | 8 | 0.381
L4b2a | 11944 | 1 | 0.048
L4b2a | 12173 | 2 | 0.095
L4b2a | 12681 | 2 | 0.095
L4b2a | 13272 | 1 | 0.048
L4b2a | 13834 | 1 | 0.048
L4b2a | 14257 | 8 | 0.381
L4b2a | 14470 | 8 | 0.381
L4b2a | 14569 | 1 | 0.048
L4b2a | 14573 | 1 | 0.048
L4b2a | 150 | 9 | 0.429
L4b2a | 15139 | 1 | 0.048
L4b2a | 152 | 3 | 0.143
L4b2a | 15217 | 2 | 0.095
L4b2a | 16025 | 1 | 0.048
L4b2a | 16075 | 9 | 0.429
L4b2a | 16233 | 1 | 0.048
L4b2a | 16271 | 1 | 0.048

L4b2a | 16274 | 17 | 0.81
L4b2a | 16301 | 2 | 0.095
L4b2a | 16367C | 1 | 0.048
L4b2a | 16368 | 6 | 0.286
L4b2a | 263T | 1 | 0.048
L4b2a | 2863 | 2 | 0.095
L4b2a | 309d | 1 | 0.048
L4b2a | 33.1C | 1 | 0.048
L4b2a | 3333 | 2 | 0.095
L4b2a | 3531 | 8 | 0.381
L4b2a | 368 | 2 | 0.095
L4b2a | 44.1C | 2 | 0.095
L4b2a | 455.1T | 2 | 0.095
L4b2a | 4823 | 1 | 0.048
L4b2a | 4838 | 1 | 0.048
L4b2a | 513 | 2 | 0.095
L4b2a | 5162 | 1 | 0.048
L4b2a | 525d | 1 | 0.048
L4b2a | 5580 | 9 | 0.429
L4b2a | 5746 | 9 | 0.429
L4b2a | 7129 | 2 | 0.095
L4b2a | 7844 | 8 | 0.381
L4b2a | 8843 | 2 | 0.095
L4b2a | 9123 | 2 | 0.095
L4b2a | 9287 | 2 | 0.095
L4b2a | 980 | 1 | 0.048
L4b2a1 | 10750 | 1 | 0.111
L4b2a1 | 12295 | 1 | 0.111
L4b2a1 | 12891 | 2 | 0.222
L4b2a1 | 14182 | 1 | 0.111
L4b2a1 | 14861 | 1 | 0.111
L4b2a1 | 16075 | 1 | 0.111
L4b2a1 | 16086 | 1 | 0.111
L4b2a1 | 16093 | 1 | 0.111
L4b2a1 | 16104 | 1 | 0.111
L4b2a1 | 16184 | 1 | 0.111
L4b2a1 | 16192 | 1 | 0.111
L4b2a1 | 16201 | 1 | 0.111
L4b2a1 | 16255 | 1 | 0.111
L4b2a1 | 16293 | 1 | 0.111
L4b2a1 | 16532d | 2 | 0.222
L4b2a1 | 573.2C | 1 | 0.111
L4b2a1 | 648 | 1 | 0.111
L4b2a1 | 723 | 1 | 0.111
L4b2a1 | 7241 | 2 | 0.222
L4b2a2 | 10381 | 1 | 0.024
L4b2a2 | 10646C | 1 | 0.024
L4b2a2 | 10652G | 1 | 0.024
L4b2a2 | 11151 | 1 | 0.024
L4b2a2 | 11179 | 1 | 0.024
L4b2a2 | 11236G | 2 | 0.049
L4b2a2 | 11608 | 1 | 0.024
L4b2a2 | 11914 | 1 | 0.024
L4b2a2 | 12234 | 1 | 0.024
L4b2a2 | 12240 | 1 | 0.024
L4b2a2 | 12249T | 1 | 0.024
L4b2a2 | 12300T | 1 | 0.024
L4b2a2 | 12341 | 8 | 0.195
L4b2a2 | 12664A | 1 | 0.024
L4b2a2 | 12664T | 1 | 0.024
L4b2a2 | 12684 | 1 | 0.024
L4b2a2 | 13105 | 2 | 0.049
L4b2a2 | 13224 | 1 | 0.024
L4b2a2 | 13602 | 1 | 0.024
L4b2a2 | 13695 | 1 | 0.024
L4b2a2 | 14016 | 5 | 0.122
L4b2a2 | 14179 | 9 | 0.22
L4b2a2 | 14182 | 1 | 0.024
L4b2a2 | 14221 | 1 | 0.024
L4b2a2 | 14767 | 1 | 0.024
L4b2a2 | 15019 | 3 | 0.073
L4b2a2 | 152 | 2 | 0.049
L4b2a2 | 15298 | 1 | 0.024
L4b2a2 | 15448 | 3 | 0.073
L4b2a2 | 15687C | 1 | 0.024
L4b2a2 | 15712 | 1 | 0.024
L4b2a2 | 15778 | 1 | 0.024
L4b2a2 | 15784 | 1 | 0.024
L4b2a2 | 15851C | 1 | 0.024
L4b2a2 | 15924 | 3 | 0.073
L4b2a2 | 15930 | 2 | 0.049
L4b2a2 | 15970 | 1 | 0.024
L4b2a2 | 16004 | 1 | 0.024
L4b2a2 | 16071 | 11 | 0.268
L4b2a2 | 16093 | 1 | 0.024
L4b2a2 | 16111 | 1 | 0.024
L4b2a2 | 16168 | 1 | 0.024
L4b2a2 | 16189 | 1 | 0.024
L4b2a2 | 16193 | 1 | 0.024
L4b2a2 | 16249 | 1 | 0.024
L4b2a2 | 16278 | 2 | 0.049
L4b2a2 | 16294 | 2 | 0.049
L4b2a2 | 16319 | 3 | 0.073
L4b2a2 | 16327 | 1 | 0.024
L4b2a2 | 16344 | 1 | 0.024
L4b2a2 | 16532d | 4 | 0.098
L4b2a2 | 16542G | 1 | 0.024
L4b2a2 | 16547 | 1 | 0.024
L4b2a2 | 182 | 6 | 0.146
L4b2a2 | 183 | 6 | 0.146
L4b2a2 | 185 | 2 | 0.049
L4b2a2 | 189 | 5 | 0.122
L4b2a2 | 2010 | 2 | 0.049
L4b2a2 | 2232.1A | 1 | 0.024
L4b2a2 | 291.1A | 1 | 0.024
L4b2a2 | 3396 | 1 | 0.024
L4b2a2 | 3546 | 1 | 0.024
L4b2a2 | 3766 | 5 | 0.122
L4b2a2 | 3909 | 5 | 0.122
L4b2a2 | 398 | 1 | 0.024
L4b2a2 | 410 | 1 | 0.024

L4b2a2 | 4227 | 1 | 0.024
L4b2a2 | 4232 | 1 | 0.024
L4b2a2 | 4491 | 1 | 0.024
L4b2a2 | 456 | 1 | 0.024
L4b2a2 | 4772 | 1 | 0.024
L4b2a2 | 4820 | 1 | 0.024
L4b2a2 | 5048 | 3 | 0.073
L4b2a2 | 513 | 3 | 0.073
L4b2a2 | 5243 | 5 | 0.122
L4b2a2 | 5558 | 1 | 0.024
L4b2a2 | 5585 | 1 | 0.024
L4b2a2 | 5894 | 1 | 0.024
L4b2a2 | 5899.1C | 1 | 0.024
L4b2a2 | 5910 | 3 | 0.073
L4b2a2 | 606 | 1 | 0.024
L4b2a2 | 6227 | 1 | 0.024
L4b2a2 | 6329 | 6 | 0.146
L4b2a2 | 6366 | 2 | 0.049
L4b2a2 | 6680 | 3 | 0.073
L4b2a2 | 6719 | 2 | 0.049
L4b2a2 | 6956 | 2 | 0.049
L4b2a2 | 7278 | 1 | 0.024
L4b2a2 | 742 | 1 | 0.024
L4b2a2 | 7664 | 1 | 0.024
L4b2a2 | 7987 | 1 | 0.024
L4b2a2 | 8276d | 1 | 0.024
L4b2a2 | 8292 | 1 | 0.024
L4b2a2 | 8435 | 1 | 0.024
L4b2a2 | 8485d | 1 | 0.024
L4b2a2 | 8574 | 1 | 0.024
L4b2a2 | 9101 | 1 | 0.024
L4b2a2 | 9145 | 1 | 0.024
L4b2a2 | 9821 | 1 | 0.024
L4b2a2a | 10304 | 1 | 0.01
L4b2a2a | 10348 | 1 | 0.01
L4b2a2a | 12348 | 1 | 0.01
L4b2a2a | 12361 | 1 | 0.01
L4b2a2a | 12406 | 1 | 0.01
L4b2a2a | 13029 | 1 | 0.01
L4b2a2a | 13708 | 4 | 0.042
L4b2a2a | 14319 | 1 | 0.01
L4b2a2a | 14422 | 4 | 0.042
L4b2a2a | 15067 | 1 | 0.01
L4b2a2a | 152 | 1 | 0.01
L4b2a2a | 15203 | 1 | 0.01
L4b2a2a | 15299 | 1 | 0.01
L4b2a2a | 15530 | 1 | 0.01
L4b2a2a | 15670 | 1 | 0.01
L4b2a2a | 16024 | 7 | 0.073
L4b2a2a | 16025 | 14 | 0.146
L4b2a2a | 16025d | 1 | 0.01
L4b2a2a | 16025G | 1 | 0.01
L4b2a2a | 16147 | 1 | 0.01
L4b2a2a | 16170 | 1 | 0.01
L4b2a2a | 16189 | 1 | 0.01
L4b2a2a | 16209 | 1 | 0.01
L4b2a2a | 16234 | 1 | 0.01
L4b2a2a | 16278 | 45 | 0.469
L4b2a2a | 16294 | 45 | 0.469
L4b2a2a | 16546 | 1 | 0.01
L4b2a2a | 16560G | 1 | 0.01
L4b2a2a | 16564C | 1 | 0.01
L4b2a2a | 195 | 1 | 0.01
L4b2a2a | 199 | 18 | 0.188
L4b2a2a | 214T | 1 | 0.01
L4b2a2a | 224A | 1 | 0.01
L4b2a2a | 224G | 2 | 0.021
L4b2a2a | 226G | 3 | 0.031
L4b2a2a | 246 | 1 | 0.01
L4b2a2a | 247T | 1 | 0.01
L4b2a2a | 257 | 4 | 0.042
L4b2a2a | 263T | 1 | 0.01
L4b2a2a | 291.1A | 1 | 0.01
L4b2a2a | 3213 | 1 | 0.01
L4b2a2a | 340 | 1 | 0.01
L4b2a2a | 349A | 1 | 0.01
L4b2a2a | 36.1G | 8 | 0.083
L4b2a2a | 3666 | 1 | 0.01
L4b2a2a | 397T | 1 | 0.01
L4b2a2a | 42.1T | 2 | 0.021
L4b2a2a | 4219 | 1 | 0.01
L4b2a2a | 430G | 3 | 0.031
L4b2a2a | 438A | 1 | 0.01
L4b2a2a | 44.1C | 1 | 0.01
L4b2a2a | 456 | 1 | 0.01
L4b2a2a | 460 | 1 | 0.01
L4b2a2a | 4639 | 1 | 0.01
L4b2a2a | 483 | 1 | 0.01
L4b2a2a | 483A | 1 | 0.01
L4b2a2a | 49T | 2 | 0.021
L4b2a2a | 525d | 6 | 0.062
L4b2a2a | 525G | 1 | 0.01
L4b2a2a | 527d | 1 | 0.01
L4b2a2a | 527G | 7 | 0.073
L4b2a2a | 53T | 2 | 0.021
L4b2a2a | 573.1C | 1 | 0.01
L4b2a2a | 5824 | 1 | 0.01
L4b2a2a | 64d | 1 | 0.01
L4b2a2a | 64G | 1 | 0.01
L4b2a2a | 66C | 1 | 0.01
L4b2a2a | 68C | 1 | 0.01
L4b2a2a | 6A | 1 | 0.01
L4b2a2a | 75T | 1 | 0.01
L4b2a2a | 79 | 2 | 0.021
L4b2a2a | 81 | 2 | 0.021
L4b2a2a | 8128 | 1 | 0.01
L4b2a2a | 8774 | 1 | 0.01
L4b2a2a | 9 | 1 | 0.01
L4b2a2a | 9422 | 1 | 0.01
L4b2a2a | 9667 | 1 | 0.01
L4b2a2b | 10535 | 2 | 0.118

L4b2a2b | 10589 | 1 | 0.059
L4b2a2b | 10978 | 1 | 0.059
L4b2a2b | 11608 | 1 | 0.059
L4b2a2b | 12396 | 1 | 0.059
L4b2a2b | 12630 | 1 | 0.059
L4b2a2b | 13759 | 1 | 0.059
L4b2a2b | 14572 | 2 | 0.118
L4b2a2b | 152 | 1 | 0.059
L4b2a2b | 15204 | 1 | 0.059
L4b2a2b | 15314 | 1 | 0.059
L4b2a2b | 15355 | 1 | 0.059
L4b2a2b | 15712 | 1 | 0.059
L4b2a2b | 16183 | 2 | 0.118
L4b2a2b | 16189 | 1 | 0.059
L4b2a2b | 16261 | 2 | 0.118
L4b2a2b | 16301 | 2 | 0.118
L4b2a2b | 16532d | 8 | 0.471
L4b2a2b | 1842 | 2 | 0.118
L4b2a2b | 189 | 1 | 0.059
L4b2a2b | 1943 | 1 | 0.059
L4b2a2b | 210 | 1 | 0.059
L4b2a2b | 228 | 1 | 0.059
L4b2a2b | 257 | 3 | 0.176
L4b2a2b | 294 | 2 | 0.118
L4b2a2b | 356.1C | 1 | 0.059
L4b2a2b | 398 | 1 | 0.059
L4b2a2b | 401d | 1 | 0.059
L4b2a2b | 513 | 2 | 0.118
L4b2a2b | 515-524d | 1 | 0.059
L4b2a2b | 5573 | 1 | 0.059
L4b2a2b | 742 | 1 | 0.059
L4b2a2b | 7976 | 1 | 0.059
L4b2a2b | 9371 | 1 | 0.059
L4b2a2c | 10325 | 2 | 0.042
L4b2a2c | 10532 | 4 | 0.083
L4b2a2c | 10589 | 1 | 0.021
L4b2a2c | 10724 | 1 | 0.021
L4b2a2c | 11698G | 1 | 0.021
L4b2a2c | 11854 | 1 | 0.021
L4b2a2c | 13443 | 1 | 0.021
L4b2a2c | 13533T | 1 | 0.021
L4b2a2c | 152 | 15 | 0.312
L4b2a2c | 15758 | 1 | 0.021
L4b2a2c | 16004 | 1 | 0.021
L4b2a2c | 16162 | 12 | 0.25
L4b2a2c | 16168 | 1 | 0.021
L4b2a2c | 16169 | 6 | 0.125
L4b2a2c | 16180 | 2 | 0.042
L4b2a2c | 16192 | 1 | 0.021
L4b2a2c | 16209 | 4 | 0.083
L4b2a2c | 16249 | 1 | 0.021
L4b2a2c | 16266 | 1 | 0.021
L4b2a2c | 16295 | 1 | 0.021
L4b2a2c | 16327 | 15 | 0.312
L4b2a2c | 16356 | 12 | 0.25
L4b2a2c | 16391 | 1 | 0.021
L4b2a2c | 16488 | 1 | 0.021
L4b2a2c | 16532d | 7 | 0.146
L4b2a2c | 1832 | 1 | 0.021
L4b2a2c | 189 | 2 | 0.042
L4b2a2c | 195 | 1 | 0.021
L4b2a2c | 2463d | 1 | 0.021
L4b2a2c | 3242C | 1 | 0.021
L4b2a2c | 3394 | 1 | 0.021
L4b2a2c | 3435 | 3 | 0.062
L4b2a2c | 4021 | 4 | 0.083
L4b2a2c | 5483G | 14 | 0.292
L4b2a2c | 5553 | 1 | 0.021
L4b2a2c | 7455 | 3 | 0.062
L4b2a2c | 8071C | 4 | 0.083
L4b2a2c | 8119 | 4 | 0.083
L4b2a2c | 8248 | 4 | 0.083
L4b2a2c | 8487 | 11 | 0.229
L4b2a2c | 9464 | 1 | 0.021
L4b2a2c | 9524 | 1 | 0.021
L4b2b | 10389 | 4 | 0.133
L4b2b | 10G | 1 | 0.033
L4b2b | 11204 | 1 | 0.033
L4b2b | 11914 | 6 | 0.2
L4b2b | 12354 | 5 | 0.167
L4b2b | 125G | 1 | 0.033
L4b2b | 12903 | 5 | 0.167
L4b2b | 12950 | 1 | 0.033
L4b2b | 14267 | 1 | 0.033
L4b2b | 150 | 8 | 0.267
L4b2b | 152 | 16 | 0.533
L4b2b | 15217 | 6 | 0.2
L4b2b | 15883 | 1 | 0.033
L4b2b | 15930 | 1 | 0.033
L4b2b | 16093A | 6 | 0.2
L4b2b | 16093G | 9 | 0.3
L4b2b | 16111 | 3 | 0.1
L4b2b | 16153 | 1 | 0.033
L4b2b | 16184 | 1 | 0.033
L4b2b | 16189 | 1 | 0.033
L4b2b | 16221 | 4 | 0.133
L4b2b | 16248 | 1 | 0.033
L4b2b | 16261 | 1 | 0.033
L4b2b | 16265 | 1 | 0.033
L4b2b | 16287A | 15 | 0.5
L4b2b | 16287G | 1 | 0.033
L4b2b | 16293C | 1 | 0.033
L4b2b | 16301 | 23 | 0.767
L4b2b | 16320 | 4 | 0.133
L4b2b | 16356 | 6 | 0.2
L4b2b | 16396A | 1 | 0.033
L4b2b | 16397A | 1 | 0.033
L4b2b | 16484-16489d | 1 | 0.033
L4b2b | 16560G | 1 | 0.033
L4b2b | 16562 | 1 | 0.033
L4b2b | 198 | 7 | 0.233

L4b2b | 200 | 12 | 0.4
L4b2b | 207 | 4 | 0.133
L4b2b | 214 | 1 | 0.033
L4b2b | 2163 | 1 | 0.033
L4b2b | 3945 | 1 | 0.033
L4b2b | 3970 | 1 | 0.033
L4b2b | 398 | 2 | 0.067
L4b2b | 4500 | 1 | 0.033
L4b2b | 451T | 1 | 0.033
L4b2b | 456 | 3 | 0.1
L4b2b | 460 | 1 | 0.033
L4b2b | 47C | 1 | 0.033
L4b2b | 4988 | 1 | 0.033
L4b2b | 513 | 14 | 0.467
L4b2b | 513-522d | 4 | 0.133
L4b2b | 513d | 1 | 0.033
L4b2b | 525d | 1 | 0.033
L4b2b | 527G | 1 | 0.033
L4b2b | 573.6C | 1 | 0.033
L4b2b | 6878 | 1 | 0.033
L4b2b | 8227 | 6 | 0.2
L4b2b | 8966 | 1 | 0.033
L4b2b1 | 12397 | 1 | 0.04
L4b2b1 | 14764 | 1 | 0.04
L4b2b1 | 150 | 1 | 0.04
L4b2b1 | 152 | 18 | 0.72
L4b2b1 | 15759 | 2 | 0.08
L4b2b1 | 15924 | 2 | 0.08
L4b2b1 | 16051 | 16 | 0.64
L4b2b1 | 16086 | 1 | 0.04
L4b2b1 | 16114 | 18 | 0.72
L4b2b1 | 16188 | 1 | 0.04
L4b2b1 | 16189 | 18 | 0.72
L4b2b1 | 16192 | 11 | 0.44
L4b2b1 | 16193 | 2 | 0.08
L4b2b1 | 16209 | 1 | 0.04
L4b2b1 | 16224 | 4 | 0.16
L4b2b1 | 16234 | 2 | 0.08
L4b2b1 | 16274 | 5 | 0.2
L4b2b1 | 16292 | 5 | 0.2
L4b2b1 | 16294 | 4 | 0.16
L4b2b1 | 16335 | 1 | 0.04
L4b2b1 | 1709 | 1 | 0.04
L4b2b1 | 1888 | 1 | 0.04
L4b2b1 | 226G | 1 | 0.04
L4b2b1 | 2463d | 1 | 0.04
L4b2b1 | 291.1A | 5 | 0.2
L4b2b1 | 3398 | 1 | 0.04
L4b2b1 | 4973G | 1 | 0.04
L4b2b1 | 5134 | 2 | 0.08
L4b2b1 | 5156 | 1 | 0.04
L4b2b1 | 5483 | 1 | 0.04
L4b2b1 | 7471d | 1 | 0.04
L4b2b1 | 7699 | 1 | 0.04
L4b2b1 | 7897 | 1 | 0.04
L4b2b1 | 8251 | 1 | 0.04
L4b2b1 | 8292 | 2 | 0.08
L4b2b1 | 8343 | 2 | 0.08
L4b2b1 | 9758 | 1 | 0.04
L5 | 152 | 1 | 0.333
L5 | 16532d | 4 | 1.333
L5 | 196d | 3 | 1
L5a1 | 12302 | 2 | 0.182
L5a1 | 1452 | 2 | 0.182
L5a1 | 146 | 4 | 0.364
L5a1 | 16086 | 1 | 0.091
L5a1 | 16093 | 1 | 0.091
L5a1 | 16186 | 8 | 0.727
L5a1 | 16234 | 1 | 0.091
L5a1 | 16265T | 1 | 0.091
L5a1 | 16291 | 4 | 0.364
L5a1 | 16T | 4 | 0.364
L5a1 | 2244 | 1 | 0.091
L5a1 | 310 | 1 | 0.091
L5a1 | 5074 | 2 | 0.182
L5a1 | 6995 | 2 | 0.182
L5a1 | 9899 | 3 | 0.273
L5a1a | 10310 | 1 | 0.083
L5a1a | 10463 | 1 | 0.083
L5a1a | 11015 | 1 | 0.083
L5a1a | 14374 | 1 | 0.083
L5a1a | 14470 | 2 | 0.167
L5a1a | 14861 | 1 | 0.083
L5a1a | 16093 | 1 | 0.083
L5a1a | 16183d | 1 | 0.083
L5a1a | 16186 | 3 | 0.25
L5a1a | 16188 | 3 | 0.25
L5a1a | 16193d | 6 | 0.5
L5a1a | 16274 | 1 | 0.083
L5a1a | 16354 | 1 | 0.083
L5a1a | 16399 | 1 | 0.083
L5a1a | 189 | 1 | 0.083
L5a1a | 5029 | 1 | 0.083
L5a1a | 5783 | 2 | 0.167
L5a1a | 676 | 1 | 0.083
L5a1a | 7873 | 1 | 0.083
L5a1a | 8697 | 2 | 0.167
L5a1b | 13224 | 2 | 0.118
L5a1b | 14470 | 1 | 0.059
L5a1b | 151 | 1 | 0.059
L5a1b | 15734 | 1 | 0.059
L5a1b | 16086 | 2 | 0.118
L5a1b | 16093 | 5 | 0.294
L5a1b | 16193d | 1 | 0.059
L5a1b | 16234 | 1 | 0.059
L5a1b | 16265T | 1 | 0.059
L5a1b | 204 | 3 | 0.176
L5a1b | 3438 | 3 | 0.176
L5a1b | 515-524d | 4 | 0.235
L5a1b | 537 | 3 | 0.176
L5a1b | 5375 | 1 | 0.059

L5a1c | 10006 | 1 | 0.077
L5a1c | 11770 | 1 | 0.077
L5a1c | 12303 | 7 | 0.538
L5a1c | 12780 | 4 | 0.308
L5a1c | 12873 | 1 | 0.077
L5a1c | 13752A | 7 | 0.538
L5a1c | 151 | 3 | 0.231
L5a1c | 15929 | 1 | 0.077
L5a1c | 16093 | 2 | 0.154
L5a1c | 16183d | 3 | 0.231
L5a1c | 16185 | 1 | 0.077
L5a1c | 16186 | 1 | 0.077
L5a1c | 16193d | 1 | 0.077
L5a1c | 1850 | 1 | 0.077
L5a1c | 310 | 1 | 0.077
L5a1c | 5978 | 4 | 0.308
L5a1c | 6527 | 1 | 0.077
L5a1c | 7960 | 1 | 0.077
L5a1c | 8856 | 1 | 0.077
L5a2 | 10583 | 5 | 0.147
L5a2 | 10750 | 1 | 0.029
L5a2 | 11182 | 4 | 0.118
L5a2 | 11318 | 1 | 0.029
L5a2 | 11356 | 1 | 0.029
L5a2 | 11362 | 1 | 0.029
L5a2 | 12022 | 1 | 0.029
L5a2 | 12084 | 6 | 0.176
L5a2 | 1211 | 6 | 0.176
L5a2 | 12438 | 1 | 0.029
L5a2 | 125G | 2 | 0.059
L5a2 | 12654 | 1 | 0.029
L5a2 | 13162 | 1 | 0.029
L5a2 | 13813 | 1 | 0.029
L5a2 | 13819 | 1 | 0.029
L5a2 | 14690 | 1 | 0.029
L5a2 | 15493A | 1 | 0.029
L5a2 | 1555 | 4 | 0.118
L5a2 | 15672 | 1 | 0.029
L5a2 | 15826 | 11 | 0.324
L5a2 | 15930 | 1 | 0.029
L5a2 | 16093 | 3 | 0.088
L5a2 | 16183d | 19 | 0.559
L5a2 | 16185 | 3 | 0.088
L5a2 | 16186 | 9 | 0.265
L5a2 | 16188 | 5 | 0.147
L5a2 | 16192 | 3 | 0.088
L5a2 | 16193d | 6 | 0.176
L5a2 | 16265C | 1 | 0.029
L5a2 | 16274 | 4 | 0.118
L5a2 | 16293 | 6 | 0.176
L5a2 | 16298 | 1 | 0.029
L5a2 | 16343 | 1 | 0.029
L5a2 | 16399 | 4 | 0.118
L5a2 | 1766 | 1 | 0.029
L5a2 | 2361 | 11 | 0.324
L5a2 | 3010 | 1 | 0.029
L5a2 | 309d | 7 | 0.206
L5a2 | 310 | 1 | 0.029
L5a2 | 3199A | 6 | 0.176
L5a2 | 3336 | 1 | 0.029
L5a2 | 444 | 2 | 0.059
L5a2 | 4580 | 1 | 0.029
L5a2 | 464 | 4 | 0.118
L5a2 | 499 | 5 | 0.147
L5a2 | 5321 | 10 | 0.294
L5a2 | 5460 | 4 | 0.118
L5a2 | 5580 | 1 | 0.029
L5a2 | 5582 | 1 | 0.029
L5a2 | 573.1C | 4 | 0.118
L5a2 | 573.2C | 1 | 0.029
L5a2 | 5746 | 3 | 0.088
L5a2 | 5892 | 1 | 0.029
L5a2 | 5918 | 1 | 0.029
L5a2 | 678 | 6 | 0.176
L5a2 | 8296 | 1 | 0.029
L5a2 | 8392 | 1 | 0.029
L5a2 | 8473 | 1 | 0.029
L5a2 | 8584 | 1 | 0.029
L5a2 | 8588 | 1 | 0.029
L5a2 | 8745 | 1 | 0.029
L5a2 | 8812 | 6 | 0.176
L5a2 | 9303 | 1 | 0.029
L5a2 | 980 | 1 | 0.029
L5a2 | 9884 | 6 | 0.176
L5a2 | 9893 | 5 | 0.147
L5a2 | 9966 | 4 | 0.118
L5b | 16532d | 1 | 0.5
L5b | 200 | 1 | 0.5
L5b1 | 10045 | 2 | 0.095
L5b1 | 10364 | 1 | 0.048
L5b1 | 11287 | 2 | 0.095
L5b1 | 11840 | 3 | 0.143
L5b1 | 12406 | 2 | 0.095
L5b1 | 15148 | 3 | 0.143
L5b1 | 152 | 2 | 0.095
L5b1 | 15442 | 2 | 0.095
L5b1 | 15827 | 1 | 0.048
L5b1 | 15927 | 11 | 0.524
L5b1 | 16172 | 1 | 0.048
L5b1 | 16231 | 1 | 0.048
L5b1 | 16233 | 17 | 0.81
L5b1 | 16256 | 2 | 0.095
L5b1 | 16265C | 2 | 0.095
L5b1 | 16311 | 2 | 0.095
L5b1 | 16325 | 1 | 0.048
L5b1 | 16368 | 2 | 0.095
L5b1 | 16399C | 1 | 0.048
L5b1 | 182 | 2 | 0.095
L5b1 | 189 | 5 | 0.238
L5b1 | 1969C | 1 | 0.048
L5b1 | 2380 | 2 | 0.095

L5b1 | 310 | 2 | 0.095
L5b1 | 315.2C | 2 | 0.095
L5b1 | 4233 | 2 | 0.095
L5b1 | 4529 | 2 | 0.095
L5b1 | 4907 | 2 | 0.095
L5b1 | 501 | 2 | 0.095
L5b1 | 5147 | 3 | 0.143
L5b1 | 515-524d | 7 | 0.333
L5b1 | 6173A | 2 | 0.095
L5b1 | 8829 | 2 | 0.095
L5b1 | 8937 | 2 | 0.095
L5b1 | 9025 | 2 | 0.095
L5b1 | 9966 | 2 | 0.095
L5b1a | 11260 | 2 | 0.143
L5b1a | 15383 | 1 | 0.071
L5b1a | 15400 | 1 | 0.071
L5b1a | 15924 | 1 | 0.071
L5b1a | 16176 | 1 | 0.071
L5b1a | 16290 | 1 | 0.071
L5b1a | 16311 | 7 | 0.5
L5b1a | 16319 | 3 | 0.214
L5b1a | 16526 | 1 | 0.071
L5b1a | 16532d | 5 | 0.357
L5b1a | 183 | 1 | 0.071
L5b1a | 185 | 2 | 0.143
L5b1a | 185C | 1 | 0.071
L5b1a | 308-309d | 1 | 0.071
L5b1a | 515-524d | 1 | 0.071
L5b1a | 573.3C | 1 | 0.071
L5b1a | 6317 | 1 | 0.071
L5b1a | 7076 | 1 | 0.071
L5b1a | 7702 | 1 | 0.071
L5b1b | 10172 | 1 | 0.077
L5b1b | 12879 | 1 | 0.077
L5b1b | 14260 | 1 | 0.077
L5b1b | 15930T | 1 | 0.077
L5b1b | 16176 | 5 | 0.385
L5b1b | 16291 | 4 | 0.308
L5b1b | 16335 | 4 | 0.308
L5b1b | 16362 | 1 | 0.077
L5b1b | 16526 | 1 | 0.077
L5b1b | 16532d | 4 | 0.308
L5b1b | 182 | 1 | 0.077
L5b1b | 189 | 1 | 0.077
L5b1b | 2911 | 1 | 0.077
L5b1b | 3801 | 1 | 0.077
L5b1b | 4117 | 1 | 0.077
L5b1b | 455.1T | 1 | 0.077
L5b1b | 459d | 1 | 0.077
L5b1b | 515-524d | 1 | 0.077
L5b1b | 574 | 1 | 0.077
L5b1b | 6446 | 1 | 0.077
L5b1b | 6965 | 1 | 0.077
L5b1b | 7013 | 1 | 0.077
L5b1b | 8658 | 1 | 0.077
L5b1b | 9003 | 1 | 0.077
L5b1b | 9554 | 1 | 0.077
L5b2 | 10178 | 1 | 0.1
L5b2 | 10694 | 1 | 0.1
L5b2 | 11711 | 1 | 0.1
L5b2 | 12441 | 1 | 0.1
L5b2 | 14071 | 1 | 0.1
L5b2 | 14326 | 1 | 0.1
L5b2 | 14560 | 1 | 0.1
L5b2 | 15235 | 1 | 0.1
L5b2 | 15262 | 1 | 0.1
L5b2 | 16189 | 1 | 0.1
L5b2 | 16212 | 1 | 0.1
L5b2 | 16291 | 5 | 0.5
L5b2 | 16301 | 1 | 0.1
L5b2 | 16342 | 1 | 0.1
L5b2 | 1819 | 1 | 0.1
L5b2 | 195 | 3 | 0.3
L5b2 | 200 | 4 | 0.4
L5b2 | 2581 | 1 | 0.1
L5b2 | 499 | 1 | 0.1
L5b2 | 5501 | 1 | 0.1
L5b2 | 5628 | 1 | 0.1
L5b2 | 573.1C | 1 | 0.1
L5b2 | 7076 | 3 | 0.3
L5b2 | 709 | 1 | 0.1
L5b2 | 710 | 1 | 0.1
L6 | 10142 | 1 | 0.167
L6 | 15262 | 1 | 0.167
L6 | 16129 | 1 | 0.167
L6 | 16294 | 1 | 0.167
L6 | 184 | 1 | 0.167
L6 | 228 | 1 | 0.167
L6 | 265 | 4 | 0.667
L6 | 3335 | 1 | 0.167
L6 | 3776 | 1 | 0.167
L6 | 515-524d | 1 | 0.167
L6 | 7145 | 1 | 0.167
L6 | 8656 | 1 | 0.167
L6 | 8715 | 1 | 0.167
L6 | 8874 | 1 | 0.167
L6a | 14693 | 4 | 0.5
L6a | 15479 | 4 | 0.5
L6a | 16093 | 1 | 0.125
L6a | 16173 | 4 | 0.5
L6a | 16184 | 1 | 0.125
L6a | 16243 | 1 | 0.125
L6a | 16274 | 1 | 0.125
L6a | 16362 | 4 | 0.5
L6a | 16399 | 4 | 0.5
L6a | 1943 | 1 | 0.125
L6a | 195 | 4 | 0.5
L6a | 204 | 2 | 0.25
L6a | 2407 | 1 | 0.125
L6a | 4226 | 1 | 0.125
L6a | 515-524d | 1 | 0.125

L6a | 5812 | 3 | 0.375
L6a | 6836 | 3 | 0.375
L6a | 7145 | 1 | 0.125
L6a | 7647 | 1 | 0.125
L6a | 8814 | 4 | 0.5
L6a | 961-962d | 3 | 0.375
L6b | 10253 | 1 | 0.143
L6b | 10310 | 1 | 0.143
L6b | 1187 | 1 | 0.143
L6b | 12405 | 3 | 0.429
L6b | 12714 | 3 | 0.429
L6b | 14861 | 1 | 0.143
L6b | 153 | 1 | 0.143
L6b | 15394 | 1 | 0.143
L6b | 15928 | 2 | 0.286
L6b | 16069 | 1 | 0.143
L6b | 16093 | 1 | 0.143
L6b | 16169 | 1 | 0.143
L6b | 16264 | 1 | 0.143
L6b | 16274 | 2 | 0.286
L6b | 184 | 2 | 0.286
L6b | 188 | 1 | 0.143
L6b | 5082 | 1 | 0.143
L6b | 6710 | 1 | 0.143
L6b | 7292 | 1 | 0.143
L6b | 7340 | 1 | 0.143
L6b | 9545 | 1 | 0.143
L6b | 9644 | 1 | 0.143
L6b | 965.3C | 1 | 0.143
M | 10 | 1 | 0.003
M | 10084 | 1 | 0.003
M | 10091 | 1 | 0.003
M | 10127 | 1 | 0.003
M | 10235 | 1 | 0.003
M | 10289 | 2 | 0.005
M | 103 | 1 | 0.003
M | 10310 | 11 | 0.029
M | 10410 | 1 | 0.003
M | 10586 | 1 | 0.003
M | 10598 | 1 | 0.003
M | 10619 | 1 | 0.003
M | 10646 | 3 | 0.008
M | 10670 | 3 | 0.008
M | 10685 | 1 | 0.003
M | 10739 | 1 | 0.003
M | 10754 | 2 | 0.005
M | 10790 | 3 | 0.008
M | 10915 | 1 | 0.003
M | 10977A | 1 | 0.003
M | 10986A | 1 | 0.003
M | 11084 | 3 | 0.008
M | 11089 | 1 | 0.003
M | 11197 | 2 | 0.005
M | 11233 | 3 | 0.008
M | 11239 | 2 | 0.005
M | 11278 | 1 | 0.003
M | 11314 | 1 | 0.003
M | 11362 | 1 | 0.003
M | 11404 | 1 | 0.003
M | 11410 | 2 | 0.005
M | 11437 | 10 | 0.026
M | 11470 | 1 | 0.003
M | 11611 | 1 | 0.003
M | 11617 | 2 | 0.005
M | 11803 | 2 | 0.005
M | 11864 | 1 | 0.003
M | 11908 | 2 | 0.005
M | 11914 | 18 | 0.047
M | 1192 | 1 | 0.003
M | 11963 | 10 | 0.026
M | 12016 | 1 | 0.003
M | 12067 | 1 | 0.003
M | 12285 | 3 | 0.008
M | 12362 | 1 | 0.003
M | 12366 | 3 | 0.008
M | 12375 | 1 | 0.003
M | 12414 | 1 | 0.003
M | 12426 | 2 | 0.005
M | 12477 | 2 | 0.005
M | 12498 | 1 | 0.003
M | 12535 | 16 | 0.042
M | 12727 | 1 | 0.003
M | 12783 | 1 | 0.003
M | 12816 | 1 | 0.003
M | 12867 | 10 | 0.026
M | 12918 | 2 | 0.005
M | 12945 | 1 | 0.003
M | 12954 | 1 | 0.003
M | 12961 | 1 | 0.003
M | 13035A | 1 | 0.003
M | 13092 | 1 | 0.003
M | 13105 | 14 | 0.037
M | 1313 | 3 | 0.008
M | 13135 | 1 | 0.003
M | 13254 | 1 | 0.003
M | 13395 | 8 | 0.021
M | 13437 | 1 | 0.003
M | 13461 | 1 | 0.003
M | 13623 | 1 | 0.003
M | 13681 | 1 | 0.003
M | 13708 | 14 | 0.037
M | 13713 | 1 | 0.003
M | 13801 | 1 | 0.003
M | 13824 | 1 | 0.003
M | 13849C | 1 | 0.003
M | 13899 | 1 | 0.003
M | 13928 | 1 | 0.003
M | 13992 | 2 | 0.005
M | 140 | 8 | 0.021
M | 14007 | 1 | 0.003
M | 14218 | 3 | 0.008

M | 14272 | 1 | 0.003
M | 14299 | 2 | 0.005
M | 143 | 3 | 0.008
M | 14311 | 3 | 0.008
M | 14374 | 1 | 0.003
M | 14515 | 1 | 0.003
M | 14527 | 1 | 0.003
M | 14552 | 2 | 0.005
M | 146 | 8 | 0.021
M | 14692 | 1 | 0.003
M | 14693 | 4 | 0.011
M | 14745 | 1 | 0.003
M | 14770 | 2 | 0.005
M | 14861 | 1 | 0.003
M | 14926C | 2 | 0.005
M | 14995 | 3 | 0.008
M | 15007 | 1 | 0.003
M | 151 | 12 | 0.032
M | 15110 | 1 | 0.003
M | 15172 | 1 | 0.003
M | 152 | 17 | 0.045
M | 152.1C | 1 | 0.003
M | 15213 | 3 | 0.008
M | 15217 | 3 | 0.008
M | 15244 | 1 | 0.003
M | 153 | 1 | 0.003
M | 15314 | 1 | 0.003
M | 15434 | 1 | 0.003
M | 15519 | 1 | 0.003
M | 15530 | 7 | 0.018
M | 1556 | 3 | 0.008
M | 15596 | 8 | 0.021
M | 15613 | 2 | 0.005
M | 15658 | 8 | 0.021
M | 15670 | 1 | 0.003
M | 15758 | 8 | 0.021
M | 15774 | 6 | 0.016
M | 15777 | 3 | 0.008
M | 15836 | 1 | 0.003
M | 15837 | 10 | 0.026
M | 15900 | 2 | 0.005
M | 15910 | 1 | 0.003
M | 15924 | 4 | 0.011
M | 15930 | 2 | 0.005
M | 15938 | 1 | 0.003
M | 15941 | 1 | 0.003
M | 1598 | 3 | 0.008
M | 16000 | 1 | 0.003
M | 16086 | 4 | 0.011
M | 16093 | 2 | 0.005
M | 16126 | 3 | 0.008
M | 16129 | 7 | 0.018
M | 16138 | 3 | 0.008
M | 16145 | 2 | 0.005
M | 16166 | 3 | 0.008
M | 16172 | 1 | 0.003
M | 16174 | 1 | 0.003
M | 16179 | 2 | 0.005
M | 16183 | 1 | 0.003
M | 16186 | 1 | 0.003
M | 16189 | 3 | 0.008
M | 16193 | 1 | 0.003
M | 16214 | 1 | 0.003
M | 16214A | 3 | 0.008
M | 16215 | 1 | 0.003
M | 16217 | 2 | 0.005
M | 16218 | 3 | 0.008
M | 16224 | 1 | 0.003
M | 16234 | 3 | 0.008
M | 16243 | 1 | 0.003
M | 16245 | 1 | 0.003
M | 16251 | 1 | 0.003
M | 16259 | 1 | 0.003
M | 16260 | 1 | 0.003
M | 16261 | 2 | 0.005
M | 16263 | 1 | 0.003
M | 16264 | 1 | 0.003
M | 16266G | 6 | 0.016
M | 16267 | 1 | 0.003
M | 16270 | 3 | 0.008
M | 16272 | 1 | 0.003
M | 16273 | 10 | 0.026
M | 16278 | 4 | 0.011
M | 16289 | 3 | 0.008
M | 16290 | 2 | 0.005
M | 16291 | 3 | 0.008
M | 16292 | 1 | 0.003
M | 16297 | 2 | 0.005
M | 16299 | 1 | 0.003
M | 16304 | 13 | 0.034
M | 16311 | 16 | 0.042
M | 16316 | 2 | 0.005
M | 16317 | 1 | 0.003
M | 16318 | 13 | 0.034
M | 16319 | 4 | 0.011
M | 16320 | 3 | 0.008
M | 16325 | 3 | 0.008
M | 16327 | 1 | 0.003
M | 16355 | 6 | 0.016
M | 16357 | 29 | 0.077
M | 16362 | 7 | 0.018
M | 16365 | 2 | 0.005
M | 16368 | 1 | 0.003
M | 16381 | 2 | 0.005
M | 16390 | 4 | 0.011
M | 16391 | 7 | 0.018
M | 16518T | 1 | 0.003
M | 16527 | 4 | 0.011
M | 1664 | 3 | 0.008
M | 1719 | 3 | 0.008
M | 1780 | 1 | 0.003

M | 1809 | 1 | 0.003
M | 1842 | 1 | 0.003
M | 185 | 5 | 0.013
M | 189 | 5 | 0.013
M | 195 | 25 | 0.066
M | 195A | 1 | 0.003
M | 198 | 1 | 0.003
M | 199 | 7 | 0.018
M | 200 | 1 | 0.003
M | 204 | 2 | 0.005
M | 207 | 6 | 0.016
M | 212 | 1 | 0.003
M | 214 | 1 | 0.003
M | 215 | 10 | 0.026
M | 2179T | 1 | 0.003
M | 228 | 3 | 0.008
M | 234 | 1 | 0.003
M | 235 | 1 | 0.003
M | 2392 | 3 | 0.008
M | 2416 | 1 | 0.003
M | 249d | 1 | 0.003
M | 2619 | 1 | 0.003
M | 262 | 2 | 0.005
M | 2755 | 1 | 0.003
M | 2757 | 1 | 0.003
M | 2772 | 2 | 0.005
M | 3010 | 4 | 0.011
M | 309d | 1 | 0.003
M | 3106A | 1 | 0.003
M | 316 | 1 | 0.003
M | 3173 | 10 | 0.026
M | 3290 | 1 | 0.003
M | 338 | 2 | 0.005
M | 3434 | 10 | 0.026
M | 3519 | 10 | 0.026
M | 3531 | 1 | 0.003
M | 356.1C | 7 | 0.018
M | 3606 | 5 | 0.013
M | 3714 | 1 | 0.003
M | 3753 | 1 | 0.003
M | 3849 | 2 | 0.005
M | 3865 | 1 | 0.003
M | 3918 | 10 | 0.026
M | 3921 | 1 | 0.003
M | 3994 | 1 | 0.003
M | 4012 | 1 | 0.003
M | 4093 | 1 | 0.003
M | 4232 | 1 | 0.003
M | 4248 | 1 | 0.003
M | 4254 | 1 | 0.003
M | 4529 | 1 | 0.003
M | 4554 | 2 | 0.005
M | 4562 | 4 | 0.011
M | 463 | 1 | 0.003
M | 4715 | 3 | 0.008
M | 4768 | 1 | 0.003
M | 477G | 1 | 0.003
M | 4802 | 2 | 0.005
M | 482 | 2 | 0.005
M | 4820 | 1 | 0.003
M | 485 | 1 | 0.003
M | 4850 | 1 | 0.003
M | 4943 | 2 | 0.005
M | 4973 | 1 | 0.003
M | 503 | 1 | 0.003
M | 5093 | 1 | 0.003
M | 513 | 3 | 0.008
M | 5147 | 2 | 0.005
M | 514A | 1 | 0.003
M | 5201 | 1 | 0.003
M | 5279A | 2 | 0.005
M | 5291 | 1 | 0.003
M | 5298 | 1 | 0.003
M | 5319 | 3 | 0.008
M | 54 | 1 | 0.003
M | 5432 | 1 | 0.003
M | 5460 | 10 | 0.026
M | 5466 | 3 | 0.008
M | 5471 | 1 | 0.003
M | 5492 | 2 | 0.005
M | 5493 | 1 | 0.003
M | 5558 | 1 | 0.003
M | 5580 | 1 | 0.003
M | 5683 | 1 | 0.003
M | 56T | 1 | 0.003
M | 5783 | 1 | 0.003
M | 57A | 1 | 0.003
M | 5894 | 1 | 0.003
M | 5990 | 1 | 0.003
M | 6040 | 1 | 0.003
M | 6041 | 1 | 0.003
M | 6045 | 3 | 0.008
M | 6092 | 1 | 0.003
M | 6164 | 1 | 0.003
M | 6203 | 1 | 0.003
M | 6216 | 3 | 0.008
M | 6227 | 1 | 0.003
M | 6249 | 1 | 0.003
M | 6284 | 3 | 0.008
M | 629 | 1 | 0.003
M | 63 | 9 | 0.024
M | 63.1T | 1 | 0.003
M | 6305T | 2 | 0.005
M | 64 | 25 | 0.066
M | 66 | 7 | 0.018
M | 66.1A | 4 | 0.011
M | 6620 | 1 | 0.003
M | 66T | 4 | 0.011
M | 6747 | 1 | 0.003
M | 6842 | 1 | 0.003
M | 6852 | 1 | 0.003

M | 6960 | 16 | 0.042
M | 709 | 10 | 0.026
M | 7091 | 1 | 0.003
M | 7202 | 1 | 0.003
M | 7205 | 1 | 0.003
M | 7211 | 1 | 0.003
M | 7337 | 1 | 0.003
M | 7340 | 1 | 0.003
M | 7397 | 1 | 0.003
M | 7469 | 2 | 0.005
M | 751 | 4 | 0.011
M | 7521 | 1 | 0.003
M | 7711 | 10 | 0.026
M | 7738 | 1 | 0.003
M | 7764 | 1 | 0.003
M | 783 | 4 | 0.011
M | 794 | 8 | 0.021
M | 8027 | 10 | 0.026
M | 8059 | 1 | 0.003
M | 8084 | 1 | 0.003
M | 8108 | 1 | 0.003
M | 8119 | 1 | 0.003
M | 8143 | 4 | 0.011
M | 8270 | 2 | 0.005
M | 8271 | 2 | 0.005
M | 8281 | 1 | 0.003
M | 8281-8289d | 2 | 0.005
M | 8307 | 4 | 0.011
M | 8348 | 1 | 0.003
M | 8414 | 1 | 0.003
M | 8502 | 1 | 0.003
M | 856 | 1 | 0.003
M | 8619A | 3 | 0.008
M | 8623 | 2 | 0.005
M | 8632 | 1 | 0.003
M | 8659 | 1 | 0.003
M | 8666 | 2 | 0.005
M | 8674 | 1 | 0.003
M | 8730 | 1 | 0.003
M | 8757 | 1 | 0.003
M | 8812 | 1 | 0.003
M | 8838 | 1 | 0.003
M | 8850 | 2 | 0.005
M | 8856 | 1 | 0.003
M | 8928 | 2 | 0.005
M | 8997 | 1 | 0.003
M | 9012 | 1 | 0.003
M | 9015 | 1 | 0.003
M | 9126 | 1 | 0.003
M | 9156 | 17 | 0.045
M | 9211 | 1 | 0.003
M | 930 | 1 | 0.003
M | 9300 | 1 | 0.003
M | 9329 | 1 | 0.003
M | 9359 | 8 | 0.021
M | 9644 | 3 | 0.008
M | 9656 | 1 | 0.003
M | 9725 | 1 | 0.003
M | 9797 | 1 | 0.003
M | 980 | 1 | 0.003
M | 9833 | 1 | 0.003
M | 9855 | 2 | 0.005
M | 9872 | 1 | 0.003
M | 9899 | 1 | 0.003
M10 | 12976 | 7 | 0.056
M10 | 13203 | 6 | 0.048
M10 | 13722 | 1 | 0.008
M10 | 15514 | 6 | 0.048
M10 | 16051 | 1 | 0.008
M10 | 16075 | 1 | 0.008
M10 | 16086 | 1 | 0.008
M10 | 16145 | 1 | 0.008
M10 | 16176 | 1 | 0.008
M10 | 16213 | 1 | 0.008
M10 | 16215A | 1 | 0.008
M10 | 16215T | 1 | 0.008
M10 | 16256 | 26 | 0.208
M10 | 16299 | 3 | 0.024
M10 | 16343 | 1 | 0.008
M10 | 16356 | 2 | 0.016
M10 | 16399 | 2 | 0.016
M10 | 195 | 2 | 0.016
M10 | 207 | 2 | 0.016
M10 | 316C | 1 | 0.008
M10 | 3172.1C | 6 | 0.048
M10 | 417 | 5 | 0.04
M10 | 4823 | 7 | 0.056
M10 | 574C | 1 | 0.008
M10 | 6040 | 6 | 0.048
M10 | 953 | 7 | 0.056
M10 | 9644 | 7 | 0.056
M10 | 9729 | 7 | 0.056
M10a | 10694 | 1 | 0.007
M10a | 146 | 31 | 0.22
M10a | 150 | 2 | 0.014
M10a | 151 | 5 | 0.035
M10a | 152 | 1 | 0.007
M10a | 16126 | 1 | 0.007
M10a | 16145 | 1 | 0.007
M10a | 16170 | 1 | 0.007
M10a | 16171 | 1 | 0.007
M10a | 16181 | 3 | 0.021
M10a | 16209 | 3 | 0.021
M10a | 16241C | 2 | 0.014
M10a | 16278 | 2 | 0.014
M10a | 16294 | 2 | 0.014
M10a | 16319 | 12 | 0.085
M10a | 16417d | 1 | 0.007
M10a | 16474d | 1 | 0.007
M10a | 16524 | 2 | 0.014
M10a | 16549d | 1 | 0.007

M10a | 246 | 6 | 0.043
M10a | 316 | 1 | 0.007
M10a | 316C | 18 | 0.128
M10a | 461 | 1 | 0.007
M10a | 498d | 1 | 0.007
M10a | 94 | 22 | 0.156
M10a1 | 10 | 1 | 0.007
M10a1 | 10007 | 3 | 0.02
M10a1 | 1193 | 2 | 0.014
M10a1 | 13477 | 1 | 0.007
M10a1 | 13708 | 3 | 0.02
M10a1 | 138 | 1 | 0.007
M10a1 | 13992 | 1 | 0.007
M10a1 | 14311 | 4 | 0.027
M10a1 | 14870 | 1 | 0.007
M10a1 | 152 | 2 | 0.014
M10a1 | 16031 | 1 | 0.007
M10a1 | 16126 | 1 | 0.007
M10a1 | 16140 | 3 | 0.02
M10a1 | 16148 | 13 | 0.088
M10a1 | 16226 | 1 | 0.007
M10a1 | 16238A | 1 | 0.007
M10a1 | 16248 | 2 | 0.014
M10a1 | 16254 | 2 | 0.014
M10a1 | 16271 | 6 | 0.041
M10a1 | 16298 | 1 | 0.007
M10a1 | 16312C | 1 | 0.007
M10a1 | 16362 | 5 | 0.034
M10a1 | 16437G | 1 | 0.007
M10a1 | 16460 | 1 | 0.007
M10a1 | 16493 | 1 | 0.007
M10a1 | 16525d | 1 | 0.007
M10a1 | 16542 | 1 | 0.007
M10a1 | 195 | 12 | 0.081
M10a1 | 199 | 1 | 0.007
M10a1 | 20 | 1 | 0.007
M10a1 | 271 | 1 | 0.007
M10a1 | 299 | 1 | 0.007
M10a1 | 2T | 5 | 0.034
M10a1 | 331 | 1 | 0.007
M10a1 | 366 | 1 | 0.007
M10a1 | 382A | 1 | 0.007
M10a1 | 392 | 1 | 0.007
M10a1 | 3945 | 2 | 0.014
M10a1 | 4820 | 2 | 0.014
M10a1 | 4976 | 2 | 0.014
M10a1 | 540 | 1 | 0.007
M10a1 | 574C | 1 | 0.007
M10a1 | 593 | 1 | 0.007
M10a1 | 595 | 1 | 0.007
M10a1 | 608C | 1 | 0.007
M10a1 | 609 | 1 | 0.007
M10a1 | 616G | 1 | 0.007
M10a1 | 622 | 1 | 0.007
M10a1 | 628G | 1 | 0.007
M10a1 | 631C | 1 | 0.007
M10a1 | 636C | 1 | 0.007
M10a1 | 641C | 1 | 0.007
M10a1 | 646A | 1 | 0.007
M10a1 | 659G | 1 | 0.007
M10a1 | 664C | 1 | 0.007
M10a1 | 670 | 1 | 0.007
M10a1 | 676 | 1 | 0.007
M10a1 | 683T | 1 | 0.007
M10a1 | 687 | 1 | 0.007
M10a1 | 9180 | 2 | 0.014
M10a1 | 92 | 1 | 0.007
M10a1 | 94 | 43 | 0.291
M10a1+16129 | 12070 | 3 | 0.034
M10a1+16129 | 125G | 1 | 0.011
M10a1+16129 | 13105 | 3 | 0.034
M10a1+16129 | 13934 | 3 | 0.034
M10a1+16129 | 143 | 1 | 0.011
M10a1+16129 | 146 | 3 | 0.034
M10a1+16129 | 150 | 1 | 0.011
M10a1+16129 | 15924 | 1 | 0.011
M10a1+16129 | 16093 | 4 | 0.045
M10a1+16129 | 16133 | 2 | 0.023
M10a1+16129 | 16176 | 2 | 0.023
M10a1+16129 | 16186 | 1 | 0.011
M10a1+16129 | 16189 | 1 | 0.011
M10a1+16129 | 16213 | 2 | 0.023
M10a1+16129 | 16230 | 1 | 0.011
M10a1+16129 | 16233 | 1 | 0.011
M10a1+16129 | 16249 | 3 | 0.034
M10a1+16129 | 16257A | 1 | 0.011
M10a1+16129 | 16261 | 1 | 0.011
M10a1+16129 | 16272 | 2 | 0.023
M10a1+16129 | 16297 | 2 | 0.023
M10a1+16129 | 16320 | 1 | 0.011
M10a1+16129 | 16352 | 1 | 0.011
M10a1+16129 | 16357 | 2 | 0.023
M10a1+16129 | 16362 | 10 | 0.114
M10a1+16129 | 16374 | 1 | 0.011
M10a1+16129 | 16438 | 2 | 0.023
M10a1+16129 | 16526 | 2 | 0.023
M10a1+16129 | 185 | 3 | 0.034
M10a1+16129 | 189 | 4 | 0.045
M10a1+16129 | 195 | 1 | 0.011
M10a1+16129 | 228 | 2 | 0.023
M10a1+16129 | 235 | 2 | 0.023
M10a1+16129 | 267d | 1 | 0.011
M10a1+16129 | 3253 | 3 | 0.034
M10a1+16129 | 507 | 1 | 0.011
M10a1+16129 | 70 | 1 | 0.011
M10a1+16129 | 739 | 3 | 0.034
M10a1+16129 | 94 | 1 | 0.011
M10a1a | 10245 | 4 | 0.043
M10a1a | 10454 | 1 | 0.011
M10a1a | 11732 | 1 | 0.011
M10a1a | 12534 | 3 | 0.032

M10a1a | 125G | 1 | 0.011
M10a1a | 12786 | 2 | 0.021
M10a1a | 13320 | 3 | 0.032
M10a1a | 143 | 1 | 0.011
M10a1a | 151 | 5 | 0.053
M10a1a | 15109 | 4 | 0.043
M10a1a | 152 | 6 | 0.064
M10a1a | 16083 | 1 | 0.011
M10a1a | 16086 | 15 | 0.16
M10a1a | 16172 | 1 | 0.011
M10a1a | 16192 | 2 | 0.021
M10a1a | 16219 | 1 | 0.011
M10a1a | 16233 | 1 | 0.011
M10a1a | 16249 | 1 | 0.011
M10a1a | 16257A | 1 | 0.011
M10a1a | 16261 | 1 | 0.011
M10a1a | 16272 | 2 | 0.021
M10a1a | 16357 | 2 | 0.021
M10a1a | 16362 | 1 | 0.011
M10a1a | 16438 | 2 | 0.021
M10a1a | 16526 | 4 | 0.043
M10a1a | 1694 | 3 | 0.032
M10a1a | 1892 | 3 | 0.032
M10a1a | 195 | 1 | 0.011
M10a1a | 215 | 1 | 0.011
M10a1a | 267d | 1 | 0.011
M10a1a | 3397 | 1 | 0.011
M10a1a | 507 | 1 | 0.011
M10a1a | 5319 | 3 | 0.032
M10a1a | 5471 | 1 | 0.011
M10a1a | 7440G | 3 | 0.032
M10a1a | 9205 | 1 | 0.011
M10a1a | 94 | 2 | 0.021
M10a1a | 9932 | 4 | 0.043
M10a1a1 | 143 | 2 | 0.031
M10a1a1 | 146 | 3 | 0.047
M10a1a1 | 16032.1A | 1 | 0.016
M10a1a1 | 16143 | 1 | 0.016
M10a1a1 | 16152 | 1 | 0.016
M10a1a1 | 16174 | 1 | 0.016
M10a1a1 | 16175T | 1 | 0.016
M10a1a1 | 16227 | 1 | 0.016
M10a1a1 | 16293C | 1 | 0.016
M10a1a1 | 16312 | 1 | 0.016
M10a1a1 | 16340C | 1 | 0.016
M10a1a1 | 16344 | 1 | 0.016
M10a1a1 | 16362 | 17 | 0.266
M10a1a1 | 16443G | 1 | 0.016
M10a1a1 | 16445 | 17 | 0.266
M10a1a1 | 16464C | 1 | 0.016
M10a1a1 | 16465 | 3 | 0.047
M10a1a1 | 16487 | 1 | 0.016
M10a1a1 | 16512 | 1 | 0.016
M10a1a1 | 16520A | 1 | 0.016
M10a1a1 | 16555 | 1 | 0.016
M10a1a1 | 189 | 1 | 0.016
M10a1a1 | 201 | 1 | 0.016
M10a1a1 | 206 | 1 | 0.016
M10a1a1 | 20A | 1 | 0.016
M10a1a1 | 215 | 1 | 0.016
M10a1a1 | 235 | 1 | 0.016
M10a1a1 | 335 | 1 | 0.016
M10a1a1 | 361C | 1 | 0.016
M10a1a1 | 401 | 1 | 0.016
M10a1a1 | 402 | 1 | 0.016
M10a1a1 | 411 | 1 | 0.016
M10a1a1 | 413 | 1 | 0.016
M10a1a1 | 45 | 1 | 0.016
M10a1a1 | 453 | 1 | 0.016
M10a1a1 | 464 | 1 | 0.016
M10a1a1 | 490 | 1 | 0.016
M10a1a1 | 512 | 1 | 0.016
M10a1a1 | 557 | 1 | 0.016
M10a1a1 | 574C | 1 | 0.016
M10a1a1 | 576C | 1 | 0.016
M10a1a1a | 11908 | 1 | 0.023
M10a1a1a | 11914 | 1 | 0.023
M10a1a1a | 12696 | 2 | 0.045
M10a1a1a | 14198 | 1 | 0.023
M10a1a1a | 14476 | 1 | 0.023
M10a1a1a | 14484 | 1 | 0.023
M10a1a1a | 14533 | 1 | 0.023
M10a1a1a | 146 | 1 | 0.023
M10a1a1a | 16145 | 1 | 0.023
M10a1a1a | 16146 | 1 | 0.023
M10a1a1a | 16266 | 3 | 0.068
M10a1a1a | 16269 | 1 | 0.023
M10a1a1a | 2195 | 1 | 0.023
M10a1a1a | 235 | 1 | 0.023
M10a1a1a | 310 | 1 | 0.023
M10a1a1a | 3397 | 1 | 0.023
M10a1a1a | 4181 | 1 | 0.023
M10a1a1a | 574C | 1 | 0.023
M10a1a1a | 576C | 1 | 0.023
M10a1a1a | 8764 | 1 | 0.023
M10a1a1a | 94 | 1 | 0.023
M10a1a1a | 9494 | 1 | 0.023
M10a1a1b | 1211 | 1 | 0.024
M10a1a1b | 12712 | 1 | 0.024
M10a1a1b | 13032 | 1 | 0.024
M10a1a1b | 14564 | 1 | 0.024
M10a1a1b | 151 | 2 | 0.049
M10a1a1b | 152 | 7 | 0.171
M10a1a1b | 1530 | 1 | 0.024
M10a1a1b | 15323 | 1 | 0.024
M10a1a1b | 16148 | 1 | 0.024
M10a1a1b | 16214 | 3 | 0.073
M10a1a1b | 16249 | 1 | 0.024
M10a1a1b | 16294 | 3 | 0.073
M10a1a1b | 16317 | 1 | 0.024
M10a1a1b | 16483 | 1 | 0.024

M10a1a1b | 185 | 2 | 0.049
M10a1a1b | 204 | 1 | 0.024
M10a1a1b | 226 | 1 | 0.024
M10a1a1b | 234 | 1 | 0.024
M10a1a1b | 385 | 1 | 0.024
M10a1a1b | 93 | 1 | 0.024
M10a1a1b | 9368 | 1 | 0.024
M10a1a1b1 | 10993 | 2 | 0.051
M10a1a1b1 | 11024 | 1 | 0.026
M10a1a1b1 | 11215 | 1 | 0.026
M10a1a1b1 | 11614 | 1 | 0.026
M10a1a1b1 | 152 | 2 | 0.051
M10a1a1b1 | 16075 | 1 | 0.026
M10a1a1b1 | 16192 | 1 | 0.026
M10a1a1b1 | 16249 | 1 | 0.026
M10a1a1b1 | 16381 | 1 | 0.026
M10a1a1b1 | 16526 | 1 | 0.026
M10a1a1b1 | 1656 | 2 | 0.051
M10a1a1b1 | 189 | 6 | 0.154
M10a1a1b1 | 195 | 2 | 0.051
M10a1a1b1 | 199 | 6 | 0.154
M10a1a1b1 | 4225 | 2 | 0.051
M10a1a1b1 | 5483 | 1 | 0.026
M10a1a1b1 | 574C | 4 | 0.103
M10a1a1b1 | 576C | 4 | 0.103
M10a1a1b1 | 577C | 2 | 0.051
M10a1a1b1 | 5843 | 2 | 0.051
M10a1a1b1 | 5978 | 2 | 0.051
M10a1a1b1 | 9181 | 4 | 0.103
M10a1a1b1 | 9336 | 4 | 0.103
M10a1a1b2 | 10653 | 2 | 0.056
M10a1a1b2 | 12727 | 1 | 0.028
M10a1a1b2 | 14239 | 2 | 0.056
M10a1a1b2 | 151 | 1 | 0.028
M10a1a1b2 | 152 | 4 | 0.111
M10a1a1b2 | 16051 | 1 | 0.028
M10a1a1b2 | 16109 | 1 | 0.028
M10a1a1b2 | 16231 | 1 | 0.028
M10a1a1b2 | 16258C | 4 | 0.111
M10a1a1b2 | 16294 | 1 | 0.028
M10a1a1b2 | 200 | 2 | 0.056
M10a1a1b2 | 3394 | 1 | 0.028
M10a1a1b2 | 3930A | 1 | 0.028
M10a1a1b2 | 5246 | 2 | 0.056
M10a1a1b2 | 569.1TCCC | 1 | 0.028
M10a1a1b2 | 574C | 4 | 0.111
M10a1a1b2 | 576C | 4 | 0.111
M10a1a1b2 | 577C | 2 | 0.056
M10a1a1b2 | 578 | 1 | 0.028
M10a1a1b2 | 6251 | 1 | 0.028
M10a1a1b2 | 8521 | 1 | 0.028
M10a1a1b2 | 93 | 1 | 0.028
M10a1b | 10724 | 2 | 0.015
M10a1b | 11647 | 2 | 0.015
M10a1b | 11778 | 1 | 0.007
M10a1b | 12067 | 2 | 0.015
M10a1b | 13602 | 1 | 0.007
M10a1b | 14128 | 1 | 0.007
M10a1b | 14168 | 1 | 0.007
M10a1b | 146 | 1 | 0.007
M10a1b | 14755 | 1 | 0.007
M10a1b | 150 | 4 | 0.029
M10a1b | 15050 | 1 | 0.007
M10a1b | 15172 | 1 | 0.007
M10a1b | 152 | 1 | 0.007
M10a1b | 153 | 1 | 0.007
M10a1b | 15302 | 1 | 0.007
M10a1b | 15769 | 1 | 0.007
M10a1b | 15852 | 1 | 0.007
M10a1b | 15913 | 2 | 0.015
M10a1b | 15924 | 1 | 0.007
M10a1b | 16086 | 2 | 0.015
M10a1b | 16092 | 3 | 0.022
M10a1b | 16187 | 1 | 0.007
M10a1b | 16189 | 3 | 0.022
M10a1b | 16213 | 2 | 0.015
M10a1b | 16217 | 1 | 0.007
M10a1b | 16220 | 1 | 0.007
M10a1b | 16235 | 1 | 0.007
M10a1b | 16240 | 1 | 0.007
M10a1b | 16249 | 2 | 0.015
M10a1b | 16261 | 3 | 0.022
M10a1b | 16274 | 3 | 0.022
M10a1b | 16290 | 2 | 0.015
M10a1b | 16320 | 1 | 0.007
M10a1b | 16398 | 3 | 0.022
M10a1b | 291d | 2 | 0.015
M10a1b | 4023 | 1 | 0.007
M10a1b | 4035 | 1 | 0.007
M10a1b | 516G | 1 | 0.007
M10a1b | 5426 | 2 | 0.015
M10a1b | 573.1CCTCCC | 1 | 0.007
M10a1b | 593 | 3 | 0.022
M10a1b | 70 | 2 | 0.015
M10a1b | 7428 | 1 | 0.007
M10a1b | 7660 | 1 | 0.007
M10a1b | 7888 | 2 | 0.015
M10a1b | 7897 | 1 | 0.007
M10a1b | 8410 | 2 | 0.015
M10a1b | 8820 | 1 | 0.007
M10a1b | 8886 | 1 | 0.007
M10a2 | 11272 | 1 | 0.014
M10a2 | 12360 | 1 | 0.014
M10a2 | 14569 | 2 | 0.028
M10a2 | 16145 | 1 | 0.014
M10a2 | 16150 | 1 | 0.014
M10a2 | 16168 | 1 | 0.014
M10a2 | 16175C | 2 | 0.028
M10a2 | 16179 | 1 | 0.014
M10a2 | 16192 | 12 | 0.169
M10a2 | 16248 | 1 | 0.014

M10a2 | 16317T | 1 | 0.014
M10a2 | 16320 | 2 | 0.028
M10a2 | 3882 | 1 | 0.014
M10a2 | 4012 | 1 | 0.014
M10a2 | 574C | 1 | 0.014
M10a2 | 576C | 1 | 0.014
M10a2 | 8919 | 1 | 0.014
M11 | 10 | 1 | 0.004
M11 | 150 | 2 | 0.008
M11 | 16093 | 1 | 0.004
M11 | 16172 | 4 | 0.016
M11 | 16173 | 4 | 0.016
M11 | 16175 | 5 | 0.02
M11 | 16189 | 1 | 0.004
M11 | 16194 | 1 | 0.004
M11 | 16294 | 1 | 0.004
M11 | 16298d | 1 | 0.004
[truncated: 1,065,449 more chars]
