# Supplementary material for: Mitochondrial Haplogroup Classification of Ancient DNA Samples Using Haplotracker
Source: Biomed Res Int. 2022 Mar 18;2022:5344418. doi: 10.1155/2022/5344418 (PMC8956381; doi:10.1155/2022/5344418)
Supplement: Supplementary Materials — Fig. S1: characterization of Phylotree-provided control region sequences tested for haplogroup classification by Haplotracker. Fig. S2: minimum number of amplicons required by Haplotracker in discriminating between haplogroups using mtDNA control and coding region sequences. Fig. S3: variant identification of an aDNA sample (MNW3) using an HRM real-time PCR. Table S1: haplogroups and their variant profiles extracted from Phylotree mtDNA Build 17. Table S2: haplogroup frequency carrying an extra variant in 118,869 haplotypes. Table S3: haplogroup frequency carrying a missing variant in 118,869 haplotypes. Table S4: haplogroup frequency in 118,869 haplotypes. Table S5: list of ancient human samples found in 2,000-year-old elite Xiongnu cemetery in Northeast Mongolia. Table S6: primers for the amplification of mtDNA coding region segments for haplogroup determination. Table S7: high-resolution melting real-time PCR primer design for screening variants to differentiate haplogroups G1a1, G1a1a, and G1a1b. Table S8: haplogroup classification of full-length mtGenome sequences from Phylotree (n = 8,216). Table S9: haplogroup classification with full-length and control region sequences of mtDNA using Haplotracker and HaploGrep 2. Table S10: comparison of servers using control region sequences from GenBank before December 25, 2018 (n = 45,177). Table S11: comparison details for the servers using control region sequences from GenBank before December 25, 2018 (n = 45,177). Table S12: comparison of servers using control region sequences downloaded from GenBank from December 26, 2018 to August 22, 2019. Table S13: sequences of mtDNA PCR products from Mongolian ancient DNA samples. Table S14: haplogroup classification of Mongolian ancient DNA samples using Haplotracker. Table S15: minimum number of amplicons required by Haplotracker in discriminating between haplogroups using mtDNA control and coding region sequences. Table S16: minimum number of amplicons per superhaplogroup requ [file 5344418.f1.zip › 5344418.f6.pdf]

Table S3. Haplogroup frequency carrying a missing variant in 118,869 haplotypes

HG	Missed variant	Frequency	Rate	HG	Missed variant	Frequency	Rate	HG	Missed variant	Frequency	Rate
A	235	2	0.056	A15a	16362	3	0.007	A2+(64)+@153	16111	4	0.039
A	4824	1	0.028	A15a	235	1	0.002	A2+(64)+@153	16290	2	0.02
A	663	1	0.028	A15a	663	1	0.002	A2+(64)+@153	16362	2	0.02
A+152+16362	15326	2	0.004	A15a	750	3	0.007	A2+(64)+@153	73	4	0.039
A+152+16362	16319	1	0.002	A15b	16290	1	0.04	A2+(64)+@16111	16290	2	0.005
A+152+16362	4769	1	0.002	A15b	16319	1	0.04	A2+(64)+@16111	16362	1	0.002
A+152+16362+16189	152	5	0.128	A15c	750	3	0.007	A2+(64)+@16111	4248	2	0.005
A+152+16362+16189	16290	1	0.026	A15c1	15924	1	0.002	A2+(64)+@16111	4824	3	0.007
A+152+16362+16189	16319	1	0.026	A15c1	750	3	0.007	A2+(64)+16129	146	1	0.023
A+152+16362+200	152	8	0.019	A17	16223	1	0.002	A2+(64)+16129	153	4	0.091
A+152+16362+200	16290	1	0.002	A17	16290	1	0.002	A2+(64)+16129	16111	1	0.023
A+152+16362+200	1736	1	0.002	A17	16319	1	0.002	A2+(64)+16129	16362	2	0.045
A+152+16362+200	2706	1	0.002	A17	663	1	0.002	A2+(64)+16129	73	4	0.091
A+152+16362+200	4248	1	0.002	A18	16319	1	0.002	A2+(64)+16189	153	3	0.083
A+152+16362+200	4824	1	0.002	A18	235	3	0.007	A2+(64)+16189	15326	1	0.028
A+152+16362+200	663	3	0.007	A19	152	1	0.009	A2+(64)+16189	16223	1	0.028
A+152+16362+200	8860	1	0.002	A19	16223	3	0.028	A2+(64)+16189	16319	1	0.028
A1	16319	2	0.005	A19	199	50	0.467	A2+(64)+16189	16362	1	0.028
A1	235	3	0.007	A19	235	3	0.028	A2+(64)+16189	235	1	0.028
A11	235	5	0.034	A1a	16223	1	0.025	A2+(64)+16189	73	3	0.083
A11+16234	663	1	0.009	A1a1	235	8	0.2	A20	16319	2	0.005
A11+16234	750	1	0.009	A2	12705	1	0.009	A20	235	3	0.007
A11a	16223	5	0.011	A2	16290	1	0.009	A21	16319	2	0.004
A11a	16290	2	0.005	A2	16319	1	0.009	A21	235	4	0.008
A11a	5899.XC	3	0.007	A2	16362	2	0.019	A22	152	1	0.002
A11a	8860	1	0.002	A2+(64)	146	5	0.016	A22	16319	1	0.002
A12	16362	2	0.071	A2+(64)	16223	6	0.019	A22	235	3	0.007
A12a	4769	1	0.04	A2+(64)	16290	1	0.003	A23	73	4	0.174
A13	1736	5	0.011	A2+(64)	16319	5	0.016	A2a1	153	1	0.034
A13	235	3	0.007	A2+(64)	16362	3	0.009	A2a1	16223	1	0.034
A14	200	5	0.01	A2+(64)	1736	2	0.006	A2a2	16192	5	0.041
A15	750	3	0.007	A2+(64)	235	2	0.006	A2a2	16290	3	0.024
A15a	152	2	0.004	A2+(64)	263	1	0.003	A2a2	16319	1	0.008
A15a	16290	1	0.002	A2+(64)	4248	2	0.006	A2a3	16362	5	0.1
A15a	16319	1	0.002	A2+(64)+@153	146	13	0.127	A2a5	16192	2	0.059

A2aa | 16239A | 3 | 0.094
A2aa | 16266 | 1 | 0.031
A2aa | 73 | 6 | 0.188
A2ab | 14476 | 1 | 0.05
A2ab | 146 | 2 | 0.1
A2ab | 16223 | 1 | 0.05
A2ab | 235 | 1 | 0.05
A2ab | 73 | 2 | 0.1
A2ac | 153 | 1 | 0.015
A2ac | 16111 | 1 | 0.015
A2ac | 16362 | 2 | 0.03
A2ac1 | 16362 | 3 | 0.188
A2ac1 | 73 | 1 | 0.062
A2ad | 153 | 1 | 0.111
A2ad | 16175 | 2 | 0.222
A2ad1 | 16175 | 2 | 0.286
A2ad1 | 73 | 1 | 0.143
A2ae | 3565 | 1 | 0.029
A2af1a | 106-111d | 4 | 0.571
A2ag | 153 | 2 | 0.032
A2ag | 16111 | 4 | 0.065
A2ag | 235 | 8 | 0.129
A2ag | 73 | 6 | 0.097
A2ag | 9653 | 9 | 0.145
A2ai | 16290 | 1 | 0.091
A2aj | 16223 | 1 | 0.125
A2an | 146 | 1 | 0.008
A2ao | 16290 | 1 | 0.013
A2ao | 235 | 13 | 0.169
A2ap | 153 | 9 | 0.265
A2ap | 235 | 2 | 0.059
A2b | 16290 | 1 | 0.013
A2b | 235 | 5 | 0.066
A2b | 73 | 18 | 0.237
A2b1 | 1438 | 1 | 0.004
A2b1 | 16362 | 1 | 0.004
A2c | 16290 | 1 | 0.007
A2c | 16362 | 3 | 0.021
A2d | 12007 | 1 | 0.008
A2d | 16111 | 1 | 0.008
A2d | 235 | 1 | 0.008
A2d1 | 235 | 1 | 0.008
A2d1a | 235 | 2 | 0.125
A2d2 | 16111 | 2 | 0.017
A2d2 | 235 | 1 | 0.008
A2e | 16336 | 1 | 0.167
A2e | 16362 | 1 | 0.167
A2f1 | 16362 | 1 | 0.005
A2f1a | 12092A | 1 | 0.005
A2f1a | 16362 | 1 | 0.005
A2f2 | 16111 | 2 | 0.333
A2f3 | 151 | 2 | 0.057
A2g | 16223 | 1 | 0.067
A2g | 8794 | 1 | 0.067
A2g1 | 153 | 1 | 0.056
A2g1 | 16223 | 5 | 0.278
A2h | 1438 | 1 | 0.042
A2h | 146 | 2 | 0.083
A2h1 | 12007 | 1 | 0.062
A2h1 | 153 | 1 | 0.062
A2h1 | 16362 | 6 | 0.375
A2h1 | 235 | 3 | 0.188
A2i | 16111 | 1 | 0.048
A2i | 3307.1A | 16 | 0.762
A2i | 3308 | 16 | 0.762
A2i | 960.XC | 1 | 0.048
A2j | 235 | 1 | 0.008
A2j1 | 235 | 1 | 0.008
A2l | 235 | 2 | 0.005
A2m | 153 | 6 | 0.154
A2n | 235 | 2 | 0.005
A2n | 8860 | 1 | 0.003
A2o | 146 | 1 | 0.003
A2o | 153 | 1 | 0.003
A2o | 16290 | 1 | 0.003
A2o | 235 | 2 | 0.005
A2o | 73 | 1 | 0.003
A2p2 | 16111 | 13 | 0.406
A2q | 146 | 1 | 0.036
A2q | 153 | 1 | 0.036
A2q | 15326 | 4 | 0.143
A2q | 16111 | 3 | 0.107
A2q | 16290 | 4 | 0.143
A2q | 235 | 1 | 0.036
A2q1 | 73 | 12 | 0.5
A2r | 12007 | 1 | 0.015
A2s | 16207 | 1 | 0.5
A2t | 16223 | 2 | 0.017
A2t | 235 | 1 | 0.008
A2u | 12705 | 3 | 0.136
A2u | 16111 | 6 | 0.273
A2u1 | 16290 | 1 | 0.067
A2u2 | 16223 | 1 | 0.111
A2v1 | 153 | 1 | 0.111
A2v1a | 153 | 2 | 0.143
A2w | 8027 | 1 | 0.008
A2w1 | 16111 | 4 | 0.148
A2w1 | 235 | 1 | 0.037
A2w1 | 263 | 1 | 0.037
A2w1 | 573.XC | 6 | 0.222
A2y | 16111 | 2 | 0.111
A2y | 16304 | 2 | 0.111
A2y | 189 | 1 | 0.056
A2y | 5910 | 2 | 0.111
A2y | 6641 | 2 | 0.111
A2z | 153 | 2 | 0.133
A2z | 3744 | 1 | 0.067
A3 | 16223 | 1 | 0.028
A3a | 16223 | 1 | 0.027
A3a | 1736 | 1 | 0.027
A5 | 750 | 1 | 0.036

A5a1a | 16223 | 1 | 0.026
A5a1a1 | 11536 | 1 | 0.018
A5a1a2 | 16223 | 2 | 0.061
A5a1a2a | 16187 | 1 | 0.03
A5a2 | 16187 | 1 | 0.03
A5a2 | 16223 | 1 | 0.03
A5a2 | 16290 | 1 | 0.03
A5a2 | 16319 | 1 | 0.03
A5a3a | 11536 | 1 | 0.033
A5a4 | 16319 | 1 | 0.032
A5b | 965.XC | 1 | 0.033
A5b1 | 16223 | 6 | 0.207
A5b1 | 16235 | 1 | 0.034
A5b1 | 4824 | 1 | 0.034
A5b1 | 663 | 1 | 0.034
A5b1 | 965.XC | 3 | 0.103
A5b1a | 16235 | 6 | 0.214
A5b1a | 965.XC | 2 | 0.071
A5b1b | 16235 | 26 | 0.553
A5b1b | 16319 | 1 | 0.021
A5b1b | 1736 | 1 | 0.021
A5b1b | 965.XC | 6 | 0.128
A5b1c | 965.XC | 1 | 0.038
A5b1c1 | 16235 | 1 | 0.026
A5b1c1 | 235 | 1 | 0.026
A5b1c1 | 961 | 3 | 0.079
A5b1c1 | 965.XC | 3 | 0.079
A5c | 16319 | 1 | 0.043
A5c1 | 16223 | 1 | 0.04
A6 | 16223 | 1 | 0.002
A6a | 16223 | 1 | 0.003
A6a | 16319 | 2 | 0.005
A6b | 15326 | 1 | 0.003
A7 | 10172 | 6 | 0.207
A7 | 146 | 18 | 0.621
A8a | 16223 | 12 | 0.324
A8a | 16242 | 15 | 0.405
A8a | 235 | 1 | 0.027
A8a1 | 12705 | 1 | 0.032
A8a1 | 146 | 1 | 0.032
A8a1 | 16223 | 2 | 0.065
A8a1 | 16290 | 2 | 0.065
A8a1 | 64 | 1 | 0.032
B2 | 14766 | 1 | 0.002
B2 | 16189 | 1 | 0.002
B2 | 16217 | 1 | 0.002
B2 | 263 | 1 | 0.002
B2 | 3547 | 2 | 0.004
B2 | 499 | 3 | 0.006
B2 | 73 | 48 | 0.101
B2 | 8281-8289d | 3 | 0.006
B2a2 | 16111 | 7 | 0.093
B2a2 | 8281-8289d | 1 | 0.013
B2a4a1 | 16092 | 2 | 0.167
B2a5 | 16111 | 1 | 0.053
B2a5 | 16217 | 12 | 0.632
B2b | 1438 | 1 | 0.004
B2b | 16189 | 1 | 0.004
B2b | 16217 | 2 | 0.008
B2b | 4977 | 1 | 0.004
B2b+152 | 263 | 1 | 0.008
B2b+152 | 6755 | 1 | 0.008
B2b2 | 16217 | 7 | 0.241
B2b4 | 16217 | 1 | 0.021
B2b4 | 73 | 3 | 0.062
B2c1a | 4977 | 1 | 0.009
B2c2 | 16189 | 3 | 0.032
B2c2 | 73 | 4 | 0.043
B2c2a | 13590 | 1 | 0.071
B2c2a | 263 | 1 | 0.071
B2c2b | 14757 | 1 | 0.077
B2c2b | 14766 | 1 | 0.077
B2c2b | 16189 | 1 | 0.077
B2c2b | 499 | 1 | 0.077
B2c2b | 73 | 1 | 0.077
B2d | 4122 | 1 | 0.011
B2d | 4123 | 2 | 0.022
B2d | 498d | 1 | 0.011
B2e | 15326 | 3 | 0.023
B2g1 | 16189 | 3 | 0.2
B2g1 | 9950 | 1 | 0.067
B2i2a1a | 16291 | 1 | 0.091
B2i2b | 73 | 2 | 0.028
B2i2b1 | 207 | 7 | 0.636
B2j | 16361 | 3 | 0.5
B2k | 2706 | 3 | 0.041
B2k | 4371 | 21 | 0.288
B2k | 4820 | 1 | 0.014
B2l | 827 | 1 | 0.042
B2o | 15535 | 1 | 0.014
B2o | 16189 | 1 | 0.014
B2o | 16217 | 3 | 0.041
B2o | 263 | 1 | 0.014
B2o | 3547 | 1 | 0.014
B2o | 499 | 1 | 0.014
B2o | 73 | 8 | 0.11
B2o | 8281-8289d | 1 | 0.014
B2o1 | 7270 | 2 | 0.333
B2t | 15884 | 2 | 0.154
B2t | 16259 | 4 | 0.308
B2y | 73 | 1 | 0.005
B2y1 | 16217 | 1 | 0.005
B4a | 8860 | 1 | 0.004
B4a1+16311 | 16189 | 1 | 0.01
B4a1+16311 | 16261 | 6 | 0.061
B4a1a | 146 | 1 | 0.004
B4a1a | 16189 | 12 | 0.047
B4a1a | 8281-8289d | 13 | 0.051
B4a1a1 | 1438 | 1 | 0.001
B4a1a1 | 16189 | 3 | 0.004
B4a1a1 | 5465 | 1 | 0.001

B4a1a1 | 73 | 1 | 0.001
B4a1a1 | 8281-8289d | 2 | 0.003
B4a1a1+151 | 16189 | 4 | 0.011
B4a1a1+152 | 146 | 1 | 0.003
B4a1a1+16126 | 8281-8289d | 11 | 0.458
B4a1a1a | 146 | 1 | 0.002
B4a1a1a | 16189 | 1 | 0.002
B4a1a1a | 16217 | 2 | 0.003
B4a1a1a+195 | 16189 | 4 | 0.011
B4a1a1a1 | 16217 | 1 | 0.002
B4a1a1a1 | 8281-8289d | 1 | 0.002
B4a1a1a11b | 73 | 18 | 0.212
B4a1a1a14 | 6905 | 1 | 0.053
B4a1a1a16 | 16189 | 1 | 0.026
B4a1a1a16 | 16217 | 1 | 0.026
B4a1a1a16 | 73 | 2 | 0.051
B4a1a1a16 | 8281-8289d | 6 | 0.154
B4a1a1a18 | 8281-8289d | 4 | 0.07
B4a1a1a1c | 16189 | 4 | 0.011
B4a1a1a1d | 146 | 1 | 0.003
B4a1a1a1d | 16189 | 4 | 0.011
B4a1a1a20 | 16217 | 2 | 0.077
B4a1a1a20 | 16261 | 2 | 0.077
B4a1a1a22 | 8281-8289d | 6 | 0.014
B4a1a1a23 | 16189 | 4 | 0.011
B4a1a1a2a | 16189 | 1 | 0.029
B4a1a1a2a | 16217 | 2 | 0.057
B4a1a1a2b | 8281-8289d | 1 | 0.125
B4a1a1a4 | 14022 | 1 | 0.062
B4a1a1a4 | 6905 | 2 | 0.125
B4a1a1a7 | 16217 | 1 | 0.002
B4a1a1a8 | 16189 | 4 | 0.011
B4a1a1a9 | 8281-8289d | 1 | 0.071
B4a1a1b | 10238 | 1 | 0.001
B4a1a1b | 12239 | 1 | 0.001
B4a1a1b | 16189 | 6 | 0.006
B4a1a1b | 16217 | 8 | 0.008
B4a1a1b | 16261 | 10 | 0.01
B4a1a1b | 263 | 1 | 0.001
B4a1a1b | 3423A | 1 | 0.001
B4a1a1b | 8281-8289d | 6 | 0.006
B4a1a1c | 1185 | 1 | 0.002
B4a1a1c | 16189 | 1 | 0.002
B4a1a1c | 8281-8289d | 61 | 0.122
B4a1a1h | 16261 | 1 | 0.003
B4a1a1i | 16261 | 2 | 0.005
B4a1a1j | 8281-8289d | 15 | 0.625
B4a1a1m | 16189 | 4 | 0.011
B4a1a1m1 | 16261 | 1 | 0.002
B4a1a1m1 | 8281-8289d | 51 | 0.11
B4a1a1o | 146 | 1 | 0.003
B4a1a1o | 16189 | 4 | 0.011
B4a1a1s | 16261 | 13 | 0.029
B4a1a3a | 16217 | 1 | 0.029
B4a1a3a1a | 16189 | 7 | 0.152
B4a1a3a1a | 8281-8289d | 6 | 0.13
B4a1a6 | 16261 | 4 | 0.02
B4a1a7 | 16189 | 4 | 0.02
B4a1a7 | 8281-8289d | 4 | 0.02
B4a1c | 16189 | 1 | 0.004
B4a1c | 16217 | 1 | 0.004
B4a1c | 8281-8289d | 1 | 0.004
B4a1c1a1 | 9254 | 1 | 0.005
B4a1c2 | 16317 | 22 | 0.71
B4a1c2 | 709 | 1 | 0.032
B4a1c2 | 8281-8289d | 2 | 0.065
B4a1c2 | 9822A | 3 | 0.097
B4a1c3a | 7927 | 1 | 0.077
B4a1c4 | 16217 | 1 | 0.004
B4a1c4 | 16261 | 1 | 0.004
B4a1c4 | 8281-8289d | 3 | 0.012
B4a1c5 | 16189 | 4 | 0.02
B4a1c5 | 5465 | 1 | 0.005
B4a1c5 | 8281-8289d | 4 | 0.02
B4a1e | 16189 | 1 | 0.01
B4a1e | 16261 | 1 | 0.01
B4a1e | 8281-8289d | 2 | 0.021
B4a2a | 16189 | 2 | 0.028
B4a2a | 8281-8289d | 2 | 0.028
B4a2a1 | 16189 | 7 | 0.109
B4a2a1 | 8281-8289d | 7 | 0.109
B4a2a2 | 16189 | 3 | 0.051
B4a2a2 | 8281-8289d | 3 | 0.051
B4a2a3 | 16189 | 15 | 0.155
B4a2a3 | 5465 | 1 | 0.01
B4a2a3 | 8281-8289d | 15 | 0.155
B4a2b1 | 16217 | 7 | 0.099
B4a4 | 16217 | 3 | 0.02
B4a4 | 16261 | 69 | 0.457
B4a5 | 12732 | 13 | 0.049
B4a5 | 16189 | 2 | 0.008
B4a5 | 8281-8289d | 2 | 0.008
B4b1 | 4769 | 1 | 0.006
B4b1a1 | 6023 | 1 | 0.032
B4b1a1b | 16136 | 1 | 0.05
B4b1a1b | 16217 | 2 | 0.1
B4b1a2 | 8281-8289d | 2 | 0.011
B4b1a2a | 16189 | 1 | 0.004
B4b1a2a | 16217 | 3 | 0.011
B4b1a2a | 207 | 1 | 0.004
B4b1a2a | 499 | 1 | 0.004
B4b1a2a | 5899.XC | 20 | 0.071
B4b1a2a | 6023 | 2 | 0.007
B4b1a2a | 73 | 1 | 0.004
B4b1a2a | 8281-8289d | 9 | 0.032
B4b1a2b | 207 | 2 | 0.154
B4b1a2b2 | 13105 | 1 | 0.083
B4b1a2b2 | 15326 | 1 | 0.083
B4b1a2b2 | 16189 | 5 | 0.417
B4b1a2b2 | 8281-8289d | 5 | 0.417
B4b1a2c | 16136 | 1 | 0.111

B4b1a2f | 16189 | 8 | 0.051
B4b1a2f | 8281-8289d | 8 | 0.051
B4b1a2g | 16189 | 7 | 0.292
B4b1a2g | 8281-8289d | 7 | 0.292
B4b1a2g1 | 16189 | 7 | 0.292
B4b1a2g1 | 8281-8289d | 7 | 0.292
B4b1a2h | 16189 | 4 | 0.024
B4b1a2h | 8281-8289d | 4 | 0.024
B4b1a2i | 207 | 1 | 0.04
B4b1a3 | 207 | 9 | 0.063
B4b1a3 | 6023 | 1 | 0.007
B4b1a3a | 207 | 4 | 0.1
B4b1a3a | 827 | 2 | 0.05
B4b1a3a | 8281-8289d | 1 | 0.025
B4b1b | 16218 | 5 | 0.263
B4b1b | 16362 | 1 | 0.053
B4b1b'c | 16217 | 3 | 0.044
B4b1b'c | 499 | 1 | 0.015
B4b1c | 16217 | 3 | 0.044
B4b1c | 499 | 1 | 0.015
B4b1c1 | 11914 | 1 | 0.021
B4b1c1 | 16218 | 1 | 0.021
B4b1c1 | 499 | 1 | 0.021
B4b1c1 | 8281-8289d | 1 | 0.021
B4b1c2 | 16189 | 4 | 0.08
B4b1c2 | 499 | 1 | 0.02
B4b1c2 | 73 | 1 | 0.02
B4b1c2 | 8281-8289d | 1 | 0.02
B4c | 16217 | 1 | 0.008
B4c | 73 | 5 | 0.04
B4c | 8281-8289d | 1 | 0.008
B4c1a | 73 | 3 | 0.03
B4c1a1c | 16217 | 1 | 0.02
B4c1b | 16217 | 1 | 0.016
B4c1b+16335 | 16140 | 1 | 0.007
B4c1b+16335 | 16189 | 7 | 0.049
B4c1b+16335 | 16274 | 1 | 0.007
B4c1b2 | 16140 | 8 | 0.054
B4c1b2a | 16140 | 1 | 0.006
B4c1b2a | 16335 | 1 | 0.006
B4c1b2a | 3497 | 1 | 0.006
B4c1b2a | 8281-8289d | 5 | 0.03
B4c1b2a1 | 16140 | 1 | 0.006
B4c1b2a1 | 16274 | 1 | 0.006
B4c1b2a1 | 16335 | 3 | 0.017
B4c1b2a2 | 15301 | 2 | 0.013
B4c1b2a2 | 15346 | 1 | 0.006
B4c1b2a2 | 16189 | 2 | 0.013
B4c1b2a2 | 16335 | 4 | 0.026
B4c1b2a2 | 8281-8289d | 14 | 0.09
B4c1b2a2 | 8772 | 3 | 0.019
B4c1b2a2a | 16189 | 15 | 0.104
B4c1b2a2a | 8281-8289d | 15 | 0.104
B4c1b2a2b | 16335 | 1 | 0.007
B4c1b2b | 150 | 1 | 0.007
B4c1b2b | 16189 | 1 | 0.007
B4c1b2b | 16274 | 4 | 0.029
B4c1b2b | 8281-8289d | 1 | 0.007
B4c1b2c1 | 16136 | 1 | 0.022
B4c1b2c1 | 16189 | 1 | 0.022
B4c1b2c1 | 16274 | 1 | 0.022
B4c1b2c1 | 16335 | 1 | 0.022
B4c1b2c1 | 8281-8289d | 1 | 0.022
B4c1b2c2 | 150 | 1 | 0.011
B4c1b2c2 | 16166 | 1 | 0.011
B4c1b2c2 | 16189 | 2 | 0.021
B4c1b2c2 | 16217 | 3 | 0.032
B4c1b2c2 | 16274 | 2 | 0.021
B4c1b2c2 | 16335 | 13 | 0.138
B4c1b2c2 | 8281-8289d | 1 | 0.011
B4c1c1 | 16217 | 2 | 0.053
B4c1c1 | 16311 | 1 | 0.026
B4c2 | 16184A | 3 | 0.022
B4c2 | 8281-8289d | 4 | 0.03
B4c2a | 16147 | 7 | 0.047
B4c2b | 16217 | 2 | 0.013
B4c2b | 16235 | 36 | 0.24
B4c2c | 16184A | 18 | 0.122
B4c2c | 16189 | 3 | 0.02
B4c2c | 16217 | 1 | 0.007
B4c2c | 16235 | 1 | 0.007
B4c2c | 8281-8289d | 4 | 0.027
B4d1 | 16217 | 2 | 0.015
B4d1 | 73 | 1 | 0.007
B4d3 | 151 | 2 | 0.286
B4d3a1 | 151 | 1 | 0.071
B4d3a1 | 16185 | 1 | 0.071
B4d3a1 | 197 | 5 | 0.357
B4e | 146 | 1 | 0.013
B4e | 16217 | 2 | 0.025
B4e | 195 | 14 | 0.177
B4e | 6026 | 7 | 0.089
B4f | 16168 | 1 | 0.167
B4f | 16172 | 1 | 0.167
B4f | 16189 | 2 | 0.333
B4f1 | 16217 | 1 | 0.167
B4f1 | 573.XC | 2 | 0.333
B4g1 | 16213 | 2 | 0.014
B4g1 | 16217 | 2 | 0.014
B4g1 | 16261 | 10 | 0.069
B4g1 | 16292 | 3 | 0.021
B4g1 | 263 | 1 | 0.007
B4g1a | 16189 | 1 | 0.014
B4g1a | 16213 | 1 | 0.014
B4g1a | 16261 | 1 | 0.014
B4g1a | 8281-8289d | 5 | 0.068
B4g1b | 16213 | 3 | 0.041
B4g1b | 16217 | 1 | 0.014
B4g1b | 4769 | 1 | 0.014
B4g2 | 16189 | 1 | 0.013
B4g2 | 16213 | 1 | 0.013

B4g2 | 16261 | 3 | 0.038
B4g2 | 16301 | 48 | 0.608
B4g2 | 62 | 1 | 0.013
B4g2 | 8281-8289d | 2 | 0.025
B4h | 16189 | 1 | 0.006
B4h | 16261 | 2 | 0.012
B4h | 8281-8289d | 1 | 0.006
B4h1 | 16129 | 1 | 0.01
B4i1 | 16217 | 1 | 0.004
B4j | 14248 | 1 | 0.032
B4j | 16362 | 6 | 0.194
B4k | 16189 | 1 | 0.009
B4k | 16217 | 1 | 0.009
B4k | 8281-8289d | 1 | 0.009
B4m | 16189 | 2 | 0.019
B4m | 16240 | 1 | 0.01
B4m | 8281-8289d | 4 | 0.038
B5 | 263 | 2 | 0.037
B5a | 16189 | 2 | 0.004
B5a | 210 | 4 | 0.008
B5a | 8281-8289d | 2 | 0.004
B5a1 | 16140 | 1 | 0.002
B5a1 | 8281-8289d | 5 | 0.009
B5a1a | 16140 | 4 | 0.006
B5a1a | 16189 | 1 | 0.001
B5a1a | 210 | 1 | 0.001
B5a1a | 2706 | 9 | 0.013
B5a1a | 8281-8289d | 20 | 0.03
B5a1a1 | 210 | 1 | 0.002
B5a1b1 | 263 | 2 | 0.004
B5a1b1 | 8281-8289d | 28 | 0.053
B5a1c | 16140 | 26 | 0.053
B5a1c | 16189 | 2 | 0.004
B5a1c | 73 | 1 | 0.002
B5a1c | 8281-8289d | 2 | 0.004
B5a1c1 | 16189 | 1 | 0.002
B5a1c1 | 8281-8289d | 1 | 0.002
B5a1c1a | 16189 | 3 | 0.007
B5a1c1a | 73 | 2 | 0.005
B5a1c1a | 8281-8289d | 1 | 0.002
B5a1c1a1 | 16140 | 2 | 0.039
B5a1d | 10398 | 1 | 0.003
B5a1d | 14766 | 1 | 0.003
B5a1d | 16140 | 10 | 0.034
B5a1d | 16261 | 1 | 0.003
B5a1d | 16266A | 1 | 0.003
B5a1d | 8281-8289d | 4 | 0.014
B5a2 | 11151 | 8 | 0.127
B5a2 | 16140 | 6 | 0.095
B5a2 | 2706 | 2 | 0.032
B5a2 | 8281-8289d | 2 | 0.032
B5a2 | 9962 | 8 | 0.127
B5a2a1a | 16187 | 1 | 0.026
B5a2a1a | 16189 | 1 | 0.026
B5a2a1a | 16256 | 1 | 0.026
B5a2a1a | 16266G | 1 | 0.026
B5a2a1a | 93 | 1 | 0.026
B5a2a1b | 16187 | 1 | 0.071
B5a2a1b | 16266G | 11 | 0.786
B5a2a1b | 4769 | 1 | 0.071
B5a2a1b | 709 | 1 | 0.071
B5a2a1b | 8860 | 1 | 0.071
B5a2a2a1 | 16189 | 10 | 0.556
B5a2a2a1 | 16266G | 2 | 0.111
B5a2a2a1 | 8281-8289d | 10 | 0.556
B5a2a2a2 | 16189 | 5 | 0.417
B5a2a2a2 | 16266G | 3 | 0.25
B5a2a2a2 | 8281-8289d | 5 | 0.417
B5a2a2b1 | 16189 | 2 | 0.118
B5a2a2b1 | 8281-8289d | 2 | 0.118
B5a2a2b1a | 16189 | 5 | 0.263
B5a2a2b1a | 8281-8289d | 5 | 0.263
B5a2a2b2 | 16189 | 2 | 0.667
B5a2a2b2 | 8281-8289d | 2 | 0.667
B5b | 103 | 2 | 0.012
B5b | 204 | 1 | 0.006
B5b | 8860 | 1 | 0.006
B5b1 | 103 | 11 | 0.04
B5b1 | 15662 | 1 | 0.004
B5b1 | 16189 | 2 | 0.007
B5b1 | 16243 | 1 | 0.004
B5b1 | 204 | 47 | 0.171
B5b1 | 73 | 1 | 0.004
B5b1 | 8784 | 1 | 0.004
B5b1a1 | 16140 | 1 | 0.143
B5b1a2 | 14766 | 1 | 0.056
B5b1a2 | 16140 | 1 | 0.056
B5b1a2 | 16223 | 1 | 0.056
B5b1a2 | 16319 | 1 | 0.056
B5b1c | 15851 | 1 | 0.005
B5b1c | 16140 | 2 | 0.011
B5b1c | 204 | 6 | 0.033
B5b1c | 8281-8289d | 1 | 0.005
B5b1c1 | 103 | 8 | 0.047
B5b1c1 | 15851 | 1 | 0.006
B5b1c1a | 15851 | 1 | 0.006
B5b2 | 131 | 4 | 0.182
B5b2 | 16111 | 1 | 0.045
B5b2 | 16140 | 1 | 0.045
B5b2 | 16234 | 2 | 0.091
B5b2+@204 | 131 | 1 | 0.032
B5b2+@204 | 16111 | 2 | 0.065
B5b2+@204 | 16463 | 3 | 0.097
B5b2a | 15223 | 1 | 0.059
B5b2a | 16111 | 1 | 0.059
B5b2a | 16189 | 1 | 0.059
B5b2a | 16234 | 2 | 0.118
B5b2a | 16463 | 1 | 0.059
B5b2a | 204 | 3 | 0.176
B5b2a | 8829 | 1 | 0.059
B5b2a1 | 16463 | 1 | 0.034

B5b2a1 | 204 | 5 | 0.172
B5b2a2 | 16463 | 1 | 0.059
B5b2a2a | 16463 | 2 | 0.1
B5b2a2a2 | 16463 | 1 | 0.077
B5b2b | 16111 | 1 | 0.053
B5b2b | 16463 | 3 | 0.158
B5b2b | 204 | 1 | 0.053
B5b2c | 103 | 2 | 0.083
B5b2c | 11101 | 3 | 0.125
B5b2c | 131 | 4 | 0.167
B5b2c | 16111 | 2 | 0.083
B5b2c | 16189 | 2 | 0.083
B5b2c | 750 | 1 | 0.042
B5b2c | 8281-8289d | 2 | 0.083
B5b2c1 | 103 | 6 | 0.261
B5b2c1 | 16111 | 4 | 0.174
B5b2c1 | 16140 | 1 | 0.043
B5b2c1 | 73 | 2 | 0.087
B5b3 | 103 | 7 | 0.04
B5b3 | 189 | 1 | 0.006
B5b3 | 204 | 6 | 0.034
B5b4 | 16189 | 1 | 0.062
B5b4 | 16243 | 1 | 0.062
B5b4 | 16274 | 10 | 0.625
B5b4 | 204 | 1 | 0.062
B5b5 | 103 | 1 | 0.077
B5b5 | 16166 | 6 | 0.462
B5b5 | 16189 | 1 | 0.077
B5b5 | 16243 | 1 | 0.077
B5b5 | 73 | 1 | 0.077
B5b5 | 8281-8289d | 2 | 0.154
B6a | 150 | 3 | 0.03
B6a | 16093 | 5 | 0.05
B6a | 5894C | 17 | 0.168
B6a | 8281-8289d | 7 | 0.069
B6a1 | 16093 | 17 | 0.654
B6a1 | 356.1C | 1 | 0.038
B6a1 | 5894C | 1 | 0.038
B6a1 | 8281-8289d | 1 | 0.038
B6a1a | 356.1C | 20 | 0.282
B6a1a | 8281-8289d | 20 | 0.282
C | 14318 | 1 | 0.004
C | 14766 | 1 | 0.004
C | 15326 | 1 | 0.004
C | 16223 | 1 | 0.004
C | 16298 | 1 | 0.004
C | 16327 | 1 | 0.004
C | 249d | 1 | 0.004
C | 7196A | 1 | 0.004
C1 | 16223 | 5 | 0.024
C1 | 16298 | 1 | 0.005
C1 | 16327 | 2 | 0.01
C1 | 73 | 37 | 0.18
C1a | 489 | 1 | 0.007
C1b | 12705 | 1 | 0.004
C1b | 13263 | 1 | 0.004
C1b | 15043 | 3 | 0.011
C1b | 16223 | 5 | 0.019
C1b | 16298 | 17 | 0.064
C1b | 249d | 6 | 0.023
C1b | 290-291d | 2 | 0.008
C1b | 4715 | 7 | 0.026
C1b | 489 | 1 | 0.004
C1b | 7028 | 1 | 0.004
C1b | 8584 | 1 | 0.004
C1b | 9545 | 5 | 0.019
C1b+16311 | 10398 | 1 | 0.034
C1b+16311 | 4715 | 1 | 0.034
C1b1 | 16298 | 4 | 0.055
C1b10 | 146 | 3 | 0.3
C1b10 | 16223 | 2 | 0.2
C1b10 | 263 | 2 | 0.2
C1b11 | 1438 | 1 | 0.01
C1b13a1 | 16298 | 1 | 0.026
C1b13a1 | 290-291d | 1 | 0.026
C1b13c1 | 290-291d | 1 | 0.024
C1b13d | 16327 | 2 | 0.154
C1b13d | 258 | 1 | 0.077
C1b13e | 16327 | 3 | 0.067
C1b14 | 16325 | 5 | 0.5
C1b2 | 12454 | 11 | 0.145
C1b2 | 16298 | 1 | 0.013
C1b2 | 4242 | 1 | 0.013
C1b4 | 16086 | 1 | 0.071
C1b5 | 16298 | 2 | 0.015
C1b5 | 16327 | 1 | 0.007
C1b5 | 249d | 1 | 0.007
C1b5 | 290-291d | 1 | 0.007
C1b5 | 73 | 28 | 0.206
C1b5a | 73 | 3 | 0.041
C1b7 | 73 | 8 | 0.163
C1b8 | 15613 | 7 | 0.184
C1b8 | 16223 | 1 | 0.026
C1b8 | 493 | 1 | 0.026
C1b8 | 73 | 4 | 0.105
C1b8a | 16362 | 2 | 0.2
C1b8a | 8584 | 1 | 0.1
C1b9 | 9545 | 1 | 0.017
C1c | 13263 | 22 | 0.088
C1c | 16223 | 1 | 0.004
C1c | 16298 | 3 | 0.012
C1c | 249d | 7 | 0.028
C1c | 8701 | 1 | 0.004
C1c+195 | 1438 | 1 | 0.007
C1c+195 | 16327 | 1 | 0.007
C1c+195 | 290-291d | 1 | 0.007
C1c+195 | 73 | 4 | 0.03
C1c1 | 16325 | 4 | 0.027
C1c1 | 249d | 1 | 0.007
C1c1 | 8584 | 1 | 0.007
C1c2 | 11794 | 1 | 0.007

C1c6 | 16327 | 1 | 0.009
C1c6 | 195 | 5 | 0.045
C1c7 | 8584 | 1 | 0.01
C1d | 13263 | 1 | 0.006
C1d | 16223 | 2 | 0.013
C1d+194 | 8860 | 1 | 0.009
C1d1 | 10873 | 1 | 0.006
C1d1 | 14318 | 1 | 0.006
C1d1 | 16223 | 9 | 0.05
C1d1 | 16298 | 2 | 0.011
C1d1 | 16327 | 3 | 0.017
C1d1 | 249d | 1 | 0.006
C1d1 | 290-291d | 2 | 0.011
C1d1 | 3552A | 2 | 0.011
C1d1 | 73 | 1 | 0.006
C1d1 | 8860 | 2 | 0.011
C1d1a1 | 16298 | 1 | 0.009
C1d1b | 194 | 1 | 0.059
C1d1b1 | 16223 | 1 | 0.059
C1d1b1 | 4715 | 1 | 0.059
C1d1d | 194 | 6 | 0.051
C1d2a | 16223 | 1 | 0.25
C1e | 290-291d | 1 | 0.167
C1e | 534 | 1 | 0.167
C1e | 73 | 1 | 0.167
C1f | 15326 | 2 | 0.016
C4 | 249d | 2 | 0.008
C4 | 4769 | 1 | 0.004
C4a1 | 12705 | 5 | 0.013
C4a1a | 16223 | 2 | 0.006
C4a1a+195 | 2232.1A | 2 | 0.006
C4a1a+195 | 249d | 1 | 0.003
C4a1a+195 | 263 | 11 | 0.031
C4a1a+195 | 489 | 1 | 0.003
C4a1a+195 | 8584 | 2 | 0.006
C4a1a1 | 249d | 2 | 0.006
C4a1a1 | 8508 | 2 | 0.006
C4a1a1a | 249d | 2 | 0.005
C4a1a1a | 3552A | 2 | 0.005
C4a1a1a | 73 | 1 | 0.003
C4a1a2 | 15204 | 1 | 0.003
C4a1a2 | 16223 | 16 | 0.047
C4a1a2 | 16327 | 2 | 0.006
C4a1a2 | 489 | 1 | 0.003
C4a1a2a | 489 | 1 | 0.003
C4a1a3 | 15968 | 1 | 0.003
C4a1a3 | 2232.1A | 3 | 0.008
C4a1a3 | 249d | 3 | 0.008
C4a1a3 | 489 | 1 | 0.003
C4a1a3a | 489 | 1 | 0.003
C4a1a3a1 | 489 | 1 | 0.003
C4a1a3b | 489 | 1 | 0.003
C4a1a3c | 489 | 1 | 0.003
C4a1a3d | 15607 | 3 | 0.021
C4a1a4 | 489 | 1 | 0.003
C4a1a4a | 10398 | 1 | 0.006
C4a1a4a | 12672 | 1 | 0.006
C4a1a4a | 7196A | 1 | 0.006
C4a1a5 | 2232.1A | 1 | 0.003
C4a1a6 | 16298 | 1 | 0.003
C4a1b | 16298 | 1 | 0.007
C4a1b | 16327 | 1 | 0.007
C4a2a1 | 16223 | 2 | 0.01
C4a2a1 | 2232.1A | 2 | 0.01
C4a2a1 | 249d | 1 | 0.005
C4a2a1a | 15301 | 2 | 0.012
C4a2a1a | 16327 | 1 | 0.006
C4a2a1a | 16357 | 1 | 0.006
C4a2a1b | 16327 | 1 | 0.006
C4a2a1b | 16357 | 1 | 0.006
C4a2b | 16327 | 4 | 0.02
C4a2b | 2232.1A | 5 | 0.025
C4a2b | 249d | 1 | 0.005
C4a2b1 | 2232.1A | 2 | 0.015
C4a2b1 | 3552A | 1 | 0.008
C4a2b2 | 16223 | 1 | 0.004
C4a2b2 | 16327 | 2 | 0.008
C4a2b2 | 310 | 1 | 0.004
C4a2b2 | 7196A | 2 | 0.008
C4a2b2a | 16311 | 1 | 0.004
C4a2b2a | 249d | 48 | 0.183
C4a2c | 11914 | 1 | 0.005
C4a2c | 11969 | 1 | 0.005
C4a2c | 12672 | 1 | 0.005
C4a2c | 16298 | 1 | 0.005
C4a2c | 16327 | 1 | 0.005
C4a2c | 8584 | 2 | 0.01
C4a2c1 | 10724 | 3 | 0.025
C4a2c1 | 13145 | 3 | 0.025
C4a2c1 | 16298 | 5 | 0.041
C4a2c1 | 249d | 1 | 0.008
C4a2c1 | 3394 | 2 | 0.017
C4a2c1 | 3552A | 2 | 0.017
C4a2c1 | 8584 | 2 | 0.017
C4a2c1 | 9545 | 1 | 0.008
C4a2c2 | 16327 | 9 | 0.067
C4a2c2 | 2232.1A | 1 | 0.007
C4a2c2 | 47 | 1 | 0.007
C4a2c2a | 2232.1A | 1 | 0.008
C4b | 2232.1A | 1 | 0.004
C4b | 249d | 1 | 0.004
C4b1 | 2232.1A | 5 | 0.018
C4b1 | 249d | 4 | 0.015
C4b1 | 8584 | 1 | 0.004
C4b1 | 9540 | 1 | 0.004
C4b1 | 9545 | 2 | 0.007
C4b1b | 16223 | 11 | 0.045
C4b2a | 2232.1A | 1 | 0.007
C4b3 | 2232.1A | 1 | 0.007
C4b3 | 249d | 1 | 0.007
C4b3a | 1438 | 3 | 0.019

C4c | 10873 | 1 | 0.004
C4c1 | 10873 | 1 | 0.004
C4c1 | 249d | 11 | 0.042
C4c1 | 4715 | 1 | 0.004
C4c1b | 16223 | 4 | 0.018
C4c1b | 249d | 2 | 0.009
C4c2 | 189 | 1 | 0.013
C4c2 | 473 | 1 | 0.013
C4d | 152 | 1 | 0.005
C4d | 16223 | 92 | 0.449
C4e | 249d | 2 | 0.011
C5 | 16223 | 1 | 0.022
C5 | 16327 | 1 | 0.022
C5 | 249d | 1 | 0.022
C5 | 595.1C | 6 | 0.13
C5 | 8701 | 1 | 0.022
C5 | 9540 | 1 | 0.022
C5 | 9545 | 1 | 0.022
C5+16093 | 249d | 1 | 0.027
C5+16093 | 595.1C | 1 | 0.027
C5a | 16298 | 3 | 0.058
C5a1 | 595.1C | 2 | 0.027
C5a2 | 16298 | 1 | 0.016
C5a2a | 595.1C | 2 | 0.031
C5a2b | 595.1C | 5 | 0.077
C5b1 | 1438 | 1 | 0.02
C5b1 | 595.1C | 1 | 0.02
C5c | 16327 | 1 | 0.034
C5c+16234 | 16093 | 1 | 0.038
C5c1 | 249d | 2 | 0.071
C5c1a | 16093 | 1 | 0.026
C5c1a | 595.1C | 2 | 0.053
C5d1 | 16093 | 1 | 0.016
C5d1 | 16327 | 1 | 0.016
C5d1 | 595.1C | 1 | 0.016
C7 | 1438 | 1 | 0.004
C7 | 249d | 2 | 0.008
C7 | 489 | 1 | 0.004
C7+16051 | 249d | 1 | 0.007
C7a | 1438 | 1 | 0.003
C7a | 16223 | 2 | 0.006
C7a | 16327 | 1 | 0.003
C7a | 249d | 17 | 0.052
C7a | 489 | 4 | 0.012
C7a | 7196A | 1 | 0.003
C7a1 | 249d | 17 | 0.062
C7a1 | 489 | 8 | 0.029
C7a1 | 750 | 1 | 0.004
C7a1a2 | 11914 | 1 | 0.009
C7a2 | 249d | 2 | 0.008
C7a2 | 489 | 2 | 0.008
C7a2 | 750 | 1 | 0.004
C7a2a | 16189 | 2 | 0.009
C7a2a | 16327 | 1 | 0.004
C7a2a | 2232.1A | 3 | 0.013
C7a2a | 249d | 2 | 0.009
C7b | 13263 | 1 | 0.006
C7b | 15043 | 6 | 0.038
C7b | 16051 | 1 | 0.006
C7b | 489 | 1 | 0.006
D+16189 | 16223 | 1 | 0.005
D1 | 10873 | 3 | 0.007
D1 | 12705 | 3 | 0.007
D1 | 14766 | 1 | 0.002
D1 | 14783 | 1 | 0.002
D1 | 15301 | 1 | 0.002
D1 | 15326 | 3 | 0.007
D1 | 16223 | 20 | 0.046
D1 | 16325 | 7 | 0.016
D1 | 16362 | 6 | 0.014
D1 | 2092 | 3 | 0.007
D1 | 263 | 1 | 0.002
D1 | 4769 | 1 | 0.002
D1 | 489 | 2 | 0.005
D1 | 7028 | 1 | 0.002
D1 | 750 | 1 | 0.002
D1 | 8414 | 1 | 0.002
D1 | 8701 | 1 | 0.002
D1 | 8860 | 1 | 0.002
D1a1 | 14783 | 40 | 0.248
D1a1 | 57G | 2 | 0.012
D1a2 | 16223 | 1 | 0.004
D1a2 | 73 | 26 | 0.101
D1d2 | 73 | 1 | 0.006
D1e | 16362 | 2 | 0.009
D1e | 73 | 8 | 0.036
D1f1 | 11719 | 2 | 0.011
D1f3 | 8701 | 1 | 0.006
D1g2 | 143 | 1 | 0.01
D1g2 | 16189 | 2 | 0.019
D1g2a | 15930 | 1 | 0.062
D1g5 | 499 | 1 | 0.167
D1h | 16093 | 1 | 0.006
D1h1 | 16093 | 1 | 0.009
D1i | 73 | 25 | 0.245
D1j | 16242 | 3 | 0.019
D1j | 16325 | 1 | 0.006
D1j1a | 152 | 2 | 0.013
D1j1a2 | 16325 | 3 | 0.025
D2a1 | 10873 | 1 | 0.013
D2a1 | 16271 | 2 | 0.025
D2a1 | 3010 | 1 | 0.013
D2a'b | 4769 | 1 | 0.013
D2a'b | 8860 | 1 | 0.013
D2b1 | 195 | 1 | 0.033
D2c | 73 | 1 | 0.016
D4 | 10398 | 1 | 0.001
D4 | 10400 | 1 | 0.001
D4 | 8701 | 1 | 0.001
D4+195 | 10398 | 1 | 0.002
D4+195 | 10400 | 1 | 0.002

D4+195 | 16223 | 1 | 0.002
D4+195 | 73 | 1 | 0.002
D4a | 16223 | 2 | 0.007
D4a | 489 | 1 | 0.003
D4a | 73 | 1 | 0.003
D4a1 | 16223 | 2 | 0.007
D4a1 | 489 | 1 | 0.003
D4a1 | 73 | 1 | 0.003
D4a1a | 16223 | 2 | 0.007
D4a1a | 489 | 1 | 0.003
D4a1a | 73 | 1 | 0.003
D4a1a1 | 16223 | 2 | 0.007
D4a1a1 | 3206 | 1 | 0.003
D4a1a1 | 489 | 1 | 0.003
D4a1a1 | 73 | 1 | 0.003
D4a1a1a | 16223 | 2 | 0.007
D4a1a1a | 489 | 1 | 0.004
D4a1a1a | 73 | 1 | 0.004
D4a1b | 16129 | 1 | 0.003
D4a1b | 16223 | 2 | 0.007
D4a1b | 489 | 1 | 0.003
D4a1b | 73 | 1 | 0.003
D4a1c | 16223 | 2 | 0.007
D4a1c | 489 | 1 | 0.004
D4a1c | 73 | 1 | 0.004
D4a1e | 14979 | 1 | 0.007
D4a1f | 16223 | 2 | 0.007
D4a1f | 489 | 1 | 0.003
D4a1f | 73 | 1 | 0.003
D4a1f1 | 16129 | 1 | 0.004
D4a1f1 | 16223 | 2 | 0.007
D4a1f1 | 489 | 1 | 0.004
D4a1f1 | 73 | 1 | 0.004
D4a1g | 16223 | 2 | 0.007
D4a1g | 489 | 1 | 0.004
D4a1g | 73 | 1 | 0.004
D4a1h | 16223 | 2 | 0.007
D4a1h | 489 | 1 | 0.004
D4a1h | 73 | 1 | 0.004
D4a2 | 16223 | 2 | 0.007
D4a2 | 489 | 1 | 0.003
D4a2 | 73 | 1 | 0.003
D4a2a | 16223 | 2 | 0.007
D4a2a | 489 | 1 | 0.004
D4a2a | 73 | 1 | 0.004
D4a2b | 16223 | 2 | 0.007
D4a2b | 489 | 1 | 0.003
D4a2b | 73 | 1 | 0.003
D4a3a1 | 10398 | 1 | 0.009
D4a3a1 | 16223 | 1 | 0.009
D4a3a2 | 16362 | 1 | 0.006
D4a3b | 16249 | 1 | 0.007
D4a3b | 4769 | 1 | 0.007
D4a3b2 | 489 | 2 | 0.01
D4a3b2 | 73 | 3 | 0.014
D4a5 | 16129 | 1 | 0.004
D4a5 | 16223 | 2 | 0.007
D4a5 | 489 | 1 | 0.004
D4a5 | 73 | 1 | 0.004
D4a7 | 152 | 2 | 0.009
D4a7 | 16129 | 1 | 0.005
D4a7 | 16362 | 1 | 0.005
D4a7 | 489 | 1 | 0.005
D4b1a | 15326 | 1 | 0.005
D4b1a | 263 | 1 | 0.005
D4b1a1a | 16319 | 1 | 0.005
D4b1a2 | 15326 | 1 | 0.005
D4b1a2 | 4769 | 1 | 0.005
D4b1a2 | 8860 | 1 | 0.005
D4b1a2a1 | 16223 | 1 | 0.004
D4b1a2a1 | 16319 | 1 | 0.004
D4b1a2a1 | 16362 | 1 | 0.004
D4b1b | 16223 | 3 | 0.048
D4b1b | 16287 | 1 | 0.016
D4b1b | 750 | 1 | 0.016
D4b1b1a1 | 16223 | 1 | 0.017
D4b1b1a1 | 16362 | 1 | 0.017
D4b1b1a1 | 16399 | 1 | 0.017
D4b1b1a1 | 4769 | 1 | 0.017
D4b1c | 4769 | 1 | 0.007
D4b2 | 14668 | 1 | 0.001
D4b2 | 14766 | 1 | 0.001
D4b2 | 4883 | 1 | 0.001
D4b2 | 489 | 1 | 0.001
D4b2 | 5178A | 1 | 0.001
D4b2a1 | 16362 | 1 | 0.009
D4b2a2 | 10873 | 1 | 0.001
D4b2a2 | 15524 | 1 | 0.001
D4b2a2a1 | 8860 | 1 | 0.005
D4b2b | 73 | 1 | 0.001
D4b2b | 750 | 1 | 0.001
D4b2b1 | 2706 | 1 | 0.001
D4b2b1 | 4883 | 1 | 0.001
D4b2b1 | 9824A | 1 | 0.001
D4b2b1+146 | 8860 | 1 | 0.002
D4b2b1d | 4769 | 1 | 0.002
D4b2b2a | 73 | 1 | 0.002
D4b2b2a1 | 73 | 1 | 0.002
D4b2b2b | 16172 | 2 | 0.009
D4b2b2b | 8577 | 3 | 0.014
D4b2b4 | 73 | 1 | 0.002
D4b2b5 | 8020 | 1 | 0.002
D4b2b5 | 9296 | 4 | 0.007
D4c1a1 | 16245 | 1 | 0.037
D4c1a1 | 16362 | 1 | 0.037
D4c1a1 | 199 | 1 | 0.037
D4c1a1 | 207 | 1 | 0.037
D4c1b1 | 16223 | 1 | 0.033
D4c1b1 | 16292 | 1 | 0.033
D4c1b1 | 16362 | 1 | 0.033
D4c2b | 263 | 1 | 0.012

D4e1 | 16223 | 1 | 0.001
D4e1a | 16223 | 1 | 0.002
D4e1a1 | 16093 | 5 | 0.167
D4e1a1 | 194 | 1 | 0.033
D4e1a1 | 3316 | 1 | 0.033
D4e1a1 | 5178A | 1 | 0.033
D4e1a2 | 16223 | 1 | 0.002
D4e1a2a | 94 | 1 | 0.002
D4e1a3 | 16223 | 1 | 0.002
D4e4 | 11215 | 1 | 0.002
D4e4a | 4769 | 1 | 0.002
D4e4a1 | 16189 | 1 | 0.012
D4e4a1 | 310 | 1 | 0.012
D4e5 | 489 | 1 | 0.007
D4e5a | 573.XC | 3 | 0.026
D4f | 1438 | 1 | 0.002
D4f | 263 | 1 | 0.002
D4g1 | 573.XC | 4 | 0.037
D4g1a | 573.XC | 2 | 0.021
D4g1b | 573.XC | 1 | 0.01
D4g1c | 573.XC | 2 | 0.02
D4g2a | 16223 | 2 | 0.018
D4g2a | 16362 | 2 | 0.018
D4g2a1 | 16274 | 2 | 0.016
D4g2a1 | 263 | 2 | 0.016
D4g2a1 | 750 | 1 | 0.008
D4g2a1b | 459d | 1 | 0.008
D4g2a1c | 13104 | 1 | 0.009
D4g2a1c | 14668 | 1 | 0.009
D4g2a1c | 489 | 1 | 0.009
D4g2b | 14783 | 1 | 0.002
D4g2b | 298 | 1 | 0.002
D4h1 | 263 | 1 | 0.007
D4h1a1 | 12396 | 1 | 0.083
D4h1b | 10398 | 3 | 0.034
D4h1c | 13914A | 1 | 0.011
D4h1c | 16311 | 1 | 0.011
D4h1c | 5048 | 1 | 0.011
D4h3 | 16342 | 9 | 0.29
D4h3a | 12705 | 1 | 0.025
D4h3a | 16241 | 3 | 0.075
D4h3a+@152 | 73 | 1 | 0.015
D4h3a1a1 | 16301 | 1 | 0.1
D4h3a2 | 12705 | 1 | 0.04
D4h3a3 | 16342 | 1 | 0.25
D4h3a6 | 16241 | 1 | 0.167
D4h3a6 | 73 | 1 | 0.167
D4h3a7 | 73 | 1 | 0.015
D4h3a8 | 2706 | 1 | 0.014
D4h3a9 | 73 | 1 | 0.015
D4h4 | 12705 | 1 | 0.002
D4h4a | 644 | 1 | 0.006
D4i3 | 16114 | 1 | 0.056
D4i3 | 16318 | 2 | 0.111
D4j | 8860 | 1 | 0.002
D4j+16311 | 11696 | 2 | 0.009
D4j1 | 4769 | 1 | 0.002
D4j1a | 16223 | 1 | 0.005
D4j1a1 | 489 | 1 | 0.004
D4j1a1a | 16223 | 1 | 0.005
D4j1a1b | 16223 | 1 | 0.005
D4j1a1b | 5262 | 1 | 0.005
D4j1a1b | 8414 | 1 | 0.005
D4j1b | 11696 | 1 | 0.002
D4j1b | 12705 | 1 | 0.002
D4j1b | 15043 | 2 | 0.003
D4j1b | 15301 | 2 | 0.003
D4j1b | 16223 | 1 | 0.002
D4j1b | 2706 | 2 | 0.003
D4j1b | 3010 | 2 | 0.003
D4j1b | 5262 | 2 | 0.003
D4j1b | 8701 | 2 | 0.003
D4j1b | 8860 | 2 | 0.003
D4j1b | 9540 | 1 | 0.002
D4j2 | 16291 | 1 | 0.013
D4j2 | 73 | 2 | 0.025
D4j3 | 16223 | 2 | 0.007
D4j5 | 15326 | 1 | 0.002
D4j7 | 16082 | 2 | 0.029
D4j8 | 16223 | 6 | 0.056
D4l1a | 10427 | 1 | 0.015
D4l1a1 | 16145 | 2 | 0.029
D4l1a1 | 16362 | 1 | 0.014
D4l2a | 16368 | 2 | 0.091
D4l2a1 | 16145 | 1 | 0.111
D4l2a1 | 16223 | 1 | 0.111
D4l2b | 125 | 1 | 0.05
D4m2a | 3492 | 1 | 0.012
D4o | 195 | 2 | 0.074
D4o1 | 16183 | 111 | 0.76
D4o1 | 16223 | 1 | 0.007
D4o2 | 16290 | 3 | 0.188
D4o2a | 12705 | 1 | 0.018
D4o2a | 16093 | 2 | 0.036
D4o2a | 16290 | 5 | 0.091
D4o2a | 73 | 1 | 0.018
D4o2a1 | 16093 | 1 | 0.032
D4q | 16223 | 1 | 0.008
D4q | 16256 | 2 | 0.017
D4q | 16362 | 1 | 0.008
D4q | 200 | 1 | 0.008
D4s | 12662 | 10 | 0.021
D4s | 1438 | 1 | 0.002
D5 | 10873 | 1 | 0.01
D5a | 263 | 1 | 0.009
D5a1a1 | 16223 | 1 | 0.05
D5a1a1 | 68 | 1 | 0.05
D5a2 | 10397 | 1 | 0.009
D5a2 | 10398 | 1 | 0.009
D5a2 | 10400 | 1 | 0.009
D5a2 | 150 | 4 | 0.037

D5a2 | 16172 | 2 | 0.019
D5a2 | 16189 | 7 | 0.065
D5a2 | 16362 | 1 | 0.009
D5a2 | 263 | 2 | 0.019
D5a2 | 489 | 1 | 0.009
D5a2 | 5301 | 1 | 0.009
D5a2a | 10397 | 1 | 0.005
D5a2a | 10398 | 1 | 0.005
D5a2a | 10400 | 1 | 0.005
D5a2a | 16362 | 14 | 0.064
D5a2a | 263 | 3 | 0.014
D5a2a | 4883 | 1 | 0.005
D5a2a | 5178A | 2 | 0.009
D5a2a | 5301 | 2 | 0.009
D5a2a | 9180 | 2 | 0.009
D5a2a+16092 | 150 | 1 | 0.011
D5a2a+16092 | 16172 | 19 | 0.204
D5a2a+16092 | 16223 | 1 | 0.011
D5a2a+16092 | 16362 | 1 | 0.011
D5a2a+16092 | 752 | 1 | 0.011
D5a2a1 | 16223 | 1 | 0.009
D5a2a1 | 16266 | 9 | 0.082
D5a2a1 | 16362 | 2 | 0.018
D5a2a1+@16172 | 10398 | 1 | 0.006
D5a2a1+@16172 | 16092 | 51 | 0.282
D5a2a1+@16172 | 16164 | 2 | 0.011
D5a2a1+@16172 | 16189 | 1 | 0.006
D5a2a1+@16172 | 16223 | 5 | 0.028
D5a2a1+@16172 | 16266 | 10 | 0.055
D5a2a1+@16172 | 16362 | 1 | 0.006
D5a2a1+@16172 | 263 | 6 | 0.033
D5a2a1+@16172 | 489 | 1 | 0.006
D5a2a1+@16172 | 752 | 1 | 0.006
D5a2a1+@16172 | 9180 | 1 | 0.006
D5a2a1a | 150 | 1 | 0.059
D5a2a1a | 16266 | 1 | 0.059
D5a2a1a | 44.1C | 1 | 0.059
D5a2a1a1 | 16223 | 2 | 0.154
D5a2a1a1 | 16266 | 1 | 0.077
D5a2a1a1a | 16092 | 1 | 0.077
D5a2a1a1a | 16223 | 1 | 0.077
D5a2a1a1a | 8701 | 1 | 0.077
D5a2a1a2 | 16223 | 1 | 0.05
D5a2a1b | 16092 | 4 | 0.042
D5a2a1b | 16189 | 7 | 0.073
D5a2a1b | 263 | 1 | 0.01
D5a2a1b1 | 15326 | 2 | 0.044
D5a2a1b1 | 16092 | 1 | 0.022
D5a2a1b1 | 16164 | 3 | 0.067
D5a2a1b1 | 16189 | 1 | 0.022
D5a2a1b1 | 73 | 1 | 0.022
D5a2a2 | 14766 | 1 | 0.01
D5a2a2 | 16189 | 7 | 0.069
D5a2a2 | 16266 | 6 | 0.059
D5a3 | 150 | 2 | 0.029
D5a3 | 16223 | 2 | 0.029
D5a3 | 263 | 2 | 0.029
D5a3 | 73 | 1 | 0.014
D5a3a1a | 1438 | 1 | 0.033
D5a3a1a | 14783 | 1 | 0.033
D5a3a1a | 15043 | 1 | 0.033
D5a3a1a | 15301 | 1 | 0.033
D5a3a1a | 16189 | 1 | 0.033
D5b | 16223 | 4 | 0.029
D5b | 16362 | 1 | 0.007
D5b1a | 16362 | 1 | 0.007
D5b1a1 | 10398 | 1 | 0.029
D5b1a1 | 13437 | 1 | 0.029
D5b1a1 | 16223 | 1 | 0.029
D5b1b2 | 16189 | 2 | 0.036
D5b1c | 16362 | 2 | 0.014
D5b1c | 9180 | 1 | 0.007
D5b1c1 | 16148 | 2 | 0.039
D5b1c1 | 16189 | 1 | 0.02
D5b1c1 | 16223 | 2 | 0.039
D5b1c1 | 16362 | 1 | 0.02
D5b1c1 | 185 | 14 | 0.275
D5b1c1 | 5899.1C | 2 | 0.039
D5b1c1a | 16189 | 6 | 0.044
D5b1c1a | 16223 | 3 | 0.022
D5b1c1a | 16362 | 1 | 0.007
D5b1c1a | 185 | 2 | 0.015
D5b1c1a | 4203 | 2 | 0.015
D5b1c1a | 456 | 2 | 0.015
D5b1c1a | 4883 | 1 | 0.007
D5b1c1a | 5153 | 1 | 0.007
D5b1c1a | 5301 | 1 | 0.007
D5b1c1a | 5899.1C | 11 | 0.081
D5b1d | 4048 | 2 | 0.017
D5b1d | 73 | 2 | 0.017
D5b3 | 16189 | 1 | 0.007
D5b3 | 5153 | 1 | 0.007
D5b3a | 10397 | 1 | 0.007
D5b3a | 10398 | 1 | 0.007
D5b3a | 10400 | 1 | 0.007
D5b3a | 15724 | 2 | 0.014
D5b3a | 16189 | 13 | 0.09
D5b3a1 | 15724 | 1 | 0.023
D5b3a1 | 16189 | 20 | 0.455
D5b4 | 16189 | 2 | 0.019
D5b4 | 456 | 4 | 0.038
D5b4 | 489 | 2 | 0.019
D5b4 | 681 | 2 | 0.019
D5b4 | 9992 | 1 | 0.01
D5c | 150 | 2 | 0.667
D5c | 16190 | 1 | 0.333
D5c1 | 16190 | 6 | 1
D5c1a | 151 | 1 | 0.028
D5c1a | 16189 | 2 | 0.056
D5c1a | 16190 | 4 | 0.111
D5c1a | 16362 | 2 | 0.056

D5c1a | 16390 | 2 | 0.056
D5c1a | 182 | 1 | 0.028
D5c1a | 5178A | 1 | 0.028
D5c2 | 151 | 3 | 0.176
D5c2 | 16189 | 2 | 0.118
D5c2 | 16190 | 4 | 0.235
D6 | 489 | 1 | 0.011
D6a | 16189 | 1 | 0.014
D6a | 16311 | 1 | 0.014
D6a1a | 16362 | 1 | 0.01
D6a1a | 8860 | 1 | 0.01
D6a2 | 16317 | 5 | 0.061
D6c | 16189 | 1 | 0.015
D6c1 | 16223 | 1 | 0.012
D6c1 | 16311 | 1 | 0.012
D6c1a | 15326 | 1 | 0.012
D6c1a | 16223 | 1 | 0.012
D6c1a | 16311 | 1 | 0.012
E1 | 14766 | 1 | 0.004
E1 | 16390 | 1 | 0.004
E1 | 3027 | 1 | 0.004
E1 | 4491 | 1 | 0.004
E1a1a1 | 13254 | 1 | 0.002
E1a1a1 | 13626 | 2 | 0.003
E1a1a1 | 14783 | 1 | 0.002
E1a1a1 | 16362 | 2 | 0.003
E1a1a1 | 16390 | 2 | 0.003
E1a1a1 | 3027 | 1 | 0.002
E1a1a1 | 3705 | 2 | 0.003
E1a1a1 | 4248 | 1 | 0.002
E1a1a1 | 7598 | 1 | 0.002
E1a1a1 | 8860 | 1 | 0.002
E1a1a1b1 | 131 | 1 | 0.004
E1a1b | 13254 | 1 | 0.006
E1a1b1 | 16390 | 1 | 0.006
E1a1c | 16223 | 1 | 0.004
E1a2 | 4248 | 1 | 0.01
E1a2a1 | 16362 | 1 | 0.005
E2 | 16362 | 1 | 0.005
E2a1 | 16362 | 1 | 0.006
E2a1a | 16362 | 1 | 0.005
E2a2 | 16362 | 1 | 0.006
E2b2 | 13626 | 1 | 0.008
F1a1 | 1438 | 1 | 0.003
F1a1 | 14766 | 2 | 0.006
F1a1 | 15326 | 1 | 0.003
F1a1 | 16129 | 4 | 0.012
F1a1 | 16304 | 2 | 0.006
F1a1 | 249d | 11 | 0.034
F1a1 | 263 | 4 | 0.012
F1a1 | 2706 | 1 | 0.003
F1a1 | 3970 | 1 | 0.003
F1a1 | 4086 | 1 | 0.003
F1a1 | 4769 | 1 | 0.003
F1a1 | 7028 | 1 | 0.003
F1a1 | 73 | 1 | 0.003
F1a1 | 8860 | 1 | 0.003
F1a1'4 | 16304 | 5 | 0.013
F1a1'4 | 263 | 1 | 0.003
F1a1a | 1438 | 1 | 0.002
F1a1a | 14766 | 1 | 0.002
F1a1a | 15326 | 1 | 0.002
F1a1a | 16129 | 6 | 0.009
F1a1a | 16162 | 2 | 0.003
F1a1a | 16304 | 1 | 0.002
F1a1a | 249d | 25 | 0.039
F1a1a | 263 | 2 | 0.003
F1a1a | 2706 | 1 | 0.002
F1a1a | 3970 | 1 | 0.002
F1a1a | 4086 | 1 | 0.002
F1a1a | 4769 | 1 | 0.002
F1a1a | 7028 | 1 | 0.002
F1a1a | 73 | 2 | 0.003
F1a1a | 8860 | 1 | 0.002
F1a1a1 | 1438 | 4 | 0.007
F1a1a1 | 14766 | 2 | 0.003
F1a1a1 | 15326 | 3 | 0.005
F1a1a1 | 16129 | 4 | 0.007
F1a1a1 | 16162 | 3 | 0.005
F1a1a1 | 16172 | 1 | 0.002
F1a1a1 | 16304 | 23 | 0.04
F1a1a1 | 249d | 18 | 0.031
F1a1a1 | 263 | 2 | 0.003
F1a1a1 | 2706 | 2 | 0.003
F1a1a1 | 3970 | 1 | 0.002
F1a1a1 | 4086 | 1 | 0.002
F1a1a1 | 4769 | 2 | 0.003
F1a1a1 | 7028 | 2 | 0.003
F1a1a1 | 73 | 2 | 0.003
F1a1a1 | 8860 | 2 | 0.003
F1a1b | 8860 | 1 | 0.013
F1a1c | 10609 | 1 | 0.006
F1a1c | 16129 | 4 | 0.024
F1a1c | 16172 | 1 | 0.006
F1a1c | 16304 | 1 | 0.006
F1a1c | 249d | 1 | 0.006
F1a1c1 | 10211 | 1 | 0.056
F1a1c1 | 16162 | 1 | 0.056
F1a1c1 | 9053 | 1 | 0.056
F1a1c2 | 16129 | 1 | 0.008
F1a1d | 11380 | 1 | 0.005
F1a1d | 16304 | 1 | 0.005
F1a1d | 249d | 9 | 0.043
F1a1d1 | 16129 | 2 | 0.011
F1a1d1 | 16304 | 1 | 0.005
F1a1d1 | 249d | 15 | 0.081
F1a2 | 10034 | 5 | 0.034
F1a2 | 249d | 2 | 0.014
F1a2 | 750 | 1 | 0.007
F1a2a | 9053 | 1 | 0.014
F1a3 | 16304 | 5 | 0.013

F1a3 | 249d | 2 | 0.005
F1a3+16311 | 249d | 1 | 0.005
F1a3a | 16304 | 2 | 0.012
F1a3a | 249d | 6 | 0.037
F1a3a1 | 249d | 2 | 0.043
F1a3a1a | 16172 | 1 | 0.026
F1a3a2 | 16172 | 2 | 0.167
F1a3a2 | 16311 | 4 | 0.333
F1a3a3 | 249d | 1 | 0.006
F1a3a3a | 249d | 5 | 0.032
F1a3b | 13044 | 1 | 0.005
F1a3b | 249d | 1 | 0.005
F1a3b | 53 | 1 | 0.005
F1a3b | 54C | 7 | 0.035
F1a4 | 16304 | 5 | 0.013
F1a4 | 263 | 1 | 0.003
F1a4a | 16129 | 3 | 0.058
F1a4a | 16172 | 4 | 0.077
F1a4a | 249d | 1 | 0.019
F1a4a1 | 152 | 8 | 0.027
F1a4a1 | 16304 | 2 | 0.007
F1a4a1 | 249d | 10 | 0.034
F1a4a1 | 73 | 1 | 0.003
F1a4a1 | 8277 | 14 | 0.047
F1a'c'f | 16304 | 4 | 0.043
F1b1+@152 | 249d | 3 | 0.015
F1b1+@152 | 2706 | 1 | 0.005
F1b1a | 2706 | 1 | 0.036
F1b1a1a | 16129 | 1 | 0.028
F1b1a1a | 16249 | 1 | 0.028
F1b1a1a | 16344 | 2 | 0.056
F1b1a1a1 | 16129 | 1 | 0.026
F1b1a1a1 | 16189 | 1 | 0.026
F1b1a1a1 | 16344 | 1 | 0.026
F1b1a1a1a | 16344 | 9 | 0.243
F1b1a2 | 8860 | 1 | 0.034
F1b1b | 16189 | 1 | 0.02
F1b1b | 16232A | 1 | 0.02
F1b1b | 249d | 2 | 0.04
F1b1b | 5147 | 1 | 0.02
F1b1b | 73 | 1 | 0.02
F1b1c | 152 | 2 | 0.02
F1b1c | 16232A | 1 | 0.01
F1b1c | 16249 | 7 | 0.069
F1b1f | 10310 | 2 | 0.012
F1b1f | 16311 | 3 | 0.018
F1c | 249d | 1 | 0.016
F1c1 | 709 | 1 | 0.038
F1c1a | 16129 | 15 | 0.484
F1c1a1 | 13759 | 1 | 0.006
F1c1a1 | 13928C | 1 | 0.006
F1c1a1 | 152 | 1 | 0.006
F1c1a1 | 16129 | 4 | 0.023
F1c1a1 | 249d | 2 | 0.012
F1c1a1 | 709 | 24 | 0.14
F1c1a1 | 73 | 1 | 0.006
F1c1a1a | 3970 | 1 | 0.007
F1c1a2 | 1438 | 1 | 0.009
F1c1a2 | 152 | 1 | 0.009
F1c1a2 | 16111 | 1 | 0.009
F1c1a2 | 16129 | 1 | 0.009
F1c1a2 | 249d | 5 | 0.046
F1c1a2 | 709 | 38 | 0.349
F1c1a2 | 73 | 1 | 0.009
F1d | 146 | 6 | 0.016
F1d | 14766 | 2 | 0.005
F1d | 15326 | 1 | 0.003
F1d | 15402 | 2 | 0.005
F1d | 16189 | 2 | 0.005
F1d | 249d | 4 | 0.011
F1d | 8860 | 1 | 0.003
F1d1 | 16304 | 2 | 0.005
F1d1 | 249d | 1 | 0.002
F1e | 249d | 3 | 0.008
F1e1 | 16304 | 1 | 0.002
F1e1a | 249d | 1 | 0.012
F1e1a | 73 | 1 | 0.012
F1e2 | 16234 | 7 | 0.5
F1e2 | 189 | 2 | 0.143
F1e3 | 16300 | 9 | 0.129
F1e3 | 249d | 1 | 0.014
F1f | 16129 | 1 | 0.006
F1f | 249d | 20 | 0.122
F1g | 249d | 2 | 0.005
F1g1 | 10310 | 1 | 0.002
F1g1 | 16189 | 2 | 0.005
F1g1 | 249d | 1 | 0.002
F1g1 | 3970 | 3 | 0.007
F2 | 249d | 1 | 0.005
F2a | 16304 | 1 | 0.007
F2a | 249d | 1 | 0.007
F2a | 6392 | 1 | 0.007
F2a1 | 16304 | 2 | 0.017
F2a1 | 249d | 1 | 0.009
F2b1 | 249d | 10 | 0.054
F2b1 | 73 | 1 | 0.005
F2e | 16304 | 2 | 0.01
F2e1 | 16304 | 1 | 0.5
F2e1 | 249d | 1 | 0.5
F2g | 3970 | 1 | 0.012
F2i | 16221 | 2 | 0.062
F2i | 16304 | 3 | 0.094
F2i | 249d | 1 | 0.031
F3 | 16362 | 1 | 0.03
F3a | 249d | 3 | 0.049
F3a | 263 | 1 | 0.016
F3a+207 | 249d | 1 | 0.02
F3a+207 | 73 | 1 | 0.02
F3a1 | 13928C | 2 | 0.01
F3a1 | 16260 | 6 | 0.029
F3a1 | 16355 | 3 | 0.014

F3a1 | 207 | 11 | 0.053
F3a1 | 249d | 8 | 0.038
F3a1 | 5913 | 2 | 0.01
F3a1 | 709 | 1 | 0.005
F3b | 249d | 2 | 0.047
F3b+152 | 16362 | 1 | 0.036
F3b+152 | 249d | 2 | 0.071
F3b1a | 249d | 1 | 0.2
F3b1a | 5913 | 1 | 0.2
F3b1a+16093 | 16298 | 1 | 0.023
F3b1a+16093 | 249d | 10 | 0.233
F3b1a2 | 249d | 27 | 0.45
F3b1b | 10320 | 1 | 0.006
F3b1b | 1438 | 1 | 0.006
F3b1b | 150 | 28 | 0.174
F3b1b | 152 | 28 | 0.174
F3b1b | 16265 | 4 | 0.025
F3b1b | 16298 | 3 | 0.019
F3b1b | 5899.XC | 57 | 0.354
F3b1b | 5913 | 1 | 0.006
F3b1b | 9947 | 1 | 0.006
F4a | 152 | 8 | 0.667
F4a | 16207 | 1 | 0.083
F4a | 16304 | 1 | 0.083
F4a | 16399 | 1 | 0.083
F4a1 | 146 | 1 | 0.077
F4a1a | 146 | 1 | 0.043
F4a1a | 16207 | 3 | 0.13
F4a1a | 207 | 1 | 0.043
F4a1b | 249d | 1 | 0.042
F4a1b | 7561 | 1 | 0.042
F4a2 | 13928C | 1 | 0.029
F4a2 | 14766 | 1 | 0.029
F4a2 | 152 | 10 | 0.294
F4a2 | 16304 | 9 | 0.265
F4a2 | 249d | 3 | 0.088
F4a2 | 5263 | 1 | 0.029
F4b | 16304 | 5 | 0.062
F4b | 16311 | 1 | 0.012
F4b | 573.XC | 6 | 0.075
F4b | 73 | 1 | 0.012
F4b | 8575 | 4 | 0.05
F4b1 | 16218 | 1 | 0.007
F4b1 | 16304 | 2 | 0.014
F4b1 | 249d | 26 | 0.184
F4b1 | 573.XC | 61 | 0.433
F4b1 | 6392 | 1 | 0.007
F4b1 | 73 | 1 | 0.007
G1a1 | 150 | 6 | 0.058
G1a1 | 16362 | 6 | 0.058
G1a1 | 73 | 1 | 0.01
G1a1a | 73 | 1 | 0.009
G1a1a1 | 16223 | 1 | 0.009
G1a1b | 73 | 3 | 0.032
G1a2 | 150 | 10 | 0.233
G1b | 16017 | 1 | 0.006
G1b+16129 | 16017 | 1 | 0.007
G1b1 | 16017 | 1 | 0.01
G1b2 | 16017 | 1 | 0.006
G1b3 | 16017 | 1 | 0.012
G1c1 | 593 | 1 | 0.011
G2 | 489 | 1 | 0.002
G2a | 16223 | 2 | 0.008
G2a | 263 | 4 | 0.017
G2a+152 | 16278 | 1 | 0.006
G2a+152 | 16362 | 4 | 0.023
G2a1 | 12705 | 1 | 0.003
G2a1 | 13563 | 1 | 0.003
G2a1 | 15043 | 2 | 0.006
G2a1 | 15301 | 2 | 0.006
G2a1 | 2706 | 2 | 0.006
G2a1 | 4833 | 1 | 0.003
G2a1 | 489 | 2 | 0.006
G2a1 | 5108 | 1 | 0.003
G2a1 | 5601 | 2 | 0.006
G2a1 | 709 | 1 | 0.003
G2a1 | 8860 | 2 | 0.006
G2a1+16189 | 16362 | 1 | 0.007
G2a1b | 16223 | 5 | 0.053
G2a1b | 709 | 1 | 0.011
G2a1c1 | 16223 | 1 | 0.014
G2a1c1 | 16362 | 1 | 0.014
G2a1c2 | 16189 | 1 | 0.014
G2a1d | 16223 | 3 | 0.068
G2a1d2 | 260 | 1 | 0.011
G2a1d2 | 489 | 2 | 0.021
G2a1d2 | 709 | 1 | 0.011
G2a1d2a | 709 | 1 | 0.011
G2a1d2a | 750 | 1 | 0.011
G2a1g | 16362 | 1 | 0.2
G2a2a | 16278 | 1 | 0.011
G2a4 | 16278 | 1 | 0.02
G2b1 | 12705 | 1 | 0.002
G2b1 | 13563 | 1 | 0.002
G2b1 | 14569 | 1 | 0.002
G2b1 | 14783 | 1 | 0.002
G2b1 | 15043 | 1 | 0.002
G2b1 | 15301 | 1 | 0.002
G2b1 | 489 | 1 | 0.002
G2b1a | 489 | 3 | 0.005
G2b1a1 | 489 | 2 | 0.004
G2b1a1 | 709 | 1 | 0.002
G2b1a2 | 750 | 1 | 0.021
G2b1b | 13563 | 1 | 0.008
G2b1b | 8877 | 1 | 0.008
G2b2a | 6932 | 1 | 0.002
G2b2a | 8877 | 1 | 0.002
G2b2c | 13563 | 2 | 0.027
G2c | 16223 | 2 | 0.004
G2c | 489 | 1 | 0.002
G2c | 5782 | 6 | 0.013

G2c | 709 | 1 | 0.002
G3 | 489 | 1 | 0.007
G3a | 489 | 1 | 0.007
G3a1a | 143 | 1 | 0.009
G3a1a | 16274 | 4 | 0.036
G3a1a | 263 | 1 | 0.009
G3a2 | 16362 | 6 | 0.062
G3a2+152 | 16362 | 1 | 0.01
G3a2a | 152 | 1 | 0.062
G3a2a | 573.XC | 6 | 0.375
G3b1 | 13477 | 1 | 0.008
G3b1 | 195 | 1 | 0.008
G3b1 | 4833 | 1 | 0.008
G3b1 | 489 | 5 | 0.039
G3b2 | 13477 | 1 | 0.007
G3b2 | 7028 | 2 | 0.013
G3b2 | 750 | 1 | 0.007
G4 | 194 | 1 | 0.033
H+152 | 8860 | 1 | 0.004
H+195 | 8860 | 2 | 0.026
H1+152 | 15326 | 5 | 0.017
H1+16189 | 8860 | 1 | 0.003
H1+16239 | 3010 | 1 | 0.008
H1+16278 | 3010 | 4 | 0.037
H10+(16093) | 263 | 1 | 0.001
H101 | 9230 | 1 | 0.01
H10e | 1438 | 1 | 0.008
H10e1 | 16221 | 3 | 0.031
H10e3a | 16221 | 1 | 0.011
H10g | 15326 | 1 | 0.015
H11a | 16293 | 1 | 0.014
H11a | 16311 | 1 | 0.014
H11a | 8860 | 2 | 0.027
H11a+152 | 16293 | 3 | 0.143
H11a1 | 1438 | 2 | 0.024
H11a1 | 16293 | 5 | 0.06
H11a1 | 195 | 2 | 0.024
H11a1 | 8898 | 1 | 0.012
H11a1 | 961G | 4 | 0.048
H11a2 | 13759 | 1 | 0.017
H11a2 | 16293 | 1 | 0.017
H11a2 | 195 | 5 | 0.086
H11a2 | 8448 | 1 | 0.017
H11a2 | 961G | 2 | 0.034
H11a2a1 | 16293 | 3 | 0.231
H11a2a2 | 13759 | 1 | 0.043
H12a | 195 | 2 | 0.067
H13a | 1438 | 1 | 0.001
H13a1a1 | 13326 | 1 | 0.001
H13a1a1 | 2259 | 1 | 0.001
H13a1a1a | 2259 | 1 | 0.001
H13a1a1d1 | 13680 | 2 | 0.029
H13a1a1d1 | 14872 | 6 | 0.087
H13a1a1d1 | 2259 | 4 | 0.058
H13a1a1d1 | 4745 | 5 | 0.072
H13a2 | 4769 | 1 | 0.001
H13a2 | 8860 | 1 | 0.001
H13a2a | 14872 | 1 | 0.001
H13a2b1 | 13762G | 1 | 0.003
H13a2b4 | 15326 | 1 | 0.001
H13a2c1 | 15326 | 1 | 0.048
H13a2c1 | 2259 | 1 | 0.048
H13a2c1 | 6827 | 1 | 0.048
H13a2c1 | 8860 | 1 | 0.048
H13b1 | 4107 | 1 | 0.01
H13b1a | 16261 | 8 | 0.216
H13c2 | 16300 | 1 | 0.091
H14b4 | 10217 | 1 | 0.001
H15a1 | 55 | 5 | 0.081
H15a1 | 57 | 5 | 0.081
H15a1a1 | 55 | 6 | 0.146
H15a1a1 | 57 | 3 | 0.073
H15a1b | 16184 | 1 | 0.018
H15a1b | 44.1C | 2 | 0.036
H15a1b | 55 | 3 | 0.055
H15a1b | 57 | 3 | 0.055
H15a1b | 8860 | 1 | 0.018
H15b | 55 | 4 | 0.078
H15b | 57 | 1 | 0.02
H15b1 | 55 | 8 | 0.195
H15b1 | 57 | 4 | 0.098
H15b2 | 57 | 1 | 0.027
H17 | 263 | 1 | 0.004
H17a | 16129 | 1 | 0.004
H17a | 263 | 1 | 0.004
H17a1 | 16129 | 1 | 0.015
H1a | 16162 | 1 | 0.005
H1a1 | 16162 | 1 | 0.006
H1a1 | 4769 | 1 | 0.006
H1a2 | 16162 | 1 | 0.008
H1a3 | 16162 | 2 | 0.016
H1a3c1 | 16266 | 1 | 0.009
H1a5 | 16162 | 1 | 0.01
H1ag1 | 3010 | 1 | 0.001
H1ao1 | 263 | 1 | 0.042
H1ap1 | 4769 | 1 | 0.009
H1ar | 15326 | 1 | 0.025
H1ar | 8860 | 1 | 0.025
H1ax | 1438 | 1 | 0.001
H1b | 3010 | 2 | 0.011
H1b1+16362 | 16189 | 2 | 0.014
H1b1a | 16189 | 1 | 0.009
H1b1b | 16362 | 4 | 0.05
H1b1b | 3796 | 1 | 0.012
H1b1f | 16189 | 1 | 0.012
H1b1f | 3796 | 1 | 0.012
H1bc | 15326 | 1 | 0.007
H1bo | 267 | 1 | 0.011
H1bs | 3010 | 2 | 0.018
H1c1 | 3010 | 1 | 0.007
H1c1a1 | 9150 | 1 | 0.02

H1c3 | 15326 | 1 | 0.017
H1e3 | 960.XC | 1 | 0.009
H1e5a | 15326 | 1 | 0.01
H1e5a | 263 | 1 | 0.01
H1f | 16189 | 3 | 0.014
H1f | 263 | 1 | 0.005
H1f1 | 9066 | 1 | 0.01
H1f1a | 16093 | 4 | 0.061
H1f1a | 16189 | 4 | 0.061
H1f1a | 263 | 1 | 0.015
H1n+146 | 2098 | 3 | 0.045
H1n4 | 2098 | 1 | 0.015
H1n4 | 263 | 1 | 0.015
H1q3 | 16037 | 1 | 0.009
H1z1 | 16189 | 1 | 0.031
H2 | 263 | 1 | 0.001
H24a | 16293 | 2 | 0.015
H27a | 11719 | 1 | 0.012
H28a | 186A | 1 | 0.019
H29 | 93 | 9 | 0.153
H29a | 573.XC | 2 | 0.05
H29a | 93 | 9 | 0.225
H29b | 93 | 9 | 0.321
H2a1 | 16354 | 3 | 0.013
H2a1+146 | 16354 | 1 | 0.015
H2a1a | 13095 | 1 | 0.005
H2a1a | 263 | 1 | 0.005
H2a1j | 16354 | 2 | 0.019
H2a1n | 16354 | 1 | 0.015
H2a2a2 | 6716 | 1 | 0.007
H2a3a1 | 1462 | 1 | 0.01
H2b | 152 | 1 | 0.005
H2c | 152 | 1 | 0.006
H3 | 4769 | 3 | 0.003
H3+16189 | 6776 | 2 | 0.009
H3+73 | 6776 | 1 | 0.007
H31 | 195 | 1 | 0.028
H32 | 152 | 2 | 0.034
H36 | 13056 | 1 | 0.022
H36 | 152 | 6 | 0.13
H36 | 8578 | 1 | 0.022
H3a1 | 16239G | 1 | 0.018
H3af | 1438 | 2 | 0.007
H3ak | 143 | 1 | 0.015
H3am | 6776 | 5 | 0.152
H3ao | 1438 | 1 | 0.01
H3ap | 1438 | 2 | 0.002
H3b1a | 5147 | 1 | 0.014
H3b1b1 | 153 | 1 | 0.067
H3g | 15326 | 1 | 0.006
H3h1 | 12811 | 2 | 0.006
H3h3b | 1438 | 1 | 0.003
H3h7 | 16311 | 1 | 0.008
H3n | 16104 | 1 | 0.012
H3q1 | 750 | 1 | 0.001
H3v+16093 | 408A | 1 | 0.014
H40b | 750 | 1 | 0.001
H41a | 14118 | 1 | 0.02
H41a | 15326 | 2 | 0.039
H46b | 152 | 1 | 0.007
H4a1 | 15326 | 1 | 0.001
H4a1a | 750 | 1 | 0.001
H4a1a1a | 14365 | 1 | 0.005
H4a1a1a | 4024 | 1 | 0.005
H4a1a1a | 5004 | 1 | 0.005
H4a1a1a | 8269 | 2 | 0.011
H4a1a1a1a | 14365 | 1 | 0.008
H4a1a1a2 | 5004 | 1 | 0.008
H4a1a3a | 14365 | 1 | 0.032
H4a1a4b2 | 3992 | 1 | 0.029
H5 | 456 | 1 | 0.003
H5'36 | 263 | 2 | 0.005
H55b | 153 | 5 | 0.054
H5a+152 | 1438 | 1 | 0.006
H5a+152 | 456 | 1 | 0.006
H5a1 | 1438 | 1 | 0.003
H5a1+152 | 456 | 1 | 0.006
H5a1a | 16304 | 1 | 0.003
H5a1c2 | 16304 | 1 | 0.028
H5a1f | 4336 | 1 | 0.003
H5a1j | 263 | 2 | 0.036
H5a2 | 16304 | 1 | 0.003
H5b | 263 | 2 | 0.006
H5b2 | 16304 | 1 | 0.005
H5c1a | 12127 | 1 | 0.003
H5c2 | 16304 | 2 | 0.056
H5e1a | 16304 | 1 | 0.014
H5m | 456 | 1 | 0.006
H5r1 | 16304 | 1 | 0.026
H5r2 | 16304 | 1 | 0.026
H5u1 | 16304 | 3 | 0.054
H6 | 16362 | 4 | 0.028
H6 | 239 | 1 | 0.007
H66a | 7337 | 1 | 0.01
H6a1a | 8860 | 3 | 0.012
H6a1a | 9380 | 1 | 0.004
H6a1a1 | 239 | 1 | 0.007
H6a1a4 | 263 | 1 | 0.007
H6a1a8 | 3915 | 1 | 0.05
H6a1b | 4727 | 1 | 0.006
H6a1b | 9380 | 1 | 0.006
H6a1b3 | 16193 | 1 | 0.037
H6a2 | 16362 | 5 | 0.035
H6b1 | 263 | 1 | 0.042
H6b1 | 44.1C | 3 | 0.125
H6c1 | 16362 | 1 | 0.059
H76 | 8572 | 2 | 0.006
H76a | 152 | 1 | 0.042
H76a | 8572 | 1 | 0.042
H7d3a | 4793 | 1 | 0.001
H7f | 4793 | 2 | 0.027

H7h1 | 4793 | 1 | 0.012
H8 | 16288 | 2 | 0.167
H8 | 195 | 1 | 0.083
H8+(114)+152 | 195 | 1 | 0.083
H80 | 2361 | 2 | 0.006
H8b | 195 | 2 | 0.143
H8c | 16362 | 2 | 0.08
H8c2 | 16288 | 1 | 0.333
H95a | 14025 | 1 | 0.001
H9a | 16168 | 1 | 0.022
HV+73 | 15326 | 1 | 0.006
HV+73 | 263 | 2 | 0.012
HV+73 | 7028 | 2 | 0.012
HV0+195 | 16298 | 6 | 0.037
HV0+195 | 72 | 1 | 0.006
HV0a1 | 16298 | 1 | 0.028
HV0a1 | 72 | 3 | 0.083
HV0c | 72 | 2 | 0.016
HV0d | 72 | 1 | 0.008
HV0e | 16311 | 1 | 0.022
HV0e | 72 | 1 | 0.022
HV0f | 8706 | 4 | 0.044
HV0g | 72 | 3 | 0.15
HV12a | 16292 | 1 | 0.012
HV12b1 | 150 | 18 | 0.175
HV13a | 12879 | 1 | 0.011
HV14 | 16311 | 3 | 0.011
HV14a | 15115 | 1 | 0.003
HV14a | 15326 | 2 | 0.006
HV14a | 8860 | 2 | 0.006
HV15 | 15326 | 1 | 0.011
HV18 | 9039 | 1 | 0.005
HV19 | 263 | 3 | 0.037
HV1a1a | 8277 | 3 | 0.073
HV1a2b | 8277 | 1 | 0.023
HV1a'b'c | 750 | 2 | 0.022
HV1b3 | 16067 | 1 | 0.067
HV1b3b | 14161 | 1 | 0.083
HV2 | 16217 | 2 | 0.027
HV2 | 2706 | 1 | 0.013
HV2 | 8860 | 1 | 0.013
HV2a1 | 12061 | 1 | 0.023
HV2a1 | 152 | 1 | 0.023
HV2a1 | 195 | 15 | 0.341
HV2a1 | 8860 | 1 | 0.023
HV2a2 | 195 | 7 | 0.137
HV2a3 | 263 | 1 | 0.022
HV4 | 15326 | 1 | 0.001
HV4a1+16291 | 16221 | 1 | 0.012
HV4a1a2 | 4769 | 1 | 0.013
HV4a2 | 16221 | 3 | 0.041
HV4b | 1715 | 1 | 0.009
HV6 | 6755 | 1 | 0.009
HV7 | 16278 | 1 | 0.013
HV9a | 263 | 1 | 0.008
HV9a1 | 263 | 1 | 0.008
HV9a1a | 263 | 1 | 0.008
I | 15043 | 1 | 0.014
I | 16223 | 1 | 0.014
I | 204 | 4 | 0.057
I | 263 | 2 | 0.029
I | 573.XC | 8 | 0.114
I1 | 15924 | 1 | 0.03
I1 | 16391 | 2 | 0.061
I1 | 204 | 1 | 0.03
I1 | 455.1T | 2 | 0.061
I1 | 573.XC | 3 | 0.091
I1a | 15924 | 1 | 0.037
I1a | 16129 | 1 | 0.037
I1a | 455.1T | 1 | 0.037
I1a | 573.XC | 2 | 0.074
I1a | 6734 | 2 | 0.074
I1a1 | 16391 | 1 | 0.013
I1a1 | 203 | 2 | 0.026
I1a1 | 455.1T | 2 | 0.026
I1a1 | 573.XC | 8 | 0.103
I1a1a | 11719 | 1 | 0.013
I1a1a | 15326 | 1 | 0.013
I1a1a | 16172 | 2 | 0.026
I1a1a | 16391 | 3 | 0.038
I1a1a | 3447 | 3 | 0.038
I1a1a | 455.1T | 8 | 0.103
I1a1a | 573.XC | 11 | 0.141
I1a1a | 750 | 1 | 0.013
I1a1a1 | 16391 | 3 | 0.062
I1a1a1 | 455.1T | 1 | 0.021
I1a1a1 | 573.XC | 4 | 0.083
I1a1a2 | 199 | 1 | 0.022
I1a1a2 | 455.1T | 3 | 0.065
I1a1a3 | 573.XC | 1 | 0.111
I1a1a3a | 203 | 1 | 0.143
I1a1a3a | 3447 | 1 | 0.143
I1a1a3a | 573.XC | 1 | 0.143
I1a1b | 10915 | 1 | 0.015
I1a1b | 14766 | 1 | 0.015
I1a1b | 15043 | 1 | 0.015
I1a1b | 16391 | 2 | 0.029
I1a1b | 455.1T | 2 | 0.029
I1a1b | 573.XC | 2 | 0.029
I1a1b | 6734 | 1 | 0.015
I1a1b | 7028 | 1 | 0.015
I1a1b | 8251 | 1 | 0.015
I1a1c | 203 | 1 | 0.019
I1a1c | 573.XC | 2 | 0.037
I1a1d | 573.XC | 1 | 0.111
I1a1e | 573.XC | 1 | 0.021
I1b | 16129 | 11 | 0.177
I1b | 16391 | 2 | 0.032
I1b | 204 | 2 | 0.032
I1b | 455.1T | 5 | 0.081
I1b | 573.XC | 8 | 0.129

I1c1a | 16319 | 2 | 0.154
I1c1a | 250 | 1 | 0.077
I1d | 16223 | 1 | 0.034
I1d | 250 | 1 | 0.034
I1d | 455.1T | 1 | 0.034
I1d | 573.XC | 1 | 0.034
I1e | 13780 | 1 | 0.029
I1e | 14766 | 1 | 0.029
I1e | 15924 | 1 | 0.029
I1e | 204 | 5 | 0.143
I1e | 2706 | 1 | 0.029
I1e | 4529T | 1 | 0.029
I1e | 455.1T | 1 | 0.029
I1e | 4769 | 1 | 0.029
I1e | 573.XC | 1 | 0.029
I1e | 7028 | 1 | 0.029
I1e | 8251 | 1 | 0.029
I1f | 16171 | 1 | 0.045
I1f | 455.1T | 2 | 0.091
I1f | 573.XC | 12 | 0.545
I2 | 12705 | 1 | 0.008
I2 | 13780 | 1 | 0.008
I2 | 14766 | 1 | 0.008
I2 | 16129 | 2 | 0.016
I2 | 16391 | 4 | 0.032
I2 | 199 | 1 | 0.008
I2 | 204 | 5 | 0.04
I2 | 207 | 1 | 0.008
I2 | 263 | 1 | 0.008
I2 | 4529T | 1 | 0.008
I2 | 573.XC | 8 | 0.064
I2 | 750 | 1 | 0.008
I2'3 | 15924 | 1 | 0.021
I2'3 | 204 | 1 | 0.021
I2'3 | 573.XC | 7 | 0.149
I2a | 14766 | 1 | 0.091
I2a | 207 | 1 | 0.091
I2a1 | 750 | 1 | 0.143
I2a1a | 207 | 1 | 0.143
I2a1a | 533 | 1 | 0.143
I2a2 | 204 | 5 | 0.417
I2a3 | 573.XC | 2 | 0.5
I2b | 152 | 1 | 0.25
I2c | 152 | 2 | 0.043
I2d | 14766 | 1 | 0.019
I2d | 199 | 2 | 0.037
I2d | 573.XC | 4 | 0.074
I2e | 573.XC | 1 | 0.027
I3 | 573.XC | 6 | 0.15
I3a | 204 | 1 | 0.026
I3a | 573.XC | 7 | 0.179
I3a1 | 207 | 1 | 0.043
I3a1 | 750 | 1 | 0.043
I3a1 | 8251 | 4 | 0.174
I3b | 16491 | 1 | 0.111
I3b | 4529T | 1 | 0.111
I3b | 573.XC | 2 | 0.222
I3c | 207 | 1 | 0.029
I3d | 573.XC | 1 | 0.027
I3d1 | 207 | 1 | 0.029
I4 | 204 | 1 | 0.021
I4 | 573.XC | 2 | 0.042
I4a | 16129 | 1 | 0.008
I4a | 16391 | 1 | 0.008
I4a | 199 | 1 | 0.008
I4a | 263 | 6 | 0.051
I4a | 573.XC | 27 | 0.229
I4a1 | 1438 | 6 | 0.14
I4a1 | 15924 | 4 | 0.093
I4a1 | 204 | 5 | 0.116
I4a1 | 573.XC | 8 | 0.186
I4a2 | 573.XC | 1 | 0.021
I4b | 15924 | 1 | 0.017
I4b | 16129 | 1 | 0.017
I4b | 16391 | 6 | 0.102
I4b | 573.XC | 2 | 0.034
I5a | 573.XC | 2 | 0.118
I5a1 | 16391 | 9 | 0.45
I5a1a | 573.XC | 2 | 0.105
I5a1b | 14233 | 1 | 0.077
I5a1c | 12961 | 2 | 0.133
I5a2 | 16129 | 1 | 0.029
I5a2 | 573.XC | 21 | 0.6
I5a2+16086 | 16129 | 1 | 0.143
I5a2+16086 | 16223 | 1 | 0.143
I5a2+16086 | 199 | 2 | 0.286
I5a2+16086 | 204 | 2 | 0.286
I5a4 | 13780 | 2 | 0.071
I5a4 | 16192 | 3 | 0.107
I5a4 | 16391 | 10 | 0.357
I5a4 | 199 | 1 | 0.036
I5a4 | 204 | 1 | 0.036
I5a4 | 250 | 1 | 0.036
I5a4 | 573.XC | 2 | 0.071
I5b | 204 | 1 | 0.022
I5b1 | 573.XC | 1 | 0.048
I5c | 16391 | 3 | 0.125
I5c | 573.XC | 4 | 0.167
I5c1 | 573.XC | 1 | 0.059
I6a | 11719 | 1 | 0.02
I6a | 573.XC | 1 | 0.02
I6b | 204 | 1 | 0.125
I6b | 573.XC | 3 | 0.375
J | 489 | 1 | 0.008
J1 | 295 | 1 | 0.007
J1b | 16126 | 6 | 0.033
J1b | 16261 | 5 | 0.027
J1b | 73 | 1 | 0.005
J1b1a1 | 13879 | 1 | 0.008
J1b1a1 | 14766 | 1 | 0.008
J1b1a1 | 8557 | 1 | 0.008

J1b1a1+146 | 16126 | 1 | 0.016
J1b1a1+146 | 16261 | 3 | 0.048
J1b1a1a | 16126 | 1 | 0.012
J1b1a1a | 16261 | 1 | 0.012
J1b1a1b | 12007 | 2 | 0.024
J1b1a1b | 16069 | 2 | 0.024
J1b1a1b | 16172 | 1 | 0.012
J1b1a1b | 263 | 1 | 0.012
J1b1a1c | 16069 | 2 | 0.062
J1b1a1c | 16126 | 1 | 0.031
J1b1a1c | 8269 | 1 | 0.031
J1b1a1c | 8557 | 1 | 0.031
J1b1a2b | 16187 | 1 | 0.333
J1b1b1 | 16261 | 1 | 0.007
J1b1b1a | 15326 | 2 | 0.118
J1b1b1a | 16126 | 1 | 0.059
J1b1b1b | 16261 | 2 | 0.017
J1b1b1b | 271 | 1 | 0.008
J1b1b2 | 16261 | 1 | 0.1
J1b1b3 | 263 | 1 | 0.014
J1b1b3 | 5460 | 1 | 0.014
J1b2 | 12612 | 1 | 0.005
J1b2 | 16126 | 3 | 0.015
J1b2 | 16145 | 1 | 0.005
J1b2 | 263 | 6 | 0.029
J1b2 | 8269 | 1 | 0.005
J1b2a | 9667 | 3 | 0.077
J1b3 | 16261 | 1 | 0.006
J1b3b | 16126 | 2 | 0.011
J1b3b1 | 3010 | 1 | 0.006
J1b6a | 16261 | 1 | 0.006
J1b6a | 295 | 2 | 0.011
J1b7a | 15326 | 1 | 0.25
J1b7a | 16126 | 1 | 0.25
J1b8 | 16126 | 13 | 0.074
J1b9 | 16290 | 1 | 0.091
J1b9 | 263 | 2 | 0.182
J1c | 11251 | 4 | 0.017
J1c | 295 | 1 | 0.004
J1c | 7028 | 1 | 0.004
J1c+16261 | 13708 | 1 | 0.012
J1c+16261 | 1438 | 2 | 0.025
J1c+16261 | 3010 | 2 | 0.025
J1c+16261 | 489 | 2 | 0.025
J1c1 | 16126 | 2 | 0.008
J1c1 | 295 | 1 | 0.004
J1c10a | 295 | 1 | 0.005
J1c11 | 1438 | 1 | 0.056
J1c11 | 3010 | 1 | 0.056
J1c16 | 16256 | 7 | 0.438
J1c17a | 489 | 1 | 0.071
J1c1b | 16069 | 2 | 0.009
J1c1b1a | 295 | 1 | 0.004
J1c1b2 | 14798 | 1 | 0.005
J1c1b2 | 3394 | 1 | 0.005
J1c1b2 | 7184 | 1 | 0.005
J1c1c | 150 | 2 | 0.118
J1c1e | 295 | 1 | 0.111
J1c2 | 13708 | 1 | 0.003
J1c2 | 16069 | 1 | 0.003
J1c2 | 263 | 1 | 0.003
J1c2 | 462 | 1 | 0.003
J1c2b2 | 1438 | 1 | 0.006
J1c2b3 | 263 | 1 | 0.143
J1c2c1 | 10398 | 1 | 0.007
J1c2c1 | 13933 | 1 | 0.007
J1c2c1 | 295 | 1 | 0.007
J1c2c1 | 489 | 1 | 0.007
J1c2c1a | 222 | 3 | 0.5
J1c2c1a | 3456 | 1 | 0.167
J1c2c2a | 263 | 1 | 0.005
J1c2e | 15452A | 1 | 0.023
J1c2e | 263 | 1 | 0.023
J1c2e | 489 | 1 | 0.023
J1c2e2 | 16126 | 1 | 0.023
J1c2i | 16242 | 1 | 0.067
J1c2i | 2706 | 3 | 0.2
J1c2i | 295 | 1 | 0.067
J1c2j | 263 | 1 | 0.143
J1c2l | 16069 | 2 | 0.011
J1c2l | 16126 | 3 | 0.017
J1c2m | 16126 | 1 | 0.067
J1c2m1 | 15326 | 1 | 0.1
J1c2o | 14766 | 1 | 0.031
J1c2o | 15452A | 1 | 0.031
J1c2t | 750 | 1 | 0.005
J1c3 | 16126 | 1 | 0.003
J1c3 | 4216 | 1 | 0.003
J1c3 | 750 | 1 | 0.003
J1c3a1 | 1438 | 1 | 0.004
J1c3d | 295 | 1 | 0.005
J1c3e | 73 | 1 | 0.042
J1c3f | 16069 | 1 | 0.027
J1c3g | 263 | 1 | 0.004
J1c3j | 295 | 1 | 0.029
J1c4c | 13708 | 1 | 0.045
J1c5 | 12612 | 1 | 0.004
J1c5 | 13708 | 1 | 0.004
J1c5 | 14798 | 1 | 0.004
J1c5 | 16069 | 5 | 0.019
J1c5 | 16126 | 2 | 0.008
J1c5 | 3010 | 1 | 0.004
J1c5a | 16069 | 1 | 0.004
J1c5a1 | 16126 | 1 | 0.004
J1c5c1 | 16126 | 2 | 0.037
J1c7 | 1438 | 1 | 0.012
J1c7 | 15452A | 1 | 0.012
J1c7 | 295 | 5 | 0.06
J1c7 | 73 | 1 | 0.012
J1c7a | 14798 | 2 | 0.018
J1c7a | 16069 | 1 | 0.009

J1c7a | 16126 | 4 | 0.035
J1c7a | 16261 | 3 | 0.026
J1c7a | 2706 | 1 | 0.009
J1c7a | 3010 | 1 | 0.009
J1c8a | 16319 | 2 | 0.027
J1c9 | 13708 | 1 | 0.005
J1c9 | 2706 | 1 | 0.005
J1d | 152 | 1 | 0.013
J1d | 16069 | 1 | 0.013
J1d | 16193 | 6 | 0.08
J1d1 | 13708 | 1 | 0.059
J1d1 | 489 | 1 | 0.059
J1d1 | 7789 | 1 | 0.059
J1d1a | 152 | 2 | 0.026
J1d1a1 | 13708 | 1 | 0.012
J1d1a1 | 16069 | 1 | 0.012
J1d1a1 | 16193 | 5 | 0.059
J1d1a1 | 16300 | 4 | 0.047
J1d1a1 | 16309 | 1 | 0.012
J1d1b1 | 10398 | 1 | 0.043
J1d1b1 | 16193 | 5 | 0.217
J1d3 | 152 | 1 | 0.016
J1d3 | 16126 | 1 | 0.016
J1d4 | 16193 | 1 | 0.016
J1d5 | 16193 | 1 | 0.034
J1d5 | 295 | 1 | 0.034
J1d6 | 8860 | 1 | 0.015
J1d6a | 1438 | 1 | 0.143
J2 | 295 | 1 | 0.008
J2a | 16126 | 1 | 0.008
J2a1a1 | 152 | 1 | 0.017
J2a1a1 | 16126 | 9 | 0.15
J2a1a1 | 195 | 1 | 0.017
J2a1a1 | 489 | 1 | 0.017
J2a1a1 | 513 | 2 | 0.033
J2a1a1a | 152 | 1 | 0.023
J2a1a1a | 16231 | 1 | 0.023
J2a1a1a | 310.1T | 1 | 0.023
J2a1a1a1 | 15191 | 1 | 0.1
J2a1a1a1 | 152 | 1 | 0.1
J2a1a1a1 | 310.1T | 1 | 0.1
J2a1a1a1 | 513 | 2 | 0.2
J2a1a1a2 | 16231 | 1 | 0.013
J2a1a1a2 | 310.1T | 4 | 0.053
J2a1a1a2 | 4769 | 1 | 0.013
J2a1a1a2 | 513 | 1 | 0.013
J2a1a1a2a | 11251 | 1 | 0.029
J2a1a1a2a | 295 | 1 | 0.029
J2a1a1a2a | 310.1T | 1 | 0.029
J2a1a1a3 | 310.1T | 1 | 0.043
J2a1a1a3 | 7028 | 1 | 0.043
J2a1a1a3 | 7789 | 1 | 0.043
J2a1a1b | 16069 | 1 | 0.037
J2a1a1c | 16126 | 3 | 0.115
J2a1a1e | 152 | 1 | 0.027
J2a1a1e | 16126 | 1 | 0.027
J2a1a1e | 73 | 1 | 0.027
J2a1a2 | 263 | 1 | 0.333
J2a1a2a | 295 | 2 | 0.5
J2a2 | 16126 | 1 | 0.007
J2a2a | 11002 | 1 | 0.008
J2a2a | 13708 | 1 | 0.008
J2a2a1a1 | 16069 | 1 | 0.014
J2a2a1a1 | 16126 | 3 | 0.043
J2a2a1a1 | 195 | 2 | 0.029
J2a2b1 | 295 | 1 | 0.067
J2a2b1a | 295 | 1 | 0.067
J2a2d | 195 | 1 | 0.007
J2a2e | 16126 | 1 | 0.007
J2b | 73 | 1 | 0.015
J2b1 | 13708 | 1 | 0.01
J2b1 | 15257 | 1 | 0.01
J2b1 | 15452A | 1 | 0.01
J2b1 | 16126 | 1 | 0.01
J2b1 | 16193 | 2 | 0.02
J2b1 | 2706 | 1 | 0.01
J2b1 | 295 | 1 | 0.01
J2b1 | 5633 | 1 | 0.01
J2b1a | 15812 | 2 | 0.012
J2b1a | 16126 | 1 | 0.006
J2b1a | 16193 | 2 | 0.012
J2b1a | 263 | 1 | 0.006
J2b1a+16311 | 16193 | 1 | 0.091
J2b1a1a | 16362 | 4 | 0.308
J2b1a2 | 152 | 1 | 0.015
J2b1a2 | 16193 | 1 | 0.015
J2b1a5 | 4769 | 1 | 0.011
J2b1a6 | 16126 | 1 | 0.033
J2b1a6 | 9494 | 3 | 0.1
J2b1e1 | 16193 | 2 | 0.087
J2b1h | 13612T | 1 | 0.5
J2b1h | 159 | 1 | 0.5
J2b2 | 152 | 1 | 0.015
K1a | 10398 | 1 | 0.003
K1a | 10550 | 1 | 0.003
K1a | 11299 | 1 | 0.003
K1a | 11467 | 1 | 0.003
K1a | 11719 | 1 | 0.003
K1a | 16224 | 1 | 0.003
K1a | 3480 | 1 | 0.003
K1a | 9055 | 1 | 0.003
K1a | 9698 | 1 | 0.003
K1a+150 | 15326 | 1 | 0.005
K1a+150 | 73 | 1 | 0.005
K1a+195 | 10550 | 1 | 0.003
K1a+195 | 11467 | 1 | 0.003
K1a+195 | 16311 | 2 | 0.007
K1a+195 | 1811 | 1 | 0.003
K1a+195 | 9055 | 1 | 0.003
K1a10 | 1811 | 1 | 0.045
K1a10a | 16224 | 1 | 0.034

K1a10a | 9698 | 1 | 0.034
K1a11 | 750 | 6 | 0.207
K1a11 | 8281-8289d | 3 | 0.103
K1a11b | 16129 | 1 | 0.036
K1a12 | 12372 | 1 | 0.004
K1a12a | 1438 | 1 | 0.003
K1a12a | 750 | 1 | 0.003
K1a13a | 195 | 1 | 0.006
K1a13a | 263 | 1 | 0.006
K1a14 | 16224 | 1 | 0.005
K1a15 | 16224 | 1 | 0.005
K1a16 | 16224 | 1 | 0.005
K1a17 | 14766 | 3 | 0.016
K1a17 | 15670 | 2 | 0.011
K1a17 | 73 | 1 | 0.005
K1a17 | 9055 | 1 | 0.005
K1a17a | 1719 | 1 | 0.167
K1a19a | 12338 | 1 | 0.007
K1a1a2a | 73 | 1 | 0.003
K1a1b1 | 11299 | 1 | 0.003
K1a1b1 | 1189 | 1 | 0.003
K1a1b1 | 12372 | 1 | 0.003
K1a1b1 | 14167 | 1 | 0.003
K1a1b1 | 16224 | 5 | 0.017
K1a1b1 | 7028 | 1 | 0.003
K1a1b1a | 10978 | 1 | 0.011
K1a1b1a | 12954 | 1 | 0.011
K1a1b1a | 16234 | 1 | 0.011
K1a1b1b1 | 593 | 1 | 0.004
K1a1b2a | 73 | 2 | 0.01
K1a1b2a1 | 73 | 2 | 0.01
K1a1c | 16400 | 1 | 0.045
K1a26 | 16224 | 1 | 0.005
K1a3 | 15326 | 1 | 0.003
K1a3 | 1811 | 2 | 0.007
K1a30a | 16311 | 2 | 0.011
K1a4 | 16224 | 1 | 0.003
K1a4+146 | 73 | 2 | 0.011
K1a4a1 | 10398 | 4 | 0.012
K1a4a1 | 9055 | 1 | 0.003
K1a4a1a+195 | 16224 | 1 | 0.005
K1a4a1a1 | 16224 | 1 | 0.005
K1a4a1a2 | 15326 | 1 | 0.038
K1a4a1a2 | 6260 | 1 | 0.038
K1a4a1a2 | 9055 | 1 | 0.038
K1a4a1a2 | 9698 | 1 | 0.038
K1a4a1a2a | 14766 | 1 | 0.028
K1a4a1a2a | 16245 | 1 | 0.028
K1a4a1a3 | 16224 | 1 | 0.005
K1a4a1b1 | 1438 | 1 | 0.004
K1a4a1b2 | 16224 | 1 | 0.077
K1a4a1b2 | 16311 | 1 | 0.077
K1a4a1c1 | 10398 | 1 | 0.006
K1a4a1c1 | 11485 | 1 | 0.006
K1a4a1f | 6260 | 1 | 0.006
K1a4b1 | 73 | 4 | 0.02
K1a4c1 | 13710 | 5 | 0.116
K1a4c1 | 152 | 9 | 0.209
K1a4d | 11071 | 1 | 0.003
K1a4d | 11299 | 1 | 0.003
K1a4d | 12308 | 1 | 0.003
K1a4d | 12372 | 1 | 0.003
K1a4f | 16311 | 1 | 0.003
K1a4f1 | 16224 | 1 | 0.004
K1a4g | 263 | 1 | 0.004
K1a4j | 14167 | 1 | 0.006
K1a4j | 73 | 2 | 0.011
K1a5a | 11017 | 1 | 0.024
K1a5b | 11017 | 1 | 0.006
K1a5b | 408A | 1 | 0.006
K1a6 | 16527 | 1 | 0.008
K1a6 | 3480 | 1 | 0.008
K1a6 | 8790 | 1 | 0.008
K1a7 | 1438 | 2 | 0.009
K1a8b | 295A | 4 | 0.051
K1b1a | 11923 | 2 | 0.038
K1b1a | 16463 | 7 | 0.132
K1b1a1 | 16463 | 1 | 0.018
K1b1a1+199 | 11467 | 1 | 0.05
K1b1a1+199 | 16463 | 1 | 0.05
K1b1a1a | 16224 | 2 | 0.062
K1b1a1a | 16311 | 1 | 0.031
K1b1a1a | 5913 | 1 | 0.031
K1b1a1b | 16319 | 1 | 0.05
K1b1a1d | 15946 | 1 | 0.021
K1b1a1d1 | 152 | 1 | 0.021
K1b1c | 94 | 3 | 0.024
K1b2 | 11299 | 1 | 0.008
K1b2 | 3480 | 1 | 0.008
K1b2 | 750 | 1 | 0.008
K1b2a | 16224 | 2 | 0.015
K1b2a | 1811 | 2 | 0.015
K1b2a | 2706 | 1 | 0.007
K1b2a1 | 5913 | 1 | 0.008
K1c1 | 10550 | 1 | 0.004
K1c1 | 146 | 1 | 0.004
K1c1 | 152 | 2 | 0.008
K1c1 | 16224 | 2 | 0.008
K1c1 | 498d | 4 | 0.017
K1c1a | 498d | 1 | 0.005
K1c1c | 10398 | 3 | 0.014
K1c1c | 10550 | 2 | 0.01
K1c1c | 498d | 2 | 0.01
K1c1c | 9698 | 1 | 0.005
K1c1e | 152 | 1 | 0.029
K1c1e | 73 | 1 | 0.029
K1c1f | 498d | 1 | 0.005
K1c1h | 498d | 1 | 0.005
K1c1i | 152 | 1 | 0.005
K1c1i | 498d | 1 | 0.005
K1c2 | 12308 | 1 | 0.01

K1c2 | 152 | 1 | 0.01
K1c2 | 498d | 4 | 0.039
K1c2 | 9006 | 3 | 0.029
K1d | 573.XC | 1 | 0.043
K1d1 | 573.XC | 1 | 0.038
K2a | 10550 | 1 | 0.005
K2a | 146 | 1 | 0.005
K2a | 16311 | 1 | 0.005
K2a | 263 | 1 | 0.005
K2a | 3480 | 1 | 0.005
K2a | 73 | 1 | 0.005
K2a1 | 16224 | 2 | 0.011
K2a1a | 16224 | 2 | 0.1
K2a2 | 16224 | 2 | 0.011
K2a2a | 16224 | 2 | 0.011
K2a2a1 | 11719 | 1 | 0.006
K2a3 | 709 | 1 | 0.005
K2a3a | 16224 | 2 | 0.011
K2a3a1 | 8860 | 1 | 0.006
K2a4 | 709 | 1 | 0.006
K2a6 | 1811 | 1 | 0.005
K2a6 | 709 | 1 | 0.005
K2a7 | 73 | 1 | 0.006
K2a9 | 152 | 22 | 0.117
K2a9 | 709 | 23 | 0.122
K2b | 146 | 1 | 0.008
K2b1b | 16224 | 6 | 0.25
L0a | 13276 | 8 | 0.348
L0a | 152 | 1 | 0.043
L0a | 16223 | 1 | 0.043
L0a | 185 | 8 | 0.348
L0a | 189 | 1 | 0.043
L0a | 247 | 1 | 0.043
L0a | 263 | 1 | 0.043
L0a1 | 16187 | 4 | 0.121
L0a1 | 16188G | 4 | 0.121
L0a1 | 16189 | 4 | 0.121
L0a1 | 16230 | 1 | 0.03
L0a1 | 16311 | 1 | 0.03
L0a1+16293 | 152 | 1 | 0.143
L0a1+16293 | 16188G | 4 | 0.571
L0a1'4 | 152 | 4 | 0.16
L0a1a | 152 | 11 | 0.234
L0a1a | 16187 | 2 | 0.043
L0a1a | 16188G | 2 | 0.043
L0a1a | 16189 | 2 | 0.043
L0a1a | 185 | 3 | 0.064
L0a1a | 247 | 1 | 0.021
L0a1a | 5096 | 1 | 0.021
L0a1a+200 | 10873 | 1 | 0.022
L0a1a+200 | 10915 | 1 | 0.022
L0a1a+200 | 14308 | 1 | 0.022
L0a1a+200 | 16187 | 1 | 0.022
L0a1a+200 | 16188G | 2 | 0.043
L0a1a+200 | 16189 | 2 | 0.043
L0a1a+200 | 16230 | 1 | 0.022
L0a1a+200 | 5231 | 1 | 0.022
L0a1a+200 | 5442 | 1 | 0.022
L0a1a1 | 12720 | 1 | 0.025
L0a1a1 | 16129 | 3 | 0.075
L0a1a1 | 16223 | 1 | 0.025
L0a1a1 | 185 | 3 | 0.075
L0a1a1 | 200 | 1 | 0.025
L0a1a2 | 13276 | 1 | 0.008
L0a1a2 | 1438 | 1 | 0.008
L0a1a2 | 16129 | 2 | 0.015
L0a1a2 | 16168 | 2 | 0.015
L0a1a2 | 16172 | 1 | 0.008
L0a1a2 | 16187 | 12 | 0.09
L0a1a2 | 16188G | 17 | 0.128
L0a1a2 | 16189 | 10 | 0.075
L0a1a2 | 16223 | 1 | 0.008
L0a1a2 | 185 | 2 | 0.015
L0a1a2 | 200 | 7 | 0.053
L0a1a2 | 236 | 16 | 0.12
L0a1a3 | 13650 | 1 | 0.014
L0a1a3 | 16187 | 38 | 0.543
L0a1a3 | 16188G | 37 | 0.529
L0a1a3 | 16189 | 36 | 0.514
L0a1a3 | 16223 | 2 | 0.029
L0a1a3 | 185 | 18 | 0.257
L0a1a3 | 247 | 1 | 0.014
L0a1b | 16187 | 4 | 0.108
L0a1b | 16188G | 2 | 0.054
L0a1b | 16189 | 2 | 0.054
L0a1b | 16293 | 1 | 0.027
L0a1b1 | 16187 | 5 | 0.057
L0a1b1 | 16188G | 34 | 0.386
L0a1b1 | 16189 | 5 | 0.057
L0a1b1 | 8191 | 2 | 0.023
L0a1b1a | 16187 | 1 | 0.015
L0a1b1a | 16188G | 3 | 0.045
L0a1b1a | 16189 | 1 | 0.015
L0a1b1a | 16293 | 2 | 0.03
L0a1b1a1 | 13506 | 1 | 0.007
L0a1b1a1 | 15326 | 1 | 0.007
L0a1b1a1 | 15431 | 1 | 0.007
L0a1b1a1 | 16129 | 1 | 0.007
L0a1b1a1 | 16188G | 10 | 0.067
L0a1b1a1 | 16278 | 1 | 0.007
L0a1b1a1 | 16293 | 16 | 0.107
L0a1b1a1 | 7146 | 1 | 0.007
L0a1b1a1a | 16129 | 4 | 0.053
L0a1b1a1a | 16293 | 1 | 0.013
L0a1b1a1a | 93 | 1 | 0.013
L0a1b1a1a | 961 | 1 | 0.013
L0a1b2 | 152 | 33 | 0.688
L0a1b2 | 16093 | 21 | 0.438
L0a1b2 | 16187 | 4 | 0.083
L0a1b2 | 16188G | 15 | 0.312
L0a1b2 | 16189 | 4 | 0.083

L0a1b2	16293	2	0.042
L0a1b2	263	1	0.021
L0a1b2a	152	2	0.1
L0a1b2a	16093	3	0.15
L0a1b2a	2706	1	0.05
L0a1c	16187	2	0.182
L0a1c	16188G	2	0.182
L0a1c	16189	2	0.182
L0a1c	16320	3	0.273
L0a1c	189	1	0.091
L0a1c	247	1	0.091
L0a1c1	12705	1	0.056
L0a1c1	152	5	0.278
L0a1c1	16187	2	0.111
L0a1c1	16188G	2	0.111
L0a1c1	16189	2	0.111
L0a1c1	16230	1	0.056
L0a1c1	7256	1	0.056
L0a1d	16187	4	0.148
L0a1d	16188G	4	0.148
L0a1d	16189	4	0.148
L0a1d	16293	2	0.074
L0a1d	185	1	0.037
L0a1d	189	1	0.037
L0a1d	93	1	0.037
L0a1e	13650	2	0.091
L0a1e	16129	1	0.045
L0a2	10810	2	0.026
L0a2	10873	1	0.013
L0a2	10915	2	0.026
L0a2	11719	1	0.013
L0a2	14308	1	0.013
L0a2	152	1	0.013
L0a2	16188G	8	0.105
L0a2	16320	3	0.039
L0a2	189	21	0.276
L0a2	64	22	0.289
L0a2	9042	1	0.013
L0a2	93	1	0.013
L0a2a	10664	1	0.03
L0a2a	11641	1	0.03
L0a2a	16187	1	0.03
L0a2a	16188G	1	0.03
L0a2a	16189	1	0.03
L0a2a	16230	1	0.03
L0a2a	64	1	0.03
L0a2a	7256	1	0.03
L0a2a	7521	1	0.03
L0a2a1	64	10	0.244
L0a2a1a	152	1	0.02
L0a2a1a	16188G	3	0.06
L0a2a1a2	16188G	4	0.105
L0a2a1a2	8281-8289d	2	0.053
L0a2a1b	11172	3	0.029
L0a2a1b	152	1	0.01
L0a2a1b	16172	1	0.01
L0a2a1b	16187	2	0.019
L0a2a1b	16188G	24	0.229
L0a2a1b	16189	2	0.019
L0a2a1b	189	6	0.057
L0a2a1b	64	5	0.048
L0a2a2	93	1	0.013
L0a2a2a	10589	1	0.004
L0a2a2a	10664	1	0.004
L0a2a2a	10688	1	0.004
L0a2a2a	11914	1	0.004
L0a2a2a	12720	2	0.007
L0a2a2a	14308	1	0.004
L0a2a2a	14766	1	0.004
L0a2a2a	15136	1	0.004
L0a2a2a	152	6	0.021
L0a2a2a	16148	1	0.004
L0a2a2a	16172	1	0.004
L0a2a2a	16187	10	0.035
L0a2a2a	16188G	38	0.135
L0a2a2a	16189	6	0.021
L0a2a2a	16230	2	0.007
L0a2a2a	16311	1	0.004
L0a2a2a	16320	1	0.004
L0a2a2a	204	1	0.004
L0a2a2a	2885	1	0.004
L0a2a2a	3516A	1	0.004
L0a2a2a	3594	1	0.004
L0a2a2a	4104	1	0.004
L0a2a2a	4312	1	0.004
L0a2a2a	5147	2	0.007
L0a2a2a	5460	1	0.004
L0a2a2a	5603	1	0.004
L0a2a2a	5711	1	0.004
L0a2a2a	64	17	0.06
L0a2a2a	7146	9	0.032
L0a2a2a	7256	2	0.007
L0a2a2a	7521	2	0.007
L0a2a2a	8281-8289d	19	0.067
L0a2a2a	8460	2	0.007
L0a2a2a	8655	1	0.004
L0a2a2a	93	1	0.004
L0a2a2a	9347	1	0.004
L0a2a2a	9554	1	0.004
L0a2a2a1	8281-8289d	2	0.027
L0a2a2a2	8281-8289d	1	0.013
L0a2a2a2	93	1	0.013
L0a2b	13281	1	0.034
L0a2b	16242	1	0.034
L0a2b	2245	1	0.034
L0a2b	3372	1	0.034
L0a2b	5237	1	0.034
L0a2b	7146	1	0.034
L0a2b1	16242	3	0.143
L0a2b1	16390	4	0.19
L0a2b1	2245	2	0.095

L0a2b1 | 4312 | 1 | 0.048
L0a2b1 | 64 | 1 | 0.048
L0a2c | 11009 | 4 | 0.667
L0a2c | 13984 | 3 | 0.5
L0a2c | 194 | 5 | 0.833
L0a2d | 14308 | 1 | 0.026
L0a2d | 152 | 1 | 0.026
L0a2d | 16188G | 3 | 0.077
L0a2d | 5231 | 1 | 0.026
L0a2d | 64 | 1 | 0.026
L0a3 | 13276 | 1 | 0.056
L0a3 | 152 | 2 | 0.111
L0a3 | 16129 | 3 | 0.167
L0a3 | 16187 | 2 | 0.111
L0a3 | 16188G | 1 | 0.056
L0a3 | 16189 | 2 | 0.111
L0a3 | 93 | 3 | 0.167
L0a4 | 16192 | 4 | 0.235
L0a4 | 16259A | 4 | 0.235
L0a4 | 185 | 1 | 0.059
L0a4 | 189 | 1 | 0.059
L0a4 | 236 | 1 | 0.059
L0a4 | 247 | 1 | 0.059
L0a4 | 93 | 1 | 0.059
L0a'b'g | 16187 | 1 | 0.333
L0a'b'g | 16189 | 1 | 0.333
L0a'b'g | 16278 | 1 | 0.333
L0a'b'g | 185 | 2 | 0.667
L0a'b'g | 189 | 2 | 0.667
L0a'g | 16278 | 1 | 0.167
L0a'g | 185 | 4 | 0.667
L0a'g | 189 | 3 | 0.5
L0b | 16129 | 8 | 0.889
L0b | 16223 | 1 | 0.111
L0b | 185 | 4 | 0.444
L0b | 189 | 4 | 0.444
L0d1a | 8860 | 1 | 0.071
L0d1a1a | 498d | 2 | 0.1
L0d1a1a2 | 498d | 1 | 0.053
L0d1a1b | 195 | 2 | 0.062
L0d1a1b | 199 | 1 | 0.031
L0d1a1b1 | 195 | 2 | 0.125
L0d1a1b1a | 153 | 12 | 0.25
L0d1a1b1b | 498d | 1 | 0.056
L0d1a1c | 199 | 2 | 0.105
L0d1a1d | 498d | 1 | 0.053
L0d1a'd | 16234 | 1 | 0.083
L0d1a'd | 195 | 1 | 0.083
L0d1b1+@152 | 2484.1C | 2 | 0.4
L0d1b1a | 15217 | 1 | 0.077
L0d1b1a | 188 | 1 | 0.077
L0d1b1a1 | 188 | 3 | 0.103
L0d1b1b1 | 16129 | 29 | 0.853
L0d1b1b1 | 2484.1C | 21 | 0.618
L0d1b1b1 | 8251 | 1 | 0.029
L0d1b2 | 73 | 1 | 0.03
L0d1b2a | 152 | 1 | 0.022
L0d1b2a | 73 | 1 | 0.022
L0d1b2a1 | 16129 | 13 | 0.153
L0d1b2b | 498d | 1 | 0.016
L0d1b2b1 | 16187 | 1 | 0.016
L0d1b2b1a | 152 | 19 | 0.229
L0d1b2b1a | 16243 | 2 | 0.024
L0d1b2b1a | 73 | 1 | 0.012
L0d1b2b1b | 498d | 4 | 0.049
L0d1b2b1b1 | 498d | 1 | 0.016
L0d1b2b2 | 152 | 2 | 0.042
L0d1b2b2 | 16243 | 1 | 0.021
L0d1b2b2 | 573.XC | 2 | 0.042
L0d1b2b2a | 152 | 2 | 0.032
L0d1b2b2a | 498d | 4 | 0.065
L0d1b2b2a | 573.XC | 5 | 0.081
L0d1b2b2a | 8251 | 1 | 0.016
L0d1b2b2b | 152 | 2 | 0.047
L0d1b2b2b | 498d | 1 | 0.023
L0d1b2b2b | 573.XC | 2 | 0.047
L0d1b2b2b1 | 152 | 2 | 0.037
L0d1b2b2b1 | 573.XC | 9 | 0.167
L0d1b2b2c | 152 | 2 | 0.048
L0d1b2b2c | 16129 | 1 | 0.024
L0d1b2b2c1 | 152 | 2 | 0.041
L0d1b2b2c1 | 573.XC | 5 | 0.102
L0d1b2b2c2 | 152 | 2 | 0.045
L0d1b2b2c2 | 573.XC | 7 | 0.159
L0d1c | 12720 | 2 | 0.074
L0d1c | 13129 | 1 | 0.037
L0d1c | 13759 | 1 | 0.037
L0d1c | 15466 | 1 | 0.037
L0d1c | 15930 | 1 | 0.037
L0d1c | 15941 | 1 | 0.037
L0d1c | 15951 | 1 | 0.037
L0d1c | 16187 | 2 | 0.074
L0d1c | 16230 | 1 | 0.037
L0d1c | 16234 | 1 | 0.037
L0d1c | 195 | 1 | 0.037
L0d1c | 456 | 1 | 0.037
L0d1c | 498d | 3 | 0.111
L0d1c | 7146 | 1 | 0.037
L0d1c | 719 | 1 | 0.037
L0d1c1 | 7146 | 1 | 0.048
L0d1c1a1 | 146 | 1 | 0.009
L0d1c1a1 | 16242 | 1 | 0.009
L0d1c1a1 | 16243 | 1 | 0.009
L0d1c1a1 | 247 | 1 | 0.009
L0d1c1a1a | 10398 | 2 | 0.012
L0d1c1a1a | 16167 | 1 | 0.006
L0d1c1a1a | 198 | 2 | 0.012
L0d1c1a1a | 498d | 2 | 0.012
L0d1c1a1a2 | 16242 | 1 | 0.009
L0d1c1a1a2 | 16243 | 1 | 0.009
L0d1c1a1a2 | 247 | 1 | 0.009

L0d1c1a1a2 | 9438 | 1 | 0.009
L0d1c1a1b | 498d | 4 | 0.031
L0d1c1a1b | 719 | 1 | 0.008
L0d1c1a2 | 498d | 1 | 0.031
L0d1c2 | 152 | 5 | 0.227
L0d1c2 | 16187 | 1 | 0.045
L0d1c2a | 146 | 1 | 0.091
L0d1c2a | 152 | 2 | 0.182
L0d1c3 | 13276 | 5 | 0.156
L0d1c3 | 498d | 2 | 0.062
L0d1d | 16187 | 1 | 0.083
L0d1d | 195 | 1 | 0.083
L0d2a1 | 16129 | 1 | 0.007
L0d2a1 | 498d | 2 | 0.014
L0d2a1a | 13276 | 2 | 0.011
L0d2a1a | 152 | 1 | 0.005
L0d2a1a | 16129 | 4 | 0.021
L0d2a1a | 16187 | 2 | 0.011
L0d2a1a | 16212 | 24 | 0.128
L0d2a1a | 16230 | 2 | 0.011
L0d2a1a | 498d | 6 | 0.032
L0d2a1a1a | 152 | 4 | 0.047
L0d2a1a2 | 498d | 1 | 0.007
L0d2a1b | 16129 | 1 | 0.062
L0d2a2 | 16187 | 4 | 0.8
L0d2a'b'd | 16187 | 1 | 0.029
L0d2a'b'd | 16230 | 1 | 0.029
L0d2a'b'd | 7146 | 1 | 0.029
L0d2a'b'd | 7154 | 1 | 0.029
L0d2a'b'd | 769 | 1 | 0.029
L0d2a'b'd | 825A | 1 | 0.029
L0d2b1a1 | 146 | 1 | 0.077
L0d2b1a1 | 195 | 8 | 0.615
L0d2b1a1 | 247 | 1 | 0.077
L0d2b1a1 | 265 | 1 | 0.077
L0d2b1a1 | 73 | 1 | 0.077
L0d2b1b | 15944d | 1 | 0.056
L0d2b1b | 16258C | 5 | 0.278
L0d2b1b | 498d | 1 | 0.056
L0d2b2 | 16212 | 1 | 0.077
L0d2b2 | 498d | 1 | 0.077
L0d2c1 | 12007 | 1 | 0.03
L0d2c1 | 12705 | 1 | 0.03
L0d2c1 | 13276 | 1 | 0.03
L0d2c1 | 13506 | 1 | 0.03
L0d2c1 | 15941 | 1 | 0.03
L0d2c1 | 16187 | 2 | 0.061
L0d2c1 | 16189 | 1 | 0.03
L0d2c1 | 3516A | 1 | 0.03
L0d2c1 | 498d | 1 | 0.03
L0d2c1 | 8113A | 1 | 0.03
L0d2c1 | 8420 | 1 | 0.03
L0d2c1a1 | 13827 | 1 | 0.048
L0d2c1a1 | 498d | 1 | 0.048
L0d2c1b | 16230 | 5 | 0.417
L0d2c2 | 498d | 2 | 0.062
L0d2c2a1 | 498d | 1 | 0.033
L0d2c2b | 498d | 1 | 0.032
L0d2d | 16243 | 1 | 0.111
L0d2d | 16390T | 1 | 0.111
L0d2d | 188 | 1 | 0.111
L0d3 | 16129 | 3 | 0.429
L0d3 | 16300 | 2 | 0.286
L0d3a | 10810 | 1 | 0.077
L0d3a | 10915 | 1 | 0.077
L0d3a | 12007 | 1 | 0.077
L0d3a | 152 | 2 | 0.154
L0d3a | 15586 | 1 | 0.077
L0d3b1 | 16300 | 7 | 0.167
L0f | 10688 | 8 | 0.242
L0f | 152 | 2 | 0.061
L0f | 16129 | 9 | 0.273
L0f | 16172 | 23 | 0.697
L0f | 16187 | 5 | 0.152
L0f | 16189 | 2 | 0.061
L0f | 16327 | 3 | 0.091
L0f | 185 | 1 | 0.03
L0f | 2885 | 4 | 0.121
L0f | 8468 | 1 | 0.03
L0f | 8860 | 1 | 0.03
L0f | 9818 | 2 | 0.061
L0f1 | 10664 | 3 | 0.429
L0f1 | 151 | 2 | 0.286
L0f1 | 16129 | 4 | 0.571
L0f1 | 185 | 3 | 0.429
L0f2 | 146 | 13 | 0.765
L0f2 | 16129 | 15 | 0.882
L0f2 | 16230 | 1 | 0.059
L0f2 | 185 | 1 | 0.059
L0f2 | 189 | 1 | 0.059
L0f2a | 10810 | 1 | 0.053
L0f2a | 13506 | 10 | 0.526
L0f2a | 152 | 3 | 0.158
L0f2a | 16223 | 5 | 0.263
L0f2a | 16230 | 6 | 0.316
L0f2a | 185 | 5 | 0.263
L0f2a | 263 | 1 | 0.053
L0f2a | 769 | 1 | 0.053
L0f2a1 | 11299 | 1 | 0.067
L0f2a1 | 13276 | 1 | 0.067
L0f2a1 | 13680 | 1 | 0.067
L0f2a1 | 14109 | 1 | 0.067
L0f2a1 | 152 | 1 | 0.067
L0f2a1 | 15431 | 1 | 0.067
L0f2a1 | 15852 | 2 | 0.133
L0f2a1 | 16173 | 2 | 0.133
L0f2a1 | 16187 | 1 | 0.067
L0f2a1 | 16189 | 1 | 0.067
L0f2a1 | 16239 | 1 | 0.067
L0f2a1 | 769 | 1 | 0.067
L0f2b | 12855 | 1 | 0.1

L0f2b | 15852 | 1 | 0.1
L0f2b | 15884 | 1 | 0.1
L0f2b | 16187 | 4 | 0.4
L0f2b | 16189 | 4 | 0.4
L0f2b | 16278 | 1 | 0.1
L0f2b | 7419 | 1 | 0.1
L0f2b | 8748 | 1 | 0.1
L0g | 10039 | 3 | 0.375
L0g | 10427 | 2 | 0.25
L0g | 14002 | 3 | 0.375
L0g | 146 | 1 | 0.125
L0g | 15803 | 3 | 0.375
L0g | 16129 | 1 | 0.125
L0g | 16169 | 1 | 0.125
L0g | 195 | 1 | 0.125
L0g | 198 | 1 | 0.125
L0g | 204 | 5 | 0.625
L0g | 207 | 2 | 0.25
L0g | 4466 | 3 | 0.375
L0g | 4814 | 2 | 0.25
L0g | 4943 | 1 | 0.125
L0g | 499 | 1 | 0.125
L0g | 5141 | 3 | 0.375
L0g | 8224 | 3 | 0.375
L0k1a1 | 16291G | 2 | 0.025
L0k1a1 | 850 | 1 | 0.013
L0k1a1a | 16187 | 27 | 0.375
L0k1a1a | 207 | 1 | 0.014
L0k1a1b | 12705 | 1 | 0.015
L0k1a1b | 14020 | 1 | 0.015
L0k1a1b | 16214 | 1 | 0.015
L0k1a1b | 2758 | 5 | 0.077
L0k1a1b | 5442 | 1 | 0.015
L0k1a1b | 769 | 1 | 0.015
L0k1a1d | 16166C | 1 | 0.014
L0k1a1d | 16291G | 9 | 0.127
L0k1a2 | 198 | 1 | 0.02
L0k1a2a | 16291G | 1 | 0.029
L0k2a | 16187 | 2 | 1
L0k2a1 | 204 | 1 | 0.5
L0k2b | 13650 | 1 | 0.333
L0k2b | 15221 | 1 | 0.333
L1 | 195 | 1 | 0.111
L1'2'3'4'5'6 | 13506 | 17 | 0.654
L1'2'3'4'5'6 | 13650 | 18 | 0.692
L1'2'3'4'5'6 | 1438 | 3 | 0.115
L1'2'3'4'5'6 | 152 | 2 | 0.077
L1'2'3'4'5'6 | 16129 | 6 | 0.231
L1'2'3'4'5'6 | 16187 | 21 | 0.808
L1'2'3'4'5'6 | 16189 | 3 | 0.115
L1'2'3'4'5'6 | 16223 | 3 | 0.115
L1'2'3'4'5'6 | 182 | 21 | 0.808
L1'2'3'4'5'6 | 195 | 22 | 0.846
L1'2'3'4'5'6 | 247 | 1 | 0.038
L1'2'3'4'5'6 | 2706 | 21 | 0.808
L1'2'3'4'5'6 | 2758 | 16 | 0.615
L1'2'3'4'5'6 | 7146 | 14 | 0.538
L1'2'3'4'5'6 | 750 | 3 | 0.115
L1'2'3'4'5'6 | 769 | 3 | 0.115
L1'2'3'4'5'6 | 8468 | 3 | 0.115
L1'2'3'4'5'6 | 8701 | 1 | 0.038
L1b | 16126 | 3 | 0.037
L1b1 | 10873 | 1 | 0.012
L1b1 | 13880A | 1 | 0.012
L1b1 | 14560 | 1 | 0.012
L1b1 | 185T | 1 | 0.012
L1b1 | 2768 | 1 | 0.012
L1b1 | 357 | 2 | 0.025
L1b1 | 825A | 1 | 0.012
L1b1a | 10810 | 1 | 0.007
L1b1a | 10873 | 1 | 0.007
L1b1a | 152 | 3 | 0.022
L1b1a | 16187 | 2 | 0.015
L1b1a | 16189 | 2 | 0.015
L1b1a | 16223 | 1 | 0.007
L1b1a | 182 | 2 | 0.015
L1b1a | 185T | 1 | 0.007
L1b1a | 195 | 1 | 0.007
L1b1a | 4104 | 1 | 0.007
L1b1a | 709 | 5 | 0.036
L1b1a | 710 | 3 | 0.022
L1b1a | 7146 | 1 | 0.007
L1b1a | 7256 | 1 | 0.007
L1b1a | 7521 | 1 | 0.007
L1b1a | 8860 | 1 | 0.007
L1b1a+189 | 16187 | 1 | 0.01
L1b1a10 | 14769 | 1 | 0.017
L1b1a10 | 16189 | 1 | 0.017
L1b1a10 | 16270 | 2 | 0.034
L1b1a10 | 709 | 2 | 0.034
L1b1a10 | 710 | 2 | 0.034
L1b1a10a | 152 | 1 | 0.02
L1b1a10a | 16187 | 2 | 0.04
L1b1a10a | 16189 | 2 | 0.04
L1b1a10a | 182 | 1 | 0.02
L1b1a10a | 185T | 1 | 0.02
L1b1a10b | 16126 | 1 | 0.018
L1b1a12 | 182 | 1 | 0.062
L1b1a12 | 185T | 1 | 0.062
L1b1a12 | 247 | 1 | 0.062
L1b1a12 | 73 | 1 | 0.062
L1b1a12a | 16126 | 1 | 0.071
L1b1a12b | 152 | 3 | 0.048
L1b1a12b | 16126 | 2 | 0.032
L1b1a12b | 16189 | 55 | 0.887
L1b1a12b | 16223 | 1 | 0.016
L1b1a12b | 16270 | 2 | 0.032
L1b1a12b | 16400 | 55 | 0.887
L1b1a12b | 182 | 1 | 0.016
L1b1a12b | 7389 | 1 | 0.016
L1b1a13 | 2768 | 1 | 0.012

L1b1a15 | 152 | 1 | 0.01
L1b1a15 | 16264 | 9 | 0.092
L1b1a15 | 182 | 1 | 0.01
L1b1a16 | 14016 | 1 | 0.024
L1b1a16 | 16126 | 3 | 0.073
L1b1a16 | 16187 | 1 | 0.024
L1b1a16 | 16189 | 1 | 0.024
L1b1a16 | 16264 | 2 | 0.049
L1b1a16 | 1738 | 1 | 0.024
L1b1a16 | 182 | 1 | 0.024
L1b1a16 | 185C | 2 | 0.049
L1b1a16 | 195 | 1 | 0.024
L1b1a16 | 7256 | 1 | 0.024
L1b1a17 | 152 | 3 | 0.026
L1b1a17 | 16187 | 30 | 0.263
L1b1a17 | 16189 | 20 | 0.175
L1b1a17 | 263 | 2 | 0.018
L1b1a18 | 10873 | 1 | 0.016
L1b1a18 | 14560 | 1 | 0.016
L1b1a18 | 152 | 1 | 0.016
L1b1a18 | 16187 | 2 | 0.032
L1b1a18 | 16189 | 2 | 0.032
L1b1a18 | 185T | 1 | 0.016
L1b1a18 | 189 | 4 | 0.065
L1b1a18 | 195 | 3 | 0.048
L1b1a18 | 263 | 1 | 0.016
L1b1a18 | 73 | 1 | 0.016
L1b1a2a | 16126 | 4 | 0.174
L1b1a2a | 16187 | 8 | 0.348
L1b1a2a | 16189 | 7 | 0.304
L1b1a2a | 16264 | 3 | 0.13
L1b1a2a | 16270 | 3 | 0.13
L1b1a2a | 182 | 2 | 0.087
L1b1a2a | 263 | 1 | 0.043
L1b1a2a | 5393 | 1 | 0.043
L1b1a3 | 10810 | 1 | 0.008
L1b1a3 | 16264 | 1 | 0.008
L1b1a3 | 182 | 1 | 0.008
L1b1a3 | 185T | 1 | 0.008
L1b1a3 | 4104 | 1 | 0.008
L1b1a3 | 4769 | 1 | 0.008
L1b1a3 | 7146 | 1 | 0.008
L1b1a3 | 7256 | 1 | 0.008
L1b1a3 | 7521 | 1 | 0.008
L1b1a3 | 8248 | 1 | 0.008
L1b1a3 | 8860 | 1 | 0.008
L1b1a3a | 152 | 1 | 0.01
L1b1a3a1 | 16126 | 1 | 0.01
L1b1a3a1 | 16223 | 3 | 0.029
L1b1a3a1 | 16270 | 1 | 0.01
L1b1a3a1 | 16278 | 1 | 0.01
L1b1a3a1 | 357 | 1 | 0.01
L1b1a3b | 1018 | 1 | 0.01
L1b1a3b | 189 | 1 | 0.01
L1b1a4 | 16187 | 4 | 0.16
L1b1a4 | 16189 | 2 | 0.08
L1b1a4 | 16223 | 1 | 0.04
L1b1a4a | 16114A | 7 | 0.35
L1b1a4a | 16187 | 1 | 0.05
L1b1a5 | 13880A | 1 | 0.011
L1b1a5 | 3693 | 1 | 0.011
L1b1a5 | 6548 | 1 | 0.011
L1b1a5 | 6989 | 1 | 0.011
L1b1a5 | 710 | 1 | 0.011
L1b1a6 | 16126 | 4 | 0.04
L1b1a6 | 16311 | 1 | 0.01
L1b1a6 | 357 | 1 | 0.01
L1b1a6 | 5393 | 2 | 0.02
L1b1a6 | 6548 | 1 | 0.01
L1b1a6 | 825A | 1 | 0.01
L1b1a7 | 13506 | 1 | 0.013
L1b1a7 | 16187 | 1 | 0.013
L1b1a7 | 16270 | 1 | 0.013
L1b1a7a | 16187 | 3 | 0.086
L1b1a7a | 16189 | 1 | 0.029
L1b1a7a | 228 | 1 | 0.029
L1b1a8 | 10688 | 1 | 0.011
L1b1a8 | 10810 | 1 | 0.011
L1b1a8 | 10873 | 1 | 0.011
L1b1a8 | 12519 | 1 | 0.011
L1b1a8 | 12705 | 1 | 0.011
L1b1a8 | 13105 | 1 | 0.011
L1b1a8 | 13506 | 1 | 0.011
L1b1a8 | 152 | 13 | 0.138
L1b1a8 | 16223 | 1 | 0.011
L1b1a8 | 16270 | 10 | 0.106
L1b1a8 | 182 | 4 | 0.043
L1b1a8 | 185T | 1 | 0.011
L1b1a8 | 195 | 1 | 0.011
L1b1a8 | 247 | 1 | 0.011
L1b1a8 | 263 | 1 | 0.011
L1b1a8 | 357 | 1 | 0.011
L1b1a8 | 73 | 1 | 0.011
L1b1a9 | 152 | 8 | 0.074
L1b1a9 | 16264 | 3 | 0.028
L1b2 | 152 | 2 | 0.083
L1b2 | 16187 | 13 | 0.542
L1b2 | 16189 | 13 | 0.542
L1b2 | 16239 | 1 | 0.042
L1b2 | 16264 | 1 | 0.042
L1b2 | 16270 | 5 | 0.208
L1b2 | 185T | 1 | 0.042
L1b2'3 | 16270 | 2 | 0.071
L1b2'3 | 16311 | 1 | 0.036
L1b2'3 | 73 | 1 | 0.036
L1b2a | 16187 | 4 | 0.114
L1b2a | 16189 | 3 | 0.086
L1b2a | 16239 | 1 | 0.029
L1b2a | 16270 | 1 | 0.029
L1b2a | 182 | 1 | 0.029
L1b2a | 357 | 1 | 0.029

L1b3 | 14769 | 1 | 0.036
L1b3 | 152 | 4 | 0.143
L1b3 | 16187 | 2 | 0.071
L1b3 | 16189 | 1 | 0.036
L1b3 | 16223 | 1 | 0.036
L1b3 | 185T | 1 | 0.036
L1b3 | 247 | 1 | 0.036
L1b3 | 73 | 1 | 0.036
L1c1 | 10398 | 8 | 0.615
L1c1 | 16129 | 1 | 0.077
L1c1 | 16187 | 10 | 0.769
L1c1 | 189C | 11 | 0.846
L1c1 | 2395d | 1 | 0.077
L1c1'2'4'5'6 | 16129 | 1 | 0.2
L1c1'2'4'5'6 | 16294 | 1 | 0.2
L1c1'2'4'5'6 | 195 | 1 | 0.2
L1c1'2'4'6 | 16129 | 1 | 0.25
L1c1'2'4'6 | 16187 | 1 | 0.25
L1c1'2'4'6 | 16189 | 1 | 0.25
L1c1'2'4'6 | 16223 | 1 | 0.25
L1c1'2'4'6 | 16278 | 1 | 0.25
L1c1'2'4'6 | 16294 | 1 | 0.25
L1c1'2'4'6 | 16311 | 1 | 0.25
L1c1'2'4'6 | 16360 | 1 | 0.25
L1c1a1a1a | 16129 | 9 | 0.17
L1c1a1a1a | 16187 | 4 | 0.075
L1c1a1a1a | 16189 | 1 | 0.019
L1c1a1a1a | 316 | 1 | 0.019
L1c1a1a1a | 44.1C | 3 | 0.057
L1c1a1a1b | 16234 | 1 | 0.026
L1c1a1a1b | 16274 | 3 | 0.077
L1c1a1a1b | 16360 | 5 | 0.128
L1c1a1a1b | 189C | 1 | 0.026
L1c1a1a1b | 263 | 1 | 0.026
L1c1a1a1b | 297 | 1 | 0.026
L1c1a1a1b | 316 | 1 | 0.026
L1c1a1a1b | 44.1C | 3 | 0.077
L1c1a1a1b1 | 11899 | 1 | 0.03
L1c1a1a1b1 | 14088 | 1 | 0.03
L1c1a1a1b1 | 16293 | 2 | 0.061
L1c1a1a1b1 | 2885 | 1 | 0.03
L1c1a1a1b1 | 4454A | 1 | 0.03
L1c1a1a1b1 | 8087 | 1 | 0.03
L1c1a2 | 12768 | 1 | 0.029
L1c1a2 | 16187 | 10 | 0.294
L1c1a2 | 16274 | 3 | 0.088
L1c1a2 | 195 | 11 | 0.324
L1c1a2 | 4506 | 4 | 0.118
L1c1a2 | 7202 | 4 | 0.118
L1c1a2a | 16311 | 1 | 0.037
L1c1a2a1 | 14766 | 2 | 0.065
L1c1a2a1 | 316 | 1 | 0.032
L1c1a2a1 | 4454A | 2 | 0.065
L1c1a2a2 | 189C | 1 | 0.034
L1c1a2b | 10810 | 1 | 0.021
L1c1a2b | 12810 | 1 | 0.021
L1c1a2b | 13485 | 1 | 0.021
L1c1a2b | 13506 | 1 | 0.021
L1c1a2b | 16360 | 1 | 0.021
L1c1a2b | 316 | 1 | 0.021
L1c1a2b | 3927 | 2 | 0.042
L1c1a2b | 4104 | 2 | 0.042
L1c1a2b | 4506 | 1 | 0.021
L1c1a2b | 6752 | 1 | 0.021
L1c1a2b | 769 | 1 | 0.021
L1c1a2b | 8027 | 1 | 0.021
L1c1a2b | 8087 | 1 | 0.021
L1c1a2b | 8468 | 1 | 0.021
L1c1a2b | 8655 | 1 | 0.021
L1c1a2b | 93 | 1 | 0.021
L1c1a'b'd | 151 | 2 | 0.286
L1c1a'b'd | 152 | 2 | 0.286
L1c1a'b'd | 16187 | 4 | 0.571
L1c1a'b'd | 16189 | 4 | 0.571
L1c1a'b'd | 297 | 1 | 0.143
L1c1a'b'd | 316 | 4 | 0.571
L1c1b | 16293 | 1 | 0.019
L1c1b | 16360 | 1 | 0.019
L1c1b | 4688 | 1 | 0.019
L1c1b1 | 15025 | 1 | 0.042
L1c1b1 | 151 | 8 | 0.333
L1c1b1 | 16291 | 1 | 0.042
L1c1b1 | 16294 | 1 | 0.042
L1c1b1 | 16360 | 6 | 0.25
L1c1b1 | 198 | 2 | 0.083
L1c1b1 | 4688 | 2 | 0.083
L1c1b1 | 4824 | 1 | 0.042
L1c1b1 | 8277 | 3 | 0.125
L1c1b'd | 151 | 1 | 0.2
L1c1b'd | 16294 | 1 | 0.2
L1c1c | 14911 | 1 | 0.031
L1c1c | 151 | 7 | 0.219
L1c1c | 152 | 1 | 0.031
L1c1c | 16129 | 4 | 0.125
L1c1c | 16172 | 3 | 0.094
L1c1c | 16293 | 17 | 0.531
L1c1c | 16360 | 5 | 0.156
L1c1c | 195 | 1 | 0.031
L1c1d | 14911 | 1 | 0.043
L1c1d | 151 | 4 | 0.174
L1c1d | 152 | 4 | 0.174
L1c1d | 15626 | 1 | 0.043
L1c1d | 16086 | 17 | 0.739
L1c1d | 16129 | 2 | 0.087
L1c1d | 16187 | 1 | 0.043
L1c1d1 | 16086 | 2 | 0.077
L1c1d1 | 16129 | 5 | 0.192
L1c1d1 | 16293 | 1 | 0.038
L1c1d1 | 16311 | 10 | 0.385
L1c1d1 | 198 | 7 | 0.269
L1c1d1 | 297 | 1 | 0.038

L1c2 | 151 | 4 | 0.211
L1c2 | 16129 | 1 | 0.053
L1c2 | 16278 | 1 | 0.053
L1c2 | 198 | 5 | 0.263
L1c2'4 | 16129 | 1 | 0.25
L1c2'4 | 16187 | 1 | 0.25
L1c2'4 | 16189 | 1 | 0.25
L1c2'4 | 16223 | 1 | 0.25
L1c2'4 | 16278 | 1 | 0.25
L1c2'4 | 16294 | 1 | 0.25
L1c2'4 | 16311 | 1 | 0.25
L1c2'4 | 16360 | 1 | 0.25
L1c2a1a | 13281 | 6 | 0.079
L1c2a1a | 151 | 8 | 0.105
L1c2a1a | 152 | 4 | 0.053
L1c2a1a | 16223 | 1 | 0.013
L1c2a1a | 16286G | 1 | 0.013
L1c2a1a | 16311 | 1 | 0.013
L1c2a1a | 2156.1A | 7 | 0.092
L1c2a1a | 5899.1C | 11 | 0.145
L1c2a1a | 8251 | 6 | 0.079
L1c2a1b | 151 | 1 | 0.053
L1c2a1b | 16129 | 6 | 0.316
L1c2a1b | 16145 | 1 | 0.053
L1c2a1b | 2156.1A | 4 | 0.211
L1c2a1b | 316 | 2 | 0.105
L1c2a1b | 5899.1C | 5 | 0.263
L1c2a2 | 13437 | 1 | 0.125
L1c2a2 | 16288 | 1 | 0.125
L1c2a2 | 16357 | 3 | 0.375
L1c2a2 | 7070 | 1 | 0.125
L1c2a2 | 9018 | 3 | 0.375
L1c2a3 | 16129 | 1 | 0.091
L1c2a3 | 16527 | 1 | 0.091
L1c2a3 | 471 | 1 | 0.091
L1c2a3 | 5899.1C | 9 | 0.818
L1c2a3a | 1048 | 1 | 0.067
L1c2a3a | 13485 | 8 | 0.533
L1c2a3a | 14127 | 1 | 0.067
L1c2a3a | 151 | 1 | 0.067
L1c2a3a | 16286G | 1 | 0.067
L1c2a3a | 2156.1A | 5 | 0.333
L1c2a3a | 2395d | 5 | 0.333
L1c2a3a | 5899.1C | 5 | 0.333
L1c2b | 10810 | 1 | 0.091
L1c2b | 151 | 1 | 0.091
L1c2b | 152 | 1 | 0.091
L1c2b | 16129 | 1 | 0.091
L1c2b | 16223 | 1 | 0.091
L1c2b | 16265C | 1 | 0.091
L1c2b | 16360 | 1 | 0.091
L1c2b | 198 | 1 | 0.091
L1c2b | 5899.1C | 1 | 0.091
L1c2b1 | 16129 | 3 | 0.167
L1c2b1 | 16360 | 8 | 0.444
L1c2b1a | 13506 | 4 | 0.25
L1c2b1a | 16187 | 2 | 0.125
L1c2b1a | 16223 | 1 | 0.062
L1c2b1a | 2758 | 1 | 0.062
L1c2b1a | 5899.1C | 2 | 0.125
L1c2b1a | 6209 | 1 | 0.062
L1c2b1a1 | 182 | 1 | 0.083
L1c2b1a1 | 2395d | 1 | 0.083
L1c2b1a1 | 5899.1C | 1 | 0.083
L1c2b1a'b | 10793 | 1 | 0.056
L1c2b1a'b | 151 | 1 | 0.056
L1c2b1a'b | 152 | 1 | 0.056
L1c2b1a'b | 16278 | 9 | 0.5
L1c2b1a'b | 16286A | 1 | 0.056
L1c2b1a'b | 189C | 1 | 0.056
L1c2b1a'b | 316 | 1 | 0.056
L1c2b1a'b | 5899.1C | 4 | 0.222
L1c2b1b | 16187 | 1 | 0.038
L1c2b1b | 16527 | 1 | 0.038
L1c2b1b | 297 | 1 | 0.038
L1c2b1b | 5899.1C | 2 | 0.077
L1c2b1b1 | 151 | 1 | 0.018
L1c2b1b1 | 3275G | 1 | 0.018
L1c2b1b1 | 5899.1C | 53 | 0.946
L1c2b1c | 151 | 20 | 0.667
L1c2b1c | 16129 | 5 | 0.167
L1c2b1c | 16278 | 4 | 0.133
L1c2b1c | 16360 | 4 | 0.133
L1c2b1c | 189C | 1 | 0.033
L1c2b1c | 316 | 1 | 0.033
L1c2b1c | 5580 | 1 | 0.033
L1c2b1c | 5894d | 8 | 0.267
L1c2b1c | 5899.1C | 8 | 0.267
L1c2b2 | 151 | 2 | 0.05
L1c2b2 | 152 | 2 | 0.05
L1c2b2 | 16187 | 1 | 0.025
L1c2b2 | 16189 | 1 | 0.025
L1c2b2 | 16278 | 18 | 0.45
L1c2b2 | 16294 | 1 | 0.025
L1c2b2 | 16527 | 1 | 0.025
L1c2b2 | 182 | 17 | 0.425
L1c2b2 | 195 | 1 | 0.025
L1c2b2 | 198 | 1 | 0.025
L1c2b2 | 2395d | 1 | 0.025
L1c2b2 | 316 | 1 | 0.025
L1c2b2 | 5899.1C | 16 | 0.4
L1c3 | 10398 | 1 | 0.1
L1c3 | 10586 | 1 | 0.1
L1c3 | 10688 | 1 | 0.1
L1c3 | 10810 | 1 | 0.1
L1c3 | 12705 | 1 | 0.1
L1c3 | 13105 | 1 | 0.1
L1c3 | 13789 | 1 | 0.1
L1c3 | 151 | 5 | 0.5
L1c3 | 16187 | 5 | 0.5
L1c3 | 16360 | 1 | 0.1

L1c3 | 182 | 1 | 0.1
L1c3 | 186A | 1 | 0.1
L1c3 | 189C | 1 | 0.1
L1c3 | 2395d | 2 | 0.2
L1c3 | 247 | 1 | 0.1
L1c3 | 3666 | 1 | 0.1
L1c3 | 4104 | 2 | 0.2
L1c3 | 5951 | 1 | 0.1
L1c3 | 6917 | 1 | 0.1
L1c3 | 7389 | 1 | 0.1
L1c3 | 7521 | 1 | 0.1
L1c3 | 8655 | 1 | 0.1
L1c3 | 9072 | 1 | 0.1
L1c3a | 11719 | 1 | 0.019
L1c3a | 151 | 14 | 0.259
L1c3a | 152 | 7 | 0.13
L1c3a | 15226 | 1 | 0.019
L1c3a | 15978 | 1 | 0.019
L1c3a | 16215 | 3 | 0.056
L1c3a | 16223 | 1 | 0.019
L1c3a | 16294 | 2 | 0.037
L1c3a | 16360 | 4 | 0.074
L1c3a | 2395d | 3 | 0.056
L1c3a | 6260 | 1 | 0.019
L1c3a | 8027 | 1 | 0.019
L1c3a1 | 13650 | 1 | 0.029
L1c3a1 | 16129 | 8 | 0.229
L1c3a1 | 16189 | 11 | 0.314
L1c3a1 | 16215 | 1 | 0.029
L1c3a1 | 16223 | 4 | 0.114
L1c3a1 | 16278 | 1 | 0.029
L1c3a1 | 263 | 2 | 0.057
L1c3a1 | 316 | 12 | 0.343
L1c3a1a | 151 | 9 | 0.321
L1c3a1a | 15226 | 1 | 0.036
L1c3a1a | 16129 | 3 | 0.107
L1c3a1a | 16223 | 1 | 0.036
L1c3a1b | 151 | 1 | 0.026
L1c3a1b | 152 | 1 | 0.026
L1c3a1b | 15905 | 1 | 0.026
L1c3a1b | 16215 | 5 | 0.128
L1c3a1b | 16360 | 5 | 0.128
L1c3a1b | 6260 | 1 | 0.026
L1c3b | 15978 | 1 | 0.067
L1c3b | 16129 | 2 | 0.133
L1c3b | 16294 | 1 | 0.067
L1c3b1 | 16187 | 2 | 0.133
L1c3b1 | 16189 | 1 | 0.067
L1c3b1 | 16360 | 1 | 0.067
L1c3b1 | 316 | 1 | 0.067
L1c3b1 | 9072 | 1 | 0.067
L1c3b1a | 10586 | 1 | 0.023
L1c3b1a | 13485 | 1 | 0.023
L1c3b1a | 151 | 4 | 0.093
L1c3b1a | 15905 | 1 | 0.023
L1c3b1a | 3210 | 1 | 0.023
L1c3b1a | 6221A | 1 | 0.023
L1c3b1b | 16163 | 2 | 0.133
L1c3b1b | 16311 | 1 | 0.067
L1c3b1b | 16360 | 1 | 0.067
L1c3b2 | 151 | 1 | 0.025
L1c3b2 | 15978 | 1 | 0.025
L1c3b2 | 16086 | 1 | 0.025
L1c3b2 | 16129 | 19 | 0.475
L1c3b2 | 16163 | 1 | 0.025
L1c3b2 | 16187 | 1 | 0.025
L1c3b2 | 16189 | 1 | 0.025
L1c3b2 | 16223 | 2 | 0.05
L1c3b2 | 16293 | 2 | 0.05
L1c3b2 | 16294 | 1 | 0.025
L1c3b2 | 2395d | 1 | 0.025
L1c3b2 | 316 | 1 | 0.025
L1c3b'c | 15978 | 2 | 0.333
L1c3c | 11852 | 20 | 0.625
L1c3c | 1438 | 2 | 0.062
L1c3c | 151 | 21 | 0.656
L1c3c | 15978 | 1 | 0.031
L1c3c | 16129 | 1 | 0.031
L1c3c | 16223 | 1 | 0.031
L1c3c | 16293 | 1 | 0.031
L1c3c | 2283 | 19 | 0.594
L1c3c | 3027 | 19 | 0.594
L1c3c | 3600 | 19 | 0.594
L1c3c | 458 | 23 | 0.719
L1c3c | 745.1T | 6 | 0.188
L1c3c | 93 | 22 | 0.688
L1c4a | 10398 | 1 | 0.2
L1c4a | 16223 | 1 | 0.2
L1c4a | 5899.1C | 4 | 0.8
L1c4b | 16184 | 1 | 0.02
L1c4b | 2395d | 1 | 0.02
L1c4b | 297 | 1 | 0.02
L1c4b | 316 | 1 | 0.02
L1c4b | 5899.1C | 31 | 0.633
L1c4b | 9266 | 1 | 0.02
L1c5 | 16187 | 1 | 0.032
L1c5 | 16261 | 1 | 0.032
L1c5 | 182 | 1 | 0.032
L1c5 | 195 | 27 | 0.871
L1c5 | 247 | 1 | 0.032
L1c6 | 16187 | 1 | 0.167
L1c6 | 16360 | 2 | 0.333
L1c6 | 247 | 1 | 0.167
L2 | 16390 | 3 | 0.6
L2'3'4'5'6 | 13105 | 6 | 0.5
L2'3'4'5'6 | 16129 | 1 | 0.083
L2'3'4'5'6 | 16187 | 2 | 0.167
L2'3'4'5'6 | 16189 | 1 | 0.083
L2'3'4'5'6 | 195 | 6 | 0.5
L2'3'4'5'6 | 247 | 1 | 0.083
L2a | 146 | 1 | 0.2

L2a | 16390 | 1 | 0.2
L2a | 182 | 1 | 0.2
L2a | 9221 | 1 | 0.2
L2a1 | 1018 | 1 | 0.008
L2a1 | 13650 | 1 | 0.008
L2a1 | 13803 | 1 | 0.008
L2a1 | 14566 | 1 | 0.008
L2a1 | 146 | 1 | 0.008
L2a1 | 152 | 2 | 0.016
L2a1 | 16223 | 1 | 0.008
L2a1 | 16278 | 1 | 0.008
L2a1 | 16390 | 4 | 0.032
L2a1 | 195 | 6 | 0.048
L2a1 | 2416 | 1 | 0.008
L2a1 | 2789 | 1 | 0.008
L2a1 | 3594 | 1 | 0.008
L2a1 | 4104 | 1 | 0.008
L2a1 | 73 | 1 | 0.008
L2a1 | 769 | 1 | 0.008
L2a1+143 | 2789 | 1 | 0.019
L2a1+143 | 3594 | 1 | 0.019
L2a1+143 | 7256 | 1 | 0.019
L2a1+143+@16309 | 146 | 1 | 0.017
L2a1+143+@16309 | 152 | 1 | 0.017
L2a1+143+@16309 | 16223 | 1 | 0.017
L2a1+143+@16309 | 16294 | 3 | 0.052
L2a1+143+@16309 | 16390 | 1 | 0.017
L2a1+143+@16309 | 195 | 2 | 0.034
L2a1+143+16189_(16192) | 10398 | 1 | 0.008
L2a1+143+16189_(16192) | 16223 | 1 | 0.008
L2a1+143+16189_(16192) | 16294 | 1 | 0.008
L2a1+143+16189_(16192) | 2706 | 1 | 0.008
L2a1+143+16189_(16192) | 4104 | 1 | 0.008
L2a1+143+16189_(16192) | 73 | 1 | 0.008
L2a1+143+16189_(16192) | 7521 | 1 | 0.008
L2a1+143+16189_(16192) | 9221 | 1 | 0.008
L2a1+143+16189_(16192) | 9540 | 1 | 0.008
L2a1+143+16189_(16192)+@16309 | 10115 | 1 | 0.011
L2a1+143+16189_(16192)+@16309 | 1018 | 1 | 0.011
L2a1+143+16189_(16192)+@16309 | 13590 | 1 | 0.011
L2a1+143+16189_(16192)+@16309 | 13650 | 3 | 0.032
L2a1+143+16189_(16192)+@16309 | 143 | 21 | 0.223
L2a1+143+16189_(16192)+@16309 | 14566 | 1 | 0.011
L2a1+143+16189_(16192)+@16309 | 146 | 2 | 0.021
L2a1+143+16189_(16192)+@16309 | 152 | 1 | 0.011
L2a1+143+16189_(16192)+@16309 | 15784 | 1 | 0.011
L2a1+143+16189_(16192)+@16309 | 16223 | 2 | 0.021
L2a1+143+16189_(16192)+@16309 | 195 | 3 | 0.032
L2a1+143+16189_(16192)+@16309 | 263 | 1 | 0.011
L2a1+143+16189_(16192)+@16309 | 3594 | 1 | 0.011
L2a1+143+16189_(16192)+@16309 | 7521 | 1 | 0.011
L2a1+143+16189_(16192)+@16309 | 7771 | 1 | 0.011
L2a1+143+16189_(16192)+@16309 | 9221 | 1 | 0.011
L2a1+16189_(16192) | 152 | 2 | 0.016
L2a1'2'3'4 | 16223 | 1 | 0.1
L2a1'2'3'4 | 16278 | 2 | 0.2
L2a1'2'3'4 | 182 | 7 | 0.7
L2a1a | 152 | 1 | 0.007
L2a1a | 16309 | 3 | 0.022
L2a1a | 16390 | 2 | 0.015
L2a1a1 | 13650 | 1 | 0.01
L2a1a1 | 13803 | 1 | 0.01
L2a1a1 | 146 | 2 | 0.019
L2a1a1 | 152 | 2 | 0.019
L2a1a1 | 16223 | 1 | 0.01
L2a1a1 | 16294 | 1 | 0.01
L2a1a1 | 16309 | 3 | 0.029
L2a1a1 | 6152 | 2 | 0.019
L2a1a2 | 16223 | 1 | 0.005
L2a1a2 | 16309 | 5 | 0.027
L2a1a2 | 3918 | 3 | 0.016
L2a1a2 | 5285 | 2 | 0.011
L2a1a2 | 9221 | 1 | 0.005
L2a1a2a | 146 | 1 | 0.011
L2a1a2a | 152 | 1 | 0.011
L2a1a2a | 16294 | 1 | 0.011
L2a1a2a | 16309 | 6 | 0.068
L2a1a2a | 16390 | 1 | 0.011
L2a1a2a1a | 10454 | 1 | 0.009
L2a1a2a1a | 16286 | 6 | 0.053
L2a1a2b | 146 | 1 | 0.011
L2a1a2b | 152 | 1 | 0.011
L2a1a2b | 16294 | 1 | 0.011
L2a1a2b | 16390 | 1 | 0.011
L2a1a2c | 16286 | 1 | 0.013
L2a1a3a | 15244 | 1 | 0.018
L2a1a3a | 16309 | 1 | 0.018
L2a1a3b | 143 | 3 | 0.046
L2a1a3b | 16294 | 2 | 0.031
L2a1a3c | 16309 | 1 | 0.037
L2a1a3c | 16390 | 1 | 0.037
L2a1a3c | 2789 | 1 | 0.037
L2a1a3c | 3594 | 1 | 0.037
L2a1a3c | 3918 | 1 | 0.037
L2a1a3c | 7175 | 1 | 0.037
L2a1a3c | 750 | 1 | 0.037
L2a1a3c | 769 | 1 | 0.037
L2a1a3c | 9221 | 1 | 0.037
L2a1b | 16223 | 5 | 0.037
L2a1b | 195 | 1 | 0.007
L2a1b+143 | 16189 | 1 | 0.015
L2a1b+143 | 195 | 2 | 0.03
L2a1b1 | 152 | 1 | 0.008
L2a1b1a | 1438 | 1 | 0.003
L2a1b1a | 14766 | 1 | 0.003
L2a1b1a | 152 | 4 | 0.012
L2a1b1a | 15326 | 2 | 0.006
L2a1b1a | 16294 | 2 | 0.006
L2a1b1a | 16309 | 15 | 0.045
L2a1b1a | 16390 | 2 | 0.006
L2a1b1a | 263 | 1 | 0.003

L2a1b1a | 4104 | 2 | 0.006
L2a1b1a | 8860 | 1 | 0.003
L2a1b2 | 16309 | 1 | 0.008
L2a1b2 | 195 | 1 | 0.008
L2a1b2 | 4769 | 1 | 0.008
L2a1b2 | 7256 | 1 | 0.008
L2a1b2 | 8860 | 1 | 0.008
L2a1c | 143 | 2 | 0.027
L2a1c | 152 | 2 | 0.027
L2a1c | 16309 | 2 | 0.027
L2a1c | 195 | 6 | 0.081
L2a1c+16086 | 143 | 6 | 0.3
L2a1c+16086 | 195 | 1 | 0.05
L2a1c+16129 | 143 | 1 | 0.014
L2a1c+16129 | 16309 | 6 | 0.087
L2a1c+16129 | 263 | 1 | 0.014
L2a1c+16129 | 4104 | 1 | 0.014
L2a1c1 | 146 | 1 | 0.04
L2a1c1 | 16086 | 3 | 0.12
L2a1c1 | 198 | 1 | 0.04
L2a1c1 | 8701 | 3 | 0.12
L2a1c1a | 146 | 1 | 0.062
L2a1c1a | 16309 | 1 | 0.062
L2a1c1a1 | 143 | 2 | 0.118
L2a1c1a1 | 14566 | 3 | 0.176
L2a1c1a1 | 198 | 1 | 0.059
L2a1c2 | 143 | 2 | 0.067
L2a1c2 | 16294 | 1 | 0.033
L2a1c2 | 16309 | 5 | 0.167
L2a1c2a | 146 | 2 | 0.053
L2a1c2a | 152 | 1 | 0.026
L2a1c2a | 16193 | 2 | 0.053
L2a1c2a | 16213 | 1 | 0.026
L2a1c2a | 16239 | 1 | 0.026
L2a1c2a | 16278 | 1 | 0.026
L2a1c2a | 16294 | 2 | 0.053
L2a1c2a | 16309 | 2 | 0.053
L2a1c3 | 146 | 1 | 0.009
L2a1c3 | 152 | 1 | 0.009
L2a1c3 | 16294 | 1 | 0.009
L2a1c3 | 195 | 1 | 0.009
L2a1c3a | 143 | 3 | 0.055
L2a1c3b | 10398 | 1 | 0.05
L2a1c3b | 143 | 1 | 0.05
L2a1c3b | 152 | 1 | 0.05
L2a1c3b1 | 143 | 3 | 0.176
L2a1c3b1 | 152 | 3 | 0.176
L2a1c3b1 | 16223 | 1 | 0.059
L2a1c3b1 | 195 | 1 | 0.059
L2a1c3b2 | 16355 | 1 | 0.111
L2a1c3b2 | 16390 | 1 | 0.111
L2a1c3b2 | 195 | 1 | 0.111
L2a1c4 | 146 | 1 | 0.017
L2a1c4a | 15301 | 1 | 0.017
L2a1c4a | 16309 | 2 | 0.033
L2a1c4a1 | 14566 | 1 | 0.018
L2a1c4a1 | 152 | 1 | 0.018
L2a1c4a1 | 16309 | 1 | 0.018
L2a1c5 | 143 | 3 | 0.045
L2a1c5 | 146 | 1 | 0.015
L2a1c5 | 9221 | 1 | 0.015
L2a1c6 | 16086 | 6 | 0.75
L2a1c6 | 16169 | 1 | 0.125
L2a1c6 | 16309 | 1 | 0.125
L2a1d1 | 16223 | 1 | 0.021
L2a1d1 | 16301 | 4 | 0.085
L2a1d1 | 16354 | 2 | 0.043
L2a1d1 | 182 | 7 | 0.149
L2a1d2 | 152 | 6 | 0.167
L2a1d2 | 15301 | 1 | 0.028
L2a1d2 | 15784 | 1 | 0.028
L2a1d2 | 7028 | 1 | 0.028
L2a1e | 16223 | 1 | 0.018
L2a1e | 16309 | 1 | 0.018
L2a1e | 73 | 2 | 0.036
L2a1e1 | 10873 | 1 | 0.01
L2a1e1 | 16223 | 3 | 0.03
L2a1e1 | 16294 | 1 | 0.01
L2a1e1 | 16390 | 1 | 0.01
L2a1e1 | 2706 | 1 | 0.01
L2a1e1 | 73 | 2 | 0.02
L2a1f | 13650 | 1 | 0.005
L2a1f | 14766 | 1 | 0.005
L2a1f | 16223 | 2 | 0.009
L2a1f | 16278 | 1 | 0.005
L2a1f | 16309 | 14 | 0.065
L2a1f | 16390 | 1 | 0.005
L2a1f | 195 | 1 | 0.005
L2a1f | 4769 | 1 | 0.005
L2a1f | 7521 | 1 | 0.005
L2a1f1 | 14566 | 1 | 0.007
L2a1f1a | 195 | 1 | 0.007
L2a1f2 | 14566 | 7 | 0.043
L2a1f2 | 16223 | 2 | 0.012
L2a1f2 | 16278 | 1 | 0.006
L2a1f2 | 16294 | 2 | 0.012
L2a1f2 | 263 | 1 | 0.006
L2a1f3 | 15326 | 1 | 0.011
L2a1f3 | 16223 | 1 | 0.011
L2a1f3 | 16278 | 1 | 0.011
L2a1f3 | 16294 | 2 | 0.022
L2a1f3 | 16309 | 1 | 0.011
L2a1f3 | 16390 | 1 | 0.011
L2a1f3 | 263 | 2 | 0.022
L2a1f3 | 7521 | 1 | 0.011
L2a1f3 | 8860 | 3 | 0.033
L2a1g | 152 | 2 | 0.038
L2a1g | 16223 | 1 | 0.019
L2a1g | 16225 | 2 | 0.038
L2a1g | 195 | 14 | 0.269
L2a1h | 143 | 12 | 0.316

L2a1h | 146 | 2 | 0.053
L2a1h | 16278 | 1 | 0.026
L2a1i | 195 | 2 | 0.03
L2a1i | 263 | 2 | 0.03
L2a1i1 | 143 | 4 | 0.093
L2a1i1 | 152 | 1 | 0.023
L2a1i1 | 16294 | 1 | 0.023
L2a1k | 146 | 3 | 0.333
L2a1k | 16223 | 1 | 0.111
L2a1l1a | 195 | 3 | 0.054
L2a1l1a2 | 16309 | 1 | 0.017
L2a1l1b | 16294 | 2 | 0.032
L2a1l1b | 16309 | 1 | 0.016
L2a1l1b | 263 | 7 | 0.113
L2a1l2a | 14180 | 1 | 0.018
L2a1l2a1 | 146 | 1 | 0.016
L2a1l2a1 | 152 | 2 | 0.032
L2a1l2a1 | 16390 | 1 | 0.016
L2a1l2a1 | 195 | 1 | 0.016
L2a1l2a1 | 263 | 1 | 0.016
L2a1l3 | 16309 | 1 | 0.111
L2a1m1a | 146 | 1 | 0.014
L2a1n | 16189 | 1 | 0.014
L2a1n | 16309 | 2 | 0.028
L2a1p | 16309 | 1 | 0.014
L2a1p | 7274 | 1 | 0.014
L2a1q | 16294 | 1 | 0.125
L2a2 | 16189 | 2 | 0.222
L2a2 | 16223 | 1 | 0.111
L2a2'3 | 16189 | 2 | 0.222
L2a2'3 | 16223 | 1 | 0.111
L2a2'3'4 | 152 | 1 | 0.2
L2a2'3'4 | 16189 | 1 | 0.2
L2a2'3'4 | 182 | 1 | 0.2
L2a2'3'4 | 195 | 2 | 0.4
L2a2a1 | 16390 | 5 | 0.357
L2a2a1 | 195 | 1 | 0.071
L2a2b | 16189 | 4 | 0.444
L2a2b | 16229 | 2 | 0.222
L2a2b | 182 | 1 | 0.111
L2a2b1 | 14118 | 3 | 0.6
L2a2b1a | 16390 | 5 | 0.085
L2a2b1a | 4104 | 1 | 0.017
L2a2b2 | 16390 | 1 | 0.111
L2a4 | 16390 | 1 | 0.333
L2a4a | 152 | 2 | 0.143
L2a4a | 16170 | 2 | 0.143
L2a4a | 573.XC | 1 | 0.071
L2a4b | 15326 | 1 | 0.143
L2a4b | 16170 | 1 | 0.143
L2a5 | 146 | 15 | 0.312
L2a5 | 152 | 1 | 0.021
L2a5 | 16223 | 5 | 0.104
L2a5 | 16224 | 2 | 0.042
L2a5 | 16390 | 3 | 0.062
L2a5 | 182 | 1 | 0.021
L2a5 | 3654 | 1 | 0.021
L2a5 | 6497 | 1 | 0.021
L2a5 | 8206 | 6 | 0.125
L2a5 | 9221 | 2 | 0.042
L2b | 146 | 1 | 0.125
L2b | 16129 | 1 | 0.125
L2b | 182 | 1 | 0.125
L2b | 198 | 1 | 0.125
L2b | 204 | 1 | 0.125
L2b1 | 10828 | 1 | 0.143
L2b1 | 16129 | 2 | 0.286
L2b1 | 263 | 1 | 0.143
L2b1 | 8080 | 1 | 0.143
L2b1a2 | 14059 | 1 | 0.022
L2b1a2 | 152 | 7 | 0.156
L2b1a2 | 16129 | 1 | 0.022
L2b1a2 | 198 | 10 | 0.222
L2b1a2 | 204 | 4 | 0.089
L2b1a2 | 418 | 2 | 0.044
L2b1a3 | 1018 | 1 | 0.014
L2b1a3 | 13650 | 1 | 0.014
L2b1a3 | 150 | 3 | 0.042
L2b1a3 | 152 | 3 | 0.042
L2b1a3 | 16355 | 2 | 0.028
L2b1a3 | 16362 | 10 | 0.141
L2b1a3 | 769 | 1 | 0.014
L2b1a4 | 12236 | 1 | 0.015
L2b1a4 | 13924 | 2 | 0.03
L2b1a4 | 14059 | 2 | 0.03
L2b1a4 | 16129 | 4 | 0.06
L2b1a4 | 16362 | 1 | 0.015
L2b1a4 | 182 | 6 | 0.09
L2b1a4 | 204 | 2 | 0.03
L2b1a4 | 418 | 2 | 0.03
L2b1b | 385 | 1 | 0.024
L2b1b | 4769 | 1 | 0.024
L2b1b | 7256 | 1 | 0.024
L2b1b | 7521 | 1 | 0.024
L2b1b | 8860 | 1 | 0.024
L2b2 | 12948 | 1 | 0.019
L2b2 | 146 | 1 | 0.019
L2b2 | 150 | 1 | 0.019
L2b2 | 152 | 2 | 0.038
L2b2 | 16223 | 2 | 0.038
L2b2 | 16278 | 8 | 0.151
L2b2 | 182 | 1 | 0.019
L2b2 | 204 | 5 | 0.094
L2b2 | 73 | 1 | 0.019
L2b2 | 750 | 1 | 0.019
L2b2a | 10398 | 1 | 0.024
L2b2a | 16129 | 5 | 0.122
L2b2a | 16354 | 1 | 0.024
L2b2a | 204 | 2 | 0.049
L2b2a | 709 | 1 | 0.024
L2b3 | 263 | 1 | 0.1

L2b3a | 15944.1T | 6 | 0.146
L2b3a | 16129 | 2 | 0.049
L2b3a | 16278 | 1 | 0.024
L2b3a | 204 | 3 | 0.073
L2b3c | 12948 | 1 | 0.077
L2b3c | 16129 | 1 | 0.077
L2b3c | 182 | 2 | 0.154
L2b'c'd | 11944 | 1 | 0.25
L2b'c'd | 13590 | 1 | 0.25
L2b'c'd | 13650 | 1 | 0.25
L2b'c'd | 1438 | 1 | 0.25
L2b'c'd | 152 | 1 | 0.25
L2b'c'd | 4104 | 1 | 0.25
L2b'c'd | 7256 | 1 | 0.25
L2b'c'd | 9221 | 1 | 0.25
L2c | 10398 | 1 | 0.021
L2c | 146 | 2 | 0.042
L2c | 152 | 7 | 0.146
L2c | 15849 | 1 | 0.021
L2c | 182 | 1 | 0.021
L2c | 195 | 1 | 0.021
L2c | 198 | 4 | 0.083
L2c | 93 | 5 | 0.104
L2c1 | 146 | 1 | 0.03
L2c1 | 16223 | 2 | 0.061
L2c1a | 13928C | 1 | 0.03
L2c1a | 146 | 6 | 0.182
L2c1a | 150 | 1 | 0.03
L2c1a | 16318 | 2 | 0.061
L2c2 | 16264 | 1 | 0.023
L2c2 | 16390 | 1 | 0.023
L2c2 | 182 | 2 | 0.045
L2c2 | 198 | 1 | 0.023
L2c2a | 152 | 4 | 0.062
L2c2a | 16264 | 1 | 0.015
L2c2a | 16278 | 1 | 0.015
L2c2a | 198 | 7 | 0.108
L2c2a | 73 | 2 | 0.031
L2c2a | 93 | 9 | 0.138
L2c2b1 | 198 | 2 | 0.087
L2c2b1b | 10790 | 1 | 0.034
L2c2b1b | 152 | 2 | 0.069
L2c2b1b | 15849 | 2 | 0.069
L2c2b1b | 198 | 1 | 0.034
L2c3 | 150 | 1 | 0.022
L2c3 | 152 | 19 | 0.422
L2c3 | 16390 | 1 | 0.022
L2c3 | 182 | 2 | 0.044
L2c3 | 198 | 1 | 0.022
L2c3 | 8860 | 2 | 0.044
L2c3 | 93 | 1 | 0.022
L2c4 | 146 | 15 | 0.263
L2c4 | 93 | 1 | 0.018
L2c5 | 16223 | 1 | 0.067
L2d+16129 | 16300 | 1 | 0.1
L2d+16129 | 3254A | 1 | 0.1
L2d+16129 | 3693 | 1 | 0.1
L2d1 | 16189 | 2 | 0.069
L2d1a | 13650 | 1 | 0.048
L2d1a | 16129 | 1 | 0.048
L2d1a | 16300 | 1 | 0.048
L2d1a | 16390 | 1 | 0.048
L2d1a | 4104 | 1 | 0.048
L2d1a | 456 | 1 | 0.048
L2e | 150 | 9 | 0.265
L2e | 16184 | 6 | 0.176
L2e | 16223 | 1 | 0.029
L2e | 16239 | 4 | 0.118
L2e | 16292 | 2 | 0.059
L2e | 16400 | 1 | 0.029
L2e | 479 | 1 | 0.029
L2e1 | 150 | 1 | 0.067
L2e1 | 152 | 6 | 0.4
L2e1 | 15734 | 1 | 0.067
L2e1 | 16399 | 1 | 0.067
L2e1 | 16400 | 1 | 0.067
L2e1 | 4769 | 1 | 0.067
L2e1 | 7256 | 1 | 0.067
L2e1 | 7521 | 1 | 0.067
L2e1 | 8860 | 1 | 0.067
L2e1a | 146 | 6 | 0.316
L2e1a | 150 | 1 | 0.053
L2e1a | 152 | 1 | 0.053
L2e1a | 16111A | 1 | 0.053
L2e1a | 16292 | 3 | 0.158
L2e1a | 16399 | 1 | 0.053
L2e1a | 479 | 2 | 0.105
L2e1a | 8206 | 1 | 0.053
L2e1a | 954 | 5 | 0.263
L3 | 10873 | 1 | 0.005
L3 | 12705 | 1 | 0.005
L3 | 1438 | 3 | 0.014
L3 | 15301 | 3 | 0.014
L3 | 7028 | 1 | 0.005
L3 | 8701 | 1 | 0.005
L3'4 | 263 | 1 | 0.008
L3a+709 | 151 | 16 | 0.571
L3a1 | 151 | 2 | 0.222
L3a1 | 16311 | 2 | 0.222
L3a1 | 16316 | 5 | 0.556
L3a1 | 721 | 1 | 0.111
L3a1 | 8701 | 1 | 0.111
L3a1a | 16311 | 1 | 0.023
L3a1b | 151 | 2 | 0.222
L3a1b | 152 | 2 | 0.222
L3a1b | 16316 | 2 | 0.222
L3a2 | 151 | 1 | 0.019
L3a2 | 263 | 1 | 0.019
L3b | 16223 | 1 | 0.008
L3b1a | 10373 | 1 | 0.006
L3b1a | 10398 | 2 | 0.012

L3b1a | 15311 | 9 | 0.054
L3b1a | 15944d | 5 | 0.03
L3b1a | 16362 | 2 | 0.012
L3b1a | 73 | 1 | 0.006
L3b1a | 8860 | 1 | 0.006
L3b1a+@16124 | 10373 | 1 | 0.005
L3b1a+@16124 | 15944d | 1 | 0.005
L3b1a+@16124 | 6221 | 1 | 0.005
L3b1a+@16124 | 9449 | 1 | 0.005
L3b1a+152 | 11002 | 1 | 0.01
L3b1a+152 | 15311 | 3 | 0.031
L3b1a+152 | 15944d | 2 | 0.021
L3b1a+152 | 16124 | 2 | 0.021
L3b1a+152 | 16362 | 2 | 0.021
L3b1a+152 | 8860 | 1 | 0.01
L3b1a10 | 15944d | 1 | 0.007
L3b1a10 | 73 | 2 | 0.014
L3b1a11 | 15944d | 2 | 0.019
L3b1a11 | 16124 | 9 | 0.085
L3b1a1a | 10086 | 1 | 0.002
L3b1a1a | 15311 | 1 | 0.002
L3b1a1a | 15824 | 1 | 0.002
L3b1a1a | 15944d | 1 | 0.002
L3b1a1a | 16124 | 165 | 0.397
L3b1a1a | 16362 | 2 | 0.005
L3b1a1a | 5773 | 1 | 0.002
L3b1a1a | 6221 | 2 | 0.005
L3b1a4 | 1438 | 1 | 0.008
L3b1a4 | 15944d | 1 | 0.008
L3b1a4 | 16124 | 1 | 0.008
L3b1a6 | 12705 | 1 | 0.013
L3b1a6 | 15311 | 2 | 0.026
L3b1a6 | 15944d | 3 | 0.039
L3b1a6 | 16362 | 1 | 0.013
L3b1a7 | 5211 | 2 | 0.015
L3b1a7a | 8860 | 1 | 0.007
L3b1a8 | 12705 | 2 | 0.019
L3b1a8 | 13105 | 1 | 0.009
L3b1a8 | 3450 | 1 | 0.009
L3b1a8 | 5773 | 1 | 0.009
L3b1a8 | 9449 | 1 | 0.009
L3b1a8 | 9540 | 1 | 0.009
L3b1a9 | 16362 | 1 | 0.011
L3b1a9a | 15944d | 1 | 0.012
L3b1b | 15326 | 1 | 0.012
L3b1b | 15664 | 2 | 0.024
L3b1b | 16124 | 1 | 0.012
L3b1b1 | 73 | 2 | 0.095
L3b1b1 | 8701 | 1 | 0.048
L3b2 | 16278 | 1 | 0.012
L3b2a | 10640 | 1 | 0.012
L3b2a | 16124 | 1 | 0.012
L3b2b | 10640 | 1 | 0.011
L3b2b | 15944d | 1 | 0.011
L3b2b | 16124 | 1 | 0.011
L3b2b | 16223 | 1 | 0.011
L3b3 | 13934 | 2 | 0.08
L3b3 | 16048 | 1 | 0.04
L3b3 | 16278 | 1 | 0.04
L3b3 | 185 | 3 | 0.12
L3b3 | 6527 | 2 | 0.08
L3b3 | 8701 | 1 | 0.04
L3d1a1'2 | 152 | 1 | 0.014
L3d1a1a | 10873 | 1 | 0.006
L3d1a1a | 10915 | 1 | 0.006
L3d1a1a | 11719 | 1 | 0.006
L3d1a1a | 13105 | 1 | 0.006
L3d1a1a | 13886 | 1 | 0.006
L3d1a1a | 14284 | 1 | 0.006
L3d1a1a | 150 | 10 | 0.064
L3d1a1a | 152 | 10 | 0.064
L3d1a1a | 16319 | 1 | 0.006
L3d1a1a | 4048 | 2 | 0.013
L3d1a1a | 4203 | 1 | 0.006
L3d1a1a | 6680 | 1 | 0.006
L3d1a1a | 8618 | 1 | 0.006
L3d1a1a | 921 | 1 | 0.006
L3d1a1a1 | 1438 | 1 | 0.008
L3d1a1a1 | 150 | 7 | 0.053
L3d1a1a1 | 152 | 8 | 0.06
L3d1a1a1 | 16124 | 7 | 0.053
L3d1a1a1 | 16319 | 5 | 0.038
L3d1a1b | 16124 | 1 | 0.014
L3d1a1b | 195 | 1 | 0.014
L3d1a1b | 750 | 1 | 0.014
L3d1b | 10398 | 3 | 0.033
L3d1b | 16124 | 1 | 0.011
L3d1b1 | 152 | 1 | 0.011
L3d1b1a | 4769 | 3 | 0.033
L3d1b1a | 8618 | 3 | 0.033
L3d1b2 | 150 | 1 | 0.014
L3d1b2 | 263 | 1 | 0.014
L3d1b3 | 146 | 1 | 0.016
L3d1b3 | 8618 | 1 | 0.016
L3d1b3a | 16223 | 1 | 0.018
L3d1b3a | 263 | 2 | 0.036
L3d1c | 152 | 1 | 0.015
L3d1c | 16223 | 1 | 0.015
L3d1d | 152 | 1 | 0.008
L3d1d | 16124 | 1 | 0.008
L3d1d | 16223 | 2 | 0.017
L3d1d | 9151 | 1 | 0.008
L3d2 | 263 | 1 | 0.016
L3d2b | 152 | 3 | 0.046
L3d2b | 263 | 10 | 0.154
L3d2b | 4769 | 1 | 0.015
L3d2b | 5147 | 1 | 0.015
L3d2b | 8860 | 1 | 0.015
L3d3a | 16223 | 1 | 0.007
L3d3a1 | 152 | 6 | 0.038
L3d3a1b | 16124 | 6 | 0.049

L3d3a1b | 263 | 1 | 0.008
L3d3a1b | 73 | 3 | 0.024
L3d3b | 921 | 1 | 0.011
L3d4 | 152 | 1 | 0.017
L3d4 | 15326 | 2 | 0.034
L3d4 | 189 | 1 | 0.017
L3d4a | 13105 | 1 | 0.02
L3d4a | 152 | 1 | 0.02
L3d4a | 16124 | 5 | 0.1
L3d4a | 189 | 1 | 0.02
L3d5 | 152 | 1 | 0.016
L3d5 | 15799 | 1 | 0.016
L3d5 | 921 | 1 | 0.016
L3e1 | 189 | 4 | 0.06
L3e1 | 200 | 1 | 0.015
L3e1a1a | 16185 | 4 | 0.06
L3e1a1a | 16223 | 1 | 0.015
L3e1a1a | 200 | 27 | 0.403
L3e1a1a | 8860 | 2 | 0.03
L3e1a2 | 16185 | 1 | 0.013
L3e1a2 | 195 | 1 | 0.013
L3e1a2 | 207 | 1 | 0.013
L3e1a2 | 8860 | 1 | 0.013
L3e1a3 | 150 | 1 | 0.029
L3e1a3 | 15942 | 1 | 0.029
L3e1a3 | 16327 | 4 | 0.118
L3e1a3 | 189 | 1 | 0.029
L3e1a3 | 200 | 3 | 0.088
L3e1a3 | 73 | 1 | 0.029
L3e1a3a | 16185 | 1 | 0.007
L3e1a3a | 16223 | 1 | 0.007
L3e1a3a | 189 | 2 | 0.014
L3e1a3a | 200 | 2 | 0.014
L3e1b | 150 | 1 | 0.067
L3e1b | 16327 | 1 | 0.067
L3e1b | 189 | 1 | 0.067
L3e1b | 200 | 3 | 0.2
L3e1b1 | 16223 | 1 | 0.04
L3e1b1 | 73 | 1 | 0.04
L3e1b2 | 16325d | 5 | 0.093
L3e1b2 | 16327 | 19 | 0.352
L3e1c | 15942 | 1 | 0.022
L3e1c | 16223 | 3 | 0.065
L3e1c | 16327 | 1 | 0.022
L3e1d | 152 | 1 | 0.048
L3e1d | 73 | 1 | 0.048
L3e1d1 | 150 | 2 | 0.044
L3e1d1 | 152 | 2 | 0.044
L3e1d1 | 16223 | 1 | 0.022
L3e1d1 | 189 | 2 | 0.044
L3e1d1 | 200 | 5 | 0.111
L3e1e | 189 | 1 | 0.014
L3e1e | 200 | 18 | 0.257
L3e1e1 | 200 | 1 | 0.014
L3e1f1 | 189 | 1 | 0.043
L3e1f1 | 200 | 2 | 0.087
L3e1f1a | 16327 | 5 | 0.238
L3e1f1a | 200 | 2 | 0.095
L3e1g | 189 | 1 | 0.062
L3e2 | 14212 | 1 | 0.028
L3e2 | 14905 | 1 | 0.028
L3e2 | 7028 | 1 | 0.028
L3e2a | 4769 | 1 | 0.037
L3e2a | 8860 | 1 | 0.037
L3e2a1 | 263 | 1 | 0.029
L3e2a1a | 14869 | 1 | 0.042
L3e2a1b1 | 16223 | 2 | 0.026
L3e2a1b1 | 198 | 3 | 0.039
L3e2a1b2 | 73 | 1 | 0.056
L3e2a2 | 150 | 1 | 0.038
L3e2a3 | 16320 | 2 | 0.05
L3e2b | 10873 | 1 | 0.007
L3e2b | 14905 | 1 | 0.007
L3e2b | 150 | 7 | 0.052
L3e2b | 15326 | 1 | 0.007
L3e2b | 16223 | 1 | 0.007
L3e2b | 195 | 1 | 0.007
L3e2b | 2352 | 1 | 0.007
L3e2b | 73 | 1 | 0.007
L3e2b+152 | 1438 | 1 | 0.011
L3e2b+152 | 14905 | 1 | 0.011
L3e2b+152 | 16223 | 1 | 0.011
L3e2b1 | 195 | 1 | 0.014
L3e2b1a1 | 16223 | 2 | 0.027
L3e2b1a1 | 73 | 1 | 0.014
L3e2b1a1 | 9540 | 1 | 0.014
L3e2b1a2 | 1438 | 3 | 0.038
L3e2b2 | 263 | 2 | 0.029
L3e2b2 | 4769 | 1 | 0.014
L3e2b2 | 8860 | 1 | 0.014
L3e2b3 | 16320 | 1 | 0.016
L3e2b3 | 195 | 2 | 0.033
L3e2b4 | 16172 | 1 | 0.011
L3e2b4 | 16189 | 4 | 0.045
L3e2b4 | 16320 | 3 | 0.034
L3e2b4 | 263 | 4 | 0.045
L3e2b5 | 16172 | 1 | 0.013
L3e2b5 | 16320 | 3 | 0.039
L3e2b6 | 150 | 1 | 0.062
L3e2b6 | 195 | 1 | 0.062
L3e2b6 | 73 | 1 | 0.062
L3e2b7 | 16320 | 1 | 0.009
L3e2b7 | 195 | 2 | 0.018
L3e3'4'5 | 14212 | 1 | 0.006
L3e3'4'5 | 1438 | 1 | 0.006
L3e3'4'5 | 15301 | 1 | 0.006
L3e3a | 10667 | 2 | 0.013
L3e3a | 150 | 2 | 0.013
L3e3a | 16223 | 30 | 0.199
L3e3a | 16265T | 1 | 0.007
L3e3a | 2000 | 1 | 0.007

L3e3a | 5262 | 4 | 0.026
L3e3a | 573.XC | 81 | 0.536
L3e3b | 10816 | 1 | 0.009
L3e3b | 10819 | 2 | 0.019
L3e3b | 150 | 1 | 0.009
L3e3b | 16223 | 1 | 0.009
L3e3b | 2000 | 1 | 0.009
L3e3b | 4769 | 1 | 0.009
L3e3b | 8860 | 2 | 0.019
L3e3b | 9554 | 1 | 0.009
L3e3b1 | 10667 | 1 | 0.01
L3e3b1 | 10816 | 2 | 0.019
L3e3b1 | 12248 | 2 | 0.019
L3e3b1 | 13101C | 1 | 0.01
L3e3b1 | 13651 | 1 | 0.01
L3e3b1 | 16265T | 2 | 0.019
L3e3b1 | 5262 | 2 | 0.019
L3e3b2 | 1438 | 2 | 0.071
L3e3b3 | 10816 | 1 | 0.014
L3e3b3 | 10819 | 1 | 0.014
L3e3b3 | 13651 | 1 | 0.014
L3e3b3 | 195 | 9 | 0.129
L3e4 | 150 | 1 | 0.023
L3e4 | 16051 | 1 | 0.023
L3e4 | 16223 | 1 | 0.023
L3e4 | 263 | 1 | 0.023
L3e4 | 73 | 1 | 0.023
L3e4a | 16051 | 1 | 0.016
L3e4a1 | 5262 | 2 | 0.036
L3e5 | 263 | 1 | 0.019
L3e5a | 10398 | 1 | 0.022
L3e5a1a | 73 | 2 | 0.05
L3e5b | 16223 | 1 | 0.019
L3e5b | 263 | 1 | 0.019
L3f | 15514 | 1 | 0.012
L3f | 16209 | 2 | 0.025
L3f | 4769 | 1 | 0.012
L3f1a | 15514 | 1 | 0.014
L3f1b | 11440 | 1 | 0.043
L3f1b+16292 | 16311 | 1 | 0.019
L3f1b+16292 | 189 | 2 | 0.038
L3f1b+16292 | 3396 | 1 | 0.019
L3f1b+16292 | 5601 | 1 | 0.019
L3f1b+16292+150 | 10873 | 1 | 0.016
L3f1b+16292+150 | 14766 | 1 | 0.016
L3f1b+16292+150 | 14769 | 1 | 0.016
L3f1b+16292+150 | 15514 | 1 | 0.016
L3f1b+16292+150 | 15944d | 11 | 0.18
L3f1b1 | 15944d | 1 | 0.036
L3f1b1 | 189 | 1 | 0.036
L3f1b1a | 15944d | 3 | 0.031
L3f1b1a | 16223 | 1 | 0.01
L3f1b1a | 16292 | 2 | 0.021
L3f1b1a | 16295 | 2 | 0.021
L3f1b1a | 189 | 7 | 0.073
L3f1b1a | 263 | 1 | 0.01
L3f1b1a | 8527 | 1 | 0.01
L3f1b1a1 | 16295 | 6 | 0.222
L3f1b1a1 | 189 | 1 | 0.037
L3f1b2 | 189 | 9 | 0.129
L3f1b2 | 73 | 1 | 0.014
L3f1b2a | 15944d | 1 | 0.1
L3f1b4 | 16292 | 2 | 0.057
L3f1b4a1 | 1438 | 1 | 0.016
L3f1b4a1 | 15944d | 2 | 0.031
L3f1b4a1 | 189 | 1 | 0.016
L3f1b4b | 189 | 1 | 0.025
L3f1b4c | 15944d | 4 | 0.083
L3f1b4c | 16292 | 1 | 0.021
L3f1b4c | 189 | 10 | 0.208
L3f1b4c | 4769 | 1 | 0.021
L3f1b4c | 73 | 4 | 0.083
L3f1b4c | 8860 | 1 | 0.021
L3f1b5 | 15944d | 2 | 0.069
L3f2 | 15944d | 1 | 0.009
L3f2 | 16311 | 1 | 0.009
L3f2a1 | 152 | 5 | 0.074
L3f2a1 | 15944d | 1 | 0.015
L3f2a1 | 3396 | 1 | 0.015
L3f2a1 | 4218 | 1 | 0.015
L3f2a1a | 16209 | 1 | 0.021
L3f3 | 15944d | 4 | 0.222
L3f3 | 16234 | 2 | 0.111
L3f3 | 189 | 1 | 0.056
L3f3 | 318 | 2 | 0.111
L3f3a | 189 | 1 | 0.2
L3f3b | 15944d | 1 | 0.1
L3f3b | 16176 | 1 | 0.1
L3f3b | 16234 | 2 | 0.2
L3h1a1 | 16192 | 1 | 0.012
L3h1a1 | 16223 | 13 | 0.155
L3h1a1 | 263 | 4 | 0.048
L3h1a1 | 2706 | 3 | 0.036
L3h1a1 | 73 | 1 | 0.012
L3h1a2a | 9575 | 1 | 0.029
L3h1a2a1 | 16399 | 4 | 0.077
L3h1b1 | 16256A | 2 | 0.286
L3h1b1 | 195 | 1 | 0.143
L3h1b1 | 4388 | 1 | 0.143
L3h1b1a | 14410 | 1 | 0.027
L3h1b1a | 195 | 1 | 0.027
L3h1b2 | 151 | 1 | 0.05
L3h1b2 | 152 | 1 | 0.05
L3h1b2 | 15301 | 1 | 0.05
L3h1b2 | 16129 | 1 | 0.05
L3h1b2 | 606 | 2 | 0.1
L3h1b2 | 990 | 1 | 0.05
L3h2 | 195 | 1 | 0.077
L3i1a | 150 | 1 | 0.022
L3i2 | 16260 | 1 | 0.033
L3i2 | 189 | 1 | 0.033

L3i2 | 5441 | 3 | 0.1
L3k | 150 | 1 | 0.007
L3k | 152 | 1 | 0.007
L3k | 235 | 1 | 0.007
L3k | 2706 | 1 | 0.007
L3k1 | 150 | 5 | 0.179
L3k1 | 152 | 5 | 0.179
L3k1 | 8649 | 1 | 0.036
L3k1 | 8701 | 1 | 0.036
L3k1 | 9329 | 1 | 0.036
L3x1 | 204 | 1 | 0.125
L3x1+16311 | 16223 | 1 | 0.111
L3x1+16311 | 204 | 3 | 0.333
L3x1+16311 | 5899.XC | 1 | 0.111
L3x1a1 | 204 | 1 | 0.2
L3x1a2 | 16278 | 1 | 0.05
L3x1a2 | 5899.XC | 1 | 0.05
L3x1a2 | 750 | 1 | 0.05
L3x1b | 1438 | 2 | 0.222
L3x1b | 16223 | 1 | 0.111
L3x2a | 16169 | 6 | 0.462
L3x2a | 5899.XC | 2 | 0.154
L3x2a1 | 249d | 1 | 0.5
L3x2a1 | 5899.XC | 1 | 0.5
L3x2a1a | 5899.XC | 2 | 0.4
L3x2b | 16193 | 3 | 0.273
L3x2b | 16195 | 1 | 0.091
L3x2b | 3483 | 1 | 0.091
L4a1a | 13174 | 1 | 0.091
L4a1a | 16207T | 1 | 0.091
L4a1a | 16362 | 2 | 0.182
L4a1a | 198 | 2 | 0.182
L4a1a | 8631 | 2 | 0.182
L4a2 | 4769 | 3 | 0.429
L4b1 | 204 | 9 | 0.089
L4b1 | 3918 | 1 | 0.01
L4b1 | 709 | 1 | 0.01
L4b1 | 8701 | 1 | 0.01
L4b1a | 12661T | 1 | 0.04
L4b1a | 16179 | 1 | 0.04
L4b1a | 16189 | 1 | 0.04
L4b1a | 16320 | 1 | 0.04
L4b1a | 1804 | 1 | 0.04
L4b1a | 204 | 3 | 0.12
L4b1a | 263 | 1 | 0.04
L4b1a | 513 | 2 | 0.08
L4b1a | 7624 | 1 | 0.04
L4b2 | 146 | 1 | 0.056
L4b2 | 16355 | 1 | 0.056
L4b2 | 16362 | 1 | 0.056
L4b2 | 195 | 7 | 0.389
L4b2 | 244 | 3 | 0.167
L4b2a | 16399 | 1 | 0.048
L4b2a | 195 | 3 | 0.143
L4b2a | 263 | 1 | 0.048
L4b2a | 6260 | 9 | 0.429
L4b2a1 | 16293T | 1 | 0.111
L4b2a2 | 10398 | 1 | 0.024
L4b2a2 | 11719 | 1 | 0.024
L4b2a2 | 12609 | 1 | 0.024
L4b2a2 | 1438 | 2 | 0.049
L4b2a2 | 146 | 1 | 0.024
L4b2a2 | 16172 | 3 | 0.073
L4b2a2 | 16362 | 1 | 0.024
L4b2a2 | 769 | 1 | 0.024
L4b2a2a | 16172 | 5 | 0.052
L4b2a2a | 16311 | 1 | 0.01
L4b2a2a | 16362 | 48 | 0.5
L4b2a2a | 16399 | 2 | 0.021
L4b2a2a | 244 | 6 | 0.062
L4b2a2a | 263 | 3 | 0.031
L4b2a2a | 73 | 2 | 0.021
L4b2a2b | 16172 | 2 | 0.118
L4b2a2b | 16287 | 1 | 0.059
L4b2a2b | 16362 | 1 | 0.059
L4b2a2c | 13470 | 1 | 0.021
L4b2a2c | 16293T | 1 | 0.021
L4b2b | 146 | 1 | 0.033
L4b2b | 16293T | 1 | 0.033
L4b2b | 195 | 6 | 0.2
L4b2b | 73 | 1 | 0.033
L4b2b1 | 16311 | 1 | 0.04
L4b2b1 | 4206 | 1 | 0.04
L5 | 195 | 1 | 0.333
L5a | 16166 | 1 | 0.2
L5a1 | 15884 | 1 | 0.091
L5a1 | 16187 | 6 | 0.545
L5a1a | 12950 | 1 | 0.083
L5a1a | 152 | 1 | 0.083
L5a1a | 16148 | 1 | 0.083
L5a1a | 16187 | 4 | 0.333
L5a1a | 7972 | 1 | 0.083
L5a1b | 15884 | 1 | 0.059
L5a1b | 16187 | 12 | 0.706
L5a1b | 16189 | 4 | 0.235
L5a1b | 16223 | 1 | 0.059
L5a1b | 16355 | 1 | 0.059
L5a1b | 182 | 1 | 0.059
L5a1b | 455.2T | 3 | 0.176
L5a1b | 459.1C | 3 | 0.176
L5a1c | 13105 | 2 | 0.154
L5a1c | 16187 | 4 | 0.308
L5a1c | 7424 | 1 | 0.077
L5a1c | 9329 | 1 | 0.077
L5a2 | 16187 | 8 | 0.235
L5a2 | 16223 | 2 | 0.059
L5a2 | 195 | 14 | 0.412
L5b1 | 16187 | 7 | 0.333
L5b1 | 16189 | 7 | 0.333
L5b1 | 16360 | 1 | 0.048
L5b1 | 2417G | 2 | 0.095

L5b1 | 247 | 1 | 0.048
L5b1 | 249d | 3 | 0.143
L5b1 | 3027 | 2 | 0.095
L5b1 | 459.1C | 10 | 0.476
L5b1 | 4976 | 2 | 0.095
L5b1 | 5213 | 2 | 0.095
L5b1 | 535 | 2 | 0.095
L5b1a | 16187 | 2 | 0.143
L5b1a | 16189 | 1 | 0.071
L5b1a | 459.1C | 1 | 0.071
L5b1b | 16187 | 1 | 0.077
L5b1b | 16189 | 1 | 0.077
L5b1b | 16360 | 1 | 0.077
L5b1b | 459.1C | 1 | 0.077
L5b2 | 16148 | 9 | 0.9
L6 | 16223 | 1 | 0.167
L6a | 770 | 1 | 0.125
L6a | 961 | 3 | 0.375
L6b | 152 | 2 | 0.286
L6b | 2706 | 2 | 0.286
M | 10398 | 1 | 0.003
M | 10400 | 1 | 0.003
M | 12705 | 1 | 0.003
M | 15043 | 1 | 0.003
M | 15301 | 1 | 0.003
M | 16223 | 3 | 0.008
M | 263 | 1 | 0.003
M | 2706 | 1 | 0.003
M | 489 | 4 | 0.011
M | 8701 | 1 | 0.003
M | 8860 | 1 | 0.003
M | 9540 | 1 | 0.003
M10 | 573.XC | 6 | 0.048
M10a1 | 3172.1C | 2 | 0.014
M10a1+16129 | 263 | 1 | 0.011
M10a1+16129 | 489 | 3 | 0.034
M10a1+16129 | 573.XC | 1 | 0.011
M10a1+16129 | 73 | 2 | 0.023
M10a1a | 263 | 1 | 0.011
M10a1a | 3172.1C | 1 | 0.011
M10a1a | 573.XC | 7 | 0.074
M10a1a1 | 16093 | 20 | 0.312
M10a1a1 | 16129 | 1 | 0.016
M10a1a1 | 16497 | 1 | 0.016
M10a1a1 | 573.XC | 3 | 0.047
M10a1a1a | 16093 | 1 | 0.023
M10a1a1a | 16357 | 2 | 0.045
M10a1a1a | 3172.1C | 2 | 0.045
M10a1a1a | 573.XC | 2 | 0.045
M10a1a1b | 146 | 1 | 0.024
M10a1a1b | 16093 | 22 | 0.537
M10a1a1b | 16497 | 1 | 0.024
M10a1a1b | 263 | 2 | 0.049
M10a1a1b | 573.XC | 1 | 0.024
M10a1a1b | 7250 | 1 | 0.024
M10a1a1b1 | 16093 | 13 | 0.333
M10a1a1b1 | 16223 | 1 | 0.026
M10a1a1b1 | 16357 | 1 | 0.026
M10a1a1b1 | 16497 | 3 | 0.077
M10a1a1b1 | 573.XC | 7 | 0.179
M10a1a1b2 | 16193 | 1 | 0.028
M10a1a1b2 | 16497 | 7 | 0.194
M10a1a1b2 | 3172.1C | 1 | 0.028
M10a1a1b2 | 573.XC | 10 | 0.278
M10a1a1b2 | 73 | 1 | 0.028
M10a1b | 15040 | 1 | 0.007
M10a1b | 15301 | 1 | 0.007
M10a1b | 3172.1C | 3 | 0.022
M10a1b | 573.XC | 6 | 0.044
M10a1b | 73 | 1 | 0.007
M10a2 | 573.XC | 2 | 0.028
M11+200 | 326 | 1 | 0.004
M11a | 16223 | 1 | 0.004
M11a | 200 | 3 | 0.012
M11a | 326 | 1 | 0.004
M11a1 | 16223 | 1 | 0.004
M11a1 | 200 | 3 | 0.012
M11a1 | 215 | 3 | 0.012
M11a1 | 326 | 1 | 0.004
M11a2 | 16173 | 1 | 0.006
M11a2 | 200 | 11 | 0.064
M11a2 | 215 | 15 | 0.088
M11a2 | 73 | 1 | 0.006
M11a'b | 326 | 1 | 0.004
M11b1 | 16223 | 1 | 0.004
M11b1 | 200 | 1 | 0.004
M11b2 | 10398 | 1 | 0.007
M11b2 | 10400 | 1 | 0.007
M11b2 | 73 | 1 | 0.007
M11c | 215 | 1 | 0.009
M1'20'51 | 489 | 1 | 0.003
M12a | 318 | 1 | 0.009
M12a | 489 | 2 | 0.019
M12a1a | 125 | 1 | 0.006
M12a1a | 127 | 3 | 0.018
M12a1a | 128 | 2 | 0.012
M12a1a1 | 16234 | 9 | 0.136
M12a1a1 | 318 | 1 | 0.015
M12a1b | 11353 | 1 | 0.007
M12a1b | 125 | 2 | 0.014
M12a1b | 128 | 7 | 0.049
M12a1b | 16234 | 1 | 0.007
M12a2 | 318 | 1 | 0.036
M12a2 | 463 | 1 | 0.036
M12a2 | 573.XC | 3 | 0.107
M12b | 16223 | 1 | 0.006
M12b1a | 15951 | 2 | 0.012
M12b1a2a | 16129 | 1 | 0.004
M12b1a2a | 16172 | 1 | 0.004
M12b1b | 16129 | 5 | 0.038
M12b1b | 489 | 1 | 0.008

M12b1b | 5336 | 2 | 0.015
M12b2 | 16290 | 7 | 0.047
M12b2 | 16305 | 14 | 0.093
M12b2 | 489 | 1 | 0.007
M12b2a | 16305 | 3 | 0.027
M12b2a | 7337 | 1 | 0.009
M13 | 152 | 2 | 0.008
M13'46'61+16362 | 15301 | 1 | 0.002
M13'46'61+16362 | 2706 | 1 | 0.002
M13'46'61+16362 | 6253 | 2 | 0.003
M13a | 152 | 1 | 0.008
M13a | 16189 | 1 | 0.008
M13a1 | 16145 | 1 | 0.008
M13a1 | 16188 | 1 | 0.008
M13a1 | 750 | 1 | 0.008
M13a1b | 16188 | 6 | 0.026
M13a1b | 16189 | 2 | 0.009
M13a1b | 16223 | 3 | 0.013
M13a2 | 16188 | 14 | 0.059
M13a2 | 16189 | 210 | 0.882
M13a2 | 16223 | 1 | 0.004
M13a2 | 16257 | 22 | 0.092
M13a2 | 513 | 1 | 0.004
M13b1 | 16129 | 4 | 0.057
M13c | 152 | 6 | 0.038
M15 | 11002 | 1 | 0.01
M15 | 16249 | 1 | 0.01
M15 | 183 | 1 | 0.01
M15 | 64 | 1 | 0.01
M17a | 16129 | 5 | 0.042
M17a | 16223 | 2 | 0.017
M17a | 489 | 2 | 0.017
M17a | 862 | 1 | 0.008
M17c | 16209 | 1 | 0.011
M17c | 489 | 2 | 0.022
M17c1a | 143 | 1 | 0.2
M17c1a1 | 15853 | 2 | 0.154
M17c1a1 | 16233 | 1 | 0.077
M17c1a1 | 489 | 1 | 0.077
M17c1a1a | 16209 | 1 | 0.077
M18 | 12498 | 1 | 0.009
M18 | 12705 | 1 | 0.009
M18 | 15043 | 1 | 0.009
M18 | 15301 | 1 | 0.009
M18 | 16223 | 1 | 0.009
M18 | 16318T | 2 | 0.017
M18 | 2706 | 1 | 0.009
M18 | 8860 | 1 | 0.009
M18a | 12498 | 1 | 0.007
M18a | 13135 | 2 | 0.014
M18a | 14766 | 1 | 0.007
M18a | 16318T | 3 | 0.021
M18b | 10398 | 1 | 0.009
M18b | 12498 | 1 | 0.009
M18b | 15043 | 1 | 0.009
M18b | 15301 | 1 | 0.009
M18b | 16223 | 1 | 0.009
M18b | 2706 | 1 | 0.009
M18b | 8277 | 1 | 0.009
M18b | 8701 | 1 | 0.009
M18b | 8860 | 1 | 0.009
M18b | 9540 | 1 | 0.009
M18c | 1438 | 1 | 0.01
M18c | 16318T | 1 | 0.01
M19 | 12465 | 1 | 0.024
M19 | 152 | 1 | 0.024
M19 | 16249 | 1 | 0.024
M19 | 3828 | 1 | 0.024
M19 | 5250 | 1 | 0.024
M19 | 527 | 7 | 0.171
M1a | 16189 | 10 | 0.133
M1a | 195 | 11 | 0.147
M1a1 | 10873 | 1 | 0.024
M1a1 | 12403 | 1 | 0.024
M1a1 | 14110 | 1 | 0.024
M1a1 | 16129 | 1 | 0.024
M1a1 | 489 | 1 | 0.024
M1a1+16093 | 16223 | 1 | 0.083
M1a1a1 | 10398 | 1 | 0.043
M1a1a1 | 16223 | 1 | 0.043
M1a1b1a | 16129 | 1 | 0.016
M1a1b1a | 195 | 11 | 0.18
M1a1b1a | 73 | 1 | 0.016
M1a1b1a | 7853 | 1 | 0.016
M1a1b2 | 6671 | 1 | 0.025
M1a1d | 16093 | 5 | 0.294
M1a1d | 73 | 1 | 0.059
M1a1d | 8270 | 1 | 0.059
M1a1e | 16189 | 18 | 0.367
M1a1f | 16129 | 1 | 0.023
M1a1h | 16189 | 5 | 0.278
M1a1h | 16249 | 1 | 0.056
M1a1h | 16359 | 4 | 0.222
M1a1h | 195 | 1 | 0.056
M1a1i | 16129 | 1 | 0.043
M1a1i | 16311 | 1 | 0.043
M1a1i | 16527 | 2 | 0.087
M1a2 | 1438 | 1 | 0.036
M1a2 | 73 | 1 | 0.036
M1a2a | 10873 | 1 | 0.032
M1a2a | 195 | 2 | 0.065
M1a2a | 73 | 1 | 0.032
M1a2b | 16129 | 6 | 0.375
M1a3 | 16189 | 1 | 0.042
M1a3 | 263 | 1 | 0.042
M1a3 | 73 | 1 | 0.042
M1a3a | 16189 | 1 | 0.03
M1a3a | 16249 | 2 | 0.061
M1a3a | 195 | 5 | 0.152
M1a3a | 813 | 1 | 0.03
M1a3b | 12705 | 1 | 0.034

M1a3b | 13637 | 1 | 0.034
M1a4 | 16129 | 1 | 0.018
M1a4a | 16129 | 1 | 0.02
M1a4a | 16311 | 1 | 0.02
M1a5 | 10694 | 1 | 0.048
M1a5 | 14110 | 1 | 0.048
M1a5 | 15770 | 1 | 0.048
M1a5 | 16249 | 3 | 0.143
M1a5 | 489 | 1 | 0.048
M1a6 | 16249 | 2 | 0.034
M1a7 | 16249 | 1 | 0.062
M1a7 | 195 | 1 | 0.062
M1a8a | 489 | 1 | 0.017
M1b1 | 16129 | 1 | 0.083
M1b1 | 4936 | 1 | 0.083
M1b1a | 16185 | 1 | 0.053
M1b1a | 16189 | 2 | 0.105
M1b1a | 73 | 1 | 0.053
M1b1b | 15247G | 1 | 0.083
M1b2 | 16249 | 1 | 0.008
M1b2 | 2706 | 1 | 0.008
M1b2a | 2706 | 1 | 0.009
M1b2c | 16189 | 3 | 0.023
M20 | 14110 | 1 | 0.004
M20 | 152 | 1 | 0.004
M20 | 16129 | 4 | 0.014
M20 | 16209 | 1 | 0.004
M20 | 249d | 2 | 0.007
M20 | 263 | 1 | 0.004
M20 | 316 | 1 | 0.004
M20 | 489 | 1 | 0.004
M21a | 10202 | 10 | 0.097
M21a | 16271 | 10 | 0.097
M21a | 16362 | 13 | 0.126
M21a | 489 | 4 | 0.039
M21a | 709 | 1 | 0.01
M21a | 73 | 1 | 0.01
M21b | 3915 | 4 | 0.013
M21b | 489 | 2 | 0.006
M21b+210 | 3915 | 1 | 0.004
M21b2 | 16181 | 3 | 0.081
M21b2 | 16223 | 4 | 0.108
M21b2 | 16304 | 1 | 0.027
M21b2 | 1763 | 1 | 0.027
M21b2 | 2080 | 1 | 0.027
M21b2 | 3819 | 1 | 0.027
M21b2 | 489 | 1 | 0.027
M21b2 | 6231 | 1 | 0.027
M23 | 10142 | 1 | 0.004
M23 | 10295 | 2 | 0.008
M23 | 10400 | 2 | 0.008
M23 | 11569 | 1 | 0.004
M23 | 11899 | 1 | 0.004
M23 | 12618 | 2 | 0.008
M23 | 12705 | 1 | 0.004
M23 | 1438 | 1 | 0.004
M23 | 14783 | 1 | 0.004
M23 | 15025 | 1 | 0.004
M23 | 15043 | 1 | 0.004
M23 | 152 | 3 | 0.012
M23 | 16263 | 1 | 0.004
M23 | 16311 | 2 | 0.008
M23 | 195 | 1 | 0.004
M23 | 204 | 4 | 0.016
M23 | 8701 | 1 | 0.004
M23 | 8860 | 1 | 0.004
M24a | 1438 | 1 | 0.005
M24a | 146 | 1 | 0.005
M24a | 195 | 1 | 0.005
M24b | 16086 | 3 | 0.019
M24b | 195 | 3 | 0.019
M27a2a | 234 | 1 | 0.1
M27a2b | 15193 | 1 | 0.091
M27a2b | 189 | 8 | 0.727
M27b1 | 16299 | 2 | 0.4
M27b2a1 | 4769 | 1 | 0.037
M27c | 16301 | 1 | 0.007
M28a1 | 16468 | 1 | 0.009
M28a2 | 16468 | 1 | 0.25
M28a2 | 195 | 3 | 0.75
M28a2 | 204 | 1 | 0.25
M28a2a | 152 | 1 | 0.2
M28a2a | 16468 | 1 | 0.2
M28a2a | 6281 | 1 | 0.2
M28a3 | 16148 | 1 | 0.009
M28a5 | 152 | 1 | 0.009
M28a5 | 16362 | 1 | 0.009
M28a6a | 15481A | 1 | 0.1
M28a7a | 16086 | 2 | 0.018
M28a7a | 16320 | 1 | 0.009
M28a7b | 16471 | 2 | 0.018
M28b | 16318T | 1 | 0.01
M2a1 | 10873 | 1 | 0.014
M2a1 | 11083 | 2 | 0.027
M2a1 | 12810 | 2 | 0.027
M2a1 | 15670 | 2 | 0.027
M2a1 | 16274 | 3 | 0.041
M2a1 | 1780 | 1 | 0.014
M2a1 | 204 | 1 | 0.014
M2a1 | 447G | 1 | 0.014
M2a1 | 7961 | 2 | 0.027
M2a1a | 10873 | 1 | 0.011
M2a1a | 12705 | 1 | 0.011
M2a1a | 12810 | 1 | 0.011
M2a1a | 14783 | 1 | 0.011
M2a1a | 15043 | 1 | 0.011
M2a1a | 15301 | 1 | 0.011
M2a1a | 15670 | 1 | 0.011
M2a1a | 16223 | 1 | 0.011
M2a1a | 1780 | 1 | 0.011
M2a1a | 204 | 3 | 0.033

M2a1a | 2706 | 1 | 0.011
M2a1a | 447G | 1 | 0.011
M2a1a | 489 | 1 | 0.011
M2a1a | 8860 | 1 | 0.011
M2a1a+207 | 12810 | 1 | 0.014
M2a1a+207 | 16352 | 1 | 0.014
M2a1a+207 | 195 | 1 | 0.014
M2a1a+207 | 204 | 1 | 0.014
M2a1a+207 | 8701 | 1 | 0.014
M2a1a1 | 11083 | 1 | 0.012
M2a1a1 | 12810 | 1 | 0.012
M2a1a1 | 15670 | 1 | 0.012
M2a1a1 | 195 | 1 | 0.012
M2a1a1 | 7961 | 1 | 0.012
M2a1a1b1 | 14693 | 1 | 0.018
M2a1a1b1 | 7472 | 1 | 0.018
M2a1a2a1 | 16352 | 1 | 0.018
M2a1a2a1a | 11083 | 1 | 0.016
M2a1a2a1a | 12501 | 1 | 0.016
M2a1a2a1a | 5252 | 1 | 0.016
M2a1a2a1a | 9758 | 1 | 0.016
M2a1a3 | 16223 | 1 | 0.053
M2a1a3 | 16319 | 3 | 0.158
M2a1a3b | 14783 | 1 | 0.2
M2a1b | 11083 | 1 | 0.016
M2a1c | 5252 | 1 | 0.036
M2a1c | 526 | 1 | 0.036
M2a2 | 9540 | 1 | 0.011
M2a3a | 8860 | 3 | 0.045
M2a'b | 11083 | 1 | 0.011
M2a'b | 15670 | 1 | 0.011
M2b | 10398 | 1 | 0.018
M2b | 10400 | 1 | 0.018
M2b | 10873 | 1 | 0.018
M2b | 11083 | 1 | 0.018
M2b | 12705 | 1 | 0.018
M2b | 14783 | 1 | 0.018
M2b | 15043 | 1 | 0.018
M2b | 15301 | 1 | 0.018
M2b | 15670 | 1 | 0.018
M2b | 16169.1C | 1 | 0.018
M2b | 16189 | 1 | 0.018
M2b | 16223 | 1 | 0.018
M2b | 16274 | 1 | 0.018
M2b | 16319 | 1 | 0.018
M2b | 16320 | 2 | 0.035
M2b | 2706 | 1 | 0.018
M2b | 5744 | 1 | 0.018
M2b | 8860 | 1 | 0.018
M2b1 | 10873 | 1 | 0.016
M2b1 | 13254 | 1 | 0.016
M2b1 | 14783 | 1 | 0.016
M2b1 | 16169.1C | 4 | 0.062
M2b1 | 16223 | 1 | 0.016
M2b1 | 16274 | 1 | 0.016
M2b1 | 16319 | 1 | 0.016
M2b1 | 16320 | 1 | 0.016
M2b1 | 2831T | 1 | 0.016
M2b1 | 5744 | 1 | 0.016
M2b1 | 7028 | 1 | 0.016
M2b1a | 16320 | 1 | 0.019
M2b1b | 16169.1C | 4 | 0.286
M2b1b | 16319 | 1 | 0.071
M2b1b | 16320 | 1 | 0.071
M2b2 | 152 | 3 | 0.053
M2b3 | 10400 | 1 | 0.017
M2b3 | 12705 | 1 | 0.017
M2b3 | 13254 | 1 | 0.017
M2b3 | 15043 | 1 | 0.017
M2b3 | 15301 | 1 | 0.017
M2b3 | 15670 | 1 | 0.017
M2b3 | 16169.1C | 1 | 0.017
M2b3 | 16189 | 1 | 0.017
M2b3 | 16223 | 1 | 0.017
M2b3 | 16274 | 1 | 0.017
M2b3 | 16319 | 2 | 0.034
M2b3 | 16320 | 2 | 0.034
M2b3 | 1780 | 1 | 0.017
M2b3 | 182 | 1 | 0.017
M2b3 | 195 | 1 | 0.017
M2b3 | 2831T | 1 | 0.017
M2b3 | 489 | 1 | 0.017
M2b3 | 5744 | 1 | 0.017
M2b3 | 8860 | 1 | 0.017
M2b3a | 16169.1C | 64 | 0.542
M2b3a | 16223 | 1 | 0.008
M2b3a | 1780 | 1 | 0.008
M2b3a | 5744 | 1 | 0.008
M2c | 16274 | 2 | 0.019
M3 | 14766 | 1 | 0.004
M3 | 16223 | 1 | 0.004
M3 | 2706 | 1 | 0.004
M30 | 14783 | 1 | 0.004
M30+16234 | 8701 | 1 | 0.005
M30a | 2706 | 1 | 0.007
M30b | 16278 | 1 | 0.008
M30b | 16311 | 17 | 0.139
M30b | 263 | 1 | 0.008
M30c1 | 489 | 1 | 0.008
M30c1a | 195A | 1 | 0.01
M30d1 | 10400 | 1 | 0.004
M30d1 | 15043 | 1 | 0.004
M30d1 | 15301 | 1 | 0.004
M30d1 | 16223 | 1 | 0.004
M30d1 | 195A | 1 | 0.004
M30d1 | 2706 | 1 | 0.004
M30d1 | 8860 | 1 | 0.004
M30f | 16223 | 2 | 0.017
M30g | 12007 | 1 | 0.004
M30g | 16223 | 1 | 0.004
M31a1b | 10873 | 1 | 0.005

M31a1b | 11719 | 1 | 0.005
M31a1b | 249d | 1 | 0.005
M31a2 | 16093 | 7 | 0.241
M31a2 | 195 | 2 | 0.069
M31b1 | 10873 | 2 | 0.057
M31b1 | 16136 | 1 | 0.029
M31b1 | 16223 | 2 | 0.057
M32'56 | 16223 | 1 | 0.003
M32a | 195 | 1 | 0.009
M32a | 207 | 1 | 0.009
M32c | 10400 | 1 | 0.003
M32c | 13308 | 1 | 0.003
M32c | 13590 | 1 | 0.003
M32c | 150 | 5 | 0.017
M32c | 15301 | 1 | 0.003
M32c | 15625 | 2 | 0.007
M32c | 16086 | 2 | 0.007
M32c | 16148 | 1 | 0.003
M32c | 16223 | 2 | 0.007
M32c | 16259 | 3 | 0.01
M32c | 16319 | 1 | 0.003
M32c | 16399 | 1 | 0.003
M32c | 16526 | 1 | 0.003
M32c | 200 | 14 | 0.046
M32c | 3588 | 1 | 0.003
M32c | 7960 | 1 | 0.003
M33+16362 | 12705 | 1 | 0.001
M33+16362 | 15043 | 1 | 0.001
M33+16362 | 15301 | 1 | 0.001
M33+16362 | 2706 | 1 | 0.001
M33+16362 | 8860 | 1 | 0.001
M33a | 15043 | 1 | 0.003
M33a | 15301 | 1 | 0.003
M33a | 16223 | 2 | 0.007
M33a | 2361 | 1 | 0.003
M33a | 2706 | 1 | 0.003
M33a | 8860 | 1 | 0.003
M33a1a | 573.XC | 1 | 0.038
M33a1b | 10598 | 2 | 0.008
M33a1b | 12153 | 1 | 0.004
M33a1b | 199 | 1 | 0.004
M33a2a | 15908 | 1 | 0.027
M33a2a | 4769 | 1 | 0.027
M33a3a | 146 | 4 | 0.037
M33a3a | 16172 | 2 | 0.018
M33b1 | 16223 | 1 | 0.008
M33b1 | 6293 | 1 | 0.008
M33b1 | 676 | 1 | 0.008
M33b2 | 10873 | 1 | 0.011
M33c | 263 | 1 | 0.004
M33c | 4769 | 1 | 0.004
M34a1a | 5108 | 1 | 0.017
M34a1a | 569 | 1 | 0.017
M35 | 16223 | 1 | 0.003
M35a | 16093 | 1 | 0.007
M35a | 5432 | 1 | 0.007
M35a1 | 16093 | 3 | 0.017
M35a1a | 10670 | 1 | 0.008
M35a1a | 16093 | 12 | 0.097
M35b+16304 | 10873 | 1 | 0.006
M35b+16304 | 12561 | 2 | 0.012
M35b+16304 | 15301 | 1 | 0.006
M35b+16304 | 199 | 1 | 0.006
M35b+16304 | 4769 | 2 | 0.012
M35b1 | 13500 | 1 | 0.007
M35b1 | 14783 | 1 | 0.007
M36b | 153 | 4 | 0.038
M36d1 | 151 | 1 | 0.004
M37a | 10556 | 1 | 0.004
M37e | 16111 | 5 | 0.028
M37e | 489 | 2 | 0.011
M37e | 73 | 2 | 0.011
M37e2 | 14783 | 1 | 0.006
M37e2 | 16295 | 2 | 0.012
M37e2 | 489 | 1 | 0.006
M38 | 4099 | 1 | 0.004
M38a | 189 | 1 | 0.004
M38a | 4099 | 1 | 0.004
M38a | 6899 | 1 | 0.004
M39 | 55.1T | 2 | 0.008
M39a | 1811 | 1 | 0.004
M39a1 | 207 | 1 | 0.004
M39b | 15043 | 1 | 0.007
M39b | 15301 | 1 | 0.007
M39b | 15326 | 1 | 0.007
M39b1 | 153 | 1 | 0.007
M39b1 | 1811 | 1 | 0.007
M39b1 | 55.1T | 2 | 0.014
M39b2 | 1811 | 2 | 0.015
M39c | 16270 | 1 | 0.01
M39c | 182 | 1 | 0.01
M3a1+204 | 16126 | 1 | 0.005
M3a1b | 16126 | 7 | 0.029
M3a1b | 16223 | 2 | 0.008
M3a1b | 204 | 2 | 0.008
M3a2a | 482 | 1 | 0.005
M3c | 16223 | 2 | 0.009
M3c1 | 152 | 1 | 0.008
M3c1a | 10873 | 1 | 0.008
M3c1a | 14440 | 1 | 0.008
M3c1a | 15043 | 1 | 0.008
M3c1a | 15301 | 1 | 0.008
M3c1a | 16189 | 2 | 0.015
M3c1a | 16223 | 2 | 0.015
M3c1a | 16294 | 1 | 0.008
M3c1a | 2706 | 1 | 0.008
M3c1a | 482 | 2 | 0.015
M3c1a | 8860 | 1 | 0.008
M3c1b | 10632 | 1 | 0.008
M3c1b | 16189 | 1 | 0.008
M3c1b | 16294 | 1 | 0.008

M3c2 | 10398 | 1 | 0.006
M3c2 | 10400 | 1 | 0.006
M3c2 | 12705 | 1 | 0.006
M3c2 | 15043 | 1 | 0.006
M3c2 | 15301 | 1 | 0.006
M3c2 | 2706 | 1 | 0.006
M3c2 | 482 | 1 | 0.006
M3c2 | 8860 | 1 | 0.006
M3d | 16126 | 2 | 0.013
M3d | 482 | 2 | 0.013
M3d1a | 1438 | 11 | 0.064
M40a | 13542 | 1 | 0.009
M40a | 15954 | 1 | 0.009
M40a | 200 | 2 | 0.018
M40a1 | 16463 | 1 | 0.009
M40a1 | 200 | 1 | 0.009
M40a1 | 489 | 1 | 0.009
M40a1 | 8925 | 1 | 0.009
M40a1a | 16463 | 3 | 0.026
M41a1 | 8701 | 1 | 0.2
M41b | 15601 | 1 | 0.143
M41c | 2706 | 1 | 0.143
M41c | 870 | 1 | 0.143
M42'74 | 4769 | 1 | 0.003
M42a | 14783 | 1 | 0.005
M42a | 15043 | 1 | 0.005
M42a | 16287 | 1 | 0.005
M42a | 16356 | 1 | 0.005
M42a | 8251 | 7 | 0.033
M42a | 8860 | 2 | 0.009
M42a | 9156 | 1 | 0.005
M42b1 | 16223 | 1 | 0.008
M42b1 | 2880 | 1 | 0.008
M42b1 | 8251 | 1 | 0.008
M42b1 | 8597 | 1 | 0.008
M42b1 | 9156 | 1 | 0.008
M42b1 | 9165 | 1 | 0.008
M42b1a | 143 | 1 | 0.008
M42b1a | 6131 | 1 | 0.008
M42b2 | 234 | 1 | 0.005
M43+16311 | 263 | 1 | 0.007
M43a | 16223 | 2 | 0.012
M43a | 16311 | 1 | 0.006
M43b | 709 | 2 | 0.01
M44a | 489 | 1 | 0.083
M45 | 16189 | 7 | 0.05
M45a | 12007 | 1 | 0.008
M45a | 143 | 5 | 0.039
M45a | 146 | 3 | 0.024
M45a | 15043 | 1 | 0.008
M45a | 15301 | 1 | 0.008
M45a | 16223 | 1 | 0.008
M45a | 4734 | 1 | 0.008
M45a | 4769 | 1 | 0.008
M45a | 489 | 1 | 0.008
M45a | 7049 | 1 | 0.008
M45a | 8701 | 1 | 0.008
M45a | 8860 | 1 | 0.008
M46 | 152 | 1 | 0.002
M4"67 | 10873 | 1 | 0.003
M4"67 | 14766 | 1 | 0.003
M4"67 | 15301 | 1 | 0.003
M4"67 | 15326 | 1 | 0.003
M4"67 | 8701 | 1 | 0.003
M4"67+16311 | 750 | 1 | 0.004
M46a | 13434 | 1 | 0.009
M46a | 14766 | 1 | 0.009
M46a | 16300 | 3 | 0.027
M46a | 16362 | 3 | 0.027
M46a | 263 | 3 | 0.027
M46a | 8886 | 1 | 0.009
M46a | 9115 | 1 | 0.009
M48 | 16223 | 2 | 0.015
M48 | 16225 | 2 | 0.015
M49 | 16223 | 2 | 0.009
M49a | 12346 | 2 | 0.011
M49a | 14384 | 2 | 0.011
M49c | 3780 | 1 | 0.005
M49d | 3780 | 1 | 0.006
M49d | 4769 | 2 | 0.012
M49d | 489 | 1 | 0.006
M4a | 15043 | 1 | 0.004
M4a | 15301 | 1 | 0.004
M4a | 16261 | 1 | 0.004
M4a | 2706 | 1 | 0.004
M4a | 6620 | 1 | 0.004
M4a | 8701 | 1 | 0.004
M4a | 8860 | 1 | 0.004
M4a | 9540 | 1 | 0.004
M4b | 489 | 2 | 0.016
M5 | 12705 | 1 | 0.004
M5 | 14783 | 1 | 0.004
M5 | 15043 | 1 | 0.004
M5 | 15301 | 1 | 0.004
M5 | 16223 | 1 | 0.004
M5 | 2706 | 1 | 0.004
M5 | 489 | 1 | 0.004
M5 | 8860 | 1 | 0.004
M5 | 9540 | 1 | 0.004
M50 | 12705 | 1 | 0.005
M50 | 1438 | 1 | 0.005
M50 | 14766 | 1 | 0.005
M50 | 15326 | 1 | 0.005
M50 | 15663 | 1 | 0.005
M50 | 263 | 1 | 0.005
M50 | 2706 | 1 | 0.005
M50 | 4769 | 1 | 0.005
M50 | 489 | 1 | 0.005
M50 | 7028 | 1 | 0.005
M50 | 73 | 1 | 0.005
M50 | 8860 | 1 | 0.005

M50a1 | 16129 | 1 | 0.077
M50a2 | 204 | 1 | 0.01
M51 | 73 | 1 | 0.007
M51a | 73 | 1 | 0.005
M51a1 | 14687 | 1 | 0.042
M51a1 | 14766 | 1 | 0.042
M51a1 | 14783 | 1 | 0.042
M51a1b | 16223 | 15 | 0.224
M51a1b | 16278 | 5 | 0.075
M51b | 73 | 1 | 0.007
M51b1a | 2833 | 1 | 0.008
M51b1b | 4697 | 1 | 0.008
M52a1 | 12705 | 1 | 0.024
M52a1 | 573.XC | 2 | 0.048
M52a1 | 73 | 1 | 0.024
M52a1a | 15349 | 1 | 0.019
M52a1a | 16327A | 1 | 0.019
M52a1a | 16390 | 2 | 0.037
M52a1b | 73 | 1 | 0.024
M52a1b1 | 13135 | 1 | 0.02
M52a1b1 | 15349 | 2 | 0.039
M52a1b1 | 16093 | 1 | 0.02
M52a1b1 | 3591 | 1 | 0.02
M52a1b1 | 489 | 1 | 0.02
M52a1b1 | 573.XC | 16 | 0.314
M52a1b1 | 614 | 2 | 0.039
M52b | 1462T | 1 | 0.003
M52b1a | 63 | 1 | 0.009
M53 | 10873 | 1 | 0.111
M53 | 11167 | 1 | 0.111
M53 | 11560 | 1 | 0.111
M53 | 14766 | 1 | 0.111
M53 | 14783 | 1 | 0.111
M53 | 16189 | 5 | 0.556
M53 | 16316 | 1 | 0.111
M53 | 240 | 1 | 0.111
M53 | 390T | 1 | 0.111
M53 | 489 | 1 | 0.111
M53 | 572 | 1 | 0.111
M53 | 9302 | 1 | 0.111
M53 | 9540 | 1 | 0.111
M54 | 12414 | 1 | 0.004
M54 | 1438 | 1 | 0.004
M54 | 16304 | 1 | 0.004
M54 | 9374 | 7 | 0.031
M55 | 10398 | 3 | 0.086
M55 | 16136 | 19 | 0.543
M55 | 204 | 5 | 0.143
M55 | 4655 | 9 | 0.257
M55 | 5899.1C | 11 | 0.314
M55 | 6752 | 11 | 0.314
M55 | 709 | 6 | 0.171
M55 | 8279.1T | 5 | 0.143
M57+152 | 16223 | 5 | 0.022
M57+152 | 263 | 1 | 0.004
M57a | 8701 | 1 | 0.007
M57b1 | 4132 | 1 | 0.014
M58 | 750 | 2 | 0.007
M59 | 11140 | 1 | 0.009
M59 | 14766 | 1 | 0.009
M59 | 16223 | 1 | 0.009
M59 | 16278 | 1 | 0.009
M59 | 249 | 1 | 0.009
M59 | 263 | 1 | 0.009
M5a | 16129 | 2 | 0.011
M5a | 1888 | 1 | 0.006
M5a | 73 | 1 | 0.006
M5a | 8860 | 3 | 0.017
M5a1a | 10873 | 1 | 0.011
M5a1a | 12705 | 1 | 0.011
M5a1a | 16223 | 1 | 0.011
M5a1a | 3921 | 1 | 0.011
M5a1a | 73 | 1 | 0.011
M5a1a | 9064 | 1 | 0.011
M5a1b | 1303 | 1 | 0.006
M5a1b | 16223 | 1 | 0.006
M5a1b | 16291 | 1 | 0.006
M5a1b | 1888 | 1 | 0.006
M5a1b | 263 | 1 | 0.006
M5a1b | 709 | 1 | 0.006
M5a1b | 73 | 2 | 0.011
M5a2a | 12477 | 1 | 0.006
M5a2a | 16129 | 1 | 0.006
M5a2a1 | 16129 | 1 | 0.006
M5a2a1 | 1888 | 1 | 0.006
M5a2a1a | 73 | 1 | 0.01
M5a2a1a1 | 73 | 1 | 0.01
M5a2a2 | 15262 | 1 | 0.012
M5a2a2 | 15301 | 1 | 0.012
M5a2a2 | 3921 | 1 | 0.012
M5a2a2 | 4454 | 1 | 0.012
M5a3 | 194 | 2 | 0.133
M5a4 | 14323 | 1 | 0.1
M5a'd | 12705 | 1 | 0.007
M5a'd | 14323 | 1 | 0.007
M5a'd | 15043 | 1 | 0.007
M5a'd | 15301 | 1 | 0.007
M5a'd | 2706 | 1 | 0.007
M5a'd | 709 | 1 | 0.007
M5a'd | 8860 | 1 | 0.007
M5b1 | 13368 | 1 | 0.006
M5b1 | 5147 | 1 | 0.006
M5b2a | 1888 | 1 | 0.006
M5b2b | 13368 | 1 | 0.005
M5b2b | 15301 | 1 | 0.005
M5b2b | 489 | 1 | 0.005
M5b2b1 | 15301 | 1 | 0.016
M5b2b1a | 16129 | 2 | 0.03
M5b2b1a | 455.1T | 6 | 0.09
M5c1 | 12705 | 1 | 0.008
M5c1 | 150 | 1 | 0.008

M5c1 | 15043 | 2 | 0.017
M5c1 | 15301 | 2 | 0.017
M5c1 | 16129 | 1 | 0.008
M5c1 | 2706 | 2 | 0.017
M5c1 | 575 | 6 | 0.05
M5c1 | 8860 | 2 | 0.017
M5c1 | 9540 | 1 | 0.008
M5c2 | 13368 | 2 | 0.037
M5c2 | 16240 | 1 | 0.019
M5c2 | 16291 | 3 | 0.056
M5c2 | 575 | 7 | 0.13
M5d | 146 | 1 | 0.036
M5d | 16311 | 2 | 0.071
M60 | 16223 | 1 | 0.003
M60a2 | 16223 | 3 | 0.018
M61 | 152 | 14 | 0.088
M61 | 489 | 1 | 0.006
M61a | 16362 | 3 | 0.016
M61a | 2706 | 1 | 0.005
M61a | 3438 | 1 | 0.005
M62a | 13708 | 1 | 0.008
M62a | 146 | 1 | 0.008
M62a | 16295 | 1 | 0.008
M62a | 310 | 1 | 0.008
M62a | 4561 | 2 | 0.017
M62b1a | 16295 | 15 | 0.114
M62b1a1 | 12705 | 1 | 0.006
M62b1a1 | 16223 | 1 | 0.006
M62b1a1 | 16260 | 8 | 0.05
M62b1a1 | 489 | 1 | 0.006
M62b1a1 | 7028 | 1 | 0.006
M62b2 | 150 | 1 | 0.007
M62b2 | 16295 | 1 | 0.007
M62b2 | 204 | 1 | 0.007
M64 | 2706 | 1 | 0.008
M65 | 16223 | 1 | 0.006
M65 | 16311 | 1 | 0.006
M65a | 14766 | 1 | 0.011
M65a+@16311 | 16223 | 10 | 0.074
M65a2 | 511 | 1 | 0.007
M65b | 12007 | 1 | 0.008
M65b | 241 | 3 | 0.023
M66 | 1438 | 1 | 0.009
M66 | 1888 | 1 | 0.009
M66 | 263 | 1 | 0.009
M66 | 4541 | 1 | 0.009
M66 | 489 | 1 | 0.009
M66 | 73 | 1 | 0.009
M68 | 16223 | 1 | 0.008
M68 | 4561 | 1 | 0.008
M68a | 14233 | 1 | 0.01
M68a | 16255 | 2 | 0.019
M68a | 7664 | 2 | 0.019
M68a1a | 16259 | 2 | 0.017
M68a1a | 16278 | 1 | 0.009
M6a1 | 10873 | 1 | 0.011
M6a1 | 11719 | 1 | 0.011
M6a1 | 12705 | 1 | 0.011
M6a1 | 13966 | 1 | 0.011
M6a1 | 14766 | 1 | 0.011
M6a1 | 15301 | 1 | 0.011
M6a1 | 15326 | 1 | 0.011
M6a1 | 16223 | 1 | 0.011
M6a1 | 4769 | 1 | 0.011
M6a1 | 5082 | 1 | 0.011
M6a1a | 3486 | 1 | 0.008
M6a1a | 9329 | 1 | 0.008
M6a1b | 13966 | 1 | 0.013
M6a1b | 14128 | 1 | 0.013
M6a1b | 152 | 13 | 0.165
M6a1b | 16188 | 1 | 0.013
M6a1b | 16223 | 1 | 0.013
M6a2 | 14128 | 1 | 0.018
M6b | 14128 | 1 | 0.01
M6b | 14766 | 1 | 0.01
M6b | 461 | 1 | 0.01
M6b | 5301 | 1 | 0.01
M6b | 8281-8289d | 1 | 0.01
M70 | 16297 | 2 | 0.013
M71+151 | 16271 | 1 | 0.006
M71+151 | 489 | 1 | 0.006
M71a1 | 151 | 1 | 0.009
M71a1a | 151 | 2 | 0.014
M71a2 | 10873 | 1 | 0.007
M71a2 | 12705 | 1 | 0.007
M71a2 | 13759 | 7 | 0.048
M71a2 | 143 | 1 | 0.007
M71a2 | 1438 | 1 | 0.007
M71a2 | 146 | 1 | 0.007
M71a2 | 14605 | 1 | 0.007
M71a2 | 14766 | 1 | 0.007
M71a2 | 151 | 9 | 0.062
M71a2 | 15326 | 1 | 0.007
M71a2 | 16129 | 2 | 0.014
M71a2 | 16223 | 18 | 0.124
M71a2 | 263 | 1 | 0.007
M71a2 | 2706 | 1 | 0.007
M71a2 | 4769 | 1 | 0.007
M71a2 | 489 | 1 | 0.007
M71a2 | 7028 | 1 | 0.007
M71a2 | 73 | 1 | 0.007
M71a2 | 8860 | 1 | 0.007
M71b | 151 | 1 | 0.008
M71b | 16260 | 1 | 0.008
M71b | 16264 | 1 | 0.008
M71b | 489 | 1 | 0.008
M71c | 16271 | 1 | 0.008
M72 | 16166d | 1 | 0.005
M72a | 16166d | 1 | 0.005
M73b | 16354 | 3 | 0.158
M73b | 489 | 1 | 0.053

M74 | 16311 | 1 | 0.004
M74a | 12850 | 1 | 0.005
M74a | 16381 | 3 | 0.016
M74a | 64 | 2 | 0.011
M74a | 66 | 1 | 0.005
M74b | 489 | 1 | 0.003
M74b2 | 489 | 1 | 0.004
M75 | 10400 | 1 | 0.007
M75 | 150 | 2 | 0.014
M75 | 263 | 1 | 0.007
M75 | 489 | 1 | 0.007
M76 | 14766 | 1 | 0.019
M76 | 489 | 2 | 0.038
M76 | 513 | 10 | 0.189
M76a | 11009 | 4 | 0.167
M76a | 15547 | 2 | 0.083
M76a | 16293C | 5 | 0.208
M76a | 234 | 1 | 0.042
M76a | 3438 | 4 | 0.167
M76a | 513 | 2 | 0.083
M76a | 8470 | 4 | 0.167
M76a | 9055 | 4 | 0.167
M77 | 16274 | 3 | 0.023
M79 | 14034 | 3 | 0.429
M79 | 151 | 3 | 0.429
M79 | 16260 | 5 | 0.714
M79 | 16262 | 3 | 0.429
M79 | 489 | 1 | 0.143
M79 | 499 | 1 | 0.143
M79 | 7661 | 3 | 0.429
M7a1a | 16209 | 1 | 0.005
M7a1a | 16324 | 1 | 0.005
M7a1a | 5899.XC | 1 | 0.005
M7a1a2 | 16324 | 3 | 0.027
M7a1a2 | 5899.XC | 3 | 0.027
M7a1a4a | 5899.XC | 2 | 0.01
M7a1a6 | 5899.XC | 1 | 0.005
M7a1a7 | 16209 | 1 | 0.005
M7a1a7 | 8701 | 1 | 0.005
M7a1b1 | 16209 | 2 | 0.039
M7a2a2 | 146 | 3 | 0.027
M7b | 489 | 1 | 0.003
M7b1a1 | 16129 | 1 | 0.004
M7b1a1 | 16297 | 1 | 0.004
M7b1a1 | 489 | 2 | 0.008
M7b1a1+(16192) | 1438 | 1 | 0.001
M7b1a1+(16192) | 16223 | 8 | 0.012
M7b1a1+(16192) | 16297 | 1 | 0.001
M7b1a1+(16192) | 199 | 2 | 0.003
M7b1a1+(16192) | 489 | 1 | 0.001
M7b1a1+(16192) | 73 | 1 | 0.001
M7b1a1a | 16223 | 1 | 0.004
M7b1a1a | 16297 | 1 | 0.004
M7b1a1a | 263 | 2 | 0.009
M7b1a1a | 489 | 6 | 0.027
M7b1a1a | 73 | 2 | 0.009
M7b1a1a1 | 16129 | 1 | 0.004
M7b1a1a1 | 16189 | 1 | 0.004
M7b1a1a1 | 16223 | 3 | 0.013
M7b1a1a1 | 16297 | 2 | 0.009
M7b1a1a1b | 150 | 1 | 0.006
M7b1a1a1c | 4048 | 1 | 0.008
M7b1a1a2 | 16129 | 5 | 0.017
M7b1a1a2 | 16223 | 1 | 0.003
M7b1a1a2 | 199 | 1 | 0.003
M7b1a1a2 | 263 | 1 | 0.003
M7b1a1a3 | 16129 | 4 | 0.011
M7b1a1a3 | 16189 | 1 | 0.003
M7b1a1a3 | 16223 | 5 | 0.014
M7b1a1a3 | 199 | 1 | 0.003
M7b1a1a3 | 204 | 2 | 0.005
M7b1a1a3 | 263 | 1 | 0.003
M7b1a1a3 | 489 | 6 | 0.016
M7b1a1a3 | 5351 | 2 | 0.005
M7b1a1a3 | 73 | 5 | 0.014
M7b1a1b | 150 | 6 | 0.018
M7b1a1b | 15326 | 31 | 0.091
M7b1a1b | 204 | 5 | 0.015
M7b1a1b | 489 | 3 | 0.009
M7b1a1c | 16092 | 4 | 0.033
M7b1a1c | 16129 | 1 | 0.008
M7b1a1d | 16223 | 1 | 0.002
M7b1a1d1 | 16223 | 1 | 0.002
M7b1a1d1 | 489 | 1 | 0.002
M7b1a1e | 16223 | 1 | 0.002
M7b1a1e1 | 16129 | 7 | 0.014
M7b1a1e1 | 489 | 2 | 0.004
M7b1a1e2 | 16223 | 3 | 0.011
M7b1a1e2 | 16527 | 2 | 0.007
M7b1a1f | 16129 | 1 | 0.002
M7b1a1f | 16297 | 3 | 0.006
M7b1a1f | 199 | 20 | 0.038
M7b1a1f | 489 | 3 | 0.006
M7b1a1g | 16223 | 1 | 0.002
M7b1a1h | 16223 | 1 | 0.002
M7b1a1h | 8860 | 3 | 0.006
M7b1a1i | 16297 | 1 | 0.007
M7b1a2a | 10497 | 1 | 0.006
M7b1a2a | 16086 | 1 | 0.006
M7b1a2a | 16129 | 35 | 0.202
M7b1a2a | 9468 | 3 | 0.017
M7b1a2a1 | 16086 | 6 | 0.029
M7b1a2a1 | 16129 | 2 | 0.01
M7b1a2a1 | 8701 | 1 | 0.005
M7b1a2a1b1 | 16297 | 1 | 0.017
M7b1b | 16129 | 1 | 0.006
M7c | 10398 | 1 | 0.004
M7c | 12705 | 1 | 0.004
M7c | 16223 | 3 | 0.012
M7c1 | 199 | 1 | 0.003
M7c1a | 10398 | 1 | 0.002

M7c1a | 14766 | 1 | 0.002
M7c1a | 199 | 9 | 0.022
M7c1a | 489 | 2 | 0.005
M7c1a | 73 | 1 | 0.002
M7c1a1a | 9540 | 1 | 0.003
M7c1a1b | 199 | 1 | 0.006
M7c1a1b | 6455 | 1 | 0.006
M7c1a2 | 199 | 4 | 0.03
M7c1a3 | 16223 | 28 | 0.184
M7c1a3 | 199 | 1 | 0.007
M7c1a3a | 146 | 2 | 0.067
M7c1a3a | 16223 | 1 | 0.033
M7c1a3a | 263 | 1 | 0.033
M7c1b2a | 146 | 1 | 0.007
M7c1b2a | 263 | 1 | 0.007
M7c1b2a | 73 | 2 | 0.014
M7c1b2b | 1438 | 1 | 0.003
M7c1c | 11665 | 1 | 0.003
M7c1c | 16223 | 3 | 0.009
M7c1c | 199 | 1 | 0.003
M7c1c2 | 199 | 3 | 0.013
M7c1c2 | 489 | 1 | 0.004
M7c1c3 | 10398 | 1 | 0.001
M7c1c3 | 10400 | 1 | 0.001
M7c1c3 | 10873 | 1 | 0.001
M7c1c3 | 11665 | 4 | 0.003
M7c1c3 | 11719 | 1 | 0.001
M7c1c3 | 146 | 1 | 0.001
M7c1c3 | 15236 | 1 | 0.001
M7c1c3 | 16223 | 3 | 0.002
M7c1c3 | 3606 | 1 | 0.001
M7c1c3 | 4071 | 2 | 0.002
M7c1c3 | 6455 | 2 | 0.002
M7c1c3 | 73 | 1 | 0.001
M7c1c3 | 9824 | 1 | 0.001
M7c1c3i | 16223 | 7 | 0.01
M7c1c3i | 263 | 40 | 0.056
M7c3 | 16223 | 3 | 0.012
M8a1 | 16223 | 3 | 0.014
M8a2+152 | 16298 | 13 | 0.058
M8a2+152 | 16319 | 3 | 0.013
M8a2+152 | 2835 | 1 | 0.004
M8a2a | 152 | 1 | 0.007
M8a2a | 16184 | 1 | 0.007
M8a2a1 | 152 | 68 | 0.27
M8a2a1 | 16184 | 1 | 0.004
M8a2a1 | 16298 | 6 | 0.024
M8a2a1 | 16319 | 1 | 0.004
M8a2a1 | 489 | 3 | 0.012
M8a2b | 152 | 1 | 0.006
M8a2b | 16223 | 1 | 0.006
M8a2e | 16184 | 1 | 0.004
M8a2e | 8584 | 1 | 0.004
M8a3a | 16298 | 3 | 0.014
M8a3a | 489 | 1 | 0.005
M8a3a | 8584 | 1 | 0.005
M8a3a1 | 16184 | 1 | 0.005
M8a3a1 | 16223 | 1 | 0.005
M91a | 16223 | 1 | 0.007
M91a | 16287 | 5 | 0.036
M91a | 200 | 1 | 0.007
M91a | 489 | 3 | 0.022
M91b | 3438 | 1 | 0.1
M91b | 3447 | 2 | 0.2
M91b | 647 | 1 | 0.1
M9a | 153 | 4 | 0.018
M9a1a | 16362 | 5 | 0.005
M9a1a | 73 | 1 | 0.001
M9a1a | 8860 | 1 | 0.001
M9a1a1 | 153 | 1 | 0.001
M9a1a1a | 11963 | 6 | 0.005
M9a1a1a | 16223 | 2 | 0.002
M9a1a1b | 153 | 1 | 0.009
M9a1a1c1 | 16234 | 26 | 0.165
M9a1a1c1a | 153 | 6 | 0.036
M9a1a1c1a | 16291 | 1 | 0.006
M9a1a1c1a | 16362 | 2 | 0.012
M9a1a1c1b | 153 | 1 | 0.001
M9a1a1c1b1 | 16223 | 2 | 0.002
M9a1a1c1b1 | 5899.XC | 1 | 0.001
M9a1a1c1b1a | 14417 | 2 | 0.002
M9a1a1c1b1a | 16234 | 1 | 0.001
M9a1a1c1b1a | 16316 | 5 | 0.004
M9a1a1c1b1a | 16362 | 1 | 0.001
M9a1a1c1b1a | 5899.XC | 5 | 0.004
M9a1a1c1b1a1 | 16316 | 1 | 0.001
M9a1a1c1b1a1 | 5899.XC | 1 | 0.001
M9a1a1c1c | 16223 | 1 | 0.007
M9a1a1d | 153 | 1 | 0.001
M9a1a2 | 153 | 1 | 0.005
M9a1a2 | 16316 | 1 | 0.005
M9a1a2 | 16362 | 96 | 0.455
M9a1b+150 | 152 | 1 | 0.004
M9a1b1 | 14308 | 1 | 0.002
M9a1b1 | 150 | 2 | 0.004
M9a1b1 | 152 | 1 | 0.002
M9a1b1 | 153 | 20 | 0.04
M9a1b1 | 15671 | 1 | 0.002
M9a1b1 | 16223 | 2 | 0.004
M9a1b1 | 16362 | 1 | 0.002
M9a1b1 | 489 | 1 | 0.002
M9a1b1a | 16362 | 1 | 0.003
M9a1b1c | 153 | 1 | 0.003
M9a1b1c | 15671 | 1 | 0.003
M9a1b1c | 16223 | 15 | 0.039
M9a1b2 | 153 | 1 | 0.005
M9a1b2 | 16234 | 4 | 0.018
M9a4a | 153 | 2 | 0.018
M9a4a2 | 15326 | 1 | 0.009
M9a5 | 153 | 3 | 0.013
M9a5 | 16234 | 1 | 0.004

M9a5 | 385 | 1 | 0.004
M9a5 | 489 | 1 | 0.004
M9a'b | 16223 | 1 | 0.002
M9b | 573.XC | 4 | 0.121
N10 | 16223 | 6 | 0.053
N10 | 16362 | 6 | 0.053
N10 | 263 | 6 | 0.053
N10 | 73 | 7 | 0.062
N10a | 15211 | 1 | 0.013
N10a | 16223 | 3 | 0.039
N10a | 189 | 2 | 0.026
N10b | 152 | 8 | 0.4
N10b | 16069 | 2 | 0.1
N10b | 16223 | 1 | 0.05
N10b | 16278 | 2 | 0.1
N10b | 16298 | 2 | 0.1
N10b | 16362 | 2 | 0.1
N11a1 | 750 | 1 | 0.023
N11a2 | 6674 | 1 | 0.071
N11a2 | 8618 | 1 | 0.071
N13 | 16261 | 1 | 0.1
N13 | 16290 | 2 | 0.2
N13 | 178 | 1 | 0.1
N13 | 195 | 2 | 0.2
N13 | 263 | 1 | 0.1
N1a1'2 | 16223 | 1 | 0.007
N1a1'2 | 263 | 1 | 0.007
N1a1a | 16248 | 1 | 0.083
N1a1a | 573.XC | 1 | 0.083
N1a1a+152 | 13780 | 1 | 0.077
N1a1a+152 | 199 | 1 | 0.077
N1a1a+152 | 204 | 1 | 0.077
N1a1a1a | 152 | 3 | 0.176
N1a1a1a | 16172 | 2 | 0.118
N1a1a1a | 199 | 2 | 0.118
N1a1a1a | 204 | 2 | 0.118
N1a1a1a | 573.XC | 3 | 0.176
N1a1a1a1 | 152 | 1 | 0.042
N1a1a1a1 | 573.XC | 8 | 0.333
N1a1a1a1a | 16248 | 1 | 0.111
N1a1a1a1a | 204 | 1 | 0.111
N1a1a1a1a | 573.XC | 2 | 0.222
N1a1a1a2 | 16086 | 1 | 0.032
N1a1a1a2 | 16248 | 1 | 0.032
N1a1a1a2 | 573.XC | 5 | 0.161
N1a1a1a3 | 573.XC | 3 | 0.143
N1a1a1a3 | 669 | 1 | 0.048
N1a1a1b | 16093 | 2 | 0.5
N1a1a1b | 573.XC | 2 | 0.5
N1a1a2 | 15043 | 1 | 0.1
N1a1a2 | 15326 | 1 | 0.1
N1a1a2 | 573.XC | 1 | 0.1
N1a1a3 | 13780 | 1 | 0.04
N1a1a3 | 15043 | 1 | 0.04
N1a1a3 | 16147G | 3 | 0.12
N1a1a3 | 16248 | 3 | 0.12
N1a1a3 | 573.XC | 8 | 0.32
N1a1b | 15924 | 1 | 0.04
N1a1b | 573.XC | 4 | 0.16
N1a1b1 | 10790 | 1 | 0.042
N1a1b1 | 16309 | 6 | 0.25
N1a1b1 | 16311 | 10 | 0.417
N1a1b1 | 16391 | 1 | 0.042
N1a1b1 | 204 | 6 | 0.25
N1a1b1 | 573.XC | 4 | 0.167
N1a1b1 | 710 | 1 | 0.042
N1a2 | 16274 | 10 | 0.833
N1a2 | 3783A | 2 | 0.167
N1a2 | 6713 | 2 | 0.167
N1a3 | 195 | 2 | 0.167
N1a3a | 11914 | 2 | 0.019
N1a3a | 12705 | 1 | 0.009
N1a3a | 16223 | 1 | 0.009
N1a3a | 16265 | 7 | 0.065
N1a3a | 1719 | 1 | 0.009
N1a3a | 189 | 2 | 0.019
N1a3a | 195 | 20 | 0.187
N1a3a | 204 | 1 | 0.009
N1a3a | 207 | 9 | 0.084
N1a3a | 73 | 1 | 0.009
N1a3a | 8860 | 1 | 0.009
N1a3a3 | 11914 | 1 | 0.25
N1b1 | 152 | 4 | 0.038
N1b1a | 152 | 3 | 0.023
N1b1a | 16390 | 1 | 0.008
N1b1a | 8836 | 1 | 0.008
N1b1a1 | 152 | 25 | 0.248
N1b1a1 | 16145 | 1 | 0.01
N1b1a1 | 16176G | 4 | 0.04
N1b1a1 | 16223 | 2 | 0.02
N1b1a1 | 16390 | 2 | 0.02
N1b1a1 | 455.1T | 2 | 0.02
N1b1a2 | 152 | 9 | 0.067
N1b1a2 | 16223 | 1 | 0.007
N1b1a2 | 16390 | 1 | 0.007
N1b1a2 | 73 | 1 | 0.007
N1b1a2 | 9335 | 1 | 0.007
N1b1a2a | 8251 | 1 | 0.009
N1b1a2b | 1598 | 1 | 0.009
N1b1a3 | 11362 | 1 | 0.009
N1b1a3 | 12501 | 1 | 0.009
N1b1a3 | 16223 | 1 | 0.009
N1b1a3 | 1703 | 1 | 0.009
N1b1a3 | 1719 | 1 | 0.009
N1b1a3 | 8836 | 1 | 0.009
N1b1a4 | 16390 | 1 | 0.111
N1b1a5 | 12705 | 1 | 0.009
N1b1a5 | 1438 | 1 | 0.009
N1b1a5 | 14766 | 1 | 0.009
N1b1a5 | 16145 | 1 | 0.009
N1b1a5 | 16176G | 1 | 0.009

N1b1a5 | 16223 | 1 | 0.009
N1b1a5 | 16390 | 1 | 0.009
N1b1a5 | 1719 | 1 | 0.009
N1b1a5 | 5471 | 1 | 0.009
N1b1a5 | 7028 | 1 | 0.009
N1b1a5 | 8251 | 1 | 0.009
N1b1a5 | 8472 | 1 | 0.009
N1b1a5 | 9335 | 1 | 0.009
N1b1a6 | 9335 | 1 | 0.01
N1b1a7 | 16223 | 2 | 0.047
N1b1a7 | 73 | 1 | 0.023
N1b1a8a | 16176G | 4 | 0.308
N1b1a8b | 195 | 1 | 0.077
N1b1b | 10238 | 1 | 0.02
N1b1b | 11928 | 1 | 0.02
N1b1b | 12501 | 1 | 0.02
N1b1b | 12705 | 1 | 0.02
N1b1b | 12822 | 1 | 0.02
N1b1b | 13129 | 1 | 0.02
N1b1b | 14766 | 1 | 0.02
N1b1b | 152 | 2 | 0.039
N1b1b | 1703 | 1 | 0.02
N1b1b | 1719 | 1 | 0.02
N1b1b | 2639 | 1 | 0.02
N1b1b | 5471 | 1 | 0.02
N1b1b | 8251 | 1 | 0.02
N1b1b | 8836 | 1 | 0.02
N1b1b | 8860 | 1 | 0.02
N1b1b1 | 11719 | 3 | 0.046
N1b1b1 | 16390 | 1 | 0.015
N21+195 | 16193 | 4 | 0.033
N21+195 | 337d | 4 | 0.033
N21a | 16193 | 6 | 0.24
N21a | 16223 | 9 | 0.36
N21a | 16291 | 4 | 0.16
N21a | 337d | 1 | 0.04
N22 | 150 | 1 | 0.02
N2a | 189 | 2 | 0.08
N2a1 | 16223 | 2 | 0.222
N3 | 16086 | 2 | 0.105
N3a | 16086 | 1 | 0.033
N3a | 16217 | 2 | 0.067
N5 | 1438 | 1 | 0.012
N5 | 14766 | 1 | 0.012
N5 | 1719 | 1 | 0.012
N5a | 11626 | 1 | 0.167
N5a | 11719 | 1 | 0.167
N5a | 131 | 1 | 0.167
N5a | 16185 | 1 | 0.167
N5a | 16319 | 1 | 0.167
N5a | 1719 | 1 | 0.167
N5a | 199 | 1 | 0.167
N5a | 374 | 1 | 0.167
N5a | 6974 | 1 | 0.167
N5a | 9545 | 1 | 0.167
N7a1 | 5261 | 1 | 0.333
N7a2 | 16129 | 1 | 0.125
N7a2 | 3396 | 1 | 0.125
N8 | 16263 | 1 | 0.009
N8 | 16343 | 1 | 0.009
N9a | 150 | 1 | 0.011
N9a | 15326 | 3 | 0.034
N9a | 16257A | 1 | 0.011
N9a | 16261 | 4 | 0.046
N9a1 | 12007 | 1 | 0.007
N9a1 | 16129 | 1 | 0.007
N9a1 | 4386 | 1 | 0.007
N9a10 | 150 | 2 | 0.019
N9a10 | 16257A | 1 | 0.009
N9a10+16311 | 150 | 1 | 0.007
N9a10b | 16311 | 1 | 0.037
N9a11 | 453A | 3 | 0.214
N9a1'3 | 16223 | 1 | 0.01
N9a1a | 12358 | 1 | 0.007
N9a1a | 12372 | 1 | 0.007
N9a1a | 150 | 6 | 0.04
N9a1a | 16223 | 1 | 0.007
N9a1a | 16257A | 1 | 0.007
N9a1a | 16261 | 1 | 0.007
N9a2'4'5'11 | 150 | 1 | 0.015
N9a2'4'5'11 | 16257A | 1 | 0.015
N9a2a | 961 | 1 | 0.014
N9a2a1 | 338 | 6 | 0.462
N9a2a1 | 961 | 1 | 0.077
N9a2a2 | 961 | 2 | 0.043
N9a2a3 | 16223 | 1 | 0.02
N9a2a3 | 16261 | 1 | 0.02
N9a2c | 4769 | 1 | 0.023
N9a2c | 8860 | 1 | 0.023
N9a2c | 961 | 1 | 0.023
N9a2d | 16172 | 1 | 0.016
N9a2d | 16257A | 6 | 0.095
N9a2d | 961 | 2 | 0.032
N9a3 | 12354 | 1 | 0.01
N9a3 | 12358 | 1 | 0.01
N9a3 | 12372 | 1 | 0.01
N9a3 | 2706 | 1 | 0.01
N9a3 | 5417 | 1 | 0.01
N9a3 | 8860 | 1 | 0.01
N9a4b | 16261 | 3 | 0.067
N9a4b1 | 8860 | 1 | 0.022
N9a5 | 16172 | 1 | 0.028
N9a6 | 16261 | 2 | 0.023
N9a6 | 750 | 1 | 0.012
N9a6a | 16257A | 1 | 0.014
N9a6a | 16261 | 1 | 0.014
N9a6a | 263 | 1 | 0.014
N9a6a | 4856 | 1 | 0.014
N9a6a | 5231 | 1 | 0.014
N9a9 | 16223 | 2 | 0.022
N9b | 5147 | 1 | 0.01

N9b1a | 12705 | 2 | 0.02
N9b1c1 | 16189 | 1 | 0.05
N9b2 | 73 | 1 | 0.013
N9b2a | 16223 | 1 | 0.013
N9b4 | 10607 | 1 | 0.012
O1 | 263 | 1 | 0.059
O1 | 9140 | 1 | 0.059
O1a | 152 | 1 | 0.059
O1a | 16223 | 2 | 0.118
P | 1438 | 1 | 0.01
P | 14766 | 1 | 0.01
P1 | 212 | 5 | 0.357
P1+152 | 16176 | 18 | 0.562
P1+152 | 212 | 9 | 0.281
P1d | 16176 | 4 | 0.1
P1d | 16266 | 27 | 0.675
P1d | 212 | 2 | 0.05
P1d1 | 212 | 3 | 0.158
P1d1a | 16266 | 1 | 0.022
P1d1a | 16357 | 1 | 0.022
P1d2 | 16176 | 2 | 0.118
P1d2 | 16266 | 1 | 0.059
P1d2 | 212 | 2 | 0.118
P1d2 | 8860 | 1 | 0.059
P1d2a | 16176 | 1 | 0.1
P1d2a | 16266 | 1 | 0.1
P1f | 16357 | 1 | 0.333
P3b1 | 16399 | 2 | 0.286
P3b1 | 573.XC | 1 | 0.143
P4b | 1719 | 1 | 0.05
P4b1 | 11016 | 1 | 0.037
P4b1 | 16337 | 9 | 0.333
P5 | 12406 | 19 | 0.271
P5 | 13368 | 1 | 0.014
P5 | 16192 | 29 | 0.414
P5 | 16311 | 19 | 0.271
P5 | 7419 | 19 | 0.271
P6 | 6719 | 1 | 0.029
P8 | 15110 | 1 | 0.028
P8 | 15511 | 1 | 0.028
P8 | 15521C | 1 | 0.028
P8 | 16241T | 2 | 0.056
P9 | 8860 | 3 | 0.036
Q1 | 16241 | 3 | 0.018
Q1 | 16343 | 3 | 0.018
Q1 | 5843 | 2 | 0.012
Q1 | 92 | 3 | 0.018
Q1a | 16129 | 1 | 0.009
Q1a | 92 | 4 | 0.036
Q1a1 | 16144 | 6 | 0.045
Q1a1 | 92 | 8 | 0.06
Q1a1a | 16241 | 9 | 0.055
Q1a1a | 16311 | 1 | 0.006
Q1a1a | 16343 | 33 | 0.201
Q1a1a | 208 | 3 | 0.018
Q1a1a | 92 | 34 | 0.207
Q1c | 92 | 1 | 0.009
Q1c1a | 13368 | 1 | 0.007
Q1d | 92 | 6 | 0.034
Q1e | 16343 | 3 | 0.02
Q1e1 | 14783 | 1 | 0.007
Q1e1a | 16241 | 1 | 0.009
Q1e1b | 15326 | 1 | 0.009
Q1e1b | 16129 | 1 | 0.009
Q1e1b | 16241 | 3 | 0.027
Q1e1b | 92 | 1 | 0.009
Q1e1b1 | 263 | 1 | 0.01
Q1e1c | 146 | 4 | 0.023
Q1e1c | 16129 | 2 | 0.012
Q1e1c | 16144 | 2 | 0.012
Q1e1c | 16148 | 1 | 0.006
Q1e1c | 16241 | 4 | 0.023
Q1e1c | 16343 | 8 | 0.047
Q1e1c | 89 | 9 | 0.052
Q1e1c | 92 | 27 | 0.157
Q1f1 | 16148 | 1 | 0.009
Q1f1 | 16311 | 2 | 0.017
Q1f1 | 92 | 3 | 0.026
Q2a2a | 16223 | 1 | 0.009
Q3 | 16241 | 1 | 0.006
Q3 | 73 | 1 | 0.006
Q3a | 7028 | 1 | 0.009
Q3a | 73 | 1 | 0.009
Q3a1 | 15172 | 1 | 0.008
Q3a1 | 16223 | 1 | 0.008
R | 2706 | 2 | 0.014
R | 4769 | 2 | 0.014
R | 8860 | 2 | 0.014
R0a | 64 | 1 | 0.017
R0a+60.1T | 14766 | 1 | 0.009
R0a+60.1T | 16126 | 23 | 0.213
R0a+60.1T | 16362 | 1 | 0.009
R0a+60.1T | 2706 | 1 | 0.009
R0a+60.1T | 7028 | 1 | 0.009
R0a1 | 64 | 1 | 0.009
R0a1a | 16126 | 3 | 0.021
R0a1a | 16355 | 4 | 0.029
R0a1a | 16362 | 2 | 0.014
R0a1a | 64 | 3 | 0.021
R0a1a2 | 12741 | 1 | 0.012
R0a1a3 | 146 | 3 | 0.033
R0a1b | 16093 | 2 | 0.087
R0a2 | 16126 | 3 | 0.02
R0a2 | 60.1T | 6 | 0.041
R0a2 | 64 | 1 | 0.007
R0a2+195 | 60.1T | 3 | 0.059
R0a2+195 | 64 | 1 | 0.02
R0a2'3 | 60.1T | 1 | 0.013
R0a2c | 14766 | 1 | 0.007
R0a2c | 60.1T | 3 | 0.021
R0a2f | 60.1T | 7 | 0.084

R0a2f1a | 131 | 1 | 0.018
R0a2g | 60.1T | 1 | 0.012
R0a2i | 16126 | 7 | 0.184
R0a2i | 16362 | 2 | 0.053
R0a2i | 60.1T | 1 | 0.026
R0a2k | 60.1T | 1 | 0.012
R0a2n | 11914 | 3 | 0.081
R0a4 | 2351 | 1 | 0.017
R0b | 10775 | 2 | 0.125
R0b | 15628 | 2 | 0.125
R0b | 4924 | 2 | 0.125
R0b | 9055 | 2 | 0.125
R0b | 93 | 3 | 0.188
R1 | 1391 | 1 | 0.006
R1 | 14766 | 1 | 0.006
R11 | 185 | 2 | 0.049
R11 | 8278.XC | 2 | 0.049
R11a | 1438 | 1 | 0.016
R11a | 15326 | 1 | 0.016
R11a | 8278.XC | 4 | 0.063
R11b | 8278.XC | 3 | 0.125
R11b1 | 16189 | 1 | 0.032
R11b1 | 185 | 2 | 0.065
R11b1 | 8277 | 1 | 0.032
R11b1 | 8278.XC | 3 | 0.097
R11b1a | 8278.XC | 2 | 0.222
R11b1b | 1438 | 1 | 0.03
R11b1b | 8278.XC | 4 | 0.121
R12 | 10398 | 1 | 0.05
R14 | 16187 | 4 | 0.308
R14 | 16362 | 11 | 0.846
R14 | 182 | 2 | 0.154
R14 | 56T | 2 | 0.154
R1a | 15326 | 5 | 0.044
R1a | 2706 | 1 | 0.009
R1a | 73 | 2 | 0.018
R1a1 | 3360 | 4 | 0.035
R1a1 | 6671 | 1 | 0.009
R1a1 | 8887 | 1 | 0.009
R1a1a | 15326 | 1 | 0.009
R1b1 | 513 | 9 | 0.085
R21 | 16168 | 1 | 0.038
R21 | 16295 | 4 | 0.154
R21 | 16296 | 1 | 0.038
R21 | 9109 | 1 | 0.038
R22 | 152 | 26 | 0.139
R22 | 16249 | 5 | 0.027
R22 | 16304 | 1 | 0.005
R22 | 329 | 1 | 0.005
R23 | 10987 | 1 | 0.043
R23 | 13885A | 1 | 0.043
R23 | 14687 | 1 | 0.043
R23 | 16256 | 1 | 0.043
R23 | 16465 | 1 | 0.043
R23 | 7388 | 1 | 0.043
R23 | 8614 | 1 | 0.043
R24a | 16193d | 8 | 0.533
R2b1 | 152 | 3 | 0.12
R2d | 152 | 5 | 0.072
R30a | 12714 | 1 | 0.012
R30a | 8860 | 1 | 0.012
R30a1 | 2056 | 1 | 0.008
R30a1b1 | 11735 | 1 | 0.018
R30a1b1 | 15326 | 1 | 0.018
R30a1b1 | 16209 | 1 | 0.018
R30a1b1 | 263 | 1 | 0.018
R30a1b1 | 4225 | 1 | 0.018
R30a1b1 | 9242 | 1 | 0.018
R30a1c | 4232 | 1 | 0.01
R30b | 2706 | 1 | 0.03
R30b | 8860 | 1 | 0.03
R30b1 | 152 | 2 | 0.095
R30b1 | 480 | 5 | 0.238
R30b1 | 7268G | 1 | 0.048
R30b1 | 9174 | 2 | 0.095
R30b2 | 14766 | 1 | 0.018
R30b2 | 15148 | 2 | 0.036
R30b2 | 373 | 2 | 0.036
R31a | 15334 | 1 | 0.029
R31a | 15884 | 1 | 0.029
R31a | 16172 | 2 | 0.059
R31a | 338 | 4 | 0.118
R31a | 6293 | 1 | 0.029
R31a1 | 237 | 1 | 0.1
R31b | 16093 | 1 | 0.143
R32 | 16185 | 1 | 0.027
R32 | 16311 | 3 | 0.081
R32 | 8281-8289d | 2 | 0.054
R32 | 8743 | 2 | 0.054
R5a | 10754 | 1 | 0.022
R5a | 2706 | 1 | 0.022
R5a | 8860 | 1 | 0.022
R5a1 | 200 | 2 | 0.182
R5a2 | 10754 | 1 | 0.026
R5a2 | 14040 | 1 | 0.026
R5a2 | 14544 | 2 | 0.053
R5a2 | 14766 | 2 | 0.053
R5a2 | 16356 | 1 | 0.026
R5a2 | 16524 | 2 | 0.053
R5a2 | 750 | 1 | 0.026
R5a2 | 8860 | 1 | 0.026
R5a2a | 10754 | 1 | 0.013
R5a2a | 11293 | 2 | 0.025
R5a2a | 13635 | 1 | 0.013
R5a2a | 14040 | 1 | 0.013
R5a2a | 14544 | 2 | 0.025
R5a2a | 152 | 1 | 0.013
R5a2a | 15385 | 2 | 0.025
R5a2a | 16524 | 2 | 0.025
R5a2a | 263 | 1 | 0.013
R5a2a | 2706 | 4 | 0.051

R5a2a | 4769 | 2 | 0.025
R5a2a | 8594 | 1 | 0.013
R5a2a | 8860 | 4 | 0.051
R5a2b | 10754 | 1 | 0.034
R5a2b | 11293 | 1 | 0.034
R5a2b | 14040 | 1 | 0.034
R5a2b | 16524 | 6 | 0.207
R5a2b | 597.1T | 1 | 0.034
R5a2b1 | 16309 | 7 | 0.28
R5a2b1 | 16524 | 5 | 0.2
R5a2b2 | 16309 | 1 | 0.059
R5a2b2 | 16325 | 1 | 0.059
R5a2b2 | 16356 | 1 | 0.059
R5a2b2 | 16524 | 3 | 0.176
R5a2b3 | 152 | 1 | 0.05
R6+16129 | 12285 | 2 | 0.023
R6+16129 | 16362 | 1 | 0.011
R6a1 | 16362 | 2 | 0.095
R6a1 | 228 | 2 | 0.095
R6a2 | 11719 | 1 | 0.022
R6a2 | 16129 | 2 | 0.044
R6a2 | 228 | 4 | 0.089
R6b | 16227 | 1 | 0.048
R7 | 16362 | 1 | 0.016
R7 | 73 | 1 | 0.016
R7a1 | 10915 | 1 | 0.017
R7a1 | 14766 | 1 | 0.017
R7a1 | 2706 | 1 | 0.017
R7a1 | 7870 | 1 | 0.017
R7a1b2 | 73 | 1 | 0.018
R7b | 16261 | 1 | 0.143
R7b1 | 16261 | 1 | 0.143
R7b1a | 15942 | 2 | 0.125
R7b1a | 16261 | 2 | 0.125
R7b1a | 16362 | 1 | 0.062
R7b1a | 7870 | 1 | 0.062
R7b2 | 10289 | 2 | 0.2
R7b2 | 12246 | 1 | 0.1
R7b2 | 146 | 3 | 0.3
R7b2 | 14766 | 2 | 0.2
R7b2 | 16311 | 1 | 0.1
R7b2 | 16362 | 1 | 0.1
R7b2 | 4769 | 1 | 0.1
R8a | 195 | 2 | 0.105
R8a1a1 | 195 | 1 | 0.062
R8a1a1a1 | 195 | 1 | 0.062
R8a1a1a1a | 6131 | 1 | 0.062
R8a1a1c | 13782 | 1 | 0.048
R8a1a1d | 195 | 2 | 0.091
R8a1a2a | 12127 | 1 | 0.043
R8a1a3 | 709 | 1 | 0.028
R8a1b | 16093 | 1 | 0.02
R8b1 | 73 | 1 | 0.04
R8b1a | 6485 | 1 | 0.027
R8b1a | 7028 | 1 | 0.027
R9b | 16304 | 1 | 0.005
R9b1 | 16304 | 1 | 0.008
R9b1 | 16309 | 2 | 0.016
R9b1a | 16304 | 1 | 0.023
R9b1a1a | 16192 | 41 | 0.432
R9b1a1a | 16309 | 6 | 0.063
R9b1a1a | 16390 | 1 | 0.011
R9b1a1a | 183 | 1 | 0.011
R9b1a2 | 16192 | 2 | 0.044
R9b1a2a | 16192 | 2 | 0.065
R9b1a2a | 16309 | 4 | 0.129
R9b1a2a | 183 | 3 | 0.097
R9b1a2a | 750 | 1 | 0.032
R9b1a2b | 16192 | 4 | 0.058
R9b1a3 | 16192 | 3 | 0.018
R9b1a3 | 16309 | 2 | 0.012
R9b1a3 | 183 | 99 | 0.604
R9b1a3 | 7028 | 2 | 0.012
R9b1b | 16309 | 1 | 0.008
R9c1a | 249d | 2 | 0.021
R9c1a | 8860 | 1 | 0.011
R9c1a1 | 16157 | 3 | 0.031
R9c1a1 | 249d | 5 | 0.052
R9c1a1 | 8440 | 1 | 0.01
R9c1a2 | 16157 | 1 | 0.011
R9c1a2 | 249d | 12 | 0.126
R9c1a2 | 263 | 1 | 0.011
R9c1a2 | 73 | 1 | 0.011
R9c1b1 | 151 | 4 | 0.042
R9c1b1 | 16157 | 1 | 0.011
R9c1b1 | 3970 | 1 | 0.011
R9c1b1 | 4769 | 2 | 0.021
S2 | 16223 | 10 | 0.044
S3 | 16140 | 1 | 0.2
S3 | 195 | 1 | 0.2
S4 | 16192 | 3 | 0.065
S4 | 750 | 3 | 0.065
S5 | 249d | 1 | 0.333
T | 15607 | 1 | 0.009
T | 15928 | 1 | 0.009
T | 16126 | 1 | 0.009
T | 4216 | 1 | 0.009
T | 4917 | 1 | 0.009
T1 | 16189 | 3 | 0.04
T1a | 16163 | 3 | 0.013
T1a | 7028 | 1 | 0.004
T1a | 8697 | 1 | 0.004
T1a+152 | 11251 | 1 | 0.006
T1a+152 | 13368 | 1 | 0.006
T1a+152 | 15452A | 1 | 0.006
T1a+152 | 15607 | 1 | 0.006
T1a+152 | 16186 | 1 | 0.006
T1a+152 | 4917 | 1 | 0.006
T1a+152 | 709 | 1 | 0.006
T1a1 | 1438 | 1 | 0.003
T1a1 | 16163 | 5 | 0.014

T1a1 | 1888 | 1 | 0.003
T1a1 | 2706 | 1 | 0.003
T1a1+@152 | 16189 | 1 | 0.008
T1a10 | 16126 | 1 | 0.005
T1a10 | 16163 | 1 | 0.005
T1a10 | 16294 | 2 | 0.01
T1a10 | 263 | 1 | 0.005
T1a10a | 13708 | 2 | 0.018
T1a11 | 16189 | 1 | 0.009
T1a11 | 16294 | 14 | 0.121
T1a13 | 1888 | 1 | 0.024
T1a1b | 152 | 1 | 0.005
T1a1b | 16163 | 5 | 0.023
T1a1b | 16189 | 1 | 0.005
T1a1b | 195 | 2 | 0.009
T1a1b | 750 | 1 | 0.005
T1a1b1 | 14281 | 1 | 0.005
T1a1c | 16186 | 3 | 0.016
T1a1c | 16294 | 3 | 0.016
T1a1d | 1438 | 1 | 0.006
T1a1d | 4917 | 1 | 0.006
T1a1h | 1888 | 1 | 0.053
T1a1i | 152 | 2 | 0.011
T1a1k1 | 16189 | 1 | 0.009
T1a1k1 | 8697 | 1 | 0.009
T1a1k2 | 16126 | 2 | 0.016
T1a1k2 | 16163 | 1 | 0.008
T1a1p | 12633A | 1 | 0.005
T1a1q | 16186 | 1 | 0.006
T1a2 | 16163 | 3 | 0.016
T1a2 | 16294 | 9 | 0.048
T1a2 | 73 | 1 | 0.005
T1a2a | 7853 | 1 | 0.053
T1a2b | 16163 | 3 | 0.067
T1a2b | 16186 | 1 | 0.022
T1a2b | 16189 | 1 | 0.022
T1a4 | 750 | 1 | 0.021
T1a4 | 8697 | 1 | 0.021
T1a5 | 15928 | 1 | 0.006
T1a6 | 16186 | 1 | 0.005
T1a6 | 16294 | 1 | 0.005
T1a7 | 16189 | 2 | 0.033
T1a8 | 15928 | 1 | 0.006
T1a8 | 73 | 4 | 0.022
T1a8b | 15928 | 1 | 0.006
T1a8b | 16163 | 2 | 0.011
T1a9 | 1438 | 1 | 0.014
T1b1 | 15928 | 2 | 0.023
T1b1 | 16163 | 3 | 0.034
T1b3 | 1888 | 1 | 0.011
T1b4 | 11251 | 1 | 0.011
T1b4 | 16294 | 1 | 0.011
T2 | 2706 | 1 | 0.004
T2+150 | 10463 | 1 | 0.011
T2+150 | 11812 | 1 | 0.011
T2+150 | 16126 | 1 | 0.011
T2a1a2 | 14905 | 1 | 0.005
T2a1a2 | 709 | 1 | 0.005
T2a1b | 15326 | 1 | 0.009
T2a1b1a | 1888 | 1 | 0.009
T2a1b1a1 | 16324 | 1 | 0.009
T2a1b1a1a | 16126 | 2 | 0.02
T2a1b1a1b | 13965 | 1 | 0.009
T2a1b1a1b | 13966 | 1 | 0.009
T2a1b2 | 16126 | 2 | 0.02
T2a3 | 1888 | 1 | 0.011
T2b | 11251 | 3 | 0.006
T2b | 14766 | 1 | 0.002
T2b | 15326 | 1 | 0.002
T2b | 15452A | 2 | 0.004
T2b | 16126 | 6 | 0.013
T2b | 16294 | 1 | 0.002
T2b | 16304 | 1 | 0.002
T2b | 1888 | 4 | 0.009
T2b | 263 | 7 | 0.015
T2b | 2706 | 1 | 0.002
T2b | 7028 | 1 | 0.002
T2b | 709 | 2 | 0.004
T2b | 8697 | 2 | 0.004
T2b+150 | 16126 | 1 | 0.01
T2b+152 | 15326 | 1 | 0.007
T2b+152 | 16304 | 1 | 0.007
T2b+152 | 73 | 1 | 0.007
T2b+16362 | 14233 | 1 | 0.011
T2b+16362 | 16304 | 1 | 0.011
T2b+16362 | 1888 | 1 | 0.011
T2b11 | 3398 | 1 | 0.011
T2b16 | 16304 | 1 | 0.014
T2b19 | 16304 | 1 | 0.004
T2b19b | 15928 | 1 | 0.016
T2b2 | 11812 | 1 | 0.004
T2b21 | 152 | 1 | 0.008
T2b21 | 73 | 1 | 0.008
T2b21 | 8697 | 2 | 0.016
T2b21b | 73 | 1 | 0.008
T2b22 | 73 | 1 | 0.008
T2b23 | 16297 | 1 | 0.012
T2b23 | 16304 | 2 | 0.024
T2b23a | 11812 | 2 | 0.027
T2b23a | 4769 | 1 | 0.014
T2b23a | 5147 | 1 | 0.014
T2b24 | 14905 | 1 | 0.008
T2b24 | 4216 | 1 | 0.008
T2b24 | 5147 | 2 | 0.017
T2b24 | 7028 | 1 | 0.008
T2b24 | 709 | 1 | 0.008
T2b24 | 8697 | 1 | 0.008
T2b24 | 930 | 1 | 0.008
T2b24a | 16126 | 4 | 0.033
T2b25 | 16294 | 1 | 0.004
T2b26 | 263 | 1 | 0.004

T2b28 | 11914 | 2 | 0.009
T2b2b | 11242G | 1 | 0.005
T2b2b | 709 | 1 | 0.005
T2b3+151 | 2706 | 1 | 0.008
T2b3+151 | 5147 | 1 | 0.008
T2b3+151 | 8860 | 1 | 0.008
T2b33 | 15928 | 1 | 0.034
T2b33 | 16126 | 1 | 0.034
T2b37 | 709 | 1 | 0.004
T2b3a | 16304 | 4 | 0.143
T2b3a | 5147 | 1 | 0.036
T2b3a1 | 13368 | 5 | 0.081
T2b3a1 | 151 | 3 | 0.048
T2b3a1 | 16126 | 2 | 0.032
T2b3a1 | 16292 | 1 | 0.016
T2b3a1 | 55.1T | 40 | 0.645
T2b3a1 | 73 | 7 | 0.113
T2b3b | 16304 | 2 | 0.009
T2b3d | 151 | 1 | 0.011
T2b3d | 16126 | 1 | 0.011
T2b3e | 151 | 2 | 0.087
T2b4+152 | 13368 | 1 | 0.007
T2b4+152 | 14233 | 1 | 0.007
T2b4+152 | 14766 | 1 | 0.007
T2b4+152 | 16294 | 12 | 0.082
T2b4a1 | 15928 | 1 | 0.011
T2b4c | 73 | 1 | 0.008
T2b4d | 73 | 1 | 0.008
T2b4e | 73 | 1 | 0.008
T2b4f | 16126 | 2 | 0.016
T2b4f | 16304 | 1 | 0.008
T2b4f | 73 | 1 | 0.008
T2b4g | 73 | 1 | 0.008
T2b5 | 8697 | 1 | 0.004
T2b6b | 73 | 1 | 0.077
T2b7 | 14766 | 1 | 0.004
T2b7a1 | 263 | 1 | 0.024
T2b7a3 | 11251 | 1 | 0.012
T2b7a3 | 14905 | 1 | 0.012
T2b7a3 | 15928 | 1 | 0.012
T2b7a3 | 16126 | 2 | 0.025
T2b7a3 | 16292 | 1 | 0.012
T2b7a3 | 16294 | 1 | 0.012
T2b7a3 | 16304 | 1 | 0.012
T2b7a3 | 1888 | 1 | 0.012
T2b7a3 | 2706 | 1 | 0.012
T2b7a3 | 7028 | 1 | 0.012
T2b7a3 | 73 | 1 | 0.012
T2b7a3 | 8697 | 1 | 0.012
T2b8 | 14233 | 1 | 0.004
T2b8 | 14905 | 1 | 0.004
T2b8 | 16294 | 1 | 0.004
T2b9 | 16126 | 1 | 0.009
T2c | 4769 | 1 | 0.005
T2c | 8860 | 1 | 0.005
T2c1 | 16126 | 1 | 0.01
T2c1+146 | 15928 | 2 | 0.042
T2c1+146 | 73 | 1 | 0.021
T2c1a | 16292 | 1 | 0.015
T2c1a | 573.XC | 3 | 0.045
T2c1a | 7028 | 1 | 0.015
T2c1a1 | 573.XC | 1 | 0.036
T2c1a2 | 16292 | 1 | 0.015
T2c1a2 | 573.XC | 2 | 0.03
T2c1a3 | 73 | 2 | 0.031
T2c1c | 16292 | 2 | 0.024
T2c1c | 6261 | 1 | 0.012
T2c1c1 | 11719 | 5 | 0.089
T2c1c2 | 16146 | 1 | 0.015
T2c1c2 | 6261 | 1 | 0.015
T2c1d | 16126 | 1 | 0.029
T2c1d+152 | 14766 | 1 | 0.02
T2c1d+152 | 16292 | 4 | 0.08
T2c1d+152 | 4769 | 1 | 0.02
T2c1d1 | 16292 | 5 | 0.122
T2c1d1 | 16294 | 1 | 0.024
T2c1d1a | 14233 | 1 | 0.025
T2c1d2 | 15928 | 1 | 0.023
T2c1d2a | 14766 | 1 | 0.014
T2c1e | 16126 | 1 | 0.02
T2c1f | 16126 | 1 | 0.023
T2c1f | 16292 | 1 | 0.023
T2d1 | 15928 | 1 | 0.008
T2d1b | 152 | 1 | 0.013
T2d1b1 | 152 | 5 | 0.078
T2d1b1 | 15607 | 1 | 0.016
T2d1b2 | 200 | 1 | 0.015
T2d2 | 16294 | 1 | 0.005
T2d2 | 263 | 1 | 0.005
T2e | 13368 | 1 | 0.009
T2e1 | 10463 | 1 | 0.015
T2e1 | 16126 | 1 | 0.015
T2e1 | 8697 | 1 | 0.015
T2e2 | 15928 | 1 | 0.015
T2e2 | 16294 | 1 | 0.015
T2f | 263 | 1 | 0.013
T2f1 | 15928 | 1 | 0.02
T2f1 | 16294 | 2 | 0.04
T2f1 | 8281-8289d | 3 | 0.06
T2f1a | 8270 | 1 | 0.038
T2f1a | 8281-8289d | 1 | 0.038
T2f1a1 | 14233 | 1 | 0.011
T2f1a1 | 15928 | 1 | 0.011
T2f1a1 | 16126 | 2 | 0.021
T2f1a1 | 16189 | 1 | 0.011
T2f1a1 | 16298 | 2 | 0.021
T2f1a1 | 4917 | 1 | 0.011
T2f1a1 | 5426 | 1 | 0.011
T2f1a1 | 6489A | 2 | 0.021
T2f1a1 | 8270 | 10 | 0.105
T2f1a1 | 8281-8289d | 17 | 0.179

T2f1a1 | 8697 | 1 | 0.011
T2f2 | 16126 | 1 | 0.012
T2f2 | 16189 | 1 | 0.012
T2f3 | 8270 | 2 | 0.029
T2f4 | 8270 | 1 | 0.013
T2f5 | 8270 | 1 | 0.019
T2f6 | 16294 | 1 | 0.013
T2f7 | 8281-8289d | 5 | 0.064
T2f8a | 8281-8289d | 4 | 0.049
T2g1 | 16126 | 2 | 0.011
T2g1a | 14839 | 1 | 0.005
T2g1a | 16126 | 1 | 0.005
T2g2a | 14798 | 1 | 0.012
T2h2 | 16294 | 2 | 0.026
T2i | 15326 | 1 | 0.013
T2k | 16126 | 2 | 0.027
T2k | 16294 | 1 | 0.013
T3 | 15928 | 1 | 0.03
U1a | 11467 | 1 | 0.023
U1a | 2218 | 1 | 0.023
U1a | 2706 | 1 | 0.023
U1a | 4769 | 1 | 0.023
U1a | 7028 | 1 | 0.023
U1a1a | 14070 | 1 | 0.011
U1a1a | 16189 | 1 | 0.011
U1a1a | 16249 | 4 | 0.043
U1a1a | 263 | 1 | 0.011
U1a1a | 2706 | 1 | 0.011
U1a1a | 285 | 2 | 0.022
U1a1a | 3158.1T | 6 | 0.065
U1a1a | 385 | 1 | 0.011
U1a1a+16129 | 11467 | 3 | 0.111
U1a1a+16129 | 12372 | 1 | 0.037
U1a1a+16129 | 16189 | 3 | 0.111
U1a1a+16129 | 16249 | 3 | 0.111
U1a1a+16129 | 285 | 2 | 0.074
U1a1a+16129 | 3158.1T | 2 | 0.074
U1a1a+16129 | 385 | 1 | 0.037
U1a1a1a | 3158.1T | 1 | 0.023
U1a1a1a | 385 | 2 | 0.045
U1a1a1a | 573.XC | 1 | 0.023
U1a1a2 | 16249 | 4 | 0.078
U1a1a2 | 3158.1T | 1 | 0.02
U1a1a2 | 6026 | 1 | 0.02
U1a1a3 | 285 | 1 | 0.062
U1a1b | 16166d | 4 | 0.182
U1a1c1 | 16249 | 1 | 0.019
U1a1c1c1 | 12879 | 1 | 0.05
U1a1c1c1 | 13104 | 1 | 0.05
U1a1c1c1 | 285 | 1 | 0.05
U1a1c1d | 12879 | 2 | 0.033
U1a1c1d | 15326 | 1 | 0.016
U1a1c1d | 2218 | 2 | 0.033
U1a1c1d | 7581 | 1 | 0.016
U1a1c1d1 | 285 | 2 | 0.154
U1a1d | 12308 | 1 | 0.111
U1a1d | 12372 | 1 | 0.111
U1a1d | 12879 | 1 | 0.111
U1a1d | 14766 | 1 | 0.111
U1a1d | 16362 | 1 | 0.111
U1a1d | 285 | 1 | 0.111
U1a1d | 4769 | 1 | 0.111
U1a1d | 7028 | 1 | 0.111
U1a1d | 7581 | 1 | 0.111
U1a1d | 8860 | 1 | 0.111
U1a2 | 16249 | 2 | 0.077
U1b1 | 14070 | 3 | 0.188
U1b1 | 16327 | 4 | 0.25
U1b2 | 11566 | 1 | 0.05
U1b2 | 16327 | 1 | 0.05
U1b3 | 1438 | 1 | 0.038
U1b3 | 2387 | 1 | 0.038
U1b3 | 285 | 2 | 0.077
U1b3 | 7028 | 1 | 0.038
U1b3 | 750 | 1 | 0.038
U1b3 | 8395 | 1 | 0.038
U2 | 15326 | 1 | 0.023
U2+152 | 16051 | 1 | 0.017
U2+152 | 73 | 1 | 0.017
U2a | 16051 | 1 | 0.026
U2a1 | 73 | 1 | 0.015
U2a1a | 16051 | 2 | 0.028
U2a1a | 8860 | 1 | 0.014
U2a1b | 12308 | 1 | 0.016
U2a2 | 11151 | 4 | 0.148
U2a2 | 15734 | 4 | 0.148
U2a2 | 16271 | 16 | 0.593
U2a2 | 194 | 2 | 0.074
U2a2 | 7382 | 4 | 0.148
U2a2 | 7853 | 4 | 0.148
U2b | 16051 | 4 | 0.045
U2b1 | 146 | 1 | 0.014
U2b1 | 3915 | 1 | 0.014
U2b1 | 5186T | 1 | 0.014
U2b1 | 73 | 1 | 0.014
U2b2 | 11719 | 1 | 0.012
U2b2 | 12106 | 1 | 0.012
U2b2 | 12308 | 1 | 0.012
U2b2 | 12793 | 1 | 0.012
U2b2 | 13656 | 1 | 0.012
U2b2 | 1438 | 1 | 0.012
U2b2 | 146 | 2 | 0.025
U2b2 | 14766 | 2 | 0.025
U2b2 | 15049 | 1 | 0.012
U2b2 | 152 | 4 | 0.05
U2b2 | 15930 | 1 | 0.012
U2b2 | 16051 | 3 | 0.038
U2b2 | 16209 | 4 | 0.05
U2b2 | 16239 | 1 | 0.012
U2b2 | 16352 | 2 | 0.025
U2b2 | 16353 | 2 | 0.025

U2b2 | 1811 | 1 | 0.012
U2b2 | 1888 | 2 | 0.025
U2b2 | 5186T | 1 | 0.012
U2b2 | 7028 | 2 | 0.025
U2b2 | 8860 | 3 | 0.038
U2b2 | 9094 | 1 | 0.012
U2c | 152 | 1 | 0.02
U2c1 | 152 | 1 | 0.019
U2c1 | 16051 | 1 | 0.019
U2c1a | 152 | 1 | 0.02
U2c1a | 16234 | 4 | 0.082
U2c'd | 12308 | 1 | 0.015
U2c'd | 12372 | 1 | 0.015
U2c'd | 16051 | 1 | 0.015
U2c'd | 2706 | 1 | 0.015
U2c'd | 8860 | 1 | 0.015
U2d | 16051 | 1 | 0.053
U2d | 16234 | 1 | 0.053
U2d1 | 12308 | 1 | 0.071
U2d1 | 16189 | 3 | 0.214
U2d1 | 16234 | 1 | 0.071
U2d2 | 16234 | 1 | 0.059
U2d2 | 199 | 3 | 0.176
U2d2 | 6956 | 1 | 0.059
U2d2 | 8296 | 1 | 0.059
U2d2 | 8938 | 1 | 0.059
U2d2a | 12308 | 1 | 0.071
U2d3 | 12308 | 1 | 0.071
U2d3 | 16234 | 1 | 0.071
U2e | 10876 | 1 | 0.02
U2e | 13734 | 1 | 0.02
U2e | 1438 | 1 | 0.02
U2e | 152 | 3 | 0.06
U2e | 16051 | 2 | 0.04
U2e | 16129C | 3 | 0.06
U2e | 16362 | 1 | 0.02
U2e | 1811 | 1 | 0.02
U2e | 508 | 1 | 0.02
U2e | 6045 | 1 | 0.02
U2e | 6152 | 1 | 0.02
U2e | 7028 | 1 | 0.02
U2e | 73 | 1 | 0.02
U2e | 8860 | 1 | 0.02
U2e1a | 16051 | 1 | 0.013
U2e1a | 16129C | 1 | 0.013
U2e1a1 | 11197 | 1 | 0.009
U2e1a1 | 16189 | 1 | 0.009
U2e1a1 | 16362 | 2 | 0.018
U2e1a1 | 217 | 2 | 0.018
U2e1a1 | 340 | 1 | 0.009
U2e1a1 | 508 | 2 | 0.018
U2e1a1a | 11197 | 1 | 0.015
U2e1a1a | 16362 | 1 | 0.015
U2e1a1a | 1811 | 1 | 0.015
U2e1a1a | 217 | 3 | 0.045
U2e1a1a | 340 | 2 | 0.03
U2e1a1a | 3720 | 2 | 0.03
U2e1a1a | 508 | 1 | 0.015
U2e1a1b | 508 | 1 | 0.031
U2e1a1c | 1811 | 1 | 0.015
U2e1b | 217 | 1 | 0.077
U2e1b1 | 14766 | 1 | 0.027
U2e1b1 | 15784 | 2 | 0.054
U2e1b1 | 16129C | 2 | 0.054
U2e1b1 | 16256 | 1 | 0.027
U2e1b1 | 217 | 2 | 0.054
U2e1b2 | 16129C | 1 | 0.083
U2e1b2 | 16189 | 1 | 0.083
U2e1c1 | 13734 | 1 | 0.015
U2e1c1 | 16129C | 1 | 0.015
U2e1d | 15907 | 1 | 0.015
U2e1e | 16145 | 4 | 0.364
U2e1e | 16189 | 1 | 0.091
U2e1g | 217 | 3 | 0.045
U2e1h | 16051 | 3 | 0.042
U2e1h | 16129C | 2 | 0.028
U2e1h | 217 | 1 | 0.014
U2e1h | 340 | 2 | 0.028
U2e1h | 508 | 3 | 0.042
U2e2 | 10876 | 1 | 0.015
U2e2 | 13020 | 1 | 0.015
U2e2 | 13734 | 1 | 0.015
U2e2 | 16189 | 3 | 0.045
U2e2 | 508 | 1 | 0.015
U2e2a | 16189 | 3 | 0.045
U2e2a1 | 508 | 1 | 0.016
U2e2a1a1 | 152 | 1 | 0.012
U2e2a1a1 | 15907 | 1 | 0.012
U2e2a1a1 | 16129C | 1 | 0.012
U2e2a1a2 | 16362 | 1 | 0.014
U2e2a1a2 | 217 | 3 | 0.041
U2e2a1a2 | 5390 | 1 | 0.014
U2e2a1b | 16362 | 2 | 0.051
U2e2a1d | 7028 | 1 | 0.014
U2e3 | 16189 | 1 | 0.143
U2e3 | 16356 | 1 | 0.143
U2e3 | 217 | 2 | 0.286
U2e3a | 16181 | 1 | 0.2
U2e3a | 575 | 1 | 0.2
U3a | 12308 | 1 | 0.016
U3a | 150 | 1 | 0.016
U3a | 1811 | 1 | 0.016
U3a | 2294 | 1 | 0.016
U3a | 6518 | 2 | 0.031
U3a1 | 12372 | 1 | 0.013
U3a1 | 15454 | 1 | 0.013
U3a1 | 16343 | 2 | 0.025
U3a1 | 16390 | 1 | 0.013
U3a1 | 6518 | 1 | 0.013
U3a1b | 6518 | 1 | 0.021
U3a1c | 2294 | 2 | 0.05

U3a1c | 263 | 1 | 0.025
U3a2a | 16343 | 1 | 0.032
U3a2a1 | 150 | 1 | 0.043
U3a2a1 | 16343 | 2 | 0.087
U3a3 | 10143 | 4 | 0.065
U3a3 | 16189 | 1 | 0.016
U3a3 | 16343 | 2 | 0.032
U3b | 13743 | 1 | 0.01
U3b1 | 13743 | 1 | 0.008
U3b1 | 4188 | 1 | 0.008
U3b1 | 4640A | 1 | 0.008
U3b1a1 | 16343 | 5 | 0.238
U3b1a1 | 750 | 1 | 0.048
U3b2a1 | 16343 | 2 | 0.019
U3b2a1 | 2707 | 1 | 0.009
U3b3 | 12372 | 1 | 0.012
U3b3 | 1438 | 1 | 0.012
U3b3 | 14766 | 11 | 0.128
U3c | 11467 | 1 | 0.027
U3c | 11719 | 1 | 0.027
U3c | 150 | 1 | 0.027
U3c | 15613 | 8 | 0.216
U3c | 16193 | 2 | 0.054
U3c | 16249 | 1 | 0.027
U3c | 4703 | 1 | 0.027
U3c | 9266 | 1 | 0.027
U4a | 195 | 8 | 0.06
U4a1 | 14620 | 1 | 0.007
U4a1 | 152 | 7 | 0.05
U4a1 | 15693 | 2 | 0.014
U4a1 | 2706 | 1 | 0.007
U4a1 | 4646 | 1 | 0.007
U4a1 | 8818 | 1 | 0.007
U4a1 | 8860 | 1 | 0.007
U4a1a | 11332 | 1 | 0.008
U4a1a | 12308 | 1 | 0.008
U4a1a | 12937 | 1 | 0.008
U4a1a | 152 | 1 | 0.008
U4a1a1 | 152 | 1 | 0.01
U4a1b1 | 16356 | 1 | 0.037
U4a1b2 | 152 | 1 | 0.111
U4a1e | 16134 | 2 | 0.222
U4a1e | 16311 | 1 | 0.111
U4a2 | 11332 | 1 | 0.007
U4a2 | 11467 | 1 | 0.007
U4a2 | 195 | 1 | 0.007
U4a2 | 4646 | 1 | 0.007
U4a2 | 5999 | 1 | 0.007
U4a2 | 6047 | 1 | 0.007
U4a2 | 8818 | 1 | 0.007
U4a2a | 11332 | 2 | 0.054
U4a2a | 1811 | 1 | 0.027
U4a2a | 310 | 4 | 0.108
U4a2a | 4769 | 1 | 0.027
U4a2b | 4646 | 1 | 0.026
U4a2c | 14766 | 2 | 0.021
U4a2f | 11467 | 1 | 0.01
U4a2f | 15172 | 2 | 0.021
U4a2g | 4646 | 1 | 0.01
U4a3a | 4646 | 1 | 0.056
U4b1+146_152 | 195 | 1 | 0.014
U4b1a1a1 | 1438 | 2 | 0.032
U4b1a3 | 195 | 1 | 0.015
U4b1a3a | 5752d | 1 | 0.013
U4b1b | 195 | 1 | 0.014
U4b1b1 | 146 | 2 | 0.023
U4b1b1 | 16356 | 4 | 0.045
U4b1b1 | 6047 | 2 | 0.023
U4b1b1a | 16356 | 2 | 0.2
U4b1b1d | 195 | 1 | 0.014
U4b2a | 195 | 1 | 0.071
U4b3 | 1438 | 1 | 0.011
U4b3 | 1811 | 1 | 0.011
U4b3 | 195 | 1 | 0.011
U4b3 | 8860 | 1 | 0.011
U4c1 | 11009 | 1 | 0.013
U4c1 | 16179 | 1 | 0.013
U4c1 | 7028 | 1 | 0.013
U4c1a | 11009 | 1 | 0.022
U4c1a | 73 | 1 | 0.022
U4d | 15693 | 1 | 0.008
U4d | 16356 | 1 | 0.008
U4d1a1 | 11332 | 1 | 0.008
U4d1a1 | 629 | 1 | 0.008
U4d1a1a | 15693 | 2 | 0.016
U4d2 | 16189 | 15 | 0.3
U4d2 | 16356 | 2 | 0.04
U4d2 | 1811 | 1 | 0.02
U4d2 | 2405.1C | 1 | 0.02
U4d3 | 573.XC | 1 | 0.009
U5a | 13617 | 1 | 0.021
U5a | 16192 | 2 | 0.043
U5a | 73 | 1 | 0.021
U5a1+@16192 | 1438 | 1 | 0.01
U5a1+@16192 | 16256 | 1 | 0.01
U5a1+@16192 | 16270 | 2 | 0.019
U5a1a1 | 16270 | 4 | 0.024
U5a1a1+152 | 15218 | 1 | 0.009
U5a1a1+152 | 16256 | 1 | 0.009
U5a1a1+152 | 9477 | 1 | 0.009
U5a1a1+16362 | 16256 | 1 | 0.031
U5a1a1c | 15218 | 2 | 0.018
U5a1a1c | 15326 | 1 | 0.009
U5a1a1c | 16256 | 1 | 0.009
U5a1a1c | 16270 | 1 | 0.009
U5a1a1c | 16399 | 1 | 0.009
U5a1a1d | 16270 | 1 | 0.032
U5a1a1d1 | 15218 | 1 | 0.04
U5a1a1e | 14793 | 1 | 0.009
U5a1a1e | 16399 | 1 | 0.009
U5a1a1i | 16270 | 1 | 0.011

U5a1a2a | 16270 | 5 | 0.065
U5a1a2a | 573.XC | 5 | 0.065
U5a1a2a1 | 73 | 1 | 0.017
U5a1a2a1a | 73 | 1 | 0.016
U5a1a2b | 16256 | 4 | 0.058
U5a1a2b | 573.XC | 3 | 0.043
U5a1b | 16256 | 1 | 0.014
U5a1b | 16270 | 1 | 0.014
U5a1b1 | 16192 | 11 | 0.131
U5a1b1a | 15218 | 1 | 0.018
U5a1b1a | 16192 | 4 | 0.07
U5a1b1a1 | 16256 | 1 | 0.019
U5a1b1a1 | 16270 | 1 | 0.019
U5a1b1a2 | 16256 | 1 | 0.019
U5a1b1a2 | 16270 | 1 | 0.019
U5a1b1b1 | 16192 | 1 | 0.045
U5a1b1b1 | 16270 | 4 | 0.182
U5a1b1c | 16256 | 1 | 0.019
U5a1b1c | 16270 | 1 | 0.019
U5a1b1c1 | 16192 | 1 | 0.019
U5a1b1d | 16256 | 1 | 0.019
U5a1b1d | 16270 | 1 | 0.019
U5a1b1d+16093 | 16270 | 1 | 0.036
U5a1b1d1 | 16399 | 1 | 0.071
U5a1b1e | 16192 | 2 | 0.029
U5a1b1g | 16192 | 1 | 0.021
U5a1b1g | 73 | 1 | 0.021
U5a1b1h | 16291 | 1 | 0.018
U5a1b3a1 | 15218 | 1 | 0.067
U5a1b3a1 | 16362 | 2 | 0.133
U5a1b3a1 | 16399 | 2 | 0.133
U5a1b3a1 | 16428 | 6 | 0.4
U5a1b3a1 | 2706 | 1 | 0.067
U5a1b3a1 | 3197 | 1 | 0.067
U5a1b4 | 16362 | 1 | 0.032
U5a1c | 16270 | 1 | 0.042
U5a1c1 | 16192 | 4 | 0.182
U5a1c1a | 16192 | 5 | 0.333
U5a1c2 | 16270 | 2 | 0.1
U5a1c2a1 | 16399 | 1 | 0.045
U5a1c2a1 | 961 | 12 | 0.545
U5a1d2a | 16192 | 15 | 0.429
U5a1d2a | 16399 | 4 | 0.114
U5a1d2a | 573.XC | 9 | 0.257
U5a1d2a1 | 16145 | 1 | 0.048
U5a1d2a1 | 16192 | 1 | 0.048
U5a1d2a1 | 573.XC | 1 | 0.048
U5a1d2b | 16256 | 1 | 0.04
U5a1d2b | 16399 | 1 | 0.04
U5a1d2b | 573.XC | 9 | 0.36
U5a1e | 16399 | 1 | 0.021
U5a1f1 | 16192 | 2 | 0.067
U5a1f1a | 16399 | 1 | 0.125
U5a1g1 | 16399 | 2 | 0.059
U5a1g2 | 16399 | 2 | 0.051
U5a1h | 16270 | 2 | 0.057
U5a1i | 14893 | 1 | 0.021
U5a1i | 16192 | 3 | 0.062
U5a1i1 | 16399 | 1 | 0.016
U5a1j | 16270 | 1 | 0.022
U5a2 | 14766 | 1 | 0.018
U5a2 | 7028 | 1 | 0.018
U5a2a | 12372 | 1 | 0.029
U5a2a | 1438 | 1 | 0.029
U5a2a | 4769 | 1 | 0.029
U5a2a | 7028 | 1 | 0.029
U5a2a | 8860 | 1 | 0.029
U5a2a1 | 15326 | 1 | 0.013
U5a2a1 | 16192 | 2 | 0.026
U5a2a1 | 16256 | 1 | 0.013
U5a2a1 | 16526 | 2 | 0.026
U5a2a1a | 1438 | 1 | 0.024
U5a2a1a | 16114A | 2 | 0.048
U5a2a1a | 263 | 2 | 0.048
U5a2a1b | 16192 | 1 | 0.026
U5a2a1b | 263 | 1 | 0.026
U5a2a1c | 16270 | 3 | 0.062
U5a2a1c | 16526 | 6 | 0.125
U5a2a1d | 16192 | 2 | 0.056
U5a2a2 | 16270 | 1 | 0.048
U5a2a2a | 750 | 1 | 0.038
U5a2b | 11719 | 1 | 0.012
U5a2b | 13617 | 1 | 0.012
U5a2b | 16192 | 2 | 0.025
U5a2b1 | 16192 | 3 | 0.039
U5a2b1a | 16256 | 2 | 0.031
U5a2b1a | 16270 | 1 | 0.016
U5a2b1b | 16192 | 1 | 0.026
U5a2b2 | 16192 | 1 | 0.017
U5a2b3 | 455d | 2 | 0.105
U5a2b3a | 14684 | 1 | 0.062
U5a2b3a | 455d | 1 | 0.062
U5a2b4a | 16192 | 2 | 0.033
U5a2c1 | 16192 | 1 | 0.015
U5a2c2 | 16526 | 1 | 0.017
U5a2c3a | 16270 | 1 | 0.024
U5a2c4 | 16192 | 2 | 0.033
U5a2c4 | 16256 | 1 | 0.017
U5a2c4 | 16270 | 1 | 0.017
U5a2c4 | 73 | 1 | 0.017
U5a2d | 16192 | 1 | 0.016
U5a2d | 16270 | 2 | 0.033
U5a2d1 | 3750 | 2 | 0.049
U5a2d1a | 3750 | 1 | 0.023
U5a2e | 152 | 1 | 0.031
U5a2e | 15289 | 1 | 0.031
U5a2e | 16192 | 6 | 0.188
U5a2e | 16270 | 2 | 0.062
U5a2e | 16526 | 4 | 0.125
U5a'b | 16192 | 1 | 0.036
U5a'b | 3197 | 1 | 0.036

U5b1 | 16192 | 2 | 0.05
U5b1 | 73 | 1 | 0.025
U5b1a | 15326 | 6 | 0.12
U5b1a | 16192 | 1 | 0.02
U5b1b1 | 16189 | 1 | 0.032
U5b1b1+@16192 | 16189 | 2 | 0.021
U5b1b1+@16192 | 7385 | 2 | 0.021
U5b1b1a | 10927 | 1 | 0.008
U5b1b1a | 150 | 1 | 0.008
U5b1b1a | 16189 | 1 | 0.008
U5b1b1a | 16270 | 1 | 0.008
U5b1b1a | 7385 | 4 | 0.034
U5b1b1a1 | 7385 | 2 | 0.029
U5b1b1a1 | 9477 | 1 | 0.015
U5b1b1a1a | 16144 | 1 | 0.014
U5b1b1a1a | 7385 | 1 | 0.014
U5b1b1a2 | 150 | 1 | 0.015
U5b1b1a2 | 3197 | 1 | 0.015
U5b1b1a3 | 16270 | 1 | 0.026
U5b1b1a3 | 1850 | 1 | 0.026
U5b1b1a3 | 7385 | 1 | 0.026
U5b1b1b | 16192 | 1 | 0.026
U5b1b1e | 16192 | 1 | 0.05
U5b1b1g1 | 16192 | 1 | 0.04
U5b1b1g1a | 10927 | 1 | 0.333
U5b1b1g1a | 16192 | 1 | 0.333
U5b1b2 | 12618 | 1 | 0.021
U5b1b2 | 16189 | 1 | 0.021
U5b1b2 | 16192 | 8 | 0.17
U5b1b2 | 16270 | 1 | 0.021
U5b1b2 | 2706 | 1 | 0.021
U5b1b2a | 16192 | 3 | 0.167
U5b1b2b | 16192 | 1 | 0.1
U5b1c | 16192 | 3 | 0.094
U5b1c1 | 16192 | 1 | 0.034
U5b1c1a | 16192 | 6 | 0.429
U5b1c1a1 | 15191 | 1 | 0.053

U5b1c1a1 | 16192 | 2 | 0.105
U5b1c1a1 | 55A | 1 | 0.053
U5b1c2 | 16192 | 8 | 0.25
U5b1d1 | 16192 | 1 | 0.029
U5b1d1 | 7028 | 1 | 0.029
U5b1d1a | 14766 | 1 | 0.014
U5b1d1b | 16192 | 3 | 0.13
U5b1d1c | 16270 | 1 | 0.034
U5b1d2 | 16192 | 6 | 0.222
U5b1d2 | 16270 | 2 | 0.074
U5b1e1 | 152 | 1 | 0.014
U5b1e1 | 16465 | 5 | 0.068
U5b1e1 | 2706 | 1 | 0.014
U5b1e1 | 5656 | 1 | 0.014
U5b1f1a | 12308 | 1 | 0.005
U5b1f1a | 13617 | 1 | 0.005
U5b1f1a | 1438 | 1 | 0.005
U5b1f1a | 16192 | 1 | 0.005
U5b1f1a | 16270 | 2 | 0.01
U5b1f1a | 7768 | 1 | 0.005
U5b1g | 150 | 1 | 0.04
U5b1g | 2706 | 1 | 0.04
U5b1g | 573.XC | 3 | 0.12
U5b1i | 16192 | 44 | 0.611
U5b1i | 3105 | 37 | 0.514
U5b2 | 13617 | 1 | 0.021
U5b2a+@16192 | 16189 | 1 | 0.017
U5b2a1a | 16192 | 1 | 0.026
U5b2a1a | 73 | 1 | 0.026
U5b2a1a1 | 16192 | 13 | 0.232
U5b2a1a1 | 4769 | 1 | 0.018
U5b2a1a1 | 8860 | 1 | 0.018
U5b2a1a1a | 16192 | 1 | 0.029
U5b2a1a1b | 16192 | 5 | 0.185
U5b2a1a1b | 896 | 1 | 0.037
U5b2a1a1d | 150 | 1 | 0.026
U5b2a1a2 | 8705 | 1 | 0.017

U5b2a1b | 152 | 1 | 0.034
U5b2a1b | 16325 | 1 | 0.034
U5b2a2 | 16192 | 1 | 0.026
U5b2a2a1 | 16192 | 2 | 0.045
U5b2a2b | 16192 | 2 | 0.047
U5b2a2b1 | 16192 | 5 | 0.1
U5b2a2b1 | 16270 | 2 | 0.04
U5b2a2c | 16261 | 2 | 0.08
U5b2a3 | 1438 | 2 | 0.061
U5b2a5a | 16189 | 1 | 0.033
U5b2a5a | 16270 | 1 | 0.033
U5b2a5a | 16311 | 1 | 0.033
U5b2a5a | 73 | 1 | 0.033
U5b2b | 150 | 1 | 0.011
U5b2b | 16270 | 2 | 0.023
U5b2b1a | 12634 | 1 | 0.037
U5b2b1a | 13630 | 1 | 0.037
U5b2b1a | 14766 | 1 | 0.037
U5b2b1a | 8860 | 1 | 0.037
U5b2b1a | 9477 | 1 | 0.037
U5b2b1a1 | 13630 | 1 | 0.032
U5b2b2 | 11467 | 1 | 0.014
U5b2b2 | 12372 | 1 | 0.014
U5b2b2 | 13617 | 1 | 0.014
U5b2b2 | 13630 | 1 | 0.014
U5b2b2 | 13637 | 1 | 0.014
U5b2b2 | 1721 | 1 | 0.014
U5b2b2 | 7768 | 1 | 0.014
U5b2b4a | 150 | 1 | 0.028
U5b2b4a | 16270 | 1 | 0.028
U5b2b4a | 750 | 1 | 0.028
U5b2c | 16192 | 1 | 0.031
U5b2c1 | 16192 | 1 | 0.026
U5b2c1 | 16270 | 1 | 0.026
U5b2c2b | 16192 | 2 | 0.05
U5b3 | 16304 | 2 | 0.038
U5b3a1a | 14182 | 1 | 0.01

U5b3a1a | 16235 | 3 | 0.031
U5b3a1a | 16270 | 3 | 0.031
U5b3a2 | 16235 | 3 | 0.075
U5b3b | 16192 | 1 | 0.024
U5b3b2 | 16270 | 1 | 0.037
U5b3g | 16192 | 2 | 0.049
U5b3h | 12308 | 1 | 0.03
U5b3h | 12372 | 1 | 0.03
U5b3h | 13617 | 2 | 0.061
U5b3h | 14182 | 1 | 0.03
U5b3h | 14766 | 1 | 0.03
U5b3h | 6527 | 2 | 0.061
U6a | 16219 | 1 | 0.043
U6a1a | 73 | 1 | 0.027
U6a1a1 | 11938 | 1 | 0.016
U6a1b1b | 16278 | 1 | 0.043
U6a2+195 | 73 | 1 | 0.048
U6a2a2 | 960d | 1 | 0.067
U6a2b1 | 16354 | 1 | 0.045
U6a3 | 12308 | 1 | 0.029
U6a3b | 152 | 1 | 0.043
U6a3b | 16189 | 1 | 0.043
U6a3b1 | 11467 | 1 | 0.091
U6a3b1 | 16172 | 1 | 0.091
U6a3c | 263 | 1 | 0.045
U6a3c | 291.1A | 1 | 0.045
U6a3d1a | 14766 | 1 | 0.167
U6a3f1 | 185 | 1 | 0.091
U6a3f2 | 16189 | 4 | 0.364
U6a3f2 | 185 | 2 | 0.182
U6a5 | 11467 | 1 | 0.033
U6a5 | 13590 | 1 | 0.033
U6a5c | 16172 | 1 | 0.028
U6a5c | 7805 | 1 | 0.028
U6a6a1 | 16079 | 1 | 0.048
U6a6a1 | 16274 | 1 | 0.048
U6a7a1 | 1193 | 1 | 0.018
U6a7a1 | 8473 | 1 | 0.018
U6a7a1+@152 | 16278 | 1 | 0.043
U6a7a1b | 15530 | 1 | 0.037
U6a7a2 | 2706 | 1 | 0.029
U6a7b | 195 | 2 | 0.167
U6a7b1 | 16172 | 1 | 0.062
U6a7b1 | 195 | 1 | 0.062
U6a7b1 | 960.1C | 3 | 0.188
U6a8b | 16172 | 1 | 0.028
U6a8b | 16189 | 7 | 0.194
U6a8b | 5894C | 7 | 0.194
U6a8b | 9100 | 7 | 0.194
U6a'b'd | 7028 | 1 | 0.053
U6c1 | 16169 | 1 | 0.043
U6c2 | 13879 | 1 | 0.091
U6c2 | 16189 | 1 | 0.091
U6d3a | 14766 | 1 | 0.038
U7 | 14569 | 1 | 0.007
U7 | 152 | 5 | 0.033
U7 | 16318T | 2 | 0.013
U7 | 5360 | 1 | 0.007
U7a | 15326 | 1 | 0.004
U7a | 16318T | 57 | 0.239
U7a | 3741 | 1 | 0.004
U7a | 4769 | 13 | 0.055
U7a1 | 16318T | 1 | 0.125
U7a1a | 5486 | 2 | 0.125
U7a2a | 151 | 5 | 0.152
U7a2a | 16318T | 1 | 0.03
U7a3 | 10142 | 1 | 0.009
U7a3 | 14569 | 1 | 0.009
U7a3 | 14766 | 1 | 0.009
U7a3 | 980 | 1 | 0.009
U7a3a | 12308 | 1 | 0.005
U7a3a | 12372 | 1 | 0.005
U7a3a | 12618 | 2 | 0.009
U7a3a | 13500 | 1 | 0.005
U7a3a | 151 | 6 | 0.028
U7a3a | 152 | 2 | 0.009
U7a3a | 16318T | 13 | 0.062
U7a3a | 1811 | 1 | 0.005
U7a3a | 263 | 1 | 0.005
U7a3a | 2706 | 1 | 0.005
U7a3a | 3741 | 1 | 0.005
U7a3a | 5360 | 1 | 0.005
U7a3a | 8860 | 1 | 0.005
U7a3a | 980 | 1 | 0.005
U7a3b | 151 | 1 | 0.011
U7a3b | 16207 | 4 | 0.045
U7a3b | 16318T | 18 | 0.205
U7a4 | 151 | 4 | 0.103
U7a4 | 152 | 3 | 0.077
U7a4 | 16318T | 5 | 0.128
U7a4a | 16318T | 2 | 0.125
U7a4a1 | 16126 | 2 | 0.059
U7a4a1 | 16318T | 1 | 0.029
U7a4a1a | 151 | 1 | 0.028
U7a4a1a | 16318T | 14 | 0.389
U7b | 10142 | 1 | 0.004
U7b | 11467 | 1 | 0.004
U7b | 11719 | 1 | 0.004
U7b | 1438 | 1 | 0.004
U7b | 14569 | 1 | 0.004
U7b | 152 | 4 | 0.017
U7b | 16318T | 22 | 0.094
U7b | 1811 | 1 | 0.004
U7b | 4769 | 1 | 0.004
U7b | 7028 | 1 | 0.004
U7b | 73 | 1 | 0.004
U7b | 8684 | 1 | 0.004
U7b1 | 16318T | 13 | 0.213
U7b2 | 13500 | 2 | 0.2
U7b2 | 14569 | 2 | 0.2
U7b2 | 291.1A | 2 | 0.2

U7b2 | 3741 | 2 | 0.2
U7b2 | 8137 | 3 | 0.3
U7b2 | 8684 | 2 | 0.2
U7b2 | 980 | 1 | 0.1
U8a | 9365 | 6 | 0.207
U8a1a1 | 263 | 1 | 0.023
U8a1a1 | 5240 | 1 | 0.023
U8a1a1 | 6392 | 1 | 0.023
U8a1a1 | 7055 | 1 | 0.023
U8a1a1 | 9365 | 1 | 0.023
U8a1a1b1 | 12135A | 1 | 0.022
U8a1a1b1 | 5240 | 1 | 0.022
U8a1a2 | 4769 | 2 | 0.061
U8a2 | 195 | 2 | 0.125
U8b1a1 | 12308 | 3 | 0.064
U8b1a1 | 16234 | 12 | 0.255
U8b1a1 | 3480 | 1 | 0.021
U8b1a1 | 750 | 1 | 0.021
U8b1a2+16311 | 16189 | 1 | 0.062
U8b1a2a | 15326 | 1 | 0.071
U8b1a2a | 1811 | 1 | 0.071
U8b1a2a | 195 | 1 | 0.071
U8b1a2a | 3480 | 1 | 0.071
U8b1a2b | 16234 | 9 | 0.281
U8b1b | 16189 | 1 | 0.033
U8b1b | 195 | 5 | 0.167
U8b1b1 | 11719 | 1 | 0.034
U8b1b1 | 263 | 1 | 0.034
U8b1b2 | 195 | 1 | 0.036
U8b1b2 | 263 | 1 | 0.036
U8c | 16189 | 1 | 0.017
U9a | 16051 | 1 | 0.023
U9a1 | 16051 | 1 | 0.111
V | 15326 | 1 | 0.003
V+@16298 | 4580 | 4 | 0.062
V+@72 | 16298 | 1 | 0.005
V18a | 508 | 1 | 0.008
V19 | 150 | 10 | 0.357
V1a | 72 | 7 | 0.032
V1a1 | 1438 | 1 | 0.004
V1a1 | 263 | 3 | 0.013
V1a1 | 72 | 5 | 0.021
V1a1a | 263 | 1 | 0.071
V1a1a1 | 16183 | 2 | 0.118
V1a1a1 | 4639 | 1 | 0.059
V1a1a1 | 8869 | 1 | 0.059
V1a1b | 72 | 3 | 0.015
V2 | 15326 | 1 | 0.005
V2 | 16298 | 1 | 0.005
V2 | 4769 | 1 | 0.005
V2 | 72 | 1 | 0.005
V2 | 750 | 1 | 0.005
V22 | 72 | 2 | 0.016
V2b | 72 | 1 | 0.005
V2c | 72 | 1 | 0.005
V3c | 72 | 5 | 0.106
V7 | 72 | 6 | 0.061
V7a | 15326 | 1 | 0.014
V7a | 16298 | 1 | 0.014
V7a | 72 | 1 | 0.014
V7a1 | 16153 | 3 | 0.042
V7a1 | 7444 | 1 | 0.014
V9a2 | 204 | 15 | 0.682
V9a2 | 263 | 1 | 0.045
V9a2 | 72 | 11 | 0.5
W | 12414 | 1 | 0.017
W | 1243 | 1 | 0.017
W | 1438 | 1 | 0.017
W | 16292 | 6 | 0.102
W | 189 | 2 | 0.034
W | 195 | 6 | 0.102
W | 207 | 3 | 0.051
W | 2706 | 1 | 0.017
W | 3505 | 1 | 0.017
W | 5046 | 1 | 0.017
W | 5460 | 1 | 0.017
W | 709 | 1 | 0.017
W | 73 | 1 | 0.017
W | 8251 | 1 | 0.017
W | 8860 | 1 | 0.017
W+194 | 189 | 3 | 0.048
W+194 | 7028 | 1 | 0.016
W+194 | 8860 | 1 | 0.016
W1 | 16223 | 2 | 0.024
W1 | 16292 | 5 | 0.059
W1 | 195 | 2 | 0.024
W1 | 204 | 6 | 0.071
W1 | 207 | 1 | 0.012
W1 | 263 | 3 | 0.035
W1 | 750 | 1 | 0.012
W1+119 | 204 | 3 | 0.064
W1+119 | 207 | 1 | 0.021
W1a | 11947 | 1 | 0.014
W1a | 15326 | 1 | 0.014
W1a | 16223 | 2 | 0.029
W1a | 195 | 1 | 0.014
W1a | 204 | 1 | 0.014
W1a | 207 | 1 | 0.014
W1a | 2706 | 1 | 0.014
W1b | 16292 | 3 | 0.111
W1b | 195 | 4 | 0.148
W1b1 | 12414 | 1 | 0.029
W1b1 | 15884C | 2 | 0.057
W1b1 | 227 | 1 | 0.029
W1b1 | 8251 | 1 | 0.029
W1c | 16223 | 1 | 0.028
W1c | 16292 | 7 | 0.194
W1c | 189 | 1 | 0.028
W1c | 207 | 2 | 0.056
W1c1 | 119 | 3 | 0.273
W1c1 | 16184 | 1 | 0.091

W1c1 | 207 | 1 | 0.091
W1e | 16223 | 2 | 0.049
W1e1 | 12414 | 1 | 0.031
W1e1 | 16292 | 1 | 0.031
W1e1 | 207 | 3 | 0.094
W1e1 | 5046 | 2 | 0.062
W1f | 207 | 4 | 0.108
W1g | 16292 | 1 | 0.067
W1h | 16292 | 3 | 0.3
W1h1 | 1243 | 1 | 0.1
W1h1 | 5046 | 1 | 0.1
W1i | 119 | 1 | 0.031
W1i | 16223 | 5 | 0.156
W1i | 195 | 3 | 0.094
W3 | 189 | 2 | 0.043
W3 | 207 | 1 | 0.022
W3 | 8251 | 1 | 0.022
W3a | 207 | 1 | 0.019
W3a1 | 16292 | 5 | 0.062
W3a1 | 194 | 1 | 0.012
W3a1 | 195 | 4 | 0.049
W3a1 | 263 | 1 | 0.012
W3a1+199 | 16223 | 3 | 0.081
W3a1+199 | 16292 | 2 | 0.054
W3a1a | 207 | 1 | 0.019
W3a1a1 | 16292 | 1 | 0.022
W3a1a1 | 194 | 2 | 0.043
W3a1a1 | 207 | 1 | 0.022
W3a1a2 | 16223 | 1 | 0.022
W3a1a2 | 16292 | 1 | 0.022
W3a1a2 | 207 | 1 | 0.022
W3a1a3 | 207 | 1 | 0.022
W3a1b | 11947 | 1 | 0.014
W3a1b | 16223 | 2 | 0.028
W3a1b | 16292 | 2 | 0.028
W3a1b | 194 | 1 | 0.014
W3a1b | 195 | 1 | 0.014
W3a1b | 204 | 2 | 0.028
W3a1b | 207 | 1 | 0.014
W3a1b | 5460 | 1 | 0.014
W3a1c | 195 | 2 | 0.057
W3a1d | 12705 | 1 | 0.024
W3a1d | 207 | 2 | 0.048
W3a2 | 194 | 9 | 0.5
W3a2 | 207 | 5 | 0.278
W3b | 16292 | 5 | 0.054
W3b | 194 | 2 | 0.022
W3b | 204 | 1 | 0.011
W3b | 3505 | 2 | 0.022
W3b1 | 16292 | 1 | 0.037
W4 | 16292 | 1 | 0.025
W4 | 194 | 5 | 0.125
W4 | 195 | 3 | 0.075
W4a | 196 | 1 | 0.036
W4a | 204 | 1 | 0.036
W4a1 | 16223 | 2 | 0.014
W4a1 | 3505 | 1 | 0.007
W4b | 1438 | 1 | 0.036
W4c | 8251 | 1 | 0.037
W4d | 207 | 1 | 0.083
W5 | 16223 | 1 | 0.017
W5 | 16292 | 1 | 0.017
W5a | 1243 | 1 | 0.059
W5a | 189 | 1 | 0.059
W5a | 194 | 1 | 0.059
W5a | 207 | 1 | 0.059
W5a | 5046 | 1 | 0.059
W5a | 7028 | 1 | 0.059
W5a | 709 | 1 | 0.059
W5a1a | 16292 | 3 | 0.083
W5a1a | 195 | 1 | 0.028
W5a1a | 204 | 1 | 0.028
W5a1a | 207 | 1 | 0.028
W5a2 | 189 | 1 | 0.059
W5a2 | 194 | 4 | 0.235
W5a2 | 207 | 2 | 0.118
W5a2b | 194 | 1 | 0.5
W5b1a | 58 | 1 | 0.038
W5b1a | 60.2T | 1 | 0.038
W5b1a | 64-65d | 2 | 0.077
W5b1a | 66C | 1 | 0.038
W6 | 11947 | 1 | 0.01
W6 | 12414 | 1 | 0.01
W6 | 1243 | 1 | 0.01
W6 | 16292 | 21 | 0.208
W6 | 16325 | 5 | 0.05
W6 | 189 | 1 | 0.01
W6 | 194 | 12 | 0.119
W6 | 195 | 1 | 0.01
W6 | 207 | 1 | 0.01
W6 | 8251 | 1 | 0.01
W6a | 195 | 1 | 0.016
W6a | 207 | 1 | 0.016
W6b | 16292 | 1 | 0.022
W6b | 194 | 3 | 0.067
W6b | 204 | 3 | 0.067
W6c1 | 189 | 1 | 0.015
W6c1 | 194 | 2 | 0.03
W7 | 204 | 1 | 0.037
W8 | 16223 | 1 | 0.017
W8 | 16292 | 3 | 0.051
W8 | 195 | 1 | 0.017
W8 | 3505 | 1 | 0.017
W9 | 16292 | 1 | 0.019
W9 | 204 | 2 | 0.038
X1a | 153 | 1 | 0.034
X1c | 16223 | 25 | 0.49
X1c | 263 | 1 | 0.02
X1c | 5302 | 1 | 0.02
X2 | 153 | 1 | 0.008
X2 | 16278 | 1 | 0.008

X2+225 | 16189 | 2 | 0.014
X2+225+@153 | 225 | 2 | 0.02
X2+225+@153 | 73 | 1 | 0.01
X2+225+@16223 | 16278 | 5 | 0.278
X2+225+@16223 | 225 | 3 | 0.167
X2+225+@16223 | 750 | 1 | 0.056
X2a1 | 143 | 1 | 0.059
X2a1 | 16093 | 4 | 0.235
X2a1 | 16213 | 1 | 0.059
X2a1a | 16213 | 2 | 0.25
X2a1a1 | 16357 | 1 | 0.067
X2a1a1 | 2706 | 1 | 0.067
X2a1b | 16093 | 1 | 0.071
X2a1c | 16189 | 1 | 0.5
X2b | 15927 | 1 | 0.009
X2b | 225 | 5 | 0.043
X2b+226 | 153 | 6 | 0.036
X2b+226 | 16278 | 4 | 0.024
X2b+226+16192 | 153 | 1 | 0.111
X2b11 | 153 | 1 | 0.01
X2b11 | 226 | 2 | 0.02
X2b11 | 73 | 1 | 0.01
X2b2 | 16278 | 3 | 0.024
X2b2 | 226 | 1 | 0.008
X2b3 | 14818C | 3 | 0.024
X2b4 | 153 | 1 | 0.007
X2b4 | 16189 | 1 | 0.007
X2b4 | 16278 | 1 | 0.007
X2b4 | 195 | 1 | 0.007
X2b4 | 225 | 1 | 0.007
X2b4a | 16278 | 3 | 0.024
X2b4a1 | 153 | 2 | 0.016
X2b4a1 | 16278 | 3 | 0.024
X2b5 | 16223 | 1 | 0.008
X2b6 | 16278 | 3 | 0.024
X2b6a | 153 | 1 | 0.008
X2b6a | 15927 | 1 | 0.008
X2b6a | 16278 | 3 | 0.024
X2b7 | 16278 | 3 | 0.024
X2b8 | 195 | 2 | 0.016
X2b8 | 225 | 1 | 0.008
X2b8 | 226 | 1 | 0.008
X2b9 | 16278 | 3 | 0.024
X2c | 16223 | 2 | 0.091
X2c | 16255 | 1 | 0.045
X2c1 | 153 | 5 | 0.085
X2c1 | 16223 | 1 | 0.017
X2c1 | 227 | 2 | 0.034
X2c1 | 73 | 1 | 0.017
X2c1a | 11719 | 1 | 0.056
X2c1a | 153 | 2 | 0.111
X2c1a | 16189 | 1 | 0.056
X2c1a | 16223 | 1 | 0.056
X2c1a | 16255 | 1 | 0.056
X2c1a | 16278 | 1 | 0.056
X2c1a | 225 | 1 | 0.056
X2c1a | 227 | 2 | 0.111
X2c2 | 16189 | 22 | 0.579
X2c2 | 227 | 1 | 0.026
X2c2 | 73 | 3 | 0.079
X2d1 | 16189 | 1 | 0.009
X2d1 | 16223 | 2 | 0.019
X2d1 | 204 | 1 | 0.009
X2d1 | 207 | 2 | 0.019
X2d1a | 16278 | 1 | 0.008
X2e1a | 153 | 1 | 0.062
X2e1a | 225 | 1 | 0.062
X2e1b | 153 | 22 | 0.71
X2e1b | 16126 | 1 | 0.032
X2e1b | 7028 | 1 | 0.032
X2e2a | 16189 | 1 | 0.009
X2f | 153 | 1 | 0.009
X2h | 153 | 1 | 0.053
X2h | 195 | 1 | 0.053
X2i+@225 | 153 | 3 | 0.15
X2i+@225 | 16189 | 1 | 0.05
X2l | 16223 | 1 | 0.01
X2m1 | 226 | 1 | 0.125
X2m2 | 143 | 1 | 0.143
X2m2 | 6713 | 2 | 0.286
X2o1 | 1656d | 1 | 0.009
X2p | 153 | 7 | 0.061
X4 | 16189 | 1 | 0.034
X4 | 16266 | 5 | 0.172
X4 | 16390 | 1 | 0.034
Y1 | 146 | 1 | 0.01
Y1a | 1438 | 1 | 0.017
Y1a+16189 | 146 | 2 | 0.016
Y1a+16189 | 16231 | 4 | 0.031
Y1b | 16126 | 10 | 0.167
Y1b1 | 16231 | 1 | 0.014
Y2 | 4769 | 1 | 0.007
Y2 | 8392 | 1 | 0.007
Y2 | 8860 | 1 | 0.007
Y2a1 | 16126 | 1 | 0.007
Z | 16185 | 1 | 0.004
Z | 16223 | 3 | 0.012
Z | 249d | 1 | 0.004
Z+152 | 16223 | 1 | 0.004
Z+152 | 73 | 1 | 0.004
Z+152 | 750 | 1 | 0.004
Z1 | 750 | 1 | 0.005
Z1a | 16185 | 1 | 0.008
Z1a1a | 16185 | 4 | 0.073
Z1a1a | 16223 | 1 | 0.018
Z1a1a | 489 | 1 | 0.018
Z1a1a | 8584 | 1 | 0.018
Z1a2a | 151 | 1 | 0.007
Z1a2a | 16260 | 1 | 0.007
Z1a3 | 16298 | 3 | 0.022
Z2 | 16185 | 1 | 0.004

Z2 | 750 | 1 | 0.004
Z3 | 16185 | 9 | 0.031
Z3 | 16223 | 10 | 0.035
Z3 | 4715 | 5 | 0.017
Z3+709 | 152 | 1 | 0.006
Z3+709 | 15301 | 1 | 0.006
Z3+709 | 16298 | 3 | 0.019
Z3a | 152 | 1 | 0.005
Z3a | 16223 | 4 | 0.018
Z3a | 207 | 1 | 0.005
Z3a1 | 16185 | 1 | 0.005
Z3a1 | 16260 | 1 | 0.005
Z3a1a | 207 | 1 | 0.005
Z3a1a | 8931 | 1 | 0.005
Z3a1a | 9090 | 1 | 0.005
Z3a2 | 16185 | 1 | 0.005
Z3a2 | 16260 | 1 | 0.005
Z3a2 | 9090 | 1 | 0.005
Z3b | 146 | 8 | 0.038
Z3b | 16185 | 8 | 0.038
Z3b | 16260 | 2 | 0.009
Z3b | 16298 | 1 | 0.005
Z3c | 16185 | 3 | 0.02
Z3c | 16298 | 5 | 0.034
Z3c | 73 | 1 | 0.007
Z3d | 16185 | 1 | 0.005
Z3d | 16260 | 1 | 0.005
Z3d | 249d | 2 | 0.011
Z3d | 489 | 1 | 0.005
Z4 | 151 | 1 | 0.004
Z4 | 16185 | 1 | 0.004
Z4 | 16260 | 5 | 0.021
Z4 | 16298 | 1 | 0.004
Z4 | 249d | 1 | 0.004
Z4a | 151 | 2 | 0.011
Z4a | 15475 | 1 | 0.006
Z4a | 15944d | 1 | 0.006
Z4a1 | 16189 | 2 | 0.017
Z4a1 | 16302 | 1 | 0.009
Z4a1a | 15944d | 1 | 0.009
Z4a1a | 16189 | 1 | 0.009
Z4a1a | 7196A | 1 | 0.009
Z4a1a1 | 73 | 1 | 0.009
Z7 | 7196A | 1 | 0.005
Z7 | 7471d | 2 | 0.009
Z7 | 8348 | 2 | 0.009

HG, haplogroup
