# Supplementary material for: Mitochondrial Haplogroup Classification of Ancient DNA Samples Using Haplotracker
Source: Biomed Res Int. 2022 Mar 18;2022:5344418. doi: 10.1155/2022/5344418 (PMC8956381; doi:10.1155/2022/5344418)
Supplement: Supplementary Materials — Fig. S1: characterization of Phylotree-provided control region sequences tested for haplogroup classification by Haplotracker. Fig. S2: minimum number of amplicons required by Haplotracker in discriminating between haplogroups using mtDNA control and coding region sequences. Fig. S3: variant identification of an aDNA sample (MNW3) using an HRM real-time PCR. Table S1: haplogroups and their variant profiles extracted from Phylotree mtDNA Build 17. Table S2: haplogroup frequency carrying an extra variant in 118,869 haplotypes. Table S3: haplogroup frequency carrying a missing variant in 118,869 haplotypes. Table S4: haplogroup frequency in 118,869 haplotypes. Table S5: list of ancient human samples found in 2,000-year-old elite Xiongnu cemetery in Northeast Mongolia. Table S6: primers for the amplification of mtDNA coding region segments for haplogroup determination. Table S7: high-resolution melting real-time PCR primer design for screening variants to differentiate haplogroups G1a1, G1a1a, and G1a1b. Table S8: haplogroup classification of full-length mtGenome sequences from Phylotree (n = 8,216). Table S9: haplogroup classification with full-length and control region sequences of mtDNA using Haplotracker and HaploGrep 2. Table S10: comparison of servers using control region sequences from GenBank before December 25, 2018 (n = 45,177). Table S11: comparison details for the servers using control region sequences from GenBank before December 25, 2018 (n = 45,177). Table S12: comparison of servers using control region sequences downloaded from GenBank from December 26, 2018 to August 22, 2019. Table S13: sequences of mtDNA PCR products from Mongolian ancient DNA samples. Table S14: haplogroup classification of Mongolian ancient DNA samples using Haplotracker. Table S15: minimum number of amplicons required by Haplotracker in discriminating between haplogroups using mtDNA control and coding region sequences. Table S16: minimum number of amplicons per superhaplogroup requ [file 5344418.f1.zip › 5344418.f7.pdf]

Table S4. Haplogroup frequency in 118,869 haplotypes

HG	Frequency	HG	Frequency	HG	Frequency
A	36	A23	23	A2am	29
A+152	60	A24	384	A2an	122
A+152+16362	516	A25	25	A2ao	77
A+152+16362+16189	39	A26	14	A2ao1	3
A+152+16362+200	432	A2a	135	A2ap	34
A1	411	A2a1	29	A2aq	3
A10	9	A2a2	123	A2b	76
A11	149	A2a3	50	A2b1	252
A11+16234	106	A2a4	9	A2c	145
A11a	441	A2a5	34	A2d	125
A11b	92	A2aa	32	A2d1	120
A12	28	A2ab	20	A2d1a	16
A12a	25	A2ac	67	A2d2	118
A13	444	A2ac1	16	A2e	6
A14	501	A2ad	9	A2f	125
A15	410	A2ad1	7	A2f1	215
A15a	454	A2ad2	8	A2f1a	222
A15b	25	A2ae	34	A2f2	6
A15c	414	A2af	1	A2f3	35
A15c1	425	A2af1	1	A2g	15
A16	17	A2af1a	7	A2g1	18
A17	582	A2af1a1	8	A2h	24
A18	408	A2af1a2	2	A2h1	16
A19	107	A2af1b	4	A2i	21
A1a	40	A2af1b1	2	A2j	120
A1a1	40	A2af1b1a	2	A2j1	119
A2	108	A2af1b1b	2	A2k	125
A2+(64)	319	A2af1b2	3	A2k1	115
A2+(64)+@153	102	A2af2	1	A2k1a	129
A2+(64)+@16111	441	A2ag	62	A2l	391
A2+(64)+16129	44	A2ah	11	A2m	39
A2+(64)+16189	36	A2ai	11	A2n	385
A20	411	A2aj	8	A2o	396
A21	514	A2ak	10	A2p	29
A22	402	A2al	17	A2p1	25

A2p2 | 32
A2q | 28
A2q1 | 24
A2r | 67
A2r1 | 37
A2s | 2
A2t | 121
A2u | 22
A2u1 | 15
A2u2 | 9
A2v | 4
A2v1 | 9
A2v1+152 | 10
A2v1a | 14
A2v1b | 3
A2w | 128
A2w1 | 27
A2x | 114
A2y | 18
A2z | 15
A3 | 36
A3a | 37
A5 | 28
A5a | 36
A5a1 | 30
A5a1a | 38
A5a1a1 | 55
A5a1a1a | 30
A5a1a1b | 30
A5a1a2 | 33
A5a1a2a | 33
A5a1b | 6
A5a2 | 33
A5a3 | 32
A5a3a | 30
A5a4 | 31
A5a5 | 32
A5b | 30
A5b1 | 29
A5b1a | 28
A5b1b | 47
A5b1c | 26
A5b1c1 | 38
A5c | 23
A5c1 | 25
A6 | 432
A6a | 392
A6b | 378
A7 | 29
A8 | 16
A8a | 37
A8a1 | 31
A9 | 42
B2 | 475
B2+16278 | 41
B2a | 72
B2a1 | 91
B2a1a | 75
B2a1a1 | 77
B2a1b | 96
B2a2 | 75
B2a3 | 5
B2a4 | 8
B2a4a | 7
B2a4a1 | 12
B2a5 | 19
B2b | 246
B2b+152 | 132
B2b1 | 2
B2b2 | 29
B2b2a | 2
B2b3 | 107
B2b3a | 39
B2b4 | 48
B2c | 104
B2c1 | 96
B2c1a | 117
B2c1b | 93
B2c1c | 92
B2c2 | 93
B2c2a | 14
B2c2b | 13
B2d | 89
B2e | 129
B2f | 98
B2g | 44
B2g1 | 15
B2g2 | 45
B2h | 28
B2i | 127
B2i1 | 30
B2i2 | 52
B2i2a | 5
B2i2a1 | 9
B2i2a1a | 11
B2i2a1b | 9
B2i2b | 71
B2i2b1 | 11
B2j | 6
B2k | 73
B2l | 24
B2m | 5
B2n | 116
B2o | 73
B2o1 | 6
B2o1a | 21
B2p | 92
B2q | 119
B2r | 93
B2s | 8
B2t | 13

B2u | 5
B2v | 11
B2w | 8
B2x | 6
B2y | 192
B2y1 | 215
B4 | 123
B4+16261 | 266
B4'5 | 122
B4a | 281
B4a1 | 263
B4a1+16311 | 98
B4a1a | 253
B4a1a1 | 777
B4a1a1+151 | 357
B4a1a1+152 | 373
B4a1a1+16126 | 24
B4a1a1a | 585
B4a1a1a+195 | 359
B4a1a1a1 | 435
B4a1a1a10 | 428
B4a1a1a11 | 427
B4a1a1a11a | 427
B4a1a1a11b | 85
B4a1a1a12 | 12
B4a1a1a13 | 428
B4a1a1a14 | 19
B4a1a1a15 | 428
B4a1a1a16 | 39
B4a1a1a17 | 428
B4a1a1a18 | 57
B4a1a1a19 | 9
B4a1a1a1a | 372
B4a1a1a1a1 | 377
B4a1a1a1b | 430
B4a1a1a1c | 354
B4a1a1a1d | 352
B4a1a1a2 | 34
B4a1a1a20 | 26
B4a1a1a21 | 428
B4a1a1a22 | 431
B4a1a1a23 | 358
B4a1a1a2a | 35
B4a1a1a2b | 8
B4a1a1a3 | 427
B4a1a1a4 | 16
B4a1a1a5 | 431
B4a1a1a6 | 425
B4a1a1a7 | 468
B4a1a1a8 | 354
B4a1a1a9 | 14
B4a1a1aa | 53
B4a1a1ab | 435
B4a1a1ac | 432
B4a1a1ad | 431
B4a1a1ae | 428
B4a1a1af | 7
B4a1a1b | 1013
B4a1a1c | 502
B4a1a1d | 437
B4a1a1e | 430
B4a1a1f | 429
B4a1a1g | 435
B4a1a1h | 392
B4a1a1i | 427
B4a1a1j | 24
B4a1a1k | 26
B4a1a1k1 | 9
B4a1a1m | 354
B4a1a1m1 | 465
B4a1a1n | 430
B4a1a1o | 366
B4a1a1p | 429
B4a1a1q | 7
B4a1a1r | 7
B4a1a1s | 445
B4a1a1t | 428
B4a1a1u | 427
B4a1a1v | 431
B4a1a1w | 438
B4a1a1x | 404
B4a1a1y | 427
B4a1a1z | 425
B4a1a2 | 201
B4a1a3 | 200
B4a1a3a | 34
B4a1a3a1 | 36
B4a1a3a1a | 46
B4a1a4 | 197
B4a1a5 | 197
B4a1a5a | 303
B4a1a6 | 200
B4a1a6a | 197
B4a1a7 | 203
B4a1b | 53
B4a1b1 | 62
B4a1b1a | 61
B4a1c | 247
B4a1c+146 | 199
B4a1c1 | 239
B4a1c1a | 242
B4a1c1a1 | 186
B4a1c2 | 31
B4a1c3 | 62
B4a1c3a | 13
B4a1c3b | 68
B4a1c4 | 253
B4a1c5 | 201
B4a1d | 259
B4a1e | 96
B4a2 | 263

B4a2a | 72
B4a2a1 | 64
B4a2a2 | 59
B4a2a3 | 97
B4a2b | 73
B4a2b1 | 71
B4a2b1a | 67
B4a3 | 87
B4a4 | 151
B4a5 | 266
B4b | 109
B4b1 | 173
B4b1a | 157
B4b1a+207 | 162
B4b1a1 | 31
B4b1a1a | 23
B4b1a1b | 20
B4b1a1c | 12
B4b1a2 | 190
B4b1a2a | 281
B4b1a2b | 13
B4b1a2b1 | 12
B4b1a2b2 | 12
B4b1a2c | 9
B4b1a2d | 8
B4b1a2e | 166
B4b1a2f | 157
B4b1a2g | 24
B4b1a2g1 | 24
B4b1a2h | 166
B4b1a2i | 25
B4b1a3 | 143
B4b1a3a | 40
B4b1b | 19
B4b1b'c | 68
B4b1c | 68
B4b1c1 | 48
B4b1c2 | 50
B4b'd'e'j | 83
B4c | 126
B4c1 | 88
B4c1a | 101
B4c1a1 | 43
B4c1a1a | 30
B4c1a1a1 | 31
B4c1a1a1a | 33
B4c1a1a2 | 31
B4c1a1b | 79
B4c1a1c | 51
B4c1a2 | 67
B4c1a2a | 32
B4c1a'b | 76
B4c1b | 63
B4c1b+16335 | 144
B4c1b1 | 1
B4c1b1a | 3
B4c1b2 | 148
B4c1b2a | 168
B4c1b2a1 | 174
B4c1b2a2 | 156
B4c1b2a2a | 144
B4c1b2a2b | 140
B4c1b2b | 138
B4c1b2c | 144
B4c1b2c1 | 46
B4c1b2c2 | 94
B4c1c | 81
B4c1c+16311 | 31
B4c1c1 | 38
B4c2 | 134
B4c2a | 150
B4c2b | 150
B4c2c | 148
B4d | 65
B4d1 | 134
B4d1'2'3 | 157
B4d1a | 65
B4d2 | 65
B4d3 | 7
B4d3a | 3
B4d3a1 | 14
B4d4 | 22
B4e | 79
B4f | 6
B4f1 | 6
B4g | 55
B4g1 | 145
B4g1a | 74
B4g1b | 73
B4g2 | 79
B4h | 170
B4h1 | 101
B4i | 272
B4i1 | 253
B4j | 31
B4k | 117
B4m | 104
B5 | 54
B5a | 512
B5a1 | 536
B5a1a | 674
B5a1a1 | 454
B5a1b | 473
B5a1b1 | 528
B5a1c | 486
B5a1c1 | 454
B5a1c1a | 439
B5a1c1a1 | 51
B5a1c2 | 456
B5a1d | 290
B5a2 | 63

B5a2a | 15
B5a2a1 | 1
B5a2a1+16129 | 11
B5a2a1a | 38
B5a2a1b | 14
B5a2a2 | 18
B5a2a2a | 5
B5a2a2a1 | 18
B5a2a2a2 | 12
B5a2a2b | 15
B5a2a2b1 | 17
B5a2a2b1a | 19
B5a2a2b2 | 3
B5b | 170
B5b1 | 275
B5b1a | 171
B5b1a1 | 7
B5b1a2 | 18
B5b1a2a | 12
B5b1c | 182
B5b1c1 | 172
B5b1c1a | 178
B5b2 | 22
B5b2+@204 | 31
B5b2a | 17
B5b2a1 | 29
B5b2a2 | 17
B5b2a2a | 20
B5b2a2a1 | 12
B5b2a2a2 | 13
B5b2b | 19
B5b2c | 24
B5b2c1 | 23
B5b3 | 176
B5b3a | 7
B5b3b | 175
B5b4 | 16
B5b5 | 13
B6 | 81
B6a | 101
B6a1 | 26
B6a1a | 71
C | 253
C1 | 206
C1a | 134
C1b | 266
C1b+16311 | 29
C1b1 | 73
C1b10 | 10
C1b11 | 100
C1b12 | 72
C1b13 | 38
C1b13a | 39
C1b13a1 | 38
C1b13b | 37
C1b13c | 36
C1b13c1 | 41
C1b13d | 13
C1b13e | 45
C1b14 | 10
C1b2 | 76
C1b3 | 88
C1b4 | 14
C1b5 | 136
C1b5a | 73
C1b5b | 13
C1b6 | 3
C1b7 | 49
C1b7a | 17
C1b8 | 38
C1b8a | 10
C1b9 | 60
C1c | 251
C1c+195 | 135
C1c1 | 150
C1c1a | 138
C1c1b | 138
C1c2 | 145
C1c3 | 108
C1c4 | 108
C1c5 | 129
C1c6 | 111
C1c7 | 103
C1c8 | 103
C1d | 157
C1d+194 | 115
C1d1 | 181
C1d1a | 110
C1d1a1 | 113
C1d1b | 17
C1d1b1 | 17
C1d1c | 105
C1d1c1 | 116
C1d1d | 118
C1d2 | 110
C1d2a | 4
C1d3 | 105
C1e | 6
C1f | 122
C4 | 247
C4+152 | 210
C4+152+16093 | 127
C4a | 251
C4a1 | 394
C4a1a | 363
C4a1a+195 | 358
C4a1a1 | 361
C4a1a1a | 393
C4a1a2 | 344
C4a1a2a | 319
C4a1a3 | 364

C4a1a3a | 332
C4a1a3a1 | 321
C4a1a3b | 319
C4a1a3c | 328
C4a1a3d | 143
C4a1a4 | 331
C4a1a4a | 162
C4a1a5 | 356
C4a1a6 | 359
C4a1b | 147
C4a2 | 192
C4a2a | 132
C4a2a1 | 204
C4a2a1a | 164
C4a2a1b | 164
C4a2b | 201
C4a2b1 | 132
C4a2b2 | 238
C4a2b2a | 263
C4a2c | 192
C4a2c1 | 121
C4a2c2 | 134
C4a2c2a | 128
C4a'b'c | 251
C4b | 281
C4b1 | 273
C4b1a | 20
C4b1b | 242
C4b2 | 137
C4b2a | 151
C4b3 | 152
C4b3a | 155
C4b3a1 | 133
C4b3b | 132
C4b5 | 253
C4b6 | 254
C4b7 | 248
C4b8 | 252
C4b8a | 188
C4c | 252
C4c1 | 263
C4c1a | 132
C4c1b | 223
C4c2 | 78
C4d | 205
C4e | 182
C5 | 46
C5+16093 | 37
C5a | 52
C5a1 | 73
C5a2 | 62
C5a2a | 65
C5a2b | 65
C5a2b1 | 59
C5b | 38
C5b1 | 50
C5b1a | 45
C5b1a1 | 50
C5b1b | 38
C5b1b1 | 45
C5c | 29
C5c+16234 | 26
C5c1 | 28
C5c1a | 38
C5d | 29
C5d1 | 62
C5d2 | 33
C7 | 264
C7+16051 | 137
C7a | 324
C7a1 | 273
C7a1a | 180
C7a1a1 | 112
C7a1a2 | 116
C7a1c | 243
C7a1d | 110
C7a2 | 240
C7a2a | 223
C7b | 156
CZ | 147
D | 711
D+16189 | 192
D1 | 437
D1a | 226
D1a1 | 161
D1a2 | 257
D1b | 220
D1c | 219
D1d | 219
D1d1 | 220
D1d2 | 181
D1e | 223
D1f | 202
D1f+16189 | 172
D1f1 | 177
D1f2 | 127
D1f3 | 171
D1g | 175
D1g+16189 | 181
D1g1 | 107
D1g1a | 111
D1g1b | 115
D1g2 | 104
D1g2a | 16
D1g3 | 179
D1g4 | 102
D1g5 | 6
D1g6 | 104
D1h | 170
D1h1 | 108
D1h2 | 165

D1i | 102
D1i1 | 79
D1i2 | 48
D1j | 156
D1j1 | 146
D1j1a | 151
D1j1a1 | 148
D1j1a2 | 122
D1k | 227
D1m | 165
D1n | 219
D2 | 146
D2a | 81
D2a1 | 79
D2a1a | 109
D2a1b | 73
D2a2 | 89
D2a'b | 79
D2b | 24
D2b1 | 30
D2b1a | 28
D2b2 | 24
D2c | 64
D3 | 70
D4 | 836
D4+195 | 473
D4a | 290
D4a+16294 | 129
D4a1 | 299
D4a1a | 289
D4a1a1 | 289
D4a1a1a | 277
D4a1b | 292
D4a1b1 | 130
D4a1c | 279
D4a1d | 129
D4a1e | 145
D4a1e1 | 143
D4a1f | 289
D4a1f1 | 282
D4a1g | 282
D4a1h | 284
D4a2 | 290
D4a2a | 284
D4a2b | 290
D4a3 | 136
D4a3a | 136
D4a3a1 | 109
D4a3a2 | 160
D4a3b | 139
D4a3b1 | 129
D4a3b2 | 207
D4a4 | 24
D4a5 | 274
D4a6 | 165
D4a7 | 222
D4a8 | 126
D4b | 712
D4b1 | 203
D4b1a | 204
D4b1a1 | 178
D4b1a1a | 194
D4b1a2 | 205
D4b1a2a | 205
D4b1a2a1 | 237
D4b1a2a2 | 205
D4b1b | 62
D4b1b1 | 51
D4b1b1a | 43
D4b1b1a1 | 60
D4b1b2 | 58
D4b1b'd | 167
D4b1c | 143
D4b1d | 132
D4b2 | 714
D4b2a | 712
D4b2a1 | 113
D4b2a2 | 714
D4b2a2a | 252
D4b2a2a1 | 220
D4b2a2a2 | 249
D4b2a2b | 716
D4b2b | 851
D4b2b1 | 821
D4b2b1+146 | 539
D4b2b1a | 800
D4b2b1b | 796
D4b2b1c | 795
D4b2b1d | 534
D4b2b2 | 801
D4b2b2a | 564
D4b2b2a1 | 566
D4b2b2b | 211
D4b2b2c | 182
D4b2b3 | 811
D4b2b4 | 565
D4b2b5 | 534
D4b2b6 | 790
D4b2b7 | 173
D4b2d | 188
D4c | 79
D4c1 | 75
D4c1a | 27
D4c1a1 | 27
D4c1b | 71
D4c1b1 | 30
D4c1b2 | 69
D4c2 | 111
D4c2a | 72
D4c2b | 86
D4c2c | 87

D4d | 221
D4e | 622
D4e1 | 682
D4e1'3 | 621
D4e1a | 525
D4e1a1 | 30
D4e1a2 | 483
D4e1a2a | 474
D4e1a3 | 513
D4e1c | 668
D4e2 | 654
D4e2a | 516
D4e2b | 622
D4e2c | 625
D4e2d | 622
D4e3 | 636
D4e4 | 625
D4e4a | 640
D4e4a1 | 83
D4e4b | 623
D4e5 | 138
D4e5a | 114
D4e5b | 83
D4f | 622
D4f1 | 636
D4g | 621
D4g1 | 107
D4g1a | 97
D4g1b | 102
D4g1c | 102
D4g2 | 443
D4g2a | 111
D4g2a1 | 129
D4g2a1a | 11
D4g2a1b | 124
D4g2a1c | 117
D4g2b | 437
D4g2b1 | 438
D4g2b1a | 427
D4h | 627
D4h1 | 136
D4h1a | 15
D4h1a1 | 12
D4h1a2 | 16
D4h1b | 87
D4h1c | 92
D4h1c1 | 34
D4h1d | 68
D4h2 | 67
D4h3 | 31
D4h3a | 40
D4h3a+@152 | 67
D4h3a1 | 26
D4h3a1a | 23
D4h3a1a1 | 10
D4h3a1a2 | 29
D4h3a2 | 25
D4h3a3 | 4
D4h3a3a | 9
D4h3a4 | 28
D4h3a5 | 37
D4h3a6 | 6
D4h3a7 | 66
D4h3a8 | 69
D4h3a9 | 68
D4h3b | 24
D4h4 | 625
D4h4a | 164
D4i | 151
D4i1 | 69
D4i2 | 161
D4i3 | 18
D4j | 647
D4j+(16286) | 663
D4j+146 | 462
D4j+16311 | 214
D4j1 | 627
D4j10 | 629
D4j11 | 193
D4j12 | 631
D4j13 | 434
D4j14 | 71
D4j15 | 623
D4j16 | 623
D4j1a | 189
D4j1a1 | 234
D4j1a1a | 190
D4j1a1b | 189
D4j1a2 | 98
D4j1b | 634
D4j1b2 | 436
D4j2 | 79
D4j2a | 46
D4j3 | 271
D4j3a | 252
D4j3a1 | 133
D4j4 | 625
D4j4a | 72
D4j5 | 623
D4j5a | 634
D4j6 | 446
D4j7 | 70
D4j7a | 71
D4j8 | 107
D4j9 | 643
D4k | 177
D4l | 77
D4l1 | 81
D4l1a | 66
D4l1a1 | 70
D4l2 | 26

D4l2a | 22
D4l2a1 | 9
D4l2a2 | 11
D4l2b | 20
D4m | 630
D4m1 | 71
D4m2 | 157
D4m2a | 84
D4m2a1 | 72
D4m2a1a | 76
D4n | 98
D4n1 | 95
D4n1a | 97
D4n2 | 61
D4o | 27
D4o1 | 146
D4o1a | 29
D4o2 | 16
D4o2a | 55
D4o2a1 | 31
D4p | 434
D4p1 | 431
D4q | 120
D4q1 | 3
D4q1a | 8
D4s | 473
D4t | 449
D5 | 101
D5a | 113
D5a1 | 16
D5a1a | 38
D5a1a1 | 20
D5a1a2 | 17
D5a2 | 108
D5a2a | 218
D5a2a+16092 | 93
D5a2a1 | 110
D5a2a1+@16172 | 181
D5a2a1a | 17
D5a2a1a1 | 13
D5a2a1a1a | 13
D5a2a1a2 | 20
D5a2a1b | 96
D5a2a1b1 | 45
D5a2a2 | 101
D5a2b | 59
D5a3 | 69
D5a3a | 53
D5a3a1 | 30
D5a3a1a | 30
D5a'b | 100
D5b | 138
D5b1 | 139
D5b1a | 140
D5b1a1 | 35
D5b1a2 | 20
D5b1b | 138
D5b1b1 | 28
D5b1b2 | 56
D5b1c | 147
D5b1c1 | 51
D5b1c1a | 136
D5b1d | 115
D5b2 | 4
D5b3 | 143
D5b3a | 145
D5b3a1 | 44
D5b4 | 105
D5c | 3
D5c+16311 | 14
D5c1 | 6
D5c1a | 36
D5c2 | 17
D6 | 93
D6a | 70
D6a1 | 98
D6a1a | 99
D6a2 | 82
D6c | 67
D6c1 | 83
D6c1a | 81
E | 223
E1 | 225
E1a | 223
E1a1 | 227
E1a1a | 229
E1a1a1 | 600
E1a1a1a | 348
E1a1a1b | 249
E1a1a1b1 | 246
E1a1a1b2 | 250
E1a1a1c | 254
E1a1b | 171
E1a1b1 | 171
E1a1b2 | 105
E1a1b3 | 105
E1a1b4 | 102
E1a1c | 233
E1a2 | 101
E1a2+(16261) | 190
E1a2a | 188
E1a2a1 | 188
E1a2a2 | 24
E1a2a3 | 188
E1a2a4 | 162
E2 | 182
E2a | 239
E2a1 | 181
E2a1a | 182
E2a2 | 180
E2b | 142

E2b1 | 120
E2b2 | 121
F | 192
F1 | 192
F1+16189 | 385
F1a | 361
F1a1 | 328
F1a1'4 | 376
F1a1a | 638
F1a1a1 | 580
F1a1b | 75
F1a1c | 164
F1a1c1 | 18
F1a1c2 | 122
F1a1c3 | 120
F1a1d | 209
F1a1d1 | 185
F1a2 | 148
F1a2a | 69
F1a3 | 399
F1a3+16311 | 184
F1a3a | 163
F1a3a1 | 46
F1a3a1a | 39
F1a3a2 | 12
F1a3a3 | 168
F1a3a3a | 154
F1a3b | 200
F1a4 | 382
F1a4a | 52
F1a4a1 | 297
F1a4b | 216
F1a'c'f | 93
F1b | 84
F1b1 | 84
F1b1+@152 | 195
F1b1a | 28
F1b1a1 | 31
F1b1a1a | 36
F1b1a1a1 | 38
F1b1a1a1a | 37
F1b1a1a2 | 32
F1b1a1a3 | 29
F1b1a2 | 29
F1b1b | 50
F1b1c | 101
F1b1d | 92
F1b1e | 191
F1b1e1 | 174
F1b1f | 169
F1c | 62
F1c1 | 26
F1c1a | 31
F1c1a1 | 171
F1c1a1a | 150
F1c1a1b | 161
F1c1a2 | 109
F1d | 364
F1d1 | 430
F1e | 354
F1e1 | 406
F1e1a | 81
F1e2 | 14
F1e3 | 70
F1f | 164
F1g | 382
F1g1 | 410
F2 | 184
F2+16291 | 78
F2+195 | 155
F2a | 138
F2a+@16291 | 55
F2a1 | 117
F2b | 45
F2b1 | 186
F2c | 193
F2c1 | 383
F2c2 | 37
F2d | 59
F2e | 204
F2e1 | 2
F2f | 189
F2g | 86
F2h | 165
F2i | 32
F3 | 33
F3a | 61
F3a+207 | 51
F3a1 | 209
F3b | 43
F3b+152 | 28
F3b1 | 27
F3b1a | 5
F3b1a+16093 | 43
F3b1a1 | 4
F3b1a2 | 60
F3b1b | 161
F3b1b1 | 27
F4 | 192
F4a | 12
F4a1 | 13
F4a1a | 23
F4a1b | 24
F4a2 | 34
F4b | 80
F4b1 | 141
G | 632
G1 | 628
G1a | 439
G1a1 | 103
G1a1a | 111

G1a1a1 | 106
G1a1a2 | 90
G1a1a3 | 25
G1a1a4 | 86
G1a1b | 94
G1a2 | 43
G1a2'3 | 28
G1a3 | 69
G1b | 164
G1b+16129 | 152
G1b1 | 96
G1b2 | 164
G1b3 | 82
G1b4 | 164
G1c | 611
G1c1 | 91
G1c2 | 617
G2 | 628
G2a | 241
G2a+152 | 175
G2a1 | 342
G2a1+16189 | 146
G2a1+16189+16194 | 66
G2a1b | 94
G2a1c | 81
G2a1c1 | 71
G2a1c2 | 72
G2a1d | 44
G2a1d1 | 32
G2a1d1a | 45
G2a1d2 | 95
G2a1d2a | 95
G2a1e | 72
G2a1f | 86
G2a1f1 | 8
G2a1g | 5
G2a1h | 98
G2a2 | 175
G2a2a | 87
G2a3 | 2
G2a3a | 4
G2a4 | 51
G2a5 | 75
G2a'c | 600
G2b | 633
G2b1 | 625
G2b1a | 604
G2b1a1 | 447
G2b1a2 | 48
G2b1b | 120
G2b2 | 632
G2b2a | 626
G2b2b | 89
G2b2c | 74
G2c | 446
G3 | 142
G3a | 142
G3a1 | 113
G3a1'2 | 117
G3a1a | 112
G3a2 | 97
G3a2+152 | 101
G3a2a | 16
G3a3 | 90
G3b | 189
G3b1 | 127
G3b2 | 153
G4 | 30
H | 1075
H+13708 | 788
H+152 | 235
H+16129 | 268
H+16291 | 164
H+195 | 78
H+195+146 | 41
H1 | 1200
H1+152 | 302
H1+16189 | 306
H1+16239 | 127
H1+16278 | 108
H1+16311 | 409
H1+16355 | 91
H10 | 794
H10+(16093) | 881
H100 | 780
H101 | 100
H102 | 774
H103 | 24
H104 | 773
H104a | 50
H105 | 773
H105a | 754
H106 | 30
H107 | 25
H108 | 32
H10a | 792
H10a1 | 80
H10a1a | 70
H10a1a1 | 71
H10a1b | 40
H10b | 793
H10b1 | 787
H10c | 779
H10c1 | 783
H10d | 384
H10e | 124
H10e1 | 98
H10e1a | 64
H10e2 | 91
H10e3 | 85
H10e3a | 87

H10f | 872
H10g | 65
H10h | 788
H11 | 142
H11a | 74
H11a+152 | 21
H11a1 | 84
H11a2 | 58
H11a2a | 23
H11a2a1 | 13
H11a2a2 | 23
H11a2a3 | 12
H11a3 | 11
H11a4 | 10
H11a5 | 38
H11a6 | 11
H11a7 | 14
H11a8 | 38
H11b | 141
H11b1 | 145
H12 | 31
H12a | 30
H13 | 790
H13a | 786
H13a1 | 785
H13a1+152 | 143
H13a1a | 817
H13a1a1 | 820
H13a1a1a | 815
H13a1a1b | 788
H13a1a1c | 791
H13a1a1d | 68
H13a1a1d1 | 69
H13a1a1e | 791
H13a1a2 | 790
H13a1a2+16311 | 325
H13a1a2a | 103
H13a1a2b | 48
H13a1a3 | 790
H13a1a4 | 789
H13a1a5 | 786
H13a1a6 | 13
H13a1b | 30
H13a1c | 73
H13a1d | 50
H13a2 | 780
H13a2a | 800
H13a2a1 | 781
H13a2b | 780
H13a2b1 | 331
H13a2b2 | 784
H13a2b2a | 789
H13a2b3 | 9
H13a2b4 | 784
H13a2b5 | 92
H13a2c | 54
H13a2c1 | 21
H13b | 854
H13b1 | 98
H13b1+200 | 30
H13b1a | 37
H13b1b | 9
H13b2 | 857
H13c | 785
H13c1 | 788
H13c1a | 789
H13c2 | 11
H14 | 794
H14a | 109
H14a+146 | 23
H14a1 | 30
H14a2 | 82
H14a2a | 59
H14a2b | 81
H14a2c | 82
H14b | 931
H14b1 | 98
H14b2 | 922
H14b2a | 8
H14b3 | 98
H14b4 | 922
H15 | 35
H15a | 35
H15a1 | 62
H15a1a | 37
H15a1a1 | 41
H15a1b | 55
H15b | 51
H15b1 | 41
H15b2 | 37
H16 | 802
H16+152 | 157
H16a | 151
H16a1 | 144
H16b | 808
H16c | 150
H16d | 148
H16e | 794
H17 | 266
H17a | 274
H17a1 | 66
H17a2 | 253
H17b | 259
H17c | 73
H18 | 803
H18b | 800
H19 | 13
H1a | 200
H1a1 | 163
H1a1a | 98
H1a1a1 | 104

H1a1b | 104
H1a1c | 91
H1a2 | 133
H1a3 | 123
H1a3a | 92
H1a3a1 | 81
H1a3a2 | 30
H1a3a3 | 78
H1a3a4 | 45
H1a3b | 104
H1a3b1 | 83
H1a3c | 91
H1a3c1 | 109
H1a3d | 82
H1a4 | 106
H1a5 | 104
H1a6 | 20
H1a7 | 106
H1a8 | 106
H1a8a | 106
H1a9 | 74
H1aa | 271
H1aa1 | 211
H1ab | 213
H1ab1 | 93
H1ac | 201
H1ad | 202
H1ae | 797
H1ae1 | 801
H1ae2 | 797
H1ae2a | 798
H1ae3 | 797
H1ae3a | 798
H1af | 94
H1af1 | 84
H1af1a | 80
H1af1b | 86
H1af2 | 79
H1ag | 797
H1ag1 | 809
H1ag1a | 80
H1ag1b | 797
H1ah | 795
H1ah1 | 90
H1ah2 | 71
H1ai | 794
H1ai1 | 799
H1aj | 803
H1aj1 | 83
H1aj1a | 5
H1ak | 800
H1ak1 | 797
H1ak2 | 801
H1am | 796
H1am1 | 797
H1an | 145
H1an1 | 146
H1an1a | 153
H1an2 | 20
H1ao | 30
H1ao1 | 24
H1ap | 796
H1ap1 | 106
H1aq | 799
H1aq1 | 81
H1ar | 40
H1ar1 | 3
H1as | 797
H1as1 | 77
H1as1a | 31
H1as2 | 797
H1at | 796
H1at1 | 799
H1at1a | 801
H1au | 90
H1au1 | 77
H1au1a | 79
H1au1b | 80
H1av | 796
H1av1 | 84
H1av1a | 73
H1aw | 144
H1aw1 | 150
H1ax | 800
H1ax1 | 798
H1ay | 796
H1az | 816
H1b | 175
H1b1 | 136
H1b1+16362 | 139
H1b1a | 116
H1b1b | 80
H1b1c | 100
H1b1d | 41
H1b1e | 135
H1b1e1 | 135
H1b1f | 82
H1b1g | 26
H1b1h | 101
H1b1i | 135
H1b2 | 104
H1b2a | 3
H1b2a1 | 3
H1b3 | 87
H1b4 | 135
H1b5 | 125
H1ba | 169
H1ba1 | 83
H1bb | 177
H1bc | 151
H1bd | 797

H1be | 799
H1bf | 88
H1bf1 | 74
H1bg | 80
H1bh | 89
H1bi | 797
H1bj | 796
H1bk | 802
H1bm | 797
H1bn | 799
H1bo | 91
H1bp | 797
H1bq | 794
H1br | 798
H1bs | 111
H1bt | 150
H1bt1 | 143
H1bu | 796
H1bv | 795
H1bv1 | 173
H1bw | 799
H1bx | 782
H1bz | 79
H1c | 492
H1c+152 | 92
H1c1 | 145
H1c1+16093 | 49
H1c10 | 398
H1c11 | 399
H1c12 | 398
H1c13 | 403
H1c14 | 397
H1c15 | 396
H1c16 | 398
H1c17 | 397
H1c18 | 398
H1c19 | 399
H1c1a | 51
H1c1a1 | 51
H1c1b | 39
H1c1c | 41
H1c1d | 50
H1c2 | 405
H1c20 | 397
H1c21 | 397
H1c22 | 399
H1c2a | 398
H1c3 | 59
H1c3a | 18
H1c3b | 87
H1c4 | 397
H1c4a | 397
H1c4a1 | 400
H1c4b | 40
H1c4b1 | 38
H1c5 | 398
H1c5a | 26
H1c6 | 399
H1c7 | 400
H1c8 | 399
H1c9 | 72
H1c9a | 80
H1ca | 805
H1cc | 25
H1cd | 90
H1cf | 98
H1cg | 86
H1ch | 88
H1ci | 796
H1cj | 266
H1ck | 78
H1e | 819
H1e+16129 | 261
H1e1 | 811
H1e1a | 839
H1e1a+16278 | 82
H1e1a1 | 84
H1e1a2 | 798
H1e1a3 | 83
H1e1a4 | 328
H1e1a5 | 83
H1e1a6 | 82
H1e1a7 | 801
H1e1a8 | 25
H1e1b | 384
H1e1b1 | 389
H1e1b1a | 386
H1e1b1b | 373
H1e1c | 810
H1e2 | 814
H1e2a | 803
H1e2b | 796
H1e2c | 139
H1e2d | 796
H1e3 | 116
H1e4 | 797
H1e4a | 79
H1e5 | 97
H1e5a | 103
H1e5b | 98
H1e6 | 796
H1e7 | 795
H1e8 | 796
H1e8a | 800
H1f | 209
H1f+16093 | 90
H1f1 | 104
H1f1a | 66
H1g | 271
H1g1 | 237
H1g2 | 213

H1h | 794
H1h1 | 811
H1h2 | 796
H1i | 152
H1i1 | 162
H1i2 | 147
H1i2a | 149
H1j | 813
H1j1 | 277
H1j1a | 272
H1j1a1 | 146
H1j1a2 | 265
H1j1b | 276
H1j1c | 268
H1j2 | 136
H1j2a | 29
H1j3 | 799
H1j4 | 83
H1j5 | 796
H1j6 | 781
H1j7 | 795
H1j8 | 76
H1j9 | 942
H1k | 82
H1k1 | 74
H1k1a | 76
H1m | 806
H1m1 | 63
H1n | 794
H1n+146 | 66
H1n+146+195 | 10
H1n1 | 63
H1n1a | 64
H1n1b | 36
H1n2 | 55
H1n3 | 62
H1n4 | 66
H1n5 | 7
H1n6 | 383
H1o | 43
H1p | 796
H1q | 823
H1q1 | 804
H1q1a | 99
H1q2 | 759
H1q3 | 106
H1r | 796
H1r1 | 35
H1s | 797
H1s1 | 798
H1t | 799
H1t1 | 797
H1t1a | 825
H1t1a1 | 96
H1t2 | 797
H1u | 805
H1u1 | 807
H1u2 | 86
H1v | 805
H1v1 | 794
H1v1a | 797
H1v1b | 796
H1w | 797
H1x | 71
H1y | 210
H1z | 89
H1z1 | 32
H2 | 798
H2+152_16311 | 176
H20 | 80
H20a | 76
H20a1 | 21
H20a1a | 30
H20a2 | 77
H20b | 86
H20c | 83
H21 | 26
H22 | 65
H23 | 812
H24 | 112
H24a | 137
H24a1 | 65
H24a2 | 95
H24b | 92
H25 | 787
H26 | 790
H26a | 789
H26a1 | 813
H26a1a | 786
H26a1a1 | 18
H26a1b | 61
H26b | 87
H26c | 73
H27 | 103
H27+16093 | 80
H27a | 84
H27b | 61
H27c | 65
H27d | 18
H27e | 65
H27f | 63
H28 | 21
H28a | 54
H28a1 | 8
H28a2 | 7
H29 | 59
H29a | 40
H29b | 28
H2a | 817
H2a1 | 235
H2a1+146 | 65

H2a1a | 220
H2a1a1 | 171
H2a1a2 | 170
H2a1b | 167
H2a1b1 | 50
H2a1b2 | 33
H2a1c | 185
H2a1d | 171
H2a1e | 109
H2a1e1 | 103
H2a1e1a | 45
H2a1e1a1 | 40
H2a1e1b | 107
H2a1f | 82
H2a1f1 | 19
H2a1f2 | 21
H2a1g | 172
H2a1i | 17
H2a1j | 106
H2a1k | 169
H2a1m | 50
H2a1n | 65
H2a2 | 780
H2a2+(16235) | 786
H2a2a | 971
H2a2a1 | 148
H2a2a1a | 12
H2a2a1b | 13
H2a2a1c | 20
H2a2a1d | 42
H2a2a1e | 14
H2a2a1f | 29
H2a2a1g | 94
H2a2a1h | 33
H2a2a2 | 152
H2a2b | 152
H2a2b1 | 56
H2a2b1a | 52
H2a2b1a1 | 58
H2a2b2 | 128
H2a2b3 | 134
H2a2b4 | 129
H2a2b5 | 134
H2a2b5a | 131
H2a3 | 108
H2a3a | 103
H2a3a1 | 103
H2a3b | 101
H2a4 | 797
H2a5 | 802
H2a5a | 794
H2a5a1 | 146
H2a5a1a | 155
H2a5a1b | 138
H2a5b | 806
H2a5b1 | 18
H2a5b2 | 75
H2b | 199
H2c | 166
H2c1 | 115
H3 | 1168
H3+152 | 253
H3+16189 | 230
H3+16311 | 344
H3+73 | 152
H30 | 785
H30a | 788
H30b | 785
H30b1 | 148
H31 | 36
H31a | 19
H31b | 9
H32 | 59
H33 | 792
H33a | 790
H33b | 72
H33c | 85
H34 | 147
H35 | 802
H35a | 792
H36 | 46
H39 | 73
H39a | 63
H39a1 | 63
H39b | 71
H39c | 70
H3a | 56
H3a1 | 56
H3a1a | 55
H3aa | 796
H3ab | 799
H3ac | 797
H3ad | 796
H3ae | 796
H3af | 271
H3ag | 796
H3ag1 | 796
H3ah | 796
H3ai | 796
H3aj | 796
H3ak | 66
H3am | 33
H3an | 15
H3ao | 100
H3ao1 | 81
H3ap | 811
H3aq | 796
H3ar | 797
H3as | 798
H3at | 795
H3at1 | 799

H3au | 795
H3av | 118
H3b | 800
H3b+16129 | 282
H3b1 | 69
H3b1a | 73
H3b1b | 11
H3b1b1 | 15
H3b1b1a | 13
H3b2 | 264
H3b3 | 266
H3b4 | 152
H3b4a | 132
H3b5 | 263
H3b6 | 151
H3b6a | 152
H3b7 | 265
H3c | 806
H3c1 | 802
H3c2 | 48
H3c2a | 60
H3c2a1 | 50
H3c2b | 48
H3c2b1 | 48
H3c2c | 49
H3c3 | 22
H3d | 114
H3e | 799
H3g | 157
H3g1 | 174
H3g1a | 147
H3g1b | 150
H3g2 | 151
H3g3 | 152
H3g4 | 153
H3h | 341
H3h1 | 354
H3h2 | 358
H3h2a | 74
H3h3 | 334
H3h3a | 334
H3h3b | 333
H3h4 | 119
H3h5 | 338
H3h6 | 334
H3h7 | 128
H3i | 148
H3i1 | 150
H3j | 149
H3k | 154
H3k1 | 147
H3k1a | 153
H3m | 338
H3n | 80
H3p | 93
H3q | 798
H3q1 | 798
H3r | 798
H3r1 | 796
H3s | 9
H3t | 799
H3u | 817
H3u1 | 78
H3v | 208
H3v+16093 | 72
H3v1 | 207
H3v2 | 62
H3w | 88
H3x | 108
H3x1 | 81
H3y | 802
H3z | 92
H3z1 | 92
H3z2 | 91
H4 | 801
H40 | 790
H40a | 790
H40b | 788
H41 | 785
H41a | 51
H42 | 786
H42a | 86
H42a1 | 88
H42a2 | 86
H43 | 787
H44 | 786
H44a | 792
H44a1 | 787
H44b | 43
H45 | 786
H45a | 797
H45b | 790
H46 | 151
H46a | 152
H46b | 149
H47 | 793
H47a | 152
H48 | 798
H49 | 803
H49a | 797
H49a1 | 790
H49a2 | 55
H49b | 789
H4a | 795
H4a1 | 822
H4a1a | 803
H4a1a+195 | 33
H4a1a1 | 812
H4a1a1a | 185
H4a1a1a1 | 127
H4a1a1a1a | 131

H4a1a1a1a1 | 136
H4a1a1a2 | 132
H4a1a1a3 | 78
H4a1a1a4 | 130
H4a1a2 | 794
H4a1a2a | 799
H4a1a2a1 | 795
H4a1a3 | 30
H4a1a3a | 31
H4a1a4 | 33
H4a1a4a | 53
H4a1a4b | 61
H4a1a4b1 | 13
H4a1a4b2 | 35
H4a1a5 | 794
H4a1c | 789
H4a1c1 | 789
H4a1c1a | 794
H4a1c2 | 67
H4a1d | 804
H4a2 | 71
H4b | 796
H4b1 | 54
H4c | 794
H4c1 | 77
H4d | 375
H5 | 364
H5+16192 | 49
H5+16311 | 82
H5+709 | 294
H50 | 794
H51 | 792
H51a | 789
H52 | 158
H53 | 792
H5'36 | 384
H54 | 789
H55 | 785
H55+153 | 11
H55a | 789
H55b | 93
H56 | 801
H56a | 786
H56a1 | 787
H56b | 786
H56c | 787
H56d | 21
H57 | 42
H58 | 789
H58a | 791
H59 | 792
H59a | 790
H5a | 300
H5a+152 | 181
H5a1 | 388
H5a1+152 | 178
H5a1+16093 | 49
H5a1a | 321
H5a1b | 294
H5a1c | 291
H5a1c1 | 291
H5a1c1a | 295
H5a1c2 | 36
H5a1d | 294
H5a1e | 40
H5a1f | 297
H5a1g | 47
H5a1g1 | 43
H5a1g1a | 48
H5a1g2 | 38
H5a1h | 292
H5a1i | 40
H5a1j | 55
H5a1k | 294
H5a1m | 170
H5a1n | 166
H5a1p | 65
H5a1q | 295
H5a2 | 331
H5a2a | 294
H5a3 | 246
H5a3a | 254
H5a3a+152 | 161
H5a3a1 | 167
H5a3a2 | 162
H5a3a3 | 252
H5a3b | 254
H5a4 | 69
H5a4a | 41
H5a4a1 | 43
H5a4a1a | 41
H5a5 | 155
H5a6 | 177
H5a6a | 167
H5a7 | 297
H5a8 | 295
H5a9 | 293
H5b | 328
H5b1 | 166
H5b2 | 194
H5b3 | 294
H5b4 | 168
H5b5 | 294
H5c | 294
H5c1 | 305
H5c1a | 293
H5c2 | 36
H5d | 306
H5e | 67
H5e1 | 73
H5e1a | 71

H5e1a1 | 78
H5e1b | 76
H5f | 295
H5g | 42
H5h | 40
H5j | 297
H5k | 293
H5m | 176
H5n | 201
H5p | 293
H5q | 52
H5r | 34
H5r1 | 38
H5r2 | 38
H5s | 53
H5t | 36
H5u | 42
H5u1 | 56
H5v | 298
H6 | 143
H60 | 785
H60a | 794
H61 | 789
H61a | 91
H62 | 786
H63 | 792
H63a | 252
H64 | 131
H65 | 789
H65a | 787
H66 | 785
H66a | 104
H66a1 | 61
H67 | 779
H67a | 782
H69 | 152
H6a | 145
H6a1 | 138
H6a1a | 250
H6a1a1 | 139
H6a1a10 | 140
H6a1a1a | 22
H6a1a2 | 136
H6a1a2a | 69
H6a1a2b | 138
H6a1a2b1 | 140
H6a1a3 | 146
H6a1a3a | 136
H6a1a4 | 147
H6a1a5 | 141
H6a1a6 | 89
H6a1a7 | 96
H6a1a8 | 20
H6a1a8a | 9
H6a1a9 | 139
H6a1b | 169
H6a1b1 | 14
H6a1b2 | 177
H6a1b2a | 139
H6a1b2b | 137
H6a1b2c | 144
H6a1b2d | 14
H6a1b2e | 136
H6a1b3 | 27
H6a1b3a | 11
H6a1b3b | 10
H6a1b4 | 50
H6a2 | 144
H6a2a | 133
H6b | 30
H6b1 | 24
H6b2 | 50
H6c | 149
H6c1 | 17
H7 | 822
H70 | 783
H71 | 780
H72 | 779
H73 | 781
H73a | 781
H73a1 | 783
H74 | 13
H75 | 782
H76 | 339
H76a | 24
H77 | 89
H78 | 781
H79 | 783
H79a | 780
H7a | 798
H7a1 | 115
H7a1a | 47
H7a1b | 104
H7a1c | 95
H7a1d | 96
H7a2 | 75
H7b | 818
H7b1 | 804
H7b2 | 801
H7b2a | 786
H7b3 | 790
H7b4 | 791
H7b5 | 790
H7b6 | 758
H7c | 788
H7c1 | 116
H7c2 | 791
H7c3 | 790
H7c4 | 69
H7c5 | 788
H7c6 | 789

H7d | 812
H7d1 | 790
H7d2 | 90
H7d2a | 91
H7d3 | 787
H7d3a | 788
H7d4 | 787
H7d5 | 61
H7e | 809
H7f | 73
H7g | 793
H7h | 80
H7h1 | 81
H7i | 29
H7i1 | 13
H8 | 12
H8+(114) | 9
H8+(114)+152 | 12
H80 | 338
H81 | 780
H81a | 779
H82 | 81
H83 | 59
H84 | 780
H85 | 83
H86 | 784
H87 | 784
H88 | 779
H89 | 780
H8a | 5
H8a1 | 11
H8b | 14
H8b1 | 21
H8c | 25
H8c1 | 8
H8c2 | 3
H9 | 150
H90 | 779
H91 | 24
H92 | 780
H93 | 780
H94 | 67
H95 | 779
H95a | 780
H96 | 74
H9a | 45
HV | 831
HV+16311 | 393
HV+73 | 169
HV0 | 201
HV0+195 | 164
HV0a | 218
HV0a1 | 36
HV0a1a | 8
HV0b | 87
HV0c | 127
HV0d | 131
HV0e | 46
HV0f | 91
HV0g | 20
HV1 | 88
HV10 | 337
HV11 | 337
HV11a | 334
HV12 | 795
HV12a | 83
HV12a1 | 29
HV12b | 80
HV12b1 | 103
HV12b1a | 68
HV13 | 82
HV13a | 89
HV13b | 93
HV14 | 276
HV14a | 324
HV15 | 87
HV16 | 347
HV17 | 78
HV17a | 40
HV18 | 218
HV19 | 82
HV1a | 88
HV1a1 | 33
HV1a1a | 41
HV1a1b | 34
HV1a2 | 94
HV1a2a | 84
HV1a2b | 43
HV1a3 | 76
HV1a3a | 70
HV1a'b'c | 93
HV1b | 83
HV1b+152 | 44
HV1b1 | 76
HV1b1a | 76
HV1b1b | 79
HV1b2 | 42
HV1b3 | 15
HV1b3a | 25
HV1b3b | 12
HV1c | 93
HV1d | 2
HV2 | 75
HV20 | 132
HV21 | 108
HV22 | 18
HV23 | 334
HV24 | 334
HV2a | 32
HV2a1 | 44
HV2a2 | 51

HV2a3 | 46
HV4 | 811
HV4a | 80
HV4a1 | 96
HV4a1+16291 | 80
HV4a1a | 94
HV4a1a1 | 26
HV4a1a2 | 78
HV4a1a3 | 77
HV4a1a4 | 88
HV4a2 | 73
HV4a2a | 71
HV4a2b | 73
HV4b | 113
HV4c | 796
HV5 | 798
HV5a | 801
HV5b | 77
HV6 | 113
HV6a | 70
HV7 | 79
HV8 | 88
HV9 | 347
HV9+152 | 170
HV9a | 124
HV9a1 | 129
HV9a1a | 125
HV9b | 104
HV9c | 797
I | 70
I1 | 33
I1a | 27
I1a1 | 78
I1a1a | 78
I1a1a1 | 48
I1a1a2 | 46
I1a1a3 | 9
I1a1a3a | 7
I1a1b | 68
I1a1c | 54
I1a1d | 9
I1a1e | 47
I1b | 62
I1c | 7
I1c1 | 11
I1c1a | 13
I1d | 29
I1e | 35
I1f | 22
I2 | 125
I2'3 | 47
I2a | 11
I2a1 | 7
I2a1a | 7
I2a2 | 12
I2a3 | 4
I2b | 4
I2c | 46
I2d | 54
I2e | 37
I2f | 37
I3 | 40
I3a | 39
I3a1 | 23
I3b | 9
I3c | 34
I3d | 37
I3d1 | 35
I4 | 48
I4a | 118
I4a1 | 43
I4a2 | 48
I4b | 59
I5 | 50
I5a | 17
I5a1 | 20
I5a1a | 19
I5a1b | 13
I5a1c | 15
I5a2 | 35
I5a2+16086 | 7
I5a2a | 11
I5a3 | 2
I5a4 | 28
I5b | 46
I5b1 | 21
I5c | 24
I5c1 | 17
I6 | 46
I6a | 50
I6b | 8
I7 | 15
J | 133
J1 | 150
J1+16193 | 72
J1b | 182
J1b1 | 178
J1b1a | 119
J1b1a1 | 123
J1b1a1+146 | 62
J1b1a1a | 83
J1b1a1b | 83
J1b1a1c | 32
J1b1a1d | 29
J1b1a1e | 48
J1b1a2 | 8
J1b1a2a | 3
J1b1a2b | 3
J1b1a3 | 131
J1b1b | 119
J1b1b1 | 141

J1b1b1a | 17
J1b1b1b | 121
J1b1b1c | 8
J1b1b2 | 10
J1b1b3 | 74
J1b2 | 204
J1b2a | 39
J1b3 | 172
J1b3a | 18
J1b3b | 186
J1b3b1 | 177
J1b4 | 178
J1b4a | 9
J1b4a1 | 16
J1b4a2 | 9
J1b5 | 21
J1b5a | 5
J1b5a1 | 7
J1b6 | 180
J1b6a | 180
J1b6b | 3
J1b7 | 119
J1b7a | 4
J1b8 | 175
J1b9 | 11
J1c | 234
J1c+16261 | 81
J1c+16261+189 | 16
J1c1 | 240
J1c10 | 213
J1c10a | 217
J1c11 | 18
J1c11a | 15
J1c12 | 13
J1c12a | 15
J1c12b | 13
J1c13 | 15
J1c14 | 8
J1c15 | 213
J1c15a | 214
J1c15a1 | 215
J1c15b | 99
J1c16 | 16
J1c17 | 213
J1c17a | 14
J1c1a | 208
J1c1b | 232
J1c1b1 | 207
J1c1b1a | 245
J1c1b1a1 | 203
J1c1b2 | 207
J1c1b2a | 205
J1c1c | 17
J1c1d | 29
J1c1e | 9
J1c1f | 207
J1c1g | 206
J1c1g1 | 207
J1c1h | 9
J1c2 | 292
J1c2a | 179
J1c2a1 | 177
J1c2a1a | 183
J1c2a2 | 12
J1c2a3 | 179
J1c2b | 180
J1c2b1 | 177
J1c2b2 | 177
J1c2b3 | 7
J1c2b4 | 13
J1c2b5 | 180
J1c2c | 188
J1c2c1 | 149
J1c2c1a | 6
J1c2c2 | 193
J1c2c2a | 193
J1c2c3 | 187
J1c2d | 177
J1c2e | 44
J1c2e1 | 24
J1c2e2 | 43
J1c2f | 180
J1c2g | 177
J1c2h | 138
J1c2i | 15
J1c2j | 7
J1c2k | 178
J1c2l | 181
J1c2m | 15
J1c2m1 | 10
J1c2n | 180
J1c2n1 | 181
J1c2o | 32
J1c2p | 177
J1c2q | 177
J1c2q1 | 182
J1c2r | 15
J1c2s | 179
J1c2s1 | 118
J1c2t | 183
J1c3 | 303
J1c3+189 | 151
J1c3a | 212
J1c3a1 | 226
J1c3a2 | 216
J1c3b | 216
J1c3b1 | 212
J1c3b1a | 212
J1c3b2 | 214
J1c3c | 21
J1c3c1 | 17

J1c3c2 | 17
J1c3d | 216
J1c3e | 24
J1c3e1 | 25
J1c3e2 | 27
J1c3f | 37
J1c3g | 231
J1c3h | 251
J1c3i | 212
J1c3j | 34
J1c3k | 216
J1c3m | 5
J1c4 | 240
J1c4b | 217
J1c4c | 22
J1c5 | 265
J1c5a | 229
J1c5a1 | 240
J1c5b | 212
J1c5c | 213
J1c5c1 | 54
J1c5d | 212
J1c5e | 211
J1c5f | 214
J1c6 | 217
J1c6a | 216
J1c7 | 83
J1c7a | 114
J1c8 | 213
J1c8a | 75
J1c8a1 | 13
J1c8a1a | 12
J1c8a2 | 12
J1c8b | 214
J1c9 | 219
J1d | 75
J1d1 | 17
J1d1a | 78
J1d1a1 | 85
J1d1a1a | 43
J1d1b | 19
J1d1b1 | 23
J1d2 | 11
J1d2a | 11
J1d3 | 64
J1d3a | 65
J1d3a1 | 7
J1d3a2 | 67
J1d4 | 64
J1d5 | 29
J1d5a | 20
J1d6 | 66
J1d6a | 7
J2 | 125
J2a | 130
J2a1 | 125
J2a1a | 3
J2a1a1 | 60
J2a1a1a | 44
J2a1a1a1 | 10
J2a1a1a2 | 76
J2a1a1a2a | 34
J2a1a1a3 | 23
J2a1a1b | 27
J2a1a1c | 26
J2a1a1d | 86
J2a1a1e | 37
J2a1a2 | 3
J2a1a2a | 4
J2a1a2a1 | 3
J2a1a2a1a | 4
J2a2 | 137
J2a2a | 131
J2a2a1 | 128
J2a2a1+16311 | 12
J2a2a1a | 14
J2a2a1a1 | 69
J2a2a2 | 14
J2a2b | 31
J2a2b1 | 15
J2a2b1a | 15
J2a2b2 | 16
J2a2b3 | 14
J2a2c | 20
J2a2c1 | 10
J2a2d | 148
J2a2e | 142
J2b | 67
J2b1 | 99
J2b1a | 160
J2b1a+16311 | 11
J2b1a1 | 33
J2b1a1a | 13
J2b1a2 | 67
J2b1a2a | 37
J2b1a3 | 34
J2b1a4 | 54
J2b1a5 | 91
J2b1a6 | 30
J2b1b | 67
J2b1b1 | 72
J2b1c | 67
J2b1c1 | 7
J2b1d | 71
J2b1e | 67
J2b1e1 | 23
J2b1f | 69
J2b1g | 66
J2b1h | 2
J2b2 | 68
JT | 64

K | 191
K1 | 194
K1+16362 | 74
K1a | 316
K1a+150 | 205
K1a+195 | 298
K1a1 | 297
K1a10 | 22
K1a10a | 29
K1a11 | 29
K1a11a | 8
K1a11a1 | 8
K1a11b | 28
K1a12 | 277
K1a12a | 315
K1a12a1 | 278
K1a12a1a | 282
K1a13 | 174
K1a13a | 177
K1a14 | 195
K1a15 | 198
K1a16 | 198
K1a17 | 186
K1a17a | 6
K1a18 | 280
K1a19 | 285
K1a19a | 148
K1a1a | 291
K1a1a1 | 285
K1a1a2 | 282
K1a1a2a | 289
K1a1a2a1 | 286
K1a1b | 274
K1a1b1 | 290
K1a1b1a | 92
K1a1b1b | 270
K1a1b1b1 | 266
K1a1b1c | 17
K1a1b1d | 9
K1a1b1e | 280
K1a1b1f | 270
K1a1b1g | 270
K1a1b2 | 268
K1a1b2a | 198
K1a1b2a1 | 198
K1a1b2a1a | 198
K1a1b2b | 151
K1a1c | 22
K1a2 | 298
K1a23 | 278
K1a24 | 183
K1a24a | 18
K1a25 | 203
K1a26 | 201
K1a27 | 19
K1a28 | 231
K1a29 | 279
K1a29a | 9
K1a2a | 295
K1a2a1 | 34
K1a2a2 | 280
K1a2b | 292
K1a2c | 281
K1a3 | 300
K1a30 | 184
K1a30a | 182
K1a31 | 183
K1a3a | 290
K1a3a1 | 284
K1a3a1a | 277
K1a3a1b | 281
K1a3a2 | 279
K1a3a3 | 280
K1a3a4 | 279
K1a4 | 301
K1a4+146 | 182
K1a4a | 285
K1a4a1 | 321
K1a4a1a | 270
K1a4a1a+195 | 213
K1a4a1a1 | 195
K1a4a1a2 | 26
K1a4a1a2a | 36
K1a4a1a2b | 43
K1a4a1a3 | 203
K1a4a1b | 278
K1a4a1b1 | 281
K1a4a1b2 | 13
K1a4a1c | 278
K1a4a1c1 | 173
K1a4a1d | 279
K1a4a1e | 53
K1a4a1f | 162
K1a4a1f1 | 158
K1a4a1g | 9
K1a4a1h | 281
K1a4a1i | 266
K1a4b | 197
K1a4b1 | 201
K1a4c | 203
K1a4c1 | 43
K1a4d | 303
K1a4e | 283
K1a4f | 287
K1a4f1 | 280
K1a4g | 282
K1a4h | 277
K1a4h1 | 279
K1a4i | 289
K1a4j | 180
K1a4j1 | 204

K1a5 | 279
K1a5a | 42
K1a5b | 158
K1a6 | 119
K1a7 | 231
K1a8 | 190
K1a8a | 9
K1a8a1 | 9
K1a8b | 78
K1a9 | 115
K1b | 191
K1b1 | 189
K1b1+(16093) | 226
K1b1a | 53
K1b1a1 | 56
K1b1a1+199 | 20
K1b1a1a | 32
K1b1a1b | 20
K1b1a1c | 50
K1b1a1c1 | 47
K1b1a1d | 48
K1b1a1d1 | 48
K1b1a2 | 14
K1b1b | 227
K1b1b1 | 230
K1b1c | 127
K1b2 | 125
K1b2a | 134
K1b2a1 | 132
K1b2a1a | 127
K1b2a1a1 | 68
K1b2a2 | 139
K1b2a2a | 124
K1b2a3 | 124
K1b2b | 133
K1b2b1 | 114
K1c | 195
K1c1 | 239
K1c1a | 197
K1c1b | 211
K1c1c | 210
K1c1d | 198
K1c1e | 34
K1c1f | 201
K1c1g | 196
K1c1h | 203
K1c1i | 197
K1c2 | 102
K1c2a | 54
K1d | 23
K1d1 | 26
K1e | 44
K1e1 | 9
K1f | 28
K2 | 129
K2a | 199
K2a1 | 176
K2a10 | 175
K2a11 | 16
K2a1a | 20
K2a2 | 177
K2a2a | 175
K2a2a1 | 159
K2a3 | 185
K2a3a | 177
K2a3a1 | 169
K2a4 | 175
K2a5 | 144
K2a5a | 128
K2a5a1 | 5
K2a5b | 134
K2a6 | 209
K2a7 | 175
K2a8 | 27
K2a9 | 188
K2b | 125
K2b1 | 128
K2b1a | 129
K2b1a1 | 29
K2b1a1a | 29
K2b1a2 | 129
K2b1a3 | 133
K2b1a4 | 107
K2b1b | 24
K2b2 | 127
K2c | 130
K3 | 5
L0a | 23
L0a1 | 33
L0a1+16293 | 7
L0a1'4 | 25
L0a1a | 47
L0a1a+200 | 46
L0a1a1 | 40
L0a1a2 | 133
L0a1a3 | 70
L0a1b | 37
L0a1b1 | 88
L0a1b1a | 66
L0a1b1a1 | 150
L0a1b1a1a | 75
L0a1b2 | 48
L0a1b2a | 20
L0a1c | 11
L0a1c1 | 18
L0a1d | 27
L0a1e | 22
L0a2 | 76
L0a2a | 33
L0a2a1 | 41
L0a2a1a | 50

L0a2a1a1 | 34
L0a2a1a2 | 38
L0a2a1b | 105
L0a2a2 | 75
L0a2a2a | 282
L0a2a2a1 | 74
L0a2a2a2 | 75
L0a2b | 29
L0a2b1 | 21
L0a2c | 6
L0a2d | 39
L0a3 | 18
L0a4 | 17
L0a'b'g | 3
L0a'g | 6
L0b | 9
L0d1 | 49
L0d1'2 | 47
L0d1a | 14
L0d1a1 | 21
L0d1a1a | 20
L0d1a1a1 | 28
L0d1a1a2 | 19
L0d1a1a3 | 19
L0d1a1b | 32
L0d1a1b1 | 16
L0d1a1b1a | 48
L0d1a1b1b | 18
L0d1a1c | 19
L0d1a1d | 19
L0d1a'c'd | 33
L0d1a'd | 12
L0d1b | 47
L0d1b1 | 12
L0d1b1+@152 | 5
L0d1b1a | 13
L0d1b1a1 | 29
L0d1b1b | 11
L0d1b1b1 | 34
L0d1b1c | 6
L0d1b2 | 33
L0d1b2a | 45
L0d1b2a1 | 85
L0d1b2a2 | 55
L0d1b2b | 63
L0d1b2b1 | 63
L0d1b2b1a | 83
L0d1b2b1b | 81
L0d1b2b1b1 | 64
L0d1b2b2 | 48
L0d1b2b2a | 62
L0d1b2b2b | 43
L0d1b2b2b1 | 54
L0d1b2b2c | 42
L0d1b2b2c1 | 49
L0d1b2b2c2 | 44
L0d1c | 27
L0d1c1 | 21
L0d1c1a | 17
L0d1c1a1 | 116
L0d1c1a1a | 162
L0d1c1a1a1 | 55
L0d1c1a1a2 | 110
L0d1c1a1b | 131
L0d1c1a2 | 32
L0d1c2 | 22
L0d1c2a | 11
L0d1c2a1 | 8
L0d1c3 | 32
L0d1d | 12
L0d2 | 47
L0d2a | 103
L0d2a1 | 140
L0d2a1a | 187
L0d2a1a1 | 155
L0d2a1a1a | 86
L0d2a1a2 | 136
L0d2a1a3 | 91
L0d2a1b | 16
L0d2a1c | 137
L0d2a2 | 5
L0d2a'b'd | 34
L0d2b | 9
L0d2b1 | 5
L0d2b1a | 6
L0d2b1a1 | 13
L0d2b1a1a | 20
L0d2b1b | 18
L0d2b2 | 13
L0d2c | 19
L0d2c1 | 33
L0d2c1a | 22
L0d2c1a1 | 21
L0d2c1b | 12
L0d2c2 | 32
L0d2c2a | 30
L0d2c2a1 | 30
L0d2c2a1a | 32
L0d2c2b | 31
L0d2d | 9
L0d3 | 7
L0d3a | 13
L0d3b | 24
L0d3b1 | 42
L0d3b2 | 23
L0f | 33
L0f1 | 7
L0f2 | 17
L0f2a | 19
L0f2a1 | 15
L0f2b | 10

L0g | 8
L0k1 | 22
L0k1a | 29
L0k1a1 | 79
L0k1a1a | 72
L0k1a1b | 65
L0k1a1c | 65
L0k1a1d | 71
L0k1a2 | 51
L0k1a2a | 34
L0k1a3 | 8
L0k1b | 28
L0k2 | 1
L0k2a | 2
L0k2a1 | 2
L0k2a1a | 6
L0k2b | 3
L1 | 9
L1'2'3'4'5'6 | 26
L1b | 81
L1b1 | 81
L1b1a | 137
L1b1a+189 | 98
L1b1a1 | 2
L1b1a10 | 59
L1b1a10a | 50
L1b1a10b | 57
L1b1a12 | 16
L1b1a12a | 14
L1b1a12b | 62
L1b1a13 | 81
L1b1a1'4 | 14
L1b1a14 | 75
L1b1a15 | 98
L1b1a15a | 6
L1b1a16 | 41
L1b1a17 | 114
L1b1a18 | 62
L1b1a2 | 74
L1b1a2a | 23
L1b1a3 | 123
L1b1a3a | 99
L1b1a3a1 | 102
L1b1a3b | 104
L1b1a4 | 25
L1b1a4a | 20
L1b1a5 | 87
L1b1a6 | 99
L1b1a7 | 78
L1b1a7a | 35
L1b1a8 | 94
L1b1a9 | 108
L1b2 | 24
L1b2'3 | 28
L1b2a | 35
L1b3 | 28
L1c | 2
L1c1 | 13
L1c1'2'4'5'6 | 5
L1c1'2'4'6 | 4
L1c1a | 7
L1c1a+@198 | 2
L1c1a1 | 1
L1c1a1a1a | 53
L1c1a1a1b | 39
L1c1a1a1b1 | 33
L1c1a1a2 | 3
L1c1a1b | 3
L1c1a2 | 34
L1c1a2a | 27
L1c1a2a1 | 31
L1c1a2a2 | 29
L1c1a2b | 48
L1c1a2c | 21
L1c1a'b'd | 7
L1c1b | 52
L1c1b1 | 24
L1c1b'd | 5
L1c1c | 32
L1c1d | 23
L1c1d1 | 26
L1c2 | 19
L1c2'4 | 4
L1c2a | 7
L1c2a1 | 14
L1c2a1a | 76
L1c2a1b | 19
L1c2a2 | 8
L1c2a3 | 11
L1c2a3a | 15
L1c2b | 11
L1c2b1 | 18
L1c2b1a | 16
L1c2b1a1 | 12
L1c2b1a'b | 18
L1c2b1b | 26
L1c2b1b1 | 56
L1c2b1c | 30
L1c2b2 | 40
L1c3 | 10
L1c3a | 54
L1c3a1 | 35
L1c3a1a | 28
L1c3a1b | 39
L1c3b | 15
L1c3b1 | 15
L1c3b1a | 43
L1c3b1b | 15
L1c3b2 | 40
L1c3b'c | 6
L1c3c | 32

L1c4 | 2
L1c4a | 5
L1c4b | 49
L1c5 | 31
L1c6 | 6
L2 | 5
L2'3'4'5'6 | 12
L2'3'4'6 | 29
L2a | 5
L2a1 | 124
L2a1+143 | 52
L2a1+143+@16309 | 58
L2a1+143+16189_(16192) | 121
L2a1+143+16189_(16192)+@16309 | 94
L2a1+16189_(16192) | 128
L2a1'2'3'4 | 10
L2a1a | 137
L2a1a1 | 104
L2a1a2 | 186
L2a1a2a | 88
L2a1a2a1 | 85
L2a1a2a1a | 114
L2a1a2b | 93
L2a1a2c | 79
L2a1a3 | 58
L2a1a3a | 56
L2a1a3b | 65
L2a1a3c | 27
L2a1b | 134
L2a1b+143 | 66
L2a1b1 | 126
L2a1b1a | 333
L2a1b2 | 130
L2a1b3 | 48
L2a1c | 74
L2a1c+16086 | 20
L2a1c+16129 | 69
L2a1c1 | 25
L2a1c1a | 16
L2a1c1a1 | 17
L2a1c1a2 | 14
L2a1c2 | 30
L2a1c2a | 38
L2a1c3 | 110
L2a1c3a | 55
L2a1c3a1 | 10
L2a1c3b | 20
L2a1c3b1 | 17
L2a1c3b2 | 9
L2a1c4 | 59
L2a1c4a | 60
L2a1c4a1 | 57
L2a1c5 | 66
L2a1c6 | 8
L2a1d | 9
L2a1d1 | 47
L2a1d2 | 36
L2a1e | 55
L2a1e1 | 100
L2a1f | 214
L2a1f1 | 138
L2a1f1a | 137
L2a1f2 | 163
L2a1f3 | 90
L2a1g | 52
L2a1h | 38
L2a1i | 66
L2a1i1 | 43
L2a1j | 74
L2a1k | 9
L2a1l | 58
L2a1l1 | 56
L2a1l1a | 56
L2a1l1a1 | 58
L2a1l1a2 | 58
L2a1l1b | 62
L2a1l2 | 56
L2a1l2a | 56
L2a1l2a1 | 62
L2a1l3 | 9
L2a1m | 74
L2a1m1 | 74
L2a1m1a | 69
L2a1n | 71
L2a1o | 74
L2a1p | 74
L2a1q | 8
L2a2 | 9
L2a2'3 | 9
L2a2'3'4 | 5
L2a2a | 8
L2a2a1 | 14
L2a2b | 9
L2a2b1 | 5
L2a2b1a | 59
L2a2b2 | 9
L2a3 | 2
L2a4 | 3
L2a4a | 14
L2a4b | 7
L2a5 | 48
L2a'b'c'd | 7
L2b | 8
L2b1 | 7
L2b1a | 35
L2b1a2 | 45
L2b1a3 | 71
L2b1a4 | 67
L2b1b | 41
L2b2 | 53
L2b2a | 41

L2b3 | 10
L2b3a | 41
L2b3b | 5
L2b3c | 13
L2b'c | 4
L2b'c'd | 4
L2c | 48
L2c1 | 33
L2c1a | 33
L2c2 | 44
L2c2a | 65
L2c2a1 | 34
L2c2b | 7
L2c2b1 | 23
L2c2b1a | 7
L2c2b1b | 29
L2c2b2 | 7
L2c3 | 45
L2c3a | 25
L2c4 | 57
L2c5 | 15
L2d | 1
L2d+16129 | 10
L2d1 | 29
L2d1a | 21
L2e | 34
L2e1 | 15
L2e1a | 19
L3 | 222
L3'4 | 127
L3'4'6 | 29
L3a | 15
L3a+709 | 28
L3a1 | 9
L3a1a | 43
L3a1b | 9
L3a2 | 54
L3a2a | 25
L3b | 133
L3b1 | 138
L3b1a | 168
L3b1a+@16124 | 202
L3b1a+152 | 96
L3b1a1 | 126
L3b1a10 | 141
L3b1a11 | 106
L3b1a1a | 416
L3b1a2 | 137
L3b1a3 | 110
L3b1a4 | 125
L3b1a5 | 125
L3b1a5a | 133
L3b1a6 | 76
L3b1a7 | 131
L3b1a7a | 136
L3b1a8 | 108
L3b1a9 | 87
L3b1a9a | 83
L3b1b | 83
L3b1b1 | 21
L3b2 | 86
L3b2a | 83
L3b2b | 91
L3b3 | 25
L3b'f | 211
L3c | 141
L3c'd | 182
L3d | 91
L3d1 | 95
L3d1'2'3'4'5'6 | 91
L3d1a | 90
L3d1a1 | 65
L3d1a1'2 | 71
L3d1a1a | 157
L3d1a1a1 | 133
L3d1a1b | 74
L3d1a2 | 71
L3d1b | 92
L3d1b1 | 92
L3d1b1a | 90
L3d1b1b | 34
L3d1b2 | 69
L3d1b3 | 63
L3d1b3a | 56
L3d1c | 67
L3d1c1 | 61
L3d1d | 121
L3d2 | 62
L3d2a | 71
L3d2b | 65
L3d3 | 90
L3d3a | 135
L3d3a1 | 159
L3d3a1a | 210
L3d3a1b | 123
L3d3b | 92
L3d4 | 59
L3d4a | 50
L3d5 | 62
L3d5a | 66
L3d6 | 90
L3e | 164
L3e1 | 67
L3e1a | 31
L3e1a1 | 26
L3e1a1a | 67
L3e1a2 | 75
L3e1a3 | 34
L3e1a3a | 142
L3e1a3b | 12
L3e1b | 15

L3e1b1 | 25
L3e1b2 | 54
L3e1c | 46
L3e1d | 21
L3e1d1 | 45
L3e1d1a | 16
L3e1e | 70
L3e1e1 | 69
L3e1e2 | 19
L3e1f | 53
L3e1f1 | 23
L3e1f1a | 21
L3e1f2 | 17
L3e1g | 16
L3e2 | 36
L3e2a | 27
L3e2a1 | 34
L3e2a1a | 24
L3e2a1b | 21
L3e2a1b1 | 77
L3e2a1b2 | 18
L3e2a1b3 | 41
L3e2a2 | 26
L3e2a3 | 40
L3e2b | 134
L3e2b+152 | 95
L3e2b1 | 72
L3e2b1a | 68
L3e2b1a1 | 74
L3e2b1a2 | 79
L3e2b2 | 70
L3e2b3 | 61
L3e2b4 | 89
L3e2b5 | 77
L3e2b6 | 16
L3e2b7 | 111
L3e2b8 | 68
L3e3 | 48
L3e3'4 | 150
L3e3'4'5 | 157
L3e3a | 151
L3e3b | 106
L3e3b1 | 104
L3e3b2 | 28
L3e3b3 | 70
L3e4 | 43
L3e4a | 61
L3e4a1 | 56
L3e5 | 52
L3e5+195 | 34
L3e5a | 46
L3e5a1 | 62
L3e5a1a | 40
L3e5b | 52
L3e5c | 35
L3e5d | 49
L3e5e | 35
L3e5f | 8
L3e'i'k'x | 164
L3f | 81
L3f1 | 73
L3f1a | 74
L3f1a1 | 82
L3f1b | 23
L3f1b+16292 | 52
L3f1b+16292+150 | 61
L3f1b1 | 28
L3f1b1a | 96
L3f1b1a1 | 27
L3f1b2 | 70
L3f1b2a | 10
L3f1b3 | 37
L3f1b4 | 35
L3f1b4a | 121
L3f1b4a1 | 64
L3f1b4b | 40
L3f1b4c | 48
L3f1b5 | 29
L3f2 | 116
L3f2a | 57
L3f2a1 | 68
L3f2a1a | 48
L3f2b | 23
L3f3 | 18
L3f3a | 5
L3f3b | 10
L3h | 213
L3h1 | 138
L3h1a | 138
L3h1a1 | 84
L3h1a2 | 136
L3h1a2a | 34
L3h1a2a1 | 52
L3h1a2b | 79
L3h1b | 1
L3h1b1 | 7
L3h1b1a | 37
L3h1b2 | 20
L3h2 | 13
L3i | 164
L3i1 | 44
L3i1a | 45
L3i1b | 36
L3i2 | 30
L3k | 134
L3k1 | 28
L3x | 49
L3x1 | 8
L3x1+16311 | 9
L3x1a | 4
L3x1a1 | 5

L3x1a2 | 20
L3x1b | 9
L3x2 | 3
L3x2a | 13
L3x2a1 | 2
L3x2a1a | 5
L3x2b | 11
L4 | 122
L4a | 3
L4a1 | 17
L4a1a | 11
L4a2 | 7
L4b | 104
L4b1 | 101
L4b1a | 25
L4b2 | 18
L4b2a | 21
L4b2a1 | 9
L4b2a2 | 41
L4b2a2a | 96
L4b2a2b | 17
L4b2a2c | 48
L4b2b | 30
L4b2b1 | 25
L5 | 3
L5a | 5
L5a1 | 11
L5a1a | 12
L5a1b | 17
L5a1c | 13
L5a2 | 34
L5b | 2
L5b1 | 21
L5b1a | 14
L5b1b | 13
L5b2 | 10
L6 | 6
L6a | 8
L6b | 7
M | 379
M1 | 152
M10 | 125
M10a | 141
M10a1 | 148
M10a1+16129 | 88
M10a1a | 94
M10a1a1 | 64
M10a1a1a | 44
M10a1a1b | 41
M10a1a1b1 | 39
M10a1a1b2 | 36
M10a1b | 137
M10a2 | 71
M11 | 244
M11+200 | 250
M11a | 256
M11a1 | 256
M11a2 | 171
M11a'b | 250
M11b | 231
M11b1 | 233
M11b1a | 230
M11b1a1 | 191
M11b2 | 136
M11c | 107
M11d | 49
M12 | 166
M1'20'51 | 341
M12a | 106
M12a1 | 104
M12a1a | 163
M12a1a1 | 66
M12a1a2 | 112
M12a1b | 144
M12a2 | 28
M12b | 172
M12b1 | 169
M12b1a | 171
M12b1a1 | 171
M12b1a2 | 214
M12b1a2a | 236
M12b1a2b | 178
M12b1b | 131
M12b2 | 150
M12b2a | 112
M12'G | 296
M13 | 253
M13'46'61 | 251
M13'46'61+16362 | 602
M13a | 131
M13a1 | 133
M13a1a | 130
M13a1b | 230
M13a1b1 | 228
M13a2 | 238
M13a'b | 252
M13b | 248
M13b1 | 70
M13b2 | 177
M13c | 156
M14 | 237
M15 | 102
M17 | 73
M17a | 119
M17c | 90
M17c1 | 5
M17c1a | 5
M17c1a1 | 13
M17c1a1a | 13
M18 | 117
M18'38 | 234

M18a | 141
M18b | 113
M18c | 104
M19 | 41
M19'53 | 296
M1a | 75
M1a1 | 42
M1a1+16093 | 12
M1a1a | 34
M1a1a1 | 23
M1a1b | 35
M1a1b1 | 37
M1a1b1a | 61
M1a1b1b | 39
M1a1b1b1 | 34
M1a1b1c | 2
M1a1b2 | 40
M1a1c | 11
M1a1d | 17
M1a1e | 49
M1a1e1 | 35
M1a1e2 | 34
M1a1f | 44
M1a1g | 11
M1a1h | 18
M1a1i | 23
M1a2 | 28
M1a2a | 31
M1a2b | 16
M1a3 | 24
M1a3a | 33
M1a3b | 29
M1a3b1 | 28
M1a3b2 | 25
M1a4 | 56
M1a4a | 51
M1a5 | 21
M1a6 | 58
M1a7 | 16
M1a8 | 59
M1a8a | 59
M1b | 153
M1b1 | 12
M1b1a | 19
M1b1b | 12
M1b2 | 133
M1b2a | 112
M1b2b | 111
M1b2c | 128
M2 | 107
M20 | 283
M21 | 296
M21a | 103
M21b | 314
M21b+210 | 228
M21b1 | 297
M21b1a | 306
M21b2 | 37
M22 | 198
M22a | 85
M22b | 206
M23 | 255
M23'75 | 299
M24 | 237
M24'41 | 297
M24a | 195
M24b | 159
M25 | 7
M26 | 130
M27 | 296
M27a1a1 | 2
M27a1a2 | 2
M27a1b | 16
M27a2a | 10
M27a2b | 11
M27a3 | 7
M27b | 24
M27b1 | 5
M27b2 | 34
M27b2a | 25
M27b2a1 | 27
M27b2b | 25
M27b2b1 | 52
M27b2c | 39
M27c | 139
M28 | 108
M28a | 105
M28a+204 | 100
M28a1 | 111
M28a2 | 4
M28a2a | 5
M28a3 | 108
M28a4 | 103
M28a5 | 107
M28a5a | 117
M28a5b | 102
M28a6 | 9
M28a6a | 10
M28a7 | 106
M28a7a | 109
M28a7b | 111
M28b | 103
M28b1 | 102
M29 | 37
M29a | 28
M29b | 10
M29b1 | 6
M29'Q | 301
M2a | 88
M2a1 | 74
M2a1a | 91

M2a1a+207 | 71
M2a1a1 | 81
M2a1a1a | 80
M2a1a1a1 | 54
M2a1a1b | 80
M2a1a1b1 | 57
M2a1a2 | 53
M2a1a2a | 53
M2a1a2a1 | 56
M2a1a2a1a | 61
M2a1a3 | 19
M2a1a3+16093 | 2
M2a1a3a | 2
M2a1a3a1 | 4
M2a1a3b | 5
M2a1b | 61
M2a1c | 28
M2a2 | 90
M2a2a | 94
M2a3 | 63
M2a3a | 67
M2a'b | 94
M2b | 57
M2b1 | 64
M2b1a | 54
M2b1b | 14
M2b2 | 57
M2b3 | 59
M2b3a | 118
M2b4 | 2
M2c | 104
M3 | 229
M30 | 278
M30+16234 | 194
M30a | 140
M30a1 | 140
M30a2 | 139
M30b | 122
M30c | 235
M30c1 | 129
M30c1a | 102
M30c1a1 | 101
M30d | 240
M30d1 | 267
M30d2 | 244
M30e | 142
M30f | 116
M30g | 236
M31 | 296
M31a | 296
M31a1 | 196
M31a1a | 199
M31a1b | 198
M31a2 | 29
M31b | 28
M31b1 | 35
M31b2 | 28
M31b'c | 172
M31c | 113
M32 | 150
M32'56 | 298
M32a | 111
M32c | 302
M33 | 296
M33+16362 | 715
M33a | 301
M33a1 | 297
M33a1a | 26
M33a1b | 240
M33a2 | 171
M33a2'3 | 168
M33a2a | 37
M33a3 | 121
M33a3a | 109
M33b | 97
M33b1 | 122
M33b2 | 95
M33c | 279
M33d | 9
M34 | 89
M34'57 | 297
M34a | 89
M34a1 | 57
M34a1a | 59
M34a2 | 56
M34b | 95
M35 | 302
M35+199 | 233
M35a | 141
M35a1 | 172
M35a1a | 124
M35a2 | 177
M35b | 250
M35b+16304 | 170
M35b1 | 136
M35b2 | 151
M35b3 | 142
M35b4 | 238
M35c | 167
M36 | 227
M36a | 151
M36b | 105
M36c | 108
M36d | 229
M36d1 | 252
M37 | 296
M37+152 | 253
M37+152+151 | 233
M37a | 237
M37a1 | 237
M37d | 239

M37e | 180
M37e2 | 171
M38 | 248
M38+195 | 227
M38+199 | 230
M38a | 232
M38b | 35
M38c | 137
M38d | 102
M38e | 74
M39 | 236
M39'70 | 296
M39a | 225
M39a1 | 227
M39a2 | 105
M39b | 136
M39b1 | 143
M39b2 | 133
M39c | 105
M3a | 229
M3a1 | 234
M3a1+204 | 214
M3a1a | 188
M3a1b | 243
M3a2 | 235
M3a2a | 188
M3b | 228
M3c | 231
M3c+152 | 192
M3c1 | 124
M3c1a | 133
M3c1b | 119
M3c1b1 | 112
M3c1b1a | 132
M3c1b1b | 116
M3c2 | 162
M3d | 156
M3d1 | 151
M3d1a | 171
M3d1a1 | 153
M4 | 213
M40 | 213
M40a | 114
M40a1 | 108
M40a1a | 114
M40a1b | 106
M41 | 8
M41a | 7
M41a1 | 5
M41b | 7
M41c | 7
M42 | 296
M42'74 | 298
M42a | 213
M42b | 123
M42b1 | 122
M42b1a | 123
M42b2 | 193
M43 | 199
M43+16311 | 144
M43a | 161
M43a1 | 207
M43b | 201
M44 | 105
M44a | 12
M44a1 | 12
M45 | 141
M45a | 127
M46 | 528
M4"67 | 299
M4"67+16311 | 232
M46a | 111
M47 | 171
M48 | 130
M49 | 220
M49a | 179
M49a1 | 190
M49a2 | 174
M49c | 211
M49c1 | 219
M49d | 171
M49e | 200
M49e1 | 212
M4a | 237
M4b | 128
M5 | 261
M50 | 189
M50a | 100
M50a1 | 13
M50a2 | 102
M51 | 145
M51a | 184
M51a1 | 24
M51a1a | 15
M51a1b | 67
M51a2 | 215
M51b | 146
M51b1 | 124
M51b1a | 129
M51b1b | 130
M52 | 296
M52a | 48
M52a1 | 42
M52a1a | 54
M52a1b | 41
M52a1b1 | 51
M52b | 308
M52b1 | 196
M52b1a | 107
M53 | 9
M53b | 28

M54 | 228
M55 | 35
M55'77 | 296
M56 | 167
M57 | 230
M57+152 | 230
M57a | 142
M57b | 161
M57b1 | 70
M58 | 301
M59 | 117
M5a | 179
M5a1 | 92
M5a1a | 95
M5a1b | 176
M5a2 | 166
M5a2a | 177
M5a2a1 | 158
M5a2a1a | 98
M5a2a1a1 | 98
M5a2a1a2 | 86
M5a2a2 | 80
M5a2a3 | 82
M5a2a4 | 129
M5a3 | 15
M5a3a | 10
M5a3b | 28
M5a4 | 10
M5a5 | 163
M5a'd | 152
M5b | 252
M5b1 | 165
M5b2 | 190
M5b2a | 171
M5b2b | 206
M5b2b1 | 64
M5b2b1a | 67
M5b'c | 249
M5c | 149
M5c1 | 120
M5c2 | 54
M5d | 28
M6 | 534
M60 | 298
M60a | 169
M60a1 | 169
M60a2 | 169
M60b | 111
M61 | 159
M61a | 191
M62 | 152
M62'68 | 248
M62a | 120
M62b | 142
M62b+204 | 136
M62b1 | 125
M62b1a | 132
M62b1a1 | 161
M62b2 | 136
M63 | 113
M64 | 125
M65 | 157
M65a | 91
M65a+@16311 | 135
M65a1 | 92
M65a2 | 150
M65b | 130
M66 | 111
M66a | 115
M66b | 127
M67 | 103
M68 | 122
M68a | 104
M68a1 | 104
M68a1a | 115
M68a2 | 6
M68a2a | 6
M69 | 297
M69a | 23
M6a | 91
M6a1 | 92
M6a1a | 128
M6a1b | 79
M6a2 | 55
M6b | 105
M7 | 296
M70 | 157
M71 | 169
M71+151 | 162
M71a | 118
M71a1 | 113
M71a1a | 146
M71a2 | 145
M71b | 126
M71c | 119
M72 | 197
M72a | 195
M73 | 199
M73'79 | 186
M73a | 178
M73a1 | 182
M73b | 19
M74 | 279
M74a | 189
M74b | 296
M74b1 | 188
M74b2 | 282
M75 | 139
M76 | 53
M76a | 24
M77 | 129

M79 | 7
M7a | 165
M7a+16324 | 202
M7a1 | 202
M7a1a | 214
M7a1a1 | 203
M7a1a1a | 205
M7a1a2 | 112
M7a1a3 | 205
M7a1a4 | 202
M7a1a4a | 206
M7a1a5 | 202
M7a1a5a | 205
M7a1a6 | 203
M7a1a6a | 125
M7a1a7 | 205
M7a1a8 | 167
M7a1a9 | 205
M7a1b | 201
M7a1b1 | 51
M7a1b2 | 147
M7a2 | 166
M7a2a | 109
M7a2a1 | 109
M7a2a2 | 110
M7a2a3 | 107
M7a2a3a | 110
M7b | 300
M7b1 | 248
M7b1a | 136
M7b1a1 | 263
M7b1a1+(16192) | 694
M7b1a1a | 225
M7b1a1a1 | 229
M7b1a1a1a | 160
M7b1a1a1b | 156
M7b1a1a1b1 | 156
M7b1a1a1c | 125
M7b1a1a1d | 164
M7b1a1a2 | 289
M7b1a1a3 | 369
M7b1a1b | 339
M7b1a1c | 120
M7b1a1c1 | 106
M7b1a1d | 497
M7b1a1d1 | 499
M7b1a1e | 494
M7b1a1e1 | 514
M7b1a1e2 | 274
M7b1a1f | 524
M7b1a1g | 512
M7b1a1h | 507
M7b1a1i | 141
M7b1a1i1 | 125
M7b1a2 | 105
M7b1a2a | 173
M7b1a2a1 | 204
M7b1a2a1a | 58
M7b1a2a1b | 138
M7b1a2a1b1 | 58
M7b1b | 172
M7b2 | 90
M7b2a | 72
M7b'c | 296
M7c | 243
M7c1 | 327
M7c1a | 410
M7c1a1 | 294
M7c1a1a | 302
M7c1a1a1 | 299
M7c1a1b | 158
M7c1a1b1 | 110
M7c1a2 | 134
M7c1a2a | 133
M7c1a2a1 | 130
M7c1a3 | 152
M7c1a3a | 30
M7c1a4 | 294
M7c1a4a | 796
M7c1a4b | 267
M7c1a5 | 263
M7c1b | 302
M7c1b1 | 297
M7c1b2 | 294
M7c1b2a | 148
M7c1b2b | 316
M7c1c | 323
M7c1c1 | 128
M7c1c1a | 112
M7c1c1a1 | 122
M7c1c2 | 232
M7c1c2a | 116
M7c1c3 | 1251
M7c1c3a | 112
M7c1c3a1 | 119
M7c1c3b | 711
M7c1c3c | 767
M7c1c3d | 768
M7c1c3e | 181
M7c1c3f | 115
M7c1c3g | 771
M7c1c3h | 136
M7c1c3i | 718
M7c2 | 141
M7c2a | 160
M7c2b | 139
M7c3 | 249
M8 | 169
M80 | 1
M80'D | 296
M81 | 110

M8a | 209
M8a1 | 219
M8a1a | 175
M8a2 | 233
M8a2+152 | 224
M8a2'3 | 234
M8a2a | 138
M8a2a1 | 252
M8a2b | 180
M8a2c | 241
M8a2d | 134
M8a2e | 237
M8a3 | 245
M8a3a | 217
M8a3a1 | 205
M9 | 711
M91 | 100
M91a | 138
M91b | 10
M9a | 220
M9a1 | 213
M9a1a | 968
M9a1a1 | 932
M9a1a1a | 1093
M9a1a1b | 112
M9a1a1c | 133
M9a1a1c1 | 158
M9a1a1c1a | 169
M9a1a1c1b | 928
M9a1a1c1b1 | 1043
M9a1a1c1b1a | 1125
M9a1a1c1b1a1 | 1059
M9a1a1c1b1a2 | 872
M9a1a1c1b2 | 929
M9a1a1c1c | 139
M9a1a1d | 1009
M9a1a2 | 211
M9a1a3 | 114
M9a1b | 210
M9a1b+150 | 229
M9a1b1 | 499
M9a1b1a | 381
M9a1b1a1 | 357
M9a1b1b | 350
M9a1b1c | 388
M9a1b2 | 220
M9a4 | 212
M9a4a | 111
M9a4a1 | 108
M9a4a2 | 110
M9a4b | 214
M9a5 | 224
M9a'b | 535
M9b | 33
N | 213
N1 | 213
N10 | 113
N10a | 76
N10b | 20
N11 | 213
N11a | 14
N11a1 | 44
N11a2 | 14
N11b | 15
N13 | 10
N14 | 212
N1'5 | 213
N1a | 133
N1a1 | 143
N1a1'2 | 134
N1a1a | 12
N1a1a+152 | 13
N1a1a1 | 11
N1a1a1a | 17
N1a1a1a1 | 24
N1a1a1a1a | 9
N1a1a1a2 | 31
N1a1a1a3 | 21
N1a1a1b | 4
N1a1a2 | 10
N1a1a3 | 25
N1a1b | 25
N1a1b1 | 24
N1a2 | 12
N1a3 | 12
N1a3a | 107
N1a3a1 | 2
N1a3a1a | 4
N1a3a2 | 12
N1a3a3 | 4
N1b | 42
N1b1 | 105
N1b1a | 130
N1b1a+16129 | 50
N1b1a+195 | 54
N1b1a1 | 101
N1b1a2 | 134
N1b1a2a | 108
N1b1a2b | 106
N1b1a3 | 113
N1b1a4 | 9
N1b1a4a | 8
N1b1a5 | 109
N1b1a6 | 105
N1b1a7 | 43
N1b1a8 | 13
N1b1a8a | 13
N1b1a8b | 13
N1b1b | 51
N1b1b1 | 65
N1b2 | 46

N2 | 133
N21 | 47
N21+195 | 123
N21a | 25
N22 | 50
N22a | 12
N2a | 25
N2a1 | 9
N2a2 | 5
N3 | 19
N3a | 30
N3a1 | 12
N3b | 14
N5 | 83
N5a | 6
N7 | 136
N7a | 15
N7a1 | 3
N7a2 | 8
N7b | 141
N8 | 111
N9 | 213
N9a | 87
N9a1 | 143
N9a10 | 107
N9a10+16311 | 142
N9a10a | 49
N9a10a1 | 49
N9a10a2 | 47
N9a10a2a | 39
N9a10b | 27
N9a11 | 14
N9a1'3 | 102
N9a1a | 150
N9a2 | 53
N9a2'4'5'11 | 67
N9a2a | 69
N9a2a1 | 13
N9a2a2 | 46
N9a2a3 | 50
N9a2c | 43
N9a2d | 63
N9a3 | 104
N9a4 | 41
N9a4a | 44
N9a4b | 45
N9a4b1 | 45
N9a5 | 36
N9a6 | 86
N9a6a | 70
N9a6b | 61
N9a7 | 36
N9a8 | 79
N9a9 | 93
N9b | 99
N9b1 | 97
N9b1a | 98
N9b1b | 73
N9b1c | 28
N9b1c1 | 20
N9b2 | 76
N9b2a | 78
N9b3 | 90
N9b4 | 85
O | 87
O1 | 17
O1a | 17
P | 100
P+16176 | 48
P1 | 14
P1+152 | 32
P10 | 20
P1d | 40
P1d1 | 19
P1d1a | 46
P1d2 | 17
P1d2a | 10
P1f | 3
P2 | 87
P2'10 | 27
P3 | 2
P3a | 8
P3b | 5
P3b1 | 7
P4 | 81
P4a | 35
P4a1 | 19
P4b | 20
P4b1 | 27
P5 | 70
P6 | 35
P7 | 2
P8 | 36
P9 | 84
P9a | 92
Q | 162
Q1 | 165
Q1+@16223 | 106
Q1'2 | 162
Q1a | 110
Q1a1 | 133
Q1a1a | 164
Q1b | 110
Q1c | 109
Q1c1 | 112
Q1c1a | 142
Q1c2 | 112
Q1c2a | 109
Q1d | 176
Q1e | 153
Q1e1 | 151

Q1e1a | 106
Q1e1a1 | 103
Q1e1b | 110
Q1e1b1 | 105
Q1e1c | 172
Q1f | 150
Q1f1 | 117
Q1f2 | 163
Q2 | 114
Q2a | 112
Q2a1 | 104
Q2a2 | 114
Q2a2a | 110
Q2a2b | 2
Q2a3 | 114
Q2a3a | 110
Q2a3b | 103
Q2a4 | 111
Q2b | 1
Q3 | 164
Q3a | 109
Q3a+61_62 | 125
Q3a1 | 129
Q3b | 164
R | 143
R+16189 | 118
R0 | 794
R0a | 60
R0a+60.1T | 108
R0a1 | 112
R0a1+152 | 61
R0a1a | 140
R0a1a1 | 79
R0a1a1a | 13
R0a1a2 | 85
R0a1a3 | 90
R0a1a4 | 79
R0a1b | 23
R0a2 | 147
R0a2+195 | 51
R0a2'3 | 79
R0a2a | 78
R0a2a1 | 77
R0a2b | 28
R0a2c | 140
R0a2d | 86
R0a2e | 79
R0a2f | 83
R0a2f1 | 49
R0a2f1a | 55
R0a2f1b | 12
R0a2g | 81
R0a2h | 27
R0a2i | 38
R0a2j | 44
R0a2k | 86
R0a2k1 | 27
R0a2l | 77
R0a2m | 77
R0a2n | 37
R0a3 | 77
R0a3a | 79
R0a4 | 58
R0a'b | 105
R0b | 16
R1 | 161
R11 | 41
R11a | 63
R11b | 24
R11b1 | 31
R11b1a | 9
R11b1b | 33
R11'B6 | 120
R12 | 20
R12'21 | 19
R14 | 13
R1a | 114
R1a1 | 113
R1a1a | 109
R1a1a1 | 112
R1a1a1a | 111
R1a1a2 | 111
R1a1b | 112
R1a1c | 111
R1b | 111
R1b1 | 106
R2 | 77
R2+13500 | 68
R2+13500+195 | 19
R21 | 26
R22 | 187
R23 | 23
R24 | 10
R24a | 15
R2a | 20
R2b | 70
R2b1 | 25
R2c | 11
R2d | 69
R2'JT | 113
R30 | 85
R30a | 83
R30a1 | 123
R30a1a | 124
R30a1b | 90
R30a1b1 | 57
R30a1c | 100
R30b | 33
R30b1 | 21
R30b2 | 55
R30b2a | 25

R31 | 123
R31a | 34
R31a1 | 10
R31b | 7
R32 | 37
R5 | 116
R5a | 45
R5a1 | 11
R5a1a | 21
R5a2 | 38
R5a2a | 79
R5a2b | 29
R5a2b1 | 25
R5a2b2 | 17
R5a2b3 | 20
R5a2b4 | 20
R6 | 144
R6+16129 | 87
R6a | 8
R6a1 | 21
R6a2 | 45
R6b | 21
R7 | 64
R7a | 58
R7a1 | 58
R7a1a | 58
R7a1b | 56
R7a1b1 | 56
R7a1b2 | 55
R7a'b | 56
R7b | 7
R7b1 | 7
R7b1a | 16
R7b1a1 | 10
R7b2 | 10
R8 | 21
R8a | 19
R8a1 | 17
R8a1+16093 | 50
R8a1a | 15
R8a1a1 | 16
R8a1a1a | 17
R8a1a1a1 | 16
R8a1a1a1a | 16
R8a1a1a2 | 22
R8a1a1b | 24
R8a1a1c | 21
R8a1a1d | 22
R8a1a2 | 23
R8a1a2a | 23
R8a1a3 | 36
R8a1b | 49
R8a2 | 16
R8b | 20
R8b1 | 25
R8b1a | 37
R8b2 | 12
R9 | 208
R9b | 186
R9b1 | 123
R9b1a | 43
R9b1a1 | 45
R9b1a1a | 95
R9b1a2 | 45
R9b1a2a | 31
R9b1a2b | 69
R9b1a3 | 164
R9b1b | 128
R9b2 | 153
R9c | 205
R9c1 | 91
R9c1a | 95
R9c1a1 | 97
R9c1a2 | 95
R9c1a3 | 100
R9c1b | 91
R9c1b1 | 95
R9c1b2 | 75
S | 214
S+152 | 175
S1 | 127
S1a | 31
S2 | 229
S3 | 5
S4 | 46
S5 | 3
T | 111
T1 | 75
T1a | 236
T1a+152 | 171
T1a1 | 365
T1a1+@152 | 132
T1a10 | 198
T1a10a | 109
T1a11 | 116
T1a12 | 35
T1a1'3 | 171
T1a13 | 42
T1a1a | 177
T1a1a1 | 179
T1a1b | 217
T1a1b1 | 191
T1a1c | 183
T1a1d | 177
T1a1e | 9
T1a1f | 179
T1a1g | 179
T1a1h | 19
T1a1i | 181
T1a1j | 182
T1a1k | 125

T1a1k1 | 107
T1a1k2 | 128
T1a1l | 120
T1a1m | 19
T1a1m1 | 24
T1a1n | 19
T1a1p | 183
T1a1q | 180
T1a1r | 178
T1a2 | 186
T1a2a | 19
T1a2b | 45
T1a3 | 176
T1a3a | 189
T1a4 | 48
T1a5 | 181
T1a5a | 64
T1a6 | 192
T1a7 | 61
T1a8 | 179
T1a8a | 65
T1a8b | 178
T1a9 | 72
T1b | 95
T1b1 | 88
T1b2 | 85
T1b3 | 87
T1b4 | 87
T2 | 243
T2+150 | 88
T2+16189 | 78
T2a | 177
T2a+195 | 94
T2a1 | 193
T2a1a | 224
T2a1a1 | 186
T2a1a2 | 200
T2a1a3 | 185
T2a1a3a | 180
T2a1a5 | 177
T2a1a6 | 180
T2a1a7 | 182
T2a1a8 | 180
T2a1b | 114
T2a1b1 | 102
T2a1b1a | 116
T2a1b1a1 | 115
T2a1b1a1a | 101
T2a1b1a1a1 | 102
T2a1b1a1a2 | 104
T2a1b1a1b | 107
T2a1b1a1b1 | 103
T2a1b1a2 | 65
T2a1b2 | 101
T2a1b2a | 101
T2a1b2b | 68
T2a2 | 73
T2a2a | 71
T2a3 | 92
T2b | 465
T2b+150 | 103
T2b+152 | 139
T2b+16362 | 92
T2b1 | 235
T2b11 | 90
T2b13 | 233
T2b13a | 66
T2b13b | 227
T2b15 | 227
T2b16 | 71
T2b17 | 225
T2b17a | 235
T2b19 | 233
T2b19b | 64
T2b2 | 227
T2b21 | 124
T2b21a | 36
T2b21b | 123
T2b22 | 123
T2b23 | 82
T2b23a | 73
T2b24 | 118
T2b24a | 122
T2b25 | 241
T2b26 | 227
T2b27 | 227
T2b28 | 231
T2b29 | 226
T2b2b | 189
T2b2b1 | 76
T2b3 | 229
T2b3+151 | 132
T2b30 | 226
T2b31 | 227
T2b32 | 225
T2b33 | 29
T2b34 | 107
T2b35 | 227
T2b36 | 235
T2b37 | 225
T2b3a | 28
T2b3a1 | 62
T2b3b | 232
T2b3c | 99
T2b3d | 88
T2b3e | 23
T2b4 | 266
T2b4+152 | 147
T2b4a | 98
T2b4a1 | 88
T2b4b | 50

T2b4c | 123
T2b4d | 123
T2b4e | 124
T2b4f | 125
T2b4g | 125
T2b4h | 88
T2b4i | 63
T2b5 | 260
T2b5a | 227
T2b5a1 | 167
T2b6 | 172
T2b6+146 | 93
T2b6a | 168
T2b6b | 13
T2b7 | 225
T2b7a | 224
T2b7a1 | 42
T2b7a2 | 69
T2b7a3 | 80
T2b8 | 231
T2b9 | 110
T2c | 183
T2c1 | 96
T2c1+146 | 48
T2c1a | 67
T2c1a1 | 28
T2c1a2 | 66
T2c1a3 | 65
T2c1c | 84
T2c1c1 | 56
T2c1c2 | 66
T2c1d | 35
T2c1d+152 | 50
T2c1d1 | 41
T2c1d1a | 40
T2c1d2 | 44
T2c1d2a | 74
T2c1e | 49
T2c1f | 43
T2d | 177
T2d1 | 129
T2d1a | 39
T2d1b | 79
T2d1b1 | 64
T2d1b2 | 65
T2d2 | 188
T2e | 114
T2e+152 | 36
T2e1 | 67
T2e1a | 55
T2e1a1 | 35
T2e1a1a | 30
T2e1a1b | 7
T2e1a1b1 | 3
T2e1b | 37
T2e1b1 | 36
T2e2 | 67
T2e2a | 56
T2e5 | 26
T2e6 | 27
T2e7 | 38
T2f | 78
T2f1 | 50
T2f1a | 26
T2f1a1 | 95
T2f2 | 80
T2f3 | 70
T2f4 | 77
T2f5 | 52
T2f6 | 77
T2f7 | 78
T2f7a | 65
T2f8 | 78
T2f8a | 82
T2g | 187
T2g1 | 187
T2g1a | 205
T2g1a1 | 74
T2g1b | 74
T2g2 | 122
T2g2a | 80
T2h | 195
T2h1 | 180
T2h2 | 78
T2i | 77
T2i1 | 70
T2i2 | 103
T2j | 64
T2j1 | 65
T2k | 75
T2l | 181
T2m | 7
T2n | 121
T3 | 33
U | 83
U1 | 23
U1a | 44
U1a1 | 52
U1a1a | 92
U1a1a+16129 | 27
U1a1a1 | 44
U1a1a1a | 44
U1a1a2 | 51
U1a1a3 | 16
U1a1b | 22
U1a1c | 41
U1a1c1 | 54
U1a1c1a | 16
U1a1c1b | 42
U1a1c1c | 42
U1a1c1c1 | 20

U1a1c1d | 61
U1a1c1d1 | 13
U1a1d | 9
U1a2 | 26
U1a3 | 49
U1b | 14
U1b1 | 16
U1b2 | 20
U1b3 | 26
U2 | 44
U2+152 | 60
U2'3'4'7'8'9 | 81
U2a | 39
U2a1 | 67
U2a1a | 71
U2a1b | 62
U2a2 | 27
U2b | 88
U2b1 | 70
U2b1a | 36
U2b2 | 80
U2c | 50
U2c1 | 53
U2c1a | 49
U2c1b | 44
U2c'd | 65
U2d | 19
U2d1 | 14
U2d2 | 17
U2d2a | 14
U2d3 | 14
U2e | 50
U2e1 | 83
U2e1'2'3 | 77
U2e1a | 79
U2e1a1 | 109
U2e1a1a | 67
U2e1a1b | 32
U2e1a1c | 67
U2e1b | 13
U2e1b1 | 37
U2e1b2 | 12
U2e1c | 62
U2e1c1 | 66
U2e1d | 65
U2e1e | 11
U2e1f | 4
U2e1f1 | 6
U2e1g | 66
U2e1h | 71
U2e2 | 67
U2e2a | 67
U2e2a1 | 62
U2e2a1a | 80
U2e2a1a1 | 85
U2e2a1a2 | 74
U2e2a1b | 39
U2e2a1c | 73
U2e2a1d | 74
U2e3 | 7
U2e3a | 5
U3 | 89
U3a | 64
U3a1 | 79
U3a1a | 62
U3a1a1 | 61
U3a1b | 48
U3a1c | 40
U3a1c1 | 25
U3a2 | 48
U3a2a | 31
U3a2a1 | 23
U3a2a1a | 12
U3a3 | 62
U3a'c | 88
U3b | 98
U3b1 | 118
U3b1a | 26
U3b1a1 | 21
U3b1b | 98
U3b2 | 113
U3b2a | 90
U3b2a1 | 108
U3b2a1a | 58
U3b2b | 22
U3b2c | 92
U3b3 | 86
U3c | 37
U4 | 126
U4'9 | 13
U4a | 133
U4a1 | 140
U4a1a | 120
U4a1a1 | 102
U4a1a2 | 91
U4a1a3 | 91
U4a1b | 85
U4a1b1 | 27
U4a1b1a | 6
U4a1b2 | 9
U4a1c | 9
U4a1d | 38
U4a1e | 9
U4a2 | 146
U4a2a | 37
U4a2a1 | 13
U4a2a2 | 14
U4a2a3 | 33
U4a2b | 38
U4a2c | 97
U4a2c1 | 5

U4a2d | 5
U4a2e | 96
U4a2f | 97
U4a2g | 100
U4a2h | 98
U4a2h1 | 6
U4a3 | 34
U4a3a | 18
U4b | 130
U4b1 | 126
U4b1+146_152 | 73
U4b1a | 122
U4b1a1 | 122
U4b1a1a | 128
U4b1a1a1 | 62
U4b1a2 | 120
U4b1a2a | 124
U4b1a2b | 122
U4b1a3 | 67
U4b1a3a | 76
U4b1a4 | 141
U4b1b | 73
U4b1b1 | 88
U4b1b1+16311 | 8
U4b1b1a | 10
U4b1b1b | 82
U4b1b1c | 12
U4b1b1d | 74
U4b1b2 | 91
U4b2 | 21
U4b2a | 14
U4b2a1 | 15
U4b2a1a | 13
U4b3 | 93
U4c | 126
U4c1 | 78
U4c1a | 46
U4c2 | 122
U4c2a | 25
U4d | 120
U4d1 | 123
U4d1a | 122
U4d1a1 | 127
U4d1a1a | 123
U4d1b | 120
U4d2 | 50
U4d3 | 113
U5 | 29
U5a | 47
U5a1 | 54
U5a1+@16192 | 103
U5a1a | 117
U5a1a1 | 169
U5a1a1+152 | 113
U5a1a1+16362 | 32
U5a1a1a | 97
U5a1a1b | 98
U5a1a1c | 109
U5a1a1d | 31
U5a1a1d1 | 25
U5a1a1e | 107
U5a1a1g | 99
U5a1a1h | 53
U5a1a1i | 93
U5a1a2 | 56
U5a1a2a | 77
U5a1a2a1 | 60
U5a1a2a1a | 62
U5a1a2b | 69
U5a1a2b1 | 57
U5a1b | 69
U5a1b+16362 | 28
U5a1b1 | 84
U5a1b1a | 57
U5a1b1a1 | 54
U5a1b1a2 | 54
U5a1b1b | 48
U5a1b1b1 | 22
U5a1b1c | 54
U5a1b1c1 | 52
U5a1b1c2 | 23
U5a1b1d | 52
U5a1b1d+16093 | 28
U5a1b1d1 | 14
U5a1b1e | 68
U5a1b1f | 22
U5a1b1g | 48
U5a1b1h | 56
U5a1b2 | 47
U5a1b3 | 29
U5a1b3a | 23
U5a1b3a1 | 15
U5a1b4 | 31
U5a1c | 24
U5a1c1 | 22
U5a1c1a | 15
U5a1c2 | 20
U5a1c2a | 4
U5a1c2a1 | 22
U5a1d | 48
U5a1d1 | 60
U5a1d2 | 47
U5a1d2a | 35
U5a1d2a1 | 21
U5a1d2b | 25
U5a1e | 47
U5a1f | 48
U5a1f1 | 30
U5a1f1a | 8
U5a1f1a1 | 20
U5a1f2 | 32

U5a1g | 106
U5a1g1 | 34
U5a1g2 | 39
U5a1h | 35
U5a1i | 48
U5a1i1 | 64
U5a1j | 46
U5a2 | 57
U5a2+16294 | 32
U5a2+16362 | 25
U5a2a | 34
U5a2a1 | 78
U5a2a1+152 | 44
U5a2a1a | 42
U5a2a1b | 38
U5a2a1b1 | 15
U5a2a1c | 48
U5a2a1d | 36
U5a2a1e | 39
U5a2a2 | 21
U5a2a2a | 26
U5a2b | 81
U5a2b1 | 77
U5a2b1a | 64
U5a2b1b | 38
U5a2b1c | 61
U5a2b1d | 56
U5a2b2 | 59
U5a2b2a | 61
U5a2b2a1 | 19
U5a2b3 | 19
U5a2b3a | 16
U5a2b3a1 | 23
U5a2b4 | 60
U5a2b4a | 60
U5a2b5 | 33
U5a2c | 60
U5a2c1 | 66
U5a2c2 | 60
U5a2c3 | 40
U5a2c3a | 42
U5a2c4 | 60
U5a2d | 61
U5a2d1 | 41
U5a2d1a | 44
U5a2e | 32
U5a'b | 28
U5b | 34
U5b1 | 40
U5b1+16189 | 35
U5b1+16189+@16192 | 91
U5b1a | 50
U5b1b | 33
U5b1b1 | 31
U5b1b1+@16192 | 95
U5b1b1+152 | 19
U5b1b1a | 118
U5b1b1a1 | 68
U5b1b1a1a | 73
U5b1b1a1a1 | 31
U5b1b1a1b | 67
U5b1b1a2 | 68
U5b1b1a3 | 38
U5b1b1b | 39
U5b1b1d | 58
U5b1b1e | 20
U5b1b1f | 59
U5b1b1g | 30
U5b1b1g1 | 25
U5b1b1g1a | 3
U5b1b2 | 47
U5b1b2a | 18
U5b1b2b | 10
U5b1c | 32
U5b1c1 | 29
U5b1c1a | 14
U5b1c1a1 | 19
U5b1c2 | 32
U5b1c2a | 20
U5b1c2b | 28
U5b1d | 34
U5b1d1 | 34
U5b1d1a | 72
U5b1d1b | 23
U5b1d1c | 29
U5b1d2 | 27
U5b1e | 36
U5b1e1 | 73
U5b1e1a | 32
U5b1f | 36
U5b1f1 | 27
U5b1f1a | 192
U5b1g | 25
U5b1h | 35
U5b1i | 72
U5b2 | 47
U5b2a | 30
U5b2a+@16192 | 60
U5b2a1 | 27
U5b2a1a | 39
U5b2a1a+16311 | 38
U5b2a1a1 | 56
U5b2a1a1a | 34
U5b2a1a1b | 27
U5b2a1a1d | 38
U5b2a1a2 | 59
U5b2a1b | 29
U5b2a2 | 38
U5b2a2a | 35
U5b2a2a1 | 44
U5b2a2a2 | 36

U5b2a2b | 43
U5b2a2b1 | 50
U5b2a2c | 25
U5b2a3 | 33
U5b2a3a | 32
U5b2a4 | 60
U5b2a4a | 59
U5b2a5 | 30
U5b2a5a | 30
U5b2a6 | 59
U5b2b | 87
U5b2b1 | 82
U5b2b1a | 27
U5b2b1a1 | 31
U5b2b1a2 | 21
U5b2b1b | 50
U5b2b2 | 74
U5b2b3 | 21
U5b2b3a | 12
U5b2b3a1 | 7
U5b2b3a1a | 5
U5b2b3b | 34
U5b2b4 | 67
U5b2b4a | 36
U5b2b5 | 43
U5b2c | 32
U5b2c1 | 38
U5b2c2 | 35
U5b2c2a | 34
U5b2c2b | 40
U5b3 | 53
U5b3a | 21
U5b3a1 | 21
U5b3a1a | 98
U5b3a1b | 23
U5b3a2 | 40
U5b3b | 42
U5b3b1 | 17
U5b3b2 | 27
U5b3c | 41
U5b3d | 23
U5b3e | 43
U5b3f | 27
U5b3g | 41
U5b3h | 33
U6 | 49
U6+16311 | 19
U6a | 23
U6a+16189 | 34
U6a+16189+(103) | 35
U6a1 | 26
U6a1a | 37
U6a1a1 | 61
U6a1a2 | 35
U6a1b | 19
U6a1b1 | 26
U6a1b1a | 9
U6a1b1b | 23
U6a1b2 | 16
U6a1b3 | 19
U6a1b4 | 19
U6a2 | 36
U6a2+195 | 21
U6a2a | 34
U6a2a1 | 39
U6a2a2 | 15
U6a2a2a | 2
U6a2b | 19
U6a2b1 | 22
U6a2c | 20
U6a3 | 35
U6a3+185 | 11
U6a3a | 35
U6a3a1 | 37
U6a3a1a | 37
U6a3a2 | 6
U6a3a2a | 7
U6a3b | 23
U6a3b1 | 11
U6a3c | 22
U6a3d | 21
U6a3d1 | 21
U6a3d1a | 6
U6a3e | 7
U6a3f | 11
U6a3f1 | 11
U6a3f2 | 11
U6a4 | 27
U6a5 | 30
U6a5a | 28
U6a5a1 | 26
U6a5b | 21
U6a5c | 36
U6a6 | 24
U6a6a | 19
U6a6a1 | 21
U6a6b | 23
U6a6b1 | 32
U6a6b2 | 25
U6a7 | 25
U6a7a | 43
U6a7a1 | 56
U6a7a1+@152 | 23
U6a7a1a | 59
U6a7a1b | 27
U6a7a1c | 24
U6a7a2 | 35
U6a7a2a | 36
U6a7b | 12
U6a7b1 | 16
U6a7c | 35

U6a7c1 | 38
U6a8 | 34
U6a8a | 16
U6a8b | 36
U6a'b'd | 19
U6b | 34
U6b1 | 18
U6b1a | 29
U6b1a1 | 26
U6b1b | 19
U6b2 | 21
U6b3 | 19
U6b3a | 7
U6c | 34
U6c1 | 23
U6c2 | 11
U6d | 19
U6d1 | 21
U6d1a | 20
U6d1b | 19
U6d2 | 21
U6d3 | 19
U6d3a | 26
U7 | 152
U7a | 238
U7a1 | 8
U7a1a | 16
U7a2 | 129
U7a2a | 33
U7a3 | 114
U7a3a | 211
U7a3b | 88
U7a4 | 39
U7a4a | 16
U7a4a1 | 34
U7a4a1a | 36
U7a5 | 6
U7b | 234
U7b1 | 61
U7b2 | 10
U8 | 83
U8a | 29
U8a1 | 22
U8a1a | 36
U8a1a1 | 44
U8a1a1a | 38
U8a1a1a1 | 39
U8a1a1b | 34
U8a1a1b1 | 46
U8a1a2 | 33
U8a1a3 | 32
U8a1a4 | 6
U8a1b | 4
U8a2 | 16
U8b | 83
U8b1 | 27
U8b1a | 27
U8b1a1 | 47
U8b1a2 | 31
U8b1a2+16311 | 16
U8b1a2a | 14
U8b1a2b | 32
U8b1b | 30
U8b1b1 | 29
U8b1b2 | 28
U8b'c | 83
U8c | 60
U9 | 13
U9a | 44
U9a1 | 9
U9b | 12
U9b1 | 22
V | 382
V+@16298 | 64
V+@72 | 184
V+150 | 116
V1 | 195
V10 | 195
V10a | 39
V10b | 200
V10b1 | 189
V10b2 | 192
V11 | 194
V12 | 196
V13 | 206
V14 | 192
V15 | 189
V15a | 199
V16 | 40
V17 | 192
V18 | 136
V18a | 132
V19 | 28
V1a | 220
V1a1 | 239
V1a1a | 14
V1a1a1 | 17
V1a1b | 197
V1b | 30
V2 | 205
V20 | 37
V21 | 92
V22 | 124
V23 | 191
V24 | 189
V25 | 210
V26 | 193
V27 | 140
V28 | 190
V2a | 189
V2a1 | 195

V2a1a | 192
V2b | 195
V2b1 | 189
V2b2 | 192
V2c | 205
V3 | 202
V3a | 43
V3a1 | 43
V3b | 192
V3c | 47
V4 | 192
V5 | 30
V6 | 28
V7 | 98
V7a | 74
V7a1 | 72
V7b | 37
V8 | 205
V9 | 79
V9a | 16
V9a1 | 3
V9a2 | 22
W | 59
W+194 | 63
W1 | 85
W1+119 | 47
W1a | 69
W1b | 27
W1b1 | 35
W1c | 36
W1c1 | 11
W1d | 12
W1e | 41
W1e1 | 32
W1e1a | 16
W1f | 37
W1g | 15
W1h | 10
W1h1 | 10
W1i | 32
W3 | 46
W3a | 53
W3a1 | 81
W3a1+199 | 37
W3a1a | 53
W3a1a1 | 46
W3a1a2 | 46
W3a1a3 | 46
W3a1b | 72
W3a1c | 35
W3a1d | 42
W3a2 | 18
W3b | 92
W3b1 | 27
W4 | 40
W4a | 28
W4a1 | 146
W4b | 28
W4c | 27
W4d | 12
W5 | 58
W5a | 17
W5a1 | 24
W5a1a | 36
W5a1a1 | 19
W5a1a1a | 14
W5a2 | 17
W5a2b | 2
W5b | 27
W5b1 | 23
W5b1a | 26
W6 | 101
W6a | 63
W6b | 45
W6b1 | 47
W6c | 42
W6c1 | 67
W6c1a | 9
W6d | 45
W7 | 27
W8 | 59
W9 | 52
X | 159
X1 | 29
X1'2'3 | 99
X1'3 | 100
X1a | 29
X1c | 51
X2 | 123
X2+225 | 139
X2+225+@153 | 99
X2+225+@16223 | 18
X2a | 8
X2a1 | 17
X2a1a | 8
X2a1a1 | 15
X2a1b | 14
X2a1b1 | 8
X2a1b1a | 11
X2a1c | 2
X2a2 | 5
X2a'j | 106
X2b | 116
X2b+226 | 166
X2b+226+16192 | 9
X2b1 | 9
X2b10 | 94
X2b10a | 6
X2b11 | 101
X2b12 | 5
X2b13 | 6

X2b2 | 124
X2b3 | 126
X2b4 | 138
X2b4a | 125
X2b4a1 | 125
X2b5 | 125
X2b6 | 126
X2b6a | 125
X2b7 | 124
X2b8 | 124
X2b9 | 124
X2b'd | 106
X2c | 22
X2c1 | 59
X2c1a | 18
X2c1b | 7
X2c1c | 6
X2c1c1 | 6
X2c1d | 21
X2c1e | 3
X2c2 | 38
X2d | 117
X2d1 | 106
X2d1a | 119
X2d2 | 116
X2e | 106
X2e1 | 30
X2e1a | 16
X2e1a1 | 4
X2e1b | 31
X2e2 | 116
X2e2a | 117
X2e2a1 | 113
X2e2a2 | 102
X2e2b | 19
X2e2b1 | 11
X2e2c | 14
X2e2c1 | 16
X2f | 108
X2f1 | 11
X2g | 102
X2h | 19
X2i | 16
X2i+@225 | 20
X2i1 | 13
X2j | 3
X2k | 113
X2l | 102
X2m | 98
X2m1 | 8
X2m2 | 7
X2m'n | 126
X2n | 12
X2o | 107
X2o1 | 113
X2p | 114
X2p1 | 6
X3 | 98
X3a | 106
X4 | 29
Y | 74
Y1 | 99
Y1a | 58
Y1a+16189 | 128
Y1a1 | 61
Y1a2 | 66
Y1b | 60
Y1b1 | 72
Y1b1a | 54
Y2 | 142
Y2a | 140
Y2a1 | 151
Y2a1a | 132
Y2b | 88
Z | 247
Z+152 | 237
Z1 | 222
Z1a | 124
Z1a1 | 120
Z1a1a | 55
Z1a1b | 124
Z1a2 | 118
Z1a2a | 137
Z1a3 | 137
Z2 | 231
Z3 | 289
Z3+709 | 159
Z3a | 220
Z3a1 | 203
Z3a1a | 218
Z3a2 | 204
Z3b | 213
Z3c | 147
Z3d | 189
Z4 | 234
Z4a | 181
Z4a1 | 117
Z4a1a | 109
Z4a1a1 | 110
Z5 | 98
Z7 | 218

HG, haplogroup
