# Supplementary material for: Mitochondrial Haplogroup Classification of Ancient DNA Samples Using Haplotracker
Source: Biomed Res Int. 2022 Mar 18;2022:5344418. doi: 10.1155/2022/5344418 (PMC8956381; doi:10.1155/2022/5344418)
Supplement: Supplementary Materials — Fig. S1: characterization of Phylotree-provided control region sequences tested for haplogroup classification by Haplotracker. Fig. S2: minimum number of amplicons required by Haplotracker in discriminating between haplogroups using mtDNA control and coding region sequences. Fig. S3: variant identification of an aDNA sample (MNW3) using an HRM real-time PCR. Table S1: haplogroups and their variant profiles extracted from Phylotree mtDNA Build 17. Table S2: haplogroup frequency carrying an extra variant in 118,869 haplotypes. Table S3: haplogroup frequency carrying a missing variant in 118,869 haplotypes. Table S4: haplogroup frequency in 118,869 haplotypes. Table S5: list of ancient human samples found in 2,000-year-old elite Xiongnu cemetery in Northeast Mongolia. Table S6: primers for the amplification of mtDNA coding region segments for haplogroup determination. Table S7: high-resolution melting real-time PCR primer design for screening variants to differentiate haplogroups G1a1, G1a1a, and G1a1b. Table S8: haplogroup classification of full-length mtGenome sequences from Phylotree (n = 8,216). Table S9: haplogroup classification with full-length and control region sequences of mtDNA using Haplotracker and HaploGrep 2. Table S10: comparison of servers using control region sequences from GenBank before December 25, 2018 (n = 45,177). Table S11: comparison details for the servers using control region sequences from GenBank before December 25, 2018 (n = 45,177). Table S12: comparison of servers using control region sequences downloaded from GenBank from December 26, 2018 to August 22, 2019. Table S13: sequences of mtDNA PCR products from Mongolian ancient DNA samples. Table S14: haplogroup classification of Mongolian ancient DNA samples using Haplotracker. Table S15: minimum number of amplicons required by Haplotracker in discriminating between haplogroups using mtDNA control and coding region sequences. Table S16: minimum number of amplicons per superhaplogroup requ [file 5344418.f1.zip › 5344418.f8.pdf]

Table S5. List of ancient human samples found in 2,000-year-old elite Xiongnu cemetery in Northeast Mongolia

No.	Code	Excavation site	Archeological date	Sample	Note
1	MNX2	Duurlig Nars, Mongolia	100BC-100AD, Xiongnu	Molar teeth	[30]
2	MNX3	Duurlig Nars, Mongolia	300BC-100BC, Xiongnu	Tibia and fibula	[30]
3	MNX4	Duurlig Nars, Mongolia	300BC-100AD, Xiongnu	Femur	[30]
4	MNE1	Duurlig Nars, Mongolia	300BC–100AD, Xiongnu	Femur	[31]
5	MNE2	Duurlig Nars, Mongolia	100BC–100AD, Xiongnu	Femur	[31]
6	MNE3	Duurlig Nars, Mongolia	100BC–100AD, Xiongnu	Tibia	[31]
7	MNW1	Duurlig Nars, Mongolia	100BC–100AD, Xiongnu	Femur	[31]
8	MNW3	Duurlig Nars, Mongolia	100BC–100AD, Xiongnu	Humerus	[31]
9	MNW4	Duurlig Nars, Mongolia	100BC–100AD, Xiongnu	Molar teeth	[31]
