# Supplementary material for: Mitochondrial Haplogroup Classification of Ancient DNA Samples Using Haplotracker
Source: Biomed Res Int. 2022 Mar 18;2022:5344418. doi: 10.1155/2022/5344418 (PMC8956381; doi:10.1155/2022/5344418)
Supplement: Supplementary Materials — Fig. S1: characterization of Phylotree-provided control region sequences tested for haplogroup classification by Haplotracker. Fig. S2: minimum number of amplicons required by Haplotracker in discriminating between haplogroups using mtDNA control and coding region sequences. Fig. S3: variant identification of an aDNA sample (MNW3) using an HRM real-time PCR. Table S1: haplogroups and their variant profiles extracted from Phylotree mtDNA Build 17. Table S2: haplogroup frequency carrying an extra variant in 118,869 haplotypes. Table S3: haplogroup frequency carrying a missing variant in 118,869 haplotypes. Table S4: haplogroup frequency in 118,869 haplotypes. Table S5: list of ancient human samples found in 2,000-year-old elite Xiongnu cemetery in Northeast Mongolia. Table S6: primers for the amplification of mtDNA coding region segments for haplogroup determination. Table S7: high-resolution melting real-time PCR primer design for screening variants to differentiate haplogroups G1a1, G1a1a, and G1a1b. Table S8: haplogroup classification of full-length mtGenome sequences from Phylotree (n = 8,216). Table S9: haplogroup classification with full-length and control region sequences of mtDNA using Haplotracker and HaploGrep 2. Table S10: comparison of servers using control region sequences from GenBank before December 25, 2018 (n = 45,177). Table S11: comparison details for the servers using control region sequences from GenBank before December 25, 2018 (n = 45,177). Table S12: comparison of servers using control region sequences downloaded from GenBank from December 26, 2018 to August 22, 2019. Table S13: sequences of mtDNA PCR products from Mongolian ancient DNA samples. Table S14: haplogroup classification of Mongolian ancient DNA samples using Haplotracker. Table S15: minimum number of amplicons required by Haplotracker in discriminating between haplogroups using mtDNA control and coding region sequences. Table S16: minimum number of amplicons per superhaplogroup requ [file 5344418.f1.zip › 5344418.f9.pdf]

Table S6. Primers for the amplification of mtDNA coding region segments for haplogroup determination

Haplogroup	Variant[1]	Region[2]	Primer name[3]	Sequence (5' → 3')	T_m (°C)[4]	Size (bp)	Note
A	1736	1690-1815	F1652	CTTAACTTGACCGCTCTGAGCTAAAC	66.9	201	[31]
			R1852	GCAGAAGGTATAGGGGTTAGTCCTTG	67.3		[31]
			FN1670	AGCTAAACCTAGCCCCAAAC	64.5	171	[31]
			RN1840	GGGGTTAGTCCTTGCTATATTATGC	64.0		[31]
A12	12720	12652-12800	F12601	TTCATCCCTGTAGCATTGTTCGT	65.7	226	Present study
			R12826	CATCTGCTCGGGCGTATCAT	66.8		Present study
			FN12628	TGGTCCATCATAGAATTCTCACTG	63.1	179	Present study
			RN12818	CGGGCGTATCATCAACTG	62.8		Present study
A12	14290	14276-14368	F14243	GCCCCCGCACCAATAG	64.6	184	Present study
			R14426	GGTCAGGGGTTGAGGTCTT	65.4		Present study
			FN14258	GGATCCTCCCGAATCAAC	61.0	130	Present study
			RN14387	TAGCGATGGAGGTAGGATT	61.0		Present study
C	3552A	3493-3598	F3453	CGCTGACGCCATAAAACTCT	65.4	188	[31]
			R3640	CTAGGCTAGAGGTGGCTAGAAT	64.7		[31]
			FN3473	TCACCAAAGAGCCCCTAAAA	61.5	146	[31]
			RN3618	AAATAGGAGGCCTAGGTTGA	61.5		[31]
C4	15204	15133-15207	F15076	AGAAACCTGAAACATCGGCATTAT	65.5	195	[31]
			R15270	AGGGTGGGACTGTCTACTGA	65.5		[31]
			FN15113	ACTATAGCAACAGCCTTCAT	61.7	115	[31]
			RN15227	CTAGGTCTGTCCCAATGTAT	60.6		[31]
C4a	12672	12652-12800	F12601	TTCATCCCTGTAGCATTGTTCGT	63.8	226	Present study
			R12826	CATCTGCTCGGGCGTATCAT	65.0		Present study
			FN12628	TGGTCCATCATAGAATTCTCACTG	61.2	191	Present study
			RN12818	CGGGCGTATCATCAACTG	61.0		Present study
C4a1	7999	7951-8070	F7918	CGACTACGGCGGACTAATC	63.9	203	Same as for haplogroup D4b
			R8120	GACGTCCGGGAATTGCAT	64.8		Same as for haplogroup D4b
			FN7950	GGACTAATCTTCAACTCCTACAT	61.1	159	Same as for haplogroup D4b

			RN8086	TGTGGGGACAGCTCAT	61.5		Same as for haplogroup D4b
C4a1a	1715	1649-1757	F1600	ACCAGAGTGTAGCTTAACACAAAG	65.1	200	Present study
			R1799	ATCTTTCCCTTGCGGTACTATATC	63.9		Present study
			FN1626	CCCAACTTACACTTAGGAGATTT	62.1	151	Present study
			RN1776	CTATTGCGCCAGGTTTCAA	62.0		Present study
C4a1a2	10891	10802-10939	F10761	TGCTAAAACTAATCGTCCCAACAAT	64.3	220	Present study
			R10982	GGGGTAGGAGTCAGGTAGTTAG	64.3		Present study
			FN10766	TCCCAACAATTATATTACTACCACTG	61.6	183	Present study
			RN10958	TTAGGAGGGGGGTTGTTAG	61.6		Present study
C4a1a3	15607	15561-15686	F15536	ACCCCTCCCCACATCAAG	66.3	200	Present study
			R15735	GCGGCTAGGAGTCAATAAAGTG	64.8		Present study
			FN15544	CCACATCAAGCCCGAAT	62.3	165	Present study
			RN15708	CTTAGTGGGCGAAATATTATGC	61.2		Present study
C4a1a4	12940	12868-13002	F12835	GCAGCCATTCAAGCAATCCTATAC	65.3	199	Present study
			R13033	GTCAGGGGTGGAGACCTAATTG	65.8		Present study
			FN12846	AGCAATCCTATACAACCGTATC	61.9	177	Present study
			RN13022	AGACCTAATTGGGCTGATTT	61.9		Present study
D	5178A	5080-5305	F5047	TTCTACCGTACAACCCTAACATAAC	62.9	286	[30]
			R5313	AGGAGGGTGATGGTGGCTAT	67.9		[30]
			FN5055	TACAACCCTAACATAACCATTCTTA	59.7	270	[30]
			RN5324	GATGGTGGCTATGATGGTG	62.0		[30]
D	5178A	5051-5179	F5007	GCATACTCCTCAATTACCCACATAG	64.2	213	Same as for haplogroup G
			R5219	GGAGAGGAGGGTGGATGGAATTA	65.6		Same as for haplogroup G
			FN5026	ACATAGGATGAATAATAGCAGTTCT	61.9	178	Same as for haplogroup G
			RN5203	GGAATTAAGGGTGTTAGTCATGTT	62.5		Same as for haplogroup G
D3	4023	3999-4098	F3960	CCCCTTCGCCCTATTCTTC	63.9	181	Present study
			R4140	GGGGTATGCTGTTCGAATTCAT	64.7		Present study
			FN3979	ATAGCCGAATACACAAACAT	60.1	139	Present study
			RN4117	AGAACAGGGAGGTTAGAAG	60.7		Present study

D3	9785	9692-9828	F9636	ATCACCTGAGCTCACCATAGTC	65.7	228	Present study
			R9863	GAAGCAGATAGTGAGGAAAGTTGAG	64.5		Present study
			FN9670	ACCGAAACCAAATAATTCAAGC	62.2	183	Present study
			RN9852	TGAGGAAAGTTGAGCCAATAATGA	62.5		Present study
D4	3010	2956-3217	F2907	TTGACCAACGGAACAAGTTACC	64.1	342	[30]
			R3229	CGGGCTCTGCCATCTTAACA	65.6		[30]
			FN2934	GATAACAGCGCAATCCTATTCT	62.4	306	[30]
			RN3239	CCATCTTAACAAACCCTGTTCT	62.7		[30]
D4	8414	8408-8503	F8358	ACAGTGAAATGCCCCAACTAAATAC	65.5	190	[31]
			R8547	AGCGAACAGATTTTCGTTCATTTTG	65.4		[31]
			FN8388	TATGGCCCACCATAATTACC	60.2	140	[31]
			RN8527	TTTTGGTTCTCAGGGTTTGTTATA	60.6		[31]
D4b	8020	7951-8070	F7918	CGACTACGGCGGACTAATC	63.9	203	Same as for haplogroup C4a1
			R8120	GACGTCCGGGAATTGCAT	64.8		Same as for haplogroup C4a1
			FN7928	GGACTAATCTTCAACTCCTACAT	61.1	159	Same as for haplogroup C4a1
			RN8086	TGTGGGGACAGCTCAT	61.5		Same as for haplogroup C4a1
D4b1c	951	894-1044	F868	CCCAGGGTTGGTCAATTTCG	65.7	221	Present study
			R1088	GTGGGGTATCTAATCCCAGTTTG	64.1		Present study
			FN877	GGTCAATTTCGTGCCAG	60.6	190	Present study
			RN1066	GGGTCTTAGCTATTGTGTGTTC	62.3		Present study
D4e	11215	11157-11276	F11117	ATCTTCTTCGAAACCACACTTATC	62.9	199	Same as for haplogroup D4j11
			R11315	GTTCTTGGGCAGTGAGAGT	63.8		Same as for haplogroup D4j11
			FN11141	CCCACCTTGGCTATCA	58.8	159	Same as for haplogroup D4j11
			RN11299	AGTGAGTAGTAGAATGTTTAGTG	59.7		Same as for haplogroup D4j11
D4e1	3316	3262-3370	F3212	CACCCAAGAACAGGGTTTGTTAA	64.2	205	Present study
			R3416	GGGCCTTTGCGTAGTTGTAT	64.7		Present study
			FN3244	GCCCGGTAATCGCATAAA	60.8	149	Present study
			RN3392	CCTAGAATTTTTCGTTCGGTAA	59.6		Present study
D4e2	15874	15819-15929	F15772	AGTAAGCTACCCTTTTACCATCATTG	65.0	198	Present study

			R15969	GACTTTTTCTCTGATTTGTCCTTGGAA	64.2		Present study
			FN15798	GACAAGTAGCATCCGTACTAT	60.9	153	Present study
			RN15950	CCTTGGAAAAAGGTTTTCATC	59.7		Present study
D4e4	1935	1888-1992	F1837	CCCCTATACCTTCTGCATAATGAAT	64.1	196	Present study
			R2032	CTATCTTGGACAACCAGCTATCAC	64.3		Present study
			FN1865	CTAGAAATAACTTTGCAAGGAGA	59.4	144	Present study
			RN2008	CAGGCTCGGTAGGTTT	60.8		Present study
D4e4a	8683	8619-8739	F8580	CGCCGCAGTACTGATCATT	65.1	203	Present study
			R8782	CGAGGAGGTTAGTTGTGGCAATA	65.3		Present study
			FN8597	TTCTATTTCCCCCTCTATTGAT	59.5	170	Present study
			RN8766	GGCAATAAAAATGATTAAGGATACTAG	60.0		Present study
D4e4b	12882	12826-12935	F12774	CGTAGGAATTATATCCTTCTTGCTCAT	64.3	200	Present study
			R12973	GTGGGGTGAGGCTTGGATTA	64.6		Present study
			FN12811	TACGCCCGAGCAGAT	61.4	143	Present study
			RN12953	GCGTTTAGAAGGGCTATT	59.2		Present study
D4j	11696	11628-11743	F11586	GCCTACGACAAACAGACCTAAAATC	65.0	204	Present study
			R11789	GGATTATGATGCGACTGTGAGTG	64.9		Present study
			FN11610	CGCTCATTGCATACTCTT	60.0	154	Present study
			RN11763	TCGTAGTTTGAGTTTGCTAG	58.9		Present study
D4j11	11218	11157-11276	F11117	ATCTTCTTCGAAACCACACTTATC	62.9	199	Same as for haplogroup D4e
			R11315	GTTCTTGGGCAGTGAGAGT	63.8		Same as for haplogroup D4e
			FN11141	CCCACCTTGGCTATCA	58.8	159	Same as for haplogroup D4e
			RN11299	AGTGAGTAGTAGAATGTTTAGTG	59.7		Same as for haplogroup D4e
G	5108	5051-5167	F5007	GCATACTCCTCAATTACCCACATAG	64.2	213	[31]
			R5210	GGTGGATGGAATTAAGGGTGTTAG	64.5		[31]
			FN5026	ACATAGGATGAATAATAGCAGTTCT	61.9	160	[31]
			RN5191	GTTAGTCATGTTAGCTTGTTTCAG	61.6		[31]
G1	15323	15280-15390	F15242	GGAGGCTACTCAGTAGACAGT	64.3	196	[31]
			R15437	CGAGGGCGTCTTTGATTGT	65.2		[31]

			FN15264	CCACCCTCACACGATT	61.1	147	[31]
			RN15410	GGTGGAAGGTGATTTTATCG	60.4		[31]
G1a	7867	7813-7902	F7744	TAACATCTCAGACGCTCAGGAAATAG	65.1	196	Present study
			R7939	GAAGATTAGTCCGCCGTAGT	63.3		Present study
			FN7794	CCATCATCCTAGTCCTCAT	60.0	126	Present study
			RN7919	CGGTGTACTCGTAGGTT	60.5		Present study
G1a1	15860	15728-15901	F15699	GCCCACTAAGCCAATCACTT	64.7	235	Present study
			R15933	CATCTCCGGTTTACAAGACTGG	64.8		Present study
			FN15706	AAGCCAATCACTTTATTGACTC	61.1	221	Present study
			RN15926	GGTTTACAAGACTGGTGTATTAGTT	62.4		Present study
G1a1a	11914	11867-11960	F11836	ACTTCTAGCAAGCCTCGCTAA	65.0	179	Present study
			R12014	GTGAGCCCCATTGTGTTGTG	65.8		Present study
			FN11850	TCGCTAACCTCGCCTTA	60.1	131	Present study
			RN11980	GGAGTATAGGGCTGTGACTA	60.5		Present study
G1a1b	12178	12135-12243	F12102	CCCTCAACCCCGACATCA	64.7	204	Present study
			R12305	GGGGCCTAAGACCAATGGA	64.2		Present study
			FN12115	CATCATTACCGGGTTTTCCT	62.4	150	Present study
			RN12264	GAGAAAGCCATGTTGTTAGAC	60.6		Present study
G2	13563	13507-13605	F13463	GCAGCCTAGCATTAGCAGGAATA	65.1	186	[31]
			R13648	GGAAGCGAGGTTGACCTGTTA	64.9		[31]
			FN13486	CCTTTCCTCACAGGTTTCTAC	62.0	142	[31]
			RN13627	GGGTGAGAAGAATTATTCGAGT	61.7		[31]
G2a	7600	7491-7644	F7466	CCCCCCAAAGCTGGTTT	65.0	214	Present study
			R7679	AAATGATTATGAGGGCGTGATCATG	65.7		Present study
			FN7471	CAAAGCTGGTTTCAAGCCAA	62.9	196	Present study
			RN7666	GGCGTGATCATGAAAGGTGATA	63.2		Present study
G2a1	14200	14153-14248	F14115	CCCACTCATCCTAACCCTACTC	64.9	162	Present study
			R14276	GGTTGATTCGGGAGGATCCTAT	65.2		Present study
			FN14127	AACCCTACTCCTAATCACATAACCTA	63.6	141	Present study

			RN14267	GGGAGGATCCTATTGGTGC	63.3		Present study
U	11467	11426-11518	F11384	CTTTACGGACTCCACTTATGACTC	64.0	181	Present study
			R11564	TGCCTCATAGGGATAGTACAAGGAA	64.6		Present study
			FN11408	CCTAAAGCCCATGTCGAA	60.4	131	Present study
			RN11538	GGGTAGGCTATGTGTTTTGT	61.9		Present study
U2e	6045	5992-6112	F5961	CTATTATTCGGCGCATGAGC	63.4	183	Present study
			R6143	GTTGCCAAAGCCTCCGATTA	63.6		Present study
			FN5973	GCATGAGCTGGAGTCCTAG	63.5	161	Present study
			RN6133	CCTCCGATTATGATGGGTATT	61.4		Present study
U2e1a	11197	11114-11273	F11079	TTATAACATTCACAGCCACAGAACT	63.8	234	Present study
			R11312	CTTGGGCAGTGAGAGTGAG	64.4		Present study
			FN11091	CAGCCACAGAACTAATCATATTT	60.9	208	Present study
			RN11298	GTGAGTAGTAGAATGTTTAGTGAGC	62.1		Present study
U2e1a1	3116	3050-3197	F3016	GCAGCCGCTATTAAAGGTTC	63.1	203	Present study
			R3218	TTGGGTGGGTGTGGGTAT	64.6		Present study
			FN3026	TTAAAGGTTCGTTTGTTCAACGAT	63.0	191	Present study
			RN3216	GGGTGGGTGTGGGTATAAT	63.1		Present study
U2e1a1c	10127	10034-10162	F9976	ATTGATGAGGGTCTTACTCTTTTAG	62.4	209	Present study
			R10184	GTCGAAGCCGCACTCGTAA	65.5		Present study
			FN10009	GTACCGTTAACTTCCAATTAACTAG	61.0	170	Present study
			RN10178	GCCGCACTCGTAAGGG	64.5		Present study
W	15884C	15728-15901	F15699	GCCCACTAAGCCAATCACTT	64.7	235	Same as for W3a and W5 [31]
			R15933	CATCTCCGGTTTACAAGACTGG	64.8		Same as for W3a and W5 [31]
			FN15706	AAGCCAATCACTTTATTGACTC	61.1	221	Same as for W3a and W5 [31]
			RN15926	GGTTTACAAGACTGGTGTATTAGTT	62.4		Same as for W3a and W5 [31]
W3	1406	1339-1467	F1301	GCGCAAGTACCCACGTAAAG	65.5	193	Present study
			R1493	GTGACGGGCGGTGTGTA	65.0		Present study
			FN1318	AAGACGTTAGGTCAAGGTGTA	62.2	167	Present study
			RN1484	GGTGTGTACGCGCTTCA	63.3		Present study

W3a	15784	15728-15901	F15699	GCCCACTAAGCCAATCACTT	64.7	235	Same as for W and W5 [31]
			R15933	CATCTCCGGTTTACAAGACTGG	64.8		Same as for W and W5 [31]
			FN15706	AAGCCAATCACTTTATTGACTC	61.1	221	Same as for W and W5 [31]
			RN15926	GGTTTACAAGACTGGTGTATTAGTT	62.4		Same as for W and W5 [31]
W3a1	13263	13188-13306	F13145	GCCCACTAATCCAAACTCTAACAC	64.6	196	Present study
			R13340	AAGAAGGCGTGGGTACAGAT	66.3		Present study
			FN13171	TGCTTAGGCGCTATCAC	60.1	155	Present study
			RN13325	CAGATGTGCAGGAATGCTA	60.8		Present study
W3a1a	7151	7093-7219	F7052	AGGAGCTGTATTTGCCATCATAG	64.8	206	Present study
			R7257	TGTTTCATGTGGTGTATGCATCG	64.9		Present study
			FN7074	GGAGGCTTCATTCACTGAT	61.4	163	Present study
			RN7236	CGGGGTAGTCCGAGTAA	60.7		Present study
W3a1b	10245	10193-10281	F10136	ACTCAACGGCTACATAGAAAAATCC	65.1	185	Present study
			R10320	CTATTAGTGGCAGGTTAGTTGTTTGT	65.1		Present study
			FN10175	CGGCTTCGACCCTATATC	61.0	127	Present study
			RN10301	TGTTTGTAGGGCTCATGGTA	61.0		Present study
W5	15775	15728-15901	F15699	GCCCACTAAGCCAATCACTT	64.7	235	Same as for W and W3a [31]
			R15933	CATCTCCGGTTTACAAGACTGG	64.8		Same as for W and W3a [31]
			FN15706	AAGCCAATCACTTTATTGACTC	61.1	221	Same as for W and W3a [31]
			RN15926	GGTTTACAAGACTGGTGTATTAGTT	62.4		Same as for W and W3a [31]

[1] Shown in PhyloTree format.

[2] Numbers indicate nucleotide positions based on the revised Cambridge reference sequence [29].

[3] F, forward primer; R, reverse primer; N, primers for nested PCR and sequencing.

[4] Primer melting temperature was calculated by using LC PDS software V 2.0.
