# Supplementary material for: Mitochondrial Haplogroup Classification of Ancient DNA Samples Using Haplotracker
Source: Biomed Res Int. 2022 Mar 18;2022:5344418. doi: 10.1155/2022/5344418 (PMC8956381; doi:10.1155/2022/5344418)
Supplement: Supplementary Materials — Fig. S1: characterization of Phylotree-provided control region sequences tested for haplogroup classification by Haplotracker. Fig. S2: minimum number of amplicons required by Haplotracker in discriminating between haplogroups using mtDNA control and coding region sequences. Fig. S3: variant identification of an aDNA sample (MNW3) using an HRM real-time PCR. Table S1: haplogroups and their variant profiles extracted from Phylotree mtDNA Build 17. Table S2: haplogroup frequency carrying an extra variant in 118,869 haplotypes. Table S3: haplogroup frequency carrying a missing variant in 118,869 haplotypes. Table S4: haplogroup frequency in 118,869 haplotypes. Table S5: list of ancient human samples found in 2,000-year-old elite Xiongnu cemetery in Northeast Mongolia. Table S6: primers for the amplification of mtDNA coding region segments for haplogroup determination. Table S7: high-resolution melting real-time PCR primer design for screening variants to differentiate haplogroups G1a1, G1a1a, and G1a1b. Table S8: haplogroup classification of full-length mtGenome sequences from Phylotree (n = 8,216). Table S9: haplogroup classification with full-length and control region sequences of mtDNA using Haplotracker and HaploGrep 2. Table S10: comparison of servers using control region sequences from GenBank before December 25, 2018 (n = 45,177). Table S11: comparison details for the servers using control region sequences from GenBank before December 25, 2018 (n = 45,177). Table S12: comparison of servers using control region sequences downloaded from GenBank from December 26, 2018 to August 22, 2019. Table S13: sequences of mtDNA PCR products from Mongolian ancient DNA samples. Table S14: haplogroup classification of Mongolian ancient DNA samples using Haplotracker. Table S15: minimum number of amplicons required by Haplotracker in discriminating between haplogroups using mtDNA control and coding region sequences. Table S16: minimum number of amplicons per superhaplogroup requ [file 5344418.f14.pdf › 5344418.f14.pdf]

Table S11. Comparison details for the servers using control region sequences from GenBank before December 25, 2018 (n=45,177).

Serial No.	Accession No.	HaploGrep 2				Haplotracker					
		mtGenome variants	mtGenome-assignment	Control region variants	Rank	mtGenome variants	mtGenome-assignment	Control region variants	Rank Group 1	Rank Group 2	Rank
1	>MH981685.1 H	64Y 73G 151T 152C 182T 186A 189C 195C 198T 247A 263G 297G 316A 385G 471C 750G 769A 825A 1018A 1048T 1420C 1438G 2394M 2395M 2706G 2758A 2885C 3106d 3107C 3594T 3666A 4104G 4769G 5951G 6071C 6150A 6253C 6723A 7028T 7055G 7076G 7146G 7256T 7337A 7389C 7521A 7741C 8027A 8468T 8655T 8701G 8784G 8860G 8877C 9072G 9540C 10321C 10398G 10586A 10688A 10792G 10793T 10810C 10873C 11329G 11654G 11719A 12049T 12705T 12810G 13105G 13149G 13506T 13650T 13789C 14000A 14127G 14178C 14560A 14766T 14861A 14911T 15016T 15326G 15784C 16172C 16184T 16187T 16189C 16223T 16265C 16278T 16286G 16294T 16311C 16360T 16519C 16527T	L1c2a3a	64Y 73G 151T 152C 182T 186A 189C 195C 198T 247A 263G 297G 316A 385G 471C 16172C 16184T 16187T 16189C 16223T 16265C 16278T 16286G 16294T 16311C 16360T 16519C 16527T	1	64Y 73 151 152 182 186A 189C 195 198 247 263 297 316 385 471 750 769 825A 1018 1048 1420 1438 2394M 2395M 2706 2758 2885 3594 3666 4104 4769 5951 6071 6150 6253 6723 7028 7055 7076 7146 7256 7337 7389 7521 7741 8027 8468 8655 8701 8784 8860 8877 9072 9540 10321 10398 10586 10688 10792 10793 10810 10873 11329 11654 11719 12049 12705 12810 13105 13149 13506 13650 13789 14000A 14127 14178 14560 14766 14861 14911 15016 15326 15784 16172 16184 16187 16189 16223 16265C 16278 16286G 16294 16311 16360 16527	L1c2a3a	64Y 73 151 152 182 186A 189C 195 198 247 263 297 316 385 471 16172 16184 16187 16189 16223 16265C 16278 16286G 16294 16311 16360 16527	[0.134] L1c2a3a (1)	[0.091] L1c2a3 (2)	1
2	>MH981683.1 H	73G 150T 152C 195C 215G 263G 295T 319C 489C 513A 750G 1438G 2706G 3106d 3107C 4216C 4769G 7028T 7080C 7476T 7789A 8270T 8860G 9548S 10398G 10499G 11251G 11377A 11719A 12612G 13708A 13722G 14133G 14766T 15257A 15326G 15452A 16069T 16126C 16145A 16183C 16189C 16231C 16261T	J2a1a1e	73G 150T 152C 195C 215G 263G 295T 319C 489C 513A 16069T 16126C 16145A 16183C 16189C 16231C 16261T	1	73 150 152 195 215 263 295 319 489 513 750 1438 2706 4216 4769 7028 7080 7476 7789 8270 8860 9548S 10398 10499 11251 11377 11719 12612 13708 13722 14133 14766 15257 15326 15452A 16069 16126 16145 16189 16231 16261	J2a1a1e	73 150 152 195 215 263 295 319 489 513 16069 16126 16145 16189 16231 16261	[0.270] J2a1a1e (1) [0.000] J2a1a1 J2a1a1b J2a1a1c J2a1a1d (5)	[0.270] J2a1a1e (6) [0.053] J2a1a1a2 (7) [0.029] J2a1a1a2a (8) [0.023] J2a1a1a (9) [0.000] J2a1a J2a1a1 J2a1a1b J2a1a1c J2a1a1d (14)	1
3	>MH981682.1 H	73G 146C 152C 195C 263G 750G 769A 1018A 1438G 2416C 2706G 2789T 3106d 3107C 3594T 4104G 4769G 5090C 7028T 7175C 7256T 7274T 7521A 7771G 8206A 8701G 8860G 9221G 9540C 10115C 10143A 10398G 10873C 11719A 11914A 11944C 12693G 12705T 13590A 13650T 13803G 14566G 14766T 15301A 15326G 15735T 15784C 16182M 16183C 16189C 16223T 16278T 16290T 16294T 16309G 16390A	L2a1b1a	73G 146C 152C 195C 263G 16182M 16183C 16189C 16223T 16278T 16290T 16294T 16309G 16390A	1	73 146 152 195 263 750 769 1018 1438 2416 2706 2789 3594 4104 4769 5090 7028 7175 7256 7274 7521 7771 8206 8701 8860 9221 9540 10115 10143 10398 10873 11719 11914 11944 12693 12705 13590 13650 13803 14566 14766 15301 15326 15735 15784 16182M 16189 16223 16278 16290 16294 16309 16390	L2a1b1a	73 146 152 195 263 16182M 16189 16223 16278 16290 16294 16309 16390	[0.000] L2a1b1a (1)	[0.005] L2a1f (2) [0.000] L2a1+16189_(16192) L2a1b L2a1b1 L2a1b1a L2a1b2 L2a1f1 L2a1f1a L2a1f2 (10)	1

4	>MH981680.1 H	73G 146C 152C 195C 263G 750G 769A 1018A 1438G 2416C 2706G 2789T 3106d 3107C 3594T 4104G 4769G 5090C 7028T 7175C 7256T 7274T 7521A 7771G 8206A 8701G 8860G 9221G 9540C 9548S 10115C 10143A 10398G 10873C 11719A 11914A 11944C 12693G 12705T 13590A 13650T 13803G 14566G 14766T 15301A 15326G 15735T 15784C 16182C 16183C 16189C 16223T 16278T 16290T 16294T 16309G 16390A	L2a1b1a	73G 146C 152C 195C 263G 16182C 16183C 16189C 16223T 16278T 16290T 16294T 16309G 16390A	1	73 146 152 195 263 750 769 1018 1438 2416 2706 2789 3594 4104 4769 5090 7028 7175 7256 7274 7521 7771 8206 8701 8860 9221 9540 9548S 10115 10143 10398 10873 11719 11914 11944 12693 12705 13590 13650 13803 14566 14766 15301 15326 15735 15784 16189 16223 16278 16290 16294 16309 16390	L2a1b1a	73 146 152 195 263 16189 16223 16278 16290 16294 16309 16390	[0.000] L2a1b1a (1)	[0.005] L2a1f (2) [0.000] L2a1+16189_(16192) L2a1b L2a1b1 L2a1b1a L2a1b2 L2a1f1 L2a1f1a L2a1f2 (10)	1
5	>MH981676.1 H	73G 146C 152C 195C 263G 750G 769A 1018A 1438G 2416C 2706G 2789T 3106d 3107C 3594T 4104G 4769G 5090C 7028T 7175C 7256T 7274T 7521A 7705Y 7771G 8206A 8701G 8860G 9221G 9540C 10115C 10143A 10398G 10873C 11719A 11914A 11944C 12397G 12693G 12705T 13590A 13650T 13803G 14566G 14766T 15301A 15326G 15735T 15784C 16182M 16183C 16189C 16223T 16278T 16290T 16294T 16309G 16390A	L2a1b1a	73G 146C 152C 195C 263G 16182M 16183C 16189C 16223T 16278T 16290T 16294T 16309G 16390A	1	73 146 152 195 263 750 769 1018 1438 2416 2706 2789 3594 4104 4769 5090 7028 7175 7256 7274 7521 7705Y 7771 8206 8701 8860 9221 9540 10115 10143 10398 10873 11719 11914 11944 12397 12693 12705 13590 13650 13803 14566 14766 15301 15326 15735 15784 16182M 16189 16223 16278 16290 16294 16309 16390	L2a1b1a	73 146 152 195 263 16182M 16189 16223 16278 16290 16294 16309 16390	[0.000] L2a1b1a (1)	[0.005] L2a1f (2) [0.000] L2a1+16189_(16192) L2a1b L2a1b1 L2a1b1a L2a1b2 L2a1f1 L2a1f1a L2a1f2 (10)	1
6	>MH981672.1 H	73G 146C 152C 195C 263G 750G 769A 1018A 1438G 2416C 2706G 2789T 3106d 3107C 3594T 4104G 4769G 5090C 7028T 7175C 7256T 7274T 7521A 7771G 8206A 8701G 8860G 9221G 9540C 10115C 10143A 10398G 10873C 11719A 11914A 11944C 12693G 12705T 13590A 13650T 13803G 14566G 14766T 15301A 15326G 15735T 15784C 16182M 16183C 16189C 16192Y 16223T 16278T 16290T 16294T 16309G 16390A	L2a1b1a	73G 146C 152C 195C 263G 16182M 16183C 16189C 16192Y 16223T 16278T 16290T 16294T 16309G 16390A	1	73 146 152 195 263 750 769 1018 1438 2416 2706 2789 3594 4104 4769 5090 7028 7175 7256 7274 7521 7771 8206 8701 8860 9221 9540 10115 10143 10398 10873 11719 11914 11944 12693 12705 13590 13650 13803 14566 14766 15301 15326 15735 15784 16182M 16189 16192Y 16223 16278 16290 16294 16309 16390	L2a1b1a	73 146 152 195 263 16182M 16189 16192Y 16223 16278 16290 16294 16309 16390	[0.015] L2a1b1a (1) [0.000] L2a1b1a	[0.005] L2a1f (3) [0.000] L2a1+16189_(16192) L2a1b L2a1b1 L2a1b2 L2a1f1 L2a1f1a L2a1f2 (10)	1

7	>MH981668.1 H	73G 146C 152C 195C 263G 750G 769A 1018A 1438G 2304A 2416C 2706G 2789T 3106d 3107C 3594T 4104G 4769G 5090C 7028T 7175C 7256T 7274T 7521A 7771G 8206A 8701G 8860G 9221G 9540C 10115C 10143A 10398G 10873C 11719A 11914A 11944C 12693G 12705T 13590A 13650T 13803G 14566G 14766T 15301A 15326G 15735T 15784C 16182M 16183C 16189C 16223T 16278T 16290T 16294T 16309G 16380Y 16390A	L2a1b1a	73G 146C 152C 195C 263G 16182M 16183C 16189C 16223T 16278T 16290T 16294T 16309G 16380Y 16390A	1	73 146 152 195 263 750 769 1018 1438 2304 2416 2706 2789 3594 4104 4769 5090 7028 7175 7256 7274 7521 7771 8206 8701 8860 9221 9540 10115 10143 10398 10873 11719 11914 11944 12693 12705 13590 13650 13803 14566 14766 15301 15326 15735 15784 16182M 16189 16223 16278 16290 16294 16309 16380Y 16390	L2a1b1a	73 146 152 195 263 16182M 16189 16223 16278 16290 16294 16309 16380Y 16390	[0.003] L2a1b1a (1)	[0.005] L2a1f (2) [0.003] L2a1b1a (3) [0.000] L2a1+16189_(16192) L2a1b L2a1b1 L2a1b2 L2a1f1 L2a1f1a L2a1f2 (10)	1
8	>MH981665.1 H	73G 146C 152C 195C 263G 750G 769A 1018A 1438G 2416C 2706G 2789T 3106d 3107C 3594T 4104G 4769G 5090C 7028T 7175C 7256T 7274T 7521A 7771G 8206A 8701G 8860G 9221G 9540C 10115C 10143A 10398G 10873C 11719A 11914A 11944C 12693G 12705T 13590A 13650T 13803G 14566G 14766T 15301A 15326G 15735T 15784C 16051G 16182M 16183C 16189C 16223T 16278T 16290T 16294T 16309G 16390A	L2a1b1a	73G 146C 152C 195C 263G 16051G 16182M 16183C 16189C 16223T 16278T 16290T 16294T 16309G 16390A	1	73 146 152 195 263 750 769 1018 1438 2416 2706 2789 3594 4104 4769 5090 7028 7175 7256 7274 7521 7771 8206 8701 8860 9221 9540 10115 10143 10398 10873 11719 11914 11944 12693 12705 13590 13650 13803 14566 14766 15301 15326 15735 15784 16051 16182M 16189 16223 16278 16290 16294 16309 16390	L2a1b1a	73 146 152 195 263 16051 16182M 16189 16223 16278 16290 16294 16309 16390	[0.006] L2a1b1a (1)	[0.006] L2a1b1a (2) [0.005] L2a1f (3) [0.000] L2a1+16189_(16192) L2a1b L2a1b1 L2a1b2 L2a1f1 L2a1f1a L2a1f2 (10)	1
9	>MH981659.1 H	73G 151T 152C 182T 186A 189C 195C 198T 247A 263G 297G 316A 385G 471C 750G 769A 825A 1018A 1048T 1420C 1438G 2394M 2395M 2706G 2758A 2885C 3106d 3107C 3594T 3666A 4104G 4769G 5951G 6071C 6150A 6203G 6253C 6723A 7028T 7055G 7076G 7146G 7256T 7337A 7389C 7521A 7741C 8027A 8468T 8655T 8701G 8784G 8860G 8877C 9072G 9540C 10321C 10398G 10586A 10688A 10792G 10793T 10810C 10873C 11329G 11654G 11719A 12049T 12705T 12810G 13105G 13149G 13506T 13650T 13789C 14000A 14127G 14178C 14560A 14766T 14861A 14911T 15016T 15326G 15784C 16172C 16187T 16189C 16223T 16265C 16278T 16286G 16294T 16311C 16360T 16519C 16527T	L1c2a3a	73G 151T 152C 182T 186A 189C 195C 198T 247A 263G 297G 316A 385G 471C 16172C 16187T 16189C 16223T 16265C 16278T 16286G 16294T 16311C 16360T 16519C 16527T	1	73 151 152 182 186A 189C 195 198 247 263 297 316 385 471 750 769 825A 1018 1048 1420 1438 2394M 2395M 2706 2758 2885 3594 3666 4104 4769 5951 6071 6150 6203 6253 6723 7028 7055 7076 7146 7256 7337 7389 7521 7741 8027 8468 8655 8701 8784 8860 8877 9072 9540 10321 10398 10586 10688 10792 10793 10810 10873 11329 11654 11719 12049 12705 12810 13105 13149 13506 13650 13789 14000A 14127 14178 14560 14766 14861 14911 15016 15326 15784 16172 16187 16189 16223 16265C 16278 16286G 16294 16311 16360 16527	L1c2a3a	73 151 152 182 186A 189C 195 198 247 263 297 316 385 471 16172 16187 16189 16223 16265C 16278 16286G 16294 16311 16360 16527	[0.000] L1c2a3a (1)	[0.091] L1c2a3 (2)	1

10 >MH981657.1 H	73G 146C 152C 195C 263G 750G 769A 1018A 1120T 1438G 2416C 2706G 2789T 3106d 3107C 3594T 4104G 4769G 5090C 7028T 7175C 7256T 7274T 7521A 7771G 8206A 8270T 8701G 8860G 9221G 9540C 10115C 10143A 10398G 10873C 11719A 11914A 11944C 12693G 12705T 13590A 13650T 13803G 14566G 14766T 15301A 15326G 15735T 15784C 16183C 16189C 16223T 16278T 16290T 16294T 16309G 16390A	L2a1b1a	73G 146C 152C 195C 263G 16183C 16189C 16223T 16278T 16290T 16294T 16309G 16390A	1	73 146 152 195 263 750 769 1018 1120 1438 2416 2706 2789 3594 4104 4769 5090 7028 7175 7256 7274 7521 7771 8206 8270 8701 8860 9221 9540 10115 10143 10398 10873 11719 11914 11944 12693 12705 13590 13650 13803 14566 14766 15301 15326 15735 15784 16189 16223 16278 16290 16294 16309 16390	L2a1b1a	73 146 152 195 263 16189 16223 16278 16290 16294 16309 16390	[0.000] L2a1b1a (1)	[0.005] L2a1f (2) [0.000] L2a1+16189_(16192) L2a1b L2a1b1 L2a1b1a L2a1b2 L2a1f1 L2a1f1a L2a1f2 (10)	1
11 >MH981654.1 H	73G 151T 152C 182T 186A 189C 195C 198T 247A 263G 297G 316A 385G 471C 750G 769A 825A 1018A 1048T 1420C 1438G 2394M 2395M 2706G 2758A 2885C 3106d 3107C 3594T 3666A 4104G 4769G 5951G 6071C 6150A 6253C 6723A 7028T 7055G 7076G 7146G 7256T 7337A 7389C 7521A 7741C 8027A 8468T 8473C 8655T 8701G 8784G 8860G 8877C 9072G 9540C 10321C 10398G 10586A 10688A 10792G 10793T 10810C 10873C 11329G 11654G 11719A 12049T 12705T 12810G 13105G 13149G 13506T 13650T 13789C 14000A 14127G 14178C 14560A 14766T 14861A 14911T 15016T 15326G 15784C 16169Y 16172C 16187T 16189C 16223T 16265C 16278T 16286G 16294T 16311C 16360T 16519C 16527T	L1c2a3a	73G 151T 152C 182T 186A 189C 195C 198T 247A 263G 297G 316A 385G 471C 16169Y 16172C 16187T 16189C 16223T 16265C 16278T 16286G 16294T 16311C 16360T 16519C 16527T	1	73 151 152 182 186A 189C 195 198 247 263 297 316 385 471 750 769 825A 1018 1048 1420 1438 2394M 2395M 2706 2758 2885 3594 3666 4104 4769 5951 6071 6150 6253 6723 7028 7055 7076 7146 7256 7337 7389 7521 7741 8027 8468 8473 8655 8701 8784 8860 8877 9072 9540 10321 10398 10586 10688 10792 10793 10810 10873 11329 11654 11719 12049 12705 12810 13105 13149 13506 13650 13789 14000A 14127 14178 14560 14766 14861 14911 15016 15326 15784 16169Y 16172 16187 16189 16223 16265C 16278 16286G 16294 16311 16360 16527	L1c2a3a	73 151 152 182 186A 189C 195 198 247 263 297 316 385 471 16169Y 16172 16187 16189 16223 16265C 16278 16286G 16294 16311 16360 16527	[0.067] L1c2a3a (1)	[0.273] L1c2a3 (2)	1
12 >MH981645.1 H	73G 146C 152C 195C 263G 750G 769A 1018A 1438G 2416C 2706G 2789T 3106d 3107C 3594T 4104G 4769G 5090C 7028T 7175C 7256T 7274T 7521A 7771G 8206A 8701G 8860G 9221G 9540C 10115C 10143A 10398G 10873C 11719A 11914A 11944C 12693G 12705T 13590A 13650T 13803G 14566G 14766T 15301A 15326G 15735T 15784C 16126C 16182C 16183C 16189C 16223T 16278T 16290T 16294T 16309G 16390A	L2a1b1a	73G 146C 152C 195C 263G 16126C 16182C 16183C 16189C 16223T 16278T 16290T 16294T 16309G 16390A	1	73 146 152 195 263 750 769 1018 1438 2416 2706 2789 3594 4104 4769 5090 7028 7175 7256 7274 7521 7771 8206 8701 8860 9221 9540 10115 10143 10398 10873 11719 11914 11944 12693 12705 13590 13650 13803 14566 14766 15301 15326 15735 15784 16126 16189 16223 16278 16290 16294 16309 16390	L2a1b1a	73 146 152 195 263 16126 16189 16223 16278 16290 16294 16309 16390	[0.003] L2a1b1a (1)	[0.005] L2a1f (2) [0.003] L2a1b1a (3) [0.000] L2a1+16189_(16192) L2a1b L2a1b1 L2a1b2 L2a1f1 L2a1f1a L2a1f2 (10)	1

13	>MH981642.1 H	73G 146C 152C 195C 263G 750G 769A 1018A 1438G 2416C 2706G 2789T 3106d 3107C 3594T 4104G 4769G 5090C 7028T 7175C 7256T 7274T 7521A 7771G 8206A 8701G 8860G 9221G 9540C 10115C 10143A 10398G 10873C 11719A 11914A 11944C 12693G 12705T 13590A 13650T 13803G 14566G 14766T 15301A 15326G 15735T 15784C 16182M 16183C 16189C 16223T 16278T 16290T 16294T 16309R 16390A	L2a1b1a	73G 146C 152C 195C 263G 16182M 16183C 16189C 16223T 16278T 16290T 16294T 16309R 16390A	1	73 146 152 195 263 750 769 1018 1438 2416 2706 2789 3594 4104 4769 5090 7028 7175 7256 7274 7521 7771 8206 8701 8860 9221 9540 10115 10143 10398 10873 11719 11914 11944 12693 12705 13590 13650 13803 14566 14766 15301 15326 15735 15784 16182M 16189 16223 16278 16290 16294 16309R 16390	L2a1b1a	73 146 152 195 263 16182M 16189 16223 16278 16290 16294 16309R 16390	[0.000] L2a1b1a (1)	[0.005] L2a1f (2) [0.000] L2a1+16189_(16192) L2a1b L2a1b1 L2a1b1a L2a1b2 L2a1f1 L2a1f1a L2a1f2 (10)	1
14	>MH981629.1 H	73G 146C 152C 195C 244G 263G 340T 709A 750G 769A 1018A 1438G 2220T 2706G 3106d 3107C 3918A 4206G 4769G 5128G 6260A 7028T 7805A 7897A 8104C 8292A 8343G 8701G 8860G 9540C 9855G 10265C 10398G 10873C 11719A 12438C 12609C 12705T 13470G 14766T 15301A 15326G 16051G 16114T 16189C 16223T 16293T 16311C 16316G 16355T 16362C 16399G 16519C	L4b2b1	73G 146C 152C 195C 244G 263G 340T 16051G 16114T 16189C 16223T 16293T 16311C 16316G 16355T 16362C 16399G 16519C	1	73 146 152 195 244 263 340 709 750 769 1018 1438 2220T 2706 3918 4206 4769 5128 6260 7028 7805 7897 8104 8292 8343 8701 8860 9540 9855 10265 10398 10873 11719 12438 12609 12705 13470 14766 15301 15326 16051 16114 16189 16223 16293T 16311 16316 16355 16362 16399	L4b2b1	73 146 152 195 244 263 340 16051 16114 16189 16223 16293T 16311 16316 16355 16362 16399	[2.800] L4b2b1 (1)	[0.566] L4b2b (2) [0.500] L4b2 (3) [0.143] L4b2a	1
15	>MH981628.1 H	73G 146C 152C 195C 263G 750G 769A 1018A 1438G 2416C 2706G 2789T 3106d 3107C 3505G 3594T 4104G 4769G 5090C 7028T 7175C 7256T 7274T 7521A 7771G 8206A 8701G 8860G 9221G 9540C 10115C 10143A 10398G 10873C 11719A 11914A 11944C 12693G 12705T 13590A 13650T 13803G 14566G 14766T 15301A 15326G 15735T 15784C 16182M 16183C 16189C 16223T 16278T 16290T 16294T 16309G 16390A	L2a1b1a	73G 146C 152C 195C 263G 16182M 16183C 16189C 16223T 16278T 16290T 16294T 16309G 16390A	1	73 146 152 195 263 750 769 1018 1438 2416 2706 2789 3505 3594 4104 4769 5090 7028 7175 7256 7274 7521 7771 8206 8701 8860 9221 9540 10115 10143 10398 10873 11719 11914 11944 12693 12705 13590 13650 13803 14566 14766 15301 15326 15735 15784 16182M 16189 16223 16278 16290 16294 16309 16390	L2a1b1a	73 146 152 195 263 16182M 16189 16223 16278 16290 16294 16309 16390	[0.000] L2a1b1a (1)	[0.005] L2a1f (2) [0.000] L2a1+16189_(16192) L2a1b L2a1b1 L2a1b1a L2a1b2 L2a1f1 L2a1f1a L2a1f2 (10)	1
16	>MH981623.1 H	73G 146C 185A 188G 222T 228A 263G 295T 462T 489C 750G 1438G 2706G 3010A 3106d 3107C 4216C 4769G 7028T 8860G 10398G 10685A 11251G 11719A 12612G 13281C 13708A 13933G 14766T 14798C 15326G 15452A 16069T 16126C 16519C	J1c2c1	73G 146C 185A 188G 222T 228A 263G 295T 462T 489C 16069T 16126C 16519C	1	73 146 185 188 222 228 263 295 462 489 750 1438 2706 3010 4216 4769 7028 8860 10398 10685 11251 11719 12612 13281 13708 13933 14766 14798 15326 15452A 16069 16126	J1c2c1	73 146 185 188 222 228 263 295 462 489 16069 16126	[0.000] J1c2c1 (1)	[0.005] J1c2c (2) [0.000] J1c2c1 J1c2c1a J1c2c2 J1c2c2a J1c2c3 J1c2h (8)	1

17 >MH981618.1 H	73G 125G 151T 152C 182T 186A 189C 195C 198T 247A 263G 297G 316A 385G 471C 750G 769A 825A 1018A 1048T 1420C 1438G 2395N 2706G 2758A 2885C 3106d 3107C 3594T 3666A 4104G 4769G 5951G 6071C 6150A 6253C 6723A 7028T 7055G 7076G 7146G 7256T 7337A 7389C 7521A 7741C 8027A 8468T 8655T 8701G 8784G 8860G 8877C 9072G 9540C 10321C 10398G 10586A 10688A 10792G 10793T 10810C 10873C 11329G 11654G 11719A 12049T 12705T 12810G 13105G 13149G 13506T 13650T 13789C 14000A 14127G 14178C 14560A 14766T 14861A 14911T 15016T 15326G 15784C 16172C 16187T 16189C 16223T 16265C 16278T 16286G 16294T 16311C 16360T 16519C 16527T	L1c2a3a	73G 125G 151T 152C 182T 186A 189C 195C 198T 247A 263G 297G 316A 385G 471C 16172C 16187T 16189C 16223T 16265C 16278T 16286G 16294T 16311C 16360T 16519C 16527T	1	73 125G 151 152 182 186A 189C 195 198 247 263 297 316 385 471 750 769 825A 1018 1048 1420 1438 2395N 2706 2758 2885 3594 3666 4104 4769 5951 6071 6150 6253 6723 7028 7055 7076 7146 7256 7337 7389 7521 7741 8027 8468 8655 8701 8784 8860 8877 9072 9540 10321 10398 10586 10688 10792 10793 10810 10873 11329 11654 11719 12049 12705 12810 13105 13149 13506 13650 13789 14000A 14127 14178 14560 14766 14861 14911 15016 15326 15784 16172 16187 16189 16223 16265C 16278 16286G 16294 16311 16360 16527	L1c2a3a	73 125G 151 152 182 186A 189C 195 198 247 263 297 316 385 471 16172 16187 16189 16223 16265C 16278 16286G 16294 16311 16360 16527	[0.067] L1c2a3a (1)	[0.091] L1c2a3 (2)	1
18 >MH981617.1 H	73G 150T 152C 195C 215G 263G 295T 319C 489C 513A 750G 1438G 2706G 3106d 3107C 4216C 4769G 7028T 7080C 7476T 7789A 8270T 8860G 10398G 10499G 11251G 11377A 11719A 12612G 13708A 13722G 14133G 14305A 14766T 15257A 15326G 15452A 16069T 16126C 16145A 16183C 16189C 16231C 16261T	J2a1a1e	73G 150T 152C 195C 215G 263G 295T 319C 489C 513A 16069T 16126C 16145A 16183C 16189C 16231C 16261T	1	73 150 152 195 215 263 295 319 489 513 750 1438 2706 4216 4769 7028 7080 7476 7789 8270 8860 10398 10499 11251 11377 11719 12612 13708 13722 14133 14305 14766 15257 15326 15452A 16069 16126 16145 16189 16231 16261	J2a1a1e	73 150 152 195 215 263 295 319 489 513 16069 16126 16145 16189 16231 16261	[0.270] J2a1a1e (1) [0.000] J2a1a1 J2a1a1b J2a1a1c J2a1a1d (5)	[0.270] J2a1a1e (6) [0.053] J2a1a1a2 (7) [0.029] J2a1a1a2a (8) [0.023] J2a1a1a (9) [0.000] J2a1a J2a1a1 J2a1a1b J2a1a1c J2a1a1d (14)	1
19 >MH981615.1 H	73G 146C 152C 195C 263G 750G 769A 1018A 1438G 2416C 2706G 2789T 3106d 3107C 3594T 4104G 4769G 5090C 7028T 7175C 7256T 7274T 7521A 7771G 8206A 8701G 8860G 9221G 9540C 10115C 10143A 10398G 10873C 11719A 11914A 11944C 12693G 12705T 13590A 13650T 13803G 14053G 14566G 14766T 15301A 15326G 15735T 15784C 16183C 16189C 16223T 16278T 16290T 16294T 16309G 16390A	L2a1b1a	73G 146C 152C 195C 263G 16183C 16189C 16223T 16278T 16290T 16294T 16309G 16390A	1	73 146 152 195 263 750 769 1018 1438 2416 2706 2789 3594 4104 4769 5090 7028 7175 7256 7274 7521 7771 8206 8701 8860 9221 9540 10115 10143 10398 10873 11719 11914 11944 12693 12705 13590 13650 13803 14053 14566 14766 15301 15326 15735 15784 16189 16223 16278 16290 16294 16309 16390	L2a1b1a	73 146 152 195 263 16189 16223 16278 16290 16294 16309 16390	[0.000] L2a1b1a (1)	[0.005] L2a1f (2) [0.000] L2a1+16189_(16192) L2a1b L2a1b1 L2a1b1a L2a1b2 L2a1f1 L2a1f1a L2a1f2 (10)	1

20 >MH981611.1 H	73G 146C 152C 195C 247A 456T 719A 750G 769A 825A 1018A 1048T 2758A 2885C 3106d 3107C 3438A 3516A 3594T 3756G 4104G 4197T 4232C 4312T 4769G 5442C 6185C 6266G 6815C 7028T 7146G 7256T 7521A 8113A 8152A 8251A 8468T 8655T 8701G 8860G 8994A 9042T 9150G 9347G 9540C 9755A 10398G 10589A 10664T 10688A 10810C 10873C 10915C 11167G 11437C 11719A 11914A 12007A 12121C 12235C 12705T 12720G 13105G 13129T 13276G 13473G 13506T 13650T 13759A 14766T 15315T 15326G 15466A 15930A 15941C 15951G 16187T 16189C 16223T 16230G 16234T 16243C 16249C 16311C 16519C	L0d1c	73G 146C 152C 195C 247A 456T 16187T 16189C 16223T 16230G 16234T 16243C 16249C 16311C 16519C	1	73 146 152 195 247 456 719 750 769 825A 1018 1048 2758 2885 3438 3516A 3594 3756 4104 4197 4232 4312 4769 5442 6185 6266 6815 7028 7146 7256 7521 8113A 8152 8251 8468 8655 8701 8860 8994 9042 9150 9347 9540 9755 10398 10589 10664 10688 10810 10873 10915 11167 11437 11719 11914 12007 12121 12235 12705 12720 13105 13129 13276 13473 13506 13650 13759 14766 15315 15326 15466 15930 15941 15951 16187 16189 16223 16230 16234 16243 16249 16311	L0d1c	73 146 152 195 247 456 16187 16189 16223 16230 16234 16243 16249 16311	[0.407] L0d1c (1)	[0.000] L0d1c1 L0d1c1a L0d1c2 (4)	1
21 >MH981610.1 H	73G 151T 152C 182T 186A 189C 195C 198T 247A 263G 297G 316A 385G 471C 750G 769A 825A 1018A 1048T 1420C 1438G 1822C 2395M 2706G 2758A 2885C 3106d 3107C 3594T 3666A 4104G 4769G 5951G 6071C 6150A 6253C 6723A 7028T 7055G 7076G 7146G 7256T 7337A 7389C 7521A 7741C 8027A 8468T 8655T 8701G 8784G 8860G 8877C 9072G 9540C 10321C 10398G 10586A 10688A 10792G 10793T 10810C 10873C 11329G 11654G 11719A 12049T 12705T 12810G 13105G 13149G 13506T 13650T 13789C 14000A 14127G 14178C 14560A 14766T 14861A 14911T 15016T 15326G 15784C 16172C 16187T 16189C 16223T 16265C 16278T 16286G 16294T 16311C 16360T 16519C 16527T	L1c2a3a	73G 151T 152C 182T 186A 189C 195C 198T 247A 263G 297G 316A 385G 471C 16172C 16187T 16189C 16223T 16265C 16278T 16286G 16294T 16311C 16360T 16519C 16527T	1	73 151 152 182 186A 189C 195 198 247 263 297 316 385 471 750 769 825A 1018 1048 1420 1438 1822 2395M 2706 2758 2885 3594 3666 4104 4769 5951 6071 6150 6253 6723 7028 7055 7076 7146 7256 7337 7389 7521 7741 8027 8468 8655 8701 8784 8860 8877 9072 9540 10321 10398 10586 10688 10792 10793 10810 10873 11329 11654 11719 12049 12705 12810 13105 13149 13506 13650 13789 14000A 14127 14178 14560 14766 14861 14911 15016 15326 15784 16172 16187 16189 16223 16265C 16278 16286G 16294 16311 16360 16527	L1c2a3a	73 151 152 182 186A 189C 195 198 247 263 297 316 385 471 16172 16187 16189 16223 16265C 16278 16286G 16294 16311 16360 16527	[0.000] L1c2a3a (1)	[0.091] L1c2a3 (2)	1

22 >MH981609.1 H	73G 146C 150T 152C 182T 195C 198T 204C 263G 709A 750G 769A 1018A 1438G 1442A 1706T 2332T 2358G 2416C 2706G 3106d 3107C 3594T 4104G 4158G 4370C 4767G 4769G 5027T 5331A 5814C 6614C 6710G 6713T 6806G 7028T 7256T 7521A 7624A 8080T 8206A 8387A 8503C 8701G 8790A 8860G 9221G 9350G 9540C 10115C 10398G 10873C 11719A 11944C 12236A 12705T 12948G 13590A 13650T 13966G 14059G 14407T 14766T 15110A 15217A 15301A 15326G 16114A 16129A 16213A 16223T 16278T 16354T 16390A 16519C	L2b2a	73G 146C 150T 152C 182T 195C 198T 204C 263G 16114A 16129A 16213A 16223T 16278T 16354T 16390A 16519C	1	73 146 150 152 182 195 198 204 263 709 750 769 1018 1438 1442 1706 2332 2358 2416 2706 3594 4104 4158 4370 4767 4769 5027 5331A 5814 6614 6710 6713 6806 7028 7256 7521 7624A 8080 8206 8387 8503 8701 8790 8860 9221 9350 9540 10115 10398 10873 11719 11944 12236 12705 12948 13590 13650 13966 14059 14407 14766 15110 15217 15301 15326 16114A 16129 16213 16223 16278 16354 16390	L2b2a	73 146 150 152 182 195 198 204 263 16114A 16129 16213 16223 16278 16354 16390	[0.000] L2b2a (1)	[0.000] L2b L2b2 L2b3 L2b3a L2b3b L2b3c (7)	1
23 >MH981607.1 H	151T 152C 189G 207A 247A 263G 482C 750G 769A 825A 1018A 1048T 1438G 2245G 2706G 2758A 2885C 3106d 3107C 3516A 3594T 3644C 3699G 4104G 4312T 4586C 4769G 4964T 5108C 5442C 5492C 5603T 5984G 6185C 7028T 7146G 7148C 7256T 7364G 7521A 7543G 8468T 8655T 8701G 8860G 8911C 9042T 9347G 9540C 9581C 9620T 9818T 10310A 10398G 10580R 10589A 10664T 10688A 10692T 10694G 10810C 10873C 10915C 11084G 11314G 11641G 11719A 11914A 12007A 12346T 12705T 12720G 13105G 13145A 13276G 13470G 13506T 13650T 14091G 14109T 14178C 14364A 14620T 14766T 15136T 15236G 15326G 15431A 15852C 16129A 16169T 16172C 16174T 16183C 16189C 16223T 16230G 16278T 16311C 16327T 16354T 16368C 16519C	L0f1	151T 152C 189G 207A 247A 263G 482C 16129A 16169T 16172C 16174T 16183C 16189C 16223T 16230G 16278T 16311C 16327T 16354T 16368C 16519C	1	151 152 189 207 247 263 482 750 769 825A 1018 1048 1438 2245 2706 2758 2885 3516A 3594 3644 3699G 4104 4312 4586 4769 4964 5108 5442 5492 5603 5984 6185 7028 7146 7148 7256 7364 7521 7543 8468 8655 8701 8860 8911 9042 9347 9540 9581 9620 9818 10310 10398 10580R 10589 10664 10688 10692 10694 10810 10873 10915 11084 11314 11641 11719 11914 12007 12346 12705 12720 13105 13145 13276 13470 13506 13650 14091 14109 14178 14364 14620 14766 15136 15236 15326 15431 15852 16129 16169 16172 16174 16189 16223 16230 16278 16311 16327 16354 16368	L0f1	151 152 189 207 247 263 482 16129 16169 16172 16174 16189 16223 16230 16278 16311 16327 16354 16368	[1.287] L0f1 (1)	[0.824] L0f2 (2) [0.758] L0f (3) [0.631] L0f2a	1

24 >MH981605.1 H	73G 146C 152C 195C 263G 750G 769A 1018A 1438G 2416C 2706G 2789T 3106d 3107C 3594T 4104G 4769G 5090C 7028T 7175C 7256T 7274T 7521A 7771G 8206A 8701G 8860G 9221G 9540C 10115C 10143A 10398G 10873C 11719A 11914A 11944C 12693G 12705T 13590A 13650T 13803G 14566G 14766T 15301A 15326G 15735T 15784C 16182C 16183C 16189C 16223T 16278T 16290T 16294T 16309G 16390A	L2a1b1a	73G 146C 152C 195C 263G 16182C 16183C 16189C 16223T 16278T 16290T 16294T 16309G 16390A	1	73 146 152 195 263 750 769 1018 1438 2416 2706 2789 3594 4104 4769 5090 7028 7175 7256 7274 7521 7771 8206 8701 8860 9221 9540 10115 10143 10398 10873 11719 11914 11944 12693 12705 13590 13650 13803 14566 14766 15301 15326 15735 15784 16189 16223 16278 16290 16294 16309 16390	L2a1b1a	73 146 152 195 263 16189 16223 16278 16290 16294 16309 16390	[0.000] L2a1b1a (1)	[0.005] L2a1f (2) [0.000] L2a1+16189_(16192) L2a1b L2a1b1 L2a1b1a L2a1b2 L2a1f1 L2a1f1a L2a1f2 (10)	1
25 >MH981604.1 H	73G 146C 152C 195C 247A 456T 719A 750G 769A 825A 980C 1018A 1048T 2758A 2885C 3106d 3107C 3438A 3516A 3594T 3756G 4104G 4197T 4232C 4312T 4769G 5442C 6185C 6266G 6815C 7028T 7146G 7256T 7521A 7696G 7699T 8113A 8152A 8251A 8468T 8655T 8701G 8860G 8994A 9042T 9150G 9347G 9449T 9540C 9755A 10398G 10589A 10664T 10688A 10810C 10873C 10915C 11167G 11437C 11719A 11914A 12007A 12121C 12235C 12705T 13105G 13129T 13276G 13473G 13506T 13650T 13759A 14766T 15315T 15326G 15466A 15930A 15941C 15951G 16187T 16189C 16223T 16230G 16234T 16243C 16249C 16311C 16519C	L0d1c	73G 146C 152C 195C 247A 456T 16187T 16189C 16223T 16230G 16234T 16243C 16249C 16311C 16519C	1	73 146 152 195 247 456 719 750 769 825A 980 1018 1048 2758 2885 3438 3516A 3594 3756 4104 4197 4232 4312 4769 5442 6185 6266 6815 7028 7146 7256 7521 7696 7699 8113A 8152 8251 8468 8655 8701 8860 8994 9042 9150 9347 9449 9540 9755 10398 10589 10664 10688 10810 10873 10915 11167 11437 11719 11914 12007 12121 12235 12705 13105 13129 13276 13473 13506 13650 13759 14766 15315 15326 15466 15930 15941 15951 16187 16189 16223 16230 16234 16243 16249 16311	L0d1c	73 146 152 195 247 456 16187 16189 16223 16230 16234 16243 16249 16311	[0.407] L0d1c (1)	[0.000] L0d1c1 L0d1c1a L0d1c2 (4)	1
26 >MH981600.1 H	73G 146C 152C 195C 263G 750G 769A 1018A 1438G 2416C 2706G 2789T 3106d 3107C 3594T 4104G 4769G 5090C 7028T 7175C 7256T 7274T 7521A 7771G 8206A 8701G 8860G 9221G 9540C 10115C 10143A 10398G 10873C 11719A 11914A 11944C 12693G 12705T 13590A 13650T 13803G 14566G 14766T 15301A 15326G 15735T 15784C 16167T 16182M 16183C 16189C 16223T 16278T 16290T 16294T 16309G 16390A 16519C	L2a1b1a	73G 146C 152C 195C 263G 16167T 16182M 16183C 16189C 16223T 16278T 16290T 16294T 16309G 16390A 16519C	1	73 146 152 195 263 750 769 1018 1438 2416 2706 2789 3594 4104 4769 5090 7028 7175 7256 7274 7521 7771 8206 8701 8860 9221 9540 10115 10143 10398 10873 11719 11914 11944 12693 12705 13590 13650 13803 14566 14766 15301 15326 15735 15784 16167 16182M 16189 16223 16278 16290 16294 16309 16390	L2a1b1a	73 146 152 195 263 16167 16182M 16189 16223 16278 16290 16294 16309 16390	[0.003] L2a1b1a (1)	[0.005] L2a1f (2) [0.003] L2a1b1a (3) [0.000] L2a1+16189_(16192) L2a1b L2a1b1 L2a1b2 L2a1f1 L2a1f1a L2a1f2 (10)	1

27	>MK114116.1 H	73G 150T 263G 309.1CT 310C 750G 1438G 1721T 1834C 2706G 3106d 3107C 3197C 4732G 4769G 5452T 7028T 7377T 7768G 8705C 8860G 9477A 11467G 11719A 12308G 12372A 13617C 13637G 14182C 14766T 15326G 15511C 15924G 16519C	U5b2a1a2	73G 150T 263G 309.1CT 310C 16519C	1	73 150 263 750 1438 1721 1834 2706 3197 4732 4769 5452 7028 7377 7768 8705 8860 9477 11467 11719 12308 12372 13617 13637 14182 14766 15326 15511 15924	U5b2a1a2	73 150 263	[0.000] U5b2a1a2 (1)	[0.031] H4a1a1a1a (2) [0.026] U5b2a1a (3) [0.023] U5b2b (4) [0.021] R (5) [0.019] U3b2a1 (6) [0.000] L3e'i'k'x L3e L3e3'4'5 L3e3'4 L3i HV+73 HV20 HV5b HV12b H1e1a6 H1e2c H1as1 H1av1 H1av1a H3+73 H3d H4a1a1a H4a1a1a1 H4a1a1a1a1 H4a1a1a2 H4a1a1a4 R2'JT B6 R30 R30a R30a1 R30a1a R31 P P2 P4 P9 P9a U U5b1d1a U5b2b1 U5b2b2 U2'3'4'7'8'9 U3 U3a'c U3b U3b1 U3b1b U3b2 U3b2a U3b2c U8 U8b'c U8b (55)	1
28	>MK114115.1 H	73G 150T 189G 195C 204C 263G 310.1C 709A 750G 1243C 1438G 2706G 3106d 3107C 3505G 4769G 5046A 5460A 6528T 7028T 8251A 8860G 8994A 10097G 11674T 11719A 11947G 12414C 12705T 14766T 15326G 15775G 15884C 16223T 16292T 16293G 16362C 16519C	W5a2	73G 150T 189G 195C 204C 263G 310.1C 16223T 16292T 16293G 16362C 16519C	1	73 150 189 195 204 263 709 750 1243 1438 2706 3505 4769 5046 5460 6528 7028 8251 8860 8994 10097 11674 11719 11947 12414 12705 14766 15326 15775 15884C 16223 16292 16293 16362	W5a2	73 150 189 195 204 263 16223 16292 16293 16362	[0.412] W5a2 (1)	[0.500] W5a2b (2) [0.162] W1f (3) [0.118] W5a (4) [0.107] W1 (5) [0.102] W (6) [0.028] W5a1a (7) [0.014] W1a (8) [0.000] W1e W5a1 W5a1a1 (11)	1
29	>MK114114.1 H	251A 263G 310.1C 750G 1438G 3106d 3107C 4769G 6776C 7976A 8860G 15326G 16519C	H3an	251A 263G 310.1C 16519C	1	251 263 750 1438 4769 6776 7976 8860 15326	H3an	251 263	[0.000] H3an (1)	[0.000] R0 HV HV4 HV4c HV5 HV5a HV9c HV12 H H1 H1e H1e1 H1e1a H1e1a2 H1e1a7 H1e1c H1e2 H1e2a H1e2b H1e2d H1e4 H1e6 H1e7 H1e8 H1e8a H1h H1h1 H1h2 H1j H1j3 H1j5 H1j6 H1j7 H1j9 H1m H1n H1p H1q H1q1 H1q2 H1r H1s H1s1 H1t H1t1 H1t1a H1t2 H1u H1u1 H1v H1v1 H1v1a H1v1b H1w H1ae H1ae1 H1ae2 H1ae2a H1ae3 H1ae3a H1ag H1ag1 H1ag1b H1ah H1ai H1ai1 H1aj H1ak H1ak1 H1ak2 H1am H1am1 H1ap H1aq H1as H1as2 H1at H1at1 H1at1a H1av H1ax H1ax1 H1ay H1az H1bd H1be H1bi H1bj H1bk H1bm H1bn H1bp H1bq H1br H1bu H1bv H1bw H1bx H1ca H1ci H2 H2a H2a2 H2a2a H2a2+(16235) H2a4 H2a5 H2a5a H2a5b H3 H3b H3c H3c1 H3e H3q H3q1 H3r H3r1 H3t H3u H3y H3aa H3ab H3ac H3ad H3ae H3ag H3ag1 H3ah H3ai H3aj H3ap H3aq H3ar H3as H3at H3at1 H3au H4 H4a H4a1 H4a1a H4a1a1 H4a1a2 H4a1a2a H4a1a2a1 H4a1a5 H4a1c H4a1c1 H4a1c1a H4a1d H4b H4c H7 H7a H7b H7b1 H7b2 H7b2a H7b3 H7b4 H7b5 H7b6 H7c H7c2 H7c3 H7c5 [illegible]	1

30 >MH763841.1 H	153G 204C 263G 309.1CT 310C 750G 1126G 1438G 3106d 3107C 4769G 8860G 10646A 15326G 16189C 16519C	H55b	153G 204C 263G 309.1CT 310C 16189C 16519C	1	153 204 263 750 1126 1438 4769 8860 10646 15326 16189	H55b	153 204 263 16189	[0.366] H55b (1)	[0.103] H1g H1aa (3) [0.091] H55+153 (4) [0.019] H4b1 (5) [0.005] H1j9 (6) [0.000] HV18 H1+16189 H1f H1g1 H1g2 H1y H1aa1 H1ab H1ac H1ad H3+16189 H13a1b H56d (19)	1
31 >MH176338.1 H	73G 143A 152C 195C 263G 310C 750G 1438G 2706G 3107C 4769G 6221C 6227C 6371T 7028T 8860G 9438A 11719A 12406A 12696C 12705T 13966G 14470C 14766T 15326G 16182C 16183C 16189C 16223T 16266T 16274A 16278T 16301T 16390A 16519C	X4	73G 143A 152C 195C 263G 310C 16182C 16183C 16189C 16223T 16266T 16274A 16278T 16301T 16390A 16519C	1	73 143 152 195 263 310 750 1438 2706 4769 6221 6227 6371 7028 8860 9438 11719 12406 12696 12705 13966 14470 14766 15326 16189 16223 16266 16274 16278 16301 16390	X4	73 143 152 195 263 310 16189 16223 16266 16274 16278 16301 16390	[0.379] X4 (1)	[0.064] L2a1+143+16189_(16192)+@ 16309 (2)	1
32 >MH777996.1 H	73G 146C 152C 263G 497T 750G 990C 1189C 1438G 1811G 2706G 3106d 3107C 3480G 4769G 7028T 8856A 8860G 9055A 9698C 10398G 10550G 11167G 11299C 11467G 11485C 11719A 12308G 12372A 12681C 14167T 14766T 14798C 15317A 15326G 16224C 16311C 16360T 16519C	K1a4j1	73G 146C 152C 263G 497T 16224C 16311C 16360T 16519C	1	73 146 152 263 497 750 990 1189 1438 1811 2706 3480 4769 7028 8856 8860 9055 9698 10398 10550 11167 11299 11467 11485 11719 12308 12372 12681 14167 14766 14798 15317 15326 16224 16311 16360	K1a4j1	73 146 152 263 497 16224 16311 16360	[0.044] K1a4j1 (1)	[0.063] K2a2a (2) [0.062] K2a1 K2a2 K2a3a (5) [0.044] K1a4j1 (6) [0.000] K1a1b2a K1a1b2a1 K1a1b2a1a K1a4c K1a4+146 K1a4j K1a25 K2a K2a3 K2a3a1 K2a4 K2a6 K2a7 K2a9 K2a10 (21)	1
33 >MH444427.1 H	73G 146C 199C 263G 482C 489C 750G 1438G 2706G 3106d 3107C 4769G 5426C 5432G 7028T 8701G 8860G 9540C 10398G 10400T 10670T 10873C 11719A 12561A 12705T 13928C 14766T 14783C 15043A 15301A 15326G 15924G 16189C 16223T 16319A 16519C	M35a1a	73G 146C 199C 263G 482C 489C 16189C 16223T 16319A 16519C	1	73 146 199 263 482 489 750 1438 2706 4769 5426 5432 7028 8701 8860 9540 10398 10400 10670 10873 11719 12561 12705 13928C 14766 14783 15043 15301 15326 15924 16189 16223 16319	M35a1a	73 146 199 263 482 489 16189 16223 16319	[0.194] M35a1a (1) [0.000] M7c1a3 (2)	[0.064] M45 (3) [0.049] M7c1 (4) [0.007] M7c1a M7c1a1a M7c1b1 (7) [0.003] M7c1b M7c1c (9) [0.000] M7c1a1 M7c1a1a1 M7c1a3 M7c1a3a M7c1a4 M7c1b2 M7c1b2b M7c1c1 (17)	1
34 >MH444426.1 H	73G 146C 199C 263G 482C 489C 750G 1438G 2706G 3106d 3107C 4769G 5426C 5432G 7028T 8701G 8860G 9540C 10398G 10400T 10670T 10873C 11719A 12561A 12705T 13928C 14766T 14783C 15043A 15301A 15326G 15924G 16189C 16223T 16319A 16519C	M35a1a	73G 146C 199C 263G 482C 489C 16189C 16223T 16319A 16519C	1	73 146 199 263 482 489 750 1438 2706 4769 5426 5432 7028 8701 8860 9540 10398 10400 10670 10873 11719 12561 12705 13928C 14766 14783 15043 15301 15326 15924 16189 16223 16319	M35a1a	73 146 199 263 482 489 16189 16223 16319	[0.194] M35a1a (1) [0.000] M7c1a3 (2)	[0.064] M45 (3) [0.049] M7c1 (4) [0.007] M7c1a M7c1a1a M7c1b1 (7) [0.003] M7c1b M7c1c (9) [0.000] M7c1a1 M7c1a1a1 M7c1a3 M7c1a3a M7c1a4 M7c1b2 M7c1b2b M7c1c1 (17)	1
35 >MH444425.1 H	73G 146C 152C 199C 263G 310.1C 482C 489C 750G 1438G 2706G 3106d 3107C 4769G 5426C 5432G 7028T 8701G 8860G 9540C 10398G 10400T 10670T 10873C 11719A 12561A 12705T 13928C 14766T 14783C 15043A 15301A 15326G 15924G 16189C 16223T 16319A 16519C	M35a1a	73G 146C 152C 199C 263G 310.1C 482C 489C 16189C 16223T 16319A 16519C	1	73 146 152 199 263 482 489 750 1438 2706 4769 5426 5432 7028 8701 8860 9540 10398 10400 10670 10873 11719 12561 12705 13928C 14766 14783 15043 15301 15326 15924 16189 16223 16319	M35a1a	73 146 152 199 263 482 489 16189 16223 16319	[0.210] M35a1a (1) [0.007] M7c1a3 (2)	[0.231] M7c1a (3) [0.121] M45 (4) [0.061] M7c1 (5) [0.057] M7c1b2b (6) [0.023] M3c1a (7) [0.008] M3c1 (8) [0.007] M7c1a1a M7c1a3 M7c1b1 (11) [0.006] M7c1c (12) [0.003] M7c1b (13) [0.000] M7c1a1 M7c1a1a1 M7c1a3a M7c1a4 M7c1b2 M7c1c1 (19)	1

36	>MH444424.1 H	73G 146C 199C 263G 310.1C 482C 489C 750G 1438G 2706G 3106d 3107C 4769G 5426C 5432G 7028T 8701G 8860G 9540C 10398G 10400T 10670T 10873C 11719A 12561A 12705T 13928C 14766T 14783C 15043A 15301A 15326G 15869T 15870C 15924G 16189C 16223T 16319A 16519C	M35a1a	73G 146C 199C 263G 310.1C 482C 489C 16189C 16223T 16319A 16519C	1	73 146 199 263 482 489 750 1438 2706 4769 5426 5432 7028 8701 8860 9540 10398 10400 10670 10873 11719 12561 12705 13928C 14766 14783 15043 15301 15326 15869T 15870C 15924 16189 16223 16319	M35a1a	73 146 199 263 482 489 16189 16223 16319	[0.194] M35a1a (1) [0.000] M7c1a3 (2)	[0.064] M45 (3) [0.049] M7c1 (4) [0.007] M7c1a M7c1a1a M7c1b1 (7) [0.003] M7c1b M7c1c (9) [0.000] M7c1a1 M7c1a1a1 M7c1a3 M7c1a3a M7c1a4 M7c1b2 M7c1b2b M7c1c1 (17)	1
37	>MH444423.1 H	73G 146C 152C 199C 263G 310.1C 482C 489C 750G 1438G 2706G 3106d 3107C 4769G 5426C 5432G 7028T 8701G 8860G 9540C 10398G 10400T 10873C 11719A 12561A 12705T 13928C 14766T 14783C 15043A 15301A 15326G 15869T 15870C 15924G 16093C 16223T 16319A 16519C	M35a1a	73G 146C 152C 199C 263G 310.1C 482C 489C 16093C 16223T 16319A 16519C	1	73 146 152 199 263 482 489 750 1438 2706 4769 5426 5432 7028 8701 8860 9540 10398 10400 10873 11719 12561 12705 13928C 14766 14783 15043 15301 15326 15869T 15870C 15924 16093 16223 16319	M35a1a	73 146 152 199 263 482 489 16093 16223 16319	[0.016] M35a1a (1)	[0.014] M7c1a3 (2) [0.006] M35a2 (3) [0.000] M35a M35a1 (5)	1
38	>MH444419.1 H	73G 146C 150T 263G 309.1CT 310C 482C 489C 750G 1438G 1736T 2706G 3106d 3107C 4562G 4580A 4769G 5783A 7028T 8701G 8860G 8988G 9540C 9554A 10398G 10400T 10727T 10873C 11719A 12705T 14766T 14783C 15043A 15301A 15326G 16126C 16192T 16223T 16519C	M3a2a	73G 146C 150T 263G 309.1CT 310C 482C 489C 16126C 16192T 16223T 16519C	1	73 146 150 263 482 489 750 1438 1736T 2706 4562 4580 4769 5783 7028 8701 8860 8988 9540 9554 10398 10400 10727 10873 11719 12705 14766 14783 15043 15301 15326 16126 16192 16223	M3a2a	73 146 150 263 482 489 16126 16192 16223	[0.048] M3a2a (1)	[0.008] M3 M3a M3a1 M3a2 M3b M3c (7)	1
39	>MH444418.1 H	73G 146C 150T 263G 309.1CT 310C 482C 489C 750G 1438G 1736T 2706G 3106d 3107C 4562G 4580A 4769G 5783A 7028T 8701G 8860G 8988G 9540C 9554A 10398G 10400T 10727T 10873C 11719A 12705T 14766T 14783C 15043A 15301A 15326G 16126C 16192T 16223T 16519C	M3a2a	73G 146C 150T 263G 309.1CT 310C 482C 489C 16126C 16192T 16223T 16519C	1	73 146 150 263 482 489 750 1438 1736T 2706 4562 4580 4769 5783 7028 8701 8860 8988 9540 9554 10398 10400 10727 10873 11719 12705 14766 14783 15043 15301 15326 16126 16192 16223	M3a2a	73 146 150 263 482 489 16126 16192 16223	[0.048] M3a2a (1)	[0.008] M3 M3a M3a1 M3a2 M3b M3c (7)	1
40	>MH444417.1 H	73G 146C 150T 263G 309.1CT 310C 482C 489C 750G 1438G 1736T 2706G 3106d 3107C 4562G 4580A 4769G 5783A 7028T 8701G 8860G 8988G 9540C 9554A 10398G 10400T 10727T 10873C 11719A 12705T 14766T 14783C 15043A 15301A 15326G 16126C 16192T 16223T 16519C	M3a2a	73G 146C 150T 263G 309.1CT 310C 482C 489C 16126C 16192T 16223T 16519C	1	73 146 150 263 482 489 750 1438 1736T 2706 4562 4580 4769 5783 7028 8701 8860 8988 9540 9554 10398 10400 10727 10873 11719 12705 14766 14783 15043 15301 15326 16126 16192 16223	M3a2a	73 146 150 263 482 489 16126 16192 16223	[0.048] M3a2a (1)	[0.008] M3 M3a M3a1 M3a2 M3b M3c (7)	1
41	>MH444416.1 H	73G 146C 150T 263G 309.1CT 310C 482C 489C 750G 1438G 1736T 2706G 3106d 3107C 4562G 4580A 4769G 5783A 7028T 8701G 8860G 8988G 9540C 9554A 10398G 10400T 10727T 10873C 11719A 12705T 14766T 14783C 15043A 15301A 15326G 16126C 16192T 16223T 16519C	M3a2a	73G 146C 150T 263G 309.1CT 310C 482C 489C 16126C 16192T 16223T 16519C	1	73 146 150 263 482 489 750 1438 1736T 2706 4562 4580 4769 5783 7028 8701 8860 8988 9540 9554 10398 10400 10727 10873 11719 12705 14766 14783 15043 15301 15326 16126 16192 16223	M3a2a	73 146 150 263 482 489 16126 16192 16223	[0.048] M3a2a (1)	[0.008] M3 M3a M3a1 M3a2 M3b M3c (7)	1

42 >MH444415.1 H	73G 146C 152C 182T 195C 263G 309.1CT 310C 447G 489C 514d 515d 750G 1438G 1453G 1719A 1780C 2706G 2831T 3106d 3107C 3630T 4769G 5744A 6647G 7028T 8502G 8701G 8860G 9540C 9899C 10398G 10400T 10873C 11083G 11377A 11719A 12705T 13254C 14783C 15043A 15301A 15326G 15670C 16136C 16183C 16189C 16223T 16274A 16319A 16320T 16519C	M2b3a	73G 146C 152C 182T 195C 263G 309.1CT 310C 447G 489C 514d 515d 16136C 16183C 16189C 16223T 16274A 16319A 16320T 16519C	1	73 146 152 182 195 263 447G 489 750 1438 1453 1719 1780 2706 2831T 3630 4769 5744 6647 7028 8502 8701 8860 9540 9899 10398 10400 10873 11083 11377 11719 12705 13254 14783 15043 15301 15326 15670 16136 16189 16223 16274 16319 16320	M2b3a	73 146 152 182 195 263 447G 489 16136 16189 16223 16274 16319 16320	[0.542] M2b3a (1)	[0.062] M2b1 (2) [0.053] M2b (3) [0.017] M2b3	1
43 >MH444414.1 H	73G 146C 152C 182T 195C 263G 310.1C 447G 489C 514d 515d 750G 1438G 1453G 1780C 2706G 2831T 3630T 4769G 5744A 6647G 7028T 8502G 8701G 8860G 9540C 9899C 10398G 10400T 10873C 11083G 11377A 11719A 12705T 13254C 13488C 14783C 15043A 15301A 15326G 15670C 16136C 16183C 16189C 16223T 16274A 16319A 16320T 16519C	M2b3a	73G 146C 152C 182T 195C 263G 310.1C 447G 489C 514d 515d 16136C 16183C 16189C 16223T 16274A 16319A 16320T 16519C	1	73 146 152 182 195 263 447G 489 750 1438 1453 1780 2706 2831T 3630 4769 5744 6647 7028 8502 8701 8860 9540 9899 10398 10400 10873 11083 11377 11719 12705 13254 13488 14783 15043 15301 15326 15670 16136 16189 16223 16274 16319 16320	M2b3a	73 146 152 182 195 263 447G 489 16136 16189 16223 16274 16319 16320	[0.542] M2b3a (1)	[0.062] M2b1 (2) [0.053] M2b (3) [0.017] M2b3	1
44 >MH444413.1 H	73G 146C 152C 182T 195C 263G 310.1C 447G 489C 514d 515d 750G 1438G 1453G 1780C 2706G 2831T 3630T 4769G 5744A 6647G 7028T 8502G 8701G 8860G 9540C 9899C 10398G 10400T 10873C 11083G 11377A 11719A 12705T 13254C 13488C 14783C 15043A 15301A 15326G 15670C 16136C 16183C 16189C 16223T 16274A 16319A 16320T 16519C	M2b3a	73G 146C 152C 182T 195C 263G 310.1C 447G 489C 514d 515d 16136C 16183C 16189C 16223T 16274A 16319A 16320T 16519C	1	73 146 152 182 195 263 447G 489 750 1438 1453 1780 2706 2831T 3630 4769 5744 6647 7028 8502 8701 8860 9540 9899 10398 10400 10873 11083 11377 11719 12705 13254 13488 14783 15043 15301 15326 15670 16136 16189 16223 16274 16319 16320	M2b3a	73 146 152 182 195 263 447G 489 16136 16189 16223 16274 16319 16320	[0.542] M2b3a (1)	[0.062] M2b1 (2) [0.053] M2b (3) [0.017] M2b3	1
45 >MH444412.1 H	73G 146C 152C 182T 195C 263G 447G 489C 750G 1438G 1453G 1780C 2706G 2831T 3630T 4769G 5744A 6647G 7028T 8502G 8701G 8860G 9540C 9899C 10398G 10400T 10873C 11083G 11377A 11719A 12705T 13254C 13488C 14783C 15043A 15301A 15326G 15670C 16136C 16183C 16189C 16223T 16274A 16319A 16320T 16519C	M2b3a	73G 146C 152C 182T 195C 263G 447G 489C 16136C 16183C 16189C 16223T 16274A 16319A 16320T 16519C	1	73 146 152 182 195 263 447G 489 750 1438 1453 1780 2706 2831T 3630 4769 5744 6647 7028 8502 8701 8860 9540 9899 10398 10400 10873 11083 11377 11719 12705 13254 13488 14783 15043 15301 15326 15670 16136 16189 16223 16274 16319 16320	M2b3a	73 146 152 182 195 263 447G 489 16136 16189 16223 16274 16319 16320	[0.542] M2b3a (1)	[0.062] M2b1 (2) [0.053] M2b (3) [0.017] M2b3	1

46	>MH444411.1 H	73G 146C 152C 182T 195C 263G 310.1C 345T 447G 489C 514d 515d 750G 1438G 1453G 1780C 2706G 2831T 3630T 4769G 6437G 6647G 7028T 8502G 8701G 8860G 9272A 9540C 9899C 10398G 10400T 10873C 11083G 11377A 11719A 12705T 13254C 14783C 15043A 15301A 15326G 15670C 16136C 16145A 16183C 16189C 16274A 16319A 16320T 16519C	M2b3a	73G 146C 152C 182T 195C 263G 310.1C 345T 447G 489C 514d 515d 16136C 16145A 16183C 16189C 16274A 16319A 16320T 16519C	1	73 146 152 182 195 263 345 447G 489 750 1438 1453 1780 2706 2831T 3630 4769 6437 6647 7028 8502 8701 8860 9272A 9540 9899 10398 10400 10873 11083 11377 11719 12705 13254 14783 15043 15301 15326 15670 16136 16145 16189 16274 16319 16320	M2b3a	73 146 152 182 195 263 345 447G 489 16136 16145 16189 16274 16319 16320	[0.668] M2b3a (1)	[0.078] M2b1 (2) [0.071] M2b (3) [0.034] M2b3	1
47	>MH444410.1 H	73G 146C 152C 182T 195C 263G 310.1C 447G 489C 514d 515d 750G 1438G 1453G 1780C 2706G 2831T 3106d 3107C 3630T 4769G 5744A 6437G 6647G 7028T 8502G 8701G 8860G 9540C 9899C 10398G 10400T 10873C 11083G 11377A 11719A 12705T 13254C 14783C 15043A 15301A 15326G 15670C 16136C 16145A 16183C 16189C 16223T 16274A 16319A 16320T 16519C	M2b3a	73G 146C 152C 182T 195C 263G 310.1C 447G 489C 514d 515d 16136C 16145A 16183C 16189C 16223T 16274A 16319A 16320T 16519C	1	73 146 152 182 195 263 447G 489 750 1438 1453 1780 2706 2831T 3630 4769 5744 6437 6647 7028 8502 8701 8860 9540 9899 10398 10400 10873 11083 11377 11719 12705 13254 14783 15043 15301 15326 15670 16136 16145 16189 16223 16274 16319 16320	M2b3a	73 146 152 182 195 263 447G 489 16136 16145 16189 16223 16274 16319 16320	[0.652] M2b3a (1)	[0.062] M2b1 (2) [0.053] M2b (3) [0.017] M2b3	1
48	>MH444409.1 H	73G 146C 152C 182T 195C 263G 310.1C 447G 489C 514d 515d 750G 1438G 1453G 1780C 2706G 2831T 3106d 3107C 3630T 4769G 5744A 6437G 6647G 7028T 8502G 8701G 8860G 9540C 9899C 10398G 10400T 10873C 11083G 11377A 11719A 12705T 13254C 14783C 15043A 15301A 15326G 15670C 16136C 16145A 16183C 16189C 16223T 16274A 16319A 16320T 16519C	M2b3a	73G 146C 152C 182T 195C 263G 310.1C 447G 489C 514d 515d 16136C 16145A 16183C 16189C 16223T 16274A 16319A 16320T 16519C	1	73 146 152 182 195 263 447G 489 750 1438 1453 1780 2706 2831T 3630 4769 5744 6437 6647 7028 8502 8701 8860 9540 9899 10398 10400 10873 11083 11377 11719 12705 13254 14783 15043 15301 15326 15670 16136 16145 16189 16223 16274 16319 16320	M2b3a	73 146 152 182 195 263 447G 489 16136 16145 16189 16223 16274 16319 16320	[0.652] M2b3a (1)	[0.062] M2b1 (2) [0.053] M2b (3) [0.017] M2b3	1
49	>MH444408.1 H	73G 146C 152C 182T 195C 263G 310.1C 447G 489C 514d 515d 750G 1438G 1453G 1780C 2706G 2831T 3106d 3107C 3630T 4769G 5744A 6437G 6647G 7028T 8502G 8701G 8860G 9540C 9899C 10398G 10400T 10873C 11083G 11377A 11719A 12705T 13254C 14783C 15043A 15301A 15326G 15670C 16136C 16145A 16183C 16189C 16223T 16274A 16319A 16320T 16519C	M2b3a	73G 146C 152C 182T 195C 263G 310.1C 447G 489C 514d 515d 16136C 16145A 16183C 16189C 16223T 16274A 16319A 16320T 16519C	1	73 146 152 182 195 263 447G 489 750 1438 1453 1780 2706 2831T 3630 4769 5744 6437 6647 7028 8502 8701 8860 9540 9899 10398 10400 10873 11083 11377 11719 12705 13254 14783 15043 15301 15326 15670 16136 16145 16189 16223 16274 16319 16320	M2b3a	73 146 152 182 195 263 447G 489 16136 16145 16189 16223 16274 16319 16320	[0.652] M2b3a (1)	[0.062] M2b1 (2) [0.053] M2b (3) [0.017] M2b3	1

50	>MH444407.1 H	73G 146C 152C 182T 195C 263G 310.1C 447G 489C 514d 515d 750G 1438G 1453G 1780C 2706G 2831T 3106d 3107C 3630T 4769G 5744A 6437G 6647G 7028T 8502G 8701G 8860G 9540C 9899C 10398G 10400T 10873C 11083G 11377A 11719A 12705T 13254C 14783C 15043A 15301A 15326G 15670C 16136C 16145A 16183C 16189C 16223T 16274A 16319A 16320T 16519C	M2b3a	73G 146C 152C 182T 195C 263G 310.1C 447G 489C 514d 515d 16136C 16145A 16183C 16189C 16223T 16274A 16319A 16320T 16519C	1	73 146 152 182 195 263 447G 489 750 1438 1453 1780 2706 2831T 3630 4769 5744 6437 6647 7028 8502 8701 8860 9540 9899 10398 10400 10873 11083 11377 11719 12705 13254 14783 15043 15301 15326 15670 16136 16145 16189 16223 16274 16319 16320	M2b3a	73 146 152 182 195 263 447G 489 16136 16145 16189 16223 16274 16319 16320	[0.652] M2b3a (1)	[0.062] M2b1 (2) [0.053] M2b (3) [0.017] M2b3	1
51	>MH444406.1 H	73G 146C 152C 182T 195C 263G 310.1C 447G 489C 514d 515d 750G 1438G 1453G 1780C 2706G 2831T 3106d 3107C 3630T 4769G 5744A 6437G 6647G 7028T 8502G 8701G 8860G 9540C 9899C 10398G 10400T 10873C 11083G 11377A 11719A 12705T 13254C 14783C 15043A 15301A 15326G 15670C 16136C 16145A 16183C 16189C 16223T 16274A 16319A 16320T 16519C	M2b3a	73G 146C 152C 182T 195C 263G 310.1C 447G 489C 514d 515d 16136C 16145A 16183C 16189C 16223T 16274A 16319A 16320T 16519C	1	73 146 152 182 195 263 447G 489 750 1438 1453 1780 2706 2831T 3630 4769 5744 6437 6647 7028 8502 8701 8860 9540 9899 10398 10400 10873 11083 11377 11719 12705 13254 14783 15043 15301 15326 15670 16136 16145 16189 16223 16274 16319 16320	M2b3a	73 146 152 182 195 263 447G 489 16136 16145 16189 16223 16274 16319 16320	[0.652] M2b3a (1)	[0.062] M2b1 (2) [0.053] M2b (3) [0.017] M2b3	1
52	>MH444405.1 H	73G 146C 152C 182T 195C 263G 310.1C 447G 489C 514d 515d 750G 1438G 1453G 2706G 2831T 3106d 3107C 3630T 4769G 5744A 6437G 6647G 7028T 8502G 8701G 8860G 9540C 9899C 10398G 10400T 10873C 11083G 11377A 11719A 12705T 13254C 14783C 15043A 15301A 15326G 15670C 16136C 16145A 16183C 16189C 16223T 16274A 16319A 16320T 16519C	M2b3a	73G 146C 152C 182T 195C 263G 310.1C 447G 489C 514d 515d 16136C 16145A 16183C 16189C 16223T 16274A 16319A 16320T 16519C	1	73 146 152 182 195 263 447G 489 750 1438 1453 2706 2831T 3630 4769 5744 6437 6647 7028 8502 8701 8860 9540 9899 10398 10400 10873 11083 11377 11719 12705 13254 14783 15043 15301 15326 15670 16136 16145 16189 16223 16274 16319 16320	M2b3a	73 146 152 182 195 263 447G 489 16136 16145 16189 16223 16274 16319 16320	[0.652] M2b3a (1)	[0.062] M2b1 (2) [0.053] M2b (3) [0.017] M2b3	1
53	>MH444404.1 H	73G 146C 152C 182T 195C 263G 310.1C 447G 489C 514d 515d 750G 1438G 1453G 1780C 2706G 2831T 3106d 3107C 3630T 4769G 5744A 6437G 6647G 7028T 8502G 8701G 8860G 9540C 9899C 10398G 10400T 10873C 11083G 11377A 11719A 12705T 13254C 14783C 15043A 15301A 15326G 15670C 16136C 16145A 16183C 16189C 16223T 16274A 16319A 16320T 16519C	M2b3a	73G 146C 152C 182T 195C 263G 310.1C 447G 489C 514d 515d 16136C 16145A 16183C 16189C 16223T 16274A 16319A 16320T 16519C	1	73 146 152 182 195 263 447G 489 750 1438 1453 1780 2706 2831T 3630 4769 5744 6437 6647 7028 8502 8701 8860 9540 9899 10398 10400 10873 11083 11377 11719 12705 13254 14783 15043 15301 15326 15670 16136 16145 16189 16223 16274 16319 16320	M2b3a	73 146 152 182 195 263 447G 489 16136 16145 16189 16223 16274 16319 16320	[0.652] M2b3a (1)	[0.062] M2b1 (2) [0.053] M2b (3) [0.017] M2b3	1

54	>MH444403.1 H[illegible]	73G 146C 152C 182T 195C 263G 310.1C 447G 489C 514d 515d 750G 1438G 1453G 1780C 2706G 2831T 3106d 3107C 3630T 4769G 5744A 6437G 6647G 7028T 8502G 8701G 8860G 9540C 9899C 10398G 10400T 10873C 11083G 11377A 11719A 12705T 13254C 14783C 15043A 15301A 15326G 15670C 16136C 16145A 16183C 16189C 16223T 16274A 16319A 16320T 16519C	M2b3a	73G 146C 152C 182T 195C 263G 310.1C 447G 489C 514d 515d 16136C 16145A 16183C 16189C 16223T 16274A 16319A 16320T 16519C	1	73 146 152 182 195 263 447G 489 750 1438 1453 1780 2706 2831T 3630 4769 5744 6437 6647 7028 8502 8701 8860 9540 9899 10398 10400 10873 11083 11377 11719 12705 13254 14783 15043 15301 15326 15670 16136 16145 16189 16223 16274 16319 16320	M2b3a	73 146 152 182 195 263 447G 489 16136 16145 16189 16223 16274 16319 16320	[0.652] M2b3a (1)	[0.062] M2b1 (2) [0.053] M2b (3) [0.017] M2b3	1
55	>MH444402.1 H[illegible]	73G 146C 152C 182T 195C 263G 310.1C 447G 489C 514d 515d 750G 1438G 1453G 1780C 2706G 2831T 3106d 3107C 3630T 4769G 5744A 6437G 6647G 7028T 8502G 8701G 8860G 9540C 9899C 10398G 10400T 10873C 11083G 11377A 11719A 12705T 13254C 14783C 15043A 15301A 15326G 15670C 16136C 16145A 16183C 16189C 16223T 16274A 16319A 16320T 16519C	M2b3a	73G 146C 152C 182T 195C 263G 310.1C 447G 489C 514d 515d 16136C 16145A 16183C 16189C 16223T 16274A 16319A 16320T 16519C	1	73 146 152 182 195 263 447G 489 750 1438 1453 1780 2706 2831T 3630 4769 5744 6437 6647 7028 8502 8701 8860 9540 9899 10398 10400 10873 11083 11377 11719 12705 13254 14783 15043 15301 15326 15670 16136 16145 16189 16223 16274 16319 16320	M2b3a	73 146 152 182 195 263 447G 489 16136 16145 16189 16223 16274 16319 16320	[0.652] M2b3a (1)	[0.062] M2b1 (2) [0.053] M2b (3) [0.017] M2b3	1
56	>MH444401.1 H[illegible]	73G 146C 152C 182T 195C 263G 310.1C 447G 489C 514d 515d 750G 1438G 1453G 1780C 2706G 2831T 3106d 3107C 3630T 4769G 5744A 6647G 7028T 8502G 8701G 8860G 9540C 9899C 10398G 10400T 10873C 11083G 11377A 11719A 12705T 13254C 14783C 15043A 15301A 15326G 15670C 16136C 16145A 16183C 16189C 16223T 16274A 16319A 16320T 16519C	M2b3a	73G 146C 152C 182T 195C 263G 310.1C 447G 489C 514d 515d 16136C 16145A 16183C 16189C 16223T 16274A 16319A 16320T 16519C	1	73 146 152 182 195 263 447G 489 750 1438 1453 1780 2706 2831T 3630 4769 5744 6647 7028 8502 8701 8860 9540 9899 10398 10400 10873 11083 11377 11719 12705 13254 14783 15043 15301 15326 15670 16136 16145 16189 16223 16274 16319 16320	M2b3a	73 146 152 182 195 263 447G 489 16136 16145 16189 16223 16274 16319 16320	[0.652] M2b3a (1)	[0.062] M2b1 (2) [0.053] M2b (3) [0.017] M2b3	1
57	>MH444400.1 H[illegible]	73G 146C 152C 182T 195C 263G 310.1C 447G 489C 514d 515d 750G 1438G 1453G 1780C 2706G 2831T 3106d 3107C 3630T 4769G 5744A 6647G 7028T 8502G 8701G 8860G 9540C 9899C 10398G 10400T 10873C 11083G 11377A 11719A 12705T 13254C 14783C 15043A 15301A 15326G 15670C 16136C 16145A 16183C 16189C 16223T 16274A 16319A 16320T 16519C	M2b3a	73G 146C 152C 182T 195C 263G 310.1C 447G 489C 514d 515d 16136C 16145A 16183C 16189C 16223T 16274A 16319A 16320T 16519C	1	73 146 152 182 195 263 447G 489 750 1438 1453 1780 2706 2831T 3630 4769 5744 6647 7028 8502 8701 8860 9540 9899 10398 10400 10873 11083 11377 11719 12705 13254 14783 15043 15301 15326 15670 16136 16145 16189 16223 16274 16319 16320	M2b3a	73 146 152 182 195 263 447G 489 16136 16145 16189 16223 16274 16319 16320	[0.652] M2b3a (1)	[0.062] M2b1 (2) [0.053] M2b (3) [0.017] M2b3	1

58 >MH444399.1 H	73G 146C 152C 182T 195C 263G 310.1C 447G 489C 514d 515d 750G 1438G 1453G 1780C 2706G 2831T 3106d 3107C 3630T 4769G 5744A 6647G 7028T 8502G 8701G 8860G 9540C 9899C 10398G 10400T 10873C 11083G 11377A 11719A 12705T 13254C 14783C 15043A 15301A 15326G 15670C 16136C 16183C 16189C 16223T 16274A 16319A 16320T 16519C	M2b3a	73G 146C 152C 182T 195C 263G 310.1C 447G 489C 514d 515d 16136C 16183C 16189C 16223T 16274A 16319A 16320T 16519C	1	73 146 152 182 195 263 447G 489 750 1438 1453 1780 2706 2831T 3630 4769 5744 6647 7028 8502 8701 8860 9540 9899 10398 10400 10873 11083 11377 11719 12705 13254 14783 15043 15301 15326 15670 16136 16189 16223 16274 16319 16320	M2b3a	73 146 152 182 195 263 447G 489 16136 16189 16223 16274 16319 16320	[0.542] M2b3a (1)	[0.062] M2b1 (2) [0.053] M2b (3) [0.017] M2b3	1
59 >MH444398.1 H	73G 146C 152C 182T 195C 263G 310.1C 447G 489C 514d 515d 750G 1438G 1453G 1780C 2706G 2831T 3106d 3107C 3630T 4769G 5744A 6647G 7028T 8502G 8701G 8860G 9540C 9899C 10398G 10400T 10873C 11083G 11377A 11719A 12705T 13254C 14783C 15043A 15301A 15326G 15670C 16136C 16183C 16189C 16223T 16274A 16319A 16320T 16519C	M2b3a	73G 146C 152C 182T 195C 263G 310.1C 447G 489C 514d 515d 16136C 16183C 16189C 16223T 16274A 16319A 16320T 16519C	1	73 146 152 182 195 263 447G 489 750 1438 1453 1780 2706 2831T 3630 4769 5744 6647 7028 8502 8701 8860 9540 9899 10398 10400 10873 11083 11377 11719 12705 13254 14783 15043 15301 15326 15670 16136 16189 16223 16274 16319 16320	M2b3a	73 146 152 182 195 263 447G 489 16136 16189 16223 16274 16319 16320	[0.542] M2b3a (1)	[0.062] M2b1 (2) [0.053] M2b (3) [0.017] M2b3	1
60 >MH444397.1 H	73G 146C 152C 182T 195C 263G 310.1C 447G 489C 514d 515d 750G 1438G 1453G 1780C 2706G 2831T 3106d 3107C 3630T 4769G 5744A 6647G 7028T 8502G 8701G 8860G 9540C 9899C 10398G 10400T 10873C 11083G 11377A 11719A 12705T 13254C 14783C 15043A 15301A 15326G 15670C 16136C 16183C 16189C 16223T 16274A 16319A 16320T 16519C	M2b3a	73G 146C 152C 182T 195C 263G 310.1C 447G 489C 514d 515d 16136C 16183C 16189C 16223T 16274A 16319A 16320T 16519C	1	73 146 152 182 195 263 447G 489 750 1438 1453 1780 2706 2831T 3630 4769 5744 6647 7028 8502 8701 8860 9540 9899 10398 10400 10873 11083 11377 11719 12705 13254 14783 15043 15301 15326 15670 16136 16189 16223 16274 16319 16320	M2b3a	73 146 152 182 195 263 447G 489 16136 16189 16223 16274 16319 16320	[0.542] M2b3a (1)	[0.062] M2b1 (2) [0.053] M2b (3) [0.017] M2b3	1
61 >MH444396.1 H	73G 146C 152C 182T 195C 263G 310.1C 447G 489C 514d 515d 750G 1438G 1453G 1780C 2706G 2831T 3106d 3107C 3630T 4769G 5744A 6647G 7028T 8502G 8701G 8860G 9540C 9899C 10398G 10400T 10873C 11083G 11377A 11719A 12705T 13254C 14783C 15043A 15301A 15326G 15670C 16136C 16183C 16189C 16223T 16274A 16319A 16320T 16519C	M2b3a	73G 146C 152C 182T 195C 263G 310.1C 447G 489C 514d 515d 16136C 16183C 16189C 16223T 16274A 16319A 16320T 16519C	1	73 146 152 182 195 263 447G 489 750 1438 1453 1780 2706 2831T 3630 4769 5744 6647 7028 8502 8701 8860 9540 9899 10398 10400 10873 11083 11377 11719 12705 13254 14783 15043 15301 15326 15670 16136 16189 16223 16274 16319 16320	M2b3a	73 146 152 182 195 263 447G 489 16136 16189 16223 16274 16319 16320	[0.542] M2b3a (1)	[0.062] M2b1 (2) [0.053] M2b (3) [0.017] M2b3	1

62	>MH444395.1 H	73G 146C 152C 182T 195C 263G 310.1C 447G 489C 514d 515d 750G 1438G 1453G 1780C 2706G 2831T 3106d 3107C 3630T 4769G 5744A 6647G 7028T 8502G 8701G 8860G 9540C 9899C 10398G 10400T 10873C 11083G 11377A 11719A 12705T 13254C 14783C 15043A 15301A 15326G 15670C 16136C 16183C 16189C 16223T 16274A 16319A 16320T 16519C	M2b3a	73G 146C 152C 182T 195C 263G 310.1C 447G 489C 514d 515d 16136C 16183C 16189C 16223T 16274A 16319A 16320T 16519C	1	73 146 152 182 195 263 447G 489 750 1438 1453 1780 2706 2831T 3630 4769 5744 6647 7028 8502 8701 8860 9540 9899 10398 10400 10873 11083 11377 11719 12705 13254 14783 15043 15301 15326 15670 16136 16189 16223 16274 16319 16320	M2b3a	73 146 152 182 195 263 447G 489 16136 16189 16223 16274 16319 16320	[0.542] M2b3a (1)	[0.062] M2b1 (2) [0.053] M2b (3) [0.017] M2b3	1
63	>MH444394.1 H	73G 146C 152C 182T 195C 263G 310.1C 447G 489C 514d 515d 750G 1438G 1453G 1780C 2706G 2831T 3106d 3107C 3630T 4769G 5744A 6647G 7028T 8502G 8701G 8860G 9540C 9899C 10398G 10400T 10873C 11083G 11377A 11719A 12705T 13254C 14783C 15043A 15301A 15326G 15670C 16136C 16183C 16189C 16223T 16274A 16319A 16320T 16519C	M2b3a	73G 146C 152C 182T 195C 263G 310.1C 447G 489C 514d 515d 16136C 16183C 16189C 16223T 16274A 16319A 16320T 16519C	1	73 146 152 182 195 263 447G 489 750 1438 1453 1780 2706 2831T 3630 4769 5744 6647 7028 8502 8701 8860 9540 9899 10398 10400 10873 11083 11377 11719 12705 13254 14783 15043 15301 15326 15670 16136 16189 16223 16274 16319 16320	M2b3a	73 146 152 182 195 263 447G 489 16136 16189 16223 16274 16319 16320	[0.542] M2b3a (1)	[0.062] M2b1 (2) [0.053] M2b (3) [0.017] M2b3	1
64	>MH444393.1 H	73G 146C 152C 182T 195C 263G 310.1C 447G 489C 514d 515d 750G 1438G 1453G 1780C 2706G 2831T 3106d 3107C 3630T 4769G 5744A 6647G 7028T 8502G 8701G 8860G 9540C 9899C 10398G 10400T 10873C 11083G 11377A 11719A 12705T 13254C 14783C 15043A 15301A 15326G 15670C 16136C 16183C 16189C 16223T 16274A 16319A 16320T 16519C	M2b3a	73G 146C 152C 182T 195C 263G 310.1C 447G 489C 514d 515d 16136C 16183C 16189C 16223T 16274A 16319A 16320T 16519C	1	73 146 152 182 195 263 447G 489 750 1438 1453 1780 2706 2831T 3630 4769 5744 6647 7028 8502 8701 8860 9540 9899 10398 10400 10873 11083 11377 11719 12705 13254 14783 15043 15301 15326 15670 16136 16189 16223 16274 16319 16320	M2b3a	73 146 152 182 195 263 447G 489 16136 16189 16223 16274 16319 16320	[0.542] M2b3a (1)	[0.062] M2b1 (2) [0.053] M2b (3) [0.017] M2b3	1
65	>MH444392.1 H	73G 146C 152C 182T 195C 263G 310.1C 447G 489C 514d 515d 750G 1438G 1453G 1780C 2706G 2831T 3106d 3107C 3630T 4769G 5744A 6647G 7028T 8502G 8701G 8860G 9540C 9899C 10398G 10400T 10873C 11083G 11377A 11719A 12705T 13254C 14783C 15043A 15301A 15326G 15670C 16136C 16183C 16189C 16223T 16274A 16319A 16320T 16519C	M2b3a	73G 146C 152C 182T 195C 263G 310.1C 447G 489C 514d 515d 16136C 16183C 16189C 16223T 16274A 16319A 16320T 16519C	1	73 146 152 182 195 263 447G 489 750 1438 1453 1780 2706 2831T 3630 4769 5744 6647 7028 8502 8701 8860 9540 9899 10398 10400 10873 11083 11377 11719 12705 13254 14783 15043 15301 15326 15670 16136 16189 16223 16274 16319 16320	M2b3a	73 146 152 182 195 263 447G 489 16136 16189 16223 16274 16319 16320	[0.542] M2b3a (1)	[0.062] M2b1 (2) [0.053] M2b (3) [0.017] M2b3	1

66	>MH444391.1 H	73G 146C 152C 182T 195C 263G 310.1C 447G 489C 514d 515d 750G 1438G 1453G 1780C 2706G 2831T 3106d 3107C 3630T 4769G 5744A 6647G 7028T 8502G 8701G 8860G 9540C 9899C 10398G 10400T 10873C 11083G 11377A 11719A 12705T 13254C 14783C 15043A 15301A 15326G 15670C 16136C 16183C 16189C 16223T 16274A 16319A 16320T 16519C	M2b3a	73G 146C 152C 182T 195C 263G 310.1C 447G 489C 514d 515d 16136C 16183C 16189C 16223T 16274A 16319A 16320T 16519C	1	73 146 152 182 195 263 447G 489 750 1438 1453 1780 2706 2831T 3630 4769 5744 6647 7028 8502 8701 8860 9540 9899 10398 10400 10873 11083 11377 11719 12705 13254 14783 15043 15301 15326 15670 16136 16189 16223 16274 16319 16320	M2b3a	73 146 152 182 195 263 447G 489 16136 16189 16223 16274 16319 16320	[0.542] M2b3a (1)	[0.062] M2b1 (2) [0.053] M2b (3) [0.017] M2b3	1
67	>MH444390.1 H	73G 146C 152C 182T 195C 263G 310.1C 447G 489C 514d 515d 750G 1438G 1453G 1780C 2706G 2831T 3106d 3107C 3630T 4769G 5744A 6647G 7028T 8502G 8701G 8860G 9540C 9899C 10398G 10400T 10873C 11083G 11377A 11719A 12705T 13254C 14783C 15043A 15301A 15326G 15670C 16136C 16183C 16189C 16223T 16274A 16319A 16320T 16519C	M2b3a	73G 146C 152C 182T 195C 263G 310.1C 447G 489C 514d 515d 16136C 16183C 16189C 16223T 16274A 16319A 16320T 16519C	1	73 146 152 182 195 263 447G 489 750 1438 1453 1780 2706 2831T 3630 4769 5744 6647 7028 8502 8701 8860 9540 9899 10398 10400 10873 11083 11377 11719 12705 13254 14783 15043 15301 15326 15670 16136 16189 16223 16274 16319 16320	M2b3a	73 146 152 182 195 263 447G 489 16136 16189 16223 16274 16319 16320	[0.542] M2b3a (1)	[0.062] M2b1 (2) [0.053] M2b (3) [0.017] M2b3	1
68	>MH444389.1 H	73G 146C 152C 182T 195C 263G 310.1C 447G 489C 514d 515d 750G 1438G 1453G 1780C 2706G 2831T 3106d 3107C 3630T 4769G 5744A 6647G 7028T 8502G 8701G 8860G 9540C 9899C 10398G 10400T 10873C 11083G 11377A 11719A 12705T 13254C 14783C 15043A 15301A 15326G 15670C 16136C 16183C 16189C 16223T 16274A 16319A 16320T 16519C	M2b3a	73G 146C 152C 182T 195C 263G 310.1C 447G 489C 514d 515d 16136C 16183C 16189C 16223T 16274A 16319A 16320T 16519C	1	73 146 152 182 195 263 447G 489 750 1438 1453 1780 2706 2831T 3630 4769 5744 6647 7028 8502 8701 8860 9540 9899 10398 10400 10873 11083 11377 11719 12705 13254 14783 15043 15301 15326 15670 16136 16189 16223 16274 16319 16320	M2b3a	73 146 152 182 195 263 447G 489 16136 16189 16223 16274 16319 16320	[0.542] M2b3a (1)	[0.062] M2b1 (2) [0.053] M2b (3) [0.017] M2b3	1
69	>MH444388.1 H	73G 146C 152C 182T 195C 263G 310.1C 447G 489C 514d 515d 750G 1438G 1453G 1780C 2706G 2831T 3106d 3107C 3630T 4769G 5744A 6647G 7028T 8502G 8701G 8860G 9540C 9899C 10398G 10400T 10873C 11083G 11377A 11719A 12705T 13254C 14783C 15043A 15301A 15326G 15670C 16136C 16183C 16189C 16223T 16274A 16319A 16320T 16519C	M2b3a	73G 146C 152C 182T 195C 263G 310.1C 447G 489C 514d 515d 16136C 16183C 16189C 16223T 16274A 16319A 16320T 16519C	1	73 146 152 182 195 263 447G 489 750 1438 1453 1780 2706 2831T 3630 4769 5744 6647 7028 8502 8701 8860 9540 9899 10398 10400 10873 11083 11377 11719 12705 13254 14783 15043 15301 15326 15670 16136 16189 16223 16274 16319 16320	M2b3a	73 146 152 182 195 263 447G 489 16136 16189 16223 16274 16319 16320	[0.542] M2b3a (1)	[0.062] M2b1 (2) [0.053] M2b (3) [0.017] M2b3	1

70	>MH444387.1 H	73G 146C 152C 182T 195C 263G 310.1C 447G 489C 514d 515d 750G 1438G 1453G 1780C 2706G 2831T 3106d 3107C 3630T 4769G 5744A 6647G 7028T 8502G 8701G 8860G 9540C 9899C 10398G 10400T 10873C 11083G 11377A 11719A 12705T 13254C 14783C 15043A 15301A 15326G 15670C 16136C 16183C 16189C 16223T 16274A 16319A 16320T 16519C	M2b3a	73G 146C 152C 182T 195C 263G 310.1C 447G 489C 514d 515d 16136C 16183C 16189C 16223T 16274A 16319A 16320T 16519C	1	73 146 152 182 195 263 447G 489 750 1438 1453 1780 2706 2831T 3630 4769 5744 6647 7028 8502 8701 8860 9540 9899 10398 10400 10873 11083 11377 11719 12705 13254 14783 15043 15301 15326 15670 16136 16189 16223 16274 16319 16320	M2b3a	73 146 152 182 195 263 447G 489 16136 16189 16223 16274 16319 16320	[0.542] M2b3a (1)	[0.062] M2b1 (2) [0.053] M2b (3) [0.017] M2b3	1
71	>MH444386.1 H	73G 146C 152C 182T 195C 263G 310.1C 447G 489C 514d 515d 750G 1438G 1453G 1780C 2706G 2831T 3106d 3107C 3630T 4769G 5744A 6647G 7028T 8502G 8701G 8860G 9540C 9899C 10398G 10400T 10873C 11083G 11377A 11719A 12705T 13254C 14783C 15043A 15301A 15326G 15670C 16136C 16183C 16189C 16223T 16274A 16319A 16320T 16519C	M2b3a	73G 146C 152C 182T 195C 263G 310.1C 447G 489C 514d 515d 16136C 16183C 16189C 16223T 16274A 16319A 16320T 16519C	1	73 146 152 182 195 263 447G 489 750 1438 1453 1780 2706 2831T 3630 4769 5744 6647 7028 8502 8701 8860 9540 9899 10398 10400 10873 11083 11377 11719 12705 13254 14783 15043 15301 15326 15670 16136 16189 16223 16274 16319 16320	M2b3a	73 146 152 182 195 263 447G 489 16136 16189 16223 16274 16319 16320	[0.542] M2b3a (1)	[0.062] M2b1 (2) [0.053] M2b (3) [0.017] M2b3	1
72	>MH444385.1 H	73G 146C 152C 182T 195C 263G 447G 489C 514d 515d 750G 1438G 1453G 1780C 2706G 2831T 3106d 3107C 3630T 4769G 5744A 6647G 7028T 8502G 8701G 8860G 9540C 9899C 10398G 10400T 10873C 11083G 11377A 11719A 12705T 13254C 14783C 15043A 15301A 15326G 15670C 16136C 16183C 16189C 16223T 16274A 16319A 16320T 16519C	M2b3a	73G 146C 152C 182T 195C 263G 447G 489C 514d 515d 16136C 16183C 16189C 16223T 16274A 16319A 16320T 16519C	1	73 146 152 182 195 263 447G 489 750 1438 1453 1780 2706 2831T 3630 4769 5744 6647 7028 8502 8701 8860 9540 9899 10398 10400 10873 11083 11377 11719 12705 13254 14783 15043 15301 15326 15670 16136 16189 16223 16274 16319 16320	M2b3a	73 146 152 182 195 263 447G 489 16136 16189 16223 16274 16319 16320	[0.542] M2b3a (1)	[0.062] M2b1 (2) [0.053] M2b (3) [0.017] M2b3	1
73	>MH444384.1 H	73G 146C 152C 182T 195C 263G 447G 489C 514d 515d 750G 1438G 1453G 1780C 2706G 2831T 3106d 3107C 3630T 4769G 5744A 6647G 7028T 8502G 8701G 8860G 9540C 9899C 10398G 10400T 10873C 11083G 11377A 11719A 12705T 13254C 14783C 15043A 15301A 15326G 15670C 16136C 16183C 16189C 16223T 16274A 16319A 16320T 16519C	M2b3a	73G 146C 152C 182T 195C 263G 447G 489C 514d 515d 16136C 16183C 16189C 16223T 16274A 16319A 16320T 16519C	1	73 146 152 182 195 263 447G 489 750 1438 1453 1780 2706 2831T 3630 4769 5744 6647 7028 8502 8701 8860 9540 9899 10398 10400 10873 11083 11377 11719 12705 13254 14783 15043 15301 15326 15670 16136 16189 16223 16274 16319 16320	M2b3a	73 146 152 182 195 263 447G 489 16136 16189 16223 16274 16319 16320	[0.542] M2b3a (1)	[0.062] M2b1 (2) [0.053] M2b (3) [0.017] M2b3	1

74	>MH444383.1 H	73G 146C 152C 182T 195C 263G 310.1C 447G 489C 514d 515d 750G 1438G 1453G 1780C 2706G 2831T 3106d 3107C 3630T 4769G 5744A 6647G 7028T 8502G 8701G 8860G 9540C 9899C 10398G 10400T 10873C 11083G 11377A 11719A 12705T 13254C 14783C 15043A 15301A 15326G 15670C 16136C 16183C 16189C 16223T 16274A 16319A 16320T 16519C	M2b3a	73G 146C 152C 182T 195C 263G 310.1C 447G 489C 514d 515d 16136C 16183C 16189C 16223T 16274A 16319A 16320T 16519C	1	73 146 152 182 195 263 447G 489 750 1438 1453 1780 2706 2831T 3630 4769 5744 6647 7028 8502 8701 8860 9540 9899 10398 10400 10873 11083 11377 11719 12705 13254 14783 15043 15301 15326 15670 16136 16189 16223 16274 16319 16320	M2b3a	73 146 152 182 195 263 447G 489 16136 16189 16223 16274 16319 16320	[0.542] M2b3a (1)	[0.062] M2b1 (2) [0.053] M2b (3) [0.017] M2b3	1
75	>MH444382.1 H	73G 146C 152C 182T 195C 263G 310.1C 447G 489C 750G 1438G 1453G 1780C 2706G 2831T 3106d 3107C 3630T 4769G 5744A 6647G 7028T 8502G 8701G 8860G 9540C 9899C 10398G 10400T 10873C 11083G 11377A 11719A 12705T 13254C 14783C 15043A 15301A 15326G 15670C 16136C 16183C 16189C 16223T 16274A 16319A 16320T 16519C	M2b3a	73G 146C 152C 182T 195C 263G 310.1C 447G 489C 16136C 16183C 16189C 16223T 16274A 16319A 16320T 16519C	1	73 146 152 182 195 263 447G 489 750 1438 1453 1780 2706 2831T 3630 4769 5744 6647 7028 8502 8701 8860 9540 9899 10398 10400 10873 11083 11377 11719 12705 13254 14783 15043 15301 15326 15670 16136 16189 16223 16274 16319 16320	M2b3a	73 146 152 182 195 263 447G 489 16136 16189 16223 16274 16319 16320	[0.542] M2b3a (1)	[0.062] M2b1 (2) [0.053] M2b (3) [0.017] M2b3	1
76	>MH444381.1 H	73G 146C 152C 182T 195C 263G 310.1C 447G 489C 514d 515d 750G 1438G 1453G 1780C 2706G 2831T 3106d 3107C 3630T 4769G 5744A 6647G 7028T 8502G 8701G 8860G 9540C 9899C 10398G 10400T 10873C 11083G 11377A 11719A 12705T 13254C 14783C 15043A 15301A 15326G 15670C 16136C 16183C 16189C 16223T 16274A 16319A 16320T 16519C	M2b3a	73G 146C 152C 182T 195C 263G 310.1C 447G 489C 514d 515d 16136C 16183C 16189C 16223T 16274A 16319A 16320T 16519C	1	73 146 152 182 195 263 447G 489 750 1438 1453 1780 2706 2831T 3630 4769 5744 6647 7028 8502 8701 8860 9540 9899 10398 10400 10873 11083 11377 11719 12705 13254 14783 15043 15301 15326 15670 16136 16189 16223 16274 16319 16320	M2b3a	73 146 152 182 195 263 447G 489 16136 16189 16223 16274 16319 16320	[0.542] M2b3a (1)	[0.062] M2b1 (2) [0.053] M2b (3) [0.017] M2b3	1
77	>MH444380.1 H	73G 146C 152C 182T 195C 263G 310.1C 447G 489C 514d 515d 750G 1438G 1453G 1780C 2706G 2831T 3106d 3107C 3630T 4769G 5744A 6647G 7028T 8502G 8701G 8860G 9540C 9899C 10398G 10400T 10873C 11083G 11377A 11719A 12705T 13254C 14783C 15043A 15301A 15326G 15670C 16136C 16183C 16189C 16223T 16274A 16319A 16320T 16519C	M2b3a	73G 146C 152C 182T 195C 263G 310.1C 447G 489C 514d 515d 16136C 16183C 16189C 16223T 16274A 16319A 16320T 16519C	1	73 146 152 182 195 263 447G 489 750 1438 1453 1780 2706 2831T 3630 4769 5744 6647 7028 8502 8701 8860 9540 9899 10398 10400 10873 11083 11377 11719 12705 13254 14783 15043 15301 15326 15670 16136 16189 16223 16274 16319 16320	M2b3a	73 146 152 182 195 263 447G 489 16136 16189 16223 16274 16319 16320	[0.542] M2b3a (1)	[0.062] M2b1 (2) [0.053] M2b (3) [0.017] M2b3	1

78 >MH444379.1 H	73G 146C 152C 182T 195C 263G 447G 489C 514d 515d 750G 1438G 1453G 1780C 2706G 2831T 3106d 3107C 3630T 4769G 5744A 6647G 7028T 8502G 8701G 8860G 9540C 9899C 10398G 10400T 10873C 11083G 11377A 11719A 12705T 13254C 14783C 15043A 15301A 15326G 15670C 16136C 16183C 16189C 16223T 16274A 16319A 16320T 16519C	M2b3a	73G 146C 152C 182T 195C 263G 447G 489C 514d 515d 16136C 16183C 16189C 16223T 16274A 16319A 16320T 16519C	1	73 146 152 182 195 263 447G 489 750 1438 1453 1780 2706 2831T 3630 4769 5744 6647 7028 8502 8701 8860 9540 9899 10398 10400 10873 11083 11377 11719 12705 13254 14783 15043 15301 15326 15670 16136 16189 16223 16274 16319 16320	M2b3a	73 146 152 182 195 263 447G 489 16136 16189 16223 16274 16319 16320	[0.542] M2b3a (1)	[0.062] M2b1 (2) [0.053] M2b (3) [0.017] M2b3	1
79 >MH444378.1 H	73G 146C 152C 182T 195C 263G 310.1C 447G 489C 514d 515d 750G 1438G 1453G 1780C 2706G 2831T 3106d 3107C 3630T 4769G 5744A 6647G 7028T 8502G 8701G 8860G 9540C 9899C 10398G 10400T 10873C 11083G 11377A 11719A 12705T 13254C 14783C 15043A 15301A 15326G 15670C 16136C 16183C 16189C 16223T 16274A 16319A 16320T 16519C	M2b3a	73G 146C 152C 182T 195C 263G 310.1C 447G 489C 514d 515d 16136C 16183C 16189C 16223T 16274A 16319A 16320T 16519C	1	73 146 152 182 195 263 447G 489 750 1438 1453 1780 2706 2831T 3630 4769 5744 6647 7028 8502 8701 8860 9540 9899 10398 10400 10873 11083 11377 11719 12705 13254 14783 15043 15301 15326 15670 16136 16189 16223 16274 16319 16320	M2b3a	73 146 152 182 195 263 447G 489 16136 16189 16223 16274 16319 16320	[0.542] M2b3a (1)	[0.062] M2b1 (2) [0.053] M2b (3) [0.017] M2b3	1
80 >MH444377.1 H	73G 146C 152C 182T 195C 263G 447G 489C 514d 515d 750G 1438G 1453G 1780C 2706G 2831T 3106d 3107C 3630T 4769G 5744A 6647G 7028T 8502G 8701G 8860G 9540C 9899C 10398G 10400T 10873C 11083G 11377A 11719A 12705T 13254C 14783C 15043A 15301A 15326G 15670C 16136C 16183C 16189C 16223T 16274A 16319A 16320T 16519C	M2b3a	73G 146C 152C 182T 195C 263G 447G 489C 514d 515d 16136C 16183C 16189C 16223T 16274A 16319A 16320T 16519C	1	73 146 152 182 195 263 447G 489 750 1438 1453 1780 2706 2831T 3630 4769 5744 6647 7028 8502 8701 8860 9540 9899 10398 10400 10873 11083 11377 11719 12705 13254 14783 15043 15301 15326 15670 16136 16189 16223 16274 16319 16320	M2b3a	73 146 152 182 195 263 447G 489 16136 16189 16223 16274 16319 16320	[0.542] M2b3a (1)	[0.062] M2b1 (2) [0.053] M2b (3) [0.017] M2b3	1
81 >MH444376.1 H	73G 146C 152C 182T 195C 263G 447G 489C 514d 515d 750G 1438G 1453G 1780C 2706G 2831T 3106d 3107C 3630T 4769G 5744A 6647G 7028T 8502G 8701G 8860G 9540C 9899C 10398G 10400T 10873C 11083G 11377A 11719A 12705T 13254C 14783C 15043A 15301A 15326G 15670C 16136C 16183C 16189C 16223T 16274A 16319A 16320T 16519C	M2b3a	73G 146C 152C 182T 195C 263G 447G 489C 514d 515d 16136C 16183C 16189C 16223T 16274A 16319A 16320T 16519C	1	73 146 152 182 195 263 447G 489 750 1438 1453 1780 2706 2831T 3630 4769 5744 6647 7028 8502 8701 8860 9540 9899 10398 10400 10873 11083 11377 11719 12705 13254 14783 15043 15301 15326 15670 16136 16189 16223 16274 16319 16320	M2b3a	73 146 152 182 195 263 447G 489 16136 16189 16223 16274 16319 16320	[0.542] M2b3a (1)	[0.062] M2b1 (2) [0.053] M2b (3) [0.017] M2b3	1

82	>MH444375.1 H	73G 146C 152C 182T 195C 263G 447G 489C 514d 515d 750G 1438G 1453G 1780C 2706G 2831T 3106d 3107C 3630T 4769G 5744A 6647G 7028T 8502G 8701G 8860G 9540C 9899C 10398G 10400T 10873C 11083G 11377A 11719A 12705T 13254C 14783C 15043A 15301A 15326G 15670C 16136C 16183C 16189C 16223T 16274A 16319A 16320T 16519C	M2b3a	73G 146C 152C 182T 195C 263G 447G 489C 514d 515d 16136C 16183C 16189C 16223T 16274A 16319A 16320T 16519C	1	73 146 152 182 195 263 447G 489 750 1438 1453 1780 2706 2831T 3630 4769 5744 6647 7028 8502 8701 8860 9540 9899 10398 10400 10873 11083 11377 11719 12705 13254 14783 15043 15301 15326 15670 16136 16189 16223 16274 16319 16320	M2b3a	73 146 152 182 195 263 447G 489 16136 16189 16223 16274 16319 16320	[0.542] M2b3a (1)	[0.062] M2b1 (2) [0.053] M2b (3) [0.017] M2b3	1
83	>MH444374.1 H	73G 146C 152C 182T 195C 263G 447G 489C 514d 515d 750G 1438G 1453G 1780C 2706G 2831T 3106d 3107C 3630T 4769G 5744A 6647G 7028T 8502G 8701G 8860G 9540C 9899C 10398G 10400T 10873C 11083G 11377A 11719A 12705T 13254C 14783C 15043A 15301A 15326G 15670C 16136C 16183C 16189C 16223T 16274A 16319A 16320T 16519C	M2b3a	73G 146C 152C 182T 195C 263G 447G 489C 514d 515d 16136C 16183C 16189C 16223T 16274A 16319A 16320T 16519C	1	73 146 152 182 195 263 447G 489 750 1438 1453 1780 2706 2831T 3630 4769 5744 6647 7028 8502 8701 8860 9540 9899 10398 10400 10873 11083 11377 11719 12705 13254 14783 15043 15301 15326 15670 16136 16189 16223 16274 16319 16320	M2b3a	73 146 152 182 195 263 447G 489 16136 16189 16223 16274 16319 16320	[0.542] M2b3a (1)	[0.062] M2b1 (2) [0.053] M2b (3) [0.017] M2b3	1
84	>MH444373.1 H	73G 146C 152C 182T 195C 263G 447G 489C 514d 515d 750G 1438G 1453G 1780C 2706G 2831T 3106d 3107C 3630T 4769G 5744A 6647G 7028T 8502G 8701G 8860G 9540C 9899C 10398G 10400T 10873C 11083G 11377A 11719A 12705T 13254C 14783C 15043A 15301A 15326G 15670C 16136C 16183C 16189C 16223T 16274A 16319A 16320T 16519C	M2b3a	73G 146C 152C 182T 195C 263G 447G 489C 514d 515d 16136C 16183C 16189C 16223T 16274A 16319A 16320T 16519C	1	73 146 152 182 195 263 447G 489 750 1438 1453 1780 2706 2831T 3630 4769 5744 6647 7028 8502 8701 8860 9540 9899 10398 10400 10873 11083 11377 11719 12705 13254 14783 15043 15301 15326 15670 16136 16189 16223 16274 16319 16320	M2b3a	73 146 152 182 195 263 447G 489 16136 16189 16223 16274 16319 16320	[0.542] M2b3a (1)	[0.062] M2b1 (2) [0.053] M2b (3) [0.017] M2b3	1
85	>MH444372.1 H	73G 146C 152C 182T 195C 263G 447G 489C 514d 515d 750G 1438G 1453G 1780C 2706G 2831T 3106d 3107C 3630T 4769G 5744A 6647G 7028T 8502G 8701G 8860G 9540C 9899C 10398G 10400T 10873C 11083G 11377A 11719A 12705T 13254C 14783C 15043A 15301A 15326G 15670C 16136C 16183C 16189C 16223T 16274A 16319A 16320T 16519C	M2b3a	73G 146C 152C 182T 195C 263G 447G 489C 514d 515d 16136C 16183C 16189C 16223T 16274A 16319A 16320T 16519C	1	73 146 152 182 195 263 447G 489 750 1438 1453 1780 2706 2831T 3630 4769 5744 6647 7028 8502 8701 8860 9540 9899 10398 10400 10873 11083 11377 11719 12705 13254 14783 15043 15301 15326 15670 16136 16189 16223 16274 16319 16320	M2b3a	73 146 152 182 195 263 447G 489 16136 16189 16223 16274 16319 16320	[0.542] M2b3a (1)	[0.062] M2b1 (2) [0.053] M2b (3) [0.017] M2b3	1

86 >MH444371.1 H	73G 146C 152C 182T 195C 263G 447G 489C 514d 515d 750G 1438G 1453G 1780C 2706G 2831T 3106d 3107C 3630T 4769G 5744A 6647G 7028T 8502G 8701G 8860G 9540C 9899C 10398G 10400T 10873C 11083G 11377A 11719A 12705T 13254C 14783C 15043A 15301A 15326G 15670C 16136C 16183C 16189C 16223T 16274A 16319A 16320T 16519C	M2b3a	73G 146C 152C 182T 195C 263G 447G 489C 514d 515d 16136C 16183C 16189C 16223T 16274A 16319A 16320T 16519C	1	73 146 152 182 195 263 447G 489 750 1438 1453 1780 2706 2831T 3630 4769 5744 6647 7028 8502 8701 8860 9540 9899 10398 10400 10873 11083 11377 11719 12705 13254 14783 15043 15301 15326 15670 16136 16189 16223 16274 16319 16320	M2b3a	73 146 152 182 195 263 447G 489 16136 16189 16223 16274 16319 16320	[0.542] M2b3a (1)	[0.062] M2b1 (2) [0.053] M2b (3) [0.017] M2b3	1
87 >MH444370.1 H	73G 146C 152C 182T 195C 263G 447G 489C 514d 515d 750G 1438G 1453G 1780C 2706G 2831T 3106d 3107C 3630T 4769G 5744A 6647G 7028T 8502G 8701G 8860G 9540C 9899C 10398G 10400T 10873C 11083G 11377A 11719A 12705T 13254C 14783C 15043A 15301A 15326G 15670C 16136C 16183C 16189C 16223T 16274A 16319A 16320T 16519C	M2b3a	73G 146C 152C 182T 195C 263G 447G 489C 514d 515d 16136C 16183C 16189C 16223T 16274A 16319A 16320T 16519C	1	73 146 152 182 195 263 447G 489 750 1438 1453 1780 2706 2831T 3630 4769 5744 6647 7028 8502 8701 8860 9540 9899 10398 10400 10873 11083 11377 11719 12705 13254 14783 15043 15301 15326 15670 16136 16189 16223 16274 16319 16320	M2b3a	73 146 152 182 195 263 447G 489 16136 16189 16223 16274 16319 16320	[0.542] M2b3a (1)	[0.062] M2b1 (2) [0.053] M2b (3) [0.017] M2b3	1
88 >MH444369.1 H	73G 146C 152C 182T 195C 263G 447G 489C 514d 515d 750G 1438G 1453G 1780C 2706G 2831T 3106d 3107C 3630T 4769G 5744A 6647G 7028T 8502G 8701G 8860G 9540C 9899C 10398G 10400T 10873C 11083G 11377A 11719A 12705T 13254C 14783C 15043A 15301A 15326G 15670C 16136C 16183C 16189C 16223T 16274A 16319A 16320T 16519C	M2b3a	73G 146C 152C 182T 195C 263G 447G 489C 514d 515d 16136C 16183C 16189C 16223T 16274A 16319A 16320T 16519C	1	73 146 152 182 195 263 447G 489 750 1438 1453 1780 2706 2831T 3630 4769 5744 6647 7028 8502 8701 8860 9540 9899 10398 10400 10873 11083 11377 11719 12705 13254 14783 15043 15301 15326 15670 16136 16189 16223 16274 16319 16320	M2b3a	73 146 152 182 195 263 447G 489 16136 16189 16223 16274 16319 16320	[0.542] M2b3a (1)	[0.062] M2b1 (2) [0.053] M2b (3) [0.017] M2b3	1
89 >MH444368.1 H	73G 146C 152C 182T 195C 263G 310.1C 447G 489C 514d 515d 750G 1438G 1453G 1780C 2706G 2831T 3106d 3107C 3630T 4769G 5744A 6647G 7028T 8502G 8701G 8860G 9540C 9899C 10398G 10400T 10873C 11083G 11377A 11719A 12705T 13254C 14783C 15043A 15301A 15326G 15670C 16136C 16183C 16189C 16223T 16274A 16319A 16320T 16519C	M2b3a	73G 146C 152C 182T 195C 263G 310.1C 447G 489C 514d 515d 16136C 16183C 16189C 16223T 16274A 16319A 16320T 16519C	1	73 146 152 182 195 263 447G 489 750 1438 1453 1780 2706 2831T 3630 4769 5744 6647 7028 8502 8701 8860 9540 9899 10398 10400 10873 11083 11377 11719 12705 13254 14783 15043 15301 15326 15670 16136 16189 16223 16274 16319 16320	M2b3a	73 146 152 182 195 263 447G 489 16136 16189 16223 16274 16319 16320	[0.542] M2b3a (1)	[0.062] M2b1 (2) [0.053] M2b (3) [0.017] M2b3	1
90 >MH000027.1 H	263G 310.1C 575T 750G 751G 951A 3106d 3107C 8860G 9052G 15326G 16124C 16354T	H2a1e1a	263G 310.1C 575T 16124C 16354T	1	263 575 750 751 951 8860 9052 15326 16124 16354	H2a1e1a	263 575 16124 16354	[0.000] H2a1e1a (1)	[0.000] H2a1e H2a1e1 H2a1e1b (4)	1
91 >MH029802.1 H	257G 263G 309.1CCT 310C 477C 750G 1438G 3010A 3106d 3107C 4769G 8473C 8860G 12795A 13933G 15326G 16182C 16183C 16189C 16362C 16519C	H1c3b	257G 263G 309.1CCT 310C 477C 16182C 16183C 16189C 16362C 16519C	1	257 263 477 750 1438 3010 4769 8473 8860 12795 13933 15326 16189 16362	H1c3b	257 263 477 16189 16362	[0.000] H1c3b (1)	[0.034] H1c3 (2)	1

92 >MH025373.1 H	72C 195C 198T 263G 309.1CT 310C 750G 1438G 1888A 2706G 3106d 3107C 4769G 7028T 8628T 8860G 15326G 16093Y 16298C 16519C	HV0b	72C 195C 198T 263G 309.1CT 310C 16093Y 16298C 16519C	1	72 195 198 263 750 1438 1888 2706 4769 7028 8628 8860 15326 16093Y 16298	HV0b	72 195 198 263 16093Y 16298	[0.034] HV0b (1)	[0.000] HV0+195 HV0c HV0d (4)	1
93 >MG273135.1 H	73G 263G 302.1C 750G 1438G 1541C 2322A 2706G 3106d 3107C 3970T 4769G 7028T 8860G 11719A 12714C 13928C 14766T 15326G 16124C 16148T 16275G 16304C 16309G 16327T 16390A 16519C	R9b1b	73G 263G 302.1C 16124C 16148T 16275G 16304C 16309G 16327T 16390A 16519C	1	73 263 750 1438 1541 2322A 2706 3970 4769 7028 8860 11719 12714 13928C 14766 15326 16124 16148 16275 16304 16309 16327 16390	R9b1b	73 263 16124 16148 16275 16304 16309 16327 16390	[0.289] R9b1b (1)	[0.008] R9b1 (2)	1
94 >MG273133.1 H	73G 263G 302.1C 750G 1438G 2706G 2835T 3106d 3107C 3714G 4715G 4769G 6179A 6216C 7028T 7196A 8584A 8684T 8701G 8860G 9540C 10398G 10400T 10873C 11176A 11719A 12705T 14470C 14766T 14783C 15043A 15301A 15326G 15487T 16184T 16189C 16192T 16223T 16298C 16311C 16319A 16390A 16443C 16468C 16470A 16471A 16473A	M8a2a1	73G 263G 302.1C 16184T 16189C 16192T 16223T 16298C 16311C 16319A 16390A 16443C 16468C 16470A 16471A 16473A	1	73 263 750 1438 2706 2835 3714 4715 4769 6179 6216 7028 7196A 8584 8684 8701 8860 9540 10398 10400 10873 11176 11719 12705 14470 14766 14783 15043 15301 15326 15487T 16184 16189 16192 16223 16298 16311 16319 16390 16443 16468 16470 16471 16473	M8a2a1	73 263 16184 16189 16192 16223 16298 16311 16319 16390 16443 16468 16470 16471 16473	[1.523] M8a2a1 (1)	[0.131] L3h1a1 (2)	1
95 >MG273130.1 H	73G 263G 489C 750G 1438G 2706G 2835T 3106d 3107C 3714G 4715G 4769G 6179A 6216C 7028T 7196A 8584A 8684T 8701G 8860G 9540C 10398G 10400T 10873C 11176A 11719A 12705T 14470C 14766T 14783C 15043A 15301A 15326G 15487T 16184T 16189C 16223T 16298C 16311C 16319A 16390A 16443C 16468C 16470A 16471A 16473A	M8a2a1	73G 263G 489C 16184T 16189C 16223T 16298C 16311C 16319A 16390A 16443C 16468C 16470A 16471A 16473A	1	73 263 489 750 1438 2706 2835 3714 4715 4769 6179 6216 7028 7196A 8584 8684 8701 8860 9540 10398 10400 10873 11176 11719 12705 14470 14766 14783 15043 15301 15326 15487T 16184 16189 16223 16298 16311 16319 16390 16443 16468 16470 16471 16473	M8a2a1	73 263 489 16184 16189 16223 16298 16311 16319 16390 16443 16468 16470 16471 16473	[1.479] M8a2a1 (1)	[0.046] M8a1a (2) [0.042] M8a2a (3) [0.037] M8a3 (4) [0.033] M8a2c (5) [0.000] M8a2'3 M8a2 M8a2e (8)	1
96 >MG273126.1 H	73G 207A 263G 750G 1438G 2706G 3106d 3107C 3434G 3970T 4769G 4824G 4991A 5585A 5894G 5913A 5978G 6392C 7028T 8860G 10310A 10320A 11065G 11719A 12621T 13928C 14766T 14971C 15326G 15412G 16260T 16298C 16355T 16362C	F3a1	73G 207A 263G 16260T 16298C 16355T 16362C	1	73 207 263 750 1438 2706 3434 3970 4769 4824 4991 5585 5894 5913 5978 6392 7028 8860 10310 10320 11065 11719 12621 13928C 14766 14971 15326 15412G 16260 16298 16355 16362	F3a1	73 207 263 16260 16298 16355 16362	[0.038] F3a1 (1)	[0.020] F3a+207 (2)	1
97 >MG273125.1 H	73G 263G 302.1C 750G 1438G 2706G 2835T 3106d 3107C 3714G 4715G 4769G 6179A 6216C 7028T 7196A 8584A 8684T 8701G 8860G 9540C 10398G 10400T 10873C 11176A 11719A 12705T 14470C 14766T 14783C 15043A 15301A 15326G 15487T 16184T 16189C 16192T 16223T 16298C 16311C 16319A 16390A 16443C 16468C 16470A 16471A 16473A	M8a2a1	73G 263G 302.1C 16184T 16189C 16192T 16223T 16298C 16311C 16319A 16390A 16443C 16468C 16470A 16471A 16473A	1	73 263 750 1438 2706 2835 3714 4715 4769 6179 6216 7028 7196A 8584 8684 8701 8860 9540 10398 10400 10873 11176 11719 12705 14470 14766 14783 15043 15301 15326 15487T 16184 16189 16192 16223 16298 16311 16319 16390 16443 16468 16470 16471 16473	M8a2a1	73 263 16184 16189 16192 16223 16298 16311 16319 16390 16443 16468 16470 16471 16473	[1.523] M8a2a1 (1)	[0.131] L3h1a1 (2)	1

98 >MG273119.1 H	73G 263G 310.1C 750G 1438G 2706G 2835T 3106d 3107C 3714G 4715G 4769G 6179A 6216C 7028T 7196A 8584A 8684T 8701G 8860G 9540C 10398G 10400T 10873C 11176A 11719A 12705T 14470C 14766T 14783C 15043A 15301A 15326G 15487T 16183.1TT 16188T 16189C 16223T 16298C 16311C 16319A 16390A 16443C 16468C 16470A 16471A 16473A	M8a2a1	73G 263G 310.1C 16183.1TT 16188T 16189C 16223T 16298C 16311C 16319A 16390A 16443C 16468C 16470A 16471A 16473A	1	73 263 750 1438 2706 2835 3714 4715 4769 6179 6216 7028 7196A 8584 8684 8701 8860 9540 10398 10400 10873 11176 11719 12705 14470 14766 14783 15043 15301 15326 15487T 16185 16189 16190 16223 16298 16311 16319 16390 16443 16468 16470 16471 16473	M8a2a1	73 263 16185 16189 16190 16223 16298 16311 16319 16390 16443 16468 16470 16471 16473	[1.535] M8a2a1 (1) [0.000] M8a1a (2)	[0.143] L3h1a1 (3) [0.091] N9b (4) [0.087] L3'4 (5) [0.051] N9b1a (6) [0.014] M8a1 (7) [0.010] N9b1 (8) [0.007] L3h1a2 (9) [0.000] L3h1 L3h1a M8a M8a1a N9b1b N9b3 (15)	1
99 >MG273118.1 H	73G 263G 489C 750G 1438G 2706G 2835T 3106d 3107C 3714G 4715G 4769G 6179A 6216C 7028T 7196A 8584A 8684T 8701G 8860G 9540C 10398G 10400T 10873C 11176A 11719A 12705T 14470C 14766T 14783C 15043A 15301A 15326G 15487T 16184T 16189C 16192T 16223T 16298C 16311C 16319A 16390A 16443C 16468C 16470A 16471A 16473A	M8a2a1	73G 263G 489C 16184T 16189C 16192T 16223T 16298C 16311C 16319A 16390A 16443C 16468C 16470A 16471A 16473A	1	73 263 489 750 1438 2706 2835 3714 4715 4769 6179 6216 7028 7196A 8584 8684 8701 8860 9540 10398 10400 10873 11176 11719 12705 14470 14766 14783 15043 15301 15326 15487T 16184 16189 16192 16223 16298 16311 16319 16390 16443 16468 16470 16471 16473	M8a2a1	73 263 489 16184 16189 16192 16223 16298 16311 16319 16390 16443 16468 16470 16471 16473	[1.511] M8a2a1 (1)	[0.049] M8a2a (2) [0.046] M8a1a (3) [0.037] M8a3 (4) [0.033] M8a2c (5) [0.000] M8a2'3 M8a2 M8a2e (8)	1
100 >MG273112.1 H	73G 263G 489C 750G 1438G 2706G 2835T 3106d 3107C 3714G 4715G 4769G 6179A 6216C 7028T 7196A 8584A 8684T 8701G 8860G 9540C 10398G 10400T 10873C 11176A 11719A 12705T 14470C 14766T 14783C 15043A 15301A 15326G 15487T 16184T 16189C 16223T 16298C 16311C 16319A 16390A 16443C 16468C 16470A 16471A 16473A	M8a2a1	73G 263G 489C 16184T 16189C 16223T 16298C 16311C 16319A 16390A 16443C 16468C 16470A 16471A 16473A	1	73 263 489 750 1438 2706 2835 3714 4715 4769 6179 6216 7028 7196A 8584 8684 8701 8860 9540 10398 10400 10873 11176 11719 12705 14470 14766 14783 15043 15301 15326 15487T 16184 16189 16223 16298 16311 16319 16390 16443 16468 16470 16471 16473	M8a2a1	73 263 489 16184 16189 16223 16298 16311 16319 16390 16443 16468 16470 16471 16473	[1.479] M8a2a1 (1)	[0.046] M8a1a (2) [0.042] M8a2a (3) [0.037] M8a3 (4) [0.033] M8a2c (5) [0.000] M8a2'3 M8a2 M8a2e (8)	1
101 >MG273097.1 H	73G 151T 263G 479G 750G 1438G 2706G 3106d 3107C 3970T 4769G 7028T 7169C 7684C 7861C 8860G 11002G 11719A 12618A 13928C 14766T 15326G 15479C 15637T 16093C 16157C 16304C	R9c1b1	73G 151T 263G 479G 16093C 16157C 16304C	1	73 151 263 479 750 1438 2706 3970 4769 7028 7169 7684 7861 8860 11002 11719 12618 13928C 14766 15326 15479 15637 16093 16157 16304	R9c1b1	73 151 263 479 16093 16157 16304	[0.316] R9c1b1 (1)	[0.040] R9c1b2 (2) [0.000] R9c1 R9c1b (4)	1
102 >MG273093.1 H	73G 146A 199C 263G 489C 750G 1438G 2706G 3106d 3107C 3606G 4071T 4769G 4850T 5442C 6455T 7028T 8701G 8860G 9540C 9824C 10398G 10400T 10873C 11665T 11719A 12091C 12705T 14319C 14766T 14783C 15043A 15236G 15301A 15326G 16519C	M7c1c2	73G 146A 199C 263G 489C 16519C	1	73 146A 199 263 489 750 1438 2706 3606 4071 4769 4850 5442 6455 7028 8701 8860 9540 9824 10398 10400 10873 11665 11719 12091 12705 14319 14766 14783 15043 15236 15301 15326	M7c1c2	73 146A 199 263 489	[0.000] M7c1c2 (1)	[0.000] M7c1c2a (2)	1

103 >MG273084.1 H	73G 146C 152C 263G 489C 750G 1438G 1719A 2706G 3106d 3107C 3316A 3453T 3666A 4769G 5147A 6480A 7028T 7313T 7765G 8701G 8860G 9540C 9755A 10398G 10400T 10873C 11719A 12279G 12609C 12705T 13269G 13768C 14110C 14224T 14356T 14766T 14783C 15043A 15109C 15301A 15326G 15893C 16068C 16126C 16182C 16183C 16189C 16223T 16319A 16325C	M75	73G 146C 152C 263G 489C 16068C 16126C 16182C 16183C 16189C 16223T 16319A 16325C	1	73 146 152 263 489 750 1438 1719 2706 3316 3453 3666 4769 5147 6480 7028 7313 7765 8701 8860 9540 9755 10398 10400 10873 11719 12279 12609 12705 13269 13768 14110 14224T 14356 14766 14783 15043 15109 15301 15326 15893 16068 16126 16189 16223 16319 16325	M75	73 146 152 263 489 16068 16126 16189 16223 16319 16325	[0.397] M75 (1) [0.057] M45 (2)	[0.057] M3c+152 (3) [0.047] M45a (4) [0.045] D1e (5) [0.014] M7c1a3 (6) [0.012] M13a'b (7) [0.007] M8a2a (8) [0.006] D4b1a1 (9) [0.005] D4b1a1a (10) [0.004] M7c3 (11) [0.003] M21b1a (12) [0.002] M46 (13) [0.000] M37+152 M7c M13'46'61 M13 (17)	1
104 >MG273080.1 H	73G 94A 173C 204C 263G 482C 489C 709A 750G 1393A 1438G 2706G 3106d 3107C 3540C 4491A 4769G 5563A 7028T 8701G 8860G 9477A 9540C 9938C 10398G 10400T 10873C 11314G 11719A 12705T 14766T 14783C 15043A 15301A 15326G 16217C 16223T 16319A 16381C 16519C	M55	73G 94A 173C 204C 263G 482C 489C 16217C 16223T 16319A 16381C 16519C	1	73 94 173 204 263 482 489 709 750 1393 1438 2706 3540 4491 4769 5563 7028 8701 8860 9477 9540 9938 10398 10400 10873 11314 11719 12705 14766 14783 15043 15301 15326 16217 16223 16319 16381	M55	73 94 173 204 263 482 489 16217 16223 16319 16381	[0.543] M55 (1)	[0.029] M3a1b (2) [0.007] M21b1a (3) [0.005] M3a1+204 (4) [0.000] M3a1a	1
105 >MG273079.1 H	73G 94A 173C 204C 263G 482C 489C 750G 1393A 1438G 2706G 3106d 3107C 3540C 4491A 4769G 5563A 7028T 8701G 8860G 9477A 9540C 9938C 10398G 10400T 10873C 11314G 11719A 12705T 14766T 14783C 15043A 15301A 15326G 16217C 16223T 16319A 16381C 16519C	M55	73G 94A 173C 204C 263G 482C 489C 16217C 16223T 16319A 16381C 16519C	1	73 94 173 204 263 482 489 750 1393 1438 2706 3540 4491 4769 5563 7028 8701 8860 9477 9540 9938 10398 10400 10873 11314 11719 12705 14766 14783 15043 15301 15326 16217 16223 16319 16381	M55	73 94 173 204 263 482 489 16217 16223 16319 16381	[0.543] M55 (1)	[0.029] M3a1b (2) [0.007] M21b1a (3) [0.005] M3a1+204 (4) [0.000] M3a1a	1
106 >MG273076.1 H	73G 150T 263G 335G 750G 942G 1438G 1503A 2706G 3106d 3107C 4769G 4820A 5130C 7028T 7158G 8860G 9254G 11365C 11719A 12705T 13819C 13980A 14766T 15326G 16168T 16223T 16249C	N22	73G 150T 263G 335G 16168T 16223T 16249C	1	73 150 263 335 750 942 1438 1503 2706 4769 4820 5130 7028 7158 8860 9254 11365 11719 12705 13819 13980 14766 15326 16168 16223 16249	N22	73 150 263 335 16168 16223 16249	[0.140] N22 (1)	[0.000] N22a (2)	1
107 >MG273075.1 H	73G 263G 750G 1438G 1541C 2706G 3106d 3107C 3591A 3666A 3970T 4232C 4769G 6647G 7028T 8724G 8860G 11299C 11353C 11422T 11719A 13191C 13722G 13928C 14766T 15314A 15326G 15883A 16304C 16362C 16519C	R9b2	73G 263G 16304C 16362C 16519C	1	73 263 750 1438 1541 2706 3591 3666 3970 4232 4769 6647 7028 8724 8860 11299 11353 11422 11719 13191 13722 13928C 14766 15314 15326 15883 16304 16362	R9b2	73 263 16304 16362	[0.000] R9b2 (1)	[0.010] R9c (2) [0.000] R6 R9 R9b R31 (6)	1
108 >MG273073.1 H	73G 152C 185A 189G 263G 489C 750G 1438G 2706G 3106d 3107C 3897T 4170T 4769G 5580C 7028T 8701G 8860G 9540C 10398G 10400T 10873C 11359G 11719A 12030G 12372A 12705T 14569A 14727C 14766T 14783C 14788C 14869A 15010G 15043A 15301A 15326G 16108T 16129A 16172C 16223T 16234T 16250T 16290T 16311C	M12b1b	73G 152C 185A 189G 263G 489C 16108T 16129A 16172C 16223T 16234T 16250T 16290T 16311C	1	73 152 185 189 263 489 750 1438 2706 3897 4170 4769 5580 7028 8701 8860 9540 10398 10400 10873 11359 11719 12030 12372 12705 14569 14727 14766 14783 14788 14869 15010 15043 15301 15326 16108 16129 16172 16223 16234 16250 16290 16311	M12b1b	73 152 185 189 263 489 16108 16129 16172 16223 16234 16250 16290 16311	[0.038] M12b1b (1)	[0.006] M12b1a (2) [0.000] M12b M12b1 M12b1a1 (5)	1

109 >MG273066.1 H	73G 146A 199C 204C 263G 489C 750G 1438G 2706G 3106d 3107C 3606G 4071T 4137T 4769G 4850T 5442C 6455T 7028T 8701G 8860G 9540C 9824C 10398G 10400T 10750G 10873C 11665T 11719A 12091C 12705T 14766T 14783C 15043A 15236G 15301A 15326G 16519C	M7c1c2	73G 146A 199C 204C 263G 489C 16519C	1	73 146A 199 204 263 489 750 1438 2706 3606 4071 4137 4769 4850 5442 6455 7028 8701 8860 9540 9824 10398 10400 10750 10873 11665 11719 12091 12705 14766 14783 15043 15236 15301 15326	M7c1c2	73 146A 199 204 263 489	[0.198] M7c1c2 (1)	[0.000] M7c1c2a (2)	1
110 >MG273065.1 H	73G 128.1T 146C 152C 263G 318C 326G 489C 750G 1438G 1819C 2706G 3106d 3107C 4170T 4769G 5580C 6293C 6338G 7028T 8701G 8860G 9064A 9540C 10235C 10398G 10400T 10873C 11719A 12030G 12358G 12372A 12705T 14569A 14727C 14766T 14783C 15010G 15043A 15301A 15326G 15463G 15562G 15651T 16223T 16234T 16266T 16290T 16311C	M12a	73G 128.1T 146C 152C 263G 318C 326G 489C 16223T 16234T 16266T 16290T 16311C	1	73 129.1T 146 152 263 318 326 489 750 1438 1819 2706 4170 4769 5580 6293 6338 7028 8701 8860 9064 9540 10235 10398 10400 10873 11719 12030 12358 12372 12705 14569 14727 14766 14783 15010 15043 15301 15326 15463 15562 15651 16223 16234 16266 16290 16311	M12a	73 129.1T 146 152 263 318 326 489 16223 16234 16266 16290 16311	[0.140] M12a (1)	[0.017] M57+152 (2) [0.005] M24a (3) [0.000] M12	1
111 >MG273064.1 H	73G 263G 750G 1438G 2706G 3106d 3107C 3970T 4086T 4769G 6392C 6962A 7028T 8860G 9053A 9548A 10310A 10609C 11176A 11719A 12406A 12561A 12882T 13759A 13928C 14766T 15326G 15884A 16129A 16162G 16172C 16304C 16519C	F1a1	73G 263G 16129A 16162G 16172C 16304C 16519C	1	73 263 750 1438 2706 3970 4086 4769 6392 6962 7028 8860 9053 9548 10310 10609 11176 11719 12406 12561 12882 13759 13928C 14766 15326 15884 16129 16162 16172 16304	F1a1	73 263 16129 16162 16172 16304	[0.034] F1a1 (1)	[0.081] F1a1d1 (2) [0.043] F1a1d (3) [0.039] F1a1a (4) [0.031] F1a1a1 (5) [0.006] F1a1c (6) [0.005] F1a3 (7) [0.000] F1a F1a1'4 F1a1b F1a1c2 F1a1c3 F1a4 (13)	1
112 >MG273062.1 H	73G 151T 263G 302.1C 489C 523.1C 750G 1438G 2706G 3106d 3107C 3777C 4769G 5471A 7028T 7055G 8521G 8701G 8718G 8860G 9540C 9548A 10398G 10400T 10873C 11719A 12705T 13149G 14766T 14783C 15043A 15301A 15326G 15458C 16172C 16223T 16260T 16264T 16271C 16519C	M71b	73G 151T 263G 302.1C 489C 523.1C 16172C 16223T 16260T 16264T 16271C 16519C	1	73 151 263 489 525.1C 750 1438 2706 3777 4769 5471 7028 7055 8521 8701 8718 8860 9540 9548 10398 10400 10873 11719 12705 13149 14766 14783 15043 15301 15326 15458 16172 16223 16260 16264 16271	M71b	73 151 263 489 525.1C 16172 16223 16260 16264 16271	[0.111] M71b (1)	[0.006] M71+151 (2) [0.000] M71a	1
113 >MG273051.1 H	73G 143A 146C 263G 489C 750G 1284C 1438G 2706G 3106d 3107C 4769G 7028T 8440G 8701G 8718G 8838A 8860G 9540C 9615C 10398G 10400T 10873C 11719A 12681C 12705T 13407G 14605G 14766T 14783C 15043A 15301A 15326G 15458C 16093C 16129A 16140C 16223T 16271C 16295T 16304C	M71a2	73G 143A 146C 263G 489C 16093C 16129A 16140C 16223T 16271C 16295T 16304C	1	73 143 146 263 489 750 1284 1438 2706 4769 7028 8440 8701 8718 8838 8860 9540 9615 10398 10400 10873 11719 12681 12705 13407 14605 14766 14783 15043 15301 15326 15458 16093 16129 16140 16223 16271 16295 16304	M71a2	73 143 146 263 489 16093 16129 16140 16223 16271 16295 16304	[0.262] M71a2 (1)	[0.028] M7a2a1 (2) [0.007] G1b+16129 (3) [0.006] G1b2 G1b4 (5)	1

114 >MG273048.1 H	73G 263G 302.1C 489C 750G 1193C 1342T 1438G 2706G 3106d 3107C 4769G 5812G 6253C 7028T 7759C 8269A 8701G 8860G 9540C 10398G 10400T 10873C 11719A 11810T 12705T 12732C 13468T 14334T 14766T 14783C 15043A 15301A 15326G 16223T 16304C 16344T 16362C 16381C 16519C	M61	73G 263G 302.1C 489C 16223T 16304C 16344T 16362C 16381C 16519C	1	73 263 489 750 1193 1342 1438 2706 4769 5812 6253 7028 7759 8269 8701 8860 9540 10398 10400 10873 11719 11810 12705 12732 13468 14334 14766 14783 15043 15301 15326 16223 16304 16344 16362 16381	M61	73 263 489 16223 16304 16344 16362 16381	[0.326] M61 (1) [0.044] M54 (2) [0.002] D4e2 (3) [0.001] D4b2b (4) [0.000] M9 G G1 G1c G1c2 G2 G2a'c G2b G2b1 G2b1a G2b2 G2b2a M33+16362 D D4 D4b D4b2 D4b2a D4b2a2 D4b2a2b D4b2b1 D4b2b1a D4b2b1b D4b2b1c D4b2b2 D4b2b3 D4b2b6 D4e D4e1'3 D4e1 D4e1c D4e3 D4e2b D4e2c D4e2d D4e4 D4e4a D4e4b D4f D4f1 D4g D4h D4h4 D4j D4j1 D4j1b D4j4 D4j5 D4j5a D4j+(16286) D4j9 D4j10 D4j12 D4j15 D4j16 D4m (60)	[0.086] M35b2 (61) [0.057] M (62) [0.037] G2a (63) [0.029] D4j7 (64) [0.025] D1 (65) [0.019] M7c1a1b (66) [0.018] G1b (67) [0.014] D4i (68) [0.006] M35b+16304 (69) [0.005] M43a1 G3b M61a D4d D4j1a D4j1a1a D4j1a1b D4j+16311 (77) [0.004] E1 M33c M74 (80) [0.003] M7b M32'56 M33a M35 M42'74 (85) [0.002] D4b2b2a1 D4g2 D4g2b (88) [0.001] D4b2b (89) [0.000] M1'20'51 M4"67 M37 M43 M43b M6 M7 M7b'c M9a'b E E1a E1a1 E1a1a E1a1c M12'G G1a G1c1 G2a1 G2b1a1 G2b1b G2b2b G2b2c G3 G3a G3b2 M13'46'61+16362 M19'53 M21 M21b M21b1 M21b1a M22 M22b M23'75 M24'41 M27 M29'Q M31 M31a M33 M33a1 M33b M33b2 M34'57 M35b3 M39'70 M42 M74b M74b2 M52 M52b M55'77 M58 M60 M69 M80'D D1a D1a2 D1b D1c D1d D1d1 D1k D1n D4b1 D4b1a D4b1a2 D4b1a2a D4b1a2a2 D4b2a2a D4b2a2a2 D4b2b1 D4b2b1a D4b2b1b D4b2b1c D4b2b1+146 D4b2b1d D4b2b2 D4b2b2a D4b2b3 D4b2b4 D4b2b5 D4b2b6 D4b2d D4c D4c2 [illegible]	1
115 >MG273047.1 H	73G 150T 199C 204C 263G 302.1C 489C 750G 1438G 2706G 3106d 3107C 3483A 4048A 4071T 4164G 4769G 5351G 5460A 6455T 6680C 7028T 7684C 7853A 8701G 8860G 9540C 9824C 10398G 10400T 10873C 11719A 12405T 12705T 12811C 14766T 14783C 15043A 15301A 15326G 16223T 16297C	M7b1a1b	73G 150T 199C 204C 263G 302.1C 489C 16223T 16297C	1	73 150 199 204 263 489 750 1438 2706 3483 4048 4071 4164 4769 5351 5460 6455 6680 7028 7684 7853 8701 8860 9540 9824 10398 10400 10873 11719 12405 12705 12811 14766 14783 15043 15301 15326 16223 16297	M7b1a1b	73 150 199 204 263 489 16223 16297	[0.000] M7b1a1b (1)	[0.042] M7b1a1+(16192) (2) [0.018] M7b1a1e1 (3) [0.015] M7b1a1 (4) [0.014] M7b1a1a3 (5) [0.010] M7b1a1f (6) [0.004] M7b1a1h (7) [0.002] M7b1a1g (8) [0.000] M7b1a1d M7b1a1d1 M7b1a1e (11)	1
116 >MG273045.1 H	73G 150T 199C 204C 263G 489C 750G 1438G 2706G 3106d 3107C 3483A 4048A 4071T 4164G 4769G 5351G 5460A 6455T 6680C 7028T 7684C 7853A 8149G 8701G 8860G 9540C 9824C 10398G 10400T 10873C 11719A 12405T 12705T 12811C 14766T 14783C 15043A 15301A 15326G 16223T 16297C	M7b1a1b	73G 150T 199C 204C 263G 489C 16223T 16297C	1	73 150 199 204 263 489 750 1438 2706 3483 4048 4071 4164 4769 5351 5460 6455 6680 7028 7684 7853 8149 8701 8860 9540 9824 10398 10400 10873 11719 12405 12705 12811 14766 14783 15043 15301 15326 16223 16297	M7b1a1b	73 150 199 204 263 489 16223 16297	[0.000] M7b1a1b (1)	[0.042] M7b1a1+(16192) (2) [0.018] M7b1a1e1 (3) [0.015] M7b1a1 (4) [0.014] M7b1a1a3 (5) [0.010] M7b1a1f (6) [0.004] M7b1a1h (7) [0.002] M7b1a1g (8) [0.000] M7b1a1d M7b1a1d1 M7b1a1e (11)	1

117 >MG273041.1 H	73G 150T 263G 1438G 2706G 3106d 3107C 4769G 4856C 5231A 5417A 7028T 8573A 8860G 9506T 10084C 11399C 11719A 12358G 12372A 12705T 14766T 15080G 15326G 16223T 16257A 16261T 16266G 16292T 16318G 16519C	N9a6	73G 150T 263G 16223T 16257A 16261T 16266G 16292T 16318G 16519C	1	73 150 263 1438 2706 4769 4856 5231 5417 7028 8573 8860 9506 10084 11399 11719 12358 12372 12705 14766 15080 15326 16223 16257A 16261 16266G 16292 16318	N9a6	73 150 263 16223 16257A 16261 16266G 16292 16318	[0.046] N9a6 (1)	[0.000] N9a N9a6a N9a6b N9a8 N9a9 N9a10 (7)	1
118 >MG273040.1 H	73G 146C 226C 263G 750G 1438G 2706G 3106d 3107C 3548C 4343G 4769G 5465C 5892C 7028T 8860G 9123A 9477A 9740T 10238C 11719A 13145A 14766T 15326G 16168T 16182C 16183C 16189C 16217C 16261T 16311C 16519C	B4a1e	73G 146C 226C 263G 16168T 16182C 16183C 16189C 16217C 16261T 16311C 16519C	1	73 146 226 263 750 1438 2706 3548 4343 4769 5465 5892 7028 8860 9123 9477 9740 10238 11719 13145 14766 15326 16168 16189 16217 16261 16311	B4a1e	73 146 226 263 16168 16189 16217 16261 16311	[0.125] B4a1e (1) [0.000] B4a1a1aa (2)	[0.034] B4a1a7 (3) [0.026] B4a1a1a (4) [0.020] B4a1a6 (5) [0.009] B4a1a1 (6) [0.004] B4a1a (7) [0.002] B4a1a1e (8) [0.000] B4a1a1a1 B4a1a1a1b B4a1a1a3 B4a1a1a5 B4a1a1a6 B4a1a1a7 B4a1a1a10 B4a1a1a11 B4a1a1a11a B4a1a1a13 B4a1a1a15 B4a1a1a17 B4a1a1a21 B4a1a1a22 B4a1a1b B4a1a1c B4a1a1d B4a1a1f B4a1a1g B4a1a1i B4a1a1n B4a1a1p B4a1a1s B4a1a1t B4a1a1u B4a1a1v B4a1a1w B4a1a1x B4a1a1y B4a1a1z B4a1a1aa B4a1a1ab B4a1a1ac B4a1a1ad B4a1a1ae B4a1a2 B4a1a3 B4a1a4 B4a1a5 B4a1a5a B4a1a6a B4a1+16311 B4a1c+146 B4a1c4 B4a1c5 (53)	1
119 >MG273025.1 H	73G 207A 263G 709A 750G 1438G 2706G 3106d 3107C 3434G 3970T 4769G 4824G 4991A 5585A 5894G 5913A 5978G 6392C 7028T 8860G 10310A 10320A 11065G 11719A 12621T 13145A 13928C 14766T 14971C 15326G 15412G 16260T 16298C 16355T 16362C	F3a1	73G 207A 263G 16260T 16298C 16355T 16362C	1	73 207 263 709 750 1438 2706 3434 3970 4769 4824 4991 5585 5894 5913 5978 6392 7028 8860 10310 10320 11065 11719 12621 13145 13928C 14766 14971 15326 15412G 16260 16298 16355 16362	F3a1	73 207 263 16260 16298 16355 16362	[0.038] F3a1 (1)	[0.020] F3a+207 (2)	1
120 >MG273019.1 H	73G 150T 159C 199C 263G 302.1C 489C 750G 1438G 2706G 3106d 3107C 4048A 4071T 4164G 4769G 5351G 5460A 6455T 6680C 7028T 7684C 7853A 8245G 8701G 8860G 9196A 9540C 9824C 10398G 10400T 10873C 11719A 12405T 12705T 12811C 14766T 14783C 15043A 15301A 15326G 16129A 16223T 16297C	M7b1a1	73G 150T 159C 199C 263G 302.1C 489C 16129A 16223T 16297C	1	73 150 159 199 263 489 750 1438 2706 4048 4071 4164 4769 5351 5460 6455 6680 7028 7684 7853 8245 8701 8860 9196 9540 9824 10398 10400 10873 11719 12405 12705 12811 14766 14783 15043 15301 15326 16129 16223 16297	M7b1a1	73 150 159 199 263 489 16129 16223 16297	[0.232] M7b1a1 (1) [0.033] M7b1a1c (2) [0.000] M7b1a1+(16192) M7b1a1d M7b1a1d1 M7b1a1e M7b1a1e1 M7b1a1f M7b1a1g M7b1a1h (10)	[0.033] M7b1a1c (11) [0.007] M7b1a1e2 (12) [0.003] M7b1a1a2 (13) [0.000] M7b1a M7b1a1a M7b1a1+(16192) M7b1a1d M7b1a1d1 M7b1a1e M7b1a1e1 M7b1a1f M7b1a1g M7b1a1h M7b1a1i (24)	1
121 >MG273013.1 H	61A 62A 73G 263G 750G 1438G 2378T 2706G 3106d 3107C 4769G 6482T 7028T 8215T 8860G 9968T 11719A 14766T 15326G 16189C 16213A 16217C 16261T 16292T 16519C	B4g2	61A 62A 73G 263G 16189C 16213A 16217C 16261T 16292T 16519C	1	61A 62 73 263 750 1438 2378 2706 4769 6482 7028 8215 8860 9968 11719 14766 15326 16189 16213 16217 16261 16292	B4g2	61A 62 73 263 16189 16213 16217 16261 16292	[1.507] B4g2 (1) [0.007] B4g1 (2) [0.000] B4g B4g1a (4)	[0.000] B4g1b (5)	1

122	>MG273012.1 H	73G 146C 234G 263G 489C 750G 1438G 1709A 2706G 3106d 3107C 3753C 4769G 7028T 7353G 7521A 7793T 8701G 8860G 9540C 10325A 10398G 10400T 10873C 11002G 11252G 11719A 12406A 12603T 12705T 14341T 14766T 14783C 15043A 15055C 15301A 15326G 16124C 16183C 16189C 16293T 16325C 16362C	M76a	73G 146C 234G 263G 489C 16124C 16183C 16189C 16293T 16325C 16362C	1	73 146 234 263 489 750 1438 1709 2706 3753 4769 7028 7353 7521 7793T 8701 8860 9540 10325 10398 10400 10873 11002 11252 11719 12406 12603 12705 14341 14766 14783 15043 15055 15301 15326 16124 16189 16293T 16325 16362	M76a	73 146 234 263 489 16124 16189 16293T 16325 16362	[0.916] M76a (1)	[0.018] D1e (2)	1
123	>MG273010.1 H	73G 152C 263G 750G 1438G 1811G 2392C 2706G 3106d 3107C 3434G 3504C 3535C 3970T 4769G 4802C 5585A 5913A 5978G 6392C 7028T 8860G 9456G 9947A 10310A 10320A 11065G 11719A 13044T 13928C 14766T 15326G 15910T 16220C 16227G 16298C 16362C	F3b+152	73G 152C 263G 16220C 16227G 16298C 16362C	1	73 152 263 750 1438 1811 2392 2706 3434 3504 3535 3970 4769 4802 5585 5913 5978 6392 7028 8860 9456 9947 10310 10320 11065 11719 13044 13928C 14766 15326 15910 16220C 16227 16298 16362	F3b+152	73 152 263 16220C 16227 16298 16362	[0.964] F3b+152 (1)	[0.047] F3b (2)	1
124	>MG273005.1 H	73G 152C 263G 309.1CT 310C 750G 1438G 1811G 2392C 2706G 3106d 3107C 3434G 3535C 3970T 4769G 4802C 5585A 5913A 5978G 6392C 7028T 8860G 9947A 10310A 10320A 11065G 11719A 13044T 13928C 14766T 15326G 15910T 16126C 16220C 16227G 16298C 16362C	F3b+152	73G 152C 263G 309.1CT 310C 16126C 16220C 16227G 16298C 16362C	1	73 152 263 750 1438 1811 2392 2706 3434 3535 3970 4769 4802 5585 5913 5978 6392 7028 8860 9947 10310 10320 11065 11719 13044 13928C 14766 15326 15910 16126 16220C 16227 16298 16362	F3b+152	73 152 263 16126 16220C 16227 16298 16362	[1.035] F3b+152 (1)	[0.047] F3b (2) [0.000] T2i2	1
125	>MG273004.1 H	73G 143A 154C 183G 263G 302.1C 567.1C 750G 1438G 1541C 2706G 3106d 3107C 3970T 4017T 4769G 7028T 7849T 8860G 11719A 12714C 13928C 14766T 15326G 16192T 16288C 16304C 16309G 16390A 16519C	R9b1a1a	73G 143A 154C 183G 263G 302.1C 567.1C 16192T 16288C 16304C 16309G 16390A 16519C	1	73 143 154 183 263 573.1C 750 1438 1541 2706 3970 4017 4769 7028 7849 8860 11719 12714 13928C 14766 15326 16192 16288 16304 16309 16390	R9b1a1a	73 143 154 183 263 573.1C 16192 16288 16304 16309 16390	[0.011] R9b1a1a (1)	[0.000] R9b1a1 (2)	1
126	>MG273002.1 H	73G 151T 263G 479G 750G 1048T 1438G 2706G 3106d 3107C 3970T 4769G 7028T 7684C 7861C 8843C 8860G 9612A 11002G 11719A 12414C 12618A 13928C 14766T 15326G 15479C 16157C 16243C 16304C	R9c1b1	73G 151T 263G 479G 16157C 16243C 16304C	1	73 151 263 479 750 1048 1438 2706 3970 4769 7028 7684 7861 8843 8860 9612 11002 11719 12414 12618 13928C 14766 15326 15479 16157 16243 16304	R9c1b1	73 151 263 479 16157 16243 16304	[0.105] R9c1b1 (1)	[0.040] R9c1b2 (2) [0.000] R9c1 R9c1b (4)	1
127	>MG273000.1 H	73G 150T 199C 204C 263G 489C 750G 1438G 2706G 3106d 3107C 3335C 3483A 4048A 4071T 4164G 4769G 5351G 5460A 6455T 6680C 7028T 7684C 7853A 8701G 8860G 9540C 9824C 10398G 10400T 10873C 11361C 11719A 12405T 12705T 12811C 14766T 14783C 15043A 15301A 16223T 16297C	M7b1a1b	73G 150T 199C 204C 263G 489C 16223T 16297C	1	73 150 199 204 263 489 750 1438 2706 3335 3483 4048 4071 4164 4769 5351 5460 6455 6680 7028 7684 7853 8701 8860 9540 9824 10398 10400 10873 11361 11719 12405 12705 12811 14766 14783 15043 15301 16223 16297	M7b1a1b	73 150 199 204 263 489 16223 16297	[0.000] M7b1a1b (1)	[0.042] M7b1a1+(16192) (2) [0.018] M7b1a1e1 (3) [0.015] M7b1a1 (4) [0.014] M7b1a1a3 (5) [0.010] M7b1a1f (6) [0.004] M7b1a1h (7) [0.002] M7b1a1g (8) [0.000] M7b1a1d M7b1a1d1 M7b1a1e (11)	1

128	>MG272996.1 H	73G 150T 199C 204C 263G 750G 1438G 2706G 3106d 3107C 3335C 3483A 4048A 4071T 4164G 4769G 5351G 5460A 6455T 6680C 7028T 7684C 7853A 8701G 8860G 9540C 9824C 10398G 10400T 10873C 11361C 11719A 12405T 12705T 12811C 14766T 14783C 15043A 15301A 16223T 16297C	M7b1a1b	73G 150T 199C 204C 263G 16223T 16297C	1	73 150 199 204 263 750 1438 2706 3335 3483 4048 4071 4164 4769 5351 5460 6455 6680 7028 7684 7853 8701 8860 9540 9824 10398 10400 10873 11361 11719 12405 12705 12811 14766 14783 15043 15301 16223 16297	M7b1a1b	73 150 199 204 263 16223 16297	[0.009] M7b1a1b (1)	[0.000] N1a1'2 (2)	1
129	>MG272995.1 H	73G 146C 234G 263G 489C 750G 1438G 1709A 2706G 3106d 3107C 3753C 4769G 7028T 7353G 7521A 7793T 8701G 8860G 9540C 10325A 10398G 10400T 10873C 11002G 11252G 11719A 12406A 12603T 12705T 14341T 14766T 14783C 15043A 15055C 15301A 15326G 16124C 16183C 16189C 16293T 16325C 16362C	M76a	73G 146C 234G 263G 489C 16124C 16183C 16189C 16293T 16325C 16362C	1	73 146 234 263 489 750 1438 1709 2706 3753 4769 7028 7353 7521 7793T 8701 8860 9540 10325 10398 10400 10873 11002 11252 11719 12406 12603 12705 14341 14766 14783 15043 15055 15301 15326 16124 16189 16293T 16325 16362	M76a	73 146 234 263 489 16124 16189 16293T 16325 16362	[0.916] M76a (1)	[0.018] D1e (2)	1
130	>MG272984.1 H	73G 263G 750G 1438G 1541C 2706G 3106d 3107C 3621C 3666A 3970T 4769G 6647G 7028T 8724G 8860G 11299C 11422T 11545C 11719A 13191C 13722G 13928C 14766T 15314A 15326G 15883A 16304C 16362C 16519C	R9b2	73G 263G 16304C 16362C 16519C	1	73 263 750 1438 1541 2706 3621 3666 3970 4769 6647 7028 8724 8860 11299 11422 11545 11719 13191 13722 13928C 14766 15314 15326 15883 16304 16362	R9b2	73 263 16304 16362	[0.000] R9b2 (1)	[0.010] R9c (2) [0.000] R6 R9 R9b R31 (6)	1
131	>MG272970.1 H	73G 263G 750G 1005C 1438G 1824C 2706G 3106d 3107C 3970T 4643A 4769G 4811G 6392C 7028T 7828G 8697A 8860G 10310A 10535C 10586A 11719A 12338C 13708A 13928C 14766T 15326G 16092A 16291T 16304C	F2b1	73G 263G 16092A 16291T 16304C	1	73 263 750 1005 1438 1824 2706 3970 4643 4769 4811 6392 7028 7828 8697 8860 10310 10535 10586 11719 12338 13708 13928C 14766 15326 16092A 16291 16304	F2b1	73 263 16092A 16291 16304	[0.054] F2b1 (1)	[0.013] F2+16291 (2) [0.000] R9 R9b R9c F2b F2g (7)	1
132	>MG272969.1 H	73G 263G 310.1C 750G 1005C 1438G 1824C 2706G 3106d 3107C 3970T 4643A 4769G 4811G 6392C 7028T 7828G 8697A 8860G 9210G 10310A 10535C 10586A 11719A 12338C 13708A 13928C 14766T 15326G 16092A 16291T 16304C	F2b1	73G 263G 310.1C 16092A 16291T 16304C	1	73 263 750 1005 1438 1824 2706 3970 4643 4769 4811 6392 7028 7828 8697 8860 9210 10310 10535 10586 11719 12338 13708 13928C 14766 15326 16092A 16291 16304	F2b1	73 263 16092A 16291 16304	[0.054] F2b1 (1)	[0.013] F2+16291 (2) [0.000] R9 R9b R9c F2b F2g (7)	1
133	>MG272967.1 H	73G 263G 310.1C 750G 1005C 1438G 1824C 2706G 3106d 3107C 3970T 4643A 4769G 4811G 6392C 7028T 7828G 8697A 8860G 10310A 10535C 10586A 11719A 12338C 13708A 13928C 14766T 15326G 16092A 16291T 16304C	F2b1	73G 263G 310.1C 16092A 16291T 16304C	1	73 263 750 1005 1438 1824 2706 3970 4643 4769 4811 6392 7028 7828 8697 8860 10310 10535 10586 11719 12338 13708 13928C 14766 15326 16092A 16291 16304	F2b1	73 263 16092A 16291 16304	[0.054] F2b1 (1)	[0.013] F2+16291 (2) [0.000] R9 R9b R9c F2b F2g (7)	1

134	>MG272966.1 H	73G 263G 750G 1005C 1438G 1824C 2706G 3106d 3107C 3970T 4643A 4769G 4811G 6392C 7028T 7828G 8697A 8860G 10310A 10535C 10586A 11719A 12338C 13708A 13928C 14766T 15326G 16092A 16291T 16304C	F2b1	73G 263G 16092A 16291T 16304C	1	73 263 750 1005 1438 1824 2706 3970 4643 4769 4811 6392 7028 7828 8697 8860 10310 10535 10586 11719 12338 13708 13928C 14766 15326 16092A 16291 16304	F2b1	73 263 16092A 16291 16304	[0.054] F2b1 (1)	[0.013] F2+16291 (2) [0.000] R9 R9b R9c F2b F2g (7)	1
135	>MG272963.1 H	73G 150T 199C 204C 263G 489C 750G 942G 1438G 2706G 3106d 3107C 3483A 3531A 4048A 4071T 4164G 4769G 5351G 5460A 6455T 6680C 7028T 7684C 7853A 8701G 8860G 9540C 9824C 10398G 10400T 10873C 11719A 12405T 12705T 12811C 14766T 14783C 15043A 15301A 16086C 16223T 16297C	M7b1a1b	73G 150T 199C 204C 263G 489C 16086C 16223T 16297C	1	73 150 199 204 263 489 750 942 1438 2706 3483 3531 4048 4071 4164 4769 5351 5460 6455 6680 7028 7684 7853 8701 8860 9540 9824 10398 10400 10873 11719 12405 12705 12811 14766 14783 15043 15301 16086 16223 16297	M7b1a1b	73 150 199 204 263 489 16086 16223 16297	[0.050] M7b1a1b (1)	[0.059] M7b1a1+(16192) (2) [0.018] M7b1a1e1 (3) [0.017] M7b1a1a3 (4) [0.015] M7b1a1 (5) [0.010] M7b1a1f (6) [0.004] M7b1a1h (7) [0.002] M7b1a1g (8) [0.000] M7b1a1d M7b1a1d1 M7b1a1e (11)	1
136	>MG272961.1 H	73G 150T 199C 204C 263G 750G 942G 1438G 2706G 3106d 3107C 3483A 3531A 4048A 4071T 4164G 4769G 5351G 5460A 6455T 6680C 7028T 7684C 7853A 8701G 8860G 9540C 9824C 10398G 10400T 10688A 10873C 11719A 12405T 12705T 12811C 14766T 14783C 15043A 15301A 16086C 16223T 16297C	M7b1a1b	73G 150T 199C 204C 263G 16086C 16223T 16297C	1	73 150 199 204 263 750 942 1438 2706 3483 3531 4048 4071 4164 4769 5351 5460 6455 6680 7028 7684 7853 8701 8860 9540 9824 10398 10400 10688 10873 11719 12405 12705 12811 14766 14783 15043 15301 16086 16223 16297	M7b1a1b	73 150 199 204 263 16086 16223 16297	[0.059] M7b1a1b (1)	[0.000] N1a1'2 (2)	1
137	>MG272959.1 H	73G 263G 310.1C 750G 1005C 1438G 1824C 2706G 3106d 3107C 3970T 4643A 4769G 4811G 6392C 7028T 7828G 8697A 8860G 10310A 10535C 10586A 11719A 12338C 13641C 13708A 13928C 14766T 15326G 16092A 16167T 16291T 16304C	F2b1	73G 263G 310.1C 16092A 16167T 16291T 16304C	1	73 263 750 1005 1438 1824 2706 3970 4643 4769 4811 6392 7028 7828 8697 8860 10310 10535 10586 11719 12338 13641 13708 13928C 14766 15326 16092A 16167 16291 16304	F2b1	73 263 16092A 16167 16291 16304	[0.059] F2b1 (1)	[0.093] R9c (2) [0.013] F2+16291 (3) [0.000] R9 R9b F2b F2g (7)	1
138	>MG272958.1 H	73G 263G 750G 1005C 1438G 1824C 2706G 3106d 3107C 3970T 4643A 4769G 4811G 6392C 7028T 7828G 8697A 8860G 10310A 10535C 10586A 11719A 12338C 13708A 13928C 14766T 15326G 16092A 16291T 16304C	F2b1	73G 263G 16092A 16291T 16304C	1	73 263 750 1005 1438 1824 2706 3970 4643 4769 4811 6392 7028 7828 8697 8860 10310 10535 10586 11719 12338 13708 13928C 14766 15326 16092A 16291 16304	F2b1	73 263 16092A 16291 16304	[0.054] F2b1 (1)	[0.013] F2+16291 (2) [0.000] R9 R9b R9c F2b F2g (7)	1
139	>MG272956.1 H	63C 66A 73G 215G 263G 489C 750G 1438G 2706G 3106d 3107C 4769G 5054A 6185C 6575G 7028T 8251A 8701G 8860G 9540C 10268T 10398G 10400T 10873C 11719A 12705T 12850G 14311C 14766T 14783C 15043A 15301A 15326G 16223T 16311C 16362C 16381C	M74a	63C 66A 73G 215G 263G 489C 16223T 16311C 16362C 16381C	1	63 66 73 215 263 489 750 1438 2706 4769 5054 6185 6575 7028 8251 8701 8860 9540 10268 10398 10400 10873 11719 12705 12850 14311 14766 14783 15043 15301 15326 16223 16311 16362 16381	M74a	63 66 73 215 263 489 16223 16311 16362 16381	[0.186] M74a (1)	[0.019] D4j+16311 (2) [0.000] M43a1 M74 M74b M74b2 D4j11 (7)	1

140 >MG272955.1 H	73G 150T 199C 204C 263G 489C 750G 942G 1438G 2706G 3106d 3107C 3483A 3531A 4048A 4071T 4164G 4769G 5351G 5460A 6455T 6680C 7028T 7684C 7853A 8701G 8860G 9540C 9824C 10398G 10400T 10873C 11719A 12405T 12705T 12811C 14766T 14783C 15043A 15301A 16086C 16223T 16297C	M7b1a1b	73G 150T 199C 204C 263G 489C 16086C 16223T 16297C	1	73 150 199 204 263 489 750 942 1438 2706 3483 3531 4048 4071 4164 4769 5351 5460 6455 6680 7028 7684 7853 8701 8860 9540 9824 10398 10400 10873 11719 12405 12705 12811 14766 14783 15043 15301 16086 16223 16297	M7b1a1b	73 150 199 204 263 489 16086 16223 16297	[0.050] M7b1a1b (1)	[0.059] M7b1a1+(16192) (2) [0.018] M7b1a1e1 (3) [0.017] M7b1a1a3 (4) [0.015] M7b1a1 (5) [0.010] M7b1a1f (6) [0.004] M7b1a1h (7) [0.002] M7b1a1g (8) [0.000] M7b1a1d M7b1a1d1 M7b1a1e (11)	1
141 >MG272950.1 H	73G 146C 263G 310.1C 750G 1005C 1438G 1824C 2706G 3106d 3107C 3970T 4643A 4769G 4811G 6392C 7028T 7828G 8697A 8860G 10310A 10535C 10586A 11719A 12338C 13708A 13928C 14766T 15326G 16092A 16291T 16304C	F2b1	73G 146C 263G 310.1C 16092A 16291T 16304C	1	73 146 263 750 1005 1438 1824 2706 3970 4643 4769 4811 6392 7028 7828 8697 8860 10310 10535 10586 11719 12338 13708 13928C 14766 15326 16092A 16291 16304	F2b1	73 146 263 16092A 16291 16304	[0.065] F2b1 (1)	[0.200] F2b (2) [0.013] F2+16291 (3) [0.005] R9b R9c (5) [0.000] R9 F2g (7)	1
142 >MG272948.1 H	73G 263G 310.1C 750G 1005C 1438G 1824C 2706G 3106d 3107C 3970T 4643A 4769G 4811G 6392C 7028T 7828G 8697A 8860G 10310A 10535C 10586A 11719A 12338C 13708A 13928C 14766T 15326G 16092A 16291T 16304C	F2b1	73G 263G 310.1C 16092A 16291T 16304C	1	73 263 750 1005 1438 1824 2706 3970 4643 4769 4811 6392 7028 7828 8697 8860 10310 10535 10586 11719 12338 13708 13928C 14766 15326 16092A 16291 16304	F2b1	73 263 16092A 16291 16304	[0.054] F2b1 (1)	[0.013] F2+16291 (2) [0.000] R9 R9b R9c F2b F2g (7)	1
143 >MG272938.1 H	73G 146C 263G 281G 567.1C 750G 1438G 2706G 3106d 3107C 3290C 3970T 4769G 5069G 5263T 5354T 6392C 7028T 8860G 10310A 10909C 10915C 11719A 11776C 12612G 12630A 13368A 13759A 13928C 14016A 14328T 14766T 15326G 15670C 15908C 16129A 16185T 16207G 16399G 16519C	F4a2	73G 146C 263G 281G 567.1C 16129A 16185T 16207G 16399G 16519C	1	73 146 263 281 573.1C 750 1438 2706 3290 3970 4769 5069 5263 5354 6392 7028 8860 10310 10909 10915 11719 11776 12612 12630 13368 13759 13928C 14016 14328 14766 15326 15670 15908 16129 16185 16207 16399	F4a2	73 146 263 281 573.1C 16129 16185 16207 16399	[1.206] F4a2 (1) [0.000] H3b6 H3b6a (3)	[0.750] F4a (4) [0.458] K2b1b (5) [0.136] U2b (6) [0.130] U5a1a2b (7) [0.096] H26c (8) [0.090] P (9) [0.086] U2b1 (10) [0.076] P9a (11) [0.065] H1n3 U5a1a2a (13) [0.063] H1m1 H1n1 (15) [0.062] H1n1a (16) [0.059] H4a1a1a (17) [0.056] R6+16129 (18) [0.034] P2 (19) [0.028] R (20) [0.019] L3h1a2a1 (21) [0.015] H1n4 (22) [0.008] H17b (23) [0.007] N7 H1j1a (25) [0.000] L3h1a2a N7b HV+73 HV20 H1e2c H1e+16129 H1j1 H1j1a2 H1j1b H1j1c H1n+146 H1n2 H1cj H3b+16129 H3b2 H3b3 H3b5 H3b7 H3+73 H3d H3af H4a1a1a1 H4a1a1a1a H4a1a1a1a1 H4a1a1a2 H4a1a1a4 H13a1a2b H+16129 H17 H17a H17a2 H17c H63a R2'JT R8a1a1a2 R30 R30a R30a1 R30a1a R31 P4 P9 U U5a1a2 U5a1a2a1 U5a1a2a1a U5a1a2b1 U2'3'4'7'8'9 U2b1a U8 U8b'c U8b (77)	1

144 >MG272934.1 H	73G 143A 146C 263G 489C 750G 1284C 1438G 2706G 3106d 3107C 4769G 7028T 8440G 8701G 8718G 8838A 8860G 9540C 9615C 10398G 10400T 10873C 11719A 12681C 12705T 13407G 14605G 14766T 14783C 15043A 15301A 15326G 15458C 16093C 16129A 16140C 16223T 16271C 16295T 16304C	M71a2	73G 143A 146C 263G 489C 16093C 16129A 16140C 16223T 16271C 16295T 16304C	1	73 143 146 263 489 750 1284 1438 2706 4769 7028 8440 8701 8718 8838 8860 9540 9615 10398 10400 10873 11719 12681 12705 13407 14605 14766 14783 15043 15301 15326 15458 16093 16129 16140 16223 16271 16295 16304	M71a2	73 143 146 263 489 16093 16129 16140 16223 16271 16295 16304	[0.262] M71a2 (1)	[0.028] M7a2a1 (2) [0.007] G1b+16129 (3) [0.006] G1b2 G1b4 (5)	1
145 >MG272929.1 H	73G 151T 263G 489C 709A 750G 1438G 2706G 3106d 3107C 3777C 4769G 7028T 7055G 8521G 8701G 8718G 8860G 9540C 9548A 10398G 10400T 10873C 11719A 12705T 13149G 14766T 14783C 15043A 15301A 15326G 15458C 16172C 16223T 16260T 16264T 16271C 16519C	M71b	73G 151T 263G 489C 16172C 16223T 16260T 16264T 16271C 16519C	1	73 151 263 489 709 750 1438 2706 3777 4769 7028 7055 8521 8701 8718 8860 9540 9548 10398 10400 10873 11719 12705 13149 14766 14783 15043 15301 15326 15458 16172 16223 16260 16264 16271	M71b	73 151 263 489 16172 16223 16260 16264 16271	[0.095] M71b (1)	[0.006] M71+151 (2) [0.000] M71a	1
146 >MG272923.1 H	73G 207A 263G 709A 750G 1438G 2706G 3106d 3107C 3434G 3970T 4769G 4824G 4991A 5423G 5585A 5894G 5913A 5978G 6392C 7028T 8860G 9851A 10310A 10320A 11065G 11719A 12621T 13928C 14766T 14971C 15326G 15412G 16260T 16298C 16355T 16362C	F3a1	73G 207A 263G 16260T 16298C 16355T 16362C	1	73 207 263 709 750 1438 2706 3434 3970 4769 4824 4991 5423 5585 5894 5913 5978 6392 7028 8860 9851A 10310 10320 11065 11719 12621 13928C 14766 14971 15326 15412G 16260 16298 16355 16362	F3a1	73 207 263 16260 16298 16355 16362	[0.038] F3a1 (1)	[0.020] F3a+207 (2)	1
147 >MG272922.1 H	73G 207A 263G 302.1C 709A 750G 1438G 2706G 3106d 3107C 3434G 3970T 4769G 4824G 4991A 5423G 5585A 5894G 5913A 5978G 6392C 7028T 8860G 9851A 10310A 10320A 11065G 11719A 12621T 13928C 14766T 14971C 15326G 15412G 16260T 16298C 16355T 16362C	F3a1	73G 207A 263G 302.1C 16260T 16298C 16355T 16362C	1	73 207 263 709 750 1438 2706 3434 3970 4769 4824 4991 5423 5585 5894 5913 5978 6392 7028 8860 9851A 10310 10320 11065 11719 12621 13928C 14766 14971 15326 15412G 16260 16298 16355 16362	F3a1	73 207 263 16260 16298 16355 16362	[0.038] F3a1 (1)	[0.020] F3a+207 (2)	1
148 >MG272918.1 H	73G 146C 185A 263G 329A 750G 1040C 1438G 1654C 2706G 3106d 3107C 4769G 5048C 6770G 6917A 7028T 8860G 9773A 10316G 10609C 10877T 11719A 13359A 14514C 14569A 14766T 15326G 15885T 16232T 16249C 16288C 16295T 16304C 16519C	R22	73G 146C 185A 263G 329A 16232T 16249C 16288C 16295T 16304C 16519C	1	73 146 185 263 329 750 1040 1438 1654 2706 4769 5048 6770 6917 7028 8860 9773A 10316 10609 10877 11719 13359 14514 14569 14766 15326 15885 16232 16249 16288 16295 16304	R22	73 146 185 263 329 16232 16249 16288 16295 16304	[0.181] R22 (1)	[0.048] R9 (2) [0.005] R9b R9c (4) [0.000] R12'21 R12 (6)	1

149 >MG272915.1 H	59C 64T 73G 93G 189G 200G 263G 750G 1438G 1842G 2706G 3106d 3107C 3447G 4164G 4769G 5821A 5999C 7028T 7064C 8701G 8860G 9509C 9540C 9554A 9770C 10398G 10400T 10873C 11719A 11887A 12705T 13101G 13194A 14325C 14766T 14783C 14788C 14902T 15043A 15301A 15326G 15622C 15652G 16129A 16223T 16287T 16311C 16327A	M91a	59C 64T 73G 93G 189G 200G 263G 16129A 16223T 16287T 16311C 16327A	1	59 64 73 93 189 200 263 750 1438 1842 2706 3447 4164 4769 5821 5999 7028 7064 8701 8860 9509 9540 9554 9770 10398 10400 10873 11719 11887 12705 13101 13194 14325 14766 14783 14788 14902 15043 15301 15326 15622 15652 16129 16223 16287 16311 16327A	M91a	59 64 73 93 189 200 263 16129 16223 16287 16311 16327A	[0.616] M91a (1)	[0.000] L3f1b M91 (3)	1
150 >MG272911.1 H	61A 62A 73G 146C 152C 263G 489C 709A 750G 1438G 2706G 3106d 3107C 3666A 4643A 4769G 5910A 6023A 6221C 6253C 7028T 7403G 8092T 8701G 8860G 9144T 9214C 9540C 9548A 10398G 10400T 10873C 11719A 11944C 12189C 12406A 12585T 12705T 12952A 14766T 14783C 15043A 15301A 15326G 16223T 16381C 16390A	M13c	61A 62A 73G 146C 152C 263G 489C 16223T 16381C 16390A	1	61A 62 73 146 152 263 489 709 750 1438 2706 3666 4643 4769 5910 6023 6221 6253 7028 7403 8092 8701 8860 9144 9214C 9540 9548 10398 10400 10873 11719 11944 12189 12406 12585 12705 12952 14766 14783 15043 15301 15326 16223 16381 16390	M13c	61A 62 73 146 152 263 489 16223 16381 16390	[0.519] M13c (1)	[0.012] M13a'b (2) [0.008] M13 (3) [0.006] M61 (4) [0.005] E1a2a1 (5) [0.000] M37+152 M7c M7c3 E1a2 E1a2+(16261) E1a2a E1a2a3 M13'46'61 M46 (14)	1
151 >MG272909.1 H	61A 62A 73G 146C 152C 263G 310.1C 489C 709A 750G 1438G 2706G 3106d 3107C 3666A 4643A 4769G 5910A 6023A 6221C 6253C 7028T 7403G 8092T 8701G 8860G 9144T 9214C 9540C 9548A 10398G 10400T 10873C 11719A 11944C 12189C 12406A 12585T 12705T 12952A 14766T 14783C 15043A 15301A 15326G 16223T 16381C 16390A	M13c	61A 62A 73G 146C 152C 263G 310.1C 489C 16223T 16381C 16390A	1	61A 62 73 146 152 263 489 709 750 1438 2706 3666 4643 4769 5910 6023 6221 6253 7028 7403 8092 8701 8860 9144 9214C 9540 9548 10398 10400 10873 11719 11944 12189 12406 12585 12705 12952 14766 14783 15043 15301 15326 16223 16381 16390	M13c	61A 62 73 146 152 263 489 16223 16381 16390	[0.519] M13c (1)	[0.012] M13a'b (2) [0.008] M13 (3) [0.006] M61 (4) [0.005] E1a2a1 (5) [0.000] M37+152 M7c M7c3 E1a2 E1a2+(16261) E1a2a E1a2a3 M13'46'61 M46 (14)	1
152 >MG272905.1 H	61A 62A 73G 146C 152C 263G 489C 709A 750G 1438G 2706G 3106d 3107C 3666A 4643A 4769G 5910A 6023A 6221C 6253C 7028T 7403G 8092T 8701G 8860G 9144T 9214C 9540C 9548A 10398G 10400T 10873C 11719A 11944C 12189C 12406A 12585T 12705T 12952A 14766T 14783C 15043A 15301A 15326G 16223T 16381C 16390A	M13c	61A 62A 73G 146C 152C 263G 489C 16223T 16381C 16390A	1	61A 62 73 146 152 263 489 709 750 1438 2706 3666 4643 4769 5910 6023 6221 6253 7028 7403 8092 8701 8860 9144 9214C 9540 9548 10398 10400 10873 11719 11944 12189 12406 12585 12705 12952 14766 14783 15043 15301 15326 16223 16381 16390	M13c	61A 62 73 146 152 263 489 16223 16381 16390	[0.519] M13c (1)	[0.012] M13a'b (2) [0.008] M13 (3) [0.006] M61 (4) [0.005] E1a2a1 (5) [0.000] M37+152 M7c M7c3 E1a2 E1a2+(16261) E1a2a E1a2a3 M13'46'61 M46 (14)	1
153 >MG272904.1 H	73G 263G 310.1C 461T 489C 750G 1438G 2706G 3106d 3107C 3486T 3537G 4769G 5082C 5301G 5558G 7028T 8701G 8860G 8865A 9329A 9540C 10398G 10400T 10640C 10873C 11719A 12507G 12705T 13966G 14128G 14766T 14783C 15043A 15301A 15326G 16218T 16223T 16231C 16356C 16362C 16519C	M6a1a	73G 263G 310.1C 461T 489C 16218T 16223T 16231C 16356C 16362C 16519C	1	73 263 461 489 750 1438 2706 3486 3537 4769 5082 5301 5558 7028 8701 8860 8865 9329 9540 10398 10400 10640 10873 11719 12507 12705 13966 14128 14766 14783 15043 15301 15326 16218 16223 16231 16356 16362	M6a1a	73 263 461 489 16218 16223 16231 16356 16362	[0.055] M6a1a (1)	[0.011] M6a1 (2) [0.000] M6a	1

154	>MG272897.1 H	73G 263G 750G 1438G 1541C 2706G 3106d 3107C 3970T 4769G 5459T 7028T 7679C 8860G 9055A 11719A 12714C 13928C 14001G 14696G 14766T 15326G 16169T 16189C 16294T 16304C 16309G 16390A 16519C	R9b1	73G 263G 16169T 16189C 16294T 16304C 16309G 16390A 16519C	1	73 263 750 1438 1541 2706 3970 4769 5459 7028 7679 8860 9055 11719 12714 13928C 14001 14696 14766 15326 16169 16189 16294 16304 16309 16390	R9b1	73 263 16169 16189 16294 16304 16309 16390	[0.130] R9b1 (1)	[0.013] N9b2a (2)	1
155	>MG272895.1 H	73G 152C 263G 310.1C 750G 1438G 2706G 2760G 3027C 3106d 3107C 3483A 3633C 3666A 4769G 7028T 7885C 7961C 8188G 8860G 10166C 10398G 11719A 12705T 13710G 14766T 15211T 15326G 16223T 16263C 16274A 16311C 16343G 16357C 16519C	N8	73G 152C 263G 310.1C 16223T 16263C 16274A 16311C 16343G 16357C 16519C	1	73 152 263 750 1438 2706 2760 3027 3483 3633 3666 4769 7028 7885 7961 8188 8860 10166 10398 11719 12705 13710 14766 15211 15326 16223 16263 16274 16311 16343 16357	N8	73 152 263 16223 16263 16274 16311 16343 16357	[0.459] N8 (1)	[0.065] L3h1 L3h1a (3) [0.057] L3d3a1b (4) [0.043] M57+152 (5) [0.023] L3a1a (6) [0.015] L3d3a (7) [0.000] L3'4 L3f2a L3f2a1 L3c'd L3d3a1 L3d3a1a L3h1a2 S+152 (15)	1
156	>MG272891.1 H	73G 207A 263G 302.1C 489C 709A 750G 1438G 2706G 3106d 3107C 3398C 4048A 4769G 4833G 4853A 5108C 5601T 7028T 8701G 8860G 8877C 9540C 10398G 10400T 10873C 11151T 11719A 12705T 13191C 13563G 14182C 14569A 14766T 14783C 15043A 15301A 15326G 16223T 16239T 16362C	G2b1a1	73G 207A 263G 302.1C 489C 16223T 16239T 16362C	1	73 207 263 489 709 750 1438 2706 3398 4048 4769 4833 4853 5108 5601 7028 8701 8860 8877 9540 10398 10400 10873 11151 11719 12705 13191 13563 14182 14569 14766 14783 15043 15301 15326 16223 16239 16362	G2b1a1	73 207 263 489 16223 16239 16362	[0.009] G2b1a1 (1)	[0.006] D4b2b2 (2) [0.002] D4e4a D4f (4) [0.001] D4b2a D4b2b (6) [0.000] M9 G G1 G1b G1c G1c2 G2 G2a'c G2b G2b1 G2b1a G2b2 G2b2a M33+16362 D D4 D4b D4b2 D4b2a2 D4b2a2b D4b2b1 D4b2b1a D4b2b1b D4b2b1c D4b2b3 D4b2b6 D4e D4e1'3 D4e1 D4e1c D4e3 D4e2 D4e2b D4e2c D4e2d D4e4 D4e4b D4f1 D4g D4h D4h4 D4j D4j1 D4j1b D4j4 D4j5 D4j5a D4j+(16286) D4j9 D4j10 D4j12 D4j15 D4j16 D4m (60)	1
157	>MG272889.1 H	73G 152C 263G 750G 1438G 2706G 2760G 3027C 3106d 3107C 3483A 3633C 3666A 4769G 7028T 7885C 7961C 8188G 8860G 10166C 10398G 11719A 12705T 13710G 14766T 15211T 15326G 16223T 16263C 16274A 16311C 16343G 16357C 16519C	N8	73G 152C 263G 16223T 16263C 16274A 16311C 16343G 16357C 16519C	1	73 152 263 750 1438 2706 2760 3027 3483 3633 3666 4769 7028 7885 7961 8188 8860 10166 10398 11719 12705 13710 14766 15211 15326 16223 16263 16274 16311 16343 16357	N8	73 152 263 16223 16263 16274 16311 16343 16357	[0.459] N8 (1)	[0.065] L3h1 L3h1a (3) [0.057] L3d3a1b (4) [0.043] M57+152 (5) [0.023] L3a1a (6) [0.015] L3d3a (7) [0.000] L3'4 L3f2a L3f2a1 L3c'd L3d3a1 L3d3a1a L3h1a2 S+152 (15)	1
158	>MG272871.1 H	73G 152C 263G 489C 750G 1438G 2706G 2786C 3106d 3107C 3644C 4769G 5773A 6023A 6253C 6293C 6620C 7028T 8405G 8701G 8860G 9540C 10373A 10398G 10400T 10411G 10790C 10873C 11719A 11908G 12705T 13395G 14766T 14783C 15043A 15301A 15326G 15924G 16145A 16168T 16188T 16223T 16257T 16311C 16320T 16519C	M13a2	73G 152C 263G 489C 16145A 16168T 16188T 16223T 16257T 16311C 16320T 16519C	1	73 152 263 489 750 1438 2706 2786 3644 4769 5773 6023 6253 6293 6620 7028 8405 8701 8860 9540 10373 10398 10400 10411 10790 10873 11719 11908 12705 13395 14766 14783 15043 15301 15326 15924 16145 16168 16188 16223 16257 16311 16320	M13a2	73 152 263 489 16145 16168 16188 16223 16257 16311 16320	[0.890] M13a2 (1)	[0.034] M57+152 (2) [0.008] M13a (3) [0.000] M13a1a	1

159 >MG272868.1 H	73G 93G 200G 263G 309.1CT 310C 750G 1438G 2706G 3106d 3107C 3447G 3732C 4113A 4164G 4769G 5999C 7028T 7064C 8701G 8860G 9540C 10398G 10400T 10873C 11719A 11887A 12705T 13194A 13434G 14766T 14783C 14902T 15043A 15301A 15326G 15622C 15652G 16129A 16223T 16278T 16287T 16311C 16327A 16456A	M91a	73G 93G 200G 263G 309.1CT 310C 16129A 16223T 16278T 16287T 16311C 16327A 16456A	1	73 93 200 263 750 1438 2706 3447 3732 4113 4164 4769 5999 7028 7064 8701 8860 9540 10398 10400 10873 11719 11887 12705 13194 13434 14766 14783 14902 15043 15301 15326 15622 15652 16129 16223 16278 16287 16311 16327A 16456	M91a	73 93 200 263 16129 16223 16278 16287 16311 16327A 16456	[0.478] M91a (1)	[0.000] M91 (2)	1
160 >MG272866.1 H	73G 93G 200G 263G 489C 520T 750G 1438G 2706G 3106d 3107C 3447G 3732C 4113A 4164G 4769G 5999C 7028T 7064C 8701G 8860G 9540C 10398G 10400T 10873C 11719A 11887A 12705T 13194A 14766T 14783C 14902T 15043A 15301A 15326G 15622C 15652G 16129A 16223T 16278T 16287T 16311C 16327A 16456A	M91a	73G 93G 200G 263G 489C 520T 16129A 16223T 16278T 16287T 16311C 16327A 16456A	1	73 93 200 263 489 520 750 1438 2706 3447 3732 4113 4164 4769 5999 7028 7064 8701 8860 9540 10398 10400 10873 11719 11887 12705 13194 14766 14783 14902 15043 15301 15326 15622 15652 16129 16223 16278 16287 16311 16327A 16456	M91a	73 93 200 263 489 520 16129 16223 16278 16287 16311 16327A 16456	[0.478] M91a (1)	[0.000] M91 (2)	1
161 >MG272858.1 H	73G 128.1T 263G 318C 750G 1438G 2706G 3106d 3107C 3813G 4170T 4769G 4853A 5580C 7028T 8286C 8701G 8860G 9100G 9540C 9966A 10398G 10400T 10873C 11719A 12030G 12358G 12372A 12705T 13712T 14293G 14569A 14727C 14766T 14783C 15010G 15043A 15301A 15326G 15463G 15651T 16051G 16129A 16189C 16223T 16234T 16290T	M12a	73G 128.1T 263G 318C 16051G 16129A 16189C 16223T 16234T 16290T	1	73 129.1T 263 318 750 1438 2706 3813 4170 4769 4853 5580 7028 8286 8701 8860 9100 9540 9966 10398 10400 10873 11719 12030 12358 12372 12705 13712 14293 14569 14727 14766 14783 15010 15043 15301 15326 15463 15651 16051 16129 16189 16223 16234 16290	M12a	73 129.1T 263 318 16051 16129 16189 16223 16234 16290	[0.084] M12a (1)	[0.010] N9b N9b1 (3) [0.006] M12b M12b1 M12b1a M12b1a1 (7) [0.000] M12 N7 N7b N9b1a N9b1b N9b3 U8b1a1 (14)	1
162 >MG272853.1 H	73G 204C 263G 750G 1438G 1541C 2322A 2706G 3106d 3107C 3970T 4769G 7028T 8860G 11719A 12714C 13928C 14766T 15326G 16124C 16148T 16304C 16309G 16327T 16390A 16519C	R9b1b	73G 204C 263G 16124C 16148T 16304C 16309G 16327T 16390A 16519C	1	73 204 263 750 1438 1541 2322A 2706 3970 4769 7028 8860 11719 12714 13928C 14766 15326 16124 16148 16304 16309 16327 16390	R9b1b	73 204 263 16124 16148 16304 16309 16327 16390	[0.265] R9b1b (1)	[0.008] R9b1 (2)	1
163 >MG272849.1 H	73G 150T 199C 204C 263G 750G 1438G 2706G 3106d 3107C 3483A 4048A 4071T 4164G 4769G 5351G 5460A 6455T 6680C 7028T 7647C 7684C 7853A 8701G 8860G 9540C 9824C 10398G 10400T 10873C 11404G 11719A 12405T 12705T 12811C 14766T 14783C 15043A 15301A 16223T 16297C	M7b1a1b	73G 150T 199C 204C 263G 16223T 16297C	1	73 150 199 204 263 750 1438 2706 3483 4048 4071 4164 4769 5351 5460 6455 6680 7028 7647 7684 7853 8701 8860 9540 9824 10398 10400 10873 11404 11719 12405 12705 12811 14766 14783 15043 15301 16223 16297	M7b1a1b	73 150 199 204 263 16223 16297	[0.009] M7b1a1b (1)	[0.000] N1a1'2 (2)	1

164 >MG272836.1 H	73G 152C 263G 750G 1438G 2706G 2760G 3027C 3106d 3107C 3483A 3633C 3666A 4769G 7028T 7885C 7961C 8188G 8860G 10166C 10398G 11719A 12705T 13710G 14766T 15211T 15326G 16223T 16263C 16274A 16311C 16343G 16357C 16519C	N8	73G 152C 263G 16223T 16263C 16274A 16311C 16343G 16357C 16519C	1	73 152 263 750 1438 2706 2760 3027 3483 3633 3666 4769 7028 7885 7961 8188 8860 10166 10398 11719 12705 13710 14766 15211 15326 16223 16263 16274 16311 16343 16357	N8	73 152 263 16223 16263 16274 16311 16343 16357	[0.459] N8 (1)	[0.065] L3h1 L3h1a (3) [0.057] L3d3a1b (4) [0.043] M57+152 (5) [0.023] L3a1a (6) [0.015] L3d3a (7) [0.000] L3'4 L3f2a L3f2a1 L3c'd L3d3a1 L3d3a1a L3h1a2 S+152 (15)	1
165 >MG272835.1 H	73G 152C 263G 750G 1438G 2706G 2760G 3027C 3106d 3107C 3483A 3633C 3666A 4769G 7028T 7885C 7961C 8188G 8774G 8860G 10166C 10398G 11719A 12705T 13710G 14766T 15211T 15326G 16223T 16263C 16274A 16311C 16343G 16357C 16519C	N8	73G 152C 263G 16223T 16263C 16274A 16311C 16343G 16357C 16519C	1	73 152 263 750 1438 2706 2760 3027 3483 3633 3666 4769 7028 7885 7961 8188 8774 8860 10166 10398 11719 12705 13710 14766 15211 15326 16223 16263 16274 16311 16343 16357	N8	73 152 263 16223 16263 16274 16311 16343 16357	[0.459] N8 (1)	[0.065] L3h1 L3h1a (3) [0.057] L3d3a1b (4) [0.043] M57+152 (5) [0.023] L3a1a (6) [0.015] L3d3a (7) [0.000] L3'4 L3f2a L3f2a1 L3c'd L3d3a1 L3d3a1a L3h1a2 S+152 (15)	1
166 >MG272834.1 H	73G 143A 183G 263G 567.1C 750G 1438G 1541C 2706G 3106d 3107C 3970T 4017T 4092A 4769G 7028T 7849T 8860G 11719A 12714C 13928C 14766T 15326G 15673G 16172C 16192T 16259T 16288C 16304C 16309G 16390A 16519C	R9b1a1a	73G 143A 183G 263G 567.1C 16172C 16192T 16259T 16288C 16304C 16309G 16390A 16519C	1	73 143 183 263 573.1C 750 1438 1541 2706 3970 4017 4092 4769 7028 7849 8860 11719 12714 13928C 14766 15326 15673 16172 16192 16259 16288 16304 16309 16390	R9b1a1a	73 143 183 263 573.1C 16172 16192 16259 16288 16304 16309 16390	[0.137] R9b1a1a (1)	[0.000] R9b1a1 (2)	1
167 >MG272832.1 H	73G 143A 183G 259G 263G 302.1C 750G 1438G 1541C 2706G 3106d 3107C 3970T 4017T 4769G 7028T 7849T 8860G 11719A 12714C 13928C 14766T 15326G 16288C 16304C 16309G 16390A 16519C	R9b1a1a	73G 143A 183G 259G 263G 302.1C 16288C 16304C 16309G 16390A 16519C	1	73 143 183 259 263 750 1438 1541 2706 3970 4017 4769 7028 7849 8860 11719 12714 13928C 14766 15326 16288 16304 16309 16390	R9b1a1a	73 143 183 259 263 16288 16304 16309 16390	[0.569] R9b1a1a (1)	[0.000] R9b1a1 (2)	1
168 >MG272830.1 H	73G 143A 183G 263G 750G 1438G 1541C 2706G 3106d 3107C 3970T 4017T 4092A 4769G 7028T 7849T 8860G 11719A 12714C 13928C 14766T 15326G 15673G 16172C 16192T 16259T 16288C 16304C 16309G 16390A 16519C	R9b1a1a	73G 143A 183G 263G 16172C 16192T 16259T 16288C 16304C 16309G 16390A 16519C	1	73 143 183 263 750 1438 1541 2706 3970 4017 4092 4769 7028 7849 8860 11719 12714 13928C 14766 15326 15673 16172 16192 16259 16288 16304 16309 16390	R9b1a1a	73 143 183 263 16172 16192 16259 16288 16304 16309 16390	[0.137] R9b1a1a (1)	[0.000] R9b1a1 (2)	1
169 >MG272829.1 H	73G 143A 183G 259G 263G 750G 1438G 1541C 2706G 3106d 3107C 3970T 4017T 4769G 7028T 7849T 8860G 11719A 12714C 13928C 14766T 15326G 16288C 16304C 16309G 16390A 16519C	R9b1a1a	73G 143A 183G 259G 263G 16288C 16304C 16309G 16390A 16519C	1	73 143 183 259 263 750 1438 1541 2706 3970 4017 4769 7028 7849 8860 11719 12714 13928C 14766 15326 16288 16304 16309 16390	R9b1a1a	73 143 183 259 263 16288 16304 16309 16390	[0.569] R9b1a1a (1)	[0.000] R9b1a1 (2)	1

170	>MG272812.1 H	73G 150T 199C 201G 263G 302.1C 750G 1438G 2706G 3106d 3107C 4048A 4071T 4164G 4769G 5351G 5460A 6455T 6680C 7028T 7684C 7853A 8701G 8860G 9540C 9824C 10398G 10400T 10873C 11719A 12405T 12705T 12811C 13053T 13395G 14766T 14783C 15043A 15236G 15301A 15326G 16129A 16223T 16297C 16389A	M7b1a1	73G 150T 199C 201G 263G 302.1C 16129A 16223T 16297C 16389A	1	73 150 199 201 263 750 1438 2706 4048 4071 4164 4769 5351 5460 6455 6680 7028 7684 7853 8701 8860 9540 9824 10398 10400 10873 11719 12405 12705 12811 13053 13395 14766 14783 15043 15236 15301 15326 16129 16223 16297 16389	M7b1a1	73 150 199 201 263 16129 16223 16297 16389	[0.016] M7b1a1 (1) [0.006] M7b1a1f (2) [0.004] M7b1a1e1 (3) [0.002] M7b1a1d1 (4) [0.001] M7b1a1+(16192) (5) [0.000] M7b1a1d M7b1a1e M7b1a1g M7b1a1h (9)	[0.027] M7b1a1a (10) [0.007] M7b1a1e2 (11) [0.006] M7b1a1f (12) [0.004] M7b1a1e1 (13) [0.002] M7b1a1d1 (14) [0.001] M7b1a1+(16192) (15) [0.000] M7b1a M7b1a1a2 M7b1a1d M7b1a1e M7b1a1g M7b1a1h M7b1a1i (22)	1
171	>MG272801.1 H	73G 143A 183G 259G 263G 750G 1438G 1541C 2706G 3106d 3107C 3970T 4017T 4769G 6752G 7028T 7849T 8860G 11719A 12714C 13928C 14766T 15326G 16288C 16304C 16309G 16390A 16519C	R9b1a1a	73G 143A 183G 259G 263G 16288C 16304C 16309G 16390A 16519C	1	73 143 183 259 263 750 1438 1541 2706 3970 4017 4769 6752 7028 7849 8860 11719 12714 13928C 14766 15326 16288 16304 16309 16390	R9b1a1a	73 143 183 259 263 16288 16304 16309 16390	[0.569] R9b1a1a (1)	[0.000] R9b1a1 (2)	1
172	>MG272797.1 H	73G 143A 183G 263G 750G 1438G 1541C 2706G 3106d 3107C 3970T 4017T 4092A 4769G 7028T 7055G 7849T 8860G 11719A 12714C 13928C 14766T 15326G 15673G 16172C 16192T 16259T 16288C 16304C 16309G 16390A 16519C	R9b1a1a	73G 143A 183G 263G 16172C 16192T 16259T 16288C 16304C 16309G 16390A 16519C	1	73 143 183 263 750 1438 1541 2706 3970 4017 4092 4769 7028 7055 7849 8860 11719 12714 13928C 14766 15326 15673 16172 16192 16259 16288 16304 16309 16390	R9b1a1a	73 143 183 263 16172 16192 16259 16288 16304 16309 16390	[0.137] R9b1a1a (1)	[0.000] R9b1a1 (2)	1
173	>MG272791.1 H	73G 143A 183G 259G 263G 302.1C 567.1C 750G 1438G 1541C 2706G 3106d 3107C 3711G 3970T 4017T 4769G 7028T 7849T 8860G 11719A 12714C 13928C 14016A 14766T 15326G 16288C 16304C 16309G 16390A 16519C	R9b1a1a	73G 143A 183G 259G 263G 302.1C 567.1C 16288C 16304C 16309G 16390A 16519C	1	73 143 183 259 263 573.1C 750 1438 1541 2706 3711 3970 4017 4769 7028 7849 8860 11719 12714 13928C 14016 14766 15326 16288 16304 16309 16390	R9b1a1a	73 143 183 259 263 573.1C 16288 16304 16309 16390	[0.569] R9b1a1a (1)	[0.000] R9b1a1 (2)	1
174	>MG272788.1 H	73G 143A 183G 259G 263G 302.1C 750G 1438G 1541C 2706G 3106d 3107C 3970T 4017T 4769G 7028T 7849T 8860G 11719A 12714C 13928C 14766T 15326G 16288C 16304C 16309G 16390A 16519C	R9b1a1a	73G 143A 183G 259G 263G 302.1C 16288C 16304C 16309G 16390A 16519C	1	73 143 183 259 263 750 1438 1541 2706 3970 4017 4769 7028 7849 8860 11719 12714 13928C 14766 15326 16288 16304 16309 16390	R9b1a1a	73 143 183 259 263 16288 16304 16309 16390	[0.569] R9b1a1a (1)	[0.000] R9b1a1 (2)	1
175	>MG272783.1 H	73G 263G 310.1C 750G 1438G 2706G 3106d 3107C 4769G 4859C 4977C 7028T 8410T 8701G 8860G 9540C 10398G 10400T 10556T 10873C 11050C 11719A 12007A 12705T 14581C 14696G 14766T 14783C 15043A 15301A 15326G 15497A 16111T 16183C 16189C 16223T 16224C 16519C	M37e2	73G 263G 310.1C 16111T 16183C 16189C 16223T 16224C 16519C	1	73 263 750 1438 2706 4769 4859 4977 7028 8410 8701 8860 9540 10398 10400 10556 10873 11050 11719 12007 12705 14581 14696 14766 14783 15043 15301 15326 15497 16111 16189 16223 16224	M37e2	73 263 16111 16189 16223 16224	[0.018] M37e2 (1) [0.000] N9b N9b1 N9b1a N9b3 (5)	[0.011] M37e (6) [0.008] R+16189 (7) [0.004] S2 (8) [0.000] L3 L3b'f L3d2a L3h N N1'5 N1 N5 N9 N9b1c N9b2 N11 N14 S X R11'B6 B4'5 (25)	1

176	>MG272775.1 H	73G 143A 195C 204C 207A 263G 489C 750G 1438G 2706G 3106d 3107C 3894T 4769G 7028T 8701G 8718G 8860G 9055A 9380A 9540C 10398G 10400T 10873C 11017C 11719A 12705T 13401C 14766T 14783C 15043A 15301A 15326G 15458C 15734A 16223T 16271C 16362C 16519C	M71c	73G 143A 195C 204C 207A 263G 489C 16223T 16271C 16362C 16519C	1	73 143 195 204 207 263 489 750 1438 2706 3894 4769 7028 8701 8718 8860 9055 9380 9540 10398 10400 10873 11017 11719 12705 13401 14766 14783 15043 15301 15326 15458 15734 16223 16271 16362	M71c	73 143 195 204 207 263 489 16223 16271 16362	[0.042] M71c (1)	[0.008] D4+195 (2) [0.007] G2b1a1 (3) [0.000] D2b D2b1 D2b1a D2b2 (7)	1
177	>MG272773.1 H	73G 150T 263G 750G 1438G 2706G 3010A 3106d 3107C 4769G 4856C 5231A 5417A 7028T 8860G 11719A 12358G 12372A 12705T 14766T 15326G 16223T 16257A 16261T 16292T 16519C	N9a6	73G 150T 263G 16223T 16257A 16261T 16292T 16519C	1	73 150 263 750 1438 2706 3010 4769 4856 5231 5417 7028 8860 11719 12358 12372 12705 14766 15326 16223 16257A 16261 16292	N9a6	73 150 263 16223 16257A 16261 16292	[0.000] N9a6 (1)	[0.000] N9a N9a6a N9a6b N9a8 N9a9 N9a10 (7)	1
178	>MG272772.1 H	73G 150T 750G 1048T 1107C 1438G 2706G 3106d 3107C 4048A 4769G 4883T 5153G 5178A 5301G 7028T 8860G 9180G 9540C 9667G 9992T 10397G 10398G 10400T 10873C 11176A 11719A 12705T 13954T 14766T 14783C 15043A 15301A 15326G 15724G 16183C 16189C 16223T 16362C 16519C	D5b4	73G 150T 16183C 16189C 16223T 16362C 16519C	1	73 150 750 1048 1107 1438 2706 4048 4769 4883 5153 5178A 5301 7028 8860 9180 9540 9667 9992 10397 10398 10400 10873 11176 11719 12705 13954 14766 14783 15043 15301 15326 15724 16189 16223 16362	D5b4	73 150 16189 16223 16362	[0.057] D5b4 (1) [0.009] D5a (2) [0.000] D5 D5a'b (4)	[0.061] N9b (5) [0.047] D5a2 (6) [0.032] B4j (7) [0.029] D5a3 (8) [0.004] D4b2b4 (9) [0.002] D4t (10) [0.000] L3e'i'k'x L3e L3e3'4'5 L3e3'4 L3i G1a D4b2a2a D4b2a2a2 D4b2b2a D4b2b2a1 D+16189 D5a2b D5a3a D5a3a1 D5b D5b1 D5b1a D5b1b D5b1c D5b3 D5b3a N9b1 N9b1a N9b3 B6 (35)	1
179	>MG272768.1 H	73G 143A 146C 151T 263G 338T 489C 750G 1438G 2706G 3106d 3107C 4769G 5493C 7028T 8440G 8485A 8701G 8718G 8838A 8860G 9540C 9615C 10398G 10400T 10873C 11719A 12705T 13759A 14605G 14766T 14783C 15043A 15301A 15326G 15458C 16093C 16140C 16223T 16271C 16360T 16381C	M71a2	73G 143A 146C 151T 263G 338T 489C 16093C 16140C 16223T 16271C 16360T 16381C	1	73 143 146 151 263 338 489 750 1438 2706 4769 5493 7028 8440 8485 8701 8718 8838 8860 9540 9615 10398 10400 10873 11719 12705 13759 14605 14766 14783 15043 15301 15326 15458 16093 16140 16223 16271 16360 16381	M71a2	73 143 146 151 263 338 489 16093 16140 16223 16271 16360 16381	[0.111] M71a2 (1)	[0.080] M71+151 (2) [0.008] M71a (3) [0.000] M7a2a1	1
180	>MG272766.1 H	73G 152C 263G 750G 1438G 1541C 2623G 2706G 3106d 3107C 3666A 3970T 4769G 6647G 7028T 8724G 8860G 11299C 11422T 11719A 13191C 13722G 13928C 14766T 15314A 15326G 15883A 16304C 16362C 16519C	R9b2	73G 152C 263G 16304C 16362C 16519C	1	73 152 263 750 1438 1541 2623 2706 3666 3970 4769 6647 7028 8724 8860 11299 11422 11719 13191 13722 13928C 14766 15314 15326 15883 16304 16362	R9b2	73 152 263 16304 16362	[0.059] R9b2 (1)	[0.048] R9 (2) [0.015] R9c (3) [0.008] R30a1 (4) [0.007] R6 (5) [0.000] H32 R9b R30a1a R31 (9)	1

181	>MG272762.1 H	73G 146C 263G 281G 567.1C 750G 1438G 2706G 3106d 3107C 3290C 3970T 4769G 5069G 5263T 6392C 7028T 8860G 10310A 10909C 10915C 11719A 11776C 12612G 12630A 13368A 13928C 14016A 14766T 15326G 15670C 15908C 16129A 16185T 16207G 16399G 16519C	F4a2	73G 146C 263G 281G 567.1C 16129A 16185T 16207G 16399G 16519C	1	73 146 263 281 573.1C 750 1438 2706 3290 3970 4769 5069 5263 6392 7028 8860 10310 10909 10915 11719 11776 12612 12630 13368 13928C 14016 14766 15326 15670 15908 16129 16185 16207 16399	F4a2	73 146 263 281 573.1C 16129 16185 16207 16399	[1.206] F4a2 (1) [0.000] H3b6 H3b6a (3)	[0.750] F4a (4) [0.458] K2b1b (5) [0.136] U2b (6) [0.130] U5a1a2b (7) [0.096] H26c (8) [0.090] P (9) [0.086] U2b1 (10) [0.076] P9a (11) [0.065] H1n3 U5a1a2a (13) [0.063] H1m1 H1n1 (15) [0.062] H1n1a (16) [0.059] H4a1a1a (17) [0.056] R6+16129 (18) [0.034] P2 (19) [0.028] R (20) [0.019] L3h1a2a1 (21) [0.015] H1n4 (22) [0.008] H17b (23) [0.007] N7 H1j1a (25) [0.000] L3h1a2a N7b HV+73 HV20 H1e2c H1e+16129 H1j1 H1j1a2 H1j1b H1j1c H1n+146 H1n2 H1cj H3b+16129 H3b2 H3b3 H3b5 H3b7 H3+73 H3d H3af H4a1a1a1 H4a1a1a1a H4a1a1a1a1 H4a1a1a2 H4a1a1a4 H13a1a2b H+16129 H17 H17a H17a2 H17c H63a R2'JT R8a1a1a2 R30 R30a R30a1 R30a1a R31 P4 P9 U U5a1a2 U5a1a2a1 U5a1a2a1a U5a1a2b1 U2'3'4'7'8'9 U2b1a U8 U8b'c U8b (77)	1
182	>MG272761.1 H	73G 143A 146C 151T 263G 489C 750G 1438G 2706G 3106d 3107C 4769G 5493C 7028T 8440G 8485A 8701G 8718G 8838A 8860G 9540C 9615C 10398G 10400T 10873C 11719A 12705T 13759A 14605G 14766T 14783C 15043A 15301A 15326G 15458C 16093C 16140C 16223T 16271C 16360T 16381C	M71a2	73G 143A 146C 151T 263G 489C 16093C 16140C 16223T 16271C 16360T 16381C	1	73 143 146 151 263 489 750 1438 2706 4769 5493 7028 8440 8485 8701 8718 8838 8860 9540 9615 10398 10400 10873 11719 12705 13759 14605 14766 14783 15043 15301 15326 15458 16093 16140 16223 16271 16360 16381	M71a2	73 143 146 151 263 489 16093 16140 16223 16271 16360 16381	[0.104] M71a2 (1)	[0.080] M71+151 (2) [0.008] M71a (3) [0.000] M7a2a1	1
183	>MG272759.1 H	73G 150T 195C 263G 750G 1438G 2706G 3106d 3107C 4769G 6752G 7028T 7220C 8701G 8860G 10583G 11719A 12705T 13437C 14153C 14560A 14766T 14769G 15326G 15929G 16193T 16223T 16519C	N21+195	73G 150T 195C 263G 16193T 16223T 16519C	1	73 150 195 263 750 1438 2706 4769 6752 7028 7220 8701 8860 10583 11719 12705 13437 14153 14560 14766 14769 15326 15929 16193 16223	N21+195	73 150 195 263 16193 16223	[0.033] N21+195 (1)	[0.200] N21a (2) [0.075] L3e2a3 (3) [0.030] L3e'i'k'x L3e L3i (6) [0.019] L3e3b1 (7) [0.006] L3e3'4'5 (8) [0.000] L3e2 L3e2a L3e2a2 L3e3'4 L3e3 L3e3b N21 (15)	1
184	>MG272754.1 H	73G 152C 263G 302.1C 567.1C 750G 1438G 2706G 3106d 3107C 3337A 4170T 4769G 5580C 5773A 7028T 7337A 8344G 8701G 8860G 9540C 10398G 10400T 10873C 10923T 11359G 11719A 12030G 12372A 12705T 14569A 14727C 14766T 14783C 15010G 15043A 15301A 15326G 15884T 16129A 16172C 16223T 16290T 16305G 16325C 16354T 16519C	M12b2	73G 152C 263G 302.1C 567.1C 16129A 16172C 16223T 16290T 16305G 16325C 16354T 16519C	1	73 152 263 573.1C 750 1438 2706 3337 4170 4769 5580 5773 7028 7337 8344 8701 8860 9540 10398 10400 10873 10923 11359 11719 12030 12372 12705 14569 14727 14766 14783 15010 15043 15301 15326 15884T 16129 16172 16223 16290 16305 16325 16354	M12b2	73 152 263 573.1C 16129 16172 16223 16290 16305 16325 16354	[0.101] M12b2 (1)	[0.000] M12b2a (2)	1

185	>MG272749.1 H	73G 152C 263G 329A 750G 1040C 1438G 2706G 2836T 3106d 3107C 3210T 3398C 4634C 4639C 4769G 6770G 7028T 8387A 8860G 9542G 10316G 10609C 11719A 12026G 13359A 14034C 14766T 15115C 15326G 16093C 16249C 16288C 16304C 16519C	R22	73G 152C 263G 329A 16093C 16249C 16288C 16304C 16519C	1	73 152 263 329 750 1040 1438 2706 2836 3210 3398 4634 4639 4769 6770 7028 8387 8860 9542 10316 10609 11719 12026 13359 14034 14766 15115 15326 16093 16249 16288 16304	R22	73 152 263 329 16093 16249 16288 16304 [0.005] R22 (1)	[0.048] R9 (2) [0.011] R9b (3) [0.010] R9c (4) [0.000] H32 R30a1 R30a1a (7)	1
186	>MG272745.1 H	73G 146C 263G 489C 750G 1438G 1878C 2706G 3106d 3107C 3912G 4071T 4769G 4850T 5378G 6455T 7028T 8701G 8860G 9449T 9540C 9824C 10398G 10400T 10873C 10897T 11665T 11719A 11932T 12091C 12705T 14053G 14766T 14783C 15043A 15301A 15326G 15338T 15924G 16172C 16223T 16274A 16291T 16311C 16519C	M7c2a	73G 146C 263G 489C 16172C 16223T 16274A 16291T 16311C 16519C	1	73 146 263 489 750 1438 1878 2706 3912 4071 4769 4850 5378 6455 7028 8701 8860 9449 9540 9824 10398 10400 10873 10897 11665 11719 11932 12091 12705 14053 14766 14783 15043 15301 15326 15338 15924 16172 16223 16274 16291 16311	M7c2a	73 146 263 489 16172 16223 16274 16291 16311 [0.100] M7c2a (1)	[0.000] M7c2 M7c2b (3)	1
187	>MG272727.1 H	73G 152C 200G 263G 310.1C 750G 1438G 2706G 3106d 3107C 3970T 4086T 4769G 5978G 5985A 6392C 6962A 7028T 8860G 9053A 9548A 10310A 10609C 11719A 12406A 12882T 13759A 13928C 14766T 15326G 15445C 16129A 16172C 16304C 16311C 16362C 16519C	F1a4a	73G 152C 200G 263G 310.1C 16129A 16172C 16304C 16311C 16362C 16519C	1	73 152 200 263 750 1438 2706 3970 4086 4769 5978 5985 6392 6962 7028 8860 9053 9548 10310 10609 11719 12406 12882 13759 13928C 14766 15326 15445 16129 16172 16304 16311 16362	F1a4a	73 152 200 263 16129 16172 16304 16311 16362 [0.807] F1a4a (1)	[0.054] F1a4a1 (2) [0.037] F1a3a (3) [0.032] F1a3a3a (4) [0.027] F1a3+16311 (5) [0.024] F1a3a3	1
188	>MG272725.1 H	73G 152C 210G 263G 310.1C 709A 750G 1438G 2706G 3106d 3107C 3537G 4086T 4769G 5366A 6392C 6960T 7028T 8584A 8860G 9950C 10398G 11465C 11719A 14766T 15235G 15326G 16140C 16182C 16183C 16189C 16234T 16261T 16266A 16519C	B5a1d	73G 152C 210G 263G 310.1C 16140C 16182C 16183C 16189C 16234T 16261T 16266A 16519C	1	73 152 210 263 709 750 1438 2706 3537 4086 4769 5366A 6392 6960 7028 8584 8860 9950 10398 11465 11719 14766 15235 15326 16140 16189 16234 16261 16266A	B5a1d	73 152 210 263 16140 16189 16234 16261 16266A [0.003] B5a1d (1)	[0.083] B5a1a (2) [0.032] B5a1 (3) [0.010] B5a (4) [0.006] B5a1b1 (5) [0.002] B5a1c1 B5a1c1a (7) [0.000] B5a1a1 B5a1b B5a1c B5a1c2 (11)	1
189	>MG272724.1 H	73G 263G 310.1C 318C 463T 489C 567.1C 750G 1438G 1719A 2706G 3106d 3107C 4170T 4769G 5580C 7028T 8155A 8701G 8860G 9540C 10398G 10400T 10865C 10873C 11719A 12030G 12358G 12372A 12705T 14569A 14727C 14766T 14783C 15010G 15043A 15244G 15301A 15326G 16223T 16234T 16261T 16290T	M12a2	73G 263G 310.1C 318C 463T 489C 567.1C 16223T 16234T 16261T 16290T	1	73 263 318 463 489 573.1C 750 1438 1719 2706 4170 4769 5580 7028 8155 8701 8860 9540 10398 10400 10865C 10873 11719 12030 12358 12372 12705 14569 14727 14766 14783 15010 15043 15244 15301 15326 16223 16234 16261 16290	M12a2	73 263 318 463 489 573.1C 16223 16234 16261 16290 [0.000] M12a2 (1)	[0.000] M12a (2)	1

190 >MG272723.1 H	73G 150T 199C 263G 750G 1438G 2706G 3106d 3107C 4048A 4071T 4164G 4742C 4769G 5351G 5460A 6455T 6680C 7028T 7684C 7853A 8701G 8860G 9540C 9824C 10398G 10400T 10837T 10873C 11719A 12397G 12405T 12705T 12811C 13053T 13395G 14766T 14783C 15043A 15244G 15301A 15326G 15734A 16129A 16223T 16297C 16319A	M7b1a1	73G 150T 199C 263G 16129A 16223T 16297C 16319A	1	73 150 199 263 750 1438 2706 4048 4071 4164 4742 4769 5351 5460 6455 6680 7028 7684 7853 8701 8860 9540 9824 10398 10400 10837 10873 11719 12397 12405 12705 12811 13053 13395 14766 14783 15043 15244 15301 15326 15734 16129 16223 16297 16319	M7b1a1	73 150 199 263 16129 16223 16297 16319	[0.019] M7b1a1 (1) [0.008] M7b1a1f (2) [0.004] M7b1a1+(16192) M7b1a1e1 (4) [0.002] M7b1a1d1 M7b1a1h (6) [0.000] M7b1a1d M7b1a1e M7b1a1g (9)	[0.027] M7b1a1a (10) [0.008] M7b1a1f (11) [0.007] M7b1a1a2 M7b1a1e2 (13) [0.004] M7b1a1+(16192) M7b1a1e1 (15) [0.002] M7b1a1d1 M7b1a1h (17) [0.000] M7b1a M7b1a1d M7b1a1e M7b1a1g M7b1a1i (22)	1
191 >MG272722.1 H	73G 152C 210G 263G 709A 750G 1438G 2706G 3106d 3107C 3537G 4086T 4769G 6960T 7028T 8584A 8860G 9950C 10398G 11465C 11719A 13928C 14766T 15235G 15326G 16129A 16140C 16182C 16183C 16189C 16261T 16266A 16519C	B5a1d	73G 152C 210G 263G 16129A 16140C 16182C 16183C 16189C 16261T 16266A 16519C	1	73 152 210 263 709 750 1438 2706 3537 4086 4769 6960 7028 8584 8860 9950 10398 11465 11719 13928C 14766 15235 15326 16129 16140 16189 16261 16266A	B5a1d	73 152 210 263 16129 16140 16189 16261 16266A	[0.400] B5a1d (1)	[0.047] B5a1a (2) [0.041] B5a1 (3) [0.010] B5a (4) [0.004] B5a1b1 B5a1c1a (6) [0.000] B5a1a1 B5a1b B5a1c B5a1c1 B5a1c2 (11)	1
192 >MG272719.1 H	73G 263G 310.1C 750G 1438G 1541C 2706G 3106d 3107C 3666A 3970T 4769G 5074C 6647G 7028T 8724G 8860G 11299C 11422T 11719A 13191C 13722G 13928C 14766T 15314A 15326G 15431A 15883A 16304C 16362C 16519C	R9b2	73G 263G 310.1C 16304C 16362C 16519C	1	73 263 750 1438 1541 2706 3666 3970 4769 5074 6647 7028 8724 8860 11299 11422 11719 13191 13722 13928C 14766 15314 15326 15431 15883 16304 16362	R9b2	73 263 16304 16362	[0.000] R9b2 (1)	[0.010] R9c (2) [0.000] R6 R9 R9b R31 (6)	1
193 >MG272717.1 H	73G 125G 143A 146C 234G 263G 489C 750G 1284C 1438G 2706G 3106d 3107C 4769G 7028T 7372C 8440G 8701G 8718G 8838A 8860G 9540C 9615C 10398G 10400T 10873C 11719A 12681C 12705T 13407G 13547T 14605G 14766T 14783C 15043A 15301A 15326G 15458C 16129A 16140C 16223T 16271C 16295T 16304C	M71a2	73G 125G 143A 146C 234G 263G 489C 16129A 16140C 16223T 16271C 16295T 16304C	1	73 125G 143 146 234 263 489 750 1284 1438 2706 4769 7028 7372 8440 8701 8718 8838 8860 9540 9615 10398 10400 10873 11719 12681 12705 13407 13547 14605 14766 14783 15043 15301 15326 15458 16129 16140 16223 16271 16295 16304	M71a2	73 125G 143 146 234 263 489 16129 16140 16223 16271 16295 16304	[0.221] M71a2 (1)	[0.200] M17c1a (2) [0.133] M5a3 (3) [0.071] M5d (4) [0.040] M5a (5) [0.031] M7c1a (6) [0.021] M7c1c (7) [0.012] M5 M7c1a1b (9) [0.009] M54 M7a2a M7a2a2 (12) [0.008] M14 (13) [0.007] G1b+16129 M27c (15) [0.006] G1b2 G1b4 (17) [0.004] M5b (18) [0.003] M7c1 M7c1b2b (20) [0.000] M5a'd M5a2 M5a2a M5a2a1 M5a3a M5a5 M5b'c M7b1 M7c M7c1a1 M7c1a1a M7c1a1a1 M7c1a4 M7c1b M7c1b1 M7c1b2 M7c3 M13b M17c1 M71 D2a'b D2a D2a1 D2a1a D2a2 (45)	1
194 >MG272715.1 H	73G 143A 183G 263G 567.1C 750G 1438G 1541C 2706G 3106d 3107C 3970T 4017T 4769G 6962A 7028T 7849T 8860G 11719A 12714C 13928C 14766T 15237C 15326G 16192T 16274A 16288C 16304C 16390A 16519C	R9b1a1a	73G 143A 183G 263G 567.1C 16192T 16274A 16288C 16304C 16390A 16519C	1	73 143 183 263 573.1C 750 1438 1541 2706 3970 4017 4769 6962 7028 7849 8860 11719 12714 13928C 14766 15237 15326 16192 16274 16288 16304 16390	R9b1a1a	73 143 183 263 573.1C 16192 16274 16288 16304 16390	[0.105] R9b1a1a (1)	[0.000] R9b1a1 (2)	1

195 >MG272712.1 H	73G 125G 143A 146C 263G 489C 750G 1284C 1438G 2706G 3106d 3107C 4769G 7028T 7372C 8440G 8701G 8718G 8838A 8860G 9540C 9615C 10398G 10400T 10873C 11719A 12681C 12705T 13407G 13547T 14605G 14766T 14783C 15043A 15301A 15326G 15458C 16129A 16140C 16223T 16271C 16295T 16304C	M71a2	73G 125G 143A 146C 263G 489C 16129A 16140C 16223T 16271C 16295T 16304C	1	73 125G 143 146 263 489 750 1284 1438 2706 4769 7028 7372 8440 8701 8718 8838 8860 9540 9615 10398 10400 10873 11719 12681 12705 13407 13547 14605 14766 14783 15043 15301 15326 15458 16129 16140 16223 16271 16295 16304	M71a2	73 125G 143 146 263 489 16129 16140 16223 16271 16295 16304	[0.214] M71a2 (1)	[0.200] M17c1a (2) [0.133] M5a3 (3) [0.071] M5d (4) [0.040] M5a (5) [0.029] M7c1a (6) [0.021] M7c1c (7) [0.012] M5 M7c1a1b (9) [0.009] M54 M7a2a M7a2a2 (12) [0.007] G1b+16129 M27c (14) [0.006] G1b2 G1b4 (16) [0.004] M5b (17) [0.003] M7c1 (18) [0.000] M5a'd M5a2 M5a2a M5a2a1 M5a3a M5a5 M5b'c M7b1 M7c M7c1a1 M7c1a1a M7c1a1a1 M7c1a4 M7c1b M7c1b1 M7c1b2 M7c1b2b M7c3 M13b M17c1 M71 D2a'b D2a D2a1 D2a1a D2a2 (44)	1
196 >MG272708.1 H	73G 263G 310.1C 318C 463T 489C 750G 1438G 1719A 2706G 2831A 3106d 3107C 4170T 4769G 5580C 7028T 7142C 8348G 8701G 8860G 9380A 9540C 10398G 10400T 10873C 11719A 12030G 12177G 12358G 12372A 12705T 14569A 14727C 14766T 14783C 15010G 15043A 15301A 15326G 16223T 16234T 16261T 16290T	M12a2	73G 263G 310.1C 318C 463T 489C 16223T 16234T 16261T 16290T	1	73 263 318 463 489 750 1438 1719 2706 2831 4170 4769 5580 7028 7142 8348 8701 8860 9380 9540 10398 10400 10873 11719 12030 12177 12358 12372 12705 14569 14727 14766 14783 15010 15043 15301 15326 16223 16234 16261 16290	M12a2	73 263 318 463 489 16223 16234 16261 16290	[0.107] M12a2 (1)	[0.000] M12a (2)	1
197 >MG272705.1 H	73G 152C 210G 263G 310.1C 709A 750G 1438G 2706G 3106d 3107C 3537G 4086T 4769G 6960T 7028T 8584A 8860G 9950C 10398G 11465C 11719A 13928C 14766T 15235G 15326G 16129A 16140C 16182C 16183C 16189C 16261T 16266A 16519C	B5a1d	73G 152C 210G 263G 310.1C 16129A 16140C 16182C 16183C 16189C 16261T 16266A 16519C	1	73 152 210 263 709 750 1438 2706 3537 4086 4769 6960 7028 8584 8860 9950 10398 11465 11719 13928C 14766 15235 15326 16129 16140 16189 16261 16266A	B5a1d	73 152 210 263 16129 16140 16189 16261 16266A	[0.400] B5a1d (1)	[0.047] B5a1a (2) [0.041] B5a1 (3) [0.010] B5a (4) [0.004] B5a1b1 B5a1c1a (6) [0.000] B5a1a1 B5a1b B5a1c B5a1c1 B5a1c2 (11)	1
198 >MG272688.1 H	73G 152C 263G 750G 1438G 2706G 3106d 3107C 3970T 4086T 4769G 6392C 6791G 6962A 7028T 8860G 9053A 9548A 10310A 10420G 10609C 10972G 11719A 12406A 12882T 12963T 13759A 13928C 14766T 15326G 16129A 16162G 16172C 16292T 16304C 16519C	F1a1	73G 152C 263G 16129A 16162G 16172C 16292T 16304C 16519C	1	73 152 263 750 1438 2706 3970 4086 4769 6392 6791 6962 7028 8860 9053 9548 10310 10420 10609 10972 11719 12406 12882 12963 13759 13928C 14766 15326 16129 16162 16172 16292 16304	F1a1	73 152 263 16129 16162 16172 16292 16304	[0.177] F1a1 (1)	[0.346] F1c1 (2) [0.192] F1a1d (3) [0.081] F1a1d1 (4) [0.079] F1a1c (5) [0.060] F1a1a1 (6) [0.057] F1a4a (7) [0.056] F1a1a (8) [0.027] F1a1b (9) [0.011] F1a1'4 (10) [0.008] F1a3 (11) [0.000] F1a F1a1c2 F1a1c3 F1a4 (15)	1
199 >MG272687.1 H	73G 263G 750G 1438G 2706G 3106d 3107C 3579G 3970T 4086T 4769G 6392C 6962A 7028T 8301G 8860G 9053A 9141C 9548A 10310A 10609C 11719A 12406A 12882T 13759A 13928C 14766T 15326G 16129A 16162G 16172C 16304C 16519C	F1a1	73G 263G 16129A 16162G 16172C 16304C 16519C	1	73 263 750 1438 2706 3579 3970 4086 4769 6392 6962 7028 8301 8860 9053 9141 9548 10310 10609 11719 12406 12882 13759 13928C 14766 15326 16129 16162 16172 16304	F1a1	73 263 16129 16162 16172 16304	[0.034] F1a1 (1)	[0.081] F1a1d1 (2) [0.043] F1a1d (3) [0.039] F1a1a (4) [0.031] F1a1a1 (5) [0.006] F1a1c (6) [0.005] F1a3 (7) [0.000] F1a F1a1'4 F1a1b F1a1c2 F1a1c3 F1a4 (13)	1

200 >MG272682.1 H	73G 263G 310.1C 489C 750G 1438G 2706G 3106d 3107C 3434G 3510T 3970T 4047C 4769G 5585A 5913A 5978G 6392C 7028T 8860G 9438A 10310A 10320A 11065G 11719A 12477C 12621T 13105G 13681G 13928C 14356T 14766T 15326G 16183C 16189C 16221T 16298C 16355T 16362C 16519C	F3a	73G 263G 310.1C 489C 16183C 16189C 16221T 16298C 16355T 16362C 16519C	1	73 263 489 750 1438 2706 3434 3510 3970 4047 4769 5585 5913 5978 6392 7028 8860 9438 10310 10320 11065 11719 12477 12621 13105 13681 13928C 14356 14766 15326 16189 16221 16298 16355 16362	F3a	73 263 489 16189 16221 16298 16355 16362	[0.295] F3a (1) [0.005] D+16189 (2) [0.000] D4b2a2a D4b2a2a2 (4)	[0.294] F3a+207 (5) [0.189] M76 (6) [0.118] R6 (7) [0.032] B4'5 (8) [0.025] R11'B6 (9) [0.024] M8 (10) [0.023] D4b2b2b (11) [0.017] R+16189 (12) [0.016] R31 (13) [0.013] G1c G1c2 (15) [0.011] D6 (16) [0.005] D4 (17) [0.004] D4b2b (18) [0.002] G G1 G2 G2b G2b1 G2b2 G2b2a D4e1 D4j1b (27) [0.001] D4b2b1 D4b2b1a D4e1c (30) [0.000] M9 G2a'c G2a1+16189 G2b1a M33+16362 D D4b D4b2 D4b2a D4b2a1 D4b2a2 D4b2a2a1 D4b2a2b D4b2b1b D4b2b1c D4b2b2 D4b2b2c D4b2b3 D4b2b6 D4c1 D4e D4e1'3 D4e3 D4e2 D4e2b D4e2c D4e2d D4e4 D4e4a D4e4b D4f D4f1 D4g D4h D4h4 D4j D4j1 D4j4 D4j5 D4j5a D4j+(16286) D4j9 D4j10 D4j12 D4j15 D4j16 D4m D4m1 D5 D5a'b D5a D6a1 D6a1a F3 (84)	1
201 >MG272680.1 H	73G 263G 302.1C 750G 1438G 1541C 2706G 3106d 3107C 3970T 4769G 7028T 8614C 8860G 11719A 12342T 12714C 13654T 13928C 14766T 15326G 16122G 16153A 16209C 16304C 16309G 16390A	R9b1	73G 263G 302.1C 16122G 16153A 16209C 16304C 16309G 16390A	1	73 263 750 1438 1541 2706 3970 4769 7028 8614 8860 11719 12342 12714 13654 13928C 14766 15326 16122 16153 16209 16304 16309 16390	R9b1	73 263 16122 16153 16209 16304 16309 16390	[0.155] R9b1 (1)	[0.628] R9b1a3 (2) [0.058] R9b1a2b (3) [0.044] R9b1a2 (4) [0.011] R30a1b (5) [0.000] R9 R9b R9b1a R9b1b R9c (10)	1
202 >MG272679.1 H	73G 152C 263G 310.1C 329A 750G 1040C 1438G 2706G 2836T 3106d 3107C 3210T 4634C 4639C 4769G 6770G 7028T 8387A 8860G 9300A 9360T 10316G 10609C 11419C 11719A 12026G 12810G 13302T 13359A 14766T 15326G 16249C 16288C 16304C 16519C	R22	73G 152C 263G 310.1C 329A 16249C 16288C 16304C 16519C	1	73 152 263 329 750 1040 1438 2706 2836 3210 4634 4639 4769 6770 7028 8387 8860 9300 9360 10316 10609 11419 11719 12026 12810 13302T 13359 14766 15326 16249 16288 16304	R22	73 152 263 329 16249 16288 16304	[0.000] R22 (1)	[0.048] R9 (2) [0.005] R9c (3) [0.000] H32 R9b R30a1 R30a1a (7)	1
203 >MG272676.1 H	73G 204C 207A 263G 466C 489C 750G 1438G 2706G 3106d 3107C 4688G 4769G 5054A 6293C 6620C 6962A 7028T 7226A 7642A 8297T 8467T 8701G 8860G 9540C 10398G 10400T 10873C 11719A 12705T 13802T 14766T 14783C 15043A 15301A 15326G 15663C 16129A 16153A 16223T 16263C 16309G 16526A	M50a1	73G 204C 207A 263G 466C 489C 16129A 16153A 16223T 16263C 16309G 16526A	1	73 204 207 263 466 489 750 1438 2706 4688G 4769 5054 6293 6620 6962 7028 7226 7642 8297 8467 8701 8860 9540 10398 10400 10873 11719 12705 13802 14766 14783 15043 15301 15326 15663 16129 16153 16223 16263 16309 16526	M50a1	73 204 207 263 466 489 16129 16153 16223 16263 16309 16526	[0.000] M50a1 (1)	[0.000] M50a M50a2 (3)	1

204	>MG272666.1 H	73G 146C 263G 489C 750G 1438G 2706G 3106d 3107C 3753C 4769G 7028T 7521A 7624C 8701G 8860G 9540C 10325A 10398G 10400T 10873C 11719A 12361G 12406A 12705T 12738C 13135A 13281C 14341T 14766T 14783C 15043A 15301A 15326G 16093C 16124C 16183C 16189C 16278T 16293C 16362C 16366T	M76	73G 146C 263G 489C 16093C 16124C 16183C 16189C 16278T 16293C 16362C 16366T	1	73 146 263 489 750 1438 2706 3753 4769 7028 7521 7624 8701 8860 9540 10325 10398 10400 10873 11719 12361 12406 12705 12738 13135 13281 14341 14766 14783 15043 15301 15326 16093 16124 16189 16278 16293C 16362 16366	M76	73 146 263 489 16093 16124 16189 16278 16293C 16362 16366	[0.850] M76 (1) [0.048] G2a1+16189 (2)	[0.586] L3b1a1a (3) [0.542] M76a (4) [0.182] G2a (5) [0.113] G2a1d (6) [0.085] M45 (7) [0.064] G2a1b (8) [0.048] G2a1+16189 (9) [0.040] L3b1a1 (10) [0.038] L3b (11) [0.036] L3b2a (12) [0.030] G2a1 (13) [0.015] G2a1+16189+16194 (14) [0.014] L3b1a10 (15) [0.010] D+16189 (16) [0.008] L3b1a5a (17) [0.007] L3b1a2 (18) [0.006] L3b1a (19) [0.004] D4b2a2a D4j6 (21) [0.002] D4j+146 (22) [0.000] L3b1 L3b1a4 L3b1a5 G2a1d1 G2a1d1a G2a1g D4b2a2a2 D4b2b1+146 D4b2b1d (31)	1
205	>MG272659.1 H	73G 152C 210G 263G 489C 709A 750G 1438G 1598A 1763G 2080C 2706G 3106d 3107C 3663G 3819T 3915A 4769G 5108C 6231T 7028T 7861C 8509T 8701G 8860G 9540C 10398G 10400T 10873C 11482C 11719A 12705T 14766T 14783C 15043A 15236G 15301A 15326G 16145A 16181G 16192T 16223T 16291T 16304C 16519C	M21b2	73G 152C 210G 263G 489C 16145A 16181G 16192T 16223T 16291T 16304C 16519C	1	73 152 210 263 489 709 750 1438 1598 1763 2080 2706 3663 3819 3915 4769 5108 6231 7028 7861 8509 8701 8860 9540 10398 10400 10873 11482 11719 12705 14766 14783 15043 15236 15301 15326 16145 16181 16192 16223 16291 16304	M21b2	73 152 210 263 489 16145 16181 16192 16223 16291 16304	[1.838] M21b2 (1)	[0.118] M54 (2) [0.054] M7c1a1b1 (3) [0.022] D4j2a (4) [0.004] M37+152 (5) [0.000] M13'46'61 M13 M13a'b M21b+210 D4j2 (10)	1
206	>MG272653.1 H	73G 93G 200G 263G 489C 750G 999T 1438G 2706G 3106d 3107C 3447G 3453T 4113A 4164G 4769G 5999C 7028T 7064C 8701G 8860G 9540C 10398G 10400T 10873C 11719A 11887A 12705T 13194A 14766T 14783C 14902T 15043A 15301A 15326G 15622C 15652G 16129A 16223T 16287T 16311C 16327A 16519C	M91a	73G 93G 200G 263G 489C 16129A 16223T 16287T 16311C 16327A 16519C	1	73 93 200 263 489 750 999 1438 2706 3447 3453 4113 4164 4769 5999 7028 7064 8701 8860 9540 10398 10400 10873 11719 11887 12705 13194 14766 14783 14902 15043 15301 15326 15622 15652 16129 16223 16287 16311 16327A	M91a	73 93 200 263 489 16129 16223 16287 16311 16327A	[0.384] M91a (1)	[0.000] M91 (2)	1
207	>MG272642.1 H	64T 73G 93G 200G 263G 485C 489C 750G 1438G 2706G 3106d 3107C 3447G 4164G 4769G 5999C 7028T 7064C 8701G 8860G 9509C 9540C 9554A 9770C 10398G 10400T 10873C 11719A 11887A 12705T 13101G 13194A 14325C 14766T 14783C 14902T 15043A 15301A 15326G 15622C 15652G 16129A 16223T 16287T 16311C 16327A	M91a	64T 73G 93G 200G 263G 485C 489C 16129A 16223T 16287T 16311C 16327A	1	64 73 93 200 263 485 489 750 1438 2706 3447 4164 4769 5999 7028 7064 8701 8860 9509 9540 9554 9770 10398 10400 10873 11719 11887 12705 13101 13194 14325 14766 14783 14902 15043 15301 15326 15622 15652 16129 16223 16287 16311 16327A	M91a	64 73 93 200 263 485 489 16129 16223 16287 16311 16327A	[0.623] M91a (1)	[0.000] M91 (2)	1

208 >MG272639.1 H	73G 94A 214G 263G 489C 750G 1438G 2706G 3010A 3106d 3107C 3316A 4769G 4883T 5178A 5964C 7028T 8414T 8701G 8860G 9536T 9540C 10398G 10400T 10873C 10915C 11215T 11719A 12705T 12879C 14470C 14668T 14766T 14783C 15043A 15301A 15326G 15924G 16092C 16223T 16311C 16362C	D4e1a2	73G 94A 214G 263G 489C 16092C 16223T 16311C 16362C	1	73 94 214 263 489 750 1438 2706 3010 3316 4769 4883 5178A 5964 7028 8414 8701 8860 9536 9540 10398 10400 10873 10915 11215 11719 12705 12879 14470 14668 14766 14783 15043 15301 15326 15924 16092 16223 16311 16362	D4e1a2	73 94 214 263 489 16092 16223 16311 16362	[0.048] D4e1a2 (1)	[0.048] D4e1a2 (2) [0.015] D4e1a (3) [0.002] D4e1a2a (4) [0.000] D4e1a3	1
209 >MG272632.1 H	73G 152C 210G 263G 709A 750G 1438G 2706G 3106d 3107C 3537G 4086T 4769G 6960T 7028T 8584A 8860G 9950C 10398G 11465C 11719A 14766T 15235G 15326G 16140C 16182C 16183C 16189C 16261T 16266A 16304C 16519C	B5a1d	73G 152C 210G 263G 16140C 16182C 16183C 16189C 16261T 16266A 16304C 16519C	1	73 152 210 263 709 750 1438 2706 3537 4086 4769 6960 7028 8584 8860 9950 10398 11465 11719 14766 15235 15326 16140 16189 16261 16266A 16304	B5a1d	73 152 210 263 16140 16189 16261 16266A 16304	[0.021] B5a1d (1)	[0.032] B5a1 (2) [0.017] B5a1a (3) [0.012] B5a (4) [0.009] B5a1a1 B5a1c1 B5a1c2 (7) [0.008] B5a1b (8) [0.004] B5a1b1 (9) [0.002] B5a1c1a (10) [0.000] B5a1c	1
210 >MG272626.1 H	73G 146C 263G 489C 750G 1438G 2706G 3106d 3107C 3912G 4071T 4769G 4850T 5378G 6455T 7028T 8701G 8860G 9449T 9540C 9824C 10398G 10400T 10873C 10897T 11665T 11719A 11932T 12091C 12705T 14053G 14766T 14783C 15043A 15301A 15326G 15338T 16172C 16223T 16291T 16311C 16519C	M7c2a	73G 146C 263G 489C 16172C 16223T 16291T 16311C 16519C	1	73 146 263 489 750 1438 2706 3912 4071 4769 4850 5378 6455 7028 8701 8860 9449 9540 9824 10398 10400 10873 10897 11665 11719 11932 12091 12705 14053 14766 14783 15043 15301 15326 15338 16172 16223 16291 16311	M7c2a	73 146 263 489 16172 16223 16291 16311	[0.000] M7c2a (1)	[0.000] M7c2 M7c2b (3)	1
211 >MG272624.1 H	73G 152C 195C 238G 263G 750G 1438G 2706G 3106d 3107C 3442T 3588T 3669G 3972G 4769G 5471C 7028T 8437T 8701G 8860G 9540C 10398G 10400T 10873C 11719A 12705T 14766T 14783C 15043A 15301A 15326G 16086C 16223T 16243C 16262T 16278T 16311C 16319A 16519C	M79	73G 152C 195C 238G 263G 16086C 16223T 16243C 16262T 16278T 16311C 16319A 16519C	1	73 152 195 238 263 750 1438 2706 3442 3588 3669 3972 4769 5471C 7028 8437 8701 8860 9540 10398 10400 10873 11719 12705 14766 14783 15043 15301 15326 16086 16223 16243 16262 16278 16311 16319	M79	73 152 195 238 263 16086 16223 16243 16262 16278 16311 16319	[2.429] M79 (1) [0.057] L3d3a1a L3d3a1b (3) [0.025] L3d3a1 (4) [0.000] L3d3a (5)	[0.209] L3a1a (6) [0.069] L2'3'4'6 L3'4'6 (8) [0.057] L3d3a1a L3d3a1b (10) [0.048] M57+152 (11) [0.032] L4 (12) [0.030] L3d1a1 (13) [0.028] L3d1a1b (14) [0.025] L3d3a1 (15) [0.022] L3c'd L3h1 L3h1a (18) [0.020] L4b (19) [0.018] X2d (20) [0.014] L3d1a2 (21) [0.011] S+152 (22) [0.010] X2e2a2 (23) [0.009] X2d2 (24) [0.008] M30b (25) [0.007] L3c (26) [0.005] M24a (27) [0.000] L3'4 L3f2a L3f2a1 L3d1a1'2 L3d3a L3h1a2 M8a1a (34)	1
212 >MG272621.1 H	73G 210G 263G 709A 750G 1438G 1598A 1763G 2080C 2706G 3106d 3107C 3254T 3663G 3819T 3915A 4769G 5096C 5108C 6231T 7028T 7861C 8509T 8701G 8860G 9540C 10398G 10400T 10873C 11482C 11719A 12705T 14569A 14766T 14783C 15043A 15236G 15301A 15326G 16145A 16181G 16192T 16223T 16278T 16291T 16304C 16519C	M21b2	73G 210G 263G 16145A 16181G 16192T 16223T 16278T 16291T 16304C 16519C	1	73 210 263 709 750 1438 1598 1763 2080 2706 3254 3663 3819 3915 4769 5096 5108 6231 7028 7861 8509 8701 8860 9540 10398 10400 10873 11482 11719 12705 14569 14766 14783 15043 15236 15301 15326 16145 16181 16192 16223 16278 16291 16304	M21b2	73 210 263 16145 16181 16192 16223 16278 16291 16304	[1.784] M21b2 (1)	[0.009] L3b1a8 (2)	1

213	>MG272620.1 H	73G 263G 318C 463T 489C 750G 1438G 1719A 2706G 3106d 3107C 4170T 4769G 5046A 5580C 7028T 8701G 8860G 9540C 10398G 10400T 10873C 11719A 12030G 12358G 12372A 12705T 14470C 14569A 14727C 14766T 14783C 15010G 15043A 15301A 15326G 16223T 16234T 16261T 16290T	M12a2	73G 263G 318C 463T 489C 16223T 16234T 16261T 16290T	1	73 263 318 463 489 750 1438 1719 2706 4170 4769 5046 5580 7028 8701 8860 9540 10398 10400 10873 11719 12030 12358 12372 12705 14470 14569 14727 14766 14783 15010 15043 15301 15326 16223 16234 16261 16290	M12a2	73 263 318 463 489 16223 16234 16261 16290	[0.107] M12a2 (1)	[0.000] M12a (2)	1
214	>MG272617.1 H	73G 150T 263G 273T 489C 732G 750G 1438G 2045G 2706G 3105d 3106G 3107C 4697T 4769G 4973C 7028T 8701G 8860G 9509C 9540C 10398G 10400T 10873C 11719A 12236A 12705T 12715G 14110C 14356T 14687G 14766T 14783C 15043A 15301A 15317A 15326G 16129A 16223T 16278T 16294T 16304C	M51a1	73G 150T 263G 273T 489C 16129A 16223T 16278T 16294T 16304C	1	73 150 263 273 489 732 750 1438 2045 2706 3105 4697 4769 4973 7028 8701 8860 9509 9540 10398 10400 10873 11719 12236 12705 12715 14110 14356 14687 14766 14783 15043 15301 15317 15326 16129 16223 16278 16294 16304	M51a1	73 150 263 273 489 16129 16223 16278 16294 16304	[0.208] M51a1 (1) [0.067] M51a1a (2)	[0.090] M51a1b (3) [0.000] M51 M51a M51b (6)	1
215	>MG272615.1 H	73G 194T 263G 310.1C 750G 1438G 1811G 2706G 3106d 3107C 3316A 4769G 4970G 5201C 7028T 7258C 7382A 7853A 7859A 8860G 9053A 9770C 10373A 11151T 11299C 11467G 11719A 12308G 12372A 12477C 12561A 14766T 14883T 15326G 15734A 16051G 16206C 16271C 16328T	U2a2	73G 194T 263G 310.1C 16051G 16206C 16271C 16328T	1	73 194 263 750 1438 1811 2706 3316 4769 4970 5201 7028 7258 7382 7853 7859 8860 9053 9770 10373 11151 11299 11467 11719 12308 12372 12477 12561 14766 14883 15326 15734 16051 16206C 16271 16328	U2a2	73 194 263 16051 16206C 16271 16328	[0.037] U2a2 (1)	[0.000] U2a (2)	1
216	>MG272612.1 H	73G 146C 152C 199C 249G 263G 750G 1438G 1709A 1719A 2397T 2706G 3106d 3107C 4711T 4769G 7028T 7942T 8279C 8860G 9067G 9077C 9109G 10398G 10610T 11404G 11719A 12234G 12510T 13145A 14766T 15326G 15613G 16168T 16295T 16296T 16304C	R21	73G 146C 152C 199C 249G 263G 16168T 16295T 16296T 16304C	1	73 146 152 199 249 263 750 1438 1709 1719 2397 2706 4711 4769 7028 7942 8279 8860 9067 9077 9109 10398 10610T 11404 11719 12234 12510 13145 14766 15326 15613 16168 16295 16296 16304	R21	73 146 152 199 249 263 16168 16295 16296 16304	[0.000] R21 (1)	[0.231] M7c1a (2) [0.150] K2a1a (3) [0.089] T2b4+152 (4) [0.083] U2b1a (5) [0.057] M7c1b2b U2b1 (7) [0.048] R9 (8) [0.040] R2b1 (9) [0.032] M7c1a1b (10) [0.029] T2b+152 (11) [0.021] M7c1c (12) [0.016] T2b4f R9b (14) [0.012] M7c1 (15) [0.010] R9c (16) [0.000] M7c1a1 M7c1a1a M7c1a1a1 M7c1a4 M7c1b M7c1b1 M7c1b2 M7c1c3a M7c1c3a1 H9a H32 H103 T2b4c T2b4d T2b4e T2b4g T2b21 T2b21b T2b22 R12'21 R12 R30a1 R30a1a (39)	1

217 >MG272607.1 H	73G 263G 489C 750G 1438G 2263A 2352C 2706G 3106d 3107C 3396C 3399T 3533T 4769G 4856C 5655C 6902G 7028T 8701G 8860G 9540C 10398G 10400T 10586A 10685A 10873C 11476T 11719A 12705T 12950G 14034C 14305A 14766T 14783C 15043A 15301A 15326G 16093C 16148T 16172C 16184A 16223T 16243C 16278T 16519C	M73a1	73G 263G 489C 16093C 16148T 16172C 16184A 16223T 16243C 16278T 16519C	1	73 263 489 750 1438 2263A 2352 2706 3396 3399T 3533 4769 4856 5655 6902 7028 8701 8860 9540 10398 10400 10586 10685 10873 11476 11719 12705 12950 14034 14305 14766 14783 15043 15301 15326 16093 16148 16172 16184A 16223 16243 16278	M73a1	73 263 489 16093 16148 16172 16184A 16223 16243 16278	[0.059] M73a1 (1)	[0.000] M73a (2)	1
218 >MG272603.1 H	41T 73G 204C 207A 263G 302.1C 709A 750G 1438G 2706G 3106d 3107C 3434G 3970T 4769G 5585A 5894G 5913A 5978G 6392C 7028T 8860G 9854C 10310A 10320A 10499G 11053G 11065G 11719A 12621T 13928C 14766T 14971C 15326G 15943C 16260T 16298C 16355T 16362C	F3a1	41T 73G 204C 207A 263G 302.1C 16260T 16298C 16355T 16362C	1	41 73 204 207 263 709 750 1438 2706 3434 3970 4769 5585 5894 5913 5978 6392 7028 8860 9854 10310 10320 10499 11053 11065 11719 12621 13928C 14766 14971 15326 15943 16260 16298 16355 16362	F3a1	41 73 204 207 263 16260 16298 16355 16362	[0.163] F3a1 (1)	[0.059] F3a+207 (2)	1
219 >MG272602.1 H	56.1T 73G 263G 489C 750G 1438G 2263A 2352C 2706G 3106d 3107C 3396C 3399T 3533T 4769G 4856C 5655C 7028T 8701G 8860G 9540C 10398G 10400T 10685A 10873C 11476T 11569C 11719A 12705T 12950G 14034C 14766T 14783C 15043A 15301A 15326G 16093C 16184A 16223T 16278T 16519C	M73a1	56.1T 73G 263G 489C 16093C 16184A 16223T 16278T 16519C	1	60.1T 73 263 489 750 1438 2263A 2352 2706 3396 3399T 3533 4769 4856 5655 7028 8701 8860 9540 10398 10400 10685 10873 11476 11569 11719 12705 12950 14034 14766 14783 15043 15301 15326 16093 16184A 16223 16278	M73a1	60.1T 73 263 489 16093 16184A 16223 16278	[0.011] M73a1 (1)	[0.000] M73a (2)	1
220 >MG272600.1 H	73G 143A 183G 263G 750G 1438G 1541C 2706G 3106d 3107C 3970T 4017T 4769G 7028T 7849T 8860G 9055A 11719A 12714C 13928C 14766T 15326G 16192T 16288C 16304C 16309G 16390A 16519C	R9b1a1a	73G 143A 183G 263G 16192T 16288C 16304C 16309G 16390A 16519C	1	73 143 183 263 750 1438 1541 2706 3970 4017 4769 7028 7849 8860 9055 11719 12714 13928C 14766 15326 16192 16288 16304 16309 16390	R9b1a1a	73 143 183 263 16192 16288 16304 16309 16390	[0.000] R9b1a1a (1)	[0.000] R9b1a1 (2)	1
221 >MG272598.1 H	73G 146A 199C 263G 750G 1438G 2706G 3106d 3107C 3606G 4071T 4769G 4850T 5442C 6455T 7028T 8701G 8860G 9540C 9824C 10398G 10400T 10873C 11665T 11719A 12091C 12705T 14766T 14783C 15043A 15236G 15301A 15326G 16181G 16519C	M7c1c2	73G 146A 199C 263G 16181G 16519C	1	73 146A 199 263 750 1438 2706 3606 4071 4769 4850 5442 6455 7028 8701 8860 9540 9824 10398 10400 10873 11665 11719 12091 12705 14766 14783 15043 15236 15301 15326 16181	M7c1c2	73 146A 199 263 16181	[0.073] M7c1c2 (1)	[0.007] R (2) [0.000] M7c1c2a HV+73 HV20 H1e2c H3+73 H3d H4a1a1a H4a1a1a1 H4a1a1a1a H4a1a1a1a1 H4a1a1a2 H4a1a1a4 R2'JT R30 R30a R30a1 R30a1a R31 P P2 P4 P9 P9a U U2'3'4'7'8'9 U8 U8b'c U8b (30)	1
222 >MG272597.1 H	73G 150T 195C 263G 310.1C 750G 1438G 2706G 3106d 3107C 3970T 4435G 4649T 4769G 5587C 6392C 6962A 7028T 8860G 10310A 10609C 11719A 12406A 12882T 13928C 14766T 15024A 15326G 16183C 16189C 16300G 16304C 16519C	F1e3	73G 150T 195C 263G 310.1C 16183C 16189C 16300G 16304C 16519C	1	73 150 195 263 750 1438 2706 3970 4435 4649 4769 5587 6392 6962 7028 8860 10310 10609 11719 12406 12882 13928C 14766 15024 15326 16189 16300 16304	F1e3	73 150 195 263 16189 16300 16304	[0.743] F1e3 (1)	[0.012] B4c1c (2) [0.000] B6	1

223	>MG272596.1 H[illegible]	73G 185A 189G 263G 302.1C 722T 1438G 2706G 3106d 3107C 3543T 3873G 4562G 4769G 5581G 6314T 7028T 7853A 8860G 9899C 10373A 11719A 12136C 12705T 13434G 13500C 13928C 14022G 14766T 14953T 15211T 15326G 16172C 16183C 16189C 16223T 16362C 16519C	N10a	73G 185A 189G 263G 302.1C 16172C 16183C 16189C 16223T 16362C 16519C	1	73 185 189 263 722 1438 2706 3543 3873 4562 4769 5581 6314 7028 7853 8860 9899 10373 11719 12136 12705 13434 13500 13928C 14022 14766 14953 15211 15326 16172 16189 16223 16362	N10a	73 185 189 263 16172 16189 16223 16362 [0.000] N10a (1)	[0.071] N10 (2)	1
224	>MG272594.1 H[illegible]	73G 185A 189G 263G 722T 1438G 2706G 3106d 3107C 3543T 3873G 4562G 4769G 5581G 6314T 7028T 7853A 8860G 9899C 10373A 11719A 12136C 12705T 13434G 13500C 13928C 14022G 14766T 14953T 15211T 15326G 16172C 16183C 16189C 16223T 16362C 16519C	N10a	73G 185A 189G 263G 16172C 16183C 16189C 16223T 16362C 16519C	1	73 185 189 263 722 1438 2706 3543 3873 4562 4769 5581 6314 7028 7853 8860 9899 10373 11719 12136 12705 13434 13500 13928C 14022 14766 14953 15211 15326 16172 16189 16223 16362	N10a	73 185 189 263 16172 16189 16223 16362 [0.000] N10a (1)	[0.071] N10 (2)	1
225	>MG272593.1 H[illegible]	73G 185A 189G 263G 722T 1438G 2706G 3106d 3107C 3543T 3873G 4562G 4769G 5581G 6314T 7028T 7853A 8860G 9899C 10373A 11719A 12136C 12705T 13434G 13500C 13928C 14022G 14766T 14953T 15211T 15326G 16172C 16183C 16189C 16223T 16362C 16519C	N10a	73G 185A 189G 263G 16172C 16183C 16189C 16223T 16362C 16519C	1	73 185 189 263 722 1438 2706 3543 3873 4562 4769 5581 6314 7028 7853 8860 9899 10373 11719 12136 12705 13434 13500 13928C 14022 14766 14953 15211 15326 16172 16189 16223 16362	N10a	73 185 189 263 16172 16189 16223 16362 [0.000] N10a (1)	[0.071] N10 (2)	1
226	>MG272592.1 H[illegible]	73G 185A 189G 263G 722T 1438G 2706G 3106d 3107C 3543T 3873G 4562G 4769G 5581G 6314T 7028T 7853A 8860G 9899C 10373A 11719A 12136C 12705T 13434G 13500C 13928C 14022G 14766T 14953T 15211T 15326G 16172C 16183C 16189C 16223T 16362C 16519C	N10a	73G 185A 189G 263G 16172C 16183C 16189C 16223T 16362C 16519C	1	73 185 189 263 722 1438 2706 3543 3873 4562 4769 5581 6314 7028 7853 8860 9899 10373 11719 12136 12705 13434 13500 13928C 14022 14766 14953 15211 15326 16172 16189 16223 16362	N10a	73 185 189 263 16172 16189 16223 16362 [0.000] N10a (1)	[0.071] N10 (2)	1
227	>MG272590.1 H[illegible]	73G 185A 189G 263G 750G 1438G 2706G 3106d 3107C 3897T 4170T 4769G 5580C 7028T 8701G 8860G 9540C 10398G 10400T 10873C 11359G 11719A 12030G 12372A 12705T 14040A 14569A 14727C 14766T 14783C 14788C 14869A 15010G 15043A 15301A 15326G 16108T 16172C 16223T 16234T 16290T 16428A	M12b1b	73G 185A 189G 263G 16108T 16172C 16223T 16234T 16290T 16428A	1	73 185 189 263 750 1438 2706 3897 4170 4769 5580 7028 8701 8860 9540 10398 10400 10873 11359 11719 12030 12372 12705 14040 14569 14727 14766 14783 14788 14869 15010 15043 15301 15326 16108 16172 16223 16234 16290 16428	M12b1b	73 185 189 263 16108 16172 16223 16234 16290 16428 [0.061] M12b1b (1)	[0.062] R8a2 (2) [0.000] M12 M12b M12b1 M12b1a M12b1a1 N2 N10a (9)	1

228 >MG272582.1 H	63C 64.1T 66A 73G 215G 263G 489C 750G 1438G 2706G 3106d 3107C 4769G 5054A 6185C 6575G 7028T 8251A 8701G 8860G 9540C 10268T 10398G 10400T 10873C 11719A 12705T 12850G 14311C 14766T 14783C 15043A 15301A 15326G 16093C 16223T 16311C 16362C 16381C	M74a	63C 64.1T 66A 73G 215G 263G 489C 16093C 16223T 16311C 16362C 16381C	1	63 66T 66.1A 73 215 263 489 750 1438 2706 4769 5054 6185 6575 7028 8251 8701 8860 9540 10268 10398 10400 10873 11719 12705 12850 14311 14766 14783 15043 15301 15326 16093 16223 16311 16362 16381	M74a	63 66T 66.1A 73 215 263 489 16093 16223 16311 16362 16381	[0.439] M74a (1) [0.048] M43a1 (2) [0.029] D4j+16311 (3) [0.000] M74 M74b M74b2 D4j11 (7)	[0.088] M61 (8) [0.056] M43+16311 (9) [0.051] G2a1h (10) [0.033] G2a'c (11) [0.030] D4h4a (12) [0.028] D4j3a (13) [0.026] D4j3 (14) [0.025] D4b2b (15) [0.013] D4 (16) [0.012] M43a G1b D4e2 (19) [0.011] G1c (20) [0.009] M81 (21) [0.007] D4j1b (22) [0.005] G1 G1c2 D4h4 (25) [0.002] G2b2 D4e2c (27) [0.001] D4b2 D4b2a2 D4b2b1a D4b2b6 (31) [0.000] M4"67+16311 M9 G G2 G2b G2b1 G2b1a G2b2a M33+16362 M57 D D1d2 D4b D4b2a D4b2a2b D4b2b1 D4b2b1b D4b2b1c D4b2b2 D4b2b3 D4e D4e1'3 D4e1 D4e1c D4e3 D4e2b D4e2d D4e4 D4e4a D4e4b D4f D4f1 D4g D4h D4j D4j1 D4j4 D4j5 D4j5a D4j+(16286) D4j9 D4j10 D4j12 D4j15 D4j16 D4m D6 (78)	1
229 >MG272580.1 H	73G 150T 159C 199C 263G 489C 750G 1438G 2706G 3106d 3107C 4048A 4071T 4164G 4769G 5351G 5460A 6455T 6680C 7028T 7684C 7853A 8701G 8860G 9540C 9824C 10398G 10400T 10873C 11719A 12405T 12705T 12811C 14766T 14783C 15043A 15301A 15326G 16129A 16223T 16294G 16297C	M7b1a1	73G 150T 159C 199C 263G 489C 16129A 16223T 16294G 16297C	1	73 150 159 199 263 489 750 1438 2706 4048 4071 4164 4769 5351 5460 6455 6680 7028 7684 7853 8701 8860 9540 9824 10398 10400 10873 11719 12405 12705 12811 14766 14783 15043 15301 15326 16129 16223 16294G 16297	M7b1a1	73 150 159 199 263 489 16129 16223 16294G 16297	[0.236] M7b1a1 (1) [0.033] M7b1a1c (2) [0.002] M7b1a1f (3) [0.000] M7b1a1+(16192) M7b1a1d M7b1a1d1 M7b1a1e M7b1a1e1 M7b1a1g M7b1a1h (10)	[0.033] M7b1a1c (11) [0.007] M7b1a1e2 (12) [0.003] M7b1a1a2 (13) [0.002] M7b1a1f (14) [0.000] M7b1a M7b1a1a M7b1a1+(16192) M7b1a1d M7b1a1d1 M7b1a1e M7b1a1e1 M7b1a1g M7b1a1h M7b1a1i (24)	1
230 >MG736653.1 H	73G 150T 263G 310.1C 750G 1438G 1721T 2706G 3106d 3107C 3197C 4769G 7028T 7768G 8860G 8975C 9477A 11467G 11653G 11719A 12308G 12372A 12634G 13161C 13617C 13630G 13637G 14182C 14766T 15326G 15538T 16187T 16260T 16270T 16296T	U5b2b5	73G 150T 263G 310.1C 16187T 16260T 16270T 16296T	1	73 150 263 750 1438 1721 2706 3197 4769 7028 7768 8860 8975 9477 11467 11653 11719 12308 12372 12634 13161 13617 13630 13637 14182 14766 15326 15538 16187 16260 16270 16296	U5b2b5	73 150 263 16187 16260 16270 16296	[0.046] U5b2b5 (1)	[0.023] U5b2b (2) [0.000] U5b1d1a U5b2b1 U5b2b2 (5)	1
231 >MK041084.1 H	73G 195C 204C 207A 263G 309.1CT 310C 470G 499A 750G 827G 1438G 2706G 3106d 3107C 3547G 4769G 4820A 4977C 6272G 6473T 7028T 8271d 8272d 8273d 8274d 8275d 8276d 8277d 8278d 8279d 8860G 9950C 11177T 11611A 11719A 13590A 14766T 15077A 15326G 15535T 15778T 16183C 16189C 16217C 16519C	B2i2b	73G 195C 204C 207A 263G 309.1CT 310C 470G 499A 16183C 16189C 16217C 16519C	1	73 195 204 207 263 470 499 750 827 1438 2706 3547 4769 4820 4977 6272 6473 7028 8281-8289d 8860 9950 11177 11611 11719 13590 14766 15077 15326 15535 15778 16189 16217	B2i2b	73 195 204 207 263 470 499 16189 16217	[0.056] B2i2b (1)	[0.000] B2i2 (2)	1

232	>MK049277.1 H	73G 199C 204C 250C 263G 309.1CT 310C 513.1CA 567.1CC 750G 1438G 1719A 2044G 2706G 3106d 3107C 4529T 4769G 5471A 7028T 8251A 8269A 8860G 10034C 10238C 10398G 11719A 11914A 12501A 12705T 13780G 14233G 14766T 15043A 15119A 15326G 15924G 16129A 16169T 16223T 16391A 16519C	I5c	73G 199C 204C 250C 263G 309.1CT 310C 513.1CA 567.1CC 16129A 16169T 16223T 16391A 16519C	1	73 199 204 250 263 573.2C 750 1438 1719 2044 2706 4529T 4769 5471 7028 8251 8269 8860 10034 10238 10398 11719 11914 12501 12705 13780 14233 14766 15043 15119 15326 15924 16129 16169 16223 16391	I5c	73 199 204 250 263 573.2C 16129 16169 16223 16391	[0.000] I5c (1)	[0.000] I I4 I4a I4a2 I4b I5 I5b I6 I6a (10)	1
233	>MK036913.1 H	72C 263G 309.1CCT 310C 750G 1438G 2706G 3106d 3107C 3310T 4580A 4769G 6570T 7028T 8860G 9368G 11914A 15326G 15904T 15928A 16261T 16298C 16311C 16519C	V10a	72C 263G 309.1CCT 310C 16261T 16298C 16311C 16519C	1	72 263 750 1438 2706 3310 4580 4769 6570T 7028 8860 9368 11914 15326 15904 15928 16261 16298 16311	V10a	72 263 16261 16298 16311	[0.000] V10a (1)	[0.117] V2c V13 (3) [0.050] V (4) [0.014] V1a (5) [0.005] V2 V10b1 V12 (8) [0.004] V1a1 (9) [0.000] HV0 HV0a V1 V1a1b V2a V2a1 V2a1a V2b V2b1 V2b2 V3 V3b V4 V8 V10 V10b V10b2 V11 V14 V15 V15a V17 V23 V24 V25 V26 V28 HV0e H13a1a1d H13a1a1d1 (39)	1
234	>MK080157.1 H	73G 150T 263G 309.1CCT 310C 489C 750G 752T 1107C 1438G 2706G 3106d 3107C 3702G 4769G 4883T 5178A 5301G 7028T 8701G 8838A 8860G 9180G 9540C 10397G 10398G 10400T 10873C 11719A 11944C 12026G 12705T 13759A 14766T 14783C 15043A 15301A 15326G 16126C 16136C 16182C 16183C 16189C 16223T 16360T 16362C	D5a3a1a	73G 150T 263G 309.1CCT 310C 489C 16126C 16136C 16182C 16183C 16189C 16223T 16360T 16362C	1	73 150 263 489 750 752 1107 1438 2706 3702 4769 4883 5178A 5301 7028 8701 8838 8860 9180 9540 10397 10398 10400 10873 11719 11944 12026 12705 13759 14766 14783 15043 15301 15326 16126 16136 16189 16223 16360 16362	D5a3a1a	73 150 263 489 16126 16136 16189 16223 16360 16362	[0.000] D5a3a1a (1)	[0.033] D5a3a1 (2) [0.000] D5a3 D5a3a (4)	1
235	>MK049276.1 H	73G 152C 263G 310.1C 514d 515d 573T 709A 750G 1438G 1812T 2706G 3106d 3107C 3996T 4769G 7028T 7364G 8701G 8860G 9254G 9458T 9540C 10398G 10873C 11719A 12705T 12816T 13887G 14766T 15301A 15326G 15442G 16254G 16256T 16316G 16519C	L3a2a	73G 152C 263G 310.1C 514d 515d 573T 16254G 16256T 16316G 16519C	1	73 152 263 573 709 750 1438 1812 2706 3996 4769 7028 7364 8701 8860 9254 9458 9540 10398 10873 11719 12705 12816 13887 14766 15301 15326 15442 16254 16256 16316	L3a2a	73 152 263 573 16254 16256 16316	[0.040] L3a2a (1)	[0.019] L3a2 (2)	1
236	>MK007328.1 H	73G 143A 152C 182T 199C 204C 234G 263G 309.1CT 310C 567.1CCCCC 750G 1438G 1719A 2702A 2706G 2955C 3106d 3107C 3336C 4769G 4772C 5315G 7028T 8860G 8901G 9103C 10238C 10398G 11719A 12501A 12705T 13780G 14766T 15043A 15326G 16092C 16129A 16147A 16154C 16172C 16223T 16248T 16320T 16355T 16519C	N1a1a1a3	73G 143A 152C 182T 199C 204C 234G 263G 309.1CT 310C 567.1CCCCC 16092C 16129A 16147A 16154C 16172C 16223T 16248T 16320T 16355T 16519C	1	73 143 152 182 199 204 234 263 573.5C 750 1438 1719 2702 2706 2955 3336 4769 4772 5315 7028 8860 8901 9103 10238 10398 11719 12501 12705 13780 14766 15043 15326 16092 16129 16147A 16154 16172 16223 16248 16320 16355	N1a1a1a3	73 143 152 182 199 204 234 263 573.5C 16092 16129 16147A 16154 16172 16223 16248 16320 16355	[1.428] N1a1a1a3 (1)	[0.000] N1a1a1a N1a1a1a1 (3)	1

237 >MH782168.1 H	93G 152C 263G 750G 1438G 3106d 3107C 3591A 4310G 4769G 8860G 13020C 14593G 15326G 16168T 16519C	H9a	93G 152C 263G 16168T 16519C	1	93 152 263 750 1438 3591 4310 4769 8860 13020 14593 15326 16168	H9a	93 152 263 16168	[0.044] H9a (1)	[0.036] H49a2 (2) [0.032] H3+152 (3) [0.026] H46 (4) [0.013] H+152 (5) [0.007] H13a1+152 H47a (7) [0.006] H16+152 (8) [0.001] H14b2 (9) [0.000] H1e1a1 H1+152 H1i H1i1 H1i2 H1i2a H1an H1an1 H1an1a H1bb H1bc H2a2a2 H3g H3g1 H3g1a H3g1b H3g2 H3g3 H3g4 H3i H3i1 H3j H3k H3k1 H3k1a H7f H9 H46a H46b H52 H69 H14b H14b4 H16a H16a1 H16c H16d H30b1 (47)	1
238 >MH177819.1 H	73G 152C 225A 263G 292C 309.1CT 310C 489C 750G 1438G 2706G 2835T 3106d 3107C 4715G 4769G 6179A 7028T 7196A 7319C 8410T 8584A 8684T 8701G 8860G 9540C 10398G 10400T 10873C 11176A 11719A 12705T 14470C 14766T 14783C 15043A 15301A 15326G 15487T 16184T 16189C 16223T 16298C 16319A 16470A 16471A 16473A	M8a2a1	73G 152C 225A 263G 292C 309.1CT 310C 489C 16184T 16189C 16223T 16298C 16319A 16470A 16471A 16473A	1	73 152 225 263 292 489 750 1438 2706 2835 4715 4769 6179 7028 7196A 7319 8410 8584 8684 8701 8860 9540 10398 10400 10873 11176 11719 12705 14470 14766 14783 15043 15301 15326 15487T 16184 16189 16223 16298 16319 16470 16471 16473	M8a2a1	73 152 225 263 292 489 16184 16189 16223 16298 16319 16470 16471 16473	[0.472] M8a2a1 (1)	[0.014] M8a2a (2)	1
239 >MH177799.1 H	73G 93G 210G 263G 310.1C 438T 514d 515d 709A 750G 1438G 2706G 3106d 3107C 3537G 4769G 7028T 8271d 8272d 8273d 8274d 8275d 8276d 8277d 8278d 8279d 8584A 8614C 8860G 9950C 9962A 10398G 11149A 11151T 11719A 14149T 14766T 15046G 15235G 15326G 16140C 16183C 16189C 16245T 16266G 16362C 16519C	B5a2a2a2	73G 93G 210G 263G 310.1C 438T 514d 515d 16140C 16183C 16189C 16245T 16266G 16362C 16519C	1	73 93 210 263 438 709 750 1438 2706 3537 4769 7028 8281-8289d 8584 8614 8860 9950 9962 10398 11149 11151 11719 14149 14766 15046 15235 15326 16140 16189 16245 16266G 16362	B5a2a2a2	73 93 210 263 438 16140 16189 16245 16266G 16362	[0.083] B5a2a2a2 (1)	[0.083] B5a2a2a2 (2) [0.000] B5a2a2a B5a2a2a1 (4)	1
240 >MH177790.1 H	73G 248d 263G 309.1CT 310C 514d 515d 750G 1438G 2706G 3106d 3107C 3531A 3970T 4086T 4769G 6392C 6962A 7028T 8860G 9053A 9554A 9944C 10310A 10609C 11719A 12406A 12882T 13748G 13759A 13928C 14766T 15326G 15565C 16172C 16189C 16304C 16311C 16390A 16519C	F1a3a1	73G 248d 263G 309.1CT 310C 514d 515d 16172C 16189C 16304C 16311C 16390A 16519C	1	73 249d 263 750 1438 2706 3531 3970 4086 4769 6392 6962 7028 8860 9053 9554 9944 10310 10609 11719 12406 12882 13748 13759 13928C 14766 15326 15565 16172 16189 16304 16311 16390	F1a3a1	73 249d 263 16172 16189 16304 16311 16390	[0.043] F1a3a1 (1)	[0.000] F1a3a1a (2)	1
241 >MH816937.1 H	73G 150T 152C 263G 295T 309.1CT 310C 489C 750G 1438G 2706G 3106d 3107C 3766C 4216C 4769G 5633T 6830A 7028T 7476T 8020A 8860G 10172A 10398G 11050C 11251G 11383C 11719A 12612G 12969G 13708A 14766T 15257A 15326G 15452A 15812A 16069T 16126C 16193T 16195C 16242A 16526A	J2b1h	73G 150T 152C 263G 295T 309.1CT 310C 489C 16069T 16126C 16193T 16195C 16242A 16526A	1	73 150 152 263 295 489 750 1438 2706 3766 4216 4769 5633 6830A 7028 7476 8020 8860 10172 10398 11050 11251 11383 11719 12612 12969G 13708 14766 15257 15326 15452A 15812 16069 16126 16193 16195 16242A 16526	J2b1h	73 150 152 263 295 489 16069 16126 16193 16195 16242A 16526	[0.500] J2b1h (1)	[0.000] J2b J2b1 J2b1b J2b1b1 J2b1c J2b1d J2b1e J2b1f J2b1g J2b2 (11)	1

242	>MH499996.1 H	73G 263G 309.1CT 310C 514d 515d 516d 517d 750G 1438G 2056A 2706G 3106d 3107C 3316A 4232C 4769G 5442C 6764A 7028T 8584A 8860G 9142A 9156G 9242G 9869G 11047A 11719A 12714C 13161C 13773G 14766T 15055C 15272G 15326G 16172C 16278T 16519C	R30a1c	73G 263G 309.1CT 310C 514d 515d 516d 517d 16172C 16278T 16519C	1	73 263 750 1438 2056 2706 3316 4232 4769 5442 6764 7028 8584 8860 9142 9156 9242 9869G 11047A 11719 12714 13161 13773 14766 15055 15272 15326 16172 16278	R30a1c	73 263 16172 16278	[0.000] R30a1c (1)	[0.043] U6a (2) [0.000] U6 U6a1 U6a5 U6a5a U6a5a1 U6a6 U6a6b U6a6b1 U6a6b2 U6a7 U6a7a1+@152 U6a7a1c (14)	1
243	>MH499995.1 H	73G 263G 310.1CC 513.1CACA 750G 1438G 2056A 2706G 3106d 3107C 3316A 4232C 4769G 5442C 6764A 7028T 8584A 8860G 9142A 9156G 9242G 9869G 11047A 11719A 12714C 13161C 13773G 14766T 15055C 15272G 15326G 16172C 16278T 16519C	R30a1c	73G 263G 310.1CC 513.1CACA 16172C 16278T 16519C	1	73 263 315.2C 750 1438 2056 2706 3316 4232 4769 5442 6764 7028 8584 8860 9142 9156 9242 9869G 11047A 11719 12714 13161 13773 14766 15055 15272 15326 16172 16278	R30a1c	73 263 315.2C 16172 16278	[0.040] R30a1c (1)	[0.043] U6a (2) [0.000] U6 U6a1 U6a5 U6a5a U6a5a1 U6a6 U6a6b U6a6b1 U6a6b2 U6a7 U6a7a1+@152 U6a7a1c (14)	1
244	>MH499994.1 H	73G 263G 309.1CCT 310C 750G 1438G 2056A 2706G 3106d 3107C 3316A 4232C 4769G 5442C 6764A 7028T 8584A 8860G 9142A 9156G 9242G 9869G 11047A 11719A 12714C 13161C 13773G 14766T 15055C 15272G 15326G 16172C 16278T 16519C	R30a1c	73G 263G 309.1CCT 310C 16172C 16278T 16519C	1	73 263 750 1438 2056 2706 3316 4232 4769 5442 6764 7028 8584 8860 9142 9156 9242 9869G 11047A 11719 12714 13161 13773 14766 15055 15272 15326 16172 16278	R30a1c	73 263 16172 16278	[0.000] R30a1c (1)	[0.043] U6a (2) [0.000] U6 U6a1 U6a5 U6a5a U6a5a1 U6a6 U6a6b U6a6b1 U6a6b2 U6a7 U6a7a1+@152 U6a7a1c (14)	1
245	>MH499993.1 H	73G 263G 514d 515d 750G 1438G 2056A 2706G 3106d 3107C 3316A 4232C 4769G 5442C 6764A 7028T 8584A 8860G 9142A 9156G 9242G 9869G 11047A 11719A 12714C 13161C 13773G 14766T 15055C 15272G 15326G 16172C 16278T 16519C	R30a1c	73G 263G 514d 515d 16172C 16278T 16519C	1	73 263 750 1438 2056 2706 3316 4232 4769 5442 6764 7028 8584 8860 9142 9156 9242 9869G 11047A 11719 12714 13161 13773 14766 15055 15272 15326 16172 16278	R30a1c	73 263 16172 16278	[0.000] R30a1c (1)	[0.043] U6a (2) [0.000] U6 U6a1 U6a5 U6a5a U6a5a1 U6a6 U6a6b U6a6b1 U6a6b2 U6a7 U6a7a1+@152 U6a7a1c (14)	1
246	>MH499992.1 H	73G 263G 309.1CCT 310C 750G 1438G 2056A 2706G 3106d 3107C 3316A 4232C 4769G 5442C 6764A 7028T 8584A 8860G 9142A 9156G 9242G 9869G 11047A 11719A 12714C 13161C 13773G 14766T 15055C 15272G 15326G 16172C 16278T 16519C	R30a1c	73G 263G 309.1CCT 310C 16172C 16278T 16519C	1	73 263 750 1438 2056 2706 3316 4232 4769 5442 6764 7028 8584 8860 9142 9156 9242 9869G 11047A 11719 12714 13161 13773 14766 15055 15272 15326 16172 16278	R30a1c	73 263 16172 16278	[0.000] R30a1c (1)	[0.043] U6a (2) [0.000] U6 U6a1 U6a5 U6a5a U6a5a1 U6a6 U6a6b U6a6b1 U6a6b2 U6a7 U6a7a1+@152 U6a7a1c (14)	1
247	>MH499991.1 H	73G 263G 309.1CT 310C 513.1CA 750G 1438G 2056A 2706G 3106d 3107C 3316A 4232C 4769G 5442C 6764A 7028T 8584A 8860G 9142A 9156G 9242G 9869G 11047A 11719A 12714C 13161C 13773G 14766T 15055C 15272G 15326G 16172C 16278T 16519C	R30a1c	73G 263G 309.1CT 310C 513.1CA 16172C 16278T 16519C	1	73 263 750 1438 2056 2706 3316 4232 4769 5442 6764 7028 8584 8860 9142 9156 9242 9869G 11047A 11719 12714 13161 13773 14766 15055 15272 15326 16172 16278	R30a1c	73 263 16172 16278	[0.000] R30a1c (1)	[0.043] U6a (2) [0.000] U6 U6a1 U6a5 U6a5a U6a5a1 U6a6 U6a6b U6a6b1 U6a6b2 U6a7 U6a7a1+@152 U6a7a1c (14)	1

248 >MH499990.1 H	73G 263G 309.1CT 310C 513.1CA 750G 1438G 2056A 2706G 3106d 3107C 3316A 4232C 4769G 5442C 6764A 7028T 8584A 8860G 9142A 9156G 9242G 9869G 11047A 11719A 12714C 13161C 13773G 14766T 15055C 15272G 15326G 16172C 16278T 16519C	R30a1c	73G 263G 309.1CT 310C 513.1CA 16172C 16278T 16519C	1	73 263 750 1438 2056 2706 3316 4232 4769 5442 6764 7028 8584 8860 9142 9156 9242 9869G 11047A 11719 12714 13161 13773 14766 15055 15272 15326 16172 16278	R30a1c	73 263 16172 16278	[0.000] R30a1c (1)	[0.043] U6a (2) [0.000] U6 U6a1 U6a5 U6a5a U6a5a1 U6a6 U6a6b U6a6b1 U6a6b2 U6a7 U6a7a1+@152 U6a7a1c (14)	1
249 >MH499989.1 H	73G 263G 309.1CT 310C 513.1CA 750G 1438G 2056A 2706G 3106d 3107C 3316A 4232C 4769G 5442C 6764A 7028T 8584A 8860G 9142A 9156G 9242G 9869G 11047A 11719A 12714C 13161C 13773G 14766T 15055C 15272G 15326G 16172C 16278T 16519C	R30a1c	73G 263G 309.1CT 310C 513.1CA 16172C 16278T 16519C	1	73 263 750 1438 2056 2706 3316 4232 4769 5442 6764 7028 8584 8860 9142 9156 9242 9869G 11047A 11719 12714 13161 13773 14766 15055 15272 15326 16172 16278	R30a1c	73 263 16172 16278	[0.000] R30a1c (1)	[0.043] U6a (2) [0.000] U6 U6a1 U6a5 U6a5a U6a5a1 U6a6 U6a6b U6a6b1 U6a6b2 U6a7 U6a7a1+@152 U6a7a1c (14)	1
250 >MH499988.1 H	73G 263G 309.1CT 310C 513.1CA 750G 1438G 2056A 2706G 3106d 3107C 3316A 4232C 4769G 5442C 6764A 7028T 8584A 8860G 9142A 9156G 9242G 9869G 11047A 11719A 12714C 13161C 13773G 14766T 15055C 15272G 15326G 16172C 16278T 16519C	R30a1c	73G 263G 309.1CT 310C 513.1CA 16172C 16278T 16519C	1	73 263 750 1438 2056 2706 3316 4232 4769 5442 6764 7028 8584 8860 9142 9156 9242 9869G 11047A 11719 12714 13161 13773 14766 15055 15272 15326 16172 16278	R30a1c	73 263 16172 16278	[0.000] R30a1c (1)	[0.043] U6a (2) [0.000] U6 U6a1 U6a5 U6a5a U6a5a1 U6a6 U6a6b U6a6b1 U6a6b2 U6a7 U6a7a1+@152 U6a7a1c (14)	1
251 >MH499987.1 H	73G 263G 750G 1438G 2056A 2706G 3106d 3107C 3316A 4232C 4769G 5442C 6764A 7028T 8584A 8860G 9142A 9156G 9242G 9869G 11047A 11719A 12714C 13161C 13773G 14766T 15055C 15272G 15326G 16172C 16278T 16519C	R30a1c	73G 263G 16172C 16278T 16519C	1	73 263 750 1438 2056 2706 3316 4232 4769 5442 6764 7028 8584 8860 9142 9156 9242 9869G 11047A 11719 12714 13161 13773 14766 15055 15272 15326 16172 16278	R30a1c	73 263 16172 16278	[0.000] R30a1c (1)	[0.043] U6a (2) [0.000] U6 U6a1 U6a5 U6a5a U6a5a1 U6a6 U6a6b U6a6b1 U6a6b2 U6a7 U6a7a1+@152 U6a7a1c (14)	1
252 >MH499986.1 H	73G 263G 750G 1438G 2056A 2706G 3106d 3107C 3316A 4232C 4769G 5442C 6764A 7028T 8584A 8860G 9142A 9156G 9242G 9869G 11047A 11719A 12714C 13161C 13773G 14766T 15055C 15272G 15326G 16172C 16278T 16519C	R30a1c	73G 263G 16172C 16278T 16519C	1	73 263 750 1438 2056 2706 3316 4232 4769 5442 6764 7028 8584 8860 9142 9156 9242 9869G 11047A 11719 12714 13161 13773 14766 15055 15272 15326 16172 16278	R30a1c	73 263 16172 16278	[0.000] R30a1c (1)	[0.043] U6a (2) [0.000] U6 U6a1 U6a5 U6a5a U6a5a1 U6a6 U6a6b U6a6b1 U6a6b2 U6a7 U6a7a1+@152 U6a7a1c (14)	1
253 >MH499985.1 H	73G 263G 750G 1438G 2056A 2706G 3106d 3107C 3316A 4232C 4769G 5442C 6764A 7028T 8584A 8860G 9142A 9156G 9242G 9869G 11047A 11719A 12714C 13161C 13773G 14766T 15055C 15272G 15326G 16172C 16278T 16519C	R30a1c	73G 263G 16172C 16278T 16519C	1	73 263 750 1438 2056 2706 3316 4232 4769 5442 6764 7028 8584 8860 9142 9156 9242 9869G 11047A 11719 12714 13161 13773 14766 15055 15272 15326 16172 16278	R30a1c	73 263 16172 16278	[0.000] R30a1c (1)	[0.043] U6a (2) [0.000] U6 U6a1 U6a5 U6a5a U6a5a1 U6a6 U6a6b U6a6b1 U6a6b2 U6a7 U6a7a1+@152 U6a7a1c (14)	1

254	>MH499984.1 H	73G 263G 750G 1438G 2056A 2706G 3106d 3107C 3316A 4232C 4769G 5442C 6764A 7028T 8584A 8860G 9142A 9156G 9242G 9869G 11047A 11719A 12714C 13161C 13773G 14766T 15055C 15272G 15326G 16172C 16278T 16519C	R30a1c	73G 263G 16172C 16278T 16519C	1	73 263 750 1438 2056 2706 3316 4232 4769 5442 6764 7028 8584 8860 9142 9156 9242 9869G 11047A 11719 12714 13161 13773 14766 15055 15272 15326 16172 16278	R30a1c	73 263 16172 16278	[0.000] R30a1c (1)	[0.043] U6a (2) [0.000] U6 U6a1 U6a5 U6a5a U6a5a1 U6a6 U6a6b U6a6b1 U6a6b2 U6a7 U6a7a1+@152 U6a7a1c (14)	1
255	>MH499983.1 H	73G 263G 750G 1438G 2056A 2706G 3106d 3107C 3316A 4232C 4769G 5442C 6764A 7028T 8584A 8860G 9142A 9156G 9242G 9869G 11047A 11719A 12714C 13161C 13773G 14766T 15055C 15272G 15326G 16172C 16278T 16519C	R30a1c	73G 263G 16172C 16278T 16519C	1	73 263 750 1438 2056 2706 3316 4232 4769 5442 6764 7028 8584 8860 9142 9156 9242 9869G 11047A 11719 12714 13161 13773 14766 15055 15272 15326 16172 16278	R30a1c	73 263 16172 16278	[0.000] R30a1c (1)	[0.043] U6a (2) [0.000] U6 U6a1 U6a5 U6a5a U6a5a1 U6a6 U6a6b U6a6b1 U6a6b2 U6a7 U6a7a1+@152 U6a7a1c (14)	1
256	>MH499982.1 H	73G 263G 308.1CCT 310C 513.1CA 750G 1438G 2056A 2706G 3106d 3107C 3316A 4232C 4769G 5442C 6764A 7028T 8584A 8860G 9142A 9156G 9242G 9869G 11047A 11719A 12714C 13161C 13773G 14766T 15055C 15272G 15326G 16172C 16278T 16519C	R30a1c	73G 263G 308.1CCT 310C 513.1CA 16172C 16278T 16519C	1	73 263 315.2C 750 1438 2056 2706 3316 4232 4769 5442 6764 7028 8584 8860 9142 9156 9242 9869G 11047A 11719 12714 13161 13773 14766 15055 15272 15326 16172 16278	R30a1c	73 263 315.2C 16172 16278	[0.040] R30a1c (1)	[0.043] U6a (2) [0.000] U6 U6a1 U6a5 U6a5a U6a5a1 U6a6 U6a6b U6a6b1 U6a6b2 U6a7 U6a7a1+@152 U6a7a1c (14)	1
257	>MH499981.1 H	73G 263G 309.1CCT 310C 513.1CA 750G 1438G 2056A 2706G 3106d 3107C 3316A 4232C 4769G 5442C 6764A 7028T 8584A 8860G 9142A 9156G 9242G 9869G 11047A 11719A 12714C 13161C 13773G 14766T 15055C 15326G 16172C 16278T 16519C	R30a1c	73G 263G 309.1CCT 310C 513.1CA 16172C 16278T 16519C	1	73 263 750 1438 2056 2706 3316 4232 4769 5442 6764 7028 8584 8860 9142 9156 9242 9869G 11047A 11719 12714 13161 13773 14766 15055 15326 16172 16278	R30a1c	73 263 16172 16278	[0.000] R30a1c (1)	[0.043] U6a (2) [0.000] U6 U6a1 U6a5 U6a5a U6a5a1 U6a6 U6a6b U6a6b1 U6a6b2 U6a7 U6a7a1+@152 U6a7a1c (14)	1
258	>MH499980.1 H	73G 263G 309.1CT 310C 513.1CACA 750G 1438G 2056A 2706G 3106d 3107C 3316A 4232C 4769G 5442C 6764A 7028T 8584A 8860G 9142A 9156G 9242G 9869G 11047A 11719A 12714C 13161C 13773G 14766T 15055C 15272G 15326G 16172C 16278T 16519C	R30a1c	73G 263G 309.1CT 310C 513.1CACA 16172C 16278T 16519C	1	73 263 750 1438 2056 2706 3316 4232 4769 5442 6764 7028 8584 8860 9142 9156 9242 9869G 11047A 11719 12714 13161 13773 14766 15055 15272 15326 16172 16278	R30a1c	73 263 16172 16278	[0.000] R30a1c (1)	[0.043] U6a (2) [0.000] U6 U6a1 U6a5 U6a5a U6a5a1 U6a6 U6a6b U6a6b1 U6a6b2 U6a7 U6a7a1+@152 U6a7a1c (14)	1
259	>MH499979.1 H	73G 263G 309.1CT 310C 513.1CA 750G 1438G 2056A 2706G 3106d 3107C 3316A 4769G 5442C 6764A 7028T 8584A 8860G 9142A 9156G 9242G 9869G 11047A 11719A 12714C 13161C 13773G 14766T 15055C 15272G 15326G 16172C 16278T 16519C	R30a1c	73G 263G 309.1CT 310C 513.1CA 16172C 16278T 16519C	1	73 263 750 1438 2056 2706 3316 4769 5442 6764 7028 8584 8860 9142 9156 9242 9869G 11047A 11719 12714 13161 13773 14766 15055 15272 15326 16172 16278	R30a1c	73 263 16172 16278	[0.000] R30a1c (1)	[0.043] U6a (2) [0.000] U6 U6a1 U6a5 U6a5a U6a5a1 U6a6 U6a6b U6a6b1 U6a6b2 U6a7 U6a7a1+@152 U6a7a1c (14)	1

260	>MH499978.1 H(	73G 263G 309.1CT 310C 513.1CACA 750G 1438G 2056A 2706G 3106d 3107C 3316A 4232C 4769G 5442C 6764A 7028T 8584A 8860G 9142A 9156G 9242G 9869G 11047A 11719A 12714C 13161C 13773G 14766T 15055C 15272G 15326G 16172C 16278T 16519C	R30a1c	73G 263G 309.1CT 310C 513.1CACA 16172C 16278T 16519C	1	73 263 750 1438 2056 2706 3316 4232 4769 5442 6764 7028 8584 8860 9142 9156 9242 9869G 11047A 11719 12714 13161 13773 14766 15055 15272 15326 16172 16278	R30a1c	73 263 16172 16278	[0.000] R30a1c (1)	[0.043] U6a (2) [0.000] U6 U6a1 U6a5 U6a5a U6a5a1 U6a6 U6a6b U6a6b1 U6a6b2 U6a7 U6a7a1+@152 U6a7a1c (14)	1
261	>MH499977.1 H(	73G 263G 513.1CA 750G 1438G 2056A 2706G 3106d 3107C 3316A 4232C 4769G 5442C 6764A 7028T 8584A 8860G 9142A 9156G 9242G 9869G 11047A 11719A 12714C 13161C 13773G 14766T 15055C 15326G 16172C 16278T 16519C	R30a1c	73G 263G 513.1CA 16172C 16278T 16519C	1	73 263 750 1438 2056 2706 3316 4232 4769 5442 6764 7028 8584 8860 9142 9156 9242 9869G 11047A 11719 12714 13161 13773 14766 15055 15326 16172 16278	R30a1c	73 263 16172 16278	[0.000] R30a1c (1)	[0.043] U6a (2) [0.000] U6 U6a1 U6a5 U6a5a U6a5a1 U6a6 U6a6b U6a6b1 U6a6b2 U6a7 U6a7a1+@152 U6a7a1c (14)	1
262	>MH499976.1 H(	73G 263G 513.1CACA 750G 1438G 2056A 2706G 3106d 3107C 3316A 4232C 4769G 5442C 6764A 7028T 8584A 8860G 9142A 9156G 9242G 9869G 11047A 11719A 12714C 13161C 13773G 14766T 15055C 15272G 15326G 16172C 16278T 16519C	R30a1c	73G 263G 513.1CACA 16172C 16278T 16519C	1	73 263 750 1438 2056 2706 3316 4232 4769 5442 6764 7028 8584 8860 9142 9156 9242 9869G 11047A 11719 12714 13161 13773 14766 15055 15272 15326 16172 16278	R30a1c	73 263 16172 16278	[0.000] R30a1c (1)	[0.043] U6a (2) [0.000] U6 U6a1 U6a5 U6a5a U6a5a1 U6a6 U6a6b U6a6b1 U6a6b2 U6a7 U6a7a1+@152 U6a7a1c (14)	1
263	>MH499975.1 H(	73G 263G 513.1CA 750G 1438G 2056A 2706G 3106d 3107C 3316A 4232C 4769G 5442C 6764A 7028T 8584A 8860G 9142A 9156G 9242G 9869G 11047A 11719A 12714C 13161C 13773G 14766T 15055C 15272G 15326G 16172C 16278T 16519C	R30a1c	73G 263G 513.1CA 16172C 16278T 16519C	1	73 263 750 1438 2056 2706 3316 4232 4769 5442 6764 7028 8584 8860 9142 9156 9242 9869G 11047A 11719 12714 13161 13773 14766 15055 15272 15326 16172 16278	R30a1c	73 263 16172 16278	[0.000] R30a1c (1)	[0.043] U6a (2) [0.000] U6 U6a1 U6a5 U6a5a U6a5a1 U6a6 U6a6b U6a6b1 U6a6b2 U6a7 U6a7a1+@152 U6a7a1c (14)	1
264	>MH499974.1 H(	73G 263G 750G 1438G 2056A 2706G 3106d 3107C 3316A 4232C 4769G 5442C 6764A 7028T 8584A 8860G 9142A 9156G 9242G 9869G 11047A 11719A 12714C 13161C 13773G 14766T 15055C 15272G 15326G 16172C 16278T 16519C	R30a1c	73G 263G 16172C 16278T 16519C	1	73 263 750 1438 2056 2706 3316 4232 4769 5442 6764 7028 8584 8860 9142 9156 9242 9869G 11047A 11719 12714 13161 13773 14766 15055 15272 15326 16172 16278	R30a1c	73 263 16172 16278	[0.000] R30a1c (1)	[0.043] U6a (2) [0.000] U6 U6a1 U6a5 U6a5a U6a5a1 U6a6 U6a6b U6a6b1 U6a6b2 U6a7 U6a7a1+@152 U6a7a1c (14)	1
265	>MH499973.1 H(	73G 263G 750G 1438G 2056A 2706G 3106d 3107C 3316A 3525T 4232C 4769G 5442C 6764A 7028T 8584A 8860G 9142A 9156G 9242G 9869G 11047A 11719A 12714C 13161C 13773G 14766T 15055C 15272G 15326G 16172C 16278T 16519C	R30a1c	73G 263G 16172C 16278T 16519C	1	73 263 750 1438 2056 2706 3316 3525 4232 4769 5442 6764 7028 8584 8860 9142 9156 9242 9869G 11047A 11719 12714 13161 13773 14766 15055 15272 15326 16172 16278	R30a1c	73 263 16172 16278	[0.000] R30a1c (1)	[0.043] U6a (2) [0.000] U6 U6a1 U6a5 U6a5a U6a5a1 U6a6 U6a6b U6a6b1 U6a6b2 U6a7 U6a7a1+@152 U6a7a1c (14)	1

266 >MH499972.1 H	73G 263G 309.1CT 310C 513.1CACA 750G 1438G 2056A 2706G 3106d 3107C 3316A 4232C 4769G 5442C 6764A 7028T 8584A 8860G 9142A 9156G 9242G 9869G 11047A 11719A 12714C 13161C 13773G 14766T 15055C 15272G 15326G 16172C 16278T 16519C	R30a1c	73G 263G 309.1CT 310C 513.1CACA 16172C 16278T 16519C	1	73 263 750 1438 2056 2706 3316 4232 4769 5442 6764 7028 8584 8860 9142 9156 9242 9869G 11047A 11719 12714 13161 13773 14766 15055 15272 15326 16172 16278	R30a1c	73 263 16172 16278	[0.000] R30a1c (1)	[0.043] U6a (2) [0.000] U6 U6a1 U6a5 U6a5a U6a5a1 U6a6 U6a6b U6a6b1 U6a6b2 U6a7 U6a7a1+@152 U6a7a1c (14)	1
267 >MH499971.1 H	73G 263G 309.1CT 310C 513.1CACA 750G 1438G 2056A 2706G 3106d 3107C 3316A 4232C 4769G 5442C 6764A 7028T 8584A 8860G 9142A 9156G 9242G 9869G 11047A 11719A 12714C 13161C 13773G 14766T 15055C 15272G 15326G 16172C 16278T 16519C	R30a1c	73G 263G 309.1CT 310C 513.1CACA 16172C 16278T 16519C	1	73 263 750 1438 2056 2706 3316 4232 4769 5442 6764 7028 8584 8860 9142 9156 9242 9869G 11047A 11719 12714 13161 13773 14766 15055 15272 15326 16172 16278	R30a1c	73 263 16172 16278	[0.000] R30a1c (1)	[0.043] U6a (2) [0.000] U6 U6a1 U6a5 U6a5a U6a5a1 U6a6 U6a6b U6a6b1 U6a6b2 U6a7 U6a7a1+@152 U6a7a1c (14)	1
268 >MH499970.1 H	73G 263G 309.1CCT 310C 513.1CACA 750G 1438G 2056A 2706G 3106d 3107C 3316A 4232C 4769G 5442C 6764A 7028T 8584A 8860G 9142A 9156G 9242G 9869G 11047A 11719A 12714C 13161C 13773G 14766T 15055C 15272G 15326G 16172C 16278T 16519C	R30a1c	73G 263G 309.1CCT 310C 513.1CACA 16172C 16278T 16519C	1	73 263 750 1438 2056 2706 3316 4232 4769 5442 6764 7028 8584 8860 9142 9156 9242 9869G 11047A 11719 12714 13161 13773 14766 15055 15272 15326 16172 16278	R30a1c	73 263 16172 16278	[0.000] R30a1c (1)	[0.043] U6a (2) [0.000] U6 U6a1 U6a5 U6a5a U6a5a1 U6a6 U6a6b U6a6b1 U6a6b2 U6a7 U6a7a1+@152 U6a7a1c (14)	1
269 >MH499969.1 H	73G 263G 309.1CCT 310C 513.1CACA 750G 1438G 2056A 2706G 3106d 3107C 3316A 4232C 4769G 5442C 6764A 7028T 8584A 8860G 9142A 9156G 9242G 9869G 11047A 11719A 12714C 13161C 13773G 14766T 15055C 15272G 15326G 16172C 16278T 16519C	R30a1c	73G 263G 309.1CCT 310C 513.1CACA 16172C 16278T 16519C	1	73 263 750 1438 2056 2706 3316 4232 4769 5442 6764 7028 8584 8860 9142 9156 9242 9869G 11047A 11719 12714 13161 13773 14766 15055 15272 15326 16172 16278	R30a1c	73 263 16172 16278	[0.000] R30a1c (1)	[0.043] U6a (2) [0.000] U6 U6a1 U6a5 U6a5a U6a5a1 U6a6 U6a6b U6a6b1 U6a6b2 U6a7 U6a7a1+@152 U6a7a1c (14)	1
270 >MH499968.1 H	73G 263G 309.1CT 310C 513.1CACA 750G 1438G 2056A 2706G 3106d 3107C 3316A 4232C 4769G 5442C 6764A 7028T 8584A 8860G 9142A 9156G 9242G 9869G 11047A 11719A 12714C 13161C 13773G 14766T 15055C 15272G 15326G 16172C 16278T 16519C	R30a1c	73G 263G 309.1CT 310C 513.1CACA 16172C 16278T 16519C	1	73 263 750 1438 2056 2706 3316 4232 4769 5442 6764 7028 8584 8860 9142 9156 9242 9869G 11047A 11719 12714 13161 13773 14766 15055 15272 15326 16172 16278	R30a1c	73 263 16172 16278	[0.000] R30a1c (1)	[0.043] U6a (2) [0.000] U6 U6a1 U6a5 U6a5a U6a5a1 U6a6 U6a6b U6a6b1 U6a6b2 U6a7 U6a7a1+@152 U6a7a1c (14)	1
271 >MH499967.1 H	73G 263G 310.1CC 513.1CACA 750G 1438G 2056A 2706G 3106d 3107C 3316A 4232C 4769G 5442C 6764A 7028T 8584A 8860G 9142A 9156G 9242G 9869G 11047A 11719A 12714C 13161C 13773G 14766T 15055C 15272G 15326G 16172C 16278T 16519C	R30a1c	73G 263G 310.1CC 513.1CACA 16172C 16278T 16519C	1	73 263 315.2C 750 1438 2056 2706 3316 4232 4769 5442 6764 7028 8584 8860 9142 9156 9242 9869G 11047A 11719 12714 13161 13773 14766 15055 15272 15326 16172 16278	R30a1c	73 263 315.2C 16172 16278	[0.040] R30a1c (1)	[0.043] U6a (2) [0.000] U6 U6a1 U6a5 U6a5a U6a5a1 U6a6 U6a6b U6a6b1 U6a6b2 U6a7 U6a7a1+@152 U6a7a1c (14)	1

272	>MH499966.1 H	73G 263G 513.1CACA 750G 1438G 2056A 2706G 3106d 3107C 3316A 3525T 4232C 4769G 5442C 6764A 7028T 8584A 8860G 9142A 9156G 9242G 9869G 11047A 11719A 12714C 13161C 13773G 14766T 15055C 15272G 15326G 16172C 16278T 16519C	R30a1c	73G 263G 513.1CACA 16172C 16278T 16519C	1	73 263 750 1438 2056 2706 3316 3525 4232 4769 5442 6764 7028 8584 8860 9142 9156 9242 9869G 11047A 11719 12714 13161 13773 14766 15055 15272 15326 16172 16278	R30a1c	73 263 16172 16278	[0.000] R30a1c (1)	[0.043] U6a (2) [0.000] U6 U6a1 U6a5 U6a5a U6a5a1 U6a6 U6a6b U6a6b1 U6a6b2 U6a7 U6a7a1+@152 U6a7a1c (14)	1
273	>MH499965.1 H	73G 263G 513.1CACA 750G 1438G 2056A 2706G 3106d 3107C 3316A 4232C 4769G 5442C 6764A 7028T 8584A 8860G 9142A 9156G 9242G 9869G 11047A 11719A 12714C 13161C 13773G 14766T 15055C 15272G 15326G 16172C 16278T 16519C	R30a1c	73G 263G 513.1CACA 16172C 16278T 16519C	1	73 263 750 1438 2056 2706 3316 4232 4769 5442 6764 7028 8584 8860 9142 9156 9242 9869G 11047A 11719 12714 13161 13773 14766 15055 15272 15326 16172 16278	R30a1c	73 263 16172 16278	[0.000] R30a1c (1)	[0.043] U6a (2) [0.000] U6 U6a1 U6a5 U6a5a U6a5a1 U6a6 U6a6b U6a6b1 U6a6b2 U6a7 U6a7a1+@152 U6a7a1c (14)	1
274	>MH499964.1 H	73G 263G 513.1CA 750G 1438G 2056A 2706G 3106d 3107C 3316A 4232C 4769G 5442C 6764A 7028T 8584A 8860G 9142A 9156G 9242G 9869G 11047A 11719A 12714C 13161C 13773G 14766T 15055C 15272G 15326G 16172C 16278T 16519C	R30a1c	73G 263G 513.1CA 16172C 16278T 16519C	1	73 263 750 1438 2056 2706 3316 4232 4769 5442 6764 7028 8584 8860 9142 9156 9242 9869G 11047A 11719 12714 13161 13773 14766 15055 15272 15326 16172 16278	R30a1c	73 263 16172 16278	[0.000] R30a1c (1)	[0.043] U6a (2) [0.000] U6 U6a1 U6a5 U6a5a U6a5a1 U6a6 U6a6b U6a6b1 U6a6b2 U6a7 U6a7a1+@152 U6a7a1c (14)	1
275	>MH499963.1 H	73G 263G 513.1CA 750G 1438G 2056A 2706G 3106d 3107C 3316A 4232C 4769G 5442C 6764A 7028T 8584A 8860G 9142A 9156G 9242G 9869G 11047A 11719A 12714C 13161C 13773G 14766T 15055C 15272G 15326G 16172C 16278T 16519C	R30a1c	73G 263G 513.1CA 16172C 16278T 16519C	1	73 263 750 1438 2056 2706 3316 4232 4769 5442 6764 7028 8584 8860 9142 9156 9242 9869G 11047A 11719 12714 13161 13773 14766 15055 15272 15326 16172 16278	R30a1c	73 263 16172 16278	[0.000] R30a1c (1)	[0.043] U6a (2) [0.000] U6 U6a1 U6a5 U6a5a U6a5a1 U6a6 U6a6b U6a6b1 U6a6b2 U6a7 U6a7a1+@152 U6a7a1c (14)	1
276	>MH499962.1 H	73G 263G 309.1CT 310C 513.1CACA 750G 1438G 2056A 2706G 3106d 3107C 3316A 4232C 4769G 5442C 6764A 7028T 8584A 8860G 9142A 9156G 9242G 9869G 11047A 11719A 12714C 13161C 13773G 14766T 15055C 15272G 15326G 16172C 16278T 16519C	R30a1c	73G 263G 309.1CT 310C 513.1CACA 16172C 16278T 16519C	1	73 263 750 1438 2056 2706 3316 4232 4769 5442 6764 7028 8584 8860 9142 9156 9242 9869G 11047A 11719 12714 13161 13773 14766 15055 15272 15326 16172 16278	R30a1c	73 263 16172 16278	[0.000] R30a1c (1)	[0.043] U6a (2) [0.000] U6 U6a1 U6a5 U6a5a U6a5a1 U6a6 U6a6b U6a6b1 U6a6b2 U6a7 U6a7a1+@152 U6a7a1c (14)	1
277	>MH499934.1 H	73G 89C 152C 185A 189G 195C 204C 263G 310.1C 750G 1438G 2706G 2755G 3106d 3107C 3384G 4769G 5510G 7028T 7759C 8860G 9449T 11719A 11864C 13215C 14766T 16249A 16519C	R8a2	73G 89C 152C 185A 189G 195C 204C 263G 310.1C 16249A 16519C	1	73 89 152 185 189 195 204 263 750 1438 2706 2755 3384 4769 5510 7028 7759 8860 9449 11719 11864 13215 14766 16249A	R8a2	73 89 152 185 189 195 204 263 16249A	[1.437] R8a2 (1)	[0.176] R8a1 R8a1a1a (3) [0.100] L3d4a (4) [0.057] R30a1 (5) [0.056] R30a1a (6) [0.053] R8a (7) [0.050] R2a (8) [0.048] R8a1a1c (9) [0.034] L3d4 (10) [0.017] H32 (11) [0.000] HV2a HV2a2 HV2a3 R2+13500+195 R8 R8a1a R8a1a1 (18)	1

278 >MH499933.1 H	73G 89C 152C 170A 185A 189G 195C 204C 263G 310.1C 750G 1438G 1842G 2706G 2755G 3106d 3107C 3384G 4769G 5510G 7028T 7759C 8860G 9449T 9716C 11719A 11864C 13215C 14766T 16519C	R8a2	73G 89C 152C 170A 185A 189G 195C 204C 263G 310.1C 16519C	1	73 89 152 170A 185 189 195 204 263 750 1438 1842 2706 2755 3384 4769 5510 7028 7759 8860 9449 9716 11719 11864 13215 14766	R8a2	73 89 152 170A 185 189 195 204 263	[1.500] R8a2 (1)	[0.176] R8a1 R8a1a1a (3) [0.100] L3d4a (4) [0.057] R30a1 (5) [0.056] R30a1a (6) [0.053] R8a (7) [0.048] R8a1a1c (8) [0.034] L3d4 (9) [0.017] H32 (10) [0.000] HV2a HV2a2 HV2a3 R2+13500+195 R2a R8 R8a1a R8a1a1 (18)	1
279 >MH499932.1 H	73G 89C 152C 170A 185A 189G 195C 204C 263G 310.1C 750G 1438G 1842G 2706G 2755G 3106d 3107C 3384G 4769G 5510G 7028T 7759C 8860G 9386C 9449T 11719A 11864C 13215C 14766T 16519C	R8a2	73G 89C 152C 170A 185A 189G 195C 204C 263G 310.1C 16519C	1	73 89 152 170A 185 189 195 204 263 750 1438 1842 2706 2755 3384 4769 5510 7028 7759 8860 9386 9449 11719 11864 13215 14766	R8a2	73 89 152 170A 185 189 195 204 263	[1.500] R8a2 (1)	[0.176] R8a1 R8a1a1a (3) [0.100] L3d4a (4) [0.057] R30a1 (5) [0.056] R30a1a (6) [0.053] R8a (7) [0.048] R8a1a1c (8) [0.034] L3d4 (9) [0.017] H32 (10) [0.000] HV2a HV2a2 HV2a3 R2+13500+195 R2a R8 R8a1a R8a1a1 (18)	1
280 >MH499931.1 H	73G 89C 152C 185A 189G 195C 204C 263G 310.1C 750G 1438G 1842G 2706G 2755G 3106d 3107C 3384G 4769G 5510G 7028T 7759C 8860G 9449T 11719A 11864C 13215C 14766T 16519C	R8a2	73G 89C 152C 185A 189G 195C 204C 263G 310.1C 16519C	1	73 89 152 185 189 195 204 263 750 1438 1842 2706 2755 3384 4769 5510 7028 7759 8860 9449 11719 11864 13215 14766	R8a2	73 89 152 185 189 195 204 263	[1.375] R8a2 (1)	[0.176] R8a1 R8a1a1a (3) [0.100] L3d4a (4) [0.057] R30a1 (5) [0.056] R30a1a (6) [0.053] R8a (7) [0.048] R8a1a1c (8) [0.034] L3d4 (9) [0.017] H32 (10) [0.000] HV2a HV2a2 HV2a3 R2+13500+195 R2a R8 R8a1a R8a1a1 (18)	1
281 >MH631155.1 H	73G 150T 195C 263G 310.1C 723G 750G 955.1C 1438G 1721T 2706G 3106d 3107C 3197C 3861G 4769G 5836G 7028T 7768G 8860G 9477A 10262G 11467G 11719A 11929C 12308G 12372A 13017G 13617C 13637G 14182C 14766T 15326G 16189C 16192T 16249C 16270T	U5b2c2b	73G 150T 195C 263G 310.1C 16189C 16192T 16249C 16270T	1	73 150 195 263 723 750 960.1C 1438 1721 2706 3197 3861 4769 5836 7028 7768 8860 9477 10262 11467 11719 11929 12308 12372 13017 13617 13637 14182 14766 15326 16189 16192 16249 16270	U5b2c2b	73 150 195 263 16189 16192 16249 16270	[0.425] U5b2c2b (1) [0.029] U5b1+16189 (2) [0.000] U5b1b U5b1b1 U5b1b1g U5b2a U5b2a3 U5b2a3a (8)	[0.080] U5b1b1g1 (9) [0.062] U5b1c (10) [0.043] U5b2 (11) [0.040] U5b1a (12) [0.037] U5b2a1 (13) [0.034] U5b1b1d (14) [0.033] U5b2a+@16192 U5b2a4 (16) [0.031] U5b2c (17) [0.029] U5b2c2a (18) [0.026] U5b1b1b (19) [0.023] U5b2a2a1 (20) [0.017] U5b2a4a (21) [0.011] U5b1b1+@16192 (22) [0.000] U5b U5b1 U5b1b1f U5b1b1+152 U5b1b1e U5b1b2 U5b1b2a U5b1b2b U5b1+16189+@16192 U5b1d U5b1d1 U5b1f U5b2a2 U5b2a2a U5b2a2a2 U5b2a2b U5b2a2b1 U5b2a6 U5b2b4 U5b2c1 U5b2c2 (43)	1

282 >MH631062.1 H	73G 150T 195C 263G 310.1C 723G 750G 955.1C 1438G 1721T 2706G 3106d 3107C 3197C 3861G 4769G 5836G 7028T 7768G 8860G 9477A 10262G 11467G 11719A 11929C 12308G 12372A 13017G 13617C 13637G 14182C 14766T 15326G 16189C 16192T 16249C 16270T	U5b2c2b	73G 150T 195C 263G 310.1C 16189C 16192T 16249C 16270T	1	73 150 195 263 723 750 960.1C 1438 1721 2706 3197 3861 4769 5836 7028 7768 8860 9477 10262 11467 11719 11929 12308 12372 13017 13617 13637 14182 14766 15326 16189 16192 16249 16270	U5b2c2b	73 150 195 263 16189 16192 16249 16270	[0.425] U5b2c2b (1) [0.029] U5b1+16189 (2) [0.000] U5b1b U5b1b1 U5b1b1g U5b2a U5b2a3 U5b2a3a (8)	[0.080] U5b1b1g1 (9) [0.062] U5b1c (10) [0.043] U5b2 (11) [0.040] U5b1a (12) [0.037] U5b2a1 (13) [0.034] U5b1b1d (14) [0.033] U5b2a+@16192 U5b2a4 (16) [0.031] U5b2c (17) [0.029] U5b2c2a (18) [0.026] U5b1b1b (19) [0.023] U5b2a2a1 (20) [0.017] U5b2a4a (21) [0.011] U5b1b1+@16192 (22) [0.000] U5b U5b1 U5b1b1f U5b1b1+152 U5b1b1e U5b1b2 U5b1b2a U5b1b2b U5b1+16189+@16192 U5b1d U5b1d1 U5b1f U5b2a2 U5b2a2a U5b2a2a2 U5b2a2b U5b2a2b1 U5b2a6 U5b2b4 U5b2c1 U5b2c2 (43)	1
283 >MG182531.1 H	73G 183G 263G 750G 961C 1438G 2706G 3108N 3197C 4769G 5147A 5746N 7028T 8860G 9477A 10544T 10946N 11467G 11719A 12308G 12372A 13230N 13617C 14766T 14793G 15218G 15326G 16192T 16256T 16270T 16286T 16320T 16399G	U5a1c2a1	73G 183G 263G 16192T 16256T 16270T 16286T 16320T 16399G	1	73 183 263 750 961 1438 2706 3108N 3197 4769 5147 5746N 7028 8860 9477 10544 10946N 11467 11719 12308 12372 13230N 13617 14766 14793 15218 15326 16192 16256 16270 16286 16320 16399	U5a1c2a1	73 183 263 16192 16256 16270 16286 16320 16399	[0.000] U5a1c2a1 (1)	[0.000] U5a1c2a (2)	1
284 >MG182529.1 H	73G 263G 303N 304N 305N 306N 307N 308N 309N 311N 312N 313N 314N 315N 709A 750G 1438G 1888A 2706G 4216C 4769G 4823C 4917G 6261A 7028T 8697A 8860G 10463C 10822T 11251G 11719A 11812G 13368A 14233G 14766T 14905A 15326G 15452A 15607G 15758G 15928A 16126C 16146G 16147T 16292T 16294T 16296T 16519C	T2c1c	73G 263G 303N 304N 305N 306N 307N 308N 309N 311N 312N 313N 314N 315N 16126C 16146G 16147T 16292T 16294T 16296T 16519C	1	73 263 303N 304N 305N 306N 307N 308N 309N 311N 312N 313N 314N 315N 709 750 1438 1888 2706 4216 4769 4823 4917 6261 7028 8697 8860 10463 10822 11251 11719 11812 13368 14233 14766 14905 15326 15452A 15607 15758 15928 16126 16146 16147 16292 16294 16296	T2c1c	73 263 303N 304N 305N 306N 307N 308N 309N 311N 312N 313N 314N 315N 16126 16146 16147 16292 16294 16296	[0.048] T2c1c (1)	[0.048] T2c1c (2) [0.000] T2c1 T2c1c1 T2c1c2 (5)	1
285 >MG182500.1 H	73G 185A 242T 263G 295T 302N 462T 489C 750G 992N 1438G 2158C 2706G 3010A 3106N 3108N 3109N 4216C 4604N 4769G 5460A 5746N 6345C 7028T 7299G 8269A 8557A 8860G 9794N 10398G 10813N 11031N 11251G 11719A 12007A 12417N 12612G 13708A 13879C 14766T 15326G 15452A 16069T 16126C 16145A 16172C 16222T 16261T	J1b1a1d	73G 185A 242T 263G 295T 302N 462T 489C 16069T 16126C 16145A 16172C 16222T 16261T	1	73 185 242 263 295 302N 462 489 750 992N 1438 2158 2706 3010 3106N 3108N 3109N 4216 4604N 4769 5460 5746N 6345 7028 7299 8269 8557 8860 9794N 10398 10813N 11031N 11251 11719 12007 12417N 12612 13708 13879 14766 15326 15452A 16069 16126 16145 16172 16222 16261	J1b1a1d	73 185 242 263 295 302N 462 489 16069 16126 16145 16172 16222 16261	[0.000] J1b1a1d (1)	[0.016] J1b1a1 (2) [0.000] J1b1a1b J1b1a1d (4)	1

286 >MG182487.1 H	263G 750G 1438G 4769G 5746N 7337A 8860G 9794N 10879N 11031N 11866N 12384N 15326G 16172C 16519C	H66a	263G 16172C 16519C	1	263 750 1438 4769 5746N 7337 8860 9794N 10879N 11031N 11866N 12384N 15326 16172	H66a	263 16172	[0.000] H66a (1)	[0.157] H50 (2) [0.143] H2a2a1d (3) [0.008] H (4) [0.004] H3 (5) [0.003] H1 (6) [0.001] H1e1a H1q H2a2 H2a2+(16235) H13a H14b H16b H56 H65 (15) [0.000] R0 HV HV4 HV4c HV5 HV5a HV6 HV9c HV12 H1e H1e1 H1e1a2 H1e1a7 H1e1c H1e2 H1e2a H1e2b H1e2d H1e4 H1e6 H1e7 H1e8 H1e8a H1h H1h1 H1h2 H1j H1j3 H1j5 H1j6 H1j7 H1j9 H1m H1n H1p H1q1 H1q2 H1r H1s H1s1 H1t H1t1 H1t1a H1t2 H1u H1u1 H1v H1v1 H1v1a H1v1b H1w H1ae H1ae1 H1ae2 H1ae2a H1ae3 H1ae3a H1ag H1ag1 H1ag1b H1ah H1ai H1ai1 H1aj H1ak H1ak1 H1ak2 H1am H1am1 H1ap H1aq H1as H1as2 H1at H1at1 H1at1a H1av H1ax H1ax1 H1ay H1az H1bd H1be H1bi H1bj H1bk H1bm H1bn H1bp H1bq H1br H1bu H1bv H1bw H1bx H1ca H1ci H2 H2a H2a2a H2a4 H2a5 H2a5a H2a5b H3b H3c H3c1 H3e H3q H3q1 H3r H3r1 H3t H3u H3y H3aa H3ab H3ac H3ad H3ae H3ag H3ag1 H3ah H3ai H3aj H3ap H3aq H3ar H3as H3at H3at1 H3au H4 H4a H4a1 H4a1a H4a1a1 H4a1a2 H4a1a2a H4a1a2a1 H4a1a5 [illegible]	1
287 >MG182486.1 H	73G 195C 263G 499A 750G 1438G 1811G 2706G 3106N 3108N 4646C 4769G 5088N 5089N 5090N 5091N 5092N 5093N 5094N 5095N 5508N 5999C 6047G 7028T 7269A 7298N 7299N 7300N 7301N 7302N 7303N 7304N 7305N 7306N 8705C 8860G 10373A 10676T 10907C 11332T 11467G 11719A 12308G 12372A 12417N 14504N 14505N 14506N 14507N 14508N 14509N 14510N 14620T 14766T 15326G 15693C 16261T 16356C	U4c2a	73G 195C 263G 499A 16261T 16356C	1	73 195 263 499 750 1438 1811 2706 3106N 3108N 4646 4769 5088N 5089N 5090N 5091N 5092N 5093N 5094N 5095N 5508N 5999 6047 7028 7269 7298N 7299N 7300N 7301N 7302N 7303N 7304N 7305N 7306N 8705 8860 10373 10676 10907 11332 11467 11719 12308 12372 12417N 14504N 14505N 14506N 14507N 14508N 14509N 14510N 14620 14766 15326 15693 16261 16356	U4c2a	73 195 263 499 16261 16356	[0.000] U4c2a (1)	[0.000] U4 U4a U4b U4b1 U4b1a U4b1a1 U4b1a1a U4b1a2 U4b1a2a U4b1a2b U4b1a4 U4c U4c2 U4d U4d1 U4d1a U4d1a1 U4d1a1a U4d1b (20)	1
288 >MG182485.1 H	73G 150T 228A 263G 750G 1438G 2706G 3108N 3197C 4769G 7028T 7226A 7465N 7768G 8860G 9196A 9477A 11467G 11719A 12308G 12372A 13617C 14182C 14766T 15326G 16192T 16270T 16304C 16526A	U5b3b2	73G 150T 228A 263G 16192T 16270T 16304C 16526A	1	73 150 228 263 750 1438 2706 3108N 3197 4769 7028 7226 7465N 7768 8860 9196 9477 11467 11719 12308 12372 13617 14182 14766 15326 16192 16270 16304 16526	U5b3b2	73 150 228 263 16192 16270 16304 16526	[0.000] U5b3b2 (1)	[0.019] U5b3 (2) [0.000] U5b3b U5b3b2 U5b3c U5b3e U5b3g (7)	1

289	>MG182484.1 H	65N 263G 311N 312N 313N 314N 315N 432N 456T 524N 750G 1438G 2129N 3108N 3109N 3397G 4336C 4547N 4769G 5281N 5746N 7465N 8727A 8856A 8860G 9477N 9794N 11506C 15326G 15833T 16209C 16304C 16519C	H5a1j	65N 263G 311N 312N 313N 314N 315N 432N 456T 524N 16209C 16304C 16519C	1	65N 263 311N 312N 313N 314N 315N 432N 456 524N 750 1438 2129N 3108N 3109N 3397 4336 4547N 4769 5281N 5746N 7465N 8727A 8856 8860 9477N 9794N 11506 15326 15833 16209 16304	H5a1j	65N 263 311N 312N 313N 314N 315N 432N 456 524N 16209 16304	[0.000] H5a1j (1)	[0.000] H5 H5a H5a1 H5a1a H5a1b H5a1c H5a1c1 H5a1c1a H5a1d H5a1f H5a1h H5a1k H5a1q H5a2 H5a2a H5a7 H5a8 H5a9 H5b H5b3 H5b5 H5c H5c1 H5c1a H5d H5f H5j H5k H5p H5+709 H5v (32)	1
290	>MG182483.1 H	72G 152C 263G 304N 305N 306N 307N 308N 309N 750G 1438G 3108N 4769G 8860G 9932A 12753G 13299G 14488C 15326G 16519C	H74	72G 152C 263G 304N 305N 306N 307N 308N 309N 16519C	1	72G 152 263 304N 305N 306N 307N 308N 309N 750 1438 3108N 4769 8860 9932 12753 13299 14488 15326	H74	72G 152 263 304N 305N 306N 307N 308N 309N	[0.154] H74 (1) [0.000] H1+152 H1i H1i1 H1i2 H1i2a H1an H1an1 H1an1a H1bb H1bc H2a2a2 H3+152 H3g H3g1 H3g1a H3g1b H3g2 H3g3 H3g4 H3i H3i1 H3j H3k H3k1 H3k1a H+152 H9 H46 H46a H46b H52 H69 H13a1+152 H14b H14b2 H14b4 H16+152 H16a H16a1 H16c H16d H30b1 H47a (44)	[0.031] H13a1a1a (45) [0.023] H2a2a (46) [0.022] H9a (47) [0.018] H3a1 (48) [0.012] HV (49) [0.009] H4a1 H26a1 (51) [0.005] H1h1 (52) [0.004] H3q1 H7b (54) [0.003] HV5 H4b H16e H58a H75 (59) [0.002] H1 H1e1a H1e2 H2a H3 H13a1a1 H13a2a H18 H18b (68) [0.001] HV4 H1e1a2 H1e1c H1h2 H1q H1t1a H1ag1 H1aj H1at1a H4 H4a1a H4a1a1 H4a1c H7b1 H7c6 H7d H10b H10+(16093) H13a2b4 H35 H40b H45a (90) [0.000] R0 HV1b+152 HV4c HV5a HV9+152 HV9c HV12 H H1c+152 H1c9 H1c9a H1e H1e1 H1e1a7 H1e2a H1e2b H1e2d H1e4 H1e6 H1e7 H1e8 H1e8a H1h H1j H1j3 H1j5 H1j6 H1j7 H1j9 H1m H1n H1p H1q1 H1q2 H1r H1s H1s1 H1t H1t1 H1t2 H1u H1u1 H1v H1v1 H1v1a H1v1b H1w H1ae H1ae1 H1ae2 H1ae2a H1ae3 H1ae3a H1ag H1ag1b H1ah H1ai H1ai1 H1ak H1ak1 H1ak2 H1am H1am1 H1ap H1ap1 H1aq H1as H1as2 H1at H1at1 H1av H1ax H1ax1 H1ay H1az H1bd H1be H1bi H1bj H1bk H1bm H1bn H1bp H1bq H1br H1bu H1bv H1bw H1bx H1ca H1ci H2 H2a2 [illegible]	1
291	>MG182473.1 H	73G 195C 263G 499A 750G 1438G 1811G 2083C 2456N 2706G 3106N 3108N 3672G 4604N 4646C 4769G 5746N 5999C 6047G 6691N 7028T 7705C 8642G 8860G 9377G 10946N 11031N 11332T 11339C 11467G 11719A 12297C 12308G 12372A 12406A 14620T 14766T 15326G 15693C 15789T 16356C 16362C 16519C	U4b1a1a1	73G 195C 263G 499A 16356C 16362C 16519C	1	73 195 263 499 750 1438 1811 2083 2456N 2706 3106N 3108N 3672 4604N 4646 4769 5746N 5999 6047 6691N 7028 7705 8642 8860 9377 10946N 11031N 11332 11339 11467 11719 12297 12308 12372 12406 14620 14766 15326 15693 15789 16356 16362	U4b1a1a1	73 195 263 499 16356 16362	[0.000] U4b1a1a1 (1)	[0.015] U4a (2) [0.000] U4 U4a3 U4b U4b1 U4b1a U4b1a1 U4b1a1a U4b1a2 U4b1a2a U4b1a2b U4b1a4 U4c U4c2 U4d U4d1 U4d1a U4d1a1 U4d1a1a U4d1b (21)	1

292	>MG182456.1 H	93G 263G 302N 306N 307N 308N 309N 311N 312N 313N 314N 315N 750G 1438G 2706G 2848G 3106N 3108N 3109N 4596A 4769G 5505N 5506N 5508N 5509N 5510N 7028T 8014T 8272N 8273N 8274N 8275N 8276N 8277N 8278N 8279N 8860G 10473N 10474N 10475N 10476N 10477N 10478N 10479N 10480N 10481N 10482N 10483N 10484N 10485N 10486N 10487N 10488N 10489N 10490N 12417N 15218G 15326G 16067T	HV1a2b	93G 263G 302N 306N 307N 308N 309N 311N 312N 313N 314N 315N 16067T	1	93 263 302N 306N 307N 308N 309N 311N 312N 313N 314N 315N 750 1438 2706 2848 3106N 3108N 3109N 4596 4769 5505N 5506N 5508N 5509N 5510N 7028 8014T 8272N 8273N 8274N 8275N 8276N 8277N 8278N 8279N 8860 10473N 10474N 10475N 10476N 10477N 10478N 10479N 10480N 10481N 10482N 10483N 10484N 10485N 10486N 10487N 10488N 10489N 10490N 12417N 15218 15326 16067	HV1a2b	93 263 302N 306N 307N 308N 309N 311N 312N 313N 314N 315N 16067	[0.000] HV1a2b (1)	[0.000] HV1 HV1a'b'c HV1a HV1a2 HV1a2a HV1b HV1c H1e1a1 H49a2 (10)	1
293	>MH605021.1 H	73G 185A 228A 263G 295T 309.1CT 310C 462T 482C 489C 750G 1438G 2706G 3010A 3106d 3107C 3394C 4216C 4769G 7028T 8860G 10398G 11251G 11719A 11884G 12612G 13708A 14766T 14798C 15326G 15452A 16069T 16126C 16213A	J1c1d	73G 185A 228A 263G 295T 309.1CT 310C 462T 482C 489C 16069T 16126C 16213A	1	73 185 228 263 295 462 482 489 750 1438 2706 3010 3394 4216 4769 7028 8860 10398 11251 11719 11884 12612 13708 14766 14798 15326 15452A 16069 16126 16213	J1c1d	73 185 228 263 295 462 482 489 16069 16126 16213	[0.000] J1c1d (1)	[0.000] J1c1 J1c1a J1c1b J1c1b1 J1c1b1a J1c1b1a1 J1c1b2 J1c1b2a J1c1d J1c1f J1c1g J1c1g1 (13)	1
294	>MH546077.1 H	73G 198T 263G 310.1C 750G 1438G 2706G 3106d 3107C 3197C 4769G 5474G 7028T 8860G 9477A 9667G 11467G 11719A 12308G 12358G 12372A 13617C 14766T 14793G 15218G 15326G 16093Y 16192T 16256T 16270T 16291T 16399G	U5a1b1d1	73G 198T 263G 310.1C 16093Y 16192T 16256T 16270T 16291T 16399G	1	73 198 263 750 1438 2706 3197 4769 5474 7028 8860 9477 9667 11467 11719 12308 12358 12372 13617 14766 14793 15218 15326 16093Y 16192 16256 16270 16291 16399	U5a1b1d1	73 198 263 16093Y 16192 16256 16270 16291 16399	[0.000] U5a1b1d1 (1)	[0.000] U5a1b1d+16093 (2)	1
295	>MH542465.1 H	127C 263G 309.1CCT 310C 750G 951A 3106d 3107C 8860G 15326G 16193T 16354T	H2a1f1	127C 263G 309.1CCT 310C 16193T 16354T	1	127 263 750 951 8860 15326 16193 16354	H2a1f1	127 263 16193 16354	[0.000] H2a1f1 (1)	[0.000] H2a1f (2)	1
296	>MG952872.1 H	146C 195C 263G 309.1CCT 310C 456T 514d 515d 750G 1438G 3106d 3107C 4769G 5471A 8860G 14497G 15326G 16304C 16311C	H5b1	146C 195C 263G 309.1CCT 310C 456T 514d 515d 16304C 16311C	1	146 195 263 456 750 1438 4769 5471 8860 14497 15326 16304 16311	H5b1	146 195 263 456 16304 16311	[0.006] H5b1 (1)	[0.012] H5+16311 (2) [0.006] H5a5 (3) [0.000] H5m	1
297	>MG952849.1 H	93G 195C 263G 310.1C 750G 1438G 3106d 3107C 3936T 4769G 8860G 14552G 15326G 16287T 16519C	H12a	93G 195C 263G 310.1C 16287T 16519C	1	93 195 263 750 1438 3936 4769 8860 14552 15326 16287	H12a	93 195 263 16287	[0.133] H12a (1)	[0.016] H4a1a4b (2) [0.000] H1e1a1 H4a1a+195 H4a1a3 H4a1a3a H4a1a4 H4a1a4a H4a1a4b2 H+195 H12 H108 H44b H49a2 (14)	1
298	>MG952846.1 H	73G 152C 199C 204C 263G 310.1C 574C 669C 750G 1438G 1719A 2687T 2702A 2706G 3106d 3107C 3336C 4769G 5315G 7028T 8860G 8901G 9974T 10238C 10398G 11719A 11800G 12501A 12705T 13780G 14305A 14766T 15043A 15326G 16147A 16154C 16172C 16223T 16248T 16294T 16320T 16355T 16519C	N1a1a1a3	73G 152C 199C 204C 263G 310.1C 574C 16147A 16154C 16172C 16223T 16248T 16294T 16320T 16355T 16519C	1	73 152 199 204 263 574C 669 750 1438 1719 2687 2702 2706 3336 4769 5315 7028 8860 8901 9974 10238 10398 11719 11800 12501 12705 13780 14305 14766 15043 15326 16147A 16154 16172 16223 16248 16294 16320 16355	N1a1a1a3	73 152 199 204 263 574C 16147A 16154 16172 16223 16248 16294 16320 16355	[0.239] N1a1a1a3 (1)	[0.416] N1a1a1a1 (2) [0.235] N1a1a1a	1

299 >MG952845.1 H	73G 146C 263G 285T 309.1CCT 310C 750G 1438G 2387C 2706G 2858G 3106d 3107C 4769G 5894.1C 7028T 8395T 8581A 8860G 10885C 11467G 11566G 11719A 12308G 12372A 12879C 13104G 14070G 14766T 15148A 15172A 15326G 15954C 16111T 16214A 16249C 16327T	U1b2	73G 146C 263G 285T 309.1CCT 310C 16111T 16214A 16249C 16327T	1	73 146 263 285 750 1438 2387 2706 2858 4769 5899.1C 7028 8395 8581 8860 10885 11467 11566 11719 12308 12372 12879 13104 14070 14766 15148 15172 15326 15954C 16111 16214A 16249 16327	U1b2	73 146 263 285 16111 16214A 16249 16327	[0.000] U1b2 (1)	[0.000] U1b U1b1 U1b2 (4)	1
300 >MG952835.1 H	73G 146C 263G 310.1C 488G 750G 1438G 1811G 2217T 2626C 2706G 3106d 3107C 3480G 4769G 5231A 7028T 8860G 9055A 9698C 9716C 10550G 11299C 11467G 11719A 11869A 12308G 12372A 13135A 14037G 14167T 14766T 14798C 15326G 16224C 16270T 16311C 16519C	K2b1a1	73G 146C 263G 310.1C 488G 16224C 16270T 16311C 16519C	1	73 146 263 488 750 1438 1811 2217 2626 2706 3480 4769 5231 7028 8860 9055 9698 9716 10550 11299 11467 11719 11869A 12308 12372 13135 14037 14167 14766 14798 15326 16224 16270 16311	K2b1a1	73 146 263 488 16224 16270 16311	[0.103] K2b1a1 (1)	[0.000] K2 K2b K2b1 K2b1a K2b1a1a K2b1a2 K2b1a3 K2b2 K2c (10)	1
301 >MG952810.1 H	152C 263G 309.1CT 310C 750G 1438G 3106d 3107C 3591A 4310G 4769G 8860G 13020C 13734C 15326G 16168T 16269G 16519C	H9a	152C 263G 309.1CT 310C 16168T 16269G 16519C	1	152 263 750 1438 3591 4310 4769 8860 13020 13734 15326 16168 16269	H9a	152 263 16168 16269	[0.022] H9a (1)	[0.004] H+152 (2) [0.000] H1+152 H1i H1i1 H1i2 H1i2a H1an H1an1 H1an1a H1bb H1bc H2a2a2 H3+152 H3g H3g1 H3g1a H3g1b H3g2 H3g3 H3g4 H3i H3i1 H3j H3k H3k1 H3k1a H7f H9 H46 H46a H46b H52 H69 H13a1+152 H14b H14b2 H14b4 H16+152 H16a H16a1 H16c H16d H30b1 H47a (45)	1
302 >MG952807.1 H	73G 150T 263G 295T 309.1CT 310C 489C 750G 1438G 2706G 3106d 3107C 4216C 4769G 5633T 7028T 7476T 8860G 9016G 9139A 10172A 10398G 11251G 11719A 12612G 13708A 14766T 15257A 15326G 15452A 15662G 15812A 16069T 16126C 16193T 16278T 16519C	J2b1a6	73G 150T 263G 295T 309.1CT 310C 489C 16069T 16126C 16193T 16278T 16519C	1	73 150 263 295 489 750 1438 2706 4216 4769 5633 7028 7476 8860 9016 9139 10172 10398 11251 11719 12612 13708 14766 15257 15326 15452A 15662 15812 16069 16126 16193 16278	J2b1a6	73 150 263 295 489 16069 16126 16193 16278	[0.000] J2b1a6 (1)	[0.015] J2b1a2 (2) [0.000] J2b1a J2b1a1 J2b1a2a J2b1a3 J2b1a4 J2b1a6 (8)	1
303 >MG952804.1 H	73G 153G 195C 225A 227G 263G 310.1C 750G 1438G 1719A 2120A 2706G 3106d 3107C 4769G 6221C 6371T 7028T 8463G 8705C 8860G 9477A 11719A 12705T 13966G 14470C 14766T 15326G 16182C 16183C 16189C 16223T 16255A 16278T 16300G 16519C	X2c1b	73G 153G 195C 225A 227G 263G 310.1C 16182C 16183C 16189C 16223T 16255A 16278T 16300G 16519C	1	73 153 195 225 227 263 750 1438 1719 2120 2706 4769 6221 6371 7028 8463 8705 8860 9477 11719 12705 13966 14470 14766 15326 16189 16223 16255 16278 16300	X2c1b	73 153 195 225 227 263 16189 16223 16255 16278 16300	[0.000] X2c1b (1)	[0.000] X2c X2c1 X2c1d X2c2 (5)	1
304 >MG952803.1 H	73G 195C 225A 226C 263G 310.1C 750G 1048T 1438G 1719A 2706G 3106d 3107C 4059T 4769G 6221C 6371T 7028T 8473C 8860G 11719A 12705T 13966G 14470C 14766T 15326G 16189C 16217C 16223T 16270T 16278T 16519C	X2m2	73G 195C 225A 226C 263G 310.1C 16189C 16217C 16223T 16270T 16278T 16519C	1	73 195 225 226 263 750 1048 1438 1719 2706 4059 4769 6221 6371 7028 8473 8860 11719 12705 13966 14470 14766 15326 16189 16217 16223 16270 16278	X2m2	73 195 225 226 263 16189 16217 16223 16270 16278	[0.286] X2m2 (1)	[0.036] X2b+226 (2) [0.016] X2b4a1 (3) [0.008] X2b6a (4) [0.007] X2b4 (5) [0.000] X2b2 X2b3 X2b4a X2b5 X2b6 X2b7 X2b8 X2b9 X2+225+@153 X2g X2l X2m'n X2m (18)	1

305	>MG952795.1 H[illegible]	263G 309T 310C 514d 515d 516d 517d 750G 1438G 3010A 3106d 3107C 3796G 4769G 8860G 15326G 16129A 16189C 16355T 16356C 16362C 16519C	H1b1b	263G 309T 310C 514d 515d 516d 517d 16129A 16189C 16355T 16356C 16362C 16519C	1	263 309d 750 1438 3010 3796 4769 8860 15326 16129 16189 16355 16356 16362	H1b1b	263 309d 16129 16189 16355 16356 16362	[0.012] H1b1b (1)	[0.014] H1b1+16362 (2) [0.009] H1b1a (3) [0.000] H1b1c H1b1h (5)	1
306	>MG952794.1 H[illegible]	73G 150T 263G 295T 309.1CT 310C 489C 750G 1438G 2706G 3106d 3107C 4216C 4769G 5633T 7028T 7476T 8860G 9016G 10172A 10398G 11251G 11719A 11767T 12612G 13708A 14245A 14766T 15257A 15326G 15380G 15452A 15662G 15812A 16069T 16126C 16193T 16278T 16519C	J2b1a6	73G 150T 263G 295T 309.1CT 310C 489C 16069T 16126C 16193T 16278T 16519C	1	73 150 263 295 489 750 1438 2706 4216 4769 5633 7028 7476 8860 9016 10172 10398 11251 11719 11767 12612 13708 14245A 14766 15257 15326 15380 15452A 15662 15812 16069 16126 16193 16278	J2b1a6	73 150 263 295 489 16069 16126 16193 16278	[0.000] J2b1a6 (1)	[0.015] J2b1a2 (2) [0.000] J2b1a J2b1a1 J2b1a2a J2b1a3 J2b1a4 J2b1a6 (8)	1
307	>MG952788.1 H[illegible]	73G 146C 195C 263G 310.1C 750G 1438G 1811G 2217T 2706G 3106d 3107C 3480G 4769G 5054A 5231A 5460A 7028T 8860G 9055A 9698C 9716C 10550G 11016A 11299C 11467G 11719A 11869A 12308G 12372A 12501A 14037G 14167T 14766T 14798C 15326G 16129A 16224C 16311C 16519C	K2b1b	73G 146C 195C 263G 310.1C 16129A 16224C 16311C 16519C	1	73 146 195 263 750 1438 1811 2217 2706 3480 4769 5054 5231 5460 7028 8860 9055 9698 9716 10550 11016 11299 11467 11719 11869A 12308 12372 12501 14037 14167 14766 14798 15326 16129 16224 16311	K2b1b	73 146 195 263 16129 16224 16311	[0.417] K2b1b (1) [0.000] K1b2 K1b2a K1b2a1 K1b2a1a K1b2a2 K1b2a2a K1b2a3 K1b2b (9)	[0.008] K2b1 K2b2 (11) [0.000] K1a13 K1a13a K1b2a1a1 K1b2b1 K2 K2b K2b1a K2b1a2 K2b1a3 K2c (21)	1
308	>MG952783.1 H[illegible]	73G 152C 235G 263G 310.1C 514d 515d 663G 750G 1438G 1709A 1736G 2706G 3106d 3107C 4248C 4769G 4824G 7028T 7308G 8794T 8839A 8860G 9754G 11719A 12705T 12720G 14290C 14766T 15326G 16039A 16176T 16188T 16189C 16223T 16290T 16319A 16356C 16362C	A12a	73G 152C 235G 263G 310.1C 514d 515d 16039A 16176T 16188T 16189C 16223T 16290T 16319A 16356C 16362C	1	73 152 235 263 663 750 1438 1709 1736 2706 4248 4769 4824 7028 7308 8794 8839 8860 9754 11719 12705 12720 14290 14766 15326 16039 16176 16188 16189 16223 16290 16319 16356 16362	A12a	73 152 235 263 16039 16176 16188 16189 16223 16290 16319 16356 16362	[0.320] A12a (1)	[0.036] A12 (2) [0.000] A+152+16362+16189	1
309	>MH178741.1 H[illegible]	73G 199C 263G 310.1C 750G 1438G 2706G 3106d 3107C 3197C 4769G 5585A 6023A 7028T 7569G 8251A 8860G 9477A 11467G 11719A 12308G 12372A 13617C 14766T 14793G 15218G 15326G 16192T 16256T 16270T 16311C 16399G 16526A	U5a1f1a1	73G 199C 263G 310.1C 16192T 16256T 16270T 16311C 16399G 16526A	1	73 199 263 750 1438 2706 3197 4769 5585 6023 7028 7569 8251 8860 9477 11467 11719 12308 12372 13617 14766 14793 15218 15326 16192 16256 16270 16311 16399 16526	U5a1f1a1	73 199 263 16192 16256 16270 16311 16399 16526	[0.000] U5a1f1a1 (1)	[0.000] U5a1f1a (2)	1
310	>MH161389.1 H[illegible]	73G 152C 225A 248d 263G 309.1CT 310C 316A 489C 514d 515d 750G 1438G 2706G 3106d 3107C 3200C 3714G 4385T 4769G 4772C 7028T 7433T 8701G 8853G 8860G 9127G 9512T 9540C 10274C 10398G 10400T 10679G 10873C 11719A 11914A 12354C 12705T 14110C 14142T 14766T 14783C 14974T 15043A 15301A 15326G 15691G 16129A 16209C 16223T 16272G 16519C	M20	73G 152C 225A 248d 263G 309.1CT 310C 316A 489C 514d 515d 16129A 16209C 16223T 16272G 16519C	1	73 152 225 249d 263 316 489 750 1438 2706 3200 3714 4385T 4769 4772 7028 7433 8701 8853 8860 9127 9512 9540 10274 10398 10400 10679 10873 11719 11914 12354 12705 14110 14142 14766 14783 14974 15043 15301 15326 15691 16129 16209 16223 16272	M20	73 152 225 249d 263 316 489 16129 16209 16223 16272	[0.307] M20 (1)	[0.068] M17 (2) [0.067] M17a (3) [0.059] G1b+16129 (4) [0.028] M5a (5) [0.021] D4a1a1 (6) [0.019] M5 (7) [0.017] M5a2a (8) [0.007] M5a'd (9) [0.004] M37+152 (10) [0.003] D4a1 (11) [0.000] M5a2 M5a2a1 M5a5 M5b'c M5b M7a M7a1b2 M7a2 M7b1 G1b2 G1b4 M13'46'61 M13 M13a'b M13b M17c D4a D4a1a D4a1a1a D4a1b D4a1c D4a1f D4a1f1 D4a1g D4a1h D4a2 D4a2a D4a2b D4a5 (40)	1

311	>MH158239.1 H	73G 152C 217C 263G 309.1CCT 310C 340T 508G 514d 515d 750G 1438G 1811G 2526T 2706G 3106d 3107C 3720G 4769G 5390G 5426C 5951G 6045T 6152C 7028T 8860G 10876G 11467G 11719A 12308G 12372A 12618A 13020C 13734C 14766T 15326G 15907G 16051G 16129C 16145A 16183C 16189C 16223T 16362C 16519C 16527T	U2e1e	73G 152C 217C 263G 309.1CCT 310C 340T 508G 514d 515d 16051G 16129C 16145A 16183C 16189C 16223T 16362C 16519C 16527T	1	73 152 217 263 340 508 750 1438 1811 2526 2706 3720 4769 5390 5426 5951 6045 6152 7028 8860 10876 11467 11719 12308 12372 12618 13020 13734 14766 15326 15907 16051 16129C 16145 16189 16223 16362 16527	U2e1e	73 152 217 263 340 508 16051 16129C 16145 16189 16223 16362 16527	[0.182] U2e1e (1)	[0.000] U2e1 U2e1a U2e1a1 U2e1a1a U2e1a1c U2e1c U2e1c1 U2e1d U2e1g (10)	1
312	>MH161390.1 H	73G 151T 152C 263G 310.1C 750G 1438G 2706G 3106d 3107C 3197C 3768G 4769G 6182A 7028T 8860G 9477A 11467G 11719A 12308G 12372A 13617C 14766T 14793G 15289C 15326G 16148Y 16189C 16192T 16234T 16256T 16270T 16311C 16362C 16526A	U5a2e	73G 151T 152C 263G 310.1C 16148Y 16189C 16192T 16234T 16256T 16270T 16311C 16362C 16526A	1	73 151 152 263 750 1438 2706 3197 3768 4769 6182 7028 8860 9477 11467 11719 12308 12372 13617 14766 14793 15289 15326 16148Y 16189 16192 16234 16256 16270 16311 16362 16526	U5a2e	73 151 152 263 16148Y 16189 16192 16234 16256 16270 16311 16362 16526	[1.124] U5a2e (1)	[0.040] U5a2+16362 (2) [0.000] U5a2b2a1	1
313	>MH158238.1 H	73G 146C 243G 263G 310.1C 750G 1438G 1811G 2217T 2706G 3106d 3107C 3480G 4769G 5231A 7028T 8860G 9055A 9698C 9716C 10550G 11299C 11467G 11719A 11869A 12308G 12372A 13135A 14037G 14167T 14766T 14798C 15326G 16222T 16224C 16270T 16304C 16311C 16519C	K2b1a1a	73G 146C 243G 263G 310.1C 16222T 16224C 16270T 16304C 16311C 16519C	1	73 146 243 263 750 1438 1811 2217 2706 3480 4769 5231 7028 8860 9055 9698 9716 10550 11299 11467 11719 11869A 12308 12372 13135 14037 14167 14766 14798 15326 16222 16224 16270 16304 16311	K2b1a1a	73 146 243 263 16222 16224 16270 16304 16311	[0.068] K2b1a1a (1)	[0.000] K2b1a1 (2)	1
314	>KY782247.1 H	73G 114T 263G 310.1C 497T 750G 1189C 1438G 1811G 2706G 3106d 3107C 3480G 4769G 7028T 8860G 9055A 9698C 10398G 10550G 10978G 11299C 11467G 11470G 11719A 11914A 12308G 12372A 12954C 14040A 14167T 14766T 14798C 15326G 15924G 16223T 16224C 16234T 16311C 16519C	K1a1b1a	73G 114T 263G 310.1C 497T 16223T 16224C 16234T 16311C 16519C	1	73 114 263 497 750 1189 1438 1811 2706 3480 4769 7028 8860 9055 9698 10398 10550 10978 11299 11467 11470 11719 11914 12308 12372 12954 14040 14167 14766 14798 15326 15924 16223 16224 16234 16311	K1a1b1a	73 114 263 497 16223 16224 16234 16311	[0.435] K1a1b1a (1)	[0.435] K1a1b1a (2) [0.072] K1a1b1 (3) [0.000] K1a1 K1a1a K1a1a1 K1a1a2 K1a1a2a K1a1a2a1 K1a1b K1a1b1b K1a1b1b1 K1a1b1d K1a1b1e K1a1b1f K1a1b1g K1a1b2 (17)	1
315	>KY782237.1 H	73G 195C 263G 310C 499A 513.1CACACA 750G 1438G 1811G 2706G 3106d 3107C 4646C 4769G 5999C 6047G 7028T 8818T 8860G 11332T 11467G 11719A 12308G 12372A 14620T 14766T 15326G 15693C 16223T 16356C 16519C	U4a2b	73G 195C 263G 310C 499A 513.1CACACA 16223T 16356C 16519C	1	73 195 263 310 499 750 1438 1811 2706 4646 4769 5999 6047 7028 8818 8860 11332 11467 11719 12308 12372 14620 14766 15326 15693 16223 16356	U4a2b	73 195 263 310 499 16223 16356	[0.000] U4a2b (1)	[0.000] U4a2 U4a2c U4a2e U4a2f U4a2g U4a2h (7)	1

316 >KY782228.1 Hc	41T 73G 263G 310.1C 319C 709A 750G 930A 1438G 1888A 2706G 3106d 3107C 4216C 4769G 4917G 5147A 6845T 7028T 8697A 8860G 9861C 10463C 11251G 11719A 11812G 13368A 13547T 14233G 14766T 14905A 15326G 15452A 15607G 15928A 16126C 16294T 16296T 16304C 16519C	T2b34	41T 73G 263G 310.1C 319C 16126C 16294T 16296T 16304C 16519C	1	41 73 263 319 709 750 930 1438 1888 2706 4216 4769 4917 5147 6845 7028 8697 8860 9861 10463 11251 11719 11812 13368 13547 14233 14766 14905 15326 15452A 15607 15928 16126 16294 16296 16304	T2b34	41 73 263 319 16126 16294 16296 16304	[0.000] T2b34 (1)	[0.004] T2b (2) [0.000] T2b1 T2b2 T2b3 T2b3b T2b4 T2b5 T2b5a T2b7 T2b7a T2b8 T2b13 T2b13b T2b15 T2b17 T2b17a T2b19 T2b25 T2b26 T2b27 T2b28 T2b29 T2b30 T2b31 T2b32 T2b35 T2b36 T2b37 (29)	1
317 >KY782226.1 Hc	195C 263G 310.1C 750G 961G 1438G 3106d 3107C 4769G 5585A 8448C 8860G 12235C 13759A 14587G 15326G 15670C 16140C 16265G 16293G 16311C	H11a2a2	195C 263G 310.1C 16140C 16265G 16293G 16311C	1	195 263 750 961G 1438 4769 5585 8448 8860 12235 13759 14587 15326 15670 16140 16265 16293 16311	H11a2a2	195 263 16140 16265 16293 16311	[0.000] H11a2a2 (1)	[0.000] H11a2a H11a2a1 H11a2a2 H11a2a3 (5)	1
318 >KY782221.1 Hc	263G 309.1CT 310C 750G 1438G 3010A 3106d 3107C 4769G 8251A 8860G 10003C 11152C 14052T 15326G 16080G 16189C 16356C	H1b2	263G 309.1CT 310C 16080G 16189C 16356C	1	263 750 1438 3010 4769 8251 8860 10003 11152 14052 15326 16080 16189 16356	H1b2	263 16080 16189 16356	[0.000] H1b2 (1)	[0.000] H1b H1b1 H1b1e H1b1e1 H1b1i H1b4 H1b5 (8)	1
319 >KY782217.1 Hc	73G 153G 195C 225A 227G 263G 310.1C 750G 1438G 1719A 2706G 3106d 3107C 4769G 6221C 6371T 7028T 8463G 8705C 8860G 11719A 12705T 13966G 14470C 14766T 15326G 16183C 16189C 16223T 16255A 16278T 16300G 16519C	X2c1b	73G 153G 195C 225A 227G 263G 310.1C 16183C 16189C 16223T 16255A 16278T 16300G 16519C	1	73 153 195 225 227 263 750 1438 1719 2706 4769 6221 6371 7028 8463 8705 8860 11719 12705 13966 14470 14766 15326 16189 16223 16255 16278 16300	X2c1b	73 153 195 225 227 263 16189 16223 16255 16278 16300	[0.000] X2c1b (1)	[0.000] X2c X2c1 X2c1d X2c2 (5)	1
320 >KY782213.1 Hc	73G 151T 152C 263G 309.1CT 310C 750G 1438G 2706G 3106d 3107C 3197C 3768G 4769G 7028T 7229T 8860G 9477A 11467G 11719A 12308G 12372A 13617C 14766T 14793G 15289C 15326G 16189C 16192T 16234T 16256T 16270T 16311C 16362C 16526A	U5a2e	73G 151T 152C 263G 309.1CT 310C 16189C 16192T 16234T 16256T 16270T 16311C 16362C 16526A	1	73 151 152 263 750 1438 2706 3197 3768 4769 7028 7229 8860 9477 11467 11719 12308 12372 13617 14766 14793 15289 15326 16189 16192 16234 16256 16270 16311 16362 16526	U5a2e	73 151 152 263 16189 16192 16234 16256 16270 16311 16362 16526	[1.093] U5a2e (1)	[0.040] U5a2+16362 (2) [0.000] U5a2b2a1	1
321 >KY782208.1 Hc	73G 195C 263G 310C 499A 513.1CA 750G 1438G 1811G 2706G 3106d 3107C 4646C 4769G 5999C 6047G 7028T 8818T 8860G 11332T 11467G 11719A 12308G 12372A 14620T 14766T 15326G 15693C 16223T 16356C 16519C	U4a2b	73G 195C 263G 310C 499A 513.1CA 16223T 16356C 16519C	1	73 195 263 310 499 750 1438 1811 2706 4646 4769 5999 6047 7028 8818 8860 11332 11467 11719 12308 12372 14620 14766 15326 15693 16223 16356	U4a2b	73 195 263 310 499 16223 16356	[0.000] U4a2b (1)	[0.000] U4a2 U4a2c U4a2e U4a2f U4a2g U4a2h (7)	1
322 >KY782207.1 Hc	239C 263G 310.1C 750G 1438G 3106d 3107C 4769G 6869T 8860G 9804A 14185T 15326G 16362C 16400T 16482G	H6c1	239C 263G 310.1C 16362C 16400T 16482G	1	239 263 750 1438 4769 6869 8860 9804 14185T 15326 16362 16400 16482	H6c1	239 263 16362 16400 16482	[0.000] H6c1 (1)	[0.000] H6 H6a H6a1 H6a1a H6a1a1 H6a1a2 H6a1a2b H6a1a2b1 H6a1a3 H6a1a3a H6a1a4 H6a1a5 H6a1a9 H6a1a10 H6a1b H6a1b2 H6a1b2a H6a1b2b H6a1b2c H6a1b2e H6a2 H6a2a H6c H6c1 (25)	1

323 >KY782204.1 Hc	64T 73G 146C 235G 263G 310.1C 514d 515d 663G 722T 750G 1438G 1736G 2706G 3106d 3107C 3395G 4248C 4769G 4824G 6779G 7028T 7762A 7859A 8794T 8860G 11719A 12705T 14766T 15326G 16242T 16290T 16293C 16319A	A8a	64T 73G 146C 235G 263G 310.1C 514d 515d 16242T 16290T 16293C 16319A	1	64 73 146 235 263 663 722 750 1438 1736 2706 3395 4248 4769 4824 6779 7028 7762 7859 8794 8860 11719 12705 14766 15326 16242 16290 16293C 16319	A8a	64 73 146 235 263 16242 16290 16293C 16319	[0.702] A8a (1)	[0.130] A8a1 (2)	1
324 >KY782199.1 Hc	73G 150T 263G 309.1CT 310C 516T 750G 1438G 2706G 2833G 3106d 3107C 3197C 4769G 5656G 5894.1C 7028T 7768G 8860G 9477A 9707C 11467G 11719A 12308G 12372A 13617C 14182C 14766T 15191C 15326G 16140C 16174T 16189C 16270T 16288C 16311C	U5b1c2a	73G 150T 263G 309.1CT 310C 516T 16140C 16174T 16189C 16270T 16288C 16311C	1	73 150 263 516 750 1438 2706 2833 3197 4769 5656 5899.1C 7028 7768 8860 9477 9707 11467 11719 12308 12372 13617 14182 14766 15191 15326 16140 16174 16189 16270 16288 16311	U5b1c2a	73 150 263 516 16140 16174 16189 16270 16288 16311	[0.000] U5b1c2a (1)	[0.250] U5b1c2 (2) [0.000] U5b1c2b	1
325 >KY782198.1 Hc	73G 263G 310.1C 750G 1438G 3010A 3106d 3107C 4769G 6188T 8860G 15326G 16051G 16162G 16266T 16519C	H1a3c	73G 263G 310.1C 16051G 16162G 16266T 16519C	1	73 263 750 1438 3010 4769 6188 8860 15326 16051 16162 16266	H1a3c	73 263 16051 16162 16266	[0.000] H1a3c (1)	[0.000] H1a3 H1a3a H1a3a1 H1a3c1 H1a3d (6)	1
326 >KY782193.1 Hc	263G 309.1CT 310C 750G 1438G 3106d 3107C 4769G 4793G 6296A 8395T 8503C 8860G 15326G 16265G 16519C	H7c1	263G 309.1CT 310C 16265G 16519C	1	263 750 1438 4769 4793 6296A 8395 8503 8860 15326 16265	H7c1	263 16265	[0.000] H7c1 (1)	[0.001] H1h1 H2a2a (3) [0.000] R0 HV HV4 HV4c HV5 HV5a HV9c HV12 H H1 H1e H1e1 H1e1a H1e1a2 H1e1a7 H1e1c H1e2 H1e2a H1e2b H1e2d H1e4 H1e6 H1e7 H1e8 H1e8a H1h H1h2 H1j H1j3 H1j5 H1j6 H1j7 H1j9 H1m H1n H1p H1q H1q1 H1q2 H1r H1s H1s1 H1t H1t1 H1t1a H1t2 H1u H1u1 H1v H1v1 H1v1a H1v1b H1w H1ae H1ae1 H1ae2 H1ae2a H1ae3 H1ae3a H1ag H1ag1 H1ag1b H1ah H1ai H1ai1 H1aj H1ak H1ak1 H1ak2 H1am H1am1 H1ap H1aq H1as H1as2 H1at H1at1 H1at1a H1av H1ax H1ax1 H1ay H1az H1bd H1be H1bi H1bj H1bk H1bm H1bn H1bp H1bq H1br H1bu H1bv H1bw H1bx H1ca H1ci H2 H2a H2a2 H2a2+(16235) H2a4 H2a5 H2a5a H2a5b H3 H3b H3c H3c1 H3e H3q H3q1 H3r H3r1 H3t H3u H3y H3aa H3ab H3ac H3ad H3ae H3ag H3ag1 H3ah H3ai H3aj H3ap H3aq H3ar H3as H3at H3at1 H3au H4 H4a H4a1 H4a1a H4a1a1 H4a1a2 H4a1a2a H4a1a2a1 H4a1a5 H4a1c H4a1c1 H4a1c1a H4a1d H4b H4c H7 H7a H7b H7b1 H7b2 H7b2a H7b3 H7b4 H7b5 H7b6 H7c [illegible]	1

327 >KY782187.1 Ho	73G 195C 263G 310.1C 513.1CA 750G 1438G 2706G 3106d 3107C 3197C 4769G 7028T 8860G 9477A 11467G 11719A 11809C 12308G 12372A 13617C 13802T 14766T 14793G 15218G 15326G 16192T 16256T 16270T 16320T 16399G	U5a1c1	73G 195C 263G 310.1C 513.1CA 16192T 16256T 16270T 16320T 16399G	1	73 195 263 750 1438 2706 3197 4769 7028 8860 9477 11467 11719 11809 12308 12372 13617 13802 14766 14793 15218 15326 16192 16256 16270 16320 16399	U5a1c1	73 195 263 16192 16256 16270 16320 16399	[0.000] U5a1c1 (1)	[0.000] U5a1c U5a1c1a U5a1c2 (4)	1
328 >KY782181.1 Ho	143A 228A 263G 309.1CT 310C 750G 1438G 3106d 3107C 3984T 4769G 4793G 8860G 11016A 11971T 15326G 16213A 16519C	H7i1	143A 228A 263G 309.1CT 310C 16213A 16519C	1	143 228 263 750 1438 3984 4769 4793 8860 11016 11971 15326 16213	H7i1	143 228 263 16213	[0.077] H7i1 (1)	[0.000] H7h H7h1 H7i (4)	1
329 >KY782178.1 Ho	239C 263G 309.1CT 310C 750G 1438G 3106d 3107C 3915A 4727G 4769G 8860G 9380A 10589A 15326G 16249C 16362C 16482G	H6a1b4	239C 263G 309.1CT 310C 16249C 16362C 16482G	1	239 263 750 1438 3915 4727 4769 8860 9380 10589 15326 16249 16362 16482	H6a1b4	239 263 16249 16362 16482	[0.000] H6a1b4 (1)	[0.000] H6 H6a H6a1 H6a1a H6a1a1 H6a1a2 H6a1a2b H6a1a2b1 H6a1a3 H6a1a3a H6a1a4 H6a1a5 H6a1a9 H6a1a10 H6a1b H6a1b2 H6a1b2a H6a1b2b H6a1b2c H6a1b2e H6a1b4 H6a2 H6a2a H6c (25)	1
330 >KY782165.1 Ho	152C 263G 309.1CT 310C 750G 1438G 2706G 3106d 3107C 3547G 4769G 6023A 7028T 8014T 8860G 12696C 15218G 15326G 16067T 16183C 16189C 16519C	HV1b2	152C 263G 309.1CT 310C 16067T 16183C 16189C 16519C	1	152 263 750 1438 2706 3547 4769 6023 7028 8014T 8860 12696 15218 15326 16067 16189	HV1b2	152 263 16067 16189	[0.000] HV1b2 (1)	[0.000] HV1b+152 H1ap1 H3av (4)	1
331 >KY782151.1 Ho	73G 152C 217C 263G 310.1C 340T 508G 513.1CA 546G 750G 988A 1438G 1811G 2706G 3106d 3107C 3720G 4769G 5199G 5390G 5426C 6045T 6152C 7028T 7109T 8860G 10876G 11467G 11719A 12308G 12372A 13020C 13734C 14766T 15326G 15784C 15907G 16051G 16129C 16189C 16256T 16519C	U2e1b1	73G 152C 217C 263G 310.1C 340T 508G 513.1CA 546G 16051G 16129C 16189C 16256T 16519C	1	73 152 217 263 340 508 546 750 988 1438 1811 2706 3720 4769 5199 5390 5426 6045 6152 7028 7109 8860 10876 11467 11719 12308 12372 13020 13734 14766 15326 15784 15907 16051 16129C 16189 16256	U2e1b1	73 152 217 263 340 508 546 16051 16129C 16189 16256	[0.054] U2e1b1 (1)	[0.000] U2e1b U2e1b2 (3)	1
332 >KY782150.1 Ho	73G 151T 152C 263G 309.1CT 310C 750G 1438G 2706G 3106d 3107C 3197C 3768G 4769G 7028T 7229T 8860G 9477A 11467G 11719A 12308G 12372A 13617C 14766T 14793G 15289C 15326G 16189C 16192T 16234T 16256T 16270T 16311C 16362C 16526A	U5a2e	73G 151T 152C 263G 309.1CT 310C 16189C 16192T 16234T 16256T 16270T 16311C 16362C 16526A	1	73 151 152 263 750 1438 2706 3197 3768 4769 7028 7229 8860 9477 11467 11719 12308 12372 13617 14766 14793 15289 15326 16189 16192 16234 16256 16270 16311 16362 16526	U5a2e	73 151 152 263 16189 16192 16234 16256 16270 16311 16362 16526	[1.093] U5a2e (1)	[0.040] U5a2+16362 (2) [0.000] U5a2b2a1	1
333 >MG672511.1 Ho	73G 146C 152C 199C 249G 263G 750G 1438G 1709A 1719A 2397T 2706G 3106d 3107C 4711T 4769G 7028T 8279C 8860G 9067G 9077C 9109G 10398G 10610T 11404G 11719A 12234G 12510T 13145A 14766T 15326G 15613G 16051G 16168T 16295T 16296T 16304C	R21	73G 146C 152C 199C 249G 263G 16051G 16168T 16295T 16296T 16304C	1	73 146 152 199 249 263 750 1438 1709 1719 2397 2706 4711 4769 7028 8279 8860 9067 9077 9109 10398 10610T 11404 11719 12234 12510 13145 14766 15326 15613 16051 16168 16295 16296 16304	R21	73 146 152 199 249 263 16051 16168 16295 16296 16304	[0.038] R21 (1)	[0.083] U2b1a (2) [0.057] U2b1	1

334 >MG672510.1 H	73G 263G 489C 709A 750G 1048T 1438G 2706G 3106d 3107C 3796G 4769G 7028T 8701G 8860G 9540C 10202T 10398G 10400T 10873C 11287C 11482C 11611A 11719A 12705T 14766T 14783C 15043A 15301A 15326G 15924G 16129A 16223T 16256T 16271C 16362C	M21a	73G 263G 489C 16129A 16223T 16256T 16271C 16362C	1	73 263 489 709 750 1048 1438 2706 3796 4769 7028 8701 8860 9540 10202 10398 10400 10873 11287 11482 11611 11719 12705 14766 14783 15043 15301 15326 15924 16129 16223 16256 16271 16362	M21a	73 263 489 16129 16223 16256 16271 16362	[0.000] M21a (1)	[0.000] D2a'b D2a D2a1 D2a1a D2a2 (6)	1
335 >MG672509.1 H	73G 263G 489C 709A 750G 1048T 1438G 2706G 3106d 3107C 3796G 4769G 7028T 8701G 8860G 9540C 10202T 10398G 10400T 10873C 11287C 11482C 11611A 11719A 12705T 14766T 14783C 15043A 15301A 15326G 15924G 16129A 16223T 16256T 16271C 16362C	M21a	73G 263G 489C 16129A 16223T 16256T 16271C 16362C	1	73 263 489 709 750 1048 1438 2706 3796 4769 7028 8701 8860 9540 10202 10398 10400 10873 11287 11482 11611 11719 12705 14766 14783 15043 15301 15326 15924 16129 16223 16256 16271 16362	M21a	73 263 489 16129 16223 16256 16271 16362	[0.000] M21a (1)	[0.000] D2a'b D2a D2a1 D2a1a D2a2 (6)	1
336 >MG672508.1 H	64T 73G 150T 263G 310.1C 489C 750G 862G 930C 1438G 2706G 3106d 3107C 4769G 7028T 7170T 8701G 8860G 9540C 10324C 10398G 10400T 10873C 11016A 11719A 11908G 12705T 12711G 12804C 12973T 14716T 14766T 14783C 15043A 15301A 15326G 15530C 15802G 15941C 16093C 16129A 16209C 16223T 16261T 16278T 16325C 16519C	M17a	64T 73G 150T 263G 310.1C 489C 16093C 16129A 16209C 16223T 16261T 16278T 16325C 16519C	1	64 73 150 263 489 750 862 930C 1438 2706 4769 7028 7170 8701 8860 9540 10324 10398 10400 10873 11016 11719 11908 12705 12711 12804 12973 14716 14766 14783 15043 15301 15326 15530 15802 15941 16093 16129 16209 16223 16261 16278 16325	M17a	64 73 150 263 489 16093 16129 16209 16223 16261 16278 16325	[0.412] M17a (1)	[0.169] M51a (2) [0.024] G1b2 G1b4 (4) [0.007] G1b+16129 (5) [0.000] M51 M51b (7)	1
337 >MG672507.1 H	64T 73G 150T 263G 489C 750G 862G 930C 1438G 2706G 3106d 3107C 4769G 7028T 7170T 8701G 8860G 9540C 10324C 10398G 10400T 10873C 11016A 11719A 11908G 12705T 12711G 12804C 12973T 14716T 14766T 14783C 15043A 15301A 15326G 15530C 15802G 15941C 16093C 16129A 16209C 16223T 16261T 16278T 16325C 16519C	M17a	64T 73G 150T 263G 489C 16093C 16129A 16209C 16223T 16261T 16278T 16325C 16519C	1	64 73 150 263 489 750 862 930C 1438 2706 4769 7028 7170 8701 8860 9540 10324 10398 10400 10873 11016 11719 11908 12705 12711 12804 12973 14716 14766 14783 15043 15301 15326 15530 15802 15941 16093 16129 16209 16223 16261 16278 16325	M17a	64 73 150 263 489 16093 16129 16209 16223 16261 16278 16325	[0.412] M17a (1)	[0.169] M51a (2) [0.024] G1b2 G1b4 (4) [0.007] G1b+16129 (5) [0.000] M51 M51b (7)	1
338 >MG672506.1 H	64T 73G 150T 263G 489C 750G 862G 930C 1438G 2706G 3106d 3107C 4769G 7028T 7170T 8701G 8860G 9540C 10324C 10398G 10400T 10873C 11016A 11719A 11908G 12705T 12711G 12804C 12973T 14716T 14766T 14783C 15043A 15301A 15326G 15530C 15802G 15941C 16093C 16129A 16209C 16223T 16261T 16278T 16325C 16519C	M17a	64T 73G 150T 263G 489C 16093C 16129A 16209C 16223T 16261T 16278T 16325C 16519C	1	64 73 150 263 489 750 862 930C 1438 2706 4769 7028 7170 8701 8860 9540 10324 10398 10400 10873 11016 11719 11908 12705 12711 12804 12973 14716 14766 14783 15043 15301 15326 15530 15802 15941 16093 16129 16209 16223 16261 16278 16325	M17a	64 73 150 263 489 16093 16129 16209 16223 16261 16278 16325	[0.412] M17a (1)	[0.169] M51a (2) [0.024] G1b2 G1b4 (4) [0.007] G1b+16129 (5) [0.000] M51 M51b (7)	1

339 >MG672505.1 H	73G 263G 489C 750G 1048T 1438G 2706G 3106d 3107C 3796G 4769G 7028T 8701G 8860G 9540C 10202T 10398G 10400T 10873C 11287C 11482C 11611A 11719A 12705T 14766T 14783C 15043A 15301A 15326G 15924G 16129A 16223T 16256T 16271C 16362C	M21a	73G 263G 489C 16129A 16223T 16256T 16271C 16362C	1	73 263 489 750 1048 1438 2706 3796 4769 7028 8701 8860 9540 10202 10398 10400 10873 11287 11482 11611 11719 12705 14766 14783 15043 15301 15326 15924 16129 16223 16256 16271 16362	M21a	73 263 489 16129 16223 16256 16271 16362	[0.000] M21a (1)	[0.000] D2a'b D2a D2a1 D2a1a D2a2 (6)	1
340 >MG672504.1 H	73G 263G 489C 709A 750G 1048T 1438G 2706G 3106d 3107C 3796G 4769G 7028T 8701G 8860G 9540C 10202T 10398G 10400T 10873C 11287C 11482C 11611A 11719A 12705T 14766T 14783C 15043A 15301A 15326G 15924G 16129A 16223T 16256T 16271C 16362C	M21a	73G 263G 489C 16129A 16223T 16256T 16271C 16362C	1	73 263 489 709 750 1048 1438 2706 3796 4769 7028 8701 8860 9540 10202 10398 10400 10873 11287 11482 11611 11719 12705 14766 14783 15043 15301 15326 15924 16129 16223 16256 16271 16362	M21a	73 263 489 16129 16223 16256 16271 16362	[0.000] M21a (1)	[0.000] D2a'b D2a D2a1 D2a1a D2a2 (6)	1
341 >MG672503.1 H	73G 146C 152C 199C 249G 263G 750G 1438G 1709A 1719A 2397T 2706G 3106d 3107C 4711T 4769G 7028T 8279C 8860G 9067G 9077C 9109G 10398G 10610T 11404G 11719A 12234G 12510T 13145A 14766T 15326G 15613G 16168T 16295T 16296T 16304C	R21	73G 146C 152C 199C 249G 263G 16168T 16295T 16296T 16304C	1	73 146 152 199 249 263 750 1438 1709 1719 2397 2706 4711 4769 7028 8279 8860 9067 9077 9109 10398 10610T 11404 11719 12234 12510 13145 14766 15326 15613 16168 16295 16296 16304	R21	73 146 152 199 249 263 16168 16295 16296 16304	[0.000] R21 (1)	[0.231] M7c1a (2) [0.150] K2a1a (3) [0.089] T2b4+152 (4) [0.083] U2b1a (5) [0.057] M7c1b2b U2b1 (7) [0.048] R9 (8) [0.040] R2b1 (9) [0.032] M7c1a1b (10) [0.029] T2b+152 (11) [0.021] M7c1c (12) [0.016] T2b4f R9b (14) [0.012] M7c1 (15) [0.010] R9c (16) [0.000] M7c1a1 M7c1a1a M7c1a1a1 M7c1a4 M7c1b M7c1b1 M7c1b2 M7c1c3a M7c1c3a1 H9a H32 H103 T2b4c T2b4d T2b4e T2b4g T2b21 T2b21b T2b22 R12'21 R12 R30a1 R30a1a (39)	1
342 >MG672502.1 H	73G 146C 152C 199C 249G 263G 750G 1438G 1709A 1719A 2397T 2706G 3106d 3107C 4711T 4769G 7028T 8279C 8860G 9067G 9077C 9109G 10398G 10610T 11404G 11719A 12234G 12510T 13145A 14766T 15326G 15613G 16168T 16295T 16296T 16304C	R21	73G 146C 152C 199C 249G 263G 16168T 16295T 16296T 16304C	1	73 146 152 199 249 263 750 1438 1709 1719 2397 2706 4711 4769 7028 8279 8860 9067 9077 9109 10398 10610T 11404 11719 12234 12510 13145 14766 15326 15613 16168 16295 16296 16304	R21	73 146 152 199 249 263 16168 16295 16296 16304	[0.000] R21 (1)	[0.231] M7c1a (2) [0.150] K2a1a (3) [0.089] T2b4+152 (4) [0.083] U2b1a (5) [0.057] M7c1b2b U2b1 (7) [0.048] R9 (8) [0.040] R2b1 (9) [0.032] M7c1a1b (10) [0.029] T2b+152 (11) [0.021] M7c1c (12) [0.016] T2b4f R9b (14) [0.012] M7c1 (15) [0.010] R9c (16) [0.000] M7c1a1 M7c1a1a M7c1a1a1 M7c1a4 M7c1b M7c1b1 M7c1b2 M7c1c3a M7c1c3a1 H9a H32 H103 T2b4c T2b4d T2b4e T2b4g T2b21 T2b21b T2b22 R12'21 R12 R30a1 R30a1a (39)	1

343	>MG672501.1 H	64T 73G 150T 263G 489C 750G 862G 930C 1438G 2706G 3106d 3107C 4769G 7028T 7170T 8701G 8860G 9540C 10324C 10398G 10400T 10873C 11016A 11719A 11908G 12705T 12711G 12804C 12973T 14716T 14766T 14783C 15043A 15301A 15326G 15530C 15802G 15941C 16093C 16129A 16209C 16223T 16261T 16278T 16325C 16519C	M17a	64T 73G 150T 263G 489C 16093C 16129A 16209C 16223T 16261T 16278T 16325C 16519C	1	64 73 150 263 489 750 862 930C 1438 2706 4769 7028 7170 8701 8860 9540 10324 10398 10400 10873 11016 11719 11908 12705 12711 12804 12973 14716 14766 14783 15043 15301 15326 15530 15802 15941 16093 16129 16209 16223 16261 16278 16325	M17a	64 73 150 263 489 16093 16129 16209 16223 16261 16278 16325	[0.412] M17a (1)	[0.169] M51a (2) [0.024] G1b2 G1b4 (4) [0.007] G1b+16129 (5) [0.000] M51 M51b (7)	1
344	>MF696136.1 H	73G 189G 200G 263G 272G 309.1CT 310C 750G 1438G 1822C 2706G 3396C 4218C 4769G 5601T 7028T 7819A 8410T 8527G 8701G 8860G 8932T 9540C 9950C 10070T 10398G 10873C 11440A 11719A 12705T 14766T 14769G 15301A 15326G 15514C 15940d 16129A 16209C 16223T 16292T 16311C 16519C	L3f1b1a1	73G 189G 200G 263G 272G 309.1CT 310C 16129A 16209C 16223T 16292T 16311C 16519C	1	73 189 200 263 272 750 1438 1822 2706 3396 4218 4769 5601 7028 7819A 8410 8527 8701 8860 8932 9540 9950 10070 10398 10873 11440 11719 12705 14766 14769 15301 15326 15514 15944d 16129 16209 16223 16292 16311	L3f1b1a1	73 189 200 263 272 16129 16209 16223 16292 16311	[0.222] L3f1b1a1 (1)	[0.222] L3f1b1a1 (2) [0.096] L3f1b+16292 (3) [0.021] L3f1b1a (4) [0.000] L3f1b2 L3f1b5 (6)	1
345	>MF696135.1 H	73G 189G 200G 263G 272G 309.1CCT 310C 750G 1438G 1822C 2706G 3396C 4218C 4769G 4843T 5601T 7028T 7819A 8410T 8527G 8701G 8860G 8932T 9540C 9950C 10070T 10398G 10873C 11440A 11719A 12705T 14766T 14769G 15301A 15326G 15514C 15940d 16129A 16209C 16223T 16292T 16311C 16519C	L3f1b1a1	73G 189G 200G 263G 272G 309.1CCT 310C 16129A 16209C 16223T 16292T 16311C 16519C	1	73 189 200 263 272 750 1438 1822 2706 3396 4218 4769 4843 5601 7028 7819A 8410 8527 8701 8860 8932 9540 9950 10070 10398 10873 11440 11719 12705 14766 14769 15301 15326 15514 15944d 16129 16209 16223 16292 16311	L3f1b1a1	73 189 200 263 272 16129 16209 16223 16292 16311	[0.222] L3f1b1a1 (1)	[0.222] L3f1b1a1 (2) [0.096] L3f1b+16292 (3) [0.021] L3f1b1a (4) [0.000] L3f1b2 L3f1b5 (6)	1
346	>MF696134.1 H	73G 189G 200G 263G 272G 309.1CT 310C 750G 1438G 1822C 2706G 3396C 4218C 4769G 5601T 7028T 7819A 8410T 8527G 8701G 8860G 8932T 9540C 9950C 10070T 10398G 10873C 11440A 11719A 12705T 14766T 14769G 15301A 15326G 15514C 15940d 16129A 16209C 16223T 16292T 16311C 16519C	L3f1b1a1	73G 189G 200G 263G 272G 309.1CT 310C 16129A 16209C 16223T 16292T 16311C 16519C	1	73 189 200 263 272 750 1438 1822 2706 3396 4218 4769 5601 7028 7819A 8410 8527 8701 8860 8932 9540 9950 10070 10398 10873 11440 11719 12705 14766 14769 15301 15326 15514 15944d 16129 16209 16223 16292 16311	L3f1b1a1	73 189 200 263 272 16129 16209 16223 16292 16311	[0.222] L3f1b1a1 (1)	[0.222] L3f1b1a1 (2) [0.096] L3f1b+16292 (3) [0.021] L3f1b1a (4) [0.000] L3f1b2 L3f1b5 (6)	1
347	>MF696131.1 H	73G 146C 152C 195C 198T 263G 309.1CT 310C 750G 769A 1018A 1438G 2416C 2706G 2789T 3594T 4104G 4769G 5042G 5581G 6413C 7028T 7175C 7256T 7274T 7521A 7771G 8206A 8701G 8860G 9221G 9540C 10115C 10398G 10873C 11719A 11914A 11944C 12693G 12705T 13590A 13650T 13803G 14566G 14766T 15301A 15326G 15784C 16189C 16223T 16278T 16294T 16309G 16390A 16519C	L2a1f3	73G 146C 152C 195C 198T 263G 309.1CT 310C 16189C 16223T 16278T 16294T 16309G 16390A 16519C	1	73 146 152 195 198 263 750 769 1018 1438 2416 2706 2789 3594 4104 4769 5042 5581 6413 7028 7175 7256 7274 7521 7771 8206 8701 8860 9221 9540 10115 10398 10873 11719 11914 11944 12693 12705 13590 13650 13803 14566 14766 15301 15326 15784 16189 16223 16278 16294 16309 16390	L2a1f3	73 146 152 195 198 263 16189 16223 16278 16294 16309 16390	[0.000] L2a1f3 (1)	[0.007] L2a1b (2) [0.005] L2a1f (3) [0.000] L2a1+16189_(16192) L2a1b1 L2a1b2 L2a1f1 L2a1f1a L2a1f2 L2a1f3 (10)	1

348	>MF696129.1 Hc	73G 146C 152C 195C 263G 309.1CCT 310C 750G 769A 1018A 1438G 2416C 2706G 2789T 3594T 4769G 5090C 7028T 7175C 7256T 7274T 7521A 7771G 8206A 8701G 8860G 9221G 9540C 10115C 10143A 10398G 10873C 11719A 11887A 11914A 11944C 12693G 12705T 13590A 13650T 13803G 14566G 14766T 15301A 15326G 15735T 15784C 16182C 16183C 16189C 16192T 16223T 16278T 16290T 16294T 16309G 16390A	L2a1b1a	73G 146C 152C 195C 263G 309.1CCT 310C 16182C 16183C 16189C 16192T 16223T 16278T 16290T 16294T 16309G 16390A	1	73 146 152 195 263 750 769 1018 1438 2416 2706 2789 3594 4769 5090 7028 7175 7256 7274 7521 7771 8206 8701 8860 9221 9540 10115 10143 10398 10873 11719 11887 11914 11944 12693 12705 13590 13650 13803 14566 14766 15301 15326 15735 15784 16189 16192 16223 16278 16290 16294 16309 16390	L2a1b1a	73 146 152 195 263 16189 16192 16223 16278 16290 16294 16309 16390	[0.000] L2a1b1a (1)	[0.015] L2a1b1a (2) [0.005] L2a1f (3) [0.000] L2a1+16189_(16192) L2a1b L2a1b1 L2a1b2 L2a1f1 L2a1f1a L2a1f2 (10)	1
349	>MF696128.1 Hc	73G 146C 152C 263G 310.1C 750G 769A 1018A 1438G 2416C 2706G 2789T 3594T 4104G 4769G 7028T 7175C 7256T 7274T 7521A 7771G 8014G 8206A 8701G 8860G 9221G 9540C 10115C 10398G 10873C 11719A 11914A 11944C 12693G 12705T 13590A 13650T 13803G 14281T 14566G 14766T 15301A 15326G 15784C 16131C 16189C 16223T 16225T 16234T 16278T 16294T 16309G 16390A	L2a1g	73G 146C 152C 263G 310.1C 16131C 16189C 16223T 16225T 16234T 16278T 16294T 16309G 16390A	1	73 146 152 263 750 769 1018 1438 2416 2706 2789 3594 4104 4769 7028 7175 7256 7274 7521 7771 8014 8206 8701 8860 9221 9540 10115 10398 10873 11719 11914 11944 12693 12705 13590 13650 13803 14281 14566 14766 15301 15326 15784 16131 16189 16223 16225 16234 16278 16294 16309 16390	L2a1g	73 146 152 263 16131 16189 16223 16225 16234 16278 16294 16309 16390	[0.269] L2a1g (1)	[0.023] L2a1b2 (2) [0.022] L2a1b L2a1f1a (4) [0.010] L2a1f (5) [0.000] L2a1+16189_(16192) L2a1b1 L2a1f1 L2a1f2 (9)	1
350	>MF696126.1 Hc	73G 146C 152C 195C 263G 310.1C 750G 769A 1018A 1438G 2416C 2706G 2789T 3594T 4104G 4769G 5090C 7028T 7175C 7256T 7274T 7521A 7771G 8206A 8701G 8860G 9221G 9540C 10115C 10143A 10398G 10873C 11719A 11914A 11944C 12693G 12705T 13590A 13650T 13803G 14566G 14766T 15301A 15326G 15735T 15784C 16189C 16223T 16278T 16290T 16294T 16309G 16390A	L2a1b1a	73G 146C 152C 195C 263G 310.1C 16189C 16223T 16278T 16290T 16294T 16309G 16390A	1	73 146 152 195 263 750 769 1018 1438 2416 2706 2789 3594 4104 4769 5090 7028 7175 7256 7274 7521 7771 8206 8701 8860 9221 9540 10115 10143 10398 10873 11719 11914 11944 12693 12705 13590 13650 13803 14566 14766 15301 15326 15735 15784 16189 16223 16278 16290 16294 16309 16390	L2a1b1a	73 146 152 195 263 16189 16223 16278 16290 16294 16309 16390	[0.000] L2a1b1a (1)	[0.005] L2a1f (2) [0.000] L2a1+16189_(16192) L2a1b L2a1b1 L2a1b1a L2a1b2 L2a1f1 L2a1f1a L2a1f2 (10)	1

351	>MF696125.1 Hc	73G 146C 152C 189G 195C 199C 204C 247A 310.1C 514d 515d 750G 769A 825A 850C 1018A 1048T 1243C 1438G 2706G 2758A 2885C 3516A 3594T 3882A 4104G 4312T 4541A 4586C 4767G 4769G 4907C 5442C 5705G 5811G 6053T 6185C 7028T 7146G 7256T 7257G 7521A 8468T 8655T 8701G 8860G 8911C 8922T 8994A 9042T 9136G 9347G 9540C 9818T 9884T 10398G 10499G 10589A 10664T 10688A 10810C 10873C 10876G 10915C 10920T 11061T 11296T 11299C 11653G 11719A 11914A 12007A 12651A 12705T 12720G 13105G 13276G 13506T 13535G 13590A 13819C 13928C 14020C 14182C 14371C 14374C 14766T 15221A 15326G 15467G 16129A 16166C 16172C 16187T 16189C 16214T 16230G 16278T 16287T 16291A 16311C 16519C	L0k2b	73G 146C 152C 189G 195C 199C 204C 247A 310.1C 514d 515d 16129A 16166C 16172C 16187T 16189C 16214T 16230G 16278T 16287T 16291A 16311C 16519C	1	73 146 152 189 195 199 204 247 750 769 825A 850 1018 1048 1243 1438 2706 2758 2885 3516A 3594 3882 4104 4312 4541 4586 4767 4769 4907 5442 5705 5811 6053 6185 7028 7146 7256 7257 7521 8468 8655 8701 8860 8911 8922 8994 9042 9136 9347 9540 9818 9884 10398 10499 10589 10664 10688 10810 10873 10876 10915 10920 11061 11296 11299 11653 11719 11914 12007 12651 12705 12720 13105 13276 13506 13535 13590 13819 13928C 14020 14182 14371 14374 14766 15221 15326 15467 16129 16166C 16172 16187 16189 16214 16230 16278 16287 16291A 16311	L0k2b	73 146 152 189 195 199 204 247 16129 16166C 16172 16187 16189 16214 16230 16278 16287 16291A 16311	[0.667] L0k2b (1)	[0.000] L0k2 (2)	1
352	>MF696124.1 Hc	73G 150T 195C 263G 310.1C 514d 515d 1438G 2000T 2352C 2706G 4655A 4769G 5262A 6524C 7028T 8701G 8860G 9540C 9554A 10398G 10667C 10816G 10819G 10873C 11719A 11963A 12248G 12705T 13101C 13197T 13651G 14212C 14766T 14953T 15029C 15301A 15326G 15812A 15924G 16223T 16265T 16519C 16527T	L3e3b2	73G 150T 195C 263G 310.1C 514d 515d 16223T 16265T 16519C 16527T	1	73 150 195 263 1438 2000 2352 2706 4655 4769 5262 6524 7028 8701 8860 9540 9554 10398 10667 10816 10819 10873 11719 11963 12248 12705 13101C 13197 13651 14212 14766 14953 15029 15301 15326 15812 15924 16223 16265T 16527	L3e3b2	73 150 195 263 16223 16265T 16527	[0.000] L3e3b2 (1)	[0.000] L3e3 L3e3b L3e3b1 (4)	1

353 >MF696123.1 Hc	93G 146C 152C 185A 189G 195C 236C 247A 263G 309.1CT 310C 499A 514d 515d 750G 769A 825A 1018A 1048T 1415A 1438G 2245G 2706G 2758A 2885C 3165.1T 3516A 3594T 4104G 4312T 4586C 4769G 4943G 5252A 5442C 5656G 5782C 6185C 7028T 7146G 7256T 7444A 7521A 7598A 8047C 8251A 8428T 8468T 8566G 8655T 8701G 8860G 9042T 9347G 9540C 9755A 9818T 10398G 10589A 10664T 10688A 10810C 10873C 10915C 11176A 11641G 11719A 11914A 12007A 12528A 12720G 13105G 13276G 13306T 13431T 13434G 13506T 13650T 13767A 14110C 14766T 15136T 15301A 15326G 15431A 16129A 16148T 16169T 16171G 16172C 16187T 16188G 16189C 16223T 16230G 16278T 16311C 16320T 16519C	L0g	93G 146C 152C 185A 189G 195C 236C 247A 263G 309.1CT 310C 499A 514d 515d 16129A 16148T 16169T 16171G 16172C 16187T 16188G 16189C 16223T 16230G 16278T 16311C 16320T 16519C	1	93 146 152 185 189 195 236 247 263 499 750 769 825A 1018 1048 1415 1438 2245 2706 2758 2885 3167.1T 3516A 3594 4104 4312 4586 4769 4943 5252 5442 5656 5782 6185 7028 7146 7256 7444 7521 7598 8047 8251 8428 8468 8566 8655 8701 8860 9042 9347 9540 9755 9818 10398 10589 10664 10688 10810 10873 10915 11176 11641 11719 11914 12007 12528 12720 13105 13276 13306 13431 13434 13506 13650 13767A 14110 14766 15136 15301 15326 15431 16129 16148 16169 16171 16172 16187 16188G 16189 16223 16230 16278 16311 16320	L0g	93 146 152 185 189 195 236 247 263 499 16129 16148 16169 16171 16172 16187 16188G 16189 16223 16230 16278 16311 16320	[1.375] L0g (1) [0.833] L0a'g (2)	[1.375] L0g (3) [0.833] L0a'g (4) [0.333] L0a'b'g (5) [0.278] L0a3 (6) [0.043] L0a	1
354 >MF696116.1 Hc	73G 146C 152C 195C 263G 310.1C 750G 769A 1018A 1438G 2416C 2706G 2789T 3594T 4104G 4769G 5090C 7028T 7175C 7256T 7274T 7521A 7771G 8206A 8701G 8860G 9221G 9540C 10115C 10143A 10398G 10873C 11719A 11914A 11944C 12693G 12705T 13590A 13650T 13803G 14566G 14766T 15301A 15326G 15735T 15784C 16189C 16223T 16278T 16290T 16294T 16309G 16390A	L2a1b1a	73G 146C 152C 195C 263G 310.1C 16189C 16223T 16278T 16290T 16294T 16309G 16390A	1	73 146 152 195 263 750 769 1018 1438 2416 2706 2789 3594 4104 4769 5090 7028 7175 7256 7274 7521 7771 8206 8701 8860 9221 9540 10115 10143 10398 10873 11719 11914 11944 12693 12705 13590 13650 13803 14566 14766 15301 15326 15735 15784 16189 16223 16278 16290 16294 16309 16390	L2a1b1a	73 146 152 195 263 16189 16223 16278 16290 16294 16309 16390	[0.000] L2a1b1a (1)	[0.005] L2a1f (2) [0.000] L2a1+16189_(16192) L2a1b L2a1b1 L2a1b1a L2a1b2 L2a1f1 L2a1f1a L2a1f2 (10)	1
355 >MF696113.1 Hc	73G 146C 152C 195C 263G 309.1CT 310C 750G 769A 1018A 1438G 2416C 2706G 2789T 3594T 4104G 4769G 5090C 7028T 7175C 7256T 7274T 7521A 7771G 8206A 8701G 8860G 9221G 9540C 10115C 10143A 10398G 10873C 10943G 11719A 11914A 11944C 12693G 12705T 13590A 13650T 13803G 14566G 14766T 15301A 15326G 15735T 15784C 16182C 16183C 16189C 16192T 16223T 16278T 16290T 16294T 16309G 16390A	L2a1b1a	73G 146C 152C 195C 263G 309.1CT 310C 16182C 16183C 16189C 16192T 16223T 16278T 16290T 16294T 16309G 16390A	1	73 146 152 195 263 750 769 1018 1438 2416 2706 2789 3594 4104 4769 5090 7028 7175 7256 7274 7521 7771 8206 8701 8860 9221 9540 10115 10143 10398 10873 10943 11719 11914 11944 12693 12705 13590 13650 13803 14566 14766 15301 15326 15735 15784 16189 16192 16223 16278 16290 16294 16309 16390	L2a1b1a	73 146 152 195 263 16189 16192 16223 16278 16290 16294 16309 16390	[0.000] L2a1b1a (1)	[0.015] L2a1b1a (2) [0.005] L2a1f (3) [0.000] L2a1+16189_(16192) L2a1b L2a1b1 L2a1b2 L2a1f1 L2a1f1a L2a1f2 (10)	1

356	>MF696107.1 Hc	73G 146C 152C 182T 183G 263G 310.1C 373G 511T 750G 769A 1018A 1438G 1719A 2416C 2626C 2706G 3594T 3654T 4104G 4380T 4769G 4823C 5183G 5246A 5263T 6040C 6080G 6248C 6497C 7028T 7175C 7256T 7521A 8206A 8251A 8701G 8723A 8860G 9221G 9540C 10115C 10398G 10873C 11719A 11944C 12705T 12879C 12967G 12972G 13590A 13650T 13810A 14088C 14314G 14557T 14766T 14890G 15301A 15326G 15796C 16183C 16189C 16223T 16224C 16278T 16390A 16519C	L2a5	73G 146C 152C 182T 183G 263G 310.1C 373G 511T 16183C 16189C 16223T 16224C 16278T 16390A 16519C	1	73 146 152 182 183 263 373 511 750 769 1018 1438 1719 2416 2626 2706 3594 3654 4104 4380 4769 4823 5183 5246A 5263 6040C 6080 6248 6497 7028 7175 7256 7521 8206 8251 8701 8723 8860 9221 9540 10115 10398 10873 11719 11944 12705 12879 12967 12972 13590 13650 13810 14088 14314 14557 14766 14890 15301 15326 15796 16189 16223 16224 16278 16390	L2a5	73 146 152 182 183 263 373 511 16189 16223 16224 16278 16390	[1.708] L2a5 (1)	[0.000] L2 L2a (3)	1
357	>MF696106.1 Hc	73G 93G 146C 150T 182T 183G 195C 198T 263G 309.1CT 310C 325T 514d 515d 680C 709A 750G 769A 1018A 1040C 1438G 1442A 2332T 2416C 2706G 3200A 3594T 4104G 4769G 7028T 7256T 7521A 7624A 8206A 8567C 8701G 8772C 8860G 9063G 9139A 9165C 9221G 9540C 10115C 10398G 10790C 10873C 11719A 11944C 12236A 12705T 13590A 13650T 13928C 13958C 14766T 15110A 15217A 15301A 15313C 15326G 15849T 16223T 16264T 16278T 16311C 16390A	L2c2b1b	73G 93G 146C 150T 182T 183G 195C 198T 263G 309.1CT 310C 325T 514d 515d 16223T 16264T 16278T 16311C 16390A	1	73 93 146 150 182 183 195 198 263 325 680 709 750 769 1018 1040 1438 1442 2332 2416 2706 3200A 3594 4104 4769 7028 7256 7521 7624A 8206 8567 8701 8772 8860 9063 9139 9165 9221 9540 10115 10398 10790 10873 11719 11944 12236 12705 13590 13650 13928C 13958C 14766 15110 15217 15301 15313 15326 15849 16223 16264 16278 16311 16390	L2c2b1b	73 93 146 150 182 183 195 198 263 325 16223 16264 16278 16311 16390	[0.069] L2c2b1b (1)	[0.000] L2c2b L2c2b1 L2c2b1a L2c2b2 (5)	1
358	>MF696100.1 Hc	73G 146C 152C 195C 198T 263G 309.1CCCT 310C 750G 769A 1018A 1438G 2416C 2706G 2789T 3594T 4104G 4769G 5581G 7028T 7175C 7256T 7274T 7521A 7771G 8206A 8701G 8860G 9221G 9540C 10115C 10398G 10873C 11719A 11914A 11944C 12693G 12705T 13590A 13650T 13803G 14566G 14766T 15301A 15326G 15784C 16183.1C 16189C 16223T 16278T 16294T 16309G 16390A 16519C	L2a1f3	73G 146C 152C 195C 198T 263G 309.1CCCT 310C 16183.1C 16189C 16223T 16278T 16294T 16309G 16390A 16519C	1	73 146 152 195 198 263 309.3C 750 769 1018 1438 2416 2706 2789 3594 4104 4769 5581 7028 7175 7256 7274 7521 7771 8206 8701 8860 9221 9540 10115 10398 10873 11719 11914 11944 12693 12705 13590 13650 13803 14566 14766 15301 15326 15784 16189 16223 16278 16294 16309 16390	L2a1f3	73 146 152 195 198 263 309.3C 16189 16223 16278 16294 16309 16390	[0.011] L2a1f3 (1)	[0.011] L2a1f3 (2) [0.007] L2a1b (3) [0.005] L2a1f (4) [0.000] L2a1+16189_(16192) L2a1b1 L2a1b2 L2a1f1 L2a1f1a L2a1f2 (10)	1

359 >MF696098.1 Hc	73G 146C 152C 182T 183G 263G 310.1C 373G 511T 750G 769A 1018A 1438G 1719A 2416C 2626C 2706G 3594T 3654T 4104G 4380T 4769G 4823C 5183G 5246A 5263T 6040C 6080G 6248C 6497C 7028T 7175C 7256T 7521A 8206A 8251A 8701G 8723A 8860G 9221G 9540C 10115C 10398G 10873C 11719A 11944C 12705T 12879C 12967G 12972G 13590A 13650T 13810A 14088C 14314G 14557T 14766T 14890G 15301A 15326G 15796C 16182.1C 16183C 16189C 16223T 16224C 16278T 16390A 16519C	L2a5	73G 146C 152C 182T 183G 263G 310.1C 373G 511T 16182.1C 16183C 16189C 16223T 16224C 16278T 16390A 16519C	1	73 146 152 182 183 263 373 511 750 769 1018 1438 1719 2416 2626 2706 3594 3654 4104 4380 4769 4823 5183 5246A 5263 6040C 6080 6248 6497 7028 7175 7256 7521 8206 8251 8701 8723 8860 9221 9540 10115 10398 10873 11719 11944 12705 12879 12967 12972 13590 13650 13810 14088 14314 14557 14766 14890 15301 15326 15796 16189 16223 16224 16278 16390	L2a5	73 146 152 182 183 263 373 511 16189 16223 16224 16278 16390	[1.708] L2a5 (1)	[0.000] L2 L2a (3)	1
360 >MF696097.1 Hc	73G 146C 152C 182T 183G 263G 310.1C 373G 511T 750G 769A 1018A 1438G 1719A 2416C 2626C 2706G 3594T 3654T 4104G 4380T 4769G 4823C 5183G 5246A 5263T 6040C 6080G 6248C 6497C 7028T 7175C 7256T 7521A 8206A 8251A 8701G 8723A 8860G 9221G 9540C 10115C 10398G 10873C 11719A 11944C 12705T 12879C 12967G 12972G 13590A 13650T 13810A 14088C 14314G 14557T 14766T 14890G 15301A 15326G 15796C 16183C 16189C 16223T 16224C 16278T 16390A 16519C	L2a5	73G 146C 152C 182T 183G 263G 310.1C 373G 511T 16183C 16189C 16223T 16224C 16278T 16390A 16519C	1	73 146 152 182 183 263 373 511 750 769 1018 1438 1719 2416 2626 2706 3594 3654 4104 4380 4769 4823 5183 5246A 5263 6040C 6080 6248 6497 7028 7175 7256 7521 8206 8251 8701 8723 8860 9221 9540 10115 10398 10873 11719 11944 12705 12879 12967 12972 13590 13650 13810 14088 14314 14557 14766 14890 15301 15326 15796 16189 16223 16224 16278 16390	L2a5	73 146 152 182 183 263 373 511 16189 16223 16224 16278 16390	[1.708] L2a5 (1)	[0.000] L2 L2a (3)	1
361 >MF696096.1 Hc	73G 93G 146C 150T 182T 183G 195C 198T 263G 309.1CCT 310C 325T 514d 515d 680C 709A 750G 769A 1018A 1040C 1438G 1442A 2332T 2416C 2706G 3200A 3594T 4104G 4769G 7028T 7256T 7521A 7624A 8206A 8567C 8701G 8772C 8860G 9063G 9139A 9165C 9221G 9540C 10115C 10398G 10790C 10873C 11719A 11944C 12236A 12705T 13590A 13650T 13928C 13958C 14766T 15110A 15217A 15301A 15313C 15326G 15849T 16223T 16264T 16278T 16311C 16390A	L2c2b1b	73G 93G 146C 150T 182T 183G 195C 198T 263G 309.1CCT 310C 325T 514d 515d 16223T 16264T 16278T 16311C 16390A	1	73 93 146 150 182 183 195 198 263 325 680 709 750 769 1018 1040 1438 1442 2332 2416 2706 3200A 3594 4104 4769 7028 7256 7521 7624A 8206 8567 8701 8772 8860 9063 9139 9165 9221 9540 10115 10398 10790 10873 11719 11944 12236 12705 13590 13650 13928C 13958C 14766 15110 15217 15301 15313 15326 15849 16223 16264 16278 16311 16390	L2c2b1b	73 93 146 150 182 183 195 198 263 325 16223 16264 16278 16311 16390	[0.069] L2c2b1b (1)	[0.000] L2c2b L2c2b1 L2c2b1a L2c2b2 (5)	1

362	>MF696092.1 Ho	73G 151T 152C 182T 186A 189C 195C 198T 247A 263G 297G 310.1C 316A 750G 769A 825A 1018A 1420C 1438G 2150.1A 2394d 2706G 2758A 2885C 3594T 3666A 4104G 4769G 5894.1C 5951G 6071C 6150A 6253C 7028T 7055G 7076G 7146G 7256T 7337A 7389C 7521A 7744C 8027A 8251A 8468T 8655T 8701G 8784G 8860G 8877C 9072G 9540C 10321C 10398G 10586A 10688A 10792G 10793T 10810C 10873C 11654G 11719A 12049T 12705T 12810G 13105G 13149G 13212T 13281C 13485G 13506T 13650T 13789C 14000A 14178C 14560A 14766T 14812T 14911T 15016T 15326G 15784C 16071T 16129A 16145A 16187T 16189C 16213A 16223T 16234T 16265C 16278T 16286G 16294T 16311C 16360T 16527T	L1c2a1a	73G 151T 152C 182T 186A 189C 195C 198T 247A 263G 297G 310.1C 316A 16071T 16129A 16145A 16187T 16189C 16213A 16223T 16234T 16265C 16278T 16286G 16294T 16311C 16360T 16527T	1	73 151 152 182 186A 189C 195 198 247 263 297 316 750 769 825A 1018 1420 1438 2156.1A 2395d 2706 2758 2885 3594 3666 4104 4769 5899.1C 5951 6071 6150 6253 7028 7055 7076 7146 7256 7337 7389 7521 7744 8027 8251 8468 8655 8701 8784 8860 8877 9072 9540 10321 10398 10586 10688 10792 10793 10810 10873 11654 11719 12049 12705 12810 13105 13149 13212 13281 13485 13506 13650 13789 14000A 14178 14560 14766 14812 14911 15016 15326 15784 16071 16129 16145 16187 16189 16213 16223 16234 16265C 16278 16286G 16294 16311 16360 16527	L1c2a1a	73 151 152 182 186A 189C 195 198 247 263 297 316 16071 16129 16145 16187 16189 16213 16223 16234 16265C 16278 16286G 16294 16311 16360 16527	[0.000] L1c2a1a (1)	[0.105] L1c2a1b (2) [0.000] L1c2a1	1
363	>MF696086.1 Ho	73G 195C 247A 263G 310.1C 514d 515d 750G 769A 825A 1018A 1438G 1717C 1719A 2080C 2484T 2706G 3106d 3107C 3423C 3564G 3594T 3918A 4092A 4104G 4655A 4676T 4769G 4967C 6527G 7028T 7256T 7521A 7765G 7789A 8655T 8701G 8860G 9018C 9540C 9653C 10101C 10398G 10688A 10694G 10810C 10873C 11266T 11287C 11719A 11809C 12432T 12507G 12705T 12940A 13050G 13383T 13506T 13602C 13650T 14071G 14766T 15236G 15326G 15418T 16129A 16166G 16187T 16223T 16254G 16278T 16291T 16368C 16527T	L5b2	73G 195C 247A 263G 310.1C 514d 515d 16129A 16166G 16187T 16223T 16254G 16278T 16291T 16368C 16527T	1	73 195 247 263 750 769 825A 1018 1438 1717 1719 2080 2484 2706 3423 3564 3594 3918 4092 4104 4655 4676 4769 4967 6527 7028 7256 7521 7765 7789 8655 8701 8860 9018 9540 9653 10101 10398 10688 10694 10810 10873 11266 11287 11719 11809 12432 12507 12705 12940 13050 13383 13506 13602 13650 14071 14766 15236 15326 15418 16129 16166 16187 16223 16254 16278 16291 16368 16527	L5b2	73 195 247 263 16129 16166 16187 16223 16254 16278 16291 16368 16527	[1.700] L5b2 (1)	[0.000] L5b (2)	1
364	>MF696083.1 Ho	73G 146C 152C 195C 263G 309T 310C 750G 769A 1018A 1438G 2416C 2706G 2789T 3106d 3107C 3594T 4104G 4769G 5090C 6233G 7028T 7175C 7256T 7274T 7521A 7771G 8206A 8701G 8860G 9221G 9540C 10115C 10143A 10398G 10873C 11719A 11914A 11944C 12693G 12705T 13590A 13650T 13803G 14566G 14766T 15301A 15326G 15735T 15784C 16182C 16183C 16189C 16223T 16278T 16290T 16294T 16309G 16390A	L2a1b1a	73G 146C 152C 195C 263G 309T 310C 16182C 16183C 16189C 16223T 16278T 16290T 16294T 16309G 16390A	1	73 146 152 195 263 309d 750 769 1018 1438 2416 2706 2789 3594 4104 4769 5090 6233 7028 7175 7256 7274 7521 7771 8206 8701 8860 9221 9540 10115 10143 10398 10873 11719 11914 11944 12693 12705 13590 13650 13803 14566 14766 15301 15326 15735 15784 16189 16223 16278 16290 16294 16309 16390	L2a1b1a	73 146 152 195 263 309d 16189 16223 16278 16290 16294 16309 16390	[0.033] L2a1b1a (1)	[0.052] L2a1f (2) [0.033] L2a1b1a (3) [0.029] L2a1f1 (4) [0.007] L2a1b (5) [0.000] L2a1+16189_(16192) L2a1b1 L2a1b2 L2a1f1a L2a1f2 (10)	1

365	>MF696082.1 Hc	73G 150T 152C 189G 195C 200G 207A 263G 309.1CT 310C 750G 1438G 2352C 2706G 3106d 3107C 4173G 4769G 5774A 6221C 6587T 7028T 8701G 8860G 9254G 9540C 10398G 10688A 10819G 10873C 11024T 11719A 12358G 12705T 13822T 14152G 14212C 14569A 14766T 15301A 15326G 15670C 15942C 16185T 16209C 16223T 16327T	L3e1a2	73G 150T 152C 189G 195C 200G 207A 263G 309.1CT 310C 16185T 16209C 16223T 16327T	1	73 150 152 189 195 200 207 263 750 1438 2352 2706 4173 4769 5774A 6221 6587 7028 8701 8860 9254 9540 10398 10688 10819 10873 11024 11719 12358 12705 13822 14152 14212 14569 14766 15301 15326 15670 15942 16185 16209 16223 16327	L3e1a2	73 150 152 189 195 200 207 263 16185 16209 16223 16327	[0.000] L3e1a2 (1)	[0.000] L3e1f2 (2)	1
366	>MF696071.1 Hc	73G 151T 152C 189C 195C 263G 294C 310.1C 514d 515d 606G 750G 990C 1438G 1664A 1719A 2706G 3106d 3107C 4388G 4769G 5300T 7028T 7278C 7861C 8701G 8842G 8860G 9509C 9540C 9575A 9758C 10044G 10398G 10754G 10873C 11287C 11590G 11719A 12705T 12882T 13105G 13194A 13437C 14410A 14766T 14793G 15301A 15326G 16129A 16172C 16174T 16192T 16218T 16223T 16256A 16311C 16362C	L3h1b2	73G 151T 152C 189C 195C 263G 294C 310.1C 514d 515d 16129A 16172C 16174T 16192T 16218T 16223T 16256A 16311C 16362C	1	73 151 152 189C 195 263 294 606 750 990 1438 1664 1719 2706 4388 4769 5300 7028 7278 7861 8701 8842 8860 9509 9540 9575 9758 10044 10398 10754 10873 11287 11590 11719 12705 12882 13105 13194 13437 14410 14766 14793 15301 15326 16129 16172 16174 16192 16218 16223 16256A 16311 16362	L3h1b2	73 151 152 189C 195 263 294 16129 16172 16174 16192 16218 16223 16256A 16311 16362	[2.200] L3h1b2 (1)	[0.000] L3h1b (2)	1
367	>MF696068.1 Hc	73G 146C 244G 263G 310.1C 391C 750G 769A 1018A 1413C 1438G 1694C 2706G 3106d 3107C 3918A 4769G 4949G 5483G 6260A 7028T 8104C 8478T 8701G 8860G 9540C 9855G 10325A 10398G 10783C 10873C 11719A 12609C 12705T 13470G 14766T 15301A 15326G 15454C 16172C 16209C 16223T 16293T 16311C 16355T 16362C 16399G 16519C	L4b2a2c	73G 146C 244G 263G 310.1C 391C 16172C 16209C 16223T 16293T 16311C 16355T 16362C 16399G 16519C	1	73 146 244 263 391 750 769 1018 1413 1438 1694 2706 3918 4769 4949 5483G 6260 7028 8104 8478 8701 8860 9540 9855 10325 10398 10783C 10873 11719 12609 12705 13470 14766 15301 15326 15454 16172 16209 16223 16293T 16311 16355 16362 16399	L4b2a2c	73 146 244 263 391 16172 16209 16223 16293T 16311 16355 16362 16399	[0.083] L4b2a2c (1)	[0.010] L4b2a2a (2) [0.000] L4b2a2	1

368 >MF696056.1 Ho	73G 151T 152C 182T 186A 189C 195C 198T 247A 263G 297G 310.1C 316A 750G 769A 825A 1018A 1420C 1438G 2150.1A 2394d 2706G 2758A 2885C 3106d 3107C 3594T 3666A 4104G 4769G 5894.1C 5951G 6071C 6150A 6253C 7028T 7055G 7076G 7146G 7256T 7337A 7389C 7521A 7744C 8027A 8251A 8468T 8655T 8701G 8784G 8860G 8877C 9072G 9540C 10321C 10398G 10586A 10688A 10792G 10793T 10810C 10873C 11654G 11719A 12049T 12705T 12810G 13105G 13149G 13212T 13281C 13485G 13506T 13650T 13789C 14000A 14178C 14560A 14766T 14812T 14911T 15016T 15326G 15784C 16071T 16129A 16145A 16187T 16189C 16213A 16223T 16234T 16265C 16278T 16286G 16294T 16311C 16360T 16527T	L1c2a1a	73G 151T 152C 182T 186A 189C 195C 198T 247A 263G 297G 310.1C 316A 16071T 16129A 16145A 16187T 16189C 16213A 16223T 16234T 16265C 16278T 16286G 16294T 16311C 16360T 16527T	1	73 151 152 182 186A 189C 195 198 247 263 297 316 750 769 825A 1018 1420 1438 2156.1A 2395d 2706 2758 2885 3594 3666 4104 4769 5899.1C 5951 6071 6150 6253 7028 7055 7076 7146 7256 7337 7389 7521 7744 8027 8251 8468 8655 8701 8784 8860 8877 9072 9540 10321 10398 10586 10688 10792 10793 10810 10873 11654 11719 12049 12705 12810 13105 13149 13212 13281 13485 13506 13650 13789 14000A 14178 14560 14766 14812 14911 15016 15326 15784 16071 16129 16145 16187 16189 16213 16223 16234 16265C 16278 16286G 16294 16311 16360 16527	L1c2a1a	73 151 152 182 186A 189C 195 198 247 263 297 316 16071 16129 16145 16187 16189 16213 16223 16234 16265C 16278 16286G 16294 16311 16360 16527	[0.000] L1c2a1a (1)	[0.105] L1c2a1b (2) [0.000] L1c2a1	1
369 >MF696054.1 Ho	73G 146C 152C 195C 198T 228A 247A 310.1C 456T 494d 514d 515d 719A 750G 769A 825A 1018A 1048T 2000T 2758A 2885C 3083C 3106d 3107C 3438A 3516A 3594T 3756G 4104G 4197T 4232C 4312T 4769G 5263T 5442C 5773A 6060C 6185C 6266G 6815C 7028T 7146G 7256T 7265G 7521A 8113A 8152A 8251A 8468T 8655T 8701G 8860G 9042T 9144T 9150G 9347G 9540C 9755A 10373A 10398G 10589A 10664T 10688A 10810C 10873C 10915C 11150A 11437C 11719A 11809C 11914A 12007A 12121C 12235C 12615G 12705T 12720G 13105G 13129T 13276G 13506T 13650T 13759A 14224G 14384A 14766T 15187G 15326G 15466A 15930A 15941C 15951G 16187T 16189C 16223T 16230G 16234T 16243C 16311C	L0d1c	73G 146C 152C 195C 198T 228A 247A 310.1C 456T 494d 514d 515d 16187T 16189C 16223T 16230G 16234T 16243C 16311C	1	73 146 152 195 198 228 247 456 498d 719 750 769 825A 1018 1048 2000 2758 2885 3083 3438 3516A 3594 3756 4104 4197 4232 4312 4769 5263 5442 5773 6060C 6185 6266 6815 7028 7146 7256 7265 7521 8113A 8152 8251 8468 8655 8701 8860 9042 9144 9150 9347 9540 9755 10373 10398 10589 10664 10688 10810 10873 10915 11150 11437 11719 11809 11914 12007 12121 12235 12615 12705 12720 13105 13129 13276 13506 13650 13759 14224 14384 14766 15187 15326 15466 15930 15941 15951 16187 16189 16223 16230 16234 16243 16311	L0d1c	73 146 152 195 198 228 247 456 498d 16187 16189 16223 16230 16234 16243 16311	[0.148] L0d1c (1)	[0.009] L0d1c1a1 L0d1c1a1a2 (3) [0.006] L0d1c1a1a (4) [0.000] L0d1c1 L0d1c1a L0d1c1a1b L0d1c2 (8)	1

370	>MF696047.1 Hc	73G 151T 152C 182T 186A 189C 195C 198T 247A 263G 297G 310.1C 316A 750G 769A 825A 1018A 1420C 1438G 2150.1A 2394d 2706G 2758A 2885C 3106d 3107C 3594T 3666A 4104G 4769G 5894.1C 5951G 6071C 6150A 6253C 7028T 7055G 7076G 7146G 7256T 7337A 7389C 7521A 7744C 8027A 8251A 8468T 8655T 8701G 8784G 8860G 8877C 9072G 9540C 10321C 10398G 10586A 10688A 10792G 10793T 10810C 10873C 11654G 11719A 12049T 12705T 12810G 13105G 13149G 13212T 13281C 13485G 13506T 13650T 13789C 14000A 14178C 14560A 14766T 14812T 14911T 15016T 15326G 15784C 16071T 16129A 16145A 16187T 16189C 16213A 16223T 16234T 16265C 16278T 16286G 16294T 16311C 16360T 16527T	L1c2a1a	73G 151T 152C 182T 186A 189C 195C 198T 247A 263G 297G 310.1C 316A 16071T 16129A 16145A 16187T 16189C 16213A 16223T 16234T 16265C 16278T 16286G 16294T 16311C 16360T 16527T	1	73 151 152 182 186A 189C 195 198 247 263 297 316 750 769 825A 1018 1420 1438 2156.1A 2395d 2706 2758 2885 3594 3666 4104 4769 5899.1C 5951 6071 6150 6253 7028 7055 7076 7146 7256 7337 7389 7521 7744 8027 8251 8468 8655 8701 8784 8860 8877 9072 9540 10321 10398 10586 10688 10792 10793 10810 10873 11654 11719 12049 12705 12810 13105 13149 13212 13281 13485 13506 13650 13789 14000A 14178 14560 14766 14812 14911 15016 15326 15784 16071 16129 16145 16187 16189 16213 16223 16234 16265C 16278 16286G 16294 16311 16360 16527	L1c2a1a	73 151 152 182 186A 189C 195 198 247 263 297 316 16071 16129 16145 16187 16189 16213 16223 16234 16265C 16278 16286G 16294 16311 16360 16527	[0.000] L1c2a1a (1)	[0.105] L1c2a1b (2) [0.000] L1c2a1	1
371	>MF696046.1 Hc	73G 150T 152C 189G 195C 200G 207A 263G 309.1CT 310C 750G 1438G 2352C 2706G 3106d 3107C 4769G 5774A 6221C 6587T 7028T 8701G 8860G 9254G 9540C 10398G 10819G 10873C 11024T 11719A 12705T 14152G 14212C 14569A 14766T 15301A 15326G 15670C 15942C 16185T 16209C 16223T 16327T	L3e1a2	73G 150T 152C 189G 195C 200G 207A 263G 309.1CT 310C 16185T 16209C 16223T 16327T	1	73 150 152 189 195 200 207 263 750 1438 2352 2706 4769 5774A 6221 6587 7028 8701 8860 9254 9540 10398 10819 10873 11024 11719 12705 14152 14212 14569 14766 15301 15326 15670 15942 16185 16209 16223 16327	L3e1a2	73 150 152 189 195 200 207 263 16185 16209 16223 16327	[0.000] L3e1a2 (1)	[0.000] L3e1f2 (2)	1
372	>MF696045.1 Hc	73G 146C 152C 195C 263G 310.1C 750G 769A 1018A 1438G 2416C 2706G 2789T 3106d 3107C 3594T 4104G 4314C 4769G 5090C 7028T 7175C 7256T 7274T 7521A 7771G 8206A 8701G 8860G 9221G 9540C 10115C 10143A 10398G 10873C 11719A 11914A 11944C 12693G 12705T 13590A 13650T 13803G 14566G 14766T 15301A 15326G 15735T 15784C 16182C 16183C 16189C 16192T 16223T 16278T 16290T 16294T 16309G 16390A	L2a1b1a	73G 146C 152C 195C 263G 310.1C 16182C 16183C 16189C 16192T 16223T 16278T 16290T 16294T 16309G 16390A	1	73 146 152 195 263 750 769 1018 1438 2416 2706 2789 3594 4104 4314 4769 5090 7028 7175 7256 7274 7521 7771 8206 8701 8860 9221 9540 10115 10143 10398 10873 11719 11914 11944 12693 12705 13590 13650 13803 14566 14766 15301 15326 15735 15784 16189 16192 16223 16278 16290 16294 16309 16390	L2a1b1a	73 146 152 195 263 16189 16192 16223 16278 16290 16294 16309 16390	[0.000] L2a1b1a (1)	[0.015] L2a1b1a (2) [0.005] L2a1f (3) [0.000] L2a1+16189_(16192) L2a1b L2a1b1 L2a1b2 L2a1f1 L2a1f1a L2a1f2 (10)	1

373 >MF696043.1 Ho	73G 152C 195C 263G 310.1C 489C 750G 813G 1438G 2706G 3106d 3107C 4769G 6446A 6671C 6680C 7028T 8701G 8860G 9540C 10398G 10400T 10694G 10873C 11719A 12403T 12705T 12950C 13215C 13722G 14110C 14323A 14515C 14766T 14783C 15043A 15301A 15326G 15770T 15799G 16183C 16189C 16223T 16249C 16311C 16519C	M1a5	73G 152C 195C 263G 310.1C 489C 16183C 16189C 16223T 16249C 16311C 16519C	1	73 152 195 263 489 750 813 1438 2706 4769 6446 6671 6680 7028 8701 8860 9540 10398 10400 10694 10873 11719 12403 12705 12950C 13215 13722 14110 14323 14515 14766 14783 15043 15301 15326 15770 15799 16189 16223 16249 16311	M1a5	73 152 195 263 489 16189 16223 16249 16311	[0.048] M1a5 (1) [0.000] M1a2a (2)	[0.133] M1a (3) [0.018] M1a4 (4) [0.000] M1 M1a2 M1a2a M1a3b1 M1a6 M1a8 M1a8a M1b (12)	1
374 >MF696042.1 Ho	73G 146C 152C 182T 183G 263G 309.1CCT 310C 466C 511T 514d 515d 709A 750G 769A 1018A 1438G 2416C 2626C 2706G 3106d 3107C 3594T 3654T 4104G 4769G 4823C 5246A 5263T 6040C 6080G 6248C 6497C 7028T 7175C 7256T 7521A 8206A 8701G 8723A 8860G 9221G 9540C 10115C 10398G 10873C 11719A 11815A 11944C 12172G 12705T 12879C 12967G 13470G 13590A 13650T 13810A 14088C 14766T 14890G 15301A 15326G 16183C 16189C 16223T 16224C 16278T 16309G 16390A 16519C	L2a5	73G 146C 152C 182T 183G 263G 309.1CCT 310C 466C 511T 514d 515d 16183C 16189C 16223T 16224C 16278T 16309G 16390A 16519C	1	73 146 152 182 183 263 466 511 709 750 769 1018 1438 2416 2626 2706 3594 3654 4104 4769 4823 5246A 5263 6040C 6080 6248 6497 7028 7175 7256 7521 8206 8701 8723 8860 9221 9540 10115 10398 10873 11719 11815A 11944 12172 12705 12879 12967 13470 13590 13650 13810 14088 14766 14890 15301 15326 16189 16223 16224 16278 16309 16390	L2a5	73 146 152 182 183 263 466 511 16189 16223 16224 16278 16309 16390	[2.167] L2a5 (1)	[0.023] L2a1b2 (2) [0.022] L2a1b L2a1f1a (4) [0.012] L2a1f2 (5) [0.005] L2a1f (6) [0.000] L2 L2a L2a1+16189_(16192) L2a1b1 L2a1f1 (11)	1
375 >MF696020.1 Ho	73G 146C 152C 195C 263G 310.1C 750G 769A 1018A 1438G 2416C 2706G 2789T 3106d 3107C 3594T 4104G 4769G 5090C 6734A 7028T 7175C 7245G 7256T 7274T 7521A 7771G 8206A 8701G 8860G 9221G 9540C 10115C 10143A 10398G 10873C 11719A 11914A 11944C 12693G 12705T 13590A 13650T 13803G 14566G 14766T 15301A 15326G 15735T 15784C 16180d 16181d 16189C 16223T 16278T 16290T 16294T 16309G 16390A	L2a1b1a	73G 146C 152C 195C 263G 310.1C 16180d 16181d 16189C 16223T 16278T 16290T 16294T 16309G 16390A	1	73 146 152 195 263 750 769 1018 1438 2416 2706 2789 3594 4104 4769 5090 6734 7028 7175 7245 7256 7274 7521 7771 8206 8701 8860 9221 9540 10115 10143 10398 10873 11719 11914 11944 12693 12705 13590 13650 13803 14566 14766 15301 15326 15735 15784 16189 16192-16193d 16223 16278 16290 16294 16309 16390	L2a1b1a	73 146 152 195 263 16189 16192-16193d 16223 16278 16290 16294 16309 16390	[0.030] L2a1b1a (1)	[0.030] L2a1b1a (2) [0.005] L2a1f (3) [0.000] L2a1+16189_(16192) L2a1b L2a1b1 L2a1b2 L2a1f1 L2a1f1a L2a1f2 (10)	1
376 >MF696016.1 Ho	73G 150T 152C 189G 195C 200G 207A 263G 309.1CT 310C 750G 1438G 2352C 2706G 3106d 3107C 4769G 5774A 6221C 6587T 7028T 8701G 8860G 9254G 9540C 10398G 10819G 10873C 11024T 11719A 12705T 14152G 14212C 14569A 14766T 15301A 15326G 15670C 15942C 16185T 16209C 16223T 16327T	L3e1a2	73G 150T 152C 189G 195C 200G 207A 263G 309.1CT 310C 16185T 16209C 16223T 16327T	1	73 150 152 189 195 200 207 263 750 1438 2352 2706 4769 5774A 6221 6587 7028 8701 8860 9254 9540 10398 10819 10873 11024 11719 12705 14152 14212 14569 14766 15301 15326 15670 15942 16185 16209 16223 16327	L3e1a2	73 150 152 189 195 200 207 263 16185 16209 16223 16327	[0.000] L3e1a2 (1)	[0.000] L3e1f2 (2)	1

377 >MF695995.1 Hc	73G 146C 152C 195C 263G 310.1C 750G 769A 1018A 1438G 2416C 2706G 2789T 3106d 3107C 3594T 4104G 4769G 5090C 5662T 7028T 7175C 7256T 7274T 7521A 7771G 8206A 8701G 8860G 9221G 9540C 10115C 10143A 10398G 10873C 11719A 11914A 11944C 12693G 12705T 13590A 13650T 13803G 14566G 14766T 15301A 15326G 15735T 15784C 16189C 16223T 16278T 16290T 16294T 16309G 16390A	L2a1b1a	73G 146C 152C 195C 263G 310.1C 16189C 16223T 16278T 16290T 16294T 16309G 16390A	1	73 146 152 195 263 750 769 1018 1438 2416 2706 2789 3594 4104 4769 5090 5662 7028 7175 7256 7274 7521 7771 8206 8701 8860 9221 9540 10115 10143 10398 10873 11719 11914 11944 12693 12705 13590 13650 13803 14566 14766 15301 15326 15735 15784 16189 16223 16278 16290 16294 16309 16390	L2a1b1a	73 146 152 195 263 16189 16223 16278 16290 16294 16309 16390	[0.000] L2a1b1a (1)	[0.005] L2a1f (2) [0.000] L2a1+16189_(16192) L2a1b L2a1b1 L2a1b1a L2a1b2 L2a1f1 L2a1f1a L2a1f2 (10)	1
378 >MF695991.1 Hc	73G 146C 152C 182T 183G 263G 310.1C 511T 514d 515d 709A 750G 769A 1018A 1438G 2416C 2626C 2706G 3106d 3107C 3594T 3654T 4104G 4769G 4823C 5246A 5263T 6040C 6080G 6248C 6497C 7028T 7175C 7256T 7521A 8206A 8701G 8723A 8860G 9221G 9540C 10115C 10398G 10873C 11719A 11815A 11944C 12172G 12705T 12879C 12967G 13470G 13590A 13650T 13810A 14088C 14410A 14766T 14890G 15301A 15326G 16189C 16223T 16224C 16278T 16309G 16390A 16519C	L2a5	73G 146C 152C 182T 183G 263G 310.1C 511T 514d 515d 16189C 16223T 16224C 16278T 16309G 16390A 16519C	1	73 146 152 182 183 263 511 709 750 769 1018 1438 2416 2626 2706 3594 3654 4104 4769 4823 5246A 5263 6040C 6080 6248 6497 7028 7175 7256 7521 8206 8701 8723 8860 9221 9540 10115 10398 10873 11719 11815A 11944 12172 12705 12879 12967 13470 13590 13650 13810 14088 14410 14766 14890 15301 15326 16189 16223 16224 16278 16309 16390	L2a5	73 146 152 182 183 263 511 16189 16223 16224 16278 16309 16390	[2.084] L2a5 (1)	[0.023] L2a1b2 (2) [0.022] L2a1b L2a1f1a (4) [0.012] L2a1f2 (5) [0.005] L2a1f (6) [0.000] L2 L2a L2a1+16189_(16192) L2a1b1 L2a1f1 (11)	1
379 >MF695983.1 Hc	73G 151T 152C 182T 186A 189C 195C 198T 247A 263G 297G 309.1CT 310C 316A 471C 514d 515d 750G 769A 825A 1018A 1420C 1438G 2150.1A 2394d 2706G 2758A 2885C 3106d 3107C 3594T 3666A 4104G 4769G 5951G 6071C 6150A 6253C 6723A 7028T 7055G 7076G 7146G 7256T 7337A 7389C 7521A 7744C 8027A 8468T 8655T 8701G 8784G 8860G 8877C 9072G 9540C 9632G 10321C 10398G 10586A 10688A 10792G 10793T 10810C 10873C 11329G 11654G 11719A 12049T 12654G 12705T 12810G 13105G 13149G 13485G 13506T 13650T 13789C 14000A 14178C 14560A 14757C 14766T 14861A 14911T 15016T 15326G 15784C 16129A 16169T 16187T 16189C 16223T 16265C 16278T 16286G 16294T 16311C 16360T 16519C 16527T	L1c2a3	73G 151T 152C 182T 186A 189C 195C 198T 247A 263G 297G 309.1CT 310C 316A 471C 514d 515d 16129A 16169T 16187T 16189C 16223T 16265C 16278T 16286G 16294T 16311C 16360T 16519C 16527T	1	73 151 152 182 186A 189C 195 198 247 263 297 316 471 750 769 825A 1018 1420 1438 2156.1A 2395d 2706 2758 2885 3594 3666 4104 4769 5951 6071 6150 6253 6723 7028 7055 7076 7146 7256 7337 7389 7521 7744 8027 8468 8655 8701 8784 8860 8877 9072 9540 9632 10321 10398 10586 10688 10792 10793 10810 10873 11329 11654 11719 12049 12654 12705 12810 13105 13149 13485 13506 13650 13789 14000A 14178 14560 14757 14766 14861 14911 15016 15326 15784 16129 16169 16187 16189 16223 16265C 16278 16286G 16294 16311 16360 16527	L1c2a3	73 151 152 182 186A 189C 195 198 247 263 297 316 471 16129 16169 16187 16189 16223 16265C 16278 16286G 16294 16311 16360 16527	[0.182] L1c2a3 (1)	[0.000] L1c2 L1c2a L1c2b L1c2b1 L1c2b1a'b L1c2b1a L1c2b1a1 L1c2b1b (9)	1

380 >MF695958.1 Hc	73G 146C 152C 182T 185T 189G 204C 247A 263G 310.1C 357G 514d 515d 709A 710C 750G 769A 825A 1018A 1738C 2352C 2706G 2758A 2768G 2885C 3106d 3107C 3308C 3594T 3666A 3693A 4104G 4769G 5581G 6548T 6827C 6989G 7028T 7055G 7146G 7256T 7389C 7521A 7867T 8248G 8282T 8468T 8655T 8701G 8860G 9540C 10398G 10688A 10810C 10873C 11719A 12171G 12519C 12705T 12891T 13105G 13506T 13650T 13789C 13893G 14178C 14323A 14560A 14766T 14769G 15115C 15326G 16111T 16126C 16187T 16189C 16223T 16239T 16270T 16278T 16292T 16293G 16311C 16519C	L1b2a	73G 146C 152C 182T 185T 189G 204C 247A 263G 310.1C 357G 514d 515d 16111T 16126C 16187T 16189C 16223T 16239T 16270T 16278T 16292T 16293G 16311C 16519C	1	73 146 152 182 185T 189 204 247 263 357 709 710 750 769 825A 1018 1738 2352 2706 2758 2768 2885 3308 3594 3666 3693 4104 4769 5581 6548 6827 6989 7028 7055 7146 7256 7389 7521 7867 8248 8282 8468 8655 8701 8860 9540 10398 10688 10810 10873 11719 12171 12519 12705 12891 13105 13506 13650 13789 13893 14178 14323 14560 14766 14769 15115 15326 16111 16126 16187 16189 16223 16239 16270 16278 16292 16293 16311	L1b2a	73 146 152 182 185T 189 204 247 263 357 16111 16126 16187 16189 16223 16239 16270 16278 16292 16293 16311	[0.115] L1b2a (1)	[0.375] L1b2 (2)	1
381 >MF695952.1 Hc	73G 150T 195C 263G 318C 513d 514d 750G 1438G 2706G 3106d 3107C 3879G 4769G 5460A 5813G 5930C 6018A 7028T 7861C 8020A 8155A 8701G 8860G 9098C 9380A 9540C 9575A 9965C 10398G 10873C 11440A 11719A 12469G 12705T 13080T 13753C 13755T 14766T 15301A 15326G 16111T 16184T 16223T 16304C 16519C	L3h2	73G 150T 195C 263G 318C 513d 514d 16111T 16184T 16223T 16304C 16519C	1	73 150 195 263 318 513 750 1438 2706 3879 4769 5460 5813 5930 6018 7028 7861 8020 8155 8701 8860 9098 9380 9540 9575 9965 10398 10873 11440 11719 12469 12705 13080 13753 13755 14766 15301 15326 16111 16184 16223 16304	L3h2	73 150 195 263 318 513 16111 16184 16223 16304	[0.154] L3h2 (1)	[0.050] L3e2a3 (2) [0.042] L3e'i'k'x L3e L3i (5) [0.019] L3e3b1 (6) [0.009] M66 (7) [0.006] L3e3'4'5 (8) [0.000] L3e2 L3e2a L3e2a2 L3e3'4 L3e3 L3e3b M66a (15)	1
382 >MF695949.1 Hc	73G 152C 263G 309.1CT 310C 514d 515d 750G 921C 1438G 2706G 3106d 3107C 4769G 5147A 6164T 6680C 7028T 7424G 7765G 8618C 8701G 8860G 9540C 10398G 10873C 11719A 12705T 13105G 13886C 14284T 14659T 14766T 15301A 15326G 15672C 16124C 16223T 16256T 16311C 16368C	L3d1d	73G 152C 263G 309.1CT 310C 514d 515d 16124C 16223T 16256T 16311C 16368C	1	73 152 263 750 921 1438 2706 4769 5147 6164 6680 7028 7424 7765 8618 8701 8860 9540 10398 10873 11719 12705 13105 13886 14284 14659 14766 15301 15326 15672 16124 16223 16256 16311 16368	L3d1d	73 152 263 16124 16223 16256 16311 16368	[0.017] L3d1d (1)	[0.000] L3d3a L3d3a1 L3d3a1a L3d3a1b (5)	1
383 >MF695938.1 Hc	73G 146C 152C 195C 263G 310.1C 750G 769A 1018A 1438G 2416C 2706G 2789T 3106d 3107C 3594T 4104G 4769G 5090C 7028T 7175C 7256T 7274T 7521A 7771G 8206A 8701G 8860G 9221G 9540C 10115C 10143A 10398G 10873C 11719A 11914A 11944C 12693G 12705T 13590A 13650T 13803G 14566G 14766T 15151G 15301A 15326G 15735T 15784C 16182C 16183C 16189C 16192T 16223T 16278T 16290T 16294T 16309G 16390A	L2a1b1a	73G 146C 152C 195C 263G 310.1C 16182C 16183C 16189C 16192T 16223T 16278T 16290T 16294T 16309G 16390A	1	73 146 152 195 263 750 769 1018 1438 2416 2706 2789 3594 4104 4769 5090 7028 7175 7256 7274 7521 7771 8206 8701 8860 9221 9540 10115 10143 10398 10873 11719 11914 11944 12693 12705 13590 13650 13803 14566 14766 15151 15301 15326 15735 15784 16189 16192 16223 16278 16290 16294 16309 16390	L2a1b1a	73 146 152 195 263 16189 16192 16223 16278 16290 16294 16309 16390	[0.000] L2a1b1a (1)	[0.015] L2a1b1a (2) [0.005] L2a1f (3) [0.000] L2a1+16189_(16192) L2a1b L2a1b1 L2a1b2 L2a1f1 L2a1f1a L2a1f2 (10)	1

384 >MF695929.1 Hc	73G 146C 150T 152C 182T 195C 198T 204C 263G 310.1C 456T 709A 750G 769A 1018A 1438G 1442A 1706T 2332T 2358G 2416C 2706G 3106d 3107C 3594T 4104G 4158G 4370C 4767G 4769G 5027T 5331A 5814C 6614C 6713T 6806G 7028T 7256T 7521A 7624A 8080T 8206A 8387A 8503C 8701G 8790A 8860G 9221G 9350G 9540C 10115C 10398G 10873C 11654G 11719A 11944C 12236A 12705T 12948G 13590A 13650T 13966G 14059G 14407T 14766T 15110A 15217A 15301A 15326G 15872G 16114A 16129A 16213A 16223T 16278T 16354T 16390A	L2b2a	73G 146C 150T 152C 182T 195C 198T 204C 263G 310.1C 456T 16114A 16129A 16213A 16223T 16278T 16354T 16390A	1	73 146 150 152 182 195 198 204 263 456 709 750 769 1018 1438 1442 1706 2332 2358 2416 2706 3594 4104 4158 4370 4767 4769 5027 5331A 5814 6614 6713 6806 7028 7256 7521 7624A 8080 8206 8387 8503 8701 8790 8860 9221 9350 9540 10115 10398 10873 11654 11719 11944 12236 12705 12948 13590 13650 13966 14059 14407 14766 15110 15217 15301 15326 15872 16114A 16129 16213 16223 16278 16354 16390	L2b2a	73 146 150 152 182 195 198 204 263 456 16114A 16129 16213 16223 16278 16354 16390	[0.024] L2b2a (1)	[0.000] L2b L2b2 L2b3 L2b3a L2b3b L2b3c (7)	1
385 >MF695917.1 Hc	73G 146C 152C 195C 263G 310.1C 750G 769A 1018A 1438G 2416C 2706G 2789T 3106d 3107C 3594T 4104G 4769G 5090C 7028T 7175C 7256T 7274T 7521A 7771G 8206A 8701G 8860G 9221G 9540C 10115C 10143A 10398G 10873C 11719A 11914A 11944C 12693G 12705T 13590A 13650T 13803G 14566G 14766T 15301A 15326G 15735T 15784C 16182C 16183C 16189C 16192T 16223T 16278T 16290T 16294T 16309G 16390A	L2a1b1a	73G 146C 152C 195C 263G 310.1C 16182C 16183C 16189C 16192T 16223T 16278T 16290T 16294T 16309G 16390A	1	73 146 152 195 263 750 769 1018 1438 2416 2706 2789 3594 4104 4769 5090 7028 7175 7256 7274 7521 7771 8206 8701 8860 9221 9540 10115 10143 10398 10873 11719 11914 11944 12693 12705 13590 13650 13803 14566 14766 15301 15326 15735 15784 16189 16192 16223 16278 16290 16294 16309 16390	L2a1b1a	73 146 152 195 263 16189 16192 16223 16278 16290 16294 16309 16390	[0.000] L2a1b1a (1)	[0.015] L2a1b1a (2) [0.005] L2a1f (3) [0.000] L2a1+16189_(16192) L2a1b L2a1b1 L2a1b2 L2a1f1 L2a1f1a L2a1f2 (10)	1
386 >MF695916.1 Hc	73G 146C 152C 195C 263G 310.1C 750G 769A 1018A 1438G 2416C 2706G 2789T 3106d 3107C 3594T 4104G 4769G 5090C 7028T 7175C 7256T 7274T 7521A 7771G 8206A 8701G 8860G 9221G 9540C 10115C 10143A 10398G 10873C 11719A 11914A 11944C 12693G 12705T 13590A 13650T 13803G 14566G 14766T 15301A 15326G 15735T 15784C 16182C 16183C 16189C 16192T 16223T 16247G 16278T 16290T 16294T 16309G 16390A	L2a1b1a	73G 146C 152C 195C 263G 310.1C 16182C 16183C 16189C 16192T 16223T 16247G 16278T 16290T 16294T 16309G 16390A	1	73 146 152 195 263 750 769 1018 1438 2416 2706 2789 3594 4104 4769 5090 7028 7175 7256 7274 7521 7771 8206 8701 8860 9221 9540 10115 10143 10398 10873 11719 11914 11944 12693 12705 13590 13650 13803 14566 14766 15301 15326 15735 15784 16189 16192 16223 16247 16278 16290 16294 16309 16390	L2a1b1a	73 146 152 195 263 16189 16192 16223 16247 16278 16290 16294 16309 16390	[0.003] L2a1b1a (1)	[0.018] L2a1b1a (2) [0.005] L2a1f (3) [0.000] L2a1+16189_(16192) L2a1b L2a1b1 L2a1b2 L2a1f1 L2a1f1a L2a1f2 (10)	1

387	>MF695894.1 Hc	143A 146C 152C 185A 189G 247A 263G 309.1CT 310C 750G 769A 825A 961C 978G 1018A 1048T 1438G 1719A 2245G 2706G 2758A 2772T 2789T 2885C 3106d 3107C 3516A 3594T 3852T 4104G 4194T 4312T 4562G 4586C 4769G 4964T 5321T 5442C 5603T 6185C 7028T 7146G 7148C 7256T 7521A 8468T 8655T 8701G 8860G 9042T 9347G 9540C 9581C 9620T 9818T 10143A 10398G 10589A 10664T 10688A 10790C 10810C 10873C 10915C 11212T 11287C 11299C 11641G 11719A 11914A 12007A 12705T 13105G 13276G 13470G 13506T 13650T 13680T 13708A 13928C 14109T 14620T 14766T 15136T 15326G 15431A 15852C 16129A 16169T 16172C 16173T 16187T 16189C 16223T 16230G 16239T 16278T 16311C 16327T 16368C 16519C	L0f2a1	143A 146C 152C 185A 189G 247A 263G 309.1CT 310C 16129A 16169T 16172C 16173T 16187T 16189C 16223T 16230G 16239T 16278T 16311C 16327T 16368C 16519C	1	143 146 152 185 189 247 263 750 769 825A 961 978 1018 1048 1438 1719 2245 2706 2758 2772 2789 2885 3516A 3594 3852 4104 4194 4312 4562 4586 4769 4964 5321 5442 5603 6185 7028 7146 7148 7256 7521 8468 8655 8701 8860 9042 9347 9540 9581 9620 9818 10143 10398 10589 10664 10688 10790 10810 10873 10915 11212 11287 11299 11641 11719 11914 12007 12705 13105 13276 13470 13506 13650 13680 13708 13928C 14109 14620 14766 15136 15326 15431 15852 16129 16169 16172 16173 16187 16189 16223 16230 16239 16278 16311 16327 16368	L0f2a1	143 146 152 185 189 247 263 16129 16169 16172 16173 16187 16189 16223 16230 16239 16278 16311 16327 16368	[0.000] L0f2a1 (1)	[0.454] L0f (2) [0.053] L0f2a (3) [0.000] L0f2	1
388	>MF695893.1 Hc	93G 152C 185A 189G 236C 247A 263G 310.1C 514d 515d 553T 750G 769A 825A 1018A 1048T 1438G 2245G 2706G 2758A 2885C 3106d 3107C 3516A 3594T 4104G 4312T 4586C 4769G 5096C 5231A 5442C 5460A 5603T 6185C 7028T 7146G 7256T 7521A 8428T 8468T 8566G 8655T 8701G 8860G 9042T 9347G 9540C 9755A 9818T 10398G 10589A 10664T 10688A 10810C 10873C 10915C 11176A 11641G 11719A 11914A 12007A 12557T 12705T 12720G 13105G 13276G 13506T 13650T 14308C 14766T 15136T 15326G 15431A 16129A 16148T 16168T 16172C 16187T 16188G 16189C 16223T 16230G 16293G 16311C	L0a1d	93G 152C 185A 189G 236C 247A 263G 310.1C 514d 515d 553T 16129A 16148T 16168T 16172C 16187T 16188G 16189C 16223T 16230G 16293G 16311C	1	93 152 185 189 236 247 263 553 750 769 825A 1018 1048 1438 2245 2706 2758 2885 3516A 3594 4104 4312 4586 4769 5096 5231 5442 5460 5603 6185 7028 7146 7256 7521 8428 8468 8566 8655 8701 8860 9042 9347 9540 9755 9818 10398 10589 10664 10688 10810 10873 10915 11176 11641 11719 11914 12007 12557 12705 12720 13105 13276 13506 13650 14308 14766 15136 15326 15431 16129 16148 16168 16172 16187 16188G 16189 16223 16230 16293 16311	L0a1d	93 152 185 189 236 247 263 553 16129 16148 16168 16172 16187 16188G 16189 16223 16230 16293 16311	[0.000] L0a1d (1)	[0.714] L0a1+16293 (2) [0.273] L0a1c	1

389 >MF695869.1 H	93G 152C 185A 189G 236C 247A 263G 310.1C 514d 515d 553T 750G 769A 825A 1018A 1048T 1438G 2245G 2706G 2758A 2885C 3106d 3107C 3516A 3594T 4104G 4312T 4586C 4769G 5096C 5231A 5442C 5460A 5603T 6185C 7028T 7146G 7256T 7521A 8428T 8468T 8566G 8655T 8701G 8860G 9042T 9347G 9540C 9755A 9818T 10398G 10589A 10664T 10688A 10810C 10873C 10915C 11176A 11641G 11719A 11914A 12007A 12557T 12705T 12720G 13105G 13276G 13506T 13650T 14308C 14766T 15136T 15326G 15431A 16129A 16148T 16168T 16172C 16187T 16188G 16189C 16223T 16230G 16293G 16311C	L0a1d	93G 152C 185A 189G 236C 247A 263G 310.1C 514d 515d 553T 16129A 16148T 16168T 16172C 16187T 16188G 16189C 16223T 16230G 16293G 16311C	1	93 152 185 189 236 247 263 553 750 769 825A 1018 1048 1438 2245 2706 2758 2885 3516A 3594 4104 4312 4586 4769 5096 5231 5442 5460 5603 6185 7028 7146 7256 7521 8428 8468 8566 8655 8701 8860 9042 9347 9540 9755 9818 10398 10589 10664 10688 10810 10873 10915 11176 11641 11719 11914 12007 12557 12705 12720 13105 13276 13506 13650 14308 14766 15136 15326 15431 16129 16148 16168 16172 16187 16188G 16189 16223 16230 16293 16311	L0a1d	93 152 185 189 236 247 263 553 16129 16148 16168 16172 16187 16188G 16189 16223 16230 16293 16311	[0.000] L0a1d (1)	[0.714] L0a1+16293 (2) [0.273] L0a1c	1
390 >MG646275.1 H	55C 56.1C 263G 302.1CC 310C 750G 1438G 3106d 3107C 4769G 6253C 8860G 11143T 11410C 14953T 15326G 16366T	H15a1a1	55C 56.1C 263G 302.1CC 310C 16366T	1	55 57 60.1T 263 310 315.2C 750 1438 4769 6253 8860 11143 11410 14953 15326 16366	H15a1a1	55 57 60.1T 263 310 315.2C 16366	[0.243] H15a1a1 (1)	[0.313] H15b (2) [0.146] H15b1 (3) [0.064] H15a1 (4) [0.027] H15a1a (5) [0.000] H15 H15a H15b2 (8)	1
391 >MG646273.1 H	73G 195C 263G 310.1C 514d 515d 750G 1438G 1811G 2706G 3106d 3107C 3480G 4769G 6546T 6599G 7028T 7567T 8860G 9055A 9650T 9698C 11467G 11719A 12308G 12372A 12477C 12771A 14167T 14766T 14831A 15031T 15097C 15326G 16172C 16183.1C 16189C 16234T 16311C 16519C	U8b1a2b	73G 195C 263G 310.1C 514d 515d 16172C 16183.1C 16189C 16234T 16311C 16519C	1	73 195 263 750 1438 1811 2706 3480 4769 6546 6599 7028 7567 8860 9055 9650 9698 11467 11719 12308 12372 12477 12771 14167 14766 14831 15031 15097 15326 16172 16189 16234 16311	U8b1a2b	73 195 263 16172 16189 16234 16311	[0.000] U8b1a2b (1)	[0.000] U8b1a2+16311 (2)	1
392 >MG646268.1 H	73G 195C 263G 308d 309d 310d 499A 513.1CACA 750G 1438G 1811G 2706G 3106d 3107C 4646C 4769G 5999C 6047G 7028T 8818T 8860G 11332T 11467G 11719A 12308G 12372A 14620T 14766T 15326G 15693C 16223T 16356C 16519C	U4a2b	73G 195C 263G 308d 309d 310d 499A 513.1CACA 16223T 16356C 16519C	1	73 195 263 310 313-315d 499 750 1438 1811 2706 4646 4769 5999 6047 7028 8818 8860 11332 11467 11719 12308 12372 14620 14766 15326 15693 16223 16356	U4a2b	73 195 263 310 313-315d 499 16223 16356	[0.026] U4a2b (1)	[0.007] U4a2 (2) [0.000] U4a2c U4a2e U4a2f U4a2g U4a2h (7)	1
393 >MG646251.1 H	195C 263G 309.1CT 310C 750G 961G 1438G 3106d 3107C 4769G 8448C 8860G 8898T 11503A 13434G 13759A 15326G 15639C 16278T 16293G 16301T 16311C	H11a1	195C 263G 309.1CT 310C 16278T 16293G 16301T 16311C	1	195 263 750 961G 1438 4769 8448 8860 8898 11503A 13434 13759 15326 15639 16278 16293 16301 16311	H11a1	195 263 16278 16293 16301 16311	[0.012] H11a1 (1)	[0.052] H11a2 (2) [0.000] H11a H11a5 H11a8 (5)	1

394 >MG646232.1 H	73G 152C 217C 263G 302.1C 310C 340T 508G 513.1CA 750G 988A 1438G 1811G 2706G 3106d 3107C 3720G 4769G 5390G 5426C 6045T 6152C 7028T 7109T 8860G 10876G 11467G 11719A 12308G 12372A 13020C 13734C 14766T 15326G 15784C 15907G 16051G 16129C 16183.1C 16189C 16256T 16519C	U2e1b1	73G 152C 217C 263G 302.1C 310C 340T 508G 513.1CA 16051G 16129C 16183.1C 16189C 16256T 16519C	1	73 152 217 263 310 340 508 750 988 1438 1811 2706 3720 4769 5390 5426 6045 6152 7028 7109 8860 10876 11467 11719 12308 12372 13020 13734 14766 15326 15784 15907 16051 16129C 16189 16256	U2e1b1	73 152 217 263 310 340 508 16051 16129C 16189 16256	[0.081] U2e1b1 (1)	[0.083] U2e1b2 (2) [0.000] U2e1b	1
395 >MG646231.1 H	195C 263G 302.1CC 310C 750G 961G 1438G 3106d 3107C 4769G 8448C 8860G 8898T 11503A 13434G 13759A 15326G 16278T 16293G 16311C	H11a1	195C 263G 302.1CC 310C 16278T 16293G 16311C	1	195 263 310 315.2C 750 961G 1438 4769 8448 8860 8898 11503A 13434 13759 15326 16278 16293 16311	H11a1	195 263 310 315.2C 16278 16293 16311	[0.072] H11a1 (1)	[0.149] H11a (2) [0.017] H11a2 (3) [0.000] H11a5 H11a8 (5)	1
396 >MG646217.1 H	146C 195C 263G 302.1CC 310C 456T 750G 1438G 3106d 3107C 4769G 5471A 5492C 8860G 10589A 14497G 15326G 16304C	H5b1	146C 195C 263G 302.1CC 310C 456T 16304C	1	146 195 263 310 315.2C 456 750 1438 4769 5471 5492 8860 10589 14497 15326 16304	H5b1	146 195 263 310 315.2C 456 16304	[0.018] H5b1 (1)	[0.000] H5a5 H5m (3)	1
397 >MG646215.1 H	73G 146C 185A 188G 222T 228A 263G 295T 310.1C 462T 489C 750G 1438G 2706G 3010A 3106d 3107C 4216C 4769G 7028T 8860G 9647A 10398G 10685A 11158T 11251G 11719A 12612G 13281C 13708A 13933G 14766T 14798C 15326G 15452A 16069T 16126C 16519C	J1c2c1	73G 146C 185A 188G 222T 228A 263G 295T 310.1C 462T 489C 16069T 16126C 16519C	1	73 146 185 188 222 228 263 295 462 489 750 1438 2706 3010 4216 4769 7028 8860 9647A 10398 10685 11158 11251 11719 12612 13281 13708 13933 14766 14798 15326 15452A 16069 16126	J1c2c1	73 146 185 188 222 228 263 295 462 489 16069 16126	[0.000] J1c2c1 (1)	[0.005] J1c2c (2) [0.000] J1c2c1 J1c2c1a J1c2c2 J1c2c2a J1c2c3 J1c2h (8)	1
398 >MG646212.1 H	73G 150T 185A 263G 310.1C 513.1CACA 750G 1438G 1721T 2706G 3106d 3107C 3197C 4769G 7028T 7768G 8860G 9477A 9670G 11467G 11653G 11719A 12308G 12372A 12634G 13617C 13630G 13637G 14182C 14766T 15326G 16182.1C 16183C 16189C 16270T 16519C	U5b2b4a	73G 150T 185A 263G 310.1C 513.1CACA 16182.1C 16183C 16189C 16270T 16519C	1	73 150 185 263 750 1438 1721 2706 3197 4769 7028 7768 8860 9477 9670 11467 11653 11719 12308 12372 12634 13617 13630 13637 14182 14766 15326 16189 16270	U5b2b4a	73 150 185 263 16189 16270	[0.000] U5b2b4a (1)	[0.000] U5b1b1+@16192 U5b1b1d U5b1b1f U5b1+16189+@16192 U5b2a+@16192 U5b2a4 U5b2a4a U5b2a6 U5b2b4 (10)	1
399 >MG646208.1 H	73G 185A 188G 263G 295T 302.1CC 310C 462T 489C 514d 515d 729C 750G 1438G 2706G 3010A 3106d 3107C 4216C 4769G 7028T 7830A 8860G 10398G 11251G 11719A 12007A 12612G 13708A 14766T 14798C 15326G 15452A 16069T 16126C 16366T 16390A 16519C	J1c2e1	73G 185A 188G 263G 295T 302.1CC 310C 462T 489C 514d 515d 16069T 16126C 16366T 16390A 16519C	1	73 185 188 263 295 310 315.2C 462 489 729 750 1438 2706 3010 4216 4769 7028 7830 8860 10398 11251 11719 12007 12612 13708 14766 14798 15326 15452A 16069 16126 16366 16390	J1c2e1	73 185 188 263 295 310 315.2C 462 489 16069 16126 16366 16390	[0.250] J1c2e1 (1)	[0.250] J1c2e1 (2) [0.000] J1c2e	1
400 >MG646207.1 H	146C 189G 193G 194T 249G 263G 302.1C 310C 316A 709A 750G 1438G 2259T 3106d 3107C 4577T 4769G 6827C 8860G 10373A 14028G 14872T 15218G 15326G 15412C 16147T 16148T 16256T 16319A	H13a2c1	146C 189G 193G 194T 249G 263G 302.1C 310C 316A 16147T 16148T 16256T 16319A	1	146 189 193 194 249 263 310 316 709 750 1438 2259 4577 4769 6827 8860 10373 14028 14872 15218 15326 15412 16147 16148 16256 16319	H13a2c1	146 189 193 194 249 263 310 316 16147 16148 16256 16319	[2.001] H13a2c1 (1)	[0.000] H13a2c (2)	1

401	>MG646182.1 H[illegible]	262T 263G 302.1CC 310C 750G 1438G 3106d 3107C 4769G 5460A 8860G 10124C 14118G 15326G 15617A 16246C 16264A 16519C	H41a	262T 263G 302.1CC 310C 16246C 16264A 16519C	1	262 263 310 315.2C 750 1438 4769 5460 8860 10124 14118 15326 15617 16246C 16264A	H41a	262 263 310 315.2C 16246C 16264A	[0.099] H41a (1)	[0.026] H (2) [0.015] H26a1 (3) [0.006] H1e1a H13a2b2a (5) [0.005] H2a2a H14 (7) [0.004] HV H1az H3 H7d H13a1a1 H48 H49 H59 (15) [0.003] H1 H10+(16093) H16 (18) [0.002] H1e2 H1e8a H1bk H2a2+(16235) H7b1 H7c3 H13a1a1a H13a1a3 H13a1a4 H16b H18b H23 H49b H56 H75 (33) [0.001] HV4 H1e H1j H1s H1s1 H1v H1ai1 H1ap H1av H2a H2a5 H3c1 H3r H4 H4a1 H7 H7b H7c6 H10a H10b H10c1 H10f H10h H13a1a H13a1a1e H35 H40 H44a H49a H55a H58a H86 (65) [0.000] R0 HV4c HV5 HV5a HV9c HV12 H1e1 H1e1a2 H1e1a7 H1e1c H1e2a H1e2b H1e2d H1e4 H1e6 H1e7 H1e8 H1h H1h1 H1h2 H1j3 H1j5 H1j6 H1j7 H1j9 H1m H1n H1p H1q H1q1 H1q2 H1r H1t H1t1 H1t1a H1t2 H1u H1u1 H1v1 H1v1a H1v1b H1w H1ae H1ae1 H1ae2 H1ae2a H1ae3 H1ae3a H1ag H1ag1 H1ag1b H1ah H1ai H1aj H1ak H1ak1 H1ak2 H1am H1am1 H1aq H1as H1as2 H1at H1at1 H1at1a H1ax H1ax1 H1ay H1bd H1be H1bi H1bj H1bm H1bn H1bp H1bq H1br H1bu H1bv H1bw [illegible]	1
402	>MG646177.1 H[illegible]	263G 310.1C 750G 1438G 2706G 3106d 3107C 4769G 7028T 8860G 15326G 16311C 16354T	HV8	263G 310.1C 16311C 16354T	1	263 750 1438 2706 4769 7028 8860 15326 16311 16354	HV8	263 16311 16354	[0.000] HV8 (1)	[0.051] H2a1 (2) [0.009] H2a1a (3) [0.000] HV+16311 HV9 HV10 HV11 HV11a HV16 HV23 HV24 H1e1a4 H1+16311 H2a1a1 H2a1a2 H2a1b H2a1c H2a1d H2a1g H2a1k H3+16311 H3h H3h1 H3h2 H3h3 H3h3a H3h3b H3h5 H3h6 H3m H13a1a2+16311 H13a2b1 H76 H80 (34)	1
403	>MG646168.1 H[illegible]	73G 195C 263G 310d 499A 750G 1438G 1811G 2706G 3106d 3107C 4646C 4769G 5999C 6047G 7028T 8818T 8860G 11332T 11467G 11719A 12308G 12372A 14620T 14766T 15326G 15693C 16223T 16304C 16356C 16519C	U4a2b	73G 195C 263G 310d 499A 16223T 16304C 16356C 16519C	1	73 195 263 310 315d 499 750 1438 1811 2706 4646 4769 5999 6047 7028 8818 8860 11332 11467 11719 12308 12372 14620 14766 15326 15693 16223 16304 16356	U4a2b	73 195 263 310 315d 499 16223 16304 16356	[0.158] U4a2b (1)	[0.137] U4a2 (2) [0.031] U4a2c U4a2f (4) [0.030] U4a2g (5) [0.021] U4a2e (6) [0.020] U4a2h	1

404 >MG646165.1 H	73G 199C 204C 250C 263G 310.1C 567.1CCCC 750G 1438G 1719A 2706G 3106d 3107C 3504C 4529T 4769G 7028T 8251A 8519A 8860G 10034C 10238C 10398G 10819G 11719A 12501A 12705T 13780G 14766T 15043A 15326G 15924G 16129A 16223T 16304C 16391A 16519C	I4a1	73G 199C 204C 250C 263G 310.1C 567.1CCCC 16129A 16223T 16304C 16391A 16519C	1	73 199 204 250 263 573.4C 750 1438 1719 2706 3504 4529T 4769 7028 8251 8519 8860 10034 10238 10398 10819 11719 12501 12705 13780 14766 15043 15326 15924 16129 16223 16304 16391	I4a1	73 199 204 250 263 573.4C 16129 16223 16304 16391	[0.000] I4a1 (1)	[0.000] I I4 I4a I4a2 I4b I5 I5b I6 I6a (10)	1
405 >MG646158.1 H	143A 189G 193G 249G 263G 310.1C 709A 750G 1438G 2259T 3106d 3107C 4577T 4769G 5772A 6827C 8860G 12819G 14872T 15218G 15326G 15412C 16148T 16256T 16319A	H13a2c1	143A 189G 193G 249G 263G 310.1C 16148T 16256T 16319A	1	143 189 193 249 263 709 750 1438 2259 4577 4769 5772 6827 8860 12819 14872 15218 15326 15412 16148 16256 16319	H13a2c1	143 189 193 249 263 16148 16256 16319	[1.525] H13a2c1 (1)	[0.000] H13a2c (2)	1
406 >MG646148.1 H	195C 263G 302.1C 310C 750G 961G 1438G 3106d 3107C 4769G 8448C 8860G 8898T 13759A 15326G 16261T 16278T 16293G 16311C 16390A	H11a1	195C 263G 302.1C 310C 16261T 16278T 16293G 16311C 16390A	1	195 263 310 750 961G 1438 4769 8448 8860 8898 13759 15326 16261 16278 16293 16311 16390	H11a1	195 263 310 16261 16278 16293 16311 16390	[0.120] H11a1 (1)	[0.135] H11a (2) [0.086] H11a2 (3) [0.000] H11a5 H11a8 (5)	1
407 >MG646146.1 H	195C 263G 309.1CT 310C 750G 961G 1438G 3106d 3107C 4769G 6848T 8448C 8860G 8898T 11503A 13434G 13759A 15326G 16278T 16293G 16311C	H11a1	195C 263G 309.1CT 310C 16278T 16293G 16311C	1	195 263 750 961G 1438 4769 6848 8448 8860 8898 11503A 13434 13759 15326 16278 16293 16311	H11a1	195 263 16278 16293 16311	[0.000] H11a1 (1)	[0.000] H11a H11a2 H11a5 H11a8 (5)	1
408 >MG646137.1 H	263G 302.1CC 310C 514d 515d 519G 750G 1438G 3010A 3106d 3107C 3796G 4769G 8860G 15326G 16129A 16189C 16355T 16356C 16362C 16519C	H1b1b	263G 302.1CC 310C 514d 515d 519G 16129A 16189C 16355T 16356C 16362C 16519C	1	263 310 315.2C 517 750 1438 3010 3796 4769 8860 15326 16129 16189 16355 16356 16362	H1b1b	263 310 315.2C 517 16129 16189 16355 16356 16362	[0.049] H1b1b (1)	[0.020] H1b1c (2) [0.009] H1b1a (3) [0.000] H1b1+16362 H1b1h (5)	1
409 >MG646132.1 H	152C 263G 302.1CCC 310C 456T 750G 1438G 1787A 2765G 3106d 3107C 3525A 4769G 8578T 8860G 13056T 15326G 16070G 16343T	H36	152C 263G 302.1CCC 310C 456T 16070G 16343T	1	152 263 310 315.3C 456 750 1438 1787 2765 3525A 4769 8578 8860 13056 15326 16070 16343T	H36	152 263 310 315.3C 456 16070 16343T	[0.216] H36 (1)	[0.021] H+152 (2) [0.020] H3+152 (3) [0.017] H3g1 (4) [0.014] H46 (5) [0.011] H5a6 (6) [0.007] H1+152 H1i H2a2a2 H3g2 H9 H46a H46b H69 H16a H16c H16d H30b1 H47a (19) [0.006] H1bb H3g H5a1+152 H16+152 (23) [0.000] H1i1 H1i2 H1i2a H1an H1an1 H1an1a H1bc H3g1a H3g1b H3g3 H3g4 H3i H3i1 H3j H3k H3k1 H3k1a H5'36 H5a1m H5a1n H5a+152 H5a6a H52 H13a1+152 H14b H14b2 H14b4 H16a1 (51)	1
410 >MG646128.1 H	73G 185A 188G 228A 263G 295T 309.1CT 310C 462T 489C 514d 515d 750G 1438G 1555G 2706G 3010A 3106d 3107C 4216C 4769G 7028T 8860G 10398G 11251G 11719A 12612G 13708A 14502C 14766T 14798C 15326G 15452A 16069T 16126C 16324C 16366T 16390A 16519C	J1c2e1	73G 185A 188G 228A 263G 295T 309.1CT 310C 462T 489C 514d 515d 16069T 16126C 16324C 16366T 16390A 16519C	1	73 185 188 228 263 295 462 489 750 1438 1555 2706 3010 4216 4769 7028 8860 10398 11251 11719 12612 13708 14502 14766 14798 15326 15452A 16069 16126 16324 16366 16390	J1c2e1	73 185 188 228 263 295 462 489 16069 16126 16324 16366 16390	[0.375] J1c2e1 (1)	[0.375] J1c2e1 (2) [0.000] J1c2e	1

411	>MG646125.1 H	72C 263G 302.1CCC 310C 750G 1438G 2706G 3106d 3107C 4580A 4769G 7028T 8860G 8869G 15326G 15904T 16092C 16290T 16298C 16355T 16519C	V1b	72C 263G 302.1CCC 310C 16092C 16290T 16298C 16355T 16519C	1	72 263 310 315.3C 750 1438 2706 4580 4769 7028 8860 8869 15326 15904 16092 16290 16298 16355	V1b	72 263 310 315.3C 16092 16290 16298 16355	[0.166] V1b (1)	[0.050] V (2) [0.029] V1a1 V13 (4) [0.015] V2b V3 (6) [0.010] V23 (7) [0.005] HV0 V3b V10b2 V11 V15a V25 V26 (14) [0.000] HV0a V1 V1a V1a1b V2 V2a V2a1 V2a1a V2b1 V2b2 V2c V4 V8 V10 V10b V10b1 V12 V14 V15 V17 V24 V28 (36)	1
412	>MG646119.1 H	152C 263G 302.1CCC 310C 456T 750G 1438G 2765G 3106d 3107C 3525A 4769G 8578T 8860G 9370G 13056T 15191C 15326G 16070G	H36	152C 263G 302.1CCC 310C 456T 16070G	1	152 263 310 315.3C 456 750 1438 2765 3525A 4769 8578 8860 9370 13056 15191 15326 16070	H36	152 263 310 315.3C 456 16070	[0.173] H36 (1)	[0.021] H+152 (2) [0.020] H3+152 (3) [0.017] H3g1 (4) [0.014] H46 (5) [0.011] H5a6 (6) [0.007] H1+152 H1i H2a2a2 H3g2 H9 H46a H46b H69 H16a H16c H16d H30b1 H47a (19) [0.006] H1bb H3g H5a1+152 H16+152 (23) [0.000] H1i1 H1i2 H1i2a H1an H1an1 H1an1a H1bc H3g1a H3g1b H3g3 H3g4 H3i H3i1 H3j H3k H3k1 H3k1a H5'36 H5a1m H5a1n H5a+152 H5a6a H52 H13a1+152 H14b H14b2 H14b4 H16a1 (51)	1
413	>MG646089.1 H	195C 263G 309.1CT 310C 750G 961G 1438G 1542C 3106d 3107C 4769G 8448C 8860G 8898T 13434G 13759A 15326G 16278T 16293G 16311C	H11a1	195C 263G 309.1CT 310C 16278T 16293G 16311C	1	195 263 750 961G 1438 1542 4769 8448 8860 8898 13434 13759 15326 16278 16293 16311	H11a1	195 263 16278 16293 16311	[0.000] H11a1 (1)	[0.000] H11a H11a2 H11a5 H11a8 (5)	1
414	>MG244208.1 H	73G 89C 92A 146C 263G 309.1CT 310C 489C 514d 515d 750G 1407A 1438G 2706G 3106d 3107C 3834A 4117C 4769G 4913C 5460A 5843G 7028T 8701G 8790A 8860G 8964T 9101C 9540C 10398G 10400T 10873C 11719A 11884G 12705T 12940A 13047G 13500C 14025C 14766T 14783C 14798C 15043A 15301A 15326G 16129A 16144C 16148T 16223T 16241G 16265C 16293G 16311C 16343G 16526A	Q1f1	73G 89C 92A 146C 263G 309.1CT 310C 489C 514d 515d 16129A 16144C 16148T 16223T 16241G 16265C 16293G 16311C 16343G 16526A	1	73 89 92 146 263 489 750 1407A 1438 2706 3834 4117 4769 4913C 5460 5843 7028 8701 8790 8860 8964 9101 9540 10398 10400 10873 11719 11884 12705 12940 13047 13500 14025 14766 14783 14798 15043 15301 15326 16129 16144 16148 16223 16241 16265C 16293 16311 16343 16526	Q1f1	73 89 92 146 263 489 16129 16144 16148 16223 16241 16265C 16293 16311 16343 16526	[0.068] Q1f1 (1)	[0.000] Q1 Q1d Q1e Q1e1 Q1e1c Q1f Q1f2 (8)	1
415	>MG244204.1 H	73G 146C 263G 309.1CT 310C 514d 515d 750G 1438G 2706G 3106d 3107C 4769G 5465C 6719C 7028T 8271d 8272d 8273d 8274d 8275d 8276d 8277d 8278d 8279d 8860G 9123A 10238C 11719A 12239T 14022G 14766T 15326G 15746G 16182C 16183C 16189C 16213A 16217C 16247G 16261T 16399G 16519C	B4a1a1k	73G 146C 263G 309.1CT 310C 514d 515d 16182C 16183C 16189C 16213A 16217C 16247G 16261T 16399G 16519C	1	73 146 263 750 1438 2706 4769 5465 6719 7028 8281-8289d 8860 9123 10238 11719 12239 14022 14766 15326 15746 16189 16213 16217 16247 16261 16399	B4a1a1k	73 146 263 16189 16213 16217 16247 16261 16399	[0.038] B4a1a1k (1)	[0.038] B4a1a1k (2) [0.000] B4a1a1 B4a1a1a B4a1a1a1 B4a1a1a1b B4a1a1a3 B4a1a1a5 B4a1a1a6 B4a1a1a7 B4a1a1a10 B4a1a1a11 B4a1a1a11a B4a1a1a13 B4a1a1a15 B4a1a1a17 B4a1a1a21 B4a1a1a22 B4a1a1b B4a1a1c B4a1a1d B4a1a1e B4a1a1f B4a1a1g B4a1a1i B4a1a1n B4a1a1p B4a1a1s B4a1a1t B4a1a1u B4a1a1v B4a1a1w B4a1a1x B4a1a1y B4a1a1z B4a1a1ab B4a1a1ac B4a1a1ad B4a1a1ae (39)	1

416 >KY671125.1 Hc	195C 263G 310.1C 750G 961G 1438G 3106d 3107C 4769G 8448C 8860G 8898T 13759A 15326G 16224C 16278T 16293G 16311C	H11a1	195C 263G 310.1C 16224C 16278T 16293G 16311C	1	195 263 750 961G 1438 4769 8448 8860 8898 13759 15326 16224 16278 16293 16311	H11a1	195 263 16224 16278 16293 16311	[0.083] H11a1 (1)	[0.000] H11a H11a2 H11a5 H11a8 (5)	1
417 >KY671118.1 Hc	262T 263G 309.1CCT 310C 750G 1438G 3106d 3107C 4769G 5460A 8860G 10124C 11899C 14055T 14118G 15326G 15617A	H41a	262T 263G 309.1CCT 310C	1	262 263 750 1438 4769 5460 8860 10124 11899 14055 14118 15326 15617	H41a	262 263	[0.000] H41a (1)	[0.000] R0 HV HV4 HV4c HV5 HV5a HV9c HV12 H H1 H1e H1e1 H1e1a H1e1a2 H1e1a7 H1e1c H1e2 H1e2a H1e2b H1e2d H1e4 H1e6 H1e7 H1e8 H1e8a H1h H1h1 H1h2 H1j H1j3 H1j5 H1j6 H1j7 H1j9 H1m H1n H1p H1q H1q1 H1q2 H1r H1s H1s1 H1t H1t1 H1t1a H1t2 H1u H1u1 H1v H1v1 H1v1a H1v1b H1w H1ae H1ae1 H1ae2 H1ae2a H1ae3 H1ae3a H1ag H1ag1 H1ag1b H1ah H1ai H1ai1 H1aj H1ak H1ak1 H1ak2 H1am H1am1 H1ap H1aq H1as H1as2 H1at H1at1 H1at1a H1av H1ax H1ax1 H1ay H1az H1bd H1be H1bi H1bj H1bk H1bm H1bn H1bp H1bq H1br H1bu H1bv H1bw H1bx H1ca H1ci H2 H2a H2a2 H2a2a H2a2+(16235) H2a4 H2a5 H2a5a H2a5b H3 H3b H3c H3c1 H3e H3q H3q1 H3r H3r1 H3t H3u H3y H3aa H3ab H3ac H3ad H3ae H3ag H3ag1 H3ah H3ai H3aj H3ap H3aq H3ar H3as H3at H3at1 H3au H4 H4a H4a1 H4a1a H4a1a1 H4a1a2 H4a1a2a H4a1a2a1 H4a1a5 H4a1c H4a1c1 H4a1c1a H4a1d H4b H4c H7 H7a H7b H7b1 H7b2 H7b2a H7b3 H7b4 H7b5 H7b6 H7c H7c2 H7c3 H7c5 [illegible]	1
418 >KY671072.1 Hc	73G 189G 195C 204C 207A 263G 310.1C 709A 750G 1243C 1438G 2706G 3106d 3107C 3505G 3645C 4769G 5046A 5460A 7028T 7864T 8251A 8516C 8659G 8860G 8887G 8989A 8994A 10398G 11674T 11719A 11947G 12414C 12705T 14766T 15326G 15884C 16093C 16223T 16292T 16295T 16519C	W1e1	73G 189G 195C 204C 207A 263G 310.1C 16093C 16223T 16292T 16295T 16519C	1	73 189 195 204 207 263 709 750 1243 1438 2706 3505 3645 4769 5046 5460 7028 7864 8251 8516 8659 8860 8887 8989 8994 10398 11674 11719 11947 12414 12705 14766 15326 15884C 16093 16223 16292 16295	W1e1	73 189 195 204 207 263 16093 16223 16292 16295	[0.094] W1e1 (1)	[0.051] W (2) [0.012] W1 (3) [0.000] W1a W1e W1e1a W1f (7)	1
419 >KY671059.1 Hc	195C 207A 263G 309.1CT 310C 750G 961G 1438G 3106d 3107C 4769G 5585A 6386T 8448C 8860G 13759A 14587G 15326G 15670C 16092C 16140C 16265G 16293G 16311C	H11a2a2	195C 207A 263G 309.1CT 310C 16092C 16140C 16265G 16293G 16311C	1	195 207 263 750 961G 1438 4769 5585 6386 8448 8860 13759 14587 15326 15670 16092 16140 16265 16293 16311	H11a2a2	195 207 263 16092 16140 16265 16293 16311	[0.043] H11a2a2 (1)	[0.043] H11a2a2 (2) [0.000] H11a2a H11a2a1 H11a2a3 (5)	1
420 >KY671056.1 Hc	152C 263G 310.1C 456T 750G 1438G 2765G 3106d 3107C 3525A 4769G 5147A 8485A 8578T 8860G 13056T 15326G 16070G 16093C 16311C	H36	152C 263G 310.1C 456T 16070G 16093C 16311C	1	152 263 456 750 1438 2765 3525A 4769 5147 8485 8578 8860 13056 15326 16070 16093 16311	H36	152 263 456 16070 16093 16311	[0.044] H36 (1) [0.000] H76a (2)	[0.101] H3h2 (3) [0.006] HV9+152 H2+152_16311 H2c (6) [0.005] H2b	1

421 >KY671049.1 Hc	195C 198T 263G 310.1C 750G 961G 1438G 3106d 3107C 4769G 5585A 8448C 8860G 13759A 14566G 14587G 15326G 15670C 16092C 16140C 16265G 16293G 16311C	H11a2a2	195C 198T 263G 310.1C 16092C 16140C 16265G 16293G 16311C	1	195 198 263 750 961G 1438 4769 5585 8448 8860 13759 14566 14587 15326 15670 16092 16140 16265 16293 16311	H11a2a2	195 198 263 16092 16140 16265 16293 16311	[0.043] H11a2a2 (1)	[0.043] H11a2a2 (2) [0.000] H11a2a H11a2a1 H11a2a3 (5)	1
422 >KY671036.1 Hc	263G 310.1C 750G 1438G 3010A 3106d 3107C 4769G 8860G 15326G 16075C 16111T 16183C 16189C 16209C 16356C 16519C	H1b3	263G 310.1C 16075C 16111T 16183C 16189C 16209C 16356C 16519C	1	263 750 1438 3010 4769 8860 15326 16075 16111 16189 16209 16356	H1b3	263 16075 16111 16189 16209 16356	[0.080] H1b3 (1)	[0.022] H1b1e H1b1e1 H1b1i H1b4 (5) [0.007] H1b1 (6) [0.000] H1b H1b5 (8)	1
423 >KY671035.1 Hc	72C 263G 309.1CT 310C 514d 515d 750G 1438G 2706G 3106d 3107C 4769G 7028T 8860G 10196T 15326G 15596A 15904T 16126C 16189C 16298C	HV0a1	72C 263G 309.1CT 310C 514d 515d 16126C 16189C 16298C	1	72 263 750 1438 2706 4769 7028 8860 10196 15326 15596 15904 16126 16189 16298	HV0a1	72 263 16126 16189 16298	[0.028] HV0a1 (1)	[0.068] V (2) [0.009] HV0a (3) [0.005] HV0 V1a1b V8 V11 (7) [0.000] V1 V1a V1a1 V2 V2a V2a1 V2a1a V2b V2b1 V2b2 V2c V3 V3b V4 V10 V10b V10b1 V10b2 V12 V13 V14 V15 V15a V17 V23 V24 V25 V26 V28 (36)	1
424 >KY671031.1 Hc	73G 263G 309.1CT 310C 497T 750G 1189C 1438G 1811G 2706G 3106d 3107C 3480G 4748T 4769G 5773A 7028T 8350G 8860G 9055A 9698C 10398G 10550G 11025C 11084G 11299C 11467G 11719A 12308G 12372A 13368A 14167T 14766T 14798C 15326G 16180G 16189C 16224C 16233G 16311C 16519C	K1a2a1	73G 263G 309.1CT 310C 497T 16180G 16189C 16224C 16233G 16311C 16519C	1	73 263 497 750 1189 1438 1811 2706 3480 4748 4769 5773 7028 8350 8860 9055 9698 10398 10550 11025 11084 11299 11467 11719 12308 12372 13368 14167 14766 14798 15326 16180 16189 16224 16233 16311	K1a2a1	73 263 497 16180 16189 16224 16233 16311	[0.058] K1a2a1 (1)	[0.058] K1a2a1 (2) [0.012] K1a (3) [0.004] K1a4g (4) [0.003] K1a2a K1a3a K1a4a1 K1a4f K1a4i K1a12a (10) [0.000] K1a1 K1a1a K1a1a1 K1a1a2 K1a1a2a K1a1a2a1 K1a1b K1a1b1 K1a1b1b K1a1b1b1 K1a1b1e K1a1b1f K1a1b1g K1a1b2 K1a2 K1a2a2 K1a2b K1a2c K1a3 K1a3a1 K1a3a1a K1a3a1b K1a3a2 K1a3a3 K1a3a4 K1a4 K1a4a K1a4a1a K1a4a1b K1a4a1b1 K1a4a1c K1a4a1d K1a4a1h K1a4a1i K1a4d K1a4e K1a4f1 K1a4h K1a4h1 K1a5 K1a12 K1a12a1 K1a12a1a K1a18 K1a19 K1a23 K1a29 (57)	1
425 >KY671025.1 Hc	263G 309.1CCT 310C 750G 961G 1438G 3106d 3107C 4769G 8448C 8860G 8898T 13759A 15326G 16134T 16278T 16293G 16311C	H11a1	263G 309.1CCT 310C 16134T 16278T 16293G 16311C	1	263 750 961G 1438 4769 8448 8860 8898 13759 15326 16134 16278 16293 16311	H11a1	263 16134 16278 16293 16311	[0.036] H11a1 (1) [0.000] HV7 H13a1a2a (3)	[0.086] H11a2 (4) [0.065] H1+16278 (5) [0.006] H76 (6) [0.003] HV+16311 H13a2b1 (8) [0.000] HV9 HV10 HV11 HV11a HV16 HV23 HV24 H1e1a4 H1e1a+16278 H1cg H1+16311 H3+16311 H3h H3h1 H3h2 H3h3 H3h3a H3h3b H3h5 H3h6 H3m H11a H11a5 H11a8 H13a1a2+16311 H13a2b5 H24 H24a H24a2 H24b H80 (39)	1

426 >KY671013.1 Hc	152C 195C 263G 309.1CT 310C 750G 1438G 2259T 3106d 3107C 4745G 4769G 8860G 14872T 15326G 16234T 16519C	H13a1d	152C 195C 263G 309.1CT 310C 16234T 16519C	1	152 195 263 750 1438 2259 4745 4769 8860 14872 15326 16234	H13a1d	152 195 263 16234	[0.020] H13a1d (1)	[0.090] H+195 (2) [0.075] H4a1a4a (3) [0.067] HV1b3 (4) [0.013] H3k (5) [0.011] H3g1 (6) [0.007] H16c (7) [0.006] H1bb (8) [0.004] H3+152 H+152 (10) [0.003] H1+152 (11) [0.000] H1i H1i1 H1i2 H1i2a H1an H1an1 H1an1a H1bc H2a2a2 H3g H3g1a H3g1b H3g2 H3g3 H3g4 H3i H3i1 H3j H3k1 H3k1a H4a1a+195 H4a1a3 H4a1a3a H4a1a4 H4a1a4b H4a1a4b2 H12 H108 H9 H46 H46a H46b H52 H69 H13a1+152 H14b H14b2 H14b4 H16+152 H16a H16a1 H16d H30b1 H44b H47a (56)	1
427 >KY671012.1 Hc	195C 263G 310.1C 750G 961A 1438G 3106d 3107C 4215G 4769G 7389C 8448C 8860G 8898T 12678C 13759A 15326G 16278T 16293G 16311C	H11a1	195C 263G 310.1C 16278T 16293G 16311C	1	195 263 750 961A 1438 4215 4769 7389 8448 8860 8898 12678 13759 15326 16278 16293 16311	H11a1	195 263 16278 16293 16311	[0.000] H11a1 (1)	[0.000] H11a H11a2 H11a5 H11a8 (5)	1
428 >KY671011.1 Hc	195C 263G 310.1C 750G 961G 1438G 3106d 3107C 4769G 8448C 8860G 8898T 13759A 15326G 16224C 16278T 16293G 16311C	H11a1	195C 263G 310.1C 16224C 16278T 16293G 16311C	1	195 263 750 961G 1438 4769 8448 8860 8898 13759 15326 16224 16278 16293 16311	H11a1	195 263 16224 16278 16293 16311	[0.083] H11a1 (1)	[0.000] H11a H11a2 H11a5 H11a8 (5)	1
429 >KY671006.1 Hc	73G 185A 188G 228A 263G 295T 309.1CT 310C 462T 489C 750G 1438G 2706G 3010A 3106d 3107C 4216C 4769G 7028T 8860G 10398G 11251G 11719A 12612G 13708A 14766T 14798C 15326G 15452A 16069T 16126C 16163G 16519C	J1c2o	73G 185A 188G 228A 263G 295T 309.1CT 310C 462T 489C 16069T 16126C 16163G 16519C	1	73 185 188 228 263 295 462 489 750 1438 2706 3010 4216 4769 7028 8860 10398 11251 11719 12612 13708 14766 14798 15326 15452A 16069 16126 16163	J1c2o	73 185 188 228 263 295 462 489 16069 16126 16163	[0.000] J1c2o (1)	[0.000] J1c2 J1c2a J1c2a1 J1c2a1a J1c2a3 J1c2b J1c2b1 J1c2b2 J1c2b5 J1c2c J1c2c2 J1c2c2a J1c2c3 J1c2d J1c2f J1c2g J1c2k J1c2l J1c2n J1c2n1 J1c2o J1c2p J1c2q J1c2q1 J1c2s J1c2t (27)	1
430 >KY670995.1 Hc	195C 263G 310.1C 750G 961G 1438G 3106d 3107C 4769G 5585A 6386T 8448C 8860G 13759A 14587G 15326G 15670C 16092C 16140C 16265G 16293G 16311C	H11a2a2	195C 263G 310.1C 16092C 16140C 16265G 16293G 16311C	1	195 263 750 961G 1438 4769 5585 6386 8448 8860 13759 14587 15326 15670 16092 16140 16265 16293 16311	H11a2a2	195 263 16092 16140 16265 16293 16311	[0.000] H11a2a2 (1)	[0.000] H11a2a H11a2a1 H11a2a2 H11a2a3 (5)	1
431 >KY670994.1 Hc	73G 185A 188G 228A 263G 295T 309.1CCT 310C 462T 489C 750G 1438G 2706G 3010A 3106d 3107C 4216C 4769G 7028T 8860G 10398G 11002G 11251G 11719A 12612G 13032G 13708A 14325C 14766T 14798C 15326G 15452A 16069T 16126C 16186T 16362C 16519C	J1c2r	73G 185A 188G 228A 263G 295T 309.1CCT 310C 462T 489C 16069T 16126C 16186T 16362C 16519C	1	73 185 188 228 263 295 462 489 750 1438 2706 3010 4216 4769 7028 8860 10398 11002 11251 11719 12612 13032 13708 14325 14766 14798 15326 15452A 16069 16126 16186 16362	J1c2r	73 185 188 228 263 295 462 489 16069 16126 16186 16362	[0.067] J1c2r (1)	[0.067] J1c2r (2) [0.005] J1c2a1a (3) [0.000] J1c2 J1c2a J1c2a1 J1c2a3 J1c2b J1c2b1 J1c2b2 J1c2b5 J1c2c J1c2c2 J1c2c2a J1c2c3 J1c2d J1c2f J1c2g J1c2j J1c2k J1c2l J1c2n J1c2n1 J1c2p J1c2q J1c2q1 J1c2s J1c2t (28)	1
432 >KY670985.1 Hc	263G 309.1CT 310C 750G 1438G 3010A 3106d 3107C 4769G 8251A 8860G 10527T 15326G 16080G 16129A 16189C 16356C	H1b2	263G 309.1CT 310C 16080G 16129A 16189C 16356C	1	263 750 1438 3010 4769 8251 8860 10527 15326 16080 16129 16189 16356	H1b2	263 16080 16129 16189 16356	[0.029] H1b2 (1)	[0.052] H1b1e H1b1e1 H1b1i H1b4 (5) [0.011] H1b (6) [0.000] H1b1 H1b5 (8)	1

433	>KY670974.1 Hc	73G 150T 152C 263G 309.1CT 310C 750G 1438G 1811G 2706G 3106d 3107C 3397G 4188G 4640A 4769G 7028T 7711C 8860G 9426T 9656C 10775A 11467G 11719A 12308G 12372A 13743C 14139G 14766T 15326G 15454C 15954G 16168T 16189C 16343G	U3b3	73G 150T 152C 263G 309.1CT 310C 16168T 16189C 16343G	1	73 150 152 263 750 1438 1811 2706 3397 4188 4640A 4769 7028 7711 8860 9426 9656 10775 11467 11719 12308 12372 13743 14139 14766 15326 15454 15954 16168 16189 16343	U3b3	73 150 152 263 16168 16189 16343	[0.245] U3b3 (1)	[0.239] U3b2 (2) [0.092] U3b (3) [0.034] U5b2a1b (4) [0.032] U3a3 (5) [0.025] B6 (6) [0.019] U3b2a1 (7) [0.011] U3b2a (8) [0.010] U3b1b (9) [0.008] U3b1 (10) [0.000] U3 U3a'c U3b1a1 U3b2c (14)	1
434	>KY670966.1 Hc	195C 263G 310.1C 750G 961G 1438G 3106d 3107C 4769G 8448C 8860G 8898T 13635C 13759A 15326G 16224C 16278T 16293G 16311C	H11a1	195C 263G 310.1C 16224C 16278T 16293G 16311C	1	195 263 750 961G 1438 4769 8448 8860 8898 13635 13759 15326 16224 16278 16293 16311	H11a1	195 263 16224 16278 16293 16311	[0.083] H11a1 (1)	[0.000] H11a H11a2 H11a5 H11a8 (5)	1
435	>KY670946.1 Hc	73G 263G 309.1CT 310C 709A 750G 1438G 1888A 2706G 3106d 3107C 4216C 4769G 4917G 7028T 8155A 8697A 8860G 9422C 10463C 11251G 11719A 11812G 13368A 14233G 14766T 14905A 15326G 15452A 15607G 15928A 16126C 16264T 16286T 16294T 16296T 16362C 16519C	T2i1	73G 263G 309.1CT 310C 16126C 16264T 16286T 16294T 16296T 16362C 16519C	1	73 263 709 750 1438 1888 2706 4216 4769 4917 7028 8155 8697 8860 9422C 10463 11251 11719 11812 13368 14233 14766 14905 15326 15452A 15607 15928 16126 16264 16286 16294 16296 16362	T2i1	73 263 16126 16264 16286 16294 16296 16362	[0.014] T2i1 (1)	[0.014] T2i1 (2) [0.000] T2i	1
436	>KY670943.1 Hc	73G 150T 195C 207A 262T 263G 295T 310.1C 456T 489C 513.1CACACA 750G 1438G 2706G 3106d 3107C 4216C 4769G 6671C 7028T 7476T 8860G 10398G 10499G 11002G 11251G 11377A 11719A 12441C 12570G 12612G 13015C 13708A 13830C 14766T 15257A 15326G 15452A 15543T 15679G 16069T 16126C 16241G	J2a2b2	73G 150T 195C 207A 262T 263G 295T 310.1C 456T 489C 513.1CACACA 16069T 16126C 16241G	1	73 150 195 207 262 263 295 456 489 750 1438 2706 4216 4769 6671 7028 7476 8860 10398 10499 11002 11251 11377 11719 12441 12570 12612 13015 13708 13830 14766 15257 15326 15452A 15543 15679 16069 16126 16241	J2a2b2	73 150 195 207 262 263 295 456 489 16069 16126 16241	[0.124] J2a2b2 (1)	[0.124] J2a2b2 (2) [0.000] J2a2b J2a2b3 (4)	1
437	>KY670941.1 Hc	239C 263G 309.1CT 310C 750G 1438G 3106d 3107C 4769G 6467T 8860G 9100G 14040A 15326G 16300G 16325C 16362C	H6b2	239C 263G 309.1CT 310C 16300G 16325C 16362C	1	239 263 750 1438 4769 6467 8860 9100 14040 15326 16300 16325 16362	H6b2	239 263 16300 16325 16362	[0.000] H6b2 (1)	[0.000] H6b H6b2 (3)	1

438 >KY670939.1 Hc	152C 263G 309.1CCT 310C 750G 1438G 3106d 3107C 4769G 4793G 6296A 8860G 15326G 16265G 16519C	H7c1	152C 263G 309.1CCT 310C 16265G 16519C	1	152 263 750 1438 4769 4793 6296A 8860 15326 16265	H7c1	152 263 16265	[0.043] H7c1 (1) [0.004] H+152 (2) [0.000] H1+152 H1i H1i1 H1i2 H1i2a H1an H1an1 H1an1a H1bb H1bc H2a2a2 H3+152 H3g H3g1 H3g1a H3g1b H3g2 H3g3 H3g4 H3i H3i1 H3j H3k H3k1 H3k1a H9 H46 H46a H46b H52 H69 H13a1+152 H14b H14b2 H14b4 H16+152 H16a H16a1 H16c H16d H30b1 H47a (44)	[0.217] H1c+152 (45) [0.031] H13a1a1a (46) [0.024] H2a2a (47) [0.022] H9a (48) [0.018] H3a1 (49) [0.012] HV (50) [0.009] H4a1 H26a1 (52) [0.006] H1h1 (53) [0.004] H3q1 H7b (55) [0.003] HV5 H4b H16e H58a H75 (60) [0.002] H1 H1e1a H1e2 H2a H13a1a1 H13a2a H18 H18b (68) [0.001] HV4 H1e1a2 H1e1c H1h2 H1q H1t1a H1ag1 H1aj H1at1a H3 H4 H4a1a H4a1a1 H4a1c H7b1 H7c6 H7d H10b H10+(16093) H13a2b4 H35 H40b H45a (91) [0.000] R0 HV1b+152 HV4c HV5a HV9+152 HV9c HV12 H H1c9 H1c9a H1e H1e1 H1e1a7 H1e2a H1e2b H1e2d H1e4 H1e6 H1e7 H1e8 H1e8a H1h H1j H1j3 H1j5 H1j6 H1j7 H1j9 H1m H1n H1p H1q1 H1q2 H1r H1s H1s1 H1t H1t1 H1t2 H1u H1u1 H1v H1v1 H1v1a H1v1b H1w H1ae H1ae1 H1ae2 H1ae2a H1ae3 H1ae3a H1ag H1ag1b H1ah H1ai H1ai1 H1ak H1ak1 H1ak2 H1am H1am1 H1ap H1ap1 H1aq H1as H1as2 H1at H1at1 H1av H1ax H1ax1 H1ay H1az H1bd H1be H1bi H1bj H1bk H1bm H1bn H1bp H1bq H1br H1bu H1bv H1bw H1bx [illegible]	1
439 >KY670934.1 Hc	73G 263G 309.1CT 310C 750G 1438G 3010A 3106d 3107C 4769G 8860G 14020C 15326G 16051G 16162G 16213A 16266T 16519C	H1a3c1	73G 263G 309.1CT 310C 16051G 16162G 16213A 16266T 16519C	1	73 263 750 1438 3010 4769 8860 14020 15326 16051 16162 16213 16266	H1a3c1	73 263 16051 16162 16213 16266	[0.000] H1a3c1 (1)	[0.000] H1a3c (2)	1
440 >KY670926.1 Hc	151T 263G 309.1CCT 310C 750G 1438G 3010A 3106d 3107C 4769G 8251A 8860G 12501A 15326G 16080G 16182.1C 16183C 16189C 16356C	H1b2	151T 263G 309.1CCT 310C 16080G 16182.1C 16183C 16189C 16356C	1	151 263 750 1438 3010 4769 8251 8860 12501 15326 16080 16189 16356	H1b2	151 263 16080 16189 16356	[0.048] H1b2 (1)	[0.000] H1b H1b1 H1b1e H1b1e1 H1b1i H1b4 H1b5 (8)	1
441 >KY670924.1 Hc	150T 263G 309.1CT 310C 750G 951A 3106d 3107C 8860G 15326G 16193T 16354T	H2a1f2	150T 263G 309.1CT 310C 16193T 16354T	1	150 263 750 951 8860 15326 16193 16354	H2a1f2	150 263 16193 16354	[0.000] H2a1f2 (1)	[0.000] H2a1f (2)	1
442 >KY670923.1 Hc	195C 263G 310.1C 750G 961G 1438G 3106d 3107C 4769G 5585A 8448C 8860G 13759A 14587G 15326G 15670C 16092C 16140C 16265G 16293G 16311C	H11a2a2	195C 263G 310.1C 16092C 16140C 16265G 16293G 16311C	1	195 263 750 961G 1438 4769 5585 8448 8860 13759 14587 15326 15670 16092 16140 16265 16293 16311	H11a2a2	195 263 16092 16140 16265 16293 16311	[0.000] H11a2a2 (1)	[0.000] H11a2a H11a2a1 H11a2a2 H11a2a3 (5)	1

443 >KY670912.1 Hc	73G 189G 195C 204C 207A 263G 310.1C 709A 750G 1243C 1438G 2706G 3106d 3107C 3505G 4769G 5046A 5460A 7028T 7864T 8251A 8659G 8860G 8887G 8994A 10398G 11674T 11719A 11947G 12414C 12705T 14766T 15326G 15884C 16189C 16223T 16292T 16295T 16519C	W1e1	73G 189G 195C 204C 207A 263G 310.1C 16189C 16223T 16292T 16295T 16519C	1	73 189 195 204 207 263 709 750 1243 1438 2706 3505 4769 5046 5460 7028 7864 8251 8659 8860 8887 8994 10398 11674 11719 11947 12414 12705 14766 15326 15884C 16189 16223 16292 16295	W1e1	73 189 195 204 207 263 16189 16223 16292 16295	[0.094] W1e1 (1)	[0.098] W1e (2) [0.024] W1 (3) [0.000] W W1a W1e1a W1f (7)	1
444 >KY670871.1 Hc	195C 263G 310.1C 750G 961A 1438G 2430G 3106d 3107C 4215G 4769G 8448C 8860G 8898T 12678C 13759A 15326G 16278T 16293G 16311C	H11a1	195C 263G 310.1C 16278T 16293G 16311C	1	195 263 750 961A 1438 2430 4215 4769 8448 8860 8898 12678 13759 15326 16278 16293 16311	H11a1	195 263 16278 16293 16311	[0.000] H11a1 (1)	[0.000] H11a H11a2 H11a5 H11a8 (5)	1
445 >KY670856.1 Hc	195C 263G 309.1CT 310C 750G 961G 1438G 3106d 3107C 4769G 8448C 8860G 8898T 13759A 15326G 16278T 16293G 16311C	H11a1	195C 263G 309.1CT 310C 16278T 16293G 16311C	1	195 263 750 961G 1438 4769 8448 8860 8898 13759 15326 16278 16293 16311	H11a1	195 263 16278 16293 16311	[0.000] H11a1 (1)	[0.000] H11a H11a2 H11a5 H11a8 (5)	1
446 >KU683593.1 Hc	64T 73G 146C 152C 235G 263G 514d 515d 663G 750G 1438G 1736G 2706G 3106d 3107C 4248C 4769G 4824G 6962A 7028T 8794T 8860G 8865A 11719A 12705T 12777G 14766T 15326G 16223T 16242T 16278T 16290T 16319A	A8a1	64T 73G 146C 152C 235G 263G 514d 515d 16223T 16242T 16278T 16290T 16319A	1	64 73 146 152 235 263 663 750 1438 1736 2706 4248 4769 4824 6962 7028 8794 8860 8865 11719 12705 12777 14766 15326 16223 16242 16278 16290 16319	A8a1	64 73 146 152 235 263 16223 16242 16278 16290 16319	[0.161] A8a1 (1)	[0.000] A8a (2)	1
447 >KU683592.1 Hc	73G 153G 263G 310N 489C 513A 750G 1041G 1438G 2706G 3106d 3107C 3394C 4491A 4769G 5090C 7028T 7256T 8701G 8860G 9540C 10398G 10400T 10873C 11719A 12705T 14142A 14180C 14308C 14766T 14783C 15043A 15301A 15326G 16145A 16189C 16223T 16234T 16293G 16316G	M9a1a2	73G 153G 263G 310N 489C 513A 16145A 16189C 16223T 16234T 16293G 16316G	1	73 153 263 310N 489 513 750 1041 1438 2706 3394 4491 4769 5090 7028 7256 8701 8860 9540 10398 10400 10873 11719 12705 14142A 14180 14308 14766 14783 15043 15301 15326 16145 16189 16223 16234 16293 16316	M9a1a2	73 153 263 310N 489 513 16145 16189 16223 16234 16293 16316	[0.654] M9a1a2 (1)	[0.007] M9a1a (2) [0.000] M9a1a1 M9a1a1c1b M9a1a1c1b2 M9a1a1d (6)	1
448 >KU683588.1 Hc	73G 150T 263G 456T 489C 681C 750G 1048T 1107C 1438G 2706G 3106d 3107C 3759G 4769G 4883T 5147A 5153G 5178A 5301G 6253C 6465A 7028T 7403G 8701G 8860G 8886A 9180G 9531G 9540C 10397G 10398G 10400T 10873C 11719A 11869T 12705T 14766T 14783C 15043A 15301A 15326G 15724G 16183N 16189C 16223T 16259T 16357C 16362C 16519C	D5b1b2	73G 150T 263G 456T 489C 16183N 16189C 16223T 16259T 16357C 16362C 16519C	1	73 150 263 456 489 681 750 1048 1107 1438 2706 3759 4769 4883 5147 5153 5178A 5301 6253 6465 7028 7403 8701 8860 8886 9180 9531 9540 10397 10398 10400 10873 11719 11869 12705 14766 14783 15043 15301 15326 15724 16183N 16189 16223 16259 16357 16362	D5b1b2	73 150 263 456 489 16183N 16189 16223 16259 16357 16362	[0.036] D5b1b2 (1)	[0.000] D5b D5b1 D5b1a D5b1b D5b1c D5b3 D5b3a (8)	1
449 >KU683581.1 Hc	263G 310N 750G 1438G 3106d 3107C 4769G 8860G 11447A 15326G 16172C 16400T	H33b	263G 310N 16172C 16400T	1	263 310N 750 1438 4769 8860 11447 15326 16172 16400	H33b	263 310N 16172 16400	[0.000] H33b (1)	[0.000] H66a (2)	1

450 >KU683568.1 Hc	73G 152N 204C 248d 263G 514d 515d 516d 517d 750G 1438G 2706G 3106d 3107C 3970T 4732G 4769G 5147A 6392C 6962A 7028T 8772C 8860G 9180G 10310A 10609C 10976T 11719A 12406A 12633T 12882T 13928C 14476A 14766T 15326G 16172C 16183N 16189C 16232A 16249C 16304C 16311C	F1b1b	73G 152N 204C 248d 263G 514d 515d 516d 517d 16172C 16183N 16189C 16232A 16249C 16304C 16311C	1	73 152N 204 249d 263 750 1438 2706 3970 4732 4769 5147 6392 6962 7028 8772 8860 9180 10310 10609 10976 11719 12406 12633 12882 13928C 14476 14766 15326 16172 16183N 16189 16232A 16249 16304 16311	F1b1b	73 152N 204 249d 263 16172 16183N 16189 16232A 16249 16304 16311	[0.000] F1b1b (1)	[0.251] F1b1+@152 (2) [0.048] F1b F1b1 (4) [0.000] F1b1e F1b1e1 F1b1f (7)	1
451 >KU683563.1 Hc	72C 263G 310N 750G 1438G 2706G 3106d 3107C 4580A 4769G 7028T 8860G 15326G 15904T	V+@16298	72C 263G 310N	1	72 263 310N 750 1438 2706 4580 4769 7028 8860 15326 15904	V+@16298	72 263 310N	[0.000] V+@16298 (1)	[0.005] V2 H2a2a (3) [0.003] H1as H13a2b2a (5) [0.002] H7 (6) [0.001] H H1 H2a H3 H3c1 H16 H23 H54 (14) [0.000] R0 HV HV0 HV0a V V1 V1a V1a1 V1a1b V2a V2a1 V2a1a V2b V2b1 V2b2 V2c V3 V3b V4 V8 V10 V10b V10b1 V10b2 V11 V12 V13 V14 V15 V15a V17 V20 V23 V24 V25 V26 V28 HV4 HV4c HV5 HV5a HV9c HV12 H1e H1e1 H1e1a H1e1a2 H1e1a7 H1e1c H1e2 H1e2a H1e2b H1e2d H1e4 H1e6 H1e7 H1e8 H1e8a H1h H1h1 H1h2 H1j H1j3 H1j5 H1j6 H1j7 H1j9 H1m H1n H1p H1q H1q1 H1q2 H1r H1s H1s1 H1t H1t1 H1t1a H1t2 H1u H1u1 H1v H1v1 H1v1a H1v1b H1w H1ae H1ae1 H1ae2 H1ae2a H1ae3 H1ae3a H1ag H1ag1 H1ag1b H1ah H1ai H1ai1 H1aj H1ak H1ak1 H1ak2 H1am H1am1 H1ap H1aq H1as2 H1at H1at1 H1at1a H1av H1ax H1ax1 H1ay H1az H1bd H1be H1bi H1bj H1bk H1bm H1bn H1bp H1bq H1br H1bu H1bv H1bw H1bx H1ca H1ci H2 H2a2 H2a2+(16235) H2a4 H2a5 H2a5a H2a5b H3b H3c H3e H3q H3q1 H3r H3r1 H3t H3u H3y H3aa H3ab H3ac H3ad [illegible]	1

452	>KU683562.1 Hc	72C 263G 750G 1438G 2706G 3106d 3107C 4580A 4769G 7028T 8860G 15326G 15904T	V+@16298	72C 263G	1	72 263 750 1438 2706 4580 4769 7028 8860 15326 15904	V+@16298	72 263	[0.000] V+@16298 (1)	[0.005] V2 H2a2a (3) [0.003] H1as H13a2b2a (5) [0.002] H7 (6) [0.001] H H1 H2a H3 H3c1 H16 H23 H54 (14) [0.000] R0 HV HV0 HV0a V V1 V1a V1a1 V1a1b V2a V2a1 V2a1a V2b V2b1 V2b2 V2c V3 V3b V4 V8 V10 V10b V10b1 V10b2 V11 V12 V13 V14 V15 V15a V17 V20 V23 V24 V25 V26 V28 HV4 HV4c HV5 HV5a HV9c HV12 H1e H1e1 H1e1a H1e1a2 H1e1a7 H1e1c H1e2 H1e2a H1e2b H1e2d H1e4 H1e6 H1e7 H1e8 H1e8a H1h H1h1 H1h2 H1j H1j3 H1j5 H1j6 H1j7 H1j9 H1m H1n H1p H1q H1q1 H1q2 H1r H1s H1s1 H1t H1t1 H1t1a H1t2 H1u H1u1 H1v H1v1 H1v1a H1v1b H1w H1ae H1ae1 H1ae2 H1ae2a H1ae3 H1ae3a H1ag H1ag1 H1ag1b H1ah H1ai H1ai1 H1aj H1ak H1ak1 H1ak2 H1am H1am1 H1ap H1aq H1as2 H1at H1at1 H1at1a H1av H1ax H1ax1 H1ay H1az H1bd H1be H1bi H1bj H1bk H1bm H1bn H1bp H1bq H1br H1bu H1bv H1bw H1bx H1ca H1ci H2 H2a2 H2a2+(16235) H2a4 H2a5 H2a5a H2a5b H3b H3c H3e H3q H3q1 H3r H3r1 H3t H3u H3y H3aa H3ab H3ac H3ad [illegible]	1
453	>KU683551.1 Hc	263G 310.1C 750G 1438G 2706G 3106d 3107C 4769G 6755A 7028T 8772C 8860G 15326G 16172C 16189C 16311C	HV6	263G 310.1C 16172C 16189C 16311C	1	263 750 1438 2706 4769 6755 7028 8772 8860 15326 16172 16189 16311	HV6	263 16172 16189 16311	[0.044] HV6 (1)	[0.020] H3h2 (2) [0.013] HV+16311 (3) [0.009] H3+16189 (4) [0.007] H1g H1aa (6) [0.003] H1+16189 H3h3b (8) [0.002] H1+16311 (9) [0.000] HV6a HV9 HV10 HV11 HV11a HV16 HV23 HV24 HV18 H1f H1g1 H1g2 H1y H1z1 H1aa1 H1ab H1ac H1ad H1e1a4 H1j9 H3+16311 H3h H3h1 H3h3 H3h3a H3h5 H3h6 H3m H13a1a2+16311 H13a2b1 H66a H76 H80 U6b1b (43)	1

454 >KU683540.1 H	73G 152C 263G 489C 750G 789C 1222G 1438G 1719A 2706G 3010A 3106d 3107C 3492G 4769G 4883T 5178A 7028T 8414T 8701G 8762C 8860G 8868C 9540C 10398G 10400T 10873C 11719A 12651C 12705T 14668T 14766T 14783C 15043A 15229C 15301A 15326G 15927A 16042A 16172C 16223T 16362C	D4m2	73G 152C 263G 489C 16042A 16172C 16223T 16362C	1	73 152 263 489 750 789 1222 1438 1719 2706 3010 3492 4769 4883 5178A 7028 8414 8701 8762 8860 8868 9540 10398 10400 10873 11719 12651C 12705 14668 14766 14783 15043 15229 15301 15326 15927 16042 16172 16223 16362	D4m2	73 152 263 489 16042 16172 16223 16362	[0.089] D4m2 (1) [0.002] M13'46'61+16362 (2)	[0.125] G2b1a2 (3) [0.057] D4b2b2b (4) [0.027] N10 (5) [0.020] G2a'c (6) [0.017] D4a1b (7) [0.013] D4 D4j2 (9) [0.009] D4b2b (10) [0.006] G2a+152 M33+16362 D4h4a (13) [0.005] D4b1a1a (14) [0.004] M37+152 M46 D4a1f1 D4a5 (18) [0.003] G2b2 D4e1c D4e2 D4f1 D4h4 D4j1b (24) [0.002] G1c G1c2 D4e2d D4j D4j1 D4j+(16286) (30) [0.001] D4b2 D4b2b1 (32) [0.000] M9 E1a2 E1a2+(16261) E1a2a E1a2a1 E1a2a3 G G1 G2 G2a2 G2c G2b G2b1 G2b1a G2b2a M13'46'61 M13 M13a'b M61 M33a2'3 D D4a D4a1 D4a1a D4a1a1 D4a1a1a D4a1c D4a1f D4a1g D4a1h D4a2 D4a2a D4a2b D4b D4b1a1 D4b2a D4b2a2 D4b2a2b D4b2b1a D4b2b1b D4b2b1c D4b2b2 D4b2b3 D4b2b6 D4e D4e1'3 D4e1 D4e3 D4e2b D4e2c D4e4 D4e4a D4e4b D4f D4g D4h D4j2a D4j4 D4j5 D4j5a D4j9 D4j10 D4j12 D4j15 D4j16 D4m D4m2a D4m2a1 D4m2a1a (101)	1
455 >KU683533.1 H	73G 150N 263G 334C 514d 515d 750G 1438G 1811G 2706G 2707G 3106d 3107C 4188G 4640A 4769G 7028T 8860G 8950A 9656C 9770C 11467G 11719A 12308G 12372A 13174C 13743C 14139G 14766T 15326G 15454C 15940d 16131C 16343G	U3b2a1a	73G 150N 263G 334C 514d 515d 16131C 16343G	1	73 150N 263 334 750 1438 1811 2706 2707 4188 4640A 4769 7028 8860 8950 9656 9770 11467 11719 12308 12372 13174 13743 14139 14766 15326 15454 15944d 16131 16343	U3b2a1a	73 150N 263 334 16131 16343	[0.034] U3b2a1a (1)	[0.000] U3 U3a'c U3b U3b1 U3b1b U3b2 U3b2a U3b2a1 U3b2c (10)	1
456 >KU683530.1 H	73G 146C 152C 153G 195C 234G 263G 750G 1438G 1811G 1888A 3106d 3107C 4317G 4769G 5186T 5773A 7028T 8860G 9094T 9614G 11467G 11719A 12106T 12308G 12372A 12406A 12793C 13194A 13656C 14766T 15049T 15326G 15930A 16051G 16209C 16239T 16311C 16352C 16353T	U2b2	73G 146C 152C 153G 195C 234G 263G 16051G 16209C 16239T 16311C 16352C 16353T	1	73 146 152 153 195 234 263 750 1438 1811 1888 4317 4769 5186T 5773 7028 8860 9094 9614 11467 11719 12106 12308 12372 12406 12793 13194 13656 14766 15049 15326 15930 16051 16209 16239 16311 16352 16353	U2b2	73 146 152 153 195 234 263 16051 16209 16239 16311 16352 16353	[0.275] U2b2 (1)	[0.989] U2b (2) [0.585] U2b1 (3) [0.389] U2b1a (4) [0.151] U2+152 (5) [0.043] K2a6 (6) [0.033] R30a1b (7) [0.022] K1b2a (8) [0.017] K2a2a (9) [0.014] K1b2a2 (10) [0.011] K2a1 K2a2 K2a3a (13) [0.010] K2a (14) [0.008] K1b2b (15) [0.005] M24a (16) [0.000] L3f2a L3f2a1 U4b1b1+16311 U4b1b1c K1b2 K1b2a1 K1b2a1a K1b2a2a K1b2a3 K2a3 K2a3a1 K2a4 K2a7 K2a9 K2a10 (31)	1

457	>KU683529.1 Hc	73G 263G 489C 750G 789C 1222G 1438G 1719A 2706G 3010A 3106d 3107C 3492G 4769G 4883T 5178A 7028T 8414T 8701G 8762C 8860G 8868C 9540C 10398G 10400T 10873C 11353C 11719A 12651C 12705T 14668T 14766T 14783C 15043A 15229C 15301A 15326G 16042A 16172C 16223T 16362C	D4m2	73G 263G 489C 16042A 16172C 16223T 16362C	1	73 263 489 750 789 1222 1438 1719 2706 3010 3492 4769 4883 5178A 7028 8414 8701 8762 8860 8868 9540 10398 10400 10873 11353 11719 12651C 12705 14668 14766 14783 15043 15229 15301 15326 16042 16172 16223 16362	D4m2	73 263 489 16042 16172 16223 16362	[0.070] D4m2 (1)	[0.125] G2b1a2 (2) [0.024] D4b2b2b (3) [0.003] D4e1c D4j1b (5) [0.002] D4j D4j+(16286) (7) [0.001] D4 D4b2 D4b2b (10) [0.000] M9 G G1 G1c G1c2 G2 G2a'c G2b G2b1 G2b1a G2b2 G2b2a M33a2'3 M33+16362 D D4b D4b2a D4b2a2 D4b2a2b D4b2b1 D4b2b1a D4b2b1b D4b2b1c D4b2b2 D4b2b3 D4b2b6 D4e D4e1'3 D4e1 D4e3 D4e2 D4e2b D4e2c D4e2d D4e4 D4e4a D4e4b D4f D4f1 D4g D4h D4h4 D4j1 D4j4 D4j5 D4j5a D4j9 D4j10 D4j12 D4j15 D4j16 D4m D4m2a D4m2a1 D4m2a1a N10 (66)	1
458	>KU683525.1 Hc	73G 152C 263G 489C 750G 789C 1222G 1438G 1719A 2706G 3010A 3106d 3107C 3492G 4769G 4883T 5178A 7028T 8414T 8701G 8762C 8860G 8868C 9540C 10398G 10400T 10873C 11719A 12651C 12705T 14668T 14766T 14783C 15043A 15229C 15301A 15326G 16042A 16172C 16223T 16362C	D4m2	73G 152C 263G 489C 16042A 16172C 16223T 16362C	1	73 152 263 489 750 789 1222 1438 1719 2706 3010 3492 4769 4883 5178A 7028 8414 8701 8762 8860 8868 9540 10398 10400 10873 11719 12651C 12705 14668 14766 14783 15043 15229 15301 15326 16042 16172 16223 16362	D4m2	73 152 263 489 16042 16172 16223 16362	[0.089] D4m2 (1) [0.002] M13'46'61+16362 (2)	[0.125] G2b1a2 (3) [0.057] D4b2b2b (4) [0.027] N10 (5) [0.020] G2a'c (6) [0.017] D4a1b (7) [0.013] D4 D4j2 (9) [0.009] D4b2b (10) [0.006] G2a+152 M33+16362 D4h4a (13) [0.005] D4b1a1a (14) [0.004] M37+152 M46 D4a1f1 D4a5 (18) [0.003] G2b2 D4e1c D4e2 D4f1 D4h4 D4j1b (24) [0.002] G1c G1c2 D4e2d D4j D4j1 D4j+(16286) (30) [0.001] D4b2 D4b2b1 (32) [0.000] M9 E1a2 E1a2+(16261) E1a2a E1a2a1 E1a2a3 G G1 G2 G2a2 G2c G2b G2b1 G2b1a G2b2a M13'46'61 M13 M13a'b M61 M33a2'3 D D4a D4a1 D4a1a D4a1a1 D4a1a1a D4a1c D4a1f D4a1g D4a1h D4a2 D4a2a D4a2b D4b D4b1a1 D4b2a D4b2a2 D4b2a2b D4b2b1a D4b2b1b D4b2b1c D4b2b2 D4b2b3 D4b2b6 D4e D4e1'3 D4e1 D4e3 D4e2b D4e2c D4e4 D4e4a D4e4b D4f D4g D4h D4j2a D4j4 D4j5 D4j5a D4j9 D4j10 D4j12 D4j15 D4j16 D4m D4m2a D4m2a1 D4m2a1a (101)	1
459	>KU683523.1 Hc	152C 239C 263G 310N 750G 1438G 3106d 3107C 4769G 8860G 14040A 15326G 16186T 16300G 16325C 16362C 16482G	H6b2	152C 239C 263G 310N 16186T 16300G 16325C 16362C 16482G	1	152 239 263 310N 750 1438 4769 8860 14040 15326 16186 16300 16325 16362 16482	H6b2	152 239 263 310N 16186 16300 16325 16362 16482	[0.380] H6b2 (1)	[0.380] H6b2 (2) [0.125] H6b1 (3) [0.033] H6b	1

460	>KU683519.1 Hc	73G 150T 152C 263G 750G 1438G 1811G 2706G 3106d 3107C 4188G 4640A 4769G 7028T 8382T 8860G 9656C 11467G 11719A 12308G 12372A 13743C 14139G 14766T 15326G 15454C 16157C 16168T 16343G 16390A	U3b3	73G 150T 152C 263G 16157C 16168T 16343G 16390A	1	73 150 152 263 750 1438 1811 2706 4188 4640A 4769 7028 8382 8860 9656 11467 11719 12308 12372 13743 14139 14766 15326 15454 16157 16168 16343 16390	U3b3	73 150 152 263 16157 16168 16343 16390	[0.349] U3b3 (1) [0.089] U3a1 (2) [0.021] U3a2 (3) [0.016] U3a (4) [0.000] U3a1a U3a1a1 U3a1b (7)	[0.345] U3b2 (8) [0.092] U3b (9) [0.048] U3a3 (10) [0.032] U3a2a (11) [0.028] U3b2a1 (12) [0.025] U3a1c (13) [0.010] U3b1b (14) [0.000] U3 U3a'c U3b1 U3b1a1 U3b2a U3b2c (20)	1
461	>KU683513.1 Hc	73G 152N 195C 263G 489C 750G 1438G 2706G 3010A 3106d 3107C 4769G 4883T 5178A 7028T 8414T 8701G 8860G 9540C 10398G 10400T 10873C 11719A 12092A 12705T 14668T 14766T 14783C 15043A 15301A 15326G 16192T 16223T	D4k	73G 152N 195C 263G 489C 16192T 16223T	1	73 152N 195 263 489 750 1438 2706 3010 4769 4883 5178A 7028 8414 8701 8860 9540 10398 10400 10873 11719 12092A 12705 14668 14766 14783 15043 15301 15326 16192 16223	D4k	73 152N 195 263 489 16192 16223	[0.203] D4k (1)	[0.066] M (2) [0.019] M52b (3) [0.018] G1b (4) [0.008] M13 (5) [0.006] M1'20'51 D4+195 (7) [0.005] M22b (8) [0.004] M37+152 (9) [0.003] M7b M21b M21b1a M24'41 M33a M35 (15) [0.000] M4"67 M38+195 M38c M37 M43 M43b M66 M66a M7 M7b'c M12'G G2c M13'46'61 M13a'b M19'53 M21 M21b1 M22 M23'75 M24 M27 M29'Q M31 M31a M32'56 M33 M33a1 M34'57 M39'70 M42'74 M42 M52 M55'77 M58 M60 M69 M80'D (52)	1
462	>KU683371.1 Hc	73G 143A 189G 195C 204C 207A 210G 263G 310N 750G 1438G 1719A 2706G 3106d 3107C 4769G 5054A 6297C 7028T 8222C 8595T 8706G 8860G 10238C 11025C 11140T 11437C 11719A 11914A 12501A 12705T 13637G 13780G 14766T 15326G 16201T 16223T 16265G 16311C 16519C	N1a3a	73G 143A 189G 195C 204C 207A 210G 263G 310N 16201T 16223T 16265G 16311C 16519C	1	73 143 189 195 204 207 210 263 310N 750 1438 1719 2706 4769 5054 6297 7028 8222 8595 8706 8860 10238 11025 11140 11437 11719 11914 12501 12705 13637 13780 14766 15326 16201 16223 16265 16311	N1a3a	73 143 189 195 204 207 210 263 310N 16201 16223 16265 16311	[0.140] N1a3a (1)	[0.000] N1a3 N1a3a2 (3)	1

463	>KU683358.1 Hc	72C 263G 750G 1438G 2706G 3106d 3107C 4580A 4769G 7028T 8860G 15326G 15904T	V+@16298	72C 263G	1	72 263 750 1438 2706 4580 4769 7028 8860 15326 15904	V+@16298	72 263	[0.000] V+@16298 (1)	[0.005] V2 H2a2a (3) [0.003] H1as H13a2b2a (5) [0.002] H7 (6) [0.001] H H1 H2a H3 H3c1 H16 H23 H54 (14) [0.000] R0 HV HV0 HV0a V V1 V1a V1a1 V1a1b V2a V2a1 V2a1a V2b V2b1 V2b2 V2c V3 V3b V4 V8 V10 V10b V10b1 V10b2 V11 V12 V13 V14 V15 V15a V17 V20 V23 V24 V25 V26 V28 HV4 HV4c HV5 HV5a HV9c HV12 H1e H1e1 H1e1a H1e1a2 H1e1a7 H1e1c H1e2 H1e2a H1e2b H1e2d H1e4 H1e6 H1e7 H1e8 H1e8a H1h H1h1 H1h2 H1j H1j3 H1j5 H1j6 H1j7 H1j9 H1m H1n H1p H1q H1q1 H1q2 H1r H1s H1s1 H1t H1t1 H1t1a H1t2 H1u H1u1 H1v H1v1 H1v1a H1v1b H1w H1ae H1ae1 H1ae2 H1ae2a H1ae3 H1ae3a H1ag H1ag1 H1ag1b H1ah H1ai H1ai1 H1aj H1ak H1ak1 H1ak2 H1am H1am1 H1ap H1aq H1as2 H1at H1at1 H1at1a H1av H1ax H1ax1 H1ay H1az H1bd H1be H1bi H1bj H1bk H1bm H1bn H1bp H1bq H1br H1bu H1bv H1bw H1bx H1ca H1ci H2 H2a2 H2a2+(16235) H2a4 H2a5 H2a5a H2a5b H3b H3c H3e H3q H3q1 H3r H3r1 H3t H3u H3y H3aa H3ab H3ac H3ad [illegible]	1
464	>KU683357.1 Hc	73G 200G 263G 310N 489C 750G 1438G 2706G 3010A 3106d 3107C 4769G 4883T 5178A 7028T 8414T 8701G 8794T 8860G 9540C 10398G 10400T 10873C 11719A 12705T 13899C 14668T 14766T 14783C 14827T 15043A 15301A 15326G 16223T 16256T 16311C 16362C 16519C	D4q	73G 200G 263G 310N 489C 16223T 16256T 16311C 16362C 16519C	1	73 200 263 310N 489 750 1438 2706 3010 4769 4883 5178A 7028 8414 8701 8794 8860 9540 10398 10400 10873 11719 12705 13899 14668 14766 14783 14827 15043 15301 15326 16223 16256 16311 16362	D4q	73 200 263 310N 489 16223 16256 16311 16362	[0.000] D4q (1)	[0.000] D4q1 D4q1a (3)	1
465	>KU683351.1 Hc	73G 195C 215G 263G 310.1C 499A 750G 1438G 1811G 2706G 3106d 3107C 4646C 4769G 5999C 6047G 7028T 7705C 7859A 8860G 11332T 11467G 11719A 12308G 12372A 14620T 14766T 15217A 15326G 15693C 16356C 16519C	U4b3	73G 195C 215G 263G 310.1C 499A 16356C 16519C	1	73 195 215 263 499 750 1438 1811 2706 4646 4769 5999 6047 7028 7705 7859 8860 11332 11467 11719 12308 12372 14620 14766 15217 15326 15693 16356	U4b3	73 195 215 263 499 16356	[0.000] U4b3 (1)	[0.000] U4 U4a U4b U4b1 U4b1a U4b1a1 U4b1a1a U4b1a2 U4b1a2a U4b1a2b U4b1a4 U4c U4c2 U4d U4d1 U4d1a U4d1a1 U4d1a1a U4d1b (20)	1

466 >KU683345.1 Hc	72C 263G 310N 750G 1438G 2706G 3106d 3107C 4580A 4769G 7028T 8860G 15326G 15904T	V+@16298	72C 263G 310N	1	72 263 310N 750 1438 2706 4580 4769 7028 8860 15326 15904	V+@16298	72 263 310N	[0.000] V+@16298 (1)	[0.005] V2 H2a2a (3) [0.003] H1as H13a2b2a (5) [0.002] H7 (6) [0.001] H H1 H2a H3 H3c1 H16 H23 H54 (14) [0.000] R0 HV HV0 HV0a V V1 V1a V1a1 V1a1b V2a V2a1 V2a1a V2b V2b1 V2b2 V2c V3 V3b V4 V8 V10 V10b V10b1 V10b2 V11 V12 V13 V14 V15 V15a V17 V20 V23 V24 V25 V26 V28 HV4 HV4c HV5 HV5a HV9c HV12 H1e H1e1 H1e1a H1e1a2 H1e1a7 H1e1c H1e2 H1e2a H1e2b H1e2d H1e4 H1e6 H1e7 H1e8 H1e8a H1h H1h1 H1h2 H1j H1j3 H1j5 H1j6 H1j7 H1j9 H1m H1n H1p H1q H1q1 H1q2 H1r H1s H1s1 H1t H1t1 H1t1a H1t2 H1u H1u1 H1v H1v1 H1v1a H1v1b H1w H1ae H1ae1 H1ae2 H1ae2a H1ae3 H1ae3a H1ag H1ag1 H1ag1b H1ah H1ai H1ai1 H1aj H1ak H1ak1 H1ak2 H1am H1am1 H1ap H1aq H1as2 H1at H1at1 H1at1a H1av H1ax H1ax1 H1ay H1az H1bd H1be H1bi H1bj H1bk H1bm H1bn H1bp H1bq H1br H1bu H1bv H1bw H1bx H1ca H1ci H2 H2a2 H2a2+(16235) H2a4 H2a5 H2a5a H2a5b H3b H3c H3e H3q H3q1 H3r H3r1 H3t H3u H3y H3aa H3ab H3ac H3ad [illegible]	1
467 >KU683342.1 Hc	73G 151T 152C 248d 263G 489C 750G 1438G 2706G 3106d 3107C 4715G 4769G 6752G 7028T 7196A 8584A 8701G 8860G 9090C 9540C 10398G 10400T 10873C 11719A 11923G 12705T 14766T 14783C 15043A 15301A 15326G 15475G 15487T 15784C 15940d 16185T 16223T 16260T 16298C 16302G 16362C	Z4a	73G 151T 152C 248d 263G 489C 16185T 16223T 16260T 16298C 16302G 16362C	1	73 151 152 249d 263 489 750 1438 2706 4715 4769 6752 7028 7196A 8584 8701 8860 9090 9540 10398 10400 10873 11719 11923 12705 14766 14783 15043 15301 15326 15475 15487T 15784 15944d 16185 16223 16260 16298 16302 16362	Z4a	73 151 152 249d 263 489 16185 16223 16260 16298 16302 16362	[0.022] Z4a (1)	[0.004] Z4 (2)	1
468 >KU683340.1 Hc	73G 195C 215G 263G 499A 750G 1438G 1811G 2706G 3106d 3107C 4646C 4769G 5999C 6047G 7028T 7705C 7859A 8860G 11332T 11467G 11719A 12308G 12372A 14620T 14766T 15217A 15326G 15693C 16356C 16519C	U4b3	73G 195C 215G 263G 499A 16356C 16519C	1	73 195 215 263 499 750 1438 1811 2706 4646 4769 5999 6047 7028 7705 7859 8860 11332 11467 11719 12308 12372 14620 14766 15217 15326 15693 16356	U4b3	73 195 215 263 499 16356	[0.000] U4b3 (1)	[0.000] U4 U4a U4b U4b1 U4b1a U4b1a1 U4b1a1a U4b1a2 U4b1a2a U4b1a2b U4b1a4 U4c U4c2 U4d U4d1 U4d1a U4d1a1 U4d1a1a U4d1b (20)	1

469	>KU683339.1 Ho	72C 263G 750G 1438G 2706G 3106d 3107C 4580A 4769G 7028T 8860G 15326G 15904T	V+@16298	72C 263G	1	72 263 750 1438 2706 4580 4769 7028 8860 15326 15904	V+@16298	72 263	[0.000] V+@16298 (1)	[0.005] V2 H2a2a (3) [0.003] H1as H13a2b2a (5) [0.002] H7 (6) [0.001] H H1 H2a H3 H3c1 H16 H23 H54 (14) [0.000] R0 HV HV0 HV0a V V1 V1a V1a1 V1a1b V2a V2a1 V2a1a V2b V2b1 V2b2 V2c V3 V3b V4 V8 V10 V10b V10b1 V10b2 V11 V12 V13 V14 V15 V15a V17 V20 V23 V24 V25 V26 V28 HV4 HV4c HV5 HV5a HV9c HV12 H1e H1e1 H1e1a H1e1a2 H1e1a7 H1e1c H1e2 H1e2a H1e2b H1e2d H1e4 H1e6 H1e7 H1e8 H1e8a H1h H1h1 H1h2 H1j H1j3 H1j5 H1j6 H1j7 H1j9 H1m H1n H1p H1q H1q1 H1q2 H1r H1s H1s1 H1t H1t1 H1t1a H1t2 H1u H1u1 H1v H1v1 H1v1a H1v1b H1w H1ae H1ae1 H1ae2 H1ae2a H1ae3 H1ae3a H1ag H1ag1 H1ag1b H1ah H1ai H1ai1 H1aj H1ak H1ak1 H1ak2 H1am H1am1 H1ap H1aq H1as2 H1at H1at1 H1at1a H1av H1ax H1ax1 H1ay H1az H1bd H1be H1bi H1bj H1bk H1bm H1bn H1bp H1bq H1br H1bu H1bv H1bw H1bx H1ca H1ci H2 H2a2 H2a2+(16235) H2a4 H2a5 H2a5a H2a5b H3b H3c H3e H3q H3q1 H3r H3r1 H3t H3u H3y H3aa H3ab H3ac H3ad [illegible]	1
470	>KU683331.1 Ho	73G 152C 263G 417A 489C 750G 1438G 2706G 3106d 3107C 3644C 4769G 5295T 5773A 6023A 6253C 6620C 7028T 8701G 8860G 9540C 10181T 10398G 10400T 10411G 10790C 10873C 11719A 12297C 12705T 13135A 13590A 14766T 14783C 15043A 15301A 15326G 15924G 16145A 16188T 16189C 16223T 16381C	M13a1	73G 152C 263G 417A 489C 16145A 16188T 16189C 16223T 16381C	1	73 152 263 417 489 750 1438 2706 3644 4769 5295 5773 6023 6253 6620 7028 8701 8860 9540 10181 10398 10400 10411 10790 10873 11719 12297 12705 13135 13590 14766 14783 15043 15301 15326 15924 16145 16188 16189 16223 16381	M13a1	73 152 263 417 489 16145 16188 16189 16223 16381	[0.023] M13a1 (1)	[0.000] M13a M13a1a M13a1b M13a1b1 (5)	1
471	>KU683326.1 Ho	73G 195C 215G 263G 499A 750G 1438G 1811G 2706G 3106d 3107C 4646C 4769G 5999C 6047G 7028T 7705C 7859A 8860G 11332T 11467G 11719A 12308G 12372A 14620T 14766T 15217A 15326G 15693C 16356C 16519C	U4b3	73G 195C 215G 263G 499A 16356C 16519C	1	73 195 215 263 499 750 1438 1811 2706 4646 4769 5999 6047 7028 7705 7859 8860 11332 11467 11719 12308 12372 14620 14766 15217 15326 15693 16356	U4b3	73 195 215 263 499 16356	[0.000] U4b3 (1)	[0.000] U4 U4a U4b U4b1 U4b1a U4b1a1 U4b1a1a U4b1a2 U4b1a2a U4b1a2b U4b1a4 U4c U4c2 U4d U4d1 U4d1a U4d1a1 U4d1a1a U4d1b (20)	1

472	>KU683325.1 Hc	64T 73G 235G 263G 514d 515d 663G 750G 1438G 1736G 2706G 3106d 3107C 4248C 4769G 4824G 5393C 7028T 7468T 8794T 8860G 9948A 10094T 10289G 11719A 12705T 14766T 15326G 16223T 16227C 16290T 16295T 16311C 16319A	A10	64T 73G 235G 263G 514d 515d 16223T 16227C 16290T 16295T 16311C 16319A	1	64 73 235 263 663 750 1438 1736 2706 4248 4769 4824 5393 7028 7468 8794 8860 9948 10094 10289 11719 12705 14766 15326 16223 16227C 16290 16295 16311 16319	A10	64 73 235 263 16223 16227C 16290 16295 16311 16319	[0.555] A10 (1)	[0.389] A (2) [0.000] A5 A5b (4)	1
473	>KU683324.1 Hc	72C 263G 310N 750G 1438G 2706G 3106d 3107C 4580A 4769G 7028T 8860G 15326G 15904T	V+@16298	72C 263G 310N	1	72 263 310N 750 1438 2706 4580 4769 7028 8860 15326 15904	V+@16298	72 263 310N	[0.000] V+@16298 (1)	[0.005] V2 H2a2a (3) [0.003] H1as H13a2b2a (5) [0.002] H7 (6) [0.001] H H1 H2a H3 H3c1 H16 H23 H54 (14) [0.000] R0 HV HV0 HV0a V V1 V1a V1a1 V1a1b V2a V2a1 V2a1a V2b V2b1 V2b2 V2c V3 V3b V4 V8 V10 V10b V10b1 V10b2 V11 V12 V13 V14 V15 V15a V17 V20 V23 V24 V25 V26 V28 HV4 HV4c HV5 HV5a HV9c HV12 H1e H1e1 H1e1a H1e1a2 H1e1a7 H1e1c H1e2 H1e2a H1e2b H1e2d H1e4 H1e6 H1e7 H1e8 H1e8a H1h H1h1 H1h2 H1j H1j3 H1j5 H1j6 H1j7 H1j9 H1m H1n H1p H1q H1q1 H1q2 H1r H1s H1s1 H1t H1t1 H1t1a H1t2 H1u H1u1 H1v H1v1 H1v1a H1v1b H1w H1ae H1ae1 H1ae2 H1ae2a H1ae3 H1ae3a H1ag H1ag1 H1ag1b H1ah H1ai H1ai1 H1aj H1ak H1ak1 H1ak2 H1am H1am1 H1ap H1aq H1as2 H1at H1at1 H1at1a H1av H1ax H1ax1 H1ay H1az H1bd H1be H1bi H1bj H1bk H1bm H1bn H1bp H1bq H1br H1bu H1bv H1bw H1bx H1ca H1ci H2 H2a2 H2a2+(16235) H2a4 H2a5 H2a5a H2a5b H3b H3c H3e H3q H3q1 H3r H3r1 H3t H3u H3y H3aa H3ab H3ac H3ad [illegible]	1
474	>KU683322.1 Hc	73G 151T 152C 200G 235G 263G 514d 515d 663G 735G 750G 1438G 1736G 2706G 3106d 3107C 4248C 4769G 4824G 7028T 8794T 8860G 11719A 12705T 14766T 15326G 16223T 16290T 16319A 16362C 16365T	A14	73G 151T 152C 200G 235G 263G 514d 515d 16223T 16290T 16319A 16362C 16365T	1	73 151 152 200 235 263 663 735 750 1438 1736 2706 4248 4769 4824 7028 8794 8860 11719 12705 14766 15326 16223 16290 16319 16362 16365	A14	73 151 152 200 235 263 16223 16290 16319 16362 16365	[0.006] A14 (1)	[0.009] A+152+16362+200 (2) [0.005] A13	1
475	>KU683314.1 Hc	64T 73G 146C 152C 199C 235G 263G 514d 515d 663G 750G 1438G 1736G 2706G 3106d 3107C 4248C 4769G 4824G 6962A 7028T 8794T 8860G 8865A 11719A 12705T 12777G 14766T 15301A 15326G 16066G 16223T 16242T 16290T 16319A 16519C	A8a1	64T 73G 146C 152C 199C 235G 263G 514d 515d 16066G 16223T 16242T 16290T 16319A 16519C	1	64 73 146 152 199 235 263 663 750 1438 1736 2706 4248 4769 4824 6962 7028 8794 8860 8865 11719 12705 12777 14766 15301 15326 16066 16223 16242 16290 16319	A8a1	64 73 146 152 199 235 263 16066 16223 16242 16290 16319	[0.452] A8a1 (1)	[0.000] A8a (2)	1

476	>KU683312.1 Hc	72C 263G 310N 750G 1438G 2706G 3106d 3107C 4580A 4769G 7028T 8860G 15326G 15904T	V+@16298	72C 263G 310N	1	72 263 310N 750 1438 2706 4580 4769 7028 8860 15326 15904	V+@16298	72 263 310N	[0.000] V+@16298 (1)	[0.005] V2 H2a2a (3) [0.003] H1as H13a2b2a (5) [0.002] H7 (6) [0.001] H H1 H2a H3 H3c1 H16 H23 H54 (14) [0.000] R0 HV HV0 HV0a V V1 V1a V1a1 V1a1b V2a V2a1 V2a1a V2b V2b1 V2b2 V2c V3 V3b V4 V8 V10 V10b V10b1 V10b2 V11 V12 V13 V14 V15 V15a V17 V20 V23 V24 V25 V26 V28 HV4 HV4c HV5 HV5a HV9c HV12 H1e H1e1 H1e1a H1e1a2 H1e1a7 H1e1c H1e2 H1e2a H1e2b H1e2d H1e4 H1e6 H1e7 H1e8 H1e8a H1h H1h1 H1h2 H1j H1j3 H1j5 H1j6 H1j7 H1j9 H1m H1n H1p H1q H1q1 H1q2 H1r H1s H1s1 H1t H1t1 H1t1a H1t2 H1u H1u1 H1v H1v1 H1v1a H1v1b H1w H1ae H1ae1 H1ae2 H1ae2a H1ae3 H1ae3a H1ag H1ag1 H1ag1b H1ah H1ai H1ai1 H1aj H1ak H1ak1 H1ak2 H1am H1am1 H1ap H1aq H1as2 H1at H1at1 H1at1a H1av H1ax H1ax1 H1ay H1az H1bd H1be H1bi H1bj H1bk H1bm H1bn H1bp H1bq H1br H1bu H1bv H1bw H1bx H1ca H1ci H2 H2a2 H2a2+(16235) H2a4 H2a5 H2a5a H2a5b H3b H3c H3e H3q H3q1 H3r H3r1 H3t H3u H3y H3aa H3ab H3ac H3ad [illegible]	1
477	>KU683302.1 Hc	263G 310N 489N 750G 1438G 3106d 3107C 4769G 8860G 11447A 15326G 16172C 16400T	H33b	263G 310N 489N 16172C 16400T	1	263 310N 489N 750 1438 4769 8860 11447 15326 16172 16400	H33b	263 310N 489N 16172 16400	[0.000] H33b (1)	[0.000] H66a (2)	1
478	>KU683298.1 Hc	73G 152C 204C 248d 263G 310N 514d 515d 750G 1438G 2706G 3106d 3107C 3970T 4732G 4769G 5147A 6392C 6962A 7028T 7966T 8772C 8860G 10310A 10609C 10976T 11710T 11719A 12406A 12633T 12882T 13928C 14476A 14766T 15326G 15924G 16172C 16183C 16189C 16232A 16249C 16304C 16311C	F1b1b	73G 152C 204C 248d 263G 310N 514d 515d 16172C 16183C 16189C 16232A 16249C 16304C 16311C	1	73 152 204 249d 263 310N 750 1438 2706 3970 4732 4769 5147 6392 6962 7028 7966 8772 8860 10310 10609 10976 11710 11719 12406 12633 12882 13928C 14476 14766 15326 15924 16172 16189 16232A 16249 16304 16311	F1b1b	73 152 204 249d 263 310N 16172 16189 16232A 16249 16304 16311	[0.000] F1b1b (1)	[0.048] F1b F1b1 (3)	1
479	>KU683296.1 Hc	73G 152C 263G 451.1T 750G 1438G 2248C 2706G 3106d 3107C 3311T 4615G 4769G 7028T 8843C 8860G 9554A 11167G 12681C 13269G 13708A 13768C 15326G 16217C 16519C	HV2	73G 152C 263G 451.1T 16217C 16519C	1	73 152 263 455.1T 750 1438 2248 2706 3311 4615 4769 7028 8843 8860 9554 11167 12681 13269 13708 13768 15326 16217	HV2	73 152 263 455.1T 16217	[0.213] HV2 (1)	[0.137] HV2a2 (2) [0.000] HV2a HV2a3 H32 R30a1 R30a1a (7)	1

480	>KU683295.1 Hc	73G 150T 159C 199C 263G 489C 750G 1438G 2706G 3106d 3107C 4048A 4071T 4164G 4769G 5351G 5460A 6455T 6680C 7028T 7684C 7853A 8701G 8860G 9494G 9540C 9824C 9959C 10159T 10398G 10400T 10873C 11719A 11944C 12405T 12705T 12811C 14766T 14783C 15043A 15301A 15326G 15479C 16092C 16129A 16192T 16223T 16297C	M7b1a1c	73G 150T 159C 199C 263G 489C 16092C 16129A 16192T 16223T 16297C	1	73 150 159 199 263 489 750 1438 2706 4048 4071 4164 4769 5351 5460 6455 6680 7028 7684 7853 8701 8860 9494 9540 9824 9959 10159 10398 10400 10873 11719 11944 12405 12705 12811 14766 14783 15043 15301 15326 15479 16092 16129 16192 16223 16297	M7b1a1c	73 150 159 199 263 489 16092 16129 16192 16223 16297	[0.000] M7b1a1c (1)	[0.000] M7b1a1+(16192) M7b1a1c1 M7b1a1d M7b1a1d1 M7b1a1e M7b1a1e1 M7b1a1f M7b1a1g M7b1a1h (10)	1
481	>KU683286.1 Hc	152C 239C 263G 750G 1438G 3106d 3107C 4769G 8860G 14040A 15326G 16186T 16300G 16325C 16362C 16482G	H6b2	152C 239C 263G 16186T 16300G 16325C 16362C 16482G	1	152 239 263 750 1438 4769 8860 14040 15326 16186 16300 16325 16362 16482	H6b2	152 239 263 16186 16300 16325 16362 16482	[0.380] H6b2 (1)	[0.380] H6b2 (2) [0.125] H6b1 (3) [0.033] H6b	1
482	>KU683284.1 Hc	73G 217C 263G 310N 340T 451.1T 508G 750G 1438G 1811G 2706G 3106d 3107C 3720G 4769G 5390G 5426C 6045T 6152C 7028T 7424G 8271d 8272d 8273d 8274d 8275d 8276d 8277d 8278d 8279d 8860G 10876G 11467G 11719A 12308G 12372A 13020C 13734C 14470C 14766T 15326G 15907G 16051G 16129C 16183C 16189.1C 16362C 16519C	U2e1h	73G 217C 263G 310N 340T 451.1T 508G 16051G 16129C 16183C 16189.1C 16362C 16519C	1	73 217 263 310N 340 455.1T 508 750 1438 1811 2706 3720 4769 5390 5426 6045 6152 7028 7424 8281-8289d 8860 10876 11467 11719 12308 12372 13020 13734 14470 14766 15326 15907 16051 16129C 16362	U2e1h	73 217 263 310N 340 455.1T 508 16051 16129C 16362	[0.028] U2e1h (1)	[0.009] U2e1a1 (2) [0.000] U2e1 U2e1a U2e1a1a U2e1a1c U2e1c U2e1c1 U2e1d U2e1g (10)	1
483	>KU683280.1 Hc	73G 195C 215G 263G 310N 499A 750G 1438G 1811G 2706G 3106d 3107C 4646C 4769G 5999C 6047G 7028T 7705C 7859A 8860G 11332T 11467G 11719A 12308G 12372A 14620T 14766T 15217A 15326G 15693C 16356C 16519C	U4b3	73G 195C 215G 263G 310N 499A 16356C 16519C	1	73 195 215 263 310N 499 750 1438 1811 2706 4646 4769 5999 6047 7028 7705 7859 8860 11332 11467 11719 12308 12372 14620 14766 15217 15326 15693 16356	U4b3	73 195 215 263 310N 499 16356	[0.000] U4b3 (1)	[0.000] U4 U4a U4a2 U4a2c U4a2e U4a2f U4a2g U4a2h U4b U4b1 U4b1a U4b1a1 U4b1a1a U4b1a2 U4b1a2a U4b1a2b U4b1a4 U4c U4c2 U4d U4d1 U4d1a U4d1a1 U4d1a1a U4d1b (26)	1
484	>KU683278.1 Hc	151T 152C 195C 263G 335G 478G 750G 1438G 3106d 3107C 4769G 8860G 15326G 16519C	H91	151T 152C 195C 263G 335G 478G 16519C	1	151 152 195 263 335 478 750 1438 4769 8860 15326	H91	151 152 195 263 335 478	[0.458] H91 (1)	[0.334] HV1b3 (2) [0.136] H3g1a (3) [0.090] H+195 (4) [0.075] H4a1a4a (5) [0.062] H1i1 (6) [0.021] H+152 (7) [0.019] H3k (8) [0.011] H3g1 (9) [0.010] H1+152 (10) [0.008] H3+152 (11) [0.007] H16c (12) [0.006] H1bb (13) [0.001] H14b4 (14) [0.000] HV1b3a H1i H1i2 H1i2a H1an H1an1 H1an1a H1an2 H1bc H2a2a2 H3g H3g1b H3g2 H3g3 H3g4 H3i H3i1 H3j H3k1 H3k1a H4a1a+195 H4a1a3 H4a1a3a H4a1a4 H4a1a4b H4a1a4b2 H12 H108 H9 H46 H46a H46b H52 H69 H13a1+152 H14b H14b2 H16+152 H16a H16a1 H16d H30b1 H44b H47a (58)	1

485 >KU683277.1 Hc	73G 146N 189G 199C 263G 489C 514d 515d 573.1N 709A 750G 1438G 2706G 3106d 3107C 3167.1C 4140T 4769G 7028T 7250G 8701G 8793C 8856A 8860G 9181G 9336G 9540C 10398G 10400T 10529G 10646A 10873C 11719A 12549T 12705T 13135A 13152G 14502C 14766T 14783C 15040T 15043A 15071C 15218G 15301A 15326G 16093C 16193T 16223T 16311C 16357C 16497G	M10a1a1b1	73G 146N 189G 199C 263G 489C 514d 515d 573.1N 16093C 16193T 16223T 16311C 16357C 16497G	1	73 146N 189 199 263 489 573.1N 709 750 1438 2706 3172.1C 4140 4769 7028 7250 8701 8793 8856 8860 9181 9336 9540 10398 10400 10529 10646 10873 11719 12549 12705 13135 13152 14502 14766 14783 15040 15043 15071 15218 15301 15326 16093 16193 16223 16311 16357 16497	M10a1a1b1	73 146N 189 199 263 489 573.1N 16093 16193 16223 16311 16357 16497	[0.308] M10a1a1b1 (1)	[0.000] M10a1a1b M10a1a1b2 (3)	1
486 >KU683265.1 Hc	73G 152N 263G 514d 515d 750G 980C 1438G 1811G 2706G 3106d 3107C 3741T 3834A 4769G 5360T 7028T 8137T 8684T 8860G 10142T 10237C 11467G 11719A 12308G 12372A 12618A 13500C 14569A 14766T 15326G 16207G 16309G 16318C 16519C	U7a3b	73G 152N 263G 514d 515d 16207G 16309G 16318C 16519C	1	73 152N 263 750 980 1438 1811 2706 3741 3834 4769 5360 7028 8137 8684 8860 10142 10237 11467 11719 12308 12372 12618 13500 14569 14766 15326 16207 16309 16318C	U7a3b	73 152N 263 16207 16309 16318C	[0.421] U7a3b (1) [0.184] U7b (2) [0.068] H32 (3) [0.032] R31 (4) [0.026] U7 (5) [0.020] P (6) [0.007] R (7) [0.000] HV+73 HV20 H1e2c H3+73 H3d H4a1a1a H4a1a1a1 H4a1a1a1a H4a1a1a1a1 H4a1a1a2 H4a1a1a4 R2'JT R30 R30a R30a1 R30a1a P2 P4 P9 P9a U U2'3'4'7'8'9 U8 U8b'c U8b (32)	[0.496] U7a (33) [0.421] U7a3b (34) [0.213] U7b1 (35) [0.184] U7b (36) [0.162] H7b6 (37) [0.155] H18b (38) [0.152] U7a3a (39) [0.132] H3g2 (40) [0.130] H1i1 (41) [0.105] R8a (42) [0.067] H2a2a (43) [0.062] R8a1a1 (44) [0.051] U5b2a1a2 (45) [0.044] S2 (46) [0.032] R31 (47) [0.027] HV2 (48) [0.026] U7 (49) [0.020] H1+152 (50) [0.017] U2+152 (51) [0.013] H+152 R2 (53) [0.010] H H1a5 (55) [0.008] H1a2 R9b1 R+16189 (58) [0.007] H1an1a (59) [0.006] H1e8a H1bb H2a H7b H16+152 (64) [0.005] H1a H13a1a1 R9b (67) [0.004] H1 H10c1 (69) [0.003] H61 (70) [0.002] H1az H1bd (72) [0.001] HV H1aj H1ax H1br H3ap H4a1 H4a1a2 H7 H26a H59 H60a H70 (84) [0.000] L3 L3b'f L3c'd L3h G1b1 N N1'5 N1 N9 N11 N14 S S+152 R0 HV4 HV4c HV5 HV5a HV9c HV12 H1a4 H1a7 H1a8 H1a8a H1e H1e1 H1e1a H1e1a2 H1e1a7 H1e1c H1e2 H1e2a H1e2b H1e2d H1e4 H1e6 H1e7 H1e8 H1h H1h1 H1h2 H1i H1i2 H1i2a H1an H1an1 H1j H1j3 H1j5 H1j6 H1j7 H1j9 H1m H1n H1p H1q H1q1 [illegible]	1
487 >KU683258.1 Hc	73G 152C 204C 263G 298d 373G 480C 750G 1438G 1598A 2706G 3106d 3107C 4062C 4225G 4769G 5773A 5836G 7028T 7082T 7268G 7406T 7490G 8584A 8805G 8860G 10631N 11009C 11719A 12406A 13236G 14194T 14305A 14766T 15289C 15307T 15326G 16183N 16189C 16298C 16299G	R30b1	73G 152C 204C 263G 298d 373G 480C 16183N 16189C 16298C 16299G	1	73 152 204 263 299d 373 480 750 1438 1598 2706 4062 4225 4769 5773 5836 7028 7082 7268G 7406 7490 8584 8805 8860 10631N 11009 11719 12406 13236 14194 14305 14766 15289 15307 15326 16183N 16189 16298 16299	R30b1	73 152 204 263 299d 373 480 16183N 16189 16298 16299	[0.095] R30b1 (1)	[0.065] R30a1 (2) [0.064] R30a1a (3) [0.061] R30b (4) [0.034] R+16189 (5) [0.017] H32 (6) [0.016] B4'5 (7) [0.008] R11'B6 (8) [0.000] H1ap1 H3av (10)	1

488 >KU683253.1 Hc	73G 263G 489C 709A 750G 1438G 2706G 3106d 3107C 4769G 4833G 4853A 5108C 5601T 6422T 7028T 8701G 8860G 8877C 9540C 10398G 10400T 10873C 11719A 12375C 12705T 13563G 14569A 14766T 14783C 15043A 15301A 15326G 16223T 16269G 16362C	G2b1b	73G 263G 489C 16223T 16269G 16362C	1	73 263 489 709 750 1438 2706 4769 4833 4853 5108 5601 6422 7028 8701 8860 8877 9540 10398 10400 10873 11719 12375 12705 13563 14569 14766 14783 15043 15301 15326 16223 16269 16362	G2b1b	73 263 489 16223 16269 16362	[0.000] G2b1b (1)	[0.002] G2b1 (2) [0.000] M9 G G1 G1c G1c2 G2 G2a'c G2b G2b1a G2b2 G2b2a M33+16362 D D4 D4b D4b2 D4b2a D4b2a2 D4b2a2b D4b2b D4b2b1 D4b2b1a D4b2b1b D4b2b1c D4b2b2 D4b2b3 D4b2b6 D4e D4e1'3 D4e1 D4e1c D4e3 D4e2 D4e2b D4e2c D4e2d D4e4 D4e4a D4e4b D4f D4f1 D4g D4h D4h4 D4j D4j1 D4j1b D4j4 D4j5 D4j5a D4j+(16286) D4j9 D4j10 D4j12 D4j15 D4j16 D4m (59)	1
489 >KU683252.1 Hc	73G 263G 489C 709A 750G 1438G 2706G 3106d 3107C 4769G 4833G 4853A 5108C 5601T 6422T 7028T 8701G 8860G 8877C 9540C 10398G 10400T 10873C 11719A 12375C 12705T 13563G 14569A 14766T 14783C 15043A 15301A 15326G 16223T 16269G 16362C	G2b1b	73G 263G 489C 16223T 16269G 16362C	1	73 263 489 709 750 1438 2706 4769 4833 4853 5108 5601 6422 7028 8701 8860 8877 9540 10398 10400 10873 11719 12375 12705 13563 14569 14766 14783 15043 15301 15326 16223 16269 16362	G2b1b	73 263 489 16223 16269 16362	[0.000] G2b1b (1)	[0.002] G2b1 (2) [0.000] M9 G G1 G1c G1c2 G2 G2a'c G2b G2b1a G2b2 G2b2a M33+16362 D D4 D4b D4b2 D4b2a D4b2a2 D4b2a2b D4b2b D4b2b1 D4b2b1a D4b2b1b D4b2b1c D4b2b2 D4b2b3 D4b2b6 D4e D4e1'3 D4e1 D4e1c D4e3 D4e2 D4e2b D4e2c D4e2d D4e4 D4e4a D4e4b D4f D4f1 D4g D4h D4h4 D4j D4j1 D4j1b D4j4 D4j5 D4j5a D4j+(16286) D4j9 D4j10 D4j12 D4j15 D4j16 D4m (59)	1
490 >KU683250.1 Hc	73G 152C 204C 263G 298d 373G 480C 750G 1438G 1598A 2706G 3106d 3107C 4062C 4225G 4769G 5773A 5836G 7028T 7082T 7268G 7406T 7490G 8584A 8805G 8860G 10631N 11009C 11719A 12406A 13236G 14194T 14305A 14766T 15289C 15307T 15326G 16183N 16189C 16298C 16299G	R30b1	73G 152C 204C 263G 298d 373G 480C 16183N 16189C 16298C 16299G	1	73 152 204 263 299d 373 480 750 1438 1598 2706 4062 4225 4769 5773 5836 7028 7082 7268G 7406 7490 8584 8805 8860 10631N 11009 11719 12406 13236 14194 14305 14766 15289 15307 15326 16183N 16189 16298 16299	R30b1	73 152 204 263 299d 373 480 16183N 16189 16298 16299	[0.095] R30b1 (1)	[0.065] R30a1 (2) [0.064] R30a1a (3) [0.061] R30b (4) [0.034] R+16189 (5) [0.017] H32 (6) [0.016] B4'5 (7) [0.008] R11'B6 (8) [0.000] H1ap1 H3av (10)	1
491 >KU683246.1 Hc	73G 263G 489C 709A 750G 1438G 2706G 3106d 3107C 4769G 4833G 4853A 5108C 5601T 6422T 7028T 8701G 8860G 8877C 9540C 10398G 10400T 10873C 11719A 12375C 12705T 13563G 14569A 14766T 14783C 15043A 15301A 15326G 16223T 16269G 16362C	G2b1b	73G 263G 489C 16223T 16269G 16362C	1	73 263 489 709 750 1438 2706 4769 4833 4853 5108 5601 6422 7028 8701 8860 8877 9540 10398 10400 10873 11719 12375 12705 13563 14569 14766 14783 15043 15301 15326 16223 16269 16362	G2b1b	73 263 489 16223 16269 16362	[0.000] G2b1b (1)	[0.002] G2b1 (2) [0.000] M9 G G1 G1c G1c2 G2 G2a'c G2b G2b1a G2b2 G2b2a M33+16362 D D4 D4b D4b2 D4b2a D4b2a2 D4b2a2b D4b2b D4b2b1 D4b2b1a D4b2b1b D4b2b1c D4b2b2 D4b2b3 D4b2b6 D4e D4e1'3 D4e1 D4e1c D4e3 D4e2 D4e2b D4e2c D4e2d D4e4 D4e4a D4e4b D4f D4f1 D4g D4h D4h4 D4j D4j1 D4j1b D4j4 D4j5 D4j5a D4j+(16286) D4j9 D4j10 D4j12 D4j15 D4j16 D4m (59)	1

492 >KU683099.1 Hc	73G 152C 263G 489C 573.1N 750G 1438G 2706G 3010A 3106d 3107C 4769G 4883T 5021C 5178A 7028T 8414T 8701G 8860G 9540C 10398G 10400T 10873C 11215T 11719A 12705T 14668T 14755G 14766T 14783C 15043A 15106A 15184C 15301A 15326G 16173T 16223T 16274A 16291T 16362C	D4e5a	73G 152C 263G 489C 573.1N 16173T 16223T 16274A 16291T 16362C	1	73 152 263 489 573.1N 750 1438 2706 3010 4769 4883 5021 5178A 7028 8414 8701 8860 9540 10398 10400 10873 11215 11719 12705 14668 14755 14766 14783 15043 15106 15184 15301 15326 16173 16223 16274 16291 16362	D4e5a	73 152 263 489 573.1N 16173 16223 16274 16291 16362	[0.167] D4e5a (1)	[0.072] D4e5b (2) [0.013] D4j2 (3) [0.000] D4j2a	1
493 >KU683097.1 Hc	151N 152N 195C 263G 335G 478G 750G 1438G 3106d 3107C 4769G 8860G 15326G 16519C	H91	151N 152N 195C 263G 335G 478G 16519C	1	151N 152N 195 263 335 478 750 1438 4769 8860 15326	H91	151N 152N 195 263 335 478	[0.208] H91 (1)	[0.000] H4a1a+195 H4a1a3 H4a1a3a H4a1a4 H4a1a4a H4a1a4b H4a1a4b2 H+195 H12 H108 H44b (12)	1
494 >KU683093.1 Hc	64T 73G 146C 152C 199C 235G 263G 514d 515d 663G 750G 1438G 1736G 2706G 3106d 3107C 4248C 4769G 4824G 6962A 7028T 8794T 8860G 8865A 11719A 12705T 12777G 14766T 15301A 15326G 16066G 16223T 16242T 16290T 16319A 16519C	A8a1	64T 73G 146C 152C 199C 235G 263G 514d 515d 16066G 16223T 16242T 16290T 16319A 16519C	1	64 73 146 152 199 235 263 663 750 1438 1736 2706 4248 4769 4824 6962 7028 8794 8860 8865 11719 12705 12777 14766 15301 15326 16066 16223 16242 16290 16319	A8a1	64 73 146 152 199 235 263 16066 16223 16242 16290 16319	[0.452] A8a1 (1)	[0.000] A8a (2)	1
495 >KU683082.1 Hc	73G 235G 263G 310N 514d 515d 663G 750G 961C 1438G 1709A 1736G 2706G 3106d 3107C 4248C 4769G 4824G 7028T 8563G 8794T 8860G 9797C 11536T 11719A 12705T 14207C 14766T 15326G 16126C 16223T 16234T 16248T 16290T 16311C 16319A 16519C	A5b1b	73G 235G 263G 310N 514d 515d 16126C 16223T 16234T 16248T 16290T 16311C 16319A 16519C	1	73 235 263 310N 663 750 961 1438 1709 1736 2706 4248 4769 4824 7028 8563 8794 8860 9797 11536 11719 12705 14207C 14766 15326 16126 16223 16234 16248 16290 16311 16319	A5b1b	73 235 263 310N 16126 16223 16234 16248 16290 16311 16319	[0.702] A5b1b (1)	[0.417] A (2) [0.214] A5b1a (3) [0.038] A5b1c (4) [0.036] A5 (5) [0.034] A5b1 (6) [0.000] A5b A10 (8)	1
496 >KU683080.1 Hc	73G 263G 408A 499A 750G 827G 1438G 2706G 3106d 3107C 4769G 4820A 5843G 6023A 6413C 7028T 8271d 8272d 8273d 8274d 8275d 8276d 8277d 8278d 8279d 8860G 9055A 9338T 9615C 11719A 12945C 13590A 14766T 15326G 15535T 16136C 16183N 16189C 16217C 16360T 16519C	B4b1a3	73G 263G 408A 499A 16136C 16183N 16189C 16217C 16360T 16519C	1	73 263 408A 499 750 827 1438 2706 4769 4820 5843 6023 6413 7028 8281-8289d 8860 9055 9338T 9615 11719 12945 13590 14766 15326 15535 16136 16183N 16189 16217 16360	B4b1a3	73 263 408A 499 16136 16183N 16189 16217 16360	[0.091] B4b1a3 (1) [0.012] B4b1 (2) [0.006] B4b1a (3)	[0.160] B2q (4) [0.042] B4b1c1 (5) [0.027] B2 (6) [0.011] B2p (7) [0.008] B4b1a2a (8) [0.000] B4b B2b B2b3 B2c B2c1 B2c1a B2c1b B2c1c B2e B2f B2i B2n B2r B4b1a+207 B4b1a2 B4b1a2e B4b1a2f B4b1a2h B4b1b'c B4b1c B4b1c2 (29)	1
497 >KU683071.1 Hc	64T 73G 146C 152C 235G 263G 514d 515d 663G 750G 1438G 1736G 2706G 3106d 3107C 4248C 4769G 4824G 6962A 7028T 8794T 8860G 8865A 11719A 12705T 12777G 13708A 14766T 15326G 16223T 16242T 16290T 16319A	A8a1	64T 73G 146C 152C 235G 263G 514d 515d 16223T 16242T 16290T 16319A	1	64 73 146 152 235 263 663 750 1438 1736 2706 4248 4769 4824 6962 7028 8794 8860 8865 11719 12705 12777 13708 14766 15326 16223 16242 16290 16319	A8a1	64 73 146 152 235 263 16223 16242 16290 16319	[0.000] A8a1 (1)	[0.000] A8a (2)	1

498 >KU683059.1 Hc	73G 152C 263G 489C 573.1N 750G 1438G 2706G 3010A 3106d 3107C 4769G 4883T 5021C 5178A 7028T 8414T 8701G 8860G 9540C 10398G 10400T 10873C 11215T 11719A 12705T 14668T 14755G 14766T 14783C 15043A 15106A 15184C 15301A 15326G 16173T 16223T 16274A 16291T 16362C	D4e5a	73G 152C 263G 489C 573.1N 16173T 16223T 16274A 16291T 16362C	1	73 152 263 489 573.1N 750 1438 2706 3010 4769 4883 5021 5178A 7028 8414 8701 8860 9540 10398 10400 10873 11215 11719 12705 14668 14755 14766 14783 15043 15106 15184 15301 15326 16173 16223 16274 16291 16362	D4e5a	73 152 263 489 573.1N 16173 16223 16274 16291 16362	[0.167] D4e5a (1)	[0.072] D4e5b (2) [0.013] D4j2 (3) [0.000] D4j2a	1
499 >KU683055.1 Hc	151T 152C 195C 263G 335G 478G 750G 1438G 3106d 3107C 4769G 8860G 15326G 16519C	H91	151T 152C 195C 263G 335G 478G 16519C	1	151 152 195 263 335 478 750 1438 4769 8860 15326	H91	151 152 195 263 335 478	[0.458] H91 (1)	[0.334] HV1b3 (2) [0.136] H3g1a (3) [0.090] H+195 (4) [0.075] H4a1a4a (5) [0.062] H1i1 (6) [0.021] H+152 (7) [0.019] H3k (8) [0.011] H3g1 (9) [0.010] H1+152 (10) [0.008] H3+152 (11) [0.007] H16c (12) [0.006] H1bb (13) [0.001] H14b4 (14) [0.000] HV1b3a H1i H1i2 H1i2a H1an H1an1 H1an1a H1an2 H1bc H2a2a2 H3g H3g1b H3g2 H3g3 H3g4 H3i H3i1 H3j H3k1 H3k1a H4a1a+195 H4a1a3 H4a1a3a H4a1a4 H4a1a4b H4a1a4b2 H12 H108 H9 H46 H46a H46b H52 H69 H13a1+152 H14b H14b2 H16+152 H16a H16a1 H16d H30b1 H44b H47a (58)	1
500 >KU683051.1 Hc	263G 750G 1438G 3106d 3107C 4769G 8860G 11447A 15326G 16172C 16400T	H33b	263G 16172C 16400T	1	263 750 1438 4769 8860 11447 15326 16172 16400	H33b	263 16172 16400	[0.000] H33b (1)	[0.000] H66a (2)	1
501 >KU683046.1 Hc	73G 152C 248d 263G 489C 750G 1438G 2706G 3106d 3107C 3552A 4715G 4742C 4769G 6026A 7028T 7196A 8152A 8584A 8701G 8860G 9540C 9545G 10398G 10400T 10873C 11719A 11914A 11969A 12705T 13263G 14318C 14766T 14783C 15043A 15204C 15301A 15326G 15487T 16223T 16297C 16298C 16327T 16519C	C4+152	73G 152C 248d 263G 489C 16223T 16297C 16298C 16327T 16519C	1	73 152 249d 263 489 750 1438 2706 3552A 4715 4742 4769 6026 7028 7196A 8152 8584 8701 8860 9540 9545 10398 10400 10873 11719 11914 11969 12705 13263 14318 14766 14783 15043 15204 15301 15326 15487T 16223 16297 16298 16327	C4+152	73 152 249d 263 489 16223 16297 16298 16327	[0.052] C4+152 (1)	[0.102] C4+152+16093 (2) [0.020] C4a1a1 (3) [0.019] C7 (4) [0.014] C4a1a C4a1a5 C4a1a6 (7) [0.009] C7a (8) [0.004] C4b (9) [0.003] C4a1 (10) [0.000] C C4 C4a'b'c C4a C4a1a1a C4b5 C4b6 C4b7 C4b8 C4c C4c1 C4d C4e (23)	1

502	>KU683042.1 Hc	151T 152C 195C 263G 335G 478G 750G 1438G 3106d 3107C 4769G 8860G 15326G 16519C	H91	151T 152C 195C 263G 335G 478G 16519C	1	151 152 195 263 335 478 750 1438 4769 8860 15326	H91	151 152 195 263 335 478	[0.458] H91 (1)	[0.334] HV1b3 (2) [0.136] H3g1a (3) [0.090] H+195 (4) [0.075] H4a1a4a (5) [0.062] H1i1 (6) [0.021] H+152 (7) [0.019] H3k (8) [0.011] H3g1 (9) [0.010] H1+152 (10) [0.008] H3+152 (11) [0.007] H16c (12) [0.006] H1bb (13) [0.001] H14b4 (14) [0.000] HV1b3a H1i H1i2 H1i2a H1an H1an1 H1an1a H1an2 H1bc H2a2a2 H3g H3g1b H3g2 H3g3 H3g4 H3i H3i1 H3j H3k1 H3k1a H4a1a+195 H4a1a3 H4a1a3a H4a1a4 H4a1a4b H4a1a4b2 H12 H108 H9 H46 H46a H46b H52 H69 H13a1+152 H14b H14b2 H16+152 H16a H16a1 H16d H30b1 H44b H47a (58)	1
503	>KU683039.1 Hc	73G 150T 152C 263G 456T 489C 681C 750G 1048T 1107C 1438G 2706G 3106d 3107C 3759G 4769G 4883T 5147A 5153G 5178A 5301G 6253C 7028T 7403G 8701G 8860G 9180G 9540C 10397G 10398G 10400T 10873C 11719A 12705T 14766T 14783C 15043A 15301A 15326G 15724G 16183N 16189C 16223T 16357C 16362C 16519C	D5b1b2	73G 150T 152C 263G 456T 489C 16183N 16189C 16223T 16357C 16362C 16519C	1	73 150 152 263 456 489 681 750 1048 1107 1438 2706 3759 4769 4883 5147 5153 5178A 5301 6253 7028 7403 8701 8860 9180 9540 10397 10398 10400 10873 11719 12705 14766 14783 15043 15301 15326 15724 16183N 16189 16223 16357 16362	D5b1b2	73 150 152 263 456 489 16183N 16189 16223 16357 16362	[0.071] D5b1b2 (1)	[0.151] D5b1 (2) [0.007] D5b3 (3) [0.000] D5b D5b1a D5b1b D5b1c D5b3a (8)	1
504	>KU683038.1 Hc	263G 750G 1438G 3106d 3107C 4769G 8860G 11447A 15326G 16172C 16400T	H33b	263G 16172C 16400T	1	263 750 1438 4769 8860 11447 15326 16172 16400	H33b	263 16172 16400	[0.000] H33b (1)	[0.000] H66a (2)	1
505	>KU683035.1 Hc	152C 239C 263G 310N 750G 1438G 3106d 3107C 4769G 8860G 14040A 15326G 16186T 16300G 16325C 16362C 16482G	H6b2	152C 239C 263G 310N 16186T 16300G 16325C 16362C 16482G	1	152 239 263 310N 750 1438 4769 8860 14040 15326 16186 16300 16325 16362 16482	H6b2	152 239 263 310N 16186 16300 16325 16362 16482	[0.380] H6b2 (1)	[0.380] H6b2 (2) [0.125] H6b1 (3) [0.033] H6b	1
506	>KU683030.1 Hc	263G 750G 1438G 2706G 3106d 3107C 4769G 6755A 7028T 8772C 8860G 15326G 16172C 16189C 16311C	HV6	263G 16172C 16189C 16311C	1	263 750 1438 2706 4769 6755 7028 8772 8860 15326 16172 16189 16311	HV6	263 16172 16189 16311	[0.044] HV6 (1)	[0.020] H3h2 (2) [0.013] HV+16311 (3) [0.009] H3+16189 (4) [0.007] H1g H1aa (6) [0.003] H1+16189 H3h3b (8) [0.002] H1+16311 (9) [0.000] HV6a HV9 HV10 HV11 HV11a HV16 HV23 HV24 HV18 H1f H1g1 H1g2 H1y H1z1 H1aa1 H1ab H1ac H1ad H1e1a4 H1j9 H3+16311 H3h H3h1 H3h3 H3h3a H3h5 H3h6 H3m H13a1a2+16311 H13a2b1 H66a H76 H80 U6b1b (43)	1

507 >KU683020.1 Hc	73G 152C 235G 263G 298A 514d 515d 663G 750G 1438G 1736G 3106d 3107C 4248C 4769G 4824G 6023A 7028T 8794T 8860G 11557G 11719A 12705T 14766T 14929T 15326G 16223T 16290T 16292A 16319A 16362C	A16	73G 152C 235G 263G 298A 514d 515d 16223T 16290T 16292A 16319A 16362C	1	73 152 235 263 298A 663 750 1438 1736 4248 4769 4824 6023 7028 8794 8860 11557 11719 12705 14766 14929 15326 16223 16290 16292A 16319 16362	A16	73 152 235 263 298A 16223 16290 16292A 16319 16362	[0.000] A16 (1)	[0.000] A+152+16362 A1 A6 A6a A17 A18 A20 A21 A22 (10)	1
508 >KU683015.1 Hc	73G 152C 195C 263G 489C 709A 750G 1438G 2706G 3106d 3107C 4769G 4833G 5108C 5601T 7028T 7502d 8626C 8701G 8860G 9540C 9575A 10398G 10400T 10873C 11719A 12441C 12705T 13145A 13563G 14569A 14766T 14783C 14839G 15043A 15301A 15326G 16223T 16362C	G2c	73G 152C 195C 263G 489C 16223T 16362C	1	73 152 195 263 489 709 750 1438 2706 4769 4833 5108 5601 7028 7503d 8626 8701 8860 9540 9575 10398 10400 10873 11719 12441 12705 13145 13563 14569 14766 14783 14839 15043 15301 15326 16223 16362	G2c	73 152 195 263 489 16223 16362	[0.000] G2c (1)	[0.021] D4+195 (2) [0.000] M13'46'61+16362	1
509 >KU683013.1 Hc	263G 750G 1438G 3106d 3107C 4769G 8860G 11447A 15326G 16172C 16400T	H33b	263G 16172C 16400T	1	263 750 1438 4769 8860 11447 15326 16172 16400	H33b	263 16172 16400	[0.000] H33b (1)	[0.000] H66a (2)	1
510 >KU683012.1 Hc	73G 152C 204C 248d 263G 514d 515d 516d 517d 750G 1438G 2706G 3106d 3107C 3970T 4732G 4769G 5147A 6392C 6962A 7028T 8772C 8860G 9180G 10310A 10609C 10976T 11719A 12406A 12633T 12882T 13928C 14476A 14766T 15326G 16172C 16183C 16189C 16232A 16249C 16304C 16311C	F1b1b	73G 152C 204C 248d 263G 514d 515d 516d 517d 16172C 16183C 16189C 16232A 16249C 16304C 16311C	1	73 152 204 249d 263 750 1438 2706 3970 4732 4769 5147 6392 6962 7028 8772 8860 9180 10310 10609 10976 11719 12406 12633 12882 13928C 14476 14766 15326 16172 16189 16232A 16249 16304 16311	F1b1b	73 152 204 249d 263 16172 16189 16232A 16249 16304 16311	[0.000] F1b1b (1)	[0.048] F1b F1b1 (3)	1
511 >KU683009.1 Hc	73G 152N 204C 248d 263G 514d 515d 516d 517d 750G 1438G 2706G 3106d 3107C 3970T 4732G 4769G 5147A 6392C 6962A 7028T 8772C 8860G 9180G 10310A 10609C 10976T 11719A 12406A 12633T 12882T 13590A 13928C 14476A 14766T 15326G 16172C 16183C 16189C 16232A 16249C 16304C 16311C	F1b1b	73G 152N 204C 248d 263G 514d 515d 516d 517d 16172C 16183C 16189C 16232A 16249C 16304C 16311C	1	73 152N 204 249d 263 750 1438 2706 3970 4732 4769 5147 6392 6962 7028 8772 8860 9180 10310 10609 10976 11719 12406 12633 12882 13590 13928C 14476 14766 15326 16172 16189 16232A 16249 16304 16311	F1b1b	73 152N 204 249d 263 16172 16189 16232A 16249 16304 16311	[0.000] F1b1b (1)	[0.251] F1b1+@152 (2) [0.048] F1b F1b1 (4) [0.000] F1b1e F1b1e1 F1b1f (7)	1
512 >KU683005.1 Hc	73G 152N 185A 189G 195C 204C 263G 310.1C 709A 750G 1243C 1406N 1438G 2706G 3106d 3107C 3505G 4769G 5046A 5291C 5460A 7028T 8251A 8784G 8860G 8994A 11674T 11719A 11947G 12414C 12705T 14766T 15326G 15784C 15884C 16209C 16223T 16255A 16292T 16381G 16519C	W3a2	73G 152N 185A 189G 195C 204C 263G 310.1C 16209C 16223T 16255A 16292T 16381G 16519C	1	73 152N 185 189 195 204 263 709 750 1243 1406N 1438 2706 3505 4769 5046 5291 5460 7028 8251 8784 8860 8994 11674 11719 11947 12414 12705 14766 15326 15784 15884C 16209 16223 16255 16292 16381G	W3a2	73 152N 185 189 195 204 263 16209 16223 16255 16292 16381G	[1.333] W3a2 (1)	[0.135] W1f (2) [0.068] W (3) [0.014] W1a (4) [0.012] W1 (5) [0.000] W1e W7 (7)	1

513 >KU682998.1 Hc	73G 214G 263G 390G 489C 709A 750G 1438G 2263A 2706G 3106d 3107C 4769G 4833G 5108C 7028T 8701G 8860G 9540C 10398G 10400T 10873C 11050C 11719A 12408C 12705T 14218C 14569A 14766T 14783C 15043A 15301A 15326G 15746G 16156A 16223T 16274A 16362C 16390A	G3a3	73G 214G 263G 390G 489C 16156A 16223T 16274A 16362C 16390A	1	73 214 263 390 489 709 750 1438 2263A 2706 4769 4833 5108 7028 8701 8860 9540 10398 10400 10873 11050 11719 12408 12705 14218 14569 14766 14783 15043 15301 15326 15746 16156 16223 16274 16362 16390	G3a3	73 214 263 390 489 16156 16223 16274 16362 16390	[0.133] G3a3 (1)	[0.000] E E1 E1a E1a1 E1a1a E1a1c G3 G3a G3b G3b2 D4e5 (12)	1
514 >KU682993.1 Hc	263G 711C 750G 1438G 2706G 3106d 3107C 3360G 4769G 6755A 7028T 8860G 11152C 15326G 16172C 16311C	HV6	263G 16172C 16311C	1	263 711 750 1438 2706 3360 4769 6755 7028 8860 11152 15326 16172 16311	HV6	263 16172 16311	[0.000] HV6 (1)	[0.010] HV+16311 (2) [0.003] H3h3b (3) [0.002] H1+16311 (4) [0.000] HV6a HV9 HV10 HV11 HV11a HV16 HV23 HV24 H1e1a4 H3+16311 H3h H3h1 H3h2 H3h3 H3h3a H3h5 H3h6 H3m H13a1a2+16311 H13a2b1 H66a H76 H80 (27)	1
515 >KU682985.1 Hc	73G 152N 207A 263G 310.1C 489C 514d 515d 709A 711C 750G 1438G 2706G 3106d 3107C 4769G 4833G 5108C 5601T 7028T 7600A 8701G 8860G 8943T 9377G 9540C 9575A 10398G 10400T 10873C 11719A 12705T 13395G 13563G 14569A 14766T 14783C 15043A 15110A 15301A 15326G 16051G 16093C 16223T 16227G 16278T 16362C	G2a2a	73G 152N 207A 263G 310.1C 489C 514d 515d 16051G 16093C 16223T 16227G 16278T 16362C	1	73 152N 207 263 489 709 711 750 1438 2706 4769 4833 5108 5601 7028 7600 8701 8860 8943 9377 9540 9575 10398 10400 10873 11719 12705 13395 13563 14569 14766 14783 15043 15110 15301 15326 16051 16093 16223 16227 16278 16362	G2a2a	73 152N 207 263 489 16051 16093 16223 16227 16278 16362	[0.022] G2a2a (1)	[0.108] G2a (2) [0.040] G2a+152 (3) [0.022] G2a2a (4) [0.015] G2a1 (5) [0.011] G2a1b (6) [0.000] G2a1e G2a2 (8)	1
516 >KU682977.1 Hc	73G 146N 207A 263G 408A 499A 750G 827G 1438G 2706G 3106d 3107C 4769G 4820A 5892N 6023A 6413C 7028T 8271d 8272d 8273d 8274d 8275d 8276d 8277d 8278d 8279d 8860G 9055A 9338T 9615C 11084G 11719A 13590A 14133G 14766T 15326G 15535T 15813C 16086C 16136C 16183N 16189C 16217C 16519C	B4b1a3a	73G 146N 207A 263G 408A 499A 16086C 16136C 16183N 16189C 16217C 16519C	1	73 146N 207 263 408A 499 750 827 1438 2706 4769 4820 5892N 6023 6413 7028 8281-8289d 8860 9055 9338T 9615 11084 11719 13590 14133 14766 15326 15535 15813 16086 16136 16183N 16189 16217	B4b1a3a	73 146N 207 263 408A 499 16086 16136 16183N 16189 16217	[0.000] B4b1a3a (1)	[0.000] B4b1a3 (2)	1
517 >KU682971.1 Hc	73G 248d 263G 514d 515d 750G 1438G 1896C 2706G 3106d 3107C 3970T 4086T 4769G 6392C 6962A 7028T 8860G 9053A 9548A 10310A 10609C 11719A 12406A 12882T 13759A 13928C 14766T 15326G 16129A 16162G 16172C 16303A 16304C 16497G 16519C	F1a1	73G 248d 263G 514d 515d 16129A 16162G 16172C 16303A 16304C 16497G 16519C	1	73 249d 263 750 1438 1896 2706 3970 4086 4769 6392 6962 7028 8860 9053 9548 10310 10609 11719 12406 12882 13759 13928C 14766 15326 16129 16162 16172 16303 16304 16497	F1a1	73 249d 263 16129 16162 16172 16303 16304 16497	[0.103] F1a1 (1)	[0.017] F1a1c3 (2) [0.005] F1a1'4 F1a1d1 (4) [0.003] F1a3 (5) [0.002] F1a1a (6) [0.000] F1a F1a1a1 F1a1b F1a1c F1a1c2 F1a1d F1a4 (13)	1

518	>KU682970.1 H[illegible]	73G 248d 263G 514d 515d 750G 1438G 1896C 2706G 3106d 3107C 3970T 4086T 4769G 6392C 6962A 7028T 8860G 9053A 9548A 10310A 10609C 11719A 12406A 12882T 13759A 13928C 14766T 15326G 16129A 16162G 16172C 16303A 16304C 16497G 16519C	F1a1	73G 248d 263G 514d 515d 16129A 16162G 16172C 16303A 16304C 16497G 16519C	1	73 249d 263 750 1438 1896 2706 3970 4086 4769 6392 6962 7028 8860 9053 9548 10310 10609 11719 12406 12882 13759 13928C 14766 15326 16129 16162 16172 16303 16304 16497	F1a1	73 249d 263 16129 16162 16172 16303 16304 16497	[0.103] F1a1 (1)	[0.017] F1a1c3 (2) [0.005] F1a1'4 F1a1d1 (4) [0.003] F1a3 (5) [0.002] F1a1a (6) [0.000] F1a F1a1a1 F1a1b F1a1c F1a1c2 F1a1d F1a4 (13)	1
519	>KU682967.1 H[illegible]	73G 263G 310N 489C 514d 515d 750G 1438G 2706G 3010A 3106d 3107C 4769G 4883T 5178A 6881G 7028T 8020A 8414T 8701G 8860G 9540C 10181T 10398G 10400T 10873C 11719A 12705T 13720T 14668T 14766T 14783C 14815T 14927G 15043A 15301A 15326G 15440C 15951G 16093C 16129A 16173T 16223T 16319A 16362C	D4b1a2a1	73G 263G 310N 489C 514d 515d 16093C 16129A 16173T 16223T 16319A 16362C	1	73 263 310N 489 750 1438 2706 3010 4769 4883 5178A 6881 7028 8020 8414 8701 8860 9540 10181 10398 10400 10873 11719 12705 13720 14668 14766 14783 14815 14927 15043 15301 15326 15440 15951 16093 16129 16173 16223 16319 16362	D4b1a2a1	73 263 310N 489 16093 16129 16173 16223 16319 16362	[0.308] D4b1a2a1 (1)	[0.203] D4d (2) [0.021] D2 (3) [0.015] D4b1a D4b1a2 D4b1a2a (6) [0.005] D4b1 (7) [0.000] G1b+16129 G1b2 G1b4 D4b1a2a2 D2c (12)	1
520	>KU682963.1 H[illegible]	73G 151T 152C 248d 263G 489C 750G 1438G 2706G 3106d 3107C 4715G 4769G 6752G 7028T 7196A 8584A 8701G 8860G 9090C 9540C 10398G 10400T 10873C 11719A 11923G 12705T 14766T 14783C 15043A 15301A 15326G 15475G 15487T 15784C 15940d 16185T 16223T 16260T 16298C 16302G 16362C	Z4a	73G 151T 152C 248d 263G 489C 16185T 16223T 16260T 16298C 16302G 16362C	1	73 151 152 249d 263 489 750 1438 2706 4715 4769 6752 7028 7196A 8584 8701 8860 9090 9540 10398 10400 10873 11719 11923 12705 14766 14783 15043 15301 15326 15475 15487T 15784 15944d 16185 16223 16260 16298 16302 16362	Z4a	73 151 152 249d 263 489 16185 16223 16260 16298 16302 16362	[0.022] Z4a (1)	[0.004] Z4 (2)	1
521	>KU682946.1 H[illegible]	73G 152N 263G 310.1C 417A 489C 750G 1438G 2706G 3106d 3107C 3644C 4769G 5295T 5773A 6023A 6253C 6620C 7028T 8701G 8860G 9540C 10181T 10398G 10400T 10411G 10790C 10873C 11719A 12297C 12705T 13135A 13590A 14766T 14783C 15043A 15301A 15326G 15924G 16145A 16188T 16189C 16223T 16381C	M13a1	73G 152N 263G 310.1C 417A 489C 16145A 16188T 16189C 16223T 16381C	1	73 152N 263 417 489 750 1438 2706 3644 4769 5295 5773 6023 6253 6620 7028 8701 8860 9540 10181 10398 10400 10411 10790 10873 11719 12297 12705 13135 13590 14766 14783 15043 15301 15326 15924 16145 16188 16189 16223 16381	M13a1	73 152N 263 417 489 16145 16188 16189 16223 16381	[0.023] M13a1 (1)	[0.000] M13a M13a1a M13a1b M13a1b1 (5)	1
522	>KU682942.1 H[illegible]	73G 263G 750G 827G 1438G 2706G 3106d 3107C 3548C 4080C 4769G 5300T 6122T 6179A 7028T 8271d 8272d 8273d 8274d 8275d 8276d 8277d 8278d 8279d 8860G 9007G 11719A 11893G 11941G 12358G 13896C 13911G 14274G 14766T 15172A 15326G 15535T 16183N 16189C 16217C 16223T 16519C	B4j	73G 263G 16183N 16189C 16217C 16223T 16519C	1	73 263 750 827 1438 2706 3548 4080 4769 5300 6122 6179 7028 8281-8289d 8860 9007 11719 11893 11941 12358 13896 13911 14274 14766 15172 15326 15535 16183N 16189 16217 16223	B4j	73 263 16183N 16189 16217 16223	[0.194] B4j (1) [0.000] N9b N9b1 N9b1a N9b3 B4 B4b'd'e'j B4d B4d1'2'3 B4d1 B4d1a B4d2 B4c B4c1 B4c1a'b B4c1a (16)	[0.059] B4a1a1a11b (17) [0.034] B2c1a (18) [0.013] B4c1a1b (19) [0.008] R+16189 (20) [0.006] B2 (21) [0.004] S2 B2b (23) [0.000] L3 L3b'f L3h N N1'5 N1 N9 N9b1c N9b2 N11 N14 S X R11'B6 B4'5 B4+16261 B4a B4a1 B4a1a3a B4a1a3a1 B4a1a5a B4a1c B4a1c1 B4a1c1a B4a1d B4a2 B4a5 B4i B4i1 B4b B2b3 B2c B2c1 B2c1b B2c1c B2e B2f B2i B2n B2p B2q B2r B4c1a1 B4c1a2 (67)	1

523 >KU682940.1 Ho	73G 150T 241G 263G 310N 750G 1095C 1391C 1438G 2706G 3106d 3107C 4769G 4936T 6960T 7028T 8860G 9894G 10160T 11151T 11719A 12245C 12720G 13167G 13359A 14239T 14766T 15326G 15940C 16172C 16224C 16311C 16390A 16519C	R1b	73G 150T 241G 263G 310N 16172C 16224C 16311C 16390A 16519C	1	73 150 241 263 310N 750 1095 1391 1438 2706 4769 4936 6960 7028 8860 9894 10160 11151 11719 12245 12720 13167 13359 14239 14766 15326 15940 16172 16224 16311 16390	R1b	73 150 241 263 310N 16172 16224 16311 16390	[0.126] R1b (1)	[0.085] R1b1 (2) [0.000] K K1 K1a+150 K1a24 K1a30 K1a30a K1a31 K1b K1b1 K1b1+(16093) K1b1b K1b1b1 (14)	1
524 >KU682938.1 Ho	73G 150T 263G 489C 750G 1438G 2626C 2706G 2772T 3106d 3107C 4386N 4769G 4958G 5894.1C 6455T 7028T 8701G 8860G 9540C 9824C 10398G 10400T 10873C 11017C 11084G 11719A 12705T 12771A 14364A 14581C 14766T 14783C 15043A 15301A 15326G 16093C 16209C 16223T 16291T 16324C	M7a1a7	73G 150T 263G 489C 16093C 16209C 16223T 16291T 16324C	1	73 150 263 489 750 1438 2626 2706 2772 4386N 4769 4958 5899.1C 6455 7028 8701 8860 9540 9824 10398 10400 10873 11017 11084 11719 12705 12771 14364 14581 14766 14783 15043 15301 15326 16093 16209 16223 16291 16324	M7a1a7	73 150 263 489 16093 16209 16223 16291 16324	[0.044] M7a1a7 (1)	[0.010] M7a+16324 M7a1 M7a1a1 M7a1a1a M7a1a3 M7a1a4 M7a1a4a M7a1a5 M7a1a5a M7a1a6 M7a1a9 M7a1b (13) [0.009] M7a1a	1
525 >KU682935.1 Ho	73G 150N 263G 489C 750G 1438G 2626C 2706G 2772T 3106d 3107C 4386C 4769G 4958G 5894.1C 6455T 7028T 8701G 8860G 9540C 9824C 10398G 10400T 10873C 11017C 11084G 11719A 12705T 12771A 14364A 14581C 14766T 14783C 15043A 15301A 15326G 16209C 16223T 16291T 16324C	M7a1a7	73G 150N 263G 489C 16209C 16223T 16291T 16324C	1	73 150N 263 489 750 1438 2626 2706 2772 4386 4769 4958 5899.1C 6455 7028 8701 8860 9540 9824 10398 10400 10873 11017 11084 11719 12705 12771 14364 14581 14766 14783 15043 15301 15326 16209 16223 16291 16324	M7a1a7	73 150N 263 489 16209 16223 16291 16324	[0.000] M7a1a7 (1)	[0.000] M7a+16324 M7a1 M7a1a M7a1a1 M7a1a1a M7a1a3 M7a1a4 M7a1a4a M7a1a5 M7a1a5a M7a1a6 M7a1a9 M7a1b (14)	1
526 >KU682932.1 Ho	73G 150N 263G 750G 961C 1438G 2706G 3106d 3107C 4769G 5231A 5417A 7028T 8860G 10685A 11719A 12358G 12372A 12705T 13857G 14766T 15067C 15326G 16172C 16223T 16250T 16257A 16261T	N9a2c	73G 150N 263G 16172C 16223T 16250T 16257A 16261T	1	73 150N 263 750 961 1438 2706 4769 5231 5417 7028 8860 10685 11719 12358 12372 12705 13857 14766 15067 15326 16172 16223 16250 16257A 16261	N9a2c	73 150N 263 16172 16223 16250 16257A 16261	[0.000] N9a2c (1)	[0.000] N9a2'4'5'11 N9a2 N9a2d (4)	1
527 >KU682929.1 Ho	73G 114T 204C 207A 248d 263G 750G 961C 1438G 1719A 2706G 3106d 3107C 3434G 3970T 4769G 5585A 5913A 5978G 6392C 6480A 7028T 8860G 9182A 9962A 10310A 10320A 11065G 11719A 12621T 12810G 13928C 14766T 15326G 15586C 15854G 16187T 16298C 16355T 16362C 16390A 16519C	F3a+207	73G 114T 204C 207A 248d 263G 16187T 16298C 16355T 16362C 16390A 16519C	1	73 114 204 207 249d 263 750 961 1438 1719 2706 3434 3970 4769 5585 5913 5978 6392 6480 7028 8860 9182 9962 10310 10320 11065 11719 12621 12810 13928C 14766 15326 15586 15854G 16187 16298 16355 16362 16390	F3a+207	73 114 204 207 249d 263 16187 16298 16355 16362 16390	[0.784] F3a+207 (1)	[0.178] F3a1 (2) [0.066] F3a	1

528 >KU682923.1 Hc	73G 263G 514d 515d 750G 1438G 1541C 2706G 3106d 3107C 3970T 4769G 5147A 7028T 8271d 8272d 8273d 8274d 8275d 8276d 8277d 8278d 8279d 8614C 8860G 11719A 12342T 12714C 13654T 13928C 14766T 15326G 16209C 16278T 16309G 16390A	R9b1	73G 263G 514d 515d 16209C 16278T 16309G 16390A	1	73 263 750 1438 1541 2706 3970 4769 5147 7028 8281-8289d 8614 8860 11719 12342 12714 13654 13928C 14766 15326 16209 16278 16309 16390	R9b1	73 263 16209 16278 16309 16390	[0.163] R9b1 (1) [0.000] R30a1b (2)	[0.136] R8a1a1d (3) [0.130] P (4) [0.103] P2 (5) [0.054] H13a2b5 (6) [0.025] L3f (7) [0.023] HV20 (8) [0.022] P9a (9) [0.019] H1+16278 (10) [0.016] R31 (11) [0.012] H1a1 R30 (13) [0.010] H1a1a H1a1a1 (15) [0.000] L3f1 L3f1a L3f1a1 R HV+73 H1a1b H1e1a+16278 H1e2c H1cg H3+73 H3d H4a1a1a H4a1a1a1 H4a1a1a1a H4a1a1a1a1 H4a1a1a2 H4a1a1a4 R2'JT R30a R30a1 R30a1a R30a1b R30a1b1 R30a1c P4 P9 U U2'3'4'7'8'9 U8 U8b'c U8b (46)	1
529 >KU682921.1 Hc	73G 150T 263G 334C 514d 515d 750G 1438G 1811G 2706G 2707G 3106d 3107C 4188G 4640A 4769G 7028T 8860G 8950A 9656C 9770C 11467G 11719A 12308G 12372A 13174C 13743C 14139G 14766T 15326G 15454C 15940d 16131C 16343G	U3b2a1a	73G 150T 263G 334C 514d 515d 16131C 16343G	1	73 150 263 334 750 1438 1811 2706 2707 4188 4640A 4769 7028 8860 8950 9656 9770 11467 11719 12308 12372 13174 13743 14139 14766 15326 15454 15944d 16131 16343	U3b2a1a	73 150 263 334 16131 16343	[0.034] U3b2a1a (1)	[0.000] U3 U3a'c U3b U3b1 U3b1b U3b2 U3b2a U3b2a1 U3b2c (10)	1
530 >KU682919.1 Hc	73G 199C 263G 489C 750G 813G 1438G 2706G 3010A 3106d 3107C 4769G 4883T 5178A 7028T 8414T 8701G 8860G 9540C 10398G 10400T 10873C 11719A 12662G 12705T 14668T 14766T 14783C 15043A 15301A 15326G 15734A 16093C 16223T 16362C 16519C	D4s	73G 199C 263G 489C 16093C 16223T 16362C 16519C	1	73 199 263 489 750 813 1438 2706 3010 4769 4883 5178A 7028 8414 8701 8860 9540 10398 10400 10873 11719 12662 12705 14668 14766 14783 15043 15301 15326 15734 16093 16223 16362	D4s	73 199 263 489 16093 16223 16362	[0.006] D4s (1)	[0.226] M35a2 (2) [0.025] D4b2b (3) [0.017] M7c1c3 M35b4 (5) [0.014] D4 (6) [0.012] G1b (7) [0.010] G2a'c (8) [0.009] G1c (9) [0.006] D4e2 (10) [0.005] G1 (11) [0.003] M7c1a4a D4b2a2 D4h4 (14) [0.002] G2b2 D4e2c D4j1b (17) [0.001] D4b2 D4b2b1 D4b2b2 D4b2b6 (21) [0.000] M7c1c3b M7c1c3c M7c1c3d M7c1c3g M9 G G1c2 G2 G2b G2b1 G2b1a G2b2a M33a1b M33+16362 M35+199 M35a M35a1 M35b D D1d2 D4b D4b2a D4b2a2a1 D4b2a2b D4b2b1a D4b2b1b D4b2b1c D4b2b3 D4e D4e1'3 D4e1 D4e1c D4e3 D4e2b D4e2d D4e4 D4e4a D4e4b D4f D4f1 D4g D4h D4j D4j1 D4j4 D4j5 D4j5a D4j+(16286) D4j9 D4j10 D4j12 D4j15 D4j16 D4m (75)	1
531 >KU682912.1 Hc	73G 153G 263G 310.1C 489C 750G 1041G 1438G 2706G 3106d 3107C 3394C 4491A 4769G 7028T 7256T 8701G 8860G 9540C 10398G 10400T 10873C 11204C 11719A 12705T 14308C 14766T 14783C 15043A 15301A 15326G 16145A 16148T 16223T 16234T 16316G	M9a1a2	73G 153G 263G 310.1C 489C 16145A 16148T 16223T 16234T 16316G	1	73 153 263 489 750 1041 1438 2706 3394 4491 4769 7028 7256 8701 8860 9540 10398 10400 10873 11204 11719 12705 14308 14766 14783 15043 15301 15326 16145 16148 16223 16234 16316	M9a1a2	73 153 263 489 16145 16148 16223 16234 16316	[0.498] M9a1a2 (1)	[0.005] M9a1a (2) [0.000] M9a1a1 M9a1a1c1b M9a1a1c1b2 M9a1a1d (6)	1

532 >KU682909.1 Hc	73G 235G 248d 263G 310.1C 750G 1005C 1009T 1438G 1824C 2706G 2875G 3106d 3107C 3970T 4538T 4769G 5790A 6392C 7028T 7828G 8860G 10310A 10535C 10586A 11719A 12338C 13708A 13928C 14766T 15326G	F2d	73G 235G 248d 263G 310.1C	1	73 235 249d 263 750 1005 1009 1438 1824 2706 2875 3970 4538 4769 5790A 6392 7028 7828 8860 10310 10535 10586 11719 12338 13708 13928C 14766 15326	F2d	73 235 249d 263	[0.000] F2d (1)	[0.111] H2a5b1 (2) [0.010] F2e (3) [0.006] HV+73 (4) [0.000] R HV20 H1e2c H3+73 H3d H4a1a1a H4a1a1a1 H4a1a1a1a H4a1a1a1a1 H4a1a1a2 H4a1a1a4 R2'JT F F1 F2 F2c F2f F4 R30 R30a R30a1 R30a1a R31 P P2 P4 P9 P9a U U2'3'4'7'8'9 U8 U8b'c U8b (37)	1
533 >KU682907.1 Hc	73G 235G 248d 263G 750G 1005C 1009T 1438G 1824C 2706G 2875G 3106d 3107C 3970T 4538T 4769G 5790A 6392C 7028T 7828G 8860G 10310A 10535C 10586A 11719A 12338C 13708A 13800N 13928C 14766T 15326G	F2d	73G 235G 248d 263G	1	73 235 249d 263 750 1005 1009 1438 1824 2706 2875 3970 4538 4769 5790A 6392 7028 7828 8860 10310 10535 10586 11719 12338 13708 13800N 13928C 14766 15326	F2d	73 235 249d 263	[0.000] F2d (1)	[0.111] H2a5b1 (2) [0.010] F2e (3) [0.006] HV+73 (4) [0.000] R HV20 H1e2c H3+73 H3d H4a1a1a H4a1a1a1 H4a1a1a1a H4a1a1a1a1 H4a1a1a2 H4a1a1a4 R2'JT F F1 F2 F2c F2f F4 R30 R30a R30a1 R30a1a R31 P P2 P4 P9 P9a U U2'3'4'7'8'9 U8 U8b'c U8b (37)	1
534 >KU682898.1 Hc	73G 150N 263G 489C 523G 750G 1438G 2626C 2706G 2772T 3106d 3107C 4386C 4768C 4769G 4958G 5894.1C 6455T 7028T 8701G 8860G 9540C 9824C 10398G 10400T 10873C 11017C 11084G 11719A 12705T 12771A 14364A 14581C 14766T 14783C 15043A 15301A 15326G 16209C 16223T 16291T 16311C 16324C	M7a1a7	73G 150N 263G 489C 523G 16209C 16223T 16291T 16311C 16324C	1	73 150N 263 489 523 750 1438 2626 2706 2772 4386 4768 4769 4958 5899.1C 6455 7028 8701 8860 9540 9824 10398 10400 10873 11017 11084 11719 12705 12771 14364 14581 14766 14783 15043 15301 15326 16209 16223 16291 16311 16324	M7a1a7	73 150N 263 489 523 16209 16223 16291 16311 16324	[0.010] M7a1a7 (1)	[0.000] M7a+16324 M7a1 M7a1a M7a1a1 M7a1a1a M7a1a3 M7a1a4 M7a1a4a M7a1a5 M7a1a5a M7a1a6 M7a1a9 M7a1b (14)	1
535 >KU682892.1 Hc	73G 235G 248d 263G 310.1C 750G 1005C 1009T 1438G 1824C 2706G 2875G 3106d 3107C 3970T 4538T 4769G 5790A 6392C 7028T 7828G 8860G 10310A 10535C 10586A 11719A 12338C 13708A 13928C 14766T 15326G	F2d	73G 235G 248d 263G 310.1C	1	73 235 249d 263 750 1005 1009 1438 1824 2706 2875 3970 4538 4769 5790A 6392 7028 7828 8860 10310 10535 10586 11719 12338 13708 13928C 14766 15326	F2d	73 235 249d 263	[0.000] F2d (1)	[0.111] H2a5b1 (2) [0.010] F2e (3) [0.006] HV+73 (4) [0.000] R HV20 H1e2c H3+73 H3d H4a1a1a H4a1a1a1 H4a1a1a1a H4a1a1a1a1 H4a1a1a2 H4a1a1a4 R2'JT F F1 F2 F2c F2f F4 R30 R30a R30a1 R30a1a R31 P P2 P4 P9 P9a U U2'3'4'7'8'9 U8 U8b'c U8b (37)	1
536 >KU682890.1 Hc	73G 248d 263G 514d 515d 750G 1438G 1896C 2706G 3106d 3107C 3970T 4086T 4769G 6392C 6962A 7028T 8860G 9053A 9548A 10310A 10609C 11719A 12406A 12882T 13759A 13928C 14766T 15326G 16129A 16162G 16172C 16303A 16304C 16497G 16519C	F1a1	73G 248d 263G 514d 515d 16129A 16162G 16172C 16303A 16304C 16497G 16519C	1	73 249d 263 750 1438 1896 2706 3970 4086 4769 6392 6962 7028 8860 9053 9548 10310 10609 11719 12406 12882 13759 13928C 14766 15326 16129 16162 16172 16303 16304 16497	F1a1	73 249d 263 16129 16162 16172 16303 16304 16497	[0.103] F1a1 (1)	[0.017] F1a1c3 (2) [0.005] F1a1'4 F1a1d1 (4) [0.003] F1a3 (5) [0.002] F1a1a (6) [0.000] F1a F1a1a1 F1a1b F1a1c F1a1c2 F1a1d F1a4 (13)	1
537 >KU682884.1 Hc	73G 150T 263G 310N 750G 961C 1438G 2706G 3106d 3107C 4769G 5231A 5417A 7028T 8860G 10685A 11719A 12358G 12372A 12705T 13857G 14766T 15067C 15326G 16172C 16223T 16250T 16257A 16261T	N9a2c	73G 150T 263G 310N 16172C 16223T 16250T 16257A 16261T	1	73 150 263 310N 750 961 1438 2706 4769 5231 5417 7028 8860 10685 11719 12358 12372 12705 13857 14766 15067 15326 16172 16223 16250 16257A 16261	N9a2c	73 150 263 310N 16172 16223 16250 16257A 16261	[0.000] N9a2c (1)	[0.000] N9a2'4'5'11 N9a2 N9a2d (4)	1

538 >KU682878.1 Hc	73G 193G 263G 310N 480C 514d 515d 709A 750G 1438G 2706G 3106d 3107C 4769G 5465C 5498G 7028T 8271d 8272d 8273d 8274d 8275d 8276d 8277d 8278d 8279d 8860G 9123A 11719A 13834G 14751T 14766T 15262C 15326G 16158G 16182N 16183C 16189C 16217C 16299G 16519C	B4a4	73G 193G 263G 310N 480C 514d 515d 16158G 16182N 16183C 16189C 16217C 16299G 16519C	1	73 193 263 310N 480 709 750 1438 2706 4769 5465 5498 7028 8281-8289d 8860 9123 11719 13834 14751 14766 15262 15326 16158 16182N 16189 16217 16299	B4a4	73 193 263 310N 480 16158 16182N 16189 16217 16299	[0.471] B4a4 (1)	[0.013] B4d1'2'3 (2) [0.000] B4 B4b'd'e'j B4d B4d1 B4d1a B4d2 B4c B4c1 B4c1a'b B4c1a (12)	1
539 >MF039862.1 Hc	73G 146C 152C 195C 263G 310.1C 513.1CACA 750G 769A 1018A 1438G 1901T 2416C 2706G 2789T 3106d 3107C 3594T 3918A 4104G 4161T 4769G 5285G 5774Y 7028T 7175C 7256T 7274T 7521A 7771G 8206A 8701G 8860G 9221G 9540C 10115C 10398G 10873C 11719A 11914A 11944C 12693G 12705T 13590A 13650T 13803G 14258R 14484C 14566G 14766T 15244G 15301A 15326G 15391T 15629C 15784C 16223T 16278T 16294T 16309G 16368C 16390A 16519C	L2a1a1	73G 146C 152C 195C 263G 310.1C 513.1CACA 16223T 16278T 16294T 16309G 16368C 16390A 16519C	1	73 146 152 195 263 750 769 1018 1438 1901 2416 2706 2789 3594 3918 4104 4161 4769 5285 5774Y 7028 7175 7256 7274 7521 7771 8206 8701 8860 9221 9540 10115 10398 10873 11719 11914 11944 12693 12705 13590 13650 13803 14258R 14484 14566 14766 15244 15301 15326 15391 15629 15784 16223 16278 16294 16309 16368 16390	L2a1a1	73 146 152 195 263 16223 16278 16294 16309 16368 16390	[0.000] L2a1a1 (1)	[0.000] L2a1 L2a1a L2a1e1 (4)	1
540 >MF621127.1 Hc	73G 146C 152C 182T 185T 189G 247A 263G 310.1C 357G 514d 515d 709A 710C 750G 769A 825A 1018A 1738C 2352C 2706G 2758A 2768G 2885C 3106d 3107C 3308C 3594T 3666A 3693A 4104G 4769G 6066G 6548T 6827C 6989G 7028T 7055G 7146G 7256T 7389C 7521A 7867T 8248G 8282T 8468T 8655T 8701G 8860G 9540C 10381G 10398G 10688A 10810C 10873C 11719A 12171G 12519C 12705T 12891T 13105G 13506T 13650T 13789C 13893G 14178C 14323A 14560A 14766T 14769G 15115C 15326G 16093C 16111T 16126C 16187T 16189C 16223T 16239T 16278T 16293G 16311C 16519C	L1b2a	73G 146C 152C 182T 185T 189G 247A 263G 310.1C 357G 514d 515d 16093C 16111T 16126C 16187T 16189C 16223T 16239T 16278T 16293G 16311C 16519C	1	73 146 152 182 185T 189 247 263 357 709 710 750 769 825A 1018 1738 2352 2706 2758 2768 2885 3308 3594 3666 3693 4104 4769 6066 6548 6827 6989 7028 7055 7146 7256 7389 7521 7867 8248 8282 8468 8655 8701 8860 9540 10381 10398 10688 10810 10873 11719 12171 12519 12705 12891 13105 13506 13650 13789 13893 14178 14323 14560 14766 14769 15115 15326 16093 16111 16126 16187 16189 16223 16239 16278 16293 16311	L1b2a	73 146 152 182 185T 189 247 263 357 16093 16111 16126 16187 16189 16223 16239 16278 16293 16311	[0.143] L1b2a (1)	[0.250] L1b2 (2)	1

541	>MF621120.1 Hc	73G 143A 146C 152C 195C 263G 309.1CT 310C 513A 750G 769A 1018A 1438G 2416C 2706G 2789T 3010A 3106d 3107C 3594T 4104G 4143G 4769G 6663G 7028T 7175C 7256T 7274T 7521A 7771G 8206A 8701G 8860G 9221G 9540C 10115C 10385G 10398G 10873C 10903T 11719A 11914A 11944C 12236A 12693G 12705T 13590A 13650T 13803G 14267T 14566G 14766T 15301A 15326G 15784C 15924G 16124A 16193T 16213A 16223T 16239T 16278T 16294T 16309G 16319A 16327T 16390A	L2a1c2a	73G 143A 146C 152C 195C 263G 309.1CT 310C 513A 16124A 16193T 16213A 16223T 16239T 16278T 16294T 16309G 16319A 16327T 16390A	1	73 143 146 152 195 263 513 750 769 1018 1438 2416 2706 2789 3010 3594 4104 4143 4769 6663 7028 7175 7256 7274 7521 7771 8206 8701 8860 9221 9540 10115 10385 10398 10873 10903 11719 11914 11944 12236 12693 12705 13590 13650 13803 14267 14566 14766 15301 15326 15784 15924 16124A 16193 16213 16223 16239 16278 16294 16309 16319 16327 16390	L2a1c2a	73 143 146 152 195 263 513 16124A 16193 16213 16223 16239 16278 16294 16309 16319 16327 16390	[0.078] L2a1c2a (1)	[0.067] L2a1c2 (2)	1
542	>MF621118.1 Hc	73G 146C 152C 195C 244G 263G 310.1C 471C 547G 750G 769A 1018A 1413C 1438G 2706G 3106d 3107C 3918A 4769G 5471A 5580C 5746A 7028T 7241G 8104C 8701G 8860G 9540C 9855G 10398G 10873C 11719A 12609C 12705T 12891T 13470G 14766T 15301A 15326G 16223T 16274A 16293T 16311C 16355T 16362C 16399G	L4b2a1	73G 146C 152C 195C 244G 263G 310.1C 471C 547G 16223T 16274A 16293T 16311C 16355T 16362C 16399G	1	73 146 152 195 244 263 471 547 750 769 1018 1413 1438 2706 3918 4769 5471 5580 5746 7028 7241 8104 8701 8860 9540 9855 10398 10873 11719 12609 12705 12891 13470 14766 15301 15326 16223 16274 16293T 16311 16355 16362 16399	L4b2a1	73 146 152 195 244 263 471 547 16223 16274 16293T 16311 16355 16362 16399	[0.000] L4b2a1 (1)	[0.953] L4b2a (2) [0.533] L4b2b (3) [0.000] L4b2	1
543	>MF621117.1 Hc	73G 150T 263G 310.1C 514d 515d 709A 750G 769A 1018A 1438G 1914G 2706G 3106d 3107C 3918A 4137T 4248C 4769G 4977C 5460A 7028T 7389C 7849T 8392A 8577G 8860G 8928C 9168T 9540C 10398G 10754G 10813T 10873C 11719A 12705T 12988T 14766T 14793G 15007T 15301A 15326G 16223T 16311C 16362C	L4b1	73G 150T 263G 310.1C 514d 515d 16223T 16311C 16362C	1	73 150 263 709 750 769 1018 1438 1914 2706 3918 4137 4248 4769 4977 5460 7028 7389 7849 8392 8577 8860 8928 9168 9540 10398 10754 10813 10873 11719 12705 12988 14766 14793 15007 15301 15326 16223 16311 16362	L4b1	73 150 263 16223 16311 16362	[0.089] L4b1 (1)	[0.019] D4j+16311 (2) [0.004] M74b2 (3) [0.003] M74b (4) [0.002] D4b2b4 (5) [0.000] L3'4 L4 L4b L3e'i'k'x L3e L3e3'4'5 L3e3'4 L3i L3h1 L3h1a L3h1a2 M43a1 G1a M74 D4b2b2a D4b2b2a1 D4j11 P6 (23)	1
544	>MF621113.1 Hc	73G 150T 263G 309.1CT 310C 709A 750G 769A 1018A 1438G 1914G 2706G 3106d 3107C 3918A 4137T 4769G 4977C 5460A 7028T 7389C 7849T 7909T 8270.1ACCCCCTCT 8392A 8587A 8701G 8860G 8928C 9168T 9540C 10398G 10813T 10873C 11719A 12028C 12705T 12855G 12988T 14766T 14793G 15301A 15326G 15924G 16223T 16311C 16362C	L4b1	73G 150T 263G 309.1CT 310C 16223T 16311C 16362C	1	73 150 263 709 750 769 1018 1438 1914 2706 3918 4137 4769 4977 5460 7028 7389 7849 7909 8289.1CCCCCTCTA 8392 8587 8701 8860 8928 9168 9540 10398 10813 10873 11719 12028 12705 12855 12988 14766 14793 15301 15326 15924 16223 16311 16362	L4b1	73 150 263 16223 16311 16362	[0.089] L4b1 (1)	[0.019] D4j+16311 (2) [0.004] M74b2 (3) [0.003] M74b (4) [0.002] D4b2b4 (5) [0.000] L3'4 L4 L4b L3e'i'k'x L3e L3e3'4'5 L3e3'4 L3i L3h1 L3h1a L3h1a2 M43a1 G1a M74 D4b2b2a D4b2b2a1 D4j11 P6 (23)	1

545 >MF621111.1 Hc	73G 150T 199C 204C 263G 309.1CT 310C 513A 709A 750G 769A 789C 1018A 1438G 1804G 2706G 3010A 3106d 3107C 3505G 3918A 4017T 4029A 4216C 4232C 4769G 4977C 5460A 7028T 7166G 7624C 8614C 8701G 8860G 8974G 9248T 9540C 9986A 10398G 10813T 10873C 11719A 12661T 12705T 13497G 14016A 14766T 14905A 14935C 15301A 15326G 16111T 16179T 16183C 16189C 16223T 16239T 16311C 16320T 16362C 16519C	L4b1a	73G 150T 199C 204C 263G 309.1CT 310C 513A 16111T 16179T 16183C 16189C 16223T 16239T 16311C 16320T 16362C 16519C	1	73 150 199 204 263 513 709 750 769 789 1018 1438 1804 2706 3010 3505 3918 4017 4029A 4216 4232 4769 4977 5460 7028 7166 7624 8614 8701 8860 8974G 9248 9540 9986 10398 10813 10873 11719 12661T 12705 13497 14016 14766 14905 14935 15301 15326 16111 16179 16189 16223 16239 16311 16320 16362	L4b1a	73 150 199 204 263 513 16111 16179 16189 16223 16239 16311 16320 16362	[0.040] L4b1a (1)	[0.010] L4b1 (2)	1
546 >MF621110.1 Hc	64T 73G 152C 182T 195C 198T 263G 310.1C 514d 515d 750G 769A 1018A 1438G 2706G 3106d 3107C 3357A 4769G 5333C 5460A 5471A 5889.1CC 7028T 8701G 8838A 8860G 9081T 9540C 9758C 10373A 10398G 10873C 11253C 11344G 11485C 11719A 12361G 12414C 12705T 13174C 13194A 14302C 14766T 15301A 15326G 15514C 16223T 16260T 16264T 16311C 16362C 16519C	L4a2	64T 73G 152C 182T 195C 198T 263G 310.1C 514d 515d 16223T 16260T 16264T 16311C 16362C 16519C	1	64 73 152 182 195 198 263 750 769 1018 1438 2706 3357 4769 5333 5460 5471 5891.2C 7028 8701 8838 8860 9081 9540 9758 10373 10398 10873 11253 11344 11485 11719 12361 12414 12705 13174 13194 14302 14766 15301 15326 15514 16223 16260 16264 16311 16362	L4a2	64 73 152 182 195 198 263 16223 16260 16264 16311 16362	[0.857] L4a2 (1)	[0.000] L4a (2)	1
547 >MF621109.1 Hc	73G 152C 195C 198T 200G 263G 309.1CT 310C 750G 769A 1018A 1438G 2706G 3106d 3107C 3357A 5460A 7028T 8701G 8860G 9221G 9344T 9540C 9827T 10032G 10373A 10398G 10873C 11253C 11344G 11485C 11719A 12361G 12414C 12705T 13174C 13392C 14302C 14766T 15301A 15326G 15924G 16182C 16183C 16189C 16223T 16260T 16264T 16311C 16362C 16519C	L4a2	73G 152C 195C 198T 200G 263G 309.1CT 310C 16182C 16183C 16189C 16223T 16260T 16264T 16311C 16362C 16519C	1	73 152 195 198 200 263 750 769 1018 1438 2706 3357 5460 7028 8701 8860 9221 9344 9540 9827 10032 10373 10398 10873 11253 11344 11485 11719 12361 12414 12705 13174 13392 14302 14766 15301 15326 15924 16189 16223 16260 16264 16311 16362	L4a2	73 152 195 198 200 263 16189 16223 16260 16264 16311 16362	[1.571] L4a2 (1)	[0.000] L4a (2)	1
548 >MF621108.1 Hc	73G 150T 195C 198T 263G 309.1CT 310C 325T 750G 769A 921C 1018A 1438G 2416C 2706G 3106d 3107C 3357A 4769G 5460A 6167C 7028T 7376T 7762A 7775A 8133T 8473C 8701G 8860G 9540C 10373A 10398G 10873C 11025C 11143T 11253C 11344G 11485C 11653G 11719A 12414C 12705T 12814A 14000A 14302C 14766T 15301A 15326G 16207T 16223T 16260T 16311C 16362C 16519C	L4a1a	73G 150T 195C 198T 263G 309.1CT 310C 325T 16207T 16223T 16260T 16311C 16362C 16519C	1	73 150 195 198 263 325 750 769 921 1018 1438 2416 2706 3357 4769 5460 6167 7028 7376 7762 7775 8133 8473 8701 8860 9540 10373 10398 10873 11025 11143 11253 11344 11485 11653 11719 12414 12705 12814 14000A 14302 14766 15301 15326 16207T 16223 16260 16311 16362	L4a1a	73 150 195 198 263 325 16207T 16223 16260 16311 16362	[0.000] L4a1a (1)	[0.000] L4a1 L4a1a (3)	1

549	>MF621107.1 Ho	73G 146C 189C 195C 198T 263G 294C 309.1CT 310C 750G 769A 1438G 1719A 2706G 3106d 3107C 4388G 4769G 5300T 7028T 7424G 7861C 8701G 8842G 8860G 9509C 9540C 9575A 9758C 10044G 10398G 10873C 11380G 11590G 11719A 12705T 12882T 12912T 13194A 13437C 13476G 14410A 14766T 15326G 16129A 16192T 16218T 16223T 16256A 16311C 16362C	L3h1b2	73G 146C 189C 195C 198T 263G 294C 309.1CT 310C 16129A 16192T 16218T 16223T 16256A 16311C 16362C	1	73 146 189C 195 198 263 294 750 769 1438 1719 2706 4388 4769 5300 7028 7424 7861 8701 8842 8860 9509 9540 9575 9758 10044 10398 10873 11380 11590 11719 12705 12882 12912 13194 13437 13476 14410 14766 15326 16129 16192 16218 16223 16256A 16311 16362	L3h1b2	73 146 189C 195 198 263 294 16129 16192 16218 16223 16256A 16311 16362	[1.450] L3h1b2 (1)	[0.000] L3h1b (2)	1
550	>MF621106.1 Ho	73G 189C 195C 263G 310.1C 514d 515d 750G 1438G 1719A 2706G 3106d 3107C 3777C 4388G 4769G 5300T 6137C 7028T 7861C 8701G 8860G 9509C 9540C 9575A 9776T 10044G 10398G 10873C 11590G 11719A 12705T 14410A 14766T 14950T 15301A 15326G 15924G 16179T 16223T 16256A 16284G 16311C	L3h1b1a	73G 189C 195C 263G 310.1C 514d 515d 16179T 16223T 16256A 16284G 16311C	1	73 189C 195 263 750 1438 1719 2706 3777 4388 4769 5300 6137 7028 7861 8701 8860 9509 9540 9575 9776 10044 10398 10873 11590 11719 12705 14410 14766 14950 15301 15326 15924 16179 16223 16256A 16284 16311	L3h1b1a	73 189C 195 263 16179 16223 16256A 16284 16311	[0.000] L3h1b1a (1)	[0.000] L3h1b1 (2)	1
551	>MF621105.1 Ho	73G 204C 263G 309.1CT 310C 750G 1438G 1719A 2706G 3106d 3107C 3396C 4218C 4388G 4769G 5300T 5492C 7028T 7310C 7861C 8701G 8781A 8860G 9509C 9540C 9575A 9824A 10398G 10873C 11466C 11590G 11719A 12351C 12705T 13153G 14167T 14407T 14664T 14766T 15301A 15326G 16188G 16223T 16270T 16311C	L3h1a2b	73G 204C 263G 309.1CT 310C 16188G 16223T 16270T 16311C	1	73 204 263 750 1438 1719 2706 3396 4218 4388 4769 5300 5492 7028 7310 7861 8701 8781A 8860 9509 9540 9575 9824A 10398 10873 11466 11590 11719 12351 12705 13153 14167 14407 14664 14766 15301 15326 16188G 16223 16270 16311	L3h1a2b	73 204 263 16188G 16223 16270 16311	[0.026] L3h1a2b (1)	[0.000] L3'4 L3h1 L3h1a L3h1a2 N1a (6)	1
552	>MF621104.1 Ho	73G 263G 309.1CT 310C 750G 1438G 1719A 2706G 3106d 3107C 4388G 4769G 5300T 5492C 7028T 7310C 7861C 8701G 8781A 8860G 9509C 9540C 9575A 9824A 10398G 10873C 11590G 11719A 12627G 12705T 12952A 13153G 14407T 14766T 15301A 15326G 16223T 16270T 16311C	L3h1a2b	73G 263G 309.1CT 310C 16223T 16270T 16311C	1	73 263 750 1438 1719 2706 4388 4769 5300 5492 7028 7310 7861 8701 8781A 8860 9509 9540 9575 9824A 10398 10873 11590 11719 12627 12705 12952 13153 14407 14766 15301 15326 16223 16270 16311	L3h1a2b	73 263 16223 16270 16311	[0.000] L3h1a2b (1)	[0.000] L3'4 L3h1 L3h1a L3h1a2 (5)	1
553	>MF621097.1 Ho	73G 195C 263G 309.1CT 310C 709A 750G 1242T 1438G 1719A 1888A 2706G 2833G 3106d 3107C 4388G 4769G 5300T 5492C 7028T 7861C 8047C 8485A 8701G 8860G 9509C 9540C 9575A 9845C 10343T 10398G 10873C 11590G 11719A 12432T 12705T 13708A 13926C 14110C 14766T 15301A 15326G 16192T 16298C 16311C	L3h1a1	73G 195C 263G 309.1CT 310C 16192T 16298C 16311C	1	73 195 263 709 750 1242 1438 1719 1888 2706 2833 4388 4769 5300 5492 7028 7861 8047 8485 8701 8860 9509 9540 9575 9845 10343 10398 10873 11590 11719 12432 12705 13708 13926 14110 14766 15301 15326 16192 16298 16311	L3h1a1	73 195 263 16192 16298 16311	[0.179] L3h1a1 (1)	[0.158] R8a (2) [0.132] HV0f (3) [0.057] P5 (4) [0.052] U5b2a1a1d (5) [0.022] HV0e (6) [0.006] R1 (7) [0.000] H11 H11b H11b1 R8 R8a1 R8a1a R8a1a1 R8a1a1a R8a1a1c U5b2a1a+16311 U5b2a1a1 U5b2a1a1a (19)	1

554 >MF621094.1 Hc	73G 150T 200G 248d 263G 310.1C 494T 650C 750G 1438G 2706G 3106d 3107C 3483A 4104G 4769G 5894.1C 6401G 7028T 7933G 8158G 8311C 8701G 8817G 8860G 9254G 9540C 9941G 10398G 10819G 10873C 11719A 12705T 13708A 14766T 15301A 15326G 15519C 15758G 15928A 16169T 16193T 16195C 16223T 16243C 16261T	L3x2b	73G 150T 200G 248d 263G 310.1C 494T 16169T 16193T 16195C 16223T 16243C 16261T	1	73 150 200 249d 263 494 650 750 1438 2706 3483 4104 4769 5899.1C 6401 7028 7933 8158 8311 8701 8817 8860 9254 9540 9941 10398 10819 10873 11719 12705 13708 14766 15301 15326 15519 15758 15928 16169 16193 16195 16223 16243 16261	L3x2b	73 150 200 249d 263 494 16169 16193 16195 16223 16243 16261	[0.455] L3x2b (1)	[0.455] L3x2b (2) [0.000] L3x2	1
555 >MF621092.1 Hc	73G 150T 189G 199C 204C 263G 309.1CT 310C 750G 1438G 2706G 3106d 3107C 3483A 4216C 4490T 4769G 5460A 5894.1C 6401G 7028T 8311C 8701G 8817G 8860G 9540C 10398G 10819G 10873C 11719A 12705T 13708A 14750G 14766T 15172A 15301A 15326G 15718C 16169T 16223T 16278T 16298C 16311C 16519C	L3x1a1	73G 150T 189G 199C 204C 263G 309.1CT 310C 16169T 16223T 16278T 16298C 16311C 16519C	1	73 150 189 199 204 263 750 1438 2706 3483 4216 4490 4769 5460 5899.1C 6401 7028 8311 8701 8817 8860 9540 10398 10819 10873 11719 12705 13708 14750 14766 15172 15301 15326 15718 16169 16223 16278 16298 16311	L3x1a1	73 150 189 199 204 263 16169 16223 16278 16298 16311	[0.400] L3x1a1 (1)	[0.333] L3x1+16311 (2)	1
556 >MF621091.1 Hc	73G 150T 152C 189G 263G 309.1CT 310C 750G 1438G 2706G 3106d 3107C 3209G 4216C 4769G 5441G 7028T 7645C 8222C 8701G 8860G 9540C 10398G 10819G 10873C 11719A 12630A 12680G 12705T 14197C 14766T 14818G 15301A 15326G 15388C 16093C 16223T 16260T 16278T 16311C 16519C	L3i2	73G 150T 152C 189G 263G 309.1CT 310C 16093C 16223T 16260T 16278T 16311C 16519C	1	73 150 152 189 263 750 1438 2706 3209 4216 4769 5441 7028 7645 8222 8701 8860 9540 10398 10819 10873 11719 12630 12680 12705 14197 14766 14818 15301 15326 15388 16093 16223 16260 16278 16311	L3i2	73 150 152 189 263 16093 16223 16260 16278 16311	[0.333] L3i2 (1)	[0.065] L3d3a1b (2) [0.047] L3f1b4a1 (3) [0.016] M51b1 M51b1a (5) [0.000] L3f1b4a L3d3a L3d3a1 L3d3a1a (9)	1
557 >MF621090.1 Hc	73G 263G 310.1C 514d 515d 750G 1438G 2706G 3106d 3107C 4769G 7028T 7645C 8701G 8860G 9540C 10398G 10679G 10819G 10873C 11260C 11719A 11914A 12705T 13650T 13800A 14766T 15301A 15326G 15734A 15758G 16129A 16153A 16318T	L3i1a	73G 263G 310.1C 514d 515d 16129A 16153A 16318T	1	73 263 750 1438 2706 4769 7028 7645 8701 8860 9540 10398 10679 10819 10873 11260 11719 11914 12705 13650 13800A 14766 15301 15326 15734 15758 16129 16153 16318T	L3i1a	73 263 16129 16153 16318T	[0.111] L3i1a (1) [0.007] H3b6 H3b6a (3)	[0.152] U7a2a (4) [0.070] P (5) [0.043] U7b (6) [0.040] U7 (7) [0.029] H1e2c (8) [0.027] H4a1a1a (9) [0.014] R (10) [0.011] R6+16129 (11) [0.007] H1j1a (12) [0.000] N7 N7b HV+73 HV20 H1e+16129 H1j1 H1j1a2 H1j1b H1j1c H1cj H3b+16129 H3b2 H3b3 H3b5 H3b7 H3+73 H3d H3af H4a1a1a1 H4a1a1a1a H4a1a1a1a1 H4a1a1a2 H4a1a1a4 H+16129 H17 H17a H17a2 H17b H17c H63a R2'JT R8a1a1b R30 R30a R30a1 R30a1a R31 P2 P4 P9 P9a U U2'3'4'7'8'9 U8 U8b'c U8b (58)	1

558 >MF621082.1 Ho	73G 150T 152C 189G 200G 263G 310.1C 750G 1438G 2352C 2706G 3106d 3107C 4769G 6221C 6587T 7028T 8701G 8860G 9540C 10172A 10819G 10873C 11719A 12438C 12705T 14152G 14212C 14766T 15301A 15326G 15670C 15942C 16093C 16185T 16223T 16327T	L3e1a3b	73G 150T 152C 189G 200G 263G 310.1C 16093C 16185T 16223T 16327T	1	73 150 152 189 200 263 750 1438 2352 2706 4769 6221 6587 7028 8701 8860 9540 10172 10819 10873 11719 12438 12705 14152 14212 14766 15301 15326 15670 15942 16093 16185 16223 16327	L3e1a3b	73 150 152 189 200 263 16093 16185 16223 16327	[0.250] L3e1a3b (1)	[0.258] L3e1a (2) [0.014] L3e1a3a (3) [0.000] L3e1a3	1
559 >MF621078.1 Ho	73G 146C 152C 263G 310.1C 514d 515d 750G 921C 1438G 1842G 2438G 2706G 3106d 3107C 4769G 5046A 5147A 6680C 7028T 7424G 8618C 8701G 8860G 8910T 9540C 10398G 10873C 11719A 12705T 13105G 13681G 13886C 14040A 14284T 14569A 14634C 14766T 15110A 15113G 15301A 15326G 15596A 16124C 16223T 16256T 16311C	L3d1b3a	73G 146C 152C 263G 310.1C 514d 515d 16124C 16223T 16256T 16311C	1	73 146 152 263 750 921 1438 1842 2438 2706 4769 5046 5147 6680 7028 7424 8618 8701 8860 8910 9540 10398 10873 11719 12705 13105 13681 13886 14040 14284 14569 14634 14766 15110 15113 15301 15326 15596 16124 16223 16256 16311	L3d1b3a	73 146 152 263 16124 16223 16256 16311	[0.018] L3d1b3a (1)	[0.017] L3d1d (2) [0.013] L3d3a1 (3) [0.008] L3d3a1b (4) [0.000] L3d1b3 L3d3a L3d3a1a (7)	1
560 >MF621077.1 Ho	73G 151T 152C 195C 263G 310.1C 514d 515d 750G 921C 1438G 2706G 3106d 3107C 3360G 3957G 4769G 5046A 5147A 5238T 5567C 6272G 6680C 7028T 7424G 8618C 8701G 8860G 9540C 10398G 10873C 11719A 12705T 13105G 13886C 14284T 14766T 15301A 15326G 15924G 16124C 16223T 16519C	L3d1b1b	73G 151T 152C 195C 263G 310.1C 514d 515d 16124C 16223T 16519C	1	73 151 152 195 263 750 921 1438 2706 3360 3957 4769 5046 5147 5238 5567 6272 6680 7028 7424 8618 8701 8860 9540 10398 10873 11719 12705 13105 13886 14284 14766 15301 15326 15924 16124 16223	L3d1b1b	73 151 152 195 263 16124 16223	[0.000] L3d1b1b (1)	[0.077] L3d1'2'3'4'5'6 (2) [0.065] L3d1b (3) [0.057] L3d3a1a (4) [0.031] L3d3a1 (5) [0.020] L3d4a (6) [0.017] L3d4 (7) [0.016] L3d1d (8) [0.000] L3d L3d1 L3d1a L3d1b1 L3d1b1a L3d3 L3d3a L3d3a1b L3d3b L3d6 (18)	1
561 >MF621074.1 Ho	73G 152C 263G 310.1C 414G 513.1CA 745.1T 750G 1438G 1842G 2706G 3106d 3107C 3396C 4218C 4769G 5821A 7028T 8701G 8860G 9365T 9540C 10398G 10873C 11466C 11719A 12705T 13269G 14167T 14766T 15301A 15314A 15326G 15479C 15514C 15518T 15940d 16223T 16266T 16274A 16293G 16311C 16519C	L3f2a1a	73G 152C 263G 310.1C 414G 513.1CA 16223T 16266T 16274A 16293G 16311C 16519C	1	73 152 263 414G 745.1T 750 1438 1842 2706 3396 4218 4769 5821 7028 8701 8860 9365 9540 10398 10873 11466 11719 12705 13269 14167 14766 15301 15314 15326 15479 15514 15518 15944d 16223 16266 16274 16293 16311	L3f2a1a	73 152 263 414G 16223 16266 16274 16293 16311	[0.084] L3f2a1a (1)	[0.065] L3h1 L3h1a (3) [0.057] L3d3a1b (4) [0.047] L3'4 L3a1a (6) [0.022] L3d3a (7) [0.000] L3f2a L3f2a1 L3c'd L3d3a1 L3d3a1a L3h1a2 M57+152 S+152 (15)	1
562 >MF621073.1 Ho	73G 189G 200G 263G 310.1C 514d 515d 750G 1438G 1822C 2438G 2706G 3106d 3107C 3396C 4218C 4769G 5601T 7028T 7819A 8527G 8701G 8860G 8932T 8994A 9540C 9950C 10398G 10873C 11719A 12705T 14766T 14769G 15301A 15326G 15514C 15940d 16093C 16209C 16223T 16311C 16519C	L3f1b	73G 189G 200G 263G 310.1C 514d 515d 16093C 16209C 16223T 16311C 16519C	1	73 189 200 263 750 1438 1822 2438 2706 3396 4218 4769 5601 7028 7819A 8527 8701 8860 8932 8994 9540 9950 10398 10873 11719 12705 14766 14769 15301 15326 15514 15944d 16093 16209 16223 16311	L3f1b	73 189 200 263 16093 16209 16223 16311	[0.087] L3f1b (1)	[0.043] L3f1b2 (2) [0.038] L3f1b+16292 (3) [0.000] L3f1b4a L3f1b4a1 L3f1b5 (6)	1

563 >MF621066.1 Ho	73G 263G 310.1C 514d 515d 750G 1438G 2706G 3106d 3107C 3450T 4769G 5773A 6221C 7028T 8701G 8860G 9449T 9540C 10086G 10373A 10398G 10819G 10873C 11002G 11719A 12705T 13105G 13914A 14267T 14670C 14766T 15301A 15311G 15326G 15824G 15940d 16051G 16223T 16278T 16362C 16519C	L3b1a9	73G 263G 310.1C 514d 515d 16051G 16223T 16278T 16362C 16519C	1	73 263 750 1438 2706 3450 4769 5773 6221 7028 8701 8860 9449 9540 10086 10373 10398 10819 10873 11002 11719 12705 13105 13914A 14267 14670 14766 15301 15311 15326 15824 15944d 16051 16223 16278 16362	L3b1a9	73 263 16051 16223 16278 16362	[0.000] L3b1a9 (1)	[0.000] L3b1a+@16124 L3b1a7 L3b1a7a L3b1a9a (5)	1
564 >MF621063.1 Ho	73G 152C 263G 309.1CT 310C 514T 721C 750G 1438G 2150.1A 2702A 2706G 3106d 3107C 3796G 4088T 4733C 4769G 6500T 7028T 8701G 8860G 9540C 9966A 10314T 10398G 10873C 11719A 12705T 12816T 14461C 14766T 14851G 14927G 15109C 15301A 15326G 15553A 16223T 16254G 16293G 16311C 16316G	L3a1a	73G 152C 263G 309.1CT 310C 514T 16223T 16254G 16293G 16311C 16316G	1	73 152 263 514 721 750 1438 2156.1A 2702 2706 3796 4088 4733 4769 6500 7028 8701 8860 9540 9966 10314 10398 10873 11719 12705 12816 14461 14766 14851 14927 15109 15301 15326 15553 16223 16254 16293 16311 16316	L3a1a	73 152 263 514 16223 16254 16293 16311 16316	[0.070] L3a1a (1)	[0.222] L3a1 (2) [0.070] L3a1a	1
565 >KU683243.1 Ho	73G 150T 152C 263G 310.1C 750G 1438G 1811G 2706G 3106d 3107C 4188G 4640A 4769G 7028T 8382T 8860G 9656C 11467G 11719A 12308G 12372A 13743C 14139G 14766T 15326G 15454C 16157C 16168T 16343G 16390A	U3b3	73G 150T 152C 263G 310.1C 16157C 16168T 16343G 16390A	1	73 150 152 263 750 1438 1811 2706 4188 4640A 4769 7028 8382 8860 9656 11467 11719 12308 12372 13743 14139 14766 15326 15454 16157 16168 16343 16390	U3b3	73 150 152 263 16157 16168 16343 16390	[0.349] U3b3 (1) [0.089] U3a1 (2) [0.021] U3a2 (3) [0.016] U3a (4) [0.000] U3a1a U3a1a1 U3a1b (7)	[0.345] U3b2 (8) [0.092] U3b (9) [0.048] U3a3 (10) [0.032] U3a2a (11) [0.028] U3b2a1 (12) [0.025] U3a1c (13) [0.010] U3b1b (14) [0.000] U3 U3a'c U3b1 U3b1a1 U3b2a U3b2c (20)	1
566 >KU683241.1 Ho	73G 151T 152C 248d 263G 310N 489C 750G 1438G 2706G 3106d 3107C 4715G 4769G 6752G 7028T 7196A 8584A 8701G 8860G 9090C 9329A 9540C 10398G 10400T 10873C 11719A 12705T 14329T 14766T 14783C 14968C 15043A 15301A 15326G 15475G 15487T 15784C 15940d 16185T 16223T 16260T 16265C 16298C 16302G 16519C	Z4a	73G 151T 152C 248d 263G 310N 489C 16185T 16223T 16260T 16265C 16298C 16302G 16519C	1	73 151 152 249d 263 310N 489 750 1438 2706 4715 4769 6752 7028 7196A 8584 8701 8860 9090 9329 9540 10398 10400 10873 11719 12705 14329 14766 14783 14968 15043 15301 15326 15475 15487T 15784 15944d 16185 16223 16260 16265C 16298 16302	Z4a	73 151 152 249d 263 310N 489 16185 16223 16260 16265C 16298 16302	[0.017] Z4a (1)	[0.000] Z4 (2)	1
567 >KU683236.1 Ho	73G 152C 185A 189G 195C 204C 263G 709A 750G 1243C 1406C 1438G 2706G 3106d 3107C 3505G 4769G 5046A 5291C 5460A 7028T 8251A 8784G 8860G 8994A 11674T 11719A 11947G 12414C 12705T 14766T 15326G 15784C 15884C 16209C 16223T 16255A 16292T 16381G 16519C	W3a2	73G 152C 185A 189G 195C 204C 263G 16209C 16223T 16255A 16292T 16381G 16519C	1	73 152 185 189 195 204 263 709 750 1243 1406 1438 2706 3505 4769 5046 5291 5460 7028 8251 8784 8860 8994 11674 11719 11947 12414 12705 14766 15326 15784 15884C 16209 16223 16255 16292 16381G	W3a2	73 152 185 189 195 204 263 16209 16223 16255 16292 16381G	[1.666] W3a2 (1)	[0.136] W (2) [0.135] W1f (3) [0.014] W1a (4) [0.012] W1 (5) [0.000] W1e W7 (7)	1

568 >KU683230.1 Hc	73G 150T 152C 263G 456T 489C 681C 750G 1048T 1107C 1438G 2706G 3106d 3107C 3759G 4769G 4883T 5147A 5153G 5178A 5301G 6253C 7028T 7403G 8348N 8701G 8860G 9180G 9540C 10397G 10398G 10400T 10873C 11719A 12705T 14766T 14783C 15043A 15301A 15326G 15724G 16183N 16189C 16223T 16357C 16362C 16519C	D5b1b2	73G 150T 152C 263G 456T 489C 16183N 16189C 16223T 16357C 16362C 16519C	1	73 150 152 263 456 489 681 750 1048 1107 1438 2706 3759 4769 4883 5147 5153 5178A 5301 6253 7028 7403 8348N 8701 8860 9180 9540 10397 10398 10400 10873 11719 12705 14766 14783 15043 15301 15326 15724 16183N 16189 16223 16357 16362	D5b1b2	73 150 152 263 456 489 16183N 16189 16223 16357 16362	[0.071] D5b1b2 (1)	[0.151] D5b1 (2) [0.007] D5b3 (3) [0.000] D5b D5b1a D5b1b D5b1c D5b3a (8)	1
569 >KU683228.1 Hc	73G 143A 152C 263G 489C 709A 750G 1438G 2706G 3106d 3107C 4769G 4833G 5108C 7028T 7621C 8701G 8860G 9054T 9540C 10398G 10400T 10873C 11719A 12705T 13209T 13350G 14569A 14766T 14783C 15043A 15301A 15326G 15746G 16223T 16274A 16362C	G3a2+152	73G 143A 152C 263G 489C 16223T 16274A 16362C	1	73 143 152 263 489 709 750 1438 2706 4769 4833 5108 7028 7621 8701 8860 9054 9540 10398 10400 10873 11719 12705 13209 13350 14569 14766 14783 15043 15301 15326 15746 16223 16274 16362	G3a2+152	73 143 152 263 489 16223 16274 16362	[0.000] G3a2+152 (1)	[0.000] G3a1'2 G3a2 (3)	1
570 >KU683225.1 Hc	73G 263G 489C 534T 750G 1438G 1462A 1958A 2706G 3010A 3106d 3107C 4769G 4883T 5178A 7028T 8383C 8414T 8701G 8860G 9431T 9540C 10398G 10400T 10873C 11719A 12705T 14668T 14766T 14783C 15043A 15301A 15326G 16223T 16245T 16362C	D4c2b	73G 263G 489C 534T 16223T 16245T 16362C	1	73 263 489 534 750 1438 1462 1958 2706 3010 4769 4883 5178A 7028 8383 8414 8701 8860 9431 9540 10398 10400 10873 11719 12705 14668 14766 14783 15043 15301 15326 16223 16245 16362	D4c2b	73 263 489 534 16223 16245 16362	[0.000] D4c2b (1)	[0.000] D4c D4c2 D4c2c (4)	1
571 >KU683222.1 Hc	73G 152C 263G 489C 573.1N 750G 1438G 2706G 3010A 3106d 3107C 4769G 4883T 5021C 5178A 7028T 8414T 8701G 8860G 9540C 10398G 10400T 10873C 11215T 11719A 12705T 14668T 14755G 14766T 14783C 15043A 15106A 15184C 15301A 15326G 16173T 16223T 16274A 16291T 16362C	D4e5a	73G 152C 263G 489C 573.1N 16173T 16223T 16274A 16291T 16362C	1	73 152 263 489 573.1N 750 1438 2706 3010 4769 4883 5021 5178A 7028 8414 8701 8860 9540 10398 10400 10873 11215 11719 12705 14668 14755 14766 14783 15043 15106 15184 15301 15326 16173 16223 16274 16291 16362	D4e5a	73 152 263 489 573.1N 16173 16223 16274 16291 16362	[0.167] D4e5a (1)	[0.072] D4e5b (2) [0.013] D4j2 (3) [0.000] D4j2a	1
572 >KU683220.1 Hc	73G 263G 310N 408A 499A 750G 827G 1438G 2706G 3106d 3107C 4769G 4820A 5843G 6023A 6413C 7028T 8271d 8272d 8273d 8274d 8275d 8276d 8277d 8278d 8279d 8860G 9055A 9338T 9615C 11719A 12945C 13590A 14766T 15326G 15535T 16136C 16183C 16189C 16217C 16360T 16519C	B4b1a3	73G 263G 310N 408A 499A 16136C 16183C 16189C 16217C 16360T 16519C	1	73 263 310N 408A 499 750 827 1438 2706 4769 4820 5843 6023 6413 7028 8281-8289d 8860 9055 9338T 9615 11719 12945 13590 14766 15326 15535 16136 16189 16217 16360	B4b1a3	73 263 310N 408A 499 16136 16189 16217 16360	[0.091] B4b1a3 (1) [0.012] B4b1 (2) [0.006] B4b1a (3)	[0.160] B2q (4) [0.042] B4b1c1 (5) [0.027] B2 (6) [0.011] B2p (7) [0.008] B4b1a2a (8) [0.000] B4b B2b B2b3 B2c B2c1 B2c1a B2c1b B2c1c B2e B2f B2i B2n B2r B4b1a+207 B4b1a2 B4b1a2e B4b1a2f B4b1a2h B4b1b'c B4b1c B4b1c2 (29)	1

573	>KU683218.1 Hc	73G 217C 263G 310N 340T 451.1T 508G 750G 1438G 1811G 2706G 3106d 3107C 3720G 4769G 5390G 5426C 6045T 6152C 7028T 8271d 8272d 8273d 8274d 8275d 8276d 8277d 8278d 8279d 8860G 10876G 11467G 11719A 12308G 12372A 13020C 13734C 14470C 14766T 15326G 15907G 16051G 16129C 16183C 16189.1C 16362C 16519C	U2e1h	73G 217C 263G 310N 340T 451.1T 508G 16051G 16129C 16183C 16189.1C 16362C 16519C	1	73 217 263 310N 340 455.1T 508 750 1438 1811 2706 3720 4769 5390 5426 6045 6152 7028 8281-8289d 8860 10876 11467 11719 12308 12372 13020 13734 14470 14766 15326 15907 16051 16129C 16362	U2e1h	73 217 263 310N 340 455.1T 508 16051 16129C 16362	[0.028] U2e1h (1)	[0.009] U2e1a1 (2) [0.000] U2e1 U2e1a U2e1a1a U2e1a1c U2e1c U2e1c1 U2e1d U2e1g (10)	1
574	>KU683217.1 Hc	73G 94A 150T 263G 456T 489C 681C 750G 1048T 1107C 1438G 2706G 3106d 3107C 3759G 4769G 4883T 5147A 5153G 5178A 5206G 5301G 6253C 7028T 7403G 8701G 8860G 9180G 9540C 10397G 10398G 10400T 10873C 11719A 12705T 14766T 14783C 15043A 15301A 15326G 15724G 16183C 16189C 16223T 16357C 16362C 16519C	D5b1b2	73G 94A 150T 263G 456T 489C 16183C 16189C 16223T 16357C 16362C 16519C	1	73 94 150 263 456 489 681 750 1048 1107 1438 2706 3759 4769 4883 5147 5153 5178A 5206G 5301 6253 7028 7403 8701 8860 9180 9540 10397 10398 10400 10873 11719 12705 14766 14783 15043 15301 15326 15724 16189 16223 16357 16362	D5b1b2	73 94 150 263 456 489 16189 16223 16357 16362	[0.054] D5b1b2 (1)	[0.007] D5b D5b1a D5b1b D5b1c D5b3 D5b3a (7) [0.000] D5b1	1
575	>KU683212.1 Hc	73G 195C 263G 310N 489C 750G 1438G 2706G 3010A 3106d 3107C 4769G 4883T 5178A 7028T 8414T 8701G 8860G 9077C 9540C 10398G 10400T 10646A 10873C 11719A 12705T 13812C 14668T 14766T 14783C 14883T 15043A 15301A 15326G 16093C 16223T 16232T 16290T 16362C	D4o2a	73G 195C 263G 310N 489C 16093C 16223T 16232T 16290T 16362C	1	73 195 263 310N 489 750 1438 2706 3010 4769 4883 5178A 7028 8414 8701 8860 9077 9540 10398 10400 10646 10873 11719 12705 13812 14668 14766 14783 14883 15043 15301 15326 16093 16223 16232 16290 16362	D4o2a	73 195 263 310N 489 16093 16223 16232 16290 16362	[0.000] D4o2a (1)	[0.000] D4o2 (2)	1
576	>KU683210.1 Hc	153G 204C 263G 750G 1438G 3106d 3107C 4769G 7861C 8185C 8860G 9966A 10646A 15326G 16189C 16519C	H55b	153G 204C 263G 16189C 16519C	1	153 204 263 750 1438 4769 7861 8185 8860 9966 10646 15326 16189	H55b	153 204 263 16189	[0.366] H55b (1)	[0.103] H1g H1aa (3) [0.091] H55+153 (4) [0.019] H4b1 (5) [0.005] H1j9 (6) [0.000] HV18 H1+16189 H1f H1g1 H1g2 H1y H1aa1 H1ab H1ac H1ad H3+16189 H13a1b H56d (19)	1
577	>KU683208.1 Hc	64T 73G 146N 152C 199C 235G 263G 514d 515d 663G 750G 1438G 1736G 2706G 3106d 3107C 4248C 4769G 4824G 6962A 7028T 8794T 8860G 8865A 11719A 12777G 14766T 15326G 15814G 15828N 16223T 16242T 16290T 16319A 16519C	A8a1	64T 73G 146N 152C 199C 235G 263G 514d 515d 16223T 16242T 16290T 16319A 16519C	1	64 73 146N 152 199 235 263 663 750 1438 1736 2706 4248 4769 4824 6962 7028 8794 8860 8865 11719 12777 14766 15326 15814 15828N 16223 16242 16290 16319	A8a1	64 73 146N 152 199 235 263 16223 16242 16290 16319	[0.226] A8a1 (1)	[0.000] A8a (2)	1
578	>KU683207.1 Hc	73G 152C 204C 248d 263G 514d 515d 516d 517d 750G 1438G 2706G 3106d 3107C 3970T 4732G 4769G 5147A 6392C 6962A 7028T 8772C 8860G 9180G 10310A 10609C 10976T 11719A 12406A 12633T 12882T 13928C 14476A 14766T 15326G 16172C 16183N 16189C 16232A 16249C 16304C 16311C	F1b1b	73G 152C 204C 248d 263G 514d 515d 516d 517d 16172C 16183N 16189C 16232A 16249C 16304C 16311C	1	73 152 204 249d 263 750 1438 2706 3970 4732 4769 5147 6392 6962 7028 8772 8860 9180 10310 10609 10976 11719 12406 12633 12882 13928C 14476 14766 15326 16172 16183N 16189 16232A 16249 16304 16311	F1b1b	73 152 204 249d 263 16172 16183N 16189 16232A 16249 16304 16311	[0.000] F1b1b (1)	[0.048] F1b F1b1 (3)	1

579 >KU683204.1 Hc	73G 151T 152C 200G 235G 263G 471C 514d 515d 663G 709A 735G 750G 1438G 1736G 2706G 3106d 3107C 4248C 4769G 4824G 7028T 8765T 8794T 8860G 9182A 11719A 12705T 14766T 15326G 16223T 16290T 16319A 16362C	A14	73G 151T 152C 200G 235G 263G 471C 514d 515d 16223T 16290T 16319A 16362C	1	73 151 152 200 235 263 471 663 709 735 750 1438 1736 2706 4248 4769 4824 7028 8765 8794 8860 9182 11719 12705 14766 15326 16223 16290 16319 16362	A14	73 151 152 200 235 263 471 16223 16290 16319 16362	[0.002] A14 (1)	[0.009] A+152+16362+200 (2) [0.005] A13	1
580 >KU683202.1 Hc	73G 195C 263G 489C 750G 1438G 2706G 3010A 3106d 3107C 3789T 4769G 4883T 5178A 7028T 8414T 8701G 8860G 9077C 9540C 10398G 10400T 10646A 10873C 11719A 12705T 13812N 14668T 14766T 14783C 15043A 15301A 15326G 16093C 16223T 16232T 16290T 16362C 16519C	D4o2a	73G 195C 263G 489C 16093C 16223T 16232T 16290T 16362C 16519C	1	73 195 263 489 750 1438 2706 3010 3789 4769 4883 5178A 7028 8414 8701 8860 9077 9540 10398 10400 10646 10873 11719 12705 13812N 14668 14766 14783 15043 15301 15326 16093 16223 16232 16290 16362	D4o2a	73 195 263 489 16093 16223 16232 16290 16362	[0.000] D4o2a (1)	[0.000] D4o2 (2)	1
581 >KU683197.1 Hc	73G 263G 489C 534T 750G 1438G 1462A 1958A 2706G 3010A 3106d 3107C 4769G 4883T 5178A 7028T 8383C 8414T 8701G 8860G 9431T 9540C 10398G 10400T 10873C 11719A 12705T 14668T 14766T 14783C 15043A 15301A 15326G 16223T 16245T 16362C	D4c2b	73G 263G 489C 534T 16223T 16245T 16362C	1	73 263 489 534 750 1438 1462 1958 2706 3010 4769 4883 5178A 7028 8383 8414 8701 8860 9431 9540 10398 10400 10873 11719 12705 14668 14766 14783 15043 15301 15326 16223 16245 16362	D4c2b	73 263 489 534 16223 16245 16362	[0.000] D4c2b (1)	[0.000] D4c D4c2 D4c2c (4)	1
582 >KU683195.1 Hc	73G 146C 151T 152C 199C 263G 514d 515d 750G 980C 1438G 1811G 2523T 2706G 3106d 3107C 3338C 3741T 4769G 5360T 7028T 8137T 8684T 8860G 10142T 11255C 11467G 11719A 12308G 12372A 13145A 13500C 14569A 14766T 15326G 16126C 16309G 16318T 16519C	U7a4	73G 146C 151T 152C 199C 263G 514d 515d 16126C 16309G 16318T 16519C	1	73 146 151 152 199 263 750 980 1438 1811 2523 2706 3338 3741 4769 5360 7028 8137 8684 8860 10142 11255 11467 11719 12308 12372 13145 13500 14569 14766 15326 16126 16309 16318T	U7a4	73 146 151 152 199 263 16126 16309 16318T	[0.026] U7a4 (1)	[0.000] U7a4a (2)	1
583 >KU683194.1 Hc	263G 310.1C 750G 1438G 3106d 3107C 4769G 8860G 11447A 15326G 16172C 16400T	H33b	263G 310.1C 16172C 16400T	1	263 750 1438 4769 8860 11447 15326 16172 16400	H33b	263 16172 16400	[0.000] H33b (1)	[0.000] H66a (2)	1
584 >KU683192.1 Hc	73G 263G 310N 408A 499A 750G 827G 1438G 2706G 3106d 3107C 4769G 4820A 5843G 6023A 6413C 7028T 8271d 8272d 8273d 8274d 8275d 8276d 8277d 8278d 8279d 8860G 9055A 9338T 9615C 11719A 12945C 13590A 14766T 15326G 15535T 16136C 16183C 16189C 16217C 16360T 16519C	B4b1a3	73G 263G 310N 408A 499A 16136C 16183C 16189C 16217C 16360T 16519C	1	73 263 310N 408A 499 750 827 1438 2706 4769 4820 5843 6023 6413 7028 8281-8289d 8860 9055 9338T 9615 11719 12945 13590 14766 15326 15535 16136 16189 16217 16360	B4b1a3	73 263 310N 408A 499 16136 16189 16217 16360	[0.091] B4b1a3 (1) [0.012] B4b1 (2) [0.006] B4b1a (3)	[0.160] B2q (4) [0.042] B4b1c1 (5) [0.027] B2 (6) [0.011] B2p (7) [0.008] B4b1a2a (8) [0.000] B4b B2b B2b3 B2c B2c1 B2c1a B2c1b B2c1c B2e B2f B2i B2n B2r B4b1a+207 B4b1a2 B4b1a2e B4b1a2f B4b1a2h B4b1b'c B4b1c B4b1c2 (29)	1

585	>KU683191.1 H[illegible]	73G 195C 263G 302N 489C 750G 1438G 2487C 2706G 3010A 3106d 3107C 3488N 3492N 4769G 4883T 5178A 7028T 8126N 8127N 8128N 8129N 8130N 8131N 8414T 8701G 8860G 8897T 9077C 9540C 10398G 10400T 10646A 10873C 11620N 11719A 11728N 11729N 11982N 11983N 11984N 11985N 11986N 11987N 11988N 11989N 11990N 11991N 11992N 11993N 11994N 11995N 11996N 11997N 11998N 11999N 12000N 12001N 12002N 12003N 12004N 12005N 12006N 12007N 12008N 12009N 12010N 12013T 12705T 13812C 14668T 14766T 14783C 14872N 14873N 14874N 14875N 14876N 14877N 14878N 14879N 14880N 14881N 14882N 14883N 15043A 15301A 15326G 16093C 16223T 16232T 16290T 16362C	D4o2a	73G 195C 263G 302N 489C 16093C 16223T 16232T 16290T 16362C	1	73 195 263 302N 489 750 1438 2487C 2706 3010 3488N 3492N 4769 4883 5178A 7028 8126N 8127N 8128N 8129N 8130N 8131N 8414 8701 8860 8897 9077 9540 10398 10400 10646 10873 11620N 11719 11728N 11729N 11982N 11983N 11984N 11985N 11986N 11987N 11988N 11989N 11990N 11991N 11992N 11993N 11994N 11995N 11996N 11997N 11998N 11999N 12000N 12001N 12002N 12003N 12004N 12005N 12006N 12007N 12008N 12009N 12010N 12013T 12705 13812 14668 14766 14783 14872N 14873N 14874N 14875N 14876N 14877N 14878N 14879N 14880N 14881N 14882N 14883N 15043 15301 15326 16093 16223 16232 16290 16362	D4o2a	73 195 263 302N 489 16093 16223 16232 16290 16362	[0.000] D4o2a (1)	[0.000] D4o2 (2)	1
586	>KU683186.1 H[illegible]	73G 146C 207A 263G 310N 408A 499A 750G 827G 1438G 2706G 3106d 3107C 4769G 4820A 6023A 6089G 6413C 7028T 8271d 8272d 8273d 8274d 8275d 8276d 8277d 8278d 8279d 8860G 9055A 9338T 9615C 11719A 13590A 14133G 14766T 15326G 15535T 16086C 16136C 16183C 16189C 16217C 16519C	B4b1a3a	73G 146C 207A 263G 310N 408A 499A 16086C 16136C 16183C 16189C 16217C 16519C	1	73 146 207 263 310N 408A 499 750 827 1438 2706 4769 4820 6023 6089 6413 7028 8281-8289d 8860 9055 9338T 9615 11719 13590 14133 14766 15326 15535 16086 16136 16189 16217	B4b1a3a	73 146 207 263 310N 408A 499 16086 16136 16189 16217	[0.000] B4b1a3a (1)	[0.000] B4b1a3 (2)	1
587	>KU683182.1 H[illegible]	73G 152C 204C 248d 263G 514d 515d 516d 517d 750G 1438G 2706G 3106d 3107C 3970T 4732G 4769G 5147A 6392C 6962A 7028T 8772C 8860G 9180G 10310A 10609C 10976T 11719A 12406A 12633T 12882T 13928C 14476A 14766T 15326G 16172C 16183C 16189C 16232A 16249C 16304C 16311C	F1b1b	73G 152C 204C 248d 263G 514d 515d 516d 517d 16172C 16183C 16189C 16232A 16249C 16304C 16311C	1	73 152 204 249d 263 750 1438 2706 3970 4732 4769 5147 6392 6962 7028 8772 8860 9180 10310 10609 10976 11719 12406 12633 12882 13928C 14476 14766 15326 16172 16189 16232A 16249 16304 16311	F1b1b	73 152 204 249d 263 16172 16189 16232A 16249 16304 16311	[0.000] F1b1b (1)	[0.048] F1b F1b1 (3)	1
588	>KU683177.1 H[illegible]	152C 239C 263G 750G 1438G 3106d 3107C 4769G 8860G 14040A 15326G 16186T 16300G 16325C 16362C 16482G	H6b2	152C 239C 263G 16186T 16300G 16325C 16362C 16482G	1	152 239 263 750 1438 4769 8860 14040 15326 16186 16300 16325 16362 16482	H6b2	152 239 263 16186 16300 16325 16362 16482	[0.380] H6b2 (1)	[0.380] H6b2 (2) [0.125] H6b1 (3) [0.033] H6b	1

589 >KU683173.1 Hc	73G 263G 408A 499A 750G 827G 1438G 2706G 3106d 3107C 4769G 4820A 5843G 6023A 6413C 7028T 8271d 8272d 8273d 8274d 8275d 8276d 8277d 8278d 8279d 8860G 9055A 9338T 9615C 11719A 12945C 13590A 14766T 15326G 15535T 16136C 16183N 16189C 16217C 16360T 16519C	B4b1a3	73G 263G 408A 499A 16136C 16183N 16189C 16217C 16360T 16519C	1	73 263 408A 499 750 827 1438 2706 4769 4820 5843 6023 6413 7028 8281-8289d 8860 9055 9338T 9615 11719 12945 13590 14766 15326 15535 16136 16183N 16189 16217 16360	B4b1a3	73 263 408A 499 16136 16183N 16189 16217 16360	[0.091] B4b1a3 (1) [0.012] B4b1 (2) [0.006] B4b1a (3)	[0.160] B2q (4) [0.042] B4b1c1 (5) [0.027] B2 (6) [0.011] B2p (7) [0.008] B4b1a2a (8) [0.000] B4b B2b B2b3 B2c B2c1 B2c1a B2c1b B2c1c B2e B2f B2i B2n B2r B4b1a+207 B4b1a2 B4b1a2e B4b1a2f B4b1a2h B4b1b'c B4b1c B4b1c2 (29)	1
590 >KU683172.1 Hc	151N 152N 195C 263G 335G 478G 750G 1438G 3106d 3107C 4769G 8860G 15326G 16519C	H91	151N 152N 195C 263G 335G 478G 16519C	1	151N 152N 195 263 335 478 750 1438 4769 8860 15326	H91	151N 152N 195 263 335 478	[0.208] H91 (1)	[0.000] H4a1a+195 H4a1a3 H4a1a3a H4a1a4 H4a1a4a H4a1a4b H4a1a4b2 H+195 H12 H108 H44b (12)	1
591 >KU683168.1 Hc	73G 152C 225N 235G 263G 310N 514d 515d 663G 750G 1438G 1736G 2706G 3106d 3107C 4248C 4769G 4824G 5794C 7028T 8794T 8860G 11086G 11719A 12705T 13708A 14766T 15326G 15346A 15562G 15856T 16179T 16223T 16290T 16319A 16362C	A25	73G 152C 225N 235G 263G 310N 514d 515d 16179T 16223T 16290T 16319A 16362C	1	73 152 225N 235 263 310N 663 750 1438 1736 2706 4248 4769 4824 5794 7028 8794 8860 11086 11719 12705 13708 14766 15326 15346 15562 15856 16179 16223 16290 16319 16362	A25	73 152 225N 235 263 310N 16179 16223 16290 16319 16362	[0.000] A25 (1)	[0.000] A+152+16362 A1 A6 A6a A17 A18 A20 A21 A22 (10)	1
592 >KU683158.1 Hc	73G 150T 263G 456T 489C 681C 750G 1048T 1107C 1438G 2706G 3106d 3107C 3759G 4769G 4883T 5147A 5153G 5178A 5301G 6253C 6465A 7028T 7403G 8701G 8860G 8886A 9180G 9531G 9540C 10397G 10398G 10400T 10873C 11719A 11869T 12705T 14766T 14783C 15043A 15301A 15326G 15724G 16189C 16223T 16259T 16357C 16362C 16519C	D5b1b2	73G 150T 263G 456T 489C 16189C 16223T 16259T 16357C 16362C 16519C	1	73 150 263 456 489 681 750 1048 1107 1438 2706 3759 4769 4883 5147 5153 5178A 5301 6253 6465 7028 7403 8701 8860 8886 9180 9531 9540 10397 10398 10400 10873 11719 11869 12705 14766 14783 15043 15301 15326 15724 16189 16223 16259 16357 16362	D5b1b2	73 150 263 456 489 16189 16223 16259 16357 16362	[0.036] D5b1b2 (1)	[0.000] D5b D5b1 D5b1a D5b1b D5b1c D5b3 D5b3a (8)	1
593 >KU683153.1 Hc	73G 150T 152C 263G 750G 1438G 1811G 2706G 3106d 3107C 4188G 4640A 4769G 7028T 8382T 8860G 9656C 11467G 11719A 12308G 12372A 13743C 14139G 14766T 15326G 15454C 16157C 16168T 16343G 16390A	U3b3	73G 150T 152C 263G 16157C 16168T 16343G 16390A	1	73 150 152 263 750 1438 1811 2706 4188 4640A 4769 7028 8382 8860 9656 11467 11719 12308 12372 13743 14139 14766 15326 15454 16157 16168 16343 16390	U3b3	73 150 152 263 16157 16168 16343 16390	[0.349] U3b3 (1) [0.089] U3a1 (2) [0.021] U3a2 (3) [0.016] U3a (4) [0.000] U3a1a U3a1a1 U3a1b (7)	[0.345] U3b2 (8) [0.092] U3b (9) [0.048] U3a3 (10) [0.032] U3a2a (11) [0.028] U3b2a1 (12) [0.025] U3a1c (13) [0.010] U3b1b (14) [0.000] U3 U3a'c U3b1 U3b1a1 U3b2a U3b2c (20)	1
594 >KU683150.1 Hc	263G 310N 750G 1438G 3106d 3107C 4769G 6323G 8860G 15326G 16287T 16291T 16390A 16519C	H85	263G 310N 16287T 16291T 16390A 16519C	1	263 310N 750 1438 4769 6323 8860 15326 16287 16291 16390	H85	263 310N 16287 16291 16390	[0.036] H85 (1)	[0.012] H+16291 (2) [0.007] H2a2b (3) [0.000] H1j2 H2a2b2 H2a2b3 H2a2b4 H2a2b5 H2a2b5a H2a5a1 H2a5a1a H2a5a1b H34 H64 (14)	1

595 >KU683142.1 Hc	152C 263G 750G 1438G 3106d 3107C 4769G 5480G 8860G 15326G 16209C 16261T	H107	152C 263G 16209C 16261T	1	152 263 750 1438 4769 5480 8860 15326 16209 16261	H107	152 263 16209 16261	[0.000] H107 (1)	[0.187] H+152 (2) [0.071] H13b1 (3) [0.011] H7a1c (4) [0.010] H7a1b (5) [0.007] H1+152 (6) [0.006] H52 (7) [0.000] H1i H1i1 H1i2 H1i2a H1an H1an1 H1an1a H1bb H1bc H2a2a2 H3+152 H3g H3g1 H3g1a H3g1b H3g2 H3g3 H3g4 H3i H3i1 H3j H3k H3k1 H3k1a H7a1 H7a1d H9 H46 H46a H46b H69 H13a1+152 H14b H14b2 H14b4 H16+152 H16a H16a1 H16c H16d H30b1 H47a R30a1b (50)	1
596 >KU683134.1 Hc	73G 152N 204C 248d 263G 514d 515d 516d 517d 750G 1438G 2706G 3106d 3107C 3970T 4732G 4769G 5147A 6392C 6962A 7028T 8772C 8860G 9180G 10310A 10609C 10976T 11719A 12406A 12633T 12882T 13928C 14476A 14766T 15326G 16172C 16183N 16189C 16232A 16249C 16304C 16311C	F1b1b	73G 152N 204C 248d 263G 514d 515d 516d 517d 16172C 16183N 16189C 16232A 16249C 16304C 16311C	1	73 152N 204 249d 263 750 1438 2706 3970 4732 4769 5147 6392 6962 7028 8772 8860 9180 10310 10609 10976 11719 12406 12633 12882 13928C 14476 14766 15326 16172 16183N 16189 16232A 16249 16304 16311	F1b1b	73 152N 204 249d 263 16172 16183N 16189 16232A 16249 16304 16311	[0.000] F1b1b (1)	[0.251] F1b1+@152 (2) [0.048] F1b F1b1 (4) [0.000] F1b1e F1b1e1 F1b1f (7)	1
597 >KU683121.1 Hc	73G 152C 235G 263G 298A 514d 515d 663G 750G 1438G 1736G 3106d 3107C 4248C 4769G 4824G 6023A 7028T 8794T 8860G 11557G 11719A 12705T 14766T 14929T 15326G 16223T 16290T 16292A 16319A 16362C	A16	73G 152C 235G 263G 298A 514d 515d 16223T 16290T 16292A 16319A 16362C	1	73 152 235 263 298A 663 750 1438 1736 4248 4769 4824 6023 7028 8794 8860 11557 11719 12705 14766 14929 15326 16223 16290 16292A 16319 16362	A16	73 152 235 263 298A 16223 16290 16292A 16319 16362	[0.000] A16 (1)	[0.000] A+152+16362 A1 A6 A6a A17 A18 A20 A21 A22 (10)	1
598 >KU683118.1 Hc	73G 146C 189G 199C 263G 310N 489C 514d 515d 709A 750G 1438G 2706G 3106d 3107C 3167.1C 4140T 4769G 7028T 7250G 8701G 8793C 8856A 8860G 9181G 9336G 9540C 10398G 10400T 10529G 10646A 10873C 11719A 12549T 12705T 13135A 13152G 14502C 14766T 14783C 15040T 15043A 15071C 15218G 15301A 15326G 16093C 16193T 16223T 16311C 16357C 16497G	M10a1a1b1	73G 146C 189G 199C 263G 310N 489C 514d 515d 16093C 16193T 16223T 16311C 16357C 16497G	1	73 146 189 199 263 310N 489 709 750 1438 2706 3172.1C 4140 4769 7028 7250 8701 8793 8856 8860 9181 9336 9540 10398 10400 10529 10646 10873 11719 12549 12705 13135 13152 14502 14766 14783 15040 15043 15071 15218 15301 15326 16093 16193 16223 16311 16357 16497	M10a1a1b1	73 146 189 199 263 310N 489 16093 16193 16223 16311 16357 16497	[0.487] M10a1a1b1 (1)	[0.278] M10a1a1b2 (2) [0.024] M10a1a1b	1
599 >KU683116.1 Hc	73G 248d 263G 514d 515d 750G 1438G 1896C 2706G 3106d 3107C 3970T 4086T 4769G 6392C 6962A 7028T 8860G 9053A 9548A 10310A 10609C 11719A 12406A 12882T 13759A 13928C 14766T 15326G 16129A 16162G 16172C 16303A 16304C 16497G 16519C	F1a1	73G 248d 263G 514d 515d 16129A 16162G 16172C 16303A 16304C 16497G 16519C	1	73 249d 263 750 1438 1896 2706 3970 4086 4769 6392 6962 7028 8860 9053 9548 10310 10609 11719 12406 12882 13759 13928C 14766 15326 16129 16162 16172 16303 16304 16497	F1a1	73 249d 263 16129 16162 16172 16303 16304 16497	[0.103] F1a1 (1)	[0.017] F1a1c3 (2) [0.005] F1a1'4 F1a1d1 (4) [0.003] F1a3 (5) [0.002] F1a1a (6) [0.000] F1a F1a1a1 F1a1b F1a1c F1a1c2 F1a1d F1a4 (13)	1

600 >KU683115.1 Ho	263G 750G 1438G 3106d 3107C 4769G 6323G 8860G 15326G 16287T 16291T 16362C 16390A 16391A 16519C	H85	263G 16287T 16291T 16362C 16390A 16391A 16519C	1	263 750 1438 4769 6323 8860 15326 16287 16291 16362 16390 16391	H85	263 16287 16291 16362 16390 16391	[0.072] H85 (1)	[0.040] H1bv1 (2) [0.012] H+16291 (3) [0.007] H2a2b (4) [0.000] H1j2 H2a2b2 H2a2b3 H2a2b4 H2a2b5 H2a2b5a H2a5a1 H2a5a1a H2a5a1b H13b H13b2 H34 H64 (17)	1
601 >KU683114.1 Ho	73G 150T 152C 263G 456T 489C 681C 750G 1048T 1107C 1438G 2706G 3106d 3107C 3759G 4769G 4883T 5147A 5153G 5178A 5301G 6253C 7028T 7403G 8701G 8860G 9180G 9540C 10397G 10398G 10400T 10873C 11719A 12705T 14766T 14783C 15043A 15301A 15326G 15724G 16183N 16189C 16223T 16357C 16362C 16519C	D5b1b2	73G 150T 152C 263G 456T 489C 16183N 16189C 16223T 16357C 16362C 16519C	1	73 150 152 263 456 489 681 750 1048 1107 1438 2706 3759 4769 4883 5147 5153 5178A 5301 6253 7028 7403 8701 8860 9180 9540 10397 10398 10400 10873 11719 12705 14766 14783 15043 15301 15326 15724 16183N 16189 16223 16357 16362	D5b1b2	73 150 152 263 456 489 16183N 16189 16223 16357 16362	[0.071] D5b1b2 (1)	[0.151] D5b1 (2) [0.007] D5b3 (3) [0.000] D5b D5b1a D5b1b D5b1c D5b3a (8)	1
602 >KU683113.1 Ho	73G 146C 189G 199C 263G 489C 514d 515d 709A 750G 1438G 2706G 3106d 3107C 3167.1C 4140T 4769G 7028T 7250G 8701G 8793C 8856A 8860G 9181G 9336G 9540C 10398G 10400T 10529G 10646A 10873C 11719A 12549T 12705T 13135A 13152G 14502C 14766T 14783C 15040T 15043A 15071C 15218G 15301A 15326G 16093C 16193T 16223T 16311C 16357C 16497G	M10a1a1b1	73G 146C 189G 199C 263G 489C 514d 515d 16093C 16193T 16223T 16311C 16357C 16497G	1	73 146 189 199 263 489 709 750 1438 2706 3172.1C 4140 4769 7028 7250 8701 8793 8856 8860 9181 9336 9540 10398 10400 10529 10646 10873 11719 12549 12705 13135 13152 14502 14766 14783 15040 15043 15071 15218 15301 15326 16093 16193 16223 16311 16357 16497	M10a1a1b1	73 146 189 199 263 489 16093 16193 16223 16311 16357 16497	[0.487] M10a1a1b1 (1)	[0.278] M10a1a1b2 (2) [0.024] M10a1a1b	1
603 >KU683105.1 Ho	73G 150T 152C 263G 750G 1438G 1811G 2706G 3106d 3107C 4188G 4640A 4769G 7028T 7496C 8382T 8860G 9656C 11467G 11719A 12308G 12372A 13743C 14139G 14745T 14766T 15326G 15454C 16157C 16168T 16343G 16390A	U3b3	73G 150T 152C 263G 16157C 16168T 16343G 16390A	1	73 150 152 263 750 1438 1811 2706 4188 4640A 4769 7028 7496 8382 8860 9656 11467 11719 12308 12372 13743 14139 14745 14766 15326 15454 16157 16168 16343 16390	U3b3	73 150 152 263 16157 16168 16343 16390	[0.349] U3b3 (1) [0.089] U3a1 (2) [0.021] U3a2 (3) [0.016] U3a (4) [0.000] U3a1a U3a1a1 U3a1b (7)	[0.345] U3b2 (8) [0.092] U3b (9) [0.048] U3a3 (10) [0.032] U3a2a (11) [0.028] U3b2a1 (12) [0.025] U3a1c (13) [0.010] U3b1b (14) [0.000] U3 U3a'c U3b1 U3b1a1 U3b2a U3b2c (20)	1
604 >KU683499.1 Ho	73G 146C 150T 151T 152C 182T 195C 217C 263G 489C 750G 1107C 1438G 2706G 3106d 3107C 3221G 3546T 4200T 4216C 4769G 4883T 5153G 5178A 5261A 5301G 6614C 7028T 8701G 8860G 9540C 10352G 10397G 10398G 10400T 10873C 11719A 12705T 13105G 14766T 14783C 14927G 15043A 15301A 15326G 15622C 16189.1C 16290T 16362C 16390A 16519C	D5c1a	73G 146C 150T 151T 152C 182T 195C 217C 263G 489C 16189.1C 16290T 16362C 16390A 16519C	1	73 146 150 151 152 182 195 217 263 489 750 1107 1438 2706 3221 3546 4200T 4216 4769 4883 5153 5178A 5261 5301 6614 7028 8701 8860 9540 10352 10397 10398 10400 10873 11719 12705 13105 14766 14783 14927 15043 15301 15326 15622 16290 16362 16390	D5c1a	73 146 150 151 152 182 195 217 263 489 16290 16362 16390	[0.833] D5c1a (1)	[1.000] D5c1 (2) [0.000] L2a'b'c'd L2b'c'd (4)	1

605	>KU683497.1 Hc	73G 195C 240G 263G 514d 515d 750G 813G 1438G 2706G 3106d 3107C 4769G 6674C 7028T 7975G 8618C 8860G 10289G 10908C 11581A 11719A 12634G 12705T 14311C 14431C 14502C 14766T 15326G 15514C 15900C 16145A 16189C 16223T 16355T 16519C	N11a1	73G 195C 240G 263G 514d 515d 16145A 16189C 16223T 16355T 16519C	1	73 195 240 263 750 813 1438 2706 4769 6674 7028 7975 8618 8860 10289 10908 11581A 11719 12634 12705 14311 14431 14502 14766 15326 15514 15900 16145 16189 16223 16355	N11a1	73 195 240 263 16145 16189 16223 16355	[1.022] N11a1 (1)	[0.000] N11a (2)	1
606	>KU683487.1 Hc	73G 235G 263G 310.1C 514d 515d 663G 750G 961C 1438G 1709A 1736G 2706G 3106d 3107C 3900N 4248C 4769G 4824G 7028T 8563G 8794T 8860G 9797C 11536T 11719A 12705T 14207C 14766T 15326G 16126C 16223T 16234T 16248T 16290T 16311C 16319A 16519C	A5b1b	73G 235G 263G 310.1C 514d 515d 16126C 16223T 16234T 16248T 16290T 16311C 16319A 16519C	1	73 235 263 663 750 961 1438 1709 1736 2706 3900N 4248 4769 4824 7028 8563 8794 8860 9797 11536 11719 12705 14207C 14766 15326 16126 16223 16234 16248 16290 16311 16319	A5b1b	73 235 263 16126 16223 16234 16248 16290 16311 16319	[0.702] A5b1b (1)	[0.417] A (2) [0.214] A5b1a (3) [0.038] A5b1c (4) [0.036] A5 (5) [0.034] A5b1 (6) [0.000] A5b A10 (8)	1
607	>KU683485.1 Hc	73G 150T 152C 263G 750G 1438G 1811G 2706G 3106d 3107C 4188G 4640A 4769G 7028T 8382T 8860G 9656C 11467G 11719A 12308G 12372A 13743C 14139G 14766T 15326G 15454C 16157C 16168T 16343G 16390A	U3b3	73G 150T 152C 263G 16157C 16168T 16343G 16390A	1	73 150 152 263 750 1438 1811 2706 4188 4640A 4769 7028 8382 8860 9656 11467 11719 12308 12372 13743 14139 14766 15326 15454 16157 16168 16343 16390	U3b3	73 150 152 263 16157 16168 16343 16390	[0.349] U3b3 (1) [0.089] U3a1 (2) [0.021] U3a2 (3) [0.016] U3a (4) [0.000] U3a1a U3a1a1 U3a1b (7)	[0.345] U3b2 (8) [0.092] U3b (9) [0.048] U3a3 (10) [0.032] U3a2a (11) [0.028] U3b2a1 (12) [0.025] U3a1c (13) [0.010] U3b1b (14) [0.000] U3 U3a'c U3b1 U3b1a1 U3b2a U3b2c (20)	1
608	>KU683481.1 Hc	152C 239C 263G 310N 750G 1438G 3106d 3107C 4769G 8860G 14040A 15326G 16186T 16300G 16325C 16362C 16482G	H6b2	152C 239C 263G 310N 16186T 16300G 16325C 16362C 16482G	1	152 239 263 310N 750 1438 4769 8860 14040 15326 16186 16300 16325 16362 16482	H6b2	152 239 263 310N 16186 16300 16325 16362 16482	[0.380] H6b2 (1)	[0.380] H6b2 (2) [0.125] H6b1 (3) [0.033] H6b	1
609	>KU683480.1 Hc	73G 235G 263G 310N 514d 515d 663G 750G 961C 1438G 1709A 1736G 2706G 3106d 3107C 4248C 4769G 4824G 7028T 8563G 8794T 8860G 9797C 11536N 11719A 12705T 14207C 14766T 15326G 16126C 16223T 16234T 16248T 16290T 16311C 16319A 16519C	A5b1b	73G 235G 263G 310N 514d 515d 16126C 16223T 16234T 16248T 16290T 16311C 16319A 16519C	1	73 235 263 310N 663 750 961 1438 1709 1736 2706 4248 4769 4824 7028 8563 8794 8860 9797 11536N 11719 12705 14207C 14766 15326 16126 16223 16234 16248 16290 16311 16319	A5b1b	73 235 263 310N 16126 16223 16234 16248 16290 16311 16319	[0.702] A5b1b (1)	[0.417] A (2) [0.214] A5b1a (3) [0.038] A5b1c (4) [0.036] A5 (5) [0.034] A5b1 (6) [0.000] A5b A10 (8)	1
610	>KU683477.1 Hc	263G 750G 1438G 3106d 3107C 4769G 6323G 8860G 15326G 16287T 16291T 16390A 16519C	H85	263G 16287T 16291T 16390A 16519C	1	263 750 1438 4769 6323 8860 15326 16287 16291 16390	H85	263 16287 16291 16390	[0.036] H85 (1)	[0.012] H+16291 (2) [0.007] H2a2b (3) [0.000] H1j2 H2a2b2 H2a2b3 H2a2b4 H2a2b5 H2a2b5a H2a5a1 H2a5a1a H2a5a1b H34 H64 (14)	1
611	>KU683472.1 Hc	263G 750G 1438G 3106d 3107C 4769G 8860G 11447A 15326G 16172C 16400T	H33b	263G 16172C 16400T	1	263 750 1438 4769 8860 11447 15326 16172 16400	H33b	263 16172 16400	[0.000] H33b (1)	[0.000] H66a (2)	1
612	>KU683468.1 Hc	263G 750G 951A 3106d 3107C 4312T 8860G 15326G 16193T 16354T	H2a1f	263G 16193T 16354T	1	263 750 951 4312 8860 15326 16193 16354	H2a1f	263 16193 16354	[0.000] H2a1f (1)	[0.000] H2a1 H2a1a H2a1a1 H2a1a2 H2a1b H2a1c H2a1d H2a1f1 H2a1f2 H2a1g H2a1k (12)	1

613 >KU683463.1 Hc	263G 750G 1438G 2706G 3106d 3107C 4769G 6755A 7028T 8772C 8860G 15326G 16172C 16189C 16311C	HV6	263G 16172C 16189C 16311C	1	263 750 1438 2706 4769 6755 7028 8772 8860 15326 16172 16189 16311	HV6	263 16172 16189 16311	[0.044] HV6 (1)	[0.020] H3h2 (2) [0.013] HV+16311 (3) [0.009] H3+16189 (4) [0.007] H1g H1aa (6) [0.003] H1+16189 H3h3b (8) [0.002] H1+16311 (9) [0.000] HV6a HV9 HV10 HV11 HV11a HV16 HV23 HV24 HV18 H1f H1g1 H1g2 H1y H1z1 H1aa1 H1ab H1ac H1ad H1e1a4 H1j9 H3+16311 H3h H3h1 H3h3 H3h3a H3h5 H3h6 H3m H13a1a2+16311 H13a2b1 H66a H76 H80 U6b1b (43)	1
614 >KU683456.1 Hc	72C 263G 750G 1438G 2706G 3106d 3107C 4025T 4580A 4769G 7028T 8860G 15326G 15904T	V+@16298	72C 263G	1	72 263 750 1438 2706 4025 4580 4769 7028 8860 15326 15904	V+@16298	72 263	[0.000] V+@16298 (1)	[0.005] V2 H2a2a (3) [0.003] H1as H13a2b2a (5) [0.002] H7 (6) [0.001] H H1 H2a H3 H3c1 H16 H23 H54 (14) [0.000] R0 HV HV0 HV0a V V1 V1a V1a1 V1a1b V2a V2a1 V2a1a V2b V2b1 V2b2 V2c V3 V3b V4 V8 V10 V10b V10b1 V10b2 V11 V12 V13 V14 V15 V15a V17 V20 V23 V24 V25 V26 V28 HV4 HV4c HV5 HV5a HV9c HV12 H1e H1e1 H1e1a H1e1a2 H1e1a7 H1e1c H1e2 H1e2a H1e2b H1e2d H1e4 H1e6 H1e7 H1e8 H1e8a H1h H1h1 H1h2 H1j H1j3 H1j5 H1j6 H1j7 H1j9 H1m H1n H1p H1q H1q1 H1q2 H1r H1s H1s1 H1t H1t1 H1t1a H1t2 H1u H1u1 H1v H1v1 H1v1a H1v1b H1w H1ae H1ae1 H1ae2 H1ae2a H1ae3 H1ae3a H1ag H1ag1 H1ag1b H1ah H1ai H1ai1 H1aj H1ak H1ak1 H1ak2 H1am H1am1 H1ap H1aq H1as2 H1at H1at1 H1at1a H1av H1ax H1ax1 H1ay H1az H1bd H1be H1bi H1bj H1bk H1bm H1bn H1bp H1bq H1br H1bu H1bv H1bw H1bx H1ca H1ci H2 H2a2 H2a2+(16235) H2a4 H2a5 H2a5a H2a5b H3b H3c H3e H3q H3q1 H3r H3r1 H3t H3u H3y H3aa H3ab H3ac H3ad [illegible]	1
615 >KU683454.1 Hc	73G 146C 207A 263G 310N 408A 499A 513.1CA 750G 827G 1438G 2706G 3106d 3107C 4435G 4769G 4820A 6023A 6413C 7028T 7389C 8271d 8272d 8273d 8274d 8275d 8276d 8277d 8278d 8279d 8473C 8860G 9055A 9338T 9615C 11719A 13590A 14133G 14766T 15326G 15535T 16086C 16136C 16183C 16189C 16217C 16519C	B4b1a3a	73G 146C 207A 263G 310N 408A 499A 513.1CA 16086C 16136C 16183C 16189C 16217C 16519C	1	73 146 207 263 310N 408A 499 750 827 1438 2706 4435 4769 4820 6023 6413 7028 7389 8281-8289d 8473 8860 9055 9338T 9615 11719 13590 14133 14766 15326 15535 16086 16136 16189 16217	B4b1a3a	73 146 207 263 310N 408A 499 16086 16136 16189 16217	[0.000] B4b1a3a (1)	[0.000] B4b1a3 (2)	1

616	>KU683453.1 Hc	73G 152C 185A 189G 195C 204C 263G 709A 750G 1243C 1406C 1438G 2706G 3106d 3107C 3505G 4769G 5046A 5291C 5460A 7028T 8251A 8784G 8860G 8994A 11674T 11719A 11947G 12414C 12705T 14766T 15326G 15784C 15884C 16209C 16223T 16255A 16292T 16381G 16519C	W3a2	73G 152C 185A 189G 195C 204C 263G 16209C 16223T 16255A 16292T 16381G 16519C	1	73 152 185 189 195 204 263 709 750 1243 1406 1438 2706 3505 4769 5046 5291 5460 7028 8251 8784 8860 8994 11674 11719 11947 12414 12705 14766 15326 15784 15884C 16209 16223 16255 16292 16381G	W3a2	73 152 185 189 195 204 263 16209 16223 16255 16292 16381G	[1.666] W3a2 (1)	[0.136] W (2) [0.135] W1f (3) [0.014] W1a (4) [0.012] W1 (5) [0.000] W1e W7 (7)	1
617	>KU683452.1 Hc	73G 146N 263G 489C 514d 515d 573.1N 709A 750G 1438G 2706G 3106d 3107C 3167.1C 4140T 4769G 5483C 7028T 7250N 8701G 8793C 8856A 8860G 9540C 10398G 10400T 10529G 10646A 10873C 11719A 12549T 12705T 13135A 13152G 14502C 14766T 14783C 15040T 15043A 15071C 15218G 15301A 15326G 16193T 16223T 16311C 16357C 16497G 16519C	M10a1a1b1	73G 146N 263G 489C 514d 515d 573.1N 16193T 16223T 16311C 16357C 16497G 16519C	1	73 146N 263 489 573.1N 709 750 1438 2706 3172.1C 4140 4769 5483 7028 7250N 8701 8793 8856 8860 9540 10398 10400 10529 10646 10873 11719 12549 12705 13135 13152 14502 14766 14783 15040 15043 15071 15218 15301 15326 16193 16223 16311 16357 16497	M10a1a1b1	73 146N 263 489 573.1N 16193 16223 16311 16357 16497	[0.333] M10a1a1b1 (1)	[0.537] M10a1a1b (2) [0.000] M10a1a1b2	1
618	>KU683450.1 Hc	73G 150T 152C 263G 456T 489C 681C 750G 1048T 1107C 1438G 2706G 3106d 3107C 3759G 4769G 4883T 5147A 5153G 5178A 5301G 6253C 7028T 7403G 8701G 8860G 9180G 9540C 10397G 10398G 10400T 10873C 11719A 12705T 14766T 14783C 15043A 15301A 15326G 15724G 16183N 16189C 16223T 16357C 16362C 16519C	D5b1b2	73G 150T 152C 263G 456T 489C 16183N 16189C 16223T 16357C 16362C 16519C	1	73 150 152 263 456 489 681 750 1048 1107 1438 2706 3759 4769 4883 5147 5153 5178A 5301 6253 7028 7403 8701 8860 9180 9540 10397 10398 10400 10873 11719 12705 14766 14783 15043 15301 15326 15724 16183N 16189 16223 16357 16362	D5b1b2	73 150 152 263 456 489 16183N 16189 16223 16357 16362	[0.071] D5b1b2 (1)	[0.151] D5b1 (2) [0.007] D5b3 (3) [0.000] D5b D5b1a D5b1b D5b1c D5b3a (8)	1
619	>KU683444.1 Hc	73G 143A 189G 195C 204C 207A 210G 263G 750G 1438G 1719A 2706G 3106d 3107C 4769G 5054A 6297C 7028T 8222C 8595T 8706G 8860G 10238C 11025C 11140T 11437C 11719A 11914A 12501A 12705T 13637G 13780G 14766T 15326G 16201T 16223T 16265G 16311C 16519C	N1a3a	73G 143A 189G 195C 204C 207A 210G 263G 16201T 16223T 16265G 16311C 16519C	1	73 143 189 195 204 207 210 263 750 1438 1719 2706 4769 5054 6297 7028 8222 8595 8706 8860 10238 11025 11140 11437 11719 11914 12501 12705 13637 13780 14766 15326 16201 16223 16265 16311	N1a3a	73 143 189 195 204 207 210 263 16201 16223 16265 16311	[0.140] N1a3a (1)	[0.000] N1a3 N1a3a2 (3)	1
620	>KU683442.1 Hc	41T 73G 263G 319C 709A 750G 930A 1438G 1888A 2706G 3106d 3107C 4216C 4769G 4917G 5147A 7028T 8697A 8860G 9861C 10463C 11251G 11719A 11812G 13368A 14233G 14766T 14905A 15326G 15452A 15607G 15928A 16126C 16227G 16294T 16304C 16519C	T2b34	41T 73G 263G 319C 16126C 16227G 16294T 16304C 16519C	1	41 73 263 319 709 750 930 1438 1888 2706 4216 4769 4917 5147 7028 8697 8860 9861 10463 11251 11719 11812 13368 14233 14766 14905 15326 15452A 15607 15928 16126 16227 16294 16304	T2b34	41 73 263 319 16126 16227 16294 16304	[0.009] T2b34 (1)	[0.004] T2b (2) [0.000] T2b1 T2b2 T2b3 T2b3b T2b4 T2b5 T2b5a T2b7 T2b7a T2b8 T2b13 T2b13b T2b15 T2b17 T2b17a T2b19 T2b25 T2b26 T2b27 T2b28 T2b29 T2b30 T2b31 T2b32 T2b35 T2b36 T2b37 (29)	1

621 >KU683437.1 Hc	73G 153G 263G 489C 750G 1041G 1438G 2706G 3106d 3107C 3394C 4491A 4769G 7028T 7256T 8701G 8860G 9540C 10398G 10400T 10873C 11204C 11719A 12705T 14308C 14766T 14783C 15043A 15301A 15326G 16145A 16148T 16223T 16234T 16316G	M9a1a2	73G 153G 263G 489C 16145A 16148T 16223T 16234T 16316G	1	73 153 263 489 750 1041 1438 2706 3394 4491 4769 7028 7256 8701 8860 9540 10398 10400 10873 11204 11719 12705 14308 14766 14783 15043 15301 15326 16145 16148 16223 16234 16316	M9a1a2	73 153 263 489 16145 16148 16223 16234 16316	[0.498] M9a1a2 (1)	[0.005] M9a1a (2) [0.000] M9a1a1 M9a1a1c1b M9a1a1c1b2 M9a1a1d (6)	1
622 >KU683434.1 Hc	152C 239C 263G 310N 750G 1438G 3106d 3107C 4769G 8860G 14040A 15326G 16186T 16300G 16325C 16362C 16482G	H6b2	152C 239C 263G 310N 16186T 16300G 16325C 16362C 16482G	1	152 239 263 310N 750 1438 4769 8860 14040 15326 16186 16300 16325 16362 16482	H6b2	152 239 263 310N 16186 16300 16325 16362 16482	[0.380] H6b2 (1)	[0.380] H6b2 (2) [0.125] H6b1 (3) [0.033] H6b	1
623 >KU683433.1 Hc	73G 152C 185A 189G 195C 204C 263G 709A 750G 1243C 1406C 1438G 2706G 3106d 3107C 3505G 4769G 5046A 5291C 5460A 7028T 8251A 8784G 8860G 8994A 11674T 11719A 11947G 12414C 12705T 14766T 15326G 15784C 15884C 16209C 16223T 16255A 16292T 16381G 16519C	W3a2	73G 152C 185A 189G 195C 204C 263G 16209C 16223T 16255A 16292T 16381G 16519C	1	73 152 185 189 195 204 263 709 750 1243 1406 1438 2706 3505 4769 5046 5291 5460 7028 8251 8784 8860 8994 11674 11719 11947 12414 12705 14766 15326 15784 15884C 16209 16223 16255 16292 16381G	W3a2	73 152 185 189 195 204 263 16209 16223 16255 16292 16381G	[1.666] W3a2 (1)	[0.136] W (2) [0.135] W1f (3) [0.014] W1a (4) [0.012] W1 (5) [0.000] W1e W7 (7)	1
624 >KU683430.1 Hc	73G 263G 489C 534T 750G 1438G 1462A 1958A 2706G 3010A 3106d 3107C 4769G 4883T 5178A 7028T 8383C 8414T 8701G 8860G 9431T 9540C 10398G 10400T 10873C 11719A 12705T 14668T 14766T 14783C 15043A 15301A 15326G 16223T 16245T 16362C	D4c2b	73G 263G 489C 534T 16223T 16245T 16362C	1	73 263 489 534 750 1438 1462 1958 2706 3010 4769 4883 5178A 7028 8383 8414 8701 8860 9431 9540 10398 10400 10873 11719 12705 14668 14766 14783 15043 15301 15326 16223 16245 16362	D4c2b	73 263 489 534 16223 16245 16362	[0.000] D4c2b (1)	[0.000] D4c D4c2 D4c2c (4)	1
625 >KU683424.1 Hc	73G 146C 263G 485C 489C 750G 1438G 2706G 3010A 3106d 3107C 4655A 4769G 4883T 5075C 5178A 7028T 8414T 8701G 8860G 9269T 9540C 10398G 10400T 10658G 10873C 11696A 11719A 12705T 14582G 14668T 14766T 14783C 15043A 15301A 15326G 16223T 16362C	D4j13	73G 146C 263G 485C 489C 16223T 16362C	1	73 146 263 485 489 750 1438 2706 3010 4655 4769 4883 5075 5178A 7028 8414 8701 8860 9269 9540 10398 10400 10658 10873 11696 11719 12705 14582 14668 14766 14783 15043 15301 15326 16223 16362	D4j13	73 146 263 485 489 16223 16362	[0.000] D4j13 (1)	[0.000] D4b2b1+146 D4b2b1d D4j+146 D4j6 (5)	1
626 >KU683423.1 Hc	73G 150T 241G 263G 750G 1095C 1391C 1438G 2706G 3106d 3107C 4769G 4936T 6960T 7028T 8860G 9894G 10160T 11151T 11719A 12245C 12720G 13167G 13359A 14239T 14766T 15326G 15940C 16172C 16224C 16311C 16390A 16519C	R1b	73G 150T 241G 263G 16172C 16224C 16311C 16390A 16519C	1	73 150 241 263 750 1095 1391 1438 2706 4769 4936 6960 7028 8860 9894 10160 11151 11719 12245 12720 13167 13359 14239 14766 15326 15940 16172 16224 16311 16390	R1b	73 150 241 263 16172 16224 16311 16390	[0.126] R1b (1)	[0.085] R1b1 (2) [0.000] K K1 K1a+150 K1a24 K1a30 K1a30a K1a31 K1b K1b1 K1b1+(16093) K1b1b K1b1b1 (14)	1
627 >KU683413.1 Hc	73G 152C 235G 263G 298A 514d 515d 663G 750G 1438G 1736G 3106d 3107C 4248C 4769G 4824G 6023A 7028T 8794T 8860G 11557G 11719A 12705T 14766T 14929T 15326G 16223T 16290T 16292A 16319A 16362C	A16	73G 152C 235G 263G 298A 514d 515d 16223T 16290T 16292A 16319A 16362C	1	73 152 235 263 298A 663 750 1438 1736 4248 4769 4824 6023 7028 8794 8860 11557 11719 12705 14766 14929 15326 16223 16290 16292A 16319 16362	A16	73 152 235 263 298A 16223 16290 16292A 16319 16362	[0.000] A16 (1)	[0.000] A+152+16362 A1 A6 A6a A17 A18 A20 A21 A22 (10)	1

628 >KU683412.1 Hc	73G 150T 263G 310N 489C 750G 1438G 2626C 2706G 2772T 3106d 3107C 4386C 4769G 4958G 5894.1C 6455T 7028T 8701G 8860G 9540C 9824C 10398G 10400T 10873C 11017C 11084G 11719A 12705T 12771A 14364A 14581C 14766T 14783C 15043A 15301A 15326G 16093C 16209C 16223T 16291T 16324C	M7a1a7	73G 150T 263G 310N 489C 16093C 16209C 16223T 16291T 16324C	1	73 150 263 310N 489 750 1438 2626 2706 2772 4386 4769 4958 5899.1C 6455 7028 8701 8860 9540 9824 10398 10400 10873 11017 11084 11719 12705 12771 14364 14581 14766 14783 15043 15301 15326 16093 16209 16223 16291 16324	M7a1a7	73 150 263 310N 489 16093 16209 16223 16291 16324	[0.044] M7a1a7 (1)	[0.010] M7a+16324 M7a1 M7a1a1 M7a1a1a M7a1a3 M7a1a4 M7a1a4a M7a1a5 M7a1a5a M7a1a6 M7a1a9 M7a1b (13) [0.009] M7a1a	1
629 >KU683409.1 Hc	73G 150T 263G 310N 489C 750G 1438G 2626C 2706G 2772T 3106d 3107C 4386C 4769G 4958G 5894.1C 6455T 7028T 8701G 8860G 9540C 9824C 10398G 10400T 10873C 11017C 11084G 11719A 12705T 12771A 14364A 14581C 14766T 14783C 15043A 15301A 15326G 16093C 16209C 16223T 16291T 16324C	M7a1a7	73G 150T 263G 310N 489C 16093C 16209C 16223T 16291T 16324C	1	73 150 263 310N 489 750 1438 2626 2706 2772 4386 4769 4958 5899.1C 6455 7028 8701 8860 9540 9824 10398 10400 10873 11017 11084 11719 12705 12771 14364 14581 14766 14783 15043 15301 15326 16093 16209 16223 16291 16324	M7a1a7	73 150 263 310N 489 16093 16209 16223 16291 16324	[0.044] M7a1a7 (1)	[0.010] M7a+16324 M7a1 M7a1a1 M7a1a1a M7a1a3 M7a1a4 M7a1a4a M7a1a5 M7a1a5a M7a1a6 M7a1a9 M7a1b (13) [0.009] M7a1a	1
630 >KU683403.1 Hc	73G 94A 150T 263G 310N 456T 489C 681C 750G 1048T 1107C 1438G 2706G 3106d 3107C 3759G 4769G 4883T 5147A 5153G 5178A 5206G 5301G 6253C 7028T 7403G 8701G 8860G 9180G 9540C 10397G 10398G 10400T 10873C 11719A 12705T 14766T 14783C 15043A 15301A 15326G 15724G 16183C 16189C 16223T 16357C 16362C 16519C	D5b1b2	73G 94A 150T 263G 310N 456T 489C 16183C 16189C 16223T 16357C 16362C 16519C	1	73 94 150 263 310N 456 489 681 750 1048 1107 1438 2706 3759 4769 4883 5147 5153 5178A 5206G 5301 6253 7028 7403 8701 8860 9180 9540 10397 10398 10400 10873 11719 12705 14766 14783 15043 15301 15326 15724 16189 16223 16357 16362	D5b1b2	73 94 150 263 310N 456 489 16189 16223 16357 16362	[0.054] D5b1b2 (1)	[0.007] D5b D5b1a D5b1b D5b1c D5b3 D5b3a (7) [0.000] D5b1	1
631 >KU683392.1 Hc	73G 195C 240G 263G 310N 514d 515d 750G 813G 1438G 2706G 3106d 3107C 4769G 6674C 7028T 7975G 8618C 8860G 10289G 10908C 11581A 11719A 12634G 12705T 14311C 14431C 14502C 14766T 15326G 15514C 15900C 16145A 16189C 16223T 16355T 16519C	N11a1	73G 195C 240G 263G 310N 514d 515d 16145A 16189C 16223T 16355T 16519C	1	73 195 240 263 310N 750 813 1438 2706 4769 6674 7028 7975 8618 8860 10289 10908 11581A 11719 12634 12705 14311 14431 14502 14766 15326 15514 15900 16145 16189 16223 16355	N11a1	73 195 240 263 310N 16145 16189 16223 16355	[1.022] N11a1 (1)	[0.000] N11a (2)	1
632 >KU683383.1 Hc	73G 152C 263G 417A 489C 750G 1438G 2706G 3106d 3107C 3644C 4769G 5295T 5773A 6023A 6253C 6620C 7028T 8701G 8860G 9540C 10181T 10398G 10400T 10411G 10790C 10873C 11719A 12297C 12705T 13135A 13590A 14766T 14783C 15043A 15301A 15326G 15924G 16145A 16188T 16189C 16223T 16381C	M13a1	73G 152C 263G 417A 489C 16145A 16188T 16189C 16223T 16381C	1	73 152 263 417 489 750 1438 2706 3644 4769 5295 5773 6023 6253 6620 7028 8701 8860 9540 10181 10398 10400 10411 10790 10873 11719 12297 12705 13135 13590 14766 14783 15043 15301 15326 15924 16145 16188 16189 16223 16381	M13a1	73 152 263 417 489 16145 16188 16189 16223 16381	[0.023] M13a1 (1)	[0.000] M13a M13a1a M13a1b M13a1b1 (5)	1

633 >KU683380.1 H[illegible]	73G 151T 152C 200G 235G 263G 310N 514d 515d 663G 735G 750G 1438G 1736G 2706G 3106d 3107C 4248C 4769G 4824G 7028T 8794T 8860G 11719A 12705T 14766T 15326G 16223T 16290T 16319A 16362C 16365T	A14	73G 151T 152C 200G 235G 263G 310N 514d 515d 16223T 16290T 16319A 16362C 16365T	1	73 151 152 200 235 263 310N 663 735 750 1438 1736 2706 4248 4769 4824 7028 8794 8860 11719 12705 14766 15326 16223 16290 16319 16362 16365	A14	73 151 152 200 235 263 310N 16223 16290 16319 16362 16365	[0.006] A14 (1)	[0.009] A+152+16362+200 (2) [0.005] A13	1
634 >KU683379.1 H[illegible]	73G 200G 263G 489C 750G 1438G 2706G 3010A 3106d 3107C 4769G 4883T 5178A 7028T 8414T 8701G 8794T 8860G 9540C 10398G 10400T 10873C 11719A 12705T 13899C 14668T 14766T 14783C 14827T 15043A 15301A 15326G 16223T 16256T 16311C 16362C 16519C	D4q	73G 200G 263G 489C 16223T 16256T 16311C 16362C 16519C	1	73 200 263 489 750 1438 2706 3010 4769 4883 5178A 7028 8414 8701 8794 8860 9540 10398 10400 10873 11719 12705 13899 14668 14766 14783 14827 15043 15301 15326 16223 16256 16311 16362	D4q	73 200 263 489 16223 16256 16311 16362	[0.000] D4q (1)	[0.000] D4q1 D4q1a (3)	1
635 >KU683375.1 H[illegible]	73G 103A 263G 310N 481T 514d 515d 709A 750G 1438G 1598A 2361A 2706G 3106d 3107C 4161T 4769G 4895G 7028T 8271d 8272d 8273d 8274d 8275d 8276d 8277d 8278d 8279d 8584A 8829T 8860G 9950C 10398G 10427A 11101G 11539T 11719A 12361G 12451G 14766T 15223T 15326G 15508T 15662G 15850C 15851G 15927A 16051G 16111T 16140C 16183C 16189C 16234T 16243C 16463G 16519C	B5b2c1	73G 103A 263G 310N 481T 514d 515d 16051G 16111T 16140C 16183C 16189C 16234T 16243C 16463G 16519C	1	73 103 263 310N 481 709 750 1438 1598 2361 2706 4161 4769 4895 7028 8281-8289d 8584 8829 8860 9950 10398 10427 11101 11539 11719 12361 12451 14766 15223 15326 15508 15662 15850 15851 15927 16051 16111 16140 16189 16234 16243 16463	B5b2c1	73 103 263 310N 481 16051 16111 16140 16189 16234 16243 16463	[0.130] B5b2c1 (1)	[0.167] B5b2c (2)	1
636 >MG429045.1 H[illegible]	73G 195C 263G 303N 304N 305N 306N 307N 308N 309N 310N 311N 312N 313N 314N 315N 515N 516N 517N 518N 519N 520N 521N 522N 750G 1438G 2706G 3197C 4769G 7028T 8860G 9477A 11467G 11719A 12308G 12372A 13617C 13802T 14766T 14793G 15218G 15326G 16256T 16270T 16320T 16399G 16519N	U5a1c1	73G 195C 263G 303N 304N 305N 306N 307N 308N 309N 310N 311N 312N 313N 314N 315N 515N 516N 517N 518N 519N 520N 521N 522N 16256T 16270T 16320T 16399G 16519N	1	73 195 263 303N 304N 305N 306N 307N 308N 309N 310N 311N 312N 313N 314N 315N 515N 516N 517N 518N 519N 520N 521N 522N 750 1438 2706 3197 4769 7028 8860 9477 11467 11719 12308 12372 13617 13802 14766 14793 15218 15326 16256 16270 16320 16399 16519N	U5a1c1	73 195 263 303N 304N 305N 306N 307N 308N 309N 310N 311N 312N 313N 314N 315N 515N 516N 517N 518N 519N 520N 521N 522N 16256 16270 16320 16399 16519N	[0.182] U5a1c1 (1)	[0.333] U5a1c1a (2) [0.041] U5a1a1 (3) [0.010] U5a1+@16192 (4) [0.009] U5a1a1c (5) [0.000] U5a1a U5a1a1e U5a1a1g U5a1a1i U5a1g U5a1c U5a1c2 (12)	1
637 >MG429042.1 H[illegible]	263G 303N 304N 305N 306N 307N 308N 309N 310N 311N 312N 313N 314N 315N 515N 516N 517N 518N 519N 520N 521N 522N 750G 1438G 3010A 4769G 8251A 8860G 15326G 16080G 16189C 16356C 16519N	H1b2	263G 303N 304N 305N 306N 307N 308N 309N 310N 311N 312N 313N 314N 315N 515N 516N 517N 518N 519N 520N 521N 522N 16080G 16189C 16356C 16519N	1	263 303N 304N 305N 306N 307N 308N 309N 310N 311N 312N 313N 314N 315N 515N 516N 517N 518N 519N 520N 521N 522N 750 1438 3010 4769 8251 8860 15326 16080 16189 16356 16519N	H1b2	263 303N 304N 305N 306N 307N 308N 309N 310N 311N 312N 313N 314N 315N 515N 516N 517N 518N 519N 520N 521N 522N 16080 16189 16356 16519N	[0.000] H1b2 (1)	[0.000] H1b H1b1 H1b1e H1b1e1 H1b1i H1b4 H1b5 (8)	1

638 >MG429000.1 H	73G 151T 263G 303N 304N 305N 306N 307N 308N 309N 310N 311N 312N 313N 314N 315N 515N 516N 517N 518N 519N 520N 521N 522N 750G 1438G 2706G 3197C 3768G 4769G 7028T 8860G 9477A 11467G 11719A 12308G 12372A 13617C 14766T 14793G 15326G 16192T 16256T 16270T 16311C 16362C 16519N 16526A	U5a2e	73G 151T 263G 303N 304N 305N 306N 307N 308N 309N 310N 311N 312N 313N 314N 315N 515N 516N 517N 518N 519N 520N 521N 522N 16192T 16256T 16270T 16311C 16362C 16519N 16526A	1	73 151 263 303N 304N 305N 306N 307N 308N 309N 310N 311N 312N 313N 314N 315N 515N 516N 517N 518N 519N 520N 521N 522N 750 1438 2706 3197 3768 4769 7028 8860 9477 11467 11719 12308 12372 13617 14766 14793 15326 16192 16256 16270 16311 16362 16519N 16526	U5a2e	73 151 263 303N 304N 305N 306N 307N 308N 309N 310N 311N 312N 313N 314N 315N 515N 516N 517N 518N 519N 520N 521N 522N 16192 16256 16270 16311 16362 16519N 16526	[0.031] U5a2e (1)	[0.000] U5a2+16362 (2)	1
639 >MG428995.1 H	54T 73G 146C 150T 241G 263G 303N 304N 305N 306N 307N 308N 309N 310N 311N 312N 313N 314N 315N 515N 516N 517N 518N 519N 520N 521N 522N 750G 1095C 1391C 1438G 2706G 4769G 7028T 8860G 10492N 11151T 11654G 11719A 12245C 13167G 13359A 14239T 14766T 15326G 15422G 15940C 16235G 16311C 16362C 16519N	R1b	54T 73G 146C 150T 241G 263G 303N 304N 305N 306N 307N 308N 309N 310N 311N 312N 313N 314N 315N 515N 516N 517N 518N 519N 520N 521N 522N 16235G 16311C 16362C 16519N	1	54T 73 146 150 241 263 303N 304N 305N 306N 307N 308N 309N 310N 311N 312N 313N 314N 315N 515N 516N 517N 518N 519N 520N 521N 522N 750 1095 1391 1438 2706 4769 7028 8860 10492N 11151 11654 11719 12245 13167 13359 14239 14766 15326 15422 15940 16235 16311 16362 16519N	R1b	54T 73 146 150 241 263 303N 304N 305N 306N 307N 308N 309N 310N 311N 312N 313N 314N 315N 515N 516N 517N 518N 519N 520N 521N 522N 16235 16311 16362 16519N	[0.036] R1b (1)	[0.132] R1b1 (2) [0.000] P6	1
640 >MG812519.1 H	73G 152C 246C 263G 309.1CT 310C 567.1CCCC 750G 1438G 2706G 3106d 3107C 4580A 4769G 5147A 6491T 7028T 7193C 8860G 9336G 9804A 11935C 12061T 15326G 16140C 16214T 16217C 16335G 16519C	HV2a1	73G 152C 246C 263G 309.1CT 310C 567.1CCCC 16140C 16214T 16217C 16335G 16519C	1	73 152 246 263 573.4C 750 1438 2706 4580 4769 5147 6491 7028 7193 8860 9336 9804 11935 12061 15326 16140 16214 16217 16335	HV2a1	73 152 246 263 573.4C 16140 16214 16217 16335	[1.000] HV2a1 (1)	[1.000] HV2a1 (2) [0.000] HV2	1
641 >MG817370.1 H	263G 309.1CT 310C 750G 1438G 3010A 3106d 3107C 4769G 8251A 8860G 11152C 15326G 16080G 16189C 16356C	H1b2	263G 309.1CT 310C 16080G 16189C 16356C	1	263 750 1438 3010 4769 8251 8860 11152 15326 16080 16189 16356	H1b2	263 16080 16189 16356	[0.000] H1b2 (1)	[0.000] H1b H1b1 H1b1e H1b1e1 H1b1i H1b4 H1b5 (8)	1
642 >MG807321.1 H	73G 199C 204C 250C 263G 310.1C 567.1CC 750G 1719A 2706G 3106d 3107C 4529T 4695C 4769G 7028T 8251A 8860G 9007G 10034C 10238C 10398G 11719A 12501A 12705T 13780G 14233G 14574T 14766T 15043A 15326G 15924G 16075C 16129A 16169T 16223T 16260T 16298C 16391A 16519C	I5c1	73G 199C 204C 250C 263G 310.1C 567.1CC 16075C 16129A 16169T 16223T 16260T 16298C 16391A 16519C	1	73 199 204 250 263 573.2C 750 1719 2706 4529T 4695 4769 7028 8251 8860 9007 10034 10238 10398 11719 12501 12705 13780 14233 14574 14766 15043 15326 15924 16075 16129 16169 16223 16260 16298 16391	I5c1	73 199 204 250 263 573.2C 16075 16129 16169 16223 16260 16298 16391	[0.059] I5c1 (1)	[0.000] I5c (2)	1
643 >MG767173.1 H	72C 263G 309.1CT 310C 750G 1438G 2706G 3106d 3107C 4580A 4769G 7028T 8860G 9368G 10586R 15326G 15904T 16261T 16298C 16311C 16519C	V10a	72C 263G 309.1CT 310C 16261T 16298C 16311C 16519C	1	72 263 750 1438 2706 4580 4769 7028 8860 9368 10586R 15326 15904 16261 16298 16311	V10a	72 263 16261 16298 16311	[0.000] V10a (1)	[0.117] V2c V13 (3) [0.050] V (4) [0.014] V1a (5) [0.005] V2 V10b1 V12 (8) [0.004] V1a1 (9) [0.000] HV0 HV0a V1 V1a1b V2a V2a1 V2a1a V2b V2b1 V2b2 V3 V3b V4 V8 V10 V10b V10b2 V11 V14 V15 V15a V17 V23 V24 V25 V26 V28 HV0e H13a1a1d H13a1a1d1 (39)	1

644 >MG787294.1 H	73G 152C 199C 204C 207A 250C 263G 310.1C 335G 451.1T 460C 567.1CC 750G 1438G 1719A 2706G 3106d 3107C 4529T 4712T 4769G 7028T 8251A 8860G 9438A 10034C 10238C 10398G 11719A 12501A 12705T 13780G 14766T 15043A 15326G 15758G 15924G 16129A 16218T 16223T 16263C 16391A 16519C	I2c	73G 152C 199C 204C 207A 250C 263G 310.1C 335G 451.1T 460C 567.1CC 16129A 16218T 16223T 16263C 16391A 16519C	1	73 152 199 204 207 250 263 335 455.1T 460 573.2C 750 1438 1719 2706 4529T 4712 4769 7028 8251 8860 9438 10034 10238 10398 11719 12501 12705 13780 14766 15043 15326 15758 15924 16129 16218 16223 16263 16391	I2c	73 152 199 204 207 250 263 335 455.1T 460 573.2C 16129 16218 16223 16263 16391	[0.326] I2c (1)	[0.008] I2 (2) [0.000] I2'3 I2d I2e I2f (6)	1
645 >MF381579.1 H	73G 150T 152C 189G 195C 200G 207A 263G 309.1CT 310C 750G 1438G 2352C 2706G 3106d 3107C 4769G 5774A 6221C 6587T 7028T 8701G 9254G 9533T 9540C 10398G 10819G 10873C 11024T 11719A 12705T 14152G 14212C 14569A 14766T 15301A 15326G 15670C 15942C 16185T 16209C 16223T 16327T	L3e1a2	73G 150T 152C 189G 195C 200G 207A 263G 309.1CT 310C 16185T 16209C 16223T 16327T	1	73 150 152 189 195 200 207 263 750 1438 2352 2706 4769 5774A 6221 6587 7028 8701 9254 9533 9540 10398 10819 10873 11024 11719 12705 14152 14212 14569 14766 15301 15326 15670 15942 16185 16209 16223 16327	L3e1a2	73 150 152 189 195 200 207 263 16185 16209 16223 16327	[0.000] L3e1a2 (1)	[0.000] L3e1f2 (2)	1
646 >MF381564.1 H	73G 146C 152C 189G 195C 198T 204C 207A 247A 514d 515d 750G 769A 825A 850C 1018A 1048T 1243C 1438G 2706G 2758A 2836A 2885C 3106d 3107C 3516A 3594T 4104G 4312T 4541A 4586C 4769G 4907C 5442C 5811G 6185C 6938T 7028T 7146G 7256T 7257G 7521A 8251A 8468T 8655T 8701G 8860G 8911C 8994A 9042T 9136G 9347G 9540C 9818T 10398G 10499G 10589A 10664T 10688A 10810C 10873C 10876G 10915C 10920T 10939T 11296T 11299C 11653G 11719A 11914A 12007A 12070A 12705T 13020C 13105G 13276G 13506T 13590A 13650T 13819C 13928C 14020C 14182C 14371C 14374C 14766T 15326G 16166C 16172C 16189C 16209C 16214T 16223T 16230G 16278T 16291G 16311C 16519C	L0k1a1a	73G 146C 152C 189G 195C 198T 204C 207A 247A 514d 515d 16166C 16172C 16189C 16209C 16214T 16223T 16230G 16278T 16291G 16311C 16519C	1	73 146 152 189 195 198 204 207 247 750 769 825A 850 1018 1048 1243 1438 2706 2758 2836A 2885 3516A 3594 4104 4312 4541 4586 4769 4907 5442 5811 6185 6938 7028 7146 7256 7257 7521 8251 8468 8655 8701 8860 8911 8994 9042 9136 9347 9540 9818 10398 10499 10589 10664 10688 10810 10873 10876 10915 10920 10939 11296 11299 11653 11719 11914 12007 12070 12705 13020 13105 13276 13506 13590 13650 13819 13928C 14020 14182 14371 14374 14766 15326 16166C 16172 16189 16209 16214 16223 16230 16278 16291G 16311	L0k1a1a	73 146 152 189 195 198 204 207 247 16166C 16172 16189 16209 16214 16223 16230 16278 16291G 16311	[0.375] L0k1a1a (1)	[0.025] L0k1a1 (2) [0.000] L0k1a1b L0k1a1c L0k1a1d (5)	1

647 >MF381562.1 Hc	73G 146C 152C 189G 195C 198T 204C 207A 247A 309.1CT 310C 514d 515d 750G 769A 825A 850C 1018A 1048T 1243C 1438G 2706G 2758A 2836A 2885C 3106d 3107C 3516A 3594T 4104G 4312T 4541A 4586C 4769G 4907C 5442C 5811G 6185C 6938T 7028T 7146G 7256T 7257G 7521A 8251A 8468T 8655T 8701G 8860G 8911C 8994A 9042T 9136G 9347G 9540C 9818T 10398G 10499G 10589A 10664T 10688A 10810C 10873C 10876G 10915C 10920T 10939T 11296T 11299C 11653G 11719A 11914A 12007A 12070A 12705T 13020C 13105G 13276G 13506T 13590A 13650T 13819C 13928C 14020C 14182C 14371C 14374C 14766T 15326G 16166C 16172C 16189C 16209C 16214T 16223T 16230G 16278T 16291G 16311C 16519C	L0k1a1a	73G 146C 152C 189G 195C 198T 204C 207A 247A 309.1CT 310C 514d 515d 16166C 16172C 16189C 16209C 16214T 16223T 16230G 16278T 16291G 16311C 16519C	1	73 146 152 189 195 198 204 207 247 750 769 825A 850 1018 1048 1243 1438 2706 2758 2836A 2885 3516A 3594 4104 4312 4541 4586 4769 4907 5442 5811 6185 6938 7028 7146 7256 7257 7521 8251 8468 8655 8701 8860 8911 8994 9042 9136 9347 9540 9818 10398 10499 10589 10664 10688 10810 10873 10876 10915 10920 10939 11296 11299 11653 11719 11914 12007 12070 12705 13020 13105 13276 13506 13590 13650 13819 13928C 14020 14182 14371 14374 14766 15326 16166C 16172 16189 16209 16214 16223 16230 16278 16291G 16311	L0k1a1a	73 146 152 189 195 198 204 207 247 16166C 16172 16189 16209 16214 16223 16230 16278 16291G 16311	[0.375] L0k1a1a (1)	[0.025] L0k1a1 (2) [0.000] L0k1a1b L0k1a1c L0k1a1d (5)	1
648 >MF381539.1 Hc	73G 146C 152C 189G 195C 198T 204C 207A 247A 309.1CCT 310C 514d 515d 750G 769A 825A 850C 1018A 1048T 1243C 1438G 2706G 2758A 2836A 2885C 3106d 3107C 3516A 3594T 4104G 4312T 4541A 4586C 4769G 4907C 5442C 5811G 6185C 6938T 7028T 7146G 7256T 7257G 7521A 8251A 8468T 8655T 8701G 8860G 8911C 8994A 9042T 9136G 9347G 9540C 9818T 10398G 10499G 10589A 10664T 10688A 10810C 10873C 10876G 10915C 10920T 10939T 11296T 11299C 11653G 11719A 11914A 12007A 12070A 12705T 13020C 13105G 13276G 13506T 13590A 13650T 13819C 13928C 14020C 14182C 14371C 14374C 14766T 15326G 16166C 16172C 16189C 16209C 16214T 16223T 16230G 16278T 16291G 16311C 16519C	L0k1a1a	73G 146C 152C 189G 195C 198T 204C 207A 247A 309.1CCT 310C 514d 515d 16166C 16172C 16189C 16209C 16214T 16223T 16230G 16278T 16291G 16311C 16519C	1	73 146 152 189 195 198 204 207 247 750 769 825A 850 1018 1048 1243 1438 2706 2758 2836A 2885 3516A 3594 4104 4312 4541 4586 4769 4907 5442 5811 6185 6938 7028 7146 7256 7257 7521 8251 8468 8655 8701 8860 8911 8994 9042 9136 9347 9540 9818 10398 10499 10589 10664 10688 10810 10873 10876 10915 10920 10939 11296 11299 11653 11719 11914 12007 12070 12705 13020 13105 13276 13506 13590 13650 13819 13928C 14020 14182 14371 14374 14766 15326 16166C 16172 16189 16209 16214 16223 16230 16278 16291G 16311	L0k1a1a	73 146 152 189 195 198 204 207 247 16166C 16172 16189 16209 16214 16223 16230 16278 16291G 16311	[0.375] L0k1a1a (1)	[0.025] L0k1a1 (2) [0.000] L0k1a1b L0k1a1c L0k1a1d (5)	1

649	>MF381536.1 Hc	73G 150T 152C 189G 195C 200G 207A 263G 309.1CT 310C 750G 1438G 2352C 2706G 3106d 3107C 4769G 5774A 6221C 6587T 7028T 8701G 8860G 9254G 9540C 10398G 10819G 10873C 11024T 11719A 12705T 14152G 14212C 14569A 14766T 15301A 15326G 15670C 15942C 16185T 16209C 16223T 16327T	L3e1a2	73G 150T 152C 189G 195C 200G 207A 263G 309.1CT 310C 16185T 16209C 16223T 16327T	1	73 150 152 189 195 200 207 263 750 1438 2352 2706 4769 5774A 6221 6587 7028 8701 8860 9254 9540 10398 10819 10873 11024 11719 12705 14152 14212 14569 14766 15301 15326 15670 15942 16185 16209 16223 16327	L3e1a2	73 150 152 189 195 200 207 263 16185 16209 16223 16327	[0.000] L3e1a2 (1)	[0.000] L3e1f2 (2)	1
650	>MF381532.1 Hc	73G 146C 152C 188G 195C 204C 207A 247A 494d 514d 515d 719A 750G 769A 825A 1018A 1048T 2758A 2885C 3106d 3107C 3438A 3516A 3594T 3618C 3756G 4104G 4232C 4312T 4562C 4769G 5442C 6185C 6266G 6815C 7028T 7146G 7256T 7521A 7705C 8113A 8152A 8251A 8468T 8655T 8701G 8860G 9042T 9347G 9540C 9755A 10589A 10664T 10688A 10810C 10873C 10915C 11341G 11719A 11914A 12007A 12121C 12705T 12720G 13105G 13276G 13506T 13650T 13759A 14766T 15326G 15355A 15466A 15930A 15941C 16140C 16179T 16187T 16189C 16223T 16230G 16243C 16293G 16311C 16519C	L0d1b1b1	73G 146C 152C 188G 195C 204C 207A 247A 494d 514d 515d 16140C 16179T 16187T 16189C 16223T 16230G 16243C 16293G 16311C 16519C	1	73 146 152 188 195 204 207 247 498d 719 750 769 825A 1018 1048 2758 2885 3438 3516A 3594 3618 3756 4104 4232 4312 4562C 4769 5442 6185 6266 6815 7028 7146 7256 7521 7705 8113A 8152 8251 8468 8655 8701 8860 9042 9347 9540 9755 10589 10664 10688 10810 10873 10915 11341 11719 11914 12007 12121 12705 12720 13105 13276 13506 13650 13759 14766 15326 15355 15466 15930 15941 16140 16179 16187 16189 16223 16230 16243 16293 16311	L0d1b1b1	73 146 152 188 195 204 207 247 498d 16140 16179 16187 16189 16223 16230 16243 16293 16311	[0.853] L0d1b1b1 (1)	[0.000] L0d1b1 L0d1b1a L0d1b1a1 L0d1b1b (5)	1
651	>MF381531.1 Hc	73G 146C 152C 188G 195C 204C 207A 247A 494d 514d 515d 719A 750G 769A 825A 1018A 1048T 2758A 2885C 3106d 3107C 3438A 3516A 3594T 3618C 3756G 4104G 4232C 4312T 4562C 4769G 5442C 6185C 6266G 6815C 7028T 7146G 7256T 7521A 8113A 8152A 8251A 8468T 8655T 8701G 8860G 9042T 9347G 9540C 9755A 10589A 10664T 10688A 10810C 10873C 10915C 11341G 11719A 11914A 12007A 12121C 12705T 12720G 13105G 13276G 13506T 13650T 13676G 13759A 14766T 15326G 15466A 15930A 15941C 16140C 16179T 16187T 16189C 16223T 16230G 16243C 16293G 16311C 16519C	L0d1b1b1	73G 146C 152C 188G 195C 204C 207A 247A 494d 514d 515d 16140C 16179T 16187T 16189C 16223T 16230G 16243C 16293G 16311C 16519C	1	73 146 152 188 195 204 207 247 498d 719 750 769 825A 1018 1048 2758 2885 3438 3516A 3594 3618 3756 4104 4232 4312 4562C 4769 5442 6185 6266 6815 7028 7146 7256 7521 8113A 8152 8251 8468 8655 8701 8860 9042 9347 9540 9755 10589 10664 10688 10810 10873 10915 11341 11719 11914 12007 12121 12705 12720 13105 13276 13506 13650 13676 13759 14766 15326 15466 15930 15941 16140 16179 16187 16189 16223 16230 16243 16293 16311	L0d1b1b1	73 146 152 188 195 204 207 247 498d 16140 16179 16187 16189 16223 16230 16243 16293 16311	[0.853] L0d1b1b1 (1)	[0.000] L0d1b1 L0d1b1a L0d1b1a1 L0d1b1b (5)	1

652	>MF381529.1 Hc	73G 146C 152C 188G 195C 204C 207A 247A 494d 514d 515d 719A 750G 769A 825A 1018A 1048T 2758A 2885C 3106d 3107C 3438A 3516A 3594T 3618C 3756G 4104G 4232C 4312T 4562C 4769G 5442C 6185C 6266G 6815C 7028T 7146G 7256T 7521A 8113A 8152A 8251A 8468T 8655T 8701G 8860G 9042T 9347G 9540C 9755A 10589A 10664T 10688A 10810C 10873C 10915C 11341G 11719A 11914A 12007A 12121C 12705T 12720G 13105G 13276G 13506T 13650T 13759A 14766T 15326G 15466A 15930A 15941C 16140C 16179T 16187T 16189C 16223T 16230G 16243C 16293G 16311C 16519C	L0d1b1b1	73G 146C 152C 188G 195C 204C 207A 247A 494d 514d 515d 16140C 16179T 16187T 16189C 16223T 16230G 16243C 16293G 16311C 16519C	1	73 146 152 188 195 204 207 247 498d 719 750 769 825A 1018 1048 2758 2885 3438 3516A 3594 3618 3756 4104 4232 4312 4562C 4769 5442 6185 6266 6815 7028 7146 7256 7521 8113A 8152 8251 8468 8655 8701 8860 9042 9347 9540 9755 10589 10664 10688 10810 10873 10915 11341 11719 11914 12007 12121 12705 12720 13105 13276 13506 13650 13759 14766 15326 15466 15930 15941 16140 16179 16187 16189 16223 16230 16243 16293 16311	L0d1b1b1	73 146 152 188 195 204 207 247 498d 16140 16179 16187 16189 16223 16230 16243 16293 16311	[0.853] L0d1b1b1 (1)	[0.000] L0d1b1 L0d1b1a L0d1b1a1 L0d1b1b (5)	1
653	>MF381526.1 Hc	73G 151T 152C 182T 186A 189C 247A 263G 316A 514d 515d 629C 750G 769A 825A 1018A 1438G 2283T 2394d 2706G 2758A 2885C 3106d 3107C 3210T 3434G 3594T 3666A 4104G 4755C 4769G 5951G 6071C 6221A 6917A 7028T 7146G 7256T 7389C 7521A 8027A 8251A 8417T 8468T 8655T 8701G 8860G 9072G 9540C 10398G 10586A 10688A 10810C 10873C 11302T 11317G 11719A 12400G 12542T 12705T 12810G 13105G 13485G 13506T 13650T 13708A 13789C 13981T 14000A 14178C 14560A 14766T 14794T 14911T 15226G 15326G 15905C 15978T 16017C 16129A 16163G 16187T 16189C 16209C 16223T 16278T 16293G 16294T 16311C 16360T 16519C	L1c3b1a	73G 151T 152C 182T 186A 189C 247A 263G 316A 514d 515d 16129A 16163G 16187T 16189C 16209C 16223T 16278T 16293G 16294T 16311C 16360T 16519C	1	73 151 152 182 186A 189C 247 263 316 629 750 769 825A 1018 1438 2283 2395d 2706 2758 2885 3210 3434 3594 3666 4104 4755 4769 5951 6071 6221A 6917 7028 7146 7256 7389 7521 8027 8251 8417 8468 8655 8701 8860 9072 9540 10398 10586 10688 10810 10873 11302 11317 11719 12400 12542 12705 12810 13105 13485 13506 13650 13708 13789 13981 14000A 14178 14560 14766 14794 14911 15226 15326 15905 15978 16017 16129 16163 16187 16189 16209 16223 16278 16293 16294 16311 16360	L1c3b1a	73 151 152 182 186A 189C 247 263 316 16129 16163 16187 16189 16209 16223 16278 16293 16294 16311 16360	[0.000] L1c3b1a (1)	[0.000] L1c3b L1c3b1 L1c3b1b (4)	1

654	>MF381524.1 Hc	73G 146C 152C 188G 195C 204C 207A 247A 494d 514d 515d 719A 750G 769A 825A 1018A 1048T 2758A 2885C 3106d 3107C 3438A 3516A 3594T 3618C 3756G 4104G 4232C 4312T 4562C 4769G 5442C 6185C 6266G 6815C 7028T 7146G 7256T 7521A 8113A 8152A 8251A 8468T 8655T 8701G 8860G 9042T 9347G 9540C 9755A 10589A 10664T 10688A 10810C 10873C 10915C 11341G 11719A 11914A 12007A 12121C 12705T 12720G 13105G 13276G 13506T 13650T 13759A 14766T 15326G 15466A 15930A 15941C 16140C 16179T 16187T 16189C 16223T 16230G 16243C 16293G 16311C 16519C	L0d1b1b1	73G 146C 152C 188G 195C 204C 207A 247A 494d 514d 515d 16140C 16179T 16187T 16189C 16223T 16230G 16243C 16293G 16311C 16519C	1	73 146 152 188 195 204 207 247 498d 719 750 769 825A 1018 1048 2758 2885 3438 3516A 3594 3618 3756 4104 4232 4312 4562C 4769 5442 6185 6266 6815 7028 7146 7256 7521 8113A 8152 8251 8468 8655 8701 8860 9042 9347 9540 9755 10589 10664 10688 10810 10873 10915 11341 11719 11914 12007 12121 12705 12720 13105 13276 13506 13650 13759 14766 15326 15466 15930 15941 16140 16179 16187 16189 16223 16230 16243 16293 16311	L0d1b1b1	73 146 152 188 195 204 207 247 498d 16140 16179 16187 16189 16223 16230 16243 16293 16311	[0.853] L0d1b1b1 (1)	[0.000] L0d1b1 L0d1b1a L0d1b1a1 L0d1b1b (5)	1
655	>MF381523.1 Hc	73G 93G 146C 150T 152C 182T 183G 195C 198T 263G 325T 514d 515d 680C 709A 750G 769A 1018A 1040C 1438G 1442A 2332T 2416C 2706G 3106d 3107C 3200A 3594T 4104G 4769G 7028T 7256T 7521A 7624A 8206A 8567C 8701G 8772C 8860G 9063G 9221G 9540C 10115C 10398G 10790C 10873C 11719A 11944C 12236A 12705T 13590A 13650T 13928C 13958C 14766T 15110A 15217A 15301A 15313C 15326G 15849T 16223T 16264T 16278T 16311C 16390A	L2c2b1b	73G 93G 146C 150T 152C 182T 183G 195C 198T 263G 325T 514d 515d 16223T 16264T 16278T 16311C 16390A	1	73 93 146 150 152 182 183 195 198 263 325 680 709 750 769 1018 1040 1438 1442 2332 2416 2706 3200A 3594 4104 4769 7028 7256 7521 7624A 8206 8567 8701 8772 8860 9063 9221 9540 10115 10398 10790 10873 11719 11944 12236 12705 13590 13650 13928C 13958C 14766 15110 15217 15301 15313 15326 15849 16223 16264 16278 16311 16390	L2c2b1b	73 93 146 150 152 182 183 195 198 263 325 16223 16264 16278 16311 16390	[0.000] L2c2b1b (1)	[0.000] L2c2b L2c2b1 L2c2b1a L2c2b2 (5)	1
656	>MF381522.1 Hc	73G 93G 146C 150T 152C 182T 183G 195C 198T 263G 325T 514d 515d 680C 709A 750G 769A 1018A 1040C 1438G 1442A 2332T 2416C 2706G 3106d 3107C 3200A 3594T 4104G 4769G 7028T 7256T 7521A 7624A 8206A 8567C 8701G 8772C 8860G 9063G 9221G 9540C 10115C 10398G 10790C 10873C 11719A 11944C 12236A 12705T 13590A 13650T 13928C 13958C 14766T 15110A 15217A 15301A 15313C 15326G 15849T 16223T 16264T 16278T 16311C 16390A	L2c2b1b	73G 93G 146C 150T 152C 182T 183G 195C 198T 263G 325T 514d 515d 16223T 16264T 16278T 16311C 16390A	1	73 93 146 150 152 182 183 195 198 263 325 680 709 750 769 1018 1040 1438 1442 2332 2416 2706 3200A 3594 4104 4769 7028 7256 7521 7624A 8206 8567 8701 8772 8860 9063 9221 9540 10115 10398 10790 10873 11719 11944 12236 12705 13590 13650 13928C 13958C 14766 15110 15217 15301 15313 15326 15849 16223 16264 16278 16311 16390	L2c2b1b	73 93 146 150 152 182 183 195 198 263 325 16223 16264 16278 16311 16390	[0.000] L2c2b1b (1)	[0.000] L2c2b L2c2b1 L2c2b1a L2c2b2 (5)	1

657	>MF381518.1 Hc	73G 146C 152C 188G 195C 204C 207A 247A 494d 514d 515d 719A 750G 769A 825A 1018A 1048T 2758A 2885C 3106d 3107C 3438A 3516A 3594T 3618C 3756G 4104G 4232C 4312T 4562C 4769G 5442C 6185C 6266G 6815C 7028T 7146G 7256T 7521A 8113A 8152A 8251A 8468T 8655T 8701G 8860G 9042T 9347G 9540C 9755A 10589A 10664T 10688A 10810C 10873C 10915C 11341G 11719A 11914A 12007A 12121C 12705T 12720G 13105G 13276G 13506T 13650T 13759A 14766T 15326G 15466A 15930A 15941C 16140C 16179T 16187T 16189C 16223T 16230G 16243C 16293G 16311C 16519C	L0d1b1b1	73G 146C 152C 188G 195C 204C 207A 247A 494d 514d 515d 16140C 16179T 16187T 16189C 16223T 16230G 16243C 16293G 16311C 16519C	1	73 146 152 188 195 204 207 247 498d 719 750 769 825A 1018 1048 2758 2885 3438 3516A 3594 3618 3756 4104 4232 4312 4562C 4769 5442 6185 6266 6815 7028 7146 7256 7521 8113A 8152 8251 8468 8655 8701 8860 9042 9347 9540 9755 10589 10664 10688 10810 10873 10915 11341 11719 11914 12007 12121 12705 12720 13105 13276 13506 13650 13759 14766 15326 15466 15930 15941 16140 16179 16187 16189 16223 16230 16243 16293 16311	L0d1b1b1	73 146 152 188 195 204 207 247 498d 16140 16179 16187 16189 16223 16230 16243 16293 16311	[0.853] L0d1b1b1 (1)	[0.000] L0d1b1 L0d1b1a L0d1b1a1 L0d1b1b (5)	1
658	>MF381513.1 Hc	73G 146C 152C 188G 195C 204C 207A 247A 494d 514d 515d 719A 750G 769A 825A 1018A 1048T 2758A 2885C 3106d 3107C 3438A 3516A 3594T 3618C 3756G 4104G 4232C 4312T 4562C 4769G 5442C 6185C 6266G 6815C 7028T 7146G 7256T 7521A 8113A 8152A 8251A 8468T 8655T 8701G 8860G 9042T 9347G 9540C 9755A 10589A 10664T 10688A 10810C 10873C 10915C 11341G 11719A 11914A 12007A 12121C 12705T 12720G 13105G 13276G 13506T 13650T 13759A 14766T 15326G 15466A 15930A 15941C 16140C 16179T 16187T 16189C 16223T 16230G 16243C 16293G 16311C 16519C	L0d1b1b1	73G 146C 152C 188G 195C 204C 207A 247A 494d 514d 515d 16140C 16179T 16187T 16189C 16223T 16230G 16243C 16293G 16311C 16519C	1	73 146 152 188 195 204 207 247 498d 719 750 769 825A 1018 1048 2758 2885 3438 3516A 3594 3618 3756 4104 4232 4312 4562C 4769 5442 6185 6266 6815 7028 7146 7256 7521 8113A 8152 8251 8468 8655 8701 8860 9042 9347 9540 9755 10589 10664 10688 10810 10873 10915 11341 11719 11914 12007 12121 12705 12720 13105 13276 13506 13650 13759 14766 15326 15466 15930 15941 16140 16179 16187 16189 16223 16230 16243 16293 16311	L0d1b1b1	73 146 152 188 195 204 207 247 498d 16140 16179 16187 16189 16223 16230 16243 16293 16311	[0.853] L0d1b1b1 (1)	[0.000] L0d1b1 L0d1b1a L0d1b1a1 L0d1b1b (5)	1

659	>MF381510.1 Hc	73G 152C 182T 186A 189C 195C 198T 247A 263G 297G 316A 709A 750G 769A 825A 1018A 1420C 1438G 2394d 2706G 2758A 2885C 3106d 3107C 3594T 3666A 4104G 4769G 5951G 6071C 6150A 6253C 7028T 7055G 7076G 7146G 7256T 7337A 7389C 7521A 7744C 8027A 8468T 8655T 8701G 8784G 8860G 8877C 9072G 9181G 9540C 10321C 10398G 10586A 10688A 10792G 10793T 10810C 10873C 11654G 11719A 12049T 12705T 12810G 13105G 13149G 13212T 13485G 13506T 13650T 13789C 13866G 14000A 14178C 14560A 14766T 14812T 14911T 15016T 15326G 15784C 16071T 16129A 16145A 16187T 16189C 16213A 16223T 16234T 16265C 16278T 16286G 16294T 16311C 16360T 16527T	L1c2a1a	73G 152C 182T 186A 189C 195C 198T 247A 263G 297G 316A 16071T 16129A 16145A 16187T 16189C 16213A 16223T 16234T 16265C 16278T 16286G 16294T 16311C 16360T 16527T	1	73 152 182 186A 189C 195 198 247 263 297 316 709 750 769 825A 1018 1420 1438 2395d 2706 2758 2885 3594 3666 4104 4769 5951 6071 6150 6253 7028 7055 7076 7146 7256 7337 7389 7521 7744 8027 8468 8655 8701 8784 8860 8877 9072 9181 9540 10321 10398 10586 10688 10792 10793 10810 10873 11654 11719 12049 12705 12810 13105 13149 13212 13485 13506 13650 13789 13866 14000A 14178 14560 14766 14812 14911 15016 15326 15784 16071 16129 16145 16187 16189 16213 16223 16234 16265C 16278 16286G 16294 16311 16360 16527	L1c2a1a	73 152 182 186A 189C 195 198 247 263 297 316 16071 16129 16145 16187 16189 16213 16223 16234 16265C 16278 16286G 16294 16311 16360 16527	[0.105] L1c2a1a (1)	[0.158] L1c2a1b (2) [0.000] L1c2a1	1
660	>MF381509.1 Hc	73G 146C 152C 188G 195C 204C 207A 247A 494d 514d 515d 719A 750G 769A 825A 1018A 1048T 2758A 2885C 3106d 3107C 3438A 3516A 3594T 3618C 3756G 4104G 4232C 4312T 4562C 4769G 5442C 6185C 6266G 6815C 7028T 7146G 7256T 7521A 8113A 8152A 8251A 8468T 8655T 8701G 8860G 9042T 9347G 9540C 9755A 10589A 10664T 10688A 10810C 10873C 10915C 11341G 11719A 11914A 12007A 12121C 12662G 12705T 12720G 13105G 13276G 13506T 13650T 13759A 14766T 15326G 15466A 15930A 15941C 16140C 16179T 16187T 16189C 16223T 16230G 16243C 16293G 16311C 16519C	L0d1b1b1	73G 146C 152C 188G 195C 204C 207A 247A 494d 514d 515d 16140C 16179T 16187T 16189C 16223T 16230G 16243C 16293G 16311C 16519C	1	73 146 152 188 195 204 207 247 498d 719 750 769 825A 1018 1048 2758 2885 3438 3516A 3594 3618 3756 4104 4232 4312 4562C 4769 5442 6185 6266 6815 7028 7146 7256 7521 8113A 8152 8251 8468 8655 8701 8860 9042 9347 9540 9755 10589 10664 10688 10810 10873 10915 11341 11719 11914 12007 12121 12662 12705 12720 13105 13276 13506 13650 13759 14766 15326 15466 15930 15941 16140 16179 16187 16189 16223 16230 16243 16293 16311	L0d1b1b1	73 146 152 188 195 204 207 247 498d 16140 16179 16187 16189 16223 16230 16243 16293 16311	[0.853] L0d1b1b1 (1)	[0.000] L0d1b1 L0d1b1a L0d1b1a1 L0d1b1b (5)	1

661	>MF381506.1 Ho	73G 146C 152C 188G 195C 204C 207A 247A 494d 514d 515d 719A 750G 769A 825A 1018A 1048T 2758A 2885C 3106d 3107C 3438A 3516A 3594T 3618C 3756G 4104G 4232C 4312T 4562C 4769G 5442C 6185C 6266G 6815C 7028T 7146G 7256T 7521A 8113A 8152A 8251A 8468T 8655T 8701G 8860G 9042T 9347G 9540C 9755A 10589A 10664T 10688A 10810C 10873C 10915C 11341G 11719A 11914A 12007A 12121C 12705T 12720G 13105G 13276G 13506T 13650T 13759A 14766T 15326G 15466A 15930A 15941C 16140C 16179T 16187T 16189C 16223T 16230G 16243C 16293G 16311C 16519C	L0d1b1b1	73G 146C 152C 188G 195C 204C 207A 247A 494d 514d 515d 16140C 16179T 16187T 16189C 16223T 16230G 16243C 16293G 16311C 16519C	1	73 146 152 188 195 204 207 247 498d 719 750 769 825A 1018 1048 2758 2885 3438 3516A 3594 3618 3756 4104 4232 4312 4562C 4769 5442 6185 6266 6815 7028 7146 7256 7521 8113A 8152 8251 8468 8655 8701 8860 9042 9347 9540 9755 10589 10664 10688 10810 10873 10915 11341 11719 11914 12007 12121 12705 12720 13105 13276 13506 13650 13759 14766 15326 15466 15930 15941 16140 16179 16187 16189 16223 16230 16243 16293 16311	L0d1b1b1	73 146 152 188 195 204 207 247 498d 16140 16179 16187 16189 16223 16230 16243 16293 16311	[0.853] L0d1b1b1 (1)	[0.000] L0d1b1 L0d1b1a L0d1b1a1 L0d1b1b (5)	1
662	>MF381505.1 Ho	73G 151T 152C 182T 186A 189C 195C 198T 247A 263G 297G 316A 416A 514d 515d 520T 750G 769A 825A 1018A 1291C 1438G 2394d 2581G 2706G 2758A 2885C 3106d 3107C 3594T 3666A 3796T 3834A 3843G 4104G 4589C 4688C 4769G 5480G 5553C 5951G 6071C 6179A 7028T 7055G 7146G 7196T 7256T 7389C 7521A 7660C 7714T 8027A 8468T 8619T 8655T 8701G 8860G 9072G 9540C 9861C 10084C 10321C 10398G 10586A 10688A 10810C 10873C 11353C 11440A 11719A 11899C 12681C 12705T 12810G 13105G 13485G 13506T 13650T 13789C 13874C 14000A 14148G 14178C 14393G 14560A 14766T 14911T 15326G 16086C 16129A 16187T 16189C 16223T 16241G 16278T 16293G 16294T 16311C 16360T 16519C	L1c1b	73G 151T 152C 182T 186A 189C 195C 198T 247A 263G 297G 316A 416A 514d 515d 520T 16086C 16129A 16187T 16189C 16223T 16241G 16278T 16293G 16294T 16311C 16360T 16519C	1	73 151 152 182 186A 189C 195 198 247 263 297 316 416A 518 750 769 825A 1018 1291 1438 2395d 2581 2706 2758 2885 3594 3666 3796T 3834 3843 4104 4589 4688 4769 5480 5553 5951 6071 6179 7028 7055 7146 7196 7256 7389 7521 7660 7714 8027 8468 8619 8655 8701 8860 9072 9540 9861 10084 10321 10398 10586 10688 10810 10873 11353 11440 11719 11899 12681 12705 12810 13105 13485 13506 13650 13789 13874 14000A 14148 14178 14393 14560 14766 14911 15326 16086 16129 16187 16189 16223 16241 16278 16293 16294 16311 16360	L1c1b	73 151 152 182 186A 189C 195 198 247 263 297 316 416A 518 16086 16129 16187 16189 16223 16241 16278 16293 16294 16311 16360	[1.673] L1c1b (1)	[0.000] L1c1b'd (2)	1

663 >MF381499.1 Hc	73G 146C 152C 188G 195C 204C 207A 247A 494d 514d 515d 719A 750G 769A 825A 1018A 1048T 2758A 2885C 3106d 3107C 3438A 3516A 3594T 3618C 3756G 4104G 4232C 4312T 4562C 4769G 5442C 6185C 6266G 6815C 7028T 7146G 7256T 7521A 8113A 8152A 8251A 8468T 8655T 8701G 8860G 9042T 9347G 9540C 9755A 10589A 10664T 10688A 10810C 10873C 10915C 11341G 11719A 11914A 12007A 12121C 12705T 12720G 13105G 13276G 13506T 13650T 13759A 14766T 15040T 15326G 15466A 15930A 15941C 16140C 16179T 16187T 16189C 16223T 16230G 16243C 16293G 16311C 16519C	L0d1b1b1	73G 146C 152C 188G 195C 204C 207A 247A 494d 514d 515d 16140C 16179T 16187T 16189C 16223T 16230G 16243C 16293G 16311C 16519C	1	73 146 152 188 195 204 207 247 498d 719 750 769 825A 1018 1048 2758 2885 3438 3516A 3594 3618 3756 4104 4232 4312 4562C 4769 5442 6185 6266 6815 7028 7146 7256 7521 8113A 8152 8251 8468 8655 8701 8860 9042 9347 9540 9755 10589 10664 10688 10810 10873 10915 11341 11719 11914 12007 12121 12705 12720 13105 13276 13506 13650 13759 14766 15040 15326 15466 15930 15941 16140 16179 16187 16189 16223 16230 16243 16293 16311	L0d1b1b1	73 146 152 188 195 204 207 247 498d 16140 16179 16187 16189 16223 16230 16243 16293 16311	[0.853] L0d1b1b1 (1)	[0.000] L0d1b1 L0d1b1a L0d1b1a1 L0d1b1b (5)	1
664 >MF381498.1 Hc	73G 146C 152C 188G 195C 204C 207A 247A 494d 514d 515d 719A 750G 769A 825A 1018A 1048T 2758A 2885C 3106d 3107C 3438A 3516A 3594T 3618C 3756G 4104G 4232C 4312T 4562C 4769G 5442C 6185C 6266G 6815C 7028T 7146G 7256T 7521A 8113A 8152A 8251A 8468T 8655T 8701G 8860G 9042T 9347G 9540C 9755A 10589A 10664T 10688A 10810C 10873C 10915C 11341G 11719A 11914A 12007A 12121C 12705T 12720G 13105G 13276G 13506T 13650T 13759A 14766T 15326G 15466A 15930A 15941C 16140C 16179T 16187T 16189C 16223T 16230G 16243C 16293G 16311C 16519C	L0d1b1b1	73G 146C 152C 188G 195C 204C 207A 247A 494d 514d 515d 16140C 16179T 16187T 16189C 16223T 16230G 16243C 16293G 16311C 16519C	1	73 146 152 188 195 204 207 247 498d 719 750 769 825A 1018 1048 2758 2885 3438 3516A 3594 3618 3756 4104 4232 4312 4562C 4769 5442 6185 6266 6815 7028 7146 7256 7521 8113A 8152 8251 8468 8655 8701 8860 9042 9347 9540 9755 10589 10664 10688 10810 10873 10915 11341 11719 11914 12007 12121 12705 12720 13105 13276 13506 13650 13759 14766 15326 15466 15930 15941 16140 16179 16187 16189 16223 16230 16243 16293 16311	L0d1b1b1	73 146 152 188 195 204 207 247 498d 16140 16179 16187 16189 16223 16230 16243 16293 16311	[0.853] L0d1b1b1 (1)	[0.000] L0d1b1 L0d1b1a L0d1b1a1 L0d1b1b (5)	1

665	>MF381497.1 Hc	73G 146C 152C 188G 195C 204C 207A 247A 494d 514d 515d 719A 750G 769A 825A 1018A 1048T 2758A 2885C 3106d 3107C 3438A 3516A 3594T 3618C 3756G 4104G 4232C 4312T 4562C 4769G 5442C 6185C 6266G 6815C 7028T 7146G 7256T 7521A 8113A 8152A 8251A 8468T 8655T 8701G 8860G 9042T 9347G 9540C 9755A 10589A 10664T 10688A 10810C 10873C 10915C 11341G 11719A 11914A 12007A 12121C 12705T 12720G 13105G 13276G 13506T 13650T 13759A 14766T 15326G 15466A 15930A 15941C 16140C 16179T 16187T 16189C 16223T 16230G 16243C 16293G 16311C 16519C	L0d1b1b1	73G 146C 152C 188G 195C 204C 207A 247A 494d 514d 515d 16140C 16179T 16187T 16189C 16223T 16230G 16243C 16293G 16311C 16519C	1	73 146 152 188 195 204 207 247 498d 719 750 769 825A 1018 1048 2758 2885 3438 3516A 3594 3618 3756 4104 4232 4312 4562C 4769 5442 6185 6266 6815 7028 7146 7256 7521 8113A 8152 8251 8468 8655 8701 8860 9042 9347 9540 9755 10589 10664 10688 10810 10873 10915 11341 11719 11914 12007 12121 12705 12720 13105 13276 13506 13650 13759 14766 15326 15466 15930 15941 16140 16179 16187 16189 16223 16230 16243 16293 16311	L0d1b1b1	73 146 152 188 195 204 207 247 498d 16140 16179 16187 16189 16223 16230 16243 16293 16311	[0.853] L0d1b1b1 (1)	[0.000] L0d1b1 L0d1b1a L0d1b1a1 L0d1b1b (5)	1
666	>MF381495.1 Hc	73G 146C 188G 195C 200G 247A 494d 719A 750G 769A 825A 1018A 1048T 1842G 2758A 2885C 3106d 3107C 3438A 3516A 3594T 3618C 3756G 4104G 4232C 4312T 4562C 4769G 5442C 6185C 6266G 6815C 7028T 7146G 7256T 7521A 8113A 8152A 8251A 8468T 8655T 8701G 8860G 9042T 9347G 9540C 9755A 10589A 10664T 10688A 10810C 10873C 10915C 11404G 11719A 11914A 12007A 12121C 12136C 12705T 12720G 13105G 13276G 13506T 13650T 13759A 14766T 15326G 15466A 15930A 15941C 16129A 16179T 16187T 16189C 16223T 16230G 16243C 16311C 16519C	L0d1b1+@152	73G 146C 188G 195C 200G 247A 494d 16129A 16179T 16187T 16189C 16223T 16230G 16243C 16311C 16519C	1	73 146 188 195 200 247 498d 719 750 769 825A 1018 1048 1842 2758 2885 3438 3516A 3594 3618 3756 4104 4232 4312 4562C 4769 5442 6185 6266 6815 7028 7146 7256 7521 8113A 8152 8251 8468 8655 8701 8860 9042 9347 9540 9755 10589 10664 10688 10810 10873 10915 11404 11719 11914 12007 12121 12136 12705 12720 13105 13276 13506 13650 13759 14766 15326 15466 15930 15941 16129 16179 16187 16189 16223 16230 16243 16311	L0d1b1+@152	73 146 188 195 200 247 498d 16129 16179 16187 16189 16223 16230 16243 16311	[0.600] L0d1b1+@152 (1)	[0.333] L0d1b1c (2) [0.000] L0d1b1 L0d1b1a L0d1b1a1 L0d1b1b (6)	1

667 >MF381494.1 Hc	73G 146C 152C 188G 195C 204C 207A 247A 494d 514d 515d 719A 750G 769A 825A 1018A 1048T 2758A 2885C 3106d 3107C 3438A 3516A 3594T 3618C 3756G 4104G 4232C 4312T 4562C 4769G 5442C 6185C 6266G 6815C 7028T 7146G 7256T 7521A 8113A 8152A 8251A 8468T 8655T 8701G 8860G 9042T 9347G 9540C 9755A 10589A 10664T 10688A 10810C 10873C 10915C 11341G 11719A 11914A 12007A 12121C 12705T 12720G 13105G 13276G 13506T 13650T 13759A 14766T 15326G 15466A 15930A 15941C 16140C 16179T 16187T 16189C 16223T 16230G 16243C 16293G 16311C 16519C	L0d1b1b1	73G 146C 152C 188G 195C 204C 207A 247A 494d 514d 515d 16140C 16179T 16187T 16189C 16223T 16230G 16243C 16293G 16311C 16519C	1	73 146 152 188 195 204 207 247 498d 719 750 769 825A 1018 1048 2758 2885 3438 3516A 3594 3618 3756 4104 4232 4312 4562C 4769 5442 6185 6266 6815 7028 7146 7256 7521 8113A 8152 8251 8468 8655 8701 8860 9042 9347 9540 9755 10589 10664 10688 10810 10873 10915 11341 11719 11914 12007 12121 12705 12720 13105 13276 13506 13650 13759 14766 15326 15466 15930 15941 16140 16179 16187 16189 16223 16230 16243 16293 16311	L0d1b1b1	73 146 152 188 195 204 207 247 498d 16140 16179 16187 16189 16223 16230 16243 16293 16311	[0.853] L0d1b1b1 (1)	[0.000] L0d1b1 L0d1b1a L0d1b1a1 L0d1b1b (5)	1
668 >MF381492.1 Hc	73G 146C 152C 188G 195C 204C 207A 247A 494d 514d 515d 719A 750G 769A 825A 1018A 1048T 2758A 2885C 3106d 3107C 3438A 3516A 3594T 3618C 3756G 4104G 4232C 4312T 4562C 4769G 5442C 6185C 6266G 6815C 7028T 7146G 7256T 7521A 8113A 8152A 8251A 8468T 8655T 8701G 8860G 9042T 9347G 9540C 9755A 10589A 10664T 10688A 10810C 10873C 10915C 11341G 11719A 11914A 12007A 12121C 12705T 12720G 13105G 13276G 13506T 13650T 13759A 14766T 15326G 15466A 15930A 15941C 16140C 16179T 16187T 16189C 16223T 16230G 16243C 16293G 16311C 16519C	L0d1b1b1	73G 146C 152C 188G 195C 204C 207A 247A 494d 514d 515d 16140C 16179T 16187T 16189C 16223T 16230G 16243C 16293G 16311C 16519C	1	73 146 152 188 195 204 207 247 498d 719 750 769 825A 1018 1048 2758 2885 3438 3516A 3594 3618 3756 4104 4232 4312 4562C 4769 5442 6185 6266 6815 7028 7146 7256 7521 8113A 8152 8251 8468 8655 8701 8860 9042 9347 9540 9755 10589 10664 10688 10810 10873 10915 11341 11719 11914 12007 12121 12705 12720 13105 13276 13506 13650 13759 14766 15326 15466 15930 15941 16140 16179 16187 16189 16223 16230 16243 16293 16311	L0d1b1b1	73 146 152 188 195 204 207 247 498d 16140 16179 16187 16189 16223 16230 16243 16293 16311	[0.853] L0d1b1b1 (1)	[0.000] L0d1b1 L0d1b1a L0d1b1a1 L0d1b1b (5)	1

669 >MF381491.1 Ho	73G 151T 152C 182T 186A 189C 195C 198T 247A 263G 297G 316A 416A 514d 515d 520T 750G 769A 825A 1018A 1291C 1438G 2394d 2581G 2706G 2758A 2885C 3106d 3107C 3594T 3666A 3796T 3834A 3843G 4104G 4589C 4688C 4769G 5480G 5553C 5951G 6071C 6179A 7028T 7055G 7146G 7196T 7256T 7389C 7521A 7660C 7714T 7979A 8027A 8468T 8619T 8655T 8701G 8860G 9072G 9540C 9861C 10084C 10321C 10398G 10586A 10688A 10810C 10873C 11353C 11440A 11719A 11899C 12681C 12705T 12810G 13105G 13485G 13506T 13650T 13789C 13874C 14000A 14148G 14178C 14393G 14560A 14766T 14911T 15326G 15930A 16086C 16129A 16187T 16189C 16223T 16241G 16278T 16293G 16294T 16311C 16360T 16519C	L1c1b	73G 151T 152C 182T 186A 189C 195C 198T 247A 263G 297G 316A 416A 514d 515d 520T 16086C 16129A 16187T 16189C 16223T 16241G 16278T 16293G 16294T 16311C 16360T 16519C	1	73 151 152 182 186A 189C 195 198 247 263 297 316 416A 518 750 769 825A 1018 1291 1438 2395d 2581 2706 2758 2885 3594 3666 3796T 3834 3843 4104 4589 4688 4769 5480 5553 5951 6071 6179 7028 7055 7146 7196 7256 7389 7521 7660 7714 7979 8027 8468 8619 8655 8701 8860 9072 9540 9861 10084 10321 10398 10586 10688 10810 10873 11353 11440 11719 11899 12681 12705 12810 13105 13485 13506 13650 13789 13874 14000A 14148 14178 14393 14560 14766 14911 15326 15930 16086 16129 16187 16189 16223 16241 16278 16293 16294 16311 16360	L1c1b	73 151 152 182 186A 189C 195 198 247 263 297 316 416A 518 16086 16129 16187 16189 16223 16241 16278 16293 16294 16311 16360	[1.673] L1c1b (1)	[0.000] L1c1b'd (2)	1
670 >MF381488.1 Ho	73G 151T 152C 182T 186A 189C 195C 198T 247A 263G 297G 316A 416A 514d 515d 520T 750G 769A 825A 1018A 1291C 1438G 2394d 2581G 2706G 2758A 2885C 3106d 3107C 3594T 3666A 3796T 3834A 3843G 4104G 4589C 4688C 4769G 5480G 5553C 5951G 6071C 6179A 7028T 7055G 7146G 7196T 7256T 7389C 7521A 7660C 7714T 8027A 8468T 8619T 8655T 8701G 8860G 9072G 9540C 9861C 10084C 10321C 10398G 10586A 10688A 10810C 10873C 11353C 11440A 11719A 11899C 12681C 12705T 12810G 13105G 13485G 13506T 13650T 13789C 13874C 14000A 14148G 14178C 14393G 14560A 14766T 14911T 15326G 16086C 16129A 16187T 16189C 16223T 16241G 16278T 16293G 16294T 16311C 16360T 16519C	L1c1b	73G 151T 152C 182T 186A 189C 195C 198T 247A 263G 297G 316A 416A 514d 515d 520T 16086C 16129A 16187T 16189C 16223T 16241G 16278T 16293G 16294T 16311C 16360T 16519C	1	73 151 152 182 186A 189C 195 198 247 263 297 316 416A 518 750 769 825A 1018 1291 1438 2395d 2581 2706 2758 2885 3594 3666 3796T 3834 3843 4104 4589 4688 4769 5480 5553 5951 6071 6179 7028 7055 7146 7196 7256 7389 7521 7660 7714 8027 8468 8619 8655 8701 8860 9072 9540 9861 10084 10321 10398 10586 10688 10810 10873 11353 11440 11719 11899 12681 12705 12810 13105 13485 13506 13650 13789 13874 14000A 14148 14178 14393 14560 14766 14911 15326 16086 16129 16187 16189 16223 16241 16278 16293 16294 16311 16360	L1c1b	73 151 152 182 186A 189C 195 198 247 263 297 316 416A 518 16086 16129 16187 16189 16223 16241 16278 16293 16294 16311 16360	[1.673] L1c1b (1)	[0.000] L1c1b'd (2)	1

671 >MF381486.1 Ho	73G 146C 152C 188G 195C 204C 207A 247A 494d 514d 515d 719A 750G 769A 825A 1018A 1048T 2758A 2885C 3106d 3107C 3438A 3516A 3594T 3618C 3756G 4104G 4232C 4312T 4562C 4769G 5442C 6185C 6266G 6815C 7028T 7146G 7256T 7521A 8113A 8152A 8251A 8468T 8655T 8701G 8860G 9042T 9347G 9540C 9755A 10589A 10664T 10688A 10810C 10873C 10915C 11341G 11719A 11914A 12007A 12121C 12705T 12720G 13105G 13276G 13506T 13650T 13759A 14766T 15326G 15466A 15930A 15941C 16140C 16179T 16187T 16189C 16223T 16230G 16243C 16293G 16311C 16519C	L0d1b1b1	73G 146C 152C 188G 195C 204C 207A 247A 494d 514d 515d 16140C 16179T 16187T 16189C 16223T 16230G 16243C 16293G 16311C 16519C	1	73 146 152 188 195 204 207 247 498d 719 750 769 825A 1018 1048 2758 2885 3438 3516A 3594 3618 3756 4104 4232 4312 4562C 4769 5442 6185 6266 6815 7028 7146 7256 7521 8113A 8152 8251 8468 8655 8701 8860 9042 9347 9540 9755 10589 10664 10688 10810 10873 10915 11341 11719 11914 12007 12121 12705 12720 13105 13276 13506 13650 13759 14766 15326 15466 15930 15941 16140 16179 16187 16189 16223 16230 16243 16293 16311	L0d1b1b1	73 146 152 188 195 204 207 247 498d 16140 16179 16187 16189 16223 16230 16243 16293 16311	[0.853] L0d1b1b1 (1)	[0.000] L0d1b1 L0d1b1a L0d1b1a1 L0d1b1b (5)	1
672 >MF381472.1 Ho	73G 146C 152C 188G 195C 204C 207A 247A 494d 514d 515d 719A 735G 750G 769A 825A 1018A 1048T 2758A 2885C 3106d 3107C 3438A 3516A 3594T 3618C 3756G 4104G 4232C 4312T 4562C 4769G 5442C 6185C 6266G 6815C 7028T 7146G 7256T 7521A 8113A 8152A 8251A 8468T 8655T 8701G 8860G 9042T 9347G 9540C 9755A 10589A 10664T 10688A 10810C 10873C 10915C 11341G 11719A 11914A 12007A 12121C 12705T 12720G 13105G 13276G 13506T 13650T 13759A 14766T 15326G 15466A 15930A 15941C 16140C 16179T 16187T 16189C 16223T 16230G 16243C 16293G 16311C 16519C	L0d1b1b1	73G 146C 152C 188G 195C 204C 207A 247A 494d 514d 515d 16140C 16179T 16187T 16189C 16223T 16230G 16243C 16293G 16311C 16519C	1	73 146 152 188 195 204 207 247 498d 719 735 750 769 825A 1018 1048 2758 2885 3438 3516A 3594 3618 3756 4104 4232 4312 4562C 4769 5442 6185 6266 6815 7028 7146 7256 7521 8113A 8152 8251 8468 8655 8701 8860 9042 9347 9540 9755 10589 10664 10688 10810 10873 10915 11341 11719 11914 12007 12121 12705 12720 13105 13276 13506 13650 13759 14766 15326 15466 15930 15941 16140 16179 16187 16189 16223 16230 16243 16293 16311	L0d1b1b1	73 146 152 188 195 204 207 247 498d 16140 16179 16187 16189 16223 16230 16243 16293 16311	[0.853] L0d1b1b1 (1)	[0.000] L0d1b1 L0d1b1a L0d1b1a1 L0d1b1b (5)	1

673	>MF381467.1 Hc	73G 151T 152C 182T 186A 189C 195C 198T 247A 263G 297G 316A 416A 514d 515d 520T 750G 769A 825A 1018A 1291C 1438G 2394d 2581G 2706G 2758A 2885C 3106d 3107C 3594T 3666A 3796T 3834A 3843G 4104G 4589C 4688C 4769G 5480G 5553C 5951G 6071C 6179A 7028T 7055G 7146G 7196T 7256T 7389C 7521A 7660C 7714T 8027A 8468T 8619T 8655T 8701G 8860G 9072G 9540C 9861C 10084C 10321C 10398G 10586A 10688A 10810C 10873C 11353C 11440A 11719A 11899C 12681C 12705T 12810G 13105G 13485G 13506T 13650T 13789C 13874C 14000A 14148G 14178C 14393G 14560A 14766T 14911T 15326G 16086C 16129A 16187T 16189C 16223T 16241G 16278T 16293G 16294T 16311C 16360T 16519C	L1c1b	73G 151T 152C 182T 186A 189C 195C 198T 247A 263G 297G 316A 416A 514d 515d 520T 16086C 16129A 16187T 16189C 16223T 16241G 16278T 16293G 16294T 16311C 16360T 16519C	1	73 151 152 182 186A 189C 195 198 247 263 297 316 416A 518 750 769 825A 1018 1291 1438 2395d 2581 2706 2758 2885 3594 3666 3796T 3834 3843 4104 4589 4688 4769 5480 5553 5951 6071 6179 7028 7055 7146 7196 7256 7389 7521 7660 7714 8027 8468 8619 8655 8701 8860 9072 9540 9861 10084 10321 10398 10586 10688 10810 10873 11353 11440 11719 11899 12681 12705 12810 13105 13485 13506 13650 13789 13874 14000A 14148 14178 14393 14560 14766 14911 15326 16086 16129 16187 16189 16223 16241 16278 16293 16294 16311 16360	L1c1b	73 151 152 182 186A 189C 195 198 247 263 297 316 416A 518 16086 16129 16187 16189 16223 16241 16278 16293 16294 16311 16360	[1.673] L1c1b (1)	[0.000] L1c1b'd (2)	1
674	>MF381460.1 Hc	73G 151T 152C 182T 186A 189C 195C 198T 247A 263G 297G 316A 416A 514d 515d 520T 750G 769A 825A 1018A 1291C 1438G 2394d 2581G 2706G 2758A 2885C 3106d 3107C 3594T 3666A 3796T 3834A 3843G 4104G 4589C 4688C 4769G 5480G 5553C 5951G 6071C 6179A 7028T 7055G 7146G 7196T 7256T 7389C 7521A 7660C 7714T 8027A 8468T 8619T 8655T 8701G 8860G 9072G 9540C 9861C 10084C 10321C 10398G 10586A 10688A 10810C 10873C 11353C 11440A 11719A 11899C 12681C 12705T 12810G 13105G 13485G 13506T 13650T 13789C 13874C 14000A 14148G 14178C 14393G 14560A 14766T 14911T 15326G 16086C 16129A 16187T 16189C 16223T 16241G 16278T 16293G 16294T 16311C 16360T 16519C	L1c1b	73G 151T 152C 182T 186A 189C 195C 198T 247A 263G 297G 316A 416A 514d 515d 520T 16086C 16129A 16187T 16189C 16223T 16241G 16278T 16293G 16294T 16311C 16360T 16519C	1	73 151 152 182 186A 189C 195 198 247 263 297 316 416A 518 750 769 825A 1018 1291 1438 2395d 2581 2706 2758 2885 3594 3666 3796T 3834 3843 4104 4589 4688 4769 5480 5553 5951 6071 6179 7028 7055 7146 7196 7256 7389 7521 7660 7714 8027 8468 8619 8655 8701 8860 9072 9540 9861 10084 10321 10398 10586 10688 10810 10873 11353 11440 11719 11899 12681 12705 12810 13105 13485 13506 13650 13789 13874 14000A 14148 14178 14393 14560 14766 14911 15326 16086 16129 16187 16189 16223 16241 16278 16293 16294 16311 16360	L1c1b	73 151 152 182 186A 189C 195 198 247 263 297 316 416A 518 16086 16129 16187 16189 16223 16241 16278 16293 16294 16311 16360	[1.673] L1c1b (1)	[0.000] L1c1b'd (2)	1

675 >MF381452.1 Hc	73G 150T 152C 189G 195C 200G 207A 263G 750G 1438G 2352C 2706G 3106d 3107C 4769G 5774A 6221C 6587T 7028T 8701G 8860G 9254G 9540C 10398G 10819G 10873C 11024T 11719A 12705T 14152G 14212C 14569A 14693G 14766T 15301A 15326G 15670C 15942C 16185T 16209C 16223T 16327T	L3e1a2	73G 150T 152C 189G 195C 200G 207A 263G 16185T 16209C 16223T 16327T	1	73 150 152 189 195 200 207 263 750 1438 2352 2706 4769 5774A 6221 6587 7028 8701 8860 9254 9540 10398 10819 10873 11024 11719 12705 14152 14212 14569 14693 14766 15301 15326 15670 15942 16185 16209 16223 16327	L3e1a2	73 150 152 189 195 200 207 263 16185 16209 16223 16327	[0.000] L3e1a2 (1)	[0.000] L3e1f2 (2)	1
676 >MF381451.1 Hc	73G 150T 152C 189G 195C 200G 207A 263G 750G 1438G 2352C 2706G 3106d 3107C 4769G 5774A 6221C 6587T 7028T 8701G 8860G 9254G 9540C 10398G 10819G 10873C 11024T 11719A 12705T 14152G 14212C 14569A 14766T 15301A 15326G 15670C 15942C 16185T 16209C 16223T 16327T	L3e1a2	73G 150T 152C 189G 195C 200G 207A 263G 16185T 16209C 16223T 16327T	1	73 150 152 189 195 200 207 263 750 1438 2352 2706 4769 5774A 6221 6587 7028 8701 8860 9254 9540 10398 10819 10873 11024 11719 12705 14152 14212 14569 14766 15301 15326 15670 15942 16185 16209 16223 16327	L3e1a2	73 150 152 189 195 200 207 263 16185 16209 16223 16327	[0.000] L3e1a2 (1)	[0.000] L3e1f2 (2)	1
677 >MF381449.1 Hc	73G 151T 152C 182T 186A 189C 195C 198T 247A 263G 297G 316A 416A 514d 515d 520T 750G 769A 825A 1018A 1291C 1438G 2394d 2581G 2706G 2758A 2885C 3106d 3107C 3594T 3666A 3796T 3834A 3843G 4104G 4589C 4688C 4769G 5480G 5553C 5951G 6071C 6179A 7028T 7055G 7146G 7196T 7256T 7389C 7521A 7660C 7714T 8027A 8468T 8619T 8655T 8701G 8860G 9072G 9540C 9861C 10084C 10321C 10398G 10586A 10688A 10810C 10873C 11353C 11440A 11719A 11899C 12681C 12705T 12810G 13105G 13485G 13506T 13650T 13789C 13874C 14000A 14148G 14178C 14393G 14560A 14766T 14911T 15326G 16086C 16129A 16187T 16189C 16223T 16241G 16278T 16293G 16294T 16311C 16360T 16519C	L1c1b	73G 151T 152C 182T 186A 189C 195C 198T 247A 263G 297G 316A 416A 514d 515d 520T 16086C 16129A 16187T 16189C 16223T 16241G 16278T 16293G 16294T 16311C 16360T 16519C	1	73 151 152 182 186A 189C 195 198 247 263 297 316 416A 518 750 769 825A 1018 1291 1438 2395d 2581 2706 2758 2885 3594 3666 3796T 3834 3843 4104 4589 4688 4769 5480 5553 5951 6071 6179 7028 7055 7146 7196 7256 7389 7521 7660 7714 8027 8468 8619 8655 8701 8860 9072 9540 9861 10084 10321 10398 10586 10688 10810 10873 11353 11440 11719 11899 12681 12705 12810 13105 13485 13506 13650 13789 13874 14000A 14148 14178 14393 14560 14766 14911 15326 16086 16129 16187 16189 16223 16241 16278 16293 16294 16311 16360	L1c1b	73 151 152 182 186A 189C 195 198 247 263 297 316 416A 518 16086 16129 16187 16189 16223 16241 16278 16293 16294 16311 16360	[1.673] L1c1b (1)	[0.000] L1c1b'd (2)	1
678 >MF381443.1 Hc	73G 150T 152C 189G 195C 200G 207A 263G 750G 1438G 2352C 2706G 3106d 3107C 4769G 5774A 6221C 6587T 7028T 8701G 8860G 9254G 9540C 10398G 10819G 10873C 11024T 11719A 12705T 14152G 14212C 14569A 14693G 14766T 15301A 15326G 15670C 15942C 16185T 16209C 16223T 16327T	L3e1a2	73G 150T 152C 189G 195C 200G 207A 263G 16185T 16209C 16223T 16327T	1	73 150 152 189 195 200 207 263 750 1438 2352 2706 4769 5774A 6221 6587 7028 8701 8860 9254 9540 10398 10819 10873 11024 11719 12705 14152 14212 14569 14693 14766 15301 15326 15670 15942 16185 16209 16223 16327	L3e1a2	73 150 152 189 195 200 207 263 16185 16209 16223 16327	[0.000] L3e1a2 (1)	[0.000] L3e1f2 (2)	1

679 >MF381440.1 Hc	73G 150T 152C 189G 195C 200G 207A 263G 750G 1438G 2352C 2706G 3106d 3107C 4769G 5774A 6221C 6587T 7028T 8701G 8860G 9254G 9540C 10398G 10819G 10873C 11024T 11719A 12705T 14152G 14212C 14569A 14766T 15301A 15326G 15670C 15942C 16185T 16209C 16223T 16327T	L3e1a2	73G 150T 152C 189G 195C 200G 207A 263G 16185T 16209C 16223T 16327T	1	73 150 152 189 195 200 207 263 750 1438 2352 2706 4769 5774A 6221 6587 7028 8701 8860 9254 9540 10398 10819 10873 11024 11719 12705 14152 14212 14569 14766 15301 15326 15670 15942 16185 16209 16223 16327	L3e1a2	73 150 152 189 195 200 207 263 16185 16209 16223 16327	[0.000] L3e1a2 (1)	[0.000] L3e1f2 (2)	1
680 >MF381436.1 Hc	73G 150T 152C 189G 195C 200G 207A 263G 750G 1438G 2352C 2706G 3106d 3107C 4769G 5774A 6221C 6587T 7028T 8701G 8860G 9254G 9540C 10398G 10819G 10873C 11024T 11719A 12705T 14152G 14212C 14569A 14766T 15301A 15326G 15670C 15942C 16185T 16209C 16223T 16327T	L3e1a2	73G 150T 152C 189G 195C 200G 207A 263G 16185T 16209C 16223T 16327T	1	73 150 152 189 195 200 207 263 750 1438 2352 2706 4769 5774A 6221 6587 7028 8701 8860 9254 9540 10398 10819 10873 11024 11719 12705 14152 14212 14569 14766 15301 15326 15670 15942 16185 16209 16223 16327	L3e1a2	73 150 152 189 195 200 207 263 16185 16209 16223 16327	[0.000] L3e1a2 (1)	[0.000] L3e1f2 (2)	1
681 >MF381435.1 Hc	73G 150T 152C 189G 195C 200G 207A 263G 750G 1438G 2352C 2706G 3106d 3107C 4769G 5774A 6221C 6587T 7028T 8701G 8860G 9254G 9540C 10398G 10819G 10873C 11024T 11719A 12705T 14152G 14212C 14569A 14766T 15301A 15326G 15670C 15942C 16185T 16209C 16223T 16327T	L3e1a2	73G 150T 152C 189G 195C 200G 207A 263G 16185T 16209C 16223T 16327T	1	73 150 152 189 195 200 207 263 750 1438 2352 2706 4769 5774A 6221 6587 7028 8701 8860 9254 9540 10398 10819 10873 11024 11719 12705 14152 14212 14569 14766 15301 15326 15670 15942 16185 16209 16223 16327	L3e1a2	73 150 152 189 195 200 207 263 16185 16209 16223 16327	[0.000] L3e1a2 (1)	[0.000] L3e1f2 (2)	1
682 >MF381433.1 Hc	73G 151T 152C 182T 186A 189C 195C 198T 247A 263G 297G 316A 416A 514d 515d 520T 750G 769A 825A 1018A 1291C 1438G 2394d 2581G 2706G 2758A 2885C 3106d 3107C 3594T 3666A 3796T 3834A 3843G 4104G 4589C 4688C 4769G 5480G 5553C 5951G 6071C 6179A 7028T 7055G 7146G 7196T 7256T 7389C 7521A 7660C 7714T 7854C 8027A 8468T 8619T 8655T 8701G 8860G 9072G 9540C 9861C 10084C 10321C 10398G 10586A 10688A 10810C 10873C 11353C 11440A 11719A 11899C 12681C 12705T 12810G 13105G 13485G 13506T 13650T 13789C 13874C 14000A 14148G 14178C 14393G 14560A 14766T 14911T 15326G 16086C 16129A 16187T 16189C 16223T 16241G 16278T 16293G 16294T 16311C 16360T 16519C	L1c1b	73G 151T 152C 182T 186A 189C 195C 198T 247A 263G 297G 316A 416A 514d 515d 520T 16086C 16129A 16187T 16189C 16223T 16241G 16278T 16293G 16294T 16311C 16360T 16519C	1	73 151 152 182 186A 189C 195 198 247 263 297 316 416A 518 750 769 825A 1018 1291 1438 2395d 2581 2706 2758 2885 3594 3666 3796T 3834 3843 4104 4589 4688 4769 5480 5553 5951 6071 6179 7028 7055 7146 7196 7256 7389 7521 7660 7714 7854 8027 8468 8619 8655 8701 8860 9072 9540 9861 10084 10321 10398 10586 10688 10810 10873 11353 11440 11719 11899 12681 12705 12810 13105 13485 13506 13650 13789 13874 14000A 14148 14178 14393 14560 14766 14911 15326 16086 16129 16187 16189 16223 16241 16278 16293 16294 16311 16360	L1c1b	73 151 152 182 186A 189C 195 198 247 263 297 316 416A 518 16086 16129 16187 16189 16223 16241 16278 16293 16294 16311 16360	[1.673] L1c1b (1)	[0.000] L1c1b'd (2)	1

683 >MF381428.1 Hc	73G 151T 152C 182T 186A 189C 195C 198T 247A 263G 297G 316A 416A 514d 515d 520T 750G 769A 825A 1018A 1291C 1438G 2394d 2581G 2706G 2758A 2885C 3106d 3107C 3594T 3666A 3796T 3834A 3843G 4104G 4589C 4688C 4769G 5480G 5553C 5951G 6071C 6179A 7028T 7055G 7146G 7196T 7256T 7389C 7521A 7660C 7714T 7854C 8027A 8468T 8619T 8655T 8701G 8860G 9072G 9540C 9861C 10084C 10321C 10398G 10586A 10688A 10810C 10873C 11353C 11440A 11719A 11899C 12681C 12705T 12810G 13105G 13485G 13506T 13650T 13789C 13874C 14000A 14148G 14178C 14393G 14560A 14766T 14911T 15326G 16086C 16129A 16187T 16189C 16223T 16241G 16278T 16293G 16294T 16311C 16360T 16519C	L1c1b	73G 151T 152C 182T 186A 189C 195C 198T 247A 263G 297G 316A 416A 514d 515d 520T 16086C 16129A 16187T 16189C 16223T 16241G 16278T 16293G 16294T 16311C 16360T 16519C	1	73 151 152 182 186A 189C 195 198 247 263 297 316 416A 518 750 769 825A 1018 1291 1438 2395d 2581 2706 2758 2885 3594 3666 3796T 3834 3843 4104 4589 4688 4769 5480 5553 5951 6071 6179 7028 7055 7146 7196 7256 7389 7521 7660 7714 7854 8027 8468 8619 8655 8701 8860 9072 9540 9861 10084 10321 10398 10586 10688 10810 10873 11353 11440 11719 11899 12681 12705 12810 13105 13485 13506 13650 13789 13874 14000A 14148 14178 14393 14560 14766 14911 15326 16086 16129 16187 16189 16223 16241 16278 16293 16294 16311 16360	L1c1b	73 151 152 182 186A 189C 195 198 247 263 297 316 416A 518 16086 16129 16187 16189 16223 16241 16278 16293 16294 16311 16360	[1.673] L1c1b (1)	[0.000] L1c1b'd (2)	1
684 >MF381425.1 Hc	73G 151T 152C 182T 186A 189C 195C 198T 247A 263G 297G 316A 416A 514d 515d 520T 750G 769A 825A 1018A 1291C 1438G 2394d 2581G 2706G 2758A 2885C 3106d 3107C 3594T 3666A 3796T 3834A 3843G 4104G 4589C 4688C 4769G 5480G 5553C 5951G 6071C 6179A 7028T 7055G 7146G 7196T 7256T 7389C 7521A 7660C 7714T 7854C 8027A 8468T 8619T 8655T 8701G 8860G 9072G 9540C 9861C 10084C 10321C 10398G 10586A 10688A 10810C 10873C 11353C 11440A 11719A 11899C 12681C 12705T 12810G 13105G 13485G 13506T 13650T 13789C 13874C 14000A 14148G 14178C 14393G 14560A 14766T 14911T 15326G 16086C 16129A 16187T 16189C 16223T 16241G 16278T 16293G 16294T 16311C 16360T 16519C	L1c1b	73G 151T 152C 182T 186A 189C 195C 198T 247A 263G 297G 316A 416A 514d 515d 520T 16086C 16129A 16187T 16189C 16223T 16241G 16278T 16293G 16294T 16311C 16360T 16519C	1	73 151 152 182 186A 189C 195 198 247 263 297 316 416A 518 750 769 825A 1018 1291 1438 2395d 2581 2706 2758 2885 3594 3666 3796T 3834 3843 4104 4589 4688 4769 5480 5553 5951 6071 6179 7028 7055 7146 7196 7256 7389 7521 7660 7714 7854 8027 8468 8619 8655 8701 8860 9072 9540 9861 10084 10321 10398 10586 10688 10810 10873 11353 11440 11719 11899 12681 12705 12810 13105 13485 13506 13650 13789 13874 14000A 14148 14178 14393 14560 14766 14911 15326 16086 16129 16187 16189 16223 16241 16278 16293 16294 16311 16360	L1c1b	73 151 152 182 186A 189C 195 198 247 263 297 316 416A 518 16086 16129 16187 16189 16223 16241 16278 16293 16294 16311 16360	[1.673] L1c1b (1)	[0.000] L1c1b'd (2)	1

685	>MF381422.1 Ho	73G 151T 152C 182T 186A 189C 195C 198T 247A 263G 297G 316A 416A 514d 515d 520T 750G 769A 825A 1018A 1291C 1438G 2394d 2581G 2706G 2758A 2885C 3106d 3107C 3594T 3666A 3796T 3834A 3843G 4104G 4589C 4688C 4769G 5480G 5553C 5951G 6071C 6179A 7028T 7055G 7146G 7196T 7256T 7389C 7521A 7660C 7714T 7854C 8027A 8468T 8619T 8655T 8701G 8860G 9072G 9540C 9861C 10084C 10321C 10398G 10586A 10688A 10810C 10873C 11353C 11440A 11719A 11899C 12681C 12705T 12810G 13105G 13485G 13506T 13650T 13789C 13874C 14000A 14148G 14178C 14393G 14560A 14766T 14911T 15326G 16086C 16129A 16187T 16189C 16223T 16241G 16278T 16293G 16294T 16311C 16360T 16519C	L1c1b	73G 151T 152C 182T 186A 189C 195C 198T 247A 263G 297G 316A 416A 514d 515d 520T 16086C 16129A 16187T 16189C 16223T 16241G 16278T 16293G 16294T 16311C 16360T 16519C	1	73 151 152 182 186A 189C 195 198 247 263 297 316 416A 518 750 769 825A 1018 1291 1438 2395d 2581 2706 2758 2885 3594 3666 3796T 3834 3843 4104 4589 4688 4769 5480 5553 5951 6071 6179 7028 7055 7146 7196 7256 7389 7521 7660 7714 7854 8027 8468 8619 8655 8701 8860 9072 9540 9861 10084 10321 10398 10586 10688 10810 10873 11353 11440 11719 11899 12681 12705 12810 13105 13485 13506 13650 13789 13874 14000A 14148 14178 14393 14560 14766 14911 15326 16086 16129 16187 16189 16223 16241 16278 16293 16294 16311 16360	L1c1b	73 151 152 182 186A 189C 195 198 247 263 297 316 416A 518 16086 16129 16187 16189 16223 16241 16278 16293 16294 16311 16360	[1.673] L1c1b (1)	[0.000] L1c1b'd (2)	1
686	>MF381415.1 Ho	73G 150T 152C 189G 195C 200G 207A 263G 750G 1438G 2352C 2706G 3106d 3107C 4769G 5774A 5998N 5999N 6000N 6001N 6002N 6003N 6004N 6005N 6006N 6007N 6008N 6221C 6587T 7028T 7775A 8701G 8860G 9254G 9540C 10398G 10819G 10873C 11024T 11719A 12705T 14152G 14212C 14569A 14766T 15301A 15326G 15670C 15942C 16185T 16209C 16223T 16327T	L3e1a2	73G 150T 152C 189G 195C 200G 207A 263G 16185T 16209C 16223T 16327T	1	73 150 152 189 195 200 207 263 750 1438 2352 2706 4769 5774A 5998N 5999N 6000N 6001N 6002N 6003N 6004N 6005N 6006N 6007N 6008N 6221 6587 7028 7775 8701 8860 9254 9540 10398 10819 10873 11024 11719 12705 14152 14212 14569 14766 15301 15326 15670 15942 16185 16209 16223 16327	L3e1a2	73 150 152 189 195 200 207 263 16185 16209 16223 16327	[0.000] L3e1a2 (1)	[0.000] L3e1f2 (2)	1

687 >MF381407.1 Ho	73G 146C 152C 188G 195C 204C 207A 247A 494d 514d 515d 719A 750G 769A 825A 1018A 1048T 2758A 2885C 3106d 3107C 3438A 3516A 3594T 3618C 3756G 4104G 4232C 4312T 4562C 4769G 5442C 6185C 6266G 6815C 7028T 7146G 7256T 7521A 8113A 8152A 8251A 8468T 8655T 8701G 8860G 9042T 9347G 9540C 9755A 10589A 10664T 10688A 10810C 10873C 10915C 11341G 11719A 11914A 12007A 12121C 12705T 12720G 13105G 13276G 13506T 13650T 13759A 14766T 15326G 15466A 15930A 15941C 16140C 16179T 16187T 16189C 16223T 16230G 16243C 16293G 16311C 16519C	L0d1b1b1	73G 146C 152C 188G 195C 204C 207A 247A 494d 514d 515d 16140C 16179T 16187T 16189C 16223T 16230G 16243C 16293G 16311C 16519C	1	73 146 152 188 195 204 207 247 498d 719 750 769 825A 1018 1048 2758 2885 3438 3516A 3594 3618 3756 4104 4232 4312 4562C 4769 5442 6185 6266 6815 7028 7146 7256 7521 8113A 8152 8251 8468 8655 8701 8860 9042 9347 9540 9755 10589 10664 10688 10810 10873 10915 11341 11719 11914 12007 12121 12705 12720 13105 13276 13506 13650 13759 14766 15326 15466 15930 15941 16140 16179 16187 16189 16223 16230 16243 16293 16311	L0d1b1b1	73 146 152 188 195 204 207 247 498d 16140 16179 16187 16189 16223 16230 16243 16293 16311	[0.853] L0d1b1b1 (1)	[0.000] L0d1b1 L0d1b1a L0d1b1a1 L0d1b1b (5)	1
688 >MF381402.1 Ho	73G 151T 152C 182T 186A 189C 195C 198T 247A 263G 297G 316A 416A 514d 515d 520T 750G 769A 825A 1018A 1291C 1438G 2394d 2581G 2706G 2758A 2885C 3106d 3107C 3594T 3666A 3796T 3834A 3843G 4104G 4589C 4688C 4769G 5480G 5553C 5951G 6071C 6179A 7028T 7055G 7146G 7196T 7256T 7389C 7521A 7660C 7714T 8027A 8468T 8619T 8655T 8701G 8860G 9072G 9540C 9861C 10084C 10321C 10398G 10586A 10688A 10810C 10873C 11353C 11440A 11719A 11899C 12681C 12705T 12810G 13105G 13485G 13506T 13650T 13789C 13874C 14000A 14148G 14178C 14393G 14560A 14766T 14911T 15326G 16086C 16129A 16187T 16189C 16223T 16241G 16278T 16293G 16294T 16311C 16360T 16519C	L1c1b	73G 151T 152C 182T 186A 189C 195C 198T 247A 263G 297G 316A 416A 514d 515d 520T 16086C 16129A 16187T 16189C 16223T 16241G 16278T 16293G 16294T 16311C 16360T 16519C	1	73 151 152 182 186A 189C 195 198 247 263 297 316 416A 518 750 769 825A 1018 1291 1438 2395d 2581 2706 2758 2885 3594 3666 3796T 3834 3843 4104 4589 4688 4769 5480 5553 5951 6071 6179 7028 7055 7146 7196 7256 7389 7521 7660 7714 8027 8468 8619 8655 8701 8860 9072 9540 9861 10084 10321 10398 10586 10688 10810 10873 11353 11440 11719 11899 12681 12705 12810 13105 13485 13506 13650 13789 13874 14000A 14148 14178 14393 14560 14766 14911 15326 16086 16129 16187 16189 16223 16241 16278 16293 16294 16311 16360	L1c1b	73 151 152 182 186A 189C 195 198 247 263 297 316 416A 518 16086 16129 16187 16189 16223 16241 16278 16293 16294 16311 16360	[1.673] L1c1b (1)	[0.000] L1c1b'd (2)	1

689 >MF381400.1 Ho	73G 146C 152C 188G 195C 204C 207A 247A 494d 514d 515d 719A 750G 769A 825A 1018A 1048T 2758A 2885C 3106d 3107C 3438A 3516A 3594T 3618C 3756G 4104G 4232C 4312T 4562C 4769G 5442C 6185C 6266G 6815C 7028T 7146G 7256T 7521A 8113A 8152A 8251A 8468T 8655T 8701G 8860G 9042T 9347G 9540C 9755A 10589A 10664T 10688A 10810C 10873C 10915C 11341G 11719A 11914A 12007A 12121C 12705T 12720G 13105G 13276G 13506T 13650T 13759A 14766T 15326G 15466A 15930A 15941C 16140C 16179T 16187T 16189C 16223T 16230G 16243C 16293G 16311C 16519C	L0d1b1b1	73G 146C 152C 188G 195C 204C 207A 247A 494d 514d 515d 16140C 16179T 16187T 16189C 16223T 16230G 16243C 16293G 16311C 16519C	1	73 146 152 188 195 204 207 247 498d 719 750 769 825A 1018 1048 2758 2885 3438 3516A 3594 3618 3756 4104 4232 4312 4562C 4769 5442 6185 6266 6815 7028 7146 7256 7521 8113A 8152 8251 8468 8655 8701 8860 9042 9347 9540 9755 10589 10664 10688 10810 10873 10915 11341 11719 11914 12007 12121 12705 12720 13105 13276 13506 13650 13759 14766 15326 15466 15930 15941 16140 16179 16187 16189 16223 16230 16243 16293 16311	L0d1b1b1	73 146 152 188 195 204 207 247 498d 16140 16179 16187 16189 16223 16230 16243 16293 16311	[0.853] L0d1b1b1 (1)	[0.000] L0d1b1 L0d1b1a L0d1b1a1 L0d1b1b (5)	1
690 >MF381398.1 Ho	73G 146C 152C 188G 195C 204C 207A 247A 494d 514d 515d 719A 750G 769A 825A 1018A 1048T 2758A 2885C 3106d 3107C 3438A 3516A 3594T 3618C 3756G 4104G 4232C 4312T 4562C 4769G 5442C 6185C 6266G 6815C 7028T 7146G 7256T 7521A 8113A 8152A 8251A 8468T 8655T 8701G 8860G 9042T 9347G 9540C 9755A 10589A 10664T 10688A 10810C 10873C 10915C 11341G 11719A 11914A 12007A 12121C 12705T 12720G 13105G 13276G 13506T 13650T 13759A 14766T 15326G 15466A 15930A 15941C 16140C 16179T 16187T 16189C 16223T 16230G 16243C 16293G 16311C 16519C	L0d1b1b1	73G 146C 152C 188G 195C 204C 207A 247A 494d 514d 515d 16140C 16179T 16187T 16189C 16223T 16230G 16243C 16293G 16311C 16519C	1	73 146 152 188 195 204 207 247 498d 719 750 769 825A 1018 1048 2758 2885 3438 3516A 3594 3618 3756 4104 4232 4312 4562C 4769 5442 6185 6266 6815 7028 7146 7256 7521 8113A 8152 8251 8468 8655 8701 8860 9042 9347 9540 9755 10589 10664 10688 10810 10873 10915 11341 11719 11914 12007 12121 12705 12720 13105 13276 13506 13650 13759 14766 15326 15466 15930 15941 16140 16179 16187 16189 16223 16230 16243 16293 16311	L0d1b1b1	73 146 152 188 195 204 207 247 498d 16140 16179 16187 16189 16223 16230 16243 16293 16311	[0.853] L0d1b1b1 (1)	[0.000] L0d1b1 L0d1b1a L0d1b1a1 L0d1b1b (5)	1

691 >MF381394.1 Ho	73G 146C 152C 188G 195C 204C 207A 247A 494d 514d 515d 719A 750G 769A 825A 1018A 1048T 2758A 2885C 3106d 3107C 3438A 3516A 3594T 3618C 3756G 4104G 4232C 4312T 4562C 4769G 5442C 6185C 6266G 6815C 7028T 7146G 7256T 7521A 8113A 8152A 8251A 8468T 8655T 8701G 8860G 9042T 9347G 9540C 9755A 10589A 10664T 10688A 10810C 10873C 10915C 11341G 11719A 11914A 12007A 12121C 12705T 12720G 13105G 13276G 13506T 13650T 13759A 14766T 15326G 15466A 15930A 15941C 16140C 16179T 16187T 16189C 16223T 16230G 16243C 16293G 16311C 16519C	L0d1b1b1	73G 146C 152C 188G 195C 204C 207A 247A 494d 514d 515d 16140C 16179T 16187T 16189C 16223T 16230G 16243C 16293G 16311C 16519C	1	73 146 152 188 195 204 207 247 498d 719 750 769 825A 1018 1048 2758 2885 3438 3516A 3594 3618 3756 4104 4232 4312 4562C 4769 5442 6185 6266 6815 7028 7146 7256 7521 8113A 8152 8251 8468 8655 8701 8860 9042 9347 9540 9755 10589 10664 10688 10810 10873 10915 11341 11719 11914 12007 12121 12705 12720 13105 13276 13506 13650 13759 14766 15326 15466 15930 15941 16140 16179 16187 16189 16223 16230 16243 16293 16311	L0d1b1b1	73 146 152 188 195 204 207 247 498d 16140 16179 16187 16189 16223 16230 16243 16293 16311	[0.853] L0d1b1b1 (1)	[0.000] L0d1b1 L0d1b1a L0d1b1a1 L0d1b1b (5)	1
692 >MF381390.1 Ho	73G 152C 182T 186A 189C 195C 198T 247A 263G 297G 316A 709A 750G 769A 825A 1018A 1420C 1438G 2394d 2706G 2758A 2885C 3106d 3107C 3594T 3666A 4104G 4769G 5951G 6071C 6150A 6253C 7028T 7055G 7076G 7146G 7256T 7337A 7389C 7521A 7744C 8027A 8468T 8655T 8701G 8784G 8860G 8877C 9072G 9181G 9540C 10321C 10398G 10586A 10688A 10792G 10793T 10810C 10873C 11654G 11719A 12049T 12705T 12810G 13105G 13149G 13212T 13485G 13506T 13650T 13789C 14000A 14178C 14560A 14766T 14812T 14911T 15016T 15326G 15784C 16071T 16129A 16145A 16187T 16189C 16213A 16223T 16234T 16265C 16278T 16286G 16294T 16311C 16360T 16527T	L1c2a1a	73G 152C 182T 186A 189C 195C 198T 247A 263G 297G 316A 16071T 16129A 16145A 16187T 16189C 16213A 16223T 16234T 16265C 16278T 16286G 16294T 16311C 16360T 16527T	1	73 152 182 186A 189C 195 198 247 263 297 316 709 750 769 825A 1018 1420 1438 2395d 2706 2758 2885 3594 3666 4104 4769 5951 6071 6150 6253 7028 7055 7076 7146 7256 7337 7389 7521 7744 8027 8468 8655 8701 8784 8860 8877 9072 9181 9540 10321 10398 10586 10688 10792 10793 10810 10873 11654 11719 12049 12705 12810 13105 13149 13212 13485 13506 13650 13789 14000A 14178 14560 14766 14812 14911 15016 15326 15784 16071 16129 16145 16187 16189 16213 16223 16234 16265C 16278 16286G 16294 16311 16360 16527	L1c2a1a	73 152 182 186A 189C 195 198 247 263 297 316 16071 16129 16145 16187 16189 16213 16223 16234 16265C 16278 16286G 16294 16311 16360 16527	[0.105] L1c2a1a (1)	[0.158] L1c2a1b (2) [0.000] L1c2a1	1

693	>MF381389.1 Hc	73G 146C 152C 188G 195C 204C 207A 247A 494d 514d 515d 719A 750G 769A 825A 1018A 1048T 2758A 2885C 3106d 3107C 3438A 3516A 3594T 3618C 3756G 4104G 4232C 4312T 4562C 4769G 5442C 6185C 6266G 6815C 7028T 7146G 7256T 7521A 8113A 8152A 8251A 8468T 8655T 8701G 8860G 9042T 9347G 9540C 9755A 10589A 10664T 10688A 10810C 10873C 10915C 11341G 11719A 11914A 12007A 12121C 12705T 12720G 13105G 13276G 13506T 13650T 13759A 14766T 15326G 15466A 15930A 15941C 16140C 16179T 16187T 16189C 16223T 16230G 16243C 16293G 16311C 16519C	L0d1b1b1	73G 146C 152C 188G 195C 204C 207A 247A 494d 514d 515d 16140C 16179T 16187T 16189C 16223T 16230G 16243C 16293G 16311C 16519C	1	73 146 152 188 195 204 207 247 498d 719 750 769 825A 1018 1048 2758 2885 3438 3516A 3594 3618 3756 4104 4232 4312 4562C 4769 5442 6185 6266 6815 7028 7146 7256 7521 8113A 8152 8251 8468 8655 8701 8860 9042 9347 9540 9755 10589 10664 10688 10810 10873 10915 11341 11719 11914 12007 12121 12705 12720 13105 13276 13506 13650 13759 14766 15326 15466 15930 15941 16140 16179 16187 16189 16223 16230 16243 16293 16311	L0d1b1b1	73 146 152 188 195 204 207 247 498d 16140 16179 16187 16189 16223 16230 16243 16293 16311	[0.853] L0d1b1b1 (1)	[0.000] L0d1b1 L0d1b1a L0d1b1a1 L0d1b1b (5)	1
694	>MF381388.1 Hc	73G 152C 182T 186A 189C 195C 198T 247A 263G 297G 316A 709A 750G 769A 825A 1018A 1420C 1438G 1949A 2394d 2706G 2758A 2885C 3106d 3107C 3594T 3666A 4104G 4769G 5951G 6071C 6150A 6253C 7028T 7055G 7076G 7146G 7256T 7337A 7389C 7521A 7744C 8027A 8468T 8655T 8701G 8784G 8860G 8877C 9072G 9181G 9540C 10321C 10398G 10586A 10688A 10792G 10793T 10810C 10873C 11654G 11719A 12049T 12705T 12810G 13105G 13149G 13212T 13485G 13506T 13650T 13789C 14000A 14178C 14560A 14766T 14812T 14911T 15016T 15326G 15784C 16071T 16129A 16145A 16187T 16189C 16213A 16223T 16234T 16265C 16278T 16286G 16294T 16311C 16360T 16527T	L1c2a1a	73G 152C 182T 186A 189C 195C 198T 247A 263G 297G 316A 16071T 16129A 16145A 16187T 16189C 16213A 16223T 16234T 16265C 16278T 16286G 16294T 16311C 16360T 16527T	1	73 152 182 186A 189C 195 198 247 263 297 316 709 750 769 825A 1018 1420 1438 1949 2395d 2706 2758 2885 3594 3666 4104 4769 5951 6071 6150 6253 7028 7055 7076 7146 7256 7337 7389 7521 7744 8027 8468 8655 8701 8784 8860 8877 9072 9181 9540 10321 10398 10586 10688 10792 10793 10810 10873 11654 11719 12049 12705 12810 13105 13149 13212 13485 13506 13650 13789 14000A 14178 14560 14766 14812 14911 15016 15326 15784 16071 16129 16145 16187 16189 16213 16223 16234 16265C 16278 16286G 16294 16311 16360 16527	L1c2a1a	73 152 182 186A 189C 195 198 247 263 297 316 16071 16129 16145 16187 16189 16213 16223 16234 16265C 16278 16286G 16294 16311 16360 16527	[0.105] L1c2a1a (1)	[0.158] L1c2a1b (2) [0.000] L1c2a1	1

695	>MF381387.1 H[illegible]	73G 151T 152C 182T 186A 189C 195C 198T 247A 263G 297G 316A 416A 514d 515d 520T 750G 769A 825A 1018A 1291C 1438G 2394d 2581G 2706G 2758A 2885C 3106d 3107C 3594T 3666A 3796T 3834A 3843G 4104G 4589C 4688C 4769G 5480G 5553C 5951G 6071C 6179A 7028T 7055G 7146G 7196T 7256T 7389C 7521A 7660C 7714T 7854C 8027A 8468T 8619T 8655T 8701G 8860G 9072G 9540C 9861C 10084C 10321C 10398G 10586A 10688A 10810C 10873C 11353C 11440A 11719A 11899C 12681C 12705T 12810G 13105G 13485G 13506T 13650T 13789C 13874C 14000A 14148G 14178C 14393G 14560A 14766T 14911T 15326G 16086C 16129A 16187T 16189C 16223T 16241G 16278T 16293G 16294T 16311C 16360T 16519C	L1c1b	73G 151T 152C 182T 186A 189C 195C 198T 247A 263G 297G 316A 416A 514d 515d 520T 16086C 16129A 16187T 16189C 16223T 16241G 16278T 16293G 16294T 16311C 16360T 16519C	1	73 151 152 182 186A 189C 195 198 247 263 297 316 416A 518 750 769 825A 1018 1291 1438 2395d 2581 2706 2758 2885 3594 3666 3796T 3834 3843 4104 4589 4688 4769 5480 5553 5951 6071 6179 7028 7055 7146 7196 7256 7389 7521 7660 7714 7854 8027 8468 8619 8655 8701 8860 9072 9540 9861 10084 10321 10398 10586 10688 10810 10873 11353 11440 11719 11899 12681 12705 12810 13105 13485 13506 13650 13789 13874 14000A 14148 14178 14393 14560 14766 14911 15326 16086 16129 16187 16189 16223 16241 16278 16293 16294 16311 16360	L1c1b	73 151 152 182 186A 189C 195 198 247 263 297 316 416A 518 16086 16129 16187 16189 16223 16241 16278 16293 16294 16311 16360	[1.673] L1c1b (1)	[0.000] L1c1b'd (2)	1
696	>MF381385.1 H[illegible]	73G 151T 152C 182T 186A 189C 195C 198T 247A 263G 297G 316A 416A 514d 515d 520T 750G 769A 825A 1018A 1291C 1438G 2394d 2581G 2706G 2758A 2885C 3106d 3107C 3594T 3666A 3796T 3834A 3843G 4104G 4589C 4688C 4769G 5480G 5553C 5951G 6071C 6179A 7028T 7055G 7146G 7196T 7256T 7389C 7521A 7660C 7714T 8027A 8468T 8619T 8655T 8701G 8860G 9072G 9540C 9861C 10084C 10321C 10398G 10586A 10688A 10810C 10873C 11353C 11440A 11719A 11899C 12681C 12705T 12810G 13105G 13485G 13506T 13650T 13789C 13874C 14000A 14148G 14178C 14393G 14560A 14766T 14911T 15326G 16086C 16129A 16187T 16189C 16223T 16241G 16278T 16293G 16294T 16311C 16360T 16519C	L1c1b	73G 151T 152C 182T 186A 189C 195C 198T 247A 263G 297G 316A 416A 514d 515d 520T 16086C 16129A 16187T 16189C 16223T 16241G 16278T 16293G 16294T 16311C 16360T 16519C	1	73 151 152 182 186A 189C 195 198 247 263 297 316 416A 518 750 769 825A 1018 1291 1438 2395d 2581 2706 2758 2885 3594 3666 3796T 3834 3843 4104 4589 4688 4769 5480 5553 5951 6071 6179 7028 7055 7146 7196 7256 7389 7521 7660 7714 8027 8468 8619 8655 8701 8860 9072 9540 9861 10084 10321 10398 10586 10688 10810 10873 11353 11440 11719 11899 12681 12705 12810 13105 13485 13506 13650 13789 13874 14000A 14148 14178 14393 14560 14766 14911 15326 16086 16129 16187 16189 16223 16241 16278 16293 16294 16311 16360	L1c1b	73 151 152 182 186A 189C 195 198 247 263 297 316 416A 518 16086 16129 16187 16189 16223 16241 16278 16293 16294 16311 16360	[1.673] L1c1b (1)	[0.000] L1c1b'd (2)	1

697 >MF381383.1 Hc	73G 151T 152C 182T 186A 189C 195C 198T 247A 263G 297G 316A 416A 514d 515d 520T 750G 769A 825A 1018A 1291C 1438G 2394d 2581G 2706G 2758A 2885C 3106d 3107C 3594T 3666A 3796T 3834A 3843G 4104G 4589C 4688C 4769G 5480G 5553C 5951G 6071C 6179A 7028T 7055G 7146G 7196T 7256T 7389C 7521A 7660C 7714T 8027A 8468T 8619T 8655T 8701G 8860G 9072G 9540C 9861C 10084C 10321C 10398G 10586A 10688A 10810C 10873C 11353C 11440A 11719A 11899C 12681C 12705T 12810G 13105G 13485G 13506T 13650T 13789C 13874C 14000A 14148G 14178C 14393G 14560A 14766T 14911T 15326G 16086C 16129A 16187T 16189C 16223T 16241G 16278T 16293G 16294T 16311C 16360T 16519C	L1c1b	73G 151T 152C 182T 186A 189C 195C 198T 247A 263G 297G 316A 416A 514d 515d 520T 16086C 16129A 16187T 16189C 16223T 16241G 16278T 16293G 16294T 16311C 16360T 16519C	1	73 151 152 182 186A 189C 195 198 247 263 297 316 416A 518 750 769 825A 1018 1291 1438 2395d 2581 2706 2758 2885 3594 3666 3796T 3834 3843 4104 4589 4688 4769 5480 5553 5951 6071 6179 7028 7055 7146 7196 7256 7389 7521 7660 7714 8027 8468 8619 8655 8701 8860 9072 9540 9861 10084 10321 10398 10586 10688 10810 10873 11353 11440 11719 11899 12681 12705 12810 13105 13485 13506 13650 13789 13874 14000A 14148 14178 14393 14560 14766 14911 15326 16086 16129 16187 16189 16223 16241 16278 16293 16294 16311 16360	L1c1b	73 151 152 182 186A 189C 195 198 247 263 297 316 416A 518 16086 16129 16187 16189 16223 16241 16278 16293 16294 16311 16360	[1.673] L1c1b (1)	[0.000] L1c1b'd (2)	1
698 >MF381381.1 Hc	73G 151T 152C 182T 186A 189C 195C 198T 247A 263G 297G 316A 416A 514d 515d 520T 750G 769A 825A 1018A 1291C 1438G 2394d 2581G 2706G 2758A 2885C 3106d 3107C 3594T 3666A 3796T 3834A 3843G 4104G 4589C 4688C 4769G 5480G 5553C 5951G 6071C 6179A 7028T 7055G 7146G 7196T 7256T 7389C 7521A 7660C 7714T 8027A 8468T 8619T 8655T 8701G 8860G 9072G 9540C 9861C 10084C 10321C 10398G 10586A 10688A 10810C 10873C 11353C 11440A 11719A 11899C 12681C 12705T 12810G 13105G 13485G 13506T 13650T 13789C 13874C 14000A 14148G 14178C 14393G 14560A 14766T 14911T 15326G 16086C 16129A 16187T 16189C 16223T 16241G 16278T 16293G 16294T 16311C 16360T 16519C	L1c1b	73G 151T 152C 182T 186A 189C 195C 198T 247A 263G 297G 316A 416A 514d 515d 520T 16086C 16129A 16187T 16189C 16223T 16241G 16278T 16293G 16294T 16311C 16360T 16519C	1	73 151 152 182 186A 189C 195 198 247 263 297 316 416A 518 750 769 825A 1018 1291 1438 2395d 2581 2706 2758 2885 3594 3666 3796T 3834 3843 4104 4589 4688 4769 5480 5553 5951 6071 6179 7028 7055 7146 7196 7256 7389 7521 7660 7714 8027 8468 8619 8655 8701 8860 9072 9540 9861 10084 10321 10398 10586 10688 10810 10873 11353 11440 11719 11899 12681 12705 12810 13105 13485 13506 13650 13789 13874 14000A 14148 14178 14393 14560 14766 14911 15326 16086 16129 16187 16189 16223 16241 16278 16293 16294 16311 16360	L1c1b	73 151 152 182 186A 189C 195 198 247 263 297 316 416A 518 16086 16129 16187 16189 16223 16241 16278 16293 16294 16311 16360	[1.673] L1c1b (1)	[0.000] L1c1b'd (2)	1

699 >MF381378.1 Ho	73G 151T 152C 182T 186A 189C 195C 198T 247A 263G 297G 316A 416A 514d 515d 520T 750G 769A 825A 1018A 1291C 1438G 2394d 2581G 2706G 2758A 2885C 3106d 3107C 3594T 3666A 3796T 3834A 3843G 4104G 4589C 4688C 4769G 5480G 5553C 5951G 6071C 6179A 7028T 7055G 7146G 7196T 7256T 7389C 7521A 7660C 7714T 8027A 8468T 8619T 8655T 8701G 8860G 9072G 9540C 9861C 10084C 10321C 10398G 10586A 10688A 10810C 10873C 11353C 11440A 11719A 11899C 12681C 12705T 12810G 13105G 13485G 13506T 13650T 13789C 13874C 14000A 14148G 14178C 14393G 14560A 14766T 14911T 15326G 16086C 16129A 16187T 16189C 16223T 16241G 16278T 16293G 16294T 16311C 16360T 16519C	L1c1b	73G 151T 152C 182T 186A 189C 195C 198T 247A 263G 297G 316A 416A 514d 515d 520T 16086C 16129A 16187T 16189C 16223T 16241G 16278T 16293G 16294T 16311C 16360T 16519C	1	73 151 152 182 186A 189C 195 198 247 263 297 316 416A 518 750 769 825A 1018 1291 1438 2395d 2581 2706 2758 2885 3594 3666 3796T 3834 3843 4104 4589 4688 4769 5480 5553 5951 6071 6179 7028 7055 7146 7196 7256 7389 7521 7660 7714 8027 8468 8619 8655 8701 8860 9072 9540 9861 10084 10321 10398 10586 10688 10810 10873 11353 11440 11719 11899 12681 12705 12810 13105 13485 13506 13650 13789 13874 14000A 14148 14178 14393 14560 14766 14911 15326 16086 16129 16187 16189 16223 16241 16278 16293 16294 16311 16360	L1c1b	73 151 152 182 186A 189C 195 198 247 263 297 316 416A 518 16086 16129 16187 16189 16223 16241 16278 16293 16294 16311 16360	[1.673] L1c1b (1)	[0.000] L1c1b'd (2)	1
700 >MF381375.1 Ho	73G 151T 152C 182T 186A 189C 195C 198T 247A 263G 297G 316A 416A 514d 515d 520T 750G 769A 825A 1018A 1291C 1438G 2394d 2581G 2706G 2758A 2885C 3106d 3107C 3594T 3666A 3796T 3834A 3843G 4104G 4589C 4688C 4769G 5480G 5553C 5951G 6071C 6179A 7028T 7055G 7146G 7196T 7256T 7389C 7521A 7660C 7714T 7979A 8027A 8468T 8619T 8655T 8701G 8860G 9072G 9540C 9861C 10084C 10321C 10398G 10586A 10688A 10810C 10873C 11353C 11440A 11719A 11899C 12681C 12705T 12810G 13105G 13485G 13506T 13650T 13789C 13874C 14000A 14148G 14178C 14393G 14560A 14766T 14911T 15326G 15930A 16086C 16129A 16187T 16189C 16223T 16241G 16278T 16293G 16294T 16311C 16360T 16519C	L1c1b	73G 151T 152C 182T 186A 189C 195C 198T 247A 263G 297G 316A 416A 514d 515d 520T 16086C 16129A 16187T 16189C 16223T 16241G 16278T 16293G 16294T 16311C 16360T 16519C	1	73 151 152 182 186A 189C 195 198 247 263 297 316 416A 518 750 769 825A 1018 1291 1438 2395d 2581 2706 2758 2885 3594 3666 3796T 3834 3843 4104 4589 4688 4769 5480 5553 5951 6071 6179 7028 7055 7146 7196 7256 7389 7521 7660 7714 7979 8027 8468 8619 8655 8701 8860 9072 9540 9861 10084 10321 10398 10586 10688 10810 10873 11353 11440 11719 11899 12681 12705 12810 13105 13485 13506 13650 13789 13874 14000A 14148 14178 14393 14560 14766 14911 15326 15930 16086 16129 16187 16189 16223 16241 16278 16293 16294 16311 16360	L1c1b	73 151 152 182 186A 189C 195 198 247 263 297 316 416A 518 16086 16129 16187 16189 16223 16241 16278 16293 16294 16311 16360	[1.673] L1c1b (1)	[0.000] L1c1b'd (2)	1

701 >MF381374.1 Hc	73G 151T 152C 182T 186A 189C 195C 198T 247A 263G 297G 316A 416A 514d 515d 520T 750G 769A 825A 1018A 1291C 1438G 2394d 2581G 2706G 2758A 2885C 3106d 3107C 3594T 3666A 3796T 3834A 3843G 4104G 4589C 4688C 4769G 5480G 5553C 5951G 6071C 6179A 7028T 7055G 7146G 7196T 7256T 7389C 7521A 7660C 7714T 8027A 8468T 8619T 8655T 8701G 8860G 9072G 9540C 9861C 10084C 10321C 10398G 10586A 10688A 10810C 10873C 11353C 11440A 11719A 11899C 12681C 12705T 12810G 13105G 13485G 13506T 13650T 13789C 13874C 14000A 14148G 14178C 14393G 14560A 14766T 14911T 15326G 16086C 16129A 16187T 16189C 16223T 16241G 16278T 16293G 16294T 16311C 16360T 16519C	L1c1b	73G 151T 152C 182T 186A 189C 195C 198T 247A 263G 297G 316A 416A 514d 515d 520T 16086C 16129A 16187T 16189C 16223T 16241G 16278T 16293G 16294T 16311C 16360T 16519C	1	73 151 152 182 186A 189C 195 198 247 263 297 316 416A 518 750 769 825A 1018 1291 1438 2395d 2581 2706 2758 2885 3594 3666 3796T 3834 3843 4104 4589 4688 4769 5480 5553 5951 6071 6179 7028 7055 7146 7196 7256 7389 7521 7660 7714 8027 8468 8619 8655 8701 8860 9072 9540 9861 10084 10321 10398 10586 10688 10810 10873 11353 11440 11719 11899 12681 12705 12810 13105 13485 13506 13650 13789 13874 14000A 14148 14178 14393 14560 14766 14911 15326 16086 16129 16187 16189 16223 16241 16278 16293 16294 16311 16360	L1c1b	73 151 152 182 186A 189C 195 198 247 263 297 316 416A 518 16086 16129 16187 16189 16223 16241 16278 16293 16294 16311 16360	[1.673] L1c1b (1)	[0.000] L1c1b'd (2)	1
702 >KX457667.1 Hc	73G 146C 195C 263G 309.1CT 310C 489C 514d 515d 750G 951A 1438G 2140A 2706G 3106d 3107C 4769G 5773A 7028T 8701G 8860G 9540C 10101C 10398G 10400T 10873C 11719A 12651A 12705T 13359A 13812C 14766T 14783C 15043A 15301A 15326G 15601C 16086C 16223T 16519C	M24b	73G 146C 195C 263G 309.1CT 310C 489C 514d 515d 16086C 16223T 16519C	1	73 146 195 263 489 750 951 1438 2140 2706 4769 5773 7028 8701 8860 9540 10101 10398 10400 10873 11719 12651 12705 13359 13812 14766 14783 15043 15301 15326 15601 16086 16223	M24b	73 146 195 263 489 16086 16223	[0.000] M24b (1)	[0.000] M24 (2)	1
703 >KX457666.1 Hc	73G 152C 225A 248d 263G 310.1C 316A 489C 514d 515d 750G 956T 1438G 2706G 3106d 3107C 3200C 3714G 4385T 4769G 4772C 7028T 7433T 8701G 8853G 8860G 9127G 9512T 9540C 10274C 10398G 10400T 10679G 10873C 11719A 11914A 12354C 12705T 14110C 14766T 14783C 14974T 15043A 15301A 15326G 15691G 16086C 16129A 16209C 16223T 16272G 16519C	M20	73G 152C 225A 248d 263G 310.1C 316A 489C 514d 515d 16086C 16129A 16209C 16223T 16272G 16519C	1	73 152 225 249d 263 316 489 750 956 1438 2706 3200 3714 4385T 4769 4772 7028 7433 8701 8853 8860 9127 9512 9540 10274 10398 10400 10679 10873 11719 11914 12354 12705 14110 14766 14783 14974 15043 15301 15326 15691 16086 16129 16209 16223 16272	M20	73 152 225 249d 263 316 489 16086 16129 16209 16223 16272	[0.607] M20 (1)	[0.130] M5a2a (2) [0.068] M17 (3) [0.067] M17a (4) [0.059] G1b+16129 (5) [0.028] M5a (6) [0.021] D4a1a1 (7) [0.019] M5 M27b2b1 (9) [0.007] M5a'd (10) [0.006] G1b2 G1b4 D4a1 (13) [0.004] M37+152 D4a1f1 (15) [0.000] M5a2 M5a2a1 M5a5 M5b'c M5b M7a M7a1b2 M7a2 M7b1 M13'46'61 M13 M13a'b M13b M17c M27b2 M27b2a M27b2b M27b2c D4a D4a1a D4a1a1a D4a1b D4a1c D4a1f D4a1g D4a1h D4a2 D4a2a D4a2b D4a5 (45)	1

704	>KX457664.1 Ho	73G 152C 210G 263G 514d 515d 709A 750G 1438G 2706G 3106d 3107C 3537G 4086T 4769G 6960T 7028T 8271d 8272d 8273d 8274d 8275d 8276d 8277d 8278d 8279d 8584A 8860G 9053A 9950C 10398G 11465C 11719A 14198A 14766T 15235G 15326G 16093C 16140C 16182C 16183C 16189C 16261T 16266A 16519C	B5a1d	73G 152C 210G 263G 514d 515d 16093C 16140C 16182C 16183C 16189C 16261T 16266A 16519C	1	73 152 210 263 709 750 1438 2706 3537 4086 4769 6960 7028 8281-8289d 8584 8860 9053 9950 10398 11465 11719 14198 14766 15235 15326 16093 16140 16189 16261 16266A	B5a1d	73 152 210 263 16093 16140 16189 16261 16266A	[0.028] B5a1d (1)	[0.102] B5a (2) [0.032] B5a1 (3) [0.019] B5a1a (4) [0.004] B5a1b1 (5) [0.002] B5a1c1a (6) [0.000] B5a1a1 B5a1b B5a1c B5a1c1 B5a1c2 (11)	1
705	>KX457662.1 Ho	73G 150T 263G 309.1CT 310C 385G 489C 750G 1438G 2706G 3106d 3107C 3394C 4491A 4769G 7028T 8155A 8701G 8860G 9540C 10398G 10400T 10873C 11719A 12237T 12414C 12705T 13098G 14308C 14766T 14783C 15043A 15301A 15326G 16093C 16223T 16234T 16362C 16519C	M9a5	73G 150T 263G 309.1CT 310C 385G 489C 16093C 16223T 16234T 16362C 16519C	1	73 150 263 385 489 750 1438 2706 3394 4491 4769 7028 8155 8701 8860 9540 10398 10400 10873 11719 12237 12414 12705 13098 14308 14766 14783 15043 15301 15326 16093 16223 16234 16362	M9a5	73 150 263 385 489 16093 16223 16234 16362	[0.125] M9a5 (1) [0.000] G1a D4b2b2a D4b2b2a1 D4b2b4 (5)	[0.067] M9a1a1c1b1a (6) [0.025] D4b2b (7) [0.020] D4 (8) [0.018] M9a G1a1a1 (10) [0.014] M49c M49c1 M49e1 (13) [0.010] M9a1b2 G2a'c (15) [0.007] G1 G1c (17) [0.006] G1b D4e2 (19) [0.005] M9a4 (20) [0.004] M9a1b+150 G2b2 (22) [0.003] D4h4 (23) [0.002] G G2 G2b G2b1 G2b2a D4b2b1 D4e2c D4j1b (31) [0.001] M9a1a1a M9a1a1c1b1 M9a1a1c1b1a1 D4b D4b2 D4b2b2 D4b2b6 (38) [0.000] M9 M9a1 M9a4b G1a1 G1a1a G1a1a2 G1a1a4 G1a1b G1a2'3 G1c2 G2b1a M33+16362 M49 M49e M62'68 D D1d2 D4b2a D4b2a2 D4b2a2b D4b2b1a D4b2b1b D4b2b1c D4b2b2a D4b2b2a1 D4b2b3 D4b2b4 D4e D4e1'3 D4e1 D4e1c D4e3 D4e2b D4e2d D4e4 D4e4a D4e4b D4f D4f1 D4g D4h D4j D4j1 D4j4 D4j5 D4j5a D4j+(16286) D4j9 D4j10 D4j12 D4j15 D4j16 D4m D5 D5a'b D5a (94)	1
706	>KX457658.1 Ho	73G 263G 310.1C 329A 513A 750G 1040C 1438G 2706G 3106d 3107C 4491A 4769G 6770G 7028T 7861C 8860G 9653C 10316G 10398G 10609C 10685A 10877T 10927C 11719A 12173C 12366G 13145A 13359A 14766T 15163G 15326G 15885T 16249C 16257T 16288C 16304C 16390A 16519C	R22	73G 263G 310.1C 329A 513A 16249C 16257T 16288C 16304C 16390A 16519C	1	73 263 329 513 750 1040 1438 2706 4491 4769 6770 7028 7861 8860 9653 10316 10398 10609 10685 10877 10927 11719 12173 12366 13145 13359 14766 15163 15326 15885 16249 16257 16288 16304 16390	R22	73 263 329 513 16249 16257 16288 16304 16390	[0.983] R22 (1)	[0.016] R9b1 (2) [0.000] R9 R9b R9c (5)	1

707	>KX457657.1 Hc	73G 152C 225A 248d 263G 310.1C 316A 489C 514d 515d 750G 956T 1438G 2706G 3106d 3107C 3200C 3714G 4385T 4769G 4772C 7028T 7433T 8701G 8853G 8860G 9127G 9512T 9540C 10274C 10398G 10400T 10679G 10873C 11719A 11914A 12354C 12705T 14110C 14766T 14783C 14974T 15043A 15301A 15326G 15691G 16086C 16129A 16209C 16223T 16272G 16519C	M20	73G 152C 225A 248d 263G 310.1C 316A 489C 514d 515d 16086C 16129A 16209C 16223T 16272G 16519C	1	73 152 225 249d 263 316 489 750 956 1438 2706 3200 3714 4385T 4769 4772 7028 7433 8701 8853 8860 9127 9512 9540 10274 10398 10400 10679 10873 11719 11914 12354 12705 14110 14766 14783 14974 15043 15301 15326 15691 16086 16129 16209 16223 16272	M20	73 152 225 249d 263 316 489 16086 16129 16209 16223 16272	[0.607] M20 (1)	[0.130] M5a2a (2) [0.068] M17 (3) [0.067] M17a (4) [0.059] G1b+16129 (5) [0.028] M5a (6) [0.021] D4a1a1 (7) [0.019] M5 M27b2b1 (9) [0.007] M5a'd (10) [0.006] G1b2 G1b4 D4a1 (13) [0.004] M37+152 D4a1f1 (15) [0.000] M5a2 M5a2a1 M5a5 M5b'c M5b M7a M7a1b2 M7a2 M7b1 M13'46'61 M13 M13a'b M13b M17c M27b2 M27b2a M27b2b M27b2c D4a D4a1a D4a1a1a D4a1b D4a1c D4a1f D4a1g D4a1h D4a2 D4a2a D4a2b D4a5 (45)	1
708	>KX457655.1 Hc	73G 248d 263G 309.1CT 310C 514d 515d 750G 1438G 2706G 3106d 3107C 3970T 4086T 4769G 6392C 6962A 7028T 7444A 8659G 8860G 9053A 10034C 10310A 10609C 11719A 12406A 12882T 13759A 13928C 14766T 15326G 16172C 16304C 16465T 16519C	F1a2a	73G 248d 263G 309.1CT 310C 514d 515d 16172C 16304C 16465T 16519C	1	73 249d 263 750 1438 2706 3970 4086 4769 6392 6962 7028 7444 8659 8860 9053 10034 10310 10609 11719 12406 12882 13759 13928C 14766 15326 16172 16304 16465	F1a2a	73 249d 263 16172 16304 16465	[0.000] F1a2a (1)	[0.000] F1a2 (2)	1
709	>KX457653.1 Hc	73G 263G 310.1C 329A 513A 750G 1040C 1438G 2706G 3106d 3107C 4491A 4769G 6770G 7028T 7861C 8860G 9653C 10316G 10398G 10609C 10685A 10877T 10927C 11719A 12173C 12366G 13145A 13359A 14766T 15163G 15326G 15885T 16249C 16257T 16288C 16304C 16390A 16519C	R22	73G 263G 310.1C 329A 513A 16249C 16257T 16288C 16304C 16390A 16519C	1	73 263 329 513 750 1040 1438 2706 4491 4769 6770 7028 7861 8860 9653 10316 10398 10609 10685 10877 10927 11719 12173 12366 13145 13359 14766 15163 15326 15885 16249 16257 16288 16304 16390	R22	73 263 329 513 16249 16257 16288 16304 16390	[0.983] R22 (1)	[0.016] R9b1 (2) [0.000] R9 R9b R9c (5)	1
710	>KX457652.1 Hc	73G 152C 225A 248d 263G 310.1C 316A 489C 514d 515d 750G 956T 1438G 2706G 3106d 3107C 3200C 3714G 4385T 4769G 4772C 7028T 7433T 8701G 8853G 8860G 9127G 9512T 9540C 10274C 10398G 10400T 10679G 10873C 11719A 11914A 12354C 12705T 14110C 14766T 14783C 14974T 15043A 15301A 15326G 15691G 16086C 16129A 16209C 16223T 16272G 16519C	M20	73G 152C 225A 248d 263G 310.1C 316A 489C 514d 515d 16086C 16129A 16209C 16223T 16272G 16519C	1	73 152 225 249d 263 316 489 750 956 1438 2706 3200 3714 4385T 4769 4772 7028 7433 8701 8853 8860 9127 9512 9540 10274 10398 10400 10679 10873 11719 11914 12354 12705 14110 14766 14783 14974 15043 15301 15326 15691 16086 16129 16209 16223 16272	M20	73 152 225 249d 263 316 489 16086 16129 16209 16223 16272	[0.607] M20 (1)	[0.130] M5a2a (2) [0.068] M17 (3) [0.067] M17a (4) [0.059] G1b+16129 (5) [0.028] M5a (6) [0.021] D4a1a1 (7) [0.019] M5 M27b2b1 (9) [0.007] M5a'd (10) [0.006] G1b2 G1b4 D4a1 (13) [0.004] M37+152 D4a1f1 (15) [0.000] M5a2 M5a2a1 M5a5 M5b'c M5b M7a M7a1b2 M7a2 M7b1 M13'46'61 M13 M13a'b M13b M17c M27b2 M27b2a M27b2b M27b2c D4a D4a1a D4a1a1a D4a1b D4a1c D4a1f D4a1g D4a1h D4a2 D4a2a D4a2b D4a5 (45)	1

711	>KX457649.1 Hc	73G 248d 263G 309.1CT 310C 514d 515d 750G 1438G 2706G 3106d 3107C 3970T 4086T 4769G 6392C 6962A 7028T 7444A 8659G 8860G 9053A 10034C 10310A 10609C 11719A 12406A 12882T 13759A 13928C 14766T 15326G 16172C 16304C 16465T 16519C	F1a2a	73G 248d 263G 309.1CT 310C 514d 515d 16172C 16304C 16465T 16519C	1	73 249d 263 750 1438 2706 3970 4086 4769 6392 6962 7028 7444 8659 8860 9053 10034 10310 10609 11719 12406 12882 13759 13928C 14766 15326 16172 16304 16465	F1a2a	73 249d 263 16172 16304 16465	[0.000] F1a2a (1)	[0.000] F1a2 (2)	1
712	>KX457632.1 Hc	73G 207A 248d 263G 309.1CT 310C 709A 750G 1438G 2706G 3106d 3107C 3434G 3970T 4769G 4824G 4991A 5585A 5894G 5913A 5978G 6392C 7028T 8860G 10310A 10320A 11065G 11719A 12621T 13928C 14766T 14971C 15326G 15412G 16260T 16298C 16355T 16362C	F3a1	73G 207A 248d 263G 309.1CT 310C 16260T 16298C 16355T 16362C	1	73 207 249d 263 709 750 1438 2706 3434 3970 4769 4824 4991 5585 5894 5913 5978 6392 7028 8860 10310 10320 11065 11719 12621 13928C 14766 14971 15326 15412G 16260 16298 16355 16362	F3a1	73 207 249d 263 16260 16298 16355 16362	[0.000] F3a1 (1)	[0.000] F3a+207 (2)	1
713	>KX457630.1 Hc	73G 150T 199C 204C 263G 456T 489C 750G 1438G 2706G 3106d 3107C 4048A 4071T 4164G 4769G 5351G 5460A 6455T 6680C 7028T 7684C 7853A 8701G 8860G 9540C 9824C 10398G 10400T 10873C 11719A 12405T 12705T 12811C 14377T 14766T 14783C 14978G 15043A 15301A 15326G 16129A 16182C 16183C 16189C 16223T 16297C 16468C	M7b1a1a3	73G 150T 199C 204C 263G 456T 489C 16129A 16182C 16183C 16189C 16223T 16297C 16468C	1	73 150 199 204 263 456 489 750 1438 2706 4048 4071 4164 4769 5351 5460 6455 6680 7028 7684 7853 8701 8860 9540 9824 10398 10400 10873 11719 12405 12705 12811 14377 14766 14783 14978 15043 15301 15326 16129 16189 16223 16297 16468	M7b1a1a3	73 150 199 204 263 456 489 16129 16189 16223 16297 16468	[0.016] M7b1a1a3 (1)	[0.003] M7b1a1a2 (2) [0.000] M7b1a1a	1
714	>KX457628.1 Hc	73G 146C 263G 310.1C 489C 513d 514d 750G 1438G 2706G 3106d 3107C 3912G 4071T 4769G 4850T 5378G 6455T 7028T 8701G 8860G 9180G 9449T 9540C 9824C 10398G 10400T 10873C 10897T 11665T 11719A 11932T 12091C 12705T 14053G 14766T 14783C 15043A 15301A 15326G 15338T 16172C 16223T 16274A 16291T 16311C 16519C	M7c2a	73G 146C 263G 310.1C 489C 513d 514d 16172C 16223T 16274A 16291T 16311C 16519C	1	73 146 263 489 513 750 1438 2706 3912 4071 4769 4850 5378 6455 7028 8701 8860 9180 9449 9540 9824 10398 10400 10873 10897 11665 11719 11932 12091 12705 14053 14766 14783 15043 15301 15326 15338 16172 16223 16274 16291 16311	M7c2a	73 146 263 489 513 16172 16223 16274 16291 16311	[0.119] M7c2a (1)	[0.000] M7c2 M7c2b (3)	1
715	>KX457627.1 Hc	73G 146C 263G 310.1C 489C 513d 514d 750G 1438G 2706G 3106d 3107C 3912G 4071T 4769G 4850T 5378G 6455T 7028T 8701G 8860G 9180G 9449T 9540C 9824C 10398G 10400T 10873C 10897T 11665T 11719A 11932T 12091C 12705T 14053G 14766T 14783C 15043A 15301A 15326G 15338T 16172C 16223T 16274A 16291T 16311C 16519C	M7c2a	73G 146C 263G 310.1C 489C 513d 514d 16172C 16223T 16274A 16291T 16311C 16519C	1	73 146 263 489 513 750 1438 2706 3912 4071 4769 4850 5378 6455 7028 8701 8860 9180 9449 9540 9824 10398 10400 10873 10897 11665 11719 11932 12091 12705 14053 14766 14783 15043 15301 15326 15338 16172 16223 16274 16291 16311	M7c2a	73 146 263 489 513 16172 16223 16274 16291 16311	[0.119] M7c2a (1)	[0.000] M7c2 M7c2b (3)	1

716 >KX457618.1 Ho	73G 146C 150T 152C 248d 263G 281G 310.1C 750G 1438G 2706G 3106d 3107C 3290C 3970T 4703C 4769G 5263T 6392C 7028T 8860G 10310A 10915C 11719A 11776C 12612G 12630A 13928C 14016A 14766T 15314A 15326G 15670C 15908C 16207G 16304C 16399G	F4a2	73G 146C 150T 152C 248d 263G 281G 310.1C 16207G 16304C 16399G	1	73 146 150 152 249d 263 281 750 1438 2706 3290 3970 4703 4769 5263 6392 7028 8860 10310 10915 11719 11776 12612 12630 13928C 14016 14766 15314 15326 15670 15908 16207 16304 16399	F4a2	73 146 150 152 249d 263 281 16207 16304 16399	[0.059] F4a2 (1)	[0.000] F4a (2)	1
717 >KX457615.1 Ho	73G 152C 248d 263G 309.1CCT 310C 316A 489C 514d 515d 619C 750G 1438G 2706G 3106d 3107C 3200C 3714G 4385T 4769G 4772C 7028T 7433T 8701G 8853G 8860G 9127G 9512T 9540C 10274C 10398G 10400T 10679G 10873C 11719A 11914A 12354C 12705T 14001G 14110C 14766T 14783C 14883T 14974T 15043A 15301A 15326G 15691G 16129A 16209C 16223T 16272G 16519C	M20	73G 152C 248d 263G 309.1CCT 310C 316A 489C 514d 515d 16129A 16209C 16223T 16272G 16519C	1	73 152 249d 263 316 489 619 750 1438 2706 3200 3714 4385T 4769 4772 7028 7433 8701 8853 8860 9127 9512 9540 10274 10398 10400 10679 10873 11719 11914 12354 12705 14001 14110 14766 14783 14883 14974 15043 15301 15326 15691 16129 16209 16223 16272	M20	73 152 249d 263 316 489 16129 16209 16223 16272	[0.000] M20 (1)	[0.068] M17 (2) [0.067] M17a (3) [0.059] G1b+16129 (4) [0.028] M5a (5) [0.021] D4a1a1 (6) [0.019] M5 (7) [0.017] M5a2a (8) [0.007] M5a'd (9) [0.004] M37+152 (10) [0.003] D4a1 (11) [0.000] M5a2 M5a2a1 M5a5 M5b'c M5b M7a M7a1b2 M7a2 M7b1 G1b2 G1b4 M13'46'61 M13 M13a'b M13b M17c D4a D4a1a D4a1a1a D4a1b D4a1c D4a1f D4a1f1 D4a1g D4a1h D4a2 D4a2a D4a2b D4a5 (40)	1
718 >KX457614.1 Ho	73G 146C 150T 185A 189G 196C 199C 263G 309.1CCT 310C 513A 750G 827G 1438G 2706G 3106d 3107C 4769G 6614C 7028T 7657C 8271d 8272d 8273d 8274d 8275d 8276d 8277d 8278d 8279d 8860G 9758C 9921A 10410C 10750G 11719A 13135A 14766T 15326G 15535T 15940d 16129A 16182C 16183C 16189C 16217C 16223T 16311C 16519C	B4e	73G 146C 150T 185A 189G 196C 199C 263G 309.1CCT 310C 513A 16129A 16182C 16183C 16189C 16217C 16223T 16311C 16519C	1	73 146 150 185 189 196 199 263 513 750 827 1438 2706 4769 6614 7028 7657 8281-8289d 8860 9758 9921 10410 10750 11719 13135 14766 15326 15535 15944d 16129 16189 16217 16223 16311	B4e	73 146 150 185 189 196 199 263 513 16129 16189 16217 16223 16311	[1.354] B4e (1)	[0.084] R11b (2) [0.073] R11 (3) [0.048] R11a	1
719 >KX457609.1 Ho	73G 263G 309.1CT 310C 750G 1438G 1541C 2706G 3106d 3107C 3666A 3970T 4769G 5486T 6647G 7028T 8724G 8860G 11299C 11422T 11719A 13191C 13722G 13759A 13928C 14766T 15314A 15326G 15883A 16092C 16304C 16362C 16519C	R9b2	73G 263G 309.1CT 310C 16092C 16304C 16362C 16519C	1	73 263 750 1438 1541 2706 3666 3970 4769 5486 6647 7028 8724 8860 11299 11422 11719 13191 13722 13759 13928C 14766 15314 15326 15883 16092 16304 16362	R9b2	73 263 16092 16304 16362	[0.007] R9b2 (1)	[0.010] R9c (2) [0.000] R6 R9 R9b R31 (6)	1
720 >KX457608.1 Ho	73G 195C 263G 310.1C 329A 512.1AC 513A 750G 1040C 1438G 2706G 3106d 3107C 4491A 4769G 6770G 7028T 8545A 8860G 9099T 10166C 10316G 10609C 10877T 11719A 13145A 13287T 13359A 14766T 14790G 15326G 15885T 16249C 16259T 16288C 16301T 16304C 16390A 16519C	R22	73G 195C 263G 310.1C 329A 512.1AC 513A 16249C 16259T 16288C 16301T 16304C 16390A 16519C	1	73 195 263 329 513 750 1040 1438 2706 4491 4769 6770 7028 8545 8860 9099 10166 10316 10609 10877 11719 13145 13287 13359 14766 14790 15326 15885 16249 16259 16288 16301 16304 16390	R22	73 195 263 329 513 16249 16259 16288 16301 16304 16390	[1.626] R22 (1)	[0.091] R8a1a1d (2)	1

721 >KX457607.1 Ho	73G 150T 199C 204C 207A 263G 309.1CT 310C 456T 489C 750G 1438G 2706G 3106d 3107C 4048A 4071T 4164G 4769G 5351G 5460A 6455T 6680C 7028T 7684C 7853A 8701G 8860G 9540C 9824C 10398G 10400T 10873C 11719A 12405T 12705T 12811C 13651G 14766T 14783C 14978G 15043A 15148A 15301A 15326G 16129A 16189C 16223T 16297C	M7b1a1a3	73G 150T 199C 204C 207A 263G 309.1CT 310C 456T 489C 16129A 16189C 16223T 16297C	1	73 150 199 204 207 263 456 489 750 1438 2706 4048 4071 4164 4769 5351 5460 6455 6680 7028 7684 7853 8701 8860 9540 9824 10398 10400 10873 11719 12405 12705 12811 13651 14766 14783 14978 15043 15148 15301 15326 16129 16189 16223 16297	M7b1a1a3	73 150 199 204 207 263 456 489 16129 16189 16223 16297	[0.238] M7b1a1a3 (1)	[0.004] M7b1a1a (2) [0.003] M7b1a1a2	1
722 >KX457603.1 Ho	73G 248d 263G 309.1CCT 310C 417A 514d 515d 750G 1438G 2706G 3106d 3107C 3970T 4086T 4769G 6392C 6962A 7028T 8860G 9053A 9548A 10310A 10609C 11719A 12406A 12624C 12882T 13759A 13928C 14766T 15326G 16129A 16162G 16172C 16304C 16519C	F1a1	73G 248d 263G 309.1CCT 310C 417A 514d 515d 16129A 16162G 16172C 16304C 16519C	1	73 249d 263 417 750 1438 2706 3970 4086 4769 6392 6962 7028 8860 9053 9548 10310 10609 11719 12406 12624 12882 13759 13928C 14766 15326 16129 16162 16172 16304	F1a1	73 249d 263 417 16129 16162 16172 16304	[0.003] F1a1 (1)	[0.000] F1a F1a1'4 F1a1a F1a1a1 F1a1b F1a1c F1a1c2 F1a1c3 F1a1d F1a1d1 F1a4 F1a3 (13)	1
723 >KX457602.1 Ho	73G 151T 263G 309.1CT 310C 750G 1438G 1541C 2706G 3106d 3107C 3970T 4769G 6293C 6713T 7028T 7080C 8860G 9722C 11719A 12714C 13105G 13928C 14766T 15326G 16304C 16309G 16390A 16519C	R9b1	73G 151T 263G 309.1CT 310C 16304C 16309G 16390A 16519C	1	73 151 263 750 1438 1541 2706 3970 4769 6293 6713 7028 7080 8860 9722 11719 12714 13105 13928C 14766 15326 16304 16309 16390	R9b1	73 151 263 16304 16309 16390	[0.033] R9b1 (1)	[0.628] R9b1a3 (2) [0.058] R9b1a2b (3) [0.044] R9b1a2 (4) [0.005] R9c (5) [0.000] R9 R9b R9b1a R9b1b (9)	1
724 >KX457598.1 Ho	73G 146C 150T 185A 189G 196C 199C 263G 513A 750G 827G 1438G 2706G 3106d 3107C 4769G 6614C 7028T 7657C 8271d 8272d 8273d 8274d 8275d 8276d 8277d 8278d 8279d 8860G 9758C 9921A 10410C 10750G 11719A 13135A 14766T 15326G 15535T 15940d 16129A 16182C 16183C 16189C 16217C 16223T 16311C 16519C	B4e	73G 146C 150T 185A 189G 196C 199C 263G 513A 16129A 16182C 16183C 16189C 16217C 16223T 16311C 16519C	1	73 146 150 185 189 196 199 263 513 750 827 1438 2706 4769 6614 7028 7657 8281-8289d 8860 9758 9921 10410 10750 11719 13135 14766 15326 15535 15944d 16129 16189 16217 16223 16311	B4e	73 146 150 185 189 196 199 263 513 16129 16189 16217 16223 16311	[1.354] B4e (1)	[0.084] R11b (2) [0.073] R11 (3) [0.048] R11a	1
725 >KX457596.1 Ho	73G 263G 489C 750G 1438G 2361A 2706G 3106d 3107C 3316A 4079G 4769G 5220T 5492C 5894G 7028T 8227C 8701G 8848C 8860G 9540C 10398G 10400T 10873C 11719A 12705T 14259A 14766T 14783C 15043A 15301A 15326G 16111T 16223T 16362C 16519C	M33c	73G 263G 489C 16111T 16223T 16362C 16519C	1	73 263 489 750 1438 2361 2706 3316 4079 4769 5220 5492 5894 7028 8227 8701 8848 8860 9540 10398 10400 10873 11719 12705 14259 14766 14783 15043 15301 15326 16111 16223 16362	M33c	73 263 489 16111 16223 16362	[0.000] M33c (1)	[0.002] D4b2b (2) [0.001] M33+16362 D4 D4b2b1 (5) [0.000] M9 G G1 G1c G1c2 G2 G2a'c G2b G2b1 G2b1a G2b2 G2b2a D D4b D4b2 D4b2a D4b2a2 D4b2a2b D4b2b1a D4b2b1b D4b2b1c D4b2b2 D4b2b3 D4b2b6 D4e D4e1'3 D4e1 D4e1c D4e3 D4e2 D4e2b D4e2c D4e2d D4e4 D4e4a D4e4b D4f D4f1 D4g D4h D4h4 D4j D4j1 D4j1b D4j4 D4j5 D4j5a D4j+(16286) D4j9 D4j10 D4j12 D4j15 D4j16 D4m (59)	1

726 >KX457594.1 Hc	73G 152C 263G 310.1C 750G 1438G 2706G 2760G 3027C 3106d 3107C 3483A 3666A 4769G 7028T 7119A 7885C 7961C 8188G 8271.1C 8860G 10166C 10398G 11719A 12705T 13710G 14766T 15211T 15326G 16193T 16223T 16263C 16274A 16311C 16343G 16357C 16519C	N8	73G 152C 263G 310.1C 16193T 16223T 16263C 16274A 16311C 16343G 16357C 16519C	1	73 152 263 750 1438 2706 2760 3027 3483 3666 4769 7028 7119 7885 7961 8188 8276.1C 8860 10166 10398 11719 12705 13710 14766 15211 15326 16193 16223 16263 16274 16311 16343 16357	N8	73 152 263 16193 16223 16263 16274 16311 16343 16357	[0.495] N8 (1)	[0.065] L3h1 L3h1a (3) [0.057] L3d3a1b (4) [0.043] M57+152 (5) [0.023] L3a1a (6) [0.015] L3d3a (7) [0.000] L3'4 L3f2a L3f2a1 L3c'd L3d3a1 L3d3a1a L3h1a2 S+152 (15)	1
727 >KX457589.1 Hc	73G 152C 263G 310.1C 750G 1438G 2706G 2760G 3027C 3106d 3107C 3483A 3666A 4769G 7028T 7119A 7885C 7961C 8188G 8271.1C 8860G 10166C 10398G 11009C 11719A 12705T 13710G 14766T 15211T 15326G 15833T 16193T 16223T 16263C 16274A 16311C 16343G 16357C 16519C	N8	73G 152C 263G 310.1C 16193T 16223T 16263C 16274A 16311C 16343G 16357C 16519C	1	73 152 263 750 1438 2706 2760 3027 3483 3666 4769 7028 7119 7885 7961 8188 8276.1C 8860 10166 10398 11009 11719 12705 13710 14766 15211 15326 15833 16193 16223 16263 16274 16311 16343 16357	N8	73 152 263 16193 16223 16263 16274 16311 16343 16357	[0.495] N8 (1)	[0.065] L3h1 L3h1a (3) [0.057] L3d3a1b (4) [0.043] M57+152 (5) [0.023] L3a1a (6) [0.015] L3d3a (7) [0.000] L3'4 L3f2a L3f2a1 L3c'd L3d3a1 L3d3a1a L3h1a2 S+152 (15)	1
728 >KX457588.1 Hc	73G 207A 263G 309.1CT 310C 489C 709A 750G 1438G 2706G 3106d 3107C 3398C 4048A 4769G 4833G 4853A 5108C 5601T 7028T 8701G 8860G 8877C 9540C 10398G 10400T 10873C 11151T 11719A 12705T 13191C 13563G 14167T 14182C 14569A 14766T 14783C 15043A 15301A 15326G 16223T 16362C	G2b1a1	73G 207A 263G 309.1CT 310C 489C 16223T 16362C	1	73 207 263 489 709 750 1438 2706 3398 4048 4769 4833 4853 5108 5601 7028 8701 8860 8877 9540 10398 10400 10873 11151 11719 12705 13191 13563 14167 14182 14569 14766 14783 15043 15301 15326 16223 16362	G2b1a1	73 207 263 489 16223 16362	[0.000] G2b1a1 (1)	[0.006] D4b2b2 (2) [0.002] D4e4a D4f (4) [0.001] D4b2a D4b2b (6) [0.000] M9 G G1 G1b G1c G1c2 G2 G2a'c G2b G2b1 G2b1a G2b2 G2b2a M33+16362 D D4 D4b D4b2 D4b2a2 D4b2a2b D4b2b1 D4b2b1a D4b2b1b D4b2b1c D4b2b3 D4b2b6 D4e D4e1'3 D4e1 D4e1c D4e3 D4e2 D4e2b D4e2c D4e2d D4e4 D4e4b D4f1 D4g D4h D4h4 D4j D4j1 D4j1b D4j4 D4j5 D4j5a D4j+(16286) D4j9 D4j10 D4j12 D4j15 D4j16 D4m (60)	1
729 >KX457587.1 Hc	73G 146C 185A 189G 195C 200G 263G 309.1CT 310C 513A 750G 827G 1438G 2706G 3106d 3107C 4769G 6026A 6614C 7028T 8271d 8272d 8273d 8274d 8275d 8276d 8277d 8278d 8279d 8764A 8860G 9921A 10750G 10912T 11719A 13194A 14766T 15218G 15326G 15514C 15535T 15824G 16182C 16183C 16189C 16217C 16223T 16519C	B4e	73G 146C 185A 189G 195C 200G 263G 309.1CT 310C 513A 16182C 16183C 16189C 16217C 16223T 16519C	1	73 146 185 189 195 200 263 513 750 827 1438 2706 4769 6026 6614 7028 8281-8289d 8764 8860 9921 10750 10912 11719 13194 14766 15218 15326 15514 15535 15824 16189 16217 16223	B4e	73 146 185 189 195 200 263 513 16189 16217 16223	[0.469] B4e (1)	[0.000] B4a1a3a1a (2)	1
730 >KX457581.1 Hc	73G 152C 263G 310.1C 750G 1438G 2706G 2760G 3027C 3106d 3107C 3483A 3666A 4769G 7028T 7119A 7885C 7961C 8188G 8271.1C 8860G 10166C 10398G 11719A 12705T 13710G 14766T 15211T 15326G 15833T 16193T 16223T 16263C 16274A 16311C 16343G 16357C 16519C	N8	73G 152C 263G 310.1C 16193T 16223T 16263C 16274A 16311C 16343G 16357C 16519C	1	73 152 263 750 1438 2706 2760 3027 3483 3666 4769 7028 7119 7885 7961 8188 8276.1C 8860 10166 10398 11719 12705 13710 14766 15211 15326 15833 16193 16223 16263 16274 16311 16343 16357	N8	73 152 263 16193 16223 16263 16274 16311 16343 16357	[0.495] N8 (1)	[0.065] L3h1 L3h1a (3) [0.057] L3d3a1b (4) [0.043] M57+152 (5) [0.023] L3a1a (6) [0.015] L3d3a (7) [0.000] L3'4 L3f2a L3f2a1 L3c'd L3d3a1 L3d3a1a L3h1a2 S+152 (15)	1

731	>KX457579.1 Ho	73G 152C 263G 310.1C 750G 1438G 2706G 2760G 3027C 3106d 3107C 3483A 3666A 4769G 7028T 7119A 7885C 7961C 8188G 8271.1C 8860G 10166C 10398G 11719A 12705T 13710G 14766T 15211T 15326G 16193T 16223T 16263C 16274A 16311C 16343G 16357C 16519C	N8	73G 152C 263G 310.1C 16193T 16223T 16263C 16274A 16311C 16343G 16357C 16519C	1	73 152 263 750 1438 2706 2760 3027 3483 3666 4769 7028 7119 7885 7961 8188 8276.1C 8860 10166 10398 11719 12705 13710 14766 15211 15326 16193 16223 16263 16274 16311 16343 16357	N8	73 152 263 16193 16223 16263 16274 16311 16343 16357	[0.495] N8 (1)	[0.065] L3h1 L3h1a (3) [0.057] L3d3a1b (4) [0.043] M57+152 (5) [0.023] L3a1a (6) [0.015] L3d3a (7) [0.000] L3'4 L3f2a L3f2a1 L3c'd L3d3a1 L3d3a1a L3h1a2 S+152 (15)	1
732	>KX457568.1 Ho	73G 152C 210G 263G 514d 515d 709A 750G 1438G 2706G 3106d 3107C 3537G 4086T 4769G 6249A 6960T 7028T 8271d 8272d 8273d 8274d 8275d 8276d 8277d 8278d 8279d 8584A 8860G 9950C 10398G 11465C 11719A 14766T 15235G 15326G 15465C 16140C 16182C 16183C 16189C 16261T 16266A 16519C	B5a1d	73G 152C 210G 263G 514d 515d 16140C 16182C 16183C 16189C 16261T 16266A 16519C	1	73 152 210 263 709 750 1438 2706 3537 4086 4769 6249 6960 7028 8281-8289d 8584 8860 9950 10398 11465 11719 14766 15235 15326 15465 16140 16189 16261 16266A	B5a1d	73 152 210 263 16140 16189 16261 16266A	[0.000] B5a1d (1)	[0.032] B5a1 (2) [0.016] B5a1a (3) [0.010] B5a (4) [0.004] B5a1b1 (5) [0.002] B5a1c1a (6) [0.000] B5a1a1 B5a1b B5a1c B5a1c1 B5a1c2 (11)	1
733	>KX457559.1 Ho	73G 146C 234G 263G 309.1CT 310C 489C 513A 750G 1438G 1709A 2706G 3106d 3107C 3753C 4769G 7028T 7353G 7521A 7793T 8701G 8860G 9540C 10325A 10398G 10400T 10873C 11002G 11252G 11719A 12406A 12603T 12705T 14341T 14766T 14783C 15043A 15055C 15301A 15326G 15547T 16124C 16183C 16189C 16293d 16297C 16298C 16325C 16362C	M76a	73G 146C 234G 263G 309.1CT 310C 489C 513A 16124C 16183C 16189C 16293d 16297C 16298C 16325C 16362C	1	73 146 234 263 489 513 750 1438 1709 2706 3753 4769 7028 7353 7521 7793T 8701 8860 9540 10325 10398 10400 10873 11002 11252 11719 12406 12603 12705 14341 14766 14783 15043 15055 15301 15326 15547 16124 16189 16293C 16297 16298d 16325 16362	M76a	73 146 234 263 489 513 16124 16189 16293C 16297 16298d 16325 16362	[0.751] M76a (1)	[0.321] M76 (2)	1
734	>KX457556.1 Ho	73G 195C 263G 310.1C 489C 750G 1438G 2706G 3106d 3107C 4769G 5054A 5912T 6040G 7028T 8080A 8155A 8167C 8251A 8616A 8701G 8860G 9540C 10268T 10398G 10400T 10873C 11453A 11719A 12651A 12705T 13434G 14766T 14783C 15043A 15226G 15301A 15326G 15758G 15908C 15969A 16223T 16246T 16311C 16362C 16400T 16519C	M74b1	73G 195C 263G 310.1C 489C 16223T 16246T 16311C 16362C 16400T 16519C	1	73 195 263 489 750 1438 2706 4769 5054 5912 6040 7028 8080A 8155 8167 8251 8616 8701 8860 9540 10268 10398 10400 10873 11453 11719 12651 12705 13434 14766 14783 15043 15226 15301 15326 15758 15908 15969A 16223 16246T 16311 16362 16400	M74b1	73 195 263 489 16223 16246T 16311 16362 16400	[0.016] M74b1 (1)	[0.071] M74b (2) [0.039] M74 (3) [0.018] M74b2 (4) [0.000] L4 L4b M43a1 D4j+16311 D4j11 D4+195 (10)	1
735	>KX457555.1 Ho	61A 62A 73G 182T 263G 514d 515d 750G 1438G 2378T 2706G 3106d 3107C 4769G 6482T 7028T 8215T 8271d 8272d 8273d 8274d 8275d 8276d 8277d 8278d 8279d 8860G 8931G 9968T 11719A 14766T 15326G 16181C 16182C 16183C 16189C 16213A 16217C 16261T 16292T 16362C 16519C	B4g2	61A 62A 73G 182T 263G 514d 515d 16181C 16182C 16183C 16189C 16213A 16217C 16261T 16292T 16362C 16519C	1	61A 62 73 182 263 750 1438 2378 2706 4769 6482 7028 8215 8281-8289d 8860 8931 9968 11719 14766 15326 16181C 16189 16213 16217 16261 16292 16362	B4g2	61A 62 73 182 263 16181C 16189 16213 16217 16261 16292 16362	[2.696] B4g2 (1) [0.641] B4g1 (2) [0.149] B4g1a (3) [0.000] B4g (4)	[0.452] B4g1b (5)	1

736 >MF381361.1 Ho	73G 150T 152C 189G 195C 200G 207A 263G 750G 1438G 2352C 2706G 3106d 3107C 4769G 5774A 6221C 6587T 7028T 8701G 8860G 9254G 9533T 9540C 10398G 10819G 10873C 11024T 11719A 12705T 14152G 14212C 14569A 14766T 15301A 15326G 15670C 15942C 16185T 16209C 16223T 16327T	L3e1a2	73G 150T 152C 189G 195C 200G 207A 263G 16185T 16209C 16223T 16327T	1	73 150 152 189 195 200 207 263 750 1438 2352 2706 4769 5774A 6221 6587 7028 8701 8860 9254 9533 9540 10398 10819 10873 11024 11719 12705 14152 14212 14569 14766 15301 15326 15670 15942 16185 16209 16223 16327	L3e1a2	73 150 152 189 195 200 207 263 16185 16209 16223 16327	[0.000] L3e1a2 (1)	[0.000] L3e1f2 (2)	1
737 >MF381360.1 Ho	73G 151T 152C 182T 186A 189C 195C 198T 247A 263G 297G 316A 416A 514d 515d 520T 750G 769A 825A 1018A 1291C 1438G 2394d 2581G 2706G 2758A 2885C 3106d 3107C 3594T 3666A 3796T 3834A 3843G 4104G 4589C 4688C 4769G 5480G 5553C 5951G 6071C 6179A 7028T 7055G 7146G 7196T 7256T 7389C 7521A 7660C 7714T 7854C 8027A 8468T 8619T 8655T 8701G 8860G 9072G 9540C 9861C 10084C 10321C 10398G 10586A 10688A 10810C 10873C 11353C 11440A 11719A 11899C 12681C 12705T 12810G 13105G 13485G 13506T 13650T 13789C 13874C 14000A 14148G 14178C 14393G 14560A 14766T 14911T 15326G 16086C 16129A 16187T 16189C 16223T 16241G 16278T 16293G 16294T 16311C 16360T 16519C	L1c1b	73G 151T 152C 182T 186A 189C 195C 198T 247A 263G 297G 316A 416A 514d 515d 520T 16086C 16129A 16187T 16189C 16223T 16241G 16278T 16293G 16294T 16311C 16360T 16519C	1	73 151 152 182 186A 189C 195 198 247 263 297 316 416A 518 750 769 825A 1018 1291 1438 2395d 2581 2706 2758 2885 3594 3666 3796T 3834 3843 4104 4589 4688 4769 5480 5553 5951 6071 6179 7028 7055 7146 7196 7256 7389 7521 7660 7714 7854 8027 8468 8619 8655 8701 8860 9072 9540 9861 10084 10321 10398 10586 10688 10810 10873 11353 11440 11719 11899 12681 12705 12810 13105 13485 13506 13650 13789 13874 14000A 14148 14178 14393 14560 14766 14911 15326 16086 16129 16187 16189 16223 16241 16278 16293 16294 16311 16360	L1c1b	73 151 152 182 186A 189C 195 198 247 263 297 316 416A 518 16086 16129 16187 16189 16223 16241 16278 16293 16294 16311 16360	[1.673] L1c1b (1)	[0.000] L1c1b'd (2)	1
738 >MF381359.1 Ho	73G 150T 152C 189G 195C 200G 207A 263G 750G 1438G 2352C 2706G 3106d 3107C 4769G 5774A 6221C 6587T 7028T 8701G 8860G 9254G 9533T 9540C 10398G 10819G 10873C 11024T 11719A 12705T 14152G 14212C 14569A 14766T 15301A 15326G 15670C 15942C 16185T 16209C 16223T 16327T	L3e1a2	73G 150T 152C 189G 195C 200G 207A 263G 16185T 16209C 16223T 16327T	1	73 150 152 189 195 200 207 263 750 1438 2352 2706 4769 5774A 6221 6587 7028 8701 8860 9254 9533 9540 10398 10819 10873 11024 11719 12705 14152 14212 14569 14766 15301 15326 15670 15942 16185 16209 16223 16327	L3e1a2	73 150 152 189 195 200 207 263 16185 16209 16223 16327	[0.000] L3e1a2 (1)	[0.000] L3e1f2 (2)	1
739 >MF381356.1 Ho	73G 150T 152C 189G 195C 200G 207A 263G 750G 1438G 2352C 2706G 3106d 3107C 4769G 5774A 6221C 6587T 7028T 8701G 8860G 9254G 9540C 10398G 10819G 10873C 11024T 11719A 12705T 14152G 14212C 14569A 14766T 15301A 15326G 15670C 15942C 16185T 16209C 16223T 16327T	L3e1a2	73G 150T 152C 189G 195C 200G 207A 263G 16185T 16209C 16223T 16327T	1	73 150 152 189 195 200 207 263 750 1438 2352 2706 4769 5774A 6221 6587 7028 8701 8860 9254 9540 10398 10819 10873 11024 11719 12705 14152 14212 14569 14766 15301 15326 15670 15942 16185 16209 16223 16327	L3e1a2	73 150 152 189 195 200 207 263 16185 16209 16223 16327	[0.000] L3e1a2 (1)	[0.000] L3e1f2 (2)	1

740 >MF381350.1 Hc	73G 93G 146C 150T 152C 182T 183G 195C 198T 263G 325T 514d 515d 680C 709A 750G 769A 1018A 1040C 1438G 1442A 2332T 2416C 2706G 3106d 3107C 3200A 3594T 4104G 4769G 7028T 7256T 7521A 7624A 8206A 8567C 8701G 8772C 8860G 9063G 9221G 9540C 10115C 10398G 10790C 10873C 11719A 11944C 12236A 12705T 13590A 13650T 13928C 13958C 14766T 15110A 15217A 15301A 15313C 15326G 15849T 16223T 16264T 16278T 16311C 16390A	L2c2b1b	73G 93G 146C 150T 152C 182T 183G 195C 198T 263G 325T 514d 515d 16223T 16264T 16278T 16311C 16390A	1	73 93 146 150 152 182 183 195 198 263 325 680 709 750 769 1018 1040 1438 1442 2332 2416 2706 3200A 3594 4104 4769 7028 7256 7521 7624A 8206 8567 8701 8772 8860 9063 9221 9540 10115 10398 10790 10873 11719 11944 12236 12705 13590 13650 13928C 13958C 14766 15110 15217 15301 15313 15326 15849 16223 16264 16278 16311 16390	L2c2b1b	73 93 146 150 152 182 183 195 198 263 325 16223 16264 16278 16311 16390	[0.000] L2c2b1b (1)	[0.000] L2c2b L2c2b1 L2c2b1a L2c2b2 (5)	1
741 >MF381349.1 Hc	73G 152C 189G 200G 263G 310C 750G 1438G 1822C 2706G 3106d 3107C 3396C 4218C 4769G 5601T 7028T 7819A 8410T 8527G 8701G 8860G 8932T 9540C 9950C 10070T 10235C 10398G 10873C 11440A 11719A 12705T 14766T 14769G 15301A 15326G 15514C 15940d 16129A 16209C 16223T 16292T 16295T 16311C 16519C	L3f1b1a	73G 152C 189G 200G 263G 310C 16129A 16209C 16223T 16292T 16295T 16311C 16519C	1	73 152 189 200 263 310 750 1438 1822 2706 3396 4218 4769 5601 7028 7819A 8410 8527 8701 8860 8932 9540 9950 10070 10235 10398 10873 11440 11719 12705 14766 14769 15301 15326 15514 15944d 16129 16209 16223 16292 16295 16311	L3f1b1a	73 152 189 200 263 310 16129 16209 16223 16292 16295 16311	[0.084] L3f1b1a (1)	[0.111] L3f1b1a1 (2) [0.094] L3f1b1a (3) [0.071] L3f1b1	1
742 >MF381347.1 Hc	73G 151T 152C 182T 183G 186A 189C 204C 247A 257G 263G 316A 514d 515d 750G 769A 825A 1018A 1438G 2394d 2706G 2758A 2885C 3105d 3106G 3107C 3552C 3594T 3666A 4104G 4769G 5033G 5821A 5951G 6071C 6221A 6260A 6917A 6932G 7028T 7146G 7256T 7389C 7498A 7521A 7789A 8027A 8468T 8655T 8668C 8701G 8860G 8987C 9072G 9540C 9966A 10398G 10586A 10688A 10810C 10873C 11302T 11506C 11719A 12019T 12501A 12705T 12810G 13020C 13105G 13485G 13506T 13650T 13768C 13789C 14000A 14178C 14560A 14581C 14766T 14911T 15226G 15326G 15905C 15978T 16129A 16183C 16189C 16215G 16223T 16278T 16294T 16311C 16355T 16360T 16519C	L1c3a1b	73G 151T 152C 182T 183G 186A 189C 204C 247A 257G 263G 316A 514d 515d 16129A 16183C 16189C 16215G 16223T 16278T 16294T 16311C 16355T 16360T 16519C	1	73 151 152 182 183 186A 189C 204 247 257 263 316 750 769 825A 1018 1438 2395d 2706 2758 2885 3105 3552 3594 3666 4104 4769 5033 5821 5951 6071 6221A 6260 6917 6932 7028 7146 7256 7389 7498 7521 7789 8027 8468 8655 8668 8701 8860 8987 9072 9540 9966 10398 10586 10688 10810 10873 11302 11506 11719 12019 12501 12705 12810 13020 13105 13485 13506 13650 13768 13789 14000A 14178 14560 14581 14766 14911 15226 15326 15905 15978 16129 16189 16215 16223 16278 16294 16311 16355 16360	L1c3a1b	73 151 152 182 183 186A 189C 204 247 257 263 316 16129 16189 16215 16223 16278 16294 16311 16355 16360	[0.897] L1c3a1b (1)	[0.000] L1c3a L1c3a1 L1c3a1a (4)	1

743 >MF381339.1 Hc	73G 150T 152C 189G 195C 200G 207A 263G 750G 1438G 2352C 2706G 3106d 3107C 4769G 5774A 6221C 6587T 7028T 8701G 8860G 9254G 9540C 10398G 10643C 10819G 10873C 11024T 11719A 12705T 14152G 14212C 14569A 14766T 15301A 15326G 15670C 15942C 16185T 16209C 16223T 16327T	L3e1a2	73G 150T 152C 189G 195C 200G 207A 263G 16185T 16209C 16223T 16327T	1	73 150 152 189 195 200 207 263 750 1438 2352 2706 4769 5774A 6221 6587 7028 8701 8860 9254 9540 10398 10643 10819 10873 11024 11719 12705 14152 14212 14569 14766 15301 15326 15670 15942 16185 16209 16223 16327	L3e1a2	73 150 152 189 195 200 207 263 16185 16209 16223 16327	[0.000] L3e1a2 (1)	[0.000] L3e1f2 (2)	1
744 >MF381334.1 Hc	73G 150T 152C 189G 195C 200G 207A 263G 750G 1438G 2352C 2706G 3106d 3107C 4769G 5774A 6221C 6587T 7028T 8701G 8860G 9254G 9540C 10398G 10819G 10873C 11024T 11719A 12705T 14152G 14212C 14569A 14766T 15301A 15326G 15670C 15942C 16185T 16209C 16223T 16327T	L3e1a2	73G 150T 152C 189G 195C 200G 207A 263G 16185T 16209C 16223T 16327T	1	73 150 152 189 195 200 207 263 750 1438 2352 2706 4769 5774A 6221 6587 7028 8701 8860 9254 9540 10398 10819 10873 11024 11719 12705 14152 14212 14569 14766 15301 15326 15670 15942 16185 16209 16223 16327	L3e1a2	73 150 152 189 195 200 207 263 16185 16209 16223 16327	[0.000] L3e1a2 (1)	[0.000] L3e1f2 (2)	1
745 >MF381332.1 Hc	73G 150T 152C 189G 195C 200G 207A 263G 750G 1438G 2352C 2706G 3106d 3107C 4769G 5774A 6221C 6587T 7028T 8701G 8860G 9254G 9540C 10398G 10819G 10873C 11024T 11719A 12705T 14152G 14212C 14569A 14766T 15301A 15326G 15670C 15942C 16185T 16209C 16223T 16327T	L3e1a2	73G 150T 152C 189G 195C 200G 207A 263G 16185T 16209C 16223T 16327T	1	73 150 152 189 195 200 207 263 750 1438 2352 2706 4769 5774A 6221 6587 7028 8701 8860 9254 9540 10398 10819 10873 11024 11719 12705 14152 14212 14569 14766 15301 15326 15670 15942 16185 16209 16223 16327	L3e1a2	73 150 152 189 195 200 207 263 16185 16209 16223 16327	[0.000] L3e1a2 (1)	[0.000] L3e1f2 (2)	1
746 >MF381329.1 Hc	73G 150T 152C 189G 195C 200G 207A 263G 750G 1438G 2352C 2706G 3106d 3107C 4769G 5774A 6221C 6587T 7028T 8701G 8860G 9254G 9540C 10398G 10819G 10873C 11024T 11719A 12705T 14152G 14212C 14569A 14766T 15301A 15326G 15670C 15942C 16185T 16209C 16223T 16327T 16519C	L3e1a2	73G 150T 152C 189G 195C 200G 207A 263G 16185T 16209C 16223T 16327T 16519C	1	73 150 152 189 195 200 207 263 750 1438 2352 2706 4769 5774A 6221 6587 7028 8701 8860 9254 9540 10398 10819 10873 11024 11719 12705 14152 14212 14569 14766 15301 15326 15670 15942 16185 16209 16223 16327	L3e1a2	73 150 152 189 195 200 207 263 16185 16209 16223 16327	[0.000] L3e1a2 (1)	[0.000] L3e1f2 (2)	1
747 >MF381328.1 Hc	73G 150T 152C 189G 195C 200G 207A 263G 750G 1438G 2352C 2706G 3106d 3107C 4769G 5774A 6221C 6587T 7028T 8701G 8860G 9254G 9540C 10398G 10819G 10873C 11024T 11719A 12705T 14152G 14212C 14569A 14766T 15301A 15326G 15670C 15942C 16185T 16209C 16223T 16327T	L3e1a2	73G 150T 152C 189G 195C 200G 207A 263G 16185T 16209C 16223T 16327T	1	73 150 152 189 195 200 207 263 750 1438 2352 2706 4769 5774A 6221 6587 7028 8701 8860 9254 9540 10398 10819 10873 11024 11719 12705 14152 14212 14569 14766 15301 15326 15670 15942 16185 16209 16223 16327	L3e1a2	73 150 152 189 195 200 207 263 16185 16209 16223 16327	[0.000] L3e1a2 (1)	[0.000] L3e1f2 (2)	1

748 >MF381327.1 Hc	73G 151T 152C 182T 186A 189C 195C 198T 247A 263G 297G 316A 416A 514d 515d 520T 750G 769A 825A 1018A 1291C 1438G 2394d 2581G 2706G 2758A 2885C 3106d 3107C 3472N 3473N 3474N 3475N 3476N 3477N 3481N 3493N 3494N 3495N 3496N 3509N 3511N 3594T 3666A 3796T 3834A 3843G 4104G 4589C 4688C 4769G 5480G 5553C 5951G 6071C 6179A 7028T 7055G 7146G 7196T 7256T 7389C 7521A 7660C 7714T 8027A 8468T 8619T 8655T 8701G 8860G 9072G 9540C 9861C 10084C 10321C 10398G 10586A 10688A 10810C 10873C 11353C 11440A 11719A 11899C 12681C 12705T 12810G 13105G 13485G 13506T 13650T 13789C 13874C 14000A 14148G 14178C 14393G 14560A 14766T 14911T 15326G 16086C 16129A 16187T 16189C 16223T 16241G 16278T 16293G 16294T 16311C 16360T 16519C	L1c1b	73G 151T 152C 182T 186A 189C 195C 198T 247A 263G 297G 316A 416A 514d 515d 520T 16086C 16129A 16187T 16189C 16223T 16241G 16278T 16293G 16294T 16311C 16360T 16519C	1	73 151 152 182 186A 189C 195 198 247 263 297 316 416A 518 750 769 825A 1018 1291 1438 2395d 2581 2706 2758 2885 3472N 3473N 3474N 3475N 3476N 3477N 3481N 3493N 3494N 3495N 3496N 3509N 3511N 3594 3666 3796T 3834 3843 4104 4589 4688 4769 5480 5553 5951 6071 6179 7028 7055 7146 7196 7256 7389 7521 7660 7714 8027 8468 8619 8655 8701 8860 9072 9540 9861 10084 10321 10398 10586 10688 10810 10873 11353 11440 11719 11899 12681 12705 12810 13105 13485 13506 13650 13789 13874 14000A 14148 14178 14393 14560 14766 14911 15326 16086 16129 16187 16189 16223 16241 16278 16293 16294 16311 16360	L1c1b	73 151 152 182 186A 189C 195 198 247 263 297 316 416A 518 16086 16129 16187 16189 16223 16241 16278 16293 16294 16311 16360	[1.673] L1c1b (1)	[0.000] L1c1b'd (2)	1
749 >MF381326.1 Hc	73G 151T 152C 182T 186A 189C 195C 198T 247A 263G 297G 316A 416A 514d 515d 520T 750G 769A 825A 1018A 1291C 1438G 2394d 2581G 2706G 2758A 2885C 3106d 3107C 3594T 3666A 3796T 3834A 3843G 4104G 4589C 4688C 4769G 5480G 5553C 5951G 6071C 6179A 7028T 7055G 7146G 7196T 7256T 7389C 7521A 7660C 7714T 8027A 8468T 8619T 8655T 8701G 8860G 9072G 9540C 9861C 10084C 10321C 10398G 10586A 10688A 10810C 10873C 11353C 11440A 11719A 11899C 12681C 12705T 12810G 13105G 13485G 13506T 13650T 13789C 13874C 14000A 14148G 14178C 14393G 14560A 14766T 14911T 15326G 16086C 16129A 16187T 16189C 16223T 16241G 16278T 16293G 16294T 16311C 16360T 16519C	L1c1b	73G 151T 152C 182T 186A 189C 195C 198T 247A 263G 297G 316A 416A 514d 515d 520T 16086C 16129A 16187T 16189C 16223T 16241G 16278T 16293G 16294T 16311C 16360T 16519C	1	73 151 152 182 186A 189C 195 198 247 263 297 316 416A 518 750 769 825A 1018 1291 1438 2395d 2581 2706 2758 2885 3594 3666 3796T 3834 3843 4104 4589 4688 4769 5480 5553 5951 6071 6179 7028 7055 7146 7196 7256 7389 7521 7660 7714 8027 8468 8619 8655 8701 8860 9072 9540 9861 10084 10321 10398 10586 10688 10810 10873 11353 11440 11719 11899 12681 12705 12810 13105 13485 13506 13650 13789 13874 14000A 14148 14178 14393 14560 14766 14911 15326 16086 16129 16187 16189 16223 16241 16278 16293 16294 16311 16360	L1c1b	73 151 152 182 186A 189C 195 198 247 263 297 316 416A 518 16086 16129 16187 16189 16223 16241 16278 16293 16294 16311 16360	[1.673] L1c1b (1)	[0.000] L1c1b'd (2)	1

750 >MF381325.1 Hc	73G 150T 152C 189G 195C 200G 207A 263G 750G 1438G 2352C 2706G 3106d 3107C 4769G 5774A 6221C 6587T 7028T 8701G 8860G 9254G 9540C 10398G 10643C 10819G 10873C 11024T 11719A 12705T 14152G 14212C 14569A 14766T 15301A 15326G 15670C 15942C 16185T 16209C 16223T 16327T	L3e1a2	73G 150T 152C 189G 195C 200G 207A 263G 16185T 16209C 16223T 16327T	1	73 150 152 189 195 200 207 263 750 1438 2352 2706 4769 5774A 6221 6587 7028 8701 8860 9254 9540 10398 10643 10819 10873 11024 11719 12705 14152 14212 14569 14766 15301 15326 15670 15942 16185 16209 16223 16327	L3e1a2	73 150 152 189 195 200 207 263 16185 16209 16223 16327	[0.000] L3e1a2 (1)	[0.000] L3e1f2 (2)	1
751 >MF381323.1 Hc	73G 151T 152C 182T 186A 189C 195C 198T 247A 263G 297G 316A 416A 514d 515d 520T 750G 769A 825A 1018A 1291C 1438G 2394d 2581G 2706G 2758A 2885C 3106d 3107C 3594T 3666A 3796T 3834A 3843G 4104G 4589C 4688C 4769G 5480G 5553C 5951G 6071C 6179A 7028T 7055G 7146G 7196T 7256T 7389C 7521A 7660C 7714T 8027A 8468T 8619T 8655T 8701G 8860G 9072G 9540C 9861C 10084C 10321C 10398G 10586A 10688A 10810C 10873C 11353C 11440A 11719A 11899C 12681C 12705T 12810G 13105G 13485G 13506T 13650T 13789C 13874C 14000A 14148G 14178C 14393G 14560A 14766T 14911T 15326G 16086C 16129A 16187T 16189C 16223T 16241G 16278T 16293G 16294T 16311C 16360T 16519C	L1c1b	73G 151T 152C 182T 186A 189C 195C 198T 247A 263G 297G 316A 416A 514d 515d 520T 16086C 16129A 16187T 16189C 16223T 16241G 16278T 16293G 16294T 16311C 16360T 16519C	1	73 151 152 182 186A 189C 195 198 247 263 297 316 416A 518 750 769 825A 1018 1291 1438 2395d 2581 2706 2758 2885 3594 3666 3796T 3834 3843 4104 4589 4688 4769 5480 5553 5951 6071 6179 7028 7055 7146 7196 7256 7389 7521 7660 7714 8027 8468 8619 8655 8701 8860 9072 9540 9861 10084 10321 10398 10586 10688 10810 10873 11353 11440 11719 11899 12681 12705 12810 13105 13485 13506 13650 13789 13874 14000A 14148 14178 14393 14560 14766 14911 15326 16086 16129 16187 16189 16223 16241 16278 16293 16294 16311 16360	L1c1b	73 151 152 182 186A 189C 195 198 247 263 297 316 416A 518 16086 16129 16187 16189 16223 16241 16278 16293 16294 16311 16360	[1.673] L1c1b (1)	[0.000] L1c1b'd (2)	1
752 >MF381321.1 Hc	73G 150T 152C 189G 195C 200G 207A 263G 750G 1438G 2352C 2706G 3106d 3107C 4769G 5774A 6221C 6587T 7028T 8701G 8860G 9254G 9540C 10398G 10643C 10819G 10873C 11024T 11719A 12705T 14152G 14212C 14569A 14766T 15301A 15326G 15670C 15942C 16185T 16209C 16223T 16327T	L3e1a2	73G 150T 152C 189G 195C 200G 207A 263G 16185T 16209C 16223T 16327T	1	73 150 152 189 195 200 207 263 750 1438 2352 2706 4769 5774A 6221 6587 7028 8701 8860 9254 9540 10398 10643 10819 10873 11024 11719 12705 14152 14212 14569 14766 15301 15326 15670 15942 16185 16209 16223 16327	L3e1a2	73 150 152 189 195 200 207 263 16185 16209 16223 16327	[0.000] L3e1a2 (1)	[0.000] L3e1f2 (2)	1

753 >MF381320.1 Hc	73G 146C 152C 188G 195C 204C 207A 247A 494d 514d 515d 719A 735G 750G 769A 825A 1018A 1048T 2758A 2885C 3106d 3107C 3438A 3516A 3594T 3618C 3756G 4104G 4232C 4312T 4562C 4769G 5442C 6185C 6266G 6815C 7028T 7146G 7256T 7521A 8113A 8152A 8251A 8468T 8655T 8701G 8860G 9042T 9347G 9540C 9755A 10589A 10664T 10688A 10810C 10873C 10915C 11341G 11719A 11914A 12007A 12121C 12705T 12720G 13105G 13276G 13506T 13650T 13759A 14766T 15326G 15466A 15930A 15941C 16140C 16179T 16187T 16189C 16223T 16230G 16243C 16293G 16311C 16519C	L0d1b1b1	73G 146C 152C 188G 195C 204C 207A 247A 494d 514d 515d 16140C 16179T 16187T 16189C 16223T 16230G 16243C 16293G 16311C 16519C	1	73 146 152 188 195 204 207 247 498d 719 735 750 769 825A 1018 1048 2758 2885 3438 3516A 3594 3618 3756 4104 4232 4312 4562C 4769 5442 6185 6266 6815 7028 7146 7256 7521 8113A 8152 8251 8468 8655 8701 8860 9042 9347 9540 9755 10589 10664 10688 10810 10873 10915 11341 11719 11914 12007 12121 12705 12720 13105 13276 13506 13650 13759 14766 15326 15466 15930 15941 16140 16179 16187 16189 16223 16230 16243 16293 16311	L0d1b1b1	73 146 152 188 195 204 207 247 498d 16140 16179 16187 16189 16223 16230 16243 16293 16311	[0.853] L0d1b1b1 (1)	[0.000] L0d1b1 L0d1b1a L0d1b1a1 L0d1b1b (5)	1
754 >MF381316.1 Hc	73G 151T 152C 182T 186A 189C 195C 198T 247A 263G 297G 316A 416A 514d 515d 520T 750G 769A 825A 1018A 1291C 1438G 2394d 2581G 2706G 2758A 2885C 3106d 3107C 3594T 3666A 3796T 3834A 3843G 4104G 4589C 4688C 4769G 5480G 5553C 5951G 6071C 6179A 7028T 7055G 7146G 7196T 7256T 7389C 7521A 7660C 7714T 8027A 8468T 8619T 8655T 8701G 8860G 9072G 9540C 9861C 10084C 10321C 10398G 10586A 10688A 10810C 10873C 11353C 11440A 11719A 11899C 12681C 12705T 12810G 13105G 13485G 13506T 13650T 13789C 13874C 14000A 14148G 14178C 14393G 14560A 14766T 14911T 15326G 16086C 16129A 16187T 16189C 16223T 16241G 16278T 16293G 16294T 16311C 16360T 16519C	L1c1b	73G 151T 152C 182T 186A 189C 195C 198T 247A 263G 297G 316A 416A 514d 515d 520T 16086C 16129A 16187T 16189C 16223T 16241G 16278T 16293G 16294T 16311C 16360T 16519C	1	73 151 152 182 186A 189C 195 198 247 263 297 316 416A 518 750 769 825A 1018 1291 1438 2395d 2581 2706 2758 2885 3594 3666 3796T 3834 3843 4104 4589 4688 4769 5480 5553 5951 6071 6179 7028 7055 7146 7196 7256 7389 7521 7660 7714 8027 8468 8619 8655 8701 8860 9072 9540 9861 10084 10321 10398 10586 10688 10810 10873 11353 11440 11719 11899 12681 12705 12810 13105 13485 13506 13650 13789 13874 14000A 14148 14178 14393 14560 14766 14911 15326 16086 16129 16187 16189 16223 16241 16278 16293 16294 16311 16360	L1c1b	73 151 152 182 186A 189C 195 198 247 263 297 316 416A 518 16086 16129 16187 16189 16223 16241 16278 16293 16294 16311 16360	[1.673] L1c1b (1)	[0.000] L1c1b'd (2)	1
755 >MF381315.1 Hc	73G 150T 152C 189G 195C 200G 207A 263G 750G 1438G 2352C 2706G 3106d 3107C 4769G 5774A 6221C 6587T 7028T 8701G 8860G 9254G 9540C 10398G 10819G 10873C 11024T 11719A 12705T 14152G 14212C 14569A 14766T 15301A 15326G 15670C 15942C 16185T 16209C 16223T 16327T	L3e1a2	73G 150T 152C 189G 195C 200G 207A 263G 16185T 16209C 16223T 16327T	1	73 150 152 189 195 200 207 263 750 1438 2352 2706 4769 5774A 6221 6587 7028 8701 8860 9254 9540 10398 10819 10873 11024 11719 12705 14152 14212 14569 14766 15301 15326 15670 15942 16185 16209 16223 16327	L3e1a2	73 150 152 189 195 200 207 263 16185 16209 16223 16327	[0.000] L3e1a2 (1)	[0.000] L3e1f2 (2)	1

756	>MF381314.1 Hc	73G 151T 152C 182T 186A 189C 195C 198T 247A 263G 297G 316A 416A 514d 515d 520T 750G 769A 825A 1018A 1291C 1438G 2394d 2581G 2706G 2758A 2885C 3106d 3107C 3594T 3666A 3796T 3834A 3843G 4104G 4589C 4688C 4769G 5480G 5553C 5951G 6071C 6179A 7028T 7055G 7146G 7196T 7256T 7389C 7521A 7660C 7714T 8027A 8468T 8619T 8655T 8701G 8860G 9072G 9540C 9861C 10084C 10321C 10398G 10586A 10688A 10810C 10873C 11353C 11440A 11719A 11899C 12681C 12705T 12810G 13105G 13485G 13506T 13650T 13789C 13874C 14000A 14148G 14178C 14393G 14560A 14766T 14911T 15326G 16086C 16129A 16187T 16189C 16223T 16241G 16278T 16293G 16294T 16311C 16360T 16519C	L1c1b	73G 151T 152C 182T 186A 189C 195C 198T 247A 263G 297G 316A 416A 514d 515d 520T 16086C 16129A 16187T 16189C 16223T 16241G 16278T 16293G 16294T 16311C 16360T 16519C	1	73 151 152 182 186A 189C 195 198 247 263 297 316 416A 518 750 769 825A 1018 1291 1438 2395d 2581 2706 2758 2885 3594 3666 3796T 3834 3843 4104 4589 4688 4769 5480 5553 5951 6071 6179 7028 7055 7146 7196 7256 7389 7521 7660 7714 8027 8468 8619 8655 8701 8860 9072 9540 9861 10084 10321 10398 10586 10688 10810 10873 11353 11440 11719 11899 12681 12705 12810 13105 13485 13506 13650 13789 13874 14000A 14148 14178 14393 14560 14766 14911 15326 16086 16129 16187 16189 16223 16241 16278 16293 16294 16311 16360	L1c1b	73 151 152 182 186A 189C 195 198 247 263 297 316 416A 518 16086 16129 16187 16189 16223 16241 16278 16293 16294 16311 16360	[1.673] L1c1b (1)	[0.000] L1c1b'd (2)	1
757	>MF381311.1 Hc	73G 150T 152C 189G 195C 200G 207A 263G 750G 1438G 2352C 2706G 3106d 3107C 4769G 5774A 6221C 6587T 7028T 8701G 8860G 9254G 9540C 10398G 10819G 10873C 11024T 11719A 12705T 14152G 14212C 14569A 14766T 15301A 15326G 15670C 15942C 16185T 16209C 16223T 16327T	L3e1a2	73G 150T 152C 189G 195C 200G 207A 263G 16185T 16209C 16223T 16327T	1	73 150 152 189 195 200 207 263 750 1438 2352 2706 4769 5774A 6221 6587 7028 8701 8860 9254 9540 10398 10819 10873 11024 11719 12705 14152 14212 14569 14766 15301 15326 15670 15942 16185 16209 16223 16327	L3e1a2	73 150 152 189 195 200 207 263 16185 16209 16223 16327	[0.000] L3e1a2 (1)	[0.000] L3e1f2 (2)	1

758 >MF381309.1 Hc	73G 151T 152C 182T 186A 189C 195C 198T 247A 263G 297G 316A 416A 514d 515d 520T 750G 769A 825A 1018A 1291C 1438G 2394d 2581G 2706G 2758A 2885C 3106d 3107C 3594T 3666A 3796T 3834A 3843G 4104G 4589C 4688C 4769G 5480G 5553C 5951G 6071C 6179A 7028T 7055G 7146G 7196T 7256T 7389C 7521A 7660C 7714T 7854C 8027A 8468T 8619T 8655T 8701G 8860G 9072G 9540C 9861C 10084C 10321C 10398G 10586A 10688A 10810C 10873C 11353C 11440A 11719A 11899C 12681C 12705T 12810G 13105G 13485G 13506T 13650T 13789C 13874C 14000A 14148G 14178C 14393G 14560A 14766T 14911T 15326G 16086C 16129A 16187T 16189C 16223T 16241G 16278T 16293G 16294T 16311C 16360T 16519C	L1c1b	73G 151T 152C 182T 186A 189C 195C 198T 247A 263G 297G 316A 416A 514d 515d 520T 16086C 16129A 16187T 16189C 16223T 16241G 16278T 16293G 16294T 16311C 16360T 16519C	1	73 151 152 182 186A 189C 195 198 247 263 297 316 416A 518 750 769 825A 1018 1291 1438 2395d 2581 2706 2758 2885 3594 3666 3796T 3834 3843 4104 4589 4688 4769 5480 5553 5951 6071 6179 7028 7055 7146 7196 7256 7389 7521 7660 7714 7854 8027 8468 8619 8655 8701 8860 9072 9540 9861 10084 10321 10398 10586 10688 10810 10873 11353 11440 11719 11899 12681 12705 12810 13105 13485 13506 13650 13789 13874 14000A 14148 14178 14393 14560 14766 14911 15326 16086 16129 16187 16189 16223 16241 16278 16293 16294 16311 16360	L1c1b	73 151 152 182 186A 189C 195 198 247 263 297 316 416A 518 16086 16129 16187 16189 16223 16241 16278 16293 16294 16311 16360	[1.673] L1c1b (1)	[0.000] L1c1b'd (2)	1
759 >MF381308.1 Hc	73G 151T 152C 182T 186A 189C 195C 198T 247A 263G 297G 316A 416A 514d 515d 520T 750G 769A 825A 1018A 1291C 1438G 2394d 2581G 2706G 2758A 2885C 3106d 3107C 3594T 3666A 3796T 3834A 3843G 4104G 4589C 4688C 4769G 5480G 5553C 5951G 6071C 6179A 7028T 7055G 7146G 7196T 7256T 7389C 7521A 7660C 7714T 7854C 8027A 8468T 8619T 8655T 8701G 8860G 9072G 9540C 9861C 10084C 10321C 10398G 10586A 10688A 10810C 10873C 11353C 11440A 11719A 11899C 12681C 12705T 12810G 13105G 13485G 13506T 13650T 13789C 13874C 14000A 14148G 14178C 14393G 14560A 14766T 14911T 15326G 16086C 16129A 16187T 16189C 16223T 16241G 16278T 16293G 16294T 16311C 16360T 16519C	L1c1b	73G 151T 152C 182T 186A 189C 195C 198T 247A 263G 297G 316A 416A 514d 515d 520T 16086C 16129A 16187T 16189C 16223T 16241G 16278T 16293G 16294T 16311C 16360T 16519C	1	73 151 152 182 186A 189C 195 198 247 263 297 316 416A 518 750 769 825A 1018 1291 1438 2395d 2581 2706 2758 2885 3594 3666 3796T 3834 3843 4104 4589 4688 4769 5480 5553 5951 6071 6179 7028 7055 7146 7196 7256 7389 7521 7660 7714 7854 8027 8468 8619 8655 8701 8860 9072 9540 9861 10084 10321 10398 10586 10688 10810 10873 11353 11440 11719 11899 12681 12705 12810 13105 13485 13506 13650 13789 13874 14000A 14148 14178 14393 14560 14766 14911 15326 16086 16129 16187 16189 16223 16241 16278 16293 16294 16311 16360	L1c1b	73 151 152 182 186A 189C 195 198 247 263 297 316 416A 518 16086 16129 16187 16189 16223 16241 16278 16293 16294 16311 16360	[1.673] L1c1b (1)	[0.000] L1c1b'd (2)	1

760 >MF381304.1 Hc	73G 150T 152C 189G 195C 200G 207A 263G 750G 1438G 2352C 2706G 3106d 3107C 4002C 4769G 5774A 6221C 6587T 7028T 8701G 8860G 9254G 9540C 10398G 10819G 10873C 11024T 11719A 12705T 14152G 14212C 14569A 14766T 15301A 15326G 15670C 15942C 16185T 16209C 16223T 16327T	L3e1a2	73G 150T 152C 189G 195C 200G 207A 263G 16185T 16209C 16223T 16327T	1	73 150 152 189 195 200 207 263 750 1438 2352 2706 4002 4769 5774A 6221 6587 7028 8701 8860 9254 9540 10398 10819 10873 11024 11719 12705 14152 14212 14569 14766 15301 15326 15670 15942 16185 16209 16223 16327	L3e1a2	73 150 152 189 195 200 207 263 16185 16209 16223 16327	[0.000] L3e1a2 (1)	[0.000] L3e1f2 (2)	1
761 >MF381299.1 Hc	73G 146C 188G 195C 200G 247A 494d 719A 750G 769A 825A 1018A 1048T 1842G 2758A 2885C 3106d 3107C 3438A 3516A 3594T 3618C 3756G 4104G 4232C 4312T 4562C 4769G 5442C 6185C 6266G 6815C 7028T 7146G 7256T 7521A 8113A 8152A 8251A 8468T 8655T 8701G 8860G 9042T 9347G 9540C 9755A 10589A 10664T 10688A 10810C 10873C 10915C 11404G 11719A 11914A 12007A 12121C 12136C 12705T 12720G 13105G 13276G 13506T 13650T 13759A 14766T 15326G 15466A 15930A 15941C 16129A 16179T 16187T 16189C 16223T 16230G 16243C 16311C 16519C	L0d1b1+@152	73G 146C 188G 195C 200G 247A 494d 16129A 16179T 16187T 16189C 16223T 16230G 16243C 16311C 16519C	1	73 146 188 195 200 247 498d 719 750 769 825A 1018 1048 1842 2758 2885 3438 3516A 3594 3618 3756 4104 4232 4312 4562C 4769 5442 6185 6266 6815 7028 7146 7256 7521 8113A 8152 8251 8468 8655 8701 8860 9042 9347 9540 9755 10589 10664 10688 10810 10873 10915 11404 11719 11914 12007 12121 12136 12705 12720 13105 13276 13506 13650 13759 14766 15326 15466 15930 15941 16129 16179 16187 16189 16223 16230 16243 16311	L0d1b1+@152	73 146 188 195 200 247 498d 16129 16179 16187 16189 16223 16230 16243 16311	[0.600] L0d1b1+@152 (1)	[0.333] L0d1b1c (2) [0.000] L0d1b1 L0d1b1a L0d1b1a1 L0d1b1b (6)	1
762 >MF381298.1 Hc	73G 151T 152C 182T 186A 189C 195C 198T 247A 263G 297G 316A 416A 514d 515d 520T 750G 769A 825A 1018A 1291C 1438G 2394d 2581G 2706G 2758A 2885C 3106d 3107C 3594T 3666A 3796T 3834A 3843G 4104G 4589C 4688C 4769G 5480G 5553C 5951G 6071C 6179A 7028T 7055G 7146G 7196T 7256T 7389C 7521A 7660C 7714T 8027A 8468T 8619T 8655T 8701G 8860G 9072G 9540C 9861C 10084C 10321C 10398G 10586A 10688A 10810C 10873C 11353C 11440A 11719A 11899C 12681C 12705T 12810G 13105G 13485G 13506T 13650T 13789C 13874C 14000A 14148G 14178C 14393G 14560A 14766T 14911T 15326G 16086C 16129A 16187T 16189C 16223T 16241G 16278T 16293G 16294T 16311C 16360T 16519C	L1c1b	73G 151T 152C 182T 186A 189C 195C 198T 247A 263G 297G 316A 416A 514d 515d 520T 16086C 16129A 16187T 16189C 16223T 16241G 16278T 16293G 16294T 16311C 16360T 16519C	1	73 151 152 182 186A 189C 195 198 247 263 297 316 416A 518 750 769 825A 1018 1291 1438 2395d 2581 2706 2758 2885 3594 3666 3796T 3834 3843 4104 4589 4688 4769 5480 5553 5951 6071 6179 7028 7055 7146 7196 7256 7389 7521 7660 7714 8027 8468 8619 8655 8701 8860 9072 9540 9861 10084 10321 10398 10586 10688 10810 10873 11353 11440 11719 11899 12681 12705 12810 13105 13485 13506 13650 13789 13874 14000A 14148 14178 14393 14560 14766 14911 15326 16086 16129 16187 16189 16223 16241 16278 16293 16294 16311 16360	L1c1b	73 151 152 182 186A 189C 195 198 247 263 297 316 416A 518 16086 16129 16187 16189 16223 16241 16278 16293 16294 16311 16360	[1.673] L1c1b (1)	[0.000] L1c1b'd (2)	1

763	>MF381297.1 Hc	73G 151T 152C 182T 186A 189C 195C 198T 247A 263G 297G 316A 416A 514d 515d 520T 750G 769A 825A 1018A 1291C 1438G 2394d 2581G 2706G 2758A 2885C 3106d 3107C 3594T 3666A 3796T 3834A 3843G 4104G 4589C 4688C 4769G 5480G 5553C 5951G 6071C 6179A 7028T 7055G 7146G 7196T 7256T 7389C 7521A 7660C 7714T 7854C 8027A 8468T 8619T 8655T 8701G 8860G 9072G 9540C 9861C 10084C 10321C 10398G 10586A 10688A 10810C 10873C 11353C 11440A 11719A 11899C 12681C 12705T 12810G 13105G 13485G 13506T 13650T 13789C 13874C 14000A 14148G 14178C 14393G 14560A 14766T 14911T 15326G 16086C 16129A 16187T 16189C 16223T 16241G 16278T 16293G 16294T 16311C 16360T 16519C	L1c1b	73G 151T 152C 182T 186A 189C 195C 198T 247A 263G 297G 316A 416A 514d 515d 520T 16086C 16129A 16187T 16189C 16223T 16241G 16278T 16293G 16294T 16311C 16360T 16519C	1	73 151 152 182 186A 189C 195 198 247 263 297 316 416A 518 750 769 825A 1018 1291 1438 2395d 2581 2706 2758 2885 3594 3666 3796T 3834 3843 4104 4589 4688 4769 5480 5553 5951 6071 6179 7028 7055 7146 7196 7256 7389 7521 7660 7714 7854 8027 8468 8619 8655 8701 8860 9072 9540 9861 10084 10321 10398 10586 10688 10810 10873 11353 11440 11719 11899 12681 12705 12810 13105 13485 13506 13650 13789 13874 14000A 14148 14178 14393 14560 14766 14911 15326 16086 16129 16187 16189 16223 16241 16278 16293 16294 16311 16360	L1c1b	73 151 152 182 186A 189C 195 198 247 263 297 316 416A 518 16086 16129 16187 16189 16223 16241 16278 16293 16294 16311 16360	[1.673] L1c1b (1)	[0.000] L1c1b'd (2)	1
764	>MF381291.1 Hc	73G 146C 152C 195C 247A 265C 456T 494d 514d 515d 719A 750G 769A 825A 1018A 1048T 2758A 2885C 3106d 3107C 3438A 3516A 3591A 3594T 3756G 4104G 4197T 4232C 4312T 4769G 5442C 6185C 6266G 6815C 7028T 7146G 7256T 7521A 8113A 8152A 8251A 8468T 8655T 8701G 8860G 9042T 9150G 9347G 9540C 9755A 10398G 10589A 10664T 10688A 10810C 10873C 10915C 11437C 11719A 11914A 12007A 12121C 12235C 12705T 12720G 13105G 13129T 13276G 13506T 13650T 13759A 14766T 15326G 15466A 15519C 15930A 15941C 15951G 16093C 16187T 16189C 16223T 16230G 16234T 16243C 16311C 16519C	L0d1c3	73G 146C 152C 195C 247A 265C 456T 494d 514d 515d 16093C 16187T 16189C 16223T 16230G 16234T 16243C 16311C 16519C	1	73 146 152 195 247 265 456 498d 719 750 769 825A 1018 1048 2758 2885 3438 3516A 3591 3594 3756 4104 4197 4232 4312 4769 5442 6185 6266 6815 7028 7146 7256 7521 8113A 8152 8251 8468 8655 8701 8860 9042 9150 9347 9540 9755 10398 10589 10664 10688 10810 10873 10915 11437 11719 11914 12007 12121 12235 12705 12720 13105 13129 13276 13506 13650 13759 14766 15326 15466 15519 15930 15941 15951 16093 16187 16189 16223 16230 16234 16243 16311	L0d1c3	73 146 152 195 247 265 456 498d 16093 16187 16189 16223 16230 16234 16243 16311	[0.000] L0d1c3 (1)	[0.000] L0d1c (2)	1

765 >MF381290.1 H	73G 146C 152C 195C 247A 265C 456T 494d 514d 515d 719A 750G 769A 825A 1018A 1048T 2758A 2885C 3106d 3107C 3438A 3516A 3591A 3594T 3756G 4104G 4197T 4232C 4312T 4769G 5442C 6185C 6266G 6815C 7028T 7146G 7256T 7521A 8113A 8152A 8251A 8468T 8655T 8701G 8860G 9042T 9150G 9347G 9540C 9755A 10398G 10589A 10664T 10688A 10810C 10873C 10915C 11437C 11719A 11914A 12007A 12121C 12235C 12705T 12720G 13105G 13129T 13276G 13506T 13650T 13759A 14766T 15326G 15466A 15519C 15930A 15941C 15951G 16093C 16187T 16189C 16223T 16230G 16234T 16243C 16311C 16519C	L0d1c3	73G 146C 152C 195C 247A 265C 456T 494d 514d 515d 16093C 16187T 16189C 16223T 16230G 16234T 16243C 16311C 16519C	1	73 146 152 195 247 265 456 498d 719 750 769 825A 1018 1048 2758 2885 3438 3516A 3591 3594 3756 4104 4197 4232 4312 4769 5442 6185 6266 6815 7028 7146 7256 7521 8113A 8152 8251 8468 8655 8701 8860 9042 9150 9347 9540 9755 10398 10589 10664 10688 10810 10873 10915 11437 11719 11914 12007 12121 12235 12705 12720 13105 13129 13276 13506 13650 13759 14766 15326 15466 15519 15930 15941 15951 16093 16187 16189 16223 16230 16234 16243 16311	L0d1c3	73 146 152 195 247 265 456 498d 16093 16187 16189 16223 16230 16234 16243 16311	[0.000] L0d1c3 (1)	[0.000] L0d1c (2)	1
766 >MF381289.1 H	73G 150T 152C 189G 195C 200G 207A 263G 750G 1438G 2352C 2706G 3106d 3107C 4769G 5774A 6221C 6587T 7028T 8701G 8860G 9254G 9540C 10398G 10819G 10873C 11024T 11719A 12705T 14152G 14212C 14569A 14766T 15301A 15326G 15670C 15942C 16209C 16223T 16327T	L3e1a2	73G 150T 152C 189G 195C 200G 207A 263G 16209C 16223T 16327T	1	73 150 152 189 195 200 207 263 750 1438 2352 2706 4769 5774A 6221 6587 7028 8701 8860 9254 9540 10398 10819 10873 11024 11719 12705 14152 14212 14569 14766 15301 15326 15670 15942 16209 16223 16327	L3e1a2	73 150 152 189 195 200 207 263 16209 16223 16327	[0.013] L3e1a2 (1)	[0.000] L3e1f2 (2)	1
767 >MG571127.1 H	64T 73G 146C 153G 235G 263G 310.1C 514d 515d 663G 750G 1438G 1736G 1888A 2706G 3106d 3107C 4248C 4769G 4824G 7028T 7762A 8027A 8794T 8860G 11719A 12007A 12705T 14530C 14766T 15326G 16048A 16111T 16223T 16239A 16266T 16290T 16300G 16319A 16325C 16362C 16519C	A2aa	64T 73G 146C 153G 235G 263G 310.1C 514d 515d 16048A 16111T 16223T 16239A 16266T 16290T 16300G 16319A 16325C 16362C 16519C	1	64 73 146 153 235 263 663 750 1438 1736 1888 2706 4248 4769 4824 7028 7762 8027 8794 8860 11719 12007 12705 14530 14766 15326 16048 16111 16223 16239A 16266 16290 16300 16319 16325 16362	A2aa	64 73 146 153 235 263 16048 16111 16223 16239A 16266 16290 16300 16319 16325 16362	[0.313] A2aa (1)	[0.222] A2ad (2) [0.012] A2+(64) (3) [0.000] A2c A2d A2d1 A2d2 A2f A2f1 A2f1a A2i A2j A2j1 A2k A2k1 A2k1a A2t A2w A2x A2ad2 A2an (21)	1
768 >MG571120.1 H	64T 73G 146C 153G 235G 263G 309.1CT 310C 508G 514d 515d 663G 750G 1438G 1736G 2706G 3106d 3107C 4248C 4769G 4824G 7028T 8027A 8286C 8794T 8860G 11719A 12007A 12705T 14766T 15326G 16111T 16223T 16239A 16266T 16290T 16319A 16362C	A2aa	64T 73G 146C 153G 235G 263G 309.1CT 310C 508G 514d 515d 16111T 16223T 16239A 16266T 16290T 16319A 16362C	1	64 73 146 153 235 263 508 663 750 1438 1736 2706 4248 4769 4824 7028 8027 8286 8794 8860 11719 12007 12705 14766 15326 16111 16223 16239A 16266 16290 16319 16362	A2aa	64 73 146 153 235 263 508 16111 16223 16239A 16266 16290 16319 16362	[0.094] A2aa (1)	[0.006] A2+(64) (2) [0.000] A2c A2d A2d1 A2d2 A2f A2f1 A2f1a A2j A2j1 A2k A2k1 A2k1a A2t A2w A2x A2an (18)	1

769 >MG571118.1 H	64T 73G 146C 153G 235G 263G 309.1CT 310C 508G 514d 515d 663G 750G 1438G 1736G 2706G 3106d 3107C 4248C 4769G 4824G 7028T 8027A 8286C 8794T 8860G 11719A 12007A 12705T 14766T 15110A 15326G 16111T 16223T 16239A 16266T 16290T 16319A 16362C	A2aa	64T 73G 146C 153G 235G 263G 309.1CT 310C 508G 514d 515d 16111T 16223T 16239A 16266T 16290T 16319A 16362C	1	64 73 146 153 235 263 508 663 750 1438 1736 2706 4248 4769 4824 7028 8027 8286 8794 8860 11719 12007 12705 14766 15110 15326 16111 16223 16239A 16266 16290 16319 16362	A2aa	64 73 146 153 235 263 508 16111 16223 16239A 16266 16290 16319 16362	[0.094] A2aa (1)	[0.006] A2+(64) (2) [0.000] A2c A2d A2d1 A2d2 A2f A2f1 A2f1a A2j A2j1 A2k A2k1 A2k1a A2t A2w A2x A2an (18)	1
770 >MG182041.1 H	73G 150T 183G 263G 592T 750G 1438G 1811G 2706G 3107C 3546A 4188G 4640A 4769G 6950T 7028T 8654C 8812G 8860G 9656C 11467G 11719A 12308G 12372A 13743C 14139G 14374C 14766T 15326G 15454C 15872T 16086C 16129A 16343G	U3b1a	73G 150T 183G 263G 16086C 16129A 16343G	1	73 150 183 263 592 750 1438 1811 2706 3546A 4188 4640A 4769 6950 7028 8654 8812 8860 9656 11467 11719 12308 12372 13743 14139 14374 14766 15326 15454 15872T 16086 16129 16343	U3b1a	73 150 183 263 16086 16129 16343	[0.076] U3b1a (1)	[0.009] U3b2 (2) [0.000] U3 U3a'c U3b U3b1 U3b1a1 U3b1b U3b2a U3b2a1 U3b2c (11)	1
771 >MG182032.1 H	73G 152C 263G 310C 384G 709A 750G 980C 1438G 1888A 2706G 3107C 4216C 4769G 4917G 4959A 5558G 5563A 7028T 7853A 8697A 8860G 9300A 10463C 11251G 11719A 12633A 13368A 14766T 14905A 15326G 15452A 15607G 15928A 16126C 16163G 16186T 16189C 16261T 16294T 16519C	T1a2a	73G 152C 263G 310C 384G 16126C 16163G 16186T 16189C 16261T 16294T 16519C	1	73 152 263 310 384 709 750 980 1438 1888 2706 4216 4769 4917 4959 5558 5563 7028 7853 8697 8860 9300 10463 11251 11719 12633A 13368 14766 14905 15326 15452A 15607 15928 16126 16163 16186 16189 16261 16294	T1a2a	73 152 263 310 384 16126 16163 16186 16189 16261 16294	[0.053] T1a2a (1)	[0.016] T1a2 (2) [0.006] T1a+152	1
772 >MG182027.1 H	73G 195C 263G 310C 489C 750G 1438G 2706G 3107C 4769G 6446A 6680C 7028T 8701G 8860G 9327G 9540C 10398G 10400T 10873C 10895G 11377A 11719A 12403T 12705T 12950C 13111C 13239T 14110C 14569A 14766T 14783C 15043A 15301A 15326G 16129A 16182C 16183C 16189C 16223T 16249C 16291T 16311C 16399G 16519C	M1b2a	73G 195C 263G 310C 489C 16129A 16182C 16183C 16189C 16223T 16249C 16291T 16311C 16399G 16519C	1	73 195 263 310 489 750 1438 2706 4769 6446 6680 7028 8701 8860 9327 9540 10398 10400 10873 10895 11377 11719 12403 12705 12950C 13111 13239 14110 14569 14766 14783 15043 15301 15326 16129 16189 16223 16249 16291 16311 16399	M1b2a	73 195 263 310 489 16129 16189 16223 16249 16291 16311 16399	[0.009] M1b2a (1)	[0.000] M1b2 M1b2c (3)	1
773 >MF177157.1 H	152C 263G 310.1C 456T 750G 1438G 3106d 3107C 3525A 4769G 8578T 8860G 13056T 15326G 16070G	H36	152C 263G 310.1C 456T 16070G	1	152 263 456 750 1438 3525A 4769 8578 8860 13056 15326 16070	H36	152 263 456 16070	[0.000] H36 (1)	[0.007] H2a2a2 H3g2 H9 H46 H46a H46b H69 H16a H16c H30b1 H47a (12) [0.000] H1+152 H1i H1i1 H1i2 H1i2a H1an H1an1 H1an1a H1bb H1bc H3+152 H3g H3g1 H3g1a H3g1b H3g3 H3g4 H3i H3i1 H3j H3k H3k1 H3k1a H5'36 H5a1+152 H5a1m H5a1n H5a+152 H5a6 H5a6a H+152 H52 H13a1+152 H14b H14b2 H14b4 H16+152 H16a1 H16d (51)	1

774	>MF177138.1 Hc	73G 152Y 189G 194T 195Y 204C 207A 263G 310.1C 513.1CA 709A 750G 1243C 1438G 2706G 3106d 3107C 3505G 4093G 4769G 5046A 5460A 7028T 8002T 8149G 8251A 8614C 8658T 8860G 8994A 11674T 11719A 11947G 12414C 12705T 13374T 14766T 15326G 15884C 16145R 16192T 16211T 16223T 16292T 16325C 16519C	W6c1a	73G 152Y 189G 194T 195Y 204C 207A 263G 310.1C 513.1CA 16145R 16192T 16211T 16223T 16292T 16325C 16519C	1	73 152Y 189 194 195Y 204 207 263 709 750 1243 1438 2706 3505 4093 4769 5046 5460 7028 8002 8149 8251 8614 8658 8860 8994 11674 11719 11947 12414 12705 13374 14766 15326 15884C 16145R 16192 16211 16223 16292 16325	W6c1a	73 152Y 189 194 195Y 204 207 263 16145R 16192 16211 16223 16292 16325	[0.222] W6c1a (1)	[0.222] W6c1a (2) [0.156] W6d (3) [0.030] W6 (4) [0.022] W6b (5) [0.016] W6a (6) [0.000] W6b1 W6c W6c1 (9)	1
775	>MF177136.1 Hc	239C 263G 309.1CT 310C 750G 1438G 3106d 3107C 4769G 6869T 8860G 9233C 9804A 14185T 15326G 16400T 16482G	H6c1	239C 263G 309.1CT 310C 16400T 16482G	1	239 263 750 1438 4769 6869 8860 9233 9804 14185T 15326 16400 16482	H6c1	239 263 16400 16482	[0.059] H6c1 (1)	[0.059] H6c1 (2) [0.035] H6a2 (3) [0.028] H6 (4) [0.000] H6a H6a1 H6a1a H6a1a1 H6a1a2 H6a1a2b H6a1a2b1 H6a1a3 H6a1a3a H6a1a4 H6a1a5 H6a1a9 H6a1a10 H6a1b H6a1b2 H6a1b2a H6a1b2b H6a1b2c H6a1b2e H6a2a H6c (25)	1
776	>KY797236.1 Hc	73G 195C 263G 310.1C 512G 709A 750G 1438G 1888A 2706G 4216C 4769G 4917G 7028T 8697A 8860G 10463C 11251G 11719A 12633A 12684A 13368A 14500G 14587G 14766T 14905A 15326G 15452A 15607G 15928A 16126C 16163G 16186T 16274A 16294T 16519C	T1a7	73G 195C 263G 310.1C 512G 16126C 16163G 16186T 16274A 16294T 16519C	1	73 195 263 512 709 750 1438 1888 2706 4216 4769 4917 7028 8697 8860 10463 11251 11719 12633A 12684 13368 14500 14587 14766 14905 15326 15452A 15607 15928 16126 16163 16186 16274 16294	T1a7	73 195 263 512 16126 16163 16186 16274 16294	[0.066] T1a7 (1)	[0.008] T1a1+@152 (2) [0.000] T1a11	1
777	>KY797232.1 Hc	73G 152C 263G 310.1C 750G 1438G 1598A 1703T 1719A 2639T 2706G 3921A 4769G 4960T 5471A 7028T 8020A 8251A 8472T 8836G 8860G 9335T 9438A 10238C 11362G 11719A 12501A 12705T 12822G 12891T 13768C 14766T 15071C 15326G 16093C 16129A 16145A 16176G 16223T 16390A 16519C	N1b1a+16129	73G 152C 263G 310.1C 16093C 16129A 16145A 16176G 16223T 16390A 16519C	1	73 152 263 750 1438 1598 1703 1719 2639 2706 3921A 4769 4960 5471 7028 8020 8251 8472 8836 8860 9335 9438 10238 11362 11719 12501 12705 12822 12891 13768 14766 15071 15326 16093 16129 16145 16176G 16223 16390	N1b1a+16129	73 152 263 16093 16129 16145 16176G 16223 16390	[0.040] N1b1a+16129 (1)	[0.052] N1b1a2 (2) [0.009] N1b1a2a (3) [0.008] N1b1a (4) [0.000] N1b1 N1b1a2b N1b1a3 N1b1a5 N1b1a6 (9)	1
778	>KY797228.1 Hc	73G 150T 263G 310.1C 750G 1438G 1811G 2706G 4188G 4640A 4688C 4769G 5999C 7028T 7906T 8860G 9656C 11467G 11719A 12308G 12372A 13743C 14139G 14560A 15326G 15454C 16168T 16192T 16343G	U3b3	73G 150T 263G 310.1C 16168T 16192T 16343G	1	73 150 263 750 1438 1811 2706 4188 4640A 4688 4769 5999 7028 7906 8860 9656 11467 11719 12308 12372 13743 14139 14560 15326 15454 16168 16192 16343	U3b3	73 150 263 16168 16192 16343	[0.209] U3b3 (1)	[0.000] U5b2a1a U3 U3a'c U3b U3b1 U3b1b U3b2 U3b2a U3b2a1 U3b2c (11)	1
779	>KY797227.1 Hc	73G 146C 152C 195C 263G 310.1C 750G 2387C 2706G 4769G 5060T 7028T 8395T 8860G 10885C 11467G 11566G 11719A 12308G 12372A 12879C 13104G 14070G 14766T 15148A 15172A 15326G 15763G 15954C 16249C 16311C 16327T	U1b3	73G 146C 152C 195C 263G 310.1C 16249C 16311C 16327T	1	73 146 152 195 263 750 2387 2706 4769 5060 7028 8395 8860 10885 11467 11566 11719 12308 12372 12879 13104 14070 14766 15148 15172 15326 15763 15954C 16249 16311 16327	U1b3	73 146 152 195 263 16249 16311 16327	[0.577] U1b3 (1)	[0.357] U1b (2) [0.250] U1b1 (3) [0.022] K1b2a (4) [0.017] K2a2a (5) [0.015] K2a6 (6) [0.011] K2a1 K2a2 K2a3a (9) [0.010] M24a K2a (11) [0.008] K1b2b (12) [0.007] K1b2a2 (13) [0.000] U4b1b1+16311 U4b1b1c K1b2 K1b2a1 K1b2a1a K1b2a2a K1b2a3 K2a3 K2a3a1 K2a4 K2a7 K2a9 K2a10 (26)	1

780	>KY797222.1 Hc	152C 263G 310.1C 750G 1438G 3591A 4310G 4769G 8860G 12358G 13020C 15326G 16168T	H9a	152C 263G 310.1C 16168T	1	152 263 750 1438 3591 4310 4769 8860 12358 13020 15326 16168	H9a	152 263 16168	[0.000] H9a (1)	[0.004] H+152 (2) [0.000] H1+152 H1i H1i1 H1i2 H1i2a H1an H1an1 H1an1a H1bb H1bc H2a2a2 H3+152 H3g H3g1 H3g1a H3g1b H3g2 H3g3 H3g4 H3i H3i1 H3j H3k H3k1 H3k1a H7f H9 H46 H46a H46b H52 H69 H13a1+152 H14b H14b2 H14b4 H16+152 H16a H16a1 H16c H16d H30b1 H47a (45)	1
781	>KY797216.1 Hc	146C 225A 263G 310.1C 709A 750G 1438G 4769G 4820A 8860G 12490G 13101C 15326G 16288C 16362C	H8b	146C 225A 263G 310.1C 16288C 16362C	1	146 225 263 709 750 1438 4769 4820 8860 12490 13101C 15326 16288 16362	H8b	146 225 263 16288 16362	[0.214] H8b (1) [0.166] H8 (2) [0.000] H8+(114) H8b1 (4)	[0.214] H8b (5) [0.083] H8+(114)+152 (6) [0.015] H1n4 (7) [0.006] H1bv1 (8) [0.000] H1m1 H1n+146 H1n1 H1n1a H1n2 H1n3 H8a H8+(114) H8b1 H8c H8c1 H13a1a2b H13b H13b2 H26c (23)	1
782	>KY797215.1 Hc	73G 150T 263G 310.1C 750G 1438G 1811G 2206T 2706G 4188G 4640A 4769G 6603T 7028T 7853A 8860G 9656C 11467G 11719A 12308G 12372A 13743C 14139G 14180C 14766T 15326G 15454C 16168T 16223T 16343G 16355T	U3b3	73G 150T 263G 310.1C 16168T 16223T 16343G 16355T	1	73 150 263 750 1438 1811 2206 2706 4188 4640A 4769 6603 7028 7853 8860 9656 11467 11719 12308 12372 13743 14139 14180 14766 15326 15454 16168 16223 16343 16355	U3b3	73 150 263 16168 16223 16343 16355	[0.232] U3b3 (1)	[0.000] L3e'i'k'x L3e L3e3'4'5 L3e3'4 L3i N22 U3 U3a'c U3b U3b1 U3b1b U3b2 U3b2a U3b2a1 U3b2c (16)	1
783	>KY797213.1 Hc	73G 185A 228A 263G 295T 310.1C 462T 489C 750G 1438G 2706G 3010A 4216C 4769G 7028T 8860G 10398G 11251G 11719A 12612G 13708A 14766T 14798C 15326G 15452A 16069T 16126C 16145A 16261T	J1c+16261	73G 185A 228A 263G 295T 310.1C 462T 489C 16069T 16126C 16145A 16261T	1	73 185 228 263 295 462 489 750 1438 2706 3010 4216 4769 7028 8860 10398 11251 11719 12612 13708 14766 14798 15326 15452A 16069 16126 16145 16261	J1c+16261	73 185 228 263 295 462 489 16069 16126 16145 16261	[0.062] J1c+16261 (1) [0.000] J1c7 J1c7a (3)	[0.111] J1c6 (4) [0.062] J1c+16261 (5) [0.006] J1b3 J1b3b1 J1b4 J1b6 J1b6a (10) [0.004] J1c J1c3g J1c4 (13) [0.000] J1b J1b1 J1b2 J1b3b J1c3 J1c3a J1c3a1 J1c3a2 J1c3b J1c3b1 J1c3b1a J1c3b2 J1c3d J1c3h J1c3i J1c3k J1c4b J1c5 J1c5a J1c5a1 J1c5b J1c5c J1c5d J1c5e J1c5f J1c6a J1c7 J1c7a J1c+16261+189 J1c12 J1c12a J1c12b J1c8 J1c8b J1c9 J1c10 J1c10a J1c15 J1c15a J1c15a1 J1c17 (54)	1
784	>KY797206.1 Hc	73G 195C 263G 310.1C 512G 709A 750G 1438G 1888A 2706G 4216C 4769G 4917G 7028T 8697A 8860G 10463C 10831G 11251G 11719A 12633A 12684A 13368A 14500G 14587G 14766T 14905A 15326G 15452A 15607G 15928A 16126C 16163G 16186T 16274A 16294T 16519C	T1a7	73G 195C 263G 310.1C 512G 16126C 16163G 16186T 16274A 16294T 16519C	1	73 195 263 512 709 750 1438 1888 2706 4216 4769 4917 7028 8697 8860 10463 10831 11251 11719 12633A 12684 13368 14500 14587 14766 14905 15326 15452A 15607 15928 16126 16163 16186 16274 16294	T1a7	73 195 263 512 16126 16163 16186 16274 16294	[0.066] T1a7 (1)	[0.008] T1a1+@152 (2) [0.000] T1a1l	1
785	>KY797201.1 Hc	72C 73G 152C 195C 246C 263G 310.1C 750G 1438G 2706G 4769G 5147A 6632C 7028T 7193C 9336G 9591A 11935C 15326G 16214T 16217C 16274A 16335G 16519C	HV2a1	72C 73G 152C 195C 246C 263G 310.1C 16214T 16217C 16274A 16335G 16519C	1	72 73 152 195 246 263 750 1438 2706 4769 5147 6632 7028 7193 9336 9591 11935 15326 16214 16217 16274 16335	HV2a1	72 73 152 195 246 263 16214 16217 16274 16335	[0.681] HV2a1 (1)	[0.022] HV2a3 (2) [0.000] HV2a HV2a2 (4)	1

786	>KY797198.1 Hc	73G 153G 195C 263G 310.1C 750G 1438G 1719A 1888A 2706G 3483A 4769G 6221C 6371T 6791G 7028T 8485C 8820G 8860G 8966C 11719A 12705T 13966G 14470C 14766T 15326G 16223T 16248T 16278T 16519C	X2i+@225	73G 153G 195C 263G 310.1C 16223T 16248T 16278T 16519C	1	73 153 195 263 750 1438 1719 1888 2706 3483 4769 6221 6371 6791 7028 8485C 8820 8860 8966 11719 12705 13966 14470 14766 15326 16223 16248 16278	X2i+@225	73 153 195 263 16223 16248 16278	[0.050] X2i+@225 (1)	[0.009] X2e2a (2) [0.000] X2 X2e2 X2e2a1 X2i X2i1 X2o1 X2k X2p (10)	1
787	>KY797173.1 Hc	73G 146C 152C 195C 263G 310.1C 750G 1438G 2387C 2706G 4769G 5060T 7028T 8395T 8615C 8860G 10885C 11467G 11566G 11719A 12308G 12372A 12879C 13104G 14070G 14766T 15148A 15172A 15326G 15763G 15954C 16234T 16249C 16311C 16327T	U1b3	73G 146C 152C 195C 263G 310.1C 16234T 16249C 16311C 16327T	1	73 146 152 195 263 750 1438 2387 2706 4769 5060 7028 8395 8615 8860 10885 11467 11566 11719 12308 12372 12879 13104 14070 14766 15148 15172 15326 15763 15954C 16234 16249 16311 16327	U1b3	73 146 152 195 263 16234 16249 16311 16327	[0.615] U1b3 (1)	[0.357] U1b (2) [0.250] U1b1 (3) [0.186] U8b1a2+16311 (4) [0.022] K1b2a (5) [0.017] K2a2a (6) [0.015] K2a6 (7) [0.011] K2a1 K2a2 K2a3a (10) [0.010] M24a K2a (12) [0.008] K1b2b (13) [0.007] K1b2a2 (14) [0.000] U4b1b1+16311 U4b1b1c K1b2 K1b2a1 K1b2a1a K1b2a2a K1b2a3 K2a3 K2a3a1 K2a4 K2a7 K2a9 K2a10 (27)	1
788	>KY797172.1 Hc	73G 150T 263G 310.1C 750G 1438G 1721T 2706G 3197C 4769G 7028T 7768G 8860G 9477A 11467G 11653G 11719A 12308G 12372A 12634G 13420T 13617C 13630G 13637G 14110C 14182C 14766T 15326G 15497A 16270T 16526A	U5b2b1b	73G 150T 263G 310.1C 16270T 16526A	1	73 150 263 750 1438 1721 2706 3197 4769 7028 7768 8860 9477 11467 11653 11719 12308 12372 12634 13420 13617 13630 13637 14110 14182 14766 15326 15497 16270 16526	U5b2b1b	73 150 263 16270 16526	[0.000] U5b2b1b (1)	[0.000] U5b1d1a U5b2b U5b2b1 U5b2b2 (5)	1
789	>KY797257.2 Hc	73G 228A 263G 295T 310.1C 462T 522N 523N 524N 525N 526N 527N 528N 529N 530N 531N 534N 536N 542N 543N 750G 1438G 2706G 3010A 4216C 4769G 7028T 7154G 8860G 10398G 10749N 10750N 10751N 10752N 10755N 11251G 11719A 12612G 13708A 14766T 14798C 15326G 15452A 16069T 16092N 16093N 16094N 16095N 16096N 16097N 16098N 16099N 16100N 16101N 16102N 16103N 16104N 16105N 16106N 16107N 16108N 16109N 16110N 16111N 16112N 16113N 16114N 16126C 16228N 16261T	J1c+16261	73G 228A 263G 295T 310.1C 462T 522N 523N 524N 525N 526N 527N 528N 529N 530N 531N 534N 536N 542N 543N 16069T 16092N 16093N 16094N 16095N 16096N 16097N 16098N 16099N 16100N 16101N 16102N 16103N 16104N 16105N 16106N 16107N 16108N 16109N 16110N 16111N 16112N 16113N 16114N 16126C 16228N 16261T	1	73 228 263 295 462 522N 523N 524N 525N 526N 527N 528N 529N 530N 531N 534N 536N 542N 543N 750 1438 2706 3010 4216 4769 7028 7154 8860 10398 10749N 10750N 10751N 10752N 10755N 11251 11719 12612 13708 14766 14798 15326 15452A 16069 16092N 16093N 16094N 16095N 16096N 16097N 16098N 16099N 16100N 16101N 16102N 16103N 16104N 16105N 16106N 16107N 16108N 16109N 16110N 16111N 16112N 16113N 16114N 16126 16228N 16261	J1c+16261	73 228 263 295 462 522N 523N 524N 525N 526N 527N 528N 529N 530N 531N 534N 536N 542N 543N 16069 16092N 16093N 16094N 16095N 16096N 16097N 16098N 16099N 16100N 16101N 16102N 16103N 16104N 16105N 16106N 16107N 16108N 16109N 16110N 16111N 16112N 16113N 16114N 16126 16228N 16261	[0.025] J1c+16261 (1) [0.000] J1c7 J1c7a (3)	[0.025] J1c+16261 (4) [0.000] J1c J1c3 J1c3a J1c3a1 J1c3a2 J1c3b J1c3b1 J1c3b1a J1c3b2 J1c3d J1c3g J1c3h J1c3i J1c3k J1c4 J1c4b J1c5 J1c5a J1c5a1 J1c5b J1c5c J1c5d J1c5e J1c5f J1c6 J1c6a J1c7 J1c7a J1c+16261+189 J1c12 J1c12a J1c12b J1c8 J1c8b J1c9 J1c10 J1c10a J1c15 J1c15a J1c15a1 J1c17 (45)	1

790 >MG709130.1 H	263G 309.1CT 310C 709A 750G 1438G 3010A 3106d 3107C 4769G 5466R 8860G 14861A 15122G 15326G 15924G 16220C 16519C	H1bs	263G 309.1CT 310C 16220C 16519C	1	263 709 750 1438 3010 4769 5466R 8860 14861 15122 15326 15924 16220C	H1bs	263 16220C	[0.000] H1bs (1)	[0.012] HV12a (2) [0.001] H2a2a (3) [0.000] R0 HV HV4 HV4c HV5 HV5a HV9c HV12 H H1 H1e H1e1 H1e1a H1e1a2 H1e1a7 H1e1c H1e2 H1e2a H1e2b H1e2d H1e4 H1e6 H1e7 H1e8 H1e8a H1h H1h1 H1h2 H1j H1j3 H1j5 H1j6 H1j7 H1j9 H1m H1n H1p H1q H1q1 H1q2 H1r H1s H1s1 H1t H1t1 H1t1a H1t2 H1u H1u1 H1v H1v1 H1v1a H1v1b H1w H1ae H1ae1 H1ae2 H1ae2a H1ae3 H1ae3a H1ag H1ag1 H1ag1b H1ah H1ai H1ai1 H1aj H1ak H1ak1 H1ak2 H1am H1am1 H1ap H1aq H1as H1as2 H1at H1at1 H1at1a H1av H1ax H1ax1 H1ay H1az H1bd H1be H1bi H1bj H1bk H1bm H1bn H1bp H1bq H1br H1bu H1bv H1bw H1bx H1ca H1ci H2 H2a H2a2 H2a2+(16235) H2a4 H2a5 H2a5a H2a5b H3 H3b H3c H3c1 H3e H3q H3q1 H3r H3r1 H3t H3u H3y H3aa H3ab H3ac H3ad H3ae H3ag H3ag1 H3ah H3ai H3aj H3ap H3aq H3ar H3as H3at H3at1 H3au H4 H4a H4a1 H4a1a H4a1a1 H4a1a2 H4a1a2a H4a1a2a1 H4a1a5 H4a1c H4a1c1 H4a1c1a H4a1d H4b H4c H7 H7a H7b H7b1 H7b2 H7b2a H7b3 H7b4 H7b5 [illegible]	1
791 >MG706447.1 H	73G 152C 263G 309.1CT 310C 709A 750G 930A 1438G 1888A 2706G 3106d 3107C 4216C 4769G 4917G 4958G 5147A 7028T 8697A 8860G 9254G 10463C 11251G 11719A 11812G 13368A 14233G 14766T 14905A 15326G 15452A 15607G 15928A 16104T 16126C 16294T 16304C 16519C	T2b4b	73G 152C 263G 309.1CT 310C 16104T 16126C 16294T 16304C 16519C	1	73 152 263 709 750 930 1438 1888 2706 4216 4769 4917 4958 5147 7028 8697 8860 9254 10463 11251 11719 11812 13368 14233 14766 14905 15326 15452A 15607 15928 16104 16126 16294 16304	T2b4b	73 152 263 16104 16126 16294 16304	[0.000] T2b4b (1)	[0.000] T2b4+152 T2b4b T2b4c T2b4d T2b4e T2b4f T2b4g T2b+152 T2b21 T2b21b T2b22 (12)	1
792 >MG678791.1 H	73G 185A 228A 263G 295T 309.1CT 310C 462T 489C 513.1CA 750G 1438G 2706G 3010A 3106d 3107C 4216C 4769G 6570T 7028T 8860G 9052G 10084C 11251G 11719A 12612G 13708A 14766T 14798C 15326G 15452A 16069T 16126C 16265G 16319A	J1c8a1	73G 185A 228A 263G 295T 309.1CT 310C 462T 489C 513.1CA 16069T 16126C 16265G 16319A	1	73 185 228 263 295 462 489 750 1438 2706 3010 4216 4769 6570T 7028 8860 9052 10084 11251 11719 12612 13708 14766 14798 15326 15452A 16069 16126 16265 16319	J1c8a1	73 185 228 263 295 462 489 16069 16126 16265 16319	[0.000] J1c8a1 (1)	[0.000] J1c8a J1c8a1 J1c8a1a (4)	1

793 >MG669000.1 H	73G 150T 263G 310.1C 750G 1438G 1721T 2706G 3106d 3107C 3197C 3661G 3861G 4769G 7028T 7768G 8860G 9477A 11467G 11653G 11719A 12308G 12372A 12634G 13617C 13630G 13637G 14182C 14766T 15326G 15497A 16270T 16292T 16362C	U5b2b1a1	73G 150T 263G 310.1C 16270T 16292T 16362C	1	73 150 263 750 1438 1721 2706 3197 3661G 3861 4769 7028 7768 8860 9477 11467 11653 11719 12308 12372 12634 13617 13630 13637 14182 14766 15326 15497 16270 16292 16362	U5b2b1a1	73 150 263 16270 16292 16362	[0.000] U5b2b1a1 (1)	[0.000] U5b2b1a (2)	1
794 >MG571214.1 H	73G 263G 309.1CT 310C 489C 750G 789C 1438G 2092T 2706G 3010A 3106d 3107C 3531A 4769G 4883T 4886T 5178A 5964C 6827C 7028T 8414T 8701G 8860G 9540C 10084C 10328G 10398G 10400T 10873C 11719A 12358G 12705T 14668T 14766T 14783C 15043A 15301A 15326G 16142T 16223T 16325C 16362C 16456A 16519C	D1f	73G 263G 309.1CT 310C 489C 16142T 16223T 16325C 16362C 16456A 16519C	1	73 263 489 750 789 1438 2092 2706 3010 3531 4769 4883 4886 5178A 5964 6827 7028 8414 8701 8860 9540 10084 10328 10398 10400 10873 11719 12358 12705 14668 14766 14783 15043 15301 15326 16142 16223 16325 16362 16456	D1f	73 263 489 16142 16223 16325 16362 16456	[0.010] D1f (1)	[0.008] D1f2 (2) [0.000] D1 D1a D1a2 D1b D1c D1d D1d1 D1f+16189 D1f3 D1k D1n (13)	1
795 >MG571209.1 H	73G 150T 152C 263G 309.1CT 310C 499A 750G 827G 1438G 2706G 3106d 3107C 3547G 4025T 4769G 4820A 4977C 6473T 7028T 7270C 8271d 8272d 8273d 8274d 8275d 8276d 8277d 8278d 8279d 8860G 9950C 11177T 11719A 13590A 14500G 14766T 15326G 15535T 16092C 16145A 16183C 16189C 16217C 16519C	B2o1a	73G 150T 152C 263G 309.1CT 310C 499A 16092C 16145A 16183C 16189C 16217C 16519C	1	73 150 152 263 499 750 827 1438 2706 3547 4025 4769 4820 4977 6473 7028 7270 8281-8289d 8860 9950 11177 11719 13590 14500 14766 15326 15535 16092 16145 16189 16217	B2o1a	73 150 152 263 499 16092 16145 16189 16217	[0.095] B2o1a (1)	[0.000] B2o1 (2)	1
796 >MG571183.1 H	64T 73G 146C 153G 235G 263G 508G 514d 515d 663G 750G 1438G 1736G 2706G 3106d 3107C 4248C 4769G 4824G 7028T 8027A 8286C 8794T 8860G 11719A 12007A 12705T 14766T 15326G 16111T 16223T 16239A 16266T 16274A 16290T 16319A 16362C	A2aa	64T 73G 146C 153G 235G 263G 508G 514d 515d 16111T 16223T 16239A 16266T 16274A 16290T 16319A 16362C	1	64 73 146 153 235 263 508 663 750 1438 1736 2706 4248 4769 4824 7028 8027 8286 8794 8860 11719 12007 12705 14766 15326 16111 16223 16239A 16266 16274 16290 16319 16362	A2aa	64 73 146 153 235 263 508 16111 16223 16239A 16266 16274 16290 16319 16362	[0.156] A2aa (1)	[0.156] A2aa (2) [0.000] A2d1a	1
797 >MF057940.1 H	73G 146C 152C 195C 204C 263G 310.1C 750G 769A 1018A 1438G 2416C 2706G 2789T 3106d 3107C 3594T 4104G 4769G 5090C 7028T 7175C 7256T 7274T 7521A 7771G 8206A 8701G 8860G 9221G 9540C 10115C 10143A 10398G 10873C 11719A 11914A 11944C 12693G 12705T 13590A 13650T 13803G 14560A 14566G 14766T 15301A 15326G 15735T 15784C 16182C 16183C 16189C 16223T 16278T 16290T 16294T 16309G 16390A	L2a1b1a	73G 146C 152C 195C 204C 263G 310.1C 16182C 16183C 16189C 16223T 16278T 16290T 16294T 16309G 16390A	1	73 146 152 195 204 263 750 769 1018 1438 2416 2706 2789 3594 4104 4769 5090 7028 7175 7256 7274 7521 7771 8206 8701 8860 9221 9540 10115 10143 10398 10873 11719 11914 11944 12693 12705 13590 13650 13803 14560 14566 14766 15301 15326 15735 15784 16189 16223 16278 16290 16294 16309 16390	L2a1b1a	73 146 152 195 204 263 16189 16223 16278 16290 16294 16309 16390	[0.015] L2a1b1a (1)	[0.037] L2a1f2 (2) [0.015] L2a1b1a (3) [0.010] L2a1f (4) [0.000] L2a1+16189_(16192) L2a1b L2a1b1 L2a1b2 L2a1f1 L2a1f1a (10)	1

798	>MF057938.1 Hc	73G 146C 152C 195C 263G 750G 769A 1018A 1438G 2416C 2706G 2789T 3106d 3107C 3594T 4104G 4769G 5090C 7028T 7175C 7256T 7274T 7521A 7771G 8206A 8701G 8860G 9221G 9540C 10115C 10143A 10398G 10873C 11719A 11914A 11944C 12693G 12705T 13590A 13650T 13803G 14566G 14766T 15301A 15326G 15735T 15784C 16182C 16183C 16189C 16223T 16278T 16290T 16294T 16309G 16356C 16390A	L2a1b1a	73G 146C 152C 195C 263G 16182C 16183C 16189C 16223T 16278T 16290T 16294T 16309G 16356C 16390A	1	73 146 152 195 263 750 769 1018 1438 2416 2706 2789 3594 4104 4769 5090 7028 7175 7256 7274 7521 7771 8206 8701 8860 9221 9540 10115 10143 10398 10873 11719 11914 11944 12693 12705 13590 13650 13803 14566 14766 15301 15326 15735 15784 16189 16223 16278 16290 16294 16309 16356 16390	L2a1b1a	73 146 152 195 263 16189 16223 16278 16290 16294 16309 16356 16390	[0.015] L2a1b1a (1)	[0.015] L2a1b1a (2) [0.005] L2a1f (3) [0.000] L2a1+16189_(16192) L2a1b L2a1b1 L2a1b2 L2a1f1 L2a1f1a L2a1f2 (10)	1
799	>MF057936.1 Hc	73G 150T 200G 263G 310.1C 489C 750G 2706G 3106d 3107C 3588T 4769G 7028T 7569G 7960G 8701G 8768T 8854A 8860G 9540C 10398G 10400T 10873C 11719A 12705T 13308G 13590A 13708A 14130T 14260G 14527G 14766T 14783C 15043A 15172A 15301A 15326G 15625T 16086C 16148T 16223T 16259T 16278T 16319A 16399G 16526A	M32c	73G 150T 200G 263G 310.1C 489C 16086C 16148T 16223T 16259T 16278T 16319A 16399G 16526A	1	73 150 200 263 489 750 2706 3588 4769 7028 7569 7960 8701 8768 8854 8860 9540 10398 10400 10873 11719 12705 13308 13590 13708 14130 14260 14527 14766 14783 15043 15172 15301 15326 15625 16086 16148 16223 16259 16278 16319 16399 16526	M32c	73 150 200 263 489 16086 16148 16223 16259 16278 16319 16399 16526	[0.000] M32c (1)	[0.022] M51a (2) [0.007] M32 (3) [0.000] M51 M51b (5)	1
800	>MF057931.1 Hc	73G 152C 195C 204C 263G 310.1C 417A 489C 533G 750G 1438G 3106d 3107C 4769G 7028T 8188G 8360G 8701G 8860G 9438A 9540C 9545G 10142T 10295G 10398G 10400T 10873C 11569C 11719A 11899C 12279G 12618A 12705T 14766T 14783C 15025T 15043A 15301A 15326G 16223T 16263C 16311C 16519C	M23	73G 152C 195C 204C 263G 310.1C 417A 489C 533G 16223T 16263C 16311C 16519C	1	73 152 195 204 263 417 489 533 750 1438 4769 7028 8188 8360 8701 8860 9438 9540 9545 10142 10295 10398 10400 10873 11569 11719 11899 12279 12618 12705 14766 14783 15025 15043 15301 15326 16223 16263 16311	M23	73 152 195 204 263 417 489 533 16223 16263 16311	[0.000] M23 (1)	[0.072] M24a (2) [0.053] M57+152	1
801	>MF057915.1 Hc	73G 146C 152C 195C 263G 309.1CT 310C 750G 769A 1018A 1438G 2416C 2706G 2789T 3106d 3107C 3594T 4104G 4769G 5090C 7028T 7175C 7256T 7274T 7521A 7771G 8206A 8701G 8860G 9221G 9540C 10115C 10143A 10398G 10873C 11719A 11914A 11944C 12693G 12705T 13590A 13650T 13803G 14566G 14766T 15301A 15326G 15735T 15784C 16182C 16183C 16189C 16223T 16278T 16290T 16294T 16309G 16390A	L2a1b1a	73G 146C 152C 195C 263G 309.1CT 310C 16182C 16183C 16189C 16223T 16278T 16290T 16294T 16309G 16390A	1	73 146 152 195 263 750 769 1018 1438 2416 2706 2789 3594 4104 4769 5090 7028 7175 7256 7274 7521 7771 8206 8701 8860 9221 9540 10115 10143 10398 10873 11719 11914 11944 12693 12705 13590 13650 13803 14566 14766 15301 15326 15735 15784 16189 16223 16278 16290 16294 16309 16390	L2a1b1a	73 146 152 195 263 16189 16223 16278 16290 16294 16309 16390	[0.000] L2a1b1a (1)	[0.005] L2a1f (2) [0.000] L2a1+16189_(16192) L2a1b L2a1b1 L2a1b1a L2a1b2 L2a1f1 L2a1f1a L2a1f2 (10)	1

802 >MF057909.1 Hc	73G 152C 225A 248d 263G 309.1CT 310C 316A 489C 514d 515d 750G 1438G 2706G 3106d 3107C 3200C 3714G 4385T 4769G 4772C 7028T 7433T 8701G 8853G 8860G 9127G 9512T 9540C 10274C 10398G 10400T 10679G 10873C 11719A 11914A 12354C 12705T 14110C 14142T 14766T 14783C 14974T 15043A 15301A 15326G 15691G 16129A 16209C 16223T 16272G 16519C	M20	73G 152C 225A 248d 263G 309.1CT 310C 316A 489C 514d 515d 16129A 16209C 16223T 16272G 16519C	1	73 152 225 249d 263 316 489 750 1438 2706 3200 3714 4385T 4769 4772 7028 7433 8701 8853 8860 9127 9512 9540 10274 10398 10400 10679 10873 11719 11914 12354 12705 14110 14142 14766 14783 14974 15043 15301 15326 15691 16129 16209 16223 16272	M20	73 152 225 249d 263 316 489 16129 16209 16223 16272	[0.307] M20 (1)	[0.068] M17 (2) [0.067] M17a (3) [0.059] G1b+16129 (4) [0.028] M5a (5) [0.021] D4a1a1 (6) [0.019] M5 (7) [0.017] M5a2a (8) [0.007] M5a'd (9) [0.004] M37+152 (10) [0.003] D4a1 (11) [0.000] M5a2 M5a2a1 M5a5 M5b'c M5b M7a M7a1b2 M7a2 M7b1 G1b2 G1b4 M13'46'61 M13 M13a'b M13b M17c D4a D4a1a D4a1a1a D4a1b D4a1c D4a1f D4a1f1 D4a1g D4a1h D4a2 D4a2a D4a2b D4a5 (40)	1
803 >MF057905.1 Hc	73G 150T 199C 204C 263G 310.1C 513d 514d 709A 750G 769A 789A 1018A 1438G 1804G 2706G 3010A 3106d 3107C 3505G 3693C 3918A 4017T 4029A 4216C 4232C 4769G 4977C 5460A 7028T 7624C 8206A 8614C 8701G 8860G 8974G 9248T 9540C 9986A 10398G 10813T 10873C 11719A 12661T 12705T 13497G 13518T 14016A 14766T 14905A 14935C 15301A 15326G 15344C 16179T 16183C 16189C 16223T 16239T 16311C 16320T 16362C 16519C	L4b1a	73G 150T 199C 204C 263G 310.1C 513d 514d 16179T 16183C 16189C 16223T 16239T 16311C 16320T 16362C 16519C	1	73 150 199 204 263 513 709 750 769 789A 1018 1438 1804 2706 3010 3505 3693C 3918 4017 4029A 4216 4232 4769 4977 5460 7028 7624 8206 8614 8701 8860 8974G 9248 9540 9986 10398 10813 10873 11719 12661T 12705 13497 13518 14016 14766 14905 14935 15301 15326 15344 16179 16189 16223 16239 16311 16320 16362	L4b1a	73 150 199 204 263 513 16179 16189 16223 16239 16311 16320 16362	[0.000] L4b1a (1)	[0.010] L4b1 (2)	1
804 >MF057903.1 Hc	73G 152C 182T 186A 189C 195C 247A 248G 263G 310.1C 316A 514d 515d 745.1T 750G 769A 825A 1018A 1438G 2394d 2706G 2758A 2885C 3106d 3107C 3525T 3594T 3666A 4104G 4769G 4853A 5951G 6071C 6221A 6227C 6917A 7028T 7146G 7247T 7256T 7389C 7521A 8027A 8468T 8655T 8701G 8860G 9072G 9540C 10398G 10586A 10688A 10810C 10873C 11302T 11719A 11984C 12507G 12705T 12810G 13105G 13485G 13506T 13650T 13789C 14000A 14178C 14560A 14669G 14766T 14911T 14926G 15226G 15326G 15905C 15978T 16129A 16187T 16189C 16223T 16278T 16293G 16294T 16311C 16360T 16519C	L1c3c	73G 152C 182T 186A 189C 195C 247A 248G 263G 310.1C 316A 514d 515d 16129A 16187T 16189C 16223T 16278T 16293G 16294T 16311C 16360T 16519C	1	73 152 182 186A 189C 195 247 248 263 316 745.1T 750 769 825A 1018 1438 2395d 2706 2758 2885 3525 3594 3666 4104 4769 4853 5951 6071 6221A 6227 6917 7028 7146 7247 7256 7389 7521 8027 8468 8655 8701 8860 9072 9540 10398 10586 10688 10810 10873 11302 11719 11984 12507 12705 12810 13105 13485 13506 13650 13789 14000A 14178 14560 14669 14766 14911 14926 15226 15326 15905 15978 16129 16187 16189 16223 16278 16293 16294 16311 16360	L1c3c	73 152 182 186A 189C 195 247 248 263 316 16129 16187 16189 16223 16278 16293 16294 16311 16360	[2.063] L1c3c (1) [0.000] L1c L1c1a+@198 L1c3b'c (4)	[0.700] L1c3 (5) [0.572] L1c1a'b'd (6) [0.267] L1c3b1 (7) [0.133] L1c3b1b (8) [0.000] L1c1'2'4'5'6 L1c1 L1c1a L1c1a1 L1c3b (13)	1

805	>MF057898.1 Hc	73G 152C 195C 204C 263G 310.1C 417A 489C 533G 750G 1438G 2483C 3106d 3107C 4769G 7028T 8188G 8360G 8701G 8860G 9438A 9540C 9545G 10142T 10295G 10398G 10400T 10873C 11569C 11719A 11899C 12279G 12618A 12705T 14766T 14783C 15025T 15043A 15301A 15326G 16223T 16263C 16311C 16519C	M23	73G 152C 195C 204C 263G 310.1C 417A 489C 533G 16223T 16263C 16311C 16519C	1	73 152 195 204 263 417 489 533 750 1438 2483 4769 7028 8188 8360 8701 8860 9438 9540 9545 10142 10295 10398 10400 10873 11569 11719 11899 12279 12618 12705 14766 14783 15025 15043 15301 15326 16223 16263 16311	M23	73 152 195 204 263 417 489 533 16223 16263 16311	[0.000] M23 (1)	[0.072] M24a (2) [0.053] M57+152	1
806	>MF057895.1 Hc	73G 152C 195C 204C 263G 309.1CT 310C 417A 489C 533G 750G 1438G 3106d 3107C 4769G 5134G 7028T 8188G 8360G 8701G 8860G 9438A 9540C 9545G 10142T 10295G 10398G 10400T 10873C 11569C 11719A 11899C 12279G 12618A 12705T 14766T 14783C 15025T 15043A 15301A 15326G 16223T 16263C 16311C 16519C	M23	73G 152C 195C 204C 263G 309.1CT 310C 417A 489C 533G 16223T 16263C 16311C 16519C	1	73 152 195 204 263 417 489 533 750 1438 4769 5134 7028 8188 8360 8701 8860 9438 9540 9545 10142 10295 10398 10400 10873 11569 11719 11899 12279 12618 12705 14766 14783 15025 15043 15301 15326 16223 16263 16311	M23	73 152 195 204 263 417 489 533 16223 16263 16311	[0.000] M23 (1)	[0.072] M24a (2) [0.053] M57+152	1
807	>MF057890.1 Hc	73G 152C 195C 204C 263G 310.1C 417A 489C 524T 533G 750G 1438G 3106d 3107C 4769G 5252A 7028T 8188G 8360G 8701G 8860G 9438A 9540C 9545G 10142T 10295G 10398G 10400T 10873C 11569C 11719A 11899C 12279G 12618A 12705T 14766T 14783C 15025T 15043A 15301A 15326G 16223T 16263C 16311C 16519C	M23	73G 152C 195C 204C 263G 310.1C 417A 489C 524T 533G 16223T 16263C 16311C 16519C	1	73 152 195 204 263 417 489 524 533 750 1438 4769 5252 7028 8188 8360 8701 8860 9438 9540 9545 10142 10295 10398 10400 10873 11569 11719 11899 12279 12618 12705 14766 14783 15025 15043 15301 15326 16223 16263 16311	M23	73 152 195 204 263 417 489 524 533 16223 16263 16311	[0.008] M23 (1)	[0.072] M24a (2) [0.053] M57+152	1
808	>MF057888.1 Hc	73G 152C 195C 204C 263G 310.1C 417A 489C 533G 750G 1438G 3106d 3107C 4769G 7028T 8188G 8360G 8701G 8860G 9438A 9540C 9545G 10142T 10295G 10398G 10400T 10873C 11569C 11719A 11899C 12279G 12375C 12618A 12705T 14766T 14783C 15025T 15043A 15301A 15326G 16036.1A 16223T 16263C 16311C 16519C	M23	73G 152C 195C 204C 263G 310.1C 417A 489C 533G 16036.1A 16223T 16263C 16311C 16519C	1	73 152 195 204 263 417 489 533 750 1438 4769 7028 8188 8360 8701 8860 9438 9540 9545 10142 10295 10398 10400 10873 11569 11719 11899 12279 12375 12618 12705 14766 14783 15025 15043 15301 15326 16038.1A 16223 16263 16311	M23	73 152 195 204 263 417 489 533 16038.1A 16223 16263 16311	[0.004] M23 (1)	[0.072] M24a (2) [0.053] M57+152	1

809	>MF057879.1 Hc	73G 143A 146C 152C 195C 263G 310.1C 750G 769A 1018A 1438G 2416C 2706G 2789T 3106d 3107C 3197C 3594T 3918A 4104G 4769G 5285G 7028T 7175C 7256T 7274T 7521A 7771G 8206A 8701G 8860G 9221G 9449T 9540C 10115C 10398G 10873C 11719A 11914A 11944C 12693G 12705T 13590A 13650T 13803G 14566G 14766T 15244G 15301A 15326G 15629C 15734A 15784C 16223T 16241G 16256T 16278T 16294T 16309G 16390A 16519C	L2a1a3c	73G 143A 146C 152C 195C 263G 310.1C 16223T 16241G 16256T 16278T 16294T 16309G 16390A 16519C	1	73 143 146 152 195 263 750 769 1018 1438 2416 2706 2789 3197 3594 3918 4104 4769 5285 7028 7175 7256 7274 7521 7771 8206 8701 8860 9221 9449 9540 10115 10398 10873 11719 11914 11944 12693 12705 13590 13650 13803 14566 14766 15244 15301 15326 15629 15734 15784 16223 16241 16256 16278 16294 16309 16390	L2a1a3c	73 143 146 152 195 263 16223 16241 16256 16278 16294 16309 16390	[0.037] L2a1a3c (1)	[0.037] L2a1a3c (2) [0.000] L2a1a3 L2a1a3a L2a1a3b L2a1+143 L2a1c L2a1c3 L2a1c3a L2a1c4 L2a1c4a L2a1c4a1 L2a1e (13)	1
810	>MF057878.1 Hc	73G 150T 200G 204C 263G 310.1C 489C 750G 2706G 3106d 3107C 3588T 4769G 7028T 7960G 8701G 8768T 8854A 8860G 9540C 10398G 10400T 10873C 11719A 12705T 13308G 13590A 13708A 14130T 14260G 14527G 14766T 14783C 15043A 15172A 15301A 15326G 15625T 16086C 16148T 16223T 16259T 16278T 16319A 16399G 16526A	M32c	73G 150T 200G 204C 263G 310.1C 489C 16086C 16148T 16223T 16259T 16278T 16319A 16399G 16526A	1	73 150 200 204 263 489 750 2706 3588 4769 7028 7960 8701 8768 8854 8860 9540 10398 10400 10873 11719 12705 13308 13590 13708 14130 14260 14527 14766 14783 15043 15172 15301 15326 15625 16086 16148 16223 16259 16278 16319 16399 16526	M32c	73 150 200 204 263 489 16086 16148 16223 16259 16278 16319 16399 16526	[0.003] M32c (1)	[0.022] M51a (2) [0.007] M32 (3) [0.000] M51 M51b (5)	1
811	>MF057872.1 Hc	73G 146C 152C 195C 310.1C 750G 769A 1018A 1438G 2416C 2706G 2789T 3106d 3107C 3594T 4104G 4769G 5090C 7028T 7175C 7256T 7274T 7521A 7771G 8206A 8701G 8860G 9221G 9540C 10115C 10143A 10398G 10873C 11719A 11914A 11944C 12693G 12705T 13590A 13650T 13803G 14566G 14766T 15301A 15326G 15735T 15784C 16182C 16183C 16189C 16223T 16278T 16290T 16294T 16309G 16390A	L2a1b1a	73G 146C 152C 195C 310.1C 16182C 16183C 16189C 16223T 16278T 16290T 16294T 16309G 16390A	1	73 146 152 195 750 769 1018 1438 2416 2706 2789 3594 4104 4769 5090 7028 7175 7256 7274 7521 7771 8206 8701 8860 9221 9540 10115 10143 10398 10873 11719 11914 11944 12693 12705 13590 13650 13803 14566 14766 15301 15326 15735 15784 16189 16223 16278 16290 16294 16309 16390	L2a1b1a	73 146 152 195 16189 16223 16278 16290 16294 16309 16390	[0.003] L2a1b1a (1)	[0.006] L2a1f2 (2) [0.005] L2a1f (3) [0.003] L2a1b1a (4) [0.000] L2a1+16189_(16192) L2a1b L2a1b1 L2a1b2 L2a1f1 L2a1f1a (10)	1
812	>MF057867.1 Hc	73G 152C 195C 204C 263G 310.1C 417A 489C 524T 533G 750G 1438G 3106d 3107C 4769G 5252A 7028T 8188G 8360G 8701G 8860G 9438A 9540C 9545G 10142T 10295G 10398G 10400T 10873C 11569C 11719A 11899C 12279G 12618A 12705T 14766T 14783C 15025T 15043A 15301A 15326G 16223T 16263C 16311C 16519C	M23	73G 152C 195C 204C 263G 310.1C 417A 489C 524T 533G 16223T 16263C 16311C 16519C	1	73 152 195 204 263 417 489 524 533 750 1438 4769 5252 7028 8188 8360 8701 8860 9438 9540 9545 10142 10295 10398 10400 10873 11569 11719 11899 12279 12618 12705 14766 14783 15025 15043 15301 15326 16223 16263 16311	M23	73 152 195 204 263 417 489 524 533 16223 16263 16311	[0.008] M23 (1)	[0.072] M24a (2) [0.053] M57+152	1

813 >MF057865.1 Ho	73G 152C 195C 204C 263G 310.1C 417A 489C 533G 750G 1438G 3106d 3107C 4769G 7028T 8188G 8360G 8701G 8860G 9438A 9540C 9545G 10142T 10295G 10398G 10400T 10873C 11569C 11719A 11899C 12279G 12618A 12705T 14766T 14783C 15025T 15043A 15301A 15326G 16223T 16263C 16311C 16519C	M23	73G 152C 195C 204C 263G 310.1C 417A 489C 533G 16223T 16263C 16311C 16519C	1	73 152 195 204 263 417 489 533 750 1438 4769 7028 8188 8360 8701 8860 9438 9540 9545 10142 10295 10398 10400 10873 11569 11719 11899 12279 12618 12705 14766 14783 15025 15043 15301 15326 16223 16263 16311	M23	73 152 195 204 263 417 489 533 16223 16263 16311	[0.000] M23 (1)	[0.072] M24a (2) [0.053] M57+152	1
814 >KX457069.1 Ho	73G 248d 263G 309.1CT 310C 514d 515d 516d 517d 750G 1438G 2706G 3106d 3107C 3970T 4086T 4769G 6392C 6852A 6962A 7028T 8860G 9053A 10034C 10310A 10609C 11719A 12406A 12882T 13759A 13928C 14766T 15097C 15326G 16172C 16304C 16465T 16519C	F1a2a	73G 248d 263G 309.1CT 310C 514d 515d 516d 517d 16172C 16304C 16465T 16519C	1	73 249d 263 750 1438 2706 3970 4086 4769 6392 6852 6962 7028 8860 9053 10034 10310 10609 11719 12406 12882 13759 13928C 14766 15097 15326 16172 16304 16465	F1a2a	73 249d 263 16172 16304 16465	[0.000] F1a2a (1)	[0.000] F1a2 (2)	1
815 >KX457059.1 Ho	73G 185A 189G 210G 263G 285.1A 309.1CT 310C 514d 515d 1438G 2706G 3106d 3107C 3543T 3873G 4562G 4769G 5581G 6314T 7028T 7853A 8860G 9899C 10373A 11719A 12136C 12705T 13434G 13500C 13928C 14022G 14766T 14953T 15211T 15229C 15326G 15765A 16134T 16172C 16182C 16183C 16189C 16223T 16362C	N10a	73G 185A 189G 210G 263G 285.1A 309.1CT 310C 514d 515d 16134T 16172C 16182C 16183C 16189C 16223T 16362C	1	73 185 189 210 263 291.1A 1438 2706 3543 3873 4562 4769 5581 6314 7028 7853 8860 9899 10373 11719 12136 12705 13434 13500 13928C 14022 14766 14953 15211 15229 15326 15765 16134 16172 16189 16223 16362	N10a	73 185 189 210 263 291.1A 16134 16172 16189 16223 16362	[0.131] N10a (1)	[0.071] N10 (2)	1
816 >KX457055.1 Ho	73G 151T 152C 182T 200G 235G 263G 309.1CT 310C 514d 515d 663G 735G 750G 1438G 1736G 2706G 3106d 3107C 4248C 4769G 4824G 7028T 8167C 8794T 8860G 8975C 11719A 12705T 14766T 15326G 16223T 16290T 16319A 16362C	A14	73G 151T 152C 182T 200G 235G 263G 309.1CT 310C 514d 515d 16223T 16290T 16319A 16362C	1	73 151 152 182 200 235 263 663 735 750 1438 1736 2706 4248 4769 4824 7028 8167 8794 8860 8975 11719 12705 14766 15326 16223 16290 16319 16362	A14	73 151 152 182 200 235 263 16223 16290 16319 16362	[0.008] A14 (1)	[0.014] A+152+16362+200 (2) [0.007] A13	1
817 >KX457049.1 Ho	73G 151T 152C 182T 200G 235G 263G 309.1CT 310C 514d 515d 663G 735G 750G 1438G 1736G 2706G 3106d 3107C 4248C 4769G 4824G 7028T 8167C 8794T 8860G 8975C 11719A 12705T 14766T 15326G 16223T 16290T 16319A 16362C	A14	73G 151T 152C 182T 200G 235G 263G 309.1CT 310C 514d 515d 16223T 16290T 16319A 16362C	1	73 151 152 182 200 235 263 663 735 750 1438 1736 2706 4248 4769 4824 7028 8167 8794 8860 8975 11719 12705 14766 15326 16223 16290 16319 16362	A14	73 151 152 182 200 235 263 16223 16290 16319 16362	[0.008] A14 (1)	[0.014] A+152+16362+200 (2) [0.007] A13	1

818	>KX457048.1 Hc	73G 185A 189G 210G 263G 285.1A 309.1CT 310C 514d 515d 1438G 2706G 3106d 3107C 3543T 3873G 4562G 4769G 5581G 6314T 7028T 7853A 8860G 9899C 10373A 11719A 12136C 12705T 13434G 13500C 13928C 14022G 14766T 14953T 15211T 15229C 15326G 15765A 16134T 16172C 16180d 16183C 16189C 16223T 16362C	N10a	73G 185A 189G 210G 263G 285.1A 309.1CT 310C 514d 515d 16134T 16172C 16180d 16183C 16189C 16223T 16362C	1	73 185 189 210 263 291.1A 1438 2706 3543 3873 4562 4769 5581 6314 7028 7853 8860 9899 10373 11719 12136 12705 13434 13500 13928C 14022 14766 14953 15211 15229 15326 15765 16134 16172 16189 16193d 16223 16362	N10a	73 185 189 210 263 291.1A 16134 16172 16189 16193d 16223 16362	[0.144] N10a (1)	[0.071] N10 (2)	1
819	>KX457046.1 Hc	73G 185A 189G 210G 263G 514d 515d 1438G 2706G 3106d 3107C 3543T 3873G 4562G 4769G 5581G 6314T 7028T 7853A 8860G 9899C 10373A 11719A 12136C 12705T 13434G 13500C 13928C 14022G 14766T 14953T 15211T 15229C 15326G 15765A 16134T 16172C 16183C 16189C 16223T 16362C	N10a	73G 185A 189G 210G 263G 514d 515d 16134T 16172C 16183C 16189C 16223T 16362C	1	73 185 189 210 263 1438 2706 3543 3873 4562 4769 5581 6314 7028 7853 8860 9899 10373 11719 12136 12705 13434 13500 13928C 14022 14766 14953 15211 15229 15326 15765 16134 16172 16189 16223 16362	N10a	73 185 189 210 263 16134 16172 16189 16223 16362	[0.078] N10a (1)	[0.071] N10 (2)	1
820	>KX457045.1 Hc	73G 151T 152C 182T 200G 235G 263G 309.1CT 310C 514d 515d 663G 735G 750G 1438G 1736G 2706G 3106d 3107C 4248C 4769G 4824G 7028T 8167C 8794T 8860G 8975C 11719A 12705T 14766T 15326G 16223T 16290T 16319A 16362C	A14	73G 151T 152C 182T 200G 235G 263G 309.1CT 310C 514d 515d 16223T 16290T 16319A 16362C	1	73 151 152 182 200 235 263 663 735 750 1438 1736 2706 4248 4769 4824 7028 8167 8794 8860 8975 11719 12705 14766 15326 16223 16290 16319 16362	A14	73 151 152 182 200 235 263 16223 16290 16319 16362	[0.008] A14 (1)	[0.014] A+152+16362+200 (2) [0.007] A13	1
821	>KX457044.1 Hc	56.1C 73G 93G 103A 150T 152C 225A 227G 248d 263G 310.1C 316A 489C 514d 515d 750G 1438G 2706G 3106d 3107C 3200C 3714G 4385T 4769G 4772C 7028T 7433T 8701G 8853G 8860G 9127G 9512T 9540C 10274C 10398G 10400T 10679G 10873C 11719A 11914A 12354C 12705T 14110C 14766T 14783C 14974T 15043A 15301A 15326G 15691G 16086C 16129A 16184T 16209C 16223T 16272G 16519C	M20	56.1C 73G 93G 103A 150T 152C 225A 227G 248d 263G 310.1C 316A 489C 514d 515d 16086C 16129A 16184T 16209C 16223T 16272G 16519C	1	57 60.1T 73 93 103 150 152 225 227 249d 263 316 489 750 1438 2706 3200 3714 4385T 4769 4772 7028 7433 8701 8853 8860 9127 9512 9540 10274 10398 10400 10679 10873 11719 11914 12354 12705 14110 14766 14783 14974 15043 15301 15326 15691 16086 16129 16184 16209 16223 16272	M20	57 60.1T 73 93 103 150 152 225 227 249d 263 316 489 16086 16129 16184 16209 16223 16272	[0.703] M20 (1)	[0.159] M17a (2) [0.130] M5a2a (3) [0.100] M5c1 (4) [0.068] M17 (5) [0.066] G1b+16129 (6) [0.028] M5a D4a1a1 (8) [0.024] G1b2 G1b4 (10) [0.022] M17c (11) [0.019] M5 M27b2b1 (13) [0.014] M5a'd (14) [0.013] D4a1 (15) [0.011] M7b2 D4a1f1 (17) [0.007] D4a D4a1a D4a1a1a D4a1b D4a1c D4a1f D4a1g D4a1h D4a2 D4a2a D4a2b D4a5 (29) [0.004] M37+152 M62'68 (31) [0.000] M5a2 M5a2a1 M5a5 M5b'c M5b M7a M7a1b2 M7a2 M7b1 M7b2a G1a2'3 M13'46'61 M13 M13a'b M13b M27b2 M27b2a M27b2b M27b2c (50)	1

822	>KX457041.1 Hc	60C 73G 146C 152C 225A 248d 263G 310.1C 316A 454C 489C 514d 515d 750G 1438G 2706G 3106d 3107C 3200C 3714G 4385T 4769G 4772C 7028T 7433T 8701G 8853G 8860G 9127G 9512T 9540C 10274C 10398G 10400T 10679G 10873C 11150A 11719A 11914A 12354C 12705T 14110C 14766T 14783C 14974T 15043A 15301A 15326G 15691G 16129A 16209C 16223T 16272G 16519C	M20	60C 73G 146C 152C 225A 248d 263G 310.1C 316A 454C 489C 514d 515d 16129A 16209C 16223T 16272G 16519C	1	60 73 146 152 225 249d 263 316 454 489 750 1438 2706 3200 3714 4385T 4769 4772 7028 7433 8701 8853 8860 9127 9512 9540 10274 10398 10400 10679 10873 11150 11719 11914 12354 12705 14110 14766 14783 14974 15043 15301 15326 15691 16129 16209 16223 16272	M20	60 73 146 152 225 249d 263 316 454 489 16129 16209 16223 16272	[0.405] M20 (1)	[0.178] M5d (2) [0.095] M17 (3) [0.067] M17a (4) [0.059] G1b+16129 (5) [0.034] M5a (6) [0.021] D4a1a1 (7) [0.019] M5 (8) [0.017] M5a2a (9) [0.016] D4a1 (10) [0.014] D4a1c (11) [0.012] M13a'b (12) [0.009] M7a2a M7a2a2 (14) [0.007] M5a'd (15) [0.004] M37+152 D4a1f1 D4a5 (18) [0.000] M5a2 M5a2a1 M5a5 M5b'c M5b M7a M7a1b2 M7a2 M7b1 M7c M7c3 G1b2 G1b4 M13'46'61 M13 M13b M46 M17c D4a D4a1a D4a1a1a D4a1b D4a1f D4a1g D4a1h D4a2 D4a2a D4a2b D4a8 (47)	1
823	>KX457040.1 Hc	73G 150T 263G 309.1CT 310C 750G 1438G 1709A 2706G 3106d 3107C 4769G 4856C 5231A 5417A 7028T 8860G 11719A 12358G 12372A 12705T 14766T 15080G 15326G 16223T 16257A 16261T 16292T 16519C	N9a6	73G 150T 263G 309.1CT 310C 16223T 16257A 16261T 16292T 16519C	1	73 150 263 750 1438 1709 2706 4769 4856 5231 5417 7028 8860 11719 12358 12372 12705 14766 15080 15326 16223 16257A 16261 16292	N9a6	73 150 263 16223 16257A 16261 16292	[0.000] N9a6 (1)	[0.000] N9a N9a6a N9a6b N9a8 N9a9 N9a10 (7)	1
824	>KX457039.1 Hc	60C 73G 146C 152C 225A 248d 263G 310.1C 316A 454C 489C 514d 515d 750G 1438G 2706G 3106d 3107C 3200C 3714G 4385T 4769G 4772C 7028T 7433T 8701G 8853G 8860G 9127G 9512T 9540C 10274C 10398G 10400T 10679G 10873C 11150A 11719A 11914A 12354C 12705T 14110C 14766T 14783C 14974T 15043A 15301A 15326G 15691G 16129A 16209C 16223T 16272G 16519C	M20	60C 73G 146C 152C 225A 248d 263G 310.1C 316A 454C 489C 514d 515d 16129A 16209C 16223T 16272G 16519C	1	60 73 146 152 225 249d 263 316 454 489 750 1438 2706 3200 3714 4385T 4769 4772 7028 7433 8701 8853 8860 9127 9512 9540 10274 10398 10400 10679 10873 11150 11719 11914 12354 12705 14110 14766 14783 14974 15043 15301 15326 15691 16129 16209 16223 16272	M20	60 73 146 152 225 249d 263 316 454 489 16129 16209 16223 16272	[0.405] M20 (1)	[0.178] M5d (2) [0.095] M17 (3) [0.067] M17a (4) [0.059] G1b+16129 (5) [0.034] M5a (6) [0.021] D4a1a1 (7) [0.019] M5 (8) [0.017] M5a2a (9) [0.016] D4a1 (10) [0.014] D4a1c (11) [0.012] M13a'b (12) [0.009] M7a2a M7a2a2 (14) [0.007] M5a'd (15) [0.004] M37+152 D4a1f1 D4a5 (18) [0.000] M5a2 M5a2a1 M5a5 M5b'c M5b M7a M7a1b2 M7a2 M7b1 M7c M7c3 G1b2 G1b4 M13'46'61 M13 M13b M46 M17c D4a D4a1a D4a1a1a D4a1b D4a1f D4a1g D4a1h D4a2 D4a2a D4a2b D4a8 (47)	1

825	>KX457035.1 H[illegible]	60C 73G 146C 152C 225A 248d 263G 309.1CT 310C 316A 454C 489C 514d 515d 750G 1438G 2706G 3106d 3107C 3200C 3714G 4385T 4769G 4772C 7028T 7433T 8701G 8853G 8860G 9127G 9512T 9540C 10274C 10398G 10400T 10679G 10873C 11150A 11719A 11914A 12354C 12705T 14110C 14766T 14783C 14974T 15043A 15301A 15326G 15691G 16129A 16209C 16223T 16272G 16519C	M20	60C 73G 146C 152C 225A 248d 263G 309.1CT 310C 316A 454C 489C 514d 515d 16129A 16209C 16223T 16272G 16519C	1	60 73 146 152 225 249d 263 316 454 489 750 1438 2706 3200 3714 4385T 4769 4772 7028 7433 8701 8853 8860 9127 9512 9540 10274 10398 10400 10679 10873 11150 11719 11914 12354 12705 14110 14766 14783 14974 15043 15301 15326 15691 16129 16209 16223 16272	M20	60 73 146 152 225 249d 263 316 454 489 16129 16209 16223 16272	[0.405] M20 (1)	[0.178] M5d (2) [0.095] M17 (3) [0.067] M17a (4) [0.059] G1b+16129 (5) [0.034] M5a (6) [0.021] D4a1a1 (7) [0.019] M5 (8) [0.017] M5a2a (9) [0.016] D4a1 (10) [0.014] D4a1c (11) [0.012] M13a'b (12) [0.009] M7a2a M7a2a2 (14) [0.007] M5a'd (15) [0.004] M37+152 D4a1f1 D4a5 (18) [0.000] M5a2 M5a2a1 M5a5 M5b'c M5b M7a M7a1b2 M7a2 M7b1 M7c M7c3 G1b2 G1b4 M13'46'61 M13 M13b M46 M17c D4a D4a1a D4a1a1a D4a1b D4a1f D4a1g D4a1h D4a2 D4a2a D4a2b D4a8 (47)	1
826	>KX457033.1 H[illegible]	60C 73G 146C 152C 225A 248d 263G 310.1C 316A 454C 489C 514d 515d 750G 1438G 2706G 3106d 3107C 3200C 3714G 4385T 4769G 4772C 7028T 7433T 8701G 8853G 8860G 9127G 9512T 9540C 10274C 10398G 10400T 10679G 10873C 11150A 11719A 11914A 12354C 12705T 14110C 14766T 14783C 14974T 15043A 15301A 15326G 15691G 16129A 16209C 16223T 16272G 16519C	M20	60C 73G 146C 152C 225A 248d 263G 310.1C 316A 454C 489C 514d 515d 16129A 16209C 16223T 16272G 16519C	1	60 73 146 152 225 249d 263 316 454 489 750 1438 2706 3200 3714 4385T 4769 4772 7028 7433 8701 8853 8860 9127 9512 9540 10274 10398 10400 10679 10873 11150 11719 11914 12354 12705 14110 14766 14783 14974 15043 15301 15326 15691 16129 16209 16223 16272	M20	60 73 146 152 225 249d 263 316 454 489 16129 16209 16223 16272	[0.405] M20 (1)	[0.178] M5d (2) [0.095] M17 (3) [0.067] M17a (4) [0.059] G1b+16129 (5) [0.034] M5a (6) [0.021] D4a1a1 (7) [0.019] M5 (8) [0.017] M5a2a (9) [0.016] D4a1 (10) [0.014] D4a1c (11) [0.012] M13a'b (12) [0.009] M7a2a M7a2a2 (14) [0.007] M5a'd (15) [0.004] M37+152 D4a1f1 D4a5 (18) [0.000] M5a2 M5a2a1 M5a5 M5b'c M5b M7a M7a1b2 M7a2 M7b1 M7c M7c3 G1b2 G1b4 M13'46'61 M13 M13b M46 M17c D4a D4a1a D4a1a1a D4a1b D4a1f D4a1g D4a1h D4a2 D4a2a D4a2b D4a8 (47)	1
827	>KX457025.1 H[illegible]	60C 73G 146C 152C 225A 248d 263G 310.1C 316A 454C 489C 514d 515d 750G 1438G 2706G 3106d 3107C 3200C 3714G 4385T 4769G 4772C 7028T 7433T 8701G 8853G 8860G 9127G 9512T 9540C 10274C 10398G 10400T 10679G 10873C 11150A 11719A 11914A 12354C 12705T 14110C 14766T 14783C 14974T 15043A 15301A 15326G 15691G 16129A 16209C 16223T 16272G 16519C	M20	60C 73G 146C 152C 225A 248d 263G 310.1C 316A 454C 489C 514d 515d 16129A 16209C 16223T 16272G 16519C	1	60 73 146 152 225 249d 263 316 454 489 750 1438 2706 3200 3714 4385T 4769 4772 7028 7433 8701 8853 8860 9127 9512 9540 10274 10398 10400 10679 10873 11150 11719 11914 12354 12705 14110 14766 14783 14974 15043 15301 15326 15691 16129 16209 16223 16272	M20	60 73 146 152 225 249d 263 316 454 489 16129 16209 16223 16272	[0.405] M20 (1)	[0.178] M5d (2) [0.095] M17 (3) [0.067] M17a (4) [0.059] G1b+16129 (5) [0.034] M5a (6) [0.021] D4a1a1 (7) [0.019] M5 (8) [0.017] M5a2a (9) [0.016] D4a1 (10) [0.014] D4a1c (11) [0.012] M13a'b (12) [0.009] M7a2a M7a2a2 (14) [0.007] M5a'd (15) [0.004] M37+152 D4a1f1 D4a5 (18) [0.000] M5a2 M5a2a1 M5a5 M5b'c M5b M7a M7a1b2 M7a2 M7b1 M7c M7c3 G1b2 G1b4 M13'46'61 M13 M13b M46 M17c D4a D4a1a D4a1a1a D4a1b D4a1f D4a1g D4a1h D4a2 D4a2a D4a2b D4a8 (47)	1

828 >KX457023.1 Ho	60C 73G 146C 152C 225A 248d 263G 310.1C 316A 454C 489C 514d 515d 750G 1438G 2706G 3106d 3107C 3200C 3714G 4385T 4769G 4772C 7028T 7433T 8701G 8853G 8860G 9127G 9512T 9540C 10274C 10398G 10400T 10679G 10873C 11150A 11719A 11914A 12354C 12705T 14110C 14766T 14783C 14974T 15043A 15301A 15326G 15691G 16129A 16209C 16223T 16272G 16519C	M20	60C 73G 146C 152C 225A 248d 263G 310.1C 316A 454C 489C 514d 515d 16129A 16209C 16223T 16272G 16519C	1	60 73 146 152 225 249d 263 316 454 489 750 1438 2706 3200 3714 4385T 4769 4772 7028 7433 8701 8853 8860 9127 9512 9540 10274 10398 10400 10679 10873 11150 11719 11914 12354 12705 14110 14766 14783 14974 15043 15301 15326 15691 16129 16209 16223 16272	M20	60 73 146 152 225 249d 263 316 454 489 16129 16209 16223 16272	[0.405] M20 (1)	[0.178] M5d (2) [0.095] M17 (3) [0.067] M17a (4) [0.059] G1b+16129 (5) [0.034] M5a (6) [0.021] D4a1a1 (7) [0.019] M5 (8) [0.017] M5a2a (9) [0.016] D4a1 (10) [0.014] D4a1c (11) [0.012] M13a'b (12) [0.009] M7a2a M7a2a2 (14) [0.007] M5a'd (15) [0.004] M37+152 D4a1f1 D4a5 (18) [0.000] M5a2 M5a2a1 M5a5 M5b'c M5b M7a M7a1b2 M7a2 M7b1 M7c M7c3 G1b2 G1b4 M13'46'61 M13 M13b M46 M17c D4a D4a1a D4a1a1a D4a1b D4a1f D4a1g D4a1h D4a2 D4a2a D4a2b D4a8 (47)	1
829 >KX457012.1 Ho	73G 93G 103A 150T 152C 225A 227G 248d 263G 310.1C 316A 489C 514d 515d 750G 1438G 2706G 3106d 3107C 3200C 3714G 4385T 4769G 4772C 7028T 7433T 8701G 8853G 8860G 9127G 9512T 9540C 10274C 10398G 10400T 10679G 10873C 11719A 11914A 12354C 12705T 14110C 14766T 14783C 14974T 15043A 15301A 15326G 15691G 16086C 16129A 16209C 16223T 16272G 16519C	M20	73G 93G 103A 150T 152C 225A 227G 248d 263G 310.1C 316A 489C 514d 515d 16086C 16129A 16209C 16223T 16272G 16519C	1	73 93 103 150 152 225 227 249d 263 316 489 750 1438 2706 3200 3714 4385T 4769 4772 7028 7433 8701 8853 8860 9127 9512 9540 10274 10398 10400 10679 10873 11719 11914 12354 12705 14110 14766 14783 14974 15043 15301 15326 15691 16086 16129 16209 16223 16272	M20	73 93 103 150 152 225 227 249d 263 316 489 16086 16129 16209 16223 16272	[0.681] M20 (1)	[0.159] M17a (2) [0.130] M5a2a (3) [0.100] M5c1 (4) [0.068] M17 (5) [0.059] G1b+16129 (6) [0.028] M5a D4a1a1 (8) [0.024] G1b2 G1b4 (10) [0.022] M17c (11) [0.019] M5 M27b2b1 (13) [0.014] M5a'd (14) [0.013] D4a1 (15) [0.011] D4a1f1 (16) [0.007] D4a D4a1a D4a1a1a D4a1b D4a1c D4a1f D4a1g D4a1h D4a2 D4a2a D4a2b D4a5 (28) [0.004] M37+152 M62'68 (30) [0.000] M5a2 M5a2a1 M5a5 M5b'c M5b M7a M7a1b2 M7a2 M7b1 M13'46'61 M13 M13a'b M13b M27b2 M27b2a M27b2b M27b2c (47)	1
830 >KX457004.1 Ho	73G 263G 310.1C 489C 750G 862G 930C 1438G 2706G 3106d 3107C 4769G 5301G 5585A 6227C 7028T 8701G 8860G 9540C 10324C 10398G 10400T 10873C 11016A 11719A 11908G 11914A 12705T 12711G 12804C 12973T 13708A 14502C 14766T 14783C 15043A 15301A 15326G 15530C 15692G 15802G 16129A 16209C 16223T 16311C 16325C	M17a	73G 263G 310.1C 489C 16129A 16209C 16223T 16311C 16325C	1	73 263 489 750 862 930C 1438 2706 4769 5301 5585 6227 7028 8701 8860 9540 10324 10398 10400 10873 11016 11719 11908 11914 12705 12711 12804 12973 13708 14502 14766 14783 15043 15301 15326 15530 15692 15802 16129 16209 16223 16311 16325	M17a	73 263 489 16129 16209 16223 16311 16325	[0.050] M17a (1)	[0.142] M5a2a (2) [0.075] M43a (3) [0.074] M10a1a (4) [0.068] M17 (5) [0.036] M5d (6) [0.028] M5a (7) [0.022] M17c (8) [0.013] G1b+16129 (9) [0.011] M10a1+16129 (10) [0.008] M5 (11) [0.006] Q3 (12) [0.000] L3f2 M4"67+16311 M43+16311 M5a'd M5a2 M5a2a1 M5a5 M5b'c M5b M7a M7a2 M7b1 G1b2 G1b4 M13b Q Q1'2 M57 M81 (31)	1

831 >KX456999.1 Hc	73G 93G 103A 150T 152C 225A 227G 248d 263G 310.1C 316A 489C 514d 515d 750G 1438G 2706G 3106d 3107C 3200C 3714G 4385T 4769G 4772C 7028T 7433T 8701G 8853G 8860G 9127G 9512T 9540C 10274C 10398G 10400T 10679G 10873C 11719A 11914A 12354C 12705T 14110C 14766T 14783C 14974T 15043A 15301A 15326G 15691G 16086C 16129A 16209C 16223T 16272G 16519C	M20	73G 93G 103A 150T 152C 225A 227G 248d 263G 310.1C 316A 489C 514d 515d 16086C 16129A 16209C 16223T 16272G 16519C	1	73 93 103 150 152 225 227 249d 263 316 489 750 1438 2706 3200 3714 4385T 4769 4772 7028 7433 8701 8853 8860 9127 9512 9540 10274 10398 10400 10679 10873 11719 11914 12354 12705 14110 14766 14783 14974 15043 15301 15326 15691 16086 16129 16209 16223 16272	M20	73 93 103 150 152 225 227 249d 263 316 489 16086 16129 16209 16223 16272	[0.681] M20 (1)	[0.159] M17a (2) [0.130] M5a2a (3) [0.100] M5c1 (4) [0.068] M17 (5) [0.059] G1b+16129 (6) [0.028] M5a D4a1a1 (8) [0.024] G1b2 G1b4 (10) [0.022] M17c (11) [0.019] M5 M27b2b1 (13) [0.014] M5a'd (14) [0.013] D4a1 (15) [0.011] D4a1f1 (16) [0.007] D4a D4a1a D4a1a1a D4a1b D4a1c D4a1f D4a1g D4a1h D4a2 D4a2a D4a2b D4a5 (28) [0.004] M37+152 M62'68 (30) [0.000] M5a2 M5a2a1 M5a5 M5b'c M5b M7a M7a1b2 M7a2 M7b1 M13'46'61 M13 M13a'b M13b M27b2 M27b2a M27b2b M27b2c (47)	1
832 >KX456994.1 Hc	73G 151T 153G 263G 310.1C 489C 750G 1438G 1719A 2706G 3106d 3107C 3394C 4491A 4769G 6366A 7028T 8679G 8701G 8860G 9332T 9540C 10398G 10400T 10873C 11719A 12705T 14308C 14766T 14783C 15043A 15301A 15326G 16223T 16234T 16271C 16362C	M9a4a2	73G 151T 153G 263G 310.1C 489C 16223T 16234T 16271C 16362C	1	73 151 153 263 489 750 1438 1719 2706 3394 4491 4769 6366 7028 8679 8701 8860 9332 9540 10398 10400 10873 11719 12705 14308 14766 14783 15043 15301 15326 16223 16234 16271 16362	M9a4a2	73 151 153 263 489 16223 16234 16271 16362	[0.000] M9a4a2 (1)	[0.000] M9a4a M9a4a1 (3)	1
833 >KX456991.1 Hc	73G 150T 263G 309.1CT 310C 750G 1438G 2706G 3106d 3107C 3918A 4769G 5231A 5417A 5780A 7028T 8433C 8860G 9738A 11719A 12358G 12372A 12705T 12771A 14766T 15326G 16086C 16223T 16257A 16261T 16311C	N9a10+16311	73G 150T 263G 309.1CT 310C 16086C 16223T 16257A 16261T 16311C	1	73 150 263 750 1438 2706 3918 4769 5231 5417 5780 7028 8433 8860 9738 11719 12358 12372 12705 12771 14766 15326 16086 16223 16257A 16261 16311	N9a10+16311	73 150 263 16086 16223 16257A 16261 16311	[0.099] N9a10+16311 (1)	[0.038] N9a8 (2) [0.022] N9a9 (3) [0.011] N9a (4) [0.009] N9a10 (5) [0.000] N9a10b	1
834 >KX456989.1 Hc	73G 150T 199C 204C 263G 309.1CT 310C 489C 750G 1438G 2706G 3106d 3107C 3483A 4048A 4071T 4164G 4769G 5351G 5460A 6455T 6680C 7028T 7684C 7853A 8701G 8860G 9540C 9824C 10398G 10400T 10873C 11719A 12405T 12705T 12811C 14766T 14783C 15043A 15301A 15326G 16223T 16297C	M7b1a1b	73G 150T 199C 204C 263G 309.1CT 310C 489C 16223T 16297C	1	73 150 199 204 263 489 750 1438 2706 3483 4048 4071 4164 4769 5351 5460 6455 6680 7028 7684 7853 8701 8860 9540 9824 10398 10400 10873 11719 12405 12705 12811 14766 14783 15043 15301 15326 16223 16297	M7b1a1b	73 150 199 204 263 489 16223 16297	[0.000] M7b1a1b (1)	[0.042] M7b1a1+(16192) (2) [0.018] M7b1a1e1 (3) [0.015] M7b1a1 (4) [0.014] M7b1a1a3 (5) [0.010] M7b1a1f (6) [0.004] M7b1a1h (7) [0.002] M7b1a1g (8) [0.000] M7b1a1d M7b1a1d1 M7b1a1e (11)	1
835 >KX456980.1 Hc	73G 150T 199C 204C 263G 456T 489C 750G 1438G 2706G 3106d 3107C 4048A 4071T 4164G 4769G 5351G 5393C 5460A 6455T 6680C 7028T 7684C 7853A 8701G 8860G 9540C 9824C 10398G 10400T 10873C 11719A 12405T 12705T 12811C 14766T 14783C 14978G 15043A 15301A 15326G 16129A 16189C 16223T 16297C	M7b1a1a3	73G 150T 199C 204C 263G 456T 489C 16129A 16189C 16223T 16297C	1	73 150 199 204 263 456 489 750 1438 2706 4048 4071 4164 4769 5351 5393 5460 6455 6680 7028 7684 7853 8701 8860 9540 9824 10398 10400 10873 11719 12405 12705 12811 14766 14783 14978 15043 15301 15326 16129 16189 16223 16297	M7b1a1a3	73 150 199 204 263 456 489 16129 16189 16223 16297	[0.000] M7b1a1a3 (1)	[0.003] M7b1a1a2 (2) [0.000] M7b1a1a	1

836	>KX456976.1 Hc	73G 150T 152C 263G 310.1C 489C 514d 515d 650C 750G 862G 930C 1438G 2706G 3106d 3107C 4769G 7028T 8701G 8860G 8930T 9540C 10324C 10398G 10400T 10873C 11016A 11719A 11908G 12234G 12705T 12711G 12804C 12973T 14766T 14783C 15043A 15301A 15326G 15530C 15802G 15884A 16093C 16129A 16209C 16223T 16278T 16290T 16325C 16519C	M17a	73G 150T 152C 263G 310.1C 489C 514d 515d 16093C 16129A 16209C 16223T 16278T 16290T 16325C 16519C	1	73 150 152 263 489 650 750 862 930C 1438 2706 4769 7028 8701 8860 8930 9540 10324 10398 10400 10873 11016 11719 11908 12234 12705 12711 12804 12973 14766 14783 15043 15301 15326 15530 15802 15884 16093 16129 16209 16223 16278 16290 16325	M17a	73 150 152 263 489 16093 16129 16209 16223 16278 16290 16325	[0.436] M17a (1)	[0.212] M51a (2) [0.066] G1b+16129 (3) [0.024] G1b2 G1b4 (5) [0.014] M51b (6) [0.000] M51	1
837	>KX456975.1 Hc	73G 146C 150T 152C 248d 263G 281G 309.1CT 310C 750G 1438G 2706G 3106d 3107C 3290C 3970T 4703C 4769G 5263T 6392C 7028T 8860G 10310A 10915C 11719A 11776C 12612G 12630A 13928C 14016A 14766T 15314A 15326G 15670C 15908C 16207G 16304C 16399G	F4a2	73G 146C 150T 152C 248d 263G 281G 309.1CT 310C 16207G 16304C 16399G	1	73 146 150 152 249d 263 281 750 1438 2706 3290 3970 4703 4769 5263 6392 7028 8860 10310 10915 11719 11776 12612 12630 13928C 14016 14766 15314 15326 15670 15908 16207 16304 16399	F4a2	73 146 150 152 249d 263 281 16207 16304 16399	[0.059] F4a2 (1)	[0.000] F4a (2)	1
838	>KX456973.1 Hc	73G 152C 210G 263G 309.1CCT 310C 514d 515d 709A 750G 1438G 2706G 3106d 3107C 3537G 4086T 4769G 6960T 7028T 8271d 8272d 8273d 8274d 8275d 8276d 8277d 8278d 8279d 8584A 8860G 9950C 10398G 11465C 11719A 14766T 15235G 15326G 16140C 16182C 16183C 16189C 16261T 16266A 16519C	B5a1d	73G 152C 210G 263G 309.1CCT 310C 514d 515d 16140C 16182C 16183C 16189C 16261T 16266A 16519C	1	73 152 210 263 709 750 1438 2706 3537 4086 4769 6960 7028 8281-8289d 8584 8860 9950 10398 11465 11719 14766 15235 15326 16140 16189 16261 16266A	B5a1d	73 152 210 263 16140 16189 16261 16266A	[0.000] B5a1d (1)	[0.032] B5a1 (2) [0.016] B5a1a (3) [0.010] B5a (4) [0.004] B5a1b1 (5) [0.002] B5a1c1a (6) [0.000] B5a1a1 B5a1b B5a1c B5a1c1 B5a1c2 (11)	1
839	>KX456972.1 Hc	73G 150T 152C 263G 310.1C 489C 514d 515d 650C 750G 862G 930C 1438G 2706G 3106d 3107C 4769G 7028T 8701G 8860G 8930T 9540C 10324C 10398G 10400T 10873C 11016A 11719A 11908G 12234G 12705T 12711G 12804C 12973T 14766T 14783C 15043A 15301A 15326G 15530C 15802G 15884A 16093C 16129A 16209C 16223T 16278T 16290T 16325C 16519C	M17a	73G 150T 152C 263G 310.1C 489C 514d 515d 16093C 16129A 16209C 16223T 16278T 16290T 16325C 16519C	1	73 150 152 263 489 650 750 862 930C 1438 2706 4769 7028 8701 8860 8930 9540 10324 10398 10400 10873 11016 11719 11908 12234 12705 12711 12804 12973 14766 14783 15043 15301 15326 15530 15802 15884 16093 16129 16209 16223 16278 16290 16325	M17a	73 150 152 263 489 16093 16129 16209 16223 16278 16290 16325	[0.436] M17a (1)	[0.212] M51a (2) [0.066] G1b+16129 (3) [0.024] G1b2 G1b4 (5) [0.014] M51b (6) [0.000] M51	1
840	>KX456969.1 Hc	73G 146C 150T 263G 309.1CT 310C 750G 961C 1438G 2706G 3106d 3107C 4769G 4856C 5231A 5417A 7028T 8860G 8963T 11719A 12358G 12372A 12705T 14766T 15326G 16188T 16223T 16257A 16261T 16292T 16519C	N9a6	73G 146C 150T 263G 309.1CT 310C 16188T 16223T 16257A 16261T 16292T 16519C	1	73 146 150 263 750 961 1438 2706 4769 4856 5231 5417 7028 8860 8963 11719 12358 12372 12705 14766 15326 16188 16223 16257A 16261 16292	N9a6	73 146 150 263 16188 16223 16257A 16261 16292	[0.058] N9a6 (1)	[0.022] N9a N9a9 (3) [0.000] N9a6a N9a6b N9a8 N9a10 (7)	1

841 >KX456967.1 Ho	73G 146C 150T 185A 189G 196C 199C 263G 513A 750G 827G 1438G 2706G 3106d 3107C 4769G 6614C 7028T 7657C 8271d 8272d 8273d 8274d 8275d 8276d 8277d 8278d 8279d 8860G 9758C 9921A 10410C 10750G 11719A 13135A 14766T 15326G 15535T 15940d 16129A 16182C 16183C 16189C 16217C 16223T 16311C 16519C	B4e	73G 146C 150T 185A 189G 196C 199C 263G 513A 16129A 16182C 16183C 16189C 16217C 16223T 16311C 16519C	1	73 146 150 185 189 196 199 263 513 750 827 1438 2706 4769 6614 7028 7657 8281-8289d 8860 9758 9921 10410 10750 11719 13135 14766 15326 15535 15944d 16129 16189 16217 16223 16311	B4e	73 146 150 185 189 196 199 263 513 16129 16189 16217 16223 16311	[1.354] B4e (1)	[0.084] R11b (2) [0.073] R11 (3) [0.048] R11a	1
842 >KX456964.1 Ho	73G 146C 150T 185A 189G 196C 199C 263G 309.1CCT 310C 513A 750G 827G 1438G 2706G 3106d 3107C 4769G 6614C 7028T 7657C 8271d 8272d 8273d 8274d 8275d 8276d 8277d 8278d 8279d 8860G 9758C 9921A 10410C 10750G 11719A 13135A 14766T 15326G 15535T 15940d 16129A 16182C 16183C 16189C 16217C 16223T 16311C 16519C	B4e	73G 146C 150T 185A 189G 196C 199C 263G 309.1CCT 310C 513A 16129A 16182C 16183C 16189C 16217C 16223T 16311C 16519C	1	73 146 150 185 189 196 199 263 513 750 827 1438 2706 4769 6614 7028 7657 8281-8289d 8860 9758 9921 10410 10750 11719 13135 14766 15326 15535 15944d 16129 16189 16217 16223 16311	B4e	73 146 150 185 189 196 199 263 513 16129 16189 16217 16223 16311	[1.354] B4e (1)	[0.084] R11b (2) [0.073] R11 (3) [0.048] R11a	1
843 >KX456959.1 Ho	73G 146C 248d 263G 281G 310.1C 750G 1438G 2706G 3106d 3107C 3290C 3970T 4769G 5069G 5263T 6392C 7028T 8860G 10310A 10909C 10915C 11719A 11776C 11825A 12612G 12630A 13368A 13928C 14016A 14766T 15326G 15670C 15908C 16129A 16185T 16207G 16399G 16519C	F4a2	73G 146C 248d 263G 281G 310.1C 16129A 16185T 16207G 16399G 16519C	1	73 146 249d 263 281 750 1438 2706 3290 3970 4769 5069 5263 6392 7028 8860 10310 10909 10915 11719 11776 11825 12612 12630 13368 13928C 14016 14766 15326 15670 15908 16129 16185 16207 16399	F4a2	73 146 249d 263 281 16129 16185 16207 16399	[1.118] F4a2 (1)	[0.750] F4a (2)	1
844 >KX456957.1 Ho	73G 146C 150T 263G 309.1CT 310C 750G 961C 1438G 2706G 3106d 3107C 4769G 4856C 5231A 5417A 7028T 8860G 8963T 11719A 12358G 12372A 12705T 14766T 15326G 16223T 16257A 16261T 16292T 16519C	N9a6	73G 146C 150T 263G 309.1CT 310C 16223T 16257A 16261T 16292T 16519C	1	73 146 150 263 750 961 1438 2706 4769 4856 5231 5417 7028 8860 8963 11719 12358 12372 12705 14766 15326 16223 16257A 16261 16292	N9a6	73 146 150 263 16223 16257A 16261 16292	[0.035] N9a6 (1)	[0.011] N9a N9a9 (3) [0.000] N9a6a N9a6b N9a8 N9a10 (7)	1
845 >KX456956.1 Ho	73G 146C 150T 263G 309.1CT 310C 750G 961C 1438G 2706G 3106d 3107C 4769G 4856C 5231A 5417A 7028T 8860G 8963T 11719A 12358G 12372A 12705T 14766T 15326G 16188T 16223T 16257A 16261T 16292T 16519C	N9a6	73G 146C 150T 263G 309.1CT 310C 16188T 16223T 16257A 16261T 16292T 16519C	1	73 146 150 263 750 961 1438 2706 4769 4856 5231 5417 7028 8860 8963 11719 12358 12372 12705 14766 15326 16188 16223 16257A 16261 16292	N9a6	73 146 150 263 16188 16223 16257A 16261 16292	[0.058] N9a6 (1)	[0.022] N9a N9a9 (3) [0.000] N9a6a N9a6b N9a8 N9a10 (7)	1
846 >KX456948.1 Ho	73G 146C 195C 263G 310.1C 489C 514d 515d 750G 951A 1438G 2140A 2706G 3106d 3107C 4769G 5773A 7028T 8701G 8860G 9540C 10101C 10398G 10400T 10873C 11719A 12651A 12705T 13359A 13812C 14766T 14783C 15043A 15301A 15326G 15601C 16086C 16223T 16519C	M24b	73G 146C 195C 263G 310.1C 489C 514d 515d 16086C 16223T 16519C	1	73 146 195 263 489 750 951 1438 2140 2706 4769 5773 7028 8701 8860 9540 10101 10398 10400 10873 11719 12651 12705 13359 13812 14766 14783 15043 15301 15326 15601 16086 16223	M24b	73 146 195 263 489 16086 16223	[0.000] M24b (1)	[0.000] M24 (2)	1

847 >KX456947.1 Hc	73G 150T 195C 248d 263G 514d 515d 750G 1053G 1438G 2706G 3106d 3107C 3970T 4769G 5587C 6392C 6962A 7028T 7909T 8860G 10310A 10609C 11719A 12406A 12882T 13359A 13928C 14766T 15024A 15326G 16189C 16300G 16304C 16356C 16519C	F1e3	73G 150T 195C 248d 263G 514d 515d 16189C 16300G 16304C 16356C 16519C	1	73 150 195 249d 263 750 1053 1438 2706 3970 4769 5587 6392 6962 7028 7909 8860 10310 10609 11719 12406 12882 13359 13928C 14766 15024 15326 16189 16300 16304 16356	F1e3	73 150 195 249d 263 16189 16300 16304 16356	[0.743] F1e3 (1)	[0.073] F2h (2) [0.036] F1g (3) [0.007] F1e1 (4) [0.005] F1+16189 F1g1 F2c1 (7) [0.000] F1e F2+195 (9)	1
848 >KX456946.1 Hc	73G 146C 263G 310.1C 489C 513d 514d 750G 1438G 2706G 3106d 3107C 3912G 4071T 4769G 4850T 5378G 6455T 7028T 8701G 8860G 9180G 9449T 9540C 9824C 10398G 10400T 10789G 10873C 10897T 11665T 11719A 11932T 12091C 12705T 14053G 14766T 14783C 15043A 15301A 15326G 15338T 16172C 16223T 16274A 16291T 16311C 16519C	M7c2a	73G 146C 263G 310.1C 489C 513d 514d 16172C 16223T 16274A 16291T 16311C 16519C	1	73 146 263 489 513 750 1438 2706 3912 4071 4769 4850 5378 6455 7028 8701 8860 9180 9449 9540 9824 10398 10400 10789 10873 10897 11665 11719 11932 12091 12705 14053 14766 14783 15043 15301 15326 15338 16172 16223 16274 16291 16311	M7c2a	73 146 263 489 513 16172 16223 16274 16291 16311	[0.119] M7c2a (1)	[0.000] M7c2 M7c2b (3)	1
849 >KX456943.1 Hc	73G 152C 210G 263G 309.1CCT 310C 514d 515d 709A 750G 930A 1438G 2706G 3106d 3107C 3537G 4086T 4769G 6960T 7028T 8271d 8272d 8273d 8274d 8275d 8276d 8277d 8278d 8279d 8584A 8860G 9950C 10398G 10550G 11465C 11719A 12172G 14766T 15235G 15326G 15911G 16140C 16182C 16183C 16189C 16261T 16266A 16519C	B5a1d	73G 152C 210G 263G 309.1CCT 310C 514d 515d 16140C 16182C 16183C 16189C 16261T 16266A 16519C	1	73 152 210 263 709 750 930 1438 2706 3537 4086 4769 6960 7028 8281-8289d 8584 8860 9950 10398 10550 11465 11719 12172 14766 15235 15326 15911 16140 16189 16261 16266A	B5a1d	73 152 210 263 16140 16189 16261 16266A	[0.000] B5a1d (1)	[0.032] B5a1 (2) [0.016] B5a1a (3) [0.010] B5a (4) [0.004] B5a1b1 (5) [0.002] B5a1c1a (6) [0.000] B5a1a1 B5a1b B5a1c B5a1c1 B5a1c2 (11)	1
850 >KX456939.1 Hc	73G 185A 189G 263G 309.1CCT 310C 514d 515d 1438G 2706G 3106d 3107C 3543T 3873G 4562G 4769G 5581G 6314T 7028T 7711C 7853A 8860G 9899C 10373A 11719A 12136C 12705T 13434G 13500C 13928C 14022G 14766T 14953T 15326G 16172C 16183C 16189C 16223T 16362C	N10a	73G 185A 189G 263G 309.1CCT 310C 514d 515d 16172C 16183C 16189C 16223T 16362C	1	73 185 189 263 1438 2706 3543 3873 4562 4769 5581 6314 7028 7711 7853 8860 9899 10373 11719 12136 12705 13434 13500 13928C 14022 14766 14953 15326 16172 16189 16223 16362	N10a	73 185 189 263 16172 16189 16223 16362	[0.000] N10a (1)	[0.071] N10 (2)	1
851 >KX456937.1 Hc	73G 146C 263G 310.1C 489C 514d 515d 750G 1438G 2706G 3106d 3107C 3912G 4071T 4769G 4850T 5378G 5393C 6455T 6927T 7028T 8701G 8860G 9449T 9540C 9824C 10398G 10400T 10873C 10897T 11665T 11719A 11932T 12091C 12705T 14053G 14693G 14766T 14783C 15043A 15301A 15326G 15338T 16172C 16223T 16274A 16291T 16311C 16519C	M7c2a	73G 146C 263G 310.1C 489C 514d 515d 16172C 16223T 16274A 16291T 16311C 16519C	1	73 146 263 489 750 1438 2706 3912 4071 4769 4850 5378 5393 6455 6927 7028 8701 8860 9449 9540 9824 10398 10400 10873 10897 11665 11719 11932 12091 12705 14053 14693 14766 14783 15043 15301 15326 15338 16172 16223 16274 16291 16311	M7c2a	73 146 263 489 16172 16223 16274 16291 16311	[0.100] M7c2a (1)	[0.000] M7c2 M7c2b (3)	1

852 >KX456936.1 Ho	73G 152C 210G 263G 514d 515d 709A 750G 1438G 2706G 3106d 3107C 3537G 4086T 4769G 6960T 7028T 8271d 8272d 8273d 8274d 8275d 8276d 8277d 8278d 8279d 8584A 8860G 9950C 10398G 11465C 11719A 13928C 14766T 15235G 15326G 16129A 16140C 16182C 16183C 16189C 16261T 16266A 16519C	B5a1d	73G 152C 210G 263G 514d 515d 16129A 16140C 16182C 16183C 16189C 16261T 16266A 16519C	1	73 152 210 263 709 750 1438 2706 3537 4086 4769 6960 7028 8281-8289d 8584 8860 9950 10398 11465 11719 13928C 14766 15235 15326 16129 16140 16189 16261 16266A	B5a1d	73 152 210 263 16129 16140 16189 16261 16266A	[0.400] B5a1d (1)	[0.047] B5a1a (2) [0.041] B5a1 (3) [0.010] B5a (4) [0.004] B5a1b1 B5a1c1a (6) [0.000] B5a1a1 B5a1b B5a1c B5a1c1 B5a1c2 (11)	1
853 >MG649085.1 Ho	73G 195C 263G 310.1C 499A 750G 1438G 1664A 1811G 2706G 3106d 3107C 4646C 4769G 5999C 6047G 6776C 7028T 8705C 8860G 10676T 10907C 11332T 11467G 11719A 12308G 12372A 13759A 14097T 14620T 14766T 15326G 15569T 15693C 16261T 16356C	U4c2a	73G 195C 263G 310.1C 499A 16261T 16356C	1	73 195 263 499 750 1438 1664 1811 2706 4646 4769 5999 6047 6776 7028 8705 8860 10676 10907 11332 11467 11719 12308 12372 13759 14097 14620 14766 15326 15569 15693 16261 16356	U4c2a	73 195 263 499 16261 16356	[0.000] U4c2a (1)	[0.000] U4 U4a U4b U4b1 U4b1a U4b1a1 U4b1a1a U4b1a2 U4b1a2a U4b1a2b U4b1a4 U4c U4c2 U4d U4d1 U4d1a U4d1a1 U4d1a1a U4d1b (20)	1
854 >MG609035.1 Ho	73G 189G 200G 263G 310.1C 750G 1438G 1822C 2706G 3106d 3107C 3396C 4218C 4769G 5073G 5601T 7028T 7819A 8527G 8701G 8860G 8932T 9123A 9540C 9950C 10398G 10873C 11440A 11719A 12705T 14766T 14769G 15301A 15326G 15514C 15940d 16093C 16209C 16223T 16311C 16519C	L3f1b	73G 189G 200G 263G 310.1C 16093C 16209C 16223T 16311C 16519C	1	73 189 200 263 750 1438 1822 2706 3396 4218 4769 5073 5601 7028 7819A 8527 8701 8860 8932 9123 9540 9950 10398 10873 11440 11719 12705 14766 14769 15301 15326 15514 15944d 16093 16209 16223 16311	L3f1b	73 189 200 263 16093 16209 16223 16311	[0.087] L3f1b (1)	[0.043] L3f1b2 (2) [0.038] L3f1b+16292 (3) [0.000] L3f1b4a L3f1b4a1 L3f1b5 (6)	1
855 >MF043510.1 Ho	73G 183G 263G 309.1CT 310C 489C 523d 750G 1438G 2706G 3010A 3106d 3107C 4769G 4883T 5178A 6881G 7028T 7762A 8020A 8414T 8701G 8860G 9540C 10181T 10398G 10400T 10873C 11719A 12705T 13720T 14668T 14766T 14783C 14815T 14927G 15043A 15301A 15326G 15440C 15951G 16129A 16173T 16223T 16319A 16362C	D4b1a2a1	73G 183G 263G 309.1CT 310C 489C 523d 16129A 16173T 16223T 16319A 16362C	1	73 183 263 489 523d 750 1438 2706 3010 4769 4883 5178A 6881 7028 7762 8020 8414 8701 8860 9540 10181 10398 10400 10873 11719 12705 13720 14668 14766 14783 14815 14927 15043 15301 15326 15440 15951 16129 16173 16223 16319 16362	D4b1a2a1	73 183 263 489 523d 16129 16173 16223 16319 16362	[0.136] D4b1a2a1 (1)	[0.063] D4d (2) [0.015] D4b1a D4b1a2 D4b1a2a (5) [0.000] D4b1 D4b1a2a2 D2 (8)	1
856 >MF043499.1 Ho	73G 195C 263G 309.1CT 310C 489C 750G 1438G 2706G 3010A 3106d 3107C 3421A 4769G 4883T 5178A 6704G 7028T 8414T 8701G 8860G 9540C 10398G 10400T 10873C 11719A 12705T 14668T 14766T 14783C 15043A 15301A 15326G 16171T 16223T 16355A 16362C	D4n2	73G 195C 263G 309.1CT 310C 489C 16171T 16223T 16355A 16362C	1	73 195 263 489 750 1438 2706 3010 3421 4769 4883 5178A 6704 7028 8414 8701 8860 9540 10398 10400 10873 11719 12705 14668 14766 14783 15043 15301 15326 16171T 16223 16355A 16362	D4n2	73 195 263 489 16171T 16223 16355A 16362	[0.213] D4n2 (1)	[0.000] D4+195 D4n D4n1 D4n1a (5)	1

857	>MF043494.1 Hc	73G 263G 310.1C 489C 750G 1438G 2706G 3010A 3106d 3107C 4233C 4769G 4883T 5178A 7028T 8383C 8414T 8701G 8860G 9431T 9540C 10398G 10400T 10873C 11152C 11719A 12705T 14668T 14766T 14783C 15043A 15301A 15326G 15391A 16223T 16245T 16311C 16362C 16368C	D4c2a	73G 263G 310.1C 489C 16223T 16245T 16311C 16362C 16368C	1	73 263 489 750 1438 2706 3010 4233 4769 4883 5178A 7028 8383 8414 8701 8860 9431 9540 10398 10400 10873 11152 11719 12705 14668 14766 14783 15043 15301 15326 15391A 16223 16245 16311 16362 16368	D4c2a	73 263 489 16223 16245 16311 16362 16368	[0.000] D4c2a (1)	[0.306] D4c2 (2) [0.046] D4c2c (3) [0.000] M43a1 M74 M74b M74b2 D4c D4j+16311 D4j11 D4l D4l1 (12)	1
858	>MF043493.1 Hc	73G 263G 310.1C 489C 534T 750G 1438G 1462A 2706G 3010A 3106d 3107C 4769G 4883T 5178A 6914G 7028T 8383C 8414T 8701G 8860G 9431T 9540C 10398G 10400T 10873C 11719A 12705T 14668T 14766T 14783C 15043A 15301A 15326G 16223T 16245T 16362C	D4c2b	73G 263G 310.1C 489C 534T 16223T 16245T 16362C	1	73 263 489 534 750 1438 1462 2706 3010 4769 4883 5178A 6914 7028 8383 8414 8701 8860 9431 9540 10398 10400 10873 11719 12705 14668 14766 14783 15043 15301 15326 16223 16245 16362	D4c2b	73 263 489 534 16223 16245 16362	[0.000] D4c2b (1)	[0.000] D4c D4c2 D4c2c (4)	1
859	>MF043488.1 Hc	73G 263G 310.1C 489C 709A 750G 1438G 2706G 3106d 3107C 4769G 4833G 5108C 7028T 8701G 8860G 9540C 10398G 10400T 10873C 11719A 12408C 12705T 14569A 14766T 14783C 15043A 15301A 15326G 15746G 16093C 16223T 16274A 16362C 16390A 16519C	G3a3	73G 263G 310.1C 489C 16093C 16223T 16274A 16362C 16390A 16519C	1	73 263 489 709 750 1438 2706 4769 4833 5108 7028 8701 8860 9540 10398 10400 10873 11719 12408 12705 14569 14766 14783 15043 15301 15326 15746 16093 16223 16274 16362 16390	G3a3	73 263 489 16093 16223 16274 16362 16390	[0.011] G3a3 (1)	[0.033] G3b2 (2) [0.011] G3b (3) [0.000] E E1 E1a E1a1 E1a1a E1a1c G3 G3a D1h D4e5 (13)	1
860	>MF043481.1 Hc	73G 183G 263G 309.1CT 310C 489C 514d 515d 750G 1438G 2706G 3010A 3106d 3107C 4769G 4883T 5178A 6881G 7028T 7762A 8020A 8414T 8701G 8860G 9540C 10181T 10398G 10400T 10873C 11719A 12705T 13720T 14668T 14766T 14783C 14815T 14927G 15043A 15301A 15326G 15440C 15951G 16129A 16173T 16223T 16319A 16362C	D4b1a2a1	73G 183G 263G 309.1CT 310C 489C 514d 515d 16129A 16173T 16223T 16319A 16362C	1	73 183 263 489 750 1438 2706 3010 4769 4883 5178A 6881 7028 7762 8020 8414 8701 8860 9540 10181 10398 10400 10873 11719 12705 13720 14668 14766 14783 14815 14927 15043 15301 15326 15440 15951 16129 16173 16223 16319 16362	D4b1a2a1	73 183 263 489 16129 16173 16223 16319 16362	[0.123] D4b1a2a1 (1)	[0.063] D4d (2) [0.015] D4b1a D4b1a2 D4b1a2a (5) [0.000] D4b1 D4b1a2a2 D2 (8)	1
861	>MF043478.1 Hc	73G 263G 310.1C 489C 750G 1438G 2706G 3010A 3106d 3107C 4233C 4769G 4883T 5178A 7028T 8383C 8414T 8701G 8860G 9431T 9540C 10398G 10400T 10873C 11152C 11719A 12705T 14668T 14766T 14783C 15043A 15301A 15326G 15391A 16223T 16245T 16311C 16362C 16368C	D4c2a	73G 263G 310.1C 489C 16223T 16245T 16311C 16362C 16368C	1	73 263 489 750 1438 2706 3010 4233 4769 4883 5178A 7028 8383 8414 8701 8860 9431 9540 10398 10400 10873 11152 11719 12705 14668 14766 14783 15043 15301 15326 15391A 16223 16245 16311 16362 16368	D4c2a	73 263 489 16223 16245 16311 16362 16368	[0.000] D4c2a (1)	[0.306] D4c2 (2) [0.046] D4c2c (3) [0.000] M43a1 M74 M74b M74b2 D4c D4j+16311 D4j11 D4l D4l1 (12)	1

862 >MF043472.1 Ho	73G 248d 263G 309.1CCT 310C 514d 515d 750G 1005C 1438G 1824C 2706G 3106d 3107C 3970T 4769G 4811G 6392C 7028T 7828G 8860G 10310A 10535C 10586A 11719A 12338C 13708A 13928C 14766T 15326G 16092A 16186T 16289G 16291T 16304C	F2b1	73G 248d 263G 309.1CCT 310C 514d 515d 16092A 16186T 16289G 16291T 16304C	1	73 249d 263 750 1005 1438 1824 2706 3970 4769 4811 6392 7028 7828 8860 10310 10535 10586 11719 12338 13708 13928C 14766 15326 16092A 16186 16289 16291 16304	F2b1	73 249d 263 16092A 16186 16289 16291 16304	[0.032] F2b1 (1)	[0.013] F2+16291 (2) [0.000] F2b F2g (4)	1
863 >MF043471.1 Ho	73G 183G 263G 309.1CT 310C 489C 523d 750G 1438G 2706G 3010A 3106d 3107C 4769G 4883T 5178A 6881G 7028T 7762A 8020A 8414T 8701G 8860G 9540C 10181T 10398G 10400T 10873C 11719A 12705T 13720T 14668T 14766T 14783C 14815T 14927G 15043A 15301A 15326G 15440C 15951G 16129A 16173T 16223T 16319A 16362C	D4b1a2a1	73G 183G 263G 309.1CT 310C 489C 523d 16129A 16173T 16223T 16319A 16362C	1	73 183 263 489 523d 750 1438 2706 3010 4769 4883 5178A 6881 7028 7762 8020 8414 8701 8860 9540 10181 10398 10400 10873 11719 12705 13720 14668 14766 14783 14815 14927 15043 15301 15326 15440 15951 16129 16173 16223 16319 16362	D4b1a2a1	73 183 263 489 523d 16129 16173 16223 16319 16362	[0.136] D4b1a2a1 (1)	[0.063] D4d (2) [0.015] D4b1a D4b1a2 D4b1a2a (5) [0.000] D4b1 D4b1a2a2 D2 (8)	1
864 >MF043468.1 Ho	73G 183G 263G 309.1CT 310C 489C 514d 515d 750G 1438G 2706G 3010A 3106d 3107C 4769G 4883T 5178A 6881G 7028T 7762A 8020A 8414T 8701G 8860G 9540C 10181T 10398G 10400T 10873C 11719A 12705T 13720T 14668T 14766T 14783C 14815T 14927G 15043A 15301A 15326G 15440C 15951G 16129A 16173T 16223T 16319A 16362C	D4b1a2a1	73G 183G 263G 309.1CT 310C 489C 514d 515d 16129A 16173T 16223T 16319A 16362C	1	73 183 263 489 750 1438 2706 3010 4769 4883 5178A 6881 7028 7762 8020 8414 8701 8860 9540 10181 10398 10400 10873 11719 12705 13720 14668 14766 14783 14815 14927 15043 15301 15326 15440 15951 16129 16173 16223 16319 16362	D4b1a2a1	73 183 263 489 16129 16173 16223 16319 16362	[0.123] D4b1a2a1 (1)	[0.063] D4d (2) [0.015] D4b1a D4b1a2 D4b1a2a (5) [0.000] D4b1 D4b1a2a2 D2 (8)	1
865 >MF043458.1 Ho	73G 263G 310.1C 489C 534T 750G 1438G 1462A 2706G 3010A 3106d 3107C 4004C 4769G 4883T 5178A 6914G 7028T 7741C 8383C 8414T 8701G 8860G 8867C 9431T 9540C 10398G 10400T 10873C 11719A 12705T 14668T 14766T 14783C 15043A 15301A 15326G 16223T 16245T 16362C	D4c2b	73G 263G 310.1C 489C 534T 16223T 16245T 16362C	1	73 263 489 534 750 1438 1462 2706 3010 4004 4769 4883 5178A 6914 7028 7741 8383 8414 8701 8860 8867 9431 9540 10398 10400 10873 11719 12705 14668 14766 14783 15043 15301 15326 16223 16245 16362	D4c2b	73 263 489 534 16223 16245 16362	[0.000] D4c2b (1)	[0.000] D4c D4c2 D4c2c (4)	1
866 >MF043457.1 Ho	73G 152C 207A 263G 310.1C 489C 514d 515d 709A 711C 750G 1438G 2706G 3106d 3107C 4493T 4769G 4833G 5108C 5601T 7028T 7600A 8701G 8860G 8943T 9377G 9540C 9575A 10398G 10400T 10873C 11719A 12705T 13395G 13563G 14569A 14766T 14783C 15043A 15110A 15301A 15326G 16223T 16227G 16278T 16362C 16519C	G2a2a	73G 152C 207A 263G 310.1C 489C 514d 515d 16223T 16227G 16278T 16362C 16519C	1	73 152 207 263 489 709 711 750 1438 2706 4493 4769 4833 5108 5601 7028 7600 8701 8860 8943 9377 9540 9575 10398 10400 10873 11719 12705 13395 13563 14569 14766 14783 15043 15110 15301 15326 16223 16227 16278 16362	G2a2a	73 152 207 263 489 16223 16227 16278 16362	[0.000] G2a2a (1)	[0.000] G2a+152 G2a2 G2a2a (4)	1

867 >MF043456.1 Hc	73G 150T 152C 248d 263G 309T 310C 514d 515d 750G 1438G 2706G 3106d 3107C 3970T 4732G 4769G 5147A 5508C 6392C 6962A 7028T 8860G 9615C 10235C 10310A 10609C 10976T 11719A 12406A 12882T 13928C 14476A 14766T 15326G 16182C 16183C 16189C 16232A 16249C 16304C 16519C	F1b1c	73G 150T 152C 248d 263G 309T 310C 514d 515d 16182C 16183C 16189C 16232A 16249C 16304C 16519C	1	73 150 152 249d 263 309d 750 1438 2706 3970 4732 4769 5147 5508 6392 6962 7028 8860 9615 10235 10310 10609 10976 11719 12406 12882 13928C 14476 14766 15326 16189 16232A 16249 16304	F1b1c	73 150 152 249d 263 309d 16189 16232A 16249 16304	[0.080] F1b1c (1)	[0.000] F1b F1b1 (3)	1
868 >MF043455.1 Hc	73G 152C 204C 207A 263G 310.1C 489C 514d 515d 709A 711C 750G 1438G 2706G 3106d 3107C 4769G 4833G 5108C 5601T 7028T 7364G 7600A 8701G 8860G 8943T 9377G 9540C 9575A 10398G 10400T 10873C 11719A 12705T 13395G 13563G 14569A 14766T 14783C 15043A 15110A 15301A 15326G 16223T 16227G 16278T 16362C	G2a2a	73G 152C 204C 207A 263G 310.1C 489C 514d 515d 16223T 16227G 16278T 16362C	1	73 152 204 207 263 489 709 711 750 1438 2706 4769 4833 5108 5601 7028 7364 7600 8701 8860 8943 9377 9540 9575 10398 10400 10873 11719 12705 13395 13563 14569 14766 14783 15043 15110 15301 15326 16223 16227 16278 16362	G2a2a	73 152 204 207 263 489 16223 16227 16278 16362	[0.011] G2a2a (1)	[0.011] G2a2a (2) [0.006] G2a+152 (3) [0.000] G2a2	1
869 >MF043453.1 Hc	73G 195C 263G 309.1CT 310C 489C 750G 1438G 2706G 3010A 3106d 3107C 4769G 4883T 5178A 6260A 7028T 8414T 8701G 8860G 9077C 9540C 10398G 10400T 10646A 10873C 11719A 12705T 13812C 14668T 14766T 14783C 15043A 15301A 15326G 16093C 16223T 16232T 16290T 16362C	D4o2a	73G 195C 263G 309.1CT 310C 489C 16093C 16223T 16232T 16290T 16362C	1	73 195 263 489 750 1438 2706 3010 4769 4883 5178A 6260 7028 8414 8701 8860 9077 9540 10398 10400 10646 10873 11719 12705 13812 14668 14766 14783 15043 15301 15326 16093 16223 16232 16290 16362	D4o2a	73 195 263 489 16093 16223 16232 16290 16362	[0.000] D4o2a (1)	[0.000] D4o2 (2)	1
870 >MF043446.1 Hc	73G 207A 263G 310.1C 489C 709A 750G 1438G 2706G 3106d 3107C 4769G 4833G 5108C 7028T 8200C 8701G 8860G 8865A 9068C 9540C 10398G 10400T 10586C 10873C 11719A 12361G 12705T 12972G 13646C 14569A 14766T 14783C 15043A 15301A 15323A 15326G 15497A 16017C 16093C 16188T 16207G 16223T 16519C	G1b1	73G 207A 263G 310.1C 489C 16093C 16188T 16207G 16223T 16519C	1	73 207 263 489 709 750 1438 2706 4769 4833 5108 7028 8200 8701 8860 8865 9068 9540 10398 10400 10586C 10873 11719 12361 12705 12972 13646 14569 14766 14783 15043 15301 15323 15326 15497 16017 16093 16188 16207 16223	G1b1	73 207 263 489 16093 16188 16207 16223	[0.021] G1b1 (1)	[0.021] G1b1 (2) [0.000] G1b	1
871 >MF043441.1 Hc	73G 263G 309.1CT 310C 489C 514d 515d 750G 1382C 1438G 2706G 3010A 3106d 3107C 4769G 4883T 5178A 6407C 7028T 7816T 8020A 8414T 8701G 8860G 8964T 9540C 9824A 10398G 10400T 10873C 11719A 12705T 13174C 13239T 14668T 14766T 14783C 15043A 15301A 15326G 16223T 16287T 16362C	D4b2d	73G 263G 309.1CT 310C 489C 514d 515d 16223T 16287T 16362C	1	73 263 489 750 1382C 1438 2706 3010 4769 4883 5178A 6407 7028 7816 8020 8414 8701 8860 8964 9540 9824A 10398 10400 10873 11719 12705 13174 13239 14668 14766 14783 15043 15301 15326 16223 16287 16362	D4b2d	73 263 489 16223 16287 16362	[0.000] D4b2d (1)	[0.000] M9 G G1 G1c G1c2 G2 G2a'c G2b G2b1 G2b1a G2b2 G2b2a M33+16362 D D4 D4b D4b1b'd D4b2 D4b2a D4b2a2 D4b2a2b D4b2b D4b2b1 D4b2b1a D4b2b1b D4b2b1c D4b2b2 D4b2b3 D4b2b6 D4e D4e1'3 D4e1 D4e1c D4e3 D4e2 D4e2b D4e2c D4e2d D4e4 D4e4a D4e4b D4f D4f1 D4g D4h D4h4 D4j D4j1 D4j1b D4j4 D4j5 D4j5a D4j+(16286) D4j9 D4j10 D4j12 D4j15 D4j16 D4m (60)	1

872 >MF043440.1 H	73G 263G 310.1C 489C 534T 750G 1438G 1462A 2706G 3010A 3106d 3107C 4769G 4883T 5178A 7028T 8270.1ACCCCCTCT 8383C 8414T 8701G 8860G 9431T 9540C 10398G 10400T 10873C 11719A 12705T 14668T 14766T 14783C 15043A 15301A 15326G 16223T 16245T 16362C	D4c2b	73G 263G 310.1C 489C 534T 16223T 16245T 16362C	1	73 263 489 534 750 1438 1462 2706 3010 4769 4883 5178A 7028 8289.1CCCCCTCTA 8383 8414 8701 8860 9431 9540 10398 10400 10873 11719 12705 14668 14766 14783 15043 15301 15326 16223 16245 16362	D4c2b	73 263 489 534 16223 16245 16362	[0.000] D4c2b (1)	[0.000] D4c D4c2 D4c2c (4)	1
873 >MF043439.1 H	73G 263G 309.1CCT 310C 709A 750G 1119C 1438G 2706G 3106d 3107C 3497T 4769G 7028T 8271d 8272d 8273d 8274d 8275d 8276d 8277d 8278d 8279d 8860G 10310A 11719A 14766T 15326G 15346A 16182C 16183C 16189C 16217C 16519C 16527T	B4c1a2	73G 263G 309.1CCT 310C 16182C 16183C 16189C 16217C 16519C 16527T	1	73 263 709 750 1119 1438 2706 3497 4769 7028 8281-8289d 8860 10310 11719 14766 15326 15346 16189 16217 16527	B4c1a2	73 263 16189 16217 16527	[0.000] B4c1a2 (1)	[0.007] B4d1 (2) [0.000] B4 B4b'd'e'j B4d B4d1'2'3 B4d1a B4d2 B4c B4c1 B4c1a'b B4c1a B4c1a2a (13)	1
874 >MF043428.1 H	73G 146C 248d 263G 310.1C 489C 710C 750G 1438G 2226.1A 2706G 3106d 3107C 3552A 3816G 4715G 4769G 6026A 7028T 7196A 8251A 8584A 8701G 8860G 9540C 9545G 10398G 10400T 10873C 11719A 11914A 11969A 12705T 13263G 13470G 14318C 14766T 14783C 15043A 15204C 15301A 15326G 15487T 16223T 16259.1A 16298C 16327T 16519C	C4b1a	73G 146C 248d 263G 310.1C 489C 16223T 16259.1A 16298C 16327T 16519C	1	73 146 249d 263 489 710 750 1438 2232.1A 2706 3552A 3816 4715 4769 6026 7028 7196A 8251 8584 8701 8860 9540 9545 10398 10400 10873 11719 11914 11969 12705 13263 13470 14318 14766 14783 15043 15204 15301 15326 15487T 16223 16259.1A 16298 16327	C4b1a	73 146 249d 263 489 16223 16259.1A 16298 16327	[0.000] C4b1a (1)	[0.000] C4b1 C4b1b C7a1 C7a1c (5)	1
875 >MF043427.1 H	73G 150T 263G 309.1CCT 310C 489C 514d 515d 750G 752T 1107C 2706G 3106d 3107C 4769G 4883T 5178A 5301G 7028T 8701G 8860G 9180G 9540C 10397G 10398G 10400T 10873C 11719A 11944C 12026G 12705T 14766T 14783C 15043A 15301A 15326G 16093C 16164G 16182C 16183C 16189C 16223T 16266T 16362C 16519C	D5a2a1+@16172	73G 150T 263G 309.1CCT 310C 489C 514d 515d 16093C 16164G 16182C 16183C 16189C 16223T 16266T 16362C 16519C	1	73 150 263 489 750 752 1107 2706 4769 4883 5178A 5301 7028 8701 8860 9180 9540 10397 10398 10400 10873 11719 11944 12026 12705 14766 14783 15043 15301 15326 16093 16164 16189 16223 16266 16362	D5a2a1+@16172	73 150 263 489 16093 16164 16189 16223 16266 16362	[0.304] D5a2a1+@16172 (1)	[0.789] D5a2a (2) [0.160] D5a (3) [0.059] D5a2a1a (4) [0.052] D5a2a1b (5) [0.000] D5 D5a'b D5a2a1 D5a2a1a2 (9)	1
876 >MG551914.1 H	189G 263G 309.1CT 310C 750G 1438G 3106d 3107C 4769G 6323G 8860G 13857G 15326G 16291T 16390A 16519C	H85	189G 263G 309.1CT 310C 16291T 16390A 16519C	1	189 263 750 1438 4769 6323 8860 13857 15326 16291 16390	H85	189 263 16291 16390	[0.012] H85 (1)	[0.012] H+16291 (2) [0.007] H2a2b (3) [0.000] H1j2 H2a2b2 H2a2b3 H2a2b4 H2a2b5 H2a2b5a H2a5a1 H2a5a1a H2a5a1b H34 H64 (14)	1
877 >MG586885.1 H	73G 150T 263G 310.1C 750G 1341T 1438G 2706G 3106d 3107C 3197C 4059T 4769G 5656G 7028T 7385G 7768G 8860G 9477A 10927C 11467G 11719A 12308G 12372A 12618A 13617C 14182C 14766T 15326G 16144C 16189C 16270T 16301T	U5b1b1a1a1	73G 150T 263G 310.1C 16144C 16189C 16270T 16301T	1	73 150 263 750 1341 1438 2706 3197 4059 4769 5656 7028 7385 7768 8860 9477 10927 11467 11719 12308 12372 12618 13617 14182 14766 15326 16144 16189 16270 16301	U5b1b1a1a1	73 150 263 16144 16189 16270 16301	[0.000] U5b1b1a1a1 (1)	[0.000] U5b1b1a U5b1b1a1 U5b1b1a1a U5b1b1a1b U5b1b1a2 (6)	1

878 >MG519781.1 H	72C 93G 263G 309.1CT 310C 750G 1438G 2706G 3106d 3107C 4580A 4769G 7028T 8860G 14550C 15326G 15904T 16203G 16298C	V5	72C 93G 263G 309.1CT 310C 16203G 16298C	1	72 93 263 750 1438 2706 4580 4769 7028 8860 14550 15326 15904 16203 16298	V5	72 93 263 16203 16298	[0.333] V5 (1) [0.000] V7 (2)	[0.070] V7a1 (3) [0.005] V2c V8 V11 (6) [0.003] V (7) [0.000] HV0 HV0a V1 V1a V1a1 V1a1b V2 V2a V2a1 V2a1a V2b V2b1 V2b2 V3 V3b V4 V7a V10 V10b V10b1 V10b2 V12 V13 V14 V15 V15a V17 V23 V24 V25 V26 V28 (39)	1
879 >MG517557.1 H	73G 151T 263G 310.1C 750G 1438G 3010A 3106d 3107C 4769G 8860G 11893G 15326G 16162G 16189C 16519C	H1a6	73G 151T 263G 310.1C 16162G 16189C 16519C	1	73 151 263 750 1438 3010 4769 8860 11893 15326 16162 16189	H1a6	73 151 263 16162 16189	[0.000] H1a6 (1)	[0.000] U8c (2)	1
880 >MG548630.1 H	239C 263G 310.1C 513.1CA 750G 1438G 3106d 3107C 3915A 4727G 4769G 8860G 9380A 10589A 10697T 15326G 16249C 16362C 16482G	H6a1b4	239C 263G 310.1C 513.1CA 16249C 16362C 16482G	1	239 263 750 1438 3915 4727 4769 8860 9380 10589 10697 15326 16249 16362 16482	H6a1b4	239 263 16249 16362 16482	[0.000] H6a1b4 (1)	[0.000] H6 H6a H6a1 H6a1a H6a1a1 H6a1a2 H6a1a2b H6a1a2b1 H6a1a3 H6a1a3a H6a1a4 H6a1a5 H6a1a9 H6a1a10 H6a1b H6a1b2 H6a1b2a H6a1b2b H6a1b2c H6a1b2e H6a1b4 H6a2 H6a2a H6c (25)	1
881 >MG496255.1 H	73G 150T 152Y 263G 310.1C 750G 1438G 1811G 2294G 2706G 3010A 3106d 3107C 4703C 4769G 6518T 7028T 7400T 8860G 9266A 10506G 11467G 11719A 12308G 12372A 12738C 13934T 14139G 14766T 15326G 15418T 15454C 15734A 16343G 16356C 16390A 16519C	U3a1c	73G 150T 152Y 263G 310.1C 16343G 16356C 16390A 16519C	1	73 150 152Y 263 750 1438 1811 2294 2706 3010 4703 4769 6518 7028 7400 8860 9266 10506 11467 11719 12308 12372 12738 13934 14139 14766 15326 15418 15454 15734 16343 16356 16390	U3a1c	73 150 152Y 263 16343 16356 16390	[0.025] U3a1c (1)	[0.089] U3a1 (2) [0.021] U3a2 (3) [0.016] U3a (4) [0.000] U3a1a U3a1a1 U3a1b U3a1c1 (8)	1
882 >MF114212.1 H	73G 150T 263G 307N 308N 309N 310N 311N 312N 313N 314N 750G 1438G 1721T 2129N 2706G 3106N 3108N 3109N 3197C 3861G 4769G 7028T 7396N 7768G 8860G 9477A 11467G 11653G 11719A 12308G 12372A 12634G 13617C 13630G 13637G 14182C 14766T 15326G 15497A 16270T 16292T 16362C	U5b2b1a1	73G 150T 263G 307N 308N 309N 310N 311N 312N 313N 314N 16270T 16292T 16362C	1	73 150 263 307N 308N 309N 310N 311N 312N 313N 314N 750 1438 1721 2129N 2706 3106N 3108N 3109N 3197 3861 4769 7028 7396N 7768 8860 9477 11467 11653 11719 12308 12372 12634 13617 13630 13637 14182 14766 15326 15497 16270 16292 16362	U5b2b1a1	73 150 263 307N 308N 309N 310N 311N 312N 313N 314N 16270 16292 16362	[0.000] U5b2b1a1 (1)	[0.000] U5b2b1a (2)	1
883 >MG322267.1 H	73G 199C 203A 204C 250C 263G 310.1C 451.1T 567.1CC 750G 1438G 1719A 1836G 2706G 3106d 3107C 3447G 3990T 4023C 4529T 4769G 6734A 7028T 8251A 8616T 8860G 9947A 10034C 10238C 10398G 10915C 11719A 12501A 12705T 13488C 13780G 14766T 14890G 15043A 15326G 15924G 16129A 16172C 16183C 16189C 16223T 16265C 16311C 16362C 16391A 16519C	I1a1d	73G 199C 203A 204C 250C 263G 310.1C 451.1T 567.1CC 16129A 16172C 16183C 16189C 16223T 16265C 16311C 16362C 16391A 16519C	1	73 199 203 204 250 263 455.1T 573.2C 750 1438 1719 1836 2706 3447 3990 4023 4529T 4769 6734 7028 8251 8616T 8860 9947 10034 10238 10398 10915 11719 12501 12705 13488 13780 14766 14890 15043 15326 15924 16129 16172 16189 16223 16265C 16311 16362 16391	I1a1d	73 199 203 204 250 263 455.1T 573.2C 16129 16172 16189 16223 16265C 16311 16362 16391	[0.444] I1a1d (1)	[0.188] I1a1a1 (2) [0.176] I1a1b (3) [0.021] I1a1e (4) [0.013] I1a1 (5) [0.000] I1a1a I1a1a2 I1a1c (8)	1

884	>KY681016.1 Hc	73G 152C 263G 271T 309.1CCT 310C 451.1TT 460C 499A 750G 827G 1438G 2706G 3106d 3107C 3547G 3918A 4012G 4232C 4769G 4820A 4977C 6473T 6755A 7028T 8271d 8272d 8273d 8274d 8275d 8276d 8277d 8278d 8279d 8860G 9950C 11177T 11719A 13590A 13708A 14766T 15326G 15535T 15784C 16092C 16182C 16183C 16189C 16217C 16249C 16312G 16344T 16519C	B2b3a	73G 152C 263G 271T 309.1CCT 310C 451.1TT 460C 499A 16092C 16182C 16183C 16189C 16217C 16249C 16312G 16344T 16519C	1	73 152 263 271 455.2T 460 499 750 827 1438 2706 3547 3918 4012 4232 4769 4820 4977 6473 6755 7028 8281-8289d 8860 9950 11177 11719 13590 13708 14766 15326 15535 15784 16092 16189 16217 16249 16312 16344	B2b3a	73 152 263 271 455.2T 460 499 16092 16189 16217 16249 16312 16344	[1.615] B2b3a (1)	[0.165] B2o (2) [0.045] B2b+152 (3) [0.000] B2o1a	1
885	>KY681014.1 Hc	73G 263G 309.1CT 310C 489C 524.1CA 750G 1438G 2092T 2706G 3010A 3106d 3107C 4769G 4883T 5178A 7028T 8414T 8701G 8860G 9540C 10398G 10400T 10873C 10874T 11719A 12705T 14668T 14766T 14783C 15043A 15301A 15326G 16142T 16179T 16223T 16295T 16325C 16362C 16497G 16519C	D1f1	73G 263G 309.1CT 310C 489C 524.1CA 16142T 16179T 16223T 16295T 16325C 16362C 16497G 16519C	1	73 263 489 525.1AC 750 1438 2092 2706 3010 4769 4883 5178A 7028 8414 8701 8860 9540 10398 10400 10873 10874 11719 12705 14668 14766 14783 15043 15301 15326 16142 16179 16223 16295 16325 16362 16497	D1f1	73 263 489 525.1AC 16142 16179 16223 16295 16325 16362 16497	[0.006] D1f1 (1)	[0.000] D1f (2)	1
886	>KY681012.1 Hc	73G 263G 309.1CT 310C 489C 750G 1438G 2092T 2706G 3010A 3106d 3107C 4769G 4883T 5178A 7028T 8414T 8701G 8860G 9540C 10398G 10400T 10873C 10874T 11719A 12705T 14668T 14766T 14783C 15043A 15301A 15326G 16142T 16179T 16223T 16295T 16325C 16362C 16497G 16519C	D1f1	73G 263G 309.1CT 310C 489C 16142T 16179T 16223T 16295T 16325C 16362C 16497G 16519C	1	73 263 489 750 1438 2092 2706 3010 4769 4883 5178A 7028 8414 8701 8860 9540 10398 10400 10873 10874 11719 12705 14668 14766 14783 15043 15301 15326 16142 16179 16223 16295 16325 16362 16497	D1f1	73 263 489 16142 16179 16223 16295 16325 16362 16497	[0.000] D1f1 (1)	[0.000] D1f (2)	1
887	>KY681000.1 Hc	73G 97A 100d 101d 102d 103d 104d 105d 195C 263G 309.1CCT 310C 489C 750G 964A 1438G 2092T 2706G 3010A 3106d 3107C 4769G 4883T 5178A 5237A 7028T 8414T 8701G 8860G 9540C 9621A 10398G 10400T 10873C 11719A 12561A 12705T 14034C 14587G 14668T 14766T 14783C 15043A 15301A 15326G 16142T 16223T 16325C 16362C 16519C	D1f	73G 97A 100d 101d 102d 103d 104d 105d 195C 263G 309.1CCT 310C 489C 16142T 16223T 16325C 16362C 16519C	1	73 97 105-110d 195 263 489 750 964A 1438 2092 2706 3010 4769 4883 5178A 5237 7028 8414 8701 8860 9540 9621 10398 10400 10873 11719 12561 12705 14034 14587 14668 14766 14783 15043 15301 15326 16142 16223 16325 16362	D1f	73 97 105-110d 195 263 489 16142 16223 16325 16362	[0.050] D1f (1)	[0.049] D1a (2) [0.016] D1 (3) [0.008] D1a2 (4) [0.000] D1b D1c D1d D1d1 D1f2 D1f+16189 D1f3 D1k D1n D4+195 (14)	1
888	>KY680999.1 Hc	73G 195C 263G 310.1C 489C 750G 1438G 2092T 2706G 3010A 3106d 3107C 4769G 4883T 5178A 6351C 6827C 7028T 8265C 8414T 8701G 8860G 9540C 10172A 10398G 10400T 10873C 11719A 12705T 14569A 14668T 14766T 14783C 15043A 15301A 15326G 15776G 16142T 16223T 16293G 16325C 16362C 16519C	D1f	73G 195C 263G 310.1C 489C 16142T 16223T 16293G 16325C 16362C 16519C	1	73 195 263 489 750 1438 2092 2706 3010 4769 4883 5178A 6351 6827 7028 8265 8414 8701 8860 9540 10172 10398 10400 10873 11719 12705 14569 14668 14766 14783 15043 15301 15326 15776 16142 16223 16293 16325 16362	D1f	73 195 263 489 16142 16223 16293 16325 16362	[0.055] D1f (1)	[0.049] D1a (2) [0.016] D1 (3) [0.008] D1a2 (4) [0.000] D1b D1c D1d D1d1 D1f2 D1f+16189 D1f3 D1k D1n D4+195 (14)	1

889 >KY680998.1 Hc	73G 195C 263G 310.1C 489C 513.1CA 750G 1438G 2092T 2706G 3010A 3106d 3107C 4769G 4823C 4883T 5178A 6351C 6827C 7028T 7196A 8414T 8701G 8860G 9540C 10398G 10400T 10873C 11719A 12705T 14569A 14668T 14766T 14783C 15043A 15301A 15326G 15776G 16142T 16223T 16293G 16325C 16362C 16519C	D1f	73G 195C 263G 310.1C 489C 513.1CA 16142T 16223T 16293G 16325C 16362C 16519C	1	73 195 263 489 750 1438 2092 2706 3010 4769 4823 4883 5178A 6351 6827 7028 7196A 8414 8701 8860 9540 10398 10400 10873 11719 12705 14569 14668 14766 14783 15043 15301 15326 15776 16142 16223 16293 16325 16362	D1f	73 195 263 489 16142 16223 16293 16325 16362	[0.055] D1f (1)	[0.049] D1a (2) [0.016] D1 (3) [0.008] D1a2 (4) [0.000] D1b D1c D1d D1d1 D1f2 D1f+16189 D1f3 D1k D1n D4+195 (14)	1
890 >KY680997.1 Hc	73G 263G 289G 310.1C 489C 750G 989C 1438G 2092T 2706G 3010A 3106d 3107C 3666A 4769G 4883T 5178A 7028T 8414T 8701G 8860G 9540C 10398G 10400T 10873C 11719A 12705T 14668T 14766T 14783C 15043A 15301A 15326G 16086C 16142T 16223T 16292T 16325C 16362C 16400T 16519C	D1f	73G 263G 289G 310.1C 489C 16086C 16142T 16223T 16292T 16325C 16362C 16400T 16519C	1	73 263 289 489 750 989 1438 2092 2706 3010 3666 4769 4883 5178A 7028 8414 8701 8860 9540 10398 10400 10873 11719 12705 14668 14766 14783 15043 15301 15326 16086 16142 16223 16292 16325 16362 16400	D1f	73 263 289 489 16086 16142 16223 16292 16325 16362 16400	[0.040] D1f (1)	[0.018] D1k (2) [0.007] D1 (3) [0.006] D1f+16189 (4) [0.004] D1a2 (5) [0.000] D1a D1b D1c D1d D1d1 D1f2 D1f3 D1n D4j1a D4j1a1 D4j1a1a D4j1a1b (17)	1
891 >KY680996.1 Hc	57C 73G 263G 289G 310.1C 489C 750G 989C 1438G 2092T 2706G 3010A 3106d 3107C 4769G 4883T 5178A 7028T 8414T 8701G 8860G 9540C 10398G 10400T 10873C 11719A 12705T 14668T 14766T 14783C 15043A 15301A 15326G 16086C 16142T 16223T 16325C 16362C 16400T 16519C	D1f	57C 73G 263G 289G 310.1C 489C 16086C 16142T 16223T 16325C 16362C 16400T 16519C	1	57 73 263 289 489 750 989 1438 2092 2706 3010 4769 4883 5178A 7028 8414 8701 8860 9540 10398 10400 10873 11719 12705 14668 14766 14783 15043 15301 15326 16086 16142 16223 16325 16362 16400	D1f	57 73 263 289 489 16086 16142 16223 16325 16362 16400	[0.040] D1f (1)	[0.016] D1 (2) [0.006] D1f+16189 (3) [0.004] D1a2 (4) [0.000] D1a D1b D1c D1d D1d1 D1f2 D1f3 D1k D1n D4j1a D4j1a1 D4j1a1a D4j1a1b (17)	1
892 >KY680995.1 Hc	73G 263G 289G 309.1CT 310C 489C 750G 989C 1438G 2092T 2281C 2706G 3010A 3106d 3107C 4056A 4769G 4883T 5178A 7028T 8414T 8701G 8860G 9540C 9612A 10398G 10400T 10873C 11719A 12477C 12705T 14668T 14766T 14783C 15043A 15301A 15326G 16142T 16223T 16325C 16362C	D1f	73G 263G 289G 309.1CT 310C 489C 16142T 16223T 16325C 16362C	1	73 263 289 489 750 989 1438 2092 2281C 2706 3010 4056A 4769 4883 5178A 7028 8414 8701 8860 9540 9612 10398 10400 10873 11719 12477 12705 14668 14766 14783 15043 15301 15326 16142 16223 16325 16362	D1f	73 263 289 489 16142 16223 16325 16362	[0.015] D1f (1)	[0.002] D1 (2) [0.000] D1a D1a2 D1b D1c D1d D1d1 D1f2 D1f+16189 D1f3 D1k D1n (13)	1
893 >KY680994.1 Hc	73G 143A 152C 263G 309.1CT 310C 489C 750G 1438G 2706G 3010A 3106d 3107C 3336C 3396C 3644C 4025T 4363C 4769G 4883T 5048C 5178A 6285A 7028T 8414T 8701G 8860G 8946G 9458T 9540C 10398G 10400T 10873C 11719A 12705T 13135A 14668T 14766T 14783C 15043A 15301A 15326G 16129A 16185T 16192T 16223T 16241G 16301T 16342C 16362C 16463G	D4h3a6	73G 143A 152C 263G 309.1CT 310C 489C 16129A 16185T 16192T 16223T 16241G 16301T 16342C 16362C 16463G	1	73 143 152 263 489 750 1438 2706 3010 3336 3396 3644 4025 4363 4769 4883 5048 5178A 6285 7028 8414 8701 8860 8946 9458 9540 10398 10400 10873 11719 12705 13135 14668 14766 14783 15043 15301 15326 16129 16185 16192 16223 16241 16301 16342 16362 16463	D4h3a6	73 143 152 263 489 16129 16185 16192 16223 16241 16301 16342 16362 16463	[1.000] D4h3a6 (1)	[0.050] D4h3a (2) [0.000] D4h3a1 D4h3a1a D4h3a1a2 (5)	1

894	>KY680947.1 H[illegible]	73G 152C 263G 309.1CT 310C 499A 750G 827G 1438G 2352C 2706G 3106d 3107C 3547G 4769G 4820A 4977C 5656G 6473T 6755A 7028T 7082T 7964C 8271d 8272d 8273d 8274d 8275d 8276d 8277d 8278d 8279d 8860G 8883C 9950C 11177T 11719A 11977T 13590A 14163T 14766T 15326G 15535T 16183C 16189C 16217C 16291T 16519C	B2b+152	73G 152C 263G 309.1CT 310C 499A 16183C 16189C 16217C 16291T 16519C	1	73 152 263 499 750 827 1438 2352 2706 3547 4769 4820 4977 5656 6473 6755 7028 7082 7964 8281-8289d 8860 8883 9950 11177 11719 11977 13590 14163 14766 15326 15535 16189 16217 16291	B2b+152	73 152 263 499 16189 16217 16291	[0.023] B2b+152 (1)	[0.126] B2q (2) [0.093] B2 (3) [0.032] B2r (4) [0.016] B2e (5) [0.010] B2c B2c1 (7) [0.009] B2n (8) [0.004] B2b (9) [0.000] B4b B2b3 B2c1a B2c1b B2c1c B2f B2i B2p (17)	1
895	>KY680938.1 H[illegible]	73G 152C 263G 309.1CT 310C 499A 750G 827G 1438G 2706G 3106d 3107C 3547G 4025T 4769G 4820A 4977C 6473T 6755A 7028T 8271d 8272d 8273d 8274d 8275d 8276d 8277d 8278d 8279d 8860G 9950C 11177T 11719A 13590A 13788T 14470C 14766T 15326G 15535T 16169T 16183C 16189C 16217C 16519C	B2b+152	73G 152C 263G 309.1CT 310C 499A 16169T 16183C 16189C 16217C 16519C	1	73 152 263 499 750 827 1438 2706 3547 4025 4769 4820 4977 6473 6755 7028 8281-8289d 8860 9950 11177 11719 13590 13788 14470 14766 15326 15535 16169 16189 16217	B2b+152	73 152 263 499 16169 16189 16217	[0.015] B2b+152 (1)	[0.126] B2q (2) [0.112] B2 (3) [0.032] B2r (4) [0.016] B2e (5) [0.010] B2c B2c1 (7) [0.009] B2n (8) [0.000] B4b B2b B2b3 B2c1a B2c1b B2c1c B2f B2i B2p (17)	1
896	>KY680933.1 H[illegible]	73G 152C 263G 302.1C 499A 750G 827G 1438G 2706G 3106d 3107C 3547G 4769G 4820A 4977C 6473T 6755A 7028T 8271d 8272d 8273d 8274d 8275d 8276d 8277d 8278d 8279d 8860G 9950C 11177T 11719A 13590A 13788T 14470C 14766T 15326G 15535T 16182.1C 16183C 16189C 16217C 16519C	B2b+152	73G 152C 263G 302.1C 499A 16182.1C 16183C 16189C 16217C 16519C	1	73 152 263 499 750 827 1438 2706 3547 4769 4820 4977 6473 6755 7028 8281-8289d 8860 9950 11177 11719 13590 13788 14470 14766 15326 15535 16189 16217	B2b+152	73 152 263 499 16189 16217	[0.000] B2b+152 (1)	[0.126] B2q (2) [0.093] B2 (3) [0.032] B2r (4) [0.016] B2e (5) [0.010] B2c B2c1 (7) [0.009] B2n (8) [0.000] B4b B2b B2b3 B2c1a B2c1b B2c1c B2f B2i B2p (17)	1
897	>KY680917.1 H[illegible]	73G 103A 152C 183G 263G 309.1CCT 310C 499A 750G 827G 1438G 2706G 3106d 3107C 3547G 4769G 4820A 4977C 6473T 6755A 7028T 7961C 8271d 8272d 8273d 8274d 8275d 8276d 8277d 8278d 8279d 8860G 9950C 11177T 11719A 12061T 13590A 14766T 15326G 15535T 16182C 16183C 16189C 16217C 16234T 16266T 16319A 16519C	B2b+152	73G 103A 152C 183G 263G 309.1CCT 310C 499A 16182C 16183C 16189C 16217C 16234T 16266T 16319A 16519C	1	73 103 152 183 263 499 750 827 1438 2706 3547 4769 4820 4977 6473 6755 7028 7961 8281-8289d 8860 9950 11177 11719 12061 13590 14766 15326 15535 16189 16217 16234 16266 16319	B2b+152	73 103 152 183 263 499 16189 16217 16234 16266 16319	[0.250] B2b+152 (1)	[0.302] B2b (2) [0.128] B2 (3) [0.126] B2q (4) [0.032] B2r (5) [0.020] B2c (6) [0.016] B2e (7) [0.010] B2c1 (8) [0.009] B2n (9) [0.000] B4b B2b3 B2c1a B2c1b B2c1c B2c2a B2f B2i B2p (18)	1
898	>KY680875.1 H[illegible]	73G 263G 309.1CCT 310C 499A 750G 827G 1438G 2706G 3106d 3107C 3547G 4769G 4820A 4977C 6473T 7028T 8271d 8272d 8273d 8274d 8275d 8276d 8277d 8278d 8279d 8860G 9950C 11177T 11719A 13590A 14766T 15326G 15535T 16051G 16189C 16217C 16422C 16438A 16519C	B2l	73G 263G 309.1CCT 310C 499A 16051G 16189C 16217C 16422C 16438A 16519C	1	73 263 499 750 827 1438 2706 3547 4769 4820 4977 6473 7028 8281-8289d 8860 9950 11177 11719 13590 14766 15326 15535 16051 16189 16217 16422 16438	B2l	73 263 499 16051 16189 16217 16422 16438	[0.833] B2l (1)	[0.193] B2q (2) [0.063] B2 (3) [0.004] B2b (4) [0.000] B4b B2b3 B2c B2c1 B2c1a B2c1b B2c1c B2e B2f B2i B2n B2p B2r (17)	1

899	>KY680874.1 Hc	73G 263G 302.1C 499A 750G 827G 1438G 2706G 3106d 3107C 3547G 4769G 4820A 4977C 6473T 7028T 8271d 8272d 8273d 8274d 8275d 8276d 8277d 8278d 8279d 8860G 9950C 11177T 11719A 13590A 14766T 15326G 15535T 16051G 16102C 16182.1C 16183C 16189C 16217C 16257T 16422C 16438A 16519C	B2l	73G 263G 302.1C 499A 16051G 16102C 16182.1C 16183C 16189C 16217C 16257T 16422C 16438A 16519C	1	73 263 499 750 827 1438 2706 3547 4769 4820 4977 6473 7028 8281-8289d 8860 9950 11177 11719 13590 14766 15326 15535 16051 16102 16189 16217 16257 16422 16438	B2l	73 263 499 16051 16102 16189 16217 16257 16422 16438	[1.167] B2l (1)	[0.193] B2q (2) [0.063] B2 (3) [0.004] B2b (4) [0.000] B4b B2b3 B2c B2c1 B2c1a B2c1b B2c1c B2e B2f B2i B2n B2p B2r (17)	1
900	>KY680873.1 Hc	73G 263G 309.1CT 310C 499A 750G 827G 1438G 2706G 3106d 3107C 3547G 4769G 4820A 4977C 6473T 7028T 8271d 8272d 8273d 8274d 8275d 8276d 8277d 8278d 8279d 8860G 9950C 11177T 11719A 13590A 14766T 15326G 15535T 16051G 16102C 16182.1C 16183C 16189C 16217C 16302G 16422C 16438A 16519C	B2l	73G 263G 309.1CT 310C 499A 16051G 16102C 16182.1C 16183C 16189C 16217C 16302G 16422C 16438A 16519C	1	73 263 499 750 827 1438 2706 3547 4769 4820 4977 6473 7028 8281-8289d 8860 9950 11177 11719 13590 14766 15326 15535 16051 16102 16189 16217 16302 16422 16438	B2l	73 263 499 16051 16102 16189 16217 16302 16422 16438	[1.167] B2l (1)	[0.193] B2q (2) [0.063] B2 (3) [0.004] B2b (4) [0.000] B4b B2b3 B2c B2c1 B2c1a B2c1b B2c1c B2e B2f B2i B2n B2p B2r (17)	1
901	>KY680872.1 Hc	73G 263G 309.1CT 310C 499A 750G 827G 1438G 2706G 3106d 3107C 3547G 4769G 4820A 4977C 6473T 7028T 8271d 8272d 8273d 8274d 8275d 8276d 8277d 8278d 8279d 8860G 9950C 11177T 11719A 13590A 14766T 15326G 15535T 16051G 16102C 16111T 16181.1C 16182C 16183C 16189C 16217C 16422C 16438A 16519C	B2l	73G 263G 309.1CT 310C 499A 16051G 16102C 16111T 16181.1C 16182C 16183C 16189C 16217C 16422C 16438A 16519C	1	73 263 499 750 827 1438 2706 3547 4769 4820 4977 6473 7028 8281-8289d 8860 9950 11177 11719 13590 14766 15326 15535 16051 16102 16111 16189 16217 16422 16438	B2l	73 263 499 16051 16102 16111 16189 16217 16422 16438	[1.167] B2l (1)	[0.193] B2q (2) [0.065] B2 (3) [0.004] B2b (4) [0.000] B4b B2a B2a1 B2a1a B2a1a1 B2a1b B2a2 B2b3 B2c B2c1 B2c1a B2c1b B2c1c B2e B2f B2i B2n B2p B2r (23)	1
902	>KY680871.1 Hc	73G 263G 309.1CT 310C 499A 750G 827G 1438G 2706G 3106d 3107C 3547G 4769G 4820A 4977C 6473T 7028T 8271d 8272d 8273d 8274d 8275d 8276d 8277d 8278d 8279d 8860G 9950C 11177T 11719A 13590A 14766T 15326G 15535T 16051G 16102C 16182.1C 16183C 16189C 16217C 16422C 16438A 16519C	B2l	73G 263G 309.1CT 310C 499A 16051G 16102C 16182.1C 16183C 16189C 16217C 16422C 16438A 16519C	1	73 263 499 750 827 1438 2706 3547 4769 4820 4977 6473 7028 8281-8289d 8860 9950 11177 11719 13590 14766 15326 15535 16051 16102 16189 16217 16422 16438	B2l	73 263 499 16051 16102 16189 16217 16422 16438	[1.125] B2l (1)	[0.193] B2q (2) [0.063] B2 (3) [0.004] B2b (4) [0.000] B4b B2b3 B2c B2c1 B2c1a B2c1b B2c1c B2e B2f B2i B2n B2p B2r (17)	1
903	>KY680870.1 Hc	73G 263G 309.1CT 310C 499A 750G 827G 1438G 2706G 3106d 3107C 3547G 4769G 4820A 4977C 6473T 7028T 8271d 8272d 8273d 8274d 8275d 8276d 8277d 8278d 8279d 8860G 9950C 11177T 11719A 13590A 14766T 15326G 15535T 16051G 16102C 16183C 16189C 16217C 16422C 16438A 16519C	B2l	73G 263G 309.1CT 310C 499A 16051G 16102C 16183C 16189C 16217C 16422C 16438A 16519C	1	73 263 499 750 827 1438 2706 3547 4769 4820 4977 6473 7028 8281-8289d 8860 9950 11177 11719 13590 14766 15326 15535 16051 16102 16189 16217 16422 16438	B2l	73 263 499 16051 16102 16189 16217 16422 16438	[1.125] B2l (1)	[0.193] B2q (2) [0.063] B2 (3) [0.004] B2b (4) [0.000] B4b B2b3 B2c B2c1 B2c1a B2c1b B2c1c B2e B2f B2i B2n B2p B2r (17)	1

904 >KY680869.1 H	73G 263G 309.1CT 310C 499A 750G 827G 1438G 2706G 3106d 3107C 3547G 4769G 4820A 4977C 6473T 7028T 8271d 8272d 8273d 8274d 8275d 8276d 8277d 8278d 8279d 8860G 9950C 11177T 11719A 13590A 14766T 15326G 15535T 16051G 16102C 16182.1C 16183C 16189C 16217C 16422C 16438A 16519C	B2l	73G 263G 309.1CT 310C 499A 16051G 16102C 16182.1C 16183C 16189C 16217C 16422C 16438A 16519C	1	73 263 499 750 827 1438 2706 3547 4769 4820 4977 6473 7028 8281-8289d 8860 9950 11177 11719 13590 14766 15326 15535 16051 16102 16189 16217 16422 16438	B2l	73 263 499 16051 16102 16189 16217 16422 16438	[1.125] B2l (1)	[0.193] B2q (2) [0.063] B2 (3) [0.004] B2b (4) [0.000] B4b B2b3 B2c B2c1 B2c1a B2c1b B2c1c B2e B2f B2i B2n B2p B2r (17)	1
905 >KY680868.1 H	73G 263G 499A 750G 827G 1438G 2706G 3106d 3107C 3547G 4769G 4820A 4977C 6473T 7028T 8271d 8272d 8273d 8274d 8275d 8276d 8277d 8278d 8279d 8860G 9950C 11177T 11719A 13590A 14766T 15326G 15535T 16051G 16102C 16182.1C 16183C 16189C 16217C 16422C 16438A 16519C	B2l	73G 263G 499A 16051G 16102C 16182.1C 16183C 16189C 16217C 16422C 16438A 16519C	1	73 263 499 750 827 1438 2706 3547 4769 4820 4977 6473 7028 8281-8289d 8860 9950 11177 11719 13590 14766 15326 15535 16051 16102 16189 16217 16422 16438	B2l	73 263 499 16051 16102 16189 16217 16422 16438	[1.125] B2l (1)	[0.193] B2q (2) [0.063] B2 (3) [0.004] B2b (4) [0.000] B4b B2b3 B2c B2c1 B2c1a B2c1b B2c1c B2e B2f B2i B2n B2p B2r (17)	1
906 >KY680846.1 H	73G 152C 263G 309.1CCT 310C 499A 750G 827G 1438G 2706G 3106d 3107C 3547G 4769G 4820A 4977C 6473T 6755A 7028T 8271d 8272d 8273d 8274d 8275d 8276d 8277d 8278d 8279d 8860G 9950C 11177T 11719A 13590A 14041T 14587G 14766T 15326G 15535T 16153A 16183C 16189C 16217C 16519C	B2b+152	73G 152C 263G 309.1CCT 310C 499A 16153A 16183C 16189C 16217C 16519C	1	73 152 263 499 750 827 1438 2706 3547 4769 4820 4977 6473 6755 7028 8281-8289d 8860 9950 11177 11719 13590 14041 14587 14766 15326 15535 16153 16189 16217	B2b+152	73 152 263 499 16153 16189 16217	[0.008] B2b+152 (1)	[0.126] B2q (2) [0.093] B2 (3) [0.032] B2r (4) [0.016] B2e (5) [0.010] B2c B2c1 (7) [0.009] B2n (8) [0.004] B2b (9) [0.000] B4b B2b3 B2c1a B2c1b B2c1c B2f B2i B2p (17)	1
907 >KY680808.1 H	73G 146C 153G 189G 207A 235G 263G 310.1C 514d 515d 663G 750G 1438G 1736G 2706G 3106d 3107C 4248C 4769G 4824G 5093C 7028T 8027A 8794T 8860G 11719A 12007A 12705T 14668T 14766T 15262C 15326G 16111T 16223T 16290T 16293G 16304C 16319A 16362C	A2y	73G 146C 153G 189G 207A 235G 263G 310.1C 514d 515d 16111T 16223T 16290T 16293G 16304C 16319A 16362C	1	73 146 153 189 207 235 263 663 750 1438 1736 2706 4248 4769 4824 5093 7028 8027 8794 8860 11719 12007 12705 14668 14766 15262 15326 16111 16223 16290 16293 16304 16319 16362	A2y	73 146 153 189 207 235 263 16111 16223 16290 16293 16304 16319 16362	[0.000] A2y (1)	[0.019] A2 (2) [0.012] A2+(64) (3) [0.005] A2f1 A2f1a (5) [0.000] A2b A2c A2d A2d1 A2d2 A2f A2j A2j1 A2k A2k1 A2k1a A2t A2w A2x A2an A2r (21)	1
908 >MG022095.1 H	73G 150T 214G 263G 309.1CT 310C 567.1CC 709A 750G 1438G 1888A 2706G 3106d 3107C 4216C 4769G 4917G 7028T 8697A 8860G 10463C 11251G 11465C 11719A 12633A 13368A 13470G 13708A 14766T 14905A 15326G 15452A 15607G 15928A 16126C 16163G 16186T 16189C 16294T 16390R 16519C	T1a10a	73G 150T 214G 263G 309.1CT 310C 567.1CC 16126C 16163G 16186T 16189C 16294T 16390R 16519C	1	73 150 214 263 573.2C 709 750 1438 1888 2706 4216 4769 4917 7028 8697 8860 10463 11251 11465 11719 12633A 13368 13470 13708 14766 14905 15326 15452A 15607 15928 16126 16163 16186 16189 16294 16390R	T1a10a	73 150 214 263 573.2C 16126 16163 16186 16189 16294 16390R	[0.009] T1a10a (1)	[0.172] T1a10 (2) [0.017] T1a5 (3) [0.011] T1a8b (4) [0.000] T1a T1a6 T1a8 (7)	1

909 >MG201857.1 H	73G 153G 189G 195C 225A 226C 263G 309.1CCT 310C 513.1CA 750G 1438G 1719A 2706G 3106d 3107C 4769G 6221C 6371T 7028T 8393T 8860G 8910T 11719A 12705T 13708A 13966G 14470C 14766T 15326G 15927A 16183C 16189C 16223Y 16278T 16311C 16519C	X2b11	73G 153G 189G 195C 225A 226C 263G 309.1CCT 310C 513.1CA 16183C 16189C 16223Y 16278T 16311C 16519C	1	73 153 189 195 225 226 263 750 1438 1719 2706 4769 6221 6371 7028 8393 8860 8910 11719 12705 13708 13966 14470 14766 15326 15927 16189 16223Y 16278 16311	X2b11	73 153 189 195 225 226 263 16189 16223Y 16278 16311	[0.020] X2b11 (1)	[0.006] X2b+226 (2) [0.000] X2b2 X2b3 X2b4 X2b4a X2b4a1 X2b5 X2b6 X2b6a X2b7 X2b8 X2b9 X2m'n (14)	1
910 >MG053504.1 H	73G 185A 188G 228A 263G 295T 309.1CT 310C 462T 489C 750G 1438G 2706G 3010A 3106d 3107C 4216C 4769G 7028T 8860G 10398G 11002G 11251G 11719A 12612G 13032G 13708A 14325C 14766T 14798C 15326G 15452A 16069T 16126C 16186T 16519C	J1c2r	73G 185A 188G 228A 263G 295T 309.1CT 310C 462T 489C 16069T 16126C 16186T 16519C	1	73 185 188 228 263 295 462 489 750 1438 2706 3010 4216 4769 7028 8860 10398 11002 11251 11719 12612 13032 13708 14325 14766 14798 15326 15452A 16069 16126 16186	J1c2r	73 185 188 228 263 295 462 489 16069 16126 16186	[0.000] J1c2r (1)	[0.000] J1c2 J1c2a J1c2a1 J1c2a1a J1c2a3 J1c2b J1c2b1 J1c2b2 J1c2b5 J1c2c J1c2c2 J1c2c2a J1c2c3 J1c2d J1c2f J1c2g J1c2k J1c2l J1c2n J1c2n1 J1c2p J1c2q J1c2q1 J1c2r J1c2s J1c2t (27)	1
911 >MG201867.1 H	150T 263G 310.1C 408A 750G 1438G 3106d 3107C 4769G 6776C 8860G 15326G 16093C 16519C	H3v+16093	150T 263G 310.1C 408A 16093C 16519C	1	150 263 408A 750 1438 4769 6776 8860 15326 16093	H3v+16093	150 263 408A 16093	[0.028] H3v+16093 (1)	[0.012] H1e1a6 (2) [0.005] H3v (3) [0.002] H10+(16093) (4) [0.000] HV5b HV12b H1as1 H1av1 H1av1a H3v1 H3v2 H10f (12)	1
912 >MG022094.1 H	73G 150T 152C 263G 310.1C 514d 515d 750G 921C 1438G 2706G 3106d 3107C 4553C 4769G 5046A 5147A 6680C 7028T 7424G 8618C 8701G 8860G 9540C 10398G 10873C 11719A 12705T 13105G 13886C 14284T 14766T 15301A 15326G 15514C 16124C 16223T	L3d1b2	73G 150T 152C 263G 310.1C 514d 515d 16124C 16223T	1	73 150 152 263 750 921 1438 2706 4553 4769 5046 5147 6680 7028 7424 8618 8701 8860 9540 10398 10873 11719 12705 13105 13886 14284 14766 15301 15326 15514 16124 16223	L3d1b2	73 150 152 263 16124 16223	[0.000] L3d1b2 (1)	[0.043] L3d1b1 (2) [0.038] L3d1a1a1 (3) [0.006] L3d1a1a (4) [0.000] L3d L3d1'2'3'4'5'6 L3d1 L3d1a L3d1b L3d1b1a L3d1d L3d3 L3d3a L3d3a1 L3d3a1a L3d3a1b L3d3b L3d6 (18)	1
913 >MG182427.1 H	55C 57C 146C 152C 263G 309.1CT 310C 750G 1438G 3106d 3107C 4769G 5442C 6253C 8270.1ACCCCCTCT 8860G 11143T 11410C 14548G 14953T 15326G 16189C 16366T	H15a1a1	55C 57C 146C 152C 263G 309.1CT 310C 16189C 16366T	1	55 57 146 152 263 750 1438 4769 5442 6253 8289.1CCCCCTCTA 8860 11143 11410 14548 14953 15326 16189 16366	H15a1a1	55 57 146 152 263 16189 16366	[0.414] H15a1a1 (1)	[0.097] H15a1 (2) [0.081] H15a1a H15b2 (4) [0.059] H15b (5) [0.057] H15 (6) [0.034] H3av (7) [0.029] H15a (8) [0.000] H1ap1 H15b1 (10)	1
914 >MF523221.1 H	239C 263G 309.1CT 310C 750G 1438G 4769G 8860G 14040A 15326G 16186T 16300G 16325C 16362C 16482G	H6b2	239C 263G 309.1CT 310C 16186T 16300G 16325C 16362C 16482G	1	239 263 750 1438 4769 8860 14040 15326 16186 16300 16325 16362 16482	H6b2	239 263 16186 16300 16325 16362 16482	[0.240] H6b2 (1)	[0.240] H6b2 (2) [0.000] H6b	1
915 >MF523219.1 H	73G 195A 263G 309.1CT 310C 489C 514d 515d 750G 1438G 2706G 4769G 7028T 8701G 8860G 9540C 10398G 10400T 10873C 11437C 11719A 12007A 12705T 14766T 14783C 15043A 15301A 15326G 15431A 16223T 16274A 16519C	M30	73G 195A 263G 309.1CT 310C 489C 514d 515d 16223T 16274A 16519C	1	73 195A 263 489 750 1438 2706 4769 7028 8701 8860 9540 10398 10400 10873 11437 11719 12007 12705 14766 14783 15043 15301 15326 15431 16223 16274	M30	73 195A 263 489 16223 16274	[0.004] M30 (1) [0.000] M30d M30d1 M30d2 (4)	[0.057] M30+16234 (5) [0.007] M23'75 (6) [0.006] G1b (7) [0.005] M22b (8) [0.003] M M32'56 (10) [0.000] M1'20'51 M2 M2c M4"67 M30a M30a1 M30a2 M30c M30d M30d1 M30d2 M30f M30g M37 M43 M43b M7 M7b'c M7b M12'G G3 G3a G3b G3b2 M19'53 M21 M21b M21b1 M21b1a M22 M24'41 M27 M29'Q M31 M31a M33 M33a M33a1 M34'57 M35 M39'70 M42'74 M42 M52 M52b M55'77 M58 M60 M69 M80'D D4e5 (61)	1

916 >MF523170.1 Hc	73G 146C 152C 153G 195C 234G 263G 309.1CT 310C 750G 1438G 1811G 1888A 4317G 4769G 5186T 5773A 7028T 8860G 9094T 9614G 11467G 11719A 12106T 12308G 12372A 12406A 12793C 13194A 13656C 14766T 15049T 15326G 15930A 16051G 16209C 16239T 16311C 16352C 16353T	U2b2	73G 146C 152C 153G 195C 234G 263G 309.1CT 310C 16051G 16209C 16239T 16311C 16352C 16353T	1	73 146 152 153 195 234 263 750 1438 1811 1888 4317 4769 5186T 5773 7028 8860 9094 9614 11467 11719 12106 12308 12372 12406 12793 13194 13656 14766 15049 15326 15930 16051 16209 16239 16311 16352 16353	U2b2	73 146 152 153 195 234 263 16051 16209 16239 16311 16352 16353	[0.275] U2b2 (1)	[0.989] U2b (2) [0.585] U2b1 (3) [0.389] U2b1a (4) [0.151] U2+152 (5) [0.043] K2a6 (6) [0.033] R30a1b (7) [0.022] K1b2a (8) [0.017] K2a2a (9) [0.014] K1b2a2 (10) [0.011] K2a1 K2a2 K2a3a (13) [0.010] K2a (14) [0.008] K1b2b (15) [0.005] M24a (16) [0.000] L3f2a L3f2a1 U4b1b1+16311 U4b1b1c K1b2 K1b2a1 K1b2a1a K1b2a2a K1b2a3 K2a3 K2a3a1 K2a4 K2a7 K2a9 K2a10 (31)	1
917 >MF523163.1 Hc	239C 263G 309.1CT 310C 750G 1438G 4769G 8860G 14040A 15326G 15758R 16186T 16300G 16325C 16362C 16482G	H6b2	239C 263G 309.1CT 310C 16186T 16300G 16325C 16362C 16482G	1	239 263 750 1438 4769 8860 14040 15326 15758R 16186 16300 16325 16362 16482	H6b2	239 263 16186 16300 16325 16362 16482	[0.240] H6b2 (1)	[0.240] H6b2 (2) [0.000] H6b	1
918 >MF523162.1 Hc	239C 263G 309.1CT 310C 750G 1438G 4769G 8860G 13154C 14040A 15326G 16186T 16300G 16325C 16362C 16482G	H6b2	239C 263G 309.1CT 310C 16186T 16300G 16325C 16362C 16482G	1	239 263 750 1438 4769 8860 13154 14040 15326 16186 16300 16325 16362 16482	H6b2	239 263 16186 16300 16325 16362 16482	[0.240] H6b2 (1)	[0.240] H6b2 (2) [0.000] H6b	1
919 >MF523154.1 Hc	73G 185A 188G 228A 263G 295T 309.1CT 310C 462T 489C 750G 1438G 2706G 3010A 4216C 4769G 7028T 8860G 10398G 11251G 11719A 12612G 13708A 14766T 14798C 15326G 15452A 16069T 16126C 16163G 16519C	J1c2o	73G 185A 188G 228A 263G 295T 309.1CT 310C 462T 489C 16069T 16126C 16163G 16519C	1	73 185 188 228 263 295 462 489 750 1438 2706 3010 4216 4769 7028 8860 10398 11251 11719 12612 13708 14766 14798 15326 15452A 16069 16126 16163	J1c2o	73 185 188 228 263 295 462 489 16069 16126 16163	[0.000] J1c2o (1)	[0.000] J1c2 J1c2a J1c2a1 J1c2a1a J1c2a3 J1c2b J1c2b1 J1c2b2 J1c2b5 J1c2c J1c2c2 J1c2c2a J1c2c3 J1c2d J1c2f J1c2g J1c2k J1c2l J1c2n J1c2n1 J1c2o J1c2p J1c2q J1c2q1 J1c2s J1c2t (27)	1
920 >MF523148.1 Hc	73G 146C 152C 153G 195C 234G 263G 309.1CT 310C 750G 1438G 1811G 1888A 4317G 4769G 5186T 5773A 7028T 8860G 9094T 9614G 11467G 11719A 12106T 12308G 12372A 12406A 12793C 13194A 13656C 14766T 15049T 15326G 15930A 16051G 16209C 16239T 16311C 16352C 16353T	U2b2	73G 146C 152C 153G 195C 234G 263G 309.1CT 310C 16051G 16209C 16239T 16311C 16352C 16353T	1	73 146 152 153 195 234 263 750 1438 1811 1888 4317 4769 5186T 5773 7028 8860 9094 9614 11467 11719 12106 12308 12372 12406 12793 13194 13656 14766 15049 15326 15930 16051 16209 16239 16311 16352 16353	U2b2	73 146 152 153 195 234 263 16051 16209 16239 16311 16352 16353	[0.275] U2b2 (1)	[0.989] U2b (2) [0.585] U2b1 (3) [0.389] U2b1a (4) [0.151] U2+152 (5) [0.043] K2a6 (6) [0.033] R30a1b (7) [0.022] K1b2a (8) [0.017] K2a2a (9) [0.014] K1b2a2 (10) [0.011] K2a1 K2a2 K2a3a (13) [0.010] K2a (14) [0.008] K1b2b (15) [0.005] M24a (16) [0.000] L3f2a L3f2a1 U4b1b1+16311 U4b1b1c K1b2 K1b2a1 K1b2a1a K1b2a2a K1b2a3 K2a3 K2a3a1 K2a4 K2a7 K2a9 K2a10 (31)	1
921 >MF523147.1 Hc	73G 146C 152C 153G 195C 234G 263G 309.1CT 310C 750G 1438G 1811G 1888A 4317G 4769G 5186T 5773A 7028T 8860G 9094T 9614G 11467G 11719A 12106T 12308G 12372A 12406A 12793C 13194A 13656C 14766T 15049T 15326G 15930A 16051G 16209C 16239T 16311C 16352C 16353T	U2b2	73G 146C 152C 153G 195C 234G 263G 309.1CT 310C 16051G 16209C 16239T 16311C 16352C 16353T	1	73 146 152 153 195 234 263 750 1438 1811 1888 4317 4769 5186T 5773 7028 8860 9094 9614 11467 11719 12106 12308 12372 12406 12793 13194 13656 14766 15049 15326 15930 16051 16209 16239 16311 16352 16353	U2b2	73 146 152 153 195 234 263 16051 16209 16239 16311 16352 16353	[0.275] U2b2 (1)	[0.989] U2b (2) [0.585] U2b1 (3) [0.389] U2b1a (4) [0.151] U2+152 (5) [0.043] K2a6 (6) [0.033] R30a1b (7) [0.022] K1b2a (8) [0.017] K2a2a (9) [0.014] K1b2a2 (10) [0.011] K2a1 K2a2 K2a3a (13) [0.010] K2a (14) [0.008] K1b2b (15) [0.005] M24a (16) [0.000] L3f2a L3f2a1 U4b1b1+16311 U4b1b1c K1b2 K1b2a1 K1b2a1a K1b2a2a K1b2a3 K2a3 K2a3a1 K2a4 K2a7 K2a9 K2a10 (31)	1

922	>MF523146.1 Hc	73G 151T 152C 207A 263G 309.1CT 310C 514d 515d 750G 980C 1438G 1811G 2706G 3741T 3834A 4529G 4769G 5360T 7028T 8137T 8684T 8860G 10142T 11467G 11719A 12308G 12372A 12618A 13500C 14218C 14569A 14766T 15109C 15326G 16207G 16309G 16318T 16519C	U7a3b	73G 151T 152C 207A 263G 309.1CT 310C 514d 515d 16207G 16309G 16318T 16519C	1	73 151 152 207 263 750 980 1438 1811 2706 3741 3834 4529 4769 5360 7028 8137 8684 8860 10142 11467 11719 12308 12372 12618 13500 14218 14569 14766 15109 15326 16207 16309 16318T	U7a3b	73 151 152 207 263 16207 16309 16318T	[0.011] U7a3b (1)	[0.013] U7a (2) [0.011] U7a3b (3) [0.000] U7a2 U7a3 U7a3a (6)	1
923	>MF523133.1 Hc	73G 152C 248d 263G 309.1CT 310C 489C 750G 1438G 2706G 3552A 4715G 4742C 4769G 6026A 7028T 7196A 8584A 8602C 8701G 8860G 9102A 9540C 9545G 9966A 10398G 10400T 10873C 11719A 11914A 11969A 12705T 13263G 14318C 14384A 14766T 14783C 15043A 15204C 15301A 15326G 15487T 16223T 16297C 16298C 16301T 16327T 16519C	C4+152	73G 152C 248d 263G 309.1CT 310C 489C 16223T 16297C 16298C 16301T 16327T 16519C	1	73 152 249d 263 489 750 1438 2706 3552A 4715 4742 4769 6026 7028 7196A 8584 8602 8701 8860 9102A 9540 9545 9966 10398 10400 10873 11719 11914 11969 12705 13263 14318 14384 14766 14783 15043 15204 15301 15326 15487T 16223 16297 16298 16301 16327	C4+152	73 152 249d 263 489 16223 16297 16298 16301 16327	[0.076] C4+152 (1)	[0.102] C4+152+16093 (2) [0.020] C4a1a1 (3) [0.019] C7 (4) [0.014] C4a1a C4a1a5 C4a1a6 (7) [0.009] C7a (8) [0.005] C4d (9) [0.004] C4b (10) [0.003] C4a1 (11) [0.000] C C4 C4a'b'c C4a C4a1a1a C4b5 C4b6 C4b7 C4b8 C4c C4c1 C4e (23)	1
924	>MF523127.1 Hc	73G 217C 228A 263G 309.1CT 310C 340T 508G 750G 1438G 1811G 2706G 3720G 4769G 5390G 5426C 6045T 6152C 7028T 7996T 8860G 10876G 11467G 11719A 12308G 12372A 13020C 13734C 14766T 15326G 15907G 16051G 16129C 16183C 16362C 16519C	U2e1h	73G 217C 228A 263G 309.1CT 310C 340T 508G 16051G 16129C 16183C 16362C 16519C	1	73 217 228 263 340 508 750 1438 1811 2706 3720 4769 5390 5426 6045 6152 7028 7996 8860 10876 11467 11719 12308 12372 13020 13734 14766 15326 15907 16051 16129C 16362	U2e1h	73 217 228 263 340 508 16051 16129C 16362	[0.338] U2e1h (1)	[0.009] U2e1a1 (2) [0.000] U2e1 U2e1a U2e1a1a U2e1a1c U2e1c U2e1c1 U2e1d U2e1g (10)	1
925	>MF523117.1 Hc	73G 146C 152C 234G 263G 309.1CT 310C 750G 1438G 1811G 1888A 4769G 5186T 7028T 8860G 9094T 9614G 11467G 11719A 12106T 12308G 12372A 12793C 13194A 13305T 13656C 13980A 14766T 15049T 15326G 15930A 16051G 16129A 16209C 16239T 16311C 16352C 16353T	U2b2	73G 146C 152C 234G 263G 309.1CT 310C 16051G 16129A 16209C 16239T 16311C 16352C 16353T	1	73 146 152 234 263 750 1438 1811 1888 4769 5186T 7028 8860 9094 9614 11467 11719 12106 12308 12372 12793 13194 13305 13656 13980 14766 15049 15326 15930 16051 16129 16209 16239 16311 16352 16353	U2b2	73 146 152 234 263 16051 16129 16209 16239 16311 16352 16353	[0.176] U2b2 (1)	[1.046] U2b (2) [0.642] U2b1 (3) [0.334] K2b1b (4) [0.222] U2b1a (5) [0.168] U2+152 (6) [0.033] K2a6 (7) [0.022] R30a1b (8) [0.018] L3f2a (9) [0.017] K2a4 (10) [0.014] H17c (11) [0.011] K2a1 K2a2 K2a2a K2a3a (15) [0.005] K2a (16) [0.000] L3f2a1 K2a3 K2a3a1 K2a7 K2a9 K2a10 (22)	1
926	>MF523106.1 Hc	41T 73G 263G 309.1CT 310C 319C 709A 750G 930A 1438G 1888A 2706G 4216C 4769G 4917G 5147A 7028T 8697A 8860G 9861C 10463C 11251G 11719A 11812G 12793C 13368A 14233G 14766T 14905A 15326G 15452A 15497A 15607G 15928A 16126C 16294T 16296T 16304C 16519C	T2b34	41T 73G 263G 309.1CT 310C 319C 16126C 16294T 16296T 16304C 16519C	1	41 73 263 319 709 750 930 1438 1888 2706 4216 4769 4917 5147 7028 8697 8860 9861 10463 11251 11719 11812 12793 13368 14233 14766 14905 15326 15452A 15497 15607 15928 16126 16294 16296 16304	T2b34	41 73 263 319 16126 16294 16296 16304	[0.000] T2b34 (1)	[0.004] T2b (2) [0.000] T2b1 T2b2 T2b3 T2b3b T2b4 T2b5 T2b5a T2b7 T2b7a T2b8 T2b13 T2b13b T2b15 T2b17 T2b17a T2b19 T2b25 T2b26 T2b27 T2b28 T2b29 T2b30 T2b31 T2b32 T2b35 T2b36 T2b37 (29)	1

927 >MF523103.1 Hc	73G 146C 152C 234G 263G 309.1CT 310C 750G 1438G 1811G 1888A 4769G 5186T 7028T 8860G 9094T 9614G 11467G 11719A 12106T 12308G 12372A 12793C 13194A 13305T 13656C 13980A 14766T 15049T 15326G 15930A 16051G 16129A 16209C 16239T 16311C 16352C 16353T	U2b2	73G 146C 152C 234G 263G 309.1CT 310C 16051G 16129A 16209C 16239T 16311C 16352C 16353T	1	73 146 152 234 263 750 1438 1811 1888 4769 5186T 7028 8860 9094 9614 11467 11719 12106 12308 12372 12793 13194 13305 13656 13980 14766 15049 15326 15930 16051 16129 16209 16239 16311 16352 16353	U2b2	73 146 152 234 263 16051 16129 16209 16239 16311 16352 16353	[0.176] U2b2 (1)	[1.046] U2b (2) [0.642] U2b1 (3) [0.334] K2b1b (4) [0.222] U2b1a (5) [0.168] U2+152 (6) [0.033] K2a6 (7) [0.022] R30a1b (8) [0.018] L3f2a (9) [0.017] K2a4 (10) [0.014] H17c (11) [0.011] K2a1 K2a2 K2a2a K2a3a (15) [0.005] K2a (16) [0.000] L3f2a1 K2a3 K2a3a1 K2a7 K2a9 K2a10 (22)	1
928 >MF523102.1 Hc	210G 239C 263G 309.1CT 310C 373G 750G 1438G 4769G 8167C 8860G 14040A 15326G 16300G 16362C 16482G	H6b	210G 239C 263G 309.1CT 310C 373G 16300G 16362C 16482G	1	210 239 263 373 750 1438 4769 8167 8860 14040 15326 16300 16362 16482	H6b	210 239 263 373 16300 16362 16482	[0.066] H6b (1)	[0.066] H6b (2) [0.000] H6 H6a H6a1 H6a1a H6a1a1 H6a1a2 H6a1a2b H6a1a2b1 H6a1a3 H6a1a3a H6a1a4 H6a1a5 H6a1a9 H6a1a10 H6a1b H6a1b2 H6a1b2a H6a1b2b H6a1b2c H6a1b2e H6a2 H6a2a H6b2 H6c (26)	1
929 >MF523097.1 Hc	72C 73G 151T 152C 195C 246C 263G 309.1CT 310C 596C 629C 750G 1438G 2706G 4706C 4769G 5147A 7028T 7193C 8860G 9336G 9617G 11935C 12061T 13204A 15326G 16214T 16217C 16335G 16519C	HV2a1	72C 73G 151T 152C 195C 246C 263G 309.1CT 310C 16214T 16217C 16335G 16519C	1	72 73 151 152 195 246 263 596 629 750 1438 2706 4706C 4769 5147 7028 7193 8860 9336 9617 11935 12061 13204 15326 16214 16217 16335	HV2a1	72 73 151 152 195 246 263 16214 16217 16335	[0.795] HV2a1 (1)	[0.022] HV2a3 (2) [0.000] HV2a HV2a2 (4)	1
930 >MF523093.1 Hc	73G 195A 263G 309.1CT 310C 489C 514d 515d 750G 1438G 2706G 4769G 7028T 8701G 8860G 9540C 10398G 10400T 10873C 11719A 12007A 12705T 14766T 14783C 15043A 15301A 15326G 15431A 16153A 16223T 16234T 16519C	M30+16234	73G 195A 263G 309.1CT 310C 489C 514d 515d 16153A 16223T 16234T 16519C	1	73 195A 263 489 750 1438 2706 4769 7028 8701 8860 9540 10398 10400 10873 11719 12007 12705 14766 14783 15043 15301 15326 15431 16153 16223 16234	M30+16234	73 195A 263 489 16153 16223 16234	[0.005] M30+16234 (1) [0.000] M49a M49a1 M49a2 (4)	[0.000] M30 M30d M30d1 M30d2 M30e M49 M49c M49c1 M49e M49e1 (14)	1
931 >MF523085.1 Hc	73G 146C 152C 153G 234G 263G 309.1CT 310C 750G 1438G 1811G 1888A 4317G 4769G 5186T 7028T 8860G 9094T 9614G 11467G 11719A 12106T 12308G 12372A 12406A 12793C 13194A 13656C 14766T 15049T 15218G 15326G 15930A 16051G 16209C 16239T 16311C 16352C 16353T	U2b2	73G 146C 152C 153G 234G 263G 309.1CT 310C 16051G 16209C 16239T 16311C 16352C 16353T	1	73 146 152 153 234 263 750 1438 1811 1888 4317 4769 5186T 7028 8860 9094 9614 11467 11719 12106 12308 12372 12406 12793 13194 13656 14766 15049 15218 15326 15930 16051 16209 16239 16311 16352 16353	U2b2	73 146 152 153 234 263 16051 16209 16239 16311 16352 16353	[0.213] U2b2 (1)	[0.955] U2b (2) [0.556] U2b1 (3) [0.222] U2b1a (4) [0.151] U2+152 (5) [0.033] K2a6 (6) [0.022] R30a1b (7) [0.011] K2a1 K2a2 K2a2a K2a3a (11) [0.000] L3f2a L3f2a1 K2a K2a3 K2a3a1 K2a4 K2a7 K2a9 K2a10 (20)	1
932 >MF523080.1 Hc	239C 263G 309.1CT 310C 750G 1438G 3766C 4769G 7749Y 8860G 14040A 15326G 16186T 16300G 16325C 16362C 16482G	H6b2	239C 263G 309.1CT 310C 16186T 16300G 16325C 16362C 16482G	1	239 263 750 1438 3766 4769 7749Y 8860 14040 15326 16186 16300 16325 16362 16482	H6b2	239 263 16186 16300 16325 16362 16482	[0.240] H6b2 (1)	[0.240] H6b2 (2) [0.000] H6b	1
933 >MF523075.1 Hc	73G 143A 199C 250C 263G 309.1CT 310C 567.1C 710C 750G 1438G 1719A 2706G 4529T 4769G 6392C 7028T 8251A 8860G 10238C 10398G 10790C 11719A 12501A 12705T 13752C 13780G 14766T 15043A 15326G 15924G 16223T 16274A 16295T 16309G 16319A 16391A 16482G 16519C	N1a1b1	73G 143A 199C 250C 263G 309.1CT 310C 567.1C 16223T 16274A 16295T 16309G 16319A 16391A 16482G 16519C	1	73 143 199 250 263 573.1C 710 750 1438 1719 2706 4529T 4769 6392 7028 8251 8860 10238 10398 10790 11719 12501 12705 13752 13780 14766 15043 15326 15924 16223 16274 16295 16309 16319 16391 16482	N1a1b1	73 143 199 250 263 573.1C 16223 16274 16295 16309 16319 16391 16482	[1.709] N1a1b1 (1)	[0.040] N1a1b (2)	1

934	>MF523073.1 Hc	73G 143A 199C 250C 263G 309.1CT 310C 567.1C 710C 750G 1438G 1719A 2706G 4529T 4769G 6392C 7028T 8251A 8860G 10238C 10398G 10790C 11719A 12501A 12705T 13780G 14766T 15043A 15326G 15924G 16223T 16274A 16295T 16309G 16319A 16391A 16482G 16519C	N1a1b1	73G 143A 199C 250C 263G 309.1CT 310C 567.1C 16223T 16274A 16295T 16309G 16319A 16391A 16482G 16519C	1	73 143 199 250 263 573.1C 710 750 1438 1719 2706 4529T 4769 6392 7028 8251 8860 10238 10398 10790 11719 12501 12705 13780 14766 15043 15326 15924 16223 16274 16295 16309 16319 16391 16482	N1a1b1	73 143 199 250 263 573.1C 16223 16274 16295 16309 16319 16391 16482	[1.709] N1a1b1 (1)	[0.040] N1a1b (2)	1
935	>MF523062.1 Hc	263G 309.1CT 310C 750G 951A 8860G 10256C 15326G 16193T 16354T 16519C	H2a1f	263G 309.1CT 310C 16193T 16354T 16519C	1	263 750 951 8860 10256 15326 16193 16354	H2a1f	263 16193 16354	[0.000] H2a1f (1)	[0.000] H2a1 H2a1a H2a1a1 H2a1a2 H2a1b H2a1c H2a1d H2a1f1 H2a1f2 H2a1g H2a1k (12)	1
936	>MF523060.1 Hc	263G 309.1CT 310C 750G 951A 8860G 10256C 15326G 16193T 16354T 16519C	H2a1f	263G 309.1CT 310C 16193T 16354T 16519C	1	263 750 951 8860 10256 15326 16193 16354	H2a1f	263 16193 16354	[0.000] H2a1f (1)	[0.000] H2a1 H2a1a H2a1a1 H2a1a2 H2a1b H2a1c H2a1d H2a1f1 H2a1f2 H2a1g H2a1k (12)	1
937	>MF523054.1 Hc	239C 263G 309.1CT 310C 750G 1438G 4769G 8860G 14040A 15326G 16186T 16300G 16325C 16362C 16482G	H6b2	239C 263G 309.1CT 310C 16186T 16300G 16325C 16362C 16482G	1	239 263 750 1438 4769 8860 14040 15326 16186 16300 16325 16362 16482	H6b2	239 263 16186 16300 16325 16362 16482	[0.240] H6b2 (1)	[0.240] H6b2 (2) [0.000] H6b	1
938	>MF523050.1 Hc	57C 204Y 263G 309.1CT 310C 750G 1438G 4769G 6253C 8860G 11143T 11410C 14953T 15326G 16366T	H15a1a1	57C 204Y 263G 309.1CT 310C 16366T	1	57 204Y 263 750 1438 4769 6253 8860 11143 11410 14953 15326 16366	H15a1a1	57 204Y 263 16366	[0.195] H15a1a1 (1)	[0.195] H15b1 (2) [0.113] H15a1 (3) [0.078] H15b (4) [0.011] H2a2a (5) [0.005] H1j9 (6) [0.004] H1e1a H1bd (8) [0.001] H H1q H4 H10+(16093) H51 (13) [0.000] R0 HV HV4 HV4c HV5 HV5a HV9c HV12 H1 H1e H1e1 H1e1a2 H1e1a7 H1e1c H1e2 H1e2a H1e2b H1e2d H1e4 H1e6 H1e7 H1e8 H1e8a H1h H1h1 H1h2 H1j H1j3 H1j5 H1j6 H1j7 H1m H1n H1p H1q1 H1q2 H1r H1s H1s1 H1t H1t1 H1t1a H1t2 H1u H1u1 H1v H1v1 H1v1a H1v1b H1w H1ae H1ae1 H1ae2 H1ae2a H1ae3 H1ae3a H1ag H1ag1 H1ag1b H1ah H1ai H1ai1 H1aj H1ak H1ak1 H1ak2 H1am H1am1 H1ap H1aq H1as H1as2 H1at H1at1 H1at1a H1av H1ax H1ax1 H1ay H1az H1be H1bi H1bj H1bk H1bm H1bn H1bp H1bq H1br H1bu H1bv H1bw H1bx H1ca H1ci H2 H2a H2a2 H2a2+(16235) H2a4 H2a5 H2a5a H2a5b H3 H3b H3c H3c1 H3e H3q H3q1 H3r H3r1 H3t H3u H3y H3aa H3ab H3ac H3ad H3ae H3ag H3ag1 H3ah H3ai H3aj H3ap H3aq H3ar H3as H3at H3at1 H3au H4a H4a1 H4a1a H4a1a1 H4a1a2 H4a1a2a H4a1a2a1 [illegible]	1

939 >MF523047.1 Hc	262T 263G 309.1CT 310C 750G 1438G 3039Y 4769G 5460A 6392C 8860G 10124C 12630A 14118G 15326G 15617A 16274A	H41a	262T 263G 309.1CT 310C 16274A	1	262 263 750 1438 3039Y 4769 5460 6392 8860 10124 12630 14118 15326 15617 16274	H41a	262 263 16274	[0.020] H41a (1) [0.019] H2a3 (2) [0.000] H2a3a H2a3a1 H2a3b (5)	[0.004] H H1ae3 (7) [0.002] H1 H3 (9) [0.001] H2a2a H16b H56 (12) [0.000] R0 HV HV1b1 HV1b1a HV1b1b HV4 HV4c HV5 HV5a HV9c HV12 H1e H1e1 H1e1a H1e1a2 H1e1a7 H1e1c H1e2 H1e2a H1e2b H1e2d H1e4 H1e6 H1e7 H1e8 H1e8a H1h H1h1 H1h2 H1j H1j3 H1j5 H1j6 H1j7 H1j9 H1m H1n H1p H1q H1q1 H1q2 H1r H1s H1s1 H1t H1t1 H1t1a H1t2 H1u H1u1 H1v H1v1 H1v1a H1v1b H1w H1ae H1ae1 H1ae2 H1ae2a H1ae3a H1ag H1ag1 H1ag1b H1ah H1ai H1ai1 H1aj H1ak H1ak1 H1ak2 H1am H1am1 H1ap H1aq H1as H1as2 H1at H1at1 H1at1a H1av H1ax H1ax1 H1ay H1az H1bd H1be H1bi H1bj H1bk H1bm H1bn H1bp H1bq H1br H1bu H1bv H1bw H1bx H1ca H1ci H2 H2a H2a2 H2a2+(16235) H2a4 H2a5 H2a5a H2a5b H3b H3c H3c1 H3e H3q H3q1 H3r H3r1 H3t H3u H3y H3aa H3ab H3ac H3ad H3ae H3ag H3ag1 H3ah H3ai H3aj H3ap H3aq H3ar H3as H3at H3at1 H3au H4 H4a H4a1 H4a1a H4a1a1 H4a1a2 H4a1a2a H4a1a2a1 H4a1a5 H4a1c H4a1c1 H4a1c1a H4a1d H4b H4c H7 H7a H7b H7b1 [illegible]	1
940 >MF523042.1 Hc	55C 57C 263G 309.1CT 310C 750G 1438G 3565G 4769G 6253C 8860G 11143T 11152Y 11410C 14953T 15326G 16366T	H15a1a1	55C 57C 263G 309.1CT 310C 16366T	1	55 57 263 750 1438 3565 4769 6253 8860 11143 11152Y 11410 14953 15326 16366	H15a1a1	55 57 263 16366	[0.000] H15a1a1 (1)	[0.000] H15 H15a H15a1 H15a1a H15b H15b1 H15b2 (8)	1
941 >MF523041.1 Hc	55C 57C 263G 309.1CT 310C 750G 1438G 3565G 4769G 6253C 8860G 11143T 11152Y 11410C 14953T 15326G 16366T	H15a1a1	55C 57C 263G 309.1CT 310C 16366T	1	55 57 263 750 1438 3565 4769 6253 8860 11143 11152Y 11410 14953 15326 16366	H15a1a1	55 57 263 16366	[0.000] H15a1a1 (1)	[0.000] H15 H15a H15a1 H15a1a H15b H15b1 H15b2 (8)	1
942 >MF523038.1 Hc	55C 57C 263G 309.1CT 310C 750G 1438G 3565G 4769G 6253C 8860G 11143T 11152C 11410C 14953T 15326G 16366T	H15a1a1	55C 57C 263G 309.1CT 310C 16366T	1	55 57 263 750 1438 3565 4769 6253 8860 11143 11152 11410 14953 15326 16366	H15a1a1	55 57 263 16366	[0.000] H15a1a1 (1)	[0.000] H15 H15a H15a1 H15a1a H15b H15b1 H15b2 (8)	1
943 >MF523034.1 Hc	73G 152C 248d 263G 310.1C 489C 750G 1438G 2706G 3552A 4715G 4742C 4769G 6026A 7028T 7196A 8584A 8602C 8701G 8860G 9102A 9540C 9545G 9966A 10398G 10400T 10873C 11176A 11719A 11914A 11969A 12705T 13263G 14318C 14384A 14766T 14783C 15043A 15204C 15301A 15326G 15487T 16223T 16297C 16298C 16301T 16327T 16519C	C4+152	73G 152C 248d 263G 310.1C 489C 16223T 16297C 16298C 16301T 16327T 16519C	1	73 152 249d 263 489 750 1438 2706 3552A 4715 4742 4769 6026 7028 7196A 8584 8602 8701 8860 9102A 9540 9545 9966 10398 10400 10873 11176 11719 11914 11969 12705 13263 14318 14384 14766 14783 15043 15204 15301 15326 15487T 16223 16297 16298 16301 16327	C4+152	73 152 249d 263 489 16223 16297 16298 16301 16327	[0.076] C4+152 (1)	[0.102] C4+152+16093 (2) [0.020] C4a1a1 (3) [0.019] C7 (4) [0.014] C4a1a C4a1a5 C4a1a6 (7) [0.009] C7a (8) [0.005] C4d (9) [0.004] C4b (10) [0.003] C4a1 (11) [0.000] C C4 C4a'b'c C4a C4a1a1a C4b5 C4b6 C4b7 C4b8 C4c C4c1 C4e (23)	1

944 >MF523030.1 Hc	73G 217C 228A 263G 309.1CT 310C 340T 508G 750G 1438G 1811G 2706G 3720G 4769G 5390G 5426C 6045T 6152C 7028T 8860G 10876G 11467G 11719A 12308G 12372A 12396C 13020C 13734C 14766T 15326G 15907G 16051G 16129C 16183C 16189.1C 16235G 16362C 16519C	U2e1h	73G 217C 228A 263G 309.1CT 310C 340T 508G 16051G 16129C 16183C 16189.1C 16235G 16362C 16519C	1	73 217 228 263 340 508 750 1438 1811 2706 3720 4769 5390 5426 6045 6152 7028 8860 10876 11467 11719 12308 12372 12396 13020 13734 14766 15326 15907 16051 16129C 16235 16362	U2e1h	73 217 228 263 340 508 16051 16129C 16235 16362	[0.437] U2e1h (1)	[0.009] U2e1a1 (2) [0.000] U2e1 U2e1a U2e1a1a U2e1a1c U2e1c U2e1c1 U2e1d U2e1g (10)	1
945 >MF523027.1 Hc	47A 73G 207A 248d 263G 309.1CT 310C 489C 750G 1438G 2226.1A 2706G 3394C 3552A 4715G 4769G 6026A 6929G 7028T 7196A 8584A 8701G 8860G 9540C 9545G 10398G 10400T 10873C 11719A 11914A 11969A 12672G 12705T 13263G 13967T 14318C 14766T 14783C 15043A 15204C 15301A 15326G 15487T 16223T 16239T 16298C 16327T 16357C 16519C	C4a2c1	47A 73G 207A 248d 263G 309.1CT 310C 489C 16223T 16239T 16298C 16327T 16357C 16519C	1	47 73 207 249d 263 489 750 1438 2232.1A 2706 3394 3552A 4715 4769 6026 6929 7028 7196A 8584 8701 8860 9540 9545 10398 10400 10873 11719 11914 11969 12672 12705 13263 13967 14318 14766 14783 15043 15204 15301 15326 15487T 16223 16239 16298 16327 16357	C4a2c1	47 73 207 249d 263 489 16223 16239 16298 16327 16357	[0.000] C4a2c1 (1)	[0.036] C4a2c (2)	1
946 >MF523019.1 Hc	55C 56T 57C 263G 309.1CT 310C 750G 1438G 4769G 6253C 8860G 11143T 11410C 14364A 14953T 15217A 15326G 16366T	H15a1a1	55C 56T 57C 263G 309.1CT 310C 16366T	1	55 56T 57 263 750 1438 4769 6253 8860 11143 11410 14364 14953 15217 15326 16366	H15a1a1	55 56T 57 263 16366	[0.024] H15a1a1 (1)	[0.029] H15 (2) [0.027] H15b2 (3) [0.016] H15a1 (4) [0.000] H15a H15a1a H15b H15b1 (8)	1
947 >MF523015.1 Hc	73G 152C 247A 263G 309.1CT 310C 750G 1438G 2706G 3311T 4615G 4769G 5894.1C 7028T 8843C 8860G 12681C 13708A 13768C 14016A 15326G 16217C 16519C	HV2	73G 152C 247A 263G 309.1CT 310C 16217C 16519C	1	73 152 247 263 750 1438 2706 3311 4615 4769 5899.1C 7028 8843 8860 12681 13708 13768 14016 15326 16217	HV2	73 152 247 263 16217	[0.013] HV2 (1)	[0.137] HV2a2 (2) [0.000] HV2a HV2a3 H32 R30a1 R30a1a (7)	1
948 >MF523010.1 Hc	73G 152C 185A 263G 309.1CT 310C 489C 709A 750G 1438G 2706G 3882A 4769G 4833G 5108C 5601T 7028T 7600A 7805A 8701G 8860G 9377G 9540C 9575A 10398G 10400T 10873C 11719A 12705T 13563G 14155T 14569A 14766T 14783C 15043A 15301A 15326G 16223T 16227G 16262T 16278T 16294T 16356C 16362C	G2a3	73G 152C 185A 263G 309.1CT 310C 489C 16223T 16227G 16262T 16278T 16294T 16356C 16362C	1	73 152 185 263 489 709 750 1438 2706 3882 4769 4833 5108 5601 7028 7600 7805 8701 8860 9377 9540 9575 10398 10400 10873 11719 12705 13563 14155 14569 14766 14783 15043 15301 15326 16223 16227 16262 16278 16294 16356 16362	G2a3	73 152 185 263 489 16223 16227 16262 16278 16294 16356 16362	[0.500] G2a3 (1)	[0.023] G2a+152 (2) [0.000] G2a2 G2a3a (4)	1
949 >MF523004.1 Hc	73G 150T 159C 199C 263G 309.1CT 310C 489C 750G 1438G 2706G 4048A 4071T 4164G 4769G 5351G 5460A 6455T 6680C 7028T 7684C 7853A 8701G 8860G 9540C 9824C 10159T 10398G 10400T 10873C 11719A 11944C 12405T 12705T 12811C 14766T 14783C 15043A 15301A 15326G 15479C 15498R 16129A 16192T 16223T 16297C	M7b1a1c	73G 150T 159C 199C 263G 309.1CT 310C 489C 16129A 16192T 16223T 16297C	1	73 150 159 199 263 489 750 1438 2706 4048 4071 4164 4769 5351 5460 6455 6680 7028 7684 7853 8701 8860 9540 9824 10159 10398 10400 10873 11719 11944 12405 12705 12811 14766 14783 15043 15301 15326 15479 15498R 16129 16192 16223 16297	M7b1a1c	73 150 159 199 263 489 16129 16192 16223 16297	[0.033] M7b1a1c (1) [0.000] M7b1a1+(16192) M7b1a1d M7b1a1d1 M7b1a1e M7b1a1e1 M7b1a1f M7b1a1g M7b1a1h (9)	[0.232] M7b1a1 (10) [0.033] M7b1a1c (11) [0.007] M7b1a1e2 (12) [0.000] M7b1a1+(16192) M7b1a1d M7b1a1d1 M7b1a1e M7b1a1e1 M7b1a1f M7b1a1g M7b1a1h M7b1a1i (21)	1

950	>MF522996.1 Hc	73G 263G 309.1CT 310C 489C 709A 750G 1438G 2706G 4769G 4833G 5108C 7028T 8680T 8701G 8860G 9540C 10398G 10400T 10492Y 10873C 11050C 11719A 12408C 12705T 14218C 14569A 14766T 14783C 15043A 15301A 15326G 15746G 16156A 16189C 16223T 16274A 16299G 16362C 16390A	G3a3	73G 263G 309.1CT 310C 489C 16156A 16189C 16223T 16274A 16299G 16362C 16390A	1	73 263 489 709 750 1438 2706 4769 4833 5108 7028 8680 8701 8860 9540 10398 10400 10492Y 10873 11050 11719 12408 12705 14218 14569 14766 14783 15043 15301 15326 15746 16156 16189 16223 16274 16299 16362 16390	G3a3	73 263 489 16156 16189 16223 16274 16299 16362 16390	[0.100] G3a3 (1) [0.000] D6a1 D6a1a (3)	[0.014] D6a (4) [0.000] E E1 E1a E1a1 E1a1a E1a1c G3 G3a G3b G3b2 D4b2a2a D4b2a2a2 D4e5 D+16189 (18)	1
951	>MF522985.1 Hc	239C 263G 309.1CT 310C 750G 1438G 4769G 8860G 14040A 15326G 16145A 16300G 16311C 16362C 16482G	H6b	239C 263G 309.1CT 310C 16145A 16300G 16311C 16362C 16482G	1	239 263 750 1438 4769 8860 14040 15326 16145 16300 16311 16362 16482	H6b	239 263 16145 16300 16311 16362 16482	[0.066] H6b (1) [0.000] H6a1a1a (2)	[0.066] H6b (3) [0.062] H6a1a3 (4) [0.020] H6a1a (5) [0.011] H6a1b2 (6) [0.000] H6 H6a H6a1 H6a1a1 H6a1a1a H6a1a2 H6a1a2b H6a1a2b1 H6a1a3a H6a1a4 H6a1a5 H6a1a8a H6a1a9 H6a1a10 H6a1b H6a1b2a H6a1b2b H6a1b2c H6a1b2e H6a2 H6a2a H6b2 H6c (29)	1
952	>MF522981.1 Hc	239C 263G 309.1CT 310C 750G 1438G 4769G 6467T 8860G 14040A 15326G 16129A 16300G 16325C 16362C	H6b2	239C 263G 309.1CT 310C 16129A 16300G 16325C 16362C	1	239 263 750 1438 4769 6467 8860 14040 15326 16129 16300 16325 16362	H6b2	239 263 16129 16300 16325 16362	[0.020] H6b2 (1)	[0.020] H6b2 (2) [0.000] H6b	1
953	>MF522971.1 Hc	73G 200G 263G 309.1CT 310C 709A 750G 1438G 1888A 2706G 3204T 3834A 4216C 4769G 4917G 4973C 7028T 8697A 8860G 10463C 11251G 11719A 11812G 13368A 13708A 14233G 14766T 14798C 14839G 14905A 15326G 15452A 15607G 15928A 16051G 16126C 16234T 16294T 16296T 16519C	T2g1b	73G 200G 263G 309.1CT 310C 16051G 16126C 16234T 16294T 16296T 16519C	1	73 200 263 709 750 1438 1888 2706 3204 3834 4216 4769 4917 4973 7028 8697 8860 10463 11251 11719 11812 13368 13708 14233 14766 14798 14839 14905 15326 15452A 15607 15928 16051 16126 16234 16294 16296	T2g1b	73 200 263 16051 16126 16234 16294 16296	[0.014] T2g1b (1)	[0.014] T2g1b (2) [0.000] T2g1 T2g1a (4)	1
954	>MF522963.1 Hc	73G 152C 263G 309.1CT 310C 750G 1438G 2706G 3311T 4615G 4769G 5894.1CC 7028T 8270.1ACCCCCTCT 8843C 8860G 12681C 13708A 13768C 15326G 16217C 16519C	HV2	73G 152C 263G 309.1CT 310C 16217C 16519C	1	73 152 263 750 1438 2706 3311 4615 4769 5899.2C 7028 8289.1CCCCCTCTA 8843 8860 12681 13708 13768 15326 16217	HV2	73 152 263 16217	[0.000] HV2 (1)	[0.137] HV2a2 (2) [0.000] HV2a HV2a3 H32 R30a1 R30a1a (7)	1
955	>MF522959.1 Hc	73G 153G 195C 225A 263G 309.1CT 310C 750G 1438G 1719A 2403A 2706G 4769G 6221C 6371T 7028T 8860G 8966C 9716C 11719A 12705T 13966G 14470C 14766T 15326G 16183C 16189C 16223T 16248T 16278T 16302G 16519C	X2i	73G 153G 195C 225A 263G 309.1CT 310C 16183C 16189C 16223T 16248T 16278T 16302G 16519C	1	73 153 195 225 263 750 1438 1719 2403 2706 4769 6221 6371 7028 8860 8966 9716 11719 12705 13966 14470 14766 15326 16189 16223 16248 16278 16302	X2i	73 153 195 225 263 16189 16223 16248 16278 16302	[0.062] X2i (1)	[0.000] X2+225 X2a'j X2b'd X2b X2b1 X2e X2i+@225 X2o (9)	1

956	>MF522956.1 Hc	73G 195C 248d 263G 309.1CT 310C 489C 750G 1438G 1715T 2226.1A 2706G 3552A 4715G 4769G 6026A 7028T 7196A 7999C 8584A 8639C 8701G 8860G 9540C 9545G 10398G 10400T 10873C 11719A 11914A 11969A 12672G 12705T 12940A 13263G 14318C 14757Y 14766T 14783C 15043A 15204C 15301A 15326G 15487T 15968C 16129A 16150T 16223T 16298C 16327T 16519C	C4a1a4a	73G 195C 248d 263G 309.1CT 310C 489C 16129A 16150T 16223T 16298C 16327T 16519C	1	73 195 249d 263 489 750 1438 1715 2232.1A 2706 3552A 4715 4769 6026 7028 7196A 7999 8584 8639 8701 8860 9540 9545 10398 10400 10873 11719 11914 11969 12672 12705 12940 13263 14318 14757Y 14766 14783 15043 15204 15301 15326 15487T 15968 16129 16150 16223 16298 16327	C4a1a4a	73 195 249d 263 489 16129 16150 16223 16298 16327	[0.000] C4a1a4a (1)	[0.000] C4a1a+195 C4a1a2 C4a1a2a C4a1a3 C4a1a3a C4a1a3a1 C4a1a3b C4a1a3c C4a1a4 C4a1a4a (11)	1
957	>MF522954.1 Hc	73G 195C 248d 263G 309.1CT 310C 489C 750G 1438G 1715T 2226.1A 2706G 3552A 4715G 4769G 6026A 7028T 7196A 7999C 8584A 8639C 8701G 8860G 9540C 9545G 10398G 10400T 10873C 11719A 11914A 11969A 12672G 12705T 12940A 13263G 14318C 14757Y 14766T 14783C 15043A 15204C 15301A 15326G 15487T 15968C 16129A 16150T 16223T 16298C 16327T 16519C	C4a1a4a	73G 195C 248d 263G 309.1CT 310C 489C 16129A 16150T 16223T 16298C 16327T 16519C	1	73 195 249d 263 489 750 1438 1715 2232.1A 2706 3552A 4715 4769 6026 7028 7196A 7999 8584 8639 8701 8860 9540 9545 10398 10400 10873 11719 11914 11969 12672 12705 12940 13263 14318 14757Y 14766 14783 15043 15204 15301 15326 15487T 15968 16129 16150 16223 16298 16327	C4a1a4a	73 195 249d 263 489 16129 16150 16223 16298 16327	[0.000] C4a1a4a (1)	[0.000] C4a1a+195 C4a1a2 C4a1a2a C4a1a3 C4a1a3a C4a1a3a1 C4a1a3b C4a1a3c C4a1a4 C4a1a4a (11)	1
958	>MF522953.1 Hc	73G 146C 263G 309.1CT 310C 485C 489C 750G 1438G 2706G 3010A 4655A 4769G 4883T 5075C 5178A 7028T 8414T 8701G 8860G 9540C 10398G 10400T 10873C 11696A 11719A 12705T 14668T 14766T 14783C 15043A 15301A 15326G 16223T 16362C	D4j13	73G 146C 263G 309.1CT 310C 485C 489C 16223T 16362C	1	73 146 263 485 489 750 1438 2706 3010 4655 4769 4883 5075 5178A 7028 8414 8701 8860 9540 10398 10400 10873 11696 11719 12705 14668 14766 14783 15043 15301 15326 16223 16362	D4j13	73 146 263 485 489 16223 16362	[0.000] D4j13 (1)	[0.000] D4b2b1+146 D4b2b1d D4j+146 D4j6 (5)	1
959	>MF522951.1 Hc	47A 73G 248d 263G 309.1CT 310C 489C 750G 951A 1438G 2226.1A 2706G 3394C 3552A 4715G 4769G 6026A 6665T 7028T 7196A 8443G 8584A 8701G 8860G 9540C 9545G 10398G 10400T 10873C 11654G 11719A 11914A 11969A 12672G 12705T 13263G 13967T 14318C 14766T 14783C 15043A 15204C 15301A 15326G 15487T 16223T 16298C 16327T 16357C 16519C	C4a2c	47A 73G 248d 263G 309.1CT 310C 489C 16223T 16298C 16327T 16357C 16519C	1	47 73 249d 263 489 750 951 1438 2232.1A 2706 3394 3552A 4715 4769 6026 6665 7028 7196A 8443 8584 8701 8860 9540 9545 10398 10400 10873 11654 11719 11914 11969 12672 12705 13263 13967 14318 14766 14783 15043 15204 15301 15326 15487T 16223 16298 16327 16357	C4a2c	47 73 249d 263 489 16223 16298 16327 16357	[0.000] C4a2c (1)	[0.000] C4a2 C4a2b C4a2c2 C4a2c2a (5)	1
960	>MF522938.1 Hc	73G 146C 263G 309.1CT 310C 485C 489C 750G 1438G 2706G 3010A 4655A 4769G 4883T 5075C 5178A 7028T 8414T 8701G 8860G 9540C 10398G 10400T 10873C 11696A 11719A 12705T 14668T 14766T 14783C 15043A 15301A 15326G 16223T 16362C	D4j13	73G 146C 263G 309.1CT 310C 485C 489C 16223T 16362C	1	73 146 263 485 489 750 1438 2706 3010 4655 4769 4883 5075 5178A 7028 8414 8701 8860 9540 10398 10400 10873 11696 11719 12705 14668 14766 14783 15043 15301 15326 16223 16362	D4j13	73 146 263 485 489 16223 16362	[0.000] D4j13 (1)	[0.000] D4b2b1+146 D4b2b1d D4j+146 D4j6 (5)	1

961 >MF522928.1 Hc	73G 151T 152C 200G 235G 263G 309.1CT 310C 514d 515d 663G 735G 750G 1438G 1736G 2706G 4248C 4769G 4824G 7028T 8334A 8794T 8860G 11719A 12004G 12705T 12855G 12990G 14766T 15326G 16093C 16223T 16290T 16292T 16309R 16319A 16362C	A14	73G 151T 152C 200G 235G 263G 309.1CT 310C 514d 515d 16093C 16223T 16290T 16292T 16309R 16319A 16362C	1	73 151 152 200 235 263 663 735 750 1438 1736 2706 4248 4769 4824 7028 8334 8794 8860 11719 12004 12705 12855 12990 14766 15326 16093 16223 16290 16292 16309R 16319 16362	A14	73 151 152 200 235 263 16093 16223 16290 16292 16309R 16319 16362	[0.026] A14 (1)	[0.025] A13 (2) [0.009] A+152+16362+200	1
962 >MF522924.1 Hc	73G 263G 309.1CT 310C 489C 750G 1438G 2706G 3010A 4769G 4883T 5021C 5178A 7028T 8414T 8701G 8860G 9540C 10398G 10400T 10873C 11215T 11719A 12705T 14384A 14668T 14766T 14783C 15043A 15106A 15184C 15301A 15326G 16223T 16274A 16291T 16362C	D4e5b	73G 263G 309.1CT 310C 489C 16223T 16274A 16291T 16362C	1	73 263 489 750 1438 2706 3010 4769 4883 5021 5178A 7028 8414 8701 8860 9540 10398 10400 10873 11215 11719 12705 14384 14668 14766 14783 15043 15106 15184 15301 15326 16223 16274 16291 16362	D4e5b	73 263 489 16223 16274 16291 16362	[0.000] D4e5b (1)	[0.000] G3 G3a G3b G3b2 D4e5 (6)	1
963 >MF522922.1 Hc	73G 217C 228A 263G 309.1CT 310C 340T 508G 750G 1438G 1811G 2706G 3720G 4769G 5390G 5426C 6045T 6152C 7028T 8860G 10876G 11467G 11719A 12308G 12372A 13020C 13734C 14766T 15326G 15907G 16051G 16129C 16183C 16362C 16519C	U2e1h	73G 217C 228A 263G 309.1CT 310C 340T 508G 16051G 16129C 16183C 16362C 16519C	1	73 217 228 263 340 508 750 1438 1811 2706 3720 4769 5390 5426 6045 6152 7028 8860 10876 11467 11719 12308 12372 13020 13734 14766 15326 15907 16051 16129C 16362	U2e1h	73 217 228 263 340 508 16051 16129C 16362	[0.338] U2e1h (1)	[0.009] U2e1a1 (2) [0.000] U2e1 U2e1a U2e1a1a U2e1a1c U2e1c U2e1c1 U2e1d U2e1g (10)	1
964 >MF522919.1 Hc	73G 263G 309.1CT 310C 709A 750G 1119C 1438G 2706G 3497T 4769G 7028T 8271d 8272d 8273d 8274d 8275d 8276d 8277d 8278d 8279d 8860G 10310A 11719A 12373G 14766T 15326G 15346A 16092C 16182C 16183C 16189C 16217C 16519C 16527T	B4c1a2a	73G 263G 309.1CT 310C 16092C 16182C 16183C 16189C 16217C 16519C 16527T	1	73 263 709 750 1119 1438 2706 3497 4769 7028 8281-8289d 8860 10310 11719 12373 14766 15326 15346 16092 16189 16217 16527	B4c1a2a	73 263 16092 16189 16217 16527	[0.000] B4c1a2a (1)	[0.000] B4c1a2 (2)	1
965 >MF522918.1 Hc	73G 263G 309.1CT 310C 709A 750G 1119C 1438G 2706G 3497T 4769G 7028T 8271d 8272d 8273d 8274d 8275d 8276d 8277d 8278d 8279d 8860G 10310A 11719A 12373G 14766T 15326G 15346A 16092C 16182C 16183C 16189C 16217C 16519C 16527T	B4c1a2a	73G 263G 309.1CT 310C 16092C 16182C 16183C 16189C 16217C 16519C 16527T	1	73 263 709 750 1119 1438 2706 3497 4769 7028 8281-8289d 8860 10310 11719 12373 14766 15326 15346 16092 16189 16217 16527	B4c1a2a	73 263 16092 16189 16217 16527	[0.000] B4c1a2a (1)	[0.000] B4c1a2 (2)	1
966 >MF522916.1 Hc	73G 150T 199C 204C 239C 263G 309.1CT 310C 489C 750G 1438G 2706G 3483A 4048A 4071T 4164G 4769G 5351G 5460A 6455T 6680C 7028T 7684C 7853A 8701G 8860G 9540C 9824C 10398G 10400T 10873C 11719A 12405T 12705T 12811C 14766T 14783C 15043A 15301A 15326G 16223T 16256T 16272G 16297C	M7b1a1b	73G 150T 199C 204C 239C 263G 309.1CT 310C 489C 16223T 16256T 16272G 16297C	1	73 150 199 204 239 263 489 750 1438 2706 3483 4048 4071 4164 4769 5351 5460 6455 6680 7028 7684 7853 8701 8860 9540 9824 10398 10400 10873 11719 12405 12705 12811 14766 14783 15043 15301 15326 16223 16256 16272 16297	M7b1a1b	73 150 199 204 239 263 489 16223 16256 16272 16297	[0.021] M7b1a1b (1)	[0.046] M7b1a1+(16192) (2) [0.042] M7b1a1 (3) [0.033] M7b1a1a3 (4) [0.018] M7b1a1e1 (5) [0.010] M7b1a1f (6) [0.008] M7b1a1h (7) [0.006] M7b1a1g (8) [0.004] M7b1a1d M7b1a1d1 M7b1a1e (11)	1

967 >MF522897.1 Hc	73G 146C 234G 263G 309.1CT 310C 750G 1438G 1811G 1888A 4129G 4769G 5105C 5186T 5237A 7028T 8110C 8860G 9094T 11467G 11719A 12106T 12308G 12372A 12507G 13194A 14766T 15049T 15283C 15326G 15940d 16051G 16086C 16129A 16353T 16519C	U2b	73G 146C 234G 263G 309.1CT 310C 16051G 16086C 16129A 16353T 16519C	1	73 146 234 263 750 1438 1811 1888 4129 4769 5105 5186T 5237 7028 8110 8860 9094 11467 11719 12106 12308 12372 12507 13194 14766 15049 15283 15326 15944d 16051 16086 16129 16353	U2b	73 146 234 263 16051 16086 16129 16353	[0.864] U2b (1) [0.100] U2b1 (2) [0.000] U2b1a (3)	[0.100] U2b1 (4) [0.069] U2 (5) [0.000] H3b6 H3b6a U2b1a (8)	1
968 >MF522880.1 Hc	73G 189G 195C 248d 263G 309.1CT 310C 514d 515d 750G 1438G 2706G 3970T 4769G 5587C 6392C 6962A 7028T 8860G 9078C 10310A 10609C 10653A 11326T 11719A 12406A 12882T 13928C 14218C 14766T 15024A 15326G 16182C 16183C 16189C 16234T 16242T 16304C 16311C 16519C	F1e2	73G 189G 195C 248d 263G 309.1CT 310C 514d 515d 16182C 16183C 16189C 16234T 16242T 16304C 16311C 16519C	1	73 189 195 249d 263 750 1438 2706 3970 4769 5587 6392 6962 7028 8860 9078 10310 10609 10653 11326 11719 12406 12882 13928C 14218 14766 15024 15326 16189 16234 16242 16304 16311	F1e2	73 189 195 249d 263 16189 16234 16242 16304 16311	[0.571] F1e2 (1)	[0.000] U8b1a2+16311 (2)	1
969 >MF522877.1 Hc	73G 195A 263G 309.1CT 310C 489C 514d 515d 750G 1438G 2706G 4394T 4491A 4769G 7028T 8701G 8860G 9540C 10398G 10400T 10873C 11719A 12007A 12451G 12705T 14766T 14783C 15043A 15301A 15326G 15431A 16093C 16223T 16519C	M30	73G 195A 263G 309.1CT 310C 489C 514d 515d 16093C 16223T 16519C	1	73 195A 263 489 750 1438 2706 4394 4491 4769 7028 8701 8860 9540 10398 10400 10873 11719 12007 12451 12705 14766 14783 15043 15301 15326 15431 16093 16223	M30	73 195A 263 489 16093 16223	[0.018] M30 (1) [0.004] M30d1 M30d2 (3) [0.000] M30d G1b (5)	[0.031] M30+16234 (6) [0.019] M21b (7) [0.012] M1'20'51 (8) [0.008] M (9) [0.007] G1b+16129 (10) [0.006] G1b2 G1b4 (12) [0.005] M22b (13) [0.004] M30d1 M30d2 (15) [0.003] M21b1a M24'41 (17) [0.000] M4"67 M30a M30a1 M30a2 M30c M30d M30f M30g M37 M43 M43b M7 M7b'c M7b M12'G G1b G1b1 M19'53 M21 M21b1 M22 M23'75 M27 M29'Q M31 M31a M31b'c M32'56 M33 M33a M33a1 M34'57 M35 M39'70 M42'74 M42 M52 M52b M55'77 M58 M60 M69 M80'D (60)	1
970 >MF522875.1 Hc	73G 204C 207A 248d 263G 309.1CT 310C 709A 750G 1438G 2706G 3434G 3970T 4769G 5585A 5894G 5913A 5978G 6392C 7028T 8860G 9854C 10310A 10320A 10499G 11065G 11719A 12621T 13928C 14766T 14971C 15326G 15943C 16260T 16298C 16355T 16362C	F3a1	73G 204C 207A 248d 263G 309.1CT 310C 16260T 16298C 16355T 16362C	1	73 204 207 249d 263 709 750 1438 2706 3434 3970 4769 5585 5894 5913 5978 6392 7028 8860 9854 10310 10320 10499 11065 11719 12621 13928C 14766 14971 15326 15943 16260 16298 16355 16362	F3a1	73 204 207 249d 263 16260 16298 16355 16362	[0.120] F3a1 (1)	[0.039] F3a+207 (2)	1
971 >MF522860.1 Hc	263G 309.1CT 310C 711C 750G 1438G 2706G 3360G 4769G 6755A 7028T 8860G 15326G 16172C 16311C	HV6	263G 309.1CT 310C 16172C 16311C	1	263 711 750 1438 2706 3360 4769 6755 7028 8860 15326 16172 16311	HV6	263 16172 16311	[0.000] HV6 (1)	[0.010] HV+16311 (2) [0.003] H3h3b (3) [0.002] H1+16311 (4) [0.000] HV6a HV9 HV10 HV11 HV11a HV16 HV23 HV24 H1e1a4 H3+16311 H3h H3h1 H3h2 H3h3 H3h3a H3h5 H3h6 H3m H13a1a2+16311 H13a2b1 H66a H76 H80 (27)	1

972 >MF522855.1 Hc	73G 204C 207A 248d 263G 309.1CT 310C 709A 750G 1438G 2706G 3434G 3970T 4769G 5585A 5894G 5913A 5978G 6392C 7028T 8860G 9854C 10310A 10320A 10499G 11065G 11719A 12621T 13928C 14766T 14971C 15326G 15943C 16260T 16298C 16355T 16362C	F3a1	73G 204C 207A 248d 263G 309.1CT 310C 16260T 16298C 16355T 16362C	1	73 204 207 249d 263 709 750 1438 2706 3434 3970 4769 5585 5894 5913 5978 6392 7028 8860 9854 10310 10320 10499 11065 11719 12621 13928C 14766 14971 15326 15943 16260 16298 16355 16362	F3a1	73 204 207 249d 263 16260 16298 16355 16362	[0.120] F3a1 (1)	[0.039] F3a+207 (2)	1
973 >MF522854.1 Hc	73G 195C 263G 309.1CT 310C 489C 750G 1438G 2706G 3010A 3421A 4769G 4883T 5178A 6704G 7028T 8414T 8701G 8860G 9540C 10398G 10400T 10873C 11719A 12705T 14668T 14766T 14783C 15043A 15301A 15326G 16171T 16223T 16355A 16362C	D4n2	73G 195C 263G 309.1CT 310C 489C 16171T 16223T 16355A 16362C	1	73 195 263 489 750 1438 2706 3010 3421 4769 4883 5178A 6704 7028 8414 8701 8860 9540 10398 10400 10873 11719 12705 14668 14766 14783 15043 15301 15326 16171T 16223 16355A 16362	D4n2	73 195 263 489 16171T 16223 16355A 16362	[0.213] D4n2 (1)	[0.000] D4+195 D4n D4n1 D4n1a (5)	1
974 >MF522852.1 Hc	73G 195A 263G 309.1CT 310C 489C 514d 515d 750G 1438G 2706G 4394T 4491A 4769G 7028T 8701G 8860G 9540C 10398G 10400T 10873C 11719A 12007A 12451G 12705T 14766T 14783C 15043A 15301A 15326G 15431A 16093C 16223T 16519C	M30	73G 195A 263G 309.1CT 310C 489C 514d 515d 16093C 16223T 16519C	1	73 195A 263 489 750 1438 2706 4394 4491 4769 7028 8701 8860 9540 10398 10400 10873 11719 12007 12451 12705 14766 14783 15043 15301 15326 15431 16093 16223	M30	73 195A 263 489 16093 16223	[0.018] M30 (1) [0.004] M30d1 M30d2 (3) [0.000] M30d G1b (5)	[0.031] M30+16234 (6) [0.019] M21b (7) [0.012] M1'20'51 (8) [0.008] M (9) [0.007] G1b+16129 (10) [0.006] G1b2 G1b4 (12) [0.005] M22b (13) [0.004] M30d1 M30d2 (15) [0.003] M21b1a M24'41 (17) [0.000] M4"67 M30a M30a1 M30a2 M30c M30d M30f M30g M37 M43 M43b M7 M7b'c M7b M12'G G1b G1b1 M19'53 M21 M21b1 M22 M23'75 M27 M29'Q M31 M31a M31b'c M32'56 M33 M33a M33a1 M34'57 M35 M39'70 M42'74 M42 M52 M52b M55'77 M58 M60 M69 M80'D (60)	1

975	>MF522851.1 H[illegible]	262T 263G 309.1CT 310C 750G 1438G 3208T 4769G 5460A 6392C 8860G 10124C 12630A 15326G 15617A	H41a	262T 263G 309.1CT 310C	1	262 263 750 1438 3208 4769 5460 6392 8860 10124 12630 15326 15617	H41a	262 263	[0.000] H41a (1)	[0.000] R0 HV HV4 HV4c HV5 HV5a HV9c HV12 H H1 H1e H1e1 H1e1a H1e1a2 H1e1a7 H1e1c H1e2 H1e2a H1e2b H1e2d H1e4 H1e6 H1e7 H1e8 H1e8a H1h H1h1 H1h2 H1j H1j3 H1j5 H1j6 H1j7 H1j9 H1m H1n H1p H1q H1q1 H1q2 H1r H1s H1s1 H1t H1t1 H1t1a H1t2 H1u H1u1 H1v H1v1 H1v1a H1v1b H1w H1ae H1ae1 H1ae2 H1ae2a H1ae3 H1ae3a H1ag H1ag1 H1ag1b H1ah H1ai H1ai1 H1aj H1ak H1ak1 H1ak2 H1am H1am1 H1ap H1aq H1as H1as2 H1at H1at1 H1at1a H1av H1ax H1ax1 H1ay H1az H1bd H1be H1bi H1bj H1bk H1bm H1bn H1bp H1bq H1br H1bu H1bv H1bw H1bx H1ca H1ci H2 H2a H2a2 H2a2a H2a2+(16235) H2a4 H2a5 H2a5a H2a5b H3 H3b H3c H3c1 H3e H3q H3q1 H3r H3r1 H3t H3u H3y H3aa H3ab H3ac H3ad H3ae H3ag H3ag1 H3ah H3ai H3aj H3ap H3aq H3ar H3as H3at H3at1 H3au H4 H4a H4a1 H4a1a H4a1a1 H4a1a2 H4a1a2a H4a1a2a1 H4a1a5 H4a1c H4a1c1 H4a1c1a H4a1d H4b H4c H7 H7a H7b H7b1 H7b2 H7b2a H7b3 H7b4 H7b5 H7b6 H7c H7c2 H7c3 H7c5 [illegible]	1
976	>MG015877.1 H[illegible]	73G 199C 204C 250C 263G 310.1C 567.1CCCC 750G 1438G 1719A 2706G 3106d 3107C 3528T 4025T 4529T 4769G 7028T 8251A 8519A 8860G 10034C 10238C 10398G 10819G 11050C 11253C 11719A 12501A 12705T 13780G 14766T 15043A 15326G 15924G 16129A 16223T 16304C 16391A 16519C	I4a1	73G 199C 204C 250C 263G 310.1C 567.1CCCC 16129A 16223T 16304C 16391A 16519C	1	73 199 204 250 263 573.4C 750 1438 1719 2706 3528 4025 4529T 4769 7028 8251 8519 8860 10034 10238 10398 10819 11050 11253 11719 12501 12705 13780 14766 15043 15326 15924 16129 16223 16304 16391	I4a1	73 199 204 250 263 573.4C 16129 16223 16304 16391	[0.000] I4a1 (1)	[0.000] I I4 I4a I4a2 I4b I5 I5b I6 I6a (10)	1
977	>MG011475.1 H[illegible]	73G 150T 152C 263G 310.1C 750G 1438G 1721T 2706G 3106d 3107C 3197C 4732G 4769G 5918C 7028T 7768G 8860G 9477A 11467G 11719A 12308G 12372A 13617C 13637G 14182C 14323A 14766T 15326G 16189C 16325C 16362C	U5b2a1b	73G 150T 152C 263G 310.1C 16189C 16325C 16362C	1	73 150 152 263 750 1438 1721 2706 3197 4732 4769 5918 7028 7768 8860 9477 11467 11719 12308 12372 13617 13637 14182 14323 14766 15326 16189 16325 16362	U5b2a1b	73 150 152 263 16189 16325 16362	[0.103] U5b2a1b (1)	[0.025] B6 (2)	1

978 >MF997504.1 Ho	73G 146C 152C 195C 263G 309.1CT 310C 513.1CACACACA 750G 769A 1018A 1438G 2416C 2706G 2789T 3106d 3107C 3594T 3918A 4104G 4769G 5285G 6152C 7028T 7175C 7256T 7274T 7521A 7598A 7771G 8020A 8206A 8701G 8860G 9221G 9540C 10115C 10398G 10873C 11719A 11914A 11944C 12693G 12705T 13376Y 13590A 13650T 13803G 14566G 14766T 15244G 15301A 15326G 15391T 15629C 15784C 16223T 16278T 16290T 16294T 16309G 16368C 16390A 16519C	L2a1a1	73G 146C 152C 195C 263G 309.1CT 310C 513.1CACACACA 16223T 16278T 16290T 16294T 16309G 16368C 16390A 16519C	1	73 146 152 195 263 750 769 1018 1438 2416 2706 2789 3594 3918 4104 4769 5285 6152 7028 7175 7256 7274 7521 7598 7771 8020 8206 8701 8860 9221 9540 10115 10398 10873 11719 11914 11944 12693 12705 13376Y 13590 13650 13803 14566 14766 15244 15301 15326 15391 15629 15784 16223 16278 16290 16294 16309 16368 16390	L2a1a1	73 146 152 195 263 16223 16278 16290 16294 16309 16368 16390	[0.010] L2a1a1 (1)	[0.008] L2a1 (2) [0.003] L2a1b1a (3) [0.000] L2a1a L2a1e1 (5)	1
979 >MG011474.1 Ho	263G 309.1CCT 310C 514d 515d 750G 1438G 3010A 3106d 3107C 3796G 4769G 8860G 13536T 15326G 16129A 16189C 16355T 16356C 16362C 16519C	H1b1b	263G 309.1CCT 310C 514d 515d 16129A 16189C 16355T 16356C 16362C 16519C	1	263 750 1438 3010 3796 4769 8860 13536 15326 16129 16189 16355 16356 16362	H1b1b	263 16129 16189 16355 16356 16362	[0.000] H1b1b (1)	[0.009] H1b1a (2) [0.000] H1b1+16362 H1b1c H1b1h (5)	1
980 >MG015876.1 Ho	73G 146C 153G 195C 225A 226C 263G 309.1CT 310C 750G 1438G 1719A 2706G 3106d 3107C 4769G 6221C 6371T 7028T 8393T 8425G 8860G 11719A 12705T 13708A 13966G 14470C 14766T 15326G 15466A 15927A 16183C 16189C 16223T 16274A 16278T 16355T 16365T 16519C	X2b10a	73G 146C 153G 195C 225A 226C 263G 309.1CT 310C 16183C 16189C 16223T 16274A 16278T 16355T 16365T 16519C	1	73 146 153 195 225 226 263 750 1438 1719 2706 4769 6221 6371 7028 8393 8425 8860 11719 12705 13708 13966 14470 14766 15326 15466 15927 16189 16223 16274 16278 16355 16365	X2b10a	73 146 153 195 225 226 263 16189 16223 16274 16278 16355 16365	[0.334] X2b10a (1)	[0.000] X2b10 (2)	1
981 >MF158630.1 Ho	73G 195C 198T 263G 310.1C 489C 750G 1438G 2706G 3010A 3106d 3107C 4769G 4883T 5004C 5178A 7028T 8414T 8701G 8730G 8860G 9077C 9540C 10398G 10400T 10646A 10873C 11719A 12705T 13812C 14668T 14766T 14783C 15043A 15301A 15326G 16093C 16223T 16232T 16290T 16362C	D4o2a	73G 195C 198T 263G 310.1C 489C 16093C 16223T 16232T 16290T 16362C	1	73 195 198 263 489 750 1438 2706 3010 4769 4883 5004 5178A 7028 8414 8701 8730 8860 9077 9540 10398 10400 10646 10873 11719 12705 13812 14668 14766 14783 15043 15301 15326 16093 16223 16232 16290 16362	D4o2a	73 195 198 263 489 16093 16223 16232 16290 16362	[0.055] D4o2a (1)	[0.000] D4o2 (2)	1
982 >MF926446.1 Ho	73G 146C 263G 309.1CT 310C 514d 515d 750G 1438G 3106d 3107C 3992T 4024G 4769G 5004C 8269A 8860G 9123A 9422G 10044G 14365T 14582G 15244G 15326G 16234T 16287T	H4a1a1a3	73G 146C 263G 309.1CT 310C 514d 515d 16234T 16287T	1	73 146 263 750 1438 3992 4024 4769 5004 8269 8860 9123 9422 10044 14365 14582 15244 15326 16234 16287	H4a1a1a3	73 146 263 16234 16287	[0.077] H4a1a1a3 (1)	[0.076] P9a (2) [0.056] U2b1a (3) [0.045] U2b (4) [0.037] H4a1a1a (5) [0.023] P2 (6) [0.016] H1m1 H1n1 H1n1a H1n3 (10) [0.015] H1n+146 (11) [0.014] R (12) [0.010] P (13) [0.008] R30a1 R30a1a (15) [0.000] HV+73 HV20 H1e2c H1n2 H1n4 H3+73 H3d H4a1a1a1 H4a1a1a1a H4a1a1a1a1 H4a1a1a2 H4a1a1a4 H13a1a2b H26c R2'JT R30 R30a R31 P4 P9 U U2'3'4'7'8'9 U2b1 U8 U8b'c U8b (41)	1

983 >MF774675.1 Ho	73G 146C 263G 309.1CT 310C 497T 513.1CA 750G 1189C 1438G 1811G 2706G 3106d 3107C 3480G 4295G 4769G 6260A 7028T 8860G 9055A 9698C 10398G 10550G 11299C 11467G 11485C 11719A 11840T 12308G 12372A 13401C 13740C 14167T 14766T 14798C 15326G 15884A 16224C 16245T 16311C 16519C	K1a4a1a2b	73G 146C 263G 309.1CT 310C 497T 513.1CA 16224C 16245T 16311C 16519C	1	73 146 263 497 750 1189 1438 1811 2706 3480 4295 4769 6260 7028 8860 9055 9698 10398 10550 11299 11467 11485 11719 11840 12308 12372 13401 13740 14167 14766 14798 15326 15884 16224 16245 16311	K1a4a1a2b	73 146 263 497 16224 16245 16311	[0.000] K1a4a1a2b (1)	[0.038] K1a4a1a2 (2) [0.028] K1a4a1a2a (3) [0.000] K1a4a1a2b K1a4+146 K1a4j (6)	1
984 >KY645936.1 Ho	73G 263G 310.1C 489C 750G 1438G 2092T 2706G 3010A 3106d 3107C 4769G 4883T 5178A 7028T 8414T 8701G 8860G 9540C 10398G 10400T 10873C 11719A 12530G 12705T 14668T 14766T 14783C 15043A 15301A 15326G 16142T 16223T 16325C 16362C 16519C	D1f	73G 263G 310.1C 489C 16142T 16223T 16325C 16362C 16519C	1	73 263 489 750 1438 2092 2706 3010 4769 4883 5178A 7028 8414 8701 8860 9540 10398 10400 10873 11719 12530 12705 14668 14766 14783 15043 15301 15326 16142 16223 16325 16362	D1f	73 263 489 16142 16223 16325 16362	[0.000] D1f (1)	[0.000] D1 D1a D1a2 D1b D1c D1d D1d1 D1f2 D1f+16189 D1f3 D1k D1n (13)	1
985 >KY645933.1 Ho	73G 263G 310.1C 489C 750G 1438G 2092T 2706G 3010A 3106d 3107C 4769G 4883T 5178A 7028T 8414T 8701G 8860G 9540C 10398G 10400T 10873C 11719A 12530G 12705T 14668T 14766T 14783C 15043A 15301A 15326G 16142T 16223T 16325C 16362C 16519C	D1f	73G 263G 310.1C 489C 16142T 16223T 16325C 16362C 16519C	1	73 263 489 750 1438 2092 2706 3010 4769 4883 5178A 7028 8414 8701 8860 9540 10398 10400 10873 11719 12530 12705 14668 14766 14783 15043 15301 15326 16142 16223 16325 16362	D1f	73 263 489 16142 16223 16325 16362	[0.000] D1f (1)	[0.000] D1 D1a D1a2 D1b D1c D1d D1d1 D1f2 D1f+16189 D1f3 D1k D1n (13)	1
986 >KY645930.1 Ho	73G 194T 248d 263G 286d 287d 309.1CT 310C 489C 514d 515d 750G 1438G 1452C 2706G 3106d 3107C 3552A 4715G 4769G 5836G 6272G 7028T 7196A 7609C 7697A 8425G 8584A 8701G 8860G 9540C 9545G 10398G 10400T 10873C 11719A 11914A 12705T 13263G 13770T 14318C 14766T 14783C 15043A 15301A 15326G 15487T 15852C 15884A 16051G 16172C 16223T 16298C 16325C 16327T 16519C	C1d1d	73G 194T 248d 263G 286d 287d 309.1CT 310C 489C 514d 515d 16051G 16172C 16223T 16298C 16325C 16327T 16519C	1	73 194 249d 263 290-291d 489 750 1438 1452 2706 3552A 4715 4769 5836 6272 7028 7196A 7609 7697 8425 8584 8701 8860 9540 9545 10398 10400 10873 11719 11914 12705 13263 13770 14318 14766 14783 15043 15301 15326 15487T 15852 15884 16051 16172 16223 16298 16325 16327	C1d1d	73 194 249d 263 290-291d 489 16051 16172 16223 16298 16325 16327	[0.000] C1d1d (1)	[0.000] C1d+194 C1d1 C1d2 (4)	1
987 >KY645920.1 Ho	73G 263G 310.1C 489C 750G 1438G 2092T 2706G 3010A 3106d 3107C 4769G 4883T 5178A 7028T 8414T 8701G 8860G 9196A 9540C 10398G 10400T 10873C 11719A 12530G 12705T 14668T 14766T 14783C 15043A 15301A 15326G 16142T 16223T 16290T 16325C 16362C 16519C	D1f	73G 263G 310.1C 489C 16142T 16223T 16290T 16325C 16362C 16519C	1	73 263 489 750 1438 2092 2706 3010 4769 4883 5178A 7028 8414 8701 8860 9196 9540 10398 10400 10873 11719 12530 12705 14668 14766 14783 15043 15301 15326 16142 16223 16290 16325 16362	D1f	73 263 489 16142 16223 16290 16325 16362	[0.010] D1f (1)	[0.004] D1a2 (2) [0.000] D1 D1a D1b D1c D1d D1d1 D1f2 D1f+16189 D1f3 D1k D1n (13)	1

988	>KY645913.1 Hc	73G 263G 310.1C 489C 750G 1438G 2092T 2706G 3010A 3106d 3107C 4769G 4883T 5178A 7028T 8414T 8701G 8860G 9196A 9540C 10398G 10400T 10873C 11719A 12530G 12705T 14668T 14766T 14783C 15043A 15301A 15326G 16142T 16223T 16290T 16325C 16362C 16519C	D1f	73G 263G 310.1C 489C 16142T 16223T 16290T 16325C 16362C 16519C	1	73 263 489 750 1438 2092 2706 3010 4769 4883 5178A 7028 8414 8701 8860 9196 9540 10398 10400 10873 11719 12530 12705 14668 14766 14783 15043 15301 15326 16142 16223 16290 16325 16362	D1f	73 263 489 16142 16223 16290 16325 16362	[0.010] D1f (1)	[0.004] D1a2 (2) [0.000] D1 D1a D1b D1c D1d D1d1 D1f2 D1f+16189 D1f3 D1k D1n (13)	1
989	>KY645908.1 Hc	73G 151T 263G 310.1C 489C 750G 1438G 2092T 2706G 3010A 3106d 3107C 4491A 4769G 4883T 5178A 7028T 8414T 8701G 8860G 9540C 10398G 10400T 10873C 11719A 12530G 12705T 14668T 14766T 14783C 15043A 15301A 15326G 16142T 16223T 16325C 16362C 16519C	D1f	73G 151T 263G 310.1C 489C 16142T 16223T 16325C 16362C 16519C	1	73 151 263 489 750 1438 2092 2706 3010 4491 4769 4883 5178A 7028 8414 8701 8860 9540 10398 10400 10873 11719 12530 12705 14668 14766 14783 15043 15301 15326 16142 16223 16325 16362	D1f	73 151 263 489 16142 16223 16325 16362	[0.010] D1f (1)	[0.006] D1f+16189 D1f3 (3) [0.000] D1 D1a D1a2 D1b D1c D1d D1d1 D1f2 D1k D1n (13)	1
990	>KY645883.1 Hc	73G 263G 310.1C 489C 513.1CA 750G 1438G 2092T 2706G 3010A 3106d 3107C 4769G 4883T 5178A 7028T 8414T 8701G 8860G 9540C 10398G 10400T 10873C 11719A 12530G 12705T 14668T 14766T 14783C 15043A 15301A 15326G 16142T 16223T 16231C 16325C 16362C 16519C	D1f	73G 263G 310.1C 489C 513.1CA 16142T 16223T 16231C 16325C 16362C 16519C	1	73 263 489 750 1438 2092 2706 3010 4769 4883 5178A 7028 8414 8701 8860 9540 10398 10400 10873 11719 12530 12705 14668 14766 14783 15043 15301 15326 16142 16223 16231 16325 16362	D1f	73 263 489 16142 16223 16231 16325 16362	[0.005] D1f (1)	[0.000] D1 D1a D1a2 D1b D1c D1d D1d1 D1f2 D1f+16189 D1f3 D1k D1n D4j14 (14)	1
991	>KY645867.1 Hc	73G 152C 263G 309.1CT 310C 499A 750G 827G 1438G 2706G 3106d 3107C 3547G 4769G 4820A 4977C 6473T 6755A 7028T 8271d 8272d 8273d 8274d 8275d 8276d 8277d 8278d 8279d 8545A 8860G 9950C 10685A 11177T 11719A 13590A 13788T 14470C 14766T 15326G 15535T 15758G 16183C 16189C 16217C 16519C	B2b+152	73G 152C 263G 309.1CT 310C 499A 16183C 16189C 16217C 16519C	1	73 152 263 499 750 827 1438 2706 3547 4769 4820 4977 6473 6755 7028 8281-8289d 8545 8860 9950 10685 11177 11719 13590 13788 14470 14766 15326 15535 15758 16189 16217	B2b+152	73 152 263 499 16189 16217	[0.000] B2b+152 (1)	[0.126] B2q (2) [0.093] B2 (3) [0.032] B2r (4) [0.016] B2e (5) [0.010] B2c B2c1 (7) [0.009] B2n (8) [0.000] B4b B2b B2b3 B2c1a B2c1b B2c1c B2f B2i B2p (17)	1
992	>KX457406.1 Hc	66A 73G 150T 199C 204C 252C 263G 309.1CT 310C 489C 750G 1438G 2706G 3106d 3107C 3483A 4048A 4071T 4164G 4769G 5351G 5460A 6455T 6680C 7028T 7684C 7853A 8699C 8701G 8860G 9540C 9824C 10398G 10400T 10873C 11266T 11719A 12405T 12705T 12811C 14766T 14783C 15043A 15301A 15326G 16176T 16223T 16271C 16297C 16309G 16519C	M7b1a1b	66A 73G 150T 199C 204C 252C 263G 309.1CT 310C 489C 16176T 16223T 16271C 16297C 16309G 16519C	1	66 73 150 199 204 252 263 489 750 1438 2706 3483 4048 4071 4164 4769 5351 5460 6455 6680 7028 7684 7853 8699 8701 8860 9540 9824 10398 10400 10873 11266 11719 12405 12705 12811 14766 14783 15043 15301 15326 16176 16223 16271 16297 16309	M7b1a1b	66 73 150 199 204 252 263 489 16176 16223 16271 16297 16309	[0.051] M7b1a1b (1)	[0.060] M7b1a1+(16192) (2) [0.034] M7b1a1 (3) [0.018] M7b1a1e1 (4) [0.014] M7b1a1f M7b1a1a3 (6) [0.004] M7b1a1h (7) [0.002] M7b1a1g (8) [0.000] M7b1a1d M7b1a1d1 M7b1a1e (11)	1

993	>KX457404.1 Ho	73G 185A 189G 263G 309.1CT 310C 514d 515d 1438G 2308G 2413T 2706G 3106d 3107C 3543T 3873G 4562G 4769G 5192G 5581G 6314T 7028T 7853A 8860G 9899C 10373A 11719A 12136C 12705T 13434G 13500C 13928C 14022G 14581C 14766T 14953T 15211T 15326G 16172C 16183C 16189C 16223T 16362C	N10a	73G 185A 189G 263G 309.1CT 310C 514d 515d 16172C 16183C 16189C 16223T 16362C	1	73 185 189 263 1438 2308 2413 2706 3543 3873 4562 4769 5192 5581 6314 7028 7853 8860 9899 10373 11719 12136 12705 13434 13500 13928C 14022 14581 14766 14953 15211 15326 16172 16189 16223 16362	N10a	73 185 189 263 16172 16189 16223 16362	[0.000] N10a (1)	[0.071] N10 (2)	1
994	>KX457403.1 Ho	73G 152C 210G 257G 263G 514d 515d 709A 750G 1438G 2706G 3106d 3107C 3537G 4086T 4769G 6960T 7028T 8271d 8272d 8273d 8274d 8275d 8276d 8277d 8278d 8279d 8584A 8860G 9950C 10398G 10589A 11465C 11719A 14696G 14766T 15235G 15326G 16140C 16182C 16183C 16189C 16261T 16266A 16519C	B5a1d	73G 152C 210G 257G 263G 514d 515d 16140C 16182C 16183C 16189C 16261T 16266A 16519C	1	73 152 210 257 263 709 750 1438 2706 3537 4086 4769 6960 7028 8281-8289d 8584 8860 9950 10398 10589 11465 11719 14696 14766 15235 15326 16140 16189 16261 16266A	B5a1d	73 152 210 257 263 16140 16189 16261 16266A	[0.028] B5a1d (1)	[0.032] B5a1 (2) [0.016] B5a1a (3) [0.010] B5a (4) [0.004] B5a1b1 (5) [0.002] B5a1c1a (6) [0.000] B5a1a1 B5a1b B5a1c B5a1c1 B5a1c2 (11)	1
995	>KX457402.1 Ho	73G 152C 195C 210G 263G 309.1CCT 310C 514d 515d 709A 750G 1438G 2706G 3106d 3107C 3537G 4086T 4769G 5460A 6960T 7028T 8271d 8272d 8273d 8274d 8275d 8276d 8277d 8278d 8279d 8584A 8860G 8897T 9950C 10398G 11465C 11719A 14766T 15235G 15326G 15828T 16129A 16140C 16182C 16183C 16189C 16261T 16266A 16519C	B5a1d	73G 152C 195C 210G 263G 309.1CCT 310C 514d 515d 16129A 16140C 16182C 16183C 16189C 16261T 16266A 16519C	1	73 152 195 210 263 709 750 1438 2706 3537 4086 4769 5460 6960 7028 8281-8289d 8584 8860 8897 9950 10398 11465 11719 14766 15235 15326 15828 16129 16140 16189 16261 16266A	B5a1d	73 152 195 210 263 16129 16140 16189 16261 16266A	[0.452] B5a1d (1)	[0.069] B5a1 (2) [0.050] B5a1a (3) [0.010] B5a (4) [0.004] B5a1b1 B5a1c1a (6) [0.000] B5a1a1 B5a1b B5a1c B5a1c1 B5a1c2 (11)	1
996	>KX457399.1 Ho	73G 152C 195C 210G 263G 514d 515d 709A 750G 1438G 2706G 3106d 3107C 3537G 4086T 4769G 5460A 6960T 7028T 8271d 8272d 8273d 8274d 8275d 8276d 8277d 8278d 8279d 8584A 8860G 8897T 9950C 10398G 11465C 11719A 14766T 15235G 15326G 15828T 16129A 16140C 16182C 16183C 16189C 16261T 16266A 16519C	B5a1d	73G 152C 195C 210G 263G 514d 515d 16129A 16140C 16182C 16183C 16189C 16261T 16266A 16519C	1	73 152 195 210 263 709 750 1438 2706 3537 4086 4769 5460 6960 7028 8281-8289d 8584 8860 8897 9950 10398 11465 11719 14766 15235 15326 15828 16129 16140 16189 16261 16266A	B5a1d	73 152 195 210 263 16129 16140 16189 16261 16266A	[0.452] B5a1d (1)	[0.069] B5a1 (2) [0.050] B5a1a (3) [0.010] B5a (4) [0.004] B5a1b1 B5a1c1a (6) [0.000] B5a1a1 B5a1b B5a1c B5a1c1 B5a1c2 (11)	1
997	>KX457398.1 Ho	73G 152C 195C 210G 263G 309.1CCT 310C 514d 515d 709A 750G 1438G 2706G 3106d 3107C 3537G 4086T 4769G 5460A 6960T 7028T 8271d 8272d 8273d 8274d 8275d 8276d 8277d 8278d 8279d 8584A 8860G 8897T 9950C 10398G 11465C 11719A 14766T 15235G 15326G 15828T 16129A 16140C 16182C 16183C 16189C 16261T 16266A 16519C	B5a1d	73G 152C 195C 210G 263G 309.1CCT 310C 514d 515d 16129A 16140C 16182C 16183C 16189C 16261T 16266A 16519C	1	73 152 195 210 263 709 750 1438 2706 3537 4086 4769 5460 6960 7028 8281-8289d 8584 8860 8897 9950 10398 11465 11719 14766 15235 15326 15828 16129 16140 16189 16261 16266A	B5a1d	73 152 195 210 263 16129 16140 16189 16261 16266A	[0.452] B5a1d (1)	[0.069] B5a1 (2) [0.050] B5a1a (3) [0.010] B5a (4) [0.004] B5a1b1 B5a1c1a (6) [0.000] B5a1a1 B5a1b B5a1c B5a1c1 B5a1c2 (11)	1

998	>KX457397.1 Ho	73G 152C 195C 210G 263G 514d 515d 709A 750G 1438G 2706G 3106d 3107C 3537G 4086T 4769G 5460A 6960T 7028T 8271d 8272d 8273d 8274d 8275d 8276d 8277d 8278d 8279d 8584A 8860G 8897T 9950C 10398G 11465C 11719A 14766T 15235G 15326G 15828T 16129A 16140C 16182C 16183C 16189C 16261T 16266A 16519C	B5a1d	73G 152C 195C 210G 263G 514d 515d 16129A 16140C 16182C 16183C 16189C 16261T 16266A 16519C	1	73 152 195 210 263 709 750 1438 2706 3537 4086 4769 5460 6960 7028 8281-8289d 8584 8860 8897 9950 10398 11465 11719 14766 15235 15326 15828 16129 16140 16189 16261 16266A	B5a1d	73 152 195 210 263 16129 16140 16189 16261 16266A	[0.452] B5a1d (1)	[0.069] B5a1 (2) [0.050] B5a1a (3) [0.010] B5a (4) [0.004] B5a1b1 B5a1c1a (6) [0.000] B5a1a1 B5a1b B5a1c B5a1c1 B5a1c2 (11)	1
999	>KX457396.1 Ho	73G 152C 195C 210G 263G 309.1CCT 310C 514d 515d 709A 750G 1438G 2706G 3106d 3107C 3537G 4086T 4769G 5460A 6960T 7028T 8271d 8272d 8273d 8274d 8275d 8276d 8277d 8278d 8279d 8584A 8860G 8897T 9950C 10398G 11465C 11719A 14766T 15235G 15326G 15828T 16129A 16140C 16182C 16183C 16189C 16261T 16266A 16519C	B5a1d	73G 152C 195C 210G 263G 309.1CCT 310C 514d 515d 16129A 16140C 16182C 16183C 16189C 16261T 16266A 16519C	1	73 152 195 210 263 709 750 1438 2706 3537 4086 4769 5460 6960 7028 8281-8289d 8584 8860 8897 9950 10398 11465 11719 14766 15235 15326 15828 16129 16140 16189 16261 16266A	B5a1d	73 152 195 210 263 16129 16140 16189 16261 16266A	[0.452] B5a1d (1)	[0.069] B5a1 (2) [0.050] B5a1a (3) [0.010] B5a (4) [0.004] B5a1b1 B5a1c1a (6) [0.000] B5a1a1 B5a1b B5a1c B5a1c1 B5a1c2 (11)	1
1000	>KX457395.1 Ho	73G 152C 195C 210G 263G 514d 515d 709A 750G 1438G 2706G 3106d 3107C 3537G 4086T 4769G 5460A 6960T 7028T 8271d 8272d 8273d 8274d 8275d 8276d 8277d 8278d 8279d 8584A 8860G 8897T 9950C 10398G 11465C 11719A 14766T 15235G 15326G 15828T 16129A 16140C 16182C 16183C 16189C 16261T 16266A 16519C	B5a1d	73G 152C 195C 210G 263G 514d 515d 16129A 16140C 16182C 16183C 16189C 16261T 16266A 16519C	1	73 152 195 210 263 709 750 1438 2706 3537 4086 4769 5460 6960 7028 8281-8289d 8584 8860 8897 9950 10398 11465 11719 14766 15235 15326 15828 16129 16140 16189 16261 16266A	B5a1d	73 152 195 210 263 16129 16140 16189 16261 16266A	[0.452] B5a1d (1)	[0.069] B5a1 (2) [0.050] B5a1a (3) [0.010] B5a (4) [0.004] B5a1b1 B5a1c1a (6) [0.000] B5a1a1 B5a1b B5a1c B5a1c1 B5a1c2 (11)	1
1001	>KX457394.1 Ho	73G 146C 263G 309.1CCT 310C 489C 750G 1438G 2706G 2835T 3106d 3107C 3714G 4715G 4769G 6179A 7028T 7196A 8584A 8684T 8701G 8860G 9540C 10398G 10400T 10873C 11176A 11719A 12705T 14470C 14766T 14783C 15043A 15301A 15326G 15487T 16184T 16189C 16223T 16298C 16311C 16319A 16390A 16468C 16470A 16471A 16473A	M8a2a1	73G 146C 263G 309.1CCT 310C 489C 16184T 16189C 16223T 16298C 16311C 16319A 16390A 16468C 16470A 16471A 16473A	1	73 146 263 489 750 1438 2706 2835 3714 4715 4769 6179 7028 7196A 8584 8684 8701 8860 9540 10398 10400 10873 11176 11719 12705 14470 14766 14783 15043 15301 15326 15487T 16184 16189 16223 16298 16311 16319 16390 16468 16470 16471 16473	M8a2a1	73 146 263 489 16184 16189 16223 16298 16311 16319 16390 16468 16470 16471 16473	[1.459] M8a2a1 (1)	[0.049] M8a3 (2) [0.046] M8a1a (3) [0.035] M8a2a (4) [0.033] M8a2c (5) [0.000] M8a2'3 M8a2 M8a2e (8)	1
1002	>KX457393.1 Ho	73G 150T 152C 260A 263G 310.1C 489C 750G 1193C 1342T 1438G 2706G 3106d 3107C 4769G 6253C 7028T 8269A 8701G 8860G 9540C 10398G 10400T 10873C 11719A 11810T 12705T 12732C 13468T 14557T 14766T 14783C 14883T 15043A 15301A 15326G 15949A 16223T 16362C 16381C 16519C	M61	73G 150T 152C 260A 263G 310.1C 489C 16223T 16362C 16381C 16519C	1	73 150 152 260 263 489 750 1193 1342 1438 2706 4769 6253 7028 8269 8701 8860 9540 10398 10400 10873 11719 11810 12705 12732 13468 14557 14766 14783 14883 15043 15301 15326 15949 16223 16362 16381	M61	73 150 152 260 263 489 16223 16362 16381	[0.170] M61 (1)	[0.003] M13'46'61+16362 (2) [0.002] D4b2b2a D4b2b2a1 D4b2b4 (5) [0.000] G1a M61a (7)	1

1003	>KX457390.1 Hc	73G 152C 195C 210G 263G 514d 515d 709A 750G 1438G 2706G 3106d 3107C 3537G 4086T 4769G 5460A 6960T 7028T 8271d 8272d 8273d 8274d 8275d 8276d 8277d 8278d 8279d 8584A 8860G 8897T 9950C 10398G 11465C 11719A 14766T 15235G 15326G 15828T 16129A 16140C 16182C 16183C 16189C 16261T 16266A 16519C	B5a1d	73G 152C 195C 210G 263G 514d 515d 16129A 16140C 16182C 16183C 16189C 16261T 16266A 16519C	1	73 152 195 210 263 709 750 1438 2706 3537 4086 4769 5460 6960 7028 8281-8289d 8584 8860 8897 9950 10398 11465 11719 14766 15235 15326 15828 16129 16140 16189 16261 16266A	B5a1d	73 152 195 210 263 16129 16140 16189 16261 16266A	[0.452] B5a1d (1)	[0.069] B5a1 (2) [0.050] B5a1a (3) [0.010] B5a (4) [0.004] B5a1b1 B5a1c1a (6) [0.000] B5a1a1 B5a1b B5a1c B5a1c1 B5a1c2 (11)	1
1004	>KX457389.1 Hc	73G 152C 195C 210G 263G 309.1CCT 310C 514d 515d 709A 750G 1438G 2706G 3106d 3107C 3537G 4086T 4769G 5460A 6960T 7028T 8271d 8272d 8273d 8274d 8275d 8276d 8277d 8278d 8279d 8584A 8860G 8897T 9950C 10398G 11465C 11719A 14766T 15235G 15326G 15828T 16129A 16140C 16182C 16183C 16189C 16261T 16266A 16519C	B5a1d	73G 152C 195C 210G 263G 309.1CCT 310C 514d 515d 16129A 16140C 16182C 16183C 16189C 16261T 16266A 16519C	1	73 152 195 210 263 709 750 1438 2706 3537 4086 4769 5460 6960 7028 8281-8289d 8584 8860 8897 9950 10398 11465 11719 14766 15235 15326 15828 16129 16140 16189 16261 16266A	B5a1d	73 152 195 210 263 16129 16140 16189 16261 16266A	[0.452] B5a1d (1)	[0.069] B5a1 (2) [0.050] B5a1a (3) [0.010] B5a (4) [0.004] B5a1b1 B5a1c1a (6) [0.000] B5a1a1 B5a1b B5a1c B5a1c1 B5a1c2 (11)	1
1005	>KX457388.1 Hc	73G 152C 195C 210G 263G 309.1CCT 310C 514d 515d 709A 750G 1438G 2706G 3106d 3107C 3537G 4086T 4769G 5460A 6960T 7028T 8271d 8272d 8273d 8274d 8275d 8276d 8277d 8278d 8279d 8584A 8860G 8897T 9950C 10398G 11465C 11719A 14766T 15235G 15326G 15828T 16129A 16140C 16182C 16183C 16189C 16261T 16266A 16519C	B5a1d	73G 152C 195C 210G 263G 309.1CCT 310C 514d 515d 16129A 16140C 16182C 16183C 16189C 16261T 16266A 16519C	1	73 152 195 210 263 709 750 1438 2706 3537 4086 4769 5460 6960 7028 8281-8289d 8584 8860 8897 9950 10398 11465 11719 14766 15235 15326 15828 16129 16140 16189 16261 16266A	B5a1d	73 152 195 210 263 16129 16140 16189 16261 16266A	[0.452] B5a1d (1)	[0.069] B5a1 (2) [0.050] B5a1a (3) [0.010] B5a (4) [0.004] B5a1b1 B5a1c1a (6) [0.000] B5a1a1 B5a1b B5a1c B5a1c1 B5a1c2 (11)	1
1006	>KX457387.1 Hc	73G 152C 195C 210G 263G 514d 515d 709A 750G 1438G 2706G 3106d 3107C 3537G 4086T 4769G 5460A 6960T 7028T 8271d 8272d 8273d 8274d 8275d 8276d 8277d 8278d 8279d 8584A 8860G 8897T 9950C 10398G 11465C 11719A 14766T 15235G 15326G 15828T 16129A 16140C 16182C 16183C 16189C 16261T 16266A 16519C	B5a1d	73G 152C 195C 210G 263G 514d 515d 16129A 16140C 16182C 16183C 16189C 16261T 16266A 16519C	1	73 152 195 210 263 709 750 1438 2706 3537 4086 4769 5460 6960 7028 8281-8289d 8584 8860 8897 9950 10398 11465 11719 14766 15235 15326 15828 16129 16140 16189 16261 16266A	B5a1d	73 152 195 210 263 16129 16140 16189 16261 16266A	[0.452] B5a1d (1)	[0.069] B5a1 (2) [0.050] B5a1a (3) [0.010] B5a (4) [0.004] B5a1b1 B5a1c1a (6) [0.000] B5a1a1 B5a1b B5a1c B5a1c1 B5a1c2 (11)	1
1007	>KX457385.1 Hc	73G 152C 195C 210G 263G 514d 515d 709A 750G 1438G 2706G 3106d 3107C 3537G 4086T 4769G 5460A 6960T 7028T 8271d 8272d 8273d 8274d 8275d 8276d 8277d 8278d 8279d 8584A 8860G 8897T 9950C 10398G 11465C 11719A 14766T 15235G 15326G 15828T 16129A 16140C 16182C 16183C 16189C 16261T 16266A 16519C	B5a1d	73G 152C 195C 210G 263G 514d 515d 16129A 16140C 16182C 16183C 16189C 16261T 16266A 16519C	1	73 152 195 210 263 709 750 1438 2706 3537 4086 4769 5460 6960 7028 8281-8289d 8584 8860 8897 9950 10398 11465 11719 14766 15235 15326 15828 16129 16140 16189 16261 16266A	B5a1d	73 152 195 210 263 16129 16140 16189 16261 16266A	[0.452] B5a1d (1)	[0.069] B5a1 (2) [0.050] B5a1a (3) [0.010] B5a (4) [0.004] B5a1b1 B5a1c1a (6) [0.000] B5a1a1 B5a1b B5a1c B5a1c1 B5a1c2 (11)	1

1008	>KX457384.1 Hc	73G 152C 195C 210G 263G 514d 515d 709A 750G 1438G 2706G 3106d 3107C 3537G 4086T 4769G 5460A 6960T 7028T 8271d 8272d 8273d 8274d 8275d 8276d 8277d 8278d 8279d 8584A 8860G 8897T 9950C 10398G 11465C 11719A 14766T 15235G 15326G 15828T 16129A 16140C 16182C 16183C 16189C 16261T 16266A 16519C	B5a1d	73G 152C 195C 210G 263G 514d 515d 16129A 16140C 16182C 16183C 16189C 16261T 16266A 16519C	1	73 152 195 210 263 709 750 1438 2706 3537 4086 4769 5460 6960 7028 8281-8289d 8584 8860 8897 9950 10398 11465 11719 14766 15235 15326 15828 16129 16140 16189 16261 16266A	B5a1d	73 152 195 210 263 16129 16140 16189 16261 16266A	[0.452] B5a1d (1)	[0.069] B5a1 (2) [0.050] B5a1a (3) [0.010] B5a (4) [0.004] B5a1b1 B5a1c1a (6) [0.000] B5a1a1 B5a1b B5a1c B5a1c1 B5a1c2 (11)	1
1009	>KX457382.1 Hc	73G 152C 195C 210G 263G 514d 515d 709A 750G 1438G 2706G 3106d 3107C 3537G 4086T 4769G 5460A 6960T 7028T 8271d 8272d 8273d 8274d 8275d 8276d 8277d 8278d 8279d 8584A 8860G 8897T 9950C 10398G 11465C 11719A 14766T 15235G 15326G 15828T 16129A 16140C 16182C 16183C 16189C 16261T 16266A 16519C	B5a1d	73G 152C 195C 210G 263G 514d 515d 16129A 16140C 16182C 16183C 16189C 16261T 16266A 16519C	1	73 152 195 210 263 709 750 1438 2706 3537 4086 4769 5460 6960 7028 8281-8289d 8584 8860 8897 9950 10398 11465 11719 14766 15235 15326 15828 16129 16140 16189 16261 16266A	B5a1d	73 152 195 210 263 16129 16140 16189 16261 16266A	[0.452] B5a1d (1)	[0.069] B5a1 (2) [0.050] B5a1a (3) [0.010] B5a (4) [0.004] B5a1b1 B5a1c1a (6) [0.000] B5a1a1 B5a1b B5a1c B5a1c1 B5a1c2 (11)	1
1010	>KX457381.1 Hc	73G 152C 195C 210G 263G 514d 515d 709A 750G 1438G 2706G 3106d 3107C 3537G 4086T 4769G 5460A 6960T 7028T 8271d 8272d 8273d 8274d 8275d 8276d 8277d 8278d 8279d 8584A 8860G 8897T 9950C 10398G 11465C 11719A 14766T 15235G 15326G 15828T 16129A 16140C 16182C 16183C 16189C 16261T 16266A 16519C	B5a1d	73G 152C 195C 210G 263G 514d 515d 16129A 16140C 16182C 16183C 16189C 16261T 16266A 16519C	1	73 152 195 210 263 709 750 1438 2706 3537 4086 4769 5460 6960 7028 8281-8289d 8584 8860 8897 9950 10398 11465 11719 14766 15235 15326 15828 16129 16140 16189 16261 16266A	B5a1d	73 152 195 210 263 16129 16140 16189 16261 16266A	[0.452] B5a1d (1)	[0.069] B5a1 (2) [0.050] B5a1a (3) [0.010] B5a (4) [0.004] B5a1b1 B5a1c1a (6) [0.000] B5a1a1 B5a1b B5a1c B5a1c1 B5a1c2 (11)	1
1011	>KX457377.1 Hc	73G 152C 195C 210G 263G 514d 515d 709A 750G 1438G 2706G 3106d 3107C 3537G 4086T 4769G 5460A 6960T 7028T 8271d 8272d 8273d 8274d 8275d 8276d 8277d 8278d 8279d 8584A 8860G 8897T 9950C 10398G 11465C 11719A 14766T 15235G 15326G 15828T 16129A 16140C 16182C 16183C 16189C 16261T 16266A 16519C	B5a1d	73G 152C 195C 210G 263G 514d 515d 16129A 16140C 16182C 16183C 16189C 16261T 16266A 16519C	1	73 152 195 210 263 709 750 1438 2706 3537 4086 4769 5460 6960 7028 8281-8289d 8584 8860 8897 9950 10398 11465 11719 14766 15235 15326 15828 16129 16140 16189 16261 16266A	B5a1d	73 152 195 210 263 16129 16140 16189 16261 16266A	[0.452] B5a1d (1)	[0.069] B5a1 (2) [0.050] B5a1a (3) [0.010] B5a (4) [0.004] B5a1b1 B5a1c1a (6) [0.000] B5a1a1 B5a1b B5a1c B5a1c1 B5a1c2 (11)	1
1012	>KX457358.1 Hc	73G 146C 152C 195C 263G 309.1CT 310C 489C 514d 515d 750G 951A 2706G 3106d 3107C 4769G 5773A 7028T 7040C 8701G 8860G 9540C 10398G 10400T 10873C 11719A 12705T 13359A 14766T 14783C 15043A 15301A 15326G 15601C 16223T 16298C 16311C 16519C	M24a	73G 146C 152C 195C 263G 309.1CT 310C 489C 514d 515d 16223T 16298C 16311C 16519C	1	73 146 152 195 263 489 750 951 2706 4769 5773 7028 7040 8701 8860 9540 10398 10400 10873 11719 12705 13359 14766 14783 15043 15301 15326 15601 16223 16298 16311	M24a	73 146 152 195 263 489 16223 16298 16311	[0.005] M24a (1)	[0.022] M57+152 (2) [0.016] M24	1

1013 >KX457353.1 Ho	73G 152C 210G 263G 514d 515d 709A 750G 1438G 2706G 2756T 3106d 3107C 3392C 3537G 4086T 4769G 6960T 7028T 8271d 8272d 8273d 8274d 8275d 8276d 8277d 8278d 8279d 8584A 8860G 9950C 10398G 11465C 11719A 14766T 15235G 15326G 16140C 16182C 16183C 16189C 16261T 16266A 16519C	B5a1d	73G 152C 210G 263G 514d 515d 16140C 16182C 16183C 16189C 16261T 16266A 16519C	1	73 152 210 263 709 750 1438 2706 2756 3392C 3537 4086 4769 6960 7028 8281-8289d 8584 8860 9950 10398 11465 11719 14766 15235 15326 16140 16189 16261 16266A	B5a1d	73 152 210 263 16140 16189 16261 16266A	[0.000] B5a1d (1)	[0.032] B5a1 (2) [0.016] B5a1a (3) [0.010] B5a (4) [0.004] B5a1b1 (5) [0.002] B5a1c1a (6) [0.000] B5a1a1 B5a1b B5a1c B5a1c1 B5a1c2 (11)	1
1014 >KX457347.1 Ho	73G 150T 199C 204C 263G 309.1CT 310C 456T 489C 750G 1438G 2706G 3106d 3107C 4048A 4071T 4164G 4318T 4769G 5351G 5460A 6455T 6680C 7028T 7684C 7853A 8701G 8860G 9540C 9824C 10398G 10400T 10873C 11719A 12405T 12705T 12811C 14766T 14783C 14978G 15043A 15301A 15326G 16129A 16189C 16223T 16297C	M7b1a1a3	73G 150T 199C 204C 263G 309.1CT 310C 456T 489C 16129A 16189C 16223T 16297C	1	73 150 199 204 263 456 489 750 1438 2706 4048 4071 4164 4318 4769 5351 5460 6455 6680 7028 7684 7853 8701 8860 9540 9824 10398 10400 10873 11719 12405 12705 12811 14766 14783 14978 15043 15301 15326 16129 16189 16223 16297	M7b1a1a3	73 150 199 204 263 456 489 16129 16189 16223 16297	[0.000] M7b1a1a3 (1)	[0.003] M7b1a1a2 (2) [0.000] M7b1a1a	1
1015 >KX457338.1 Ho	73G 152C 263G 309.1CT 310C 489C 750G 1438G 2706G 3106d 3107C 3483A 3720G 4020T 4117C 4769G 7028T 8701G 8860G 9540C 10398G 10400T 10873C 11101G 11719A 12705T 13651G 14766T 14783C 15043A 15301A 15326G 16111T 16223T 16311C 16354T 16519C	M57a	73G 152C 263G 309.1CT 310C 489C 16111T 16223T 16311C 16354T 16519C	1	73 152 263 489 750 1438 2706 3483 3720 4020 4117 4769 7028 8701 8860 9540 10398 10400 10873 11101 11719 12705 13651 14766 14783 15043 15301 15326 16111 16223 16311 16354	M57a	73 152 263 489 16111 16223 16311 16354	[0.014] M57a (1)	[0.004] M57+152 (2)	1
1016 >KX457336.1 Ho	73G 150T 199C 204C 263G 309.1CT 310C 456T 489C 750G 1438G 2706G 3106d 3107C 4048A 4071T 4164G 4318T 4769G 5351G 5460A 6455T 6680C 7028T 7684C 7853A 8701G 8860G 9540C 9824C 10398G 10400T 10873C 11719A 12405T 12705T 12811C 14766T 14783C 14978G 15043A 15301A 15326G 16129A 16189C 16223T 16297C	M7b1a1a3	73G 150T 199C 204C 263G 309.1CT 310C 456T 489C 16129A 16189C 16223T 16297C	1	73 150 199 204 263 456 489 750 1438 2706 4048 4071 4164 4318 4769 5351 5460 6455 6680 7028 7684 7853 8701 8860 9540 9824 10398 10400 10873 11719 12405 12705 12811 14766 14783 14978 15043 15301 15326 16129 16189 16223 16297	M7b1a1a3	73 150 199 204 263 456 489 16129 16189 16223 16297	[0.000] M7b1a1a3 (1)	[0.003] M7b1a1a2 (2) [0.000] M7b1a1a	1
1017 >KX457331.1 Ho	73G 152C 263G 489C 750G 1438G 2706G 3106d 3107C 3483A 3720G 4020T 4117C 4769G 7028T 8701G 8860G 9540C 10398G 10400T 10873C 11101G 11719A 12705T 13651G 14766T 14783C 15043A 15301A 15326G 16111T 16223T 16311C 16354T 16519C	M57a	73G 152C 263G 489C 16111T 16223T 16311C 16354T 16519C	1	73 152 263 489 750 1438 2706 3483 3720 4020 4117 4769 7028 8701 8860 9540 10398 10400 10873 11101 11719 12705 13651 14766 14783 15043 15301 15326 16111 16223 16311 16354	M57a	73 152 263 489 16111 16223 16311 16354	[0.014] M57a (1)	[0.004] M57+152 (2)	1

1018	>KX457328.1 Hc	73G 195C 263G 310.1C 489C 514d 515d 750G 1438G 2706G 3106d 3107C 4769G 5054A 5585A 7028T 8080A 8155A 8167C 8251A 8616A 8701G 8860G 9540C 10268T 10398G 10400T 10873C 11719A 12280G 12651A 12705T 13434G 14766T 14783C 15043A 15226G 15301A 15326G 15908C 15969A 16223T 16246T 16311C 16362C 16519C	M74b1	73G 195C 263G 310.1C 489C 514d 515d 16223T 16246T 16311C 16362C 16519C	1	73 195 263 489 750 1438 2706 4769 5054 5585 7028 8080A 8155 8167 8251 8616 8701 8860 9540 10268 10398 10400 10873 11719 12280 12651 12705 13434 14766 14783 15043 15226 15301 15326 15908 15969A 16223 16246T 16311 16362	M74b1	73 195 263 489 16223 16246T 16311 16362	[0.000] M74b1 (1)	[0.039] M74 (2) [0.018] M74b2 (3) [0.000] L4 L4b M43a1 M74b D4j+16311 D4j11 D4+195 (10)	1
1019	>KX457327.1 Hc	73G 152C 210G 263G 309.1CCT 310C 514d 515d 709A 750G 1438G 2069C 2706G 3106d 3107C 3537G 4086T 4769G 6960T 7028T 8271d 8272d 8273d 8274d 8275d 8276d 8277d 8278d 8279d 8584A 8860G 9950C 10398G 11465C 11719A 14766T 15235G 15326G 16140C 16182C 16183C 16189C 16261T 16266A 16519C	B5a1d	73G 152C 210G 263G 309.1CCT 310C 514d 515d 16140C 16182C 16183C 16189C 16261T 16266A 16519C	1	73 152 210 263 709 750 1438 2069 2706 3537 4086 4769 6960 7028 8281-8289d 8584 8860 9950 10398 11465 11719 14766 15235 15326 16140 16189 16261 16266A	B5a1d	73 152 210 263 16140 16189 16261 16266A	[0.000] B5a1d (1)	[0.032] B5a1 (2) [0.016] B5a1a (3) [0.010] B5a (4) [0.004] B5a1b1 (5) [0.002] B5a1c1a (6) [0.000] B5a1a1 B5a1b B5a1c B5a1c1 B5a1c2 (11)	1
1020	>KX457325.1 Hc	73G 150T 152C 199C 204C 207A 263G 310.1C 456T 489C 750G 827G 1438G 2706G 3106d 3107C 4048A 4071T 4164G 4769G 5351G 5460A 6455T 6680C 7028T 7684C 7853A 8701G 8860G 9540C 9824C 10398G 10400T 10873C 11719A 12405T 12705T 12811C 14766T 14783C 14978G 15043A 15301A 15326G 16129A 16189C 16223T 16297C	M7b1a1a3	73G 150T 152C 199C 204C 207A 263G 310.1C 456T 489C 16129A 16189C 16223T 16297C	1	73 150 152 199 204 207 263 456 489 750 827 1438 2706 4048 4071 4164 4769 5351 5460 6455 6680 7028 7684 7853 8701 8860 9540 9824 10398 10400 10873 11719 12405 12705 12811 14766 14783 14978 15043 15301 15326 16129 16189 16223 16297	M7b1a1a3	73 150 152 199 204 207 263 456 489 16129 16189 16223 16297	[0.284] M7b1a1a3 (1)	[0.022] M7b1a1a (2) [0.006] M7b1a1a2	1
1021	>KX457321.1 Hc	61A 62A 73G 263G 309.1CCT 310C 514d 515d 750G 1438G 2378T 2706G 3106d 3107C 4769G 6482T 7028T 8215T 8271d 8272d 8273d 8274d 8275d 8276d 8277d 8278d 8279d 8860G 9968T 11242T 11719A 13926C 14766T 15326G 16180d 16182C 16183C 16189C 16213A 16217C 16261T 16292T 16519C	B4g2	61A 62A 73G 263G 309.1CCT 310C 514d 515d 16180d 16182C 16183C 16189C 16213A 16217C 16261T 16292T 16519C	1	61A 62 73 263 750 1438 2378 2706 4769 6482 7028 8215 8281-8289d 8860 9968 11242 11719 13926 14766 15326 16181C 16189 16193d 16213 16217 16261 16292	B4g2	61A 62 73 263 16181C 16189 16193d 16213 16217 16261 16292	[2.570] B4g2 (1) [0.641] B4g1 (2) [0.243] B4g1a (3) [0.000] B4g (4)	[0.246] B4g1b (5)	1
1022	>KX457320.1 Hc	73G 150T 263G 750G 1438G 2706G 3106d 3107C 4769G 4856C 5231A 5417A 7028T 8860G 11719A 12358G 12372A 12705T 13812G 14766T 15326G 16223T 16257A 16292T 16361A 16519C	N9a6	73G 150T 263G 16223T 16257A 16292T 16361A 16519C	1	73 150 263 750 1438 2706 4769 4856 5231 5417 7028 8860 11719 12358 12372 12705 13812G 14766 15326 16223 16257A 16292 16361	N9a6	73 150 263 16223 16257A 16292 16361	[0.035] N9a6 (1) [0.000] N9a10a N9a10a1 N9a10a2 N9a10a2a (5)	[0.046] N9a (6) [0.014] N9a6a (7) [0.007] L3e3'4 (8) [0.000] L3e'i'k'x L3e L3e3'4'5 L3i N9a6b N9a8 N9a9 N9a10 (16)	1

1023	>KX457319.1 H[illegible]	73G 152C 248d 263G 309.1CT 310C 514d 515d 750G 1438G 2706G 3106d 3107C 3970T 4086T 4769G 6392C 6962A 7028T 7444A 8659G 8860G 9053A 10034C 10310A 10609C 11719A 12406A 12882T 13759A 13928C 14766T 15326G 16172C 16304C 16465T 16519C	F1a2a	73G 152C 248d 263G 309.1CT 310C 514d 515d 16172C 16304C 16465T 16519C	1	73 152 249d 263 750 1438 2706 3970 4086 4769 6392 6962 7028 7444 8659 8860 9053 10034 10310 10609 11719 12406 12882 13759 13928C 14766 15326 16172 16304 16465	F1a2a	73 152 249d 263 16172 16304 16465	[0.014] F1a2a (1)	[0.169] F1a2 (2)	1
1024	>KX457317.1 H[illegible]	73G 195C 263G 310.1C 489C 514d 515d 750G 1438G 2706G 3106d 3107C 4769G 5054A 5585A 7028T 8080A 8155A 8167C 8251A 8616A 8701G 8860G 9540C 10268T 10398G 10400T 10873C 11719A 12280G 12651A 12705T 13434G 14766T 14783C 15043A 15226G 15301A 15326G 15908C 15969A 16223T 16246T 16311C 16362C 16519C	M74b1	73G 195C 263G 310.1C 489C 514d 515d 16223T 16246T 16311C 16362C 16519C	1	73 195 263 489 750 1438 2706 4769 5054 5585 7028 8080A 8155 8167 8251 8616 8701 8860 9540 10268 10398 10400 10873 11719 12280 12651 12705 13434 14766 14783 15043 15226 15301 15326 15908 15969A 16223 16246T 16311 16362	M74b1	73 195 263 489 16223 16246T 16311 16362	[0.000] M74b1 (1)	[0.039] M74 (2) [0.018] M74b2 (3) [0.000] L4 L4b M43a1 M74b D4j+16311 D4j11 D4+195 (10)	1
1025	>KX457315.1 H[illegible]	73G 195C 263G 310.1C 489C 514d 515d 750G 1438G 2706G 3106d 3107C 4769G 5054A 5585A 7028T 8080A 8155A 8167C 8251A 8616A 8701G 8860G 9540C 10268T 10398G 10400T 10873C 11719A 12280G 12651A 12705T 13434G 14766T 14783C 15043A 15226G 15301A 15326G 15908C 15969A 16223T 16246T 16311C 16362C 16519C	M74b1	73G 195C 263G 310.1C 489C 514d 515d 16223T 16246T 16311C 16362C 16519C	1	73 195 263 489 750 1438 2706 4769 5054 5585 7028 8080A 8155 8167 8251 8616 8701 8860 9540 10268 10398 10400 10873 11719 12280 12651 12705 13434 14766 14783 15043 15226 15301 15326 15908 15969A 16223 16246T 16311 16362	M74b1	73 195 263 489 16223 16246T 16311 16362	[0.000] M74b1 (1)	[0.039] M74 (2) [0.018] M74b2 (3) [0.000] L4 L4b M43a1 M74b D4j+16311 D4j11 D4+195 (10)	1
1026	>KX457313.1 H[illegible]	73G 152C 248d 263G 310.1C 316A 489C 514d 515d 750G 1438G 2706G 3106d 3107C 3200C 3714G 4385T 4769G 4772C 7028T 7433T 8701G 8853G 8860G 9127G 9512T 9540C 10274C 10398G 10400T 10679G 10873C 11719A 11914A 12354C 12705T 14110C 14766T 14783C 14974T 15043A 15301A 15326G 15691G 16086C 16129A 16209C 16223T 16263C 16272G 16519C	M20	73G 152C 248d 263G 310.1C 316A 489C 514d 515d 16086C 16129A 16209C 16223T 16263C 16272G 16519C	1	73 152 249d 263 316 489 750 1438 2706 3200 3714 4385T 4769 4772 7028 7433 8701 8853 8860 9127 9512 9540 10274 10398 10400 10679 10873 11719 11914 12354 12705 14110 14766 14783 14974 15043 15301 15326 15691 16086 16129 16209 16223 16263 16272	M20	73 152 249d 263 316 489 16086 16129 16209 16223 16263 16272	[0.342] M20 (1)	[0.010] D4a7 (2) [0.000] M13b1	1
1027	>KX457310.1 H[illegible]	61A 62A 73G 263G 514d 515d 750G 1438G 2378T 2706G 3106d 3107C 4769G 6482T 7028T 8215T 8271d 8272d 8273d 8274d 8275d 8276d 8277d 8278d 8279d 8860G 9968T 11242T 11719A 13926C 14766T 15326G 16180d 16181C 16182C 16183C 16189C 16213A 16217C 16261T 16292T 16519C	B4g2	61A 62A 73G 263G 514d 515d 16180d 16181C 16182C 16183C 16189C 16213A 16217C 16261T 16292T 16519C	1	61A 62 73 263 750 1438 2378 2706 4769 6482 7028 8215 8281-8289d 8860 9968 11242 11719 13926 14766 15326 16180C 16181C 16189 16193d 16213 16217 16261 16292	B4g2	61A 62 73 263 16180C 16181C 16189 16193d 16213 16217 16261 16292	[2.633] B4g2 (1) [0.682] B4g1 (2) [0.270] B4g1a (3) [0.000] B4g (4)	[0.246] B4g1b (5)	1

1028 >KX457308.1 Hc	73G 150T 199C 204C 263G 456T 489C 750G 1438G 2706G 3106d 3107C 4048A 4071T 4164G 4769G 5351G 5460A 6455T 6680C 7028T 7684C 7853A 8701G 8860G 9540C 9824C 10310A 10398G 10400T 10873C 11719A 11914A 12405T 12705T 12811C 14766T 14783C 14978G 15043A 15301A 15326G 16129A 16189C 16223T 16297C	M7b1a1a3	73G 150T 199C 204C 263G 456T 489C 16129A 16189C 16223T 16297C	1	73 150 199 204 263 456 489 750 1438 2706 4048 4071 4164 4769 5351 5460 6455 6680 7028 7684 7853 8701 8860 9540 9824 10310 10398 10400 10873 11719 11914 12405 12705 12811 14766 14783 14978 15043 15301 15326 16129 16189 16223 16297	M7b1a1a3	73 150 199 204 263 456 489 16129 16189 16223 16297	[0.000] M7b1a1a3 (1)	[0.003] M7b1a1a2 (2) [0.000] M7b1a1a	1
1029 >KX457304.1 Hc	73G 93G 263G 309.1CT 310C 489C 750G 1438G 2706G 3106d 3107C 4164G 4674G 4769G 5147A 5592G 7028T 8701G 8860G 9540C 10398G 10400T 10586A 10873C 11389T 11719A 11914A 12705T 14766T 14783C 15043A 15236G 15301A 15326G 15652G 15940C 16069T 16223T 16287T 16299G 16327A	M91b	73G 93G 263G 309.1CT 310C 489C 16069T 16223T 16287T 16299G 16327A	1	73 93 263 489 750 1438 2706 4164 4674 4769 5147 5592 7028 8701 8860 9540 10398 10400 10586 10873 11389 11719 11914 12705 14766 14783 15043 15236 15301 15326 15652 15940 16069 16223 16287 16299 16327A	M91b	73 93 263 489 16069 16223 16287 16299 16327A	[0.000] M91b (1)	[0.000] M91 (2)	1
1030 >KX457303.1 Hc	73G 151T 263G 310.1C 479G 750G 1048T 1438G 2706G 3106d 3107C 3970T 4769G 7028T 7419A 7684C 7861C 8860G 11002G 11719A 12618A 13928C 14766T 15326G 15479C 16157C 16243C 16304C	R9c1b1	73G 151T 263G 310.1C 479G 16157C 16243C 16304C	1	73 151 263 479 750 1048 1438 2706 3970 4769 7028 7419 7684 7861 8860 11002 11719 12618 13928C 14766 15326 15479 16157 16243 16304	R9c1b1	73 151 263 479 16157 16243 16304	[0.105] R9c1b1 (1)	[0.040] R9c1b2 (2) [0.000] R9c1 R9c1b (4)	1
1031 >KX457295.1 Hc	73G 150T 263G 309.1CCT 310C 750G 1041G 1438G 2706G 3106d 3107C 4386C 4769G 5231A 5417A 5585A 7028T 8860G 11719A 12358G 12372A 12705T 12771A 13488C 14766T 15094T 15326G 16183C 16189C 16223T 16257A 16261T 16311C 16390A	N9a10+16311	73G 150T 263G 309.1CCT 310C 16183C 16189C 16223T 16257A 16261T 16311C 16390A	1	73 150 263 750 1041 1438 2706 4386 4769 5231 5417 5585 7028 8860 11719 12358 12372 12705 12771 13488 14766 15094 15326 16189 16223 16257A 16261 16311 16390	N9a10+16311	73 150 263 16189 16223 16257A 16261 16311 16390	[0.373] N9a10+16311 (1)	[0.327] N9a10 (2) [0.068] N9a (3) [0.022] N9a9 (4) [0.013] N9a8 (5) [0.000] N9a10b	1
1032 >KX457293.1 Hc	61A 62A 73G 150T 263G 308T 310d 514d 515d 750G 1438G 2378T 2706G 3106d 3107C 4769G 6482T 7028T 8271d 8272d 8273d 8274d 8275d 8276d 8277d 8278d 8279d 8860G 9968T 11719A 13752C 14766T 15326G 16180d 16182C 16183C 16189C 16213A 16217C 16242T 16261T 16292T 16301T 16519C	B4g2	61A 62A 73G 150T 263G 308T 310d 514d 515d 16180d 16182C 16183C 16189C 16213A 16217C 16242T 16261T 16292T 16301T 16519C	1	61A 62 73 150 263 308-309d 750 1438 2378 2706 4769 6482 7028 8281-8289d 8860 9968 11719 13752 14766 15326 16181C 16189 16193d 16213 16217 16242 16261 16292 16301	B4g2	61A 62 73 150 263 308-309d 16181C 16189 16193d 16213 16217 16242 16261 16292 16301	[2.291] B4g2 (1)	[0.655] B4g1 (2) [0.243] B4g1a (3) [0.000] B4g	1
1033 >KX457292.1 Hc	73G 150T 263G 750G 1041G 1438G 2706G 3106d 3107C 4386C 4769G 5231A 5417A 5585A 7028T 8860G 11719A 12358G 12372A 12705T 12771A 13488C 14766T 15094T 15326G 16183C 16189C 16223T 16257A 16261T 16311C 16390A	N9a10+16311	73G 150T 263G 16183C 16189C 16223T 16257A 16261T 16311C 16390A	1	73 150 263 750 1041 1438 2706 4386 4769 5231 5417 5585 7028 8860 11719 12358 12372 12705 12771 13488 14766 15094 15326 16189 16223 16257A 16261 16311 16390	N9a10+16311	73 150 263 16189 16223 16257A 16261 16311 16390	[0.373] N9a10+16311 (1)	[0.327] N9a10 (2) [0.068] N9a (3) [0.022] N9a9 (4) [0.013] N9a8 (5) [0.000] N9a10b	1

1034	>KX457288.1 Hc	61A 62A 73G 150T 263G 308T 310d 514d 515d 750G 1438G 2378T 2706G 3106d 3107C 4769G 6482T 7028T 8271d 8272d 8273d 8274d 8275d 8276d 8277d 8278d 8279d 8860G 9968T 11719A 13752C 14766T 15326G 16181C 16182C 16183C 16189C 16213A 16217C 16242T 16261T 16292T 16301T 16519C	B4g2	61A 62A 73G 150T 263G 308T 310d 514d 515d 16181C 16182C 16183C 16189C 16213A 16217C 16242T 16261T 16292T 16301T 16519C	1	61A 62 73 150 263 308-309d 750 1438 2378 2706 4769 6482 7028 8281-8289d 8860 9968 11719 13752 14766 15326 16181C 16189 16213 16217 16242 16261 16292 16301	B4g2	61A 62 73 150 263 308-309d 16181C 16189 16213 16217 16242 16261 16292 16301	[2.177] B4g2 (1)	[0.607] B4g1 (2) [0.135] B4g1a (3) [0.000] B4g	1
1035	>KX457285.1 Hc	73G 150T 199C 203A 204C 263G 310.1C 489C 750G 1438G 2706G 3106d 3107C 3483A 4048A 4071T 4164G 4769G 5351G 5460A 6455T 6680C 7028T 7684C 7853A 8027A 8701G 8860G 9540C 9824C 10398G 10400T 10873C 11719A 12405T 12705T 12811C 14766T 14783C 15043A 15301A 16223T 16297C	M7b1a1b	73G 150T 199C 203A 204C 263G 310.1C 489C 16223T 16297C	1	73 150 199 203 204 263 489 750 1438 2706 3483 4048 4071 4164 4769 5351 5460 6455 6680 7028 7684 7853 8027 8701 8860 9540 9824 10398 10400 10873 11719 12405 12705 12811 14766 14783 15043 15301 16223 16297	M7b1a1b	73 150 199 203 204 263 489 16223 16297	[0.024] M7b1a1b (1)	[0.042] M7b1a1+(16192) (2) [0.018] M7b1a1e1 (3) [0.015] M7b1a1 (4) [0.014] M7b1a1a3 (5) [0.010] M7b1a1f (6) [0.004] M7b1a1h (7) [0.002] M7b1a1g (8) [0.000] M7b1a1d M7b1a1d1 M7b1a1e (11)	1
1036	>KX457278.1 Hc	73G 150T 159C 199C 263G 310.1C 489C 750G 1438G 2706G 3106d 3107C 4048A 4071T 4164G 4769G 5351G 5460A 6455T 6680C 7028T 7684C 7853A 8701G 8860G 9540C 9824C 10398G 10400T 10873C 11440A 11719A 12405T 12705T 12811C 14766T 14783C 15043A 15301A 15326G 16129A 16223T 16297C	M7b1a1	73G 150T 159C 199C 263G 310.1C 489C 16129A 16223T 16297C	1	73 150 159 199 263 489 750 1438 2706 4048 4071 4164 4769 5351 5460 6455 6680 7028 7684 7853 8701 8860 9540 9824 10398 10400 10873 11440 11719 12405 12705 12811 14766 14783 15043 15301 15326 16129 16223 16297	M7b1a1	73 150 159 199 263 489 16129 16223 16297	[0.232] M7b1a1 (1) [0.033] M7b1a1c (2) [0.000] M7b1a1+(16192) M7b1a1d M7b1a1d1 M7b1a1e M7b1a1e1 M7b1a1f M7b1a1g M7b1a1h (10)	[0.033] M7b1a1c (11) [0.007] M7b1a1e2 (12) [0.003] M7b1a1a2 (13) [0.000] M7b1a M7b1a1a M7b1a1+(16192) M7b1a1d M7b1a1d1 M7b1a1e M7b1a1e1 M7b1a1f M7b1a1g M7b1a1h M7b1a1i (24)	1
1037	>KX457276.1 Hc	73G 150T 159C 199C 263G 310.1C 489C 750G 1438G 2706G 3106d 3107C 4048A 4071T 4164G 4769G 5351G 5460A 6455T 6680C 7028T 7684C 7853A 8701G 8860G 9540C 9824C 10398G 10400T 10873C 11440A 11719A 12405T 12705T 12811C 14766T 14783C 15043A 15301A 15326G 16129A 16223T 16297C	M7b1a1	73G 150T 159C 199C 263G 310.1C 489C 16129A 16223T 16297C	1	73 150 159 199 263 489 750 1438 2706 4048 4071 4164 4769 5351 5460 6455 6680 7028 7684 7853 8701 8860 9540 9824 10398 10400 10873 11440 11719 12405 12705 12811 14766 14783 15043 15301 15326 16129 16223 16297	M7b1a1	73 150 159 199 263 489 16129 16223 16297	[0.232] M7b1a1 (1) [0.033] M7b1a1c (2) [0.000] M7b1a1+(16192) M7b1a1d M7b1a1d1 M7b1a1e M7b1a1e1 M7b1a1f M7b1a1g M7b1a1h (10)	[0.033] M7b1a1c (11) [0.007] M7b1a1e2 (12) [0.003] M7b1a1a2 (13) [0.000] M7b1a M7b1a1a M7b1a1+(16192) M7b1a1d M7b1a1d1 M7b1a1e M7b1a1e1 M7b1a1f M7b1a1g M7b1a1h M7b1a1i (24)	1
1038	>KX457272.1 Hc	73G 150T 159C 199C 263G 310.1C 489C 750G 1438G 2706G 3106d 3107C 4048A 4071T 4164G 4769G 5351G 5460A 6455T 6680C 7028T 7684C 7853A 8701G 8860G 9540C 9824C 10398G 10400T 10873C 11440A 11719A 12405T 12705T 12811C 14766T 14783C 15043A 15301A 15326G 16129A 16223T 16297C	M7b1a1	73G 150T 159C 199C 263G 310.1C 489C 16129A 16223T 16297C	1	73 150 159 199 263 489 750 1438 2706 4048 4071 4164 4769 5351 5460 6455 6680 7028 7684 7853 8701 8860 9540 9824 10398 10400 10873 11440 11719 12405 12705 12811 14766 14783 15043 15301 15326 16129 16223 16297	M7b1a1	73 150 159 199 263 489 16129 16223 16297	[0.232] M7b1a1 (1) [0.033] M7b1a1c (2) [0.000] M7b1a1+(16192) M7b1a1d M7b1a1d1 M7b1a1e M7b1a1e1 M7b1a1f M7b1a1g M7b1a1h (10)	[0.033] M7b1a1c (11) [0.007] M7b1a1e2 (12) [0.003] M7b1a1a2 (13) [0.000] M7b1a M7b1a1a M7b1a1+(16192) M7b1a1d M7b1a1d1 M7b1a1e M7b1a1e1 M7b1a1f M7b1a1g M7b1a1h M7b1a1i (24)	1

1039	>KX457267.1 Ho	73G 207A 249G 263G 309.1CT 310C 489C 514d 515d 750G 1438G 2706G 3106d 3107C 4021G 4769G 4901G 5894.1C 6026T 7028T 7289G 8701G 8860G 8970T 9380A 9449T 9540C 9591A 10256C 10398G 10400T 10873C 11140T 11719A 12705T 13708A 14040A 14766T 14783C 15043A 15236G 15301A 15326G 16140C 16172C 16183C 16189C 16223T 16278T	M59	73G 207A 249G 263G 309.1CT 310C 489C 514d 515d 16140C 16172C 16183C 16189C 16223T 16278T	1	73 207 249 263 489 750 1438 2706 4021 4769 4901 5899.1C 6026T 7028 7289 8701 8860 8970 9380 9449 9540 9591 10256 10398 10400 10873 11140 11719 12705 13708 14040 14766 14783 15043 15236 15301 15326 16140 16172 16189 16223 16278	M59	73 207 249 263 489 16140 16172 16189 16223 16278	[0.249] M59 (1)	[0.062] G2a1+16189 (2) [0.012] G1b (3) [0.005] M42b2 (4) [0.000] M51a2 M33a2'3 M73'79 M73 X (9)	1
1040	>KX457261.1 Ho	73G 146C 150T 185A 189G 196C 199C 263G 310.1C 513A 750G 827G 1438G 2706G 3106d 3107C 4769G 6614C 7028T 7657C 8271d 8272d 8273d 8274d 8275d 8276d 8277d 8278d 8279d 8860G 9758C 9921A 10410C 10750G 11719A 13135A 14766T 15326G 15535T 15940d 16129A 16182C 16183C 16189C 16217C 16223T 16311C 16519C	B4e	73G 146C 150T 185A 189G 196C 199C 263G 310.1C 513A 16129A 16182C 16183C 16189C 16217C 16223T 16311C 16519C	1	73 146 150 185 189 196 199 263 513 750 827 1438 2706 4769 6614 7028 7657 8281-8289d 8860 9758 9921 10410 10750 11719 13135 14766 15326 15535 15944d 16129 16189 16217 16223 16311	B4e	73 146 150 185 189 196 199 263 513 16129 16189 16217 16223 16311	[1.354] B4e (1)	[0.084] R11b (2) [0.073] R11 (3) [0.048] R11a	1
1041	>KY645623.1 Ho	73G 133C 152C 263G 309.1CT 310C 489C 750G 1438G 2092T 2177C 2706G 3010A 3106d 3107C 3203G 3834A 4769G 4883T 5178A 7028T 8414T 8701G 8860G 9540C 10398G 10400T 10873C 11719A 12705T 14668T 14766T 14783C 15043A 15221A 15301A 15326G 16142T 16223T 16256T 16325C 16362C 16519C	D1f2	73G 133C 152C 263G 309.1CT 310C 489C 16142T 16223T 16256T 16325C 16362C 16519C	1	73 133 152 263 489 750 1438 2092 2177 2706 3010 3203 3834 4769 4883 5178A 7028 8414 8701 8860 9540 10398 10400 10873 11719 12705 14668 14766 14783 15043 15221 15301 15326 16142 16223 16256 16325 16362	D1f2	73 133 152 263 489 16142 16223 16256 16325 16362	[0.016] D1f2 (1)	[0.084] D1f (2)	1
1042	>KY645590.1 Ho	73G 146C 263G 309T 310C 499A 750G 827G 1375T 1438G 2706G 3106d 3107C 3547G 4375T 4769G 4820A 4977C 6473T 7028T 8271d 8272d 8273d 8274d 8275d 8276d 8277d 8278d 8279d 8381G 8531G 8860G 9950C 11177T 11719A 13590A 14766T 15326G 15535T 16051G 16092C 16182C 16183C 16189C 16217C 16519C	B2o	73G 146C 263G 309T 310C 499A 16051G 16092C 16182C 16183C 16189C 16217C 16519C	1	73 146 263 309d 499 750 827 1375 1438 2706 3547 4375 4769 4820 4977 6473 7028 8281-8289d 8381 8531 8860 9950 11177 11719 13590 14766 15326 15535 16051 16092 16189 16217	B2o	73 146 263 309d 499 16051 16092 16189 16217	[0.246] B2o (1) [0.000] B2c2 B2k (3)	[0.193] B2q (4) [0.167] B2o1 (5) [0.120] B2 (6) [0.057] B2b (7) [0.036] B4a1a1a11b (8) [0.009] B2c1a (9) [0.000] B4a1a1a18 B4b B2b3 B2c B2c1 B2c1b B2c1c B2c2a B2e B2f B2i B2n B2p B2r (23)	1
1043	>KY645583.1 Ho	71A 73G 146C 150T 263G 309.1CT 310C 499A 750G 827G 1438G 2706G 3106d 3107C 3547G 3553T 4769G 4820A 4977C 5954C 6473T 7028T 8271d 8272d 8273d 8274d 8275d 8276d 8277d 8278d 8279d 8812G 8860G 9950C 11177T 11719A 13590A 13651G 14766T 15326G 15535T 16092C 16129A 16183C 16189C 16217C 16257T 16296T 16348T 16519C	B2o	71A 73G 146C 150T 263G 309.1CT 310C 499A 16092C 16129A 16183C 16189C 16217C 16257T 16296T 16348T 16519C	1	71 73 146 150 263 499 750 827 1438 2706 3547 3553 4769 4820 4977 5954C 6473 7028 8281-8289d 8812 8860 9950 11177 11719 13590 13651 14766 15326 15535 16092 16129 16189 16217 16257 16296 16348	B2o	71 73 146 150 263 499 16092 16129 16189 16217 16257 16296 16348	[0.409] B2o (1) [0.041] B2k (2) [0.000] B2c2 (3)	[0.236] B4a1a1a11b (4) [0.167] B2o1 (5) [0.143] B2b (6) [0.057] B2 (7) [0.043] B2n (8) [0.008] B2q (9) [0.000] B4b B2b3 B2c B2c1 B2c1a B2c1b B2c1c B2c2a B2e B2f B2i B2p B2r (22)	1

1044	>KY645581.1 Hc	71A 73G 146C 150T 263G 309.1CT 310C 499A 750G 827G 1438G 2706G 3106d 3107C 3547G 3553T 4769G 4820A 4977C 5954C 6473T 7028T 8271d 8272d 8273d 8274d 8275d 8276d 8277d 8278d 8279d 8812G 8860G 9950C 11177T 11719A 13590A 13651G 14766T 15326G 15535T 16092C 16129A 16183C 16189C 16217C 16257T 16296T 16348T 16519C	B2o	71A 73G 146C 150T 263G 309.1CT 310C 499A 16092C 16129A 16183C 16189C 16217C 16257T 16296T 16348T 16519C	1	71 73 146 150 263 499 750 827 1438 2706 3547 3553 4769 4820 4977 5954C 6473 7028 8281-8289d 8812 8860 9950 11177 11719 13590 13651 14766 15326 15535 16092 16129 16189 16217 16257 16296 16348	B2o	71 73 146 150 263 499 16092 16129 16189 16217 16257 16296 16348	[0.409] B2o (1) [0.041] B2k (2) [0.000] B2c2 (3)	[0.236] B4a1a1a11b (4) [0.167] B2o1 (5) [0.143] B2b (6) [0.057] B2 (7) [0.043] B2n (8) [0.008] B2q (9) [0.000] B4b B2b3 B2c B2c1 B2c1a B2c1b B2c1c B2c2a B2e B2f B2i B2p B2r (22)	1
1045	>KY645579.1 Hc	73G 263G 309.1CT 310C 489C 750G 789C 1438G 2092T 2706G 3010A 3106d 3107C 3531A 4769G 4883T 4886T 4937C 5178A 5964C 6827C 7028T 8414T 8701G 8860G 9540C 10084C 10328G 10398G 10400T 10873C 11719A 12358G 12705T 14668T 14766T 14783C 15043A 15301A 15326G 16142T 16223T 16325C 16362C 16519C	D1f	73G 263G 309.1CT 310C 489C 16142T 16223T 16325C 16362C 16519C	1	73 263 489 750 789 1438 2092 2706 3010 3531 4769 4883 4886 4937 5178A 5964 6827 7028 8414 8701 8860 9540 10084 10328 10398 10400 10873 11719 12358 12705 14668 14766 14783 15043 15301 15326 16142 16223 16325 16362	D1f	73 263 489 16142 16223 16325 16362	[0.000] D1f (1)	[0.000] D1 D1a D1a2 D1b D1c D1d D1d1 D1f2 D1f+16189 D1f3 D1k D1n (13)	1
1046	>KY645568.1 Hc	64T 73G 146C 152C 153G 235G 263G 309.1CCT 310C 514d 515d 663G 750G 1438G 1736G 2706G 3106d 3107C 4248C 4769G 4824G 5063C 7028T 8027A 8794T 8860G 11719A 12007A 12705T 14766T 15326G 16111T 16223T 16239A 16266T 16290T 16319A 16362C	A2aa	64T 73G 146C 152C 153G 235G 263G 309.1CCT 310C 514d 515d 16111T 16223T 16239A 16266T 16290T 16319A 16362C	1	64 73 146 152 153 235 263 663 750 1438 1736 2706 4248 4769 4824 5063 7028 8027 8794 8860 11719 12007 12705 14766 15326 16111 16223 16239A 16266 16290 16319 16362	A2aa	64 73 146 152 153 235 263 16111 16223 16239A 16266 16290 16319 16362	[0.062] A2aa (1)	[0.094] A2+(64) (2) [0.041] A2an (3) [0.008] A2f A2k (5) [0.007] A2c (6) [0.000] A2d A2d1 A2d2 A2f1 A2f1a A2j A2j1 A2k1 A2k1a A2t A2v1+152 A2v1a A2w A2x A2ae (21)	1
1047	>KY645562.1 Hc	73G 152C 263G 309.1CT 310C 499A 750G 827G 1438G 2706G 3106d 3107C 3547G 4769G 4820A 4977C 6473T 7028T 7270C 8271d 8272d 8273d 8274d 8275d 8276d 8277d 8278d 8279d 8860G 9950C 11177T 11719A 13590A 14500G 14766T 15326G 15535T 16092C 16145A 16183C 16189C 16217C 16271C 16519C	B2o1a	73G 152C 263G 309.1CT 310C 499A 16092C 16145A 16183C 16189C 16217C 16271C 16519C	1	73 152 263 499 750 827 1438 2706 3547 4769 4820 4977 6473 7028 7270 8281-8289d 8860 9950 11177 11719 13590 14500 14766 15326 15535 16092 16145 16189 16217 16271	B2o1a	73 152 263 499 16092 16145 16189 16217 16271	[0.143] B2o1a (1)	[0.000] B2o1 (2)	1
1048	>KY645558.1 Hc	64T 73G 146C 153G 235G 263G 309.1CT 310C 514d 515d 663G 750G 1438G 1736G 2706G 3106d 3107C 3535C 4248C 4769G 4824G 7028T 7854C 8027A 8794T 8860G 11719A 12007A 12705T 13967T 14766T 15326G 16111T 16140C 16223T 16290T 16319A 16362C 16526A	A2h	64T 73G 146C 153G 235G 263G 309.1CT 310C 514d 515d 16111T 16140C 16223T 16290T 16319A 16362C 16526A	1	64 73 146 153 235 263 663 750 1438 1736 2706 3535 4248 4769 4824 7028 7854 8027 8794 8860 11719 12007 12705 13967 14766 15326 16111 16140 16223 16290 16319 16362 16526	A2h	64 73 146 153 235 263 16111 16140 16223 16290 16319 16362 16526	[0.042] A2h (1)	[0.042] A2h (2) [0.000] A2+(64) A2c A2d A2d1 A2d2 A2f A2f1 A2f1a A2h1 A2j A2j1 A2k A2k1 A2k1a A2t A2w A2x A2an (20)	1

1049	>KY645557.1 Hc	73G 152C 263G 309.1CCT 310C 499A 750G 827G 1438G 2706G 3106d 3107C 3547G 4769G 4820A 4977C 6473T 7028T 7270C 8271d 8272d 8273d 8274d 8275d 8276d 8277d 8278d 8279d 8860G 9950C 11177T 11719A 13590A 14066T 14500G 14766T 15326G 15535T 16092C 16145A 16183C 16189C 16217C 16519C	B2o1a	73G 152C 263G 309.1CCT 310C 499A 16092C 16145A 16183C 16189C 16217C 16519C	1	73 152 263 499 750 827 1438 2706 3547 4769 4820 4977 6473 7028 7270 8281-8289d 8860 9950 11177 11719 13590 14066 14500 14766 15326 15535 16092 16145 16189 16217	B2o1a	73 152 263 499 16092 16145 16189 16217	[0.000] B2o1a (1)	[0.000] B2o1 (2)	1
1050	>KY645552.1 Hc	73G 152C 263G 309.1CT 310C 499A 750G 827G 1438G 2706G 3106d 3107C 3547G 4769G 4820A 4977C 6473T 7028T 7270C 8271d 8272d 8273d 8274d 8275d 8276d 8277d 8278d 8279d 8860G 9950C 11177T 11719A 13590A 14500G 14766T 15326G 15535T 16092C 16145A 16183C 16189C 16217C 16271C 16519C	B2o1a	73G 152C 263G 309.1CT 310C 499A 16092C 16145A 16183C 16189C 16217C 16271C 16519C	1	73 152 263 499 750 827 1438 2706 3547 4769 4820 4977 6473 7028 7270 8281-8289d 8860 9950 11177 11719 13590 14500 14766 15326 15535 16092 16145 16189 16217 16271	B2o1a	73 152 263 499 16092 16145 16189 16217 16271	[0.143] B2o1a (1)	[0.000] B2o1 (2)	1
1051	>MF497518.1 Hc	263G 310.1C 456T 514d 515d 750G 1438G 3106d 3107C 4336C 4769G 7465.1C 8860G 15326G 15833T 16166G 16304C	H5a1e	263G 310.1C 456T 514d 515d 16166G 16304C	1	263 456 750 1438 4336 4769 7471.1C 8860 15326 15833 16166 16304	H5a1e	263 456 16166 16304	[0.000] H5a1e (1)	[0.005] H5 (2) [0.000] H5a H5a1 H5a1a H5a1b H5a1c H5a1c1 H5a1c1a H5a1d H5a1f H5a1h H5a1k H5a1q H5a2 H5a2a H5a7 H5a8 H5a9 H5b H5b3 H5b5 H5c H5c1 H5c1a H5d H5f H5j H5k H5p H5+709 H5v (32)	1
1052	>MF497508.1 Hc	263G 309.1CT 310C 750G 1438G 3106d 3107C 4769G 8860G 14470A 15326G 16093C 16272G 16519C	H10g	263G 309.1CT 310C 16093C 16272G 16519C	1	263 750 1438 4769 8860 14470A 15326 16093 16272	H10g	263 16093 16272	[0.000] H10g (1)	[0.000] H10+(16093) H10f H10g (4)	1
1053	>MF497498.1 Hc	73G 263G 309.1CT 310C 750G 1438G 2706G 3106d 3107C 3197C 3206Y 4769G 7028T 8227Y 8860G 9477A 11467G 11719A 12308G 12372A 12630A 13617C 13827G 13928C 14766T 14793G 15326G 16114A 16192T 16256T 16270T 16271C 16294T 16526A	U5a2a1a	73G 263G 309.1CT 310C 16114A 16192T 16256T 16270T 16271C 16294T 16526A	1	73 263 750 1438 2706 3197 3206Y 4769 7028 8227Y 8860 9477 11467 11719 12308 12372 12630 13617 13827 13928C 14766 14793 15326 16114A 16192 16256 16270 16271 16294 16526	U5a2a1a	73 263 16114A 16192 16256 16270 16271 16294 16526	[0.000] U5a2a1a (1)	[0.000] U5a2a U5a2a1 U5a2a1b U5a2a1c U5a2a1d (6)	1
1054	>MF497491.1 Hc	73G 150T 263G 309.1CT 310C 750G 1438G 1850C 2706G 3106d 3107C 3197C 4769G 5656G 7028T 7768G 8860G 9477A 10927C 11467G 11719A 12308G 12372A 12618A 13617C 14182C 14766T 15326G 16144C 16148T 16189C 16270T	U5b1b1a3	73G 150T 263G 309.1CT 310C 16144C 16148T 16189C 16270T	1	73 150 263 750 1438 1850 2706 3197 4769 5656 7028 7768 8860 9477 10927 11467 11719 12308 12372 12618 13617 14182 14766 15326 16144 16148 16189 16270	U5b1b1a3	73 150 263 16144 16148 16189 16270	[0.000] U5b1b1a3 (1)	[0.000] U5b1b1a U5b1b1a1 U5b1b1a1a U5b1b1a1b U5b1b1a2 (6)	1
1055	>MF497486.1 Hc	146C 195C 263G 309.1CT 310C 750G 961G 3106d 3107C 4769G 8448C 8860G 8898T 10376G 13759A 14097T 15326G 16086Y 16278T 16293G 16311C	H11a1	146C 195C 263G 309.1CT 310C 16086Y 16278T 16293G 16311C	1	146 195 263 750 961G 4769 8448 8860 8898 10376 13759 14097 15326 16086Y 16278 16293 16311	H11a1	146 195 263 16086Y 16278 16293 16311	[0.167] H11a1 (1)	[0.241] H11a2 (2) [0.027] H11a (3) [0.000] H11a5 H11a8 (5)	1

1056	>MF497485.1 Hc	73G 152C 263G 310.1C 325T 497T 514d 515d 750G 1189C 1438G 1811G 2706G 3106d 3107C 3480G 4769G 7028T 8860G 9055A 9698C 10029G 10398G 10550G 11299C 11467G 11485C 11719A 11840T 12308G 12372A 13740C 14167T 14766T 14798C 15326G 16224C 16311C 16519C 16566.1N	K1a4a1f	73G 152C 263G 310.1C 325T 497T 514d 515d 16224C 16311C 16519C 16566.1N	1	73 152 263 325 497 750 1189 1438 1811 2706 3480 4769 7028 8860 9055 9698 10029 10398 10550 11299 11467 11485 11719 11840 12308 12372 13740 14167 14766 14798 15326 16224 16311 16566.1N	K1a4a1f	73 152 263 325 497 16224 16311 16566.1N	[0.000] K1a4a1f (1)	[0.000] K1a1b2a K1a1b2a1 K1a1b2a1a K1a4a1f K1a4a1f1 K1a4c K1a25 (8)	1
1057	>MF521309.1 Hc	73G 152C 199C 204C 207A 250C 263G 310.1C 460C 567.1CCCC 750G 1438G 1719A 2706G 3106d 3107C 4529T 4769G 7028T 8251A 8860G 9438A 10034C 10238C 10398G 11719A 12501A 12705T 13780G 14766T 15043A 15326G 15758G 15924G 16129A 16218T 16223T 16391A 16519C	I2c	73G 152C 199C 204C 207A 250C 263G 310.1C 460C 567.1CCCC 16129A 16218T 16223T 16391A 16519C	1	73 152 199 204 207 250 263 460 573.4C 750 1438 1719 2706 4529T 4769 7028 8251 8860 9438 10034 10238 10398 11719 12501 12705 13780 14766 15043 15326 15758 15924 16129 16218 16223 16391	I2c	73 152 199 204 207 250 263 460 573.4C 16129 16218 16223 16391	[0.109] I2c (1)	[0.008] I2 (2) [0.000] I2'3 I2d I2e I2f (6)	1
1058	>MF765798.1 Hc	73G 195C 263G 309.1CCT 310C 514d 515d 750G 1438G 1811G 2706G 3106d 3107C 3480G 4769G 6221C 6546T 6599G 7028T 8860G 9055A 9698C 9948A 10454C 11467G 11719A 11914A 12372A 12771A 14167T 14766T 15326G 16129A 16182C 16183C 16189C 16234T 16304C	U8b1a1	73G 195C 263G 309.1CCT 310C 514d 515d 16129A 16182C 16183C 16189C 16234T 16304C	1	73 195 263 750 1438 1811 2706 3480 4769 6221 6546 6599 7028 8860 9055 9698 9948 10454 11467 11719 11914 12372 12771 14167 14766 15326 16129 16189 16234 16304	U8b1a1	73 195 263 16129 16189 16234 16304	[0.043] U8b1a1 (1)	[0.000] U8b1 U8b1a U8b1a2 (4)	1
1059	>MF765800.1 Hc	72C 152C 263G 310.1C 750G 1438G 2706G 3106d 3107C 3398C 4580A 4769G 7028T 8860G 10646A 12189C 12810G 15299Y 15326G 15613G 15904T 16216G 16298C 16301T 16355T	V3c	72C 152C 263G 310.1C 16216G 16298C 16301T 16355T	1	72 152 263 750 1438 2706 3398 4580 4769 7028 8860 10646 12189 12810 15299Y 15326 15613 15904 16216 16298 16301 16355	V3c	72 152 263 16216 16298 16301 16355	[0.063] V3c (1) [0.000] V16 (2)	[0.019] V1a (3) [0.010] V2 V10b V25 (6) [0.008] V1a1 (7) [0.005] V (8) [0.000] HV0 HV0a V1 V1a1b V2a V2a1 V2a1a V2b V2b1 V2b2 V2c V3 V3b V4 V8 V10 V10b1 V10b2 V11 V12 V13 V14 V15 V15a V17 V23 V24 V26 V28 [illegible]	1
1060	>MF693157.1 Hc	73G 151T 199C 263G 309.1CCT 310C 709A 750G 930A 1438G 1888A 2706G 3106d 3107C 4216C 4769G 4917G 5147A 7028T 8697A 8860G 10463C 10750G 11251G 11719A 11812G 13368A 14233G 14476A 14544A 14766T 14905A 15326G 15452A 15607G 15928A 16126C 16172C 16294T 16296T 16304C 16519C	T2b3d	73G 151T 199C 263G 309.1CCT 310C 16126C 16172C 16294T 16296T 16304C 16519C	1	73 151 199 263 709 750 930 1438 1888 2706 4216 4769 4917 5147 7028 8697 8860 10463 10750 11251 11719 11812 13368 14233 14476 14544 14766 14905 15326 15452A 15607 15928 16126 16172 16294 16296 16304	T2b3d	73 151 199 263 16126 16172 16294 16296 16304	[0.023] T2b3d (1)	[0.023] T2b3d (2) [0.010] T2b4a (3) [0.000] T2b3+151 T2b3c T2b4a1 (6)	1

1061	>MF686412.1 Hc	73G 146C 195C 263G 310.1C 513.1CACA 750G 1189C 1438G 1811G 2706G 3106d 3107C 3480G 4769G 5913A 7028T 8860G 9055A 9698C 10398G 10550G 11299C 11467G 11719A 12019T 12308G 12372A 12738G 12771A 13759A 14167T 14766T 14798C 15326G 16224C 16278T 16311C 16519C 16524G	K1b2a1a1	73G 146C 195C 263G 310.1C 513.1CACA 16224C 16278T 16311C 16519C 16524G	1	73 146 195 263 750 1189 1438 1811 2706 3480 4769 5913 7028 8860 9055 9698 10398 10550 11299 11467 11719 12019 12308 12372 12738G 12771 13759 14167 14766 14798 15326 16224 16278 16311 16524	K1b2a1a1	73 146 195 263 16224 16278 16311 16524	[0.029] K1b2a1a1 (1)	[0.000] K1b2 K1b2a K1b2a1 K1b2a1a K1b2a2 K1b2a2a K1b2a3 K1b2b (9)	1
1062	>MF498884.1 Hc	73G 263G 285T 310.1C 385G 750G 1438G 2218T 2706G 3106d 3107C 3158.1T 3591A 4769G 4991A 5480G 6026A 7028T 7581C 8573A 8860G 11719A 12308G 12372A 12879C 13104G 13422G 14070G 14364A 14766T 15148A 15326G 15954C 16129A 16183C 16189C 16249C	U1a1a+16129	73G 263G 285T 310.1C 385G 16129A 16183C 16189C 16249C	1	73 263 285 385 750 1438 2218 2706 3158.1T 3591 4769 4991 5480 6026 7028 7581 8573 8860 11719 12308 12372 12879 13104 13422 14070 14364 14766 15148 15326 15954C 16129 16189 16249	U1a1a+16129	73 263 285 385 16129 16189 16249	[0.000] U1a1a+16129 (1)	[0.000] U1a1a U1a1c1d1 (3)	1
1063	>KY941897.1 Hc	73G 248d 263G 309.1CT 310C 750G 1005C 1438G 1824C 2706G 3106d 3107C 3970T 4452C 4769G 6392C 7028T 7828G 8860G 9128C 9722C 10310A 10535C 10586A 11719A 12338C 13708A 13928C 14766T 15326G 16203G 16252d 16291T 16304C 16311C 16335G 16519C	F2a	73G 248d 263G 309.1CT 310C 16203G 16252d 16291T 16304C 16311C 16335G 16519C	1	73 249d 263 750 1005 1438 1824 2706 3970 4452 4769 6392 7028 7828 8860 9128 9722 10310 10535 10586 11719 12338 13708 13928C 14766 15326 16203 16254d 16291 16304 16311 16335	F2a	73 249d 263 16203 16254d 16291 16304 16311 16335	[0.297] F2a (1)	[0.679] F2+16291 (2) [0.017] F2a1 (3) [0.000] F2a+@16291 F2b F2g (6)	1
1064	>MF621131.1 Hc	73G 195A 263G 309.1CT 310C 489C 514d 515d 750G 1438G 2706G 3106d 3107C 4769G 7028T 8701G 8860G 9540C 10398G 10400T 10873C 11719A 12007A 12705T 14766T 14783C 15043A 15287C 15301A 15326G 15431A 16223T 16234T 16519C	M30+16234	73G 195A 263G 309.1CT 310C 489C 514d 515d 16223T 16234T 16519C	1	73 195A 263 489 750 1438 2706 4769 7028 8701 8860 9540 10398 10400 10873 11719 12007 12705 14766 14783 15043 15287 15301 15326 15431 16223 16234	M30+16234	73 195A 263 489 16223 16234	[0.000] M30+16234 (1)	[0.000] M30 M30d M30d1 M30d2 M30e M49 M49c M49c1 M49e M49e1 (11)	1
1065	>MF615390.1 Hc	195C 200G 263G 309.1CT 310C 750G 1438G 2706G 3106d 3107C 4769G 5746A 7028T 8860G 10581T 15326G 16234T 16311C 16362C	HV15	195C 200G 263G 309.1CT 310C 16234T 16311C 16362C	1	195 200 263 750 1438 2706 4769 5746 7028 8860 10581 15326 16234 16311 16362	HV15	195 200 263 16234 16311 16362	[0.148] HV15 (1) [0.000] H11 H11b H11b1 (4)	[0.243] H13b1a (5) [0.107] HV9 (6) [0.082] H4a1a4b (7) [0.062] U8b1a2+16311 (8) [0.029] P6 (9) [0.028] HV+16311 (10) [0.026] H+195 (11) [0.017] H11a2 (12) [0.014] H11a (13) [0.009] H1+16311 (14) [0.003] HV10 H3h2 H3m H76 (18) [0.000] L4 L4b HV11 HV11a HV16 HV23 HV24 H1e1a4 H1bv1 H3+16311 H3h H3h1 H3h3 H3h3a H3h3b H3h5 H3h6 H4a1a+195 H4a1a3 H4a1a3a H4a1a4 H4a1a4a H4a1a4b2 H6a1a1a H11a5 H11a8 H12 H108 H13a1a2+16311 H13a2b1 H13b H13b1+200 H13b2 H44b H80 R6 (54)	1

1066	>JQ703789.1 Ho	73G 185A 188G 228A 263G 295T 309.1CT 310C 462T 489C 750G 1438G 2706G 3010A 3106d 3107C 4216C 4769G 5000G 7028T 8860G 10398G 11251G 11719A 12612G 13708A 14766T 14798C 15326G 15452A 16069T 16126C 16163G 16519C	J1c2o	73G 185A 188G 228A 263G 295T 309.1CT 310C 462T 489C 16069T 16126C 16163G 16519C	1	73 185 188 228 263 295 462 489 750 1438 2706 3010 4216 4769 5000 7028 8860 10398 11251 11719 12612 13708 14766 14798 15326 15452A 16069 16126 16163	J1c2o	73 185 188 228 263 295 462 489 16069 16126 16163	[0.000] J1c2o (1)	[0.000] J1c2 J1c2a J1c2a1 J1c2a1a J1c2a3 J1c2b J1c2b1 J1c2b2 J1c2b5 J1c2c J1c2c2 J1c2c2a J1c2c3 J1c2d J1c2f J1c2g J1c2k J1c2l J1c2n J1c2n1 J1c2o J1c2p J1c2q J1c2q1 J1c2s J1c2t (27)	1
1067	>JQ703782.1 Ho	73G 146C 195C 263G 310.1C 497T 750G 1189C 1438G 1811G 2706G 3106d 3107C 3480G 4295G 4655A 4769G 6260A 7028T 8860G 9055A 9214G 9698C 10398G 10550G 11299C 11467G 11485C 11719A 11840T 12308G 12372A 13401C 13740C 14167T 14766T 14798C 15326G 15884A 16224C 16245T 16311C 16519C	K1a4a1a2b	73G 146C 195C 263G 310.1C 497T 16224C 16245T 16311C 16519C	1	73 146 195 263 497 750 1189 1438 1811 2706 3480 4295 4655 4769 6260 7028 8860 9055 9214 9698 10398 10550 11299 11467 11485 11719 11840 12308 12372 13401 13740 14167 14766 14798 15326 15884 16224 16245 16311	K1a4a1a2b	73 146 195 263 497 16224 16245 16311	[0.023] K1a4a1a2b (1) [0.000] K1a13 K1a13a (3)	[0.038] K1a4a1a2 (4) [0.028] K1a4a1a2a (5) [0.023] K1a4a1a2b (6) [0.007] K1a+195 (7) [0.005] K1a4a1a+195 K1a4+146 (9) [0.000] K1a4a1a1 K1a4a1a3 K1a4j K1a13 K1a13a K1a14 K1a15 K1a16 K1a26 K1b2 K1b2a K1b2a1 K1b2a1a K1b2a2 K1b2a2a K1b2a3 K1b2b (26)	1
1068	>JQ703764.1 Ho	73G 195C 263G 309.1CT 310C 497T 513.1CACA 750G 1189C 1438G 1811G 2706G 3106d 3107C 3480G 4769G 7028T 8860G 9055A 9698C 10398G 10550G 11299C 11467G 11719A 12308G 12372A 14167T 14766T 14798C 15326G 16048A 16224C 16291T 16311C 16519C	K1a10a	73G 195C 263G 309.1CT 310C 497T 513.1CACA 16048A 16224C 16291T 16311C 16519C	1	73 195 263 497 750 1189 1438 1811 2706 3480 4769 7028 8860 9055 9698 10398 10550 11299 11467 11719 12308 12372 14167 14766 14798 15326 16048 16224 16291 16311	K1a10a	73 195 263 497 16048 16224 16291 16311	[0.000] K1a10a (1)	[0.000] K1a10 K1a10a (3)	1
1069	>JQ703763.1 Ho	73G 146C 153G 263G 309.1CT 310C 497T 750G 1189C 1438G 1811G 2706G 3106d 3107C 3480G 4295G 4769G 6260A 7028T 8860G 9055A 9698C 10398G 10550G 11299C 11467G 11485C 11719A 11840T 12308G 12372A 13401C 13740C 14167T 14766T 14798C 15326G 15884A 16224C 16245T 16311C 16519C	K1a4a1a2b	73G 146C 153G 263G 309.1CT 310C 497T 16224C 16245T 16311C 16519C	1	73 146 153 263 497 750 1189 1438 1811 2706 3480 4295 4769 6260 7028 8860 9055 9698 10398 10550 11299 11467 11485 11719 11840 12308 12372 13401 13740 14167 14766 14798 15326 15884 16224 16245 16311	K1a4a1a2b	73 146 153 263 497 16224 16245 16311	[0.023] K1a4a1a2b (1)	[0.038] K1a4a1a2 (2) [0.028] K1a4a1a2a (3) [0.023] K1a4a1a2b (4) [0.000] K1a4+146 K1a4j (6)	1
1070	>JQ703762.1 Ho	73G 263G 309.1CT 310C 709A 750G 930A 1438G 1888A 2706G 3106d 3107C 3254T 4216C 4769G 4917G 5147A 7028T 8697A 8860G 10463C 11251G 11719A 11812G 13368A 14233G 14766T 14861A 14905A 15313C 15326G 15452A 15607G 15928A 16051G 16126C 16180G 16294T 16296T 16304C 16519C	T2b13a	73G 263G 309.1CT 310C 16051G 16126C 16180G 16294T 16296T 16304C 16519C	1	73 263 709 750 930 1438 1888 2706 3254 4216 4769 4917 5147 7028 8697 8860 10463 11251 11719 11812 13368 14233 14766 14861 14905 15313 15326 15452A 15607 15928 16051 16126 16180 16294 16296 16304	T2b13a	73 263 16051 16126 16180 16294 16296 16304	[0.015] T2b13a (1)	[0.015] T2b13a (2) [0.006] T2b (3) [0.004] T2b5 (4) [0.000] T2b1 T2b2 T2b3 T2b3b T2b4 T2b5a T2b7 T2b7a T2b8 T2b13 T2b13b T2b15 T2b17 T2b17a T2b19 T2b25 T2b26 T2b27 T2b28 T2b29 T2b30 T2b31 T2b32 T2b35 T2b36 T2b37 T2g1b (31)	1

1071	>JQ703755.1 Ho	73G 150T 263G 310.1C 384G 513.1CA 750G 1438G 2706G 3106d 3107C 3197C 4769G 5656G 7028T 7768G 8860G 9477A 11467G 11719A 12308G 12372A 13617C 14182C 14766T 15326G 16183C 16189C 16270T 16519C	U5b1h	73G 150T 263G 310.1C 384G 513.1CA 16183C 16189C 16270T 16519C	1	73 150 263 384 750 1438 2706 3197 4769 5656 7028 7768 8860 9477 11467 11719 12308 12372 13617 14182 14766 15326 16189 16270	U5b1h	73 150 263 384 16189 16270	[0.000] U5b1h (1)	[0.017] U5b1b1f (2) [0.000] U5b1b1+@16192 U5b1b1d U5b1+16189+@16192 U5b2a+@16192 U5b2a4 U5b2a4a U5b2a6 U5b2b4 (10)	1
1072	>JQ703754.1 Ho	73G 152C 263G 309.1CT 310C 709A 750G 1438G 1888A 2706G 3106d 3107C 4216C 4769G 4917G 5747G 7028T 8697A 8860G 10398G 10463C 11251G 11719A 11812G 13260C 13368A 13708A 13934T 14233G 14766T 14905A 15326G 15452A 15607G 15928A 16086C 16126C 16294T 16296T 16368C 16519C	T2d1a	73G 152C 263G 309.1CT 310C 16086C 16126C 16294T 16296T 16368C 16519C	1	73 152 263 709 750 1438 1888 2706 4216 4769 4917 5747 7028 8697 8860 10398 10463 11251 11719 11812 13260 13368 13708 13934 14233 14766 14905 15326 15452A 15607 15928 16086 16126 16294 16296 16368	T2d1a	73 152 263 16086 16126 16294 16296 16368	[0.026] T2d1a (1)	[0.026] T2d1a (2) [0.000] T2d1 T2n (4)	1
1073	>JQ703743.1 Ho	73G 152C 214G 248d 263G 286d 287d 309.1CT 310C 489C 513.1CA 709A 750G 1438G 1888A 2706G 3106d 3107C 3552A 4715G 4769G 7028T 7196A 8584A 8701G 8860G 9540C 9545G 9617G 10398G 10400T 10873C 11719A 11914A 12705T 13263G 14318C 14766T 14783C 15043A 15301A 15326G 15487T 15715G 15930A 16104T 16223T 16274A 16298C 16325C 16327T 16519C	C1c4	73G 152C 214G 248d 263G 286d 287d 309.1CT 310C 489C 513.1CA 16104T 16223T 16274A 16298C 16325C 16327T 16519C	1	73 152 214 249d 263 290-291d 489 709 750 1438 1888 2706 3552A 4715 4769 7028 7196A 8584 8701 8860 9540 9545 9617 10398 10400 10873 11719 11914 12705 13263 14318 14766 14783 15043 15301 15326 15487T 15715 15930 16104 16223 16274 16298 16325 16327	C1c4	73 152 214 249d 263 290-291d 489 16104 16223 16274 16298 16325 16327	[0.047] C1c4 (1)	[0.183] C1c (2) [0.015] C1 (3) [0.007] C1c2	1
1074	>JQ703740.1 Ho	73G 185A 263G 295T 310.1C 408A 462T 489C 750G 1438G 2706G 3010A 3106d 3107C 4216C 4452C 4769G 5198G 7028T 8860G 10398G 11251G 11719A 12612G 13708A 14766T 14798C 15326G 15452A 15916C 16069T 16126C 16309G	J1c5e	73G 185A 263G 295T 310.1C 408A 462T 489C 16069T 16126C 16309G	1	73 185 263 295 408A 462 489 750 1438 2706 3010 4216 4452 4769 5198 7028 8860 10398 11251 11719 12612 13708 14766 14798 15326 15452A 15916 16069 16126 16309	J1c5e	73 185 263 295 408A 462 489 16069 16126 16309	[0.005] J1c5e (1)	[0.005] J1c5e (2) [0.004] J1c J1c5 (4) [0.000] J1c3 J1c3a J1c3a1 J1c3a2 J1c3b J1c3b1 J1c3b1a J1c3b2 J1c3d J1c3g J1c3h J1c3i J1c3k J1c4 J1c4b J1c5a J1c5a1 J1c5b J1c5c J1c5d J1c5f J1c6 J1c6a J1c8 J1c8b J1c9 J1c10 J1c10a J1c15 J1c15a J1c15a1 J1c17 (36)	1
1075	>JQ703719.1 Ho	195C 257G 263G 309.1CCT 310C 477C 750G 1438G 3010A 3106d 3107C 4769G 7598A 8473C 8860G 15261A 15326G 16519C	H1c3	195C 257G 263G 309.1CCT 310C 477C 16519C	1	195 257 263 477 750 1438 3010 4769 7598 8473 8860 15261 15326	H1c3	195 257 263 477	[0.610] H1c3 (1)	[0.030] H4a1a4 (2) [0.029] H4a1a4b2 (3) [0.013] H+195 (4) [0.007] H1c2 (5) [0.006] H1c (6) [0.003] H1c4a (7) [0.000] H1c2a H1c4 H1c4a1 H1c5 H1c6 H1c7 H1c8 H1c10 H1c11 H1c12 H1c13 H1c14 H1c15 H1c16 H1c17 H1c18 H1c19 H1c20 H1c21 H1c22 H4a1a+195 H4a1a3 H4a1a3a H4a1a4a H4a1a4b H12 H108 H44b (35)	1

1076	>JQ703718.1 Ho	73G 146C 185A 188G 228A 263G 295T 310.1C 462T 489C 750G 1438G 2706G 3010A 3106d 3107C 3456C 4216C 4769G 7028T 8860G 9145A 10398G 10685A 11251G 11719A 12612G 13281C 13708A 13933G 14766T 14798C 15326G 15452A 16069T 16126C 16261A 16519C	J1c2c1a	73G 146C 185A 188G 228A 263G 295T 310.1C 462T 489C 16069T 16126C 16261A 16519C	1	73 146 185 188 228 263 295 462 489 750 1438 2706 3010 3456 4216 4769 7028 8860 9145 10398 10685 11251 11719 12612 13281 13708 13933 14766 14798 15326 15452A 16069 16126 16261A	J1c2c1a	73 146 185 188 228 263 295 462 489 16069 16126 16261A	[0.500] J1c2c1a (1) [0.000] J1c2c J1c2c2 J1c2c2a J1c2c3 (5)	[0.500] J1c2c1a (6) [0.090] J1c2p (7) [0.006] J1c2a1 (8) [0.005] J1c2a1a (9) [0.000] J1c2 J1c2a J1c2a3 J1c2b J1c2b1 J1c2b2 J1c2b5 J1c2c J1c2c1 J1c2c2 J1c2c2a J1c2c3 J1c2d J1c2f J1c2g J1c2k J1c2l J1c2n J1c2n1 J1c2q J1c2q1 J1c2s J1c2t (32)	1
1077	>JQ703713.1 Ho	73G 207A 263G 309.1CT 310C 709A 750G 930A 1438G 1888A 2706G 3106d 3107C 3398C 4216C 4243G 4769G 4917G 5147A 7028T 8697A 8860G 10463C 11251G 11719A 11812G 13368A 14233G 14766T 14905A 15326G 15452A 15607G 15928A 16126C 16294T 16296T 16304C 16519C	T2b11	73G 207A 263G 309.1CT 310C 16126C 16294T 16296T 16304C 16519C	1	73 207 263 709 750 930 1438 1888 2706 3398 4216 4243 4769 4917 5147 7028 8697 8860 10463 11251 11719 11812 13368 14233 14766 14905 15326 15452A 15607 15928 16126 16294 16296 16304	T2b11	73 207 263 16126 16294 16296 16304	[0.000] T2b11 (1)	[0.000] T2b T2b1 T2b2 T2b3 T2b3b T2b4 T2b5 T2b5a T2b7 T2b7a T2b8 T2b11 T2b13 T2b13b T2b15 T2b17 T2b17a T2b19 T2b25 T2b26 T2b27 T2b28 T2b29 T2b30 T2b31 T2b32 T2b35 T2b36 T2b37 (30)	1
1078	>JQ703694.1 Ho	73G 150T 152C 263G 309.1CT 310C 750G 1438G 1811G 2706G 3106d 3107C 4188G 4640A 4742C 4769G 7028T 8257G 8860G 9656C 11467G 11719A 12308G 12372A 13743C 14139G 14766T 15326G 15454C 16168T 16343G 16390A	U3b3	73G 150T 152C 263G 309.1CT 310C 16168T 16343G 16390A	1	73 150 152 263 750 1438 1811 2706 4188 4640A 4742 4769 7028 8257 8860 9656 11467 11719 12308 12372 13743 14139 14766 15326 15454 16168 16343 16390	U3b3	73 150 152 263 16168 16343 16390	[0.291] U3b3 (1) [0.089] U3a1 (2) [0.021] U3a2 (3) [0.016] U3a (4) [0.000] U3a1a U3a1a1 U3a1b (7)	[0.345] U3b2 (8) [0.092] U3b (9) [0.048] U3a3 (10) [0.032] U3a2a (11) [0.028] U3b2a1 (12) [0.025] U3a1c (13) [0.010] U3b1b (14) [0.000] U3 U3a'c U3b1 U3b1a1 U3b2a U3b2c (20)	1
1079	>JQ703688.1 Ho	146C 195C 263G 310.1C 750G 961G 1438G 3106d 3107C 4769G 8448C 8860G 8898T 10785Y 13759A 15326G 16278T 16293G 16311C	H11a1	146C 195C 263G 310.1C 16278T 16293G 16311C	1	146 195 263 750 961G 1438 4769 8448 8860 8898 10785Y 13759 15326 16278 16293 16311	H11a1	146 195 263 16278 16293 16311	[0.155] H11a1 (1)	[0.241] H11a2 (2) [0.027] H11a (3) [0.000] H11a5 H11a8 (5)	1
1080	>MF577010.1 Hc	73G 263G 309.1CT 310C 507C 709A 750G 1438G 1766C 1888A 2706G 3106d 3107C 4216C 4769G 4917G 7028T 7337A 8697A 8860G 10463C 11251G 11719A 11812G 13368A 13834G 14148G 14233G 14766T 14798C 14905A 15326G 15452A 15607G 15928A 16126C 16188T 16257T 16294T 16296T 16519C	T2g2a	73G 263G 309.1CT 310C 507C 16126C 16188T 16257T 16294T 16296T 16519C	1	73 263 507 709 750 1438 1766 1888 2706 4216 4769 4917 7028 7337 8697 8860 10463 11251 11719 11812 13368 13834 14148 14233 14766 14798 14905 15326 15452A 15607 15928 16126 16188 16257 16294 16296	T2g2a	73 263 507 16126 16188 16257 16294 16296	[0.000] T2g2a (1)	[0.000] T2g2 (2)	1
1081	>MF058594.1 Hc	73G 152C 195C 204C 263G 310.1C 417A 489C 533G 750G 1438G 3106d 3107C 4769G 7028T 8188G 8360G 8701G 8860G 9438A 9540C 9545G 10142T 10295G 10398G 10400T 10873C 11569C 11719A 11899C 12279G 12618A 12705T 14766T 14783C 15025T 15043A 15299C 15301A 15326G 16051G 16176T 16223T 16263C 16311C 16519C	M23	73G 152C 195C 204C 263G 310.1C 417A 489C 533G 16051G 16176T 16223T 16263C 16311C 16519C	1	73 152 195 204 263 417 489 533 750 1438 4769 7028 8188 8360 8701 8860 9438 9540 9545 10142 10295 10398 10400 10873 11569 11719 11899 12279 12618 12705 14766 14783 15025 15043 15299 15301 15326 16051 16176 16223 16263 16311	M23	73 152 195 204 263 417 489 533 16051 16176 16223 16263 16311	[0.040] M23 (1)	[0.072] M24a (2) [0.053] M57+152	1

1082 >MF058592.1 Hc	73G 152C 195C 204C 263G 310.1C 417A 489C 533G 750G 1438G 3106d 3107C 4769G 7028T 8188G 8360G 8701G 8860G 9438A 9540C 9545G 10142T 10295G 10398G 10400T 10873C 11569C 11719A 11899C 12279G 12618A 12705T 14766T 14783C 15025T 15043A 15301A 15326G 16223T 16263C 16311C 16519C	M23	73G 152C 195C 204C 263G 310.1C 417A 489C 533G 16223T 16263C 16311C 16519C	1	73 152 195 204 263 417 489 533 750 1438 4769 7028 8188 8360 8701 8860 9438 9540 9545 10142 10295 10398 10400 10873 11569 11719 11899 12279 12618 12705 14766 14783 15025 15043 15301 15326 16223 16263 16311	M23	73 152 195 204 263 417 489 533 16223 16263 16311	[0.000] M23 (1)	[0.072] M24a (2) [0.053] M57+152	1
1083 >MF058584.1 Hc	73G 152C 195C 204C 263G 310.1C 417A 489C 533G 750G 1438G 3106d 3107C 4769G 7028T 8188G 8360G 8701G 8860G 9438A 9540C 9545G 10142T 10295G 10398G 10400T 10873C 11569C 11719A 11899C 12279G 12618A 12705T 14766T 14783C 15025T 15043A 15301A 15326G 16223T 16263C 16311C 16519C	M23	73G 152C 195C 204C 263G 310.1C 417A 489C 533G 16223T 16263C 16311C 16519C	1	73 152 195 204 263 417 489 533 750 1438 4769 7028 8188 8360 8701 8860 9438 9540 9545 10142 10295 10398 10400 10873 11569 11719 11899 12279 12618 12705 14766 14783 15025 15043 15301 15326 16223 16263 16311	M23	73 152 195 204 263 417 489 533 16223 16263 16311	[0.000] M23 (1)	[0.072] M24a (2) [0.053] M57+152	1
1084 >MF058574.1 Hc	73G 150T 200G 263G 310.1C 489C 750G 2706G 3106d 3107C 3588T 4769G 7028T 7960G 8701G 8768T 8854A 8860G 9540C 10398G 10400T 10873C 11719A 12705T 13308G 13590A 13708A 13747T 14130T 14260G 14527G 14766T 14783C 15043A 15172A 15301A 15326G 15625T 16086C 16148T 16223T 16259T 16278T 16319A 16399G 16526A	M32c	73G 150T 200G 263G 310.1C 489C 16086C 16148T 16223T 16259T 16278T 16319A 16399G 16526A	1	73 150 200 263 489 750 2706 3588 4769 7028 7960 8701 8768 8854 8860 9540 10398 10400 10873 11719 12705 13308 13590 13708 13747T 14130 14260 14527 14766 14783 15043 15172 15301 15326 15625 16086 16148 16223 16259 16278 16319 16399 16526	M32c	73 150 200 263 489 16086 16148 16223 16259 16278 16319 16399 16526	[0.000] M32c (1)	[0.022] M51a (2) [0.007] M32 (3) [0.000] M51 M51b (5)	1
1085 >MF058562.1 Hc	73G 150T 200G 263G 310.1C 489C 750G 1694C 2706G 3106d 3107C 3588T 4769G 7028T 7960G 8701G 8768T 8854A 8860G 9540C 10398G 10400T 10873C 11719A 12705T 13308G 13590A 13708A 14130T 14260G 14527G 14766T 14783C 15043A 15172A 15301A 15326G 15625T 16086C 16148T 16223T 16259T 16278T 16319A 16399G 16526A	M32c	73G 150T 200G 263G 310.1C 489C 16086C 16148T 16223T 16259T 16278T 16319A 16399G 16526A	1	73 150 200 263 489 750 1694 2706 3588 4769 7028 7960 8701 8768 8854 8860 9540 10398 10400 10873 11719 12705 13308 13590 13708 14130 14260 14527 14766 14783 15043 15172 15301 15326 15625 16086 16148 16223 16259 16278 16319 16399 16526	M32c	73 150 200 263 489 16086 16148 16223 16259 16278 16319 16399 16526	[0.000] M32c (1)	[0.022] M51a (2) [0.007] M32 (3) [0.000] M51 M51b (5)	1

1086	>MF058560.1 Hc	73G 152C 182T 186A 189C 195C 247A 248G 263G 310.1C 316A 514d 515d 745.1T 750G 769A 825A 1018A 1438G 2394d 2706G 2758A 2885C 3106d 3107C 3525T 3594T 3666A 4104G 4769G 4853A 5951G 6071C 6221A 6227C 6917A 7028T 7146G 7247T 7256T 7389C 7521A 8027A 8468T 8655T 8701G 8860G 9072G 9540C 10398G 10586A 10688A 10810C 10873C 11302T 11719A 11984C 12507G 12705T 12810G 13105G 13485G 13506T 13650T 13789C 14000A 14178C 14560A 14669G 14766T 14911T 14926G 15226G 15326G 15905C 15978T 16129A 16187T 16189C 16223T 16278T 16293G 16294T 16311C 16360T 16519C	L1c3c	73G 152C 182T 186A 189C 195C 247A 248G 263G 310.1C 316A 514d 515d 16129A 16187T 16189C 16223T 16278T 16293G 16294T 16311C 16360T 16519C	1	73 152 182 186A 189C 195 247 248 263 316 745.1T 750 769 825A 1018 1438 2395d 2706 2758 2885 3525 3594 3666 4104 4769 4853 5951 6071 6221A 6227 6917 7028 7146 7247 7256 7389 7521 8027 8468 8655 8701 8860 9072 9540 10398 10586 10688 10810 10873 11302 11719 11984 12507 12705 12810 13105 13485 13506 13650 13789 14000A 14178 14560 14669 14766 14911 14926 15226 15326 15905 15978 16129 16187 16189 16223 16278 16293 16294 16311 16360	L1c3c	73 152 182 186A 189C 195 247 248 263 316 16129 16187 16189 16223 16278 16293 16294 16311 16360	[2.063] L1c3c (1) [0.000] L1c L1c1a+@198 L1c3b'c (4)	[0.700] L1c3 (5) [0.572] L1c1a'b'd (6) [0.267] L1c3b1 (7) [0.133] L1c3b1b (8) [0.000] L1c1'2'4'5'6 L1c1 L1c1a L1c1a1 L1c3b (13)	1
1087	>MF058548.1 Hc	73G 152C 195C 204C 263G 310.1C 417A 489C 533G 750G 1438G 3106d 3107C 4769G 7028T 8188G 8360G 8701G 8860G 9438A 9540C 9545G 10142T 10295G 10398G 10400T 10873C 11569C 11719A 11899C 12279G 12618A 12705T 14766T 14783C 15025T 15043A 15301A 15326G 16223T 16263C 16311C 16519C	M23	73G 152C 195C 204C 263G 310.1C 417A 489C 533G 16223T 16263C 16311C 16519C	1	73 152 195 204 263 417 489 533 750 1438 4769 7028 8188 8360 8701 8860 9438 9540 9545 10142 10295 10398 10400 10873 11569 11719 11899 12279 12618 12705 14766 14783 15025 15043 15301 15326 16223 16263 16311	M23	73 152 195 204 263 417 489 533 16223 16263 16311	[0.000] M23 (1)	[0.072] M24a (2) [0.053] M57+152	1
1088	>MF058543.1 Hc	73G 150T 200G 263G 310.1C 489C 750G 2706G 3106d 3107C 3588T 4769G 7028T 7960G 8701G 8768T 8854A 8860G 9540C 10398G 10400T 10873C 11719A 12705T 13308G 13590A 13708A 14130T 14260G 14527G 14766T 14783C 15043A 15172A 15301A 15326G 15625T 16086C 16148T 16223T 16259T 16278T 16319A 16399G 16526A	M32c	73G 150T 200G 263G 310.1C 489C 16086C 16148T 16223T 16259T 16278T 16319A 16399G 16526A	1	73 150 200 263 489 750 2706 3588 4769 7028 7960 8701 8768 8854 8860 9540 10398 10400 10873 11719 12705 13308 13590 13708 14130 14260 14527 14766 14783 15043 15172 15301 15326 15625 16086 16148 16223 16259 16278 16319 16399 16526	M32c	73 150 200 263 489 16086 16148 16223 16259 16278 16319 16399 16526	[0.000] M32c (1)	[0.022] M51a (2) [0.007] M32 (3) [0.000] M51 M51b (5)	1
1089	>MF058542.1 Hc	73G 152C 195C 204C 263G 310.1C 417A 489C 533G 750G 1438G 3106d 3107C 4769G 7028T 8188G 8360G 8701G 8860G 9438A 9540C 9545G 10142T 10295G 10398G 10400T 10873C 11569C 11719A 11899C 12279G 12618A 12705T 14766T 14783C 15025T 15043A 15301A 15326G 16176T 16223T 16263C 16311C 16519C	M23	73G 152C 195C 204C 263G 310.1C 417A 489C 533G 16176T 16223T 16263C 16311C 16519C	1	73 152 195 204 263 417 489 533 750 1438 4769 7028 8188 8360 8701 8860 9438 9540 9545 10142 10295 10398 10400 10873 11569 11719 11899 12279 12618 12705 14766 14783 15025 15043 15301 15326 16176 16223 16263 16311	M23	73 152 195 204 263 417 489 533 16176 16223 16263 16311	[0.024] M23 (1)	[0.072] M24a (2) [0.053] M57+152	1

1090	>MF058536.1 Hc	73G 152C 182T 186A 189C 195C 247A 248G 263G 310.1C 316A 514d 515d 745.1T 750G 769A 825A 1018A 1438G 2394d 2706G 2758A 2885C 3106d 3107C 3525T 3594T 3666A 4104G 4448C 4769G 4853A 5951G 6071C 6221A 6227C 6917A 7028T 7146G 7247T 7256T 7389C 7521A 8027A 8468T 8655T 8701G 8860G 9072G 9540C 10398G 10586A 10688A 10810C 10873C 11302T 11719A 11984C 12507G 12705T 12810G 13105G 13485G 13506T 13650T 13789C 14000A 14178C 14560A 14669G 14766T 14911T 14926G 15226G 15326G 15905C 15978T 16129A 16187T 16189C 16223T 16278T 16293G 16294T 16311C 16360T 16519C	L1c3c	73G 152C 182T 186A 189C 195C 247A 248G 263G 310.1C 316A 514d 515d 16129A 16187T 16189C 16223T 16278T 16293G 16294T 16311C 16360T 16519C	1	73 152 182 186A 189C 195 247 248 263 316 745.1T 750 769 825A 1018 1438 2395d 2706 2758 2885 3525 3594 3666 4104 4448 4769 4853 5951 6071 6221A 6227 6917 7028 7146 7247 7256 7389 7521 8027 8468 8655 8701 8860 9072 9540 10398 10586 10688 10810 10873 11302 11719 11984 12507 12705 12810 13105 13485 13506 13650 13789 14000A 14178 14560 14669 14766 14911 14926 15226 15326 15905 15978 16129 16187 16189 16223 16278 16293 16294 16311 16360	L1c3c	73 152 182 186A 189C 195 247 248 263 316 16129 16187 16189 16223 16278 16293 16294 16311 16360	[2.063] L1c3c (1) [0.000] L1c L1c1a+@198 L1c3b'c (4)	[0.700] L1c3 (5) [0.572] L1c1a'b'd (6) [0.267] L1c3b1 (7) [0.133] L1c3b1b (8) [0.000] L1c1'2'4'5'6 L1c1 L1c1a L1c1a1 L1c3b (13)	1
1091	>MF058525.1 Hc	73G 152C 182T 186A 189C 195C 247A 248G 263G 310.1C 316A 514d 515d 745.1T 750G 769A 825A 1018A 1438G 2394d 2706G 2758A 2885C 3106d 3107C 3525T 3594T 3666A 4104G 4769G 4853A 5951G 6071C 6221A 6227C 6917A 7028T 7146G 7247T 7256T 7389C 7521A 8027A 8468T 8655T 8701G 8860G 9072G 9540C 10398G 10586A 10688A 10810C 10873C 11302T 11719A 11984C 12507G 12705T 12810G 13105G 13485G 13506T 13650T 13789C 14000A 14178C 14560A 14669G 14766T 14911T 14926G 15226G 15326G 15905C 15978T 16129A 16187T 16189C 16223T 16278T 16293G 16294T 16311C 16360T 16519C	L1c3c	73G 152C 182T 186A 189C 195C 247A 248G 263G 310.1C 316A 514d 515d 16129A 16187T 16189C 16223T 16278T 16293G 16294T 16311C 16360T 16519C	1	73 152 182 186A 189C 195 247 248 263 316 745.1T 750 769 825A 1018 1438 2395d 2706 2758 2885 3525 3594 3666 4104 4769 4853 5951 6071 6221A 6227 6917 7028 7146 7247 7256 7389 7521 8027 8468 8655 8701 8860 9072 9540 10398 10586 10688 10810 10873 11302 11719 11984 12507 12705 12810 13105 13485 13506 13650 13789 14000A 14178 14560 14669 14766 14911 14926 15226 15326 15905 15978 16129 16187 16189 16223 16278 16293 16294 16311 16360	L1c3c	73 152 182 186A 189C 195 247 248 263 316 16129 16187 16189 16223 16278 16293 16294 16311 16360	[2.063] L1c3c (1) [0.000] L1c L1c1a+@198 L1c3b'c (4)	[0.700] L1c3 (5) [0.572] L1c1a'b'd (6) [0.267] L1c3b1 (7) [0.133] L1c3b1b (8) [0.000] L1c1'2'4'5'6 L1c1 L1c1a L1c1a1 L1c3b (13)	1
1092	>MF058523.1 Hc	73G 152C 195C 204C 263G 310.1C 417A 489C 533G 750G 1438G 3106d 3107C 4769G 7028T 8188G 8360G 8701G 8860G 9438A 9540C 9545G 10142T 10295G 10398G 10400T 10873C 11569C 11719A 11899C 12279G 12618A 12705T 14766T 14783C 15025T 15043A 15301A 15326G 16223T 16263C 16311C 16519C	M23	73G 152C 195C 204C 263G 310.1C 417A 489C 533G 16223T 16263C 16311C 16519C	1	73 152 195 204 263 417 489 533 750 1438 4769 7028 8188 8360 8701 8860 9438 9540 9545 10142 10295 10398 10400 10873 11569 11719 11899 12279 12618 12705 14766 14783 15025 15043 15301 15326 16223 16263 16311	M23	73 152 195 204 263 417 489 533 16223 16263 16311	[0.000] M23 (1)	[0.072] M24a (2) [0.053] M57+152	1

1093	>MF058514.1 Ho	73G 150T 200G 263G 310.1C 489C 750G 2706G 3106d 3107C 3588T 4769G 7028T 7960G 8701G 8768T 8854A 8860G 9540C 10398G 10400T 10873C 11719A 12705T 13308G 13590A 13708A 14130T 14260G 14527G 14766T 14783C 15043A 15172A 15301A 15326G 15625T 16086C 16148T 16223T 16259T 16278T 16319A 16399G 16526A	M32c	73G 150T 200G 263G 310.1C 489C 16086C 16148T 16223T 16259T 16278T 16319A 16399G 16526A	1	73 150 200 263 489 750 2706 3588 4769 7028 7960 8701 8768 8854 8860 9540 10398 10400 10873 11719 12705 13308 13590 13708 14130 14260 14527 14766 14783 15043 15172 15301 15326 15625 16086 16148 16223 16259 16278 16319 16399 16526	M32c	73 150 200 263 489 16086 16148 16223 16259 16278 16319 16399 16526	[0.000] M32c (1)	[0.022] M51a (2) [0.007] M32 (3) [0.000] M51 M51b (5)	1
1094	>MF058511.1 Ho	73G 146C 152C 195C 263G 310.1C 750G 769A 1018A 1438G 2416C 2706G 2789T 3106d 3107C 3594T 4104G 4769G 5090C 5319G 7028T 7175C 7256T 7274T 7521A 7771G 8206A 8701G 8860G 8867C 9221G 9540C 10115C 10143A 10398G 10873C 11719A 11914A 11944C 12693G 12705T 13590A 13650T 13803G 14566G 14766T 15301A 15326G 15735T 15784C 16182C 16183C 16189C 16223T 16266d 16278T 16290T 16294T 16309G 16390A	L2a1b1a	73G 146C 152C 195C 263G 310.1C 16182C 16183C 16189C 16223T 16266d 16278T 16290T 16294T 16309G 16390A	1	73 146 152 195 263 750 769 1018 1438 2416 2706 2789 3594 4104 4769 5090 5319 7028 7175 7256 7274 7521 7771 8206 8701 8860 8867 9221 9540 10115 10143 10398 10873 11719 11914 11944 12693 12705 13590 13650 13803 14566 14766 15301 15326 15735 15784 16189 16223 16268d 16278 16290 16294 16309 16390	L2a1b1a	73 146 152 195 263 16189 16223 16268d 16278 16290 16294 16309 16390	[0.003] L2a1b1a (1)	[0.005] L2a1f (2) [0.003] L2a1b1a (3) [0.000] L2a1+16189_(16192) L2a1b L2a1b1 L2a1b2 L2a1f1 L2a1f1a L2a1f2 (10)	1
1095	>MF058510.1 Ho	73G 152C 195C 204C 263G 309.1CT 310C 417A 489C 533G 750G 1438G 3106d 3107C 4769G 7028T 8188G 8360G 8701G 8860G 9438A 9540C 9545G 10142T 10295G 10398G 10400T 10873C 11569C 11719A 11899C 12279G 12618A 12705T 14766T 14783C 15025T 15043A 15301A 15326G 16223T 16263C 16311C 16519C	M23	73G 152C 195C 204C 263G 309.1CT 310C 417A 489C 533G 16223T 16263C 16311C 16519C	1	73 152 195 204 263 417 489 533 750 1438 4769 7028 8188 8360 8701 8860 9438 9540 9545 10142 10295 10398 10400 10873 11569 11719 11899 12279 12618 12705 14766 14783 15025 15043 15301 15326 16223 16263 16311	M23	73 152 195 204 263 417 489 533 16223 16263 16311	[0.000] M23 (1)	[0.072] M24a (2) [0.053] M57+152	1
1096	>MF058500.1 Ho	73G 152C 195C 204C 263G 310.1C 417A 489C 533G 750G 1438G 3106d 3107C 4769G 7028T 8188G 8360G 8701G 8860G 9438A 9540C 9545G 10142T 10295G 10398G 10400T 10873C 11569C 11719A 11899C 12279G 12618A 12705T 14766T 14783C 15025T 15043A 15301A 15326G 16223T 16263C 16311C 16519C	M23	73G 152C 195C 204C 263G 310.1C 417A 489C 533G 16223T 16263C 16311C 16519C	1	73 152 195 204 263 417 489 533 750 1438 4769 7028 8188 8360 8701 8860 9438 9540 9545 10142 10295 10398 10400 10873 11569 11719 11899 12279 12618 12705 14766 14783 15025 15043 15301 15326 16223 16263 16311	M23	73 152 195 204 263 417 489 533 16223 16263 16311	[0.000] M23 (1)	[0.072] M24a (2) [0.053] M57+152	1

1097	>MF058499.1 Hc	73G 152C 195C 204C 263G 310.1C 417A 489C 533G 750G 1438G 3106d 3107C 4769G 7028T 8188G 8360G 8701G 8860G 9438A 9540C 9545G 10142T 10295G 10398G 10400T 10873C 11569C 11719A 11899C 12279G 12618A 12705T 14766T 14783C 15025T 15043A 15301A 15326G 16223T 16263C 16311C 16519C	M23	73G 152C 195C 204C 263G 310.1C 417A 489C 533G 16223T 16263C 16311C 16519C	1	73 152 195 204 263 417 489 533 750 1438 4769 7028 8188 8360 8701 8860 9438 9540 9545 10142 10295 10398 10400 10873 11569 11719 11899 12279 12618 12705 14766 14783 15025 15043 15301 15326 16223 16263 16311	M23	73 152 195 204 263 417 489 533 16223 16263 16311	[0.000] M23 (1)	[0.072] M24a (2) [0.053] M57+152	1
1098	>MF058491.1 Hc	73G 150T 200G 263G 310.1C 489C 750G 2706G 3106d 3107C 3588T 4769G 7028T 7960G 8701G 8768T 8854A 8860G 9540C 10398G 10400T 10873C 11719A 12705T 13308G 13590A 13708A 14130T 14260G 14527G 14766T 14783C 15043A 15172A 15301A 15326G 15625T 16086C 16148T 16223T 16259T 16278T 16319A 16399G 16526A	M32c	73G 150T 200G 263G 310.1C 489C 16086C 16148T 16223T 16259T 16278T 16319A 16399G 16526A	1	73 150 200 263 489 750 2706 3588 4769 7028 7960 8701 8768 8854 8860 9540 10398 10400 10873 11719 12705 13308 13590 13708 14130 14260 14527 14766 14783 15043 15172 15301 15326 15625 16086 16148 16223 16259 16278 16319 16399 16526	M32c	73 150 200 263 489 16086 16148 16223 16259 16278 16319 16399 16526	[0.000] M32c (1)	[0.022] M51a (2) [0.007] M32 (3) [0.000] M51 M51b (5)	1
1099	>MF058485.1 Hc	73G 150T 200G 263G 310.1C 489C 750G 2706G 3106d 3107C 3588T 4769G 7028T 7960G 8701G 8768T 8854A 8860G 9540C 10398G 10400T 10873C 11719A 12705T 13308G 13590A 13708A 14130T 14260G 14527G 14766T 14783C 15043A 15172A 15301A 15326G 15625T 16086C 16148T 16223T 16259T 16278T 16319A 16399G 16526A	M32c	73G 150T 200G 263G 310.1C 489C 16086C 16148T 16223T 16259T 16278T 16319A 16399G 16526A	1	73 150 200 263 489 750 2706 3588 4769 7028 7960 8701 8768 8854 8860 9540 10398 10400 10873 11719 12705 13308 13590 13708 14130 14260 14527 14766 14783 15043 15172 15301 15326 15625 16086 16148 16223 16259 16278 16319 16399 16526	M32c	73 150 200 263 489 16086 16148 16223 16259 16278 16319 16399 16526	[0.000] M32c (1)	[0.022] M51a (2) [0.007] M32 (3) [0.000] M51 M51b (5)	1
1100	>MF058484.1 Hc	73G 152C 195C 204C 263G 310.1C 417A 489C 533G 750G 1438G 3106d 3107C 4769G 7028T 8188G 8360G 8701G 8860G 9438A 9540C 9545G 10142T 10295G 10398G 10400T 10873C 11569C 11719A 11899C 12279G 12618A 12705T 14766T 14783C 15025T 15043A 15301A 15326G 16223T 16263C 16311C 16519C	M23	73G 152C 195C 204C 263G 310.1C 417A 489C 533G 16223T 16263C 16311C 16519C	1	73 152 195 204 263 417 489 533 750 1438 4769 7028 8188 8360 8701 8860 9438 9540 9545 10142 10295 10398 10400 10873 11569 11719 11899 12279 12618 12705 14766 14783 15025 15043 15301 15326 16223 16263 16311	M23	73 152 195 204 263 417 489 533 16223 16263 16311	[0.000] M23 (1)	[0.072] M24a (2) [0.053] M57+152	1
1101	>MF058481.1 Hc	73G 150T 200G 263G 310.1C 489C 750G 2706G 3106d 3107C 3588T 4769G 7028T 7960G 8701G 8768T 8854A 8860G 9540C 10398G 10400T 10873C 11719A 12705T 13308G 13590A 13708A 14130T 14260G 14527G 14766T 14783C 15043A 15172A 15301A 15326G 15625T 16086C 16148T 16223T 16259T 16278T 16319A 16399G 16526A	M32c	73G 150T 200G 263G 310.1C 489C 16086C 16148T 16223T 16259T 16278T 16319A 16399G 16526A	1	73 150 200 263 489 750 2706 3588 4769 7028 7960 8701 8768 8854 8860 9540 10398 10400 10873 11719 12705 13308 13590 13708 14130 14260 14527 14766 14783 15043 15172 15301 15326 15625 16086 16148 16223 16259 16278 16319 16399 16526	M32c	73 150 200 263 489 16086 16148 16223 16259 16278 16319 16399 16526	[0.000] M32c (1)	[0.022] M51a (2) [0.007] M32 (3) [0.000] M51 M51b (5)	1

1102	>MF058480.1 Ho	73G 150T 200G 263G 310.1C 489C 750G 2706G 3106d 3107C 3588T 4769G 7028T 7960G 8701G 8768T 8854A 8860G 9540C 10398G 10400T 10873C 11719A 12705T 13308G 13590A 13708A 14130T 14260G 14527G 14766T 14783C 15043A 15172A 15301A 15326G 15625T 16086C 16148T 16223T 16259T 16278T 16319A 16399G 16526A	M32c	73G 150T 200G 263G 310.1C 489C 16086C 16148T 16223T 16259T 16278T 16319A 16399G 16526A	1	73 150 200 263 489 750 2706 3588 4769 7028 7960 8701 8768 8854 8860 9540 10398 10400 10873 11719 12705 13308 13590 13708 14130 14260 14527 14766 14783 15043 15172 15301 15326 15625 16086 16148 16223 16259 16278 16319 16399 16526	M32c	73 150 200 263 489 16086 16148 16223 16259 16278 16319 16399 16526	[0.000] M32c (1)	[0.022] M51a (2) [0.007] M32 (3) [0.000] M51 M51b (5)	1
1103	>MF058478.1 Ho	73G 150T 200G 263G 310.1C 489C 750G 2706G 3106d 3107C 3588T 4769G 7028T 7960G 8701G 8768T 8854A 8860G 9540C 10398G 10400T 10873C 11719A 12705T 13308G 13590A 13708A 14130T 14260G 14527G 14766T 14783C 15043A 15172A 15301A 15326G 15625T 16086C 16148T 16223T 16259T 16278T 16319A 16399G 16526A	M32c	73G 150T 200G 263G 310.1C 489C 16086C 16148T 16223T 16259T 16278T 16319A 16399G 16526A	1	73 150 200 263 489 750 2706 3588 4769 7028 7960 8701 8768 8854 8860 9540 10398 10400 10873 11719 12705 13308 13590 13708 14130 14260 14527 14766 14783 15043 15172 15301 15326 15625 16086 16148 16223 16259 16278 16319 16399 16526	M32c	73 150 200 263 489 16086 16148 16223 16259 16278 16319 16399 16526	[0.000] M32c (1)	[0.022] M51a (2) [0.007] M32 (3) [0.000] M51 M51b (5)	1
1104	>MF058477.1 Ho	73G 152C 195C 204C 263G 310.1C 417A 489C 533G 750G 1438G 3106d 3107C 4769G 7028T 8188G 8360G 8701G 8860G 9438A 9540C 9545G 10142T 10295G 10398G 10400T 10873C 11569C 11719A 11899C 12279G 12618A 12705T 14766T 14783C 15025T 15043A 15301A 15326G 16223T 16263C 16311C 16519C	M23	73G 152C 195C 204C 263G 310.1C 417A 489C 533G 16223T 16263C 16311C 16519C	1	73 152 195 204 263 417 489 533 750 1438 4769 7028 8188 8360 8701 8860 9438 9540 9545 10142 10295 10398 10400 10873 11569 11719 11899 12279 12618 12705 14766 14783 15025 15043 15301 15326 16223 16263 16311	M23	73 152 195 204 263 417 489 533 16223 16263 16311	[0.000] M23 (1)	[0.072] M24a (2) [0.053] M57+152	1
1105	>MF058475.1 Ho	73G 152C 195C 204C 263G 310.1C 417A 489C 533G 750G 1438G 3106d 3107C 4769G 7028T 8188G 8360G 8701G 8860G 9438A 9540C 9545G 10142T 10295G 10398G 10400T 10873C 11569C 11719A 11899C 12279G 12618A 12705T 14129d 14135d 14362T 14766T 14783C 15025T 15043A 15301A 15326G 16086C 16148T 16223T 16263C 16311C 16519C	M23	73G 152C 195C 204C 263G 310.1C 417A 489C 533G 16086C 16148T 16223T 16263C 16311C 16519C	1	73 152 195 204 263 417 489 533 750 1438 4769 7028 8188 8360 8701 8860 9438 9540 9545 10142 10295 10398 10400 10873 11569 11719 11899 12279 12618 12705 14131d 14135d 14362 14766 14783 15025 15043 15301 15326 16086 16148 16223 16263 16311	M23	73 152 195 204 263 417 489 533 16086 16148 16223 16263 16311	[0.024] M23 (1)	[0.079] M57+152 (2) [0.072] M24a (3) [0.000] M28a+204	1
1106	>MF058473.1 Ho	73G 152C 195C 204C 263G 310.1C 417A 489C 533G 750G 1438G 3106d 3107C 4769G 7028T 8188G 8360G 8701G 8860G 9438A 9540C 9545G 10142T 10295G 10398G 10400T 10873C 11569C 11719A 11899C 12279G 12618A 12705T 14766T 14783C 15025T 15043A 15301A 15326G 16223T 16263C 16311C 16519C	M23	73G 152C 195C 204C 263G 310.1C 417A 489C 533G 16223T 16263C 16311C 16519C	1	73 152 195 204 263 417 489 533 750 1438 4769 7028 8188 8360 8701 8860 9438 9540 9545 10142 10295 10398 10400 10873 11569 11719 11899 12279 12618 12705 14766 14783 15025 15043 15301 15326 16223 16263 16311	M23	73 152 195 204 263 417 489 533 16223 16263 16311	[0.000] M23 (1)	[0.072] M24a (2) [0.053] M57+152	1

1107	>MF058470.1 Hc	73G 150T 200G 263G 310.1C 489C 750G 2706G 3106d 3107C 3588T 4769G 7028T 7960G 8701G 8768T 8854A 8860G 9540C 10398G 10400T 10873C 11719A 12705T 13308G 13590A 13708A 14130T 14260G 14527G 14766T 14783C 15043A 15172A 15301A 15326G 15625T 16086C 16148T 16223T 16259T 16278T 16319A 16399G 16526A	M32c	73G 150T 200G 263G 310.1C 489C 16086C 16148T 16223T 16259T 16278T 16319A 16399G 16526A	1	73 150 200 263 489 750 2706 3588 4769 7028 7960 8701 8768 8854 8860 9540 10398 10400 10873 11719 12705 13308 13590 13708 14130 14260 14527 14766 14783 15043 15172 15301 15326 15625 16086 16148 16223 16259 16278 16319 16399 16526	M32c	73 150 200 263 489 16086 16148 16223 16259 16278 16319 16399 16526	[0.000] M32c (1)	[0.022] M51a (2) [0.007] M32 (3) [0.000] M51 M51b (5)	1
1108	>MF058469.1 Hc	73G 143A 146C 152C 182T 183G 263G 310.1C 466C 511T 514d 515d 709A 750G 769A 1018A 1438G 2416C 2626C 2706G 3106d 3107C 3594T 3654T 4104G 4769G 4823C 5246A 5263T 6040C 6080G 6248C 6497C 7028T 7175C 7256T 7521A 8206A 8701G 8723A 8860G 9221G 9540C 10115C 10398G 10873C 11719A 11815A 11944C 12172G 12425G 12705T 12879C 12967G 13470G 13590A 13650T 13810A 14088C 14766T 14890G 15301A 15326G 16183C 16189C 16223T 16224C 16278T 16309G 16390A 16519C	L2a5	73G 143A 146C 152C 182T 183G 263G 310.1C 466C 511T 514d 515d 16183C 16189C 16223T 16224C 16278T 16309G 16390A 16519C	1	73 143 146 152 182 183 263 466 511 709 750 769 1018 1438 2416 2626 2706 3594 3654 4104 4769 4823 5246A 5263 6040C 6080 6248 6497 7028 7175 7256 7521 8206 8701 8723 8860 9221 9540 10115 10398 10873 11719 11815A 11944 12172 12425 12705 12879 12967 13470 13590 13650 13810 14088 14766 14890 15301 15326 16189 16223 16224 16278 16309 16390	L2a5	73 143 146 152 182 183 263 466 511 16189 16223 16224 16278 16309 16390	[2.188] L2a5 (1)	[0.030] L2a1b+143 (2) [0.028] L2a1n (3) [0.008] L2a1+143+16189_(16192) (4) [0.000] L2a1j L2a1m L2a1ml L2a1m1a L2a1o L2a1p (10)	1
1109	>MF058466.1 Hc	73G 150T 200G 263G 310.1C 489C 750G 2706G 3106d 3107C 3588T 4769G 7028T 7960G 8701G 8768T 8854A 8860G 9540C 10398G 10400T 10873C 11719A 12705T 13308G 13590A 13708A 14130T 14260G 14527G 14766T 14783C 15043A 15172A 15301A 15326G 15625T 16086C 16148T 16223T 16259T 16278T 16319A 16399G 16526A	M32c	73G 150T 200G 263G 310.1C 489C 16086C 16148T 16223T 16259T 16278T 16319A 16399G 16526A	1	73 150 200 263 489 750 2706 3588 4769 7028 7960 8701 8768 8854 8860 9540 10398 10400 10873 11719 12705 13308 13590 13708 14130 14260 14527 14766 14783 15043 15172 15301 15326 15625 16086 16148 16223 16259 16278 16319 16399 16526	M32c	73 150 200 263 489 16086 16148 16223 16259 16278 16319 16399 16526	[0.000] M32c (1)	[0.022] M51a (2) [0.007] M32 (3) [0.000] M51 M51b (5)	1
1110	>MF057143.1 Hc	73G 146C 152C 195C 263G 750G 769A 1018A 1438G 2416C 2706G 2789T 3106d 3107C 3594T 4104G 4769G 5090C 7028T 7175C 7256T 7274T 7521A 7771G 8206A 8701G 8860G 9221G 9540C 10115C 10143A 10398G 10873C 11719A 11914A 11944C 12693G 12705T 13590A 13650T 13803G 14566G 14766T 15301A 15326G 15735T 15784C 16182C 16183C 16189C 16223T 16278T 16290T 16294T 16309G 16390A	L2a1b1a	73G 146C 152C 195C 263G 16182C 16183C 16189C 16223T 16278T 16290T 16294T 16309G 16390A	1	73 146 152 195 263 750 769 1018 1438 2416 2706 2789 3594 4104 4769 5090 7028 7175 7256 7274 7521 7771 8206 8701 8860 9221 9540 10115 10143 10398 10873 11719 11914 11944 12693 12705 13590 13650 13803 14566 14766 15301 15326 15735 15784 16189 16223 16278 16290 16294 16309 16390	L2a1b1a	73 146 152 195 263 16189 16223 16278 16290 16294 16309 16390	[0.000] L2a1b1a (1)	[0.005] L2a1f (2) [0.000] L2a1+16189_(16192) L2a1b L2a1b1 L2a1b1a L2a1b2 L2a1f1 L2a1f1a L2a1f2 (10)	1

1111 >MF057136.1 Hc	73G 150T 200G 263G 489C 750G 2706G 3106d 3107C 3588T 4769G 7028T 7960G 8701G 8768T 8854A 8860G 9540C 10398G 10400T 10873C 11719A 12705T 13308G 13590A 13708A 14130T 14260G 14527G 14766T 14783C 15043A 15172A 15301A 15326G 15625T 16086C 16148T 16223T 16259T 16278T 16319A 16399G 16526A	M32c	73G 150T 200G 263G 489C 16086C 16148T 16223T 16259T 16278T 16319A 16399G 16526A	1	73 150 200 263 489 750 2706 3588 4769 7028 7960 8701 8768 8854 8860 9540 10398 10400 10873 11719 12705 13308 13590 13708 14130 14260 14527 14766 14783 15043 15172 15301 15326 15625 16086 16148 16223 16259 16278 16319 16399 16526	M32c	73 150 200 263 489 16086 16148 16223 16259 16278 16319 16399 16526	[0.000] M32c (1)	[0.022] M51a (2) [0.007] M32 (3) [0.000] M51 M51b (5)	1
1112 >MF057135.1 Hc	73G 146C 152C 195C 263G 750G 769A 1018A 1438G 2416C 2706G 2789T 3106d 3107C 3594T 4104G 4769G 5090C 7028T 7175C 7256T 7274T 7521A 7771G 8206A 8701G 8860G 9221G 9540C 10115C 10143A 10398G 10873C 11719A 11914A 11944C 12693G 12705T 13590A 13650T 13803G 14566G 14766T 15301A 15326G 15735T 15784C 16183C 16189C 16223T 16278T 16290T 16294T 16309G 16390A	L2a1b1a	73G 146C 152C 195C 263G 16183C 16189C 16223T 16278T 16290T 16294T 16309G 16390A	1	73 146 152 195 263 750 769 1018 1438 2416 2706 2789 3594 4104 4769 5090 7028 7175 7256 7274 7521 7771 8206 8701 8860 9221 9540 10115 10143 10398 10873 11719 11914 11944 12693 12705 13590 13650 13803 14566 14766 15301 15326 15735 15784 16189 16223 16278 16290 16294 16309 16390	L2a1b1a	73 146 152 195 263 16189 16223 16278 16290 16294 16309 16390	[0.000] L2a1b1a (1)	[0.005] L2a1f (2) [0.000] L2a1+16189_(16192) L2a1b L2a1b1 L2a1b1a L2a1b2 L2a1f1 L2a1f1a L2a1f2 (10)	1
1113 >MF057133.1 Hc	73G 146C 152C 195C 263G 750G 769A 1018A 1438G 2416C 2706G 2789T 3106d 3107C 3594T 4104G 4769G 5090C 7028T 7175C 7256T 7274T 7521A 7771G 8206A 8701G 8860G 9221G 9540C 10115C 10143A 10398G 10873C 11719A 11914A 11944C 12693G 12705T 13590A 13650T 13803G 14566G 14766T 15301A 15326G 15735T 15784C 16183C 16189C 16223T 16278T 16290T 16294T 16309G 16390A	L2a1b1a	73G 146C 152C 195C 263G 16183C 16189C 16223T 16278T 16290T 16294T 16309G 16390A	1	73 146 152 195 263 750 769 1018 1438 2416 2706 2789 3594 4104 4769 5090 7028 7175 7256 7274 7521 7771 8206 8701 8860 9221 9540 10115 10143 10398 10873 11719 11914 11944 12693 12705 13590 13650 13803 14566 14766 15301 15326 15735 15784 16189 16223 16278 16290 16294 16309 16390	L2a1b1a	73 146 152 195 263 16189 16223 16278 16290 16294 16309 16390	[0.000] L2a1b1a (1)	[0.005] L2a1f (2) [0.000] L2a1+16189_(16192) L2a1b L2a1b1 L2a1b1a L2a1b2 L2a1f1 L2a1f1a L2a1f2 (10)	1
1114 >MF057129.1 Hc	73G 146C 152C 195C 263G 750G 769A 1018A 1438G 2416C 2706G 2789T 3106d 3107C 3593C 3594T 4104G 4769G 5090C 7028T 7175C 7256T 7274T 7521A 7771G 8206A 8701G 8860G 9221G 9540C 10115C 10143A 10398G 10873C 11719A 11914A 11944C 12693G 12705T 13590A 13650T 13803G 14566G 14766T 15301A 15326G 15735T 15784C 16182C 16183C 16189C 16223T 16278T 16290T 16294T 16309G 16390A	L2a1b1a	73G 146C 152C 195C 263G 16182C 16183C 16189C 16223T 16278T 16290T 16294T 16309G 16390A	1	73 146 152 195 263 750 769 1018 1438 2416 2706 2789 3593 3594 4104 4769 5090 7028 7175 7256 7274 7521 7771 8206 8701 8860 9221 9540 10115 10143 10398 10873 11719 11914 11944 12693 12705 13590 13650 13803 14566 14766 15301 15326 15735 15784 16189 16223 16278 16290 16294 16309 16390	L2a1b1a	73 146 152 195 263 16189 16223 16278 16290 16294 16309 16390	[0.000] L2a1b1a (1)	[0.005] L2a1f (2) [0.000] L2a1+16189_(16192) L2a1b L2a1b1 L2a1b1a L2a1b2 L2a1f1 L2a1f1a L2a1f2 (10)	1

1115	>MF057127.1 Hc	73G 146C 152C 195C 263G 750G 769A 1018A 1438G 2416C 2706G 2789T 3106d 3107C 3594T 4104G 4769G 5090C 7028T 7175C 7256T 7274T 7521A 7771G 8206A 8701G 8860G 9221G 9540C 10115C 10143A 10398G 10873C 11719A 11914A 11944C 12693G 12705T 13590A 13650T 13803G 14566G 14766T 15301A 15326G 15735T 15784C 16182C 16183C 16189C 16223T 16278T 16290T 16294T 16309G 16390A	L2a1b1a	73G 146C 152C 195C 263G 16182C 16183C 16189C 16223T 16278T 16290T 16294T 16309G 16390A	1	73 146 152 195 263 750 769 1018 1438 2416 2706 2789 3594 4104 4769 5090 7028 7175 7256 7274 7521 7771 8206 8701 8860 9221 9540 10115 10143 10398 10873 11719 11914 11944 12693 12705 13590 13650 13803 14566 14766 15301 15326 15735 15784 16189 16223 16278 16290 16294 16309 16390	L2a1b1a	73 146 152 195 263 16189 16223 16278 16290 16294 16309 16390	[0.000] L2a1b1a (1)	[0.005] L2a1f (2) [0.000] L2a1+16189_(16192) L2a1b L2a1b1 L2a1b1a L2a1b2 L2a1f1 L2a1f1a L2a1f2 (10)	1
1116	>MF057126.1 Hc	73G 146C 152C 195C 263G 750G 769A 1018A 1438G 2416C 2706G 2789T 3106d 3107C 3594T 4104G 4769G 5090C 7028T 7175C 7256T 7274T 7521A 7771G 8206A 8701G 8860G 9221G 9540C 10115C 10143A 10398G 10873C 11719A 11914A 11944C 12693G 12705T 13590A 13650T 13803G 14566G 14766T 15301A 15326G 15735T 15784C 16182C 16183C 16189C 16223T 16278T 16290T 16294T 16309G 16390A	L2a1b1a	73G 146C 152C 195C 263G 16182C 16183C 16189C 16223T 16278T 16290T 16294T 16309G 16390A	1	73 146 152 195 263 750 769 1018 1438 2416 2706 2789 3594 4104 4769 5090 7028 7175 7256 7274 7521 7771 8206 8701 8860 9221 9540 10115 10143 10398 10873 11719 11914 11944 12693 12705 13590 13650 13803 14566 14766 15301 15326 15735 15784 16189 16223 16278 16290 16294 16309 16390	L2a1b1a	73 146 152 195 263 16189 16223 16278 16290 16294 16309 16390	[0.000] L2a1b1a (1)	[0.005] L2a1f (2) [0.000] L2a1+16189_(16192) L2a1b L2a1b1 L2a1b1a L2a1b2 L2a1f1 L2a1f1a L2a1f2 (10)	1
1117	>MF057123.1 Hc	73G 152C 195C 204C 263G 417A 489C 533G 750G 1438G 3106d 3107C 4769G 7028T 8188G 8360G 8701G 8860G 9438A 9540C 9545G 10142T 10295G 10398G 10400T 10873C 11569C 11719A 11899C 12279G 12618A 12705T 14766T 14783C 15025T 15043A 15301A 15326G 16223T 16263C 16311C 16519C	M23	73G 152C 195C 204C 263G 417A 489C 533G 16223T 16263C 16311C 16519C	1	73 152 195 204 263 417 489 533 750 1438 4769 7028 8188 8360 8701 8860 9438 9540 9545 10142 10295 10398 10400 10873 11569 11719 11899 12279 12618 12705 14766 14783 15025 15043 15301 15326 16223 16263 16311	M23	73 152 195 204 263 417 489 533 16223 16263 16311	[0.000] M23 (1)	[0.072] M24a (2) [0.053] M57+152	1
1118	>MF057122.1 Hc	73G 146C 152C 195C 263G 750G 769A 1018A 1438G 2416C 2706G 2789T 3106d 3107C 3594T 4104G 4769G 5090C 7028T 7175C 7256T 7274T 7521A 7771G 8206A 8701G 8860G 9221G 9540C 10115C 10143A 10398G 10873C 11719A 11914A 11944C 12693G 12705T 13590A 13650T 13803G 14566G 14766T 15301A 15326G 15735T 15784C 16183C 16189C 16223T 16278T 16290T 16294T 16309G 16390A	L2a1b1a	73G 146C 152C 195C 263G 16183C 16189C 16223T 16278T 16290T 16294T 16309G 16390A	1	73 146 152 195 263 750 769 1018 1438 2416 2706 2789 3594 4104 4769 5090 7028 7175 7256 7274 7521 7771 8206 8701 8860 9221 9540 10115 10143 10398 10873 11719 11914 11944 12693 12705 13590 13650 13803 14566 14766 15301 15326 15735 15784 16189 16223 16278 16290 16294 16309 16390	L2a1b1a	73 146 152 195 263 16189 16223 16278 16290 16294 16309 16390	[0.000] L2a1b1a (1)	[0.005] L2a1f (2) [0.000] L2a1+16189_(16192) L2a1b L2a1b1 L2a1b1a L2a1b2 L2a1f1 L2a1f1a L2a1f2 (10)	1

1119 >MF057119.1 Hc	73G 263G 279C 514d 515d 750G 1438G 2332T 2706G 3106d 3107C 3450T 4769G 5773A 6221C 6340T 7028T 8701G 8860G 9449T 9540C 10086G 10373A 10398G 10873C 11002G 11719A 12705T 13105G 13914A 14766T 15301A 15311G 15326G 15824G 15940d 16145A 16223T 16278T 16362C 16519C	L3b1a8	73G 263G 279C 514d 515d 16145A 16223T 16278T 16362C 16519C	1	73 263 279 750 1438 2332 2706 3450 4769 5773 6221 6340 7028 8701 8860 9449 9540 10086 10373 10398 10873 11002 11719 12705 13105 13914A 14766 15301 15311 15326 15824 15944d 16145 16223 16278 16362	L3b1a8	73 263 279 16145 16223 16278 16362	[0.213] L3b1a8 (1)	[0.000] L3b1a+@16124 L3b1a7 L3b1a7a (4)	1
1120 >MF057117.1 Hc	73G 152C 195C 204C 263G 417A 489C 533G 750G 1438G 3106d 3107C 3394C 4769G 7028T 8188G 8360G 8701G 8860G 9438A 9540C 9545G 10142T 10295G 10398G 10400T 10873C 11569C 11719A 11899C 12279G 12618A 12705T 14766T 14783C 15025T 15043A 15301A 15326G 16223T 16263C 16311C 16519C	M23	73G 152C 195C 204C 263G 417A 489C 533G 16223T 16263C 16311C 16519C	1	73 152 195 204 263 417 489 533 750 1438 3394 4769 7028 8188 8360 8701 8860 9438 9540 9545 10142 10295 10398 10400 10873 11569 11719 11899 12279 12618 12705 14766 14783 15025 15043 15301 15326 16223 16263 16311	M23	73 152 195 204 263 417 489 533 16223 16263 16311	[0.000] M23 (1)	[0.072] M24a (2) [0.053] M57+152	1
1121 >MF057103.1 Hc	73G 146C 152C 195C 263G 750G 769A 1018A 1438G 2416C 2706G 2789T 3106d 3107C 3594T 4104G 4769G 5090C 7028T 7175C 7256T 7274T 7521A 7771G 8206A 8701G 8860G 9221G 9540C 10115C 10143A 10398G 10873C 11719A 11914A 11944C 12693G 12705T 13590A 13650T 13803G 14566G 14766T 15301A 15326G 15735T 15784C 16183C 16189C 16223T 16278T 16290T 16294T 16309G 16390A 16519C	L2a1b1a	73G 146C 152C 195C 263G 16183C 16189C 16223T 16278T 16290T 16294T 16309G 16390A 16519C	1	73 146 152 195 263 750 769 1018 1438 2416 2706 2789 3594 4104 4769 5090 7028 7175 7256 7274 7521 7771 8206 8701 8860 9221 9540 10115 10143 10398 10873 11719 11914 11944 12693 12705 13590 13650 13803 14566 14766 15301 15326 15735 15784 16189 16223 16278 16290 16294 16309 16390	L2a1b1a	73 146 152 195 263 16189 16223 16278 16290 16294 16309 16390	[0.000] L2a1b1a (1)	[0.005] L2a1f (2) [0.000] L2a1+16189_(16192) L2a1b L2a1b1 L2a1b1a L2a1b2 L2a1f1 L2a1f1a L2a1f2 (10)	1
1122 >MF057102.1 Hc	73G 150T 200G 263G 489C 750G 2706G 3106d 3107C 3588T 4769G 7028T 7960G 8701G 8768T 8854A 8860G 9540C 10398G 10400T 10873C 11719A 12705T 13308G 13590A 13708A 14130T 14260G 14527G 14766T 14783C 15043A 15172A 15301A 15326G 15625T 16086C 16148T 16223T 16259T 16278T 16319A 16399G 16526A	M32c	73G 150T 200G 263G 489C 16086C 16148T 16223T 16259T 16278T 16319A 16399G 16526A	1	73 150 200 263 489 750 2706 3588 4769 7028 7960 8701 8768 8854 8860 9540 10398 10400 10873 11719 12705 13308 13590 13708 14130 14260 14527 14766 14783 15043 15172 15301 15326 15625 16086 16148 16223 16259 16278 16319 16399 16526	M32c	73 150 200 263 489 16086 16148 16223 16259 16278 16319 16399 16526	[0.000] M32c (1)	[0.022] M51a (2) [0.007] M32 (3) [0.000] M51 M51b (5)	1

1123	>MF057100.1 Hc	73G 152C 195C 204C 263G 417A 489C 533G 750G 1438G 3106d 3107C 4769G 7028T 8188G 8360G 8701G 8860G 9438A 9540C 9545G 10142T 10295G 10398G 10400T 10873C 11569C 11719A 11899C 12279G 12618A 12705T 14766T 14783C 15025T 15043A 15301A 15326G 16129A 16223T 16263C 16311C 16519C	M23	73G 152C 195C 204C 263G 417A 489C 533G 16129A 16223T 16263C 16311C 16519C	1	73 152 195 204 263 417 489 533 750 1438 4769 7028 8188 8360 8701 8860 9438 9540 9545 10142 10295 10398 10400 10873 11569 11719 11899 12279 12618 12705 14766 14783 15025 15043 15301 15326 16129 16223 16263 16311	M23	73 152 195 204 263 417 489 533 16129 16223 16263 16311	[0.004] M23 (1)	[0.077] M24a (2) [0.053] M57+152 (3) [0.042] D4a7 (4) [0.000] M1a2a M13b1 (6)	1
1124	>MF057096.1 Hc	73G 146C 152C 195C 263G 750G 769A 1018A 1438G 2416C 2706G 2789T 3106d 3107C 3594T 4104G 4769G 5090C 7028T 7175C 7256T 7274T 7521A 7771G 8206A 8701G 8860G 9221G 9540C 10115C 10143A 10398G 10873C 11719A 11914A 11944C 12693G 12705T 13590A 13650T 13803G 14566G 14766T 15301A 15326G 15735T 15784C 16182C 16183C 16189C 16223T 16278T 16290T 16294T 16309G 16390A	L2a1b1a	73G 146C 152C 195C 263G 16182C 16183C 16189C 16223T 16278T 16290T 16294T 16309G 16390A	1	73 146 152 195 263 750 769 1018 1438 2416 2706 2789 3594 4104 4769 5090 7028 7175 7256 7274 7521 7771 8206 8701 8860 9221 9540 10115 10143 10398 10873 11719 11914 11944 12693 12705 13590 13650 13803 14566 14766 15301 15326 15735 15784 16189 16223 16278 16290 16294 16309 16390	L2a1b1a	73 146 152 195 263 16189 16223 16278 16290 16294 16309 16390	[0.000] L2a1b1a (1)	[0.005] L2a1f (2) [0.000] L2a1+16189_(16192) L2a1b L2a1b1 L2a1b1a L2a1b2 L2a1f1 L2a1f1a L2a1f2 (10)	1
1125	>MF057095.1 Hc	73G 150T 200G 263G 489C 750G 2706G 3106d 3107C 3588T 4769G 7028T 7960G 8701G 8768T 8854A 8860G 9540C 10398G 10400T 10873C 11719A 12705T 13308G 13590A 13708A 14130T 14260G 14527G 14766T 14783C 15043A 15172A 15301A 15326G 15625T 16086C 16148T 16223T 16259T 16278T 16319A 16399G 16526A	M32c	73G 150T 200G 263G 489C 16086C 16148T 16223T 16259T 16278T 16319A 16399G 16526A	1	73 150 200 263 489 750 2706 3588 4769 7028 7960 8701 8768 8854 8860 9540 10398 10400 10873 11719 12705 13308 13590 13708 14130 14260 14527 14766 14783 15043 15172 15301 15326 15625 16086 16148 16223 16259 16278 16319 16399 16526	M32c	73 150 200 263 489 16086 16148 16223 16259 16278 16319 16399 16526	[0.000] M32c (1)	[0.022] M51a (2) [0.007] M32 (3) [0.000] M51 M51b (5)	1
1126	>MF057094.1 Hc	73G 150T 200G 263G 489C 750G 2706G 3106d 3107C 3588T 4769G 7028T 7960G 8701G 8768T 8854A 8860G 9540C 10398G 10400T 10873C 11719A 12705T 13308G 13590A 13708A 14130T 14260G 14527G 14766T 14783C 15043A 15172A 15301A 15326G 15625T 16086C 16148T 16223T 16259T 16278T 16319A 16399G 16526A	M32c	73G 150T 200G 263G 489C 16086C 16148T 16223T 16259T 16278T 16319A 16399G 16526A	1	73 150 200 263 489 750 2706 3588 4769 7028 7960 8701 8768 8854 8860 9540 10398 10400 10873 11719 12705 13308 13590 13708 14130 14260 14527 14766 14783 15043 15172 15301 15326 15625 16086 16148 16223 16259 16278 16319 16399 16526	M32c	73 150 200 263 489 16086 16148 16223 16259 16278 16319 16399 16526	[0.000] M32c (1)	[0.022] M51a (2) [0.007] M32 (3) [0.000] M51 M51b (5)	1

1127 >MF057092.1 Hc	73G 152C 195C 204C 263G 417A 489C 533G 750G 1438G 3106d 3107C 4769G 7028T 8188G 8360G 8701G 8860G 9438A 9540C 9545G 10142T 10295G 10398G 10400T 10873C 11569C 11719A 11899C 12279G 12618A 12705T 13708A 14766T 14783C 15025T 15043A 15301A 15326G 16223T 16263C 16311C 16519C	M23	73G 152C 195C 204C 263G 417A 489C 533G 16223T 16263C 16311C 16519C	1	73 152 195 204 263 417 489 533 750 1438 4769 7028 8188 8360 8701 8860 9438 9540 9545 10142 10295 10398 10400 10873 11569 11719 11899 12279 12618 12705 13708 14766 14783 15025 15043 15301 15326 16223 16263 16311	M23	73 152 195 204 263 417 489 533 16223 16263 16311	[0.000] M23 (1)	[0.072] M24a (2) [0.053] M57+152	1
1128 >MF057076.1 Hc	73G 152C 195C 204C 263G 417A 489C 533G 750G 1438G 3106d 3107C 4769G 7028T 8188G 8360G 8701G 8860G 9438A 9540C 9545G 10142T 10295G 10398G 10400T 10873C 11569C 11719A 11899C 12279G 12618A 12705T 14766T 14783C 15025T 15043A 15301A 15326G 16223T 16263C 16311C 16519C	M23	73G 152C 195C 204C 263G 417A 489C 533G 16223T 16263C 16311C 16519C	1	73 152 195 204 263 417 489 533 750 1438 4769 7028 8188 8360 8701 8860 9438 9540 9545 10142 10295 10398 10400 10873 11569 11719 11899 12279 12618 12705 14766 14783 15025 15043 15301 15326 16223 16263 16311	M23	73 152 195 204 263 417 489 533 16223 16263 16311	[0.000] M23 (1)	[0.072] M24a (2) [0.053] M57+152	1
1129 >MF057075.1 Hc	73G 152C 195C 204C 263G 417A 489C 533G 750G 1438G 3106d 3107C 4769G 7028T 8188G 8360G 8701G 8860G 9438A 9540C 9545G 10142T 10295G 10398G 10400T 10873C 11569C 11719A 11899C 12279G 12618A 12705T 14766T 14783C 15025T 15043A 15301A 15326G 16223T 16263C 16311C 16519C	M23	73G 152C 195C 204C 263G 417A 489C 533G 16223T 16263C 16311C 16519C	1	73 152 195 204 263 417 489 533 750 1438 4769 7028 8188 8360 8701 8860 9438 9540 9545 10142 10295 10398 10400 10873 11569 11719 11899 12279 12618 12705 14766 14783 15025 15043 15301 15326 16223 16263 16311	M23	73 152 195 204 263 417 489 533 16223 16263 16311	[0.000] M23 (1)	[0.072] M24a (2) [0.053] M57+152	1
1130 >MF057070.1 Hc	73G 152C 195C 204C 263G 417A 489C 533G 750G 1438G 3106d 3107C 4769G 7028T 8188G 8360G 8701G 8860G 9438A 9540C 9545G 10142T 10295G 10398G 10400T 10873C 11569C 11719A 11899C 12279G 12618A 12705T 14766T 14783C 15025T 15043A 15301A 15326G 16223T 16263C 16311C 16519C	M23	73G 152C 195C 204C 263G 417A 489C 533G 16223T 16263C 16311C 16519C	1	73 152 195 204 263 417 489 533 750 1438 4769 7028 8188 8360 8701 8860 9438 9540 9545 10142 10295 10398 10400 10873 11569 11719 11899 12279 12618 12705 14766 14783 15025 15043 15301 15326 16223 16263 16311	M23	73 152 195 204 263 417 489 533 16223 16263 16311	[0.000] M23 (1)	[0.072] M24a (2) [0.053] M57+152	1
1131 >MF057056.1 Hc	73G 152d 235G 263G 494T 735G 750G 1438G 2706G 3106d 3107C 3918A 4313C 4769G 6620G 7028T 7202G 8649G 8701G 8860G 9007T 9329A 9467C 9540C 10398G 10819G 10873C 11719A 12705T 13135A 13542G 13862G 13992T 14013G 14766T 15301A 15314A 15326G 16093C 16223T 16355T	L3k1	73G 152d 235G 263G 494T 16093C 16223T 16355T	1	73 152d 235 263 494 735 750 1438 2706 3918 4313 4769 6620G 7028 7202 8649 8701 8860 9007T 9329 9467 9540 10398 10819 10873 11719 12705 13135 13542 13862 13992 14013 14766 15301 15314 15326 16093 16223 16355	L3k1	73 152d 235 263 494 16093 16223 16355	[1.037] L3k1 (1)	[0.071] N11a (2) [0.021] L3k (3) [0.014] L3 (4) [0.000] L3b'f L3h G1b N N1'5 N1 N9 N11 N14 S S2 (15)	1

1132 >MF057053.1 Hc	73G 146C 152C 195C 263G 514d 515d 750G 769A 1018A 1438G 2416C 2706G 2789T 3106d 3107C 3594T 4104G 4769G 5090C 7028T 7175C 7256T 7274T 7521A 7771G 8206A 8701G 8860G 9221G 9540C 10115C 10143A 10398G 10873C 11719A 11914A 11944C 12693G 12705T 13590A 13650T 13803G 14566G 14766T 15301A 15326G 15735T 15784C 16182C 16183C 16189C 16223T 16278T 16290T 16294T 16309G 16390A	L2a1b1a	73G 146C 152C 195C 263G 514d 515d 16182C 16183C 16189C 16223T 16278T 16290T 16294T 16309G 16390A	1	73 146 152 195 263 750 769 1018 1438 2416 2706 2789 3594 4104 4769 5090 7028 7175 7256 7274 7521 7771 8206 8701 8860 9221 9540 10115 10143 10398 10873 11719 11914 11944 12693 12705 13590 13650 13803 14566 14766 15301 15326 15735 15784 16189 16223 16278 16290 16294 16309 16390	L2a1b1a	73 146 152 195 263 16189 16223 16278 16290 16294 16309 16390	[0.000] L2a1b1a (1)	[0.005] L2a1f (2) [0.000] L2a1+16189_(16192) L2a1b L2a1b1 L2a1b1a L2a1b2 L2a1f1 L2a1f1a L2a1f2 (10)	1
1133 >MF057043.1 Hc	73G 146C 152C 195C 263G 750G 769A 1018A 1438G 2416C 2706G 2789T 3106d 3107C 3594T 4104G 4769G 5090C 7028T 7175C 7256T 7274T 7521A 7771G 8206A 8701G 8860G 9221G 9540C 10115C 10143A 10398G 10873C 11719A 11914A 11944C 12693G 12705T 13590A 13650T 13803G 14566G 14766T 15301A 15326G 15735T 15784C 16182C 16183C 16189C 16223T 16278T 16290T 16294T 16309G 16390A	L2a1b1a	73G 146C 152C 195C 263G 16182C 16183C 16189C 16223T 16278T 16290T 16294T 16309G 16390A	1	73 146 152 195 263 750 769 1018 1438 2416 2706 2789 3594 4104 4769 5090 7028 7175 7256 7274 7521 7771 8206 8701 8860 9221 9540 10115 10143 10398 10873 11719 11914 11944 12693 12705 13590 13650 13803 14566 14766 15301 15326 15735 15784 16189 16223 16278 16290 16294 16309 16390	L2a1b1a	73 146 152 195 263 16189 16223 16278 16290 16294 16309 16390	[0.000] L2a1b1a (1)	[0.005] L2a1f (2) [0.000] L2a1+16189_(16192) L2a1b L2a1b1 L2a1b1a L2a1b2 L2a1f1 L2a1f1a L2a1f2 (10)	1
1134 >MF057030.1 Hc	73G 152C 195C 204C 263G 417A 489C 533G 750G 1438G 3106d 3107C 4769G 7028T 8188G 8360G 8701G 8860G 9438A 9540C 9545G 10142T 10295G 10398G 10400T 10873C 11569C 11719A 11899C 12279G 12618A 12705T 14766T 14783C 15025T 15043A 15301A 15326G 16223T 16263C 16311C 16519C	M23	73G 152C 195C 204C 263G 417A 489C 533G 16223T 16263C 16311C 16519C	1	73 152 195 204 263 417 489 533 750 1438 4769 7028 8188 8360 8701 8860 9438 9540 9545 10142 10295 10398 10400 10873 11569 11719 11899 12279 12618 12705 14766 14783 15025 15043 15301 15326 16223 16263 16311	M23	73 152 195 204 263 417 489 533 16223 16263 16311	[0.000] M23 (1)	[0.072] M24a (2) [0.053] M57+152	1
1135 >MF057028.1 Hc	73G 146C 152C 195C 263G 750G 769A 1018A 1438G 2416C 2706G 2789T 3106d 3107C 3594T 4104G 4769G 5090C 6707C 7028T 7175C 7256T 7274T 7521A 7771G 8206A 8701G 8860G 9221G 9540C 10115C 10143A 10398G 10873C 11719A 11914A 11944C 12693G 12705T 13590A 13650T 13803G 14566G 15301A 15326G 15735T 15784C 16182C 16183C 16189C 16223T 16278T 16290T 16294T 16309G 16390A	L2a1b1a	73G 146C 152C 195C 263G 16182C 16183C 16189C 16223T 16278T 16290T 16294T 16309G 16390A	1	73 146 152 195 263 750 769 1018 1438 2416 2706 2789 3594 4104 4769 5090 6707 7028 7175 7256 7274 7521 7771 8206 8701 8860 9221 9540 10115 10143 10398 10873 11719 11914 11944 12693 12705 13590 13650 13803 14566 15301 15326 15735 15784 16189 16223 16278 16290 16294 16309 16390	L2a1b1a	73 146 152 195 263 16189 16223 16278 16290 16294 16309 16390	[0.000] L2a1b1a (1)	[0.005] L2a1f (2) [0.000] L2a1+16189_(16192) L2a1b L2a1b1 L2a1b1a L2a1b2 L2a1f1 L2a1f1a L2a1f2 (10)	1

1136	>MF057026.1 H[illegible]	73G 146C 152C 195C 263G 750G 769A 1018A 1438G 2416C 2706G 2789T 3106d 3107C 3594T 4104G 4769G 5090C 7028T 7175C 7256T 7274T 7521A 7771G 8206A 8701G 8860G 9221G 9540C 10115C 10143A 10398G 10873C 11719A 11914A 11944C 12693G 12705T 13590A 13650T 13803G 14566G 14766T 15301A 15326G 15735T 15784C 15907G 16182C 16183C 16189C 16223T 16278T 16290T 16294T 16309G 16390A 16519C	L2a1b1a	73G 146C 152C 195C 263G 16182C 16183C 16189C 16223T 16278T 16290T 16294T 16309G 16390A 16519C	1	73 146 152 195 263 750 769 1018 1438 2416 2706 2789 3594 4104 4769 5090 7028 7175 7256 7274 7521 7771 8206 8701 8860 9221 9540 10115 10143 10398 10873 11719 11914 11944 12693 12705 13590 13650 13803 14566 14766 15301 15326 15735 15784 15907 16189 16223 16278 16290 16294 16309 16390	L2a1b1a	73 146 152 195 263 16189 16223 16278 16290 16294 16309 16390	[0.000] L2a1b1a (1)	[0.005] L2a1f (2) [0.000] L2a1+16189_(16192) L2a1b L2a1b1 L2a1b1a L2a1b2 L2a1f1 L2a1f1a L2a1f2 (10)	1
1137	>MF057021.1 H[illegible]	73G 152C 195C 204C 263G 417A 489C 533G 750G 1438G 3106d 3107C 4769G 7028T 8188G 8360G 8701G 8860G 9007G 9438A 9540C 9545G 10142T 10295G 10398G 10400T 10873C 11569C 11719A 11899C 12279G 12618A 12705T 14766T 14783C 15025T 15043A 15301A 15326G 16223T 16263C 16311C 16519C	M23	73G 152C 195C 204C 263G 417A 489C 533G 16223T 16263C 16311C 16519C	1	73 152 195 204 263 417 489 533 750 1438 4769 7028 8188 8360 8701 8860 9007 9438 9540 9545 10142 10295 10398 10400 10873 11569 11719 11899 12279 12618 12705 14766 14783 15025 15043 15301 15326 16223 16263 16311	M23	73 152 195 204 263 417 489 533 16223 16263 16311	[0.000] M23 (1)	[0.072] M24a (2) [0.053] M57+152	1
1138	>MF057003.1 H[illegible]	73G 150T 200G 263G 489C 750G 2706G 3106d 3107C 3588T 4769G 7028T 7960G 8701G 8768T 8854A 8860G 9540C 10398G 10400T 10873C 11719A 12705T 13308G 13590A 13708A 14130T 14260G 14527G 14766T 14783C 15043A 15172A 15301A 15326G 15625T 16086C 16148T 16223T 16259T 16278T 16319A 16399G 16526A	M32c	73G 150T 200G 263G 489C 16086C 16148T 16223T 16259T 16278T 16319A 16399G 16526A	1	73 150 200 263 489 750 2706 3588 4769 7028 7960 8701 8768 8854 8860 9540 10398 10400 10873 11719 12705 13308 13590 13708 14130 14260 14527 14766 14783 15043 15172 15301 15326 15625 16086 16148 16223 16259 16278 16319 16399 16526	M32c	73 150 200 263 489 16086 16148 16223 16259 16278 16319 16399 16526	[0.000] M32c (1)	[0.022] M51a (2) [0.007] M32 (3) [0.000] M51 M51b (5)	1
1139	>KX457500.1 H[illegible]	73G 146A 199C 263G 309.1CT 310C 489C 514d 515d 750G 1438G 2706G 3106d 3107C 3606G 4071T 4769G 4850T 5442C 6293C 6455T 7028T 8701G 8860G 9540C 9824C 10398G 10400T 10873C 11665T 11719A 12091C 12705T 14766T 14783C 15043A 15236G 15301A 15326G 16519C	M7c1c2	73G 146A 199C 263G 309.1CT 310C 489C 514d 515d 16519C	1	73 146A 199 263 489 750 1438 2706 3606 4071 4769 4850 5442 6293 6455 7028 8701 8860 9540 9824 10398 10400 10873 11665 11719 12091 12705 14766 14783 15043 15236 15301 15326	M7c1c2	73 146A 199 263 489	[0.000] M7c1c2 (1)	[0.000] M7c1c2a (2)	1

1140 >KX457499.1 Hc	73G 185A 189G 263G 310.1C 489C 514d 515d 750G 1438G 2706G 3106d 3107C 3897T 4170T 4769G 5336T 5580C 7028T 8701G 8860G 9540C 9590G 10398G 10400T 10873C 11359G 11719A 12030G 12372A 12705T 14569A 14727C 14766T 14783C 14788C 14869A 15010G 15043A 15301A 15326G 16108T 16129A 16172C 16223T 16234T 16290T	M12b1b	73G 185A 189G 263G 310.1C 489C 514d 515d 16108T 16129A 16172C 16223T 16234T 16290T	1	73 185 189 263 489 750 1438 2706 3897 4170 4769 5336 5580 7028 8701 8860 9540 9590 10398 10400 10873 11359 11719 12030 12372 12705 14569 14727 14766 14783 14788 14869 15010 15043 15301 15326 16108 16129 16172 16223 16234 16290	M12b1b	73 185 189 263 489 16108 16129 16172 16223 16234 16290	[0.000] M12b1b (1)	[0.000] M12b M12b1 M12b1a M12b1a1 (5)	1
1141 >KX457498.1 Hc	73G 152C 263G 310.1C 329A 750G 1040C 1438G 2706G 2836T 3106d 3107C 3210T 4634C 4639C 4769G 6770G 7028T 8387A 8537G 8860G 9542G 10316G 10609C 11719A 12026G 13359A 14180C 14766T 15115C 15326G 16234T 16249C 16288C 16304C 16519C	R22	73G 152C 263G 310.1C 329A 16234T 16249C 16288C 16304C 16519C	1	73 152 263 329 750 1040 1438 2706 2836 3210 4634 4639 4769 6770 7028 8387 8537 8860 9542 10316 10609 11719 12026 13359 14180 14766 15115 15326 16234 16249 16288 16304	R22	73 152 263 329 16234 16249 16288 16304	[0.048] R22 (1)	[0.091] U2c1b (2) [0.048] R9 (3) [0.040] H13a1d (4) [0.030] U2c'd (5) [0.020] U2c (6) [0.019] U2c1 (7) [0.008] R30a1 R30a1a (9) [0.005] R9c (10) [0.000] H32 R9b (12)	1
1142 >KX457497.1 Hc	73G 185A 189G 263G 309.1CT 310C 489C 514d 515d 750G 1438G 2706G 3106d 3107C 3897T 4170T 4769G 5336T 5580C 7028T 8701G 8860G 9540C 9590G 10398G 10400T 10873C 11359G 11719A 12030G 12372A 12705T 14569A 14727C 14766T 14783C 14788C 14869A 15010G 15043A 15301A 15326G 16108T 16129A 16172C 16223T 16234T 16290T	M12b1b	73G 185A 189G 263G 309.1CT 310C 489C 514d 515d 16108T 16129A 16172C 16223T 16234T 16290T	1	73 185 189 263 489 750 1438 2706 3897 4170 4769 5336 5580 7028 8701 8860 9540 9590 10398 10400 10873 11359 11719 12030 12372 12705 14569 14727 14766 14783 14788 14869 15010 15043 15301 15326 16108 16129 16172 16223 16234 16290	M12b1b	73 185 189 263 489 16108 16129 16172 16223 16234 16290	[0.000] M12b1b (1)	[0.000] M12b M12b1 M12b1a M12b1a1 (5)	1
1143 >KX457491.1 Hc	73G 146C 195C 263G 309.1CT 310C 489C 514d 515d 750G 951A 1438G 2140A 2706G 3106d 3107C 4769G 5773A 7028T 8701G 8860G 9540C 10101C 10398G 10400T 10873C 11719A 12651A 12705T 13359A 13812C 14766T 14783C 15043A 15301A 15326G 15601C 16086C 16223T 16519C	M24b	73G 146C 195C 263G 309.1CT 310C 489C 514d 515d 16086C 16223T 16519C	1	73 146 195 263 489 750 951 1438 2140 2706 4769 5773 7028 8701 8860 9540 10101 10398 10400 10873 11719 12651 12705 13359 13812 14766 14783 15043 15301 15326 15601 16086 16223	M24b	73 146 195 263 489 16086 16223	[0.000] M24b (1)	[0.000] M24 (2)	1
1144 >KX457490.1 Hc	73G 152C 263G 310.1C 329A 750G 1040C 1438G 2706G 2836T 3106d 3107C 3210T 4634C 4639C 4769G 6770G 7028T 8387A 8537G 8860G 9542G 10316G 10609C 11719A 12026G 13359A 14180C 14766T 15115C 15326G 16234T 16249C 16288C 16304C 16519C	R22	73G 152C 263G 310.1C 329A 16234T 16249C 16288C 16304C 16519C	1	73 152 263 329 750 1040 1438 2706 2836 3210 4634 4639 4769 6770 7028 8387 8537 8860 9542 10316 10609 11719 12026 13359 14180 14766 15115 15326 16234 16249 16288 16304	R22	73 152 263 329 16234 16249 16288 16304	[0.048] R22 (1)	[0.091] U2c1b (2) [0.048] R9 (3) [0.040] H13a1d (4) [0.030] U2c'd (5) [0.020] U2c (6) [0.019] U2c1 (7) [0.008] R30a1 R30a1a (9) [0.005] R9c (10) [0.000] H32 R9b (12)	1

1145 >KX457488.1 Ho	73G 195C 263G 309.1CCT 310C 329A 512.1ACAC 513A 750G 1040C 1438G 2706G 3106d 3107C 4491A 4769G 6770G 7028T 8296G 8545A 8860G 10166C 10316G 10609C 10877T 11009C 11719A 12498T 12842C 13145A 13287T 13359A 14766T 14790G 15326G 15885T 16249C 16259T 16288C 16301T 16304C 16390A 16519C	R22	73G 195C 263G 309.1CCT 310C 329A 512.1ACAC 513A 16249C 16259T 16288C 16301T 16304C 16390A 16519C	1	73 195 263 329 513 750 1040 1438 2706 4491 4769 6770 7028 8296 8545 8860 10166 10316 10609 10877 11009 11719 12498 12842 13145 13287 13359 14766 14790 15326 15885 16249 16259 16288 16301 16304 16390	R22	73 195 263 329 513 16249 16259 16288 16301 16304 16390	[1.626] R22 (1)	[0.091] R8a1a1d (2)	1
1146 >KX457487.1 Ho	73G 195C 263G 309.1CCT 310C 329A 512.1ACAC 513A 750G 1040C 1438G 2706G 3106d 3107C 4491A 4769G 6770G 7028T 8296G 8545A 8860G 10166C 10316G 10609C 10877T 11009C 11719A 12498T 12842C 13145A 13287T 13359A 14766T 14790G 15326G 15885T 16249C 16259T 16288C 16301T 16304C 16390A 16519C	R22	73G 195C 263G 309.1CCT 310C 329A 512.1ACAC 513A 16249C 16259T 16288C 16301T 16304C 16390A 16519C	1	73 195 263 329 513 750 1040 1438 2706 4491 4769 6770 7028 8296 8545 8860 10166 10316 10609 10877 11009 11719 12498 12842 13145 13287 13359 14766 14790 15326 15885 16249 16259 16288 16301 16304 16390	R22	73 195 263 329 513 16249 16259 16288 16301 16304 16390	[1.626] R22 (1)	[0.091] R8a1a1d (2)	1
1147 >KX457483.1 Ho	73G 195C 263G 309.1CCT 310C 329A 512.1ACAC 513A 750G 1040C 1438G 2706G 3106d 3107C 4491A 4769G 6770G 7028T 8296G 8545A 8860G 10166C 10316G 10609C 10877T 11009C 11719A 12498T 12842C 13145A 13287T 13359A 14766T 14790G 15326G 15885T 16249C 16259T 16288C 16301T 16304C 16390A 16519C	R22	73G 195C 263G 309.1CCT 310C 329A 512.1ACAC 513A 16249C 16259T 16288C 16301T 16304C 16390A 16519C	1	73 195 263 329 513 750 1040 1438 2706 4491 4769 6770 7028 8296 8545 8860 10166 10316 10609 10877 11009 11719 12498 12842 13145 13287 13359 14766 14790 15326 15885 16249 16259 16288 16301 16304 16390	R22	73 195 263 329 513 16249 16259 16288 16301 16304 16390	[1.626] R22 (1)	[0.091] R8a1a1d (2)	1
1148 >KX457482.1 Ho	73G 185A 189G 263G 309.1CT 310C 489C 514d 515d 750G 1438G 2706G 3106d 3107C 3897T 4170T 4769G 5336T 5580C 7028T 8701G 8860G 9540C 10398G 10400T 10873C 11359G 11719A 12030G 12372A 12705T 14569A 14727C 14766T 14783C 14788C 14869A 15010G 15043A 15301A 15326G 16108T 16129A 16172C 16223T 16234T 16290T 16300G	M12b1b	73G 185A 189G 263G 309.1CT 310C 489C 514d 515d 16108T 16129A 16172C 16223T 16234T 16290T 16300G	1	73 185 189 263 489 750 1438 2706 3897 4170 4769 5336 5580 7028 8701 8860 9540 10398 10400 10873 11359 11719 12030 12372 12705 14569 14727 14766 14783 14788 14869 15010 15043 15301 15326 16108 16129 16172 16223 16234 16290 16300	M12b1b	73 185 189 263 489 16108 16129 16172 16223 16234 16290 16300	[0.015] M12b1b (1)	[0.000] M12b M12b1 M12b1a M12b1a1 (5)	1
1149 >KX457476.1 Ho	73G 150T 152C 260A 263G 310.1C 489C 750G 1193C 1342T 1438G 2706G 3106d 3107C 4769G 6253C 7028T 8269A 8701G 8860G 9540C 10398G 10400T 10873C 11719A 11810T 12705T 12732C 13468T 14766T 14783C 14883T 15043A 15301A 15326G 16223T 16362C 16381C 16519C	M61	73G 150T 152C 260A 263G 310.1C 489C 16223T 16362C 16381C 16519C	1	73 150 152 260 263 489 750 1193 1342 1438 2706 4769 6253 7028 8269 8701 8860 9540 10398 10400 10873 11719 11810 12705 12732 13468 14766 14783 14883 15043 15301 15326 16223 16362 16381	M61	73 150 152 260 263 489 16223 16362 16381	[0.170] M61 (1)	[0.003] M13'46'61+16362 (2) [0.002] D4b2b2a D4b2b2a1 D4b2b4 (5) [0.000] G1a M61a (7)	1

1150	>KX457473.1 Hc	73G 152C 225A 248d 263G 309.1CT 310C 316A 489C 514d 515d 750G 1438G 2706G 3106d 3107C 3200C 3714G 4385T 4769G 4772C 7028T 7270C 7433T 8701G 8853G 8860G 9127G 9512T 9540C 10274C 10398G 10400T 10679G 10873C 11719A 11914A 12354C 12705T 14110C 14766T 14783C 14974T 15043A 15301A 15326G 15691G 16086C 16129A 16209C 16223T 16263C 16272G 16519C	M20	73G 152C 225A 248d 263G 309.1CT 310C 316A 489C 514d 515d 16086C 16129A 16209C 16223T 16263C 16272G 16519C	1	73 152 225 249d 263 316 489 750 1438 2706 3200 3714 4385T 4769 4772 7028 7270 7433 8701 8853 8860 9127 9512 9540 10274 10398 10400 10679 10873 11719 11914 12354 12705 14110 14766 14783 14974 15043 15301 15326 15691 16086 16129 16209 16223 16263 16272	M20	73 152 225 249d 263 316 489 16086 16129 16209 16223 16263 16272	[0.649] M20 (1)	[0.010] D4a7 (2) [0.000] M13b1	1
1151	>KX457468.1 Hc	73G 152C 225A 248d 263G 310.1C 316A 489C 514d 515d 750G 1438G 2706G 3106d 3107C 3200C 3714G 4385T 4769G 4772C 7028T 7433T 8701G 8853G 8860G 9127G 9512T 9540C 10274C 10398G 10400T 10679G 10873C 11719A 11914A 12354C 12705T 14110C 14766T 14783C 14974T 15043A 15301A 15326G 15691G 15884A 16086C 16129A 16209C 16223T 16272G 16519C	M20	73G 152C 225A 248d 263G 310.1C 316A 489C 514d 515d 16086C 16129A 16209C 16223T 16272G 16519C	1	73 152 225 249d 263 316 489 750 1438 2706 3200 3714 4385T 4769 4772 7028 7433 8701 8853 8860 9127 9512 9540 10274 10398 10400 10679 10873 11719 11914 12354 12705 14110 14766 14783 14974 15043 15301 15326 15691 15884 16086 16129 16209 16223 16272	M20	73 152 225 249d 263 316 489 16086 16129 16209 16223 16272	[0.607] M20 (1)	[0.130] M5a2a (2) [0.068] M17 (3) [0.067] M17a (4) [0.059] G1b+16129 (5) [0.028] M5a (6) [0.021] D4a1a1 (7) [0.019] M5 M27b2b1 (9) [0.007] M5a'd (10) [0.006] G1b2 G1b4 D4a1 (13) [0.004] M37+152 D4a1f1 (15) [0.000] M5a2 M5a2a1 M5a5 M5b'c M5b M7a M7a1b2 M7a2 M7b1 M13'46'61 M13 M13a'b M13b M17c M27b2 M27b2a M27b2b M27b2c D4a D4a1a D4a1a1a D4a1b D4a1c D4a1f D4a1g D4a1h D4a2 D4a2a D4a2b D4a5 (45)	1
1152	>KX457467.1 Hc	73G 185A 189G 263G 309.1CT 310C 514d 515d 1438G 2706G 3106d 3107C 3543T 3873G 4562G 4769G 5581G 6314T 7028T 7051C 7853A 8860G 9899C 10373A 11719A 12136C 12705T 13434G 13500C 13928C 14022G 14766T 14953T 15211T 15326G 16172C 16178C 16183C 16189C 16223T 16362C	N10a	73G 185A 189G 263G 309.1CT 310C 514d 515d 16172C 16178C 16183C 16189C 16223T 16362C	1	73 185 189 263 1438 2706 3543 3873 4562 4769 5581 6314 7028 7051 7853 8860 9899 10373 11719 12136 12705 13434 13500 13928C 14022 14766 14953 15211 15326 16172 16178 16189 16223 16362	N10a	73 185 189 263 16172 16178 16189 16223 16362	[0.013] N10a (1)	[0.071] N10 (2)	1
1153	>KX457464.1 Hc	73G 150T 159C 199C 263G 310.1C 489C 750G 1438G 2706G 3106d 3107C 4048A 4071T 4164G 4769G 5351G 5460A 6455T 6680C 7028T 7684C 7853A 8701G 8860G 9540C 9824C 10398G 10400T 10873C 11440A 11719A 12405T 12705T 12811C 14766T 14783C 15043A 15301A 15326G 16129A 16223T 16297C	M7b1a1	73G 150T 159C 199C 263G 310.1C 489C 16129A 16223T 16297C	1	73 150 159 199 263 489 750 1438 2706 4048 4071 4164 4769 5351 5460 6455 6680 7028 7684 7853 8701 8860 9540 9824 10398 10400 10873 11440 11719 12405 12705 12811 14766 14783 15043 15301 15326 16129 16223 16297	M7b1a1	73 150 159 199 263 489 16129 16223 16297	[0.232] M7b1a1 (1) [0.033] M7b1a1c (2) [0.000] M7b1a1+(16192) M7b1a1d M7b1a1d1 M7b1a1e M7b1a1e1 M7b1a1f M7b1a1g M7b1a1h (10)	[0.033] M7b1a1c (11) [0.007] M7b1a1e2 (12) [0.003] M7b1a1a2 (13) [0.000] M7b1a M7b1a1a M7b1a1+(16192) M7b1a1d M7b1a1d1 M7b1a1e M7b1a1e1 M7b1a1f M7b1a1g M7b1a1h M7b1a1i (24)	1

1154 >KX457460.1 Ho	73G 263G 310.1C 489C 513.1CA 750G 1438G 2706G 3106d 3107C 3456C 3588T 4216C 4769G 7028T 7960G 8701G 8768T 8854A 8860G 9540C 10236G 10398G 10400T 10873C 11719A 11836G 12705T 13308G 13708A 14130T 14260G 14527G 14552G 14560A 14766T 14783C 15043A 15172A 15326G 16086C 16209C 16223T 16259T 16278T 16319A 16399G 16526A	M32c	73G 263G 310.1C 489C 513.1CA 16086C 16209C 16223T 16259T 16278T 16319A 16399G 16526A	1	73 263 489 750 1438 2706 3456 3588 4216 4769 7028 7960 8701 8768 8854 8860 9540 10236 10398 10400 10873 11719 11836 12705 13308 13708 14130 14260 14527 14552 14560 14766 14783 15043 15172 15326 16086 16209 16223 16259 16278 16319 16399 16526	M32c	73 263 489 16086 16209 16223 16259 16278 16319 16399 16526	[0.073] M32c (1)	[0.000] M32 (2)	1
1155 >KX457459.1 Ho	73G 152C 225A 248d 263G 310.1C 316A 489C 514d 515d 750G 1438G 2706G 3106d 3107C 3200C 3714G 4385T 4769G 4772C 7028T 7433T 8701G 8853G 8860G 9127G 9317A 9512T 9540C 10274C 10398G 10400T 10679G 10873C 11719A 11914A 12354C 12362T 12705T 14110C 14766T 14783C 14974T 15043A 15301A 15326G 15691G 15884A 16086C 16129A 16209C 16223T 16272G 16519C	M20	73G 152C 225A 248d 263G 310.1C 316A 489C 514d 515d 16086C 16129A 16209C 16223T 16272G 16519C	1	73 152 225 249d 263 316 489 750 1438 2706 3200 3714 4385T 4769 4772 7028 7433 8701 8853 8860 9127 9317A 9512 9540 10274 10398 10400 10679 10873 11719 11914 12354 12362 12705 14110 14766 14783 14974 15043 15301 15326 15691 15884 16086 16129 16209 16223 16272	M20	73 152 225 249d 263 316 489 16086 16129 16209 16223 16272	[0.607] M20 (1)	[0.130] M5a2a (2) [0.068] M17 (3) [0.067] M17a (4) [0.059] G1b+16129 (5) [0.028] M5a (6) [0.021] D4a1a1 (7) [0.019] M5 M27b2b1 (9) [0.007] M5a'd (10) [0.006] G1b2 G1b4 D4a1 (13) [0.004] M37+152 D4a1f1 (15) [0.000] M5a2 M5a2a1 M5a5 M5b'c M5b M7a M7a1b2 M7a2 M7b1 M13'46'61 M13 M13a'b M13b M17c M27b2 M27b2a M27b2b M27b2c D4a D4a1a D4a1a1a D4a1b D4a1c D4a1f D4a1g D4a1h D4a2 D4a2a D4a2b D4a5 (45)	1
1156 >KX457457.1 Ho	73G 153G 263G 310.1C 489C 709A 750G 1438G 2706G 3106d 3107C 3394C 4491A 4769G 5964C 6842C 6956C 7028T 7609C 7789A 8701G 8860G 9540C 10398G 10400T 10873C 11719A 12705T 12864C 14766T 14783C 15043A 15301A 15326G 16051G 16209C 16223T 16362C 16519C	M9b	73G 153G 263G 310.1C 489C 16051G 16209C 16223T 16362C 16519C	1	73 153 263 489 709 750 1438 2706 3394 4491 4769 5964 6842 6956 7028 7609 7789 8701 8860 9540 10398 10400 10873 11719 12705 12864 14766 14783 15043 15301 15326 16051 16209 16223 16362	M9b	73 153 263 489 16051 16209 16223 16362	[0.121] M9b (1)	[0.000] M9a'b (2)	1
1157 >KX457456.1 Ho	73G 150T 239C 263G 309.1CT 310C 750G 1438G 2706G 3106d 3107C 4386C 4769G 5231A 5417A 7028T 8860G 9214G 11719A 12358G 12372A 12705T 12771A 14766T 15094T 15326G 16223T 16257A 16261T 16311C 16343G	N9a10+16311	73G 150T 239C 263G 309.1CT 310C 16223T 16257A 16261T 16311C 16343G	1	73 150 239 263 750 1438 2706 4386 4769 5231 5417 7028 8860 9214 11719 12358 12372 12705 12771 14766 15094 15326 16223 16257A 16261 16311 16343	N9a10+16311	73 150 239 263 16223 16257A 16261 16311 16343	[0.063] N9a10+16311 (1)	[0.022] N9a9 (2) [0.011] N9a (3) [0.000] N9a8 N9a10 N9a10b (6)	1
1158 >KX457454.1 Ho	73G 146C 150T 185A 189G 196C 199C 263G 309.1CCT 310C 513A 750G 827G 1438G 2706G 3106d 3107C 4769G 6614C 7028T 7657C 8271d 8272d 8273d 8274d 8275d 8276d 8277d 8278d 8279d 8860G 9758C 9921A 10410C 10750G 11719A 13135A 14766T 15326G 15535T 15940d 16129A 16182C 16183C 16189C 16217C 16223T 16311C 16519C	B4e	73G 146C 150T 185A 189G 196C 199C 263G 309.1CCT 310C 513A 16129A 16182C 16183C 16189C 16217C 16223T 16311C 16519C	1	73 146 150 185 189 196 199 263 513 750 827 1438 2706 4769 6614 7028 7657 8281-8289d 8860 9758 9921 10410 10750 11719 13135 14766 15326 15535 15944d 16129 16189 16217 16223 16311	B4e	73 146 150 185 189 196 199 263 513 16129 16189 16217 16223 16311	[1.354] B4e (1)	[0.084] R11b (2) [0.073] R11 (3) [0.048] R11a	1

1159 >KX457452.1 Hc	73G 150T 151T 263G 309.1CT 310C 489C 750G 1438G 2706G 3106d 3107C 4769G 7028T 7202G 7649G 8440G 8701G 8718G 8860G 9449T 9540C 9554A 10398G 10400T 10873C 11719A 12705T 14766T 14783C 15043A 15257A 15301A 15326G 15458C 16223T 16269G 16271C	M71a1a	73G 150T 151T 263G 309.1CT 310C 489C 16223T 16269G 16271C	1	73 150 151 263 489 750 1438 2706 4769 7028 7202 7649 8440 8701 8718 8860 9449 9540 9554 10398 10400 10873 11719 12705 14766 14783 15043 15257 15301 15326 15458 16223 16269 16271	M71a1a	73 150 151 263 489 16223 16269 16271	[0.000] M71a1a (1)	[0.000] M71a1 (2)	1
1160 >KX457451.1 Hc	73G 150T 152C 260A 263G 310.1C 489C 750G 1193C 1342T 1438G 2706G 3106d 3107C 4769G 6253C 7028T 8269A 8701G 8860G 9540C 10398G 10400T 10873C 11719A 11810T 12705T 12732C 13468T 14766T 14783C 14883T 15043A 15301A 15326G 16223T 16362C 16381C 16519C	M61	73G 150T 152C 260A 263G 310.1C 489C 16223T 16362C 16381C 16519C	1	73 150 152 260 263 489 750 1193 1342 1438 2706 4769 6253 7028 8269 8701 8860 9540 10398 10400 10873 11719 11810 12705 12732 13468 14766 14783 14883 15043 15301 15326 16223 16362 16381	M61	73 150 152 260 263 489 16223 16362 16381	[0.170] M61 (1)	[0.003] M13'46'61+16362 (2) [0.002] D4b2b2a D4b2b2a1 D4b2b4 (5) [0.000] G1a M61a (7)	1
1161 >KX457445.1 Hc	73G 150T 152C 260A 263G 310.1C 489C 750G 1193C 1342T 1438G 2706G 3106d 3107C 4769G 6253C 7028T 8269A 8701G 8860G 9540C 10398G 10400T 10873C 11719A 11810T 12705T 12732C 13468T 14766T 14783C 14883T 15043A 15301A 15326G 16223T 16362C 16381C 16519C	M61	73G 150T 152C 260A 263G 310.1C 489C 16223T 16362C 16381C 16519C	1	73 150 152 260 263 489 750 1193 1342 1438 2706 4769 6253 7028 8269 8701 8860 9540 10398 10400 10873 11719 11810 12705 12732 13468 14766 14783 14883 15043 15301 15326 16223 16362 16381	M61	73 150 152 260 263 489 16223 16362 16381	[0.170] M61 (1)	[0.003] M13'46'61+16362 (2) [0.002] D4b2b2a D4b2b2a1 D4b2b4 (5) [0.000] G1a M61a (7)	1
1162 >KX457444.1 Hc	73G 207A 248d 263G 309.1CT 310C 709A 750G 1438G 2706G 3106d 3107C 3434G 3970T 4769G 4824G 4991A 5585A 5894G 5913A 5978G 6392C 7028T 8860G 10310A 10320A 11065G 11719A 12621T 13928C 14766T 14971C 15326G 15412G 16260T 16298C 16355T 16362C	F3a1	73G 207A 248d 263G 309.1CT 310C 16260T 16298C 16355T 16362C	1	73 207 249d 263 709 750 1438 2706 3434 3970 4769 4824 4991 5585 5894 5913 5978 6392 7028 8860 10310 10320 11065 11719 12621 13928C 14766 14971 15326 15412G 16260 16298 16355 16362	F3a1	73 207 249d 263 16260 16298 16355 16362	[0.000] F3a1 (1)	[0.000] F3a+207 (2)	1
1163 >KX457443.1 Hc	73G 207A 248d 263G 309.1CT 310C 709A 750G 1438G 2706G 3106d 3107C 3434G 3970T 4769G 4824G 4991A 5585A 5894G 5913A 5978G 6392C 7028T 8860G 10310A 10320A 11065G 11719A 12621T 13928C 14766T 14971C 15326G 15412G 16260T 16298C 16355T 16362C	F3a1	73G 207A 248d 263G 309.1CT 310C 16260T 16298C 16355T 16362C	1	73 207 249d 263 709 750 1438 2706 3434 3970 4769 4824 4991 5585 5894 5913 5978 6392 7028 8860 10310 10320 11065 11719 12621 13928C 14766 14971 15326 15412G 16260 16298 16355 16362	F3a1	73 207 249d 263 16260 16298 16355 16362	[0.000] F3a1 (1)	[0.000] F3a+207 (2)	1
1164 >KX457442.1 Hc	73G 207A 248d 263G 309.1CT 310C 709A 750G 1438G 2706G 3106d 3107C 3434G 3970T 4769G 4824G 4991A 5585A 5894G 5913A 5978G 6392C 7028T 8860G 10310A 10320A 11065G 11719A 12621T 13928C 14766T 14971C 15326G 15412G 16260T 16298C 16355T 16362C	F3a1	73G 207A 248d 263G 309.1CT 310C 16260T 16298C 16355T 16362C	1	73 207 249d 263 709 750 1438 2706 3434 3970 4769 4824 4991 5585 5894 5913 5978 6392 7028 8860 10310 10320 11065 11719 12621 13928C 14766 14971 15326 15412G 16260 16298 16355 16362	F3a1	73 207 249d 263 16260 16298 16355 16362	[0.000] F3a1 (1)	[0.000] F3a+207 (2)	1

1165	>KX457438.1 Ho	73G 151T 152C 248d 263G 309.1CT 310C 489C 750G 1438G 2706G 3106d 3107C 4715G 4769G 6752G 7028T 7196A 8584A 8701G 8860G 9090C 9540C 10398G 10400T 10873C 11719A 12705T 14766T 14783C 15043A 15301A 15326G 15475G 15487T 15773A 15784C 15940d 16185T 16223T 16260T	Z4	73G 151T 152C 248d 263G 309.1CT 310C 489C 16185T 16223T 16260T	1	73 151 152 249d 263 489 750 1438 2706 4715 4769 6752 7028 7196A 8584 8701 8860 9090 9540 10398 10400 10873 11719 12705 14766 14783 15043 15301 15326 15475 15487T 15773 15784 15944d 16185 16223 16260	Z4	73 151 152 249d 263 489 16185 16223 16260	[0.004] Z4 (1)	[0.034] Z3c (2) [0.019] Z3+709 (3) [0.000] Z+152 Z1 Z2 Z3 Z3d Z4a Z7 (10)	1
1166	>KX457436.1 Ho	73G 150T 152C 260A 263G 310.1C 489C 750G 1193C 1342T 1438G 2706G 3106d 3107C 4769G 6253C 7028T 8269A 8701G 8860G 9540C 10398G 10400T 10873C 11719A 11810T 12705T 12732C 13468T 14766T 14783C 14883T 15043A 15301A 15326G 16223T 16362C 16381C 16519C	M61	73G 150T 152C 260A 263G 310.1C 489C 16223T 16362C 16381C 16519C	1	73 150 152 260 263 489 750 1193 1342 1438 2706 4769 6253 7028 8269 8701 8860 9540 10398 10400 10873 11719 11810 12705 12732 13468 14766 14783 14883 15043 15301 15326 16223 16362 16381	M61	73 150 152 260 263 489 16223 16362 16381	[0.170] M61 (1)	[0.003] M13'46'61+16362 (2) [0.002] D4b2b2a D4b2b2a1 D4b2b4 (5) [0.000] G1a M61a (7)	1
1167	>KX457432.1 Ho	73G 150T 153G 263G 309.1CT 310C 385G 489C 750G 1438G 2706G 3106d 3107C 3394C 4491A 4769G 7028T 8155A 8701G 8860G 9540C 10398G 10400T 10873C 11719A 11778A 12237T 12705T 14308C 14766T 14783C 15043A 15301A 15326G 16148T 16223T 16234T 16355T 16356C 16362C	M9a5	73G 150T 153G 263G 309.1CT 310C 385G 489C 16148T 16223T 16234T 16355T 16356C 16362C	1	73 150 153 263 385 489 750 1438 2706 3394 4491 4769 7028 8155 8701 8860 9540 10398 10400 10873 11719 11778 12237 12705 14308 14766 14783 15043 15301 15326 16148 16223 16234 16355 16356 16362	M9a5	73 150 153 263 385 489 16148 16223 16234 16355 16356 16362	[0.156] M9a5 (1)	[0.039] M9a1b+150 (2) [0.005] M9a4 (3) [0.000] M9a M9a1 M9a1b2 M9a4b (7)	1
1168	>KX457431.1 Ho	73G 207A 248d 263G 309.1CT 310C 709A 750G 1438G 2706G 3106d 3107C 3434G 3970T 4769G 4824G 4991A 5585A 5894G 5913A 5978G 6392C 7028T 8860G 10310A 10320A 11065G 11719A 12621T 13928C 14766T 14971C 15326G 15412G 16260T 16298C 16355T 16362C	F3a1	73G 207A 248d 263G 309.1CT 310C 16260T 16298C 16355T 16362C	1	73 207 249d 263 709 750 1438 2706 3434 3970 4769 4824 4991 5585 5894 5913 5978 6392 7028 8860 10310 10320 11065 11719 12621 13928C 14766 14971 15326 15412G 16260 16298 16355 16362	F3a1	73 207 249d 263 16260 16298 16355 16362	[0.000] F3a1 (1)	[0.000] F3a+207 (2)	1
1169	>KX457429.1 Ho	73G 150T 152C 260A 263G 310.1C 489C 750G 1193C 1342T 1438G 2706G 3106d 3107C 4769G 6253C 7028T 8269A 8701G 8860G 9540C 10398G 10400T 10873C 11719A 11810T 12705T 12732C 13468T 14766T 14783C 14883T 15043A 15301A 15326G 16223T 16362C 16381C 16519C	M61	73G 150T 152C 260A 263G 310.1C 489C 16223T 16362C 16381C 16519C	1	73 150 152 260 263 489 750 1193 1342 1438 2706 4769 6253 7028 8269 8701 8860 9540 10398 10400 10873 11719 11810 12705 12732 13468 14766 14783 14883 15043 15301 15326 16223 16362 16381	M61	73 150 152 260 263 489 16223 16362 16381	[0.170] M61 (1)	[0.003] M13'46'61+16362 (2) [0.002] D4b2b2a D4b2b2a1 D4b2b4 (5) [0.000] G1a M61a (7)	1

1170 >KX457426.1 Hc	73G 152C 248d 263G 310.1C 316A 489C 514d 515d 750G 1438G 2706G 3106d 3107C 3200C 3714G 4385T 4769G 4772C 7028T 7433T 8701G 8853G 8860G 9127G 9512T 9540C 10274C 10398G 10400T 10679G 10873C 11719A 11914A 12354C 12705T 14110C 14766T 14783C 14974T 15043A 15301A 15326G 15691G 16086C 16129A 16209C 16223T 16263C 16272G 16519C	M20	73G 152C 248d 263G 310.1C 316A 489C 514d 515d 16086C 16129A 16209C 16223T 16263C 16272G 16519C	1	73 152 249d 263 316 489 750 1438 2706 3200 3714 4385T 4769 4772 7028 7433 8701 8853 8860 9127 9512 9540 10274 10398 10400 10679 10873 11719 11914 12354 12705 14110 14766 14783 14974 15043 15301 15326 15691 16086 16129 16209 16223 16263 16272	M20	73 152 249d 263 316 489 16086 16129 16209 16223 16263 16272	[0.342] M20 (1)	[0.010] D4a7 (2) [0.000] M13b1	1
1171 >KX457422.1 Hc	73G 152C 248d 263G 310.1C 316A 489C 514d 515d 750G 1438G 2706G 3106d 3107C 3200C 3714G 4385T 4769G 4772C 7028T 7433T 8701G 8853G 8860G 9127G 9512T 9540C 10274C 10398G 10400T 10679G 10873C 11719A 11914A 12354C 12705T 14110C 14766T 14783C 14974T 15043A 15301A 15326G 15691G 16086C 16129A 16209C 16223T 16263C 16272G 16519C	M20	73G 152C 248d 263G 310.1C 316A 489C 514d 515d 16086C 16129A 16209C 16223T 16263C 16272G 16519C	1	73 152 249d 263 316 489 750 1438 2706 3200 3714 4385T 4769 4772 7028 7433 8701 8853 8860 9127 9512 9540 10274 10398 10400 10679 10873 11719 11914 12354 12705 14110 14766 14783 14974 15043 15301 15326 15691 16086 16129 16209 16223 16263 16272	M20	73 152 249d 263 316 489 16086 16129 16209 16223 16263 16272	[0.342] M20 (1)	[0.010] D4a7 (2) [0.000] M13b1	1
1172 >KX457421.1 Hc	73G 152C 248d 263G 310.1C 316A 489C 514d 515d 750G 1438G 2706G 3106d 3107C 3200C 3714G 4385T 4769G 4772C 7028T 7433T 8701G 8853G 8860G 9127G 9512T 9540C 10274C 10398G 10400T 10679G 10873C 11719A 11914A 12354C 12705T 14110C 14766T 14783C 14974T 15043A 15301A 15326G 15691G 16086C 16129A 16209C 16223T 16263C 16272G 16519C	M20	73G 152C 248d 263G 310.1C 316A 489C 514d 515d 16086C 16129A 16209C 16223T 16263C 16272G 16519C	1	73 152 249d 263 316 489 750 1438 2706 3200 3714 4385T 4769 4772 7028 7433 8701 8853 8860 9127 9512 9540 10274 10398 10400 10679 10873 11719 11914 12354 12705 14110 14766 14783 14974 15043 15301 15326 15691 16086 16129 16209 16223 16263 16272	M20	73 152 249d 263 316 489 16086 16129 16209 16223 16263 16272	[0.342] M20 (1)	[0.010] D4a7 (2) [0.000] M13b1	1
1173 >KX457420.1 Hc	73G 152C 210G 257G 263G 309.1CT 310C 514d 515d 709A 750G 1438G 2706G 3106d 3107C 3537G 4086T 4769G 6960T 7028T 8271d 8272d 8273d 8274d 8275d 8276d 8277d 8278d 8279d 8584A 8860G 9950C 10398G 10589A 11465C 11719A 14696G 14766T 15235G 15326G 16140C 16182C 16183C 16189C 16261T 16266A 16519C	B5a1d	73G 152C 210G 257G 263G 309.1CT 310C 514d 515d 16140C 16182C 16183C 16189C 16261T 16266A 16519C	1	73 152 210 257 263 709 750 1438 2706 3537 4086 4769 6960 7028 8281-8289d 8584 8860 9950 10398 10589 11465 11719 14696 14766 15235 15326 16140 16189 16261 16266A	B5a1d	73 152 210 257 263 16140 16189 16261 16266A	[0.028] B5a1d (1)	[0.032] B5a1 (2) [0.016] B5a1a (3) [0.010] B5a (4) [0.004] B5a1b1 (5) [0.002] B5a1c1a (6) [0.000] B5a1a1 B5a1b B5a1c B5a1c1 B5a1c2 (11)	1

1174	>KX457419.1 Hc	73G 152C 210G 257G 263G 309.1CT 310C 514d 515d 709A 750G 1438G 2706G 3106d 3107C 3537G 4086T 4769G 6960T 7028T 8271d 8272d 8273d 8274d 8275d 8276d 8277d 8278d 8279d 8584A 8860G 9950C 10398G 10589A 11465C 11719A 14696G 14766T 15235G 15326G 16140C 16182C 16183C 16189C 16261T 16266A 16519C	B5a1d	73G 152C 210G 257G 263G 309.1CT 310C 514d 515d 16140C 16182C 16183C 16189C 16261T 16266A 16519C	1	73 152 210 257 263 709 750 1438 2706 3537 4086 4769 6960 7028 8281-8289d 8584 8860 9950 10398 10589 11465 11719 14696 14766 15235 15326 16140 16189 16261 16266A	B5a1d	73 152 210 257 263 16140 16189 16261 16266A	[0.028] B5a1d (1)	[0.032] B5a1 (2) [0.016] B5a1a (3) [0.010] B5a (4) [0.004] B5a1b1 (5) [0.002] B5a1c1a (6) [0.000] B5a1a1 B5a1b B5a1c B5a1c1 B5a1c2 (11)	1
1175	>KX457412.1 Hc	73G 152C 210G 257G 263G 514d 515d 709A 750G 1438G 2706G 3106d 3107C 3537G 4086T 4769G 6960T 7028T 8271d 8272d 8273d 8274d 8275d 8276d 8277d 8278d 8279d 8584A 8860G 9950C 10398G 10589A 11465C 11719A 14696G 14766T 15235G 15326G 16140C 16182C 16183C 16189C 16261T 16266A 16519C	B5a1d	73G 152C 210G 257G 263G 514d 515d 16140C 16182C 16183C 16189C 16261T 16266A 16519C	1	73 152 210 257 263 709 750 1438 2706 3537 4086 4769 6960 7028 8281-8289d 8584 8860 9950 10398 10589 11465 11719 14696 14766 15235 15326 16140 16189 16261 16266A	B5a1d	73 152 210 257 263 16140 16189 16261 16266A	[0.028] B5a1d (1)	[0.032] B5a1 (2) [0.016] B5a1a (3) [0.010] B5a (4) [0.004] B5a1b1 (5) [0.002] B5a1c1a (6) [0.000] B5a1a1 B5a1b B5a1c B5a1c1 B5a1c2 (11)	1
1176	>KX457409.1 Hc	73G 263G 291d 292d 293d 294d 309.1CT 310C 489C 514d 515d 750G 1438G 2626C 2706G 3106d 3107C 3828G 4561C 4688C 4769G 4902C 5147A 5489A 6359C 7028T 8093C 8701G 8860G 9540C 9644G 10398G 10400T 10873C 11719A 12705T 12950G 13401C 13806T 14766T 14783C 15043A 15301A 15326G 16223T 16259T 16278T 16291T 16362C 16519C	M68a	73G 263G 291d 292d 293d 294d 309.1CT 310C 489C 514d 515d 16223T 16259T 16278T 16291T 16362C 16519C	1	73 263 291-294d 489 750 1438 2626 2706 3828 4561 4688 4769 4902C 5147 5489A 6359C 7028 8093 8701 8860 9540 9644 10398 10400 10873 11719 12705 12950 13401 13806 14766 14783 15043 15301 15326 16223 16259 16278 16291 16362	M68a	73 263 291-294d 489 16223 16259 16278 16291 16362	[0.029] M68a (1)	[0.000] G2a G2a1 M68a2 (4)	1
1177	>KX457408.1 Hc	73G 152C 263G 310.1C 750G 1438G 1541C 2706G 3106d 3107C 3666A 3970T 4769G 6647G 7028T 8724G 8860G 11299C 11422T 11659T 11719A 13191C 13722G 13928C 14766T 15314A 15326G 15883A 16304C 16362C 16519C	R9b2	73G 152C 263G 310.1C 16304C 16362C 16519C	1	73 152 263 750 1438 1541 2706 3666 3970 4769 6647 7028 8724 8860 11299 11422 11659 11719 13191 13722 13928C 14766 15314 15326 15883 16304 16362	R9b2	73 152 263 16304 16362	[0.059] R9b2 (1)	[0.048] R9 (2) [0.015] R9c (3) [0.008] R30a1 (4) [0.007] R6 (5) [0.000] H32 R9b R30a1a R31 (9)	1
1178	>MF058290.1 Hc	73G 150T 200G 263G 310.1C 489C 750G 2706G 3106d 3107C 3588T 4769G 6345C 7028T 7960G 8701G 8768T 8854A 8860G 9540C 10398G 10400T 10873C 11719A 12705T 13308G 13590A 13708A 14130T 14260G 14527G 14766T 14783C 15043A 15172A 15301A 15326G 15625T 16086C 16148T 16223T 16259T 16278T 16319A 16399G 16526A	M32c	73G 150T 200G 263G 310.1C 489C 16086C 16148T 16223T 16259T 16278T 16319A 16399G 16526A	1	73 150 200 263 489 750 2706 3588 4769 6345 7028 7960 8701 8768 8854 8860 9540 10398 10400 10873 11719 12705 13308 13590 13708 14130 14260 14527 14766 14783 15043 15172 15301 15326 15625 16086 16148 16223 16259 16278 16319 16399 16526	M32c	73 150 200 263 489 16086 16148 16223 16259 16278 16319 16399 16526	[0.000] M32c (1)	[0.022] M51a (2) [0.007] M32 (3) [0.000] M51 M51b (5)	1

1179	>MF058286.1 Hc	73G 152C 195C 204C 263G 310.1C 417A 489C 533G 750G 1438G 3106d 3107C 3915A 4769G 7028T 8188G 8360G 8701G 8860G 9438A 9540C 9545G 10142T 10295G 10398G 10400T 10873C 11569C 11719A 11899C 12279G 12618A 12705T 14766T 14783C 15025T 15043A 15301A 15326G 16223T 16263C 16311C 16519C	M23	73G 152C 195C 204C 263G 310.1C 417A 489C 533G 16223T 16263C 16311C 16519C	1	73 152 195 204 263 417 489 533 750 1438 3915 4769 7028 8188 8360 8701 8860 9438 9540 9545 10142 10295 10398 10400 10873 11569 11719 11899 12279 12618 12705 14766 14783 15025 15043 15301 15326 16223 16263 16311	M23	73 152 195 204 263 417 489 533 16223 16263 16311	[0.000] M23 (1)	[0.072] M24a (2) [0.053] M57+152	1
1180	>MF058283.1 Hc	73G 152C 195C 204C 263G 310.1C 417A 489C 533G 750G 1438G 3106d 3107C 4769G 7028T 8188G 8360G 8701G 8860G 9438A 9540C 9545G 10142T 10295G 10398G 10400T 10873C 11569C 11719A 11899C 12279G 12618A 12705T 14766T 14783C 15025T 15043A 15301A 15326G 16223T 16263C 16311C 16519C	M23	73G 152C 195C 204C 263G 310.1C 417A 489C 533G 16223T 16263C 16311C 16519C	1	73 152 195 204 263 417 489 533 750 1438 4769 7028 8188 8360 8701 8860 9438 9540 9545 10142 10295 10398 10400 10873 11569 11719 11899 12279 12618 12705 14766 14783 15025 15043 15301 15326 16223 16263 16311	M23	73 152 195 204 263 417 489 533 16223 16263 16311	[0.000] M23 (1)	[0.072] M24a (2) [0.053] M57+152	1
1181	>MF058279.1 Hc	73G 152C 195C 204C 263G 310.1C 417A 489C 533G 750G 1438G 3106d 3107C 3438A 4769G 7028T 8188G 8360G 8701G 8860G 9438A 9540C 9545G 10142T 10295G 10398G 10400T 10873C 11569C 11719A 11899C 12279G 12618A 12705T 14766T 14783C 15025T 15043A 15301A 15326G 16223T 16263C 16311C 16519C	M23	73G 152C 195C 204C 263G 310.1C 417A 489C 533G 16223T 16263C 16311C 16519C	1	73 152 195 204 263 417 489 533 750 1438 3438 4769 7028 8188 8360 8701 8860 9438 9540 9545 10142 10295 10398 10400 10873 11569 11719 11899 12279 12618 12705 14766 14783 15025 15043 15301 15326 16223 16263 16311	M23	73 152 195 204 263 417 489 533 16223 16263 16311	[0.000] M23 (1)	[0.072] M24a (2) [0.053] M57+152	1
1182	>MF058278.1 Hc	73G 150T 200G 263G 310.1C 489C 750G 2706G 3106d 3107C 3588T 4769G 7028T 7960G 8701G 8768T 8854A 8860G 9540C 10398G 10400T 10873C 11719A 12705T 13308G 13590A 13708A 14130T 14260G 14527G 14766T 14783C 15043A 15172A 15301A 15326G 15625T 16086C 16148T 16223T 16259T 16278T 16319A 16399G 16526A	M32c	73G 150T 200G 263G 310.1C 489C 16086C 16148T 16223T 16259T 16278T 16319A 16399G 16526A	1	73 150 200 263 489 750 2706 3588 4769 7028 7960 8701 8768 8854 8860 9540 10398 10400 10873 11719 12705 13308 13590 13708 14130 14260 14527 14766 14783 15043 15172 15301 15326 15625 16086 16148 16223 16259 16278 16319 16399 16526	M32c	73 150 200 263 489 16086 16148 16223 16259 16278 16319 16399 16526	[0.000] M32c (1)	[0.022] M51a (2) [0.007] M32 (3) [0.000] M51 M51b (5)	1
1183	>MF058277.1 Hc	73G 152C 195C 204C 263G 310.1C 417A 489C 533G 750G 1438G 3106d 3107C 3438A 4769G 7028T 8188G 8360G 8701G 8860G 9438A 9540C 9545G 10142T 10295G 10398G 10400T 10873C 11569C 11719A 11899C 12279G 12618A 12705T 14766T 14783C 15025T 15043A 15301A 15326G 16223T 16263C 16311C 16519C	M23	73G 152C 195C 204C 263G 310.1C 417A 489C 533G 16223T 16263C 16311C 16519C	1	73 152 195 204 263 417 489 533 750 1438 3438 4769 7028 8188 8360 8701 8860 9438 9540 9545 10142 10295 10398 10400 10873 11569 11719 11899 12279 12618 12705 14766 14783 15025 15043 15301 15326 16223 16263 16311	M23	73 152 195 204 263 417 489 533 16223 16263 16311	[0.000] M23 (1)	[0.072] M24a (2) [0.053] M57+152	1

1184	>MF058271.1 Hc	73G 150T 200G 263G 310.1C 489C 750G 2706G 3106d 3107C 3588T 4769G 7028T 7960G 8701G 8768T 8854A 8860G 9540C 10398G 10400T 10873C 11719A 12634G 12705T 13308G 13434G 13590A 13708A 14130T 14260G 14527G 14766T 14783C 15043A 15172A 15301A 15326G 15625T 16086C 16148T 16223T 16259T 16278T 16319A 16399G 16526A	M32c	73G 150T 200G 263G 310.1C 489C 16086C 16148T 16223T 16259T 16278T 16319A 16399G 16526A	1	73 150 200 263 489 750 2706 3588 4769 7028 7960 8701 8768 8854 8860 9540 10398 10400 10873 11719 12634 12705 13308 13434 13590 13708 14130 14260 14527 14766 14783 15043 15172 15301 15326 15625 16086 16148 16223 16259 16278 16319 16399 16526	M32c	73 150 200 263 489 16086 16148 16223 16259 16278 16319 16399 16526	[0.000] M32c (1)	[0.022] M51a (2) [0.007] M32 (3) [0.000] M51 M51b (5)	1
1185	>MF058270.1 Hc	73G 150T 200G 263G 310.1C 489C 750G 2706G 3106d 3107C 3588T 4769G 7028T 7960G 8701G 8768T 8854A 8860G 9540C 10398G 10400T 10873C 11719A 12634G 12705T 13308G 13434G 13590A 13708A 14130T 14260G 14527G 14766T 14783C 15043A 15172A 15301A 15326G 15625T 16086C 16148T 16223T 16259T 16278T 16319A 16399G 16526A	M32c	73G 150T 200G 263G 310.1C 489C 16086C 16148T 16223T 16259T 16278T 16319A 16399G 16526A	1	73 150 200 263 489 750 2706 3588 4769 7028 7960 8701 8768 8854 8860 9540 10398 10400 10873 11719 12634 12705 13308 13434 13590 13708 14130 14260 14527 14766 14783 15043 15172 15301 15326 15625 16086 16148 16223 16259 16278 16319 16399 16526	M32c	73 150 200 263 489 16086 16148 16223 16259 16278 16319 16399 16526	[0.000] M32c (1)	[0.022] M51a (2) [0.007] M32 (3) [0.000] M51 M51b (5)	1
1186	>MF058261.1 Hc	73G 146C 195C 263G 750G 769A 1018A 1438G 2416C 2706G 2789T 3106d 3107C 3594T 4104G 4769G 5090C 7028T 7175C 7256T 7274T 7521A 7771G 8206A 8701G 8860G 9221G 9540C 9604G 10115C 10143A 10398G 10873C 11719A 11914A 11944C 12693G 12705T 13590A 13650T 13803G 14566G 14766T 15301A 15326G 15735T 15784C 16183C 16189C 16223T 16278T 16290T 16294T 16309G 16390A	L2a1b1a	73G 146C 195C 263G 16183C 16189C 16223T 16278T 16290T 16294T 16309G 16390A	1	73 146 195 263 750 769 1018 1438 2416 2706 2789 3594 4104 4769 5090 7028 7175 7256 7274 7521 7771 8206 8701 8860 9221 9540 9604 10115 10143 10398 10873 11719 11914 11944 12693 12705 13590 13650 13803 14566 14766 15301 15326 15735 15784 16189 16223 16278 16290 16294 16309 16390	L2a1b1a	73 146 195 263 16189 16223 16278 16290 16294 16309 16390	[0.012] L2a1b1a (1)	[0.016] L2a1+16189_(16192) (2) [0.012] L2a1b1a (3) [0.008] L2a1b1 (4) [0.005] L2a1f (5) [0.000] L2a1b L2a1b2 L2a1f1 L2a1f1a L2a1f2 (10)	1
1187	>MF058253.1 Hc	73G 150T 200G 263G 310.1C 489C 750G 2706G 3106d 3107C 3588T 4769G 7028T 7960G 8701G 8768T 8854A 8860G 9540C 10398G 10400T 10873C 11719A 12705T 13308G 13590A 13708A 14130T 14260G 14527G 14766T 14783C 15043A 15172A 15301A 15326G 15625T 16086C 16148T 16223T 16259T 16278T 16319A 16399G 16526A	M32c	73G 150T 200G 263G 310.1C 489C 16086C 16148T 16223T 16259T 16278T 16319A 16399G 16526A	1	73 150 200 263 489 750 2706 3588 4769 7028 7960 8701 8768 8854 8860 9540 10398 10400 10873 11719 12705 13308 13590 13708 14130 14260 14527 14766 14783 15043 15172 15301 15326 15625 16086 16148 16223 16259 16278 16319 16399 16526	M32c	73 150 200 263 489 16086 16148 16223 16259 16278 16319 16399 16526	[0.000] M32c (1)	[0.022] M51a (2) [0.007] M32 (3) [0.000] M51 M51b (5)	1

1188 >MF058252.1 Ho	73G 152C 225A 248d 263G 309.1CT 310C 316A 489C 514d 515d 750G 1438G 2706G 3106d 3107C 3200C 3714G 4385T 4769G 4772C 7028T 7433T 7785C 8701G 8853G 8860G 9127G 9512T 9540C 10274C 10398G 10400T 10679G 10873C 11719A 11914A 12354C 12705T 14110C 14142T 14766T 14783C 14974T 15043A 15301A 15326G 15691G 16129A 16189C 16209C 16223T 16272G 16519C	M20	73G 152C 225A 248d 263G 309.1CT 310C 316A 489C 514d 515d 16129A 16189C 16209C 16223T 16272G 16519C	1	73 152 225 249d 263 316 489 750 1438 2706 3200 3714 4385T 4769 4772 7028 7433 7785 8701 8853 8860 9127 9512 9540 10274 10398 10400 10679 10873 11719 11914 12354 12705 14110 14142 14766 14783 14974 15043 15301 15326 15691 16129 16189 16209 16223 16272	M20	73 152 225 249d 263 316 489 16129 16189 16209 16223 16272	[0.311] M20 (1)	[0.122] M17c (2) [0.068] M17 (3) [0.067] M17a (4) [0.059] G1b+16129 (5) [0.046] M5 (6) [0.028] M5a D4a1a1 (8) [0.017] M5a2a (9) [0.012] M5a5 (10) [0.007] M5a'd D4a1c (12) [0.006] G1b2 G1b4 (14) [0.004] M37+152 (15) [0.003] D4a1 (16) [0.000] M5a2 M5a2a1 M5b'c M5b M7a M7a1b2 M7a2 M7b1 M13'46'61 M13 M13a'b M13b D4a D4a1a D4a1a1a D4a1b D4a1f D4a1f1 D4a1g D4a1h D4a2 D4a2a D4a2b D4a5 (40)	1
1189 >MF058250.1 Ho	73G 152C 195C 204C 263G 310.1C 417A 489C 533G 750G 1438G 3106d 3107C 4769G 7028T 8188G 8360G 8701G 8860G 9438A 9540C 9545G 10142T 10295G 10398G 10400T 10873C 11569C 11719A 11899C 12279G 12618A 12705T 14766T 14783C 15025T 15043A 15301A 15326G 16223T 16263C 16311C 16519C	M23	73G 152C 195C 204C 263G 310.1C 417A 489C 533G 16223T 16263C 16311C 16519C	1	73 152 195 204 263 417 489 533 750 1438 4769 7028 8188 8360 8701 8860 9438 9540 9545 10142 10295 10398 10400 10873 11569 11719 11899 12279 12618 12705 14766 14783 15025 15043 15301 15326 16223 16263 16311	M23	73 152 195 204 263 417 489 533 16223 16263 16311	[0.000] M23 (1)	[0.072] M24a (2) [0.053] M57+152	1
1190 >MF058248.1 Ho	73G 150T 263G 310.1C 489C 750G 2706G 3106d 3107C 3588T 4769G 7028T 7960G 8701G 8768T 8854A 8860G 9540C 10398G 10400T 10873C 11719A 12705T 13308G 13590A 13708A 14130T 14260G 14527G 14766T 14783C 15043A 15172A 15301A 15326G 15625T 16086C 16148T 16223T 16259T 16278T 16319A 16399G 16526A	M32c	73G 150T 263G 310.1C 489C 16086C 16148T 16223T 16259T 16278T 16319A 16399G 16526A	1	73 150 263 489 750 2706 3588 4769 7028 7960 8701 8768 8854 8860 9540 10398 10400 10873 11719 12705 13308 13590 13708 14130 14260 14527 14766 14783 15043 15172 15301 15326 15625 16086 16148 16223 16259 16278 16319 16399 16526	M32c	73 150 263 489 16086 16148 16223 16259 16278 16319 16399 16526	[0.046] M32c (1)	[0.022] M51a (2) [0.000] M51 M51b M32 (5)	1
1191 >MF058246.1 Ho	73G 146C 195C 263G 310.1C 750G 769A 1018A 1438G 2416C 2706G 2789T 3106d 3107C 3594T 4104G 4769G 5090C 7028T 7175C 7256T 7274T 7521A 7771G 8206A 8701G 8860G 9221G 9540C 9604G 10115C 10143A 10398G 10873C 11719A 11914A 11944C 12693G 12705T 13590A 13650T 13803G 14566G 14766T 15301A 15326G 15735T 15784C 16182C 16183C 16189C 16223T 16278T 16290T 16294T 16309G 16390A	L2a1b1a	73G 146C 195C 263G 310.1C 16182C 16183C 16189C 16223T 16278T 16290T 16294T 16309G 16390A	1	73 146 195 263 750 769 1018 1438 2416 2706 2789 3594 4104 4769 5090 7028 7175 7256 7274 7521 7771 8206 8701 8860 9221 9540 9604 10115 10143 10398 10873 11719 11914 11944 12693 12705 13590 13650 13803 14566 14766 15301 15326 15735 15784 16189 16223 16278 16290 16294 16309 16390	L2a1b1a	73 146 195 263 16189 16223 16278 16290 16294 16309 16390	[0.012] L2a1b1a (1)	[0.016] L2a1+16189_(16192) (2) [0.012] L2a1b1a (3) [0.008] L2a1b1 (4) [0.005] L2a1f (5) [0.000] L2a1b L2a1b2 L2a1f1 L2a1f1a L2a1f2 (10)	1

1192 >MF058245.1 Hc	73G 150T 199C 204C 263G 310.1C 513d 514d 709A 750G 769A 789A 1018A 1438G 1804G 2706G 3010A 3106d 3107C 3505G 3693C 3918A 4017T 4029A 4216C 4232C 4769G 4977C 5460A 7028T 7624C 8206A 8614C 8701G 8860G 8974G 9248T 9540C 9986A 10398G 10688A 10813T 10873C 11719A 12661T 12705T 13497G 13518T 14016A 14766T 14905A 14935C 15301A 15326G 15344C 16179T 16183C 16189C 16223T 16239T 16311C 16320T 16362C 16519C	L4b1a	73G 150T 199C 204C 263G 310.1C 513d 514d 16179T 16183C 16189C 16223T 16239T 16311C 16320T 16362C 16519C	1	73 150 199 204 263 513 709 750 769 789A 1018 1438 1804 2706 3010 3505 3693C 3918 4017 4029A 4216 4232 4769 4977 5460 7028 7624 8206 8614 8701 8860 8974G 9248 9540 9986 10398 10688 10813 10873 11719 12661T 12705 13497 13518 14016 14766 14905 14935 15301 15326 15344 16179 16189 16223 16239 16311 16320 16362	L4b1a	73 150 199 204 263 513 16179 16189 16223 16239 16311 16320 16362	[0.000] L4b1a (1)	[0.010] L4b1 (2)	1
1193 >MF058241.1 Hc	73G 146C 152C 195C 263G 310.1C 750G 769A 1018A 1438G 2416C 2706G 2789T 3106d 3107C 3594T 4104G 4769G 5090C 7028T 7175C 7256T 7274T 7521A 7771G 8206A 8701G 8860G 9221G 9540C 10115C 10143A 10398G 10873C 11719A 11914A 11944C 12693G 12705T 13590A 13650T 13803G 14566G 14766T 15301A 15326G 15735T 15784C 16180d 16189C 16223T 16278T 16290T 16294T 16309G 16356C 16390A	L2a1b1a	73G 146C 152C 195C 263G 310.1C 16180d 16189C 16223T 16278T 16290T 16294T 16309G 16356C 16390A	1	73 146 152 195 263 750 769 1018 1438 2416 2706 2789 3594 4104 4769 5090 7028 7175 7256 7274 7521 7771 8206 8701 8860 9221 9540 10115 10143 10398 10873 11719 11914 11944 12693 12705 13590 13650 13803 14566 14766 15301 15326 15735 15784 16189 16193d 16223 16278 16290 16294 16309 16356 16390	L2a1b1a	73 146 152 195 263 16189 16193d 16223 16278 16290 16294 16309 16356 16390	[0.039] L2a1b1a (1)	[0.039] L2a1b1a (2) [0.010] L2a1f (3) [0.008] L2a1b2 (4) [0.000] L2a1+16189_(16192) L2a1b L2a1b1 L2a1f1 L2a1f1a L2a1f2 (10)	1
1194 >MF058239.1 Hc	73G 152C 195C 204C 263G 310.1C 417A 489C 533G 750G 1438G 3106d 3107C 4769G 7028T 8188G 8360G 8701G 8860G 9438A 9540C 9545G 10142T 10295G 10398G 10400T 10873C 11569C 11719A 11899C 12279G 12618A 12705T 14766T 14783C 15025T 15043A 15301A 15326G 16223T 16263C 16311C 16519C	M23	73G 152C 195C 204C 263G 310.1C 417A 489C 533G 16223T 16263C 16311C 16519C	1	73 152 195 204 263 417 489 533 750 1438 4769 7028 8188 8360 8701 8860 9438 9540 9545 10142 10295 10398 10400 10873 11569 11719 11899 12279 12618 12705 14766 14783 15025 15043 15301 15326 16223 16263 16311	M23	73 152 195 204 263 417 489 533 16223 16263 16311	[0.000] M23 (1)	[0.072] M24a (2) [0.053] M57+152	1
1195 >MF058236.1 Hc	73G 150T 263G 310.1C 489C 750G 2706G 3106d 3107C 3588T 4769G 7028T 7960G 8701G 8768T 8854A 8860G 9540C 10398G 10400T 10873C 11719A 12705T 13308G 13590A 13708A 14130T 14260G 14527G 14766T 14783C 15043A 15172A 15301A 15326G 15625T 16086C 16148T 16223T 16259T 16278T 16319A 16399G 16526A	M32c	73G 150T 263G 310.1C 489C 16086C 16148T 16223T 16259T 16278T 16319A 16399G 16526A	1	73 150 263 489 750 2706 3588 4769 7028 7960 8701 8768 8854 8860 9540 10398 10400 10873 11719 12705 13308 13590 13708 14130 14260 14527 14766 14783 15043 15172 15301 15326 15625 16086 16148 16223 16259 16278 16319 16399 16526	M32c	73 150 263 489 16086 16148 16223 16259 16278 16319 16399 16526	[0.046] M32c (1)	[0.022] M51a (2) [0.000] M51 M51b M32 (5)	1

1196	>MF058235.1 Ho	73G 150T 199C 204C 263G 310.1C 513d 514d 709A 750G 769A 789A 1018A 1438G 1804G 2706G 3010A 3106d 3107C 3505G 3693C 3918A 4017T 4029A 4216C 4232C 4769G 4977C 5460A 7028T 7624C 8206A 8614C 8701G 8860G 8974G 9248T 9540C 9986A 10398G 10688A 10813T 10873C 11719A 12661T 12705T 13497G 13518T 14016A 14766T 14905A 14935C 15301A 15326G 15344C 16179T 16183C 16189C 16223T 16239T 16311C 16320T 16362C 16519C	L4b1a	73G 150T 199C 204C 263G 310.1C 513d 514d 16179T 16183C 16189C 16223T 16239T 16311C 16320T 16362C 16519C	1	73 150 199 204 263 513 709 750 769 789A 1018 1438 1804 2706 3010 3505 3693C 3918 4017 4029A 4216 4232 4769 4977 5460 7028 7624 8206 8614 8701 8860 8974G 9248 9540 9986 10398 10688 10813 10873 11719 12661T 12705 13497 13518 14016 14766 14905 14935 15301 15326 15344 16179 16189 16223 16239 16311 16320 16362	L4b1a	73 150 199 204 263 513 16179 16189 16223 16239 16311 16320 16362	[0.000] L4b1a (1)	[0.010] L4b1 (2)	1
1197	>MF058233.1 Ho	73G 146C 152C 182T 183G 200G 263G 310.1C 511T 750G 769A 1018A 1438G 1719A 2416C 2626C 2706G 3106d 3107C 3594T 3654T 4104G 4380T 4769G 4823C 5183G 5246A 5263T 6040C 6080G 6248C 6497C 7028T 7175C 7256T 7521A 8206A 8251A 8701G 8723A 8860G 9221G 9540C 10115C 10398G 10873C 11719A 11944C 12705T 12879C 12967G 12972G 13590A 13650T 13810A 14088C 14314G 14557T 14766T 14890G 15301A 15326G 15796C 16183C 16189C 16224C 16278T 16390A 16519C	L2a5	73G 146C 152C 182T 183G 200G 263G 310.1C 511T 16183C 16189C 16224C 16278T 16390A 16519C	1	73 146 152 182 183 200 263 511 750 769 1018 1438 1719 2416 2626 2706 3594 3654 4104 4380 4769 4823 5183 5246A 5263 6040C 6080 6248 6497 7028 7175 7256 7521 8206 8251 8701 8723 8860 9221 9540 10115 10398 10873 11719 11944 12705 12879 12967 12972 13590 13650 13810 14088 14314 14557 14766 14890 15301 15326 15796 16189 16224 16278 16390	L2a5	73 146 152 182 183 200 263 511 16189 16224 16278 16390	[1.771] L2a5 (1)	[0.000] L2 L2a K2a1a (4)	1
1198	>MF058221.1 Ho	73G 152C 195C 204C 263G 310.1C 417A 489C 533G 750G 1438G 3106d 3107C 4769G 7028T 8188G 8360G 8701G 8860G 9438A 9540C 9545G 10142T 10295G 10398G 10400T 10873C 11569C 11719A 11899C 12279G 12618A 12705T 14766T 14783C 15025T 15043A 15301A 15326G 16223T 16263C 16311C 16519C	M23	73G 152C 195C 204C 263G 310.1C 417A 489C 533G 16223T 16263C 16311C 16519C	1	73 152 195 204 263 417 489 533 750 1438 4769 7028 8188 8360 8701 8860 9438 9540 9545 10142 10295 10398 10400 10873 11569 11719 11899 12279 12618 12705 14766 14783 15025 15043 15301 15326 16223 16263 16311	M23	73 152 195 204 263 417 489 533 16223 16263 16311	[0.000] M23 (1)	[0.072] M24a (2) [0.053] M57+152	1
1199	>MF058214.1 Ho	73G 152C 195C 204C 263G 310.1C 417A 489C 533G 750G 1438G 3106d 3107C 4769G 7028T 8188G 8360G 8701G 8860G 9438A 9540C 9545G 10142T 10295G 10398G 10400T 10873C 11569C 11719A 11899C 12279G 12618A 12705T 14766T 14783C 15025T 15043A 15301A 15326G 16223T 16263C 16311C 16519C	M23	73G 152C 195C 204C 263G 310.1C 417A 489C 533G 16223T 16263C 16311C 16519C	1	73 152 195 204 263 417 489 533 750 1438 4769 7028 8188 8360 8701 8860 9438 9540 9545 10142 10295 10398 10400 10873 11569 11719 11899 12279 12618 12705 14766 14783 15025 15043 15301 15326 16223 16263 16311	M23	73 152 195 204 263 417 489 533 16223 16263 16311	[0.000] M23 (1)	[0.072] M24a (2) [0.053] M57+152	1

1200	>MF058213.1 Hc	73G 152C 195C 204C 263G 310.1C 417A 489C 533G 750G 1438G 3106d 3107C 4769G 7028T 8188G 8360G 8701G 8860G 9438A 9540C 9545G 10142T 10295G 10398G 10400T 10873C 11569C 11719A 11899C 12279G 12618A 12705T 14766T 14783C 15025T 15043A 15301A 15326G 16086C 16148T 16223T 16263C 16311C 16519C	M23	73G 152C 195C 204C 263G 310.1C 417A 489C 533G 16086C 16148T 16223T 16263C 16311C 16519C	1	73 152 195 204 263 417 489 533 750 1438 4769 7028 8188 8360 8701 8860 9438 9540 9545 10142 10295 10398 10400 10873 11569 11719 11899 12279 12618 12705 14766 14783 15025 15043 15301 15326 16086 16148 16223 16263 16311	M23	73 152 195 204 263 417 489 533 16086 16148 16223 16263 16311	[0.024] M23 (1)	[0.079] M57+152 (2) [0.072] M24a (3) [0.000] M28a+204	1
1201	>MF058210.1 Hc	73G 146C 152C 182T 185T 189G 204C 247A 263G 310.1C 357G 514d 515d 709A 710C 750G 769A 825A 1018A 1738C 2352C 2706G 2758A 2768G 2885C 3106d 3107C 3308C 3594T 3666A 3693A 4104G 4769G 6548T 6827C 6989G 7028T 7055G 7146G 7256T 7389C 7521A 7867T 8248G 8282T 8468T 8655T 8701G 8860G 9540C 10398G 10688A 10810C 10873C 11719A 12171G 12519C 12705T 12891T 13105G 13506T 13650T 13789C 13893G 14178C 14323A 14560A 14766T 14769G 14861A 15115C 15326G 16111T 16126C 16187T 16189C 16223T 16239T 16270T 16278T 16293G 16311C 16519C	L1b2a	73G 146C 152C 182T 185T 189G 204C 247A 263G 310.1C 357G 514d 515d 16111T 16126C 16187T 16189C 16223T 16239T 16270T 16278T 16293G 16311C 16519C	1	73 146 152 182 185T 189 204 247 263 357 709 710 750 769 825A 1018 1738 2352 2706 2758 2768 2885 3308 3594 3666 3693 4104 4769 6548 6827 6989 7028 7055 7146 7256 7389 7521 7867 8248 8282 8468 8655 8701 8860 9540 10398 10688 10810 10873 11719 12171 12519 12705 12891 13105 13506 13650 13789 13893 14178 14323 14560 14766 14769 14861 15115 15326 16111 16126 16187 16189 16223 16239 16270 16278 16293 16311	L1b2a	73 146 152 182 185T 189 204 247 263 357 16111 16126 16187 16189 16223 16239 16270 16278 16293 16311	[0.086] L1b2a (1)	[0.292] L1b2 (2)	1
1202	>MF058202.1 Hc	73G 152C 195C 204C 263G 310.1C 417A 489C 533G 750G 1438G 3106d 3107C 4769G 7028T 8188G 8360G 8701G 8860G 9007G 9438A 9540C 9545G 10142T 10295G 10398G 10400T 10873C 11569C 11719A 11899C 12279G 12618A 12705T 14766T 14783C 15025T 15043A 15301A 15326G 16223T 16263C 16311C 16519C	M23	73G 152C 195C 204C 263G 310.1C 417A 489C 533G 16223T 16263C 16311C 16519C	1	73 152 195 204 263 417 489 533 750 1438 4769 7028 8188 8360 8701 8860 9007 9438 9540 9545 10142 10295 10398 10400 10873 11569 11719 11899 12279 12618 12705 14766 14783 15025 15043 15301 15326 16223 16263 16311	M23	73 152 195 204 263 417 489 533 16223 16263 16311	[0.000] M23 (1)	[0.072] M24a (2) [0.053] M57+152	1

1203	>MF058198.1 Hc	73G 146C 150T 152C 182T 195C 198T 204C 263G 310.1C 709A 750G 769A 1018A 1438G 1442A 1706T 2332T 2358G 2416C 2706G 3106d 3107C 3594T 4104G 4158G 4370C 4767G 4769G 5027T 5331A 5814C 6614C 6713T 6806G 7028T 7256T 7521A 7624A 8080T 8206A 8387A 8503C 8701G 8790A 8860G 9221G 9350G 9540C 10115C 10398G 10873C 11719A 11944C 12236A 12705T 12948G 13590A 13650T 13966G 14059G 14407T 14766T 15110A 15217A 15301A 15326G 16114A 16129A 16213A 16218T 16223T 16278T 16354T 16390A	L2b2a	73G 146C 150T 152C 182T 195C 198T 204C 263G 310.1C 16114A 16129A 16213A 16218T 16223T 16278T 16354T 16390A	1	73 146 150 152 182 195 198 204 263 709 750 769 1018 1438 1442 1706 2332 2358 2416 2706 3594 4104 4158 4370 4767 4769 5027 5331A 5814 6614 6713 6806 7028 7256 7521 7624A 8080 8206 8387 8503 8701 8790 8860 9221 9350 9540 10115 10398 10873 11719 11944 12236 12705 12948 13590 13650 13966 14059 14407 14766 15110 15217 15301 15326 16114A 16129 16213 16218 16223 16278 16354 16390	L2b2a	73 146 150 152 182 195 198 204 263 16114A 16129 16213 16218 16223 16278 16354 16390	[0.073] L2b2a (1)	[0.024] L2b3a (2) [0.000] L2b L2b2 L2b3 L2b3b L2b3c (7)	1
1204	>MF058196.1 Hc	64T 93G 185A 189G 200G 236C 247A 263G 310.1C 514d 515d 750G 769A 825A 1018A 1048T 1438G 2245C 2706G 2758A 2885C 3106d 3107C 3516A 3594T 3866C 4104G 4312T 4586C 4769G 5096C 5231A 5442C 5460A 5603T 6185C 7028T 7146G 7256T 7521A 8428T 8468T 8566G 8655T 8701G 8860G 9042T 9347G 9540C 9755A 9818T 10398G 10589A 10664T 10688A 10810C 10873C 10915C 11176A 11641G 11719A 11914A 12007A 12705T 12720G 13105G 13276G 13506T 13650T 13977T 14308C 14766T 15136T 15326G 15431A 16129A 16148T 16168T 16172C 16187T 16188G 16189C 16223T 16230G 16271C 16311C 16320T	L0a1a2	64T 93G 185A 189G 200G 236C 247A 263G 310.1C 514d 515d 16129A 16148T 16168T 16172C 16187T 16188G 16189C 16223T 16230G 16271C 16311C 16320T	1	64 93 185 189 200 236 247 263 750 769 825A 1018 1048 1438 2245C 2706 2758 2885 3516A 3594 3866 4104 4312 4586 4769 5096 5231 5442 5460 5603 6185 7028 7146 7256 7521 8428 8468 8566 8655 8701 8860 9042 9347 9540 9755 9818 10398 10589 10664 10688 10810 10873 10915 11176 11641 11719 11914 12007 12705 12720 13105 13276 13506 13650 13977 14308 14766 15136 15326 15431 16129 16148 16168 16172 16187 16188G 16189 16223 16230 16271 16311 16320	L0a1a2	64 93 185 189 200 236 247 263 16129 16148 16168 16172 16187 16188G 16189 16223 16230 16271 16311 16320	[0.008] L0a1a2 (1)	[0.022] L0a1a+200 (2) [0.014] L0a1a3 (3) [0.008] L0a1a2 (4) [0.000] L0a1a1	1
1205	>MF058194.1 Hc	73G 150T 152C 235G 263G 310.1C 494T 735G 750G 1438G 2706G 3106d 3107C 3918A 4313C 4769G 6620G 7028T 7202G 7775A 8649G 8701G 8860G 9007T 9329A 9467C 9540C 10398G 10819G 10873C 11719A 12705T 13135A 13542G 13862G 13992T 14013G 14766T 15301A 15314A 15326G 16093C 16223T 16355T	L3k1	73G 150T 152C 235G 263G 310.1C 494T 16093C 16223T 16355T	1	73 150 152 235 263 494 735 750 1438 2706 3918 4313 4769 6620G 7028 7202 7775 8649 8701 8860 9007T 9329 9467 9540 10398 10819 10873 11719 12705 13135 13542 13862 13992 14013 14766 15301 15314 15326 16093 16223 16355	L3k1	73 150 152 235 263 494 16093 16223 16355	[0.536] L3k1 (1)	[0.000] L3k (2)	1

1206	>MF058193.1 Hc	73G 146C 150T 152C 182T 195C 198T 204C 263G 310.1C 709A 750G 769A 1018A 1438G 1442A 1706T 2332T 2358G 2416C 2706G 3106d 3107C 3594T 4104G 4158G 4370C 4767G 4769G 5027T 5331A 5814C 6614C 6713T 6806G 7028T 7256T 7521A 7624A 8080T 8206A 8387A 8503C 8701G 8790A 8860G 9221G 9350G 9540C 10115C 10398G 10873C 11719A 11944C 12236A 12705T 12948G 13590A 13650T 13966G 14059G 14407T 14766T 15110A 15217A 15301A 15326G 16114A 16129A 16189C 16213A 16218T 16223T 16278T 16354T 16390A	L2b2a	73G 146C 150T 152C 182T 195C 198T 204C 263G 310.1C 16114A 16129A 16189C 16213A 16218T 16223T 16278T 16354T 16390A	1	73 146 150 152 182 195 198 204 263 709 750 769 1018 1438 1442 1706 2332 2358 2416 2706 3594 4104 4158 4370 4767 4769 5027 5331A 5814 6614 6713 6806 7028 7256 7521 7624A 8080 8206 8387 8503 8701 8790 8860 9221 9350 9540 10115 10398 10873 11719 11944 12236 12705 12948 13590 13650 13966 14059 14407 14766 15110 15217 15301 15326 16114A 16129 16189 16213 16218 16223 16278 16354 16390	L2b2a	73 146 150 152 182 195 198 204 263 16114A 16129 16189 16213 16218 16223 16278 16354 16390	[0.122] L2b2a (1)	[0.077] L2b3c (2) [0.024] L2b3a (3) [0.019] L2b2 (4) [0.000] L2b L2b3 L2b3b (7)	1
1207	>MF058186.1 Hc	73G 152C 195C 204C 263G 310.1C 417A 489C 533G 750G 1438G 3106d 3107C 4769G 7028T 8188G 8360G 8701G 8860G 9438A 9540C 9545G 10142T 10295G 10398G 10400T 10873C 11569C 11719A 11899C 12279G 12618A 12705T 14766T 14783C 15025T 15043A 15301A 15326G 16223T 16263C 16311C 16519C	M23	73G 152C 195C 204C 263G 310.1C 417A 489C 533G 16223T 16263C 16311C 16519C	1	73 152 195 204 263 417 489 533 750 1438 4769 7028 8188 8360 8701 8860 9438 9540 9545 10142 10295 10398 10400 10873 11569 11719 11899 12279 12618 12705 14766 14783 15025 15043 15301 15326 16223 16263 16311	M23	73 152 195 204 263 417 489 533 16223 16263 16311	[0.000] M23 (1)	[0.072] M24a (2) [0.053] M57+152	1
1208	>MF058180.1 Hc	73G 152C 195C 204C 263G 310.1C 417A 489C 533G 750G 1438G 3106d 3107C 4769G 7028T 8188G 8360G 8701G 8860G 9438A 9540C 9545G 10142T 10295G 10398G 10400T 10873C 11569C 11719A 11899C 12279G 12618A 12705T 14766T 14783C 15025T 15043A 15301A 15326G 16223T 16263C 16311C 16519C	M23	73G 152C 195C 204C 263G 310.1C 417A 489C 533G 16223T 16263C 16311C 16519C	1	73 152 195 204 263 417 489 533 750 1438 4769 7028 8188 8360 8701 8860 9438 9540 9545 10142 10295 10398 10400 10873 11569 11719 11899 12279 12618 12705 14766 14783 15025 15043 15301 15326 16223 16263 16311	M23	73 152 195 204 263 417 489 533 16223 16263 16311	[0.000] M23 (1)	[0.072] M24a (2) [0.053] M57+152	1
1209	>MF058178.1 Hc	73G 152C 195C 204C 263G 310.1C 417A 489C 533G 750G 1438G 3106d 3107C 4769G 7028T 8188G 8360G 8701G 8860G 9438A 9540C 9545G 10142T 10295G 10398G 10400T 10873C 11569C 11719A 11899C 12279G 12618A 12705T 14766T 14783C 15025T 15043A 15301A 15326G 16223T 16263C 16311C 16519C	M23	73G 152C 195C 204C 263G 310.1C 417A 489C 533G 16223T 16263C 16311C 16519C	1	73 152 195 204 263 417 489 533 750 1438 4769 7028 8188 8360 8701 8860 9438 9540 9545 10142 10295 10398 10400 10873 11569 11719 11899 12279 12618 12705 14766 14783 15025 15043 15301 15326 16223 16263 16311	M23	73 152 195 204 263 417 489 533 16223 16263 16311	[0.000] M23 (1)	[0.072] M24a (2) [0.053] M57+152	1

1210	>MF058177.1 Hc	73G 151T 152C 182T 186A 189C 195C 198T 247A 263G 297G 316A 750G 769A 825A 1018A 1420C 1438G 2150.1A 2394d 2706G 2758A 2885C 3106d 3107C 3594T 3666A 4104G 4769G 5894.1C 5951G 6071C 6150A 6253C 7028T 7055G 7076G 7146G 7256T 7337A 7389C 7521A 7744C 8027A 8251A 8468T 8655T 8701G 8784G 8860G 8877C 9072G 9540C 10321C 10398G 10586A 10688A 10792G 10793T 10810C 10873C 11654G 11719A 12049T 12705T 12810G 13105G 13149G 13212T 13281C 13485G 13506T 13650T 13789C 14000A 14178C 14560A 14766T 14812T 14911T 15016T 15326G 15784C 16071T 16129A 16145A 16187T 16189C 16213A 16223T 16234T 16265C 16278T 16286G 16294T 16311C 16320T 16360T 16527T	L1c2a1a	73G 151T 152C 182T 186A 189C 195C 198T 247A 263G 297G 316A 16071T 16129A 16145A 16187T 16189C 16213A 16223T 16234T 16265C 16278T 16286G 16294T 16311C 16320T 16360T 16527T	1	73 151 152 182 186A 189C 195 198 247 263 297 316 750 769 825A 1018 1420 1438 2156.1A 2395d 2706 2758 2885 3594 3666 4104 4769 5899.1C 5951 6071 6150 6253 7028 7055 7076 7146 7256 7337 7389 7521 7744 8027 8251 8468 8655 8701 8784 8860 8877 9072 9540 10321 10398 10586 10688 10792 10793 10810 10873 11654 11719 12049 12705 12810 13105 13149 13212 13281 13485 13506 13650 13789 14000A 14178 14560 14766 14812 14911 15016 15326 15784 16071 16129 16145 16187 16189 16213 16223 16234 16265C 16278 16286G 16294 16311 16320 16360 16527	L1c2a1a	73 151 152 182 186A 189C 195 198 247 263 297 316 16071 16129 16145 16187 16189 16213 16223 16234 16265C 16278 16286G 16294 16311 16320 16360 16527	[0.053] L1c2a1a (1)	[0.158] L1c2a1b (2) [0.000] L1c2a1	1
1211	>MF058171.1 Hc	73G 152C 195C 204C 263G 310.1C 417A 489C 533G 750G 1438G 3106d 3107C 4769G 7028T 8188G 8360G 8701G 8860G 9438A 9540C 9545G 10142T 10295G 10398G 10400T 10873C 11569C 11719A 11899C 12279G 12618A 12705T 14766T 14783C 15025T 15043A 15301A 15326G 16223T 16263C 16311C 16519C	M23	73G 152C 195C 204C 263G 310.1C 417A 489C 533G 16223T 16263C 16311C 16519C	1	73 152 195 204 263 417 489 533 750 1438 4769 7028 8188 8360 8701 8860 9438 9540 9545 10142 10295 10398 10400 10873 11569 11719 11899 12279 12618 12705 14766 14783 15025 15043 15301 15326 16223 16263 16311	M23	73 152 195 204 263 417 489 533 16223 16263 16311	[0.000] M23 (1)	[0.072] M24a (2) [0.053] M57+152	1

1212 >MF058166.1 Hc	73G 151T 152C 182T 186A 189C 195C 198T 247A 263G 297G 316A 750G 769A 825A 1018A 1420C 1438G 2150.1A 2394d 2706G 2758A 2885C 3106d 3107C 3594T 3666A 4104G 4769G 5894.1C 5951G 6071C 6150A 6253C 7028T 7055G 7076G 7146G 7256T 7337A 7389C 7521A 7744C 8027A 8251A 8468T 8655T 8701G 8784G 8860G 8877C 9072G 9540C 10321C 10398G 10586A 10688A 10792G 10793T 10810C 10873C 11182G 11654G 11719A 12049T 12705T 12810G 13105G 13149G 13212T 13281C 13485G 13506T 13650T 13789C 14000A 14178C 14560A 14766T 14812T 14911T 15016T 15326G 15784C 16071T 16129A 16145A 16187T 16189C 16213A 16223T 16234T 16265C 16278T 16286G 16294T 16311C 16360T 16527T	L1c2a1a	73G 151T 152C 182T 186A 189C 195C 198T 247A 263G 297G 316A 16071T 16129A 16145A 16187T 16189C 16213A 16223T 16234T 16265C 16278T 16286G 16294T 16311C 16360T 16527T	1	73 151 152 182 186A 189C 195 198 247 263 297 316 750 769 825A 1018 1420 1438 2156.1A 2395d 2706 2758 2885 3594 3666 4104 4769 5899.1C 5951 6071 6150 6253 7028 7055 7076 7146 7256 7337 7389 7521 7744 8027 8251 8468 8655 8701 8784 8860 8877 9072 9540 10321 10398 10586 10688 10792 10793 10810 10873 11182 11654 11719 12049 12705 12810 13105 13149 13212 13281 13485 13506 13650 13789 14000A 14178 14560 14766 14812 14911 15016 15326 15784 16071 16129 16145 16187 16189 16213 16223 16234 16265C 16278 16286G 16294 16311 16360 16527	L1c2a1a	73 151 152 182 186A 189C 195 198 247 263 297 316 16071 16129 16145 16187 16189 16213 16223 16234 16265C 16278 16286G 16294 16311 16360 16527	[0.000] L1c2a1a (1)	[0.105] L1c2a1b (2) [0.000] L1c2a1	1
1213 >MF058159.1 Hc	73G 146C 152C 195C 263G 309.1CT 310C 750G 769A 1018A 1438G 2416C 2706G 2789T 3106d 3107C 3594T 4104G 4769G 6040G 7028T 7175C 7256T 7274T 7521A 7771G 8014G 8206A 8701G 8860G 9221G 9540C 10115C 10398G 10873C 11719A 11914A 11944C 12693G 12705T 13590A 13650T 13803G 14281T 14566G 14766T 15301A 15326G 15784C 16131C 16189C 16223T 16225T 16234T 16278T 16294T 16309G 16390A	L2a1g	73G 146C 152C 195C 263G 309.1CT 310C 16131C 16189C 16223T 16225T 16234T 16278T 16294T 16309G 16390A	1	73 146 152 195 263 750 769 1018 1438 2416 2706 2789 3594 4104 4769 6040 7028 7175 7256 7274 7521 7771 8014 8206 8701 8860 9221 9540 10115 10398 10873 11719 11914 11944 12693 12705 13590 13650 13803 14281 14566 14766 15301 15326 15784 16131 16189 16223 16225 16234 16278 16294 16309 16390	L2a1g	73 146 152 195 263 16131 16189 16223 16225 16234 16278 16294 16309 16390	[0.000] L2a1g (1)	[0.015] L2a1b L2a1b2 L2a1f1a (4) [0.005] L2a1f (5) [0.000] L2a1+16189_(16192) L2a1b1 L2a1f1 L2a1f2 (9)	1
1214 >MF058158.1 Hc	73G 146C 152C 182T 263G 310.1C 511T 750G 769A 1018A 1438G 1719A 2416C 2626C 2706G 3106d 3107C 3594T 3654T 4104G 4380T 4769G 4823C 5183G 5246A 5263T 6040C 6080G 6248C 6497C 7028T 7175C 7256T 7521A 8206A 8251A 8701G 8723A 8860G 9221G 9540C 10115C 10398G 10873C 11719A 11944C 12705T 12879C 12967G 12972G 13590A 13650T 13810A 14088C 14314G 14557T 14766T 14890G 15301A 15326G 15796C 16092C 16183C 16189C 16223T 16224C 16278T 16390A 16519C	L2a5	73G 146C 152C 182T 263G 310.1C 511T 16092C 16183C 16189C 16223T 16224C 16278T 16390A 16519C	1	73 146 152 182 263 511 750 769 1018 1438 1719 2416 2626 2706 3594 3654 4104 4380 4769 4823 5183 5246A 5263 6040C 6080 6248 6497 7028 7175 7256 7521 8206 8251 8701 8723 8860 9221 9540 10115 10398 10873 11719 11944 12705 12879 12967 12972 13590 13650 13810 14088 14314 14557 14766 14890 15301 15326 15796 16092 16189 16223 16224 16278 16390	L2a5	73 146 152 182 263 511 16092 16189 16223 16224 16278 16390	[1.271] L2a5 (1)	[0.000] L2 L2a (3)	1

1215	>MF058157.1 Hc	73G 150T 152C 235G 263G 310.1C 494T 735G 750G 1438G 2706G 3106d 3107C 3918A 4313C 4769G 6620G 7028T 7202G 8649G 8701G 8860G 9007T 9329A 9467C 9540C 10398G 10819G 10873C 11719A 12705T 13135A 13542G 13862G 13992T 14013G 14766T 15301A 15314A 15326G 16093C 16223T 16355T	L3k1	73G 150T 152C 235G 263G 310.1C 494T 16093C 16223T 16355T	1	73 150 152 235 263 494 735 750 1438 2706 3918 4313 4769 6620G 7028 7202 8649 8701 8860 9007T 9329 9467 9540 10398 10819 10873 11719 12705 13135 13542 13862 13992 14013 14766 15301 15314 15326 16093 16223 16355	L3k1	73 150 152 235 263 494 16093 16223 16355	[0.536] L3k1 (1)	[0.000] L3k (2)	1
1216	>MF058155.1 Hc	73G 146C 152C 195C 263G 310.1C 750G 769A 1018A 1438G 2416C 2706G 2789T 3106d 3107C 3594T 4104G 4769G 5090C 7028T 7175C 7256T 7274T 7521A 7771G 8206A 8701G 8860G 9221G 9540C 10115C 10143A 10398G 10873C 11719A 11914A 11944C 12693G 12705T 13590A 13650T 13803G 14566G 14766T 15301A 15326G 15735T 15784C 16182C 16183C 16189C 16192T 16223T 16278T 16290T 16294T 16309G 16390A	L2a1b1a	73G 146C 152C 195C 263G 310.1C 16182C 16183C 16189C 16192T 16223T 16278T 16290T 16294T 16309G 16390A	1	73 146 152 195 263 750 769 1018 1438 2416 2706 2789 3594 4104 4769 5090 7028 7175 7256 7274 7521 7771 8206 8701 8860 9221 9540 10115 10143 10398 10873 11719 11914 11944 12693 12705 13590 13650 13803 14566 14766 15301 15326 15735 15784 16189 16192 16223 16278 16290 16294 16309 16390	L2a1b1a	73 146 152 195 263 16189 16192 16223 16278 16290 16294 16309 16390	[0.000] L2a1b1a (1)	[0.015] L2a1b1a (2) [0.005] L2a1f (3) [0.000] L2a1+16189_(16192) L2a1b L2a1b1 L2a1b2 L2a1f1 L2a1f1a L2a1f2 (10)	1
1217	>MF058147.1 Hc	73G 146C 152C 195C 263G 310.1C 750G 769A 1018A 1438G 2416C 2706G 2789T 3106d 3107C 3594T 4104G 4769G 5090C 7028T 7175C 7256T 7274T 7521A 7771G 8206A 8701G 8860G 9221G 9540C 10115C 10143A 10398G 10873C 11719A 11914A 11944C 12693G 12705T 13590A 13650T 13803G 14566G 14766T 15301A 15326G 15735T 15784C 16182C 16183C 16189C 16223T 16235G 16278T 16290T 16294T 16309G 16390A	L2a1b1a	73G 146C 152C 195C 263G 310.1C 16182C 16183C 16189C 16223T 16235G 16278T 16290T 16294T 16309G 16390A	1	73 146 152 195 263 750 769 1018 1438 2416 2706 2789 3594 4104 4769 5090 7028 7175 7256 7274 7521 7771 8206 8701 8860 9221 9540 10115 10143 10398 10873 11719 11914 11944 12693 12705 13590 13650 13803 14566 14766 15301 15326 15735 15784 16189 16223 16235 16278 16290 16294 16309 16390	L2a1b1a	73 146 152 195 263 16189 16223 16235 16278 16290 16294 16309 16390	[0.006] L2a1b1a (1)	[0.006] L2a1b1a (2) [0.005] L2a1f (3) [0.000] L2a1+16189_(16192) L2a1b L2a1b1 L2a1b2 L2a1f1 L2a1f1a L2a1f2 (10)	1

1218	>MF058146.1 Hc	73G 151T 152C 182T 186A 189C 195C 198T 247A 263G 297G 309.1CT 310C 316A 750G 769A 825A 1018A 1420C 1438G 2150.1A 2394d 2706G 2758A 2885C 2955A 2968G 3009T 3019A 3027C 3106d 3107C 3594T 3666A 4104G 4769G 5894.1C 5951G 6071C 6150A 6253C 7028T 7055G 7076G 7146G 7256T 7337A 7389C 7521A 7744C 8027A 8251A 8468T 8655T 8701G 8784G 8860G 8877C 9072G 9540C 10321C 10398G 10586A 10688A 10792G 10793T 10810C 10873C 11182G 11654G 11719A 12049T 12705T 12810G 13105G 13149G 13212T 13281C 13485G 13506T 13650T 13789C 14000A 14178C 14560A 14766T 14812T 14911T 15016T 15326G 15784C 16071T 16129A 16145A 16187T 16189C 16213A 16223T 16234T 16265C 16278T 16286G 16294T 16311C 16360T 16527T	L1c2a1a	73G 151T 152C 182T 186A 189C 195C 198T 247A 263G 297G 309.1CT 310C 316A 16071T 16129A 16145A 16187T 16189C 16213A 16223T 16234T 16265C 16278T 16286G 16294T 16311C 16360T 16527T	1	73 151 152 182 186A 189C 195 198 247 263 297 316 750 769 825A 1018 1420 1438 2156.1A 2395d 2706 2758 2885 2955A 2968 3009 3019 3027 3594 3666 4104 4769 5899.1C 5951 6071 6150 6253 7028 7055 7076 7146 7256 7337 7389 7521 7744 8027 8251 8468 8655 8701 8784 8860 8877 9072 9540 10321 10398 10586 10688 10792 10793 10810 10873 11182 11654 11719 12049 12705 12810 13105 13149 13212 13281 13485 13506 13650 13789 14000A 14178 14560 14766 14812 14911 15016 15326 15784 16071 16129 16145 16187 16189 16213 16223 16234 16265C 16278 16286G 16294 16311 16360 16527	L1c2a1a	73 151 152 182 186A 189C 195 198 247 263 297 316 16071 16129 16145 16187 16189 16213 16223 16234 16265C 16278 16286G 16294 16311 16360 16527	[0.000] L1c2a1a (1)	[0.105] L1c2a1b (2) [0.000] L1c2a1	1
1219	>MF058144.1 Hc	73G 146C 152C 195C 263G 310.1C 750G 769A 1018A 1438G 2416C 2706G 2789T 3106d 3107C 3594T 4104G 4769G 5090C 7028T 7175C 7256T 7274T 7521A 7771G 8206A 8701G 8860G 9055A 9221G 9540C 10115C 10143A 10398G 10873C 11719A 11914A 11944C 12693G 12705T 13590A 13650T 13803G 14566G 14766T 15301A 15326G 15735T 15784C 16182C 16183C 16189C 16192T 16223T 16274A 16278T 16290T 16294T 16309G 16390A	L2a1b1a	73G 146C 152C 195C 263G 310.1C 16182C 16183C 16189C 16192T 16223T 16274A 16278T 16290T 16294T 16309G 16390A	1	73 146 152 195 263 750 769 1018 1438 2416 2706 2789 3594 4104 4769 5090 7028 7175 7256 7274 7521 7771 8206 8701 8860 9055 9221 9540 10115 10143 10398 10873 11719 11914 11944 12693 12705 13590 13650 13803 14566 14766 15301 15326 15735 15784 16189 16192 16223 16274 16278 16290 16294 16309 16390	L2a1b1a	73 146 152 195 263 16189 16192 16223 16274 16278 16290 16294 16309 16390	[0.012] L2a1b1a (1)	[0.027] L2a1b1a (2) [0.008] L2a1b2 (3) [0.007] L2a1b L2a1f1a (5) [0.005] L2a1f (6) [0.000] L2a1+16189_(16192) L2a1b1 L2a1f1 L2a1f2 (10)	1
1220	>MF058130.1 Hc	73G 152C 195C 204C 263G 417A 489C 533G 750G 1438G 3106d 3107C 4769G 7028T 8188G 8360G 8701G 8860G 9438A 9540C 9545G 10142T 10295G 10398G 10400T 10873C 11569C 11719A 11899C 12279G 12618A 12705T 14766T 14783C 15025T 15043A 15301A 15326G 16223T 16263C 16311C 16519C	M23	73G 152C 195C 204C 263G 417A 489C 533G 16223T 16263C 16311C 16519C	1	73 152 195 204 263 417 489 533 750 1438 4769 7028 8188 8360 8701 8860 9438 9540 9545 10142 10295 10398 10400 10873 11569 11719 11899 12279 12618 12705 14766 14783 15025 15043 15301 15326 16223 16263 16311	M23	73 152 195 204 263 417 489 533 16223 16263 16311	[0.000] M23 (1)	[0.072] M24a (2) [0.053] M57+152	1

1221	>MF056488.1 H[illegible]	73G 152C 182T 186A 189C 195C 247A 248G 263G 316A 514d 515d 750G 769A 825A 1018A 1438G 2394d 2706G 2758A 2885C 3106d 3107C 3525T 3594T 3666A 4104G 4769G 4853A 5951G 6071C 6221A 6227C 6917A 7028T 7146G 7247T 7256T 7389C 7521A 8027A 8468T 8655T 8701G 8860G 9072G 9540C 10398G 10586A 10688A 10810C 10873C 11302T 11719A 11984C 12507G 12705T 12810G 13105G 13485G 13506T 13650T 13789C 14000A 14178C 14560A 14669G 14766T 14911T 14926G 15226G 15326G 15905C 15978T 16129A 16187T 16189C 16223T 16278T 16293G 16294T 16311C 16360T 16368C 16519C	L1c3c	73G 152C 182T 186A 189C 195C 247A 248G 263G 316A 514d 515d 16129A 16187T 16189C 16223T 16278T 16293G 16294T 16311C 16360T 16368C 16519C	1	73 152 182 186A 189C 195 247 248 263 316 750 769 825A 1018 1438 2395d 2706 2758 2885 3525 3594 3666 4104 4769 4853 5951 6071 6221A 6227 6917 7028 7146 7247 7256 7389 7521 8027 8468 8655 8701 8860 9072 9540 10398 10586 10688 10810 10873 11302 11719 11984 12507 12705 12810 13105 13485 13506 13650 13789 14000A 14178 14560 14669 14766 14911 14926 15226 15326 15905 15978 16129 16187 16189 16223 16278 16293 16294 16311 16360 16368	L1c3c	73 152 182 186A 189C 195 247 248 263 316 16129 16187 16189 16223 16278 16293 16294 16311 16360 16368	[2.125] L1c3c (1) [1.000] L1c1a+@198 (2) [0.000] L1c L1c3b'c (4)	[0.900] L1c3 (5) [0.769] L1c1 (6) [0.714] L1c1a (7) [0.572] L1c1a'b'd (8) [0.267] L1c3b1 (9) [0.133] L1c3b1b (10) [0.000] L1c1'2'4'5'6 L1c1a1 L1c3b (13)	1
1222	>MF056484.1 H[illegible]	73G 146C 152C 195C 263G 750G 769A 1018A 1438G 2416C 2706G 2789T 3106d 3107C 3594T 4104G 4769G 5090C 7028T 7175C 7256T 7274T 7521A 7771G 8206A 8701G 8860G 9221G 9540C 10115C 10143A 10398G 10873C 11719A 11914A 11944C 12693G 12705T 13590A 13650T 13803G 14566G 14766T 15301A 15326G 15735T 15784C 16182C 16183C 16189C 16223T 16278T 16290T 16294T 16309G 16390A	L2a1b1a	73G 146C 152C 195C 263G 16182C 16183C 16189C 16223T 16278T 16290T 16294T 16309G 16390A	1	73 146 152 195 263 750 769 1018 1438 2416 2706 2789 3594 4104 4769 5090 7028 7175 7256 7274 7521 7771 8206 8701 8860 9221 9540 10115 10143 10398 10873 11719 11914 11944 12693 12705 13590 13650 13803 14566 14766 15301 15326 15735 15784 16189 16223 16278 16290 16294 16309 16390	L2a1b1a	73 146 152 195 263 16189 16223 16278 16290 16294 16309 16390	[0.000] L2a1b1a (1)	[0.005] L2a1f (2) [0.000] L2a1+16189_(16192) L2a1b L2a1b1 L2a1b1a L2a1b2 L2a1f1 L2a1f1a L2a1f2 (10)	1
1223	>MF056476.1 H[illegible]	73G 146C 152C 195C 263G 750G 769A 1018A 1438G 2416C 2706G 2762T 2789T 3106d 3107C 3594T 4104G 4769G 5090C 7028T 7175C 7256T 7274T 7521A 7771G 8206A 8701G 8860G 9221G 9540C 10115C 10143A 10398G 10873C 11719A 11914A 11944C 12693G 12705T 13590A 13650T 13803G 14566G 14766T 15301A 15326G 15735T 15784C 16182C 16183C 16189C 16223T 16278T 16290T 16294T 16390A	L2a1b1a	73G 146C 152C 195C 263G 16182C 16183C 16189C 16223T 16278T 16290T 16294T 16390A	1	73 146 152 195 263 750 769 1018 1438 2416 2706 2762 2789 3594 4104 4769 5090 7028 7175 7256 7274 7521 7771 8206 8701 8860 9221 9540 10115 10143 10398 10873 11719 11914 11944 12693 12705 13590 13650 13803 14566 14766 15301 15326 15735 15784 16189 16223 16278 16290 16294 16390	L2a1b1a	73 146 152 195 263 16189 16223 16278 16290 16294 16390	[0.045] L2a1b1a (1)	[0.234] L2a1+143+16189_(16192)+@16309 (2) [0.070] L2a1f (3) [0.045] L2a1b1a (4) [0.008] L2a1b2 (5) [0.000] L2a1+16189_(16192) L2a1b L2a1b1 L2a1f1 L2a1f1a L2a1f2 (11)	1

1224 >MF056469.1 Ho	73G 189G 195C 204C 263G 417A 489C 533G 750G 1438G 3106d 3107C 4769G 5460A 7028T 8188G 8360G 8701G 8860G 9438A 9540C 9545G 10142T 10295G 10398G 10400T 10873C 11569C 11719A 11899C 12279G 12618A 12705T 14766T 14783C 15025T 15043A 15301A 15326G 16223T 16263C 16311C 16519C	M23	73G 189G 195C 204C 263G 417A 489C 533G 16223T 16263C 16311C 16519C	1	73 189 195 204 263 417 489 533 750 1438 4769 5460 7028 8188 8360 8701 8860 9438 9540 9545 10142 10295 10398 10400 10873 11569 11719 11899 12279 12618 12705 14766 14783 15025 15043 15301 15326 16223 16263 16311	M23	73 189 195 204 263 417 489 533 16223 16263 16311	[0.016] M23 (1)	[0.031] M43a (2) [0.026] M4"67+16311 (3) [0.007] M43+16311 (4) [0.006] D4k (5) [0.005] M50 (6) [0.000] M57 M57b (8)	1
1225 >MF056464.1 Ho	73G 146C 152C 195C 263G 750G 769A 1018A 1438G 2416C 2706G 2789T 3106d 3107C 3594T 4104G 4769G 5090C 7028T 7175C 7256T 7274T 7521A 7771G 8206A 8701G 8860G 9221G 9540C 10115C 10143A 10398G 10873C 11719A 11914A 11944C 12693G 12705T 13590A 13650T 13803G 14566G 14766T 15301A 15326G 15735T 15784C 16182C 16183C 16189C 16223T 16278T 16290T 16294T 16309G 16390A	L2a1b1a	73G 146C 152C 195C 263G 16182C 16183C 16189C 16223T 16278T 16290T 16294T 16309G 16390A	1	73 146 152 195 263 750 769 1018 1438 2416 2706 2789 3594 4104 4769 5090 7028 7175 7256 7274 7521 7771 8206 8701 8860 9221 9540 10115 10143 10398 10873 11719 11914 11944 12693 12705 13590 13650 13803 14566 14766 15301 15326 15735 15784 16189 16223 16278 16290 16294 16309 16390	L2a1b1a	73 146 152 195 263 16189 16223 16278 16290 16294 16309 16390	[0.000] L2a1b1a (1)	[0.005] L2a1f (2) [0.000] L2a1+16189_(16192) L2a1b L2a1b1 L2a1b1a L2a1b2 L2a1f1 L2a1f1a L2a1f2 (10)	1
1226 >MF056463.1 Ho	73G 152C 182T 186A 189C 195C 247A 248G 263G 316A 514d 515d 750G 769A 825A 1018A 1438G 2394d 2706G 2758A 2885C 3106d 3107C 3525T 3594T 3666A 4104G 4769G 4853A 5951G 6071C 6221A 6227C 6917A 7028T 7146G 7247T 7256T 7389C 7521A 8027A 8468T 8655T 8701G 8860G 9072G 9540C 10398G 10586A 10688A 10810C 10873C 11302T 11719A 11984C 12507G 12705T 12810G 13105G 13485G 13506T 13650T 13789C 14000A 14178C 14560A 14669G 14766T 14911T 14926G 15226G 15326G 15905C 15978T 16129A 16187T 16189C 16223T 16278T 16293G 16294T 16311C 16360T 16519C	L1c3c	73G 152C 182T 186A 189C 195C 247A 248G 263G 316A 514d 515d 16129A 16187T 16189C 16223T 16278T 16293G 16294T 16311C 16360T 16519C	1	73 152 182 186A 189C 195 247 248 263 316 750 769 825A 1018 1438 2395d 2706 2758 2885 3525 3594 3666 4104 4769 4853 5951 6071 6221A 6227 6917 7028 7146 7247 7256 7389 7521 8027 8468 8655 8701 8860 9072 9540 10398 10586 10688 10810 10873 11302 11719 11984 12507 12705 12810 13105 13485 13506 13650 13789 14000A 14178 14560 14669 14766 14911 14926 15226 15326 15905 15978 16129 16187 16189 16223 16278 16293 16294 16311 16360	L1c3c	73 152 182 186A 189C 195 247 248 263 316 16129 16187 16189 16223 16278 16293 16294 16311 16360	[2.063] L1c3c (1) [0.000] L1c L1c1a+@198 L1c3b'c (4)	[0.700] L1c3 (5) [0.572] L1c1a'b'd (6) [0.267] L1c3b1 (7) [0.133] L1c3b1b (8) [0.000] L1c1'2'4'5'6 L1c1 L1c1a L1c1a1 L1c3b (13)	1

1227	>MF056456.1 Hc	73G 146C 152C 195C 263G 750G 769A 1018A 1438G 2416C 2706G 2789T 3106d 3107C 3594T 4104G 4769G 5090C 7028T 7175C 7256T 7274T 7521A 7771G 8206A 8701G 8860G 9221G 9540C 10115C 10143A 10398G 10873C 11719A 11914A 11944C 12471C 12693G 12705T 13590A 13650T 13803G 14566G 14766T 15301A 15326G 15735T 15784C 16183C 16189C 16223T 16278T 16290T 16294T 16309G 16390A	L2a1b1a	73G 146C 152C 195C 263G 16183C 16189C 16223T 16278T 16290T 16294T 16309G 16390A	1	73 146 152 195 263 750 769 1018 1438 2416 2706 2789 3594 4104 4769 5090 7028 7175 7256 7274 7521 7771 8206 8701 8860 9221 9540 10115 10143 10398 10873 11719 11914 11944 12471 12693 12705 13590 13650 13803 14566 14766 15301 15326 15735 15784 16189 16223 16278 16290 16294 16309 16390	L2a1b1a	73 146 152 195 263 16189 16223 16278 16290 16294 16309 16390	[0.000] L2a1b1a (1)	[0.005] L2a1f (2) [0.000] L2a1+16189_(16192) L2a1b L2a1b1 L2a1b1a L2a1b2 L2a1f1 L2a1f1a L2a1f2 (10)	1
1228	>MF056428.1 Hc	73G 146C 152C 195C 263G 750G 769A 1018A 1438G 2416C 2706G 2789T 3106d 3107C 3594T 4104G 4769G 5090C 7028T 7175C 7256T 7274T 7521A 7771G 8206A 8701G 8860G 9221G 9540C 10115C 10143A 10398G 10873C 11719A 11914A 11944C 12693G 12705T 13590A 13650T 13803G 14566G 14766T 15301A 15326G 15735T 15784C 16182C 16183C 16189C 16223T 16278T 16290T 16294T 16309G 16390A	L2a1b1a	73G 146C 152C 195C 263G 16182C 16183C 16189C 16223T 16278T 16290T 16294T 16309G 16390A	1	73 146 152 195 263 750 769 1018 1438 2416 2706 2789 3594 4104 4769 5090 7028 7175 7256 7274 7521 7771 8206 8701 8860 9221 9540 10115 10143 10398 10873 11719 11914 11944 12693 12705 13590 13650 13803 14566 14766 15301 15326 15735 15784 16189 16223 16278 16290 16294 16309 16390	L2a1b1a	73 146 152 195 263 16189 16223 16278 16290 16294 16309 16390	[0.000] L2a1b1a (1)	[0.005] L2a1f (2) [0.000] L2a1+16189_(16192) L2a1b L2a1b1 L2a1b1a L2a1b2 L2a1f1 L2a1f1a L2a1f2 (10)	1
1229	>MF056416.1 Hc	73G 150T 185A 189G 263G 568T 750G 1438G 2352C 2706G 3106d 3107C 4769G 6221C 6587T 7028T 8577G 8701G 8860G 9540C 10398G 10819G 10873C 11719A 12192A 12705T 14152G 14212C 14766T 14926G 15301A 15326G 15670C 15942C 16223T 16323d 16327T	L3e1b2	73G 150T 185A 189G 263G 568T 16223T 16323d 16327T	1	73 150 185 189 263 568 750 1438 2352 2706 4769 6221 6587 7028 8577 8701 8860 9540 10398 10819 10873 11719 12192 12705 14152 14212 14766 14926 15301 15326 15670 15942 16223 16325d 16327	L3e1b2	73 150 185 189 263 568 16223 16325d 16327	[0.019] L3e1b2 (1)	[0.200] L3e1b (2)	1
1230	>MF056414.1 Hc	73G 152C 195C 204C 263G 417A 489C 533G 750G 1438G 3106d 3107C 4769G 7028T 8188G 8360G 8701G 8860G 9438A 9540C 9545G 10142T 10295G 10398G 10400T 10873C 11569C 11719A 11899C 12279G 12469G 12618A 12705T 14766T 14783C 15025T 15043A 15301A 15326G 16223T 16263C 16311C 16519C	M23	73G 152C 195C 204C 263G 417A 489C 533G 16223T 16263C 16311C 16519C	1	73 152 195 204 263 417 489 533 750 1438 4769 7028 8188 8360 8701 8860 9438 9540 9545 10142 10295 10398 10400 10873 11569 11719 11899 12279 12469 12618 12705 14766 14783 15025 15043 15301 15326 16223 16263 16311	M23	73 152 195 204 263 417 489 533 16223 16263 16311	[0.000] M23 (1)	[0.072] M24a (2) [0.053] M57+152	1

1231	>MF056413.1 Hc	73G 150T 200G 263G 489C 750G 2706G 3106d 3107C 3588T 4769G 7028T 7960G 8701G 8768T 8854A 8860G 9540C 10398G 10400T 10873C 11719A 12705T 13308G 13590A 13708A 14130T 14260G 14527G 14766T 14783C 15043A 15172A 15301A 15326G 15625T 16086C 16148T 16223T 16259T 16278T 16319A 16399G 16526A	M32c	73G 150T 200G 263G 489C 16086C 16148T 16223T 16259T 16278T 16319A 16399G 16526A	1	73 150 200 263 489 750 2706 3588 4769 7028 7960 8701 8768 8854 8860 9540 10398 10400 10873 11719 12705 13308 13590 13708 14130 14260 14527 14766 14783 15043 15172 15301 15326 15625 16086 16148 16223 16259 16278 16319 16399 16526	M32c	73 150 200 263 489 16086 16148 16223 16259 16278 16319 16399 16526	[0.000] M32c (1)	[0.022] M51a (2) [0.007] M32 (3) [0.000] M51 M51b (5)	1
1232	>MF056410.1 Hc	73G 146C 152C 195C 263G 750G 769A 1018A 1438G 2416C 2706G 2789T 3106d 3107C 3594T 4104G 4769G 5090C 7028T 7175C 7256T 7274T 7521A 7771G 8206A 8701G 8860G 9221G 9540C 10115C 10143A 10398G 10873C 11719A 11914A 11944C 12557T 12693G 12705T 13590A 13650T 13803G 14566G 14766T 15301A 15326G 15735T 15784C 16182C 16183C 16189C 16223T 16278T 16290T 16294T 16309G 16390A 16519C	L2a1b1a	73G 146C 152C 195C 263G 16182C 16183C 16189C 16223T 16278T 16290T 16294T 16309G 16390A 16519C	1	73 146 152 195 263 750 769 1018 1438 2416 2706 2789 3594 4104 4769 5090 7028 7175 7256 7274 7521 7771 8206 8701 8860 9221 9540 10115 10143 10398 10873 11719 11914 11944 12557 12693 12705 13590 13650 13803 14566 14766 15301 15326 15735 15784 16189 16223 16278 16290 16294 16309 16390	L2a1b1a	73 146 152 195 263 16189 16223 16278 16290 16294 16309 16390	[0.000] L2a1b1a (1)	[0.005] L2a1f (2) [0.000] L2a1+16189_(16192) L2a1b L2a1b1 L2a1b1a L2a1b2 L2a1f1 L2a1f1a L2a1f2 (10)	1
1233	>MF056400.1 Hc	73G 152C 195C 204C 263G 417A 489C 533G 750G 1438G 3106d 3107C 4769G 7028T 8188G 8360G 8701G 8860G 9438A 9540C 9545G 10142T 10295G 10398G 10400T 10873C 11569C 11719A 11899C 12279G 12618A 12705T 14766T 14783C 15025T 15043A 15301A 15326G 16223T 16263C 16311C 16519C	M23	73G 152C 195C 204C 263G 417A 489C 533G 16223T 16263C 16311C 16519C	1	73 152 195 204 263 417 489 533 750 1438 4769 7028 8188 8360 8701 8860 9438 9540 9545 10142 10295 10398 10400 10873 11569 11719 11899 12279 12618 12705 14766 14783 15025 15043 15301 15326 16223 16263 16311	M23	73 152 195 204 263 417 489 533 16223 16263 16311	[0.000] M23 (1)	[0.072] M24a (2) [0.053] M57+152	1
1234	>MF056399.1 Hc	73G 152C 195C 204C 263G 417A 489C 533G 750G 1438G 3106d 3107C 4769G 7028T 8188G 8360G 8701G 8860G 9007G 9438A 9540C 9545G 10142T 10295G 10398G 10400T 10873C 11569C 11719A 11899C 12279G 12618A 12705T 14766T 14783C 15025T 15043A 15301A 15326G 16223T 16263C 16311C 16519C	M23	73G 152C 195C 204C 263G 417A 489C 533G 16223T 16263C 16311C 16519C	1	73 152 195 204 263 417 489 533 750 1438 4769 7028 8188 8360 8701 8860 9007 9438 9540 9545 10142 10295 10398 10400 10873 11569 11719 11899 12279 12618 12705 14766 14783 15025 15043 15301 15326 16223 16263 16311	M23	73 152 195 204 263 417 489 533 16223 16263 16311	[0.000] M23 (1)	[0.072] M24a (2) [0.053] M57+152	1

1235 >MF056397.1 Hc	73G 146C 152C 195C 263G 750G 769A 1018A 1438G 2416C 2706G 2789T 3106d 3107C 3594T 4104G 4769G 5090C 7028T 7175C 7256T 7274T 7521A 7771G 8206A 8701G 8860G 9221G 9540C 10115C 10143A 10398G 10873C 11719A 11914A 11944C 12693G 12705T 13590A 13650T 13803G 14566G 14766T 15301A 15326G 15735T 15784C 16182C 16183C 16189C 16223T 16278T 16290T 16294T 16309G 16390A	L2a1b1a	73G 146C 152C 195C 263G 16182C 16183C 16189C 16223T 16278T 16290T 16294T 16309G 16390A	1	73 146 152 195 263 750 769 1018 1438 2416 2706 2789 3594 4104 4769 5090 7028 7175 7256 7274 7521 7771 8206 8701 8860 9221 9540 10115 10143 10398 10873 11719 11914 11944 12693 12705 13590 13650 13803 14566 14766 15301 15326 15735 15784 16189 16223 16278 16290 16294 16309 16390	L2a1b1a	73 146 152 195 263 16189 16223 16278 16290 16294 16309 16390	[0.000] L2a1b1a (1)	[0.005] L2a1f (2) [0.000] L2a1+16189_(16192) L2a1b L2a1b1 L2a1b1a L2a1b2 L2a1f1 L2a1f1a L2a1f2 (10)	1
1236 >MF056396.1 Hc	73G 152C 195C 204C 263G 417A 489C 533G 750G 1438G 3106d 3107C 4769G 5393C 7028T 8188G 8360G 8701G 8860G 9007G 9438A 9540C 9545G 10142T 10295G 10398G 10400T 10873C 11569C 11719A 11899C 12279G 12618A 12705T 14766T 14783C 15025T 15043A 15301A 15326G 16223T 16263C 16311C 16519C	M23	73G 152C 195C 204C 263G 417A 489C 533G 16223T 16263C 16311C 16519C	1	73 152 195 204 263 417 489 533 750 1438 4769 5393 7028 8188 8360 8701 8860 9007 9438 9540 9545 10142 10295 10398 10400 10873 11569 11719 11899 12279 12618 12705 14766 14783 15025 15043 15301 15326 16223 16263 16311	M23	73 152 195 204 263 417 489 533 16223 16263 16311	[0.000] M23 (1)	[0.072] M24a (2) [0.053] M57+152	1
1237 >MF056395.1 Hc	73G 150d 182T 186A 189C 195C 198T 247A 263G 297G 316A 750G 769A 825A 1018A 1420C 1438G 2394d 2706G 2758A 2885C 3106d 3107C 3594T 3666A 4104G 4769G 5951G 6071C 6150A 6253C 7028T 7055G 7076G 7146G 7256T 7337A 7389C 7521A 7744C 8027A 8251A 8468T 8655T 8701G 8784G 8860G 8877C 9072G 9540C 10321C 10398G 10586A 10688A 10792G 10793T 10810C 10873C 11654G 11719A 12049T 12705T 12810G 13105G 13149G 13212T 13281C 13485G 13506T 13650T 13789C 14000A 14129T 14178C 14325C 14560A 14766T 14812T 14911T 15016T 15326G 15784C 16071T 16129A 16145A 16187T 16189C 16213A 16234T 16265C 16278T 16286G 16294T 16311C 16360T 16527T	L1c2a1a	73G 150d 182T 186A 189C 195C 198T 247A 263G 297G 316A 16071T 16129A 16145A 16187T 16189C 16213A 16234T 16265C 16278T 16286G 16294T 16311C 16360T 16527T	1	73 151d 182 186A 189C 195 198 247 263 297 316 750 769 825A 1018 1420 1438 2395d 2706 2758 2885 3594 3666 4104 4769 5951 6071 6150 6253 7028 7055 7076 7146 7256 7337 7389 7521 7744 8027 8251 8468 8655 8701 8784 8860 8877 9072 9540 10321 10398 10586 10688 10792 10793 10810 10873 11654 11719 12049 12705 12810 13105 13149 13212 13281 13485 13506 13650 13789 14000A 14129 14178 14325 14560 14766 14812 14911 15016 15326 15784 16071 16129 16145 16187 16189 16213 16234 16265C 16278 16286G 16294 16311 16360 16527	L1c2a1a	73 151d 182 186A 189C 195 198 247 263 297 316 16071 16129 16145 16187 16189 16213 16234 16265C 16278 16286G 16294 16311 16360 16527	[0.197] L1c2a1a (1)	[0.158] L1c2a1b (2) [0.000] L1c2a1	1

1238	>MF056389.1 Hc	73G 152C 195C 204C 263G 417A 489C 533G 750G 1438G 3106d 3107C 4769G 5393C 7028T 8188G 8360G 8701G 8860G 9007G 9438A 9540C 9545G 10142T 10295G 10398G 10400T 10873C 11569C 11719A 11899C 12279G 12618A 12705T 14766T 14783C 15025T 15043A 15301A 15326G 16223T 16263C 16311C 16519C	M23	73G 152C 195C 204C 263G 417A 489C 533G 16223T 16263C 16311C 16519C	1	73 152 195 204 263 417 489 533 750 1438 4769 5393 7028 8188 8360 8701 8860 9007 9438 9540 9545 10142 10295 10398 10400 10873 11569 11719 11899 12279 12618 12705 14766 14783 15025 15043 15301 15326 16223 16263 16311	M23	73 152 195 204 263 417 489 533 16223 16263 16311	[0.000] M23 (1)	[0.072] M24a (2) [0.053] M57+152	1
1239	>MF056379.1 Hc	73G 150T 200G 263G 489C 750G 2706G 3106d 3107C 3588T 4769G 7028T 7960G 8701G 8768T 8854A 8860G 9540C 10398G 10400T 10873C 11719A 12705T 13308G 13590A 13708A 14130T 14260G 14527G 14766T 14783C 15043A 15172A 15301A 15326G 15625T 16086C 16148T 16223T 16259T 16278T 16319A 16399G 16526A	M32c	73G 150T 200G 263G 489C 16086C 16148T 16223T 16259T 16278T 16319A 16399G 16526A	1	73 150 200 263 489 750 2706 3588 4769 7028 7960 8701 8768 8854 8860 9540 10398 10400 10873 11719 12705 13308 13590 13708 14130 14260 14527 14766 14783 15043 15172 15301 15326 15625 16086 16148 16223 16259 16278 16319 16399 16526	M32c	73 150 200 263 489 16086 16148 16223 16259 16278 16319 16399 16526	[0.000] M32c (1)	[0.022] M51a (2) [0.007] M32 (3) [0.000] M51 M51b (5)	1
1240	>MF056378.1 Hc	73G 150T 200G 263G 489C 750G 2706G 3106d 3107C 3588T 4769G 7028T 7960G 8701G 8768T 8854A 8860G 9540C 10398G 10400T 10873C 11719A 12705T 13308G 13590A 13708A 14130T 14260G 14325C 14527G 14766T 14783C 15043A 15172A 15301A 15326G 15625T 16086C 16148T 16223T 16259T 16278T 16319A 16399G 16526A	M32c	73G 150T 200G 263G 489C 16086C 16148T 16223T 16259T 16278T 16319A 16399G 16526A	1	73 150 200 263 489 750 2706 3588 4769 7028 7960 8701 8768 8854 8860 9540 10398 10400 10873 11719 12705 13308 13590 13708 14130 14260 14325 14527 14766 14783 15043 15172 15301 15326 15625 16086 16148 16223 16259 16278 16319 16399 16526	M32c	73 150 200 263 489 16086 16148 16223 16259 16278 16319 16399 16526	[0.000] M32c (1)	[0.022] M51a (2) [0.007] M32 (3) [0.000] M51 M51b (5)	1
1241	>MF056377.1 Hc	73G 152C 195C 204C 263G 417A 456T 489C 533G 750G 1438G 3106d 3107C 4769G 7028T 8188G 8360G 8701G 8860G 9438A 9540C 9545G 10142T 10295G 10398G 10400T 10873C 11569C 11719A 11899C 12279G 12618A 12705T 14766T 14783C 15025T 15043A 15301A 15326G 16223T 16263C 16311C 16519C	M23	73G 152C 195C 204C 263G 417A 456T 489C 533G 16223T 16263C 16311C 16519C	1	73 152 195 204 263 417 456 489 533 750 1438 4769 7028 8188 8360 8701 8860 9438 9540 9545 10142 10295 10398 10400 10873 11569 11719 11899 12279 12618 12705 14766 14783 15025 15043 15301 15326 16223 16263 16311	M23	73 152 195 204 263 417 456 489 533 16223 16263 16311	[0.004] M23 (1)	[0.072] M24a (2) [0.053] M57+152	1

1242	>MF056373.1 H	73G 150d 195C 198T 263G 514d 515d 721C 750G 1005C 1438G 2357T 2706G 3106d 3107C 4769G 5310G 7028T 8701G 8860G 9540C 10184T 10314T 10398G 10873C 11719A 12618A 12705T 12816T 13443C 13708A 14461C 14566G 14766T 14851G 15301A 15326G 15553A 16178C 16223T 16254G 16311C	L3a1b	73G 150d 195C 198T 263G 514d 515d 16178C 16223T 16254G 16311C	1	73 151d 195 198 263 721 750 1005 1438 2357 2706 4769 5310 7028 8701 8860 9540 10184 10314 10398 10873 11719 12618 12705 12816 13443 13708 14461 14566 14766 14851 15301 15326 15553 16178 16223 16254 16311	L3a1b	73 151d 195 198 263 16178 16223 16254 16311	[1.221] L3a1b (1) [0.000] L3'4 L4 L4a L4b L3e2a1b2 L3h1 L3h1a L3h1a2 (9)	[1.221] L3a1b (10) [0.284] L3f2 (11) [0.209] L3a1a (12) [0.158] R8a (13) [0.157] M66b (14) [0.143] L3e2a1b (15) [0.133] R8a1a (16) [0.052] S2 (17) [0.042] L3e2a1a (18) [0.030] M4"67+16311 (19) [0.028] H11b (20) [0.025] M43a (21) [0.024] N5 (22) [0.010] L3 (23) [0.007] H11 (24) [0.000] L4a2 L3b'f L3e2a1 L3e2a1b3 L3h L3h1a2b L3h1b M43+16311 M57 D4k D4p D4p1 N N1'5 N1 N9 N11 N11a N14 S H11b1 R1 R8 R8a1 R8a1a1 R8a1a1a R8a1a1c (51)	1
1243	>MF056372.1 H	73G 150T 200G 263G 489C 750G 2706G 3106d 3107C 3588T 4769G 7028T 7960G 8701G 8768T 8854A 8860G 9540C 10398G 10400T 10873C 11719A 12705T 13308G 13590A 13708A 14130T 14260G 14527G 14766T 14783C 15043A 15172A 15301A 15326G 15625T 16086C 16148T 16223T 16259T 16278T 16319A 16399G 16526A	M32c	73G 150T 200G 263G 489C 16086C 16148T 16223T 16259T 16278T 16319A 16399G 16526A	1	73 150 200 263 489 750 2706 3588 4769 7028 7960 8701 8768 8854 8860 9540 10398 10400 10873 11719 12705 13308 13590 13708 14130 14260 14527 14766 14783 15043 15172 15301 15326 15625 16086 16148 16223 16259 16278 16319 16399 16526	M32c	73 150 200 263 489 16086 16148 16223 16259 16278 16319 16399 16526	[0.000] M32c (1)	[0.022] M51a (2) [0.007] M32 (3) [0.000] M51 M51b (5)	1
1244	>MF056371.1 H	73G 150d 195C 198T 263G 514d 515d 721C 750G 1005C 1438G 2357T 2706G 3106d 3107C 4769G 5310G 7028T 8701G 8860G 9540C 10184T 10314T 10398G 10873C 11719A 12618A 12705T 12816T 13443C 13708A 14461C 14566G 14766T 14851G 15301A 15326G 15553A 16178C 16223T 16254G 16311C	L3a1b	73G 150d 195C 198T 263G 514d 515d 16178C 16223T 16254G 16311C	1	73 151d 195 198 263 721 750 1005 1438 2357 2706 4769 5310 7028 8701 8860 9540 10184 10314 10398 10873 11719 12618 12705 12816 13443 13708 14461 14566 14766 14851 15301 15326 15553 16178 16223 16254 16311	L3a1b	73 151d 195 198 263 16178 16223 16254 16311	[1.221] L3a1b (1) [0.000] L3'4 L4 L4a L4b L3e2a1b2 L3h1 L3h1a L3h1a2 (9)	[1.221] L3a1b (10) [0.284] L3f2 (11) [0.209] L3a1a (12) [0.158] R8a (13) [0.157] M66b (14) [0.143] L3e2a1b (15) [0.133] R8a1a (16) [0.052] S2 (17) [0.042] L3e2a1a (18) [0.030] M4"67+16311 (19) [0.028] H11b (20) [0.025] M43a (21) [0.024] N5 (22) [0.010] L3 (23) [0.007] H11 (24) [0.000] L4a2 L3b'f L3e2a1 L3e2a1b3 L3h L3h1a2b L3h1b M43+16311 M57 D4k D4p D4p1 N N1'5 N1 N9 N11 N11a N14 S H11b1 R1 R8 R8a1 R8a1a1 R8a1a1a R8a1a1c (51)	1

1245	>MF056365.1 H	73G 146C 152C 195C 198T 263G 750G 769A 1018A 1438G 2416C 2706G 2789T 3106d 3107C 3594T 4104G 4769G 5581G 7028T 7175C 7256T 7274T 7521A 7771G 8206A 8701G 8860G 9221G 9540C 10115C 10398G 10873C 11719A 11914A 11944C 12693G 12705T 13590A 13650T 13803G 14566G 14766T 15301A 15326G 15784C 16189C 16223T 16278T 16294T 16309G 16390A 16519C	L2a1f3	73G 146C 152C 195C 198T 263G 16189C 16223T 16278T 16294T 16309G 16390A 16519C	1	73 146 152 195 198 263 750 769 1018 1438 2416 2706 2789 3594 4104 4769 5581 7028 7175 7256 7274 7521 7771 8206 8701 8860 9221 9540 10115 10398 10873 11719 11914 11944 12693 12705 13590 13650 13803 14566 14766 15301 15326 15784 16189 16223 16278 16294 16309 16390	L2a1f3	73 146 152 195 198 263 16189 16223 16278 16294 16309 16390	[0.000] L2a1f3 (1)	[0.007] L2a1b (2) [0.005] L2a1f (3) [0.000] L2a1+16189_(16192) L2a1b1 L2a1b2 L2a1f1 L2a1f1a L2a1f2 L2a1f3 (10)	1
1246	>MF056351.1 H	73G 150T 195C 263G 514d 515d 2000T 2352C 2706G 3106d 3107C 4655A 4769G 5262A 6524C 7028T 8701G 8860G 9540C 9554A 10398G 10667C 10816G 10819G 10873C 11719A 12248G 12705T 13101C 13197T 13651G 14212C 14766T 14953T 15301A 15326G 15812A 16223T 16265T 16519C 16527T	L3e3b2	73G 150T 195C 263G 514d 515d 16223T 16265T 16519C 16527T	1	73 150 195 263 2000 2352 2706 4655 4769 5262 6524 7028 8701 8860 9540 9554 10398 10667 10816 10819 10873 11719 12248 12705 13101C 13197 13651 14212 14766 14953 15301 15326 15812 16223 16265T 16527	L3e3b2	73 150 195 263 16223 16265T 16527	[0.000] L3e3b2 (1)	[0.000] L3e3 L3e3b L3e3b1 (4)	1
1247	>MF056350.1 H	73G 152C 195C 204C 263G 417A 489C 533G 750G 1438G 3106d 3107C 4769G 7028T 8188G 8360G 8701G 8860G 9438A 9540C 9545G 10142T 10295G 10398G 10400T 10873C 11569C 11719A 11899C 12279G 12618A 12705T 14766T 14783C 15025T 15043A 15301A 15326G 16223T 16263C 16311C 16519C	M23	73G 152C 195C 204C 263G 417A 489C 533G 16223T 16263C 16311C 16519C	1	73 152 195 204 263 417 489 533 750 1438 4769 7028 8188 8360 8701 8860 9438 9540 9545 10142 10295 10398 10400 10873 11569 11719 11899 12279 12618 12705 14766 14783 15025 15043 15301 15326 16223 16263 16311	M23	73 152 195 204 263 417 489 533 16223 16263 16311	[0.000] M23 (1)	[0.072] M24a (2) [0.053] M57+152	1
1248	>MF056348.1 H	73G 146C 152C 195C 263G 750G 769A 1018A 1438G 2416C 2706G 2789T 3106d 3107C 3594T 4104G 4769G 5090C 7028T 7175C 7256T 7274T 7521A 7771G 8206A 8701G 8860G 9221G 9540C 10115C 10143A 10398G 10873C 11719A 11914A 11944C 12693G 12705T 13590A 13650T 13803G 14566G 14766T 15301A 15326G 15735T 15784C 16183C 16189C 16223T 16278T 16290T 16294T 16309G 16390A	L2a1b1a	73G 146C 152C 195C 263G 16183C 16189C 16223T 16278T 16290T 16294T 16309G 16390A	1	73 146 152 195 263 750 769 1018 1438 2416 2706 2789 3594 4104 4769 5090 7028 7175 7256 7274 7521 7771 8206 8701 8860 9221 9540 10115 10143 10398 10873 11719 11914 11944 12693 12705 13590 13650 13803 14566 14766 15301 15326 15735 15784 16189 16223 16278 16290 16294 16309 16390	L2a1b1a	73 146 152 195 263 16189 16223 16278 16290 16294 16309 16390	[0.000] L2a1b1a (1)	[0.005] L2a1f (2) [0.000] L2a1+16189_(16192) L2a1b L2a1b1 L2a1b1a L2a1b2 L2a1f1 L2a1f1a L2a1f2 (10)	1

1249	>MF056342.1 Hc	73G 146C 152C 195C 263G 750G 769A 1018A 1438G 2416C 2706G 2789T 3106d 3107C 3594T 4104G 4769G 5090C 7028T 7175C 7256T 7274T 7521A 7771G 8206A 8701G 8860G 9221G 9540C 10115C 10143A 10398G 10873C 11719A 11914A 11944C 12693G 12705T 13590A 13650T 13803G 14566G 14766T 15301A 15326G 15735T 15784C 16182C 16183C 16189C 16223T 16278T 16290T 16294T 16309G 16390A	L2a1b1a	73G 146C 152C 195C 263G 16182C 16183C 16189C 16223T 16278T 16290T 16294T 16309G 16390A	1	73 146 152 195 263 750 769 1018 1438 2416 2706 2789 3594 4104 4769 5090 7028 7175 7256 7274 7521 7771 8206 8701 8860 9221 9540 10115 10143 10398 10873 11719 11914 11944 12693 12705 13590 13650 13803 14566 14766 15301 15326 15735 15784 16189 16223 16278 16290 16294 16309 16390	L2a1b1a	73 146 152 195 263 16189 16223 16278 16290 16294 16309 16390	[0.000] L2a1b1a (1)	[0.005] L2a1f (2) [0.000] L2a1+16189_(16192) L2a1b L2a1b1 L2a1b1a L2a1b2 L2a1f1 L2a1f1a L2a1f2 (10)	1
1250	>MF056822.1 Hc	73G 152C 195C 204C 263G 417A 489C 533G 750G 1438G 3106d 3107C 4769G 7028T 8188G 8360G 8701G 8860G 9438A 9540C 9545G 10142T 10295G 10398G 10400T 10873C 11569C 11719A 11899C 12279G 12618A 12705T 14766T 14783C 15025T 15043A 15301A 15326G 16037G 16223T 16263C 16311C 16519C	M23	73G 152C 195C 204C 263G 417A 489C 533G 16037G 16223T 16263C 16311C 16519C	1	73 152 195 204 263 417 489 533 750 1438 4769 7028 8188 8360 8701 8860 9438 9540 9545 10142 10295 10398 10400 10873 11569 11719 11899 12279 12618 12705 14766 14783 15025 15043 15301 15326 16037 16223 16263 16311	M23	73 152 195 204 263 417 489 533 16037 16223 16263 16311	[0.008] M23 (1)	[0.072] M24a (2) [0.053] M57+152	1
1251	>MF056820.1 Hc	73G 152C 182T 186A 189C 247A 263G 316A 514d 515d 629C 750G 769A 825A 1018A 1438G 2283T 2394d 2706G 2758A 2885C 3106d 3107C 3210T 3434G 3594T 3666A 4104G 4755C 4758N 4759N 4769G 5951G 6071C 6221A 6917A 7028T 7146G 7256T 7389C 7521A 8027A 8251A 8417T 8468T 8655T 8701G 8860G 9072G 9540C 9581C 10398G 10400T 10688A 10810C 10873C 11302T 11317G 11719A 12400G 12542T 12705T 12810G 13105G 13485G 13506T 13650T 13708A 13789C 13981T 14000A 14178C 14560A 14766T 14794T 14911T 15226G 15326G 15905C 15978T 16017C 16129A 16163G 16187T 16189C 16209C 16223T 16278T 16293G 16294T 16311C 16360T 16519C	L1c3b1a	73G 152C 182T 186A 189C 247A 263G 316A 514d 515d 16129A 16163G 16187T 16189C 16209C 16223T 16278T 16293G 16294T 16311C 16360T 16519C	1	73 152 182 186A 189C 247 263 316 629 750 769 825A 1018 1438 2283 2395d 2706 2758 2885 3210 3434 3594 3666 4104 4755 4758N 4759N 4769 5951 6071 6221A 6917 7028 7146 7256 7389 7521 8027 8251 8417 8468 8655 8701 8860 9072 9540 9581 10398 10400 10688 10810 10873 11302 11317 11719 12400 12542 12705 12810 13105 13485 13506 13650 13708 13789 13981 14000A 14178 14560 14766 14794 14911 15226 15326 15905 15978 16017 16129 16163 16187 16189 16209 16223 16278 16293 16294 16311 16360	L1c3b1a	73 152 182 186A 189C 247 263 316 16129 16163 16187 16189 16209 16223 16278 16293 16294 16311 16360	[0.093] L1c3b1a (1)	[0.000] L1c3b L1c3b1 L1c3b1b (4)	1

1252 >MF056818.1 Hc	73G 263G 279C 514d 515d 750G 1438G 2332T 2706G 3106d 3107C 3450T 4769G 5773A 6221C 6340T 7028T 8701G 8860G 9449T 9540C 10086G 10373A 10398G 10873C 11002G 11719A 12705T 13105G 13914A 14766T 15301A 15311G 15326G 15824G 15940d 16145A 16223T 16278T 16362C 16519C	L3b1a8	73G 263G 279C 514d 515d 16145A 16223T 16278T 16362C 16519C	1	73 263 279 750 1438 2332 2706 3450 4769 5773 6221 6340 7028 8701 8860 9449 9540 10086 10373 10398 10873 11002 11719 12705 13105 13914A 14766 15301 15311 15326 15824 15944d 16145 16223 16278 16362	L3b1a8	73 263 279 16145 16223 16278 16362	[0.213] L3b1a8 (1)	[0.000] L3b1a+@16124 L3b1a7 L3b1a7a (4)	1
1253 >MF056817.1 Hc	73G 152C 195C 204C 263G 417A 489C 533G 750G 1438G 3106d 3107C 4769G 7028T 8188G 8360G 8701G 8860G 9438A 9540C 9545G 10142T 10295G 10398G 10400T 10873C 11569C 11719A 11899C 12279G 12618A 12705T 14766T 14783C 15025T 15043A 15301A 15326G 16223T 16263C 16311C 16519C	M23	73G 152C 195C 204C 263G 417A 489C 533G 16223T 16263C 16311C 16519C	1	73 152 195 204 263 417 489 533 750 1438 4769 7028 8188 8360 8701 8860 9438 9540 9545 10142 10295 10398 10400 10873 11569 11719 11899 12279 12618 12705 14766 14783 15025 15043 15301 15326 16223 16263 16311	M23	73 152 195 204 263 417 489 533 16223 16263 16311	[0.000] M23 (1)	[0.072] M24a (2) [0.053] M57+152	1
1254 >MF056811.1 Hc	73G 146C 152C 195C 263G 750G 769A 1018A 1438G 2416C 2706G 2789T 3106d 3107C 3594T 4104G 4769G 5090C 7028T 7175C 7256T 7274T 7521A 7771G 8206A 8701G 8860G 9221G 9540C 10115C 10143A 10398G 10873C 11719A 11914A 11944C 12693G 12705T 13590A 13650T 13803G 14566G 14766T 15301A 15326G 15735T 15784C 16183C 16189C 16223T 16278T 16290T 16294T 16309G 16390A	L2a1b1a	73G 146C 152C 195C 263G 16183C 16189C 16223T 16278T 16290T 16294T 16309G 16390A	1	73 146 152 195 263 750 769 1018 1438 2416 2706 2789 3594 4104 4769 5090 7028 7175 7256 7274 7521 7771 8206 8701 8860 9221 9540 10115 10143 10398 10873 11719 11914 11944 12693 12705 13590 13650 13803 14566 14766 15301 15326 15735 15784 16189 16223 16278 16290 16294 16309 16390	L2a1b1a	73 146 152 195 263 16189 16223 16278 16290 16294 16309 16390	[0.000] L2a1b1a (1)	[0.005] L2a1f (2) [0.000] L2a1+16189_(16192) L2a1b L2a1b1 L2a1b1a L2a1b2 L2a1f1 L2a1f1a L2a1f2 (10)	1
1255 >MF056810.1 Hc	73G 263G 279C 514d 515d 750G 1438G 2332T 2706G 3106d 3107C 3450T 4769G 5773A 6221C 6340T 7028T 8701G 8860G 9449T 9540C 10086G 10373A 10398G 10873C 11002G 11719A 12705T 13105G 13914A 14766T 15301A 15311G 15326G 15824G 15940d 16145A 16223T 16278T 16362C 16519C	L3b1a8	73G 263G 279C 514d 515d 16145A 16223T 16278T 16362C 16519C	1	73 263 279 750 1438 2332 2706 3450 4769 5773 6221 6340 7028 8701 8860 9449 9540 10086 10373 10398 10873 11002 11719 12705 13105 13914A 14766 15301 15311 15326 15824 15944d 16145 16223 16278 16362	L3b1a8	73 263 279 16145 16223 16278 16362	[0.213] L3b1a8 (1)	[0.000] L3b1a+@16124 L3b1a7 L3b1a7a (4)	1
1256 >MF056809.1 Hc	73G 152C 195C 204C 263G 417A 489C 533G 750G 1438G 3106d 3107C 4769G 7028T 8188G 8360G 8701G 8853N 8854N 8856N 8857N 8860G 9438A 9540C 9545G 10142T 10295G 10398G 10400T 10873C 11569C 11719A 11899C 12279G 12618A 12705T 13708A 14766T 14783C 15025T 15043A 15301A 15326G 16223T 16263C 16311C 16519C	M23	73G 152C 195C 204C 263G 417A 489C 533G 16223T 16263C 16311C 16519C	1	73 152 195 204 263 417 489 533 750 1438 4769 7028 8188 8360 8701 8853N 8854N 8856N 8857N 8860 9438 9540 9545 10142 10295 10398 10400 10873 11569 11719 11899 12279 12618 12705 13708 14766 14783 15025 15043 15301 15326 16223 16263 16311	M23	73 152 195 204 263 417 489 533 16223 16263 16311	[0.000] M23 (1)	[0.072] M24a (2) [0.053] M57+152	1

1257	>MF056806.1 Hc	73G 152C 195C 204C 263G 417A 489C 533G 750G 1438G 3106d 3107C 4769G 7028T 8188G 8360G 8701G 8860G 9438A 9540C 9545G 10142T 10295G 10398G 10400T 10873C 11569C 11719A 11899C 12279G 12618A 12705T 13708A 14766T 14783C 15025T 15043A 15301A 15326G 16223T 16263C 16311C 16519C	M23	73G 152C 195C 204C 263G 417A 489C 533G 16223T 16263C 16311C 16519C	1	73 152 195 204 263 417 489 533 750 1438 4769 7028 8188 8360 8701 8860 9438 9540 9545 10142 10295 10398 10400 10873 11569 11719 11899 12279 12618 12705 13708 14766 14783 15025 15043 15301 15326 16223 16263 16311	M23	73 152 195 204 263 417 489 533 16223 16263 16311	[0.000] M23 (1)	[0.072] M24a (2) [0.053] M57+152	1
1258	>MF056785.1 Hc	73G 248d 263G 750G 1438G 2332T 2706G 3106d 3107C 3434G 4769G 5585A 6221C 7028T 8701G 8860G 10086G 10373A 10398G 10873C 11002G 11719A 12705T 13914A 14766T 15301A 15311G 15326G 15824G 15940d 16145A 16223T 16278T 16362C 16519C	L3b1a8	73G 248d 263G 16145A 16223T 16278T 16362C 16519C	1	73 249d 263 750 1438 2332 2706 3434 4769 5585 6221 7028 8701 8860 10086 10373 10398 10873 11002 11719 12705 13914A 14766 15301 15311 15326 15824 15944d 16145 16223 16278 16362	L3b1a8	73 249d 263 16145 16223 16278 16362	[0.009] L3b1a8 (1)	[0.000] L3b1a+@16124 L3b1a7 L3b1a7a (4)	1
1259	>MF056778.1 Hc	73G 150T 200G 263G 489C 750G 2706G 3106d 3107C 3588T 4769G 7028T 7960G 8701G 8768T 8854A 8860G 9540C 10398G 10400T 10544T 10873C 11719A 12705T 13308G 13590A 13708A 14130T 14260G 14527G 14766T 14783C 15043A 15172A 15301A 15326G 15625T 16086C 16148T 16223T 16259T 16278T 16319A 16399G 16526A	M32c	73G 150T 200G 263G 489C 16086C 16148T 16223T 16259T 16278T 16319A 16399G 16526A	1	73 150 200 263 489 750 2706 3588 4769 7028 7960 8701 8768 8854 8860 9540 10398 10400 10544 10873 11719 12705 13308 13590 13708 14130 14260 14527 14766 14783 15043 15172 15301 15326 15625 16086 16148 16223 16259 16278 16319 16399 16526	M32c	73 150 200 263 489 16086 16148 16223 16259 16278 16319 16399 16526	[0.000] M32c (1)	[0.022] M51a (2) [0.007] M32 (3) [0.000] M51 M51b (5)	1
1260	>MF056770.1 Hc	73G 152C 195C 204C 263G 417A 489C 533G 750G 1438G 3106d 3107C 4769G 6149G 6719C 7028T 8188G 8360G 8701G 8860G 9438A 9540C 9545G 10398G 10873C 11569C 11719A 11899C 12279G 14766T 14783C 15025T 15043A 15301A 15326G 16223T 16263C 16311C 16519C	M23	73G 152C 195C 204C 263G 417A 489C 533G 16223T 16263C 16311C 16519C	1	73 152 195 204 263 417 489 533 750 1438 4769 6149 6719 7028 8188 8360 8701 8860 9438 9540 9545 10398 10873 11569 11719 11899 12279 14766 14783 15025 15043 15301 15326 16223 16263 16311	M23	73 152 195 204 263 417 489 533 16223 16263 16311	[0.000] M23 (1)	[0.072] M24a (2) [0.053] M57+152	1
1261	>MF056768.1 Hc	73G 263G 279C 514d 515d 750G 1438G 2332T 2706G 3106d 3107C 3450T 4769G 5773A 6221C 6340T 7028T 8701G 8821N 8860G 9449T 9540C 10086G 10373A 10398G 10873C 11002G 11719A 12705T 13105G 13914A 14766T 15301A 15311G 15326G 15824G 15940d 16145A 16223T 16278T 16362C 16519C	L3b1a8	73G 263G 279C 514d 515d 16145A 16223T 16278T 16362C 16519C	1	73 263 279 750 1438 2332 2706 3450 4769 5773 6221 6340 7028 8701 8821N 8860 9449 9540 10086 10373 10398 10873 11002 11719 12705 13105 13914A 14766 15301 15311 15326 15824 15944d 16145 16223 16278 16362	L3b1a8	73 263 279 16145 16223 16278 16362	[0.213] L3b1a8 (1)	[0.000] L3b1a+@16124 L3b1a7 L3b1a7a (4)	1

1262	>MF056764.1 Hc	73G 146C 152C 195C 263G 750G 769A 1018A 1438G 2416C 2706G 2789T 3106d 3107C 3594T 4104G 4769G 5090C 7028T 7175C 7256T 7274T 7521A 7771G 8206A 8701G 8860G 9221G 9540C 10115C 10143A 10398G 10873C 11719A 11914A 11944C 12693G 12705T 13590A 13650T 13803G 14566G 14766T 15301A 15326G 15735T 15784C 16182C 16183C 16189C 16223T 16278T 16290T 16294T 16309G 16390A	L2a1b1a	73G 146C 152C 195C 263G 16182C 16183C 16189C 16223T 16278T 16290T 16294T 16309G 16390A	1	73 146 152 195 263 750 769 1018 1438 2416 2706 2789 3594 4104 4769 5090 7028 7175 7256 7274 7521 7771 8206 8701 8860 9221 9540 10115 10143 10398 10873 11719 11914 11944 12693 12705 13590 13650 13803 14566 14766 15301 15326 15735 15784 16189 16223 16278 16290 16294 16309 16390	L2a1b1a	73 146 152 195 263 16189 16223 16278 16290 16294 16309 16390	[0.000] L2a1b1a (1)	[0.005] L2a1f (2) [0.000] L2a1+16189_(16192) L2a1b L2a1b1 L2a1b1a L2a1b2 L2a1f1 L2a1f1a L2a1f2 (10)	1
1263	>MF056762.1 Hc	73G 152C 195C 204C 263G 417A 489C 533G 750G 1438G 3106d 3107C 4769G 6149G 7028T 8188G 8360G 8701G 8860G 9438A 9540C 9545G 10142T 10295G 10398G 10400T 10873C 11569C 11719A 11899C 12279G 12618A 12705T 14766T 14783C 15025T 15043A 15301A 15326G 16223T 16263C 16311C 16519C	M23	73G 152C 195C 204C 263G 417A 489C 533G 16223T 16263C 16311C 16519C	1	73 152 195 204 263 417 489 533 750 1438 4769 6149 7028 8188 8360 8701 8860 9438 9540 9545 10142 10295 10398 10400 10873 11569 11719 11899 12279 12618 12705 14766 14783 15025 15043 15301 15326 16223 16263 16311	M23	73 152 195 204 263 417 489 533 16223 16263 16311	[0.000] M23 (1)	[0.072] M24a (2) [0.053] M57+152	1
1264	>MF056761.1 Hc	73G 152C 195C 204C 263G 417A 489C 533G 750G 1438G 3106d 3107C 4769G 7028T 8188G 8360G 8701G 8860G 9007G 9438A 9540C 9545G 10142T 10295G 10398G 10400T 10873C 11569C 11719A 11899C 12279G 12618A 12705T 14766T 14783C 15025T 15043A 15301A 15326G 16223T 16263C 16311C 16519C	M23	73G 152C 195C 204C 263G 417A 489C 533G 16223T 16263C 16311C 16519C	1	73 152 195 204 263 417 489 533 750 1438 4769 7028 8188 8360 8701 8860 9007 9438 9540 9545 10142 10295 10398 10400 10873 11569 11719 11899 12279 12618 12705 14766 14783 15025 15043 15301 15326 16223 16263 16311	M23	73 152 195 204 263 417 489 533 16223 16263 16311	[0.000] M23 (1)	[0.072] M24a (2) [0.053] M57+152	1
1265	>MF056760.1 Hc	73G 152C 195C 204C 263G 417A 489C 533G 750G 1438G 3106d 3107C 4769G 7028T 8188G 8360G 8701G 8860G 9438A 9540C 9545G 10142T 10295G 10398G 10400T 10873C 11569C 11719A 11899C 12279G 12618A 12705T 14766T 14783C 15025T 15043A 15301A 15326G 15747C 16223T 16263C 16311C 16519C	M23	73G 152C 195C 204C 263G 417A 489C 533G 16223T 16263C 16311C 16519C	1	73 152 195 204 263 417 489 533 750 1438 4769 7028 8188 8360 8701 8860 9438 9540 9545 10142 10295 10398 10400 10873 11569 11719 11899 12279 12618 12705 14766 14783 15025 15043 15301 15326 15747 16223 16263 16311	M23	73 152 195 204 263 417 489 533 16223 16263 16311	[0.000] M23 (1)	[0.072] M24a (2) [0.053] M57+152	1

1266	>MF056749.1 Hc	73G 146C 152C 195C 263G 750G 769A 1018A 1438G 2416C 2706G 2789T 3106d 3107C 3594T 4104G 4769G 5090C 7028T 7175C 7256T 7274T 7521A 7771G 8206A 8701G 8860G 9221G 9540C 10115C 10143A 10398G 10873C 11719A 11914A 11944C 12693G 12705T 13590A 13650T 13803G 14566G 14766T 15301A 15326G 15735T 15784C 16183C 16189C 16223T 16278T 16290T 16294T 16309G 16390A	L2a1b1a	73G 146C 152C 195C 263G 16183C 16189C 16223T 16278T 16290T 16294T 16309G 16390A	1	73 146 152 195 263 750 769 1018 1438 2416 2706 2789 3594 4104 4769 5090 7028 7175 7256 7274 7521 7771 8206 8701 8860 9221 9540 10115 10143 10398 10873 11719 11914 11944 12693 12705 13590 13650 13803 14566 14766 15301 15326 15735 15784 16189 16223 16278 16290 16294 16309 16390	L2a1b1a	73 146 152 195 263 16189 16223 16278 16290 16294 16309 16390	[0.000] L2a1b1a (1)	[0.005] L2a1f (2) [0.000] L2a1+16189_(16192) L2a1b L2a1b1 L2a1b1a L2a1b2 L2a1f1 L2a1f1a L2a1f2 (10)	1
1267	>MF056747.1 Hc	73G 150T 199C 204C 263G 513d 514d 709A 750G 769A 789A 1018A 1438G 1804G 2706G 3010A 3106d 3107C 3505G 3693C 3918A 4017T 4029A 4216C 4232C 4769G 4977C 5460A 7028T 7624C 8206A 8614C 8701G 8860G 8974G 9248T 9540C 9986A 10398G 10813T 10873C 11719A 12661T 12705T 13497G 13518T 14016A 14259A 14766T 14905A 14935C 15301A 15326G 15344C 16179T 16183C 16189C 16223T 16239T 16311C 16320T 16362C 16519C	L4b1a	73G 150T 199C 204C 263G 513d 514d 16179T 16183C 16189C 16223T 16239T 16311C 16320T 16362C 16519C	1	73 150 199 204 263 513 709 750 769 789A 1018 1438 1804 2706 3010 3505 3693C 3918 4017 4029A 4216 4232 4769 4977 5460 7028 7624 8206 8614 8701 8860 8974G 9248 9540 9986 10398 10813 10873 11719 12661T 12705 13497 13518 14016 14259 14766 14905 14935 15301 15326 15344 16179 16189 16223 16239 16311 16320 16362	L4b1a	73 150 199 204 263 513 16179 16189 16223 16239 16311 16320 16362	[0.000] L4b1a (1)	[0.010] L4b1 (2)	1
1268	>MF056746.1 Hc	73G 150T 263G 489C 750G 2706G 3106d 3107C 3588T 4769G 7028T 7960G 8701G 8768T 8854A 8860G 9540C 10398G 10400T 10873C 11719A 12705T 13308G 13590A 13708A 14130T 14260G 14527G 14766T 14783C 15043A 15172A 15301A 15326G 15625T 16086C 16148T 16223T 16259T 16278T 16319A 16399G 16526A	M32c	73G 150T 263G 489C 16086C 16148T 16223T 16259T 16278T 16319A 16399G 16526A	1	73 150 263 489 750 2706 3588 4769 7028 7960 8701 8768 8854 8860 9540 10398 10400 10873 11719 12705 13308 13590 13708 14130 14260 14527 14766 14783 15043 15172 15301 15326 15625 16086 16148 16223 16259 16278 16319 16399 16526	M32c	73 150 263 489 16086 16148 16223 16259 16278 16319 16399 16526	[0.046] M32c (1)	[0.022] M51a (2) [0.000] M51 M51b M32 (5)	1
1269	>MF056739.1 Hc	73G 150T 200G 263G 489C 750G 2706G 3106d 3107C 3588T 4769G 6947T 7028T 7960G 8701G 8715C 8768T 8854A 8860G 9540C 10398G 10400T 10873C 11719A 11914A 12705T 13308G 13590A 13708A 14130T 14260G 14527G 14766T 14783C 15043A 15172A 15301A 15326G 15625T 16086C 16148T 16223T 16259T 16278T 16319A 16399G 16526A	M32c	73G 150T 200G 263G 489C 16086C 16148T 16223T 16259T 16278T 16319A 16399G 16526A	1	73 150 200 263 489 750 2706 3588 4769 6947 7028 7960 8701 8715 8768 8854 8860 9540 10398 10400 10873 11719 11914 12705 13308 13590 13708 14130 14260 14527 14766 14783 15043 15172 15301 15326 15625 16086 16148 16223 16259 16278 16319 16399 16526	M32c	73 150 200 263 489 16086 16148 16223 16259 16278 16319 16399 16526	[0.000] M32c (1)	[0.022] M51a (2) [0.007] M32 (3) [0.000] M51 M51b (5)	1

1270 >MF056737.1 Hc	73G 152C 195C 204C 263G 417A 489C 533G 750G 1438G 3106d 3107C 4769G 7028T 8188G 8360G 8701G 8860G 9438A 9540C 9545G 10142T 10295G 10398G 10400T 10873C 11569C 11719A 11899C 11963A 12279G 12618A 12705T 14766T 14783C 15025T 15043A 15301A 15326G 16223T 16263C 16311C 16519C	M23	73G 152C 195C 204C 263G 417A 489C 533G 16223T 16263C 16311C 16519C	1	73 152 195 204 263 417 489 533 750 1438 4769 7028 8188 8360 8701 8860 9438 9540 9545 10142 10295 10398 10400 10873 11569 11719 11899 11963 12279 12618 12705 14766 14783 15025 15043 15301 15326 16223 16263 16311	M23	73 152 195 204 263 417 489 533 16223 16263 16311 [0.000] M23 (1)	[0.072] M24a (2) [0.053] M57+152	1
1271 >MF056736.1 Hc	73G 150T 263G 489C 750G 2706G 3106d 3107C 3588T 4769G 7028T 7960G 8701G 8768T 8854A 8860G 9540C 10398G 10400T 10873C 11719A 12705T 13308G 13590A 13708A 14130T 14260G 14527G 14766T 14783C 15043A 15172A 15301A 15326G 15625T 16086C 16148T 16223T 16259T 16278T 16319A 16355T 16399G 16526A	M32c	73G 150T 263G 489C 16086C 16148T 16223T 16259T 16278T 16319A 16355T 16399G 16526A	1	73 150 263 489 750 2706 3588 4769 7028 7960 8701 8768 8854 8860 9540 10398 10400 10873 11719 12705 13308 13590 13708 14130 14260 14527 14766 14783 15043 15172 15301 15326 15625 16086 16148 16223 16259 16278 16319 16355 16399 16526	M32c	73 150 263 489 16086 16148 16223 16259 16278 16319 16355 16399 16526 [0.049] M32c (1)	[0.022] M51a (2) [0.000] M51 M51b M32 (5)	1
1272 >MF056727.1 Hc	73G 150T 263G 489C 750G 2706G 3106d 3107C 3588T 4769G 7028T 7960G 8701G 8768T 8854A 8860G 9540C 10398G 10400T 10873C 11719A 12705T 13308G 13590A 13708A 14130T 14260G 14527G 14766T 14783C 15043A 15172A 15301A 15326G 15625T 16086C 16148T 16223T 16259T 16278T 16319A 16399G 16526A	M32c	73G 150T 263G 489C 16086C 16148T 16223T 16259T 16278T 16319A 16399G 16526A	1	73 150 263 489 750 2706 3588 4769 7028 7960 8701 8768 8854 8860 9540 10398 10400 10873 11719 12705 13308 13590 13708 14130 14260 14527 14766 14783 15043 15172 15301 15326 15625 16086 16148 16223 16259 16278 16319 16399 16526	M32c	73 150 263 489 16086 16148 16223 16259 16278 16319 16399 16526 [0.046] M32c (1)	[0.022] M51a (2) [0.000] M51 M51b M32 (5)	1
1273 >MF056723.1 Hc	73G 152C 195C 204C 263G 417A 489C 533G 750G 1438G 3106d 3107C 4769G 7028T 8188G 8360G 8701G 8860G 9438A 9540C 9545G 10142T 10295G 10398G 10400T 10873C 11569C 11719A 11899C 12279G 12618A 12705T 14766T 14783C 15025T 15043A 15301A 15326G 16223T 16263C 16311C 16519C	M23	73G 152C 195C 204C 263G 417A 489C 533G 16223T 16263C 16311C 16519C	1	73 152 195 204 263 417 489 533 750 1438 4769 7028 8188 8360 8701 8860 9438 9540 9545 10142 10295 10398 10400 10873 11569 11719 11899 12279 12618 12705 14766 14783 15025 15043 15301 15326 16223 16263 16311	M23	73 152 195 204 263 417 489 533 16223 16263 16311 [0.000] M23 (1)	[0.072] M24a (2) [0.053] M57+152	1
1274 >MF056722.1 Hc	73G 150T 263G 489C 750G 2706G 3106d 3107C 3588T 4769G 7028T 7960G 8701G 8768T 8854A 8860G 9540C 10398G 10400T 10873C 11719A 12705T 13308G 13590A 13708A 14130T 14260G 14527G 14766T 14783C 15043A 15172A 15301A 15326G 15625T 16086C 16148T 16223T 16259T 16278T 16319A 16399G 16526A	M32c	73G 150T 263G 489C 16086C 16148T 16223T 16259T 16278T 16319A 16399G 16526A	1	73 150 263 489 750 2706 3588 4769 7028 7960 8701 8768 8854 8860 9540 10398 10400 10873 11719 12705 13308 13590 13708 14130 14260 14527 14766 14783 15043 15172 15301 15326 15625 16086 16148 16223 16259 16278 16319 16399 16526	M32c	73 150 263 489 16086 16148 16223 16259 16278 16319 16399 16526 [0.046] M32c (1)	[0.022] M51a (2) [0.000] M51 M51b M32 (5)	1

1275	>MF056715.1 Ho	73G 146C 152C 195C 263G 750G 769A 1018A 1438G 2416C 2706G 2789T 3106d 3107C 3594T 4104G 4769G 5090C 7028T 7175C 7256T 7274T 7521A 7771G 8206A 8701G 8860G 9221G 9540C 10115C 10143A 10398G 10873C 11719A 11914A 11944C 12693G 12705T 13590A 13650T 13803G 14566G 14766T 15301A 15326G 15735T 15784C 16182C 16183C 16189C 16223T 16278T 16290T 16294T 16309G 16390A	L2a1b1a	73G 146C 152C 195C 263G 16182C 16183C 16189C 16223T 16278T 16290T 16294T 16309G 16390A	1	73 146 152 195 263 750 769 1018 1438 2416 2706 2789 3594 4104 4769 5090 7028 7175 7256 7274 7521 7771 8206 8701 8860 9221 9540 10115 10143 10398 10873 11719 11914 11944 12693 12705 13590 13650 13803 14566 14766 15301 15326 15735 15784 16189 16223 16278 16290 16294 16309 16390	L2a1b1a	73 146 152 195 263 16189 16223 16278 16290 16294 16309 16390	[0.000] L2a1b1a (1)	[0.005] L2a1f (2) [0.000] L2a1+16189_(16192) L2a1b L2a1b1 L2a1b1a L2a1b2 L2a1f1 L2a1f1a L2a1f2 (10)	1
1276	>MF057585.1 Ho	73G 152C 195C 204C 263G 309.1CT 310C 417A 489C 533G 750G 1438G 1842G 3106d 3107C 4769G 7028T 8188G 8360G 8701G 8860G 9438A 9540C 9545G 10142T 10295G 10398G 10400T 10873C 11569C 11719A 11899C 12279G 12618A 12705T 14766T 14783C 15025T 15043A 15301A 15326G 16223T 16263C 16311C 16519C	M23	73G 152C 195C 204C 263G 309.1CT 310C 417A 489C 533G 16223T 16263C 16311C 16519C	1	73 152 195 204 263 417 489 533 750 1438 1842 4769 7028 8188 8360 8701 8860 9438 9540 9545 10142 10295 10398 10400 10873 11569 11719 11899 12279 12618 12705 14766 14783 15025 15043 15301 15326 16223 16263 16311	M23	73 152 195 204 263 417 489 533 16223 16263 16311	[0.000] M23 (1)	[0.072] M24a (2) [0.053] M57+152	1
1277	>MF057584.1 Ho	73G 152C 195C 204C 263G 310.1C 417A 489C 533G 750G 1438G 3106d 3107C 4769G 7028T 8188G 8360G 8701G 8860G 9438A 9540C 9545G 10142T 10295G 10398G 10400T 10873C 11176A 11569C 11719A 11899C 12279G 12618A 12705T 14766T 14783C 15025T 15043A 15301A 15326G 16223T 16263C 16311C 16519C	M23	73G 152C 195C 204C 263G 310.1C 417A 489C 533G 16223T 16263C 16311C 16519C	1	73 152 195 204 263 417 489 533 750 1438 4769 7028 8188 8360 8701 8860 9438 9540 9545 10142 10295 10398 10400 10873 11176 11569 11719 11899 12279 12618 12705 14766 14783 15025 15043 15301 15326 16223 16263 16311	M23	73 152 195 204 263 417 489 533 16223 16263 16311	[0.000] M23 (1)	[0.072] M24a (2) [0.053] M57+152	1
1278	>MF057580.1 Ho	73G 152C 195C 204C 263G 310.1C 417A 489C 533G 750G 1438G 3106d 3107C 4769G 7028T 8188G 8360G 8701G 8860G 9438A 9540C 9545G 10142T 10295G 10398G 10400T 10704A 10873C 11176A 11569C 11719A 11899C 12279G 12618A 12705T 14766T 14783C 15025T 15043A 15301A 15326G 16223T 16263C 16311C 16519C	M23	73G 152C 195C 204C 263G 310.1C 417A 489C 533G 16223T 16263C 16311C 16519C	1	73 152 195 204 263 417 489 533 750 1438 4769 7028 8188 8360 8701 8860 9438 9540 9545 10142 10295 10398 10400 10704 10873 11176 11569 11719 11899 12279 12618 12705 14766 14783 15025 15043 15301 15326 16223 16263 16311	M23	73 152 195 204 263 417 489 533 16223 16263 16311	[0.000] M23 (1)	[0.072] M24a (2) [0.053] M57+152	1

1279 >MF057579.1 Hc	73G 152C 195C 204C 263G 310.1C 417A 489C 533G 750G 1438G 3106d 3107C 4769G 7028T 8188G 8360G 8701G 8860G 9438A 9540C 9545G 10142T 10295G 10398G 10400T 10873C 11176A 11569C 11719A 11899C 12279G 12618A 12705T 14766T 14783C 15025T 15043A 15301A 15326G 16223T 16263C 16311C 16519C	M23	73G 152C 195C 204C 263G 310.1C 417A 489C 533G 16223T 16263C 16311C 16519C	1	73 152 195 204 263 417 489 533 750 1438 4769 7028 8188 8360 8701 8860 9438 9540 9545 10142 10295 10398 10400 10873 11176 11569 11719 11899 12279 12618 12705 14766 14783 15025 15043 15301 15326 16223 16263 16311	M23	73 152 195 204 263 417 489 533 16223 16263 16311	[0.000] M23 (1)	[0.072] M24a (2) [0.053] M57+152	1
1280 >MF057578.1 Hc	73G 152C 195C 204C 263G 310.1C 417A 489C 533G 750G 1438G 3106d 3107C 4769G 7028T 8188G 8360G 8701G 8860G 9438A 9540C 9545G 10142T 10295G 10398G 10400T 10873C 11176A 11569C 11719A 11899C 12279G 12618A 12705T 14766T 14783C 15025T 15043A 15301A 15326G 16223T 16263C 16311C 16519C	M23	73G 152C 195C 204C 263G 310.1C 417A 489C 533G 16223T 16263C 16311C 16519C	1	73 152 195 204 263 417 489 533 750 1438 4769 7028 8188 8360 8701 8860 9438 9540 9545 10142 10295 10398 10400 10873 11176 11569 11719 11899 12279 12618 12705 14766 14783 15025 15043 15301 15326 16223 16263 16311	M23	73 152 195 204 263 417 489 533 16223 16263 16311	[0.000] M23 (1)	[0.072] M24a (2) [0.053] M57+152	1
1281 >MF057576.1 Hc	73G 150T 200G 263G 310.1C 489C 750G 2706G 3106d 3107C 3588T 4769G 5231A 7028T 7960G 8701G 8768T 8854A 8860G 9540C 10398G 10400T 10873C 11719A 12705T 13308G 13590A 13708A 14130T 14260G 14527G 14766T 14783C 15043A 15172A 15301A 15326G 15625T 16086C 16148T 16192T 16223T 16259T 16278T 16319A 16399G 16526A	M32c	73G 150T 200G 263G 310.1C 489C 16086C 16148T 16192T 16223T 16259T 16278T 16319A 16399G 16526A	1	73 150 200 263 489 750 2706 3588 4769 5231 7028 7960 8701 8768 8854 8860 9540 10398 10400 10873 11719 12705 13308 13590 13708 14130 14260 14527 14766 14783 15043 15172 15301 15326 15625 16086 16148 16192 16223 16259 16278 16319 16399 16526	M32c	73 150 200 263 489 16086 16148 16192 16223 16259 16278 16319 16399 16526	[0.003] M32c (1)	[0.022] M51a (2) [0.007] M32 (3) [0.000] M51 M51b (5)	1
1282 >MF057556.1 Hc	73G 150T 200G 263G 310.1C 489C 750G 2706G 3106d 3107C 3588T 4769G 7028T 7960G 8701G 8768T 8854A 8860G 9540C 10398G 10400T 10873C 11719A 12705T 13308G 13590A 13708A 14130T 14260G 14527G 14766T 14783C 15043A 15172A 15301A 15326G 15625T 16086C 16148T 16223T 16259T 16278T 16311C 16319A 16399G 16526A	M32c	73G 150T 200G 263G 310.1C 489C 16086C 16148T 16223T 16259T 16278T 16311C 16319A 16399G 16526A	1	73 150 200 263 489 750 2706 3588 4769 7028 7960 8701 8768 8854 8860 9540 10398 10400 10873 11719 12705 13308 13590 13708 14130 14260 14527 14766 14783 15043 15172 15301 15326 15625 16086 16148 16223 16259 16278 16311 16319 16399 16526	M32c	73 150 200 263 489 16086 16148 16223 16259 16278 16311 16319 16399 16526	[0.007] M32c (1)	[0.000] M51b1 M51b1a (3)	1
1283 >MF057551.1 Hc	73G 152C 195C 204C 263G 310.1C 417A 489C 533G 750G 1438G 3106d 3107C 4769G 7028T 8188G 8360G 8701G 8860G 9438A 9540C 9545G 10142T 10295G 10398G 10400T 10873C 11569C 11719A 11899C 12279G 12618A 12705T 14766T 14783C 15025T 15043A 15301A 15326G 16223T 16263C 16311C 16519C	M23	73G 152C 195C 204C 263G 310.1C 417A 489C 533G 16223T 16263C 16311C 16519C	1	73 152 195 204 263 417 489 533 750 1438 4769 7028 8188 8360 8701 8860 9438 9540 9545 10142 10295 10398 10400 10873 11569 11719 11899 12279 12618 12705 14766 14783 15025 15043 15301 15326 16223 16263 16311	M23	73 152 195 204 263 417 489 533 16223 16263 16311	[0.000] M23 (1)	[0.072] M24a (2) [0.053] M57+152	1

1284	>MF057546.1 Hc	73G 152C 195C 204C 263G 310.1C 417A 489C 533G 750G 1438G 3106d 3107C 4769G 7028T 8188G 8360G 8701G 8860G 9438A 9540C 9545G 10142T 10295G 10398G 10400T 10873C 11176A 11569C 11719A 11899C 12279G 12618A 12705T 14766T 14783C 15025T 15043A 15301A 15326G 16223T 16263C 16311C 16519C	M23	73G 152C 195C 204C 263G 310.1C 417A 489C 533G 16223T 16263C 16311C 16519C	1	73 152 195 204 263 417 489 533 750 1438 4769 7028 8188 8360 8701 8860 9438 9540 9545 10142 10295 10398 10400 10873 11176 11569 11719 11899 12279 12618 12705 14766 14783 15025 15043 15301 15326 16223 16263 16311	M23	73 152 195 204 263 417 489 533 16223 16263 16311	[0.000] M23 (1)	[0.072] M24a (2) [0.053] M57+152	1
1285	>MF057543.1 Hc	73G 152C 195C 204C 263G 310.1C 417A 489C 533G 750G 1438G 3106d 3107C 3394C 4769G 7028T 8188G 8360G 8701G 8860G 9438A 9540C 9545G 10142T 10295G 10398G 10400T 10873C 11569C 11719A 11899C 12279G 12618A 12705T 14766T 14783C 15025T 15043A 15301A 15326G 16223T 16263C 16311C 16519C	M23	73G 152C 195C 204C 263G 310.1C 417A 489C 533G 16223T 16263C 16311C 16519C	1	73 152 195 204 263 417 489 533 750 1438 3394 4769 7028 8188 8360 8701 8860 9438 9540 9545 10142 10295 10398 10400 10873 11569 11719 11899 12279 12618 12705 14766 14783 15025 15043 15301 15326 16223 16263 16311	M23	73 152 195 204 263 417 489 533 16223 16263 16311	[0.000] M23 (1)	[0.072] M24a (2) [0.053] M57+152	1
1286	>MF057538.1 Hc	73G 152C 195C 204C 263G 310.1C 417A 489C 533G 750G 1438G 3106d 3107C 4769G 7028T 8188G 8360G 8701G 8860G 9438A 9540C 9545G 10142T 10295G 10398G 10400T 10873C 11569C 11719A 11899C 12279G 12618A 12705T 14766T 14783C 15025T 15043A 15301A 15326G 16223T 16263C 16311C 16519C	M23	73G 152C 195C 204C 263G 310.1C 417A 489C 533G 16223T 16263C 16311C 16519C	1	73 152 195 204 263 417 489 533 750 1438 4769 7028 8188 8360 8701 8860 9438 9540 9545 10142 10295 10398 10400 10873 11569 11719 11899 12279 12618 12705 14766 14783 15025 15043 15301 15326 16223 16263 16311	M23	73 152 195 204 263 417 489 533 16223 16263 16311	[0.000] M23 (1)	[0.072] M24a (2) [0.053] M57+152	1
1287	>MF057532.1 Hc	73G 146C 152C 195C 263G 750G 769A 1018A 1438G 2416C 2706G 2789T 3106d 3107C 3594T 4104G 4769G 5090C 7028T 7175C 7256T 7274T 7521A 7771G 8206A 8701G 8860G 9221G 9540C 10115C 10143A 10398G 10873C 11719A 11914A 11944C 12693G 12705T 13590A 13650T 13803G 14566G 14766T 15301A 15326G 15735T 15784C 15907G 16182C 16183C 16189C 16223T 16278T 16290T 16294T 16309G 16390A 16519C	L2a1b1a	73G 146C 152C 195C 263G 16182C 16183C 16189C 16223T 16278T 16290T 16294T 16309G 16390A 16519C	1	73 146 152 195 263 750 769 1018 1438 2416 2706 2789 3594 4104 4769 5090 7028 7175 7256 7274 7521 7771 8206 8701 8860 9221 9540 10115 10143 10398 10873 11719 11914 11944 12693 12705 13590 13650 13803 14566 14766 15301 15326 15735 15784 15907 16189 16223 16278 16290 16294 16309 16390	L2a1b1a	73 146 152 195 263 16189 16223 16278 16290 16294 16309 16390	[0.000] L2a1b1a (1)	[0.005] L2a1f (2) [0.000] L2a1+16189_(16192) L2a1b L2a1b1 L2a1b1a L2a1b2 L2a1f1 L2a1f1a L2a1f2 (10)	1

1288	>MF057527.1 Ho	73G 152C 182T 186A 189C 195C 247A 248G 263G 310.1C 316A 514d 515d 745.1T 750G 769A 825A 1018A 1438G 2394d 2706G 2758A 2885C 3106d 3107C 3525T 3594T 3666A 4104G 4769G 4853A 5951G 6071C 6221A 6227C 6917A 7028T 7146G 7247T 7256T 7389C 7521A 8027A 8468T 8655T 8701G 8860G 9072G 9540C 10398G 10586A 10688A 10810C 10873C 11302T 11719A 11984C 12507G 12705T 12810G 13105G 13485G 13506T 13650T 13789C 14000A 14178C 14560A 14669G 14766T 14911T 14926G 15226G 15326G 15905C 15978T 16129A 16187T 16189C 16223T 16278T 16293G 16294T 16311C 16360T 16519C	L1c3c	73G 152C 182T 186A 189C 195C 247A 248G 263G 310.1C 316A 514d 515d 16129A 16187T 16189C 16223T 16278T 16293G 16294T 16311C 16360T 16519C	1	73 152 182 186A 189C 195 247 248 263 316 745.1T 750 769 825A 1018 1438 2395d 2706 2758 2885 3525 3594 3666 4104 4769 4853 5951 6071 6221A 6227 6917 7028 7146 7247 7256 7389 7521 8027 8468 8655 8701 8860 9072 9540 10398 10586 10688 10810 10873 11302 11719 11984 12507 12705 12810 13105 13485 13506 13650 13789 14000A 14178 14560 14669 14766 14911 14926 15226 15326 15905 15978 16129 16187 16189 16223 16278 16293 16294 16311 16360	L1c3c	73 152 182 186A 189C 195 247 248 263 316 16129 16187 16189 16223 16278 16293 16294 16311 16360	[2.063] L1c3c (1) [0.000] L1c L1c1a+@198 L1c3b'c (4)	[0.700] L1c3 (5) [0.572] L1c1a'b'd (6) [0.267] L1c3b1 (7) [0.133] L1c3b1b (8) [0.000] L1c1'2'4'5'6 L1c1 L1c1a L1c1a1 L1c3b (13)	1
1289	>MF057513.1 Ho	73G 150T 200G 263G 310.1C 489C 750G 2706G 3106d 3107C 3588T 4769G 7028T 7960G 8701G 8768T 8854A 8860G 9540C 10398G 10400T 10873C 11719A 12705T 13308G 13590A 13708A 14130T 14260G 14527G 14766T 14783C 15043A 15172A 15301A 15326G 15625T 16086C 16148T 16223T 16259T 16278T 16319A 16399G 16526A	M32c	73G 150T 200G 263G 310.1C 489C 16086C 16148T 16223T 16259T 16278T 16319A 16399G 16526A	1	73 150 200 263 489 750 2706 3588 4769 7028 7960 8701 8768 8854 8860 9540 10398 10400 10873 11719 12705 13308 13590 13708 14130 14260 14527 14766 14783 15043 15172 15301 15326 15625 16086 16148 16223 16259 16278 16319 16399 16526	M32c	73 150 200 263 489 16086 16148 16223 16259 16278 16319 16399 16526	[0.000] M32c (1)	[0.022] M51a (2) [0.007] M32 (3) [0.000] M51 M51b (5)	1
1290	>MF057512.1 Ho	73G 152C 182T 186A 189C 195C 247A 248G 263G 310.1C 316A 514d 515d 745.1T 750G 769A 825A 1018A 1438G 2394d 2706G 2758A 2885C 3106d 3107C 3204T 3525T 3594T 3666A 4104G 4769G 4853A 5951G 6071C 6221A 6227C 6917A 7028T 7146G 7247T 7256T 7389C 7521A 8027A 8468T 8655T 8701G 8860G 9072G 9540C 10398G 10586A 10688A 10810C 10873C 11302T 11719A 11984C 12507G 12705T 12810G 13105G 13485G 13506T 13650T 13789C 14000A 14178C 14560A 14669G 14766T 14911T 14926G 15226G 15326G 15905C 15978T 16129A 16187T 16189C 16223T 16278T 16293G 16294T 16311C 16360T 16519C	L1c3c	73G 152C 182T 186A 189C 195C 247A 248G 263G 310.1C 316A 514d 515d 16129A 16187T 16189C 16223T 16278T 16293G 16294T 16311C 16360T 16519C	1	73 152 182 186A 189C 195 247 248 263 316 745.1T 750 769 825A 1018 1438 2395d 2706 2758 2885 3204 3525 3594 3666 4104 4769 4853 5951 6071 6221A 6227 6917 7028 7146 7247 7256 7389 7521 8027 8468 8655 8701 8860 9072 9540 10398 10586 10688 10810 10873 11302 11719 11984 12507 12705 12810 13105 13485 13506 13650 13789 14000A 14178 14560 14669 14766 14911 14926 15226 15326 15905 15978 16129 16187 16189 16223 16278 16293 16294 16311 16360	L1c3c	73 152 182 186A 189C 195 247 248 263 316 16129 16187 16189 16223 16278 16293 16294 16311 16360	[2.063] L1c3c (1) [0.000] L1c L1c1a+@198 L1c3b'c (4)	[0.700] L1c3 (5) [0.572] L1c1a'b'd (6) [0.267] L1c3b1 (7) [0.133] L1c3b1b (8) [0.000] L1c1'2'4'5'6 L1c1 L1c1a L1c1a1 L1c3b (13)	1

1291 >MF057493.1 Hc	73G 150T 200G 263G 310.1C 489C 750G 2706G 3106d 3107C 3588T 4769G 7028T 7960G 8701G 8768T 8854A 8860G 8995T 9540C 10398G 10400T 10873C 11719A 12705T 13308G 13590A 13708A 14130T 14260G 14527G 14766T 14783C 15043A 15172A 15301A 15326G 15625T 16086C 16148T 16223T 16259T 16278T 16319A 16399G 16526A	M32c	73G 150T 200G 263G 310.1C 489C 16086C 16148T 16223T 16259T 16278T 16319A 16399G 16526A	1	73 150 200 263 489 750 2706 3588 4769 7028 7960 8701 8768 8854 8860 8995T 9540 10398 10400 10873 11719 12705 13308 13590 13708 14130 14260 14527 14766 14783 15043 15172 15301 15326 15625 16086 16148 16223 16259 16278 16319 16399 16526	M32c	73 150 200 263 489 16086 16148 16223 16259 16278 16319 16399 16526	[0.000] M32c (1)	[0.022] M51a (2) [0.007] M32 (3) [0.000] M51 M51b (5)	1
1292 >MF056153.1 Hc	73G 152C 195C 204C 263G 417A 489C 533G 750G 1438G 3106d 3107C 4769G 7028T 8188G 8360G 8701G 8860G 9438A 9540C 9545G 10142T 10295G 10398G 10400T 10873C 11569C 11719A 11899C 12279G 12618A 12705T 14766T 14783C 15025T 15043A 15301A 15326G 16223T 16263C 16311C 16519C	M23	73G 152C 195C 204C 263G 417A 489C 533G 16223T 16263C 16311C 16519C	1	73 152 195 204 263 417 489 533 750 1438 4769 7028 8188 8360 8701 8860 9438 9540 9545 10142 10295 10398 10400 10873 11569 11719 11899 12279 12618 12705 14766 14783 15025 15043 15301 15326 16223 16263 16311	M23	73 152 195 204 263 417 489 533 16223 16263 16311	[0.000] M23 (1)	[0.072] M24a (2) [0.053] M57+152	1
1293 >MF056151.1 Hc	73G 146C 152C 182T 185T 189G 247A 263G 357G 514d 515d 709A 710C 750G 769A 825A 1018A 1738C 2352C 2706G 2758A 2768G 2885C 3106d 3107C 3308C 3594T 3666A 3693A 4104G 4769G 6548T 6827C 6989G 7028T 7055G 7146G 7256T 7389C 7521A 7867T 8248G 8282T 8468T 8655T 8701G 8860G 9540C 10398G 10688A 10810C 10873C 11719A 12171G 12519C 12705T 12891T 13105G 13506T 13602C 13650T 13789C 13893G 14178C 14323A 14560A 14766T 14769G 15115C 15326G 16111T 16126C 16187T 16189C 16223T 16239T 16270T 16278T 16293G 16311C 16519C	L1b2a	73G 146C 152C 182T 185T 189G 247A 263G 357G 514d 515d 16111T 16126C 16187T 16189C 16223T 16239T 16270T 16278T 16293G 16311C 16519C	1	73 146 152 182 185T 189 247 263 357 709 710 750 769 825A 1018 1738 2352 2706 2758 2768 2885 3308 3594 3666 3693 4104 4769 6548 6827 6989 7028 7055 7146 7256 7389 7521 7867 8248 8282 8468 8655 8701 8860 9540 10398 10688 10810 10873 11719 12171 12519 12705 12891 13105 13506 13602 13650 13789 13893 14178 14323 14560 14766 14769 15115 15326 16111 16126 16187 16189 16223 16239 16270 16278 16293 16311	L1b2a	73 146 152 182 185T 189 247 263 357 16111 16126 16187 16189 16223 16239 16270 16278 16293 16311	[0.000] L1b2a (1)	[0.042] L1b2 (2)	1
1294 >MF056147.1 Hc	73G 150T 200G 263G 489C 750G 2706G 3106d 3107C 3588T 4769G 7028T 7960G 8701G 8768T 8854A 8860G 9540C 10398G 10400T 10873C 11719A 12705T 13308G 13590A 13708A 14130T 14260G 14527G 14766T 14783C 15043A 15172A 15301A 15326G 15625T 16086C 16148T 16223T 16259T 16278T 16319A 16399G 16526A	M32c	73G 150T 200G 263G 489C 16086C 16148T 16223T 16259T 16278T 16319A 16399G 16526A	1	73 150 200 263 489 750 2706 3588 4769 7028 7960 8701 8768 8854 8860 9540 10398 10400 10873 11719 12705 13308 13590 13708 14130 14260 14527 14766 14783 15043 15172 15301 15326 15625 16086 16148 16223 16259 16278 16319 16399 16526	M32c	73 150 200 263 489 16086 16148 16223 16259 16278 16319 16399 16526	[0.000] M32c (1)	[0.022] M51a (2) [0.007] M32 (3) [0.000] M51 M51b (5)	1

1295	>MF056145.1 Hc	73G 146C 152C 195C 263G 750G 769A 1018A 1438G 2416C 2706G 2789T 3106d 3107C 3594T 4104G 4769G 5090C 7028T 7175C 7256T 7274T 7521A 7771G 8206A 8701G 8860G 9221G 9540C 10115C 10143A 10398G 10873C 11719A 11914A 11944C 12693G 12705T 13590A 13650T 13803G 14566G 14766T 15301A 15326G 15735T 15784C 16182C 16183C 16189C 16223T 16278T 16290T 16294T 16309G 16390A	L2a1b1a	73G 146C 152C 195C 263G 16182C 16183C 16189C 16223T 16278T 16290T 16294T 16309G 16390A	1	73 146 152 195 263 750 769 1018 1438 2416 2706 2789 3594 4104 4769 5090 7028 7175 7256 7274 7521 7771 8206 8701 8860 9221 9540 10115 10143 10398 10873 11719 11914 11944 12693 12705 13590 13650 13803 14566 14766 15301 15326 15735 15784 16189 16223 16278 16290 16294 16309 16390	L2a1b1a	73 146 152 195 263 16189 16223 16278 16290 16294 16309 16390	[0.000] L2a1b1a (1)	[0.005] L2a1f (2) [0.000] L2a1+16189_(16192) L2a1b L2a1b1 L2a1b1a L2a1b2 L2a1f1 L2a1f1a L2a1f2 (10)	1
1296	>MF056135.1 Hc	73G 152C 195C 204C 263G 417A 489C 533G 750G 1438G 3106d 3107C 4769G 7028T 8188G 8360G 8701G 8860G 9438A 9540C 9545G 10142T 10295G 10398G 10400T 10873C 11569C 11719A 11899C 12279G 12618A 12705T 14766T 14783C 15025T 15043A 15301A 15326G 16223T 16263C 16311C 16519C	M23	73G 152C 195C 204C 263G 417A 489C 533G 16223T 16263C 16311C 16519C	1	73 152 195 204 263 417 489 533 750 1438 4769 7028 8188 8360 8701 8860 9438 9540 9545 10142 10295 10398 10400 10873 11569 11719 11899 12279 12618 12705 14766 14783 15025 15043 15301 15326 16223 16263 16311	M23	73 152 195 204 263 417 489 533 16223 16263 16311	[0.000] M23 (1)	[0.072] M24a (2) [0.053] M57+152	1
1297	>MF056133.1 Hc	73G 152C 195C 204C 263G 417A 489C 533G 750G 1438G 3106d 3107C 4769G 7028T 8188G 8360G 8701G 8860G 9438A 9540C 9545G 10142T 10295G 10398G 10400T 10873C 11569C 11719A 11899C 12279G 12618A 12705T 14766T 14783C 15025T 15043A 15299C 15301A 15326G 16051G 16176T 16223T 16263C 16311C 16519C	M23	73G 152C 195C 204C 263G 417A 489C 533G 16051G 16176T 16223T 16263C 16311C 16519C	1	73 152 195 204 263 417 489 533 750 1438 4769 7028 8188 8360 8701 8860 9438 9540 9545 10142 10295 10398 10400 10873 11569 11719 11899 12279 12618 12705 14766 14783 15025 15043 15299 15301 15326 16051 16176 16223 16263 16311	M23	73 152 195 204 263 417 489 533 16051 16176 16223 16263 16311	[0.040] M23 (1)	[0.072] M24a (2) [0.053] M57+152	1
1298	>MF056131.1 Hc	64T 93G 185A 189G 200G 236C 247A 263G 310C 514d 515d 750G 769A 825A 1018A 1048T 1438G 2245C 2706G 2758A 2885C 3106d 3107C 3516A 3594T 3866C 4104G 4312T 4586C 4769G 5096C 5231A 5442C 5460A 5603T 6185C 7028T 7146G 7256T 7521A 8428T 8468T 8566G 8655T 8701G 8860G 9042T 9347G 9540C 9755A 9818T 10237C 10398G 10589A 10664T 10688A 10810C 10873C 10915C 11176A 11641G 11719A 11914A 12007A 12237d 12705T 12720G 13105G 13276G 13506T 13650T 14308C 14766T 15136T 15326G 15431A 16129A 16148T 16168T 16172C 16184d 16185d 16223T 16230G 16311C 16320T	L0a1a2	64T 93G 185A 189G 200G 236C 247A 263G 310C 514d 515d 16129A 16148T 16168T 16172C 16184d 16185d 16223T 16230G 16311C 16320T	1	64 93 185 189 200 236 247 263 310 750 769 825A 1018 1048 1438 2245C 2706 2758 2885 3516A 3594 3866 4104 4312 4586 4769 5096 5231 5442 5460 5603 6185 7028 7146 7256 7521 8428 8468 8566 8655 8701 8860 9042 9347 9540 9755 9818 10237 10398 10589 10664 10688 10810 10873 10915 11176 11641 11719 11914 12007 12241d 12705 12720 13105 13276 13506 13650 14308 14766 15136 15326 15431 16129 16148 16168 16172 16187 16189 16192-16193d 16223 16230 16311 16320	L0a1a2	64 93 185 189 200 236 247 263 310 16129 16148 16168 16172 16187 16189 16192-16193d 16223 16230 16311 16320	[0.173] L0a1a2 (1)	[0.529] L0a1a3 (2) [0.173] L0a1a2 (3) [0.065] L0a1a+200 (4) [0.000] L0a1a1	1

1299 >MF056115.1 Ho	73G 150T 200G 263G 489C 750G 2706G 3106d 3107C 3588T 4682G 4769G 7028T 7960G 8701G 8768T 8854A 8860G 9540C 10398G 10400T 10873C 11719A 12705T 13308G 13590A 13708A 14130T 14260G 14527G 14766T 14783C 15043A 15172A 15301A 15326G 15625T 16086C 16148T 16223T 16259T 16278T 16319A 16399G 16526A	M32c	73G 150T 200G 263G 489C 16086C 16148T 16223T 16259T 16278T 16319A 16399G 16526A	1	73 150 200 263 489 750 2706 3588 4682 4769 7028 7960 8701 8768 8854 8860 9540 10398 10400 10873 11719 12705 13308 13590 13708 14130 14260 14527 14766 14783 15043 15172 15301 15326 15625 16086 16148 16223 16259 16278 16319 16399 16526	M32c	73 150 200 263 489 16086 16148 16223 16259 16278 16319 16399 16526	[0.000] M32c (1)	[0.022] M51a (2) [0.007] M32 (3) [0.000] M51 M51b (5)	1
1300 >MF056111.1 Ho	73G 150T 200G 263G 489C 750G 2706G 3106d 3107C 3588T 4769G 7028T 7960G 8701G 8768T 8854A 8860G 9540C 10398G 10400T 10873C 11719A 12705T 13308G 13590A 13708A 14130T 14260G 14527G 14766T 14783C 15043A 15172A 15301A 15326G 15625T 16086C 16148T 16223T 16278T 16319A 16399G 16526A	M32c	73G 150T 200G 263G 489C 16086C 16148T 16223T 16278T 16319A 16399G 16526A	1	73 150 200 263 489 750 2706 3588 4769 7028 7960 8701 8768 8854 8860 9540 10398 10400 10873 11719 12705 13308 13590 13708 14130 14260 14527 14766 14783 15043 15172 15301 15326 15625 16086 16148 16223 16278 16319 16399 16526	M32c	73 150 200 263 489 16086 16148 16223 16278 16319 16399 16526	[0.010] M32c (1)	[0.022] M51a (2) [0.007] M32 (3) [0.000] M51 M51b (5)	1
1301 >MF056102.1 Ho	73G 150T 200G 263G 489C 750G 2706G 3106d 3107C 3588T 4769G 5744N 5745N 5746N 5747N 5750N 7028T 7960G 8701G 8768T 8854A 8860G 9540C 10398G 10400T 10873C 11719A 12705T 13308G 13590A 13708A 14130T 14260G 14527G 14766T 14783C 15043A 15172A 15301A 15326G 15625T 16086C 16148T 16223T 16278T 16319A 16399G 16526A	M32c	73G 150T 200G 263G 489C 16086C 16148T 16223T 16278T 16319A 16399G 16526A	1	73 150 200 263 489 750 2706 3588 4769 5744N 5745N 5746N 5747N 5750N 7028 7960 8701 8768 8854 8860 9540 10398 10400 10873 11719 12705 13308 13590 13708 14130 14260 14527 14766 14783 15043 15172 15301 15326 15625 16086 16148 16223 16278 16319 16399 16526	M32c	73 150 200 263 489 16086 16148 16223 16278 16319 16399 16526	[0.010] M32c (1)	[0.022] M51a (2) [0.007] M32 (3) [0.000] M51 M51b (5)	1
1302 >MF056100.1 Ho	73G 146C 152C 195C 263G 750G 769A 1018A 1438G 2416C 2706G 2789T 3106d 3107C 3594T 4104G 4769G 5090C 7028T 7175C 7256T 7274T 7521A 7771G 8206A 8701G 8860G 9221G 9540C 10115C 10143A 10398G 10873C 11719A 11914A 11944C 12693G 12705T 13590A 13650T 13803G 14566G 14766T 15301A 15326G 15735T 15784C 16183C 16189C 16223T 16278T 16290T 16294T 16309G 16390A	L2a1b1a	73G 146C 152C 195C 263G 16183C 16189C 16223T 16278T 16290T 16294T 16309G 16390A	1	73 146 152 195 263 750 769 1018 1438 2416 2706 2789 3594 4104 4769 5090 7028 7175 7256 7274 7521 7771 8206 8701 8860 9221 9540 10115 10143 10398 10873 11719 11914 11944 12693 12705 13590 13650 13803 14566 14766 15301 15326 15735 15784 16189 16223 16278 16290 16294 16309 16390	L2a1b1a	73 146 152 195 263 16189 16223 16278 16290 16294 16309 16390	[0.000] L2a1b1a (1)	[0.005] L2a1f (2) [0.000] L2a1+16189_(16192) L2a1b L2a1b1 L2a1b1a L2a1b2 L2a1f1 L2a1f1a L2a1f2 (10)	1

1303	>MF056099.1 Hc	73G 146C 152C 195C 263G 750G 769A 1018A 1438G 2416C 2706G 2789T 3106d 3107C 3594T 4104G 4769G 5090C 7028T 7175C 7256T 7274T 7521A 7771G 8206A 8701G 8860G 9221G 9540C 10115C 10143A 10398G 10873C 11719A 11914A 11944C 12693G 12705T 13590A 13650T 13803G 14566G 14766T 15301A 15326G 15735T 15784C 16182C 16183C 16189C 16223T 16278T 16290T 16294T 16309G 16390A	L2a1b1a	73G 146C 152C 195C 263G 16182C 16183C 16189C 16223T 16278T 16290T 16294T 16309G 16390A	1	73 146 152 195 263 750 769 1018 1438 2416 2706 2789 3594 4104 4769 5090 7028 7175 7256 7274 7521 7771 8206 8701 8860 9221 9540 10115 10143 10398 10873 11719 11914 11944 12693 12705 13590 13650 13803 14566 14766 15301 15326 15735 15784 16189 16223 16278 16290 16294 16309 16390	L2a1b1a	73 146 152 195 263 16189 16223 16278 16290 16294 16309 16390	[0.000] L2a1b1a (1)	[0.005] L2a1f (2) [0.000] L2a1+16189_(16192) L2a1b L2a1b1 L2a1b1a L2a1b2 L2a1f1 L2a1f1a L2a1f2 (10)	1
1304	>MF056095.1 Hc	73G 152C 195C 204C 263G 417A 489C 533G 750G 1438G 3106d 3107C 4769G 7028T 8188G 8360G 8701G 8860G 9438A 9540C 9545G 10142T 10295G 10398G 10400T 10873C 11569C 11719A 11899C 12279G 12618A 12705T 14766T 14783C 15025T 15043A 15301A 15326G 16223T 16263C 16311C 16519C	M23	73G 152C 195C 204C 263G 417A 489C 533G 16223T 16263C 16311C 16519C	1	73 152 195 204 263 417 489 533 750 1438 4769 7028 8188 8360 8701 8860 9438 9540 9545 10142 10295 10398 10400 10873 11569 11719 11899 12279 12618 12705 14766 14783 15025 15043 15301 15326 16223 16263 16311	M23	73 152 195 204 263 417 489 533 16223 16263 16311	[0.000] M23 (1)	[0.072] M24a (2) [0.053] M57+152	1
1305	>MF056082.1 Hc	73G 150T 200G 263G 489C 750G 2706G 3106d 3107C 3588T 4769G 7028T 7960G 8701G 8768T 8854A 8860G 9540C 10398G 10400T 10873C 11719A 12705T 13308G 13590A 13708A 14130T 14260G 14527G 14766T 14783C 15043A 15172A 15301A 15326G 15625T 16086C 16148T 16223T 16259T 16278T 16319A 16399G 16526A	M32c	73G 150T 200G 263G 489C 16086C 16148T 16223T 16259T 16278T 16319A 16399G 16526A	1	73 150 200 263 489 750 2706 3588 4769 7028 7960 8701 8768 8854 8860 9540 10398 10400 10873 11719 12705 13308 13590 13708 14130 14260 14527 14766 14783 15043 15172 15301 15326 15625 16086 16148 16223 16259 16278 16319 16399 16526	M32c	73 150 200 263 489 16086 16148 16223 16259 16278 16319 16399 16526	[0.000] M32c (1)	[0.022] M51a (2) [0.007] M32 (3) [0.000] M51 M51b (5)	1
1306	>MF056080.1 Hc	73G 152d 200G 263G 489C 750G 2706G 3106d 3107C 3588T 4769G 7028T 7960G 8158G 8701G 8768T 8854A 8860G 9540C 10398G 10400T 10873C 11719A 12705T 12963N 12964N 12966N 12967N 12968N 13308G 13590A 13708A 14130T 14260G 14527G 14766T 14783C 14915N 14916N 14917N 14918N 14919N 14920N 15043A 15172A 15301A 15326G 15625T 16086C 16148T 16223T 16259T 16278T 16319A 16399G 16526A	M32c	73G 152d 200G 263G 489C 16086C 16148T 16223T 16259T 16278T 16319A 16399G 16526A	1	73 152d 200 263 489 750 2706 3588 4769 7028 7960 8158 8701 8768 8854 8860 9540 10398 10400 10873 11719 12705 12963N 12964N 12966N 12967N 12968N 13308 13590 13708 14130 14260 14527 14766 14783 14915N 14916N 14917N 14918N 14919N 14920N 15043 15172 15301 15326 15625 16086 16148 16223 16259 16278 16319 16399 16526	M32c	73 152d 200 263 489 16086 16148 16223 16259 16278 16319 16399 16526	[0.030] M32c (1)	[0.007] M32 (2)	1

1307	>MF056074.1 Ho	73G 152C 195C 204C 263G 417A 489C 533G 750G 1438G 3106d 3107C 4769G 7028T 8188G 8360G 8701G 8860G 9438A 9540C 9545G 10142T 10295G 10398G 10400T 10873C 11569C 11719A 11899C 12279G 12618A 12705T 14766T 14783C 15025T 15043A 15301A 15326G 16223T 16263C 16311C 16519C	M23	73G 152C 195C 204C 263G 417A 489C 533G 16223T 16263C 16311C 16519C	1	73 152 195 204 263 417 489 533 750 1438 4769 7028 8188 8360 8701 8860 9438 9540 9545 10142 10295 10398 10400 10873 11569 11719 11899 12279 12618 12705 14766 14783 15025 15043 15301 15326 16223 16263 16311	M23	73 152 195 204 263 417 489 533 16223 16263 16311	[0.000] M23 (1)	[0.072] M24a (2) [0.053] M57+152	1
1308	>MF056072.1 Ho	73G 152C 195C 204C 263G 417A 489C 533G 750G 1438G 3106d 3107C 4769G 7028T 8188G 8360G 8701G 8860G 9438A 9540C 9545G 10142T 10295G 10398G 10400T 10873C 11569C 11719A 11899C 12279G 12618A 12705T 14766T 14783C 15025T 15043A 15301A 15326G 16223T 16263C 16311C 16519C	M23	73G 152C 195C 204C 263G 417A 489C 533G 16223T 16263C 16311C 16519C	1	73 152 195 204 263 417 489 533 750 1438 4769 7028 8188 8360 8701 8860 9438 9540 9545 10142 10295 10398 10400 10873 11569 11719 11899 12279 12618 12705 14766 14783 15025 15043 15301 15326 16223 16263 16311	M23	73 152 195 204 263 417 489 533 16223 16263 16311	[0.000] M23 (1)	[0.072] M24a (2) [0.053] M57+152	1
1309	>MF058459.1 Ho	73G 152C 195C 263G 310.1C 417A 489C 533G 750G 1438G 3106d 3107C 4769G 7028T 8188G 8360G 8701G 8860G 9438A 9540C 9545G 10142T 10295G 10398G 10400T 10873C 11569C 11719A 11899C 12279G 12618A 12705T 14766T 14783C 15025T 15043A 15301A 15326G 16223T 16263C 16311C 16519C	M23	73G 152C 195C 263G 310.1C 417A 489C 533G 16223T 16263C 16311C 16519C	1	73 152 195 263 417 489 533 750 1438 4769 7028 8188 8360 8701 8860 9438 9540 9545 10142 10295 10398 10400 10873 11569 11719 11899 12279 12618 12705 14766 14783 15025 15043 15301 15326 16223 16263 16311	M23	73 152 195 263 417 489 533 16223 16263 16311	[0.016] M23 (1)	[0.031] M57+152 (2) [0.005] M24a	1
1310	>MF058451.1 Ho	73G 152C 195C 204C 263G 310.1C 417A 489C 533G 750G 1438G 3106d 3107C 4769G 7028T 8188G 8360G 8701G 8860G 9438A 9540C 9545G 10142T 10295G 10398G 10400T 10873C 11569C 11719A 11899C 12279G 12618A 12705T 14766T 14783C 15025T 15043A 15301A 15326G 16223T 16263C 16311C 16519C	M23	73G 152C 195C 204C 263G 310.1C 417A 489C 533G 16223T 16263C 16311C 16519C	1	73 152 195 204 263 417 489 533 750 1438 4769 7028 8188 8360 8701 8860 9438 9540 9545 10142 10295 10398 10400 10873 11569 11719 11899 12279 12618 12705 14766 14783 15025 15043 15301 15326 16223 16263 16311	M23	73 152 195 204 263 417 489 533 16223 16263 16311	[0.000] M23 (1)	[0.072] M24a (2) [0.053] M57+152	1
1311	>MF058447.1 Ho	73G 152C 195C 204C 263G 417A 489C 533G 750G 1438G 3106d 3107C 4769G 7028T 8188G 8360G 8701G 8860G 9438A 9540C 9545G 10142T 10295G 10398G 10400T 10873C 11569C 11719A 11899C 12279G 12618A 12705T 14766T 14783C 15025T 15043A 15301A 15326G 16223T 16263C 16311C 16362C 16519C	M23	73G 152C 195C 204C 263G 417A 489C 533G 16223T 16263C 16311C 16362C 16519C	1	73 152 195 204 263 417 489 533 750 1438 4769 7028 8188 8360 8701 8860 9438 9540 9545 10142 10295 10398 10400 10873 11569 11719 11899 12279 12618 12705 14766 14783 15025 15043 15301 15326 16223 16263 16311 16362	M23	73 152 195 204 263 417 489 533 16223 16263 16311 16362	[0.016] M23 (1)	[0.002] G2c (2) [0.000] D4h4a	1

1312 >MF058441.1 Ho	73G 151T 152C 182T 195C 215G 263G 309.1CT 310C 511T 513.1CACA 537T 750G 769A 1018A 1438G 1900G 2416C 2626C 2706G 3106d 3107C 3594T 3654T 4104G 4769G 4823C 5263T 5316A 6040C 6366A 6497C 7028T 7175C 7256T 7521A 8206A 8701G 8860G 9221G 9540C 10115C 10398G 10873C 11009C 11719A 11944C 12705T 12879C 12967G 13590A 13650T 13810A 14766T 14890G 15301A 15326G 15862C 16223T 16224C 16265C 16278T 16291T 16295T 16309G 16390A 16519C	L2a5	73G 151T 152C 182T 195C 215G 263G 309.1CT 310C 511T 513.1CACA 537T 16223T 16224C 16265C 16278T 16291T 16295T 16309G 16390A 16519C	1	73 151 152 182 195 215 263 511 537 750 769 1018 1438 1900 2416 2626 2706 3594 3654 4104 4769 4823 5263 5316 6040C 6366 6497 7028 7175 7256 7521 8206 8701 8860 9221 9540 10115 10398 10873 11009 11719 11944 12705 12879 12967 13590 13650 13810 14766 14890 15301 15326 15862 16223 16224 16265C 16278 16291 16295 16309 16390	L2a5	73 151 152 182 195 215 263 511 537 16223 16224 16265C 16278 16291 16295 16309 16390	[2.563] L2a5 (1) [0.200] L2a (2)	[0.100] L2a1'2'3'4 (3) [0.020] L2a1e1 (4) [0.008] L2a1 (5) [0.000] L2a'b'c'd L2a1a L2b'c'd (8)	1
1313 >MF058439.1 Ho	73G 152C 195C 204C 263G 417A 489C 533G 750G 1438G 3106d 3107C 4769G 7028T 8188G 8360G 8701G 8860G 9438A 9540C 9545G 10142T 10295G 10398G 10400T 10873C 11569C 11719A 11899C 12279G 12618A 12705T 14766T 14783C 15025T 15043A 15301A 15326G 16223T 16263C 16311C 16362C 16519C	M23	73G 152C 195C 204C 263G 417A 489C 533G 16223T 16263C 16311C 16362C 16519C	1	73 152 195 204 263 417 489 533 750 1438 4769 7028 8188 8360 8701 8860 9438 9540 9545 10142 10295 10398 10400 10873 11569 11719 11899 12279 12618 12705 14766 14783 15025 15043 15301 15326 16223 16263 16311 16362	M23	73 152 195 204 263 417 489 533 16223 16263 16311 16362	[0.016] M23 (1)	[0.002] G2c (2) [0.000] D4h4a	1
1314 >MF058435.1 Ho	73G 152C 182T 186A 189C 195C 247A 248G 263G 310.1C 316A 514d 515d 745.1T 750G 769A 825A 1018A 1438G 2394d 2706G 2758A 2885C 3106d 3107C 3525T 3594T 3666A 4104G 4769G 4853A 5951G 6071C 6221A 6227C 6917A 7028T 7146G 7247T 7256T 7389C 7521A 8027A 8468T 8655T 8701G 8860G 9072G 9540C 10398G 10586A 10688A 10810C 10873C 11302T 11719A 11984C 12507G 12705T 12810G 13105G 13485G 13506T 13650T 13789C 14000A 14178C 14560A 14669G 14766T 14911T 14926G 15226G 15326G 15905C 15978T 16129A 16187T 16189C 16223T 16278T 16293G 16294T 16311C 16360T 16519C	L1c3c	73G 152C 182T 186A 189C 195C 247A 248G 263G 310.1C 316A 514d 515d 16129A 16187T 16189C 16223T 16278T 16293G 16294T 16311C 16360T 16519C	1	73 152 182 186A 189C 195 247 248 263 316 745.1T 750 769 825A 1018 1438 2395d 2706 2758 2885 3525 3594 3666 4104 4769 4853 5951 6071 6221A 6227 6917 7028 7146 7247 7256 7389 7521 8027 8468 8655 8701 8860 9072 9540 10398 10586 10688 10810 10873 11302 11719 11984 12507 12705 12810 13105 13485 13506 13650 13789 14000A 14178 14560 14669 14766 14911 14926 15226 15326 15905 15978 16129 16187 16189 16223 16278 16293 16294 16311 16360	L1c3c	73 152 182 186A 189C 195 247 248 263 316 16129 16187 16189 16223 16278 16293 16294 16311 16360	[2.063] L1c3c (1) [0.000] L1c L1c1a+@198 L1c3b'c (4)	[0.700] L1c3 (5) [0.572] L1c1a'b'd (6) [0.267] L1c3b1 (7) [0.133] L1c3b1b (8) [0.000] L1c1'2'4'5'6 L1c1 L1c1a L1c1a1 L1c3b (13)	1

1315 >MF058430.1 Hc	73G 146C 152C 195C 263G 310.1C 750G 769A 1018A 1438G 2416C 2706G 2789T 3106d 3107C 3594T 4104G 4769G 5090C 7028T 7175C 7256T 7274T 7521A 7771G 8206A 8701G 8860G 9221G 9540C 10115C 10143A 10398G 10873C 11719A 11914A 11944C 12693G 12705T 13590A 13650T 13803G 14566G 14766T 15301A 15326G 15735T 15784C 16183C 16189C 16223T 16278T 16290T 16294T 16309G 16390A	L2a1b1a	73G 146C 152C 195C 263G 310.1C 16183C 16189C 16223T 16278T 16290T 16294T 16309G 16390A	1	73 146 152 195 263 750 769 1018 1438 2416 2706 2789 3594 4104 4769 5090 7028 7175 7256 7274 7521 7771 8206 8701 8860 9221 9540 10115 10143 10398 10873 11719 11914 11944 12693 12705 13590 13650 13803 14566 14766 15301 15326 15735 15784 16189 16223 16278 16290 16294 16309 16390	L2a1b1a	73 146 152 195 263 16189 16223 16278 16290 16294 16309 16390	[0.000] L2a1b1a (1)	[0.005] L2a1f (2) [0.000] L2a1+16189_(16192) L2a1b L2a1b1 L2a1b1a L2a1b2 L2a1f1 L2a1f1a L2a1f2 (10)	1
1316 >MF058425.1 Hc	73G 152C 195C 204C 263G 310.1C 417A 489C 533G 750G 1438G 3106d 3107C 4769G 7028T 8188G 8360G 8701G 8860G 9438A 9540C 9545G 10142T 10295G 10398G 10400T 10873C 11569C 11719A 11899C 12279G 12618A 12705T 14766T 14783C 15025T 15043A 15301A 15326G 16223T 16263C 16311C 16519C	M23	73G 152C 195C 204C 263G 310.1C 417A 489C 533G 16223T 16263C 16311C 16519C	1	73 152 195 204 263 417 489 533 750 1438 4769 7028 8188 8360 8701 8860 9438 9540 9545 10142 10295 10398 10400 10873 11569 11719 11899 12279 12618 12705 14766 14783 15025 15043 15301 15326 16223 16263 16311	M23	73 152 195 204 263 417 489 533 16223 16263 16311	[0.000] M23 (1)	[0.072] M24a (2) [0.053] M57+152	1
1317 >MF058422.1 Hc	73G 151T 152C 182T 186A 189C 247A 263G 309.1CT 310C 316A 514d 515d 629C 750G 769A 825A 1018A 1438G 2283T 2394d 2706G 2758A 2885C 3106d 3107C 3210T 3434G 3594T 3666A 4104G 4755C 4769G 5951G 6071C 6221A 6917A 7028T 7146G 7256T 7389C 7521A 7897A 8027A 8251A 8417T 8468T 8655T 8701G 8860G 9072G 9540C 9581C 10398G 10586A 10688A 10810C 10873C 11302T 11317G 11719A 12400G 12542T 12705T 12810G 13105G 13485G 13506T 13650T 13708A 13789C 13981T 14000A 14178C 14560A 14766T 14794T 14911T 15226G 15326G 15905C 15978T 16017C 16129A 16163G 16187T 16189C 16209C 16223T 16278T 16293G 16294T 16311C 16360T 16519C	L1c3b1a	73G 151T 152C 182T 186A 189C 247A 263G 309.1CT 310C 316A 514d 515d 16129A 16163G 16187T 16189C 16209C 16223T 16278T 16293G 16294T 16311C 16360T 16519C	1	73 151 152 182 186A 189C 247 263 316 629 750 769 825A 1018 1438 2283 2395d 2706 2758 2885 3210 3434 3594 3666 4104 4755 4769 5951 6071 6221A 6917 7028 7146 7256 7389 7521 7897 8027 8251 8417 8468 8655 8701 8860 9072 9540 9581 10398 10586 10688 10810 10873 11302 11317 11719 12400 12542 12705 12810 13105 13485 13506 13650 13708 13789 13981 14000A 14178 14560 14766 14794 14911 15226 15326 15905 15978 16017 16129 16163 16187 16189 16209 16223 16278 16293 16294 16311 16360	L1c3b1a	73 151 152 182 186A 189C 247 263 316 16129 16163 16187 16189 16209 16223 16278 16293 16294 16311 16360	[0.000] L1c3b1a (1)	[0.000] L1c3b L1c3b1 L1c3b1b (4)	1

1318 >MF058419.1 Hc	73G 146C 152C 195C 198T 228A 247A 310.1C 456T 494d 514d 515d 719A 750G 769A 825A 1018A 1048T 2000T 2758A 2885C 3083C 3106d 3107C 3438A 3516A 3594T 3756G 4104G 4197T 4232C 4312T 4769G 5263T 5442C 5773A 6060C 6185C 6266G 6815C 7028T 7146G 7256T 7265G 7521A 8113A 8152A 8251A 8468T 8655T 8701G 8860G 9042T 9144T 9150G 9347G 9540C 9755A 10373A 10398G 10589A 10664T 10688A 10810C 10873C 10915C 11150A 11437C 11719A 11809C 11914A 12007A 12121C 12235C 12615G 12705T 12720G 13105G 13129T 13276G 13506T 13650T 13759A 14224G 14384A 14766T 15187G 15326G 15466A 15930A 15941C 15951G 16187T 16189C 16223T 16230G 16234T 16243C 16311C	L0d1c	73G 146C 152C 195C 198T 228A 247A 310.1C 456T 494d 514d 515d 16187T 16189C 16223T 16230G 16234T 16243C 16311C	1	73 146 152 195 198 228 247 456 498d 719 750 769 825A 1018 1048 2000 2758 2885 3083 3438 3516A 3594 3756 4104 4197 4232 4312 4769 5263 5442 5773 6060C 6185 6266 6815 7028 7146 7256 7265 7521 8113A 8152 8251 8468 8655 8701 8860 9042 9144 9150 9347 9540 9755 10373 10398 10589 10664 10688 10810 10873 10915 11150 11437 11719 11809 11914 12007 12121 12235 12615 12705 12720 13105 13129 13276 13506 13650 13759 14224 14384 14766 15187 15326 15466 15930 15941 15951 16187 16189 16223 16230 16234 16243 16311	L0d1c	73 146 152 195 198 228 247 456 498d 16187 16189 16223 16230 16234 16243 16311	[0.148] L0d1c (1)	[0.009] L0d1c1a1 L0d1c1a1a2 (3) [0.006] L0d1c1a1a (4) [0.000] L0d1c1 L0d1c1a L0d1c1a1b L0d1c2 (8)	1
1319 >MF058414.1 Hc	73G 151T 152C 182T 186A 189C 195C 198T 247A 263G 297G 309.1CT 310C 316A 750G 769A 825A 1018A 1420C 1438G 2150.1A 2394d 2706G 2758A 2885C 3106d 3107C 3594T 3666A 4104G 4769G 5894.1C 5951G 6071C 6150A 6253C 7028T 7055G 7076G 7146G 7256T 7337A 7389C 7521A 7744C 8027A 8251A 8468T 8655T 8701G 8784G 8860G 8877C 9072G 9540C 10321C 10398G 10586A 10688A 10792G 10793T 10810C 10873C 11654G 11719A 12049T 12705T 12810G 13105G 13149G 13212T 13281C 13485G 13506T 13650T 13789C 14000A 14178C 14560A 14766T 14812T 14911T 15016T 15326G 15784C 16071T 16129A 16145A 16187T 16189C 16213A 16223T 16234T 16265C 16278T 16286G 16294T 16311C 16360T 16527T	L1c2a1a	73G 151T 152C 182T 186A 189C 195C 198T 247A 263G 297G 309.1CT 310C 316A 16071T 16129A 16145A 16187T 16189C 16213A 16223T 16234T 16265C 16278T 16286G 16294T 16311C 16360T 16527T	1	73 151 152 182 186A 189C 195 198 247 263 297 316 750 769 825A 1018 1420 1438 2156.1A 2395d 2706 2758 2885 3594 3666 4104 4769 5899.1C 5951 6071 6150 6253 7028 7055 7076 7146 7256 7337 7389 7521 7744 8027 8251 8468 8655 8701 8784 8860 8877 9072 9540 10321 10398 10586 10688 10792 10793 10810 10873 11654 11719 12049 12705 12810 13105 13149 13212 13281 13485 13506 13650 13789 14000A 14178 14560 14766 14812 14911 15016 15326 15784 16071 16129 16145 16187 16189 16213 16223 16234 16265C 16278 16286G 16294 16311 16360 16527	L1c2a1a	73 151 152 182 186A 189C 195 198 247 263 297 316 16071 16129 16145 16187 16189 16213 16223 16234 16265C 16278 16286G 16294 16311 16360 16527	[0.000] L1c2a1a (1)	[0.105] L1c2a1b (2) [0.000] L1c2a1	1

1320 >MF058411.1 Hc	73G 189G 200G 263G 272G 309.1CT 310C 750G 1438G 1822C 2706G 3106d 3107C 3396C 4218C 4769G 5601T 7028T 7819A 8410T 8527G 8701G 8860G 8932T 9540C 9950C 10070T 10398G 10873C 11440A 11719A 12705T 14766T 14769G 15301A 15326G 15514C 15940d 16129A 16209C 16223T 16292T 16295T 16311C 16519C	L3f1b1a1	73G 189G 200G 263G 272G 309.1CT 310C 16129A 16209C 16223T 16292T 16295T 16311C 16519C	1	73 189 200 263 272 750 1438 1822 2706 3396 4218 4769 5601 7028 7819A 8410 8527 8701 8860 8932 9540 9950 10070 10398 10873 11440 11719 12705 14766 14769 15301 15326 15514 15944d 16129 16209 16223 16292 16295 16311	L3f1b1a1	73 189 200 263 272 16129 16209 16223 16292 16295 16311	[0.000] L3f1b1a1 (1)	[0.000] L3f1b1a L3f1b1a1 (3)	1
1321 >MF058408.1 Hc	73G 152C 195C 204C 263G 310.1C 417A 489C 533G 750G 1438G 3106d 3107C 4769G 7028T 8188G 8360G 8701G 8860G 9007G 9438A 9540C 9545G 10142T 10295G 10398G 10400T 10873C 11569C 11719A 11899C 12279G 12618A 12705T 14766T 14783C 15025T 15043A 15301A 15326G 16223T 16263C 16311C 16519C	M23	73G 152C 195C 204C 263G 310.1C 417A 489C 533G 16223T 16263C 16311C 16519C	1	73 152 195 204 263 417 489 533 750 1438 4769 7028 8188 8360 8701 8860 9007 9438 9540 9545 10142 10295 10398 10400 10873 11569 11719 11899 12279 12618 12705 14766 14783 15025 15043 15301 15326 16223 16263 16311	M23	73 152 195 204 263 417 489 533 16223 16263 16311	[0.000] M23 (1)	[0.072] M24a (2) [0.053] M57+152	1
1322 >MF058396.1 Hc	73G 146C 152C 195C 263G 310.1C 750G 769A 1018A 1438G 2416C 2706G 2762T 2789T 3106d 3107C 3594T 4104G 4769G 5090C 7028T 7175C 7256T 7274T 7521A 7771G 8206A 8701G 8860G 9221G 9540C 10115C 10143A 10398G 10873C 11719A 11914A 11944C 12693G 12705T 13590A 13650T 13803G 14566G 14766T 15301A 15326G 15735T 15784C 16182C 16183C 16189C 16223T 16278T 16290T 16294T 16390A	L2a1b1a	73G 146C 152C 195C 263G 310.1C 16182C 16183C 16189C 16223T 16278T 16290T 16294T 16390A	1	73 146 152 195 263 750 769 1018 1438 2416 2706 2762 2789 3594 4104 4769 5090 7028 7175 7256 7274 7521 7771 8206 8701 8860 9221 9540 10115 10143 10398 10873 11719 11914 11944 12693 12705 13590 13650 13803 14566 14766 15301 15326 15735 15784 16189 16223 16278 16290 16294 16390	L2a1b1a	73 146 152 195 263 16189 16223 16278 16290 16294 16390	[0.045] L2a1b1a (1)	[0.234] L2a1+143+16189_(16192)+@16309 (2) [0.070] L2a1f (3) [0.045] L2a1b1a (4) [0.008] L2a1b2 (5) [0.000] L2a1+16189_(16192) L2a1b L2a1b1 L2a1f1 L2a1f1a L2a1f2 (11)	1
1323 >MF058389.1 Hc	73G 146C 152C 195C 263G 310.1C 750G 769A 1018A 1438G 2416C 2706G 2789T 3106d 3107C 3594T 4104G 4769G 5090C 7028T 7175C 7256T 7274T 7521A 7771G 8206A 8701G 8860G 9221G 9540C 10115C 10143A 10398G 10873C 11719A 11914A 11944C 12693G 12705T 13590A 13650T 13803G 14566G 14766T 15301A 15326G 15735T 15784C 16182C 16183C 16189C 16192T 16223T 16278T 16290T 16294T 16309G 16390A	L2a1b1a	73G 146C 152C 195C 263G 310.1C 16182C 16183C 16189C 16192T 16223T 16278T 16290T 16294T 16309G 16390A	1	73 146 152 195 263 750 769 1018 1438 2416 2706 2789 3594 4104 4769 5090 7028 7175 7256 7274 7521 7771 8206 8701 8860 9221 9540 10115 10143 10398 10873 11719 11914 11944 12693 12705 13590 13650 13803 14566 14766 15301 15326 15735 15784 16189 16192 16223 16278 16290 16294 16309 16390	L2a1b1a	73 146 152 195 263 16189 16192 16223 16278 16290 16294 16309 16390	[0.000] L2a1b1a (1)	[0.015] L2a1b1a (2) [0.005] L2a1f (3) [0.000] L2a1+16189_(16192) L2a1b L2a1b1 L2a1b2 L2a1f1 L2a1f1a L2a1f2 (10)	1

1324	>MF058381.1 Hc	73G 150T 200G 263G 310.1C 489C 750G 2706G 3106d 3107C 3588T 4769G 7028T 7960G 8701G 8768T 8854A 8860G 9540C 10398G 10400T 10873C 11719A 12705T 13308G 13590A 13708A 14130T 14260G 14527G 14766T 14783C 15043A 15172A 15301A 15326G 15625T 16086C 16148T 16223T 16259T 16278T 16319A 16399G 16526A	M32c	73G 150T 200G 263G 310.1C 489C 16086C 16148T 16223T 16259T 16278T 16319A 16399G 16526A	1	73 150 200 263 489 750 2706 3588 4769 7028 7960 8701 8768 8854 8860 9540 10398 10400 10873 11719 12705 13308 13590 13708 14130 14260 14527 14766 14783 15043 15172 15301 15326 15625 16086 16148 16223 16259 16278 16319 16399 16526	M32c	73 150 200 263 489 16086 16148 16223 16259 16278 16319 16399 16526	[0.000] M32c (1)	[0.022] M51a (2) [0.007] M32 (3) [0.000] M51 M51b (5)	1
1325	>MF058375.1 Hc	73G 263G 279C 310.1C 514d 515d 750G 1438G 2332T 2706G 3106d 3107C 3450T 4769G 5773A 6221C 6340T 7028T 8701G 8860G 9449T 9540C 10086G 10373A 10398G 10873C 11002G 11719A 12705T 13105G 13914A 14766T 15301A 15311G 15326G 15824G 15940d 16093C 16145A 16223T 16278T 16362C 16519C	L3b1a8	73G 263G 279C 310.1C 514d 515d 16093C 16145A 16223T 16278T 16362C 16519C	1	73 263 279 750 1438 2332 2706 3450 4769 5773 6221 6340 7028 8701 8860 9449 9540 10086 10373 10398 10873 11002 11719 12705 13105 13914A 14766 15301 15311 15326 15824 15944d 16093 16145 16223 16278 16362	L3b1a8	73 263 279 16093 16145 16223 16278 16362	[0.232] L3b1a8 (1)	[0.322] L3b1a+@16124 (2) [0.000] L3b1a7 L3b1a7a (4)	1
1326	>MF058374.1 Hc	73G 146C 152C 195C 263G 310.1C 750G 769A 1018A 1438G 2416C 2706G 2762T 2789T 3106d 3107C 3594T 4104G 4769G 5090C 7028T 7175C 7256T 7274T 7521A 7771G 8206A 8701G 8860G 9221G 9540C 10115C 10143A 10398G 10873C 11719A 11914A 11944C 12693G 12705T 13590A 13650T 13803G 14566G 14766T 15301A 15326G 15735T 15784C 16182C 16183C 16189C 16223T 16278T 16290T 16294T 16390A	L2a1b1a	73G 146C 152C 195C 263G 310.1C 16182C 16183C 16189C 16223T 16278T 16290T 16294T 16390A	1	73 146 152 195 263 750 769 1018 1438 2416 2706 2762 2789 3594 4104 4769 5090 7028 7175 7256 7274 7521 7771 8206 8701 8860 9221 9540 10115 10143 10398 10873 11719 11914 11944 12693 12705 13590 13650 13803 14566 14766 15301 15326 15735 15784 16189 16223 16278 16290 16294 16390	L2a1b1a	73 146 152 195 263 16189 16223 16278 16290 16294 16390	[0.045] L2a1b1a (1)	[0.234] L2a1+143+16189_(16192)+@ 16309 (2) [0.070] L2a1f (3) [0.045] L2a1b1a (4) [0.008] L2a1b2 (5) [0.000] L2a1+16189_(16192) L2a1b L2a1b1 L2a1f1 L2a1f1a L2a1f2 (11)	1
1327	>MF058359.1 Hc	73G 152C 195C 204C 263G 310.1C 417A 489C 533G 567.1C 750G 1438G 3106d 3107C 4769G 7028T 8188G 8360G 8701G 8860G 8964T 9438A 9540C 9545G 10142T 10295G 10398G 10400T 10873C 11569C 11719A 11899C 12279G 12618A 12705T 14766T 14783C 15025T 15043A 15301A 15326G 16223T 16263C 16311C 16519C	M23	73G 152C 195C 204C 263G 310.1C 417A 489C 533G 567.1C 16223T 16263C 16311C 16519C	1	73 152 195 204 263 417 489 533 573.1C 750 1438 4769 7028 8188 8360 8701 8860 8964 9438 9540 9545 10142 10295 10398 10400 10873 11569 11719 11899 12279 12618 12705 14766 14783 15025 15043 15301 15326 16223 16263 16311	M23	73 152 195 204 263 417 489 533 573.1C 16223 16263 16311	[0.000] M23 (1)	[0.095] M10a1 (2) [0.072] M24a (3) [0.056] M10 (4) [0.053] M57+152 (5) [0.007] M10a	1

1328	>MF058354.1 Hc	73G 152C 195C 204C 263G 310.1C 417A 489C 533G 750G 1438G 3106d 3107C 4769G 7028T 8188G 8360G 8701G 8860G 9438A 9540C 9545G 10142T 10295G 10398G 10400T 10873C 11569C 11719A 11899C 12279G 12618A 12705T 14766T 14783C 15025T 15043A 15301A 15326G 16223T 16263C 16311C 16519C	M23	73G 152C 195C 204C 263G 310.1C 417A 489C 533G 16223T 16263C 16311C 16519C	1	73 152 195 204 263 417 489 533 750 1438 4769 7028 8188 8360 8701 8860 9438 9540 9545 10142 10295 10398 10400 10873 11569 11719 11899 12279 12618 12705 14766 14783 15025 15043 15301 15326 16223 16263 16311	M23	73 152 195 204 263 417 489 533 16223 16263 16311	[0.000] M23 (1)	[0.072] M24a (2) [0.053] M57+152	1
1329	>MF058353.1 Hc	73G 152C 195C 204C 263G 310.1C 417A 489C 533G 750G 1438G 3106d 3107C 4769G 5420C 7028T 8188G 8360G 8701G 8860G 9438A 9540C 9545G 10142T 10295G 10398G 10400T 10873C 11569C 11719A 11899C 12279G 12618A 12705T 14766T 14783C 15025T 15043A 15301A 15326G 16223T 16263C 16311C 16519C	M23	73G 152C 195C 204C 263G 310.1C 417A 489C 533G 16223T 16263C 16311C 16519C	1	73 152 195 204 263 417 489 533 750 1438 4769 5420 7028 8188 8360 8701 8860 9438 9540 9545 10142 10295 10398 10400 10873 11569 11719 11899 12279 12618 12705 14766 14783 15025 15043 15301 15326 16223 16263 16311	M23	73 152 195 204 263 417 489 533 16223 16263 16311	[0.000] M23 (1)	[0.072] M24a (2) [0.053] M57+152	1
1330	>MF058345.1 Hc	73G 146C 152C 195C 263G 310.1C 750G 769A 1018A 1438G 2416C 2706G 2789T 3106d 3107C 3594T 4104G 4769G 5090C 7028T 7175C 7256T 7274T 7521A 7771G 8206A 8701G 8860G 9221G 9540C 10115C 10143A 10398G 10873C 11719A 11914A 11944C 12693G 12705T 13590A 13650T 13803G 14566G 14766T 15301A 15326G 15735T 15784C 16183C 16189C 16223T 16278T 16290T 16294T 16390A	L2a1b1a	73G 146C 152C 195C 263G 310.1C 16183C 16189C 16223T 16278T 16290T 16294T 16390A	1	73 146 152 195 263 750 769 1018 1438 2416 2706 2789 3594 4104 4769 5090 7028 7175 7256 7274 7521 7771 8206 8701 8860 9221 9540 10115 10143 10398 10873 11719 11914 11944 12693 12705 13590 13650 13803 14566 14766 15301 15326 15735 15784 16189 16223 16278 16290 16294 16390	L2a1b1a	73 146 152 195 263 16189 16223 16278 16290 16294 16390	[0.045] L2a1b1a (1)	[0.234] L2a1+143+16189_(16192)+@16309 (2) [0.070] L2a1f (3) [0.045] L2a1b1a (4) [0.008] L2a1b2 (5) [0.000] L2a1+16189_(16192) L2a1b L2a1b1 L2a1f1 L2a1f1a L2a1f2 (11)	1
1331	>MF058336.1 Hc	73G 152C 195C 204C 263G 310.1C 417A 489C 533G 750G 1438G 3106d 3107C 4769G 7028T 8188G 8360G 8701G 8860G 9438A 9540C 9545G 10142T 10295G 10398G 10400T 10873C 11569C 11719A 11899C 12279G 12618A 12705T 14766T 14783C 15025T 15043A 15301A 15326G 16223T 16263C 16311C 16519C	M23	73G 152C 195C 204C 263G 310.1C 417A 489C 533G 16223T 16263C 16311C 16519C	1	73 152 195 204 263 417 489 533 750 1438 4769 7028 8188 8360 8701 8860 9438 9540 9545 10142 10295 10398 10400 10873 11569 11719 11899 12279 12618 12705 14766 14783 15025 15043 15301 15326 16223 16263 16311	M23	73 152 195 204 263 417 489 533 16223 16263 16311	[0.000] M23 (1)	[0.072] M24a (2) [0.053] M57+152	1
1332	>MF058332.1 Hc	73G 150T 200G 263G 310.1C 489C 750G 2706G 3106d 3107C 3588T 4769G 7028T 7960G 8701G 8768T 8854A 8860G 9540C 10398G 10400T 10873C 11719A 12705T 12813T 13308G 13590A 13708A 14130T 14260G 14527G 14766T 14783C 15043A 15172A 15301A 15326G 15625T 16086C 16148T 16223T 16259T 16264T 16278T 16319A 16399G 16526A	M32c	73G 150T 200G 263G 310.1C 489C 16086C 16148T 16223T 16259T 16264T 16278T 16319A 16399G 16526A	1	73 150 200 263 489 750 2706 3588 4769 7028 7960 8701 8768 8854 8860 9540 10398 10400 10873 11719 12705 12813 13308 13590 13708 14130 14260 14527 14766 14783 15043 15172 15301 15326 15625 16086 16148 16223 16259 16264 16278 16319 16399 16526	M32c	73 150 200 263 489 16086 16148 16223 16259 16264 16278 16319 16399 16526	[0.017] M32c (1)	[0.027] M51a (2) [0.007] M51 M51b M32 (5)	1

1333 >MF058329.1 Hc	73G 152C 195C 204C 263G 310.1C 417A 489C 533G 750G 1438G 3106d 3107C 4767G 4769G 7028T 8188G 8360G 8701G 8860G 9438A 9540C 9545G 10142T 10295G 10398G 10400T 10873C 11569C 11719A 11899C 12279G 12618A 12705T 14766T 14783C 15025T 15043A 15301A 15326G 16183C 16189C 16223T 16263C 16311C 16519C	M23	73G 152C 195C 204C 263G 310.1C 417A 489C 533G 16183C 16189C 16223T 16263C 16311C 16519C	1	73 152 195 204 263 417 489 533 750 1438 4767 4769 7028 8188 8360 8701 8860 9438 9540 9545 10142 10295 10398 10400 10873 11569 11719 11899 12279 12618 12705 14766 14783 15025 15043 15301 15326 16189 16223 16263 16311	M23	73 152 195 204 263 417 489 533 16189 16223 16263 16311	[0.008] M23 (1)	[0.239] M1a5 (2) [0.119] D6c (3) [0.072] M24a (4) [0.057] M57+152 (5) [0.012] D6c1 D6c1a (7) [0.000] M1a2a	1
1334 >MF058327.1 Hc	73G 150T 200G 263G 310.1C 489C 750G 2706G 3106d 3107C 3588T 4769G 7028T 7960G 8701G 8768T 8854A 8860G 9540C 10398G 10400T 10873C 11719A 12705T 12813T 13308G 13590A 13708A 14130T 14260G 14527G 14766T 14783C 15043A 15172A 15301A 15326G 15625T 16086C 16148T 16223T 16259T 16264T 16278T 16319A 16399G 16526A	M32c	73G 150T 200G 263G 310.1C 489C 16086C 16148T 16223T 16259T 16264T 16278T 16319A 16399G 16526A	1	73 150 200 263 489 750 2706 3588 4769 7028 7960 8701 8768 8854 8860 9540 10398 10400 10873 11719 12705 12813 13308 13590 13708 14130 14260 14527 14766 14783 15043 15172 15301 15326 15625 16086 16148 16223 16259 16264 16278 16319 16399 16526	M32c	73 150 200 263 489 16086 16148 16223 16259 16264 16278 16319 16399 16526	[0.017] M32c (1)	[0.027] M51a (2) [0.007] M51 M51b M32 (5)	1
1335 >MF058325.1 Hc	73G 150T 200G 263G 310.1C 489C 750G 2706G 3106d 3107C 3588T 4769G 7028T 7960G 8701G 8768T 8854A 8860G 9540C 10398G 10400T 10873C 11719A 12705T 12813T 13308G 13590A 13708A 14130T 14260G 14527G 14766T 14783C 15043A 15172A 15301A 15326G 15625T 16086C 16148T 16223T 16259T 16264T 16278T 16319A 16399G 16526A	M32c	73G 150T 200G 263G 310.1C 489C 16086C 16148T 16223T 16259T 16264T 16278T 16319A 16399G 16526A	1	73 150 200 263 489 750 2706 3588 4769 7028 7960 8701 8768 8854 8860 9540 10398 10400 10873 11719 12705 12813 13308 13590 13708 14130 14260 14527 14766 14783 15043 15172 15301 15326 15625 16086 16148 16223 16259 16264 16278 16319 16399 16526	M32c	73 150 200 263 489 16086 16148 16223 16259 16264 16278 16319 16399 16526	[0.017] M32c (1)	[0.027] M51a (2) [0.007] M51 M51b M32 (5)	1
1336 >MF058324.1 Hc	73G 150T 200G 263G 310.1C 489C 750G 2706G 3106d 3107C 3588T 4769G 7028T 7960G 8701G 8768T 8854A 8860G 9540C 10398G 10400T 10873C 11719A 12705T 12813T 13308G 13590A 13708A 14130T 14260G 14527G 14766T 14783C 15043A 15172A 15301A 15326G 15625T 16086C 16148T 16223T 16259T 16264T 16278T 16319A 16399G 16526A	M32c	73G 150T 200G 263G 310.1C 489C 16086C 16148T 16223T 16259T 16264T 16278T 16319A 16399G 16526A	1	73 150 200 263 489 750 2706 3588 4769 7028 7960 8701 8768 8854 8860 9540 10398 10400 10873 11719 12705 12813 13308 13590 13708 14130 14260 14527 14766 14783 15043 15172 15301 15326 15625 16086 16148 16223 16259 16264 16278 16319 16399 16526	M32c	73 150 200 263 489 16086 16148 16223 16259 16264 16278 16319 16399 16526	[0.017] M32c (1)	[0.027] M51a (2) [0.007] M51 M51b M32 (5)	1

1337	>MF058319.1 Hc	73G 150T 200G 263G 310.1C 489C 750G 2706G 3106d 3107C 3588T 4769G 7028T 7960G 8701G 8768T 8854A 8860G 9540C 10398G 10400T 10873C 11719A 12705T 12813T 13308G 13590A 13708A 14130T 14260G 14527G 14766T 14783C 15043A 15172A 15301A 15326G 15625T 16086C 16148T 16223T 16259T 16264T 16278T 16319A 16399G 16526A	M32c	73G 150T 200G 263G 310.1C 489C 16086C 16148T 16223T 16259T 16264T 16278T 16319A 16399G 16526A	1	73 150 200 263 489 750 2706 3588 4769 7028 7960 8701 8768 8854 8860 9540 10398 10400 10873 11719 12705 12813 13308 13590 13708 14130 14260 14527 14766 14783 15043 15172 15301 15326 15625 16086 16148 16223 16259 16264 16278 16319 16399 16526	M32c	73 150 200 263 489 16086 16148 16223 16259 16264 16278 16319 16399 16526	[0.017] M32c (1)	[0.027] M51a (2) [0.007] M51 M51b M32 (5)	1
1338	>MF058311.1 Hc	73G 150T 152C 200G 263G 310.1C 489C 750G 2706G 3106d 3107C 3588T 4769G 6482T 7028T 7960G 8158G 8701G 8768T 8854A 8860G 9540C 10398G 10400T 10873C 11719A 12705T 13308G 13590A 13708A 14130T 14260G 14527G 14766T 14783C 15043A 15172A 15301A 15326G 15625T 16086C 16148T 16223T 16259T 16278T 16319A 16399G 16526A	M32c	73G 150T 152C 200G 263G 310.1C 489C 16086C 16148T 16223T 16259T 16278T 16319A 16399G 16526A	1	73 150 152 200 263 489 750 2706 3588 4769 6482 7028 7960 8158 8701 8768 8854 8860 9540 10398 10400 10873 11719 12705 13308 13590 13708 14130 14260 14527 14766 14783 15043 15172 15301 15326 15625 16086 16148 16223 16259 16278 16319 16399 16526	M32c	73 150 152 200 263 489 16086 16148 16223 16259 16278 16319 16399 16526	[0.017] M32c (1)	[0.065] M51a (2) [0.014] M51b (3) [0.007] M32 (4) [0.000] M51	1
1339	>MF058306.1 Hc	73G 152C 195C 204C 263G 310.1C 417A 489C 533G 750G 1438G 3106d 3107C 4769G 7028T 8188G 8360G 8701G 8860G 9438A 9540C 9545G 9742T 10142T 10295G 10398G 10400T 10873C 11569C 11719A 11899C 12279G 12618A 12705T 14766T 14783C 15025T 15043A 15301A 15326G 16223T 16263C 16311C 16519C	M23	73G 152C 195C 204C 263G 310.1C 417A 489C 533G 16223T 16263C 16311C 16519C	1	73 152 195 204 263 417 489 533 750 1438 4769 7028 8188 8360 8701 8860 9438 9540 9545 9742 10142 10295 10398 10400 10873 11569 11719 11899 12279 12618 12705 14766 14783 15025 15043 15301 15326 16223 16263 16311	M23	73 152 195 204 263 417 489 533 16223 16263 16311	[0.000] M23 (1)	[0.072] M24a (2) [0.053] M57+152	1
1340	>MF058303.1 Hc	73G 150T 200G 263G 310.1C 489C 750G 2706G 3106d 3107C 3588T 4769G 7028T 7960G 8701G 8768T 8854A 8860G 9540C 10398G 10400T 10873C 11719A 12705T 13308G 13590A 13708A 14130T 14260G 14527G 14766T 14783C 15043A 15172A 15301A 15326G 15625T 16086C 16148T 16223T 16259T 16278T 16319A 16399G 16526A	M32c	73G 150T 200G 263G 310.1C 489C 16086C 16148T 16223T 16259T 16278T 16319A 16399G 16526A	1	73 150 200 263 489 750 2706 3588 4769 7028 7960 8701 8768 8854 8860 9540 10398 10400 10873 11719 12705 13308 13590 13708 14130 14260 14527 14766 14783 15043 15172 15301 15326 15625 16086 16148 16223 16259 16278 16319 16399 16526	M32c	73 150 200 263 489 16086 16148 16223 16259 16278 16319 16399 16526	[0.000] M32c (1)	[0.022] M51a (2) [0.007] M32 (3) [0.000] M51 M51b (5)	1

1341	>MF058302.1 Hc	73G 152C 195C 204C 263G 310.1C 417A 489C 533G 750G 1438G 3106d 3107C 4769G 7028T 8188G 8360G 8701G 8860G 9438A 9540C 9545G 10142T 10295G 10398G 10400T 10873C 11569C 11719A 11899C 12279G 12618A 12705T 14766T 14783C 15025T 15043A 15301A 15326G 16223T 16263C 16311C 16519C	M23	73G 152C 195C 204C 263G 310.1C 417A 489C 533G 16223T 16263C 16311C 16519C	1	73 152 195 204 263 417 489 533 750 1438 4769 7028 8188 8360 8701 8860 9438 9540 9545 10142 10295 10398 10400 10873 11569 11719 11899 12279 12618 12705 14766 14783 15025 15043 15301 15326 16223 16263 16311	M23	73 152 195 204 263 417 489 533 16223 16263 16311	[0.000] M23 (1)	[0.072] M24a (2) [0.053] M57+152	1
1342	>MF058295.1 Hc	73G 150T 200G 263G 310.1C 489C 538G 750G 2706G 3106d 3107C 3588T 4769G 7028T 7960G 8701G 8768T 8854A 8860G 9540C 10398G 10400T 10873C 11719A 12705T 13308G 13590A 13708A 14130T 14260G 14527G 14766T 14783C 15043A 15172A 15301A 15326G 15625T 16086C 16148T 16223T 16259T 16278T 16319A 16399G 16526A	M32c	73G 150T 200G 263G 310.1C 489C 538G 16086C 16148T 16223T 16259T 16278T 16319A 16399G 16526A	1	73 150 200 263 489 538 750 2706 3588 4769 7028 7960 8701 8768 8854 8860 9540 10398 10400 10873 11719 12705 13308 13590 13708 14130 14260 14527 14766 14783 15043 15172 15301 15326 15625 16086 16148 16223 16259 16278 16319 16399 16526	M32c	73 150 200 263 489 538 16086 16148 16223 16259 16278 16319 16399 16526	[0.007] M32c (1)	[0.022] M51a (2) [0.007] M32 (3) [0.000] M51 M51b (5)	1
1343	>MF058294.1 Hc	73G 152C 195C 204C 263G 310.1C 417A 489C 533G 750G 1438G 3106d 3107C 4769G 7028T 8188G 8360G 8701G 8860G 9438A 9540C 9545G 10142T 10295G 10398G 10400T 10873C 11569C 11719A 11899C 12279G 12618A 12705T 14766T 14783C 15025T 15043A 15115C 15301A 15326G 16223T 16263C 16311C 16519C	M23	73G 152C 195C 204C 263G 310.1C 417A 489C 533G 16223T 16263C 16311C 16519C	1	73 152 195 204 263 417 489 533 750 1438 4769 7028 8188 8360 8701 8860 9438 9540 9545 10142 10295 10398 10400 10873 11569 11719 11899 12279 12618 12705 14766 14783 15025 15043 15115 15301 15326 16223 16263 16311	M23	73 152 195 204 263 417 489 533 16223 16263 16311	[0.000] M23 (1)	[0.072] M24a (2) [0.053] M57+152	1
1344	>MF057000.1 Hc	73G 152C 195C 204C 263G 417A 489C 533G 750G 1438G 3106d 3107C 4769G 7028T 8188G 8360G 8701G 8860G 9438A 9540C 9545G 10142T 10295G 10398G 10400T 10873C 11569C 11719A 11899C 12279G 12618A 12705T 14766T 14783C 15025T 15043A 15301A 15326G 16223T 16263C 16311C 16519C	M23	73G 152C 195C 204C 263G 417A 489C 533G 16223T 16263C 16311C 16519C	1	73 152 195 204 263 417 489 533 750 1438 4769 7028 8188 8360 8701 8860 9438 9540 9545 10142 10295 10398 10400 10873 11569 11719 11899 12279 12618 12705 14766 14783 15025 15043 15301 15326 16223 16263 16311	M23	73 152 195 204 263 417 489 533 16223 16263 16311	[0.000] M23 (1)	[0.072] M24a (2) [0.053] M57+152	1
1345	>MF056996.1 Hc	73G 150T 200G 263G 489C 750G 2706G 3106d 3107C 3588T 4769G 7028T 7960G 8701G 8768T 8854A 8860G 9540C 10398G 10400T 10873C 11719A 12705T 13308G 13590A 13708A 14130T 14260G 14527G 14766T 14783C 15043A 15172A 15301A 15326G 15625T 16086C 16148T 16223T 16259T 16278T 16319A 16399G 16526A	M32c	73G 150T 200G 263G 489C 16086C 16148T 16223T 16259T 16278T 16319A 16399G 16526A	1	73 150 200 263 489 750 2706 3588 4769 7028 7960 8701 8768 8854 8860 9540 10398 10400 10873 11719 12705 13308 13590 13708 14130 14260 14527 14766 14783 15043 15172 15301 15326 15625 16086 16148 16223 16259 16278 16319 16399 16526	M32c	73 150 200 263 489 16086 16148 16223 16259 16278 16319 16399 16526	[0.000] M32c (1)	[0.022] M51a (2) [0.007] M32 (3) [0.000] M51 M51b (5)	1

1346	>MF056992.1 H	73G 150T 200G 263G 489C 750G 2706G 3106d 3107C 3588T 4769G 7028T 7960G 8701G 8768T 8854A 8860G 9540C 10398G 10400T 10873C 11719A 12705T 13308G 13590A 13708A 14130T 14260G 14527G 14766T 14783C 15043A 15172A 15301A 15326G 15625T 16086C 16148T 16223T 16259T 16278T 16319A 16399G 16526A	M32c	73G 150T 200G 263G 489C 16086C 16148T 16223T 16259T 16278T 16319A 16399G 16526A	1	73 150 200 263 489 750 2706 3588 4769 7028 7960 8701 8768 8854 8860 9540 10398 10400 10873 11719 12705 13308 13590 13708 14130 14260 14527 14766 14783 15043 15172 15301 15326 15625 16086 16148 16223 16259 16278 16319 16399 16526	M32c	73 150 200 263 489 16086 16148 16223 16259 16278 16319 16399 16526	[0.000] M32c (1)	[0.022] M51a (2) [0.007] M32 (3) [0.000] M51 M51b (5)	1
1347	>MF056989.1 H	73G 150T 200G 263G 489C 750G 2706G 3106d 3107C 3588T 4769G 7028T 7960G 8701G 8768T 8854A 8860G 9540C 10398G 10400T 10873C 11719A 12705T 13308G 13590A 13708A 14130T 14260G 14527G 14766T 14783C 15043A 15172A 15301A 15326G 15625T 16086C 16148T 16223T 16259T 16278T 16319A 16399G 16526A	M32c	73G 150T 200G 263G 489C 16086C 16148T 16223T 16259T 16278T 16319A 16399G 16526A	1	73 150 200 263 489 750 2706 3588 4769 7028 7960 8701 8768 8854 8860 9540 10398 10400 10873 11719 12705 13308 13590 13708 14130 14260 14527 14766 14783 15043 15172 15301 15326 15625 16086 16148 16223 16259 16278 16319 16399 16526	M32c	73 150 200 263 489 16086 16148 16223 16259 16278 16319 16399 16526	[0.000] M32c (1)	[0.022] M51a (2) [0.007] M32 (3) [0.000] M51 M51b (5)	1
1348	>MF056986.1 H	73G 146C 152C 195C 263G 750G 769A 1018A 1438G 2416C 2706G 2789T 3106d 3107C 3594T 4104G 4769G 5090C 7028T 7175C 7256T 7274T 7521A 7771G 8206A 8701G 8860G 9221G 9540C 10115C 10143A 10398G 10873C 11719A 11914A 11944C 12693G 12705T 13590A 13650T 13803G 14566G 14766T 15301A 15326G 15735T 15784C 16182C 16183C 16189C 16223T 16278T 16290T 16294T 16309G 16390A	L2a1b1a	73G 146C 152C 195C 263G 16182C 16183C 16189C 16223T 16278T 16290T 16294T 16309G 16390A	1	73 146 152 195 263 750 769 1018 1438 2416 2706 2789 3594 4104 4769 5090 7028 7175 7256 7274 7521 7771 8206 8701 8860 9221 9540 10115 10143 10398 10873 11719 11914 11944 12693 12705 13590 13650 13803 14566 14766 15301 15326 15735 15784 16189 16223 16278 16290 16294 16309 16390	L2a1b1a	73 146 152 195 263 16189 16223 16278 16290 16294 16309 16390	[0.000] L2a1b1a (1)	[0.005] L2a1f (2) [0.000] L2a1+16189_(16192) L2a1b L2a1b1 L2a1b1a L2a1b2 L2a1f1 L2a1f1a L2a1f2 (10)	1
1349	>MF056980.1 H	73G 146C 152C 195C 263G 750G 769A 1018A 1438G 2416C 2706G 2789T 3106d 3107C 3394C 3594T 4104G 4769G 5090C 7028T 7175C 7256T 7274T 7521A 7771G 8206A 8701G 8860G 9221G 9540C 10115C 10143A 10398G 10873C 11719A 11914A 11944C 12693G 12705T 13590A 13650T 13803G 14566G 14766T 15301A 15326G 15735T 15784C 16183C 16189C 16223T 16278T 16290T 16294T 16309G 16390A	L2a1b1a	73G 146C 152C 195C 263G 16183C 16189C 16223T 16278T 16290T 16294T 16309G 16390A	1	73 146 152 195 263 750 769 1018 1438 2416 2706 2789 3394 3594 4104 4769 5090 7028 7175 7256 7274 7521 7771 8206 8701 8860 9221 9540 10115 10143 10398 10873 11719 11914 11944 12693 12705 13590 13650 13803 14566 14766 15301 15326 15735 15784 16189 16223 16278 16290 16294 16309 16390	L2a1b1a	73 146 152 195 263 16189 16223 16278 16290 16294 16309 16390	[0.000] L2a1b1a (1)	[0.005] L2a1f (2) [0.000] L2a1+16189_(16192) L2a1b L2a1b1 L2a1b1a L2a1b2 L2a1f1 L2a1f1a L2a1f2 (10)	1

1350	>MF056976.1 Hc	73G 146C 152C 195C 263G 750G 769A 1018A 1438G 2416C 2706G 2789T 3106d 3107C 3594T 4104G 4769G 5090C 7028T 7175C 7256T 7274T 7521A 7771G 8206A 8701G 8860G 9221G 9540C 10115C 10143A 10398G 10873C 11719A 11914A 11944C 12693G 12705T 13590A 13650T 13803G 14566G 14766T 15301A 15326G 15735T 15784C 16183C 16189C 16223T 16278T 16290T 16294T 16309G 16390A	L2a1b1a	73G 146C 152C 195C 263G 16183C 16189C 16223T 16278T 16290T 16294T 16309G 16390A	1	73 146 152 195 263 750 769 1018 1438 2416 2706 2789 3594 4104 4769 5090 7028 7175 7256 7274 7521 7771 8206 8701 8860 9221 9540 10115 10143 10398 10873 11719 11914 11944 12693 12705 13590 13650 13803 14566 14766 15301 15326 15735 15784 16189 16223 16278 16290 16294 16309 16390	L2a1b1a	73 146 152 195 263 16189 16223 16278 16290 16294 16309 16390	[0.000] L2a1b1a (1)	[0.005] L2a1f (2) [0.000] L2a1+16189_(16192) L2a1b L2a1b1 L2a1b1a L2a1b2 L2a1f1 L2a1f1a L2a1f2 (10)	1
1351	>MF056972.1 Hc	73G 150T 263G 489C 750G 2706G 3106d 3107C 3588T 4769G 7028T 7960G 8701G 8768T 8854A 8860G 9101C 9540C 10398G 10400T 10873C 11719A 12705T 13308G 13590A 13708A 14130T 14260G 14527G 14766T 14783C 15043A 15172A 15301A 15326G 15625T 16086C 16148T 16223T 16259T 16278T 16319A 16399G 16526A	M32c	73G 150T 263G 489C 16086C 16148T 16223T 16259T 16278T 16319A 16399G 16526A	1	73 150 263 489 750 2706 3588 4769 7028 7960 8701 8768 8854 8860 9101 9540 10398 10400 10873 11719 12705 13308 13590 13708 14130 14260 14527 14766 14783 15043 15172 15301 15326 15625 16086 16148 16223 16259 16278 16319 16399 16526	M32c	73 150 263 489 16086 16148 16223 16259 16278 16319 16399 16526	[0.046] M32c (1)	[0.022] M51a (2) [0.000] M51 M51b M32 (5)	1
1352	>MF056971.1 Hc	73G 146C 152C 195C 263G 750G 769A 1018A 1438G 2416C 2706G 2789T 3106d 3107C 3594T 4104G 4769G 5090C 7028T 7175C 7256T 7274T 7521A 7771G 8206A 8701G 8860G 9221G 9540C 10115C 10143A 10398G 10873C 11719A 11914A 11944C 12693G 12705T 13590A 13650T 13803G 14566G 14766T 15301A 15326G 15735T 15784C 16182C 16183C 16189C 16223T 16278T 16290T 16294T 16309G 16390A	L2a1b1a	73G 146C 152C 195C 263G 16182C 16183C 16189C 16223T 16278T 16290T 16294T 16309G 16390A	1	73 146 152 195 263 750 769 1018 1438 2416 2706 2789 3594 4104 4769 5090 7028 7175 7256 7274 7521 7771 8206 8701 8860 9221 9540 10115 10143 10398 10873 11719 11914 11944 12693 12705 13590 13650 13803 14566 14766 15301 15326 15735 15784 16189 16223 16278 16290 16294 16309 16390	L2a1b1a	73 146 152 195 263 16189 16223 16278 16290 16294 16309 16390	[0.000] L2a1b1a (1)	[0.005] L2a1f (2) [0.000] L2a1+16189_(16192) L2a1b L2a1b1 L2a1b1a L2a1b2 L2a1f1 L2a1f1a L2a1f2 (10)	1
1353	>MF056970.1 Hc	73G 152C 195C 204C 263G 417A 489C 533G 750G 1438G 3106d 3107C 4769G 7028T 8188G 8360G 8701G 8860G 9438A 9540C 9545G 10142T 10295G 10398G 10400T 10873C 11569C 11719A 11899C 12279G 12618A 12705T 14766T 14783C 15025T 15043A 15301A 15326G 16223T 16263C 16311C 16519C	M23	73G 152C 195C 204C 263G 417A 489C 533G 16223T 16263C 16311C 16519C	1	73 152 195 204 263 417 489 533 750 1438 4769 7028 8188 8360 8701 8860 9438 9540 9545 10142 10295 10398 10400 10873 11569 11719 11899 12279 12618 12705 14766 14783 15025 15043 15301 15326 16223 16263 16311	M23	73 152 195 204 263 417 489 533 16223 16263 16311	[0.000] M23 (1)	[0.072] M24a (2) [0.053] M57+152	1

1354	>MF056964.1 Hc	73G 152C 195C 203A 204C 263G 417A 489C 533G 750G 1438G 3106d 3107C 4769G 7028T 7785C 8188G 8360G 8701G 8860G 9438A 9540C 9545G 10142T 10248C 10295G 10398G 10400T 10873C 11569C 11719A 11899C 12279G 12618A 12705T 14766T 14783C 15025T 15043A 15301A 15326G 16223T 16263C 16311C 16519C	M23	73G 152C 195C 203A 204C 263G 417A 489C 533G 16223T 16263C 16311C 16519C	1	73 152 195 203 204 263 417 489 533 750 1438 4769 7028 7785 8188 8360 8701 8860 9438 9540 9545 10142 10248 10295 10398 10400 10873 11569 11719 11899 12279 12618 12705 14766 14783 15025 15043 15301 15326 16223 16263 16311	M23	73 152 195 203 204 263 417 489 533 16223 16263 16311	[0.004] M23 (1)	[0.072] M24a (2) [0.053] M57+152	1
1355	>MF056948.1 Hc	73G 182T 186A 189C 195C 198T 247A 263G 297G 316A 750G 769A 825A 1018A 1420C 1438G 2394d 2706G 2758A 2885C 3106d 3107C 3594T 3666A 4104G 4769G 5951G 6071C 6150A 6253C 7028T 7055G 7076G 7146G 7256T 7337A 7389C 7521A 7744C 8027A 8251A 8468T 8655T 8701G 8784G 8860G 8877C 9072G 9540C 10321C 10398G 10586A 10688A 10792G 10793T 10810C 10873C 11654G 11719A 12049T 12705T 12810G 13105G 13149G 13212T 13281C 13485G 13506T 13650T 13789C 14000A 14178C 14560A 14766T 14812T 14911T 15016T 15326G 15784C 16071T 16129A 16145A 16187T 16189C 16213A 16223T 16234T 16265C 16278T 16286G 16294T 16311C 16320T 16360T 16527T	L1c2a1a	73G 182T 186A 189C 195C 198T 247A 263G 297G 316A 16071T 16129A 16145A 16187T 16189C 16213A 16223T 16234T 16265C 16278T 16286G 16294T 16311C 16320T 16360T 16527T	1	73 182 186A 189C 195 198 247 263 297 316 750 769 825A 1018 1420 1438 2395d 2706 2758 2885 3594 3666 4104 4769 5951 6071 6150 6253 7028 7055 7076 7146 7256 7337 7389 7521 7744 8027 8251 8468 8655 8701 8784 8860 8877 9072 9540 10321 10398 10586 10688 10792 10793 10810 10873 11654 11719 12049 12705 12810 13105 13149 13212 13281 13485 13506 13650 13789 14000A 14178 14560 14766 14812 14911 15016 15326 15784 16071 16129 16145 16187 16189 16213 16223 16234 16265C 16278 16286G 16294 16311 16320 16360 16527	L1c2a1a	73 182 186A 189C 195 198 247 263 297 316 16071 16129 16145 16187 16189 16213 16223 16234 16265C 16278 16286G 16294 16311 16320 16360 16527	[0.211] L1c2a1a (1)	[0.211] L1c2a1b (2) [0.000] L1c2a1	1
1356	>MF056946.1 Hc	73G 146C 152C 195C 263G 750G 769A 1018A 1438G 2416C 2706G 2789T 3106d 3107C 3594T 4104G 4769G 5090C 7028T 7175C 7256T 7274T 7521A 7771G 8206A 8701G 8860G 9221G 9540C 10115C 10143A 10398G 10873C 11719A 11914A 11944C 12693G 12705T 13590A 13650T 13803G 14566G 14766T 15301A 15326G 15735T 15784C 16182C 16183C 16189C 16223T 16278T 16290T 16294T 16309G 16390A	L2a1b1a	73G 146C 152C 195C 263G 16182C 16183C 16189C 16223T 16278T 16290T 16294T 16309G 16390A	1	73 146 152 195 263 750 769 1018 1438 2416 2706 2789 3594 4104 4769 5090 7028 7175 7256 7274 7521 7771 8206 8701 8860 9221 9540 10115 10143 10398 10873 11719 11914 11944 12693 12705 13590 13650 13803 14566 14766 15301 15326 15735 15784 16189 16223 16278 16290 16294 16309 16390	L2a1b1a	73 146 152 195 263 16189 16223 16278 16290 16294 16309 16390	[0.000] L2a1b1a (1)	[0.005] L2a1f (2) [0.000] L2a1+16189_(16192) L2a1b L2a1b1 L2a1b1a L2a1b2 L2a1f1 L2a1f1a L2a1f2 (10)	1

1357	>MF056945.1 Hc	73G 146C 152C 195C 263G 750G 769A 1018A 1438G 2416C 2706G 2789T 3106d 3107C 3594T 4104G 4769G 5090C 6521T 7028T 7175C 7256T 7274T 7521A 7771G 8206A 8701G 8860G 9221G 9540C 10115C 10143A 10398G 10873C 11719A 11914A 11944C 12693G 12705T 13590A 13650T 13803G 14566G 14766T 15301A 15326G 15735T 15784C 16182C 16183C 16189C 16223T 16278T 16290T 16294T 16309G 16390A	L2a1b1a	73G 146C 152C 195C 263G 16182C 16183C 16189C 16223T 16278T 16290T 16294T 16309G 16390A	1	73 146 152 195 263 750 769 1018 1438 2416 2706 2789 3594 4104 4769 5090 6521 7028 7175 7256 7274 7521 7771 8206 8701 8860 9221 9540 10115 10143 10398 10873 11719 11914 11944 12693 12705 13590 13650 13803 14566 14766 15301 15326 15735 15784 16189 16223 16278 16290 16294 16309 16390	L2a1b1a	73 146 152 195 263 16189 16223 16278 16290 16294 16309 16390	[0.000] L2a1b1a (1)	[0.005] L2a1f (2) [0.000] L2a1+16189_(16192) L2a1b L2a1b1 L2a1b1a L2a1b2 L2a1f1 L2a1f1a L2a1f2 (10)	1
1358	>MF056941.1 Hc	73G 150d 195C 204C 263G 417A 489C 533G 750G 1438G 3106d 3107C 3394C 4769G 7028T 8188G 8360G 8701G 8860G 9438A 9540C 9545G 10142T 10295G 10398G 10400T 10873C 11569C 11719A 11899C 12279G 12618A 12705T 14766T 14783C 15025T 15043A 15301A 15326G 16223T 16263C 16311C 16519C	M23	73G 150d 195C 204C 263G 417A 489C 533G 16223T 16263C 16311C 16519C	1	73 151d 195 204 263 417 489 533 750 1438 3394 4769 7028 8188 8360 8701 8860 9438 9540 9545 10142 10295 10398 10400 10873 11569 11719 11899 12279 12618 12705 14766 14783 15025 15043 15301 15326 16223 16263 16311	M23	73 151d 195 204 263 417 489 533 16223 16263 16311	[0.016] M23 (1)	[0.031] M43a (2) [0.026] M4"67+16311 (3) [0.007] M43+16311 (4) [0.006] D4k (5) [0.005] M50 (6) [0.000] M57	1
1359	>MF056939.1 Hc	73G 195C 204C 263G 417A 489C 533G 750G 1438G 3106d 3107C 3394C 4769G 7028T 8188G 8360G 8701G 8855N 8856N 8857N 8860G 9438A 9540C 9545G 10142T 10295G 10398G 10400T 10873C 11569C 11719A 11899C 12279G 12618A 12705T 14766T 14783C 15025T 15043A 15301A 15326G 16223T 16263C 16311C 16519C	M23	73G 195C 204C 263G 417A 489C 533G 16223T 16263C 16311C 16519C	1	73 195 204 263 417 489 533 750 1438 3394 4769 7028 8188 8360 8701 8855N 8856N 8857N 8860 9438 9540 9545 10142 10295 10398 10400 10873 11569 11719 11899 12279 12618 12705 14766 14783 15025 15043 15301 15326 16223 16263 16311	M23	73 195 204 263 417 489 533 16223 16263 16311	[0.012] M23 (1)	[0.031] M43a (2) [0.026] M4"67+16311 (3) [0.007] M43+16311 (4) [0.006] D4k (5) [0.005] M50 (6) [0.000] M57	1
1360	>MF056935.1 Hc	73G 150T 200G 263G 489C 750G 2706G 3106d 3107C 3588T 4769G 7028T 7960G 8376C 8701G 8768T 8854A 8860G 9540C 10398G 10400T 10873C 11719A 12705T 13308G 13590A 13708A 14130T 14260G 14527G 14766T 14783C 15043A 15172A 15301A 15326G 15625T 16086C 16148T 16223T 16259T 16278T 16319A 16399G 16526A	M32c	73G 150T 200G 263G 489C 16086C 16148T 16223T 16259T 16278T 16319A 16399G 16526A	1	73 150 200 263 489 750 2706 3588 4769 7028 7960 8376 8701 8768 8854 8860 9540 10398 10400 10873 11719 12705 13308 13590 13708 14130 14260 14527 14766 14783 15043 15172 15301 15326 15625 16086 16148 16223 16259 16278 16319 16399 16526	M32c	73 150 200 263 489 16086 16148 16223 16259 16278 16319 16399 16526	[0.000] M32c (1)	[0.022] M51a (2) [0.007] M32 (3) [0.000] M51 M51b (5)	1

1361	>MF056933.1 Hc	73G 152C 195C 204C 207A 263G 417A 489C 533G 750G 1438G 3106d 3107C 4616T 4769G 7028T 8188G 8360G 8701G 8860G 9438A 9540C 9545G 10142T 10295G 10398G 10400T 10873C 11569C 11719A 11899C 12279G 12358G 12618A 12630A 12705T 14766T 14783C 15025T 15043A 15301A 15326G 16223T 16263C 16311C 16519C	M23	73G 152C 195C 204C 207A 263G 417A 489C 533G 16223T 16263C 16311C 16519C	1	73 152 195 204 207 263 417 489 533 750 1438 4616 4769 7028 8188 8360 8701 8860 9438 9540 9545 10142 10295 10398 10400 10873 11569 11719 11899 12279 12358 12618 12630 12705 14766 14783 15025 15043 15301 15326 16223 16263 16311	M23	73 152 195 204 207 263 417 489 533 16223 16263 16311	[0.016] M23 (1)	[0.077] M24a (2) [0.053] M57+152	1
1362	>MF056928.1 Hc	73G 150T 200G 263G 489C 750G 2706G 3106d 3107C 3588T 4769G 7028T 7960G 8701G 8768T 8854A 8860G 9540C 10398G 10400T 10873C 11719A 12705T 13308G 13590A 13708A 14130T 14260G 14527G 14766T 14783C 15043A 15172A 15301A 15326G 15625T 16086C 16148T 16223T 16259T 16278T 16319A 16399G 16526A	M32c	73G 150T 200G 263G 489C 16086C 16148T 16223T 16259T 16278T 16319A 16399G 16526A	1	73 150 200 263 489 750 2706 3588 4769 7028 7960 8701 8768 8854 8860 9540 10398 10400 10873 11719 12705 13308 13590 13708 14130 14260 14527 14766 14783 15043 15172 15301 15326 15625 16086 16148 16223 16259 16278 16319 16399 16526	M32c	73 150 200 263 489 16086 16148 16223 16259 16278 16319 16399 16526	[0.000] M32c (1)	[0.022] M51a (2) [0.007] M32 (3) [0.000] M51 M51b (5)	1
1363	>MF056921.1 Hc	73G 150T 200G 263G 489C 750G 2706G 3106d 3107C 3588T 4769G 7028T 7960G 8701G 8768T 8854A 8860G 9540C 10398G 10400T 10873C 11719A 12705T 13308G 13590A 13708A 14130T 14260G 14527G 14766T 14783C 15043A 15172A 15301A 15326G 15625T 16086C 16148T 16223T 16259T 16278T 16319A 16399G 16526A	M32c	73G 150T 200G 263G 489C 16086C 16148T 16223T 16259T 16278T 16319A 16399G 16526A	1	73 150 200 263 489 750 2706 3588 4769 7028 7960 8701 8768 8854 8860 9540 10398 10400 10873 11719 12705 13308 13590 13708 14130 14260 14527 14766 14783 15043 15172 15301 15326 15625 16086 16148 16223 16259 16278 16319 16399 16526	M32c	73 150 200 263 489 16086 16148 16223 16259 16278 16319 16399 16526	[0.000] M32c (1)	[0.022] M51a (2) [0.007] M32 (3) [0.000] M51 M51b (5)	1
1364	>MF056919.1 Hc	73G 152d 200G 263G 489C 750G 2706G 3106d 3107C 3588T 4769G 7028T 7960G 8158G 8701G 8768T 8854A 8860G 9540C 10398G 10400T 10873C 11719A 12705T 13308G 13590A 13708A 14130T 14260G 14527G 14766T 14783C 15043A 15172A 15301A 15326G 15625T 16086C 16148T 16223T 16259T 16278T 16319A 16399G 16526A	M32c	73G 152d 200G 263G 489C 16086C 16148T 16223T 16259T 16278T 16319A 16399G 16526A	1	73 152d 200 263 489 750 2706 3588 4769 7028 7960 8158 8701 8768 8854 8860 9540 10398 10400 10873 11719 12705 13308 13590 13708 14130 14260 14527 14766 14783 15043 15172 15301 15326 15625 16086 16148 16223 16259 16278 16319 16399 16526	M32c	73 152d 200 263 489 16086 16148 16223 16259 16278 16319 16399 16526	[0.030] M32c (1)	[0.007] M32 (2)	1

1365	>MF056913.1 Hc	73G 152C 195C 204C 263G 417A 489C 533G 750G 1438G 3106d 3107C 4769G 7028T 8188G 8360G 8701G 8860G 9438A 9540C 9545G 10142T 10295G 10398G 10400T 10873C 11569C 11719A 11899C 12279G 12618A 12705T 14766T 14783C 15025T 15043A 15301A 15326G 16223T 16263C 16311C 16519C	M23	73G 152C 195C 204C 263G 417A 489C 533G 16223T 16263C 16311C 16519C	1	73 152 195 204 263 417 489 533 750 1438 4769 7028 8188 8360 8701 8860 9438 9540 9545 10142 10295 10398 10400 10873 11569 11719 11899 12279 12618 12705 14766 14783 15025 15043 15301 15326 16223 16263 16311	M23	73 152 195 204 263 417 489 533 16223 16263 16311	[0.000] M23 (1)	[0.072] M24a (2) [0.053] M57+152	1
1366	>MF056912.1 Hc	73G 152C 195C 204C 263G 417A 489C 533G 750G 1438G 3106d 3107C 4769G 7028T 8188G 8360G 8701G 8860G 9438A 9540C 9545G 10142T 10295G 10398G 10400T 10873C 11569C 11719A 11899C 12279G 12618A 12705T 14766T 14783C 15025T 15043A 15301A 15326G 16223T 16263C 16311C 16519C	M23	73G 152C 195C 204C 263G 417A 489C 533G 16223T 16263C 16311C 16519C	1	73 152 195 204 263 417 489 533 750 1438 4769 7028 8188 8360 8701 8860 9438 9540 9545 10142 10295 10398 10400 10873 11569 11719 11899 12279 12618 12705 14766 14783 15025 15043 15301 15326 16223 16263 16311	M23	73 152 195 204 263 417 489 533 16223 16263 16311	[0.000] M23 (1)	[0.072] M24a (2) [0.053] M57+152	1
1367	>MF056906.1 Hc	73G 150T 200G 263G 489C 750G 2706G 3106d 3107C 3588T 4769G 7028T 7960G 8701G 8768T 8854A 8860G 9540C 10398G 10400T 10873C 11719A 12705T 13308G 13590A 13708A 14130T 14260G 14527G 14766T 14783C 15043A 15172A 15301A 15326G 15625T 16086C 16148T 16189C 16223T 16259T 16278T 16319A 16399G 16526A	M32c	73G 150T 200G 263G 489C 16086C 16148T 16189C 16223T 16259T 16278T 16319A 16399G 16526A	1	73 150 200 263 489 750 2706 3588 4769 7028 7960 8701 8768 8854 8860 9540 10398 10400 10873 11719 12705 13308 13590 13708 14130 14260 14527 14766 14783 15043 15172 15301 15326 15625 16086 16148 16189 16223 16259 16278 16319 16399 16526	M32c	73 150 200 263 489 16086 16148 16189 16223 16259 16278 16319 16399 16526	[0.010] M32c (1)	[0.000] M51a2 (2)	1
1368	>MF056905.1 Hc	73G 150T 200G 263G 489C 750G 2706G 3106d 3107C 3588T 4769G 7028T 7960G 8701G 8768T 8854A 8860G 9540C 10398G 10400T 10873C 11719A 12705T 13308G 13590A 13708A 14130T 14260G 14527G 14766T 14783C 15043A 15172A 15301A 15326G 15625T 16086C 16148T 16189C 16223T 16259T 16278T 16319A 16399G 16526A	M32c	73G 150T 200G 263G 489C 16086C 16148T 16189C 16223T 16259T 16278T 16319A 16399G 16526A	1	73 150 200 263 489 750 2706 3588 4769 7028 7960 8701 8768 8854 8860 9540 10398 10400 10873 11719 12705 13308 13590 13708 14130 14260 14527 14766 14783 15043 15172 15301 15326 15625 16086 16148 16189 16223 16259 16278 16319 16399 16526	M32c	73 150 200 263 489 16086 16148 16189 16223 16259 16278 16319 16399 16526	[0.010] M32c (1)	[0.000] M51a2 (2)	1

1369 >MF056904.1 Ho	73G 146C 152C 195C 263G 750G 769A 1018A 1438G 2416C 2706G 2789T 3106d 3107C 3594T 4104G 4769G 5090C 7028T 7175C 7256T 7274T 7521A 7771G 8206A 8701G 8860G 9221G 9540C 10115C 10143A 10398G 10873C 11719A 11914A 11944C 12693G 12705T 13590A 13650T 13803G 14566G 14766T 15301A 15326G 15735T 15784C 16182C 16183C 16189C 16223T 16278T 16290T 16294T 16309G 16390A	L2a1b1a	73G 146C 152C 195C 263G 16182C 16183C 16189C 16223T 16278T 16290T 16294T 16309G 16390A	1	73 146 152 195 263 750 769 1018 1438 2416 2706 2789 3594 4104 4769 5090 7028 7175 7256 7274 7521 7771 8206 8701 8860 9221 9540 10115 10143 10398 10873 11719 11914 11944 12693 12705 13590 13650 13803 14566 14766 15301 15326 15735 15784 16189 16223 16278 16290 16294 16309 16390	L2a1b1a	73 146 152 195 263 16189 16223 16278 16290 16294 16309 16390	[0.000] L2a1b1a (1)	[0.005] L2a1f (2) [0.000] L2a1+16189_(16192) L2a1b L2a1b1 L2a1b1a L2a1b2 L2a1f1 L2a1f1a L2a1f2 (10)	1
1370 >MF056884.1 Ho	73G 150T 200G 263G 489C 750G 1393A 2706G 3106d 3107C 3588T 4769G 7028T 7960G 8701G 8768T 8854A 8860G 9540C 10398G 10400T 10873C 11719A 12705T 13308G 13590A 13708A 14130T 14260G 14527G 14766T 14783C 15043A 15172A 15301A 15326G 15625T 16086C 16148T 16223T 16259T 16278T 16319A 16399G 16526A	M32c	73G 150T 200G 263G 489C 16086C 16148T 16223T 16259T 16278T 16319A 16399G 16526A	1	73 150 200 263 489 750 1393 2706 3588 4769 7028 7960 8701 8768 8854 8860 9540 10398 10400 10873 11719 12705 13308 13590 13708 14130 14260 14527 14766 14783 15043 15172 15301 15326 15625 16086 16148 16223 16259 16278 16319 16399 16526	M32c	73 150 200 263 489 16086 16148 16223 16259 16278 16319 16399 16526	[0.000] M32c (1)	[0.022] M51a (2) [0.007] M32 (3) [0.000] M51 M51b (5)	1
1371 >MF056879.1 Ho	73G 146C 152C 195C 257G 263G 750G 769A 1018A 1438G 2416C 2706G 2762T 2789T 3106d 3107C 3594T 4104G 4769G 5090C 7028T 7175C 7256T 7274T 7521A 7771G 8206A 8701G 9221G 9540C 10115C 10143A 10398G 10757G 10873C 11719A 11914A 11944C 12634G 12693G 12705T 13590A 13650T 13803G 14566G 14766T 15301A 15326G 15735T 15784C 16189C 16223T 16278T 16290T 16294T 16309G 16390A	L2a1b1a	73G 146C 152C 195C 257G 263G 16189C 16223T 16278T 16290T 16294T 16309G 16390A	1	73 146 152 195 257 263 750 769 1018 1438 2416 2706 2762 2789 3594 4104 4769 5090 7028 7175 7256 7274 7521 7771 8206 8701 9221 9540 10115 10143 10398 10757G 10873 11719 11914 11944 12634 12693 12705 13590 13650 13803 14566 14766 15301 15326 15735 15784 16189 16223 16278 16290 16294 16309 16390	L2a1b1a	73 146 152 195 257 263 16189 16223 16278 16290 16294 16309 16390	[0.003] L2a1b1a (1)	[0.005] L2a1f (2) [0.003] L2a1b1a (3) [0.000] L2a1+16189_(16192) L2a1b L2a1b1 L2a1b2 L2a1f1 L2a1f1a L2a1f2 (10)	1
1372 >MF056877.1 Ho	73G 146C 152C 195C 263G 750G 769A 824C 1018A 1438G 2416C 2706G 2762T 2789T 3106d 3107C 3594T 4104G 4769G 5090C 7028T 7175C 7256T 7274T 7521A 7771G 8206A 8701G 8860G 9221G 9540C 10115C 10143A 10398G 10873C 11719A 11914A 11944C 12693G 12705T 13590A 13650T 13803G 14566G 14766T 15301A 15326G 15735T 15784C 16183C 16189C 16223T 16278T 16290T 16294T 16390A	L2a1b1a	73G 146C 152C 195C 263G 16183C 16189C 16223T 16278T 16290T 16294T 16390A	1	73 146 152 195 263 750 769 824 1018 1438 2416 2706 2762 2789 3594 4104 4769 5090 7028 7175 7256 7274 7521 7771 8206 8701 8860 9221 9540 10115 10143 10398 10873 11719 11914 11944 12693 12705 13590 13650 13803 14566 14766 15301 15326 15735 15784 16189 16223 16278 16290 16294 16390	L2a1b1a	73 146 152 195 263 16189 16223 16278 16290 16294 16390	[0.045] L2a1b1a (1)	[0.234] L2a1+143+16189_(16192)+@16309 (2) [0.070] L2a1f (3) [0.045] L2a1b1a (4) [0.008] L2a1b2 (5) [0.000] L2a1+16189_(16192) L2a1b L2a1b1 L2a1f1 L2a1f1a L2a1f2 (11)	1

1373	>MF056876.1 Hc	73G 150T 200G 263G 489C 513A 750G 2706G 3106d 3107C 3588T 4002C 4769G 7028T 7960G 8701G 8768T 8854A 8860G 9540C 10398G 10400T 10873C 11719A 12705T 13308G 13590A 13708A 14130T 14260G 14527G 14766T 14783C 15043A 15172A 15301A 15326G 15625T 16086C 16148T 16223T 16259T 16278T 16319A 16399G 16526A	M32c	73G 150T 200G 263G 489C 513A 16086C 16148T 16223T 16259T 16278T 16319A 16399G 16526A	1	73 150 200 263 489 513 750 2706 3588 4002 4769 7028 7960 8701 8768 8854 8860 9540 10398 10400 10873 11719 12705 13308 13590 13708 14130 14260 14527 14766 14783 15043 15172 15301 15326 15625 16086 16148 16223 16259 16278 16319 16399 16526	M32c	73 150 200 263 489 513 16086 16148 16223 16259 16278 16319 16399 16526	[0.007] M32c (1)	[0.022] M51a (2) [0.007] M32 (3) [0.000] M51 M51b (5)	1
1374	>MF056873.1 Hc	73G 150T 200G 263G 489C 750G 2706G 3106d 3107C 3588T 4769G 7028T 7960G 8701G 8768T 8854A 8860G 9540C 10398G 10400T 10873C 11719A 12705T 13308G 13590A 13708A 14130T 14260G 14527G 14766T 14783C 15043A 15172A 15301A 15326G 15625T 16086C 16148T 16223T 16259T 16278T 16319A 16399G 16526A	M32c	73G 150T 200G 263G 489C 16086C 16148T 16223T 16259T 16278T 16319A 16399G 16526A	1	73 150 200 263 489 750 2706 3588 4769 7028 7960 8701 8768 8854 8860 9540 10398 10400 10873 11719 12705 13308 13590 13708 14130 14260 14527 14766 14783 15043 15172 15301 15326 15625 16086 16148 16223 16259 16278 16319 16399 16526	M32c	73 150 200 263 489 16086 16148 16223 16259 16278 16319 16399 16526	[0.000] M32c (1)	[0.022] M51a (2) [0.007] M32 (3) [0.000] M51 M51b (5)	1
1375	>MF056864.1 Hc	73G 152C 195C 204C 263G 417A 489C 533G 750G 1438G 3106d 3107C 4769G 7028T 8188G 8360G 8701G 8860G 9438A 9540C 9545G 10142T 10295G 10398G 10400T 10873C 11569C 11719A 11899C 12279G 12618A 12705T 14766T 14783C 15025T 15043A 15301A 15326G 16223T 16263C 16311C 16519C	M23	73G 152C 195C 204C 263G 417A 489C 533G 16223T 16263C 16311C 16519C	1	73 152 195 204 263 417 489 533 750 1438 4769 7028 8188 8360 8701 8860 9438 9540 9545 10142 10295 10398 10400 10873 11569 11719 11899 12279 12618 12705 14766 14783 15025 15043 15301 15326 16223 16263 16311	M23	73 152 195 204 263 417 489 533 16223 16263 16311	[0.000] M23 (1)	[0.072] M24a (2) [0.053] M57+152	1
1376	>MF056863.1 Hc	73G 152C 195C 204C 263G 417A 489C 533G 750G 1438G 3106d 3107C 4769G 6437G 7028T 8188G 8360G 8701G 8860G 9438A 9540C 9545G 10142T 10295G 10398G 10400T 10873C 11569C 11719A 11899C 12279G 12618A 12705T 14226A 14766T 14783C 15025T 15043A 15301A 15326G 16223T 16263C 16311C 16519C	M23	73G 152C 195C 204C 263G 417A 489C 533G 16223T 16263C 16311C 16519C	1	73 152 195 204 263 417 489 533 750 1438 4769 6437 7028 8188 8360 8701 8860 9438 9540 9545 10142 10295 10398 10400 10873 11569 11719 11899 12279 12618 12705 14226 14766 14783 15025 15043 15301 15326 16223 16263 16311	M23	73 152 195 204 263 417 489 533 16223 16263 16311	[0.000] M23 (1)	[0.072] M24a (2) [0.053] M57+152	1

1377	>MF056860.1 Hc	73G 150T 200G 263G 325T 489C 750G 2706G 3106d 3107C 3588T 4769G 7028T 7960G 8701G 8768T 8854A 8860G 9540C 10398G 10400T 10873C 11719A 12705T 13308G 13590A 13708A 14130T 14260G 14527G 14766T 14783C 15043A 15172A 15301A 15326G 15625T 16086C 16148T 16223T 16259T 16278T 16319A 16399G 16526A	M32c	73G 150T 200G 263G 325T 489C 16086C 16148T 16223T 16259T 16278T 16319A 16399G 16526A	1	73 150 200 263 325 489 750 2706 3588 4769 7028 7960 8701 8768 8854 8860 9540 10398 10400 10873 11719 12705 13308 13590 13708 14130 14260 14527 14766 14783 15043 15172 15301 15326 15625 16086 16148 16223 16259 16278 16319 16399 16526	M32c	73 150 200 263 325 489 16086 16148 16223 16259 16278 16319 16399 16526	[0.003] M32c (1)	[0.022] M51a (2) [0.007] M32 (3) [0.000] M51 M51b (5)	1
1378	>MF056859.1 Hc	73G 152C 195C 204C 263G 417A 489C 533G 750G 1438G 3106d 3107C 4769G 7028T 8188G 8360G 8701G 8860G 9438A 9540C 9545G 10142T 10295G 10398G 10400T 10873C 11569C 11719A 11899C 12279G 12618A 12705T 14766T 14783C 15025T 15043A 15301A 15326G 16223T 16263C 16311C 16519C	M23	73G 152C 195C 204C 263G 417A 489C 533G 16223T 16263C 16311C 16519C	1	73 152 195 204 263 417 489 533 750 1438 4769 7028 8188 8360 8701 8860 9438 9540 9545 10142 10295 10398 10400 10873 11569 11719 11899 12279 12618 12705 14766 14783 15025 15043 15301 15326 16223 16263 16311	M23	73 152 195 204 263 417 489 533 16223 16263 16311	[0.000] M23 (1)	[0.072] M24a (2) [0.053] M57+152	1
1379	>MF056855.1 Hc	73G 152C 195C 204C 263G 417A 489C 533G 750G 1438G 3106d 3107C 4769G 6437G 7028T 8188G 8360G 8701G 8860G 9438A 9540C 9545G 10142T 10295G 10398G 10400T 10873C 11569C 11719A 11899C 12279G 12618A 12705T 14766T 14783C 15025T 15043A 15301A 15326G 16223T 16263C 16311C 16519C	M23	73G 152C 195C 204C 263G 417A 489C 533G 16223T 16263C 16311C 16519C	1	73 152 195 204 263 417 489 533 750 1438 4769 6437 7028 8188 8360 8701 8860 9438 9540 9545 10142 10295 10398 10400 10873 11569 11719 11899 12279 12618 12705 14766 14783 15025 15043 15301 15326 16223 16263 16311	M23	73 152 195 204 263 417 489 533 16223 16263 16311	[0.000] M23 (1)	[0.072] M24a (2) [0.053] M57+152	1
1380	>MF056854.1 Hc	73G 150T 200G 263G 489C 538G 750G 2706G 3106d 3107C 3588T 4769G 7028T 7960G 8701G 8768T 8854A 8860G 9540C 10398G 10400T 10873C 11719A 12705T 13308G 13590A 13708A 14130T 14260G 14527G 14766T 14783C 15043A 15172A 15301A 15326G 15625T 16148T 16223T 16259T 16278T 16319A 16399G 16526A	M32c	73G 150T 200G 263G 489C 538G 16148T 16223T 16259T 16278T 16319A 16399G 16526A	1	73 150 200 263 489 538 750 2706 3588 4769 7028 7960 8701 8768 8854 8860 9540 10398 10400 10873 11719 12705 13308 13590 13708 14130 14260 14527 14766 14783 15043 15172 15301 15326 15625 16148 16223 16259 16278 16319 16399 16526	M32c	73 150 200 263 489 538 16148 16223 16259 16278 16319 16399 16526	[0.014] M32c (1)	[0.022] M51a (2) [0.007] M32 (3) [0.000] M51 M51b (5)	1
1381	>MF056853.1 Hc	73G 152C 195C 204C 263G 417A 489C 533G 750G 1438G 3106d 3107C 4769G 7028T 8188G 8360G 8701G 8860G 9438A 9540C 9545G 10142T 10295G 10398G 10400T 10403G 10873C 11569C 11719A 11899C 12279G 12618A 12705T 14766T 14783C 15025T 15043A 15301A 15326G 16223T 16263C 16311C 16519C	M23	73G 152C 195C 204C 263G 417A 489C 533G 16223T 16263C 16311C 16519C	1	73 152 195 204 263 417 489 533 750 1438 4769 7028 8188 8360 8701 8860 9438 9540 9545 10142 10295 10398 10400 10403 10873 11569 11719 11899 12279 12618 12705 14766 14783 15025 15043 15301 15326 16223 16263 16311	M23	73 152 195 204 263 417 489 533 16223 16263 16311	[0.000] M23 (1)	[0.072] M24a (2) [0.053] M57+152	1

1382	>MF056843.1 Hc	73G 263G 279C 514d 515d 750G 1438G 2332T 2706G 3106d 3107C 3450T 4769G 5773A 6221C 6340T 7028T 8701G 8860G 9449T 9540C 10086G 10373A 10398G 10873C 11002G 11719A 12705T 13105G 13914A 14766T 15301A 15311G 15326G 15824G 15940d 16145A 16223T 16278T 16362C 16519C	L3b1a8	73G 263G 279C 514d 515d 16145A 16223T 16278T 16362C 16519C	1	73 263 279 750 1438 2332 2706 3450 4769 5773 6221 6340 7028 8701 8860 9449 9540 10086 10373 10398 10873 11002 11719 12705 13105 13914A 14766 15301 15311 15326 15824 15944d 16145 16223 16278 16362	L3b1a8	73 263 279 16145 16223 16278 16362	[0.213] L3b1a8 (1)	[0.000] L3b1a+@16124 L3b1a7 L3b1a7a (4)	1
1383	>MF056839.1 Hc	73G 150T 152C 235G 263G 494T 735G 750G 1438G 2706G 3106d 3107C 3918A 4313C 4769G 6620G 7028T 7202G 8649G 8701G 8860G 9007T 9329A 9467C 9540C 10398G 10819G 10873C 11719A 12705T 13135A 13542G 13862G 13992T 14013G 14766T 15301A 15314A 15326G 16093C 16223T 16355T	L3k1	73G 150T 152C 235G 263G 494T 16093C 16223T 16355T	1	73 150 152 235 263 494 735 750 1438 2706 3918 4313 4769 6620G 7028 7202 8649 8701 8860 9007T 9329 9467 9540 10398 10819 10873 11719 12705 13135 13542 13862 13992 14013 14766 15301 15314 15326 16093 16223 16355	L3k1	73 150 152 235 263 494 16093 16223 16355	[0.536] L3k1 (1)	[0.000] L3k (2)	1
1384	>MF056833.1 Hc	73G 150T 152C 235G 263G 494T 735G 750G 1438G 2706G 3106d 3107C 3918A 4313C 4769G 6620G 7028T 7202G 8649G 8701G 8860G 9007T 9329A 9467C 9540C 10398G 10819G 10873C 11719A 12705T 13135A 13542G 13862G 13992T 14013G 14766T 15301A 15314A 15326G 16093C 16223T 16355T	L3k1	73G 150T 152C 235G 263G 494T 16093C 16223T 16355T	1	73 150 152 235 263 494 735 750 1438 2706 3918 4313 4769 6620G 7028 7202 8649 8701 8860 9007T 9329 9467 9540 10398 10819 10873 11719 12705 13135 13542 13862 13992 14013 14766 15301 15314 15326 16093 16223 16355	L3k1	73 150 152 235 263 494 16093 16223 16355	[0.536] L3k1 (1)	[0.000] L3k (2)	1
1385	>MF057767.1 Hc	73G 150T 200G 263G 489C 750G 2706G 3106d 3107C 3588T 4769G 7028T 7960G 8701G 8768T 8854A 8860G 9540C 10398G 10400T 10873C 11719A 12705T 13308G 13590A 13708A 14130T 14260G 14527G 14766T 14783C 15043A 15172A 15301A 15326G 15625T 16086C 16148T 16223T 16259T 16278T 16319A 16399G 16526A	M32c	73G 150T 200G 263G 489C 16086C 16148T 16223T 16259T 16278T 16319A 16399G 16526A	1	73 150 200 263 489 750 2706 3588 4769 7028 7960 8701 8768 8854 8860 9540 10398 10400 10873 11719 12705 13308 13590 13708 14130 14260 14527 14766 14783 15043 15172 15301 15326 15625 16086 16148 16223 16259 16278 16319 16399 16526	M32c	73 150 200 263 489 16086 16148 16223 16259 16278 16319 16399 16526	[0.000] M32c (1)	[0.022] M51a (2) [0.007] M32 (3) [0.000] M51 M51b (5)	1
1386	>MF057765.1 Hc	73G 150T 200G 263G 309.1CT 310C 489C 750G 2706G 3106d 3107C 3588T 4769G 7028T 7960G 8701G 8768T 8854A 8860G 9540C 10398G 10400T 10873C 11719A 12705T 13308G 13590A 13708A 14130T 14260G 14527G 14766T 14783C 15043A 15172A 15301A 15326G 15625T 16086C 16148T 16223T 16259T 16278T 16319A 16399G 16526A	M32c	73G 150T 200G 263G 309.1CT 310C 489C 16086C 16148T 16223T 16259T 16278T 16319A 16399G 16526A	1	73 150 200 263 489 750 2706 3588 4769 7028 7960 8701 8768 8854 8860 9540 10398 10400 10873 11719 12705 13308 13590 13708 14130 14260 14527 14766 14783 15043 15172 15301 15326 15625 16086 16148 16223 16259 16278 16319 16399 16526	M32c	73 150 200 263 489 16086 16148 16223 16259 16278 16319 16399 16526	[0.000] M32c (1)	[0.022] M51a (2) [0.007] M32 (3) [0.000] M51 M51b (5)	1

1387	>MF057764.1 Hc	73G 152C 195C 204C 263G 310.1C 417A 489C 533G 750G 1438G 3106d 3107C 4769G 7028T 8188G 8360G 8701G 8860G 9438A 9540C 9545G 10142T 10295G 10398G 10400T 10873C 11260C 11569C 11719A 11899C 12279G 12618A 12705T 14766T 14783C 15025T 15043A 15301A 15326G 16223T 16263C 16311C 16519C	M23	73G 152C 195C 204C 263G 310.1C 417A 489C 533G 16223T 16263C 16311C 16519C	1	73 152 195 204 263 417 489 533 750 1438 4769 7028 8188 8360 8701 8860 9438 9540 9545 10142 10295 10398 10400 10873 11260 11569 11719 11899 12279 12618 12705 14766 14783 15025 15043 15301 15326 16223 16263 16311	M23	73 152 195 204 263 417 489 533 16223 16263 16311	[0.000] M23 (1)	[0.072] M24a (2) [0.053] M57+152	1
1388	>MF057756.1 Hc	73G 150T 200G 263G 489C 750G 2706G 3106d 3107C 3588T 4769G 7028T 7960G 8701G 8768T 8854A 8860G 9540C 10398G 10400T 10873C 11719A 12705T 13308G 13590A 13708A 13747T 14130T 14260G 14527G 14766T 14783C 15043A 15172A 15301A 15326G 15625T 16086C 16148T 16223T 16259T 16278T 16319A 16399G 16526A	M32c	73G 150T 200G 263G 489C 16086C 16148T 16223T 16259T 16278T 16319A 16399G 16526A	1	73 150 200 263 489 750 2706 3588 4769 7028 7960 8701 8768 8854 8860 9540 10398 10400 10873 11719 12705 13308 13590 13708 13747T 14130 14260 14527 14766 14783 15043 15172 15301 15326 15625 16086 16148 16223 16259 16278 16319 16399 16526	M32c	73 150 200 263 489 16086 16148 16223 16259 16278 16319 16399 16526	[0.000] M32c (1)	[0.022] M51a (2) [0.007] M32 (3) [0.000] M51 M51b (5)	1
1389	>MF057755.1 Hc	73G 150T 200G 263G 310.1C 489C 750G 2706G 3106d 3107C 3588T 4769G 7028T 7960G 8701G 8768T 8854A 8860G 9540C 10398G 10400T 10873C 11719A 12705T 13308G 13590A 13708A 14130T 14260G 14527G 14766T 14783C 15043A 15172A 15301A 15326G 15625T 16086C 16148T 16223T 16259T 16278T 16319A 16399G 16526A	M32c	73G 150T 200G 263G 310.1C 489C 16086C 16148T 16223T 16259T 16278T 16319A 16399G 16526A	1	73 150 200 263 489 750 2706 3588 4769 7028 7960 8701 8768 8854 8860 9540 10398 10400 10873 11719 12705 13308 13590 13708 14130 14260 14527 14766 14783 15043 15172 15301 15326 15625 16086 16148 16223 16259 16278 16319 16399 16526	M32c	73 150 200 263 489 16086 16148 16223 16259 16278 16319 16399 16526	[0.000] M32c (1)	[0.022] M51a (2) [0.007] M32 (3) [0.000] M51 M51b (5)	1
1390	>MF057751.1 Hc	73G 150T 152C 200G 263G 310.1C 489C 750G 2706G 3106d 3107C 3588T 4769G 7028T 7960G 8158G 8701G 8768T 8854A 8860G 9540C 10398G 10400T 10873C 11719A 12705T 13308G 13590A 13708A 14130T 14260G 14527G 14766T 14783C 15043A 15172A 15301A 15326G 15625T 16086C 16148T 16223T 16259T 16278T 16319A 16399G 16526A	M32c	73G 150T 152C 200G 263G 310.1C 489C 16086C 16148T 16223T 16259T 16278T 16319A 16399G 16526A	1	73 150 152 200 263 489 750 2706 3588 4769 7028 7960 8158 8701 8768 8854 8860 9540 10398 10400 10873 11719 12705 13308 13590 13708 14130 14260 14527 14766 14783 15043 15172 15301 15326 15625 16086 16148 16223 16259 16278 16319 16399 16526	M32c	73 150 152 200 263 489 16086 16148 16223 16259 16278 16319 16399 16526	[0.017] M32c (1)	[0.065] M51a (2) [0.014] M51b (3) [0.007] M32 (4) [0.000] M51	1

1391	>MF057741.1 Hc	73G 146C 152C 195C 263G 309.1CT 310C 513.1CA 750G 769A 1018A 1438G 2416C 2706G 2789T 3106d 3107C 3594T 4104G 4769G 5090C 7028T 7175C 7256T 7274T 7521A 7771G 8206A 8701G 8860G 9221G 9540C 10115C 10143A 10398G 10873C 11719A 11914A 11944C 12693G 12705T 13590A 13650T 13803G 14566G 14766T 15301A 15326G 15735T 15784C 15907G 16182C 16183C 16189C 16223T 16278T 16290T 16294T 16309G 16390A 16519C	L2a1b1a	73G 146C 152C 195C 263G 309.1CT 310C 513.1CA 16182C 16183C 16189C 16223T 16278T 16290T 16294T 16309G 16390A 16519C	1	73 146 152 195 263 750 769 1018 1438 2416 2706 2789 3594 4104 4769 5090 7028 7175 7256 7274 7521 7771 8206 8701 8860 9221 9540 10115 10143 10398 10873 11719 11914 11944 12693 12705 13590 13650 13803 14566 14766 15301 15326 15735 15784 15907 16189 16223 16278 16290 16294 16309 16390	L2a1b1a	73 146 152 195 263 16189 16223 16278 16290 16294 16309 16390	[0.000] L2a1b1a (1)	[0.005] L2a1f (2) [0.000] L2a1+16189_(16192) L2a1b L2a1b1 L2a1b1a L2a1b2 L2a1f1 L2a1f1a L2a1f2 (10)	1
1392	>MF057732.1 Hc	73G 150T 200G 263G 310.1C 489C 750G 2706G 3106d 3107C 3588T 4769G 7028T 7960G 8701G 8768T 8854A 8860G 9540C 10398G 10400T 10873C 11719A 12705T 13308G 13590A 13708A 14130T 14260G 14527G 14766T 14783C 15043A 15172A 15301A 15326G 15625T 16086C 16148T 16223T 16259T 16278T 16319A 16399G 16526A	M32c	73G 150T 200G 263G 310.1C 489C 16086C 16148T 16223T 16259T 16278T 16319A 16399G 16526A	1	73 150 200 263 489 750 2706 3588 4769 7028 7960 8701 8768 8854 8860 9540 10398 10400 10873 11719 12705 13308 13590 13708 14130 14260 14527 14766 14783 15043 15172 15301 15326 15625 16086 16148 16223 16259 16278 16319 16399 16526	M32c	73 150 200 263 489 16086 16148 16223 16259 16278 16319 16399 16526	[0.000] M32c (1)	[0.022] M51a (2) [0.007] M32 (3) [0.000] M51 M51b (5)	1
1393	>MF057721.1 Hc	73G 152C 195C 204C 263G 310.1C 417A 489C 533G 750G 1438G 3106d 3107C 4769G 7028T 8188G 8360G 8701G 8860G 9438A 9540C 9545G 10142T 10295G 10398G 10400T 10873C 11569C 11719A 11899C 12279G 12618A 12705T 14766T 14783C 15025T 15043A 15301A 15326G 16223T 16263C 16311C 16519C	M23	73G 152C 195C 204C 263G 310.1C 417A 489C 533G 16223T 16263C 16311C 16519C	1	73 152 195 204 263 417 489 533 750 1438 4769 7028 8188 8360 8701 8860 9438 9540 9545 10142 10295 10398 10400 10873 11569 11719 11899 12279 12618 12705 14766 14783 15025 15043 15301 15326 16223 16263 16311	M23	73 152 195 204 263 417 489 533 16223 16263 16311	[0.000] M23 (1)	[0.072] M24a (2) [0.053] M57+152	1
1394	>MF057716.1 Hc	73G 152C 195C 204C 263G 310.1C 417A 489C 533G 750G 1438G 3106d 3107C 4769G 7028T 8188G 8360G 8701G 8860G 9438A 9540C 9545G 10142T 10295G 10398G 10400T 10873C 11569C 11719A 11899C 12279G 12618A 12705T 14766T 14783C 15025T 15043A 15301A 15326G 16223T 16263C 16311C 16519C	M23	73G 152C 195C 204C 263G 310.1C 417A 489C 533G 16223T 16263C 16311C 16519C	1	73 152 195 204 263 417 489 533 750 1438 4769 7028 8188 8360 8701 8860 9438 9540 9545 10142 10295 10398 10400 10873 11569 11719 11899 12279 12618 12705 14766 14783 15025 15043 15301 15326 16223 16263 16311	M23	73 152 195 204 263 417 489 533 16223 16263 16311	[0.000] M23 (1)	[0.072] M24a (2) [0.053] M57+152	1

1395	>MF057706.1 Hc	73G 150T 200G 263G 310.1C 489C 750G 2706G 3106d 3107C 3588T 4769G 7028T 7960G 8701G 8768T 8854A 8860G 9540C 10398G 10400T 10873C 11719A 12705T 13308G 13590A 13708A 14130T 14260G 14527G 14766T 14783C 15043A 15172A 15301A 15326G 15625T 16086C 16148T 16223T 16278T 16299G 16319A 16399G 16526A	M32c	73G 150T 200G 263G 310.1C 489C 16086C 16148T 16223T 16278T 16299G 16319A 16399G 16526A	1	73 150 200 263 489 750 2706 3588 4769 7028 7960 8701 8768 8854 8860 9540 10398 10400 10873 11719 12705 13308 13590 13708 14130 14260 14527 14766 14783 15043 15172 15301 15326 15625 16086 16148 16223 16278 16299 16319 16399 16526	M32c	73 150 200 263 489 16086 16148 16223 16278 16299 16319 16399 16526	[0.013] M32c (1)	[0.022] M51a (2) [0.007] M32 (3) [0.000] M51 M51b (5)	1
1396	>MF057704.1 Hc	73G 150T 200G 263G 310.1C 489C 750G 2706G 3106d 3107C 3588T 4769G 7028T 7960G 8701G 8768T 8854A 8860G 9540C 10398G 10400T 10873C 11719A 12705T 13308G 13590A 13708A 14130T 14260G 14527G 14766T 14783C 15043A 15172A 15301A 15326G 15625T 16086C 16148T 16223T 16259T 16278T 16319A 16399G 16526A	M32c	73G 150T 200G 263G 310.1C 489C 16086C 16148T 16223T 16259T 16278T 16319A 16399G 16526A	1	73 150 200 263 489 750 2706 3588 4769 7028 7960 8701 8768 8854 8860 9540 10398 10400 10873 11719 12705 13308 13590 13708 14130 14260 14527 14766 14783 15043 15172 15301 15326 15625 16086 16148 16223 16259 16278 16319 16399 16526	M32c	73 150 200 263 489 16086 16148 16223 16259 16278 16319 16399 16526	[0.000] M32c (1)	[0.022] M51a (2) [0.007] M32 (3) [0.000] M51 M51b (5)	1
1397	>MF057688.1 Hc	73G 152C 195C 204C 263G 310.1C 417A 489C 533G 750G 1438G 3106d 3107C 4769G 7028T 8188G 8360G 8701G 8860G 9438A 9540C 9545G 10142T 10295G 10398G 10400T 10873C 11569C 11719A 11899C 12279G 12618A 12705T 14766T 14783C 15025T 15043A 15301A 15326G 16223T 16263C 16311C 16519C	M23	73G 152C 195C 204C 263G 310.1C 417A 489C 533G 16223T 16263C 16311C 16519C	1	73 152 195 204 263 417 489 533 750 1438 4769 7028 8188 8360 8701 8860 9438 9540 9545 10142 10295 10398 10400 10873 11569 11719 11899 12279 12618 12705 14766 14783 15025 15043 15301 15326 16223 16263 16311	M23	73 152 195 204 263 417 489 533 16223 16263 16311	[0.000] M23 (1)	[0.072] M24a (2) [0.053] M57+152	1
1398	>MF057862.1 Hc	73G 152C 263G 279C 309.1CT 310C 514d 515d 750G 1438G 2332T 2706G 3106d 3107C 3450T 4769G 5773A 6221C 6340T 7028T 8701G 8860G 9449T 9540C 10086G 10117C 10373A 10398G 10873C 11002G 11719A 12705T 13105G 13914A 14766T 15301A 15311G 15326G 15824G 15940d 16145A 16223T 16278T 16362C 16519C	L3b1a8	73G 152C 263G 279C 309.1CT 310C 514d 515d 16145A 16223T 16278T 16362C 16519C	1	73 152 263 279 750 1438 2332 2706 3450 4769 5773 6221 6340 7028 8701 8860 9449 9540 10086 10117 10373 10398 10873 11002 11719 12705 13105 13914A 14766 15301 15311 15326 15824 15944d 16145 16223 16278 16362	L3b1a8	73 152 263 279 16145 16223 16278 16362	[0.269] L3b1a8 (1)	[0.035] L3b1a+@16124 (2) [0.021] L3b1a+152 (3) [0.012] L3b1b (4) [0.000] L3b1a6 L3b1a7 L3b1a7a G2a+152 G2a2 (9)	1
1399	>MF057856.1 Hc	73G 152C 263G 279C 309.1CT 310C 514d 515d 750G 1438G 2332T 2706G 3106d 3107C 3450T 4769G 5773A 6221C 6340T 7028T 8701G 8860G 9449T 9540C 10086G 10117C 10373A 10398G 10873C 11002G 11719A 12705T 13105G 13914A 14766T 15301A 15311G 15326G 15824G 15940d 16145A 16223T 16278T 16362C 16519C	L3b1a8	73G 152C 263G 279C 309.1CT 310C 514d 515d 16145A 16223T 16278T 16362C 16519C	1	73 152 263 279 750 1438 2332 2706 3450 4769 5773 6221 6340 7028 8701 8860 9449 9540 10086 10117 10373 10398 10873 11002 11719 12705 13105 13914A 14766 15301 15311 15326 15824 15944d 16145 16223 16278 16362	L3b1a8	73 152 263 279 16145 16223 16278 16362	[0.269] L3b1a8 (1)	[0.035] L3b1a+@16124 (2) [0.021] L3b1a+152 (3) [0.012] L3b1b (4) [0.000] L3b1a6 L3b1a7 L3b1a7a G2a+152 G2a2 (9)	1

1400	>MF057848.1 Ho	73G 263G 279C 310.1C 514d 515d 750G 1438G 2332T 2706G 3106d 3107C 3450T 4769G 5773A 6221C 6340T 7028T 8701G 8860G 9449T 9540C 10086G 10373A 10398G 10873C 11002G 11719A 12705T 13105G 13914A 14766T 15301A 15311G 15326G 15824G 15940d 16145A 16223T 16278T 16362C 16519C	L3b1a8	73G 263G 279C 310.1C 514d 515d 16145A 16223T 16278T 16362C 16519C	1	73 263 279 750 1438 2332 2706 3450 4769 5773 6221 6340 7028 8701 8860 9449 9540 10086 10373 10398 10873 11002 11719 12705 13105 13914A 14766 15301 15311 15326 15824 15944d 16145 16223 16278 16362	L3b1a8	73 263 279 16145 16223 16278 16362	[0.213] L3b1a8 (1)	[0.000] L3b1a+@16124 L3b1a7 L3b1a7a (4)	1
1401	>MF057843.1 Ho	73G 152C 182T 186A 189C 195C 247A 248G 263G 310.1C 316A 514d 515d 745.1T 750G 769A 825A 1018A 1438G 2394d 2706G 2758A 2885C 3106d 3107C 3525T 3594T 3666A 4104G 4769G 4853A 5951G 6071C 6221A 6227C 6917A 7028T 7146G 7247T 7256T 7389C 7521A 8027A 8468T 8655T 8701G 8860G 9072G 9540C 10398G 10586A 10688A 10810C 10873C 11302T 11719A 11984C 12507G 12705T 12810G 13105G 13485G 13506T 13650T 13789C 14000A 14178C 14560A 14669G 14766T 14911T 14926G 15226G 15326G 15905C 15978T 16129A 16187T 16189C 16223T 16278T 16293G 16294T 16311C 16360T 16368C 16519C	L1c3c	73G 152C 182T 186A 189C 195C 247A 248G 263G 310.1C 316A 514d 515d 16129A 16187T 16189C 16223T 16278T 16293G 16294T 16311C 16360T 16368C 16519C	1	73 152 182 186A 189C 195 247 248 263 316 745.1T 750 769 825A 1018 1438 2395d 2706 2758 2885 3525 3594 3666 4104 4769 4853 5951 6071 6221A 6227 6917 7028 7146 7247 7256 7389 7521 8027 8468 8655 8701 8860 9072 9540 10398 10586 10688 10810 10873 11302 11719 11984 12507 12705 12810 13105 13485 13506 13650 13789 14000A 14178 14560 14669 14766 14911 14926 15226 15326 15905 15978 16129 16187 16189 16223 16278 16293 16294 16311 16360 16368	L1c3c	73 152 182 186A 189C 195 247 248 263 316 16129 16187 16189 16223 16278 16293 16294 16311 16360 16368	[2.125] L1c3c (1) [1.000] L1c1a+@198 (2) [0.000] L1c L1c3b'c (4)	[0.900] L1c3 (5) [0.769] L1c1 (6) [0.714] L1c1a (7) [0.572] L1c1a'b'd (8) [0.267] L1c3b1 (9) [0.133] L1c3b1b (10) [0.000] L1c1'2'4'5'6 L1c1a1 L1c3b (13)	1
1402	>MF057832.1 Ho	73G 151T 152C 182T 186A 189C 247A 263G 309.1CCT 310C 316A 514d 515d 750G 769A 783G 825A 1018A 1438G 2394d 2706G 2758A 2885C 3105d 3106G 3107C 3594T 3666A 4104G 4769G 5951G 6071C 6221A 6260A 6917A 7028T 7146G 7256T 7389C 7498A 7521A 7789A 8027A 8468T 8655T 8668C 8701G 8860G 9072G 9540C 9966A 10398G 10586A 10688A 10810C 10873C 11302T 11719A 12019T 12501A 12705T 12810G 13105G 13485G 13506T 13650T 13789C 14000A 14178C 14560A 14766T 14911T 15226G 15326G 15905C 15916C 15978T 16093C 16129A 16183C 16189C 16215G 16223T 16278T 16294T 16311C 16355T 16360T 16390A 16519C	L1c3a1b	73G 151T 152C 182T 186A 189C 247A 263G 309.1CCT 310C 316A 514d 515d 16093C 16129A 16183C 16189C 16215G 16223T 16278T 16294T 16311C 16355T 16360T 16390A 16519C	1	73 151 152 182 186A 189C 247 263 316 750 769 783 825A 1018 1438 2395d 2706 2758 2885 3105 3594 3666 4104 4769 5951 6071 6221A 6260 6917 7028 7146 7256 7389 7498 7521 7789 8027 8468 8655 8668 8701 8860 9072 9540 9966 10398 10586 10688 10810 10873 11302 11719 12019 12501 12705 12810 13105 13485 13506 13650 13789 14000A 14178 14560 14766 14911 15226 15326 15905 15916 15978 16093 16129 16189 16215 16223 16278 16294 16311 16355 16360 16390	L1c3a1b	73 151 152 182 186A 189C 247 263 316 16093 16129 16189 16215 16223 16278 16294 16311 16355 16360 16390	[0.846] L1c3a1b (1)	[0.286] L1c3a1a (2) [0.093] L1c3a (3) [0.000] L1c3a1	1

1403	>MF057815.1 Hc	73G 146C 152C 195C 263G 567.1C 750G 769A 1018A 1438G 2416C 2706G 2789T 3106d 3107C 3594T 4104G 4769G 5090C 7028T 7175C 7256T 7274T 7521A 7771G 8206A 8701G 8860G 9221G 9540C 10115C 10143A 10398G 10873C 11719A 11914A 11944C 12693G 12705T 13590A 13650T 13803G 14566G 14766T 15301A 15326G 15735T 15784C 16182C 16183C 16189C 16223T 16278T 16290T 16294T 16309G 16390A	L2a1b1a	73G 146C 152C 195C 263G 567.1C 16182C 16183C 16189C 16223T 16278T 16290T 16294T 16309G 16390A	1	73 146 152 195 263 573.1C 750 769 1018 1438 2416 2706 2789 3594 4104 4769 5090 7028 7175 7256 7274 7521 7771 8206 8701 8860 9221 9540 10115 10143 10398 10873 11719 11914 11944 12693 12705 13590 13650 13803 14566 14766 15301 15326 15735 15784 16189 16223 16278 16290 16294 16309 16390	L2a1b1a	73 146 152 195 263 573.1C 16189 16223 16278 16290 16294 16309 16390	[0.000] L2a1b1a (1)	[0.005] L2a1f (2) [0.000] L2a1+16189_(16192) L2a1b L2a1b1 L2a1b1a L2a1b2 L2a1f1 L2a1f1a L2a1f2 (10)	1
1404	>MF057808.1 Hc	73G 146C 152C 195C 263G 750G 769A 1018A 1438G 2416C 2706G 2789T 3106d 3107C 3594T 4104G 4769G 5090C 7028T 7175C 7256T 7274T 7521A 7771G 8206A 8701G 8860G 9221G 9540C 10115C 10143A 10398G 10873C 11719A 11914A 11944C 12693G 12705T 13590A 13650T 13803G 14566G 14766T 15301A 15326G 15735T 15784C 16182C 16183C 16189C 16223T 16278T 16290T 16294T 16309G 16390A	L2a1b1a	73G 146C 152C 195C 263G 16182C 16183C 16189C 16223T 16278T 16290T 16294T 16309G 16390A	1	73 146 152 195 263 750 769 1018 1438 2416 2706 2789 3594 4104 4769 5090 7028 7175 7256 7274 7521 7771 8206 8701 8860 9221 9540 10115 10143 10398 10873 11719 11914 11944 12693 12705 13590 13650 13803 14566 14766 15301 15326 15735 15784 16189 16223 16278 16290 16294 16309 16390	L2a1b1a	73 146 152 195 263 16189 16223 16278 16290 16294 16309 16390	[0.000] L2a1b1a (1)	[0.005] L2a1f (2) [0.000] L2a1+16189_(16192) L2a1b L2a1b1 L2a1b1a L2a1b2 L2a1f1 L2a1f1a L2a1f2 (10)	1
1405	>MF057807.1 Hc	73G 152C 195C 204C 263G 310.1C 417A 489C 533G 750G 1438G 3106d 3107C 4769G 7028T 8188G 8360G 8701G 8860G 9438A 9540C 9545G 10142T 10295G 10398G 10400T 10873C 11569C 11719A 11899C 12279G 12618A 12705T 14766T 14783C 15025T 15043A 15301A 15326G 16223T 16263C 16311C 16519C	M23	73G 152C 195C 204C 263G 310.1C 417A 489C 533G 16223T 16263C 16311C 16519C	1	73 152 195 204 263 417 489 533 750 1438 4769 7028 8188 8360 8701 8860 9438 9540 9545 10142 10295 10398 10400 10873 11569 11719 11899 12279 12618 12705 14766 14783 15025 15043 15301 15326 16223 16263 16311	M23	73 152 195 204 263 417 489 533 16223 16263 16311	[0.000] M23 (1)	[0.072] M24a (2) [0.053] M57+152	1
1406	>MF057802.1 Hc	73G 152C 195C 204C 263G 310.1C 417A 489C 533G 750G 1438G 3106d 3107C 4769G 7028T 8188G 8360G 8701G 8860G 9438A 9540C 9545G 10142T 10295G 10398G 10400T 10873C 11569C 11719A 11899C 12279G 12618A 12705T 14766T 14783C 15025T 15043A 15301A 15326G 16223T 16263C 16311C 16519C	M23	73G 152C 195C 204C 263G 310.1C 417A 489C 533G 16223T 16263C 16311C 16519C	1	73 152 195 204 263 417 489 533 750 1438 4769 7028 8188 8360 8701 8860 9438 9540 9545 10142 10295 10398 10400 10873 11569 11719 11899 12279 12618 12705 14766 14783 15025 15043 15301 15326 16223 16263 16311	M23	73 152 195 204 263 417 489 533 16223 16263 16311	[0.000] M23 (1)	[0.072] M24a (2) [0.053] M57+152	1

1407 >MF057800.1 Hc	73G 150T 200G 263G 310.1C 489C 750G 2706G 3106d 3107C 3588T 4769G 7028T 7960G 8701G 8768T 8854A 8860G 9540C 10398G 10400T 10873C 11719A 12705T 13308G 13590A 13708A 14130T 14260G 14527G 14766T 14783C 15043A 15172A 15301A 15326G 15625T 16086C 16148T 16223T 16259T 16278T 16319A 16399G 16526A	M32c	73G 150T 200G 263G 310.1C 489C 16086C 16148T 16223T 16259T 16278T 16319A 16399G 16526A	1	73 150 200 263 489 750 2706 3588 4769 7028 7960 8701 8768 8854 8860 9540 10398 10400 10873 11719 12705 13308 13590 13708 14130 14260 14527 14766 14783 15043 15172 15301 15326 15625 16086 16148 16223 16259 16278 16319 16399 16526	M32c	73 150 200 263 489 16086 16148 16223 16259 16278 16319 16399 16526	[0.000] M32c (1)	[0.022] M51a (2) [0.007] M32 (3) [0.000] M51 M51b (5)	1
1408 >MF057796.1 Hc	93G 95C 185A 189G 236C 247A 263G 309.1CT 310C 750G 769A 825A 1018A 1048T 1438G 2245G 2706G 2758A 2885C 3106d 3107C 3516A 3594T 4104G 4312T 4586C 4769G 5096C 5147A 5231A 5442C 5460A 5603T 5608T 6185C 7028T 7146G 7256T 7521A 7568C 7861C 8428T 8468T 8566G 8655T 8701G 8860G 9042T 9347G 9540C 9755A 9818T 10398G 10589A 10664T 10688A 10810C 10873C 10915C 11176A 11641G 11719A 11914A 12007A 12705T 12720G 13105G 13276G 13506T 13650T 14308C 14766T 15136T 15326G 15431A 16129A 16148T 16172C 16187T 16188G 16189C 16223T 16230G 16311C 16320T	L0a1e	93G 95C 185A 189G 236C 247A 263G 309.1CT 310C 16129A 16148T 16172C 16187T 16188G 16189C 16223T 16230G 16311C 16320T	1	93 95C 185 189 236 247 263 750 769 825A 1018 1048 1438 2245 2706 2758 2885 3516A 3594 4104 4312 4586 4769 5096 5147 5231 5442 5460 5603 5608 6185 7028 7146 7256 7521 7568 7861 8428 8468 8566 8655 8701 8860 9042 9347 9540 9755 9818 10398 10589 10664 10688 10810 10873 10915 11176 11641 11719 11914 12007 12705 12720 13105 13276 13506 13650 14308 14766 15136 15326 15431 16129 16148 16172 16187 16188G 16189 16223 16230 16311 16320	L0a1e	93 95C 185 189 236 247 263 16129 16148 16172 16187 16188G 16189 16223 16230 16311 16320	[0.000] L0a1e (1)	[0.111] L0a3 (2) [0.043] L0a (3) [0.000] L0a1e	1
1409 >MF057789.1 Hc	73G 151T 152C 195C 204C 263G 310.1C 417A 489C 533G 750G 1438G 3106d 3107C 3394C 4769G 7028T 8188G 8360G 8701G 8860G 9438A 9540C 9545G 10142T 10295G 10398G 10400T 10873C 11569C 11719A 11899C 12279G 12618A 12705T 14766T 14783C 15025T 15043A 15301A 15326G 16223T 16263C 16311C 16519C	M23	73G 151T 152C 195C 204C 263G 310.1C 417A 489C 533G 16223T 16263C 16311C 16519C	1	73 151 152 195 204 263 417 489 533 750 1438 3394 4769 7028 8188 8360 8701 8860 9438 9540 9545 10142 10295 10398 10400 10873 11569 11719 11899 12279 12618 12705 14766 14783 15025 15043 15301 15326 16223 16263 16311	M23	73 151 152 195 204 263 417 489 533 16223 16263 16311	[0.016] M23 (1)	[0.072] M24a (2) [0.053] M57+152 (3) [0.013] M37a (4) [0.004] M37+152+151 (5) [0.000] M37a1 M37d (7)	1
1410 >MF057779.1 Hc	73G 150T 200G 263G 310.1C 489C 513A 750G 2706G 3106d 3107C 3588T 4769G 7028T 7960G 8701G 8768T 8854A 8860G 9540C 10398G 10400T 10873C 11719A 12705T 13308G 13590A 13708A 14130T 14260G 14527G 14766T 14783C 15043A 15172A 15301A 15326G 15625T 16086C 16148T 16223T 16259T 16278T 16319A 16399G 16526A	M32c	73G 150T 200G 263G 310.1C 489C 513A 16086C 16148T 16223T 16259T 16278T 16319A 16399G 16526A	1	73 150 200 263 489 513 750 2706 3588 4769 7028 7960 8701 8768 8854 8860 9540 10398 10400 10873 11719 12705 13308 13590 13708 14130 14260 14527 14766 14783 15043 15172 15301 15326 15625 16086 16148 16223 16259 16278 16319 16399 16526	M32c	73 150 200 263 489 513 16086 16148 16223 16259 16278 16319 16399 16526	[0.007] M32c (1)	[0.022] M51a (2) [0.007] M32 (3) [0.000] M51 M51b (5)	1

1411	>JQ704616.1 Ho	73G 199C 263G 310.1C 513.1CA 750G 1438G 2706G 3106d 3107C 3197C 3438A 4769G 5585A 6023A 7028T 7569G 8251A 8860G 9192A 9477A 11467G 11719A 12308G 12372A 13617C 14766T 14793G 15218G 15326G 16192T 16256T 16270T 16311C 16399G 16526A	U5a1f1a1	73G 199C 263G 310.1C 513.1CA 16192T 16256T 16270T 16311C 16399G 16526A	1	73 199 263 750 1438 2706 3197 3438 4769 5585 6023 7028 7569 8251 8860 9192 9477 11467 11719 12308 12372 13617 14766 14793 15218 15326 16192 16256 16270 16311 16399 16526	U5a1f1a1	73 199 263 16192 16256 16270 16311 16399 16526	[0.000] U5a1f1a1 (1)	[0.000] U5a1f1a (2)	1
1412	>JQ704608.1 Ho	41T 150T 239C 263G 297G 309.1CCT 310C 750G 1438G 3106d 3107C 3915A 4727G 4769G 7202G 8860G 9380A 11253C 15326G 16362C	H6a1a2a	41T 150T 239C 263G 297G 309.1CCT 310C 16362C	1	41 150 239 263 297 750 1438 3915 4727 4769 7202 8860 9380 11253 15326 16362	H6a1a2a	41 150 239 263 297 16362	[0.116] H6a1a2a (1) [0.000] H6a1a7 (2)	[0.116] H6a1a2a (3) [0.008] H6a1a (4) [0.007] H6a2 (5) [0.000] H6 H6a H6a1 H6a1a1 H6a1a2 H6a1a2b H6a1a2b1 H6a1a3 H6a1a3a H6a1a4 H6a1a5 H6a1a7 H6a1a9 H6a1a10 H6a1b H6a1b2 H6a1b2a H6a1b2b H6a1b2c H6a1b2e H6a2a H6c (27)	1
1413	>JQ704604.1 Ho	73G 185A 228A 263G 295T 309.1CT 310C 462T 489C 750G 1438G 2706G 3010A 3106d 3107C 3666R 4216C 4769G 7028T 8860G 10084C 11251G 11719A 12612G 13708A 14766T 14798C 15326G 15452A 16069T 16126C 16319A	J1c8a	73G 185A 228A 263G 295T 309.1CT 310C 462T 489C 16069T 16126C 16319A	1	73 185 228 263 295 462 489 750 1438 2706 3010 3666R 4216 4769 7028 8860 10084 11251 11719 12612 13708 14766 14798 15326 15452A 16069 16126 16319	J1c8a	73 185 228 263 295 462 489 16069 16126 16319	[0.000] J1c8a (1)	[0.097] J1c10a (2) [0.021] J1c (3) [0.013] J1c8a (4) [0.000] J1c3 J1c3a J1c3a1 J1c3a2 J1c3b J1c3b1 J1c3b1a J1c3b2 J1c3d J1c3g J1c3h J1c3i J1c3k J1c4 J1c4b J1c5 J1c5a J1c5a1 J1c5b J1c5c J1c5d J1c5e J1c5f J1c6 J1c6a J1c8 J1c8a1 J1c8a2 J1c8b J1c9 J1c10 J1c15 J1c15a J1c15a1 J1c17 (39)	1
1414	>JQ704586.1 Ho	62C 64T 66T 73G 114G 152C 207A 263G 309.1CCT 310C 499A 750G 827G 1002T 1438G 2706G 3106d 3107C 3547G 3766C 4769G 4820A 4977C 6164T 6473T 7028T 8271d 8272d 8273d 8274d 8275d 8276d 8277d 8278d 8279d 8860G 9950C 10598G 10792G 11177T 11719A 12535T 13590A 14766T 15326G 15535T 16183C 16189C 16194C 16195C 16217C 16298C 16519C	B2g1	62C 64T 66T 73G 114G 152C 207A 263G 309.1CCT 310C 499A 16183C 16189C 16194C 16195C 16217C 16298C 16519C	1	62C 64 66T 73 114G 152 207 263 499 750 827 1002 1438 2706 3547 3766 4769 4820 4977 6164 6473 7028 8281-8289d 8860 9950 10598 10792 11177 11719 12535 13590 14766 15326 15535 16189 16194C 16195 16217 16298	B2g1	62C 64 66T 73 114G 152 207 263 499 16189 16194C 16195 16217 16298	[1.667] B2g1 (1)	[0.053] B2b+152 (2) [0.000] B2g B2g2 (4)	1
1415	>JQ704582.1 Ho	73G 195C 263G 310.1C 497T 513.1CACA 750G 1189C 1438G 1811G 2706G 3106d 3107C 3480G 4769G 7028T 8860G 9055A 9698C 10398G 10550G 11299C 11467G 11719A 12308G 12372A 14167T 14766T 14798C 15326G 16048A 16093C 16201T 16224C 16291T 16311C 16519C	K1a10a	73G 195C 263G 310.1C 497T 513.1CACA 16048A 16093C 16201T 16224C 16291T 16311C 16519C	1	73 195 263 497 750 1189 1438 1811 2706 3480 4769 7028 8860 9055 9698 10398 10550 11299 11467 11719 12308 12372 14167 14766 14798 15326 16048 16093 16201 16224 16291 16311	K1a10a	73 195 263 497 16048 16093 16201 16224 16291 16311	[0.034] K1a10a (1)	[0.068] K1a10a (2) [0.000] K1a10	1

1416 >JQ704578.1 Ho	152C 194T 263G 310.1C 709A 750G 1438G 3106d 3107C 4769G 6776C 6953G 8860G 11150A 13105G 15043A 15326G 16519C	H3s	152C 194T 263G 310.1C 16519C	1	152 194 263 709 750 1438 4769 6776 6953 8860 11150 13105 15043 15326	H3s	152 194 263	[0.444] H3s (1) [0.006] H1bb H3g1 (3) [0.003] H1+152 (4) [0.000] H1i H1i1 H1i2 H1i2a H1an H1an1 H1an1a H1bc H2a2a2 H3+152 H3g H3g1a H3g1b H3g2 H3g3 H3g4 H3i H3i1 H3j H3k H3k1 H3k1a H+152 H9 H46 H46a H46b H52 H69 H13a1+152 H14b H14b2 H14b4 H16+152 H16a H16a1 H16c H16d H30b1 H47a (44)	[0.031] H13a1a1a (45) [0.024] H2a2a (46) [0.022] H9a (47) [0.018] H3a1 (48) [0.012] HV (49) [0.011] H26a1 (50) [0.010] H4a1 (51) [0.008] R30a1 R30a1a (53) [0.006] HV5 (54) [0.005] H1h1 (55) [0.004] H3q1 H7b (57) [0.003] H4b H16e H58a H75 (61) [0.002] H1 H1e1a H1e2 H2a H13a1a1 H13a2a H18 H18b (69) [0.001] HV4 H1e1a2 H1e1c H1h2 H1q H1t1a H1ag1 H1aj H1at1a H3 H4 H4a1a H4a1a1 H4a1c H7b1 H7b2a H7c6 H7d H10b H10+(16093) H13a2b4 H35 H40b H45a H62 (94) [0.000] R0 HV1b+152 HV4c HV5a HV9+152 HV9c HV12 H H1c+152 H1c9 H1c9a H1e H1e1 H1e1a7 H1e2a H1e2b H1e2d H1e4 H1e6 H1e7 H1e8 H1e8a H1h H1j H1j3 H1j5 H1j6 H1j7 H1j9 H1m H1n H1p H1q1 H1q2 H1r H1s H1s1 H1t H1t1 H1t2 H1u H1u1 H1v H1v1 H1v1a H1v1b H1w H1ae H1ae1 H1ae2 H1ae2a H1ae3 H1ae3a H1ag H1ag1b H1ah H1ai H1ai1 H1ak H1ak1 H1ak2 H1am H1am1 H1ap H1ap1 H1aq H1as H1as2 H1at H1at1 H1av H1ax H1ax1 H1ay H1az H1bd H1be H1bi H1bj H1bk H1bm [illegible]	1
1417 >JQ704576.1 Ho	73G 185A 228A 263G 295T 310.1C 462T 489C 513.1CA 750G 1438G 2706G 3010A 3106d 3107C 3981G 4216C 4769G 7028T 8860G 10084C 11251G 11719A 12612G 13708A 14766T 14798C 15326G 15452A 16069T 16126C 16319A	J1c8a	73G 185A 228A 263G 295T 310.1C 462T 489C 513.1CA 16069T 16126C 16319A	1	73 185 228 263 295 462 489 750 1438 2706 3010 3981 4216 4769 7028 8860 10084 11251 11719 12612 13708 14766 14798 15326 15452A 16069 16126 16319	J1c8a	73 185 228 263 295 462 489 16069 16126 16319	[0.000] J1c8a (1)	[0.097] J1c10a (2) [0.021] J1c (3) [0.013] J1c8a (4) [0.000] J1c3 J1c3a J1c3a1 J1c3a2 J1c3b J1c3b1 J1c3b1a J1c3b2 J1c3d J1c3g J1c3h J1c3i J1c3k J1c4 J1c4b J1c5 J1c5a J1c5a1 J1c5b J1c5c J1c5d J1c5e J1c5f J1c6 J1c6a J1c8 J1c8a1 J1c8a2 J1c8b J1c9 J1c10 J1c15 J1c15a J1c15a1 J1c17 (39)	1
1418 >JQ704569.1 Ho	73G 185A 195C 228A 263G 295T 309.1CT 310C 462T 489C 750G 1438G 2706G 3010A 3106d 3107C 4216C 4491A 4769G 7028T 8860G 8940T 9120G 10398G 11016A 11251G 11719A 12612G 13708A 14766T 14798C 15326G 15452A 16069T 16093Y 16126C 16261T 16274A 16355T	J1c14	73G 185A 195C 228A 263G 295T 309.1CT 310C 462T 489C 16069T 16093Y 16126C 16261T 16274A 16355T	1	73 185 195 228 263 295 462 489 750 1438 2706 3010 4216 4491 4769 7028 8860 8940 9120 10398 11016 11251 11719 12612 13708 14766 14798 15326 15452A 16069 16093Y 16126 16261 16274 16355	J1c14	73 185 195 228 263 295 462 489 16069 16093Y 16126 16261 16274 16355	[0.625] J1c14 (1)	[0.246] J1c7a (2) [0.012] J1c+16261 (3) [0.000] J1c7	1

1419 >JQ704568.1 Ho	73G 150T 263G 310.1C 513.1CA 750G 1438G 2706G 3106d 3107C 3197C 4769G 5437T 5656G 7028T 7768G 7912A 8860G 9477A 11467G 11719A 12308G 12372A 13617C 14182C 14766T 15326G 15631G 15721C 16192T 16218T 16270T 16320T	U5b1d1c	73G 150T 263G 310.1C 513.1CA 16192T 16218T 16270T 16320T	1	73 150 263 750 1438 2706 3197 4769 5437 5656 7028 7768 7912 8860 9477 11467 11719 12308 12372 13617 14182 14766 15326 15631 15721 16192 16218 16270 16320	U5b1d1c	73 150 263 16192 16218 16270 16320	[0.000] U5b1d1c (1)	[0.000] U5b U5b1 U5b1a U5b1b1b U5b1d U5b1d1 U5b1f U5b2 U5b2c U5b2c1 U5b2c2 U5b2c2a (13)	1
1420 >JQ704537.1 Ho	73G 195C 263G 310.1C 497T 513.1CACA 750G 1189C 1438G 1811G 2706G 3106d 3107C 3480G 4769G 7028T 8860G 9055A 9698C 10398G 10550G 11299C 11467G 11719A 12308G 12372A 14167T 14766T 14798C 15326G 16048A 16093C 16224C 16291T 16305R 16311C 16519C	K1a10a	73G 195C 263G 310.1C 497T 513.1CACA 16048A 16093C 16224C 16291T 16305R 16311C 16519C	1	73 195 263 497 750 1189 1438 1811 2706 3480 4769 7028 8860 9055 9698 10398 10550 11299 11467 11719 12308 12372 14167 14766 14798 15326 16048 16093 16224 16291 16305R 16311	K1a10a	73 195 263 497 16048 16093 16224 16291 16305R 16311	[0.034] K1a10a (1)	[0.068] K1a10a (2) [0.000] K1a10	1
1421 >JQ704529.1 Ho	198T 263G 309.1CT 310C 513.1CA 750G 1438G 1909G 3106d 3107C 4769G 4793G 5210T 6296A 8860G 15326G 16093C 16265G 16519C	H7c1	198T 263G 309.1CT 310C 513.1CA 16093C 16265G 16519C	1	198 263 750 1438 1909 4769 4793 5210 6296A 8860 15326 16093 16265	H7c1	198 263 16093 16265	[0.276] H7c1 (1) [0.000] H10+(16093) H10f (3)	[0.158] H7c3 (4) [0.157] H13a1a1c (5) [0.156] H7c (6) [0.155] H44a (7) [0.031] H10e1 (8) [0.014] H3v+16093 (9) [0.012] H (10) [0.011] H10e3a (11) [0.010] H1e1a (12) [0.008] H1 (13) [0.007] H2a2a (14) [0.005] H1e8a H60a (16) [0.003] H3h2 H63 H72 (19) [0.002] H1e H56 (21) [0.001] H1h1 H1j H1q H1bi H1br H3 H4 H4a1a H7a H7b1 H10f H13a2b2 H14b H14b2 H67 H73 (37) [0.000] R0 HV HV4 HV4c HV5 HV5a HV9c HV12 H1f+16093 H1f1 H1e1 H1e1a2 H1e1a7 H1e1c H1e2 H1e2a H1e2b H1e2d H1e4 H1e6 H1e7 H1e8 H1h H1h2 H1j3 H1j5 H1j6 H1j7 H1j9 H1m H1n H1p H1q1 H1q2 H1r H1s H1s1 H1t H1t1 H1t1a H1t2 H1u H1u1 H1v H1v1 H1v1a H1v1b H1w H1ae H1ae1 H1ae2 H1ae2a H1ae3 H1ae3a H1ag H1ag1 H1ag1b H1ah H1ai H1ai1 H1aj H1ak H1ak1 H1ak2 H1am H1am1 H1ap H1aq H1as H1as2 H1at H1at1 H1at1a H1av H1ax H1ax1 H1ay H1az H1bd H1be H1bj H1bk H1bm H1bn H1bp H1bq H1bu H1bv H1bw H1bx H1ca H1ci H2 H2a H2a2 H2a2+(16235) H2a4 H2a5 [illegible]	1
1422 >JQ704514.1 Ho	72C 150T 263G 309.1CT 310C 750G 1438G 2706G 3106d 3107C 4135C 4580A 4769G 7028T 7810T 8860G 15326G 15904T 16093C 16298C 16362C	V19	72C 150T 263G 309.1CT 310C 16093C 16298C 16362C	1	72 150 263 750 1438 2706 4135 4580 4769 7028 7810 8860 15326 15904 16093 16298 16362	V19	72 150 263 16093 16298 16362	[0.071] V19 (1)	[0.000] V+150 V22 (3)	1

1423	>JQ704513.1 Ho	263G 309.1CT 310C 750G 1438G 2792C 3010A 3106d 3107C 4769G 8860G 15047A 15326G 15758G 16189C 16234T 16317G 16519C	H1ab1	263G 309.1CT 310C 16189C 16234T 16317G 16519C	1	263 750 1438 2792C 3010 4769 8860 15047 15326 15758 16189 16234 16317	H1ab1	263 16189 16234 16317	[0.032] H1ab1 (1)	[0.000] HV18 H1+16189 H1f H1g H1g1 H1g2 H1y H1aa H1aa1 H1ab H1ac H1ad H1j9 H3+16189 (15)	1
1424	>JQ704508.1 Ho	73G 195C 263G 310.1C 497T 513.1CACA 750G 1189C 1438G 1811G 2706G 3106d 3107C 3480G 4769G 7028T 8860G 9055A 9698C 10398G 10550G 11299C 11467G 11719A 12308G 12372A 14167T 14766T 14798C 15326G 15431A 16048A 16093C 16224C 16291T 16311C 16519C	K1a10a	73G 195C 263G 310.1C 497T 513.1CACA 16048A 16093C 16224C 16291T 16311C 16519C	1	73 195 263 497 750 1189 1438 1811 2706 3480 4769 7028 8860 9055 9698 10398 10550 11299 11467 11719 12308 12372 14167 14766 14798 15326 15431 16048 16093 16224 16291 16311	K1a10a	73 195 263 497 16048 16093 16224 16291 16311	[0.000] K1a10a (1)	[0.034] K1a10a (2) [0.000] K1a10	1
1425	>JQ704494.1 Ho	263G 309.1CT 310C 750G 1438G 3106d 3107C 4769G 8860G 11314G 13830C 14470A 15326G 16093C 16221T 16266T 16519C	H10e1a	263G 309.1CT 310C 16093C 16221T 16266T 16519C	1	263 750 1438 4769 8860 11314 13830 14470A 15326 16093 16221 16266	H10e1a	263 16093 16221 16266	[0.000] H10e1a (1)	[0.016] H10e1a (2) [0.000] H10e H10e1 H10e2 H10e3 H10e3a (7)	1
1426	>JQ704475.1 Ho	200G 263G 309.1CCT 310C 750G 1438G 3106d 3107C 4107T 4769G 7403G 8860G 11440A 11485C 14872T 15326G 16261T 16291T 16311C 16519C	H13b1b	200G 263G 309.1CCT 310C 16261T 16291T 16311C 16519C	1	200 263 750 1438 4107 4769 7403 8860 11440 11485 14872 15326 16261 16291 16311	H13b1b	200 263 16261 16291 16311	[0.333] H13b1b (1)	[0.333] H13b1b (2) [0.033] H13b1+200 (3) [0.000] H13a1a1d H13a1a1d1 H13b1a (6)	1
1427	>JQ704463.1 Ho	73G 152C 195C 246C 263G 309.1CT 310C 567.1CC 750G 1438G 2706G 3106d 3107C 4769G 5147A 6632C 7028T 7193C 8860G 9336G 11002G 11935C 12061T 12280G 15326G 16214T 16217C 16335G 16519C	HV2a1	73G 152C 195C 246C 263G 309.1CT 310C 567.1CC 16214T 16217C 16335G 16519C	1	73 152 195 246 263 573.2C 750 1438 2706 4769 5147 6632 7028 7193 8860 9336 11002 11935 12061 12280 15326 16214 16217 16335	HV2a1	73 152 195 246 263 573.2C 16214 16217 16335	[0.636] HV2a1 (1)	[0.022] HV2a3 (2) [0.000] HV2a HV2a2 (4)	1
1428	>JQ704458.1 Ho	152C 239C 263G 310.1C 750G 1438G 3106d 3107C 3915A 4727G 4769G 8860G 9380A 10589A 11779T 14180C 15326G 16249C 16362C 16482G	H6a1b4	152C 239C 263G 310.1C 16249C 16362C 16482G	1	152 239 263 750 1438 3915 4727 4769 8860 9380 10589 11779 14180 15326 16249 16362 16482	H6a1b4	152 239 263 16249 16362 16482	[0.100] H6a1b4 (1)	[0.176] H6a1a (2) [0.100] H6a1b4 (3) [0.023] H6a1b2 (4) [0.014] H6a1a4 (5) [0.008] H6a2a (6) [0.007] H6a2 H6c (8) [0.006] H6a1b (9) [0.000] H6 H6a H6a1 H6a1a1 H6a1a2 H6a1a2b H6a1a2b1 H6a1a3 H6a1a3a H6a1a5 H6a1a9 H6a1a10 H6a1b2a H6a1b2b H6a1b2c H6a1b2e (25)	1
1429	>MF058127.1 Hc	73G 146C 152C 195C 263G 310.1C 750G 769A 1018A 1438G 2416C 2706G 2789T 3106d 3107C 3594T 4104G 4769G 5090C 7028T 7175C 7256T 7274T 7521A 7771G 8206A 8701G 8860G 9221G 9540C 10115C 10143A 10398G 10873C 11719A 11914A 11944C 12693G 12705T 13590A 13650T 13803G 14566G 14766T 15301A 15326G 15735T 15784C 16183C 16189C 16223T 16278T 16290T 16294T 16309G 16390A	L2a1b1a	73G 146C 152C 195C 263G 310.1C 16183C 16189C 16223T 16278T 16290T 16294T 16309G 16390A	1	73 146 152 195 263 750 769 1018 1438 2416 2706 2789 3594 4104 4769 5090 7028 7175 7256 7274 7521 7771 8206 8701 8860 9221 9540 10115 10143 10398 10873 11719 11914 11944 12693 12705 13590 13650 13803 14566 14766 15301 15326 15735 15784 16189 16223 16278 16290 16294 16309 16390	L2a1b1a	73 146 152 195 263 16189 16223 16278 16290 16294 16309 16390	[0.000] L2a1b1a (1)	[0.005] L2a1f (2) [0.000] L2a1+16189_(16192) L2a1b L2a1b1 L2a1b1a L2a1b2 L2a1f1 L2a1f1a L2a1f2 (10)	1

1430 >MF058121.1 Hc	73G 152C 195C 204C 263G 310.1C 417A 489C 533G 750G 1055C 1438G 3106d 3107C 4769G 7028T 8188G 8360G 8701G 8860G 9438A 9540C 9545G 10142T 10295G 10398G 10400T 10873C 11569C 11719A 11899C 12279G 12618A 12705T 14766T 14783C 15025T 15043A 15301A 15326G 16223T 16263C 16311C 16362C 16519C	M23	73G 152C 195C 204C 263G 310.1C 417A 489C 533G 16223T 16263C 16311C 16362C 16519C	1	73 152 195 204 263 417 489 533 750 1055 1438 4769 7028 8188 8360 8701 8860 9438 9540 9545 10142 10295 10398 10400 10873 11569 11719 11899 12279 12618 12705 14766 14783 15025 15043 15301 15326 16223 16263 16311 16362	M23	73 152 195 204 263 417 489 533 16223 16263 16311 16362	[0.016] M23 (1)	[0.002] G2c (2) [0.000] D4h4a	1
1431 >MF058120.1 Hc	73G 152C 195C 204C 263G 310.1C 417A 489C 533G 750G 1438G 3106d 3107C 4769G 7028T 8188G 8360G 8701G 8860G 9438A 9540C 9545G 10142T 10295G 10398G 10400T 10873C 11569C 11719A 11899C 12279G 12618A 12705T 14766T 14783C 15025T 15043A 15301A 15326G 16223T 16263C 16311C 16519C	M23	73G 152C 195C 204C 263G 310.1C 417A 489C 533G 16223T 16263C 16311C 16519C	1	73 152 195 204 263 417 489 533 750 1438 4769 7028 8188 8360 8701 8860 9438 9540 9545 10142 10295 10398 10400 10873 11569 11719 11899 12279 12618 12705 14766 14783 15025 15043 15301 15326 16223 16263 16311	M23	73 152 195 204 263 417 489 533 16223 16263 16311	[0.000] M23 (1)	[0.072] M24a (2) [0.053] M57+152	1
1432 >MF058114.1 Hc	73G 150T 200G 263G 310.1C 489C 750G 2706G 3106d 3107C 3588T 4206C 4277C 4769G 7028T 7960G 8701G 8768T 8854A 8860G 9540C 10398G 10400T 10873C 11719A 12705T 12720G 13308G 13590A 13708A 14130T 14260G 14527G 14766T 14783C 15043A 15172A 15301A 15326G 15625T 16086C 16148T 16223T 16259T 16278T 16319A 16399G 16526A	M32c	73G 150T 200G 263G 310.1C 489C 16086C 16148T 16223T 16259T 16278T 16319A 16399G 16526A	1	73 150 200 263 489 750 2706 3588 4206C 4277 4769 7028 7960 8701 8768 8854 8860 9540 10398 10400 10873 11719 12705 12720 13308 13590 13708 14130 14260 14527 14766 14783 15043 15172 15301 15326 15625 16086 16148 16223 16259 16278 16319 16399 16526	M32c	73 150 200 263 489 16086 16148 16223 16259 16278 16319 16399 16526	[0.000] M32c (1)	[0.022] M51a (2) [0.007] M32 (3) [0.000] M51 M51b (5)	1
1433 >MF058112.1 Hc	73G 93G 146C 150T 152C 182T 183G 195C 198T 263G 310.1C 325T 514d 515d 680C 709A 750G 769A 1018A 1040C 1438G 1442A 2332T 2416C 2706G 3106d 3107C 3200A 3594T 4104G 4769G 7028T 7256T 7521A 7624A 8014G 8206A 8567C 8701G 8772C 8860G 9063G 9221G 9540C 10115C 10398G 10790C 10873C 11719A 11944C 12236A 12705T 13590A 13650T 13928C 13958C 14766T 15110A 15217A 15301A 15313C 15326G 15849T 16223T 16264T 16278T 16311C 16390A	L2c2b1b	73G 93G 146C 150T 152C 182T 183G 195C 198T 263G 310.1C 325T 514d 515d 16223T 16264T 16278T 16311C 16390A	1	73 93 146 150 152 182 183 195 198 263 325 680 709 750 769 1018 1040 1438 1442 2332 2416 2706 3200A 3594 4104 4769 7028 7256 7521 7624A 8014 8206 8567 8701 8772 8860 9063 9221 9540 10115 10398 10790 10873 11719 11944 12236 12705 13590 13650 13928C 13958C 14766 15110 15217 15301 15313 15326 15849 16223 16264 16278 16311 16390	L2c2b1b	73 93 146 150 152 182 183 195 198 263 325 16223 16264 16278 16311 16390	[0.000] L2c2b1b (1)	[0.000] L2c2b L2c2b1 L2c2b1a L2c2b2 (5)	1

1434 >MF058108.1 Hc	73G 151T 152C 182T 186A 189C 195C 198T 247A 263G 297G 316A 750G 769A 825A 1018A 1420C 1438G 2150.1A 2394d 2706G 2758A 2885C 3106d 3107C 3543T 3594T 3666A 4104G 4769G 5894.1C 5951G 6071C 6150A 6253C 7028T 7055G 7076G 7146G 7256T 7337A 7389C 7521A 7744C 8027A 8251A 8468T 8655T 8701G 8784G 8860G 8877C 9072G 9540C 10321C 10398G 10586A 10688A 10792G 10793T 10810C 10873C 11654G 11719A 12049T 12705T 12810G 13105G 13149G 13212T 13281C 13485G 13506T 13650T 13789C 14000A 14178C 14560A 14766T 14812T 14911T 15016T 15326G 15784C 16071T 16129A 16145A 16187T 16189C 16213A 16223T 16234T 16265C 16278T 16286G 16294T 16311C 16320T 16360T 16527T	L1c2a1a	73G 151T 152C 182T 186A 189C 195C 198T 247A 263G 297G 316A 16071T 16129A 16145A 16187T 16189C 16213A 16223T 16234T 16265C 16278T 16286G 16294T 16311C 16320T 16360T 16527T	1	73 151 152 182 186A 189C 195 198 247 263 297 316 750 769 825A 1018 1420 1438 2156.1A 2395d 2706 2758 2885 3543 3594 3666 4104 4769 5899.1C 5951 6071 6150 6253 7028 7055 7076 7146 7256 7337 7389 7521 7744 8027 8251 8468 8655 8701 8784 8860 8877 9072 9540 10321 10398 10586 10688 10792 10793 10810 10873 11654 11719 12049 12705 12810 13105 13149 13212 13281 13485 13506 13650 13789 14000A 14178 14560 14766 14812 14911 15016 15326 15784 16071 16129 16145 16187 16189 16213 16223 16234 16265C 16278 16286G 16294 16311 16320 16360 16527	L1c2a1a	73 151 152 182 186A 189C 195 198 247 263 297 316 16071 16129 16145 16187 16189 16213 16223 16234 16265C 16278 16286G 16294 16311 16320 16360 16527	[0.053] L1c2a1a (1)	[0.158] L1c2a1b (2) [0.000] L1c2a1	1
1435 >MF058106.1 Hc	73G 93G 146C 150T 152C 182T 183G 195C 198T 263G 310.1C 325T 514d 515d 680C 709A 750G 769A 1018A 1040C 1438G 1442A 2332T 2416C 2706G 3106d 3107C 3200A 3594T 4104G 4769G 7028T 7256T 7521A 7624A 8014G 8206A 8567C 8701G 8772C 8860G 9063G 9221G 9540C 10115C 10398G 10790C 10873C 11719A 11944C 12236A 12705T 13590A 13650T 13928C 13958C 14766T 15110A 15217A 15301A 15313C 15326G 15849T 16223T 16264T 16278T 16311C 16390A	L2c2b1b	73G 93G 146C 150T 152C 182T 183G 195C 198T 263G 310.1C 325T 514d 515d 16223T 16264T 16278T 16311C 16390A	1	73 93 146 150 152 182 183 195 198 263 325 680 709 750 769 1018 1040 1438 1442 2332 2416 2706 3200A 3594 4104 4769 7028 7256 7521 7624A 8014 8206 8567 8701 8772 8860 9063 9221 9540 10115 10398 10790 10873 11719 11944 12236 12705 13590 13650 13928C 13958C 14766 15110 15217 15301 15313 15326 15849 16223 16264 16278 16311 16390	L2c2b1b	73 93 146 150 152 182 183 195 198 263 325 16223 16264 16278 16311 16390	[0.000] L2c2b1b (1)	[0.000] L2c2b L2c2b1 L2c2b1a L2c2b2 (5)	1
1436 >MF058099.1 Hc	73G 146C 152C 195C 263G 310.1C 513A 750G 769A 1018A 1438G 2416C 2706G 2789T 3106d 3107C 3594T 4104G 4769G 5090C 7028T 7175C 7256T 7274T 7521A 7771G 8206A 8701G 8860G 9221G 9540C 10115C 10143A 10398G 10873C 11719A 11914A 11944C 12693G 12705T 13225A 13590A 13650T 13803G 14566G 14766T 15301A 15326G 15735T 15784C 16182C 16183C 16189C 16223T 16278T 16290T 16294T 16309G 16356C 16390A	L2a1b1a	73G 146C 152C 195C 263G 310.1C 513A 16182C 16183C 16189C 16223T 16278T 16290T 16294T 16309G 16356C 16390A	1	73 146 152 195 263 513 750 769 1018 1438 2416 2706 2789 3594 4104 4769 5090 7028 7175 7256 7274 7521 7771 8206 8701 8860 9221 9540 10115 10143 10398 10873 11719 11914 11944 12693 12705 13225 13590 13650 13803 14566 14766 15301 15326 15735 15784 16189 16223 16278 16290 16294 16309 16356 16390	L2a1b1a	73 146 152 195 263 513 16189 16223 16278 16290 16294 16309 16356 16390	[0.024] L2a1b1a (1)	[0.024] L2a1b1a (2) [0.010] L2a1f (3) [0.006] L2a1f2 (4) [0.000] L2a1+16189_(16192) L2a1b L2a1b1 L2a1b2 L2a1f1 L2a1f1a (10)	1

1437 >MF058098.1 Hc	73G 151T 152C 182T 186A 189C 195C 198T 247A 263G 297G 316A 750G 769A 825A 1018A 1420C 1438G 2150.1A 2394d 2706G 2758A 2885C 3106d 3107C 3594T 3666A 4104G 4769G 5894.1C 5951G 6071C 6150A 6253C 7028T 7055G 7076G 7146G 7256T 7337A 7389C 7521A 7744C 8027A 8251A 8468T 8655T 8701G 8784G 8860G 8877C 9072G 9540C 10321C 10398G 10586A 10688A 10792G 10793T 10810C 10873C 11654G 11719A 12049T 12705T 12810G 13105G 13149G 13212T 13281C 13485G 13506T 13650T 13789C 14000A 14178C 14560A 14766T 14812T 14911T 15016T 15326G 15784C 16071T 16129A 16145A 16187T 16189C 16213A 16223T 16234T 16258C 16265C 16278T 16286G 16294T 16311C 16360T 16527T	L1c2a1a	73G 151T 152C 182T 186A 189C 195C 198T 247A 263G 297G 316A 16071T 16129A 16145A 16187T 16189C 16213A 16223T 16234T 16258C 16265C 16278T 16286G 16294T 16311C 16360T 16527T	1	73 151 152 182 186A 189C 195 198 247 263 297 316 750 769 825A 1018 1420 1438 2156.1A 2395d 2706 2758 2885 3594 3666 4104 4769 5899.1C 5951 6071 6150 6253 7028 7055 7076 7146 7256 7337 7389 7521 7744 8027 8251 8468 8655 8701 8784 8860 8877 9072 9540 10321 10398 10586 10688 10792 10793 10810 10873 11654 11719 12049 12705 12810 13105 13149 13212 13281 13485 13506 13650 13789 14000A 14178 14560 14766 14812 14911 15016 15326 15784 16071 16129 16145 16187 16189 16213 16223 16234 16258C 16265C 16278 16286G 16294 16311 16360 16527	L1c2a1a	73 151 152 182 186A 189C 195 198 247 263 297 316 16071 16129 16145 16187 16189 16213 16223 16234 16258C 16265C 16278 16286G 16294 16311 16360 16527	[0.026] L1c2a1a (1)	[0.105] L1c2a1b (2) [0.000] L1c2a1	1
1438 >MF058097.1 Hc	73G 146C 152C 195C 263G 310.1C 513A 750G 769A 1018A 1438G 2416C 2706G 2789T 3106d 3107C 3594T 4104G 4769G 5090C 7028T 7175C 7256T 7274T 7521A 7771G 8206A 8701G 8860G 9221G 9540C 10115C 10143A 10398G 10873C 11719A 11914A 11944C 12693G 12705T 13225A 13590A 13650T 13803G 14566G 14766T 15301A 15326G 15735T 15784C 16182C 16183C 16189C 16223T 16278T 16290T 16294T 16309G 16356C 16390A	L2a1b1a	73G 146C 152C 195C 263G 310.1C 513A 16182C 16183C 16189C 16223T 16278T 16290T 16294T 16309G 16356C 16390A	1	73 146 152 195 263 513 750 769 1018 1438 2416 2706 2789 3594 4104 4769 5090 7028 7175 7256 7274 7521 7771 8206 8701 8860 9221 9540 10115 10143 10398 10873 11719 11914 11944 12693 12705 13225 13590 13650 13803 14566 14766 15301 15326 15735 15784 16189 16223 16278 16290 16294 16309 16356 16390	L2a1b1a	73 146 152 195 263 513 16189 16223 16278 16290 16294 16309 16356 16390	[0.024] L2a1b1a (1)	[0.024] L2a1b1a (2) [0.010] L2a1f (3) [0.006] L2a1f2 (4) [0.000] L2a1+16189_(16192) L2a1b L2a1b1 L2a1b2 L2a1f1 L2a1f1a (10)	1
1439 >MF058089.1 Hc	73G 152C 195C 204C 263G 310.1C 417A 489C 533G 750G 1438G 3106d 3107C 4769G 7028T 8188G 8360G 8701G 8860G 9438A 9540C 9545G 10142T 10295G 10398G 10400T 10873C 11569C 11719A 11899C 12279G 12618A 12705T 14766T 14783C 15025T 15043A 15301A 15326G 16223T 16263C 16311C 16362C 16519C	M23	73G 152C 195C 204C 263G 310.1C 417A 489C 533G 16223T 16263C 16311C 16362C 16519C	1	73 152 195 204 263 417 489 533 750 1438 4769 7028 8188 8360 8701 8860 9438 9540 9545 10142 10295 10398 10400 10873 11569 11719 11899 12279 12618 12705 14766 14783 15025 15043 15301 15326 16223 16263 16311 16362	M23	73 152 195 204 263 417 489 533 16223 16263 16311 16362	[0.016] M23 (1)	[0.002] G2c (2) [0.000] D4h4a	1

1440 >MF058084.1 Hc	73G 150T 200G 263G 310.1C 489C 750G 2706G 3106d 3107C 3588T 4769G 7028T 7960G 8701G 8768T 8854A 8860G 9540C 10398G 10400T 10873C 10882C 11719A 12705T 13308G 13590A 13708A 14130T 14260G 14527G 14766T 14783C 15043A 15172A 15301A 15326G 15625T 16086C 16148T 16223T 16259T 16278T 16319A 16399G 16526A	M32c	73G 150T 200G 263G 310.1C 489C 16086C 16148T 16223T 16259T 16278T 16319A 16399G 16526A	1	73 150 200 263 489 750 2706 3588 4769 7028 7960 8701 8768 8854 8860 9540 10398 10400 10873 10882 11719 12705 13308 13590 13708 14130 14260 14527 14766 14783 15043 15172 15301 15326 15625 16086 16148 16223 16259 16278 16319 16399 16526	M32c	73 150 200 263 489 16086 16148 16223 16259 16278 16319 16399 16526	[0.000] M32c (1)	[0.022] M51a (2) [0.007] M32 (3) [0.000] M51 M51b (5)	1
1441 >MF058081.1 Hc	73G 263G 279C 310.1C 514d 515d 750G 1438G 2332T 2706G 3106d 3107C 3450T 4769G 5147A 5773A 6221C 6340T 7028T 8701G 8860G 9449T 9540C 10086G 10373A 10398G 10873C 11002G 11719A 12705T 13105G 13914A 14766T 15301A 15311G 15326G 15824G 15940d 16129A 16145A 16223T 16235G 16278T 16362C 16519C	L3b1a8	73G 263G 279C 310.1C 514d 515d 16129A 16145A 16223T 16235G 16278T 16362C 16519C	1	73 263 279 750 1438 2332 2706 3450 4769 5147 5773 6221 6340 7028 8701 8860 9449 9540 10086 10373 10398 10873 11002 11719 12705 13105 13914A 14766 15301 15311 15326 15824 15944d 16129 16145 16223 16235 16278 16362	L3b1a8	73 263 279 16129 16145 16223 16235 16278 16362	[0.260] L3b1a8 (1)	[0.000] L3b1a+@16124 L3b1a7 L3b1a7a (4)	1
1442 >MF058077.1 Hc	73G 151T 152C 182T 186A 189C 247A 263G 310.1C 316A 514d 515d 629C 750G 769A 825A 1018A 1438G 2283T 2394d 2706G 2758A 2885C 3106d 3107C 3210T 3434G 3594T 3666A 4104G 4755C 4769G 5951G 6071C 6221A 6917A 7028T 7146G 7256T 7389C 7521A 8027A 8251A 8417T 8468T 8655T 8701G 8860G 9072G 9540C 10398G 10586A 10688A 10810C 10873C 11302T 11317G 11719A 12400G 12542T 12705T 12810G 13105G 13485G 13506T 13650T 13708A 13789C 13981T 14000A 14070G 14178C 14560A 14766T 14794T 14911T 15226G 15326G 15905C 15978T 16017C 16129A 16163G 16187T 16189C 16209C 16223T 16278T 16293G 16294T 16311C 16360T 16519C	L1c3b1a	73G 151T 152C 182T 186A 189C 247A 263G 310.1C 316A 514d 515d 16129A 16163G 16187T 16189C 16209C 16223T 16278T 16293G 16294T 16311C 16360T 16519C	1	73 151 152 182 186A 189C 247 263 316 629 750 769 825A 1018 1438 2283 2395d 2706 2758 2885 3210 3434 3594 3666 4104 4755 4769 5951 6071 6221A 6917 7028 7146 7256 7389 7521 8027 8251 8417 8468 8655 8701 8860 9072 9540 10398 10586 10688 10810 10873 11302 11317 11719 12400 12542 12705 12810 13105 13485 13506 13650 13708 13789 13981 14000A 14070 14178 14560 14766 14794 14911 15226 15326 15905 15978 16017 16129 16163 16187 16189 16209 16223 16278 16293 16294 16311 16360	L1c3b1a	73 151 152 182 186A 189C 247 263 316 16129 16163 16187 16189 16209 16223 16278 16293 16294 16311 16360	[0.000] L1c3b1a (1)	[0.000] L1c3b L1c3b1 L1c3b1b (4)	1
1443 >MF058075.1 Hc	73G 150T 185A 189G 263G 309.1CT 310C 750G 1438G 2352C 2706G 3106d 3107C 4769G 6221C 6587T 7028T 8577G 8701G 8860G 9540C 10398G 10819G 10873C 11719A 11893G 12192A 12705T 14152G 14212C 14766T 14926G 15140A 15301A 15326G 15670C 15942C 16223T 16323d 16327T	L3e1b2	73G 150T 185A 189G 263G 309.1CT 310C 16223T 16323d 16327T	1	73 150 185 189 263 750 1438 2352 2706 4769 6221 6587 7028 8577 8701 8860 9540 10398 10819 10873 11719 11893 12192 12705 14152 14212 14766 14926 15140 15301 15326 15670 15942 16223 16325d 16327	L3e1b2	73 150 185 189 263 16223 16325d 16327	[0.000] L3e1b2 (1)	[0.200] L3e1b (2)	1

1444	>MF058072.1 Hc	73G 146C 152C 195C 263G 309.1CCT 310C 750G 769A 1018A 1438G 2416C 2706G 2789T 3106d 3107C 3397G 3594T 4104G 4769G 5090C 7028T 7175C 7256T 7274T 7521A 7771G 8206A 8701G 8860G 9221G 9540C 10115C 10143A 10398G 10873C 11719A 11914A 11944C 12693G 12705T 13590A 13650T 13803G 14566G 14766T 15301A 15326G 15735T 15784C 16182C 16183C 16189C 16223T 16278T 16290T 16294T 16309G 16390A	L2a1b1a	73G 146C 152C 195C 263G 309.1CCT 310C 16182C 16183C 16189C 16223T 16278T 16290T 16294T 16309G 16390A	1	73 146 152 195 263 750 769 1018 1438 2416 2706 2789 3397 3594 4104 4769 5090 7028 7175 7256 7274 7521 7771 8206 8701 8860 9221 9540 10115 10143 10398 10873 11719 11914 11944 12693 12705 13590 13650 13803 14566 14766 15301 15326 15735 15784 16189 16223 16278 16290 16294 16309 16390	L2a1b1a	73 146 152 195 263 16189 16223 16278 16290 16294 16309 16390	[0.000] L2a1b1a (1)	[0.005] L2a1f (2) [0.000] L2a1+16189_(16192) L2a1b L2a1b1 L2a1b1a L2a1b2 L2a1f1 L2a1f1a L2a1f2 (10)	1
1445	>MF058069.1 Hc	73G 151T 152C 182T 186A 189C 195C 198T 247A 263G 297G 310.1C 316A 385G 471C 514d 515d 750G 769A 825A 1018A 1420C 1438G 1694C 1888A 2150.1A 2394d 2706G 2758A 2885C 3106d 3107C 3594T 3666A 4104G 4769G 5894.1C 5951G 6071C 6150A 6253C 6723A 7028T 7055G 7076G 7146G 7256T 7337A 7389C 7521A 7741C 8027A 8468T 8655T 8701G 8784G 8860G 8877C 9072G 9540C 10321C 10398G 10586A 10688A 10792G 10793T 10810C 10873C 11329G 11654G 11719A 12033G 12049T 12705T 12810G 13105G 13149G 13485G 13506T 13650T 13789C 14000A 14178C 14560A 14766T 14861A 14911T 15016T 15191C 15326G 15784C 16172C 16187T 16189C 16223T 16265C 16278T 16286G 16294T 16311C 16360T 16519C 16527T	L1c2a3a	73G 151T 152C 182T 186A 189C 195C 198T 247A 263G 297G 310.1C 316A 385G 471C 514d 515d 16172C 16187T 16189C 16223T 16265C 16278T 16286G 16294T 16311C 16360T 16519C 16527T	1	73 151 152 182 186A 189C 195 198 247 263 297 316 385 471 750 769 825A 1018 1420 1438 1694 1888 2156.1A 2395d 2706 2758 2885 3594 3666 4104 4769 5899.1C 5951 6071 6150 6253 6723 7028 7055 7076 7146 7256 7337 7389 7521 7741 8027 8468 8655 8701 8784 8860 8877 9072 9540 10321 10398 10586 10688 10792 10793 10810 10873 11329 11654 11719 12033 12049 12705 12810 13105 13149 13485 13506 13650 13789 14000A 14178 14560 14766 14861 14911 15016 15191 15326 15784 16172 16187 16189 16223 16265C 16278 16286G 16294 16311 16360 16527	L1c2a3a	73 151 152 182 186A 189C 195 198 247 263 297 316 385 471 16172 16187 16189 16223 16265C 16278 16286G 16294 16311 16360 16527	[0.000] L1c2a3a (1)	[0.091] L1c2a3 (2)	1
1446	>MF058064.1 Hc	73G 152C 195C 204C 263G 310.1C 417A 489C 533G 750G 1438G 3106d 3107C 4769G 7028T 8188G 8360G 8701G 8860G 9438A 9540C 9545G 10142T 10295G 10398G 10400T 10873C 11569C 11719A 11899C 12279G 12618A 12705T 14766T 14783C 15025T 15043A 15236G 15301A 15326G 16223T 16263C 16311C 16519C	M23	73G 152C 195C 204C 263G 310.1C 417A 489C 533G 16223T 16263C 16311C 16519C	1	73 152 195 204 263 417 489 533 750 1438 4769 7028 8188 8360 8701 8860 9438 9540 9545 10142 10295 10398 10400 10873 11569 11719 11899 12279 12618 12705 14766 14783 15025 15043 15236 15301 15326 16223 16263 16311	M23	73 152 195 204 263 417 489 533 16223 16263 16311	[0.000] M23 (1)	[0.072] M24a (2) [0.053] M57+152	1

1447	>MF058060.1 Hc	73G 150T 200G 263G 310.1C 489C 750G 2706G 3106d 3107C 3588T 4769G 7028T 7960G 8701G 8768T 8854A 8860G 9540C 10398G 10400T 10873C 11719A 12705T 13308G 13590A 13708A 14130T 14260G 14527G 14766T 14783C 15043A 15172A 15301A 15326G 15625T 16086C 16148T 16223T 16259T 16278T 16319A 16399G 16526A	M32c	73G 150T 200G 263G 310.1C 489C 16086C 16148T 16223T 16259T 16278T 16319A 16399G 16526A	1	73 150 200 263 489 750 2706 3588 4769 7028 7960 8701 8768 8854 8860 9540 10398 10400 10873 11719 12705 13308 13590 13708 14130 14260 14527 14766 14783 15043 15172 15301 15326 15625 16086 16148 16223 16259 16278 16319 16399 16526	M32c	73 150 200 263 489 16086 16148 16223 16259 16278 16319 16399 16526	[0.000] M32c (1)	[0.022] M51a (2) [0.007] M32 (3) [0.000] M51 M51b (5)	1
1448	>MF058055.1 Hc	73G 146C 195C 263G 310.1C 750G 769A 1018A 1438G 2416C 2706G 2789T 3106d 3107C 3594T 4104G 4769G 5090C 7028T 7175C 7256T 7274T 7521A 7771G 8206A 8701G 8860G 9221G 9540C 9604G 10115C 10143A 10398G 10873C 11719A 11914A 11944C 12693G 12705T 13590A 13650T 13803G 14566G 14766T 15301A 15326G 15735T 15784C 16111T 16182C 16183C 16189C 16223T 16278T 16290T 16294T 16309G 16390A	L2a1b1a	73G 146C 195C 263G 310.1C 16111T 16182C 16183C 16189C 16223T 16278T 16290T 16294T 16309G 16390A	1	73 146 195 263 750 769 1018 1438 2416 2706 2789 3594 4104 4769 5090 7028 7175 7256 7274 7521 7771 8206 8701 8860 9221 9540 9604 10115 10143 10398 10873 11719 11914 11944 12693 12705 13590 13650 13803 14566 14766 15301 15326 15735 15784 16111 16189 16223 16278 16290 16294 16309 16390	L2a1b1a	73 146 195 263 16111 16189 16223 16278 16290 16294 16309 16390	[0.024] L2a1b1a (1)	[0.024] L2a1b1a (2) [0.016] L2a1+16189_(16192) (3) [0.008] L2a1b1 (4) [0.005] L2a1f (5) [0.000] L2a1b L2a1b2 L2a1f1 L2a1f1a L2a1f2 (10)	1
1449	>MF058054.1 Hc	73G 146C 152C 195C 263G 310.1C 750G 769A 1018A 1438G 2416C 2706G 2789T 3106d 3107C 3594T 4104G 4769G 5090C 7028T 7175C 7256T 7274T 7521A 7771G 8206A 8701G 8860G 9221G 9540C 10115C 10143A 10398G 10873C 11719A 11914A 11944C 12693G 12705T 13590A 13650T 13803G 14566G 14766T 15301A 15326G 15735T 15784C 16182C 16183C 16189C 16223T 16278T 16290T 16294T 16309G	L2a1b1a	73G 146C 152C 195C 263G 310.1C 16182C 16183C 16189C 16223T 16278T 16290T 16294T 16309G	1	73 146 152 195 263 750 769 1018 1438 2416 2706 2789 3594 4104 4769 5090 7028 7175 7256 7274 7521 7771 8206 8701 8860 9221 9540 10115 10143 10398 10873 11719 11914 11944 12693 12705 13590 13650 13803 14566 14766 15301 15326 15735 15784 16189 16223 16278 16290 16294 16309	L2a1b1a	73 146 152 195 263 16189 16223 16278 16290 16294 16309	[0.006] L2a1b1a (1)	[0.010] L2a1f (2) [0.006] L2a1b1a (3) [0.000] L2a1+16189_(16192) L2a1b L2a1b1 L2a1b2 L2a1f1 L2a1f1a L2a1f2 (10)	1
1450	>MF058046.1 Hc	73G 152C 195C 204C 263G 310.1C 417A 489C 533G 750G 1438G 3106d 3107C 4769G 7028T 8188G 8360G 8701G 8860G 9438A 9540C 9545G 10142T 10295G 10398G 10400T 10873C 11569C 11719A 11899C 12279G 12618A 12705T 14207A 14766T 14783C 15025T 15043A 15301A 15326G 16223T 16263C 16311C 16519C	M23	73G 152C 195C 204C 263G 310.1C 417A 489C 533G 16223T 16263C 16311C 16519C	1	73 152 195 204 263 417 489 533 750 1438 4769 7028 8188 8360 8701 8860 9438 9540 9545 10142 10295 10398 10400 10873 11569 11719 11899 12279 12618 12705 14207 14766 14783 15025 15043 15301 15326 16223 16263 16311	M23	73 152 195 204 263 417 489 533 16223 16263 16311	[0.000] M23 (1)	[0.072] M24a (2) [0.053] M57+152	1

1451	>MF058029.1 Hc	73G 152C 195C 204C 263G 309.1CT 310C 417A 489C 533G 750G 1438G 3106d 3107C 4769G 7028T 8188G 8360G 8701G 8860G 9438A 9540C 9545G 10142T 10295G 10398G 10400T 10873C 11569C 11719A 11899C 12279G 12618A 12705T 14766T 14783C 15025T 15043A 15301A 15326G 16223T 16263C 16311C 16519C	M23	73G 152C 195C 204C 263G 309.1CT 310C 417A 489C 533G 16223T 16263C 16311C 16519C	1	73 152 195 204 263 417 489 533 750 1438 4769 7028 8188 8360 8701 8860 9438 9540 9545 10142 10295 10398 10400 10873 11569 11719 11899 12279 12618 12705 14766 14783 15025 15043 15301 15326 16223 16263 16311	M23	73 152 195 204 263 417 489 533 16223 16263 16311	[0.000] M23 (1)	[0.072] M24a (2) [0.053] M57+152	1
1452	>MF058027.1 Hc	73G 152C 195C 204C 263G 310.1C 417A 489C 533G 750G 1438G 3106d 3107C 4769G 7028T 8188G 8360G 8701G 8860G 9438A 9540C 9545G 10142T 10295G 10398G 10400T 10873C 11569C 11719A 11899C 12279G 12618A 12705T 14766T 14783C 15025T 15043A 15301A 15326G 16223T 16263C 16311C 16519C	M23	73G 152C 195C 204C 263G 310.1C 417A 489C 533G 16223T 16263C 16311C 16519C	1	73 152 195 204 263 417 489 533 750 1438 4769 7028 8188 8360 8701 8860 9438 9540 9545 10142 10295 10398 10400 10873 11569 11719 11899 12279 12618 12705 14766 14783 15025 15043 15301 15326 16223 16263 16311	M23	73 152 195 204 263 417 489 533 16223 16263 16311	[0.000] M23 (1)	[0.072] M24a (2) [0.053] M57+152	1
1453	>MF058024.1 Hc	73G 152C 195C 204C 263G 310.1C 417A 489C 533G 750G 1438G 3106d 3107C 4769G 7028T 8188G 8360G 8701G 8860G 9438A 9540C 9545G 10142T 10295G 10398G 10400T 10873C 11569C 11719A 11899C 12279G 12618A 12705T 14766T 14783C 15025T 15043A 15301A 15326G 16223T 16263C 16311C 16519C	M23	73G 152C 195C 204C 263G 310.1C 417A 489C 533G 16223T 16263C 16311C 16519C	1	73 152 195 204 263 417 489 533 750 1438 4769 7028 8188 8360 8701 8860 9438 9540 9545 10142 10295 10398 10400 10873 11569 11719 11899 12279 12618 12705 14766 14783 15025 15043 15301 15326 16223 16263 16311	M23	73 152 195 204 263 417 489 533 16223 16263 16311	[0.000] M23 (1)	[0.072] M24a (2) [0.053] M57+152	1
1454	>MF058018.1 Hc	73G 146C 152C 195C 263G 750G 769A 1018A 1438G 2416C 2706G 2762T 2789T 3106d 3107C 3594T 4104G 4769G 5090C 7028T 7175C 7256T 7274T 7521A 7771G 8206A 8701G 8860G 9221G 9540C 10115C 10143A 10398G 10873C 11719A 11914A 11944C 12693G 12705T 13590A 13650T 13803G 14566G 14766T 15301A 15326G 15735T 15784C 16182C 16183C 16189C 16223T 16278T 16290T 16294T 16309G 16359C 16390A	L2a1b1a	73G 146C 152C 195C 263G 16182C 16183C 16189C 16223T 16278T 16290T 16294T 16309G 16359C 16390A	1	73 146 152 195 263 750 769 1018 1438 2416 2706 2762 2789 3594 4104 4769 5090 7028 7175 7256 7274 7521 7771 8206 8701 8860 9221 9540 10115 10143 10398 10873 11719 11914 11944 12693 12705 13590 13650 13803 14566 14766 15301 15326 15735 15784 16189 16223 16278 16290 16294 16309 16359 16390	L2a1b1a	73 146 152 195 263 16189 16223 16278 16290 16294 16309 16359 16390	[0.003] L2a1b1a (1)	[0.005] L2a1f (2) [0.003] L2a1b1a (3) [0.000] L2a1+16189_(16192) L2a1b L2a1b1 L2a1b2 L2a1f1 L2a1f1a L2a1f2 (10)	1
1455	>MF058017.1 Hc	73G 152C 195C 204C 263G 310.1C 417A 489C 533G 750G 1438G 3106d 3107C 4769G 7028T 8188G 8360G 8701G 8860G 9438A 9540C 9545G 10142T 10295G 10398G 10400T 10873C 11569C 11719A 11899C 12279G 12618A 12705T 13395G 14766T 14783C 15025T 15043A 15301A 15326G 16223T 16263C 16311C 16519C	M23	73G 152C 195C 204C 263G 310.1C 417A 489C 533G 16223T 16263C 16311C 16519C	1	73 152 195 204 263 417 489 533 750 1438 4769 7028 8188 8360 8701 8860 9438 9540 9545 10142 10295 10398 10400 10873 11569 11719 11899 12279 12618 12705 13395 14766 14783 15025 15043 15301 15326 16223 16263 16311	M23	73 152 195 204 263 417 489 533 16223 16263 16311	[0.000] M23 (1)	[0.072] M24a (2) [0.053] M57+152	1

1456 >MF058012.1 Hc	73G 152C 182T 186A 189C 195C 247A 248G 263G 310.1C 316A 514d 515d 745.1T 750G 769A 825A 1018A 1438G 2394d 2706G 2758A 2885C 3106d 3107C 3525T 3594T 3666A 4104G 4769G 4853A 5951G 6071C 6221A 6227C 6917A 7028T 7146G 7247T 7256T 7389C 7521A 8027A 8468T 8655T 8701G 8860G 9072G 9540C 10398G 10586A 10688A 10810C 10873C 11302T 11719A 11984C 12507G 12705T 12810G 13105G 13485G 13506T 13650T 13789C 14000A 14178C 14560A 14669G 14766T 14911T 14926G 15226G 15326G 15905C 15976C 15978T 16129A 16187T 16189C 16223T 16278T 16293G 16294T 16311C 16360T 16519C	L1c3c	73G 152C 182T 186A 189C 195C 247A 248G 263G 310.1C 316A 514d 515d 16129A 16187T 16189C 16223T 16278T 16293G 16294T 16311C 16360T 16519C	1	73 152 182 186A 189C 195 247 248 263 316 745.1T 750 769 825A 1018 1438 2395d 2706 2758 2885 3525 3594 3666 4104 4769 4853 5951 6071 6221A 6227 6917 7028 7146 7247 7256 7389 7521 8027 8468 8655 8701 8860 9072 9540 10398 10586 10688 10810 10873 11302 11719 11984 12507 12705 12810 13105 13485 13506 13650 13789 14000A 14178 14560 14669 14766 14911 14926 15226 15326 15905 15976 15978 16129 16187 16189 16223 16278 16293 16294 16311 16360	L1c3c	73 152 182 186A 189C 195 247 248 263 316 16129 16187 16189 16223 16278 16293 16294 16311 16360	[2.063] L1c3c (1) [0.000] L1c L1c1a+@198 L1c3b'c (4)	[0.700] L1c3 (5) [0.572] L1c1a'b'd (6) [0.267] L1c3b1 (7) [0.133] L1c3b1b (8) [0.000] L1c1'2'4'5'6 L1c1 L1c1a L1c1a1 L1c3b (13)	1
1457 >MF057999.1 Hc	73G 150T 200G 263G 310.1C 489C 750G 2706G 3106d 3107C 3588T 4769G 7028T 7960G 8701G 8768T 8854A 8860G 9540C 10398G 10400T 10873C 11719A 12705T 13308G 13590A 13708A 14130T 14260G 14527G 14766T 14783C 15043A 15172A 15301A 15326G 15625T 16086C 16148T 16223T 16259T 16278T 16319A 16399G 16526A	M32c	73G 150T 200G 263G 310.1C 489C 16086C 16148T 16223T 16259T 16278T 16319A 16399G 16526A	1	73 150 200 263 489 750 2706 3588 4769 7028 7960 8701 8768 8854 8860 9540 10398 10400 10873 11719 12705 13308 13590 13708 14130 14260 14527 14766 14783 15043 15172 15301 15326 15625 16086 16148 16223 16259 16278 16319 16399 16526	M32c	73 150 200 263 489 16086 16148 16223 16259 16278 16319 16399 16526	[0.000] M32c (1)	[0.022] M51a (2) [0.007] M32 (3) [0.000] M51 M51b (5)	1
1458 >MF057994.1 Hc	73G 263G 279C 310.1C 514d 515d 750G 1438G 2332T 2706G 3106d 3107C 3450T 4769G 5773A 6221C 6340T 7028T 8701G 8860G 9449T 9540C 10086G 10373A 10398G 10873C 11002G 11719A 12705T 13105G 13914A 14766T 15301A 15311G 15326G 15824G 15940d 16145A 16223T 16271C 16278T 16362C 16519C	L3b1a8	73G 263G 279C 310.1C 514d 515d 16145A 16223T 16271C 16278T 16362C 16519C	1	73 263 279 750 1438 2332 2706 3450 4769 5773 6221 6340 7028 8701 8860 9449 9540 10086 10373 10398 10873 11002 11719 12705 13105 13914A 14766 15301 15311 15326 15824 15944d 16145 16223 16271 16278 16362	L3b1a8	73 263 279 16145 16223 16271 16278 16362	[0.222] L3b1a8 (1)	[0.000] L3b1a+@16124 L3b1a7 L3b1a7a (4)	1
1459 >MF057974.1 Hc	73G 152C 195C 204C 263G 310.1C 417A 489C 533G 750G 1438G 3106d 3107C 4769G 7028T 8188G 8360G 8701G 8860G 9438A 9540C 9545G 10142T 10295G 10398G 10400T 10873C 11569C 11719A 11899C 12279G 12618A 12705T 14766T 14783C 15025T 15043A 15301A 15326G 16223T 16263C 16311C 16519C	M23	73G 152C 195C 204C 263G 310.1C 417A 489C 533G 16223T 16263C 16311C 16519C	1	73 152 195 204 263 417 489 533 750 1438 4769 7028 8188 8360 8701 8860 9438 9540 9545 10142 10295 10398 10400 10873 11569 11719 11899 12279 12618 12705 14766 14783 15025 15043 15301 15326 16223 16263 16311	M23	73 152 195 204 263 417 489 533 16223 16263 16311	[0.000] M23 (1)	[0.072] M24a (2) [0.053] M57+152	1

1460	>MF057972.1 Hc	73G 152C 195C 204C 263G 310.1C 417A 489C 533G 750G 1438G 3106d 3107C 4769G 7028T 8188G 8360G 8701G 8860G 9438A 9540C 9545G 10142T 10295G 10398G 10400T 10873C 11569C 11719A 11899C 12279G 12618A 12705T 14766T 14783C 15025T 15043A 15301A 15326G 16223T 16263C 16311C 16519C	M23	73G 152C 195C 204C 263G 310.1C 417A 489C 533G 16223T 16263C 16311C 16519C	1	73 152 195 204 263 417 489 533 750 1438 4769 7028 8188 8360 8701 8860 9438 9540 9545 10142 10295 10398 10400 10873 11569 11719 11899 12279 12618 12705 14766 14783 15025 15043 15301 15326 16223 16263 16311	M23	73 152 195 204 263 417 489 533 16223 16263 16311	[0.000] M23 (1)	[0.072] M24a (2) [0.053] M57+152	1
1461	>MF057971.1 Hc	73G 152C 195C 204C 263G 310.1C 417A 489C 533G 750G 1438G 3106d 3107C 4769G 7028T 8188G 8360G 8701G 8860G 9438A 9540C 9545G 10142T 10295G 10398G 10400T 10873C 11569C 11719A 11899C 12279G 12618A 12705T 14766T 14783C 15025T 15043A 15301A 15326G 16223T 16263C 16311C 16519C	M23	73G 152C 195C 204C 263G 310.1C 417A 489C 533G 16223T 16263C 16311C 16519C	1	73 152 195 204 263 417 489 533 750 1438 4769 7028 8188 8360 8701 8860 9438 9540 9545 10142 10295 10398 10400 10873 11569 11719 11899 12279 12618 12705 14766 14783 15025 15043 15301 15326 16223 16263 16311	M23	73 152 195 204 263 417 489 533 16223 16263 16311	[0.000] M23 (1)	[0.072] M24a (2) [0.053] M57+152	1
1462	>MF057970.1 Hc	73G 152C 195C 204C 263G 310.1C 417A 489C 533G 750G 1438G 3106d 3107C 4769G 7028T 8188G 8360G 8701G 8860G 9438A 9540C 9545G 10142T 10295G 10398G 10400T 10873C 11569C 11719A 11899C 12279G 12618A 12705T 14766T 14783C 15025T 15043A 15301A 15326G 16223T 16263C 16311C 16519C	M23	73G 152C 195C 204C 263G 310.1C 417A 489C 533G 16223T 16263C 16311C 16519C	1	73 152 195 204 263 417 489 533 750 1438 4769 7028 8188 8360 8701 8860 9438 9540 9545 10142 10295 10398 10400 10873 11569 11719 11899 12279 12618 12705 14766 14783 15025 15043 15301 15326 16223 16263 16311	M23	73 152 195 204 263 417 489 533 16223 16263 16311	[0.000] M23 (1)	[0.072] M24a (2) [0.053] M57+152	1
1463	>MF057966.1 Hc	73G 152C 195C 204C 263G 310.1C 417A 489C 533G 750G 1438G 3106d 3107C 4769G 7028T 8188G 8360G 8701G 8860G 9438A 9540C 9545G 10142T 10295G 10398G 10400T 10873C 11569C 11719A 11899C 12279G 12618A 12705T 14766T 14783C 15025T 15043A 15301A 15326G 16223T 16263C 16311C 16519C	M23	73G 152C 195C 204C 263G 310.1C 417A 489C 533G 16223T 16263C 16311C 16519C	1	73 152 195 204 263 417 489 533 750 1438 4769 7028 8188 8360 8701 8860 9438 9540 9545 10142 10295 10398 10400 10873 11569 11719 11899 12279 12618 12705 14766 14783 15025 15043 15301 15326 16223 16263 16311	M23	73 152 195 204 263 417 489 533 16223 16263 16311	[0.000] M23 (1)	[0.072] M24a (2) [0.053] M57+152	1
1464	>MF057964.1 Hc	73G 152C 195C 204C 263G 310.1C 417A 489C 533G 750G 1438G 3106d 3107C 4769G 7028T 8188G 8360G 8701G 8860G 9438A 9540C 9545G 10142T 10295G 10398G 10400T 10873C 11569C 11719A 11899C 12279G 12618A 12705T 14766T 14783C 15025T 15043A 15301A 15326G 16223T 16263C 16311C 16519C	M23	73G 152C 195C 204C 263G 310.1C 417A 489C 533G 16223T 16263C 16311C 16519C	1	73 152 195 204 263 417 489 533 750 1438 4769 7028 8188 8360 8701 8860 9438 9540 9545 10142 10295 10398 10400 10873 11569 11719 11899 12279 12618 12705 14766 14783 15025 15043 15301 15326 16223 16263 16311	M23	73 152 195 204 263 417 489 533 16223 16263 16311	[0.000] M23 (1)	[0.072] M24a (2) [0.053] M57+152	1

1465	>MF057963.1 Hc	73G 152C 195C 204C 263G 310.1C 417A 489C 533G 750G 1438G 3106d 3107C 4769G 7028T 8188G 8360G 8701G 8860G 9438A 9540C 9545G 10142T 10295G 10398G 10400T 10873C 11569C 11719A 11899C 12279G 12618A 12705T 14766T 14783C 15025T 15043A 15301A 15326G 16223T 16263C 16311C 16519C	M23	73G 152C 195C 204C 263G 310.1C 417A 489C 533G 16223T 16263C 16311C 16519C	1	73 152 195 204 263 417 489 533 750 1438 4769 7028 8188 8360 8701 8860 9438 9540 9545 10142 10295 10398 10400 10873 11569 11719 11899 12279 12618 12705 14766 14783 15025 15043 15301 15326 16223 16263 16311	M23	73 152 195 204 263 417 489 533 16223 16263 16311	[0.000] M23 (1)	[0.072] M24a (2) [0.053] M57+152	1
1466	>MF057962.1 Hc	73G 152C 195C 204C 263G 310.1C 417A 489C 533G 750G 1438G 3106d 3107C 4769G 7028T 8188G 8360G 8701G 8860G 9438A 9540C 9545G 10142T 10295G 10398G 10400T 10873C 11569C 11719A 11899C 12279G 12618A 12705T 14766T 14783C 15025T 15043A 15301A 15326G 16223T 16263C 16311C 16519C	M23	73G 152C 195C 204C 263G 310.1C 417A 489C 533G 16223T 16263C 16311C 16519C	1	73 152 195 204 263 417 489 533 750 1438 4769 7028 8188 8360 8701 8860 9438 9540 9545 10142 10295 10398 10400 10873 11569 11719 11899 12279 12618 12705 14766 14783 15025 15043 15301 15326 16223 16263 16311	M23	73 152 195 204 263 417 489 533 16223 16263 16311	[0.000] M23 (1)	[0.072] M24a (2) [0.053] M57+152	1
1467	>MF056338.1 Hc	73G 146C 152C 195C 263G 750G 769A 1018A 1438G 2416C 2706G 2789T 3106d 3107C 3594T 4104G 4769G 5090C 7028T 7175C 7256T 7274T 7521A 7771G 8206A 8701G 8860G 9221G 9540C 10115C 10143A 10398G 10873C 11719A 11914A 11944C 12693G 12705T 13590A 13650T 13803G 14566G 14766T 15301A 15326G 15735T 15784C 16182C 16183C 16189C 16223T 16278T 16290T 16294T 16309G 16390A	L2a1b1a	73G 146C 152C 195C 263G 16182C 16183C 16189C 16223T 16278T 16290T 16294T 16309G 16390A	1	73 146 152 195 263 750 769 1018 1438 2416 2706 2789 3594 4104 4769 5090 7028 7175 7256 7274 7521 7771 8206 8701 8860 9221 9540 10115 10143 10398 10873 11719 11914 11944 12693 12705 13590 13650 13803 14566 14766 15301 15326 15735 15784 16189 16223 16278 16290 16294 16309 16390	L2a1b1a	73 146 152 195 263 16189 16223 16278 16290 16294 16309 16390	[0.000] L2a1b1a (1)	[0.005] L2a1f (2) [0.000] L2a1+16189_(16192) L2a1b L2a1b1 L2a1b1a L2a1b2 L2a1f1 L2a1f1a L2a1f2 (10)	1

1468	>MF056335.1 Hc	73G 150d 182T 186A 189C 247A 263G 316A 514d 515d 750G 769A 783G 825A 1018A 1438G 2394d 2706G 2758A 2885C 3105d 3106G 3107C 3594T 3666A 4104G 4769G 5951G 6071C 6221A 6260A 6917A 7028T 7146G 7256T 7296A 7389C 7498A 7521A 7789A 8027A 8468T 8555C 8655T 8668C 8701G 8860G 9072G 9540C 9966A 10398G 10586A 10688A 10810C 10873C 11302T 11719A 12019T 12501A 12705T 12810G 13105G 13485G 13506T 13650T 13789C 14000A 14022G 14178C 14560A 14766T 14911T 14927N 14928N 15226G 15326G 15905C 15978T 16129A 16183C 16189C 16215G 16223T 16278T 16294T 16311C 16355T 16360T 16390A 16519C	L1c3a1b	73G 150d 182T 186A 189C 247A 263G 316A 514d 515d 16129A 16183C 16189C 16215G 16223T 16278T 16294T 16311C 16355T 16360T 16390A 16519C	1	73 151d 182 186A 189C 247 263 316 750 769 783 825A 1018 1438 2395d 2706 2758 2885 3105 3594 3666 4104 4769 5951 6071 6221A 6260 6917 7028 7146 7256 7296 7389 7498 7521 7789 8027 8468 8555 8655 8668 8701 8860 9072 9540 9966 10398 10586 10688 10810 10873 11302 11719 12019 12501 12705 12810 13105 13485 13506 13650 13789 14000A 14022 14178 14560 14766 14911 14927N 14928N 15226 15326 15905 15978 16129 16189 16215 16223 16278 16294 16311 16355 16360 16390	L1c3a1b	73 151d 182 186A 189C 247 263 316 16129 16189 16215 16223 16278 16294 16311 16355 16360 16390	[0.565] L1c3a1b (1)	[0.482] L1c3a (2) [0.321] L1c3a1a (3) [0.000] L1c3a1	1
1469	>MF056327.1 Hc	73G 150T 200G 263G 489C 750G 2706G 3106d 3107C 3588T 4769G 7028T 7960G 8701G 8768T 8854A 8860G 9540C 10398G 10400T 10873C 11719A 12705T 13308G 13590A 13708A 14130T 14260G 14527G 14766T 14783C 15043A 15172A 15301A 15326G 15625T 16086C 16148T 16223T 16259T 16278T 16319A 16399G 16526A	M32c	73G 150T 200G 263G 489C 16086C 16148T 16223T 16259T 16278T 16319A 16399G 16526A	1	73 150 200 263 489 750 2706 3588 4769 7028 7960 8701 8768 8854 8860 9540 10398 10400 10873 11719 12705 13308 13590 13708 14130 14260 14527 14766 14783 15043 15172 15301 15326 15625 16086 16148 16223 16259 16278 16319 16399 16526	M32c	73 150 200 263 489 16086 16148 16223 16259 16278 16319 16399 16526	[0.000] M32c (1)	[0.022] M51a (2) [0.007] M32 (3) [0.000] M51 M51b (5)	1
1470	>MF056326.1 Hc	73G 152d 200G 263G 489C 750G 2706G 3106d 3107C 3588T 4769G 7028T 7960G 8158G 8701G 8768T 8854A 8860G 9540C 10398G 10400T 10873C 11719A 12705T 13308G 13590A 13708A 14130T 14260G 14527G 14766T 14783C 15043A 15172A 15301A 15326G 15625T 16086C 16148T 16223T 16259T 16278T 16319A 16399G 16526A	M32c	73G 152d 200G 263G 489C 16086C 16148T 16223T 16259T 16278T 16319A 16399G 16526A	1	73 152d 200 263 489 750 2706 3588 4769 7028 7960 8158 8701 8768 8854 8860 9540 10398 10400 10873 11719 12705 13308 13590 13708 14130 14260 14527 14766 14783 15043 15172 15301 15326 15625 16086 16148 16223 16259 16278 16319 16399 16526	M32c	73 152d 200 263 489 16086 16148 16223 16259 16278 16319 16399 16526	[0.030] M32c (1)	[0.007] M32 (2)	1

1471	>MF056322.1 Hc	73G 150T 152C 200G 263G 489C 750G 2706G 3106d 3107C 3588T 4769G 7028T 7960G 8158G 8701G 8768T 8854A 8860G 9540C 10398G 10400T 10873C 11719A 12705T 13308G 13590A 13708A 14130T 14260G 14527G 14766T 14783C 15043A 15172A 15301A 15326G 15625T 16086C 16148T 16223T 16259T 16278T 16319A 16399G 16526A	M32c	73G 150T 152C 200G 263G 489C 16086C 16148T 16223T 16259T 16278T 16319A 16399G 16526A	1	73 150 152 200 263 489 750 2706 3588 4769 7028 7960 8158 8701 8768 8854 8860 9540 10398 10400 10873 11719 12705 13308 13590 13708 14130 14260 14527 14766 14783 15043 15172 15301 15326 15625 16086 16148 16223 16259 16278 16319 16399 16526	M32c	73 150 152 200 263 489 16086 16148 16223 16259 16278 16319 16399 16526	[0.017] M32c (1)	[0.065] M51a (2) [0.014] M51b (3) [0.007] M32 (4) [0.000] M51	1
1472	>MF056319.1 Hc	73G 152C 195C 204C 263G 417A 489C 533G 750G 1438G 3106d 3107C 4769G 7028T 8188G 8360G 8701G 8860G 9438A 9540C 9545G 10142T 10295G 10398G 10400T 10873C 11569C 11719A 11899C 12279G 12618A 12705T 14766T 14783C 15025T 15043A 15301A 15326G 16223T 16263C 16311C 16519C	M23	73G 152C 195C 204C 263G 417A 489C 533G 16223T 16263C 16311C 16519C	1	73 152 195 204 263 417 489 533 750 1438 4769 7028 8188 8360 8701 8860 9438 9540 9545 10142 10295 10398 10400 10873 11569 11719 11899 12279 12618 12705 14766 14783 15025 15043 15301 15326 16223 16263 16311	M23	73 152 195 204 263 417 489 533 16223 16263 16311	[0.000] M23 (1)	[0.072] M24a (2) [0.053] M57+152	1
1473	>MF056316.1 Hc	73G 152C 195C 204C 263G 417A 489C 533G 750G 1438G 3106d 3107C 4769G 7028T 8188G 8360G 8701G 8860G 9438A 9540C 9545G 10142T 10295G 10398G 10400T 10873C 11569C 11719A 11899C 12279G 12618A 12705T 14766T 14783C 15025T 15043A 15301A 15326G 16223T 16263C 16311C 16519C	M23	73G 152C 195C 204C 263G 417A 489C 533G 16223T 16263C 16311C 16519C	1	73 152 195 204 263 417 489 533 750 1438 4769 7028 8188 8360 8701 8860 9438 9540 9545 10142 10295 10398 10400 10873 11569 11719 11899 12279 12618 12705 14766 14783 15025 15043 15301 15326 16223 16263 16311	M23	73 152 195 204 263 417 489 533 16223 16263 16311	[0.000] M23 (1)	[0.072] M24a (2) [0.053] M57+152	1
1474	>MF056315.1 Hc	73G 150T 200G 263G 489C 750G 2706G 3106d 3107C 3588T 4769G 7028T 7960G 8701G 8768T 8854A 8860G 9540C 10398G 10400T 10873C 11719A 12705T 13308G 13590A 13708A 14130T 14260G 14527G 14766T 14783C 15043A 15172A 15301A 15326G 15625T 16086C 16148T 16223T 16259T 16278T 16319A 16399G 16526A	M32c	73G 150T 200G 263G 489C 16086C 16148T 16223T 16259T 16278T 16319A 16399G 16526A	1	73 150 200 263 489 750 2706 3588 4769 7028 7960 8701 8768 8854 8860 9540 10398 10400 10873 11719 12705 13308 13590 13708 14130 14260 14527 14766 14783 15043 15172 15301 15326 15625 16086 16148 16223 16259 16278 16319 16399 16526	M32c	73 150 200 263 489 16086 16148 16223 16259 16278 16319 16399 16526	[0.000] M32c (1)	[0.022] M51a (2) [0.007] M32 (3) [0.000] M51 M51b (5)	1
1475	>MF056314.1 Hc	73G 150T 200G 263G 489C 750G 2706G 3106d 3107C 3588T 4769G 7028T 7960G 8701G 8768T 8854A 8860G 9540C 10398G 10400T 10873C 11719A 12634G 12705T 13308G 13434G 13590A 13708A 14130T 14260G 14527G 14766T 14783C 15043A 15172A 15301A 15326G 15625T 16086C 16111T 16148T 16223T 16259T 16278T 16319A 16399G 16526A	M32c	73G 150T 200G 263G 489C 16086C 16111T 16148T 16223T 16259T 16278T 16319A 16399G 16526A	1	73 150 200 263 489 750 2706 3588 4769 7028 7960 8701 8768 8854 8860 9540 10398 10400 10873 11719 12634 12705 13308 13434 13590 13708 14130 14260 14527 14766 14783 15043 15172 15301 15326 15625 16086 16111 16148 16223 16259 16278 16319 16399 16526	M32c	73 150 200 263 489 16086 16111 16148 16223 16259 16278 16319 16399 16526	[0.007] M32c (1)	[0.022] M51a (2) [0.007] M51 M51b M32 (5)	1

1476	>MF056312.1 Hc	73G 150T 200G 263G 489C 750G 2706G 3106d 3107C 3588T 4769G 7028T 7960G 8701G 8768T 8854A 8860G 9540C 10398G 10400T 10873C 11719A 12634G 12705T 13308G 13434G 13590A 13708A 14130T 14260G 14527G 14766T 14783C 15043A 15172A 15301A 15326G 15625T 16086C 16111T 16148T 16223T 16259T 16278T 16319A 16399G 16526A	M32c	73G 150T 200G 263G 489C 16086C 16111T 16148T 16223T 16259T 16278T 16319A 16399G 16526A	1	73 150 200 263 489 750 2706 3588 4769 7028 7960 8701 8768 8854 8860 9540 10398 10400 10873 11719 12634 12705 13308 13434 13590 13708 14130 14260 14527 14766 14783 15043 15172 15301 15326 15625 16086 16111 16148 16223 16259 16278 16319 16399 16526	M32c	73 150 200 263 489 16086 16111 16148 16223 16259 16278 16319 16399 16526	[0.007] M32c (1)	[0.022] M51a (2) [0.007] M51 M51b M32 (5)	1
1477	>MF056311.1 Hc	73G 150T 200G 263G 489C 750G 2706G 3106d 3107C 3588T 4769G 7028T 7960G 8701G 8768T 8854A 8860G 9540C 10398G 10400T 10873C 11719A 12705T 13308G 13590A 13708A 14130T 14260G 14527G 14766T 14783C 15043A 15172A 15301A 15326G 15625T 16086C 16148T 16223T 16259T 16278T 16319A 16399G 16526A	M32c	73G 150T 200G 263G 489C 16086C 16148T 16223T 16259T 16278T 16319A 16399G 16526A	1	73 150 200 263 489 750 2706 3588 4769 7028 7960 8701 8768 8854 8860 9540 10398 10400 10873 11719 12705 13308 13590 13708 14130 14260 14527 14766 14783 15043 15172 15301 15326 15625 16086 16148 16223 16259 16278 16319 16399 16526	M32c	73 150 200 263 489 16086 16148 16223 16259 16278 16319 16399 16526	[0.000] M32c (1)	[0.022] M51a (2) [0.007] M32 (3) [0.000] M51 M51b (5)	1
1478	>MF056294.1 Hc	73G 152C 225A 248d 263G 316A 489C 514d 515d 750G 1438G 2706G 3106d 3107C 3200C 3714G 4385T 4769G 4772C 7028T 7433T 8701G 8853G 8860G 9127G 9512T 9540C 10274C 10398G 10400T 10679G 10873C 11719A 11914A 12354C 12705T 14110C 14142T 14766T 14783C 14974T 15043A 15301A 15326G 15691G 16129A 16209C 16223T 16272G 16519C	M20	73G 152C 225A 248d 263G 316A 489C 514d 515d 16129A 16209C 16223T 16272G 16519C	1	73 152 225 249d 263 316 489 750 1438 2706 3200 3714 4385T 4769 4772 7028 7433 8701 8853 8860 9127 9512 9540 10274 10398 10400 10679 10873 11719 11914 12354 12705 14110 14142 14766 14783 14974 15043 15301 15326 15691 16129 16209 16223 16272	M20	73 152 225 249d 263 316 489 16129 16209 16223 16272	[0.307] M20 (1)	[0.068] M17 (2) [0.067] M17a (3) [0.059] G1b+16129 (4) [0.028] M5a (5) [0.021] D4a1a1 (6) [0.019] M5 (7) [0.017] M5a2a (8) [0.007] M5a'd (9) [0.004] M37+152 (10) [0.003] D4a1 (11) [0.000] M5a2 M5a2a1 M5a5 M5b'c M5b M7a M7a1b2 M7a2 M7b1 G1b2 G1b4 M13'46'61 M13 M13a'b M13b M17c D4a D4a1a D4a1a1a D4a1b D4a1c D4a1f D4a1f1 D4a1g D4a1h D4a2 D4a2a D4a2b D4a5 (40)	1
1479	>MF056293.1 Hc	73G 146C 152C 195C 263G 750G 769A 1018A 1438G 2416C 2706G 2789T 3106d 3107C 3594T 4104G 4769G 5090C 7028T 7175C 7256T 7274T 7521A 7771G 8206A 8701G 8860G 9221G 9540C 10115C 10143A 10398G 10873C 11719A 11914A 11944C 12693G 12705T 13590A 13650T 13803G 14566G 14766T 15301A 15326G 15735T 15784C 16183C 16189C 16223T 16278T 16290T 16294T 16309G 16390A	L2a1b1a	73G 146C 152C 195C 263G 16183C 16189C 16223T 16278T 16290T 16294T 16309G 16390A	1	73 146 152 195 263 750 769 1018 1438 2416 2706 2789 3594 4104 4769 5090 7028 7175 7256 7274 7521 7771 8206 8701 8860 9221 9540 10115 10143 10398 10873 11719 11914 11944 12693 12705 13590 13650 13803 14566 14766 15301 15326 15735 15784 16189 16223 16278 16290 16294 16309 16390	L2a1b1a	73 146 152 195 263 16189 16223 16278 16290 16294 16309 16390	[0.000] L2a1b1a (1)	[0.005] L2a1f (2) [0.000] L2a1+16189_(16192) L2a1b L2a1b1 L2a1b1a L2a1b2 L2a1f1 L2a1f1a L2a1f2 (10)	1

1480	>MF056292.1 Hc	73G 152C 182T 186A 189C 195C 247A 248G 263G 316A 514d 515d 750G 769A 825A 1018A 2394d 2706G 2758A 2885C 3106d 3107C 3525T 3594T 3666A 4104G 4769G 4853A 5951G 6071C 6221A 6227C 6917A 7028T 7146G 7247T 7256T 7389C 7521A 8027A 8468T 8655T 8701G 8860G 9072G 9540C 10398G 10586A 10688A 10810C 10873C 11302T 11719A 11984C 12507G 12705T 12810G 13105G 13485G 13506T 13650T 13789C 14000A 14178C 14560A 14669G 14766T 14911T 14926G 15226G 15326G 15905C 15978T 16129A 16187T 16189C 16223T 16278T 16293G 16294T 16311C 16360T 16519C	L1c3c	73G 152C 182T 186A 189C 195C 247A 248G 263G 316A 514d 515d 16129A 16187T 16189C 16223T 16278T 16293G 16294T 16311C 16360T 16519C	1	73 152 182 186A 189C 195 247 248 263 316 750 769 825A 1018 2395d 2706 2758 2885 3525 3594 3666 4104 4769 4853 5951 6071 6221A 6227 6917 7028 7146 7247 7256 7389 7521 8027 8468 8655 8701 8860 9072 9540 10398 10586 10688 10810 10873 11302 11719 11984 12507 12705 12810 13105 13485 13506 13650 13789 14000A 14178 14560 14669 14766 14911 14926 15226 15326 15905 15978 16129 16187 16189 16223 16278 16293 16294 16311 16360	L1c3c	73 152 182 186A 189C 195 247 248 263 316 16129 16187 16189 16223 16278 16293 16294 16311 16360	[2.063] L1c3c (1) [0.000] L1c L1c1a+@198 L1c3b'c (4)	[0.700] L1c3 (5) [0.572] L1c1a'b'd (6) [0.267] L1c3b1 (7) [0.133] L1c3b1b (8) [0.000] L1c1'2'4'5'6 L1c1 L1c1a L1c1a1 L1c3b (13)	1
1481	>MF056289.1 Hc	73G 146C 152C 195C 263G 750G 769A 1018A 1438G 2416C 2706G 2762T 2789T 3106d 3107C 3594T 4104G 4769G 5090C 7028T 7175C 7256T 7274T 7521A 7771G 8206A 8701G 8860G 9221G 9540C 10115C 10143A 10398G 10873C 11719A 11914A 11944C 12693G 12705T 13590A 13650T 13803G 14566G 14766T 15301A 15326G 15735T 15784C 16183C 16189C 16223T 16278T 16290T 16294T 16309G 16390A	L2a1b1a	73G 146C 152C 195C 263G 16183C 16189C 16223T 16278T 16290T 16294T 16309G 16390A	1	73 146 152 195 263 750 769 1018 1438 2416 2706 2762 2789 3594 4104 4769 5090 7028 7175 7256 7274 7521 7771 8206 8701 8860 9221 9540 10115 10143 10398 10873 11719 11914 11944 12693 12705 13590 13650 13803 14566 14766 15301 15326 15735 15784 16189 16223 16278 16290 16294 16309 16390	L2a1b1a	73 146 152 195 263 16189 16223 16278 16290 16294 16309 16390	[0.000] L2a1b1a (1)	[0.005] L2a1f (2) [0.000] L2a1+16189_(16192) L2a1b L2a1b1 L2a1b1a L2a1b2 L2a1f1 L2a1f1a L2a1f2 (10)	1
1482	>MF056281.1 Hc	73G 152C 182T 186A 189C 195C 247A 248G 263G 316A 514d 515d 750G 769A 825A 1018A 2394d 2706G 2758A 2885C 3106d 3107C 3525T 3594T 3666A 4104G 4769G 4853A 5951G 6071C 6221A 6227C 6917A 7028T 7146G 7247T 7256T 7389C 7521A 8027A 8468T 8655T 8701G 8860G 9072G 9540C 10398G 10586A 10688A 10810C 10873C 11302T 11719A 11984C 12507G 12705T 12810G 13105G 13485G 13506T 13650T 13789C 14000A 14178C 14560A 14669G 14766T 14911T 14926G 15226G 15326G 15905C 15978T 16129A 16187T 16189C 16223T 16278T 16293G 16294T 16311C 16360T 16519C	L1c3c	73G 152C 182T 186A 189C 195C 247A 248G 263G 316A 514d 515d 16129A 16187T 16189C 16223T 16278T 16293G 16294T 16311C 16360T 16519C	1	73 152 182 186A 189C 195 247 248 263 316 750 769 825A 1018 2395d 2706 2758 2885 3525 3594 3666 4104 4769 4853 5951 6071 6221A 6227 6917 7028 7146 7247 7256 7389 7521 8027 8468 8655 8701 8860 9072 9540 10398 10586 10688 10810 10873 11302 11719 11984 12507 12705 12810 13105 13485 13506 13650 13789 14000A 14178 14560 14669 14766 14911 14926 15226 15326 15905 15978 16129 16187 16189 16223 16278 16293 16294 16311 16360	L1c3c	73 152 182 186A 189C 195 247 248 263 316 16129 16187 16189 16223 16278 16293 16294 16311 16360	[2.063] L1c3c (1) [0.000] L1c L1c1a+@198 L1c3b'c (4)	[0.700] L1c3 (5) [0.572] L1c1a'b'd (6) [0.267] L1c3b1 (7) [0.133] L1c3b1b (8) [0.000] L1c1'2'4'5'6 L1c1 L1c1a L1c1a1 L1c3b (13)	1

1483	>MF056273.1 Hc	73G 150T 200G 263G 489C 750G 2706G 3106d 3107C 3588T 4769G 7028T 7960G 8701G 8768T 8854A 8860G 9540C 10398G 10400T 10873C 11719A 12705T 13308G 13590A 13708A 14130T 14260G 14527G 14766T 14783C 15043A 15172A 15301A 15326G 15625T 16086C 16148T 16223T 16259T 16278T 16319A 16399G 16526A	M32c	73G 150T 200G 263G 489C 16086C 16148T 16223T 16259T 16278T 16319A 16399G 16526A	1	73 150 200 263 489 750 2706 3588 4769 7028 7960 8701 8768 8854 8860 9540 10398 10400 10873 11719 12705 13308 13590 13708 14130 14260 14527 14766 14783 15043 15172 15301 15326 15625 16086 16148 16223 16259 16278 16319 16399 16526	M32c	73 150 200 263 489 16086 16148 16223 16259 16278 16319 16399 16526	[0.000] M32c (1)	[0.022] M51a (2) [0.007] M32 (3) [0.000] M51 M51b (5)	1
1484	>MF056271.1 Hc	73G 150T 200G 263G 489C 750G 2706G 3106d 3107C 3588T 4769G 7028T 7960G 8701G 8768T 8854A 8860G 9540C 10398G 10400T 10873C 11719A 12705T 13308G 13590A 13708A 14130T 14260G 14527G 14766T 14783C 15043A 15172A 15301A 15326G 15625T 16086C 16148T 16223T 16259T 16278T 16319A 16399G 16526A	M32c	73G 150T 200G 263G 489C 16086C 16148T 16223T 16259T 16278T 16319A 16399G 16526A	1	73 150 200 263 489 750 2706 3588 4769 7028 7960 8701 8768 8854 8860 9540 10398 10400 10873 11719 12705 13308 13590 13708 14130 14260 14527 14766 14783 15043 15172 15301 15326 15625 16086 16148 16223 16259 16278 16319 16399 16526	M32c	73 150 200 263 489 16086 16148 16223 16259 16278 16319 16399 16526	[0.000] M32c (1)	[0.022] M51a (2) [0.007] M32 (3) [0.000] M51 M51b (5)	1
1485	>MF056269.1 Hc	73G 150T 200G 263G 489C 750G 2706G 3106d 3107C 3588T 4769G 7028T 7960G 8701G 8768T 8854A 8860G 9540C 10398G 10400T 10873C 11719A 12705T 13308G 13590A 13708A 14130T 14260G 14527G 14766T 14783C 15043A 15104T 15172A 15301A 15326G 15625T 16086C 16148T 16223T 16259T 16278T 16319A 16399G 16526A	M32c	73G 150T 200G 263G 489C 16086C 16148T 16223T 16259T 16278T 16319A 16399G 16526A	1	73 150 200 263 489 750 2706 3588 4769 7028 7960 8701 8768 8854 8860 9540 10398 10400 10873 11719 12705 13308 13590 13708 14130 14260 14527 14766 14783 15043 15104 15172 15301 15326 15625 16086 16148 16223 16259 16278 16319 16399 16526	M32c	73 150 200 263 489 16086 16148 16223 16259 16278 16319 16399 16526	[0.000] M32c (1)	[0.022] M51a (2) [0.007] M32 (3) [0.000] M51 M51b (5)	1
1486	>MF056267.1 Hc	73G 150T 200G 263G 489C 750G 2706G 3106d 3107C 3588T 4769G 7028T 7960G 8701G 8768T 8854A 8860G 9540C 10398G 10400T 10873C 11719A 12705T 13308G 13590A 13708A 14130T 14260G 14527G 14766T 14783C 15043A 15172A 15301A 15326G 15625T 16086C 16148T 16223T 16259T 16278T 16319A 16399G 16526A	M32c	73G 150T 200G 263G 489C 16086C 16148T 16223T 16259T 16278T 16319A 16399G 16526A	1	73 150 200 263 489 750 2706 3588 4769 7028 7960 8701 8768 8854 8860 9540 10398 10400 10873 11719 12705 13308 13590 13708 14130 14260 14527 14766 14783 15043 15172 15301 15326 15625 16086 16148 16223 16259 16278 16319 16399 16526	M32c	73 150 200 263 489 16086 16148 16223 16259 16278 16319 16399 16526	[0.000] M32c (1)	[0.022] M51a (2) [0.007] M32 (3) [0.000] M51 M51b (5)	1

1487	>MF056265.1 Hc	73G 150T 200G 263G 489C 750G 2706G 3106d 3107C 3588T 4769G 7028T 7960G 8701G 8768T 8854A 8860G 9540C 10398G 10400T 10873C 11719A 12705T 13308G 13590A 13708A 14130T 14260G 14527G 14766T 14783C 15043A 15172A 15301A 15326G 15625T 16086C 16148T 16223T 16259T 16278T 16319A 16399G 16526A	M32c	73G 150T 200G 263G 489C 16086C 16148T 16223T 16259T 16278T 16319A 16399G 16526A	1	73 150 200 263 489 750 2706 3588 4769 7028 7960 8701 8768 8854 8860 9540 10398 10400 10873 11719 12705 13308 13590 13708 14130 14260 14527 14766 14783 15043 15172 15301 15326 15625 16086 16148 16223 16259 16278 16319 16399 16526	M32c	73 150 200 263 489 16086 16148 16223 16259 16278 16319 16399 16526	[0.000] M32c (1)	[0.022] M51a (2) [0.007] M32 (3) [0.000] M51 M51b (5)	1
1488	>MF056264.1 Hc	73G 152C 195C 204C 263G 417A 489C 533G 750G 1438G 3106d 3107C 4769G 7028T 8188G 8360G 8701G 8860G 9438A 9540C 9545G 10142T 10295G 10398G 10400T 10873C 11569C 11719A 11899C 12279G 12618A 12705T 14766T 14783C 15025T 15043A 15301A 15326G 16223T 16263C 16311C 16519C	M23	73G 152C 195C 204C 263G 417A 489C 533G 16223T 16263C 16311C 16519C	1	73 152 195 204 263 417 489 533 750 1438 4769 7028 8188 8360 8701 8860 9438 9540 9545 10142 10295 10398 10400 10873 11569 11719 11899 12279 12618 12705 14766 14783 15025 15043 15301 15326 16223 16263 16311	M23	73 152 195 204 263 417 489 533 16223 16263 16311	[0.000] M23 (1)	[0.072] M24a (2) [0.053] M57+152	1
1489	>MF056263.1 Hc	73G 152C 195C 204C 263G 417A 489C 533G 750G 1438G 3106d 3107C 4769G 7028T 8188G 8360G 8701G 8860G 9438A 9540C 9545G 10142T 10295G 10398G 10400T 10873C 11569C 11719A 11899C 12279G 12618A 12705T 14766T 14783C 15025T 15043A 15301A 15326G 16223T 16263C 16311C 16519C	M23	73G 152C 195C 204C 263G 417A 489C 533G 16223T 16263C 16311C 16519C	1	73 152 195 204 263 417 489 533 750 1438 4769 7028 8188 8360 8701 8860 9438 9540 9545 10142 10295 10398 10400 10873 11569 11719 11899 12279 12618 12705 14766 14783 15025 15043 15301 15326 16223 16263 16311	M23	73 152 195 204 263 417 489 533 16223 16263 16311	[0.000] M23 (1)	[0.072] M24a (2) [0.053] M57+152	1
1490	>MF056250.1 Hc	73G 152C 195C 204C 263G 417A 489C 533G 750G 1438G 3106d 3107C 4769G 7028T 8188G 8360G 8701G 8860G 9438A 9540C 9545G 10142T 10295G 10398G 10400T 10873C 11569C 11719A 11899C 12279G 12618A 12705T 14766T 14783C 15025T 15043A 15301A 15326G 16223T 16263C 16311C 16519C	M23	73G 152C 195C 204C 263G 417A 489C 533G 16223T 16263C 16311C 16519C	1	73 152 195 204 263 417 489 533 750 1438 4769 7028 8188 8360 8701 8860 9438 9540 9545 10142 10295 10398 10400 10873 11569 11719 11899 12279 12618 12705 14766 14783 15025 15043 15301 15326 16223 16263 16311	M23	73 152 195 204 263 417 489 533 16223 16263 16311	[0.000] M23 (1)	[0.072] M24a (2) [0.053] M57+152	1
1491	>MF056246.1 Hc	73G 152C 195C 204C 263G 417A 489C 533G 750G 1438G 3106d 3107C 4769G 7028T 8188G 8360G 8701G 8860G 9438A 9540C 9545G 10142T 10295G 10398G 10400T 10873C 11569C 11719A 11899C 12279G 12618A 12705T 14766T 14783C 15025T 15043A 15301A 15326G 16223T 16263C 16311C 16519C	M23	73G 152C 195C 204C 263G 417A 489C 533G 16223T 16263C 16311C 16519C	1	73 152 195 204 263 417 489 533 750 1438 4769 7028 8188 8360 8701 8860 9438 9540 9545 10142 10295 10398 10400 10873 11569 11719 11899 12279 12618 12705 14766 14783 15025 15043 15301 15326 16223 16263 16311	M23	73 152 195 204 263 417 489 533 16223 16263 16311	[0.000] M23 (1)	[0.072] M24a (2) [0.053] M57+152	1

1492	>MF056241.1 Hc	73G 152C 195C 204C 263G 417A 489C 533G 750G 1438G 3106d 3107C 4769G 7028T 8188G 8360G 8701G 8860G 9438A 9540C 9545G 10142T 10295G 10398G 10400T 10873C 11569C 11719A 11899C 12279G 12618A 12705T 14766T 14783C 15025T 15043A 15301A 15326G 16223T 16263C 16311C 16519C	M23	73G 152C 195C 204C 263G 417A 489C 533G 16223T 16263C 16311C 16519C	1	73 152 195 204 263 417 489 533 750 1438 4769 7028 8188 8360 8701 8860 9438 9540 9545 10142 10295 10398 10400 10873 11569 11719 11899 12279 12618 12705 14766 14783 15025 15043 15301 15326 16223 16263 16311	M23	73 152 195 204 263 417 489 533 16223 16263 16311	[0.000] M23 (1)	[0.072] M24a (2) [0.053] M57+152	1
1493	>MF056240.1 Hc	73G 152C 195C 204C 263G 417A 489C 533G 750G 1438G 3106d 3107C 4769G 7028T 8188G 8360G 8701G 8860G 9438A 9540C 9545G 10142T 10295G 10398G 10400T 10873C 11569C 11719A 11899C 12279G 12618A 12705T 14766T 14783C 15025T 15043A 15301A 15326G 16223T 16263C 16311C 16519C	M23	73G 152C 195C 204C 263G 417A 489C 533G 16223T 16263C 16311C 16519C	1	73 152 195 204 263 417 489 533 750 1438 4769 7028 8188 8360 8701 8860 9438 9540 9545 10142 10295 10398 10400 10873 11569 11719 11899 12279 12618 12705 14766 14783 15025 15043 15301 15326 16223 16263 16311	M23	73 152 195 204 263 417 489 533 16223 16263 16311	[0.000] M23 (1)	[0.072] M24a (2) [0.053] M57+152	1
1494	>MF056236.1 Hc	73G 150T 200G 263G 489C 750G 2706G 3106d 3107C 3588T 4769G 7028T 7960G 8701G 8768T 8854A 8860G 9540C 10398G 10400T 10873C 11719A 12705T 12813T 13308G 13590A 13708A 14130T 14260G 14527G 14766T 14783C 15043A 15172A 15301A 15326G 15625T 16086C 16148T 16223T 16259T 16278T 16319A 16399G 16526A	M32c	73G 150T 200G 263G 489C 16086C 16148T 16223T 16259T 16278T 16319A 16399G 16526A	1	73 150 200 263 489 750 2706 3588 4769 7028 7960 8701 8768 8854 8860 9540 10398 10400 10873 11719 12705 12813 13308 13590 13708 14130 14260 14527 14766 14783 15043 15172 15301 15326 15625 16086 16148 16223 16259 16278 16319 16399 16526	M32c	73 150 200 263 489 16086 16148 16223 16259 16278 16319 16399 16526	[0.000] M32c (1)	[0.022] M51a (2) [0.007] M32 (3) [0.000] M51 M51b (5)	1
1495	>MF056213.1 Hc	73G 146C 152C 195C 263G 750G 769A 1018A 1438G 2416C 2706G 2789T 3106d 3107C 3594T 4104G 4769G 5090C 7028T 7175C 7256T 7274T 7521A 7771G 8206A 8701G 8860G 9221G 9540C 10115C 10143A 10398G 10873C 11719A 11914A 11944C 12693G 12705T 13590A 13650T 13803G 14566G 14766T 15301A 15326G 15735T 15784C 16182C 16183C 16189C 16223T 16278T 16290T 16294T 16309G 16390A	L2a1b1a	73G 146C 152C 195C 263G 16182C 16183C 16189C 16223T 16278T 16290T 16294T 16309G 16390A	1	73 146 152 195 263 750 769 1018 1438 2416 2706 2789 3594 4104 4769 5090 7028 7175 7256 7274 7521 7771 8206 8701 8860 9221 9540 10115 10143 10398 10873 11719 11914 11944 12693 12705 13590 13650 13803 14566 14766 15301 15326 15735 15784 16189 16223 16278 16290 16294 16309 16390	L2a1b1a	73 146 152 195 263 16189 16223 16278 16290 16294 16309 16390	[0.000] L2a1b1a (1)	[0.005] L2a1f (2) [0.000] L2a1+16189_(16192) L2a1b L2a1b1 L2a1b1a L2a1b2 L2a1f1 L2a1f1a L2a1f2 (10)	1

1496	>MF056211.1 Ho	73G 150T 200G 263G 489C 750G 2706G 3106d 3107C 3588T 4769G 7028T 7960G 8701G 8768T 8854A 8860G 9540C 10398G 10400T 10873C 11719A 12705T 13308G 13590A 13708A 14130T 14260G 14527G 14766T 14783C 15043A 15172A 15301A 15326G 15625T 16086C 16148T 16223T 16259T 16278T 16319A 16399G 16526A	M32c	73G 150T 200G 263G 489C 16086C 16148T 16223T 16259T 16278T 16319A 16399G 16526A	1	73 150 200 263 489 750 2706 3588 4769 7028 7960 8701 8768 8854 8860 9540 10398 10400 10873 11719 12705 13308 13590 13708 14130 14260 14527 14766 14783 15043 15172 15301 15326 15625 16086 16148 16223 16259 16278 16319 16399 16526	M32c	73 150 200 263 489 16086 16148 16223 16259 16278 16319 16399 16526	[0.000] M32c (1)	[0.022] M51a (2) [0.007] M32 (3) [0.000] M51 M51b (5)	1
1497	>MF056197.1 Ho	73G 152C 195C 204C 263G 417A 489C 533G 750G 1438G 3106d 3107C 4769G 7028T 8188G 8360G 8701G 8860G 9438A 9540C 9545G 10142T 10295G 10398G 10400T 10873C 11569C 11719A 11899C 12279G 12618A 12705T 14766T 14783C 15025T 15043A 15301A 15326G 16223T 16263C 16311C 16519C	M23	73G 152C 195C 204C 263G 417A 489C 533G 16223T 16263C 16311C 16519C	1	73 152 195 204 263 417 489 533 750 1438 4769 7028 8188 8360 8701 8860 9438 9540 9545 10142 10295 10398 10400 10873 11569 11719 11899 12279 12618 12705 14766 14783 15025 15043 15301 15326 16223 16263 16311	M23	73 152 195 204 263 417 489 533 16223 16263 16311	[0.000] M23 (1)	[0.072] M24a (2) [0.053] M57+152	1
1498	>MF056191.1 Ho	73G 152C 195C 204C 263G 417A 489C 533G 750G 1095C 1438G 3106d 3107C 4769G 7028T 8188G 8360G 8701G 8860G 9438A 9540C 9545G 10142T 10295G 10398G 10400T 10873C 11569C 11719A 11899C 12279G 12618A 12705T 14766T 14783C 15025T 15043A 15301A 15326G 16223T 16263C 16311C 16519C	M23	73G 152C 195C 204C 263G 417A 489C 533G 16223T 16263C 16311C 16519C	1	73 152 195 204 263 417 489 533 750 1095 1438 4769 7028 8188 8360 8701 8860 9438 9540 9545 10142 10295 10398 10400 10873 11569 11719 11899 12279 12618 12705 14766 14783 15025 15043 15301 15326 16223 16263 16311	M23	73 152 195 204 263 417 489 533 16223 16263 16311	[0.000] M23 (1)	[0.072] M24a (2) [0.053] M57+152	1
1499	>MF056182.1 Ho	73G 152C 195C 204C 263G 417A 489C 533G 750G 1438G 3106d 3107C 4769G 7028T 8188G 8360G 8701G 8860G 9438A 9540C 9545G 10142T 10295G 10398G 10400T 10873C 11569C 11719A 11899C 12279G 12618A 12705T 14766T 14783C 15025T 15043A 15301A 15326G 16223T 16263C 16311C 16519C	M23	73G 152C 195C 204C 263G 417A 489C 533G 16223T 16263C 16311C 16519C	1	73 152 195 204 263 417 489 533 750 1438 4769 7028 8188 8360 8701 8860 9438 9540 9545 10142 10295 10398 10400 10873 11569 11719 11899 12279 12618 12705 14766 14783 15025 15043 15301 15326 16223 16263 16311	M23	73 152 195 204 263 417 489 533 16223 16263 16311	[0.000] M23 (1)	[0.072] M24a (2) [0.053] M57+152	1
1500	>MF056179.1 Ho	73G 152C 195C 204C 263G 417A 489C 533G 750G 1438G 3106d 3107C 4769G 7028T 8188G 8360G 8701G 8860G 9438A 9540C 9545G 10142T 10295G 10398G 10400T 10873C 11569C 11719A 11899C 12279G 12618A 12705T 14766T 14783C 15025T 15043A 15301A 15326G 16223T 16263C 16311C 16519C	M23	73G 152C 195C 204C 263G 417A 489C 533G 16223T 16263C 16311C 16519C	1	73 152 195 204 263 417 489 533 750 1438 4769 7028 8188 8360 8701 8860 9438 9540 9545 10142 10295 10398 10400 10873 11569 11719 11899 12279 12618 12705 14766 14783 15025 15043 15301 15326 16223 16263 16311	M23	73 152 195 204 263 417 489 533 16223 16263 16311	[0.000] M23 (1)	[0.072] M24a (2) [0.053] M57+152	1

1501	>MF056178.1 Hc	73G 152C 195C 204C 263G 417A 489C 533G 750G 1438G 3106d 3107C 4769G 7028T 8188G 8360G 8701G 8860G 9438A 9540C 9545G 10142T 10295G 10398G 10400T 10873C 11569C 11719A 11899C 12279G 12618A 12705T 14766T 14783C 15025T 15043A 15301A 15326G 16223T 16263C 16311C 16519C	M23	73G 152C 195C 204C 263G 417A 489C 533G 16223T 16263C 16311C 16519C	1	73 152 195 204 263 417 489 533 750 1438 4769 7028 8188 8360 8701 8860 9438 9540 9545 10142 10295 10398 10400 10873 11569 11719 11899 12279 12618 12705 14766 14783 15025 15043 15301 15326 16223 16263 16311	M23	73 152 195 204 263 417 489 533 16223 16263 16311	[0.000] M23 (1)	[0.072] M24a (2) [0.053] M57+152	1
1502	>MF056176.1 Hc	73G 143A 146C 152C 195C 263G 1018A 1438G 2416C 2706G 3106d 3107C 4104G 4435G 4769G 5285G 6524C 7028T 7256T 7274T 7521A 7771G 8206A 8701G 8860G 9449T 9540C 10115C 10398G 10873C 11719A 11914A 11944C 12693G 12705T 13590A 13650T 13803G 14566G 14766T 15244G 15301A 15326G 15629C 15734A 15784C 16223T 16256T 16278T 16294T 16309G 16519C	L2a1a3c	73G 143A 146C 152C 195C 263G 16223T 16256T 16278T 16294T 16309G 16519C	1	73 143 146 152 195 263 1018 1438 2416 2706 4104 4435 4769 5285 6524 7028 7256 7274 7521 7771 8206 8701 8860 9449 9540 10115 10398 10873 11719 11914 11944 12693 12705 13590 13650 13803 14566 14766 15244 15301 15326 15629 15734 15784 16223 16256 16278 16294 16309	L2a1a3c	73 143 146 152 195 263 16223 16256 16278 16294 16309	[0.037] L2a1a3c (1)	[0.037] L2a1a3c (2) [0.000] L2a1a3 L2a1a3a L2a1a3b L2a1+143 L2a1c L2a1c3 L2a1c3a L2a1c4 L2a1c4a L2a1c4a1 L2a1e (13)	1
1503	>MF056175.1 Hc	73G 146C 152C 195C 198T 263G 750G 769A 1018A 1438G 2416C 2706G 2789T 3106d 3107C 3594T 4104G 4769G 5581G 7028T 7175C 7256T 7274T 7521A 7771G 8206A 8701G 8860G 9221G 9540C 10115C 10398G 10873C 11719A 11914A 11944C 12693G 12705T 13590A 13650T 13803G 14566G 14766T 15301A 15326G 15784C 16189C 16223T 16278T 16294T 16309G 16390A 16519C	L2a1f3	73G 146C 152C 195C 198T 263G 16189C 16223T 16278T 16294T 16309G 16390A 16519C	1	73 146 152 195 198 263 750 769 1018 1438 2416 2706 2789 3594 4104 4769 5581 7028 7175 7256 7274 7521 7771 8206 8701 8860 9221 9540 10115 10398 10873 11719 11914 11944 12693 12705 13590 13650 13803 14566 14766 15301 15326 15784 16189 16223 16278 16294 16309 16390	L2a1f3	73 146 152 195 198 263 16189 16223 16278 16294 16309 16390	[0.000] L2a1f3 (1)	[0.007] L2a1b (2) [0.005] L2a1f (3) [0.000] L2a1+16189_(16192) L2a1b1 L2a1b2 L2a1f1 L2a1f1a L2a1f2 L2a1f3 (10)	1
1504	>MF056171.1 Hc	73G 152C 195C 204C 263G 417A 489C 533G 750G 1438G 3106d 3107C 3394C 4769G 7028T 8188G 8360G 8701G 8860G 9438A 9540C 9545G 10142T 10295G 10398G 10400T 10873C 11569C 11719A 11899C 12279G 12618A 12705T 14766T 14783C 15025T 15043A 15301A 15326G 16223T 16263C 16311C 16519C	M23	73G 152C 195C 204C 263G 417A 489C 533G 16223T 16263C 16311C 16519C	1	73 152 195 204 263 417 489 533 750 1438 3394 4769 7028 8188 8360 8701 8860 9438 9540 9545 10142 10295 10398 10400 10873 11569 11719 11899 12279 12618 12705 14766 14783 15025 15043 15301 15326 16223 16263 16311	M23	73 152 195 204 263 417 489 533 16223 16263 16311	[0.000] M23 (1)	[0.072] M24a (2) [0.053] M57+152	1

1505	>MF056170.1 Ho	73G 152C 195C 204C 263G 417A 489C 533G 750G 1438G 3106d 3107C 3394C 4769G 7028T 8188G 8360G 8701G 8860G 9438A 9540C 9545G 10142T 10295G 10398G 10400T 10873C 11569C 11719A 11899C 12279G 12618A 12705T 14766T 14783C 15025T 15043A 15301A 15326G 16223T 16263C 16311C 16519C	M23	73G 152C 195C 204C 263G 417A 489C 533G 16223T 16263C 16311C 16519C	1	73 152 195 204 263 417 489 533 750 1438 3394 4769 7028 8188 8360 8701 8860 9438 9540 9545 10142 10295 10398 10400 10873 11569 11719 11899 12279 12618 12705 14766 14783 15025 15043 15301 15326 16223 16263 16311	M23	73 152 195 204 263 417 489 533 16223 16263 16311	[0.000] M23 (1)	[0.072] M24a (2) [0.053] M57+152	1
1506	>MF056169.1 Ho	73G 146C 152C 182T 183G 263G 466C 511T 514d 515d 709A 750G 769A 1018A 1438G 2416C 2626C 2706G 3106d 3107C 3594T 3654T 4104G 4769G 4823C 5246A 5263T 6040C 6080G 6248C 6497C 7028T 7175C 7256T 7521A 8206A 8701G 8723A 8860G 9221G 9540C 10115C 10398G 10873C 11719A 11815A 11944C 12172G 12425G 12705T 12879C 12967G 13470G 13590A 13650T 13810A 14088C 14766T 14890G 15301A 15326G 16183C 16189C 16223T 16224C 16278T 16309G 16390A 16519C	L2a5	73G 146C 152C 182T 183G 263G 466C 511T 514d 515d 16183C 16189C 16223T 16224C 16278T 16309G 16390A 16519C	1	73 146 152 182 183 263 466 511 709 750 769 1018 1438 2416 2626 2706 3594 3654 4104 4769 4823 5246A 5263 6040C 6080 6248 6497 7028 7175 7256 7521 8206 8701 8723 8860 9221 9540 10115 10398 10873 11719 11815A 11944 12172 12425 12705 12879 12967 13470 13590 13650 13810 14088 14766 14890 15301 15326 16189 16223 16224 16278 16309 16390	L2a5	73 146 152 182 183 263 466 511 16189 16223 16224 16278 16309 16390	[2.167] L2a5 (1)	[0.023] L2a1b2 (2) [0.022] L2a1b L2a1f1a (4) [0.012] L2a1f2 (5) [0.005] L2a1f (6) [0.000] L2 L2a L2a1+16189_(16192) L2a1b1 L2a1f1 (11)	1
1507	>MF056164.1 Ho	73G 152C 195C 204C 263G 417A 489C 533G 750G 1438G 3106d 3107C 3394C 4769G 7028T 8188G 8360G 8701G 8860G 9438A 9540C 9545G 10142T 10295G 10398G 10400T 10873C 11569C 11719A 11899C 12279G 12618A 12705T 14766T 14783C 15025T 15043A 15301A 15326G 16223T 16263C 16311C 16519C	M23	73G 152C 195C 204C 263G 417A 489C 533G 16223T 16263C 16311C 16519C	1	73 152 195 204 263 417 489 533 750 1438 3394 4769 7028 8188 8360 8701 8860 9438 9540 9545 10142 10295 10398 10400 10873 11569 11719 11899 12279 12618 12705 14766 14783 15025 15043 15301 15326 16223 16263 16311	M23	73 152 195 204 263 417 489 533 16223 16263 16311	[0.000] M23 (1)	[0.072] M24a (2) [0.053] M57+152	1
1508	>MF056163.1 Ho	73G 146C 152C 195C 263G 750G 769A 1018A 1438G 2416C 2706G 2789T 3106d 3107C 3394C 3594T 4104G 4742C 4769G 5090C 7028T 7175C 7256T 7274T 7521A 7771G 8206A 8701G 8860G 9221G 9540C 10115C 10143A 10398G 10873C 11719A 11914A 11944C 12693G 12705T 13590A 13650T 13803G 14566G 14766T 15301A 15326G 15735T 15784C 16182C 16183C 16189C 16223T 16278T 16290T 16294T 16309G 16390A	L2a1b1a	73G 146C 152C 195C 263G 16182C 16183C 16189C 16223T 16278T 16290T 16294T 16309G 16390A	1	73 146 152 195 263 750 769 1018 1438 2416 2706 2789 3394 3594 4104 4742 4769 5090 7028 7175 7256 7274 7521 7771 8206 8701 8860 9221 9540 10115 10143 10398 10873 11719 11914 11944 12693 12705 13590 13650 13803 14566 14766 15301 15326 15735 15784 16189 16223 16278 16290 16294 16309 16390	L2a1b1a	73 146 152 195 263 16189 16223 16278 16290 16294 16309 16390	[0.000] L2a1b1a (1)	[0.005] L2a1f (2) [0.000] L2a1+16189_(16192) L2a1b L2a1b1 L2a1b1a L2a1b2 L2a1f1 L2a1f1a L2a1f2 (10)	1

1509	>MF056159.1 Hc	73G 146C 152C 195C 263G 750G 769A 1018A 1438G 2416C 2706G 2789T 3106d 3107C 3594T 4104G 4769G 5090C 7028T 7175C 7256T 7274T 7521A 7771G 8206A 8701G 8860G 9221G 9540C 10115C 10143A 10398G 10873C 11719A 11914A 11944C 12693G 12705T 13590A 13650T 13803G 14566G 14766T 15301A 15326G 15735T 15784C 16183C 16189C 16223T 16278T 16290T 16294T 16309G 16390A	L2a1b1a	73G 146C 152C 195C 263G 16183C 16189C 16223T 16278T 16290T 16294T 16309G 16390A	1	73 146 152 195 263 750 769 1018 1438 2416 2706 2789 3594 4104 4769 5090 7028 7175 7256 7274 7521 7771 8206 8701 8860 9221 9540 10115 10143 10398 10873 11719 11914 11944 12693 12705 13590 13650 13803 14566 14766 15301 15326 15735 15784 16189 16223 16278 16290 16294 16309 16390	L2a1b1a	73 146 152 195 263 16189 16223 16278 16290 16294 16309 16390	[0.000] L2a1b1a (1)	[0.005] L2a1f (2) [0.000] L2a1+16189_(16192) L2a1b L2a1b1 L2a1b1a L2a1b2 L2a1f1 L2a1f1a L2a1f2 (10)	1
1510	>MF056158.1 Hc	73G 146C 152C 195C 204C 263G 750G 769A 1018A 1438G 2416C 2706G 2789T 3106d 3107C 3594T 4104G 4769G 5090C 7028T 7175C 7256T 7274T 7521A 7771G 8206A 8701G 8860G 9221G 9540C 10115C 10143A 10398G 10873C 11719A 11914A 11944C 12693G 12705T 13590A 13650T 13803G 14566G 14766T 15301A 15326G 15735T 15784C 16182C 16183C 16189C 16223T 16278T 16290T 16294T 16309G 16390A	L2a1b1a	73G 146C 152C 195C 204C 263G 16182C 16183C 16189C 16223T 16278T 16290T 16294T 16309G 16390A	1	73 146 152 195 204 263 750 769 1018 1438 2416 2706 2789 3594 4104 4769 5090 7028 7175 7256 7274 7521 7771 8206 8701 8860 9221 9540 10115 10143 10398 10873 11719 11914 11944 12693 12705 13590 13650 13803 14566 14766 15301 15326 15735 15784 16189 16223 16278 16290 16294 16309 16390	L2a1b1a	73 146 152 195 204 263 16189 16223 16278 16290 16294 16309 16390	[0.015] L2a1b1a (1)	[0.037] L2a1f2 (2) [0.015] L2a1b1a (3) [0.010] L2a1f (4) [0.000] L2a1+16189_(16192) L2a1b L2a1b1 L2a1b2 L2a1f1 L2a1f1a (10)	1
1511	>MF056157.1 Hc	73G 146C 152C 195C 204C 263G 750G 769A 1018A 1438G 2416C 2706G 2789T 3106d 3107C 3594T 4104G 4769G 5090C 7028T 7175C 7256T 7274T 7521A 7771G 8206A 8701G 8860G 9221G 9540C 10115C 10143A 10398G 10873C 11719A 11914A 11944C 12693G 12705T 13590A 13650T 13803G 14566G 14766T 15301A 15326G 15735T 15784C 16182C 16183C 16189C 16223T 16278T 16290T 16294T 16309G 16390A	L2a1b1a	73G 146C 152C 195C 204C 263G 16182C 16183C 16189C 16223T 16278T 16290T 16294T 16309G 16390A	1	73 146 152 195 204 263 750 769 1018 1438 2416 2706 2789 3594 4104 4769 5090 7028 7175 7256 7274 7521 7771 8206 8701 8860 9221 9540 10115 10143 10398 10873 11719 11914 11944 12693 12705 13590 13650 13803 14566 14766 15301 15326 15735 15784 16189 16223 16278 16290 16294 16309 16390	L2a1b1a	73 146 152 195 204 263 16189 16223 16278 16290 16294 16309 16390	[0.015] L2a1b1a (1)	[0.037] L2a1f2 (2) [0.015] L2a1b1a (3) [0.010] L2a1f (4) [0.000] L2a1+16189_(16192) L2a1b L2a1b1 L2a1b2 L2a1f1 L2a1f1a (10)	1
1512	>MF056696.1 Hc	73G 150T 200G 263G 489C 750G 2706G 3106d 3107C 3588T 4769G 5893T 7028T 7960G 8701G 8768T 8854A 8860G 9540C 10398G 10400T 10873C 11719A 12705T 13308G 13590A 13708A 14130T 14260G 14527G 14766T 14783C 15043A 15172A 15301A 15326G 15625T 16086C 16148T 16223T 16259T 16278T 16319A 16399G 16526A	M32c	73G 150T 200G 263G 489C 16086C 16148T 16223T 16259T 16278T 16319A 16399G 16526A	1	73 150 200 263 489 750 2706 3588 4769 5893 7028 7960 8701 8768 8854 8860 9540 10398 10400 10873 11719 12705 13308 13590 13708 14130 14260 14527 14766 14783 15043 15172 15301 15326 15625 16086 16148 16223 16259 16278 16319 16399 16526	M32c	73 150 200 263 489 16086 16148 16223 16259 16278 16319 16399 16526	[0.000] M32c (1)	[0.022] M51a (2) [0.007] M32 (3) [0.000] M51 M51b (5)	1

1513	>MF056688.1 Hc	73G 150T 199C 204C 263G 513d 514d 709A 750G 769A 789A 1018A 1438G 1804G 2706G 3010A 3106d 3107C 3505G 3693C 3918A 4017T 4029A 4216C 4232C 4769G 4977C 5460A 7028T 7624C 8206A 8614C 8701G 8860G 8974G 9248T 9540C 9986A 10398G 10813T 10873C 11719A 12661T 12705T 13497G 13518T 14016A 14766T 14905A 14935C 15301A 15326G 15344C 16179T 16183C 16189C 16223T 16239T 16311C 16320T 16362C 16519C	L4b1a	73G 150T 199C 204C 263G 513d 514d 16179T 16183C 16189C 16223T 16239T 16311C 16320T 16362C 16519C	1	73 150 199 204 263 513 709 750 769 789A 1018 1438 1804 2706 3010 3505 3693C 3918 4017 4029A 4216 4232 4769 4977 5460 7028 7624 8206 8614 8701 8860 8974G 9248 9540 9986 10398 10813 10873 11719 12661T 12705 13497 13518 14016 14766 14905 14935 15301 15326 15344 16179 16189 16223 16239 16311 16320 16362	L4b1a	73 150 199 204 263 513 16179 16189 16223 16239 16311 16320 16362	[0.000] L4b1a (1)	[0.010] L4b1 (2)	1
1514	>MF056686.1 Hc	73G 146C 152C 195C 263G 750G 769A 1018A 1438G 2416C 2706G 2789T 3106d 3107C 3594T 4104G 4769G 5090C 7028T 7175C 7256T 7274T 7521A 7771G 8206A 8701G 8860G 9221G 9540C 10115C 10143A 10398G 10873C 11719A 11914A 11944C 12693G 12705T 13590A 13650T 13803G 14566G 14766T 15301A 15326G 15735T 15784C 16182C 16183C 16189C 16223T 16278T 16290T 16294T 16309G 16390A	L2a1b1a	73G 146C 152C 195C 263G 16182C 16183C 16189C 16223T 16278T 16290T 16294T 16309G 16390A	1	73 146 152 195 263 750 769 1018 1438 2416 2706 2789 3594 4104 4769 5090 7028 7175 7256 7274 7521 7771 8206 8701 8860 9221 9540 10115 10143 10398 10873 11719 11914 11944 12693 12705 13590 13650 13803 14566 14766 15301 15326 15735 15784 16189 16223 16278 16290 16294 16309 16390	L2a1b1a	73 146 152 195 263 16189 16223 16278 16290 16294 16309 16390	[0.000] L2a1b1a (1)	[0.005] L2a1f (2) [0.000] L2a1+16189_(16192) L2a1b L2a1b1 L2a1b1a L2a1b2 L2a1f1 L2a1f1a L2a1f2 (10)	1
1515	>MF056684.1 Hc	73G 150T 200G 263G 489C 750G 2706G 3106d 3107C 3588T 4769G 7028T 7960G 8701G 8768T 8854A 8860G 9540C 10398G 10400T 10873C 11719A 12705T 13308G 13590A 13708A 14130T 14260G 14527G 14766T 14783C 15043A 15172A 15301A 15326G 15625T 16086C 16148T 16223T 16241G 16259T 16278T 16319A 16399G 16526A	M32c	73G 150T 200G 263G 489C 16086C 16148T 16223T 16241G 16259T 16278T 16319A 16399G 16526A	1	73 150 200 263 489 750 2706 3588 4769 7028 7960 8701 8768 8854 8860 9540 10398 10400 10873 11719 12705 13308 13590 13708 14130 14260 14527 14766 14783 15043 15172 15301 15326 15625 16086 16148 16223 16241 16259 16278 16319 16399 16526	M32c	73 150 200 263 489 16086 16148 16223 16241 16259 16278 16319 16399 16526	[0.003] M32c (1)	[0.022] M51a (2) [0.007] M32 (3) [0.000] M51 M51b (5)	1
1516	>MF056682.1 Hc	73G 152C 204C 263G 417A 489C 533G 750G 1438G 3106d 3107C 4769G 7028T 8188G 8360G 8701G 8860G 9438A 9540C 9545G 10142T 10295G 10398G 10400T 10873C 11569C 11719A 11899C 12279G 12618A 12705T 14766T 14783C 15025T 15043A 15301A 15326G 16086C 16148T 16223T 16263C 16311C 16320T 16519C	M23	73G 152C 204C 263G 417A 489C 533G 16086C 16148T 16223T 16263C 16311C 16320T 16519C	1	73 152 204 263 417 489 533 750 1438 4769 7028 8188 8360 8701 8860 9438 9540 9545 10142 10295 10398 10400 10873 11569 11719 11899 12279 12618 12705 14766 14783 15025 15043 15301 15326 16086 16148 16223 16263 16311 16320	M23	73 152 204 263 417 489 533 16086 16148 16223 16263 16311 16320	[0.032] M23 (1)	[0.074] M57+152 (2)	1

1517	>MF056679.1 Hc	73G 152C 195C 204C 263G 417A 489C 533G 750G 1438G 3106d 3107C 4769G 7028T 8188G 8360G 8701G 8860G 9438A 9540C 9545G 10142T 10295G 10398G 10400T 10873C 11569C 11719A 11899C 12279G 12618A 12705T 14766T 14783C 15025T 15043A 15301A 15326G 16223T 16263C 16311C 16519C	M23	73G 152C 195C 204C 263G 417A 489C 533G 16223T 16263C 16311C 16519C	1	73 152 195 204 263 417 489 533 750 1438 4769 7028 8188 8360 8701 8860 9438 9540 9545 10142 10295 10398 10400 10873 11569 11719 11899 12279 12618 12705 14766 14783 15025 15043 15301 15326 16223 16263 16311	M23	73 152 195 204 263 417 489 533 16223 16263 16311	[0.000] M23 (1)	[0.072] M24a (2) [0.053] M57+152	1
1518	>MF056669.1 Hc	73G 263G 279C 514d 515d 750G 1438G 2332T 2706G 3106d 3107C 3450T 4769G 5773A 6221C 6340T 7028T 8701G 8860G 9449T 9540C 10086G 10373A 10398G 10873C 11002G 11719A 12705T 13105G 13914A 14766T 15301A 15311G 15326G 15824G 15940d 16145A 16223T 16278T 16362C 16519C	L3b1a8	73G 263G 279C 514d 515d 16145A 16223T 16278T 16362C 16519C	1	73 263 279 750 1438 2332 2706 3450 4769 5773 6221 6340 7028 8701 8860 9449 9540 10086 10373 10398 10873 11002 11719 12705 13105 13914A 14766 15301 15311 15326 15824 15944d 16145 16223 16278 16362	L3b1a8	73 263 279 16145 16223 16278 16362	[0.213] L3b1a8 (1)	[0.000] L3b1a+@16124 L3b1a7 L3b1a7a (4)	1
1519	>MF056667.1 Hc	73G 150T 200G 263G 489C 750G 2706G 3106d 3107C 3588T 4769G 7028T 7960G 8701G 8768T 8854A 8860G 9540C 10398G 10400T 10873C 11719A 12705T 13308G 13590A 13708A 14130T 14260G 14527G 14766T 14783C 15043A 15172A 15301A 15326G 15625T 16086C 16148T 16223T 16259T 16278T 16319A 16399G 16526A	M32c	73G 150T 200G 263G 489C 16086C 16148T 16223T 16259T 16278T 16319A 16399G 16526A	1	73 150 200 263 489 750 2706 3588 4769 7028 7960 8701 8768 8854 8860 9540 10398 10400 10873 11719 12705 13308 13590 13708 14130 14260 14527 14766 14783 15043 15172 15301 15326 15625 16086 16148 16223 16259 16278 16319 16399 16526	M32c	73 150 200 263 489 16086 16148 16223 16259 16278 16319 16399 16526	[0.000] M32c (1)	[0.022] M51a (2) [0.007] M32 (3) [0.000] M51 M51b (5)	1
1520	>MF056665.1 Hc	73G 146C 152C 195C 263G 750G 769A 1018A 1438G 2416C 2706G 2789T 3106d 3107C 3594T 4104G 4769G 5090C 7028T 7175C 7256T 7274T 7521A 7771G 8206A 8701G 8860G 9221G 9540C 10115C 10143A 10398G 10873C 11719A 11914A 11944C 12693G 12705T 13590A 13650T 13803G 14566G 14766T 15301A 15326G 15735T 15784C 16182C 16183C 16189C 16223T 16278T 16290T 16294T 16309G 16390A	L2a1b1a	73G 146C 152C 195C 263G 16182C 16183C 16189C 16223T 16278T 16290T 16294T 16309G 16390A	1	73 146 152 195 263 750 769 1018 1438 2416 2706 2789 3594 4104 4769 5090 7028 7175 7256 7274 7521 7771 8206 8701 8860 9221 9540 10115 10143 10398 10873 11719 11914 11944 12693 12705 13590 13650 13803 14566 14766 15301 15326 15735 15784 16189 16223 16278 16290 16294 16309 16390	L2a1b1a	73 146 152 195 263 16189 16223 16278 16290 16294 16309 16390	[0.000] L2a1b1a (1)	[0.005] L2a1f (2) [0.000] L2a1+16189_(16192) L2a1b L2a1b1 L2a1b1a L2a1b2 L2a1f1 L2a1f1a L2a1f2 (10)	1

1521 >MF056659.1 H[illegible]	73G 152C 195C 204C 263G 417A 489C 533G 750G 1438G 3106d 3107C 4769G 5582G 7028T 8188G 8360G 8701G 8860G 9438A 9540C 9545G 10142T 10295G 10398G 10400T 10873C 11569C 11719A 11899C 12279G 12618A 12705T 14766T 14783C 15025T 15043A 15301A 15326G 16223T 16263C 16311C 16519C	M23	73G 152C 195C 204C 263G 417A 489C 533G 16223T 16263C 16311C 16519C	1	73 152 195 204 263 417 489 533 750 1438 4769 5582 7028 8188 8360 8701 8860 9438 9540 9545 10142 10295 10398 10400 10873 11569 11719 11899 12279 12618 12705 14766 14783 15025 15043 15301 15326 16223 16263 16311	M23	73 152 195 204 263 417 489 533 16223 16263 16311	[0.000] M23 (1)	[0.072] M24a (2) [0.053] M57+152	1
1522 >MF056649.1 H[illegible]	73G 150T 200G 263G 489C 750G 2706G 3106d 3107C 3588T 4769G 7028T 7960G 8701G 8768T 8854A 8860G 9540C 10398G 10400T 10873C 11719A 12705T 13308G 13590A 13708A 14130T 14260G 14527G 14766T 14783C 15043A 15172A 15301A 15326G 15625T 16086C 16148T 16223T 16259T 16278T 16319A 16399G 16526A	M32c	73G 150T 200G 263G 489C 16086C 16148T 16223T 16259T 16278T 16319A 16399G 16526A	1	73 150 200 263 489 750 2706 3588 4769 7028 7960 8701 8768 8854 8860 9540 10398 10400 10873 11719 12705 13308 13590 13708 14130 14260 14527 14766 14783 15043 15172 15301 15326 15625 16086 16148 16223 16259 16278 16319 16399 16526	M32c	73 150 200 263 489 16086 16148 16223 16259 16278 16319 16399 16526	[0.000] M32c (1)	[0.022] M51a (2) [0.007] M32 (3) [0.000] M51 M51b (5)	1
1523 >MF056631.1 H[illegible]	73G 146C 152C 195C 263G 750G 769A 1018A 1438G 2416C 2706G 2789T 3106d 3107C 3594T 4104G 4769G 5090C 7028T 7175C 7256T 7274T 7521A 7771G 8206A 8701G 8860G 9221G 9540C 10115C 10143A 10398G 10873C 11719A 11914A 11944C 12693G 12705T 13590A 13650T 13803G 14566G 14766T 15301A 15326G 15735T 15784C 16182C 16183C 16189C 16223T 16278T 16290T 16291T 16294T 16309G 16390A	L2a1b1a	73G 146C 152C 195C 263G 16182C 16183C 16189C 16223T 16278T 16290T 16291T 16294T 16309G 16390A	1	73 146 152 195 263 750 769 1018 1438 2416 2706 2789 3594 4104 4769 5090 7028 7175 7256 7274 7521 7771 8206 8701 8860 9221 9540 10115 10143 10398 10873 11719 11914 11944 12693 12705 13590 13650 13803 14566 14766 15301 15326 15735 15784 16189 16223 16278 16290 16291 16294 16309 16390	L2a1b1a	73 146 152 195 263 16189 16223 16278 16290 16291 16294 16309 16390	[0.003] L2a1b1a (1)	[0.005] L2a1f (2) [0.003] L2a1b1a (3) [0.000] L2a1+16189_(16192) L2a1b L2a1b1 L2a1b2 L2a1f1 L2a1f1a L2a1f2 (10)	1
1524 >MF056619.1 H[illegible]	73G 146C 152C 195C 263G 750G 769A 1018A 1438G 2416C 2706G 2789T 3106d 3107C 3594T 4104G 4769G 5090C 7028T 7175C 7256T 7274T 7521A 7771G 8206A 8701G 8860G 9221G 9540C 10115C 10143A 10398G 10873C 11719A 11914A 11944C 12693G 12705T 13590A 13650T 13803G 14566G 14766T 15301A 15326G 15735T 15784C 16182C 16183C 16189C 16223T 16278T 16290T 16294T 16309G 16390A	L2a1b1a	73G 146C 152C 195C 263G 16182C 16183C 16189C 16223T 16278T 16290T 16294T 16309G 16390A	1	73 146 152 195 263 750 769 1018 1438 2416 2706 2789 3594 4104 4769 5090 7028 7175 7256 7274 7521 7771 8206 8701 8860 9221 9540 10115 10143 10398 10873 11719 11914 11944 12693 12705 13590 13650 13803 14566 14766 15301 15326 15735 15784 16189 16223 16278 16290 16294 16309 16390	L2a1b1a	73 146 152 195 263 16189 16223 16278 16290 16294 16309 16390	[0.000] L2a1b1a (1)	[0.005] L2a1f (2) [0.000] L2a1+16189_(16192) L2a1b L2a1b1 L2a1b1a L2a1b2 L2a1f1 L2a1f1a L2a1f2 (10)	1

1525 >MF056613.1 Hc	73G 152C 195C 204C 263G 417A 489C 533G 750G 1438G 3106d 3107C 4769G 7028T 8188G 8360G 8701G 8860G 9438A 9540C 9545G 10142T 10295G 10398G 10400T 10873C 11569C 11719A 11899C 12279G 12618A 12705T 14766T 14783C 15025T 15043A 15299C 15301A 15326G 16051G 16176T 16223T 16263C 16311C 16519C	M23	73G 152C 195C 204C 263G 417A 489C 533G 16051G 16176T 16223T 16263C 16311C 16519C	1	73 152 195 204 263 417 489 533 750 1438 4769 7028 8188 8360 8701 8860 9438 9540 9545 10142 10295 10398 10400 10873 11569 11719 11899 12279 12618 12705 14766 14783 15025 15043 15299 15301 15326 16051 16176 16223 16263 16311	M23	73 152 195 204 263 417 489 533 16051 16176 16223 16263 16311	[0.040] M23 (1)	[0.072] M24a (2) [0.053] M57+152	1
1526 >MF056612.1 Hc	73G 150T 200G 263G 489C 750G 2706G 3106d 3107C 3588T 4769G 7028T 7960G 8701G 8768T 8854A 8860G 9540C 10398G 10400T 10873C 11719A 11778A 12705T 13308G 13590A 13708A 14130T 14260G 14527G 14766T 14783C 15043A 15172A 15301A 15326G 15625T 16086C 16148T 16223T 16259T 16278T 16319A 16399G 16526A	M32c	73G 150T 200G 263G 489C 16086C 16148T 16223T 16259T 16278T 16319A 16399G 16526A	1	73 150 200 263 489 750 2706 3588 4769 7028 7960 8701 8768 8854 8860 9540 10398 10400 10873 11719 11778 12705 13308 13590 13708 14130 14260 14527 14766 14783 15043 15172 15301 15326 15625 16086 16148 16223 16259 16278 16319 16399 16526	M32c	73 150 200 263 489 16086 16148 16223 16259 16278 16319 16399 16526	[0.000] M32c (1)	[0.022] M51a (2) [0.007] M32 (3) [0.000] M51 M51b (5)	1
1527 >MF056611.1 Hc	73G 146C 152C 195C 263G 292C 750G 769A 1018A 1438G 2416C 2706G 2789T 3106d 3107C 3594T 4104G 4769G 5090C 7028T 7175C 7256T 7274T 7521A 7771G 8206A 8280G 8701G 8860G 9221G 9540C 10115C 10143A 10398G 10873C 11719A 11914A 11944C 12693G 12705T 13590A 13650T 13803G 14566G 14766T 15301A 15326G 15735T 15784C 16183C 16189C 16223T 16278T 16290T 16294T 16309G 16390A	L2a1b1a	73G 146C 152C 195C 263G 292C 16183C 16189C 16223T 16278T 16290T 16294T 16309G 16390A	1	73 146 152 195 263 292 750 769 1018 1438 2416 2706 2789 3594 4104 4769 5090 7028 7175 7256 7274 7521 7771 8206 8280 8701 8860 9221 9540 10115 10143 10398 10873 11719 11914 11944 12693 12705 13590 13650 13803 14566 14766 15301 15326 15735 15784 16189 16223 16278 16290 16294 16309 16390	L2a1b1a	73 146 152 195 263 292 16189 16223 16278 16290 16294 16309 16390	[0.009] L2a1b1a (1)	[0.009] L2a1b1a (2) [0.005] L2a1f (3) [0.000] L2a1+16189_(16192) L2a1b L2a1b1 L2a1b2 L2a1f1 L2a1f1a L2a1f2 (10)	1
1528 >MF056610.1 Hc	73G 146C 152C 195C 263G 292C 750G 769A 1018A 1438G 2416C 2706G 2789T 3106d 3107C 3594T 4104G 4769G 5090C 7028T 7175C 7256T 7274T 7521A 7771G 8206A 8280G 8701G 8860G 9221G 9540C 10115C 10143A 10398G 10873C 11719A 11914A 11944C 12693G 12705T 13590A 13650T 13803G 14566G 14766T 15301A 15326G 15735T 15784C 16182C 16183C 16189C 16223T 16278T 16290T 16294T 16309G 16390A	L2a1b1a	73G 146C 152C 195C 263G 292C 16182C 16183C 16189C 16223T 16278T 16290T 16294T 16309G 16390A	1	73 146 152 195 263 292 750 769 1018 1438 2416 2706 2789 3594 4104 4769 5090 7028 7175 7256 7274 7521 7771 8206 8280 8701 8860 9221 9540 10115 10143 10398 10873 11719 11914 11944 12693 12705 13590 13650 13803 14566 14766 15301 15326 15735 15784 16189 16223 16278 16290 16294 16309 16390	L2a1b1a	73 146 152 195 263 292 16189 16223 16278 16290 16294 16309 16390	[0.009] L2a1b1a (1)	[0.009] L2a1b1a (2) [0.005] L2a1f (3) [0.000] L2a1+16189_(16192) L2a1b L2a1b1 L2a1b2 L2a1f1 L2a1f1a L2a1f2 (10)	1

1529	>MF056609.1 Hc	73G 152C 235G 263G 494T 735G 750G 1438G 2706G 3106d 3107C 3918A 4313C 4769G 6620G 7028T 7202G 8649G 8701G 8860G 9007T 9329A 9467C 9540C 10398G 10819G 10873C 11719A 12705T 13135A 13542G 13862G 13992T 14013G 14766T 15301A 15314A 15326G 16093C 16223T 16355T	L3k1	73G 152C 235G 263G 494T 16093C 16223T 16355T	1	73 152 235 263 494 735 750 1438 2706 3918 4313 4769 6620G 7028 7202 8649 8701 8860 9007T 9329 9467 9540 10398 10819 10873 11719 12705 13135 13542 13862 13992 14013 14766 15301 15314 15326 16093 16223 16355	L3k1	73 152 235 263 494 16093 16223 16355	[0.715] L3k1 (1)	[0.007] L3k (2)	1
1530	>MF056608.1 Hc	73G 150T 199C 204C 263G 513d 514d 709A 750G 769A 789A 1018A 1438G 1804G 2706G 3010A 3106d 3107C 3505G 3693C 3918A 4017T 4029A 4216C 4232C 4769G 4977C 5460A 7028T 7624C 8206A 8614C 8701G 8860G 8974G 9248T 9540C 9986A 10398G 10813T 10873C 11719A 12661T 12705T 13497G 13518T 14016A 14766T 14905A 14935C 15301A 15326G 15344C 16179T 16183C 16189C 16223T 16239T 16311C 16320T 16362C 16519C	L4b1a	73G 150T 199C 204C 263G 513d 514d 16179T 16183C 16189C 16223T 16239T 16311C 16320T 16362C 16519C	1	73 150 199 204 263 513 709 750 769 789A 1018 1438 1804 2706 3010 3505 3693C 3918 4017 4029A 4216 4232 4769 4977 5460 7028 7624 8206 8614 8701 8860 8974G 9248 9540 9986 10398 10813 10873 11719 12661T 12705 13497 13518 14016 14766 14905 14935 15301 15326 15344 16179 16189 16223 16239 16311 16320 16362	L4b1a	73 150 199 204 263 513 16179 16189 16223 16239 16311 16320 16362	[0.000] L4b1a (1)	[0.010] L4b1 (2)	1
1531	>MF057376.1 Hc	73G 146C 152C 195C 263G 310.1C 750G 769A 1018A 1438G 2416C 2706G 2789T 3106d 3107C 3594T 4104G 4769G 5090C 7028T 7175C 7256T 7274T 7521A 7771G 8206A 8701G 8860G 9221G 9540C 10115C 10143A 10398G 10873C 11719A 11914A 11944C 12693G 12705T 13590A 13650T 13803G 14566G 14766T 15301A 15326G 15735T 15784C 16182C 16183C 16189C 16223T 16278T 16290T 16294T 16309G 16390A 16519C	L2a1b1a	73G 146C 152C 195C 263G 310.1C 16182C 16183C 16189C 16223T 16278T 16290T 16294T 16309G 16390A 16519C	1	73 146 152 195 263 750 769 1018 1438 2416 2706 2789 3594 4104 4769 5090 7028 7175 7256 7274 7521 7771 8206 8701 8860 9221 9540 10115 10143 10398 10873 11719 11914 11944 12693 12705 13590 13650 13803 14566 14766 15301 15326 15735 15784 16189 16223 16278 16290 16294 16309 16390	L2a1b1a	73 146 152 195 263 16189 16223 16278 16290 16294 16309 16390	[0.000] L2a1b1a (1)	[0.005] L2a1f (2) [0.000] L2a1+16189_(16192) L2a1b L2a1b1 L2a1b1a L2a1b2 L2a1f1 L2a1f1a L2a1f2 (10)	1
1532	>MF057353.1 Hc	73G 152C 195C 204C 263G 310.1C 417A 489C 533G 750G 1438G 3106d 3107C 4769G 7028T 8188G 8360G 8701G 8860G 9438A 9540C 9545G 10142T 10295G 10398G 10400T 10873C 11569C 11719A 11899C 12279G 12618A 12705T 14766T 14783C 15025T 15043A 15301A 15326G 16223T 16263C 16311C 16519C	M23	73G 152C 195C 204C 263G 310.1C 417A 489C 533G 16223T 16263C 16311C 16519C	1	73 152 195 204 263 417 489 533 750 1438 4769 7028 8188 8360 8701 8860 9438 9540 9545 10142 10295 10398 10400 10873 11569 11719 11899 12279 12618 12705 14766 14783 15025 15043 15301 15326 16223 16263 16311	M23	73 152 195 204 263 417 489 533 16223 16263 16311	[0.000] M23 (1)	[0.072] M24a (2) [0.053] M57+152	1

1533	>MF057332.1 Hc	73G 263G 279C 310.1C 514d 515d 750G 1438G 2332T 2706G 3106d 3107C 3450T 4769G 5147A 5773A 6221C 6340T 7028T 8701G 8860G 9449T 9540C 10086G 10373A 10398G 10873C 11002G 11719A 12705T 13105G 13914A 14766T 15301A 15311G 15326G 15824G 15940d 16129A 16145A 16223T 16235G 16278T 16362C 16519C	L3b1a8	73G 263G 279C 310.1C 514d 515d 16129A 16145A 16223T 16235G 16278T 16362C 16519C	1	73 263 279 750 1438 2332 2706 3450 4769 5147 5773 6221 6340 7028 8701 8860 9449 9540 10086 10373 10398 10873 11002 11719 12705 13105 13914A 14766 15301 15311 15326 15824 15944d 16129 16145 16223 16235 16278 16362	L3b1a8	73 263 279 16129 16145 16223 16235 16278 16362	[0.260] L3b1a8 (1)	[0.000] L3b1a+@16124 L3b1a7 L3b1a7a (4)	1
1534	>MF057330.1 Hc	73G 150T 200G 263G 310.1C 489C 750G 2706G 3106d 3107C 3588T 4769G 5231A 7028T 7960G 8701G 8768T 8854A 8860G 9540C 10398G 10400T 10873C 11719A 12705T 13308G 13590A 13708A 14130T 14260G 14527G 14766T 14783C 15043A 15172A 15301A 15326G 15625T 16086C 16148T 16223T 16259T 16278T 16319A 16399G 16526A	M32c	73G 150T 200G 263G 310.1C 489C 16086C 16148T 16223T 16259T 16278T 16319A 16399G 16526A	1	73 150 200 263 489 750 2706 3588 4769 5231 7028 7960 8701 8768 8854 8860 9540 10398 10400 10873 11719 12705 13308 13590 13708 14130 14260 14527 14766 14783 15043 15172 15301 15326 15625 16086 16148 16223 16259 16278 16319 16399 16526	M32c	73 150 200 263 489 16086 16148 16223 16259 16278 16319 16399 16526	[0.000] M32c (1)	[0.022] M51a (2) [0.007] M32 (3) [0.000] M51 M51b (5)	1
1535	>MF057328.1 Hc	73G 150T 195C 263G 310.1C 514d 515d 2000T 2352C 2706G 3106d 3107C 4655A 4769G 5262A 6524C 7028T 8701G 8860G 9540C 9554A 10398G 10667C 10816G 10819G 10873C 11719A 12248G 12705T 13101C 13197T 13651G 14212C 14766T 14953T 15301A 15326G 15812A 16223T 16265T 16519C 16527T	L3e3b2	73G 150T 195C 263G 310.1C 514d 515d 16223T 16265T 16519C 16527T	1	73 150 195 263 2000 2352 2706 4655 4769 5262 6524 7028 8701 8860 9540 9554 10398 10667 10816 10819 10873 11719 12248 12705 13101C 13197 13651 14212 14766 14953 15301 15326 15812 16223 16265T 16527	L3e3b2	73 150 195 263 16223 16265T 16527	[0.000] L3e3b2 (1)	[0.000] L3e3 L3e3b L3e3b1 (4)	1
1536	>MF057325.1 Hc	73G 146C 152C 195C 263G 310.1C 750G 769A 1018A 1438G 2416C 2706G 2789T 3106d 3107C 3594T 4104G 4769G 5090C 7028T 7175C 7256T 7274T 7521A 7771G 8206A 8701G 8860G 9221G 9540C 10115C 10143A 10398G 10873C 11719A 11914A 11944C 12693G 12705T 13590A 13650T 13803G 14566G 14766T 15301A 15326G 15735T 15784C 16182C 16183C 16189C 16223T 16235G 16274A 16278T 16290T 16294T 16309G 16390A	L2a1b1a	73G 146C 152C 195C 263G 310.1C 16182C 16183C 16189C 16223T 16235G 16274A 16278T 16290T 16294T 16309G 16390A	1	73 146 152 195 263 750 769 1018 1438 2416 2706 2789 3594 4104 4769 5090 7028 7175 7256 7274 7521 7771 8206 8701 8860 9221 9540 10115 10143 10398 10873 11719 11914 11944 12693 12705 13590 13650 13803 14566 14766 15301 15326 15735 15784 16189 16223 16235 16274 16278 16290 16294 16309 16390	L2a1b1a	73 146 152 195 263 16189 16223 16235 16274 16278 16290 16294 16309 16390	[0.018] L2a1b1a (1)	[0.018] L2a1b1a (2) [0.008] L2a1b2 (3) [0.007] L2a1b L2a1f1a (5) [0.005] L2a1f (6) [0.000] L2a1+16189_(16192) L2a1b1 L2a1f1 L2a1f2 (10)	1

1537	>MF057324.1 Hc	73G 150T 200G 263G 310.1C 489C 750G 2706G 3106d 3107C 3588T 4769G 7028T 7960G 8701G 8768T 8854A 8860G 9540C 10398G 10400T 10873C 11061T 11719A 12705T 13308G 13590A 13708A 14130T 14260G 14527G 14766T 14783C 15043A 15172A 15301A 15326G 15625T 16086C 16148T 16223T 16259T 16278T 16319A 16399G 16526A	M32c	73G 150T 200G 263G 310.1C 489C 16086C 16148T 16223T 16259T 16278T 16319A 16399G 16526A	1	73 150 200 263 489 750 2706 3588 4769 7028 7960 8701 8768 8854 8860 9540 10398 10400 10873 11061 11719 12705 13308 13590 13708 14130 14260 14527 14766 14783 15043 15172 15301 15326 15625 16086 16148 16223 16259 16278 16319 16399 16526	M32c	73 150 200 263 489 16086 16148 16223 16259 16278 16319 16399 16526	[0.000] M32c (1)	[0.022] M51a (2) [0.007] M32 (3) [0.000] M51 M51b (5)	1
1538	>MF057319.1 Hc	73G 152C 195C 204C 263G 310.1C 417A 489C 533G 750G 3106d 3107C 4769G 7028T 8188G 8360G 8701G 8860G 9438A 9540C 9545G 10142T 10295G 10398G 10400T 10873C 11569C 11719A 11899C 12279G 12618A 12705T 14766T 14783C 15025T 15043A 15301A 15326G 16223T 16263C 16311C 16519C	M23	73G 152C 195C 204C 263G 310.1C 417A 489C 533G 16223T 16263C 16311C 16519C	1	73 152 195 204 263 417 489 533 750 4769 7028 8188 8360 8701 8860 9438 9540 9545 10142 10295 10398 10400 10873 11569 11719 11899 12279 12618 12705 14766 14783 15025 15043 15301 15326 16223 16263 16311	M23	73 152 195 204 263 417 489 533 16223 16263 16311	[0.000] M23 (1)	[0.072] M24a (2) [0.053] M57+152	1
1539	>MF057317.1 Hc	73G 152C 195C 204C 263G 310.1C 417A 489C 533G 750G 1438G 3106d 3107C 4769G 7028T 8188G 8360G 8701G 8860G 9438A 9540C 9545G 10142T 10295G 10398G 10400T 10873C 11569C 11719A 11899C 12279G 12618A 12705T 14766T 14783C 15025T 15043A 15301A 15326G 16223T 16263C 16311C 16519C	M23	73G 152C 195C 204C 263G 310.1C 417A 489C 533G 16223T 16263C 16311C 16519C	1	73 152 195 204 263 417 489 533 750 1438 4769 7028 8188 8360 8701 8860 9438 9540 9545 10142 10295 10398 10400 10873 11569 11719 11899 12279 12618 12705 14766 14783 15025 15043 15301 15326 16223 16263 16311	M23	73 152 195 204 263 417 489 533 16223 16263 16311	[0.000] M23 (1)	[0.072] M24a (2) [0.053] M57+152	1
1540	>MF057315.1 Hc	73G 146C 152C 195C 263G 750G 769A 1018A 1438G 2416C 2706G 2789T 3106d 3107C 3594T 4104G 4769G 5090C 6893T 7028T 7175C 7256T 7274T 7521A 7771G 8206A 8701G 8860G 9221G 9540C 10115C 10143A 10398G 10873C 11719A 11914A 11944C 12693G 12705T 13590A 13650T 13803G 14566G 14766T 15301A 15326G 15735T 15784C 16182C 16183C 16189C 16223T 16278T 16290T 16294T 16309G 16390A 16519C	L2a1b1a	73G 146C 152C 195C 263G 16182C 16183C 16189C 16223T 16278T 16290T 16294T 16309G 16390A 16519C	1	73 146 152 195 263 750 769 1018 1438 2416 2706 2789 3594 4104 4769 5090 6893 7028 7175 7256 7274 7521 7771 8206 8701 8860 9221 9540 10115 10143 10398 10873 11719 11914 11944 12693 12705 13590 13650 13803 14566 14766 15301 15326 15735 15784 16189 16223 16278 16290 16294 16309 16390	L2a1b1a	73 146 152 195 263 16189 16223 16278 16290 16294 16309 16390	[0.000] L2a1b1a (1)	[0.005] L2a1f (2) [0.000] L2a1+16189_(16192) L2a1b L2a1b1 L2a1b1a L2a1b2 L2a1f1 L2a1f1a L2a1f2 (10)	1

1541 >MF057314.1 Ho	73G 146C 152C 195C 234G 263G 310.1C 750G 769A 1018A 1438G 2416C 2706G 2789T 3106d 3107C 3394C 3594T 4104G 4769G 5090C 7028T 7175C 7256T 7274T 7521A 7771G 8206A 8701G 8860G 9221G 9540C 10115C 10143A 10398G 10873C 11719A 11914A 11944C 12693G 12705T 13590A 13650T 13803G 14566G 14766T 15301A 15326G 15735T 15784C 16182C 16183C 16189C 16223T 16278T 16290T 16294T 16309G 16390A	L2a1b1a	73G 146C 152C 195C 234G 263G 310.1C 16182C 16183C 16189C 16223T 16278T 16290T 16294T 16309G 16390A	1	73 146 152 195 234 263 750 769 1018 1438 2416 2706 2789 3394 3594 4104 4769 5090 7028 7175 7256 7274 7521 7771 8206 8701 8860 9221 9540 10115 10143 10398 10873 11719 11914 11944 12693 12705 13590 13650 13803 14566 14766 15301 15326 15735 15784 16189 16223 16278 16290 16294 16309 16390	L2a1b1a	73 146 152 195 234 263 16189 16223 16278 16290 16294 16309 16390	[0.006] L2a1b1a (1)	[0.006] L2a1b1a (2) [0.005] L2a1f (3) [0.000] L2a1+16189_(16192) L2a1b L2a1b1 L2a1b2 L2a1f1 L2a1f1a L2a1f2 (10)	1
1542 >MF057307.1 Ho	73G 152C 195C 204C 263G 309.1CT 310C 417A 489C 533G 750G 1438G 3106d 3107C 4769G 7028T 8188G 8360G 8701G 8860G 9438A 9540C 9545G 10142T 10295G 10398G 10400T 10873C 11569C 11719A 11899C 12279G 12618A 12705T 14766T 14783C 15025T 15043A 15301A 15326G 16223T 16263C 16311C 16519C	M23	73G 152C 195C 204C 263G 309.1CT 310C 417A 489C 533G 16223T 16263C 16311C 16519C	1	73 152 195 204 263 417 489 533 750 1438 4769 7028 8188 8360 8701 8860 9438 9540 9545 10142 10295 10398 10400 10873 11569 11719 11899 12279 12618 12705 14766 14783 15025 15043 15301 15326 16223 16263 16311	M23	73 152 195 204 263 417 489 533 16223 16263 16311	[0.000] M23 (1)	[0.072] M24a (2) [0.053] M57+152	1
1543 >MF057295.1 Ho	73G 152C 195C 204C 263G 310.1C 417A 489C 533G 750G 1438G 3106d 3107C 3398C 4769G 6267A 7028T 8188G 8360G 8701G 8860G 9438A 9540C 9545G 10142T 10295G 10398G 10400T 10873C 11569C 11719A 11899C 12279G 12397G 12618A 12705T 14766T 14783C 15025T 15043A 15301A 15326G 16223T 16263C 16311C 16519C	M23	73G 152C 195C 204C 263G 310.1C 417A 489C 533G 16223T 16263C 16311C 16519C	1	73 152 195 204 263 417 489 533 750 1438 3398 4769 6267 7028 8188 8360 8701 8860 9438 9540 9545 10142 10295 10398 10400 10873 11569 11719 11899 12279 12397 12618 12705 14766 14783 15025 15043 15301 15326 16223 16263 16311	M23	73 152 195 204 263 417 489 533 16223 16263 16311	[0.000] M23 (1)	[0.072] M24a (2) [0.053] M57+152	1
1544 >MF057286.1 Ho	73G 152C 195C 204C 263G 310.1C 417A 489C 533G 750G 1438G 3106d 3107C 4769G 7028T 8188G 8360G 8701G 8860G 9438A 9540C 9545G 10142T 10295G 10398G 10400T 10873C 11569C 11719A 11899C 12279G 12618A 12705T 14766T 14783C 15025T 15043A 15301A 15326G 16223T 16263C 16311C 16519C	M23	73G 152C 195C 204C 263G 310.1C 417A 489C 533G 16223T 16263C 16311C 16519C	1	73 152 195 204 263 417 489 533 750 1438 4769 7028 8188 8360 8701 8860 9438 9540 9545 10142 10295 10398 10400 10873 11569 11719 11899 12279 12618 12705 14766 14783 15025 15043 15301 15326 16223 16263 16311	M23	73 152 195 204 263 417 489 533 16223 16263 16311	[0.000] M23 (1)	[0.072] M24a (2) [0.053] M57+152	1

1545 >MF057278.1 Hc	73G 150T 200G 263G 310.1C 489C 750G 2706G 3106d 3107C 3588T 4769G 7028T 7960G 8701G 8768T 8854A 8860G 9540C 10398G 10400T 10873C 11719A 12705T 13308G 13590A 13708A 14130T 14260G 14527G 14766T 14783C 15043A 15172A 15301A 15326G 15625T 16086C 16148T 16223T 16259T 16274A 16278T 16319A 16399G 16526A	M32c	73G 150T 200G 263G 310.1C 489C 16086C 16148T 16223T 16259T 16274A 16278T 16319A 16399G 16526A	1	73 150 200 263 489 750 2706 3588 4769 7028 7960 8701 8768 8854 8860 9540 10398 10400 10873 11719 12705 13308 13590 13708 14130 14260 14527 14766 14783 15043 15172 15301 15326 15625 16086 16148 16223 16259 16274 16278 16319 16399 16526	M32c	73 150 200 263 489 16086 16148 16223 16259 16274 16278 16319 16399 16526	[0.007] M32c (1)	[0.060] M51a (2) [0.007] M32 (3) [0.000] M51 M51b (5)	1
1546 >MF057276.1 Hc	73G 150T 200G 263G 310.1C 489C 750G 2706G 3106d 3107C 3588T 4769G 7028T 7960G 8701G 8768T 8854A 8860G 9540C 10398G 10400T 10873C 11719A 12705T 13308G 13590A 13708A 14130T 14260G 14527G 14766T 14783C 15043A 15172A 15301A 15326G 15625T 16086C 16148T 16223T 16259T 16278T 16319A 16399G 16526A	M32c	73G 150T 200G 263G 310.1C 489C 16086C 16148T 16223T 16259T 16278T 16319A 16399G 16526A	1	73 150 200 263 489 750 2706 3588 4769 7028 7960 8701 8768 8854 8860 9540 10398 10400 10873 11719 12705 13308 13590 13708 14130 14260 14527 14766 14783 15043 15172 15301 15326 15625 16086 16148 16223 16259 16278 16319 16399 16526	M32c	73 150 200 263 489 16086 16148 16223 16259 16278 16319 16399 16526	[0.000] M32c (1)	[0.022] M51a (2) [0.007] M32 (3) [0.000] M51 M51b (5)	1
1547 >MF057274.1 Hc	73G 146C 152C 182T 183G 263G 310.1C 466C 511T 514d 515d 709A 750G 769A 1018A 1438G 2416C 2626C 2706G 3106d 3107C 3594T 3654T 4104G 4769G 4823C 5246A 5263T 6040C 6080G 6248C 6497C 7028T 7175C 7256T 7521A 8206A 8701G 8723A 8860G 9221G 9540C 10115C 10398G 10873C 11719A 11815A 11944C 12172G 12425G 12705T 12879C 12967G 13470G 13590A 13650T 13810A 14088C 14766T 14890G 15301A 15326G 16183C 16189C 16223T 16224C 16278T 16309G 16390A 16519C	L2a5	73G 146C 152C 182T 183G 263G 310.1C 466C 511T 514d 515d 16183C 16189C 16223T 16224C 16278T 16309G 16390A 16519C	1	73 146 152 182 183 263 466 511 709 750 769 1018 1438 2416 2626 2706 3594 3654 4104 4769 4823 5246A 5263 6040C 6080 6248 6497 7028 7175 7256 7521 8206 8701 8723 8860 9221 9540 10115 10398 10873 11719 11815A 11944 12172 12425 12705 12879 12967 13470 13590 13650 13810 14088 14766 14890 15301 15326 16189 16223 16224 16278 16309 16390	L2a5	73 146 152 182 183 263 466 511 16189 16223 16224 16278 16309 16390	[2.167] L2a5 (1)	[0.023] L2a1b2 (2) [0.022] L2a1b L2a1f1a (4) [0.012] L2a1f2 (5) [0.005] L2a1f (6) [0.000] L2 L2a L2a1+16189_(16192) L2a1b1 L2a1f1 (11)	1
1548 >MF057272.1 Hc	73G 150T 200G 263G 310.1C 489C 750G 2706G 3106d 3107C 3588T 4769G 7028T 7960G 8701G 8768T 8854A 8860G 9540C 10398G 10400T 10873C 11719A 12705T 13308G 13590A 13708A 14130T 14260G 14527G 14766T 14783C 15043A 15172A 15301A 15326G 15625T 16086C 16148T 16223T 16259T 16274A 16278T 16319A 16399G 16526A	M32c	73G 150T 200G 263G 310.1C 489C 16086C 16148T 16223T 16259T 16274A 16278T 16319A 16399G 16526A	1	73 150 200 263 489 750 2706 3588 4769 7028 7960 8701 8768 8854 8860 9540 10398 10400 10873 11719 12705 13308 13590 13708 14130 14260 14527 14766 14783 15043 15172 15301 15326 15625 16086 16148 16223 16259 16274 16278 16319 16399 16526	M32c	73 150 200 263 489 16086 16148 16223 16259 16274 16278 16319 16399 16526	[0.007] M32c (1)	[0.060] M51a (2) [0.007] M32 (3) [0.000] M51 M51b (5)	1

1549 >MF057485.1 Hc	73G 150T 200G 263G 310.1C 489C 750G 2706G 3106d 3107C 3588T 4769G 7028T 7960G 8701G 8768T 8854A 8860G 9540C 10398G 10400T 10873C 11719A 12705T 13308G 13590A 13708A 14130T 14260G 14527G 14766T 14783C 15043A 15172A 15301A 15326G 15625T 16086C 16148T 16223T 16259T 16278T 16319A 16399G 16526A	M32c	73G 150T 200G 263G 310.1C 489C 16086C 16148T 16223T 16259T 16278T 16319A 16399G 16526A	1	73 150 200 263 489 750 2706 3588 4769 7028 7960 8701 8768 8854 8860 9540 10398 10400 10873 11719 12705 13308 13590 13708 14130 14260 14527 14766 14783 15043 15172 15301 15326 15625 16086 16148 16223 16259 16278 16319 16399 16526	M32c	73 150 200 263 489 16086 16148 16223 16259 16278 16319 16399 16526	[0.000] M32c (1)	[0.022] M51a (2) [0.007] M32 (3) [0.000] M51 M51b (5)	1
1550 >MF057480.1 Hc	73G 146C 152C 195C 263G 310.1C 750G 769A 1018A 1438G 2416C 2706G 2762T 2789T 3106d 3107C 3594T 4104G 4769G 5090C 7028T 7175C 7256T 7274T 7521A 7578G 7771G 8206A 8701G 8860G 9221G 9540C 10115C 10143A 10398G 10873C 11719A 11914A 11944C 11992C 12693G 12705T 13590A 13650T 13803G 14566G 14766T 15301A 15326G 15735T 15784C 16182C 16183C 16189C 16223T 16278T 16290T 16294T 16390A	L2a1b1a	73G 146C 152C 195C 263G 310.1C 16182C 16183C 16189C 16223T 16278T 16290T 16294T 16390A	1	73 146 152 195 263 750 769 1018 1438 2416 2706 2762 2789 3594 4104 4769 5090 7028 7175 7256 7274 7521 7578 7771 8206 8701 8860 9221 9540 10115 10143 10398 10873 11719 11914 11944 11992 12693 12705 13590 13650 13803 14566 14766 15301 15326 15735 15784 16189 16223 16278 16290 16294 16390	L2a1b1a	73 146 152 195 263 16189 16223 16278 16290 16294 16390	[0.045] L2a1b1a (1)	[0.234] L2a1+143+16189_(16192)+@16309 (2) [0.070] L2a1f (3) [0.045] L2a1b1a (4) [0.008] L2a1b2 (5) [0.000] L2a1+16189_(16192) L2a1b L2a1b1 L2a1f1 L2a1f1a L2a1f2 (11)	1
1551 >MF057477.1 Hc	73G 146C 152C 195C 263G 310.1C 750G 769A 1018A 1438G 2416C 2706G 2789T 3106d 3107C 3594T 4104G 4769G 5090C 7028T 7175C 7256T 7274T 7521A 7771G 8206A 8701G 8860G 9221G 9540C 10115C 10143A 10398G 10873C 11719A 11914A 11944C 12693G 12705T 13590A 13650T 13803G 14566G 14766T 15301A 15326G 15735T 15784C 16183C 16189C 16223T 16278T 16290T 16294T 16309G 16390A	L2a1b1a	73G 146C 152C 195C 263G 310.1C 16183C 16189C 16223T 16278T 16290T 16294T 16309G 16390A	1	73 146 152 195 263 750 769 1018 1438 2416 2706 2789 3594 4104 4769 5090 7028 7175 7256 7274 7521 7771 8206 8701 8860 9221 9540 10115 10143 10398 10873 11719 11914 11944 12693 12705 13590 13650 13803 14566 14766 15301 15326 15735 15784 16189 16223 16278 16290 16294 16309 16390	L2a1b1a	73 146 152 195 263 16189 16223 16278 16290 16294 16309 16390	[0.000] L2a1b1a (1)	[0.005] L2a1f (2) [0.000] L2a1+16189_(16192) L2a1b L2a1b1 L2a1b1a L2a1b2 L2a1f1 L2a1f1a L2a1f2 (10)	1
1552 >MF057476.1 Hc	73G 152C 195C 204C 207A 263G 310.1C 417A 489C 533G 750G 1438G 3106d 3107C 4769G 7028T 8188G 8360G 8701G 8860G 9438A 9540C 9545G 10142T 10295G 10398G 10400T 10873C 11569C 11719A 11899C 12279G 12358G 12618A 12705T 14766T 14783C 15025T 15043A 15301A 15326G 15758G 16223T 16263C 16311C 16519C	M23	73G 152C 195C 204C 207A 263G 310.1C 417A 489C 533G 16223T 16263C 16311C 16519C	1	73 152 195 204 207 263 417 489 533 750 1438 4769 7028 8188 8360 8701 8860 9438 9540 9545 10142 10295 10398 10400 10873 11569 11719 11899 12279 12358 12618 12705 14766 14783 15025 15043 15301 15326 15758 16223 16263 16311	M23	73 152 195 204 207 263 417 489 533 16223 16263 16311	[0.016] M23 (1)	[0.077] M24a (2) [0.053] M57+152	1

1553 >MF057460.1 Hc	73G 150T 185A 189G 263G 750G 1438G 2352C 2706G 3106d 3107C 4769G 6221C 6587T 7028T 8577G 8701G 8860G 9540C 10398G 10819G 10873C 11719A 12192A 12705T 14152G 14212C 14766T 14926G 15301A 15326G 15670C 15942C 16223T 16323d 16327T 16400T	L3e1b2	73G 150T 185A 189G 263G 16223T 16323d 16327T 16400T	1	73 150 185 189 263 750 1438 2352 2706 4769 6221 6587 7028 8577 8701 8860 9540 10398 10819 10873 11719 12192 12705 14152 14212 14766 14926 15301 15326 15670 15942 16223 16325d 16327 16400	L3e1b2	73 150 185 189 263 16223 16325d 16327 16400	[0.037] L3e1b2 (1)	[0.200] L3e1b (2)	1
1554 >MF057445.1 Hc	73G 150T 200G 263G 310.1C 489C 750G 2223G 2706G 3106d 3107C 3588T 4682G 4769G 7028T 7960G 8701G 8768T 8854A 8860G 9540C 10398G 10400T 10873C 11719A 12705T 13308G 13590A 13708A 14130T 14260G 14527G 14766T 14783C 15043A 15172A 15301A 15326G 15625T 16086C 16148T 16223T 16259T 16278T 16319A 16399G 16526A	M32c	73G 150T 200G 263G 310.1C 489C 16086C 16148T 16223T 16259T 16278T 16319A 16399G 16526A	1	73 150 200 263 489 750 2223 2706 3588 4682 4769 7028 7960 8701 8768 8854 8860 9540 10398 10400 10873 11719 12705 13308 13590 13708 14130 14260 14527 14766 14783 15043 15172 15301 15326 15625 16086 16148 16223 16259 16278 16319 16399 16526	M32c	73 150 200 263 489 16086 16148 16223 16259 16278 16319 16399 16526	[0.000] M32c (1)	[0.022] M51a (2) [0.007] M32 (3) [0.000] M51 M51b (5)	1
1555 >MF057444.1 Hc	73G 151T 152C 182T 186A 189C 247A 263G 310.1C 316A 514d 515d 629C 750G 769A 825A 1018A 1438G 2283T 2394d 2706G 2758A 2885C 3106d 3107C 3210T 3434G 3594T 3666A 4104G 4755C 4769G 5951G 6071C 6221A 6917A 7028T 7146G 7256T 7389C 7521A 8027A 8251A 8417T 8468T 8655T 8701G 8860G 9072G 9540C 10398G 10586A 10688A 10810C 10873C 11302T 11317G 11719A 12400G 12542T 12705T 12810G 13105G 13485G 13506T 13650T 13708A 13789C 13981T 14000A 14070G 14178C 14560A 14766T 14794T 14911T 15226G 15326G 15905C 15978T 16017C 16129A 16163G 16187T 16189C 16209C 16223T 16278T 16293G 16294T 16311C 16360T 16519C	L1c3b1a	73G 151T 152C 182T 186A 189C 247A 263G 310.1C 316A 514d 515d 16129A 16163G 16187T 16189C 16209C 16223T 16278T 16293G 16294T 16311C 16360T 16519C	1	73 151 152 182 186A 189C 247 263 316 629 750 769 825A 1018 1438 2283 2395d 2706 2758 2885 3210 3434 3594 3666 4104 4755 4769 5951 6071 6221A 6917 7028 7146 7256 7389 7521 8027 8251 8417 8468 8655 8701 8860 9072 9540 10398 10586 10688 10810 10873 11302 11317 11719 12400 12542 12705 12810 13105 13485 13506 13650 13708 13789 13981 14000A 14070 14178 14560 14766 14794 14911 15226 15326 15905 15978 16017 16129 16163 16187 16189 16209 16223 16278 16293 16294 16311 16360	L1c3b1a	73 151 152 182 186A 189C 247 263 316 16129 16163 16187 16189 16209 16223 16278 16293 16294 16311 16360	[0.000] L1c3b1a (1)	[0.000] L1c3b L1c3b1 L1c3b1b (4)	1
1556 >MF057423.1 Hc	73G 152C 195C 204C 263G 310.1C 417A 489C 533G 750G 1438G 3106d 3107C 4769G 7028T 8188G 8360G 8701G 8860G 9438A 9540C 9545G 10142T 10295G 10398G 10400T 10873C 11569C 11719A 11899C 12279G 12618A 12705T 14766T 14783C 15025T 15043A 15301A 15326G 15479C 15927A 16223T 16263C 16311C 16519C	M23	73G 152C 195C 204C 263G 310.1C 417A 489C 533G 16223T 16263C 16311C 16519C	1	73 152 195 204 263 417 489 533 750 1438 4769 7028 8188 8360 8701 8860 9438 9540 9545 10142 10295 10398 10400 10873 11569 11719 11899 12279 12618 12705 14766 14783 15025 15043 15301 15326 15479 15927 16223 16263 16311	M23	73 152 195 204 263 417 489 533 16223 16263 16311	[0.000] M23 (1)	[0.072] M24a (2) [0.053] M57+152	1

1557	>MF057422.1 Hc	73G 150T 152C 235G 263G 310.1C 494T 735G 750G 1438G 2706G 3106d 3107C 3918A 4313C 4769G 6620G 7028T 7202G 8649G 8701G 8860G 9007T 9329A 9467C 9540C 10398G 10819G 10873C 11719A 12705T 13135A 13542G 13862G 13992T 14013G 14766T 15301A 15314A 15326G 16093C 16223T 16355T	L3k1	73G 150T 152C 235G 263G 310.1C 494T 16093C 16223T 16355T	1	73 150 152 235 263 494 735 750 1438 2706 3918 4313 4769 6620G 7028 7202 8649 8701 8860 9007T 9329 9467 9540 10398 10819 10873 11719 12705 13135 13542 13862 13992 14013 14766 15301 15314 15326 16093 16223 16355	L3k1	73 150 152 235 263 494 16093 16223 16355	[0.536] L3k1 (1)	[0.000] L3k (2)	1
1558	>MF057421.1 Hc	73G 152C 195C 204C 263G 310.1C 417A 489C 533G 750G 1438G 3106d 3107C 4769G 7028T 8188G 8360G 8701G 8860G 9438A 9540C 9545G 10142T 10295G 10398G 10400T 10873C 11569C 11719A 11899C 12279G 12618A 12705T 14766T 14783C 15025T 15043A 15301A 15326G 16223T 16263C 16311C 16519C	M23	73G 152C 195C 204C 263G 310.1C 417A 489C 533G 16223T 16263C 16311C 16519C	1	73 152 195 204 263 417 489 533 750 1438 4769 7028 8188 8360 8701 8860 9438 9540 9545 10142 10295 10398 10400 10873 11569 11719 11899 12279 12618 12705 14766 14783 15025 15043 15301 15326 16223 16263 16311	M23	73 152 195 204 263 417 489 533 16223 16263 16311	[0.000] M23 (1)	[0.072] M24a (2) [0.053] M57+152	1
1559	>MF057419.1 Hc	73G 151T 152C 195C 204C 263G 310.1C 417A 489C 533G 750G 1438G 2483C 3106d 3107C 4769G 7028T 8188G 8360G 8701G 8860G 9438A 9540C 9545G 10142T 10295G 10398G 10400T 10873C 11569C 11719A 11899C 12279G 12618A 12705T 14766T 14783C 15025T 15043A 15301A 15326G 16223T 16263C 16311C 16519C	M23	73G 151T 152C 195C 204C 263G 310.1C 417A 489C 533G 16223T 16263C 16311C 16519C	1	73 151 152 195 204 263 417 489 533 750 1438 2483 4769 7028 8188 8360 8701 8860 9438 9540 9545 10142 10295 10398 10400 10873 11569 11719 11899 12279 12618 12705 14766 14783 15025 15043 15301 15326 16223 16263 16311	M23	73 151 152 195 204 263 417 489 533 16223 16263 16311	[0.016] M23 (1)	[0.072] M24a (2) [0.053] M57+152 (3) [0.013] M37a (4) [0.004] M37+152+151 (5) [0.000] M37a1 M37d (7)	1
1560	>MF057408.1 Hc	73G 150T 200G 263G 310.1C 489C 750G 2706G 3106d 3107C 3588T 4188G 4769G 7028T 7960G 8701G 8768T 8854A 8860G 9540C 10398G 10400T 10873C 11719A 12705T 13308G 13590A 13708A 14130T 14260G 14527G 14766T 14783C 15043A 15172A 15301A 15326G 15625T 16086C 16148T 16223T 16259T 16278T 16319A 16399G 16526A	M32c	73G 150T 200G 263G 310.1C 489C 16086C 16148T 16223T 16259T 16278T 16319A 16399G 16526A	1	73 150 200 263 489 750 2706 3588 4188 4769 7028 7960 8701 8768 8854 8860 9540 10398 10400 10873 11719 12705 13308 13590 13708 14130 14260 14527 14766 14783 15043 15172 15301 15326 15625 16086 16148 16223 16259 16278 16319 16399 16526	M32c	73 150 200 263 489 16086 16148 16223 16259 16278 16319 16399 16526	[0.000] M32c (1)	[0.022] M51a (2) [0.007] M32 (3) [0.000] M51 M51b (5)	1
1561	>MF057398.1 Hc	73G 150T 200G 263G 310.1C 489C 750G 2706G 3106d 3107C 3588T 4769G 7028T 7960G 8701G 8768T 8854A 8860G 9540C 10398G 10400T 10873C 11719A 12705T 13308G 13590A 13708A 14130T 14260G 14527G 14766T 14783C 15043A 15172A 15301A 15326G 15625T 16086C 16148T 16223T 16259T 16278T 16319A 16399G 16526A	M32c	73G 150T 200G 263G 310.1C 489C 16086C 16148T 16223T 16259T 16278T 16319A 16399G 16526A	1	73 150 200 263 489 750 2706 3588 4769 7028 7960 8701 8768 8854 8860 9540 10398 10400 10873 11719 12705 13308 13590 13708 14130 14260 14527 14766 14783 15043 15172 15301 15326 15625 16086 16148 16223 16259 16278 16319 16399 16526	M32c	73 150 200 263 489 16086 16148 16223 16259 16278 16319 16399 16526	[0.000] M32c (1)	[0.022] M51a (2) [0.007] M32 (3) [0.000] M51 M51b (5)	1

1562	>MF057390.1 Hc	73G 152C 195C 204C 263G 310.1C 417A 489C 533G 750G 1438G 3106d 3107C 4769G 7028T 8188G 8360G 8701G 8860G 9438A 9540C 9545G 10142T 10295G 10398G 10400T 10873C 11569C 11719A 11899C 12279G 12618A 12705T 14766T 14783C 15025T 15043A 15301A 15326G 16223T 16263C 16311C 16519C	M23	73G 152C 195C 204C 263G 310.1C 417A 489C 533G 16223T 16263C 16311C 16519C	1	73 152 195 204 263 417 489 533 750 1438 4769 7028 8188 8360 8701 8860 9438 9540 9545 10142 10295 10398 10400 10873 11569 11719 11899 12279 12618 12705 14766 14783 15025 15043 15301 15326 16223 16263 16311	M23	73 152 195 204 263 417 489 533 16223 16263 16311	[0.000] M23 (1)	[0.072] M24a (2) [0.053] M57+152	1
1563	>MF055957.1 Hc	73G 263G 279C 514d 515d 750G 1438G 2332T 2706G 3106d 3107C 3450T 4769G 5773A 6221C 6340T 7028T 8701G 8860G 9449T 9540C 10086G 10373A 10398G 10873C 11002G 11719A 12705T 13105G 13914A 14766T 15301A 15311G 15326G 15824G 15940d 16145A 16223T 16278T 16362C 16519C	L3b1a8	73G 263G 279C 514d 515d 16145A 16223T 16278T 16362C 16519C	1	73 263 279 750 1438 2332 2706 3450 4769 5773 6221 6340 7028 8701 8860 9449 9540 10086 10373 10398 10873 11002 11719 12705 13105 13914A 14766 15301 15311 15326 15824 15944d 16145 16223 16278 16362	L3b1a8	73 263 279 16145 16223 16278 16362	[0.213] L3b1a8 (1)	[0.000] L3b1a+@16124 L3b1a7 L3b1a7a (4)	1
1564	>MF055953.1 Hc	73G 150T 200G 263G 489C 750G 2706G 3106d 3107C 3588T 4769G 7028T 7960G 8376C 8701G 8768T 8854A 8860G 9540C 10398G 10400T 10873C 11719A 12705T 13308G 13590A 13708A 14130T 14260G 14527G 14766T 14783C 15043A 15172A 15301A 15326G 15625T 16086C 16148T 16223T 16259T 16278T 16319A 16399G 16526A	M32c	73G 150T 200G 263G 489C 16086C 16148T 16223T 16259T 16278T 16319A 16399G 16526A	1	73 150 200 263 489 750 2706 3588 4769 7028 7960 8376 8701 8768 8854 8860 9540 10398 10400 10873 11719 12705 13308 13590 13708 14130 14260 14527 14766 14783 15043 15172 15301 15326 15625 16086 16148 16223 16259 16278 16319 16399 16526	M32c	73 150 200 263 489 16086 16148 16223 16259 16278 16319 16399 16526	[0.000] M32c (1)	[0.022] M51a (2) [0.007] M32 (3) [0.000] M51 M51b (5)	1
1565	>MF055950.1 Hc	73G 152C 195C 204C 263G 417A 489C 533G 750G 1438G 3106d 3107C 4769G 7028T 8188G 8360G 8701G 8860G 9438A 9540C 9545G 10142T 10295G 10398G 10400T 10873C 11569C 11719A 11899C 12279G 12618A 12705T 14766T 14783C 15025T 15043A 15301A 15326G 16223T 16263C 16311C 16519C	M23	73G 152C 195C 204C 263G 417A 489C 533G 16223T 16263C 16311C 16519C	1	73 152 195 204 263 417 489 533 750 1438 4769 7028 8188 8360 8701 8860 9438 9540 9545 10142 10295 10398 10400 10873 11569 11719 11899 12279 12618 12705 14766 14783 15025 15043 15301 15326 16223 16263 16311	M23	73 152 195 204 263 417 489 533 16223 16263 16311	[0.000] M23 (1)	[0.072] M24a (2) [0.053] M57+152	1

1566 >MF055943.1 Ho	73G 146C 152C 195C 263G 750G 769A 1018A 1438G 2416C 2706G 2789T 3010A 3106d 3107C 3594T 4104G 4769G 5090C 7028T 7175C 7256T 7274T 7521A 7771G 8206A 8701G 8860G 9221G 9540C 10115C 10143A 10398G 10873C 11719A 11914A 11944C 12693G 12705T 13590A 13650T 13803G 14566G 14766T 15301A 15326G 15735T 15784C 16182C 16183C 16189C 16223T 16278T 16290T 16294T 16309G 16390A	L2a1b1a	73G 146C 152C 195C 263G 16182C 16183C 16189C 16223T 16278T 16290T 16294T 16309G 16390A	1	73 146 152 195 263 750 769 1018 1438 2416 2706 2789 3010 3594 4104 4769 5090 7028 7175 7256 7274 7521 7771 8206 8701 8860 9221 9540 10115 10143 10398 10873 11719 11914 11944 12693 12705 13590 13650 13803 14566 14766 15301 15326 15735 15784 16189 16223 16278 16290 16294 16309 16390	L2a1b1a	73 146 152 195 263 16189 16223 16278 16290 16294 16309 16390	[0.000] L2a1b1a (1)	[0.005] L2a1f (2) [0.000] L2a1+16189_(16192) L2a1b L2a1b1 L2a1b1a L2a1b2 L2a1f1 L2a1f1a L2a1f2 (10)	1
1567 >MF055941.1 Ho	73G 152C 195C 204C 263G 417A 489C 533G 750G 1438G 3106d 3107C 4769G 7028T 8188G 8360G 8701G 8860G 9438A 9540C 9545G 10142T 10295G 10398G 10400T 10873C 11569C 11719A 11899C 12279G 12618A 12705T 14766T 14783C 15025T 15043A 15301A 15326G 16223T 16263C 16311C 16519C	M23	73G 152C 195C 204C 263G 417A 489C 533G 16223T 16263C 16311C 16519C	1	73 152 195 204 263 417 489 533 750 1438 4769 7028 8188 8360 8701 8860 9438 9540 9545 10142 10295 10398 10400 10873 11569 11719 11899 12279 12618 12705 14766 14783 15025 15043 15301 15326 16223 16263 16311	M23	73 152 195 204 263 417 489 533 16223 16263 16311	[0.000] M23 (1)	[0.072] M24a (2) [0.053] M57+152	1
1568 >MF055940.1 Ho	73G 146C 152C 195C 263G 750G 769A 1018A 1438G 2416C 2706G 2789T 3106d 3107C 3594T 4104G 4769G 5090C 7028T 7175C 7256T 7274T 7521A 7771G 8206A 8701G 8860G 9221G 9540C 10115C 10143A 10398G 10873C 11002G 11719A 11914A 11944C 12693G 12705T 13590A 13650T 13803G 14566G 14766T 15301A 15326G 15735T 15784C 16182C 16183C 16189C 16223T 16278T 16290T 16294T 16309G 16390A	L2a1b1a	73G 146C 152C 195C 263G 16182C 16183C 16189C 16223T 16278T 16290T 16294T 16309G 16390A	1	73 146 152 195 263 750 769 1018 1438 2416 2706 2789 3594 4104 4769 5090 7028 7175 7256 7274 7521 7771 8206 8701 8860 9221 9540 10115 10143 10398 10873 11002 11719 11914 11944 12693 12705 13590 13650 13803 14566 14766 15301 15326 15735 15784 16189 16223 16278 16290 16294 16309 16390	L2a1b1a	73 146 152 195 263 16189 16223 16278 16290 16294 16309 16390	[0.000] L2a1b1a (1)	[0.005] L2a1f (2) [0.000] L2a1+16189_(16192) L2a1b L2a1b1 L2a1b1a L2a1b2 L2a1f1 L2a1f1a L2a1f2 (10)	1
1569 >MF055937.1 Ho	73G 146C 152C 195C 263G 750G 769A 1018A 1438G 2416C 2706G 2789T 3106d 3107C 3594T 4104G 4769G 5090C 7028T 7175C 7256T 7274T 7521A 7771G 8206A 8701G 8860G 9221G 9540C 10115C 10143A 10398G 10873C 11719A 11914A 11944C 12693G 12705T 13590A 13650T 13803G 14566G 14766T 15301A 15326G 15735T 15784C 16102C 16182C 16183C 16189C 16223T 16278T 16290T 16294T 16309G 16390A	L2a1b1a	73G 146C 152C 195C 263G 16102C 16182C 16183C 16189C 16223T 16278T 16290T 16294T 16309G 16390A	1	73 146 152 195 263 750 769 1018 1438 2416 2706 2789 3594 4104 4769 5090 7028 7175 7256 7274 7521 7771 8206 8701 8860 9221 9540 10115 10143 10398 10873 11719 11914 11944 12693 12705 13590 13650 13803 14566 14766 15301 15326 15735 15784 16102 16189 16223 16278 16290 16294 16309 16390	L2a1b1a	73 146 152 195 263 16102 16189 16223 16278 16290 16294 16309 16390	[0.003] L2a1b1a (1)	[0.005] L2a1f (2) [0.003] L2a1b1a (3) [0.000] L2a1+16189_(16192) L2a1b L2a1b1 L2a1b2 L2a1f1 L2a1f1a L2a1f2 (10)	1

1570	>MF055936.1 Hc	73G 146C 152C 195C 263G 750G 769A 1018A 1438G 2416C 2706G 2789T 3106d 3107C 3594T 4104G 4769G 5090C 7028T 7175C 7256T 7274T 7521A 7771G 8206A 8701G 8860G 9221G 9540C 10115C 10143A 10398G 10873C 11719A 11914A 11944C 12693G 12705T 13590A 13650T 13803G 14566G 14766T 15301A 15326G 15735T 15784C 16183C 16189C 16223T 16278T 16290T 16294T 16309G 16390A 16526A	L2a1b1a	73G 146C 152C 195C 263G 16183C 16189C 16223T 16278T 16290T 16294T 16309G 16390A 16526A	1	73 146 152 195 263 750 769 1018 1438 2416 2706 2789 3594 4104 4769 5090 7028 7175 7256 7274 7521 7771 8206 8701 8860 9221 9540 10115 10143 10398 10873 11719 11914 11944 12693 12705 13590 13650 13803 14566 14766 15301 15326 15735 15784 16189 16223 16278 16290 16294 16309 16390 16526	L2a1b1a	73 146 152 195 263 16189 16223 16278 16290 16294 16309 16390 16526	[0.003] L2a1b1a (1)	[0.005] L2a1f (2) [0.003] L2a1b1a (3) [0.000] L2a1+16189_(16192) L2a1b L2a1b1 L2a1b2 L2a1f1 L2a1f1a L2a1f2 (10)	1
1571	>MF055934.1 Hc	73G 152C 182T 186A 189C 195C 247A 248G 263G 310.1C 316A 514d 515d 745.1T 750G 769A 825A 1018A 1438G 2394d 2706G 2758A 2885C 3106d 3107C 3525T 3594T 3666A 4104G 4769G 4853A 5951G 6071C 6221A 6227C 6917A 7028T 7146G 7247T 7256T 7389C 7521A 8027A 8468T 8655T 8701G 8860G 9072G 9540C 10398G 10586A 10688A 10810C 10873C 11302T 11719A 11984C 12507G 12705T 12810G 13105G 13485G 13506T 13650T 13789C 14000A 14178C 14560A 14669G 14766T 14911T 14926G 15226G 15326G 15905C 15978T 16187T 16189C 16223T 16224C 16278T 16293G 16294T 16311C 16360T 16519C	L1c3c	73G 152C 182T 186A 189C 195C 247A 248G 263G 310.1C 316A 514d 515d 16187T 16189C 16223T 16224C 16278T 16293G 16294T 16311C 16360T 16519C	1	73 152 182 186A 189C 195 247 248 263 316 745.1T 750 769 825A 1018 1438 2395d 2706 2758 2885 3525 3594 3666 4104 4769 4853 5951 6071 6221A 6227 6917 7028 7146 7247 7256 7389 7521 8027 8468 8655 8701 8860 9072 9540 10398 10586 10688 10810 10873 11302 11719 11984 12507 12705 12810 13105 13485 13506 13650 13789 14000A 14178 14560 14669 14766 14911 14926 15226 15326 15905 15978 16187 16189 16223 16224 16278 16293 16294 16311 16360	L1c3c	73 152 182 186A 189C 195 247 248 263 316 16187 16189 16223 16224 16278 16293 16294 16311 16360	[2.125] L1c3c (1) [0.000] L1c L1c1a+@198 L1c3b'c (4)	[0.700] L1c3 (5) [0.572] L1c1a'b'd (6) [0.267] L1c3b1 (7) [0.200] L1c1'2'4'5'6 (8) [0.133] L1c3b L1c3b1b (10) [0.077] L1c1 (11) [0.000] L1c1a L1c1a1 (13)	1
1572	>MF055923.1 Hc	73G 152C 195C 204C 263G 310.1C 417A 489C 533G 750G 1438G 3106d 3107C 4769G 7028T 8188G 8360G 8701G 8860G 9438A 9540C 9545G 10142T 10295G 10398G 10400T 10873C 11569C 11719A 11899C 12279G 12618A 12705T 14766T 14783C 15025T 15043A 15301A 15326G 16223T 16263C 16311C 16519C	M23	73G 152C 195C 204C 263G 310.1C 417A 489C 533G 16223T 16263C 16311C 16519C	1	73 152 195 204 263 417 489 533 750 1438 4769 7028 8188 8360 8701 8860 9438 9540 9545 10142 10295 10398 10400 10873 11569 11719 11899 12279 12618 12705 14766 14783 15025 15043 15301 15326 16223 16263 16311	M23	73 152 195 204 263 417 489 533 16223 16263 16311	[0.000] M23 (1)	[0.072] M24a (2) [0.053] M57+152	1
1573	>MF055921.1 Hc	73G 152C 195C 204C 263G 310.1C 417A 489C 533G 750G 1438G 3106d 3107C 4769G 7028T 8188G 8360G 8701G 8860G 9438A 9540C 9545G 9797C 10142T 10295G 10398G 10400T 10873C 11569C 11719A 11899C 12279G 12618A 12705T 14766T 14783C 15025T 15043A 15301A 15326G 16223T 16263C 16311C 16519C	M23	73G 152C 195C 204C 263G 310.1C 417A 489C 533G 16223T 16263C 16311C 16519C	1	73 152 195 204 263 417 489 533 750 1438 4769 7028 8188 8360 8701 8860 9438 9540 9545 9797 10142 10295 10398 10400 10873 11569 11719 11899 12279 12618 12705 14766 14783 15025 15043 15301 15326 16223 16263 16311	M23	73 152 195 204 263 417 489 533 16223 16263 16311	[0.000] M23 (1)	[0.072] M24a (2) [0.053] M57+152	1

1574	>MF055899.1 Hc	73G 152C 195C 204C 263G 309.1CT 310C 417A 489C 533G 750G 1438G 1462A 3106d 3107C 4769G 7028T 8188G 8360G 8860G 9438A 9540C 9545G 10142T 10295G 10398G 10400T 10873C 11569C 11719A 11899C 12279G 12618A 12705T 14766T 14783C 15025T 15043A 15301A 15326G 16223T 16263C 16311C 16519C	M23	73G 152C 195C 204C 263G 309.1CT 310C 417A 489C 533G 16223T 16263C 16311C 16519C	1	73 152 195 204 263 417 489 533 750 1438 1462 4769 7028 8188 8360 8860 9438 9540 9545 10142 10295 10398 10400 10873 11569 11719 11899 12279 12618 12705 14766 14783 15025 15043 15301 15326 16223 16263 16311	M23	73 152 195 204 263 417 489 533 16223 16263 16311	[0.000] M23 (1)	[0.072] M24a (2) [0.053] M57+152	1
1575	>MF055898.1 Hc	73G 146C 152C 182T 185T 189G 247A 263G 310.1C 357G 514d 515d 709A 710C 750G 769A 825A 1018A 1738C 2352C 2706G 2758A 2768G 2885C 3106d 3107C 3308C 3594T 3666A 3693A 4104G 4769G 6548T 6827C 6989G 7028T 7055G 7146G 7256T 7389C 7521A 7867T 8248G 8282T 8468T 8655T 8701G 8860G 9540C 10398G 10688A 10810C 10873C 11719A 12171G 12519C 12705T 12891T 13105G 13506T 13602C 13650T 13789C 13893G 14178C 14323A 14560A 14766T 14769G 15115C 15326G 16111T 16126C 16187T 16189C 16223T 16239T 16270T 16278T 16293G 16311C 16519C	L1b2a	73G 146C 152C 182T 185T 189G 247A 263G 310.1C 357G 514d 515d 16111T 16126C 16187T 16189C 16223T 16239T 16270T 16278T 16293G 16311C 16519C	1	73 146 152 182 185T 189 247 263 357 709 710 750 769 825A 1018 1738 2352 2706 2758 2768 2885 3308 3594 3666 3693 4104 4769 6548 6827 6989 7028 7055 7146 7256 7389 7521 7867 8248 8282 8468 8655 8701 8860 9540 10398 10688 10810 10873 11719 12171 12519 12705 12891 13105 13506 13602 13650 13789 13893 14178 14323 14560 14766 14769 15115 15326 16111 16126 16187 16189 16223 16239 16270 16278 16293 16311	L1b2a	73 146 152 182 185T 189 247 263 357 16111 16126 16187 16189 16223 16239 16270 16278 16293 16311	[0.000] L1b2a (1)	[0.042] L1b2 (2)	1
1576	>MF055896.1 Hc	73G 146C 152C 182T 185T 189G 247A 263G 310.1C 357G 514d 515d 709A 710C 750G 769A 825A 1018A 1738C 2352C 2706G 2758A 2768G 2885C 3106d 3107C 3308C 3594T 3666A 3693A 4104G 4769G 6548T 6827C 6989G 7028T 7055G 7146G 7256T 7389C 7521A 7867T 8248G 8282T 8468T 8655T 8701G 8860G 9540C 10398G 10688A 10810C 10873C 11719A 12171G 12519C 12705T 12891T 13105G 13506T 13602C 13650T 13789C 13893G 14178C 14323A 14560A 14766T 14769G 15115C 15326G 16111T 16126C 16187T 16189C 16223T 16239T 16270T 16278T 16293G 16311C 16519C	L1b2a	73G 146C 152C 182T 185T 189G 247A 263G 310.1C 357G 514d 515d 16111T 16126C 16187T 16189C 16223T 16239T 16270T 16278T 16293G 16311C 16519C	1	73 146 152 182 185T 189 247 263 357 709 710 750 769 825A 1018 1738 2352 2706 2758 2768 2885 3308 3594 3666 3693 4104 4769 6548 6827 6989 7028 7055 7146 7256 7389 7521 7867 8248 8282 8468 8655 8701 8860 9540 10398 10688 10810 10873 11719 12171 12519 12705 12891 13105 13506 13602 13650 13789 13893 14178 14323 14560 14766 14769 15115 15326 16111 16126 16187 16189 16223 16239 16270 16278 16293 16311	L1b2a	73 146 152 182 185T 189 247 263 357 16111 16126 16187 16189 16223 16239 16270 16278 16293 16311	[0.000] L1b2a (1)	[0.042] L1b2 (2)	1

1577	>MF055895.1 Hc	73G 146C 152C 195C 204C 263G 310.1C 750G 769A 1018A 1438G 2416C 2706G 2789T 3106d 3107C 3594T 4104G 4769G 5090C 7028T 7175C 7256T 7274T 7521A 7771G 8206A 8701G 8860G 9221G 9540C 10115C 10143A 10398G 10873C 11719A 11914A 11944C 12693G 12705T 13590A 13650T 13803G 14566G 14766T 15301A 15326G 15735T 15784C 16182C 16183C 16189C 16192T 16223T 16278T 16290T 16294T 16309G 16390A	L2a1b1a	73G 146C 152C 195C 204C 263G 310.1C 16182C 16183C 16189C 16192T 16223T 16278T 16290T 16294T 16309G 16390A	1	73 146 152 195 204 263 750 769 1018 1438 2416 2706 2789 3594 4104 4769 5090 7028 7175 7256 7274 7521 7771 8206 8701 8860 9221 9540 10115 10143 10398 10873 11719 11914 11944 12693 12705 13590 13650 13803 14566 14766 15301 15326 15735 15784 16189 16192 16223 16278 16290 16294 16309 16390	L2a1b1a	73 146 152 195 204 263 16189 16192 16223 16278 16290 16294 16309 16390	[0.015] L2a1b1a (1)	[0.037] L2a1f2 (2) [0.030] L2a1b1a (3) [0.010] L2a1f (4) [0.000] L2a1+16189_(16192) L2a1b L2a1b1 L2a1b2 L2a1f1 L2a1f1a (10)	1
1578	>MF055875.1 Hc	73G 152C 195C 204C 263G 310.1C 417A 489C 533G 750G 1438G 3106d 3107C 4769G 5237A 7028T 8188G 8360G 8701G 8860G 9438A 9540C 9545G 10142T 10295G 10398G 10400T 10873C 11569C 11719A 11899C 12279G 12618A 12705T 14766T 14783C 15025T 15043A 15301A 15326G 16223T 16263C 16311C 16519C	M23	73G 152C 195C 204C 263G 310.1C 417A 489C 533G 16223T 16263C 16311C 16519C	1	73 152 195 204 263 417 489 533 750 1438 4769 5237 7028 8188 8360 8701 8860 9438 9540 9545 10142 10295 10398 10400 10873 11569 11719 11899 12279 12618 12705 14766 14783 15025 15043 15301 15326 16223 16263 16311	M23	73 152 195 204 263 417 489 533 16223 16263 16311	[0.000] M23 (1)	[0.072] M24a (2) [0.053] M57+152	1
1579	>MF055866.1 Hc	73G 152C 195C 204C 263G 310.1C 417A 489C 533G 750G 1438G 3106d 3107C 4769G 7028T 8188G 8360G 8701G 8860G 9438A 9540C 9545G 10142T 10295G 10398G 10400T 10873C 11569C 11719A 11899C 12279G 12618A 12705T 14766T 14783C 15025T 15043A 15301A 15326G 16223T 16263C 16311C 16519C	M23	73G 152C 195C 204C 263G 310.1C 417A 489C 533G 16223T 16263C 16311C 16519C	1	73 152 195 204 263 417 489 533 750 1438 4769 7028 8188 8360 8701 8860 9438 9540 9545 10142 10295 10398 10400 10873 11569 11719 11899 12279 12618 12705 14766 14783 15025 15043 15301 15326 16223 16263 16311	M23	73 152 195 204 263 417 489 533 16223 16263 16311	[0.000] M23 (1)	[0.072] M24a (2) [0.053] M57+152	1
1580	>MF055865.1 Hc	73G 152C 195C 204C 263G 310.1C 417A 489C 533G 750G 1438G 3106d 3107C 4769G 7028T 8188G 8360G 8701G 8860G 9438A 9540C 9545G 10142T 10295G 10398G 10400T 10873C 11569C 11719A 11899C 12279G 12618A 12705T 14766T 14783C 15025T 15043A 15301A 15326G 16223T 16263C 16311C 16519C	M23	73G 152C 195C 204C 263G 310.1C 417A 489C 533G 16223T 16263C 16311C 16519C	1	73 152 195 204 263 417 489 533 750 1438 4769 7028 8188 8360 8701 8860 9438 9540 9545 10142 10295 10398 10400 10873 11569 11719 11899 12279 12618 12705 14766 14783 15025 15043 15301 15326 16223 16263 16311	M23	73 152 195 204 263 417 489 533 16223 16263 16311	[0.000] M23 (1)	[0.072] M24a (2) [0.053] M57+152	1
1581	>MF055863.1 Hc	73G 152C 195C 204C 263G 310.1C 417A 489C 533G 750G 1438G 3106d 3107C 4769G 7028T 8188G 8360G 8701G 8860G 9438A 9540C 9545G 10142T 10295G 10398G 10400T 10873C 11569C 11719A 11899C 12279G 12618A 12705T 14766T 14783C 15025T 15043A 15301A 15326G 16223T 16263C 16311C 16519C	M23	73G 152C 195C 204C 263G 310.1C 417A 489C 533G 16223T 16263C 16311C 16519C	1	73 152 195 204 263 417 489 533 750 1438 4769 7028 8188 8360 8701 8860 9438 9540 9545 10142 10295 10398 10400 10873 11569 11719 11899 12279 12618 12705 14766 14783 15025 15043 15301 15326 16223 16263 16311	M23	73 152 195 204 263 417 489 533 16223 16263 16311	[0.000] M23 (1)	[0.072] M24a (2) [0.053] M57+152	1

1582 >MF055857.1 Hc	73G 146C 152C 195C 263G 310.1C 750G 769A 1018A 1438G 2416C 2706G 2789T 3106d 3107C 3594T 4104G 4769G 5090C 7028T 7175C 7256T 7274T 7521A 7771G 8206A 8701G 8860G 9221G 9540C 10115C 10143A 10398G 10873C 11719A 11914A 11944C 12693G 12705T 13590A 13650T 13803G 14566G 14766T 15301A 15326G 15735T 15784C 16180d 16183C 16189C 16223T 16278T 16290T 16294T 16309G 16390A	L2a1b1a	73G 146C 152C 195C 263G 310.1C 16180d 16183C 16189C 16223T 16278T 16290T 16294T 16309G 16390A	1	73 146 152 195 263 750 769 1018 1438 2416 2706 2789 3594 4104 4769 5090 7028 7175 7256 7274 7521 7771 8206 8701 8860 9221 9540 10115 10143 10398 10873 11719 11914 11944 12693 12705 13590 13650 13803 14566 14766 15301 15326 15735 15784 16189 16193d 16223 16278 16290 16294 16309 16390	L2a1b1a	73 146 152 195 263 16189 16193d 16223 16278 16290 16294 16309 16390	[0.024] L2a1b1a (1)	[0.024] L2a1b1a (2) [0.010] L2a1f (3) [0.008] L2a1b2 (4) [0.000] L2a1+16189_(16192) L2a1b L2a1b1 L2a1f1 L2a1f1a L2a1f2 (10)	1
1583 >MF056066.1 Hc	73G 152C 195C 204C 263G 417A 489C 533G 750G 1438G 3106d 3107C 4769G 7028T 8188G 8360G 8701G 8860G 9438A 9540C 9545G 10142T 10295G 10398G 10400T 10873C 11569C 11719A 11899C 12279G 12618A 12705T 14766T 14783C 15025T 15043A 15301A 15326G 16223T 16263C 16311C 16519C	M23	73G 152C 195C 204C 263G 417A 489C 533G 16223T 16263C 16311C 16519C	1	73 152 195 204 263 417 489 533 750 1438 4769 7028 8188 8360 8701 8860 9438 9540 9545 10142 10295 10398 10400 10873 11569 11719 11899 12279 12618 12705 14766 14783 15025 15043 15301 15326 16223 16263 16311	M23	73 152 195 204 263 417 489 533 16223 16263 16311	[0.000] M23 (1)	[0.072] M24a (2) [0.053] M57+152	1
1584 >MF056061.1 Hc	73G 152C 195C 204C 263G 417A 489C 533G 750G 1438G 3106d 3107C 4769G 7028T 8188G 8360G 8701G 8860G 9438A 9540C 9545G 10142T 10295G 10398G 10400T 10873C 11569C 11719A 11899C 12279G 12618A 12705T 14766T 14783C 15025T 15043A 15301A 15326G 16223T 16263C 16311C 16519C	M23	73G 152C 195C 204C 263G 417A 489C 533G 16223T 16263C 16311C 16519C	1	73 152 195 204 263 417 489 533 750 1438 4769 7028 8188 8360 8701 8860 9438 9540 9545 10142 10295 10398 10400 10873 11569 11719 11899 12279 12618 12705 14766 14783 15025 15043 15301 15326 16223 16263 16311	M23	73 152 195 204 263 417 489 533 16223 16263 16311	[0.000] M23 (1)	[0.072] M24a (2) [0.053] M57+152	1
1585 >MF056059.1 Hc	73G 152d 200G 263G 489C 750G 2706G 3106d 3107C 3588T 4769G 7028T 7960G 8158G 8701G 8768T 8854A 8860G 9540C 10398G 10400T 10873C 11719A 12705T 13308G 13590A 13708A 14130T 14260G 14527G 14766T 14783C 15043A 15172A 15301A 15326G 15625T 16086C 16148T 16223T 16259T 16278T 16319A 16399G 16526A	M32c	73G 152d 200G 263G 489C 16086C 16148T 16223T 16259T 16278T 16319A 16399G 16526A	1	73 152d 200 263 489 750 2706 3588 4769 7028 7960 8158 8701 8768 8854 8860 9540 10398 10400 10873 11719 12705 13308 13590 13708 14130 14260 14527 14766 14783 15043 15172 15301 15326 15625 16086 16148 16223 16259 16278 16319 16399 16526	M32c	73 152d 200 263 489 16086 16148 16223 16259 16278 16319 16399 16526	[0.030] M32c (1)	[0.007] M32 (2)	1

1586 >MF056053.1 Hc	73G 150T 200G 263G 489C 750G 2706G 3106d 3107C 3588T 4769G 7028T 7960G 8701G 8768T 8854A 8860G 9540C 10398G 10400T 10873C 11719A 12705T 13308G 13590A 13708A 14130T 14260G 14527G 14766T 14783C 15043A 15172A 15301A 15326G 15625T 16086C 16148T 16223T 16259T 16278T 16319A 16399G 16526A	M32c	73G 150T 200G 263G 489C 16086C 16148T 16223T 16259T 16278T 16319A 16399G 16526A	1	73 150 200 263 489 750 2706 3588 4769 7028 7960 8701 8768 8854 8860 9540 10398 10400 10873 11719 12705 13308 13590 13708 14130 14260 14527 14766 14783 15043 15172 15301 15326 15625 16086 16148 16223 16259 16278 16319 16399 16526	M32c	73 150 200 263 489 16086 16148 16223 16259 16278 16319 16399 16526	[0.000] M32c (1)	[0.022] M51a (2) [0.007] M32 (3) [0.000] M51 M51b (5)	1
1587 >MF056052.1 Hc	73G 150T 200G 263G 489C 750G 2706G 3106d 3107C 3588T 4769G 7028T 7960G 8701G 8768T 8854A 8860G 9540C 10398G 10400T 10873C 11719A 12705T 13308G 13590A 13708A 14130T 14260G 14527G 14766T 14783C 15043A 15172A 15301A 15326G 15625T 16086C 16148T 16223T 16259T 16278T 16319A 16399G 16526A	M32c	73G 150T 200G 263G 489C 16086C 16148T 16223T 16259T 16278T 16319A 16399G 16526A	1	73 150 200 263 489 750 2706 3588 4769 7028 7960 8701 8768 8854 8860 9540 10398 10400 10873 11719 12705 13308 13590 13708 14130 14260 14527 14766 14783 15043 15172 15301 15326 15625 16086 16148 16223 16259 16278 16319 16399 16526	M32c	73 150 200 263 489 16086 16148 16223 16259 16278 16319 16399 16526	[0.000] M32c (1)	[0.022] M51a (2) [0.007] M32 (3) [0.000] M51 M51b (5)	1
1588 >MF056046.1 Hc	73G 150T 200G 263G 489C 750G 2706G 3106d 3107C 3588T 4769G 7028T 7960G 8701G 8768T 8854A 8860G 9540C 10398G 10400T 10873C 11719A 12705T 13308G 13590A 13708A 14130T 14260G 14527G 14766T 14783C 15043A 15172A 15301A 15326G 15625T 16086C 16148T 16223T 16259T 16278T 16319A 16399G 16526A	M32c	73G 150T 200G 263G 489C 16086C 16148T 16223T 16259T 16278T 16319A 16399G 16526A	1	73 150 200 263 489 750 2706 3588 4769 7028 7960 8701 8768 8854 8860 9540 10398 10400 10873 11719 12705 13308 13590 13708 14130 14260 14527 14766 14783 15043 15172 15301 15326 15625 16086 16148 16223 16259 16278 16319 16399 16526	M32c	73 150 200 263 489 16086 16148 16223 16259 16278 16319 16399 16526	[0.000] M32c (1)	[0.022] M51a (2) [0.007] M32 (3) [0.000] M51 M51b (5)	1
1589 >MF056045.1 Hc	73G 146C 152C 195C 263G 750G 769A 1018A 1438G 2416C 2706G 2789T 3106d 3107C 3594T 4104G 4769G 5090C 7028T 7175C 7256T 7274T 7521A 7771G 8206A 8701G 8860G 9221G 9540C 10115C 10143A 10398G 10873C 11719A 11914A 11944C 12693G 12705T 13590A 13650T 13803G 14566G 14766T 15301A 15326G 15735T 15784C 16182C 16183C 16189C 16223T 16278T 16290T 16294T 16309G 16390A	L2a1b1a	73G 146C 152C 195C 263G 16182C 16183C 16189C 16223T 16278T 16290T 16294T 16309G 16390A	1	73 146 152 195 263 750 769 1018 1438 2416 2706 2789 3594 4104 4769 5090 7028 7175 7256 7274 7521 7771 8206 8701 8860 9221 9540 10115 10143 10398 10873 11719 11914 11944 12693 12705 13590 13650 13803 14566 14766 15301 15326 15735 15784 16189 16223 16278 16290 16294 16309 16390	L2a1b1a	73 146 152 195 263 16189 16223 16278 16290 16294 16309 16390	[0.000] L2a1b1a (1)	[0.005] L2a1f (2) [0.000] L2a1+16189_(16192) L2a1b L2a1b1 L2a1b1a L2a1b2 L2a1f1 L2a1f1a L2a1f2 (10)	1

1590	>MF056044.1 Ho	73G 152C 195C 204C 263G 417A 489C 533G 750G 1438G 3106d 3107C 4769G 7028T 8188G 8360G 8701G 8860G 9438A 9540C 9545G 10142T 10295G 10398G 10400T 10873C 11569C 11719A 11899C 12279G 12618A 12705T 14766T 14783C 15025T 15043A 15301A 15326G 16223T 16263C 16311C 16519C	M23	73G 152C 195C 204C 263G 417A 489C 533G 16223T 16263C 16311C 16519C	1	73 152 195 204 263 417 489 533 750 1438 4769 7028 8188 8360 8701 8860 9438 9540 9545 10142 10295 10398 10400 10873 11569 11719 11899 12279 12618 12705 14766 14783 15025 15043 15301 15326 16223 16263 16311	M23	73 152 195 204 263 417 489 533 16223 16263 16311	[0.000] M23 (1)	[0.072] M24a (2) [0.053] M57+152	1
1591	>MF056041.1 Ho	73G 146C 152C 195C 263G 750G 769A 1018A 1438G 2416C 2706G 2789T 3106d 3107C 3594T 4104G 4769G 5090C 7028T 7175C 7256T 7274T 7521A 7771G 8206A 8701G 8860G 9221G 9540C 10115C 10143A 10398G 10873C 11719A 11914A 11944C 12693G 12705T 13590A 13650T 13803G 14566G 14766T 15301A 15326G 15735T 15784C 16183C 16189C 16223T 16278T 16290T 16294T 16309G 16390A	L2a1b1a	73G 146C 152C 195C 263G 16183C 16189C 16223T 16278T 16290T 16294T 16309G 16390A	1	73 146 152 195 263 750 769 1018 1438 2416 2706 2789 3594 4104 4769 5090 7028 7175 7256 7274 7521 7771 8206 8701 8860 9221 9540 10115 10143 10398 10873 11719 11914 11944 12693 12705 13590 13650 13803 14566 14766 15301 15326 15735 15784 16189 16223 16278 16290 16294 16309 16390	L2a1b1a	73 146 152 195 263 16189 16223 16278 16290 16294 16309 16390	[0.000] L2a1b1a (1)	[0.005] L2a1f (2) [0.000] L2a1+16189_(16192) L2a1b L2a1b1 L2a1b1a L2a1b2 L2a1f1 L2a1f1a L2a1f2 (10)	1
1592	>MF056039.1 Ho	73G 150T 152C 200G 263G 489C 750G 2706G 3106d 3107C 3588T 4769G 7028T 7960G 8158G 8701G 8768T 8854A 8860G 9540C 10398G 10400T 10873C 11719A 12705T 13308G 13590A 13708A 14130T 14260G 14527G 14766T 14783C 15043A 15172A 15301A 15326G 15625T 16086C 16148T 16223T 16259T 16278T 16319A 16399G 16526A	M32c	73G 150T 152C 200G 263G 489C 16086C 16148T 16223T 16259T 16278T 16319A 16399G 16526A	1	73 150 152 200 263 489 750 2706 3588 4769 7028 7960 8158 8701 8768 8854 8860 9540 10398 10400 10873 11719 12705 13308 13590 13708 14130 14260 14527 14766 14783 15043 15172 15301 15326 15625 16086 16148 16223 16259 16278 16319 16399 16526	M32c	73 150 152 200 263 489 16086 16148 16223 16259 16278 16319 16399 16526	[0.017] M32c (1)	[0.065] M51a (2) [0.014] M51b (3) [0.007] M32 (4) [0.000] M51	1
1593	>MF056020.1 Ho	73G 152C 195C 204C 263G 417A 489C 533G 750G 1438G 3106d 3107C 4769G 7028T 7855A 8188G 8360G 8701G 8860G 9438A 9540C 9545G 10142T 10295G 10398G 10400T 10873C 11569C 11719A 11899C 12279G 12618A 12705T 14766T 14783C 15025T 15043A 15301A 15326G 16223T 16263C 16311C 16519C	M23	73G 152C 195C 204C 263G 417A 489C 533G 16223T 16263C 16311C 16519C	1	73 152 195 204 263 417 489 533 750 1438 4769 7028 7855A 8188 8360 8701 8860 9438 9540 9545 10142 10295 10398 10400 10873 11569 11719 11899 12279 12618 12705 14766 14783 15025 15043 15301 15326 16223 16263 16311	M23	73 152 195 204 263 417 489 533 16223 16263 16311	[0.000] M23 (1)	[0.072] M24a (2) [0.053] M57+152	1

1594	>MF056019.1 Ho	73G 146C 152C 195C 263G 750G 769A 1018A 1438G 2416C 2706G 2789T 3106d 3107C 3594T 4104G 4769G 5090C 7028T 7175C 7256T 7274T 7521A 7771G 8206A 8701G 8860G 9221G 9540C 10115C 10143A 10398G 10873C 11719A 11914A 11944C 12693G 12705T 13590A 13650T 13803G 14566G 14766T 15301A 15326G 15735T 15784C 16182C 16183C 16189C 16223T 16278T 16290T 16294T 16309G 16390A	L2a1b1a	73G 146C 152C 195C 263G 16182C 16183C 16189C 16223T 16278T 16290T 16294T 16309G 16390A	1	73 146 152 195 263 750 769 1018 1438 2416 2706 2789 3594 4104 4769 5090 7028 7175 7256 7274 7521 7771 8206 8701 8860 9221 9540 10115 10143 10398 10873 11719 11914 11944 12693 12705 13590 13650 13803 14566 14766 15301 15326 15735 15784 16189 16223 16278 16290 16294 16309 16390	L2a1b1a	73 146 152 195 263 16189 16223 16278 16290 16294 16309 16390	[0.000] L2a1b1a (1)	[0.005] L2a1f (2) [0.000] L2a1+16189_(16192) L2a1b L2a1b1 L2a1b1a L2a1b2 L2a1f1 L2a1f1a L2a1f2 (10)	1
1595	>MF056016.1 Ho	73G 146C 152C 195C 263G 750G 769A 1018A 1438G 2416C 2706G 2789T 3106d 3107C 3594T 4104G 4769G 5090C 7028T 7175C 7256T 7274T 7521A 7771G 8206A 8701G 8860G 9221G 9540C 10115C 10143A 10398G 10873C 11719A 11914A 11944C 12693G 12705T 13590A 13650T 13803G 14566G 14766T 15301A 15326G 15735T 15784C 15850C 16180d 16183C 16189C 16223T 16278T 16290T 16294T 16309G 16390A	L2a1b1a	73G 146C 152C 195C 263G 16180d 16183C 16189C 16223T 16278T 16290T 16294T 16309G 16390A	1	73 146 152 195 263 750 769 1018 1438 2416 2706 2789 3594 4104 4769 5090 7028 7175 7256 7274 7521 7771 8206 8701 8860 9221 9540 10115 10143 10398 10873 11719 11914 11944 12693 12705 13590 13650 13803 14566 14766 15301 15326 15735 15784 15850 16189 16193d 16223 16278 16290 16294 16309 16390	L2a1b1a	73 146 152 195 263 16189 16193d 16223 16278 16290 16294 16309 16390	[0.024] L2a1b1a (1)	[0.024] L2a1b1a (2) [0.010] L2a1f (3) [0.008] L2a1b2 (4) [0.000] L2a1+16189_(16192) L2a1b L2a1b1 L2a1f1 L2a1f1a L2a1f2 (10)	1
1596	>MF056010.1 Ho	73G 146C 152C 195C 263G 750G 769A 1018A 1438G 2416C 2706G 2789T 3106d 3107C 3594T 4104G 4769G 5090C 7028T 7175C 7256T 7274T 7521A 7771G 8206A 8701G 8860G 9221G 9540C 10115C 10143A 10398G 10873C 11719A 11914A 11944C 12693G 12705T 13590A 13650T 13803G 14566G 14766T 15301A 15326G 15735T 15784C 16182C 16183C 16189C 16223T 16278T 16290T 16294T 16309G 16390A	L2a1b1a	73G 146C 152C 195C 263G 16182C 16183C 16189C 16223T 16278T 16290T 16294T 16309G 16390A	1	73 146 152 195 263 750 769 1018 1438 2416 2706 2789 3594 4104 4769 5090 7028 7175 7256 7274 7521 7771 8206 8701 8860 9221 9540 10115 10143 10398 10873 11719 11914 11944 12693 12705 13590 13650 13803 14566 14766 15301 15326 15735 15784 16189 16223 16278 16290 16294 16309 16390	L2a1b1a	73 146 152 195 263 16189 16223 16278 16290 16294 16309 16390	[0.000] L2a1b1a (1)	[0.005] L2a1f (2) [0.000] L2a1+16189_(16192) L2a1b L2a1b1 L2a1b1a L2a1b2 L2a1f1 L2a1f1a L2a1f2 (10)	1

1597 >MF056005.1 Hc	73G 146C 152C 182T 185T 189G 247A 263G 357G 514d 515d 709A 710C 750G 769A 825A 1018A 1738C 2352C 2706G 2758A 2768G 2885C 3106d 3107C 3308C 3594T 3666A 3693A 4104G 4769G 6548T 6827C 6989G 7028T 7055G 7146G 7256T 7389C 7521A 7867T 8248G 8282T 8468T 8655T 8701G 8860G 9540C 10398G 10688A 10810C 10873C 11719A 12171G 12519C 12705T 12891T 13105G 13506T 13602C 13650T 13789C 13893G 14178C 14323A 14560A 14766T 14769G 15115C 15326G 16111T 16126C 16187T 16189C 16223T 16239T 16270T 16278T 16293G 16311C 16519C	L1b2a	73G 146C 152C 182T 185T 189G 247A 263G 357G 514d 515d 16111T 16126C 16187T 16189C 16223T 16239T 16270T 16278T 16293G 16311C 16519C	1	73 146 152 182 185T 189 247 263 357 709 710 750 769 825A 1018 1738 2352 2706 2758 2768 2885 3308 3594 3666 3693 4104 4769 6548 6827 6989 7028 7055 7146 7256 7389 7521 7867 8248 8282 8468 8655 8701 8860 9540 10398 10688 10810 10873 11719 12171 12519 12705 12891 13105 13506 13602 13650 13789 13893 14178 14323 14560 14766 14769 15115 15326 16111 16126 16187 16189 16223 16239 16270 16278 16293 16311	L1b2a	73 146 152 182 185T 189 247 263 357 16111 16126 16187 16189 16223 16239 16270 16278 16293 16311	[0.000] L1b2a (1)	[0.042] L1b2 (2)	1
1598 >MF055999.1 Hc	73G 152C 195C 204C 263G 417A 489C 533G 750G 1438G 3106d 3107C 4769G 7028T 8188G 8360G 8701G 8860G 9438A 9540C 9545G 10142T 10295G 10398G 10400T 10873C 11569C 11719A 11899C 12279G 12618A 12705T 14766T 14783C 15025T 15043A 15301A 15326G 16223T 16263C 16311C 16519C	M23	73G 152C 195C 204C 263G 417A 489C 533G 16223T 16263C 16311C 16519C	1	73 152 195 204 263 417 489 533 750 1438 4769 7028 8188 8360 8701 8860 9438 9540 9545 10142 10295 10398 10400 10873 11569 11719 11899 12279 12618 12705 14766 14783 15025 15043 15301 15326 16223 16263 16311	M23	73 152 195 204 263 417 489 533 16223 16263 16311	[0.000] M23 (1)	[0.072] M24a (2) [0.053] M57+152	1
1599 >MF055997.1 Hc	73G 150T 200G 263G 489C 750G 2706G 3106d 3107C 3588T 4769G 7028T 7960G 8701G 8768T 8854A 8860G 9121A 9540C 10398G 10400T 10873C 11719A 12705T 13308G 13590A 13708A 14130T 14260G 14527G 14766T 14783C 15043A 15172A 15301A 15326G 15625T 16086C 16148T 16223T 16259T 16278T 16319A 16399G 16526A	M32c	73G 150T 200G 263G 489C 16086C 16148T 16223T 16259T 16278T 16319A 16399G 16526A	1	73 150 200 263 489 750 2706 3588 4769 7028 7960 8701 8768 8854 8860 9121A 9540 10398 10400 10873 11719 12705 13308 13590 13708 14130 14260 14527 14766 14783 15043 15172 15301 15326 15625 16086 16148 16223 16259 16278 16319 16399 16526	M32c	73 150 200 263 489 16086 16148 16223 16259 16278 16319 16399 16526	[0.000] M32c (1)	[0.022] M51a (2) [0.007] M32 (3) [0.000] M51 M51b (5)	1
1600 >MF055994.1 Hc	73G 150T 200G 263G 489C 750G 2706G 3106d 3107C 3588T 4769G 7028T 7960G 8701G 8768T 8854A 8860G 9301T 9540C 10398G 10400T 10873C 11719A 12705T 13308G 13590A 13708A 14130T 14260G 14527G 14766T 14783C 15043A 15172A 15301A 15326G 15625T 16086C 16148T 16223T 16259T 16278T 16319A 16399G 16526A	M32c	73G 150T 200G 263G 489C 16086C 16148T 16223T 16259T 16278T 16319A 16399G 16526A	1	73 150 200 263 489 750 2706 3588 4769 7028 7960 8701 8768 8854 8860 9301 9540 10398 10400 10873 11719 12705 13308 13590 13708 14130 14260 14527 14766 14783 15043 15172 15301 15326 15625 16086 16148 16223 16259 16278 16319 16399 16526	M32c	73 150 200 263 489 16086 16148 16223 16259 16278 16319 16399 16526	[0.000] M32c (1)	[0.022] M51a (2) [0.007] M32 (3) [0.000] M51 M51b (5)	1

1601	>MF055982.1 Hc	73G 150T 200G 263G 489C 750G 2706G 3106d 3107C 3588T 4769G 7028T 7960G 8701G 8768T 8854A 8860G 9540C 10398G 10400T 10873C 11719A 12705T 13308G 13590A 13708A 14130T 14260G 14527G 14766T 14783C 15043A 15172A 15301A 15326G 15625T 16086C 16148T 16223T 16259T 16278T 16319A 16399G 16526A	M32c	73G 150T 200G 263G 489C 16086C 16148T 16223T 16259T 16278T 16319A 16399G 16526A	1	73 150 200 263 489 750 2706 3588 4769 7028 7960 8701 8768 8854 8860 9540 10398 10400 10873 11719 12705 13308 13590 13708 14130 14260 14527 14766 14783 15043 15172 15301 15326 15625 16086 16148 16223 16259 16278 16319 16399 16526	M32c	73 150 200 263 489 16086 16148 16223 16259 16278 16319 16399 16526	[0.000] M32c (1)	[0.022] M51a (2) [0.007] M32 (3) [0.000] M51 M51b (5)	1
1602	>MF055976.1 Hc	73G 150T 200G 263G 489C 750G 2706G 3106d 3107C 3588T 4769G 7028T 7960G 8701G 8768T 8854A 8860G 9540C 10398G 10400T 10873C 11719A 12705T 13308G 13590A 13708A 14130T 14260G 14527G 14766T 14783C 15043A 15172A 15301A 15326G 15625T 16086C 16148T 16223T 16259T 16278T 16319A 16399G 16526A	M32c	73G 150T 200G 263G 489C 16086C 16148T 16223T 16259T 16278T 16319A 16399G 16526A	1	73 150 200 263 489 750 2706 3588 4769 7028 7960 8701 8768 8854 8860 9540 10398 10400 10873 11719 12705 13308 13590 13708 14130 14260 14527 14766 14783 15043 15172 15301 15326 15625 16086 16148 16223 16259 16278 16319 16399 16526	M32c	73 150 200 263 489 16086 16148 16223 16259 16278 16319 16399 16526	[0.000] M32c (1)	[0.022] M51a (2) [0.007] M32 (3) [0.000] M51 M51b (5)	1
1603	>MF055970.1 Hc	73G 146C 152C 195C 263G 750G 769A 1018A 1438G 2416C 2706G 2762T 2789T 3106d 3107C 3594T 4104G 4769G 5090C 7028T 7175C 7256T 7274T 7521A 7771G 8206A 8701G 8860G 9221G 9540C 10115C 10143A 10398G 10873C 11719A 11914A 11944C 12693G 12705T 13590A 13650T 13803G 14566G 14766T 15301A 15326G 15735T 15784C 16182C 16183C 16189C 16223T 16278T 16290T 16294T 16311C 16390A	L2a1b1a	73G 146C 152C 195C 263G 16182C 16183C 16189C 16223T 16278T 16290T 16294T 16311C 16390A	1	73 146 152 195 263 750 769 1018 1438 2416 2706 2762 2789 3594 4104 4769 5090 7028 7175 7256 7274 7521 7771 8206 8701 8860 9221 9540 10115 10143 10398 10873 11719 11914 11944 12693 12705 13590 13650 13803 14566 14766 15301 15326 15735 15784 16189 16223 16278 16290 16294 16311 16390	L2a1b1a	73 146 152 195 263 16189 16223 16278 16290 16294 16311 16390	[0.048] L2a1b1a (1)	[0.277] L2a1+143+16189_(16192)+@ 16309 (2) [0.070] L2a1f (3) [0.048] L2a1b1a (4) [0.008] L2a1+16189_(16192) L2a1b2 (6) [0.000] L2a1b L2a1b1 L2a1f1 L2a1f1a L2a1f2 (11)	1
1604	>MF055969.1 Hc	73G 150T 152C 235G 263G 494T 735G 750G 1438G 2706G 3106d 3107C 3918A 4313C 4769G 6620G 7028T 7202G 8649G 8701G 8860G 9007T 9329A 9467C 9540C 10398G 10819G 10873C 11719A 12705T 13135A 13542G 13862G 13992T 14013G 14766T 15301A 15314A 15326G 16093C 16223T 16355T	L3k1	73G 150T 152C 235G 263G 494T 16093C 16223T 16355T	1	73 150 152 235 263 494 735 750 1438 2706 3918 4313 4769 6620G 7028 7202 8649 8701 8860 9007T 9329 9467 9540 10398 10819 10873 11719 12705 13135 13542 13862 13992 14013 14766 15301 15314 15326 16093 16223 16355	L3k1	73 150 152 235 263 494 16093 16223 16355	[0.536] L3k1 (1)	[0.000] L3k (2)	1

1605	>MF057675.1 Ho	73G 152C 195C 204C 263G 310.1C 417A 489C 533G 750G 1438G 3106d 3107C 4769G 7028T 8188G 8360G 8701G 8860G 9438A 9540C 9545G 10142T 10295G 10398G 10400T 10873C 11569C 11719A 11899C 12279G 12618A 12705T 14766T 14783C 15025T 15043A 15301A 15326G 16223T 16263C 16311C 16519C	M23	73G 152C 195C 204C 263G 310.1C 417A 489C 533G 16223T 16263C 16311C 16519C	1	73 152 195 204 263 417 489 533 750 1438 4769 7028 8188 8360 8701 8860 9438 9540 9545 10142 10295 10398 10400 10873 11569 11719 11899 12279 12618 12705 14766 14783 15025 15043 15301 15326 16223 16263 16311	M23	73 152 195 204 263 417 489 533 16223 16263 16311	[0.000] M23 (1)	[0.072] M24a (2) [0.053] M57+152	1
1606	>MF057674.1 Ho	73G 150T 200G 263G 310.1C 489C 750G 2706G 3106d 3107C 3588T 4769G 7028T 7960G 8701G 8768T 8854A 8860G 9540C 10398G 10400T 10873C 11719A 12705T 13308G 13590A 13708A 14130T 14260G 14527G 14766T 14783C 15043A 15172A 15301A 15326G 15625T 16086C 16148T 16223T 16259T 16278T 16319A 16362C 16399G 16526A	M32c	73G 150T 200G 263G 310.1C 489C 16086C 16148T 16223T 16259T 16278T 16319A 16362C 16399G 16526A	1	73 150 200 263 489 750 2706 3588 4769 7028 7960 8701 8768 8854 8860 9540 10398 10400 10873 11719 12705 13308 13590 13708 14130 14260 14527 14766 14783 15043 15172 15301 15326 15625 16086 16148 16223 16259 16278 16319 16362 16399 16526	M32c	73 150 200 263 489 16086 16148 16223 16259 16278 16319 16362 16399 16526	[0.003] M32c (1)	[0.180] D4j1a1b (2) [0.132] D4j1a (3) [0.131] D4j1a1a (4) [0.062] G2a1 (5) [0.022] M51a (6) [0.019] D4d (7) [0.014] M32 (8) [0.009] D4j1a1 (9) [0.000] M51 M51b G1a G2a D4b1 D4b1a D4b1a2 D4b1a2a D4b1a2a2 D4b2b2a D4b2b2a1 D4b2b4 (21)	1
1607	>MF057660.1 Ho	73G 150T 200G 263G 310.1C 489C 492G 750G 2706G 3106d 3107C 3588T 4769G 7028T 7960G 8701G 8768T 8854A 8860G 9540C 10398G 10400T 10873C 11719A 12705T 13308G 13590A 13708A 14130T 14260G 14527G 14766T 14783C 15043A 15172A 15301A 15326G 15625T 16086C 16148T 16223T 16259T 16278T 16319A 16399G 16526A	M32c	73G 150T 200G 263G 310.1C 489C 492G 16086C 16148T 16223T 16259T 16278T 16319A 16399G 16526A	1	73 150 200 263 489 492 750 2706 3588 4769 7028 7960 8701 8768 8854 8860 9540 10398 10400 10873 11719 12705 13308 13590 13708 14130 14260 14527 14766 14783 15043 15172 15301 15326 15625 16086 16148 16223 16259 16278 16319 16399 16526	M32c	73 150 200 263 489 492 16086 16148 16223 16259 16278 16319 16399 16526	[0.003] M32c (1)	[0.022] M51a (2) [0.007] M32 (3) [0.000] M51 M51b (5)	1
1608	>MF057656.1 Ho	73G 150T 263G 310.1C 489C 750G 2706G 3106d 3107C 3588T 4769G 7028T 7960G 8701G 8768T 8854A 8860G 9288G 9540C 10398G 10400T 10873C 11719A 12705T 13308G 13590A 13708A 14130T 14260G 14527G 14766T 14783C 15043A 15172A 15301A 15326G 15625T 16086C 16148T 16223T 16259T 16278T 16319A 16399G 16526A	M32c	73G 150T 263G 310.1C 489C 16086C 16148T 16223T 16259T 16278T 16319A 16399G 16526A	1	73 150 263 489 750 2706 3588 4769 7028 7960 8701 8768 8854 8860 9288 9540 10398 10400 10873 11719 12705 13308 13590 13708 14130 14260 14527 14766 14783 15043 15172 15301 15326 15625 16086 16148 16223 16259 16278 16319 16399 16526	M32c	73 150 263 489 16086 16148 16223 16259 16278 16319 16399 16526	[0.046] M32c (1)	[0.022] M51a (2) [0.000] M51 M51b M32 (5)	1
1609	>MF057652.1 Ho	73G 152C 195C 204C 263G 310.1C 417A 489C 533G 750G 1438G 3106d 3107C 4769G 7028T 8188G 8360G 8701G 8860G 9438A 9540C 9545G 10142T 10295G 10398G 10400T 10873C 11569C 11719A 11899C 12279G 12618A 12705T 14766T 14783C 15025T 15043A 15301A 15326G 16223T 16263C 16311C 16519C	M23	73G 152C 195C 204C 263G 310.1C 417A 489C 533G 16223T 16263C 16311C 16519C	1	73 152 195 204 263 417 489 533 750 1438 4769 7028 8188 8360 8701 8860 9438 9540 9545 10142 10295 10398 10400 10873 11569 11719 11899 12279 12618 12705 14766 14783 15025 15043 15301 15326 16223 16263 16311	M23	73 152 195 204 263 417 489 533 16223 16263 16311	[0.000] M23 (1)	[0.072] M24a (2) [0.053] M57+152	1

1610	>MF057646.1 Hc	73G 146C 152C 195C 263G 310.1C 750G 769A 1018A 1438G 2416C 2706G 2789T 3106d 3107C 3594T 4104G 4769G 5090C 7028T 7175C 7256T 7274T 7521A 7771G 8206A 8701G 8860G 9221G 9540C 10115C 10143A 10398G 10873C 11719A 11914A 11944C 12651A 12693G 12705T 13590A 13650T 13803G 14566G 14766T 15301A 15326G 15735T 15784C 16183C 16189C 16223T 16270T 16278T 16290T 16309G 16390A	L2a1b1a	73G 146C 152C 195C 263G 310.1C 16183C 16189C 16223T 16270T 16278T 16290T 16309G 16390A	1	73 146 152 195 263 750 769 1018 1438 2416 2706 2789 3594 4104 4769 5090 7028 7175 7256 7274 7521 7771 8206 8701 8860 9221 9540 10115 10143 10398 10873 11719 11914 11944 12651 12693 12705 13590 13650 13803 14566 14766 15301 15326 15735 15784 16189 16223 16270 16278 16290 16309 16390	L2a1b1a	73 146 152 195 263 16189 16223 16270 16278 16290 16309 16390	[0.009] L2a1b1a (1)	[0.012] L2a1f2 (2) [0.010] L2a1f (3) [0.009] L2a1b1a (4) [0.008] L2a1b2 (5) [0.007] L2a1b L2a1f1a (7) [0.000] L2a1+16189_(16192) L2a1b1 L2a1f1 (10)	1
1611	>MF057638.1 Hc	73G 146C 152C 195C 263G 310.1C 750G 769A 1018A 1438G 2416C 2706G 2789T 3106d 3107C 3594T 4104G 4769G 5090C 7028T 7175C 7256T 7274T 7521A 7771G 8206A 8701G 8860G 9221G 9540C 10115C 10143A 10398G 10873C 11719A 11914A 11944C 12693G 12705T 13590A 13650T 13803G 14566G 14766T 15301A 15326G 15735T 15784C 16183C 16189C 16223T 16278T 16290T 16294T 16309G 16390A	L2a1b1a	73G 146C 152C 195C 263G 310.1C 16183C 16189C 16223T 16278T 16290T 16294T 16309G 16390A	1	73 146 152 195 263 750 769 1018 1438 2416 2706 2789 3594 4104 4769 5090 7028 7175 7256 7274 7521 7771 8206 8701 8860 9221 9540 10115 10143 10398 10873 11719 11914 11944 12693 12705 13590 13650 13803 14566 14766 15301 15326 15735 15784 16189 16223 16278 16290 16294 16309 16390	L2a1b1a	73 146 152 195 263 16189 16223 16278 16290 16294 16309 16390	[0.000] L2a1b1a (1)	[0.005] L2a1f (2) [0.000] L2a1+16189_(16192) L2a1b L2a1b1 L2a1b1a L2a1b2 L2a1f1 L2a1f1a L2a1f2 (10)	1
1612	>MF057630.1 Hc	73G 152C 195C 204C 263G 310.1C 417A 489C 533G 750G 1438G 3106d 3107C 4769G 7028T 8188G 8360G 8701G 8860G 9438A 9540C 9545G 10142T 10295G 10398G 10400T 10873C 11569C 11719A 11899C 12279G 12618A 12705T 14766T 14783C 15025T 15043A 15301A 15326G 16223T 16263C 16311C 16519C	M23	73G 152C 195C 204C 263G 310.1C 417A 489C 533G 16223T 16263C 16311C 16519C	1	73 152 195 204 263 417 489 533 750 1438 4769 7028 8188 8360 8701 8860 9438 9540 9545 10142 10295 10398 10400 10873 11569 11719 11899 12279 12618 12705 14766 14783 15025 15043 15301 15326 16223 16263 16311	M23	73 152 195 204 263 417 489 533 16223 16263 16311	[0.000] M23 (1)	[0.072] M24a (2) [0.053] M57+152	1
1613	>MF057625.1 Hc	73G 152C 195C 204C 263G 310.1C 417A 489C 533G 750G 1189C 1438G 3106d 3107C 4769G 7028T 8188G 8360G 8701G 8860G 9438A 9540C 9545G 10142T 10295G 10398G 10400T 10873C 11569C 11719A 11899C 12279G 12618A 12705T 14182C 14766T 14783C 15025T 15043A 15301A 15326G 16223T 16263C 16278T 16311C 16519C	M23	73G 152C 195C 204C 263G 310.1C 417A 489C 533G 16223T 16263C 16278T 16311C 16519C	1	73 152 195 204 263 417 489 533 750 1189 1438 4769 7028 8188 8360 8701 8860 9438 9540 9545 10142 10295 10398 10400 10873 11569 11719 11899 12279 12618 12705 14182 14766 14783 15025 15043 15301 15326 16223 16263 16278 16311	M23	73 152 195 204 263 417 489 533 16223 16263 16278 16311	[0.004] M23 (1)	[0.072] M24a (2) [0.053] M57+152 (3) [0.000] M30b	1

1614 >MF057616.1 Hc	73G 150T 263G 310.1C 489C 750G 2706G 3106d 3107C 3588T 4769G 7028T 7960G 8701G 8768T 8854A 8860G 9540C 10398G 10400T 10873C 11719A 12705T 13308G 13590A 13708A 14130T 14260G 14527G 14766T 14783C 15043A 15172A 15301A 15326G 15625T 16086C 16148T 16223T 16259T 16278T 16319A 16399G 16526A	M32c	73G 150T 263G 310.1C 489C 16086C 16148T 16223T 16259T 16278T 16319A 16399G 16526A	1	73 150 263 489 750 2706 3588 4769 7028 7960 8701 8768 8854 8860 9540 10398 10400 10873 11719 12705 13308 13590 13708 14130 14260 14527 14766 14783 15043 15172 15301 15326 15625 16086 16148 16223 16259 16278 16319 16399 16526	M32c	73 150 263 489 16086 16148 16223 16259 16278 16319 16399 16526	[0.046] M32c (1)	[0.022] M51a (2) [0.000] M51 M51b M32 (5)	1
1615 >MF057593.1 Hc	73G 150T 200G 263G 310.1C 489C 750G 2706G 3106d 3107C 3588T 4769G 7028T 7960G 8701G 8768T 8854A 8860G 9540C 10398G 10400T 10873C 11719A 12705T 13308G 13590A 13708A 14130T 14260G 14527G 14766T 14783C 15043A 15172A 15301A 15326G 15625T 16086C 16148T 16223T 16259T 16278T 16319A 16399G 16526A	M32c	73G 150T 200G 263G 310.1C 489C 16086C 16148T 16223T 16259T 16278T 16319A 16399G 16526A	1	73 150 200 263 489 750 2706 3588 4769 7028 7960 8701 8768 8854 8860 9540 10398 10400 10873 11719 12705 13308 13590 13708 14130 14260 14527 14766 14783 15043 15172 15301 15326 15625 16086 16148 16223 16259 16278 16319 16399 16526	M32c	73 150 200 263 489 16086 16148 16223 16259 16278 16319 16399 16526	[0.000] M32c (1)	[0.022] M51a (2) [0.007] M32 (3) [0.000] M51 M51b (5)	1
1616 >MF057588.1 Hc	73G 152C 195C 204C 263G 310.1C 417A 489C 533G 750G 1438G 3106d 3107C 4769G 7028T 8188G 8360G 8701G 8860G 9438A 9540C 9545G 10142T 10295G 10398G 10400T 10704A 10873C 11176A 11569C 11719A 11899C 12279G 12618A 12705T 14766T 14783C 15025T 15043A 15301A 15326G 16223T 16263C 16311C 16519C	M23	73G 152C 195C 204C 263G 310.1C 417A 489C 533G 16223T 16263C 16311C 16519C	1	73 152 195 204 263 417 489 533 750 1438 4769 7028 8188 8360 8701 8860 9438 9540 9545 10142 10295 10398 10400 10704 10873 11176 11569 11719 11899 12279 12618 12705 14766 14783 15025 15043 15301 15326 16223 16263 16311	M23	73 152 195 204 263 417 489 533 16223 16263 16311	[0.000] M23 (1)	[0.072] M24a (2) [0.053] M57+152	1
1617 >MF056606.1 Hc	73G 150T 199C 204C 263G 513d 514d 709A 750G 769A 789A 1018A 1341T 1438G 2706G 3010A 3106d 3107C 3505G 3693C 3918A 4017T 4029A 4216C 4232C 4769G 4977C 5460A 7028T 8614C 8701G 8860G 8974G 9248T 9540C 9986A 10398G 10400T 10813T 10873C 11719A 12705T 13497G 13518T 14016A 14766T 14905A 14935C 15301A 15326G 15344C 16189C 16223T 16239T 16311C 16320T 16362C 16519C	L4b1a	73G 150T 199C 204C 263G 513d 514d 16189C 16223T 16239T 16311C 16320T 16362C 16519C	1	73 150 199 204 263 513 709 750 769 789A 1018 1341 1438 2706 3010 3505 3693C 3918 4017 4029A 4216 4232 4769 4977 5460 7028 8614 8701 8860 8974G 9248 9540 9986 10398 10400 10813 10873 11719 12705 13497 13518 14016 14766 14905 14935 15301 15326 15344 16189 16223 16239 16311 16320 16362	L4b1a	73 150 199 204 263 513 16189 16223 16239 16311 16320 16362	[0.040] L4b1a (1)	[0.010] L4b1 (2)	1

1618	>MF056605.1 H(	73G 152d 235G 263G 494T 735G 750G 1438G 2706G 3106d 3107C 3918A 4313C 4769G 6620G 7028T 7202G 8649G 8701G 8860G 9007T 9329A 9467C 9540C 10398G 10819G 10873C 11719A 12705T 13135A 13542G 13862G 13992T 14013G 14766T 15301A 15314A 15326G 16093C 16223T 16355T	L3k1	73G 152d 235G 263G 494T 16093C 16223T 16355T	1	73 152d 235 263 494 735 750 1438 2706 3918 4313 4769 6620G 7028 7202 8649 8701 8860 9007T 9329 9467 9540 10398 10819 10873 11719 12705 13135 13542 13862 13992 14013 14766 15301 15314 15326 16093 16223 16355	L3k1	73 152d 235 263 494 16093 16223 16355	[1.037] L3k1 (1)	[0.071] N11a (2) [0.021] L3k (3) [0.014] L3 (4) [0.000] L3b'f L3h G1b N N1'5 N1 N9 N11 N14 S S2 (15)	1
1619	>MF056604.1 H(	73G 146C 152C 195C 263G 750G 769A 1018A 1438G 2416C 2706G 2789T 3106d 3107C 3594T 4104G 4769G 5090C 7028T 7175C 7256T 7274T 7521A 7771G 8206A 8280G 8701G 8860G 9221G 9540C 10115C 10143A 10398G 10873C 11719A 11914A 11944C 12693G 12705T 13590A 13650T 13803G 14566G 14766T 15301A 15326G 15735T 15784C 16183C 16189C 16223T 16278T 16290T 16294T 16309G 16390A	L2a1b1a	73G 146C 152C 195C 263G 16183C 16189C 16223T 16278T 16290T 16294T 16309G 16390A	1	73 146 152 195 263 750 769 1018 1438 2416 2706 2789 3594 4104 4769 5090 7028 7175 7256 7274 7521 7771 8206 8280 8701 8860 9221 9540 10115 10143 10398 10873 11719 11914 11944 12693 12705 13590 13650 13803 14566 14766 15301 15326 15735 15784 16189 16223 16278 16290 16294 16309 16390	L2a1b1a	73 146 152 195 263 16189 16223 16278 16290 16294 16309 16390	[0.000] L2a1b1a (1)	[0.005] L2a1f (2) [0.000] L2a1+16189_(16192) L2a1b L2a1b1 L2a1b1a L2a1b2 L2a1f1 L2a1f1a L2a1f2 (10)	1
1620	>MF056601.1 H(	73G 152d 235G 263G 494T 735G 750G 1438G 2706G 3106d 3107C 3918A 4313C 4769G 6620G 7028T 7202G 8649G 8701G 8860G 9007T 9329A 9467C 9540C 10398G 10819G 10873C 11719A 12705T 13135A 13542G 13862G 13992T 14013G 14766T 15301A 15314A 15326G 16093C 16223T 16355T	L3k1	73G 152d 235G 263G 494T 16093C 16223T 16355T	1	73 152d 235 263 494 735 750 1438 2706 3918 4313 4769 6620G 7028 7202 8649 8701 8860 9007T 9329 9467 9540 10398 10819 10873 11719 12705 13135 13542 13862 13992 14013 14766 15301 15314 15326 16093 16223 16355	L3k1	73 152d 235 263 494 16093 16223 16355	[1.037] L3k1 (1)	[0.071] N11a (2) [0.021] L3k (3) [0.014] L3 (4) [0.000] L3b'f L3h G1b N N1'5 N1 N9 N11 N14 S S2 (15)	1
1621	>MF056598.1 H(	73G 146C 152C 195C 263G 292C 750G 769A 1018A 1438G 2416C 2706G 2789T 3106d 3107C 3594T 4104G 4769G 5090C 7028T 7175C 7256T 7274T 7521A 7771G 8206A 8280G 8701G 8860G 9221G 9540C 10115C 10143A 10398G 10873C 11719A 11914A 11944C 12693G 12705T 13590A 13650T 13803G 14566G 14766T 15301A 15326G 15735T 15784C 16182C 16183C 16189C 16223T 16278T 16290T 16294T 16309G 16390A	L2a1b1a	73G 146C 152C 195C 263G 292C 16182C 16183C 16189C 16223T 16278T 16290T 16294T 16309G 16390A	1	73 146 152 195 263 292 750 769 1018 1438 2416 2706 2789 3594 4104 4769 5090 7028 7175 7256 7274 7521 7771 8206 8280 8701 8860 9221 9540 10115 10143 10398 10873 11719 11914 11944 12693 12705 13590 13650 13803 14566 14766 15301 15326 15735 15784 16189 16223 16278 16290 16294 16309 16390	L2a1b1a	73 146 152 195 263 292 16189 16223 16278 16290 16294 16309 16390	[0.009] L2a1b1a (1)	[0.009] L2a1b1a (2) [0.005] L2a1f (3) [0.000] L2a1+16189_(16192) L2a1b L2a1b1 L2a1b2 L2a1f1 L2a1f1a L2a1f2 (10)	1

1622	>MF056596.1 Hc	73G 146C 152C 195C 263G 750G 769A 1018A 1438G 2416C 2706G 2789T 3106d 3107C 3594T 4104G 4769G 5090C 7028T 7175C 7256T 7274T 7521A 7771G 8206A 8701G 8860G 9221G 9540C 10115C 10143A 10398G 10873C 11719A 11914A 11944C 12693G 12705T 13590A 13650T 13803G 14566G 14766T 15301A 15326G 15735T 15784C 16182C 16183C 16189C 16223T 16278T 16290T 16294T 16309G 16390A	L2a1b1a	73G 146C 152C 195C 263G 16182C 16183C 16189C 16223T 16278T 16290T 16294T 16309G 16390A	1	73 146 152 195 263 750 769 1018 1438 2416 2706 2789 3594 4104 4769 5090 7028 7175 7256 7274 7521 7771 8206 8701 8860 9221 9540 10115 10143 10398 10873 11719 11914 11944 12693 12705 13590 13650 13803 14566 14766 15301 15326 15735 15784 16189 16223 16278 16290 16294 16309 16390	L2a1b1a	73 146 152 195 263 16189 16223 16278 16290 16294 16309 16390	[0.000] L2a1b1a (1)	[0.005] L2a1f (2) [0.000] L2a1+16189_(16192) L2a1b L2a1b1 L2a1b1a L2a1b2 L2a1f1 L2a1f1a L2a1f2 (10)	1
1623	>MF056587.1 Hc	73G 263G 279C 514d 515d 750G 1438G 2332T 2706G 3106d 3107C 3450T 4769G 5773A 6221C 6340T 7028T 8701G 8860G 9355G 9449T 9540C 10086G 10373A 10398G 10873C 11002G 11719A 12705T 13105G 13914A 14766T 15301A 15311G 15326G 15824G 15940d 16145A 16223T 16278T 16362C 16519C	L3b1a8	73G 263G 279C 514d 515d 16145A 16223T 16278T 16362C 16519C	1	73 263 279 750 1438 2332 2706 3450 4769 5773 6221 6340 7028 8701 8860 9355 9449 9540 10086 10373 10398 10873 11002 11719 12705 13105 13914A 14766 15301 15311 15326 15824 15944d 16145 16223 16278 16362	L3b1a8	73 263 279 16145 16223 16278 16362	[0.213] L3b1a8 (1)	[0.000] L3b1a+@16124 L3b1a7 L3b1a7a (4)	1
1624	>MF056581.1 Hc	73G 152C 195C 204C 263G 417A 489C 533G 750G 1438G 3106d 3107C 3693A 4769G 7028T 8188G 8360G 8701G 8860G 9438A 9540C 9545G 10142T 10295G 10398G 10400T 10873C 11569C 11719A 11899C 12279G 12618A 12705T 14766T 14783C 15025T 15043A 15301A 15326G 16223T 16263C 16519C	M23	73G 152C 195C 204C 263G 417A 489C 533G 16223T 16263C 16519C	1	73 152 195 204 263 417 489 533 750 1438 3693 4769 7028 8188 8360 8701 8860 9438 9540 9545 10142 10295 10398 10400 10873 11569 11719 11899 12279 12618 12705 14766 14783 15025 15043 15301 15326 16223 16263	M23	73 152 195 204 263 417 489 533 16223 16263	[0.008] M23 (1)	[0.016] M13 M50 (3) [0.006] D4k (4) [0.004] M37+152 (5) [0.000] M64 G2c M13'46'61 M13a'b (9)	1
1625	>MF056580.1 Hc	73G 150T 200G 263G 489C 750G 2706G 3106d 3107C 3588T 4769G 7028T 7960G 8701G 8768T 8854A 8860G 9540C 10398G 10400T 10873C 11719A 12705T 13308G 13590A 13708A 14130T 14260G 14527G 14766T 14783C 15043A 15172A 15301A 15326G 15625T 16086C 16148T 16223T 16259T 16278T 16319A 16399G 16526A	M32c	73G 150T 200G 263G 489C 16086C 16148T 16223T 16259T 16278T 16319A 16399G 16526A	1	73 150 200 263 489 750 2706 3588 4769 7028 7960 8701 8768 8854 8860 9540 10398 10400 10873 11719 12705 13308 13590 13708 14130 14260 14527 14766 14783 15043 15172 15301 15326 15625 16086 16148 16223 16259 16278 16319 16399 16526	M32c	73 150 200 263 489 16086 16148 16223 16259 16278 16319 16399 16526	[0.000] M32c (1)	[0.022] M51a (2) [0.007] M32 (3) [0.000] M51 M51b (5)	1

1626	>MF056571.1 Hc	73G 146C 152C 195C 263G 750G 769A 1018A 1438G 2416C 2706G 2789T 3106d 3107C 3594T 4104G 4769G 5090C 7028T 7175C 7256T 7274T 7521A 7771G 8206A 8701G 8860G 9221G 9540C 10115C 10143A 10398G 10873C 11719A 11914A 11944C 12693G 12705T 13590A 13650T 13803G 14566G 14766T 15301A 15326G 15735T 15784C 16111T 16182C 16183C 16189C 16223T 16278T 16290T 16294T 16309G 16390A	L2a1b1a	73G 146C 152C 195C 263G 16111T 16182C 16183C 16189C 16223T 16278T 16290T 16294T 16309G 16390A	1	73 146 152 195 263 750 769 1018 1438 2416 2706 2789 3594 4104 4769 5090 7028 7175 7256 7274 7521 7771 8206 8701 8860 9221 9540 10115 10143 10398 10873 11719 11914 11944 12693 12705 13590 13650 13803 14566 14766 15301 15326 15735 15784 16111 16189 16223 16278 16290 16294 16309 16390	L2a1b1a	73 146 152 195 263 16111 16189 16223 16278 16290 16294 16309 16390	[0.012] L2a1b1a (1)	[0.012] L2a1b1a (2) [0.005] L2a1f (3) [0.000] L2a1+16189_(16192) L2a1b L2a1b1 L2a1b2 L2a1f1 L2a1f1a L2a1f2 (10)	1
1627	>MF056568.1 Hc	73G 146C 152C 195C 263G 750G 769A 1018A 1438G 2416C 2706G 2789T 3106d 3107C 3594T 4104G 4769G 5090C 7028T 7175C 7256T 7274T 7521A 7771G 8206A 8701G 8860G 9221G 9540C 10115C 10143A 10398G 10873C 11719A 11914A 11944C 12693G 12705T 13590A 13650T 13803G 14566G 14766T 15301A 15326G 15735T 15784C 16111T 16182C 16183C 16189C 16223T 16278T 16290T 16294T 16309G 16390A	L2a1b1a	73G 146C 152C 195C 263G 16111T 16182C 16183C 16189C 16223T 16278T 16290T 16294T 16309G 16390A	1	73 146 152 195 263 750 769 1018 1438 2416 2706 2789 3594 4104 4769 5090 7028 7175 7256 7274 7521 7771 8206 8701 8860 9221 9540 10115 10143 10398 10873 11719 11914 11944 12693 12705 13590 13650 13803 14566 14766 15301 15326 15735 15784 16111 16189 16223 16278 16290 16294 16309 16390	L2a1b1a	73 146 152 195 263 16111 16189 16223 16278 16290 16294 16309 16390	[0.012] L2a1b1a (1)	[0.012] L2a1b1a (2) [0.005] L2a1f (3) [0.000] L2a1+16189_(16192) L2a1b L2a1b1 L2a1b2 L2a1f1 L2a1f1a L2a1f2 (10)	1
1628	>MF056567.1 Hc	73G 182T 186A 189C 195C 198T 247A 263G 297G 316A 750G 769A 825A 1018A 1420C 1438G 2394d 2706G 2758A 2885C 3106d 3107C 3594T 3666A 4104G 4769G 5951G 6071C 6150A 6253C 7028T 7055G 7076G 7146G 7256T 7337A 7389C 7521A 7744C 8027A 8251A 8468T 8655T 8701G 8784G 8860G 8877C 9072G 9540C 10321C 10398G 10586A 10688A 10792G 10793T 10810C 10873C 11654G 11719A 12049T 12705T 12810G 13105G 13149G 13212T 13281C 13485G 13506T 13650T 13789C 14000A 14178C 14544A 14560A 14766T 14812T 14911T 15016T 15326G 15784C 16071T 16129A 16145A 16187T 16189C 16213A 16223T 16234T 16265C 16278T 16286G 16294T 16311C 16360T 16527T	L1c2a1a	73G 182T 186A 189C 195C 198T 247A 263G 297G 316A 16071T 16129A 16145A 16187T 16189C 16213A 16223T 16234T 16265C 16278T 16286G 16294T 16311C 16360T 16527T	1	73 182 186A 189C 195 198 247 263 297 316 750 769 825A 1018 1420 1438 2395d 2706 2758 2885 3594 3666 4104 4769 5951 6071 6150 6253 7028 7055 7076 7146 7256 7337 7389 7521 7744 8027 8251 8468 8655 8701 8784 8860 8877 9072 9540 10321 10398 10586 10688 10792 10793 10810 10873 11654 11719 12049 12705 12810 13105 13149 13212 13281 13485 13506 13650 13789 14000A 14178 14544 14560 14766 14812 14911 15016 15326 15784 16071 16129 16145 16187 16189 16213 16223 16234 16265C 16278 16286G 16294 16311 16360 16527	L1c2a1a	73 182 186A 189C 195 198 247 263 297 316 16071 16129 16145 16187 16189 16213 16223 16234 16265C 16278 16286G 16294 16311 16360 16527	[0.158] L1c2a1a (1)	[0.158] L1c2a1b (2) [0.000] L1c2a1	1

1629	>MF056566.1 H[illegible]	73G 146C 152C 195C 263G 750G 769A 1018A 1438G 2416C 2706G 2789T 3106d 3107C 3594T 4104G 4769G 5090C 7028T 7175C 7256T 7274T 7521A 7771G 8206A 8701G 8860G 9221G 9540C 9604G 10115C 10143A 10398G 10873C 11719A 11914A 11944C 12693G 12705T 13590A 13650T 13803G 14566G 14766T 15301A 15326G 15735T 15784C 16182C 16183C 16189C 16223T 16278T 16290T 16294T 16309G 16390A	L2a1b1a	73G 146C 152C 195C 263G 16182C 16183C 16189C 16223T 16278T 16290T 16294T 16309G 16390A	1	73 146 152 195 263 750 769 1018 1438 2416 2706 2789 3594 4104 4769 5090 7028 7175 7256 7274 7521 7771 8206 8701 8860 9221 9540 9604 10115 10143 10398 10873 11719 11914 11944 12693 12705 13590 13650 13803 14566 14766 15301 15326 15735 15784 16189 16223 16278 16290 16294 16309 16390	L2a1b1a	73 146 152 195 263 16189 16223 16278 16290 16294 16309 16390	[0.000] L2a1b1a (1)	[0.005] L2a1f (2) [0.000] L2a1+16189_(16192) L2a1b L2a1b1 L2a1b1a L2a1b2 L2a1f1 L2a1f1a L2a1f2 (10)	1
1630	>MF056565.1 H[illegible]	73G 152C 182T 186A 189C 195C 247A 248G 263G 316A 514d 515d 750G 769A 825A 1018A 1438G 2394d 2706G 2758A 2885C 3106d 3107C 3525T 3594T 3666A 4104G 4769G 4853A 5951G 6071C 6221A 6227C 6917A 7028T 7146G 7247T 7256T 7389C 7521A 8027A 8468T 8655T 8701G 8860G 9072G 9540C 10398G 10586A 10688A 10810C 10873C 11302T 11719A 11984C 12507G 12705T 12810G 13105G 13485G 13506T 13650T 13789C 14000A 14178C 14560A 14669G 14766T 14911T 14926G 15226G 15326G 15905C 15976C 15978T 16129A 16187T 16189C 16223T 16278T 16293G 16294T 16311C 16360T 16519C	L1c3c	73G 152C 182T 186A 189C 195C 247A 248G 263G 316A 514d 515d 16129A 16187T 16189C 16223T 16278T 16293G 16294T 16311C 16360T 16519C	1	73 152 182 186A 189C 195 247 248 263 316 750 769 825A 1018 1438 2395d 2706 2758 2885 3525 3594 3666 4104 4769 4853 5951 6071 6221A 6227 6917 7028 7146 7247 7256 7389 7521 8027 8468 8655 8701 8860 9072 9540 10398 10586 10688 10810 10873 11302 11719 11984 12507 12705 12810 13105 13485 13506 13650 13789 14000A 14178 14560 14669 14766 14911 14926 15226 15326 15905 15976 15978 16129 16187 16189 16223 16278 16293 16294 16311 16360	L1c3c	73 152 182 186A 189C 195 247 248 263 316 16129 16187 16189 16223 16278 16293 16294 16311 16360	[2.063] L1c3c (1) [0.000] L1c L1c1a+@198 L1c3b'c (4)	[0.700] L1c3 (5) [0.572] L1c1a'b'd (6) [0.267] L1c3b1 (7) [0.133] L1c3b1b (8) [0.000] L1c1'2'4'5'6 L1c1 L1c1a L1c1a1 L1c3b (13)	1

1631	>MF056562.1 Hc	73G 150d 182T 186A 189C 195C 198T 247A 263G 297G 316A 750G 769A 825A 1018A 1420C 1438G 2394d 2706G 2758A 2885C 3106d 3107C 3594T 3666A 4104G 4769G 5951G 6071C 6150A 6253C 7028T 7055G 7076G 7146G 7256T 7337A 7389C 7521A 7744C 8027A 8251A 8468T 8655T 8701G 8784G 8860G 8877C 9072G 9540C 10321C 10398G 10586A 10688A 10792G 10793T 10810C 10873C 11654G 11719A 12049T 12705T 12810G 13105G 13149G 13212T 13281C 13485G 13506T 13650T 13789C 14000A 14178C 14560A 14766T 14812T 14911T 15016T 15326G 15784C 16071T 16129A 16145A 16187T 16189C 16213A 16223T 16234T 16258C 16265C 16278T 16286G 16294T 16311C 16360T 16527T	L1c2a1a	73G 150d 182T 186A 189C 195C 198T 247A 263G 297G 316A 16071T 16129A 16145A 16187T 16189C 16213A 16223T 16234T 16258C 16265C 16278T 16286G 16294T 16311C 16360T 16527T	1	73 151d 182 186A 189C 195 198 247 263 297 316 750 769 825A 1018 1420 1438 2395d 2706 2758 2885 3594 3666 4104 4769 5951 6071 6150 6253 7028 7055 7076 7146 7256 7337 7389 7521 7744 8027 8251 8468 8655 8701 8784 8860 8877 9072 9540 10321 10398 10586 10688 10792 10793 10810 10873 11654 11719 12049 12705 12810 13105 13149 13212 13281 13485 13506 13650 13789 14000A 14178 14560 14766 14812 14911 15016 15326 15784 16071 16129 16145 16187 16189 16213 16223 16234 16258C 16265C 16278 16286G 16294 16311 16360 16527	L1c2a1a	73 151d 182 186A 189C 195 198 247 263 297 316 16071 16129 16145 16187 16189 16213 16223 16234 16258C 16265C 16278 16286G 16294 16311 16360 16527	[0.210] L1c2a1a (1)	[0.158] L1c2a1b (2) [0.000] L1c2a1	1
1632	>MF056561.1 Hc	73G 146C 152C 195C 263G 750G 769A 1018A 1438G 2416C 2706G 2789T 3106d 3107C 3594T 4104G 4769G 5090C 7028T 7175C 7256T 7274T 7521A 7771G 8206A 8701G 8860G 9221G 9540C 10115C 10143A 10398G 10873C 11719A 11914A 11944C 12693G 12705T 13590A 13650T 13803G 14566G 14766T 15301A 15326G 15735T 15784C 16111T 16182C 16183C 16189C 16223T 16278T 16290T 16294T 16309G 16390A	L2a1b1a	73G 146C 152C 195C 263G 16111T 16182C 16183C 16189C 16223T 16278T 16290T 16294T 16309G 16390A	1	73 146 152 195 263 750 769 1018 1438 2416 2706 2789 3594 4104 4769 5090 7028 7175 7256 7274 7521 7771 8206 8701 8860 9221 9540 10115 10143 10398 10873 11719 11914 11944 12693 12705 13590 13650 13803 14566 14766 15301 15326 15735 15784 16111 16189 16223 16278 16290 16294 16309 16390	L2a1b1a	73 146 152 195 263 16111 16189 16223 16278 16290 16294 16309 16390	[0.012] L2a1b1a (1)	[0.012] L2a1b1a (2) [0.005] L2a1f (3) [0.000] L2a1+16189_(16192) L2a1b L2a1b1 L2a1b2 L2a1f1 L2a1f1a L2a1f2 (10)	1
1633	>MF056560.1 Hc	73G 152C 195C 204C 263G 417A 489C 533G 750G 1438G 3106d 3107C 4769G 7028T 8188G 8360G 8701G 8860G 9438A 9540C 9545G 10142T 10295G 10398G 10400T 10873C 11569C 11719A 11899C 12279G 12618A 12705T 14766T 14783C 15025T 15043A 15301A 15326G 16223T 16263C 16311C 16519C	M23	73G 152C 195C 204C 263G 417A 489C 533G 16223T 16263C 16311C 16519C	1	73 152 195 204 263 417 489 533 750 1438 4769 7028 8188 8360 8701 8860 9438 9540 9545 10142 10295 10398 10400 10873 11569 11719 11899 12279 12618 12705 14766 14783 15025 15043 15301 15326 16223 16263 16311	M23	73 152 195 204 263 417 489 533 16223 16263 16311	[0.000] M23 (1)	[0.072] M24a (2) [0.053] M57+152	1

1634 >MF056559.1 Hc	73G 146C 152C 195C 263G 750G 769A 1018A 1438G 2416C 2706G 2789T 3106d 3107C 3594T 4104G 4769G 5090C 7028T 7175C 7256T 7274T 7521A 7771G 8206A 8701G 8860G 9221G 9540C 10115C 10143A 10398G 10873C 11719A 11914A 11944C 12693G 12705T 13590A 13650T 13803G 14566G 14766T 15301A 15326G 15735T 15784C 16182C 16183C 16189C 16223T 16278T 16290T 16294T 16309G 16390A	L2a1b1a	73G 146C 152C 195C 263G 16182C 16183C 16189C 16223T 16278T 16290T 16294T 16309G 16390A	1	73 146 152 195 263 750 769 1018 1438 2416 2706 2789 3594 4104 4769 5090 7028 7175 7256 7274 7521 7771 8206 8701 8860 9221 9540 10115 10143 10398 10873 11719 11914 11944 12693 12705 13590 13650 13803 14566 14766 15301 15326 15735 15784 16189 16223 16278 16290 16294 16309 16390	L2a1b1a	73 146 152 195 263 16189 16223 16278 16290 16294 16309 16390	[0.000] L2a1b1a (1)	[0.005] L2a1f (2) [0.000] L2a1+16189_(16192) L2a1b L2a1b1 L2a1b1a L2a1b2 L2a1f1 L2a1f1a L2a1f2 (10)	1
1635 >MF056555.1 Hc	73G 146C 152C 195C 263G 750G 769A 1018A 1438G 2416C 2706G 2789T 3106d 3107C 3594T 4104G 4769G 5090C 7028T 7175C 7256T 7274T 7521A 7771G 8206A 8701G 8860G 9221G 9540C 10115C 10143A 10398G 10873C 11719A 11914A 11944C 12693G 12705T 13590A 13650T 13803G 14566G 14766T 15301A 15326G 15735T 15784C 16182C 16183C 16189C 16223T 16278T 16290T 16294T 16309G 16390A	L2a1b1a	73G 146C 152C 195C 263G 16182C 16183C 16189C 16223T 16278T 16290T 16294T 16309G 16390A	1	73 146 152 195 263 750 769 1018 1438 2416 2706 2789 3594 4104 4769 5090 7028 7175 7256 7274 7521 7771 8206 8701 8860 9221 9540 10115 10143 10398 10873 11719 11914 11944 12693 12705 13590 13650 13803 14566 14766 15301 15326 15735 15784 16189 16223 16278 16290 16294 16309 16390	L2a1b1a	73 146 152 195 263 16189 16223 16278 16290 16294 16309 16390	[0.000] L2a1b1a (1)	[0.005] L2a1f (2) [0.000] L2a1+16189_(16192) L2a1b L2a1b1 L2a1b1a L2a1b2 L2a1f1 L2a1f1a L2a1f2 (10)	1
1636 >MF056552.1 Hc	73G 150T 200G 263G 489C 750G 1694C 2706G 3106d 3107C 3588T 4769G 7028T 7960G 8701G 8768T 8854A 8860G 9540C 10398G 10400T 10873C 11719A 12705T 13308G 13590A 13708A 14130T 14260G 14527G 14766T 14783C 15043A 15172A 15301A 15326G 15625T 16086C 16148T 16223T 16259T 16278T 16319A 16399G 16526A	M32c	73G 150T 200G 263G 489C 16086C 16148T 16223T 16259T 16278T 16319A 16399G 16526A	1	73 150 200 263 489 750 1694 2706 3588 4769 7028 7960 8701 8768 8854 8860 9540 10398 10400 10873 11719 12705 13308 13590 13708 14130 14260 14527 14766 14783 15043 15172 15301 15326 15625 16086 16148 16223 16259 16278 16319 16399 16526	M32c	73 150 200 263 489 16086 16148 16223 16259 16278 16319 16399 16526	[0.000] M32c (1)	[0.022] M51a (2) [0.007] M32 (3) [0.000] M51 M51b (5)	1
1637 >MF056544.1 Hc	73G 146C 152C 195C 263G 750G 769A 1018A 1438G 2416C 2706G 2789T 3106d 3107C 3594T 4104G 4769G 5090C 7028T 7175C 7256T 7274T 7521A 7771G 8206A 8701G 8860G 9221G 9540C 10115C 10143A 10398G 10873C 11719A 11914A 11944C 12693G 12705T 13590A 13650T 13803G 14566G 14766T 15301A 15326G 15735T 15784C 16182C 16183C 16189C 16223T 16278T 16290T 16294T 16309G 16390A	L2a1b1a	73G 146C 152C 195C 263G 16182C 16183C 16189C 16223T 16278T 16290T 16294T 16309G 16390A	1	73 146 152 195 263 750 769 1018 1438 2416 2706 2789 3594 4104 4769 5090 7028 7175 7256 7274 7521 7771 8206 8701 8860 9221 9540 10115 10143 10398 10873 11719 11914 11944 12693 12705 13590 13650 13803 14566 14766 15301 15326 15735 15784 16189 16223 16278 16290 16294 16309 16390	L2a1b1a	73 146 152 195 263 16189 16223 16278 16290 16294 16309 16390	[0.000] L2a1b1a (1)	[0.005] L2a1f (2) [0.000] L2a1+16189_(16192) L2a1b L2a1b1 L2a1b1a L2a1b2 L2a1f1 L2a1f1a L2a1f2 (10)	1

1638	>MF056533.1 Hc	73G 146C 152C 195C 263G 750G 769A 1018A 1438G 2416C 2706G 2789T 3106d 3107C 3594T 4104G 4769G 5090C 7028T 7175C 7256T 7274T 7521A 7771G 8206A 8701G 8860G 9221G 9540C 9604G 10115C 10143A 10398G 10873C 11719A 11914A 11944C 12693G 12705T 13590A 13650T 13803G 14566G 14766T 15301A 15326G 15735T 15784C 16182C 16183C 16189C 16223T 16278T 16290T 16294T 16309G 16390A	L2a1b1a	73G 146C 152C 195C 263G 16182C 16183C 16189C 16223T 16278T 16290T 16294T 16309G 16390A	1	73 146 152 195 263 750 769 1018 1438 2416 2706 2789 3594 4104 4769 5090 7028 7175 7256 7274 7521 7771 8206 8701 8860 9221 9540 9604 10115 10143 10398 10873 11719 11914 11944 12693 12705 13590 13650 13803 14566 14766 15301 15326 15735 15784 16189 16223 16278 16290 16294 16309 16390	L2a1b1a	73 146 152 195 263 16189 16223 16278 16290 16294 16309 16390	[0.000] L2a1b1a (1)	[0.005] L2a1f (2) [0.000] L2a1+16189_(16192) L2a1b L2a1b1 L2a1b1a L2a1b2 L2a1f1 L2a1f1a L2a1f2 (10)	1
1639	>MF056532.1 Hc	73G 152C 195C 204C 263G 417A 489C 533G 750G 1438G 3106d 3107C 4769G 7028T 8188G 8360G 8701G 8860G 9438A 9540C 9545G 10142T 10295G 10398G 10400T 10873C 11569C 11719A 11899C 12279G 12618A 12705T 14766T 14783C 15025T 15043A 15301A 15326G 16223T 16263C 16311C 16519C	M23	73G 152C 195C 204C 263G 417A 489C 533G 16223T 16263C 16311C 16519C	1	73 152 195 204 263 417 489 533 750 1438 4769 7028 8188 8360 8701 8860 9438 9540 9545 10142 10295 10398 10400 10873 11569 11719 11899 12279 12618 12705 14766 14783 15025 15043 15301 15326 16223 16263 16311	M23	73 152 195 204 263 417 489 533 16223 16263 16311	[0.000] M23 (1)	[0.072] M24a (2) [0.053] M57+152	1
1640	>MF056531.1 Hc	73G 150T 200G 263G 489C 750G 2706G 3106d 3107C 3588T 4769G 7028T 7960G 8701G 8768T 8854A 8860G 9540C 10398G 10400T 10873C 10915C 11719A 12705T 13308G 13443C 13590A 13708A 14130T 14260G 14527G 14766T 14783C 15043A 15172A 15301A 15326G 15625T 16086C 16148T 16223T 16259T 16278T 16319A 16399G 16526A	M32c	73G 150T 200G 263G 489C 16086C 16148T 16223T 16259T 16278T 16319A 16399G 16526A	1	73 150 200 263 489 750 2706 3588 4769 7028 7960 8701 8768 8854 8860 9540 10398 10400 10873 10915 11719 12705 13308 13443 13590 13708 14130 14260 14527 14766 14783 15043 15172 15301 15326 15625 16086 16148 16223 16259 16278 16319 16399 16526	M32c	73 150 200 263 489 16086 16148 16223 16259 16278 16319 16399 16526	[0.000] M32c (1)	[0.022] M51a (2) [0.007] M32 (3) [0.000] M51 M51b (5)	1
1641	>MF056530.1 Hc	73G 152C 195C 204C 263G 417A 489C 533G 750G 1438G 3106d 3107C 4769G 7028T 8188G 8360G 8701G 8860G 9438A 9540C 9545G 10142T 10295G 10398G 10400T 10873C 11569C 11719A 11899C 12279G 12618A 12705T 14766T 14783C 15025T 15043A 15301A 15326G 16223T 16263C 16311C 16519C	M23	73G 152C 195C 204C 263G 417A 489C 533G 16223T 16263C 16311C 16519C	1	73 152 195 204 263 417 489 533 750 1438 4769 7028 8188 8360 8701 8860 9438 9540 9545 10142 10295 10398 10400 10873 11569 11719 11899 12279 12618 12705 14766 14783 15025 15043 15301 15326 16223 16263 16311	M23	73 152 195 204 263 417 489 533 16223 16263 16311	[0.000] M23 (1)	[0.072] M24a (2) [0.053] M57+152	1

1642	>MF056528.1 Hc	73G 152C 195C 204C 263G 417A 489C 533G 750G 1438G 3106d 3107C 4769G 7028T 8188G 8360G 8701G 8860G 9438A 9540C 9545G 10142T 10295G 10398G 10400T 10873C 11569C 11719A 11899C 12279G 12618A 12705T 14207A 14766T 14783C 15025T 15043A 15301A 15326G 16223T 16263C 16311C 16519C	M23	73G 152C 195C 204C 263G 417A 489C 533G 16223T 16263C 16311C 16519C	1	73 152 195 204 263 417 489 533 750 1438 4769 7028 8188 8360 8701 8860 9438 9540 9545 10142 10295 10398 10400 10873 11569 11719 11899 12279 12618 12705 14207 14766 14783 15025 15043 15301 15326 16223 16263 16311	M23	73 152 195 204 263 417 489 533 16223 16263 16311	[0.000] M23 (1)	[0.072] M24a (2) [0.053] M57+152	1
1643	>MF056527.1 Hc	73G 152C 195C 204C 263G 417A 489C 533G 750G 1438G 3106d 3107C 4769G 7028T 8188G 8360G 8701G 8860G 9438A 9540C 9545G 10142T 10295G 10398G 10400T 10873C 11569C 11719A 11899C 12279G 12618A 12705T 14207A 14766T 14783C 15025T 15043A 15301A 15326G 16223T 16263C 16311C 16519C	M23	73G 152C 195C 204C 263G 417A 489C 533G 16223T 16263C 16311C 16519C	1	73 152 195 204 263 417 489 533 750 1438 4769 7028 8188 8360 8701 8860 9438 9540 9545 10142 10295 10398 10400 10873 11569 11719 11899 12279 12618 12705 14207 14766 14783 15025 15043 15301 15326 16223 16263 16311	M23	73 152 195 204 263 417 489 533 16223 16263 16311	[0.000] M23 (1)	[0.072] M24a (2) [0.053] M57+152	1
1644	>MF056521.1 Hc	73G 152C 195C 263G 417A 489C 533G 750G 1438G 3106d 3107C 4769G 7028T 8188G 8360G 8701G 8860G 9438A 9540C 9545G 10142T 10295G 10398G 10400T 10873C 11569C 11719A 11899C 12279G 12618A 12705T 14766T 14783C 15025T 15043A 15301A 15326G 16223T 16263C 16311C 16519C	M23	73G 152C 195C 263G 417A 489C 533G 16223T 16263C 16311C 16519C	1	73 152 195 263 417 489 533 750 1438 4769 7028 8188 8360 8701 8860 9438 9540 9545 10142 10295 10398 10400 10873 11569 11719 11899 12279 12618 12705 14766 14783 15025 15043 15301 15326 16223 16263 16311	M23	73 152 195 263 417 489 533 16223 16263 16311	[0.016] M23 (1)	[0.031] M57+152 (2) [0.005] M24a	1
1645	>MF056520.1 Hc	73G 152C 195C 263G 417A 489C 533G 750G 1438G 3106d 3107C 4769G 7028T 8188G 8360G 8701G 8860G 9438A 9540C 9545G 10142T 10295G 10398G 10400T 10873C 11569C 11719A 11899C 12279G 12618A 12705T 14766T 14783C 15025T 15043A 15301A 15326G 16223T 16263C 16311C 16519C	M23	73G 152C 195C 263G 417A 489C 533G 16223T 16263C 16311C 16519C	1	73 152 195 263 417 489 533 750 1438 4769 7028 8188 8360 8701 8860 9438 9540 9545 10142 10295 10398 10400 10873 11569 11719 11899 12279 12618 12705 14766 14783 15025 15043 15301 15326 16223 16263 16311	M23	73 152 195 263 417 489 533 16223 16263 16311	[0.016] M23 (1)	[0.031] M57+152 (2) [0.005] M24a	1
1646	>MF056506.1 Hc	73G 150T 185A 189G 263G 750G 1438G 2352C 2706G 3106d 3107C 4769G 6221C 6587T 7028T 8577G 8701G 8860G 9540C 10398G 10819G 10873C 11719A 12192A 12705T 14152G 14212C 14766T 14926G 15301A 15326G 15670C 15942C 16223T 16323d 16327T 16400T	L3e1b2	73G 150T 185A 189G 263G 16223T 16323d 16327T 16400T	1	73 150 185 189 263 750 1438 2352 2706 4769 6221 6587 7028 8577 8701 8860 9540 10398 10819 10873 11719 12192 12705 14152 14212 14766 14926 15301 15326 15670 15942 16223 16325d 16327 16400	L3e1b2	73 150 185 189 263 16223 16325d 16327 16400	[0.037] L3e1b2 (1)	[0.200] L3e1b (2)	1

1647	>MF056501.1 Hc	73G 152C 195C 204C 263G 417A 489C 533G 750G 1438G 3106d 3107C 4769G 7028T 8188G 8360G 8701G 8860G 9438A 9540C 9545G 10142T 10295G 10398G 10400T 10873C 11569C 11719A 11899C 12279G 12618A 12705T 14766T 14783C 15025T 15043A 15236G 15301A 15326G 16223T 16263C 16311C 16519C	M23	73G 152C 195C 204C 263G 417A 489C 533G 16223T 16263C 16311C 16519C	1	73 152 195 204 263 417 489 533 750 1438 4769 7028 8188 8360 8701 8860 9438 9540 9545 10142 10295 10398 10400 10873 11569 11719 11899 12279 12618 12705 14766 14783 15025 15043 15236 15301 15326 16223 16263 16311	M23	73 152 195 204 263 417 489 533 16223 16263 16311	[0.000] M23 (1)	[0.072] M24a (2) [0.053] M57+152	1
1648	>MF056499.1 Hc	73G 152C 195C 204C 263G 417A 489C 533G 750G 1438G 3106d 3107C 4769G 7028T 8188G 8360G 8701G 8860G 9438A 9540C 9545G 10142T 10295G 10398G 10400T 10873C 11569C 11719A 11899C 12279G 12618A 12705T 14766T 14783C 15025T 15043A 15301A 15326G 16223T 16263C 16311C 16519C	M23	73G 152C 195C 204C 263G 417A 489C 533G 16223T 16263C 16311C 16519C	1	73 152 195 204 263 417 489 533 750 1438 4769 7028 8188 8360 8701 8860 9438 9540 9545 10142 10295 10398 10400 10873 11569 11719 11899 12279 12618 12705 14766 14783 15025 15043 15301 15326 16223 16263 16311	M23	73 152 195 204 263 417 489 533 16223 16263 16311	[0.000] M23 (1)	[0.072] M24a (2) [0.053] M57+152	1
1649	>MF056497.1 Hc	73G 152C 195C 204C 263G 417A 489C 533G 750G 1438G 3106d 3107C 4769G 7028T 8188G 8360G 8701G 8860G 9438A 9540C 9545G 10142T 10295G 10398G 10400T 10873C 11569C 11719A 11899C 12279G 12618A 12705T 14766T 14783C 15025T 15043A 15301A 15326G 16223T 16263C 16311C 16519C	M23	73G 152C 195C 204C 263G 417A 489C 533G 16223T 16263C 16311C 16519C	1	73 152 195 204 263 417 489 533 750 1438 4769 7028 8188 8360 8701 8860 9438 9540 9545 10142 10295 10398 10400 10873 11569 11719 11899 12279 12618 12705 14766 14783 15025 15043 15301 15326 16223 16263 16311	M23	73 152 195 204 263 417 489 533 16223 16263 16311	[0.000] M23 (1)	[0.072] M24a (2) [0.053] M57+152	1
1650	>MF057270.1 Hc	73G 152C 195C 204C 263G 310.1C 417A 489C 533G 750G 1438G 3106d 3107C 4769G 7028T 8188G 8360G 8701G 8860G 9438A 9540C 9545G 10142T 10295G 10398G 10400T 10873C 11569C 11719A 11899C 12279G 12618A 12705T 14766T 14783C 15025T 15043A 15301A 15326G 16223T 16263C 16311C 16519C	M23	73G 152C 195C 204C 263G 310.1C 417A 489C 533G 16223T 16263C 16311C 16519C	1	73 152 195 204 263 417 489 533 750 1438 4769 7028 8188 8360 8701 8860 9438 9540 9545 10142 10295 10398 10400 10873 11569 11719 11899 12279 12618 12705 14766 14783 15025 15043 15301 15326 16223 16263 16311	M23	73 152 195 204 263 417 489 533 16223 16263 16311	[0.000] M23 (1)	[0.072] M24a (2) [0.053] M57+152	1
1651	>MF057268.1 Hc	73G 146C 152C 195C 263G 310.1C 750G 769A 1018A 1438G 2416C 2706G 2789T 3106d 3107C 3594T 4104G 4769G 5090C 7028T 7175C 7256T 7274T 7521A 7771G 8206A 8701G 8860G 9221G 9540C 9604G 10115C 10143A 10398G 10634G 10873C 11719A 11914A 11944C 12693G 12705T 13590A 13650T 13803G 14566G 14766T 15301A 15326G 15735T 15784C 16182C 16183C 16189C 16192T 16223T 16278T 16290T 16294T 16309G 16390A	L2a1b1a	73G 146C 152C 195C 263G 310.1C 16182C 16183C 16189C 16192T 16223T 16278T 16290T 16294T 16309G 16390A	1	73 146 152 195 263 750 769 1018 1438 2416 2706 2789 3594 4104 4769 5090 7028 7175 7256 7274 7521 7771 8206 8701 8860 9221 9540 9604 10115 10143 10398 10634 10873 11719 11914 11944 12693 12705 13590 13650 13803 14566 14766 15301 15326 15735 15784 16189 16192 16223 16278 16290 16294 16309 16390	L2a1b1a	73 146 152 195 263 16189 16192 16223 16278 16290 16294 16309 16390	[0.000] L2a1b1a (1)	[0.015] L2a1b1a (2) [0.005] L2a1f (3) [0.000] L2a1+16189_(16192) L2a1b L2a1b1 L2a1b2 L2a1f1 L2a1f1a L2a1f2 (10)	1

1652 >MF057265.1 Hc	73G 150T 200G 263G 310.1C 489C 750G 839G 2706G 3106d 3107C 3588T 4769G 7028T 7960G 8701G 8768T 8854A 8860G 9540C 10398G 10400T 10873C 11719A 12705T 13308G 13590A 13708A 14130T 14260G 14527G 14766T 14783C 15043A 15172A 15301A 15326G 15625T 16086C 16148T 16259T 16278T 16319A 16399G 16526A	M32c	73G 150T 200G 263G 310.1C 489C 16086C 16148T 16259T 16278T 16319A 16399G 16526A	1	73 150 200 263 489 750 839 2706 3588 4769 7028 7960 8701 8768 8854 8860 9540 10398 10400 10873 11719 12705 13308 13590 13708 14130 14260 14527 14766 14783 15043 15172 15301 15326 15625 16086 16148 16259 16278 16319 16399 16526	M32c	73 150 200 263 489 16086 16148 16259 16278 16319 16399 16526	[0.007] M32c (1)	[0.022] M51a (2) [0.007] M32 (3) [0.000] M51 M51b (5)	1
1653 >MF057264.1 Hc	73G 150T 200G 263G 310.1C 489C 750G 2706G 3106d 3107C 3588T 4769G 7028T 7960G 8701G 8768T 8854A 8860G 9540C 10398G 10400T 10873C 11719A 12705T 13308G 13590A 13708A 14130T 14260G 14527G 14766T 14783C 15043A 15172A 15301A 15326G 15625T 16086C 16148T 16223T 16259T 16278T 16319A 16399G 16526A	M32c	73G 150T 200G 263G 310.1C 489C 16086C 16148T 16223T 16259T 16278T 16319A 16399G 16526A	1	73 150 200 263 489 750 2706 3588 4769 7028 7960 8701 8768 8854 8860 9540 10398 10400 10873 11719 12705 13308 13590 13708 14130 14260 14527 14766 14783 15043 15172 15301 15326 15625 16086 16148 16223 16259 16278 16319 16399 16526	M32c	73 150 200 263 489 16086 16148 16223 16259 16278 16319 16399 16526	[0.000] M32c (1)	[0.022] M51a (2) [0.007] M32 (3) [0.000] M51 M51b (5)	1
1654 >MF057263.1 Hc	73G 150T 200G 263G 310.1C 489C 750G 839G 2706G 3106d 3107C 3588T 4769G 7028T 7960G 8701G 8768T 8854A 8860G 9540C 10398G 10400T 10873C 11719A 12705T 13308G 13590A 13708A 14130T 14260G 14527G 14766T 14783C 15043A 15172A 15301A 15326G 15625T 16086C 16148T 16259T 16278T 16319A 16399G 16526A	M32c	73G 150T 200G 263G 310.1C 489C 16086C 16148T 16259T 16278T 16319A 16399G 16526A	1	73 150 200 263 489 750 839 2706 3588 4769 7028 7960 8701 8768 8854 8860 9540 10398 10400 10873 11719 12705 13308 13590 13708 14130 14260 14527 14766 14783 15043 15172 15301 15326 15625 16086 16148 16259 16278 16319 16399 16526	M32c	73 150 200 263 489 16086 16148 16259 16278 16319 16399 16526	[0.007] M32c (1)	[0.022] M51a (2) [0.007] M32 (3) [0.000] M51 M51b (5)	1
1655 >MF057262.1 Hc	73G 152C 195C 204C 263G 310.1C 417A 489C 533G 750G 1438G 3106d 3107C 4769G 7028T 8188G 8360G 8701G 8860G 9438A 9540C 9545G 10142T 10295G 10398G 10400T 10873C 11569C 11719A 11899C 12279G 12618A 12705T 14766T 14783C 15025T 15043A 15301A 15326G 16223T 16263C 16311C 16519C	M23	73G 152C 195C 204C 263G 310.1C 417A 489C 533G 16223T 16263C 16311C 16519C	1	73 152 195 204 263 417 489 533 750 1438 4769 7028 8188 8360 8701 8860 9438 9540 9545 10142 10295 10398 10400 10873 11569 11719 11899 12279 12618 12705 14766 14783 15025 15043 15301 15326 16223 16263 16311	M23	73 152 195 204 263 417 489 533 16223 16263 16311	[0.000] M23 (1)	[0.072] M24a (2) [0.053] M57+152	1
1656 >MF057260.1 Hc	73G 152C 195C 204C 263G 310.1C 417A 489C 533G 750G 1438G 3106d 3107C 4769G 7028T 8188G 8360G 8701G 8860G 9438A 9540C 9545G 10142T 10295G 10398G 10400T 10873C 11569C 11719A 11899C 12279G 12618A 12705T 14766T 14783C 15025T 15043A 15301A 15326G 16223T 16263C 16311C 16519C	M23	73G 152C 195C 204C 263G 310.1C 417A 489C 533G 16223T 16263C 16311C 16519C	1	73 152 195 204 263 417 489 533 750 1438 4769 7028 8188 8360 8701 8860 9438 9540 9545 10142 10295 10398 10400 10873 11569 11719 11899 12279 12618 12705 14766 14783 15025 15043 15301 15326 16223 16263 16311	M23	73 152 195 204 263 417 489 533 16223 16263 16311	[0.000] M23 (1)	[0.072] M24a (2) [0.053] M57+152	1

1657	>MF057257.1 Hc	73G 152C 195C 204C 263G 310.1C 417A 489C 533G 750G 1438G 3106d 3107C 4769G 7028T 8188G 8360G 8701G 8860G 9438A 9540C 9545G 10142T 10295G 10398G 10400T 10873C 11569C 11719A 11899C 12279G 12618A 12705T 14766T 14783C 15025T 15043A 15301A 15326G 16223T 16263C 16311C 16519C	M23	73G 152C 195C 204C 263G 310.1C 417A 489C 533G 16223T 16263C 16311C 16519C	1	73 152 195 204 263 417 489 533 750 1438 4769 7028 8188 8360 8701 8860 9438 9540 9545 10142 10295 10398 10400 10873 11569 11719 11899 12279 12618 12705 14766 14783 15025 15043 15301 15326 16223 16263 16311	M23	73 152 195 204 263 417 489 533 16223 16263 16311	[0.000] M23 (1)	[0.072] M24a (2) [0.053] M57+152	1
1658	>MF057249.1 Hc	73G 150T 200G 263G 310.1C 489C 750G 2706G 3106d 3107C 3588T 4769G 7028T 7960G 8701G 8768T 8854A 8860G 9540C 10398G 10400T 10873C 11719A 12705T 13308G 13590A 13708A 14130T 14260G 14527G 14766T 14783C 15043A 15172A 15301A 15326G 15625T 16086C 16148T 16223T 16259T 16278T 16319A 16399G 16526A	M32c	73G 150T 200G 263G 310.1C 489C 16086C 16148T 16223T 16259T 16278T 16319A 16399G 16526A	1	73 150 200 263 489 750 2706 3588 4769 7028 7960 8701 8768 8854 8860 9540 10398 10400 10873 11719 12705 13308 13590 13708 14130 14260 14527 14766 14783 15043 15172 15301 15326 15625 16086 16148 16223 16259 16278 16319 16399 16526	M32c	73 150 200 263 489 16086 16148 16223 16259 16278 16319 16399 16526	[0.000] M32c (1)	[0.022] M51a (2) [0.007] M32 (3) [0.000] M51 M51b (5)	1
1659	>MF057242.1 Hc	73G 146C 152C 195C 263G 750G 769A 1018A 1438G 1980G 2416C 2706G 2789T 3106d 3107C 3594T 4104G 4769G 5090C 7028T 7175C 7256T 7274T 7521A 7771G 8206A 8701G 8860G 9221G 9540C 10115C 10143A 10398G 10873C 11719A 11914A 11944C 12693G 12705T 13590A 13650T 13803G 14566G 14766T 15301A 15326G 15735T 15784C 16183C 16189C 16223T 16278T 16290T 16294T 16309G 16390A	L2a1b1a	73G 146C 152C 195C 263G 16183C 16189C 16223T 16278T 16290T 16294T 16309G 16390A	1	73 146 152 195 263 750 769 1018 1438 1980 2416 2706 2789 3594 4104 4769 5090 7028 7175 7256 7274 7521 7771 8206 8701 8860 9221 9540 10115 10143 10398 10873 11719 11914 11944 12693 12705 13590 13650 13803 14566 14766 15301 15326 15735 15784 16189 16223 16278 16290 16294 16309 16390	L2a1b1a	73 146 152 195 263 16189 16223 16278 16290 16294 16309 16390	[0.000] L2a1b1a (1)	[0.005] L2a1f (2) [0.000] L2a1+16189_(16192) L2a1b L2a1b1 L2a1b1a L2a1b2 L2a1f1 L2a1f1a L2a1f2 (10)	1
1660	>MF057241.1 Hc	64T 93G 185A 189G 200G 236C 247A 263G 514d 515d 750G 769A 825A 1018A 1048T 1438G 2245C 2706G 2758A 2885C 3106d 3107C 3516A 3594T 3866C 4104G 4312T 4586C 4769G 5096C 5231A 5442C 5460A 5603T 6185C 7028T 7146G 7256T 7521A 8428T 8468T 8566G 8655T 8701G 8860G 9042T 9347G 9540C 9755A 9818T 10237C 10398G 10589A 10664T 10688A 10810C 10873C 10915C 11176A 11641G 11719A 11914A 12007A 12237d 12705T 12720G 13105G 13215A 13276G 13506T 13650T 14308C 14766T 15136T 15326G 15431A 16129A 16148T 16168T 16172C 16184d 16185d 16223T 16230G 16311C 16320T	L0a1a2	64T 93G 185A 189G 200G 236C 247A 263G 514d 515d 16129A 16148T 16168T 16172C 16184d 16185d 16223T 16230G 16311C 16320T	1	64 93 185 189 200 236 247 263 750 769 825A 1018 1048 1438 2245C 2706 2758 2885 3516A 3594 3866 4104 4312 4586 4769 5096 5231 5442 5460 5603 6185 7028 7146 7256 7521 8428 8468 8566 8655 8701 8860 9042 9347 9540 9755 9818 10237 10398 10589 10664 10688 10810 10873 10915 11176 11641 11719 11914 12007 12241d 12705 12720 13105 13215A 13276 13506 13650 14308 14766 15136 15326 15431 16129 16148 16168 16172 16187 16189 16192-16193d 16223 16230 16311 16320	L0a1a2	64 93 185 189 200 236 247 263 16129 16148 16168 16172 16187 16189 16192-16193d 16223 16230 16311 16320	[0.143] L0a1a2 (1)	[0.529] L0a1a3 (2) [0.143] L0a1a2 (3) [0.043] L0a1a+200 (4) [0.000] L0a1a1	1

1661	>MF057238.1 Hc	73G 146C 152C 195C 263G 750G 769A 1018A 1438G 1980G 2416C 2706G 2789T 3106d 3107C 3594T 4104G 4769G 5090C 7028T 7175C 7256T 7274T 7521A 7771G 8206A 8701G 8860G 9221G 9540C 10115C 10143A 10398G 10873C 11719A 11914A 11944C 12693G 12705T 13590A 13650T 13803G 14566G 14766T 15301A 15326G 15735T 15784C 16183C 16189C 16223T 16278T 16290T 16294T 16309G 16390A	L2a1b1a	73G 146C 152C 195C 263G 16183C 16189C 16223T 16278T 16290T 16294T 16309G 16390A	1	73 146 152 195 263 750 769 1018 1438 1980 2416 2706 2789 3594 4104 4769 5090 7028 7175 7256 7274 7521 7771 8206 8701 8860 9221 9540 10115 10143 10398 10873 11719 11914 11944 12693 12705 13590 13650 13803 14566 14766 15301 15326 15735 15784 16189 16223 16278 16290 16294 16309 16390	L2a1b1a	73 146 152 195 263 16189 16223 16278 16290 16294 16309 16390	[0.000] L2a1b1a (1)	[0.005] L2a1f (2) [0.000] L2a1+16189_(16192) L2a1b L2a1b1 L2a1b1a L2a1b2 L2a1f1 L2a1f1a L2a1f2 (10)	1
1662	>MF057235.1 Hc	73G 146C 152C 195C 263G 750G 769A 1018A 1438G 2416C 2706G 2789T 3106d 3107C 3594T 4104G 4769G 5090C 7028T 7175C 7256T 7274T 7521A 7771G 8206A 8701G 8860G 9221G 9540C 10115C 10143A 10398G 10873C 11719A 11914A 11944C 12693G 12705T 13590A 13650T 13803G 14566G 14766T 15301A 15326G 15735T 15784C 16182C 16183C 16189C 16223T 16278T 16290T 16294T 16309G 16390A	L2a1b1a	73G 146C 152C 195C 263G 16182C 16183C 16189C 16223T 16278T 16290T 16294T 16309G 16390A	1	73 146 152 195 263 750 769 1018 1438 2416 2706 2789 3594 4104 4769 5090 7028 7175 7256 7274 7521 7771 8206 8701 8860 9221 9540 10115 10143 10398 10873 11719 11914 11944 12693 12705 13590 13650 13803 14566 14766 15301 15326 15735 15784 16189 16223 16278 16290 16294 16309 16390	L2a1b1a	73 146 152 195 263 16189 16223 16278 16290 16294 16309 16390	[0.000] L2a1b1a (1)	[0.005] L2a1f (2) [0.000] L2a1+16189_(16192) L2a1b L2a1b1 L2a1b1a L2a1b2 L2a1f1 L2a1f1a L2a1f2 (10)	1
1663	>MF057232.1 Hc	73G 146C 152C 195C 263G 750G 769A 1018A 1438G 2416C 2706G 2789T 3106d 3107C 3594T 4104G 4769G 5090C 7028T 7175C 7256T 7274T 7521A 7771G 8206A 8701G 8860G 9221G 9540C 10115C 10143A 10398G 10873C 11719A 11914A 11944C 12693G 12705T 13590A 13650T 13803G 14566G 14766T 15301A 15326G 15735T 15784C 16183C 16189C 16223T 16278T 16290T 16294T 16309G 16390A	L2a1b1a	73G 146C 152C 195C 263G 16183C 16189C 16223T 16278T 16290T 16294T 16309G 16390A	1	73 146 152 195 263 750 769 1018 1438 2416 2706 2789 3594 4104 4769 5090 7028 7175 7256 7274 7521 7771 8206 8701 8860 9221 9540 10115 10143 10398 10873 11719 11914 11944 12693 12705 13590 13650 13803 14566 14766 15301 15326 15735 15784 16189 16223 16278 16290 16294 16309 16390	L2a1b1a	73 146 152 195 263 16189 16223 16278 16290 16294 16309 16390	[0.000] L2a1b1a (1)	[0.005] L2a1f (2) [0.000] L2a1+16189_(16192) L2a1b L2a1b1 L2a1b1a L2a1b2 L2a1f1 L2a1f1a L2a1f2 (10)	1
1664	>MF057230.1 Hc	73G 152C 195C 204C 263G 417A 489C 533G 750G 1438G 3106d 3107C 4769G 7028T 8188G 8360G 8701G 8860G 9438A 9540C 9545G 10142T 10295G 10398G 10400T 10873C 11569C 11719A 11899C 12279G 12618A 12705T 14766T 14783C 15025T 15043A 15301A 15326G 16223T 16263C 16311C 16519C	M23	73G 152C 195C 204C 263G 417A 489C 533G 16223T 16263C 16311C 16519C	1	73 152 195 204 263 417 489 533 750 1438 4769 7028 8188 8360 8701 8860 9438 9540 9545 10142 10295 10398 10400 10873 11569 11719 11899 12279 12618 12705 14766 14783 15025 15043 15301 15326 16223 16263 16311	M23	73 152 195 204 263 417 489 533 16223 16263 16311	[0.000] M23 (1)	[0.072] M24a (2) [0.053] M57+152	1

1665	>MF057225.1 Hc	73G 146C 152C 195C 263G 750G 769A 1018A 1438G 2416C 2706G 2762T 2789T 3106d 3107C 3594T 4104G 4769G 5090C 7028T 7175C 7256T 7274T 7521A 7771G 8206A 8701G 8860G 9221G 9540C 10115C 10143A 10398G 10873C 11719A 11914A 11944C 12693G 12705T 13590A 13650T 13803G 14566G 14766T 15301A 15326G 15735T 15784C 16182C 16183C 16189C 16223T 16278T 16290T 16294T 16390A	L2a1b1a	73G 146C 152C 195C 263G 16182C 16183C 16189C 16223T 16278T 16290T 16294T 16390A	1	73 146 152 195 263 750 769 1018 1438 2416 2706 2762 2789 3594 4104 4769 5090 7028 7175 7256 7274 7521 7771 8206 8701 8860 9221 9540 10115 10143 10398 10873 11719 11914 11944 12693 12705 13590 13650 13803 14566 14766 15301 15326 15735 15784 16189 16223 16278 16290 16294 16390	L2a1b1a	73 146 152 195 263 16189 16223 16278 16290 16294 16390	[0.045] L2a1b1a (1)	[0.234] L2a1+143+16189_(16192)+@16309 (2) [0.070] L2a1f (3) [0.045] L2a1b1a (4) [0.008] L2a1b2 (5) [0.000] L2a1+16189_(16192) L2a1b L2a1b1 L2a1f1 L2a1f1a L2a1f2 (11)	1
1666	>MF057220.1 Hc	73G 150T 263G 489C 750G 2706G 3106d 3107C 3588T 4769G 7028T 7960G 8701G 8768T 8854A 8860G 9288G 9540C 10398G 10400T 10873C 11719A 12705T 13308G 13590A 13708A 14130T 14260G 14527G 14766T 14783C 15043A 15172A 15301A 15326G 15625T 16086C 16148T 16223T 16259T 16278T 16319A 16399G 16526A	M32c	73G 150T 263G 489C 16086C 16148T 16223T 16259T 16278T 16319A 16399G 16526A	1	73 150 263 489 750 2706 3588 4769 7028 7960 8701 8768 8854 8860 9288 9540 10398 10400 10873 11719 12705 13308 13590 13708 14130 14260 14527 14766 14783 15043 15172 15301 15326 15625 16086 16148 16223 16259 16278 16319 16399 16526	M32c	73 150 263 489 16086 16148 16223 16259 16278 16319 16399 16526	[0.046] M32c (1)	[0.022] M51a (2) [0.000] M51 M51b M32 (5)	1
1667	>MF057215.1 Hc	73G 152C 195C 204C 263G 417A 489C 533G 750G 1438G 3106d 3107C 4769G 7028T 8188G 8360G 8701G 8860G 9438A 9540C 9545G 10142T 10295G 10398G 10400T 10873C 11569C 11719A 11899C 12279G 12618A 12705T 14766T 14783C 15025T 15043A 15301A 15326G 16223T 16263C 16311C 16519C	M23	73G 152C 195C 204C 263G 417A 489C 533G 16223T 16263C 16311C 16519C	1	73 152 195 204 263 417 489 533 750 1438 4769 7028 8188 8360 8701 8860 9438 9540 9545 10142 10295 10398 10400 10873 11569 11719 11899 12279 12618 12705 14766 14783 15025 15043 15301 15326 16223 16263 16311	M23	73 152 195 204 263 417 489 533 16223 16263 16311	[0.000] M23 (1)	[0.072] M24a (2) [0.053] M57+152	1
1668	>MF057201.1 Hc	73G 150T 200G 263G 489C 750G 2706G 3106d 3107C 3588T 4769G 7028T 7960G 8701G 8768T 8854A 8860G 9540C 10398G 10400T 10873C 11719A 12705T 13135A 13308G 13590A 13708A 14130T 14260G 14527G 14766T 14783C 15043A 15172A 15301A 15326G 15625T 16086C 16148T 16223T 16259T 16278T 16319A 16399G 16526A	M32c	73G 150T 200G 263G 489C 16086C 16148T 16223T 16259T 16278T 16319A 16399G 16526A	1	73 150 200 263 489 750 2706 3588 4769 7028 7960 8701 8768 8854 8860 9540 10398 10400 10873 11719 12705 13135 13308 13590 13708 14130 14260 14527 14766 14783 15043 15172 15301 15326 15625 16086 16148 16223 16259 16278 16319 16399 16526	M32c	73 150 200 263 489 16086 16148 16223 16259 16278 16319 16399 16526	[0.000] M32c (1)	[0.022] M51a (2) [0.007] M32 (3) [0.000] M51 M51b (5)	1

1669	>MF057198.1 Hc	73G 146C 152C 195C 263G 750G 769A 1018A 1438G 2416C 2706G 2789T 3106d 3107C 3594T 4104G 4769G 5090C 7028T 7175C 7256T 7274T 7521A 7771G 8206A 8701G 8860G 9055A 9221G 9540C 10115C 10143A 10398G 10873C 11719A 11914A 11944C 12693G 12705T 13590A 13650T 13803G 14566G 14766T 15301A 15326G 15735T 15784C 16183C 16189C 16223T 16274A 16278T 16290T 16294T 16309G 16390A	L2a1b1a	73G 146C 152C 195C 263G 16183C 16189C 16223T 16274A 16278T 16290T 16294T 16309G 16390A	1	73 146 152 195 263 750 769 1018 1438 2416 2706 2789 3594 4104 4769 5090 7028 7175 7256 7274 7521 7771 8206 8701 8860 9055 9221 9540 10115 10143 10398 10873 11719 11914 11944 12693 12705 13590 13650 13803 14566 14766 15301 15326 15735 15784 16189 16223 16274 16278 16290 16294 16309 16390	L2a1b1a	73 146 152 195 263 16189 16223 16274 16278 16290 16294 16309 16390	[0.012] L2a1b1a (1)	[0.012] L2a1b1a (2) [0.008] L2a1b2 (3) [0.007] L2a1b L2a1f1a (5) [0.005] L2a1f (6) [0.000] L2a1+16189_(16192) L2a1b1 L2a1f1 L2a1f2 (10)	1
1670	>MF057195.1 Hc	73G 146C 152C 195C 263G 750G 769A 1018A 1438G 2416C 2706G 2789T 3106d 3107C 3594T 4104G 4769G 5090C 7028T 7175C 7256T 7274T 7521A 7771G 8206A 8701G 8860G 9221G 9540C 10115C 10143A 10398G 10873C 11719A 11914A 11944C 12693G 12705T 13590A 13650T 13803G 14566G 14766T 15301A 15326G 15735T 15784C 16183C 16189C 16223T 16278T 16290T 16294T 16309G 16390A	L2a1b1a	73G 146C 152C 195C 263G 16183C 16189C 16223T 16278T 16290T 16294T 16309G 16390A	1	73 146 152 195 263 750 769 1018 1438 2416 2706 2789 3594 4104 4769 5090 7028 7175 7256 7274 7521 7771 8206 8701 8860 9221 9540 10115 10143 10398 10873 11719 11914 11944 12693 12705 13590 13650 13803 14566 14766 15301 15326 15735 15784 16189 16223 16278 16290 16294 16309 16390	L2a1b1a	73 146 152 195 263 16189 16223 16278 16290 16294 16309 16390	[0.000] L2a1b1a (1)	[0.005] L2a1f (2) [0.000] L2a1+16189_(16192) L2a1b L2a1b1 L2a1b1a L2a1b2 L2a1f1 L2a1f1a L2a1f2 (10)	1
1671	>MF057194.1 Hc	73G 150T 200G 263G 489C 750G 2706G 3106d 3107C 3588T 4769G 7028T 7960G 8701G 8768T 8854A 8860G 9540C 10398G 10400T 10873C 11719A 12705T 13308G 13590A 13708A 14130T 14260G 14527G 14766T 14783C 15043A 15172A 15301A 15326G 15625T 16086C 16148T 16223T 16259T 16278T 16319A 16399G 16526A	M32c	73G 150T 200G 263G 489C 16086C 16148T 16223T 16259T 16278T 16319A 16399G 16526A	1	73 150 200 263 489 750 2706 3588 4769 7028 7960 8701 8768 8854 8860 9540 10398 10400 10873 11719 12705 13308 13590 13708 14130 14260 14527 14766 14783 15043 15172 15301 15326 15625 16086 16148 16223 16259 16278 16319 16399 16526	M32c	73 150 200 263 489 16086 16148 16223 16259 16278 16319 16399 16526	[0.000] M32c (1)	[0.022] M51a (2) [0.007] M32 (3) [0.000] M51 M51b (5)	1
1672	>MF057191.1 Hc	73G 152C 195C 204C 263G 417A 489C 533G 750G 1438G 3106d 3107C 4769G 7028T 8188G 8360G 8701G 8860G 9438A 9540C 9545G 10142T 10295G 10398G 10400T 10873C 11569C 11719A 11899C 12279G 12618A 12705T 14766T 14783C 15025T 15043A 15301A 15326G 16223T 16263C 16311C 16519C	M23	73G 152C 195C 204C 263G 417A 489C 533G 16223T 16263C 16311C 16519C	1	73 152 195 204 263 417 489 533 750 1438 4769 7028 8188 8360 8701 8860 9438 9540 9545 10142 10295 10398 10400 10873 11569 11719 11899 12279 12618 12705 14766 14783 15025 15043 15301 15326 16223 16263 16311	M23	73 152 195 204 263 417 489 533 16223 16263 16311	[0.000] M23 (1)	[0.072] M24a (2) [0.053] M57+152	1

1673	>MF057190.1 Hc	73G 93G 146C 150T 152C 182T 183G 195C 198T 263G 325T 499A 514d 515d 680C 709A 750G 769A 1018A 1040C 1438G 1442A 2332T 2416C 2706G 3106d 3107C 3200A 3594T 4104G 4769G 7028T 7256T 7521A 7624A 8116G 8206A 8567C 8701G 8772C 8860G 9063G 9221G 9540C 10115C 10398G 10790C 10873C 11653G 11719A 11944C 12236A 12705T 13590A 13650T 13928C 13958C 14766T 15110A 15217A 15301A 15313C 15326G 15849T 15926T 16223T 16260T 16264T 16278T 16311C 16390A 16519C	L2c2b1b	73G 93G 146C 150T 152C 182T 183G 195C 198T 263G 325T 499A 514d 515d 16223T 16260T 16264T 16278T 16311C 16390A 16519C	1	73 93 146 150 152 182 183 195 198 263 325 499 680 709 750 769 1018 1040 1438 1442 2332 2416 2706 3200A 3594 4104 4769 7028 7256 7521 7624A 8116 8206 8567 8701 8772 8860 9063 9221 9540 10115 10398 10790 10873 11653 11719 11944 12236 12705 13590 13650 13928C 13958C 14766 15110 15217 15301 15313 15326 15849 15926 16223 16260 16264 16278 16311 16390	L2c2b1b	73 93 146 150 152 182 183 195 198 263 325 499 16223 16260 16264 16278 16311 16390	[0.103] L2c2b1b (1)	[0.000] L2c2b L2c2b1 L2c2b1a L2c2b2 (5)	1
1674	>MF057189.1 Hc	73G 150T 195C 263G 514d 515d 1438G 2000T 2352C 2706G 3106d 3107C 4655A 4769G 5262A 6524C 7028T 8701G 8860G 9540C 9554A 10398G 10667C 10816G 10819G 10873C 11719A 12248G 12705T 13101C 13197T 13651G 14212C 14766T 15301A 15326G 15812A 16223T 16265T 16519C 16527T	L3e3b2	73G 150T 195C 263G 514d 515d 16223T 16265T 16519C 16527T	1	73 150 195 263 1438 2000 2352 2706 4655 4769 5262 6524 7028 8701 8860 9540 9554 10398 10667 10816 10819 10873 11719 12248 12705 13101C 13197 13651 14212 14766 15301 15326 15812 16223 16265T 16527	L3e3b2	73 150 195 263 16223 16265T 16527	[0.000] L3e3b2 (1)	[0.000] L3e3 L3e3b L3e3b1 (4)	1
1675	>MF057188.1 Hc	73G 152C 195C 204C 263G 417A 489C 533G 750G 1438G 3106d 3107C 4769G 7028T 8188G 8360G 8701G 8860G 9438A 9540C 9545G 10142T 10295G 10398G 10400T 10873C 11569C 11719A 11899C 12279G 12618A 12705T 14766T 14783C 15025T 15043A 15301A 15326G 16176T 16223T 16263C 16311C 16519C	M23	73G 152C 195C 204C 263G 417A 489C 533G 16176T 16223T 16263C 16311C 16519C	1	73 152 195 204 263 417 489 533 750 1438 4769 7028 8188 8360 8701 8860 9438 9540 9545 10142 10295 10398 10400 10873 11569 11719 11899 12279 12618 12705 14766 14783 15025 15043 15301 15326 16176 16223 16263 16311	M23	73 152 195 204 263 417 489 533 16176 16223 16263 16311	[0.024] M23 (1)	[0.072] M24a (2) [0.053] M57+152	1
1676	>MF057186.1 Hc	73G 152C 195C 204C 263G 417A 489C 533G 750G 1438G 3106d 3107C 4769G 7028T 8188G 8360G 8701G 8860G 9438A 9540C 9545G 10142T 10295G 10398G 10400T 10873C 11569C 11719A 11899C 12279G 12618A 12705T 14766T 14783C 15025T 15043A 15301A 15326G 16223T 16263C 16311C 16519C	M23	73G 152C 195C 204C 263G 417A 489C 533G 16223T 16263C 16311C 16519C	1	73 152 195 204 263 417 489 533 750 1438 4769 7028 8188 8360 8701 8860 9438 9540 9545 10142 10295 10398 10400 10873 11569 11719 11899 12279 12618 12705 14766 14783 15025 15043 15301 15326 16223 16263 16311	M23	73 152 195 204 263 417 489 533 16223 16263 16311	[0.000] M23 (1)	[0.072] M24a (2) [0.053] M57+152	1

1677	>MF057183.1 H	73G 146C 152C 195C 263G 513A 750G 769A 1018A 1438G 2416C 2706G 2789T 3106d 3107C 3594T 4104G 4769G 5090C 7028T 7175C 7256T 7274T 7521A 7771G 8206A 8701G 8860G 9163A 9221G 9540C 10115C 10143A 10398G 10873C 11719A 11914A 11944C 12399T 12693G 12705T 12843C 13590A 13650T 13803G 14566G 14766T 15301A 15326G 15735T 15784C 16086C 16182C 16183C 16189C 16223T 16278T 16290T 16294T 16309G 16390A	L2a1b1a	73G 146C 152C 195C 263G 513A 16086C 16182C 16183C 16189C 16223T 16278T 16290T 16294T 16309G 16390A	1	73 146 152 195 263 513 750 769 1018 1438 2416 2706 2789 3594 4104 4769 5090 7028 7175 7256 7274 7521 7771 8206 8701 8860 9163 9221 9540 10115 10143 10398 10873 11719 11914 11944 12399 12693 12705 12843 13590 13650 13803 14566 14766 15301 15326 15735 15784 16086 16189 16223 16278 16290 16294 16309 16390	L2a1b1a	73 146 152 195 263 513 16086 16189 16223 16278 16290 16294 16309 16390	[0.012] L2a1b1a (1)	[0.022] L2a1f1a (2) [0.012] L2a1b1a (3) [0.010] L2a1f (4) [0.006] L2a1f2 (5) [0.000] L2a1+16189_(16192) L2a1b L2a1b1 L2a1b2 L2a1f1 (10)	1
1678	>MF057182.1 H	73G 150T 185A 189G 263G 750G 1438G 2352C 2706G 3106d 3107C 4769G 6221C 6587T 7028T 8577G 8701G 8860G 9540C 10398G 10819G 10873C 11719A 12192A 12705T 14152G 14212C 14766T 14926G 15301A 15326G 15670C 15942C 16223T 16323d 16327T	L3e1b2	73G 150T 185A 189G 263G 16223T 16323d 16327T	1	73 150 185 189 263 750 1438 2352 2706 4769 6221 6587 7028 8577 8701 8860 9540 10398 10819 10873 11719 12192 12705 14152 14212 14766 14926 15301 15326 15670 15942 16223 16325d 16327	L3e1b2	73 150 185 189 263 16223 16325d 16327	[0.000] L3e1b2 (1)	[0.200] L3e1b (2)	1
1679	>MF057180.1 H	73G 146C 152C 195C 263G 750G 769A 1018A 1438G 1980G 2416C 2706G 2789T 3106d 3107C 3594T 4104G 4769G 5090C 7028T 7175C 7256T 7274T 7521A 7771G 8206A 8701G 8860G 9221G 9540C 10115C 10143A 10398G 10873C 11719A 11914A 11944C 12693G 12705T 13590A 13650T 13803G 14566G 14766T 15301A 15326G 15735T 15784C 16182C 16183C 16189C 16223T 16278T 16290T 16294T 16309G 16390A	L2a1b1a	73G 146C 152C 195C 263G 16182C 16183C 16189C 16223T 16278T 16290T 16294T 16309G 16390A	1	73 146 152 195 263 750 769 1018 1438 1980 2416 2706 2789 3594 4104 4769 5090 7028 7175 7256 7274 7521 7771 8206 8701 8860 9221 9540 10115 10143 10398 10873 11719 11914 11944 12693 12705 13590 13650 13803 14566 14766 15301 15326 15735 15784 16189 16223 16278 16290 16294 16309 16390	L2a1b1a	73 146 152 195 263 16189 16223 16278 16290 16294 16309 16390	[0.000] L2a1b1a (1)	[0.005] L2a1f (2) [0.000] L2a1+16189_(16192) L2a1b L2a1b1 L2a1b1a L2a1b2 L2a1f1 L2a1f1a L2a1f2 (10)	1
1680	>MF057174.1 H	73G 152C 195C 204C 207A 263G 417A 489C 533G 750G 1438G 3106d 3107C 4769G 7028T 8188G 8360G 8701G 8860G 9438A 9540C 9545G 10142T 10295G 10398G 10400T 10873C 11569C 11719A 11899C 12279G 12358G 12618A 12705T 14766T 14783C 15025T 15043A 15301A 15326G 15758G 16223T 16263C 16311C 16519C	M23	73G 152C 195C 204C 207A 263G 417A 489C 533G 16223T 16263C 16311C 16519C	1	73 152 195 204 207 263 417 489 533 750 1438 4769 7028 8188 8360 8701 8860 9438 9540 9545 10142 10295 10398 10400 10873 11569 11719 11899 12279 12358 12618 12705 14766 14783 15025 15043 15301 15326 15758 16223 16263 16311	M23	73 152 195 204 207 263 417 489 533 16223 16263 16311	[0.016] M23 (1)	[0.077] M24a (2) [0.053] M57+152	1

1681 >MF057173.1 Hc	73G 150T 195C 200G 263G 489C 750G 2706G 3106d 3107C 3588T 4769G 7028T 7960G 8701G 8768T 8854A 8860G 9540C 9983G 10398G 10400T 10873C 11719A 12705T 13308G 13590A 13708A 14130T 14260G 14527G 14766T 14783C 15043A 15172A 15301A 15326G 15625T 16086C 16148T 16223T 16259T 16278T 16319A 16399G 16526A	M32c	73G 150T 195C 200G 263G 489C 16086C 16148T 16223T 16259T 16278T 16319A 16399G 16526A	1	73 150 195 200 263 489 750 2706 3588 4769 7028 7960 8701 8768 8854 8860 9540 9983 10398 10400 10873 11719 12705 13308 13590 13708 14130 14260 14527 14766 14783 15043 15172 15301 15326 15625 16086 16148 16223 16259 16278 16319 16399 16526	M32c	73 150 195 200 263 489 16086 16148 16223 16259 16278 16319 16399 16526	[0.010] M32c (1)	[0.022] M51a (2) [0.007] M32 (3) [0.000] M51 M51b (5)	1
1682 >MF057169.1 Hc	73G 150T 200G 263G 489C 750G 2706G 3106d 3107C 3588T 4769G 7028T 7960G 8701G 8768T 8854A 8860G 9540C 10398G 10400T 10873C 11719A 12705T 13308G 13470G 13590A 13708A 14130T 14260G 14527G 14766T 14783C 15043A 15172A 15301A 15326G 15625T 16086C 16148T 16223T 16259T 16278T 16319A 16399G 16526A	M32c	73G 150T 200G 263G 489C 16086C 16148T 16223T 16259T 16278T 16319A 16399G 16526A	1	73 150 200 263 489 750 2706 3588 4769 7028 7960 8701 8768 8854 8860 9540 10398 10400 10873 11719 12705 13308 13470 13590 13708 14130 14260 14527 14766 14783 15043 15172 15301 15326 15625 16086 16148 16223 16259 16278 16319 16399 16526	M32c	73 150 200 263 489 16086 16148 16223 16259 16278 16319 16399 16526	[0.000] M32c (1)	[0.022] M51a (2) [0.007] M32 (3) [0.000] M51 M51b (5)	1
1683 >MF057166.1 Hc	73G 146C 152C 263G 750G 769A 1018A 1438G 2416C 2706G 2789T 3106d 3107C 3594T 4104G 4769G 7028T 7175C 7256T 7274T 7521A 7771G 8014G 8206A 8557A 8701G 8860G 9221G 9540C 10115C 10398G 10873C 11719A 11914A 11944C 12693G 12705T 13590A 13650T 13803G 14281T 14566G 14766T 15301A 15326G 15784C 16131C 16189C 16223T 16225T 16234T 16278T 16294T 16309G 16390A	L2a1g	73G 146C 152C 263G 16131C 16189C 16223T 16225T 16234T 16278T 16294T 16309G 16390A	1	73 146 152 263 750 769 1018 1438 2416 2706 2789 3594 4104 4769 7028 7175 7256 7274 7521 7771 8014 8206 8557 8701 8860 9221 9540 10115 10398 10873 11719 11914 11944 12693 12705 13590 13650 13803 14281 14566 14766 15301 15326 15784 16131 16189 16223 16225 16234 16278 16294 16309 16390	L2a1g	73 146 152 263 16131 16189 16223 16225 16234 16278 16294 16309 16390	[0.269] L2a1g (1)	[0.023] L2a1b2 (2) [0.022] L2a1b L2a1f1a (4) [0.010] L2a1f (5) [0.000] L2a1+16189_(16192) L2a1b1 L2a1f1 L2a1f2 (9)	1
1684 >MF057160.1 Hc	73G 146C 152C 195C 198T 263G 750G 769A 1018A 1438G 2416C 2706G 2789T 3106d 3107C 3594T 4104G 4769G 5581G 7028T 7175C 7256T 7274T 7521A 7771G 8206A 8701G 9221G 9540C 10115C 10398G 10873C 11719A 11914A 11944C 12693G 12705T 13590A 13650T 13803G 14566G 14766T 15301A 15326G 15784C 16189C 16223T 16278T 16294T 16309G 16390A 16519C	L2a1f3	73G 146C 152C 195C 198T 263G 16189C 16223T 16278T 16294T 16309G 16390A 16519C	1	73 146 152 195 198 263 750 769 1018 1438 2416 2706 2789 3594 4104 4769 5581 7028 7175 7256 7274 7521 7771 8206 8701 9221 9540 10115 10398 10873 11719 11914 11944 12693 12705 13590 13650 13803 14566 14766 15301 15326 15784 16189 16223 16278 16294 16309 16390	L2a1f3	73 146 152 195 198 263 16189 16223 16278 16294 16309 16390	[0.000] L2a1f3 (1)	[0.007] L2a1b (2) [0.005] L2a1f (3) [0.000] L2a1+16189_(16192) L2a1b1 L2a1b2 L2a1f1 L2a1f1a L2a1f2 L2a1f3 (10)	1

1685	>MF057158.1 Hc	73G 146C 152C 195C 198T 263G 750G 769A 1018A 1438G 2416C 2706G 2789T 3106d 3107C 3594T 4104G 4769G 5581G 7028T 7175C 7256T 7274T 7521A 7771G 8206A 8701G 9221G 9540C 10115C 10398G 10873C 11719A 11914A 11944C 12693G 12705T 13590A 13650T 13803G 14566G 14766T 15301A 15326G 15784C 16189C 16223T 16278T 16294T 16309G 16390A 16519C	L2a1f3	73G 146C 152C 195C 198T 263G 16189C 16223T 16278T 16294T 16309G 16390A 16519C	1	73 146 152 195 198 263 750 769 1018 1438 2416 2706 2789 3594 4104 4769 5581 7028 7175 7256 7274 7521 7771 8206 8701 9221 9540 10115 10398 10873 11719 11914 11944 12693 12705 13590 13650 13803 14566 14766 15301 15326 15784 16189 16223 16278 16294 16309 16390	L2a1f3	73 146 152 195 198 263 16189 16223 16278 16294 16309 16390	[0.000] L2a1f3 (1)	[0.007] L2a1b (2) [0.005] L2a1f (3) [0.000] L2a1+16189_(16192) L2a1b1 L2a1b2 L2a1f1 L2a1f1a L2a1f2 L2a1f3 (10)	1
1686	>MF057155.1 Hc	73G 146C 152C 195C 247A 456T 494d 719A 750G 769A 825A 1018A 1048T 2000T 2707G 2758A 2885C 3083C 3106d 3107C 3345C 3438A 3516A 3594T 3756G 3834A 4104G 4197T 4216C 4232C 4312T 4769G 5252A 5263T 5442C 6185C 6266G 6815C 7028T 7256T 7521A 8113A 8152A 8251A 8468T 8655T 8701G 8860G 9042T 9150G 9347G 9540C 9755A 10373A 10398G 10589A 10664T 10688A 10810C 10873C 10915C 11150A 11437C 11719A 11872T 11914A 12007A 12121C 12235C 12705T 12720G 13105G 13129T 13276G 13506T 13650T 13759A 14384A 14766T 15187G 15326G 15466A 15930A 15941C 15951G 16187T 16189C 16223T 16230G 16234T 16243C 16311C 16519C	L0d1c	73G 146C 152C 195C 247A 456T 494d 16187T 16189C 16223T 16230G 16234T 16243C 16311C 16519C	1	73 146 152 195 247 456 498d 719 750 769 825A 1018 1048 2000 2707 2758 2885 3083 3345 3438 3516A 3594 3756 3834 4104 4197 4216 4232 4312 4769 5252 5263 5442 6185 6266 6815 7028 7256 7521 8113A 8152 8251 8468 8655 8701 8860 9042 9150 9347 9540 9755 10373 10398 10589 10664 10688 10810 10873 10915 11150 11437 11719 11872 11914 12007 12121 12235 12705 12720 13105 13129 13276 13506 13650 13759 14384 14766 15187 15326 15466 15930 15941 15951 16187 16189 16223 16230 16234 16243 16311	L0d1c	73 146 152 195 247 456 498d 16187 16189 16223 16230 16234 16243 16311	[0.000] L0d1c (1)	[0.000] L0d1c1 L0d1c1a L0d1c2 (4)	1

1687 >MF057154.1 Hc	73G 146C 152C 195C 247A 385G 494d 750G 1018A 1048T 2706G 2758A 2885C 3106d 3107C 3516A 3594T 3756G 3981G 4025T 4044G 4104G 4232C 4312T 4769G 5420C 5442C 6185C 6620C 6815C 7028T 7256T 7521A 7933G 8113A 8152A 8251A 8284T 8468T 8655T 8701G 8860G 9042T 9347G 9540C 9755A 10398G 10589A 10664T 10688A 10810C 10873C 10915C 10978G 11150A 11260C 11719A 11854C 11914A 12007A 12121C 12705T 12720G 13105G 13276G 13389T 13506T 13635C 13650T 14371C 14766T 15172A 15326G 15346A 15466A 15766G 15930A 15941C 16129A 16162d 16172C 16189C 16212G 16223T 16243C 16265G 16311C 16519C	L0d2a'b'd	73G 146C 152C 195C 247A 385G 494d 16129A 16162d 16172C 16189C 16212G 16223T 16243C 16265G 16311C 16519C	1	73 146 152 195 247 385 498d 750 1018 1048 2706 2758 2885 3516A 3594 3756 3981 4025 4044 4104 4232 4312 4769 5420 5442 6185 6620 6815 7028 7256 7521 7933 8113A 8152 8251 8284 8468 8655 8701 8860 9042 9347 9540 9755 10398 10589 10664 10688 10810 10873 10915 10978 11150 11260 11719 11854 11914 12007 12121 12705 12720 13105 13276 13389 13506 13635 13650 14371 14766 15172 15326 15346 15466 15766 15930 15941 16129 16166d 16172 16189 16212 16223 16243 16265 16311	L0d2a'b'd	73 146 152 195 247 385 498d 16129 16166d 16172 16189 16212 16223 16243 16265 16311	[0.174] L0d2a'b'd (1)	[0.000] L0d1'2 L0d1 L0d1b L0d2 L0d2a L0d2a1a3 (7)	1
1688 >MF057153.1 Hc	73G 146C 152C 195C 198T 263G 750G 769A 1018A 1438G 2416C 2706G 2789T 3106d 3107C 3594T 4104G 4769G 5581G 7028T 7175C 7256T 7274T 7521A 7771G 8206A 8701G 9221G 9540C 10115C 10398G 10873C 11719A 11914A 11944C 12693G 12705T 13590A 13650T 13803G 14566G 14766T 15301A 15326G 15784C 16189C 16223T 16278T 16294T 16309G 16390A 16519C	L2a1f3	73G 146C 152C 195C 198T 263G 16189C 16223T 16278T 16294T 16309G 16390A 16519C	1	73 146 152 195 198 263 750 769 1018 1438 2416 2706 2789 3594 4104 4769 5581 7028 7175 7256 7274 7521 7771 8206 8701 9221 9540 10115 10398 10873 11719 11914 11944 12693 12705 13590 13650 13803 14566 14766 15301 15326 15784 16189 16223 16278 16294 16309 16390	L2a1f3	73 146 152 195 198 263 16189 16223 16278 16294 16309 16390	[0.000] L2a1f3 (1)	[0.007] L2a1b (2) [0.005] L2a1f (3) [0.000] L2a1+16189_(16192) L2a1b1 L2a1b2 L2a1f1 L2a1f1a L2a1f2 L2a1f3 (10)	1
1689 >MF057152.1 Hc	73G 150T 200G 263G 489C 750G 2706G 3106d 3107C 3588T 4769G 7028T 7960G 8701G 8768T 8854A 8860G 9540C 10398G 10400T 10873C 11719A 12705T 13308G 13590A 13708A 14130T 14260G 14527G 14766T 14783C 15043A 15172A 15301A 15326G 15625T 16086C 16148T 16223T 16259T 16278T 16319A 16399G 16526A	M32c	73G 150T 200G 263G 489C 16086C 16148T 16223T 16259T 16278T 16319A 16399G 16526A	1	73 150 200 263 489 750 2706 3588 4769 7028 7960 8701 8768 8854 8860 9540 10398 10400 10873 11719 12705 13308 13590 13708 14130 14260 14527 14766 14783 15043 15172 15301 15326 15625 16086 16148 16223 16259 16278 16319 16399 16526	M32c	73 150 200 263 489 16086 16148 16223 16259 16278 16319 16399 16526	[0.000] M32c (1)	[0.022] M51a (2) [0.007] M32 (3) [0.000] M51 M51b (5)	1

1690	>MF057149.1 Hc	73G 146C 152C 195C 263G 750G 769A 1018A 1438G 1476N 1482N 1484N 1486N 2416C 2561N 2578N 2579N 2580N 2581N 2582N 2583N 2584N 2585N 2586N 2587N 2588N 2589N 2590N 2591N 2592N 2593N 2594N 2595N 2596N 2597N 2598N 2604N 2605N 2606N 2607N 2608N 2609N 2610N 2611N 2612N 2613N 2614N 2615N 2616N 2617N 2618N 2619N 2620N 2621N 2622N 2658N 2660N 2662N 2695N 2706G 2789T 3106d 3107C 3594T 4104G 4769G 5090C 5705N 5711N 5712N 5717N 5718N 5719N 5721N 5722N 5726N 5727N 5728N 5729N 5731N 5732N 5733N 5734N 5735N 5736N 5737N 5738N 5739N 5740N 5741N 5742N 5743N 5744N 5745N 5746N 5747N 5748N 5749N 5750N 5751N 5752N 5753N 5754N 5755N 5756N 5757N 5758N 5759N 5760N 5761N 5762N 5763N 6185N 6236N 6237N 6240G 6242N 6243N 6244N 6245N 6246N 6247N 6248N 6249N 6250N 6251N 6252N 6253N 6254N 6255N 6256N 6257N 6258N 6259N 6260N 6261N 6262N 7028T 7175C 7256T 7274T 7521A [illegible]	L2a1b1a	73G 146C 152C 195C 263G 16182C 16183C 16189C 16223T 16278T 16290T 16294T 16309G 16390A	1	73 146 152 195 263 750 769 1018 1438 1476N 1482N 1484N 1486N 2416 2561N 2578N 2579N 2580N 2581N 2582N 2583N 2584N 2585N 2586N 2587N 2588N 2589N 2590N 2591N 2592N 2593N 2594N 2595N 2596N 2597N 2598N 2604N 2605N 2606N 2607N 2608N 2609N 2610N 2611N 2612N 2613N 2614N 2615N 2616N 2617N 2618N 2619N 2620N 2621N 2622N 2658N 2660N 2662N 2695N 2706 2789 3594 4104 4769 5090 5705N 5711N 5712N 5717N 5718N 5719N 5721N 5722N 5726N 5727N 5728N 5729N 5731N 5732N 5733N 5734N 5735N 5736N 5737N 5738N 5739N 5740N 5741N 5742N 5743N 5744N 5745N 5746N 5747N 5748N 5749N 5750N 5751N 5752N 5753N 5754N 5755N 5756N 5757N 5758N 5759N 5760N 5761N 5762N 5763N 6185N 6236N 6237N 6240G 6242N 6243N 6244N 6245N 6246N 6247N 6248N 6249N 6250N 6251N 6252N 6253N 6254N 6255N 6256N 6257N 6258N 6259N 6260N 6261N 6262N 7028 7175 7256 7274 7521 7771 8206 8701 8842N 8843N [illegible]	L2a1b1a	73 146 152 195 263 16189 16223 16278 16290 16294 16309 16390	[0.000] L2a1b1a (1)	[0.005] L2a1f (2) [0.000] L2a1+16189_(16192) L2a1b L2a1b1 L2a1b1a L2a1b2 L2a1f1 L2a1f1a L2a1f2 (10)	1
1691	>MF057144.1 Hc	73G 150T 200G 263G 489C 750G 2706G 3106d 3107C 3588T 4769G 7028T 7960G 8701G 8768T 8854A 8860G 9540C 10398G 10400T 10873C 11719A 12705T 13308G 13590A 13708A 14130T 14260G 14527G 14766T 14783C 15043A 15172A 15301A 15326G 15625T 16086C 16148T 16223T 16259T 16278T 16319A 16399G 16526A	M32c	73G 150T 200G 263G 489C 16086C 16148T 16223T 16259T 16278T 16319A 16399G 16526A	1	73 150 200 263 489 750 2706 3588 4769 7028 7960 8701 8768 8854 8860 9540 10398 10400 10873 11719 12705 13308 13590 13708 14130 14260 14527 14766 14783 15043 15172 15301 15326 15625 16086 16148 16223 16259 16278 16319 16399 16526	M32c	73 150 200 263 489 16086 16148 16223 16259 16278 16319 16399 16526	[0.000] M32c (1)	[0.022] M51a (2) [0.007] M32 (3) [0.000] M51 M51b (5)	1
1692	>MF055841.1 Hc	73G 151T 152C 195C 204C 263G 310.1C 417A 489C 533G 750G 1438G 3106d 3107C 4769G 7028T 8188G 8360G 8701G 8860G 9438A 9540C 9545G 10142T 10295G 10398G 10400T 10873C 11569C 11719A 11899C 12279G 12618A 12705T 14766T 14783C 15025T 15043A 15301A 15326G 16223T 16263C 16311C 16519C	M23	73G 151T 152C 195C 204C 263G 310.1C 417A 489C 533G 16223T 16263C 16311C 16519C	1	73 151 152 195 204 263 417 489 533 750 1438 4769 7028 8188 8360 8701 8860 9438 9540 9545 10142 10295 10398 10400 10873 11569 11719 11899 12279 12618 12705 14766 14783 15025 15043 15301 15326 16223 16263 16311	M23	73 151 152 195 204 263 417 489 533 16223 16263 16311	[0.016] M23 (1)	[0.072] M24a (2) [0.053] M57+152 (3) [0.013] M37a (4) [0.004] M37+152+151 (5) [0.000] M37a1 M37d (7)	1

1693	>MF055840.1 Hc	73G 152C 195C 204C 263G 310.1C 417A 489C 533G 750G 1438G 3106d 3107C 4769G 7028T 8188G 8360G 8701G 8860G 9438A 9540C 9545G 10142T 10295G 10398G 10400T 10403G 10873C 11569C 11719A 11899C 12279G 12618A 12705T 13105G 14766T 14783C 15025T 15043A 15301A 15326G 15924G 16223T 16263C 16311C 16519C	M23	73G 152C 195C 204C 263G 310.1C 417A 489C 533G 16223T 16263C 16311C 16519C	1	73 152 195 204 263 417 489 533 750 1438 4769 7028 8188 8360 8701 8860 9438 9540 9545 10142 10295 10398 10400 10403 10873 11569 11719 11899 12279 12618 12705 13105 14766 14783 15025 15043 15301 15326 15924 16223 16263 16311	M23	73 152 195 204 263 417 489 533 16223 16263 16311	[0.000] M23 (1)	[0.072] M24a (2) [0.053] M57+152	1
1694	>MF055839.1 Hc	73G 152C 195C 204C 263G 310.1C 417A 489C 533G 750G 1438G 3106d 3107C 4769G 7028T 8188G 8360G 8701G 8860G 9438A 9540C 9545G 10142T 10295G 10398G 10400T 10873C 11569C 11719A 11899C 12279G 12618A 12705T 14766T 14783C 15025T 15043A 15301A 15326G 16223T 16263C 16311C 16519C	M23	73G 152C 195C 204C 263G 310.1C 417A 489C 533G 16223T 16263C 16311C 16519C	1	73 152 195 204 263 417 489 533 750 1438 4769 7028 8188 8360 8701 8860 9438 9540 9545 10142 10295 10398 10400 10873 11569 11719 11899 12279 12618 12705 14766 14783 15025 15043 15301 15326 16223 16263 16311	M23	73 152 195 204 263 417 489 533 16223 16263 16311	[0.000] M23 (1)	[0.072] M24a (2) [0.053] M57+152	1
1695	>MF055838.1 Hc	73G 146C 152C 195C 263G 310.1C 750G 769A 1018A 1438G 2416C 2706G 2762T 2789T 3106d 3107C 3594T 4104G 4769G 5090C 7028T 7175C 7256T 7274T 7521A 7771G 8206A 8701G 8860G 9221G 9540C 10115C 10143A 10398G 10873C 11719A 11914A 11944C 12693G 12705T 13590A 13650T 13803G 14566G 14766T 15301A 15326G 15735T 15784C 16182C 16183C 16189C 16223T 16278T 16290T 16294T 16390A	L2a1b1a	73G 146C 152C 195C 263G 310.1C 16182C 16183C 16189C 16223T 16278T 16290T 16294T 16390A	1	73 146 152 195 263 750 769 1018 1438 2416 2706 2762 2789 3594 4104 4769 5090 7028 7175 7256 7274 7521 7771 8206 8701 8860 9221 9540 10115 10143 10398 10873 11719 11914 11944 12693 12705 13590 13650 13803 14566 14766 15301 15326 15735 15784 16189 16223 16278 16290 16294 16390	L2a1b1a	73 146 152 195 263 16189 16223 16278 16290 16294 16390	[0.045] L2a1b1a (1)	[0.234] L2a1+143+16189_(16192)+@16309 (2) [0.070] L2a1f (3) [0.045] L2a1b1a (4) [0.008] L2a1b2 (5) [0.000] L2a1+16189_(16192) L2a1b L2a1b1 L2a1f1 L2a1f1a L2a1f2 (11)	1
1696	>MF055828.1 Hc	73G 152C 195C 204C 263G 310.1C 417A 489C 533G 750G 1438G 3106d 3107C 4769G 7028T 8188G 8360G 8701G 8860G 9438A 9540C 9545G 10142T 10295G 10398G 10400T 10873C 11569C 11719A 11899C 12279G 12618A 12705T 14766T 14783C 15025T 15043A 15301A 15326G 16037G 16223T 16263C 16311C 16519C	M23	73G 152C 195C 204C 263G 310.1C 417A 489C 533G 16037G 16223T 16263C 16311C 16519C	1	73 152 195 204 263 417 489 533 750 1438 4769 7028 8188 8360 8701 8860 9438 9540 9545 10142 10295 10398 10400 10873 11569 11719 11899 12279 12618 12705 14766 14783 15025 15043 15301 15326 16037 16223 16263 16311	M23	73 152 195 204 263 417 489 533 16037 16223 16263 16311	[0.008] M23 (1)	[0.072] M24a (2) [0.053] M57+152	1

1697 >MF055827.1 Hc	73G 152C 195C 204C 263G 310.1C 417A 489C 533G 750G 1438G 3106d 3107C 4769G 7028T 8188G 8360G 8701G 8860G 9438A 9540C 9545G 10142T 10295G 10398G 10400T 10873C 11569C 11719A 11899C 12279G 12618A 12705T 14766T 14783C 15025T 15043A 15301A 15326G 16223T 16263C 16311C 16519C	M23	73G 152C 195C 204C 263G 310.1C 417A 489C 533G 16223T 16263C 16311C 16519C	1	73 152 195 204 263 417 489 533 750 1438 4769 7028 8188 8360 8701 8860 9438 9540 9545 10142 10295 10398 10400 10873 11569 11719 11899 12279 12618 12705 14766 14783 15025 15043 15301 15326 16223 16263 16311	M23	73 152 195 204 263 417 489 533 16223 16263 16311	[0.000] M23 (1)	[0.072] M24a (2) [0.053] M57+152	1
1698 >MF055822.1 Hc	73G 150T 200G 263G 310.1C 489C 513.1CA 750G 2706G 3106d 3107C 3588T 4769G 7028T 7960G 8701G 8768T 8854A 8860G 9540C 10398G 10400T 10873C 11719A 12705T 13308G 13590A 13708A 14130T 14260G 14527G 14766T 14783C 15043A 15172A 15301A 15326G 15625T 16086C 16148T 16223T 16259T 16278T 16319A 16399G 16526A	M32c	73G 150T 200G 263G 310.1C 489C 513.1CA 16086C 16148T 16223T 16259T 16278T 16319A 16399G 16526A	1	73 150 200 263 489 750 2706 3588 4769 7028 7960 8701 8768 8854 8860 9540 10398 10400 10873 11719 12705 13308 13590 13708 14130 14260 14527 14766 14783 15043 15172 15301 15326 15625 16086 16148 16223 16259 16278 16319 16399 16526	M32c	73 150 200 263 489 16086 16148 16223 16259 16278 16319 16399 16526	[0.000] M32c (1)	[0.022] M51a (2) [0.007] M32 (3) [0.000] M51 M51b (5)	1
1699 >MF055819.1 Hc	73G 152C 195C 204C 263G 310.1C 417A 489C 533G 750G 1438G 3106d 3107C 4769G 7028T 8188G 8360G 8701G 8860G 9438A 9540C 9545G 10142T 10295G 10398G 10400T 10873C 11569C 11719A 11899C 12279G 12618A 12705T 14766T 14783C 15025T 15043A 15301A 15326G 16223T 16263C 16311C 16519C	M23	73G 152C 195C 204C 263G 310.1C 417A 489C 533G 16223T 16263C 16311C 16519C	1	73 152 195 204 263 417 489 533 750 1438 4769 7028 8188 8360 8701 8860 9438 9540 9545 10142 10295 10398 10400 10873 11569 11719 11899 12279 12618 12705 14766 14783 15025 15043 15301 15326 16223 16263 16311	M23	73 152 195 204 263 417 489 533 16223 16263 16311	[0.000] M23 (1)	[0.072] M24a (2) [0.053] M57+152	1
1700 >MF055817.1 Hc	73G 152C 195C 204C 263G 310.1C 417A 489C 533G 750G 1438G 3106d 3107C 4769G 7028T 8188G 8360G 8701G 8860G 9438A 9540C 9545G 10142T 10295G 10398G 10400T 10873C 11569C 11719A 11899C 12279G 12618A 12705T 14766T 14783C 15025T 15043A 15301A 15326G 16223T 16263C 16311C 16519C	M23	73G 152C 195C 204C 263G 310.1C 417A 489C 533G 16223T 16263C 16311C 16519C	1	73 152 195 204 263 417 489 533 750 1438 4769 7028 8188 8360 8701 8860 9438 9540 9545 10142 10295 10398 10400 10873 11569 11719 11899 12279 12618 12705 14766 14783 15025 15043 15301 15326 16223 16263 16311	M23	73 152 195 204 263 417 489 533 16223 16263 16311	[0.000] M23 (1)	[0.072] M24a (2) [0.053] M57+152	1
1701 >MF055814.1 Hc	73G 152C 195C 204C 263G 310.1C 417A 489C 533G 750G 1438G 3106d 3107C 4769G 7028T 8188G 8360G 8701G 8860G 9438A 9540C 9545G 10142T 10295G 10398G 10400T 10873C 11569C 11719A 11899C 12279G 12618A 12705T 14766T 14783C 15025T 15043A 15301A 15326G 16223T 16263C 16311C 16519C	M23	73G 152C 195C 204C 263G 310.1C 417A 489C 533G 16223T 16263C 16311C 16519C	1	73 152 195 204 263 417 489 533 750 1438 4769 7028 8188 8360 8701 8860 9438 9540 9545 10142 10295 10398 10400 10873 11569 11719 11899 12279 12618 12705 14766 14783 15025 15043 15301 15326 16223 16263 16311	M23	73 152 195 204 263 417 489 533 16223 16263 16311	[0.000] M23 (1)	[0.072] M24a (2) [0.053] M57+152	1

1702	>MF055806.1 Ho	73G 152C 195C 204C 263G 310.1C 417A 489C 533G 750G 1438G 3106d 3107C 4769G 7028T 8188G 8360G 8701G 8860G 9438A 9540C 9545G 10142T 10295G 10398G 10400T 10873C 11569C 11719A 11899C 12279G 12618A 12705T 14766T 14783C 15025T 15043A 15301A 15326G 16223T 16263C 16311C 16519C	M23	73G 152C 195C 204C 263G 310.1C 417A 489C 533G 16223T 16263C 16311C 16519C	1	73 152 195 204 263 417 489 533 750 1438 4769 7028 8188 8360 8701 8860 9438 9540 9545 10142 10295 10398 10400 10873 11569 11719 11899 12279 12618 12705 14766 14783 15025 15043 15301 15326 16223 16263 16311	M23	73 152 195 204 263 417 489 533 16223 16263 16311	[0.000] M23 (1)	[0.072] M24a (2) [0.053] M57+152	1
1703	>MF055800.1 Ho	73G 152C 195C 204C 263G 310.1C 417A 489C 533G 750G 1438G 3106d 3107C 4769G 7028T 8188G 8360G 8701G 8860G 9438A 9540C 9545G 10142T 10295G 10398G 10400T 10873C 11569C 11719A 11899C 12279G 12618A 12705T 14766T 14783C 15025T 15043A 15301A 15326G 16223T 16263C 16311C 16519C	M23	73G 152C 195C 204C 263G 310.1C 417A 489C 533G 16223T 16263C 16311C 16519C	1	73 152 195 204 263 417 489 533 750 1438 4769 7028 8188 8360 8701 8860 9438 9540 9545 10142 10295 10398 10400 10873 11569 11719 11899 12279 12618 12705 14766 14783 15025 15043 15301 15326 16223 16263 16311	M23	73 152 195 204 263 417 489 533 16223 16263 16311	[0.000] M23 (1)	[0.072] M24a (2) [0.053] M57+152	1
1704	>MF055798.1 Ho	73G 150T 200G 263G 310.1C 489C 750G 2706G 3106d 3107C 3588T 4769G 7028T 7960G 8701G 8768T 8854A 8860G 9540C 10398G 10400T 10873C 11719A 12705T 13308G 13590A 13708A 14130T 14260G 14527G 14766T 14783C 15043A 15172A 15301A 15326G 15625T 16086C 16148T 16223T 16259T 16278T 16319A 16399G 16526A	M32c	73G 150T 200G 263G 310.1C 489C 16086C 16148T 16223T 16259T 16278T 16319A 16399G 16526A	1	73 150 200 263 489 750 2706 3588 4769 7028 7960 8701 8768 8854 8860 9540 10398 10400 10873 11719 12705 13308 13590 13708 14130 14260 14527 14766 14783 15043 15172 15301 15326 15625 16086 16148 16223 16259 16278 16319 16399 16526	M32c	73 150 200 263 489 16086 16148 16223 16259 16278 16319 16399 16526	[0.000] M32c (1)	[0.022] M51a (2) [0.007] M32 (3) [0.000] M51 M51b (5)	1
1705	>MF055794.1 Ho	73G 152C 195C 204C 263G 310.1C 417A 489C 533G 750G 1438G 3106d 3107C 4769G 7028T 8188G 8360G 8701G 8860G 9438A 9540C 9545G 10142T 10295G 10398G 10400T 10873C 11569C 11719A 11899C 12279G 12618A 12705T 14766T 14783C 15025T 15043A 15301A 15326G 16223T 16263C 16311C 16519C	M23	73G 152C 195C 204C 263G 310.1C 417A 489C 533G 16223T 16263C 16311C 16519C	1	73 152 195 204 263 417 489 533 750 1438 4769 7028 8188 8360 8701 8860 9438 9540 9545 10142 10295 10398 10400 10873 11569 11719 11899 12279 12618 12705 14766 14783 15025 15043 15301 15326 16223 16263 16311	M23	73 152 195 204 263 417 489 533 16223 16263 16311	[0.000] M23 (1)	[0.072] M24a (2) [0.053] M57+152	1
1706	>MF055784.1 Ho	73G 150T 200G 263G 310.1C 489C 750G 2706G 3106d 3107C 3588T 4769G 7028T 7960G 8701G 8768T 8854A 8860G 9540C 10398G 10400T 10873C 11719A 12705T 13308G 13590A 13708A 14130T 14260G 14527G 14766T 14783C 15043A 15172A 15301A 15326G 15625T 16086C 16148T 16223T 16259T 16278T 16319A 16399G 16526A	M32c	73G 150T 200G 263G 310.1C 489C 16086C 16148T 16223T 16259T 16278T 16319A 16399G 16526A	1	73 150 200 263 489 750 2706 3588 4769 7028 7960 8701 8768 8854 8860 9540 10398 10400 10873 11719 12705 13308 13590 13708 14130 14260 14527 14766 14783 15043 15172 15301 15326 15625 16086 16148 16223 16259 16278 16319 16399 16526	M32c	73 150 200 263 489 16086 16148 16223 16259 16278 16319 16399 16526	[0.000] M32c (1)	[0.022] M51a (2) [0.007] M32 (3) [0.000] M51 M51b (5)	1

1707 >MF055783.1 Hc	73G 152C 182T 186A 189C 195C 247A 248G 263G 310.1C 316A 514d 515d 745.1T 750G 769A 825A 1018A 1438G 2394d 2706G 2758A 2885C 3106d 3107C 3525T 3594T 3666A 4104G 4769G 4853A 5951G 6071C 6221A 6227C 6917A 7028T 7146G 7247T 7256T 7389C 7521A 8027A 8468T 8655T 8701G 8860G 9072G 9540C 10398G 10586A 10688A 10810C 10873C 11302T 11719A 11984C 12507G 12705T 12810G 13105G 13485G 13506T 13650T 13789C 14000A 14178C 14560A 14669G 14766T 14911T 14926G 15226G 15326G 15905C 15978T 16129A 16187T 16189C 16223T 16278T 16293G 16294T 16311C 16360T 16519C	L1c3c	73G 152C 182T 186A 189C 195C 247A 248G 263G 310.1C 316A 514d 515d 16129A 16187T 16189C 16223T 16278T 16293G 16294T 16311C 16360T 16519C	1	73 152 182 186A 189C 195 247 248 263 316 745.1T 750 769 825A 1018 1438 2395d 2706 2758 2885 3525 3594 3666 4104 4769 4853 5951 6071 6221A 6227 6917 7028 7146 7247 7256 7389 7521 8027 8468 8655 8701 8860 9072 9540 10398 10586 10688 10810 10873 11302 11719 11984 12507 12705 12810 13105 13485 13506 13650 13789 14000A 14178 14560 14669 14766 14911 14926 15226 15326 15905 15978 16129 16187 16189 16223 16278 16293 16294 16311 16360	L1c3c	73 152 182 186A 189C 195 247 248 263 316 16129 16187 16189 16223 16278 16293 16294 16311 16360	[2.063] L1c3c (1) [0.000] L1c L1c1a+@198 L1c3b'c (4)	[0.700] L1c3 (5) [0.572] L1c1a'b'd (6) [0.267] L1c3b1 (7) [0.133] L1c3b1b (8) [0.000] L1c1'2'4'5'6 L1c1 L1c1a L1c1a1 L1c3b (13)	1
1708 >MF055780.1 Hc	73G 263G 279C 310.1C 514d 515d 750G 1438G 2332T 2706G 3106d 3107C 3450T 4769G 5773A 6221C 6340T 7028T 8701G 8860G 9449T 9540C 10086G 10373A 10398G 10873C 11002G 11719A 12705T 13105G 13914A 14766T 15301A 15311G 15326G 15824G 15940d 16145A 16223T 16278T 16362C 16519C	L3b1a8	73G 263G 279C 310.1C 514d 515d 16145A 16223T 16278T 16362C 16519C	1	73 263 279 750 1438 2332 2706 3450 4769 5773 6221 6340 7028 8701 8860 9449 9540 10086 10373 10398 10873 11002 11719 12705 13105 13914A 14766 15301 15311 15326 15824 15944d 16145 16223 16278 16362	L3b1a8	73 263 279 16145 16223 16278 16362	[0.213] L3b1a8 (1)	[0.000] L3b1a+@16124 L3b1a7 L3b1a7a (4)	1
1709 >MF055776.1 Hc	73G 150T 152C 200G 263G 310.1C 489C 750G 2706G 3106d 3107C 3588T 4769G 7028T 7960G 8158G 8701G 8768T 8854A 8860G 9540C 10398G 10400T 10873C 11719A 12705T 13308G 13590A 13708A 14130T 14260G 14527G 14766T 14783C 15043A 15172A 15301A 15326G 15625T 16086C 16148T 16223T 16259T 16278T 16319A 16399G 16526A	M32c	73G 150T 152C 200G 263G 310.1C 489C 16086C 16148T 16223T 16259T 16278T 16319A 16399G 16526A	1	73 150 152 200 263 489 750 2706 3588 4769 7028 7960 8158 8701 8768 8854 8860 9540 10398 10400 10873 11719 12705 13308 13590 13708 14130 14260 14527 14766 14783 15043 15172 15301 15326 15625 16086 16148 16223 16259 16278 16319 16399 16526	M32c	73 150 152 200 263 489 16086 16148 16223 16259 16278 16319 16399 16526	[0.017] M32c (1)	[0.065] M51a (2) [0.014] M51b (3) [0.007] M32 (4) [0.000] M51	1
1710 >MF055769.1 Hc	73G 152C 195C 204C 263G 310.1C 417A 489C 533G 750G 1438G 3106d 3107C 4769G 7028T 8188G 8360G 8701G 8860G 9438A 9540C 9545G 10142T 10295G 10398G 10400T 10873C 11569C 11719A 11899C 12279G 12618A 12705T 14766T 14783C 15025T 15043A 15301A 15326G 16223T 16263C 16311C 16519C	M23	73G 152C 195C 204C 263G 310.1C 417A 489C 533G 16223T 16263C 16311C 16519C	1	73 152 195 204 263 417 489 533 750 1438 4769 7028 8188 8360 8701 8860 9438 9540 9545 10142 10295 10398 10400 10873 11569 11719 11899 12279 12618 12705 14766 14783 15025 15043 15301 15326 16223 16263 16311	M23	73 152 195 204 263 417 489 533 16223 16263 16311	[0.000] M23 (1)	[0.072] M24a (2) [0.053] M57+152	1

1711 >MF055768.1 Hc	73G 152C 195C 204C 263G 310.1C 417A 489C 533G 750G 1438G 3106d 3107C 4769G 7028T 8188G 8360G 8701G 8860G 9438A 9540C 9545G 10142T 10295G 10398G 10400T 10873C 11569C 11719A 11899C 12279G 12618A 12705T 14766T 14783C 15025T 15043A 15301A 15326G 16223T 16263C 16311C 16519C	M23	73G 152C 195C 204C 263G 310.1C 417A 489C 533G 16223T 16263C 16311C 16519C	1	73 152 195 204 263 417 489 533 750 1438 4769 7028 8188 8360 8701 8860 9438 9540 9545 10142 10295 10398 10400 10873 11569 11719 11899 12279 12618 12705 14766 14783 15025 15043 15301 15326 16223 16263 16311	M23	73 152 195 204 263 417 489 533 16223 16263 16311	[0.000] M23 (1)	[0.072] M24a (2) [0.053] M57+152	1
1712 >MF055756.1 Hc	73G 146C 152C 195C 247A 310.1C 456T 494d 593C 719A 750G 769A 825A 1018A 1048T 2000T 2758A 2885C 3083C 3106d 3107C 3438A 3516A 3594T 3756G 4104G 4197T 4232C 4312T 4769G 5263T 5442C 5460A 6185C 6266G 6815C 7028T 7146G 7256T 7521A 8113A 8152A 8251A 8468T 8655T 8701G 8860G 9042T 9150G 9347G 9540C 9755A 10373A 10398G 10589A 10664T 10688A 10810C 10873C 10915C 11150A 11437C 11719A 11872T 11914A 12007A 12121C 12235C 12705T 12720G 13105G 13129T 13276G 13506T 13650T 13759A 14384A 14766T 15187G 15326G 15466A 15930A 15941C 15951G 16184T 16187T 16189C 16223T 16230G 16234T 16243C 16311C 16519C	L0d1c	73G 146C 152C 195C 247A 310.1C 456T 494d 16184T 16187T 16189C 16223T 16230G 16234T 16243C 16311C 16519C	1	73 146 152 195 247 456 498d 593 719 750 769 825A 1018 1048 2000 2758 2885 3083 3438 3516A 3594 3756 4104 4197 4232 4312 4769 5263 5442 5460 6185 6266 6815 7028 7146 7256 7521 8113A 8152 8251 8468 8655 8701 8860 9042 9150 9347 9540 9755 10373 10398 10589 10664 10688 10810 10873 10915 11150 11437 11719 11872 11914 12007 12121 12235 12705 12720 13105 13129 13276 13506 13650 13759 14384 14766 15187 15326 15466 15930 15941 15951 16184 16187 16189 16223 16230 16234 16243 16311	L0d1c	73 146 152 195 247 456 498d 16184 16187 16189 16223 16230 16234 16243 16311	[0.037] L0d1c (1)	[0.000] L0d1c1 L0d1c1a L0d1c2 (4)	1
1713 >MF055751.1 Hc	73G 152C 195C 204C 263G 310.1C 417A 489C 533G 750G 1438G 3106d 3107C 4769G 7028T 8188G 8360G 8701G 8860G 9438A 9540C 9545G 10142T 10295G 10398G 10400T 10873C 11569C 11719A 11899C 12279G 12618A 12705T 14766T 14783C 15025T 15043A 15301A 15326G 16223T 16263C 16311C 16519C	M23	73G 152C 195C 204C 263G 310.1C 417A 489C 533G 16223T 16263C 16311C 16519C	1	73 152 195 204 263 417 489 533 750 1438 4769 7028 8188 8360 8701 8860 9438 9540 9545 10142 10295 10398 10400 10873 11569 11719 11899 12279 12618 12705 14766 14783 15025 15043 15301 15326 16223 16263 16311	M23	73 152 195 204 263 417 489 533 16223 16263 16311	[0.000] M23 (1)	[0.072] M24a (2) [0.053] M57+152	1
1714 >MF317866.1 Hc	200G 263G 310C 750G 1438G 2706G 3106d 3107C 4769G 5746A 6267A 7028T 8821C 8860G 15326G 16234T 16311C	HV15	200G 263G 310C 16234T 16311C	1	200 263 310 750 1438 2706 4769 5746 6267 7028 8821 8860 15326 16234 16311	HV15	200 263 310 16234 16311	[0.103] HV15 (1)	[0.023] HV+16311 (2) [0.012] HV9 (3) [0.006] HV10 (4) [0.003] HV11a HV16 H3+16311 H3h H3h2 (9) [0.002] H1+16311 (10) [0.000] HV11 HV23 HV24 H1e1a4 H3h1 H3h3 H3h3a H3h3b H3h5 H3h6 H3m H13a1a2+16311 H13a2b1 H76 H80 (25)	1

1715 >KY923862.1 Hc	73G 150T 263G 309.1CT 310C 750G 1438G 2706G 3106d 3107C 3197C 4769G 5656G 7028T 7385G 7768G 8413G 8860G 9477A 10927C 11467G 11719A 12308G 12372A 12618A 13617C 14182C 14766T 15326G 16189C 16192T 16270T 16320T	U5b1b1b	73G 150T 263G 309.1CT 310C 16189C 16192T 16270T 16320T	1	73 150 263 750 1438 2706 3197 4769 5656 7028 7385 7768 8413 8860 9477 10927 11467 11719 12308 12372 12618 13617 14182 14766 15326 16189 16192 16270 16320	U5b1b1b	73 150 263 16189 16192 16270 16320	[0.000] U5b1b1b (1)	[0.000] U5b1+16189 U5b1b U5b1b1 U5b1b1g U5b2a U5b2a3 U5b2a3a (8)	1
1716 >KY923861.1 Hc	73G 150T 263G 309.1CT 310C 750G 1438G 2706G 3106d 3107C 3197C 4769G 5656G 7028T 7385G 7768G 8413G 8860G 9477A 10927C 11467G 11719A 12308G 12372A 12618A 13617C 14182C 14766T 15326G 16189C 16192T 16270T 16320T	U5b1b1b	73G 150T 263G 309.1CT 310C 16189C 16192T 16270T 16320T	1	73 150 263 750 1438 2706 3197 4769 5656 7028 7385 7768 8413 8860 9477 10927 11467 11719 12308 12372 12618 13617 14182 14766 15326 16189 16192 16270 16320	U5b1b1b	73 150 263 16189 16192 16270 16320	[0.000] U5b1b1b (1)	[0.000] U5b1+16189 U5b1b U5b1b1 U5b1b1g U5b2a U5b2a3 U5b2a3a (8)	1
1717 >KY923860.1 Hc	73G 150T 263G 309.1CT 310C 750G 1438G 2706G 3106d 3107C 3197C 3357A 4769G 5656G 7028T 7385G 7768G 8413G 8860G 9477A 10927C 11467G 11719A 12308G 12372A 12618A 13617C 14182C 14766T 15326G 16189C 16192T 16270T 16320T	U5b1b1b	73G 150T 263G 309.1CT 310C 16189C 16192T 16270T 16320T	1	73 150 263 750 1438 2706 3197 3357 4769 5656 7028 7385 7768 8413 8860 9477 10927 11467 11719 12308 12372 12618 13617 14182 14766 15326 16189 16192 16270 16320	U5b1b1b	73 150 263 16189 16192 16270 16320	[0.000] U5b1b1b (1)	[0.000] U5b1+16189 U5b1b U5b1b1 U5b1b1g U5b2a U5b2a3 U5b2a3a (8)	1
1718 >KY923859.1 Hc	73G 150T 263G 309.1CT 310C 750G 1438G 2706G 3106d 3107C 3197C 4769G 5656G 7028T 7385G 7768G 8413G 8860G 9477A 10927C 11467G 11719A 12308G 12372A 12618A 13617C 14182C 14766T 15326G 16189C 16192T 16270T 16320T	U5b1b1b	73G 150T 263G 309.1CT 310C 16189C 16192T 16270T 16320T	1	73 150 263 750 1438 2706 3197 4769 5656 7028 7385 7768 8413 8860 9477 10927 11467 11719 12308 12372 12618 13617 14182 14766 15326 16189 16192 16270 16320	U5b1b1b	73 150 263 16189 16192 16270 16320	[0.000] U5b1b1b (1)	[0.000] U5b1+16189 U5b1b U5b1b1 U5b1b1g U5b2a U5b2a3 U5b2a3a (8)	1
1719 >KY923858.1 Hc	73G 150T 195C 263G 309.1CT 310C 750G 1438G 2706G 3106d 3107C 3197C 4769G 5656G 7028T 7385G 7768G 8413G 8860G 9477A 10927C 11467G 11719A 12308G 12372A 12618A 13617C 14182C 14766T 15326G 16189C 16192T 16270T 16320T	U5b1b1b	73G 150T 195C 263G 309.1CT 310C 16189C 16192T 16270T 16320T	1	73 150 195 263 750 1438 2706 3197 4769 5656 7028 7385 7768 8413 8860 9477 10927 11467 11719 12308 12372 12618 13617 14182 14766 15326 16189 16192 16270 16320	U5b1b1b	73 150 195 263 16189 16192 16270 16320	[0.026] U5b1b1b (1)	[0.029] U5b1+16189 (2) [0.000] U5b1b U5b1b1 U5b1b1g U5b2a U5b2a3 U5b2a3a (8)	1
1720 >KY923857.1 Hc	73G 150T 263G 309.1CT 310C 750G 1438G 2706G 3106d 3107C 3197C 4769G 5656G 7028T 7385G 7768G 8413G 8860G 9477A 10927C 11467G 11719A 12308G 12372A 12618A 13617C 14182C 14766T 15326G 16189C 16192T 16270T 16320T	U5b1b1b	73G 150T 263G 309.1CT 310C 16189C 16192T 16270T 16320T	1	73 150 263 750 1438 2706 3197 4769 5656 7028 7385 7768 8413 8860 9477 10927 11467 11719 12308 12372 12618 13617 14182 14766 15326 16189 16192 16270 16320	U5b1b1b	73 150 263 16189 16192 16270 16320	[0.000] U5b1b1b (1)	[0.000] U5b1+16189 U5b1b U5b1b1 U5b1b1g U5b2a U5b2a3 U5b2a3a (8)	1

1721	>KY923856.1 Hc	73G 150T 263G 309.1CT 310C 750G 1438G 2706G 3106d 3107C 3197C 4769G 5656G 7028T 7385G 7768G 8413G 8860G 9477A 10927C 11467G 11719A 12308G 12372A 12618A 13617C 14182C 14766T 15326G 16189C 16192T 16270T 16320T	U5b1b1b	73G 150T 263G 309.1CT 310C 16189C 16192T 16270T 16320T	1	73 150 263 750 1438 2706 3197 4769 5656 7028 7385 7768 8413 8860 9477 10927 11467 11719 12308 12372 12618 13617 14182 14766 15326 16189 16192 16270 16320	U5b1b1b	73 150 263 16189 16192 16270 16320	[0.000] U5b1b1b (1)	[0.000] U5b1+16189 U5b1b U5b1b1 U5b1b1g U5b2a U5b2a3 U5b2a3a (8)	1
1722	>KY923855.1 Hc	73G 150T 263G 309.1CT 310C 750G 1438G 2706G 3106d 3107C 3197C 4769G 5656G 7028T 7385G 7768G 8413G 8860G 9477A 10927C 11467G 11719A 12308G 12372A 12618A 13617C 14182C 14766T 15326G 16189C 16192T 16270T 16320T	U5b1b1b	73G 150T 263G 309.1CT 310C 16189C 16192T 16270T 16320T	1	73 150 263 750 1438 2706 3197 4769 5656 7028 7385 7768 8413 8860 9477 10927 11467 11719 12308 12372 12618 13617 14182 14766 15326 16189 16192 16270 16320	U5b1b1b	73 150 263 16189 16192 16270 16320	[0.000] U5b1b1b (1)	[0.000] U5b1+16189 U5b1b U5b1b1 U5b1b1g U5b2a U5b2a3 U5b2a3a (8)	1
1723	>KY923854.1 Hc	73G 150T 263G 310.1C 750G 1438G 2706G 3106d 3107C 3197C 4769G 5656G 7028T 7385G 7768G 8413G 8860G 9477A 9591A 10927C 11467G 11719A 12308G 12372A 12618A 13617C 14182C 14766T 15326G 16189C 16192.1T 16270T 16320T	U5b1b1b	73G 150T 263G 310.1C 16189C 16192.1T 16270T 16320T	1	73 150 263 750 1438 2706 3197 4769 5656 7028 7385 7768 8413 8860 9477 9591 10927 11467 11719 12308 12372 12618 13617 14182 14766 15326 16189 16193 16270 16320	U5b1b1b	73 150 263 16189 16193 16270 16320	[0.052] U5b1b1b (1) [0.000] U5b1b1+@16192 U5b1b1d U5b1b1f U5b1+16189+@16192 U5b2a+@16192 U5b2a4 U5b2a4a U5b2a6 U5b2b4 (10)	[0.083] U5b2b4a (11) [0.046] U5b2b (12) [0.033] U5b2a5a (13) [0.029] U5b1h (14) [0.014] U5b1b1a1a U5b1d1a U5b2b2 (17) [0.012] U5b2b1 (18) [0.000] B6 U5b1+16189 U5b1b U5b1b1 U5b1b1a U5b1b1a1 U5b1b1a1b U5b1b1a2 U5b1b1g U5b2a U5b2a3 U5b2a3a U5b2a5 (31)	1
1724	>KY923852.1 Hc	73G 150T 263G 309.1CT 310C 750G 1438G 2706G 3106d 3107C 3197C 4769G 5656G 7028T 7385G 7768G 8413G 8860G 9477A 10927C 11467G 11719A 12308G 12372A 12618A 13617C 14182C 14766T 15326G 16189C 16192T 16270T 16320T	U5b1b1b	73G 150T 263G 309.1CT 310C 16189C 16192T 16270T 16320T	1	73 150 263 750 1438 2706 3197 4769 5656 7028 7385 7768 8413 8860 9477 10927 11467 11719 12308 12372 12618 13617 14182 14766 15326 16189 16192 16270 16320	U5b1b1b	73 150 263 16189 16192 16270 16320	[0.000] U5b1b1b (1)	[0.000] U5b1+16189 U5b1b U5b1b1 U5b1b1g U5b2a U5b2a3 U5b2a3a (8)	1
1725	>KY798201.1 Hc	73G 103A 189G 199C 203A 204C 263G 514d 515d 709A 750G 1438G 1598A 2626C 2706G 3106d 3107C 3915A 4769G 6962A 7028T 8271d 8272d 8273d 8274d 8275d 8276d 8277d 8278d 8279d 8584A 8829T 8856A 8860G 9950C 10103G 10398G 11719A 12361G 13477A 14766T 15223T 15326G 15508T 15662G 15851G 15927A 16140C 16147T 16189C 16243C 16256T 16519C	B5b3a	73G 103A 189G 199C 203A 204C 263G 514d 515d 16140C 16147T 16189C 16243C 16256T 16519C	1	73 103 189 199 203 204 263 709 750 1438 1598 2626 2706 3915 4769 6962 7028 8281-8289d 8584 8829 8856 8860 9950 10103 10398 11719 12361 13477 14766 15223 15326 15508 15662 15851 15927 16140 16147 16189 16243 16256	B5b3a	73 103 189 199 203 204 263 16140 16147 16189 16243 16256	[0.143] B5b3a (1)	[0.006] B5b3b (2) [0.000] B5b3	1

1726 >KY798195.1 Hc	73G 150T 263G 310.1C 709A 750G 1119C 1438G 2706G 3106d 3107C 3394C 3435T 3497T 3571T 4769G 7028T 7119A 8271d 8272d 8273d 8274d 8275d 8276d 8277d 8278d 8279d 8860G 9128C 10728A 11440A 11719A 14766T 15326G 15346A 16129A 16140C 16166G 16180d 16189C 16217C 16274A 16335G 16519C	B4c1b2c2	73G 150T 263G 310.1C 16129A 16140C 16166G 16180d 16189C 16217C 16274A 16335G 16519C	1	73 150 263 709 750 1119 1438 2706 3394 3435 3497 3571 4769 7028 7119 8281-8289d 8860 9128 10728A 11440 11719 14766 15326 15346 16129 16140 16166 16189 16193d 16217 16274 16335	B4c1b2c2	73 150 263 16129 16140 16166 16189 16193d 16217 16274 16335	[0.011] B4c1b2c2 (1)	[0.007] B4c1b+16335 (2) [0.000] B4c1b2 B4c1b2c (4)	1
1727 >MF441747.1 Hc	152C 263G 309.1CT 310C 750G 1438G 2259T 3106d 3107C 4745G 4769G 8860G 14858R 14872T 15326G 16234T	H13a1d	152C 263G 309.1CT 310C 16234T	1	152 263 750 1438 2259 4745 4769 8860 14858R 14872 15326 16234	H13a1d	152 263 16234	[0.000] H13a1d (1)	[0.000] H1+152 H1i H1i1 H1i2 H1i2a H1an H1an1 H1an1a H1bb H1bc H2a2a2 H3+152 H3g H3g1 H3g1a H3g1b H3g2 H3g3 H3g4 H3i H3i1 H3j H3k H3k1 H3k1a H+152 H9 H46 H46a H46b H52 H69 H13a1+152 H14b H14b2 H14b4 H16+152 H16a H16a1 H16c H16d H30b1 H47a (44)	1
1728 >MF397935.1 Hc	146C 195C 263G 310.1C 750G 961G 1438G 3106d 3107C 4769G 8448C 8860G 8898T 13759A 15326G 16278T 16293G 16311C	H11a1	146C 195C 263G 310.1C 16278T 16293G 16311C	1	146 195 263 750 961G 1438 4769 8448 8860 8898 13759 15326 16278 16293 16311	H11a1	146 195 263 16278 16293 16311	[0.155] H11a1 (1)	[0.241] H11a2 (2) [0.027] H11a (3) [0.000] H11a5 H11a8 (5)	1
1729 >MF399064.1 Hc	73G 146C 263G 310.1C 497T 750G 1189C 1438G 1811G 2706G 3106d 3107C 3480G 4295G 4769G 6260A 7028T 8860G 9055A 9698C 10398G 10550G 11299C 11467G 11485C 11719A 11840T 12308G 12372A 13401C 13740C 14167T 14766T 14798C 15326G 15884A 16224C 16245T 16311C 16519C	K1a4a1a2b	73G 146C 263G 310.1C 497T 16224C 16245T 16311C 16519C	1	73 146 263 497 750 1189 1438 1811 2706 3480 4295 4769 6260 7028 8860 9055 9698 10398 10550 11299 11467 11485 11719 11840 12308 12372 13401 13740 14167 14766 14798 15326 15884 16224 16245 16311	K1a4a1a2b	73 146 263 497 16224 16245 16311	[0.000] K1a4a1a2b (1)	[0.038] K1a4a1a2 (2) [0.028] K1a4a1a2a (3) [0.000] K1a4a1a2b K1a4+146 K1a4j (6)	1
1730 >MF356230.1 Hc	73G 146C 263G 310.1C 497T 750G 1189C 1438G 1811G 2706G 3106d 3107C 3480G 4295G 4769G 6260A 7028T 8860G 9055A 9698C 10398G 10550G 11299C 11467G 11485C 11719A 11840T 12308G 12372A 13401C 13740C 14167T 14766T 14798C 15326G 15884A 16224C 16245T 16311C 16519C	K1a4a1a2b	73G 146C 263G 310.1C 497T 16224C 16245T 16311C 16519C	1	73 146 263 497 750 1189 1438 1811 2706 3480 4295 4769 6260 7028 8860 9055 9698 10398 10550 11299 11467 11485 11719 11840 12308 12372 13401 13740 14167 14766 14798 15326 15884 16224 16245 16311	K1a4a1a2b	73 146 263 497 16224 16245 16311	[0.000] K1a4a1a2b (1)	[0.038] K1a4a1a2 (2) [0.028] K1a4a1a2a (3) [0.000] K1a4a1a2b K1a4+146 K1a4j (6)	1
1731 >KX467319.1 Hc	73G 152C 263G 310C 316S 334C 489C 709A 750G 1438G 1888A 2706G 3106M 3107C 3921T 4769G 4916G 5988T 7028T 7939T 8701G 8860G 9064A 9540C 10398G 10400T 10873C 11016A 11719A 12477C 12705T 14323A 14766T 14783C 15043A 15287C 15301A 15326G 16129A 16223T 16291T 16519C	M5a1a	73G 152C 263G 310C 316S 334C 489C 16129A 16223T 16291T 16519C	1	73 152 263 310 316S 334 489 709 750 1438 1888 2706 3106M 3921 4769 4916 5988 7028 7939 8701 8860 9064 9540 10398 10400 10873 11016 11719 12477 12705 14323 14766 14783 15043 15287 15301 15326 16129 16223 16291	M5a1a	73 152 263 310 316S 334 489 16129 16223 16291	[0.106] M5a1a (1)	[0.106] M5a1a (2) [0.046] M5a1b (3) [0.000] M5a1	1

1732	>KX467307.1 Hc	73G 146C 263G 310C 461T 489C 750G 1438G 1508T 2706G 3106M 3107C 3486T 3537G 4769G 5082C 5301G 5319G 5558G 5585A 7028T 8110C 8701G 8860G 9329A 9540C 10398G 10400T 10640C 10873C 11314G 11719A 12705T 13966G 14128G 14766T 14783C 15043A 15094T 15301A 15326G 16188T 16223T 16231C 16362C 16519C	M6a1b	73G 146C 263G 310C 461T 489C 16188T 16223T 16231C 16362C 16519C	1	73 146 263 310 461 489 750 1438 1508 2706 3106M 3486 3537 4769 5082 5301 5319 5558 5585 7028 8110 8701 8860 9329 9540 10398 10400 10640 10873 11314 11719 12705 13966 14128 14766 14783 15043 15094 15301 15326 16188 16223 16231 16362	M6a1b	73 146 263 310 461 489 16188 16223 16231 16362	[0.178] M6a1b (1)	[0.000] M6a M6a1 (3)	1
1733	>KX467306.1 Hc	73G 182T 263G 310C 489C 750G 1438G 2706G 3106M 3107C 3834A 4769G 5154A 6620C 7028T 7859A 8392A 8701G 8860G 9196A 9540C 10398G 10400T 10873C 11719A 12007A 12705T 13437C 13623T 14766T 14783C 15043A 15301A 15326G 16129A 16145A 16223T 16261T 16311C 16354A 16519C	M4	73G 182T 263G 310C 489C 16129A 16145A 16223T 16261T 16311C 16354A 16519C	1	73 182 263 310 489 750 1438 2706 3106M 3834 4769 5154A 6620 7028 7859 8392 8701 8860 9196 9540 10398 10400 10873 11719 12007 12705 13437 13623 14766 14783 15043 15301 15326 16129 16145 16223 16261 16311 16354A	M4	73 182 263 310 489 16129 16145 16223 16261 16311 16354A	[0.024] M4 (1)	[0.033] M4a (2)	1
1734	>KX467301.1 Hc	73G 152C 195A 263G 310Y 316S 489C 515R 750G 1438G 2706G 3106M 3107C 4586C 4769G 5147A 7028T 8650G 8701G 8860G 9540C 10398G 10400T 10873C 11719A 11969A 12007A 12705T 13980A 14766T 14783C 15043A 15301A 15326G 15431A 16192T 16223T 16239T 16278T 16519C	M30b	73G 152C 195A 263G 310Y 316S 489C 515R 16192T 16223T 16239T 16278T 16519C	1	73 152 195A 263 310Y 316S 489 515R 750 1438 2706 3106M 4586 4769 5147 7028 8650G 8701 8860 9540 10398 10400 10873 11719 11969 12007 12705 13980 14766 14783 15043 15301 15326 15431 16192 16223 16239 16278	M30b	73 152 195A 263 310Y 316S 489 515R 16192 16223 16239 16278	[0.245] M30b (1)	[0.042] M30 (2) [0.029] G2a+152 (3) [0.016] M73'79 (4) [0.012] G2a2 (5) [0.008] M37+152 (6) [0.005] M73 (7) [0.004] M30d1 (8) [0.000] M30d M30d2 M30e M13'46'61 M13 M13a'b (14)	1
1735	>KX467300.1 Hc	73G 152C 195A 263G 310Y 316S 489C 515R 750G 1438G 2706G 3106M 3107C 4231G 4769G 7028T 7085C 8701G 8860G 9540C 10398G 10400T 10873C 11719A 12007A 12705T 14766T 14783C 15043A 15301A 15326G 15431A 16172C 16223T 16519C	M30	73G 152C 195A 263G 310Y 316S 489C 515R 16172C 16223T 16519C	1	73 152 195A 263 310Y 316S 489 515R 750 1438 2706 3106M 4231 4769 7028 7085 8701 8860 9540 10398 10400 10873 11719 12007 12705 14766 14783 15043 15301 15326 15431 16172 16223	M30	73 152 195A 263 310Y 316S 489 515R 16172 16223	[0.046] M30 (1) [0.004] M30d1 (2) [0.000] M30d M30d2 M30e M37+152 M13'46'61 M13 M13a'b M33a2'3 (10)	[0.171] M30b (11) [0.066] M30+16234 (12) [0.051] M (13) [0.029] M31b1 (14) [0.010] M43b (15) [0.009] M35 M1'20'51 (17) [0.008] M33a3 (18) [0.007] M30a1 M23'75 (20) [0.006] G1b M21b M33a2 (23) [0.004] M30c M30d1 M37a M13'46'61+16362 (27) [0.003] M32'56 M58 M69 (30) [0.000] L3c'd M4"67 M30a M30a2 M30d M30d2 M30f M30g M37 M37+152+151 M37a1 M37d M43 M7 M7b'c M7b E1a2a4 M12'G M19'53 M21 M21b1 M21b1a M22 M22b M24'41 M27 M29'Q M31 M31a M31b M31b2 M33 M33a M33a1 M34'57 M57+152 M39'70 M42'74 M42 M52 M52b M55'77 M60 M80'D S+152 (75)	1

1736	>KX467299.1 Hc	73G 195A 263G 310Y 316S 489C 515R 750G 1438G 2706G 3106M 3107C 3308C 4769G 7028T 8701G 8860G 9540C 10398G 10400T 10873C 11719A 12007A 12705T 14766T 14783C 15043A 15301A 15326G 15431A 16223T 16465T 16519C	M30	73G 195A 263G 310Y 316S 489C 515R 16223T 16465T 16519C	1	73 195A 263 310Y 316S 489 515R 750 1438 2706 3106M 3308 4769 7028 8701 8860 9540 10398 10400 10873 11719 12007 12705 14766 14783 15043 15301 15326 15431 16223 16465	M30	73 195A 263 310Y 316S 489 515R 16223 16465	[0.032] M30 (1) [0.004] M30d1 (2) [0.000] M30d M30d2 (4)	[0.040] M30+16234 (5) [0.010] M43b (6) [0.004] M30d1 (7) [0.003] M M21b M21b1a M35 (11) [0.000] M1'20'51 M4"67 M30a M30a1 M30a2 M30c M30d M30d2 M30f M30g M37 M43 M7 M7b'c M7b M12'G G1b M19'53 M21 M21b1 M22 M22b M23'75 M24'41 M27 M29'Q M31 M31a M32'56 M33 M33a M33a1 M34'57 M39'70 M42'74 M42 M52 M52b M55'77 M58 M60 M69 M80'D (54)	1
1737	>KX467298.1 Hc	73G 195A 263G 310C 489C 750G 1438G 2706G 3106M 3107C 4769G 7028T 7436G 8301G 8701G 8860G 9540C 10398G 10400T 10873C 11341G 11719A 12007A 12705T 14766T 14783C 15043A 15301A 15326G 15431A 16223T 16519C	M30	73G 195A 263G 310C 489C 16223T 16519C	1	73 195A 263 310 489 750 1438 2706 3106M 4769 7028 7436 8301 8701 8860 9540 10398 10400 10873 11341 11719 12007 12705 14766 14783 15043 15301 15326 15431 16223	M30	73 195A 263 310 489 16223	[0.014] M30 (1) [0.000] M30d M30d1 M30d2 (4)	[0.015] M30+16234 (5) [0.003] M (6) [0.000] M1'20'51 M4"67 M30a M30a1 M30a2 M30c M30d M30d1 M30d2 M30f M30g M37 M43 M43b M7 M7b'c M7b M12'G G1b M19'53 M21 M21b M21b1 M21b1a M22 M22b M23'75 M24'41 M27 M29'Q M31 M31a M32'56 M33 M33a M33a1 M34'57 M35 M39'70 M42'74 M42 M52 M52b M55'77 M58 M60 M69 M80'D (54)	1
1738	>KX467297.1 Hc	73G 195A 263G 310C 316S 489C 515R 750G 1438G 2706G 3106M 3107C 4769G 7028T 7966Y 8701G 8860G 9540C 10398G 10400T 10873C 11437C 11719A 12007A 12705T 14766T 14783C 15043A 15301A 15326G 15431A 16223T 16234T 16243C 16274A 16519C	M30+16234	73G 195A 263G 310C 316S 489C 515R 16223T 16234T 16243C 16274A 16519C	1	73 195A 263 310 316S 489 515R 750 1438 2706 3106M 4769 7028 7966Y 8701 8860 9540 10398 10400 10873 11437 11719 12007 12705 14766 14783 15043 15301 15326 15431 16223 16234 16243 16274	M30+16234	73 195A 263 310 316S 489 515R 16223 16234 16243 16274	[0.102] M30+16234 (1) [0.000] M49d (2)	[0.032] M30 (3) [0.028] M30e (4) [0.005] M49c1 (5) [0.004] M30d1 (6) [0.000] M30d M30d2 M49 M49c M49e M49e1 (12)	1
1739	>KX467296.1 Hc	73G 263G 310C 482C 489C 515R 750G 1438G 2706G 3106M 3107C 4601G 4769G 6467A 7028T 7598A 8701G 8860G 9064A 9540C 9615C 10398G 10400T 10873C 11719A 12705T 14766T 14783C 15043A 15301A 15326G 16126C 16154C 16223T 16519C	M3c2	73G 263G 310C 482C 489C 515R 16126C 16154C 16223T 16519C	1	73 263 310 482 489 515R 750 1438 2706 3106M 4601 4769 6467A 7028 7598 8701 8860 9064 9540 9615 10398 10400 10873 11719 12705 14766 14783 15043 15301 15326 16126 16154 16223	M3c2	73 263 310 482 489 515R 16126 16154 16223	[0.012] M3c2 (1)	[0.004] M3a2 (2) [0.000] M3 M3a M3a1 M3b M3c (7)	1

1740 >KX467295.1 Hc	73G 195A 204C 263G 310Y 316S 489C 750G 1438G 2706G 3106M 3107C 4216C 4769G 7028T 8701G 8860G 9540C 10398G 10400T 10873C 11719A 12007A 12705T 14766T 14783C 15043A 15301A 15326G 15431A 16188Y 16223T 16234T 16362C 16519C	M30+16234	73G 195A 204C 263G 310Y 316S 489C 16188Y 16223T 16234T 16362C 16519C	1	73 195A 204 263 310Y 316S 489 750 1438 2706 3106M 4216 4769 7028 8701 8860 9540 10398 10400 10873 11719 12007 12705 14766 14783 15043 15301 15326 15431 16188Y 16223 16234 16362	M30+16234	73 195A 204 263 310Y 316S 489 16188Y 16223 16234 16362	[0.164] M30+16234 (1) [0.000] M30g (2)	[0.054] M9a1a1c1b1a1 (3) [0.025] M30 (4) [0.018] M9a D4e1 (6) [0.016] D4e4a (7) [0.014] M30e M49c1 (9) [0.012] D4b2b (10) [0.009] M9a1a1c1b1a (11) [0.008] D4b2b2 (12) [0.006] D4j5a (13) [0.005] M9a1 M9a4 M9a4b (16) [0.004] M30d1 D4 (18) [0.003] D4b2b1 D4b2b1b D4b2b1c D4b2b6 (22) [0.002] G G1 G2 G2b G2b1 G2b2 G2b2a D4b2b1a D4b2b3 D4e3 D4e4 D4j1 D4j4 D4j10 (36) [0.001] M9a1a1a M9a1a1c1b1 D4e1c (39) [0.000] M30d M30d2 M9 G1c G1c2 G2a'c G2b1a M33+16362 M49 M49c M49e M49e1 D D4b D4b2 D4b2a D4b2a2 D4b2a2b D4e D4e1'3 D2c D4e2 D4e2b D4e2c D4e2d D4e4b D4f D4f1 D4g D4h D4h4 D4j D4j1b D4j5 D4j+(16286) D4j9 D4j12 D4j15 D4j16 D4m (79)	1
1741 >KX467294.1 Hc	73G 195A 263G 310C 316S 489C 514M 515R 750G 1438G 2706G 3106M 3107C 4769G 7028T 8701G 8860G 9540C 10398G 10400T 10873C 11719A 12007A 12705T 14766T 14783C 15043A 15301A 15326G 15431A 16223T 16234T 16519C	M30+16234	73G 195A 263G 310C 316S 489C 514M 515R 16223T 16234T 16519C	1	73 195A 263 310 316S 489 514M 515R 750 1438 2706 3106M 4769 7028 8701 8860 9540 10398 10400 10873 11719 12007 12705 14766 14783 15043 15301 15326 15431 16223 16234	M30+16234	73 195A 263 310 316S 489 514M 515R 16223 16234	[0.045] M30+16234 (1)	[0.028] M30 (2) [0.004] M30d1 (3) [0.000] M30d M30d2 M30e M49 M49c M49c1 M49e M49e1 (11)	1
1742 >KX467277.1 Hc	73G 152C 263G 299M 302M 310Y 373G 480C 709A 750G 1438G 1598A 2706G 3106M 3107C 4062C 4186T 4225G 4734G 4769G 5557C 5836G 7028T 7268G 7490G 8542C 8584A 8805G 8860G 9174C 10007C 10631A 11009C 11719A 12406A 13236G 14194T 14305A 14766T 15307T 15326G 16183C 16189C 16222T 16298C 16299G	R30b1	73G 152C 263G 299M 302M 310Y 373G 480C 16183C 16189C 16222T 16298C 16299G	1	73 152 263 299M 302M 310Y 373 480 709 750 1438 1598 2706 3106M 4062 4186 4225 4734 4769 5557 5836 7028 7268G 7490 8542 8584 8805 8860 9174 10007 10631A 11009 11719 12406 13236 14194 14305 14766 15307 15326 16189 16222 16298 16299	R30b1	73 152 263 299M 302M 310Y 373 480 16189 16222 16298 16299	[0.238] R30b1 (1)	[0.061] R30b (2) [0.034] R+16189 (3) [0.032] B4'5 (4) [0.025] R11'B6 (5) [0.017] H32 (6) [0.009] H1ap1 (7) [0.008] H3av R30a1 R30a1a (10)	1

1743	>KX467270.1 Hc	262T 263G 310C 750G 1438G 3106M 3107C 4769G 5460A 8860G 10124C 10685A 14118G 15326G 15617A 16261T	H41a	262T 263G 310C 16261T	1	262 263 310 750 1438 3106M 4769 5460 8860 10124 10685 14118 15326 15617 16261	H41a	262 263 310 16261	[0.137] H41a (1) [0.035] H7a1 (2) [0.010] H7a1b H13b1 (4) [0.000] H7a1c H7a1d (6)	[0.156] H13a1a1e (7) [0.155] H13a1a1 (8) [0.033] H13b1+200 (9) [0.025] H (10) [0.015] H26a1 (11) [0.010] H13b1 (12) [0.006] H2a2a (13) [0.005] HV H13a2b2a H14 (16) [0.004] H1e1a H1az H3 H7d H49 (21) [0.003] H1 H48 H59 (24) [0.002] H1e2 H1e8a H1bk H3u H7b1 H10+(16093) H13a1a1a H16 H16b H18b H23 H56 (36) [0.001] HV4 H1e H1h1 H1j H1s H1s1 H1v H1ai1 H1ap H1av H2a H2a2+(16235) H2a5 H3c1 H3r H4a1 H7 H7b H7c3 H7c6 H10a H10b H10c1 H10f H10h H13a1a H13a1a3 H13a1a4 H35 H40 H44a H49a H49b H55a H58a H75 H86 (73) [0.000] R0 HV4c HV5 HV5a HV9c HV12 H1e1 H1e1a2 H1e1a7 H1e1c H1e2a H1e2b H1e2d H1e4 H1e6 H1e7 H1e8 H1h H1h2 H1j3 H1j5 H1j6 H1j7 H1j9 H1m H1n H1p H1q H1q1 H1q2 H1r H1t H1t1 H1t1a H1t2 H1u H1u1 H1v1 H1v1a H1v1b H1w H1ae H1ae1 H1ae2 H1ae2a H1ae3 H1ae3a H1ag H1ag1 H1ag1b H1ah H1ai H1aj H1ak H1ak1 H1ak2 H1am H1am1 H1aq H1as H1as1a H1as2 H1at H1at1 H1at1a H1ax H1ax1 [illegible]	1
1744	>KX467265.1 Hc	73G 152C 200G 263G 310Y 316S 489C 750G 1438G 1555G 2706G 3010A 3106M 3107C 4769G 4883T 5178A 7028T 8414T 8701G 8860G 9540C 10373A 10398G 10400T 10873C 11719A 12705T 13236G 14668T 14766T 14783C 15043A 15118G 15301A 15326G 16177G 16223T 16256T 16311C 16362C 16519C	D4q	73G 152C 200G 263G 310Y 316S 489C 16177G 16223T 16256T 16311C 16362C 16519C	1	73 152 200 263 310Y 316S 489 750 1438 1555 2706 3010 3106M 4769 4883 5178A 7028 8414 8701 8860 9540 10373 10398 10400 10873 11719 12705 13236 14668 14766 14783 15043 15118 15301 15326 16177 16223 16256 16311 16362	D4q	73 152 200 263 310Y 316S 489 16177 16223 16256 16311 16362	[0.083] D4q (1)	[0.000] D4h4a D4q1 D4q1a (4)	1
1745	>MF362948.1 Hc	73G 94A 195C 263G 310.1C 750G 1189C 1438G 1811G 2706G 3106d 3107C 3337A 3480G 4769G 5913A 7028T 8860G 9055A 9698C 9962A 10289G 10398G 10550G 11299C 11467G 11719A 11914A 12308G 12372A 14167T 14766T 14798C 15326G 15946T 16224C 16311C 16519C	K1b1c	73G 94A 195C 263G 310.1C 16224C 16311C 16519C	1	73 94 195 263 750 1189 1438 1811 2706 3337 3480 4769 5913 7028 8860 9055 9698 9962 10289 10398 10550 11299 11467 11719 11914 12308 12372 14167 14766 14798 15326 15946 16224 16311	K1b1c	73 94 195 263 16224 16311	[0.016] K1b1c (1)	[0.088] K1b1+(16093) K1b1b (3) [0.087] K1b1b1 (4) [0.005] K1a4a1a+195 K1a4a1a1 K1a4a1a3 K1a14 K1a15 K1a16 K1a26 (11) [0.000] K K1 K1a+195 K1b K1b1 K1b2 K1b2a K1b2a1 K1b2a1a K1b2a2 K1b2a2a K1b2a3 K1b2b (24)	1

1746	>MF362947.1 Hc	73G 263G 310.1C 709A 750G 1438G 1888A 2706G 2834T 3106d 3107C 4216C 4769G 4823C 4917G 5788C 6261A 7028T 8697A 8860G 10463C 10822T 11129T 11251G 11719A 11812G 13368A 14233G 14766T 14905A 15326G 15452A 15607G 15928A 16126C 16146G 16292T 16294T 16296T 16519C	T2c1c	73G 263G 310.1C 16126C 16146G 16292T 16294T 16296T 16519C	1	73 263 709 750 1438 1888 2706 2834 4216 4769 4823 4917 5788 6261 7028 8697 8860 10463 10822 11129T 11251 11719 11812 13368 14233 14766 14905 15326 15452A 15607 15928 16126 16146 16292 16294 16296	T2c1c	73 263 16126 16146 16292 16294 16296	[0.000] T2c1c (1)	[0.000] T2c1 T2c1c T2c1c1 T2c1c2 (5)	1
1747	>MF362946.1 Hc	73G 94A 195C 263G 310.1C 750G 1189C 1438G 1811G 2706G 3106d 3107C 3337A 3480G 4769G 5913A 7028T 8860G 9055A 9698C 9962A 10289G 10398G 10550G 11299C 11467G 11719A 11914A 12308G 12372A 14167T 14766T 14798C 15326G 15946T 16224C 16311C 16519C	K1b1c	73G 94A 195C 263G 310.1C 16224C 16311C 16519C	1	73 94 195 263 750 1189 1438 1811 2706 3337 3480 4769 5913 7028 8860 9055 9698 9962 10289 10398 10550 11299 11467 11719 11914 12308 12372 14167 14766 14798 15326 15946 16224 16311	K1b1c	73 94 195 263 16224 16311	[0.016] K1b1c (1)	[0.088] K1b1+(16093) K1b1b (3) [0.087] K1b1b1 (4) [0.005] K1a4a1a+195 K1a4a1a1 K1a4a1a3 K1a14 K1a15 K1a16 K1a26 (11) [0.000] K K1 K1a+195 K1b K1b1 K1b2 K1b2a K1b2a1 K1b2a1a K1b2a2 K1b2a2a K1b2a3 K1b2b (24)	1
1748	>MF362945.1 Hc	73G 153G 195C 257G 263G 310.1C 750G 1438G 1719A 2706G 3106d 3107C 4769G 6221C 6371T 7028T 7792T 8860G 11719A 12705T 13966G 14470C 14766T 15326G 16189C 16223T 16278T 16519C	X2f	73G 153G 195C 257G 263G 310.1C 16189C 16223T 16278T 16519C	1	73 153 195 257 263 750 1438 1719 2706 4769 6221 6371 7028 7792 8860 11719 12705 13966 14470 14766 15326 16189 16223 16278	X2f	73 153 195 257 263 16189 16223 16278	[0.000] X2f (1)	[0.000] X2 X2e2 X2e2a X2e2a1 X2o1 X2f1 X2k X2p (9)	1
1749	>MF362940.1 Hc	146C 195C 263G 309.1CT 310C 709A 750G 1438G 3106d 3107C 4385G 4769G 8381G 8860G 13101C 15326G 16111T 16167T 16288C 16362C	H8a1	146C 195C 263G 309.1CT 310C 16111T 16167T 16288C 16362C	1	146 195 263 709 750 1438 4385 4769 8381 8860 13101C 15326 16111 16167 16288 16362	H8a1	146 195 263 16111 16167 16288 16362	[0.000] H8a1 (1)	[0.000] H8a (2)	1
1750	>MF362939.1 Hc	73G 152C 263G 310.1C 497T 513.1CA 750G 1189C 1438G 1811G 2706G 3106d 3107C 3480G 4769G 5264T 7028T 8860G 9055A 9698C 10101C 10398G 10550G 11299C 11467G 11485C 11719A 12308G 12372A 12612G 13710G 13827G 14167T 14766T 14798C 15326G 16224C 16246T 16311C 16519C	K1a4c1	73G 152C 263G 310.1C 497T 513.1CA 16224C 16246T 16311C 16519C	1	73 152 263 497 750 1189 1438 1811 2706 3480 4769 5264 7028 8860 9055 9698 10101 10398 10550 11299 11467 11485 11719 12308 12372 12612 13710 13827 14167 14766 14798 15326 16224 16246T 16311	K1a4c1	73 152 263 497 16224 16246T 16311	[0.000] K1a4c1 (1)	[0.000] K1a1b2a K1a1b2a1 K1a1b2a1a K1a4c K1a4c1 K1a25 (7)	1
1751	>MF362923.1 Hc	73G 153G 195C 257G 263G 310.1C 750G 1438G 1719A 2706G 3106d 3107C 4769G 6221C 6371T 7028T 8860G 11719A 12705T 13935T 13966G 14470C 14766T 15326G 16091G 16189C 16223T 16278T 16519C	X2f	73G 153G 195C 257G 263G 310.1C 16091G 16189C 16223T 16278T 16519C	1	73 153 195 257 263 750 1438 1719 2706 4769 6221 6371 7028 8860 11719 12705 13935 13966 14470 14766 15326 16091 16189 16223 16278	X2f	73 153 195 257 263 16091 16189 16223 16278	[0.019] X2f (1)	[0.000] X2 X2e2 X2e2a X2e2a1 X2o1 X2f1 X2k X2p (9)	1

1752	>MF362921.1 Hc	73G 263G 295T 309.1CT 310C 462T 489C 750G 1438G 2706G 3010A 3106d 3107C 4216C 4769G 7028T 8269A 8860G 10274C 10398G 11251G 11695G 11719A 12372A 12612G 13145A 13708A 14766T 15326G 15452A 16069T 16126C 16145A 16222T 16261T 16274A 16362C	J1b5	73G 263G 295T 309.1CT 310C 462T 489C 16069T 16126C 16145A 16222T 16261T 16274A 16362C	1	73 263 295 462 489 750 1438 2706 3010 4216 4769 7028 8269 8860 10274 10398 11251 11695 11719 12372 12612 13145 13708 14766 15326 15452A 16069 16126 16145 16222 16261 16274 16362	J1b5	73 263 295 462 489 16069 16126 16145 16222 16261 16274 16362	[0.190] J1b5 (1)	[0.190] J1b5 (2) [0.027] J1b (3) [0.006] J1b3 J1b6a (5) [0.000] J1b1 J1b2 J1b3b J1b3b1 J1b4 J1b5a J1b5a1 J1b6 (13)	1
1753	>MF362918.1 Hc	73G 150T 263G 309.1CCT 310C 334C 514d 515d 750G 1438G 1811G 2706G 2707G 3106d 3107C 4188G 4640A 4769G 7028T 8860G 8950A 9656C 9770C 11467G 11719A 12308G 12372A 13615G 13743C 14139G 14766T 14894C 15326G 15454C 15935T 15938A 15940C 16203G 16343G 16504A	U3b2a1a	73G 150T 263G 309.1CCT 310C 334C 514d 515d 16203G 16343G 16504A	1	73 150 263 334 750 1438 1811 2706 2707 4188 4640A 4769 7028 8860 8950 9656 9770 11467 11719 12308 12372 13615 13743 14139 14766 14894 15326 15454 15934.1T 15944d 16203 16343 16504	U3b2a1a	73 150 263 334 16203 16343 16504	[0.138] U3b2a1a (1)	[0.000] U3 U3a'c U3b U3b1 U3b1b U3b2 U3b2a U3b2a1 U3b2c (10)	1
1754	>MF362910.1 Hc	73G 263G 295T 309.1CT 310C 462T 489C 750G 1438G 2706G 3010A 3106d 3107C 4216C 4464A 4769G 7028T 8269A 8860G 10274C 10398G 11251G 11695G 11719A 12372A 12612G 13145A 13708A 14766T 15326G 15452A 16069T 16126C 16145A 16222T 16261T 16274A 16362C	J1b5	73G 263G 295T 309.1CT 310C 462T 489C 16069T 16126C 16145A 16222T 16261T 16274A 16362C	1	73 263 295 462 489 750 1438 2706 3010 4216 4464 4769 7028 8269 8860 10274 10398 11251 11695 11719 12372 12612 13145 13708 14766 15326 15452A 16069 16126 16145 16222 16261 16274 16362	J1b5	73 263 295 462 489 16069 16126 16145 16222 16261 16274 16362	[0.190] J1b5 (1)	[0.190] J1b5 (2) [0.027] J1b (3) [0.006] J1b3 J1b6a (5) [0.000] J1b1 J1b2 J1b3b J1b3b1 J1b4 J1b5a J1b5a1 J1b6 (13)	1
1755	>MF362906.1 Hc	73G 150T 185A 189G 195C 204C 207A 210G 263G 310.1C 750G 1438G 1719A 2706G 3106d 3107C 4769G 5981C 7028T 8222C 8860G 10238C 11025C 11437C 11719A 11914A 12501A 12705T 13637G 13780G 14766T 15326G 16111T 16201T 16223T 16265G 16519C	N1a3a2	73G 150T 185A 189G 195C 204C 207A 210G 263G 310.1C 16111T 16201T 16223T 16265G 16519C	1	73 150 185 189 195 204 207 210 263 750 1438 1719 2706 4769 5981 7028 8222 8860 10238 11025 11437 11719 11914 12501 12705 13637 13780 14766 15326 16111 16201 16223 16265	N1a3a2	73 150 185 189 195 204 207 210 263 16111 16201 16223 16265	[0.166] N1a3a2 (1)	[0.046] N1a3a (2)	1
1756	>MF362904.1 Hc	73G 199C 204C 250C 263G 309.1CT 310C 567.1CCCC 574C 750G 1438G 1719A 2706G 3106d 3107C 4529T 4769G 7028T 8176C 8251A 8860G 10034C 10238C 10389C 10398G 11719A 12438C 12501A 12705T 12987T 13780G 14233G 14766T 15043A 15326G 15924G 16129A 16169T 16223T 16354T 16391A 16400T 16519C	I5c	73G 199C 204C 250C 263G 309.1CT 310C 567.1CCCC 574C 16129A 16169T 16223T 16354T 16391A 16400T 16519C	1	73 199 204 250 263 574C 573.4C 750 1438 1719 2706 4529T 4769 7028 8176 8251 8860 10034 10238 10389 10398 11719 12438 12501 12705 12987 13780 14233 14766 15043 15326 15924 16129 16169 16223 16354 16391 16400	I5c	73 199 204 250 263 574C 573.4C 16129 16169 16223 16354 16391 16400	[0.126] I5c (1)	[0.080] I5 (2) [0.034] I4a (3) [0.017] I4b (4) [0.014] I (5) [0.000] I4 I4a2 I5b I6 I6a (10)	1

1757 >MF362900.1 Hc	73G 146C 263G 285T 310.1C 750G 1438G 2387C 2706G 3106d 3107C 3394C 4769G 5894.1C 7028T 8395T 8860G 10885C 11467G 11566G 11719A 12308G 12372A 12879C 13104G 14070G 14527G 14766T 15148A 15172A 15326G 15954C 16111T 16188T 16214A 16249C 16327T	U1b2	73G 146C 263G 285T 310.1C 16111T 16188T 16214A 16249C 16327T	1	73 146 263 285 750 1438 2387 2706 3394 4769 5899.1C 7028 8395 8860 10885 11467 11566 11719 12308 12372 12879 13104 14070 14527 14766 15148 15172 15326 15954C 16111 16188 16214A 16249 16327	U1b2	73 146 263 285 16111 16188 16214A 16249 16327	[0.050] U1b2 (1)	[0.050] U1b2 (2) [0.000] U1b U1b1 (4)	1
1758 >MF362899.1 Hc	73G 152C 195C 263G 309.1CT 310C 678C 750G 1438G 2237G 2706G 3106d 3107C 4769G 6221C 6227C 6371T 7028T 8860G 11719A 12406A 12705T 13966G 14470C 14766T 15326G 16182C 16183C 16189C 16223T 16266T 16274A 16278T 16390A 16519C	X4	73G 152C 195C 263G 309.1CT 310C 16182C 16183C 16189C 16223T 16266T 16274A 16278T 16390A 16519C	1	73 152 195 263 678 750 1438 2237 2706 4769 6221 6227 6371 7028 8860 11719 12406 12705 13966 14470 14766 15326 16189 16223 16266 16274 16278 16390	X4	73 152 195 263 16189 16223 16266 16274 16278 16390	[0.000] X4 (1)	[0.010] X2e2a2 (2) [0.009] X2d (3) [0.000] X2d2	1
1759 >MF362894.1 Hc	146C 195C 263G 310.1C 709A 750G 1438G 3106d 3107C 3999C 4769G 8381G 8860G 13101C 15326G 16111T 16167T 16288C 16362C	H8a1	146C 195C 263G 310.1C 16111T 16167T 16288C 16362C	1	146 195 263 709 750 1438 3999 4769 8381 8860 13101C 15326 16111 16167 16288 16362	H8a1	146 195 263 16111 16167 16288 16362	[0.000] H8a1 (1)	[0.000] H8a (2)	1
1760 >MF362891.1 Hc	73G 150T 263G 310.1C 750G 1438G 1811G 2706G 3106d 3107C 4188G 4640A 4769G 5999C 6545T 7028T 7906T 8842C 8860G 9656C 11467G 11719A 12308G 12372A 13743C 14139G 15326G 15454C 16168T 16192T 16343G	U3b3	73G 150T 263G 310.1C 16168T 16192T 16343G	1	73 150 263 750 1438 1811 2706 4188 4640A 4769 5999 6545 7028 7906 8842C 8860 9656 11467 11719 12308 12372 13743 14139 15326 15454 16168 16192 16343	U3b3	73 150 263 16168 16192 16343	[0.209] U3b3 (1)	[0.000] U5b2a1a U3 U3a'c U3b U3b1 U3b1b U3b2 U3b2a U3b2a1 U3b2c (11)	1
1761 >MF362883.1 Hc	73G 263G 295T 310.1C 462T 489C 750G 1438G 1733T 2706G 3010A 3106d 3107C 4216C 4769G 7028T 8269A 8860G 9667G 10398G 11251G 11719A 12612G 13708A 14766T 15326G 15452A 15466A 16069T 16126C 16136C 16145A 16222T 16261T 16519C	J1b2a	73G 263G 295T 310.1C 462T 489C 16069T 16126C 16136C 16145A 16222T 16261T 16519C	1	73 263 295 462 489 750 1438 1733 2706 3010 4216 4769 7028 8269 8860 9667 10398 11251 11719 12612 13708 14766 15326 15452A 15466 16069 16126 16136 16145 16222 16261	J1b2a	73 263 295 462 489 16069 16126 16136 16145 16222 16261	[0.000] J1b2a (1)	[0.000] J1b J1b1 J1b2 J1b2a J1b3 J1b3b J1b3b1 J1b4 J1b6 J1b6a (11)	1
1762 >MF362882.1 Hc	73G 195C 198T 263G 310.1C 514d 515d 750G 1438G 1811G 2706G 3106d 3107C 3480G 4769G 4772C 6546T 6599G 7028T 8308G 8860G 9012C 9055A 9698C 9948A 11467G 11719A 11914A 12308G 12358G 12372A 12771A 14167T 14766T 15326G 16066G 16129A 16182C 16183C 16189C 16234T 16244A	U8b1a1	73G 195C 198T 263G 310.1C 514d 515d 16066G 16129A 16182C 16183C 16189C 16234T 16244A	1	73 195 198 263 750 1438 1811 2706 3480 4769 4772 6546 6599 7028 8308 8860 9012 9055 9698 9948 11467 11719 11914 12308 12358 12372 12771 14167 14766 15326 16066 16129 16189 16234 16244	U8b1a1	73 195 198 263 16066 16129 16189 16234 16244	[0.191] U8b1a1 (1)	[0.000] U8b1 U8b1a U8b1a2 (4)	1

1763	>MF362879.1 Hc	73G 199C 204C 250C 263G 309.1CT 310C 750G 1438G 1719A 2706G 3106d 3107C 4113A 4529T 4769G 7028T 8251A 8860G 10034C 10238C 10398G 11719A 12501A 12705T 13780G 13968A 14233G 14766T 15043A 15326G 15924G 16114T 16129A 16169T 16223T 16391A 16519C	I5c	73G 199C 204C 250C 263G 309.1CT 310C 16114T 16129A 16169T 16223T 16391A 16519C	1	73 199 204 250 263 750 1438 1719 2706 4113 4529T 4769 7028 8251 8860 10034 10238 10398 11719 12501 12705 13780 13968 14233 14766 15043 15326 15924 16114 16129 16169 16223 16391	I5c	73 199 204 250 263 16114 16129 16169 16223 16391	[0.584] I5c (1)	[0.229] I4a (2) [0.114] I (3) [0.042] I4 (4) [0.034] I4b (5) [0.021] I4a2 (6) [0.020] I6a (7) [0.000] I5 I5b I6 (10)	1
1764	>MF362878.1 Hc	73G 150T 152C 263G 310.1C 750G 1438G 1811G 2294G 2706G 2766A 3106d 3107C 4703C 4769G 5063C 6518T 7028T 8860G 9266A 10143A 10506G 11467G 11719A 12308G 12372A 13934T 14122G 14139G 14766T 15326G 15454C 16183C 16189C 16343G 16390A 16519C	U3a3	73G 150T 152C 263G 310.1C 16183C 16189C 16343G 16390A 16519C	1	73 150 152 263 750 1438 1811 2294 2706 2766A 4703 4769 5063 6518 7028 8860 9266 10143 10506 11467 11719 12308 12372 13934 14122 14139 14766 15326 15454 16189 16343 16390	U3a3	73 150 152 263 16189 16343 16390	[0.032] U3a3 (1)	[0.114] U3a1 (2) [0.048] U3a1a (3) [0.021] U3a2 (4) [0.016] U3a (5) [0.000] U3a1a1 U3a1b (7)	1
1765	>MF362855.1 Hc	73G 150T 152C 195C 198T 215G 263G 295T 309.1CT 310C 319C 489C 513A 750G 1438G 2706G 3106d 3107C 4216C 4769G 7028T 7476T 7789A 8860G 10398G 10499G 11251G 11377A 11719A 12007A 12612G 13708A 13722G 14133G 14766T 15257A 15326G 15452A 16069T 16126C 16145A 16183C 16189C 16231C 16261T	J2a1a1e	73G 150T 152C 195C 198T 215G 263G 295T 309.1CT 310C 319C 489C 513A 16069T 16126C 16145A 16183C 16189C 16231C 16261T	1	73 150 152 195 198 215 263 295 319 489 513 750 1438 2706 4216 4769 7028 7476 7789 8860 10398 10499 11251 11377 11719 12007 12612 13708 13722 14133 14766 15257 15326 15452A 16069 16126 16145 16189 16231 16261	J2a1a1e	73 150 152 195 198 215 263 295 319 489 513 16069 16126 16145 16189 16231 16261	[0.459] J2a1a1e (1) [0.000] J2a1a1 J2a1a1b J2a1a1c J2a1a1d (5)	[0.459] J2a1a1e (6) [0.066] J2a1a1a2 (7) [0.029] J2a1a1a2a (8) [0.023] J2a1a1a (9) [0.000] J2a1a J2a1a1 J2a1a1b J2a1a1c J2a1a1d (14)	1
1766	>MF362854.1 Hc	152C 195C 263G 309.1CT 310C 750G 1438G 2706G 3106d 3107C 4769G 5250C 7028T 8014T 8754T 8860G 10295G 10750G 12696C 15218G 15326G 16067T 16311C	HV1b3b	152C 195C 263G 309.1CT 310C 16067T 16311C	1	152 195 263 750 1438 2706 4769 5250 7028 8014T 8754 8860 10295 10750 12696 15218 15326 16067 16311	HV1b3b	152 195 263 16067 16311	[0.000] HV1b3b (1)	[0.000] HV1b3 (2)	1
1767	>MF362853.1 Hc	73G 150T 152C 195C 198T 215G 263G 295T 309.1CCT 310C 319C 489C 513A 750G 1438G 2706G 2848G 3106d 3107C 4216C 4769G 7028T 7476T 7789A 8860G 10398G 10499G 11251G 11377A 11719A 12007A 12612G 13708A 13722G 14133G 14766T 15257A 15326G 15452A 16069T 16126C 16145A 16183C 16189C 16231C 16261T	J2a1a1e	73G 150T 152C 195C 198T 215G 263G 295T 309.1CCT 310C 319C 489C 513A 16069T 16126C 16145A 16183C 16189C 16231C 16261T	1	73 150 152 195 198 215 263 295 319 489 513 750 1438 2706 2848 4216 4769 7028 7476 7789 8860 10398 10499 11251 11377 11719 12007 12612 13708 13722 14133 14766 15257 15326 15452A 16069 16126 16145 16189 16231 16261	J2a1a1e	73 150 152 195 198 215 263 295 319 489 513 16069 16126 16145 16189 16231 16261	[0.459] J2a1a1e (1) [0.000] J2a1a1 J2a1a1b J2a1a1c J2a1a1d (5)	[0.459] J2a1a1e (6) [0.066] J2a1a1a2 (7) [0.029] J2a1a1a2a (8) [0.023] J2a1a1a (9) [0.000] J2a1a J2a1a1 J2a1a1b J2a1a1c J2a1a1d (14)	1
1768	>MF362847.1 Hc	73G 153G 195C 263G 309T 310C 750G 1438G 1719A 2706G 3106d 3107C 4769G 6221C 6371T 7028T 7229T 8860G 11719A 12705T 13966G 14470C 14766T 15326G 16189C 16223T 16248T 16278T 16519C	X2i+@225	73G 153G 195C 263G 309T 310C 16189C 16223T 16248T 16278T 16519C	1	73 153 195 263 309d 750 1438 1719 2706 4769 6221 6371 7028 7229 8860 11719 12705 13966 14470 14766 15326 16189 16223 16248 16278	X2i+@225	73 153 195 263 309d 16189 16223 16248 16278	[0.050] X2i+@225 (1)	[0.000] X2 X2e2 X2e2a X2e2a1 X2i X2i1 X2o1 X2k X2p (10)	1

1769	>MF362842.1 Hc	73G 263G 295T 310.1C 462T 489C 750G 1438G 2706G 3010A 3106d 3107C 4216C 4769G 6253C 7028T 8269A 8860G 10274C 10398G 11251G 11695G 11719A 12372A 12612G 13145A 13708A 14766T 15326G 15452A 16069T 16126C 16145A 16222T 16261T 16274A 16362C	J1b5	73G 263G 295T 310.1C 462T 489C 16069T 16126C 16145A 16222T 16261T 16274A 16362C	1	73 263 295 462 489 750 1438 2706 3010 4216 4769 6253 7028 8269 8860 10274 10398 11251 11695 11719 12372 12612 13145 13708 14766 15326 15452A 16069 16126 16145 16222 16261 16274 16362	J1b5	73 263 295 462 489 16069 16126 16145 16222 16261 16274 16362	[0.190] J1b5 (1)	[0.190] J1b5 (2) [0.027] J1b (3) [0.006] J1b3 J1b6a (5) [0.000] J1b1 J1b2 J1b3b J1b3b1 J1b4 J1b5a J1b5a1 J1b6 (13)	1
1770	>MF362835.1 Hc	73G 263G 310.1C 497T 513.1CA 750G 1189C 1438G 1811G 2706G 3106d 3107C 3480G 4092A 4769G 5466G 5492C 7028T 7202G 8145T 8860G 8938G 9055A 9698C 10398G 10550G 11299C 11467G 11719A 12308G 12372A 14016A 14167T 14766T 14798C 15326G 16176T 16224C 16311C 16519C	K1a27	73G 263G 310.1C 497T 513.1CA 16176T 16224C 16311C 16519C	1	73 263 497 750 1189 1438 1811 2706 3480 4092 4769 5466 5492 7028 7202 8145 8860 8938 9055 9698 10398 10550 11299 11467 11719 12308 12372 14016 14167 14766 14798 15326 16176 16224 16311	K1a27	73 263 497 16176 16224 16311	[0.000] K1a27 (1)	[0.011] K1a29 (2) [0.004] K1a4a1b (3) [0.000] K1a K1a1 K1a1a K1a1a1 K1a1a2 K1a1a2a K1a1a2a1 K1a1b K1a1b1 K1a1b1b K1a1b1b1 K1a1b1e K1a1b1f K1a1b1g K1a1b2 K1a2 K1a2a K1a2a2 K1a2b K1a2c K1a3 K1a3a K1a3a1 K1a3a1a K1a3a1b K1a3a2 K1a3a3 K1a3a4 K1a4 K1a4a K1a4a1 K1a4a1a K1a4a1b1 K1a4a1c K1a4a1d K1a4a1h K1a4a1i K1a4d K1a4e K1a4f K1a4f1 K1a4g K1a4h K1a4h1 K1a4i K1a5 K1a12 K1a12a K1a12a1 K1a12a1a K1a18 K1a19 K1a23 K1a27 (57)	1
1771	>MF362834.1 Hc	263G 310.1C 466C 482C 750G 1438G 2706G 3106d 3107C 4769G 6060G 7028T 8860G 10978G 15326G 16309G 16311C 16354T	HV8	263G 310.1C 466C 482C 16309G 16311C 16354T	1	263 466 482 750 1438 2706 4769 6060 7028 8860 10978 15326 16309 16311 16354	HV8	263 466 482 16309 16311 16354	[0.033] HV8 (1)	[0.051] H2a1 (2) [0.009] H2a1a (3) [0.000] HV+16311 HV9 HV10 HV11 HV11a HV16 HV23 HV24 H1e1a4 H1+16311 H2a1a1 H2a1a2 H2a1b H2a1c H2a1d H2a1g H2a1k H3+16311 H3h H3h1 H3h2 H3h3 H3h3a H3h3b H3h5 H3h6 H3m H4d H13a1a2+16311 H13a2b1 H76 H80 (35)	1
1772	>MF362830.1 Hc	73G 189G 204C 207A 210G 263G 309.1CT 310C 750G 1438G 1719A 2706G 3106d 3107C 4769G 6962A 7028T 8222C 8860G 10238C 11025C 11437C 11719A 11914A 12501A 12705T 13637G 13780G 14766T 15326G 16201T 16223T 16265G 16519C	N1a3a	73G 189G 204C 207A 210G 263G 309.1CT 310C 16201T 16223T 16265G 16519C	1	73 189 204 207 210 263 750 1438 1719 2706 4769 6962 7028 8222 8860 10238 11025 11437 11719 11914 12501 12705 13637 13780 14766 15326 16201 16223 16265	N1a3a	73 189 204 207 210 263 16201 16223 16265	[0.187] N1a3a (1)	[0.167] N1a3 (2) [0.000] N1a3a2	1
1773	>MF362825.1 Hc	195C 263G 310.1C 750G 961G 1438G 3106d 3107C 4769G 5585A 8448C 8860G 13759A 14587G 15326G 15670C 16092C 16140C 16265G 16293G 16311C	H11a2a2	195C 263G 310.1C 16092C 16140C 16265G 16293G 16311C	1	195 263 750 961G 1438 4769 5585 8448 8860 13759 14587 15326 15670 16092 16140 16265 16293 16311	H11a2a2	195 263 16092 16140 16265 16293 16311	[0.000] H11a2a2 (1)	[0.000] H11a2a H11a2a1 H11a2a2 H11a2a3 (5)	1
1774	>MF362823.1 Hc	93G 239C 263G 309.1CCT 310C 750G 1438G 3106d 3107C 4769G 8860G 14040A 15326G 16300G 16325C 16362C 16482G	H6b2	93G 239C 263G 309.1CCT 310C 16300G 16325C 16362C 16482G	1	93 239 263 750 1438 4769 8860 14040 15326 16300 16325 16362 16482	H6b2	93 239 263 16300 16325 16362 16482	[0.020] H6b2 (1)	[0.020] H6b2 (2) [0.000] H6b	1

1775 >MF362820.1 Hc	73G 153G 195C 263G 309.1CCT 310C 750G 1438G 1719A 2706G 3106d 3107C 4769G 6221C 6371T 7028T 7229T 7456G 8860G 11719A 12705T 13966G 14470C 14766T 15326G 16189C 16223T 16248T 16278T 16519C	X2i+@225	73G 153G 195C 263G 309.1CCT 310C 16189C 16223T 16248T 16278T 16519C	1	73 153 195 263 750 1438 1719 2706 4769 6221 6371 7028 7229 7456 8860 11719 12705 13966 14470 14766 15326 16189 16223 16248 16278	X2i+@225	73 153 195 263 16189 16223 16248 16278	[0.000] X2i+@225 (1)	[0.000] X2 X2e2 X2e2a X2e2a1 X2i X2i1 X2o1 X2k X2p (10)	1
1776 >MF362819.1 Hc	73G 263G 285T 309.1CT 310C 385G 523d 750G 1438G 2218T 2706G 3106d 3107C 3158.1T 3591A 4769G 4991A 6026A 7028T 7581C 8860G 11467G 11719A 12308G 12372A 12879C 13104G 13422G 14070G 14364A 14766T 15148A 15326G 15954C 16136C 16182C 16183C 16189C 16249C 16360T	U1a1a	73G 263G 285T 309.1CT 310C 385G 523d 16136C 16182C 16183C 16189C 16249C 16360T	1	73 263 285 385 523d 750 1438 2218 2706 3158.1T 3591 4769 4991 6026 7028 7581 8860 11467 11719 12308 12372 12879 13104 13422 14070 14364 14766 15148 15326 15954C 16136 16189 16249 16360	U1a1a	73 263 285 385 523d 16136 16189 16249 16360	[0.044] U1a1a (1)	[0.040] U1a1a2 (2) [0.023] U1a U1a1a1a (4) [0.019] U1a1c1 (5) [0.000] U1a1 U1a1a1 U1a1a+16129 U1a1c U1a1c1b U1a1c1c U1a1c1d (12)	1
1777 >MF362818.1 Hc	73G 189G 204C 207A 210G 263G 309.1CT 310C 750G 1438G 1719A 2706G 3106d 3107C 4769G 4853A 6962A 7028T 7444C 8222C 8860G 10238C 11025C 11437C 11719A 11914A 12501A 12705T 13637G 13780G 14766T 15314A 15326G 16201T 16223T 16265G 16519C	N1a3a	73G 189G 204C 207A 210G 263G 309.1CT 310C 16201T 16223T 16265G 16519C	1	73 189 204 207 210 263 750 1438 1719 2706 4769 4853 6962 7028 7444C 8222 8860 10238 11025 11437 11719 11914 12501 12705 13637 13780 14766 15314 15326 16201 16223 16265	N1a3a	73 189 204 207 210 263 16201 16223 16265	[0.187] N1a3a (1)	[0.167] N1a3 (2) [0.000] N1a3a2	1
1778 >MF362817.1 Hc	73G 263G 310.1C 497T 513.1CA 750G 1189C 1438G 1811G 2706G 3106d 3107C 3480G 4092A 4769G 5466G 5492C 7028T 7202G 8145T 8860G 8938G 9055A 9698C 10398G 10550G 11299C 11467G 11719A 12308G 12372A 14016A 14167T 14766T 14798C 15326G 16176T 16224C 16311C 16519C	K1a27	73G 263G 310.1C 497T 513.1CA 16176T 16224C 16311C 16519C	1	73 263 497 750 1189 1438 1811 2706 3480 4092 4769 5466 5492 7028 7202 8145 8860 8938 9055 9698 10398 10550 11299 11467 11719 12308 12372 14016 14167 14766 14798 15326 16176 16224 16311	K1a27	73 263 497 16176 16224 16311	[0.000] K1a27 (1)	[0.011] K1a29 (2) [0.004] K1a4a1b (3) [0.000] K1a K1a1 K1a1a K1a1a1 K1a1a2 K1a1a2a K1a1a2a1 K1a1b K1a1b1 K1a1b1b K1a1b1b1 K1a1b1e K1a1b1f K1a1b1g K1a1b2 K1a2 K1a2a K1a2a2 K1a2b K1a2c K1a3 K1a3a K1a3a1 K1a3a1a K1a3a1b K1a3a2 K1a3a3 K1a3a4 K1a4 K1a4a K1a4a1 K1a4a1a K1a4a1b1 K1a4a1c K1a4a1d K1a4a1h K1a4a1i K1a4d K1a4e K1a4f K1a4f1 K1a4g K1a4h K1a4h1 K1a4i K1a5 K1a12 K1a12a K1a12a1 K1a12a1a K1a18 K1a19 K1a23 K1a27 (57)	1
1779 >MF362811.1 Hc	73G 150T 197G 263G 309.1CT 310C 373G 750G 1438G 1811G 2294G 2706G 2766A 3106d 3107C 4703C 4769G 5663T 6518T 7028T 7746G 8860G 9266A 9830T 10506G 10790C 11467G 11719A 12308G 12372A 12870T 13934T 14139G 14766T 15326G 15454C 16129A 16189C 16343G 16390A 16519C	U3a3	73G 150T 197G 263G 309.1CT 310C 373G 16129A 16189C 16343G 16390A 16519C	1	73 150 197 263 373 750 1438 1811 2294 2706 2766A 4703 4769 5663 6518 7028 7746 8860 9266 9830 10506 10790 11467 11719 12308 12372 12870 13934 14139 14766 15326 15454 16129 16189 16343 16390	U3a3	73 150 197 263 373 16129 16189 16343 16390	[0.307] U3a3 (1)	[0.104] U3a2 (2) [0.064] U3a1a (3) [0.025] U3a1 (4) [0.000] U3a U3a1a1 U3a1b (7)	1

1780	>MF362801.1 Ho	73G 152C 263G 295T 310.1C 462T 489C 709A 750G 1438G 2706G 3010A 3106d 3107C 4164G 4216C 4769G 4853A 7028T 7789A 7963G 8860G 10398G 11251G 11719A 12612G 12811C 13708A 14766T 15326G 15452A 16069T 16093Y 16126C 16193T 16274A 16365T 16519C	J1d5	73G 152C 263G 295T 310.1C 462T 489C 16069T 16093Y 16126C 16193T 16274A 16365T 16519C	1	73 152 263 295 462 489 709 750 1438 2706 3010 4164 4216 4769 4853 7028 7789 7963 8860 10398 11251 11719 12612 12811 13708 14766 15326 15452A 16069 16093Y 16126 16193 16274 16365	J1d5	73 152 263 295 462 489 16069 16093Y 16126 16193 16274 16365	[0.103] J1d5 (1)	[0.016] J1d3 J1d4 (3) [0.015] J1d3a (4) [0.000] J1d J1d3a2 J1d5a J1d6 (8)	1
1781	>MF362800.1 Ho	152C 263G 310.1C 644G 750G 1438G 2706G 3106d 3107C 3397G 4769G 7028T 7436G 8860G 15326G 16169T 16305G 16519C	HV21	152C 263G 310.1C 16169T 16305G 16519C	1	152 263 644 750 1438 2706 3397 4769 7028 7436 8860 15326 16169 16305	HV21	152 263 16169 16305	[0.065] HV21 (1) [0.004] H+152 (2) [0.003] H1+152 (3) [0.000] H1i H1i1 H1i2 H1i2a H1an H1an1 H1an1a H1bb H1bc H2a2a2 H3+152 H3g H3g1 H3g1a H3g1b H3g2 H3g3 H3g4 H3i H3i1 H3j H3k H3k1 H3k1a H9 H46 H46a H46b H52 H69 H13a1+152 H14b H14b2 H14b4 H16+152 H16a H16a1 H16c H16d H30b1 H47a (44)	[0.154] H1m (45) [0.031] H13a1a1a (46) [0.023] H2a2a (47) [0.022] H9a (48) [0.018] H3a1 (49) [0.014] H1c9 (50) [0.012] HV (51) [0.009] H4a1 H26a1 (53) [0.005] H1h1 (54) [0.004] H3q1 H7b (56) [0.003] HV5 H1 H4b H16e H58a H75 (62) [0.002] H1e1a H1e2 H2a H7d H13a1a1 H13a2a H18 H18b (70) [0.001] HV4 H H1e1a2 H1e1c H1h2 H1q H1t1a H1ag1 H1aj H1at1a H3 H3ap H4 H4a1a H4a1a1 H4a1c H7 H7b1 H7c6 H7e H10b H10+(16093) H13a1a2 H13a2b4 H35 H40b H45a (97) [0.000] R0 HV1b+152 HV4c HV5a HV9+152 HV9c HV12 H1c+152 H1c9a H1e H1e1 H1e1a7 H1e2a H1e2b H1e2d H1e4 H1e6 H1e7 H1e8 H1e8a H1h H1j H1j3 H1j5 H1j6 H1j7 H1j9 H1n H1p H1q1 H1q2 H1r H1s H1s1 H1t H1t1 H1t2 H1u H1u1 H1v H1v1 H1v1a H1v1b H1w H1ae H1ae1 H1ae2 H1ae2a H1ae3 H1ae3a H1ag H1ag1b H1ah H1ai H1ai1 H1ak H1ak1 H1ak2 H1am H1am1 H1ap H1ap1 H1aq H1as H1as2 H1at H1at1 H1av H1ax H1ax1 H1ay H1az H1bd H1be H1bi H1bj H1bk H1bm H1bn H1bp H1bq H1br H1bu [illegible]	1
1782	>MF362799.1 Ho	73G 150T 197G 263G 309.1CCT 310C 750G 1438G 1811G 2294G 2706G 2766A 3106d 3107C 4703C 4769G 5663T 6518T 7028T 7746G 8860G 9266A 9830T 10506G 10790C 11467G 11719A 12308G 12372A 12870T 13934T 14139G 14766T 15326G 15454C 16129A 16189C 16343G 16390A 16519C	U3a3	73G 150T 197G 263G 309.1CCT 310C 16129A 16189C 16343G 16390A 16519C	1	73 150 197 263 750 1438 1811 2294 2706 2766A 4703 4769 5663 6518 7028 7746 8860 9266 9830 10506 10790 11467 11719 12308 12372 12870 13934 14139 14766 15326 15454 16129 16189 16343 16390	U3a3	73 150 197 263 16129 16189 16343 16390	[0.291] U3a3 (1)	[0.104] U3a2 (2) [0.064] U3a1a (3) [0.025] U3a1 (4) [0.000] U3a U3a1a1 U3a1b (7)	1

1783 >MF362792.1 Hc	73G 125C 127C 146C 152C 263G 309.1CCT 310C 750G 1438G 2706G 3106d 3107C 4216C 7028T 7642A 7657C 8473C 8790A 8860G 9738A 9932A 10685A 11719A 12654G 13500C 13711A 14062G 14305A 14323A 14766T 15204C 15326G 16071T 16215G 16311C	R2b1	73G 125C 127C 146C 152C 263G 309.1CCT 310C 16071T 16215G 16311C	1	73 125 127 146 152 263 750 1438 2706 4216 7028 7642 7657 8473 8790 8860 9738 9932 10685 11719 12654 13500 13711 14062 14305 14323 14766 15204 15326 16071 16215 16311	R2b1	73 125 127 146 152 263 16071 16215 16311	[0.240] R2b1 (1)	[0.221] R2 (2) [0.043] R2d (3) [0.015] R2+13500 (4) [0.011] K2a1 K2a2 K2a2a K2a3a (8) [0.000] R2b K2a K2a3 K2a3a1 K2a4 K2a6 K2a7 K2a9 K2a10 (17)	1
1784 >MF362790.1 Hc	72C 73G 151T 152C 195C 198T 246C 263G 309.1CT 310C 629C 709A 750G 1438G 2706G 3106d 3107C 4769G 5147A 7028T 7193C 8860G 9336G 11935C 12061T 15326G 16214T 16217C 16335G 16519C	HV2a1	72C 73G 151T 152C 195C 198T 246C 263G 309.1CT 310C 16214T 16217C 16335G 16519C	1	72 73 151 152 195 198 246 263 629 709 750 1438 2706 4769 5147 7028 7193 8860 9336 11935 12061 15326 16214 16217 16335	HV2a1	72 73 151 152 195 198 246 263 16214 16217 16335	[0.818] HV2a1 (1)	[0.044] HV2a3 (2) [0.000] HV2a HV2a2 (4)	1
1785 >MF362784.1 Hc	263G 309.1CT 310C 709A 750G 1438G 1715T 2706G 3106d 3107C 4769G 7028T 7094C 7278C 8860G 15326G 15777A 15910T 16069T	HV4b	263G 309.1CT 310C 16069T	1	263 709 750 1438 1715 2706 4769 7028 7094 7278 8860 15326 15777 15910 16069	HV4b	263 16069	[0.000] HV4b (1)	[0.012] H2a2a (2) [0.000] R0 HV HV4 HV4c HV5 HV5a HV9c HV12 H H1 H1e H1e1 H1e1a H1e1a2 H1e1a7 H1e1c H1e2 H1e2a H1e2b H1e2d H1e4 H1e6 H1e7 H1e8 H1e8a H1h H1h1 H1h2 H1j H1j3 H1j5 H1j6 H1j7 H1j9 H1m H1n H1p H1q H1q1 H1q2 H1r H1s H1s1 H1t H1t1 H1t1a H1t2 H1u H1u1 H1v H1v1 H1v1a H1v1b H1w H1ae H1ae1 H1ae2 H1ae2a H1ae3 H1ae3a H1ag H1ag1 H1ag1b H1ah H1ai H1ai1 H1aj H1ak H1ak1 H1ak2 H1am H1am1 H1ap H1aq H1as H1as2 H1at H1at1 H1at1a H1av H1ax H1ax1 H1ay H1az H1bd H1be H1bi H1bj H1bk H1bm H1bn H1bp H1bq H1br H1bu H1bv H1bw H1bx H1ca H1ci H2 H2a H2a2 H2a2+(16235) H2a4 H2a5 H2a5a H2a5b H3 H3b H3c H3c1 H3e H3q H3q1 H3r H3r1 H3t H3u H3y H3aa H3ab H3ac H3ad H3ae H3ag H3ag1 H3ah H3ai H3aj H3ap H3aq H3ar H3as H3at H3at1 H3au H4 H4a H4a1 H4a1a H4a1a1 H4a1a2 H4a1a2a H4a1a2a1 H4a1a5 H4a1c H4a1c1 H4a1c1a H4a1d H4b H4c H7 H7a H7b H7b1 H7b2 H7b2a H7b3 H7b4 H7b5 H7b6 H7c [illegible]	1

1786	>MF362783.1 Hc	73G 150T 195C 204C 263G 295T 310.1C 456T 489C 513.1CA 567.1CCCCC 574C 750G 1438G 2706G 3106d 3107C 4216C 4769G 6671C 7028T 7476T 8860G 10398G 10499G 11002G 11251G 11377A 11719A 12570G 12612G 13015C 13708A 13830C 14766T 15257A 15326G 15452A 15679G 16069T 16126C 16241G	J2a2b2	73G 150T 195C 204C 263G 295T 310.1C 456T 489C 513.1CA 567.1CCCCC 574C 16069T 16126C 16241G	1	73 150 195 204 263 295 456 489 574C 573.5C 750 1438 2706 4216 4769 6671 7028 7476 8860 10398 10499 11002 11251 11377 11719 12570 12612 13015 13708 13830 14766 15257 15326 15452A 15679 16069 16126 16241	J2a2b2	73 150 195 204 263 295 456 489 574C 573.5C 16069 16126 16241	[0.187] J2a2b2 (1)	[0.187] J2a2b2 (2) [0.065] J2a2b (3) [0.000] J2a2b3	1
1787	>MF362781.1 Hc	73G 152C 263G 309.1CCT 310C 513.1CA 709A 750G 1438G 1888A 2706G 3106d 3107C 4216C 4769G 4917G 5747G 7028T 8697A 8860G 8978C 10463C 11251G 11626C 11719A 12633A 12879C 13368A 14314G 14766T 14905A 15326G 15452A 15607G 15928A 16126C 16163G 16186T 16189C 16249C 16294T 16302G 16519C	T1a9	73G 152C 263G 309.1CCT 310C 513.1CA 16126C 16163G 16186T 16189C 16249C 16294T 16302G 16519C	1	73 152 263 709 750 1438 1888 2706 4216 4769 4917 5747 7028 8697 8860 8978 10463 11251 11626 11719 12633A 12879 13368 14314 14766 14905 15326 15452A 15607 15928 16126 16163 16186 16189 16249 16294 16302	T1a9	73 152 263 16126 16163 16186 16189 16249 16294 16302	[0.153] T1a9 (1) [0.012] T1a+152 (2) [0.000] T1a2 (3)	[0.017] T1a5 (4) [0.009] T1a1b (5) [0.004] T1a (6) [0.000] T1a1'3 T1a1 T1a1a T1a1a1 T1a1b1 T1a1c T1a1d T1a1f T1a1g T1a1i T1a1j T1a1p T1a1q T1a1r T1a3 T1a3a T1a4 T1a12 T1a6 T1a8 T1a8b T1a10 (28)	1
1788	>MF362778.1 Hc	73G 94A 263G 309.1CT 310C 750G 1189C 1438G 1811G 2706G 3106d 3107C 3337A 3480G 4769G 5913A 7028T 8860G 9055A 9698C 9962A 10289G 10398G 10550G 11299C 11467G 11719A 11914A 12308G 12372A 13026T 14167T 14766T 14798C 15326G 15946T 16224C 16289G 16311C 16519C	K1b1c	73G 94A 263G 309.1CT 310C 16224C 16289G 16311C 16519C	1	73 94 263 750 1189 1438 1811 2706 3337 3480 4769 5913 7028 8860 9055 9698 9962 10289 10398 10550 11299 11467 11719 11914 12308 12372 13026 14167 14766 14798 15326 15946 16224 16289 16311	K1b1c	73 94 263 16224 16289 16311	[0.047] K1b1c (1)	[0.000] K K1 K1b K1b1 K1b1+(16093) K1b1b K1b1b1 (8)	1
1789	>MF362776.1 Hc	73G 151T 152C 263G 309.1CT 310C 709A 750G 1438G 1888A 2706G 3106d 3107C 4216C 4769G 4917G 5747G 7028T 8697A 8860G 8978C 10463C 11251G 11626C 11719A 12633A 12879C 13368A 14314G 14766T 14905A 15326G 15452A 15607G 15928A 16126C 16163G 16186T 16189C 16249C 16294T 16302G 16519C	T1a9	73G 151T 152C 263G 309.1CT 310C 16126C 16163G 16186T 16189C 16249C 16294T 16302G 16519C	1	73 151 152 263 709 750 1438 1888 2706 4216 4769 4917 5747 7028 8697 8860 8978 10463 11251 11626 11719 12633A 12879 13368 14314 14766 14905 15326 15452A 15607 15928 16126 16163 16186 16189 16249 16294 16302	T1a9	73 151 152 263 16126 16163 16186 16189 16249 16294 16302	[0.195] T1a9 (1) [0.041] T1a+152 (2) [0.005] T1a2 (3)	[0.025] T1a1 (4) [0.017] T1a5 (5) [0.009] T1a1b (6) [0.004] T1a (7) [0.000] T1a1'3 T1a1a T1a1a1 T1a1b1 T1a1c T1a1d T1a1f T1a1g T1a1i T1a1j T1a1p T1a1q T1a1r T1a3 T1a3a T1a4 T1a12 T1a6 T1a8 T1a8b T1a10 (28)	1
1790	>MF362773.1 Hc	73G 263G 295A 309.1CCT 310C 497T 514d 515d 750G 1189C 1438G 1811G 2706G 3106d 3107C 3480G 4769G 7028T 7624C 8860G 9055A 9698C 10293T 10398G 10550G 11299C 11467G 11719A 11818G 12308G 12372A 14167T 14766T 14798C 15326G 16224C 16266Y 16311C 16497G 16519C	K1a8b	73G 263G 295A 309.1CCT 310C 497T 514d 515d 16224C 16266Y 16311C 16497G 16519C	1	73 263 295A 497 750 1189 1438 1811 2706 3480 4769 7028 7624 8860 9055 9698 10293 10398 10550 11299 11467 11719 11818 12308 12372 14167 14766 14798 15326 16224 16266Y 16311 16497	K1a8b	73 263 295A 497 16224 16266Y 16311 16497	[0.013] K1a8b (1)	[0.013] K1a8b (2) [0.000] K1a8	1

1791 >MF362771.1 Hc	73G 151T 152C 263G 309.1CT 310C 709A 750G 1438G 1888A 2706G 3106d 3107C 4216C 4769G 4917G 5747G 7028T 8697A 8860G 8978C 10463C 11251G 11626C 11719A 12633A 12879C 13368A 14314G 14766T 14905A 15326G 15452A 15607G 15928A 16126C 16163G 16186T 16189C 16249C 16294T 16302G 16519C	T1a9	73G 151T 152C 263G 309.1CT 310C 16126C 16163G 16186T 16189C 16249C 16294T 16302G 16519C	1	73 151 152 263 709 750 1438 1888 2706 4216 4769 4917 5747 7028 8697 8860 8978 10463 11251 11626 11719 12633A 12879 13368 14314 14766 14905 15326 15452A 15607 15928 16126 16163 16186 16189 16249 16294 16302	T1a9	73 151 152 263 16126 16163 16186 16189 16249 16294 16302	[0.195] T1a9 (1) [0.041] T1a+152 (2) [0.005] T1a2 (3)	[0.025] T1a1 (4) [0.017] T1a5 (5) [0.009] T1a1b (6) [0.004] T1a (7) [0.000] T1a1'3 T1a1a T1a1a1 T1a1b1 T1a1c T1a1d T1a1f T1a1g T1a1i T1a1j T1a1p T1a1q T1a1r T1a3 T1a3a T1a4 T1a12 T1a6 T1a8 T1a8b T1a10 (28)	1
1792 >MF362764.1 Hc	152C 263G 310.1C 644G 750G 1438G 2706G 3106d 3107C 3397G 4769G 7028T 7436G 8860G 15326G 16169T 16305G 16519C	HV21	152C 263G 310.1C 16169T 16305G 16519C	1	152 263 644 750 1438 2706 3397 4769 7028 7436 8860 15326 16169 16305	HV21	152 263 16169 16305	[0.065] HV21 (1) [0.004] H+152 (2) [0.003] H1+152 (3) [0.000] H1i H1i1 H1i2 H1i2a H1an H1an1 H1an1a H1bb H1bc H2a2a2 H3+152 H3g H3g1 H3g1a H3g1b H3g2 H3g3 H3g4 H3i H3i1 H3j H3k H3k1 H3k1a H9 H46 H46a H46b H52 H69 H13a1+152 H14b H14b2 H14b4 H16+152 H16a H16a1 H16c H16d H30b1 H47a (44)	[0.154] H1m (45) [0.031] H13a1a1a (46) [0.023] H2a2a (47) [0.022] H9a (48) [0.018] H3a1 (49) [0.014] H1c9 (50) [0.012] HV (51) [0.009] H4a1 H26a1 (53) [0.005] H1h1 (54) [0.004] H3q1 H7b (56) [0.003] HV5 H1 H4b H16e H58a H75 (62) [0.002] H1e1a H1e2 H2a H7d H13a1a1 H13a2a H18 H18b (70) [0.001] HV4 H H1e1a2 H1e1c H1h2 H1q H1t1a H1ag1 H1aj H1at1a H3 H3ap H4 H4a1a H4a1a1 H4a1c H7 H7b1 H7c6 H7e H10b H10+(16093) H13a1a2 H13a2b4 H35 H40b H45a (97) [0.000] R0 HV1b+152 HV4c HV5a HV9+152 HV9c HV12 H1c+152 H1c9a H1e H1e1 H1e1a7 H1e2a H1e2b H1e2d H1e4 H1e6 H1e7 H1e8 H1e8a H1h H1j H1j3 H1j5 H1j6 H1j7 H1j9 H1n H1p H1q1 H1q2 H1r H1s H1s1 H1t H1t1 H1t2 H1u H1u1 H1v H1v1 H1v1a H1v1b H1w H1ae H1ae1 H1ae2 H1ae2a H1ae3 H1ae3a H1ag H1ag1b H1ah H1ai H1ai1 H1ak H1ak1 H1ak2 H1am H1am1 H1ap H1ap1 H1aq H1as H1as2 H1at H1at1 H1av H1ax H1ax1 H1ay H1az H1bd H1be H1bi H1bj H1bk H1bm H1bn H1bp H1bq H1br H1bu [illegible]	1
1793 >MF362763.1 Hc	73G 150T 195C 204C 263G 295T 310.1C 456T 489C 513.1CA 567.1CCCC 750G 1438G 2706G 3106d 3107C 4216C 4769G 6671C 7028T 7476T 8860G 10398G 10499G 11002G 11251G 11377A 11719A 12570G 12612G 13015C 13708A 13830C 14766T 15257A 15326G 15452A 15679G 16069T 16126C 16241G	J2a2b2	73G 150T 195C 204C 263G 295T 310.1C 456T 489C 513.1CA 567.1CCCC 16069T 16126C 16241G	1	73 150 195 204 263 295 456 489 573.4C 750 1438 2706 4216 4769 6671 7028 7476 8860 10398 10499 11002 11251 11377 11719 12570 12612 13015 13708 13830 14766 15257 15326 15452A 15679 16069 16126 16241	J2a2b2	73 150 195 204 263 295 456 489 573.4C 16069 16126 16241	[0.125] J2a2b2 (1)	[0.125] J2a2b2 (2) [0.065] J2a2b (3) [0.000] J2a2b3	1

1794 >MF362748.1 Hc	73G 151T 152C 263G 309.1CT 310C 709A 750G 1438G 1888A 2706G 3106d 3107C 4216C 4769G 4917G 5747G 7028T 8697A 8860G 8978C 10463C 11251G 11626C 11719A 12633A 12879C 13368A 14314G 14766T 14905A 15326G 15452A 15607G 15928A 16126C 16163G 16186T 16189C 16249C 16294T 16302G 16519C	T1a9	73G 151T 152C 263G 309.1CT 310C 16126C 16163G 16186T 16189C 16249C 16294T 16302G 16519C	1	73 151 152 263 709 750 1438 1888 2706 4216 4769 4917 5747 7028 8697 8860 8978 10463 11251 11626 11719 12633A 12879 13368 14314 14766 14905 15326 15452A 15607 15928 16126 16163 16186 16189 16249 16294 16302	T1a9	73 151 152 263 16126 16163 16186 16189 16249 16294 16302	[0.195] T1a9 (1) [0.041] T1a+152 (2) [0.005] T1a2 (3)	[0.025] T1a1 (4) [0.017] T1a5 (5) [0.009] T1a1b (6) [0.004] T1a (7) [0.000] T1a1'3 T1a1a T1a1a1 T1a1b1 T1a1c T1a1d T1a1f T1a1g T1a1i T1a1j T1a1p T1a1q T1a1r T1a3 T1a3a T1a4 T1a12 T1a6 T1a8 T1a8b T1a10 (28)	1
1795 >MF362744.1 Hc	73G 153G 195C 225A 263G 310.1C 376G 750G 1438G 1719A 2706G 3106d 3107C 4769G 6221C 6371T 7028T 8860G 11719A 12705T 13443C 13966G 14470C 14766T 15326G 16092C 16182C 16183C 16189C 16223T 16248T 16278T 16519C	X2i	73G 153G 195C 225A 263G 310.1C 376G 16092C 16182C 16183C 16189C 16223T 16248T 16278T 16519C	1	73 153 195 225 263 376 750 1438 1719 2706 4769 6221 6371 7028 8860 11719 12705 13443 13966 14470 14766 15326 16092 16189 16223 16248 16278	X2i	73 153 195 225 263 376 16092 16189 16223 16248 16278	[0.187] X2i (1)	[0.009] X2b (2) [0.007] X2+225 (3) [0.000] X2a'j X2b'd X2b1 X2e X2i+@225 X2o (9)	1
1796 >MF327713.1 Hc	263G 309.1CCT 310C 750G 1438G 2581G 3010A 3106d 3107C 4769G 8860G 15326G 15646T 16129A 16357C 16519C	H1af1b	263G 309.1CCT 310C 16129A 16357C 16519C	1	263 750 1438 2581 3010 4769 8860 15326 15646 16129 16357	H1af1b	263 16129 16357	[0.000] H1af1b (1)	[0.032] HV13b (2) [0.026] H17a (3) [0.012] HV13 (4) [0.000] H1e+16129 H1j1 H1j1a H1j1a2 H1j1b H1j1c H1af H1af1 H1af1a H1af2 H1cj H3b+16129 H3b2 H3b3 H3b5 H3b7 H3af H+16129 H17 H17a2 H17b H63a (26)	1
1797 >MF172950.1 Hc	73G 150T 152C 263G 309.1CT 310C 750G 1117G 1438G 1721T 2706G 3106d 3107C 3197C 3861G 4769G 7028T 7768G 8860G 9477A 11467G 11653G 11719A 12308G 12372A 12634G 13617C 13630G 13637G 14182C 14766T 15326G 15497A 16270T 16292T 16362C	U5b2b1a1	73G 150T 152C 263G 309.1CT 310C 16270T 16292T 16362C	1	73 150 152 263 750 1117 1438 1721 2706 3197 3861 4769 7028 7768 8860 9477 11467 11653 11719 12308 12372 12634 13617 13630 13637 14182 14766 15326 15497 16270 16292 16362	U5b2b1a1	73 150 152 263 16270 16292 16362	[0.032] U5b2b1a1 (1)	[0.000] U5b2b1a (2)	1
1798 >KY411492.1 Hc	73G 150T 263G 310.1C 513.1CA 750G 1438G 1811G 2706G 3106d 3107C 4188G 4640A 4769G 5237A 7028T 8093C 8860G 9656C 11467G 11719A 12308G 12372A 13743C 14139G 14766T 15326G 15454C 16168T 16266T 16343G 16519C	U3b3	73G 150T 263G 310.1C 513.1CA 16168T 16266T 16343G 16519C	1	73 150 263 750 1438 1811 2706 4188 4640A 4769 5237 7028 8093 8860 9656 11467 11719 12308 12372 13743 14139 14766 15326 15454 16168 16266 16343	U3b3	73 150 263 16168 16266 16343	[0.023] U3b3 (1)	[0.000] U3 U3a'c U3b U3b1 U3b1b U3b2 U3b2a U3b2a1 U3b2c (10)	1
1799 >KY411491.1 Hc	73G 150T 263G 310.1C 750G 1438G 1811G 2706G 3106d 3107C 4188G 4640A 4769G 5201C 7028T 8860G 9656C 11467G 11719A 12308G 12372A 13743C 13776G 14139G 14766T 15326G 15454C 16168T 16343G 16519C	U3b3	73G 150T 263G 310.1C 16168T 16343G 16519C	1	73 150 263 750 1438 1811 2706 4188 4640A 4769 5201 7028 8860 9656 11467 11719 12308 12372 13743 13776 14139 14766 15326 15454 16168 16343	U3b3	73 150 263 16168 16343	[0.000] U3b3 (1)	[0.000] U3 U3a'c U3b U3b1 U3b1b U3b2 U3b2a U3b2a1 U3b2c (10)	1

1800	>KY411490.1 Hc	73G 150T 263G 310.1C 750G 1438G 1811G 2706G 3106d 3107C 4092A 4188G 4640A 4769G 5201C 7028T 8860G 9656C 11467G 11719A 12308G 12372A 13743C 14139G 14766T 15326G 15454C 16168T 16343G 16519C	U3b3	73G 150T 263G 310.1C 16168T 16343G 16519C	1	73 150 263 750 1438 1811 2706 4092 4188 4640A 4769 5201 7028 8860 9656 11467 11719 12308 12372 13743 14139 14766 15326 15454 16168 16343	U3b3	73 150 263 16168 16343	[0.000] U3b3 (1)	[0.000] U3 U3a'c U3b U3b1 U3b1b U3b2 U3b2a U3b2a1 U3b2c (10)	1
1801	>KY411488.1 Hc	73G 150T 263G 309.1CT 310C 750G 1438G 1811G 2706G 3106d 3107C 4188G 4640A 4688C 4769G 5291C 5961T 7028T 7076G 8860G 9656C 9926G 11467G 11719A 12308G 12372A 13743C 14139G 14766T 15326G 15454C 16168T 16183C 16189C 16343G	U3b3	73G 150T 263G 309.1CT 310C 16168T 16183C 16189C 16343G	1	73 150 263 750 1438 1811 2706 4188 4640A 4688 4769 5291 5961 7028 7076 8860 9656 9926 11467 11719 12308 12372 13743 14139 14766 15326 15454 16168 16189 16343	U3b3	73 150 263 16168 16189 16343	[0.047] U3b3 (1)	[0.011] U3b2a (2) [0.008] U3b1 (3) [0.000] B6 U3 U3a'c U3a3 U3b U3b1b U3b2 U3b2a1 U3b2c (12)	1
1802	>KY411487.1 Hc	73G 146C 150T 263G 310.1C 505T 750G 1438G 1811G 2706G 3106d 3107C 4188G 4322T 4640A 4769G 6603T 7028T 7853A 8860G 8894G 9294T 9656C 10324C 11467G 11719A 12308G 12373G 13743C 14139G 14766T 15326G 15454C 16168T 16343G	U3b3	73G 146C 150T 263G 310.1C 505T 16168T 16343G	1	73 146 150 263 505 750 1438 1811 2706 4188 4322 4640A 4769 6603 7028 7853 8860 8894 9294T 9656 10324 11467 11719 12308 12373 13743 14139 14766 15326 15454 16168 16343	U3b3	73 146 150 263 505 16168 16343	[0.024] U3b3 (1)	[0.027] U3b2 (2) [0.020] U3b (3) [0.000] U3 U3a'c U3b1 U3b1b U3b2a U3b2a1 U3b2c (10)	1
1803	>KY411479.1 Hc	73G 150T 263G 310.1C 750G 1438G 1811G 2706G 3106d 3107C 3546A 4188G 4640A 4769G 7028T 8812G 8860G 9656C 11467G 11719A 12308G 12372A 12945C 13743C 14139G 14766T 15326G 15454C 16086C 16343G	U3b1a	73G 150T 263G 310.1C 16086C 16343G	1	73 150 263 750 1438 1811 2706 3546A 4188 4640A 4769 7028 8812 8860 9656 11467 11719 12308 12372 12945 13743 14139 14766 15326 15454 16086 16343	U3b1a	73 150 263 16086 16343	[0.000] U3b1a (1)	[0.000] U3 U3a'c U3b U3b1 U3b1a1 U3b1b U3b2 U3b2a U3b2a1 U3b2c (11)	1
1804	>KY411473.1 Hc	73G 150T 263G 310.1C 750G 1438G 1811G 2294G 2706G 3106d 3107C 4703C 4769G 7028T 7382A 7543G 8860G 9266A 9617G 11204C 11467G 11719A 12308G 12372A 12843C 14139G 14766T 15326G 15454C 16093C 16193T 16311C 16343G 16519C 16526A	U3c	73G 150T 263G 310.1C 16093C 16193T 16311C 16343G 16519C 16526A	1	73 150 263 750 1438 1811 2294 2706 4703 4769 7028 7382 7543 8860 9266 9617 11204 11467 11719 12308 12372 12843 14139 14766 15326 15454 16093 16193 16311 16343 16526	U3c	73 150 263 16093 16193 16311 16343 16526	[0.243] U3c (1)	[0.241] U3b2a1 (2) [0.034] U3b1 (3) [0.020] U3b (4) [0.018] U3b2 (5) [0.011] U3b2a (6) [0.000] U3 U3a'c U3b1b U3b2c (10)	1
1805	>KY411472.1 Hc	73G 150T 263G 310.1C 750G 1438G 1811G 2294G 2706G 3106d 3107C 4703C 4769G 7028T 7382A 7543G 8860G 9266A 11257T 11467G 11719A 12308G 12372A 12843C 14139G 14766T 15326G 15454C 16037G 16126C 16193T 16248T 16249C 16343G 16519C 16526A	U3c	73G 150T 263G 310.1C 16037G 16126C 16193T 16248T 16249C 16343G 16519C 16526A	1	73 150 263 750 1438 1811 2294 2706 4703 4769 7028 7382 7543 8860 9266 11257 11467 11719 12308 12372 12843 14139 14766 15326 15454 16037 16126 16193 16248 16249 16343 16526	U3c	73 150 263 16037 16126 16193 16248 16249 16343 16526	[0.108] U3c (1)	[0.009] U3b2a1 (2) [0.000] U3 U3a'c U3b U3b1 U3b1b U3b2 U3b2a U3b2c (10)	1

1806 >KY411469.1 Hc	73G 150T 197G 263G 309.1CCT 310C 750G 1375T 1438G 1811G 2294G 2706G 2766A 3106d 3107C 4703C 4769G 6518T 7028T 8450C 8860G 9266A 10143A 10506G 11467G 11719A 12308G 12372A 13934T 14139G 14766T 15326G 15454C 16183C 16189C 16343G 16390A 16519C	U3a3	73G 150T 197G 263G 309.1CCT 310C 16183C 16189C 16343G 16390A 16519C	1	73 150 197 263 750 1375 1438 1811 2294 2706 2766A 4703 4769 6518 7028 8450 8860 9266 10143 10506 11467 11719 12308 12372 13934 14139 14766 15326 15454 16189 16343 16390	U3a3	73 150 197 263 16189 16343 16390	[0.194] U3a3 (1)	[0.048] U3a1a (2) [0.025] U3a1 (3) [0.000] U3a U3a1a1 U3a1b U3a2 (7)	1
1807 >KY411465.1 Hc	73G 150T 189G 195C 200G 263G 309.1CT 310C 750G 1438G 1811G 2294G 2706G 3106d 3107C 4703C 4769G 6050C 6452T 6518T 7028T 8860G 9266A 10506G 11050C 11467G 11719A 12308G 12372A 13934T 14139G 14766T 14921A 15326G 15454C 16245T 16343G 16390A 16519C	U3a2a1a	73G 150T 189G 195C 200G 263G 309.1CT 310C 16245T 16343G 16390A 16519C	1	73 150 189 195 200 263 750 1438 1811 2294 2706 4703 4769 6050 6452 6518 7028 8860 9266 10506 11050 11467 11719 12308 12372 13934 14139 14766 14921 15326 15454 16245 16343 16390	U3a2a1a	73 150 189 195 200 263 16245 16343 16390	[0.083] U3a2a1a (1)	[0.043] U3a2a1 (2)	1
1808 >KY411464.1 Hc	73G 150T 189G 195C 200G 263G 309.1CT 310C 750G 1438G 1811G 2109G 2294G 2706G 3106d 3107C 4703C 4769G 6050C 6518T 7028T 8860G 9266A 10463C 10506G 11050C 11467G 11719A 12308G 12372A 13934T 14139G 14766T 15326G 15454C 16343G 16390A 16519C	U3a2a1	73G 150T 189G 195C 200G 263G 309.1CT 310C 16343G 16390A 16519C	1	73 150 189 195 200 263 750 1438 1811 2109 2294 2706 4703 4769 6050 6518 7028 8860 9266 10463 10506 11050 11467 11719 12308 12372 13934 14139 14766 15326 15454 16343 16390	U3a2a1	73 150 189 195 200 263 16343 16390	[0.043] U3a2a1 (1)	[0.097] U3a2a (2) [0.083] U3a2a1a	1
1809 >KY411463.1 Hc	73G 150T 152C 195C 200G 263G 310.1C 750G 1438G 1811G 2294G 2706G 3106d 3107C 4104G 4188G 4703C 4769G 6050C 6518T 7028T 8860G 9266A 10506G 11050C 11467G 11719A 12308G 12372A 13934T 14139G 14766T 15326G 15454C 16300G 16343G 16390A 16519C	U3a2a	73G 150T 152C 195C 200G 263G 310.1C 16300G 16343G 16390A 16519C	1	73 150 152 195 200 263 750 1438 1811 2294 2706 4104 4188 4703 4769 6050 6518 7028 8860 9266 10506 11050 11467 11719 12308 12372 13934 14139 14766 15326 15454 16300 16343 16390	U3a2a	73 150 152 195 200 263 16300 16343 16390	[0.161] U3a2a (1)	[0.130] U3a2a1 (2) [0.102] U3a1 (3) [0.063] U3a2 (4) [0.016] U3a (5) [0.000] U3a1a U3a1a1 U3a1b (8)	1
1810 >KY411458.1 Hc	73G 150T 263G 309.1CT 310C 750G 1438G 1811G 2294G 2706G 3010A 3106d 3107C 4703C 4769G 6518T 7028T 7165C 8860G 9266A 10506G 10724C 11467G 11719A 12308G 12372A 13934T 14139G 14766T 15326G 15454C 15734A 16343G 16356C 16390A 16519C	U3a1c	73G 150T 263G 309.1CT 310C 16343G 16356C 16390A 16519C	1	73 150 263 750 1438 1811 2294 2706 3010 4703 4769 6518 7028 7165 8860 9266 10506 10724 11467 11719 12308 12372 13934 14139 14766 15326 15454 15734 16343 16356 16390	U3a1c	73 150 263 16343 16356 16390	[0.000] U3a1c (1)	[0.000] U3a U3a1 U3a1a U3a1a1 U3a1b U3a1c1 U3a2 (8)	1

1811	>KY411457.1 Hc	73G 150T 263G 309.1CT 310C 750G 1438G 1811G 2294G 2706G 3010A 3106d 3107C 4703C 4769G 6518T 7028T 8860G 9266A 10506G 10724C 11467G 11719A 12308G 12372A 13934T 14139G 14766T 15326G 15454C 15734A 16343G 16356C 16390A 16519C	U3a1c	73G 150T 263G 309.1CT 310C 16343G 16356C 16390A 16519C	1	73 150 263 750 1438 1811 2294 2706 3010 4703 4769 6518 7028 8860 9266 10506 10724 11467 11719 12308 12372 13934 14139 14766 15326 15454 15734 16343 16356 16390	U3a1c	73 150 263 16343 16356 16390	[0.000] U3a1c (1)	[0.000] U3a U3a1 U3a1a U3a1a1 U3a1b U3a1c1 U3a2 (8)	1
1812	>KY411456.1 Hc	73G 150T 263G 309.1CT 310C 750G 1438G 1811G 2294G 2706G 3010A 3106d 3107C 4703C 4769G 6518T 7028T 8860G 9266A 10506G 11467G 11719A 12308G 12372A 13709T 13934T 14139G 14766T 15326G 15454C 16301T 16343G 16356C 16390A 16519C	U3a1c1	73G 150T 263G 309.1CT 310C 16301T 16343G 16356C 16390A 16519C	1	73 150 263 750 1438 1811 2294 2706 3010 4703 4769 6518 7028 8860 9266 10506 11467 11719 12308 12372 13709 13934 14139 14766 15326 15454 16301 16343 16356 16390	U3a1c1	73 150 263 16301 16343 16356 16390	[0.000] U3a1c1 (1)	[0.000] U3a1c (2)	1
1813	>KY411452.1 Hc	73G 195C 263G 308.1CCT 310C 514d 515d 1438G 1811G 2706G 3106d 3107C 3480G 4769G 6065T 6515G 6546T 6599G 7028T 8860G 9055A 9698C 9948A 10362T 11467G 11719A 11914A 12308G 12372A 12771A 14167T 14766T 15326G 15734A 16129A 16180d 16187T 16189C 16234T	U8b1a1	73G 195C 263G 308.1CCT 310C 514d 515d 16129A 16180d 16187T 16189C 16234T	1	73 195 263 315.2C 1438 1811 2706 3480 4769 6065 6515G 6546 6599 7028 8860 9055 9698 9948 10362 11467 11719 11914 12308 12372 12771 14167 14766 15326 15734 16129 16183d 16187 16189 16234	U8b1a1	73 195 263 315.2C 16129 16183d 16187 16189 16234	[0.107] U8b1a1 (1)	[0.000] U8b1 U8b1a U8b1a2 (4)	1
1814	>KY411449.1 Hc	73G 263G 309.1CT 310C 499A 750G 1438G 1811G 2706G 3106d 3107C 3531A 3768G 3834A 4769G 5999C 6386T 7028T 7220C 8860G 10410C 11337G 11467G 11719A 12308G 12372A 14094C 14766T 15265T 15326G 15712G 16051G 16129A 16259T 16261T 16278T	U9a	73G 263G 309.1CT 310C 499A 16051G 16129A 16259T 16261T 16278T	1	73 263 499 750 1438 1811 2706 3531 3768 3834 4769 5999 6386 7028 7220 8860 10410 11337 11467 11719 12308 12372 14094 14766 15265 15326 15712 16051 16129 16259 16261 16278	U9a	73 263 499 16051 16129 16259 16261 16278	[1.136] U9a (1)	[0.023] U2 (2) [0.010] B4h1 (3) [0.000] H1a3a3 H3b6 H3b6a U9a1 (7)	1
1815	>KY411444.1 Hc	73G 150T 263G 310.1C 750G 1438G 1721T 1834C 2706G 3106d 3107C 3197C 4732G 4769G 5452T 7028T 7768G 8860G 9477A 11467G 11719A 12308G 12372A 12684A 13105G 13617C 13637G 14182C 14766T 15326G 15511C 15924G 16519C	U5b2a1a2	73G 150T 263G 310.1C 16519C	1	73 150 263 750 1438 1721 1834 2706 3197 4732 4769 5452 7028 7768 8860 9477 11467 11719 12308 12372 12684 13105 13617 13637 14182 14766 15326 15511 15924	U5b2a1a2	73 150 263	[0.000] U5b2a1a2 (1)	[0.031] H4a1a1a1a (2) [0.026] U5b2a1a (3) [0.023] U5b2b (4) [0.021] R (5) [0.019] U3b2a1 (6) [0.000] L3e'i'k'x L3e L3e3'4'5 L3e3'4 L3i HV+73 HV20 HV5b HV12b H1e1a6 H1e2c H1as1 H1av1 H1av1a H3+73 H3d H4a1a1a H4a1a1a1 H4a1a1a1a1 H4a1a1a2 H4a1a1a4 R2'JT B6 R30 R30a R30a1 R30a1a R31 P P2 P4 P9 P9a U U5b1d1a U5b2b1 U5b2b2 U2'3'4'7'8'9 U3 U3a'c U3b U3b1 U3b1b U3b2 U3b2a U3b2c U8 U8b'c U8b (55)	1

1816	>KY411442.1 Hc	73G 146C 152C 263G 285T 309.1CT 310C 320T 750G 1438G 2056A 2387C 2410C 2706G 3106d 3107C 4769G 7028T 8395T 8860G 10885C 11467G 11719A 12308G 12372A 12879C 13104G 14070G 14766T 15148A 15172A 15326G 15709T 15954C 16111T 16214A 16249C 16327T 16519C	U1b2	73G 146C 152C 263G 285T 309.1CT 310C 320T 16111T 16214A 16249C 16327T 16519C	1	73 146 152 263 285 320 750 1438 2056 2387 2410 2706 4769 7028 8395 8860 10885 11467 11719 12308 12372 12879 13104 14070 14766 15148 15172 15326 15709 15954C 16111 16214A 16249 16327	U1b2	73 146 152 263 285 320 16111 16214A 16249 16327	[0.300] U1b2 (1)	[0.357] U1b (2) [0.300] U1b2 (3) [0.250] U1b1	1
1817	>MF116365.1 Hc	73G 150T 152C 189G 195C 215G 263G 295T 310.1TC 319C 489C 513A 750G 1438G 1850C 2706G 3106d 3107C 4216C 4769G 7028T 7476T 7789A 8860G 10237C 10398G 10499G 11251G 11377A 11719A 12612G 13708A 13722G 14133G 14766T 15191C 15257A 15326G 15452A 16069T 16126C 16145A 16172C 16231C 16261T	J2a1a1a1	73G 150T 152C 189G 195C 215G 263G 295T 310.1TC 319C 489C 513A 16069T 16126C 16145A 16172C 16231C 16261T	1	73 150 152 189 195 215 263 295 310.1T 319 489 513 750 1438 1850 2706 4216 4769 7028 7476 7789 8860 10237 10398 10499 11251 11377 11719 12612 13708 13722 14133 14766 15191 15257 15326 15452A 16069 16126 16145 16172 16231 16261	J2a1a1a1	73 150 152 189 195 215 263 295 310.1T 319 489 513 16069 16126 16145 16172 16231 16261	[0.000] J2a1a1a1 (1)	[0.000] J2a1a1a J2a1a1a1 J2a1a1a2 J2a1a1a2a (5)	1
1818	>MF112252.1 Hc	73G 199C 204C 250C 263G 309.1CT 310C 567.1CC 750G 1438G 1719A 2706G 3106d 3107C 4025T 4529T 4769G 7028T 8251A 8519A 8860G 9380A 10034C 10238C 10398G 10819G 11719A 12501A 12705T 13780G 14766T 15043A 15326G 15924G 16129A 16223T 16304C 16391A 16519C	I4a1	73G 199C 204C 250C 263G 309.1CT 310C 567.1CC 16129A 16223T 16304C 16391A 16519C	1	73 199 204 250 263 573.2C 750 1438 1719 2706 4025 4529T 4769 7028 8251 8519 8860 9380 10034 10238 10398 10819 11719 12501 12705 13780 14766 15043 15326 15924 16129 16223 16304 16391	I4a1	73 199 204 250 263 573.2C 16129 16223 16304 16391	[0.000] I4a1 (1)	[0.000] I I4 I4a I4a2 I4b I5 I5b I6 I6a (10)	1

1819 >MF043221.1 Hc	263G 309.1CT 310C 709A 750G 1438G 3010A 3106d 3107C 4769G 8860G 14861A 15122G 15326G 15924G 16220C 16519C	H1bs	263G 309.1CT 310C 16220C 16519C	1	263 709 750 1438 3010 4769 8860 14861 15122 15326 15924 16220C	H1bs	263 16220C	[0.000] H1bs (1)	[0.012] HV12a (2) [0.001] H2a2a (3) [0.000] R0 HV HV4 HV4c HV5 HV5a HV9c HV12 H H1 H1e H1e1 H1e1a H1e1a2 H1e1a7 H1e1c H1e2 H1e2a H1e2b H1e2d H1e4 H1e6 H1e7 H1e8 H1e8a H1h H1h1 H1h2 H1j H1j3 H1j5 H1j6 H1j7 H1j9 H1m H1n H1p H1q H1q1 H1q2 H1r H1s H1s1 H1t H1t1 H1t1a H1t2 H1u H1u1 H1v H1v1 H1v1a H1v1b H1w H1ae H1ae1 H1ae2 H1ae2a H1ae3 H1ae3a H1ag H1ag1 H1ag1b H1ah H1ai H1ai1 H1aj H1ak H1ak1 H1ak2 H1am H1am1 H1ap H1aq H1as H1as2 H1at H1at1 H1at1a H1av H1ax H1ax1 H1ay H1az H1bd H1be H1bi H1bj H1bk H1bm H1bn H1bp H1bq H1br H1bu H1bv H1bw H1bx H1ca H1ci H2 H2a H2a2 H2a2+(16235) H2a4 H2a5 H2a5a H2a5b H3 H3b H3c H3c1 H3e H3q H3q1 H3r H3r1 H3t H3u H3y H3aa H3ab H3ac H3ad H3ae H3ag H3ag1 H3ah H3ai H3aj H3ap H3aq H3ar H3as H3at H3at1 H3au H4 H4a H4a1 H4a1a H4a1a1 H4a1a2 H4a1a2a H4a1a2a1 H4a1a5 H4a1c H4a1c1 H4a1c1a H4a1d H4b H4c H7 H7a H7b H7b1 H7b2 H7b2a H7b3 H7b4 H7b5 [illegible]	1
1820 >MF035492.1 Hc	93G 263G 310.1C 514d 515d 750G 1438G 3010A 3106d 3107C 3796G 4769G 8860G 15326G 16183M 16189C 16356C 16362C 16519C	H1b1d	93G 263G 310.1C 514d 515d 16183M 16189C 16356C 16362C 16519C	1	93 263 750 1438 3010 3796 4769 8860 15326 16183M 16189 16356 16362	H1b1d	93 263 16183M 16189 16356 16362	[0.000] H1b1d (1)	[0.009] H1b1a (2) [0.000] H1b1+16362 H1b1c H1b1h (5)	1
1821 >KY981527.1 Hc	73G 199C 204C 250C 263G 309.1CT 310C 567.1CCCC 750G 1719A 2706G 3106d 3107C 4529T 4769G 7028T 8027A 8251A 8860G 10034C 10238C 10398G 11719A 12501A 12705T 13780G 14233G 14766T 15043A 15326G 15924G 16075C 16129A 16169T 16223T 16260T 16391A 16519C	I5c1	73G 199C 204C 250C 263G 309.1CT 310C 567.1CCCC 16075C 16129A 16169T 16223T 16260T 16391A 16519C	1	73 199 204 250 263 573.4C 750 1719 2706 4529T 4769 7028 8027 8251 8860 10034 10238 10398 11719 12501 12705 13780 14233 14766 15043 15326 15924 16075 16129 16169 16223 16260 16391	I5c1	73 199 204 250 263 573.4C 16075 16129 16169 16223 16260 16391	[0.000] I5c1 (1)	[0.000] I5c (2)	1
1822 >KY967268.1 Hc	73G 150T 263G 310.1C 750G 1438G 2706G 3106d 3107C 3197C 4769G 5437T 5656G 7028T 7768G 7912A 8860G 9477A 11467G 11719A 12308G 12372A 13191C 13617C 14182C 14766T 15326G 15631G 15721C 16189Y 16192T 16218T 16320T	U5b1d1c	73G 150T 263G 310.1C 16189Y 16192T 16218T 16320T	1	73 150 263 750 1438 2706 3197 4769 5437 5656 7028 7768 7912 8860 9477 11467 11719 12308 12372 13191 13617 14182 14766 15326 15631 15721 16189Y 16192 16218 16320	U5b1d1c	73 150 263 16189Y 16192 16218 16320	[0.068] U5b1d1c (1) [0.000] U5b1b1b U5b2a1 (3)	[0.026] U5b2a1a (4) [0.000] B6 U5b1+16189 U5b1b U5b1b1 U5b1b1g U5b2a U5b2a3 U5b2a3a (12)	1

1823	>KY967269.1 Hc	73G 152C 217C 263G 310.1C 340T 508G 513.1CA 750G 1438G 1811G 2414A 2706G 3106d 3107C 3316A 3720G 4769G 5390G 5426C 6045T 6152C 7028T 8812T 8860G 10876G 11467G 11719A 12308G 12372A 13020C 13350G 13734C 14198A 14766T 15214C 15326G 15519C 15907G 16051G 16086C 16129C 16183C 16189C 16311C 16362C 16519C	U2e1f	73G 152C 217C 263G 310.1C 340T 508G 513.1CA 16051G 16086C 16129C 16183C 16189C 16311C 16362C 16519C	1	73 152 217 263 340 508 750 1438 1811 2414A 2706 3316 3720 4769 5390 5426 6045 6152 7028 8812T 8860 10876 11467 11719 12308 12372 13020 13350 13734 14198 14766 15214 15326 15519 15907 16051 16086 16129C 16189 16311 16362	U2e1f	73 152 217 263 340 508 16051 16086 16129C 16189 16311 16362	[0.500] U2e1f (1)	[0.000] U2e1 U2e1a U2e1a1 U2e1a1a U2e1a1c U2e1c U2e1c1 U2e1d U2e1g (10)	1
1824	>KY083711.1 Hc	73G 150T 214G 263G 310.1C 709A 750G 1438G 1888A 2706G 3106d 3107C 4216C 4769G 4917G 7028T 8697A 8860G 10463C 11016A 11251G 11719A 12633A 13368A 13470G 14766T 14905A 15326G 15452A 15607G 15928A 16126C 16163G 16186T 16189C 16294T 16519C 16524G	T1a10a	73G 150T 214G 263G 310.1C 16126C 16163G 16186T 16189C 16294T 16519C 16524G	1	73 150 214 263 709 750 1438 1888 2706 4216 4769 4917 7028 8697 8860 10463 11016 11251 11719 12633A 13368 13470 14766 14905 15326 15452A 15607 15928 16126 16163 16186 16189 16294 16524	T1a10a	73 150 214 263 16126 16163 16186 16189 16294 16524	[0.018] T1a10a (1)	[0.011] T1a8b (2) [0.000] T1a T1a5 T1a6 T1a8 T1a10 (7)	1
1825	>KY083710.1 Hc	73G 150T 214G 263G 310.1C 709A 750G 1438G 1888A 2706G 3106d 3107C 4216C 4769G 4917G 7028T 8697A 8860G 10463C 11016A 11251G 11719A 12633A 13368A 13470G 14766T 14905A 15326G 15452A 15607G 15928A 16126C 16163G 16186T 16189C 16294T 16519C 16524G	T1a10a	73G 150T 214G 263G 310.1C 16126C 16163G 16186T 16189C 16294T 16519C 16524G	1	73 150 214 263 709 750 1438 1888 2706 4216 4769 4917 7028 8697 8860 10463 11016 11251 11719 12633A 13368 13470 14766 14905 15326 15452A 15607 15928 16126 16163 16186 16189 16294 16524	T1a10a	73 150 214 263 16126 16163 16186 16189 16294 16524	[0.018] T1a10a (1)	[0.011] T1a8b (2) [0.000] T1a T1a5 T1a6 T1a8 T1a10 (7)	1
1826	>KY083704.1 Hc	73G 139C 153G 195C 257G 263G 310C 750G 1438G 1719A 2706G 3106d 3107C 4769G 6221C 6371T 7028T 8860G 9555A 10550G 11719A 11914A 12705T 13966G 14203G 14470C 14766T 15326G 16093C 16189C 16223T 16278T 16519C	X2f	73G 139C 153G 195C 257G 263G 310C 16093C 16189C 16223T 16278T 16519C	1	73 139 153 195 257 263 310 750 1438 1719 2706 4769 6221 6371 7028 8860 9555A 10550 11719 11914 12705 13966 14203 14470 14766 15326 16093 16189 16223 16278	X2f	73 139 153 195 257 263 310 16093 16189 16223 16278	[0.027] X2f (1)	[0.091] X2f1 (2) [0.034] X2e2 (3) [0.032] X2 (4) [0.009] X2k X2p (6) [0.000] X2e2a X2e2a1 X2o1 (9)	1
1827	>KY825047.1 Hc	73G 146C 151T 152C 263G 309.1CT 310C 514d 515d 750G 980C 1438G 1811G 2706G 3106d 3107C 3741T 4769G 5360T 5985A 7028T 8137T 8684T 8860G 10142T 11467G 11719A 12308G 12372A 13500C 14569A 14766T 15172A 15326G 16126C 16150T 16209C 16309G 16318T 16390A 16519C	U7a4	73G 146C 151T 152C 263G 309.1CT 310C 514d 515d 16126C 16150T 16209C 16309G 16318T 16390A 16519C	1	73 146 151 152 263 750 980 1438 1811 2706 3741 4769 5360 5985 7028 8137 8684 8860 10142 11467 11719 12308 12372 13500 14569 14766 15172 15326 16126 16150 16209 16309 16318T 16390	U7a4	73 146 151 152 263 16126 16150 16209 16309 16318T 16390	[0.821] U7a4 (1)	[0.250] U7a4a (2)	1

1828	>KY825046.1 Hc	73G 151T 152C 263G 310.1C 514d 515d 750G 980C 1438G 1811G 2706G 3106d 3107C 3741T 3834A 4769G 5360T 7028T 8137T 8684T 8860G 10142T 11467G 11719A 12308G 12372A 12585T 12618A 13500C 14569A 14766T 15326G 16092C 16207G 16309G 16318T 16519C	U7a3b	73G 151T 152C 263G 310.1C 514d 515d 16092C 16207G 16309G 16318T 16519C	1	73 151 152 263 750 980 1438 1811 2706 3741 3834 4769 5360 7028 8137 8684 8860 10142 11467 11719 12308 12372 12585 12618 13500 14569 14766 15326 16092 16207 16309 16318T	U7a3b	73 151 152 263 16092 16207 16309 16318T	[0.057] U7a3b (1)	[0.132] U7a2 (2) [0.057] U7a3b (3) [0.017] U7a (4) [0.000] U7a3 U7a3a (6)	1
1829	>KY825045.1 Hc	73G 151T 152C 263G 309.1CT 310C 514d 515d 750G 980C 1438G 1811G 2706G 3106d 3107C 3434G 3741T 3834A 4769G 5360T 5840T 6956C 7028T 8137T 8684T 8860G 10142T 11467G 11719A 12308G 12372A 12384C 12618A 13500C 14218C 14569A 14766T 15326G 16093C 16126C 16207G 16292T 16309G 16318T 16519C	U7a3b	73G 151T 152C 263G 309.1CT 310C 514d 515d 16093C 16126C 16207G 16292T 16309G 16318T 16519C	1	73 151 152 263 750 980 1438 1811 2706 3434 3741 3834 4769 5360 5840 6956 7028 8137 8684 8860 10142 11467 11719 12308 12372 12384 12618 13500 14218 14569 14766 15326 16093 16126 16207 16292 16309 16318T	U7a3b	73 151 152 263 16093 16126 16207 16292 16309 16318T	[0.341] U7a3b (1)	[0.341] U7a3b (2) [0.129] U7a4 (3) [0.046] U7a (4) [0.009] U7a3a (5) [0.000] U7a2 U7a3 (7)	1
1830	>KY825036.1 Hc	73G 151T 152C 263G 309.1CT 310C 514d 515d 750G 980C 1438G 1811G 2706G 3106d 3107C 3741T 4502C 4769G 5265G 5360T 7028T 8137T 8684T 8860G 10142T 11467G 11719A 12094T 12308G 12372A 13500C 14533T 14569A 14766T 15326G 16129A 16318T 16519C	U7a2a	73G 151T 152C 263G 309.1CT 310C 514d 515d 16129A 16318T 16519C	1	73 151 152 263 750 980 1438 1811 2706 3741 4502 4769 5265 5360 7028 8137 8684 8860 10142 11467 11719 12094 12308 12372 13500 14533 14569 14766 15326 16129 16318T	U7a2a	73 151 152 263 16129 16318T	[0.000] U7a2a (1)	[0.029] U7a (2) [0.009] U7a3a (3) [0.000] U7a2 U7a2a U7a3 (6)	1
1831	>KY825035.1 Hc	73G 151T 152C 263G 309.1CT 310C 514d 515d 750G 980C 1438G 1811G 2706G 3106d 3107C 3741T 4502C 4769G 5265G 5360T 7028T 8137T 8684T 8860G 10142T 11467G 11719A 12094T 12308G 12372A 13500C 14221C 14533T 14569A 14766T 15326G 16129A 16318T 16519C	U7a2a	73G 151T 152C 263G 309.1CT 310C 514d 515d 16129A 16318T 16519C	1	73 151 152 263 750 980 1438 1811 2706 3741 4502 4769 5265 5360 7028 8137 8684 8860 10142 11467 11719 12094 12308 12372 13500 14221 14533 14569 14766 15326 16129 16318T	U7a2a	73 151 152 263 16129 16318T	[0.000] U7a2a (1)	[0.029] U7a (2) [0.009] U7a3a (3) [0.000] U7a2 U7a2a U7a3 (6)	1
1832	>KY825034.1 Hc	73G 151T 152C 263G 270G 310.1C 514d 515d 750G 980C 1438G 1811G 2706G 3106d 3107C 3741T 4769G 5360T 5492C 6911C 7028T 8137T 8684T 8860G 10142T 11150A 11467G 11719A 12092T 12308G 12372A 13281C 13500C 13708A 14569A 14766T 15326G 16167T 16318T 16327T 16519C	U7a1a	73G 151T 152C 263G 270G 310.1C 514d 515d 16167T 16318T 16327T 16519C	1	73 151 152 263 270 750 980 1438 1811 2706 3741 4769 5360 5492 6911 7028 8137 8684 8860 10142 11150 11467 11719 12092 12308 12372 13281 13500 13708 14569 14766 15326 16167 16318T 16327	U7a1a	73 151 152 263 270 16167 16318T 16327	[0.062] U7a1a (1)	[0.062] U7a1a (2) [0.000] U7a1	1

1833	>KY825031.1 Hc	73G 151T 152C 263G 309.1CT 310C 514d 515d 567.1CCCCC 750G 980C 1438G 1811G 2706G 3106d 3107C 3741T 3834A 4769G 5360T 7028T 8137T 8684T 8860G 9067G 10142T 11467G 11719A 12308G 12372A 12585T 12618A 13500C 14569A 14766T 15326G 16189C 16207G 16309G 16318C 16519C	U7a3b	73G 151T 152C 263G 309.1CT 310C 514d 515d 567.1CCCCC 16189C 16207G 16309G 16318C 16519C	1	73 151 152 263 573.5C 750 980 1438 1811 2706 3741 3834 4769 5360 7028 8137 8684 8860 9067 10142 11467 11719 12308 12372 12585 12618 13500 14569 14766 15326 16189 16207 16309 16318C	U7a3b	73 151 152 263 573.5C 16189 16207 16309 16318C	[0.626] U7a3b (1)	[0.626] U7a3b (2) [0.513] U7a (3) [0.176] U7a3a (4) [0.033] U8c (5) [0.023] U7a2 (6) [0.000] U7a3	1
1834	>KY825029.1 Hc	73G 151T 152C 263G 309.1CT 310C 514d 515d 750G 980C 1438G 1811G 2706G 3106d 3107C 3741T 3834A 4769G 4820A 5360T 6164T 7028T 8137T 8684T 8860G 10142T 11467G 11719A 12308G 12372A 12618A 13500C 14218C 14569A 14766T 15326G 16207G 16309G 16318C 16519C	U7a3b	73G 151T 152C 263G 309.1CT 310C 514d 515d 16207G 16309G 16318C 16519C	1	73 151 152 263 750 980 1438 1811 2706 3741 3834 4769 4820 5360 6164 7028 8137 8684 8860 10142 11467 11719 12308 12372 12618 13500 14218 14569 14766 15326 16207 16309 16318C	U7a3b	73 151 152 263 16207 16309 16318C	[0.410] U7a3b (1)	[0.496] U7a (2) [0.410] U7a3b (3) [0.124] U7a3a (4) [0.000] U7a2 U7a3 (6)	1
1835	>KY825023.1 Hc	73G 151T 152C 263G 309.1CT 310C 385G 514d 515d 750G 980C 1438G 1811G 2706G 3106d 3107C 3741T 3834A 4769G 5360T 7028T 8137T 8684T 8860G 10142T 11467G 11719A 12308G 12372A 12618A 13500C 14137T 14569A 14766T 15326G 16207G 16309G 16318T 16519C	U7a3b	73G 151T 152C 263G 309.1CT 310C 385G 514d 515d 16207G 16309G 16318T 16519C	1	73 151 152 263 385 750 980 1438 1811 2706 3741 3834 4769 5360 7028 8137 8684 8860 10142 11467 11719 12308 12372 12618 13500 14137 14569 14766 15326 16207 16309 16318T	U7a3b	73 151 152 263 385 16207 16309 16318T	[0.023] U7a3b (1)	[0.023] U7a3b (2) [0.021] U7a (3) [0.000] U7a2 U7a3 U7a3a (6)	1
1836	>KY825022.1 Hc	73G 151T 152C 263G 309.1CT 310C 514d 515d 750G 980C 1438G 1811G 2706G 3106d 3107C 3741T 3834A 4529G 4769G 4772C 5360T 7028T 8137T 8684T 8860G 10101C 10142T 11467G 11719A 12308G 12372A 12618A 13500C 14218C 14569A 14766T 15326G 16207G 16309G 16318T 16519C	U7a3b	73G 151T 152C 263G 309.1CT 310C 514d 515d 16207G 16309G 16318T 16519C	1	73 151 152 263 750 980 1438 1811 2706 3741 3834 4529 4769 4772 5360 7028 8137 8684 8860 10101 10142 11467 11719 12308 12372 12618 13500 14218 14569 14766 15326 16207 16309 16318T	U7a3b	73 151 152 263 16207 16309 16318T	[0.000] U7a3b (1)	[0.013] U7a (2) [0.000] U7a2 U7a3 U7a3a U7a3b (6)	1
1837	>KY825016.1 Hc	73G 151T 152C 263G 270G 309.1CT 310C 369.1AAGAA 750G 980C 1438G 1811G 2706G 3106d 3107C 3741T 4769G 5360T 5492C 7028T 8137T 8484C 8559T 8684T 8860G 10142T 11150A 11467G 11719A 12092T 12308G 12372A 13281C 13500C 14319C 14569A 14766T 15326G 15916C 16167T 16318T 16519C	U7a1a	73G 151T 152C 263G 270G 309.1CT 310C 369.1AAGAA 16167T 16318T 16519C	1	73 151 152 263 270 369.1AAGAA 750 980 1438 1811 2706 3741 4769 5360 5492 7028 8137 8484C 8559 8684 8860 10142 11150 11467 11719 12092 12308 12372 13281 13500 14319 14569 14766 15326 15916 16167 16318T	U7a1a	73 151 152 263 270 369.1AAGAA 16167 16318T	[0.062] U7a1a (1)	[0.062] U7a1a (2) [0.000] U7a1	1

1838	>KY825010.1 Ho	73G 151T 152C 188G 263G 309.1CT 310C 514d 515d 750G 980C 1438G 1811G 2706G 3106d 3107C 3741T 3834A 4769G 5360T 6164T 7028T 7220C 8137T 8684T 8860G 10142T 11467G 11719A 12308G 12372A 12618A 13500C 14218C 14569A 14766T 15326G 16207G 16309G 16318T 16352C 16519C	U7a3b	73G 151T 152C 188G 263G 309.1CT 310C 514d 515d 16207G 16309G 16318T 16352C 16519C	1	73 151 152 188 263 750 980 1438 1811 2706 3741 3834 4769 5360 6164 7028 7220 8137 8684 8860 10142 11467 11719 12308 12372 12618 13500 14218 14569 14766 15326 16207 16309 16318T 16352	U7a3b	73 151 152 188 263 16207 16309 16318T 16352	[0.046] U7a3b (1)	[0.046] U7a3b (2) [0.043] U7a3a (3) [0.013] U7a (4) [0.000] U7a2 U7a3 (6)	1
1839	>KY824987.1 Ho	73G 151T 152C 263G 310.1C 514d 515d 750G 980C 1438G 1811G 2706G 3106d 3107C 3355G 3741T 3834A 4769G 5360T 7028T 8137T 8684T 8860G 10142T 11467G 11719A 12091C 12308G 12372A 12618A 13500C 14569A 14766T 15326G 16207G 16309G 16318T 16356C 16519C	U7a3b	73G 151T 152C 263G 310.1C 514d 515d 16207G 16309G 16318T 16356C 16519C	1	73 151 152 263 750 980 1438 1811 2706 3355 3741 3834 4769 5360 7028 8137 8684 8860 10142 11467 11719 12091 12308 12372 12618 13500 14569 14766 15326 16207 16309 16318T 16356	U7a3b	73 151 152 263 16207 16309 16318T 16356	[0.011] U7a3b (1)	[0.013] U7a (2) [0.011] U7a3b (3) [0.000] U7a2 U7a3 U7a3a (6)	1
1840	>KY824980.1 Ho	73G 151T 152C 263G 310.1C 514d 515d 750G 980C 1438G 1811G 2706G 3106d 3107C 3741T 3834A 4769G 5360T 5417A 6164T 7028T 8137T 8684T 8860G 9746A 10142T 11467G 11719A 12308G 12372A 12618A 13500C 14218C 14569A 14766T 15244G 15326G 16207G 16309G 16318C 16519C	U7a3b	73G 151T 152C 263G 310.1C 514d 515d 16207G 16309G 16318C 16519C	1	73 151 152 263 750 980 1438 1811 2706 3741 3834 4769 5360 5417 6164 7028 8137 8684 8860 9746 10142 11467 11719 12308 12372 12618 13500 14218 14569 14766 15244 15326 16207 16309 16318C	U7a3b	73 151 152 263 16207 16309 16318C	[0.410] U7a3b (1)	[0.496] U7a (2) [0.410] U7a3b (3) [0.124] U7a3a (4) [0.000] U7a2 U7a3 (6)	1
1841	>KY824979.1 Ho	73G 151T 152C 263G 309.1CT 310C 514d 515d 750G 980C 1438G 1811G 2706G 3106d 3107C 3741T 3834A 4769G 5360T 7028T 7561C 8137T 8684T 8860G 10142T 11467G 11719A 12308G 12372A 12618A 13500C 14569A 14766T 15326G 16169T 16207G 16309G 16318T 16519C	U7a3b	73G 151T 152C 263G 309.1CT 310C 514d 515d 16169T 16207G 16309G 16318T 16519C	1	73 151 152 263 750 980 1438 1811 2706 3741 3834 4769 5360 7028 7561 8137 8684 8860 10142 11467 11719 12308 12372 12618 13500 14569 14766 15326 16169 16207 16309 16318T	U7a3b	73 151 152 263 16169 16207 16309 16318T	[0.011] U7a3b (1)	[0.017] U7a (2) [0.011] U7a3b (3) [0.000] U7a2 U7a3 U7a3a (6)	1
1842	>KY824978.1 Ho	73G 151T 152C 263G 270G 310.1C 514d 515d 750G 980C 1438G 1811G 2706G 3106d 3107C 3741T 4769G 5360T 5486T 7028T 8137T 8684T 8860G 10142T 11467G 11719A 12308G 12372A 13281C 13500C 14133G 14569A 14766T 15326G 16167T 16309G 16318T 16519C	U7a1a	73G 151T 152C 263G 270G 310.1C 514d 515d 16167T 16309G 16318T 16519C	1	73 151 152 263 270 750 980 1438 1811 2706 3741 4769 5360 5486 7028 8137 8684 8860 10142 11467 11719 12308 12372 13281 13500 14133 14569 14766 15326 16167 16309 16318T	U7a1a	73 151 152 263 270 16167 16309 16318T	[0.000] U7a1a (1)	[0.000] U7a1 U7a1a (3)	1

1843 >KY824976.1 Hc	73G 151T 152C 263G 309.1CT 310C 514d 515d 750G 980C 1438G 1811G 2706G 3106d 3107C 3741T 3834A 4769G 5360T 6164T 7028T 8137T 8684T 8860G 10142T 11467G 11719A 12308G 12372A 12618A 13500C 14218C 14569A 14766T 15326G 16207G 16309G 16318T 16519C	U7a3b	73G 151T 152C 263G 309.1CT 310C 514d 515d 16207G 16309G 16318T 16519C	1	73 151 152 263 750 980 1438 1811 2706 3741 3834 4769 5360 6164 7028 8137 8684 8860 10142 11467 11719 12308 12372 12618 13500 14218 14569 14766 15326 16207 16309 16318T	U7a3b	73 151 152 263 16207 16309 16318T	[0.000] U7a3b (1)	[0.013] U7a (2) [0.000] U7a2 U7a3 U7a3a U7a3b (6)	1
1844 >KY824971.1 Hc	73G 151T 152C 263G 270G 309.1CT 310C 514d 515d 750G 980C 1438G 1811G 2706G 3106d 3107C 3741T 4769G 5360T 5486T 7028T 8137T 8684T 8860G 10142T 11467G 11719A 12308G 12372A 13281C 13500C 14569A 14766T 15326G 16092C 16167T 16172C 16294T 16318T 16519C	U7a1a	73G 151T 152C 263G 270G 309.1CT 310C 514d 515d 16092C 16167T 16172C 16294T 16318T 16519C	1	73 151 152 263 270 750 980 1438 1811 2706 3741 4769 5360 5486 7028 8137 8684 8860 10142 11467 11719 12308 12372 13281 13500 14569 14766 15326 16092 16167 16172 16294 16318T	U7a1a	73 151 152 263 270 16092 16167 16172 16294 16318T	[0.686] U7a1a (1)	[0.686] U7a1a (2) [0.000] U7a1	1
1845 >KY824970.1 Hc	73G 151T 152C 188G 263G 310.1C 514d 515d 750G 980C 1438G 1811G 2706G 3106d 3107C 3741T 3834A 4171T 4769G 5360T 6164T 7028T 7220C 8137T 8684T 8860G 10142T 11467G 11719A 12308G 12372A 12618A 13500C 14218C 14569A 14766T 15326G 16207G 16309G 16318T 16352C 16519C	U7a3b	73G 151T 152C 188G 263G 310.1C 514d 515d 16207G 16309G 16318T 16352C 16519C	1	73 151 152 188 263 750 980 1438 1811 2706 3741 3834 4171 4769 5360 6164 7028 7220 8137 8684 8860 10142 11467 11719 12308 12372 12618 13500 14218 14569 14766 15326 16207 16309 16318T 16352	U7a3b	73 151 152 188 263 16207 16309 16318T 16352	[0.046] U7a3b (1)	[0.046] U7a3b (2) [0.043] U7a3a (3) [0.013] U7a (4) [0.000] U7a2 U7a3 (6)	1
1846 >KY824969.1 Hc	73G 151T 152C 263G 309.1CT 310C 514d 515d 750G 980C 1438G 1811G 2706G 3106d 3107C 3741T 3834A 4769G 5360T 7028T 7498A 8137T 8684T 8860G 9452A 10142T 11467G 11719A 12308G 12372A 12414C 12618A 13500C 14569A 14766T 15326G 16207G 16318T 16519C	U7a3b	73G 151T 152C 263G 309.1CT 310C 514d 515d 16207G 16318T 16519C	1	73 151 152 263 750 980 1438 1811 2706 3741 3834 4769 5360 7028 7498 8137 8684 8860 9452 10142 11467 11719 12308 12372 12414 12618 13500 14569 14766 15326 16207 16318T	U7a3b	73 151 152 263 16207 16318T	[0.000] U7a3b (1)	[0.013] U7a (2) [0.000] U7a2 U7a3 U7a3a U7a3b (6)	1
1847 >KY824967.1 Hc	73G 151T 152C 263G 270G 310.1C 514d 515d 750G 980C 1438G 1811G 2706G 3106d 3107C 3399G 3741T 4769G 5360T 5486T 7028T 8137T 8684T 8860G 10142T 11467G 11719A 12308G 12372A 13281C 13500C 14569A 14766T 15326G 16167T 16172C 16262T 16318T 16519C	U7a1a	73G 151T 152C 263G 270G 310.1C 514d 515d 16167T 16172C 16262T 16318T 16519C	1	73 151 152 263 270 750 980 1438 1811 2706 3399 3741 4769 5360 5486 7028 8137 8684 8860 10142 11467 11719 12308 12372 13281 13500 14569 14766 15326 16167 16172 16262 16318T	U7a1a	73 151 152 263 270 16167 16172 16262 16318T	[0.687] U7a1a (1)	[0.687] U7a1a (2) [0.000] U7a1	1

1848	>KY824964.1 Hc	73G 151T 152C 263G 270G 310.1C 514d 515d 750G 980C 1438G 1811G 2706G 3106d 3107C 3741T 4769G 5360T 5486T 7028T 8137T 8684T 8860G 10142T 11467G 11719A 12308G 12372A 13281C 13500C 14569A 14766T 15326G 16167T 16172C 16262T 16318T 16519C	U7a1a	73G 151T 152C 263G 270G 310.1C 514d 515d 16167T 16172C 16262T 16318T 16519C	1	73 151 152 263 270 750 980 1438 1811 2706 3741 4769 5360 5486 7028 8137 8684 8860 10142 11467 11719 12308 12372 13281 13500 14569 14766 15326 16167 16172 16262 16318T	U7a1a	73 151 152 263 270 16167 16172 16262 16318T	[0.687] U7a1a (1)	[0.687] U7a1a (2) [0.000] U7a1	1
1849	>KY824962.1 Hc	73G 151T 152C 263G 309.1CT 310C 514d 515d 750G 980C 1438G 1811G 2706G 3106d 3107C 3741T 3834A 4769G 5360T 6164T 7028T 8137T 8684T 8860G 10142T 11467G 11719A 12308G 12372A 12618A 13500C 14218C 14569A 14766T 15326G 16207G 16256T 16309G 16318C 16519C	U7a3b	73G 151T 152C 263G 309.1CT 310C 514d 515d 16207G 16256T 16309G 16318C 16519C	1	73 151 152 263 750 980 1438 1811 2706 3741 3834 4769 5360 6164 7028 8137 8684 8860 10142 11467 11719 12308 12372 12618 13500 14218 14569 14766 15326 16207 16256 16309 16318C	U7a3b	73 151 152 263 16207 16256 16309 16318C	[0.433] U7a3b (1)	[0.496] U7a (2) [0.433] U7a3b (3) [0.138] U7a3a (4) [0.000] U7a2 U7a3 (6)	1
1850	>KY824961.1 Hc	73G 151T 152C 263G 310.1C 514d 515d 750G 980C 1438G 1811G 2706G 3106d 3107C 3741T 3834A 4769G 5360T 7028T 7498A 8137T 8684T 8860G 9452A 10142T 11467G 11719A 12308G 12372A 12414C 12618A 13500C 14569A 14766T 15326G 16207G 16309G 16318T 16519C	U7a3b	73G 151T 152C 263G 310.1C 514d 515d 16207G 16309G 16318T 16519C	1	73 151 152 263 750 980 1438 1811 2706 3741 3834 4769 5360 7028 7498 8137 8684 8860 9452 10142 11467 11719 12308 12372 12414 12618 13500 14569 14766 15326 16207 16309 16318T	U7a3b	73 151 152 263 16207 16309 16318T	[0.000] U7a3b (1)	[0.013] U7a (2) [0.000] U7a2 U7a3 U7a3a U7a3b (6)	1
1851	>KY824960.1 Hc	73G 151T 152C 263G 310.1C 514d 515d 750G 980C 1438G 1811G 2706G 3106d 3107C 3741T 3834A 4769G 5360T 7028T 8137T 8684T 8702T 8860G 10142T 11467G 11719A 12308G 12372A 12618A 13500C 14569A 14766T 15326G 16207G 16304C 16309G 16318T	U7a3b	73G 151T 152C 263G 310.1C 514d 515d 16207G 16304C 16309G 16318T	1	73 151 152 263 750 980 1438 1811 2706 3741 3834 4769 5360 7028 8137 8684 8702 8860 10142 11467 11719 12308 12372 12618 13500 14569 14766 15326 16207 16304 16309 16318T	U7a3b	73 151 152 263 16207 16304 16309 16318T	[0.011] U7a3b (1)	[0.013] U7a (2) [0.011] U7a3b (3) [0.000] U7a2 U7a3 U7a3a (6)	1
1852	>KY824947.1 Hc	73G 151T 152C 263G 309.1CT 310C 514d 515d 750G 980C 1438G 1811G 2706G 3106d 3107C 3741T 3834A 4769G 5360T 5814C 7028T 8137T 8684T 8860G 10142T 11467G 11719A 12308G 12372A 12576T 12618A 13500C 14569A 14766T 15326G 16207G 16309G 16318T 16519C 16525C	U7a3b	73G 151T 152C 263G 309.1CT 310C 514d 515d 16207G 16309G 16318T 16519C 16525C	1	73 151 152 263 750 980 1438 1811 2706 3741 3834 4769 5360 5814 7028 8137 8684 8860 10142 11467 11719 12308 12372 12576 12618 13500 14569 14766 15326 16207 16309 16318T 16525C	U7a3b	73 151 152 263 16207 16309 16318T 16525C	[0.011] U7a3b (1)	[0.013] U7a (2) [0.011] U7a3b (3) [0.000] U7a2 U7a3 U7a3a (6)	1

1853 >KY824946.1 Ho	73G 151T 152C 263G 309.1CT 310C 514d 515d 750G 980C 1438G 1811G 2706G 3106d 3107C 3434G 3741T 3834A 4769G 5360T 5840T 6956C 7028T 8137T 8684T 8860G 10142T 11467G 11719A 12308G 12372A 12397G 12618A 13500C 14218C 14569A 14766T 15326G 16093C 16126C 16207G 16292T 16309G 16318T 16519C	U7a3b	73G 151T 152C 263G 309.1CT 310C 514d 515d 16093C 16126C 16207G 16292T 16309G 16318T 16519C	1	73 151 152 263 750 980 1438 1811 2706 3434 3741 3834 4769 5360 5840 6956 7028 8137 8684 8860 10142 11467 11719 12308 12372 12397 12618 13500 14218 14569 14766 15326 16093 16126 16207 16292 16309 16318T	U7a3b	73 151 152 263 16093 16126 16207 16292 16309 16318T	[0.341] U7a3b (1)	[0.341] U7a3b (2) [0.129] U7a4 (3) [0.046] U7a (4) [0.009] U7a3a (5) [0.000] U7a2 U7a3 (7)	1
1854 >KY824945.1 Ho	73G 151T 152C 263G 309.1CCT 310C 514d 515d 750G 980C 1438G 1811G 2706G 3106d 3107C 3741T 3834A 3927G 4769G 5360T 7028T 8137T 8684T 8860G 10142T 11467G 11719A 12308G 12372A 12618A 13105G 13500C 14569A 14766T 15326G 15734A 16172C 16207G 16309G 16318T 16519C	U7a3b	73G 151T 152C 263G 309.1CCT 310C 514d 515d 16172C 16207G 16309G 16318T 16519C	1	73 151 152 263 750 980 1438 1811 2706 3741 3834 3927 4769 5360 7028 8137 8684 8860 10142 11467 11719 12308 12372 12618 13105 13500 14569 14766 15326 15734 16172 16207 16309 16318T	U7a3b	73 151 152 263 16172 16207 16309 16318T	[0.011] U7a3b (1)	[0.017] U7a (2) [0.011] U7a3b (3) [0.000] U7a2 U7a3 U7a3a (6)	1
1855 >KY824931.1 Ho	73G 146C 151T 152C 263G 309.1CT 310C 514d 515d 750G 980C 1438G 1811G 2706G 3106d 3107C 3741T 4769G 5360T 7028T 8137T 8684T 8860G 10142T 11467G 11719A 12308G 12372A 13500C 14569A 14766T 15172A 15326G 16126C 16150T 16189C 16209C 16309G 16318T 16390A 16519C	U7a4	73G 146C 151T 152C 263G 309.1CT 310C 514d 515d 16126C 16150T 16189C 16209C 16309G 16318T 16390A 16519C	1	73 146 151 152 263 750 980 1438 1811 2706 3741 4769 5360 7028 8137 8684 8860 10142 11467 11719 12308 12372 13500 14569 14766 15172 15326 16126 16150 16189 16209 16309 16318T 16390	U7a4	73 146 151 152 263 16126 16150 16189 16209 16309 16318T 16390	[0.847] U7a4 (1)	[0.312] U7a4a (2)	1
1856 >KY824910.1 Ho	73G 146C 151T 152C 263G 309.1CT 310C 514d 515d 750G 980C 1438G 1811G 2706G 3106d 3107C 3741T 3897A 4769G 5360T 7028T 8137T 8684T 8860G 10142T 11467G 11719A 12308G 12372A 13500C 14569A 14766T 15172A 15326G 16126C 16148T 16209C 16260T 16309G 16318T 16390A 16519C	U7a4a	73G 146C 151T 152C 263G 309.1CT 310C 514d 515d 16126C 16148T 16209C 16260T 16309G 16318T 16390A 16519C	1	73 146 151 152 263 750 980 1438 1811 2706 3741 3897A 4769 5360 7028 8137 8684 8860 10142 11467 11719 12308 12372 13500 14569 14766 15172 15326 16126 16148 16209 16260 16309 16318T 16390	U7a4a	73 146 151 152 263 16126 16148 16209 16260 16309 16318T 16390	[0.312] U7a4a (1)	[0.539] U7a4 (2) [0.312] U7a4a (3) [0.000] U7a4a1 U7a4a1a (5)	1
1857 >KY824909.1 Ho	73G 146C 151T 152C 263G 309.1CT 310C 514d 515d 750G 980C 1438G 1811G 2706G 3106d 3107C 3741T 4769G 5360T 7028T 8137T 8684T 8860G 10142T 11467G 11719A 12308G 12372A 13500C 14198A 14569A 14766T 15172A 15326G 16126C 16129A 16148T 16209C 16309G 16318T 16390A 16519C	U7a4a	73G 146C 151T 152C 263G 309.1CT 310C 514d 515d 16126C 16129A 16148T 16209C 16309G 16318T 16390A 16519C	1	73 146 151 152 263 750 980 1438 1811 2706 3741 4769 5360 7028 8137 8684 8860 10142 11467 11719 12308 12372 13500 14198 14569 14766 15172 15326 16126 16129 16148 16209 16309 16318T 16390	U7a4a	73 146 151 152 263 16126 16129 16148 16209 16309 16318T 16390	[0.312] U7a4a (1)	[0.539] U7a4 (2) [0.312] U7a4a (3) [0.000] U7a4a1 U7a4a1a (5)	1

1858	>KY824906.1 Hc	73G 151T 152C 263G 309.1CT 310C 514d 515d 567.1CCCCC 750G 980C 1438G 1811G 2706G 3106d 3107C 3741T 3834A 4769G 5360T 7028T 8137T 8684T 8860G 9067G 10142T 11467G 11719A 12308G 12372A 12585T 12618A 13500C 14569A 14766T 15326G 16189C 16207G 16309G 16318C 16519C	U7a3b	73G 151T 152C 263G 309.1CT 310C 514d 515d 567.1CCCCC 16189C 16207G 16309G 16318C 16519C	1	73 151 152 263 573.5C 750 980 1438 1811 2706 3741 3834 4769 5360 7028 8137 8684 8860 9067 10142 11467 11719 12308 12372 12585 12618 13500 14569 14766 15326 16189 16207 16309 16318C	U7a3b	73 151 152 263 573.5C 16189 16207 16309 16318C	[0.626] U7a3b (1)	[0.626] U7a3b (2) [0.513] U7a (3) [0.176] U7a3a (4) [0.033] U8c (5) [0.023] U7a2 (6) [0.000] U7a3	1
1859	>KY824904.1 Hc	73G 146C 152C 263G 309.1CT 310C 514d 515d 750G 980C 1438G 1811G 2523T 2706G 3106d 3107C 3741T 4769G 5360T 7028T 8137T 8568G 8684T 8860G 10142T 11255C 11467G 11719A 11914A 12308G 12372A 13500C 14569A 14766T 15326G 16126C 16140C 16309G 16318T 16519C	U7a4	73G 146C 152C 263G 309.1CT 310C 514d 515d 16126C 16140C 16309G 16318T 16519C	1	73 146 152 263 750 980 1438 1811 2523 2706 3741 4769 5360 7028 8137 8568G 8684 8860 10142 11255 11467 11719 11914 12308 12372 13500 14569 14766 15326 16126 16140 16309 16318T	U7a4	73 146 152 263 16126 16140 16309 16318T	[0.154] U7a4 (1)	[0.154] U7a4 (2) [0.026] U7b (3) [0.013] U7 (4) [0.000] U7a4a	1
1860	>KY824875.1 Hc	73G 151T 152C 263G 310.1C 514d 515d 750G 980C 1438G 1811G 2706G 3106d 3107C 3741T 3834A 4769G 5147A 5360T 6164T 7028T 8137T 8684T 8860G 10142T 11467G 11719A 12308G 12372A 12618A 13500C 14218C 14569A 14766T 15326G 16207G 16309G 16318T 16519C	U7a3b	73G 151T 152C 263G 310.1C 514d 515d 16207G 16309G 16318T 16519C	1	73 151 152 263 750 980 1438 1811 2706 3741 3834 4769 5147 5360 6164 7028 8137 8684 8860 10142 11467 11719 12308 12372 12618 13500 14218 14569 14766 15326 16207 16309 16318T	U7a3b	73 151 152 263 16207 16309 16318T	[0.000] U7a3b (1)	[0.013] U7a (2) [0.000] U7a2 U7a3 U7a3a U7a3b (6)	1
1861	>KY824872.1 Hc	73G 143A 146C 151T 152C 263G 309.1CT 310C 750G 980C 1438G 1811G 2523T 2706G 3106d 3107C 3741T 4769G 5360T 7028T 8137T 8684T 8860G 9336G 10142T 11255C 11467G 11719A 12308G 12372A 13359A 13500C 14569A 14766T 15326G 15776G 16126C 16293G 16309G 16318T 16519C	U7a4	73G 143A 146C 151T 152C 263G 309.1CT 310C 16126C 16293G 16309G 16318T 16519C	1	73 143 146 151 152 263 750 980 1438 1811 2523 2706 3741 4769 5360 7028 8137 8684 8860 9336 10142 11255 11467 11719 12308 12372 13359 13500 14569 14766 15326 15776 16126 16293 16309 16318T	U7a4	73 143 146 151 152 263 16126 16293 16309 16318T	[0.052] U7a4 (1)	[0.000] U7a4a (2)	1
1862	>KY824870.1 Hc	73G 146C 151T 152C 263G 310.1C 514d 515d 750G 980C 1438G 1811G 2706G 3106d 3107C 3741T 4248C 4769G 5360T 7028T 7241G 8137T 8684T 8860G 10142T 11467G 11719A 11935C 12308G 12372A 13500C 14569A 14766T 15326G 16126C 16148T 16309G 16318T 16519C	U7a4a	73G 146C 151T 152C 263G 310.1C 514d 515d 16126C 16148T 16309G 16318T 16519C	1	73 146 151 152 263 750 980 1438 1811 2706 3741 4248 4769 5360 7028 7241 8137 8684 8860 10142 11467 11719 11935 12308 12372 13500 14569 14766 15326 16126 16148 16309 16318T	U7a4a	73 146 151 152 263 16126 16148 16309 16318T	[0.000] U7a4a (1)	[0.000] U7a4 U7a4a U7a4a1 U7a4a1a (5)	1

1863	>KY824869.1 Ho	73G 146C 151T 152C 263G 309.1CT 310C 514d 515d 750G 980C 1438G 1811G 2706G 3106d 3107C 3741T 3834A 4769G 5360T 6437G 7028T 8137T 8684T 8860G 10142T 11467G 11719A 12308G 12372A 13500C 14569A 14766T 15326G 16126C 16209C 16309G 16318T 16519C	U7a4	73G 146C 151T 152C 263G 309.1CT 310C 514d 515d 16126C 16209C 16309G 16318T 16519C	1	73 146 151 152 263 750 980 1438 1811 2706 3741 3834 4769 5360 6437 7028 8137 8684 8860 10142 11467 11719 12308 12372 13500 14569 14766 15326 16126 16209 16309 16318T	U7a4	73 146 151 152 263 16126 16209 16309 16318T	[0.308] U7a4 (1)	[0.125] U7a4a (2)	1
1864	>KY824866.1 Ho	73G 146C 151T 152C 263G 310.1C 514d 515d 750G 980C 1438G 1811G 2706G 3106d 3107C 3741T 3834A 4769G 5360T 7028T 8137T 8684T 8860G 10142T 11467G 11719A 12308G 12372A 13500C 14569A 14766T 15326G 16093C 16126C 16209C 16309G 16318T 16519C	U7a4	73G 146C 151T 152C 263G 310.1C 514d 515d 16093C 16126C 16209C 16309G 16318T 16519C	1	73 146 151 152 263 750 980 1438 1811 2706 3741 3834 4769 5360 7028 8137 8684 8860 10142 11467 11719 12308 12372 13500 14569 14766 15326 16093 16126 16209 16309 16318T	U7a4	73 146 151 152 263 16093 16126 16209 16309 16318T	[0.385] U7a4 (1)	[0.125] U7a4a (2)	1
1865	>KY824861.1 Ho	73G 146C 151T 152C 263G 310.1C 514d 515d 750G 980C 1438G 1811G 2706G 3106d 3107C 3741T 3834A 4769G 5360T 7028T 8137T 8684T 8860G 10142T 11467G 11719A 12308G 12372A 13500C 14569A 14766T 15326G 16126C 16209C 16309G 16318T 16519C	U7a4	73G 146C 151T 152C 263G 310.1C 514d 515d 16126C 16209C 16309G 16318T 16519C	1	73 146 151 152 263 750 980 1438 1811 2706 3741 3834 4769 5360 7028 8137 8684 8860 10142 11467 11719 12308 12372 13500 14569 14766 15326 16126 16209 16309 16318T	U7a4	73 146 151 152 263 16126 16209 16309 16318T	[0.308] U7a4 (1)	[0.125] U7a4a (2)	1
1866	>KY824860.1 Ho	73G 146C 151T 152C 263G 310.1C 514d 515d 750G 980C 1438G 1811G 2706G 3106d 3107C 3741T 4248C 4769G 5360T 7028T 7241G 8137T 8684T 8860G 10142T 11467G 11719A 11935C 12308G 12372A 13500C 14569A 14766T 15326G 16126C 16148T 16309G 16318T 16519C	U7a4a	73G 146C 151T 152C 263G 310.1C 514d 515d 16126C 16148T 16309G 16318T 16519C	1	73 146 151 152 263 750 980 1438 1811 2706 3741 4248 4769 5360 7028 7241 8137 8684 8860 10142 11467 11719 11935 12308 12372 13500 14569 14766 15326 16126 16148 16309 16318T	U7a4a	73 146 151 152 263 16126 16148 16309 16318T	[0.000] U7a4a (1)	[0.000] U7a4 U7a4a U7a4a1 U7a4a1a (5)	1
1867	>KY824859.1 Ho	73G 146C 151T 152C 263G 310.1C 514d 515d 750G 980C 1438G 1811G 2706G 3106d 3107C 3741T 4248C 4769G 5360T 7028T 7241G 8137T 8684T 8860G 10142T 11467G 11719A 11935C 12308G 12372A 13500C 14569A 14766T 15326G 16126C 16148T 16309G 16318C 16519C	U7a4a	73G 146C 151T 152C 263G 310.1C 514d 515d 16126C 16148T 16309G 16318C 16519C	1	73 146 151 152 263 750 980 1438 1811 2706 3741 4248 4769 5360 7028 7241 8137 8684 8860 10142 11467 11719 11935 12308 12372 13500 14569 14766 15326 16126 16148 16309 16318C	U7a4a	73 146 151 152 263 16126 16148 16309 16318C	[0.250] U7a4a (1)	[0.778] U7a4a1a (2) [0.256] U7a4 (3) [0.250] U7a4a (4) [0.058] U7a4a1	1
1868	>KY824848.1 Ho	73G 146C 151T 152C 263G 309.1CT 310C 514d 515d 750G 980C 1438G 1811G 2523T 2706G 3106d 3107C 3338C 3741T 4769G 5360T 7028T 8137T 8684T 8860G 10142T 11255C 11467G 11719A 12308G 12372A 13145A 13500C 14484C 14569A 14766T 15326G 16126C 16150T 16309G 16318T 16519C	U7a4	73G 146C 151T 152C 263G 309.1CT 310C 514d 515d 16126C 16150T 16309G 16318T 16519C	1	73 146 151 152 263 750 980 1438 1811 2523 2706 3338 3741 4769 5360 7028 8137 8684 8860 10142 11255 11467 11719 12308 12372 13145 13500 14484 14569 14766 15326 16126 16150 16309 16318T	U7a4	73 146 151 152 263 16126 16150 16309 16318T	[0.282] U7a4 (1)	[0.000] U7a4a (2)	1

1869	>KY824842.1 Ho	73G 146C 151T 152C 263G 309.1CT 310C 514d 515d 750G 980C 1438G 1811G 2706G 3106d 3107C 3741T 4769G 5360T 7028T 8137T 8684T 8860G 10142T 11467G 11719A 12308G 12372A 13500C 14569A 14766T 15172A 15326G 16126C 16209C 16309G 16318C 16390A 16519C	U7a4	73G 146C 151T 152C 263G 309.1CT 310C 514d 515d 16126C 16209C 16309G 16318C 16390A 16519C	1	73 146 151 152 263 750 980 1438 1811 2706 3741 4769 5360 7028 8137 8684 8860 10142 11467 11719 12308 12372 13500 14569 14766 15172 15326 16126 16209 16309 16318C 16390	U7a4	73 146 151 152 263 16126 16209 16309 16318C 16390	[0.795] U7a4 (1)	[0.500] U7a4a (2)	1
1870	>KY934477.1 Ho	73G 195C 200G 263G 310.1C 514d 515d 750G 1438G 1811G 2361A 2706G 3027C 3106d 3107C 3480G 4769G 6546T 6599G 7028T 8860G 9055A 9698C 9948A 11467G 11719A 11914A 12308G 12372A 12771A 12957C 14167T 14766T 15326G 15482C 15776G 16129A 16183C 16189C 16234T	U8b1a1	73G 195C 200G 263G 310.1C 514d 515d 16129A 16183C 16189C 16234T	1	73 195 200 263 750 1438 1811 2361 2706 3027 3480 4769 6546 6599 7028 8860 9055 9698 9948 11467 11719 11914 12308 12372 12771 12957 14167 14766 15326 15482 15776 16129 16189 16234	U8b1a1	73 195 200 263 16129 16189 16234	[0.021] U8b1a1 (1)	[0.000] U8b1 U8b1a U8b1a2 (4)	1
1871	>KY940311.1 Ho	152C 235R 263G 309.1CT 310C 750G 1438G 2259T 3106d 3107C 4745G 4769G 8860G 14872T 15326G 16234T	H13a1d	152C 235R 263G 309.1CT 310C 16234T	1	152 235R 263 750 1438 2259 4745 4769 8860 14872 15326 16234	H13a1d	152 235R 263 16234	[0.020] H13a1d (1)	[0.004] H3+152 (2) [0.000] H1+152 H1i H1i1 H1i2 H1i2a H1an H1an1 H1an1a H1bb H1bc H2a2a2 H3g H3g1 H3g1a H3g1b H3g2 H3g3 H3g4 H3i H3i1 H3j H3k H3k1 H3k1a H+152 H9 H46 H46a H46b H52 H69 H13a1+152 H14b H14b2 H14b4 H16+152 H16a H16a1 H16c H16d H30b1 H47a (44)	1
1872	>KY930469.1 Ho	73G 263G 309.1CT 310C 373G 456T 750G 1438G 3106d 3107C 4769G 8860G 13638G 15326G 16269G 16304C	H5n	73G 263G 309.1CT 310C 373G 456T 16269G 16304C	1	73 263 373 456 750 1438 4769 8860 13638 15326 16269 16304	H5n	73 263 373 456 16269 16304	[0.010] H5n (1)	[0.025] H5 (2) [0.015] H5a2 (3) [0.007] H5v (4) [0.005] H5a1 R9c (6) [0.003] H5a H5b H5+709 (9) [0.000] H5a1a H5a1b H5a1c H5a1c1 H5a1c1a H5a1d H5a1f H5a1h H5a1k H5a1q H5a2a H5a7 H5a8 H5a9 H5b3 H5b5 H5c H5c1 H5c1a H5d H5f H5j H5k H5p R9 R9b R30b (36)	1
1873	>KY930958.1 Ho	73G 195C 263G 310d 499A 513.1CACA 750G 1438G 1811G 2706G 3106d 3107C 4646C 4769G 5999C 6047G 7028T 8818T 8860G 11332T 11467G 11719A 12308G 12372A 14620T 14766T 15326G 15693C 16223T 16356C 16519C	U4a2b	73G 195C 263G 310d 499A 513.1CACA 16223T 16356C 16519C	1	73 195 263 310 315d 499 750 1438 1811 2706 4646 4769 5999 6047 7028 8818 8860 11332 11467 11719 12308 12372 14620 14766 15326 15693 16223 16356	U4a2b	73 195 263 310 315d 499 16223 16356	[0.132] U4a2b (1)	[0.137] U4a2 (2) [0.030] U4a2g (3) [0.010] U4a2c U4a2f (5) [0.000] U4a2e U4a2h (7)	1
1874	>KY686211.1 Ho	73G 150T 152C 260A 263G 489C 750G 1193C 1342T 1438G 2706G 4769G 6253C 7028T 8269A 8701G 8805G 8860G 9540C 10398G 10400T 10873C 11719A 11810T 12705T 12732C 13468T 14766T 14783C 14883T 15043A 15301A 15326G 16223T 16362C 16381C 16519C	M61	73G 150T 152C 260A 263G 489C 16223T 16362C 16381C 16519C	1	73 150 152 260 263 489 750 1193 1342 1438 2706 4769 6253 7028 8269 8701 8805 8860 9540 10398 10400 10873 11719 11810 12705 12732 13468 14766 14783 14883 15043 15301 15326 16223 16362 16381	M61	73 150 152 260 263 489 16223 16362 16381	[0.170] M61 (1)	[0.003] M13'46'61+16362 (2) [0.002] D4b2b2a D4b2b2a1 D4b2b4 (5) [0.000] G1a M61a (7)	1

1875 >KY686207.1 Hc	73G 228A 263G 309.1CT 310C 366A 373G 389A 442C 443G 444G 507C 750G 955.1C 961C 990C 1292G 1438G 2706G 4769G 5351G 5474G 6305A 7028T 7316A 8470G 8584A 8618C 8650T 8860G 10401A 11075C 11719A 12285C 14058T 14766T 15326G 16129A 16266T 16318G 16320T 16362C 16519C	R6a1	73G 228A 263G 309.1CT 310C 366A 373G 389A 442C 443G 444G 507C 16129A 16266T 16318G 16320T 16362C 16519C	1	73 228 263 366 373 389 442 443 444 507 750 961 965.1C 990 1292 1438 2706 4769 5351 5474 6305 7028 7316 8470 8584 8618 8650 8860 10401 11075 11719 12285 14058 14766 15326 16129 16266 16318 16320 16362	R6a1	73 228 263 366 373 389 442 443 444 507 16129 16266 16318 16320 16362	[0.717] R6a1 (1)	[0.717] R6a1 (2) [0.044] R6a2 (3) [0.000] R6a	1
1876 >KY863513.1 Hc	263G 310.1C 750G 1438G 3010A 3106d 3107C 4769G 8860G 15326G 16093Y 16270T 16519C	H1ba	263G 310.1C 16093Y 16270T 16519C	1	263 750 1438 3010 4769 8860 15326 16093Y 16270	H1ba	263 16093Y 16270	[0.047] H1ba (1) [0.000] H10+(16093) H10f (3)	[0.158] H7c3 (4) [0.157] H13a1a1c (5) [0.156] H7c (6) [0.155] H44a (7) [0.034] H10e3a (8) [0.031] H10e1 (9) [0.024] H10e3 (10) [0.017] H2a2a (11) [0.014] H3v+16093 (12) [0.011] H (13) [0.010] H1e1a (14) [0.008] H1 (15) [0.005] H60a (16) [0.003] H14b H63 H72 (19) [0.002] H1e H56 (21) [0.001] H1j H1q H1bi H1br H2 H3 H4 H4a1a H7a H7b1 H10f H13a2b2 H14b2 H67 (35) [0.000] R0 HV HV4 HV4c HV5 HV5a HV9c HV12 H1f+16093 H1f1 H1e1 H1e1a2 H1e1a7 H1e1c H1e2 H1e2a H1e2b H1e2d H1e4 H1e6 H1e7 H1e8 H1e8a H1h H1h1 H1h2 H1j3 H1j5 H1j6 H1j7 H1j9 H1m H1n H1p H1q1 H1q2 H1r H1s H1s1 H1t H1t1 H1t1a H1t2 H1u H1u1 H1v H1v1 H1v1a H1v1b H1w H1ae H1ae1 H1ae2 H1ae2a H1ae3 H1ae3a H1ag H1ag1 H1ag1b H1ah H1ai H1ai1 H1aj H1ak H1ak1 H1ak2 H1am H1am1 H1ap H1aq H1as H1as2 H1at H1at1 H1at1a H1av H1ax H1ax1 H1ay H1az H1ba1 H1bd H1be H1bj H1bk H1bm H1bn H1bp H1bq H1bu H1bv H1bw H1bx H1ca H1ci H2a H2a2 H2a2+(16235) H2a4 [illegible]	1
1877 >KY595668.1 Hc	73G 152C 263G 750G 1438G 2706G 3106d 3107C 4769G 5563A 6755A 7028T 8860G 9140T 11719A 12705T 14766T 15326G 16213A 16223T	O	73G 152C 263G 16213A 16223T	1	73 152 263 750 1438 2706 4769 5563 6755 7028 8860 9140 11719 12705 14766 15326 16213 16223	O	73 152 263 16213 16223	[0.023] O (1) [0.000] L3c'd S+152 (3)	[0.049] L3d3a1b (4) [0.031] S2 (5) [0.011] L3d1b (6) [0.009] L3 (7) [0.008] L3d1d (8) [0.000] L3b'f L3d L3d1'2'3'4'5'6 L3d1 L3d1a L3d1b1 L3d1b1a L3d3 L3d3a L3d3a1 L3d3a1a L3d3b L3d6 L3h M37+152 M13'46'61 M13 M13a'b N N1'5 N1 N9 N11 N14 S H32 R30a1 R30a1a (36)	1

1878	>KY595662.1 Ho	73G 152C 212C 263G 750G 1438G 2706G 4769G 5330A 6077T 6840T 7028T 8860G 10118C 11719A 12850G 13722G 14766T 15326G 15607G 15927A 16111A 16266T 16357C 16519C	P1+152	73G 152C 212C 263G 16111A 16266T 16357C 16519C	1	73 152 212 263 750 1438 2706 4769 5330A 6077 6840T 7028 8860 10118 11719 12850 13722 14766 15326 15607 15927 16111A 16266 16357	P1+152	73 152 212 263 16111A 16266 16357	[0.906] P1+152 (1)	[0.118] P1d2 (2) [0.100] P1d (3) [0.000] P1	1
1879	>KY595661.1 Ho	73G 146C 152C 178G 195C 263G 750G 1438G 2706G 2775G 2831A 3106d 3107C 4769G 5704T 6896C 7028T 8485A 8860G 9080G 10101C 11020G 11719A 12705T 13263G 13759A 13934T 14766T 15148A 15209C 15326G 15431A 16066G 16223T 16261T 16264T 16278T 16290T 16291T 16519C	N13	73G 146C 152C 178G 195C 263G 16066G 16223T 16261T 16264T 16278T 16290T 16291T 16519C	1	73 146 152 178 195 263 750 1438 2706 2775 2831 4769 5704 6896 7028 8485 8860 9080 10101 11020 11719 12705 13263 13759 13934 14766 15148 15209 15326 15431 16066 16223 16261 16264 16278 16290 16291	N13	73 146 152 178 195 263 16066 16223 16261 16264 16278 16290 16291	[1.600] N13 (1)	[0.400] L2a (2)	1
1880	>KY595657.1 Ho	73G 263G 591A 750G 1438G 1719A 2706G 3010A 4652T 4769G 5276G 6881G 7028T 8860G 10088T 10398G 10914A 11016A 11288T 11719A 11914A 12007A 14766T 15326G 15607G 15758G 16258C 16263C 16274A 16291T 16337T 16356C 16362C	P4b1	73G 263G 16258C 16263C 16274A 16291T 16337T 16356C 16362C	1	73 263 591A 750 1438 1719 2706 3010 4652 4769 5276 6881 7028 8860 10088 10398 10914 11016 11288 11719 11914 12007 14766 15326 15607 15758 16258C 16263 16274 16291 16337 16356 16362	P4b1	73 263 16258C 16263 16274 16291 16337 16356 16362	[0.444] P4b1 (1)	[0.000] P4b (2)	1
1881	>KY595654.1 Ho	73G 125C 127C 128T 152C 195C 236C 259G 499A 567.1C 576C 750G 1438G 2706G 2755G 3106d 3107C 3645C 4769G 7028T 8078A 8860G 11719A 12036C 12121C 12684A 13934T 14053G 14338T 14766T 15326G 15607G 15748C 15937C 16086C 16172C 16189C 16319A 16327T 16399G 16519C	P3b1	73G 125C 127C 128T 152C 195C 236C 259G 499A 567.1C 576C 16086C 16172C 16189C 16319A 16327T 16399G 16519C	1	73 125 127 128 152 195 236 259 499 573.1C 576C 750 1438 2706 2755 3645 4769 7028 8078 8860 11719 12036 12121 12684 13934 14053 14338 14766 15326 15607 15748 15937C 16086 16172 16189 16319 16327 16399	P3b1	73 125 127 128 152 195 236 259 499 573.1C 576C 16086 16172 16189 16319 16327 16399	[2.288] P3b1 (1)	[1.200] P3b (2)	1
1882	>KY595652.1 Ho	73G 125C 127C 128T 146C 153G 263G 515G 567.1CCC 750G 1438G 2706G 3391A 3645C 4456T 4769G 5895d 6297C 6734A 7028T 7256T 8433G 8860G 11719A 13161C 13651G 13716G 14338T 14766T 15326G 15607G 15748C 15937T 16145A 16278T 16311C	P3a	73G 125C 127C 128T 146C 153G 263G 515G 567.1CCC 16145A 16278T 16311C	1	73 125 127 128 146 153 263 515 573.3C 750 1438 2706 3391 3645 4456 4769 5899d 6297 6734 7028 7256 8433G 8860 11719 13161 13651 13716 14338 14766 15326 15607 15748 15937T 16145 16278 16311	P3a	73 125 127 128 146 153 263 515 573.3C 16145 16278 16311	[0.875] P3a (1)	[0.000] P3 (2)	1
1883	>KY595642.1 Ho	73G 185A 263G 489C 750G 1438G 1598A 2706G 4164G 4508T 4769G 4925T 6104T 6734A 7028T 8251A 8701G 8793C 8860G 9156G 9410G 9540C 10373A 10398G 10400T 10873C 11719A 12705T 12771A 13596T 14527G 14766T 14783C 14905A 15043A 15301A 15326G 15530C 16184T 16188T 16189C 16223T 16287T 16294T 16356C	M42a	73G 185A 263G 489C 16184T 16188T 16189C 16223T 16287T 16294T 16356C	1	73 185 263 489 750 1438 1598 2706 4164 4508 4769 4925 6104 6734 7028 8251 8701 8793 8860 9156 9410 9540 10373 10398 10400 10873 11719 12705 12771 13596 14527 14766 14783 14905 15043 15301 15326 15530 16184 16188 16189 16223 16287 16294 16356	M42a	73 185 263 489 16184 16188 16189 16223 16287 16294 16356	[0.440] M42a (1)	[0.000] M7b2 D4b2b2c N9b2 (4)	1

1884	>KY595641.1 Hc	73G 185A 263G 310.1C 489C 750G 1438G 1598A 2706G 4164G 4508T 4769G 4925T 6104T 6734A 7028T 8251A 8701G 8793C 8860G 9156G 9410G 9540C 10373A 10398G 10400T 10873C 11719A 12705T 12771A 13596T 14527G 14766T 14783C 14905A 15043A 15301A 15326G 15530C 16184T 16188T 16189C 16223T 16287T 16294T 16356C	M42a	73G 185A 263G 310.1C 489C 16184T 16188T 16189C 16223T 16287T 16294T 16356C	1	73 185 263 489 750 1438 1598 2706 4164 4508 4769 4925 6104 6734 7028 8251 8701 8793 8860 9156 9410 9540 10373 10398 10400 10873 11719 12705 12771 13596 14527 14766 14783 14905 15043 15301 15326 15530 16184 16188 16189 16223 16287 16294 16356	M42a	73 185 263 489 16184 16188 16189 16223 16287 16294 16356	[0.440] M42a (1)	[0.000] M7b2 D4b2b2c N9b2 (4)	1
1885	>KY595639.1 Hc	73G 152C 260A 263G 310.1C 489C 513.1CA 750G 1438G 1598A 2706G 4508T 4769G 5442C 6104T 6734A 7028T 8251A 8269A 8701G 8793C 8860G 9156G 9410G 9540C 10237C 10398G 10400T 10586A 10873C 11719A 12705T 12771A 14527G 14766T 14783C 15043A 15301A 15326G 16179T 16184T 16188T 16189C 16223T 16239T 16287T 16356C	M42a	73G 152C 260A 263G 310.1C 489C 513.1CA 16179T 16184T 16188T 16189C 16223T 16239T 16287T 16356C	1	73 152 260 263 489 750 1438 1598 2706 4508 4769 5442 6104 6734 7028 8251 8269 8701 8793 8860 9156 9410 9540 10237 10398 10400 10586 10873 11719 12705 12771 14527 14766 14783 15043 15301 15326 16179 16184 16188 16189 16223 16239 16287 16356	M42a	73 152 260 263 489 16179 16184 16188 16189 16223 16239 16287 16356	[0.389] M42a (1) [0.000] M13a (2)	[0.008] M3c1b M13a1 (4) [0.000] M37+152 M7b2 M8a2a M13'46'61 M13 M13a'b D4b2b2c (11)	1
1886	>KY595637.1 Hc	73G 263G 310.1C 489C 750G 1438G 1598A 2706G 4508T 4769G 6104T 7028T 8251A 8701G 8793C 8860G 9156G 9410G 9540C 10398G 10400T 10786C 10873C 11719A 12705T 12771A 14527G 14766T 14783C 15043A 15301A 15326G 15865G 16111T 16223T 16266A 16287T 16356C	M42a	73G 263G 310.1C 489C 16111T 16223T 16266A 16287T 16356C	1	73 263 489 750 1438 1598 2706 4508 4769 6104 7028 8251 8701 8793 8860 9156 9410 9540 10398 10400 10786 10873 11719 12705 12771 14527 14766 14783 15043 15301 15326 15865 16111 16223 16266A 16287 16356	M42a	73 263 489 16111 16223 16266A 16287 16356	[0.037] M42a (1)	[0.027] D4b2d (2) [0.007] M29'Q M33c M35 (5) [0.006] G1b M21b M52b (8) [0.000] M M1'20'51 M4"67 M37 M43 M43b M7 M7b'c M7b M12'G M19'53 M21 M21b1 M21b1a M22 M22b M23'75 M24'41 M27 M31 M31a M32'56 M33 M33a M33a1 M34'57 M39'70 M42'74 M42 M52 M55'77 M58 M60 M69 M80'D (43)	1
1887	>KY595636.1 Hc	73G 263G 489C 750G 1438G 1598A 2706G 4508T 4769G 6104T 7028T 7211A 8251A 8701G 8793C 9156G 9410G 9540C 10398G 10400T 10786C 10873C 11110G 11719A 12705T 12715G 12771A 14527G 14766T 14783C 15043A 15301A 15326G 15927A 16223T 16266A 16287T 16294T 16356C	M42a	73G 263G 489C 16223T 16266A 16287T 16294T 16356C	1	73 263 489 750 1438 1598 2706 4508 4769 6104 7028 7211 8251 8701 8793 9156 9410 9540 10398 10400 10786 10873 11110 11719 12705 12715 12771 14527 14766 14783 15043 15301 15326 15927 16223 16266A 16287 16294 16356	M42a	73 263 489 16223 16266A 16287 16294 16356	[0.136] M42a (1)	[0.040] D4i (2) [0.006] G1b M21b (4) [0.000] M M1'20'51 M4"67 M37 M43 M43b M7 M7b'c M7b M12'G M19'53 M21 M21b1 M21b1a M22 M22b M23'75 M24'41 M27 M29'Q M31 M31a M32'56 M33 M33a M33a1 M34'57 M35 M39'70 M42'74 M42 M52 M52b M55'77 M58 M60 M69 M80'D D4b2d D4i2 (44)	1

1888	>KY595632.1 Hc	73G 150T 152C 252C 263G 482C 709A 750G 1438G 1811G 2706G 4769G 6253C 7028T 8176C 8404C 8860G 10358G 11719A 12705T 14384C 14766T 14927G 15326G 16075C 16209C 16223T 16235G 16318T 16320T	S1	73G 150T 152C 252C 263G 482C 16075C 16209C 16223T 16235G 16318T 16320T	1	73 150 152 252 263 482 709 750 1438 1811 2706 4769 6253 7028 8176 8404 8860 10358 11719 12705 14384C 14766 14927 15326 16075 16209 16223 16235 16318T 16320	S1	73 150 152 252 263 482 16075 16209 16223 16235 16318T 16320	[0.999] S1 (1) [0.308] L3e2a2 (2) [0.086] L3f (3) [0.028] L3f1a (4) [0.025] L3e2a3 (5) [0.024] L3f1a1 (6) [0.021] U7b (7) [0.020] U7 (8) [0.018] L3f2a (9) [0.015] L3f2a1 (10) [0.014] L3f1 (11) [0.011] R30a1b (12) [0.006] L3e'i'k'x L3e L3i (15) [0.000] L3c'd L3d1b2 L3e2 L3e2a L3e3'4'5 L3e3'4 S+152 (22)	[0.122] N9a10a (23) [0.111] L3i1a (24) [0.102] N9a10a1 (25) [0.091] L3d1a1a1 (26) [0.089] L3e2b4 (27) [0.085] U7a3a (28) [0.071] S2 (29) [0.066] U7b1 (30) [0.059] L3e2a1 (31) [0.052] L3e2b5 (32) [0.049] L3d3a1b (33) [0.047] L3f1b4a1 (34) [0.044] M17c (35) [0.043] L3d1b1 (36) [0.033] L3e2b3 (37) [0.027] L3f2 (38) [0.026] N9a10a2a (39) [0.025] U7a (40) [0.024] L3e2a1b3 (41) [0.023] L3e4 L3i1 (43) [0.021] U7b (44) [0.020] U7 (45) [0.017] U5b2a1a2 (46) [0.016] U7a2 (47) [0.014] L3 (48) [0.011] L3d1 L3d1b R30a1b (51) [0.008] L3d1d R30a1 (53) [0.007] L3k (54) [0.006] L3d1a1a (55) [0.005] L3b'f L3h M3a2a M3c+152 N N1'5 N1 N9 N11 N14 S (66) [0.000] L3f1b4a L3f2a1a L3d L3d1'2'3'4'5'6 L3d1a L3d1b1a L3d3 L3d3a L3d3a1 L3d3a1a L3d3b L3d6 L3e2a1a L3e2a1b L3e2b+152 L3i1b L3x M37+152 M7a M7a1b2 M7a2 M13'46'61 M13 M13a'b M17 M62'68 N9a10a2 H32 R30a1a R30a1b1 U7a3 (97)	1
1889	>KY595629.1 Hc	73G 185A 263G 489C 750G 1438G 1598A 2706G 4508T 4769G 6104T 6734A 7028T 8701G 8793C 8860G 9156G 9410G 9540C 10373A 10398G 10400T 10873C 11719A 12366G 12561A 12705T 12771A 13596T 14527G 14766T 14783C 15043A 15301A 15326G 16145A 16184T 16188T 16223T 16234T 16287T 16294T 16356C	M42a	73G 185A 263G 489C 16145A 16184T 16188T 16223T 16234T 16287T 16294T 16356C	1	73 185 263 489 750 1438 1598 2706 4508 4769 6104 6734 7028 8701 8793 8860 9156 9410 9540 10373 10398 10400 10873 11719 12366 12561 12705 12771 13596 14527 14766 14783 15043 15301 15326 16145 16184 16188 16223 16234 16287 16294 16356	M42a	73 185 263 489 16145 16184 16188 16223 16234 16287 16294 16356	[0.417] M42a (1)	[0.033] M7b2 (2) [0.014] M49 (3) [0.010] M49c1 (4) [0.000] M49c M49e M49e1 (7)	1
1890	>KY595628.1 Hc	73G 185A 263G 309.1CT 310C 489C 514T 750G 1438G 1598A 2626C 2706G 4164G 4508T 4769G 6104T 6734A 7028T 8701G 8793C 8860G 9156G 9410G 9540C 10373A 10398G 10400T 10873C 11719A 12366G 12705T 12771A 13596T 14527G 14766T 14783C 15043A 15301A 15326G 16184T 16188T 16213A 16223T 16287T 16294T 16356C	M42a	73G 185A 263G 309.1CT 310C 489C 514T 16184T 16188T 16213A 16223T 16287T 16294T 16356C	1	73 185 263 489 514 750 1438 1598 2626 2706 4164 4508 4769 6104 6734 7028 8701 8793 8860 9156 9410 9540 10373 10398 10400 10873 11719 12366 12705 12771 13596 14527 14766 14783 15043 15301 15326 16184 16188 16213 16223 16287 16294 16356	M42a	73 185 263 489 514 16184 16188 16213 16223 16287 16294 16356	[0.379] M42a (1)	[0.000] M7b2 (2)	1

1891	>KY595623.1 Hc	73G 150G 189G 204C 207A 215G 263G 271G 750G 1438G 2706G 3010A 4769G 5291C 6975C 7028T 7283C 8251A 8404C 8506C 8860G 11719A 12705T 14384C 14766T 15040T 15326G 16075C 16092C 16172C 16189C 16223T 16311C	S1a	73G 150G 189G 204C 207A 215G 263G 271G 16075C 16092C 16172C 16189C 16223T 16311C	1	73 150G 189 204 207 215 263 271G 750 1438 2706 3010 4769 5291 6975 7028 7283 8251 8404 8506 8860 11719 12705 14384C 14766 15040 15326 16075 16092 16172 16189 16223 16311	S1a	73 150G 189 204 207 215 263 271G 16075 16092 16172 16189 16223 16311	[0.710] S1a (1)	[0.424] W (2) [0.333] R11b (3) [0.197] N10a (4) [0.127] L3'4 (5) [0.122] R11 (6) [0.100] N9b1c1 (7) [0.098] W1e (8) [0.095] W1 (9) [0.092] N9b1a (10) [0.079] R11a (11) [0.054] W1f (12) [0.048] S1 (13) [0.023] N2 (14) [0.020] N9b (15) [0.014] W1a (16) [0.010] N9b1 (17) [0.009] X2d1 (18) [0.008] X2d1a (19) [0.007] L3h1a2 (20) [0.000] L3f1b L3h1 L3h1a N1a N9b3 R11b1a U6b1b (27)	1
1892	>KY595619.1 Hc	73G 150T 203A 204C 263G 750G 794C 1438G 2706G 3106d 3107C 4769G 6755A 7028T 7822G 8567C 8860G 9140T 11719A 12705T 14766T 15326G 16182C 16183C 16189C 16213A 16223T 16519C	O	73G 150T 203A 204C 263G 16182C 16183C 16189C 16213A 16223T 16519C	1	73 150 203 204 263 750 794 1438 2706 4769 6755 7028 7822 8567 8860 9140 11719 12705 14766 15326 16189 16213 16223	O	73 150 203 204 263 16189 16213 16223	[0.159] O (1) [0.051] N9b1a (2) [0.000] L3e'i'k'x L3e L3e3'4'5 L3e3'4 L3i N1a N9b N9b1 N9b3 B6 (12)	[0.114] M62b1a (13) [0.050] L3x1a2 B6a (15) [0.037] U5b2a1 (16) [0.035] S2 (17) [0.026] N9b2 (18) [0.023] L3e4 (19) [0.021] N9a10a2 (20) [0.017] U5b2a1a2 (21) [0.011] U5b1+16189+@16192 (22) [0.008] R+16189 B4'5 (24) [0.000] L4b1 L3 L3b'f L3i1 L3i1a L3i1b L3x L3x1 L3x1a L3h M51a2 M62'68 M62b1 D5 D5a'b D5a N N1'5 N1 N1a1'2 N9 N9a10a N9a10a1 N9a10a2a N9b1c N11 N14 S X R11'B6 U5b1b1+@16192 U5b1b1d U5b1b1f U5b2a+@16192 U5b2a4 U5b2a4a U5b2a6 U5b2b4 (62)	1

1893 >KY595615.1 Hc	73G 150T 152C 204C 252C 263G 709A 750G 1438G 1811G 2706G 4769G 6647G 7028T 8404C 8860G 10032G 10358G 10398G 10907C 11719A 12705T 12793C 13143C 14384C 14766T 15326G 15622C 16075C 16209C 16223T 16235G 16318T	S1	73G 150T 152C 204C 252C 263G 16075C 16209C 16223T 16235G 16318T	1	73 150 152 204 252 263 709 750 1438 1811 2706 4769 6647 7028 8404 8860 10032 10358 10398 10907 11719 12705 12793 13143 14384C 14766 15326 15622 16075 16209 16223 16235 16318T	S1	73 150 152 204 252 263 16075 16209 16223 16235 16318T	[0.991] S1 (1) [0.086] L3f (2) [0.028] L3f1a (3) [0.025] U7b (4) [0.020] U7 (5) [0.018] L3f2a (6) [0.015] L3f2a1 (7) [0.014] L3f1 (8) [0.012] L3f1a1 (9) [0.006] L3e'i'k'x L3e L3i (12) [0.000] L3c'd L3d1b2 L3e3'4'5 L3e3'4 N1a S+152 R30a1b (19)	[0.122] N9a10a (20) [0.111] L3i1a (21) [0.102] N9a10a1 (22) [0.091] L3d1a1a1 (23) [0.085] U7a3a (24) [0.071] S2 (25) [0.066] U7b1 (26) [0.057] R30a1 (27) [0.056] R30a1a (28) [0.050] L3x1a2 (29) [0.049] L3d3a1b (30) [0.047] L3f1b4a1 (31) [0.044] M17c (32) [0.043] L3d1b1 (33) [0.027] L3f2 (34) [0.026] N9a10a2a (35) [0.025] U7b (36) [0.023] L3e4 L3i1 (38) [0.021] U7a (39) [0.020] U7 (40) [0.017] U5b2a1a2 (41) [0.014] L3 (42) [0.011] L3d1b (43) [0.008] L3d1d M13 (45) [0.007] L3k (46) [0.006] L3d1a1a (47) [0.005] L3b'f L3h N N1'5 N1 N9 N11 N14 S (56) [0.004] M37+152 (57) [0.000] L4b1 L3f1b4a L3f2a1a L3d L3d1'2'3'4'5'6 L3d1 L3d1a L3d1b1a L3d3 L3d3a L3d3a1 L3d3a1a L3d3b L3d6 L3i1b L3x L3x1 L3x1a M7a M7a1b2 M7a2 M13'46'61 M13a'b M17 M62'68 N1a1'2 N9a10a2 H32 R30a1b R30a1b1 U7a2 U7a3 (89)	1
1894 >KY595612.1 Hc	73G 125C 127C 128T 151T 152C 195C 236C 263G 513A 567.1C 750G 1438G 2706G 3645C 3876G 4710A 4769G 7028T 8860G 11719A 12121C 12192A 12453C 12933G 13934T 14207A 14338T 14766T 15326G 15607G 15748C 15937T 16234T 16311C 16399G 16519C 16527T	P3b	73G 125C 127C 128T 151T 152C 195C 236C 263G 513A 567.1C 16234T 16311C 16399G 16519C 16527T	1	73 125 127 128 151 152 195 236 263 513 573.1C 750 1438 2706 3645 3876 4710A 4769 7028 8860 11719 12121 12192 12453 12933 13934 14207 14338 14766 15326 15607 15748 15937T 16234 16311 16399 16527	P3b	73 125 127 128 151 152 195 236 263 513 573.1C 16234 16311 16399 16527	[2.200] P3b (1)	[0.572] P3b1 (2) [0.000] P3	1
1895 >KY595610.1 Hc	73G 150G 189G 198A 215G 263G 271G 750G 1438G 2706G 4769G 5291C 7028T 8251A 8404C 8506C 8860G 11719A 12397G 12705T 14384C 14766T 15040T 15295T 15326G 16075C 16172C 16223T 16304C 16311C	S1a	73G 150G 189G 198A 215G 263G 271G 16075C 16172C 16223T 16304C 16311C	1	73 150G 189 198A 215 263 271G 750 1438 2706 4769 5291 7028 8251 8404 8506 8860 11719 12397 12705 14384C 14766 15040 15295 15326 16075 16172 16223 16304 16311	S1a	73 150G 189 198A 215 263 271G 16075 16172 16223 16304 16311	[0.387] S1a (1)	[0.032] L3'4 (2) [0.016] S1 (3) [0.015] N2 (4) [0.000] L3f1b L3h1 L3h1a L3h1a2 (8)	1
1896 >KY595606.1 Hc	73G 152C 263G 513A 750G 794C 1438G 2706G 3106d 3107C 4769G 6755A 7028T 8860G 9140T 11719A 12705T 14766T 15326G 16183C 16189C 16213A 16223T 16519C	O	73G 152C 263G 513A 16183C 16189C 16213A 16223T 16519C	1	73 152 263 513 750 794 1438 2706 4769 6755 7028 8860 9140 11719 12705 14766 15326 16189 16213 16223	O	73 152 263 513 16189 16213 16223	[0.160] O (1) [0.049] L3d3a1b (2) [0.010] N9b1 (3) [0.006] L3d3a1 (4) [0.000] L3c'd L3d3a L3d3a1a N9b N9b1a N9b3 S+152 (11)	[0.049] L3d3a1b (12) [0.042] R+16189 (13) [0.035] S2 (14) [0.013] N9b2 (15) [0.011] L3d1'2'3'4'5'6 L3d1b (17) [0.009] L3 H1ap1 (19) [0.008] L3d1d B4'5 (21) [0.006] L3d3a1 X (23) [0.000] L3b'f L3d L3d1 L3d1a L3d1b1 L3d1b1a L3d2a L3d3 L3d3a L3d3a1a L3d3b L3d6 L3h M37+152 M13'46'61 M13 M13a'b N N1'5 N1 N9 N9b1c N11 N14 S H3av H32 R11'B6 R30a1 R30a1a (53)	1

1897	>KY595599.1 Hc	73G 152C 212C 263G 750G 1438G 2706G 4769G 5330A 6077T 7028T 8860G 10118C 11296T 11719A 12850G 13722G 14766T 15326G 15607G 15927A 16111A 16266T 16357C 16519C	P1+152	73G 152C 212C 263G 16111A 16266T 16357C 16519C	1	73 152 212 263 750 1438 2706 4769 5330A 6077 7028 8860 10118 11296 11719 12850 13722 14766 15326 15607 15927 16111A 16266 16357	P1+152	73 152 212 263 16111A 16266 16357	[0.906] P1+152 (1)	[0.118] P1d2 (2) [0.100] P1d (3) [0.000] P1	1
1898	>KY595597.1 Hc	10C 73G 150G 189G 204C 207A 215G 263G 271G 750G 1119C 1438G 2706G 3010A 4769G 5291C 6975C 7028T 7283C 8251A 8404C 8506C 8860G 11719A 12705T 14384C 14766T 15040T 15326G 16075C 16092C 16172C 16183C 16189C 16223T 16311C	S1a	10C 73G 150G 189G 204C 207A 215G 263G 271G 16075C 16092C 16172C 16183C 16189C 16223T 16311C	1	10 73 150G 189 204 207 215 263 271G 750 1119 1438 2706 3010 4769 5291 6975 7028 7283 8251 8404 8506 8860 11719 12705 14384C 14766 15040 15326 16075 16092 16172 16189 16223 16311	S1a	10 73 150G 189 204 207 215 263 271G 16075 16092 16172 16189 16223 16311	[0.742] S1a (1)	[0.424] W (2) [0.333] R11b (3) [0.197] N10a (4) [0.127] L3'4 (5) [0.122] R11 (6) [0.100] N9b1c1 (7) [0.098] W1e (8) [0.095] W1 (9) [0.092] N9b1a (10) [0.079] R11a (11) [0.054] W1f (12) [0.048] S1 (13) [0.023] N2 (14) [0.020] N9b (15) [0.014] W1a (16) [0.010] N9b1 (17) [0.009] X2d1 (18) [0.008] X2d1a (19) [0.007] L3h1a2 (20) [0.000] L3f1b L3h1 L3h1a N1a N9b3 R11b1a U6b1b (27)	1
1899	>KY595596.1 Hc	73G 150G 189G 215G 263G 271G 750G 1438G 2706G 4098G 4769G 5147A 5291C 7028T 8251A 8404C 8506C 8860G 11719A 12397G 12705T 13197T 14384C 14766T 15040T 15295T 15326G 16075C 16172C 16223T 16304C 16311C	S1a	73G 150G 189G 215G 263G 271G 16075C 16172C 16223T 16304C 16311C	1	73 150G 189 215 263 271G 750 1438 2706 4098 4769 5147 5291 7028 8251 8404 8506 8860 11719 12397 12705 13197 14384C 14766 15040 15295 15326 16075 16172 16223 16304 16311	S1a	73 150G 189 215 263 271G 16075 16172 16223 16304 16311	[0.355] S1a (1)	[0.032] L3'4 (2) [0.016] S1 (3) [0.015] N2 (4) [0.000] L3f1b L3h1 L3h1a L3h1a2 (8)	1
1900	>KY595591.1 Hc	73G 150G 189G 215G 263G 271G 750G 1438G 2706G 3444G 4769G 5291C 7028T 8251A 8404C 8506C 8860G 9007G 10289G 10972G 11719A 12705T 13917G 14384C 14766T 15040T 15326G 16075C 16172C 16223T 16311C 16427A 16519C 16527T	S1a	73G 150G 189G 215G 263G 271G 16075C 16172C 16223T 16311C 16427A 16519C 16527T	1	73 150G 189 215 263 271G 750 1438 2706 3444 4769 5291 7028 8251 8404 8506 8860 9007 10289 10972 11719 12705 13917 14384C 14766 15040 15326 16075 16172 16223 16311 16427A 16527	S1a	73 150G 189 215 263 271G 16075 16172 16223 16311 16427A 16527	[0.064] S1a (1)	[0.032] L3'4 (2) [0.016] S1 (3) [0.015] N2 (4) [0.000] L3f1b L3h1 L3h1a L3h1a2 (8)	1
1901	>KY595588.1 Hc	73G 89C 92A 146C 208C 263G 489C 570T 750G 1438G 1719A 2706G 4117C 4769G 5460A 5843G 6272G 7028T 7379A 7681T 8701G 8790A 8860G 8964T 9540C 10398G 10400T 10873C 11719A 12705T 12940A 13500C 14025C 14766T 14783C 15043A 15301A 15326G 16129A 16144C 16148T 16223T 16241G 16265C 16311C 16343G 16519C	Q1a1	73G 89C 92A 146C 208C 263G 489C 570T 16129A 16144C 16148T 16223T 16241G 16265C 16311C 16343G 16519C	1	73 89 92 146 208 263 489 570 750 1438 1719 2706 4117 4769 5460 5843 6272 7028 7379 7681 8701 8790 8860 8964 9540 10398 10400 10873 11719 12705 12940 13500 14025 14766 14783 15043 15301 15326 16129 16144 16148 16223 16241 16265C 16311 16343	Q1a1	73 89 92 146 208 263 489 570 16129 16144 16148 16223 16241 16265C 16311 16343	[0.188] Q1a1 (1) [0.006] Q1 Q1e1c (3) [0.000] Q1d Q1e Q1e1 Q1f Q1f2 (8)	[0.010] Q1e1b1 (9) [0.009] Q1e1b (10) [0.000] Q1+@16223 Q1a Q1a1a Q1c Q1c1 Q1c2 Q1e1a Q1e1a1 Q1f1 (19)	1

1902	>KY595585.1 Hc	73G 89C 92A 146C 208C 210G 263G 489C 750G 1438G 2706G 3802G 4117C 4769G 5460A 5843G 7028T 7681T 8701G 8790A 8860G 8964T 9266A 9540C 10398G 10400T 10873C 11719A 12705T 12940A 13500C 14025C 14766T 14783C 15043A 15301A 15326G 16129A 16144C 16148T 16222T 16241G 16265C 16311C 16343G 16519C	Q1a1a	73G 89C 92A 146C 208C 210G 263G 489C 16129A 16144C 16148T 16222T 16241G 16265C 16311C 16343G 16519C	1	73 89 92 146 208 210 263 489 750 1438 2706 3802 4117 4769 5460 5843 7028 7681 8701 8790 8860 8964 9266 9540 10398 10400 10873 11719 12705 12940 13500 14025 14766 14783 15043 15301 15326 16129 16144 16148 16222 16241 16265C 16311 16343	Q1a1a	73 89 92 146 208 210 263 489 16129 16144 16148 16222 16241 16265C 16311 16343	[0.006] Q1a1a (1)	[0.000] Q1a1 (2)	1
1903	>KY595583.1 Hc	73G 152C 185A 263G 316A 489C 750G 1438G 1598A 2706G 4164G 4508T 4769G 6104T 6734A 7028T 8701G 8793C 9156G 9410G 9540C 10373A 10398G 10400T 10873C 11719A 12366G 12705T 12771A 13596T 14527G 14766T 14783C 15043A 15301A 15326G 16223T 16287T 16294T 16356C 16519C	M42a	73G 152C 185A 263G 316A 489C 16223T 16287T 16294T 16356C 16519C	1	73 152 185 263 316 489 750 1438 1598 2706 4164 4508 4769 6104 6734 7028 8701 8793 9156 9410 9540 10373 10398 10400 10873 11719 12366 12705 12771 13596 14527 14766 14783 15043 15301 15326 16223 16287 16294 16356	M42a	73 152 185 263 316 489 16223 16287 16294 16356	[0.155] M42a (1)	[0.000] M37+152 M13'46'61 M13 M13a'b (5)	1
1904	>KY595581.1 Hc	73G 263G 310.1C 489C 750G 1438G 1598A 1943G 2706G 4508T 4769G 5442C 6104T 7028T 8269A 8701G 8793C 8860G 9156G 9410G 9540C 9554A 10398G 10400T 10873C 11087C 11719A 12705T 12771A 14527G 14560A 14766T 14783C 15043A 15301A 15326G 16179T 16184T 16188T 16189C 16223T 16287T 16356C	M42a	73G 263G 310.1C 489C 16179T 16184T 16188T 16189C 16223T 16287T 16356C	1	73 263 489 750 1438 1598 1943 2706 4508 4769 5442 6104 7028 8269 8701 8793 8860 9156 9410 9540 9554 10398 10400 10873 11087 11719 12705 12771 14527 14560 14766 14783 15043 15301 15326 16179 16184 16188 16189 16223 16287 16356	M42a	73 263 489 16179 16184 16188 16189 16223 16287 16356	[0.347] M42a (1)	[0.000] M7b2 D4b2b2c (3)	1

1905 >KY595579.1 Hc	73G 150T 152C 195C 252C 263G 650C 709A 750G 1438G 1811G 2706G 4769G 7028T 8404C 8860G 10358G 11719A 12705T 14384C 14766T 15326G 16075C 16209C 16223T 16318T	S1	73G 150T 152C 195C 252C 263G 16075C 16209C 16223T 16318T	1	73 150 152 195 252 263 650 709 750 1438 1811 2706 4769 7028 8404 8860 10358 11719 12705 14384C 14766 15326 16075 16209 16223 16318T	S1	73 150 152 195 252 263 16075 16209 16223 16318T	[0.873] S1 (1) [0.308] L3e2a2 (2) [0.106] L3e3b1 (3) [0.086] L3f (4) [0.075] L3e2a3 (5) [0.049] L3f1a1 (6) [0.042] U7b (7) [0.036] L3e'i'k'x L3e L3i (10) [0.029] L3d1b2 (11) [0.028] L3f1a (12) [0.020] U7 (13) [0.018] L3f2a (14) [0.014] L3f1 (15) [0.011] L3c'd R30a1b (17) [0.006] L3e3'4'5 (18) [0.000] L3f2a1 L3e2 L3e2a L3e3'4 L3e3 L3e3b S+152 (25)	[0.609] L3e3a (26) [0.302] L3f2 (27) [0.286] R8a1a1c (28) [0.176] R8a1 R8a1a1a (30) [0.133] R8a1a (31) [0.123] L3e2b4 S2 (33) [0.122] N9a10a (34) [0.120] L3d4a (35) [0.111] L3i1a (36) [0.102] N9a10a1 (37) [0.094] U7a3a (38) [0.091] L3d1a1a1 L3e2b5 (40) [0.066] N21+195 U7b1 (42) [0.059] L3e2a1 (43) [0.057] L3d3a1a (44) [0.054] L3d1b (45) [0.049] L3d3a1b (46) [0.047] L3f1b4a1 (47) [0.044] M17c (48) [0.043] L3d1b1 (49) [0.042] U7b (50) [0.036] L3e3b2 (51) [0.034] L3d4 U7a (53) [0.026] N9a10a2a (54) [0.025] L3d3a1 (55) [0.024] L3e2a1b3 (56) [0.023] L3e4 L3i1 (58) [0.020] U7 (59) [0.017] L3f1b4a H32 U5b2a1a2 (62) [0.016] L3d1d L3e2b3 (64) [0.014] L3 (65) [0.011] L3d1'2'3'4'5'6 R30a1b (67) [0.009] U7a3 (68) [0.008] M13 (69) [0.007] L3k (70) [0.006] L3d1a1a M7a2 D4k (73) [0.005] L3b'f L3h N N1'5 N1 N9 N11 N14 S (82) [0.004] G2c (83) [0.000] L3f2a1a L3d L3d1 L3d1a L3d1b1a L3d1b1b L3d3 L3d3a L3d3b L3d6 L3e2a1a L3e2a1b L3e2b+152 L3e3b3 L3e5+195 L3e5c [illegible]	1
1906 >KY595578.1 Hc	73G 263G 489C 750G 1438G 1598A 2706G 4508T 4769G 5442C 6104T 7028T 8269A 8462C 8701G 8793C 8860G 9156G 9410G 9540C 10398G 10400T 10873C 11719A 11914A 12624C 12705T 12771A 14527G 14766T 14783C 15043A 15301A 15326G 16179T 16184T 16188T 16189C 16223T 16287T 16356C	M42a	73G 263G 489C 16179T 16184T 16188T 16189C 16223T 16287T 16356C	1	73 263 489 750 1438 1598 2706 4508 4769 5442 6104 7028 8269 8462 8701 8793 8860 9156 9410 9540 10398 10400 10873 11719 11914 12624 12705 12771 14527 14766 14783 15043 15301 15326 16179 16184 16188 16189 16223 16287 16356	M42a	73 263 489 16179 16184 16188 16189 16223 16287 16356	[0.347] M42a (1)	[0.000] M7b2 D4b2b2c (3)	1
1907 >KY595563.1 Hc	73G 125C 127C 128T 263G 567.1CCC 750G 1438G 2706G 3106d 3107C 3645C 4769G 5460A 5895d 6734A 7028T 8027A 8796T 8860G 11149A 11719A 13651G 14338T 14766T 15326G 15596A 15607G 15748C 15937T 16145A 16278T 16357C	P3a	73G 125C 127C 128T 263G 567.1CCC 16145A 16278T 16357C	1	73 125 127 128 263 573.3C 750 1438 2706 3645 4769 5460 5899d 6734 7028 8027 8796 8860 11149 11719 13651 14338 14766 15326 15596 15607 15748 15937T 16145 16278 16357	P3a	73 125 127 128 263 573.3C 16145 16278 16357	[0.375] P3a (1)	[0.000] P3 (2)	1

1908 >KY595559.1 Hc	73G 150T 152C 153G 204C 252C 263G 709A 750G 1438G 1811G 2706G 3390G 4769G 4997T 7028T 8404C 8860G 10358G 11719A 12705T 13143C 13986G 14384C 14766T 15326G 15622C 16075C 16209C 16223T 16235G 16274A 16318T 16342C 16343G	S1	73G 150T 152C 153G 204C 252C 263G 16075C 16209C 16223T 16235G 16274A 16318T 16342C 16343G	1	73 150 152 153 204 252 263 709 750 1438 1811 2706 3390 4769 4997 7028 8404 8860 10358 11719 12705 13143 13986 14384C 14766 15326 15622 16075 16209 16223 16235 16274 16318T 16342 16343	S1	73 150 152 153 204 252 263 16075 16209 16223 16235 16274 16318T 16342 16343	[1.140] S1 (1) [0.248] U3b2 (2) [0.161] U7b (3) [0.102] U3a'c (4) [0.101] U3 (5) [0.098] U3b2c (6) [0.092] U3b (7) [0.086] L3f (8) [0.037] U3b2a1 (9) [0.033] U7 (10) [0.028] L3f1a (11) [0.018] L3f2a (12) [0.017] U3b1 (13) [0.015] L3f2a1 (14) [0.014] L3f1 (15) [0.012] L3f1a1 L3e'i'k'x L3e L3i (19) [0.011] U3b2a (20) [0.010] U3b1b (21) [0.006] S+152 (22) [0.005] L3c'd (23) [0.000] L3d1b2 L3e3'4'5 L3e3'4 N1a R30a1b U3b1a1 (29)	[0.350] U7a3a (30) [0.349] U3b3 (31) [0.162] U3a1a (32) [0.161] U7b (33) [0.153] N8 (34) [0.122] N9a10a (35) [0.111] L3i1a (36) [0.102] N9a10a1 U3a1 (38) [0.099] L3d1a1a1 (39) [0.071] S2 (40) [0.066] U7b1 (41) [0.063] L3f1b4a1 (42) [0.057] L3d3a1b R30a1 (44) [0.056] R30a1a (45) [0.055] M17c (46) [0.050] L3x1a2 (47) [0.043] L3d1b1 (48) [0.042] U3a2 (49) [0.035] M9a1b+150 (50) [0.034] U5b2a1a2 (51) [0.033] L3d1d U7 (53) [0.032] U3a (54) [0.028] L3i1b (55) [0.027] L3f2 (56) [0.026] N9a10a2a (57) [0.023] L3e4 L3i1 (59) [0.021] L3f2a1a U7a (61) [0.018] M9a1b2 (62) [0.016] U3a1a1 (63) [0.015] L3d3a (64) [0.014] M17 L3 (66) [0.011] L3d1b (67) [0.010] L4b1 (68) [0.008] M13 (69) [0.007] L3k (70) [0.006] L3d1a1a (71) [0.005] L3b'f L3h N N1'5 N1 N9 N11 N14 S (80) [0.004] M37+152 (81) [0.000] L3f1b4a L3d L3d1'2'3'4'5'6 L3d1 L3d1a L3d1b1a L3d3 L3d3a1 L3d3a1a L3d3b L3d6 L3e5f L3x L3x1 L3x1a M7a M7a1b2 M7a2 M13'46'61 M13a'b [illegible]	1
1909 >KY595554.1 Hc	73G 263G 750G 794C 1438G 2706G 3106d 3107C 4769G 6755A 7028T 8860G 9140T 11719A 12705T 14766T 15326G 16183C 16189C 16213A 16223T 16519C	O	73G 263G 16183C 16189C 16213A 16223T 16519C	1	73 263 750 794 1438 2706 4769 6755 7028 8860 9140 11719 12705 14766 15326 16189 16213 16223	O	73 263 16189 16213 16223	[0.126] O (1) [0.000] N9b N9b1 N9b1a N9b3 (5)	[0.008] R+16189 (6) [0.004] S2 (7) [0.000] L3 L3b'f L3h N N1'5 N1 N9 N9b1c N9b2 N11 N14 S X R11'B6 B4'5 (22)	1

1910	>KY595551.1 Hc	73G 150T 152C 204C 252C 263G 709A 750G 1438G 1811G 2706G 3106d 3107C 3390G 4769G 4997T 7028T 8404C 8860G 10358G 11719A 12705T 13143C 13986G 14384C 14766T 15326G 15622C 16075C 16209C 16223T 16235G 16274A 16318T 16342C	S1	73G 150T 152C 204C 252C 263G 16075C 16209C 16223T 16235G 16274A 16318T 16342C	1	73 150 152 204 252 263 709 750 1438 1811 2706 3390 4769 4997 7028 8404 8860 10358 11719 12705 13143 13986 14384C 14766 15326 15622 16075 16209 16223 16235 16274 16318T 16342	S1	73 150 152 204 252 263 16075 16209 16223 16235 16274 16318T 16342	[1.093] S1 (1) [0.086] L3f (2) [0.033] U7 (3) [0.028] L3f1a (4) [0.025] U7b (5) [0.018] L3f2a (6) [0.015] L3f2a1 (7) [0.014] L3f1 (8) [0.012] L3f1a1 L3e'i'k'x L3e L3i (12) [0.000] L3c'd L3d1b2 L3e3'4'5 L3e3'4 N1a S+152 R30a1b (19)	[0.284] U7a3a (20) [0.122] N9a10a (21) [0.111] L3i1a (22) [0.102] N9a10a1 (23) [0.099] L3d1a1a1 (24) [0.071] S2 (25) [0.066] U7b1 (26) [0.057] L3d3a1b R30a1 (28) [0.056] R30a1a (29) [0.055] M17c (30) [0.050] L3x1a2 (31) [0.047] L3f1b4a1 (32) [0.043] L3d1b1 (33) [0.033] U7 (34) [0.028] L3i1b (35) [0.027] L3f2 (36) [0.026] N9a10a2a (37) [0.025] U7b (38) [0.023] L3e4 L3i1 (40) [0.021] L3f2a1a U7a (42) [0.017] U5b2a1a2 (43) [0.015] L3d3a (44) [0.014] L3 (45) [0.011] L3d1b (46) [0.010] L4b1 (47) [0.008] L3d1d M13 (49) [0.007] L3k (50) [0.006] L3d1a1a (51) [0.005] L3b'f L3h N N1'5 N1 N9 N11 N14 S (60) [0.004] M37+152 (61) [0.000] L3f1b4a L3d L3d1'2'3'4'5'6 L3d1 L3d1a L3d1b1a L3d3 L3d3a1 L3d3a1a L3d3b L3d6 L3x L3x1 L3x1a M7a M7a1b2 M7a2 M13'46'61 M13a'b M17 M62'68 N1a1'2 N9a10a2 H32 R30a1b R30a1b1 U7a2 U7a3 (89)	1
1911	>KY595550.1 Hc	73G 143A 153G 263G 456T 571T 750G 956A 990C 1438G 2706G 3106d 3107C 3387A 4769G 7028T 7124G 7129G 8404C 8860G 10972G 11719A 12705T 13105G 14384C 14766T 15326G 16051G 16075C 16176T 16223T 16256T 16311C 16343G 16390A	S1	73G 143A 153G 263G 456T 571T 16051G 16075C 16176T 16223T 16256T 16311C 16343G 16390A	1	73 143 153 263 456 571 750 956A 990 1438 2706 3387A 4769 7028 7124 7129 8404 8860 10972 11719 12705 13105 14384C 14766 15326 16051 16075 16176 16223 16256 16311 16343 16390	S1	73 143 153 263 456 571 16051 16075 16176 16223 16256 16311 16343 16390	[0.111] S1 (1) [0.008] L3'4 (2) [0.000] L3h1 L3h1a L3h1a2 M67 (6)	[0.599] U3a1a (7) [0.125] P+16176 (8) [0.108] N5 (9) [0.068] U2 (10) [0.051] U3a1 (11) [0.048] U3a (12) [0.031] M43a (13) [0.025] L3h1a2b (14) [0.024] R1 (15) [0.023] L3e4 (16) [0.022] S2 (17) [0.021] U3a2 (18) [0.019] L3 (19) [0.016] U3a1a1 (20) [0.009] N8 (21) [0.000] L3b'f L3f2 L3h M4"67+16311 M43+16311 M57 N N1'5 N1 N9 N11 N14 S H13a2b3 R8b P2'10 U3a1b (38)	1
1912	>KY849396.1 Hc	73G 194T 199C 204C 250C 263G 310.1C 567.1CC 750G 1438G 1719A 2706G 3106d 3107C 4025T 4529T 4769G 7028T 8251A 8519A 8860G 10034C 10238C 10398G 10819G 11719A 12501A 12705T 13780G 14766T 15043A 15326G 15924G 16129A 16223T 16304C 16391A 16519C	I4a1	73G 194T 199C 204C 250C 263G 310.1C 567.1CC 16129A 16223T 16304C 16391A 16519C	1	73 194 199 204 250 263 573.2C 750 1438 1719 2706 4025 4529T 4769 7028 8251 8519 8860 10034 10238 10398 10819 11719 12501 12705 13780 14766 15043 15326 15924 16129 16223 16304 16391	I4a1	73 194 199 204 250 263 573.2C 16129 16223 16304 16391	[0.023] I4a1 (1)	[0.043] I (2) [0.000] I4 I4a I4a2 I4b I5 I5b I6 I6a (10)	1

1913 >KU867629.1 Hc	182T 263G 309.1CT 310C 750G 1438G 3106d 3107C 4769G 6776C 8860G 15326G 16519C	H3am	182T 263G 309.1CT 310C 16519C	1	182 263 750 1438 4769 6776 8860 15326	H3am	182 263	[0.000] H3am (1)	[0.003] H54 (2) [0.002] H1e1a H4a1 (4) [0.001] H2a2a H13a1a1c H13a1a5 (7) [0.000] R0 HV HV4 HV4c HV5 HV5a HV9c HV12 H H1 H1e H1e1 H1e1a2 H1e1a7 H1e1c H1e2 H1e2a H1e2b H1e2d H1e4 H1e6 H1e7 H1e8 H1e8a H1h H1h1 H1h2 H1j H1j3 H1j5 H1j6 H1j7 H1j9 H1m H1n H1p H1q H1q1 H1q2 H1r H1s H1s1 H1t H1t1 H1t1a H1t2 H1u H1u1 H1v H1v1 H1v1a H1v1b H1w H1ae H1ae1 H1ae2 H1ae2a H1ae3 H1ae3a H1ag H1ag1 H1ag1b H1ah H1ai H1ai1 H1aj H1ak H1ak1 H1ak2 H1am H1am1 H1ap H1aq H1as H1as2 H1at H1at1 H1at1a H1av H1ax H1ax1 H1ay H1az H1bd H1be H1bi H1bj H1bk H1bm H1bn H1bp H1bq H1br H1bu H1bv H1bw H1bx H1ca H1ci H2 H2a H2a2 H2a2+(16235) H2a4 H2a5 H2a5a H2a5b H3 H3b H3c H3c1 H3e H3q H3q1 H3r H3r1 H3t H3u H3y H3aa H3ab H3ac H3ad H3ae H3ag H3ag1 H3ah H3ai H3aj H3ap H3aq H3ar H3as H3at H3at1 H3au H4 H4a H4a1a H4a1a1 H4a1a2 H4a1a2a H4a1a2a1 H4a1a5 H4a1c H4a1c1 H4a1c1a H4a1d H4b H4c H7 H7a H7b H7b1 [illegible]	1
1914 >KU867622.1 Hc	73G 195C 263G 309.1CT 310C 489C 750G 813G 1438G 1503A 2706G 3106d 3107C 4769G 6253C 6446A 6671C 6680C 7028T 8701G 8860G 9163R 9540C 10398G 10400T 10873C 11719A 12403T 12414C 12705T 12950C 13637G 14110C 14766T 14783C 15043A 15301A 15326G 16172C 16183C 16189C 16249C 16265C 16311C 16519C	M1a3b1	73G 195C 263G 309.1CT 310C 489C 16172C 16183C 16189C 16249C 16265C 16311C 16519C	1	73 195 263 489 750 813 1438 1503 2706 4769 6253 6446 6671 6680 7028 8701 8860 9163R 9540 10398 10400 10873 11719 12403 12414 12705 12950C 13637 14110 14766 14783 15043 15301 15326 16172 16189 16249 16265C 16311	M1a3b1	73 195 263 489 16172 16189 16249 16265C 16311	[0.072] M1a3b1 (1)	[0.040] M1a3b2 (2) [0.034] M1a3b (3) [0.000] M1a3 M1a3a M1a5 (6)	1
1915 >KU867621.1 Hc	73G 146C 263G 309.1CT 310C 458T 709A 750G 930A 1438G 1709A 1888A 2550T 2706G 3106d 3107C 4216C 4769G 4917G 5147A 7028T 8697A 8730G 8860G 9300A 10463C 11251G 11533T 11719A 11812G 13368A 14016A 14233G 14766T 14905A 15326G 15452A 15607G 15928A 16126C 16218T 16287T 16294T 16304C	T2b6b	73G 146C 263G 309.1CT 310C 458T 16126C 16218T 16287T 16294T 16304C	1	73 146 263 458 709 750 930 1438 1709 1888 2550T 2706 4216 4769 4917 5147 7028 8697 8730 8860 9300 10463 11251 11533 11719 11812 13368 14016 14233 14766 14905 15326 15452A 15607 15928 16126 16218 16287 16294 16304	T2b6b	73 146 263 458 16126 16218 16287 16294 16304	[0.000] T2b6b (1)	[0.000] T2b6+146 (2)	1

1916	>KU867610.1 Hc	73G 185A 188G 228A 234G 263G 295T 309.1CT 310C 462T 489C 514d 515d 709A 750G 1438G 2706G 3010A 3106d 3107C 4216C 4232C 4769G 7028T 8404C 8860G 10398G 11251G 11719A 12612G 13708A 14766T 14798C 15326G 15452A 16069T 16126C 16278T 16366T 16519C	J1c2e2	73G 185A 188G 228A 234G 263G 295T 309.1CT 310C 462T 489C 514d 515d 16069T 16126C 16278T 16366T 16519C	1	73 185 188 228 234 263 295 462 489 709 750 1438 2706 3010 4216 4232 4769 7028 8404 8860 10398 11251 11719 12612 13708 14766 14798 15326 15452A 16069 16126 16278 16366	J1c2e2	73 185 188 228 234 263 295 462 489 16069 16126 16278 16366	[0.023] J1c2e2 (1)	[0.023] J1c2e2 (2) [0.000] J1c2e	1
1917	>KU867606.1 Hc	73G 146C 263G 309.1CT 310C 458T 709A 750G 930A 1438G 1709A 1888A 2706G 3106d 3107C 4216C 4769G 4917G 5147A 7028T 8697A 8730G 8860G 9300A 10463C 11251G 11533T 11719A 11812G 13368A 14016A 14233G 14766T 14905A 15326G 15452A 15607G 15928A 16126C 16218T 16287T 16294T 16304C	T2b6b	73G 146C 263G 309.1CT 310C 458T 16126C 16218T 16287T 16294T 16304C	1	73 146 263 458 709 750 930 1438 1709 1888 2706 4216 4769 4917 5147 7028 8697 8730 8860 9300 10463 11251 11533 11719 11812 13368 14016 14233 14766 14905 15326 15452A 15607 15928 16126 16218 16287 16294 16304	T2b6b	73 146 263 458 16126 16218 16287 16294 16304	[0.000] T2b6b (1)	[0.000] T2b6+146 (2)	1
1918	>KU867604.1 Hc	73G 263G 310.1C 514d 515d 750G 1438G 2706G 3106d 3107C 3450T 4769G 5773A 6221C 7028T 8557A 8701G 8860G 9449T 9540C 10086G 10373A 10398G 10873C 11002G 11719A 12705T 13105G 13586T 13914A 14766T 15301A 15311G 15326G 15824G 15940d 16051G 16223T 16274A 16278T 16318G 16362C 16519C	L3b1a9a	73G 263G 310.1C 514d 515d 16051G 16223T 16274A 16278T 16318G 16362C 16519C	1	73 263 750 1438 2706 3450 4769 5773 6221 7028 8557 8701 8860 9449 9540 10086 10373 10398 10873 11002 11719 12705 13105 13586 13914A 14766 15301 15311 15326 15824 15944d 16051 16223 16274 16278 16318 16362	L3b1a9a	73 263 16051 16223 16274 16278 16318 16362	[0.024] L3b1a9a (1)	[0.000] L3b1a9 (2)	1
1919	>KU867603.1 Hc	73G 146C 263G 310.1C 750G 1438G 1811G 2217T 2706G 3106d 3107C 3480G 4020T 4769G 5231A 7028T 8860G 9055A 9698C 9716C 10550G 11299C 11467G 11719A 11869A 12308G 12372A 13135A 14037G 14167T 14766T 14798C 15326G 16222T 16224C 16270T 16311C 16519C	K2b1a1a	73G 146C 263G 310.1C 16222T 16224C 16270T 16311C 16519C	1	73 146 263 750 1438 1811 2217 2706 3480 4020 4769 5231 7028 8860 9055 9698 9716 10550 11299 11467 11719 11869A 12308 12372 13135 14037 14167 14766 14798 15326 16222 16224 16270 16311	K2b1a1a	73 146 263 16222 16224 16270 16311	[0.000] K2b1a1a (1)	[0.000] K2b1a1 (2)	1

1920	>KU867589.1 Hc	263G 309.1CCT 310C 750G 1438G 2294G 2706G 3106d 3107C 4580A 4735T 4769G 7028T 8860G 14388G 15326G 15904T 16298C 16311C 16566A	V+@72	263G 309.1CCT 310C 16298C 16311C 16566A	1	263 750 1438 2294 2706 4580 4735 4769 7028 8860 14388 15326 15904 16298 16311 16566	V+@72	263 16298 16311 16566	[0.049] V+@72 (1) [0.003] HV+16311 HV9 HV10 HV11 HV11a HV16 HV23 HV24 H1e1a4 H3+16311 H3h H3h1 H3h2 H3h3 H3h3a H3h3b H3h5 H3h6 H3m H13a1a2+16311 H13a2b1 H76 H80 (24) [0.002] H1+16311 (25)	[0.160] H72 (26) [0.156] H7b5 H59a (28) [0.155] H7c2 (29) [0.154] H1t1a H1at1a H1bi H4a1a (33) [0.153] H1q1 H14b (35) [0.151] H1q (36) [0.122] V2c (37) [0.117] V13 (38) [0.106] H3 (39) [0.077] HV0f (40) [0.046] V1a (41) [0.045] V (42) [0.032] H2a2a (43) [0.026] H (44) [0.025] V1a1 (45) [0.022] HV0e (46) [0.015] V1a1b (47) [0.013] HV7 (48) [0.011] HV15 (49) [0.010] V2 (50) [0.006] H2c R1 (52) [0.005] V2b V12 H2b H4a1 (56) [0.004] H7 (57) [0.003] H3h2 H35a H60a H87 (61) [0.002] H1e1a H3u H18 (64) [0.001] H1 H1e1a2 H1j H1u1 H1ae2a H1ag1 H1ak H1ak1 H1at1 H1av H1bk H7b H7b4 H13a1a H13a1a1 H13a2a H13a2b2 H16e H+13708 H26a1 H35 H71 H78 (87) [0.000] R0 HV HV0 HV0a V1 V2a V2a1 V2a1a V2b1 V2b2 V3 V3b V4 V8 V10 V10a V10b V10b1 V10b2 V11 V14 V15 V15a V17 V21 V23 V24 V25 V26 V28 HV4 HV4c HV5 HV5a HV6 HV8 HV9+152 HV9b HV9c HV14 HV14a HV17 HV12 HV13a H1e H1e1 H1e1a7 H1e1c H1e2 H1e2a H1e2b H1e2d H1e4 H1e6 [illegible]	1

1921 >KU867583.1 Ho	263G 310.1C 750G 1438G 3010A 3106d 3107C 4769G 8631G 8860G 11620G 13602C 15326G 16270T 16519C	H1ba	263G 310.1C 16270T 16519C	1	263 750 1438 3010 4769 8631 8860 11620 13602 15326 16270	H1ba	263 16270	[0.000] H1ba (1)	[0.013] H2a2a (2) [0.002] H14b (3) [0.001] H2 (4) [0.000] R0 HV HV4 HV4c HV5 HV5a HV9c HV12 H H1 H1e H1e1 H1e1a H1e1a2 H1e1a7 H1e1c H1e2 H1e2a H1e2b H1e2d H1e4 H1e6 H1e7 H1e8 H1e8a H1h H1h1 H1h2 H1j H1j3 H1j5 H1j6 H1j7 H1j9 H1m H1n H1p H1q H1q1 H1q2 H1r H1s H1s1 H1t H1t1 H1t1a H1t2 H1u H1u1 H1v H1v1 H1v1a H1v1b H1w H1ae H1ae1 H1ae2 H1ae2a H1ae3 H1ae3a H1ag H1ag1 H1ag1b H1ah H1ai H1ai1 H1aj H1ak H1ak1 H1ak2 H1am H1am1 H1ap H1aq H1as H1as2 H1at H1at1 H1at1a H1av H1ax H1ax1 H1ay H1az H1ba1 H1bd H1be H1bi H1bj H1bk H1bm H1bn H1bp H1bq H1br H1bu H1bv H1bw H1bx H1ca H1ci H2a H2a2 H2a2+(16235) H2a4 H2a5 H2a5a H2a5b H3 H3b H3c H3c1 H3e H3q H3q1 H3r H3r1 H3t H3u H3y H3aa H3ab H3ac H3ad H3ae H3ag H3ag1 H3ah H3ai H3aj H3ap H3aq H3ar H3as H3at H3at1 H3au H4 H4a H4a1 H4a1a H4a1a1 H4a1a2 H4a1a2a H4a1a2a1 H4a1a5 H4a1c H4a1c1 H4a1c1a H4a1d H4b H4c H7 H7a H7b H7b1 [illegible]	1
1922 >KU867582.1 Ho	73G 263G 310.1C 514d 515d 750G 1438G 2706G 3106d 3107C 3450T 4769G 5773A 6221C 7028T 8557A 8701G 8860G 9449T 9540C 10086G 10373A 10398G 10873C 11002G 11719A 12705T 13105G 13586T 13914A 14766T 15301A 15311G 15326G 15824G 15940d 16051G 16223T 16274A 16278T 16318G 16362C 16519C	L3b1a9a	73G 263G 310.1C 514d 515d 16051G 16223T 16274A 16278T 16318G 16362C 16519C	1	73 263 750 1438 2706 3450 4769 5773 6221 7028 8557 8701 8860 9449 9540 10086 10373 10398 10873 11002 11719 12705 13105 13586 13914A 14766 15301 15311 15326 15824 15944d 16051 16223 16274 16278 16318 16362	L3b1a9a	73 263 16051 16223 16274 16278 16318 16362	[0.024] L3b1a9a (1)	[0.000] L3b1a9 (2)	1
1923 >KY091895.1 Ho	73G 152C 199C 204C 263G 669C 750G 1438G 1719A 2702A 2706G 3107C 3336C 4221A 4769G 4772C 5315G 7028T 8860G 8901G 10238C 10398G 11719A 12501A 12705T 13780G 14766T 15043A 15326G 16129A 16147A 16154C 16172C 16223T 16248T 16320T 16355T 16519C 16558A	N1a1a1a3	73G 152C 199C 204C 263G 16129A 16147A 16154C 16172C 16223T 16248T 16320T 16355T 16519C 16558A	1	73 152 199 204 263 669 750 1438 1719 2702 2706 3336 4221A 4769 4772 5315 7028 8860 8901 10238 10398 11719 12501 12705 13780 14766 15043 15326 16129 16147A 16154 16172 16223 16248 16320 16355 16558	N1a1a1a3	73 152 199 204 263 16129 16147A 16154 16172 16223 16248 16320 16355 16558	[0.667] N1a1a1a3 (1)	[0.333] N1a1a1a1 (2) [0.176] N1a1a1a	1

1924 >KX440374.1 Hc	73G 263G 709A 750G 1438G 1888A 2706G 4216C 4769G 4917G 7028T 8155A 8697A 8860G 9422C 10463C 11239G 11251G 11719A 11812G 13368A 14233G 14766T 14905A 15326G 15452A 15607G 15641T 15928A 16126C 16286T 16294T 16296T 16297C 16362C 16519C	T2i1	73G 263G 16126C 16286T 16294T 16296T 16297C 16362C 16519C	1	73 263 709 750 1438 1888 2706 4216 4769 4917 7028 8155 8697 8860 9422C 10463 11239 11251 11719 11812 13368 14233 14766 14905 15326 15452A 15607 15641 15928 16126 16286 16294 16296 16297 16362	T2i1	73 263 16126 16286 16294 16296 16297 16362	[0.014] T2i1 (1)	[0.014] T2i1 (2) [0.000] T2i	1
1925 >KX440366.1 Hc	73G 150T 263G 709A 750G 1438G 1888A 2308G 2706G 4216C 4769G 4917G 7028T 8697A 8860G 10463C 11251G 11719A 11812G 13368A 14233G 14766T 14905A 15326G 15452A 15499T 15607G 15928A 16114T 16126C 16153A 16192T 16294T 16519C	T2e1a1a	73G 150T 263G 16114T 16126C 16153A 16192T 16294T 16519C	1	73 150 263 709 750 1438 1888 2308 2706 4216 4769 4917 7028 8697 8860 10463 11251 11719 11812 13368 14233 14766 14905 15326 15452A 15499 15607 15928 16114 16126 16153 16192 16294	T2e1a1a	73 150 263 16114 16126 16153 16192 16294	[0.000] T2e1a1a (1)	[0.000] T2e T2e1a1a T2e2 T2e2a (5)	1
1926 >KX440363.1 Hc	73G 263G 709A 750G 1438G 1888A 2706G 4216C 4373C 4769G 4823C 4917G 6261A 6297C 7028T 8697A 8860G 10463C 10822T 11251G 11719A 11812G 13368A 14233G 14766T 14905A 15326G 15452A 15607G 15928A 16126C 16146G 16294T 16296T 16519C	T2c1c	73G 263G 16126C 16146G 16294T 16296T 16519C	1	73 263 709 750 1438 1888 2706 4216 4373 4769 4823 4917 6261 6297 7028 8697 8860 10463 10822 11251 11719 11812 13368 14233 14766 14905 15326 15452A 15607 15928 16126 16146 16294 16296	T2c1c	73 263 16126 16146 16294 16296	[0.024] T2c1c (1) [0.000] T2 T2a T2a1 T2a1a T2a1a1 T2a1a2 T2a1a3 T2a1a3a T2a1a5 T2a1a6 T2a1a7 T2a1a8 T2b2b T2c T2d T2d2 T2g T2g1 T2g1a T2h T2h1 T2l (23)	[0.052] T2b3b (24) [0.047] T2b19 (25) [0.044] T2b3 T2b13b T2b26 T2b35 (29) [0.043] T2b1 T2b8 T2b17a T2b28 (33) [0.024] T2c1c (34) [0.011] T2+150 (35) [0.009] T T2a1b1a1 (37) [0.006] T2b (38) [0.004] T2b2 T2b4 T2b5 T2b5a T2b7 T2b7a T2b13 T2b15 T2b17 T2b25 T2b27 T2b29 T2b30 T2b31 T2b36 T2b37 (54) [0.000] T2 T2a T2a1 T2a1a T2a1a1 T2a1a2 T2a1a3 T2a1a3a T2a1a5 T2a1a6 T2a1a7 T2a1a8 T2a1b T2a1b1 T2a1b1a T2a1b1a1a T2a1b1a1a1 T2a1b1a1a2 T2a1b1a1b T2a1b1a1b1 T2a1b2 T2a1b2a T2a+195 T2a3 T2b2b T2b2b1 T2b32 T2c T2c1 T2c1c1 T2c1c2 T2d T2d1 T2d2 T2+16189 T2f T2f4 T2f6 T2f7 T2f8 T2f8a T2g T2g1 T2g1a T2g1a1 T2g1b T2g2 T2h T2h1 T2h2 T2i T2j T2j1 T2k T2l T2n (110)	1
1927 >KX440362.1 Hc	73G 146C 263G 514d 515d 709A 750G 1438G 1531T 1888A 2706G 3834A 4216C 4769G 4917G 5322C 6261A 6366A 7028T 8508G 8697A 8860G 10463C 10822T 11017C 11251G 11719A 11812G 13368A 13681G 14233G 14766T 14905A 15326G 15452A 15607G 15928A 16126C 16231C 16292T 16294T 16296T 16438A 16519C	T2c1f	73G 146C 263G 514d 515d 16126C 16231C 16292T 16294T 16296T 16438A 16519C	1	73 146 263 709 750 1438 1531 1888 2706 3834 4216 4769 4917 5322C 6261 6366 7028 8508 8697 8860 10463 10822 11017 11251 11719 11812 13368 13681 14233 14766 14905 15326 15452A 15607 15928 16126 16231 16292 16294 16296 16438	T2c1f	73 146 263 16126 16231 16292 16294 16296 16438	[0.023] T2c1f (1)	[0.023] T2c1f (2) [0.000] T2c1+146 T2c1e (4)	1

1928 >KX440353.1 Hc	73G 263G 272G 385G 709A 750G 930A 1438G 1530G 1888A 2706G 4216C 4769G 4917G 5147A 7028T 8697A 8839A 8860G 10463C 11251G 11719A 11812G 13368A 14233G 14766T 14905A 15326G 15452A 15607G 15928A 16126C 16266T 16294T 16304C 16519C	T2b33	73G 263G 272G 385G 16126C 16266T 16294T 16304C 16519C	1	73 263 272 385 709 750 930 1438 1530 1888 2706 4216 4769 4917 5147 7028 8697 8839 8860 10463 11251 11719 11812 13368 14233 14766 14905 15326 15452A 15607 15928 16126 16266 16294 16304	T2b33	73 263 272 385 16126 16266 16294 16304	[0.034] T2b33 (1)	[0.002] T2b (2) [0.000] T2b1 T2b2 T2b3 T2b3b T2b4 T2b5 T2b5a T2b7 T2b7a T2b8 T2b13 T2b13b T2b15 T2b17 T2b17a T2b19 T2b25 T2b26 T2b27 T2b28 T2b29 T2b30 T2b31 T2b32 T2b35 T2b36 T2b37 (29)	1
1929 >KX440348.1 Hc	73G 152C 263G 709A 750G 930A 1438G 1888A 2706G 4024T 4216C 4769G 4917G 5147A 7028T 8697A 8860G 9180G 9540C 9966A 10463C 11251G 11440A 11719A 11812G 13368A 14233G 14766T 14905A 15326G 15452A 15607G 15928A 16126C 16292T 16294T 16296T 16304C 16311C 16519C	T2b7a3	73G 152C 263G 16126C 16292T 16294T 16296T 16304C 16311C 16519C	1	73 152 263 709 750 930 1438 1888 2706 4024T 4216 4769 4917 5147 7028 8697 8860 9180 9540 9966 10463 11251 11440 11719 11812 13368 14233 14766 14905 15326 15452A 15607 15928 16126 16292 16294 16296 16304 16311	T2b7a3	73 152 263 16126 16292 16294 16296 16304 16311	[0.062] T2b7a3 (1) [0.014] T2b+152 (2) [0.000] T2b3a T2b4+152 T2b4c T2b4d T2b4e T2b4f T2b4g T2b21 T2b21b T2b22 (12)	[0.062] T2b7a3 (13) [0.029] T2b25 (14) [0.024] T2b7a1 (15) [0.022] T2b3b (16) [0.021] T2b17a (17) [0.014] T2b+152 (18) [0.010] T2b T2c1 (20) [0.004] T2b5 T2b26 T2b27 (23) [0.000] T2b1 T2b2 T2b3 T2b3a T2b4 T2b4+152 T2b4b T2b4c T2b4d T2b4e T2b4f T2b4g T2b4h T2b5a T2b7 T2b7a T2b8 T2b13 T2b13b T2b15 T2b17 T2b19 T2b21 T2b21a T2b21b T2b22 T2b28 T2b29 T2b30 T2b31 T2b32 T2b35 T2b36 T2b37 T2d1 T2n (59)	1
1930 >KX440345.1 Hc	41T 73G 263G 319C 709A 750G 930A 1438G 1888A 2706G 4216C 4769G 4917G 5147A 7028T 8697A 8860G 9861C 10463C 11251G 11719A 11812G 13368A 13542C 14233G 14766T 14905A 15326G 15452A 15607G 15928A 16126C 16294T 16296T 16304C 16519C	T2b34	41T 73G 263G 319C 16126C 16294T 16296T 16304C 16519C	1	41 73 263 319 709 750 930 1438 1888 2706 4216 4769 4917 5147 7028 8697 8860 9861 10463 11251 11719 11812 13368 13542C 14233 14766 14905 15326 15452A 15607 15928 16126 16294 16296 16304	T2b34	41 73 263 319 16126 16294 16296 16304	[0.000] T2b34 (1)	[0.004] T2b (2) [0.000] T2b1 T2b2 T2b3 T2b3b T2b4 T2b5 T2b5a T2b7 T2b7a T2b8 T2b13 T2b13b T2b15 T2b17 T2b17a T2b19 T2b25 T2b26 T2b27 T2b28 T2b29 T2b30 T2b31 T2b32 T2b35 T2b36 T2b37 (29)	1
1931 >KX440341.1 Hc	73G 263G 709A 750G 930A 1438G 1888A 2706G 4216C 4769G 4823C 4917G 6261A 7028T 8697A 8860G 10463C 10822T 11251G 11719A 11812G 13368A 14233G 14766T 14905A 15326G 15452A 15607G 15928A 16126C 16146G 16292T 16294T 16296T 16519C	T2c1c	73G 263G 16126C 16146G 16292T 16294T 16296T 16519C	1	73 263 709 750 930 1438 1888 2706 4216 4769 4823 4917 6261 7028 8697 8860 10463 10822 11251 11719 11812 13368 14233 14766 14905 15326 15452A 15607 15928 16126 16146 16292 16294 16296	T2c1c	73 263 16126 16146 16292 16294 16296	[0.000] T2c1c (1)	[0.000] T2c1 T2c1c T2c1c1 T2c1c2 (5)	1
1932 >KX440319.1 Hc	73G 263G 513.1CA 709A 750G 1438G 1888A 2706G 3882A 4216C 4769G 4917G 7028T 8155A 8697A 8715C 8860G 10463C 11251G 11719A 11812G 13135A 13368A 13965C 14233G 14687G 14766T 14905A 15326G 15452A 15607G 15928A 16126C 16256T 16294T 16296T 16324C 16519C	T2a1b2b	73G 263G 513.1CA 16126C 16256T 16294T 16296T 16324C 16519C	1	73 263 709 750 1438 1888 2706 3882 4216 4769 4917 7028 8155 8697 8715 8860 10463 11251 11719 11812 13135 13368 13965 14233 14687 14766 14905 15326 15452A 15607 15928 16126 16256 16294 16296 16324	T2a1b2b	73 263 16126 16256 16294 16296 16324	[0.000] T2a1b2b (1)	[0.000] T2a1b T2a1b1 T2a1b1a T2a1b1a1 T2a1b1a1a T2a1b1a1a1 T2a1b1a1a2 T2a1b1a1b T2a1b1a1b1 T2a1b2 T2a1b2a T2a1b2b (13)	1

1933 >KX440272.1 H	73G 150T 152C 263G 295T 489C 514d 515d 750G 1438G 2706G 4216C 4769G 5322C 5633T 7028T 7476T 8860G 10172A 10398G 11251G 11719A 11899C 12010T 12612G 13135A 13708A 13899C 14766T 15257A 15326G 15452A 15812A 16069T 16126C 16193T 16319A 16360T	J2b1c1	73G 150T 152C 263G 295T 489C 514d 515d 16069T 16126C 16193T 16319A 16360T	1	73 150 152 263 295 489 750 1438 2706 4216 4769 5322C 5633 7028 7476 8860 10172 10398 11251 11719 11899 12010 12612 13135 13708 13899 14766 15257 15326 15452A 15812 16069 16126 16193 16319 16360	J2b1c1	73 150 152 263 295 489 16069 16126 16193 16319 16360	[0.286] J2b1c1 (1)	[0.286] J2b1c1 (2) [0.000] J2b J2b1 J2b1b J2b1b1 J2b1c J2b1d J2b1e J2b1f J2b1g J2b2 (12)	1
1934 >KX440271.1 H	73G 150T 152C 263G 295T 489C 514d 515d 750G 1438G 2706G 4216C 4769G 5633T 7028T 7476T 8860G 10172A 10398G 11251G 11719A 12010T 12612G 13708A 13899C 14766T 15257A 15326G 15452A 15812A 16069T 16126C 16193T 16319A 16362C	J2b1c1	73G 150T 152C 263G 295T 489C 514d 515d 16069T 16126C 16193T 16319A 16362C	1	73 150 152 263 295 489 750 1438 2706 4216 4769 5633 7028 7476 8860 10172 10398 11251 11719 12010 12612 13708 13899 14766 15257 15326 15452A 15812 16069 16126 16193 16319 16362	J2b1c1	73 150 152 263 295 489 16069 16126 16193 16319 16362	[0.143] J2b1c1 (1)	[0.143] J2b1c1 (2) [0.040] J2b1 (3) [0.015] J2b1e (4) [0.000] J2b J2b1b J2b1b1 J2b1c J2b1d J2b1f J2b1g J2b2 (12)	1
1935 >KX440257.1 H	73G 150T 152C 195C 198T 215G 263G 295T 319C 489C 513A 567.1CC 750G 1438G 2706G 4216C 4769G 5780C 7028T 7476T 7789A 8860G 10398G 10499G 11251G 11377A 11719A 12612G 13708A 13722G 14133G 14766T 15257A 15326G 15452A 16069T 16126C 16145A 16182C 16183C 16189C 16231C 16261T	J2a1a1e	73G 150T 152C 195C 198T 215G 263G 295T 319C 489C 513A 567.1CC 16069T 16126C 16145A 16182C 16183C 16189C 16231C 16261T	1	73 150 152 195 198 215 263 295 319 489 513 573.2C 750 1438 2706 4216 4769 5780C 7028 7476 7789 8860 10398 10499 11251 11377 11719 12612 13708 13722 14133 14766 15257 15326 15452A 16069 16126 16145 16189 16231 16261	J2a1a1e	73 150 152 195 198 215 263 295 319 489 513 573.2C 16069 16126 16145 16189 16231 16261	[0.459] J2a1a1e (1) [0.000] J2a1a1 J2a1a1b J2a1a1c J2a1a1d (5)	[0.459] J2a1a1e (6) [0.066] J2a1a1a2 (7) [0.029] J2a1a1a2a (8) [0.023] J2a1a1a (9) [0.000] J2a1a J2a1a1 J2a1a1b J2a1a1c J2a1a1d (14)	1
1936 >KX440254.1 H	73G 152C 263G 295T 462T 489C 750G 1438G 2706G 3010A 4216C 4769G 5262A 5297T 5774C 7028T 7521A 7789A 7963G 8860G 10398G 11251G 11719A 12612G 13708A 14110C 14766T 15326G 15452A 15836G 15894A 16069T 16126C 16193T 16256T 16335G 16519C	J1d3a1	73G 152C 263G 295T 462T 489C 16069T 16126C 16193T 16256T 16335G 16519C	1	73 152 263 295 462 489 750 1438 2706 3010 4216 4769 5262 5297 5774 7028 7521 7789 7963 8860 10398 11251 11719 12612 13708 14110 14766 15326 15452A 15836 15894 16069 16126 16193 16256 16335	J1d3a1	73 152 263 295 462 489 16069 16126 16193 16256 16335	[0.571] J1d3a1 (1)	[0.000] J1d J1d3 J1d3a J1d3a2 J1d4 J1d6 (7)	1
1937 >KX440253.1 H	73G 152C 263G 295T 462T 489C 513.1CA 750G 1438G 2706G 3010A 4216C 4769G 5262A 5297T 5774C 7028T 7521A 7789A 7963G 8860G 10398G 11251G 11719A 12612G 13708A 14766T 15326G 15452A 15836G 15894A 16069T 16126C 16193T 16256T 16335G 16519C	J1d3a1	73G 152C 263G 295T 462T 489C 513.1CA 16069T 16126C 16193T 16256T 16335G 16519C	1	73 152 263 295 462 489 750 1438 2706 3010 4216 4769 5262 5297 5774 7028 7521 7789 7963 8860 10398 11251 11719 12612 13708 14766 15326 15452A 15836 15894 16069 16126 16193 16256 16335	J1d3a1	73 152 263 295 462 489 16069 16126 16193 16256 16335	[0.571] J1d3a1 (1)	[0.000] J1d J1d3 J1d3a J1d3a2 J1d4 J1d6 (7)	1

1938 >KX440247.1 Hc	73G 146C 185A 189G 228A 263G 295T 462T 489C 750G 1438G 1719A 2706G 3010A 4216C 4769G 7028T 7775A 8152A 8860G 9117C 9286C 10398G 11251G 11719A 12453C 12612G 13708A 13917G 14182C 14766T 14798C 15326G 15452A 15514C 16069T 16126C 16527T	J1c15b	73G 146C 185A 189G 228A 263G 295T 462T 489C 16069T 16126C 16527T	1	73 146 185 189 228 263 295 462 489 750 1438 1719 2706 3010 4216 4769 7028 7775 8152 8860 9117 9286 10398 11251 11719 12453 12612 13708 13917 14182 14766 14798 15326 15452A 15514 16069 16126 16527	J1c15b	73 146 185 189 228 263 295 462 489 16069 16126 16527	[0.051] J1c15b (1)	[0.051] J1c15b (2) [0.000] J1c3+189	1
1939 >KX440245.1 Hc	73G 185A 228A 263G 295T 462T 489C 513.1CA 750G 1438G 2706G 3010A 4216C 4769G 7028T 8860G 10084C 11251G 11719A 12612G 13349C 13708A 14766T 14798C 15326G 15452A 16069T 16126C 16319A	J1c8a	73G 185A 228A 263G 295T 462T 489C 513.1CA 16069T 16126C 16319A	1	73 185 228 263 295 462 489 750 1438 2706 3010 4216 4769 7028 8860 10084 11251 11719 12612 13349 13708 14766 14798 15326 15452A 16069 16126 16319	J1c8a	73 185 228 263 295 462 489 16069 16126 16319	[0.000] J1c8a (1)	[0.097] J1c10a (2) [0.021] J1c (3) [0.013] J1c8a (4) [0.000] J1c3 J1c3a J1c3a1 J1c3a2 J1c3b J1c3b1 J1c3b1a J1c3b2 J1c3d J1c3g J1c3h J1c3i J1c3k J1c4 J1c4b J1c5 J1c5a J1c5a1 J1c5b J1c5c J1c5d J1c5e J1c5f J1c6 J1c6a J1c8 J1c8a1 J1c8a2 J1c8b J1c9 J1c10 J1c15 J1c15a J1c15a1 J1c17 (39)	1
1940 >KX440244.1 Hc	73G 185A 228A 263G 295T 462T 489C 514d 515d 750G 1438G 2706G 3010A 4216C 4769G 7028T 8666G 8860G 9052G 10084C 11251G 11719A 12612G 13708A 14751T 14766T 14798C 15326G 15452A 16069T 16126C 16265G 16319A	J1c8a1	73G 185A 228A 263G 295T 462T 489C 514d 515d 16069T 16126C 16265G 16319A	1	73 185 228 263 295 462 489 750 1438 2706 3010 4216 4769 7028 8666 8860 9052 10084 11251 11719 12612 13708 14751 14766 14798 15326 15452A 16069 16126 16265 16319	J1c8a1	73 185 228 263 295 462 489 16069 16126 16265 16319	[0.000] J1c8a1 (1)	[0.000] J1c8a J1c8a1 J1c8a1a (4)	1
1941 >KX440233.1 Hc	73G 185A 263G 295T 462T 489C 750G 1438G 2706G 3010A 4216C 4769G 7028T 8860G 10398G 11251G 11719A 12477C 12612G 13708A 13934T 14766T 14798C 15326G 15344C 15452A 16063C 16069T 16126C 16294T	J1c3f	73G 185A 263G 295T 462T 489C 16063C 16069T 16126C 16294T	1	73 185 263 295 462 489 750 1438 2706 3010 4216 4769 7028 8860 10398 11251 11719 12477 12612 13708 13934 14766 14798 15326 15344 15452A 16063 16069 16126 16294	J1c3f	73 185 263 295 462 489 16063 16069 16126 16294	[0.054] J1c3f (1)	[0.088] J1c4 (2) [0.054] J1c3f (3) [0.003] J1c3 (4) [0.000] J1c J1c3a J1c3a1 J1c3a2 J1c3b J1c3b1 J1c3b1a J1c3b2 J1c3d J1c3g J1c3h J1c3i J1c3k J1c4b J1c5 J1c5a J1c5a1 J1c5b J1c5c J1c5d J1c5e J1c5f J1c6 J1c6a J1c8 J1c8b J1c9 J1c10 J1c10a J1c15 J1c15a J1c15a1 J1c17 (37)	1
1942 >KX440231.1 Hc	73G 228A 263G 295T 462T 489C 750G 1438G 2706G 3010A 4216C 4769G 7028T 8860G 10398G 11251G 11719A 12477C 12612G 13708A 13934T 14766T 14798C 15326G 15452A 16063C 16069T 16126C	J1c3f	73G 228A 263G 295T 462T 489C 16063C 16069T 16126C	1	73 228 263 295 462 489 750 1438 2706 3010 4216 4769 7028 8860 10398 11251 11719 12477 12612 13708 13934 14766 14798 15326 15452A 16063 16069 16126	J1c3f	73 228 263 295 462 489 16063 16069 16126	[0.000] J1c3f (1)	[0.000] J1c J1c3 J1c3a J1c3a1 J1c3a2 J1c3b J1c3b1 J1c3b1a J1c3b2 J1c3d J1c3f J1c3g J1c3h J1c3i J1c3k J1c4 J1c4b J1c5 J1c5a J1c5a1 J1c5b J1c5c J1c5d J1c5e J1c5f J1c6 J1c6a J1c8 J1c8b J1c9 J1c10 J1c10a J1c15 J1c15a J1c15a1 J1c17 (37)	1
1943 >KX440221.1 Hc	73G 185A 188G 228A 263G 295T 462T 489C 514d 515d 750G 1438G 2706G 3010A 4216C 4769G 7028T 8697A 8860G 10398G 11251G 11719A 12612G 13635C 13708A 14766T 14798C 15326G 15452A 16069T 16126C 16366T 16519C	J1c2e	73G 185A 188G 228A 263G 295T 462T 489C 514d 515d 16069T 16126C 16366T 16519C	1	73 185 188 228 263 295 462 489 750 1438 2706 3010 4216 4769 7028 8697 8860 10398 11251 11719 12612 13635 13708 14766 14798 15326 15452A 16069 16126 16366	J1c2e	73 185 188 228 263 295 462 489 16069 16126 16366	[0.000] J1c2e (1)	[0.000] J1c2 J1c2a J1c2a1 J1c2a1a J1c2a3 J1c2b J1c2b1 J1c2b2 J1c2b5 J1c2c J1c2c2 J1c2c2a J1c2c3 J1c2d J1c2e J1c2e1 J1c2e2 J1c2f J1c2g J1c2k J1c2l J1c2n J1c2n1 J1c2p J1c2q J1c2q1 J1c2s J1c2t (29)	1

1944 >KX440220.1 Hc	73G 185A 188G 228A 263G 295T 462T 489C 514d 515d 750G 1438G 2706G 3010A 4216C 4769G 7028T 8860G 10398G 11251G 11719A 12612G 13708A 14766T 14798C 15326G 15452A 16069T 16126C 16366T 16519C	J1c2e	73G 185A 188G 228A 263G 295T 462T 489C 514d 515d 16069T 16126C 16366T 16519C	1	73 185 188 228 263 295 462 489 750 1438 2706 3010 4216 4769 7028 8860 10398 11251 11719 12612 13708 14766 14798 15326 15452A 16069 16126 16366	J1c2e	73 185 188 228 263 295 462 489 16069 16126 16366	[0.000] J1c2e (1)	[0.000] J1c2 J1c2a J1c2a1 J1c2a1a J1c2a3 J1c2b J1c2b1 J1c2b2 J1c2b5 J1c2c J1c2c2 J1c2c2a J1c2c3 J1c2d J1c2e J1c2e1 J1c2e2 J1c2f J1c2g J1c2k J1c2l J1c2n J1c2n1 J1c2p J1c2q J1c2q1 J1c2s J1c2t (29)	1
1945 >KX440219.1 Hc	73G 185A 188G 228A 234G 263G 295T 462T 489C 514d 515d 750G 1438G 2706G 3010A 4216C 4769G 7028T 8860G 10398G 11251G 11557G 11719A 12612G 13708A 14766T 14798C 15326G 15452A 16069T 16126C 16324C 16366T 16390A 16519C	J1c2e1	73G 185A 188G 228A 234G 263G 295T 462T 489C 514d 515d 16069T 16126C 16324C 16366T 16390A 16519C	1	73 185 188 228 234 263 295 462 489 750 1438 2706 3010 4216 4769 7028 8860 10398 11251 11557 11719 12612 13708 14766 14798 15326 15452A 16069 16126 16324 16366 16390	J1c2e1	73 185 188 228 234 263 295 462 489 16069 16126 16324 16366 16390	[0.458] J1c2e1 (1)	[0.458] J1c2e1 (2) [0.000] J1c2e	1
1946 >KX440217.1 Hc	73G 185A 188G 228A 263G 295T 462T 489C 750G 1438G 2706G 3010A 4216C 4769G 7028T 8860G 10398G 11251G 11719A 12612G 13708A 14766T 14798C 15326G 15452A 16069T 16086C 16126C 16148T 16519C	J1c2m	73G 185A 188G 228A 263G 295T 462T 489C 16069T 16086C 16126C 16148T 16519C	1	73 185 188 228 263 295 462 489 750 1438 2706 3010 4216 4769 7028 8860 10398 11251 11719 12612 13708 14766 14798 15326 15452A 16069 16086 16126 16148	J1c2m	73 185 188 228 263 295 462 489 16069 16086 16126 16148	[0.133] J1c2m (1) [0.000] J1c2b4 (2)	[0.133] J1c2m (3) [0.011] J1c2q1 (4) [0.003] J1c2 (5) [0.000] J1c2a J1c2a1 J1c2a1a J1c2a3 J1c2b J1c2b1 J1c2b2 J1c2b4 J1c2b5 J1c2c J1c2c2 J1c2c2a J1c2c3 J1c2d J1c2f J1c2g J1c2k J1c2l J1c2n J1c2n1 J1c2p J1c2q J1c2s J1c2t (29)	1
1947 >KX440207.1 Hc	73G 263G 295T 462T 489C 750G 1438G 2706G 3010A 4216C 4769G 7028T 8269A 8460G 8860G 10398G 11251G 11719A 12612G 13708A 14766T 15326G 15452A 16069T 16126C 16145A 16222T 16235G 16261T 16271C 16519C	J1b3a	73G 263G 295T 462T 489C 16069T 16126C 16145A 16222T 16235G 16261T 16271C 16519C	1	73 263 295 462 489 750 1438 2706 3010 4216 4769 7028 8269 8460 8860 10398 11251 11719 12612 13708 14766 15326 15452A 16069 16126 16145 16222 16235 16261 16271	J1b3a	73 263 295 462 489 16069 16126 16145 16222 16235 16261 16271	[0.000] J1b3a (1)	[0.000] J1b3 J1b3a J1b3b J1b3b1 (5)	1
1948 >KX440204.1 Hc	64T 73G 152C 263G 271T 295T 462T 489C 750G 1438G 2706G 3010A 4216C 4769G 5460A 6071C 7028T 7964C 8251A 8269A 8860G 9083C 10398G 11251G 11719A 12612G 13708A 13879C 14766T 15326G 15452A 15511C 16069T 16126C 16145A 16235G 16261T 16291T 16519C	J1b1b2	64T 73G 152C 263G 271T 295T 462T 489C 16069T 16126C 16145A 16235G 16261T 16291T 16519C	1	64 73 152 263 271 295 462 489 750 1438 2706 3010 4216 4769 5460 6071 7028 7964 8251 8269 8860 9083 10398 11251 11719 12612 13708 13879 14766 15326 15452A 15511 16069 16126 16145 16235 16261 16291	J1b1b2	64 73 152 263 271 295 462 489 16069 16126 16145 16235 16261 16291	[0.400] J1b1b2 (1)	[0.073] J1b3b1 (2) [0.069] J1b1b3 (3) [0.017] J1b8 (4) [0.007] J1b1b1 (5) [0.000] J1b1b J1b1b1b J1b3 J1b3b (9)	1
1949 >KX440203.1 Hc	73G 242T 263G 295T 462T 489C 750G 1438G 2158C 2581G 2706G 3010A 4216C 4769G 5460A 5463T 6911C 7028T 8269A 8557A 8860G 10398G 11251G 11719A 12007A 12561A 12612G 13708A 13879C 14766T 15326G 15452A 16069T 16126C 16145A 16172C 16192T 16261T	J1b1a1a	73G 242T 263G 295T 462T 489C 16069T 16126C 16145A 16172C 16192T 16261T	1	73 242 263 295 462 489 750 1438 2158 2581 2706 3010 4216 4769 5460 5463 6911 7028 8269 8557 8860 10398 11251 11719 12007 12561 12612 13708 13879 14766 15326 15452A 16069 16126 16145 16172 16192 16261	J1b1a1a	73 242 263 295 462 489 16069 16126 16145 16172 16192 16261	[0.000] J1b1a1a (1)	[0.012] J1b1a1b (2) [0.008] J1b1a1 (3) [0.000] J1b1a1a	1

1950	>KX440202.1 Hc	73G 242T 263G 295T 462T 489C 513.1CA 750G 1438G 2158C 2706G 3010A 4216C 4769G 5460A 5463T 6911C 7028T 8269A 8557A 8860G 10398G 11251G 11719A 12007A 12612G 13708A 13879C 14766T 15326G 15452A 16069T 16126C 16145A 16172C 16192T 16222T 16261T	J1b1a1a	73G 242T 263G 295T 462T 489C 513.1CA 16069T 16126C 16145A 16172C 16192T 16222T 16261T	1	73 242 263 295 462 489 750 1438 2158 2706 3010 4216 4769 5460 5463 6911 7028 8269 8557 8860 10398 11251 11719 12007 12612 13708 13879 14766 15326 15452A 16069 16126 16145 16172 16192 16222 16261	J1b1a1a	73 242 263 295 462 489 16069 16126 16145 16172 16192 16222 16261	[0.000] J1b1a1a (1)	[0.012] J1b1a1a J1b1a1b (3) [0.008] J1b1a1	1
1951	>KX440201.1 Hc	73G 242T 263G 295T 462T 489C 750G 1438G 2158C 2706G 3010A 4216C 4769G 4892T 5460A 5463T 6911C 7028T 8269A 8557A 8860G 10398G 11251G 11719A 12007A 12612G 13708A 13879C 14766T 15326G 15452A 16069T 16126C 16145A 16172C 16192T 16222T 16261T 16311C	J1b1a1a	73G 242T 263G 295T 462T 489C 16069T 16126C 16145A 16172C 16192T 16222T 16261T 16311C	1	73 242 263 295 462 489 750 1438 2158 2706 3010 4216 4769 4892 5460 5463 6911 7028 8269 8557 8860 10398 11251 11719 12007 12612 13708 13879 14766 15326 15452A 16069 16126 16145 16172 16192 16222 16261 16311	J1b1a1a	73 242 263 295 462 489 16069 16126 16145 16172 16192 16222 16261 16311	[0.024] J1b1a1a (1)	[0.036] J1b1a1a J1b1a1b (3) [0.016] J1b1a1	1
1952	>KX440200.1 Hc	73G 242T 263G 295T 462T 489C 750G 1438G 2158C 2706G 3010A 4216C 4769G 5460A 5463T 6911C 7028T 8269A 8557A 8860G 10398G 11251G 11719A 12007A 12074C 12612G 13708A 13879C 14766T 15326G 15452A 15670C 16069T 16126C 16145A 16172C 16192T 16222T 16261T	J1b1a1a	73G 242T 263G 295T 462T 489C 16069T 16126C 16145A 16172C 16192T 16222T 16261T	1	73 242 263 295 462 489 750 1438 2158 2706 3010 4216 4769 5460 5463 6911 7028 8269 8557 8860 10398 11251 11719 12007 12074C 12612 13708 13879 14766 15326 15452A 15670 16069 16126 16145 16172 16192 16222 16261	J1b1a1a	73 242 263 295 462 489 16069 16126 16145 16172 16192 16222 16261	[0.000] J1b1a1a (1)	[0.012] J1b1a1a J1b1a1b (3) [0.008] J1b1a1	1
1953	>KX440199.1 Hc	73G 242T 263G 295T 462T 489C 750G 1438G 2158C 2706G 3010A 4216C 4769G 5460A 5463T 6911C 7028T 8269A 8557A 8860G 10398G 11251G 11719A 12007A 12612G 13708A 13879C 14766T 15326G 15452A 16069T 16126C 16145A 16172C 16192T 16222T 16261T	J1b1a1a	73G 242T 263G 295T 462T 489C 16069T 16126C 16145A 16172C 16192T 16222T 16261T	1	73 242 263 295 462 489 750 1438 2158 2706 3010 4216 4769 5460 5463 6911 7028 8269 8557 8860 10398 11251 11719 12007 12612 13708 13879 14766 15326 15452A 16069 16126 16145 16172 16192 16222 16261	J1b1a1a	73 242 263 295 462 489 16069 16126 16145 16172 16192 16222 16261	[0.000] J1b1a1a (1)	[0.012] J1b1a1a J1b1a1b (3) [0.008] J1b1a1	1
1954	>KY765534.1 Hc	73G 150T 263G 309.1CCT 310C 489C 750G 752T 1107C 1438G 2706G 3106d 3107C 3702G 4769G 4883T 5178A 5301G 7028T 8701G 8838A 8860G 9180G 9540C 10397G 10398G 10400T 10873C 11719A 11944C 12026G 12705T 13759A 14766T 14783C 15043A 15301A 15326G 16126C 16136C 16182C 16183C 16189C 16223T 16360T 16362C	D5a3a1a	73G 150T 263G 309.1CCT 310C 489C 16126C 16136C 16182C 16183C 16189C 16223T 16360T 16362C	1	73 150 263 489 750 752 1107 1438 2706 3702 4769 4883 5178A 5301 7028 8701 8838 8860 9180 9540 10397 10398 10400 10873 11719 11944 12026 12705 13759 14766 14783 15043 15301 15326 16126 16136 16189 16223 16360 16362	D5a3a1a	73 150 263 489 16126 16136 16189 16223 16360 16362	[0.000] D5a3a1a (1)	[0.033] D5a3a1 (2) [0.000] D5a3 D5a3a (4)	1

1955 >KX977309.1 Hc	73G 150T 263G 750G 1438G 1721T 1834C 2706G 2831A 3107C 3197C 4732G 4769G 5452T 7028T 7768G 8705C 8860G 9185A 9477A 11467G 11719A 12308G 12372A 13617C 13637G 14182C 14766T 15326G 15511C 15924G 16519C	U5b2a1a2	73G 150T 263G 16519C	1	73 150 263 750 1438 1721 1834 2706 2831 3197 4732 4769 5452 7028 7768 8705 8860 9185A 9477 11467 11719 12308 12372 13617 13637 14182 14766 15326 15511 15924	U5b2a1a2	73 150 263	[0.000] U5b2a1a2 (1)	[0.031] H4a1a1a1a (2) [0.026] U5b2a1a (3) [0.023] U5b2b (4) [0.021] R (5) [0.019] U3b2a1 (6) [0.000] L3e'i'k'x L3e L3e3'4'5 L3e3'4 L3i HV+73 HV20 HV5b HV12b H1e1a6 H1e2c H1as1 H1av1 H1av1a H3+73 H3d H4a1a1a H4a1a1a1 H4a1a1a1a1 H4a1a1a2 H4a1a1a4 R2'JT B6 R30 R30a R30a1 R30a1a R31 P P2 P4 P9 P9a U U5b1d1a U5b2b1 U5b2b2 U2'3'4'7'8'9 U3 U3a'c U3b U3b1 U3b1b U3b2 U3b2a U3b2c U8 U8b'c U8b (55)	1
1956 >KX977306.1 Hc	73G 150T 263G 295T 310C 489C 750G 1438G 2706G 3107C 4216C 4769G 5633T 7028T 7476T 8860G 9016G 10172A 10398G 11251G 11719A 12612G 13708A 14766T 15257A 15326G 15452A 15662G 15812A 16069T 16126C 16193T 16278T 16519C 16566A	J2b1a6	73G 150T 263G 295T 310C 489C 16069T 16126C 16193T 16278T 16519C 16566A	1	73 150 263 295 310 489 750 1438 2706 4216 4769 5633 7028 7476 8860 9016 10172 10398 11251 11719 12612 13708 14766 15257 15326 15452A 15662 15812 16069 16126 16193 16278 16566	J2b1a6	73 150 263 295 310 489 16069 16126 16193 16278 16566	[0.100] J2b1a6 (1)	[0.100] J2b1a6 (2) [0.019] J2b1a (3) [0.015] J2b1a2 (4) [0.000] J2b1a1 J2b1a2a J2b1a3 J2b1a4 (8)	1
1957 >KX977304.1 Hc	152C 263G 750G 1438G 3107C 4769G 5399T 8860G 9230C 12417T 15326G 16184T	H101	152C 263G 16184T	1	152 263 750 1438 4769 5399 8860 9230 12417 15326 16184	H101	152 263 16184	[0.050] H101 (1) [0.013] H3g3 (2) [0.004] H3+152 (3) [0.000] H1+152 H1i H1i1 H1i2 H1i2a H1an H1an1 H1an1a H1bb H1bc H2a2a2 H3g H3g1 H3g1a H3g1b H3g2 H3g4 H3i H3i1 H3j H3k H3k1 H3k1a H+152 H9 H46 H46a H46b H52 H69 H13a1+152 H14b H14b2 H14b4 H16+152 H16a H16a1 H16c H16d H30b1 H47a (44)	[0.031] H13a1a1a (45) [0.023] H2a2a (46) [0.022] H9a (47) [0.018] H3a1 (48) [0.017] H32 (49) [0.012] HV (50) [0.009] H4a1 H26a1 (52) [0.005] H1h1 H2b (54) [0.004] H3q1 H7b (56) [0.003] HV5 H1 H4b H16e H58a H75 (62) [0.002] H1e1a H1e2 H2a H13a1a1 H13a2a H18 H18b (69) [0.001] HV4 H1e1a2 H1e1c H1h2 H1q H1t1a H1ag1 H1aj H1at1a H3 H4 H4a1a H4a1a1 H4a1c H7b1 H7c6 H7d H10b H10+(16093) H13a2b4 H35 H40b H45a (92) [0.000] R0 HV1b+152 HV4c HV5a HV9+152 HV9c HV12 H H1c+152 H1c9 H1c9a H1e H1e1 H1e1a7 H1e2a H1e2b H1e2d H1e4 H1e6 H1e7 H1e8 H1e8a H1h H1j H1j3 H1j5 H1j6 H1j7 H1j9 H1m H1n H1p H1q1 H1q2 H1r H1s H1s1 H1t H1t1 H1t2 H1u H1u1 H1v H1v1 H1v1a H1v1b H1w H1ae H1ae1 H1ae2 H1ae2a H1ae3 H1ae3a H1ag H1ag1b H1ah H1ai H1ai1 H1ak H1ak1 H1ak2 H1am H1am1 H1ap H1ap1 H1aq H1as H1as2 H1at H1at1 H1av H1ax H1ax1 H1ay H1az H1bd H1be H1bi H1bj H1bk H1bm H1bn H1bp H1bq H1br H1bu H1bv H1bw H1bx H1ca H1ci H2 H2a2 [illegible]	1

1958	>KY409244.1 Hc	73G 195C 263G 285T 301N 302N 303N 304N 305N 306N 307N 308N 309N 310N 311N 312N 313N 314N 315N 316N 317N 318N 663G 750G 1438G 2218T 2706G 3106d 3107C 4769G 4991A 6026A 7028T 7403G 7581C 8860G 10700R 11116C 11467G 11719A 12308G 12372A 12879C 13104G 13656C 14070G 14364A 14766T 15115C 15148A 15217A 15326G 15954C 16145A 16182C 16183C 16189C 16249C	U1a1c1c1	73G 195C 263G 285T 301N 302N 303N 304N 305N 306N 307N 308N 309N 310N 311N 312N 313N 314N 315N 316N 317N 318N 16145A 16182C 16183C 16189C 16249C	1	73 195 263 285 301N 302N 303N 304N 305N 306N 307N 308N 309N 310N 311N 312N 313N 314N 315N 316N 317N 318N 663 750 1438 2218 2706 4769 4991 6026 7028 7403 7581 8860 10700R 11116 11467 11719 12308 12372 12879 13104 13656 14070 14364 14766 15115 15148 15217 15326 15954C 16145 16189 16249	U1a1c1c1	73 195 263 285 301N 302N 303N 304N 305N 306N 307N 308N 309N 310N 311N 312N 313N 314N 315N 316N 317N 318N 16145 16189 16249	[0.000] U1a1c1c1 (1)	[0.049] U1a1c1d (2) [0.000] U1a U1a1 U1a1c U1a1c1 U1a1c1b U1a1c1c (8)	1
1959	>KY409243.1 Hc	73G 150T 263G 301N 302N 303N 304N 305N 306N 307N 308N 309N 310N 311N 312N 313N 314N 315N 316N 317N 318N 750G 1438G 2706G 3106d 3107C 3197C 3498T 4769G 5656G 6674C 7028T 7768G 8817G 8860G 9477A 11467G 11719A 12308G 12372A 13617C 14182C 14766T 15326G 15773A 15777A 16167T 16192T 16270T 16311C 16356C	U5b1i	73G 150T 263G 301N 302N 303N 304N 305N 306N 307N 308N 309N 310N 311N 312N 313N 314N 315N 316N 317N 318N 16167T 16192T 16270T 16311C 16356C	1	73 150 263 301N 302N 303N 304N 305N 306N 307N 308N 309N 310N 311N 312N 313N 314N 315N 316N 317N 318N 750 1438 2706 3197 3498 4769 5656 6674 7028 7768 8817 8860 9477 11467 11719 12308 12372 13617 14182 14766 15326 15773 15777 16167 16192 16270 16311 16356	U5b1i	73 150 263 301N 302N 303N 304N 305N 306N 307N 308N 309N 310N 311N 312N 313N 314N 315N 316N 317N 318N 16167 16192 16270 16311 16356	[0.000] U5b1i (1)	[0.026] U5b2a1a+16311 U5b2a1a1d (3) [0.025] U5b1 (4) [0.000] U5b U5b1a U5b1c U5b1d U5b1d1 U5b1f U5b2 U5b2a1a1 U5b2a1a1a U5b2c U5b2c1 U5b2c2 U5b2c2a (17)	1
1960	>KY409237.1 Hc	73G 151T 152C 263G 301N 302N 303N 304N 305N 306N 307N 308N 309N 310N 311N 312N 313N 314N 315N 316N 317N 318N 709A 750G 930A 1438G 1888A 2706G 3106d 3107C 4216C 4562G 4769G 4917G 5147A 5656G 7028T 8602C 8697A 8860G 10463C 10750G 11251G 11719A 11812G 13368A 14233G 14766T 14905A 15326G 15452A 15607G 15928A 16126C 16292T 16294T 16296T 16304C 16519C	T2b3a	73G 151T 152C 263G 301N 302N 303N 304N 305N 306N 307N 308N 309N 310N 311N 312N 313N 314N 315N 316N 317N 318N 16126C 16292T 16294T 16296T 16304C 16519C	1	73 151 152 263 301N 302N 303N 304N 305N 306N 307N 308N 309N 310N 311N 312N 313N 314N 315N 316N 317N 318N 709 750 930 1438 1888 2706 4216 4562 4769 4917 5147 5656 7028 8602 8697 8860 10463 10750 11251 11719 11812 13368 14233 14766 14905 15326 15452A 15607 15928 16126 16292 16294 16296 16304	T2b3a	73 151 152 263 301N 302N 303N 304N 305N 306N 307N 308N 309N 310N 311N 312N 313N 314N 315N 316N 317N 318N 16126 16292 16294 16296 16304	[0.000] T2b3a (1)	[0.091] T2b3+151 (2) [0.014] T2b+152 (3) [0.012] T2b7a3 (4) [0.000] T2b3c T2b4+152 T2b4c T2b4d T2b4e T2b4f T2b4g T2b21 T2b21b T2b22 (14)	1

1961	>KY409224.1 Hc	263G 301N 302N 303N 304N 305N 306N 307N 308N 309N 310N 311N 312N 313N 314N 315N 316N 317N 318N 750G 1438G 3106d 3107C 4769G 4793G 6296A 8702A 8860G 15326G 16265G 16519C	H7c1	263G 301N 302N 303N 304N 305N 306N 307N 308N 309N 310N 311N 312N 313N 314N 315N 316N 317N 318N 16265G 16519C	1	263 301N 302N 303N 304N 305N 306N 307N 308N 309N 310N 311N 312N 313N 314N 315N 316N 317N 318N 750 1438 4769 4793 6296A 8702A 8860 15326 16265	H7c1	263 301N 302N 303N 304N 305N 306N 307N 308N 309N 310N 311N 312N 313N 314N 315N 316N 317N 318N 16265	[0.000] H7c1 (1)	[0.001] H1h1 H2a2a (3) [0.000] R0 HV HV4 HV4c HV5 HV5a HV9c HV12 H H1 H1e H1e1 H1e1a H1e1a2 H1e1a7 H1e1c H1e2 H1e2a H1e2b H1e2d H1e4 H1e6 H1e7 H1e8 H1e8a H1h H1h2 H1j H1j3 H1j5 H1j6 H1j7 H1j9 H1m H1n H1p H1q H1q1 H1q2 H1r H1s H1s1 H1t H1t1 H1t1a H1t2 H1u H1u1 H1v H1v1 H1v1a H1v1b H1w H1ae H1ae1 H1ae2 H1ae2a H1ae3 H1ae3a H1ag H1ag1 H1ag1b H1ah H1ai H1ai1 H1aj H1ak H1ak1 H1ak2 H1am H1am1 H1ap H1aq H1as H1as2 H1at H1at1 H1at1a H1av H1ax H1ax1 H1ay H1az H1bd H1be H1bi H1bj H1bk H1bm H1bn H1bp H1bq H1br H1bu H1bv H1bw H1bx H1ca H1ci H2 H2a H2a2 H2a2+(16235) H2a4 H2a5 H2a5a H2a5b H3 H3b H3c H3c1 H3e H3q H3q1 H3r H3r1 H3t H3u H3y H3aa H3ab H3ac H3ad H3ae H3ag H3ag1 H3ah H3ai H3aj H3ap H3aq H3ar H3as H3at H3at1 H3au H4 H4a H4a1 H4a1a H4a1a1 H4a1a2 H4a1a2a H4a1a2a1 H4a1a5 H4a1c H4a1c1 H4a1c1a H4a1d H4b H4c H7 H7a H7b H7b1 H7b2 H7b2a H7b3 H7b4 H7b5 H7b6 H7c [illegible]	1
1962	>KY409223.1 Hc	73G 199C 204C 250C 263G 301N 302N 303N 304N 305N 306N 307N 308N 309N 310N 311N 312N 313N 314N 315N 316N 317N 318N 709A 750G 955.1C 1438G 1719A 2706G 3106d 3107C 4529T 4769G 7028T 8251A 8860G 10034C 10238C 10398G 11719A 12438C 12501A 12705T 12987T 13780G 14233G 14766T 15043A 15326G 15924G 16129A 16169T 16223T 16391A 16519C	I5c	73G 199C 204C 250C 263G 301N 302N 303N 304N 305N 306N 307N 308N 309N 310N 311N 312N 313N 314N 315N 316N 317N 318N 16129A 16169T 16223T 16391A 16519C	1	73 199 204 250 263 301N 302N 303N 304N 305N 306N 307N 308N 309N 310N 311N 312N 313N 314N 315N 316N 317N 318N 709 750 960.1C 1438 1719 2706 4529T 4769 7028 8251 8860 10034 10238 10398 11719 12438 12501 12705 12987 13780 14233 14766 15043 15326 15924 16129 16169 16223 16391	I5c	73 199 204 250 263 301N 302N 303N 304N 305N 306N 307N 308N 309N 310N 311N 312N 313N 314N 315N 316N 317N 318N 16129 16169 16223 16391	[0.167] I5c (1)	[0.229] I4a (2) [0.114] I (3) [0.042] I4 (4) [0.034] I4b (5) [0.021] I4a2 (6) [0.020] I6a (7) [0.000] I5 I5b I6 (10)	1

1963	>KY409203.1 Hc	57C 59C 73G 151T 152C 263G 301N 302N 303N 304N 305N 306N 307N 308N 309N 310N 311N 312N 313N 314N 315N 316N 317N 318N 709A 750G 930A 1438G 1888A 2706G 3106d 3107C 4216C 4227G 4769G 4917G 5147A 5656G 7028T 8697A 8860G 10463C 10750G 11251G 11719A 11812G 13368A 14233G 14766T 14905A 15326G 15452A 15607G 15928A 16126C 16288C 16292T 16294T 16296T 16304C 16465T 16519C	T2b3a1	57C 59C 73G 151T 152C 263G 301N 302N 303N 304N 305N 306N 307N 308N 309N 310N 311N 312N 313N 314N 315N 316N 317N 318N 16126C 16288C 16292T 16294T 16296T 16304C 16465T 16519C	1	57 59 73 151 152 263 301N 302N 303N 304N 305N 306N 307N 308N 309N 310N 311N 312N 313N 314N 315N 316N 317N 318N 709 750 930 1438 1888 2706 4216 4227 4769 4917 5147 5656 7028 8697 8860 10463 10750 11251 11719 11812 13368 14233 14766 14905 15326 15452A 15607 15928 16126 16288 16292 16294 16296 16304 16465	T2b3a1	57 59 73 151 152 263 301N 302N 303N 304N 305N 306N 307N 308N 309N 310N 311N 312N 313N 314N 315N 316N 317N 318N 16126 16288 16292 16294 16296 16304 16465	[0.983] T2b3a1 (1)	[0.983] T2b3a1 (2) [0.393] T2b3a	1
1964	>KY409197.1 Hc	57C 59C 62T 73G 151T 152C 263G 301N 302N 303N 304N 305N 306N 307N 308N 309N 310N 311N 312N 313N 314N 315N 316N 317N 318N 709A 750G 930A 1438G 1888A 2706G 3106d 3107C 4216C 4227G 4769G 4917G 5147A 5656G 6953G 7028T 8697A 8860G 10463C 10750G 11251G 11719A 11812G 13368A 14233G 14766T 14905A 15326G 15452A 15607G 15928A 16126C 16288C 16292T 16294T 16296T 16304C 16465T 16519C	T2b3a1	57C 59C 62T 73G 151T 152C 263G 301N 302N 303N 304N 305N 306N 307N 308N 309N 310N 311N 312N 313N 314N 315N 316N 317N 318N 16126C 16288C 16292T 16294T 16296T 16304C 16465T 16519C	1	57 59 62T 73 151 152 263 301N 302N 303N 304N 305N 306N 307N 308N 309N 310N 311N 312N 313N 314N 315N 316N 317N 318N 709 750 930 1438 1888 2706 4216 4227 4769 4917 5147 5656 6953 7028 8697 8860 10463 10750 11251 11719 11812 13368 14233 14766 14905 15326 15452A 15607 15928 16126 16288 16292 16294 16296 16304 16465	T2b3a1	57 59 62T 73 151 152 263 301N 302N 303N 304N 305N 306N 307N 308N 309N 310N 311N 312N 313N 314N 315N 316N 317N 318N 16126 16288 16292 16294 16296 16304 16465	[1.064] T2b3a1 (1)	[1.064] T2b3a1 (2) [0.393] T2b3a	1
1965	>KY409196.1 Hc	73G 150T 263G 301N 302N 303N 304N 305N 306N 307N 308N 309N 310N 311N 312N 313N 314N 315N 316N 317N 318N 750G 1438G 2706G 3106d 3107C 3197C 3498T 4769G 5656G 5964C 6674C 7028T 7768G 8860G 9163A 9477A 11467G 11719A 12308G 12372A 13617C 14182C 14530Y 14766T 15326G 15777A 16167T 16189C 16270T 16311C 16356C	U5b1i	73G 150T 263G 301N 302N 303N 304N 305N 306N 307N 308N 309N 310N 311N 312N 313N 314N 315N 316N 317N 318N 16167T 16189C 16270T 16311C 16356C	1	73 150 263 301N 302N 303N 304N 305N 306N 307N 308N 309N 310N 311N 312N 313N 314N 315N 316N 317N 318N 750 1438 2706 3197 3498 4769 5656 5964 6674 7028 7768 8860 9163 9477 11467 11719 12308 12372 13617 14182 14530Y 14766 15326 15777 16167 16189 16270 16311 16356	U5b1i	73 150 263 301N 302N 303N 304N 305N 306N 307N 308N 309N 310N 311N 312N 313N 314N 315N 316N 317N 318N 16167 16189 16270 16311 16356	[1.222] U5b1i (1) [0.000] U5b2a5 U5b2a5a (3)	[0.094] U5b1c (4) [0.077] U5b1+16189+@16192 (5) [0.051] U5b2a6 (6) [0.017] U5b2a+@16192 (7) [0.000] U5b1b1+@16192 U5b1b1d U5b1b1f U5b2a4 U5b2a4a U5b2b4 (13)	1
1966	>KY409193.1 Hc	73G 150T 263G 301N 302N 303N 304N 305N 306N 307N 308N 309N 310N 311N 312N 313N 314N 315N 316N 317N 318N 750G 1438G 1721T 2706G 3106d 3107C 3197C 4769G 7028T 7768G 8860G 9477A 11467G 11653G 11719A 12308G 12372A 12634G 13161C 13617C 13630G 13637G 14182C 14766T 15326G 16270T 16296T 16325C	U5b2b5	73G 150T 263G 301N 302N 303N 304N 305N 306N 307N 308N 309N 310N 311N 312N 313N 314N 315N 316N 317N 318N 16270T 16296T 16325C	1	73 150 263 301N 302N 303N 304N 305N 306N 307N 308N 309N 310N 311N 312N 313N 314N 315N 316N 317N 318N 750 1438 1721 2706 3197 4769 7028 7768 8860 9477 11467 11653 11719 12308 12372 12634 13161 13617 13630 13637 14182 14766 15326 16270 16296 16325	U5b2b5	73 150 263 301N 302N 303N 304N 305N 306N 307N 308N 309N 310N 311N 312N 313N 314N 315N 316N 317N 318N 16270 16296 16325	[0.256] U5b2b5 (1)	[0.057] U5b2b (2) [0.012] U5b2b1 (3) [0.000] U5b1d1a U5b2b2 (5)	1

1967 >KY409176.1 Hc	73G 150T 263G 301N 302N 303N 304N 305N 306N 307N 308N 309N 310N 311N 312N 313N 314N 315N 316N 317N 318N 750G 1438G 2706G 3106d 3107C 3197C 3498T 4769G 5656G 5964C 6674C 7028T 7768G 8860G 9163A 9477A 11467G 11719A 12308G 12372A 13617C 14182C 14766T 15326G 15777A 16167T 16189C 16270T 16311C 16356C	U5b1i	73G 150T 263G 301N 302N 303N 304N 305N 306N 307N 308N 309N 310N 311N 312N 313N 314N 315N 316N 317N 318N 16167T 16189C 16270T 16311C 16356C	1	73 150 263 301N 302N 303N 304N 305N 306N 307N 308N 309N 310N 311N 312N 313N 314N 315N 316N 317N 318N 750 1438 2706 3197 3498 4769 5656 5964 6674 7028 7768 8860 9163 9477 11467 11719 12308 12372 13617 14182 14766 15326 15777 16167 16189 16270 16311 16356	U5b1i	73 150 263 301N 302N 303N 304N 305N 306N 307N 308N 309N 310N 311N 312N 313N 314N 315N 316N 317N 318N 16167 16189 16270 16311 16356	[1.222] U5b1i (1) [0.000] U5b2a5 U5b2a5a (3)	[0.094] U5b1c (4) [0.077] U5b1+16189+@16192 (5) [0.051] U5b2a6 (6) [0.017] U5b2a+@16192 (7) [0.000] U5b1b1+@16192 U5b1b1d U5b1b1f U5b2a4 U5b2a4a U5b2b4 (13)	1
1968 >KY409125.1 Hc	263G 301N 302N 303N 304N 305N 306N 307N 308N 309N 310N 311N 312N 313N 314N 315N 316N 317N 318N 337C 480C 750G 1438G 3106d 3107C 3615G 4769G 6776C 8860G 8937C 11914A 15326G 16293G 16368C 16519C	H3x1	263G 301N 302N 303N 304N 305N 306N 307N 308N 309N 310N 311N 312N 313N 314N 315N 316N 317N 318N 337C 480C 16293G 16368C 16519C	1	263 301N 302N 303N 304N 305N 306N 307N 308N 309N 310N 311N 312N 313N 314N 315N 316N 317N 318N 337C 480 750 1438 3615 4769 6776 8860 8937 11914 15326 16293 16368	H3x1	263 301N 302N 303N 304N 305N 306N 307N 308N 309N 310N 311N 312N 313N 314N 315N 316N 317N 318N 337C 480 16293 16368	[0.283] H3x1 (1)	[0.007] H24a (2) [0.000] H3x H24 H24a2 H24b (6)	1
1969 >KY409944.1 Hc	73G 151T 152C 263G 301N 302N 303N 304N 305N 306N 307N 308N 309N 310N 311N 312N 313N 314N 315N 316N 317N 318N 709A 750G 930A 1438G 1888A 2706G 3106d 3107C 4216C 4562G 4769G 4917G 5147A 5656G 7028T 8602C 8697A 8860G 10463C 10750G 11251G 11719A 11812G 13368A 14233G 14766T 14905A 15326G 15452A 15607G 15928A 16126C 16292T 16294T 16296T 16304C 16519C	T2b3a	73G 151T 152C 263G 301N 302N 303N 304N 305N 306N 307N 308N 309N 310N 311N 312N 313N 314N 315N 316N 317N 318N 16126C 16292T 16294T 16296T 16304C 16519C	1	73 151 152 263 301N 302N 303N 304N 305N 306N 307N 308N 309N 310N 311N 312N 313N 314N 315N 316N 317N 318N 709 750 930 1438 1888 2706 4216 4562 4769 4917 5147 5656 7028 8602 8697 8860 10463 10750 11251 11719 11812 13368 14233 14766 14905 15326 15452A 15607 15928 16126 16292 16294 16296 16304	T2b3a	73 151 152 263 301N 302N 303N 304N 305N 306N 307N 308N 309N 310N 311N 312N 313N 314N 315N 316N 317N 318N 16126 16292 16294 16296 16304	[0.000] T2b3a (1)	[0.091] T2b3+151 (2) [0.014] T2b+152 (3) [0.012] T2b7a3 (4) [0.000] T2b3c T2b4+152 T2b4c T2b4d T2b4e T2b4f T2b4g T2b21 T2b21b T2b22 (14)	1
1970 >KY409935.1 Hc	73G 146C 150T 263G 285T 301N 302N 303N 304N 305N 306N 307N 308N 309N 310N 311N 312N 313N 314N 315N 316N 317N 318N 750G 1438G 2218T 2706G 3106d 3107C 4769G 6023A 7028T 8857A 8860G 10203A 11467G 11719A 12308G 12372A 12879C 13104G 13398G 13681G 14063C 14070G 14364A 14766T 15148A 15326G 15924G 15954C 16183C 16189C 16249C	U1a3	73G 146C 150T 263G 285T 301N 302N 303N 304N 305N 306N 307N 308N 309N 310N 311N 312N 313N 314N 315N 316N 317N 318N 16183C 16189C 16249C	1	73 146 150 263 285 301N 302N 303N 304N 305N 306N 307N 308N 309N 310N 311N 312N 313N 314N 315N 316N 317N 318N 750 1438 2218 2706 4769 6023 7028 8857 8860 10203 11467 11719 12308 12372 12879 13104 13398 13681 14063 14070 14364 14766 15148 15326 15924 15954C 16189 16249	U1a3	73 146 150 263 285 301N 302N 303N 304N 305N 306N 307N 308N 309N 310N 311N 312N 313N 314N 315N 316N 317N 318N 16189 16249	[0.041] U1a3 (1)	[0.048] U1a1c1c (2) [0.019] U1a1 (3) [0.000] U1a U1a1c U1a1c1 U1a1c1b U1a1c1d (8)	1
1971 >KY409933.1 Hc	73G 150T 263G 301N 302N 303N 304N 305N 306N 307N 308N 309N 310N 311N 312N 313N 314N 315N 316N 317N 318N 750G 1438G 2706G 3106d 3107C 3197C 3498T 4769G 5656G 5964C 6674C 7028T 7768G 8860G 9163A 9477A 11467G 11719A 12308G 12372A 13617C 14182C 14766T 15326G 15777A 16167T 16189C 16270T 16311C 16356C	U5b1i	73G 150T 263G 301N 302N 303N 304N 305N 306N 307N 308N 309N 310N 311N 312N 313N 314N 315N 316N 317N 318N 16167T 16189C 16270T 16311C 16356C	1	73 150 263 301N 302N 303N 304N 305N 306N 307N 308N 309N 310N 311N 312N 313N 314N 315N 316N 317N 318N 750 1438 2706 3197 3498 4769 5656 5964 6674 7028 7768 8860 9163 9477 11467 11719 12308 12372 13617 14182 14766 15326 15777 16167 16189 16270 16311 16356	U5b1i	73 150 263 301N 302N 303N 304N 305N 306N 307N 308N 309N 310N 311N 312N 313N 314N 315N 316N 317N 318N 16167 16189 16270 16311 16356	[1.222] U5b1i (1) [0.000] U5b2a5 U5b2a5a (3)	[0.094] U5b1c (4) [0.077] U5b1+16189+@16192 (5) [0.051] U5b2a6 (6) [0.017] U5b2a+@16192 (7) [0.000] U5b1b1+@16192 U5b1b1d U5b1b1f U5b2a4 U5b2a4a U5b2b4 (13)	1

1972	>KY409932.1 H	73G 150T 189G 201G 263G 301N 302N 303N 304N 305N 306N 307N 308N 309N 310N 311N 312N 313N 314N 315N 316N 317N 318N 750G 1438G 2706G 3106d 3107C 3197C 4769G 5843G 7028T 7226A 7768G 8860G 9196A 9477A 10159A 11467G 11719A 12308G 12372A 13617C 14182C 14766T 15326G 15598T 16192T 16270T 16304C	U5b3b1	73G 150T 189G 201G 263G 301N 302N 303N 304N 305N 306N 307N 308N 309N 310N 311N 312N 313N 314N 315N 316N 317N 318N 16192T 16270T 16304C	1	73 150 189 201 263 301N 302N 303N 304N 305N 306N 307N 308N 309N 310N 311N 312N 313N 314N 315N 316N 317N 318N 750 1438 2706 3197 4769 5843 7028 7226 7768 8860 9196 9477 10159A 11467 11719 12308 12372 13617 14182 14766 15326 15598 16192 16270 16304	U5b3b1	73 150 189 201 263 301N 302N 303N 304N 305N 306N 307N 308N 309N 310N 311N 312N 313N 314N 315N 316N 317N 318N 16192 16270 16304	[0.118] U5b3b1 (1)	[0.118] U5b3b1 (2) [0.019] U5b3 (3) [0.000] U5b3b U5b3c U5b3e U5b3g (7)	1
1973	>KY409927.1 H	73G 195C 263G 285T 301N 302N 303N 304N 305N 306N 307N 308N 309N 310N 311N 312N 313N 314N 315N 316N 317N 318N 663G 750G 1438G 2218T 2706G 3106d 3107C 4769G 4991A 5262A 6026A 7028T 7403G 7581C 8860G 11116C 11467G 11719A 12308G 12372A 12879C 13104G 13656C 14070G 14364A 14766T 15115C 15148A 15217A 15326G 15954C 16145A 16182C 16183C 16189C 16249C	U1a1c1c1	73G 195C 263G 285T 301N 302N 303N 304N 305N 306N 307N 308N 309N 310N 311N 312N 313N 314N 315N 316N 317N 318N 16145A 16182C 16183C 16189C 16249C	1	73 195 263 285 301N 302N 303N 304N 305N 306N 307N 308N 309N 310N 311N 312N 313N 314N 315N 316N 317N 318N 663 750 1438 2218 2706 4769 4991 5262 6026 7028 7403 7581 8860 11116 11467 11719 12308 12372 12879 13104 13656 14070 14364 14766 15115 15148 15217 15326 15954C 16145 16189 16249	U1a1c1c1	73 195 263 285 301N 302N 303N 304N 305N 306N 307N 308N 309N 310N 311N 312N 313N 314N 315N 316N 317N 318N 16145 16189 16249	[0.000] U1a1c1c1 (1)	[0.049] U1a1c1d (2) [0.000] U1a U1a1 U1a1c U1a1c1 U1a1c1b U1a1c1c (8)	1
1974	>KY409920.1 H	73G 94A 263G 301N 302N 303N 304N 305N 306N 307N 308N 309N 310N 311N 312N 313N 314N 315N 316N 317N 318N 750G 1189C 1438G 1811G 2706G 3106d 3107C 3337A 3480G 4769G 5913A 7028T 8860G 9055A 9698C 9962A 10223T 10289G 10398G 10550G 11299C 11467G 11719A 11914A 12308G 12372A 14167T 14766T 14798C 15326G 15946T 16224C 16240T 16311C 16519C	K1b1c	73G 94A 263G 301N 302N 303N 304N 305N 306N 307N 308N 309N 310N 311N 312N 313N 314N 315N 316N 317N 318N 16224C 16240T 16311C 16519C	1	73 94 263 301N 302N 303N 304N 305N 306N 307N 308N 309N 310N 311N 312N 313N 314N 315N 316N 317N 318N 750 1189 1438 1811 2706 3337 3480 4769 5913 7028 8860 9055 9698 9962 10223 10289 10398 10550 11299 11467 11719 11914 12308 12372 14167 14766 14798 15326 15946 16224 16240T 16311	K1b1c	73 94 263 301N 302N 303N 304N 305N 306N 307N 308N 309N 310N 311N 312N 313N 314N 315N 316N 317N 318N 16224 16240T 16311	[0.031] K1b1c (1)	[0.000] K K1 K1b K1b1 K1b1+(16093) K1b1b K1b1b1 (8)	1
1975	>KY409916.1 H	73G 195C 263G 285T 301N 302N 303N 304N 305N 306N 307N 308N 309N 310N 311N 312N 313N 314N 315N 316N 317N 318N 663G 750G 1438G 2218T 2706G 3106d 3107C 4769G 4991A 6026A 7028T 7403G 7581C 8860G 11116C 11467G 11719A 12308G 12372A 12879C 13104G 13656C 14070G 14364A 14766T 15115C 15148A 15217A 15326G 15954C 16145A 16182C 16183C 16189C 16249C	U1a1c1c1	73G 195C 263G 285T 301N 302N 303N 304N 305N 306N 307N 308N 309N 310N 311N 312N 313N 314N 315N 316N 317N 318N 16145A 16182C 16183C 16189C 16249C	1	73 195 263 285 301N 302N 303N 304N 305N 306N 307N 308N 309N 310N 311N 312N 313N 314N 315N 316N 317N 318N 663 750 1438 2218 2706 4769 4991 6026 7028 7403 7581 8860 11116 11467 11719 12308 12372 12879 13104 13656 14070 14364 14766 15115 15148 15217 15326 15954C 16145 16189 16249	U1a1c1c1	73 195 263 285 301N 302N 303N 304N 305N 306N 307N 308N 309N 310N 311N 312N 313N 314N 315N 316N 317N 318N 16145 16189 16249	[0.000] U1a1c1c1 (1)	[0.049] U1a1c1d (2) [0.000] U1a U1a1 U1a1c U1a1c1 U1a1c1b U1a1c1c (8)	1

1976	>KY409914.1 Hc	73G 150T 263G 284G 301N 302N 303N 304N 305N 306N 307N 308N 309N 310N 311N 312N 313N 314N 315N 316N 317N 318N 373G 750G 1438G 2706G 3106d 3107C 3197C 4769G 7028T 7226A 7768G 8860G 9477A 11177T 11467G 11719A 11914A 12308G 12372A 13617C 14182C 14766T 15326G 16169A 16192T 16235G 16270T 16519C	U5b3a1a	73G 150T 263G 284G 301N 302N 303N 304N 305N 306N 307N 308N 309N 310N 311N 312N 313N 314N 315N 316N 317N 318N 373G 16169A 16192T 16235G 16270T 16519C	1	73 150 263 284 301N 302N 303N 304N 305N 306N 307N 308N 309N 310N 311N 312N 313N 314N 315N 316N 317N 318N 373 750 1438 2706 3197 4769 7028 7226 7768 8860 9477 11177 11467 11719 11914 12308 12372 13617 14182 14766 15326 16169A 16192 16235 16270	U5b3a1a	73 150 263 284 301N 302N 303N 304N 305N 306N 307N 308N 309N 310N 311N 312N 313N 314N 315N 316N 317N 318N 373 16169A 16192 16235 16270	[0.184] U5b3a1a (1)	[0.000] U5b3a1 U5b3a1b (3)	1
1977	>KY409909.1 Hc	57C 59C 62T 73G 151T 152C 263G 301N 302N 303N 304N 305N 306N 307N 308N 309N 310N 311N 312N 313N 314N 315N 316N 317N 318N 709A 750G 930A 1438G 1888A 2706G 3106d 3107C 4216C 4227G 4769G 4917G 5147A 5656G 6953G 7028T 8697A 8860G 10463C 10750G 11251G 11719A 11812G 13368A 14233G 14766T 14905A 15326G 15452A 15607G 15928A 16126C 16288C 16292T 16294T 16296T 16304C 16465T 16519C	T2b3a1	57C 59C 62T 73G 151T 152C 263G 301N 302N 303N 304N 305N 306N 307N 308N 309N 310N 311N 312N 313N 314N 315N 316N 317N 318N 16126C 16288C 16292T 16294T 16296T 16304C 16465T 16519C	1	57 59 62T 73 151 152 263 301N 302N 303N 304N 305N 306N 307N 308N 309N 310N 311N 312N 313N 314N 315N 316N 317N 318N 709 750 930 1438 1888 2706 4216 4227 4769 4917 5147 5656 6953 7028 8697 8860 10463 10750 11251 11719 11812 13368 14233 14766 14905 15326 15452A 15607 15928 16126 16288 16292 16294 16296 16304 16465	T2b3a1	57 59 62T 73 151 152 263 301N 302N 303N 304N 305N 306N 307N 308N 309N 310N 311N 312N 313N 314N 315N 316N 317N 318N 16126 16288 16292 16294 16296 16304 16465	[1.064] T2b3a1 (1)	[1.064] T2b3a1 (2) [0.393] T2b3a	1
1978	>KY409897.1 Hc	57C 59C 151T 152C 263G 301N 302N 303N 304N 305N 306N 307N 308N 309N 310N 311N 312N 313N 314N 315N 316N 317N 318N 709A 750G 930A 1438G 1888A 2706G 3106d 3107C 4216C 4769G 4917G 5147A 5656G 7028T 8697A 8860G 10463C 10750G 11251G 11719A 11812G 13368A 14233G 14766T 14905A 15326G 15452A 15607G 15928A 16126C 16292T 16294T 16296T 16304C 16519C	T2b3a1	57C 59C 151T 152C 263G 301N 302N 303N 304N 305N 306N 307N 308N 309N 310N 311N 312N 313N 314N 315N 316N 317N 318N 16126C 16292T 16294T 16296T 16304C 16519C	1	57 59 151 152 263 301N 302N 303N 304N 305N 306N 307N 308N 309N 310N 311N 312N 313N 314N 315N 316N 317N 318N 709 750 930 1438 1888 2706 4216 4769 4917 5147 5656 7028 8697 8860 10463 10750 11251 11719 11812 13368 14233 14766 14905 15326 15452A 15607 15928 16126 16292 16294 16296 16304	T2b3a1	57 59 151 152 263 301N 302N 303N 304N 305N 306N 307N 308N 309N 310N 311N 312N 313N 314N 315N 316N 317N 318N 16126 16292 16294 16296 16304	[0.758] T2b3a1 (1)	[0.758] T2b3a1 (2) [0.179] T2b3a	1
1979	>KY409878.1 Hc	73G 150T 263G 301N 302N 303N 304N 305N 306N 307N 308N 309N 310N 311N 312N 313N 314N 315N 316N 317N 318N 373G 750G 1438G 2706G 3106d 3107C 3197C 4769G 7028T 7226A 7768G 8860G 9477A 11177T 11467G 11719A 12308G 12372A 13617C 14182C 14766T 15326G 16169A 16192T 16235R 16270T 16371G 16519C	U5b3a1a	73G 150T 263G 301N 302N 303N 304N 305N 306N 307N 308N 309N 310N 311N 312N 313N 314N 315N 316N 317N 318N 373G 16169A 16192T 16235R 16270T 16371G 16519C	1	73 150 263 301N 302N 303N 304N 305N 306N 307N 308N 309N 310N 311N 312N 313N 314N 315N 316N 317N 318N 373 750 1438 2706 3197 4769 7028 7226 7768 8860 9477 11177 11467 11719 12308 12372 13617 14182 14766 15326 16169A 16192 16235R 16270 16371	U5b3a1a	73 150 263 301N 302N 303N 304N 305N 306N 307N 308N 309N 310N 311N 312N 313N 314N 315N 316N 317N 318N 373 16169A 16192 16235R 16270 16371	[0.010] U5b3a1a (1)	[0.000] U5b3a1 U5b3a1b (3)	1

1980 >KY409857.1 Hc	73G 103A 263G 301N 302N 303N 304N 305N 306N 307N 308N 309N 310N 311N 312N 313N 314N 315N 316N 317N 318N 750G 1438G 2706G 3106d 3107C 3348G 4769G 4936T 7028T 7805A 8860G 9128C 9335A 9416T 10172A 11467G 11719A 12308G 12372A 13147T 14179G 14766T 15326G 16172C 16183C 16219G 16278T 16295T	U6a8b	73G 103A 263G 301N 302N 303N 304N 305N 306N 307N 308N 309N 310N 311N 312N 313N 314N 315N 316N 317N 318N 16172C 16183C 16219G 16278T 16295T	1	73 103 263 301N 302N 303N 304N 305N 306N 307N 308N 309N 310N 311N 312N 313N 314N 315N 316N 317N 318N 750 1438 2706 3348 4769 4936 7028 7805 8860 9128 9335A 9416 10172 11467 11719 12308 12372 13147 14179 14766 15326 16172 16219 16278 16295	U6a8b	73 103 263 301N 302N 303N 304N 305N 306N 307N 308N 309N 310N 311N 312N 313N 314N 315N 316N 317N 318N 16172 16219 16278 16295	[0.194] U6a8b (1)	[0.194] U6a8b (2) [0.042] U6a6 (3) [0.000] U6a U6a1 U6a+16189+(103) U6a2 U6a2a U6a2a1 U6a8 U6a5 U6a5a U6a5a1 U6a6b U6a6b1 U6a6b2 U6a7 U6a7a1+@152 U6a7a1c (19)	1
1981 >KY409852.1 Hc	73G 150T 263G 301N 302N 303N 304N 305N 306N 307N 308N 309N 310N 311N 312N 313N 314N 315N 316N 317N 318N 750G 1438G 1721T 2706G 3106d 3107C 3197C 4769G 7028T 7768G 8860G 9477A 11467G 11653G 11719A 12308G 12372A 12634G 13161C 13617C 13630G 13637G 14182C 14766T 15326G 16270T 16296T 16325C	U5b2b5	73G 150T 263G 301N 302N 303N 304N 305N 306N 307N 308N 309N 310N 311N 312N 313N 314N 315N 316N 317N 318N 16270T 16296T 16325C	1	73 150 263 301N 302N 303N 304N 305N 306N 307N 308N 309N 310N 311N 312N 313N 314N 315N 316N 317N 318N 750 1438 1721 2706 3197 4769 7028 7768 8860 9477 11467 11653 11719 12308 12372 12634 13161 13617 13630 13637 14182 14766 15326 16270 16296 16325	U5b2b5	73 150 263 301N 302N 303N 304N 305N 306N 307N 308N 309N 310N 311N 312N 313N 314N 315N 316N 317N 318N 16270 16296 16325	[0.256] U5b2b5 (1)	[0.057] U5b2b (2) [0.012] U5b2b1 (3) [0.000] U5b1d1a U5b2b2 (5)	1
1982 >KY409837.1 Hc	57C 59C 73G 151T 152C 182Y 263G 301N 302N 303N 304N 305N 306N 307N 308N 309N 310N 311N 312N 313N 314N 315N 316N 317N 318N 709A 750G 930A 1438G 1888A 2706G 3106d 3107C 4216C 4769G 4917G 5147A 5656G 7028T 8697A 8860G 10463C 10750G 11251G 11719A 11812G 13368A 14233G 14766T 14905A 15326G 15452A 15607G 15928A 16126C 16292T 16294T 16304C 16519C	T2b3a1	57C 59C 73G 151T 152C 182Y 263G 301N 302N 303N 304N 305N 306N 307N 308N 309N 310N 311N 312N 313N 314N 315N 316N 317N 318N 16126C 16292T 16294T 16304C 16519C	1	57 59 73 151 152 182Y 263 301N 302N 303N 304N 305N 306N 307N 308N 309N 310N 311N 312N 313N 314N 315N 316N 317N 318N 709 750 930 1438 1888 2706 4216 4769 4917 5147 5656 7028 8697 8860 10463 10750 11251 11719 11812 13368 14233 14766 14905 15326 15452A 15607 15928 16126 16292 16294 16304	T2b3a1	57 59 73 151 152 182Y 263 301N 302N 303N 304N 305N 306N 307N 308N 309N 310N 311N 312N 313N 314N 315N 316N 317N 318N 16126 16292 16294 16304	[0.661] T2b3a1 (1)	[0.661] T2b3a1 (2) [0.179] T2b3a	1
1983 >KY409830.1 Hc	73G 199C 204C 250C 263G 301N 302N 303N 304N 305N 306N 307N 308N 309N 310N 311N 312N 313N 314N 315N 316N 317N 318N 750G 1719A 2706G 3106d 3107C 4529T 4769G 7028T 8027A 8251A 8860G 10034C 10238C 10398G 11719A 12501A 12705T 13780G 14233G 14766T 15043A 15326G 15924G 16075C 16129A 16169T 16223T 16260T 16391A 16519C	I5c1	73G 199C 204C 250C 263G 301N 302N 303N 304N 305N 306N 307N 308N 309N 310N 311N 312N 313N 314N 315N 316N 317N 318N 16075C 16129A 16169T 16223T 16260T 16391A 16519C	1	73 199 204 250 263 301N 302N 303N 304N 305N 306N 307N 308N 309N 310N 311N 312N 313N 314N 315N 316N 317N 318N 750 1719 2706 4529T 4769 7028 8027 8251 8860 10034 10238 10398 11719 12501 12705 13780 14233 14766 15043 15326 15924 16075 16129 16169 16223 16260 16391	I5c1	73 199 204 250 263 301N 302N 303N 304N 305N 306N 307N 308N 309N 310N 311N 312N 313N 314N 315N 316N 317N 318N 16075 16129 16169 16223 16260 16391	[0.059] I5c1 (1)	[0.167] I5c (2)	1

1984	>KY409829.1 Ho	73G 195C 263G 285T 301N 302N 303N 304N 305N 306N 307N 308N 309N 310N 311N 312N 313N 314N 315N 316N 317N 318N 663G 750G 1438G 2218T 2706G 3106d 3107C 4769G 4991A 6026A 7028T 7403G 7581C 8860G 11116C 11467G 11719A 12308G 12372A 12879C 13104G 13656C 14070G 14364A 14766T 15115C 15148A 15217A 15326G 15954C 16145A 16182C 16183C 16189C 16249C	U1a1c1c1	73G 195C 263G 285T 301N 302N 303N 304N 305N 306N 307N 308N 309N 310N 311N 312N 313N 314N 315N 316N 317N 318N 16145A 16182C 16183C 16189C 16249C	1	73 195 263 285 301N 302N 303N 304N 305N 306N 307N 308N 309N 310N 311N 312N 313N 314N 315N 316N 317N 318N 663 750 1438 2218 2706 4769 4991 6026 7028 7403 7581 8860 11116 11467 11719 12308 12372 12879 13104 13656 14070 14364 14766 15115 15148 15217 15326 15954C 16145 16189 16249	U1a1c1c1	73 195 263 285 301N 302N 303N 304N 305N 306N 307N 308N 309N 310N 311N 312N 313N 314N 315N 316N 317N 318N 16145 16189 16249	[0.000] U1a1c1c1 (1)	[0.049] U1a1c1d (2) [0.000] U1a U1a1 U1a1c U1a1c1 U1a1c1b U1a1c1c (8)	1
1985	>KY409824.1 Ho	73G 150T 263G 284G 301N 302N 303N 304N 305N 306N 307N 308N 309N 310N 311N 312N 313N 314N 315N 316N 317N 318N 373G 750G 1438G 2706G 3106d 3107C 3197C 4769G 7028T 7226A 7768G 8860G 9477A 11177T 11467G 11719A 11914A 12308G 12372A 13617C 14182C 14766T 15326G 16169A 16192T 16235G 16270T 16519C	U5b3a1a	73G 150T 263G 284G 301N 302N 303N 304N 305N 306N 307N 308N 309N 310N 311N 312N 313N 314N 315N 316N 317N 318N 373G 16169A 16192T 16235G 16270T 16519C	1	73 150 263 284 301N 302N 303N 304N 305N 306N 307N 308N 309N 310N 311N 312N 313N 314N 315N 316N 317N 318N 373 750 1438 2706 3197 4769 7028 7226 7768 8860 9477 11177 11467 11719 11914 12308 12372 13617 14182 14766 15326 16169A 16192 16235 16270	U5b3a1a	73 150 263 284 301N 302N 303N 304N 305N 306N 307N 308N 309N 310N 311N 312N 313N 314N 315N 316N 317N 318N 373 16169A 16192 16235 16270	[0.184] U5b3a1a (1)	[0.000] U5b3a1 U5b3a1b (3)	1
1986	>KY409814.1 Ho	73G 150T 263G 301N 302N 303N 304N 305N 306N 307N 308N 309N 310N 311N 312N 313N 314N 315N 316N 317N 318N 750G 1438G 1811G 2294G 2706G 3106d 3107C 3873G 4612C 4703C 4769G 5261A 7028T 7382A 8110C 8634C 8860G 9266A 10966C 11467G 11719A 12308G 12372A 12843C 14139G 14766T 14992C 15326G 15454C 16193T 16249C 16343G 16526A	U3c	73G 150T 263G 301N 302N 303N 304N 305N 306N 307N 308N 309N 310N 311N 312N 313N 314N 315N 316N 317N 318N 16193T 16249C 16343G 16526A	1	73 150 263 301N 302N 303N 304N 305N 306N 307N 308N 309N 310N 311N 312N 313N 314N 315N 316N 317N 318N 750 1438 1811 2294 2706 3873 4612 4703 4769 5261 7028 7382 8110 8634 8860 9266 10966 11467 11719 12308 12372 12843 14139 14766 14992 15326 15454 16193 16249 16343 16526	U3c	73 150 263 301N 302N 303N 304N 305N 306N 307N 308N 309N 310N 311N 312N 313N 314N 315N 316N 317N 318N 16193 16249 16343 16526	[0.000] U3c (1)	[0.000] U3 U3a'c U3b U3b1 U3b1b U3b2 U3b2a U3b2a1 U3b2c (10)	1
1987	>KY409812.1 Ho	57C 59C 66K 73G 151T 152C 263G 301N 302N 303N 304N 305N 306N 307N 308N 309N 310N 311N 312N 313N 314N 315N 316N 317N 318N 709A 750G 930A 1438G 1888A 2706G 3106d 3107C 4216C 4769G 4917G 5147A 5656G 7028T 8697A 8860G 10463C 10750G 11251G 11719A 11812G 13368A 14233G 14766T 14905A 15326G 15452A 15607G 15928A 16126C 16292T 16294T 16296T 16304C	T2b3a1	57C 59C 66K 73G 151T 152C 263G 301N 302N 303N 304N 305N 306N 307N 308N 309N 310N 311N 312N 313N 314N 315N 316N 317N 318N 16126C 16292T 16294T 16296T 16304C	1	57 59 66K 73 151 152 263 301N 302N 303N 304N 305N 306N 307N 308N 309N 310N 311N 312N 313N 314N 315N 316N 317N 318N 709 750 930 1438 1888 2706 4216 4769 4917 5147 5656 7028 8697 8860 10463 10750 11251 11719 11812 13368 14233 14766 14905 15326 15452A 15607 15928 16126 16292 16294 16296 16304	T2b3a1	57 59 66K 73 151 152 263 301N 302N 303N 304N 305N 306N 307N 308N 309N 310N 311N 312N 313N 314N 315N 316N 317N 318N 16126 16292 16294 16296 16304	[0.661] T2b3a1 (1)	[0.661] T2b3a1 (2) [0.179] T2b3a	1

1988	>KY409804.1 Hc	73G 150T 195C 263G 295T 301N 302N 303N 304N 305N 306N 307N 308N 309N 310N 311N 312N 313N 314N 315N 316N 317N 318N 489C 750G 1438G 2706G 3106d 3107C 4216C 4769G 6671C 7028T 7476T 8860G 10398G 10499G 10750G 11002G 11251G 11377A 11719A 12570G 12612G 12672G 13708A 14502C 14766T 15257A 15326G 15452A 15679G 16069T 16126C 16214T 16231C 16519C	J2a2c1	73G 150T 195C 263G 295T 301N 302N 303N 304N 305N 306N 307N 308N 309N 310N 311N 312N 313N 314N 315N 316N 317N 318N 489C 16069T 16126C 16214T 16231C 16519C	1	73 150 195 263 295 301N 302N 303N 304N 305N 306N 307N 308N 309N 310N 311N 312N 313N 314N 315N 316N 317N 318N 489 750 1438 2706 4216 4769 6671 7028 7476 8860 10398 10499 10750 11002 11251 11377 11719 12570 12612 12672 13708 14502 14766 15257 15326 15452A 15679 16069 16126 16214 16231	J2a2c1	73 150 195 263 295 301N 302N 303N 304N 305N 306N 307N 308N 309N 310N 311N 312N 313N 314N 315N 316N 317N 318N 489 16069 16126 16214 16231	[0.000] J2a2c1 (1)	[0.000] J2a2c J2a2c1 (3)	1
1989	>KY409803.1 Hc	73G 195C 263G 301N 302N 303N 304N 305N 306N 307N 308N 309N 310N 311N 312N 313N 314N 315N 316N 317N 318N 750G 1438G 2706G 3106d 3107C 3197C 4260T 4769G 7028T 8860G 9477A 9667G 11467G 11719A 12308G 12372A 13617C 13802T 14766T 14793G 15218G 15326G 16156A 16192T 16256T 16270T 16320T 16399G 16527T	U5a1c1	73G 195C 263G 301N 302N 303N 304N 305N 306N 307N 308N 309N 310N 311N 312N 313N 314N 315N 316N 317N 318N 16156A 16192T 16256T 16270T 16320T 16399G 16527T	1	73 195 263 301N 302N 303N 304N 305N 306N 307N 308N 309N 310N 311N 312N 313N 314N 315N 316N 317N 318N 750 1438 2706 3197 4260 4769 7028 8860 9477 9667 11467 11719 12308 12372 13617 13802 14766 14793 15218 15326 16156 16192 16256 16270 16320 16399 16527	U5a1c1	73 195 263 301N 302N 303N 304N 305N 306N 307N 308N 309N 310N 311N 312N 313N 314N 315N 316N 317N 318N 16156 16192 16256 16270 16320 16399 16527	[0.090] U5a1c1 (1)	[0.267] U5a1c1a (2) [0.000] U5a1c U5a1c2 (4)	1
1990	>KY409798.1 Hc	57C 59C 73G 151T 152C 263G 301N 302N 303N 304N 305N 306N 307N 308N 309N 310N 311N 312N 313N 314N 315N 316N 317N 318N 709A 750G 930A 1438G 1888A 2706G 3106d 3107C 4216C 4769G 4917G 5147A 5656G 7028T 8697A 8860G 10398G 10463C 10750G 11251G 11719A 11812G 11929C 13368A 14233G 14766T 14905A 15326G 15452A 15607G 15928A 16126C 16261T 16292T 16294T 16296T 16304C 16519C	T2b3a1	57C 59C 73G 151T 152C 263G 301N 302N 303N 304N 305N 306N 307N 308N 309N 310N 311N 312N 313N 314N 315N 316N 317N 318N 16126C 16261T 16292T 16294T 16296T 16304C 16519C	1	57 59 73 151 152 263 301N 302N 303N 304N 305N 306N 307N 308N 309N 310N 311N 312N 313N 314N 315N 316N 317N 318N 709 750 930 1438 1888 2706 4216 4769 4917 5147 5656 7028 8697 8860 10398 10463 10750 11251 11719 11812 11929 13368 14233 14766 14905 15326 15452A 15607 15928 16126 16261 16292 16294 16296 16304	T2b3a1	57 59 73 151 152 263 301N 302N 303N 304N 305N 306N 307N 308N 309N 310N 311N 312N 313N 314N 315N 316N 317N 318N 16126 16261 16292 16294 16296 16304	[0.661] T2b3a1 (1)	[0.661] T2b3a1 (2) [0.179] T2b3a	1
1991	>KY409797.1 Hc	73G 151T 152C 263G 301N 302N 303N 304N 305N 306N 307N 308N 309N 310N 311N 312N 313N 314N 315N 316N 317N 318N 709A 750G 930A 1438G 1888A 2706G 3106d 3107C 4216C 4769G 4917G 5147A 5656G 7028T 8697A 8860G 10463C 10750G 11251G 11719A 11812G 13368A 14233G 14766T 14905A 15326G 15452A 15607G 15928A 16126C 16292T 16294T 16296T 16304C 16519C	T2b3a	73G 151T 152C 263G 301N 302N 303N 304N 305N 306N 307N 308N 309N 310N 311N 312N 313N 314N 315N 316N 317N 318N 16126C 16292T 16294T 16296T 16304C 16519C	1	73 151 152 263 301N 302N 303N 304N 305N 306N 307N 308N 309N 310N 311N 312N 313N 314N 315N 316N 317N 318N 709 750 930 1438 1888 2706 4216 4769 4917 5147 5656 7028 8697 8860 10463 10750 11251 11719 11812 13368 14233 14766 14905 15326 15452A 15607 15928 16126 16292 16294 16296 16304	T2b3a	73 151 152 263 301N 302N 303N 304N 305N 306N 307N 308N 309N 310N 311N 312N 313N 314N 315N 316N 317N 318N 16126 16292 16294 16296 16304	[0.000] T2b3a (1)	[0.091] T2b3+151 (2) [0.014] T2b+152 (3) [0.012] T2b7a3 (4) [0.000] T2b3c T2b4+152 T2b4c T2b4d T2b4e T2b4f T2b4g T2b21 T2b21b T2b22 (14)	1

1992	>KY409792.1 Hc	57C 73G 151T 152C 263G 301N 302N 303N 304N 305N 306N 307N 308N 309N 310N 311N 312N 313N 314N 315N 316N 317N 318N 709A 750G 930A 1438G 1888A 2706G 3106d 3107C 4216C 4769G 4917G 5147A 5656G 7028T 8697A 8860G 10463C 10750G 11251G 11719A 11812G 13368A 14233G 14766T 14905A 15326G 15452A 15607G 15928A 16126C 16292T 16294T 16296T 16304C 16519C	T2b3a	57C 73G 151T 152C 263G 301N 302N 303N 304N 305N 306N 307N 308N 309N 310N 311N 312N 313N 314N 315N 316N 317N 318N 16126C 16292T 16294T 16296T 16304C 16519C	1	57 73 151 152 263 301N 302N 303N 304N 305N 306N 307N 308N 309N 310N 311N 312N 313N 314N 315N 316N 317N 318N 709 750 930 1438 1888 2706 4216 4769 4917 5147 5656 7028 8697 8860 10463 10750 11251 11719 11812 13368 14233 14766 14905 15326 15452A 15607 15928 16126 16292 16294 16296 16304	T2b3a	57 73 151 152 263 301N 302N 303N 304N 305N 306N 307N 308N 309N 310N 311N 312N 313N 314N 315N 316N 317N 318N 16126 16292 16294 16296 16304	[0.179] T2b3a (1)	[0.645] T2b3a1 (2)	1
1993	>KY409791.1 Hc	73G 150T 263G 301N 302N 303N 304N 305N 306N 307N 308N 309N 310N 311N 312N 313N 314N 315N 316N 317N 318N 750G 1438G 1721T 2706G 3106d 3107C 3197C 4769G 7028T 7768G 8860G 9477A 11467G 11653G 11719A 12308G 12372A 12634G 13161C 13617C 13630G 13637G 14182C 14766T 15326G 16270T 16296T 16325C	U5b2b5	73G 150T 263G 301N 302N 303N 304N 305N 306N 307N 308N 309N 310N 311N 312N 313N 314N 315N 316N 317N 318N 16270T 16296T 16325C	1	73 150 263 301N 302N 303N 304N 305N 306N 307N 308N 309N 310N 311N 312N 313N 314N 315N 316N 317N 318N 750 1438 1721 2706 3197 4769 7028 7768 8860 9477 11467 11653 11719 12308 12372 12634 13161 13617 13630 13637 14182 14766 15326 16270 16296 16325	U5b2b5	73 150 263 301N 302N 303N 304N 305N 306N 307N 308N 309N 310N 311N 312N 313N 314N 315N 316N 317N 318N 16270 16296 16325	[0.256] U5b2b5 (1)	[0.057] U5b2b (2) [0.012] U5b2b1 (3) [0.000] U5b1d1a U5b2b2 (5)	1
1994	>KY409776.1 Hc	73G 199C 203A 204C 250C 263G 301N 302N 303N 304N 305N 306N 307N 308N 309N 310N 311N 312N 313N 314N 315N 316N 317N 318N 750G 1438G 1719A 2706G 3106d 3107C 3645C 3915A 4529T 4769G 6116G 7028T 7051C 7804G 8251A 8286C 8288C 8860G 10034C 10238C 10398G 11719A 12501A 12705T 13779C 13780G 14766T 15043A 15287C 15326G 15924G 16129A 16223T 16293C 16391A 16519C	I6a	73G 199C 203A 204C 250C 263G 301N 302N 303N 304N 305N 306N 307N 308N 309N 310N 311N 312N 313N 314N 315N 316N 317N 318N 16129A 16223T 16293C 16391A 16519C	1	73 199 203 204 250 263 301N 302N 303N 304N 305N 306N 307N 308N 309N 310N 311N 312N 313N 314N 315N 316N 317N 318N 750 1438 1719 2706 3645 3915 4529T 4769 6116 7028 7051 7804 8251 8286 8288 8860 10034 10238 10398 11719 12501 12705 13779C 13780 14766 15043 15287 15326 15924 16129 16223 16293C 16391	I6a	73 199 203 204 250 263 301N 302N 303N 304N 305N 306N 307N 308N 309N 310N 311N 312N 313N 314N 315N 316N 317N 318N 16129 16223 16293C 16391	[0.020] I6a (1)	[0.229] I4a (2) [0.114] I (3) [0.042] I4 (4) [0.034] I4b (5) [0.021] I4a2 (6) [0.020] I6a (7) [0.000] I5 I5b I6 (10)	1
1995	>KY409750.1 Hc	73G 195C 263G 285T 301N 302N 303N 304N 305N 306N 307N 308N 309N 310N 311N 312N 313N 314N 315N 316N 317N 318N 663G 750G 1438G 2218T 2706G 3106d 3107C 4769G 4991A 6026A 7028T 7403G 7581C 8860G 11116C 11467G 11719A 12308G 12372A 12879C 13104G 13656C 14070G 14364A 14766T 15115C 15148A 15217A 15326G 15954C 16145A 16182C 16183C 16189C 16249C	U1a1c1c1	73G 195C 263G 285T 301N 302N 303N 304N 305N 306N 307N 308N 309N 310N 311N 312N 313N 314N 315N 316N 317N 318N 16145A 16182C 16183C 16189C 16249C	1	73 195 263 285 301N 302N 303N 304N 305N 306N 307N 308N 309N 310N 311N 312N 313N 314N 315N 316N 317N 318N 663 750 1438 2218 2706 4769 4991 6026 7028 7403 7581 8860 11116 11467 11719 12308 12372 12879 13104 13656 14070 14364 14766 15115 15148 15217 15326 15954C 16145 16189 16249	U1a1c1c1	73 195 263 285 301N 302N 303N 304N 305N 306N 307N 308N 309N 310N 311N 312N 313N 314N 315N 316N 317N 318N 16145 16189 16249	[0.000] U1a1c1c1 (1)	[0.049] U1a1c1d (2) [0.000] U1a U1a1 U1a1c U1a1c1 U1a1c1b U1a1c1c (8)	1

1996	>KY409734.1 H[illegible]	73G 152C 263G 301N 302N 303N 304N 305N 306N 307N 308N 309N 310N 311N 312N 313N 314N 315N 316N 317N 318N 709A 750G 1438G 1888A 2706G 3106d 3107C 3397G 4216C 4769G 4917G 7001G 7028T 8393T 8697A 8860G 9266A 10463C 11251G 11719A 12633A 13368A 14287C 14766T 14905A 15326G 15452A 15607G 15928A 16126C 16163G 16169T 16186T 16189C 16263C 16294T 16519C	T1a4	73G 152C 263G 301N 302N 303N 304N 305N 306N 307N 308N 309N 310N 311N 312N 313N 314N 315N 316N 317N 318N 16126C 16163G 16169T 16186T 16189C 16263C 16294T 16519C	1	73 152 263 301N 302N 303N 304N 305N 306N 307N 308N 309N 310N 311N 312N 313N 314N 315N 316N 317N 318N 709 750 1438 1888 2706 3397 4216 4769 4917 7001 7028 8393 8697 8860 9266 10463 11251 11719 12633A 13368 14287 14766 14905 15326 15452A 15607 15928 16126 16163 16169 16186 16189 16263 16294	T1a4	73 152 263 301N 302N 303N 304N 305N 306N 307N 308N 309N 310N 311N 312N 313N 314N 315N 316N 317N 318N 16126 16163 16169 16186 16189 16263 16294	[0.208] T1a4 (1)	[0.016] T1a2 (2) [0.000] T1a+152	1
1997	>KY409730.1 H[illegible]	73G 263G 301N 302N 303N 304N 305N 306N 307N 308N 309N 310N 311N 312N 313N 314N 315N 316N 317N 318N 750G 1438G 1719A 2706G 3106d 3107C 3450T 4769G 5773A 6221C 6260A 7028T 8701G 8860G 9449T 9540C 10086G 10373A 10398G 10873C 11002G 11719A 11962G 12705T 13105G 13914A 14766T 15301A 15311G 15326G 15824G 16051G 16223T 16278T 16301T 16318G 16362C 16519C	L3b1a9a	73G 263G 301N 302N 303N 304N 305N 306N 307N 308N 309N 310N 311N 312N 313N 314N 315N 316N 317N 318N 16051G 16223T 16278T 16301T 16318G 16362C 16519C	1	73 263 301N 302N 303N 304N 305N 306N 307N 308N 309N 310N 311N 312N 313N 314N 315N 316N 317N 318N 750 1438 1719 2706 3450 4769 5773 6221 6260 7028 8701 8860 9449 9540 10086 10373 10398 10873 11002 11719 11962 12705 13105 13914A 14766 15301 15311 15326 15824 16051 16223 16278 16301 16318 16362	L3b1a9a	73 263 301N 302N 303N 304N 305N 306N 307N 308N 309N 310N 311N 312N 313N 314N 315N 316N 317N 318N 16051 16223 16278 16301 16318 16362	[0.012] L3b1a9a (1)	[0.000] L3b1a9 (2)	1
1998	>KY409721.1 H[illegible]	73G 150T 263G 301N 302N 303N 304N 305N 306N 307N 308N 309N 310N 311N 312N 313N 314N 315N 316N 317N 318N 373G 750G 1438G 2706G 3106d 3107C 3197C 4769G 6061C 7028T 7226A 7768G 8860G 9477A 11177T 11467G 11719A 12308G 12372A 13617C 14182C 14766T 15326G 16169A 16192T 16235G 16270T 16519C	U5b3a1a	73G 150T 263G 301N 302N 303N 304N 305N 306N 307N 308N 309N 310N 311N 312N 313N 314N 315N 316N 317N 318N 373G 16169A 16192T 16235G 16270T 16519C	1	73 150 263 301N 302N 303N 304N 305N 306N 307N 308N 309N 310N 311N 312N 313N 314N 315N 316N 317N 318N 373 750 1438 2706 3197 4769 6061 7028 7226 7768 8860 9477 11177 11467 11719 12308 12372 13617 14182 14766 15326 16169A 16192 16235 16270	U5b3a1a	73 150 263 301N 302N 303N 304N 305N 306N 307N 308N 309N 310N 311N 312N 313N 314N 315N 316N 317N 318N 373 16169A 16192 16235 16270	[0.000] U5b3a1a (1)	[0.000] U5b3a1 U5b3a1b (3)	1
1999	>KY409715.1 H[illegible]	73G 150T 263G 301N 302N 303N 304N 305N 306N 307N 308N 309N 310N 311N 312N 313N 314N 315N 316N 317N 318N 373G 750G 1438G 2706G 3106d 3107C 3197C 4769G 7028T 7226A 7768G 8860G 9477A 11177T 11467G 11719A 12308G 12372A 13617C 14182C 14766T 15326G 16169A 16192T 16235G 16270T 16519C	U5b3a1a	73G 150T 263G 301N 302N 303N 304N 305N 306N 307N 308N 309N 310N 311N 312N 313N 314N 315N 316N 317N 318N 373G 16169A 16192T 16235G 16270T 16519C	1	73 150 263 301N 302N 303N 304N 305N 306N 307N 308N 309N 310N 311N 312N 313N 314N 315N 316N 317N 318N 373 750 1438 2706 3197 4769 7028 7226 7768 8860 9477 11177 11467 11719 12308 12372 13617 14182 14766 15326 16169A 16192 16235 16270	U5b3a1a	73 150 263 301N 302N 303N 304N 305N 306N 307N 308N 309N 310N 311N 312N 313N 314N 315N 316N 317N 318N 373 16169A 16192 16235 16270	[0.000] U5b3a1a (1)	[0.000] U5b3a1 U5b3a1b (3)	1

2000	>KY409714.1 Ho	73G 150T 263G 301N 302N 303N 304N 305N 306N 307N 308N 309N 310N 311N 312N 313N 314N 315N 316N 317N 318N 750G 1438G 1721T 2706G 3106d 3107C 3197C 4769G 7028T 7768G 8860G 9477A 11467G 11653G 11719A 12308G 12372A 12634G 13161C 13617C 13630G 13637G 14182C 14766T 15326G 16270T 16296T 16325C	U5b2b5	73G 150T 263G 301N 302N 303N 304N 305N 306N 307N 308N 309N 310N 311N 312N 313N 314N 315N 316N 317N 318N 16270T 16296T 16325C	1	73 150 263 301N 302N 303N 304N 305N 306N 307N 308N 309N 310N 311N 312N 313N 314N 315N 316N 317N 318N 750 1438 1721 2706 3197 4769 7028 7768 8860 9477 11467 11653 11719 12308 12372 12634 13161 13617 13630 13637 14182 14766 15326 16270 16296 16325	U5b2b5	73 150 263 301N 302N 303N 304N 305N 306N 307N 308N 309N 310N 311N 312N 313N 314N 315N 316N 317N 318N 16270 16296 16325	[0.256] U5b2b5 (1)	[0.057] U5b2b (2) [0.012] U5b2b1 (3) [0.000] U5b1d1a U5b2b2 (5)	1
2001	>KY409713.1 Ho	57C 59C 73G 151T 152C 263G 301N 302N 303N 304N 305N 306N 307N 308N 309N 310N 311N 312N 313N 314N 315N 316N 317N 318N 709A 750G 930A 1438G 1888A 2706G 3106d 3107C 4216C 4769G 4917G 5147A 5656G 7028T 8697A 8860G 10463C 10750G 11251G 11719A 11812G 13368A 14233G 14766T 14905A 15326G 15452A 15607G 15928A 16126C 16183C 16189C 16292T 16294T 16304C 16362C 16519C	T2b3a1	57C 59C 73G 151T 152C 263G 301N 302N 303N 304N 305N 306N 307N 308N 309N 310N 311N 312N 313N 314N 315N 316N 317N 318N 16126C 16183C 16189C 16292T 16294T 16304C 16362C 16519C	1	57 59 73 151 152 263 301N 302N 303N 304N 305N 306N 307N 308N 309N 310N 311N 312N 313N 314N 315N 316N 317N 318N 709 750 930 1438 1888 2706 4216 4769 4917 5147 5656 7028 8697 8860 10463 10750 11251 11719 11812 13368 14233 14766 14905 15326 15452A 15607 15928 16126 16189 16292 16294 16304 16362	T2b3a1	57 59 73 151 152 263 301N 302N 303N 304N 305N 306N 307N 308N 309N 310N 311N 312N 313N 314N 315N 316N 317N 318N 16126 16189 16292 16294 16304 16362	[0.790] T2b3a1 (1)	[0.790] T2b3a1 (2) [0.179] T2b3a	1
2002	>KY409699.1 Ho	57C 59C 151T 152C 263G 301N 302N 303N 304N 305N 306N 307N 308N 309N 310N 311N 312N 313N 314N 315N 316N 317N 318N 709A 750G 930A 1438G 1888A 2706G 2779T 3106d 3107C 4216C 4769G 4917G 5147A 5656G 7028T 8697A 8860G 10463C 10750G 11251G 11719A 11812G 13368A 14233G 14560A 14766T 14905A 15326G 15452A 15607G 15928A 16126C 16292T 16294T 16304C 16519C	T2b3a1	57C 59C 151T 152C 263G 301N 302N 303N 304N 305N 306N 307N 308N 309N 310N 311N 312N 313N 314N 315N 316N 317N 318N 16126C 16292T 16294T 16304C 16519C	1	57 59 151 152 263 301N 302N 303N 304N 305N 306N 307N 308N 309N 310N 311N 312N 313N 314N 315N 316N 317N 318N 709 750 930 1438 1888 2706 2779 4216 4769 4917 5147 5656 7028 8697 8860 10463 10750 11251 11719 11812 13368 14233 14560 14766 14905 15326 15452A 15607 15928 16126 16292 16294 16304	T2b3a1	57 59 151 152 263 301N 302N 303N 304N 305N 306N 307N 308N 309N 310N 311N 312N 313N 314N 315N 316N 317N 318N 16126 16292 16294 16304	[0.758] T2b3a1 (1)	[0.758] T2b3a1 (2) [0.179] T2b3a	1
2003	>KY409696.1 Ho	73G 150T 263G 301N 302N 303N 304N 305N 306N 307N 308N 309N 310N 311N 312N 313N 314N 315N 316N 317N 318N 750G 1438G 1721T 2706G 3106d 3107C 3197C 4769G 7028T 7768G 8860G 9477A 11467G 11653G 11719A 12308G 12372A 12634G 13161C 13617C 13630G 13637G 14182C 14766T 15326G 16234Y 16270T 16296T 16325C	U5b2b5	73G 150T 263G 301N 302N 303N 304N 305N 306N 307N 308N 309N 310N 311N 312N 313N 314N 315N 316N 317N 318N 16234Y 16270T 16296T 16325C	1	73 150 263 301N 302N 303N 304N 305N 306N 307N 308N 309N 310N 311N 312N 313N 314N 315N 316N 317N 318N 750 1438 1721 2706 3197 4769 7028 7768 8860 9477 11467 11653 11719 12308 12372 12634 13161 13617 13630 13637 14182 14766 15326 16234Y 16270 16296 16325	U5b2b5	73 150 263 301N 302N 303N 304N 305N 306N 307N 308N 309N 310N 311N 312N 313N 314N 315N 316N 317N 318N 16234Y 16270 16296 16325	[0.279] U5b2b5 (1)	[0.126] U5b2b (2) [0.012] U5b2b1 (3) [0.000] U5b1d1a U5b2b2 (5)	1

2004	>KY409692.1 Hc	72C 263G 301N 302N 303N 304N 305N 306N 307N 308N 309N 310N 311N 312N 313N 314N 315N 316N 317N 318N 750G 1438G 2706G 3106d 3107C 4580A 4769G 6506T 7028T 8860G 9034G 15326G 15904T 16189C 16298C 16301T	V16	72C 263G 301N 302N 303N 304N 305N 306N 307N 308N 309N 310N 311N 312N 313N 314N 315N 316N 317N 318N 16189C 16298C 16301T	1	72 263 301N 302N 303N 304N 305N 306N 307N 308N 309N 310N 311N 312N 313N 314N 315N 316N 317N 318N 750 1438 2706 4580 4769 6506 7028 8860 9034G 15326 15904 16189 16298 16301	V16	72 263 301N 302N 303N 304N 305N 306N 307N 308N 309N 310N 311N 312N 313N 314N 315N 316N 317N 318N 16189 16298 16301	[0.025] V16 (1)	[0.068] V (2) [0.009] HV0a (3) [0.005] HV0 V1a V1a1b V8 V11 (8) [0.004] V1a1 (9) [0.000] V1 V2 V2a V2a1 V2a1a V2b V2b1 V2b2 V2c V3 V3b V4 V10 V10b V10b1 V10b2 V12 V13 V14 V15 V15a V17 V23 V24 V25 V26 V28 (36)	1
2005	>KY409677.1 Hc	57C 59C 73G 151T 152C 263G 301N 302N 303N 304N 305N 306N 307N 308N 309N 310N 311N 312N 313N 314N 315N 316N 317N 318N 709A 750G 930A 1438G 1888A 2706G 3106d 3107C 4216C 4769G 4917G 5147A 5656G 7028T 8697A 8860G 10463C 10750G 11251G 11719A 11812G 13368A 14233G 14766T 14905A 15326G 15452A 15607G 15928A 16126C 16292T 16294T 16296T 16304C 16519C	T2b3a1	57C 59C 73G 151T 152C 263G 301N 302N 303N 304N 305N 306N 307N 308N 309N 310N 311N 312N 313N 314N 315N 316N 317N 318N 16126C 16292T 16294T 16296T 16304C 16519C	1	57 59 73 151 152 263 301N 302N 303N 304N 305N 306N 307N 308N 309N 310N 311N 312N 313N 314N 315N 316N 317N 318N 709 750 930 1438 1888 2706 4216 4769 4917 5147 5656 7028 8697 8860 10463 10750 11251 11719 11812 13368 14233 14766 14905 15326 15452A 15607 15928 16126 16292 16294 16296 16304	T2b3a1	57 59 73 151 152 263 301N 302N 303N 304N 305N 306N 307N 308N 309N 310N 311N 312N 313N 314N 315N 316N 317N 318N 16126 16292 16294 16296 16304	[0.645] T2b3a1 (1)	[0.645] T2b3a1 (2) [0.179] T2b3a	1
2006	>KY409673.1 Hc	73G 150T 263G 301N 302N 303N 304N 305N 306N 307N 308N 309N 310N 311N 312N 313N 314N 315N 316N 317N 318N 373G 750G 1438G 2706G 3106d 3107C 3197C 4769G 6061C 7028T 7226A 7768G 8860G 9477A 11177T 11467G 11719A 12308G 12372A 13617C 14182C 14766T 15326G 16169A 16192T 16235G 16270T 16519C	U5b3a1a	73G 150T 263G 301N 302N 303N 304N 305N 306N 307N 308N 309N 310N 311N 312N 313N 314N 315N 316N 317N 318N 373G 16169A 16192T 16235G 16270T 16519C	1	73 150 263 301N 302N 303N 304N 305N 306N 307N 308N 309N 310N 311N 312N 313N 314N 315N 316N 317N 318N 373 750 1438 2706 3197 4769 6061 7028 7226 7768 8860 9477 11177 11467 11719 12308 12372 13617 14182 14766 15326 16169A 16192 16235 16270	U5b3a1a	73 150 263 301N 302N 303N 304N 305N 306N 307N 308N 309N 310N 311N 312N 313N 314N 315N 316N 317N 318N 373 16169A 16192 16235 16270	[0.000] U5b3a1a (1)	[0.000] U5b3a1 U5b3a1b (3)	1
2007	>KY409670.1 Hc	73G 150T 263G 301N 302N 303N 304N 305N 306N 307N 308N 309N 310N 311N 312N 313N 314N 315N 316N 317N 318N 373G 750G 1438G 2706G 3106d 3107C 3197C 4769G 7028T 7226A 7768G 8860G 9477A 9839T 9986A 11177T 11467G 11647T 11719A 12308G 12372A 13617C 14182C 14766T 15326G 16169A 16192T 16235R 16270T 16519C	U5b3a1a	73G 150T 263G 301N 302N 303N 304N 305N 306N 307N 308N 309N 310N 311N 312N 313N 314N 315N 316N 317N 318N 373G 16169A 16192T 16235R 16270T 16519C	1	73 150 263 301N 302N 303N 304N 305N 306N 307N 308N 309N 310N 311N 312N 313N 314N 315N 316N 317N 318N 373 750 1438 2706 3197 4769 7028 7226 7768 8860 9477 9839 9986 11177 11467 11647 11719 12308 12372 13617 14182 14766 15326 16169A 16192 16235R 16270	U5b3a1a	73 150 263 301N 302N 303N 304N 305N 306N 307N 308N 309N 310N 311N 312N 313N 314N 315N 316N 317N 318N 373 16169A 16192 16235R 16270	[0.000] U5b3a1a (1)	[0.000] U5b3a1 U5b3a1b (3)	1
2008	>KY409669.1 Hc	73G 103A 263G 301N 302N 303N 304N 305N 306N 307N 308N 309N 310N 311N 312N 313N 314N 315N 316N 317N 318N 750G 1438G 2706G 3106d 3107C 3348G 4769G 4936T 7028T 7805A 8860G 9128C 9335A 9416T 10172A 11467G 11719A 12308G 12372A 14179G 14766T 15326G 16172C 16183C 16219G 16278T 16295T	U6a8b	73G 103A 263G 301N 302N 303N 304N 305N 306N 307N 308N 309N 310N 311N 312N 313N 314N 315N 316N 317N 318N 16172C 16183C 16219G 16278T 16295T	1	73 103 263 301N 302N 303N 304N 305N 306N 307N 308N 309N 310N 311N 312N 313N 314N 315N 316N 317N 318N 750 1438 2706 3348 4769 4936 7028 7805 8860 9128 9335A 9416 10172 11467 11719 12308 12372 14179 14766 15326 16172 16219 16278 16295	U6a8b	73 103 263 301N 302N 303N 304N 305N 306N 307N 308N 309N 310N 311N 312N 313N 314N 315N 316N 317N 318N 16172 16219 16278 16295	[0.194] U6a8b (1)	[0.194] U6a8b (2) [0.042] U6a6 (3) [0.000] U6a U6a1 U6a+16189+(103) U6a2 U6a2a U6a2a1 U6a8 U6a5 U6a5a U6a5a1 U6a6b U6a6b1 U6a6b2 U6a7 U6a7a1+@152 U6a7a1c (19)	1

2009 >KY409668.1 Hc	73G 150T 195C 263G 295T 301N 302N 303N 304N 305N 306N 307N 308N 309N 310N 311N 312N 313N 314N 315N 316N 317N 318N 489C 750G 1438G 2706G 3106d 3107C 4216C 4769G 6671C 7028T 7476T 8860G 10398G 10499G 10750G 11002G 11251G 11377A 11719A 12570G 12612G 12672G 13708A 14502C 14766T 15257A 15326G 15452A 15679G 16069T 16126C 16214T 16231C 16519C	J2a2c1	73G 150T 195C 263G 295T 301N 302N 303N 304N 305N 306N 307N 308N 309N 310N 311N 312N 313N 314N 315N 316N 317N 318N 489C 16069T 16126C 16214T 16231C 16519C	1	73 150 195 263 295 301N 302N 303N 304N 305N 306N 307N 308N 309N 310N 311N 312N 313N 314N 315N 316N 317N 318N 489 750 1438 2706 4216 4769 6671 7028 7476 8860 10398 10499 10750 11002 11251 11377 11719 12570 12612 12672 13708 14502 14766 15257 15326 15452A 15679 16069 16126 16214 16231	J2a2c1	73 150 195 263 295 301N 302N 303N 304N 305N 306N 307N 308N 309N 310N 311N 312N 313N 314N 315N 316N 317N 318N 489 16069 16126 16214 16231	[0.000] J2a2c1 (1)	[0.000] J2a2c J2a2c1 (3)	1
2010 >KY409655.1 Hc	73G 150T 263G 301N 302N 303N 304N 305N 306N 307N 308N 309N 310N 311N 312N 313N 314N 315N 316N 317N 318N 373G 750G 1438G 2706G 3106d 3107C 3197C 4769G 7028T 7226A 7768G 8860G 9477A 11177T 11467G 11719A 12308G 12372A 13617C 14182C 14766T 15326G 16169A 16192T 16235G 16270T 16519C	U5b3a1a	73G 150T 263G 301N 302N 303N 304N 305N 306N 307N 308N 309N 310N 311N 312N 313N 314N 315N 316N 317N 318N 373G 16169A 16192T 16235G 16270T 16519C	1	73 150 263 301N 302N 303N 304N 305N 306N 307N 308N 309N 310N 311N 312N 313N 314N 315N 316N 317N 318N 373 750 1438 2706 3197 4769 7028 7226 7768 8860 9477 11177 11467 11719 12308 12372 13617 14182 14766 15326 16169A 16192 16235 16270	U5b3a1a	73 150 263 301N 302N 303N 304N 305N 306N 307N 308N 309N 310N 311N 312N 313N 314N 315N 316N 317N 318N 373 16169A 16192 16235 16270	[0.000] U5b3a1a (1)	[0.000] U5b3a1 U5b3a1b (3)	1
2011 >KY409654.1 Hc	73G 94A 263G 301N 302N 303N 304N 305N 306N 307N 308N 309N 310N 311N 312N 313N 314N 315N 316N 317N 318N 750G 1189C 1438G 1811G 2706G 3106d 3107C 3337A 3480G 4769G 5913A 7028T 8860G 9055A 9698C 9962A 10223T 10289G 10398G 10550G 11299C 11467G 11719A 11914A 12308G 12372A 14167T 14766T 14798C 15326G 15946T 16224C 16240T 16311C 16519C	K1b1c	73G 94A 263G 301N 302N 303N 304N 305N 306N 307N 308N 309N 310N 311N 312N 313N 314N 315N 316N 317N 318N 16224C 16240T 16311C 16519C	1	73 94 263 301N 302N 303N 304N 305N 306N 307N 308N 309N 310N 311N 312N 313N 314N 315N 316N 317N 318N 750 1189 1438 1811 2706 3337 3480 4769 5913 7028 8860 9055 9698 9962 10223 10289 10398 10550 11299 11467 11719 11914 12308 12372 14167 14766 14798 15326 15946 16224 16240T 16311	K1b1c	73 94 263 301N 302N 303N 304N 305N 306N 307N 308N 309N 310N 311N 312N 313N 314N 315N 316N 317N 318N 16224 16240T 16311	[0.031] K1b1c (1)	[0.000] K K1 K1b K1b1 K1b1+(16093) K1b1b K1b1b1 (8)	1
2012 >KY409637.1 Hc	73G 195C 263G 301N 302N 303N 304N 305N 306N 307N 308N 309N 310N 311N 312N 313N 314N 315N 316N 317N 318N 489C 750G 813G 1438G 2706G 3106d 3107C 3705A 4011T 4769G 6446A 6671C 6680C 7028T 8521G 8701G 8860G 9053A 9540C 9545G 10398G 10400T 10873C 11044T 11719A 12346T 12403T 12705T 12950C 14110C 14182C 14766T 14783C 15043A 15301A 15326G 16129A 16183C 16189C 16223T 16311C 16359C 16519C	M1a1a1	73G 195C 263G 301N 302N 303N 304N 305N 306N 307N 308N 309N 310N 311N 312N 313N 314N 315N 316N 317N 318N 489C 16129A 16183C 16189C 16223T 16311C 16359C 16519C	1	73 195 263 301N 302N 303N 304N 305N 306N 307N 308N 309N 310N 311N 312N 313N 314N 315N 316N 317N 318N 489 750 813 1438 2706 3705 4011 4769 6446 6671 6680 7028 8521 8701 8860 9053 9540 9545 10398 10400 10873 11044 11719 12346 12403 12705 12950C 14110 14182 14766 14783 15043 15301 15326 16129 16189 16223 16311 16359	M1a1a1	73 195 263 301N 302N 303N 304N 305N 306N 307N 308N 309N 310N 311N 312N 313N 314N 315N 316N 317N 318N 489 16129 16189 16223 16311 16359	[0.000] M1a1a1 (1)	[0.000] M1a1 M1a1a M1a1b M1a1b1 M1a1b1a M1a1b1b M1a1b1b1 M1a1b2 M1a1e M1a1e1 M1a1e2 M1a1f (13)	1

2013 >KY409633.1 Hc	73G 103A 263G 301N 302N 303N 304N 305N 306N 307N 308N 309N 310N 311N 312N 313N 314N 315N 316N 317N 318N 750G 1438G 2706G 3106d 3107C 3348G 4655A 4769G 4936T 7028T 7805A 8860G 9128C 9335A 10172A 11467G 11719A 12308G 12372A 14179G 14766T 15326G 16172C 16183C 16219G 16278T 16295T	U6a8b	73G 103A 263G 301N 302N 303N 304N 305N 306N 307N 308N 309N 310N 311N 312N 313N 314N 315N 316N 317N 318N 16172C 16183C 16219G 16278T 16295T	1	73 103 263 301N 302N 303N 304N 305N 306N 307N 308N 309N 310N 311N 312N 313N 314N 315N 316N 317N 318N 750 1438 2706 3348 4655 4769 4936 7028 7805 8860 9128 9335A 10172 11467 11719 12308 12372 14179 14766 15326 16172 16219 16278 16295	U6a8b	73 103 263 301N 302N 303N 304N 305N 306N 307N 308N 309N 310N 311N 312N 313N 314N 315N 316N 317N 318N 16172 16219 16278 16295	[0.194] U6a8b (1)	[0.194] U6a8b (2) [0.042] U6a6 (3) [0.000] U6a U6a1 U6a+16189+(103) U6a2 U6a2a U6a2a1 U6a8 U6a5 U6a5a U6a5a1 U6a6b U6a6b1 U6a6b2 U6a7 U6a7a1+@152 U6a7a1c (19)	1
2014 >KY695247.1 Hc	263G 309.1CT 310C 750G 1438G 2706G 3106d 3107C 4580A 4769G 7028T 8860G 9656C 13395G 15326G 15904T 16298C	V+@72	263G 309.1CT 310C 16298C	1	263 750 1438 2706 4580 4769 7028 8860 9656 13395 15326 15904 16298	V+@72	263 16298	[0.000] V+@72 (1)	[0.032] V1a (2) [0.021] V1a1 (3) [0.015] V1a1b (4) [0.014] H2a2a (5) [0.005] V2 V2b V2c (8) [0.003] H H35a (10) [0.002] H3u (11) [0.001] H1 H3 (13) [0.000] R0 HV HV0 HV0a V V1 V2a V2a1 V2a1a V2b1 V2b2 V3 V3b V4 V8 V10 V10b V10b1 V10b2 V11 V12 V13 V14 V15 V15a V17 V21 V23 V24 V25 V26 V28 HV0f HV4 HV4c HV5 HV5a HV9c HV12 H1e H1e1 H1e1a H1e1a2 H1e1a7 H1e1c H1e2 H1e2a H1e2b H1e2d H1e4 H1e6 H1e7 H1e8 H1e8a H1h H1h1 H1h2 H1j H1j3 H1j5 H1j6 H1j7 H1j9 H1m H1n H1p H1q H1q1 H1q2 H1r H1s H1s1 H1t H1t1 H1t1a H1t2 H1u H1u1 H1v H1v1 H1v1a H1v1b H1w H1ae H1ae1 H1ae2 H1ae2a H1ae3 H1ae3a H1ag H1ag1 H1ag1b H1ah H1ai H1ai1 H1aj H1ak H1ak1 H1ak2 H1am H1am1 H1ap H1aq H1as H1as2 H1at H1at1 H1at1a H1av H1ax H1ax1 H1ay H1az H1bd H1be H1bi H1bj H1bk H1bm H1bn H1bp H1bq H1br H1bu H1bv H1bw H1bx H1ca H1ci H2 H2a H2a2 H2a2+(16235) H2a4 H2a5 H2a5a H2a5b H3b H3c H3c1 H3e H3q H3q1 H3r H3r1 H3t [illegible]	1

2015	>KY496887.1 Hc	263G 302.1C 316.1C 750G 1438G 3106d 3107C 4769G 8860G 9115G 9591A 14470A 16272G 16519C	H10g	263G 302.1C 316.1C 16272G 16519C	1	263 317.1C 750 1438 4769 8860 9115 9591 14470A 16272	H10g	263 317.1C 16272	[0.015] H10g (1)	[0.015] H10g (2) [0.001] H H1 H7 (5) [0.000] R0 HV HV4 HV4c HV5 HV5a HV9c HV12 H1e H1e1 H1e1a H1e1a2 H1e1a7 H1e1c H1e2 H1e2a H1e2b H1e2d H1e4 H1e6 H1e7 H1e8 H1e8a H1h H1h1 H1h2 H1j H1j3 H1j5 H1j6 H1j7 H1j9 H1m H1n H1p H1q H1q1 H1q2 H1r H1s H1s1 H1t H1t1 H1t1a H1t2 H1u H1u1 H1v H1v1 H1v1a H1v1b H1w H1ae H1ae1 H1ae2 H1ae2a H1ae3 H1ae3a H1ag H1ag1 H1ag1b H1ah H1ai H1ai1 H1aj H1ak H1ak1 H1ak2 H1am H1am1 H1ap H1aq H1as H1as2 H1at H1at1 H1at1a H1av H1ax H1ax1 H1ay H1az H1bd H1be H1bi H1bj H1bk H1bm H1bn H1bp H1bq H1br H1bu H1bv H1bw H1bx H1ca H1ci H2 H2a H2a2 H2a2a H2a2+(16235) H2a4 H2a5 H2a5a H2a5b H3 H3b H3c H3c1 H3e H3q H3q1 H3r H3r1 H3t H3u H3y H3aa H3ab H3ac H3ad H3ae H3ag H3ag1 H3ah H3ai H3aj H3ap H3aq H3ar H3as H3at H3at1 H3au H4 H4a H4a1 H4a1a H4a1a1 H4a1a2 H4a1a2a H4a1a2a1 H4a1a5 H4a1c H4a1c1 H4a1c1a H4a1d H4b H4c H7a H7b H7b1 H7b2 H7b2a H7b3 H7b4 H7b5 H7b6 [illegible]	1
2016	>KY496875.1 Hc	73G 309.1CT 310C 750G 1438G 2706G 3106d 3107C 3197C 4769G 7028T 8860G 9477A 11467G 11719A 12308G 12372A 12630A 13617C 13827G 13928C 14766T 14793G 15326G 16189C 16192T 16256T 16270T 16271C 16294T 16526A	U5a2a1a	73G 309.1CT 310C 16189C 16192T 16256T 16270T 16271C 16294T 16526A	1	73 750 1438 2706 3197 4769 7028 8860 9477 11467 11719 12308 12372 12630 13617 13827 13928C 14766 14793 15326 16189 16192 16256 16270 16271 16294 16526	U5a2a1a	73 16189 16192 16256 16270 16271 16294 16526	[0.120] U5a2a1a (1) [0.000] U5a2+16294 (2)	[0.052] U5a2a1b (3) [0.029] U5a2a (4) [0.018] U5a2 (5) [0.017] U5a2b2 (6) [0.016] U5a2b1a U5a2d (8) [0.015] U5a2c1 (9) [0.013] U5a2a1 (10) [0.000] U5a2a1c U5a2a1d U5a2b U5a2b1 U5a2b1c U5a2b1d U5a2b2a U5a2b4 U5a2b4a U5a2c U5a2c2 U5a2c4 (22)	1
2017	>KY496874.1 Hc	73G 309.1CCT 310C 750G 1438G 2706G 3106d 3107C 3197C 4769G 7028T 8860G 9477A 11467G 11719A 12308G 12372A 12630A 13617C 13827G 13928C 14766T 14793G 15326G 16114A 16192T 16256T 16270T 16271C 16294T 16526A	U5a2a1a	73G 309.1CCT 310C 16114A 16192T 16256T 16270T 16271C 16294T 16526A	1	73 750 1438 2706 3197 4769 7028 8860 9477 11467 11719 12308 12372 12630 13617 13827 13928C 14766 14793 15326 16114A 16192 16256 16270 16271 16294 16526	U5a2a1a	73 16114A 16192 16256 16270 16271 16294 16526	[0.048] U5a2a1a (1)	[0.026] U5a2a1b (2) [0.000] U5a2a U5a2a1 U5a2a1c U5a2a1d (6)	1
2018	>KY496869.1 Hc	72C 309.1CCT 310C 485C 750G 1438G 2706G 3106d 3107C 4580A 4639C 4769G 5263T 7028T 8860G 8869G 15151G 15326G 15904T 16183G 16298C	V1a1a	72C 309.1CCT 310C 485C 16183G 16298C	1	72 485 750 1438 2706 4580 4639 4769 5263 7028 8860 8869 15151 15326 15904 16183 16298	V1a1a	72 485 16183 16298	[0.071] V1a1a (1)	[0.000] V1a1a1 (2)	1

2019	>KY410225.1 Hc	73G 150T 263G 301N 302N 303N 304N 305N 306N 307N 308N 309N 310N 311N 312N 313N 314N 315N 316N 317N 318N 750G 1438G 2706G 3106d 3107C 3197C 3498T 4769G 5656G 5964C 6674C 6675R 7028T 7768G 8860G 9163A 9477A 11467G 11719A 12308G 12372A 13617C 14182C 14766T 15326G 15777A 16167T 16189C 16270T 16311C 16356C	U5b1i	73G 150T 263G 301N 302N 303N 304N 305N 306N 307N 308N 309N 310N 311N 312N 313N 314N 315N 316N 317N 318N 16167T 16189C 16270T 16311C 16356C	1	73 150 263 301N 302N 303N 304N 305N 306N 307N 308N 309N 310N 311N 312N 313N 314N 315N 316N 317N 318N 750 1438 2706 3197 3498 4769 5656 5964 6674 6675R 7028 7768 8860 9163 9477 11467 11719 12308 12372 13617 14182 14766 15326 15777 16167 16189 16270 16311 16356	U5b1i	73 150 263 301N 302N 303N 304N 305N 306N 307N 308N 309N 310N 311N 312N 313N 314N 315N 316N 317N 318N 16167 16189 16270 16311 16356	[1.222] U5b1i (1) [0.000] U5b2a5 U5b2a5a (3)	[0.094] U5b1c (4) [0.077] U5b1+16189+@16192 (5) [0.051] U5b2a6 (6) [0.017] U5b2a+@16192 (7) [0.000] U5b1b1+@16192 U5b1b1d U5b1b1f U5b2a4 U5b2a4a U5b2b4 (13)	1
2020	>KY410213.1 Hc	73G 150T 263G 301N 302N 303N 304N 305N 306N 307N 308N 309N 310N 311N 312N 313N 314N 315N 316N 317N 318N 373G 750G 1438G 2706G 3106d 3107C 3197C 4769G 7028T 7226A 7768G 8860G 9477A 11177T 11467G 11719A 12308G 12372A 13617C 14182C 14766T 15326G 15607G 16145A 16169A 16192T 16235G 16270T 16519C	U5b3a1a	73G 150T 263G 301N 302N 303N 304N 305N 306N 307N 308N 309N 310N 311N 312N 313N 314N 315N 316N 317N 318N 373G 16145A 16169A 16192T 16235G 16270T 16519C	1	73 150 263 301N 302N 303N 304N 305N 306N 307N 308N 309N 310N 311N 312N 313N 314N 315N 316N 317N 318N 373 750 1438 2706 3197 4769 7028 7226 7768 8860 9477 11177 11467 11719 12308 12372 13617 14182 14766 15326 15607 16145 16169A 16192 16235 16270	U5b3a1a	73 150 263 301N 302N 303N 304N 305N 306N 307N 308N 309N 310N 311N 312N 313N 314N 315N 316N 317N 318N 373 16145 16169A 16192 16235 16270	[0.122] U5b3a1a (1)	[0.000] U5b3a1 U5b3a1b (3)	1
2021	>KY410208.1 Hc	73G 150T 263G 301N 302N 303N 304N 305N 306N 307N 308N 309N 310N 311N 312N 313N 314N 315N 316N 317N 318N 750G 1438G 2706G 3106d 3107C 3197C 3498T 4769G 5656G 5964C 6674C 7028T 7768G 8860G 9477A 11467G 11719A 12308G 12372A 13617C 14182C 14766T 14783C 15326G 15777A 16167T 16183C 16189C 16270T 16311C 16356C	U5b1i	73G 150T 263G 301N 302N 303N 304N 305N 306N 307N 308N 309N 310N 311N 312N 313N 314N 315N 316N 317N 318N 16167T 16183C 16189C 16270T 16311C 16356C	1	73 150 263 301N 302N 303N 304N 305N 306N 307N 308N 309N 310N 311N 312N 313N 314N 315N 316N 317N 318N 750 1438 2706 3197 3498 4769 5656 5964 6674 7028 7768 8860 9477 11467 11719 12308 12372 13617 14182 14766 14783 15326 15777 16167 16189 16270 16311 16356	U5b1i	73 150 263 301N 302N 303N 304N 305N 306N 307N 308N 309N 310N 311N 312N 313N 314N 315N 316N 317N 318N 16167 16189 16270 16311 16356	[1.222] U5b1i (1) [0.000] U5b2a5 U5b2a5a (3)	[0.094] U5b1c (4) [0.077] U5b1+16189+@16192 (5) [0.051] U5b2a6 (6) [0.017] U5b2a+@16192 (7) [0.000] U5b1b1+@16192 U5b1b1d U5b1b1f U5b2a4 U5b2a4a U5b2b4 (13)	1
2022	>KY410197.1 Hc	151T 152C 195C 198T 263G 301N 302N 303N 304N 305N 306N 307N 308N 309N 310N 311N 312N 313N 314N 315N 316N 317N 318N 750G 1438G 2706G 3106d 3107C 3531R 4769G 5250C 7028T 8014T 8860G 10044G 12696C 15218G 15326G 16067T 16304Y 16356Y	HV1b3	151T 152C 195C 198T 263G 301N 302N 303N 304N 305N 306N 307N 308N 309N 310N 311N 312N 313N 314N 315N 316N 317N 318N 16067T 16304Y 16356Y	1	151 152 195 198 263 301N 302N 303N 304N 305N 306N 307N 308N 309N 310N 311N 312N 313N 314N 315N 316N 317N 318N 750 1438 2706 3531R 4769 5250 7028 8014T 8860 10044 12696 15218 15326 16067 16304Y 16356Y	HV1b3	151 152 195 198 263 301N 302N 303N 304N 305N 306N 307N 308N 309N 310N 311N 312N 313N 314N 315N 316N 317N 318N 16067 16304Y 16356Y	[0.734] HV1b3 (1) [0.000] HV1b3a (2)	[0.000] HV1b+152 HV1b3b (4)	1
2023	>KY410188.1 Hc	152C 263G 301N 302N 303N 304N 305N 306N 307N 308N 309N 310N 311N 312N 313N 314N 315N 316N 317N 318N 750G 1438G 3106d 3107C 3396C 4769G 5480G 8860G 15326G 16209C 16261T	H107	152C 263G 301N 302N 303N 304N 305N 306N 307N 308N 309N 310N 311N 312N 313N 314N 315N 316N 317N 318N 16209C 16261T	1	152 263 301N 302N 303N 304N 305N 306N 307N 308N 309N 310N 311N 312N 313N 314N 315N 316N 317N 318N 750 1438 3396 4769 5480 8860 15326 16209 16261	H107	152 263 301N 302N 303N 304N 305N 306N 307N 308N 309N 310N 311N 312N 313N 314N 315N 316N 317N 318N 16209 16261	[0.000] H107 (1)	[0.187] H+152 (2) [0.071] H13b1 (3) [0.011] H7a1c (4) [0.010] H7a1b (5) [0.007] H1+152 (6) [0.006] H52 (7) [0.000] H1i H1i1 H1i2 H1i2a H1an H1an1 H1an1a H1bb H1bc H2a2a2 H3+152 H3g H3g1 H3g1a H3g1b H3g2 H3g3 H3g4 H3i H3i1 H3j H3k H3k1 H3k1a H7a1 H7a1d H9 H46 H46a H46b H69 H13a1+152 H14b H14b2 H14b4 H16+152 H16a H16a1 H16c H16d H30b1 H47a R30a1b (50)	1

2024	>KY410186.1 H	57C 59C 73G 151T 152C 263G 301N 302N 303N 304N 305N 306N 307N 308N 309N 310N 311N 312N 313N 314N 315N 316N 317N 318N 709A 750G 930A 1438G 1888A 2706G 3106d 3107C 4216C 4769G 4917G 5147A 5656G 7028T 8697A 8860G 10463C 10750G 11251G 11719A 11812G 13368A 14233G 14766T 14905A 15326G 15452A 15607G 15928A 16126C 16292T 16294T 16296T 16304C 16519C	T2b3a1	57C 59C 73G 151T 152C 263G 301N 302N 303N 304N 305N 306N 307N 308N 309N 310N 311N 312N 313N 314N 315N 316N 317N 318N 16126C 16292T 16294T 16296T 16304C 16519C	1	57 59 73 151 152 263 301N 302N 303N 304N 305N 306N 307N 308N 309N 310N 311N 312N 313N 314N 315N 316N 317N 318N 709 750 930 1438 1888 2706 4216 4769 4917 5147 5656 7028 8697 8860 10463 10750 11251 11719 11812 13368 14233 14766 14905 15326 15452A 15607 15928 16126 16292 16294 16296 16304	T2b3a1	57 59 73 151 152 263 301N 302N 303N 304N 305N 306N 307N 308N 309N 310N 311N 312N 313N 314N 315N 316N 317N 318N 16126 16292 16294 16296 16304	[0.645] T2b3a1 (1)	[0.645] T2b3a1 (2) [0.179] T2b3a	1
2025	>KY410170.1 H	73G 150T 263G 301N 302N 303N 304N 305N 306N 307N 308N 309N 310N 311N 312N 313N 314N 315N 316N 317N 318N 373G 750G 1438G 2706G 3106d 3107C 3197C 4769G 7028T 7226A 7768G 8860G 9477A 10635R 11177T 11467G 11719A 12308G 12372A 13617C 14182C 14766T 15326G 16169A 16192T 16235G 16270T 16519C	U5b3a1a	73G 150T 263G 301N 302N 303N 304N 305N 306N 307N 308N 309N 310N 311N 312N 313N 314N 315N 316N 317N 318N 373G 16169A 16192T 16235G 16270T 16519C	1	73 150 263 301N 302N 303N 304N 305N 306N 307N 308N 309N 310N 311N 312N 313N 314N 315N 316N 317N 318N 373 750 1438 2706 3197 4769 7028 7226 7768 8860 9477 10635R 11177 11467 11719 12308 12372 13617 14182 14766 15326 16169A 16192 16235 16270	U5b3a1a	73 150 263 301N 302N 303N 304N 305N 306N 307N 308N 309N 310N 311N 312N 313N 314N 315N 316N 317N 318N 373 16169A 16192 16235 16270	[0.000] U5b3a1a (1)	[0.000] U5b3a1 U5b3a1b (3)	1
2026	>KY410157.1 H	73G 151T 152C 263G 301N 302N 303N 304N 305N 306N 307N 308N 309N 310N 311N 312N 313N 314N 315N 316N 317N 318N 709A 750G 930A 1438G 1888A 2706G 3106d 3107C 4216C 4769G 4917G 5147A 5656G 7028T 8697A 8860G 10463C 10750G 11251G 11719A 11812G 13368A 14233G 14766T 14905A 15326G 15452A 15607G 15928A 16093C 16126C 16292T 16294T 16304C 16335R 16519C	T2b3a	73G 151T 152C 263G 301N 302N 303N 304N 305N 306N 307N 308N 309N 310N 311N 312N 313N 314N 315N 316N 317N 318N 16093C 16126C 16292T 16294T 16304C 16335R 16519C	1	73 151 152 263 301N 302N 303N 304N 305N 306N 307N 308N 309N 310N 311N 312N 313N 314N 315N 316N 317N 318N 709 750 930 1438 1888 2706 4216 4769 4917 5147 5656 7028 8697 8860 10463 10750 11251 11719 11812 13368 14233 14766 14905 15326 15452A 15607 15928 16093 16126 16292 16294 16304 16335R	T2b3a	73 151 152 263 301N 302N 303N 304N 305N 306N 307N 308N 309N 310N 311N 312N 313N 314N 315N 316N 317N 318N 16093 16126 16292 16294 16304 16335R	[0.215] T2b3a (1)	[0.106] T2b3+151 (2) [0.021] T2b+152 (3) [0.012] T2b7a3 (4) [0.000] T2b3c T2b4+152 T2b4c T2b4d T2b4e T2b4f T2b4g T2b21 T2b21b T2b22 (14)	1
2027	>KY410155.1 H	73G 150T 263G 284G 301N 302N 303N 304N 305N 306N 307N 308N 309N 310N 311N 312N 313N 314N 315N 316N 317N 318N 373G 750G 1438G 2706G 3106d 3107C 3197C 4769G 7028T 7226A 7768G 8860G 9477A 11177T 11467G 11719A 11914A 12308G 12372A 13617C 14182C 14766T 15326G 16169A 16192T 16235G 16270T 16519C	U5b3a1a	73G 150T 263G 284G 301N 302N 303N 304N 305N 306N 307N 308N 309N 310N 311N 312N 313N 314N 315N 316N 317N 318N 373G 16169A 16192T 16235G 16270T 16519C	1	73 150 263 284 301N 302N 303N 304N 305N 306N 307N 308N 309N 310N 311N 312N 313N 314N 315N 316N 317N 318N 373 750 1438 2706 3197 4769 7028 7226 7768 8860 9477 11177 11467 11719 11914 12308 12372 13617 14182 14766 15326 16169A 16192 16235 16270	U5b3a1a	73 150 263 284 301N 302N 303N 304N 305N 306N 307N 308N 309N 310N 311N 312N 313N 314N 315N 316N 317N 318N 373 16169A 16192 16235 16270	[0.184] U5b3a1a (1)	[0.000] U5b3a1 U5b3a1b (3)	1

2028	>KY410146.1 Hc	73G 150T 263G 301N 302N 303N 304N 305N 306N 307N 308N 309N 310N 311N 312N 313N 314N 315N 316N 317N 318N 373G 750G 1438G 2706G 3106d 3107C 3197C 4769G 7028T 7226A 7768G 8860G 9477A 9839T 11177T 11467G 11647T 11719A 12308G 12358G 12372A 13617C 14182C 14766T 15326G 16169A 16192T 16235G 16270T 16302G 16519C	U5b3a1a	73G 150T 263G 301N 302N 303N 304N 305N 306N 307N 308N 309N 310N 311N 312N 313N 314N 315N 316N 317N 318N 373G 16169A 16192T 16235G 16270T 16302G 16519C	1	73 150 263 301N 302N 303N 304N 305N 306N 307N 308N 309N 310N 311N 312N 313N 314N 315N 316N 317N 318N 373 750 1438 2706 3197 4769 7028 7226 7768 8860 9477 9839 11177 11467 11647 11719 12308 12358 12372 13617 14182 14766 15326 16169A 16192 16235 16270 16302	U5b3a1a	73 150 263 301N 302N 303N 304N 305N 306N 307N 308N 309N 310N 311N 312N 313N 314N 315N 316N 317N 318N 373 16169A 16192 16235 16270 16302	[0.153] U5b3a1a (1)	[0.000] U5b3a1 U5b3a1b (3)	1
2029	>KY410144.1 Hc	57C 59C 73G 151T 152C 263G 301N 302N 303N 304N 305N 306N 307N 308N 309N 310N 311N 312N 313N 314N 315N 316N 317N 318N 709A 750G 930A 1438G 1888A 2706G 3106d 3107C 4216C 4227G 4769G 4917G 5147A 5656G 7028T 8697A 8860G 10463C 10750G 11251G 11719A 11812G 13368A 14233G 14766T 14905A 15326G 15452A 15607G 15928A 16126C 16288C 16292T 16294T 16296T 16304C 16465T 16519C	T2b3a1	57C 59C 73G 151T 152C 263G 301N 302N 303N 304N 305N 306N 307N 308N 309N 310N 311N 312N 313N 314N 315N 316N 317N 318N 16126C 16288C 16292T 16294T 16296T 16304C 16465T 16519C	1	57 59 73 151 152 263 301N 302N 303N 304N 305N 306N 307N 308N 309N 310N 311N 312N 313N 314N 315N 316N 317N 318N 709 750 930 1438 1888 2706 4216 4227 4769 4917 5147 5656 7028 8697 8860 10463 10750 11251 11719 11812 13368 14233 14766 14905 15326 15452A 15607 15928 16126 16288 16292 16294 16296 16304 16465	T2b3a1	57 59 73 151 152 263 301N 302N 303N 304N 305N 306N 307N 308N 309N 310N 311N 312N 313N 314N 315N 316N 317N 318N 16126 16288 16292 16294 16296 16304 16465	[0.983] T2b3a1 (1)	[0.983] T2b3a1 (2) [0.393] T2b3a	1
2030	>KY410142.1 Hc	73G 151T 152C 263G 301N 302N 303N 304N 305N 306N 307N 308N 309N 310N 311N 312N 313N 314N 315N 316N 317N 318N 709A 750G 930A 1438G 1888A 2706G 3106d 3107C 4216C 4769G 4917G 5147A 5656G 7028T 8697A 8860G 10463C 10750G 11251G 11719A 11812G 13368A 14233G 14766T 14905A 15326G 15452A 15607G 15928A 16126C 16292T 16294T 16296T 16304C 16519C	T2b3a	73G 151T 152C 263G 301N 302N 303N 304N 305N 306N 307N 308N 309N 310N 311N 312N 313N 314N 315N 316N 317N 318N 16126C 16292T 16294T 16296T 16304C 16519C	1	73 151 152 263 301N 302N 303N 304N 305N 306N 307N 308N 309N 310N 311N 312N 313N 314N 315N 316N 317N 318N 709 750 930 1438 1888 2706 4216 4769 4917 5147 5656 7028 8697 8860 10463 10750 11251 11719 11812 13368 14233 14766 14905 15326 15452A 15607 15928 16126 16292 16294 16296 16304	T2b3a	73 151 152 263 301N 302N 303N 304N 305N 306N 307N 308N 309N 310N 311N 312N 313N 314N 315N 316N 317N 318N 16126 16292 16294 16296 16304	[0.000] T2b3a (1)	[0.091] T2b3+151 (2) [0.014] T2b+152 (3) [0.012] T2b7a3 (4) [0.000] T2b3c T2b4+152 T2b4c T2b4d T2b4e T2b4f T2b4g T2b21 T2b21b T2b22 (14)	1
2031	>KY410139.1 Hc	73G 103A 263G 301N 302N 303N 304N 305N 306N 307N 308N 309N 310N 311N 312N 313N 314N 315N 316N 317N 318N 750G 1438G 2706G 3106d 3107C 3348G 4655A 4769G 4936T 7028T 7805A 8860G 9128C 9335A 10172A 11467G 11719A 12308G 12372A 14179G 14766T 15326G 16172C 16183C 16219G 16278T 16295T	U6a8b	73G 103A 263G 301N 302N 303N 304N 305N 306N 307N 308N 309N 310N 311N 312N 313N 314N 315N 316N 317N 318N 16172C 16183C 16219G 16278T 16295T	1	73 103 263 301N 302N 303N 304N 305N 306N 307N 308N 309N 310N 311N 312N 313N 314N 315N 316N 317N 318N 750 1438 2706 3348 4655 4769 4936 7028 7805 8860 9128 9335A 10172 11467 11719 12308 12372 14179 14766 15326 16172 16219 16278 16295	U6a8b	73 103 263 301N 302N 303N 304N 305N 306N 307N 308N 309N 310N 311N 312N 313N 314N 315N 316N 317N 318N 16172 16219 16278 16295	[0.194] U6a8b (1)	[0.194] U6a8b (2) [0.042] U6a6 (3) [0.000] U6a U6a1 U6a+16189+(103) U6a2 U6a2a U6a2a1 U6a8 U6a5 U6a5a U6a5a1 U6a6b U6a6b1 U6a6b2 U6a7 U6a7a1+@152 U6a7a1c (19)	1

2032	>KY410138.1 Hc	263G 301N 302N 303N 304N 305N 306N 307N 308N 309N 310N 311N 312N 313N 314N 315N 316N 317N 318N 750G 1438G 2706G 3106d 3107C 4580A 4769G 7028T 7852A 8860G 15326G 15904T 15905C 16298C	V+@72	263G 301N 302N 303N 304N 305N 306N 307N 308N 309N 310N 311N 312N 313N 314N 315N 316N 317N 318N 16298C	1	263 301N 302N 303N 304N 305N 306N 307N 308N 309N 310N 311N 312N 313N 314N 315N 316N 317N 318N 750 1438 2706 4580 4769 7028 7852 8860 15326 15904 15905 16298	V+@72	263 301N 302N 303N 304N 305N 306N 307N 308N 309N 310N 311N 312N 313N 314N 315N 316N 317N 318N 16298	[0.000] V+@72 (1)	[0.032] V1a (2) [0.021] V1a1 (3) [0.015] V1a1b (4) [0.014] H2a2a (5) [0.005] V2 V2b V2c (8) [0.003] H H35a (10) [0.002] H3u (11) [0.001] H1 H3 (13) [0.000] R0 HV HV0 HV0a V V1 V2a V2a1 V2a1a V2b1 V2b2 V3 V3b V4 V8 V10 V10b V10b1 V10b2 V11 V12 V13 V14 V15 V15a V17 V21 V23 V24 V25 V26 V28 HV0f HV4 HV4c HV5 HV5a HV9c HV12 H1e H1e1 H1e1a H1e1a2 H1e1a7 H1e1c H1e2 H1e2a H1e2b H1e2d H1e4 H1e6 H1e7 H1e8 H1e8a H1h H1h1 H1h2 H1j H1j3 H1j5 H1j6 H1j7 H1j9 H1m H1n H1p H1q H1q1 H1q2 H1r H1s H1s1 H1t H1t1 H1t1a H1t2 H1u H1u1 H1v H1v1 H1v1a H1v1b H1w H1ae H1ae1 H1ae2 H1ae2a H1ae3 H1ae3a H1ag H1ag1 H1ag1b H1ah H1ai H1ai1 H1aj H1ak H1ak1 H1ak2 H1am H1am1 H1ap H1aq H1as H1as2 H1at H1at1 H1at1a H1av H1ax H1ax1 H1ay H1az H1bd H1be H1bi H1bj H1bk H1bm H1bn H1bp H1bq H1br H1bu H1bv H1bw H1bx H1ca H1ci H2 H2a H2a2 H2a2+(16235) H2a4 H2a5 H2a5a H2a5b H3b H3c H3c1 H3e H3q H3q1 H3r H3r1 H3t [illegible]	1
2033	>KY410120.1 Hc	73G 150T 263G 301N 302N 303N 304N 305N 306N 307N 308N 309N 310N 311N 312N 313N 314N 315N 316N 317N 318N 373G 750G 1438G 2706G 3106d 3107C 3197C 4769G 7028T 7226A 7768G 8860G 9477A 11177T 11467G 11719A 12308G 12372A 13617C 14182C 14766T 15326G 16169A 16192T 16235G 16270T 16519C	U5b3a1a	73G 150T 263G 301N 302N 303N 304N 305N 306N 307N 308N 309N 310N 311N 312N 313N 314N 315N 316N 317N 318N 373G 16169A 16192T 16235G 16270T 16519C	1	73 150 263 301N 302N 303N 304N 305N 306N 307N 308N 309N 310N 311N 312N 313N 314N 315N 316N 317N 318N 373 750 1438 2706 3197 4769 7028 7226 7768 8860 9477 11177 11467 11719 12308 12372 13617 14182 14766 15326 16169A 16192 16235 16270	U5b3a1a	73 150 263 301N 302N 303N 304N 305N 306N 307N 308N 309N 310N 311N 312N 313N 314N 315N 316N 317N 318N 373 16169A 16192 16235 16270	[0.000] U5b3a1a (1)	[0.000] U5b3a1 U5b3a1b (3)	1
2034	>KY410108.1 Hc	72C 195C 198T 263G 285.1A 301N 302N 303N 304N 305N 306N 307N 308N 309N 310N 311N 312N 313N 314N 315N 316N 317N 318N 750G 1438G 2706G 3106d 3107C 4769G 7028T 8860G 15326G 16298C	HV0b	72C 195C 198T 263G 285.1A 301N 302N 303N 304N 305N 306N 307N 308N 309N 310N 311N 312N 313N 314N 315N 316N 317N 318N 16298C	1	72 195 198 263 291.1A 301N 302N 303N 304N 305N 306N 307N 308N 309N 310N 311N 312N 313N 314N 315N 316N 317N 318N 750 1438 2706 4769 7028 8860 15326 16298	HV0b	72 195 198 263 291.1A 301N 302N 303N 304N 305N 306N 307N 308N 309N 310N 311N 312N 313N 314N 315N 316N 317N 318N 16298	[0.011] HV0b (1)	[0.000] HV0+195 HV0c HV0d (4)	1

2035	>KY410107.1 Hc	73G 103A 263G 301N 302N 303N 304N 305N 306N 307N 308N 309N 310N 311N 312N 313N 314N 315N 316N 317N 318N 750G 1438G 2706G 3106d 3107C 3348G 4655A 4769G 4936T 7028T 7805A 8860G 9128C 9335A 10172A 11467G 11719A 12308G 12372A 14179G 14766T 15326G 16172C 16183C 16219G 16278T 16295T	U6a8b	73G 103A 263G 301N 302N 303N 304N 305N 306N 307N 308N 309N 310N 311N 312N 313N 314N 315N 316N 317N 318N 16172C 16183C 16219G 16278T 16295T	1	73 103 263 301N 302N 303N 304N 305N 306N 307N 308N 309N 310N 311N 312N 313N 314N 315N 316N 317N 318N 750 1438 2706 3348 4655 4769 4936 7028 7805 8860 9128 9335A 10172 11467 11719 12308 12372 14179 14766 15326 16172 16219 16278 16295	U6a8b	73 103 263 301N 302N 303N 304N 305N 306N 307N 308N 309N 310N 311N 312N 313N 314N 315N 316N 317N 318N 16172 16219 16278 16295	[0.194] U6a8b (1)	[0.194] U6a8b (2) [0.042] U6a6 (3) [0.000] U6a U6a1 U6a+16189+(103) U6a2 U6a2a U6a2a1 U6a8 U6a5 U6a5a U6a5a1 U6a6b U6a6b1 U6a6b2 U6a7 U6a7a1+@152 U6a7a1c (19)	1
2036	>KY410102.1 Hc	73G 150T 263G 284G 301N 302N 303N 304N 305N 306N 307N 308N 309N 310N 311N 312N 313N 314N 315N 316N 317N 318N 373G 750G 1438G 2706G 3106d 3107C 3197C 4769G 7028T 7226A 7768G 8860G 9477A 11177T 11467G 11719A 11914A 12308G 12372A 13617C 14182C 14766T 15244G 15326G 16093Y 16169A 16192T 16235G 16270T 16519C	U5b3a1a	73G 150T 263G 284G 301N 302N 303N 304N 305N 306N 307N 308N 309N 310N 311N 312N 313N 314N 315N 316N 317N 318N 373G 16093Y 16169A 16192T 16235G 16270T 16519C	1	73 150 263 284 301N 302N 303N 304N 305N 306N 307N 308N 309N 310N 311N 312N 313N 314N 315N 316N 317N 318N 373 750 1438 2706 3197 4769 7028 7226 7768 8860 9477 11177 11467 11719 11914 12308 12372 13617 14182 14766 15244 15326 16093Y 16169A 16192 16235 16270	U5b3a1a	73 150 263 284 301N 302N 303N 304N 305N 306N 307N 308N 309N 310N 311N 312N 313N 314N 315N 316N 317N 318N 373 16093Y 16169A 16192 16235 16270	[0.194] U5b3a1a (1)	[0.000] U5b3a1 U5b3a1b (3)	1
2037	>KY410089.1 Hc	263G 301N 302N 303N 304N 305N 306N 307N 308N 309N 310N 311N 312N 313N 314N 315N 316N 317N 318N 750G 1438G 2706G 3106d 3107C 4580A 4769G 7028T 7551R 7852A 8860G 15326G 15904T 15905C 16298C	V+@72	263G 301N 302N 303N 304N 305N 306N 307N 308N 309N 310N 311N 312N 313N 314N 315N 316N 317N 318N 16298C	1	263 301N 302N 303N 304N 305N 306N 307N 308N 309N 310N 311N 312N 313N 314N 315N 316N 317N 318N 750 1438 2706 4580 4769 7028 7551R 7852 8860 15326 15904 15905 16298	V+@72	263 301N 302N 303N 304N 305N 306N 307N 308N 309N 310N 311N 312N 313N 314N 315N 316N 317N 318N 16298	[0.000] V+@72 (1)	[0.032] V1a (2) [0.021] V1a1 (3) [0.015] V1a1b (4) [0.014] H2a2a (5) [0.005] V2 V2b V2c (8) [0.003] H H35a (10) [0.002] H3u (11) [0.001] H1 H3 (13) [0.000] R0 HV HV0 HV0a V V1 V2a V2a1 V2a1a V2b1 V2b2 V3 V3b V4 V8 V10 V10b V10b1 V10b2 V11 V12 V13 V14 V15 V15a V17 V21 V23 V24 V25 V26 V28 HV0f HV4 HV4c HV5 HV5a HV9c HV12 H1e H1e1 H1e1a H1e1a2 H1e1a7 H1e1c H1e2 H1e2a H1e2b H1e2d H1e4 H1e6 H1e7 H1e8 H1e8a H1h H1h1 H1h2 H1j H1j3 H1j5 H1j6 H1j7 H1j9 H1m H1n H1p H1q H1q1 H1q2 H1r H1s H1s1 H1t H1t1 H1t1a H1t2 H1u H1u1 H1v H1v1 H1v1a H1v1b H1w H1ae H1ae1 H1ae2 H1ae2a H1ae3 H1ae3a H1ag H1ag1 H1ag1b H1ah H1ai H1ai1 H1aj H1ak H1ak1 H1ak2 H1am H1am1 H1ap H1aq H1as H1as2 H1at H1at1 H1at1a H1av H1ax H1ax1 H1ay H1az H1bd H1be H1bi H1bj H1bk H1bm H1bn H1bp H1bq H1br H1bu H1bv H1bw H1bx H1ca H1ci H2 H2a H2a2 H2a2+(16235) H2a4 H2a5 H2a5a H2a5b H3b H3c H3c1 H3e H3q H3q1 H3r H3r1 H3t [illegible]	1

2038	>KY410075.1 Hc	73G 151T 152C 263G 301N 302N 303N 304N 305N 306N 307N 308N 309N 310N 311N 312N 313N 314N 315N 316N 317N 318N 709A 750G 930A 1438G 1888A 2706G 3106d 3107C 4216C 4769G 4917G 5656G 7028T 8697A 8860G 9448G 10463C 10750G 10899G 11251G 11719A 11812G 13368A 14233G 14766T 14905A 15326G 15452A 15607G 15928A 16126C 16292T 16294T 16296T 16304C 16519C	T2b3a	73G 151T 152C 263G 301N 302N 303N 304N 305N 306N 307N 308N 309N 310N 311N 312N 313N 314N 315N 316N 317N 318N 16126C 16292T 16294T 16296T 16304C 16519C	1	73 151 152 263 301N 302N 303N 304N 305N 306N 307N 308N 309N 310N 311N 312N 313N 314N 315N 316N 317N 318N 709 750 930 1438 1888 2706 4216 4769 4917 5656 7028 8697 8860 9448 10463 10750 10899 11251 11719 11812 13368 14233 14766 14905 15326 15452A 15607 15928 16126 16292 16294 16296 16304	T2b3a	73 151 152 263 301N 302N 303N 304N 305N 306N 307N 308N 309N 310N 311N 312N 313N 314N 315N 316N 317N 318N 16126 16292 16294 16296 16304	[0.000] T2b3a (1)	[0.091] T2b3+151 (2) [0.014] T2b+152 (3) [0.012] T2b7a3 (4) [0.000] T2b3c T2b4+152 T2b4c T2b4d T2b4e T2b4f T2b4g T2b21 T2b21b T2b22 (14)	1
2039	>KY410072.1 Hc	57C 59C 73G 151T 152C 263G 301N 302N 303N 304N 305N 306N 307N 308N 309N 310N 311N 312N 313N 314N 315N 316N 317N 318N 709A 750G 930A 1438G 1888A 2706G 3106d 3107C 4216C 4769G 4917G 5147A 5656G 7028T 8697A 8860G 10463C 10750G 11251G 11719A 11812G 12305.1A 13368A 14233G 14751T 14766T 14905A 15326G 15452A 15607G 15928A 16126C 16292T 16294T 16296T 16304C 16519C	T2b3a1	57C 59C 73G 151T 152C 263G 301N 302N 303N 304N 305N 306N 307N 308N 309N 310N 311N 312N 313N 314N 315N 316N 317N 318N 16126C 16292T 16294T 16296T 16304C 16519C	1	57 59 73 151 152 263 301N 302N 303N 304N 305N 306N 307N 308N 309N 310N 311N 312N 313N 314N 315N 316N 317N 318N 709 750 930 1438 1888 2706 4216 4769 4917 5147 5656 7028 8697 8860 10463 10750 11251 11719 11812 12310.1A 13368 14233 14751 14766 14905 15326 15452A 15607 15928 16126 16292 16294 16296 16304	T2b3a1	57 59 73 151 152 263 301N 302N 303N 304N 305N 306N 307N 308N 309N 310N 311N 312N 313N 314N 315N 316N 317N 318N 16126 16292 16294 16296 16304	[0.645] T2b3a1 (1)	[0.645] T2b3a1 (2) [0.179] T2b3a	1
2040	>KY410070.1 Hc	73G 150T 263G 301N 302N 303N 304N 305N 306N 307N 308N 309N 310N 311N 312N 313N 314N 315N 316N 317N 318N 373G 750G 1438G 2706G 3106d 3107C 3197C 4769G 7028T 7226A 7768G 8860G 9477A 11177T 11467G 11719A 12308G 12372A 13617C 14182C 14766T 15326G 16169A 16192T 16235G 16270T 16519C	U5b3a1a	73G 150T 263G 301N 302N 303N 304N 305N 306N 307N 308N 309N 310N 311N 312N 313N 314N 315N 316N 317N 318N 373G 16169A 16192T 16235G 16270T 16519C	1	73 150 263 301N 302N 303N 304N 305N 306N 307N 308N 309N 310N 311N 312N 313N 314N 315N 316N 317N 318N 373 750 1438 2706 3197 4769 7028 7226 7768 8860 9477 11177 11467 11719 12308 12372 13617 14182 14766 15326 16169A 16192 16235 16270	U5b3a1a	73 150 263 301N 302N 303N 304N 305N 306N 307N 308N 309N 310N 311N 312N 313N 314N 315N 316N 317N 318N 373 16169A 16192 16235 16270	[0.000] U5b3a1a (1)	[0.000] U5b3a1 U5b3a1b (3)	1
2041	>KY410062.1 Hc	73G 150T 263G 301N 302N 303N 304N 305N 306N 307N 308N 309N 310N 311N 312N 313N 314N 315N 316N 317N 318N 373G 750G 1438G 2706G 3106d 3107C 3197C 4769G 7028T 7226A 7768G 8860G 9477A 11177T 11467G 11719A 12308G 12372A 13617C 14182C 14766T 15326G 16169A 16192T 16235G 16270T 16519C	U5b3a1a	73G 150T 263G 301N 302N 303N 304N 305N 306N 307N 308N 309N 310N 311N 312N 313N 314N 315N 316N 317N 318N 373G 16169A 16192T 16235G 16270T 16519C	1	73 150 263 301N 302N 303N 304N 305N 306N 307N 308N 309N 310N 311N 312N 313N 314N 315N 316N 317N 318N 373 750 1438 2706 3197 4769 7028 7226 7768 8860 9477 11177 11467 11719 12308 12372 13617 14182 14766 15326 16169A 16192 16235 16270	U5b3a1a	73 150 263 301N 302N 303N 304N 305N 306N 307N 308N 309N 310N 311N 312N 313N 314N 315N 316N 317N 318N 373 16169A 16192 16235 16270	[0.000] U5b3a1a (1)	[0.000] U5b3a1 U5b3a1b (3)	1

2042	>KY410061.1 Hc	73G 151T 152C 263G 301N 302N 303N 304N 305N 306N 307N 308N 309N 310N 311N 312N 313N 314N 315N 316N 317N 318N 709A 750G 930A 1438G 1888A 2706G 3106d 3107C 4216C 4562G 4769G 4917G 5147A 5656G 7028T 8602C 8697A 8860G 9752T 10463C 10750G 11251G 11719A 11812G 13368A 14233G 14766T 14905A 15326G 15452A 15607G 15928A 16126C 16292T 16294T 16296T 16304C 16519C	T2b3a	73G 151T 152C 263G 301N 302N 303N 304N 305N 306N 307N 308N 309N 310N 311N 312N 313N 314N 315N 316N 317N 318N 16126C 16292T 16294T 16296T 16304C 16519C	1	73 151 152 263 301N 302N 303N 304N 305N 306N 307N 308N 309N 310N 311N 312N 313N 314N 315N 316N 317N 318N 709 750 930 1438 1888 2706 4216 4562 4769 4917 5147 5656 7028 8602 8697 8860 9752 10463 10750 11251 11719 11812 13368 14233 14766 14905 15326 15452A 15607 15928 16126 16292 16294 16296 16304	T2b3a	73 151 152 263 301N 302N 303N 304N 305N 306N 307N 308N 309N 310N 311N 312N 313N 314N 315N 316N 317N 318N 16126 16292 16294 16296 16304	[0.000] T2b3a (1)	[0.091] T2b3+151 (2) [0.014] T2b+152 (3) [0.012] T2b7a3 (4) [0.000] T2b3c T2b4+152 T2b4c T2b4d T2b4e T2b4f T2b4g T2b21 T2b21b T2b22 (14)	1
2043	>KY408220.1 Hc	263G 301N 302N 303N 304N 305N 306N 307N 308N 309N 310N 311N 312N 313N 314N 315N 316N 317N 318N 514d 515d 750G 1438G 3106d 3107C 4769G 4793G 6296A 8860G 15326G 16093C 16265G 16519C	H7c1	263G 301N 302N 303N 304N 305N 306N 307N 308N 309N 310N 311N 312N 313N 314N 315N 316N 317N 318N 514d 515d 16093C 16265G 16519C	1	263 301N 302N 303N 304N 305N 306N 307N 308N 309N 310N 311N 312N 313N 314N 315N 316N 317N 318N 750 1438 4769 4793 6296A 8860 15326 16093 16265	H7c1	263 301N 302N 303N 304N 305N 306N 307N 308N 309N 310N 311N 312N 313N 314N 315N 316N 317N 318N 16093 16265	[0.267] H7c1 (1) [0.000] H10+(16093) H10f (3)	[0.158] H7c3 (4) [0.157] H13a1a1c (5) [0.156] H7c (6) [0.155] H44a (7) [0.031] H10e1 (8) [0.014] H3v+16093 (9) [0.011] H H10e3a (11) [0.010] H1e1a (12) [0.008] H1 (13) [0.005] H2a2a H60a (15) [0.003] H3h2 H63 H72 (18) [0.002] H1e H56 (20) [0.001] H1h1 H1j H1q H1bi H1br H3 H4 H4a1a H7a H7b1 H10f H13a2b2 H14b H14b2 H67 (35) [0.000] R0 HV HV4 HV4c HV5 HV5a HV9c HV12 H1f+16093 H1f1 H1e1 H1e1a2 H1e1a7 H1e1c H1e2 H1e2a H1e2b H1e2d H1e4 H1e6 H1e7 H1e8 H1e8a H1h H1h2 H1j3 H1j5 H1j6 H1j7 H1j9 H1m H1n H1p H1q1 H1q2 H1r H1s H1s1 H1t H1t1 H1t1a H1t2 H1u H1u1 H1v H1v1 H1v1a H1v1b H1w H1ae H1ae1 H1ae2 H1ae2a H1ae3 H1ae3a H1ag H1ag1 H1ag1b H1ah H1ai H1ai1 H1aj H1ak H1ak1 H1ak2 H1am H1am1 H1ap H1aq H1as H1as2 H1at H1at1 H1at1a H1av H1ax H1ax1 H1ay H1az H1bd H1be H1bj H1bk H1bm H1bn H1bp H1bq H1bu H1bv H1bw H1bx H1ca H1ci H2 H2a H2a2 H2a2+(16235) H2a4 H2a5 H2a5a H2a5b H3b H3c H3c1 [illegible]	1

2044	>KY408219.1 Hc	57C 59C 151T 152C 263G 301N 302N 303N 304N 305N 306N 307N 308N 309N 310N 311N 312N 313N 314N 315N 316N 317N 318N 709A 750G 930A 1438G 1888A 2706G 3106d 3107C 4216C 4769G 4917G 5147A 5656G 7028T 8697A 8860G 10463C 10750G 11251G 11719A 11812G 12674R 13368A 14233G 14766T 14905A 15326G 15452A 15607G 15928A 16126C 16292T 16294T 16296T 16304C 16519C	T2b3a1	57C 59C 151T 152C 263G 301N 302N 303N 304N 305N 306N 307N 308N 309N 310N 311N 312N 313N 314N 315N 316N 317N 318N 16126C 16292T 16294T 16296T 16304C 16519C	1	57 59 151 152 263 301N 302N 303N 304N 305N 306N 307N 308N 309N 310N 311N 312N 313N 314N 315N 316N 317N 318N 709 750 930 1438 1888 2706 4216 4769 4917 5147 5656 7028 8697 8860 10463 10750 11251 11719 11812 12674R 13368 14233 14766 14905 15326 15452A 15607 15928 16126 16292 16294 16296 16304	T2b3a1	57 59 151 152 263 301N 302N 303N 304N 305N 306N 307N 308N 309N 310N 311N 312N 313N 314N 315N 316N 317N 318N 16126 16292 16294 16296 16304	[0.758] T2b3a1 (1)	[0.758] T2b3a1 (2) [0.179] T2b3a	1
2045	>KY408200.1 Hc	263G 301N 302N 303N 304N 305N 306N 307N 308N 309N 310N 311N 312N 313N 314N 315N 316N 317N 318N 373G 456T 750G 1438G 3106d 3107C 4769G 8860G 15326G 15646T 16274A 16304C 16327T	H5n	263G 301N 302N 303N 304N 305N 306N 307N 308N 309N 310N 311N 312N 313N 314N 315N 316N 317N 318N 373G 456T 16274A 16304C 16327T	1	263 301N 302N 303N 304N 305N 306N 307N 308N 309N 310N 311N 312N 313N 314N 315N 316N 317N 318N 373 456 750 1438 4769 8860 15326 15646 16274 16304 16327	H5n	263 301N 302N 303N 304N 305N 306N 307N 308N 309N 310N 311N 312N 313N 314N 315N 316N 317N 318N 373 456 16274 16304 16327	[0.050] H5n (1)	[0.006] H5b (2) [0.005] H5 (3) [0.000] H5a H5a1 H5a1a H5a1b H5a1c H5a1c1 H5a1c1a H5a1d H5a1f H5a1h H5a1k H5a1q H5a2 H5a2a H5a7 H5a8 H5a9 H5b3 H5b5 H5c H5c1 H5c1a H5d H5f H5j H5k H5p H5+709 H5v (32)	1
2046	>KY408196.1 Hc	151T 152C 195C 198T 263G 301N 302N 303N 304N 305N 306N 307N 308N 309N 310N 311N 312N 313N 314N 315N 316N 317N 318N 750G 1438G 2706G 3106d 3107C 4769G 5250C 7028T 8014T 8860G 10044G 12696C 15218G 15326G 16067T	HV1b3	151T 152C 195C 198T 263G 301N 302N 303N 304N 305N 306N 307N 308N 309N 310N 311N 312N 313N 314N 315N 316N 317N 318N 16067T	1	151 152 195 198 263 301N 302N 303N 304N 305N 306N 307N 308N 309N 310N 311N 312N 313N 314N 315N 316N 317N 318N 750 1438 2706 4769 5250 7028 8014T 8860 10044 12696 15218 15326 16067	HV1b3	151 152 195 198 263 301N 302N 303N 304N 305N 306N 307N 308N 309N 310N 311N 312N 313N 314N 315N 316N 317N 318N 16067	[0.467] HV1b3 (1) [0.000] HV1b3a (2)	[0.000] HV1b+152 HV1b3b (4)	1
2047	>KY408194.1 Hc	151T 152C 195C 198T 263G 301N 302N 303N 304N 305N 306N 307N 308N 309N 310N 311N 312N 313N 314N 315N 316N 317N 318N 750G 1438G 2706G 3106d 3107C 4769G 5250C 7028T 8014T 8860G 10044G 12696C 15218G 15326G 16067T	HV1b3	151T 152C 195C 198T 263G 301N 302N 303N 304N 305N 306N 307N 308N 309N 310N 311N 312N 313N 314N 315N 316N 317N 318N 16067T	1	151 152 195 198 263 301N 302N 303N 304N 305N 306N 307N 308N 309N 310N 311N 312N 313N 314N 315N 316N 317N 318N 750 1438 2706 4769 5250 7028 8014T 8860 10044 12696 15218 15326 16067	HV1b3	151 152 195 198 263 301N 302N 303N 304N 305N 306N 307N 308N 309N 310N 311N 312N 313N 314N 315N 316N 317N 318N 16067	[0.467] HV1b3 (1) [0.000] HV1b3a (2)	[0.000] HV1b+152 HV1b3b (4)	1
2048	>KY408191.1 Hc	57C 59C 73G 151T 152C 263G 301N 302N 303N 304N 305N 306N 307N 308N 309N 310N 311N 312N 313N 314N 315N 316N 317N 318N 709A 750G 930A 1438G 1888A 2706G 3106d 3107C 4216C 4769G 4917G 5147A 5656G 7028T 8697A 8860G 10463C 10750G 11251G 11719A 11812G 13368A 14233G 14766T 14905A 15326G 15452A 15607G 15928A 16126C 16288Y 16292T 16294T 16296T 16304C 16519C	T2b3a1	57C 59C 73G 151T 152C 263G 301N 302N 303N 304N 305N 306N 307N 308N 309N 310N 311N 312N 313N 314N 315N 316N 317N 318N 16126C 16288Y 16292T 16294T 16296T 16304C 16519C	1	57 59 73 151 152 263 301N 302N 303N 304N 305N 306N 307N 308N 309N 310N 311N 312N 313N 314N 315N 316N 317N 318N 709 750 930 1438 1888 2706 4216 4769 4917 5147 5656 7028 8697 8860 10463 10750 11251 11719 11812 13368 14233 14766 14905 15326 15452A 15607 15928 16126 16288Y 16292 16294 16296 16304	T2b3a1	57 59 73 151 152 263 301N 302N 303N 304N 305N 306N 307N 308N 309N 310N 311N 312N 313N 314N 315N 316N 317N 318N 16126 16288Y 16292 16294 16296 16304	[0.822] T2b3a1 (1)	[0.822] T2b3a1 (2) [0.286] T2b3a	1

2049	>KY408190.1 H[illegible]	73G 152C 263G 301N 302N 303N 304N 305N 306N 307N 308N 309N 310N 311N 312N 313N 314N 315N 316N 317N 318N 709A 750G 1438G 1888A 2706G 3106d 3107C 3397G 4216C 4769G 4917G 7001G 7028T 8393T 8697A 8860G 9266A 10463C 11251G 11719A 12633A 13368A 14287C 14766T 14905A 15326G 15452A 15607G 15928A 16126C 16163G 16169T 16186T 16189C 16263C 16294T 16519C	T1a4	73G 152C 263G 301N 302N 303N 304N 305N 306N 307N 308N 309N 310N 311N 312N 313N 314N 315N 316N 317N 318N 16126C 16163G 16169T 16186T 16189C 16263C 16294T 16519C	1	73 152 263 301N 302N 303N 304N 305N 306N 307N 308N 309N 310N 311N 312N 313N 314N 315N 316N 317N 318N 709 750 1438 1888 2706 3397 4216 4769 4917 7001 7028 8393 8697 8860 9266 10463 11251 11719 12633A 13368 14287 14766 14905 15326 15452A 15607 15928 16126 16163 16169 16186 16189 16263 16294	T1a4	73 152 263 301N 302N 303N 304N 305N 306N 307N 308N 309N 310N 311N 312N 313N 314N 315N 316N 317N 318N 16126 16163 16169 16186 16189 16263 16294	[0.208] T1a4 (1)	[0.016] T1a2 (2) [0.000] T1a+152	1
2050	>KY408183.1 H[illegible]	57C 59C 64Y 73G 151T 152C 263G 301N 302N 303N 304N 305N 306N 307N 308N 309N 310N 311N 312N 313N 314N 315N 316N 317N 318N 709A 750G 930A 1438G 1888A 2706G 3106d 3107C 4216C 4769G 4917G 5147A 5656G 7028T 8697A 8860G 10463C 10750G 11251G 11719A 11812G 13368A 14233G 14766T 14905A 15326G 15452A 15607G 15928A 16126C 16292T 16294T 16296T 16304C 16519C	T2b3a1	57C 59C 64Y 73G 151T 152C 263G 301N 302N 303N 304N 305N 306N 307N 308N 309N 310N 311N 312N 313N 314N 315N 316N 317N 318N 16126C 16292T 16294T 16296T 16304C 16519C	1	57 59 64Y 73 151 152 263 301N 302N 303N 304N 305N 306N 307N 308N 309N 310N 311N 312N 313N 314N 315N 316N 317N 318N 709 750 930 1438 1888 2706 4216 4769 4917 5147 5656 7028 8697 8860 10463 10750 11251 11719 11812 13368 14233 14766 14905 15326 15452A 15607 15928 16126 16292 16294 16296 16304	T2b3a1	57 59 64Y 73 151 152 263 301N 302N 303N 304N 305N 306N 307N 308N 309N 310N 311N 312N 313N 314N 315N 316N 317N 318N 16126 16292 16294 16296 16304	[0.661] T2b3a1 (1)	[0.661] T2b3a1 (2) [0.179] T2b3a	1
2051	>KY408180.1 H[illegible]	57C 59C 73G 152C 263G 301N 302N 303N 304N 305N 306N 307N 308N 309N 310N 311N 312N 313N 314N 315N 316N 317N 318N 709A 750G 930A 1438G 1888A 2706G 3106d 3107C 4216C 4769G 4917G 5147A 5656G 7028T 8697A 8860G 10463C 10750G 11251G 11719A 11812G 13368A 14233G 14766T 14905A 15326G 15452A 15607G 15928A 16126C 16292T 16294T 16296T 16304C 16519C	T2b3a1	57C 59C 73G 152C 263G 301N 302N 303N 304N 305N 306N 307N 308N 309N 310N 311N 312N 313N 314N 315N 316N 317N 318N 16126C 16292T 16294T 16296T 16304C 16519C	1	57 59 73 152 263 301N 302N 303N 304N 305N 306N 307N 308N 309N 310N 311N 312N 313N 314N 315N 316N 317N 318N 709 750 930 1438 1888 2706 4216 4769 4917 5147 5656 7028 8697 8860 10463 10750 11251 11719 11812 13368 14233 14766 14905 15326 15452A 15607 15928 16126 16292 16294 16296 16304	T2b3a1	57 59 73 152 263 301N 302N 303N 304N 305N 306N 307N 308N 309N 310N 311N 312N 313N 314N 315N 316N 317N 318N 16126 16292 16294 16296 16304	[0.693] T2b3a1 (1)	[0.693] T2b3a1 (2) [0.179] T2b3a (3) [0.014] T2b+152 (4) [0.012] T2b7a3 (5) [0.000] T2b4+152 T2b4c T2b4d T2b4e T2b4f T2b4g T2b21 T2b21b T2b22 (14)	1
2052	>KY408178.1 H[illegible]	57C 59C 73G 151T 152C 263G 301N 302N 303N 304N 305N 306N 307N 308N 309N 310N 311N 312N 313N 314N 315N 316N 317N 318N 709A 750G 930A 1438G 1888A 2706G 2772T 3106d 3107C 4216C 4769G 4917G 5147A 5656G 7028T 8697A 8860G 10463C 10750G 11251G 11719A 11812G 11914A 14233G 14766T 14905A 15326G 15452A 15607G 15928A 16126C 16292T 16294T 16296T 16304C 16519C	T2b3a1	57C 59C 73G 151T 152C 263G 301N 302N 303N 304N 305N 306N 307N 308N 309N 310N 311N 312N 313N 314N 315N 316N 317N 318N 16126C 16292T 16294T 16296T 16304C 16519C	1	57 59 73 151 152 263 301N 302N 303N 304N 305N 306N 307N 308N 309N 310N 311N 312N 313N 314N 315N 316N 317N 318N 709 750 930 1438 1888 2706 2772 4216 4769 4917 5147 5656 7028 8697 8860 10463 10750 11251 11719 11812 11914 14233 14766 14905 15326 15452A 15607 15928 16126 16292 16294 16296 16304	T2b3a1	57 59 73 151 152 263 301N 302N 303N 304N 305N 306N 307N 308N 309N 310N 311N 312N 313N 314N 315N 316N 317N 318N 16126 16292 16294 16296 16304	[0.645] T2b3a1 (1)	[0.645] T2b3a1 (2) [0.179] T2b3a	1

2053	>KY408161.1 Hc	57C 59C 73G 151T 152C 263G 301N 302N 303N 304N 305N 306N 307N 308N 309N 310N 311N 312N 313N 314N 315N 316N 317N 318N 709A 750G 930A 1438G 1888A 2706G 3106d 3107C 4216C 4769G 4917G 5147A 5656G 7028T 8697A 8860G 10463C 10750G 11251G 11719A 11812G 13368A 14233G 14766T 14905A 15326G 15452A 15607G 15928A 16126C 16292T 16294T 16296T 16304C 16519C	T2b3a1	57C 59C 73G 151T 152C 263G 301N 302N 303N 304N 305N 306N 307N 308N 309N 310N 311N 312N 313N 314N 315N 316N 317N 318N 16126C 16292T 16294T 16296T 16304C 16519C	1	57 59 73 151 152 263 301N 302N 303N 304N 305N 306N 307N 308N 309N 310N 311N 312N 313N 314N 315N 316N 317N 318N 709 750 930 1438 1888 2706 4216 4769 4917 5147 5656 7028 8697 8860 10463 10750 11251 11719 11812 13368 14233 14766 14905 15326 15452A 15607 15928 16126 16292 16294 16296 16304	T2b3a1	57 59 73 151 152 263 301N 302N 303N 304N 305N 306N 307N 308N 309N 310N 311N 312N 313N 314N 315N 316N 317N 318N 16126 16292 16294 16296 16304	[0.645] T2b3a1 (1)	[0.645] T2b3a1 (2) [0.179] T2b3a	1
2054	>KY408157.1 Hc	73G 185A 189G 228A 234R 263G 295T 301N 302N 303N 304N 305N 306N 307N 308N 309N 310N 311N 312N 313N 314N 315N 316N 317N 318N 462T 489C 750G 1438G 2706G 3010A 3106d 3107C 4216C 4769G 5460A 7028T 8860G 10398G 11251G 11719A 12612G 13708A 13934T 14287C 14766T 14798C 15326G 15452A 16069T 16126C 16172Y	J1c3+189	73G 185A 189G 228A 234R 263G 295T 301N 302N 303N 304N 305N 306N 307N 308N 309N 310N 311N 312N 313N 314N 315N 316N 317N 318N 462T 489C 16069T 16126C 16172Y	1	73 185 189 228 234R 263 295 301N 302N 303N 304N 305N 306N 307N 308N 309N 310N 311N 312N 313N 314N 315N 316N 317N 318N 462 489 750 1438 2706 3010 4216 4769 5460 7028 8860 10398 11251 11719 12612 13708 13934 14287 14766 14798 15326 15452A 16069 16126 16172Y	J1c3+189	73 185 189 228 234R 263 295 301N 302N 303N 304N 305N 306N 307N 308N 309N 310N 311N 312N 313N 314N 315N 316N 317N 318N 462 489 16069 16126 16172Y	[0.014] J1c3+189 (1)	[0.021] J1c (2) [0.017] J1c5a (3) [0.014] J1c3+189 (4) [0.011] J1c5 (5) [0.009] J1c3g J1c6a J1c15 (8) [0.006] J1c3 (9) [0.005] J1c3b2 J1c5b (11) [0.004] J1c3h J1c4 J1c5a1 (14) [0.000] J1c3a J1c3a1 J1c3a2 J1c3b J1c3b1 J1c3b1a J1c3d J1c3i J1c3k J1c4b J1c5c J1c5d J1c5e J1c5f J1c6 J1c+16261+189 J1c12 J1c12a J1c12b J1c8 J1c8b J1c9 J1c10 J1c10a J1c15a J1c15a1 J1c17 (41)	1
2055	>KY408153.1 Hc	73G 151T 152C 263G 301N 302N 303N 304N 305N 306N 307N 308N 309N 310N 311N 312N 313N 314N 315N 316N 317N 318N 709A 750G 930A 1438G 1888A 2706G 3106d 3107C 4216C 4769G 4917G 5147A 5656G 7028T 8697A 8860G 10463C 10750G 11251G 11719A 11812G 13368A 14233G 14766T 14905A 15326G 15452A 15607G 15928A 16093C 16126C 16292T 16294T 16296T 16304C 16519C	T2b3a	73G 151T 152C 263G 301N 302N 303N 304N 305N 306N 307N 308N 309N 310N 311N 312N 313N 314N 315N 316N 317N 318N 16093C 16126C 16292T 16294T 16296T 16304C 16519C	1	73 151 152 263 301N 302N 303N 304N 305N 306N 307N 308N 309N 310N 311N 312N 313N 314N 315N 316N 317N 318N 709 750 930 1438 1888 2706 4216 4769 4917 5147 5656 7028 8697 8860 10463 10750 11251 11719 11812 13368 14233 14766 14905 15326 15452A 15607 15928 16093 16126 16292 16294 16296 16304	T2b3a	73 151 152 263 301N 302N 303N 304N 305N 306N 307N 308N 309N 310N 311N 312N 313N 314N 315N 316N 317N 318N 16093 16126 16292 16294 16296 16304	[0.179] T2b3a (1)	[0.106] T2b3+151 (2) [0.021] T2b+152 (3) [0.012] T2b7a3 (4) [0.000] T2b3c T2b4+152 T2b4c T2b4d T2b4e T2b4f T2b4g T2b21 T2b21b T2b22 (14)	1
2056	>KY408147.1 Hc	73G 151T 152C 263G 301N 302N 303N 304N 305N 306N 307N 308N 309N 310N 311N 312N 313N 314N 315N 316N 317N 318N 709A 750G 930A 1438G 1888A 2706G 3106d 3107C 4216C 4769G 4917G 5147A 5656G 7028T 7718G 8697A 8860G 10463C 10750G 11251G 11719A 11812G 13368A 14233G 14766T 14905A 15326G 15452A 15607G 15928A 16126C 16292T 16294T 16296T 16304C 16519C	T2b3a	73G 151T 152C 263G 301N 302N 303N 304N 305N 306N 307N 308N 309N 310N 311N 312N 313N 314N 315N 316N 317N 318N 16126C 16292T 16294T 16296T 16304C 16519C	1	73 151 152 263 301N 302N 303N 304N 305N 306N 307N 308N 309N 310N 311N 312N 313N 314N 315N 316N 317N 318N 709 750 930 1438 1888 2706 4216 4769 4917 5147 5656 7028 7718 8697 8860 10463 10750 11251 11719 11812 13368 14233 14766 14905 15326 15452A 15607 15928 16126 16292 16294 16296 16304	T2b3a	73 151 152 263 301N 302N 303N 304N 305N 306N 307N 308N 309N 310N 311N 312N 313N 314N 315N 316N 317N 318N 16126 16292 16294 16296 16304	[0.000] T2b3a (1)	[0.091] T2b3+151 (2) [0.014] T2b+152 (3) [0.012] T2b7a3 (4) [0.000] T2b3c T2b4+152 T2b4c T2b4d T2b4e T2b4f T2b4g T2b21 T2b21b T2b22 (14)	1

2057	>KY662469.1 Hc	73G 150T 263G 309.1CCT 310C 750G 1303A 1438G 2706G 3106d 3107C 3192T 3197C 3591A 4592C 4769G 7028T 8860G 9477A 11296T 11467G 11719A 11938T 12308G 12372A 12618A 13617C 14766T 14793G 15218G 15326G 16192T 16239T 16256T 16270T 16399G	U5a1h	73G 150T 263G 309.1CCT 310C 16192T 16239T 16256T 16270T 16399G	1	73 150 263 750 1303 1438 2706 3192 3197 3591 4592 4769 7028 8860 9477 11296 11467 11719 11938 12308 12372 12618 13617 14766 14793 15218 15326 16192 16239 16256 16270 16399	U5a1h	73 150 263 16192 16239 16256 16270 16399	[0.000] U5a1h (1)	[0.000] U5a1 U5a1b U5a1b2 U5a1d U5a1d1 U5a1e U5a1f U5a1i U5a1i1 U5a1j U5b1d2 (12)	1
2058	>KY410051.1 Hc	73G 263G 301N 302N 303N 304N 305N 306N 307N 308N 309N 310N 311N 312N 313N 314N 315N 316N 317N 318N 750G 1438G 2158C 2706G 3106d 3107C 3348G 4769G 6227C 7028T 7805A 8860G 10336C 11176A 11467G 11719A 12308G 12372A 12376G 12727C 14034C 14179G 14766T 14927G 15244G 15326G 16145A 16172C 16219G 16235G 16278T 16519C	U6a1b1b	73G 263G 301N 302N 303N 304N 305N 306N 307N 308N 309N 310N 311N 312N 313N 314N 315N 316N 317N 318N 16145A 16172C 16219G 16235G 16278T 16519C	1	73 263 301N 302N 303N 304N 305N 306N 307N 308N 309N 310N 311N 312N 313N 314N 315N 316N 317N 318N 750 1438 2158 2706 3348 4769 6227 7028 7805 8860 10336 11176 11467 11719 12308 12372 12376G 12727 14034 14179 14766 14927 15244 15326 16145 16172 16219 16235 16278	U6a1b1b	73 263 301N 302N 303N 304N 305N 306N 307N 308N 309N 310N 311N 312N 313N 314N 315N 316N 317N 318N 16145 16172 16219 16235 16278	[0.000] U6a1b1b (1)	[0.000] U6a1b U6a1b1 U6a1b3 (4)	1
2059	>KY410042.1 Hc	73G 146Y 150T 263G 301N 302N 303N 304N 305N 306N 307N 308N 309N 310N 311N 312N 313N 314N 315N 316N 317N 318N 373G 750G 1438G 2706G 3106d 3107C 3197C 4769G 7028T 7226A 7768G 8860G 9477A 10398G 11177T 11467G 11719A 12308G 12372A 13617C 14182C 14766T 15326G 16169A 16192T 16235G 16270T 16519C	U5b3a1a	73G 146Y 150T 263G 301N 302N 303N 304N 305N 306N 307N 308N 309N 310N 311N 312N 313N 314N 315N 316N 317N 318N 373G 16169A 16192T 16235G 16270T 16519C	1	73 146Y 150 263 301N 302N 303N 304N 305N 306N 307N 308N 309N 310N 311N 312N 313N 314N 315N 316N 317N 318N 373 750 1438 2706 3197 4769 7028 7226 7768 8860 9477 10398 11177 11467 11719 12308 12372 13617 14182 14766 15326 16169A 16192 16235 16270	U5b3a1a	73 146Y 150 263 301N 302N 303N 304N 305N 306N 307N 308N 309N 310N 311N 312N 313N 314N 315N 316N 317N 318N 373 16169A 16192 16235 16270	[0.020] U5b3a1a (1)	[0.000] U5b3a1 U5b3a1b (3)	1
2060	>KY410033.1 Hc	73G 94A 263G 301N 302N 303N 304N 305N 306N 307N 308N 309N 310N 311N 312N 313N 314N 315N 316N 317N 318N 750G 1189C 1438G 1811G 2706G 3106d 3107C 3337A 3480G 4769G 5913A 7028T 8860G 9055A 9698C 9962A 10223T 10289G 10398G 10550G 11299C 11467G 11719A 11914A 12308G 12372A 14167T 14766T 14798C 15326G 15946T 16224C 16240T 16311C 16519C	K1b1c	73G 94A 263G 301N 302N 303N 304N 305N 306N 307N 308N 309N 310N 311N 312N 313N 314N 315N 316N 317N 318N 16224C 16240T 16311C 16519C	1	73 94 263 301N 302N 303N 304N 305N 306N 307N 308N 309N 310N 311N 312N 313N 314N 315N 316N 317N 318N 750 1189 1438 1811 2706 3337 3480 4769 5913 7028 8860 9055 9698 9962 10223 10289 10398 10550 11299 11467 11719 11914 12308 12372 14167 14766 14798 15326 15946 16224 16240T 16311	K1b1c	73 94 263 301N 302N 303N 304N 305N 306N 307N 308N 309N 310N 311N 312N 313N 314N 315N 316N 317N 318N 16224 16240T 16311	[0.031] K1b1c (1)	[0.000] K K1 K1b K1b1 K1b1+(16093) K1b1b K1b1b1 (8)	1

2061	>KY410030.1 Hc	57C 59C 73R 151T 152C 263G 301N 302N 303N 304N 305N 306N 307N 308N 309N 310N 311N 312N 313N 314N 315N 316N 317N 318N 709A 750G 930A 1438G 1888A 2706G 3106d 3107C 4216C 4227G 4769G 4917G 5147A 5656G 7028T 8697A 8860G 10463C 10750G 11251G 11719A 11812G 13368A 14233G 14766T 14905A 15326G 15452A 15607G 15928A 16126C 16150T 16288C 16292T 16294T 16296T 16304C 16465T 16519C	T2b3a1	57C 59C 73R 151T 152C 263G 301N 302N 303N 304N 305N 306N 307N 308N 309N 310N 311N 312N 313N 314N 315N 316N 317N 318N 16126C 16150T 16288C 16292T 16294T 16296T 16304C 16465T 16519C	1	57 59 73R 151 152 263 301N 302N 303N 304N 305N 306N 307N 308N 309N 310N 311N 312N 313N 314N 315N 316N 317N 318N 709 750 930 1438 1888 2706 4216 4227 4769 4917 5147 5656 7028 8697 8860 10463 10750 11251 11719 11812 13368 14233 14766 14905 15326 15452A 15607 15928 16126 16150 16288 16292 16294 16296 16304 16465	T2b3a1	57 59 73R 151 152 263 301N 302N 303N 304N 305N 306N 307N 308N 309N 310N 311N 312N 313N 314N 315N 316N 317N 318N 16126 16150 16288 16292 16294 16296 16304 16465	[0.999] T2b3a1 (1)	[0.999] T2b3a1 (2) [0.393] T2b3a	1
2062	>KY410029.1 Hc	73G 195C 263G 285T 301N 302N 303N 304N 305N 306N 307N 308N 309N 310N 311N 312N 313N 314N 315N 316N 317N 318N 663G 750G 1438G 2218T 2706G 3106d 3107C 4769G 4991A 6026A 7028T 7403G 7581C 8860G 11116C 11467G 11719A 12308G 12372A 12879C 13104G 13656C 14070G 14364A 14766T 15115C 15148A 15217A 15326G 15954C 16145A 16182C 16183C 16189C 16249C 16519C	U1a1c1c1	73G 195C 263G 285T 301N 302N 303N 304N 305N 306N 307N 308N 309N 310N 311N 312N 313N 314N 315N 316N 317N 318N 16145A 16182C 16183C 16189C 16249C 16519C	1	73 195 263 285 301N 302N 303N 304N 305N 306N 307N 308N 309N 310N 311N 312N 313N 314N 315N 316N 317N 318N 663 750 1438 2218 2706 4769 4991 6026 7028 7403 7581 8860 11116 11467 11719 12308 12372 12879 13104 13656 14070 14364 14766 15115 15148 15217 15326 15954C 16145 16189 16249	U1a1c1c1	73 195 263 285 301N 302N 303N 304N 305N 306N 307N 308N 309N 310N 311N 312N 313N 314N 315N 316N 317N 318N 16145 16189 16249	[0.000] U1a1c1c1 (1)	[0.049] U1a1c1d (2) [0.000] U1a U1a1 U1a1c U1a1c1 U1a1c1b U1a1c1c (8)	1
2063	>KY410024.1 Hc	185A 189G 263G 301N 302N 303N 304N 305N 306N 307N 308N 309N 310N 311N 312N 313N 314N 315N 316N 317N 318N 709A 750G 1438G 3106d 3107C 4769G 8860G 11854C 13269G 14040A 15326G 16189C 16519C	H96	185A 189G 263G 301N 302N 303N 304N 305N 306N 307N 308N 309N 310N 311N 312N 313N 314N 315N 316N 317N 318N 16189C 16519C	1	185 189 263 301N 302N 303N 304N 305N 306N 307N 308N 309N 310N 311N 312N 313N 314N 315N 316N 317N 318N 709 750 1438 4769 8860 11854 13269 14040 15326 16189	H96	185 189 263 301N 302N 303N 304N 305N 306N 307N 308N 309N 310N 311N 312N 313N 314N 315N 316N 317N 318N 16189	[0.000] H96 (1)	[0.018] H1g H1aa (3) [0.005] HV18 (4) [0.003] H1+16189 (5) [0.000] H1f H1g1 H1g2 H1y H1aa1 H1ab H1ac H1ad H1j9 H3+16189 R8a2 R11 R11a R11b (19)	1
2064	>KY410021.1 Hc	73G 150T 263G 301N 302N 303N 304N 305N 306N 307N 308N 309N 310N 311N 312N 313N 314N 315N 316N 317N 318N 373G 750G 1438G 2706G 3106d 3107C 3197C 4769G 7028T 7226A 7768G 8860G 9477A 9839T 11177T 11467G 11647T 11719A 12308G 12372A 13617C 14182C 14766T 15326G 16169A 16192T 16235G 16270T 16519C	U5b3a1a	73G 150T 263G 301N 302N 303N 304N 305N 306N 307N 308N 309N 310N 311N 312N 313N 314N 315N 316N 317N 318N 373G 16169A 16192T 16235G 16270T 16519C	1	73 150 263 301N 302N 303N 304N 305N 306N 307N 308N 309N 310N 311N 312N 313N 314N 315N 316N 317N 318N 373 750 1438 2706 3197 4769 7028 7226 7768 8860 9477 9839 11177 11467 11647 11719 12308 12372 13617 14182 14766 15326 16169A 16192 16235 16270	U5b3a1a	73 150 263 301N 302N 303N 304N 305N 306N 307N 308N 309N 310N 311N 312N 313N 314N 315N 316N 317N 318N 373 16169A 16192 16235 16270	[0.000] U5b3a1a (1)	[0.000] U5b3a1 U5b3a1b (3)	1

2065 >KY410009.1 Hc	57C 59C 73G 151T 152C 263G 301N 302N 303N 304N 305N 306N 307N 308N 309N 310N 311N 312N 313N 314N 315N 316N 317N 318N 709A 750G 930A 1438G 1888A 2706G 3106d 3107C 4216C 4769G 4917G 5147A 5656G 7028T 8697A 8860G 10463C 10750G 11251G 11719A 11812G 13368A 14233G 14766T 14905A 15326G 15452A 15607G 15928A 16126C 16183C 16189C 16292T 16294T 16304C 16362C 16519C	T2b3a1	57C 59C 73G 151T 152C 263G 301N 302N 303N 304N 305N 306N 307N 308N 309N 310N 311N 312N 313N 314N 315N 316N 317N 318N 16126C 16183C 16189C 16292T 16294T 16304C 16362C 16519C	1	57 59 73 151 152 263 301N 302N 303N 304N 305N 306N 307N 308N 309N 310N 311N 312N 313N 314N 315N 316N 317N 318N 709 750 930 1438 1888 2706 4216 4769 4917 5147 5656 7028 8697 8860 10463 10750 11251 11719 11812 13368 14233 14766 14905 15326 15452A 15607 15928 16126 16189 16292 16294 16304 16362	T2b3a1	57 59 73 151 152 263 301N 302N 303N 304N 305N 306N 307N 308N 309N 310N 311N 312N 313N 314N 315N 316N 317N 318N 16126 16189 16292 16294 16304 16362	[0.790] T2b3a1 (1)	[0.790] T2b3a1 (2) [0.179] T2b3a	1
2066 >KY409996.1 Hc	73G 150T 263G 301N 302N 303N 304N 305N 306N 307N 308N 309N 310N 311N 312N 313N 314N 315N 316N 317N 318N 373G 750G 1438G 2706G 3106d 3107C 3197C 4336C 4769G 7028T 7226A 7768G 8860G 9477A 11177T 11467G 11719A 12308G 12372A 13617C 14182C 14766T 15326G 15388C 16169A 16192T 16235G 16270Y 16519C	U5b3a1a	73G 150T 263G 301N 302N 303N 304N 305N 306N 307N 308N 309N 310N 311N 312N 313N 314N 315N 316N 317N 318N 373G 16169A 16192T 16235G 16270Y 16519C	1	73 150 263 301N 302N 303N 304N 305N 306N 307N 308N 309N 310N 311N 312N 313N 314N 315N 316N 317N 318N 373 750 1438 2706 3197 4336 4769 7028 7226 7768 8860 9477 11177 11467 11719 12308 12372 13617 14182 14766 15326 15388 16169A 16192 16235 16270Y	U5b3a1a	73 150 263 301N 302N 303N 304N 305N 306N 307N 308N 309N 310N 311N 312N 313N 314N 315N 316N 317N 318N 373 16169A 16192 16235 16270Y	[0.000] U5b3a1a (1)	[0.000] U5b3a1 U5b3a1b (3)	1
2067 >KY409995.1 Hc	73G 150T 263G 301N 302N 303N 304N 305N 306N 307N 308N 309N 310N 311N 312N 313N 314N 315N 316N 317N 318N 373G 750G 1438G 2706G 3106d 3107C 3197C 4769G 7028T 7226A 7768G 8860G 9477A 11177T 11467G 11719A 12308G 12372A 13617C 14182C 14766T 15326G 16145A 16169A 16192T 16235G 16270T 16519C	U5b3a1a	73G 150T 263G 301N 302N 303N 304N 305N 306N 307N 308N 309N 310N 311N 312N 313N 314N 315N 316N 317N 318N 373G 16145A 16169A 16192T 16235G 16270T 16519C	1	73 150 263 301N 302N 303N 304N 305N 306N 307N 308N 309N 310N 311N 312N 313N 314N 315N 316N 317N 318N 373 750 1438 2706 3197 4769 7028 7226 7768 8860 9477 11177 11467 11719 12308 12372 13617 14182 14766 15326 16145 16169A 16192 16235 16270	U5b3a1a	73 150 263 301N 302N 303N 304N 305N 306N 307N 308N 309N 310N 311N 312N 313N 314N 315N 316N 317N 318N 373 16145 16169A 16192 16235 16270	[0.122] U5b3a1a (1)	[0.000] U5b3a1 U5b3a1b (3)	1
2068 >KY409984.1 Hc	93G 195C 263G 301N 302N 303N 304N 305N 306N 307N 308N 309N 310N 311N 312N 313N 314N 315N 316N 317N 318N 750G 961G 1438G 3106d 3107C 4769G 5021C 8448C 8860G 8898T 13759A 15326G 16189C 16278T 16293G 16311C	H11a1	93G 195C 263G 301N 302N 303N 304N 305N 306N 307N 308N 309N 310N 311N 312N 313N 314N 315N 316N 317N 318N 16189C 16278T 16293G 16311C	1	93 195 263 301N 302N 303N 304N 305N 306N 307N 308N 309N 310N 311N 312N 313N 314N 315N 316N 317N 318N 750 961G 1438 4769 5021 8448 8860 8898 13759 15326 16189 16278 16293 16311	H11a1	93 195 263 301N 302N 303N 304N 305N 306N 307N 308N 309N 310N 311N 312N 313N 314N 315N 316N 317N 318N 16189 16278 16293 16311	[0.107] H11a1 (1)	[0.069] H11a2 (2) [0.014] H11a (3) [0.000] H11a5 H11a8 (5)	1
2069 >KY409977.1 Hc	73G 150T 263G 301N 302N 303N 304N 305N 306N 307N 308N 309N 310N 311N 312N 313N 314N 315N 316N 317N 318N 750G 1438G 2706G 3106d 3107C 3197C 3498T 4769G 5656G 6674C 7028T 7768G 8817G 8860G 9477A 11467G 11719A 12308G 12372A 13617C 14182C 14766T 15326G 15773A 15777A 16167T 16192T 16270T 16311C 16356C	U5b1i	73G 150T 263G 301N 302N 303N 304N 305N 306N 307N 308N 309N 310N 311N 312N 313N 314N 315N 316N 317N 318N 16167T 16192T 16270T 16311C 16356C	1	73 150 263 301N 302N 303N 304N 305N 306N 307N 308N 309N 310N 311N 312N 313N 314N 315N 316N 317N 318N 750 1438 2706 3197 3498 4769 5656 6674 7028 7768 8817 8860 9477 11467 11719 12308 12372 13617 14182 14766 15326 15773 15777 16167 16192 16270 16311 16356	U5b1i	73 150 263 301N 302N 303N 304N 305N 306N 307N 308N 309N 310N 311N 312N 313N 314N 315N 316N 317N 318N 16167 16192 16270 16311 16356	[0.000] U5b1i (1)	[0.026] U5b2a1a+16311 U5b2a1a1d (3) [0.025] U5b1 (4) [0.000] U5b U5b1a U5b1c U5b1d U5b1d1 U5b1f U5b2 U5b2a1a1 U5b2a1a1a U5b2c U5b2c1 U5b2c2 U5b2c2a (17)	1

2070 >KY409972.1 Hc	73G 151T 152C 263G 301N 302N 303N 304N 305N 306N 307N 308N 309N 310N 311N 312N 313N 314N 315N 316N 317N 318N 709A 750G 930A 1009T 1438G 1888A 2706G 3106d 3107C 4216C 4769G 4917G 5147A 5656G 7028T 8602C 8697A 8860G 10463C 10750G 11251G 11719A 11812G 13368A 14233G 14766T 14905A 15326G 15452A 15607G 15928A 16126C 16292T 16294T 16296T 16519C	T2b3a	73G 151T 152C 263G 301N 302N 303N 304N 305N 306N 307N 308N 309N 310N 311N 312N 313N 314N 315N 316N 317N 318N 16126C 16292T 16294T 16296T 16519C	1	73 151 152 263 301N 302N 303N 304N 305N 306N 307N 308N 309N 310N 311N 312N 313N 314N 315N 316N 317N 318N 709 750 930 1009 1438 1888 2706 4216 4769 4917 5147 5656 7028 8602 8697 8860 10463 10750 11251 11719 11812 13368 14233 14766 14905 15326 15452A 15607 15928 16126 16292 16294 16296	T2b3a	73 151 152 263 301N 302N 303N 304N 305N 306N 307N 308N 309N 310N 311N 312N 313N 314N 315N 316N 317N 318N 16126 16292 16294 16296	[0.143] T2b3a (1)	[0.010] T2c1 (2) [0.000] T2d1 T2n (4)	1
2071 >KY409954.1 Hc	57C 59C 62T 73G 151T 152C 263G 301N 302N 303N 304N 305N 306N 307N 308N 309N 310N 311N 312N 313N 314N 315N 316N 317N 318N 709A 750G 930A 1438G 1888A 2706G 3106d 3107C 4216C 4227G 4769G 4917G 5096C 5147A 5656G 7028T 8392A 8697A 8860G 10463C 10750G 11251G 11719A 11812G 13368A 14233G 14766T 14905A 15326G 15452A 15607G 15928A 16126C 16288C 16292T 16294T 16296T 16304C 16465T 16519C	T2b3a1	57C 59C 62T 73G 151T 152C 263G 301N 302N 303N 304N 305N 306N 307N 308N 309N 310N 311N 312N 313N 314N 315N 316N 317N 318N 16126C 16288C 16292T 16294T 16296T 16304C 16465T 16519C	1	57 59 62T 73 151 152 263 301N 302N 303N 304N 305N 306N 307N 308N 309N 310N 311N 312N 313N 314N 315N 316N 317N 318N 709 750 930 1438 1888 2706 4216 4227 4769 4917 5096 5147 5656 7028 8392 8697 8860 10463 10750 11251 11719 11812 13368 14233 14766 14905 15326 15452A 15607 15928 16126 16288 16292 16294 16296 16304 16465	T2b3a1	57 59 62T 73 151 152 263 301N 302N 303N 304N 305N 306N 307N 308N 309N 310N 311N 312N 313N 314N 315N 316N 317N 318N 16126 16288 16292 16294 16296 16304 16465	[1.064] T2b3a1 (1)	[1.064] T2b3a1 (2) [0.393] T2b3a	1
2072 >KY408656.1 Hc	73G 150T 263G 301N 302N 303N 304N 305N 306N 307N 308N 309N 310N 311N 312N 313N 314N 315N 316N 317N 318N 373G 750G 1438G 2706G 3106d 3107C 3197C 4769G 7028T 7226A 7768G 8860G 9477A 11177T 11467G 11719A 12308G 12372A 13617C 14182C 14766T 15326G 16169A 16192T 16235G 16270T 16519C	U5b3a1a	73G 150T 263G 301N 302N 303N 304N 305N 306N 307N 308N 309N 310N 311N 312N 313N 314N 315N 316N 317N 318N 373G 16169A 16192T 16235G 16270T 16519C	1	73 150 263 301N 302N 303N 304N 305N 306N 307N 308N 309N 310N 311N 312N 313N 314N 315N 316N 317N 318N 373 750 1438 2706 3197 4769 7028 7226 7768 8860 9477 11177 11467 11719 12308 12372 13617 14182 14766 15326 16169A 16192 16235 16270	U5b3a1a	73 150 263 301N 302N 303N 304N 305N 306N 307N 308N 309N 310N 311N 312N 313N 314N 315N 316N 317N 318N 373 16169A 16192 16235 16270	[0.000] U5b3a1a (1)	[0.000] U5b3a1 U5b3a1b (3)	1
2073 >KY408652.1 Hc	73G 150T 263G 301N 302N 303N 304N 305N 306N 307N 308N 309N 310N 311N 312N 313N 314N 315N 316N 317N 318N 750G 1438G 2706G 3106d 3107C 3197C 3498T 4769G 5656G 5964C 6674C 7028T 7768G 8860G 9163A 9477A 11467G 11719A 12308G 12372A 13617C 14182C 14766T 15326G 15777A 16167T 16189C 16270T 16311C 16356C	U5b1i	73G 150T 263G 301N 302N 303N 304N 305N 306N 307N 308N 309N 310N 311N 312N 313N 314N 315N 316N 317N 318N 16167T 16189C 16270T 16311C 16356C	1	73 150 263 301N 302N 303N 304N 305N 306N 307N 308N 309N 310N 311N 312N 313N 314N 315N 316N 317N 318N 750 1438 2706 3197 3498 4769 5656 5964 6674 7028 7768 8860 9163 9477 11467 11719 12308 12372 13617 14182 14766 15326 15777 16167 16189 16270 16311 16356	U5b1i	73 150 263 301N 302N 303N 304N 305N 306N 307N 308N 309N 310N 311N 312N 313N 314N 315N 316N 317N 318N 16167 16189 16270 16311 16356	[1.222] U5b1i (1) [0.000] U5b2a5 U5b2a5a (3)	[0.094] U5b1c (4) [0.077] U5b1+16189+@16192 (5) [0.051] U5b2a6 (6) [0.017] U5b2a+@16192 (7) [0.000] U5b1b1+@16192 U5b1b1d U5b1b1f U5b2a4 U5b2a4a U5b2b4 (13)	1

2074	>KY408650.1 Hc	73G 150T 263G 301N 302N 303N 304N 305N 306N 307N 308N 309N 310N 311N 312N 313N 314N 315N 316N 317N 318N 750G 1438G 2706G 3106d 3107C 3197C 3498T 4769G 5656G 5964C 6674C 7028T 7768G 8860G 9163A 9477A 11467G 11719A 12308G 12372A 13617C 14182C 14766T 15326G 15777A 16167T 16189C 16270T 16311C 16356C	U5b1i	73G 150T 263G 301N 302N 303N 304N 305N 306N 307N 308N 309N 310N 311N 312N 313N 314N 315N 316N 317N 318N 16167T 16189C 16270T 16311C 16356C	1	73 150 263 301N 302N 303N 304N 305N 306N 307N 308N 309N 310N 311N 312N 313N 314N 315N 316N 317N 318N 750 1438 2706 3197 3498 4769 5656 5964 6674 7028 7768 8860 9163 9477 11467 11719 12308 12372 13617 14182 14766 15326 15777 16167 16189 16270 16311 16356	U5b1i	73 150 263 301N 302N 303N 304N 305N 306N 307N 308N 309N 310N 311N 312N 313N 314N 315N 316N 317N 318N 16167 16189 16270 16311 16356	[1.222] U5b1i (1) [0.000] U5b2a5 U5b2a5a (3)	[0.094] U5b1c (4) [0.077] U5b1+16189+@16192 (5) [0.051] U5b2a6 (6) [0.017] U5b2a+@16192 (7) [0.000] U5b1b1+@16192 U5b1b1d U5b1b1f U5b2a4 U5b2a4a U5b2b4 (13)	1
2075	>KY408645.1 Hc	73G 152C 217C 263G 301N 302N 303N 304N 305N 306N 307N 308N 309N 310N 311N 312N 313N 314N 315N 316N 317N 318N 508G 750G 1154T 1438G 1811G 2706G 3106d 3107C 3720G 3865G 4769G 5390G 5426C 6045T 6152C 7028T 8860G 10876G 11016A 11467G 11719A 12308G 12372A 13020C 13734C 13758T 14766T 15326G 15454C 15732T 15907G 16051G 16129C 16179T 16182C 16183C 16189C 16362C 16519C	U2e1'2'3	73G 152C 217C 263G 301N 302N 303N 304N 305N 306N 307N 308N 309N 310N 311N 312N 313N 314N 315N 316N 317N 318N 508G 16051G 16129C 16179T 16182C 16183C 16189C 16362C 16519C	1	73 152 217 263 301N 302N 303N 304N 305N 306N 307N 308N 309N 310N 311N 312N 313N 314N 315N 316N 317N 318N 508 750 1154T 1438 1811 2706 3720 3865 4769 5390 5426 6045 6152 7028 8860 10876 11016 11467 11719 12308 12372 13020 13734 13758 14766 15326 15454 15732 15907 16051 16129C 16179 16189 16362	U2e1'2'3	73 152 217 263 301N 302N 303N 304N 305N 306N 307N 308N 309N 310N 311N 312N 313N 314N 315N 316N 317N 318N 508 16051 16129C 16179 16189 16362	[0.494] U2e1'2'3 (1) [0.000] U2e2 U2e2a U2e2a1 U2e2a1a U2e2a1a1 U2e2a1a2 U2e2a1c U2e2a1d (9)	[0.030] U2e1a1a (10) [0.009] U2e1a1 (11) [0.000] U2e U2e1 U2e1a U2e1a1c U2e1c U2e1c1 U2e1d U2e1g U2e2 U2e2a U2e2a1 U2e2a1a U2e2a1a1 U2e2a1a2 U2e2a1b U2e2a1c U2e2a1d (28)	1
2076	>KY408634.1 Hc	73G 152C 217C 263G 301N 302N 303N 304N 305N 306N 307N 308N 309N 310N 311N 312N 313N 314N 315N 316N 317N 318N 508G 750G 1154T 1438G 1811G 2706G 3106d 3107C 3720G 3865G 4769G 5390G 5426C 6045T 6152C 7028T 8860G 10876G 11016A 11467G 11719A 12308G 12372A 13020C 13734C 13758T 14766T 15326G 15454C 15732T 15907G 16051G 16129C 16179T 16182C 16183C 16189C 16362C 16519C	U2e1'2'3	73G 152C 217C 263G 301N 302N 303N 304N 305N 306N 307N 308N 309N 310N 311N 312N 313N 314N 315N 316N 317N 318N 508G 16051G 16129C 16179T 16182C 16183C 16189C 16362C 16519C	1	73 152 217 263 301N 302N 303N 304N 305N 306N 307N 308N 309N 310N 311N 312N 313N 314N 315N 316N 317N 318N 508 750 1154T 1438 1811 2706 3720 3865 4769 5390 5426 6045 6152 7028 8860 10876 11016 11467 11719 12308 12372 13020 13734 13758 14766 15326 15454 15732 15907 16051 16129C 16179 16189 16362	U2e1'2'3	73 152 217 263 301N 302N 303N 304N 305N 306N 307N 308N 309N 310N 311N 312N 313N 314N 315N 316N 317N 318N 508 16051 16129C 16179 16189 16362	[0.494] U2e1'2'3 (1) [0.000] U2e2 U2e2a U2e2a1 U2e2a1a U2e2a1a1 U2e2a1a2 U2e2a1c U2e2a1d (9)	[0.030] U2e1a1a (10) [0.009] U2e1a1 (11) [0.000] U2e U2e1 U2e1a U2e1a1c U2e1c U2e1c1 U2e1d U2e1g U2e2 U2e2a U2e2a1 U2e2a1a U2e2a1a1 U2e2a1a2 U2e2a1b U2e2a1c U2e2a1d (28)	1
2077	>KY408630.1 Hc	73G 152C 217C 263G 301N 302N 303N 304N 305N 306N 307N 308N 309N 310N 311N 312N 313N 314N 315N 316N 317N 318N 508G 750G 1154T 1438G 1811G 2706G 3106d 3107C 3720G 3865G 4769G 5390G 5426C 6045T 6152C 7028T 8860G 10876G 11016A 11467G 11719A 12308G 12372A 13020C 13734C 13758T 14766T 15326G 15454C 15732T 15907G 16051G 16129C 16179T 16189C 16362C 16519C	U2e1'2'3	73G 152C 217C 263G 301N 302N 303N 304N 305N 306N 307N 308N 309N 310N 311N 312N 313N 314N 315N 316N 317N 318N 508G 16051G 16129C 16179T 16189C 16362C 16519C	1	73 152 217 263 301N 302N 303N 304N 305N 306N 307N 308N 309N 310N 311N 312N 313N 314N 315N 316N 317N 318N 508 750 1154T 1438 1811 2706 3720 3865 4769 5390 5426 6045 6152 7028 8860 10876 11016 11467 11719 12308 12372 13020 13734 13758 14766 15326 15454 15732 15907 16051 16129C 16179 16189 16362	U2e1'2'3	73 152 217 263 301N 302N 303N 304N 305N 306N 307N 308N 309N 310N 311N 312N 313N 314N 315N 316N 317N 318N 508 16051 16129C 16179 16189 16362	[0.494] U2e1'2'3 (1) [0.000] U2e2 U2e2a U2e2a1 U2e2a1a U2e2a1a1 U2e2a1a2 U2e2a1c U2e2a1d (9)	[0.030] U2e1a1a (10) [0.009] U2e1a1 (11) [0.000] U2e U2e1 U2e1a U2e1a1c U2e1c U2e1c1 U2e1d U2e1g U2e2 U2e2a U2e2a1 U2e2a1a U2e2a1a1 U2e2a1a2 U2e2a1b U2e2a1c U2e2a1d (28)	1

2078	>KY408617.1 Hc	73G 150T 263G 301N 302N 303N 304N 305N 306N 307N 308N 309N 310N 311N 312N 313N 314N 315N 316N 317N 318N 750G 1438G 2706G 3106d 3107C 3197C 3498T 4769G 5656G 5964C 6674C 7028T 7768G 8860G 9163A 9477A 11467G 11719A 12308G 12372A 13617C 14182C 14766T 15326G 15777A 16167T 16189C 16270T 16311C 16356C	U5b1i	73G 150T 263G 301N 302N 303N 304N 305N 306N 307N 308N 309N 310N 311N 312N 313N 314N 315N 316N 317N 318N 16167T 16189C 16270T 16311C 16356C	1	73 150 263 301N 302N 303N 304N 305N 306N 307N 308N 309N 310N 311N 312N 313N 314N 315N 316N 317N 318N 750 1438 2706 3197 3498 4769 5656 5964 6674 7028 7768 8860 9163 9477 11467 11719 12308 12372 13617 14182 14766 15326 15777 16167 16189 16270 16311 16356	U5b1i	73 150 263 301N 302N 303N 304N 305N 306N 307N 308N 309N 310N 311N 312N 313N 314N 315N 316N 317N 318N 16167 16189 16270 16311 16356	[1.222] U5b1i (1) [0.000] U5b2a5 U5b2a5a (3)	[0.094] U5b1c (4) [0.077] U5b1+16189+@16192 (5) [0.051] U5b2a6 (6) [0.017] U5b2a+@16192 (7) [0.000] U5b1b1+@16192 U5b1b1d U5b1b1f U5b2a4 U5b2a4a U5b2b4 (13)	1
2079	>KY408603.1 Hc	73G 150T 263G 301N 302N 303N 304N 305N 306N 307N 308N 309N 310N 311N 312N 313N 314N 315N 316N 317N 318N 373G 750G 1438G 2706G 3106d 3107C 3197C 4769G 7028T 7226A 7768G 8860G 9477A 9839T 11177T 11467G 11647T 11719A 12308G 12358G 12372A 13617C 14182C 14766T 15326G 16169A 16192T 16235G 16270T 16302G 16519C	U5b3a1a	73G 150T 263G 301N 302N 303N 304N 305N 306N 307N 308N 309N 310N 311N 312N 313N 314N 315N 316N 317N 318N 373G 16169A 16192T 16235G 16270T 16302G 16519C	1	73 150 263 301N 302N 303N 304N 305N 306N 307N 308N 309N 310N 311N 312N 313N 314N 315N 316N 317N 318N 373 750 1438 2706 3197 4769 7028 7226 7768 8860 9477 9839 11177 11467 11647 11719 12308 12358 12372 13617 14182 14766 15326 16169A 16192 16235 16270 16302	U5b3a1a	73 150 263 301N 302N 303N 304N 305N 306N 307N 308N 309N 310N 311N 312N 313N 314N 315N 316N 317N 318N 373 16169A 16192 16235 16270 16302	[0.153] U5b3a1a (1)	[0.000] U5b3a1 U5b3a1b (3)	1
2080	>KY408601.1 Hc	73G 150T 263G 301N 302N 303N 304N 305N 306N 307N 308N 309N 310N 311N 312N 313N 314N 315N 316N 317N 318N 373G 750G 1438G 2706G 3106d 3107C 3197C 4769G 7028T 7226A 7768G 8860G 9477A 11177T 11467G 11719A 12308G 12372A 13617C 13760Y 14182C 14766T 15326G 16169A 16192T 16235G 16270T 16519C	U5b3a1a	73G 150T 263G 301N 302N 303N 304N 305N 306N 307N 308N 309N 310N 311N 312N 313N 314N 315N 316N 317N 318N 373G 16169A 16192T 16235G 16270T 16519C	1	73 150 263 301N 302N 303N 304N 305N 306N 307N 308N 309N 310N 311N 312N 313N 314N 315N 316N 317N 318N 373 750 1438 2706 3197 4769 7028 7226 7768 8860 9477 11177 11467 11719 12308 12372 13617 13760Y 14182 14766 15326 16169A 16192 16235 16270	U5b3a1a	73 150 263 301N 302N 303N 304N 305N 306N 307N 308N 309N 310N 311N 312N 313N 314N 315N 316N 317N 318N 373 16169A 16192 16235 16270	[0.000] U5b3a1a (1)	[0.000] U5b3a1 U5b3a1b (3)	1
2081	>KY408586.1 Hc	73G 150T 263G 301N 302N 303N 304N 305N 306N 307N 308N 309N 310N 311N 312N 313N 314N 315N 316N 317N 318N 373G 750G 1438G 2706G 3106d 3107C 3197C 4769G 7028T 7226A 7768G 8860G 9477A 9839T 11177T 11467G 11647T 11719A 12308G 12358G 12372A 13617C 14182C 14766T 15326G 16169A 16192T 16235G 16270T 16302G 16519C	U5b3a1a	73G 150T 263G 301N 302N 303N 304N 305N 306N 307N 308N 309N 310N 311N 312N 313N 314N 315N 316N 317N 318N 373G 16169A 16192T 16235G 16270T 16302G 16519C	1	73 150 263 301N 302N 303N 304N 305N 306N 307N 308N 309N 310N 311N 312N 313N 314N 315N 316N 317N 318N 373 750 1438 2706 3197 4769 7028 7226 7768 8860 9477 9839 11177 11467 11647 11719 12308 12358 12372 13617 14182 14766 15326 16169A 16192 16235 16270 16302	U5b3a1a	73 150 263 301N 302N 303N 304N 305N 306N 307N 308N 309N 310N 311N 312N 313N 314N 315N 316N 317N 318N 373 16169A 16192 16235 16270 16302	[0.153] U5b3a1a (1)	[0.000] U5b3a1 U5b3a1b (3)	1

2082 >KY408580.1 Hc	73G 150T 263G 301N 302N 303N 304N 305N 306N 307N 308N 309N 310N 311N 312N 313N 314N 315N 316N 317N 318N 750G 1438G 2706G 3106d 3107C 3197C 3498T 4769G 5656G 5964C 6674C 7028T 7768G 8860G 9163A 9477A 11467G 11719A 12308G 12372A 13617C 14182C 14766T 15326G 15777A 16167T 16189C 16270T 16311C 16356C	U5b1i	73G 150T 263G 301N 302N 303N 304N 305N 306N 307N 308N 309N 310N 311N 312N 313N 314N 315N 316N 317N 318N 16167T 16189C 16270T 16311C 16356C	1	73 150 263 301N 302N 303N 304N 305N 306N 307N 308N 309N 310N 311N 312N 313N 314N 315N 316N 317N 318N 750 1438 2706 3197 3498 4769 5656 5964 6674 7028 7768 8860 9163 9477 11467 11719 12308 12372 13617 14182 14766 15326 15777 16167 16189 16270 16311 16356	U5b1i	73 150 263 301N 302N 303N 304N 305N 306N 307N 308N 309N 310N 311N 312N 313N 314N 315N 316N 317N 318N 16167 16189 16270 16311 16356	[1.222] U5b1i (1) [0.000] U5b2a5 U5b2a5a (3)	[0.094] U5b1c (4) [0.077] U5b1+16189+@16192 (5) [0.051] U5b2a6 (6) [0.017] U5b2a+@16192 (7) [0.000] U5b1b1+@16192 U5b1b1d U5b1b1f U5b2a4 U5b2a4a U5b2b4 (13)	1
2083 >KY408578.1 Hc	73G 152C 217C 263G 301N 302N 303N 304N 305N 306N 307N 308N 309N 310N 311N 312N 313N 314N 315N 316N 317N 318N 508G 750G 1154T 1438G 1811G 2706G 3106d 3107C 3720G 3865G 4769G 5390G 5426C 6045T 6152C 7028T 8860G 10876G 11016A 11467G 11719A 12308G 12372A 13020C 13734C 13758T 14766T 15326G 15454C 15732T 15907G 16051G 16129C 16179T 16182C 16183C 16189C 16362C 16519C	U2e1'2'3	73G 152C 217C 263G 301N 302N 303N 304N 305N 306N 307N 308N 309N 310N 311N 312N 313N 314N 315N 316N 317N 318N 508G 16051G 16129C 16179T 16182C 16183C 16189C 16362C 16519C	1	73 152 217 263 301N 302N 303N 304N 305N 306N 307N 308N 309N 310N 311N 312N 313N 314N 315N 316N 317N 318N 508 750 1154T 1438 1811 2706 3720 3865 4769 5390 5426 6045 6152 7028 8860 10876 11016 11467 11719 12308 12372 13020 13734 13758 14766 15326 15454 15732 15907 16051 16129C 16179 16189 16362	U2e1'2'3	73 152 217 263 301N 302N 303N 304N 305N 306N 307N 308N 309N 310N 311N 312N 313N 314N 315N 316N 317N 318N 508 16051 16129C 16179 16189 16362	[0.494] U2e1'2'3 (1) [0.000] U2e2 U2e2a U2e2a1 U2e2a1a U2e2a1a1 U2e2a1a2 U2e2a1c U2e2a1d (9)	[0.030] U2e1a1a (10) [0.009] U2e1a1 (11) [0.000] U2e U2e1 U2e1a U2e1a1c U2e1c U2e1c1 U2e1d U2e1g U2e2 U2e2a U2e2a1 U2e2a1a U2e2a1a1 U2e2a1a2 U2e2a1b U2e2a1c U2e2a1d (28)	1
2084 >KY408564.1 Hc	73G 150T 263G 301N 302N 303N 304N 305N 306N 307N 308N 309N 310N 311N 312N 313N 314N 315N 316N 317N 318N 750G 1438G 2706G 3106d 3107C 3197C 3498T 4769G 5656G 5964C 6674C 7028T 7768G 8860G 9163A 9477A 11467G 11719A 12308G 12372A 13617C 14182C 14766T 15326G 15777A 16167T 16189C 16270T 16311C 16356C	U5b1i	73G 150T 263G 301N 302N 303N 304N 305N 306N 307N 308N 309N 310N 311N 312N 313N 314N 315N 316N 317N 318N 16167T 16189C 16270T 16311C 16356C	1	73 150 263 301N 302N 303N 304N 305N 306N 307N 308N 309N 310N 311N 312N 313N 314N 315N 316N 317N 318N 750 1438 2706 3197 3498 4769 5656 5964 6674 7028 7768 8860 9163 9477 11467 11719 12308 12372 13617 14182 14766 15326 15777 16167 16189 16270 16311 16356	U5b1i	73 150 263 301N 302N 303N 304N 305N 306N 307N 308N 309N 310N 311N 312N 313N 314N 315N 316N 317N 318N 16167 16189 16270 16311 16356	[1.222] U5b1i (1) [0.000] U5b2a5 U5b2a5a (3)	[0.094] U5b1c (4) [0.077] U5b1+16189+@16192 (5) [0.051] U5b2a6 (6) [0.017] U5b2a+@16192 (7) [0.000] U5b1b1+@16192 U5b1b1d U5b1b1f U5b2a4 U5b2a4a U5b2b4 (13)	1
2085 >KY408555.1 Hc	73G 152C 217C 263G 301N 302N 303N 304N 305N 306N 307N 308N 309N 310N 311N 312N 313N 314N 315N 316N 317N 318N 508G 750G 1154T 1438G 1811G 2706G 3106d 3107C 3720G 3865G 4769G 5390G 5426C 6045T 6152C 7028T 8860G 10876G 11016A 11467G 11719A 12308G 12372A 13020C 13734C 13758T 14766T 15326G 15454C 15732T 15907G 16051G 16129C 16179T 16182C 16183C 16189C 16362C 16519C	U2e1'2'3	73G 152C 217C 263G 301N 302N 303N 304N 305N 306N 307N 308N 309N 310N 311N 312N 313N 314N 315N 316N 317N 318N 508G 16051G 16129C 16179T 16182C 16183C 16189C 16362C 16519C	1	73 152 217 263 301N 302N 303N 304N 305N 306N 307N 308N 309N 310N 311N 312N 313N 314N 315N 316N 317N 318N 508 750 1154T 1438 1811 2706 3720 3865 4769 5390 5426 6045 6152 7028 8860 10876 11016 11467 11719 12308 12372 13020 13734 13758 14766 15326 15454 15732 15907 16051 16129C 16179 16189 16362	U2e1'2'3	73 152 217 263 301N 302N 303N 304N 305N 306N 307N 308N 309N 310N 311N 312N 313N 314N 315N 316N 317N 318N 508 16051 16129C 16179 16189 16362	[0.494] U2e1'2'3 (1) [0.000] U2e2 U2e2a U2e2a1 U2e2a1a U2e2a1a1 U2e2a1a2 U2e2a1c U2e2a1d (9)	[0.030] U2e1a1a (10) [0.009] U2e1a1 (11) [0.000] U2e U2e1 U2e1a U2e1a1c U2e1c U2e1c1 U2e1d U2e1g U2e2 U2e2a U2e2a1 U2e2a1a U2e2a1a1 U2e2a1a2 U2e2a1b U2e2a1c U2e2a1d (28)	1

2086	>KY408543.1 Hc	57C 73G 151T 152C 263G 301N 302N 303N 304N 305N 306N 307N 308N 309N 310N 311N 312N 313N 314N 315N 316N 317N 318N 709A 750G 930A 1438G 1888A 2706G 3106d 3107C 4216C 4227G 4769G 4917G 5147A 5656G 7028T 8697A 8860G 10463C 10750G 11251G 11719A 11812G 13368A 14233G 14766T 14905A 15326G 15452A 15607G 15928A 16126C 16288C 16292T 16294T 16296T 16304C 16465T 16519C	T2b3a	57C 73G 151T 152C 263G 301N 302N 303N 304N 305N 306N 307N 308N 309N 310N 311N 312N 313N 314N 315N 316N 317N 318N 16126C 16288C 16292T 16294T 16296T 16304C 16465T 16519C	1	57 73 151 152 263 301N 302N 303N 304N 305N 306N 307N 308N 309N 310N 311N 312N 313N 314N 315N 316N 317N 318N 709 750 930 1438 1888 2706 4216 4227 4769 4917 5147 5656 7028 8697 8860 10463 10750 11251 11719 11812 13368 14233 14766 14905 15326 15452A 15607 15928 16126 16288 16292 16294 16296 16304 16465	T2b3a	57 73 151 152 263 301N 302N 303N 304N 305N 306N 307N 308N 309N 310N 311N 312N 313N 314N 315N 316N 317N 318N 16126 16288 16292 16294 16296 16304 16465	[0.393] T2b3a (1)	[0.983] T2b3a1 (2)	1
2087	>KY408540.1 Hc	73G 150T 263G 301N 302N 303N 304N 305N 306N 307N 308N 309N 310N 311N 312N 313N 314N 315N 316N 317N 318N 750G 1438G 2706G 3106d 3107C 3197C 3498T 4769G 5656G 5964C 6674C 7028T 7768G 8860G 9163A 9477A 11467G 11719A 12308G 12372A 13617C 14182C 14766T 15326G 15777A 16145R 16167T 16189C 16270T 16311C 16356C	U5b1i	73G 150T 263G 301N 302N 303N 304N 305N 306N 307N 308N 309N 310N 311N 312N 313N 314N 315N 316N 317N 318N 16145R 16167T 16189C 16270T 16311C 16356C	1	73 150 263 301N 302N 303N 304N 305N 306N 307N 308N 309N 310N 311N 312N 313N 314N 315N 316N 317N 318N 750 1438 2706 3197 3498 4769 5656 5964 6674 7028 7768 8860 9163 9477 11467 11719 12308 12372 13617 14182 14766 15326 15777 16145R 16167 16189 16270 16311 16356	U5b1i	73 150 263 301N 302N 303N 304N 305N 306N 307N 308N 309N 310N 311N 312N 313N 314N 315N 316N 317N 318N 16145R 16167 16189 16270 16311 16356	[1.236] U5b1i (1) [0.000] U5b2a5 U5b2a5a (3)	[0.094] U5b1c (4) [0.077] U5b1+16189+@16192 (5) [0.051] U5b2a6 (6) [0.017] U5b2a+@16192 (7) [0.000] U5b1b1+@16192 U5b1b1d U5b1b1f U5b2a4 U5b2a4a U5b2b4 (13)	1
2088	>KY408962.1 Hc	73G 150T 263G 301N 302N 303N 304N 305N 306N 307N 308N 309N 310N 311N 312N 313N 314N 315N 316N 317N 318N 750G 1438G 2706G 3106d 3107C 3197C 3498T 4769G 5656G 5964C 6674C 7028T 7768G 8860G 9163A 9477A 11467G 11719A 12308G 12372A 13617C 14182C 14766T 15326G 15777A 16167T 16189C 16270T 16311C 16356C	U5b1i	73G 150T 263G 301N 302N 303N 304N 305N 306N 307N 308N 309N 310N 311N 312N 313N 314N 315N 316N 317N 318N 16167T 16189C 16270T 16311C 16356C	1	73 150 263 301N 302N 303N 304N 305N 306N 307N 308N 309N 310N 311N 312N 313N 314N 315N 316N 317N 318N 750 1438 2706 3197 3498 4769 5656 5964 6674 7028 7768 8860 9163 9477 11467 11719 12308 12372 13617 14182 14766 15326 15777 16167 16189 16270 16311 16356	U5b1i	73 150 263 301N 302N 303N 304N 305N 306N 307N 308N 309N 310N 311N 312N 313N 314N 315N 316N 317N 318N 16167 16189 16270 16311 16356	[1.222] U5b1i (1) [0.000] U5b2a5 U5b2a5a (3)	[0.094] U5b1c (4) [0.077] U5b1+16189+@16192 (5) [0.051] U5b2a6 (6) [0.017] U5b2a+@16192 (7) [0.000] U5b1b1+@16192 U5b1b1d U5b1b1f U5b2a4 U5b2a4a U5b2b4 (13)	1
2089	>KY408948.1 Hc	57C 73G 151T 152C 263G 301N 302N 303N 304N 305N 306N 307N 308N 309N 310N 311N 312N 313N 314N 315N 316N 317N 318N 709A 750G 930A 1438G 1888A 2706G 3106d 3107C 4216C 4227G 4769G 4917G 5147A 5656G 7028T 8697A 8860G 10310R 10463C 10750G 11251G 11719A 11812G 13368A 14233G 14766T 14905A 15326G 15452A 15607G 15928A 16126C 16288C 16292T 16294T 16296T 16304C 16465T 16519C	T2b3a	57C 73G 151T 152C 263G 301N 302N 303N 304N 305N 306N 307N 308N 309N 310N 311N 312N 313N 314N 315N 316N 317N 318N 16126C 16288C 16292T 16294T 16296T 16304C 16465T 16519C	1	57 73 151 152 263 301N 302N 303N 304N 305N 306N 307N 308N 309N 310N 311N 312N 313N 314N 315N 316N 317N 318N 709 750 930 1438 1888 2706 4216 4227 4769 4917 5147 5656 7028 8697 8860 10310R 10463 10750 11251 11719 11812 13368 14233 14766 14905 15326 15452A 15607 15928 16126 16288 16292 16294 16296 16304 16465	T2b3a	57 73 151 152 263 301N 302N 303N 304N 305N 306N 307N 308N 309N 310N 311N 312N 313N 314N 315N 316N 317N 318N 16126 16288 16292 16294 16296 16304 16465	[0.393] T2b3a (1)	[0.983] T2b3a1 (2)	1

2090	>KY408944.1 Hc	73G 150T 263G 301N 302N 303N 304N 305N 306N 307N 308N 309N 310N 311N 312N 313N 314N 315N 316N 317N 318N 373G 750G 1438G 2706G 3106d 3107C 3197C 4769G 7028T 7226A 7768G 8860G 9477A 11177T 11467G 11719A 12308G 12372A 13617C 14182C 14766T 15326G 16169A 16192T 16235G 16270T 16519C	U5b3a1a	73G 150T 263G 301N 302N 303N 304N 305N 306N 307N 308N 309N 310N 311N 312N 313N 314N 315N 316N 317N 318N 373G 16169A 16192T 16235G 16270T 16519C	1	73 150 263 301N 302N 303N 304N 305N 306N 307N 308N 309N 310N 311N 312N 313N 314N 315N 316N 317N 318N 373 750 1438 2706 3197 4769 7028 7226 7768 8860 9477 11177 11467 11719 12308 12372 13617 14182 14766 15326 16169A 16192 16235 16270	U5b3a1a	73 150 263 301N 302N 303N 304N 305N 306N 307N 308N 309N 310N 311N 312N 313N 314N 315N 316N 317N 318N 373 16169A 16192 16235 16270	[0.000] U5b3a1a (1)	[0.000] U5b3a1 U5b3a1b (3)	1
2091	>KY408934.1 Hc	73G 150T 263G 301N 302N 303N 304N 305N 306N 307N 308N 309N 310N 311N 312N 313N 314N 315N 316N 317N 318N 373G 750G 1438G 2706G 3106d 3107C 3197C 4769G 7028T 7226A 7768G 8860G 9477A 11177T 11467G 11719A 12308G 12372A 13617C 14182C 14766T 15326G 16145A 16169A 16192T 16235G 16270T 16519C	U5b3a1a	73G 150T 263G 301N 302N 303N 304N 305N 306N 307N 308N 309N 310N 311N 312N 313N 314N 315N 316N 317N 318N 373G 16145A 16169A 16192T 16235G 16270T 16519C	1	73 150 263 301N 302N 303N 304N 305N 306N 307N 308N 309N 310N 311N 312N 313N 314N 315N 316N 317N 318N 373 750 1438 2706 3197 4769 7028 7226 7768 8860 9477 11177 11467 11719 12308 12372 13617 14182 14766 15326 16145 16169A 16192 16235 16270	U5b3a1a	73 150 263 301N 302N 303N 304N 305N 306N 307N 308N 309N 310N 311N 312N 313N 314N 315N 316N 317N 318N 373 16145 16169A 16192 16235 16270	[0.122] U5b3a1a (1)	[0.000] U5b3a1 U5b3a1b (3)	1
2092	>KY408929.1 Hc	73G 150T 263G 301N 302N 303N 304N 305N 306N 307N 308N 309N 310N 311N 312N 313N 314N 315N 316N 317N 318N 750G 1438G 2706G 3106d 3107C 3197C 3498T 4769G 5656G 5964C 6674C 7028T 7768G 8860G 9163A 9477A 11467G 11719A 12308G 12372A 13617C 14182C 14766T 15326G 15777A 15856T 16167T 16189C 16270T 16311C 16356C	U5b1i	73G 150T 263G 301N 302N 303N 304N 305N 306N 307N 308N 309N 310N 311N 312N 313N 314N 315N 316N 317N 318N 16167T 16189C 16270T 16311C 16356C	1	73 150 263 301N 302N 303N 304N 305N 306N 307N 308N 309N 310N 311N 312N 313N 314N 315N 316N 317N 318N 750 1438 2706 3197 3498 4769 5656 5964 6674 7028 7768 8860 9163 9477 11467 11719 12308 12372 13617 14182 14766 15326 15777 15856 16167 16189 16270 16311 16356	U5b1i	73 150 263 301N 302N 303N 304N 305N 306N 307N 308N 309N 310N 311N 312N 313N 314N 315N 316N 317N 318N 16167 16189 16270 16311 16356	[1.222] U5b1i (1) [0.000] U5b2a5 U5b2a5a (3)	[0.094] U5b1c (4) [0.077] U5b1+16189+@16192 (5) [0.051] U5b2a6 (6) [0.017] U5b2a+@16192 (7) [0.000] U5b1b1+@16192 U5b1b1d U5b1b1f U5b2a4 U5b2a4a U5b2b4 (13)	1
2093	>KY408922.1 Hc	263G 301N 302N 303N 304N 305N 306N 307N 308N 309N 310N 311N 312N 313N 314N 315N 316N 317N 318N 337C 480C 750G 1438G 3106d 3107C 3615G 4769G 6776C 8860G 8937C 11914A 15326G 16293G 16368C 16519C	H3x1	263G 301N 302N 303N 304N 305N 306N 307N 308N 309N 310N 311N 312N 313N 314N 315N 316N 317N 318N 337C 480C 16293G 16368C 16519C	1	263 301N 302N 303N 304N 305N 306N 307N 308N 309N 310N 311N 312N 313N 314N 315N 316N 317N 318N 337C 480 750 1438 3615 4769 6776 8860 8937 11914 15326 16293 16368	H3x1	263 301N 302N 303N 304N 305N 306N 307N 308N 309N 310N 311N 312N 313N 314N 315N 316N 317N 318N 337C 480 16293 16368	[0.283] H3x1 (1)	[0.007] H24a (2) [0.000] H3x H24 H24a2 H24b (6)	1
2094	>KY408917.1 Hc	73G 150T 263G 301N 302N 303N 304N 305N 306N 307N 308N 309N 310N 311N 312N 313N 314N 315N 316N 317N 318N 750G 1438G 2706G 3106d 3107C 3197C 3498T 4769G 5656G 5964C 6674C 7028T 7768G 8860G 9163A 9477A 11467G 11719A 12308G 12372A 13617C 14182C 14766T 15326G 15777A 16167T 16189C 16270T 16311C 16356C	U5b1i	73G 150T 263G 301N 302N 303N 304N 305N 306N 307N 308N 309N 310N 311N 312N 313N 314N 315N 316N 317N 318N 16167T 16189C 16270T 16311C 16356C	1	73 150 263 301N 302N 303N 304N 305N 306N 307N 308N 309N 310N 311N 312N 313N 314N 315N 316N 317N 318N 750 1438 2706 3197 3498 4769 5656 5964 6674 7028 7768 8860 9163 9477 11467 11719 12308 12372 13617 14182 14766 15326 15777 16167 16189 16270 16311 16356	U5b1i	73 150 263 301N 302N 303N 304N 305N 306N 307N 308N 309N 310N 311N 312N 313N 314N 315N 316N 317N 318N 16167 16189 16270 16311 16356	[1.222] U5b1i (1) [0.000] U5b2a5 U5b2a5a (3)	[0.094] U5b1c (4) [0.077] U5b1+16189+@16192 (5) [0.051] U5b2a6 (6) [0.017] U5b2a+@16192 (7) [0.000] U5b1b1+@16192 U5b1b1d U5b1b1f U5b2a4 U5b2a4a U5b2b4 (13)	1

2095 >KY408912.1 Hc	73G 150T 263G 301N 302N 303N 304N 305N 306N 307N 308N 309N 310N 311N 312N 313N 314N 315N 316N 317N 318N 750G 1438G 2706G 3106d 3107C 3197C 3498T 4769G 5656G 5964C 6674C 7028T 7768G 8860G 9163A 9477A 11467G 11719A 12308G 12372A 13617C 14182C 14766T 15326G 15777A 16167T 16189C 16270T 16311C 16356C	U5b1i	73G 150T 263G 301N 302N 303N 304N 305N 306N 307N 308N 309N 310N 311N 312N 313N 314N 315N 316N 317N 318N 16167T 16189C 16270T 16311C 16356C	1	73 150 263 301N 302N 303N 304N 305N 306N 307N 308N 309N 310N 311N 312N 313N 314N 315N 316N 317N 318N 750 1438 2706 3197 3498 4769 5656 5964 6674 7028 7768 8860 9163 9477 11467 11719 12308 12372 13617 14182 14766 15326 15777 16167 16189 16270 16311 16356	U5b1i	73 150 263 301N 302N 303N 304N 305N 306N 307N 308N 309N 310N 311N 312N 313N 314N 315N 316N 317N 318N 16167 16189 16270 16311 16356	[1.222] U5b1i (1) [0.000] U5b2a5 U5b2a5a (3)	[0.094] U5b1c (4) [0.077] U5b1+16189+@16192 (5) [0.051] U5b2a6 (6) [0.017] U5b2a+@16192 (7) [0.000] U5b1b1+@16192 U5b1b1d U5b1b1f U5b2a4 U5b2a4a U5b2b4 (13)	1
2096 >KY408911.1 Hc	73G 150T 263G 301N 302N 303N 304N 305N 306N 307N 308N 309N 310N 311N 312N 313N 314N 315N 316N 317N 318N 750G 1438G 2706G 3106d 3107C 3197C 3498T 4769G 5656G 5964C 6674C 7028T 7768G 8860G 9163A 9477A 11467G 11719A 12308G 12372A 13617C 14182C 14766T 15326G 15777A 16167T 16189C 16270T 16311C 16356C	U5b1i	73G 150T 263G 301N 302N 303N 304N 305N 306N 307N 308N 309N 310N 311N 312N 313N 314N 315N 316N 317N 318N 16167T 16189C 16270T 16311C 16356C	1	73 150 263 301N 302N 303N 304N 305N 306N 307N 308N 309N 310N 311N 312N 313N 314N 315N 316N 317N 318N 750 1438 2706 3197 3498 4769 5656 5964 6674 7028 7768 8860 9163 9477 11467 11719 12308 12372 13617 14182 14766 15326 15777 16167 16189 16270 16311 16356	U5b1i	73 150 263 301N 302N 303N 304N 305N 306N 307N 308N 309N 310N 311N 312N 313N 314N 315N 316N 317N 318N 16167 16189 16270 16311 16356	[1.222] U5b1i (1) [0.000] U5b2a5 U5b2a5a (3)	[0.094] U5b1c (4) [0.077] U5b1+16189+@16192 (5) [0.051] U5b2a6 (6) [0.017] U5b2a+@16192 (7) [0.000] U5b1b1+@16192 U5b1b1d U5b1b1f U5b2a4 U5b2a4a U5b2b4 (13)	1
2097 >KY408909.1 Hc	73G 150T 263G 301N 302N 303N 304N 305N 306N 307N 308N 309N 310N 311N 312N 313N 314N 315N 316N 317N 318N 750G 1438G 2706G 3106d 3107C 3197C 3498T 4769G 5656G 5964C 6674C 7028T 7768G 8860G 9163A 9477A 11467G 11692Y 11719A 12308G 12372A 13617C 14182C 14766T 15326G 15777A 16167T 16189C 16270T 16311C 16356C	U5b1i	73G 150T 263G 301N 302N 303N 304N 305N 306N 307N 308N 309N 310N 311N 312N 313N 314N 315N 316N 317N 318N 16167T 16189C 16270T 16311C 16356C	1	73 150 263 301N 302N 303N 304N 305N 306N 307N 308N 309N 310N 311N 312N 313N 314N 315N 316N 317N 318N 750 1438 2706 3197 3498 4769 5656 5964 6674 7028 7768 8860 9163 9477 11467 11692Y 11719 12308 12372 13617 14182 14766 15326 15777 16167 16189 16270 16311 16356	U5b1i	73 150 263 301N 302N 303N 304N 305N 306N 307N 308N 309N 310N 311N 312N 313N 314N 315N 316N 317N 318N 16167 16189 16270 16311 16356	[1.222] U5b1i (1) [0.000] U5b2a5 U5b2a5a (3)	[0.094] U5b1c (4) [0.077] U5b1+16189+@16192 (5) [0.051] U5b2a6 (6) [0.017] U5b2a+@16192 (7) [0.000] U5b1b1+@16192 U5b1b1d U5b1b1f U5b2a4 U5b2a4a U5b2b4 (13)	1
2098 >KY408899.1 Hc	73G 146Y 150T 263G 301N 302N 303N 304N 305N 306N 307N 308N 309N 310N 311N 312N 313N 314N 315N 316N 317N 318N 373G 750G 1438G 2706G 3106d 3107C 3197C 4769G 7028T 7226A 7768G 8860G 9477A 11177T 11467G 11620G 11719A 12308G 12372A 13617C 14182C 14766T 15326G 16145A 16169A 16192T 16235G 16270T 16519C	U5b3a1a	73G 146Y 150T 263G 301N 302N 303N 304N 305N 306N 307N 308N 309N 310N 311N 312N 313N 314N 315N 316N 317N 318N 373G 16145A 16169A 16192T 16235G 16270T 16519C	1	73 146Y 150 263 301N 302N 303N 304N 305N 306N 307N 308N 309N 310N 311N 312N 313N 314N 315N 316N 317N 318N 373 750 1438 2706 3197 4769 7028 7226 7768 8860 9477 11177 11467 11620 11719 12308 12372 13617 14182 14766 15326 16145 16169A 16192 16235 16270	U5b3a1a	73 146Y 150 263 301N 302N 303N 304N 305N 306N 307N 308N 309N 310N 311N 312N 313N 314N 315N 316N 317N 318N 373 16145 16169A 16192 16235 16270	[0.142] U5b3a1a (1)	[0.000] U5b3a1 U5b3a1b (3)	1

2099	>KY408895.1 H[illegible]	73G 150T 263G 301N 302N 303N 304N 305N 306N 307N 308N 309N 310N 311N 312N 313N 314N 315N 316N 317N 318N 373G 750G 1438G 2706G 3106d 3107C 3197C 4769G 7028T 7226A 7768G 8860G 9477A 9839T 11177T 11467G 11647T 11719A 12308G 12358G 12372A 13617C 14182C 14766T 15326G 16169A 16192T 16235G 16270T 16302G 16519C	U5b3a1a	73G 150T 263G 301N 302N 303N 304N 305N 306N 307N 308N 309N 310N 311N 312N 313N 314N 315N 316N 317N 318N 373G 16169A 16192T 16235G 16270T 16302G 16519C	1	73 150 263 301N 302N 303N 304N 305N 306N 307N 308N 309N 310N 311N 312N 313N 314N 315N 316N 317N 318N 373 750 1438 2706 3197 4769 7028 7226 7768 8860 9477 9839 11177 11467 11647 11719 12308 12358 12372 13617 14182 14766 15326 16169A 16192 16235 16270 16302	U5b3a1a	73 150 263 301N 302N 303N 304N 305N 306N 307N 308N 309N 310N 311N 312N 313N 314N 315N 316N 317N 318N 373 16169A 16192 16235 16270 16302	[0.153] U5b3a1a (1)	[0.000] U5b3a1 U5b3a1b (3)	1
2100	>KY408894.1 H[illegible]	73G 150T 263G 301N 302N 303N 304N 305N 306N 307N 308N 309N 310N 311N 312N 313N 314N 315N 316N 317N 318N 750G 1438G 2706G 3106d 3107C 3197C 3498T 4769G 5656G 5964C 6674C 7028T 7768G 8860G 9163A 9477A 11467G 11719A 12308G 12372A 13617C 14182C 14766T 15326G 15777A 16167T 16189C 16270T 16311C 16356C	U5b1i	73G 150T 263G 301N 302N 303N 304N 305N 306N 307N 308N 309N 310N 311N 312N 313N 314N 315N 316N 317N 318N 16167T 16189C 16270T 16311C 16356C	1	73 150 263 301N 302N 303N 304N 305N 306N 307N 308N 309N 310N 311N 312N 313N 314N 315N 316N 317N 318N 750 1438 2706 3197 3498 4769 5656 5964 6674 7028 7768 8860 9163 9477 11467 11719 12308 12372 13617 14182 14766 15326 15777 16167 16189 16270 16311 16356	U5b1i	73 150 263 301N 302N 303N 304N 305N 306N 307N 308N 309N 310N 311N 312N 313N 314N 315N 316N 317N 318N 16167 16189 16270 16311 16356	[1.222] U5b1i (1) [0.000] U5b2a5 U5b2a5a (3)	[0.094] U5b1c (4) [0.077] U5b1+16189+@16192 (5) [0.051] U5b2a6 (6) [0.017] U5b2a+@16192 (7) [0.000] U5b1b1+@16192 U5b1b1d U5b1b1f U5b2a4 U5b2a4a U5b2b4 (13)	1
2101	>KY408893.1 H[illegible]	73G 150T 263G 301N 302N 303N 304N 305N 306N 307N 308N 309N 310N 311N 312N 313N 314N 315N 316N 317N 318N 373G 750G 1438G 2706G 3106d 3107C 3197C 4769G 7028T 7226A 7768G 8860G 9477A 11177T 11467G 11719A 12308G 12372A 13617C 14182C 14766T 15326G 16169A 16192T 16235G 16270T 16519C	U5b3a1a	73G 150T 263G 301N 302N 303N 304N 305N 306N 307N 308N 309N 310N 311N 312N 313N 314N 315N 316N 317N 318N 373G 16169A 16192T 16235G 16270T 16519C	1	73 150 263 301N 302N 303N 304N 305N 306N 307N 308N 309N 310N 311N 312N 313N 314N 315N 316N 317N 318N 373 750 1438 2706 3197 4769 7028 7226 7768 8860 9477 11177 11467 11719 12308 12372 13617 14182 14766 15326 16169A 16192 16235 16270	U5b3a1a	73 150 263 301N 302N 303N 304N 305N 306N 307N 308N 309N 310N 311N 312N 313N 314N 315N 316N 317N 318N 373 16169A 16192 16235 16270	[0.000] U5b3a1a (1)	[0.000] U5b3a1 U5b3a1b (3)	1
2102	>KY408892.1 H[illegible]	73G 195C 263G 285T 301N 302N 303N 304N 305N 306N 307N 308N 309N 310N 311N 312N 313N 314N 315N 316N 317N 318N 663G 750G 1438G 2218T 2706G 3106d 3107C 4769G 4991A 6026A 7028T 7403G 7581C 8860G 11116C 11467G 11719A 12308G 12372A 12879C 13104G 13656C 14070G 14364A 14766T 15115C 15148A 15217A 15326G 15954C 16145A 16182C 16183C 16189C 16249C	U1a1c1c1	73G 195C 263G 285T 301N 302N 303N 304N 305N 306N 307N 308N 309N 310N 311N 312N 313N 314N 315N 316N 317N 318N 16145A 16182C 16183C 16189C 16249C	1	73 195 263 285 301N 302N 303N 304N 305N 306N 307N 308N 309N 310N 311N 312N 313N 314N 315N 316N 317N 318N 663 750 1438 2218 2706 4769 4991 6026 7028 7403 7581 8860 11116 11467 11719 12308 12372 12879 13104 13656 14070 14364 14766 15115 15148 15217 15326 15954C 16145 16189 16249	U1a1c1c1	73 195 263 285 301N 302N 303N 304N 305N 306N 307N 308N 309N 310N 311N 312N 313N 314N 315N 316N 317N 318N 16145 16189 16249	[0.000] U1a1c1c1 (1)	[0.049] U1a1c1d (2) [0.000] U1a U1a1 U1a1c U1a1c1 U1a1c1b U1a1c1c (8)	1

2103	>KY408882.1 Hc	73G 150T 263G 301N 302N 303N 304N 305N 306N 307N 308N 309N 310N 311N 312N 313N 314N 315N 316N 317N 318N 373G 750G 1438G 2706G 3106d 3107C 3197C 4769G 7028T 7226A 7768G 8860G 9477A 9839T 11177T 11467G 11647T 11719A 12308G 12358G 12372A 13617C 14182C 14766T 15326G 16169A 16192T 16235G 16270T 16302G 16519C	U5b3a1a	73G 150T 263G 301N 302N 303N 304N 305N 306N 307N 308N 309N 310N 311N 312N 313N 314N 315N 316N 317N 318N 373G 16169A 16192T 16235G 16270T 16302G 16519C	1	73 150 263 301N 302N 303N 304N 305N 306N 307N 308N 309N 310N 311N 312N 313N 314N 315N 316N 317N 318N 373 750 1438 2706 3197 4769 7028 7226 7768 8860 9477 9839 11177 11467 11647 11719 12308 12358 12372 13617 14182 14766 15326 16169A 16192 16235 16270 16302	U5b3a1a	73 150 263 301N 302N 303N 304N 305N 306N 307N 308N 309N 310N 311N 312N 313N 314N 315N 316N 317N 318N 373 16169A 16192 16235 16270 16302	[0.153] U5b3a1a (1)	[0.000] U5b3a1 U5b3a1b (3)	1
2104	>KY408876.1 Hc	263G 301N 302N 303N 304N 305N 306N 307N 308N 309N 310N 311N 312N 313N 314N 315N 316N 317N 318N 337C 480C 750G 1438G 3106d 3107C 3615G 4769G 6776C 8860G 8937C 11914A 15326G 16293G 16368C 16519C	H3x1	263G 301N 302N 303N 304N 305N 306N 307N 308N 309N 310N 311N 312N 313N 314N 315N 316N 317N 318N 337C 480C 16293G 16368C 16519C	1	263 301N 302N 303N 304N 305N 306N 307N 308N 309N 310N 311N 312N 313N 314N 315N 316N 317N 318N 337C 480 750 1438 3615 4769 6776 8860 8937 11914 15326 16293 16368	H3x1	263 301N 302N 303N 304N 305N 306N 307N 308N 309N 310N 311N 312N 313N 314N 315N 316N 317N 318N 337C 480 16293 16368	[0.283] H3x1 (1)	[0.007] H24a (2) [0.000] H3x H24 H24a2 H24b (6)	1
2105	>KY408869.1 Hc	73G 150T 263G 301N 302N 303N 304N 305N 306N 307N 308N 309N 310N 311N 312N 313N 314N 315N 316N 317N 318N 373G 750G 1438G 2706G 3106d 3107C 3197C 4769G 7028T 7226A 7768G 8860G 9477A 11177T 11467G 11719A 11890R 12308G 12372A 13617C 14182C 14766T 15326G 16169A 16192T 16235G 16270T 16519C	U5b3a1a	73G 150T 263G 301N 302N 303N 304N 305N 306N 307N 308N 309N 310N 311N 312N 313N 314N 315N 316N 317N 318N 373G 16169A 16192T 16235G 16270T 16519C	1	73 150 263 301N 302N 303N 304N 305N 306N 307N 308N 309N 310N 311N 312N 313N 314N 315N 316N 317N 318N 373 750 1438 2706 3197 4769 7028 7226 7768 8860 9477 11177 11467 11719 11890R 12308 12372 13617 14182 14766 15326 16169A 16192 16235 16270	U5b3a1a	73 150 263 301N 302N 303N 304N 305N 306N 307N 308N 309N 310N 311N 312N 313N 314N 315N 316N 317N 318N 373 16169A 16192 16235 16270	[0.000] U5b3a1a (1)	[0.000] U5b3a1 U5b3a1b (3)	1
2106	>KY408866.1 Hc	263G 301N 302N 303N 304N 305N 306N 307N 308N 309N 310N 311N 312N 313N 314N 315N 316N 317N 318N 337C 480C 750G 1438G 3106d 3107C 3615G 4769G 6750Y 6776C 8860G 8937C 11914A 15326G 16293G 16368C 16519C	H3x1	263G 301N 302N 303N 304N 305N 306N 307N 308N 309N 310N 311N 312N 313N 314N 315N 316N 317N 318N 337C 480C 16293G 16368C 16519C	1	263 301N 302N 303N 304N 305N 306N 307N 308N 309N 310N 311N 312N 313N 314N 315N 316N 317N 318N 337C 480 750 1438 3615 4769 6750Y 6776 8860 8937 11914 15326 16293 16368	H3x1	263 301N 302N 303N 304N 305N 306N 307N 308N 309N 310N 311N 312N 313N 314N 315N 316N 317N 318N 337C 480 16293 16368	[0.283] H3x1 (1)	[0.007] H24a (2) [0.000] H3x H24 H24a2 H24b (6)	1
2107	>KY408864.1 Hc	57C 73G 151T 152C 263G 301N 302N 303N 304N 305N 306N 307N 308N 309N 310N 311N 312N 313N 314N 315N 316N 317N 318N 709A 750G 930A 1438G 1888A 2706G 3106d 3107C 4216C 4227G 4769G 4917G 5147A 5656G 7028T 8697A 8860G 10463C 10750G 11251G 11719A 11812G 13368A 14233G 14766T 14905A 15326G 15452A 15607G 15928A 16126C 16288C 16292T 16294T 16296T 16304C 16465T 16519C	T2b3a	57C 73G 151T 152C 263G 301N 302N 303N 304N 305N 306N 307N 308N 309N 310N 311N 312N 313N 314N 315N 316N 317N 318N 16126C 16288C 16292T 16294T 16296T 16304C 16465T 16519C	1	57 73 151 152 263 301N 302N 303N 304N 305N 306N 307N 308N 309N 310N 311N 312N 313N 314N 315N 316N 317N 318N 709 750 930 1438 1888 2706 4216 4227 4769 4917 5147 5656 7028 8697 8860 10463 10750 11251 11719 11812 13368 14233 14766 14905 15326 15452A 15607 15928 16126 16288 16292 16294 16296 16304 16465	T2b3a	57 73 151 152 263 301N 302N 303N 304N 305N 306N 307N 308N 309N 310N 311N 312N 313N 314N 315N 316N 317N 318N 16126 16288 16292 16294 16296 16304 16465	[0.393] T2b3a (1)	[0.983] T2b3a1 (2)	1

2108	>KY408859.1 Hc	73G 151T 152C 263G 301N 302N 303N 304N 305N 306N 307N 308N 309N 310N 311N 312N 313N 314N 315N 316N 317N 318N 709A 750G 930A 1438G 1888A 2706G 3106d 3107C 4216C 4769G 4917G 5147A 5656G 7028T 8602C 8697A 8860G 10463C 10750G 11251G 11719A 11812G 13368A 14233G 14766T 14905A 15326G 15452A 15607G 15928A 16126C 16292T 16294T 16296T 16297Y 16519C	T2b3a	73G 151T 152C 263G 301N 302N 303N 304N 305N 306N 307N 308N 309N 310N 311N 312N 313N 314N 315N 316N 317N 318N 16126C 16292T 16294T 16296T 16297Y 16519C	1	73 151 152 263 301N 302N 303N 304N 305N 306N 307N 308N 309N 310N 311N 312N 313N 314N 315N 316N 317N 318N 709 750 930 1438 1888 2706 4216 4769 4917 5147 5656 7028 8602 8697 8860 10463 10750 11251 11719 11812 13368 14233 14766 14905 15326 15452A 15607 15928 16126 16292 16294 16296 16297Y	T2b3a	73 151 152 263 301N 302N 303N 304N 305N 306N 307N 308N 309N 310N 311N 312N 313N 314N 315N 316N 317N 318N 16126 16292 16294 16296 16297Y	[0.179] T2b3a (1)	[0.023] T2d1 (2) [0.010] T2c1 (3) [0.000] T2n	1
2109	>KY408852.1 Hc	73G 150T 263G 301N 302N 303N 304N 305N 306N 307N 308N 309N 310N 311N 312N 313N 314N 315N 316N 317N 318N 750G 1438G 2706G 3106d 3107C 3197C 3498T 4769G 5656G 5964C 6674C 7028T 7768G 8860G 9163A 9477A 11467G 11719A 12308G 12372A 13617C 14182C 14766T 15326G 15777A 16167T 16189C 16234Y 16258M 16270T 16311C 16356C	U5b1i	73G 150T 263G 301N 302N 303N 304N 305N 306N 307N 308N 309N 310N 311N 312N 313N 314N 315N 316N 317N 318N 16167T 16189C 16234Y 16258M 16270T 16311C 16356C	1	73 150 263 301N 302N 303N 304N 305N 306N 307N 308N 309N 310N 311N 312N 313N 314N 315N 316N 317N 318N 750 1438 2706 3197 3498 4769 5656 5964 6674 7028 7768 8860 9163 9477 11467 11719 12308 12372 13617 14182 14766 15326 15777 16167 16189 16234Y 16258M 16270 16311 16356	U5b1i	73 150 263 301N 302N 303N 304N 305N 306N 307N 308N 309N 310N 311N 312N 313N 314N 315N 316N 317N 318N 16167 16189 16234Y 16258M 16270 16311 16356	[1.250] U5b1i (1) [0.000] U5b2a5 U5b2a5a (3)	[0.125] U5b1c (4) [0.088] U5b1+16189+@16192 (5) [0.051] U5b2a6 (6) [0.017] U5b2a+@16192 (7) [0.011] U5b1b1+@16192 (8) [0.000] U5b1b1d U5b1b1f U5b2a4 U5b2a4a U5b2b4 (13)	1
2110	>KY408847.1 Hc	73G 150T 263G 284G 301N 302N 303N 304N 305N 306N 307N 308N 309N 310N 311N 312N 313N 314N 315N 316N 317N 318N 373G 750G 1438G 2706G 3106d 3107C 3197C 4769G 7028T 7226A 7768G 8860G 9477A 11177T 11467G 11719A 11914A 12308G 12372A 13617C 14182C 14766T 15326G 16169A 16192T 16235G 16270T 16519C	U5b3a1a	73G 150T 263G 284G 301N 302N 303N 304N 305N 306N 307N 308N 309N 310N 311N 312N 313N 314N 315N 316N 317N 318N 373G 16169A 16192T 16235G 16270T 16519C	1	73 150 263 284 301N 302N 303N 304N 305N 306N 307N 308N 309N 310N 311N 312N 313N 314N 315N 316N 317N 318N 373 750 1438 2706 3197 4769 7028 7226 7768 8860 9477 11177 11467 11719 11914 12308 12372 13617 14182 14766 15326 16169A 16192 16235 16270	U5b3a1a	73 150 263 284 301N 302N 303N 304N 305N 306N 307N 308N 309N 310N 311N 312N 313N 314N 315N 316N 317N 318N 373 16169A 16192 16235 16270	[0.184] U5b3a1a (1)	[0.000] U5b3a1 U5b3a1b (3)	1
2111	>KY408832.1 Hc	73G 150T 263G 301N 302N 303N 304N 305N 306N 307N 308N 309N 310N 311N 312N 313N 314N 315N 316N 317N 318N 750G 1438G 2706G 3106d 3107C 3197C 3498T 4769G 5656G 5964C 6674C 7028T 7768G 8860G 9163A 9477A 11467G 11719A 12308G 12372A 13617C 14182C 14766T 15326G 15777A 16167T 16189C 16270T 16311C 16356C	U5b1i	73G 150T 263G 301N 302N 303N 304N 305N 306N 307N 308N 309N 310N 311N 312N 313N 314N 315N 316N 317N 318N 16167T 16189C 16270T 16311C 16356C	1	73 150 263 301N 302N 303N 304N 305N 306N 307N 308N 309N 310N 311N 312N 313N 314N 315N 316N 317N 318N 750 1438 2706 3197 3498 4769 5656 5964 6674 7028 7768 8860 9163 9477 11467 11719 12308 12372 13617 14182 14766 15326 15777 16167 16189 16270 16311 16356	U5b1i	73 150 263 301N 302N 303N 304N 305N 306N 307N 308N 309N 310N 311N 312N 313N 314N 315N 316N 317N 318N 16167 16189 16270 16311 16356	[1.222] U5b1i (1) [0.000] U5b2a5 U5b2a5a (3)	[0.094] U5b1c (4) [0.077] U5b1+16189+@16192 (5) [0.051] U5b2a6 (6) [0.017] U5b2a+@16192 (7) [0.000] U5b1b1+@16192 U5b1b1d U5b1b1f U5b2a4 U5b2a4a U5b2b4 (13)	1

2112 >KY408829.1 Hc	73G 150T 263G 301N 302N 303N 304N 305N 306N 307N 308N 309N 310N 311N 312N 313N 314N 315N 316N 317N 318N 750G 1438G 2706G 3106d 3107C 3197C 3498T 4769G 5656G 5964C 6674C 7028T 7768G 8860G 9163A 9477A 11467G 11719A 12308G 12372A 13617C 14182C 14766T 15326G 15777A 16167T 16189C 16270T 16311C 16356C	U5b1i	73G 150T 263G 301N 302N 303N 304N 305N 306N 307N 308N 309N 310N 311N 312N 313N 314N 315N 316N 317N 318N 16167T 16189C 16270T 16311C 16356C	1	73 150 263 301N 302N 303N 304N 305N 306N 307N 308N 309N 310N 311N 312N 313N 314N 315N 316N 317N 318N 750 1438 2706 3197 3498 4769 5656 5964 6674 7028 7768 8860 9163 9477 11467 11719 12308 12372 13617 14182 14766 15326 15777 16167 16189 16270 16311 16356	U5b1i	73 150 263 301N 302N 303N 304N 305N 306N 307N 308N 309N 310N 311N 312N 313N 314N 315N 316N 317N 318N 16167 16189 16270 16311 16356	[1.222] U5b1i (1) [0.000] U5b2a5 U5b2a5a (3)	[0.094] U5b1c (4) [0.077] U5b1+16189+@16192 (5) [0.051] U5b2a6 (6) [0.017] U5b2a+@16192 (7) [0.000] U5b1b1+@16192 U5b1b1d U5b1b1f U5b2a4 U5b2a4a U5b2b4 (13)	1
2113 >KY408801.1 Hc	73G 150T 263G 301N 302N 303N 304N 305N 306N 307N 308N 309N 310N 311N 312N 313N 314N 315N 316N 317N 318N 750G 1438G 2706G 3106d 3107C 3197C 3498T 4769G 5656G 5964C 6674C 7028T 7768G 8860G 9163A 9477A 11467G 11719A 12308G 12372A 13617C 14182C 14766T 15326G 15777A 16167T 16189C 16270T 16311C 16356C	U5b1i	73G 150T 263G 301N 302N 303N 304N 305N 306N 307N 308N 309N 310N 311N 312N 313N 314N 315N 316N 317N 318N 16167T 16189C 16270T 16311C 16356C	1	73 150 263 301N 302N 303N 304N 305N 306N 307N 308N 309N 310N 311N 312N 313N 314N 315N 316N 317N 318N 750 1438 2706 3197 3498 4769 5656 5964 6674 7028 7768 8860 9163 9477 11467 11719 12308 12372 13617 14182 14766 15326 15777 16167 16189 16270 16311 16356	U5b1i	73 150 263 301N 302N 303N 304N 305N 306N 307N 308N 309N 310N 311N 312N 313N 314N 315N 316N 317N 318N 16167 16189 16270 16311 16356	[1.222] U5b1i (1) [0.000] U5b2a5 U5b2a5a (3)	[0.094] U5b1c (4) [0.077] U5b1+16189+@16192 (5) [0.051] U5b2a6 (6) [0.017] U5b2a+@16192 (7) [0.000] U5b1b1+@16192 U5b1b1d U5b1b1f U5b2a4 U5b2a4a U5b2b4 (13)	1
2114 >KY408785.1 Hc	73G 150T 263G 301N 302N 303N 304N 305N 306N 307N 308N 309N 310N 311N 312N 313N 314N 315N 316N 317N 318N 750G 1438G 2706G 3106d 3107C 3197C 3498T 4769G 5656G 5964C 6674C 7028T 7768G 8860G 9163A 9477A 11467G 11719A 12308G 12372A 13617C 14182C 14766T 15326G 15777A 16167T 16189C 16270T 16311C 16356C	U5b1i	73G 150T 263G 301N 302N 303N 304N 305N 306N 307N 308N 309N 310N 311N 312N 313N 314N 315N 316N 317N 318N 16167T 16189C 16270T 16311C 16356C	1	73 150 263 301N 302N 303N 304N 305N 306N 307N 308N 309N 310N 311N 312N 313N 314N 315N 316N 317N 318N 750 1438 2706 3197 3498 4769 5656 5964 6674 7028 7768 8860 9163 9477 11467 11719 12308 12372 13617 14182 14766 15326 15777 16167 16189 16270 16311 16356	U5b1i	73 150 263 301N 302N 303N 304N 305N 306N 307N 308N 309N 310N 311N 312N 313N 314N 315N 316N 317N 318N 16167 16189 16270 16311 16356	[1.222] U5b1i (1) [0.000] U5b2a5 U5b2a5a (3)	[0.094] U5b1c (4) [0.077] U5b1+16189+@16192 (5) [0.051] U5b2a6 (6) [0.017] U5b2a+@16192 (7) [0.000] U5b1b1+@16192 U5b1b1d U5b1b1f U5b2a4 U5b2a4a U5b2b4 (13)	1
2115 >KY408782.1 Hc	73G 150T 263G 301N 302N 303N 304N 305N 306N 307N 308N 309N 310N 311N 312N 313N 314N 315N 316N 317N 318N 373G 750G 1438G 2706G 3106d 3107C 3197C 4769G 7028T 7226A 7768G 8860G 9477A 11177T 11467G 11719A 12308G 12372A 13617C 14182C 14766T 15326G 16145A 16169A 16192T 16235G 16270T 16311C 16519C 16527T	U5b3a1a	73G 150T 263G 301N 302N 303N 304N 305N 306N 307N 308N 309N 310N 311N 312N 313N 314N 315N 316N 317N 318N 373G 16145A 16169A 16192T 16235G 16270T 16311C 16519C 16527T	1	73 150 263 301N 302N 303N 304N 305N 306N 307N 308N 309N 310N 311N 312N 313N 314N 315N 316N 317N 318N 373 750 1438 2706 3197 4769 7028 7226 7768 8860 9477 11177 11467 11719 12308 12372 13617 14182 14766 15326 16145 16169A 16192 16235 16270 16311 16527	U5b3a1a	73 150 263 301N 302N 303N 304N 305N 306N 307N 308N 309N 310N 311N 312N 313N 314N 315N 316N 317N 318N 373 16145 16169A 16192 16235 16270 16311 16527	[0.152] U5b3a1a (1)	[0.000] U5b3a1 U5b3a1b (3)	1
2116 >KY408776.1 Hc	73G 150T 263G 301N 302N 303N 304N 305N 306N 307N 308N 309N 310N 311N 312N 313N 314N 315N 316N 317N 318N 750G 1438G 2706G 3106d 3107C 3197C 3498T 4769G 5656G 5964C 6674C 7028T 7768G 8860G 9163A 9477A 11467G 11719A 12308G 12372A 13617C 14182C 14766T 15326G 15777A 16167T 16189C 16270T 16311C 16356C	U5b1i	73G 150T 263G 301N 302N 303N 304N 305N 306N 307N 308N 309N 310N 311N 312N 313N 314N 315N 316N 317N 318N 16167T 16189C 16270T 16311C 16356C	1	73 150 263 301N 302N 303N 304N 305N 306N 307N 308N 309N 310N 311N 312N 313N 314N 315N 316N 317N 318N 750 1438 2706 3197 3498 4769 5656 5964 6674 7028 7768 8860 9163 9477 11467 11719 12308 12372 13617 14182 14766 15326 15777 16167 16189 16270 16311 16356	U5b1i	73 150 263 301N 302N 303N 304N 305N 306N 307N 308N 309N 310N 311N 312N 313N 314N 315N 316N 317N 318N 16167 16189 16270 16311 16356	[1.222] U5b1i (1) [0.000] U5b2a5 U5b2a5a (3)	[0.094] U5b1c (4) [0.077] U5b1+16189+@16192 (5) [0.051] U5b2a6 (6) [0.017] U5b2a+@16192 (7) [0.000] U5b1b1+@16192 U5b1b1d U5b1b1f U5b2a4 U5b2a4a U5b2b4 (13)	1

2117 >KY408774.1 Hc	73G 150T 263G 301N 302N 303N 304N 305N 306N 307N 308N 309N 310N 311N 312N 313N 314N 315N 316N 317N 318N 373G 750G 1438G 2706G 3106d 3107C 3197C 4769G 4958G 7028T 7226A 7768G 8860G 9477A 11177T 11467G 11719A 12308G 12372A 13617C 14182C 14766T 15326G 16169A 16192T 16235G 16270T 16519C	U5b3a1a	73G 150T 263G 301N 302N 303N 304N 305N 306N 307N 308N 309N 310N 311N 312N 313N 314N 315N 316N 317N 318N 373G 16169A 16192T 16235G 16270T 16519C	1	73 150 263 301N 302N 303N 304N 305N 306N 307N 308N 309N 310N 311N 312N 313N 314N 315N 316N 317N 318N 373 750 1438 2706 3197 4769 4958 7028 7226 7768 8860 9477 11177 11467 11719 12308 12372 13617 14182 14766 15326 16169A 16192 16235 16270	U5b3a1a	73 150 263 301N 302N 303N 304N 305N 306N 307N 308N 309N 310N 311N 312N 313N 314N 315N 316N 317N 318N 373 16169A 16192 16235 16270	[0.000] U5b3a1a (1)	[0.000] U5b3a1 U5b3a1b (3)	1
2118 >KY408766.1 Hc	73G 152C 217C 263G 301N 302N 303N 304N 305N 306N 307N 308N 309N 310N 311N 312N 313N 314N 315N 316N 317N 318N 508G 750G 1154T 1438G 1811G 2706G 3106d 3107C 3720G 3865G 4769G 5390G 5426C 6045T 6152C 7028T 8860G 10876G 11016A 11467G 11719A 12308G 12372A 13020C 13734C 13758T 14766T 15326G 15454C 15732T 15907G 16051G 16129C 16179T 16182C 16183C 16189C 16362C 16519C	U2e1'2'3	73G 152C 217C 263G 301N 302N 303N 304N 305N 306N 307N 308N 309N 310N 311N 312N 313N 314N 315N 316N 317N 318N 508G 16051G 16129C 16179T 16182C 16183C 16189C 16362C 16519C	1	73 152 217 263 301N 302N 303N 304N 305N 306N 307N 308N 309N 310N 311N 312N 313N 314N 315N 316N 317N 318N 508 750 1154T 1438 1811 2706 3720 3865 4769 5390 5426 6045 6152 7028 8860 10876 11016 11467 11719 12308 12372 13020 13734 13758 14766 15326 15454 15732 15907 16051 16129C 16179 16189 16362	U2e1'2'3	73 152 217 263 301N 302N 303N 304N 305N 306N 307N 308N 309N 310N 311N 312N 313N 314N 315N 316N 317N 318N 508 16051 16129C 16179 16189 16362	[0.494] U2e1'2'3 (1) [0.000] U2e2 U2e2a U2e2a1 U2e2a1a U2e2a1a1 U2e2a1a2 U2e2a1c U2e2a1d (9)	[0.030] U2e1a1a (10) [0.009] U2e1a1 (11) [0.000] U2e U2e1 U2e1a U2e1a1c U2e1c U2e1c1 U2e1d U2e1g U2e2 U2e2a U2e2a1 U2e2a1a U2e2a1a1 U2e2a1a2 U2e2a1b U2e2a1c U2e2a1d (28)	1
2119 >KY408750.1 Hc	73G 152C 217C 263G 301N 302N 303N 304N 305N 306N 307N 308N 309N 310N 311N 312N 313N 314N 315N 316N 317N 318N 508G 750G 1154T 1438G 1811G 2706G 3106d 3107C 3720G 3865G 4769G 5390G 5426C 6045T 6152C 7028T 8860G 10876G 11016A 11467G 11719A 12308G 12372A 13020C 13734C 13758T 14766T 15326G 15454C 15732T 15907G 16051G 16129C 16179T 16182C 16183C 16189C 16362C 16519C	U2e1'2'3	73G 152C 217C 263G 301N 302N 303N 304N 305N 306N 307N 308N 309N 310N 311N 312N 313N 314N 315N 316N 317N 318N 508G 16051G 16129C 16179T 16182C 16183C 16189C 16362C 16519C	1	73 152 217 263 301N 302N 303N 304N 305N 306N 307N 308N 309N 310N 311N 312N 313N 314N 315N 316N 317N 318N 508 750 1154T 1438 1811 2706 3720 3865 4769 5390 5426 6045 6152 7028 8860 10876 11016 11467 11719 12308 12372 13020 13734 13758 14766 15326 15454 15732 15907 16051 16129C 16179 16189 16362	U2e1'2'3	73 152 217 263 301N 302N 303N 304N 305N 306N 307N 308N 309N 310N 311N 312N 313N 314N 315N 316N 317N 318N 508 16051 16129C 16179 16189 16362	[0.494] U2e1'2'3 (1) [0.000] U2e2 U2e2a U2e2a1 U2e2a1a U2e2a1a1 U2e2a1a2 U2e2a1c U2e2a1d (9)	[0.030] U2e1a1a (10) [0.009] U2e1a1 (11) [0.000] U2e U2e1 U2e1a U2e1a1c U2e1c U2e1c1 U2e1d U2e1g U2e2 U2e2a U2e2a1 U2e2a1a U2e2a1a1 U2e2a1a2 U2e2a1b U2e2a1c U2e2a1d (28)	1
2120 >KY408748.1 Hc	73G 150T 263G 301N 302N 303N 304N 305N 306N 307N 308N 309N 310N 311N 312N 313N 314N 315N 316N 317N 318N 373G 750G 1438G 2706G 3106d 3107C 3197C 4769G 7028T 7226A 7768G 8860G 9477A 11177T 11467G 11719A 12308G 12372A 13617C 14182C 14766T 15326G 16169A 16192T 16235G 16270T 16519C	U5b3a1a	73G 150T 263G 301N 302N 303N 304N 305N 306N 307N 308N 309N 310N 311N 312N 313N 314N 315N 316N 317N 318N 373G 16169A 16192T 16235G 16270T 16519C	1	73 150 263 301N 302N 303N 304N 305N 306N 307N 308N 309N 310N 311N 312N 313N 314N 315N 316N 317N 318N 373 750 1438 2706 3197 4769 7028 7226 7768 8860 9477 11177 11467 11719 12308 12372 13617 14182 14766 15326 16169A 16192 16235 16270	U5b3a1a	73 150 263 301N 302N 303N 304N 305N 306N 307N 308N 309N 310N 311N 312N 313N 314N 315N 316N 317N 318N 373 16169A 16192 16235 16270	[0.000] U5b3a1a (1)	[0.000] U5b3a1 U5b3a1b (3)	1

2121	>KY408745.1 Hc	73G 150T 263G 284G 301N 302N 303N 304N 305N 306N 307N 308N 309N 310N 311N 312N 313N 314N 315N 316N 317N 318N 373G 750G 1438G 2706G 3106d 3107C 3197C 4769G 7028T 7226A 7768G 8860G 9477A 11177T 11467G 11719A 11914A 12308G 12372A 13617C 14182C 14766T 15244G 15326G 16169A 16192T 16235G 16270T 16519C	U5b3a1a	73G 150T 263G 284G 301N 302N 303N 304N 305N 306N 307N 308N 309N 310N 311N 312N 313N 314N 315N 316N 317N 318N 373G 16169A 16192T 16235G 16270T 16519C	1	73 150 263 284 301N 302N 303N 304N 305N 306N 307N 308N 309N 310N 311N 312N 313N 314N 315N 316N 317N 318N 373 750 1438 2706 3197 4769 7028 7226 7768 8860 9477 11177 11467 11719 11914 12308 12372 13617 14182 14766 15244 15326 16169A 16192 16235 16270	U5b3a1a	73 150 263 284 301N 302N 303N 304N 305N 306N 307N 308N 309N 310N 311N 312N 313N 314N 315N 316N 317N 318N 373 16169A 16192 16235 16270	[0.184] U5b3a1a (1)	[0.000] U5b3a1 U5b3a1b (3)	1
2122	>KY408742.1 Hc	73G 103A 263G 301N 302N 303N 304N 305N 306N 307N 308N 309N 310N 311N 312N 313N 314N 315N 316N 317N 318N 750G 1438G 2706G 3106d 3107C 3348G 4655A 4769G 4936T 7028T 7805A 8860G 8943T 9128C 9335A 10172A 11467G 11719A 12308G 12372A 14179G 14766T 15326G 16172C 16183C 16219G 16278T 16295T	U6a8b	73G 103A 263G 301N 302N 303N 304N 305N 306N 307N 308N 309N 310N 311N 312N 313N 314N 315N 316N 317N 318N 16172C 16183C 16219G 16278T 16295T	1	73 103 263 301N 302N 303N 304N 305N 306N 307N 308N 309N 310N 311N 312N 313N 314N 315N 316N 317N 318N 750 1438 2706 3348 4655 4769 4936 7028 7805 8860 8943 9128 9335A 10172 11467 11719 12308 12372 14179 14766 15326 16172 16219 16278 16295	U6a8b	73 103 263 301N 302N 303N 304N 305N 306N 307N 308N 309N 310N 311N 312N 313N 314N 315N 316N 317N 318N 16172 16219 16278 16295	[0.194] U6a8b (1)	[0.194] U6a8b (2) [0.042] U6a6 (3) [0.000] U6a U6a1 U6a+16189+(103) U6a2 U6a2a U6a2a1 U6a8 U6a5 U6a5a U6a5a1 U6a6b U6a6b1 U6a6b2 U6a7 U6a7a1+@152 U6a7a1c (19)	1
2123	>KY408732.1 Hc	73G 150T 263G 301N 302N 303N 304N 305N 306N 307N 308N 309N 310N 311N 312N 313N 314N 315N 316N 317N 318N 373G 750G 1438G 2706G 3106d 3107C 3197C 4769G 7028T 7226A 7768G 8860G 9477A 9839T 11177T 11467G 11647T 11719A 12308G 12358G 12372A 13617C 14182C 14766T 15326G 16169A 16192T 16235G 16270T 16302G 16519C	U5b3a1a	73G 150T 263G 301N 302N 303N 304N 305N 306N 307N 308N 309N 310N 311N 312N 313N 314N 315N 316N 317N 318N 373G 16169A 16192T 16235G 16270T 16302G 16519C	1	73 150 263 301N 302N 303N 304N 305N 306N 307N 308N 309N 310N 311N 312N 313N 314N 315N 316N 317N 318N 373 750 1438 2706 3197 4769 7028 7226 7768 8860 9477 9839 11177 11467 11647 11719 12308 12358 12372 13617 14182 14766 15326 16169A 16192 16235 16270 16302	U5b3a1a	73 150 263 301N 302N 303N 304N 305N 306N 307N 308N 309N 310N 311N 312N 313N 314N 315N 316N 317N 318N 373 16169A 16192 16235 16270 16302	[0.153] U5b3a1a (1)	[0.000] U5b3a1 U5b3a1b (3)	1
2124	>KY408721.1 Hc	73G 150T 263G 301N 302N 303N 304N 305N 306N 307N 308N 309N 310N 311N 312N 313N 314N 315N 316N 317N 318N 373G 750G 1438G 2706G 3106d 3107C 3197C 4769G 7028T 7226A 7768G 8860G 9477A 11177T 11467G 11719A 12308G 12372A 13617C 13760T 14182C 14766T 15326G 16169A 16192T 16235G 16270T 16519C	U5b3a1a	73G 150T 263G 301N 302N 303N 304N 305N 306N 307N 308N 309N 310N 311N 312N 313N 314N 315N 316N 317N 318N 373G 16169A 16192T 16235G 16270T 16519C	1	73 150 263 301N 302N 303N 304N 305N 306N 307N 308N 309N 310N 311N 312N 313N 314N 315N 316N 317N 318N 373 750 1438 2706 3197 4769 7028 7226 7768 8860 9477 11177 11467 11719 12308 12372 13617 13760 14182 14766 15326 16169A 16192 16235 16270	U5b3a1a	73 150 263 301N 302N 303N 304N 305N 306N 307N 308N 309N 310N 311N 312N 313N 314N 315N 316N 317N 318N 373 16169A 16192 16235 16270	[0.000] U5b3a1a (1)	[0.000] U5b3a1 U5b3a1b (3)	1

2125	>KY408717.1 Hc	73G 152C 217C 263G 301N 302N 303N 304N 305N 306N 307N 308N 309N 310N 311N 312N 313N 314N 315N 316N 317N 318N 508G 750G 1154T 1438G 1811G 2706G 3106d 3107C 3720G 3865G 4769G 5390G 5426C 6045T 6152C 7028T 8860G 10876G 11016A 11467G 11719A 12308G 12372A 13020C 13734C 13758T 14766T 15326G 15454C 15575R 15732T 15907G 16051G 16129C 16179T 16182C 16183C 16189C 16362C 16519C	U2e1'2'3	73G 152C 217C 263G 301N 302N 303N 304N 305N 306N 307N 308N 309N 310N 311N 312N 313N 314N 315N 316N 317N 318N 508G 16051G 16129C 16179T 16182C 16183C 16189C 16362C 16519C	1	73 152 217 263 301N 302N 303N 304N 305N 306N 307N 308N 309N 310N 311N 312N 313N 314N 315N 316N 317N 318N 508 750 1154T 1438 1811 2706 3720 3865 4769 5390 5426 6045 6152 7028 8860 10876 11016 11467 11719 12308 12372 13020 13734 13758 14766 15326 15454 15575R 15732 15907 16051 16129C 16179 16189 16362	U2e1'2'3	73 152 217 263 301N 302N 303N 304N 305N 306N 307N 308N 309N 310N 311N 312N 313N 314N 315N 316N 317N 318N 508 16051 16129C 16179 16189 16362	[0.494] U2e1'2'3 (1) [0.000] U2e2 U2e2a U2e2a1 U2e2a1a U2e2a1a1 U2e2a1a2 U2e2a1c U2e2a1d (9)	[0.030] U2e1a1a (10) [0.009] U2e1a1 (11) [0.000] U2e U2e1 U2e1a U2e1a1c U2e1c U2e1c1 U2e1d U2e1g U2e2 U2e2a U2e2a1 U2e2a1a U2e2a1a1 U2e2a1a2 U2e2a1b U2e2a1c U2e2a1d (28)	1
2126	>KY408700.1 Hc	73G 150T 263G 301N 302N 303N 304N 305N 306N 307N 308N 309N 310N 311N 312N 313N 314N 315N 316N 317N 318N 750G 1438G 2706G 3106d 3107C 3197C 3498T 4769G 5656G 5964C 6674C 7028T 7768G 8860G 9163A 9477A 11467G 11719A 12308G 12372A 13617C 14182C 14766T 15326G 15777A 16167T 16189C 16270T 16311C 16356C	U5b1i	73G 150T 263G 301N 302N 303N 304N 305N 306N 307N 308N 309N 310N 311N 312N 313N 314N 315N 316N 317N 318N 16167T 16189C 16270T 16311C 16356C	1	73 150 263 301N 302N 303N 304N 305N 306N 307N 308N 309N 310N 311N 312N 313N 314N 315N 316N 317N 318N 750 1438 2706 3197 3498 4769 5656 5964 6674 7028 7768 8860 9163 9477 11467 11719 12308 12372 13617 14182 14766 15326 15777 16167 16189 16270 16311 16356	U5b1i	73 150 263 301N 302N 303N 304N 305N 306N 307N 308N 309N 310N 311N 312N 313N 314N 315N 316N 317N 318N 16167 16189 16270 16311 16356	[1.222] U5b1i (1) [0.000] U5b2a5 U5b2a5a (3)	[0.094] U5b1c (4) [0.077] U5b1+16189+@16192 (5) [0.051] U5b2a6 (6) [0.017] U5b2a+@16192 (7) [0.000] U5b1b1+@16192 U5b1b1d U5b1b1f U5b2a4 U5b2a4a U5b2b4 (13)	1
2127	>KY408684.1 Hc	73G 150T 263G 284G 301N 302N 303N 304N 305N 306N 307N 308N 309N 310N 311N 312N 313N 314N 315N 316N 317N 318N 373G 750G 1438G 2706G 3106d 3107C 3197C 4769G 7028T 7226A 7768G 8860G 9477A 11177T 11467G 11719A 11914A 12308G 12372A 13617C 14182C 14766T 15244G 15326G 16169A 16192T 16235G 16270T 16519C	U5b3a1a	73G 150T 263G 284G 301N 302N 303N 304N 305N 306N 307N 308N 309N 310N 311N 312N 313N 314N 315N 316N 317N 318N 373G 16169A 16192T 16235G 16270T 16519C	1	73 150 263 284 301N 302N 303N 304N 305N 306N 307N 308N 309N 310N 311N 312N 313N 314N 315N 316N 317N 318N 373 750 1438 2706 3197 4769 7028 7226 7768 8860 9477 11177 11467 11719 11914 12308 12372 13617 14182 14766 15244 15326 16169A 16192 16235 16270	U5b3a1a	73 150 263 284 301N 302N 303N 304N 305N 306N 307N 308N 309N 310N 311N 312N 313N 314N 315N 316N 317N 318N 373 16169A 16192 16235 16270	[0.184] U5b3a1a (1)	[0.000] U5b3a1 U5b3a1b (3)	1
2128	>KY408683.1 Hc	73G 152C 217C 263G 301N 302N 303N 304N 305N 306N 307N 308N 309N 310N 311N 312N 313N 314N 315N 316N 317N 318N 508G 750G 1154T 1438G 1811G 2706G 3106d 3107C 3720G 3865G 4769G 5390G 5426C 6045T 6152C 7028T 8860G 10876G 11016A 11467G 11719A 12308G 12372A 13020C 13734C 13758T 14766T 15326G 15454C 15732T 15907G 16051G 16129C 16179T 16182C 16183C 16189C 16362C 16519C	U2e1'2'3	73G 152C 217C 263G 301N 302N 303N 304N 305N 306N 307N 308N 309N 310N 311N 312N 313N 314N 315N 316N 317N 318N 508G 16051G 16129C 16179T 16182C 16183C 16189C 16362C 16519C	1	73 152 217 263 301N 302N 303N 304N 305N 306N 307N 308N 309N 310N 311N 312N 313N 314N 315N 316N 317N 318N 508 750 1154T 1438 1811 2706 3720 3865 4769 5390 5426 6045 6152 7028 8860 10876 11016 11467 11719 12308 12372 13020 13734 13758 14766 15326 15454 15732 15907 16051 16129C 16179 16189 16362	U2e1'2'3	73 152 217 263 301N 302N 303N 304N 305N 306N 307N 308N 309N 310N 311N 312N 313N 314N 315N 316N 317N 318N 508 16051 16129C 16179 16189 16362	[0.494] U2e1'2'3 (1) [0.000] U2e2 U2e2a U2e2a1 U2e2a1a U2e2a1a1 U2e2a1a2 U2e2a1c U2e2a1d (9)	[0.030] U2e1a1a (10) [0.009] U2e1a1 (11) [0.000] U2e U2e1 U2e1a U2e1a1c U2e1c U2e1c1 U2e1d U2e1g U2e2 U2e2a U2e2a1 U2e2a1a U2e2a1a1 U2e2a1a2 U2e2a1b U2e2a1c U2e2a1d (28)	1

2129 >KY408681.1 Ho	263G 301N 302N 303N 304N 305N 306N 307N 308N 309N 310N 311N 312N 313N 314N 315N 316N 317N 318N 337C 480C 750G 1438G 3106d 3107C 3615G 4769G 6776C 8860G 8937C 11914A 15326G 16293G 16368C 16519C	H3x1	263G 301N 302N 303N 304N 305N 306N 307N 308N 309N 310N 311N 312N 313N 314N 315N 316N 317N 318N 337C 480C 16293G 16368C 16519C	1	263 301N 302N 303N 304N 305N 306N 307N 308N 309N 310N 311N 312N 313N 314N 315N 316N 317N 318N 337C 480 750 1438 3615 4769 6776 8860 8937 11914 15326 16293 16368	H3x1	263 301N 302N 303N 304N 305N 306N 307N 308N 309N 310N 311N 312N 313N 314N 315N 316N 317N 318N 337C 480 16293 16368	[0.283] H3x1 (1)	[0.007] H24a (2) [0.000] H3x H24 H24a2 H24b (6)	1
2130 >KY408673.1 Ho	73G 152C 217C 263G 301N 302N 303N 304N 305N 306N 307N 308N 309N 310N 311N 312N 313N 314N 315N 316N 317N 318N 508G 650Y 750G 1154T 1438G 1811G 2706G 3106d 3107C 3720G 3865G 4769G 5390G 5426C 6045T 6152C 7028T 8860G 10876G 11016A 11467G 11719A 12308G 12372A 13020C 13734C 13758T 14766T 15326G 15454C 15732T 15907G 16051G 16129C 16179T 16182C 16183C 16189C 16362C 16519C	U2e1'2'3	73G 152C 217C 263G 301N 302N 303N 304N 305N 306N 307N 308N 309N 310N 311N 312N 313N 314N 315N 316N 317N 318N 508G 16051G 16129C 16179T 16182C 16183C 16189C 16362C 16519C	1	73 152 217 263 301N 302N 303N 304N 305N 306N 307N 308N 309N 310N 311N 312N 313N 314N 315N 316N 317N 318N 508 650Y 750 1154T 1438 1811 2706 3720 3865 4769 5390 5426 6045 6152 7028 8860 10876 11016 11467 11719 12308 12372 13020 13734 13758 14766 15326 15454 15732 15907 16051 16129C 16179 16189 16362	U2e1'2'3	73 152 217 263 301N 302N 303N 304N 305N 306N 307N 308N 309N 310N 311N 312N 313N 314N 315N 316N 317N 318N 508 16051 16129C 16179 16189 16362	[0.494] U2e1'2'3 (1) [0.000] U2e2 U2e2a U2e2a1 U2e2a1a U2e2a1a1 U2e2a1a2 U2e2a1c U2e2a1d (9)	[0.030] U2e1a1a (10) [0.009] U2e1a1 (11) [0.000] U2e U2e1 U2e1a U2e1a1c U2e1c U2e1c1 U2e1d U2e1g U2e2 U2e2a U2e2a1 U2e2a1a U2e2a1a1 U2e2a1a2 U2e2a1b U2e2a1c U2e2a1d (28)	1
2131 >KY409457.1 Ho	73G 150T 204Y 263G 301N 302N 303N 304N 305N 306N 307N 308N 309N 310N 311N 312N 313N 314N 315N 316N 317N 318N 750G 1438G 2706G 3106d 3107C 3197C 3498T 4769G 5656G 6674C 7028T 7768G 8817G 8860G 9477A 11467G 11719A 12308G 12372A 13617C 14182C 14766T 15326G 15773A 15777A 16167T 16192T 16270T 16311C 16356C	U5b1i	73G 150T 204Y 263G 301N 302N 303N 304N 305N 306N 307N 308N 309N 310N 311N 312N 313N 314N 315N 316N 317N 318N 16167T 16192T 16270T 16311C 16356C	1	73 150 204Y 263 301N 302N 303N 304N 305N 306N 307N 308N 309N 310N 311N 312N 313N 314N 315N 316N 317N 318N 750 1438 2706 3197 3498 4769 5656 6674 7028 7768 8817 8860 9477 11467 11719 12308 12372 13617 14182 14766 15326 15773 15777 16167 16192 16270 16311 16356	U5b1i	73 150 204Y 263 301N 302N 303N 304N 305N 306N 307N 308N 309N 310N 311N 312N 313N 314N 315N 316N 317N 318N 16167 16192 16270 16311 16356	[0.014] U5b1i (1)	[0.280] U5b1a (2) [0.026] U5b2a1a+16311 U5b2a1a1d (4) [0.025] U5b1 (5) [0.000] U5b U5b1c U5b1d U5b1d1 U5b1f U5b2 U5b2a1a1 U5b2a1a1a U5b2c U5b2c1 U5b2c2 U5b2c2a (17)	1
2132 >KY409453.1 Ho	57C 59C 62T 151T 152C 263G 301N 302N 303N 304N 305N 306N 307N 308N 309N 310N 311N 312N 313N 314N 315N 316N 317N 318N 709A 750G 930A 1438G 1888A 2706G 2779T 3106d 3107C 4216C 4769G 4917G 5147A 5656G 7028T 8697A 8860G 10463C 10750G 11251G 11719A 11812G 13368A 14233G 14560A 14766T 14905A 15326G 15452A 15607G 15928A 16126C 16292T 16294T 16304C 16519C	T2b3a1	57C 59C 62T 151T 152C 263G 301N 302N 303N 304N 305N 306N 307N 308N 309N 310N 311N 312N 313N 314N 315N 316N 317N 318N 16126C 16292T 16294T 16304C 16519C	1	57 59 62T 151 152 263 301N 302N 303N 304N 305N 306N 307N 308N 309N 310N 311N 312N 313N 314N 315N 316N 317N 318N 709 750 930 1438 1888 2706 2779 4216 4769 4917 5147 5656 7028 8697 8860 10463 10750 11251 11719 11812 13368 14233 14560 14766 14905 15326 15452A 15607 15928 16126 16292 16294 16304	T2b3a1	57 59 62T 151 152 263 301N 302N 303N 304N 305N 306N 307N 308N 309N 310N 311N 312N 313N 314N 315N 316N 317N 318N 16126 16292 16294 16304	[0.839] T2b3a1 (1)	[0.839] T2b3a1 (2) [0.179] T2b3a	1

2133	>KY409445.1 Hc	73G 195C 263G 285T 301N 302N 303N 304N 305N 306N 307N 308N 309N 310N 311N 312N 313N 314N 315N 316N 317N 318N 663G 750G 1438G 2218T 2706G 3106d 3107C 4769G 4991A 6026A 7028T 7403G 7581C 8860G 11116C 11467G 11719A 12308G 12372A 12879C 13104G 13656C 14070G 14364A 14766T 15038G 15115C 15148A 15217A 15326G 15954C 16145A 16182C 16183C 16189C 16249C	U1a1c1c1	73G 195C 263G 285T 301N 302N 303N 304N 305N 306N 307N 308N 309N 310N 311N 312N 313N 314N 315N 316N 317N 318N 16145A 16182C 16183C 16189C 16249C	1	73 195 263 285 301N 302N 303N 304N 305N 306N 307N 308N 309N 310N 311N 312N 313N 314N 315N 316N 317N 318N 663 750 1438 2218 2706 4769 4991 6026 7028 7403 7581 8860 11116 11467 11719 12308 12372 12879 13104 13656 14070 14364 14766 15038 15115 15148 15217 15326 15954C 16145 16189 16249	U1a1c1c1	73 195 263 285 301N 302N 303N 304N 305N 306N 307N 308N 309N 310N 311N 312N 313N 314N 315N 316N 317N 318N 16145 16189 16249	[0.000] U1a1c1c1 (1)	[0.049] U1a1c1d (2) [0.000] U1a U1a1 U1a1c U1a1c1 U1a1c1b U1a1c1c (8)	1
2134	>KY409442.1 Hc	73G 150T 263G 301N 302N 303N 304N 305N 306N 307N 308N 309N 310N 311N 312N 313N 314N 315N 316N 317N 318N 373G 750G 1438G 2706G 3106d 3107C 3197C 4769G 7028T 7226A 7768G 8860G 9477A 11177T 11467G 11719A 12308G 12372A 13617C 14182C 14766T 15326G 15607G 16145A 16169A 16192T 16235G 16270T 16519C	U5b3a1a	73G 150T 263G 301N 302N 303N 304N 305N 306N 307N 308N 309N 310N 311N 312N 313N 314N 315N 316N 317N 318N 373G 16145A 16169A 16192T 16235G 16270T 16519C	1	73 150 263 301N 302N 303N 304N 305N 306N 307N 308N 309N 310N 311N 312N 313N 314N 315N 316N 317N 318N 373 750 1438 2706 3197 4769 7028 7226 7768 8860 9477 11177 11467 11719 12308 12372 13617 14182 14766 15326 15607 16145 16169A 16192 16235 16270	U5b3a1a	73 150 263 301N 302N 303N 304N 305N 306N 307N 308N 309N 310N 311N 312N 313N 314N 315N 316N 317N 318N 373 16145 16169A 16192 16235 16270	[0.122] U5b3a1a (1)	[0.000] U5b3a1 U5b3a1b (3)	1
2135	>KY409427.1 Hc	73G 150T 263G 301N 302N 303N 304N 305N 306N 307N 308N 309N 310N 311N 312N 313N 314N 315N 316N 317N 318N 373G 750G 1438G 2706G 3106d 3107C 3197C 4769G 7028T 7226A 7768G 8860G 9477A 11177T 11467G 11719A 12308G 12372A 13617C 14182C 14766T 15326G 16169A 16192T 16235R 16270T 16519C	U5b3a1a	73G 150T 263G 301N 302N 303N 304N 305N 306N 307N 308N 309N 310N 311N 312N 313N 314N 315N 316N 317N 318N 373G 16169A 16192T 16235R 16270T 16519C	1	73 150 263 301N 302N 303N 304N 305N 306N 307N 308N 309N 310N 311N 312N 313N 314N 315N 316N 317N 318N 373 750 1438 2706 3197 4769 7028 7226 7768 8860 9477 11177 11467 11719 12308 12372 13617 14182 14766 15326 16169A 16192 16235R 16270	U5b3a1a	73 150 263 301N 302N 303N 304N 305N 306N 307N 308N 309N 310N 311N 312N 313N 314N 315N 316N 317N 318N 373 16169A 16192 16235R 16270	[0.000] U5b3a1a (1)	[0.000] U5b3a1 U5b3a1b (3)	1
2136	>KY409424.1 Hc	73G 150T 263G 301N 302N 303N 304N 305N 306N 307N 308N 309N 310N 311N 312N 313N 314N 315N 316N 317N 318N 373G 750G 1438G 2706G 3106d 3107C 3197C 4336C 4769G 7028T 7226A 7768G 8860G 9477A 11177T 11467G 11719A 12308G 12372A 13617C 14182C 14766T 15326G 15388C 15790Y 16169A 16192T 16235G 16270T 16519C	U5b3a1a	73G 150T 263G 301N 302N 303N 304N 305N 306N 307N 308N 309N 310N 311N 312N 313N 314N 315N 316N 317N 318N 373G 16169A 16192T 16235G 16270T 16519C	1	73 150 263 301N 302N 303N 304N 305N 306N 307N 308N 309N 310N 311N 312N 313N 314N 315N 316N 317N 318N 373 750 1438 2706 3197 4336 4769 7028 7226 7768 8860 9477 11177 11467 11719 12308 12372 13617 14182 14766 15326 15388 15790Y 16169A 16192 16235 16270	U5b3a1a	73 150 263 301N 302N 303N 304N 305N 306N 307N 308N 309N 310N 311N 312N 313N 314N 315N 316N 317N 318N 373 16169A 16192 16235 16270	[0.000] U5b3a1a (1)	[0.000] U5b3a1 U5b3a1b (3)	1

2137 >KY409423.1 Hc	73G 150T 263G 301N 302N 303N 304N 305N 306N 307N 308N 309N 310N 311N 312N 313N 314N 315N 316N 317N 318N 373G 750G 1438G 2706G 3106d 3107C 3197C 4769G 5201Y 7028T 7226A 7768G 8860G 9477A 10398G 11177T 11467G 11719A 12308G 12372A 13617C 14182C 14766T 15326G 16169A 16192T 16235G 16270T 16519C	U5b3a1a	73G 150T 263G 301N 302N 303N 304N 305N 306N 307N 308N 309N 310N 311N 312N 313N 314N 315N 316N 317N 318N 373G 16169A 16192T 16235G 16270T 16519C	1	73 150 263 301N 302N 303N 304N 305N 306N 307N 308N 309N 310N 311N 312N 313N 314N 315N 316N 317N 318N 373 750 1438 2706 3197 4769 5201Y 7028 7226 7768 8860 9477 10398 11177 11467 11719 12308 12372 13617 14182 14766 15326 16169A 16192 16235 16270	U5b3a1a	73 150 263 301N 302N 303N 304N 305N 306N 307N 308N 309N 310N 311N 312N 313N 314N 315N 316N 317N 318N 373 16169A 16192 16235 16270	[0.000] U5b3a1a (1)	[0.000] U5b3a1 U5b3a1b (3)	1
2138 >KY409415.1 Hc	73G 94A 263G 301N 302N 303N 304N 305N 306N 307N 308N 309N 310N 311N 312N 313N 314N 315N 316N 317N 318N 750G 1189C 1438G 1811G 2706G 3106d 3107C 3337A 3480G 4769G 5913A 7028T 8860G 9055A 9698C 9962A 10289G 10398G 10550G 11299C 11467G 11719A 11914A 12308G 12372A 14167T 14766T 14798C 15326G 15946T 16129A 16148T 16224C 16240T 16311C 16519C	K1b1c	73G 94A 263G 301N 302N 303N 304N 305N 306N 307N 308N 309N 310N 311N 312N 313N 314N 315N 316N 317N 318N 16129A 16148T 16224C 16240T 16311C 16519C	1	73 94 263 301N 302N 303N 304N 305N 306N 307N 308N 309N 310N 311N 312N 313N 314N 315N 316N 317N 318N 750 1189 1438 1811 2706 3337 3480 4769 5913 7028 8860 9055 9698 9962 10289 10398 10550 11299 11467 11719 11914 12308 12372 14167 14766 14798 15326 15946 16129 16148 16224 16240T 16311	K1b1c	73 94 263 301N 302N 303N 304N 305N 306N 307N 308N 309N 310N 311N 312N 313N 314N 315N 316N 317N 318N 16129 16148 16224 16240T 16311	[0.047] K1b1c (1)	[0.000] K K1 K1b K1b1 K1b1+(16093) K1b1b K1b1b1 K2b1b (9)	1
2139 >KY409413.1 Hc	73G 195C 263G 285T 301N 302N 303N 304N 305N 306N 307N 308N 309N 310N 311N 312N 313N 314N 315N 316N 317N 318N 663G 750G 1438G 2218T 2706G 3106d 3107C 4769G 4991A 6026A 7028T 7403G 7581C 8860G 11116C 11467G 11719A 12308G 12372A 12879C 13104G 13656C 14070G 14364A 14766T 15115C 15148A 15217A 15326G 15954C 16145A 16182C 16183C 16189C 16249C	U1a1c1c1	73G 195C 263G 285T 301N 302N 303N 304N 305N 306N 307N 308N 309N 310N 311N 312N 313N 314N 315N 316N 317N 318N 16145A 16182C 16183C 16189C 16249C	1	73 195 263 285 301N 302N 303N 304N 305N 306N 307N 308N 309N 310N 311N 312N 313N 314N 315N 316N 317N 318N 663 750 1438 2218 2706 4769 4991 6026 7028 7403 7581 8860 11116 11467 11719 12308 12372 12879 13104 13656 14070 14364 14766 15115 15148 15217 15326 15954C 16145 16189 16249	U1a1c1c1	73 195 263 285 301N 302N 303N 304N 305N 306N 307N 308N 309N 310N 311N 312N 313N 314N 315N 316N 317N 318N 16145 16189 16249	[0.000] U1a1c1c1 (1)	[0.049] U1a1c1d (2) [0.000] U1a U1a1 U1a1c U1a1c1 U1a1c1b U1a1c1c (8)	1
2140 >KY409409.1 Hc	72C 195C 198T 263G 301N 302N 303N 304N 305N 306N 307N 308N 309N 310N 311N 312N 313N 314N 315N 316N 317N 318N 750G 1438G 2620S 2706G 2833R 3106d 3107C 4688C 4769G 7028T 8860G 15326G 16298C	HV0b	72C 195C 198T 263G 301N 302N 303N 304N 305N 306N 307N 308N 309N 310N 311N 312N 313N 314N 315N 316N 317N 318N 16298C	1	72 195 198 263 301N 302N 303N 304N 305N 306N 307N 308N 309N 310N 311N 312N 313N 314N 315N 316N 317N 318N 750 1438 2620S 2706 2833R 4688 4769 7028 8860 15326 16298	HV0b	72 195 198 263 301N 302N 303N 304N 305N 306N 307N 308N 309N 310N 311N 312N 313N 314N 315N 316N 317N 318N 16298	[0.000] HV0b (1)	[0.000] HV0+195 HV0c HV0d (4)	1
2141 >KY409403.1 Hc	195C 263G 301N 302N 303N 304N 305N 306N 307N 308N 309N 310N 311N 312N 313N 314N 315N 316N 317N 318N 750G 961G 1438G 3106d 3107C 4769G 5021C 8448C 8860G 8898T 11914A 13759A 15326G 16189C 16278T 16293G 16311C	H11a1	195C 263G 301N 302N 303N 304N 305N 306N 307N 308N 309N 310N 311N 312N 313N 314N 315N 316N 317N 318N 16189C 16278T 16293G 16311C	1	195 263 301N 302N 303N 304N 305N 306N 307N 308N 309N 310N 311N 312N 313N 314N 315N 316N 317N 318N 750 961G 1438 4769 5021 8448 8860 8898 11914 13759 15326 16189 16278 16293 16311	H11a1	195 263 301N 302N 303N 304N 305N 306N 307N 308N 309N 310N 311N 312N 313N 314N 315N 316N 317N 318N 16189 16278 16293 16311	[0.095] H11a1 (1)	[0.069] H11a2 (2) [0.014] H11a (3) [0.000] H11a5 H11a8 (5)	1

2142	>KY409398.1 Hc	73G 143A 195C 204C 301N 302N 303N 304N 305N 306N 307N 308N 309N 310N 311N 312N 313N 314N 315N 316N 317N 318N 489C 750G 813G 1438G 2706G 3106d 3107C 3705A 4769G 6446A 6671C 6680C 7028T 8701G 8860G 9540C 9845C 10398G 10400T 10873C 11719A 12346T 12403T 12705T 12950C 14110C 14766T 14783C 15043A 15301A 15326G 16129A 16182C 16183C 16189C 16223T 16249C 16311C 16359C 16362C 16519C 16527T	M1a1i	73G 143A 195C 204C 301N 302N 303N 304N 305N 306N 307N 308N 309N 310N 311N 312N 313N 314N 315N 316N 317N 318N 489C 16129A 16182C 16183C 16189C 16223T 16249C 16311C 16359C 16362C 16519C 16527T	1	73 143 195 204 301N 302N 303N 304N 305N 306N 307N 308N 309N 310N 311N 312N 313N 314N 315N 316N 317N 318N 489 750 813 1438 2706 3705 4769 6446 6671 6680 7028 8701 8860 9540 9845 10398 10400 10873 11719 12346 12403 12705 12950C 14110 14766 14783 15043 15301 15326 16129 16189 16223 16249 16311 16359 16362 16527	M1a1i	73 143 195 204 301N 302N 303N 304N 305N 306N 307N 308N 309N 310N 311N 312N 313N 314N 315N 316N 317N 318N 489 16129 16189 16223 16249 16311 16359 16362 16527	[0.174] M1a1i (1)	[0.016] M1a1b1a (2) [0.000] M1a1 M1a1a M1a1b M1a1b1 M1a1b1b M1a1b1b1 M1a1b2 M1a1e M1a1e1 M1a1e2 M1a1f (13)	1
2143	>KY409395.1 Hc	263G 301N 302N 303N 304N 305N 306N 307N 308N 309N 310N 311N 312N 313N 314N 315N 316N 317N 318N 750G 1438G 2706G 3106d 3107C 4769G 7028T 8715Y 8860G 9007G 15326G 16169T 16362C	HV21	263G 301N 302N 303N 304N 305N 306N 307N 308N 309N 310N 311N 312N 313N 314N 315N 316N 317N 318N 16169T 16362C	1	263 301N 302N 303N 304N 305N 306N 307N 308N 309N 310N 311N 312N 313N 314N 315N 316N 317N 318N 750 1438 2706 4769 7028 8715Y 8860 9007 15326 16169 16362	HV21	263 301N 302N 303N 304N 305N 306N 307N 308N 309N 310N 311N 312N 313N 314N 315N 316N 317N 318N 16169 16362	[0.074] HV21 (1) [0.000] H1bv1 H13b H13b2 (4)	[0.154] H1m H1be (6) [0.123] H (7) [0.017] H1e2 (8) [0.015] H2a2a H3ak (10) [0.011] H6a1b2 (11) [0.007] H6 H6a1a1 (13) [0.003] H1bw H2a2+(16235) (15) [0.002] HV H7 (17) [0.001] H1 H1j H1u1 H1ag1 H1bx H2a4 H2a5 H3 H3b H3ap H4a H4a1 H7d H10c1 H13a2a1 H47 H56 (34) [0.000] R0 R0a'b HV4 HV4c HV5 HV5a HV9c HV12 H1e H1e1 H1e1a H1e1a2 H1e1a7 H1e1c H1e2a H1e2b H1e2d H1e4 H1e6 H1e7 H1e8 H1e8a H1h H1h1 H1h2 H1j3 H1j5 H1j6 H1j7 H1j9 H1n H1p H1q H1q1 H1q2 H1r H1s H1s1 H1t H1t1 H1t1a H1t2 H1u H1v H1v1 H1v1a H1v1b H1w H1ae H1ae1 H1ae2 H1ae2a H1ae3 H1ae3a H1ag H1ag1b H1ah H1ai H1ai1 H1aj H1ak H1ak1 H1ak2 H1am H1am1 H1ap H1aq H1as H1as2 H1at H1at1 H1at1a H1av H1ax H1ax1 H1ay H1az H1bd H1bi H1bj H1bk H1bm H1bn H1bp H1bq H1br H1bu H1bv H1ca H1ci H2 H2a H2a2 H2a5a H2a5b H3c H3c1 H3e H3q H3q1 H3r H3r1 H3t H3u H3y H3aa H3ab H3ac H3ad H3ae H3ag H3ag1 H3ah H3ai H3aj H3aq H3ar H3as H3at H3at1 H3au H4 [illegible]	1

2144	>KY409393.1 Hc	263G 301N 302N 303N 304N 305N 306N 307N 308N 309N 310N 311N 312N 313N 314N 315N 316N 317N 318N 750G 1438G 3106d 3107C 4769G 4793G 6296A 8860G 15326G 16093C 16265G 16519C	H7c1	263G 301N 302N 303N 304N 305N 306N 307N 308N 309N 310N 311N 312N 313N 314N 315N 316N 317N 318N 16093C 16265G 16519C	1	263 301N 302N 303N 304N 305N 306N 307N 308N 309N 310N 311N 312N 313N 314N 315N 316N 317N 318N 750 1438 4769 4793 6296A 8860 15326 16093 16265	H7c1	263 301N 302N 303N 304N 305N 306N 307N 308N 309N 310N 311N 312N 313N 314N 315N 316N 317N 318N 16093 16265	[0.267] H7c1 (1) [0.000] H10+(16093) H10f (3)	[0.158] H7c3 (4) [0.157] H13a1a1c (5) [0.156] H7c (6) [0.155] H44a (7) [0.031] H10e1 (8) [0.014] H3v+16093 (9) [0.011] H H10e3a (11) [0.010] H1e1a (12) [0.008] H1 (13) [0.005] H2a2a H60a (15) [0.003] H3h2 H63 H72 (18) [0.002] H1e H56 (20) [0.001] H1h1 H1j H1q H1bi H1br H3 H4 H4a1a H7a H7b1 H10f H13a2b2 H14b H14b2 H67 (35) [0.000] R0 HV HV4 HV4c HV5 HV5a HV9c HV12 H1f+16093 H1f1 H1e1 H1e1a2 H1e1a7 H1e1c H1e2 H1e2a H1e2b H1e2d H1e4 H1e6 H1e7 H1e8 H1e8a H1h H1h2 H1j3 H1j5 H1j6 H1j7 H1j9 H1m H1n H1p H1q1 H1q2 H1r H1s H1s1 H1t H1t1 H1t1a H1t2 H1u H1u1 H1v H1v1 H1v1a H1v1b H1w H1ae H1ae1 H1ae2 H1ae2a H1ae3 H1ae3a H1ag H1ag1 H1ag1b H1ah H1ai H1ai1 H1aj H1ak H1ak1 H1ak2 H1am H1am1 H1ap H1aq H1as H1as2 H1at H1at1 H1at1a H1av H1ax H1ax1 H1ay H1az H1bd H1be H1bj H1bk H1bm H1bn H1bp H1bq H1bu H1bv H1bw H1bx H1ca H1ci H2 H2a H2a2 H2a2+(16235) H2a4 H2a5 H2a5a H2a5b H3b H3c H3c1 [illegible]	1
2145	>KY409391.1 Hc	73G 150T 263G 301N 302N 303N 304N 305N 306N 307N 308N 309N 310N 311N 312N 313N 314N 315N 316N 317N 318N 750G 1438G 1811G 2294G 2706G 3106d 3107C 3873G 4612C 4703C 4769G 5261A 7028T 7382A 8110C 8634C 8860G 9266A 11467G 11719A 12308G 12372A 12843C 14139G 14766T 14992C 15326G 15454C 16129A 16193T 16249C 16343G 16526A	U3c	73G 150T 263G 301N 302N 303N 304N 305N 306N 307N 308N 309N 310N 311N 312N 313N 314N 315N 316N 317N 318N 16129A 16193T 16249C 16343G 16526A	1	73 150 263 301N 302N 303N 304N 305N 306N 307N 308N 309N 310N 311N 312N 313N 314N 315N 316N 317N 318N 750 1438 1811 2294 2706 3873 4612 4703 4769 5261 7028 7382 8110 8634 8860 9266 11467 11719 12308 12372 12843 14139 14766 14992 15326 15454 16129 16193 16249 16343 16526	U3c	73 150 263 301N 302N 303N 304N 305N 306N 307N 308N 309N 310N 311N 312N 313N 314N 315N 316N 317N 318N 16129 16193 16249 16343 16526	[0.216] U3c (1)	[0.009] U3b2 (2) [0.000] U3 U3a'c U3b U3b1 U3b1b U3b2a U3b2a1 U3b2c (10)	1

2146 >KY409388.1 Hc	73G 150T 263G 301N 302N 303N 304N 305N 306N 307N 308N 309N 310N 311N 312N 313N 314N 315N 316N 317N 318N 750G 1438G 1811G 2294G 2389Y 2706G 3106d 3107C 3873G 4612C 4703C 4769G 5261A 7028T 7382A 8110C 8634C 8860G 9266A 11467G 11719A 12308G 12372A 12843C 14139G 14766T 14992C 15326G 15454C 16129A 16193T 16249C 16343G 16526A	U3c	73G 150T 263G 301N 302N 303N 304N 305N 306N 307N 308N 309N 310N 311N 312N 313N 314N 315N 316N 317N 318N 16129A 16193T 16249C 16343G 16526A	1	73 150 263 301N 302N 303N 304N 305N 306N 307N 308N 309N 310N 311N 312N 313N 314N 315N 316N 317N 318N 750 1438 1811 2294 2389Y 2706 3873 4612 4703 4769 5261 7028 7382 8110 8634 8860 9266 11467 11719 12308 12372 12843 14139 14766 14992 15326 15454 16129 16193 16249 16343 16526	U3c	73 150 263 301N 302N 303N 304N 305N 306N 307N 308N 309N 310N 311N 312N 313N 314N 315N 316N 317N 318N 16129 16193 16249 16343 16526	[0.216] U3c (1)	[0.009] U3b2 (2) [0.000] U3 U3a'c U3b U3b1 U3b1b U3b2a U3b2a1 U3b2c (10)	1
2147 >KY409387.1 Hc	73G 150T 263G 301N 302N 303N 304N 305N 306N 307N 308N 309N 310N 311N 312N 313N 314N 315N 316N 317N 318N 750G 1438G 1811G 2294G 2706G 3106d 3107C 3873G 4612C 4703C 4769G 5261A 7028T 7382A 8110C 8634C 8860G 9266A 11467G 11719A 12308G 12372A 12843C 14139G 14766T 14992C 15326G 15454C 16129A 16193T 16249C 16343G 16526A	U3c	73G 150T 263G 301N 302N 303N 304N 305N 306N 307N 308N 309N 310N 311N 312N 313N 314N 315N 316N 317N 318N 16129A 16193T 16249C 16343G 16526A	1	73 150 263 301N 302N 303N 304N 305N 306N 307N 308N 309N 310N 311N 312N 313N 314N 315N 316N 317N 318N 750 1438 1811 2294 2706 3873 4612 4703 4769 5261 7028 7382 8110 8634 8860 9266 11467 11719 12308 12372 12843 14139 14766 14992 15326 15454 16129 16193 16249 16343 16526	U3c	73 150 263 301N 302N 303N 304N 305N 306N 307N 308N 309N 310N 311N 312N 313N 314N 315N 316N 317N 318N 16129 16193 16249 16343 16526	[0.216] U3c (1)	[0.009] U3b2 (2) [0.000] U3 U3a'c U3b U3b1 U3b1b U3b2a U3b2a1 U3b2c (10)	1
2148 >KY409370.1 Hc	73G 153G 195C 263G 301N 302N 303N 304N 305N 306N 307N 308N 309N 310N 311N 312N 313N 314N 315N 316N 317N 318N 750G 1438G 1719A 2706G 3106d 3107C 4769G 6221C 6371T 7028T 8860G 9326G 11719A 12705T 13966G 14470C 14766T 14806T 15326G 16129A 16189C 16223T 16248T 16278T 16519C	X2i+@225	73G 153G 195C 263G 301N 302N 303N 304N 305N 306N 307N 308N 309N 310N 311N 312N 313N 314N 315N 316N 317N 318N 16129A 16189C 16223T 16248T 16278T 16519C	1	73 153 195 263 301N 302N 303N 304N 305N 306N 307N 308N 309N 310N 311N 312N 313N 314N 315N 316N 317N 318N 750 1438 1719 2706 4769 6221 6371 7028 8860 9326 11719 12705 13966 14470 14766 14806 15326 16129 16189 16223 16248 16278	X2i+@225	73 153 195 263 301N 302N 303N 304N 305N 306N 307N 308N 309N 310N 311N 312N 313N 314N 315N 316N 317N 318N 16129 16189 16223 16248 16278	[0.050] X2i+@225 (1)	[0.000] X2 X2e2 X2e2a X2e2a1 X2i X2i1 X2o1 X2k X2p (10)	1
2149 >KY409366.1 Hc	73G 150T 263G 301N 302N 303N 304N 305N 306N 307N 308N 309N 310N 311N 312N 313N 314N 315N 316N 317N 318N 373G 750G 1438G 2706G 3106d 3107C 3197C 4769G 7028T 7226A 7768G 8860G 9477A 9839T 11177T 11467G 11647T 11719A 12308G 12358G 12372A 13617C 14182C 14766T 15326G 16169A 16192T 16235G 16270T 16302G 16519C	U5b3a1a	73G 150T 263G 301N 302N 303N 304N 305N 306N 307N 308N 309N 310N 311N 312N 313N 314N 315N 316N 317N 318N 373G 16169A 16192T 16235G 16270T 16302G 16519C	1	73 150 263 301N 302N 303N 304N 305N 306N 307N 308N 309N 310N 311N 312N 313N 314N 315N 316N 317N 318N 373 750 1438 2706 3197 4769 7028 7226 7768 8860 9477 9839 11177 11467 11647 11719 12308 12358 12372 13617 14182 14766 15326 16169A 16192 16235 16270 16302	U5b3a1a	73 150 263 301N 302N 303N 304N 305N 306N 307N 308N 309N 310N 311N 312N 313N 314N 315N 316N 317N 318N 373 16169A 16192 16235 16270 16302	[0.153] U5b3a1a (1)	[0.000] U5b3a1 U5b3a1b (3)	1

2150	>KY409359.1 Hc	73G 150T 263G 301N 302N 303N 304N 305N 306N 307N 308N 309N 310N 311N 312N 313N 314N 315N 316N 317N 318N 373G 750G 1438G 2706G 3106d 3107C 3197C 4769G 7028T 7226A 7768G 8764A 8860G 9477A 11177T 11467G 11719A 12308G 12372A 13617C 14182C 14766T 14788C 15326G 16169A 16192T 16235G 16270T 16519C	U5b3a1a	73G 150T 263G 301N 302N 303N 304N 305N 306N 307N 308N 309N 310N 311N 312N 313N 314N 315N 316N 317N 318N 373G 16169A 16192T 16235G 16270T 16519C	1	73 150 263 301N 302N 303N 304N 305N 306N 307N 308N 309N 310N 311N 312N 313N 314N 315N 316N 317N 318N 373 750 1438 2706 3197 4769 7028 7226 7768 8764 8860 9477 11177 11467 11719 12308 12372 13617 14182 14766 14788 15326 16169A 16192 16235 16270	U5b3a1a	73 150 263 301N 302N 303N 304N 305N 306N 307N 308N 309N 310N 311N 312N 313N 314N 315N 316N 317N 318N 373 16169A 16192 16235 16270	[0.000] U5b3a1a (1)	[0.000] U5b3a1 U5b3a1b (3)	1
2151	>KY409358.1 Hc	263G 301N 302N 303N 304N 305N 306N 307N 308N 309N 310N 311N 312N 313N 314N 315N 316N 317N 318N 750G 1438G 2706G 3106d 3107C 4580A 4769G 7028T 7852A 8860G 15326G 15904T 15905C 16298C	V+@72	263G 301N 302N 303N 304N 305N 306N 307N 308N 309N 310N 311N 312N 313N 314N 315N 316N 317N 318N 16298C	1	263 301N 302N 303N 304N 305N 306N 307N 308N 309N 310N 311N 312N 313N 314N 315N 316N 317N 318N 750 1438 2706 4580 4769 7028 7852 8860 15326 15904 15905 16298	V+@72	263 301N 302N 303N 304N 305N 306N 307N 308N 309N 310N 311N 312N 313N 314N 315N 316N 317N 318N 16298	[0.000] V+@72 (1)	[0.032] V1a (2) [0.021] V1a1 (3) [0.015] V1a1b (4) [0.014] H2a2a (5) [0.005] V2 V2b V2c (8) [0.003] H H35a (10) [0.002] H3u (11) [0.001] H1 H3 (13) [0.000] R0 HV HV0 HV0a V V1 V2a V2a1 V2a1a V2b1 V2b2 V3 V3b V4 V8 V10 V10b V10b1 V10b2 V11 V12 V13 V14 V15 V15a V17 V21 V23 V24 V25 V26 V28 HV0f HV4 HV4c HV5 HV5a HV9c HV12 H1e H1e1 H1e1a H1e1a2 H1e1a7 H1e1c H1e2 H1e2a H1e2b H1e2d H1e4 H1e6 H1e7 H1e8 H1e8a H1h H1h1 H1h2 H1j H1j3 H1j5 H1j6 H1j7 H1j9 H1m H1n H1p H1q H1q1 H1q2 H1r H1s H1s1 H1t H1t1 H1t1a H1t2 H1u H1u1 H1v H1v1 H1v1a H1v1b H1w H1ae H1ae1 H1ae2 H1ae2a H1ae3 H1ae3a H1ag H1ag1 H1ag1b H1ah H1ai H1ai1 H1aj H1ak H1ak1 H1ak2 H1am H1am1 H1ap H1aq H1as H1as2 H1at H1at1 H1at1a H1av H1ax H1ax1 H1ay H1az H1bd H1be H1bi H1bj H1bk H1bm H1bn H1bp H1bq H1br H1bu H1bv H1bw H1bx H1ca H1ci H2 H2a H2a2 H2a2+(16235) H2a4 H2a5 H2a5a H2a5b H3b H3c H3c1 H3e H3q H3q1 H3r H3r1 H3t [illegible]	1
2152	>KY409356.1 Hc	73G 152C 217C 263G 301N 302N 303N 304N 305N 306N 307N 308N 309N 310N 311N 312N 313N 314N 315N 316N 317N 318N 340T 508G 750G 980C 988A 1438G 1811G 2706G 3106d 3107C 3720G 4769G 5390G 5426C 6045T 6152C 7028T 7109T 8860G 9152C 10876G 11467G 11719A 12308G 12372A 13020C 13734C 14766T 15326G 15907G 16051G 16129C 16172C 16175G 16189C 16256T 16519C	U2e1b1	73G 152C 217C 263G 301N 302N 303N 304N 305N 306N 307N 308N 309N 310N 311N 312N 313N 314N 315N 316N 317N 318N 340T 508G 16051G 16129C 16172C 16175G 16189C 16256T 16519C	1	73 152 217 263 301N 302N 303N 304N 305N 306N 307N 308N 309N 310N 311N 312N 313N 314N 315N 316N 317N 318N 340 508 750 980 988 1438 1811 2706 3720 4769 5390 5426 6045 6152 7028 7109 8860 9152 10876 11467 11719 12308 12372 13020 13734 14766 15326 15907 16051 16129C 16172 16175 16189 16256	U2e1b1	73 152 217 263 301N 302N 303N 304N 305N 306N 307N 308N 309N 310N 311N 312N 313N 314N 315N 316N 317N 318N 340 508 16051 16129C 16172 16175 16189 16256	[0.108] U2e1b1 (1)	[0.000] U2e1b U2e1b2 (3)	1

2153	>KY409350.1 H[illegible]	263G 301N 302N 303N 304N 305N 306N 307N 308N 309N 310N 311N 312N 313N 314N 315N 316N 317N 318N 444G 750G 951A 3106d 3107C 8860G 14189G 15326G 16018Y 16354T	H2a1j	263G 301N 302N 303N 304N 305N 306N 307N 308N 309N 310N 311N 312N 313N 314N 315N 316N 317N 318N 444G 16354T	1	263 301N 302N 303N 304N 305N 306N 307N 308N 309N 310N 311N 312N 313N 314N 315N 316N 317N 318N 444 750 951 8860 14189 15326 16018Y 16354	H2a1j	263 301N 302N 303N 304N 305N 306N 307N 308N 309N 310N 311N 312N 313N 314N 315N 316N 317N 318N 444 16354	[0.000] H2a1j (1)	[0.000] H2a1 H2a1a H2a1a1 H2a1a2 H2a1b H2a1c H2a1d H2a1g H2a1k (10)	1
2154	>KY409342.1 H[illegible]	263G 301N 302N 303N 304N 305N 306N 307N 308N 309N 310N 311N 312N 313N 314N 315N 316N 317N 318N 750G 1438G 3106d 3107C 4769G 4793G 6296A 8860G 15326G 16093C 16265G 16519C	H7c1	263G 301N 302N 303N 304N 305N 306N 307N 308N 309N 310N 311N 312N 313N 314N 315N 316N 317N 318N 16093C 16265G 16519C	1	263 301N 302N 303N 304N 305N 306N 307N 308N 309N 310N 311N 312N 313N 314N 315N 316N 317N 318N 750 1438 4769 4793 6296A 8860 15326 16093 16265	H7c1	263 301N 302N 303N 304N 305N 306N 307N 308N 309N 310N 311N 312N 313N 314N 315N 316N 317N 318N 16093 16265	[0.267] H7c1 (1) [0.000] H10+(16093) H10f (3)	[0.158] H7c3 (4) [0.157] H13a1a1c (5) [0.156] H7c (6) [0.155] H44a (7) [0.031] H10e1 (8) [0.014] H3v+16093 (9) [0.011] H H10e3a (11) [0.010] H1e1a (12) [0.008] H1 (13) [0.005] H2a2a H60a (15) [0.003] H3h2 H63 H72 (18) [0.002] H1e H56 (20) [0.001] H1h1 H1j H1q H1bi H1br H3 H4 H4a1a H7a H7b1 H10f H13a2b2 H14b H14b2 H67 (35) [0.000] R0 HV HV4 HV4c HV5 HV5a HV9c HV12 H1f+16093 H1f1 H1e1 H1e1a2 H1e1a7 H1e1c H1e2 H1e2a H1e2b H1e2d H1e4 H1e6 H1e7 H1e8 H1e8a H1h H1h2 H1j3 H1j5 H1j6 H1j7 H1j9 H1m H1n H1p H1q1 H1q2 H1r H1s H1s1 H1t H1t1 H1t1a H1t2 H1u H1u1 H1v H1v1 H1v1a H1v1b H1w H1ae H1ae1 H1ae2 H1ae2a H1ae3 H1ae3a H1ag H1ag1 H1ag1b H1ah H1ai H1ai1 H1aj H1ak H1ak1 H1ak2 H1am H1am1 H1ap H1aq H1as H1as2 H1at H1at1 H1at1a H1av H1ax H1ax1 H1ay H1az H1bd H1be H1bj H1bk H1bm H1bn H1bp H1bq H1bu H1bv H1bw H1bx H1ca H1ci H2 H2a H2a2 H2a2+(16235) H2a4 H2a5 H2a5a H2a5b H3b H3c H3c1 [illegible]	1
2155	>KY409331.1 H[illegible]	73G 195C 263G 285T 301N 302N 303N 304N 305N 306N 307N 308N 309N 310N 311N 312N 313N 314N 315N 316N 317N 318N 663G 750G 1438G 2218T 2706G 3106d 3107C 4769G 4991A 6026A 7028T 7403G 7581C 8860G 11116C 11467G 11719A 12308G 12372A 12879C 13104G 13656C 14070G 14364A 14766T 15115C 15148A 15217A 15326G 15954C 16145A 16182C 16183C 16189C 16249C	U1a1c1c1	73G 195C 263G 285T 301N 302N 303N 304N 305N 306N 307N 308N 309N 310N 311N 312N 313N 314N 315N 316N 317N 318N 16145A 16182C 16183C 16189C 16249C	1	73 195 263 285 301N 302N 303N 304N 305N 306N 307N 308N 309N 310N 311N 312N 313N 314N 315N 316N 317N 318N 663 750 1438 2218 2706 4769 4991 6026 7028 7403 7581 8860 11116 11467 11719 12308 12372 12879 13104 13656 14070 14364 14766 15115 15148 15217 15326 15954C 16145 16189 16249	U1a1c1c1	73 195 263 285 301N 302N 303N 304N 305N 306N 307N 308N 309N 310N 311N 312N 313N 314N 315N 316N 317N 318N 16145 16189 16249	[0.000] U1a1c1c1 (1)	[0.049] U1a1c1d (2) [0.000] U1a U1a1 U1a1c U1a1c1 U1a1c1b U1a1c1c (8)	1

2156 >KY409324.1 H	73G 150T 263G 301N 302N 303N 304N 305N 306N 307N 308N 309N 310N 311N 312N 313N 314N 315N 316N 317N 318N 750G 1438G 1721T 2706G 3106d 3107C 3197C 4769G 7028T 7768G 8860G 9477A 11467G 11653G 11719A 12308G 12372A 12634G 13161C 13617C 13630G 13637G 14182C 14766T 15326G 16270T 16296T 16325C	U5b2b5	73G 150T 263G 301N 302N 303N 304N 305N 306N 307N 308N 309N 310N 311N 312N 313N 314N 315N 316N 317N 318N 16270T 16296T 16325C	1	73 150 263 301N 302N 303N 304N 305N 306N 307N 308N 309N 310N 311N 312N 313N 314N 315N 316N 317N 318N 750 1438 1721 2706 3197 4769 7028 7768 8860 9477 11467 11653 11719 12308 12372 12634 13161 13617 13630 13637 14182 14766 15326 16270 16296 16325	U5b2b5	73 150 263 301N 302N 303N 304N 305N 306N 307N 308N 309N 310N 311N 312N 313N 314N 315N 316N 317N 318N 16270 16296 16325	[0.256] U5b2b5 (1)	[0.057] U5b2b (2) [0.012] U5b2b1 (3) [0.000] U5b1d1a U5b2b2 (5)	1
2157 >KY409323.1 H	57C 59C 151T 152C 263G 301N 302N 303N 304N 305N 306N 307N 308N 309N 310N 311N 312N 313N 314N 315N 316N 317N 318N 709A 750G 930A 1438G 1888A 2706G 3106d 3107C 4216C 4769G 4917G 5147A 5656G 7028T 8697A 8860G 10398G 10463C 10750G 11251G 11719A 11812G 13368A 14233G 14766T 14905A 15326G 15452A 15607G 15928A 16126C 16292T 16294T 16296T 16304C 16519C	T2b3a1	57C 59C 151T 152C 263G 301N 302N 303N 304N 305N 306N 307N 308N 309N 310N 311N 312N 313N 314N 315N 316N 317N 318N 16126C 16292T 16294T 16296T 16304C 16519C	1	57 59 151 152 263 301N 302N 303N 304N 305N 306N 307N 308N 309N 310N 311N 312N 313N 314N 315N 316N 317N 318N 709 750 930 1438 1888 2706 4216 4769 4917 5147 5656 7028 8697 8860 10398 10463 10750 11251 11719 11812 13368 14233 14766 14905 15326 15452A 15607 15928 16126 16292 16294 16296 16304	T2b3a1	57 59 151 152 263 301N 302N 303N 304N 305N 306N 307N 308N 309N 310N 311N 312N 313N 314N 315N 316N 317N 318N 16126 16292 16294 16296 16304	[0.758] T2b3a1 (1)	[0.758] T2b3a1 (2) [0.179] T2b3a	1
2158 >KY409300.1 H	263G 301N 302N 303N 304N 305N 306N 307N 308N 309N 310N 311N 312N 313N 314N 315N 316N 317N 318N 750G 1438G 3106d 3107C 4769G 4793G 6296A 8860G 15326G 16093C 16265G 16519C	H7c1	263G 301N 302N 303N 304N 305N 306N 307N 308N 309N 310N 311N 312N 313N 314N 315N 316N 317N 318N 16093C 16265G 16519C	1	263 301N 302N 303N 304N 305N 306N 307N 308N 309N 310N 311N 312N 313N 314N 315N 316N 317N 318N 750 1438 4769 4793 6296A 8860 15326 16093 16265	H7c1	263 301N 302N 303N 304N 305N 306N 307N 308N 309N 310N 311N 312N 313N 314N 315N 316N 317N 318N 16093 16265	[0.267] H7c1 (1) [0.000] H10+(16093) H10f (3)	[0.158] H7c3 (4) [0.157] H13a1a1c (5) [0.156] H7c (6) [0.155] H44a (7) [0.031] H10e1 (8) [0.014] H3v+16093 (9) [0.011] H H10e3a (11) [0.010] H1e1a (12) [0.008] H1 (13) [0.005] H2a2a H60a (15) [0.003] H3h2 H63 H72 (18) [0.002] H1e H56 (20) [0.001] H1h1 H1j H1q H1bi H1br H3 H4 H4a1a H7a H7b1 H10f H13a2b2 H14b H14b2 H67 (35) [0.000] R0 HV HV4 HV4c HV5 HV5a HV9c HV12 H1f+16093 H1f1 H1e1 H1e1a2 H1e1a7 H1e1c H1e2 H1e2a H1e2b H1e2d H1e4 H1e6 H1e7 H1e8 H1e8a H1h H1h2 H1j3 H1j5 H1j6 H1j7 H1j9 H1m H1n H1p H1q1 H1q2 H1r H1s H1s1 H1t H1t1 H1t1a H1t2 H1u H1u1 H1v H1v1 H1v1a H1v1b H1w H1ae H1ae1 H1ae2 H1ae2a H1ae3 H1ae3a H1ag H1ag1 H1ag1b H1ah H1ai H1ai1 H1aj H1ak H1ak1 H1ak2 H1am H1am1 H1ap H1aq H1as H1as2 H1at H1at1 H1at1a H1av H1ax H1ax1 H1ay H1az H1bd H1be H1bj H1bk H1bm H1bn H1bp H1bq H1bu H1bv H1bw H1bx H1ca H1ci H2 H2a H2a2 H2a2+(16235) H2a4 H2a5 H2a5a H2a5b H3b H3c H3c1 [illegible]	1

2159	>KY409294.1 Hc	73G 195C 263G 301N 302N 303N 304N 305N 306N 307N 308N 309N 310N 311N 312N 313N 314N 315N 316N 317N 318N 489C 750G 813G 1438G 2706G 3106d 3107C 3705A 4011T 4769G 6446A 6671C 6680C 7028T 8521G 8701G 8860G 9053A 9449Y 9540C 9545G 10398G 10400T 10873C 11044T 11719A 12346T 12403T 12705T 12950C 14110C 14182C 14766T 14783C 15043A 15301A 15326G 16129A 16183C 16189C 16223T 16311C 16359C 16519C	M1a1a1	73G 195C 263G 301N 302N 303N 304N 305N 306N 307N 308N 309N 310N 311N 312N 313N 314N 315N 316N 317N 318N 489C 16129A 16183C 16189C 16223T 16311C 16359C 16519C	1	73 195 263 301N 302N 303N 304N 305N 306N 307N 308N 309N 310N 311N 312N 313N 314N 315N 316N 317N 318N 489 750 813 1438 2706 3705 4011 4769 6446 6671 6680 7028 8521 8701 8860 9053 9449Y 9540 9545 10398 10400 10873 11044 11719 12346 12403 12705 12950C 14110 14182 14766 14783 15043 15301 15326 16129 16189 16223 16311 16359	M1a1a1	73 195 263 301N 302N 303N 304N 305N 306N 307N 308N 309N 310N 311N 312N 313N 314N 315N 316N 317N 318N 489 16129 16189 16223 16311 16359	[0.000] M1a1a1 (1)	[0.000] M1a1 M1a1a M1a1b M1a1b1 M1a1b1a M1a1b1b M1a1b1b1 M1a1b2 M1a1e M1a1e1 M1a1e2 M1a1f (13)	1
2160	>KY409293.1 Hc	72G 93G 263G 301N 302N 303N 304N 305N 306N 307N 308N 309N 310N 311N 312N 313N 314N 315N 316N 317N 318N 750G 1438G 3106d 3107C 4769G 6962A 8860G 12753G 14488C 15326G 15891T 16519C	H74	72G 93G 263G 301N 302N 303N 304N 305N 306N 307N 308N 309N 310N 311N 312N 313N 314N 315N 316N 317N 318N 16519C	1	72G 93 263 301N 302N 303N 304N 305N 306N 307N 308N 309N 310N 311N 312N 313N 314N 315N 316N 317N 318N 750 1438 4769 6962 8860 12753 14488 15326 15891	H74	72G 93 263 301N 302N 303N 304N 305N 306N 307N 308N 309N 310N 311N 312N 313N 314N 315N 316N 317N 318N	[0.154] H74 (1) [0.000] H1e1a1 H49a2 (3)	[0.276] H2a2a1f (4) [0.168] H3 (5) [0.050] H29a (6) [0.012] H H1 (8) [0.008] H3h7 (9) [0.004] H1aq (10) [0.003] H1ag H100 (12) [0.002] H1e H4 (14) [0.001] H1j H1bk H1ca H4a H4a1 H10+(16093) H13a1a1 H13a2b4 H14b2 H+13708 H18 H33 H49 H73 (28) [0.000] R0 HV HV1a2b HV4 HV4c HV5 HV5a HV9c HV12 H1e1 H1e1a H1e1a2 H1e1a7 H1e1c H1e2 H1e2a H1e2b H1e2d H1e3 H1e4 H1e6 H1e7 H1e8 H1e8a H1h H1h1 H1h2 H1j3 H1j5 H1j6 H1j7 H1j9 H1m H1n H1p H1q H1q1 H1q2 H1r H1s H1s1 H1t H1t1 H1t1a H1t2 H1u H1u1 H1v H1v1 H1v1a H1v1b H1w H1ae H1ae1 H1ae2 H1ae2a H1ae3 H1ae3a H1ag1 H1ag1b H1ah H1ai H1ai1 H1aj H1ak H1ak1 H1ak2 H1am H1am1 H1ap H1as H1as2 H1at H1at1 H1at1a H1av H1ax H1ax1 H1ay H1az H1bd H1be H1bi H1bj H1bm H1bn H1bp H1bq H1br H1bu H1bv H1bw H1bx H1ci H2 H2a H2a2 H2a2a H2a2+(16235) H2a4 H2a5 H2a5a H2a5b H3b H3c H3c1 H3e H3q H3q1 H3r H3r1 H3t H3u H3y H3aa H3ab H3ac H3ad H3ae H3ag H3ag1 H3ah [illegible]	1

2161	>KY409288.1 Hc	57C 73G 151T 152C 263G 301N 302N 303N 304N 305N 306N 307N 308N 309N 310N 311N 312N 313N 314N 315N 316N 317N 318N 709A 750G 930A 1438G 1888A 2706G 3106d 3107C 4216C 4769G 4917G 5147A 5656G 7028T 8697A 8860G 10463C 10750G 11251G 11719A 11812G 13368A 14233G 14766T 14905A 15326G 15452A 15607G 15928A 16126C 16274A 16292T 16294T 16296T 16304C 16519C	T2b3a	57C 73G 151T 152C 263G 301N 302N 303N 304N 305N 306N 307N 308N 309N 310N 311N 312N 313N 314N 315N 316N 317N 318N 16126C 16274A 16292T 16294T 16296T 16304C 16519C	1	57 73 151 152 263 301N 302N 303N 304N 305N 306N 307N 308N 309N 310N 311N 312N 313N 314N 315N 316N 317N 318N 709 750 930 1438 1888 2706 4216 4769 4917 5147 5656 7028 8697 8860 10463 10750 11251 11719 11812 13368 14233 14766 14905 15326 15452A 15607 15928 16126 16274 16292 16294 16296 16304	T2b3a	57 73 151 152 263 301N 302N 303N 304N 305N 306N 307N 308N 309N 310N 311N 312N 313N 314N 315N 316N 317N 318N 16126 16274 16292 16294 16296 16304	[0.215] T2b3a (1)	[0.645] T2b3a1 (2)	1
2162	>KY409282.1 Hc	73G 152C 217C 263G 301N 302N 303N 304N 305N 306N 307N 308N 309N 310N 311N 312N 313N 314N 315N 316N 317N 318N 340T 508G 750G 980C 988A 1438G 1811G 2706G 3106d 3107C 3720G 4769G 5390G 5426C 6045T 6152C 7028T 7109T 8860G 10876G 11467G 11719A 12308G 12372A 13020C 13734C 14766T 15326G 15907G 16051G 16129C 16172C 16175G 16189C 16256T 16519C	U2e1b1	73G 152C 217C 263G 301N 302N 303N 304N 305N 306N 307N 308N 309N 310N 311N 312N 313N 314N 315N 316N 317N 318N 340T 508G 16051G 16129C 16172C 16175G 16189C 16256T 16519C	1	73 152 217 263 301N 302N 303N 304N 305N 306N 307N 308N 309N 310N 311N 312N 313N 314N 315N 316N 317N 318N 340 508 750 980 988 1438 1811 2706 3720 4769 5390 5426 6045 6152 7028 7109 8860 10876 11467 11719 12308 12372 13020 13734 14766 15326 15907 16051 16129C 16172 16175 16189 16256	U2e1b1	73 152 217 263 301N 302N 303N 304N 305N 306N 307N 308N 309N 310N 311N 312N 313N 314N 315N 316N 317N 318N 340 508 16051 16129C 16172 16175 16189 16256	[0.108] U2e1b1 (1)	[0.000] U2e1b U2e1b2 (3)	1
2163	>KY409279.1 Hc	73G 195C 263G 285T 301N 302N 303N 304N 305N 306N 307N 308N 309N 310N 311N 312N 313N 314N 315N 316N 317N 318N 663G 750G 1438G 2218T 2706G 3106d 3107C 4769G 4991A 6026A 7028T 7403G 7581C 8860G 9768M 11116C 11467G 11719A 12308G 12372A 12879C 13104G 13656C 14070G 14364A 14766T 15115C 15148A 15217A 15326G 15954C 16145A 16182C 16183C 16189C 16249C	U1a1c1c1	73G 195C 263G 285T 301N 302N 303N 304N 305N 306N 307N 308N 309N 310N 311N 312N 313N 314N 315N 316N 317N 318N 16145A 16182C 16183C 16189C 16249C	1	73 195 263 285 301N 302N 303N 304N 305N 306N 307N 308N 309N 310N 311N 312N 313N 314N 315N 316N 317N 318N 663 750 1438 2218 2706 4769 4991 6026 7028 7403 7581 8860 9768M 11116 11467 11719 12308 12372 12879 13104 13656 14070 14364 14766 15115 15148 15217 15326 15954C 16145 16189 16249	U1a1c1c1	73 195 263 285 301N 302N 303N 304N 305N 306N 307N 308N 309N 310N 311N 312N 313N 314N 315N 316N 317N 318N 16145 16189 16249	[0.000] U1a1c1c1 (1)	[0.049] U1a1c1d (2) [0.000] U1a U1a1 U1a1c U1a1c1 U1a1c1b U1a1c1c (8)	1
2164	>KY409275.1 Hc	195C 263G 301N 302N 303N 304N 305N 306N 307N 308N 309N 310N 311N 312N 313N 314N 315N 316N 317N 318N 750G 961G 1438G 3106d 3107C 4769G 5021C 8152A 8448C 8860G 8898T 13708A 13759A 15326G 16189C 16278T 16293G 16311C	H11a1	195C 263G 301N 302N 303N 304N 305N 306N 307N 308N 309N 310N 311N 312N 313N 314N 315N 316N 317N 318N 16189C 16278T 16293G 16311C	1	195 263 301N 302N 303N 304N 305N 306N 307N 308N 309N 310N 311N 312N 313N 314N 315N 316N 317N 318N 750 961G 1438 4769 5021 8152 8448 8860 8898 13708 13759 15326 16189 16278 16293 16311	H11a1	195 263 301N 302N 303N 304N 305N 306N 307N 308N 309N 310N 311N 312N 313N 314N 315N 316N 317N 318N 16189 16278 16293 16311	[0.095] H11a1 (1)	[0.069] H11a2 (2) [0.014] H11a (3) [0.000] H11a5 H11a8 (5)	1

2165	>KY409265.1 Hc	73G 150T 263G 301N 302N 303N 304N 305N 306N 307N 308N 309N 310N 311N 312N 313N 314N 315N 316N 317N 318N 373G 750G 1438G 2706G 3106d 3107C 3197C 3317Y 4769G 7028T 7226A 7768G 8860G 8874T 9477A 11080C 11177T 11467G 11719A 12308G 12372A 13617C 14182C 14750R 14766T 15326G 16169A 16192T 16235G 16270T 16291T 16519C	U5b3a1a	73G 150T 263G 301N 302N 303N 304N 305N 306N 307N 308N 309N 310N 311N 312N 313N 314N 315N 316N 317N 318N 373G 16169A 16192T 16235G 16270T 16291T 16519C	1	73 150 263 301N 302N 303N 304N 305N 306N 307N 308N 309N 310N 311N 312N 313N 314N 315N 316N 317N 318N 373 750 1438 2706 3197 3317Y 4769 7028 7226 7768 8860 8874 9477 11080 11177 11467 11719 12308 12372 13617 14182 14750R 14766 15326 16169A 16192 16235 16270 16291	U5b3a1a	73 150 263 301N 302N 303N 304N 305N 306N 307N 308N 309N 310N 311N 312N 313N 314N 315N 316N 317N 318N 373 16169A 16192 16235 16270 16291	[0.020] U5b3a1a (1)	[0.000] U5b3a1 U5b3a1b (3)	1
2166	>KY409262.1 Hc	73G 150T 263G 301N 302N 303N 304N 305N 306N 307N 308N 309N 310N 311N 312N 313N 314N 315N 316N 317N 318N 750G 1438G 1721T 2706G 3106d 3107C 3197C 3777C 4769G 7028T 7768G 8860G 9300A 9477A 11467G 11653G 11719A 11794C 12308G 12372A 12634G 13161C 13617C 13630G 13637G 14182C 14766T 15326G 15721C 15928A 16270T 16296T	U5b2b5	73G 150T 263G 301N 302N 303N 304N 305N 306N 307N 308N 309N 310N 311N 312N 313N 314N 315N 316N 317N 318N 16270T 16296T	1	73 150 263 301N 302N 303N 304N 305N 306N 307N 308N 309N 310N 311N 312N 313N 314N 315N 316N 317N 318N 750 1438 1721 2706 3197 3777 4769 7028 7768 8860 9300 9477 11467 11653 11719 11794 12308 12372 12634 13161 13617 13630 13637 14182 14766 15326 15721 15928 16270 16296	U5b2b5	73 150 263 301N 302N 303N 304N 305N 306N 307N 308N 309N 310N 311N 312N 313N 314N 315N 316N 317N 318N 16270 16296	[0.000] U5b2b5 (1)	[0.000] U5b1d1a U5b2b U5b2b1 U5b2b2 (5)	1
2167	>KY408366.1 Hc	263G 301N 302N 303N 304N 305N 306N 307N 308N 309N 310N 311N 312N 313N 314N 315N 316N 317N 318N 337C 480C 750G 1438G 3106d 3107C 3615G 4769G 6776C 8860G 8937C 11914A 15326G 16293G 16368C 16519C	H3x1	263G 301N 302N 303N 304N 305N 306N 307N 308N 309N 310N 311N 312N 313N 314N 315N 316N 317N 318N 337C 480C 16293G 16368C 16519C	1	263 301N 302N 303N 304N 305N 306N 307N 308N 309N 310N 311N 312N 313N 314N 315N 316N 317N 318N 337C 480 750 1438 3615 4769 6776 8860 8937 11914 15326 16293 16368	H3x1	263 301N 302N 303N 304N 305N 306N 307N 308N 309N 310N 311N 312N 313N 314N 315N 316N 317N 318N 337C 480 16293 16368	[0.283] H3x1 (1)	[0.007] H24a (2) [0.000] H3x H24 H24a2 H24b (6)	1
2168	>KY408349.1 Hc	73G 150T 263G 301N 302N 303N 304N 305N 306N 307N 308N 309N 310N 311N 312N 313N 314N 315N 316N 317N 318N 373G 750G 1438G 2706G 3106d 3107C 3197C 4769G 7028T 7226A 7768G 8860G 9477A 9839T 11177T 11467G 11647T 11719A 12308G 12358G 12372A 13617C 14182C 14766T 15326G 16169A 16189C 16192T 16235G 16270T 16302G 16519C	U5b3a1a	73G 150T 263G 301N 302N 303N 304N 305N 306N 307N 308N 309N 310N 311N 312N 313N 314N 315N 316N 317N 318N 373G 16169A 16189C 16192T 16235G 16270T 16302G 16519C	1	73 150 263 301N 302N 303N 304N 305N 306N 307N 308N 309N 310N 311N 312N 313N 314N 315N 316N 317N 318N 373 750 1438 2706 3197 4769 7028 7226 7768 8860 9477 9839 11177 11467 11647 11719 12308 12358 12372 13617 14182 14766 15326 16169A 16189 16192 16235 16270 16302	U5b3a1a	73 150 263 301N 302N 303N 304N 305N 306N 307N 308N 309N 310N 311N 312N 313N 314N 315N 316N 317N 318N 373 16169A 16189 16192 16235 16270 16302	[0.163] U5b3a1a (1)	[0.000] U5b1+16189 U5b1b U5b1b1 U5b1b1g U5b2a U5b2a3 U5b2a3a U5b3a1 U5b3a1b (10)	1

2169	>KY408321.1 Ho	73G 152C 217C 263G 301N 302N 303N 304N 305N 306N 307N 308N 309N 310N 311N 312N 313N 314N 315N 316N 317N 318N 508G 750G 1154T 1438G 1811G 2706G 3106d 3107C 3720G 3865G 4769G 5390G 5426C 6045T 6152C 7028T 8860G 10876G 11016A 11467G 11719A 12308G 12372A 13020C 13734C 13758T 14766T 15326G 15454C 15732T 15907G 16051G 16129C 16179T 16182C 16183C 16189C 16362C 16519C	U2e1'2'3	73G 152C 217C 263G 301N 302N 303N 304N 305N 306N 307N 308N 309N 310N 311N 312N 313N 314N 315N 316N 317N 318N 508G 16051G 16129C 16179T 16182C 16183C 16189C 16362C 16519C	1	73 152 217 263 301N 302N 303N 304N 305N 306N 307N 308N 309N 310N 311N 312N 313N 314N 315N 316N 317N 318N 508 750 1154T 1438 1811 2706 3720 3865 4769 5390 5426 6045 6152 7028 8860 10876 11016 11467 11719 12308 12372 13020 13734 13758 14766 15326 15454 15732 15907 16051 16129C 16179 16189 16362	U2e1'2'3	73 152 217 263 301N 302N 303N 304N 305N 306N 307N 308N 309N 310N 311N 312N 313N 314N 315N 316N 317N 318N 508 16051 16129C 16179 16189 16362	[0.494] U2e1'2'3 (1) [0.000] U2e2 U2e2a U2e2a1 U2e2a1a U2e2a1a1 U2e2a1a2 U2e2a1c U2e2a1d (9)	[0.030] U2e1a1a (10) [0.009] U2e1a1 (11) [0.000] U2e U2e1 U2e1a U2e1a1c U2e1c U2e1c1 U2e1d U2e1g U2e2 U2e2a U2e2a1 U2e2a1a U2e2a1a1 U2e2a1a2 U2e2a1b U2e2a1c U2e2a1d (28)	1
2170	>KY408318.1 Ho	73G 150T 263G 301N 302N 303N 304N 305N 306N 307N 308N 309N 310N 311N 312N 313N 314N 315N 316N 317N 318N 750G 1438G 2706G 3106d 3107C 3197C 3498T 4769G 5656G 5964C 6674C 7028T 7768G 8860G 9163A 9477A 11467G 11719A 12308G 12372A 13617C 14182C 14766T 15326G 15777A 16167T 16189C 16270T 16311C 16356C	U5b1i	73G 150T 263G 301N 302N 303N 304N 305N 306N 307N 308N 309N 310N 311N 312N 313N 314N 315N 316N 317N 318N 16167T 16189C 16270T 16311C 16356C	1	73 150 263 301N 302N 303N 304N 305N 306N 307N 308N 309N 310N 311N 312N 313N 314N 315N 316N 317N 318N 750 1438 2706 3197 3498 4769 5656 5964 6674 7028 7768 8860 9163 9477 11467 11719 12308 12372 13617 14182 14766 15326 15777 16167 16189 16270 16311 16356	U5b1i	73 150 263 301N 302N 303N 304N 305N 306N 307N 308N 309N 310N 311N 312N 313N 314N 315N 316N 317N 318N 16167 16189 16270 16311 16356	[1.222] U5b1i (1) [0.000] U5b2a5 U5b2a5a (3)	[0.094] U5b1c (4) [0.077] U5b1+16189+@16192 (5) [0.051] U5b2a6 (6) [0.017] U5b2a+@16192 (7) [0.000] U5b1b1+@16192 U5b1b1d U5b1b1f U5b2a4 U5b2a4a U5b2b4 (13)	1
2171	>KY408309.1 Ho	73G 150T 263G 301N 302N 303N 304N 305N 306N 307N 308N 309N 310N 311N 312N 313N 314N 315N 316N 317N 318N 373G 750G 1438G 2706G 3106d 3107C 3197C 4769G 7028T 7226A 7768G 8860G 9477A 9839T 10481Y 11177T 11467G 11647T 11719A 12308G 12358G 12372A 13617C 14182C 14766T 15326G 16169A 16192T 16235G 16270T 16302G 16519C	U5b3a1a	73G 150T 263G 301N 302N 303N 304N 305N 306N 307N 308N 309N 310N 311N 312N 313N 314N 315N 316N 317N 318N 373G 16169A 16192T 16235G 16270T 16302G 16519C	1	73 150 263 301N 302N 303N 304N 305N 306N 307N 308N 309N 310N 311N 312N 313N 314N 315N 316N 317N 318N 373 750 1438 2706 3197 4769 7028 7226 7768 8860 9477 9839 10481Y 11177 11467 11647 11719 12308 12358 12372 13617 14182 14766 15326 16169A 16192 16235 16270 16302	U5b3a1a	73 150 263 301N 302N 303N 304N 305N 306N 307N 308N 309N 310N 311N 312N 313N 314N 315N 316N 317N 318N 373 16169A 16192 16235 16270 16302	[0.153] U5b3a1a (1)	[0.000] U5b3a1 U5b3a1b (3)	1
2172	>KY408301.1 Ho	73G 195C 263G 285T 301N 302N 303N 304N 305N 306N 307N 308N 309N 310N 311N 312N 313N 314N 315N 316N 317N 318N 663G 750G 1438G 2218T 2706G 3106d 3107C 4769G 4991A 6026A 7028T 7403G 7581C 8860G 11116C 11467G 11719A 12308G 12372A 12879C 13104G 13656C 14070G 14364A 14766T 15115C 15148A 15217A 15326G 15954C 16145A 16182C 16183C 16189C 16249C	U1a1c1c1	73G 195C 263G 285T 301N 302N 303N 304N 305N 306N 307N 308N 309N 310N 311N 312N 313N 314N 315N 316N 317N 318N 16145A 16182C 16183C 16189C 16249C	1	73 195 263 285 301N 302N 303N 304N 305N 306N 307N 308N 309N 310N 311N 312N 313N 314N 315N 316N 317N 318N 663 750 1438 2218 2706 4769 4991 6026 7028 7403 7581 8860 11116 11467 11719 12308 12372 12879 13104 13656 14070 14364 14766 15115 15148 15217 15326 15954C 16145 16189 16249	U1a1c1c1	73 195 263 285 301N 302N 303N 304N 305N 306N 307N 308N 309N 310N 311N 312N 313N 314N 315N 316N 317N 318N 16145 16189 16249	[0.000] U1a1c1c1 (1)	[0.049] U1a1c1d (2) [0.000] U1a U1a1 U1a1c U1a1c1 U1a1c1b U1a1c1c (8)	1

2173 >KY408284.1 Hc	73G 150Y 263G 301N 302N 303N 304N 305N 306N 307N 308N 309N 310N 311N 312N 313N 314N 315N 316N 317N 318N 373G 750G 1438G 2706G 3106d 3107C 3197C 4769G 7028T 7226A 7768G 8860G 9477A 9839T 11177T 11467G 11647T 11719A 12308G 12358G 12372A 13617C 14182C 14766T 15326G 16169A 16192T 16235G 16270T 16302G 16519C	U5b3a1a	73G 150Y 263G 301N 302N 303N 304N 305N 306N 307N 308N 309N 310N 311N 312N 313N 314N 315N 316N 317N 318N 373G 16169A 16192T 16235G 16270T 16302G 16519C	1	73 150Y 263 301N 302N 303N 304N 305N 306N 307N 308N 309N 310N 311N 312N 313N 314N 315N 316N 317N 318N 373 750 1438 2706 3197 4769 7028 7226 7768 8860 9477 9839 11177 11467 11647 11719 12308 12358 12372 13617 14182 14766 15326 16169A 16192 16235 16270 16302	U5b3a1a	73 150Y 263 301N 302N 303N 304N 305N 306N 307N 308N 309N 310N 311N 312N 313N 314N 315N 316N 317N 318N 373 16169A 16192 16235 16270 16302	[0.153] U5b3a1a (1)	[0.000] U5b3a1 U5b3a1b (3)	1
2174 >KY408261.1 Hc	73G 150T 263G 284G 301N 302N 303N 304N 305N 306N 307N 308N 309N 310N 311N 312N 313N 314N 315N 316N 317N 318N 373G 750G 1438G 2706G 3106d 3107C 3197C 4769G 7028T 7226A 7768G 8860G 9477A 11177T 11467G 11719A 11914A 12308G 12372A 13617C 14182C 14766T 15244G 15326G 16169A 16192T 16235G 16270T 16519C	U5b3a1a	73G 150T 263G 284G 301N 302N 303N 304N 305N 306N 307N 308N 309N 310N 311N 312N 313N 314N 315N 316N 317N 318N 373G 16169A 16192T 16235G 16270T 16519C	1	73 150 263 284 301N 302N 303N 304N 305N 306N 307N 308N 309N 310N 311N 312N 313N 314N 315N 316N 317N 318N 373 750 1438 2706 3197 4769 7028 7226 7768 8860 9477 11177 11467 11719 11914 12308 12372 13617 14182 14766 15244 15326 16169A 16192 16235 16270	U5b3a1a	73 150 263 284 301N 302N 303N 304N 305N 306N 307N 308N 309N 310N 311N 312N 313N 314N 315N 316N 317N 318N 373 16169A 16192 16235 16270	[0.184] U5b3a1a (1)	[0.000] U5b3a1 U5b3a1b (3)	1
2175 >KY408242.1 Hc	57C 59C 73G 152C 263G 301N 302N 303N 304N 305N 306N 307N 308N 309N 310N 311N 312N 313N 314N 315N 316N 317N 318N 709A 750G 930A 1438G 1888A 2706G 3106d 3107C 4216C 4769G 4917G 5147A 5656G 7028T 8697A 8860G 10463C 10750G 11251G 11719A 11812G 13368A 14233G 14766T 14905A 15326G 15452A 15607G 15928A 16126C 16292T 16294T 16296T 16304C 16519C	T2b3a1	57C 59C 73G 152C 263G 301N 302N 303N 304N 305N 306N 307N 308N 309N 310N 311N 312N 313N 314N 315N 316N 317N 318N 16126C 16292T 16294T 16296T 16304C 16519C	1	57 59 73 152 263 301N 302N 303N 304N 305N 306N 307N 308N 309N 310N 311N 312N 313N 314N 315N 316N 317N 318N 709 750 930 1438 1888 2706 4216 4769 4917 5147 5656 7028 8697 8860 10463 10750 11251 11719 11812 13368 14233 14766 14905 15326 15452A 15607 15928 16126 16292 16294 16296 16304	T2b3a1	57 59 73 152 263 301N 302N 303N 304N 305N 306N 307N 308N 309N 310N 311N 312N 313N 314N 315N 316N 317N 318N 16126 16292 16294 16296 16304	[0.693] T2b3a1 (1)	[0.693] T2b3a1 (2) [0.179] T2b3a (3) [0.014] T2b+152 (4) [0.012] T2b7a3 (5) [0.000] T2b4+152 T2b4c T2b4d T2b4e T2b4f T2b4g T2b21 T2b21b T2b22 (14)	1
2176 >KY408239.1 Hc	73G 199C 263G 301N 302N 303N 304N 305N 306N 307N 308N 309N 310N 311N 312N 313N 314N 315N 316N 317N 318N 497T 750G 1189C 1438G 1811G 2706G 3106d 3107C 3480G 4113A 4769G 6260A 7028T 8860G 9055A 9698C 10398G 10550G 11299C 11467G 11485C 11719A 11840T 12308G 12372A 13413G 13740C 14167T 14766T 14798C 15326G 16224C 16311C 16519C	K1a4a1c1	73G 199C 263G 301N 302N 303N 304N 305N 306N 307N 308N 309N 310N 311N 312N 313N 314N 315N 316N 317N 318N 497T 16224C 16311C 16519C	1	73 199 263 301N 302N 303N 304N 305N 306N 307N 308N 309N 310N 311N 312N 313N 314N 315N 316N 317N 318N 497 750 1189 1438 1811 2706 3480 4113 4769 6260 7028 8860 9055 9698 10398 10550 11299 11467 11485 11719 11840 12308 12372 13413 13740 14167 14766 14798 15326 16224 16311	K1a4a1c1	73 199 263 301N 302N 303N 304N 305N 306N 307N 308N 309N 310N 311N 312N 313N 314N 315N 316N 317N 318N 497 16224 16311	[0.000] K1a4a1c1 (1)	[0.007] K1a3a K1a5 (3) [0.006] K1a (4) [0.003] K1a1 K1a2 K1a12a (7) [0.000] K1a1a K1a1a1 K1a1a2 K1a1a2a K1a1a2a1 K1a1b K1a1b1 K1a1b1b K1a1b1b1 K1a1b1e K1a1b1f K1a1b1g K1a1b2 K1a2a K1a2a2 K1a2b K1a2c K1a3 K1a3a1 K1a3a1a K1a3a1b K1a3a2 K1a3a3 K1a3a4 K1a4 K1a4a K1a4a1 K1a4a1a K1a4a1b K1a4a1b1 K1a4a1c K1a4a1c1 K1a4a1d K1a4a1h K1a4a1i K1a4d K1a4e K1a4f K1a4f1 K1a4g K1a4h K1a4h1 K1a4i K1a12 K1a12a1 K1a12a1a K1a18 K1a19 K1a23 K1a29 (57)	1

2177 >KY408232.1 Hc	57C 59C 73G 152C 263G 301N 302N 303N 304N 305N 306N 307N 308N 309N 310N 311N 312N 313N 314N 315N 316N 317N 318N 709A 750G 930A 1438G 1888A 2706G 3106d 3107C 4216C 4769G 4917G 5147A 5656G 7028T 8697A 8860G 10463C 10750G 11251G 11719A 11812G 13368A 14233G 14766T 14905A 15326G 15452A 15607G 15928A 16126C 16292T 16294T 16296T 16304C 16519C	T2b3a1	57C 59C 73G 152C 263G 301N 302N 303N 304N 305N 306N 307N 308N 309N 310N 311N 312N 313N 314N 315N 316N 317N 318N 16126C 16292T 16294T 16296T 16304C 16519C	1	57 59 73 152 263 301N 302N 303N 304N 305N 306N 307N 308N 309N 310N 311N 312N 313N 314N 315N 316N 317N 318N 709 750 930 1438 1888 2706 4216 4769 4917 5147 5656 7028 8697 8860 10463 10750 11251 11719 11812 13368 14233 14766 14905 15326 15452A 15607 15928 16126 16292 16294 16296 16304	T2b3a1	57 59 73 152 263 301N 302N 303N 304N 305N 306N 307N 308N 309N 310N 311N 312N 313N 314N 315N 316N 317N 318N 16126 16292 16294 16296 16304	[0.693] T2b3a1 (1)	[0.693] T2b3a1 (2) [0.179] T2b3a (3) [0.014] T2b+152 (4) [0.012] T2b7a3 (5) [0.000] T2b4+152 T2b4c T2b4d T2b4e T2b4f T2b4g T2b21 T2b21b T2b22 (14)	1
2178 >KY684195.1 Hc	257G 263G 307T 309d 310d 477C 750G 1438G 3010A 3106d 3107C 4769G 8473C 8860G 15326G 16183C 16189C 16362C 16519C	H1c3b	257G 263G 307T 309d 310d 477C 16183C 16189C 16362C 16519C	1	257 263 307-309d 477 750 1438 3010 4769 8473 8860 15326 16189 16362	H1c3b	257 263 307-309d 477 16189 16362	[0.011] H1c3b (1)	[0.034] H1c3 (2)	1
2179 >KY680284.1 Hc	73G 150T 152C 189G 195C 215G 263G 295T 310.1TC 319C 489C 513A 750G 1438G 1850C 2706G 3106d 3107C 4216C 4769G 7028T 7476T 7789A 8745G 8860G 10237C 10398G 10499G 11251G 11377A 11719A 12612G 13708A 13722G 14133G 14766T 15191C 15257A 15326G 15452A 16069T 16126C 16145A 16172C 16231C 16261T	J2a1a1a1	73G 150T 152C 189G 195C 215G 263G 295T 310.1TC 319C 489C 513A 16069T 16126C 16145A 16172C 16231C 16261T	1	73 150 152 189 195 215 263 295 310.1T 319 489 513 750 1438 1850 2706 4216 4769 7028 7476 7789 8745 8860 10237 10398 10499 11251 11377 11719 12612 13708 13722 14133 14766 15191 15257 15326 15452A 16069 16126 16145 16172 16231 16261	J2a1a1a1	73 150 152 189 195 215 263 295 310.1T 319 489 513 16069 16126 16145 16172 16231 16261	[0.000] J2a1a1a1 (1)	[0.000] J2a1a1a J2a1a1a1 J2a1a1a2 J2a1a1a2a (5)	1
2180 >KY399202.1 Hc	73G 146C 152C 195C 263G 285T 301N 302N 303N 304N 305N 306N 307N 308N 309N 310N 311N 312N 313N 314N 315N 316N 317N 318N 319N 750G 1438G 2387C 2706G 3106d 3107C 4769G 7028T 8395T 8860G 10885C 11467G 11566G 11719A 12308G 12372A 12879C 13104G 14070G 14766T 15148A 15172A 15326G 15763G 15954C 16249C 16311C 16327T	U1b3	73G 146C 152C 195C 263G 285T 301N 302N 303N 304N 305N 306N 307N 308N 309N 310N 311N 312N 313N 314N 315N 316N 317N 318N 319N 16249C 16311C 16327T	1	73 146 152 195 263 285 301N 302N 303N 304N 305N 306N 307N 308N 309N 310N 311N 312N 313N 314N 315N 316N 317N 318N 319N 750 1438 2387 2706 4769 7028 8395 8860 10885 11467 11566 11719 12308 12372 12879 13104 14070 14766 15148 15172 15326 15763 15954C 16249 16311 16327	U1b3	73 146 152 195 263 285 301N 302N 303N 304N 305N 306N 307N 308N 309N 310N 311N 312N 313N 314N 315N 316N 317N 318N 319N 16249 16311 16327	[0.500] U1b3 (1)	[0.357] U1b (2) [0.250] U1b1	1
2181 >KY399182.1 Hc	73G 152C 217C 263G 301N 302N 303N 304N 305N 306N 307N 308N 309N 310N 311N 312N 313N 314N 315N 316N 317N 318N 319N 508G 750G 1438G 1811G 2706G 3106d 3107C 3720G 3849A 4553C 4769G 5390G 5426C 6045T 6152C 7028T 7235A 7418T 7427T 7624C 8473C 8860G 10876G 11467G 11719A 12308G 12372A 12557T 13020C 13734C 14766T 14788C 15326G 15907G 16051G 16092C 16129C 16183C 16189C 16362C 16519C 16525G	U2e2a1b	73G 152C 217C 263G 301N 302N 303N 304N 305N 306N 307N 308N 309N 310N 311N 312N 313N 314N 315N 316N 317N 318N 319N 508G 16051G 16092C 16129C 16183C 16189C 16362C 16519C 16525G	1	73 152 217 263 301N 302N 303N 304N 305N 306N 307N 308N 309N 310N 311N 312N 313N 314N 315N 316N 317N 318N 319N 508 750 1438 1811 2706 3720 3849 4553 4769 5390 5426 6045 6152 7028 7235A 7418 7427 7624 8473 8860 10876 11467 11719 12308 12372 12557 13020 13734 14766 14788 15326 15907 16051 16092 16129C 16189 16362 16525	U2e2a1b	73 152 217 263 301N 302N 303N 304N 305N 306N 307N 308N 309N 310N 311N 312N 313N 314N 315N 316N 317N 318N 319N 508 16051 16092 16129C 16189 16362 16525	[0.000] U2e2a1b (1)	[0.000] U2e2 U2e2a U2e2a1 U2e2a1a U2e2a1a1 U2e2a1a2 U2e2a1b U2e2a1c U2e2a1d (10)	1

2182	>KY399176.1 H	73G 150T 263G 301N 302N 303N 304N 305N 306N 307N 308N 309N 310N 311N 312N 313N 314N 315N 316N 317N 318N 319N 750G 1438G 1822C 2706G 3106d 3107C 3197C 4769G 5656G 5788C 7028T 7385G 7768G 8860G 9477A 10301G 10927C 11467G 11719A 12308G 12372A 12618A 13617C 14182C 14470C 14766T 15326G 15533G 16074G 16183C 16189C 16270T 16274A 16519C	U5b1b1g1	73G 150T 263G 301N 302N 303N 304N 305N 306N 307N 308N 309N 310N 311N 312N 313N 314N 315N 316N 317N 318N 319N 16074G 16183C 16189C 16270T 16274A 16519C	1	73 150 263 301N 302N 303N 304N 305N 306N 307N 308N 309N 310N 311N 312N 313N 314N 315N 316N 317N 318N 319N 750 1438 1822 2706 3197 4769 5656 5788 7028 7385 7768 8860 9477 10301 10927 11467 11719 12308 12372 12618 13617 14182 14470 14766 15326 15533 16074 16189 16270 16274	U5b1b1g1	73 150 263 301N 302N 303N 304N 305N 306N 307N 308N 309N 310N 311N 312N 313N 314N 315N 316N 317N 318N 319N 16074 16189 16270 16274	[0.080] U5b1b1g1 (1) [0.034] U5b1b1d (2) [0.033] U5b2a+@16192 U5b2a4 (4) [0.000] U5b1b1+@16192 U5b1b1f U5b1+16189+@16192 U5b2a4a U5b2a6 U5b2b4 (10)	[0.333] U5b1b1g1a (11) [0.066] U5b2a5a (12) [0.023] U5b2b (13) [0.014] U5b1b1a1a U5b1d1a U5b2b2 (16) [0.012] U5b2b1 (17) [0.000] B6 U5b1+16189 U5b1b U5b1b1 U5b1b1a U5b1b1a1 U5b1b1a1b U5b1b1a2 U5b1b1g U5b1h U5b2a U5b2a3 U5b2a3a U5b2a5 U5b2b4a (32)	1
2183	>KY399169.1 H	195C 263G 301N 302N 303N 304N 305N 306N 307N 308N 309N 310N 311N 312N 313N 314N 315N 316N 317N 318N 319N 750G 1438G 3106d 3107C 4769G 8860G 15326G 16220G 16519C	H82	195C 263G 301N 302N 303N 304N 305N 306N 307N 308N 309N 310N 311N 312N 313N 314N 315N 316N 317N 318N 319N 16220G 16519C	1	195 263 301N 302N 303N 304N 305N 306N 307N 308N 309N 310N 311N 312N 313N 314N 315N 316N 317N 318N 319N 750 1438 4769 8860 15326 16220	H82	195 263 301N 302N 303N 304N 305N 306N 307N 308N 309N 310N 311N 312N 313N 314N 315N 316N 317N 318N 319N 16220	[0.160] H82 (1) [0.000] H4a1a+195 H4a1a3 H4a1a3a H4a1a4 H4a1a4a H4a1a4b H4a1a4b2 H+195 H12 H108 H44b (12)	[0.022] H2a2a (13) [0.007] HV (14) [0.003] H60a (15) [0.002] H1e1a H1ak H4 H7b (19) [0.001] H H1h1 H1t H1ak1 H3b H3y H7c3 H13a1a1 H13a1a2 H13a2a1 H13c1 H47 H49a H56a1 (33) [0.000] R0 HV0f HV4 HV4c HV5 HV5a HV9c HV12 H1 H1e H1e1 H1e1a2 H1e1a7 H1e1a8 H1e1c H1e2 H1e2a H1e2b H1e2d H1e4 H1e6 H1e7 H1e8 H1e8a H1h H1h2 H1j H1j3 H1j5 H1j6 H1j7 H1j9 H1m H1n H1n+146+195 H1p H1q H1q1 H1q2 H1r H1s H1s1 H1t1 H1t1a H1t2 H1u H1u1 H1v H1v1 H1v1a H1v1b H1w H1ae H1ae1 H1ae2 H1ae2a H1ae3 H1ae3a H1ag H1ag1 H1ag1b H1ah H1ai H1ai1 H1aj H1ak2 H1am H1am1 H1ap H1aq H1as H1as2 H1at H1at1 H1at1a H1av H1ax H1ax1 H1ay H1az H1bd H1be H1bi H1bj H1bk H1bm H1bn H1bp H1bq H1br H1bu H1bv H1bw H1bx H1ca H1ci H2 H2a H2a1m H2a2 H2a2+(16235) H2a4 H2a5 H2a5a H2a5b H3 H3c H3c1 H3c2 H3c2a H3c2a1 H3c2b H3c2b1 H3c2c H3e H3q H3q1 H3r H3r1 H3t H3u H3aa H3ab H3ac H3ad H3ae H3ag H3ag1 H3ah H3ai H3aj H3ap H3aq [illegible]	1

2184 >KY399161.1 Hc	73G 150T 263G 301N 302N 303N 304N 305N 306N 307N 308N 309N 310N 311N 312N 313N 314N 315N 316N 317N 318N 319N 524.1NN 750G 1438G 1721T 2706G 3104N 3105N 3106N 3108N 3109N 3110N 3111N 3197C 4769G 7028T 7768G 8860G 9477A 11467G 11653G 11719A 12308G 12372A 12634G 13161C 13617C 13630G 13637G 14182C 14766T 15326G 16270T 16296T 16519C	U5b2b5	73G 150T 263G 301N 302N 303N 304N 305N 306N 307N 308N 309N 310N 311N 312N 313N 314N 315N 316N 317N 318N 319N 524.1NN 16270T 16296T 16519C	1	73 150 263 301N 302N 303N 304N 305N 306N 307N 308N 309N 310N 311N 312N 313N 314N 315N 316N 317N 318N 319N 524.2N 750 1438 1721 2706 3104N 3105N 3106N 3108N 3109N 3110N 3111N 3197 4769 7028 7768 8860 9477 11467 11653 11719 12308 12372 12634 13161 13617 13630 13637 14182 14766 15326 16270 16296	U5b2b5	73 150 263 301N 302N 303N 304N 305N 306N 307N 308N 309N 310N 311N 312N 313N 314N 315N 316N 317N 318N 319N 524.2N 16270 16296	[0.000] U5b2b5 (1)	[0.000] U5b1d1a U5b2b U5b2b1 U5b2b2 (5)	1
2185 >KY615004.1 Hc	73G 199C 204C 250C 263G 309.1CT 310C 567.1CC 574C 750G 1438G 1719A 2706G 3106d 3107C 4529T 4769G 5074C 7028T 8251A 8860G 10034C 10238C 10398G 11719A 12408A 12501A 12705T 14233G 14766T 15043A 15244G 15326G 15924G 16129A 16148T 16192T 16223T 16294T 16374C 16391A 16519C	I5a4	73G 199C 204C 250C 263G 309.1CT 310C 567.1CC 574C 16129A 16148T 16192T 16223T 16294T 16374C 16391A 16519C	1	73 199 204 250 263 574C 573.2C 750 1438 1719 2706 4529T 4769 5074 7028 8251 8860 10034 10238 10398 11719 12408A 12501 12705 14233 14766 15043 15244 15326 15924 16129 16148 16192 16223 16294 16374C 16391	I5a4	73 199 204 250 263 574C 573.2C 16129 16148 16192 16223 16294 16374C 16391	[0.321] I5a4 (1)	[0.158] I5a1a (2) [0.118] I5a (3) [0.000] I5a1 I5a1c I5a2 (6)	1
2186 >KX675326.1 Hc	73G 152C 263G 310.1C 489C 534T 750G 1438G 1462A 2706G 3010A 3106d 3107C 4769G 4883T 5178A 7028T 8383C 8414T 8701G 8860G 9431T 9540C 10398G 10400T 10873C 11719A 12705T 14668T 14766T 14783C 15043A 15301A 15326G 16164G 16223T 16245T 16335G 16362C 16519C	D4c2b	73G 152C 263G 310.1C 489C 534T 16164G 16223T 16245T 16335G 16362C 16519C	1	73 152 263 489 534 750 1438 1462 2706 3010 4769 4883 5178A 7028 8383 8414 8701 8860 9431 9540 10398 10400 10873 11719 12705 14668 14766 14783 15043 15301 15326 16164 16223 16245 16335 16362	D4c2b	73 152 263 489 534 16164 16223 16245 16335 16362	[0.058] D4c2b (1)	[0.063] D4c2 (2) [0.000] M13'46'61+16362 D4c D4c2c (5)	1
2187 >KX675318.1 Hc	73G 150T 263G 309T 310C 456T 489C 681C 750G 1048T 1107C 1438G 2706G 3106d 3107C 3759G 4769G 4883T 5147A 5153G 5178A 5301G 6253C 7028T 7403G 8701G 8860G 9168T 9180G 9540C 10397G 10398G 10400T 10873C 11719A 12705T 14766T 14783C 15043A 15301A 15326G 15724G 16180d 16183C 16189C 16223T 16357C 16362C 16519C	D5b1b2	73G 150T 263G 309T 310C 456T 489C 16180d 16183C 16189C 16223T 16357C 16362C 16519C	1	73 150 263 309d 456 489 681 750 1048 1107 1438 2706 3759 4769 4883 5147 5153 5178A 5301 6253 7028 7403 8701 8860 9168 9180 9540 10397 10398 10400 10873 11719 12705 14766 14783 15043 15301 15326 15724 16189 16193d 16223 16357 16362	D5b1b2	73 150 263 309d 456 489 16189 16193d 16223 16357 16362	[0.036] D5b1b2 (1)	[0.007] D5b D5b1a D5b1b D5b1c D5b3 D5b3a (7) [0.000] D5b1	1
2188 >KX675315.1 Hc	73G 152C 199C 204C 263G 309.1CCT 310C 567.1CCC 669C 750G 1438G 1719A 2706G 3106d 3107C 3336C 4769G 5315G 5744A 6641C 7028T 8164T 8860G 8901G 9300A 10238C 10398G 11719A 12501A 12705T 13780G 14766T 15043A 15326G 16147A 16172C 16189C 16223T 16248T 16320T 16355T 16519C	N1a1a1a1a	73G 152C 199C 204C 263G 309.1CCT 310C 567.1CCC 16147A 16172C 16189C 16223T 16248T 16320T 16355T 16519C	1	73 152 199 204 263 573.3C 669 750 1438 1719 2706 3336 4769 5315 5744 6641 7028 8164 8860 8901 9300 10238 10398 11719 12501 12705 13780 14766 15043 15326 16147A 16172 16189 16223 16248 16320 16355	N1a1a1a1a	73 152 199 204 263 573.3C 16147A 16172 16189 16223 16248 16320 16355	[0.000] N1a1a1a1a (1)	[0.000] N1a1a1a N1a1a1a1 (3)	1

2189	>KX675313.1 Ho	73G 150T 263G 309.1CCT 310C 456T 489C 681C 750G 1048T 1107C 1438G 2706G 3106d 3107C 3759G 4769G 4883T 5147A 5153G 5178A 5301G 6253C 7028T 7403G 8701G 8860G 9168T 9180G 9540C 10320A 10397G 10398G 10400T 10873C 11719A 12705T 14766T 14783C 15043A 15301A 15326G 15724G 16182C 16183C 16189C 16223T 16357C 16362C 16519C	D5b1b2	73G 150T 263G 309.1CCT 310C 456T 489C 16182C 16183C 16189C 16223T 16357C 16362C 16519C	1	73 150 263 456 489 681 750 1048 1107 1438 2706 3759 4769 4883 5147 5153 5178A 5301 6253 7028 7403 8701 8860 9168 9180 9540 10320 10397 10398 10400 10873 11719 12705 14766 14783 15043 15301 15326 15724 16189 16223 16357 16362	D5b1b2	73 150 263 456 489 16189 16223 16357 16362	[0.000] D5b1b2 (1)	[0.000] D5b D5b1 D5b1a D5b1b D5b1c D5b3 D5b3a (8)	1
2190	>KX675312.1 Ho	73G 200G 263G 309.1CT 310C 489C 750G 1438G 2706G 3010A 3106d 3107C 3918A 4769G 4883T 4959A 5178A 7028T 8414T 8701G 8860G 9540C 10398G 10400T 10873C 11719A 12705T 14668T 14766T 14783C 15043A 15301A 15326G 16223T 16256T 16311C 16316G 16362C	D4q	73G 200G 263G 309.1CT 310C 489C 16223T 16256T 16311C 16316G 16362C	1	73 200 263 489 750 1438 2706 3010 3918 4769 4883 4959 5178A 7028 8414 8701 8860 9540 10398 10400 10873 11719 12705 14668 14766 14783 15043 15301 15326 16223 16256 16311 16316 16362	D4q	73 200 263 489 16223 16256 16311 16316 16362	[0.008] D4q (1)	[0.000] D4q1 D4q1a (3)	1
2191	>KX675311.1 Ho	262T 263G 309.1CT 310C 750G 1438G 3106d 3107C 4769G 5460A 8860G 10124C 14118G 15326G 15617A	H41a	262T 263G 309.1CT 310C	1	262 263 750 1438 4769 5460 8860 10124 14118 15326 15617	H41a	262 263	[0.000] H41a (1)	[0.000] R0 HV HV4 HV4c HV5 HV5a HV9c HV12 H H1 H1e H1e1 H1e1a H1e1a2 H1e1a7 H1e1c H1e2 H1e2a H1e2b H1e2d H1e4 H1e6 H1e7 H1e8 H1e8a H1h H1h1 H1h2 H1j H1j3 H1j5 H1j6 H1j7 H1j9 H1m H1n H1p H1q H1q1 H1q2 H1r H1s H1s1 H1t H1t1 H1t1a H1t2 H1u H1u1 H1v H1v1 H1v1a H1v1b H1w H1ae H1ae1 H1ae2 H1ae2a H1ae3 H1ae3a H1ag H1ag1 H1ag1b H1ah H1ai H1ai1 H1aj H1ak H1ak1 H1ak2 H1am H1am1 H1ap H1aq H1as H1as2 H1at H1at1 H1at1a H1av H1ax H1ax1 H1ay H1az H1bd H1be H1bi H1bj H1bk H1bm H1bn H1bp H1bq H1br H1bu H1bv H1bw H1bx H1ca H1ci H2 H2a H2a2 H2a2a H2a2+(16235) H2a4 H2a5 H2a5a H2a5b H3 H3b H3c H3c1 H3e H3q H3q1 H3r H3r1 H3t H3u H3y H3aa H3ab H3ac H3ad H3ae H3ag H3ag1 H3ah H3ai H3aj H3ap H3aq H3ar H3as H3at H3at1 H3au H4 H4a H4a1 H4a1a H4a1a1 H4a1a2 H4a1a2a H4a1a2a1 H4a1a5 H4a1c H4a1c1 H4a1c1a H4a1d H4b H4c H7 H7a H7b H7b1 H7b2 H7b2a H7b3 H7b4 H7b5 H7b6 H7c H7c2 H7c3 H7c5 [illegible]	1

2192	>KX675310.1 Ho	73G 152C 235G 263G 309.1CT 310C 514d 515d 663G 750G 1438G 1709A 1736G 2706G 3106d 3107C 4248C 4769G 4824G 7028T 8794T 8860G 9754G 11719A 12705T 12720G 14290C 14766T 15326G 16000A 16039A 16189C 16223T 16256T 16290T 16319A 16356C 16362C	A12a	73G 152C 235G 263G 309.1CT 310C 514d 515d 16039A 16189C 16223T 16256T 16290T 16319A 16356C 16362C	1	73 152 235 263 663 750 1438 1709 1736 2706 4248 4769 4824 7028 8794 8860 9754 11719 12705 12720 14290 14766 15326 16000 16039 16189 16223 16256 16290 16319 16356 16362	A12a	73 152 235 263 16039 16189 16223 16256 16290 16319 16356 16362	[0.040] A12a (1)	[0.000] A+152+16362+16189 A12 (3)	1
2193	>KX675309.1 Ho	73G 195C 200G 263G 309.1CT 310C 489C 593C 750G 1438G 2706G 3010A 3106d 3107C 4769G 4883T 5178A 7028T 8414T 8701G 8860G 9540C 10398G 10400T 10873C 11150A 11719A 12705T 14668T 14766T 14783C 15043A 15301A 15326G 16223T 16256T 16311C 16362C 16519C	D4q	73G 195C 200G 263G 309.1CT 310C 489C 16223T 16256T 16311C 16362C 16519C	1	73 195 200 263 489 593 750 1438 2706 3010 4769 4883 5178A 7028 8414 8701 8860 9540 10398 10400 10873 11150 11719 12705 14668 14766 14783 15043 15301 15326 16223 16256 16311 16362	D4q	73 195 200 263 489 16223 16256 16311 16362	[0.067] D4q (1)	[0.000] D4q1 D4q1a (3)	1
2194	>KX675307.1 Ho	73G 263G 310C 514d 515d 709A 750G 1438G 2706G 3106d 3107C 3960T 4203G 4769G 5263T 5465C 7028T 8271d 8272d 8273d 8274d 8275d 8276d 8277d 8278d 8279d 8860G 9123A 10238C 11719A 14766T 15326G 15479C 16000A 16182C 16183C 16189C 16194C 16195C 16217C 16261T 16264T 16266G 16519C	B4a1c3a	73G 263G 310C 514d 515d 16182C 16183C 16189C 16194C 16195C 16217C 16261T 16264T 16266G 16519C	1	73 263 310 709 750 1438 2706 3960 4203 4769 5263 5465 7028 8281-8289d 8860 9123 10238 11719 14766 15326 15479 16000 16189 16194C 16195 16217 16261 16264 16266G	B4a1c3a	73 263 310 16189 16194C 16195 16217 16261 16264 16266G	[0.077] B4a1c3a (1)	[0.000] B4a1c3 B4a1c3b (3)	1
2195	>KX675305.1 Ho	73G 150T 195C 198T 200G 263G 310.1C 750G 1438G 1811G 2294G 2706G 3106d 3107C 4703C 4769G 5263T 5291C 5553C 6518T 6680C 7028T 8860G 9266A 10506G 11050C 11467G 11719A 12308G 12372A 13934T 14139G 14766T 15326G 15454C 16126C 16343G 16390A 16519C	U3a2a	73G 150T 195C 198T 200G 263G 310.1C 16126C 16343G 16390A 16519C	1	73 150 195 198 200 263 750 1438 1811 2294 2706 4703 4769 5263 5291 5553 6518 6680 7028 8860 9266 10506 11050 11467 11719 12308 12372 13934 14139 14766 15326 15454 16126 16343 16390	U3a2a	73 150 195 198 200 263 16126 16343 16390	[0.355] U3a2a (1)	[0.104] U3a2 (2) [0.043] U3a2a1 (3) [0.013] U3a1 (4) [0.000] U3a U3a1a U3a1a1 U3a1b (8)	1
2196	>KX675304.1 Ho	73G 150T 263G 309.1CT 310C 456T 489C 681C 750G 1048T 1107C 1438G 2706G 3106d 3107C 3759G 4769G 4883T 5147A 5153G 5178A 5301G 6253C 7028T 7403G 8701G 8860G 9168T 9180G 9540C 10397G 10398G 10400T 10873C 11719A 12705T 14766T 14783C 15043A 15301A 15326G 15724G 16182C 16183C 16189C 16223T 16357C 16362C 16519C	D5b1b2	73G 150T 263G 309.1CT 310C 456T 489C 16182C 16183C 16189C 16223T 16357C 16362C 16519C	1	73 150 263 456 489 681 750 1048 1107 1438 2706 3759 4769 4883 5147 5153 5178A 5301 6253 7028 7403 8701 8860 9168 9180 9540 10397 10398 10400 10873 11719 12705 14766 14783 15043 15301 15326 15724 16189 16223 16357 16362	D5b1b2	73 150 263 456 489 16189 16223 16357 16362	[0.000] D5b1b2 (1)	[0.000] D5b D5b1 D5b1a D5b1b D5b1c D5b3 D5b3a (8)	1

2197	>KX675303.1 Hc	73G 152C 204C 248d 263G 309.1CT 310C 514d 515d 750G 1438G 2706G 3106d 3107C 3970T 4732G 4769G 5147A 6392C 6962A 7028T 8772C 8860G 10310A 10609C 10976T 11719A 12406A 12633T 12882T 13928C 14476A 14766T 15326G 16172C 16183C 16189C 16232A 16249C 16304C 16311C 16342C	F1b1b	73G 152C 204C 248d 263G 309.1CT 310C 514d 515d 16172C 16183C 16189C 16232A 16249C 16304C 16311C 16342C	1	73 152 204 249d 263 750 1438 2706 3970 4732 4769 5147 6392 6962 7028 8772 8860 10310 10609 10976 11719 12406 12633 12882 13928C 14476 14766 15326 16172 16189 16232A 16249 16304 16311 16342	F1b1b	73 152 204 249d 263 16172 16189 16232A 16249 16304 16311 16342	[0.020] F1b1b (1)	[0.048] F1b F1b1 (3)	1
2198	>KX675301.1 Hc	47A 73G 248d 263G 309.1CT 310C 489C 750G 1438G 2226.1A 2706G 3106d 3107C 3394C 3552A 4715G 4769G 6026A 7028T 7196A 8443G 8584A 8701G 8860G 9540C 9545G 10398G 10400T 10873C 11654G 11719A 11914A 11969A 12672G 12705T 13263G 13759A 13967T 14318C 14766T 14783C 15043A 15204C 15301A 15326G 15487T 16223T 16298C 16327T 16357C 16519C	C4a2c	47A 73G 248d 263G 309.1CT 310C 489C 16223T 16298C 16327T 16357C 16519C	1	47 73 249d 263 489 750 1438 2232.1A 2706 3394 3552A 4715 4769 6026 7028 7196A 8443 8584 8701 8860 9540 9545 10398 10400 10873 11654 11719 11914 11969 12672 12705 13263 13759 13967 14318 14766 14783 15043 15204 15301 15326 15487T 16223 16298 16327 16357	C4a2c	47 73 249d 263 489 16223 16298 16327 16357	[0.000] C4a2c (1)	[0.000] C4a2 C4a2b C4a2c2 C4a2c2a (5)	1
2199	>KX675300.1 Hc	262T 263G 309.1CT 310C 750G 1438G 3106d 3107C 4769G 5460A 8860G 10124C 14118G 15326G 15617A	H41a	262T 263G 309.1CT 310C	1	262 263 750 1438 4769 5460 8860 10124 14118 15326 15617	H41a	262 263	[0.000] H41a (1)	[0.000] R0 HV HV4 HV4c HV5 HV5a HV9c HV12 H H1 H1e H1e1 H1e1a H1e1a2 H1e1a7 H1e1c H1e2 H1e2a H1e2b H1e2d H1e4 H1e6 H1e7 H1e8 H1e8a H1h H1h1 H1h2 H1j H1j3 H1j5 H1j6 H1j7 H1j9 H1m H1n H1p H1q H1q1 H1q2 H1r H1s H1s1 H1t H1t1 H1t1a H1t2 H1u H1u1 H1v H1v1 H1v1a H1v1b H1w H1ae H1ae1 H1ae2 H1ae2a H1ae3 H1ae3a H1ag H1ag1 H1ag1b H1ah H1ai H1ai1 H1aj H1ak H1ak1 H1ak2 H1am H1am1 H1ap H1aq H1as H1as2 H1at H1at1 H1at1a H1av H1ax H1ax1 H1ay H1az H1bd H1be H1bi H1bj H1bk H1bm H1bn H1bp H1bq H1br H1bu H1bv H1bw H1bx H1ca H1ci H2 H2a H2a2 H2a2a H2a2+(16235) H2a4 H2a5 H2a5a H2a5b H3 H3b H3c H3c1 H3e H3q H3q1 H3r H3r1 H3t H3u H3y H3aa H3ab H3ac H3ad H3ae H3ag H3ag1 H3ah H3ai H3aj H3ap H3aq H3ar H3as H3at H3at1 H3au H4 H4a H4a1 H4a1a H4a1a1 H4a1a2 H4a1a2a H4a1a2a1 H4a1a5 H4a1c H4a1c1 H4a1c1a H4a1d H4b H4c H7 H7a H7b H7b1 H7b2 H7b2a H7b3 H7b4 H7b5 H7b6 H7c H7c2 H7c3 H7c5 [illegible]	1

2200	>KX675297.1 Hc	73G 248d 263G 310.1C 750G 761G 1005C 1438G 1824C 2706G 3106d 3107C 3970T 4769G 5978G 6156T 6392C 6570T 7028T 7828G 8152A 8860G 10310A 10535C 10586A 11719A 12338C 13708A 13722G 13928C 14133G 14766T 15326G 15418T 16067T 16271C 16304C	F2c2	73G 248d 263G 310.1C 16067T 16271C 16304C	1	73 249d 263 750 761 1005 1438 1824 2706 3970 4769 5978 6156 6392 6570T 7028 7828 8152 8860 10310 10535 10586 11719 12338 13708 13722 13928C 14133 14766 15326 15418 16067 16271 16304	F2c2	73 249d 263 16067 16271 16304	[0.000] F2c2 (1)	[0.000] F F1 F2 F2c F2e F2f F4 (8)	1
2201	>KX675292.1 Hc	73G 152C 263G 310.1C 451.1T 750G 1438G 2706G 3106d 3107C 3311T 4615G 4769G 5319G 5894.1CC 7028T 8140N 8843C 8860G 12681C 13708A 13768C 15326G 16217C 16519C	HV2	73G 152C 263G 310.1C 451.1T 16217C 16519C	1	73 152 263 455.1T 750 1438 2706 3311 4615 4769 5319 5899.2C 7028 8140N 8843 8860 12681 13708 13768 15326 16217	HV2	73 152 263 455.1T 16217	[0.213] HV2 (1)	[0.137] HV2a2 (2) [0.000] HV2a HV2a3 H32 R30a1 R30a1a (7)	1
2202	>KX675287.1 Hc	73G 152C 263G 310.1C 489C 750G 958T 1438G 2706G 3106d 3107C 4071T 4769G 5351G 5460A 6455T 7028T 7684C 7853A 8701G 8860G 9540C 9824C 10398G 10400T 10873C 11719A 12358G 12405T 12705T 14053G 14314G 14766T 14783C 15043A 15301A 15326G 16129A 16152C 16179T 16192T 16223T 16362C	M7b1b	73G 152C 263G 310.1C 489C 16129A 16152C 16179T 16192T 16223T 16362C	1	73 152 263 489 750 958 1438 2706 4071 4769 5351 5460 6455 7028 7684 7853 8701 8860 9540 9824 10398 10400 10873 11719 12358 12405 12705 14053 14314 14766 14783 15043 15301 15326 16129 16152 16179 16192 16223 16362	M7b1b	73 152 263 489 16129 16152 16179 16192 16223 16362	[0.012] M7b1b (1)	[0.013] D4a1 (2) [0.003] D4a1a (3) [0.000] D4a D4a1a1 D4a1a1a D4a1b D4a1c D4a1f D4a1f1 D4a1g D4a1h D4a2 D4a2a D4a2b D4a5 (16)	1
2203	>KX675274.1 Hc	73G 195C 248d 263G 489C 750G 1438G 1715T 2226.1A 2706G 3106d 3107C 3552A 4715G 4769G 6026A 7028T 7196A 7999C 8584A 8701G 8856A 8860G 9540C 9545G 10398G 10400T 10873C 11719A 11914A 11969A 12372A 12672G 12705T 12940A 13263G 14318C 14766T 14783C 15043A 15204C 15301A 15326G 15487T 15968C 16129A 16150T 16223T 16298C 16309G 16327T 16519C	C4a1a4a	73G 195C 248d 263G 489C 16129A 16150T 16223T 16298C 16309G 16327T 16519C	1	73 195 249d 263 489 750 1438 1715 2232.1A 2706 3552A 4715 4769 6026 7028 7196A 7999 8584 8701 8856 8860 9540 9545 10398 10400 10873 11719 11914 11969 12372 12672 12705 12940 13263 14318 14766 14783 15043 15204 15301 15326 15487T 15968 16129 16150 16223 16298 16309 16327	C4a1a4a	73 195 249d 263 489 16129 16150 16223 16298 16309 16327	[0.006] C4a1a4a (1)	[0.006] C4a1a4a (2) [0.000] C4a1a+195 C4a1a2 C4a1a2a C4a1a3 C4a1a3a C4a1a3a1 C4a1a3b C4a1a3c C4a1a4 (11)	1
2204	>KX675270.1 Hc	64T 65C 73G 188G 200G 215G 263G 309N 310C 318C 326G 471C 489C 750G 1095C 1438G 2706G 3106d 3107C 3523G 3565G 4769G 6383A 6531T 7028T 7642A 8108G 8557A 8701G 8860G 9540C 9950C 10398G 10400T 10873C 11350G 11719A 11969A 12705T 14766T 14783C 14890G 15043A 15301A 15326G 16189C 16223T 16295T 16519C	M11d	64T 65C 73G 188G 200G 215G 263G 309N 310C 318C 326G 471C 489C 16189C 16223T 16295T 16519C	1	64 65 73 188 200 215 263 309N 310 318 326 471 489 750 1095 1438 2706 3523 3565 4769 6383 6531 7028 7642 8108 8557 8701 8860 9540 9950 10398 10400 10873 11350 11719 11969 12705 14766 14783 14890 15043 15301 15326 16189 16223 16295	M11d	64 65 73 188 200 215 263 309N 310 318 326 471 489 16189 16223 16295	[0.184] M11d (1)	[0.020] M11a (2) [0.012] M11a1 (3) [0.000] M11+200 M11a'b M11b M11b1 (7)	1

2205	>KY609152.1 H	73G 146C 185A 188G 222T 228A 263G 295T 310.1C 462T 489C 750G 1438G 2706G 3010A 3106d 3107C 4216C 4769G 7028T 8860G 10398G 10685A 11251G 11719A 12612G 13281C 13708A 13933G 14766T 14798C 15326G 15452A 16069T 16126C 16519C	J1c2c1	73G 146C 185A 188G 222T 228A 263G 295T 310.1C 462T 489C 16069T 16126C 16519C	1	73 146 185 188 222 228 263 295 462 489 750 1438 2706 3010 4216 4769 7028 8860 10398 10685 11251 11719 12612 13281 13708 13933 14766 14798 15326 15452A 16069 16126	J1c2c1	73 146 185 188 222 228 263 295 462 489 16069 16126	[0.000] J1c2c1 (1)	[0.005] J1c2c (2) [0.000] J1c2c1 J1c2c1a J1c2c2 J1c2c2a J1c2c3 J1c2h (8)	1
2206	>KY498015.1 H	73G 262T 263G 285T 309.1CCT 310C 323A 385G 514d 515d 750G 1438G 2218T 2706G 3106d 3107C 3158.1T 3591A 4561C 4769G 4991A 5048C 6026A 7028T 7581C 8860G 11467G 11719A 12308G 12372A 12879C 13104G 13422G 14070G 14364A 14766T 15148A 15326G 15954C 16183C 16189C	U1a1a2	73G 262T 263G 285T 309.1CCT 310C 323A 385G 514d 515d 16183C 16189C	1	73 262 263 285 323 385 750 1438 2218 2706 3158.1T 3591 4561 4769 4991 5048 6026 7028 7581 8860 11467 11719 12308 12372 12879 13104 13422 14070 14364 14766 15148 15326 15954C 16189	U1a1a2	73 262 263 285 323 385 16189	[0.156] U1a1a2 (1)	[0.043] U1a1a (2)	1
2207	>KY514166.1 H	73G 195C 263G 310.1C 499A 750G 1438G 1811G 2706G 3106d 3107C 4646C 4769G 5999C 6047G 7028T 8705C 8860G 10676T 10907C 11332T 11467G 11719A 12308G 12372A 14620T 14766T 15326G 15693C 16261T 16263C 16356C	U4c2a	73G 195C 263G 310.1C 499A 16261T 16263C 16356C	1	73 195 263 499 750 1438 1811 2706 4646 4769 5999 6047 7028 8705 8860 10676 10907 11332 11467 11719 12308 12372 14620 14766 15326 15693 16261 16263 16356	U4c2a	73 195 263 499 16261 16263 16356	[0.040] U4c2a (1)	[0.008] U4b1a2a (2) [0.000] U4 U4a U4b U4b1 U4b1a U4b1a1 U4b1a1a U4b1a2 U4b1a2b U4b1a4 U4c U4c2 U4d U4d1 U4d1a U4d1a1 U4d1a1a U4d1b (20)	1
2208	>KY474046.1 H	73G 225T 263G 310.1C 514d 515d 750G 1438G 2706G 3106d 3107C 3420T 3450T 4769G 5250C 5773A 6221C 7028T 8701G 8860G 9449T 9540C 10086G 10398G 10586A 10640C 10873C 11719A 12705T 13105G 13914A 14766T 15301A 15311G 15326G 15766G 15824G 15940d 16124C 16183C 16189C 16223T 16278T 16311C 16362C 16527T	L3b2a	73G 225T 263G 310.1C 514d 515d 16124C 16183C 16189C 16223T 16278T 16311C 16362C 16527T	1	73 225T 263 750 1438 2706 3420 3450 4769 5250 5773 6221 7028 8701 8860 9449 9540 10086 10398 10586 10640 10873 11719 12705 13105 13914A 14766 15301 15311 15326 15766 15824 15944d 16124 16189 16223 16278 16311 16362 16527	L3b2a	73 225T 263 16124 16189 16223 16278 16311 16362 16527	[0.036] L3b2a (1)	[0.035] L3b2 (2) [0.033] L3b2b (3) [0.009] L3b1a3 (4) [0.000] L3b1a11	1
2209	>KY382658.1 H	73G 146C 152Y 263G 285T 310.1C 750G 1438G 2218T 2706G 3106d 3107C 4769G 7028T 8860G 10203A 11467G 11719A 12308G 12372A 12879C 13104G 13681G 14063C 14070G 14364A 14766T 15148A 15326G 15954C 16189C 16192T 16249C 16362C	U1a3	73G 146C 152Y 263G 285T 310.1C 16189C 16192T 16249C 16362C	1	73 146 152Y 263 285 750 1438 2218 2706 4769 7028 8860 10203 11467 11719 12308 12372 12879 13104 13681 14063 14070 14364 14766 15148 15326 15954C 16189 16192 16249 16362	U1a3	73 146 152Y 263 285 16189 16192 16249 16362	[0.123] U1a3 (1)	[0.315] U1a1c1 (2) [0.099] U1a1c1d (3) [0.048] U1a1c1c (4) [0.019] U1a1 (5) [0.000] U1a U1a1c U1a1c1b U1a1d (9)	1

2210	>KT946594.1 Ho	73G 114T 215G 263G 497T 750G 1189C 1438G 1811G 2706G 3106d 3107C 3480G 4769G 7028T 8860G 9055A 9698C 10398G 10550G 10978G 11299C 11467G 11470G 11719A 11914A 12308G 12372A 12954C 14167T 14766T 14798C 15326G 15924G 16224C 16234T 16311C 16519C	K1a1b1a	73G 114T 215G 263G 497T 16224C 16234T 16311C 16519C	1	73 114 215 263 497 750 1189 1438 1811 2706 3480 4769 7028 8860 9055 9698 10398 10550 10978 11299 11467 11470 11719 11914 12308 12372 12954 14167 14766 14798 15326 15924 16224 16234 16311	K1a1b1a	73 114 215 263 497 16224 16234 16311	[0.022] K1a1b1a (1)	[0.072] K1a1b1 (2) [0.022] K1a1b1a (3) [0.000] K1a1 K1a1a K1a1a1 K1a1a2 K1a1a2a K1a1a2a1 K1a1b K1a1b1b K1a1b1b1 K1a1b1e K1a1b1f K1a1b1g K1a1b2 (16)	1
2211	>KP172385.1 Ho	73G 152C 263G 499A 750G 827G 1438G 2706G 3107C 3547G 4769G 4820A 4977C 6473T 6755A 7028T 8271d 8272d 8273d 8274d 8275d 8276d 8277d 8278d 8279d 8860G 9950C 10978G 11177T 11719A 13590A 14766T 15326G 15535T 16145A 16156A 16157C 16182C 16183C 16189C 16217C 16362C 16381C	B2b2	73G 152C 263G 499A 16145A 16156A 16157C 16182C 16183C 16189C 16217C 16362C 16381C	1	73 152 263 499 750 827 1438 2706 3547 4769 4820 4977 6473 6755 7028 8281-8289d 8860 9950 10978 11177 11719 13590 14766 15326 15535 16145 16156 16157 16189 16217 16362 16381	B2b2	73 152 263 499 16145 16156 16157 16189 16217 16362 16381	[0.584] B2b2 (1) [0.008] B2b+152 (2) [0.000] B2o1a (3)	[0.500] B2o1 (4) [0.141] B2 (5) [0.126] B2i B2q (7) [0.069] B2b (8) [0.061] B2n (9) [0.032] B2r (10) [0.024] B2e (11) [0.020] B2c1 (12) [0.010] B2c (13) [0.000] B4b B2b3 B2c1a B2c1b B2c1c B2f B2p (20)	1
2212	>KP240889.1 Ho	73G 248d 263G 310N 499A 750G 1005C 1438G 1824C 2706G 3106d 3107C 3970T 4769G 6392C 7028T 7828G 8860G 10310A 10535C 10586A 11719A 12338C 13077T 13708A 13928C 14308C 14766T 15326G 16140C 16203G 16291T 16304C 16311C 16519C	F2a	73G 248d 263G 310N 499A 16140C 16203G 16291T 16304C 16311C 16519C	1	73 249d 263 310N 499 750 1005 1438 1824 2706 3970 4769 6392 7028 7828 8860 10310 10535 10586 11719 12338 13077 13708 13928C 14308 14766 15326 16140 16203 16291 16304 16311	F2a	73 249d 263 310N 499 16140 16203 16291 16304 16311	[0.188] F2a (1)	[0.346] F2+16291 (2) [0.017] F2a1 (3) [0.000] F2a+@16291 F2b F2g (6)	1
2213	>KP240886.1 Ho	73G 146N 152C 207A 248d 263G 709A 750G 1438G 2706G 3106d 3107C 3290C 3970T 4769G 5263T 6392C 7028T 7861C 8860G 10310A 10915C 11038G 11719A 12153T 12396C 12408C 12630A 13602C 13928C 14766T 15326G 15670C 16207G 16304C 16362C 16399G 16497G	F4a1a	73G 146N 152C 207A 248d 263G 16207G 16304C 16362C 16399G 16497G	1	73 146N 152 207 249d 263 709 750 1438 2706 3290 3970 4769 5263 6392 7028 7861 8860 10310 10915 11038 11719 12153 12396 12408 12630 13602 13928C 14766 15326 15670 16207 16304 16362 16399 16497	F4a1a	73 146N 152 207 249d 263 16207 16304 16362 16399 16497	[0.000] F4a1a (1)	[0.462] F4a1 (2)	1
2214	>KP240885.1 Ho	73G 248d 263G 750G 1005C 1438G 1824C 2706G 3106d 3107C 3970T 4769G 6392C 7028T 7828G 8860G 9128C 9377G 10310A 10535C 10586A 11009C 11719A 12338C 13708A 13928C 14766T 15326G 16203G 16291T 16304C 16311C 16335G 16519C	F2a	73G 248d 263G 16203G 16291T 16304C 16311C 16335G 16519C	1	73 249d 263 750 1005 1438 1824 2706 3970 4769 6392 7028 7828 8860 9128 9377 10310 10535 10586 11009 11719 12338 13708 13928C 14766 15326 16203 16291 16304 16311 16335	F2a	73 249d 263 16203 16291 16304 16311 16335	[0.290] F2a (1)	[0.679] F2+16291 (2) [0.017] F2a1 (3) [0.000] F2a+@16291 F2b F2g (6)	1
2215	>KP240878.1 Ho	73G 94A 248d 263G 514d 515d 750G 1438G 2706G 3106d 3107C 3970T 4086T 4769G 5147C 6392C 6962A 7028T 8860G 9053A 9548A 10310A 10609C 11719A 12406A 12715G 12882T 13759A 13928C 14766T 15326G 16129A 16162G 16172C 16304C 16519C	F1a1	73G 94A 248d 263G 514d 515d 16129A 16162G 16172C 16304C 16519C	1	73 94 249d 263 750 1438 2706 3970 4086 4769 5147C 6392 6962 7028 8860 9053 9548 10310 10609 11719 12406 12715 12882 13759 13928C 14766 15326 16129 16162 16172 16304	F1a1	73 94 249d 263 16129 16162 16172 16304	[0.070] F1a1 (1)	[0.000] F1a F1a1'4 F1a1a F1a1a1 F1a1b F1a1c F1a1c2 F1a1c3 F1a1d F1a1d1 F1a4 F1a3 (13)	1

2216 >KP240874.1 Ho	73G 248d 263G 514d 515d 750G 1438G 2706G 3106d 3107C 3970T 4086T 4769G 6182A 6392C 6962A 7028T 8592A 8860G 9053A 9548A 9632G 10310A 10609C 11719A 12406A 12882T 13759A 13928C 14766T 15326G 16129A 16162G 16172C 16304C 16368C 16519C	F1a1	73G 248d 263G 514d 515d 16129A 16162G 16172C 16304C 16368C 16519C	1	73 249d 263 750 1438 2706 3970 4086 4769 6182 6392 6962 7028 8592 8860 9053 9548 9632 10310 10609 11719 12406 12882 13759 13928C 14766 15326 16129 16162 16172 16304 16368	F1a1	73 249d 263 16129 16162 16172 16304 16368	[0.009] F1a1 (1)	[0.039] F1a1a (2) [0.010] F1a1a1 (3) [0.000] F1a F1a1'4 F1a1b F1a1c F1a1c2 F1a1c3 F1a1d F1a1d1 F1a4 F1a3 (13)	1
2217 >KP240872.1 Ho	73G 151T 187T 248d 263G 514d 515d 750G 1438G 2706G 3106d 3107C 3970T 4086T 4769G 6392C 6962A 7028T 8860G 9053A 9141C 9548A 10310A 10609C 11719A 12406A 12882T 13759A 13928C 14766T 15326G 16129A 16162G 16172C 16243C 16304C	F1a1	73G 151T 187T 248d 263G 514d 515d 16129A 16162G 16172C 16243C 16304C	1	73 151 187T 249d 263 750 1438 2706 3970 4086 4769 6392 6962 7028 8860 9053 9141 9548 10310 10609 11719 12406 12882 13759 13928C 14766 15326 16129 16162 16172 16243 16304	F1a1	73 151 187T 249d 263 16129 16162 16172 16243 16304	[0.116] F1a1 (1)	[0.008] F1a1c2 (2) [0.005] F1a1a F1a1d (4) [0.003] F1a3 (5) [0.002] F1a1a1 (6) [0.000] F1a F1a1'4 F1a1b F1a1c F1a1c3 F1a1d1 F1a4 (13)	1
2218 >KP240871.1 Ho	73G 150T 199C 204C 263G 456T 489C 750G 1438G 2706G 3106d 3107C 3866C 4048A 4071T 4164G 4769G 5351G 5460A 6455T 6680C 7028T 7684C 7853A 8701G 8860G 9540C 9824C 10398G 10400T 10873C 11719A 12405T 12705T 12811C 14766T 14783C 14978G 15043A 15301A 15326G 16129A 16189C 16223T 16297C	M7b1a1a3	73G 150T 199C 204C 263G 456T 489C 16129A 16189C 16223T 16297C	1	73 150 199 204 263 456 489 750 1438 2706 3866 4048 4071 4164 4769 5351 5460 6455 6680 7028 7684 7853 8701 8860 9540 9824 10398 10400 10873 11719 12405 12705 12811 14766 14783 14978 15043 15301 15326 16129 16189 16223 16297	M7b1a1a3	73 150 199 204 263 456 489 16129 16189 16223 16297	[0.000] M7b1a1a3 (1)	[0.003] M7b1a1a2 (2) [0.000] M7b1a1a	1
2219 >KP240870.1 Ho	73G 152C 248d 263G 514d 515d 750G 1438G 2706G 3106d 3107C 3970T 4086T 4769G 6293C 6392C 6962A 7028T 7976A 8860G 9053A 9548A 10310A 10609C 11719A 12406A 12882T 13759A 13928C 14766T 15326G 16129A 16162G 16172C 16304C 16497G	F1a1	73G 152C 248d 263G 514d 515d 16129A 16162G 16172C 16304C 16497G	1	73 152 249d 263 750 1438 2706 3970 4086 4769 6293 6392 6962 7028 7976 8860 9053 9548 10310 10609 11719 12406 12882 13759 13928C 14766 15326 16129 16162 16172 16304 16497	F1a1	73 152 249d 263 16129 16162 16172 16304 16497	[0.179] F1a1 (1)	[0.384] F1c1 (2) [0.144] F1a1d (3) [0.073] F1a1c (4) [0.038] F1a4a (5) [0.027] F1a1b (6) [0.024] F1a1a1 (7) [0.017] F1a1c3 (8) [0.016] F1a1'4 (9) [0.014] F1a1a (10) [0.005] F1a1d1 (11) [0.003] F1a3 (12) [0.000] F1a F1a1c2 F1a4 (15)	1
2220 >KP240867.1 Ho	73G 248d 263G 514d 515d 750G 1005C 1438G 1824C 2706G 3106d 3107C 3970T 4769G 4811G 6392C 7028T 7828G 8697A 8860G 10310A 10535C 10586A 11719A 12338C 13708A 13928C 14766T 15316G 15326G 16092A 16274A 16291T 16304C	F2b1	73G 248d 263G 514d 515d 16092A 16274A 16291T 16304C	1	73 249d 263 750 1005 1438 1824 2706 3970 4769 4811 6392 7028 7828 8697 8860 10310 10535 10586 11719 12338 13708 13928C 14766 15316 15326 16092A 16274 16291 16304	F2b1	73 249d 263 16092A 16274 16291 16304	[0.011] F2b1 (1)	[0.013] F2+16291 (2) [0.000] F2b F2g (4)	1
2221 >KP240866.1 Ho	73G 143N 248d 263G 514d 515d 750G 1005C 1438G 1824C 2706G 3106d 3107C 3970T 4769G 4811G 6392C 7028T 7828G 8860G 10310A 10535C 10586A 11719A 12338C 12810G 13191C 13708A 13928C 14766T 15326G 16092A 16136C 16289G 16291T 16304C	F2b1	73G 143N 248d 263G 514d 515d 16092A 16136C 16289G 16291T 16304C	1	73 143N 249d 263 750 1005 1438 1824 2706 3970 4769 4811 6392 7028 7828 8860 10310 10535 10586 11719 12338 12810 13191 13708 13928C 14766 15326 16092A 16136 16289 16291 16304	F2b1	73 143N 249d 263 16092A 16136 16289 16291 16304	[0.043] F2b1 (1)	[0.013] F2+16291 (2) [0.000] F2b F2g (4)	1

2222	>KP240863.1 Ho	73G 94A 248d 263G 514d 515d 750G 1438G 2706G 3106d 3107C 3970T 4086T 4769G 5147C 6392C 6962A 7028T 8860G 9053A 9548A 10310A 10609C 11719A 12406A 12715G 12882T 13759A 13928C 14766T 15326G 16129A 16162G 16172C 16304C 16519C	F1a1	73G 94A 248d 263G 514d 515d 16129A 16162G 16172C 16304C 16519C	1	73 94 249d 263 750 1438 2706 3970 4086 4769 5147C 6392 6962 7028 8860 9053 9548 10310 10609 11719 12406 12715 12882 13759 13928C 14766 15326 16129 16162 16172 16304	F1a1	73 94 249d 263 16129 16162 16172 16304	[0.070] F1a1 (1)	[0.000] F1a F1a1'4 F1a1a F1a1a1 F1a1b F1a1c F1a1c2 F1a1c3 F1a1d F1a1d1 F1a4 F1a3 (13)	1
2223	>KP240858.1 Ho	73G 93G 210G 263G 514d 515d 709A 750G 1438G 2706G 3106d 3107C 3537G 4769G 4917G 6029T 7028T 8271d 8272d 8273d 8274d 8275d 8276d 8277d 8278d 8279d 8584A 8860G 9189T 9950C 9962A 10398G 11149A 11151T 11719A 12234G 13422G 13928C 14149T 14766T 14861A 15235G 15326G 16140C 16187T 16189C 16256T 16266G 16519C	B5a2a1a	73G 93G 210G 263G 514d 515d 16140C 16187T 16189C 16256T 16266G 16519C	1	73 93 210 263 709 750 1438 2706 3537 4769 4917 6029 7028 8281-8289d 8584 8860 9189 9950 9962 10398 11149 11151 11719 12234 13422 13928C 14149 14766 14861 15235 15326 16140 16187 16189 16256 16266G	B5a2a1a	73 93 210 263 16140 16187 16189 16256 16266G	[0.000] B5a2a1a (1)	[0.000] B5a2a1 (2)	1
2224	>KP240857.1 Ho	73G 204C 248d 263G 750G 1005C 1438G 1824C 2706G 3106d 3107C 3342T 3970T 4554G 4769G 6392C 7028T 7828G 8860G 10310A 10535C 10586A 11719A 12338C 13708A 13928C 14766T 14790G 15326G 16126C 16203G 16291T 16304C 16519C	F2a	73G 204C 248d 263G 16126C 16203G 16291T 16304C 16519C	1	73 204 249d 263 750 1005 1438 1824 2706 3342 3970 4554 4769 6392 7028 7828 8860 10310 10535 10586 11719 12338 13708 13928C 14766 14790 15326 16126 16203 16291 16304	F2a	73 204 249d 263 16126 16203 16291 16304	[0.065] F2a (1)	[0.000] F2+16291 F2a+@16291 F2a1 F2b F2g (6)	1
2225	>KP240849.1 Ho	73G 150N 159C 199C 207A 263G 310.1C 489C 750G 1438G 2706G 3106d 3107C 4048A 4071T 4164G 4769G 5351G 5460A 6455T 6680C 7028T 7684C 7853A 8404C 8701G 8860G 9540C 9824C 10159T 10398G 10400T 10873C 11719A 11944C 12405T 12705T 12811C 14766T 14783C 15043A 15301A 15326G 15479C 16092C 16129A 16192T 16223T 16297C	M7b1a1c	73G 150N 159C 199C 207A 263G 310.1C 489C 16092C 16129A 16192T 16223T 16297C	1	73 150N 159 199 207 263 489 750 1438 2706 4048 4071 4164 4769 5351 5460 6455 6680 7028 7684 7853 8404 8701 8860 9540 9824 10159 10398 10400 10873 11719 11944 12405 12705 12811 14766 14783 15043 15301 15326 15479 16092 16129 16192 16223 16297	M7b1a1c	73 150N 159 199 207 263 489 16092 16129 16192 16223 16297	[0.008] M7b1a1c (1)	[0.023] M7b1a1+(16192) (2) [0.000] M7b1a1c1 M7b1a1d M7b1a1d1 M7b1a1e M7b1a1e1 M7b1a1f M7b1a1g M7b1a1h (10)	1
2226	>KP240848.1 Ho	73G 150T 199C 263G 310N 489C 750G 1438G 2706G 3106d 3107C 4048A 4071T 4164G 4769G 5351G 5460A 6228T 6455T 6680C 7028T 7684C 7853A 8701G 8860G 9540C 9732T 9824C 10398G 10400T 10873C 11407T 11719A 12405T 12705T 12811C 14766T 14783C 15043A 15301A 15326G 16129A 16223T 16297C 16527T	M7b1a1e2	73G 150T 199C 263G 310N 489C 16129A 16223T 16297C 16527T	1	73 150 199 263 310N 489 750 1438 2706 4048 4071 4164 4769 5351 5460 6228 6455 6680 7028 7684 7853 8701 8860 9540 9732 9824 10398 10400 10873 11407 11719 12405 12705 12811 14766 14783 15043 15301 15326 16129 16223 16297 16527	M7b1a1e2	73 150 199 263 310N 489 16129 16223 16297 16527	[0.000] M7b1a1e2 (1)	[0.000] M7b1a1 M7b1a1+(16192) M7b1a1d M7b1a1d1 M7b1a1e M7b1a1e1 M7b1a1e2 M7b1a1f M7b1a1g M7b1a1h (11)	1

2227	>KP240847.1 Ho	73G 248d 263G 750G 1005C 1438G 1824C 2706G 2746C 3106d 3107C 3970T 4769G 6392C 6566T 6710G 7028T 7828G 8860G 10310A 10535C 10586A 11719A 12338C 13708A 13928C 14766T 14831A 15326G 16129A 16203G 16291T 16304C	F2a	73G 248d 263G 16129A 16203G 16291T 16304C	1	73 249d 263 750 1005 1438 1824 2706 2746 3970 4769 6392 6566 6710 7028 7828 8860 10310 10535 10586 11719 12338 13708 13928C 14766 14831 15326 16129 16203 16291 16304	F2a	73 249d 263 16129 16203 16291 16304	[0.029] F2a (1)	[0.091] F2a+@16291 (2) [0.075] F1a'c'f (3) [0.016] F1c (4) [0.000] F1f F2+16291 F2a1 F2b F2g (9)	1
2228	>KP240846.1 Ho	73G 150T 199C 204C 263G 310N 489C 750G 1438G 2706G 3106d 3107C 3483A 4048A 4071T 4164G 4769G 5351G 5460A 6455T 6680C 7028T 7684C 7853A 8701G 8817G 8860G 9540C 9824C 10398G 10400T 10873C 11719A 12405T 12705T 12811C 14766T 14783C 15043A 15301A 15326G 16223T 16297C	M7b1a1b	73G 150T 199C 204C 263G 310N 489C 16223T 16297C	1	73 150 199 204 263 310N 489 750 1438 2706 3483 4048 4071 4164 4769 5351 5460 6455 6680 7028 7684 7853 8701 8817 8860 9540 9824 10398 10400 10873 11719 12405 12705 12811 14766 14783 15043 15301 15326 16223 16297	M7b1a1b	73 150 199 204 263 310N 489 16223 16297	[0.000] M7b1a1b (1)	[0.042] M7b1a1+(16192) (2) [0.018] M7b1a1e1 (3) [0.015] M7b1a1 (4) [0.014] M7b1a1a3 (5) [0.010] M7b1a1f (6) [0.004] M7b1a1h (7) [0.002] M7b1a1g (8) [0.000] M7b1a1d M7b1a1d1 M7b1a1e (11)	1
2229	>KP240844.1 Ho	73G 152C 248d 263G 310N 750G 1005C 1438G 1824C 2706G 3106d 3107C 3970T 4769G 5147A 6392C 7028T 7828G 8292A 8473C 8860G 10310A 10535C 10586A 11719A 12338C 13708A 13928C 14766T 14831A 15326G 16203G 16291T 16304C 16352C	F2a	73G 152C 248d 263G 310N 16203G 16291T 16304C 16352C	1	73 152 249d 263 310N 750 1005 1438 1824 2706 3970 4769 5147 6392 7028 7828 8292 8473 8860 10310 10535 10586 11719 12338 13708 13928C 14766 14831 15326 16203 16291 16304 16352	F2a	73 152 249d 263 310N 16203 16291 16304 16352	[0.051] F2a (1)	[0.036] F2a+@16291 (2) [0.000] F2+16291 F2a1 F2b F2g (6)	1
2230	>KP240843.1 Ho	73G 248d 263G 514d 515d 750G 1438G 2706G 3106d 3107C 3970T 4086T 4769G 5183G 6392C 6962A 7028T 8251A 8860G 9053A 9548A 10310A 10609C 11719A 12406A 12882T 13759A 13928C 14766T 15326G 16129A 16162G 16172C 16304C 16497G 16519C	F1a1	73G 248d 263G 514d 515d 16129A 16162G 16172C 16304C 16497G 16519C	1	73 249d 263 750 1438 2706 3970 4086 4769 5183 6392 6962 7028 8251 8860 9053 9548 10310 10609 11719 12406 12882 13759 13928C 14766 15326 16129 16162 16172 16304 16497	F1a1	73 249d 263 16129 16162 16172 16304 16497	[0.091] F1a1 (1)	[0.017] F1a1c3 (2) [0.005] F1a1'4 F1a1d1 (4) [0.000] F1a F1a1a F1a1a1 F1a1b F1a1c F1a1c2 F1a1d F1a4 F1a3 (13)	1
2231	>KP240841.1 Ho	73G 150N 199C 204C 263G 310N 456T 489C 750G 1438G 2706G 3106d 3107C 4048A 4071T 4164G 4769G 5351G 5460A 6455T 6680C 7028T 7684C 7853A 8701G 8860G 9540C 9824C 10398G 10400T 10873C 11719A 12405T 12705T 12811C 14766T 14783C 14978G 15043A 15301A 15326G 16129A 16189C 16223T 16297C	M7b1a1a3	73G 150N 199C 204C 263G 310N 456T 489C 16129A 16189C 16223T 16297C	1	73 150N 199 204 263 310N 456 489 750 1438 2706 4048 4071 4164 4769 5351 5460 6455 6680 7028 7684 7853 8701 8860 9540 9824 10398 10400 10873 11719 12405 12705 12811 14766 14783 14978 15043 15301 15326 16129 16189 16223 16297	M7b1a1a3	73 150N 199 204 263 310N 456 489 16129 16189 16223 16297	[0.000] M7b1a1a3 (1)	[0.003] M7b1a1a2 (2) [0.000] M7b1a1a	1
2232	>KP240835.1 Ho	73G 94A 248d 263G 514d 515d 750G 1438G 2706G 3106d 3107C 3970T 4086T 4769G 5147C 6392C 6962A 7028T 8860G 9053A 9548A 10310A 10609C 11719A 12406A 12715G 12882T 13759A 13928C 14766T 15326G 16129A 16162G 16172C 16304C 16519C	F1a1	73G 94A 248d 263G 514d 515d 16129A 16162G 16172C 16304C 16519C	1	73 94 249d 263 750 1438 2706 3970 4086 4769 5147C 6392 6962 7028 8860 9053 9548 10310 10609 11719 12406 12715 12882 13759 13928C 14766 15326 16129 16162 16172 16304	F1a1	73 94 249d 263 16129 16162 16172 16304	[0.070] F1a1 (1)	[0.000] F1a F1a1'4 F1a1a F1a1a1 F1a1b F1a1c F1a1c2 F1a1c3 F1a1d F1a1d1 F1a4 F1a3 (13)	1

2233	>KP240834.1 Ho	73G 152N 195C 248d 263G 275A 750G 1005C 1438G 1824C 2706G 3106d 3107C 3753C 3970T 4769G 6392C 7028T 7828G 8860G 10084C 10310A 10535C 10586A 11323T 11410C 11719A 12338C 13708A 13928C 14230G 14766T 15326G 16221T 16304C 16519C	F2i	73G 152N 195C 248d 263G 275A 16221T 16304C 16519C	1	73 152N 195 249d 263 275 750 1005 1438 1824 2706 3753 3970 4769 6392 7028 7828 8860 10084 10310 10535 10586 11323 11410 11719 12338 13708 13928C 14230 14766 15326 16221 16304	F2i	73 152N 195 249d 263 275 16221 16304	[0.000] F2i (1)	[0.000] F2+195 F2h (3)	1
2234	>KP240832.1 Ho	73G 146C 215G 248d 263G 317A 750G 1391C 1438G 2706G 3106d 3107C 3290C 3316A 3970T 4769G 4820A 5263T 6392C 7028T 7561C 8860G 10310A 10915C 11719A 12153T 12630A 13602C 13928C 14766T 15326G 15670C 16126C 16189C 16207G 16304C 16362C 16399G	F4a1b	73G 146C 215G 248d 263G 317A 16126C 16189C 16207G 16304C 16362C 16399G	1	73 146 215 249d 263 317A 750 1391 1438 2706 3290 3316 3970 4769 4820 5263 6392 7028 7561 8860 10310 10915 11719 12153 12630 13602 13928C 14766 15326 15670 16126 16189 16207 16304 16362 16399	F4a1b	73 146 215 249d 263 317A 16126 16189 16207 16304 16362 16399	[0.167] F4a1b (1)	[0.000] F4a1 (2)	1
2235	>KP240831.1 Ho	73G 150T 195C 248d 263G 514d 515d 750G 1438G 2706G 3106d 3107C 3970T 4769G 5492C 5587C 6392C 6962A 7028T 8860G 10310A 10609C 11719A 12406A 12882T 13928C 14766T 15024A 15326G 15924G 16183N 16189C 16274A 16284G 16300G 16304C 16311C 16519C	F1e3	73G 150T 195C 248d 263G 514d 515d 16183N 16189C 16274A 16284G 16300G 16304C 16311C 16519C	1	73 150 195 249d 263 750 1438 2706 3970 4769 5492 5587 6392 6962 7028 8860 10310 10609 11719 12406 12882 13928C 14766 15024 15326 15924 16183N 16189 16274 16284 16300 16304 16311	F1e3	73 150 195 249d 263 16183N 16189 16274 16284 16300 16304 16311	[1.358] F1e3 (1)	[0.167] F1a3a2 (2) [0.073] F2h (3) [0.031] F1g (4) [0.024] F1+16189 (5) [0.016] F2c1 (6) [0.015] F1e1 F1g1 (8) [0.006] F1e (9) [0.000] F2+195 B4c1c+16311 (11)	1
2236	>KP240825.1 Ho	73G 248d 263G 750G 1005C 1438G 1824C 2706G 3106d 3107C 3828G 3970T 4769G 6392C 7028T 7828G 8860G 10310A 10535C 10586A 11719A 12338C 12372A 13708A 13928C 14766T 15326G 15852C 16203G 16291T 16304C 16519C	F2a	73G 248d 263G 16203G 16291T 16304C 16519C	1	73 249d 263 750 1005 1438 1824 2706 3828 3970 4769 6392 7028 7828 8860 10310 10535 10586 11719 12338 12372 13708 13928C 14766 15326 15852 16203 16291 16304	F2a	73 249d 263 16203 16291 16304	[0.000] F2a (1)	[0.000] F2+16291 F2a+@16291 F2a1 F2b F2g (6)	1
2237	>KP240824.1 Ho	73G 150T 199C 204C 263G 310d 456T 489C 750G 1438G 2706G 3106d 3107C 4048A 4071T 4164G 4769G 5351G 5460A 6455T 6680C 7028T 7684C 7853A 8701G 8860G 9540C 9824C 10398G 10400T 10873C 11719A 12405T 12705T 12811C 14766T 14783C 14978G 15043A 15301A 15326G 16129A 16189C 16223T 16297C	M7b1a1a3	73G 150T 199C 204C 263G 310d 456T 489C 16129A 16189C 16223T 16297C	1	73 150 199 204 263 310 315d 456 489 750 1438 2706 4048 4071 4164 4769 5351 5460 6455 6680 7028 7684 7853 8701 8860 9540 9824 10398 10400 10873 11719 12405 12705 12811 14766 14783 14978 15043 15301 15326 16129 16189 16223 16297	M7b1a1a3	73 150 199 204 263 310 315d 456 489 16129 16189 16223 16297	[0.011] M7b1a1a3 (1)	[0.004] M7b1a1a (2) [0.003] M7b1a1a2	1

2238	>KP240823.1 Ho	73G 150T 199C 263G 489C 750G 1438G 2706G 3106d 3107C 4048A 4071T 4164G 4769G 5351G 5460A 6455T 6680C 7028T 7684C 7853A 8701G 8860G 9540C 9824C 10398G 10400T 10873C 11719A 12405T 12705T 12811C 14256C 14766T 14783C 15043A 15301A 15326G 15779C 16129A 16145A 16223T 16297C 16327T	M7b1a1	73G 150T 199C 263G 489C 16129A 16145A 16223T 16297C 16327T	1	73 150 199 263 489 750 1438 2706 4048 4071 4164 4769 5351 5460 6455 6680 7028 7684 7853 8701 8860 9540 9824 10398 10400 10873 11719 12405 12705 12811 14256 14766 14783 15043 15301 15326 15779 16129 16145 16223 16297 16327	M7b1a1	73 150 199 263 489 16129 16145 16223 16297 16327	[0.023] M7b1a1 (1) [0.006] M7b1a1+(16192) (2) [0.000] M7b1a1d M7b1a1d1 M7b1a1e M7b1a1e1 M7b1a1f M7b1a1g M7b1a1h (9)	[0.007] M7b1a1e2 (10) [0.006] M7b1a1+(16192) (11) [0.000] M7b1a M7b1a1a M7b1a1a2 M7b1a1d M7b1a1d1 M7b1a1e M7b1a1e1 M7b1a1f M7b1a1g M7b1a1h M7b1a1i (22)	1
2239	>KP240822.1 Ho	73G 195C 248d 263G 514d 515d 750G 1438G 1709A 2706G 3106d 3107C 3970T 4086T 4769G 6392C 6962A 7028T 8697A 8860G 9053A 9548A 10310A 10609C 11719A 12406A 12882T 13759A 13928C 14258A 14766T 15326G 16129A 16162G 16172C 16304C 16311C 16519C	F1a1	73G 195C 248d 263G 514d 515d 16129A 16162G 16172C 16304C 16311C 16519C	1	73 195 249d 263 750 1438 1709 2706 3970 4086 4769 6392 6962 7028 8697 8860 9053 9548 10310 10609 11719 12406 12882 13759 13928C 14258 14766 15326 16129 16162 16172 16304 16311	F1a1	73 195 249d 263 16129 16162 16172 16304 16311	[0.162] F1a1 (1) [0.037] F1a3a (2) [0.000] F1a3+16311 F1a3a3 F1a3a3a (5)	[0.175] F1a1a (6) [0.075] F1a1d1 (7) [0.061] F1a1c (8) [0.052] F1a1a1 (9) [0.042] F1a (10) [0.013] F1a1b (11) [0.005] F1a1d (12) [0.003] F1a3 (13) [0.000] F1a1'4 F1a1c1 F1a1c2 F1a1c3 F1a4 (18)	1
2240	>KP240809.1 Ho	73G 199C 263G 489C 514d 515d 750G 1438G 2706G 3027C 3106d 3107C 3736A 3882A 4071T 4769G 4850T 5442C 5471A 5978G 6455T 7028T 8701G 8860G 9540C 9824C 10398G 10400T 10586A 10873C 11665T 11719A 12091C 12372A 12705T 13759A 14766T 14783C 15043A 15301A 15326G 16051G 16223T 16295T 16304C 16519C	M7c1a1b	73G 199C 263G 489C 514d 515d 16051G 16223T 16295T 16304C 16519C	1	73 199 263 489 750 1438 2706 3027 3736 3882 4071 4769 4850 5442 5471 5978 6455 7028 8701 8860 9540 9824 10398 10400 10586 10873 11665 11719 12091 12372 12705 13759 14766 14783 15043 15301 15326 16051 16223 16295 16304	M7c1a1b	73 199 263 489 16051 16223 16295 16304	[0.025] M7c1a1b (1)	[0.006] M35b+16304 (2) [0.000] M7c1a1b1 M35b2 M35b3 (5)	1
2241	>KP240803.1 Ho	73G 248d 263G 310N 750G 1005C 1438G 1824C 2706G 3106d 3107C 3970T 4769G 6392C 7028T 7828G 8860G 10310A 10535C 10586A 11719A 12338C 12372A 13563G 13708A 13928C 14766T 15326G 16203G 16291T 16304C 16519C	F2a	73G 248d 263G 310N 16203G 16291T 16304C 16519C	1	73 249d 263 310N 750 1005 1438 1824 2706 3970 4769 6392 7028 7828 8860 10310 10535 10586 11719 12338 12372 13563 13708 13928C 14766 15326 16203 16291 16304	F2a	73 249d 263 310N 16203 16291 16304	[0.000] F2a (1)	[0.000] F2+16291 F2a+@16291 F2a1 F2b F2g (6)	1
2242	>KP240799.1 Ho	73G 150N 199C 204C 263G 310N 456T 489C 750G 1438G 2706G 3106d 3107C 3866C 4048A 4071T 4164G 4769G 5351G 5460A 6455T 6680C 7028T 7684C 7853A 8701G 8860G 9540C 9824C 10398G 10400T 10873C 11719A 12405T 12705T 12811C 14766T 14783C 14978G 15043A 15301A 15326G 16129A 16189C 16223T 16297C	M7b1a1a3	73G 150N 199C 204C 263G 310N 456T 489C 16129A 16189C 16223T 16297C	1	73 150N 199 204 263 310N 456 489 750 1438 2706 3866 4048 4071 4164 4769 5351 5460 6455 6680 7028 7684 7853 8701 8860 9540 9824 10398 10400 10873 11719 12405 12705 12811 14766 14783 14978 15043 15301 15326 16129 16189 16223 16297	M7b1a1a3	73 150N 199 204 263 310N 456 489 16129 16189 16223 16297	[0.000] M7b1a1a3 (1)	[0.003] M7b1a1a2 (2) [0.000] M7b1a1a	1

2243	>KP240797.1 Ho	73G 152N 248d 263G 514d 515d 750G 1438G 2706G 3106d 3107C 3970T 4086T 4769G 6293C 6392C 6962A 7028T 7976A 8860G 9053A 9548A 10310A 10609C 11719A 12406A 12882T 13759A 13928C 14766T 15326G 16129A 16162G 16172C 16304C 16497G	F1a1	73G 152N 248d 263G 514d 515d 16129A 16162G 16172C 16304C 16497G	1	73 152N 249d 263 750 1438 2706 3970 4086 4769 6293 6392 6962 7028 7976 8860 9053 9548 10310 10609 11719 12406 12882 13759 13928C 14766 15326 16129 16162 16172 16304 16497	F1a1	73 152N 249d 263 16129 16162 16172 16304 16497	[0.091] F1a1 (1)	[0.017] F1a1c3 (2) [0.005] F1a1'4 F1a1d1 (4) [0.000] F1a F1a1a F1a1a1 F1a1b F1a1c F1a1c2 F1a1d F1a4 F1a3 (13)	1
2244	>KP240796.1 Ho	73G 146A 199C 263G 489C 514d 515d 750G 1438G 2706G 3106d 3107C 3606G 4071T 4769G 4850T 5442C 6455T 7028T 7150C 8701G 8860G 9540C 9824C 10398G 10400T 10873C 11665T 11719A 12091C 12705T 13015C 13734C 14766T 14783C 15043A 15236G 15301A 15326G 16092C 16147T 16153A 16171G 16519C	M7c1c2a	73G 146A 199C 263G 489C 514d 515d 16092C 16147T 16153A 16171G 16519C	1	73 146A 199 263 489 750 1438 2706 3606 4071 4769 4850 5442 6455 7028 7150 8701 8860 9540 9824 10398 10400 10873 11665 11719 12091 12705 13015 13734 14766 14783 15043 15236 15301 15326 16092 16147 16153 16171	M7c1c2a	73 146A 199 263 489 16092 16147 16153 16171	[0.027] M7c1c2a (1)	[0.027] M7c1c2a (2) [0.000] M7c1c2	1
2245	>KP240788.1 Ho	73G 152C 195C 248d 263G 275A 310N 750G 1005C 1438G 1824C 2706G 3106d 3107C 3753C 3970T 4769G 6345C 6392C 7028T 7076G 7828G 8860G 10084C 10310A 10535C 10586A 11323T 11410C 11719A 12338C 13708A 13928C 14766T 15326G 16221T 16304C 16519C	F2i	73G 152C 195C 248d 263G 275A 310N 16221T 16304C 16519C	1	73 152 195 249d 263 275 310N 750 1005 1438 1824 2706 3753 3970 4769 6345 6392 7028 7076 7828 8860 10084 10310 10535 10586 11323 11410 11719 12338 13708 13928C 14766 15326 16221 16304	F2i	73 152 195 249d 263 275 310N 16221 16304	[0.000] F2i (1)	[0.000] F2+195 F2h (3)	1
2246	>KP240786.1 Ho	66A 73G 150T 199C 204C 263G 310N 489C 750G 1438G 2706G 3106d 3107C 3398C 3483A 4048A 4071T 4164G 4769G 5351G 5460A 6455T 6491T 6680C 7028T 7684C 7853A 8701G 8860G 9540C 9824C 10398G 10400T 10873C 11266T 11719A 12405T 12705T 12811C 14766T 14783C 15043A 15301A 15326G 16176T 16223T 16297C	M7b1a1b	66A 73G 150T 199C 204C 263G 310N 489C 16176T 16223T 16297C	1	66 73 150 199 204 263 310N 489 750 1438 2706 3398 3483 4048 4071 4164 4769 5351 5460 6455 6491 6680 7028 7684 7853 8701 8860 9540 9824 10398 10400 10873 11266 11719 12405 12705 12811 14766 14783 15043 15301 15326 16176 16223 16297	M7b1a1b	66 73 150 199 204 263 310N 489 16176 16223 16297	[0.036] M7b1a1b (1)	[0.048] M7b1a1+(16192) (2) [0.018] M7b1a1e1 (3) [0.015] M7b1a1 (4) [0.014] M7b1a1a3 (5) [0.010] M7b1a1f (6) [0.004] M7b1a1h (7) [0.002] M7b1a1g (8) [0.000] M7b1a1d M7b1a1d1 M7b1a1e (11)	1
2247	>KP172331.1 Ho	73G 152C 310.1CCTC 499A 750G 827G 1438G 1949A 2706G 2755G 3547G 4769G 4820A 4977C 6473T 6485G 7028T 8271d 8272d 8273d 8274d 8275d 8276d 8277d 8278d 8279d 8860G 9950C 11177T 11719A 13590A 14766T 15326G 15535T 16092C 16126C 16182C 16183C 16189C 16214T 16217C 16355A 16519C	B2o	73G 152C 310.1CCTC 499A 16092C 16126C 16182C 16183C 16189C 16214T 16217C 16355A 16519C	1	73 152 313 315.4C 499 750 827 1438 1949 2706 2755 3547 4769 4820 4977 6473 6485 7028 8281-8289d 8860 9950 11177 11719 13590 14766 15326 15535 16092 16126 16189 16214 16217 16355A	B2o	73 152 313 315.4C 499 16092 16126 16189 16214 16217 16355A	[0.221] B2o (1) [0.053] B2b+152 (2) [0.000] B2o1a (3)	[0.134] B2q (4) [0.107] B2 (5) [0.087] B2c (6) [0.043] B2c1c (7) [0.032] B2r (8) [0.016] B2b B2e (10) [0.010] B2c1 (11) [0.009] B2n (12) [0.000] B4b B2b3 B2c1a B2c1b B2f B2i B2o1 B2p (20)	1

2248	>KU178929.1 H	73G 151T 152C 263G 310C 316S 385G 515R 750G 980C 1438G 1811G 2706G 3106M 3107C 3741T 3834A 4769G 5360T 7028T 8137T 8684T 8860G 10142T 11467G 11719A 12308G 12372A 12618A 13500C 14137T 14569A 14766T 15326G 16207G 16309G 16318T 16519C	U7a3b	73G 151T 152C 263G 310C 316S 385G 515R 16207G 16309G 16318T 16519C	1	73 151 152 263 310 316S 385 515R 750 980 1438 1811 2706 3106M 3741 3834 4769 5360 7028 8137 8684 8860 10142 11467 11719 12308 12372 12618 13500 14137 14569 14766 15326 16207 16309 16318T	U7a3b	73 151 152 263 310 316S 385 515R 16207 16309 16318T	[0.080] U7a3b (1)	[0.080] U7a3b (2) [0.038] U7a (3) [0.023] U7a3a (4) [0.000] U7a2 U7a3 (6)	1
2249	>KU178926.1 H	73G 151T 152C 263G 310C 515R 750G 980C 1438G 1811G 2706G 3106M 3107C 3741T 3834A 4529G 4769G 4772C 5360T 7028T 8137T 8684T 8860G 10101C 10142T 11467G 11719A 12308G 12372A 12618A 13500C 14218C 14569A 14766T 15326G 16207G 16309G 16318T 16519C	U7a3b	73G 151T 152C 263G 310C 515R 16207G 16309G 16318T 16519C	1	73 151 152 263 310 515R 750 980 1438 1811 2706 3106M 3741 3834 4529 4769 4772 5360 7028 8137 8684 8860 10101 10142 11467 11719 12308 12372 12618 13500 14218 14569 14766 15326 16207 16309 16318T	U7a3b	73 151 152 263 310 515R 16207 16309 16318T	[0.046] U7a3b (1)	[0.046] U7a3b (2) [0.026] U7a (3) [0.014] U7a3a (4) [0.000] U7a2 U7a3 (6)	1
2250	>KU178925.1 H	73G 146C 152C 234G 263G 310C 750G 1438G 1811G 1888A 3106M 3107C 4769G 5186T 7028T 8860G 9094T 9614G 10034C 11467G 11719A 12106T 12308G 12372A 12793C 13038T 13194A 13644A 13656C 14766T 15049T 15326G 15449C 15930A 16051G 16209C 16239T 16352C 16353T	U2b2	73G 146C 152C 234G 263G 310C 16051G 16209C 16239T 16352C 16353T	1	73 146 152 234 263 310 750 1438 1811 1888 3106M 4769 5186T 7028 8860 9094 9614 10034 11467 11719 12106 12308 12372 12793 13038 13194 13644A 13656 14766 15049 15326 15449 15930 16051 16209 16239 16352 16353	U2b2	73 146 152 234 263 310 16051 16209 16239 16352 16353	[0.012] U2b2 (1)	[0.978] U2b (2) [0.085] U2b1 (3) [0.034] U2+152 (4) [0.028] U2b1a (5) [0.011] R30a1b	1
2251	>KU178924.1 H	73G 146C 234G 263G 310C 338T 750G 1438G 1811G 1888A 3106M 3107C 4053G 4769G 5186T 7028T 8860G 9094T 9196A 9614G 11147C 11467G 11719A 11914A 12106T 12308G 12372A 13194A 14766T 14927G 15049T 15326G 16051G 16124C 16239T	U2b	73G 146C 234G 263G 310C 338T 16051G 16124C 16239T	1	73 146 234 263 310 338 750 1438 1811 1888 3106M 4053 4769 5186T 7028 8860 9094 9196 9614 11147 11467 11719 11914 12106 12308 12372 13194 14766 14927 15049 15326 16051 16124 16239	U2b	73 146 234 263 310 338 16051 16124 16239	[0.510] U2b (1) [0.028] U2b1a (2) [0.014] U2b1 (3)	[0.114] U2 (4) [0.028] U2b1a (5) [0.014] U2b1	1
2252	>KU178920.1 H	73G 146C 152C 263G 310Y 316S 750G 1438G 1811G 2706G 3106M 3107C 3732C 4769G 7028T 8572A 8860G 11467G 11719A 12308G 12372A 12585T 14766T 15326G 15629C 16051G 16154C 16206C 16230G 16311C 16519C	U2a1a	73G 146C 152C 263G 310Y 316S 16051G 16154C 16206C 16230G 16311C 16519C	1	73 146 152 263 310Y 316S 750 1438 1811 2706 3106M 3732 4769 7028 8572 8860 11467 11719 12308 12372 12585 14766 15326 15629 16051 16154 16206C 16230 16311	U2a1a	73 146 152 263 310Y 316S 16051 16154 16206C 16230 16311	[0.197] U2a1a (1)	[0.197] U2a1a (2) [0.105] U2a1 (3) [0.000] U2a1b	1
2253	>KU178918.1 H	73G 195C 263G 310C 750G 1438G 1811G 2706G 3106M 3107C 4769G 5262A 6887T 7028T 8572A 8860G 11467G 11719A 12308G 12372A 14766T 15326G 15629C 16051G 16154C 16189Y 16206C 16230G 16311C 16519C	U2a1a	73G 195C 263G 310C 16051G 16154C 16189Y 16206C 16230G 16311C 16519C	1	73 195 263 310 750 1438 1811 2706 3106M 4769 5262 6887 7028 8572 8860 11467 11719 12308 12372 14766 15326 15629 16051 16154 16189Y 16206C 16230 16311	U2a1a	73 195 263 310 16051 16154 16189Y 16206C 16230 16311	[0.056] U2a1a (1)	[0.056] U2a1a (2) [0.015] U2a1 (3) [0.000] U2a1b	1

2254	>KY295947.1 Hc	73G 93G 146C 150T 152C 182T 195C 198T 263G 309.1CT 310C 325T 514d 515d 680C 709A 750G 769A 1018A 1438G 1442A 2332T 2416C 2706G 2857C 3106d 3107C 3200A 3594T 4104G 4769G 7028T 7256T 7521A 7624A 8206A 8701G 8860G 9221G 9540C 10115C 10398G 10873C 11719A 11944C 12236A 12705T 13590A 13650T 13928C 13958C 14101T 14766T 15110A 15217A 15301A 15326G 15849T 16092C 16223T 16278T 16318G 16390A	L2c1	73G 93G 146C 150T 152C 182T 195C 198T 263G 309.1CT 310C 325T 514d 515d 16092C 16223T 16278T 16318G 16390A	1	73 93 146 150 152 182 195 198 263 325 680 709 750 769 1018 1438 1442 2332 2416 2706 2857 3200A 3594 4104 4769 7028 7256 7521 7624A 8206 8701 8860 9221 9540 10115 10398 10873 11719 11944 12236 12705 13590 13650 13928C 13958C 14101 14766 15110 15217 15301 15326 15849 16092 16223 16278 16318 16390	L2c1	73 93 146 150 152 182 195 198 263 325 16092 16223 16278 16318 16390	[0.061] L2c1 (1)	[0.000] L2c L2c1a L2c4 L2c5 (5)	1
2255	>KP240926.1 Ho	73G 263G 750G 1438G 1541C 2706G 3106d 3107C 3666A 3970T 4769G 5093C 6647G 7028T 8724G 8860G 11299C 11422T 11719A 13191C 13722G 13928C 14766T 15314A 15326G 15883A 16304C 16362C 16519C	R9b2	73G 263G 16304C 16362C 16519C	1	73 263 750 1438 1541 2706 3666 3970 4769 5093 6647 7028 8724 8860 11299 11422 11719 13191 13722 13928C 14766 15314 15326 15883 16304 16362	R9b2	73 263 16304 16362	[0.000] R9b2 (1)	[0.010] R9c (2) [0.000] R6 R9 R9b R31 (6)	1
2256	>KP240917.1 Ho	73G 146C 152C 195C 263G 489C 514d 515d 750G 951A 1438G 2706G 3106d 3107C 4769G 5773A 7028T 7040C 8262T 8701G 8860G 9540C 10398G 10400T 10873C 11719A 12705T 13359A 14766T 14783C 15043A 15301A 15326G 15473G 15601C 16223T 16311C 16519C	M24a	73G 146C 152C 195C 263G 489C 514d 515d 16223T 16311C 16519C	1	73 146 152 195 263 489 750 951 1438 2706 4769 5773 7028 7040 8262 8701 8860 9540 10398 10400 10873 11719 12705 13359 14766 14783 15043 15301 15326 15473 15601 16223 16311	M24a	73 146 152 195 263 489 16223 16311	[0.000] M24a (1)	[0.022] M57+152 (2) [0.016] M24	1
2257	>KP240911.1 Ho	73G 146C 152C 195C 263G 750G 769A 1018A 1438G 2416C 2706G 2789T 3106d 3107C 3594T 4104G 4769G 5090C 7028T 7175C 7256T 7274T 7521A 7663T 7771G 8206A 8701G 8860G 9221G 9540C 10115C 10143A 10398G 10873C 11719A 11914A 11944C 12693G 12705T 13590A 13650T 13803G 14566G 14766T 15301A 15326G 15735T 15784C 16182N 16183C 16189C 16223T 16278T 16290T 16294T 16309G 16390A	L2a1b1a	73G 146C 152C 195C 263G 16182N 16183C 16189C 16223T 16278T 16290T 16294T 16309G 16390A	1	73 146 152 195 263 750 769 1018 1438 2416 2706 2789 3594 4104 4769 5090 7028 7175 7256 7274 7521 7663 7771 8206 8701 8860 9221 9540 10115 10143 10398 10873 11719 11914 11944 12693 12705 13590 13650 13803 14566 14766 15301 15326 15735 15784 16182N 16189 16223 16278 16290 16294 16309 16390	L2a1b1a	73 146 152 195 263 16182N 16189 16223 16278 16290 16294 16309 16390	[0.000] L2a1b1a (1)	[0.005] L2a1f (2) [0.000] L2a1+16189_(16192) L2a1b L2a1b1 L2a1b1a L2a1b2 L2a1f1 L2a1f1a L2a1f2 (10)	1

2258	>KP240909.1 Ho	73G 152C 263G 709A 750G 769A 1018A 1438G 2416C 2706G 2789T 3106d 3107C 3594T 4104G 4769G 6752G 7028T 7175C 7256T 7274T 7521A 7664A 7771G 8206A 8701G 8721G 8860G 9221G 9540C 9932A 10115C 10398G 10790C 10873C 11719A 11767T 11914A 11944C 12507G 12705T 13068G 13590A 13650T 13803G 13827G 14118G 14566G 14766T 15301A 15326G 15939T 16172C 16183C 16189C 16223T 16229C 16278T 16291T 16294T 16311C 16390A 16519C	L2a2b1a	73G 152C 263G 16172C 16183C 16189C 16223T 16229C 16278T 16291T 16294T 16311C 16390A 16519C	1	73 152 263 709 750 769 1018 1438 2416 2706 2789 3594 4104 4769 6752 7028 7175 7256 7274 7521 7664 7771 8206 8701 8721 8860 9221 9540 9932 10115 10398 10790 10873 11719 11767 11914 11944 12507 12705 13068 13590 13650 13803 13827 14118 14566 14766 15301 15326 15939 16172 16189 16223 16229 16278 16291 16294 16311 16390	L2a2b1a	73 152 263 16172 16189 16223 16229 16278 16291 16294 16311 16390	[0.000] L2a2b1a (1)	[0.222] L2a2b (2) [0.000] L2a2b1	1
2259	>KP240901.1 Ho	73G 263G 489C 514d 515d 750G 1438G 2706G 3106d 3107C 3736A 3882A 4071T 4769G 4850T 5442C 7028T 8701G 8860G 9540C 9824C 10398G 10400T 10586A 10873C 11084G 11665T 11719A 12091C 12372A 12705T 13759A 14766T 14783C 15043A 15301A 15326G 16223T 16295T 16304C 16311C 16519C	M7c1a1b	73G 263G 489C 514d 515d 16223T 16295T 16304C 16311C 16519C	1	73 263 489 750 1438 2706 3736 3882 4071 4769 4850 5442 7028 8701 8860 9540 9824 10398 10400 10586 10873 11084 11665 11719 12091 12372 12705 13759 14766 14783 15043 15301 15326 16223 16295 16304 16311	M7c1a1b	73 263 489 16223 16295 16304 16311	[0.082] M7c1a1b (1) [0.009] M54 (2) [0.000] M4"67+16311 M43+16311 M43a M57 (6)	[0.082] M7c1a1b (7) [0.076] M (8) [0.048] M10 (9) [0.038] M21b (10) [0.016] M7b (11) [0.010] M58 (12) [0.009] M1'20'51 (13) [0.006] G1b M35b+16304 (15) [0.005] M43a1 M31a1b (17) [0.003] M21b1 M23'75 M29'Q M32'56 M33a M35 M42'74 (24) [0.000] L3'4 L3h1 L3h1a L3h1a2 M4"67 M65 M37 M43 M43b M7 M7b'c M7c1a1b1 M10a M10a1 M12'G M19'53 M21 M21b1a M22 M22b M24'41 M27 M31 M31a M31a1 M31a1a M56 M33 M33a1 M34'57 M57+152 M35b2 M35b3 M39'70 M42 M74 M74b M74b2 M52 M52b M55'77 M60 M62 M69 M80'D D4j+16311 D4j11 (71)	1
2260	>KP240899.1 Ho	73G 248d 263G 466C 514d 515d 516d 517d 750G 1438G 2706G 3106d 3107C 3970T 4086T 4769G 6392C 6962A 7028T 7419A 8251A 8860G 9053A 9548A 10310A 10609C 11719A 12406A 12882T 13759A 13928C 14766T 15301A 15326G 15445C 16129A 16172C 16304C 16519C	F1a4b	73G 248d 263G 466C 514d 515d 516d 517d 16129A 16172C 16304C 16519C	1	73 249d 263 466 750 1438 2706 3970 4086 4769 6392 6962 7028 7419 8251 8860 9053 9548 10310 10609 11719 12406 12882 13759 13928C 14766 15301 15326 15445 16129 16172 16304	F1a4b	73 249d 263 466 16129 16172 16304	[0.000] F1a4b (1)	[0.000] F1a F1a1'4 F1a4 F1a3 (5)	1
2261	>KP240897.1 Ho	73G 195C 198T 248d 263G 514d 515d 750G 1438G 1709A 2706G 3106d 3107C 3970T 4086T 4769G 6392C 6962A 7028T 8697A 8860G 9053A 9548A 10310A 10609C 11719A 12406A 12882T 13759A 13928C 14258A 14766T 15326G 16129A 16162G 16172C 16304C 16311C 16519C	F1a1	73G 195C 198T 248d 263G 514d 515d 16129A 16162G 16172C 16304C 16311C 16519C	1	73 195 198 249d 263 750 1438 1709 2706 3970 4086 4769 6392 6962 7028 8697 8860 9053 9548 10310 10609 11719 12406 12882 13759 13928C 14258 14766 15326 16129 16162 16172 16304 16311	F1a1	73 195 198 249d 263 16129 16162 16172 16304 16311	[0.165] F1a1 (1) [0.043] F1a3a (2) [0.006] F1a3a3 F1a3a3a (4) [0.005] F1a3+16311 (5)	[0.177] F1a1a (6) [0.075] F1a1d1 (7) [0.061] F1a1c (8) [0.054] F1a1a1 (9) [0.042] F1a (10) [0.013] F1a1b (11) [0.005] F1a1d (12) [0.003] F1a3 (13) [0.000] F1a1'4 F1a1c1 F1a1c2 F1a1c3 F1a4 (18)	1

2262	>KP240893.1 Ho	73G 146C 152C 153G 248d 263G 310N 514d 515d 516d 517d 750G 1438G 2706G 3106d 3107C 3970T 4086T 4769G 5773A 5978G 5985A 6392C 6962A 7028T 8860G 9053A 9548A 10310A 10609C 11719A 12406A 12882T 13759A 13928C 14766T 15326G 15445C 16129A 16172C 16304C 16311C 16362C 16519C	F1a4a	73G 146C 152C 153G 248d 263G 310N 514d 515d 516d 517d 16129A 16172C 16304C 16311C 16362C 16519C	1	73 146 152 153 249d 263 310N 750 1438 2706 3970 4086 4769 5773 5978 5985 6392 6962 7028 8860 9053 9548 10310 10609 11719 12406 12882 13759 13928C 14766 15326 15445 16129 16172 16304 16311 16362	F1a4a	73 146 152 153 249d 263 310N 16129 16172 16304 16311 16362	[0.865] F1a4a (1)	[0.048] F1a3a3 (2) [0.030] F1a4a1 (3) [0.022] F1a3+16311 (4) [0.006] F1a3a (5) [0.000] F1a3a3a	1
2263	>KP240770.1 Ho	73G 199C 204C 263G 489C 750G 1438G 2706G 3106d 3107C 3483A 4048A 4071T 4164G 4769G 5351G 5460A 6455T 6680C 7028T 7684C 7853A 8701G 8860G 9438A 9540C 9824C 10398G 10400T 10873C 11719A 12405T 12705T 12811C 14766T 14783C 15043A 15301A 15326G 16223T 16297C 16519C	M7b1a1b	73G 199C 204C 263G 489C 16223T 16297C 16519C	1	73 199 204 263 489 750 1438 2706 3483 4048 4071 4164 4769 5351 5460 6455 6680 7028 7684 7853 8701 8860 9438 9540 9824 10398 10400 10873 11719 12405 12705 12811 14766 14783 15043 15301 15326 16223 16297	M7b1a1b	73 199 204 263 489 16223 16297	[0.018] M7b1a1b (1)	[0.004] M33a1b (2) [0.000] M7b1a M35+199 M35b M35b1 M35b4 N1a1'2 (8)	1
2264	>KP240756.1 Ho	73G 143N 248d 263G 310N 514d 515d 750G 1005C 1438G 1824C 2706G 3106d 3107C 3970T 4769G 4811G 6392C 7028T 7828G 8860G 10310A 10535C 10586A 11719A 12338C 13191C 13708A 13928C 14766T 15326G 16092A 16136C 16289G 16291T 16304C	F2b1	73G 143N 248d 263G 310N 514d 515d 16092A 16136C 16289G 16291T 16304C	1	73 143N 249d 263 310N 750 1005 1438 1824 2706 3970 4769 4811 6392 7028 7828 8860 10310 10535 10586 11719 12338 13191 13708 13928C 14766 15326 16092A 16136 16289 16291 16304	F2b1	73 143N 249d 263 310N 16092A 16136 16289 16291 16304	[0.043] F2b1 (1)	[0.013] F2+16291 (2) [0.000] F2b F2g (4)	1
2265	>KP240750.1 Ho	73G 146N 194T 199C 263G 489C 514d 515d 750G 1438G 2706G 3083C 3106d 3107C 3398C 3882A 4071T 4769G 4850T 5196C 5442C 6455T 7028T 8701G 8860G 9540C 9824C 10398G 10400T 10873C 11665T 11719A 12091C 12705T 14766T 14783C 15043A 15301A 15326G 16295T 16319A 16519C	M7c1a3	73G 146N 194T 199C 263G 489C 514d 515d 16295T 16319A 16519C	1	73 146N 194 199 263 489 750 1438 2706 3083 3398 3882 4071 4769 4850 5196 5442 6455 7028 8701 8860 9540 9824 10398 10400 10873 11665 11719 12091 12705 14766 14783 15043 15301 15326 16295 16319	M7c1a3	73 146N 194 199 263 489 16295 16319	[0.191] M7c1a3 (1) [0.000] M7c1c2 (2)	[0.191] M7c1a3 (3) [0.049] M7c1 (4) [0.033] M7c1a3a (5) [0.009] M7c1c (6) [0.003] M7c1b (7) [0.000] M7c1a M7c1a1 M7c1a1a M7c1a1a1 M7c1a4 M7c1b1 M7c1b2 M7c1b2b M7c1c1 M7c1c2 M7c1c2a (18)	1
2266	>KP240746.1 Ho	73G 195C 248d 263G 514d 515d 750G 1438G 1709A 2706G 3106d 3107C 3970T 4086T 4769G 6392C 6962A 7028T 8697A 8860G 9053A 9548A 10310A 10609C 11719A 12406A 12882T 13759A 13928C 14258A 14766T 15326G 16129A 16162G 16172C 16304C 16311C 16519C	F1a1	73G 195C 248d 263G 514d 515d 16129A 16162G 16172C 16304C 16311C 16519C	1	73 195 249d 263 750 1438 1709 2706 3970 4086 4769 6392 6962 7028 8697 8860 9053 9548 10310 10609 11719 12406 12882 13759 13928C 14258 14766 15326 16129 16162 16172 16304 16311	F1a1	73 195 249d 263 16129 16162 16172 16304 16311	[0.162] F1a1 (1) [0.037] F1a3a (2) [0.000] F1a3+16311 F1a3a3 F1a3a3a (5)	[0.175] F1a1a (6) [0.075] F1a1d1 (7) [0.061] F1a1c (8) [0.052] F1a1a1 (9) [0.042] F1a (10) [0.013] F1a1b (11) [0.005] F1a1d (12) [0.003] F1a3 (13) [0.000] F1a1'4 F1a1c1 F1a1c2 F1a1c3 F1a4 (18)	1

2267	>KP240744.1 Ho	73G 263G 489C 514d 515d 723G 750G 1438G 2706G 3106d 3107C 3140G 4071T 4769G 5324T 6455T 7028T 8027A 8701G 8860G 9540C 9824C 9968T 10398G 10400T 10873C 11719A 12405T 12705T 14284T 14766T 14783C 15043A 15301A 15326G 16086C 16184T 16223T 16235G 16519C	M7b2a	73G 263G 489C 514d 515d 16086C 16184T 16223T 16235G 16519C	1	73 263 489 723 750 1438 2706 3140 4071 4769 5324 6455 7028 8027 8701 8860 9540 9824 9968 10398 10400 10873 11719 12405 12705 14284 14766 14783 15043 15301 15326 16086 16184 16223 16235	M7b2a	73 263 489 16086 16184 16223 16235	[0.000] M7b2a (1)	[0.000] M7b2 (2)	1
2268	>KP240743.1 Ho	73G 248d 263G 750G 1005C 1438G 1824C 2706G 2746C 3106d 3107C 3970T 4769G 6392C 6566T 7028T 7828G 8860G 10310A 10535C 10586A 11719A 12338C 13708A 13928C 14766T 14831A 15326G 16129A 16203G 16291T 16304C	F2a	73G 248d 263G 16129A 16203G 16291T 16304C	1	73 249d 263 750 1005 1438 1824 2706 2746 3970 4769 6392 6566 7028 7828 8860 10310 10535 10586 11719 12338 13708 13928C 14766 14831 15326 16129 16203 16291 16304	F2a	73 249d 263 16129 16203 16291 16304	[0.029] F2a (1)	[0.091] F2a+@16291 (2) [0.075] F1a'c'f (3) [0.016] F1c (4) [0.000] F1f F2+16291 F2a1 F2b F2g (9)	1
2269	>KP240742.1 Ho	73G 150N 199C 204C 263G 310N 489C 750G 1438G 2706G 3106d 3107C 3483A 4048A 4071T 4164G 4769G 5351G 5460A 6455T 6680C 7028T 7684C 7853A 8701G 8860G 9540C 9824C 10398G 10400T 10873C 11719A 12405T 12705T 12811C 14766T 14783C 15043A 15301A 15326G 16223T 16297C	M7b1a1b	73G 150N 199C 204C 263G 310N 489C 16223T 16297C	1	73 150N 199 204 263 310N 489 750 1438 2706 3483 4048 4071 4164 4769 5351 5460 6455 6680 7028 7684 7853 8701 8860 9540 9824 10398 10400 10873 11719 12405 12705 12811 14766 14783 15043 15301 15326 16223 16297	M7b1a1b	73 150N 199 204 263 310N 489 16223 16297	[0.000] M7b1a1b (1)	[0.042] M7b1a1+(16192) (2) [0.018] M7b1a1e1 (3) [0.015] M7b1a1 (4) [0.014] M7b1a1a3 (5) [0.010] M7b1a1f (6) [0.004] M7b1a1h M33a1b (8) [0.002] M7b1a1g (9) [0.000] M7b1a M7b1a1d M7b1a1d1 M7b1a1e M35+199 M35b M35b1 M35b4 N1a1'2 (18)	1
2270	>KP240741.1 Ho	73G 150N 199C 204C 263G 310N 489C 750G 1438G 2706G 3106d 3107C 3483A 4048A 4071T 4164G 4769G 5351G 5460A 6455T 6680C 7028T 7684C 7853A 8648A 8701G 8860G 9540C 9824C 10398G 10400T 10873C 11719A 12405T 12705T 12811C 14766T 14783C 15043A 15301A 15326G 16223T 16297C	M7b1a1b	73G 150N 199C 204C 263G 310N 489C 16223T 16297C	1	73 150N 199 204 263 310N 489 750 1438 2706 3483 4048 4071 4164 4769 5351 5460 6455 6680 7028 7684 7853 8648 8701 8860 9540 9824 10398 10400 10873 11719 12405 12705 12811 14766 14783 15043 15301 15326 16223 16297	M7b1a1b	73 150N 199 204 263 310N 489 16223 16297	[0.000] M7b1a1b (1)	[0.042] M7b1a1+(16192) (2) [0.018] M7b1a1e1 (3) [0.015] M7b1a1 (4) [0.014] M7b1a1a3 (5) [0.010] M7b1a1f (6) [0.004] M7b1a1h M33a1b (8) [0.002] M7b1a1g (9) [0.000] M7b1a M7b1a1d M7b1a1d1 M7b1a1e M35+199 M35b M35b1 M35b4 N1a1'2 (18)	1
2271	>KP240740.1 Ho	73G 152C 195C 207A 248d 263G 275A 750G 1005C 1438G 1824C 2706G 3106d 3107C 3753C 3970T 4769G 6392C 6465A 7028T 7828G 8860G 10084C 10310A 10535C 10586A 11323T 11410C 11719A 12338C 13708A 13928C 14766T 15326G 16093C 16221T 16304C 16519C	F2i	73G 152C 195C 207A 248d 263G 275A 16093C 16221T 16304C 16519C	1	73 152 195 207 249d 263 275 750 1005 1438 1824 2706 3753 3970 4769 6392 6465 7028 7828 8860 10084 10310 10535 10586 11323 11410 11719 12338 13708 13928C 14766 15326 16093 16221 16304	F2i	73 152 195 207 249d 263 275 16093 16221 16304	[0.062] F2i (1)	[0.013] F2+195 (2) [0.000] F2h	1

2272 >KP240736.1 Ho	73G 131C 204C 263G 310N 514d 515d 709A 750G 1438G 1598A 2706G 3106d 3107C 4769G 4895G 5246T 6681C 7028T 8271d 8272d 8273d 8274d 8275d 8276d 8277d 8278d 8279d 8584A 8829T 8860G 9950C 10398G 10602T 11719A 12361G 14766T 15223T 15326G 15508T 15662G 15850C 15851G 15927A 16108T 16111T 16140C 16183C 16234T 16243C 16266T 16399G 16463G 16519C	B5b2a	73G 131C 204C 263G 310N 514d 515d 16108T 16111T 16140C 16183C 16234T 16243C 16266T 16399G 16463G 16519C	1	73 131 204 263 310N 709 750 1438 1598 2706 4769 4895 5246 6681 7028 8281-8289d 8584 8829 8860 9950 10398 10602T 11719 12361 14766 15223 15326 15508 15662 15850 15851 15927 16108 16111 16140 16234 16243 16266 16399 16463	B5b2a	73 131 204 263 310N 16108 16111 16140 16234 16243 16266 16399 16463	[0.236] B5b2a (1)	[0.000] B5b2 B5b2a1 B5b2a2 B5b2a2a B5b2a2a1 B5b2a2a2 B5b2b (8)	1
2273 >KP240731.1 Ho	73G 150T 199C 204C 263G 489C 750G 1438G 2706G 3106d 3107C 3483A 4048A 4071T 4164G 4769G 5033G 5351G 5460A 6455T 6680C 7028T 7684C 7853A 8701G 8860G 9540C 9824C 10398G 10400T 10873C 11719A 12405T 12705T 12811C 14766T 14783C 15043A 15301A 16223T 16243C 16274A 16297C 16519C	M7b1a1b	73G 150T 199C 204C 263G 489C 16223T 16243C 16274A 16297C 16519C	1	73 150 199 204 263 489 750 1438 2706 3483 4048 4071 4164 4769 5033 5351 5460 6455 6680 7028 7684 7853 8701 8860 9540 9824 10398 10400 10873 11719 12405 12705 12811 14766 14783 15043 15301 16223 16243 16274 16297	M7b1a1b	73 150 199 204 263 489 16223 16243 16274 16297	[0.015] M7b1a1b (1)	[0.042] M7b1a1+(16192) (2) [0.028] M7b1a1a3 (3) [0.018] M7b1a1e1 (4) [0.015] M7b1a1 (5) [0.010] M7b1a1f (6) [0.008] M7b1a1h (7) [0.006] M7b1a1g (8) [0.004] M7b1a1d M7b1a1d1 M7b1a1e (11)	1
2274 >KP240730.1 Ho	73G 103A 146C 152C 189G 204C 263G 514d 515d 709A 750G 1438G 1598A 2706G 3106d 3107C 3951T 4703C 4769G 7028T 7170T 8271d 8272d 8273d 8274d 8275d 8276d 8277d 8278d 8279d 8584A 8829T 8860G 8930T 9950C 10398G 11017C 11719A 12361G 14766T 15001C 15223T 15326G 15508T 15662G 15851G 15927A 16093C 16140C 16183N 16189C 16243C 16274A	B5b4	73G 103A 146C 152C 189G 204C 263G 514d 515d 16093C 16140C 16183N 16189C 16243C 16274A	1	73 103 146 152 189 204 263 709 750 1438 1598 2706 3951 4703 4769 7028 7170 8281-8289d 8584 8829 8860 8930 9950 10398 11017 11719 12361 14766 15001 15223 15326 15508 15662 15851 15927 16093 16140 16183N 16189 16243 16274	B5b4	73 103 146 152 189 204 263 16093 16140 16183N 16189 16243 16274	[0.501] B5b4 (1)	[0.012] B5b1c1 (2) [0.006] B5b1c1a (3) [0.005] B5b1c	1
2275 >KP240726.1 Ho	73G 152C 248d 263G 373G 514d 515d 750G 1438G 2706G 3106d 3107C 3970T 4732G 4769G 5147A 5508C 6392C 6962A 7028T 8860G 9899C 10235C 10310A 10609C 10976T 11719A 12406A 12882T 13928C 14133G 14476A 14766T 15326G 16182N 16183C 16189C 16232A 16249C 16304C 16519C	F1b1c	73G 152C 248d 263G 373G 514d 515d 16182N 16183C 16189C 16232A 16249C 16304C 16519C	1	73 152 249d 263 373 750 1438 2706 3970 4732 4769 5147 5508 6392 6962 7028 8860 9899 10235 10310 10609 10976 11719 12406 12882 13928C 14133 14476 14766 15326 16182N 16189 16232A 16249 16304	F1b1c	73 152 249d 263 373 16182N 16189 16232A 16249 16304	[0.010] F1b1c (1)	[0.000] F1b F1b1 (3)	1
2276 >KP240725.1 Ho	54C 73G 248d 263G 310N 318C 514d 515d 750G 1438G 1462A 2706G 3106d 3107C 3970T 4086T 4732G 4769G 6392C 6962A 7028T 8718G 8860G 9053A 10310A 10609C 11719A 12406A 12882T 13759A 13928C 14766T 15326G 15565C 16129A 16172C 16304C 16519C	F1a3b	54C 73G 248d 263G 310N 318C 514d 515d 16129A 16172C 16304C 16519C	1	54C 73 249d 263 310N 318 750 1438 1462 2706 3970 4086 4732 4769 6392 6962 7028 8718 8860 9053 10310 10609 11719 12406 12882 13759 13928C 14766 15326 15565 16129 16172 16304	F1a3b	54C 73 249d 263 310N 318 16129 16172 16304	[0.005] F1a3b (1)	[0.000] F1a F1a1'4 F1a4 F1a3 (5)	1

2277 >KP240724.1 Ho	73G 248d 263G 514d 515d 750G 1005C 1438G 1824C 2706G 3106d 3107C 3970T 4643A 4769G 4811G 6392C 7028T 7828G 8010C 8697A 8860G 10310A 10535C 10586A 11719A 12338C 13708A 13928C 14766T 15326G 16092A 16291T 16304C	F2b1	73G 248d 263G 514d 515d 16092A 16291T 16304C	1	73 249d 263 750 1005 1438 1824 2706 3970 4643 4769 4811 6392 7028 7828 8010 8697 8860 10310 10535 10586 11719 12338 13708 13928C 14766 15326 16092A 16291 16304	F2b1	73 249d 263 16092A 16291 16304	[0.000] F2b1 (1)	[0.013] F2+16291 (2) [0.000] F2b F2g (4)	1
2278 >KP240723.1 Ho	73G 150T 199C 263G 489C 750G 1438G 2706G 3106d 3107C 4048A 4071T 4164G 4769G 5351G 5460A 6228T 6455T 6680C 7028T 7684C 7853A 8701G 8860G 9540C 9732T 9824C 10398G 10400T 10873C 11407T 11719A 12405T 12705T 12811C 14766T 14783C 15043A 15301A 15326G 15860G 16129A 16223T 16297C 16527T	M7b1a1e2	73G 150T 199C 263G 489C 16129A 16223T 16297C 16527T	1	73 150 199 263 489 750 1438 2706 4048 4071 4164 4769 5351 5460 6228 6455 6680 7028 7684 7853 8701 8860 9540 9732 9824 10398 10400 10873 11407 11719 12405 12705 12811 14766 14783 15043 15301 15326 15860 16129 16223 16297 16527	M7b1a1e2	73 150 199 263 489 16129 16223 16297 16527	[0.000] M7b1a1e2 (1)	[0.000] M7b1a1 M7b1a1+(16192) M7b1a1d M7b1a1d1 M7b1a1e M7b1a1e1 M7b1a1e2 M7b1a1f M7b1a1g M7b1a1h (11)	1
2279 >KP240722.1 Ho	73G 146A 199C 263G 310.1C 489C 514d 515d 750G 1438G 2706G 3106d 3107C 3606G 4071T 4679C 4769G 4850T 5442C 6455T 7028T 8701G 8860G 9540C 9824C 10398G 10400T 10873C 11665T 11719A 12091C 12705T 14766T 14783C 15043A 15236G 15301A 15326G 16519C	M7c1c2	73G 146A 199C 263G 310.1C 489C 514d 515d 16519C	1	73 146A 199 263 489 750 1438 2706 3606 4071 4679 4769 4850 5442 6455 7028 8701 8860 9540 9824 10398 10400 10873 11665 11719 12091 12705 14766 14783 15043 15236 15301 15326	M7c1c2	73 146A 199 263 489	[0.000] M7c1c2 (1)	[0.000] M7c1c2a (2)	1
2280 >KP240717.1 Ho	73G 146C 248d 263G 750G 1438G 2706G 3106d 3107C 3290C 3970T 4769G 5263T 6392C 7028T 8023C 8860G 10310A 10915C 11116C 11227A 11719A 12630A 13928C 14766T 15326G 15670C 16179T 16207G 16304C 16399G	F4a	73G 146C 248d 263G 16179T 16207G 16304C 16399G	1	73 146 249d 263 750 1438 2706 3290 3970 4769 5263 6392 7028 8023 8860 10310 10915 11116 11227A 11719 12630 13928C 14766 15326 15670 16179 16207 16304 16399	F4a	73 146 249d 263 16179 16207 16304 16399	[0.917] F4a (1)	[0.323] F4a2 (2) [0.000] F4a1	1
2281 >KP240711.1 Ho	73G 150T 195C 248d 263G 514d 515d 750G 1438G 2706G 3106d 3107C 3970T 4769G 5263T 5587C 6392C 6962A 7028T 8860G 10310A 10609C 11719A 12406A 12882T 13928C 14766T 15024A 15326G 16189C 16274A 16284G 16300G 16304C 16311C 16519C	F1e3	73G 150T 195C 248d 263G 514d 515d 16189C 16274A 16284G 16300G 16304C 16311C 16519C	1	73 150 195 249d 263 750 1438 2706 3970 4769 5263 5587 6392 6962 7028 8860 10310 10609 11719 12406 12882 13928C 14766 15024 15326 16189 16274 16284 16300 16304 16311	F1e3	73 150 195 249d 263 16189 16274 16284 16300 16304 16311	[1.358] F1e3 (1)	[0.167] F1a3a2 (2) [0.073] F2h (3) [0.031] F1g (4) [0.024] F1+16189 (5) [0.016] F2c1 (6) [0.015] F1e1 F1g1 (8) [0.006] F1e (9) [0.000] F2+195 B4c1c+16311 (11)	1
2282 >KP240707.1 Ho	73G 150T 199C 204C 263G 489C 750G 1438G 2706G 3106d 3107C 3483A 4048A 4071T 4164G 4769G 5351G 5460A 6455T 6680C 7028T 7684C 7853A 8701G 8860G 9540C 9824C 10398G 10400T 10873C 11719A 12405T 12705T 12811C 13145A 14766T 14783C 15043A 15301A 16215G 16223T 16297C	M7b1a1b	73G 150T 199C 204C 263G 489C 16215G 16223T 16297C	1	73 150 199 204 263 489 750 1438 2706 3483 4048 4071 4164 4769 5351 5460 6455 6680 7028 7684 7853 8701 8860 9540 9824 10398 10400 10873 11719 12405 12705 12811 13145 14766 14783 15043 15301 16215 16223 16297	M7b1a1b	73 150 199 204 263 489 16215 16223 16297	[0.006] M7b1a1b (1)	[0.042] M7b1a1+(16192) (2) [0.018] M7b1a1e1 (3) [0.015] M7b1a1 (4) [0.014] M7b1a1a3 (5) [0.010] M7b1a1f (6) [0.004] M7b1a1h (7) [0.002] M7b1a1g (8) [0.000] M7b1a1d M7b1a1d1 M7b1a1e (11)	1

2283 >KP240706.1 Ho	73G 146C 248d 263G 317A 750G 1438G 2706G 2763C 3106d 3107C 3290C 3316A 3970T 4769G 5263T 6392C 7028T 8860G 10310A 10915C 11719A 12153T 12630A 12882T 13602C 13928C 14766T 15326G 15670C 16126C 16140C 16207G 16304C 16362C 16399G	F4a1b	73G 146C 248d 263G 317A 16126C 16140C 16207G 16304C 16362C 16399G	1	73 146 249d 263 317A 750 1438 2706 2763 3290 3316 3970 4769 5263 6392 7028 8860 10310 10915 11719 12153 12630 12882 13602 13928C 14766 15326 15670 16126 16140 16207 16304 16362 16399	F4a1b	73 146 249d 263 317A 16126 16140 16207 16304 16362 16399	[0.417] F4a1b (1)	[0.000] F4a1 (2)	1
2284 >KP240705.1 Ho	73G 150T 159C 199C 263G 489C 750G 1438G 2706G 3106d 3107C 4048A 4071T 4164G 4769G 5351G 5460A 6455T 6641C 6680C 7028T 7684C 7853A 8701G 8860G 9540C 9824C 10055G 10398G 10400T 10873C 11719A 12405T 12705T 12811C 14766T 14783C 15043A 15301A 15326G 16129A 16223T 16255A 16297C	M7b1a1	73G 150T 159C 199C 263G 489C 16129A 16223T 16255A 16297C	1	73 150 159 199 263 489 750 1438 2706 4048 4071 4164 4769 5351 5460 6455 6641 6680 7028 7684 7853 8701 8860 9540 9824 10055 10398 10400 10873 11719 12405 12705 12811 14766 14783 15043 15301 15326 16129 16223 16255 16297	M7b1a1	73 150 159 199 263 489 16129 16223 16255 16297	[0.255] M7b1a1 (1) [0.033] M7b1a1c (2) [0.000] M7b1a1+(16192) M7b1a1d M7b1a1d1 M7b1a1e M7b1a1e1 M7b1a1f M7b1a1g M7b1a1h (10)	[0.033] M7b1a1c (11) [0.007] M7b1a1e2 (12) [0.003] M7b1a1a2 (13) [0.000] M7b1a M7b1a1a M7b1a1+(16192) M7b1a1d M7b1a1d1 M7b1a1e M7b1a1e1 M7b1a1f M7b1a1g M7b1a1h M7b1a1i (24)	1
2285 >KP240703.1 Ho	73G 248d 263G 514d 515d 750G 1005C 1438G 1824C 2706G 3106d 3107C 3970T 4769G 6392C 7028T 7828G 8860G 10310A 10535C 10586A 10927C 11719A 11969A 12338C 13708A 13860T 13928C 14766T 15326G 16111A 16203G 16291T 16304C 16519C	F2a	73G 248d 263G 514d 515d 16111A 16203G 16291T 16304C 16519C	1	73 249d 263 750 1005 1438 1824 2706 3970 4769 6392 7028 7828 8860 10310 10535 10586 10927 11719 11969 12338 13708 13860 13928C 14766 15326 16111A 16203 16291 16304	F2a	73 249d 263 16111A 16203 16291 16304	[0.080] F2a (1)	[0.013] F2+16291 (2) [0.000] F2a+@16291 F2a1 F2b F2g (6)	1
2286 >KP240701.1 Ho	73G 103A 131C 263G 481T 514d 515d 709A 1438G 1555G 1598A 2706G 3106d 3107C 4161T 4769G 4895G 7028T 8271d 8272d 8273d 8274d 8275d 8276d 8277d 8278d 8279d 8584A 8829T 8860G 9101N 9804A 9950C 10398G 11016A 11719A 12361G 13590A 14766T 15223T 15326G 15508T 15662G 15850C 15851G 15927A 16111T 16140C 16172A 16183N 16189C 16234T 16243C 16463G	B5b2c	73G 103A 131C 263G 481T 514d 515d 16111T 16140C 16172A 16183N 16189C 16234T 16243C 16463G	1	73 103 131 263 481 709 1438 1555 1598 2706 4161 4769 4895 7028 8281-8289d 8584 8829 8860 9101N 9804 9950 10398 11016 11719 12361 13590 14766 15223 15326 15508 15662 15850 15851 15927 16111 16140 16172A 16183N 16189 16234 16243 16463	B5b2c	73 103 131 263 481 16111 16140 16172A 16183N 16189 16234 16243 16463	[0.083] B5b2c (1)	[0.000] B5b2+@204 B5b2c1 (3)	1
2287 >KP240700.1 Ho	73G 200G 248d 263G 750G 1005C 1438G 1824C 2706G 3106d 3107C 3970T 4769G 6392C 7028T 7828G 8860G 10310A 10535C 10586A 11719A 12338C 12372A 13708A 13928C 14766T 15326G 16203G 16291T 16304C 16519C	F2a	73G 200G 248d 263G 16203G 16291T 16304C 16519C	1	73 200 249d 263 750 1005 1438 1824 2706 3970 4769 6392 7028 7828 8860 10310 10535 10586 11719 12338 12372 13708 13928C 14766 15326 16203 16291 16304	F2a	73 200 249d 263 16203 16291 16304	[0.022] F2a (1)	[0.000] F2+16291 F2a+@16291 F2a1 F2b F2g (6)	1

2288	>KP240697.1 Ho	73G 93G 210G 263G 514d 515d 709A 750G 1438G 2706G 3106d 3107C 3537G 4769G 6366A 7028T 8271d 8272d 8273d 8274d 8275d 8276d 8277d 8278d 8279d 8584A 8860G 9189T 9950C 9962A 10398G 11149A 11151T 11719A 12234G 13422G 14149T 14766T 14861A 15235G 15326G 16140C 16187T 16189C 16231C 16256T 16266G 16327T 16519C	B5a2a1a	73G 93G 210G 263G 514d 515d 16140C 16187T 16189C 16231C 16256T 16266G 16327T 16519C	1	73 93 210 263 709 750 1438 2706 3537 4769 6366 7028 8281-8289d 8584 8860 9189 9950 9962 10398 11149 11151 11719 12234 13422 14149 14766 14861 15235 15326 16140 16187 16189 16231 16256 16266G 16327	B5a2a1a	73 93 210 263 16140 16187 16189 16231 16256 16266G 16327	[0.052] B5a2a1a (1)	[0.000] B5a2a1 (2)	1
2289	>KP240694.1 Ho	73G 152C 248d 263G 514d 515d 516d 517d 750G 1438G 2706G 3106d 3107C 3970T 4086T 4769G 5773A 5978G 5985A 6392C 6962A 7028T 8860G 9053A 9548A 10310A 10609C 11719A 12406A 12882T 13759A 13928C 14766T 15326G 15445C 16129A 16172C 16249C 16304C 16311C 16362C 16519C	F1a4a	73G 152C 248d 263G 514d 515d 516d 517d 16129A 16172C 16249C 16304C 16311C 16362C 16519C	1	73 152 249d 263 750 1438 2706 3970 4086 4769 5773 5978 5985 6392 6962 7028 8860 9053 9548 10310 10609 11719 12406 12882 13759 13928C 14766 15326 15445 16129 16172 16249 16304 16311 16362	F1a4a	73 152 249d 263 16129 16172 16249 16304 16311 16362	[0.788] F1a4a (1)	[0.022] F1a3+16311 (2) [0.020] F1a4a1 (3) [0.018] F1a3a3 (4) [0.000] F1a3a F1a3a3a (6)	1
2290	>KP240693.1 Ho	73G 248d 263G 514d 515d 750G 1438G 1772G 2706G 3106d 3107C 3970T 4086T 4769G 5460A 6254G 6392C 6962A 7028T 8631G 8860G 9053A 9548A 10310A 10609C 11719A 12406A 12882T 13759A 13928C 14766T 15326G 16129A 16162G 16172C 16304C 16519C 16527T	F1a1	73G 248d 263G 514d 515d 16129A 16162G 16172C 16304C 16519C 16527T	1	73 249d 263 750 1438 1772 2706 3970 4086 4769 5460 6254 6392 6962 7028 8631 8860 9053 9548 10310 10609 11719 12406 12882 13759 13928C 14766 15326 16129 16162 16172 16304 16527	F1a1	73 249d 263 16129 16162 16172 16304 16527	[0.012] F1a1 (1)	[0.008] F1a1c3 (2) [0.007] F1a1a1 (3) [0.006] F1a (4) [0.005] F1a1'4 F1a4 (6) [0.002] F1a1a (7) [0.000] F1a1b F1a1c F1a1c2 F1a1d F1a1d1 F1a3 (13)	1
2291	>KP240691.1 Ho	73G 150T 199C 263G 489C 496T 750G 1438G 2706G 3106d 3107C 4048A 4071T 4164G 4769G 5351G 5460A 6228T 6455T 6680C 7028T 7684C 7853A 8701G 8860G 9540C 9732T 9824C 10398G 10400T 10873C 11407T 11719A 12405T 12705T 12811C 14766T 14783C 15043A 15301A 15326G 16129A 16223T 16297C 16527T	M7b1a1e2	73G 150T 199C 263G 489C 496T 16129A 16223T 16297C 16527T	1	73 150 199 263 489 496 750 1438 2706 4048 4071 4164 4769 5351 5460 6228 6455 6680 7028 7684 7853 8701 8860 9540 9732 9824 10398 10400 10873 11407 11719 12405 12705 12811 14766 14783 15043 15301 15326 16129 16223 16297 16527	M7b1a1e2	73 150 199 263 489 496 16129 16223 16297 16527	[0.004] M7b1a1e2 (1)	[0.004] M7b1a1e2 (2) [0.000] M7b1a1 M7b1a1+(16192) M7b1a1d M7b1a1d1 M7b1a1e M7b1a1e1 M7b1a1f M7b1a1g M7b1a1h (11)	1
2292	>KP240689.1 Ho	73G 114T 204C 207A 248d 263G 310.1C 750G 961C 1438G 1719A 2706G 3106d 3107C 3434G 3970T 4769G 5585A 5913A 5978G 6392C 7028T 8860G 9182A 10310A 10320A 11065G 11719A 12621T 13928C 14766T 15326G 15586C 15854G 16093C 16187T 16298C 16355T 16362C 16390A 16519C	F3a+207	73G 114T 204C 207A 248d 263G 310.1C 16093C 16187T 16298C 16355T 16362C 16390A 16519C	1	73 114 204 207 249d 263 750 961 1438 1719 2706 3434 3970 4769 5585 5913 5978 6392 7028 8860 9182 10310 10320 11065 11719 12621 13928C 14766 15326 15586 15854G 16093 16187 16298 16355 16362 16390	F3a+207	73 114 204 207 249d 263 16093 16187 16298 16355 16362 16390	[1.529] F3a+207 (1)	[0.369] F3a1 (2) [0.214] F3a	1

2293	>KP240685.1 Ho	73G 195C 248d 263G 750G 1438G 2706G 3106d 3107C 3390G 3434G 3970T 4769G 5585A 5913A 5978G 6392C 7028T 7094C 7741C 8860G 10310A 10320A 11065G 11719A 12621T 13928C 14766T 15326G 16209C 16298C 16311C 16355T 16362C	F3a	73G 195C 248d 263G 16209C 16298C 16311C 16355T 16362C	1	73 195 249d 263 750 1438 2706 3390 3434 3970 4769 5585 5913 5978 6392 7028 7094 7741 8860 10310 10320 11065 11719 12621 13928C 14766 15326 16209 16298 16311 16355 16362	F3a	73 195 249d 263 16209 16298 16311 16355 16362	[0.262] F3a (1)	[0.196] F3a+207 (2) [0.000] F3	1
2294	>KP240683.1 Ho	73G 228A 235G 248d 263G 310.1C 574G 750G 1005C 1009T 1438G 1824C 2706G 3106d 3107C 3970T 4769G 6392C 7028T 7492T 7828G 8860G 9099T 10310A 10535C 10586A 11719A 12338C 13161C 13708A 13928C 14766T 15040T 15326G	F2d	73G 228A 235G 248d 263G 310.1C 574G	1	73 228 235 249d 263 574 750 1005 1009 1438 1824 2706 3970 4769 6392 7028 7492 7828 8860 9099 10310 10535 10586 11719 12338 13161 13708 13928C 14766 15040 15326	F2d	73 228 235 249d 263 574	[0.136] F2d (1)	[0.111] H2a5b1 (2) [0.010] F2e (3) [0.006] HV+73 (4) [0.000] R HV20 H1e2c H3+73 H3d H4a1a1a H4a1a1a1 H4a1a1a1a H4a1a1a1a1 H4a1a1a2 H4a1a1a4 H7i R2'JT F F1 F2 F2c F2f F4 R30 R30a R30a1 R30a1a R31 P P2 P4 P9 P9a U U2'3'4'7'8'9 U8 U8b'c U8b (38)	1
2295	>KP240682.1 Ho	73G 146C 248d 263G 750G 1005C 1438G 1824C 2706G 3106d 3107C 3970T 4769G 6392C 7028T 7828G 8448C 8860G 10310A 10535C 10586A 11719A 12338C 12696C 13708A 13928C 14766T 15326G 16203G 16291T 16519C	F2a	73G 146C 248d 263G 16203G 16291T 16519C	1	73 146 249d 263 750 1005 1438 1824 2706 3970 4769 6392 7028 7828 8448 8860 10310 10535 10586 11719 12338 12696 13708 13928C 14766 15326 16203 16291	F2a	73 146 249d 263 16203 16291	[0.036] F2a (1)	[0.200] F2b (2) [0.017] F2a1 (3) [0.000] F2+16291 F2a+@16291 F2g (6)	1
2296	>KP240670.1 Ho	73G 248d 263G 750G 1005C 1438G 1824C 2706G 3106d 3107C 3970T 4769G 6392C 7028T 7828G 8538C 8860G 9128C 10310A 10535C 10586A 11719A 12338C 12603T 13708A 13928C 14766T 15326G 16203G 16291T 16304C 16311C 16335G 16519C	F2a	73G 248d 263G 16203G 16291T 16304C 16311C 16335G 16519C	1	73 249d 263 750 1005 1438 1824 2706 3970 4769 6392 7028 7828 8538 8860 9128 10310 10535 10586 11719 12338 12603 13708 13928C 14766 15326 16203 16291 16304 16311 16335	F2a	73 249d 263 16203 16291 16304 16311 16335	[0.290] F2a (1)	[0.679] F2+16291 (2) [0.017] F2a1 (3) [0.000] F2a+@16291 F2b F2g (6)	1
2297	>KP240668.1 Ho	73G 248d 263G 750G 1005C 1438G 1824C 2706G 3106d 3107C 3970T 4769G 6392C 7028T 7646G 7828G 8860G 10310A 10535C 10586A 11719A 12338C 13708A 13928C 14766T 15326G 16203G 16291T 16304C 16519C	F2a	73G 248d 263G 16203G 16291T 16304C 16519C	1	73 249d 263 750 1005 1438 1824 2706 3970 4769 6392 7028 7646 7828 8860 10310 10535 10586 11719 12338 13708 13928C 14766 15326 16203 16291 16304	F2a	73 249d 263 16203 16291 16304	[0.000] F2a (1)	[0.000] F2+16291 F2a+@16291 F2a1 F2b F2g (6)	1
2298	>KP240666.1 Ho	73G 94A 131C 263G 481T 514d 515d 709A 750G 1438G 1598A 2706G 3106d 3107C 4161T 4769G 4895G 7028T 8271d 8272d 8273d 8274d 8275d 8276d 8277d 8278d 8279d 8584A 8829T 8860G 9950C 10398G 11101G 11719A 12361G 13263G 14766T 15223T 15326G 15508T 15662G 15850C 15851G 15884A 15927A 16111T 16140C 16145A 16183N 16189C 16234T 16243C 16463G 16519C	B5b2c	73G 94A 131C 263G 481T 514d 515d 16111T 16140C 16145A 16183N 16189C 16234T 16243C 16463G 16519C	1	73 94 131 263 481 709 750 1438 1598 2706 4161 4769 4895 7028 8281-8289d 8584 8829 8860 9950 10398 11101 11719 12361 13263 14766 15223 15326 15508 15662 15850 15851 15884 15927 16111 16140 16145 16183N 16189 16234 16243 16463	B5b2c	73 94 131 263 481 16111 16140 16145 16183N 16189 16234 16243 16463	[0.292] B5b2c (1)	[0.304] B5b2c1 (2) [0.176] B5b2a (3) [0.032] B5b2+@204	1

2299	>KP240663.1 Ho	73G 146C 152C 207A 248d 263G 750G 1438G 2706G 3106d 3107C 3290C 3699G 3858G 3970T 4769G 5263T 5563A 5664G 6356N 6392C 7028T 7861C 8860G 10310A 10915C 11038G 11719A 12153T 12396C 12408C 12630A 13602C 13928C 14766T 15326G 15670C 16093C 16207G 16304C 16362C 16399G 16497G	F4a1a	73G 146C 152C 207A 248d 263G 16093C 16207G 16304C 16362C 16399G 16497G	1	73 146 152 207 249d 263 750 1438 2706 3290 3699G 3858 3970 4769 5263 5563 5664 6356N 6392 7028 7861 8860 10310 10915 11038 11719 12153 12396 12408 12630 13602 13928C 14766 15326 15670 16093 16207 16304 16362 16399 16497	F4a1a	73 146 152 207 249d 263 16093 16207 16304 16362 16399 16497	[0.087] F4a1a (1)	[0.462] F4a1 (2)	1
2300	>KP240662.1 Ho	73G 152C 195C 248d 263G 275A 750G 1005C 1438G 1824C 2706G 3106d 3107C 3753C 3970T 4769G 6392C 7028T 7828G 8860G 10084C 10310A 10535C 10586A 11323T 11410C 11719A 12338C 13708A 13710G 13928C 14766T 15326G 16221T 16304C 16519C	F2i	73G 152C 195C 248d 263G 275A 16221T 16304C 16519C	1	73 152 195 249d 263 275 750 1005 1438 1824 2706 3753 3970 4769 6392 7028 7828 8860 10084 10310 10535 10586 11323 11410 11719 12338 13708 13710 13928C 14766 15326 16221 16304	F2i	73 152 195 249d 263 275 16221 16304	[0.000] F2i (1)	[0.000] F2+195 F2h (3)	1
2301	>KP240661.1 Ho	73G 146C 248d 263G 750G 1438G 2706G 3106d 3107C 3290C 3705A 3970T 4769G 5263T 6392C 7028T 8860G 9725C 10208C 10310A 10915C 11116C 11719A 12630A 13923T 13928C 14766T 15326G 15670C 16207G 16304C 16399G	F4a	73G 146C 248d 263G 16207G 16304C 16399G	1	73 146 249d 263 750 1438 2706 3290 3705 3970 4769 5263 6392 7028 8860 9725 10208 10310 10915 11116 11719 12630 13923 13928C 14766 15326 15670 16207 16304 16399	F4a	73 146 249d 263 16207 16304 16399	[0.667] F4a (1)	[0.294] F4a2 (2) [0.000] F4a1	1
2302	>KP240659.1 Ho	73G 103A 131C 146C 263G 310.1C 709A 750G 1438G 1598A 2706G 3106d 3107C 3399G 3579G 4769G 4895G 6395T 7028T 8271d 8272d 8273d 8274d 8275d 8276d 8277d 8278d 8279d 8584A 8829T 8860G 9950C 10398G 11719A 12361G 13650T 14766T 15223T 15326G 15508T 15662G 15850C 15851G 15927A 16111T 16140C 16183C 16189C 16234T 16243C 16463G 16519C	B5b2+@204	73G 103A 131C 146C 263G 310.1C 16111T 16140C 16183C 16189C 16234T 16243C 16463G 16519C	1	73 103 131 146 263 709 750 1438 1598 2706 3399 3579 4769 4895 6395 7028 8281-8289d 8584 8829 8860 9950 10398 11719 12361 13650 14766 15223 15326 15508 15662 15850 15851 15927 16111 16140 16189 16234 16243 16463	B5b2+@204	73 103 131 146 263 16111 16140 16189 16234 16243 16463	[0.258] B5b2+@204 (1)	[0.106] B5b2b (2) [0.000] B5b2 B5b2c (4)	1
2303	>KP240658.1 Ho	73G 103A 131C 263G 514d 515d 709A 750G 1438G 1598A 2706G 3106d 3107C 4769G 4895G 6716G 7028T 8271d 8272d 8273d 8274d 8275d 8276d 8277d 8278d 8279d 8584A 8829T 8860G 9950C 10398G 11719A 12361G 14766T 15223T 15326G 15508T 15662G 15850C 15851G 15927A 16111T 16140C 16183N 16189C 16234T 16243C 16400T 16463G 16519C	B5b2+@204	73G 103A 131C 263G 514d 515d 16111T 16140C 16183N 16189C 16234T 16243C 16400T 16463G 16519C	1	73 103 131 263 709 750 1438 1598 2706 4769 4895 6716 7028 8281-8289d 8584 8829 8860 9950 10398 11719 12361 14766 15223 15326 15508 15662 15850 15851 15927 16111 16140 16183N 16189 16234 16243 16400 16463	B5b2+@204	73 103 131 263 16111 16140 16183N 16189 16234 16243 16400 16463	[0.032] B5b2+@204 (1)	[0.053] B5b2b (2) [0.000] B5b2 B5b2c (4)	1

2304	>KP240657.1 Ho	73G 248d 263G 310.1C 750G 1005C 1438G 1824C 2581G 2706G 3106d 3107C 3970T 4084A 4769G 6392C 7028T 7298G 7828G 8860G 10310A 10535C 10586A 11719A 12338C 13708A 13928C 14766T 15326G 16126C 16203G 16291T 16304C 16519C	F2a	73G 248d 263G 310.1C 16126C 16203G 16291T 16304C 16519C	1	73 249d 263 750 1005 1438 1824 2581 2706 3970 4084 4769 6392 7028 7298 7828 8860 10310 10535 10586 11719 12338 13708 13928C 14766 15326 16126 16203 16291 16304	F2a	73 249d 263 16126 16203 16291 16304	[0.058] F2a (1)	[0.000] F2+16291 F2a+@16291 F2a1 F2b F2g (6)	1
2305	>KP240656.1 Ho	73G 150T 159C 199C 263G 489C 750G 1438G 2706G 3106d 3107C 4048A 4071T 4164G 4769G 5351G 5460A 6455T 6641C 6680C 7028T 7684C 7853A 8701G 8860G 9540C 9824C 10055G 10398G 10400T 10873C 11719A 12405T 12705T 12811C 14766T 14783C 15043A 15301A 15326G 16129A 16223T 16255A 16297C	M7b1a1	73G 150T 159C 199C 263G 489C 16129A 16223T 16255A 16297C	1	73 150 159 199 263 489 750 1438 2706 4048 4071 4164 4769 5351 5460 6455 6641 6680 7028 7684 7853 8701 8860 9540 9824 10055 10398 10400 10873 11719 12405 12705 12811 14766 14783 15043 15301 15326 16129 16223 16255 16297	M7b1a1	73 150 159 199 263 489 16129 16223 16255 16297	[0.255] M7b1a1 (1) [0.033] M7b1a1c (2) [0.000] M7b1a1+(16192) M7b1a1d M7b1a1d1 M7b1a1e M7b1a1e1 M7b1a1f M7b1a1g M7b1a1h (10)	[0.033] M7b1a1c (11) [0.007] M7b1a1e2 (12) [0.003] M7b1a1a2 (13) [0.000] M7b1a M7b1a1a M7b1a1+(16192) M7b1a1d M7b1a1d1 M7b1a1e M7b1a1e1 M7b1a1f M7b1a1g M7b1a1h M7b1a1i (24)	1
2306	>KP240655.1 Ho	52C 53A 54C 66d 73G 248d 263G 310N 318C 514d 515d 750G 1438G 2706G 3106d 3107C 3970T 4086T 4769G 5894.1C 6392C 6962A 7028T 8718G 8860G 9053A 10310A 10609C 11719A 12406A 12882T 13044T 13759A 13928C 14766T 15326G 15565C 16129A 16172C 16304C 16519C	F1a3b	52C 53A 54C 66d 73G 248d 263G 310N 318C 514d 515d 16129A 16172C 16304C 16519C	1	52 53 54C 71d 73 249d 263 310N 318 750 1438 2706 3970 4086 4769 5899.1C 6392 6962 7028 8718 8860 9053 10310 10609 11719 12406 12882 13044 13759 13928C 14766 15326 15565 16129 16172 16304	F1a3b	52 53 54C 71d 73 249d 263 310N 318 16129 16172 16304	[0.195] F1a3b (1)	[0.000] F1a F1a1'4 F1a4 F1a3 (5)	1
2307	>KX456760.1 Ho	73G 146C 195C 263G 310.1C 489C 514d 515d 750G 951A 1438G 2140A 2706G 3106d 3107C 4769G 5773A 7028T 8701G 8860G 9540C 10101C 10398G 10400T 10873C 11719A 12651A 12705T 13359A 13812C 14766T 14783C 15043A 15301A 15326G 15511C 15601C 16086C 16223T 16519C	M24b	73G 146C 195C 263G 310.1C 489C 514d 515d 16086C 16223T 16519C	1	73 146 195 263 489 750 951 1438 2140 2706 4769 5773 7028 8701 8860 9540 10101 10398 10400 10873 11719 12651 12705 13359 13812 14766 14783 15043 15301 15326 15511 15601 16086 16223	M24b	73 146 195 263 489 16086 16223	[0.000] M24b (1)	[0.000] M24 (2)	1
2308	>KX456758.1 Ho	35A 36A 73G 146C 263G 309.1CT 310C 489C 750G 1438G 2706G 3106d 3107C 4769G 7028T 8701G 8860G 9540C 10398G 10400T 10556T 10873C 11050C 11719A 12007A 12705T 14302C 14766T 14783C 15043A 15148A 15301A 15326G 15930A 16111T 16184T 16185T 16189C 16223T 16295T 16519C	M37e	35A 36A 73G 146C 263G 309.1CT 310C 489C 16111T 16184T 16185T 16189C 16223T 16295T 16519C	1	35 36 73 146 263 489 750 1438 2706 4769 7028 8701 8860 9540 10398 10400 10556 10873 11050 11719 12007 12705 14302 14766 14783 15043 15148 15301 15326 15930 16111 16184 16185 16189 16223 16295	M37e	35 36 73 146 263 489 16111 16184 16185 16189 16223 16295	[0.146] M37e (1)	[0.000] M37e2 M45 (3)	1

2309 >KX456757.1 Hc	73G 152C 210G 263G 709A 750G 1438G 2706G 3106d 3107C 3537G 4086T 4769G 6960T 7028T 7934G 8271d 8272d 8273d 8274d 8275d 8276d 8277d 8278d 8279d 8584A 8860G 9950C 10398G 11465C 11719A 14766T 15235G 15326G 15857T 16140C 16182C 16183C 16189C 16240G 16261T 16266A 16519C	B5a1d	73G 152C 210G 263G 16140C 16182C 16183C 16189C 16240G 16261T 16266A 16519C	1	73 152 210 263 709 750 1438 2706 3537 4086 4769 6960 7028 7934 8281-8289d 8584 8860 9950 10398 11465 11719 14766 15235 15326 15857 16140 16189 16240 16261 16266A	B5a1d	73 152 210 263 16140 16189 16240 16261 16266A	[0.076] B5a1d (1)	[0.032] B5a1 (2) [0.016] B5a1a (3) [0.010] B5a (4) [0.004] B5a1b1 B5a1c1a (6) [0.000] B5a1a1 B5a1b B5a1c B5a1c1 B5a1c2 (11)	1
2310 >KX456753.1 Hc	73G 152C 210G 263G 309.1CT 310C 709A 750G 1438G 2706G 3106d 3107C 3537G 4086T 4769G 6960T 7028T 7934G 8271d 8272d 8273d 8274d 8275d 8276d 8277d 8278d 8279d 8584A 8860G 9950C 10398G 11465C 11719A 13005G 14766T 15235G 15326G 15857T 16140C 16182C 16183C 16189C 16240G 16261T 16266A 16519C	B5a1d	73G 152C 210G 263G 309.1CT 310C 16140C 16182C 16183C 16189C 16240G 16261T 16266A 16519C	1	73 152 210 263 709 750 1438 2706 3537 4086 4769 6960 7028 7934 8281-8289d 8584 8860 9950 10398 11465 11719 13005 14766 15235 15326 15857 16140 16189 16240 16261 16266A	B5a1d	73 152 210 263 16140 16189 16240 16261 16266A	[0.076] B5a1d (1)	[0.032] B5a1 (2) [0.016] B5a1a (3) [0.010] B5a (4) [0.004] B5a1b1 B5a1c1a (6) [0.000] B5a1a1 B5a1b B5a1c B5a1c1 B5a1c2 (11)	1
2311 >KX456752.1 Hc	73G 152C 225A 248d 263G 310.1C 316A 489C 514d 515d 750G 956T 1438G 2706G 3106d 3107C 3200C 3714G 4373C 4385T 4769G 4772C 7028T 7433T 8701G 8853G 8860G 9127G 9512T 9540C 10274C 10398G 10400T 10679G 10873C 11719A 11914A 12354C 12705T 14110C 14766T 14783C 14974T 15043A 15301A 15326G 15691G 16086C 16129A 16209C 16223T 16272G 16519C	M20	73G 152C 225A 248d 263G 310.1C 316A 489C 514d 515d 16086C 16129A 16209C 16223T 16272G 16519C	1	73 152 225 249d 263 316 489 750 956 1438 2706 3200 3714 4373 4385T 4769 4772 7028 7433 8701 8853 8860 9127 9512 9540 10274 10398 10400 10679 10873 11719 11914 12354 12705 14110 14766 14783 14974 15043 15301 15326 15691 16086 16129 16209 16223 16272	M20	73 152 225 249d 263 316 489 16086 16129 16209 16223 16272	[0.607] M20 (1)	[0.130] M5a2a (2) [0.068] M17 (3) [0.067] M17a (4) [0.059] G1b+16129 (5) [0.028] M5a (6) [0.021] D4a1a1 (7) [0.019] M5 M27b2b1 (9) [0.007] M5a'd (10) [0.006] G1b2 G1b4 D4a1 (13) [0.004] M37+152 D4a1f1 (15) [0.000] M5a2 M5a2a1 M5a5 M5b'c M5b M7a M7a1b2 M7a2 M7b1 M13'46'61 M13 M13a'b M13b M17c M27b2 M27b2a M27b2b M27b2c D4a D4a1a D4a1a1a D4a1b D4a1c D4a1f D4a1g D4a1h D4a2 D4a2a D4a2b D4a5 (45)	1
2312 >KX456749.1 Hc	73G 152C 210G 257G 263G 514d 515d 709A 750G 1438G 2706G 3106d 3107C 3537G 4086T 4769G 6960T 7028T 8271d 8272d 8273d 8274d 8275d 8276d 8277d 8278d 8279d 8584A 8860G 9950C 10398G 10589A 11465C 11719A 14696G 14766T 15235G 15326G 16140C 16182C 16183C 16189C 16261T 16266A 16519C	B5a1d	73G 152C 210G 257G 263G 514d 515d 16140C 16182C 16183C 16189C 16261T 16266A 16519C	1	73 152 210 257 263 709 750 1438 2706 3537 4086 4769 6960 7028 8281-8289d 8584 8860 9950 10398 10589 11465 11719 14696 14766 15235 15326 16140 16189 16261 16266A	B5a1d	73 152 210 257 263 16140 16189 16261 16266A	[0.028] B5a1d (1)	[0.032] B5a1 (2) [0.016] B5a1a (3) [0.010] B5a (4) [0.004] B5a1b1 (5) [0.002] B5a1c1a (6) [0.000] B5a1a1 B5a1b B5a1c B5a1c1 B5a1c2 (11)	1

2313	>KX456748.1 Hc	73G 152C 225A 248d 263G 310.1C 316A 489C 514d 515d 750G 956T 1438G 2706G 3106d 3107C 3200C 3714G 4373C 4385T 4769G 4772C 7028T 7433T 8701G 8853G 8860G 9127G 9512T 9540C 10274C 10398G 10400T 10679G 10873C 11719A 11914A 12354C 12705T 14110C 14766T 14783C 14974T 15043A 15301A 15326G 15691G 16086C 16129A 16209C 16223T 16272G 16519C	M20	73G 152C 225A 248d 263G 310.1C 316A 489C 514d 515d 16086C 16129A 16209C 16223T 16272G 16519C	1	73 152 225 249d 263 316 489 750 956 1438 2706 3200 3714 4373 4385T 4769 4772 7028 7433 8701 8853 8860 9127 9512 9540 10274 10398 10400 10679 10873 11719 11914 12354 12705 14110 14766 14783 14974 15043 15301 15326 15691 16086 16129 16209 16223 16272	M20	73 152 225 249d 263 316 489 16086 16129 16209 16223 16272	[0.607] M20 (1)	[0.130] M5a2a (2) [0.068] M17 (3) [0.067] M17a (4) [0.059] G1b+16129 (5) [0.028] M5a (6) [0.021] D4a1a1 (7) [0.019] M5 M27b2b1 (9) [0.007] M5a'd (10) [0.006] G1b2 G1b4 D4a1 (13) [0.004] M37+152 D4a1f1 (15) [0.000] M5a2 M5a2a1 M5a5 M5b'c M5b M7a M7a1b2 M7a2 M7b1 M13'46'61 M13 M13a'b M13b M17c M27b2 M27b2a M27b2b M27b2c D4a D4a1a D4a1a1a D4a1b D4a1c D4a1f D4a1g D4a1h D4a2 D4a2a D4a2b D4a5 (45)	1
2314	>KX456747.1 Hc	73G 152C 210G 257G 263G 514d 515d 709A 750G 1438G 2706G 3106d 3107C 3537G 4086T 4769G 6960T 7028T 8271d 8272d 8273d 8274d 8275d 8276d 8277d 8278d 8279d 8584A 8860G 9950C 10398G 10589A 11465C 11719A 14696G 14766T 15235G 15326G 16140C 16182C 16183C 16189C 16261T 16266A 16519C	B5a1d	73G 152C 210G 257G 263G 514d 515d 16140C 16182C 16183C 16189C 16261T 16266A 16519C	1	73 152 210 257 263 709 750 1438 2706 3537 4086 4769 6960 7028 8281-8289d 8584 8860 9950 10398 10589 11465 11719 14696 14766 15235 15326 16140 16189 16261 16266A	B5a1d	73 152 210 257 263 16140 16189 16261 16266A	[0.028] B5a1d (1)	[0.032] B5a1 (2) [0.016] B5a1a (3) [0.010] B5a (4) [0.004] B5a1b1 (5) [0.002] B5a1c1a (6) [0.000] B5a1a1 B5a1b B5a1c B5a1c1 B5a1c2 (11)	1
2315	>KX456740.1 Hc	73G 146C 195C 263G 309.1CT 310C 489C 514d 515d 750G 951A 1438G 2140A 2706G 3106d 3107C 4769G 5773A 7028T 8701G 8860G 9540C 10101C 10398G 10400T 10873C 11719A 12651A 12705T 13359A 13812C 14766T 14783C 15043A 15301A 15326G 15601C 16086C 16223T 16519C	M24b	73G 146C 195C 263G 309.1CT 310C 489C 514d 515d 16086C 16223T 16519C	1	73 146 195 263 489 750 951 1438 2140 2706 4769 5773 7028 8701 8860 9540 10101 10398 10400 10873 11719 12651 12705 13359 13812 14766 14783 15043 15301 15326 15601 16086 16223	M24b	73 146 195 263 489 16086 16223	[0.000] M24b (1)	[0.000] M24 (2)	1
2316	>KX456735.1 Hc	73G 152C 210G 257G 263G 309.1CCT 310C 514d 515d 709A 750G 1438G 2706G 3106d 3107C 3537G 4086T 4769G 6960T 7028T 8271d 8272d 8273d 8274d 8275d 8276d 8277d 8278d 8279d 8584A 8860G 9950C 10398G 10589A 11465C 11719A 14696G 14766T 15235G 15326G 16140C 16182C 16183C 16189C 16261T 16266A 16519C	B5a1d	73G 152C 210G 257G 263G 309.1CCT 310C 514d 515d 16140C 16182C 16183C 16189C 16261T 16266A 16519C	1	73 152 210 257 263 709 750 1438 2706 3537 4086 4769 6960 7028 8281-8289d 8584 8860 9950 10398 10589 11465 11719 14696 14766 15235 15326 16140 16189 16261 16266A	B5a1d	73 152 210 257 263 16140 16189 16261 16266A	[0.028] B5a1d (1)	[0.032] B5a1 (2) [0.016] B5a1a (3) [0.010] B5a (4) [0.004] B5a1b1 (5) [0.002] B5a1c1a (6) [0.000] B5a1a1 B5a1b B5a1c B5a1c1 B5a1c2 (11)	1
2317	>KX456729.1 Hc	73G 152C 210G 257G 263G 309.1CCT 310C 514d 515d 709A 750G 1438G 2706G 3106d 3107C 3537G 4086T 4769G 6960T 7028T 8271d 8272d 8273d 8274d 8275d 8276d 8277d 8278d 8279d 8584A 8860G 9950C 10398G 10589A 11465C 11719A 14696G 14766T 15235G 15326G 16140C 16182C 16183C 16189C 16261T 16266A 16519C	B5a1d	73G 152C 210G 257G 263G 309.1CCT 310C 514d 515d 16140C 16182C 16183C 16189C 16261T 16266A 16519C	1	73 152 210 257 263 709 750 1438 2706 3537 4086 4769 6960 7028 8281-8289d 8584 8860 9950 10398 10589 11465 11719 14696 14766 15235 15326 16140 16189 16261 16266A	B5a1d	73 152 210 257 263 16140 16189 16261 16266A	[0.028] B5a1d (1)	[0.032] B5a1 (2) [0.016] B5a1a (3) [0.010] B5a (4) [0.004] B5a1b1 (5) [0.002] B5a1c1a (6) [0.000] B5a1a1 B5a1b B5a1c B5a1c1 B5a1c2 (11)	1

2318 >KX456726.1 Hc	73G 150T 263G 310.1C 489C 513.1CA 732G 750G 1438G 2706G 3106d 3107C 4697T 4769G 4973C 7028T 7738C 8701G 8860G 9233C 9509C 9540C 10398G 10400T 10873C 11719A 12025T 12236A 12705T 12715G 14110C 14315T 14323A 14356T 14687G 14766T 14783C 15043A 15301A 15317A 15326G 15706G 15862C 16223T 16234T 16278T 16294T	M51a1b	73G 150T 263G 310.1C 489C 513.1CA 16223T 16234T 16278T 16294T	1	73 150 263 489 732 750 1438 2706 4697 4769 4973 7028 7738 8701 8860 9233 9509 9540 10398 10400 10873 11719 12025 12236 12705 12715 14110 14315 14323 14356 14687 14766 14783 15043 15301 15317 15326 15706 15862 16223 16234 16278 16294	M51a1b	73 150 263 489 16223 16234 16278 16294	[0.000] M51a1b (1)	[0.000] M51a1 (2)	1
2319 >KX456725.1 Hc	73G 152C 263G 329A 513A 709A 750G 1040C 1438G 2706G 3106d 3107C 4491A 4769G 6770G 7028T 8860G 10084C 10192T 10316G 10609C 10877T 11485C 11719A 12173C 13145A 13359A 14766T 15326G 15885T 16288C 16304C 16390A 16519C	R22	73G 152C 263G 329A 513A 16288C 16304C 16390A 16519C	1	73 152 263 329 513 709 750 1040 1438 2706 4491 4769 6770 7028 8860 10084 10192 10316 10609 10877 11485 11719 12173 13145 13359 14766 15326 15885 16288 16304 16390	R22	73 152 263 329 513 16288 16304 16390	[0.834] R22 (1)	[0.057] R9b1 (2) [0.048] R9 (3) [0.017] H32 (4) [0.005] R9c (5) [0.000] H5a3a+152 H5a3a2 R9b R30a1 R30a1a (10)	1
2320 >KX456724.1 Hc	73G 263G 310.1C 329A 513A 593C 750G 1040C 1117G 1438G 2706G 3106d 3107C 4491A 4769G 6770G 7028T 7325G 7598A 8860G 10316G 10609C 10877T 11719A 13145A 13359A 14766T 15326G 15885T 16221T 16249C 16288C 16301T 16304C 16390A 16519C	R22	73G 263G 310.1C 329A 513A 16221T 16249C 16288C 16301T 16304C 16390A 16519C	1	73 263 329 513 593 750 1040 1117 1438 2706 4491 4769 6770 7028 7325 7598 8860 10316 10609 10877 11719 13145 13359 14766 15326 15885 16221 16249 16288 16301 16304 16390	R22	73 263 329 513 16221 16249 16288 16301 16304 16390	[1.609] R22 (1)	[0.016] R9b1 (2) [0.000] R9 R9b R9c (5)	1
2321 >KX456719.1 Hc	73G 263G 310.1C 329A 513A 593C 750G 1040C 1117G 1438G 2706G 3106d 3107C 4491A 4769G 6770G 7028T 7325G 7598A 8860G 10316G 10609C 10877T 11719A 13145A 13359A 14766T 15326G 15885T 16221T 16249C 16288C 16301T 16304C 16390A 16519C	R22	73G 263G 310.1C 329A 513A 16221T 16249C 16288C 16301T 16304C 16390A 16519C	1	73 263 329 513 593 750 1040 1117 1438 2706 4491 4769 6770 7028 7325 7598 8860 10316 10609 10877 11719 13145 13359 14766 15326 15885 16221 16249 16288 16301 16304 16390	R22	73 263 329 513 16221 16249 16288 16301 16304 16390	[1.609] R22 (1)	[0.016] R9b1 (2) [0.000] R9 R9b R9c (5)	1
2322 >KX456716.1 Hc	73G 150T 263G 310.1C 489C 732G 750G 1438G 2706G 3106d 3107C 4697T 4769G 4973C 7028T 7738C 8701G 8860G 9233C 9509C 9540C 10398G 10400T 10873C 11719A 12025T 12236A 12705T 12715G 14110C 14315T 14323A 14356T 14687G 14766T 14783C 15043A 15301A 15317A 15326G 15706G 15862C 16223T 16234T 16278T 16294T	M51a1b	73G 150T 263G 310.1C 489C 16223T 16234T 16278T 16294T	1	73 150 263 489 732 750 1438 2706 4697 4769 4973 7028 7738 8701 8860 9233 9509 9540 10398 10400 10873 11719 12025 12236 12705 12715 14110 14315 14323 14356 14687 14766 14783 15043 15301 15317 15326 15706 15862 16223 16234 16278 16294	M51a1b	73 150 263 489 16223 16234 16278 16294	[0.000] M51a1b (1)	[0.000] M51a1 (2)	1

2323	>KX456714.1 Hc	73G 263G 310.1C 329A 513A 593C 750G 1040C 1117G 1438G 2706G 3106d 3107C 4491A 4769G 6770G 7028T 7325G 7598A 8860G 10316G 10609C 10877T 11719A 13145A 13359A 14766T 15326G 15885T 16221T 16249C 16288C 16301T 16304C 16390A 16519C	R22	73G 263G 310.1C 329A 513A 16221T 16249C 16288C 16301T 16304C 16390A 16519C	1	73 263 329 513 593 750 1040 1117 1438 2706 4491 4769 6770 7028 7325 7598 8860 10316 10609 10877 11719 13145 13359 14766 15326 15885 16221 16249 16288 16301 16304 16390	R22	73 263 329 513 16221 16249 16288 16301 16304 16390	[1.609] R22 (1)	[0.016] R9b1 (2) [0.000] R9 R9b R9c (5)	1
2324	>KX456713.1 Hc	73G 150T 263G 310.1C 489C 732G 750G 1438G 2706G 3106d 3107C 4697T 4769G 4973C 7028T 7738C 8701G 8860G 9233C 9509C 9540C 10398G 10400T 10873C 11719A 12025T 12236A 12705T 12715G 14110C 14315T 14323A 14356T 14687G 14766T 14783C 15043A 15301A 15317A 15326G 15862C 16192T 16223T 16234T 16278T 16294T	M51a1b	73G 150T 263G 310.1C 489C 16192T 16223T 16234T 16278T 16294T	1	73 150 263 489 732 750 1438 2706 4697 4769 4973 7028 7738 8701 8860 9233 9509 9540 10398 10400 10873 11719 12025 12236 12705 12715 14110 14315 14323 14356 14687 14766 14783 15043 15301 15317 15326 15862 16192 16223 16234 16278 16294	M51a1b	73 150 263 489 16192 16223 16234 16278 16294	[0.060] M51a1b (1)	[0.000] M51a1 (2)	1
2325	>KX456712.1 Hc	73G 150T 263G 310.1C 489C 732G 750G 1438G 2706G 3106d 3107C 4697T 4769G 4973C 7028T 7738C 8701G 8860G 9233C 9509C 9540C 10398G 10400T 10873C 11719A 12025T 12236A 12705T 12715G 14110C 14315T 14323A 14356T 14687G 14766T 14783C 15043A 15301A 15317A 15326G 15706G 15862C 16223T 16234T 16278T 16294T	M51a1b	73G 150T 263G 310.1C 489C 16223T 16234T 16278T 16294T	1	73 150 263 489 732 750 1438 2706 4697 4769 4973 7028 7738 8701 8860 9233 9509 9540 10398 10400 10873 11719 12025 12236 12705 12715 14110 14315 14323 14356 14687 14766 14783 15043 15301 15317 15326 15706 15862 16223 16234 16278 16294	M51a1b	73 150 263 489 16223 16234 16278 16294	[0.000] M51a1b (1)	[0.000] M51a1 (2)	1
2326	>KX456709.1 Hc	73G 150T 263G 310.1C 489C 732G 750G 1438G 2706G 3106d 3107C 4697T 4769G 4973C 7028T 7738C 8701G 8860G 9233C 9509C 9540C 10398G 10400T 10873C 11719A 12025T 12236A 12705T 12715G 14110C 14315T 14323A 14356T 14687G 14766T 14783C 15043A 15301A 15317A 15326G 15706G 15862C 16223T 16234T 16278T 16294T	M51a1b	73G 150T 263G 310.1C 489C 16223T 16234T 16278T 16294T	1	73 150 263 489 732 750 1438 2706 4697 4769 4973 7028 7738 8701 8860 9233 9509 9540 10398 10400 10873 11719 12025 12236 12705 12715 14110 14315 14323 14356 14687 14766 14783 15043 15301 15317 15326 15706 15862 16223 16234 16278 16294	M51a1b	73 150 263 489 16223 16234 16278 16294	[0.000] M51a1b (1)	[0.000] M51a1 (2)	1
2327	>KX456707.1 Hc	73G 146C 152C 248d 263G 281G 309.1CT 310C 750G 1438G 2706G 3106d 3107C 3290C 3970T 4703C 4769G 5263T 5585A 6392C 7028T 8860G 10310A 10915C 11719A 11776C 12406A 12612G 12630A 13533G 13928C 14016A 14766T 15326G 15670C 15908C 16207G 16304C 16399G	F4a2	73G 146C 152C 248d 263G 281G 309.1CT 310C 16207G 16304C 16399G	1	73 146 152 249d 263 281 750 1438 2706 3290 3970 4703 4769 5263 5585 6392 7028 8860 10310 10915 11719 11776 12406 12612 12630 13533 13928C 14016 14766 15326 15670 15908 16207 16304 16399	F4a2	73 146 152 249d 263 281 16207 16304 16399	[0.000] F4a2 (1)	[0.000] F4a (2)	1

2328 >KX456706.1 Hc	73G 150T 263G 310.1C 489C 732G 750G 1438G 2706G 3106d 3107C 4697T 4769G 4973C 7028T 7738C 8701G 8860G 9233C 9509C 9540C 10398G 10400T 10873C 11719A 12025T 12236A 12705T 12715G 14110C 14315T 14323A 14356T 14687G 14766T 14783C 15043A 15301A 15317A 15326G 15706G 15862C 16223T 16234T 16278T 16294T	M51a1b	73G 150T 263G 310.1C 489C 16223T 16234T 16278T 16294T	1	73 150 263 489 732 750 1438 2706 4697 4769 4973 7028 7738 8701 8860 9233 9509 9540 10398 10400 10873 11719 12025 12236 12705 12715 14110 14315 14323 14356 14687 14766 14783 15043 15301 15317 15326 15706 15862 16223 16234 16278 16294	M51a1b	73 150 263 489 16223 16234 16278 16294	[0.000] M51a1b (1)	[0.000] M51a1 (2)	1
2329 >KX456704.1 Hc	73G 263G 309.1CT 310C 489C 514d 515d 723G 750G 1438G 2706G 3106d 3107C 3140G 3766C 4071T 4769G 5324T 6455T 7028T 8027A 8701G 8860G 9540C 9824C 10398G 10400T 10873C 11549T 11719A 12405T 12705T 14284T 14766T 14783C 15043A 15301A 15326G 16086C 16184T 16223T 16235G 16519C	M7b2a	73G 263G 309.1CT 310C 489C 514d 515d 16086C 16184T 16223T 16235G 16519C	1	73 263 489 723 750 1438 2706 3140 3766 4071 4769 5324 6455 7028 8027 8701 8860 9540 9824 10398 10400 10873 11549 11719 12405 12705 14284 14766 14783 15043 15301 15326 16086 16184 16223 16235	M7b2a	73 263 489 16086 16184 16223 16235	[0.000] M7b2a (1)	[0.000] M7b2 (2)	1
2330 >KX456700.1 Hc	73G 185A 189G 263G 489C 514d 515d 750G 1438G 2706G 3106d 3107C 3897T 4170T 4769G 5336T 5580C 7028T 8701G 8860G 9540C 10398G 10400T 10873C 11359G 11719A 12030G 12372A 12705T 14569A 14727C 14766T 14783C 14788C 14869A 15010G 15043A 15301A 15326G 16108T 16129A 16172C 16223T 16234T 16290T	M12b1b	73G 185A 189G 263G 489C 514d 515d 16108T 16129A 16172C 16223T 16234T 16290T	1	73 185 189 263 489 750 1438 2706 3897 4170 4769 5336 5580 7028 8701 8860 9540 10398 10400 10873 11359 11719 12030 12372 12705 14569 14727 14766 14783 14788 14869 15010 15043 15301 15326 16108 16129 16172 16223 16234 16290	M12b1b	73 185 189 263 489 16108 16129 16172 16223 16234 16290	[0.000] M12b1b (1)	[0.000] M12b M12b1 M12b1a M12b1a1 (5)	1
2331 >KX456693.1 Hc	73G 263G 309.1CT 310C 489C 514d 515d 723G 750G 1438G 2706G 3106d 3107C 3140G 3766C 4071T 4769G 5324T 6455T 7028T 8027A 8701G 8860G 9540C 9824C 10398G 10400T 10873C 11549T 11719A 12405T 12705T 14284T 14766T 14783C 15043A 15301A 15326G 16086C 16184T 16223T 16235G 16519C	M7b2a	73G 263G 309.1CT 310C 489C 514d 515d 16086C 16184T 16223T 16235G 16519C	1	73 263 489 723 750 1438 2706 3140 3766 4071 4769 5324 6455 7028 8027 8701 8860 9540 9824 10398 10400 10873 11549 11719 12405 12705 14284 14766 14783 15043 15301 15326 16086 16184 16223 16235	M7b2a	73 263 489 16086 16184 16223 16235	[0.000] M7b2a (1)	[0.000] M7b2 (2)	1
2332 >KX456674.1 Hc	73G 150T 263G 513.1CA 750G 1041G 1438G 2706G 3106d 3107C 4386C 4769G 5231A 5417A 5492C 5585A 7028T 8860G 11719A 12358G 12372A 12705T 12771A 13488C 14766T 15094T 15326G 16223T 16257A 16261T 16311C 16390A	N9a10+16311	73G 150T 263G 513.1CA 16223T 16257A 16261T 16311C 16390A	1	73 150 263 750 1041 1438 2706 4386 4769 5231 5417 5492 5585 7028 8860 11719 12358 12372 12705 12771 13488 14766 15094 15326 16223 16257A 16261 16311 16390	N9a10+16311	73 150 263 16223 16257A 16261 16311 16390	[0.317] N9a10+16311 (1)	[0.022] N9a N9a9 (3) [0.000] N9a8 N9a10 N9a10b (6)	1

2333 >KX456654.1 Hc	73G 146A 199C 263G 489C 514d 515d 750G 1438G 2706G 3106d 3107C 3606G 4071T 4769G 4850T 5442C 6249A 6455T 7028T 8701G 8860G 8989A 9540C 9824C 10398G 10400T 10873C 11665T 11719A 12091C 12705T 14766T 14783C 15043A 15236G 15301A 15326G 16362C 16519C	M7c1c2	73G 146A 199C 263G 489C 514d 515d 16362C 16519C	1	73 146A 199 263 489 750 1438 2706 3606 4071 4769 4850 5442 6249 6455 7028 8701 8860 8989 9540 9824 10398 10400 10873 11665 11719 12091 12705 14766 14783 15043 15236 15301 15326 16362	M7c1c2	73 146A 199 263 489 16362	[0.060] M7c1c2 (1)	[0.000] M7c1c2a D4s (3)	1
2334 >KX456652.1 Hc	63C 64T 66A 73G 215G 263G 310.1C 489C 514d 515d 750G 1438G 2706G 3106d 3107C 4769G 5054A 6185C 6575G 7028T 8251A 8701G 8860G 9540C 10268T 10398G 10400T 10873C 11719A 12705T 12850G 14311C 14766T 14783C 15043A 15301A 15326G 16093C 16223T 16311C 16362C 16381C	M74a	63C 64T 66A 73G 215G 263G 310.1C 489C 514d 515d 16093C 16223T 16311C 16362C 16381C	1	63 64 66 73 215 263 489 750 1438 2706 4769 5054 6185 6575 7028 8251 8701 8860 9540 10268 10398 10400 10873 11719 12705 12850 14311 14766 14783 15043 15301 15326 16093 16223 16311 16362 16381	M74a	63 64 66 73 215 263 489 16093 16223 16311 16362 16381	[0.413] M74a (1)	[0.048] M43a1 (2) [0.024] D4j+16311 (3) [0.000] M74 M74b M74b2 D4j11 (7)	1
2335 >KX456649.1 Hc	73G 182T 185A 263G 309.1CT 310C 379G 514d 515d 750G 1438G 2706G 3106d 3107C 3548C 4769G 5465C 7028T 8271d 8272d 8273d 8274d 8275d 8276d 8277d 8278d 8279d 8860G 9123A 10238C 11719A 14766T 15326G 15544A 16168T 16182C 16183C 16189C 16217C 16261T 16311C 16519C	B4a1e	73G 182T 185A 263G 309.1CT 310C 379G 514d 515d 16168T 16182C 16183C 16189C 16217C 16261T 16311C 16519C	1	73 182 185 263 379 750 1438 2706 3548 4769 5465 7028 8281-8289d 8860 9123 10238 11719 14766 15326 15544A 16168 16189 16217 16261 16311	B4a1e	73 182 185 263 379 16168 16189 16217 16261 16311	[0.114] B4a1e (1)	[0.010] B4a1+16311 (2)	1
2336 >KX456648.1 Hc	73G 152C 210G 263G 514d 515d 709A 750G 1438G 2706G 3106d 3107C 3537G 4086T 4769G 6960T 7028T 8271d 8272d 8273d 8274d 8275d 8276d 8277d 8278d 8279d 8584A 8860G 9950C 10398G 11465C 11719A 14766T 15235G 15326G 16140C 16182C 16183C 16189C 16261T 16266A 16519C	B5a1d	73G 152C 210G 263G 514d 515d 16140C 16182C 16183C 16189C 16261T 16266A 16519C	1	73 152 210 263 709 750 1438 2706 3537 4086 4769 6960 7028 8281-8289d 8584 8860 9950 10398 11465 11719 14766 15235 15326 16140 16189 16261 16266A	B5a1d	73 152 210 263 16140 16189 16261 16266A	[0.000] B5a1d (1)	[0.032] B5a1 (2) [0.016] B5a1a (3) [0.010] B5a (4) [0.004] B5a1b1 (5) [0.002] B5a1c1a (6) [0.000] B5a1a1 B5a1b B5a1c B5a1c1 B5a1c2 (11)	1
2337 >KX456647.1 Hc	73G 204C 207A 248d 263G 309.1CCT 310C 709A 750G 1438G 2706G 3106d 3107C 3434G 3970T 4769G 5585A 5894G 5913A 5978G 6392C 7028T 8860G 9854C 10310A 10320A 10499G 11065G 11719A 12621T 13928C 14766T 14971C 15326G 15943C 16260T 16298C 16355T 16362C	F3a1	73G 204C 207A 248d 263G 309.1CCT 310C 16260T 16298C 16355T 16362C	1	73 204 207 249d 263 709 750 1438 2706 3434 3970 4769 5585 5894 5913 5978 6392 7028 8860 9854 10310 10320 10499 11065 11719 12621 13928C 14766 14971 15326 15943 16260 16298 16355 16362	F3a1	73 204 207 249d 263 16260 16298 16355 16362	[0.120] F3a1 (1)	[0.039] F3a+207 (2)	1
2338 >KX456646.1 Hc	73G 248d 263G 310.1C 514d 515d 750G 1005C 1438G 1824C 2706G 3106d 3107C 3579G 3970T 4065G 4769G 4811G 6392C 7028T 7828G 8860G 10310A 10535C 10586A 11719A 12338C 13708A 13928C 14766T 15326G 16092A 16189C 16291T 16304C	F2b1	73G 248d 263G 310.1C 514d 515d 16092A 16189C 16291T 16304C	1	73 249d 263 750 1005 1438 1824 2706 3579 3970 4065 4769 4811 6392 7028 7828 8860 10310 10535 10586 11719 12338 13708 13928C 14766 15326 16092A 16189 16291 16304	F2b1	73 249d 263 16092A 16189 16291 16304	[0.183] F2b1 (1)	[0.047] F2g (2) [0.026] F2+16291 (3) [0.002] F1e1 (4) [0.000] F1+16189 F1e F1g F1g1 F2b F2c1 (10)	1

2339	>KX456645.1 H	73G 207A 248d 263G 309.1CCT 310C 709A 750G 1438G 2706G 3106d 3107C 3434G 3970T 4769G 4824G 4991A 5585A 5894G 5913A 5978G 6392C 7028T 8860G 10310A 10320A 11065G 11719A 12621T 13928C 14766T 14971C 15326G 15412G 16260T 16298C 16355T 16362C	F3a1	73G 207A 248d 263G 309.1CCT 310C 16260T 16298C 16355T 16362C	1	73 207 249d 263 709 750 1438 2706 3434 3970 4769 4824 4991 5585 5894 5913 5978 6392 7028 8860 10310 10320 11065 11719 12621 13928C 14766 14971 15326 15412G 16260 16298 16355 16362	F3a1	73 207 249d 263 16260 16298 16355 16362	[0.000] F3a1 (1)	[0.000] F3a+207 (2)	1
2340	>KX456644.1 H	64T 73G 93G 189G 200G 263G 309.1CT 310C 485C 489C 750G 1438G 2706G 3106d 3107C 3447G 4164G 4769G 5894.1C 5999C 7028T 7064C 8701G 8860G 9509C 9540C 9554A 9770C 10398G 10400T 10873C 11719A 11887A 12705T 13101G 13194A 14325C 14766T 14783C 14902T 15043A 15301A 15326G 15622C 15652G 16129A 16223T 16287T 16311C 16327A	M91a	64T 73G 93G 189G 200G 263G 309.1CT 310C 485C 489C 16129A 16223T 16287T 16311C 16327A	1	64 73 93 189 200 263 485 489 750 1438 2706 3447 4164 4769 5899.1C 5999 7028 7064 8701 8860 9509 9540 9554 9770 10398 10400 10873 11719 11887 12705 13101 13194 14325 14766 14783 14902 15043 15301 15326 15622 15652 16129 16223 16287 16311 16327A	M91a	64 73 93 189 200 263 485 489 16129 16223 16287 16311 16327A	[0.710] M91a (1)	[0.000] M91 (2)	1
2341	>KX456636.1 H	73G 150T 199C 204C 263G 310.1C 489C 750G 1438G 2706G 3106d 3107C 3483A 4048A 4071T 4164G 4769G 5351G 5460A 6455T 6680C 7028T 7684C 7853A 8701G 8860G 9540C 9824C 10398G 10400T 10873C 11719A 12405T 12705T 12811C 14766T 14783C 15043A 15301A 16223T 16297C	M7b1a1b	73G 150T 199C 204C 263G 310.1C 489C 16223T 16297C	1	73 150 199 204 263 489 750 1438 2706 3483 4048 4071 4164 4769 5351 5460 6455 6680 7028 7684 7853 8701 8860 9540 9824 10398 10400 10873 11719 12405 12705 12811 14766 14783 15043 15301 16223 16297	M7b1a1b	73 150 199 204 263 489 16223 16297	[0.000] M7b1a1b (1)	[0.042] M7b1a1+(16192) (2) [0.018] M7b1a1e1 (3) [0.015] M7b1a1 (4) [0.014] M7b1a1a3 (5) [0.010] M7b1a1f (6) [0.004] M7b1a1h (7) [0.002] M7b1a1g (8) [0.000] M7b1a1d M7b1a1d1 M7b1a1e (11)	1
2342	>KX456635.1 H	73G 150T 199C 204C 263G 310.1C 489C 750G 1438G 2706G 3106d 3107C 3483A 4048A 4071T 4164G 4769G 5351G 5460A 6455T 6680C 7028T 7684C 7853A 8701G 8860G 9540C 9824C 10398G 10400T 10873C 11719A 12405T 12705T 12811C 14766T 14783C 15043A 15301A 16223T 16297C	M7b1a1b	73G 150T 199C 204C 263G 310.1C 489C 16223T 16297C	1	73 150 199 204 263 489 750 1438 2706 3483 4048 4071 4164 4769 5351 5460 6455 6680 7028 7684 7853 8701 8860 9540 9824 10398 10400 10873 11719 12405 12705 12811 14766 14783 15043 15301 16223 16297	M7b1a1b	73 150 199 204 263 489 16223 16297	[0.000] M7b1a1b (1)	[0.042] M7b1a1+(16192) (2) [0.018] M7b1a1e1 (3) [0.015] M7b1a1 (4) [0.014] M7b1a1a3 (5) [0.010] M7b1a1f (6) [0.004] M7b1a1h (7) [0.002] M7b1a1g (8) [0.000] M7b1a1d M7b1a1d1 M7b1a1e (11)	1
2343	>KX456620.1 H	73G 263G 309.1CCT 310C 489C 750G 1438G 2706G 2759C 2835T 3106d 3107C 4715G 4769G 6179A 7028T 7196A 8584A 8684T 8701G 8860G 9540C 10398G 10400T 10688A 10873C 11176A 11719A 12705T 13131T 14470C 14766T 14783C 15043A 15301A 15326G 15487T 16184T 16189C 16223T 16258C 16298C 16319A 16470A 16471A 16473A	M8a2a1	73G 263G 309.1CCT 310C 489C 16184T 16189C 16223T 16258C 16298C 16319A 16470A 16471A 16473A	1	73 263 489 750 1438 2706 2759 2835 4715 4769 6179 7028 7196A 8584 8684 8701 8860 9540 10398 10400 10688 10873 11176 11719 12705 13131 14470 14766 14783 15043 15301 15326 15487T 16184 16189 16223 16258C 16298 16319 16470 16471 16473	M8a2a1	73 263 489 16184 16189 16223 16258C 16298 16319 16470 16471 16473	[0.738] M8a2a1 (1)	[0.037] M8a3 (2) [0.033] M8a2c (3) [0.021] M8a2a (4) [0.000] M8a2'3 M8a2 M8a2e (7)	1

2344	>KX456619.1 Hc	73G 150T 199C 204C 263G 309.1CT 310C 489C 750G 1438G 2706G 3106d 3107C 3483A 4048A 4071T 4164G 4769G 5351G 5460A 6455T 6680C 7028T 7684C 7853A 8701G 8860G 9540C 9824C 10398G 10400T 10873C 11719A 12405T 12705T 12811C 14766T 14783C 15043A 15301A 15326G 16223T 16297C	M7b1a1b	73G 150T 199C 204C 263G 309.1CT 310C 489C 16223T 16297C	1	73 150 199 204 263 489 750 1438 2706 3483 4048 4071 4164 4769 5351 5460 6455 6680 7028 7684 7853 8701 8860 9540 9824 10398 10400 10873 11719 12405 12705 12811 14766 14783 15043 15301 15326 16223 16297	M7b1a1b	73 150 199 204 263 489 16223 16297	[0.000] M7b1a1b (1)	[0.042] M7b1a1+(16192) (2) [0.018] M7b1a1e1 (3) [0.015] M7b1a1 (4) [0.014] M7b1a1a3 (5) [0.010] M7b1a1f (6) [0.004] M7b1a1h (7) [0.002] M7b1a1g (8) [0.000] M7b1a1d M7b1a1d1 M7b1a1e (11)	1
2345	>KX456617.1 Hc	73G 146C 248d 263G 281G 310.1C 750G 1438G 2706G 3106d 3107C 3290C 3970T 4769G 5069G 5263T 6392C 7028T 8860G 10310A 10909C 10915C 11719A 11776C 12612G 12630A 13368A 13928C 14016A 14766T 15326G 15670C 15908C 16129A 16185T 16207G 16399G 16519C	F4a2	73G 146C 248d 263G 281G 310.1C 16129A 16185T 16207G 16399G 16519C	1	73 146 249d 263 281 750 1438 2706 3290 3970 4769 5069 5263 6392 7028 8860 10310 10909 10915 11719 11776 12612 12630 13368 13928C 14016 14766 15326 15670 15908 16129 16185 16207 16399	F4a2	73 146 249d 263 281 16129 16185 16207 16399	[1.118] F4a2 (1)	[0.750] F4a (2)	1
2346	>KX456616.1 Hc	73G 150T 152C 199C 204C 263G 310.1C 456T 489C 750G 1438G 2706G 3106d 3107C 4048A 4071T 4164G 4769G 5351G 5460A 6455T 6680C 7028T 7684C 7853A 8701G 8860G 9540C 9824C 9868A 10398G 10400T 10873C 11719A 12405T 12705T 12811C 14766T 14783C 14978G 15043A 15301A 15326G 16129A 16183C 16189C 16223T 16297C 16519C	M7b1a1a3	73G 150T 152C 199C 204C 263G 310.1C 456T 489C 16129A 16183C 16189C 16223T 16297C 16519C	1	73 150 152 199 204 263 456 489 750 1438 2706 4048 4071 4164 4769 5351 5460 6455 6680 7028 7684 7853 8701 8860 9540 9824 9868 10398 10400 10873 11719 12405 12705 12811 14766 14783 14978 15043 15301 15326 16129 16189 16223 16297	M7b1a1a3	73 150 152 199 204 263 456 489 16129 16189 16223 16297	[0.046] M7b1a1a3 (1)	[0.018] M7b1a1a (2) [0.006] M7b1a1a2	1
2347	>KX456614.1 Hc	73G 263G 489C 750G 1438G 2706G 2759C 2835T 3106d 3107C 4715G 4769G 6179A 7028T 7196A 8584A 8684T 8701G 8860G 9540C 10398G 10400T 10688A 10873C 11176A 11719A 12705T 13131T 14470C 14766T 14783C 15043A 15301A 15326G 15487T 16184T 16189C 16223T 16298C 16319A 16470A 16471A 16473A	M8a2a1	73G 263G 489C 16184T 16189C 16223T 16298C 16319A 16470A 16471A 16473A	1	73 263 489 750 1438 2706 2759 2835 4715 4769 6179 7028 7196A 8584 8684 8701 8860 9540 10398 10400 10688 10873 11176 11719 12705 13131 14470 14766 14783 15043 15301 15326 15487T 16184 16189 16223 16298 16319 16470 16471 16473	M8a2a1	73 263 489 16184 16189 16223 16298 16319 16470 16471 16473	[0.734] M8a2a1 (1)	[0.037] M8a3 (2) [0.033] M8a2c (3) [0.021] M8a2a (4) [0.000] M8a2'3 M8a2 M8a2e (7)	1
2348	>KX456613.1 Hc	73G 150T 199C 204C 263G 489C 750G 1438G 2706G 3106d 3107C 3483A 4048A 4071T 4164G 4769G 5351G 5460A 6455T 6680C 7028T 7684C 7853A 8701G 8860G 9540C 9824C 10398G 10400T 10873C 11719A 12405T 12705T 12811C 14766T 14783C 15043A 15301A 15326G 16223T 16297C	M7b1a1b	73G 150T 199C 204C 263G 489C 16223T 16297C	1	73 150 199 204 263 489 750 1438 2706 3483 4048 4071 4164 4769 5351 5460 6455 6680 7028 7684 7853 8701 8860 9540 9824 10398 10400 10873 11719 12405 12705 12811 14766 14783 15043 15301 15326 16223 16297	M7b1a1b	73 150 199 204 263 489 16223 16297	[0.000] M7b1a1b (1)	[0.042] M7b1a1+(16192) (2) [0.018] M7b1a1e1 (3) [0.015] M7b1a1 (4) [0.014] M7b1a1a3 (5) [0.010] M7b1a1f (6) [0.004] M7b1a1h (7) [0.002] M7b1a1g (8) [0.000] M7b1a1d M7b1a1d1 M7b1a1e (11)	1

2349	>KX456611.1 Hc	73G 207A 215G 248d 263G 309.1CT 310C 709A 750G 1438G 2706G 3106d 3107C 3434G 3970T 4769G 4824G 4991A 5585A 5894G 5913A 5978G 6392C 7028T 8860G 10310A 10320A 11065G 11719A 12621T 13145A 13928C 14766T 14971C 15326G 15412G 15808G 16260T 16298C 16355T 16362C	F3a1	73G 207A 215G 248d 263G 309.1CT 310C 16260T 16298C 16355T 16362C	1	73 207 215 249d 263 709 750 1438 2706 3434 3970 4769 4824 4991 5585 5894 5913 5978 6392 7028 8860 10310 10320 11065 11719 12621 13145 13928C 14766 14971 15326 15412G 15808 16260 16298 16355 16362	F3a1	73 207 215 249d 263 16260 16298 16355 16362	[0.014] F3a1 (1)	[0.000] F3a+207 (2)	1
2350	>KX456933.1 Hc	73G 125C 127C 128T 195C 263G 489C 513.1CA 750G 956d 1438G 2706G 3106d 3107C 4137T 4769G 5054A 7028T 8080A 8155A 8167C 8251A 8616A 8701G 8860G 9438A 9540C 10268T 10398G 10400T 10873C 11719A 12651A 12705T 14766T 14783C 15043A 15226G 15301A 15326G 15908C 16051G 16223T 16246T 16311C 16362C	M74b1	73G 125C 127C 128T 195C 263G 489C 513.1CA 16051G 16223T 16246T 16311C 16362C	1	73 125 127 128 195 263 489 750 960d 1438 2706 4137 4769 5054 7028 8080A 8155 8167 8251 8616 8701 8860 9438 9540 10268 10398 10400 10873 11719 12651 12705 14766 14783 15043 15226 15301 15326 15908 16051 16223 16246T 16311 16362	M74b1	73 125 127 128 195 263 489 16051 16223 16246T 16311 16362	[0.020] M74b1 (1)	[0.039] M74 (2) [0.018] M74b2 (3) [0.013] E2a (4) [0.005] D4j+16311 D4j11 (6) [0.000] L4 L4b M43a1 E2 E2a1 E2a1a E2a2 M74b D4+195 (15)	1
2351	>KX456927.1 Hc	73G 263G 309.1CCT 310C 329A 451.1T 512.1AC 513A 709A 750G 1040C 1438G 2706G 3106d 3107C 4136C 4491A 4769G 6770G 7028T 8764A 8860G 10166C 10316G 10343T 10609C 10877T 11719A 13145A 13287T 13359A 14766T 14790G 15326G 15885T 16249C 16288C 16301T 16304C 16390A 16519C	R22	73G 263G 309.1CCT 310C 329A 451.1T 512.1AC 513A 16249C 16288C 16301T 16304C 16390A 16519C	1	73 263 329 455.1T 513 709 750 1040 1438 2706 4136C 4491 4769 6770 7028 8764 8860 10166 10316 10343 10609 10877 11719 13145 13287 13359 14766 14790 15326 15885 16249 16288 16301 16304 16390	R22	73 263 329 455.1T 513 16249 16288 16301 16304 16390	[1.534] R22 (1)	[0.016] R9b1 (2) [0.000] R9 R9b R9c (5)	1
2352	>KX456917.1 Hc	73G 150T 263G 309.1CCT 310C 513.1CA 750G 1041G 1438G 2706G 3106d 3107C 4386C 4769G 5231A 5417A 5585A 7028T 7521A 8860G 11719A 12358G 12372A 12705T 12771A 13488C 14766T 15094T 15326G 16223T 16257A 16261T 16311C 16390A	N9a10+16311	73G 150T 263G 309.1CCT 310C 513.1CA 16223T 16257A 16261T 16311C 16390A	1	73 150 263 750 1041 1438 2706 4386 4769 5231 5417 5585 7028 7521 8860 11719 12358 12372 12705 12771 13488 14766 15094 15326 16223 16257A 16261 16311 16390	N9a10+16311	73 150 263 16223 16257A 16261 16311 16390	[0.317] N9a10+16311 (1)	[0.022] N9a N9a9 (3) [0.000] N9a8 N9a10 N9a10b (6)	1
2353	>KX456914.1 Hc	73G 152C 263G 310.1C 329A 513A 593C 750G 1040C 1117G 1438G 2706G 3106d 3107C 4136G 4491A 4769G 6710G 6770G 7028T 7444A 8860G 10316G 10609C 10877T 11719A 13145A 13359A 14766T 15326G 15885T 16249C 16288C 16301T 16304C 16390A 16519C	R22	73G 152C 263G 310.1C 329A 513A 16249C 16288C 16301T 16304C 16390A 16519C	1	73 152 263 329 513 593 750 1040 1117 1438 2706 4136 4491 4769 6710 6770 7028 7444 8860 10316 10609 10877 11719 13145 13359 14766 15326 15885 16249 16288 16301 16304 16390	R22	73 152 263 329 513 16249 16288 16301 16304 16390	[1.390] R22 (1)	[0.057] R9b1 (2) [0.048] R9 (3) [0.017] H32 (4) [0.005] R9c (5) [0.000] H5a3a+152 H5a3a2 R9b R30a1 R30a1a (10)	1

2354 >KX456913.1 Hc	73G 152C 263G 310.1C 329A 513A 593C 750G 1040C 1117G 1438G 2706G 3106d 3107C 4136G 4491A 4769G 6710G 6770G 7028T 7444A 8860G 10316G 10609C 10877T 11719A 13145A 13359A 14766T 15326G 15885T 16249C 16288C 16301T 16304C 16390A 16519C	R22	73G 152C 263G 310.1C 329A 513A 16249C 16288C 16301T 16304C 16390A 16519C	1	73 152 263 329 513 593 750 1040 1117 1438 2706 4136 4491 4769 6710 6770 7028 7444 8860 10316 10609 10877 11719 13145 13359 14766 15326 15885 16249 16288 16301 16304 16390	R22	73 152 263 329 513 16249 16288 16301 16304 16390	[1.390] R22 (1)	[0.057] R9b1 (2) [0.048] R9 (3) [0.017] H32 (4) [0.005] R9c (5) [0.000] H5a3a+152 H5a3a2 R9b R30a1 R30a1a (10)	1
2355 >KX456907.1 Hc	73G 152C 263G 310.1C 329A 513A 593C 750G 1040C 1117G 1438G 2706G 3106d 3107C 4136G 4491A 4769G 6710G 6770G 7028T 7444A 8860G 10316G 10609C 10877T 11719A 13145A 13359A 14766T 15326G 15885T 16249C 16288C 16301T 16304C 16390A 16519C	R22	73G 152C 263G 310.1C 329A 513A 16249C 16288C 16301T 16304C 16390A 16519C	1	73 152 263 329 513 593 750 1040 1117 1438 2706 4136 4491 4769 6710 6770 7028 7444 8860 10316 10609 10877 11719 13145 13359 14766 15326 15885 16249 16288 16301 16304 16390	R22	73 152 263 329 513 16249 16288 16301 16304 16390	[1.390] R22 (1)	[0.057] R9b1 (2) [0.048] R9 (3) [0.017] H32 (4) [0.005] R9c (5) [0.000] H5a3a+152 H5a3a2 R9b R30a1 R30a1a (10)	1
2356 >KX456900.1 Hc	73G 152C 263G 310.1C 329A 513A 593C 750G 1040C 1117G 1438G 2706G 3106d 3107C 4136G 4491A 4769G 6710G 6770G 7028T 7444A 8860G 10316G 10609C 10877T 11719A 13145A 13359A 14766T 15326G 15885T 16249C 16288C 16301T 16304C 16390A 16519C	R22	73G 152C 263G 310.1C 329A 513A 16249C 16288C 16301T 16304C 16390A 16519C	1	73 152 263 329 513 593 750 1040 1117 1438 2706 4136 4491 4769 6710 6770 7028 7444 8860 10316 10609 10877 11719 13145 13359 14766 15326 15885 16249 16288 16301 16304 16390	R22	73 152 263 329 513 16249 16288 16301 16304 16390	[1.390] R22 (1)	[0.057] R9b1 (2) [0.048] R9 (3) [0.017] H32 (4) [0.005] R9c (5) [0.000] H5a3a+152 H5a3a2 R9b R30a1 R30a1a (10)	1
2357 >KX456898.1 Hc	73G 152C 263G 310.1C 329A 513A 593C 750G 1040C 1117G 1438G 2706G 3106d 3107C 4136G 4491A 4769G 6710G 6770G 7028T 7444A 8860G 10316G 10609C 10877T 11719A 13145A 13359A 14766T 15326G 15885T 16249C 16288C 16301T 16304C 16390A 16519C	R22	73G 152C 263G 310.1C 329A 513A 16249C 16288C 16301T 16304C 16390A 16519C	1	73 152 263 329 513 593 750 1040 1117 1438 2706 4136 4491 4769 6710 6770 7028 7444 8860 10316 10609 10877 11719 13145 13359 14766 15326 15885 16249 16288 16301 16304 16390	R22	73 152 263 329 513 16249 16288 16301 16304 16390	[1.390] R22 (1)	[0.057] R9b1 (2) [0.048] R9 (3) [0.017] H32 (4) [0.005] R9c (5) [0.000] H5a3a+152 H5a3a2 R9b R30a1 R30a1a (10)	1
2358 >KX456896.1 Hc	73G 263G 309.1CT 310C 329A 512.1AC 513A 750G 1040C 1438G 2706G 3106d 3107C 3757T 4491A 4769G 6770G 7028T 8860G 10166C 10316G 10609C 10877T 11428A 11719A 12530G 13145A 13174C 13287T 13359A 14314G 14766T 14790G 15326G 15885T 16249C 16288C 16304C 16390A 16519C	R22	73G 263G 309.1CT 310C 329A 512.1AC 513A 16249C 16288C 16304C 16390A 16519C	1	73 263 329 513 750 1040 1438 2706 3757 4491 4769 6770 7028 8860 10166 10316 10609 10877 11428A 11719 12530 13145 13174 13287 13359 14314 14766 14790 15326 15885 16249 16288 16304 16390	R22	73 263 329 513 16249 16288 16304 16390	[0.946] R22 (1)	[0.016] R9b1 (2) [0.000] R9 R9b R9c (5)	1

2359	>KX456895.1 Hc	73G 195C 248d 263G 309.1CCT 310C 514d 515d 750G 1005C 1438G 1824C 2706G 2707G 3106d 3107C 3970T 4769G 4811G 5090C 5442C 6164T 6392C 6782C 7028T 7828G 8860G 10310A 10535C 10586A 11719A 12338C 12715G 13708A 13928C 14766T 15052G 15326G 15900C 16092A 16291T 16304C	F2b1	73G 195C 248d 263G 309.1CCT 310C 514d 515d 16092A 16291T 16304C	1	73 195 249d 263 750 1005 1438 1824 2706 2707 3970 4769 4811 5090 5442 6164 6392 6782 7028 7828 8860 10310 10535 10586 11719 12338 12715 13708 13928C 14766 15052 15326 15900 16092A 16291 16304	F2b1	73 195 249d 263 16092A 16291 16304	[0.070] F2b1 (1)	[0.013] F2+16291 (2) [0.000] F2b F2g F2+195 F2h (6)	1
2360	>KX456890.1 Hc	73G 152C 263G 310.1C 329A 750G 955.1C 1040C 1438G 2706G 2836T 3106d 3107C 3210T 4634C 4639C 4769G 6770G 7028T 8387A 8860G 10316G 10609C 10653A 11473G 11719A 12026G 12810G 13302T 13359A 14766T 15326G 16249C 16288C 16304C 16305G 16519C	R22	73G 152C 263G 310.1C 329A 16249C 16288C 16304C 16305G 16519C	1	73 152 263 329 750 960.1C 1040 1438 2706 2836 3210 4634 4639 4769 6770 7028 8387 8860 10316 10609 10653 11473 11719 12026 12810 13302T 13359 14766 15326 16249 16288 16304 16305	R22	73 152 263 329 16249 16288 16304 16305	[0.005] R22 (1)	[0.053] R9 (2) [0.005] R9c (3) [0.000] H32 R9b R30a1 R30a1a (7)	1
2361	>KX456887.1 Hc	73G 146C 152C 195C 263G 309.1CT 310C 489C 514d 515d 750G 951A 1438G 2706G 3106d 3107C 4769G 5773A 7028T 7040C 8701G 8860G 9540C 10398G 10400T 10873C 11719A 12705T 13359A 14766T 14783C 15043A 15301A 15326G 15601C 16223T 16311C 16519C	M24a	73G 146C 152C 195C 263G 309.1CT 310C 489C 514d 515d 16223T 16311C 16519C	1	73 146 152 195 263 489 750 951 1438 2706 4769 5773 7028 7040 8701 8860 9540 10398 10400 10873 11719 12705 13359 14766 14783 15043 15301 15326 15601 16223 16311	M24a	73 146 152 195 263 489 16223 16311	[0.000] M24a (1)	[0.022] M57+152 (2) [0.016] M24	1
2362	>KX456885.1 Hc	61A 62A 73G 263G 309.1CCT 310C 514d 515d 750G 1438G 2378T 2706G 3106d 3107C 4769G 6482T 7028T 8215T 8271d 8272d 8273d 8274d 8275d 8276d 8277d 8278d 8279d 8860G 9968T 11719A 14766T 15244G 15326G 16180d 16182C 16183C 16189C 16213A 16217C 16261T 16292T 16519C	B4g2	61A 62A 73G 263G 309.1CCT 310C 514d 515d 16180d 16182C 16183C 16189C 16213A 16217C 16261T 16292T 16519C	1	61A 62 73 263 750 1438 2378 2706 4769 6482 7028 8215 8281-8289d 8860 9968 11719 14766 15244 15326 16181C 16189 16193d 16213 16217 16261 16292	B4g2	61A 62 73 263 16181C 16189 16193d 16213 16217 16261 16292	[2.570] B4g2 (1) [0.641] B4g1 (2) [0.243] B4g1a (3) [0.000] B4g (4)	[0.246] B4g1b (5)	1
2363	>KX456883.1 Hc	73G 146C 263G 310.1C 489C 514d 515d 750G 1438G 2706G 3106d 3107C 3912G 4071T 4769G 4850T 5378G 6455T 7028T 8701G 8860G 9449T 9540C 9824C 10398G 10400T 10873C 10897T 11665T 11719A 11932T 12091C 12705T 14053G 14766T 14783C 15043A 15301A 15326G 15338T 16172C 16223T 16291T 16311C 16519C	M7c2a	73G 146C 263G 310.1C 489C 514d 515d 16172C 16223T 16291T 16311C 16519C	1	73 146 263 489 750 1438 2706 3912 4071 4769 4850 5378 6455 7028 8701 8860 9449 9540 9824 10398 10400 10873 10897 11665 11719 11932 12091 12705 14053 14766 14783 15043 15301 15326 15338 16172 16223 16291 16311	M7c2a	73 146 263 489 16172 16223 16291 16311	[0.000] M7c2a (1)	[0.000] M7c2 M7c2b (3)	1

2364	>KX456881.1 H[illegible]	73G 146C 152C 195C 263G 309.1CT 310C 489C 514d 515d 750G 951A 1438G 2706G 3106d 3107C 4769G 5773A 7028T 7040C 8701G 8860G 9540C 10398G 10400T 10873C 11719A 12705T 13359A 14766T 14783C 15043A 15301A 15326G 15601C 16223T 16311C 16519C	M24a	73G 146C 152C 195C 263G 309.1CT 310C 489C 514d 515d 16223T 16311C 16519C	1	73 146 152 195 263 489 750 951 1438 2706 4769 5773 7028 7040 8701 8860 9540 10398 10400 10873 11719 12705 13359 14766 14783 15043 15301 15326 15601 16223 16311	M24a	73 146 152 195 263 489 16223 16311	[0.000] M24a (1)	[0.022] M57+152 (2) [0.016] M24	1
2365	>KX456869.1 H[illegible]	73G 146A 152C 199C 263G 309.1CT 310C 489C 514d 515d 750G 1438G 2706G 3106d 3107C 3606G 4071T 4769G 4850T 5442C 6455T 7028T 8337C 8701G 8860G 8928C 9540C 9824C 10398G 10400T 10873C 11665T 11719A 12091C 12705T 14766T 14783C 15043A 15236G 15301A 15326G 16519C	M7c1c2	73G 146A 152C 199C 263G 309.1CT 310C 489C 514d 515d 16519C	1	73 146A 152 199 263 489 750 1438 2706 3606 4071 4769 4850 5442 6455 7028 8337 8701 8860 8928 9540 9824 10398 10400 10873 11665 11719 12091 12705 14766 14783 15043 15236 15301 15326	M7c1c2	73 146A 152 199 263 489	[0.022] M7c1c2 (1)	[0.000] M7c1c2a (2)	1
2366	>KX456868.1 H[illegible]	73G 248d 263G 309.1CT 310C 514d 515d 750G 1005C 1438G 1824C 2706G 3106d 3107C 3199A 3970T 4769G 4811G 5048C 5090C 6392C 6782C 7028T 7828G 8860G 10310A 10535C 10586A 11719A 12338C 13708A 13928C 14766T 15326G 16092A 16291T 16304C 16318G	F2b1	73G 248d 263G 309.1CT 310C 514d 515d 16092A 16291T 16304C 16318G	1	73 249d 263 750 1005 1438 1824 2706 3199A 3970 4769 4811 5048 5090 6392 6782 7028 7828 8860 10310 10535 10586 11719 12338 13708 13928C 14766 15326 16092A 16291 16304 16318	F2b1	73 249d 263 16092A 16291 16304 16318	[0.005] F2b1 (1)	[0.013] F2+16291 (2) [0.012] F2g (3) [0.000] F2b	1
2367	>KX456865.1 H[illegible]	73G 143A 183G 263G 309.1CT 310C 514d 515d 750G 1438G 1541C 2706G 3106d 3107C 3970T 4017T 4769G 6962A 7028T 7849T 8860G 11719A 12714C 13928C 14766T 15326G 16192T 16274A 16288C 16304C 16390A 16519C	R9b1a1a	73G 143A 183G 263G 309.1CT 310C 514d 515d 16192T 16274A 16288C 16304C 16390A 16519C	1	73 143 183 263 750 1438 1541 2706 3970 4017 4769 6962 7028 7849 8860 11719 12714 13928C 14766 15326 16192 16274 16288 16304 16390	R9b1a1a	73 143 183 263 16192 16274 16288 16304 16390	[0.105] R9b1a1a (1)	[0.000] R9b1a1 (2)	1
2368	>KX456864.1 H[illegible]	73G 249G 263G 310.1C 489C 514d 515d 750G 1438G 1719A 2706G 3106d 3107C 3531A 4021G 4769G 4901G 6026T 7028T 7289G 7697A 8701G 8750C 8860G 8970T 9380A 9540C 10256C 10398G 10400T 10535C 10873C 11140T 11719A 12705T 13708A 14040A 14766T 14783C 15043A 15301A 15326G 16129A 16140C 16172C 16183C 16189C 16223T 16278T	M59	73G 249G 263G 310.1C 489C 514d 515d 16129A 16140C 16172C 16183C 16189C 16223T 16278T	1	73 249 263 489 750 1438 1719 2706 3531 4021 4769 4901 6026T 7028 7289 7697 8701 8750 8860 8970 9380 9540 10256 10398 10400 10535 10873 11140 11719 12705 13708 14040 14766 14783 15043 15301 15326 16129 16140 16172 16189 16223 16278	M59	73 249 263 489 16129 16140 16172 16189 16223 16278	[0.283] M59 (1)	[0.070] M73 (2) [0.048] G2a1+16189 (3) [0.035] M5 (4) [0.017] M5a (5) [0.012] M5a5 G1b2 G1b4 (8) [0.007] G1b+16129 (9) [0.005] M42b2 (10) [0.000] M51a2 M5a'd M5a2 M5a2a M5a2a1 M5b'c M5b M7b1 G1b3 M13b M33a2'3 M73'79 X (23)	1

2369 >KX456860.1 Hc	73G 146C 150T 152C 195C 308T 310d 489C 750G 1438G 1719A 2706G 3106d 3107C 3316A 3453T 3666A 4755C 4769G 5147A 7028T 7313T 7765G 7844G 8701G 8860G 9540C 10398G 10861C 10873C 11719A 11914A 12279G 12609C 12705T 13269G 14110C 14224T 14356T 14766T 14783C 15043A 15109C 15301A 15326G 15968C 16068C 16126C 16182C 16183C 16189C 16223T 16319A 16325C	M75	73G 146C 150T 152C 195C 308T 310d 489C 16068C 16126C 16182C 16183C 16189C 16223T 16319A 16325C	1	73 146 150 152 195 308-309d 489 750 1438 1719 2706 3316 3453 3666 4755 4769 5147 7028 7313 7765 7844 8701 8860 9540 10398 10861 10873 11719 11914 12279 12609 12705 13269 14110 14224T 14356 14766 14783 15043 15109 15301 15326 15968 16068 16126 16189 16223 16319 16325	M75	73 146 150 152 195 308-309d 489 16068 16126 16189 16223 16319 16325	[0.447] M75 (1)	[0.062] L3e2b6 (2) [0.057] M45 (3) [0.042] L3d1a1b (4) [0.008] M24 (5) [0.000] M24a U5b2a1b (7)	1
2370 >KX456858.1 Hc	73G 185A 189G 263G 309.1CT 310C 489C 514d 515d 750G 1438G 2706G 3106d 3107C 3897T 4170T 4769G 5336T 5580C 7028T 8701G 8860G 9540C 10398G 10400T 10873C 11359G 11719A 12030G 12372A 12705T 14569A 14727C 14766T 14783C 14788C 14869A 15010G 15043A 15301A 15326G 16108T 16129A 16172C 16223T 16234T 16290T	M12b1b	73G 185A 189G 263G 309.1CT 310C 489C 514d 515d 16108T 16129A 16172C 16223T 16234T 16290T	1	73 185 189 263 489 750 1438 2706 3897 4170 4769 5336 5580 7028 8701 8860 9540 10398 10400 10873 11359 11719 12030 12372 12705 14569 14727 14766 14783 14788 14869 15010 15043 15301 15326 16108 16129 16172 16223 16234 16290	M12b1b	73 185 189 263 489 16108 16129 16172 16223 16234 16290	[0.000] M12b1b (1)	[0.000] M12b M12b1 M12b1a M12b1a1 (5)	1
2371 >KX456854.1 Hc	73G 263G 309.1CT 310C 489C 710C 750G 1438G 1719A 2706G 2772T 3106d 3107C 4313C 4454C 4639C 4733C 4769G 5581G 6071C 6776C 7028T 8701G 8860G 9316C 9540C 10398G 10400T 10653A 10873C 11719A 12705T 13917T 14766T 14783C 15043A 15301A 15326G 16219G 16223T 16290T 16291T 16295T 16519C	M22a	73G 263G 309.1CT 310C 489C 16219G 16223T 16290T 16291T 16295T 16519C	1	73 263 489 710 750 1438 1719 2706 2772 4313 4454 4639 4733 4769 5581 6071 6776 7028 8701 8860 9316 9540 10398 10400 10653 10873 11719 12705 13917T 14766 14783 15043 15301 15326 16219 16223 16290 16291 16295	M22a	73 263 489 16219 16223 16290 16291 16295	[0.141] M22a (1)	[0.013] M (2) [0.012] G1b (3) [0.007] M58 (4) [0.003] M21b M21b1 M42'74 (7) [0.000] M1'20'51 M4"67 M37 M43 M43b M5a1 M5a1a M5a1b M7 M7b'c M7b M12'G M12 M19'53 M21 M21b1a M22 M22b M23'75 M24'41 M27 M29'Q M31 M31a M32'56 M33 M33a M33a1 M34'57 M35 M39'70 M42 M52 M52b M55'77 M60 M62 M69 M80'D (46)	1
2372 >KX456852.1 Hc	73G 249G 263G 310.1C 489C 514d 515d 750G 1438G 1719A 2706G 3106d 3107C 3531A 4021G 4769G 4901G 6026T 7028T 7289G 7697A 8701G 8750C 8860G 8970T 9380A 9540C 10256C 10398G 10400T 10535C 10873C 11140T 11719A 12705T 13708A 14040A 14766T 14783C 15043A 15301A 15326G 16129A 16140C 16172C 16183C 16189C 16223T 16278T	M59	73G 249G 263G 310.1C 489C 514d 515d 16129A 16140C 16172C 16183C 16189C 16223T 16278T	1	73 249 263 489 750 1438 1719 2706 3531 4021 4769 4901 6026T 7028 7289 7697 8701 8750 8860 8970 9380 9540 10256 10398 10400 10535 10873 11140 11719 12705 13708 14040 14766 14783 15043 15301 15326 16129 16140 16172 16189 16223 16278	M59	73 249 263 489 16129 16140 16172 16189 16223 16278	[0.283] M59 (1)	[0.070] M73 (2) [0.048] G2a1+16189 (3) [0.035] M5 (4) [0.017] M5a (5) [0.012] M5a5 G1b2 G1b4 (8) [0.007] G1b+16129 (9) [0.005] M42b2 (10) [0.000] M51a2 M5a'd M5a2 M5a2a M5a2a1 M5b'c M5b M7b1 G1b3 M13b M33a2'3 M73'79 X (23)	1

2373	>KX456846.1 Hc	73G 195C 263G 309.1CT 310C 489C 750G 1438G 2706G 3106d 3107C 4769G 5054A 5806C 7028T 8080A 8155A 8167C 8251A 8616A 8701G 8860G 9540C 10268T 10398G 10400T 10873C 11719A 12651A 12705T 13105G 13434G 14766T 14783C 15043A 15226G 15301A 15326G 15908C 15969A 16223T 16246T 16311C 16362C 16519C	M74b1	73G 195C 263G 309.1CT 310C 489C 16223T 16246T 16311C 16362C 16519C	1	73 195 263 489 750 1438 2706 4769 5054 5806 7028 8080A 8155 8167 8251 8616 8701 8860 9540 10268 10398 10400 10873 11719 12651 12705 13105 13434 14766 14783 15043 15226 15301 15326 15908 15969A 16223 16246T 16311 16362	M74b1	73 195 263 489 16223 16246T 16311 16362	[0.000] M74b1 (1)	[0.039] M74 (2) [0.018] M74b2 (3) [0.000] L4 L4b M43a1 M74b D4j+16311 D4j11 D4+195 (10)	1
2374	>KX456845.1 Hc	73G 152C 210G 263G 309.1CT 310C 514d 515d 709A 750G 1438G 2706G 3106d 3107C 3537G 4086T 4769G 6960T 7028T 8271d 8272d 8273d 8274d 8275d 8276d 8277d 8278d 8279d 8584A 8860G 9950C 10398G 11465C 11719A 14766T 15235G 15326G 16140C 16182C 16183C 16189C 16261T 16266A 16519C	B5a1d	73G 152C 210G 263G 309.1CT 310C 514d 515d 16140C 16182C 16183C 16189C 16261T 16266A 16519C	1	73 152 210 263 709 750 1438 2706 3537 4086 4769 6960 7028 8281-8289d 8584 8860 9950 10398 11465 11719 14766 15235 15326 16140 16189 16261 16266A	B5a1d	73 152 210 263 16140 16189 16261 16266A	[0.000] B5a1d (1)	[0.032] B5a1 (2) [0.016] B5a1a (3) [0.010] B5a (4) [0.004] B5a1b1 (5) [0.002] B5a1c1a (6) [0.000] B5a1a1 B5a1b B5a1c B5a1c1 B5a1c2 (11)	1
2375	>KX456844.1 Hc	73G 152C 210G 263G 514d 515d 709A 750G 1438G 2706G 3106d 3107C 3537G 4086T 4769G 6960T 7028T 8271d 8272d 8273d 8274d 8275d 8276d 8277d 8278d 8279d 8584A 8860G 9950C 10398G 11465C 11719A 14766T 15235G 15326G 16140C 16182C 16183C 16189C 16261T 16266A 16519C	B5a1d	73G 152C 210G 263G 514d 515d 16140C 16182C 16183C 16189C 16261T 16266A 16519C	1	73 152 210 263 709 750 1438 2706 3537 4086 4769 6960 7028 8281-8289d 8584 8860 9950 10398 11465 11719 14766 15235 15326 16140 16189 16261 16266A	B5a1d	73 152 210 263 16140 16189 16261 16266A	[0.000] B5a1d (1)	[0.032] B5a1 (2) [0.016] B5a1a (3) [0.010] B5a (4) [0.004] B5a1b1 (5) [0.002] B5a1c1a (6) [0.000] B5a1a1 B5a1b B5a1c B5a1c1 B5a1c2 (11)	1
2376	>KX456841.1 Hc	73G 152C 210G 263G 310.1C 514d 515d 709A 750G 1438G 2706G 3106d 3107C 3537G 4086T 4769G 6960T 7028T 8271d 8272d 8273d 8274d 8275d 8276d 8277d 8278d 8279d 8584A 8860G 9950C 10398G 11465C 11719A 14766T 15235G 15326G 16093C 16140C 16182C 16183C 16189C 16261T 16266A 16519C	B5a1d	73G 152C 210G 263G 310.1C 514d 515d 16093C 16140C 16182C 16183C 16189C 16261T 16266A 16519C	1	73 152 210 263 709 750 1438 2706 3537 4086 4769 6960 7028 8281-8289d 8584 8860 9950 10398 11465 11719 14766 15235 15326 16093 16140 16189 16261 16266A	B5a1d	73 152 210 263 16093 16140 16189 16261 16266A	[0.028] B5a1d (1)	[0.102] B5a (2) [0.032] B5a1 (3) [0.019] B5a1a (4) [0.004] B5a1b1 (5) [0.002] B5a1c1a (6) [0.000] B5a1a1 B5a1b B5a1c B5a1c1 B5a1c2 (11)	1
2377	>KX456838.1 Hc	73G 152C 210G 263G 310.1C 514d 515d 709A 750G 1438G 2706G 2833G 3106d 3107C 3537G 4086T 4769G 6960T 7028T 8271d 8272d 8273d 8274d 8275d 8276d 8277d 8278d 8279d 8584A 8860G 9950C 10398G 11465C 11719A 14766T 15235G 15326G 16093C 16140C 16182C 16183C 16189C 16261T 16266A 16519C	B5a1d	73G 152C 210G 263G 310.1C 514d 515d 16093C 16140C 16182C 16183C 16189C 16261T 16266A 16519C	1	73 152 210 263 709 750 1438 2706 2833 3537 4086 4769 6960 7028 8281-8289d 8584 8860 9950 10398 11465 11719 14766 15235 15326 16093 16140 16189 16261 16266A	B5a1d	73 152 210 263 16093 16140 16189 16261 16266A	[0.028] B5a1d (1)	[0.102] B5a (2) [0.032] B5a1 (3) [0.019] B5a1a (4) [0.004] B5a1b1 (5) [0.002] B5a1c1a (6) [0.000] B5a1a1 B5a1b B5a1c B5a1c1 B5a1c2 (11)	1

2378	>KX456837.1 Hc	73G 152C 210G 263G 514d 515d 709A 750G 1438G 2706G 3106d 3107C 3537G 4086T 4769G 6960T 7028T 8271d 8272d 8273d 8274d 8275d 8276d 8277d 8278d 8279d 8584A 8860G 9950C 10398G 11465C 11719A 14766T 15235G 15326G 16140C 16182C 16183C 16189C 16261T 16266A 16519C	B5a1d	73G 152C 210G 263G 514d 515d 16140C 16182C 16183C 16189C 16261T 16266A 16519C	1	73 152 210 263 709 750 1438 2706 3537 4086 4769 6960 7028 8281-8289d 8584 8860 9950 10398 11465 11719 14766 15235 15326 16140 16189 16261 16266A	B5a1d	73 152 210 263 16140 16189 16261 16266A	[0.000] B5a1d (1)	[0.032] B5a1 (2) [0.016] B5a1a (3) [0.010] B5a (4) [0.004] B5a1b1 (5) [0.002] B5a1c1a (6) [0.000] B5a1a1 B5a1b B5a1c B5a1c1 B5a1c2 (11)	1
2379	>KX456834.1 Hc	73G 195C 263G 309.1CCT 310C 489C 750G 1438G 2706G 3106d 3107C 4769G 5054A 5806C 7028T 8080A 8155A 8167C 8251A 8616A 8701G 8860G 9540C 10268T 10398G 10400T 10873C 11719A 12651A 12705T 13105G 13434G 14384A 14766T 14783C 15043A 15226G 15301A 15326G 15908C 15969A 16223T 16246T 16311C 16362C 16519C	M74b1	73G 195C 263G 309.1CCT 310C 489C 16223T 16246T 16311C 16362C 16519C	1	73 195 263 489 750 1438 2706 4769 5054 5806 7028 8080A 8155 8167 8251 8616 8701 8860 9540 10268 10398 10400 10873 11719 12651 12705 13105 13434 14384 14766 14783 15043 15226 15301 15326 15908 15969A 16223 16246T 16311 16362	M74b1	73 195 263 489 16223 16246T 16311 16362	[0.000] M74b1 (1)	[0.039] M74 (2) [0.018] M74b2 (3) [0.000] L4 L4b M43a1 M74b D4j+16311 D4j11 D4+195 (10)	1
2380	>KX456832.1 Hc	73G 152C 210G 263G 514d 515d 709A 750G 1438G 2706G 3106d 3107C 3537G 4086T 4769G 6960T 7028T 8271d 8272d 8273d 8274d 8275d 8276d 8277d 8278d 8279d 8584A 8860G 9950C 10398G 11465C 11719A 14766T 15235G 15326G 16140C 16182C 16183C 16189C 16261T 16266A 16519C	B5a1d	73G 152C 210G 263G 514d 515d 16140C 16182C 16183C 16189C 16261T 16266A 16519C	1	73 152 210 263 709 750 1438 2706 3537 4086 4769 6960 7028 8281-8289d 8584 8860 9950 10398 11465 11719 14766 15235 15326 16140 16189 16261 16266A	B5a1d	73 152 210 263 16140 16189 16261 16266A	[0.000] B5a1d (1)	[0.032] B5a1 (2) [0.016] B5a1a (3) [0.010] B5a (4) [0.004] B5a1b1 (5) [0.002] B5a1c1a (6) [0.000] B5a1a1 B5a1b B5a1c B5a1c1 B5a1c2 (11)	1
2381	>KX456831.1 Hc	73G 152C 248d 263G 309.1CT 310C 514d 515d 750G 1438G 1598A 2706G 3106d 3107C 3970T 4086T 4769G 6392C 6962A 7028T 8860G 9053A 9548A 10310A 10609C 11719A 12406A 12882T 13759A 13760T 13928C 14766T 15326G 16086C 16129A 16162G 16172C 16293C 16304C 16519C	F1a1	73G 152C 248d 263G 309.1CT 310C 514d 515d 16086C 16129A 16162G 16172C 16293C 16304C 16519C	1	73 152 249d 263 750 1438 1598 2706 3970 4086 4769 6392 6962 7028 8860 9053 9548 10310 10609 11719 12406 12882 13759 13760 13928C 14766 15326 16086 16129 16162 16172 16293C 16304	F1a1	73 152 249d 263 16086 16129 16162 16172 16293C 16304	[0.100] F1a1 (1)	[0.384] F1c1 (2) [0.163] F1a1d (3) [0.078] F1a3 (4) [0.073] F1a1c (5) [0.038] F1a4a (6) [0.027] F1a1b (7) [0.026] F1a1a1 (8) [0.016] F1a1a (9) [0.011] F1a1'4 (10) [0.008] F1a1c3 (11) [0.000] F1a F1a1c2 F1a1d1 F1a4 (15)	1
2382	>KX456830.1 Hc	73G 152C 210G 263G 514d 515d 709A 750G 1438G 2706G 3106d 3107C 3537G 4086T 4769G 6960T 7028T 8271d 8272d 8273d 8274d 8275d 8276d 8277d 8278d 8279d 8584A 8860G 9950C 10398G 11465C 11719A 14766T 15235G 15326G 16140C 16182C 16183C 16189C 16261T 16266A 16519C	B5a1d	73G 152C 210G 263G 514d 515d 16140C 16182C 16183C 16189C 16261T 16266A 16519C	1	73 152 210 263 709 750 1438 2706 3537 4086 4769 6960 7028 8281-8289d 8584 8860 9950 10398 11465 11719 14766 15235 15326 16140 16189 16261 16266A	B5a1d	73 152 210 263 16140 16189 16261 16266A	[0.000] B5a1d (1)	[0.032] B5a1 (2) [0.016] B5a1a (3) [0.010] B5a (4) [0.004] B5a1b1 (5) [0.002] B5a1c1a (6) [0.000] B5a1a1 B5a1b B5a1c B5a1c1 B5a1c2 (11)	1

2383	>KX456829.1 Hc	73G 152C 210G 263G 310.1C 514d 515d 709A 750G 1438G 2706G 2833G 3106d 3107C 3537G 4086T 4769G 6960T 7028T 8271d 8272d 8273d 8274d 8275d 8276d 8277d 8278d 8279d 8584A 8860G 9950C 10398G 11465C 11719A 14766T 15235G 15326G 16093C 16140C 16182C 16183C 16189C 16261T 16266A 16519C	B5a1d	73G 152C 210G 263G 310.1C 514d 515d 16093C 16140C 16182C 16183C 16189C 16261T 16266A 16519C	1	73 152 210 263 709 750 1438 2706 2833 3537 4086 4769 6960 7028 8281-8289d 8584 8860 9950 10398 11465 11719 14766 15235 15326 16093 16140 16189 16261 16266A	B5a1d	73 152 210 263 16093 16140 16189 16261 16266A	[0.028] B5a1d (1)	[0.102] B5a (2) [0.032] B5a1 (3) [0.019] B5a1a (4) [0.004] B5a1b1 (5) [0.002] B5a1c1a (6) [0.000] B5a1a1 B5a1b B5a1c B5a1c1 B5a1c2 (11)	1
2384	>KX456827.1 Hc	73G 152C 210G 263G 309.1CT 310C 514d 515d 709A 750G 1438G 2706G 3106d 3107C 3537G 4086T 4769G 6960T 7028T 8271d 8272d 8273d 8274d 8275d 8276d 8277d 8278d 8279d 8584A 8860G 9950C 10398G 11465C 11719A 14766T 15235G 15326G 16140C 16182C 16183C 16189C 16261T 16266A 16519C	B5a1d	73G 152C 210G 263G 309.1CT 310C 514d 515d 16140C 16182C 16183C 16189C 16261T 16266A 16519C	1	73 152 210 263 709 750 1438 2706 3537 4086 4769 6960 7028 8281-8289d 8584 8860 9950 10398 11465 11719 14766 15235 15326 16140 16189 16261 16266A	B5a1d	73 152 210 263 16140 16189 16261 16266A	[0.000] B5a1d (1)	[0.032] B5a1 (2) [0.016] B5a1a (3) [0.010] B5a (4) [0.004] B5a1b1 (5) [0.002] B5a1c1a (6) [0.000] B5a1a1 B5a1b B5a1c B5a1c1 B5a1c2 (11)	1
2385	>KX456825.1 Hc	73G 152C 210G 263G 514d 515d 709A 750G 1438G 2706G 3106d 3107C 3537G 4086T 4769G 6960T 7028T 8271d 8272d 8273d 8274d 8275d 8276d 8277d 8278d 8279d 8584A 8860G 9950C 10398G 11465C 11719A 14766T 15235G 15326G 16140C 16182C 16183C 16189C 16261T 16266A 16519C	B5a1d	73G 152C 210G 263G 514d 515d 16140C 16182C 16183C 16189C 16261T 16266A 16519C	1	73 152 210 263 709 750 1438 2706 3537 4086 4769 6960 7028 8281-8289d 8584 8860 9950 10398 11465 11719 14766 15235 15326 16140 16189 16261 16266A	B5a1d	73 152 210 263 16140 16189 16261 16266A	[0.000] B5a1d (1)	[0.032] B5a1 (2) [0.016] B5a1a (3) [0.010] B5a (4) [0.004] B5a1b1 (5) [0.002] B5a1c1a (6) [0.000] B5a1a1 B5a1b B5a1c B5a1c1 B5a1c2 (11)	1
2386	>KX456819.1 Hc	73G 146C 152C 195C 198T 263G 309.1CT 310C 489C 514d 515d 750G 951A 1438G 2706G 3106d 3107C 4769G 5773A 7028T 7040C 8701G 8860G 9540C 10398G 10400T 10873C 11719A 12705T 13359A 14163T 14290C 14766T 14783C 15043A 15301A 15326G 15601C 16223T 16311C 16519C	M24a	73G 146C 152C 195C 198T 263G 309.1CT 310C 489C 514d 515d 16223T 16311C 16519C	1	73 146 152 195 198 263 489 750 951 1438 2706 4769 5773 7028 7040 8701 8860 9540 10398 10400 10873 11719 12705 13359 14163 14290 14766 14783 15043 15301 15326 15601 16223 16311	M24a	73 146 152 195 198 263 489 16223 16311	[0.041] M24a (1)	[0.022] M57+152 (2) [0.020] M24	1
2387	>KX456816.1 Hc	73G 146C 195C 263G 310.1C 489C 514d 515d 750G 951A 1438G 2140A 2706G 3106d 3107C 4769G 5773A 7028T 8701G 8860G 9540C 10101C 10398G 10400T 10873C 11719A 12651A 12705T 13359A 13812C 14766T 14783C 15043A 15301A 15326G 15601C 16086C 16223T 16519C	M24b	73G 146C 195C 263G 310.1C 489C 514d 515d 16086C 16223T 16519C	1	73 146 195 263 489 750 951 1438 2140 2706 4769 5773 7028 8701 8860 9540 10101 10398 10400 10873 11719 12651 12705 13359 13812 14766 14783 15043 15301 15326 15601 16086 16223	M24b	73 146 195 263 489 16086 16223	[0.000] M24b (1)	[0.000] M24 (2)	1
2388	>KX456815.1 Hc	73G 263G 309.1CT 310C 329A 513A 750G 980C 1040C 1438G 2706G 3106d 3107C 4491A 4769G 6770G 7028T 8860G 9458T 9921A 10316G 10609C 10877T 11719A 13145A 13359A 14766T 15326G 15885T 16172C 16249C 16288C 16301T 16304C 16390A 16519C	R22	73G 263G 309.1CT 310C 329A 513A 16172C 16249C 16288C 16301T 16304C 16390A 16519C	1	73 263 329 513 750 980 1040 1438 2706 4491 4769 6770 7028 8860 9458 9921 10316 10609 10877 11719 13145 13359 14766 15326 15885 16172 16249 16288 16301 16304 16390	R22	73 263 329 513 16172 16249 16288 16301 16304 16390	[1.561] R22 (1)	[0.043] F1a3a1 (2) [0.041] U6 (3) [0.016] R9b1 (4) [0.014] F1a2 (5) [0.005] R9b (6) [0.000] R9 R9c (8)	1

2389	>KX456814.1 Ho	73G 146C 152C 195C 198T 263G 309.1CT 310C 489C 514d 515d 750G 951A 1438G 2706G 3106d 3107C 4769G 5773A 7028T 7040C 8701G 8860G 9540C 10398G 10400T 10873C 11719A 12705T 13359A 14163T 14290C 14766T 14783C 15043A 15301A 15326G 15601C 16223T 16311C 16519C	M24a	73G 146C 152C 195C 198T 263G 309.1CT 310C 489C 514d 515d 16223T 16311C 16519C	1	73 146 152 195 198 263 489 750 951 1438 2706 4769 5773 7028 7040 8701 8860 9540 10398 10400 10873 11719 12705 13359 14163 14290 14766 14783 15043 15301 15326 15601 16223 16311	M24a	73 146 152 195 198 263 489 16223 16311	[0.041] M24a (1)	[0.022] M57+152 (2) [0.020] M24	1
2390	>KX456813.1 Ho	73G 185A 189G 263G 309.1CCT 310C 514d 515d 709A 750G 1438G 2706G 3106d 3107C 4454C 4769G 5040G 5836G 7028T 8277C 8279C 8860G 10031C 10398G 11061T 11719A 12950G 13269G 13681G 13879C 14322G 14766T 15326G 16182C 16183C 16189C 16311C 16390A 16399G 16519C	R11b1	73G 185A 189G 263G 309.1CCT 310C 514d 515d 16182C 16183C 16189C 16311C 16390A 16399G 16519C	1	73 185 189 263 709 750 1438 2706 4454 4769 5040 5836 7028 8277 8279 8860 10031 10398 11061 11719 12950 13269 13681 13879 14322 14766 15326 16189 16311 16390 16399	R11b1	73 185 189 263 16189 16311 16390 16399	[0.806] R11b1 (1)	[0.042] R11b (2) [0.000] R11 R11a R11b1a R11b1b (6)	1
2391	>KX456811.1 Ho	73G 146C 152C 195C 198T 263G 309.1CT 310C 489C 514d 515d 750G 951A 1438G 2706G 3106d 3107C 4769G 5773A 7028T 7040C 8701G 8860G 9540C 10398G 10400T 10873C 11719A 12705T 13359A 14163T 14290C 14766T 14783C 15043A 15301A 15326G 15601C 16223T 16311C 16519C	M24a	73G 146C 152C 195C 198T 263G 309.1CT 310C 489C 514d 515d 16223T 16311C 16519C	1	73 146 152 195 198 263 489 750 951 1438 2706 4769 5773 7028 7040 8701 8860 9540 10398 10400 10873 11719 12705 13359 14163 14290 14766 14783 15043 15301 15326 15601 16223 16311	M24a	73 146 152 195 198 263 489 16223 16311	[0.041] M24a (1)	[0.022] M57+152 (2) [0.020] M24	1
[truncated: 547,306 more chars]
